# A BAC/BIBAC-based physical map of chickpea, *Cicer arietinum *L

**DOI:** 10.1186/1471-2164-11-501

**Published:** 2010-09-17

**Authors:** Xiaojun Zhang, Chantel F Scheuring, Meiping Zhang, Jennifer J Dong, Yang Zhang, James J Huang, Mi-Kyung Lee, Shahal Abbo, Amir Sherman, Dani Shtienberg, Weidong Chen, Fred Muehlbauer, Hong-Bin Zhang

**Affiliations:** 1 Department of Soil and Crop Sciences, Texas A&M University, College Station, Texas 77843-2474, USA; 2 College of Life Science, Jilin Agricultural University, Changchun, Jilin 130118, China; 3 Institute of Plant Science and Genetics in Agriculture, The Hebrew University of Jerusalem, Rehovot, 76100, Israel; 4 The Volcani Center, P.O. Box 6, Bet-Dagan, 50250, Israel; 5 USDA-ARS and Department of Crop and Soil Sciences, Washington State University, Pullman, WA 99164-6434, USA; 6 The Key Laboratory of Experimental Marine Biology, Institute of Oceanology, Chinese Academy of Sciences, Qingdao 266071, China

## Abstract

**Background:**

Chickpea (*Cicer arietinum *L.) is the third most important pulse crop worldwide. Despite its importance, relatively little is known about its genome. The availability of a genome-wide physical map allows rapid fine mapping of QTL, development of high-density genome maps, and sequencing of the entire genome. However, no such a physical map has been developed in chickpea.

**Results:**

We present a genome-wide, BAC/BIBAC-based physical map of chickpea developed by fingerprint analysis. Four chickpea BAC and BIBAC libraries, two of which were constructed in this study, were used. A total of 67,584 clones were fingerprinted, and 64,211 (~11.7 ×) of the fingerprints validated and used in the physical map assembly. The physical map consists of 1,945 BAC/BIBAC contigs, with each containing an average of 28.3 clones and having an average physical length of 559 kb. The contigs collectively span approximately 1,088 Mb. By using the physical map, we identified the BAC/BIBAC contigs containing or closely linked to QTL4.1 for resistance to *Didymella rabiei *(RDR) and QTL8 for days to first flower (DTF), thus further verifying the physical map and confirming its utility in fine mapping and cloning of QTL.

**Conclusion:**

The physical map represents the first genome-wide, BAC/BIBAC-based physical map of chickpea. This map, along with other genomic resources previously developed in the species and the genome sequences of related species (soybean, *Medicago *and *Lotus*), will provide a foundation necessary for many areas of advanced genomics research in chickpea and other legume species. The inclusion of transformation-ready BIBACs in the map greatly facilitates its utility in functional analysis of the legume genomes.

## Background

Chickpea (*Cicer arietinum *L., 2*n *= 2*x *= 16) is the third most important pulse crop in the world [1,2]. It is often used as a drought-tolerant crop grown in the drier regions of India, East Africa, the Mediterranean basin, and the Americas. In the cereal-based crop rotation systems, chickpea is used as a rotation crop to break disease cycles, fix atmospheric nitrogen and improve soil fertility. It is a major source of high-quality proteins and starch for a large Asian vegetarian population and a health-conscious food in developed countries. Therefore, understanding its genome is of significance not only economically, but also for annotation, functional and evolutionary analysis of legume genomes, particularly the underlying mechanisms of plant drought tolerance and nitrogen fixation. The most significant constraints to its production are Ascochyta blight caused by *Dydimella rabiei *and cultivar adaptation to terminal drought and high temperature during pod set [3,4]. Ascochyta blight is a destructive, devastating disease of chickpea and may result in total crop loss [3,5]. Hence, combining Ascochyta blight resistance with early flowering is crucial to increasing grain yield and quality of chickpea in various rain-fed environments [6,7]. However, it has been proven difficult, without assistance of modern molecular tools, to breed for high grain yield and quality combining both Ascochyta blight resistance and early flowering [7].

To facilitate complex trait breeding, and to clone and characterize the genes and loci of agronomic importance in chickpea, some molecular tools have been developed. These include several molecular genetic maps [8-13], identification of quantitative trait loci (QTL) for a number of agronomic traits [4,9,13-19], several large-insert bacterial artificial chromosome (BAC) and plant-transformation-competent binary BAC (BIBAC) libraries [20,21], and limited tissue- or treatment-specific expressed sequence tags (ESTs) [22-24]. Nevertheless, significant efforts will be needed to make the tools suitable for chickpea breeding. QTL for controlling resistance to *Didymella rabiei *(RDR) were mapped to five of its eight chromosomes and QTL for days to first flower (DTF) to four chromosomes [9,18]. Unfortunately, the markers for most of the QTL are not sufficiently close to the loci for their effective use in marker-assisted selection for the traits.

Genome-wide integrative physical mapping has been used in several species to effectively integrate genomic tools for marker-assisted breeding, high-resolution mapping and positional cloning of genes and QTL [25,26]. Simultaneously, physical maps will also provide desirable platforms for advanced EST analysis, genome sequencing [27,28] and comparative genomics. Despite these advantages, a genome-wide physical map has not been developed for chickpea. Lack of such genomic tools and infrastructure for the species has not only limited the deeper analysis of its agronomic genes and QTL, but also prevented genomic information flow to chickpea from the model or related legumes such as *Medicago*, *Lotus *and soybean whose genomes have been sequenced [29-32]. Therefore, a comprehensive platform is needed to rapidly access the QTL for RDR, DTF and many other agronomic traits, and advance chickpea and related legume genomics research. In comparison with other major crops such as wheat, maize, soybean, cotton and tomato, chickpea has a relatively smaller genome size (740 Mb/1C). The size of the chickpea genome is comparable to those of the non-cultivated legume models, *Medicgo *[540 Mb/1C, [33]] and *Lotus *[480 Mb/1C, [34]]. The relatively small genome size of chickpea facilitates physical mapping and high-throughput sequencing of its genome, thus facilitating annotation, functional analysis and evolutionary investigation of the legume genomes.

In this study, we constructed one new BAC library and one new BIBAC library from the chickpea cultivar, Hadas, using different restriction enzymes and vectors from those used in the existing chickpea libraries. From the two new BAC and BIBAC libraries, and the two BAC and BIBAC libraries of the cultivar previously reported [21], we constructed a genome-wide, BAC/BIBAC-based physical map of the chickpea genome. Using the physical map, we identified the BAC/BIBAC contigs containing or closely linked to two QTL, one controlling RDR and the other controlling DTF. These results, especially the genome-wide BAC/BIBAC physical map, will provide a framework for many aspects of genomics and genetics research of the species and finally, for sequencing its genome by using the next-generation high-throughput genome sequencing technology.

## Results

### New BAC and BIBAC library construction

**Figure 1:**
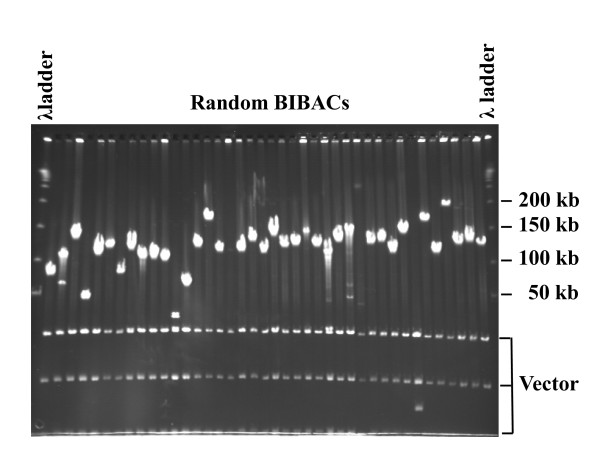
**BIBACs randomly selected from the chickpea-CHV BIBAC library (see Table 1)**. BIBAC DNA was isolated, digested with *Not *I and electrophoresed on a pulsed-field gel.

In a previous study [21], we constructed one BAC library (Chickpea-CHI) and one BIBAC library (Chickpea-CBV) for chickpea cv. Hadas using *Hin*d III and *Bam*H I, respectively (Table 1). To facilitate quality physical map development of the genome, we constructed one new BAC library (Chickpea-CME) and one new BIBAC library (Chickpea-CHV) from the nuclear DNA of the same genotype partially digested with *Mbo*I and *Hin*dIII, respectively (Table 1). The chickpea-CME BAC library contains 22,272 clones. Analysis of 84 random clones showed that it has an average insert size of 130 kb and provides a 4.0-fold coverage of the chickpea haploid genome. The chickpea-CHV BIBAC library contains 38,400 clones. Analysis of 100 random clones showed that it has an average insert size of 142 kb and provides a 7.5-fold coverage of the chickpea genome (Figure 1). For both libraries, less than 5% of their clones contain no inserts of chickpea DNA. We screened 36,864 clones of the chickpea-CHV BIBAC library using the chloroplast genes, *ndhA, rbcL *and *psbA*, as probes. A total of 49 positive clones were obtained, suggesting that approximately 0.13% (49/36,864) of the new libraries clones were derived from chloroplast DNA. This number is close to the chickpea BAC and BIBAC libraries that we constructed previously, 0.3% of whose clones were derived from chloroplast DNA [21]. Therefore, the two new libraries (11.5 ×), along with the two BAC (Chickpea-CHI) and BIBAC (Chickpea-CBV) libraries (6.1 ×) constructed previously [21], provide comprehensive and high-quality source libraries for construction of the chickpea physical map [25,35].

**Table 1:** BAC and BIBAC libraries of chickpea cv. Hadas constructed and used in chickpea genome physical mapping

							Fingerprinted clones*	
*Libraries*	*No. of clones*	*Genome coverage*	*Insert size (kb)*	*Vector*	*Type*	*Enzyme*	No. of clones	Genome coverage
Chickpea-CHI	9,216	1.5×	121	pIndigoBAC5	BAC	*Hin*d III	9,216	1.5 ×
Chickpea-CBV	23,040	4.6×	145	pCLD04541	BIBAC	*Bam*H I	6,912	1.4 ×
Chickpea-CME	22,272	4.0×	130	pECBAC1	BAC	*Mbo *I	22,272	3.9 ×
Chickpea-CHV	38,400	7.5×	142	pCLD04541	BIBAC	*Hin*d III	29,184	5.6 ×
**Total**	92,928	17.6×	137				67,584	12.4 ×

* The average insert size of fingerprinted clones was estimated to 135 kb.

### BAC/BIBAC fingerprinting

Our previous studies showed that BAC and BIBAC fingerprints generated with different restriction enzyme combinations may result in different quality BAC/BIBAC physical maps [36,37]. Therefore, we first tested twenty-four 3-, 4- and 5-enzyme combinations of *Hin*d III, *Bam*H I, *Eco*R I, *Xba *I, *Xho *I, and *Hae *III using 32 BACs randomly selected from the Chickpea-CHI library. In the combinations, only the ends produced by *Hin*d III, *Bam*H I, *Eco*R I, *Xba *I or *Xho *I digestion are labeled with a fluorescent dye (NED-ddATP or HEX-ddATP). *Hae *III is used to further digest the labeled fragments to sizes that allow separation on a capillary sequencer. According to the criteria that there are no partial digestion, no star activity, an average of 35 - 70 bands per clone and a relatively even size distribution of the bands in a window ranging from 35 - 500 bases in a single-tube single-step digestion-labeling system, the enzyme combination of *Hin*d III/*Xba *I/*Xho *I/*Hae *III was selected for generation of BAC and BIBAC fingerprints for the chickpea genome physical mapping.

A total of 67,584 (12.4-fold) BAC and BIBAC clones were fingerprinted from the four chickpea BAC and BIBAC libraries (Table 1), with 1.4-fold to 5.6-fold clones from each library, or 5.4-fold coverage BACs and 7.0-fold coverage BIBACs. Of the clones, the fingerprints of 64,211 (95.0%) clones were validated and used in the physical map assembly. The validated fingerprints represented approximately 11.7-fold of the chickpea genome (Table 2). Each clone had an average of 39.2 restriction fragment bands in the window of 35 - 500 bases, with a range from 5 to 192 bands per clone. According to our previous studies [36,38], the genome coverage of 11.7 fold should be sufficient to allow the assembly of a high-quality genome-wide physical map of the chickpea genome.

**Table 2:** Statistics of the BAC/BIBAC physical map of the chickpea genome

Total number of BAC clones fingerprinted	67,584	12.4 × genome coverage
Valid fingerprints for FPC assembly	64,211	11.7 × genome coverage
Average number of bands per BAC	39.2	
Total number of contigs assembled	1,945	
No. of contigs containing >199 clones	16	
No. of contigs containing 100-199 clones	23	
No. of contigs containing 50-99 clones	185	
No. of contigs containing 25-49 clones	357	
No. of contigs containing 10-24 clones	479	
No. of contigs containing 3-9 clones	619	
No. of contigs containing 2clones	266	
Clones contained in the 1,945 contigs	55,029	10.0 × genome coverage
Average BAC clones per contig	28.3	
Average estimated size per contig (kb)	559	
Number of singletons	9,182	
Total number of CB bands included in the contigs	265,614	
Total physical length of assembled contigs (kb)	1,087,954	

### Determination of tolerance and cutoff values

The FingerPrinted Contig (FPC) program was used to assemble the contig map from the BAC and BIBAC fingerprints, by which two parameters, tolerance and cutoff, are crucial to quality contig assembly. Tolerance is the window size in which two restriction fragments are considered as the same or equivalent bands. To determine the tolerance value to be used for the contig assembly, we selected the four pECBAC1 vector fragments generated with the enzyme combination (*Hin*d III/*Xba *I/*Xho *I/*Hae *III) of sizes 60, 161, 230 and 375 bases in the range from 35 to 500 bases released from 200 BACs randomly selected from the fingerprint dataset. We calculated the mean size deviation of each of the fragments. At a 95% confidence interval, the mean deviations of the four vector fragments, 60, 161, 230 and 375 bases, were 0.56, 0.48, 0.43 and 0.55 base, respectively, with an average of 0.505 bases. Therefore, a tolerance value of 5 (0.5 × 10) was chosen as the candidate tolerance value for FPC contig assembly, with all fragment sizes of each fingerprint multiplied by 10 [38]. Furthermore, the tolerances of 1 - 7 were tested using the entire fingerprint dataset to determine the parameters suitable for contig assembly. On the basis of these results, a tolerance of 5 was finally selected for the contig assembly.

**Figure 2:**
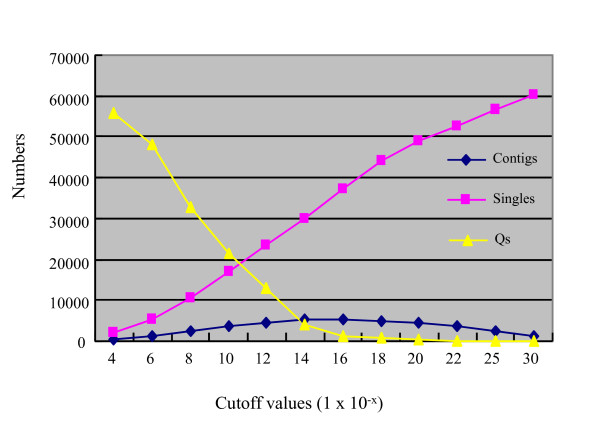
**Plot of cutoff values versus the numbers of contigs, singletons, and Q clones for physical map assembly**. The cutoff value that yielded the lowest numbers for all three factors, contigs, singletons and Q clones, was selected for the chickpea genome physical map assembly.

The cutoff value is a threshold of the probability that fingerprint bands match by coincidence. Lowering its value would increase contig assembly stringency, and therefore, increase the likelihood that overlapping BAC clones are truly overlapping. To determine the cutoff value for the chickpea physical map assembly, we tested a series of cutoff values ranging from 1e-4 to 1e-30 and a tolerance of 5 for automatic contig assembly. The resultant numbers of contigs, singletons, and Q (questionable)-clones were analyzed. At higher stringencies (1e-14 to 1e-30), "chimeric" contigs were split and Q-clones were reduced, but the number of singletons increased drastically. At lower stringencies (1e-4 - 1e-8), a smaller number of or larger contigs were obtained, but a larger number of clones were fallen in the category of Q-clones. The relationship among the three factors is plotted in Figure 2, from which it was apparent that a cutoff value of approximately 1e-12 resulted in reasonable low numbers of all three factors, suggesting the desirable quality contig assembly. While the number of Qs was 18.5% (11868/64211) when automatic contigs was initially assembled at the cutoff of 1e-12, it was reduced to 3.0% (1092/64211) after subsequent Dqer at lower cutoff values (1e-13, 1e-14 and 1e-15) that allows re-analysis of the initial assembly step-wisely to reduce the Q-clones in the contigs. We, therefore, chose 1e-12 as the cutoff value for the initial assembly of the physical map.

### Contig map assembly

**Figure 3:**
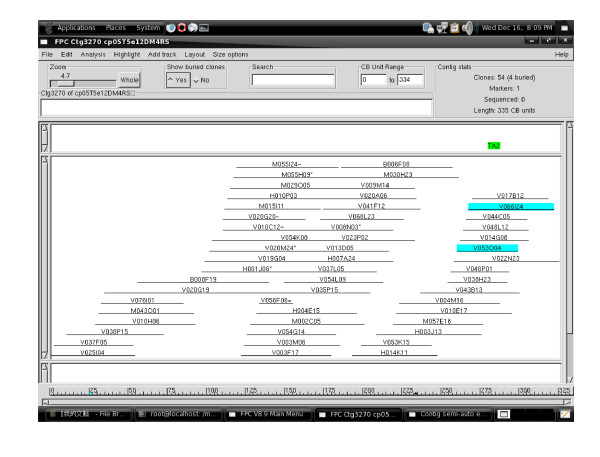
**Example of the chickpea physical map contigs**. The contig3270 (ctg3270) contains 54 clones and spans 335 CB units, thus having a physical length of 955 kb. The two highlighted clones, V066I24 and V053O04, are the positive clones of the SSR marker TA2 flanking the RDR QTL4.1.

Contigs were assembled from the fingerprint data of the clones using the computer program FPC version 8.9[39]. A total of 1,945 contigs were assembled from the validated 64,211 BAC and BIBAC fingerprints at a cutoff value of 1e-12 and a tolerance of 5, followed by Dqer, end-to-end merging and end-to-singleton merging at progressively lower stringencies (Additional files 1, 2, 3, 4 and 5; for example, see Figure 3). The 1,945 contigs contained a total of 55,029 clones whereas the remaining 9,182 clones remained as singletons.

Table 2 summarizes the resultant BAC/BIBAC contig map of the chickpea genome (Additional files 1, 2, 3, 4 and 5). The contigs each contained from 2 to over 199 clones, with the largest 225 (11.6%) contigs containing 56.0% (31,102 clones) of the total 55,029 clones. Each contig contained an average of 28.3 clones and spanned an average length of 559 kb, with a range of 200 - 6,107 kb. The 1,945 contigs contained a total of 265,614 consensus bands (CBs), thus representing a total length of approximately 1,087,954 kb. On average, each clone contributed 4.8 unique CBs to the assembly, or approximately 19.7 kb (14.6% of the 135-kb average clone insert size) to the physical length of the contig assembly.

### Assessment of the contig map and identification of the contigs containing or closely linked to RDR and DTF QTL

Several approaches were used to assess the reliability of the chickpea contig map. First, we assembled automatic contigs from the fingerprints using two different contig building strategies and compared the resultant contigs, as described by Wu et al. [40]. A sample of 100 random contigs from the two assemblies was analyzed comparatively. The result showed that 99.0% of the automated contigs resulted from the two strategies was completely consistent in both clone content and order.

In the second approach, we assembled contigs from the clones of the Chickpea-CME and, Chickpea-CHV libraries, separately. We randomly selected 100 contigs from the contigs assembled from each library and compared them with their corresponding contigs in the physical map. We found that 96% and 95% of the contigs were shown to be in complete agreement with the contigs of the chickpea physical map in terms of both clone content and order.

In the third approach, we randomly selected five contigs from the chickpea physical map, fingerprinted their BACs and BIBACs with a different enzyme combination, *Bam*HI/*Hin*dIII/*Xho*I/*Hae*III, and then reassembled them into the contigs. As a result, the same contigs as those selected from the physical map were reassembled.

**Figure 4:**
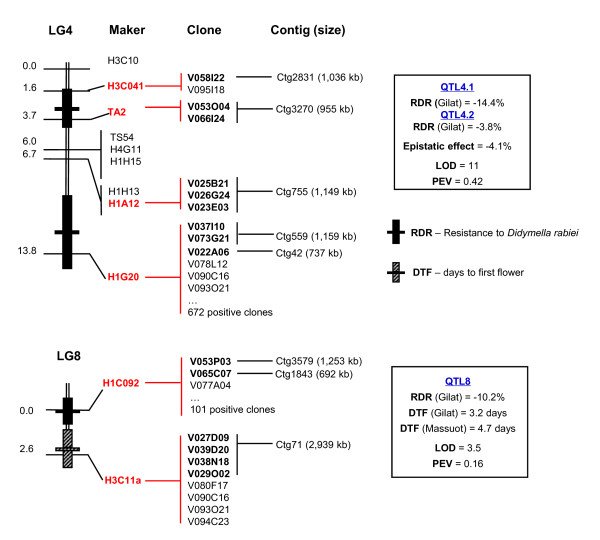
**BAC/BIBAC contigs containing the positive clones of the SSR primer pairs flanking the QTL for resistance to *Didymella rabiei *(RDR) and days to first flower (DTF)**. The clones highlighted in bold face were located to contigs and the remaining clones are not analyzed in this study. The QTL were genetically mapped using the phenotypic data collected at one or two locations (Gilat and Massuot, Israel) [18].

Finally, we verified the accuracy of the contig map by screening the source BIBAC clones of the chickpea-CHV library with SSR primers flanking the RDR and DTF QTL previously mapped to Linkage Groups LG4 and LG8 (Figure 4) and to other regions of the chickpea genetic maps [9,18,21]. If the physical map contigs were assembled properly, the positive clones of a single-copy SSR primer pair should be located to a single BAC/BIBAC contig of the map. We screened two high-density filters of the chickpea-CHV library containing a total of 36,864 double-spotted clones, of which 27,724 clones were used in the map assembly. A total of 16 SSR primer pairs were used as probes for the library screening (Additional file 6). Of the 16 SSR primer pairs, seven, H1G20, H1C092, TA3, H1A19, H1H22, H1C9-2 and H2J2, were clearly derived from multiple-copy sequences, as indicated by Southern analysis and the library screening result that they hybridized to 47 - 672 positive clones, respectively. The remaining nine SSR primer pairs were shown to be single- or low-copy by Southern analysis, with each hybridizing to 2 - 8 positive clones, being within the range of expected 6.5 positive clones per single-copy probe. The positive clones of each of all nine single- or low-copy SSR primer pairs were found to be assembled into a single contig, further suggesting that the physical map contigs are assembled properly. Of the nine single- or low-copy marker-containing contigs, contig71 (ctg71) was located to the region of DTF QTL8 and spanned a physical length of 2,939 kb, and contig3270 (ctg3270) and contig2831 (ctg2831) were located to the region of RDR QTL4.1 and spanned a physical length of 955 kb and 1,036 kb, respectively.

## Discussion

We have developed a genome-wide, BAC/BIBAC-based physical map of the cultivated chickpea, *C. arietinum*, from cultivar Hadas. The map consists of 55,029 BAC and BIBAC clones assembled into 1,945 contigs. Each contig contains 2 to >199 clones with an average of 28.3 clones per contig, spanning from 200 to 6,107 kb, with an average physical length of 559 kb (Table 2). These contigs collectively span approximately 1,088 Mb, larger than the 740-Mb estimated genome size of chickpea by approximately 47%. The longer physical length of the physical map contigs than the estimated genome size may be attributed to one or more of the following factors. Overlaps existed between contigs, but they were too small to be detected or merged unambiguously by using the FPC program alone; the average insert size of the source clones was over-estimated; and/or the genome size of chickpea was underestimated. According to previous studies [40-43], the undetected overlaps among the contigs likely account for much of the discrepancy. As described above, the current physical map has an average overlap of 85.4% (1 - 14.6%) of clone average insert size (135 kb), indicating that the clones having an overlap of <85.4% insert size might be assembled into separate contigs in this study. The existence of overlaps, even though they were undetected by the PFC program under the conditions used in this study, will allow the contigs to be further merged into larger ones. However, further unambiguously merging these contigs will require additional supporting evidence in addition to their fingerprint similarities, such as anchoring of the contigs to the genetic map by mapped DNA markers and to the related genome sequences using the end sequences of the source BACs and BIBACs as anchors.

The quality of the chickpea physical map is sufficient for its use in different aspects of chickpea genomics research. The clones equivalent to 10.0 x of the chickpea genome (55,029 clones) were assembled into the physical map contigs (Table 2). According to our previous studies [36,38,40-44], this number of clones is adequate for construction of a high-quality physical map of the chickpea genome, though it is much smaller than those used for the physical maps of human and bovine developed previously [45,46]. Moreover, the source clones of the map were randomly selected from four BAC and BIBAC libraries constructed with three restriction enzymes (*Hin*d III, *Bam*H I and *Mbo *I) in three vectors (pIndigoBAC5, pECBAC1 and pCLD04541) (Table 1). Although the distribution of the restriction sites of any particular restriction enzyme is uneven throughout a genome, the use of the clones constructed with three different restriction enzymes in three vectors would further increase the actual coverage of the resultant map for the whole genome of chickpea [25]. The accuracy of the contig physical map was confirmed using different approaches, including independent contig building methods, different fingerprinting methods and SSR marker hybridization. While the number of the DNA makers used in the verification of the physical map contigs was limited, the employment of multiple approaches that were previously proven [40,43] to the research purpose provided evidence on the reliability of the map assembly. The results of all of the approaches together have verified that the physical map contigs were assembled properly.

The physical map provides a powerful foundation for many aspects of advanced genome research of chickpea and related legume species. Using the physical map, we have identified three contigs, ctg3270, ctg2831 and ctg71, that likely contain or are closely linked to the RDR QTL4.1 contributing to 14.4% of Ascochyta blight resistance and the DTF QTL8 contributing approximately 4 days to earlier flowering (Figure 4). The ctg3270 spans 955 kb, the ctg2831 spans 1,036 kb and the ctg71 spans 2,939 kb in physical length. Assuming that the average physical/genetic ratio of the chickpea genome is from 300 kb/cM [9,12] to 630 kb/cM [10], the ctg3270 may span approximately 1.5 - 3.2 cM, the ctg2831 may span 1.6 - 3.4 cM, and the ctg71 may span 4.7 - 9.8 cM. Since the RDR QTL4.1 region spans 2.1 cM and the DTF QTL8 region spans within 2.6 cM [18], these genetic distances may cover the entire regions of the RDR QTL4.1 and DTF QTL8, respectively. Therefore, the contigs have provided powerful tools for fine mapping and cloning of the QTL. Furthermore, since the physical map was constructed from both BACs and *Agrobacterium-*mediated plant transformation-ready BIBACs, the inclusion of the BIBACs will further facilitate cloning and applications of the QTL and other agronomic genes of interest, and promote functional analysis of the chickpea genome by genetic transformation at the whole genome level [[47-49]; Chang Y-L, Chuang H-W, Meksem K, Wu F-C, Chang C-Y, Zhang M and Zhang H-B, submitted].

The physical map represents the first generation of the physical map of chickpea. Although its utility in isolation of BAC/BIBAC contigs containing or closely linked to QTL has been preliminarily demonstrated in this study, further efforts remain to optimize its effectiveness for whole genome sequencing and sequence assembly, high-density mapping of the genome (e.g., SNP mapping), and comparative genome analysis with related genome sequences such as those of soybean, *Medicago *and *Lotus*. Efforts will be also needed to integrate the contig map with the developed genetic maps of the species and to locate all mapped genes and QTL to the BAC/BIBAC contigs, as was done for RDR QTL4.1 and DTF QTL8 in this study. These include, but are not limited to, sequencing of the source BAC and BIBAC ends, alignment of the BAC/BIBAC contigs along the sequenced soybean, *Medicago *and/or *Lotus *genomes, targeted (e.g., the physical map BAC/BIBAC end sequences are used as the targeted sites) development of DNA markers and high-density genome maps, and screening of the source BACs and BIBACs using mapped DNA markers, especially those flanking the genes and QTL mapped (e.g., Figure 4). These experiments will not only allow facilitating subsequent sequencing, sequence assembly and sequence annotation of the chickpea and other legume genomes, and deciphering the evolution of the legume genomes as a whole, but also further merging the contigs, thus significantly enhancing the physical map.

## Conclusion

It has been documented that a genome-wide physical map is of significance for advanced genome research. We have developed a genome-wide quality BAC/BIBAC physical map of chickpea and using the map, identified three large contigs containing or closely linked to QTL contributing to Ascochyta blight resistance and earlier flowering in chickpea. This map, along further improvements as discussed above, will greatly advance the genomics and genetics research of chickpea and related legumes. The inclusion of plant transformation-ready BIBACs in the physical map will further promote its utility in cloning of genes and QTL of interest and functional analysis of the chickpea and related genomes.

## Methods

### Source BAC/BIBAC libraries

Two BAC and two plant transformation-ready BIBAC libraries of chickpea cv. Hadas, named Chickpea-CHI (BAC), Chickpea-CBV (BIBAC), Chickpea-CME (BAC) and Chickpea-CHV (BIBAC), were used to construct the chickpea physical map. The Chickpea-CHI and Chickpea-CBV libraries were previously constructed from the nuclear DNA of chickpea cv. Hadas with *Hin*d III and *Bam*H I in the BAC vector pIndigoBAC5 and BIBAC vector pCLD04541, respectively, and have average insert sizes of 121 kb and 145 kb [21]. The two new libraries, Chickpea-CME and Chickpea-CHV, were constructed from the nuclear DNA of chickpea cv. Hadas partially digested with *Mbo *I and *Hin*d III in the BAC vector pECBAC1 and BIBAC vector pCLD04541, respectively. The megabase-sized nuclear DNA isolation and library construction were described previously [35,50-52].

### BAC/BIBAC fingerprinting

BAC or BIBAC clones arrayed in 384-well microplates were inoculated into 96-deep well plates containing 1.0 ml TB (Terrific Broth) medium with proper antibiotics using a 96-pin replicator (BOEKEL, Feasterville, PA, USA). Thus, the clones of a 384-well plate were inoculated into four 96-deep well plates. To ensure clone tracking, the clones were always inoculated with the A01 pin of the 96-pin replicator aligned with the A01 position of the 384-well plate as the 96-deep well plate A, followed by the A01 pin of the replicator aligned with the B01, A02 and B02 of the 384-well plate as the 96-deep well plates B, C and D, respectively. The 96-deep well plates were covered with air-permeable seals (Excel Scientific, Wrightwood, CA, USA) and incubated in an orbital shaker at 320 rpm, 37°C for 20-22 h.

The overnight cultures were centrifuged at 2,500 g for 10 min in a Beckman bench-top centrifuge to harvest the bacterial cells. BAC or BIBAC DNA was isolated using a modified alkaline lysis method [53], dissolved in 15 μl TE (10 mM Tris-HCl, pH 8.0, 1 mM EDTA, pH 8.0) with 16 μg/ml RNase (Ambion, USA) and stored at -20°C before use. The DNA was digested and end-labeled in a reaction containing reaction buffer (50 mM NaCl, 10 mM Tris-HCl, 10 mM MgCl_2_, 1.0 mM dithiothreitol, pH 8.0), 6.0 μM each dTTP, dCTP and dGTP, 1.0 μg/μl BSA, 1 U each of *Hin*d III, *Xba *I, *Xho *I, and *Hae*III (New England Biolabs, Ipswich, MA, USA), 0.3 U *Taq *FS and 6.0 μM HEX-ddATP or NED-ddATP. The reaction was incubated at 37°C for 2 h, followed by further incubation at 65°C for 45 min. The clone DNAs labeled with different fluorescent dyes (HEX-ddATP or NED-ddATP) were combined, pelleted, washed, dried and dissolved in a mixture of 9.8 μl of Hi-Di formamide and 0.2 μl of the internal GeneScan-500 Rox size standard (Applied Biosystems, Foster City, CA, USA). The DNA was denatured at 95°C for 3 min, cooled on ice and then subjected to analysis on the ABI 3100 Genetic Analyzer (Applied Biosystems, Foster City, CA, USA) using the default GeneScan module (36-cm array, POP4).

### Data processing

The fragment sizes in each BAC fingerprint profile were collected by the ABI Data Collection program. The data of the ABI 3100 Genetic Analyzer were processed using the software package ABI-ExportTabularData [54] and SeqDisplayer (unpublished). The data were transformed using an automatic algorithm contained in the SeqDisplyer program into "bands" files. Several quality checks were applied to the fingerprints, with sample-empty wells being removed, fingerprints with fewer than 5 band peaks removed, the background peaks identified and removed, the off-scale bands with peak heights greater than 6,000 removed, and the vector band peaks removed. Only the band peaks falling between 35 and 500 bases were used for the contig assembly.

### Physical map contig assembly and manual editing

The program FPC version 8.9 [39] was used to assemble the clone fingerprint data into contigs. A series of tests were conducted, in which the fingerprints of a set of overlapping clones were analyzed using different tolerances (from 1 to 7) and cutoffs (1e-4 to 1e-30). On the basis of these tests, a fixed tolerance of 5 and a cutoff of 1e-12 were selected for automatic contig assembly.

### Library screening and assessment of physical map quality

Four sets of high-density filters of the Chickpea-CHV BIBAC library, with each set containing 36,864 double-spotted clones, were prepared according to Lee et al. [55]. Of these clones, 27,724 were used in the physical map contig assembly. Sixteen pairs of SSR primers were selected from published chickpea linkage maps [9,18,21] and then purchased from Sigma Genosys (Woodlands, TX, USA). The SSR primer oligos were end-labeled with ^32^P-ATP (Amersham, Piscataway, NJ, USA) at 37°C for 1 h, the unincorporated nucleotides removed using a Sephadex G50 column, the probes denatured at 95°C for 10 min, and then added to the hybridization buffer containing the high-density BIBAC filters. Hybridization was performed at 40°C for 18 h, and the filters were washed three times in 0.1% SDS, 0.5× SSC at 40°C, 30 min each wash, and exposed to X-ray film at -80°C for 2-5 days.

## Abbreviations

BAC: bacterial artificial chromosome; BIBAC: plant-transformation-competent binary BAC; QTL: quantitative trait locus; RDR: resistance to *Didymella rabiei*; DTF: days to first flower; SSR: simple sequence repeat; CB: consensus band; EST: expressed sequence tag.

## Authors' contributions

XZ conducted Chickpea-CHV library construction, fingerprinting, data processing, contig assembly, library screening, and manuscript writing; CFS constructed the Chickpea-CME BAC library; JJD arrayed the Chickpea-CHV library and provided assistance in sequencer operation; MZ and YZ conducted library hybridization; JJH provided software assistance; M-KL provided assistance for fingerprinting and FPC; SA, AS, DS, WC and FM participated in the experimental design and manuscript preparation; H-BZ designed the project, supervised its execution and wrote the manuscript. All authors read and approved the final manuscript.

## Supplementary Material

Additional file 1 **Shows the contigs constituting the physical map of the chickpea genome 1 (ctg5-904)**. Click here for file

Additional file 2 **Shows the contigs constituting the physical map of the chickpea genome 2 (ctg904-1902)**. Click here for file

Additional file 3 **Shows the contigs constituting the physical map of the chickpea genome 3 (ctg1903-2845)**. Click here for file

Additional file 4 **Shows the contigs constituting the physical map of the chickpea genome 4 (ctg2849-3727)**. Click here for file

Additional file 5 **Shows the contigs constituting the physical map of the chickpea genome 5 (ctg3731-5052)**. Click here for file

Additional file 6 **Shows the positive clones and associated contigs of SSR primers identified from 36,864 clones of the chickpea-CHV BIBAC library (384-well microplates 1 - 96)**. Click here for file
