# Supplementary material for: A BAC/BIBAC-based physical map of chickpea, Cicer arietinum L
Source: BMC Genomics. 2010 Sep 17;11:501. doi: 10.1186/1471-2164-11-501 (PMC2996997; doi:10.1186/1471-2164-11-501)
Supplement: Additional file 1 — Shows the contigs constituting the physical map of the chickpea genome 1 (ctg5-904). [file 1471-2164-11-501-S1.PDF]

A BAC/BIBAC-based physical map of chickpea, *Cicer arietinum* L.

Xiaojun Zhang[1,6], Chantel F Scheuring[1], Meiping Zhang[1,2], Jennifer J Dong[1], Yang Zhang[1], James J Huang[1], Mi-Kyung Lee[1], Shahal Abbo[3], Amir Sherman[4], Dani Shtienberg[4], Weidong Chen[5], Fred Muehlbauer[5], Hong-Bin Zhang[1,*]

[1] Department of Soil and Crop Sciences, Texas A&M University, College Station, Texas 77843-2474, USA
[2] College of Life Science, Jilin Agricultural University, Changchun, Jilin 130118, China
[3] Institute of Plant Science and Genetics in Agriculture, The Hebrew University of Jerusalem, Rehovot, 76100, Israel
[4] The Volcani Center, P.O. Box 6, Bet-Dagan, 50250, Israel
[5] USDA-ARS and Department of Crop and Soil Sciences, Washington State University, Pullman, WA 99164-6434, USA
[6] The Key Laboratory of Experimental Marine Biology, Institute of Oceanology, Chinese Academy of Sciences, Qingdao 266071, China

***Corresponding author:** H.-B. Zhang, Tel.: +1-979-862-2244; Fax: +1-979-845-0456; E-mail: hbz7049@tamu.edu

Additional file 1. The contigs constituting the physical map of the chickpea genome 1(ctg5-904)

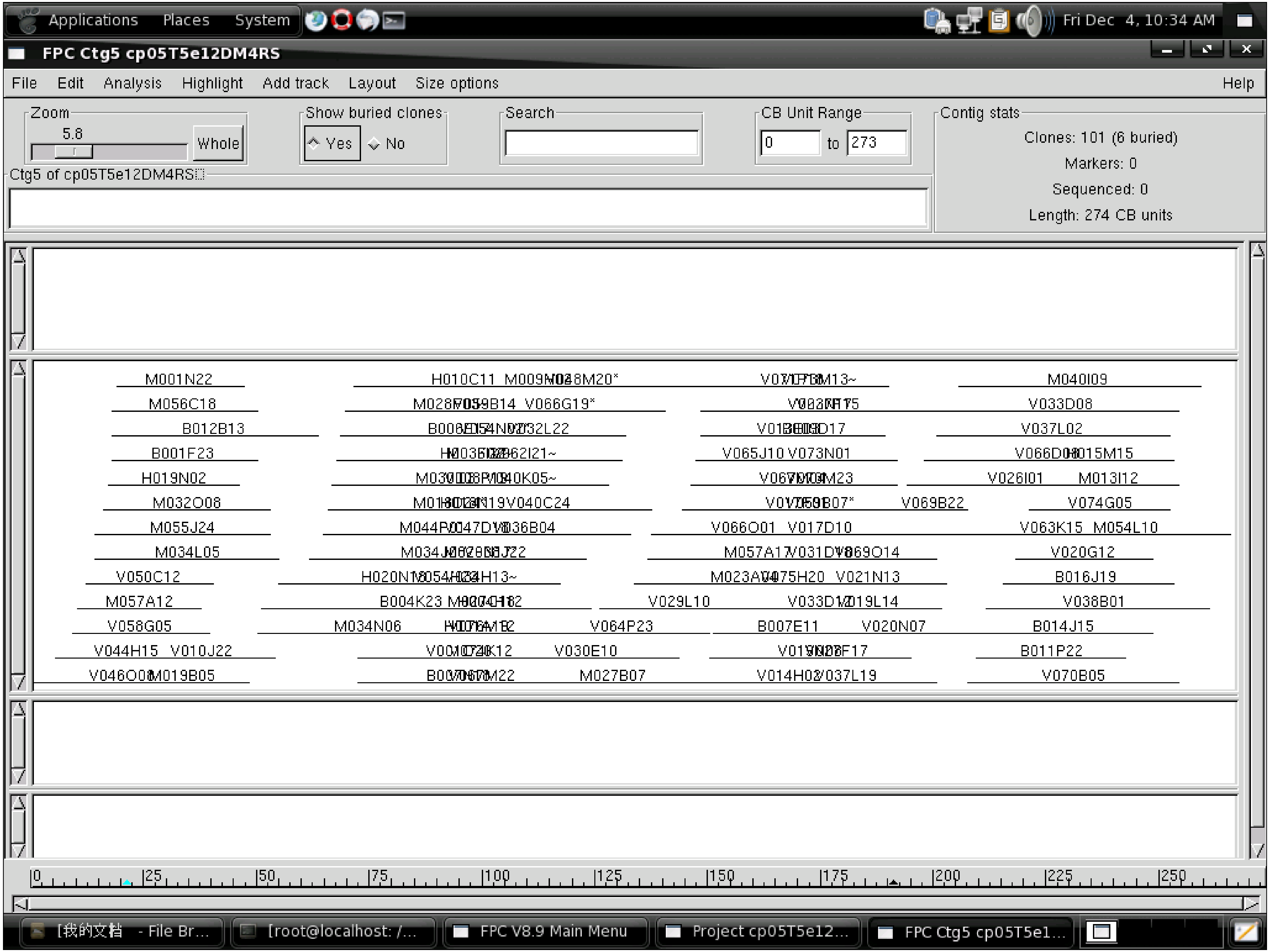
Applications Places System
Fri Dec 4, 10:34 AM
FPC Ctg5 cp05T5e12DM4RS
File Edit Analysis Highlight Add track Layout Size options Help
Zoom
5.8
Whole
Show buried clones
Yes No
Search
CB Unit Range
0 to 273
Contig stats
Clones: 101 (6 buried)
Markers: 0
Sequenced: 0
Length: 274 CB units
Ctg5 of cp05T5e12DM4RS
M001N22
M056C18
B012B13
B001F23
H019N02
M032O08
M055J24
M034L05
V050C12
M057A12
V058G05
V044H15 V010J22
H010C11
V066G19*
V065J10 V073N01
V066O01 V017D10
M040I09
V033D08
V037L02
V026I01 M013I12
V069B22
V074G05
V063K15 M054L10
V020G12
B016J19
V038B01
B014J15
B011P22
V070B05
V029L10
M034N06
V064P23
B007E11 V020N07
V030E10
M027B07
0 25 50 75 100 125 150 175 200 225 250
FPC V8.9 Main Menu
Project cp05T5e12...
FPC Ctg5 cp05T5e1...

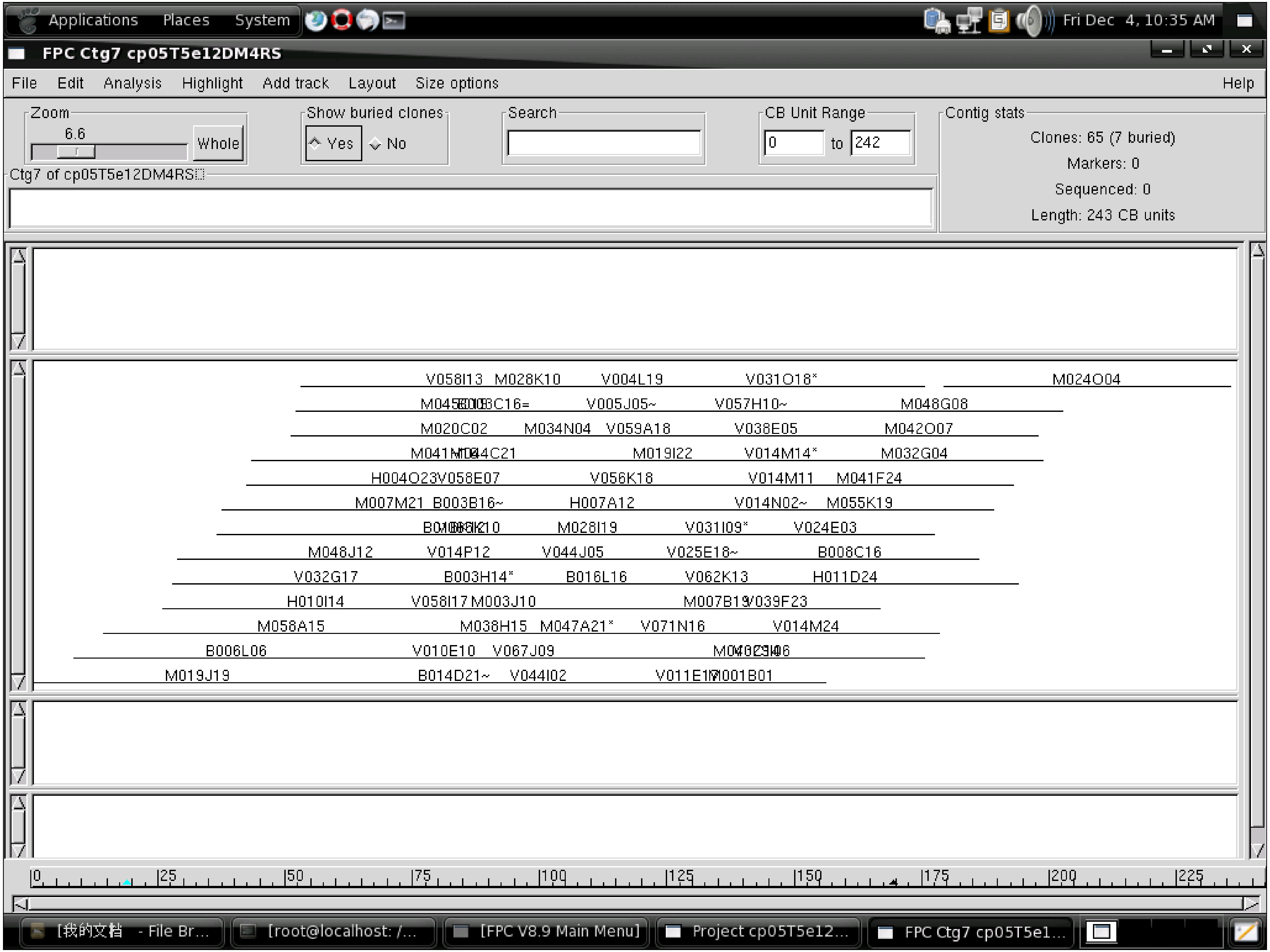
Applications Places System
Fri Dec 4, 10:35 AM
FPC Ctg7 cp05T5e12DM4RS
File Edit Analysis Highlight Add track Layout Size options Help
Zoom
6.6
Whole
Show buried clones
Yes No
Search
CB Unit Range
0 to 242
Contig stats
Clones: 65 (7 buried)
Markers: 0
Sequenced: 0
Length: 243 CB units
Ctg7 of cp05T5e12DM4RS
V058I13 M028K10 V004L19 V031O18* M024O04
V005J05~ V057H10~ M048G08
M020C02 M034N04 V059A18 V038E05 M042O07
M019I22 V014M14* M032G04
V056K18 V014M11 M041F24
M007M21 B003B16~ H007A12 V014N02~ M055K19
M028I19 V031I09* V024E03
M048J12 V014P12 V044J05 V025E18~ B008C16
V032G17 B003H14* B016L16 V062K13 H011D24
H010I14 V058I17 M003J10
M058A15 M038H15 M047A21* V071N16 V014M24
B006L06 V010E10 V067J09
M019J19 B014D21~ V044I02
0 25 50 75 100 125 150 175 200 225
[我的文档 - File Br...
[root@localhost: /...
[FPC V8.9 Main Menu]
Project cp05T5e12...
FPC Ctg7 cp05T5e1...

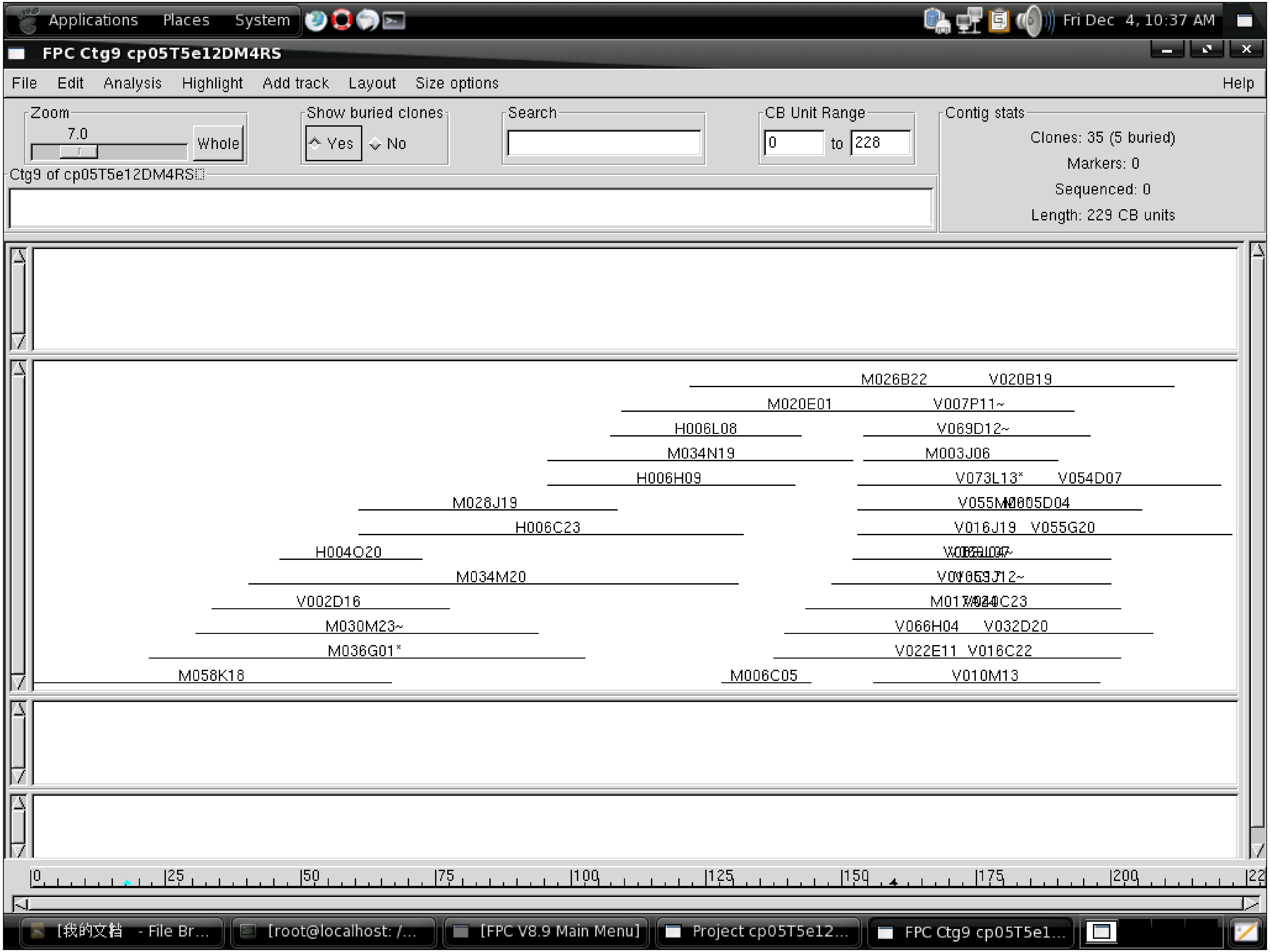

Applications Places System
Fri Dec 4, 10:37 AM
FPC Ctg9 cp05T5e12DM4RS
File Edit Analysis Highlight Add track Layout Size options Help
Zoom
7.0
Whole
Show buried clones
Yes
No
Search
CB Unit Range
0
to
228
Contig stats
Clones: 35 (5 buried)
Markers: 0
Sequenced: 0
Length: 229 CB units
Ctg9 of cp05T5e12DM4RS
M026B22
V020B19
M020E01
V007P11~
H006L08
V069D12~
M034N19
M003J06
H006H09
V073L13*
V054D07
M028J19
H006C23
V016J19
V055G20
H004O20
M034M20
V002D16
M030M23~
V066H04
V032D20
M036G01*
V022E11
V016C22
M058K18
M006C05
V010M13
0 25 50 75 100 125 150 175 200
[我的文档 - File Br...
[root@localhost: /...
[FPC V8.9 Main Menu]
Project cp05T5e12...
FPC Ctg9 cp05T5e1...

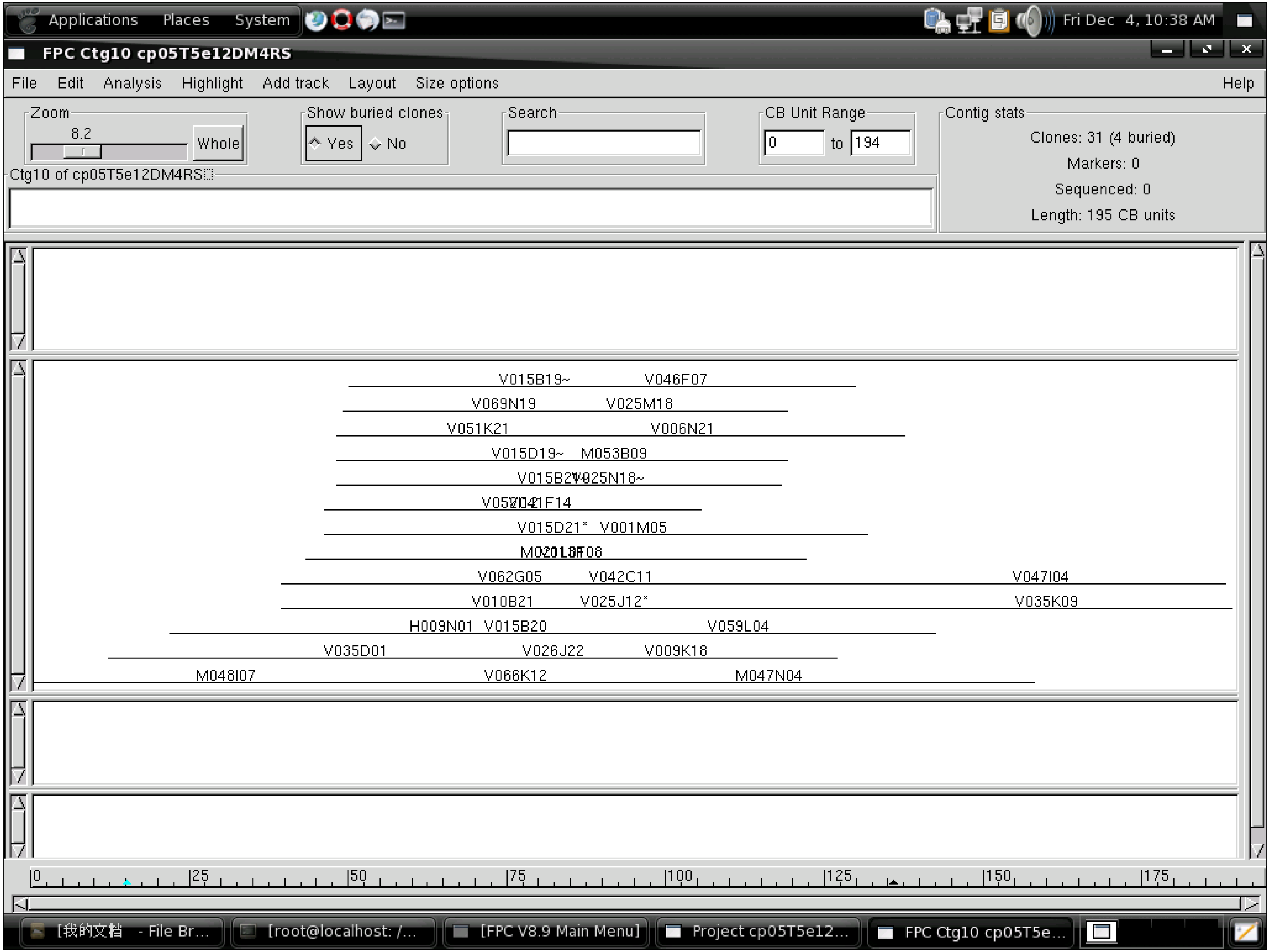
Applications Places System
Fri Dec 4, 10:38 AM
FPC Ctg10 cp05T5e12DM4RS
File Edit Analysis Highlight Add track Layout Size options Help
Zoom
8.2
Whole
Show buried clones
Yes
No
Search
CB Unit Range
0 to 194
Contig stats
Clones: 31 (4 buried)
Markers: 0
Sequenced: 0
Length: 195 CB units
Ctg10 of cp05T5e12DM4RS
V015B19~ V046F07
V069N19 V025M18
V051K21 V006N21
V015D19~ M053B09
V015D21* V001M05
V062G05 V042C11 V047I04
V010B21 V025J12* V035K09
H009N01 V015B20 V059L04
V035D01 V026J22 V009K18
M048I07 V066K12 M047N04
0 25 50 75 100 125 150 175
[我的文档 - File Br...
[root@localhost: /...
[FPC V8.9 Main Menu]
Project cp05T5e12...
FPC Ctg10 cp05T5e...

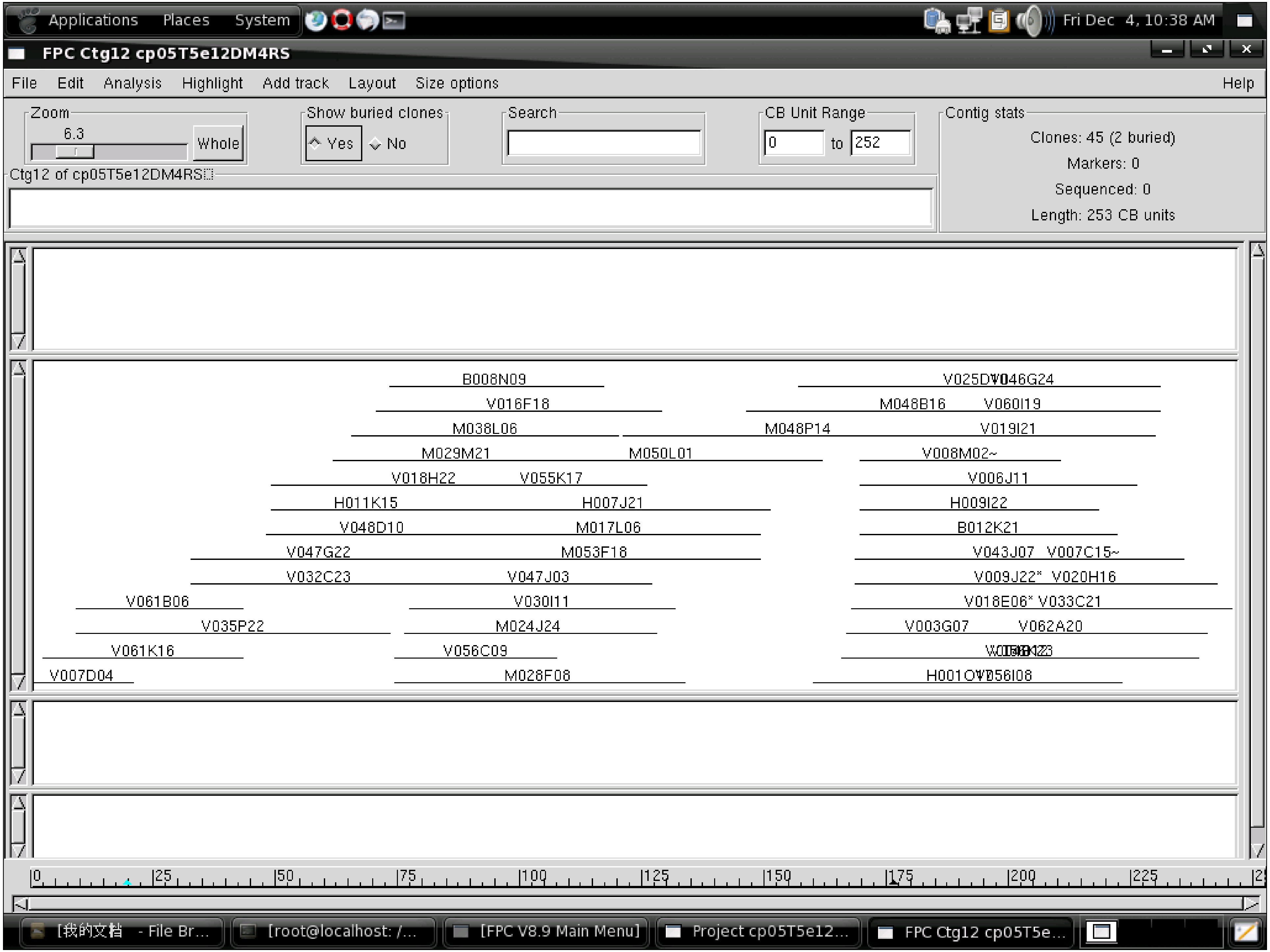
Applications Places System
Fri Dec 4, 10:38 AM
FPC Ctg12 cp05T5e12DM4RS
File Edit Analysis Highlight Add track Layout Size options Help
Zoom
6.3
Whole
Show buried clones
Yes
No
Search
CB Unit Range
0
to
252
Contig stats
Clones: 45 (2 buried)
Markers: 0
Sequenced: 0
Length: 253 CB units
Ctg12 of cp05T5e12DM4RS
B008N09
V016F18
M038L06
M029M21
V018H22
V055K17
H011K15
V048D10
V047G22
V032C23
V061B06
V035P22
V061K16
V007D04
M050L01
H007J21
M017L06
M053F18
V047J03
V030I11
M024J24
V056C09
M028F08
M048B16
V060I19
M048P14
V019I21
V008M02~
V006J11
H009I22
B012K21
V043J07
V007C15~
V009J22*
V020H16
V018E06*
V033C21
V003G07
V062A20
0 25 50 75 100 125 150 175 200 225
[FPC V8.9 Main Menu]
Project cp05T5e12...
FPC Ctg12 cp05T5e...

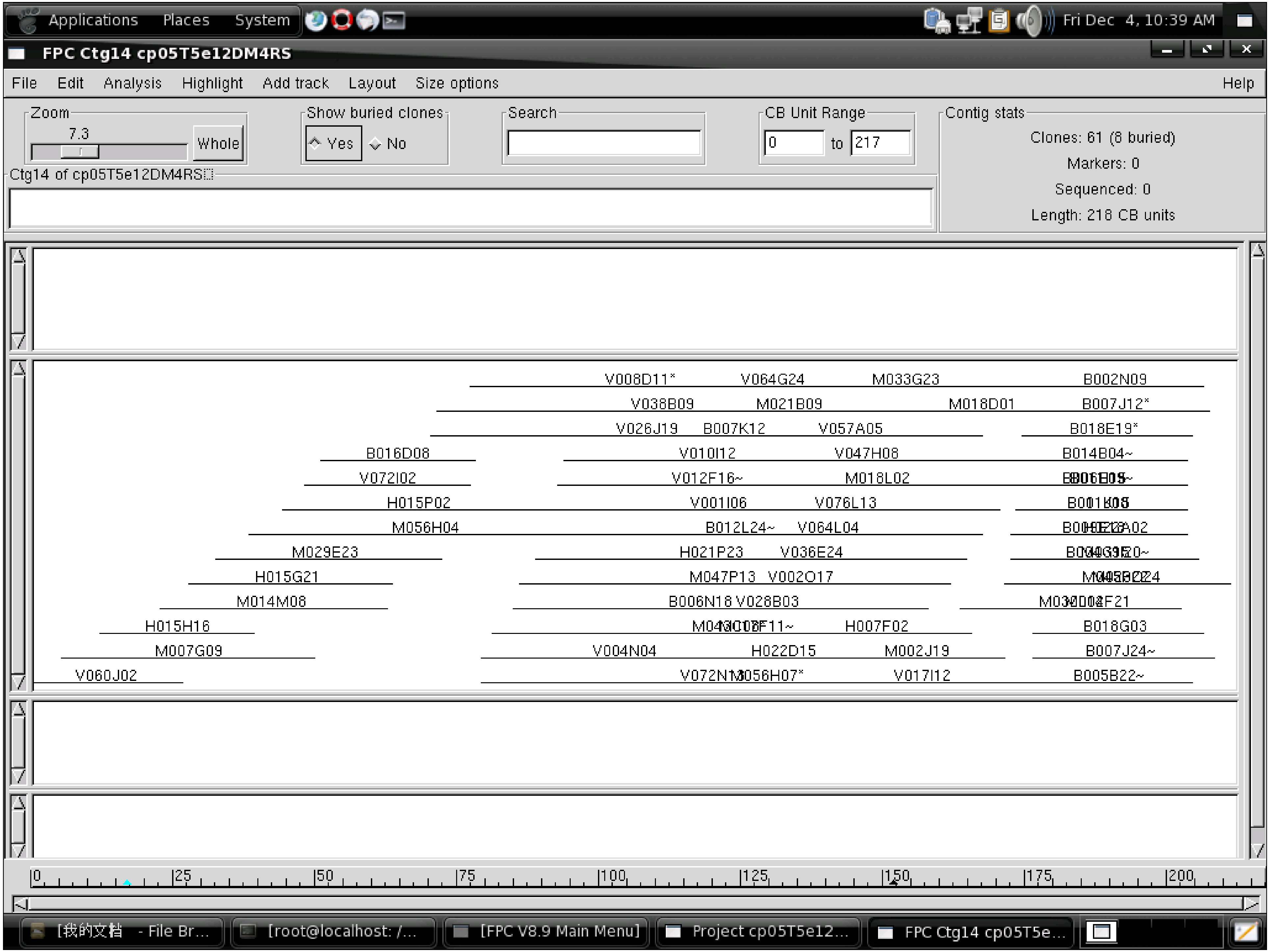
Applications Places System
Fri Dec 4, 10:39 AM
FPC Ctg14 cp05T5e12DM4RS
File Edit Analysis Highlight Add track Layout Size options Help
Zoom
7.3
Whole
Show buried clones
Yes No
Search
CB Unit Range
0 to 217
Contig stats
Clones: 61 (8 buried)
Markers: 0
Sequenced: 0
Length: 218 CB units
Ctg14 of cp05T5e12DM4RS
V008D11* V064G24 M033G23 B002N09
V038B09 M021B09 M018D01 B007J12*
V026J19 B007K12 V057A05 B018E19*
B016D08 V010I12 V047H08 B014B04~
V072I02 V012F16~ M018L02
H015P02 V001I06 V076L13
M056H04 B012L24~ V064L04
M029E23 H021P23 V036E24
H015G21 M047P13 V002O17
M014M08 B006N18 V028B03
H015H16 H007F02 B018G03
M007G09 V004N04 H022D15 M002J19 B007J24~
V060J02 V017I12 B005B22~
0 25 50 75 100 125 150 175 200
[我的文档 - File Br...
[root@localhost: /...
[FPC V8.9 Main Menu]
Project cp05T5e12...
FPC Ctg14 cp05T5e...

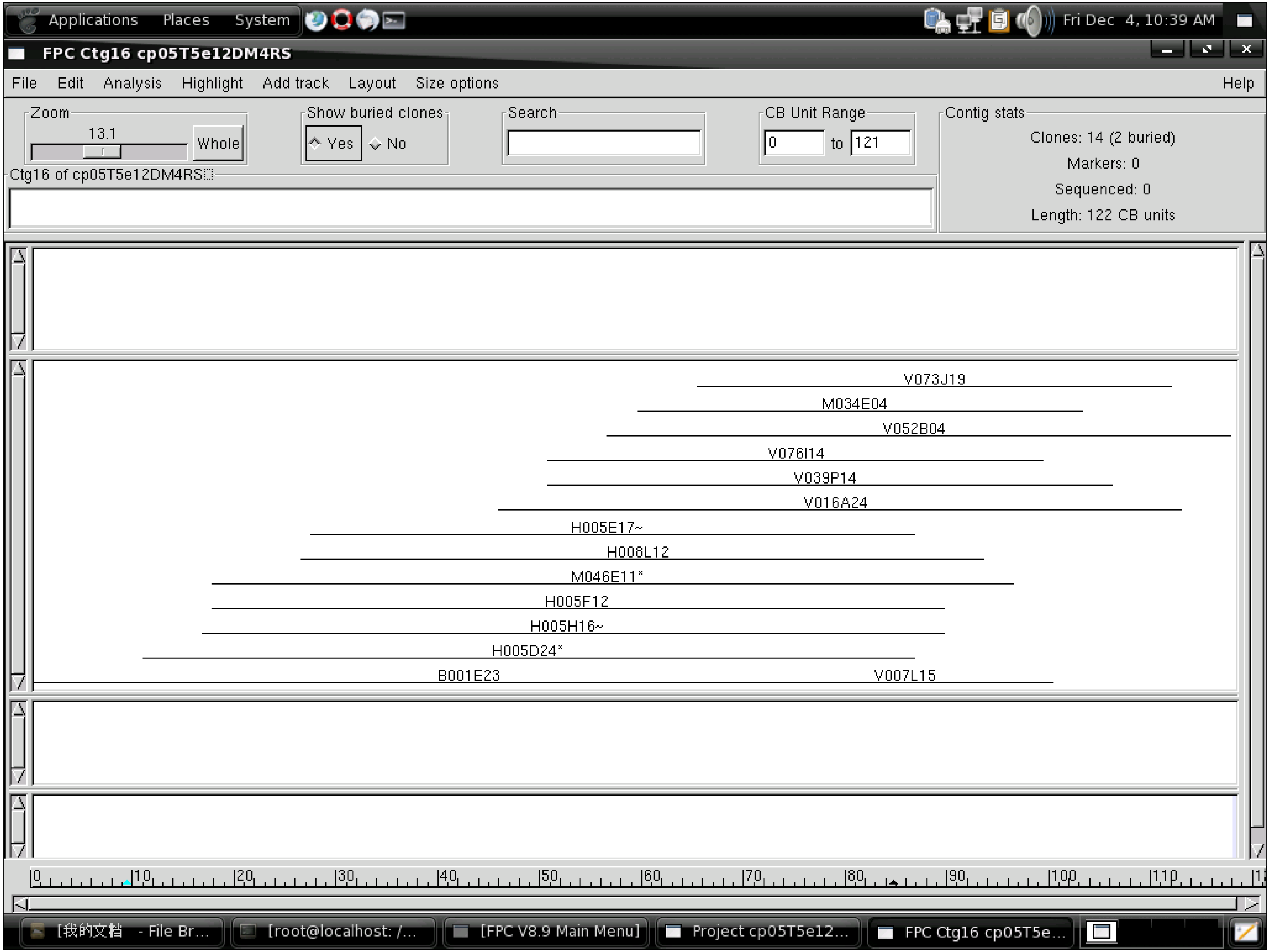
Applications Places System
Fri Dec 4, 10:39 AM
FPC Ctg16 cp05T5e12DM4RS
File Edit Analysis Highlight Add track Layout Size options Help
Zoom
13.1
Whole
Show buried clones
Yes No
Search
CB Unit Range
0 to 121
Contig stats
Clones: 14 (2 buried)
Markers: 0
Sequenced: 0
Length: 122 CB units
Ctg16 of cp05T5e12DM4RS
V073J19
M034E04
V052B04
V076I14
V039P14
V016A24
H005E17~
H008L12
M046E11*
H005F12
H005H16~
H005D24*
B001E23
V007L15
0 10 20 30 40 50 60 70 80 90 100 110
[我的文档 - File Br...
[root@localhost: /...
[FPC V8.9 Main Menu]
Project cp05T5e12...
FPC Ctg16 cp05T5e...

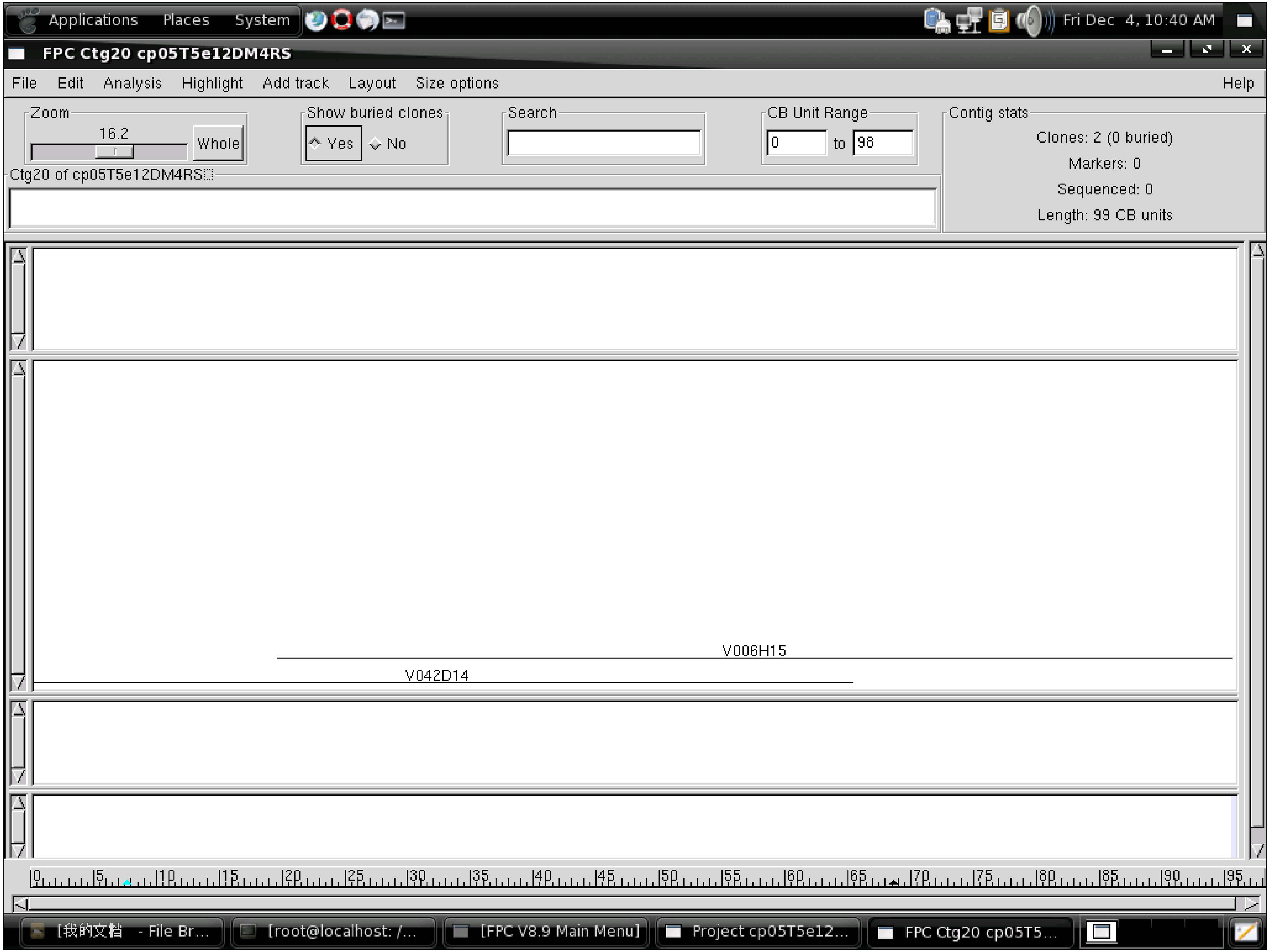
Applications
Places
System
Fri Dec 4, 10:40 AM
FPC Ctg20 cp05T5e12DM4RS
File
Edit
Analysis
Highlight
Add track
Layout
Size options
Help
Zoom
16.2
Whole
Show buried clones
Yes
No
Search
CB Unit Range
0
to
98
Contig stats
Clones: 2 (0 buried)
Markers: 0
Sequenced: 0
Length: 99 CB units
Ctg20 of cp05T5e12DM4RS
V006H15
V042D14
0 5 10 15 20 25 30 35 40 45 50 55 60 65 70 75 80 85 90 95
[我的文档 - File Br...
[root@localhost: /...
[FPC V8.9 Main Menu]
Project cp05T5e12...
FPC Ctg20 cp05T5...

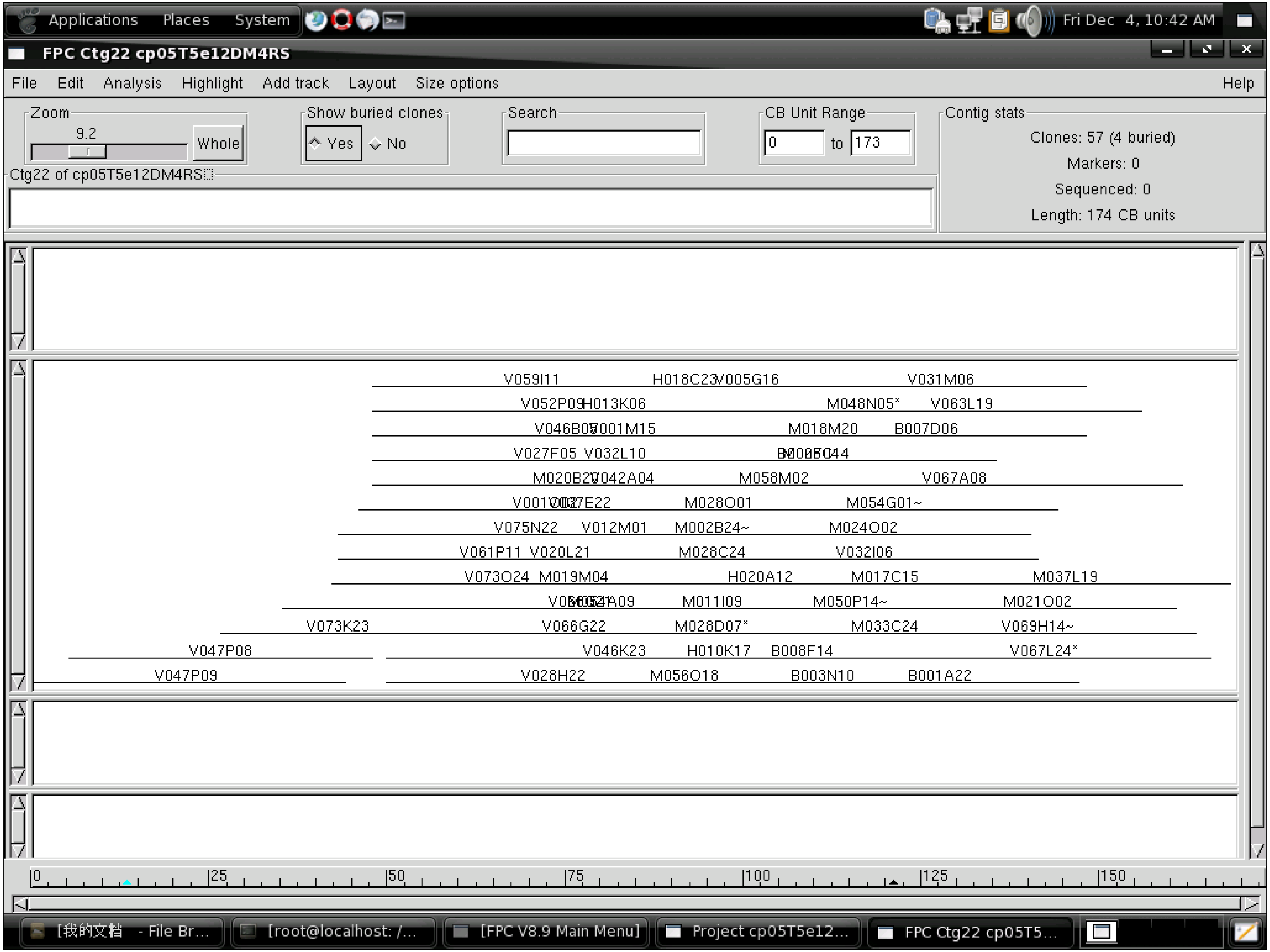
Applications Places System
Fri Dec 4, 10:42 AM
FPC Ctg22 cp05T5e12DM4RS
File Edit Analysis Highlight Add track Layout Size options Help
Zoom
9.2
Whole
Show buried clones
Yes No
Search
CB Unit Range
0 to 173
Contig stats
Clones: 57 (4 buried)
Markers: 0
Sequenced: 0
Length: 174 CB units
Ctg22 of cp05T5e12DM4RS
V059I11 H018C23 V005G16 V031M06
V052P09 H013K06 M048N05* V063L19
V046B05 V001M15 M018M20 B007D06
V027F05 V032L10
M020B2 V042A04 M058M02 V067A08
M028O01 M054G01~
V075N22 V012M01 M002B24~ M024O02
V061P11 V020L21 M028C24 V032I06
V073O24 M019M04 H020A12 M017C15 M037L19
M011I09 M050P14~ M021O02
V073K23 V066G22 M028D07* M033C24 V069H14~
V047P08 V046K23 H010K17 B008F14 V067L24*
V047P09 V028H22 M056O18 B003N10 B001A22
0 25 50 75 100 125 150
[我的文档 - File Br...
[root@localhost: /...
[FPC V8.9 Main Menu]
Project cp05T5e12...
FPC Ctg22 cp05T5...

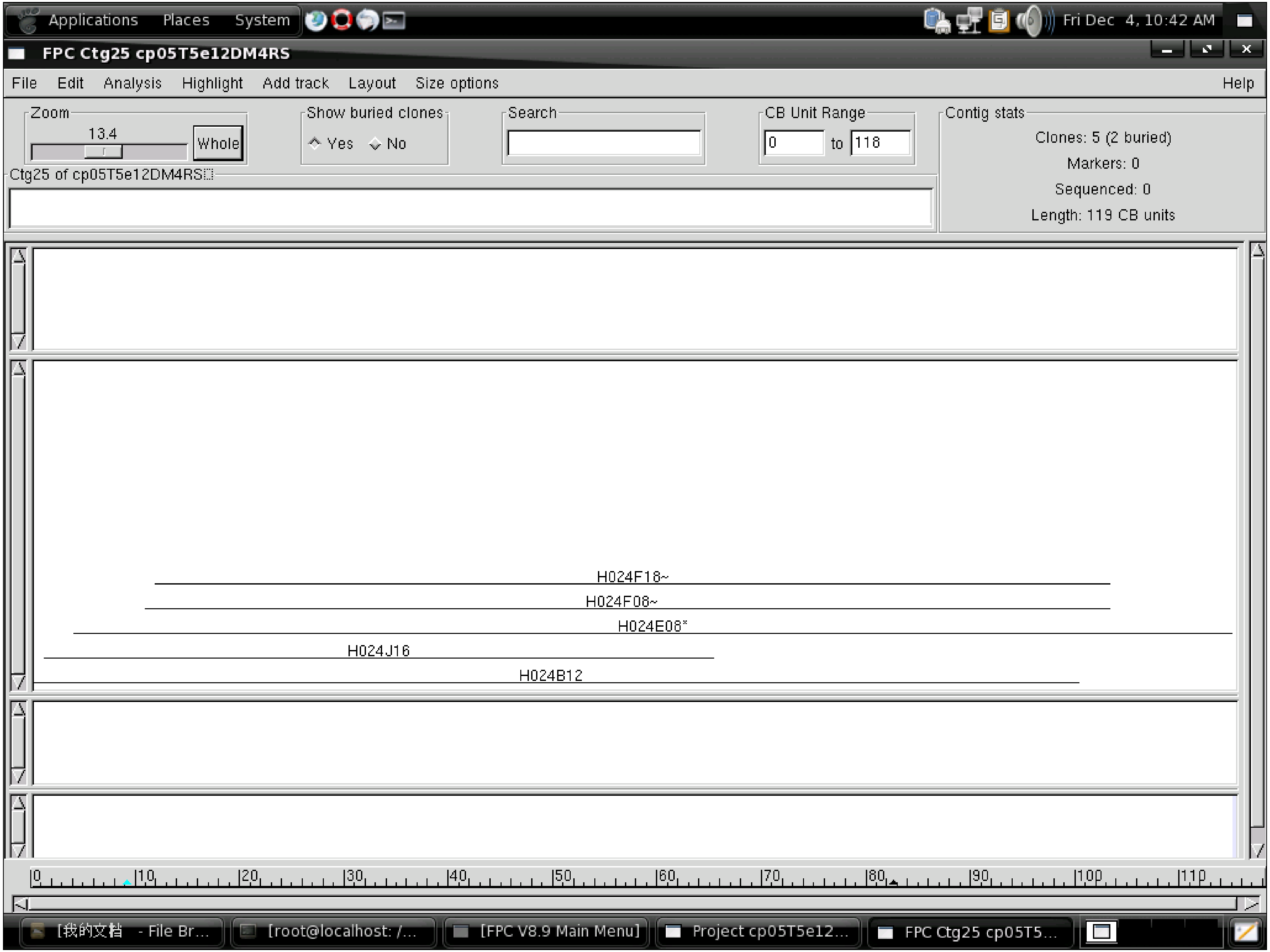
Applications Places System
Fri Dec 4, 10:42 AM
FPC Ctg25 cp05T5e12DM4RS
File Edit Analysis Highlight Add track Layout Size options Help
Zoom
13.4
Whole
Show buried clones
Yes No
Search
CB Unit Range
0 to 118
Contig stats
Clones: 5 (2 buried)
Markers: 0
Sequenced: 0
Length: 119 CB units
Ctg25 of cp05T5e12DM4RS
H024F18~
H024F08~
H024E08*
H024J16
H024B12
0 10 20 30 40 50 60 70 80 90 100 110
[我的文档 - File Br...
[root@localhost: /...
[FPC V8.9 Main Menu]
Project cp05T5e12...
FPC Ctg25 cp05T5...

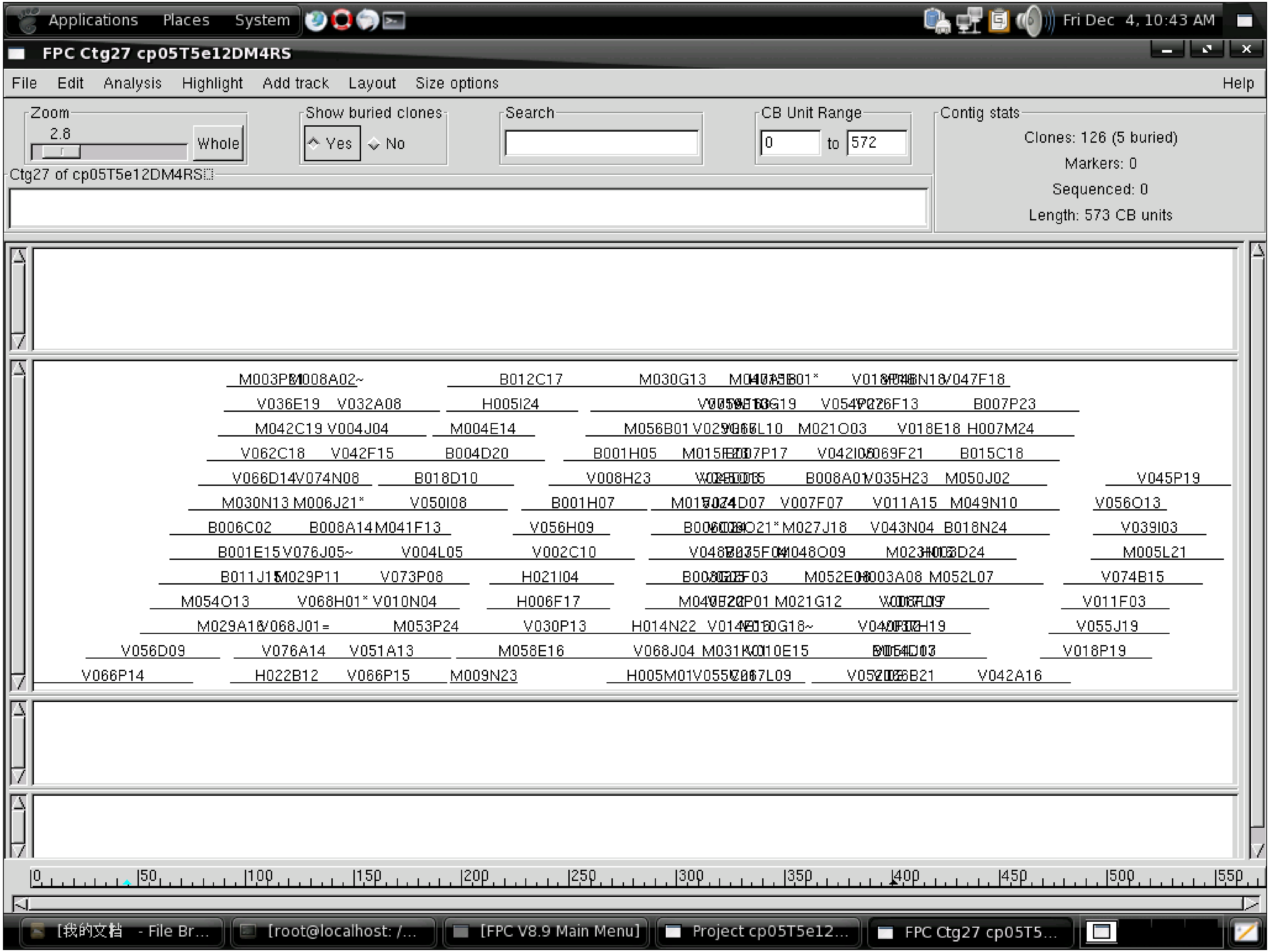
Applications Places System
Fri Dec 4, 10:43 AM
FPC Ctg27 cp05T5e12DM4RS
File Edit Analysis Highlight Add track Layout Size options Help
Zoom
2.8
Whole
Show buried clones
Yes No
Search
CB Unit Range
0 to 572
Contig stats
Clones: 126 (5 buried)
Markers: 0
Sequenced: 0
Length: 573 CB units
Ctg27 of cp05T5e12DM4RS
B012C17
M030G13
V036E19 V032A08
H005I24
B007P23
M042C19 V004J04
M004E14
M021O03
V018E18 H007M24
V062C18 V042F15
B004D20
B001H05
B015C18
V066D14V074N08
B018D10
V008H23
V035H23 M050J02
V045P19
M030N13 M006J21*
V050I08
B001H07
V007F07
V011A15 M049N10
V056O13
B006C02 B008A14M041F13
V056H09
M027J18
V043N04 B018N24
V039I03
B001E15V076J05~
V004L05
V002C10
M005L21
V073P08
H021I04
M052E08
V074B15
M054O13
V068H01* V010N04
H006F17
M021G12
V011F03
M053P24
V030P13
H014N22
V055J19
V056D09
V076A14 V051A13
M058E16
V068J04
V018P19
V066P14
H022B12 V066P15
M009N23
V042A16
0 50 100 150 200 250 300 350 400 450 500 550
[我的文档 - File Br...
[root@localhost: /...
[FPC V8.9 Main Menu]
Project cp05T5e12...
FPC Ctg27 cp05T5...

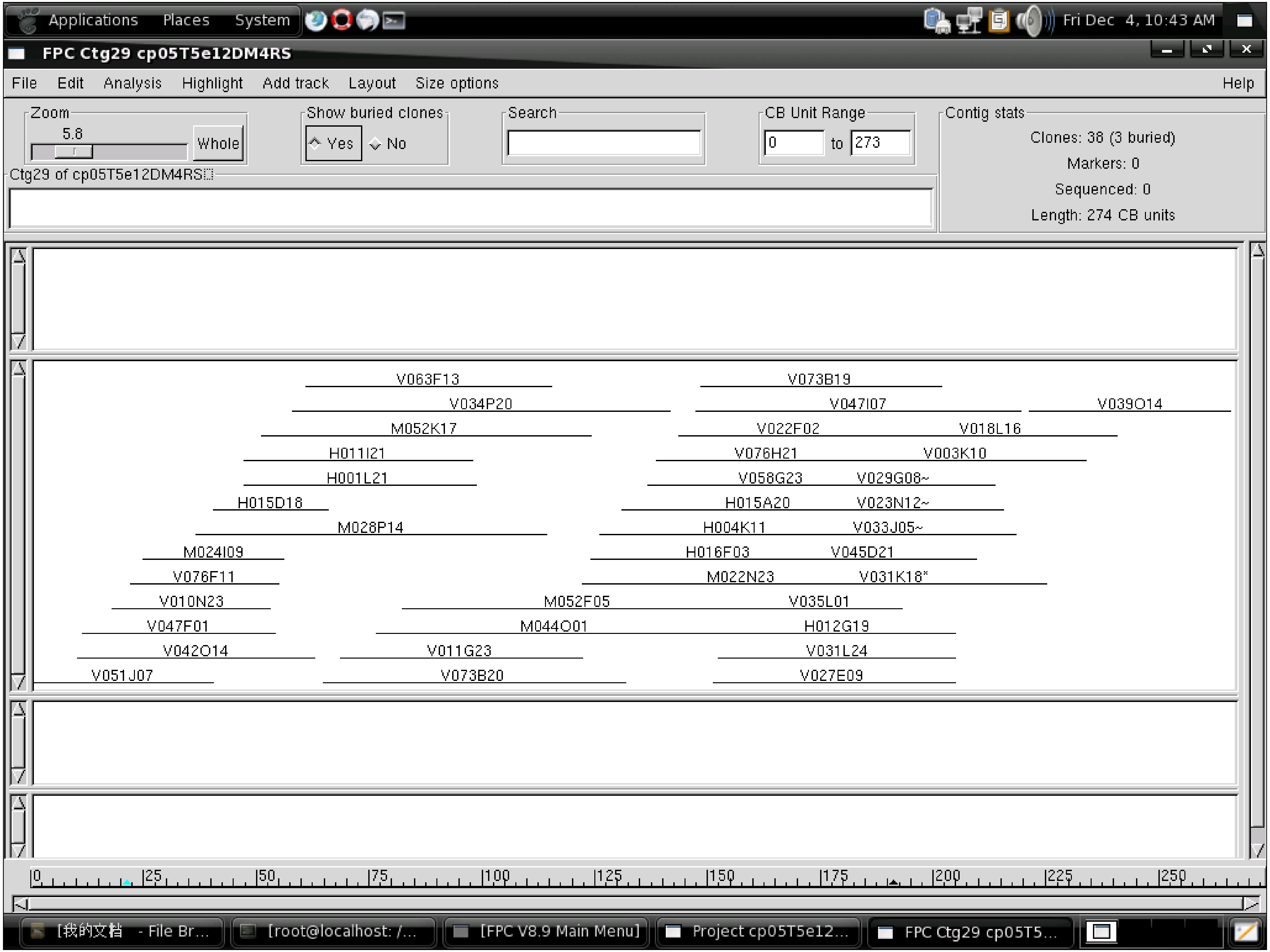
Applications Places System
Fri Dec 4, 10:43 AM
FPC Ctg29 cp05T5e12DM4RS
File Edit Analysis Highlight Add track Layout Size options Help
Zoom
5.8
Whole
Show buried clones
Yes
No
Search
CB Unit Range
0 to 273
Contig stats
Clones: 38 (3 buried)
Markers: 0
Sequenced: 0
Length: 274 CB units
Ctg29 of cp05T5e12DM4RS
V063F13
V073B19
V034P20
V047I07
V039O14
M052K17
V022F02
V018L16
H011I21
V076H21
V003K10
H001L21
V058G23
V029G08~
H015D18
H015A20
V023N12~
M028P14
H004K11
V033J05~
M024I09
H016F03
V045D21
V076F11
M022N23
V031K18*
V010N23
M052F05
V035L01
V047F01
M044O01
H012G19
V042O14
V011G23
V031L24
V051J07
V073B20
V027E09
0 25 50 75 100 125 150 175 200 225 250
[我的文档 - File Br...
[root@localhost: /...
[FPC V8.9 Main Menu]
Project cp05T5e12...
FPC Ctg29 cp05T5...

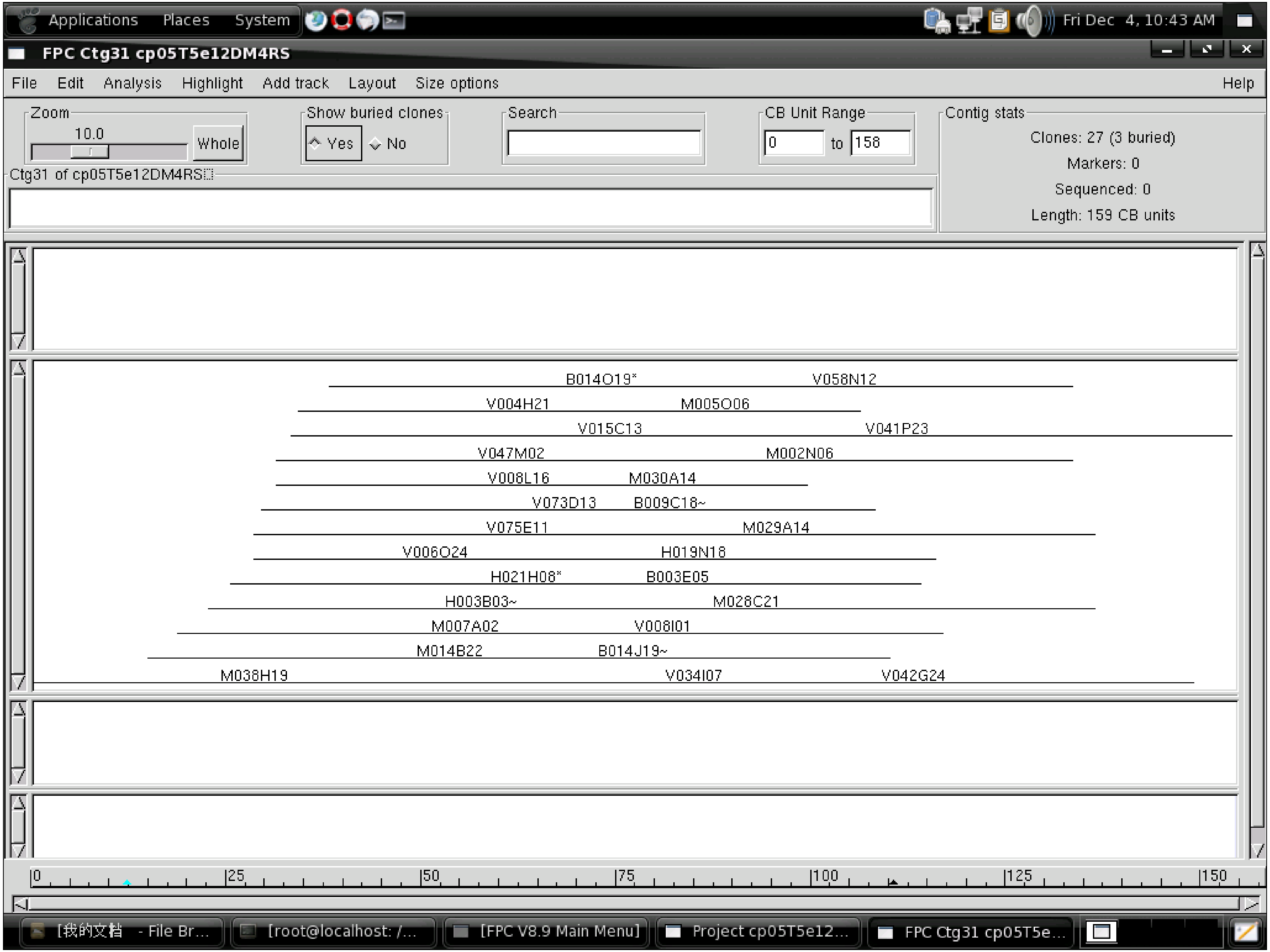
Applications Places System
Fri Dec 4, 10:43 AM
FPC Ctg31 cp05T5e12DM4RS
File Edit Analysis Highlight Add track Layout Size options Help
Zoom
10.0
Whole
Show buried clones
Yes No
Search
CB Unit Range
0 to 158
Contig stats
Clones: 27 (3 buried)
Markers: 0
Sequenced: 0
Length: 159 CB units
Ctg31 of cp05T5e12DM4RS
B014O19* V058N12
V004H21 M005O06
V015C13 V041P23
V047M02 M002N06
V008L16 M030A14
V073D13 B009C18~
V075E11 M029A14
V006O24 H019N18
H021H08* B003E05
H003B03~ M028C21
M007A02 V008I01
M014B22 B014J19~
M038H19 V034I07 V042G24
0 25 50 75 100 125 150
[我的文档 - File Br...
[root@localhost: /...
[FPC V8.9 Main Menu]
Project cp05T5e12...
FPC Ctg31 cp05T5e...

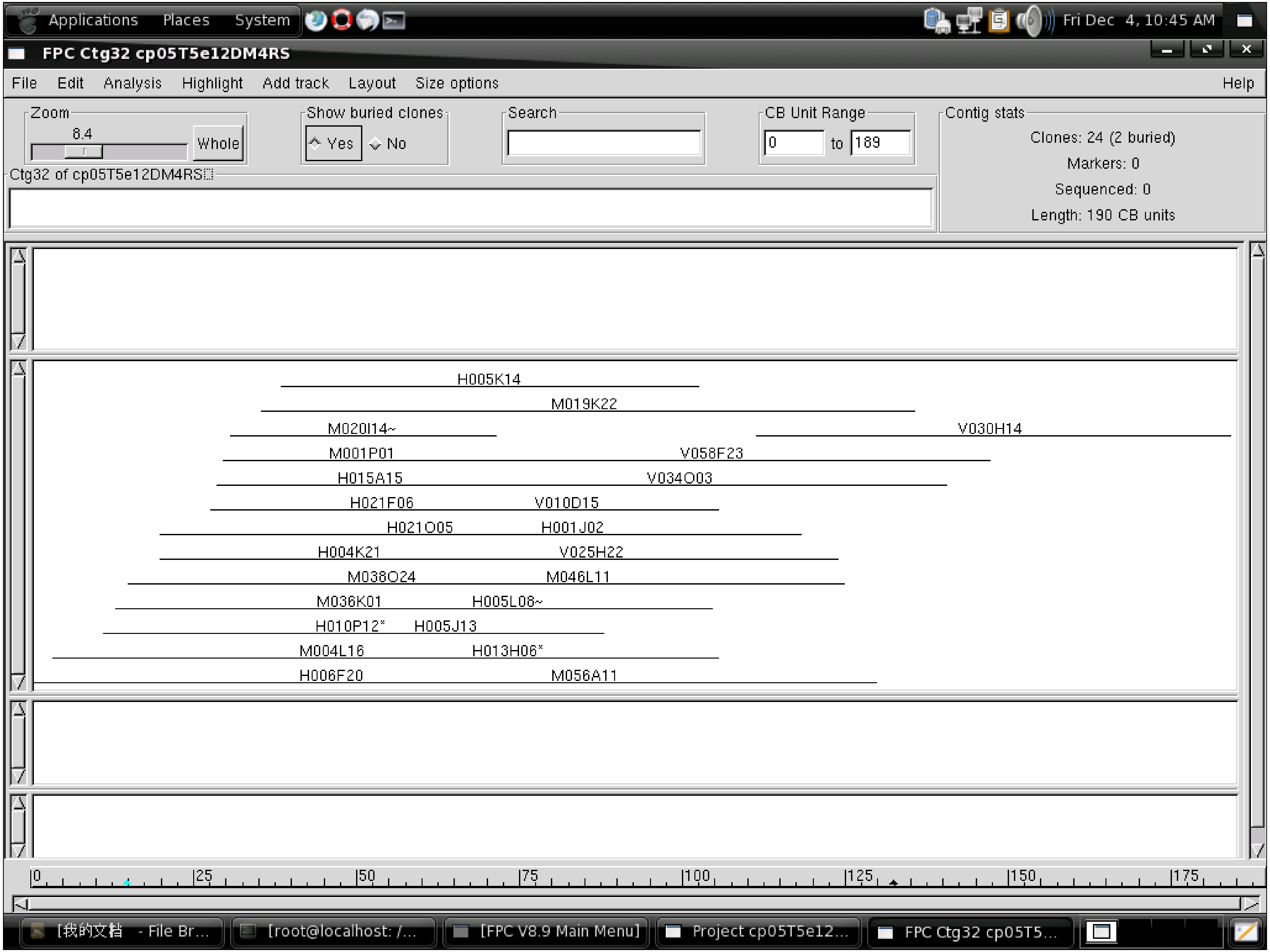
Applications
Places
System
Fri Dec 4, 10:45 AM
FPC Ctg32 cp05T5e12DM4RS
File
Edit
Analysis
Highlight
Add track
Layout
Size options
Help
Zoom
8.4
Whole
Show buried clones
Yes
No
Search
CB Unit Range
0
to
189
Contig stats
Clones: 24 (2 buried)
Markers: 0
Sequenced: 0
Length: 190 CB units
Ctg32 of cp05T5e12DM4RS
H005K14
M019K22
M020I14~
V030H14
M001P01
V058F23
H015A15
V034O03
H021F06
V010D15
H021O05
H001J02
H004K21
V025H22
M038O24
M046L11
M036K01
H005L08~
H010P12*
H005J13
M004L16
H013H06*
H006F20
M056A11
0
25
50
75
100
125
150
175
[我的文档 - File Br...
[root@localhost: /...
[FPC V8.9 Main Menu]
Project cp05T5e12...
FPC Ctg32 cp05T5...

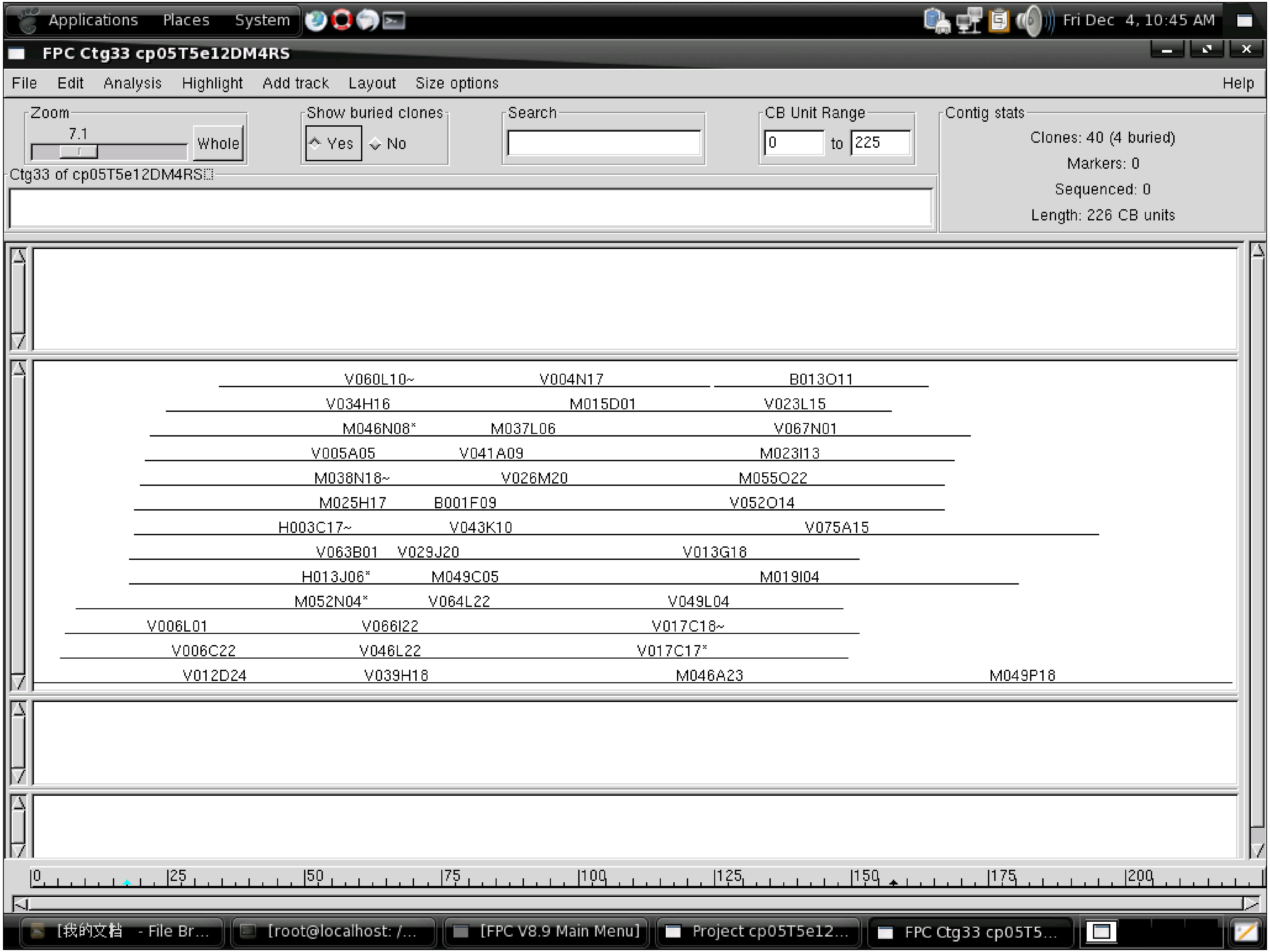
Applications Places System
Fri Dec 4, 10:45 AM
FPC Ctg33 cp05T5e12DM4RS
File Edit Analysis Highlight Add track Layout Size options Help
Zoom 7.1 Whole
Show buried clones Yes No
Search
CB Unit Range 0 to 225
Contig stats
Clones: 40 (4 buried)
Markers: 0
Sequenced: 0
Length: 226 CB units
Ctg33 of cp05T5e12DM4RS
V060L10~ V004N17 B013O11
V034H16 M015D01 V023L15
M046N08* M037L06 V067N01
V005A05 V041A09 M023I13
M038N18~ V026M20 M055O22
M025H17 B001F09 V052O14
H003C17~ V043K10 V075A15
V063B01 V029J20 V013G18
H013J06* M049C05 M019I04
M052N04* V064L22 V049L04
V006L01 V066I22 V017C18~
V006C22 V046L22 V017C17*
V012D24 V039H18 M046A23 M049P18
0 25 50 75 100 125 150 175 200
[我的文档 - File Br...
[root@localhost: /...
[FPC V8.9 Main Menu]
Project cp05T5e12...
FPC Ctg33 cp05T5...

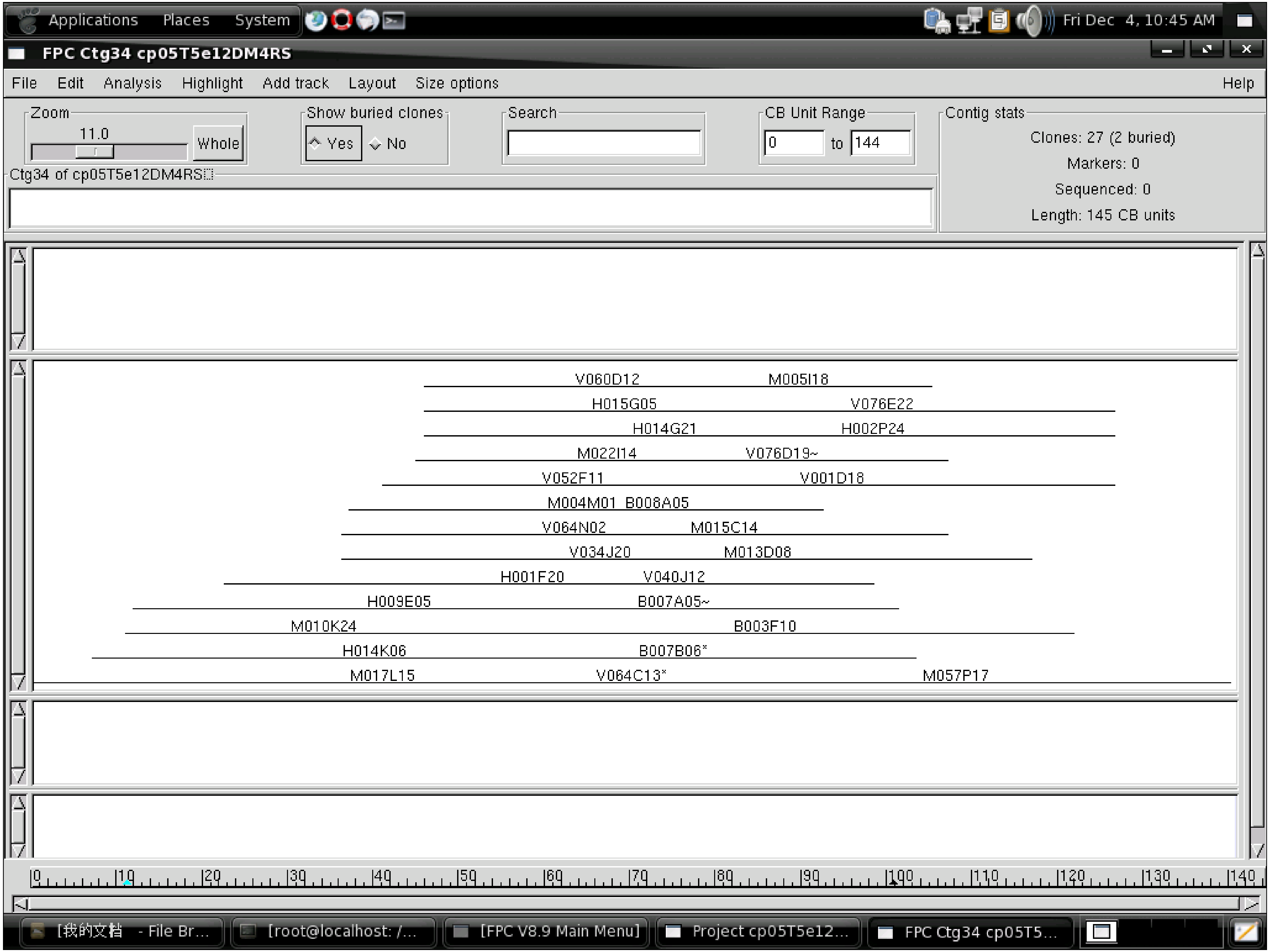
Applications Places System
Fri Dec 4, 10:45 AM
FPC Ctg34 cp05T5e12DM4RS
File Edit Analysis Highlight Add track Layout Size options Help
Zoom 11.0 Whole
Show buried clones Yes No
Search
CB Unit Range 0 to 144
Contig stats
Clones: 27 (2 buried)
Markers: 0
Sequenced: 0
Length: 145 CB units
Ctg34 of cp05T5e12DM4RS
V060D12 M005I18
H015G05 V076E22
H014G21 H002P24
M022I14 V076D19~
V052F11 V001D18
M004M01 B008A05
V064N02 M015C14
V034J20 M013D08
H001F20 V040J12
H009E05 B007A05~
M010K24 B003F10
H014K06 B007B06*
M017L15 V064C13* M057P17
0 10 20 30 40 50 60 70 80 90 100 110 120 130 140
[我的文档 - File Br...
[root@localhost: /...
[FPC V8.9 Main Menu]
Project cp05T5e12...
FPC Ctg34 cp05T5...

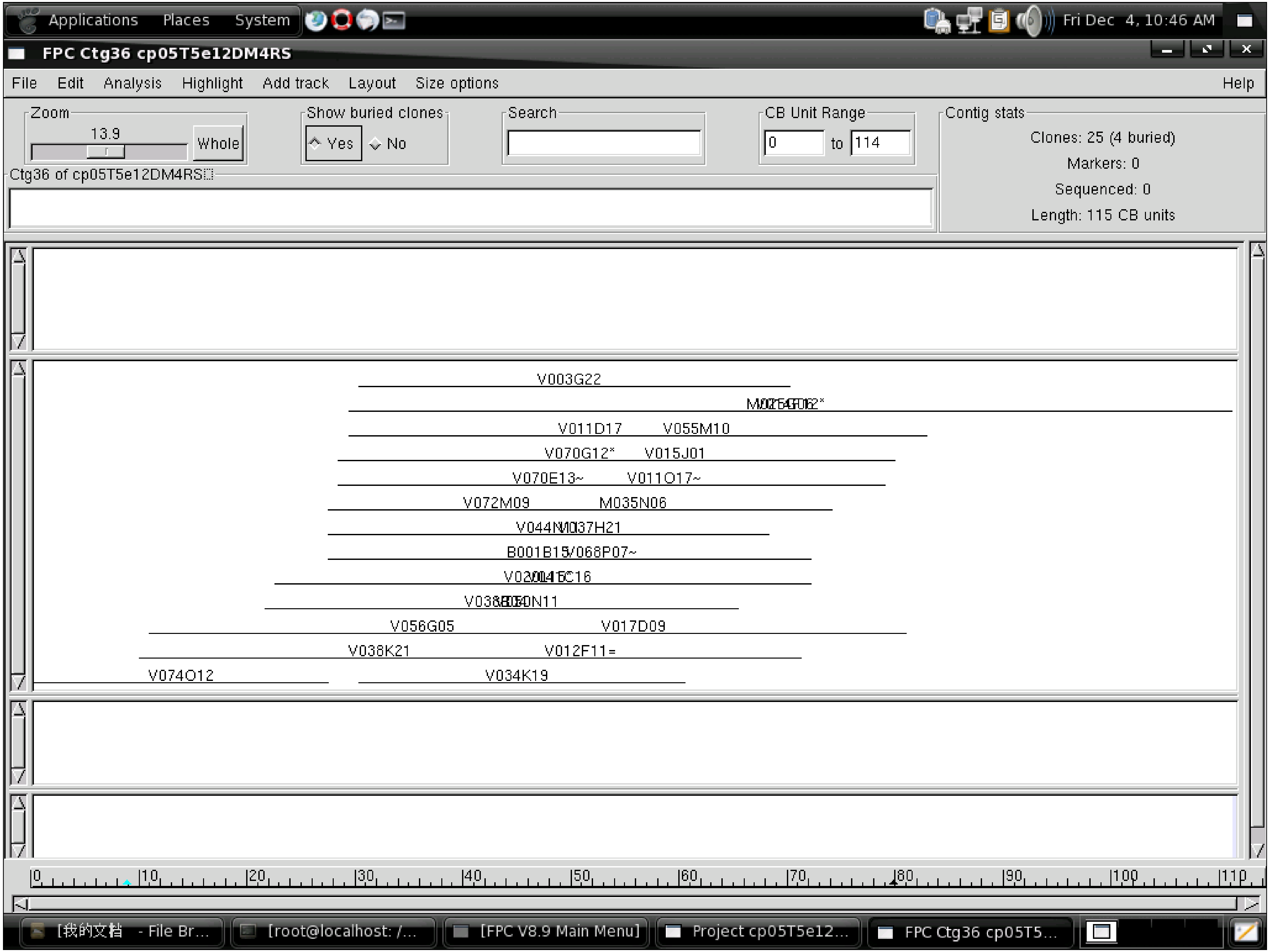
Applications
Places
System
Fri Dec 4, 10:46 AM
FPC Ctg36 cp05T5e12DM4RS
File
Edit
Analysis
Highlight
Add track
Layout
Size options
Help
Zoom
13.9
Whole
Show buried clones
Yes
No
Search
CB Unit Range
0
to
114
Contig stats
Clones: 25 (4 buried)
Markers: 0
Sequenced: 0
Length: 115 CB units
Ctg36 of cp05T5e12DM4RS
V003G22
V011D17
V055M10
V070G12*
V015J01
V070E13~
V011O17~
V072M09
M035N06
V056G05
V017D09
V038K21
V012F11=
V074O12
V034K19
0
10
20
30
40
50
60
70
80
90
100
110
[我的文档 - File Br...
[root@localhost: /...
[FPC V8.9 Main Menu]
Project cp05T5e12...
FPC Ctg36 cp05T5...

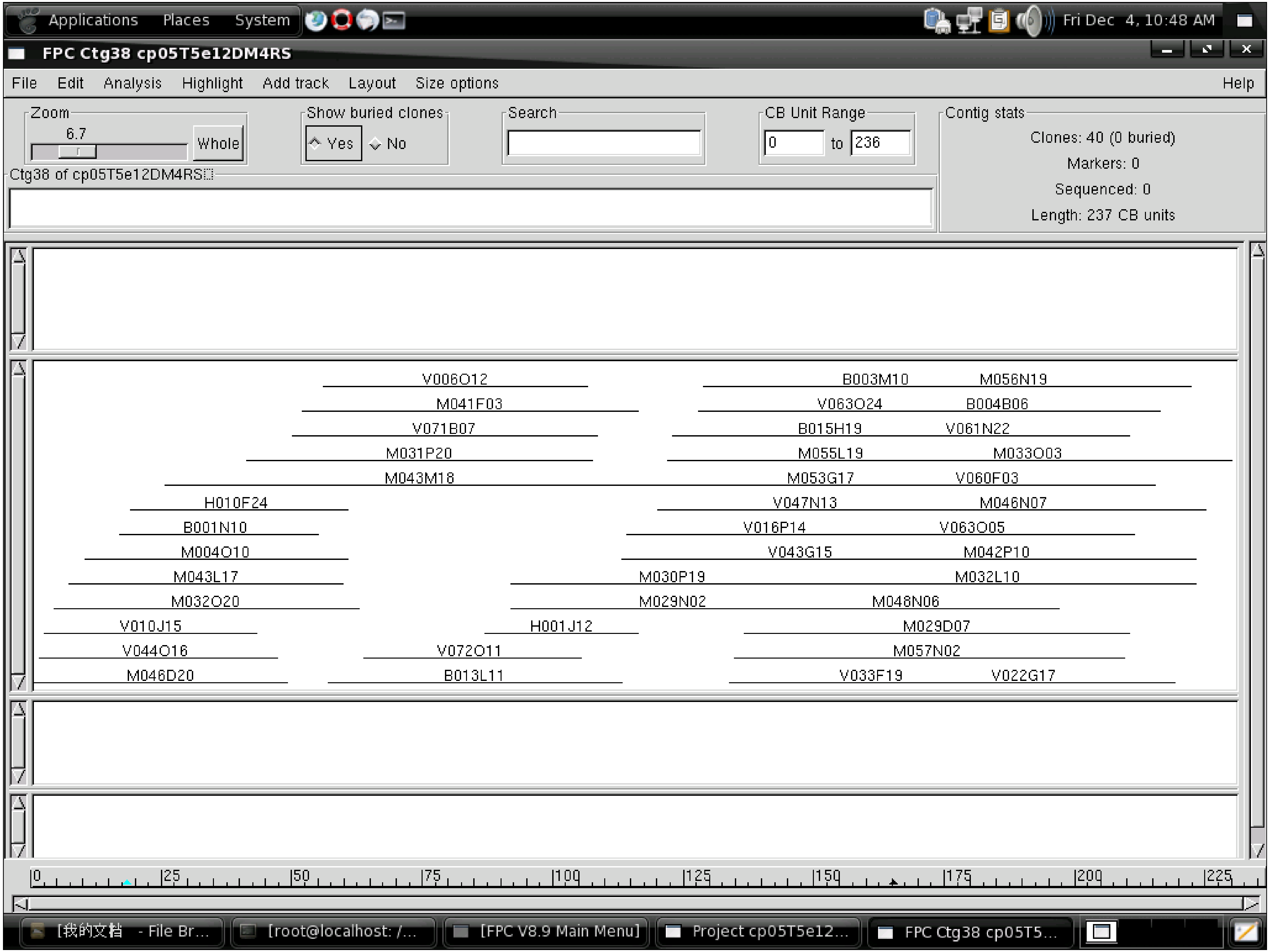
Applications
Places
System
Fri Dec 4, 10:48 AM
FPC Ctg38 cp05T5e12DM4RS
File
Edit
Analysis
Highlight
Add track
Layout
Size options
Help
Zoom
6.7
Whole
Show buried clones
Yes
No
Search
CB Unit Range
0
to
236
Contig stats
Clones: 40 (0 buried)
Markers: 0
Sequenced: 0
Length: 237 CB units
Ctg38 of cp05T5e12DM4RS
V006O12
B003M10
M056N19
M041F03
V063O24
B004B06
V071B07
B015H19
V061N22
M031P20
M055L19
M033O03
M043M18
M053G17
V060F03
H010F24
V047N13
M046N07
B001N10
V016P14
V063O05
M004O10
V043G15
M042P10
M043L17
M030P19
M032L10
M032O20
M029N02
M048N06
V010J15
H001J12
M029D07
V044O16
V072O11
M057N02
M046D20
B013L11
V033F19
V022G17
0
25
50
75
100
125
150
175
200
225
[我的文档 - File Br...
[root@localhost: /...
[FPC V8.9 Main Menu]
Project cp05T5e12...
FPC Ctg38 cp05T5...

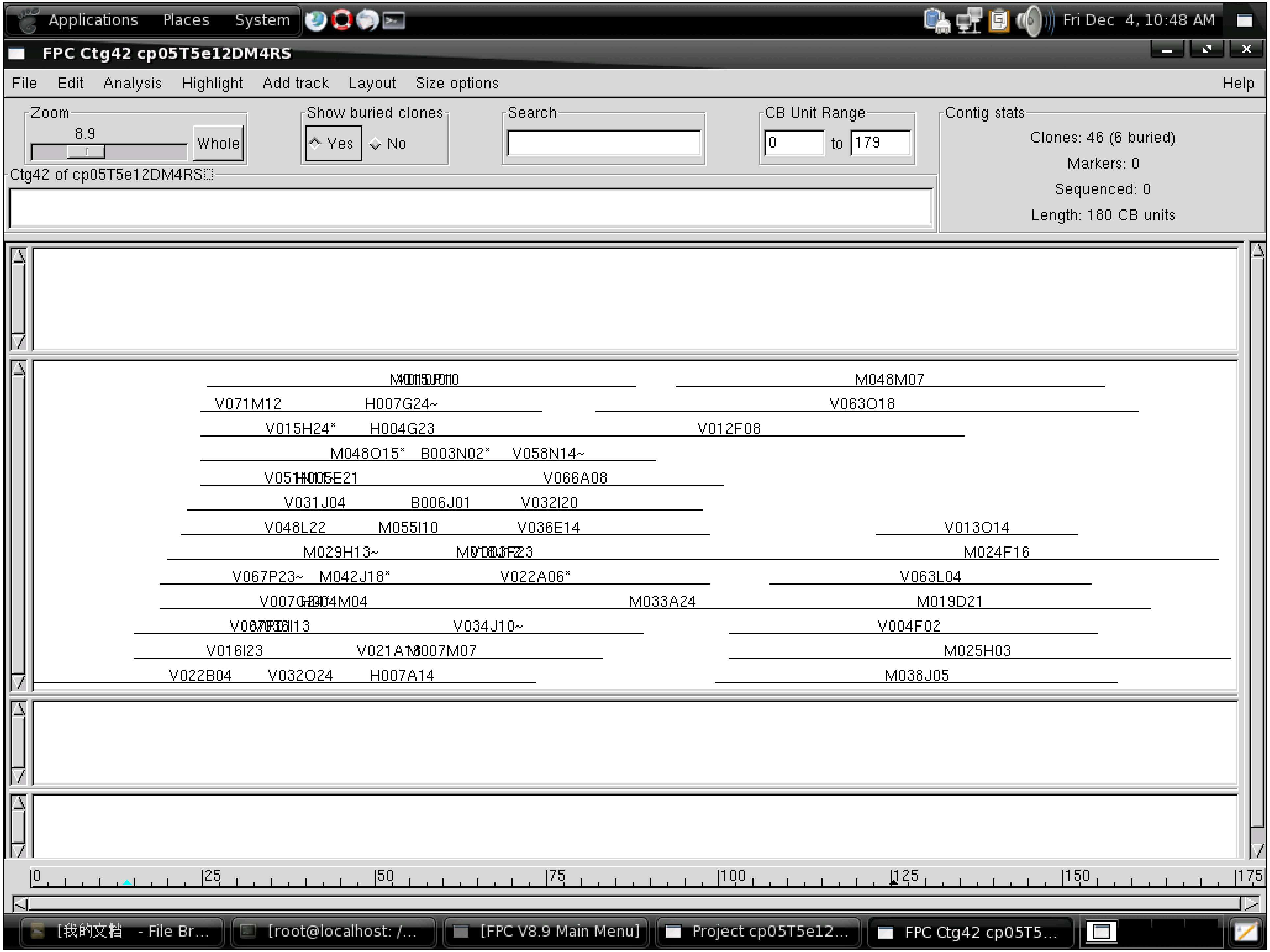
Applications Places System
Fri Dec 4, 10:48 AM
FPC Ctg42 cp05T5e12DM4RS
File Edit Analysis Highlight Add track Layout Size options Help
Zoom
8.9
Whole
Show buried clones
Yes No
Search
CB Unit Range
0 to 179
Contig stats
Clones: 46 (6 buried)
Markers: 0
Sequenced: 0
Length: 180 CB units
Ctg42 of cp05T5e12DM4RS
M048M07
V071M12 H007G24~
V063O18
V015H24* H004G23
V012F08
M048O15* B003N02* V058N14~
V066A08
V031J04 B006J01 V032I20
V048L22 M055I10 V036E14
V013O14
M029H13~
M024F16
V067P23~ M042J18* V022A06*
V063L04
M033A24
M019D21
V034J10~
V004F02
V016I23
M025H03
V022B04 V032O24 H007A14
M038J05
0 25 50 75 100 125 150 175
[我的文档 - File Br...
[root@localhost: /...
[FPC V8.9 Main Menu]
Project cp05T5e12...
FPC Ctg42 cp05T5...

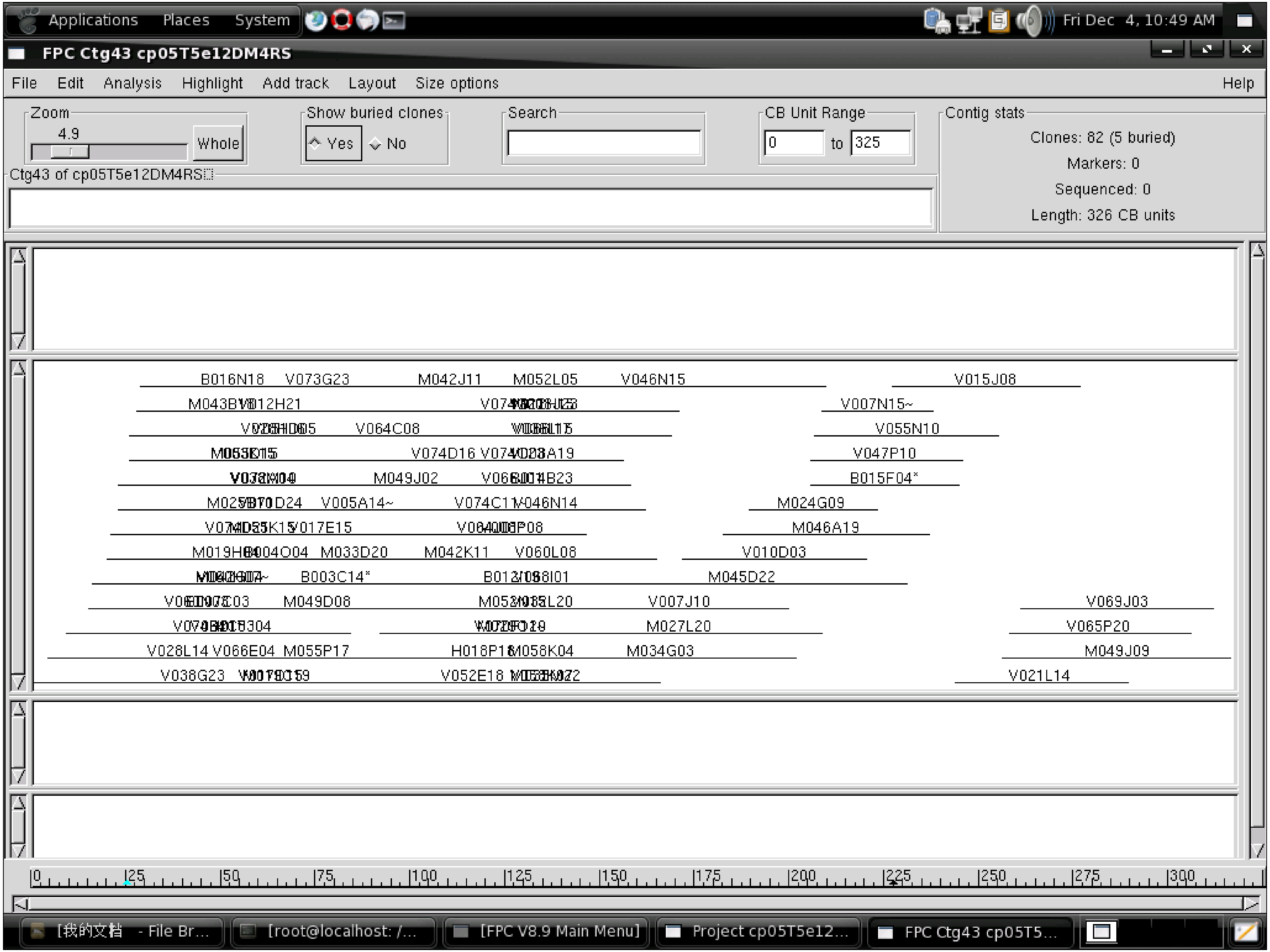
Applications Places System
Fri Dec 4, 10:49 AM
FPC Ctg43 cp05T5e12DM4RS
File Edit Analysis Highlight Add track Layout Size options Help
Zoom
4.9
Whole
Show buried clones
Yes
No
Search
CB Unit Range
0 to 325
Contig stats
Clones: 82 (5 buried)
Markers: 0
Sequenced: 0
Length: 326 CB units
Ctg43 of cp05T5e12DM4RS
B016N18 V073G23 M042J11 M052L05 V046N15 V015J08
V007N15~
V064C08
V055N10
V074D16
V047P10
M049J02
B015F04*
V005A14~
M024G09
V017E15
M046A19
M033D20 M042K11 V060L08 V010D03
B003C14*
M045D22
M049D08 V007J10 V069J03
M027L20 V065P20
V028L14 V066E04 M055P17 M034G03 M049J09
V038G23 V052E18 V021L14
0 25 50 75 100 125 150 175 200 225 250 275 300
[root@localhost: /...
[FPC V8.9 Main Menu]
Project cp05T5e12...
FPC Ctg43 cp05T5...

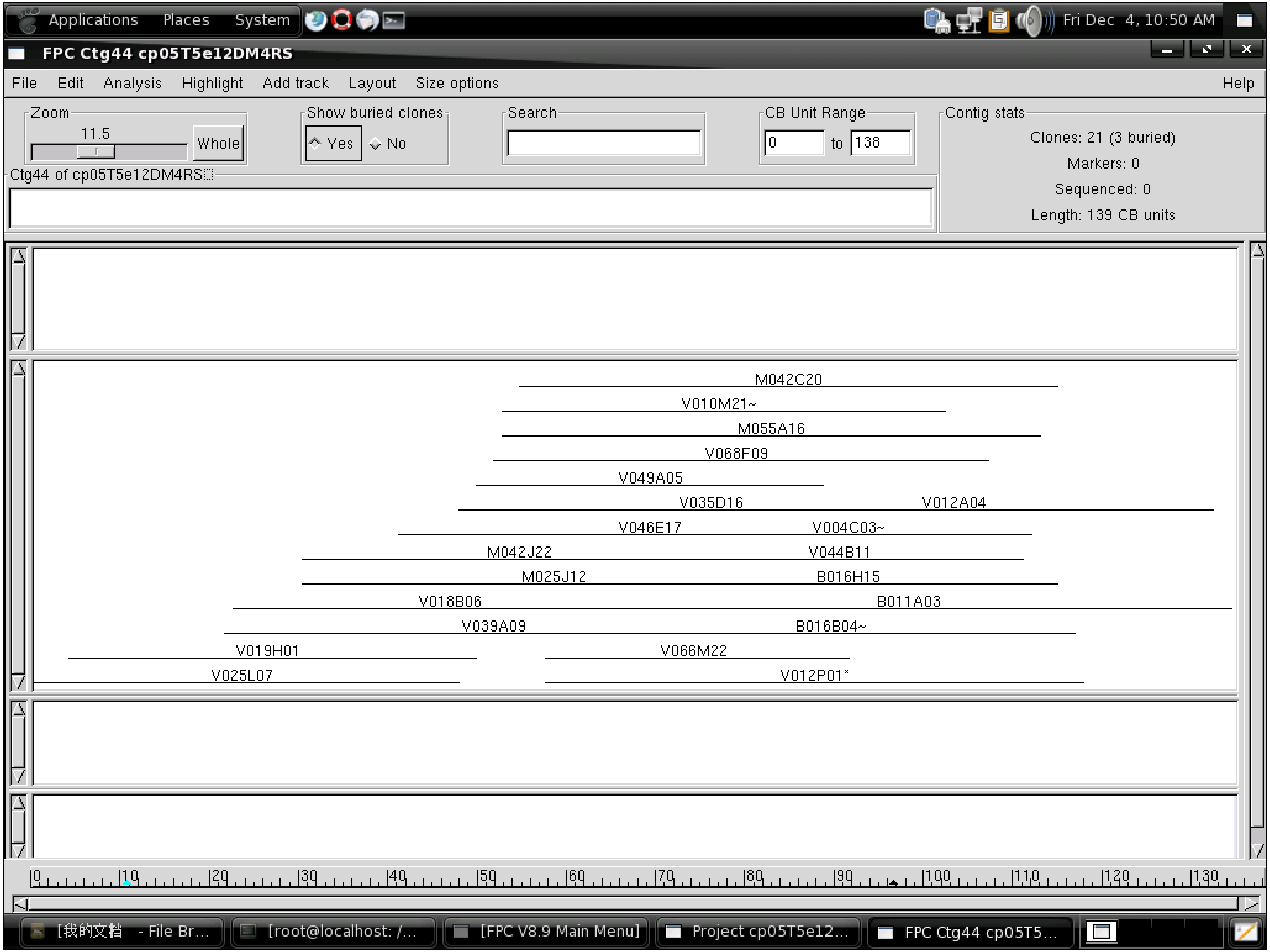
Applications Places System
Fri Dec 4, 10:50 AM
FPC Ctg44 cp05T5e12DM4RS
File Edit Analysis Highlight Add track Layout Size options Help
Zoom
11.5
Whole
Show buried clones
Yes No
Search
CB Unit Range
0 to 138
Contig stats
Clones: 21 (3 buried)
Markers: 0
Sequenced: 0
Length: 139 CB units
Ctg44 of cp05T5e12DM4RS
M042C20
V010M21~
M055A16
V068F09
V049A05
V035D16
V012A04
V046E17
V004C03~
M042J22
V044B11
M025J12
B016H15
V018B06
B011A03
V039A09
B016B04~
V019H01
V066M22
V025L07
V012P01*
0 10 20 30 40 50 60 70 80 90 100 110 120 130
[我的文档 - File Br...
[root@localhost: /...
[FPC V8.9 Main Menu]
Project cp05T5e12...
FPC Ctg44 cp05T5...

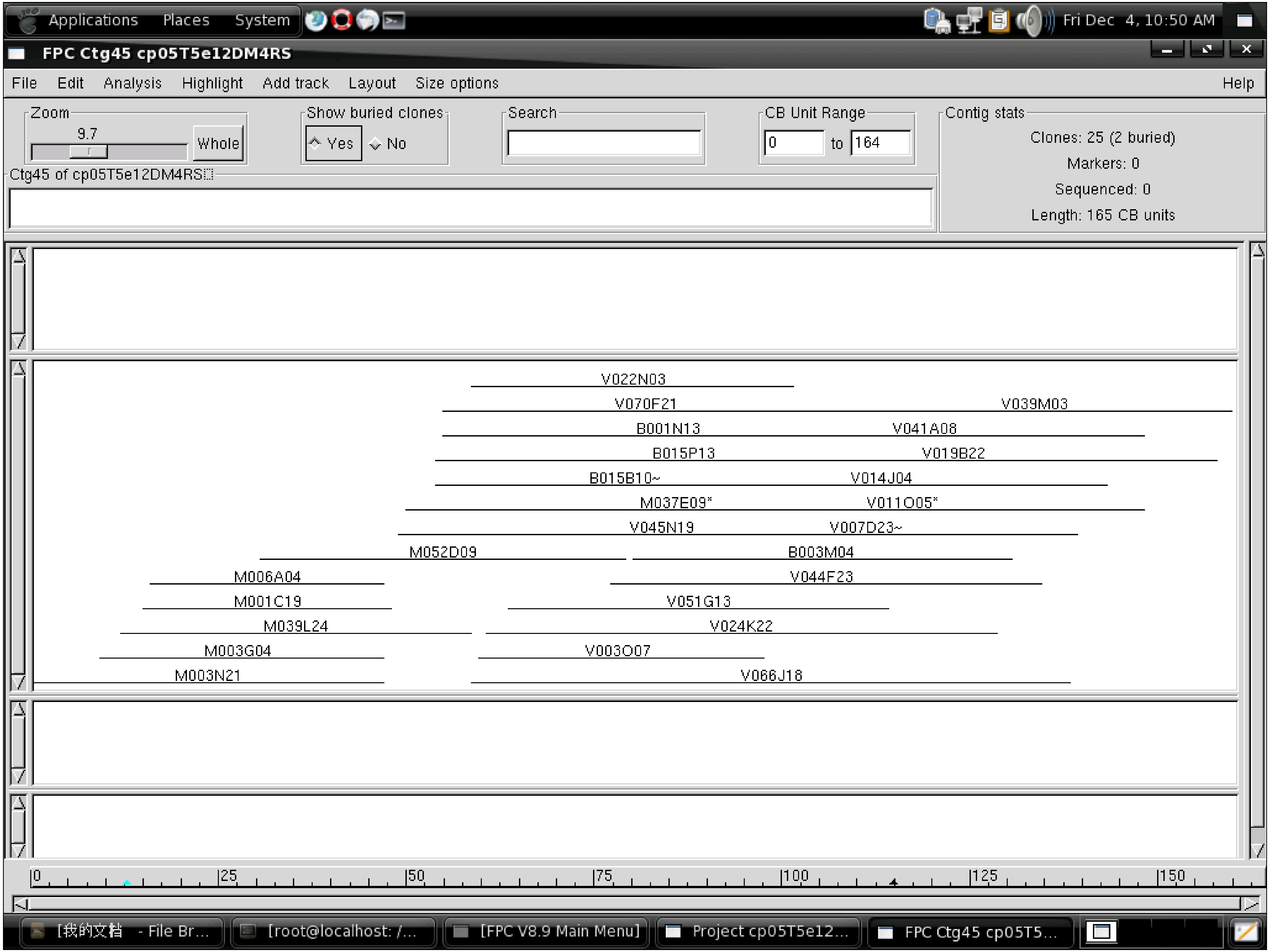
Applications Places System
Fri Dec 4, 10:50 AM
FPC Ctg45 cp05T5e12DM4RS
File Edit Analysis Highlight Add track Layout Size options Help
Zoom
9.7
Whole
Show buried clones
Yes No
Search
CB Unit Range
0 to 164
Contig stats
Clones: 25 (2 buried)
Markers: 0
Sequenced: 0
Length: 165 CB units
Ctg45 of cp05T5e12DM4RS
V022N03
V070F21
V039M03
B001N13
V041A08
B015P13
V019B22
B015B10~
V014J04
M037E09*
V011O05*
V045N19
V007D23~
M052D09
B003M04
M006A04
V044F23
M001C19
V051G13
M039L24
V024K22
M003G04
V003O07
M003N21
V066J18
0 25 50 75 100 125 150
[我的文档 - File Br...
[root@localhost: /...
[FPC V8.9 Main Menu]
Project cp05T5e12...
FPC Ctg45 cp05T5...

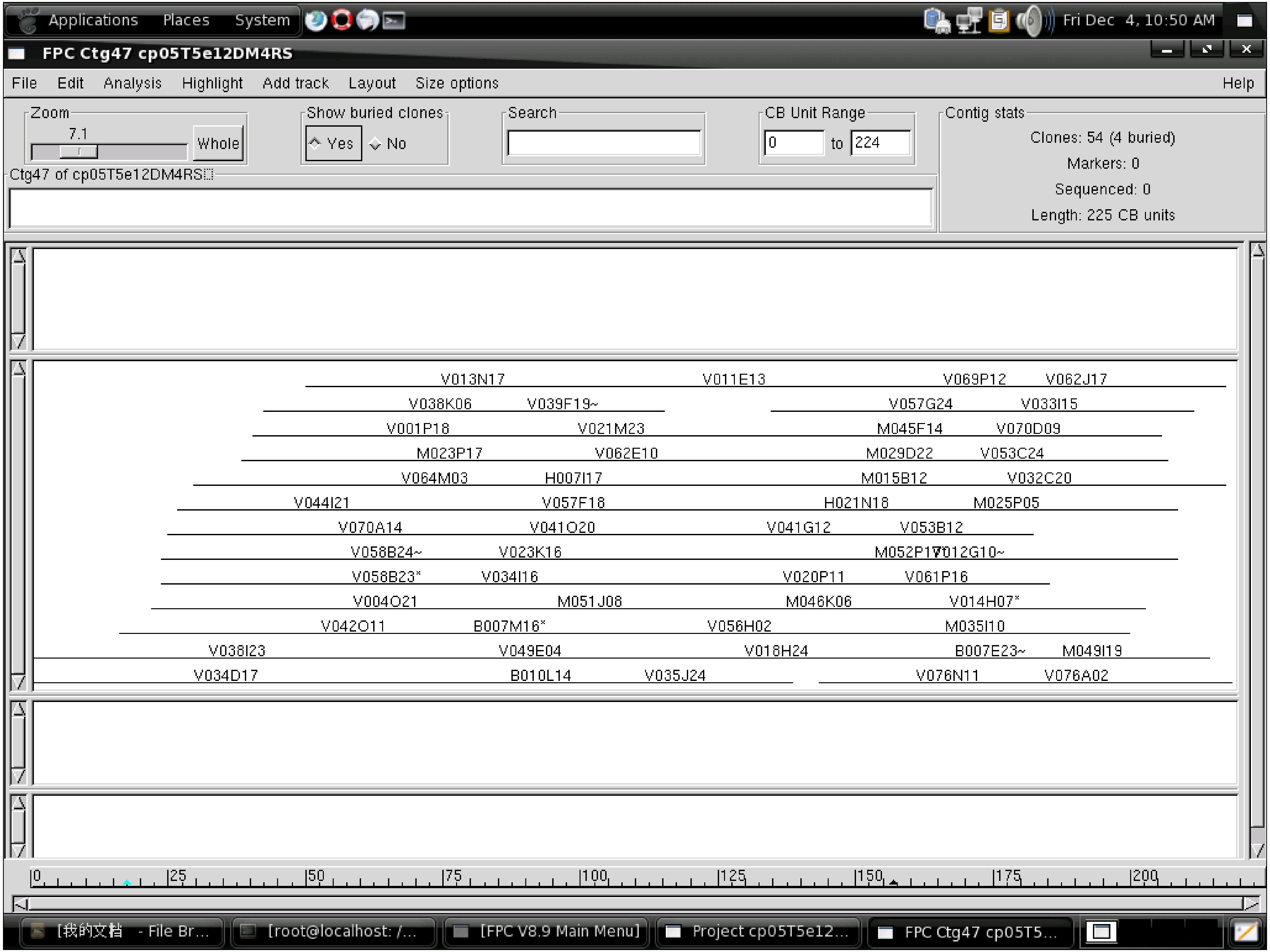
Applications Places System
Fri Dec 4, 10:50 AM
FPC Ctg47 cp05T5e12DM4RS
File Edit Analysis Highlight Add track Layout Size options Help
Zoom
7.1
Whole
Show buried clones
Yes No
Search
CB Unit Range
0 to 224
Contig stats
Clones: 54 (4 buried)
Markers: 0
Sequenced: 0
Length: 225 CB units
Ctg47 of cp05T5e12DM4RS
V013N17 V011E13 V069P12 V062J17
V038K06 V039F19~ V057G24 V033I15
V001P18 V021M23 M045F14 V070D09
M023P17 V062E10 M029D22 V053C24
V064M03 H007I17 M015B12 V032C20
V044I21 V057F18 H021N18 M025P05
V070A14 V041O20 V041G12 V053B12
V058B24~ V023K16 M052P17V012G10~
V058B23* V034I16 V020P11 V061P16
V004O21 M051J08 M046K06 V014H07*
V042O11 B007M16* V056H02 M035I10
V038I23 V049E04 V018H24 B007E23~ M049I19
V034D17 B010L14 V035J24 V076N11 V076A02
0 25 50 75 100 125 150 175 200
[我的文档 - File Br...
[root@localhost: /...
[FPC V8.9 Main Menu]
Project cp05T5e12...
FPC Ctg47 cp05T5...

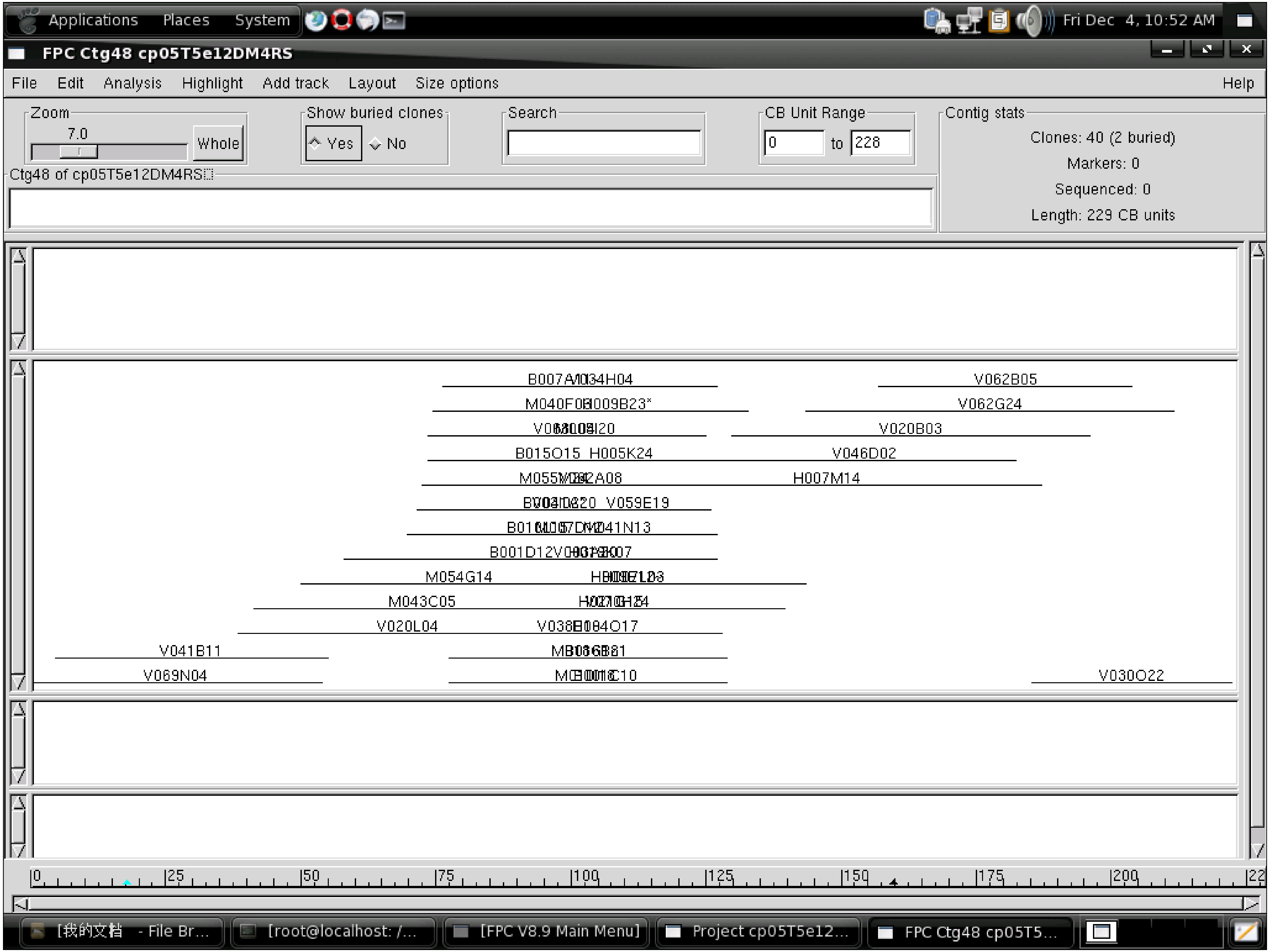

Applications Places System
Fri Dec 4, 10:52 AM
FPC Ctg48 cp05T5e12DM4RS
File Edit Analysis Highlight Add track Layout Size options Help
Zoom 7.0 Whole
Show buried clones Yes No
Search
CB Unit Range 0 to 228
Contig stats
Clones: 40 (2 buried)
Markers: 0
Sequenced: 0
Length: 229 CB units
Ctg48 of cp05T5e12DM4RS
V062B05
V062G24
V020B03
B015O15 H005K24
V046D02
H007M14
V059E19
M054G14
M043C05
V020L04
V041B11
V069N04
V030O22
0 25 50 75 100 125 150 175 200
[我的文档 - File Br...
[root@localhost: /...
[FPC V8.9 Main Menu]
Project cp05T5e12...
FPC Ctg48 cp05T5...

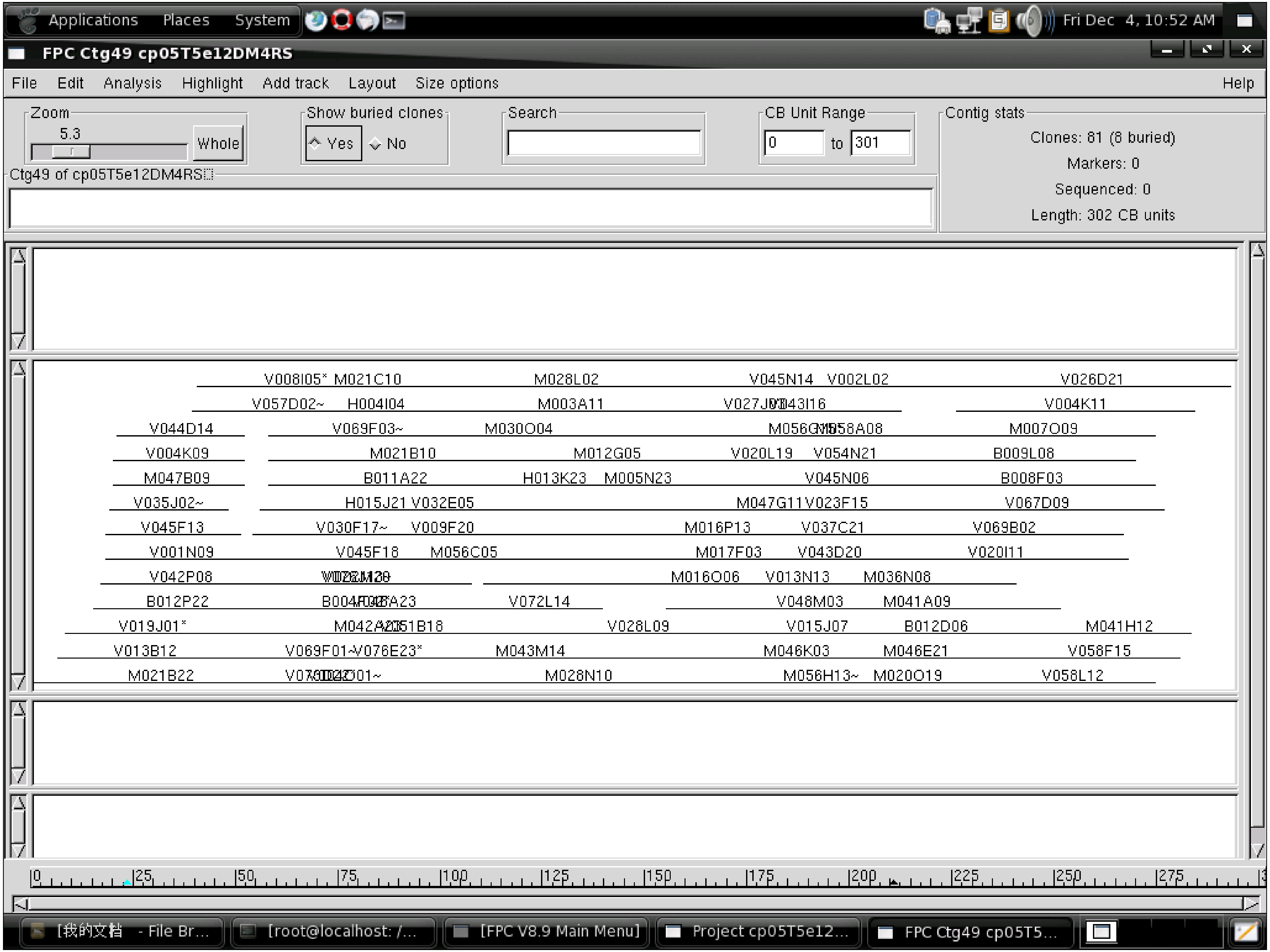
Applications Places System
Fri Dec 4, 10:52 AM
FPC Ctg49 cp05T5e12DM4RS
File Edit Analysis Highlight Add track Layout Size options Help
Zoom
5.3
Whole
Show buried clones
Yes No
Search
CB Unit Range
0 to 301
Contig stats
Clones: 81 (8 buried)
Markers: 0
Sequenced: 0
Length: 302 CB units
Ctg49 of cp05T5e12DM4RS
V008I05* M021C10 M028L02 V045N14 V002L02 V026D21
V057D02~ H004I04 M003A11 V004K11
V044D14 V069F03~ M030O04 M007O09
V004K09 M021B10 M012G05 V020L19 V054N21 B009L08
M047B09 B011A22 H013K23 M005N23 V045N06 B008F03
V035J02~ H015J21 V032E05 M047G11 V023F15 V067D09
V045F13 V030F17~ V009F20 M016P13 V037C21 V069B02
V001N09 V045F18 M056C05 M017F03 V043D20 V020I11
V042P08 M016O06 V013N13 M036N08
B012P22 V072L14 V048M03 M041A09
V019J01* V028L09 V015J07 B012D06 M041H12
V013B12 M043M14 M046K03 M046E21 V058F15
M021B22 M028N10 M056H13~ M020O19 V058L12
0 25 50 75 100 125 150 175 200 225 250 275
[我的文档 - File Br...
[root@localhost: /...
[FPC V8.9 Main Menu]
Project cp05T5e12...
FPC Ctg49 cp05T5...

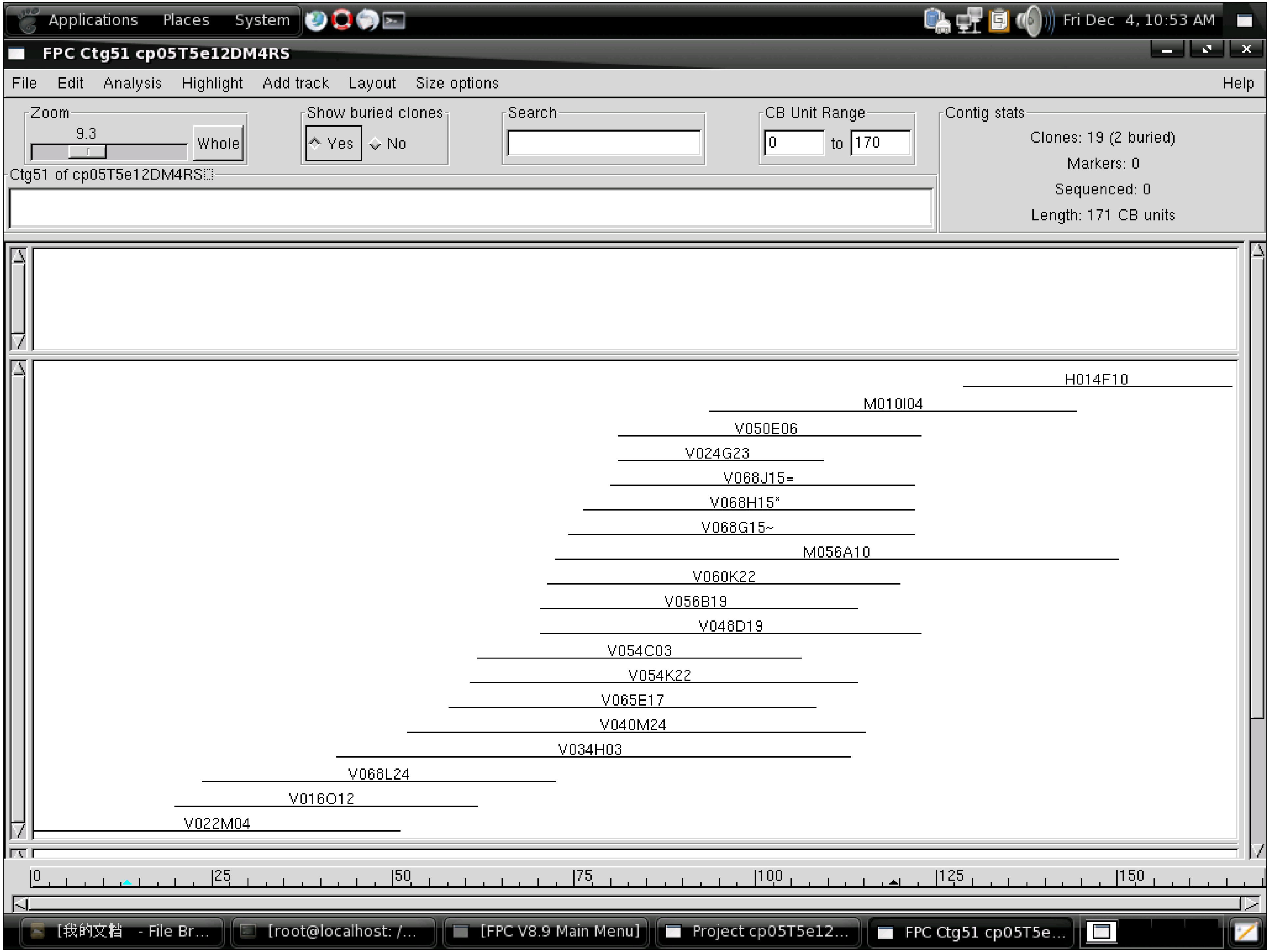
Applications Places System
Fri Dec 4, 10:53 AM
FPC Ctg51 cp05T5e12DM4RS
File Edit Analysis Highlight Add track Layout Size options Help
Zoom
9.3
Whole
Show buried clones
Yes No
Search
CB Unit Range
0 to 170
Contig stats
Clones: 19 (2 buried)
Markers: 0
Sequenced: 0
Length: 171 CB units
Ctg51 of cp05T5e12DM4RS
H014F10
M010I04
V050E06
V024G23
V068J15=
V068H15*
V068G15~
M056A10
V060K22
V056B19
V048D19
V054C03
V054K22
V065E17
V040M24
V034H03
V068L24
V016O12
V022M04
0
25
50
75
100
125
150
[我的文档 - File Br...
[root@localhost: /...
[FPC V8.9 Main Menu]
Project cp05T5e12...
FPC Ctg51 cp05T5e...

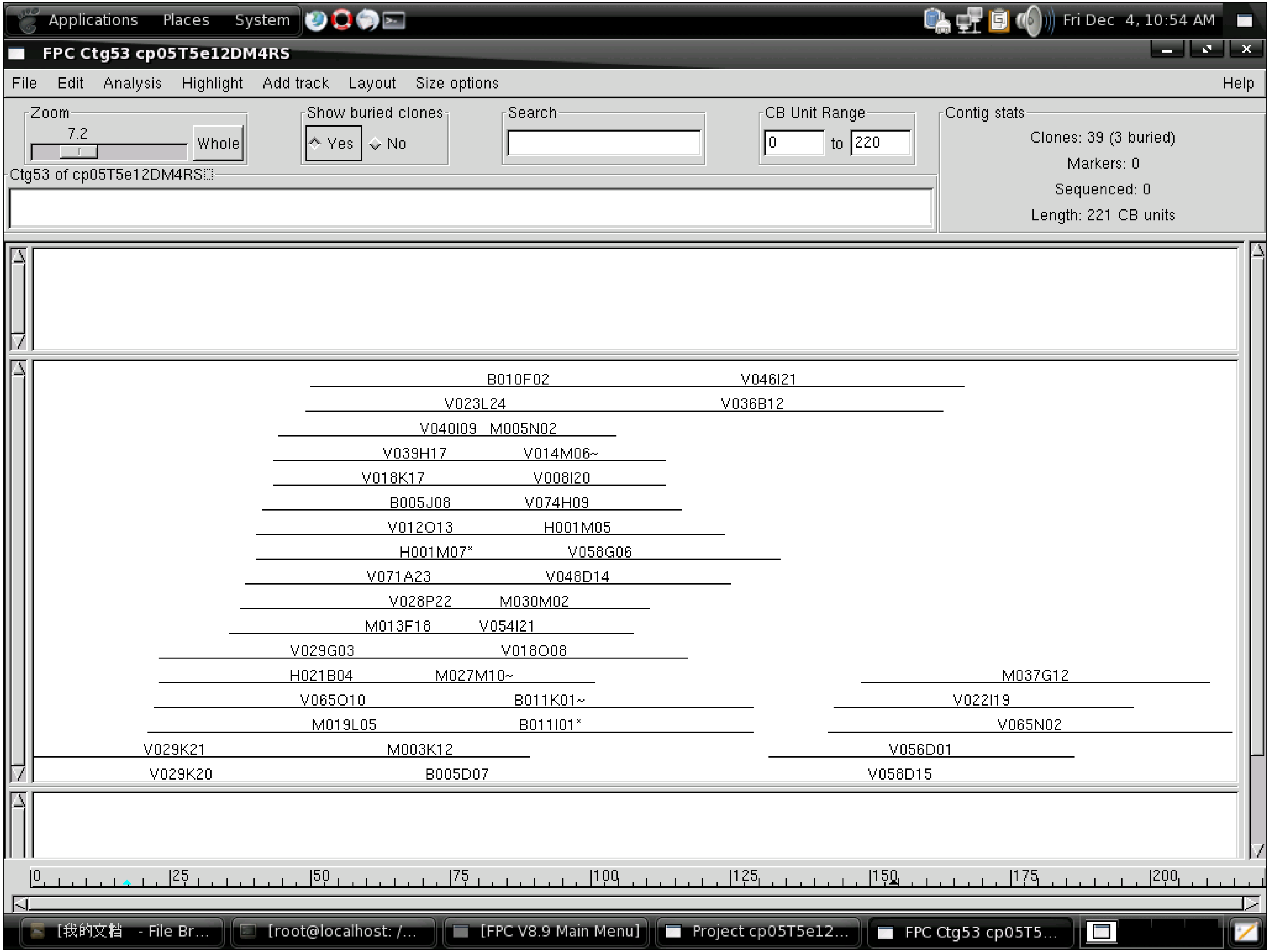
Applications Places System
Fri Dec 4, 10:54 AM
FPC Ctg53 cp05T5e12DM4RS
File Edit Analysis Highlight Add track Layout Size options Help
Zoom
7.2
Whole
Show buried clones
Yes No
Search
CB Unit Range
0 to 220
Contig stats
Clones: 39 (3 buried)
Markers: 0
Sequenced: 0
Length: 221 CB units
Ctg53 of cp05T5e12DM4RS
B010F02 V046I21
V023L24 V036B12
V040I09 M005N02
V039H17 V014M06~
V018K17 V008I20
B005J08 V074H09
V012O13 H001M05
H001M07* V058G06
V071A23 V048D14
V028P22 M030M02
M013F18 V054I21
V029G03 V018O08
H021B04 M027M10~ M037G12
V065O10 B011K01~ V022I19
M019L05 B011I01* V065N02
V029K21 M003K12 V056D01
V029K20 B005D07 V058D15
0 25 50 75 100 125 150 175 200
[我的文档 - File Br...
[root@localhost: /...
[FPC V8.9 Main Menu]
Project cp05T5e12...
FPC Ctg53 cp05T5...

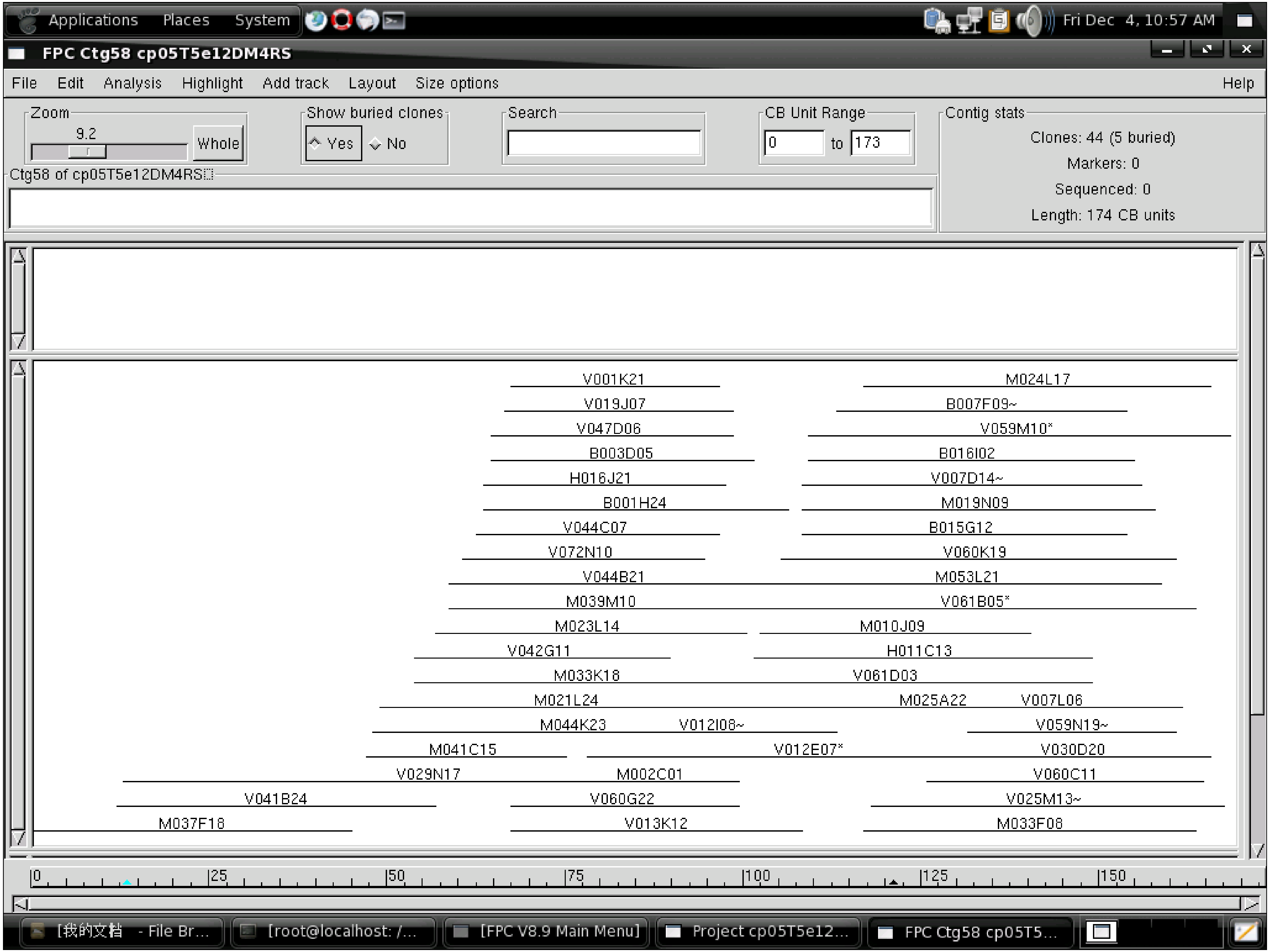
Applications Places System
Fri Dec 4, 10:57 AM
FPC Ctg58 cp05T5e12DM4RS
File Edit Analysis Highlight Add track Layout Size options Help
Zoom
9.2
Whole
Show buried clones
Yes
No
Search
CB Unit Range
0 to 173
Contig stats
Clones: 44 (5 buried)
Markers: 0
Sequenced: 0
Length: 174 CB units
Ctg58 of cp05T5e12DM4RS
V001K21
M024L17
V019J07
B007F09~
V047D06
V059M10*
B003D05
B016I02
H016J21
V007D14~
B001H24
M019N09
V044C07
B015G12
V072N10
V060K19
V044B21
M053L21
M039M10
V061B05*
M023L14
M010J09
V042G11
H011C13
M033K18
V061D03
M021L24
M025A22
V007L06
M044K23
V012I08~
V059N19~
M041C15
V012E07*
V030D20
V029N17
M002C01
V060C11
V041B24
V060G22
V025M13~
M037F18
V013K12
M033F08
0 25 50 75 100 125 150
[我的文档 - File Br...
[root@localhost: /...
[FPC V8.9 Main Menu]
Project cp05T5e12...
FPC Ctg58 cp05T5...

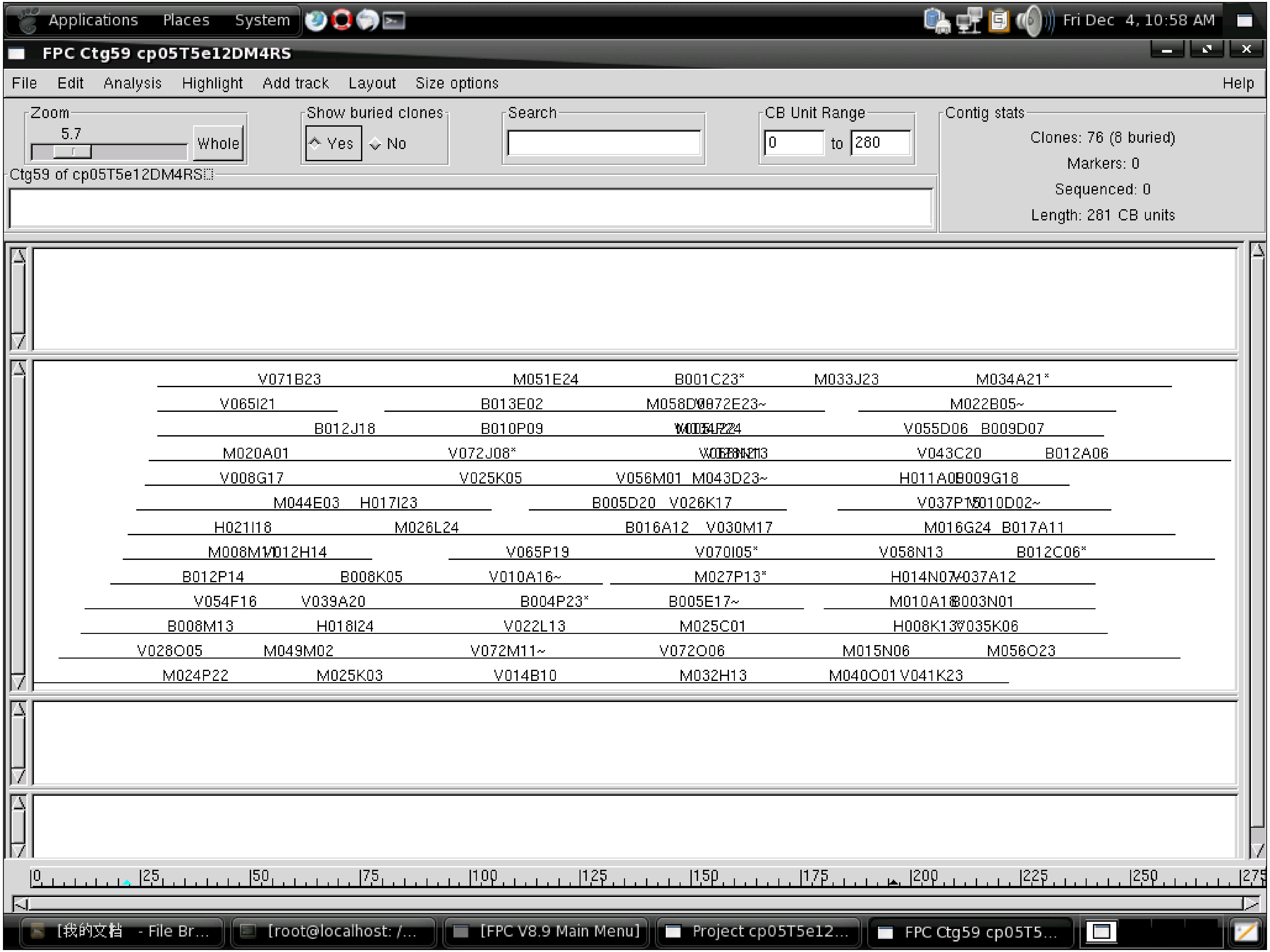
Applications Places System
Fri Dec 4, 10:58 AM
FPC Ctg59 cp05T5e12DM4RS
File Edit Analysis Highlight Add track Layout Size options Help
Zoom
5.7
Whole
Show buried clones
Yes
No
Search
CB Unit Range
0
to
280
Contig stats
Clones: 76 (8 buried)
Markers: 0
Sequenced: 0
Length: 281 CB units
Ctg59 of cp05T5e12DM4RS
V071B23 M051E24 B001C23* M033J23 M034A21*
V065I21 B013E02 M022B05~
B012J18 B010P09 V055D06 B009D07
M020A01 V072J08* V043C20 B012A06
V008G17 V025K05 V056M01 M043D23~
M044E03 H017I23 B005D20 V026K17
H021I18 M026L24 B016A12 V030M17 M016G24 B017A11
V065P19 V070I05* V058N13 B012C06*
B012P14 B008K05 V010A16~ M027P13*
V054F16 V039A20 B004P23* B005E17~
B008M13 H018I24 V022L13 M025C01
V028O05 M049M02 V072M11~ V072O06 M015N06 M056O23
M024P22 M025K03 V014B10 M032H13 M040O01 V041K23
0 25 50 75 100 125 150 175 200 225 250 275
[我的文档 - File Br...
[root@localhost: /...
[FPC V8.9 Main Menu]
Project cp05T5e12...
FPC Ctg59 cp05T5...

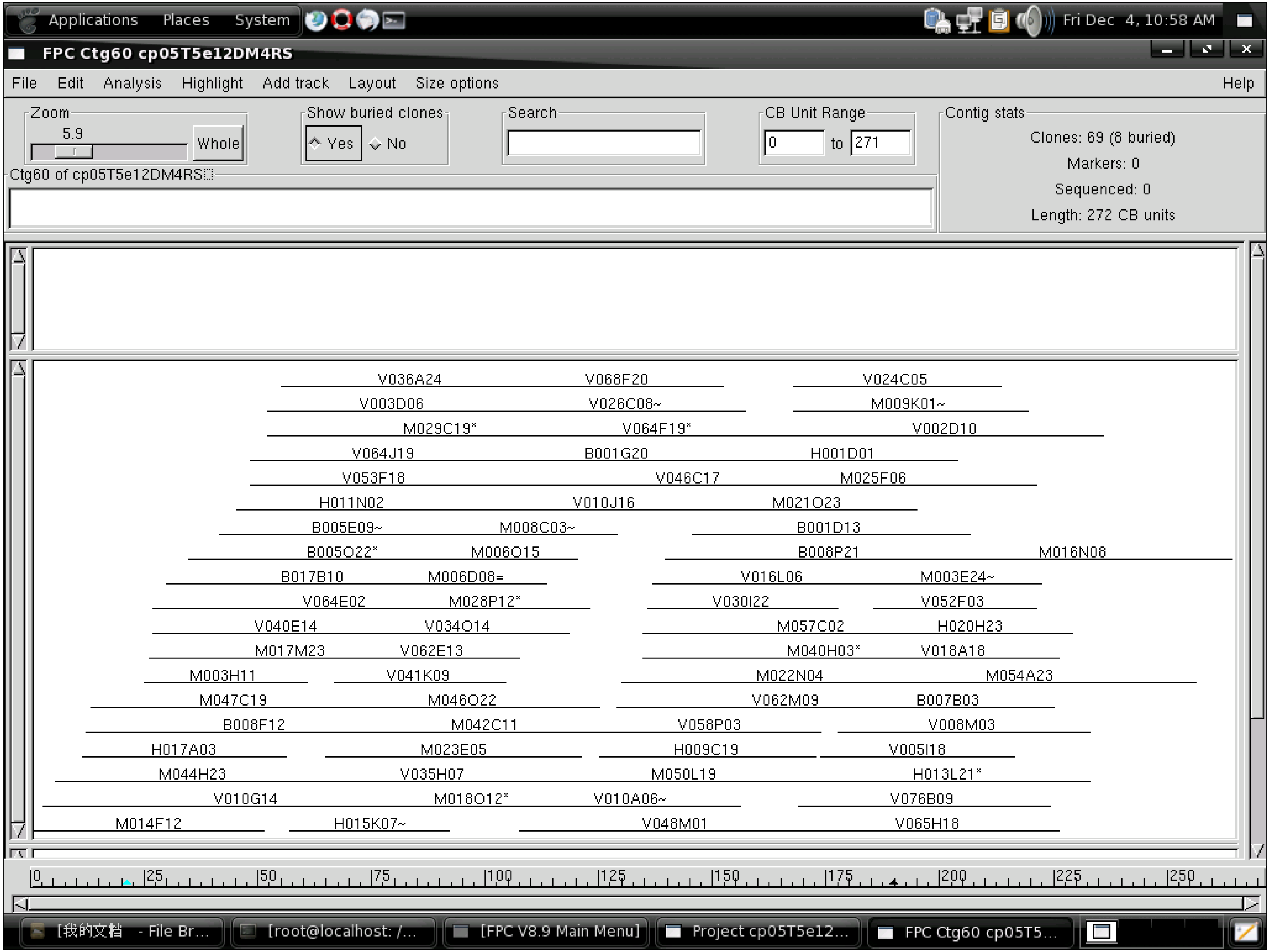
Applications Places System
Fri Dec 4, 10:58 AM
FPC Ctg60 cp05T5e12DM4RS
File Edit Analysis Highlight Add track Layout Size options Help
Zoom
5.9
Whole
Show buried clones
Yes No
Search
CB Unit Range
0 to 271
Contig stats
Clones: 69 (8 buried)
Markers: 0
Sequenced: 0
Length: 272 CB units
Ctg60 of cp05T5e12DM4RS
V036A24 V068F20 V024C05
V003D06 V026C08~ M009K01~
M029C19* V064F19* V002D10
V064J19 B001G20 H001D01
V053F18 V046C17 M025F06
H011N02 V010J16 M021O23
B005E09~ M008C03~ B001D13
B005O22* M006O15 B008P21 M016N08
B017B10 M006D08= V016L06 M003E24~
V064E02 M028P12* V030I22 V052F03
V040E14 V034O14 M057C02 H020H23
M017M23 V062E13 M040H03* V018A18
M003H11 V041K09 M022N04 M054A23
M047C19 M046O22 V062M09 B007B03
B008F12 M042C11 V058P03 V008M03
H017A03 M023E05 H009C19 V005I18
M044H23 V035H07 M050L19 H013L21*
V010G14 M018O12* V010A06~ V076B09
M014F12 H015K07~ V048M01 V065H18
0 25 50 75 100 125 150 175 200 225 250
[我的文档 - File Br...
[root@localhost: /...
[FPC V8.9 Main Menu]
Project cp05T5e12...
FPC Ctg60 cp05T5...

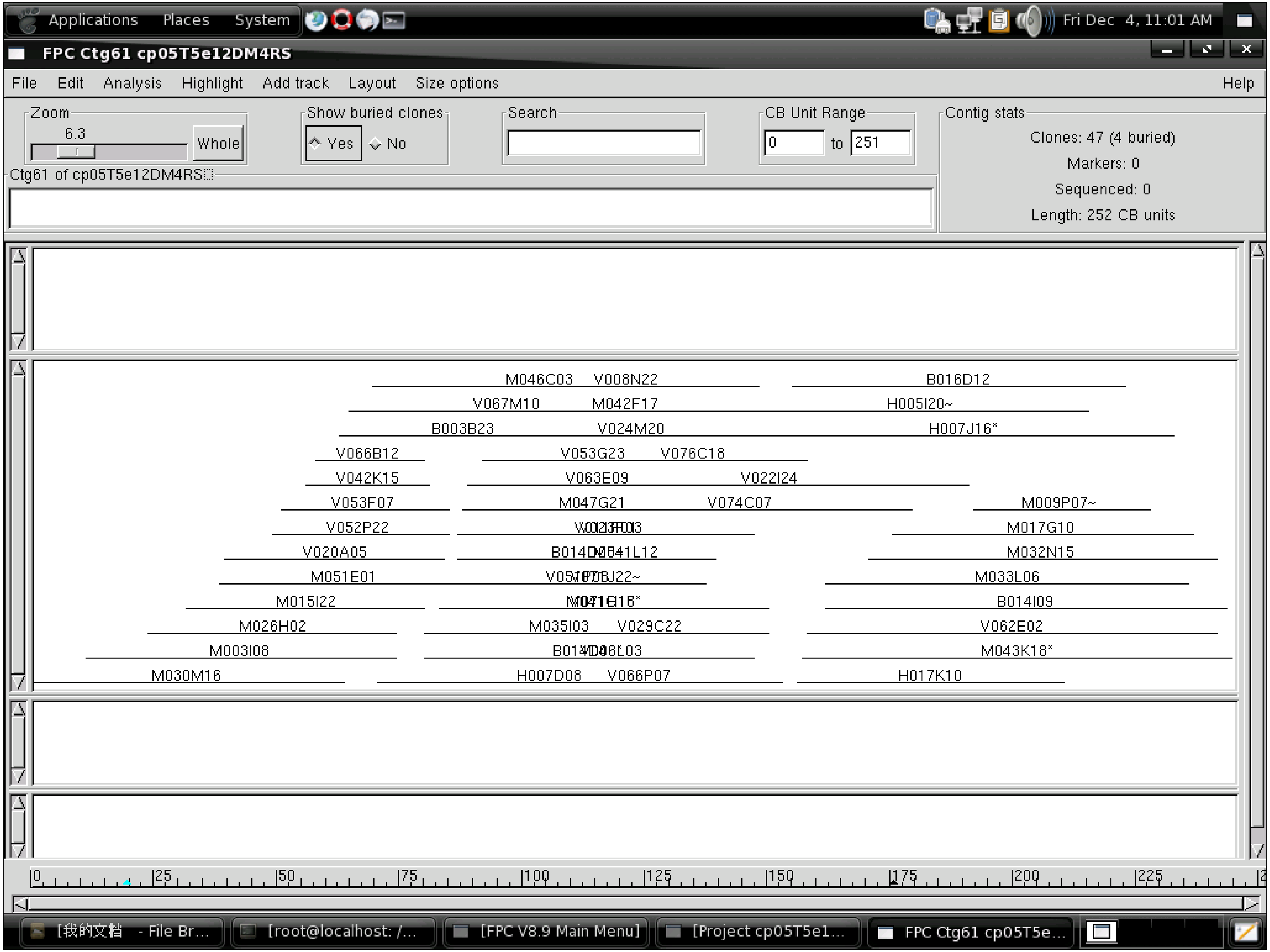
Applications Places System
Fri Dec 4, 11:01 AM
FPC Ctg61 cp05T5e12DM4RS
File Edit Analysis Highlight Add track Layout Size options Help
Zoom
6.3
Whole
Show buried clones
Yes No
Search
CB Unit Range
0 to 251
Contig stats
Clones: 47 (4 buried)
Markers: 0
Sequenced: 0
Length: 252 CB units
Ctg61 of cp05T5e12DM4RS
M046C03 V008N22 B016D12
V067M10 M042F17 H005I20~
B003B23 V024M20 H007J16*
V066B12 V053G23 V076C18
V042K15 V063E09 V022I24
V053F07 M047G21 V074C07 M009P07~
V052P22 M017G10
V020A05 M032N15
M051E01 M033L06
M015I22 B014I09
M026H02 M035I03 V029C22 V062E02
M003I08 M043K18*
M030M16 H007D08 V066P07 H017K10
0 25 50 75 100 125 150 175 200 225
[我的文档 - File Br...
[root@localhost: /...
[FPC V8.9 Main Menu]
[Project cp05T5e1...
FPC Ctg61 cp05T5e...

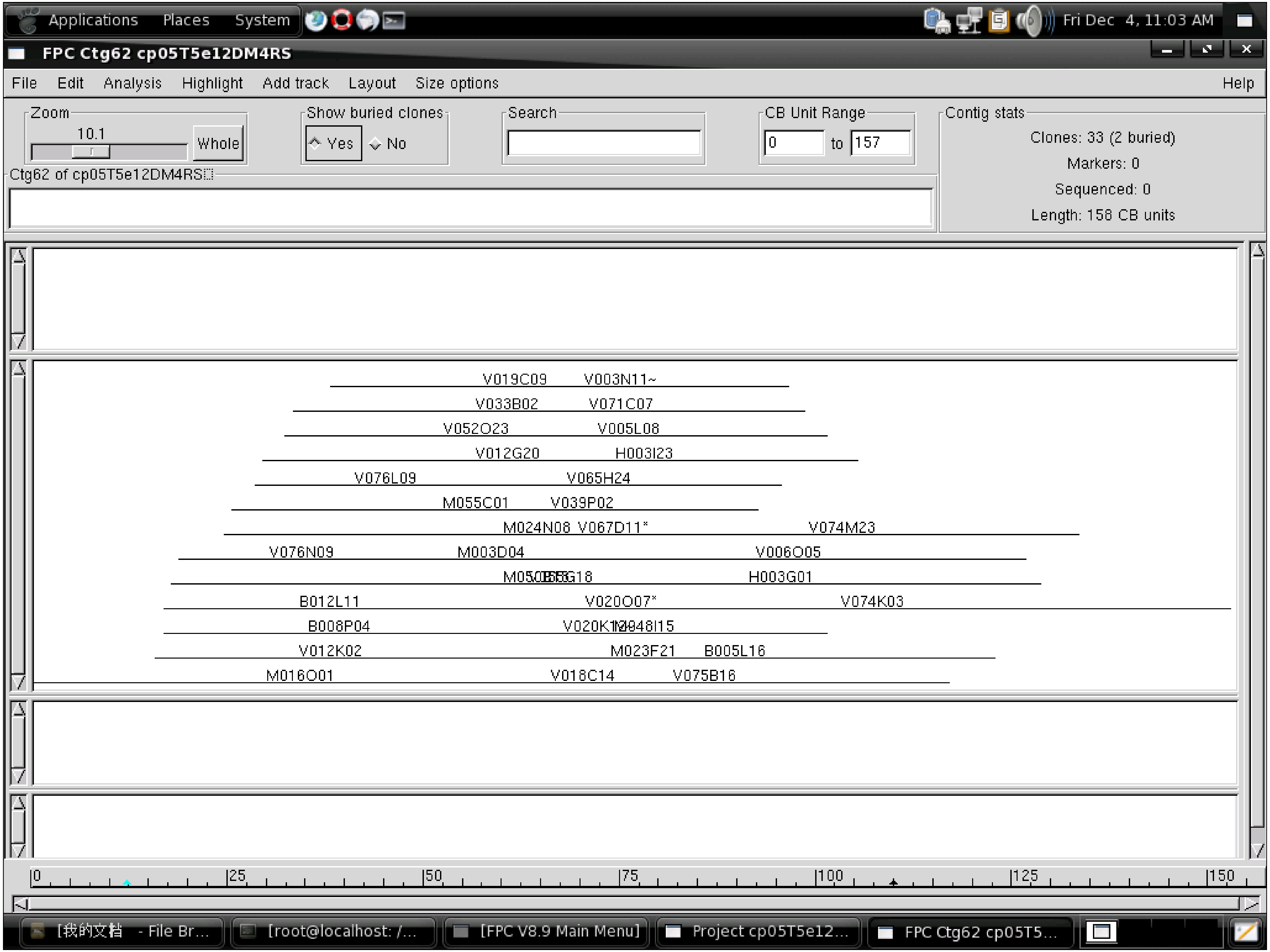
Applications Places System
Fri Dec 4, 11:03 AM
FPC Ctg62 cp05T5e12DM4RS
File Edit Analysis Highlight Add track Layout Size options Help
Zoom
10.1
Whole
Show buried clones
Yes No
Search
CB Unit Range
0 to 157
Contig stats
Clones: 33 (2 buried)
Markers: 0
Sequenced: 0
Length: 158 CB units
Ctg62 of cp05T5e12DM4RS
V019C09 V003N11~
V033B02 V071C07
V052O23 V005L08
V012G20 H003I23
V076L09 V065H24
M055C01 V039P02
M024N08 V067D11* V074M23
V076N09 M003D04 V006O05
H003G01
B012L11 V020O07* V074K03
B008P04
V012K02 M023F21 B005L16
M016O01 V018C14 V075B16
0 25 50 75 100 125 150
[我的文档 - File Br...
[root@localhost: /...
[FPC V8.9 Main Menu]
Project cp05T5e12...
FPC Ctg62 cp05T5...

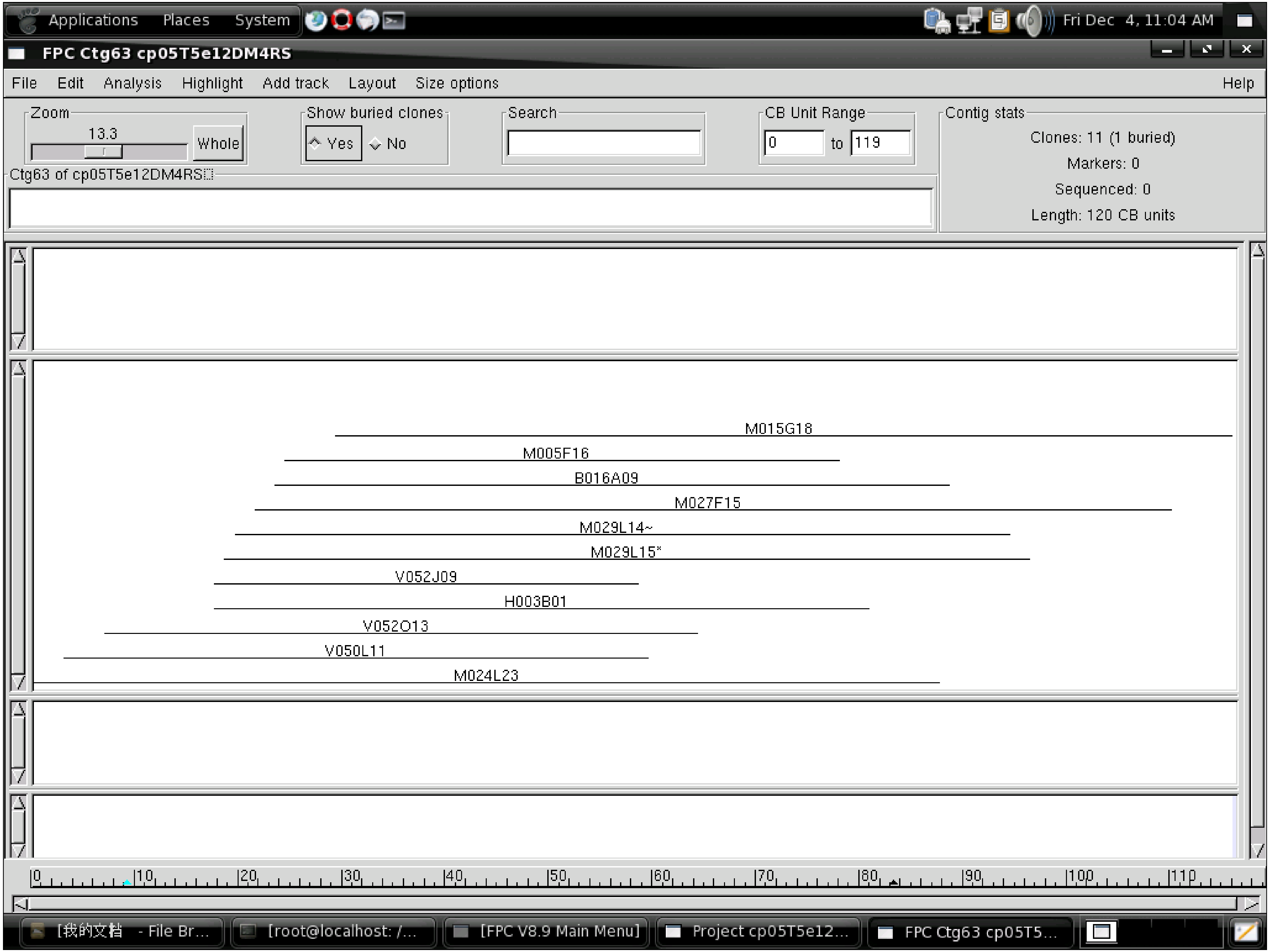
Applications Places System
Fri Dec 4, 11:04 AM
FPC Ctg63 cp05T5e12DM4RS
File Edit Analysis Highlight Add track Layout Size options Help
Zoom
13.3
Whole
Show buried clones
Yes
No
Search
CB Unit Range
0
to
119
Contig stats
Clones: 11 (1 buried)
Markers: 0
Sequenced: 0
Length: 120 CB units
Ctg63 of cp05T5e12DM4RS
M015G18
M005F16
B016A09
M027F15
M029L14~
M029L15*
V052J09
H003B01
V052O13
V050L11
M024L23
0 10 20 30 40 50 60 70 80 90 100 110
[我的文档 - File Br...
[root@localhost: /...
[FPC V8.9 Main Menu]
Project cp05T5e12...
FPC Ctg63 cp05T5...

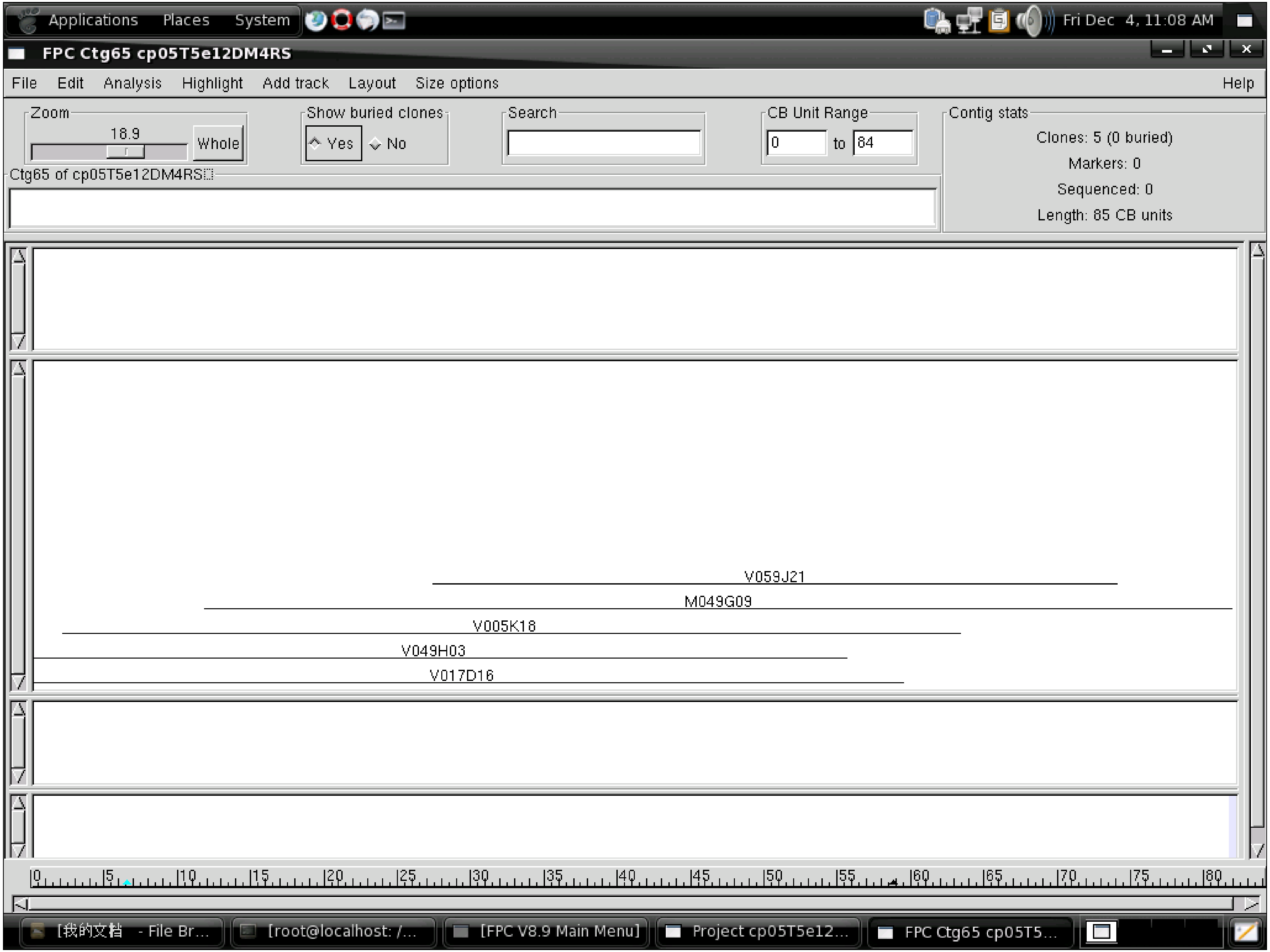
Applications Places System
Fri Dec 4, 11:08 AM
FPC Ctg65 cp05T5e12DM4RS
File Edit Analysis Highlight Add track Layout Size options Help
Zoom
18.9
Whole
Show buried clones
Yes
No
Search
CB Unit Range
0
to
84
Contig stats
Clones: 5 (0 buried)
Markers: 0
Sequenced: 0
Length: 85 CB units
Ctg65 of cp05T5e12DM4RS
V059J21
M049G09
V005K18
V049H03
V017D16
0 5 10 15 20 25 30 35 40 45 50 55 60 65 70 75 80
[我的文档 - File Br...
[root@localhost: /...
[FPC V8.9 Main Menu]
Project cp05T5e12...
FPC Ctg65 cp05T5...

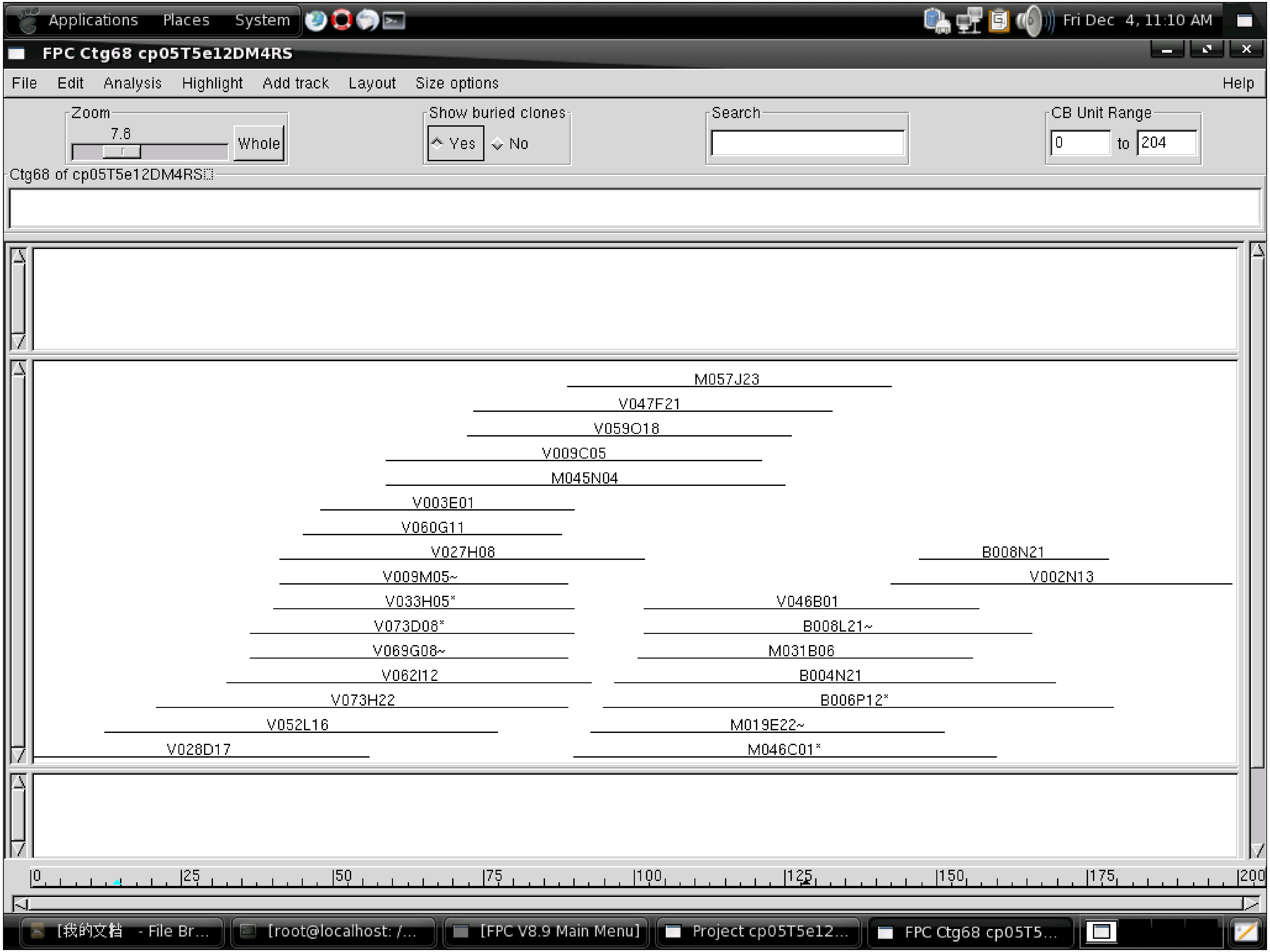
Applications
Places
System
Fri Dec 4, 11:10 AM
FPC Ctg68 cp05T5e12DM4RS
File
Edit
Analysis
Highlight
Add track
Layout
Size options
Help
Zoom
7.8
Whole
Show buried clones
Yes
No
Search
CB Unit Range
0
to
204
Ctg68 of cp05T5e12DM4RS
M057J23
V047F21
V059O18
V009C05
M045N04
V003E01
V060G11
V027H08
B008N21
V009M05~
V002N13
V033H05*
V046B01
V073D08*
B008L21~
V069G08~
M031B06
V062I12
B004N21
V073H22
B006P12*
V052L16
M019E22~
V028D17
M046C01*
0
25
50
75
100
125
150
175
200
[我的文档 - File Br...
[root@localhost: /...
[FPC V8.9 Main Menu]
Project cp05T5e12...
FPC Ctg68 cp05T5...

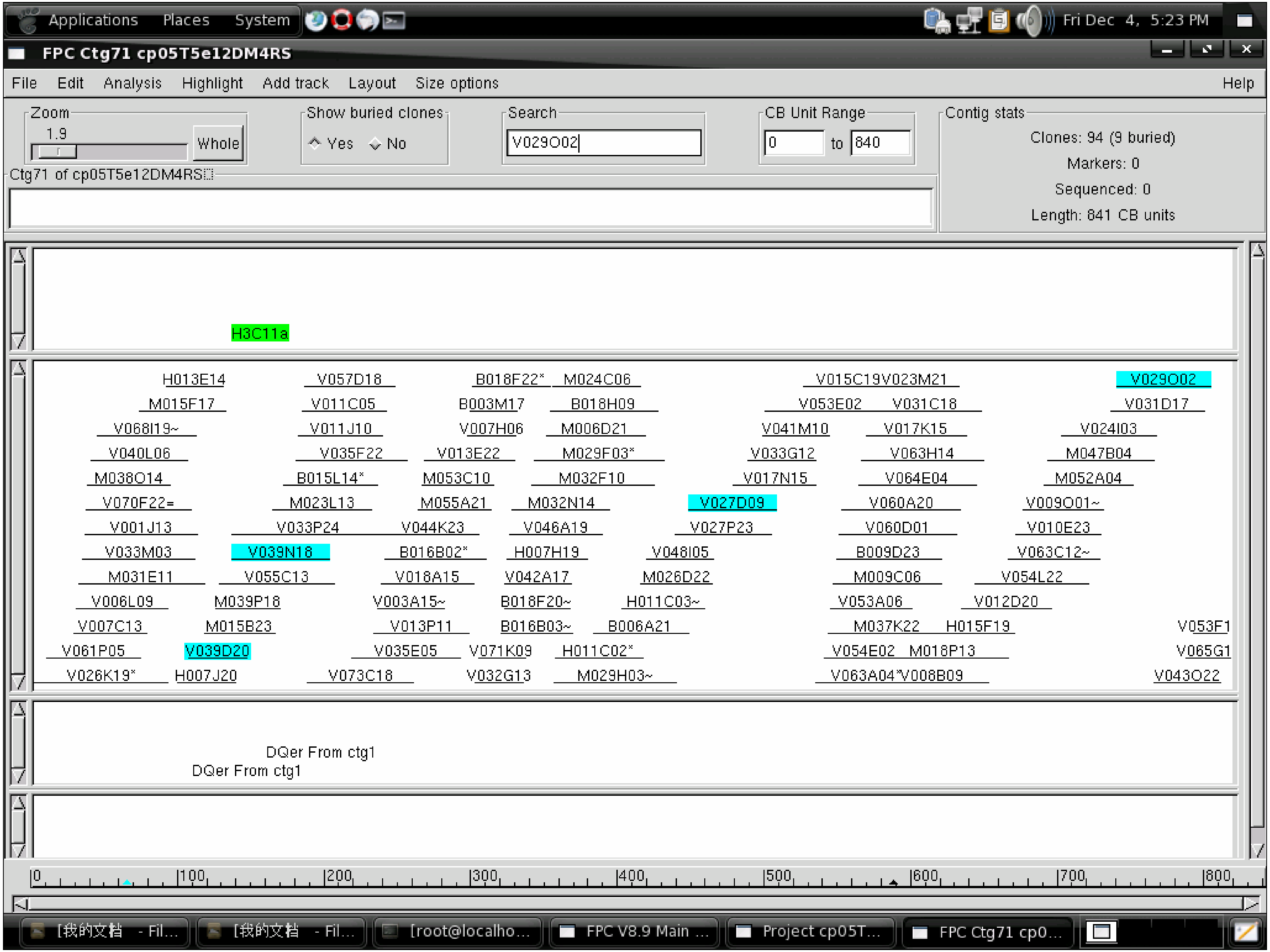
Applications Places System
Fri Dec 4, 5:23 PM
FPC Ctg71 cp05T5e12DM4RS
File Edit Analysis Highlight Add track Layout Size options Help
Zoom
1.9
Whole
Show buried clones
Yes No
Search
V029O02
CB Unit Range
0 to 840
Contig stats
Clones: 94 (9 buried)
Markers: 0
Sequenced: 0
Length: 841 CB units
Ctg71 of cp05T5e12DM4RS
H3C11a
H013E14
V057D18
B018F22*
M024C06
V015C19V023M21
V029O02
M015F17
V011C05
B003M17
B018H09
V053E02
V031C18
V031D17
V068I19~
V011J10
V007H06
M006D21
V041M10
V017K15
V024I03
V040L06
V035F22
V013E22
M029F03*
V033G12
V063H14
M047B04
M038O14
B015L14*
M053C10
M032F10
V017N15
V064E04
M052A04
V070F22=
M023L13
M055A21
M032N14
V027D09
V060A20
V009O01~
V001J13
V033P24
V044K23
V046A19
V027P23
V060D01
V010E23
V033M03
V039N18
B016B02*
H007H19
V048I05
B009D23
V063C12~
M031E11
V055C13
V018A15
V042A17
M026D22
M009C06
V054L22
V006L09
M039P18
V003A15~
B018F20~
H011C03~
V053A06
V012D20
V007C13
M015B23
V013P11
B016B03~
B006A21
M037K22
H015F19
V053F1
V061P05
V039D20
V035E05
V071K09
H011C02*
V054E02
M018P13
V065G1
V026K19*
H007J20
V073C18
V032G13
M029H03~
V063A04*V008B09
V043O22
DQer From ctg1
DQer From ctg1
0 100 200 300 400 500 600 700 800
[我的文档 - Fil...
[我的文档 - Fil...
[root@localho...
FPC V8.9 Main ...
Project cp05T...
FPC Ctg71 cp0...

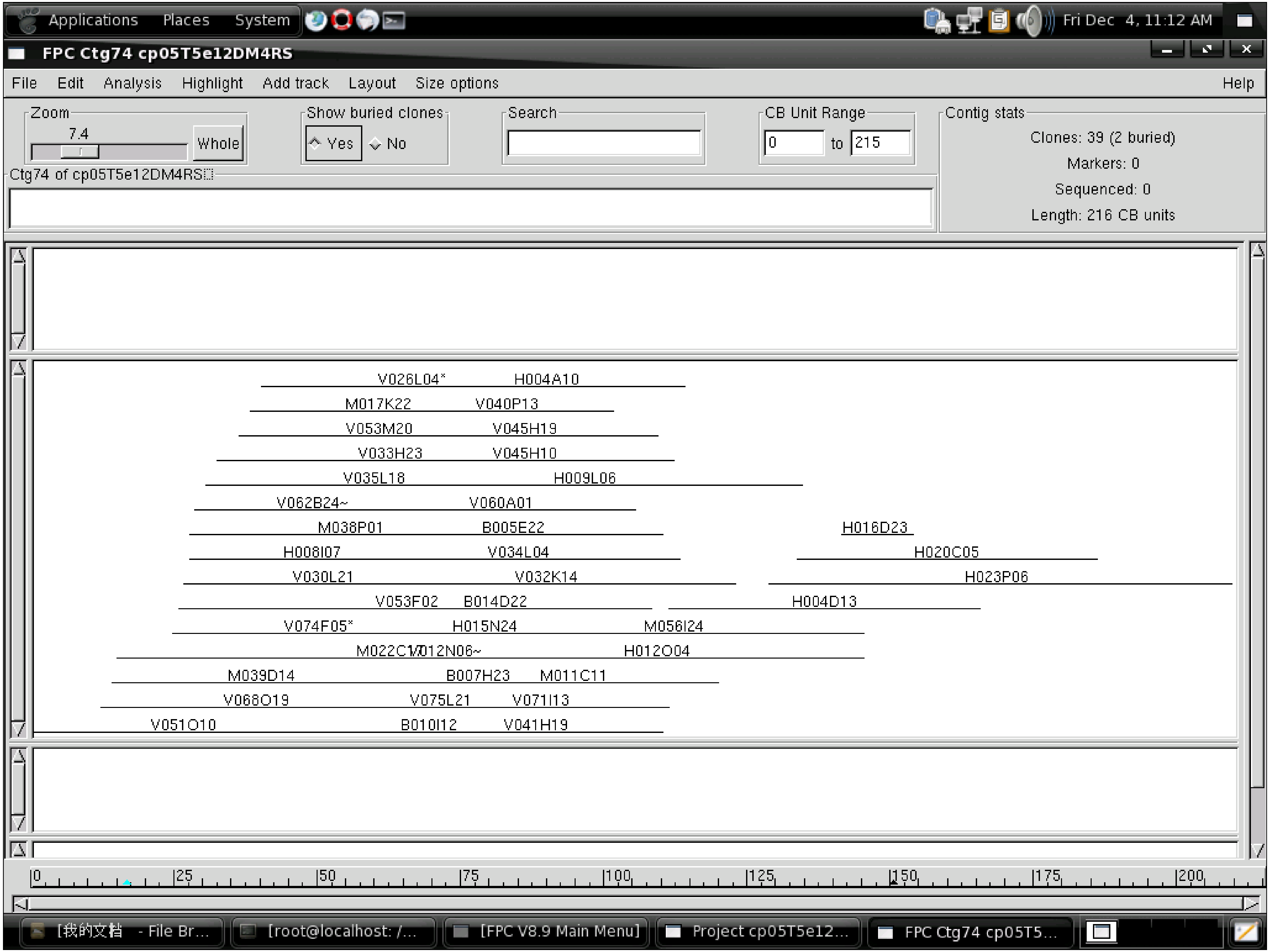
Applications Places System
Fri Dec 4, 11:12 AM
FPC Ctg74 cp05T5e12DM4RS
File Edit Analysis Highlight Add track Layout Size options Help
Zoom
7.4
Whole
Show buried clones
Yes No
Search
CB Unit Range
0 to 215
Contig stats
Clones: 39 (2 buried)
Markers: 0
Sequenced: 0
Length: 216 CB units
Ctg74 of cp05T5e12DM4RS
V026L04* H004A10
M017K22 V040P13
V053M20 V045H19
V033H23 V045H10
V035L18 H009L06
V062B24~ V060A01
M038P01 B005E22 H016D23
H008I07 V034L04 H020C05
V030L21 V032K14 H023P06
V053F02 B014D22 H004D13
V074F05* H015N24 M056I24
M022C1V012N06~ H012O04
M039D14 B007H23 M011C11
V068O19 V075L21 V071I13
V051O10 B010I12 V041H19
0 25 50 75 100 125 150 175 200
[我的文档 - File Br...
[root@localhost: /...
[FPC V8.9 Main Menu]
Project cp05T5e12...
FPC Ctg74 cp05T5...

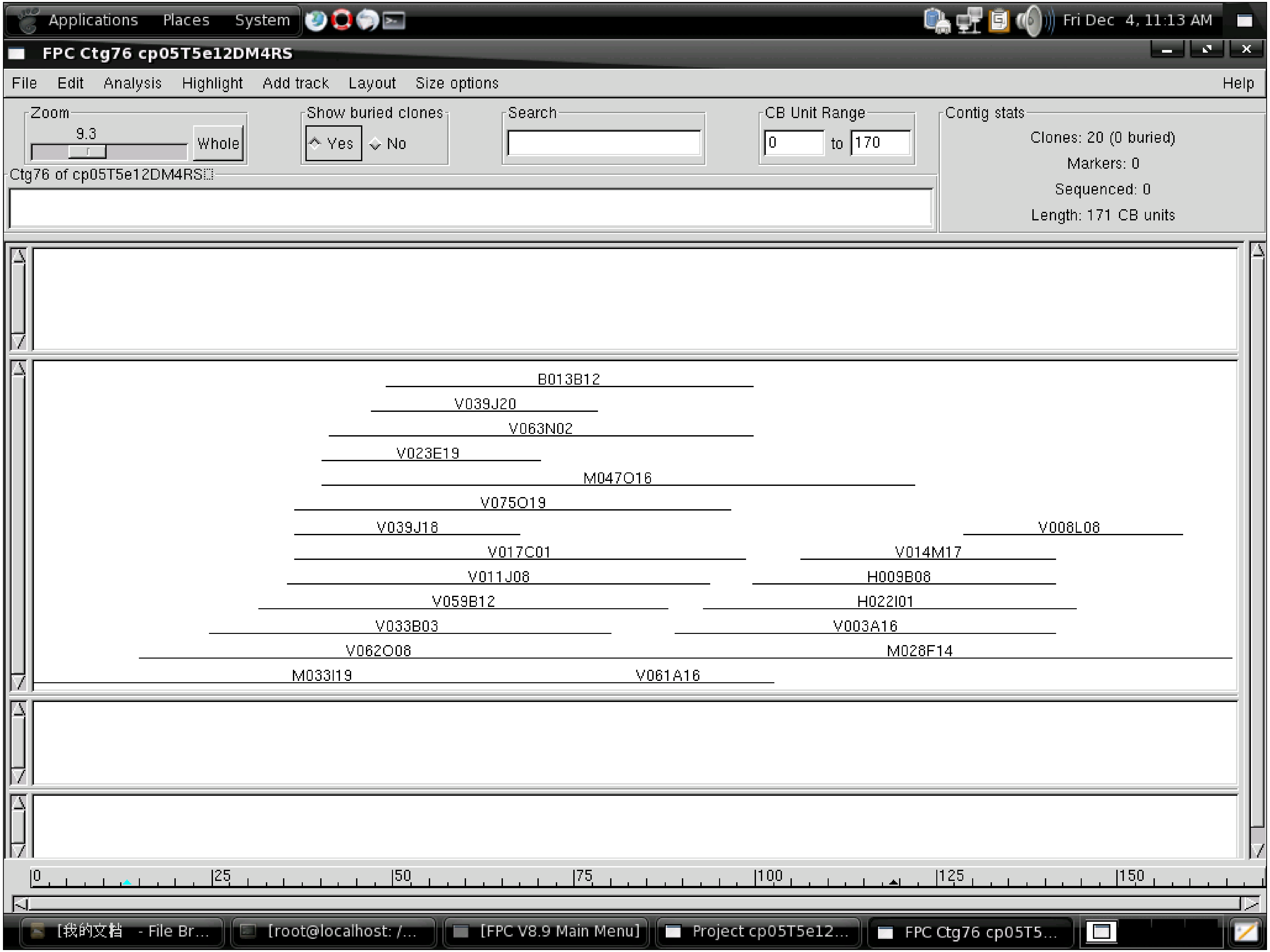
Applications Places System
Fri Dec 4, 11:13 AM
FPC Ctg76 cp05T5e12DM4RS
File Edit Analysis Highlight Add track Layout Size options Help
Zoom
9.3
Whole
Show buried clones
Yes
No
Search
CB Unit Range
0
to
170
Contig stats
Clones: 20 (0 buried)
Markers: 0
Sequenced: 0
Length: 171 CB units
Ctg76 of cp05T5e12DM4RS
B013B12
V039J20
V063N02
V023E19
M047O16
V075O19
V039J18
V008L08
V017C01
V014M17
V011J08
H009B08
V059B12
H022I01
V033B03
V003A16
V062O08
M028F14
M033I19
V061A16
0
25
50
75
100
125
150
[我的文档 - File Br...
[root@localhost: /...
[FPC V8.9 Main Menu]
Project cp05T5e12...
FPC Ctg76 cp05T5...

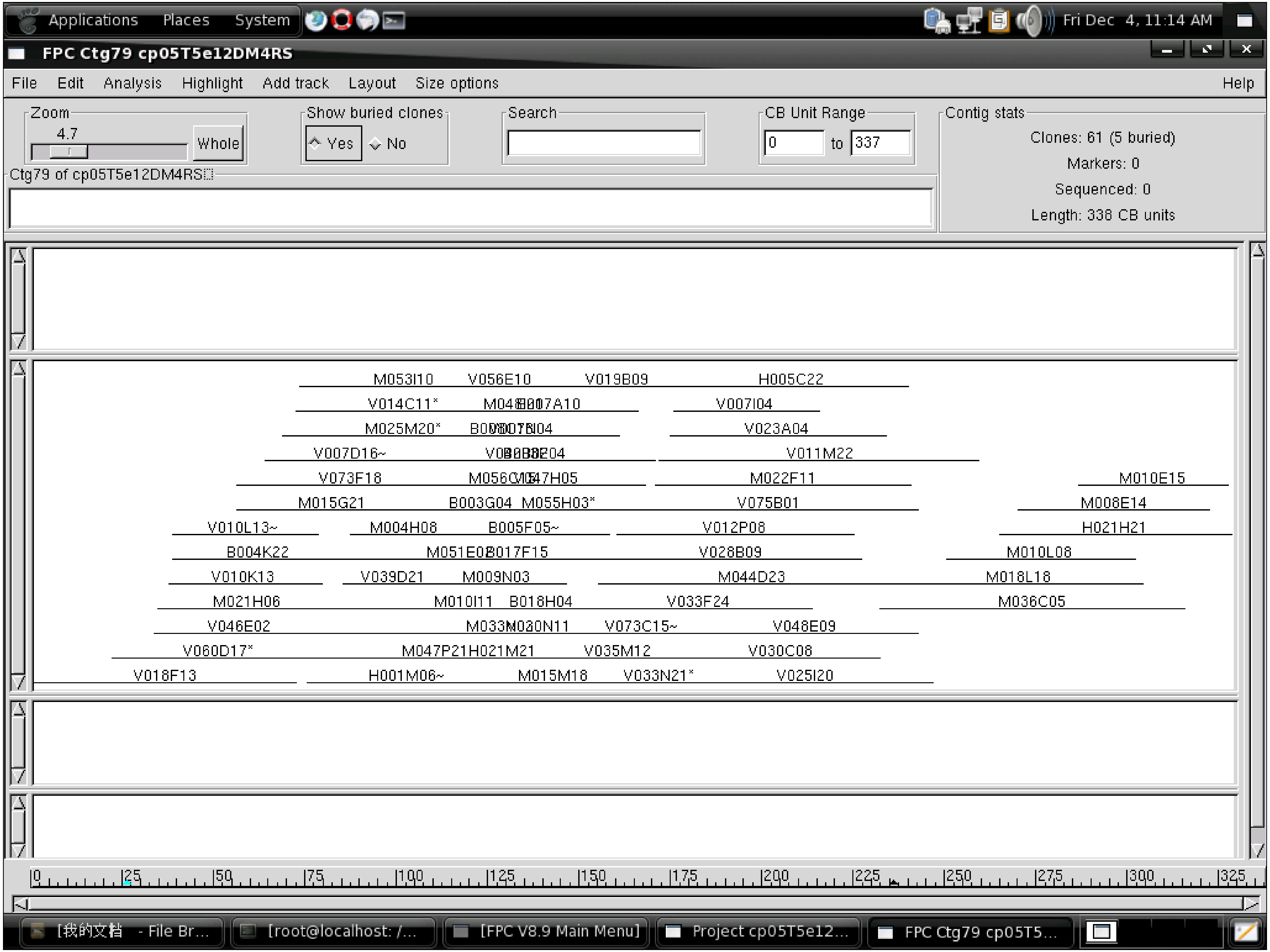
Applications Places System
Fri Dec 4, 11:14 AM
FPC Ctg79 cp05T5e12DM4RS
File Edit Analysis Highlight Add track Layout Size options Help
Zoom
4.7
Whole
Show buried clones
Yes No
Search
CB Unit Range
0 to 337
Contig stats
Clones: 61 (5 buried)
Markers: 0
Sequenced: 0
Length: 338 CB units
Ctg79 of cp05T5e12DM4RS
M053I10 V056E10 V019B09 H005C22
V014C11* V007I04
M025M20* V023A04
V007D16~ V011M22
V073F18 M022F11 M010E15
M015G21 B003G04 M055H03* V075B01 M008E14
V010L13~ M004H08 B005F05~ V012P08 H021H21
B004K22 V028B09 M010L08
V010K13 V039D21 M009N03 M044D23 M018L18
M021H06 M010I11 B018H04 V033F24 M036C05
V046E02 V073C15~ V048E09
V060D17* M047P21H021M21 V035M12 V030C08
V018F13 H001M06~ M015M18 V033N21* V025I20
0 25 50 75 100 125 150 175 200 225 250 275 300 325
[我的文档 - File Br...
[root@localhost: /...
[FPC V8.9 Main Menu]
Project cp05T5e12...
FPC Ctg79 cp05T5...

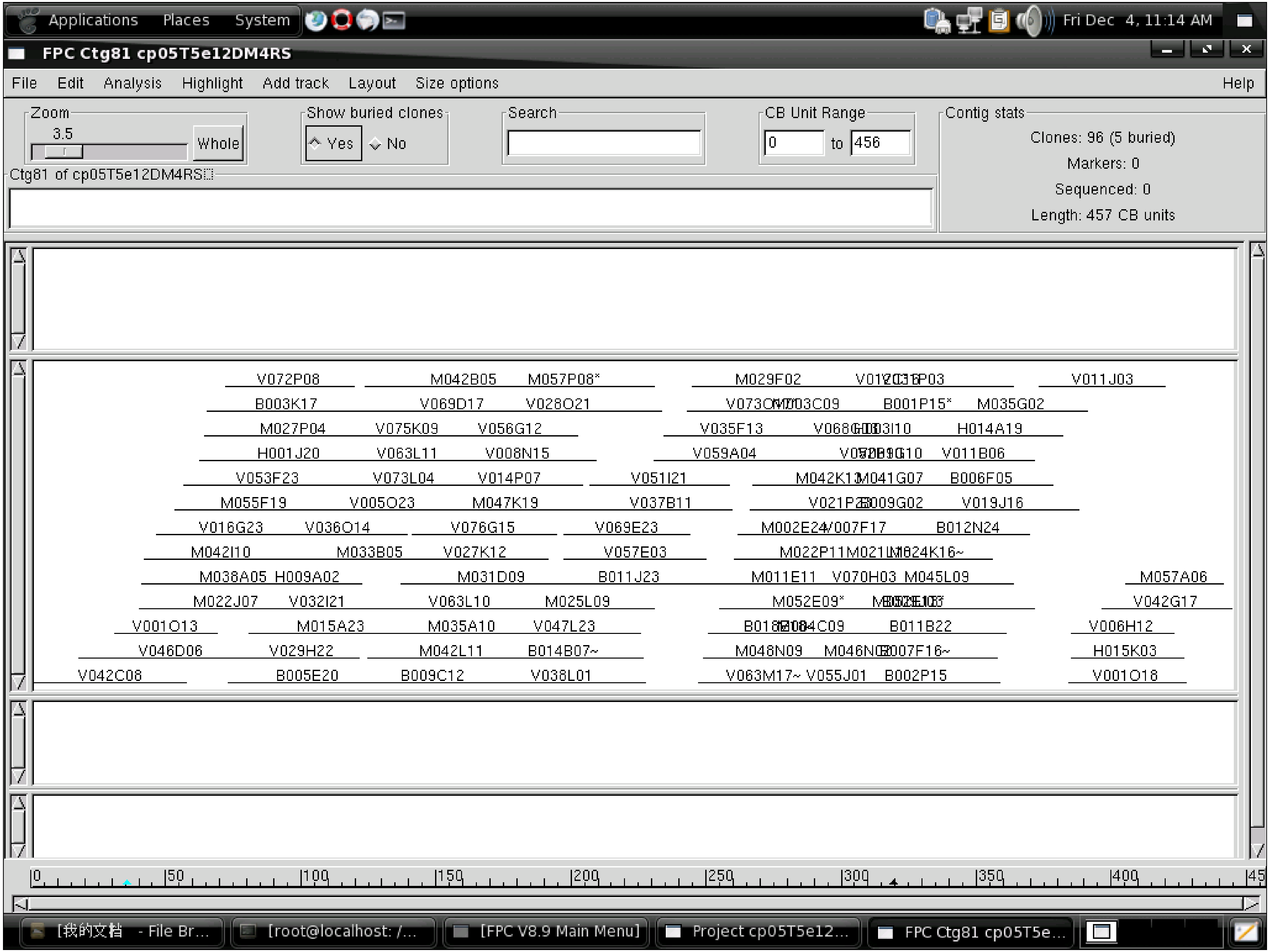
Applications Places System
Fri Dec 4, 11:14 AM
FPC Ctg81 cp05T5e12DM4RS
File Edit Analysis Highlight Add track Layout Size options Help
Zoom
3.5
Whole
Show buried clones
Yes
No
Search
CB Unit Range
0
to
456
Contig stats
Clones: 96 (5 buried)
Markers: 0
Sequenced: 0
Length: 457 CB units
Ctg81 of cp05T5e12DM4RS
V072P08 M042B05 M057P08* M029F02 V011J03
B003K17 V069D17 V028O21 B001P15* M035G02
M027P04 V075K09 V056G12 V035F13 H014A19
H001J20 V063L11 V008N15 V059A04 V011B06
V053F23 V073L04 V014P07 V051I21 B006F05
M055F19 V005O23 M047K19 V037B11 V019J16
V016G23 V036O14 V076G15 V069E23 B012N24
M042I10 M033B05 V027K12 V057E03
M038A05 H009A02 M031D09 B011J23 M011E11 V070H03 M045L09 M057A06
M022J07 V032I21 V063L10 M025L09 M052E09* V042G17
V001O13 M015A23 M035A10 V047L23 B011B22 V006H12
V046D06 V029H22 M042L11 B014B07~ M048N09 H015K03
V042C08 B005E20 B009C12 V038L01 V063M17~ V055J01 B002P15 V001O18
0 50 100 150 200 250 300 350 400
[FPC V8.9 Main Menu]

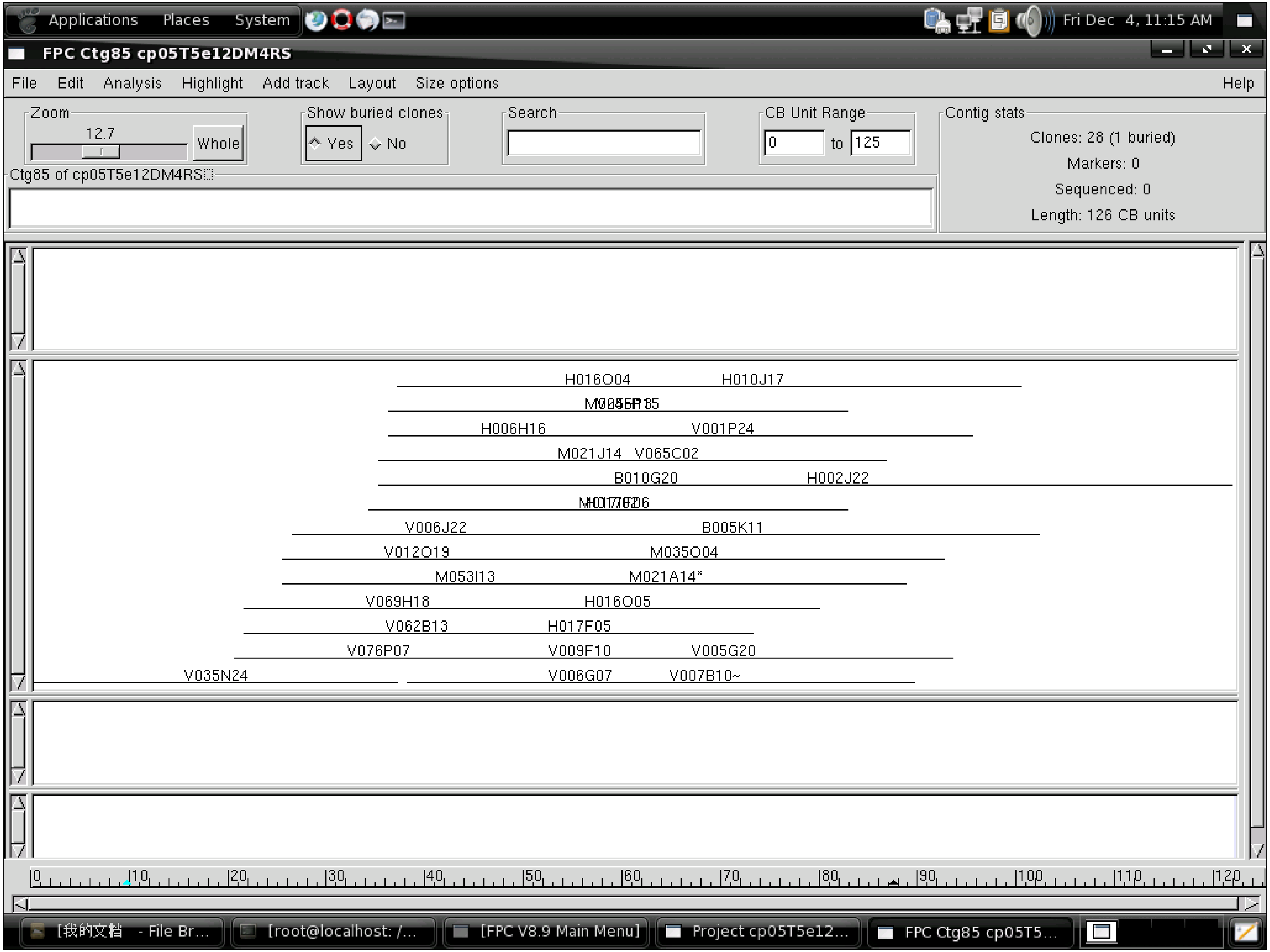
Applications Places System
Fri Dec 4, 11:15 AM
FPC Ctg85 cp05T5e12DM4RS
File Edit Analysis Highlight Add track Layout Size options Help
Zoom
12.7
Whole
Show buried clones
Yes
No
Search
CB Unit Range
0 to 125
Contig stats
Clones: 28 (1 buried)
Markers: 0
Sequenced: 0
Length: 126 CB units
Ctg85 of cp05T5e12DM4RS
H016O04 H010J17
H006H16 V001P24
M021J14 V065C02
B010G20 H002J22
V006J22 B005K11
V012O19 M035O04
M053I13 M021A14*
V069H18 H016O05
V062B13 H017F05
V076P07 V009F10 V005G20
V035N24 V006G07 V007B10~
0 10 20 30 40 50 60 70 80 90 100 110 120
[我的文档 - File Br...
[root@localhost: /...
[FPC V8.9 Main Menu]
Project cp05T5e12...
FPC Ctg85 cp05T5...

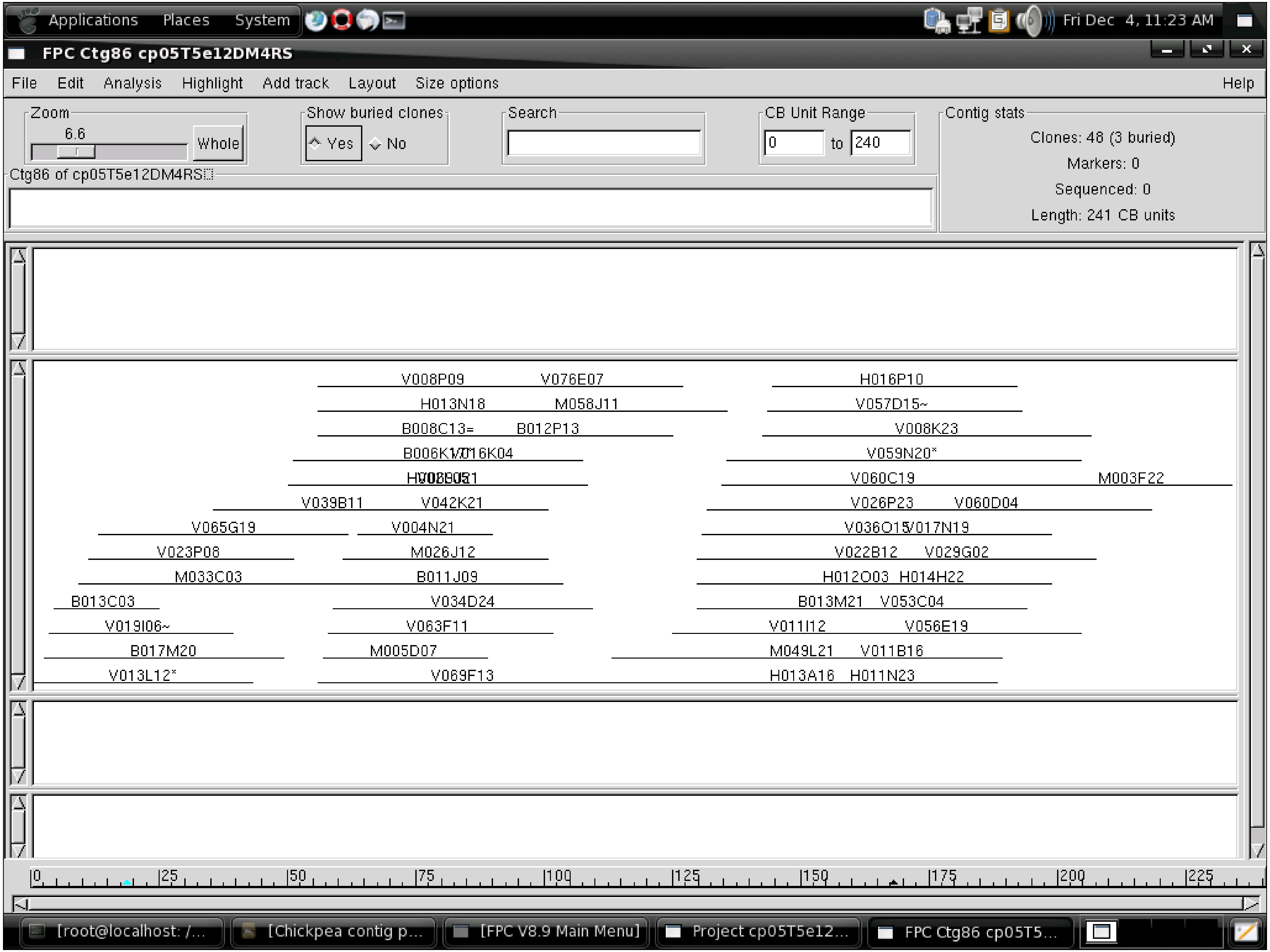
Applications Places System
Fri Dec 4, 11:23 AM
FPC Ctg86 cp05T5e12DM4RS
File Edit Analysis Highlight Add track Layout Size options Help
Zoom
6.6
Whole
Show buried clones
Yes No
Search
CB Unit Range
0 to 240
Contig stats
Clones: 48 (3 buried)
Markers: 0
Sequenced: 0
Length: 241 CB units
Ctg86 of cp05T5e12DM4RS
V008P09
V076E07
H016P10
H013N18
M058J11
V057D15~
B008C13=
B012P13
V008K23
V059N20*
V060C19
M003F22
V039B11
V042K21
V026P23
V060D04
V065G19
V004N21
V023P08
M026J12
V022B12
V029G02
M033C03
B011J09
H012O03
H014H22
B013C03
V034D24
B013M21
V053C04
V019I06~
V063F11
V011I12
V056E19
B017M20
M005D07
M049L21
V011B16
V013L12*
V069F13
H013A16
H011N23
0 25 50 75 100 125 150 175 200 225
[root@localhost: /...
[Chickpea contig p...
[FPC V8.9 Main Menu]
Project cp05T5e12...
FPC Ctg86 cp05T5...

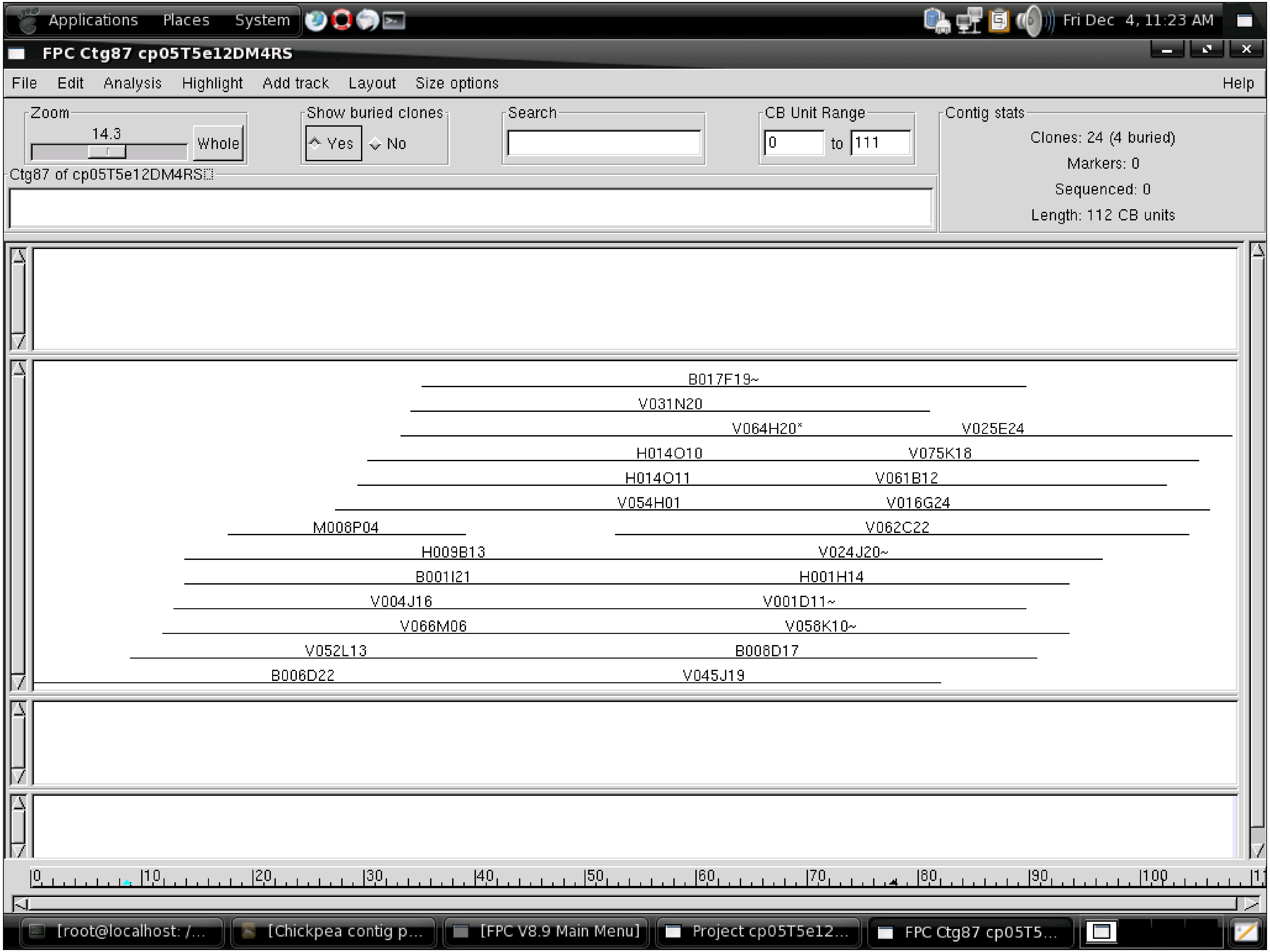
Applications Places System
Fri Dec 4, 11:23 AM
FPC Ctg87 cp05T5e12DM4RS
File Edit Analysis Highlight Add track Layout Size options Help
Zoom
14.3
Whole
Show buried clones
Yes
No
Search
CB Unit Range
0
to
111
Contig stats
Clones: 24 (4 buried)
Markers: 0
Sequenced: 0
Length: 112 CB units
Ctg87 of cp05T5e12DM4RS
B017F19~
V031N20
V064H20*
V025E24
H014O10
V075K18
H014O11
V061B12
V054H01
V016G24
M008P04
V062C22
H009B13
V024J20~
B001I21
H001H14
V004J16
V001D11~
V066M06
V058K10~
V052L13
B008D17
B006D22
V045J19
0 10 20 30 40 50 60 70 80 90 100
[root@localhost: /...
[Chickpea contig p...
[FPC V8.9 Main Menu]
Project cp05T5e12...
FPC Ctg87 cp05T5...

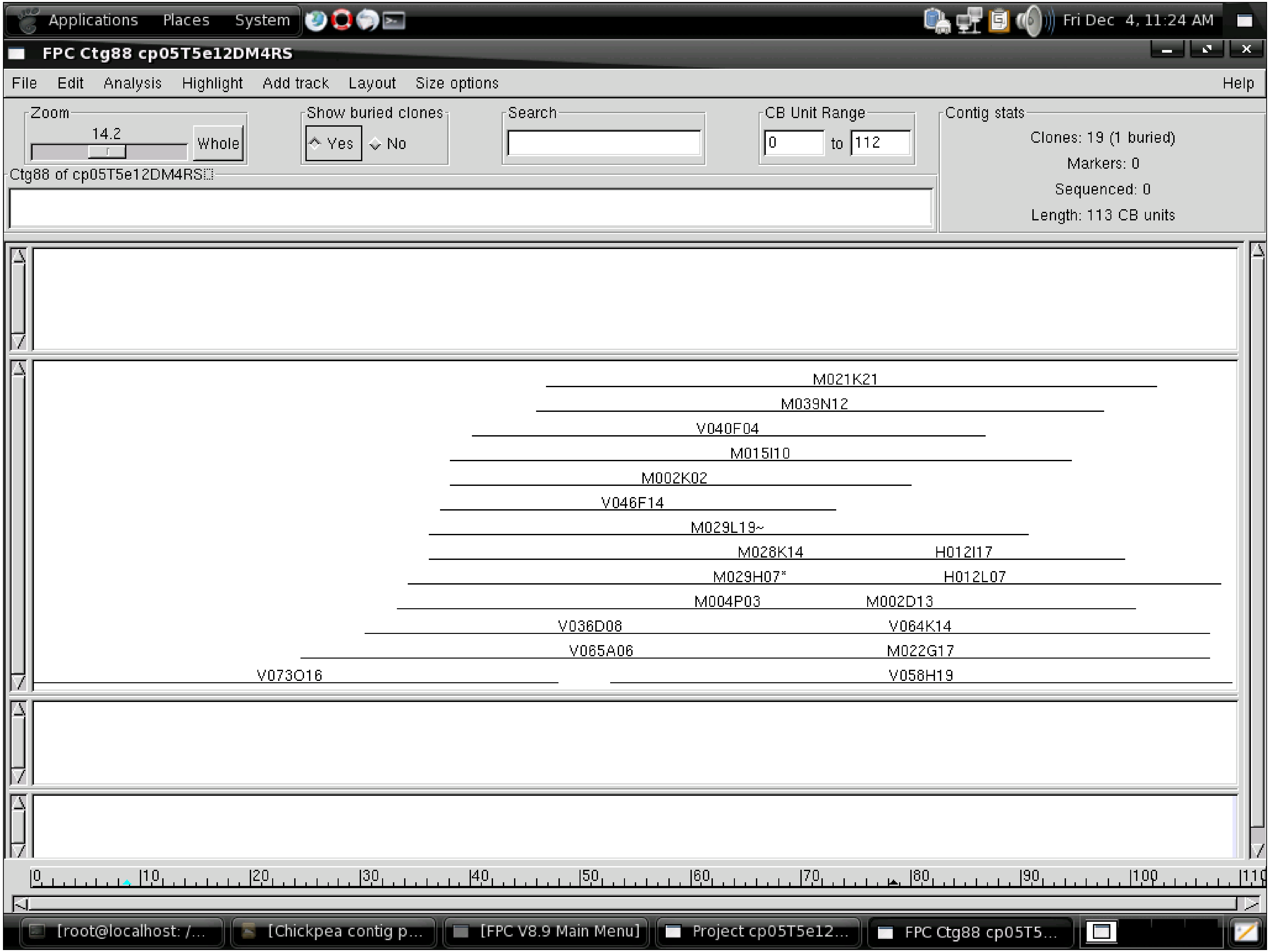
Applications Places System
Fri Dec 4, 11:24 AM
FPC Ctg88 cp05T5e12DM4RS
File Edit Analysis Highlight Add track Layout Size options Help
Zoom
14.2
Whole
Show buried clones
Yes No
Search
CB Unit Range
0 to 112
Contig stats
Clones: 19 (1 buried)
Markers: 0
Sequenced: 0
Length: 113 CB units
Ctg88 of cp05T5e12DM4RS
M021K21
M039N12
V040F04
M015I10
M002K02
V046F14
M029L19~
M028K14
H012I17
M029H07*
H012L07
M004P03
M002D13
V036D08
V064K14
V065A06
M022G17
V073O16
V058H19
0 10 20 30 40 50 60 70 80 90 100 110
[root@localhost: /...
[Chickpea contig p...
[FPC V8.9 Main Menu]
Project cp05T5e12...
FPC Ctg88 cp05T5...

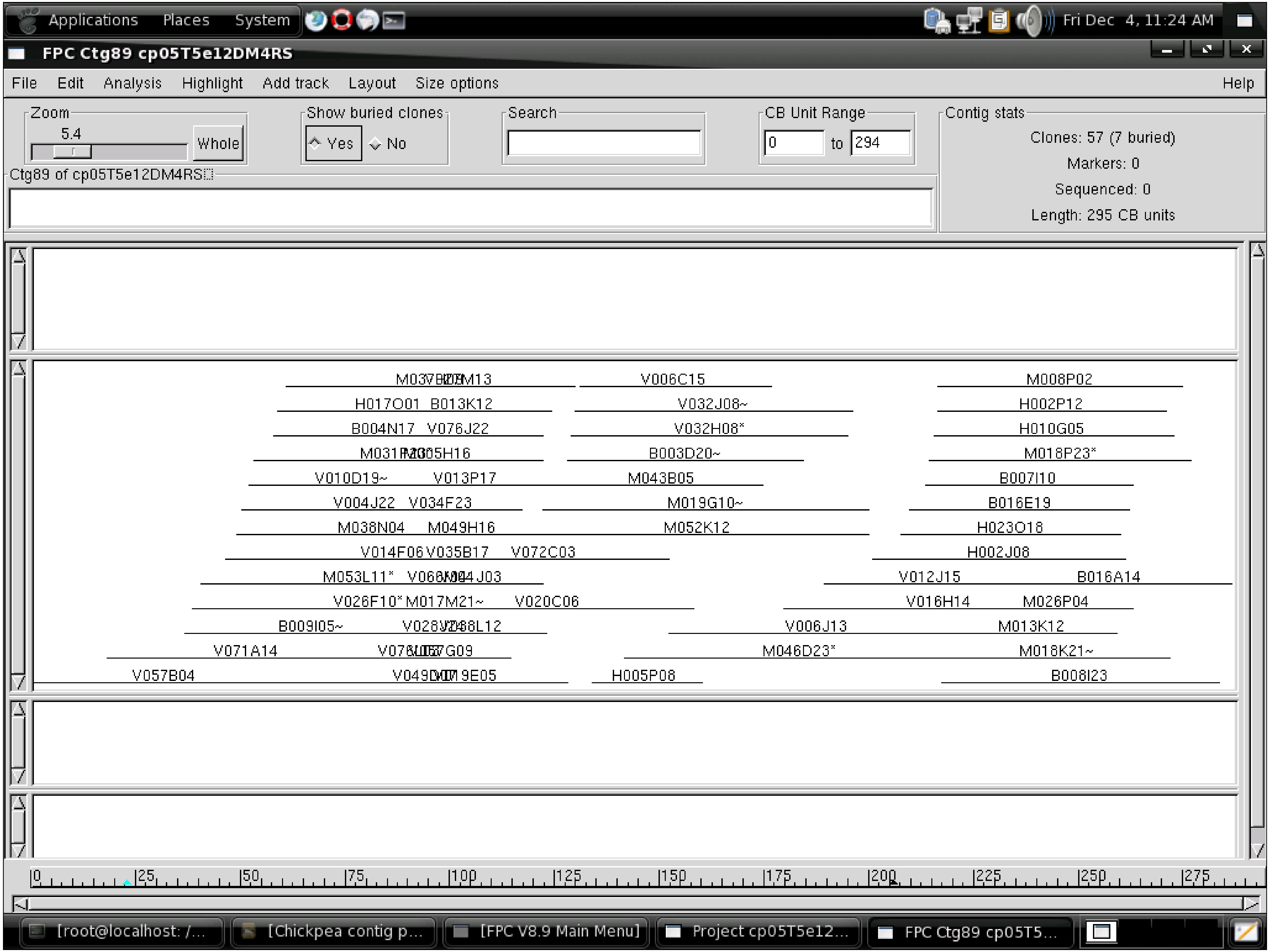
Applications Places System
Fri Dec 4, 11:24 AM
FPC Ctg89 cp05T5e12DM4RS
File Edit Analysis Highlight Add track Layout Size options Help
Zoom
5.4
Whole
Show buried clones
Yes No
Search
CB Unit Range
0 to 294
Contig stats
Clones: 57 (7 buried)
Markers: 0
Sequenced: 0
Length: 295 CB units
Ctg89 of cp05T5e12DM4RS
V006C15
M008P02
H017O01 B013K12
V032J08~
H002P12
B004N17 V076J22
V032H08*
H010G05
B003D20~
M018P23*
V010D19~ V013P17
M043B05
B007I10
V004J22 V034F23
M019G10~
B016E19
M038N04 M049H16
M052K12
H023O18
V014F06 V035B17 V072C03
H002J08
V012J15
B016A14
V026F10* M017M21~ V020C06
V016H14 M026P04
B009I05~
V006J13
M013K12
V071A14
M046D23*
M018K21~
V057B04
H005P08
B008I23
0 25 50 75 100 125 150 175 200 225 250 275
[root@localhost: /...
[Chickpea contig p...
[FPC V8.9 Main Menu]
Project cp05T5e12...
FPC Ctg89 cp05T5...

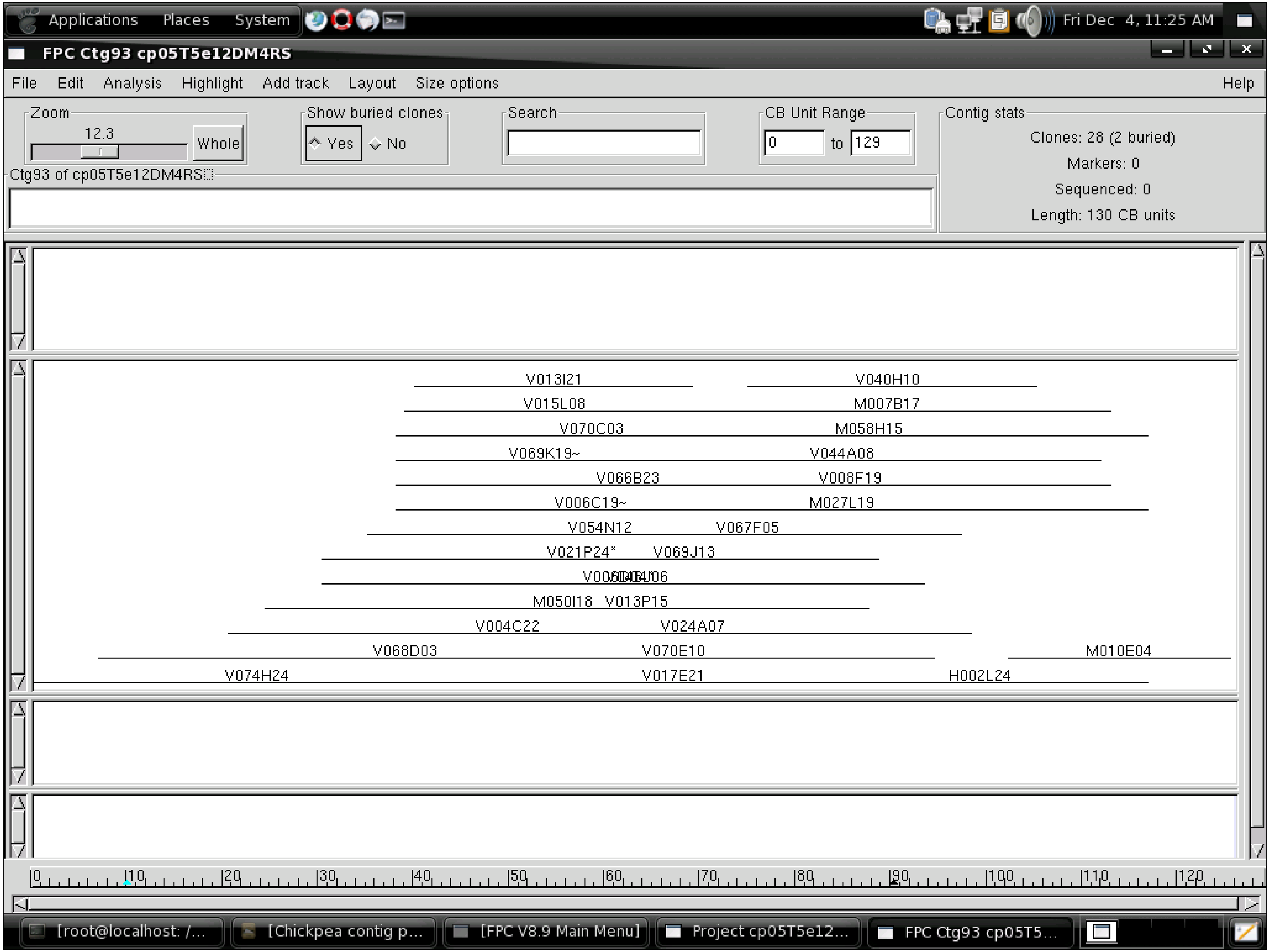
Applications Places System
Fri Dec 4, 11:25 AM
FPC Ctg93 cp05T5e12DM4RS
File Edit Analysis Highlight Add track Layout Size options Help
Zoom
12.3
Whole
Show buried clones
Yes
No
Search
CB Unit Range
0 to 129
Contig stats
Clones: 28 (2 buried)
Markers: 0
Sequenced: 0
Length: 130 CB units
Ctg93 of cp05T5e12DM4RS
V013I21
V040H10
V015L08
M007B17
V070C03
M058H15
V069K19~
V044A08
V066B23
V008F19
V006C19~
M027L19
V054N12
V067F05
V021P24*
V069J13
M050I18
V013P15
V004C22
V024A07
V068D03
V070E10
M010E04
V074H24
V017E21
H002L24
0 10 20 30 40 50 60 70 80 90 100 110 120
[root@localhost: /...
[Chickpea contig p...
[FPC V8.9 Main Menu]
Project cp05T5e12...
FPC Ctg93 cp05T5...

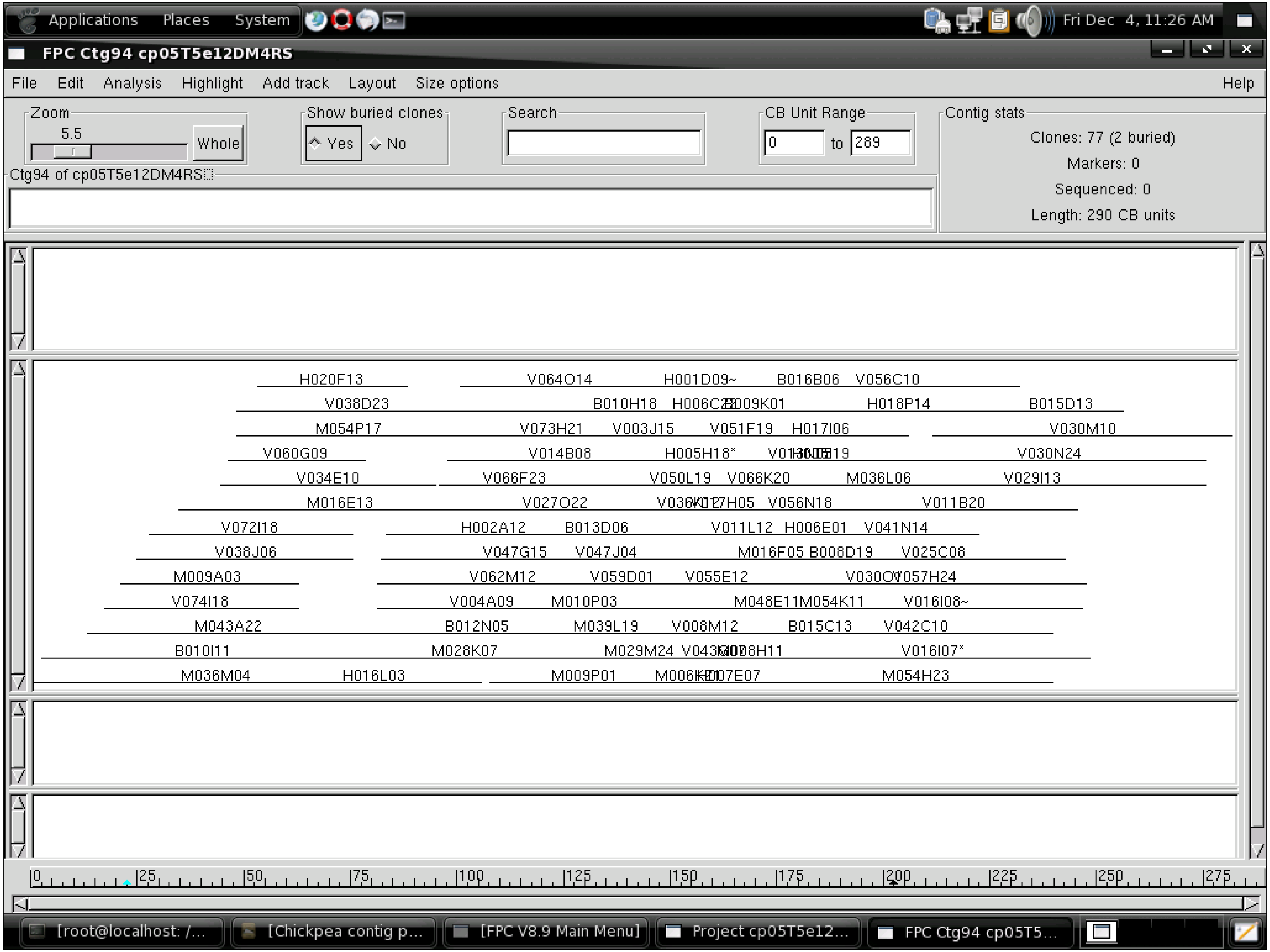
Applications Places System
Fri Dec 4, 11:26 AM
FPC Ctg94 cp05T5e12DM4RS
File Edit Analysis Highlight Add track Layout Size options Help
Zoom
5.5
Whole
Show buried clones
Yes
No
Search
CB Unit Range
0
to
289
Contig stats
Clones: 77 (2 buried)
Markers: 0
Sequenced: 0
Length: 290 CB units
Ctg94 of cp05T5e12DM4RS
H020F13 V064O14 H001D09~ B016B06 V056C10
V038D23 B010H18 H018P14 B015D13
M054P17 V073H21 V003J15 V051F19 H017I06 V030M10
V060G09 V014B08 H005H18* V030N24
V034E10 V066F23 V050L19 V066K20 M036L06 V029I13
M016E13 V027O22 V056N18 V011B20
V072I18 H002A12 B013D06 V011L12 H006E01 V041N14
V038J06 V047G15 V047J04 M016F05 B008D19 V025C08
M009A03 V062M12 V059D01 V055E12
V074I18 V004A09 M010P03 M048E11M054K11 V016I08~
M043A22 B012N05 M039L19 V008M12 B015C13 V042C10
B010I11 M028K07 M029M24 V016I07*
M036M04 H016L03 M009P01 M054H23
0 25 50 75 100 125 150 175 200 225 250 275
[root@localhost: /...
[Chickpea contig p...
[FPC V8.9 Main Menu]
Project cp05T5e12...
FPC Ctg94 cp05T5...

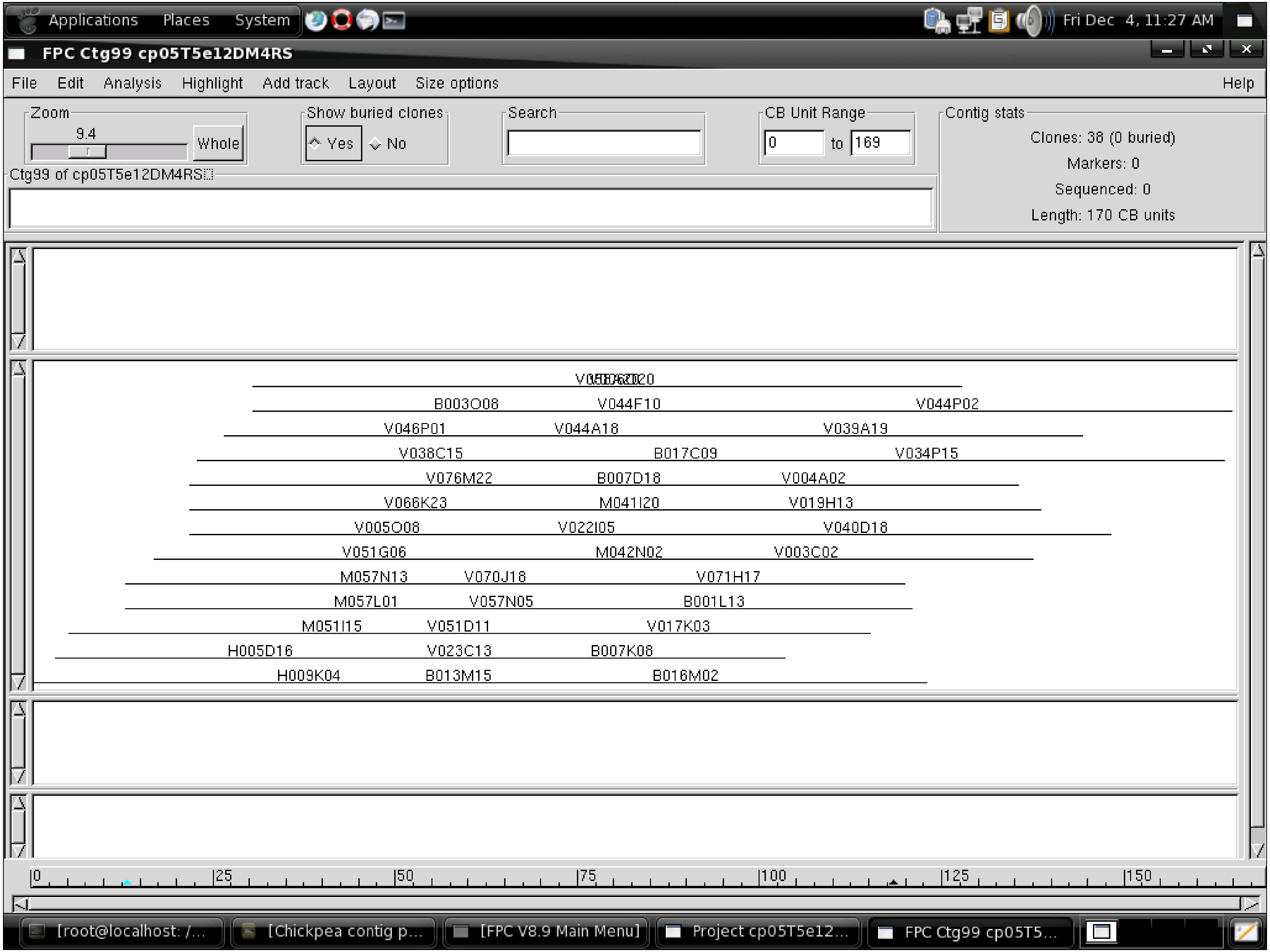

Applications
Places
System
Fri Dec 4, 11:27 AM
FPC Ctg99 cp05T5e12DM4RS
File
Edit
Analysis
Highlight
Add track
Layout
Size options
Help
Zoom
9.4
Whole
Show buried clones
Yes
No
Search
CB Unit Range
0
to
169
Contig stats
Clones: 38 (0 buried)
Markers: 0
Sequenced: 0
Length: 170 CB units
Ctg99 of cp05T5e12DM4RS
B003O08
V044F10
V044P02
V046P01
V044A18
V039A19
V038C15
B017C09
V034P15
V076M22
B007D18
V004A02
V066K23
M041I20
V019H13
V005O08
V022I05
V040D18
V051G06
M042N02
V003C02
M057N13
V070J18
V071H17
M057L01
V057N05
B001L13
M051I15
V051D11
V017K03
H005D16
V023C13
B007K08
H009K04
B013M15
B016M02
0
25
50
75
100
125
150
[root@localhost: /...
[Chickpea contig p...
[FPC V8.9 Main Menu]
Project cp05T5e12...
FPC Ctg99 cp05T5...

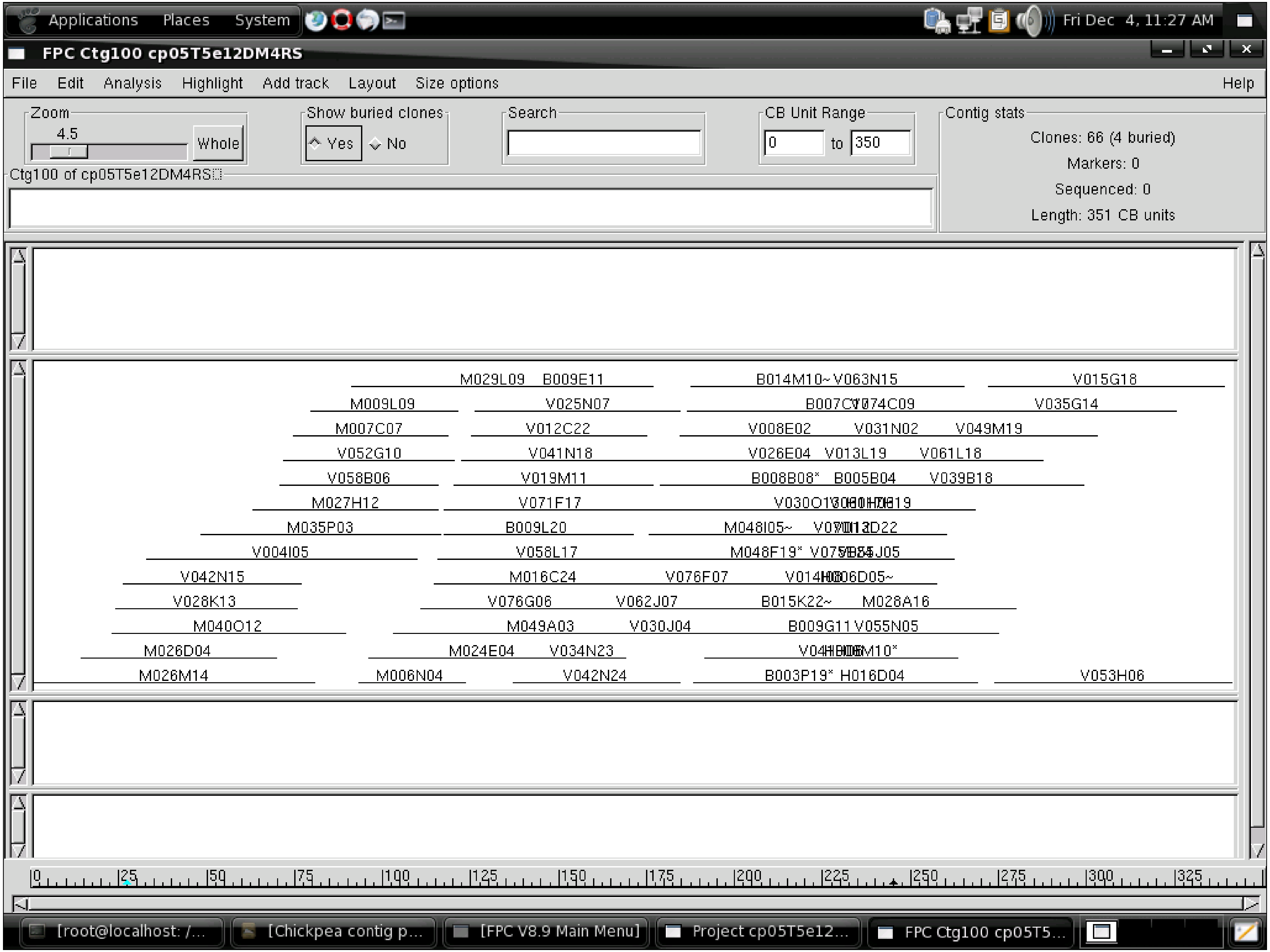

Applications Places System
Fri Dec 4, 11:27 AM
FPC Ctg100 cp05T5e12DM4RS
File Edit Analysis Highlight Add track Layout Size options Help
Zoom
4.5
Whole
Show buried clones
Yes
No
Search
CB Unit Range
0
to
350
Contig stats
Clones: 66 (4 buried)
Markers: 0
Sequenced: 0
Length: 351 CB units
Ctg100 of cp05T5e12DM4RS
M029L09 B009E11
B014M10~ V063N15
V015G18
M009L09
V025N07
V035G14
M007C07
V012C22
V008E02
V031N02
V049M19
V052G10
V041N18
V026E04
V013L19
V061L18
V058B06
V019M11
B008B08*
B005B04
V039B18
M027H12
V071F17
M035P03
B009L20
M048I05~
V004I05
V058L17
V042N15
M016C24
V076F07
V028K13
V076G06
V062J07
B015K22~
M028A16
M040O12
M049A03
V030J04
B009G11
V055N05
M026D04
M024E04
V034N23
M026M14
M006N04
V042N24
B003P19*
H016D04
V053H06
0 25 50 75 100 125 150 175 200 225 250 275 300 325
[root@localhost: /...
[Chickpea contig p...
[FPC V8.9 Main Menu]
Project cp05T5e12...
FPC Ctg100 cp05T5...

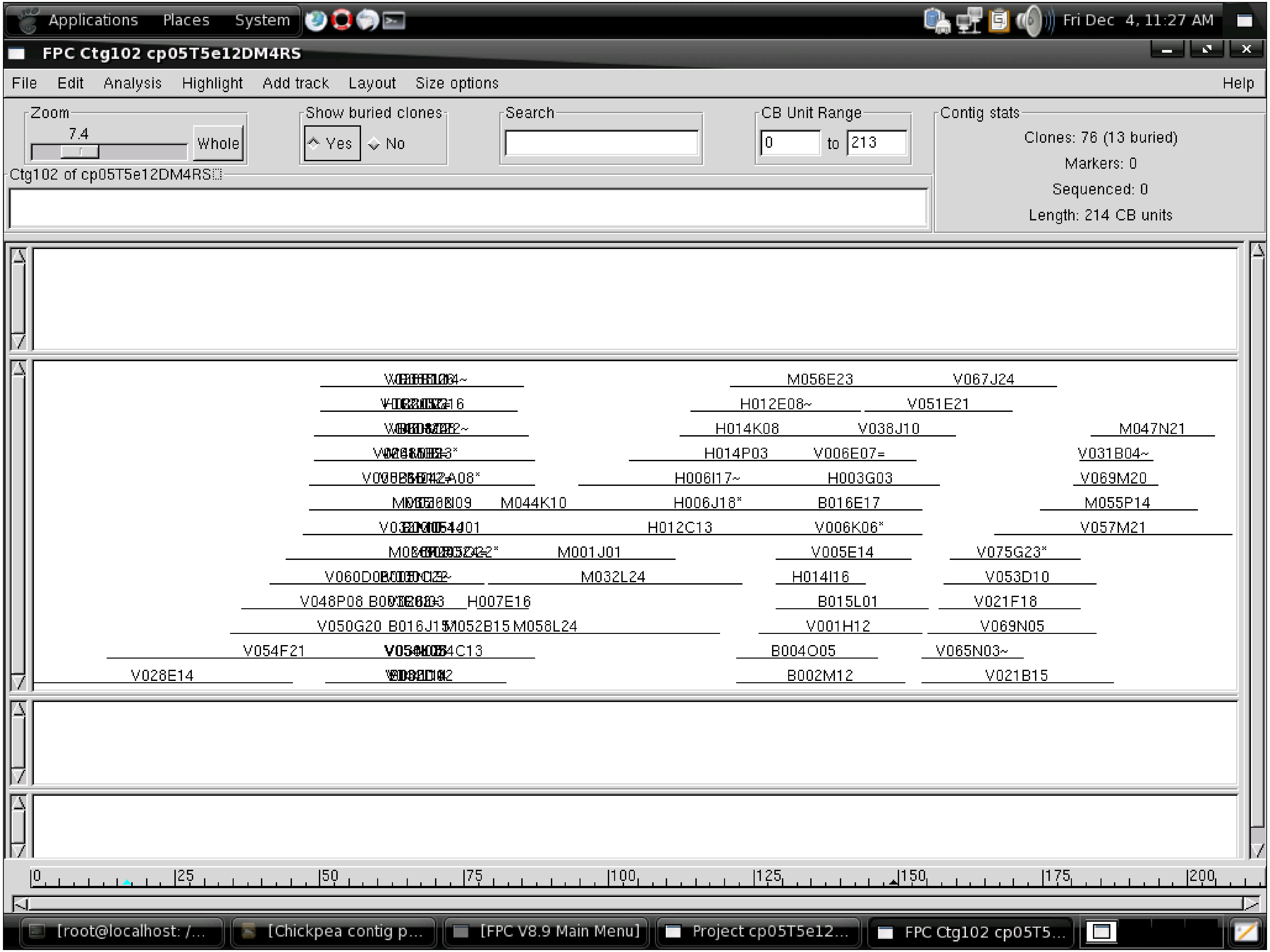
Applications Places System
Fri Dec 4, 11:27 AM
FPC Ctg102 cp05T5e12DM4RS
File Edit Analysis Highlight Add track Layout Size options Help
Zoom
7.4
Whole
Show buried clones
Yes No
Search
CB Unit Range
0 to 213
Contig stats
Clones: 76 (13 buried)
Markers: 0
Sequenced: 0
Length: 214 CB units
Ctg102 of cp05T5e12DM4RS
M056E23 V067J24
H012E08~ V051E21
H014K08 V038J10 M047N21
H014P03 V006E07= V031B04~
H006I17~ H003G03 V069M20
M044K10 H006J18* B016E17 M055P14
H012C13 V006K06* V057M21
M001J01 V005E14 V075G23*
M032L24 H014I16 V053D10
V048P08 H007E16 B015L01 V021F18
V050G20 M052B15 M058L24 V001H12 V069N05
V054F21 B004O05 V065N03~
V028E14 B002M12 V021B15
0 25 50 75 100 125 150 175 200
[root@localhost: /...
[Chickpea contig p...
[FPC V8.9 Main Menu]
Project cp05T5e12...
FPC Ctg102 cp05T5...

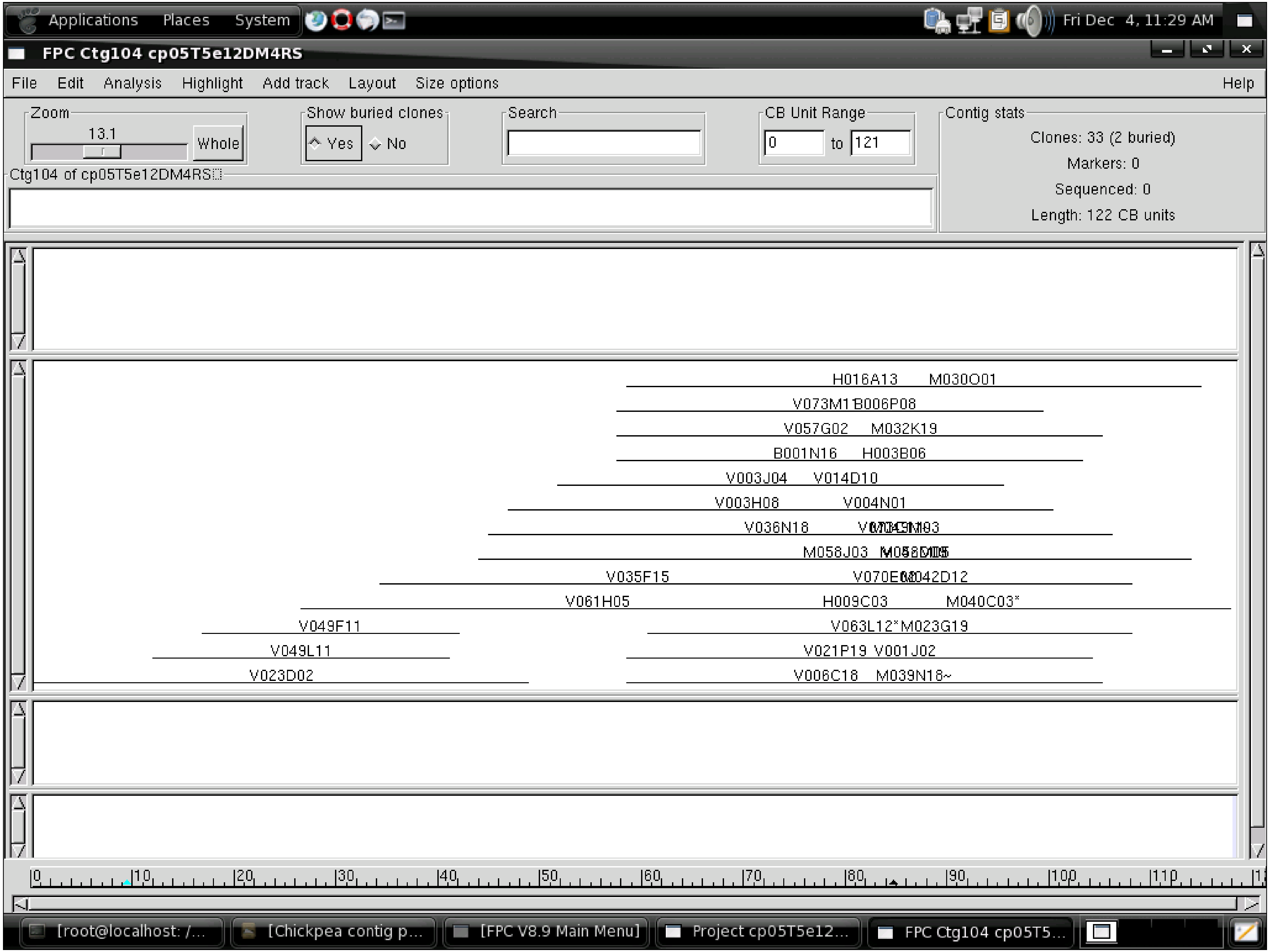

Applications Places System
Fri Dec 4, 11:29 AM
FPC Ctg104 cp05T5e12DM4RS
File Edit Analysis Highlight Add track Layout Size options Help
Zoom
13.1
Whole
Show buried clones
Yes No
Search
CB Unit Range
0 to 121
Contig stats
Clones: 33 (2 buried)
Markers: 0
Sequenced: 0
Length: 122 CB units
Ctg104 of cp05T5e12DM4RS
H016A13 M030O01
V073M11 B006P08
V057G02 M032K19
B001N16 H003B06
V003J04 V014D10
V003H08 V004N01
V036N18
M058J03
V035F15
V061H05 H009C03 M040C03*
V049F11 V063L12*M023G19
V049L11 V021P19 V001J02
V023D02 V006C18 M039N18~
0 10 20 30 40 50 60 70 80 90 100 110
[root@localhost: /...
[Chickpea contig p...
[FPC V8.9 Main Menu]
Project cp05T5e12...
FPC Ctg104 cp05T5...

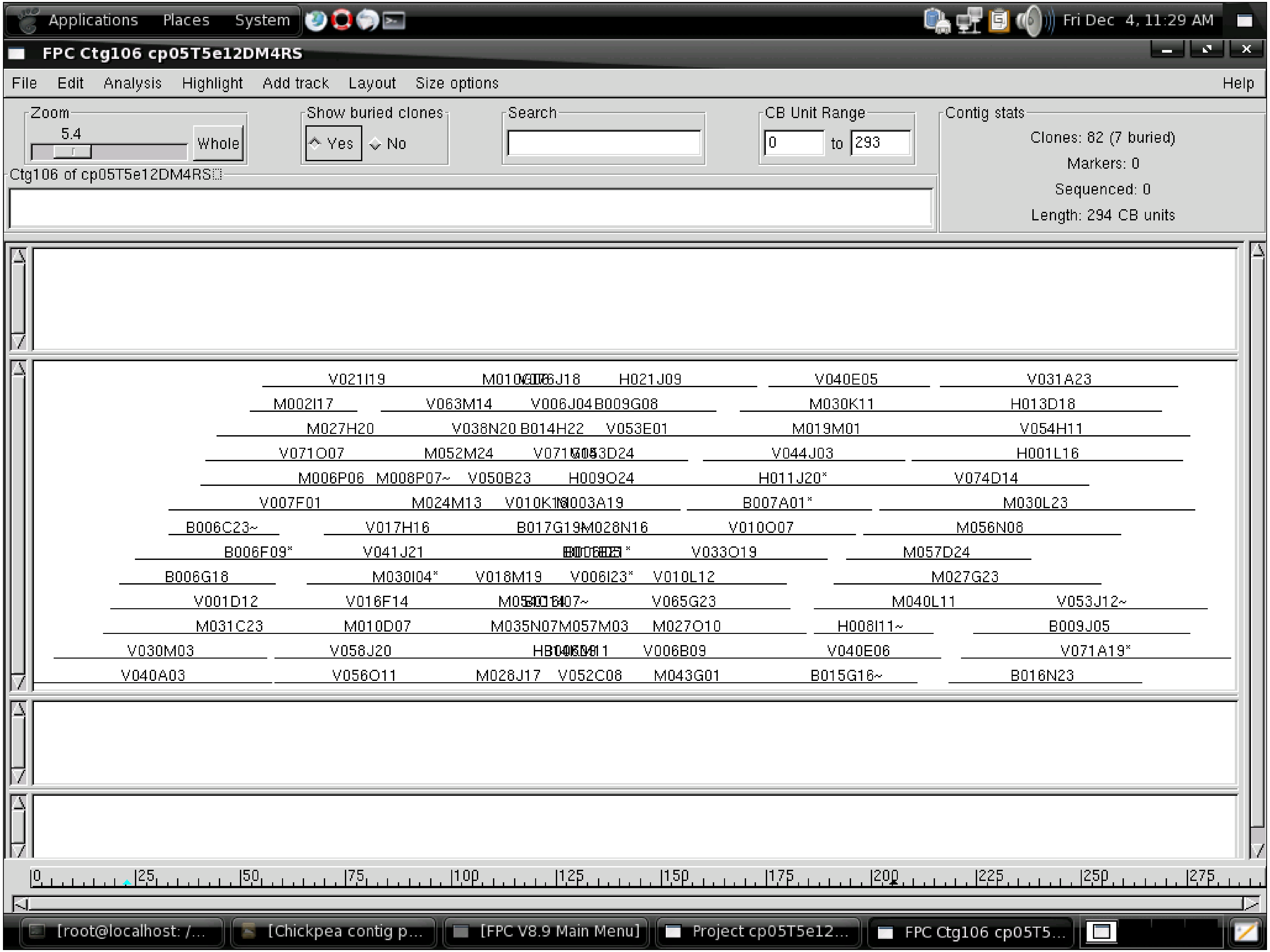

Applications Places System
Fri Dec 4, 11:29 AM
FPC Ctg106 cp05T5e12DM4RS
File Edit Analysis Highlight Add track Layout Size options Help
Zoom 5.4 Whole
Show buried clones Yes No
Search
CB Unit Range 0 to 293
Contig stats
Clones: 82 (7 buried)
Markers: 0
Sequenced: 0
Length: 294 CB units
Ctg106 of cp05T5e12DM4RS
V021I19 H021J09 V040E05 V031A23
M002I17 V063M14 V006J04 B009G08 M030K11 H013D18
M027H20 V038N20 B014H22 V053E01 M019M01 V054H11
V071O07 M052M24 V044J03 H001L16
M006P06 M008P07~ V050B23 H009O24 H011J20* V074D14
V007F01 M024M13 B007A01* M030L23
B006C23~ V017H16 M028N16 V010O07 M056N08
B006F09* V041J21 V033O19 M057D24
B006G18 M030I04* V018M19 V006I23* V010L12 M027G23
V001D12 V016F14 V065G23 M040L11 V053J12~
M031C23 M010D07 M035N07 M057M03 M027O10 H008I11~ B009J05
V030M03 V058J20 V006B09 V040E06 V071A19*
V040A03 V056O11 M028J17 V052C08 M043G01 B015G16~ B016N23
0 25 50 75 100 125 150 175 200 225 250 275
[root@localhost: /... [Chickpea contig p... [FPC V8.9 Main Menu] Project cp05T5e12... FPC Ctg106 cp05T5...

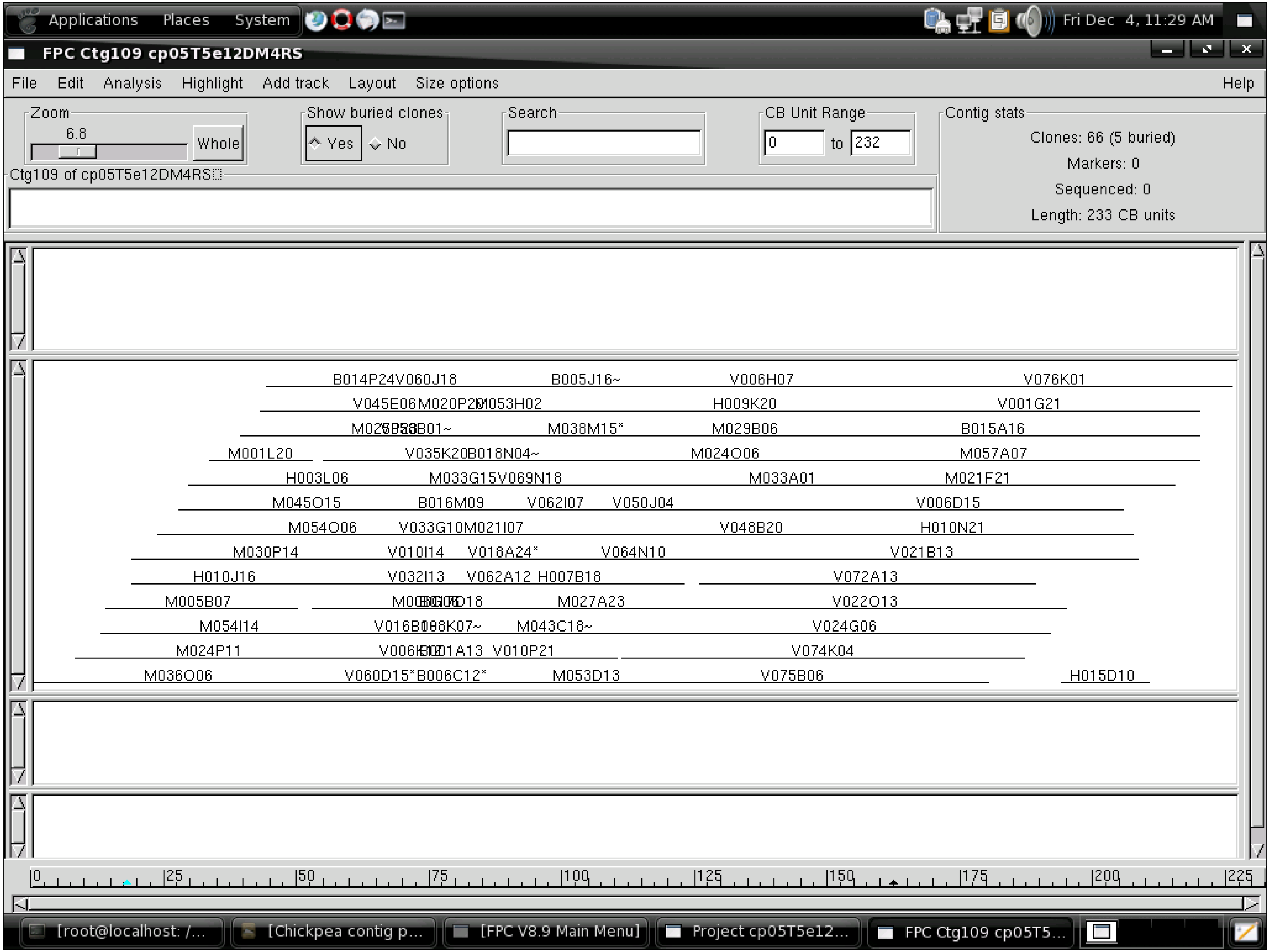
Applications Places System
Fri Dec 4, 11:29 AM
FPC Ctg109 cp05T5e12DM4RS
File Edit Analysis Highlight Add track Layout Size options Help
Zoom
6.8
Whole
Show buried clones
Yes No
Search
CB Unit Range
0 to 232
Contig stats
Clones: 66 (5 buried)
Markers: 0
Sequenced: 0
Length: 233 CB units
Ctg109 of cp05T5e12DM4RS
B014P24 V060J18 B005J16~ V006H07 V076K01
V045E06 M020P24 M053H02 H009K20 V001G21
B001~ M038M15* M029B06 B015A16
M001L20 V035K20 B018N04~ M024O06 M057A07
H003L06 M033G15 V069N18 M033A01 M021F21
M045O15 B016M09 V062I07 V050J04 V006D15
M054O06 V033G10 M021I07 V048B20 H010N21
M030P14 V010I14 V018A24* V064N10 V021B13
H010J16 V032I13 V062A12 H007B18 V072A13
M005B07 M027A23 V022O13
M054I14 M043C18~ V024G06
M024P11 V010P21 V074K04
M036O06 V060D15* B006C12* M053D13 V075B06 H015D10
0 25 50 75 100 125 150 175 200 225
[root@localhost: /...
[Chickpea contig p...
[FPC V8.9 Main Menu]
Project cp05T5e12...
FPC Ctg109 cp05T5...

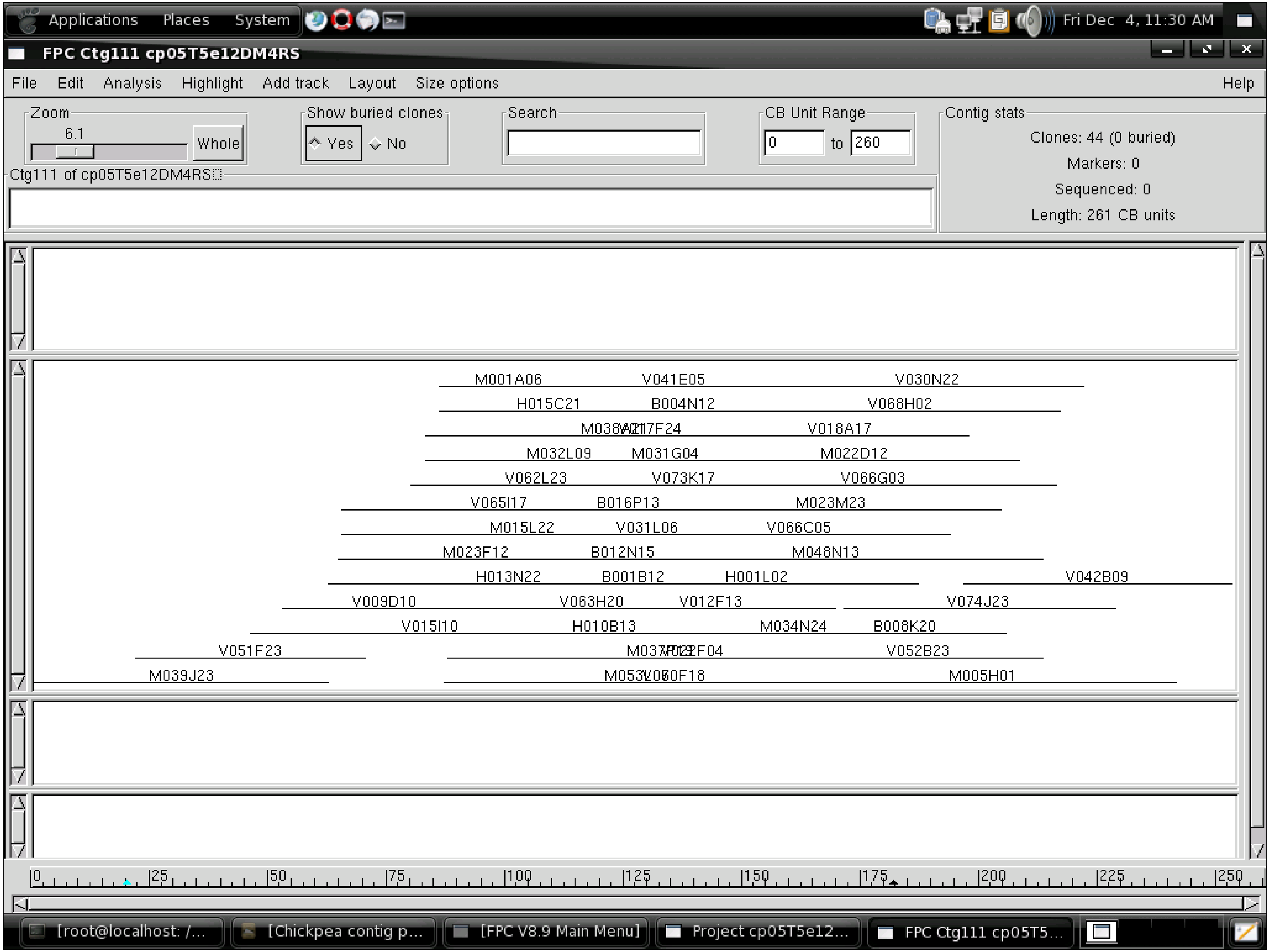
Applications Places System
Fri Dec 4, 11:30 AM
FPC Ctg111 cp05T5e12DM4RS
File Edit Analysis Highlight Add track Layout Size options Help
Zoom
6.1
Whole
Show buried clones
Yes No
Search
CB Unit Range
0 to 260
Contig stats
Clones: 44 (0 buried)
Markers: 0
Sequenced: 0
Length: 261 CB units
Ctg111 of cp05T5e12DM4RS
M001A06 V041E05 V030N22
H015C21 B004N12 V068H02
V018A17
M032L09 M031G04 M022D12
V062L23 V073K17 V066G03
V065I17 B016P13 M023M23
M015L22 V031L06 V066C05
M023F12 B012N15 M048N13
H013N22 B001B12 H001L02 V042B09
V009D10 V063H20 V012F13 V074J23
V015I10 H010B13 M034N24 B008K20
V051F23 V052B23
M039J23 M005H01
0 25 50 75 100 125 150 175 200 225 250
[root@localhost: /...
[Chickpea contig p...
[FPC V8.9 Main Menu]
Project cp05T5e12...
FPC Ctg111 cp05T5...

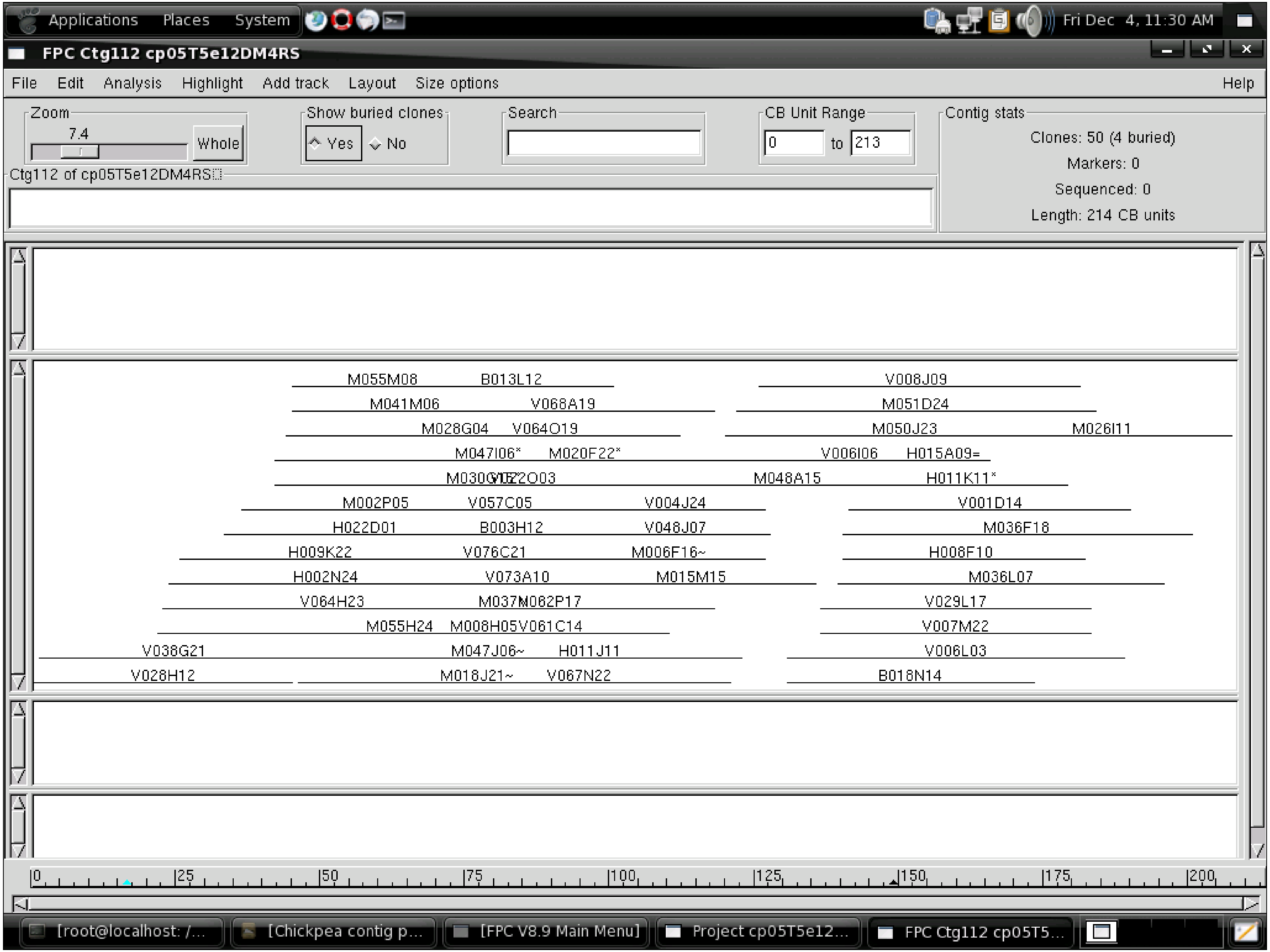
Applications Places System
Fri Dec 4, 11:30 AM
FPC Ctg112 cp05T5e12DM4RS
File Edit Analysis Highlight Add track Layout Size options Help
Zoom
7.4
Whole
Show buried clones
Yes No
Search
CB Unit Range
0 to 213
Contig stats
Clones: 50 (4 buried)
Markers: 0
Sequenced: 0
Length: 214 CB units
Ctg112 of cp05T5e12DM4RS
M055M08 B013L12 V008J09
M041M06 V068A19 M051D24
M028G04 V064O19 M050J23 M026I11
M047I06* M020F22* V006I06 H015A09=
M030G15 V072O03 M048A15 H011K11*
M002P05 V057C05 V004J24 V001D14
H022D01 B003H12 V048J07 M036F18
H009K22 V076C21 M006F16~ H008F10
H002N24 V073A10 M015M15 M036L07
V064H23 M037M06 M082P17 V029L17
M055H24 M008H05 V061C14 V007M22
V038G21 M047J06~ H011J11 V006L03
V028H12 M018J21~ V067N22 B018N14
0 25 50 75 100 125 150 175 200
[root@localhost: /... [Chickpea contig p... [FPC V8.9 Main Menu] Project cp05T5e12... FPC Ctg112 cp05T5...

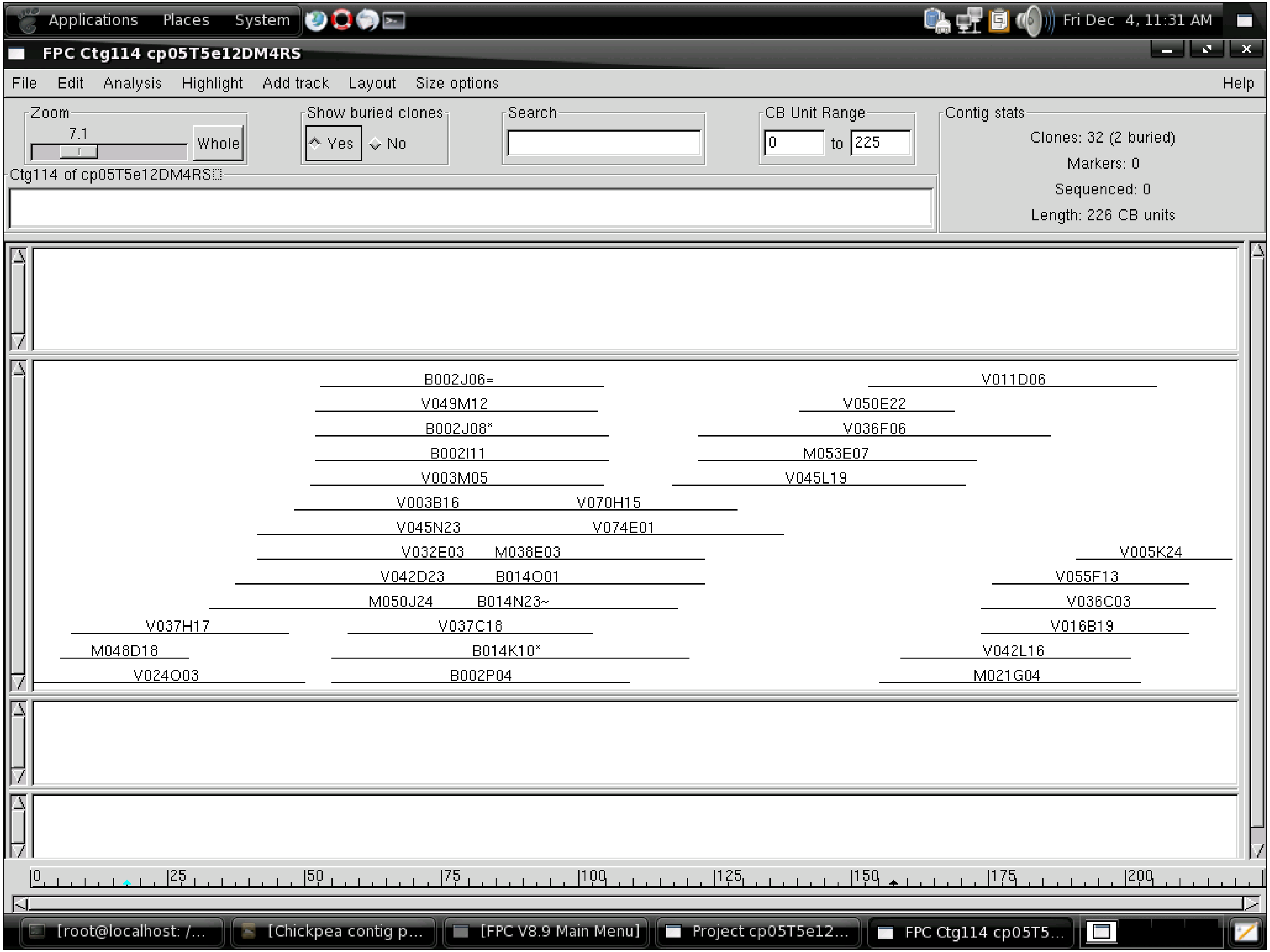
Applications Places System
Fri Dec 4, 11:31 AM
FPC Ctg114 cp05T5e12DM4RS
File Edit Analysis Highlight Add track Layout Size options Help
Zoom
7.1
Whole
Show buried clones
Yes No
Search
CB Unit Range
0 to 225
Contig stats
Clones: 32 (2 buried)
Markers: 0
Sequenced: 0
Length: 226 CB units
Ctg114 of cp05T5e12DM4RS
B002J06=
V011D06
V049M12
V050E22
B002J08*
V036F06
B002I11
M053E07
V003M05
V045L19
V003B16
V070H15
V045N23
V074E01
V032E03
M038E03
V005K24
V042D23
B014O01
V055F13
M050J24
B014N23~
V036C03
V037H17
V037C18
V016B19
M048D18
B014K10*
V042L16
V024O03
B002P04
M021G04
0 25 50 75 100 125 150 175 200
[root@localhost: /...
[Chickpea contig p...
[FPC V8.9 Main Menu]
Project cp05T5e12...
FPC Ctg114 cp05T5...

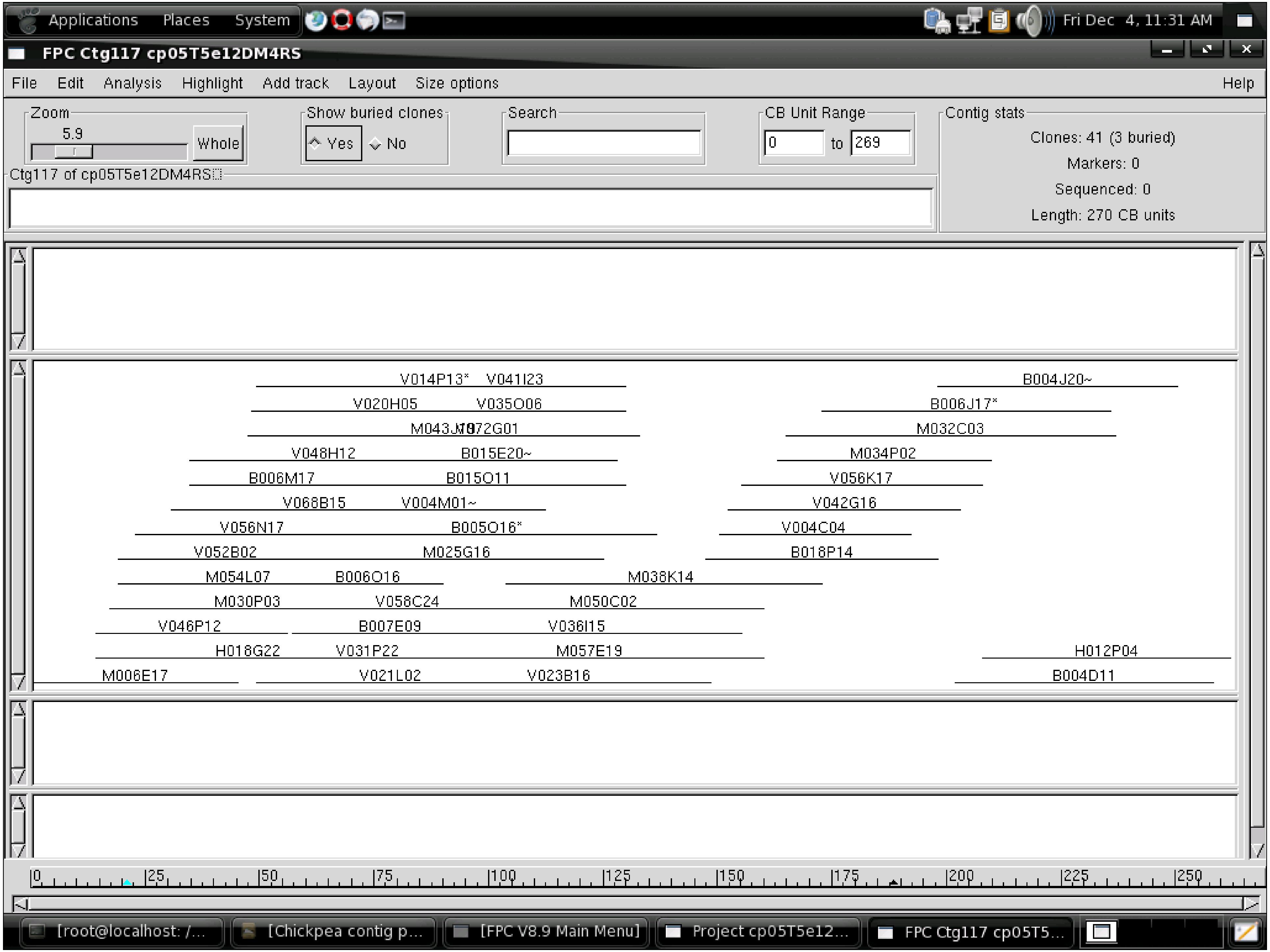
Applications Places System
Fri Dec 4, 11:31 AM
FPC Ctg117 cp05T5e12DM4RS
File Edit Analysis Highlight Add track Layout Size options Help
Zoom
5.9
Whole
Show buried clones
Yes No
Search
CB Unit Range
0 to 269
Contig stats
Clones: 41 (3 buried)
Markers: 0
Sequenced: 0
Length: 270 CB units
Ctg117 of cp05T5e12DM4RS
V014P13* V041I23
B004J20~
V020H05 V035O06
B006J17*
M043J19 V072G01
M032C03
V048H12 B015E20~
M034P02
B006M17 B015O11
V056K17
V068B15 V004M01~
V042G16
V056N17 B005O16*
V004C04
V052B02 M025G16
B018P14
M054L07 B006O16
M038K14
M030P03 V058C24 M050C02
V046P12 B007E09 V036I15
H018G22 V031P22 M057E19
H012P04
M006E17 V021L02 V023B16
B004D11
0 25 50 75 100 125 150 175 200 225 250
[root@localhost: /...
[Chickpea contig p...
[FPC V8.9 Main Menu]
Project cp05T5e12...
FPC Ctg117 cp05T5...

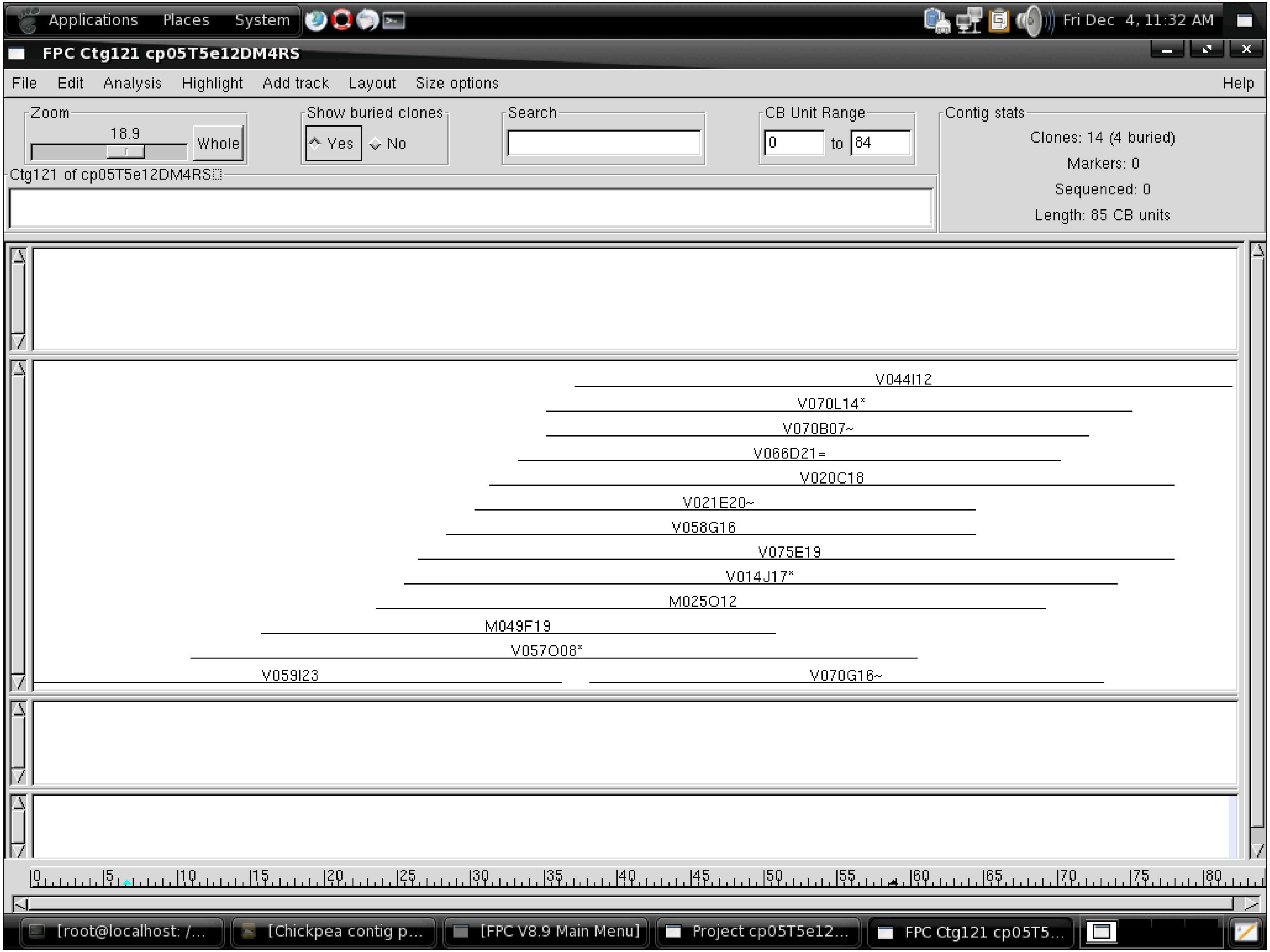
Applications
Places
System
Fri Dec 4, 11:32 AM
FPC Ctg121 cp05T5e12DM4RS
File
Edit
Analysis
Highlight
Add track
Layout
Size options
Help
Zoom
18.9
Whole
Show buried clones
Yes
No
Search
CB Unit Range
0
to
84
Contig stats
Clones: 14 (4 buried)
Markers: 0
Sequenced: 0
Length: 85 CB units
Ctg121 of cp05T5e12DM4RS
V044I12
V070L14*
V070B07~
V066D21=
V020C18
V021E20~
V058G16
V075E19
V014J17*
M025O12
M049F19
V057O08*
V059I23
V070G16~
0 5 10 15 20 25 30 35 40 45 50 55 60 65 70 75 80
[root@localhost: /...
[Chickpea contig p...
[FPC V8.9 Main Menu]
Project cp05T5e12...
FPC Ctg121 cp05T5...

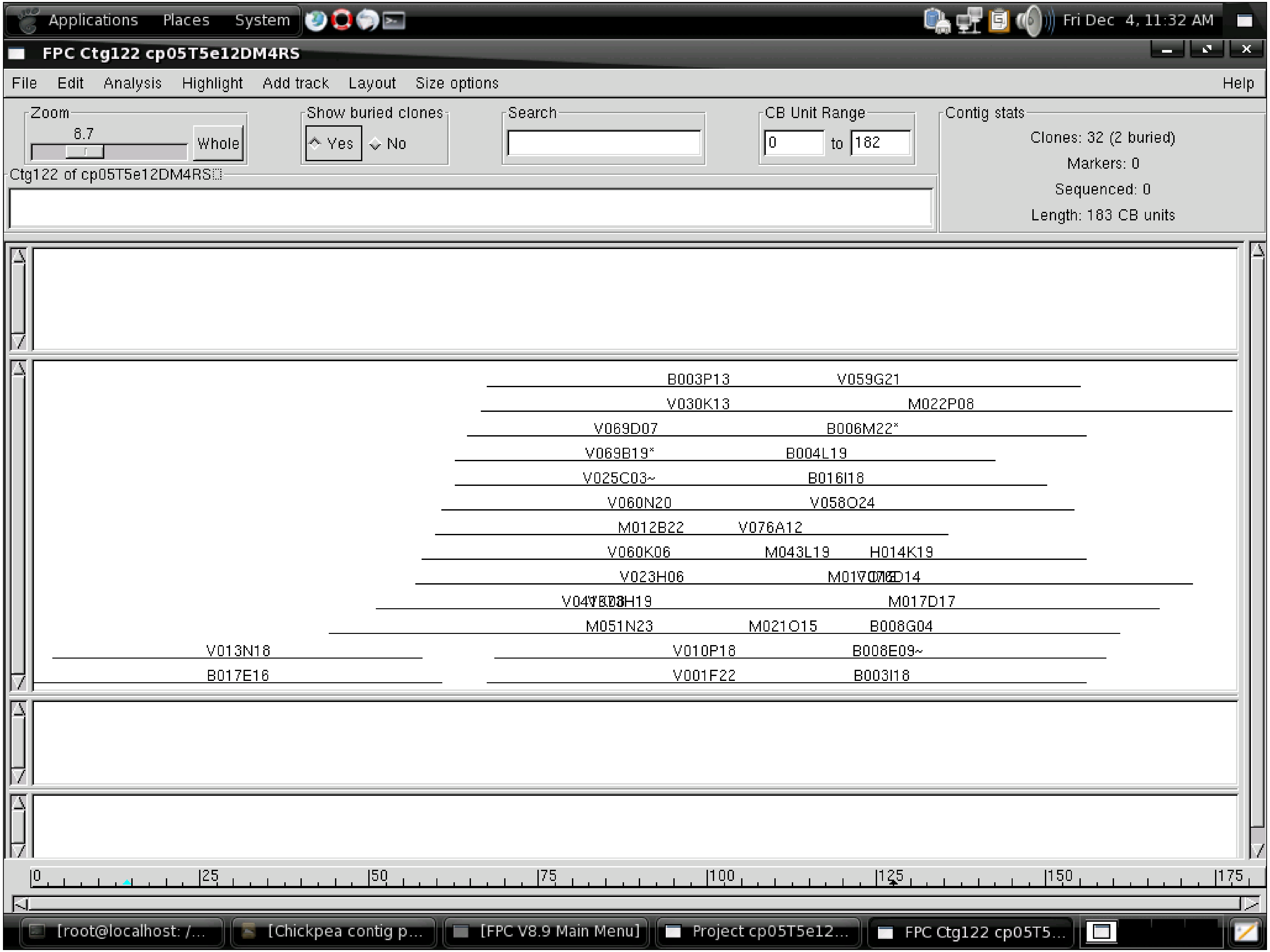
Applications Places System
Fri Dec 4, 11:32 AM
FPC Ctg122 cp05T5e12DM4RS
File Edit Analysis Highlight Add track Layout Size options Help
Zoom
8.7
Whole
Show buried clones
Yes No
Search
CB Unit Range
0 to 182
Contig stats
Clones: 32 (2 buried)
Markers: 0
Sequenced: 0
Length: 183 CB units
Ctg122 of cp05T5e12DM4RS
B003P13 V059G21
V030K13 M022P08
V069D07 B006M22*
V069B19* B004L19
V025C03~ B016I18
V060N20 V058O24
M012B22 V076A12
V060K06 M043L19 H014K19
V023H06
M017D17
M051N23 M021O15 B008G04
V013N18 V010P18 B008E09~
B017E16 V001F22 B003I18
0 25 50 75 100 125 150 175
[root@localhost: /...
[Chickpea contig p...
[FPC V8.9 Main Menu]
Project cp05T5e12...
FPC Ctg122 cp05T5...

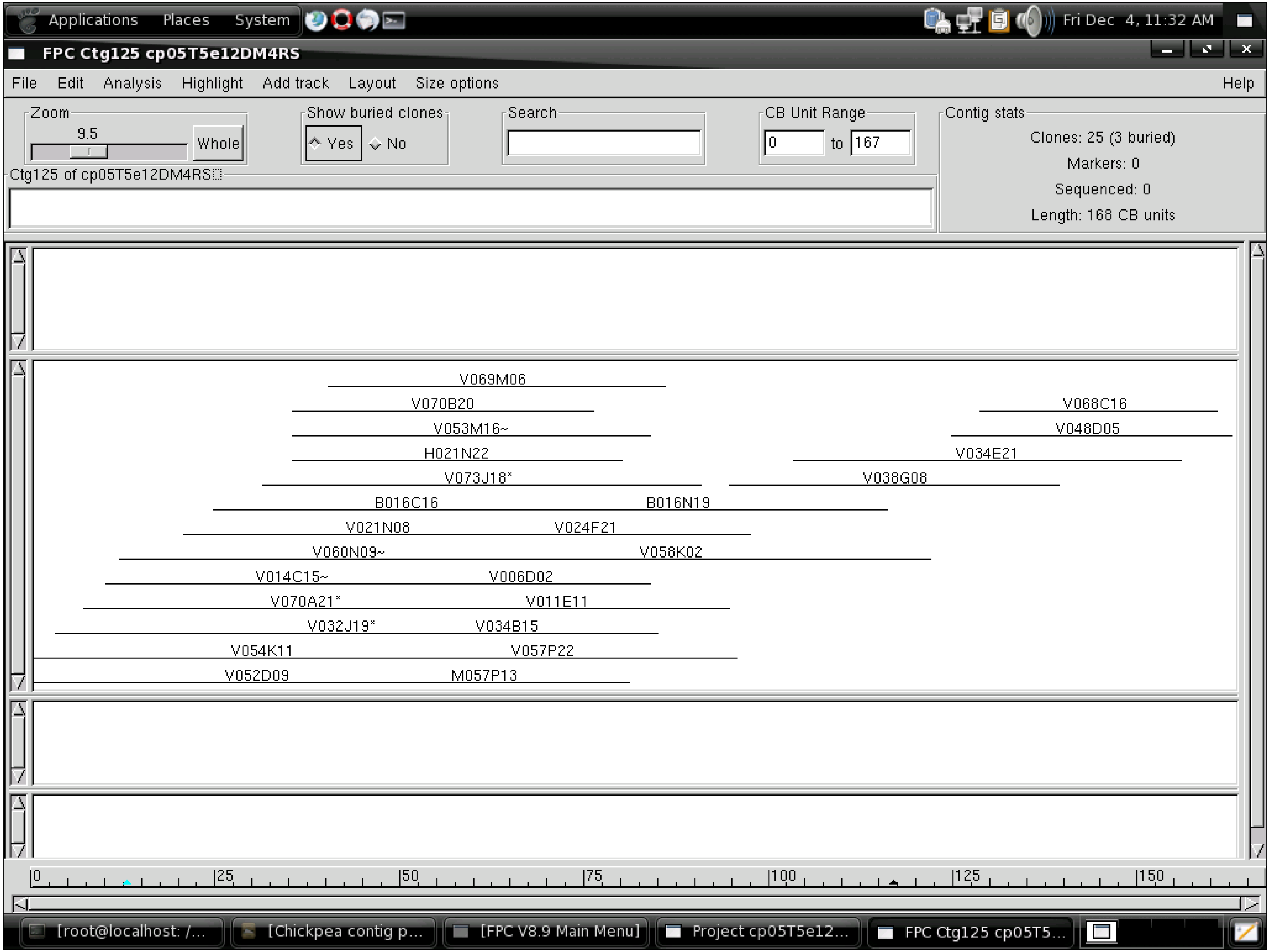
Applications Places System
Fri Dec 4, 11:32 AM
FPC Ctg125 cp05T5e12DM4RS
File Edit Analysis Highlight Add track Layout Size options Help
Zoom 9.5 Whole
Show buried clones Yes No
Search
CB Unit Range 0 to 167
Contig stats
Clones: 25 (3 buried)
Markers: 0
Sequenced: 0
Length: 168 CB units
Ctg125 of cp05T5e12DM4RS
V069M06
V070B20
V068C16
V053M16~
V048D05
H021N22
V034E21
V073J18*
V038G08
B016C16
B016N19
V021N08
V024F21
V060N09~
V058K02
V014C15~
V006D02
V070A21*
V011E11
V032J19*
V034B15
V054K11
V057P22
V052D09
M057P13
0 25 50 75 100 125 150
[root@localhost: /...
[Chickpea contig p...
[FPC V8.9 Main Menu]
Project cp05T5e12...
FPC Ctg125 cp05T5...

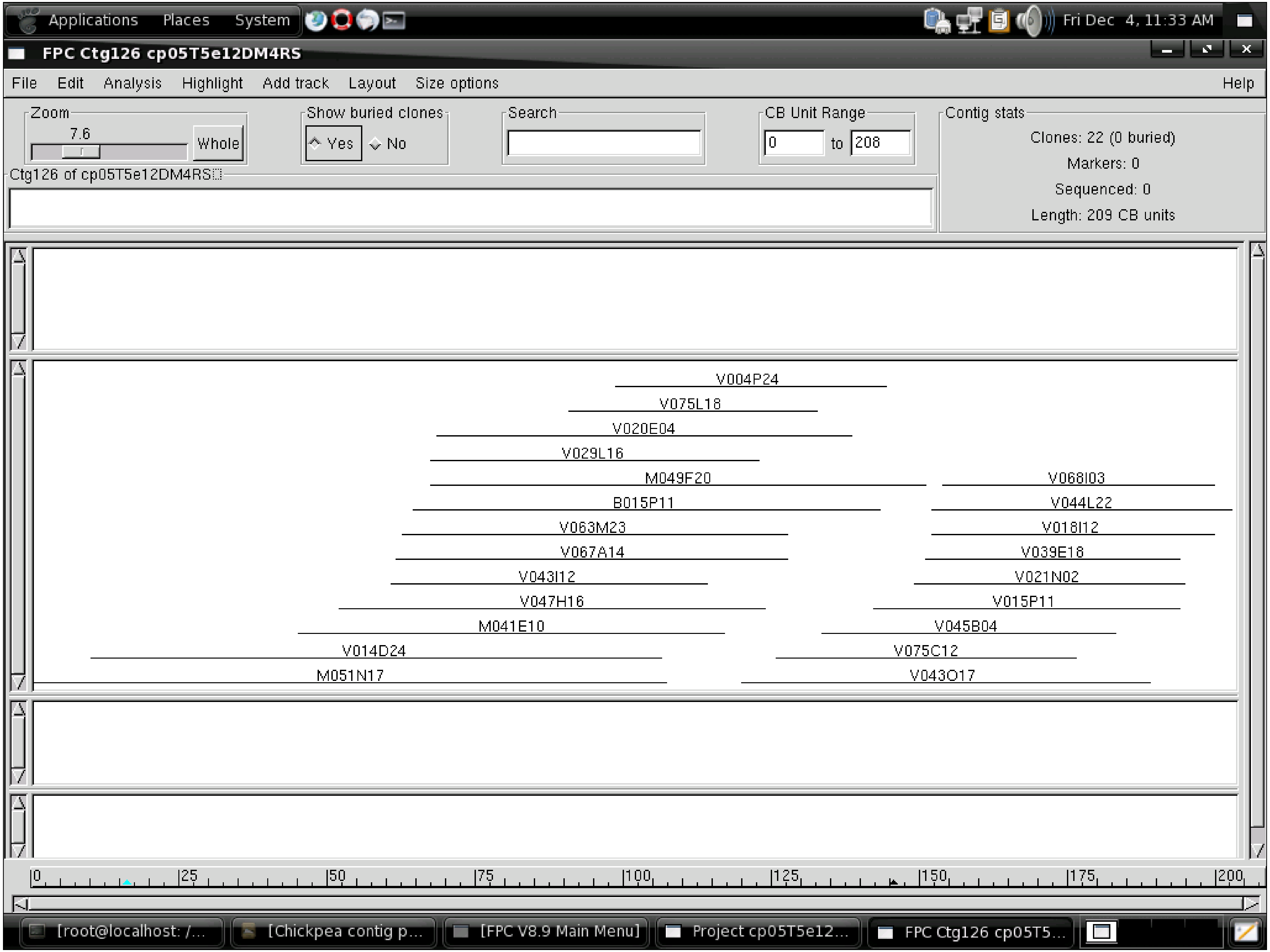
Applications Places System
Fri Dec 4, 11:33 AM
FPC Ctg126 cp05T5e12DM4RS
File Edit Analysis Highlight Add track Layout Size options Help
Zoom
7.6
Whole
Show buried clones
Yes No
Search
CB Unit Range
0 to 208
Contig stats
Clones: 22 (0 buried)
Markers: 0
Sequenced: 0
Length: 209 CB units
Ctg126 of cp05T5e12DM4RS
V004P24
V075L18
V020E04
V029L16
M049F20
V068I03
B015P11
V044L22
V063M23
V018I12
V067A14
V039E18
V043I12
V021N02
V047H16
V015P11
M041E10
V045B04
V014D24
V075C12
M051N17
V043O17
0 25 50 75 100 125 150 175 200
[root@localhost: /...
[Chickpea contig p...
[FPC V8.9 Main Menu]
Project cp05T5e12...
FPC Ctg126 cp05T5...

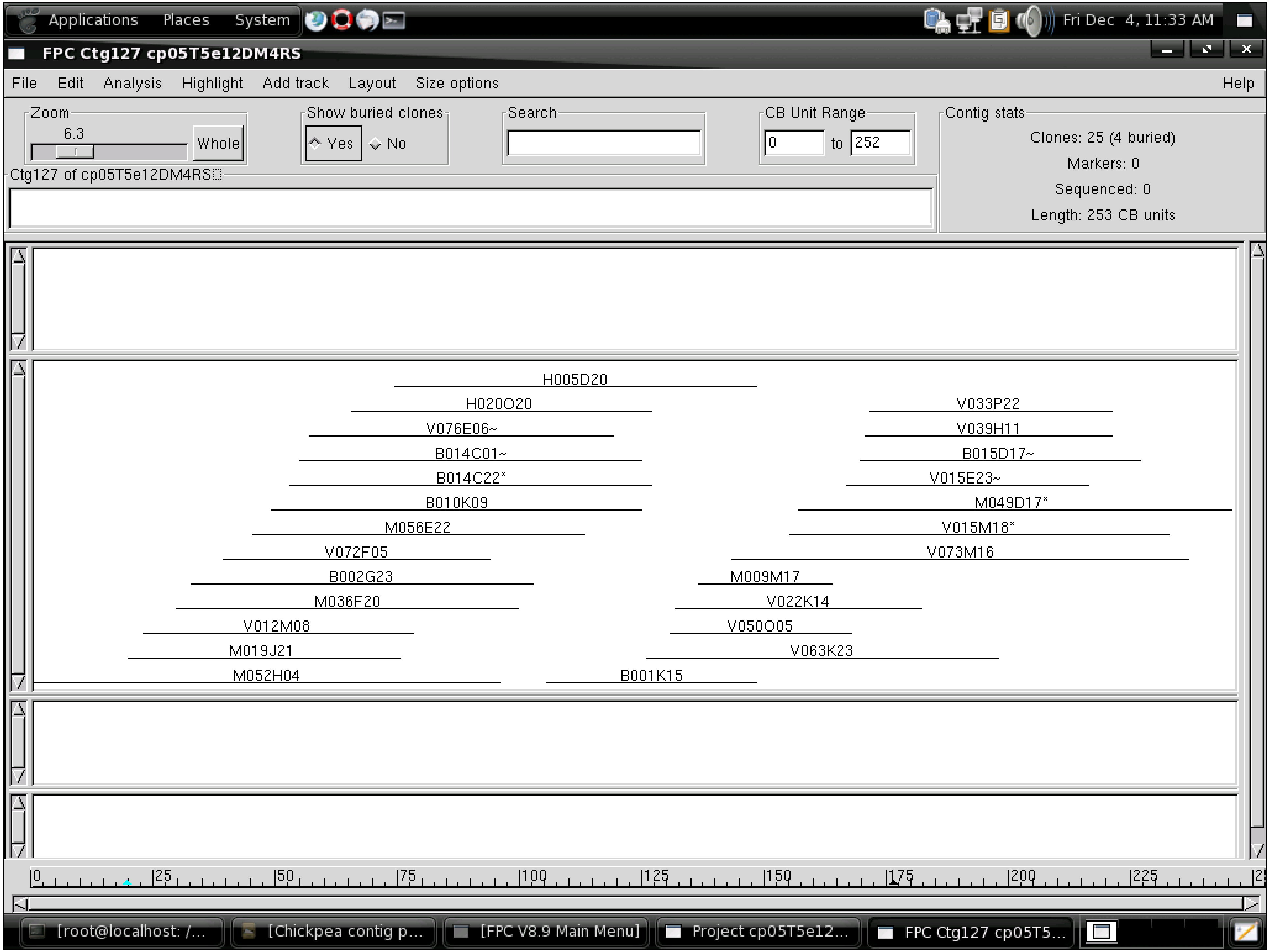

Applications Places System
Fri Dec 4, 11:33 AM
FPC Ctg127 cp05T5e12DM4RS
File Edit Analysis Highlight Add track Layout Size options Help
Zoom
6.3
Whole
Show buried clones
Yes No
Search
CB Unit Range
0 to 252
Contig stats
Clones: 25 (4 buried)
Markers: 0
Sequenced: 0
Length: 253 CB units
Ctg127 of cp05T5e12DM4RS
H005D20
H020O20
V033P22
V076E06~
V039H11
B014C01~
B015D17~
B014C22*
V015E23~
B010K09
M049D17*
M056E22
V015M18*
V072F05
V073M16
B002G23
M009M17
M036F20
V022K14
V012M08
V050O05
M019J21
V063K23
M052H04
B001K15
0 25 50 75 100 125 150 175 200 225
[root@localhost: /...
[Chickpea contig p...
[FPC V8.9 Main Menu]
Project cp05T5e12...
FPC Ctg127 cp05T5...

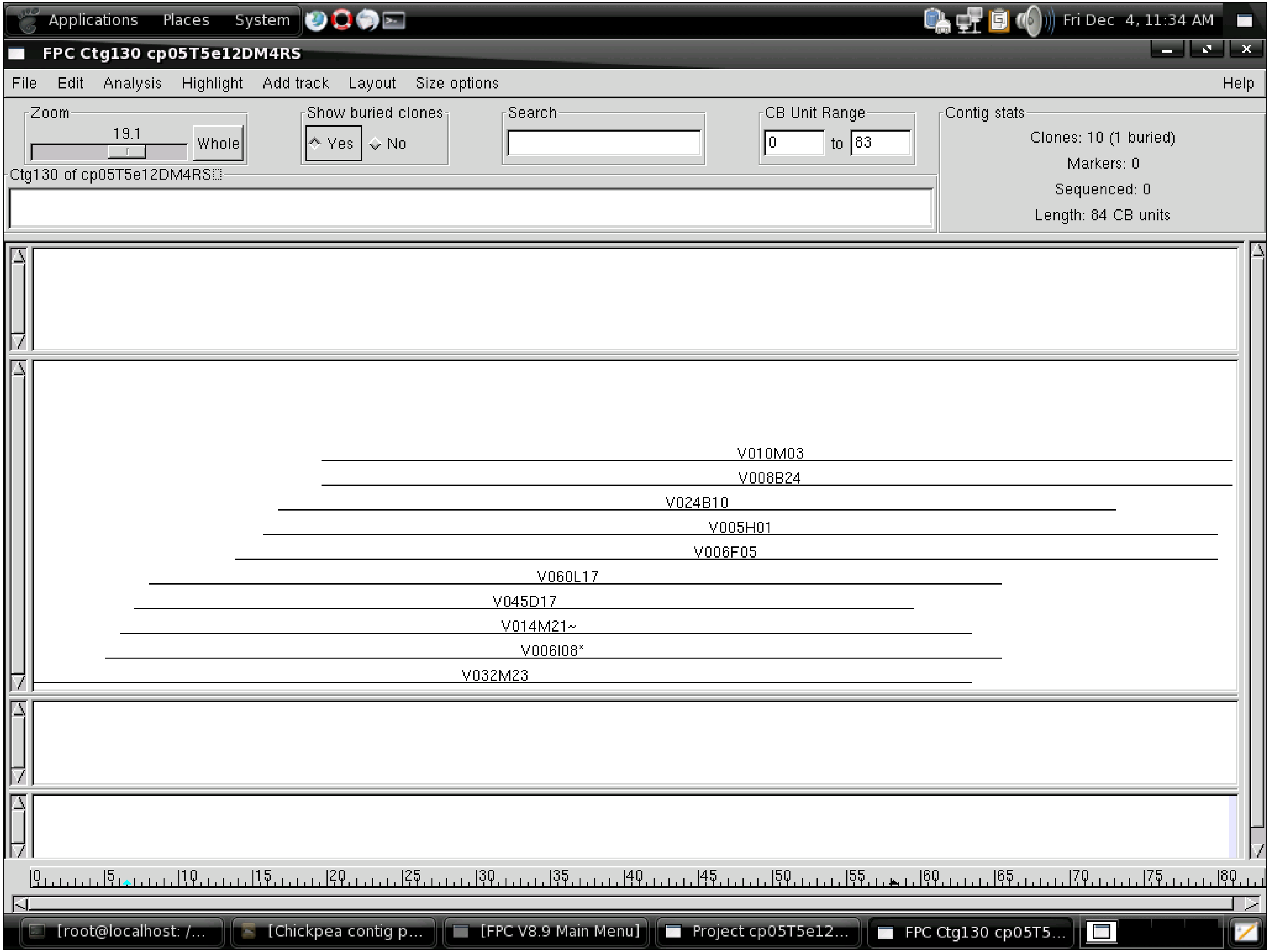
Applications Places System
Fri Dec 4, 11:34 AM
FPC Ctg130 cp05T5e12DM4RS
File Edit Analysis Highlight Add track Layout Size options Help
Zoom
19.1
Whole
Show buried clones
Yes
No
Search
CB Unit Range
0
to
83
Contig stats
Clones: 10 (1 buried)
Markers: 0
Sequenced: 0
Length: 84 CB units
Ctg130 of cp05T5e12DM4RS
V010M03
V008B24
V024B10
V005H01
V006F05
V060L17
V045D17
V014M21~
V006I08*
V032M23
0 5 10 15 20 25 30 35 40 45 50 55 60 65 70 75 80
[root@localhost: /...
[Chickpea contig p...
[FPC V8.9 Main Menu]
Project cp05T5e12...
FPC Ctg130 cp05T5...

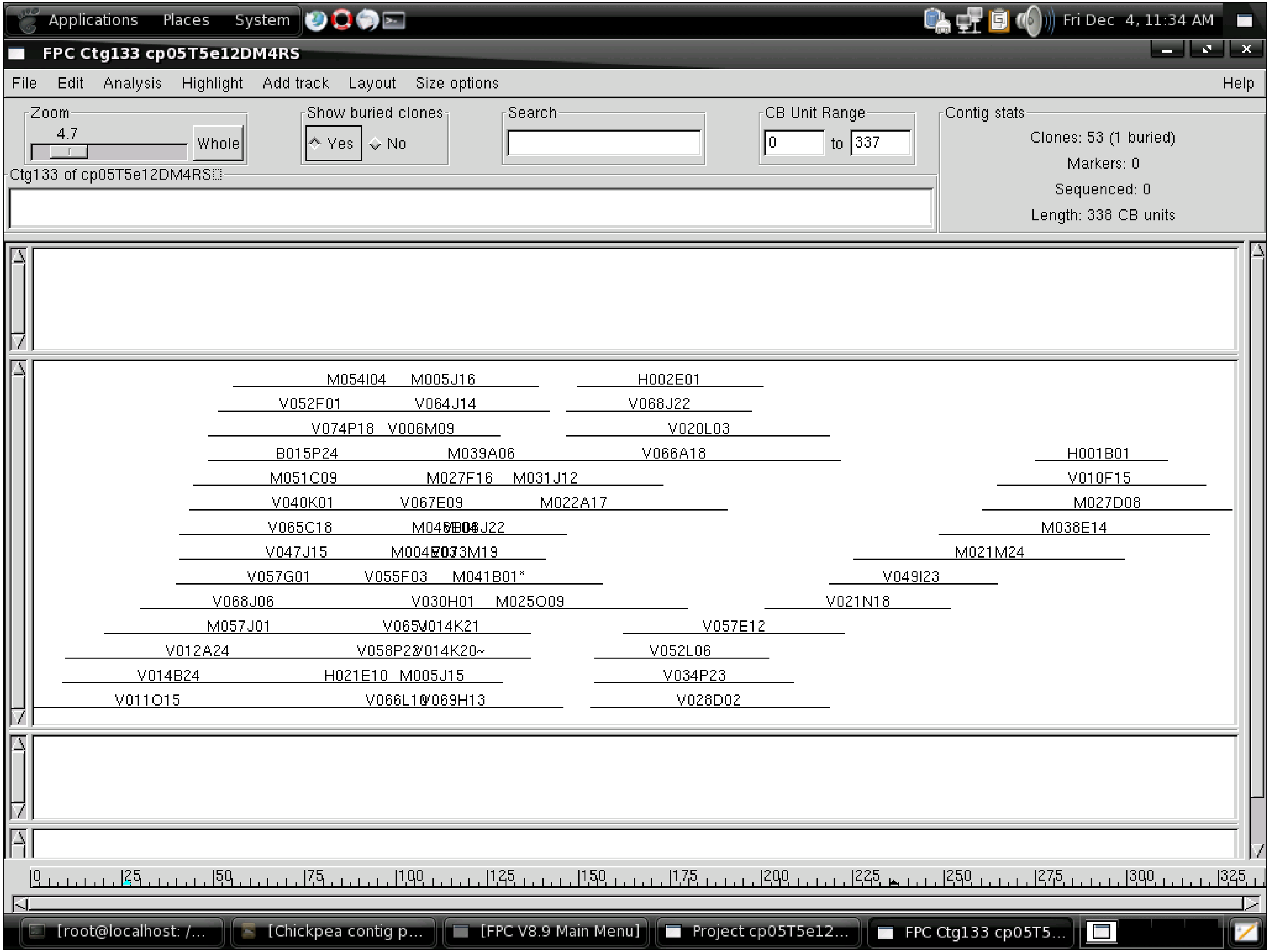
Applications Places System
Fri Dec 4, 11:34 AM
FPC Ctg133 cp05T5e12DM4RS
File Edit Analysis Highlight Add track Layout Size options Help
Zoom
4.7
Whole
Show buried clones
Yes No
Search
CB Unit Range
0 to 337
Contig stats
Clones: 53 (1 buried)
Markers: 0
Sequenced: 0
Length: 338 CB units
Ctg133 of cp05T5e12DM4RS
M054I04 M005J16 H002E01
V052F01 V064J14 V068J22
V074P18 V006M09 V020L03
B015P24 M039A06 V066A18 H001B01
M051C09 M027F16 M031J12 V010F15
V040K01 V067E09 M022A17 M027D08
V065C18 M038E14
V047J15 M021M24
V057G01 V055F03 M041B01* V049I23
V068J06 V030H01 M025O09 V021N18
M057J01 V057E12
V012A24 V052L06
V014B24 H021E10 M005J15 V034P23
V011O15 V028D02
0 25 50 75 100 125 150 175 200 225 250 275 300 325
[root@localhost: /... [Chickpea contig p... [FPC V8.9 Main Menu] Project cp05T5e12... FPC Ctg133 cp05T5...

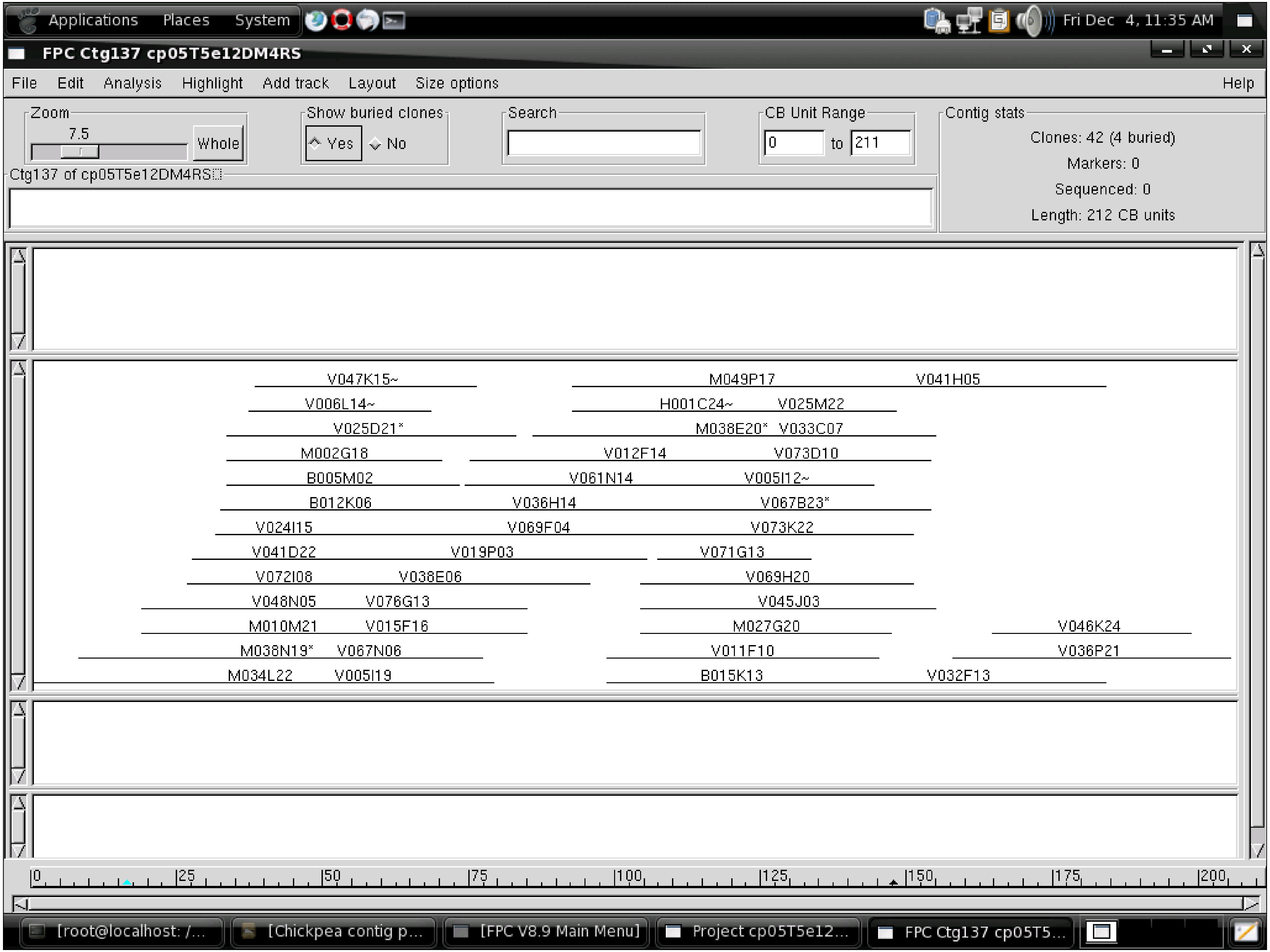
Applications Places System
Fri Dec 4, 11:35 AM
FPC Ctg137 cp05T5e12DM4RS
File Edit Analysis Highlight Add track Layout Size options Help
Zoom
7.5
Whole
Show buried clones
Yes
No
Search
CB Unit Range
0 to 211
Contig stats
Clones: 42 (4 buried)
Markers: 0
Sequenced: 0
Length: 212 CB units
Ctg137 of cp05T5e12DM4RS
V047K15~
M049P17
V041H05
V006L14~
H001C24~
V025M22
V025D21*
M038E20*
V033C07
M002G18
V012F14
V073D10
B005M02
V061N14
V005I12~
B012K06
V036H14
V067B23*
V024I15
V069F04
V073K22
V041D22
V019P03
V071G13
V072I08
V038E06
V069H20
V048N05
V076G13
V045J03
M010M21
V015F16
M027G20
V046K24
M038N19*
V067N06
V011F10
V036P21
M034L22
V005I19
B015K13
V032F13
0
25
50
75
100
125
150
175
200
[root@localhost: /...
[Chickpea contig p...
[FPC V8.9 Main Menu]
Project cp05T5e12...
FPC Ctg137 cp05T5...

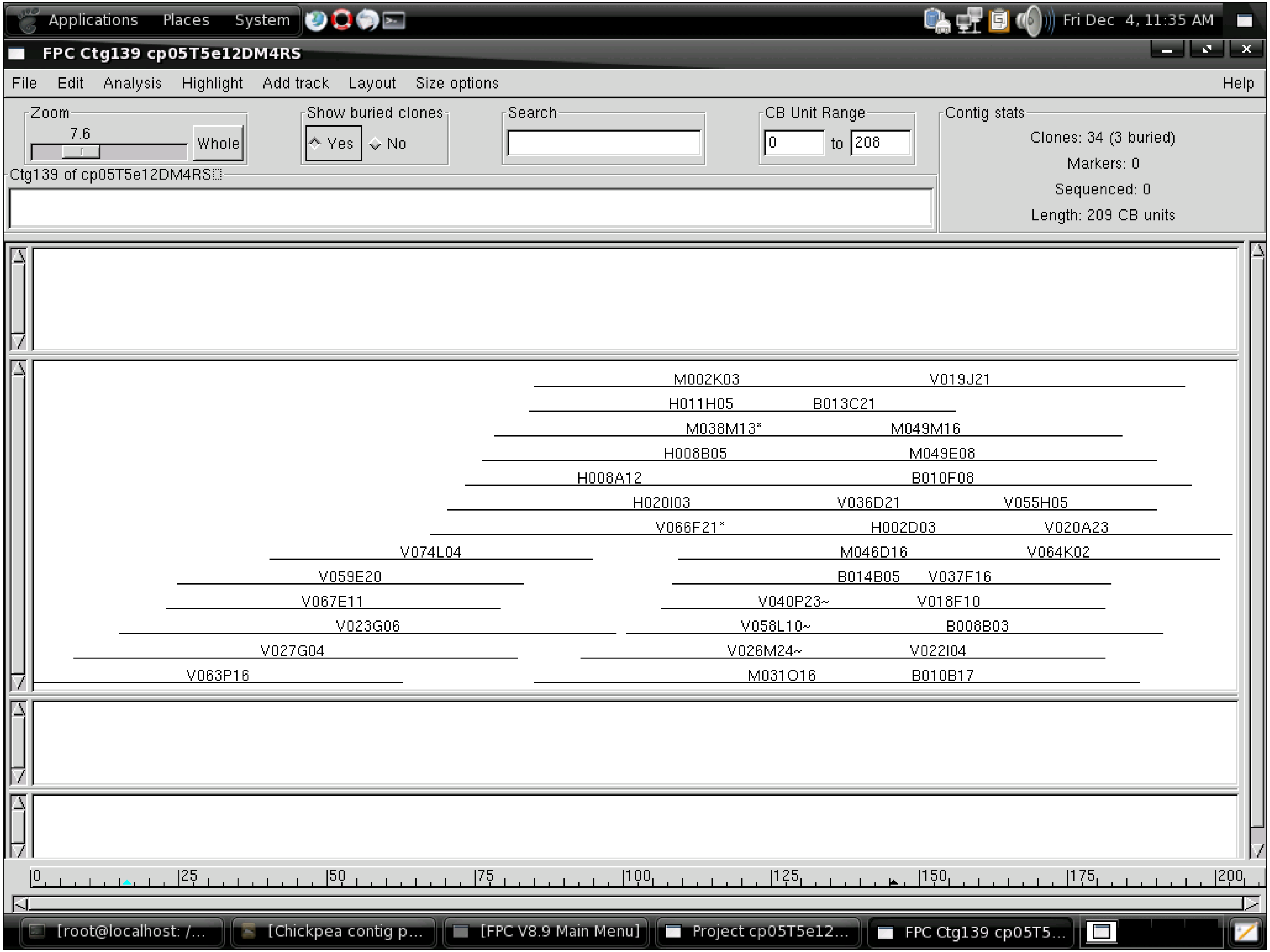
Applications Places System
Fri Dec 4, 11:35 AM
FPC Ctg139 cp05T5e12DM4RS
File Edit Analysis Highlight Add track Layout Size options Help
Zoom
7.6
Whole
Show buried clones
Yes No
Search
CB Unit Range
0 to 208
Contig stats
Clones: 34 (3 buried)
Markers: 0
Sequenced: 0
Length: 209 CB units
Ctg139 of cp05T5e12DM4RS
M002K03 V019J21
H011H05 B013C21
M038M13* M049M16
H008B05 M049E08
H008A12 B010F08
H020I03 V036D21 V055H05
V066F21* H002D03 V020A23
V074L04 M046D16 V064K02
V059E20 B014B05 V037F16
V067E11 V040P23~ V018F10
V023G06 V058L10~ B008B03
V027G04 V026M24~ V022I04
V063P16 M031O16 B010B17
0 25 50 75 100 125 150 175 200
[root@localhost: /...
[Chickpea contig p...
[FPC V8.9 Main Menu]
Project cp05T5e12...
FPC Ctg139 cp05T5...

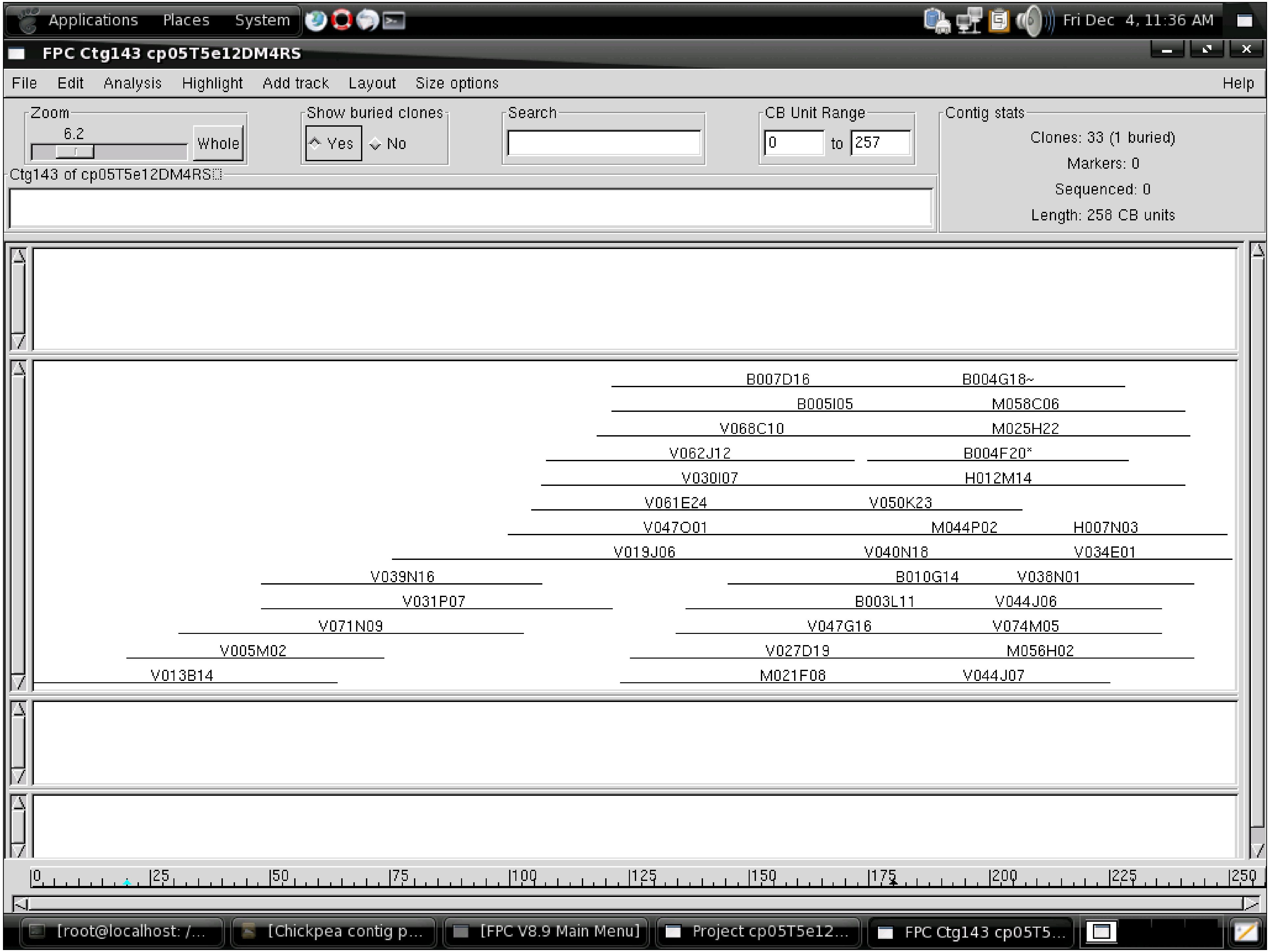
Applications Places System
Fri Dec 4, 11:36 AM
FPC Ctg143 cp05T5e12DM4RS
File Edit Analysis Highlight Add track Layout Size options Help
Zoom
6.2
Whole
Show buried clones
Yes No
Search
CB Unit Range
0 to 257
Contig stats
Clones: 33 (1 buried)
Markers: 0
Sequenced: 0
Length: 258 CB units
Ctg143 of cp05T5e12DM4RS
B007D16
B004G18~
B005I05
M058C06
V068C10
M025H22
V062J12
B004F20*
V030I07
H012M14
V061E24
V050K23
V047O01
M044P02
H007N03
V019J06
V040N18
V034E01
V039N16
B010G14
V038N01
V031P07
B003L11
V044J06
V071N09
V047G16
V074M05
V005M02
V027D19
M056H02
V013B14
M021F08
V044J07
0 25 50 75 100 125 150 175 200 225 250
[root@localhost: /...
[Chickpea contig p...
[FPC V8.9 Main Menu]
Project cp05T5e12...
FPC Ctg143 cp05T5...

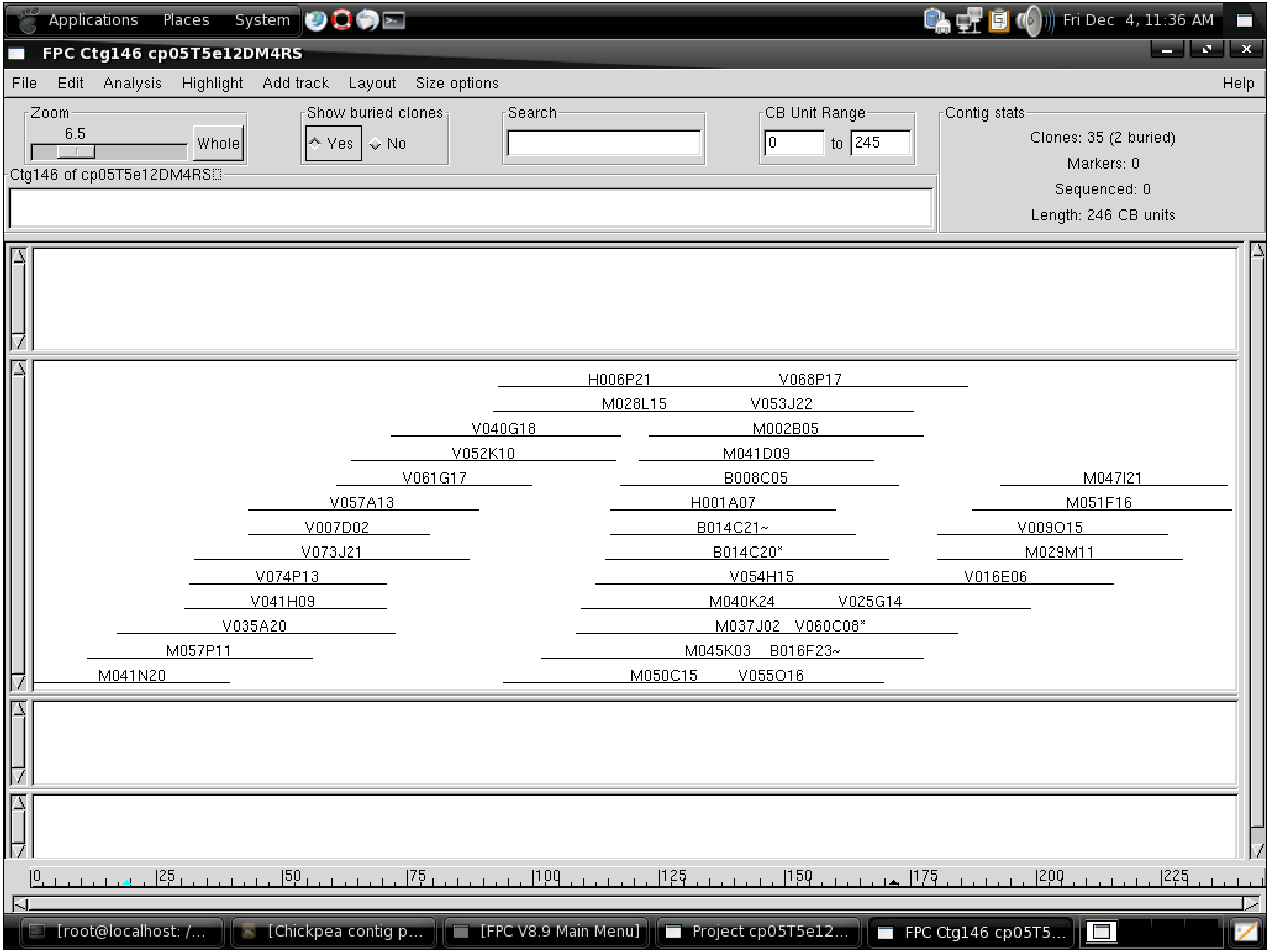
Applications Places System
Fri Dec 4, 11:36 AM
FPC Ctg146 cp05T5e12DM4RS
File Edit Analysis Highlight Add track Layout Size options Help
Zoom
6.5
Whole
Show buried clones
Yes No
Search
CB Unit Range
0 to 245
Contig stats
Clones: 35 (2 buried)
Markers: 0
Sequenced: 0
Length: 246 CB units
Ctg146 of cp05T5e12DM4RS
H006P21
V068P17
M028L15
V053J22
V040G18
M002B05
V052K10
M041D09
V061G17
B008C05
M047I21
V057A13
H001A07
M051F16
V007D02
B014C21~
V009O15
V073J21
B014C20*
M029M11
V074P13
V054H15
V016E06
V041H09
M040K24
V025G14
V035A20
M037J02
V060C08*
M057P11
M045K03
B016F23~
M041N20
M050C15
V055O16
0 25 50 75 100 125 150 175 200 225
[root@localhost: /...
[Chickpea contig p...
[FPC V8.9 Main Menu]
Project cp05T5e12...
FPC Ctg146 cp05T5...

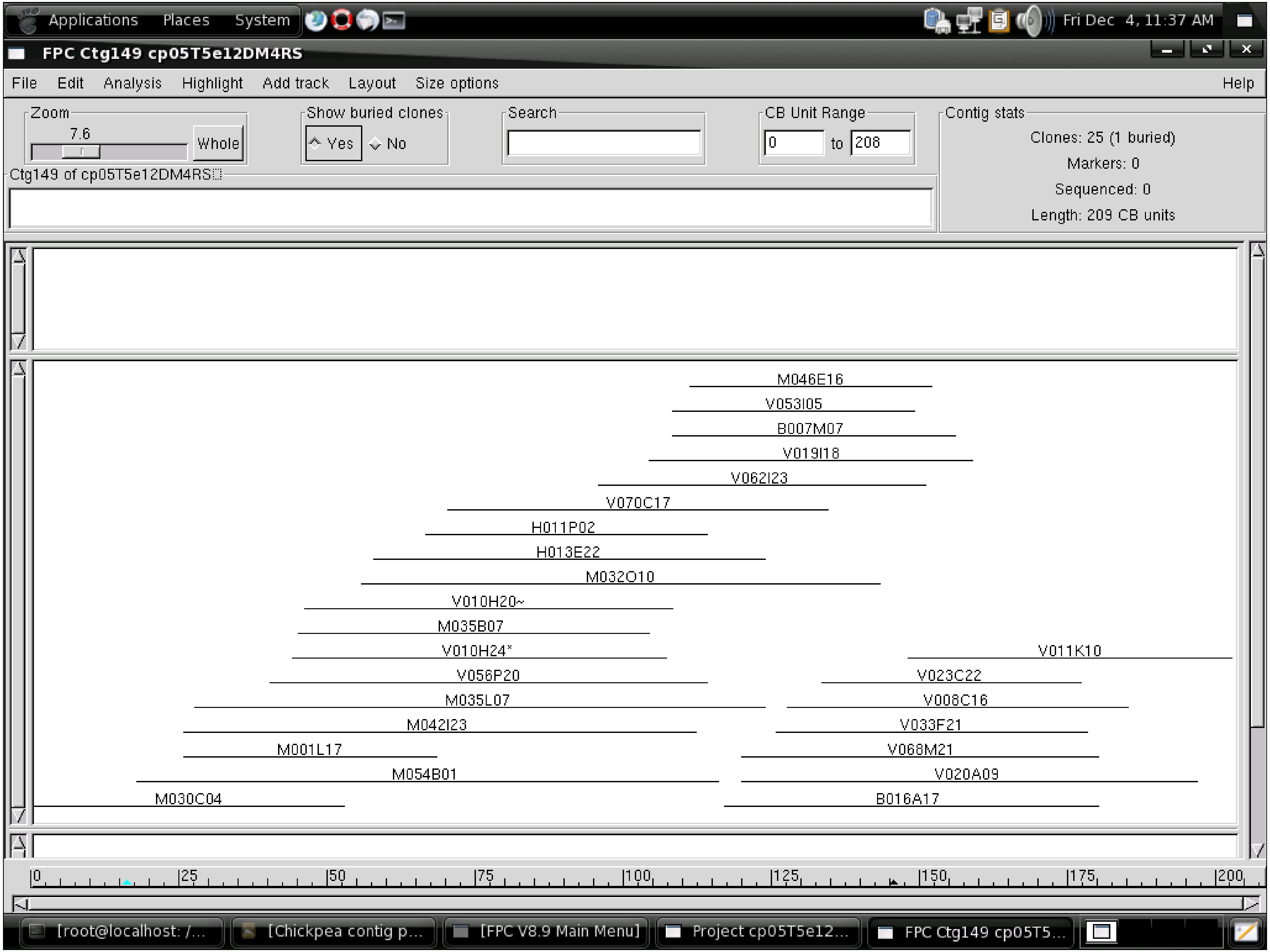

Applications Places System
Fri Dec 4, 11:37 AM
FPC Ctg149 cp05T5e12DM4RS
File Edit Analysis Highlight Add track Layout Size options Help
Zoom
7.6
Whole
Show buried clones
Yes No
Search
CB Unit Range
0 to 208
Contig stats
Clones: 25 (1 buried)
Markers: 0
Sequenced: 0
Length: 209 CB units
Ctg149 of cp05T5e12DM4RS
M046E16
V053I05
B007M07
V019I18
V062I23
V070C17
H011P02
H013E22
M032O10
V010H20~
M035B07
V010H24*
V011K10
V056P20
V023C22
M035L07
V008C16
M042I23
V033F21
M001L17
V068M21
M054B01
V020A09
M030C04
B016A17
0 25 50 75 100 125 150 175 200
[root@localhost: /...
[Chickpea contig p...
[FPC V8.9 Main Menu]
Project cp05T5e12...
FPC Ctg149 cp05T5...

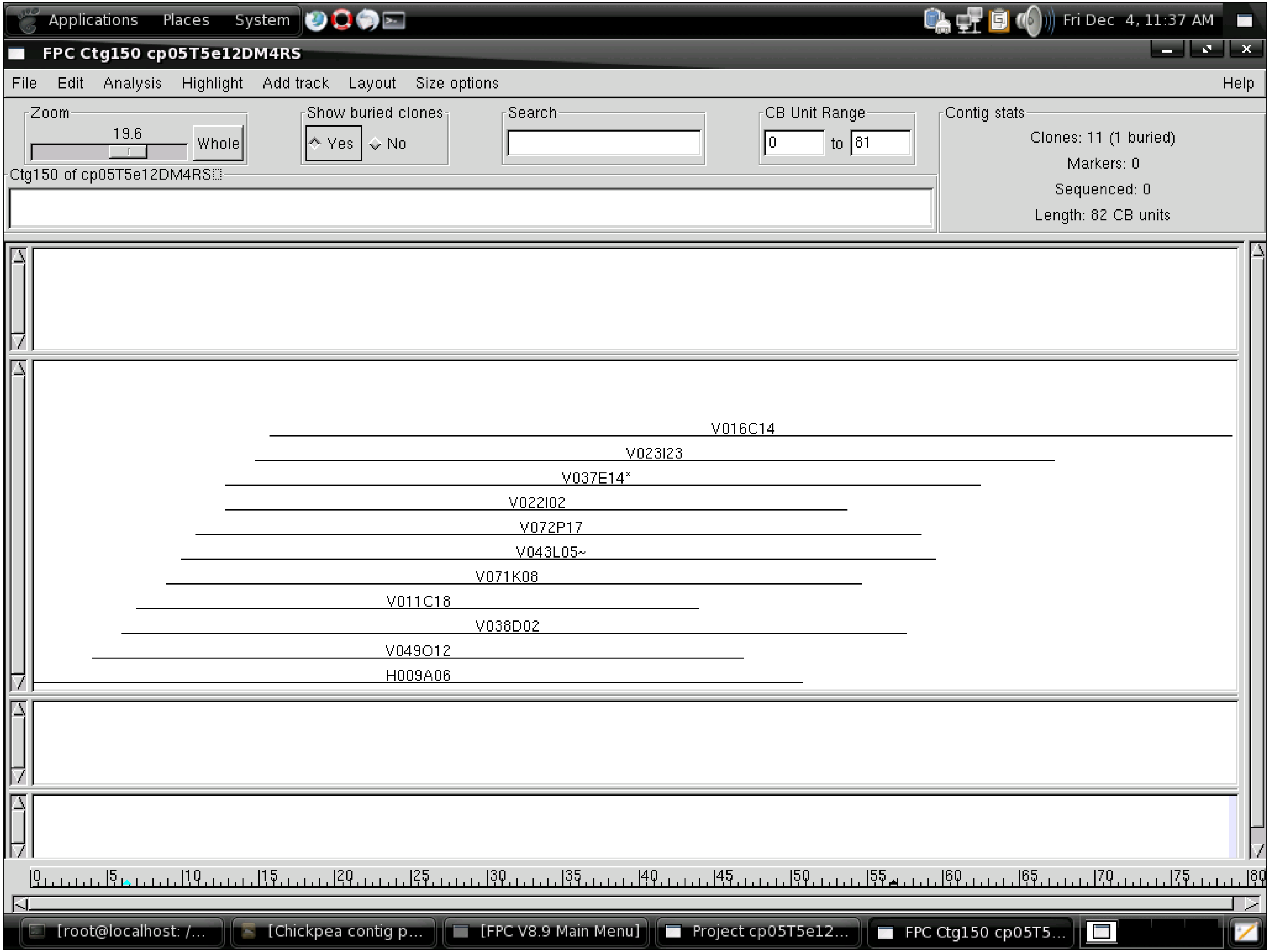
Applications
Places
System
Fri Dec 4, 11:37 AM
FPC Ctg150 cp05T5e12DM4RS
File
Edit
Analysis
Highlight
Add track
Layout
Size options
Help
Zoom
19.6
Whole
Show buried clones
Yes
No
Search
CB Unit Range
0
to
81
Contig stats
Clones: 11 (1 buried)
Markers: 0
Sequenced: 0
Length: 82 CB units
Ctg150 of cp05T5e12DM4RS
V016C14
V023I23
V037E14*
V022I02
V072P17
V043L05~
V071K08
V011C18
V038D02
V049O12
H009A06
0 5 10 15 20 25 30 35 40 45 50 55 60 65 70 75 80
[root@localhost: /...
[Chickpea contig p...
[FPC V8.9 Main Menu]
Project cp05T5e12...
FPC Ctg150 cp05T5...

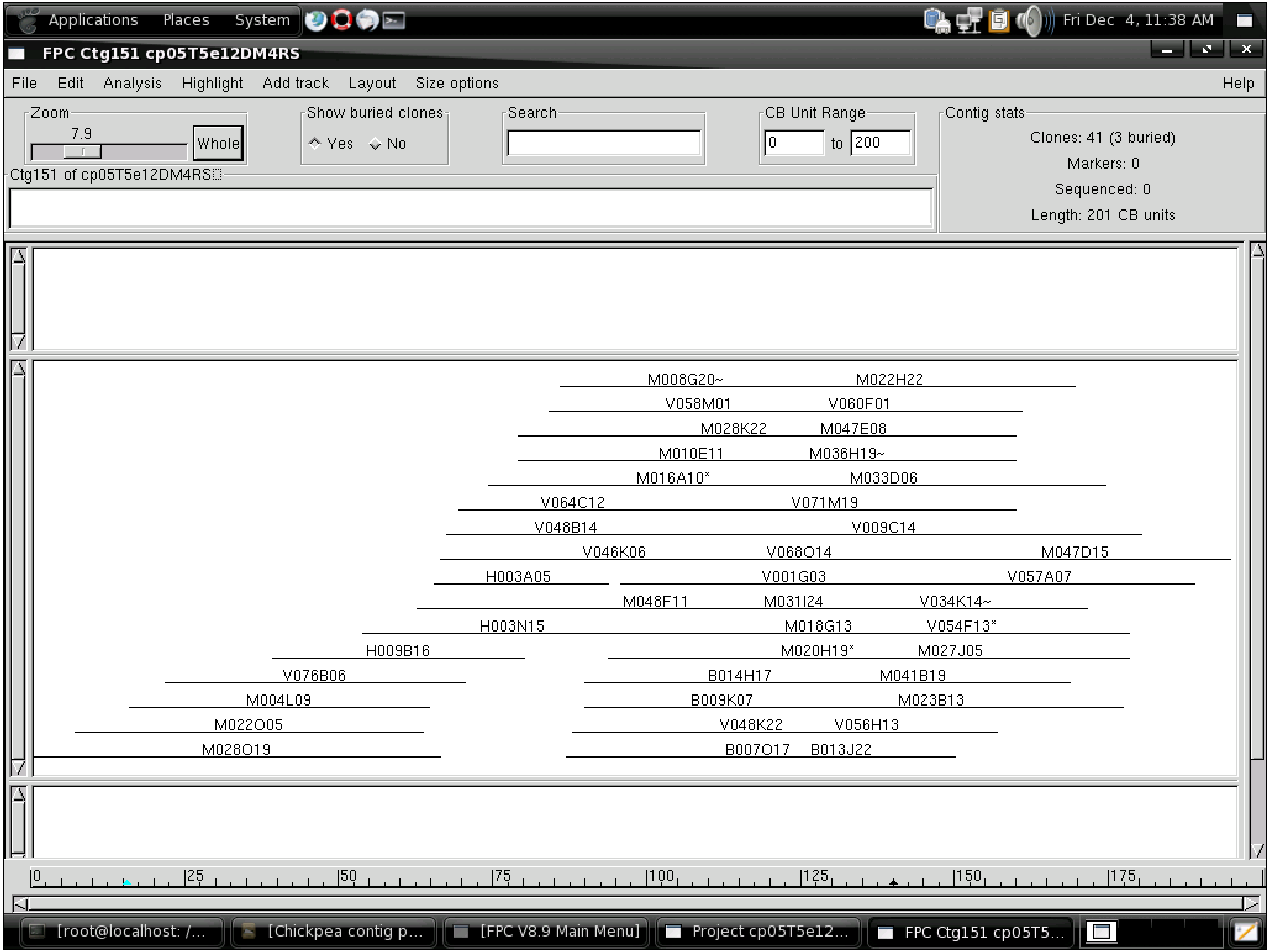
Applications
Places
System
Fri Dec 4, 11:38 AM
FPC Ctg151 cp05T5e12DM4RS
File
Edit
Analysis
Highlight
Add track
Layout
Size options
Help
Zoom
7.9
Whole
Show buried clones
Yes
No
Search
CB Unit Range
0
to
200
Contig stats
Clones: 41 (3 buried)
Markers: 0
Sequenced: 0
Length: 201 CB units
Ctg151 of cp05T5e12DM4RS
M008G20~
M022H22
V058M01
V060F01
M028K22
M047E08
M010E11
M036H19~
M016A10*
M033D06
V064C12
V071M19
V048B14
V009C14
V046K06
V068O14
M047D15
H003A05
V001G03
V057A07
M048F11
M031I24
V034K14~
H003N15
M018G13
V054F13*
H009B16
M020H19*
M027J05
V076B06
B014H17
M041B19
M004L09
B009K07
M023B13
M022O05
V048K22
V056H13
M028O19
B007O17
B013J22
0
25
50
75
100
125
150
175
[root@localhost: /...
[Chickpea contig p...
[FPC V8.9 Main Menu]
Project cp05T5e12...
FPC Ctg151 cp05T5...

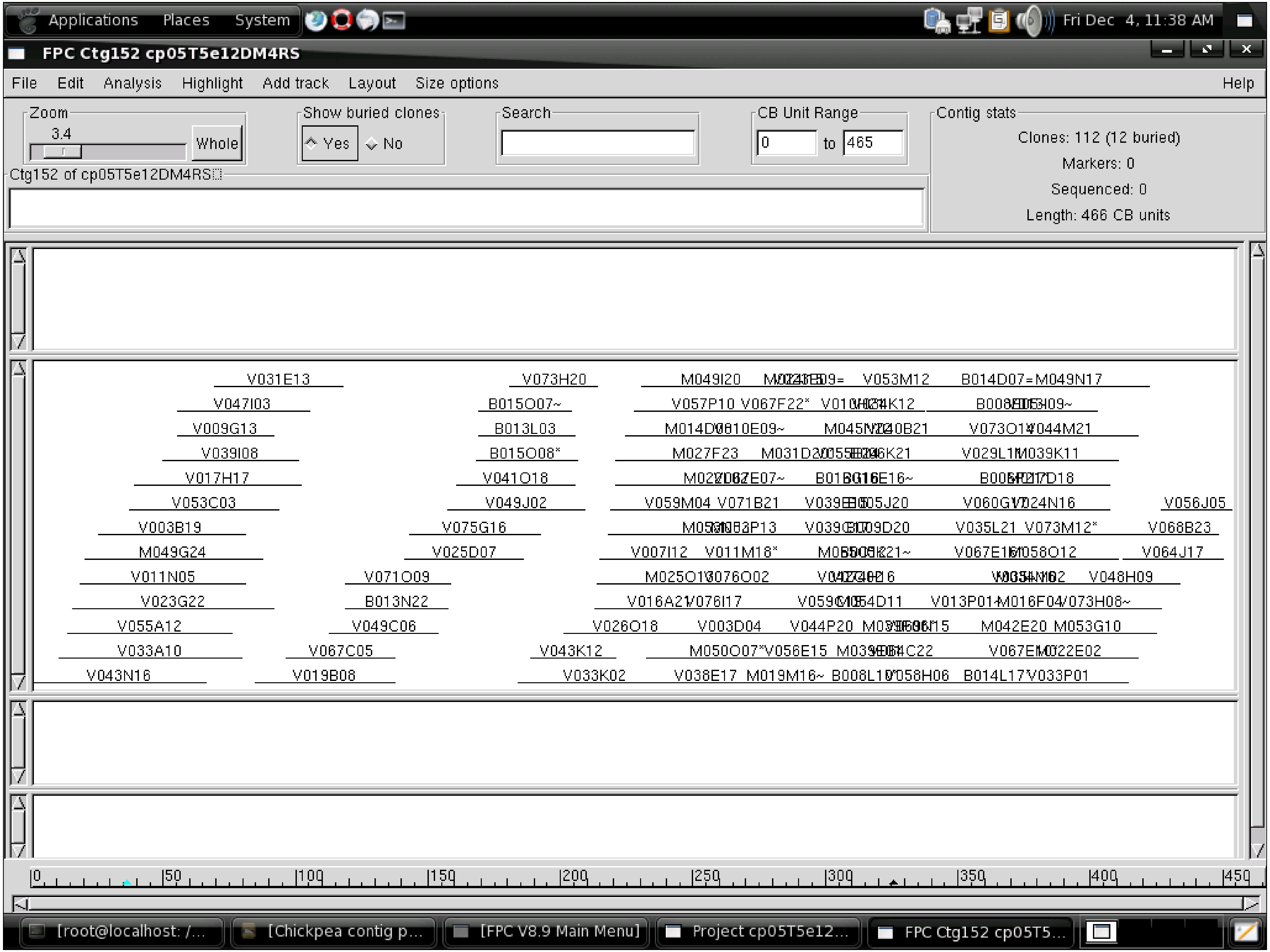
Applications Places System
Fri Dec 4, 11:38 AM
FPC Ctg152 cp05T5e12DM4RS
File Edit Analysis Highlight Add track Layout Size options Help
Zoom
3.4
Whole
Show buried clones
Yes No
Search
CB Unit Range
0 to 465
Contig stats
Clones: 112 (12 buried)
Markers: 0
Sequenced: 0
Length: 466 CB units
Ctg152 of cp05T5e12DM4RS
V031E13
V047I03
V009G13
V039I08
V017H17
V053C03
V003B19
M049G24
V011N05
V023G22
V055A12
V033A10
V043N16
V073H20
B015O07~
B013L03
B015O08*
V041O18
V049J02
V075G16
V025D07
V071O09
B013N22
V049C06
V067C05
V019B08
V043K12
V033K02
V026O18
M049I20
V053M12
B014D07=M049N17
V057P10 V067F22*
M027F23
V059M04 V071B21
V060G1
V056J05
V035L21 V073M12*
V068B23
V007I12 V011M18*
V064J17
V048H09
V003D04 V044P20
M042E20 M053G10
V038E17 M019M16~
B014L17
0 50 100 150 200 250 300 350 400 450
[root@localhost: /...
[Chickpea contig p...
[FPC V8.9 Main Menu]
Project cp05T5e12...
FPC Ctg152 cp05T5...

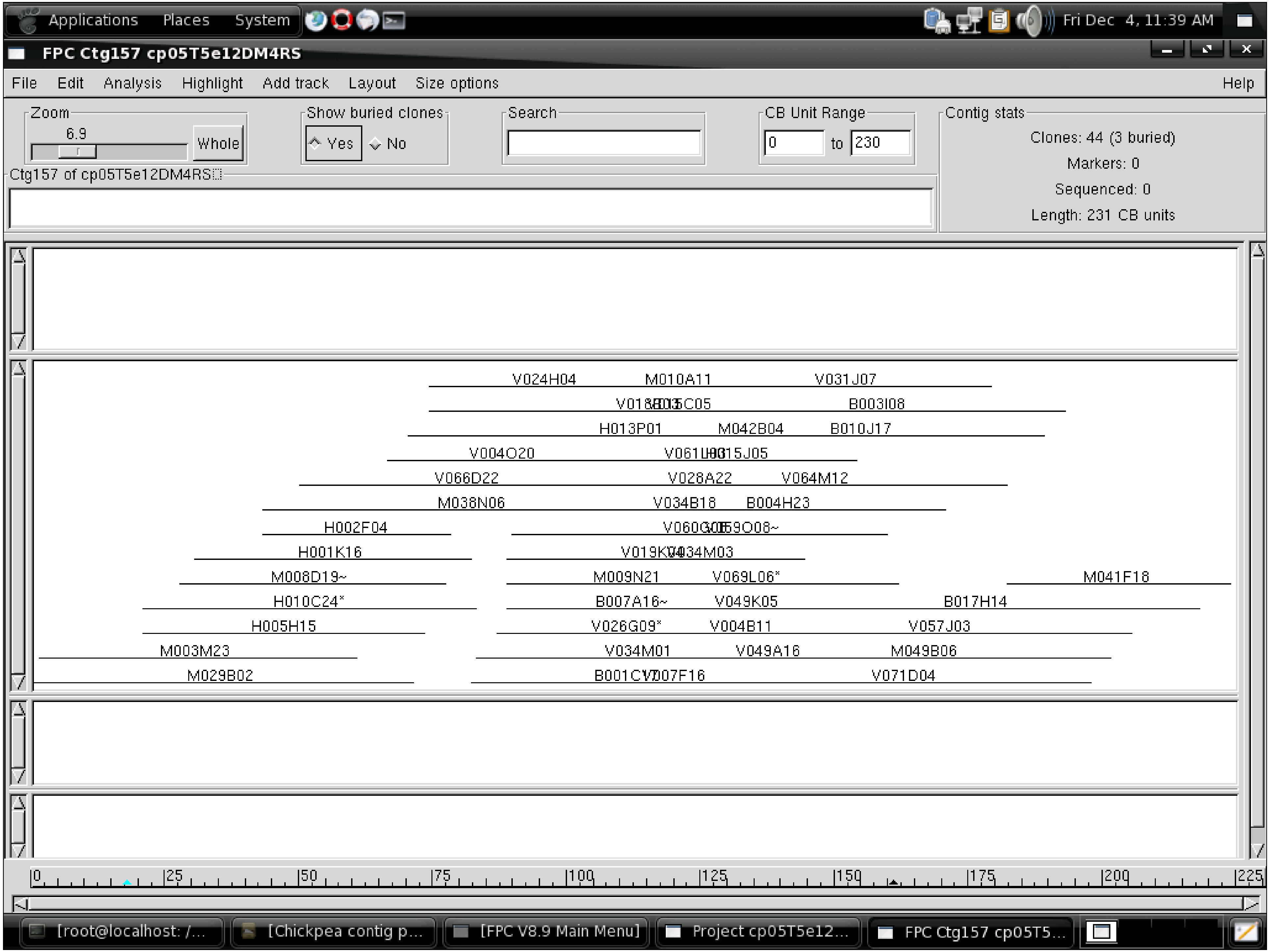

Applications
Places
System
Fri Dec 4, 11:39 AM
FPC Ctg157 cp05T5e12DM4RS
File
Edit
Analysis
Highlight
Add track
Layout
Size options
Help
Zoom
6.9
Whole
Show buried clones
Yes
No
Search
CB Unit Range
0
to
230
Contig stats
Clones: 44 (3 buried)
Markers: 0
Sequenced: 0
Length: 231 CB units
Ctg157 of cp05T5e12DM4RS
V024H04
M010A11
V031J07
B003I08
H013P01
M042B04
B010J17
V004O20
V066D22
V028A22
V064M12
M038N06
V034B18
B004H23
H002F04
H001K16
M008D19~
M009N21
V069L06*
M041F18
H010C24*
B007A16~
V049K05
B017H14
H005H15
V026G09*
V004B11
V057J03
M003M23
V034M01
V049A16
M049B06
M029B02
V071D04
0
25
50
75
100
125
150
175
200
225
[root@localhost: /...
[Chickpea contig p...
[FPC V8.9 Main Menu]
Project cp05T5e12...
FPC Ctg157 cp05T5...

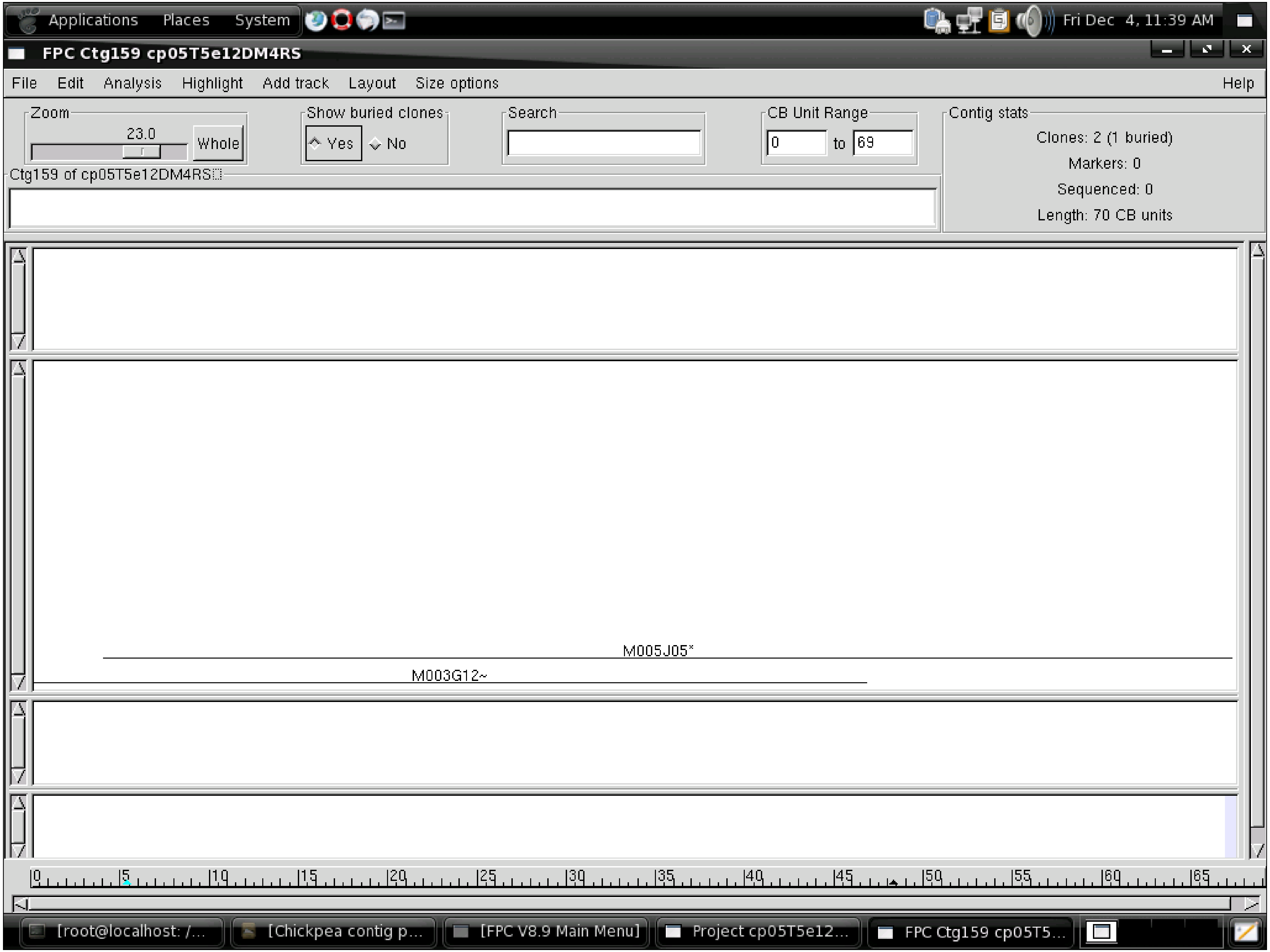
Applications Places System
Fri Dec 4, 11:39 AM
FPC Ctg159 cp05T5e12DM4RS
File Edit Analysis Highlight Add track Layout Size options Help
Zoom
23.0
Whole
Show buried clones
Yes
No
Search
CB Unit Range
0
to
69
Contig stats
Clones: 2 (1 buried)
Markers: 0
Sequenced: 0
Length: 70 CB units
Ctg159 of cp05T5e12DM4RS
M005J05*
M003G12~
0 5 10 15 20 25 30 35 40 45 50 55 60 65
[root@localhost: /...
[Chickpea contig p...
[FPC V8.9 Main Menu]
Project cp05T5e12...
FPC Ctg159 cp05T5...

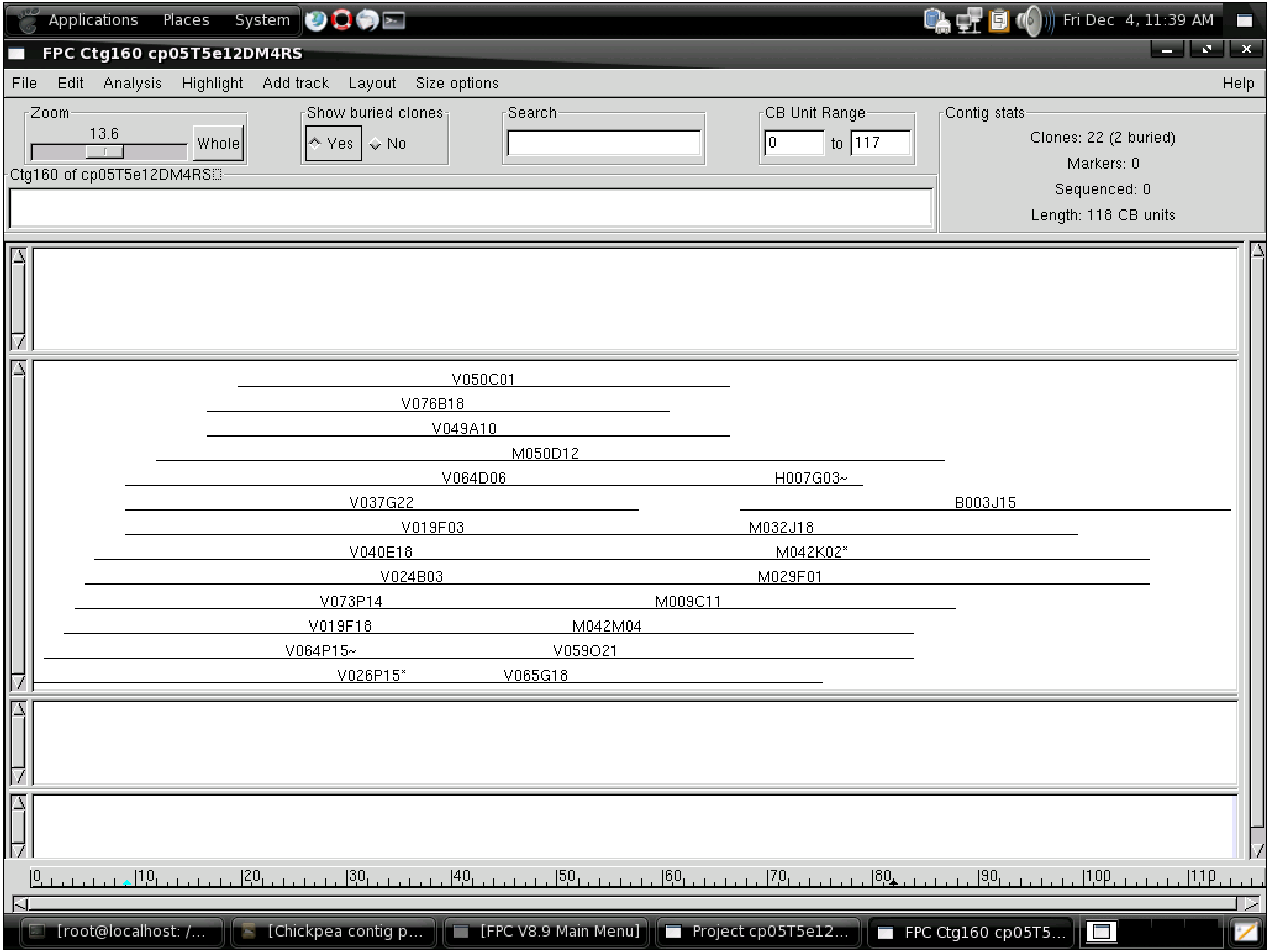
Applications Places System
Fri Dec 4, 11:39 AM
FPC Ctg160 cp05T5e12DM4RS
File Edit Analysis Highlight Add track Layout Size options Help
Zoom
13.6
Whole
Show buried clones
Yes
No
Search
CB Unit Range
0
to
117
Contig stats
Clones: 22 (2 buried)
Markers: 0
Sequenced: 0
Length: 118 CB units
Ctg160 of cp05T5e12DM4RS
V050C01
V076B18
V049A10
M050D12
V064D06
H007G03~
V037G22
B003J15
V019F03
M032J18
V040E18
M042K02*
V024B03
M029F01
V073P14
M009C11
V019F18
M042M04
V064P15~
V059O21
V026P15*
V065G18
0 10 20 30 40 50 60 70 80 90 100 110
[root@localhost: /...
[Chickpea contig p...
[FPC V8.9 Main Menu]
Project cp05T5e12...
FPC Ctg160 cp05T5...

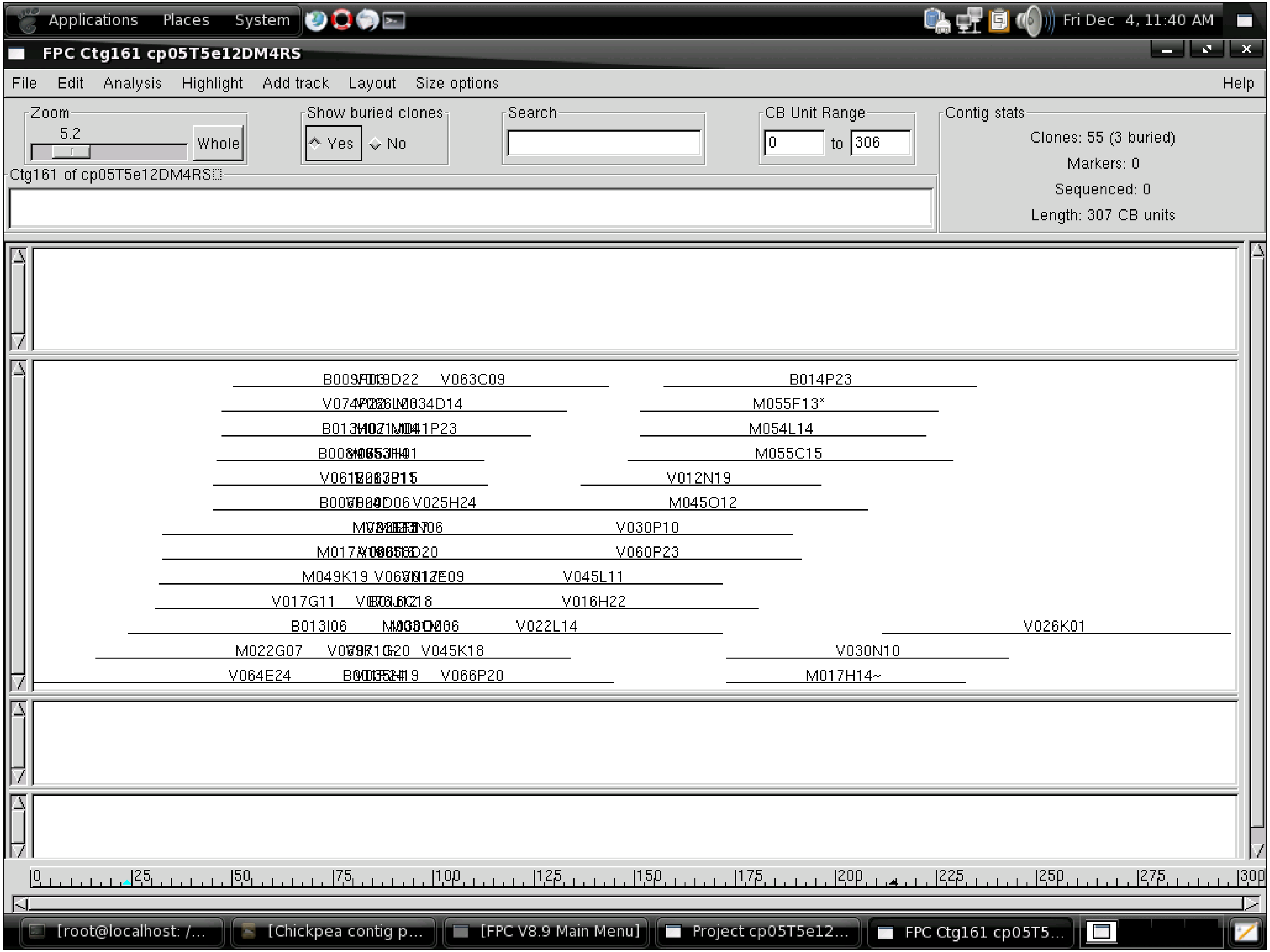
Applications Places System
Fri Dec 4, 11:40 AM
FPC Ctg161 cp05T5e12DM4RS
File Edit Analysis Highlight Add track Layout Size options Help
Zoom
5.2
Whole
Show buried clones
Yes
No
Search
CB Unit Range
0
to
306
Contig stats
Clones: 55 (3 buried)
Markers: 0
Sequenced: 0
Length: 307 CB units
Ctg161 of cp05T5e12DM4RS
V063C09
B014P23
M055F13*
M054L14
M055C15
V012N19
V025H24
M045O12
V030P10
V060P23
M049K19
V045L11
V017G11
V016H22
B013I06
V022L14
V026K01
M022G07
V045K18
V030N10
V064E24
V066P20
M017H14~
0 25 50 75 100 125 150 175 200 225 250 275 300
[root@localhost: /...
[Chickpea contig p...
[FPC V8.9 Main Menu]
Project cp05T5e12...
FPC Ctg161 cp05T5...

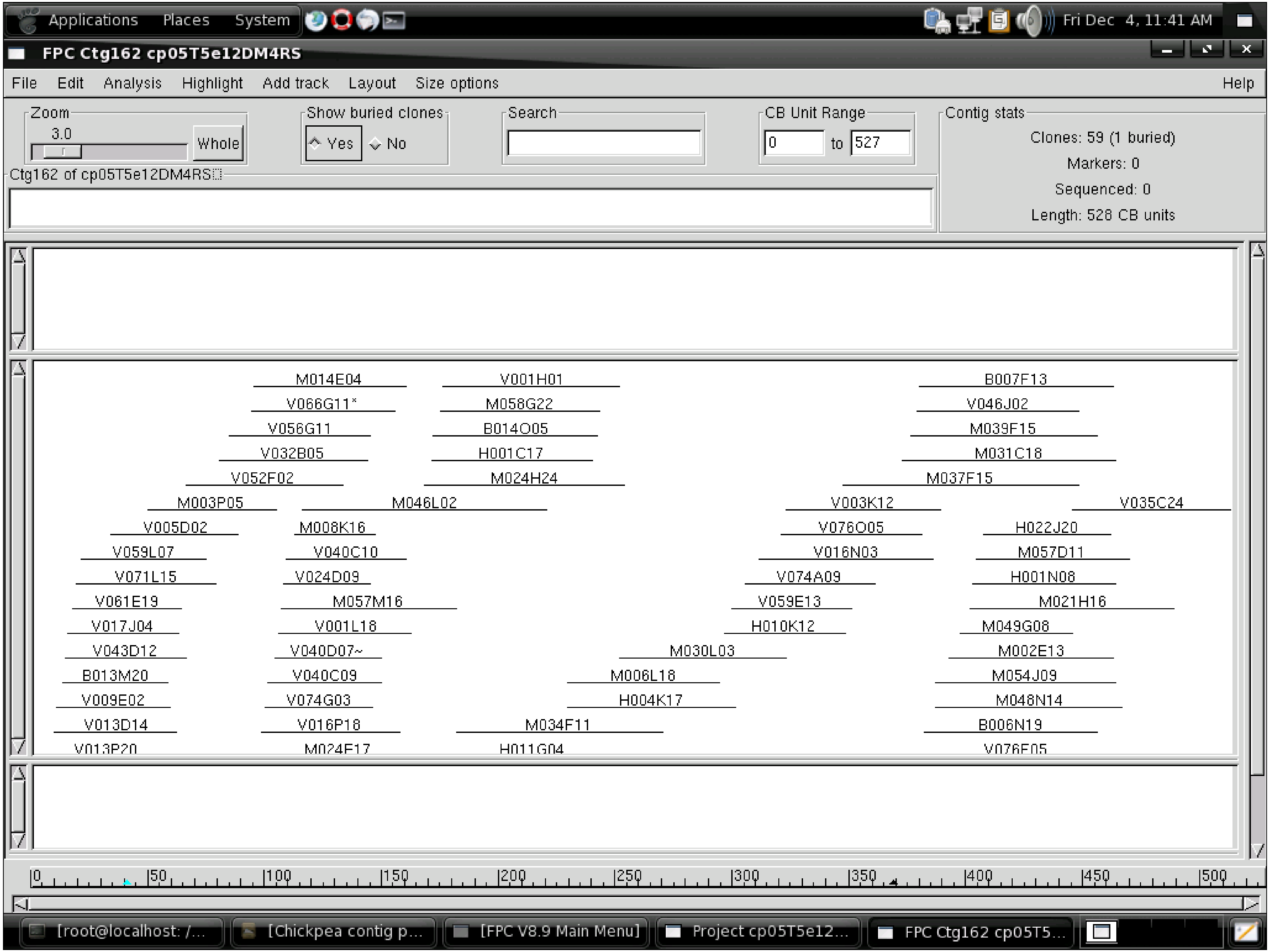
Applications Places System
Fri Dec 4, 11:41 AM
FPC Ctg162 cp05T5e12DM4RS
File Edit Analysis Highlight Add track Layout Size options Help
Zoom
3.0
Whole
Show buried clones
Yes
No
Search
CB Unit Range
0 to 527
Contig stats
Clones: 59 (1 buried)
Markers: 0
Sequenced: 0
Length: 528 CB units
Ctg162 of cp05T5e12DM4RS
M014E04
V001H01
B007F13
V066G11*
M058G22
V046J02
V056G11
B014O05
M039F15
V032B05
H001C17
M031C18
V052F02
M024H24
M037F15
M003P05
M046L02
V003K12
V035C24
V005D02
M008K16
V076O05
H022J20
V059L07
V040C10
V016N03
M057D11
V071L15
V024D09
V074A09
H001N08
V061E19
M057M16
V059E13
M021H16
V017J04
V001L18
H010K12
M049G08
V043D12
V040D07~
M030L03
M002E13
B013M20
V040C09
M006L18
M054J09
V009E02
V074G03
H004K17
M048N14
V013D14
V016P18
M034F11
B006N19
V013P20
M024F17
H011G04
V076F05
0 50 100 150 200 250 300 350 400 450 500
[root@localhost: /...
[Chickpea contig p...
[FPC V8.9 Main Menu]
Project cp05T5e12...
FPC Ctg162 cp05T5...

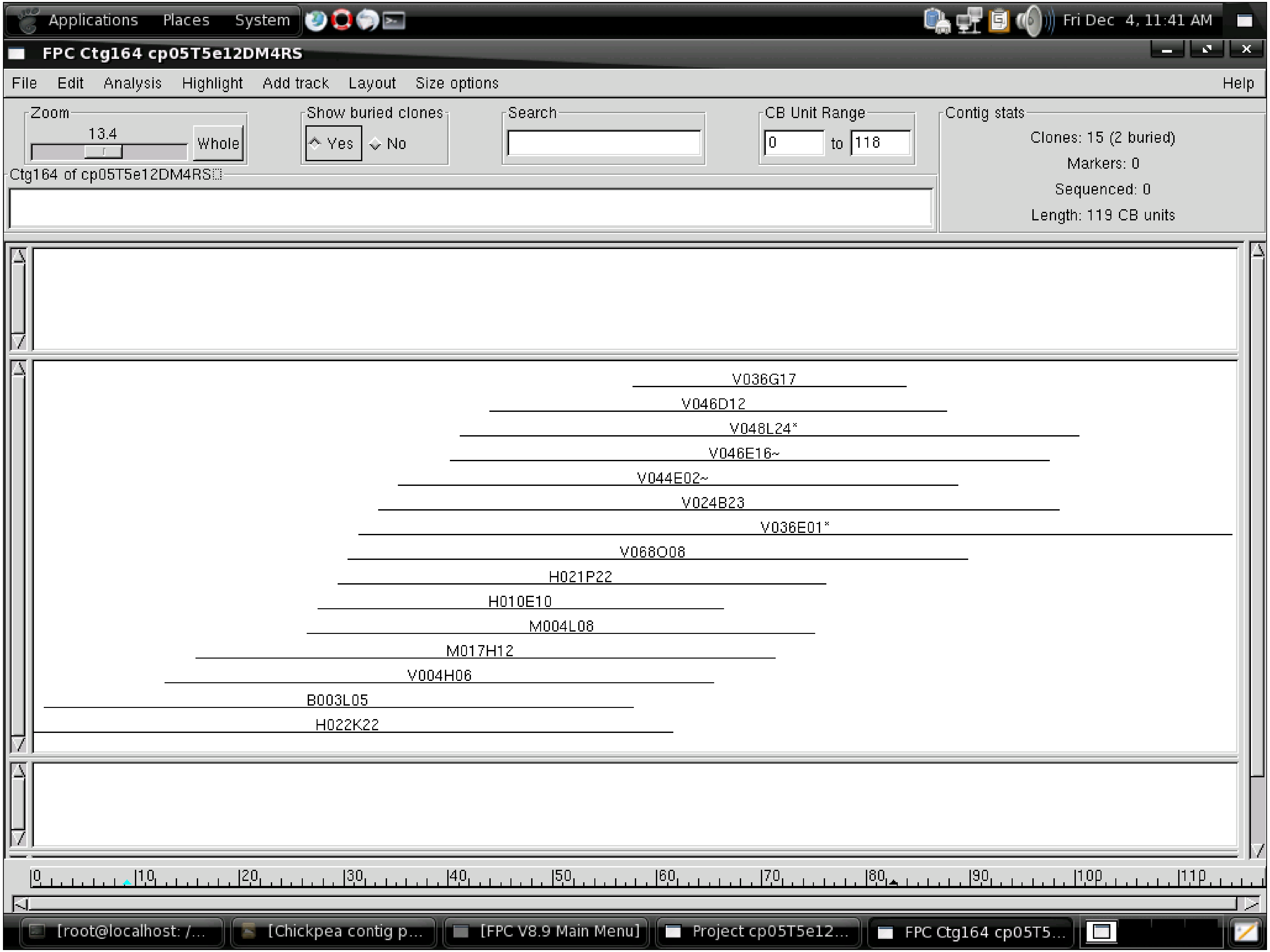
Applications
Places
System
Fri Dec 4, 11:41 AM
FPC Ctg164 cp05T5e12DM4RS
File
Edit
Analysis
Highlight
Add track
Layout
Size options
Help
Zoom
13.4
Whole
Show buried clones
Yes
No
Search
CB Unit Range
0
to
118
Contig stats
Clones: 15 (2 buried)
Markers: 0
Sequenced: 0
Length: 119 CB units
Ctg164 of cp05T5e12DM4RS
V036G17
V046D12
V048L24*
V046E16~
V044E02~
V024B23
V036E01*
V068O08
H021P22
H010E10
M004L08
M017H12
V004H06
B003L05
H022K22
0
10
20
30
40
50
60
70
80
90
100
110
[root@localhost: /...
[Chickpea contig p...
[FPC V8.9 Main Menu]
Project cp05T5e12...
FPC Ctg164 cp05T5...

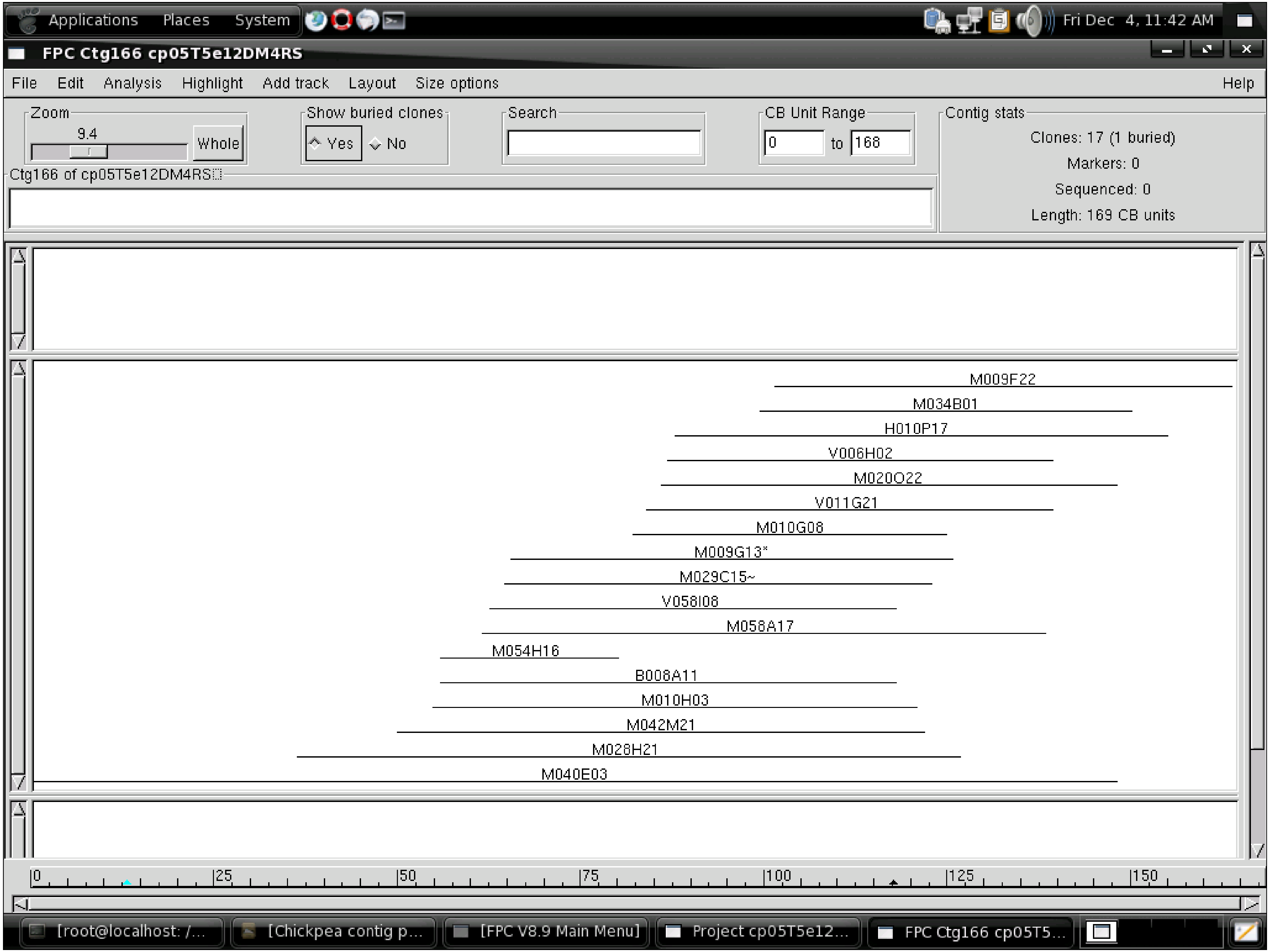
Applications Places System
Fri Dec 4, 11:42 AM
FPC Ctg166 cp05T5e12DM4RS
File Edit Analysis Highlight Add track Layout Size options Help
Zoom
9.4
Whole
Show buried clones
Yes No
Search
CB Unit Range
0 to 168
Contig stats
Clones: 17 (1 buried)
Markers: 0
Sequenced: 0
Length: 169 CB units
Ctg166 of cp05T5e12DM4RS
M009F22
M034B01
H010P17
V006H02
M020O22
V011G21
M010G08
M009G13*
M029C15~
V058I08
M058A17
M054H16
B008A11
M010H03
M042M21
M028H21
M040E03
0 25 50 75 100 125 150
[root@localhost: /...
[Chickpea contig p...
[FPC V8.9 Main Menu]
Project cp05T5e12...
FPC Ctg166 cp05T5...

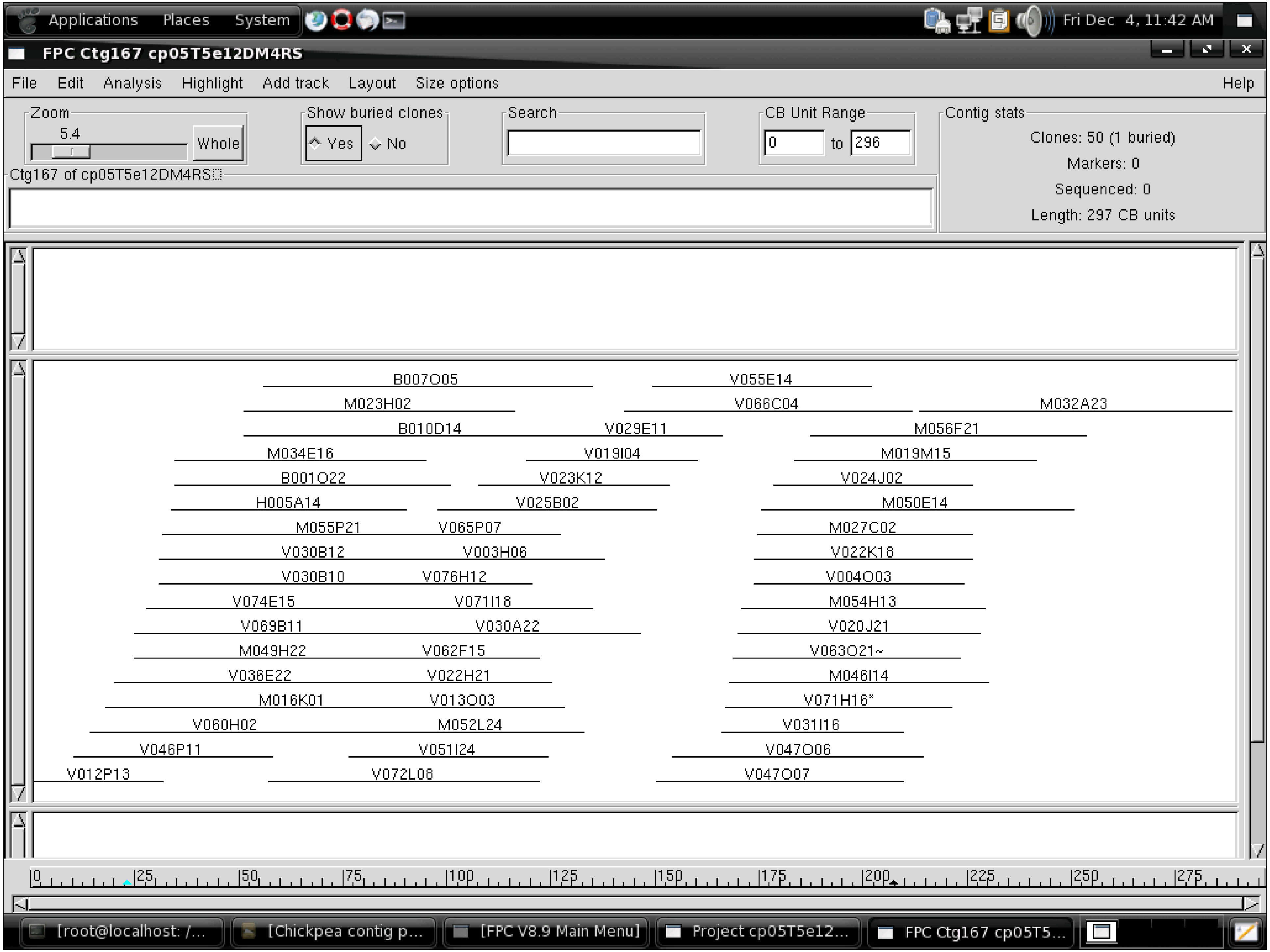
Applications Places System
Fri Dec 4, 11:42 AM
FPC Ctg167 cp05T5e12DM4RS
File Edit Analysis Highlight Add track Layout Size options Help
Zoom
5.4
Whole
Show buried clones
Yes No
Search
CB Unit Range
0 to 296
Contig stats
Clones: 50 (1 buried)
Markers: 0
Sequenced: 0
Length: 297 CB units
Ctg167 of cp05T5e12DM4RS
B007O05
V055E14
M023H02
V066C04
M032A23
B010D14
V029E11
M056F21
M034E16
V019I04
M019M15
B001O22
V023K12
V024J02
H005A14
V025B02
M050E14
M055P21
V065P07
M027C02
V030B12
V003H06
V022K18
V030B10
V076H12
V004O03
V074E15
V071I18
M054H13
V069B11
V030A22
V020J21
M049H22
V062F15
V063O21~
V036E22
V022H21
M046I14
M016K01
V013O03
V071H16*
V060H02
M052L24
V031I16
V046P11
V051I24
V047O06
V012P13
V072L08
V047O07
0 25 50 75 100 125 150 175 200 225 250 275
[root@localhost: /...
[Chickpea contig p...
[FPC V8.9 Main Menu]
Project cp05T5e12...
FPC Ctg167 cp05T5...

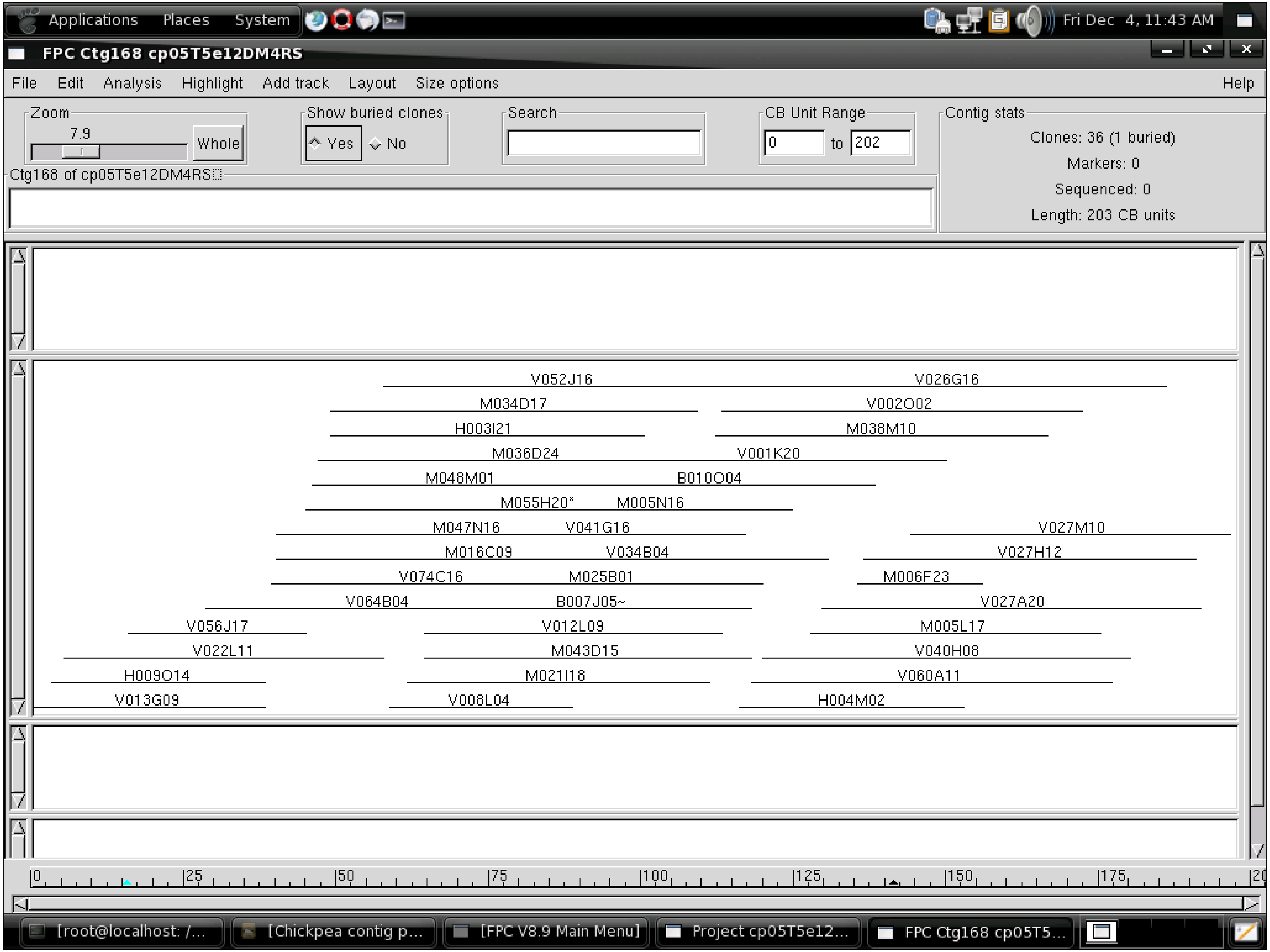
Applications Places System
Fri Dec 4, 11:43 AM
FPC Ctg168 cp05T5e12DM4RS
File Edit Analysis Highlight Add track Layout Size options Help
Zoom
7.9
Whole
Show buried clones
Yes No
Search
CB Unit Range
0 to 202
Contig stats
Clones: 36 (1 buried)
Markers: 0
Sequenced: 0
Length: 203 CB units
Ctg168 of cp05T5e12DM4RS
V052J16
V026G16
M034D17
V002O02
H003I21
M038M10
M036D24
V001K20
M048M01
B010O04
M055H20*
M005N16
M047N16
V041G16
V027M10
M016C09
V034B04
V027H12
V074C16
M025B01
M006F23
V064B04
B007J05~
V027A20
V056J17
V012L09
M005L17
V022L11
M043D15
V040H08
H009O14
M021I18
V060A11
V013G09
V008L04
H004M02
0 25 50 75 100 125 150 175
[root@localhost: /...
[Chickpea contig p...
[FPC V8.9 Main Menu]
Project cp05T5e12...
FPC Ctg168 cp05T5...

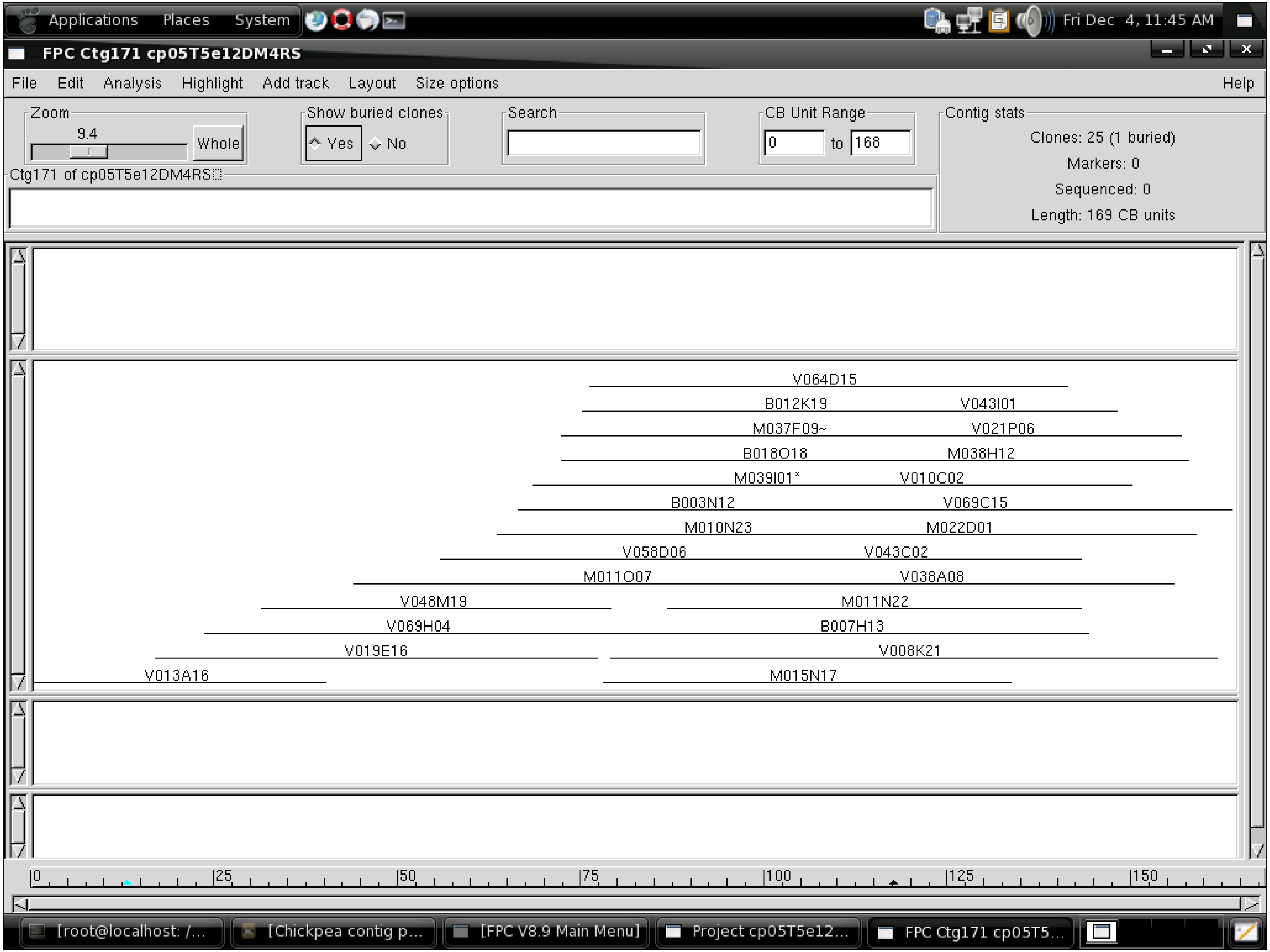
Applications Places System
Fri Dec 4, 11:45 AM
FPC Ctg171 cp05T5e12DM4RS
File Edit Analysis Highlight Add track Layout Size options Help
Zoom
9.4
Whole
Show buried clones
Yes No
Search
CB Unit Range
0 to 168
Contig stats
Clones: 25 (1 buried)
Markers: 0
Sequenced: 0
Length: 169 CB units
Ctg171 of cp05T5e12DM4RS
V064D15
B012K19
V043I01
M037F09~
V021P06
B018O18
M038H12
M039I01*
V010C02
B003N12
V069C15
M010N23
M022D01
V058D06
V043C02
M011O07
V038A08
V048M19
M011N22
V069H04
B007H13
V019E16
V008K21
V013A16
M015N17
0
25
50
75
100
125
150
[root@localhost: /...
[Chickpea contig p...
[FPC V8.9 Main Menu]
Project cp05T5e12...
FPC Ctg171 cp05T5...

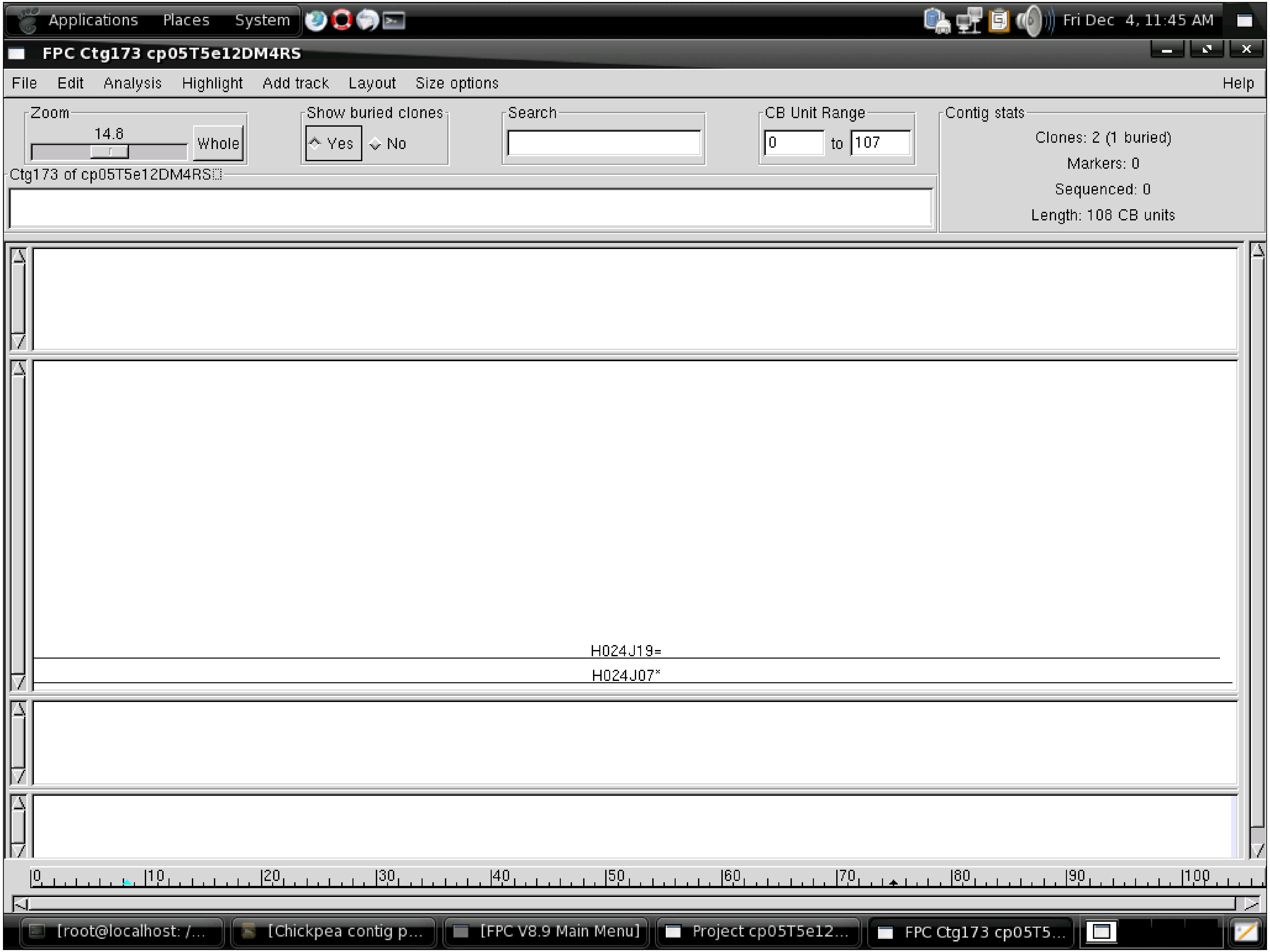

Applications Places System
Fri Dec 4, 11:45 AM
FPC Ctg173 cp05T5e12DM4RS
File Edit Analysis Highlight Add track Layout Size options Help
Zoom
14.8
Whole
Show buried clones
Yes No
Search
CB Unit Range
0 to 107
Contig stats
Clones: 2 (1 buried)
Markers: 0
Sequenced: 0
Length: 108 CB units
Ctg173 of cp05T5e12DM4RS
H024J19=
H024J07*
0 10 20 30 40 50 60 70 80 90 100
[root@localhost: /...
[Chickpea contig p...
[FPC V8.9 Main Menu]
Project cp05T5e12...
FPC Ctg173 cp05T5...

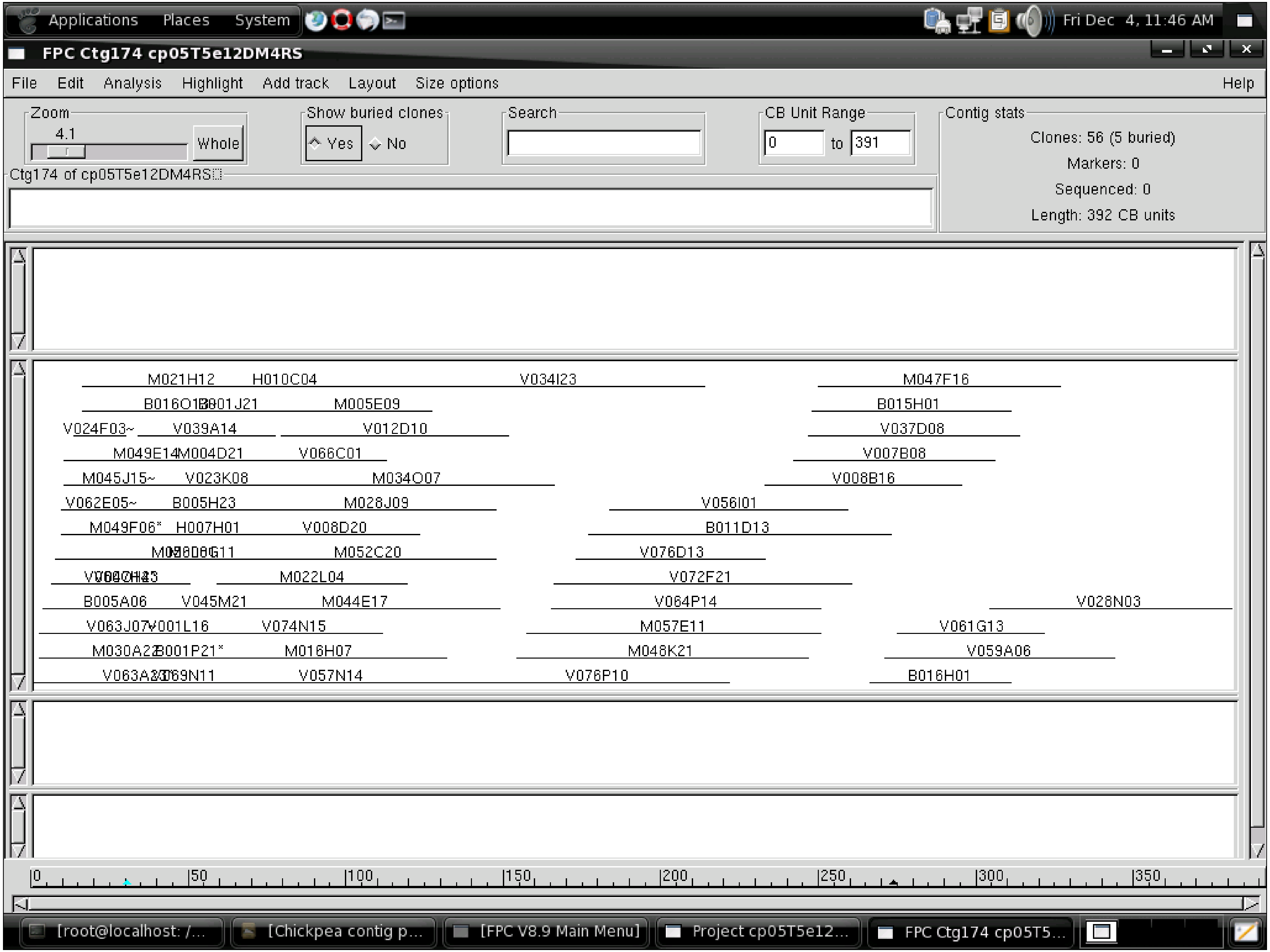
Applications Places System
Fri Dec 4, 11:46 AM
FPC Ctg174 cp05T5e12DM4RS
File Edit Analysis Highlight Add track Layout Size options Help
Zoom
4.1
Whole
Show buried clones
Yes No
Search
CB Unit Range
0 to 391
Contig stats
Clones: 56 (5 buried)
Markers: 0
Sequenced: 0
Length: 392 CB units
Ctg174 of cp05T5e12DM4RS
M021H12 H010C04 V034I23 M047F16
B015H01
V024F03~ V039A14 V012D10 V037D08
M049E14M004D21 V066C01 V007B08
M045J15~ V023K08 M034O07 V008B16
V062E05~ B005H23 M028J09 V056I01
M049F06* H007H01 V008D20 B011D13
M052C20 V076D13
M022L04 V072F21
B005A06 V045M21 M044E17 V064P14 V028N03
V074N15 M057E11 V061G13
M016H07 M048K21 V059A06
V057N14 V076P10 B016H01
M005E09
0 50 100 150 200 250 300 350
[root@localhost: /... [Chickpea contig p... [FPC V8.9 Main Menu] Project cp05T5e12... FPC Ctg174 cp05T5...

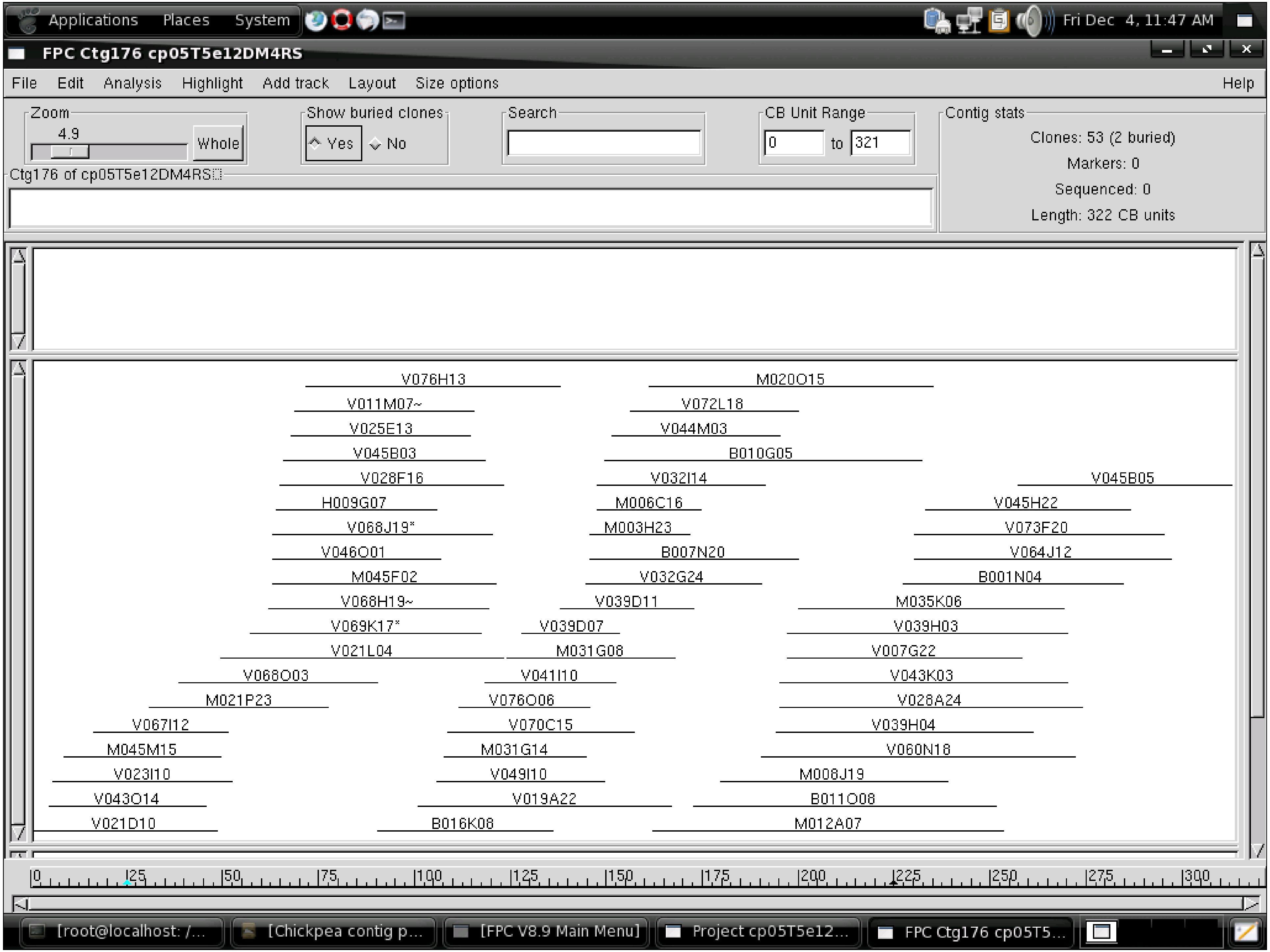
Applications Places System
Fri Dec 4, 11:47 AM
FPC Ctg176 cp05T5e12DM4RS
File Edit Analysis Highlight Add track Layout Size options Help
Zoom
4.9
Whole
Show buried clones
Yes No
Search
CB Unit Range
0 to 321
Contig stats
Clones: 53 (2 buried)
Markers: 0
Sequenced: 0
Length: 322 CB units
Ctg176 of cp05T5e12DM4RS
V076H13
M020O15
V011M07~
V072L18
V025E13
V044M03
V045B03
B010G05
V028F16
V032I14
V045B05
H009G07
M006C16
V045H22
V068J19*
M003H23
V073F20
V046O01
B007N20
V064J12
M045F02
V032G24
B001N04
V068H19~
V039D11
M035K06
V069K17*
V039D07
V039H03
V021L04
M031G08
V007G22
V068O03
V041I10
V043K03
M021P23
V076O06
V028A24
V067I12
V070C15
V039H04
M045M15
M031G14
V060N18
V023I10
V049I10
M008J19
V043O14
V019A22
B011O08
V021D10
B016K08
M012A07
0 25 50 75 100 125 150 175 200 225 250 275 300
[root@localhost: /...
[Chickpea contig p...
[FPC V8.9 Main Menu]
Project cp05T5e12...
FPC Ctg176 cp05T5...

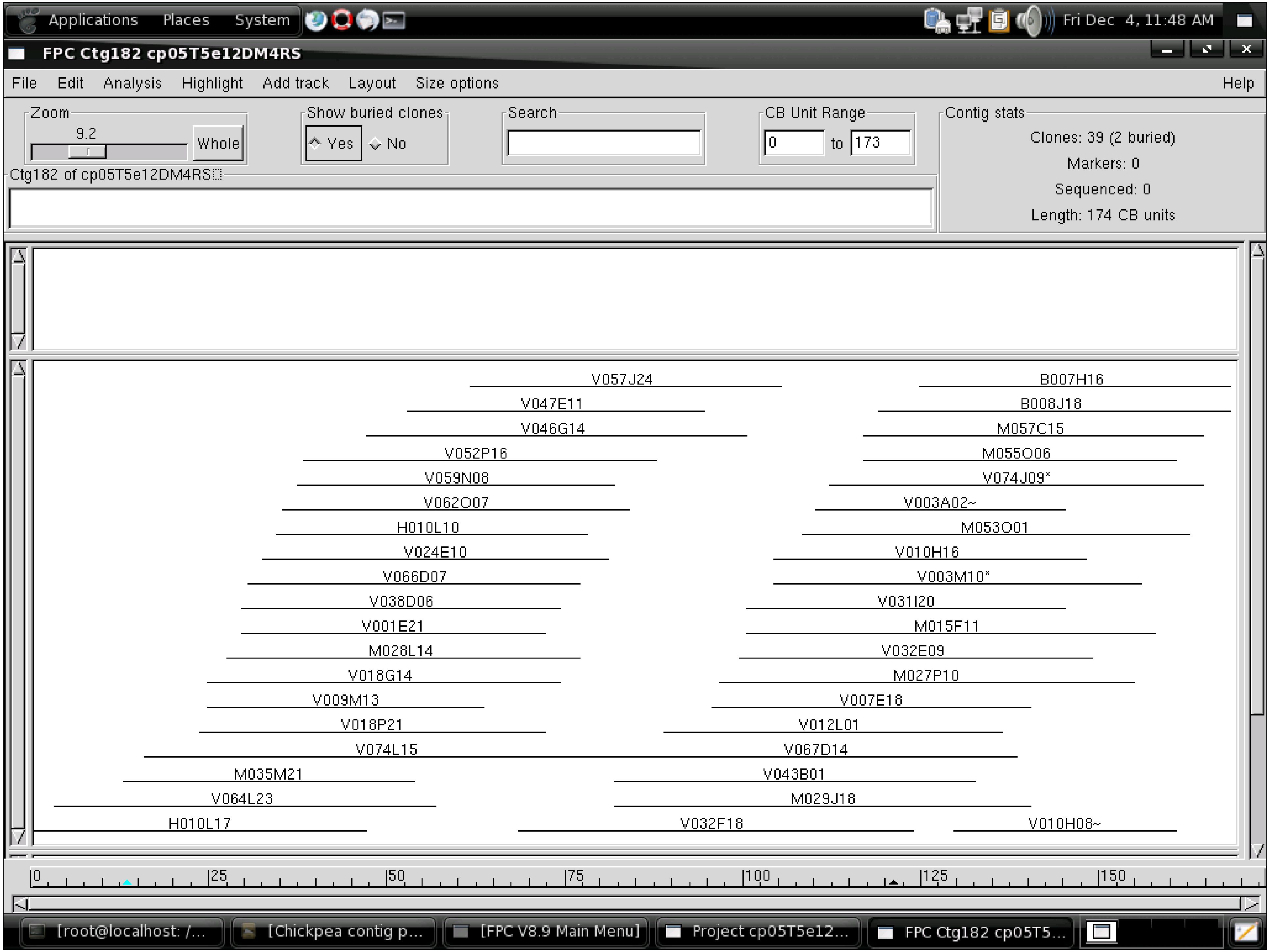
Applications Places System
Fri Dec 4, 11:48 AM
FPC Ctg182 cp05T5e12DM4RS
File Edit Analysis Highlight Add track Layout Size options Help
Zoom
9.2
Whole
Show buried clones
Yes No
Search
CB Unit Range
0 to 173
Contig stats
Clones: 39 (2 buried)
Markers: 0
Sequenced: 0
Length: 174 CB units
Ctg182 of cp05T5e12DM4RS
V057J24
B007H16
V047E11
B008J18
V046G14
M057C15
V052P16
M055O06
V059N08
V074J09*
V062O07
V003A02~
H010L10
M053O01
V024E10
V010H16
V066D07
V003M10*
V038D06
V031I20
V001E21
M015F11
M028L14
V032E09
V018G14
M027P10
V009M13
V007E18
V018P21
V012L01
V074L15
V067D14
M035M21
V043B01
V064L23
M029J18
H010L17
V032F18
V010H08~
0 25 50 75 100 125 150
[root@localhost: /...
[Chickpea contig p...
[FPC V8.9 Main Menu]
Project cp05T5e12...
FPC Ctg182 cp05T5...

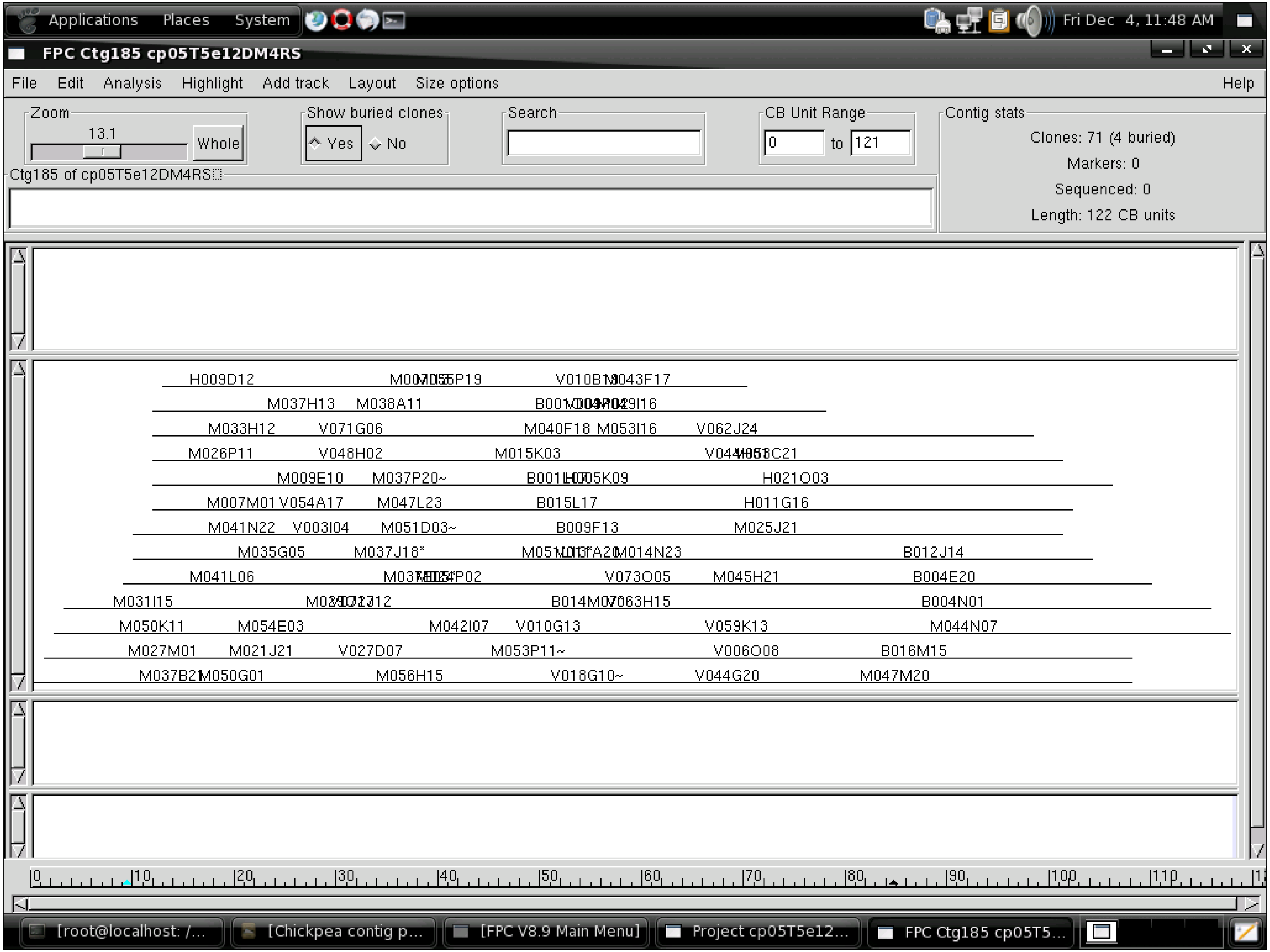
Applications Places System
Fri Dec 4, 11:48 AM
FPC Ctg185 cp05T5e12DM4RS
File Edit Analysis Highlight Add track Layout Size options Help
Zoom
13.1
Whole
Show buried clones
Yes
No
Search
CB Unit Range
0 to 121
Contig stats
Clones: 71 (4 buried)
Markers: 0
Sequenced: 0
Length: 122 CB units
Ctg185 of cp05T5e12DM4RS
H009D12
V010B1
M037H13 M038A11
M033H12 V071G06 M040F18 M053I16 V062J24
M026P11 V048H02 M015K03
M009E10 M037P20~ H021O03
M007M01 V054A17 M047L23 B015L17 H011G16
M041N22 V003I04 M051D03~ B009F13 M025J21
M035G05 M037J18* M014N23 B012J14
M041L06 V073O05 M045H21 B004E20
M031I15 B004N01
M050K11 M054E03 M042I07 V010G13 V059K13 M044N07
M027M01 M021J21 V027D07 M053P11~ V006O08 B016M15
M037B21 M050G01 M056H15 V018G10~ V044G20 M047M20
0 10 20 30 40 50 60 70 80 90 100 110
[root@localhost: /...
[Chickpea contig p...
[FPC V8.9 Main Menu]
Project cp05T5e12...
FPC Ctg185 cp05T5...

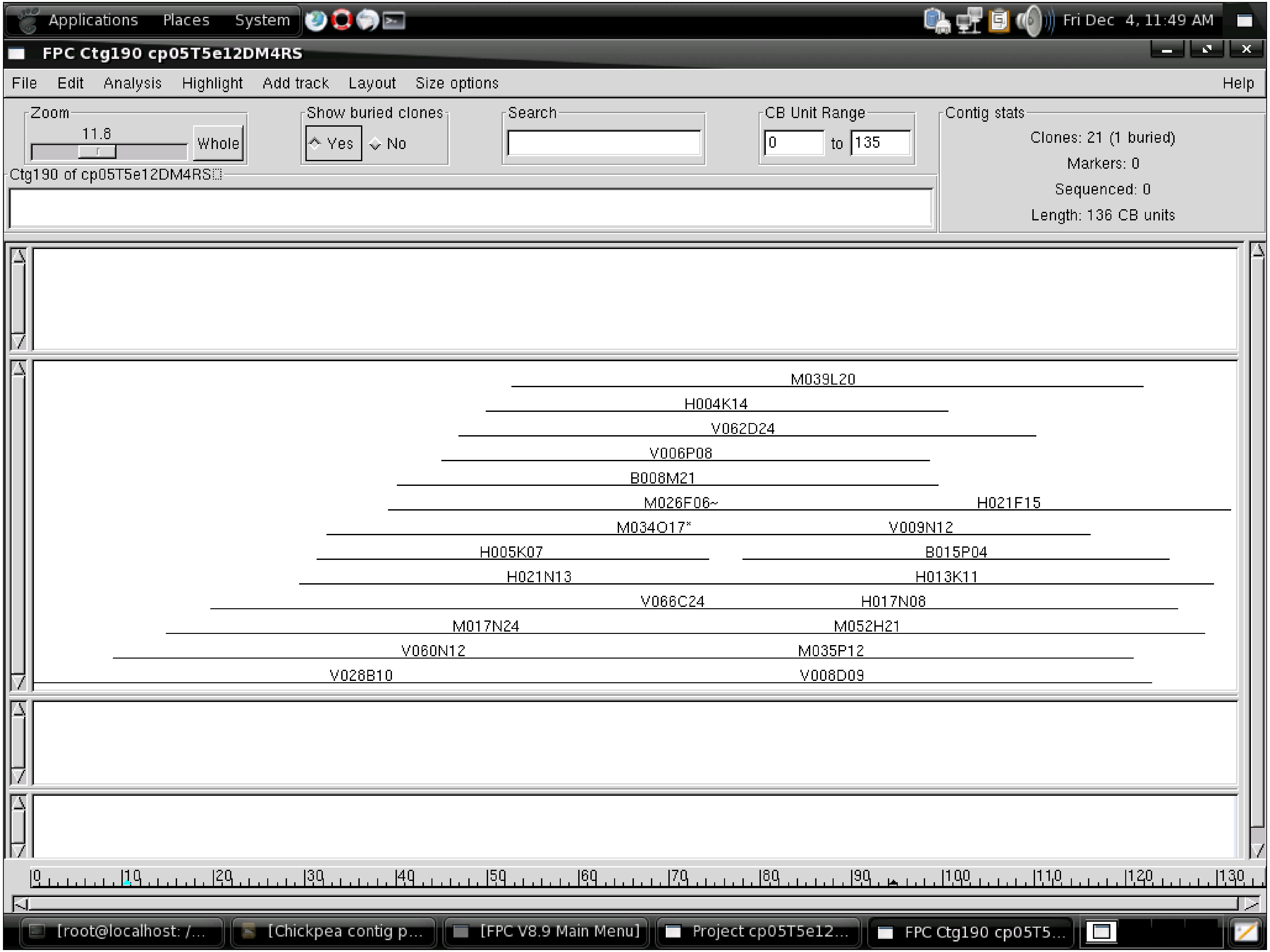
Applications Places System
Fri Dec 4, 11:49 AM
FPC Ctg190 cp05T5e12DM4RS
File Edit Analysis Highlight Add track Layout Size options Help
Zoom
11.8
Whole
Show buried clones
Yes No
Search
CB Unit Range
0 to 135
Contig stats
Clones: 21 (1 buried)
Markers: 0
Sequenced: 0
Length: 136 CB units
Ctg190 of cp05T5e12DM4RS
M039L20
H004K14
V062D24
V006P08
B008M21
M026F06~
H021F15
M034O17*
V009N12
H005K07
B015P04
H021N13
H013K11
V066C24
H017N08
M017N24
M052H21
V060N12
M035P12
V028B10
V008D09
0 10 20 30 40 50 60 70 80 90 100 110 120 130
[root@localhost: /...
[Chickpea contig p...
[FPC V8.9 Main Menu]
Project cp05T5e12...
FPC Ctg190 cp05T5...

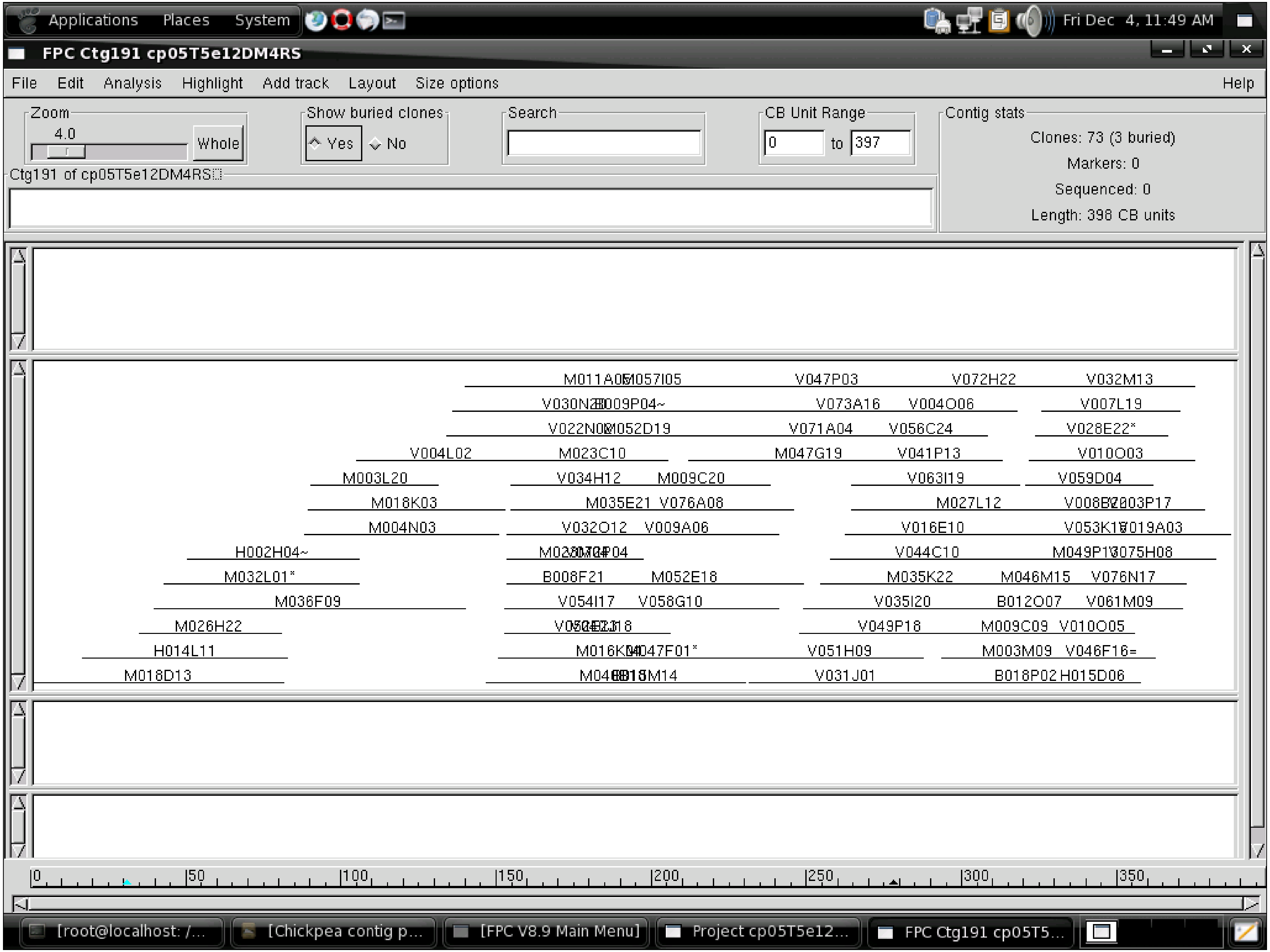
Applications Places System
Fri Dec 4, 11:49 AM
FPC Ctg191 cp05T5e12DM4RS
File Edit Analysis Highlight Add track Layout Size options Help
Zoom
4.0
Whole
Show buried clones
Yes No
Search
CB Unit Range
0 to 397
Contig stats
Clones: 73 (3 buried)
Markers: 0
Sequenced: 0
Length: 398 CB units
Ctg191 of cp05T5e12DM4RS
V047P03 V072H22 V032M13
V073A16 V004O06 V007L19
V071A04 V056C24 V028E22*
V004L02 M023C10 M047G19 V041P13 V010O03
M003L20 V034H12 M009C20 V063I19 V059D04
M018K03 M035E21 V076A08 M027L12
M004N03 V032O12 V009A06 V016E10
H002H04~ V044C10
M032L01* B008F21 M052E18 M035K22 M046M15 V076N17
M036F09 V054I17 V058G10 V035I20 B012O07 V061M09
M026H22 V049P18 M009C09 V010O05
H014L11 V051H09 M003M09 V046F16=
M018D13 V031J01 B018P02 H015D06
0 50 100 150 200 250 300 350
[root@localhost: /... [Chickpea contig p... [FPC V8.9 Main Menu] Project cp05T5e12... FPC Ctg191 cp05T5...

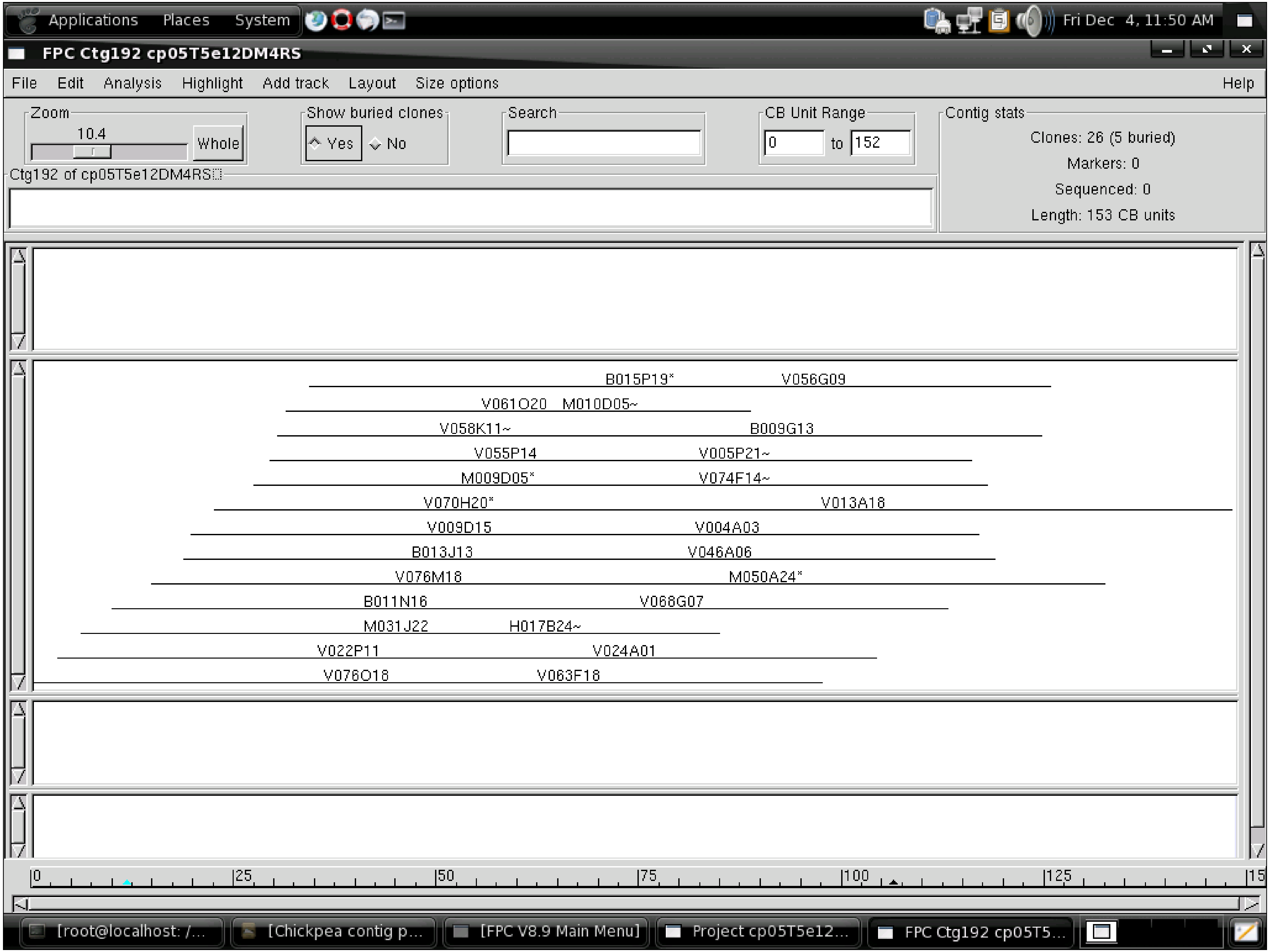
Applications Places System
Fri Dec 4, 11:50 AM
FPC Ctg192 cp05T5e12DM4RS
File Edit Analysis Highlight Add track Layout Size options Help
Zoom
10.4
Whole
Show buried clones
Yes No
Search
CB Unit Range
0 to 152
Contig stats
Clones: 26 (5 buried)
Markers: 0
Sequenced: 0
Length: 153 CB units
Ctg192 of cp05T5e12DM4RS
B015P19* V056G09
V061O20 M010D05~
V058K11~ B009G13
V055P14 V005P21~
M009D05* V074F14~
V070H20* V013A18
V009D15 V004A03
B013J13 V046A06
V076M18 M050A24*
B011N16 V068G07
M031J22 H017B24~
V022P11 V024A01
V076O18 V063F18
0 25 50 75 100 125 15
[root@localhost: /... [Chickpea contig p... [FPC V8.9 Main Menu] Project cp05T5e12... FPC Ctg192 cp05T5...

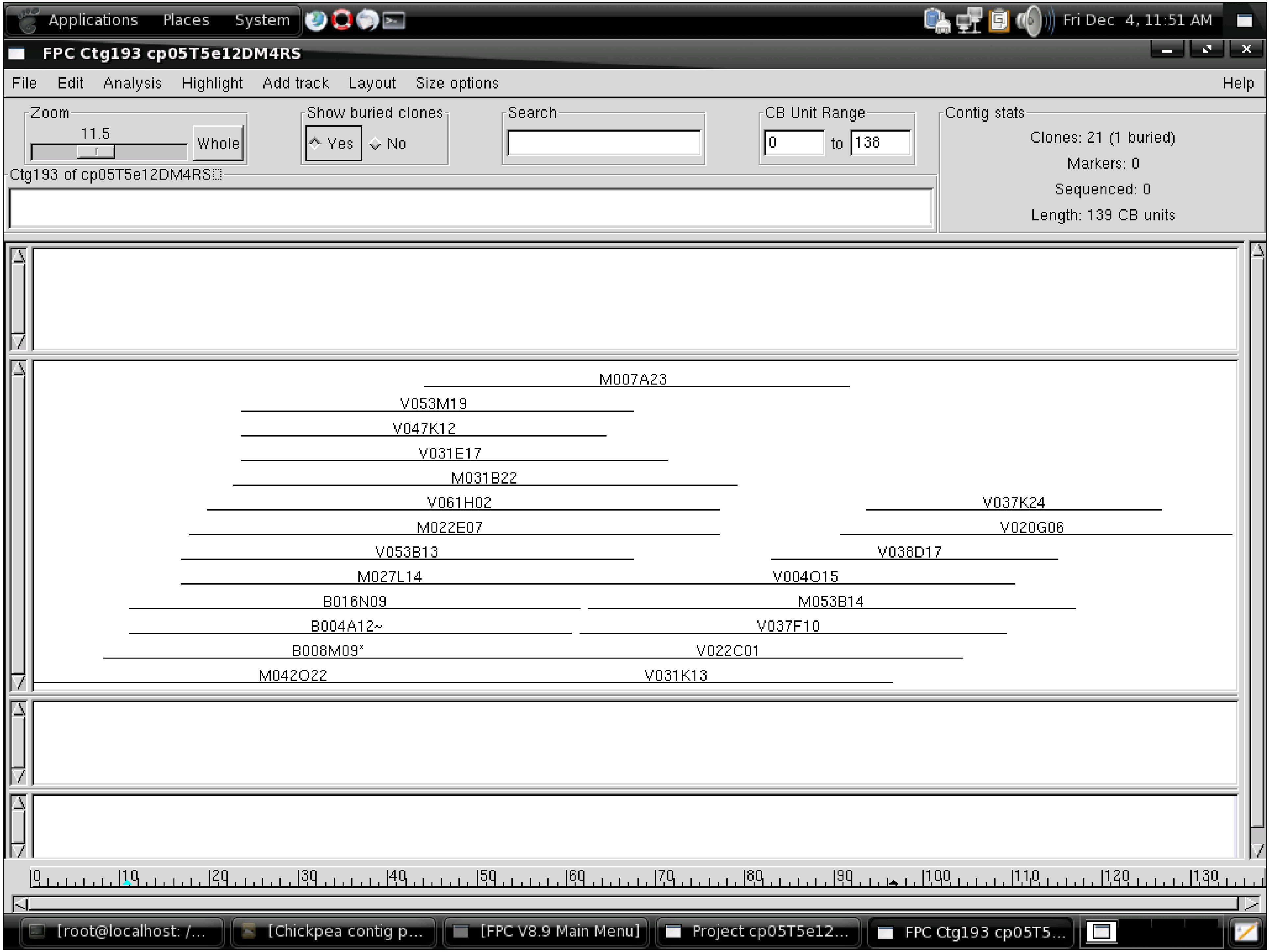
Applications Places System
Fri Dec 4, 11:51 AM
FPC Ctg193 cp05T5e12DM4RS
File Edit Analysis Highlight Add track Layout Size options Help
Zoom
11.5
Whole
Show buried clones
Yes No
Search
CB Unit Range
0 to 138
Contig stats
Clones: 21 (1 buried)
Markers: 0
Sequenced: 0
Length: 139 CB units
Ctg193 of cp05T5e12DM4RS
M007A23
V053M19
V047K12
V031E17
M031B22
V061H02
V037K24
M022E07
V020G06
V053B13
V038D17
M027L14
V004O15
B016N09
M053B14
B004A12~
V037F10
B008M09*
V022C01
M042O22
V031K13
0 10 20 30 40 50 60 70 80 90 100 110 120 130
[root@localhost: /...
[Chickpea contig p...
[FPC V8.9 Main Menu]
Project cp05T5e12...
FPC Ctg193 cp05T5...

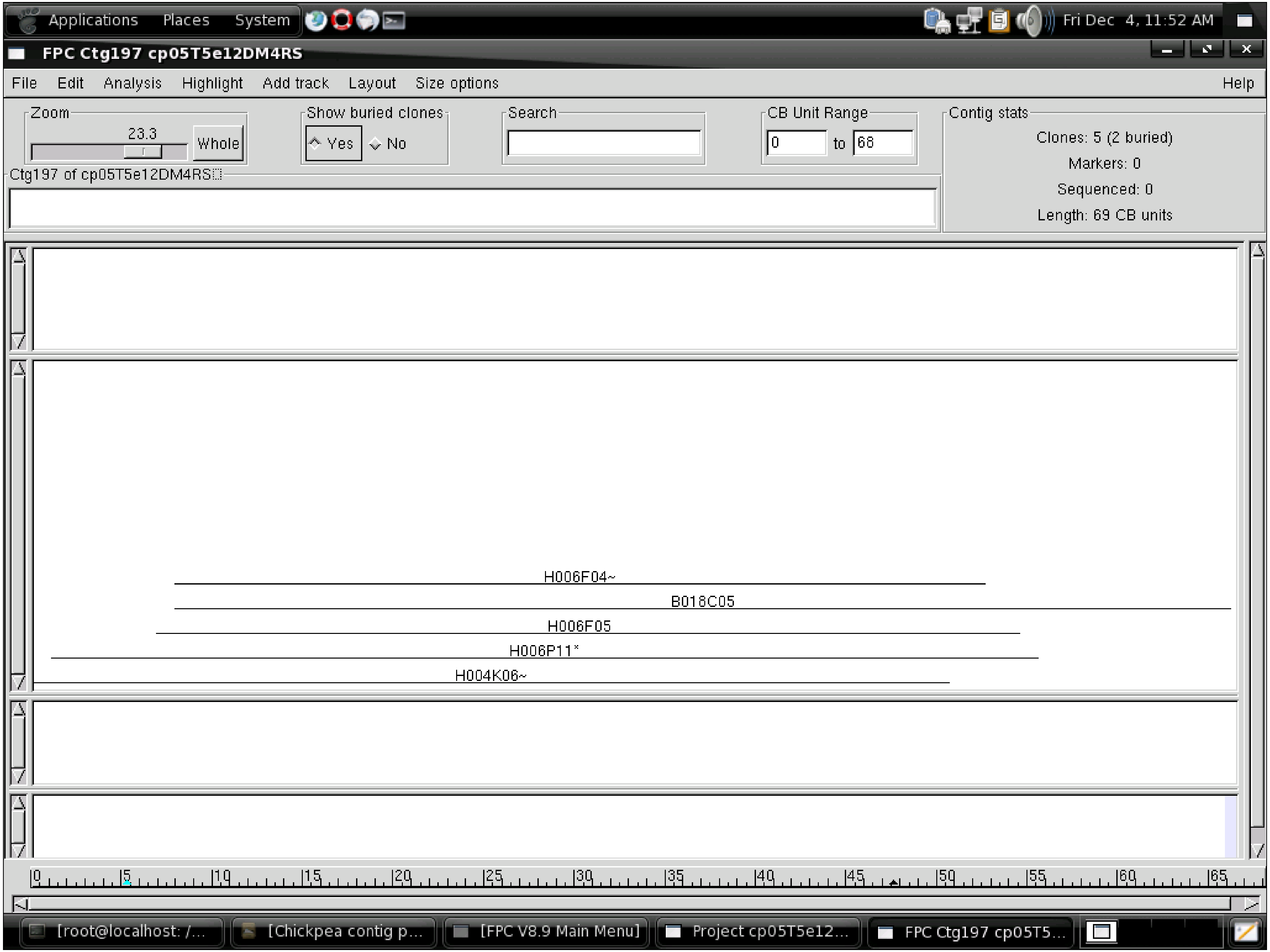
Applications Places System
Fri Dec 4, 11:52 AM
FPC Ctg197 cp05T5e12DM4RS
File Edit Analysis Highlight Add track Layout Size options Help
Zoom
23.3
Whole
Show buried clones
Yes
No
Search
CB Unit Range
0
to
68
Contig stats
Clones: 5 (2 buried)
Markers: 0
Sequenced: 0
Length: 69 CB units
Ctg197 of cp05T5e12DM4RS
H006F04~
B018C05
H006F05
H006P11*
H004K06~
0 5 10 15 20 25 30 35 40 45 50 55 60 65
[root@localhost: /...
[Chickpea contig p...
[FPC V8.9 Main Menu]
Project cp05T5e12...
FPC Ctg197 cp05T5...

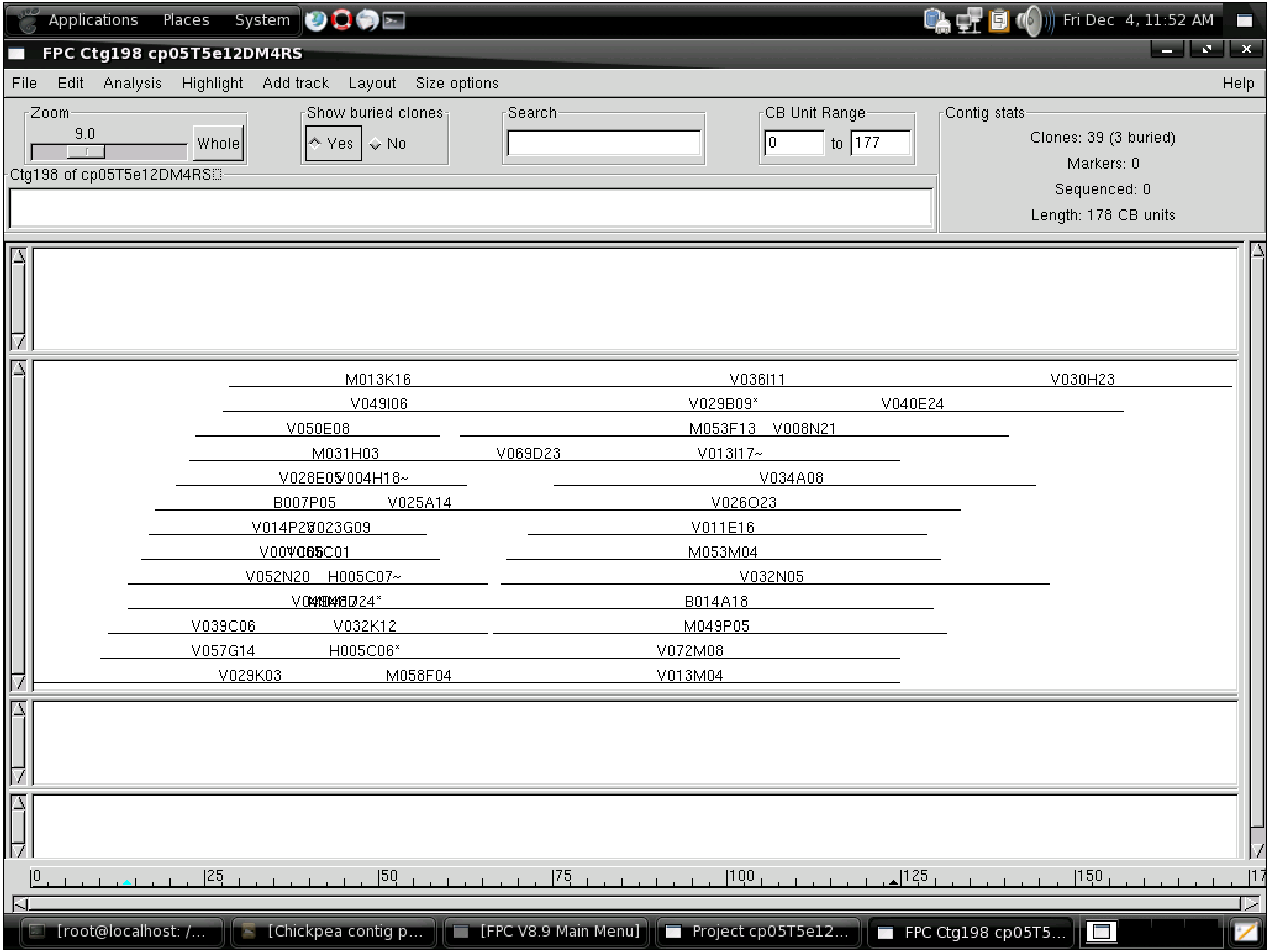
Applications Places System
Fri Dec 4, 11:52 AM
FPC Ctg198 cp05T5e12DM4RS
File Edit Analysis Highlight Add track Layout Size options Help
Zoom
9.0
Whole
Show buried clones
Yes No
Search
CB Unit Range
0 to 177
Contig stats
Clones: 39 (3 buried)
Markers: 0
Sequenced: 0
Length: 178 CB units
Ctg198 of cp05T5e12DM4RS
M013K16
V036I11
V030H23
V049I06
V029B09*
V040E24
V050E08
M053F13
V008N21
M031H03
V069D23
V013I17~
V034A08
B007P05
V025A14
V026O23
V011E16
M053M04
V052N20
H005C07~
V032N05
B014A18
V039C06
V032K12
M049P05
V057G14
H005C06*
V072M08
V029K03
M058F04
V013M04
0 25 50 75 100 125 150
[root@localhost: /...
[Chickpea contig p...
[FPC V8.9 Main Menu]
Project cp05T5e12...
FPC Ctg198 cp05T5...

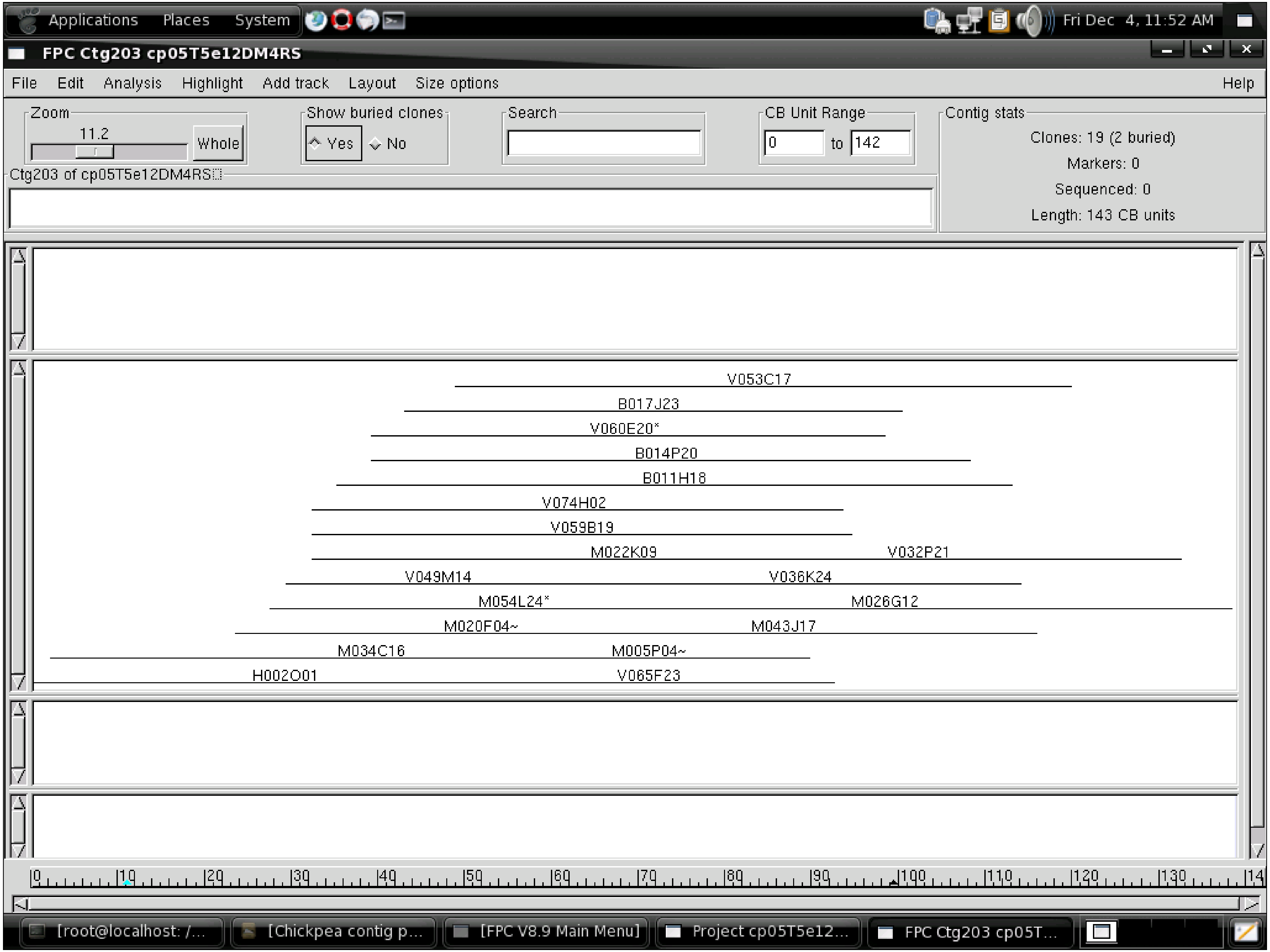
Applications Places System
Fri Dec 4, 11:52 AM
FPC Ctg203 cp05T5e12DM4RS
File Edit Analysis Highlight Add track Layout Size options Help
Zoom
11.2
Whole
Show buried clones
Yes No
Search
CB Unit Range
0 to 142
Contig stats
Clones: 19 (2 buried)
Markers: 0
Sequenced: 0
Length: 143 CB units
Ctg203 of cp05T5e12DM4RS
V053C17
B017J23
V060E20*
B014P20
B011H18
V074H02
V059B19
M022K09
V032P21
V049M14
V036K24
M054L24*
M026G12
M020F04~
M043J17
M034C16
M005P04~
H002O01
V065F23
0 10 20 30 40 50 60 70 80 90 100 110 120 130 14
[root@localhost: /...
[Chickpea contig p...
[FPC V8.9 Main Menu]
Project cp05T5e12...
FPC Ctg203 cp05T...

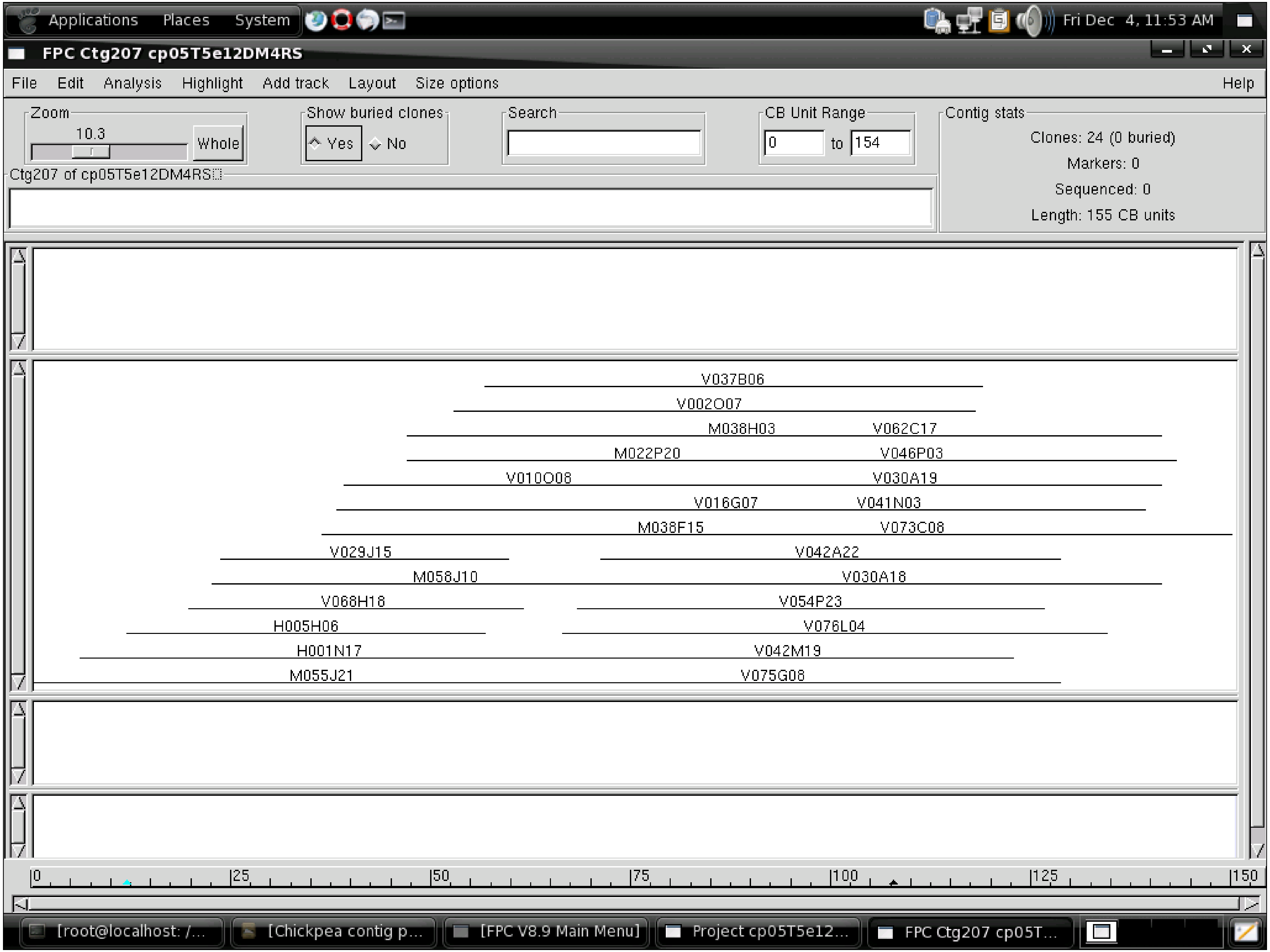
Applications Places System
Fri Dec 4, 11:53 AM
FPC Ctg207 cp05T5e12DM4RS
File Edit Analysis Highlight Add track Layout Size options Help
Zoom
10.3
Whole
Show buried clones
Yes No
Search
CB Unit Range
0 to 154
Contig stats
Clones: 24 (0 buried)
Markers: 0
Sequenced: 0
Length: 155 CB units
Ctg207 of cp05T5e12DM4RS
V037B06
V002O07
M038H03
V062C17
M022P20
V046P03
V010O08
V030A19
V016G07
V041N03
M038F15
V073C08
V029J15
V042A22
M058J10
V030A18
V068H18
V054P23
H005H06
V076L04
H001N17
V042M19
M055J21
V075G08
0
25
50
75
100
125
150
[root@localhost: /...
[Chickpea contig p...
[FPC V8.9 Main Menu]
Project cp05T5e12...
FPC Ctg207 cp05T...

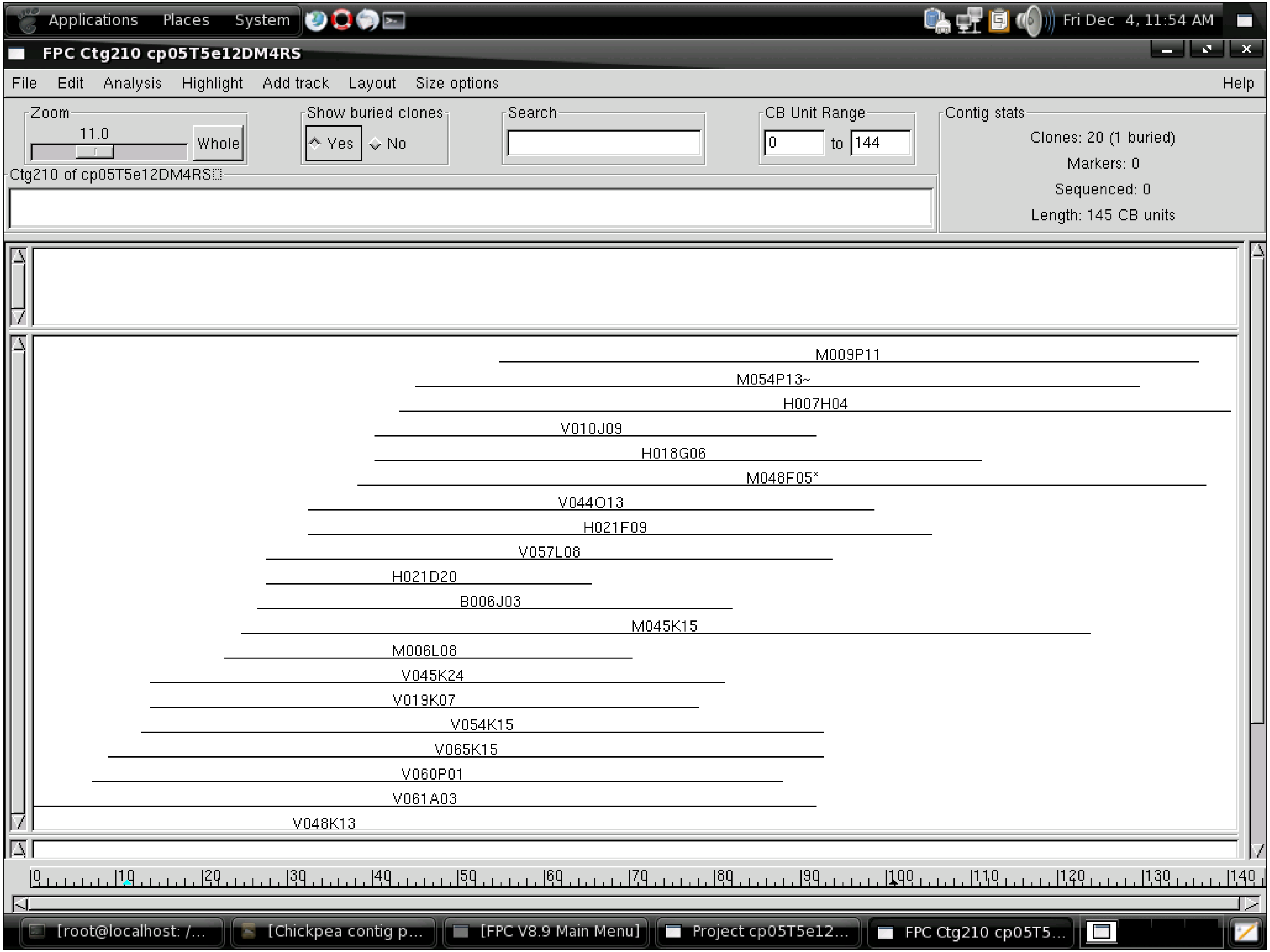
Applications Places System
Fri Dec 4, 11:54 AM
FPC Ctg210 cp05T5e12DM4RS
File Edit Analysis Highlight Add track Layout Size options Help
Zoom
11.0
Whole
Show buried clones
Yes No
Search
CB Unit Range
0 to 144
Contig stats
Clones: 20 (1 buried)
Markers: 0
Sequenced: 0
Length: 145 CB units
Ctg210 of cp05T5e12DM4RS
M009P11
M054P13~
H007H04
V010J09
H018G06
M048F05*
V044O13
H021F09
V057L08
H021D20
B006J03
M045K15
M006L08
V045K24
V019K07
V054K15
V065K15
V060P01
V061A03
V048K13
0 10 20 30 40 50 60 70 80 90 100 110 120 130 140
[root@localhost: /...
[Chickpea contig p...
[FPC V8.9 Main Menu]
Project cp05T5e12...
FPC Ctg210 cp05T5...

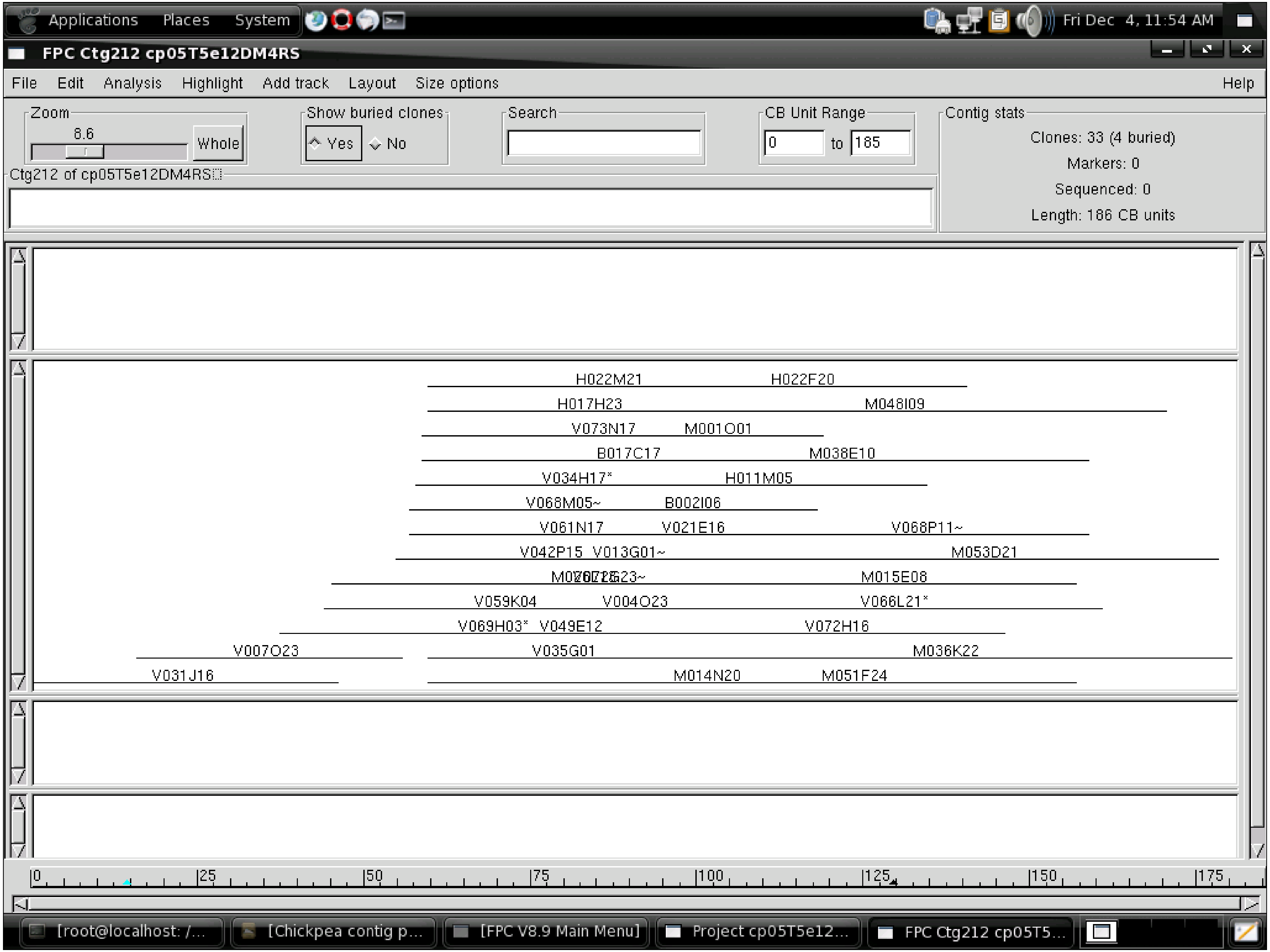
Applications Places System
Fri Dec 4, 11:54 AM
FPC Ctg212 cp05T5e12DM4RS
File Edit Analysis Highlight Add track Layout Size options Help
Zoom
8.6
Whole
Show buried clones
Yes
No
Search
CB Unit Range
0
to
185
Contig stats
Clones: 33 (4 buried)
Markers: 0
Sequenced: 0
Length: 186 CB units
Ctg212 of cp05T5e12DM4RS
H022M21
H022F20
H017H23
M048I09
V073N17
M001O01
B017C17
M038E10
V034H17*
H011M05
V068M05~
B002I06
V061N17
V021E16
V068P11~
V042P15
V013G01~
M053D21
M015E08
V059K04
V004O23
V066L21*
V069H03*
V049E12
V072H16
V007O23
V035G01
M036K22
V031J16
M014N20
M051F24
0 25 50 75 100 125 150 175
[root@localhost: /...
[Chickpea contig p...
[FPC V8.9 Main Menu]
Project cp05T5e12...
FPC Ctg212 cp05T5...

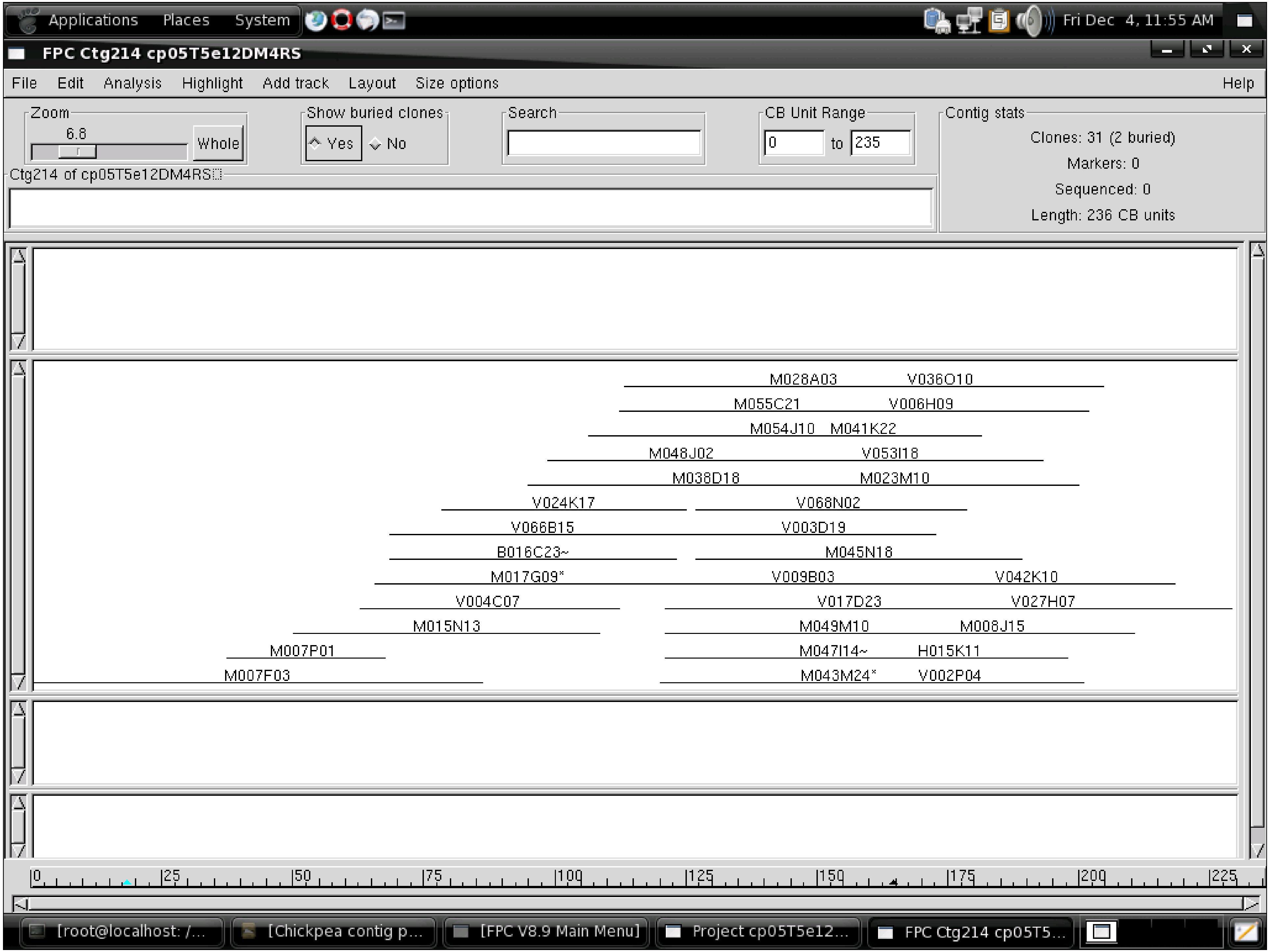
Applications
Places
System
Fri Dec 4, 11:55 AM
FPC Ctg214 cp05T5e12DM4RS
File
Edit
Analysis
Highlight
Add track
Layout
Size options
Help
Zoom
6.8
Whole
Show buried clones
Yes
No
Search
CB Unit Range
0
to
235
Contig stats
Clones: 31 (2 buried)
Markers: 0
Sequenced: 0
Length: 236 CB units
Ctg214 of cp05T5e12DM4RS
M028A03
V036O10
M055C21
V006H09
M054J10
M041K22
M048J02
V053I18
M038D18
M023M10
V024K17
V068N02
V066B15
V003D19
B016C23~
M045N18
M017G09*
V009B03
V042K10
V004C07
V017D23
V027H07
M015N13
M049M10
M008J15
M007P01
M047I14~
H015K11
M007F03
M043M24*
V002P04
0
25
50
75
100
125
150
175
200
225
[root@localhost: /...
[Chickpea contig p...
[FPC V8.9 Main Menu]
Project cp05T5e12...
FPC Ctg214 cp05T5...

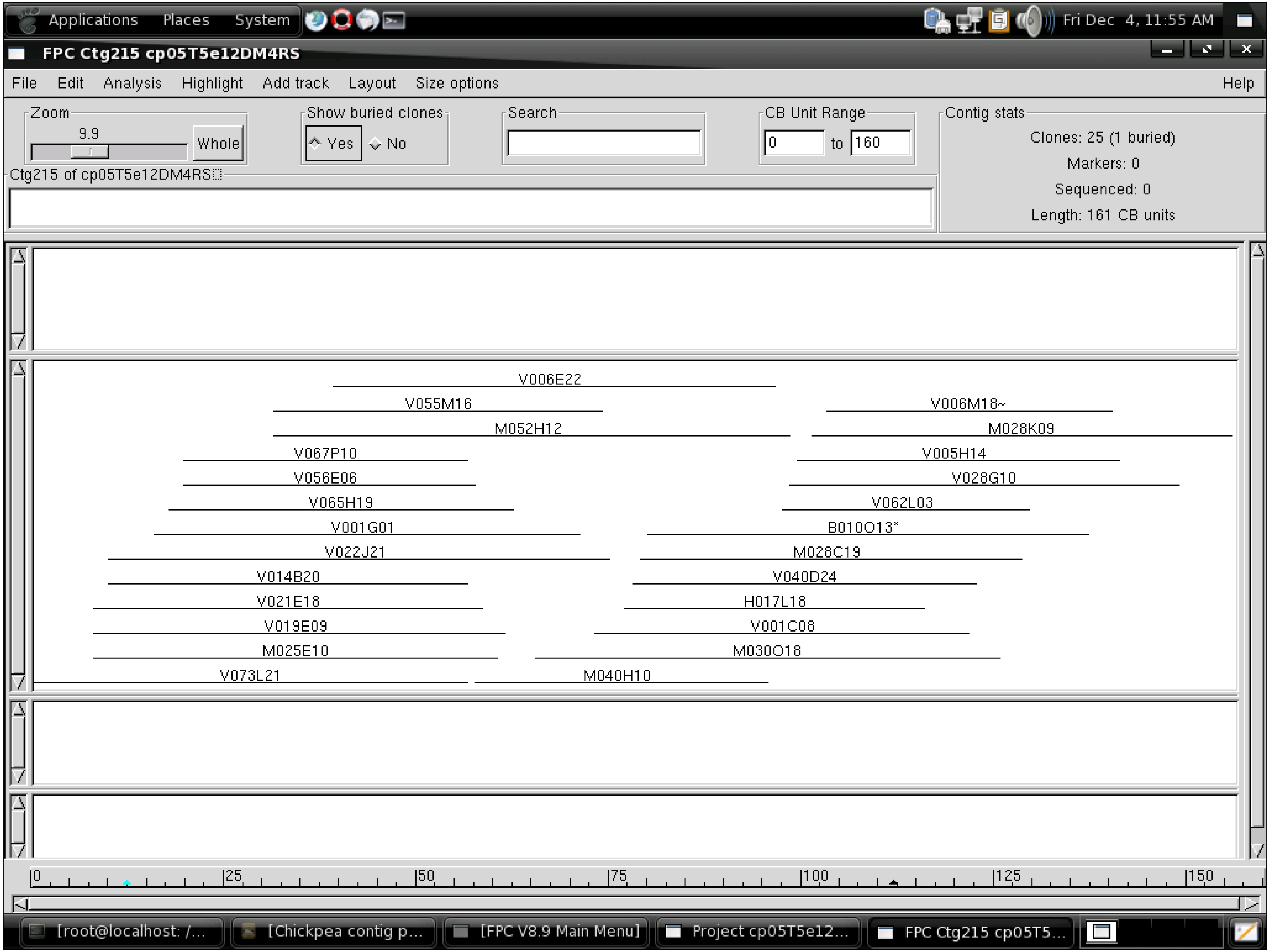
Applications Places System
Fri Dec 4, 11:55 AM
FPC Ctg215 cp05T5e12DM4RS
File Edit Analysis Highlight Add track Layout Size options Help
Zoom
9.9
Whole
Show buried clones
Yes
No
Search
CB Unit Range
0
to
160
Contig stats
Clones: 25 (1 buried)
Markers: 0
Sequenced: 0
Length: 161 CB units
Ctg215 of cp05T5e12DM4RS
V006E22
V055M16
V006M18~
M052H12
M028K09
V067P10
V005H14
V056E06
V028G10
V065H19
V062L03
V001G01
B010O13*
V022J21
M028C19
V014B20
V040D24
V021E18
H017L18
V019E09
V001C08
M025E10
M030O18
V073L21
M040H10
0
25
50
75
100
125
150
[root@localhost: /...
[Chickpea contig p...
[FPC V8.9 Main Menu]
Project cp05T5e12...
FPC Ctg215 cp05T5...

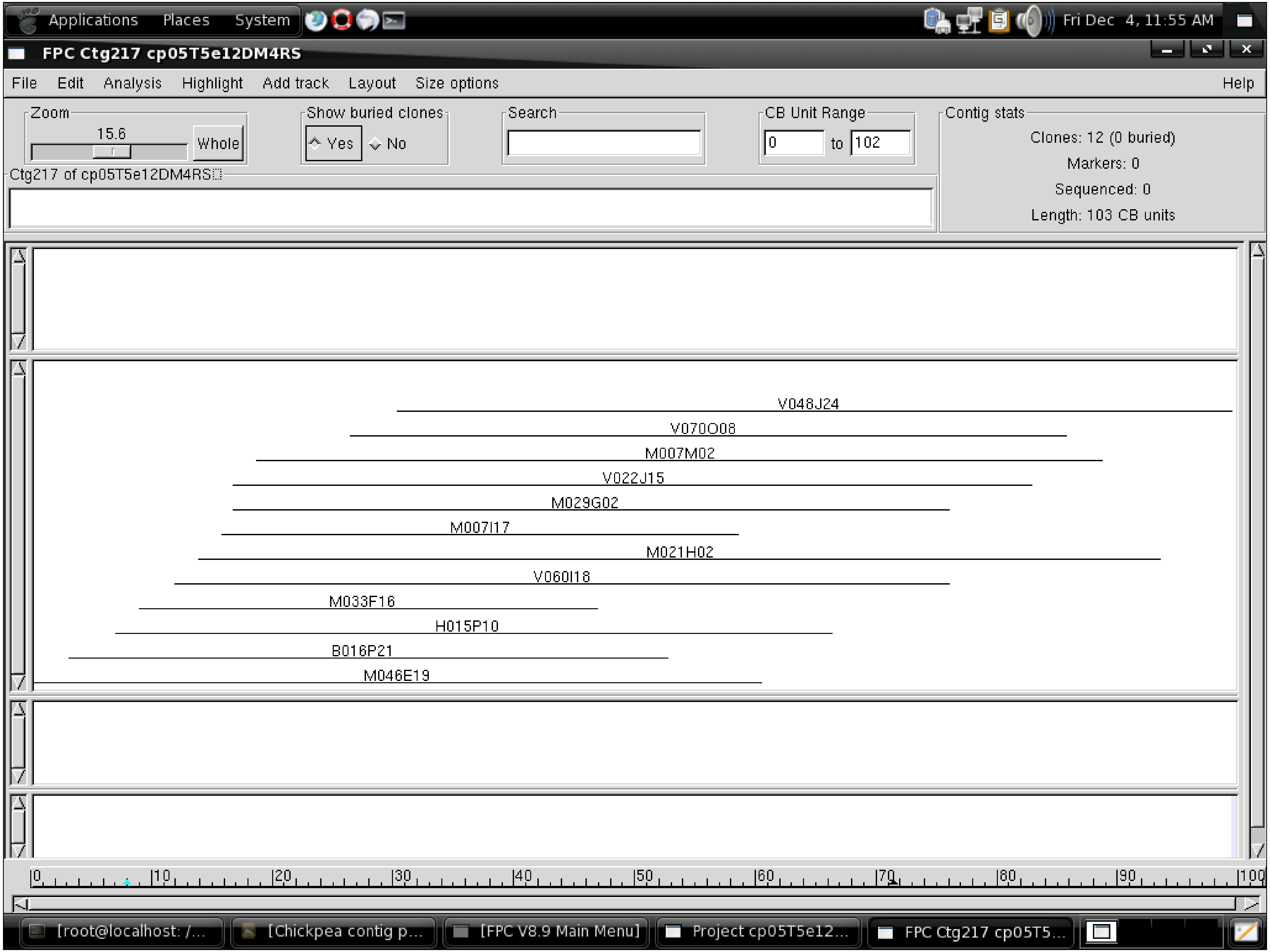
Applications
Places
System
Fri Dec 4, 11:55 AM
FPC Ctg217 cp05T5e12DM4RS
File
Edit
Analysis
Highlight
Add track
Layout
Size options
Help
Zoom
15.6
Whole
Show buried clones
Yes
No
Search
CB Unit Range
0
to
102
Contig stats
Clones: 12 (0 buried)
Markers: 0
Sequenced: 0
Length: 103 CB units
Ctg217 of cp05T5e12DM4RS
V048J24
V070O08
M007M02
V022J15
M029G02
M007I17
M021H02
V060I18
M033F16
H015P10
B016P21
M046E19
0
10
20
30
40
50
60
70
80
90
100
[root@localhost: /...
[Chickpea contig p...
[FPC V8.9 Main Menu]
Project cp05T5e12...
FPC Ctg217 cp05T5...

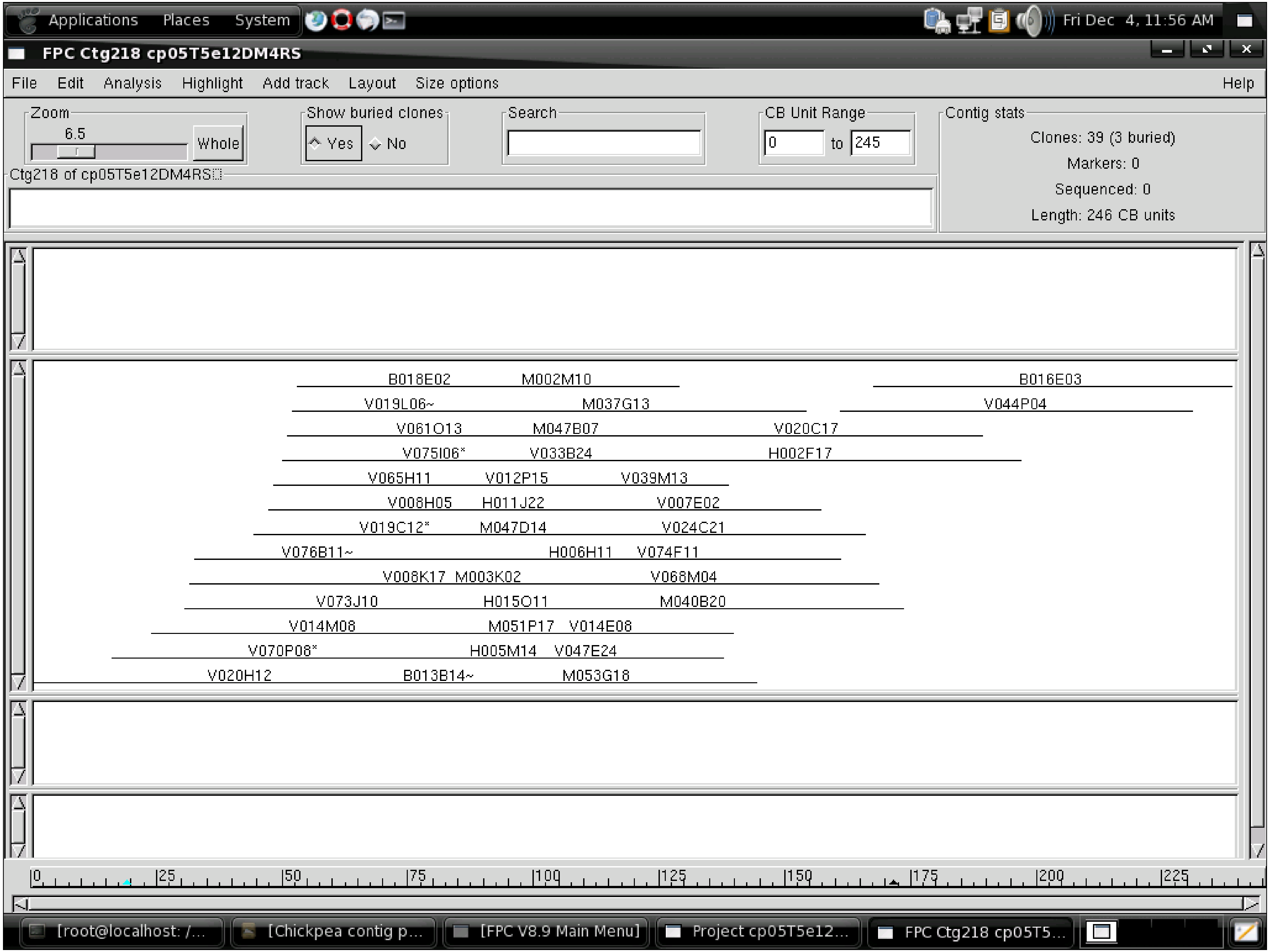
Applications Places System
Fri Dec 4, 11:56 AM
FPC Ctg218 cp05T5e12DM4RS
File Edit Analysis Highlight Add track Layout Size options Help
Zoom
6.5
Whole
Show buried clones
Yes No
Search
CB Unit Range
0 to 245
Contig stats
Clones: 39 (3 buried)
Markers: 0
Sequenced: 0
Length: 246 CB units
Ctg218 of cp05T5e12DM4RS
B018E02 M002M10 B016E03
V019L06~ M037G13 V044P04
V061O13 M047B07 V020C17
V075I06* V033B24 H002F17
V065H11 V012P15 V039M13
V008H05 H011J22 V007E02
V019C12* M047D14 V024C21
V076B11~ H006H11 V074F11
V008K17 M003K02 V068M04
V073J10 H015O11 M040B20
V014M08 M051P17 V014E08
V070P08* H005M14 V047E24
V020H12 B013B14~ M053G18
0 25 50 75 100 125 150 175 200 225
[root@localhost: /... [Chickpea contig p... [FPC V8.9 Main Menu] Project cp05T5e12... FPC Ctg218 cp05T5...

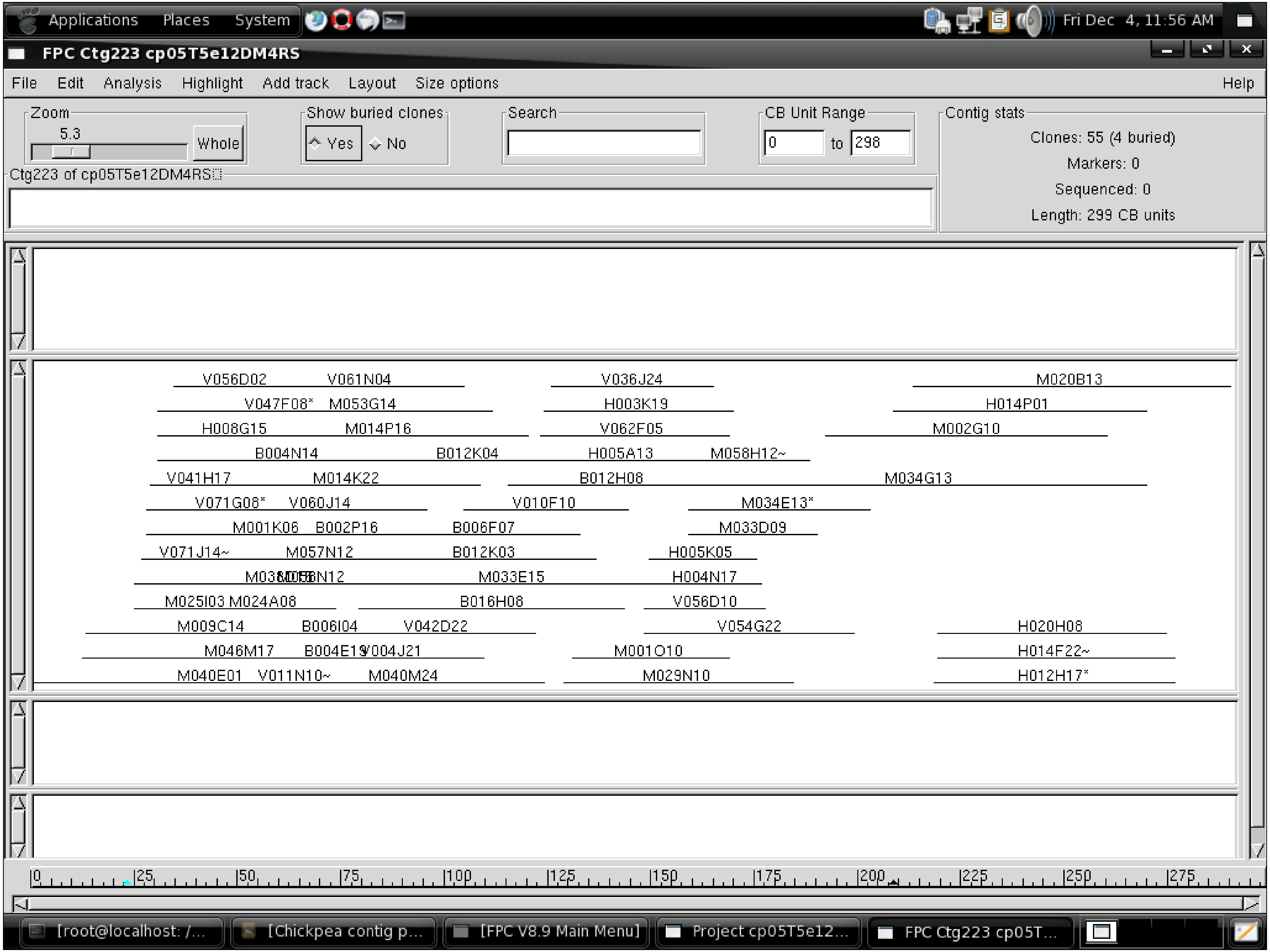
Applications Places System
Fri Dec 4, 11:56 AM
FPC Ctg223 cp05T5e12DM4RS
File Edit Analysis Highlight Add track Layout Size options Help
Zoom
5.3
Whole
Show buried clones
Yes
No
Search
CB Unit Range
0 to 298
Contig stats
Clones: 55 (4 buried)
Markers: 0
Sequenced: 0
Length: 299 CB units
Ctg223 of cp05T5e12DM4RS
V056D02 V061N04 V036J24 M020B13
V047F08* M053G14 H003K19 H014P01
H008G15 M014P16 V062F05 M002G10
B004N14 B012K04 H005A13 M058H12~
V041H17 M014K22 B012H08 M034G13
V071G08* V060J14 V010F10 M034E13*
M001K06 B002P16 B006F07 M033D09
V071J14~ M057N12 B012K03 H005K05
H004N17
M025I03 M024A08 B016H08 V056D10
M009C14 B006I04 V042D22 V054G22 H020H08
M046M17 B004E19 V004J21 M001O10 H014F22~
M040E01 V011N10~ M040M24 M029N10 H012H17*
0 25 50 75 100 125 150 175 200 225 250 275
[root@localhost: /... [Chickpea contig p... [FPC V8.9 Main Menu] Project cp05T5e12... FPC Ctg223 cp05T...

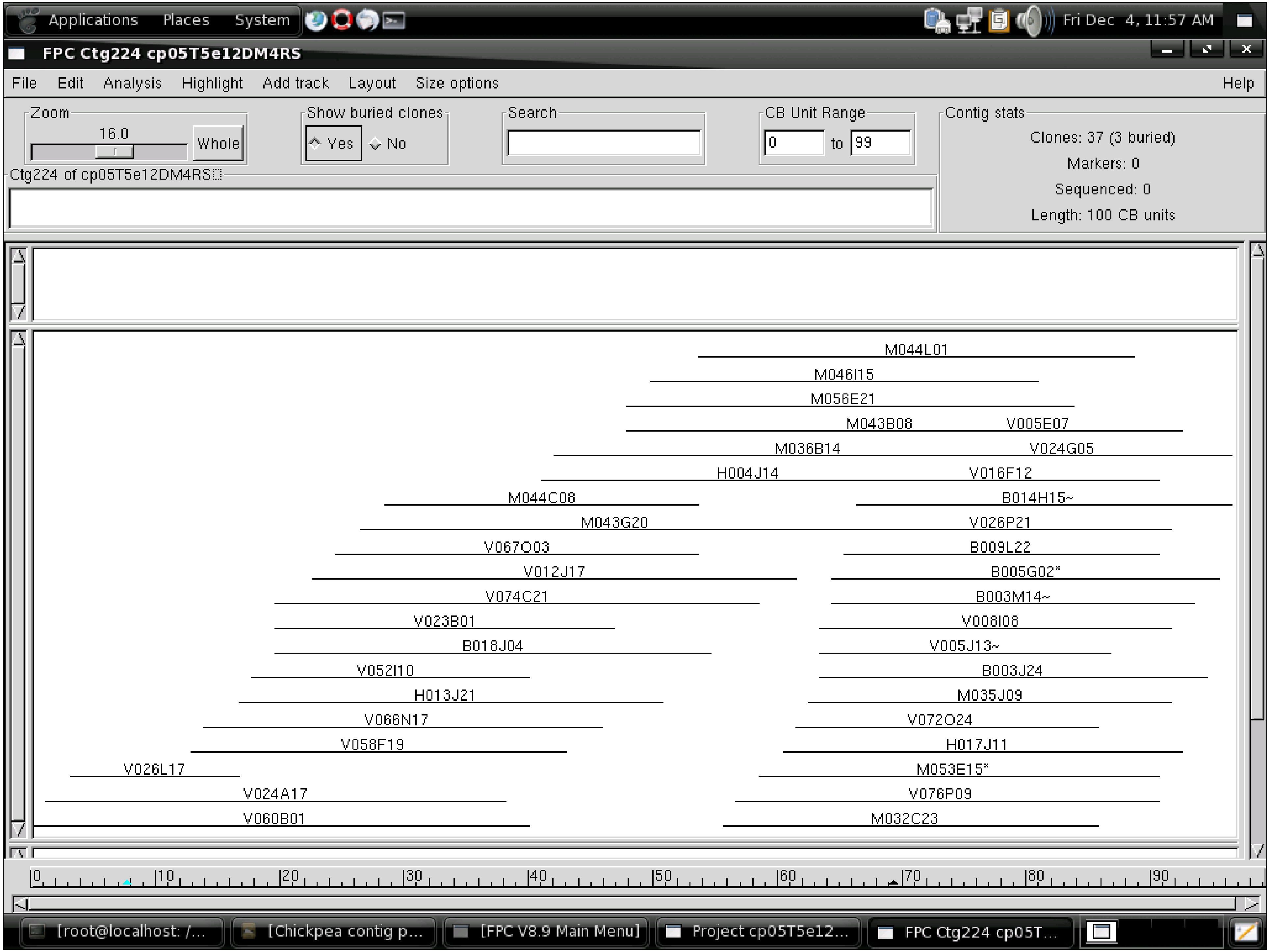
Applications
Places
System
Fri Dec 4, 11:57 AM
FPC Ctg224 cp05T5e12DM4RS
File
Edit
Analysis
Highlight
Add track
Layout
Size options
Help
Zoom
16.0
Whole
Show buried clones
Yes
No
Search
CB Unit Range
0
to
99
Contig stats
Clones: 37 (3 buried)
Markers: 0
Sequenced: 0
Length: 100 CB units
Ctg224 of cp05T5e12DM4RS
M044L01
M046I15
M056E21
M043B08
V005E07
M036B14
V024G05
H004J14
V016F12
M044C08
B014H15~
M043G20
V026P21
V067O03
B009L22
V012J17
B005G02*
V074C21
B003M14~
V023B01
V008I08
B018J04
V005J13~
V052I10
B003J24
H013J21
M035J09
V066N17
V072O24
V058F19
H017J11
V026L17
M053E15*
V024A17
V076P09
V060B01
M032C23
0
10
20
30
40
50
60
70
80
90
[root@localhost: /...
[Chickpea contig p...
[FPC V8.9 Main Menu]
Project cp05T5e12...
FPC Ctg224 cp05T...

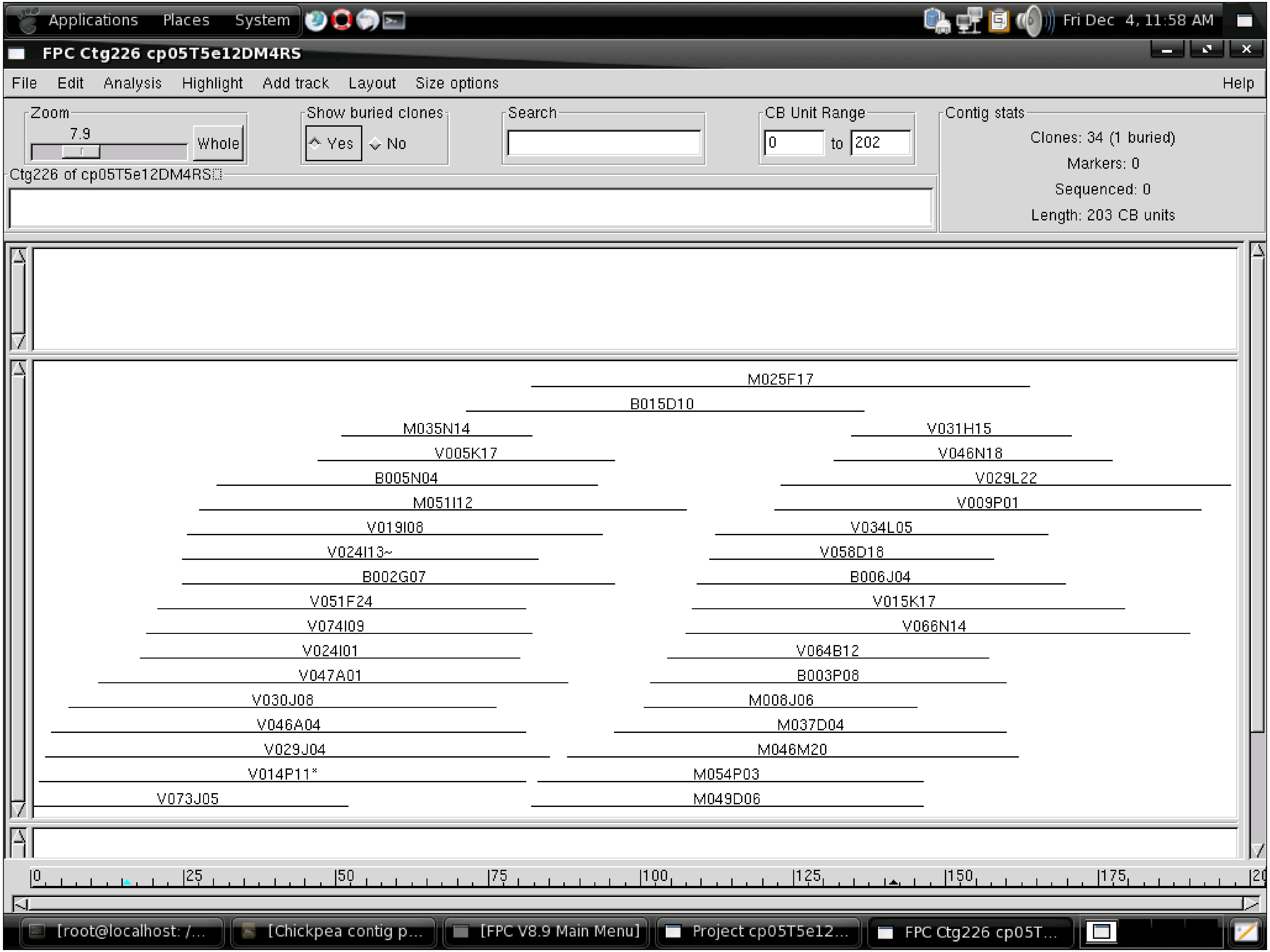
Applications Places System
Fri Dec 4, 11:58 AM
FPC Ctg226 cp05T5e12DM4RS
File Edit Analysis Highlight Add track Layout Size options Help
Zoom
7.9
Whole
Show buried clones
Yes No
Search
CB Unit Range
0 to 202
Contig stats
Clones: 34 (1 buried)
Markers: 0
Sequenced: 0
Length: 203 CB units
Ctg226 of cp05T5e12DM4RS
M025F17
B015D10
M035N14
V031H15
V005K17
V046N18
B005N04
V029L22
M051I12
V009P01
V019I08
V034L05
V024I13~
V058D18
B002G07
B006J04
V051F24
V015K17
V074I09
V066N14
V024I01
V064B12
V047A01
B003P08
V030J08
M008J06
V046A04
M037D04
V029J04
M046M20
V014P11*
M054P03
V073J05
M049D06
0 25 50 75 100 125 150 175
[root@localhost: /...
[Chickpea contig p...
[FPC V8.9 Main Menu]
Project cp05T5e12...
FPC Ctg226 cp05T...

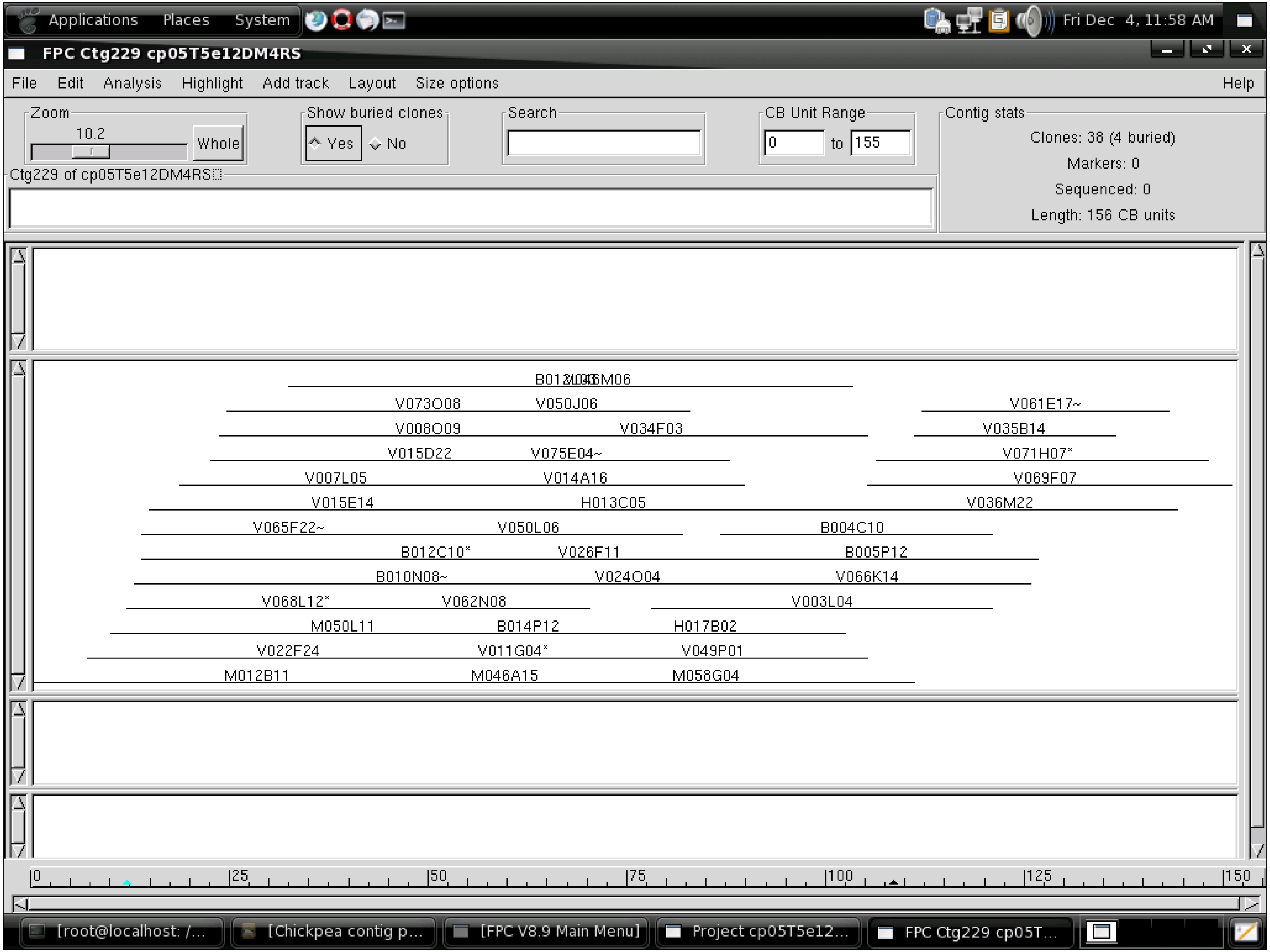
Applications Places System
Fri Dec 4, 11:58 AM
FPC Ctg229 cp05T5e12DM4RS
File Edit Analysis Highlight Add track Layout Size options Help
Zoom
10.2
Whole
Show buried clones
Yes
No
Search
CB Unit Range
0
to
155
Contig stats
Clones: 38 (4 buried)
Markers: 0
Sequenced: 0
Length: 156 CB units
Ctg229 of cp05T5e12DM4RS
V073O08
V050J06
V061E17~
V008O09
V034F03
V035B14
V015D22
V075E04~
V071H07*
V007L05
V014A16
V069F07
V015E14
H013C05
V036M22
V065F22~
V050L06
B004C10
B012C10*
V026F11
B005P12
B010N08~
V024O04
V066K14
V068L12*
V062N08
V003L04
M050L11
B014P12
H017B02
V022F24
V011G04*
V049P01
M012B11
M046A15
M058G04
0
25
50
75
100
125
150
[root@localhost: /...
[Chickpea contig p...
[FPC V8.9 Main Menu]
Project cp05T5e12...
FPC Ctg229 cp05T...

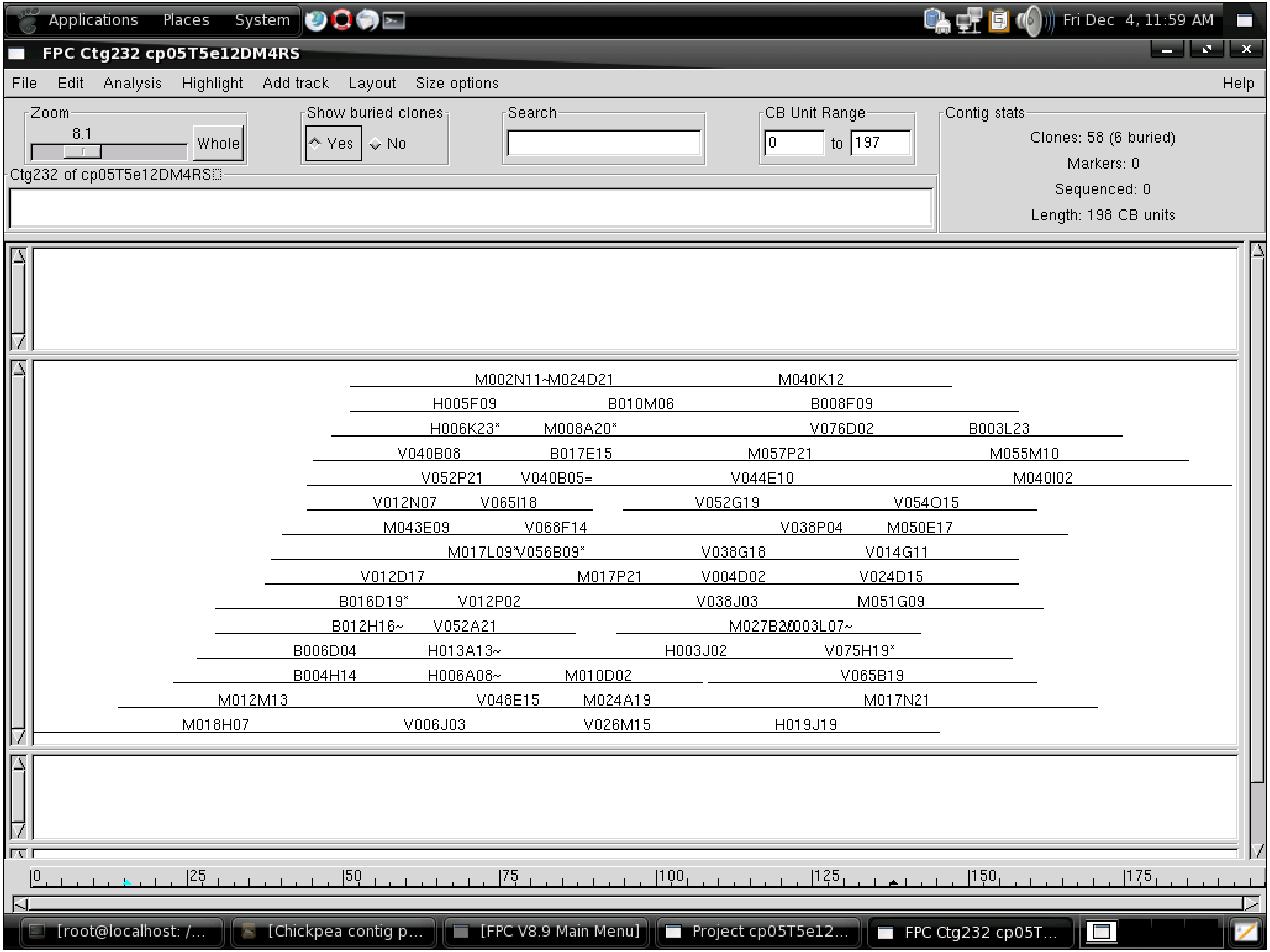
Applications Places System
Fri Dec 4, 11:59 AM
FPC Ctg232 cp05T5e12DM4RS
File Edit Analysis Highlight Add track Layout Size options Help
Zoom
8.1
Whole
Show buried clones
Yes No
Search
CB Unit Range
0 to 197
Contig stats
Clones: 58 (6 buried)
Markers: 0
Sequenced: 0
Length: 198 CB units
Ctg232 of cp05T5e12DM4RS
M002N11 M024D21 M040K12
H005F09 B010M06 B008F09
H006K23* M008A20* V076D02 B003L23
V040B08 B017E15 M057P21 M055M10
V052P21 V040B05= V044E10 M040I02
V012N07 V065I18 V052G19 V054O15
M043E09 V068F14 V038P04 M050E17
M017L09* V056B09* V038G18 V014G11
V012D17 M017P21 V004D02 V024D15
B016D19* V012P02 V038J03 M051G09
B012H16~ V052A21 M027B2 V003L07~
B006D04 H013A13~ H003J02 V075H19*
B004H14 H006A08~ M010D02 V065B19
M012M13 V048E15 M024A19 M017N21
M018H07 V006J03 V026M15 H019J19
0 25 50 75 100 125 150 175
[root@localhost: /... [Chickpea contig p... [FPC V8.9 Main Menu] Project cp05T5e12... FPC Ctg232 cp05T...

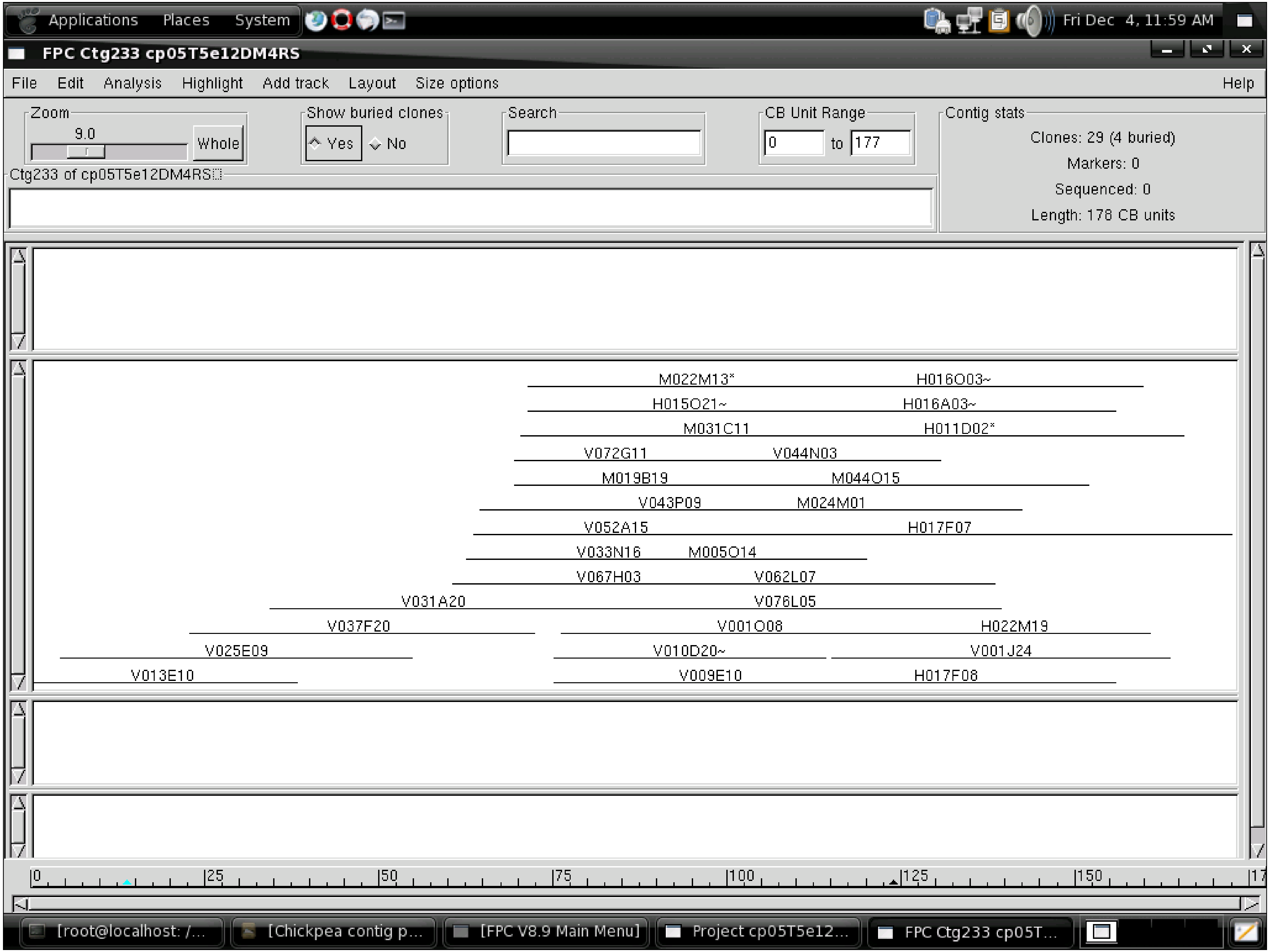
Applications Places System
Fri Dec 4, 11:59 AM
FPC Ctg233 cp05T5e12DM4RS
File Edit Analysis Highlight Add track Layout Size options Help
Zoom
9.0
Whole
Show buried clones
Yes
No
Search
CB Unit Range
0
to
177
Contig stats
Clones: 29 (4 buried)
Markers: 0
Sequenced: 0
Length: 178 CB units
Ctg233 of cp05T5e12DM4RS
M022M13*
H016O03~
H015O21~
H016A03~
M031C11
H011D02*
V072G11
V044N03
M019B19
M044O15
V043P09
M024M01
V052A15
H017F07
V033N16
M005O14
V067H03
V062L07
V031A20
V076L05
V037F20
V001O08
H022M19
V025E09
V010D20~
V001J24
V013E10
V009E10
H017F08
0
25
50
75
100
125
150
[root@localhost: /...
[Chickpea contig p...
[FPC V8.9 Main Menu]
Project cp05T5e12...
FPC Ctg233 cp05T...

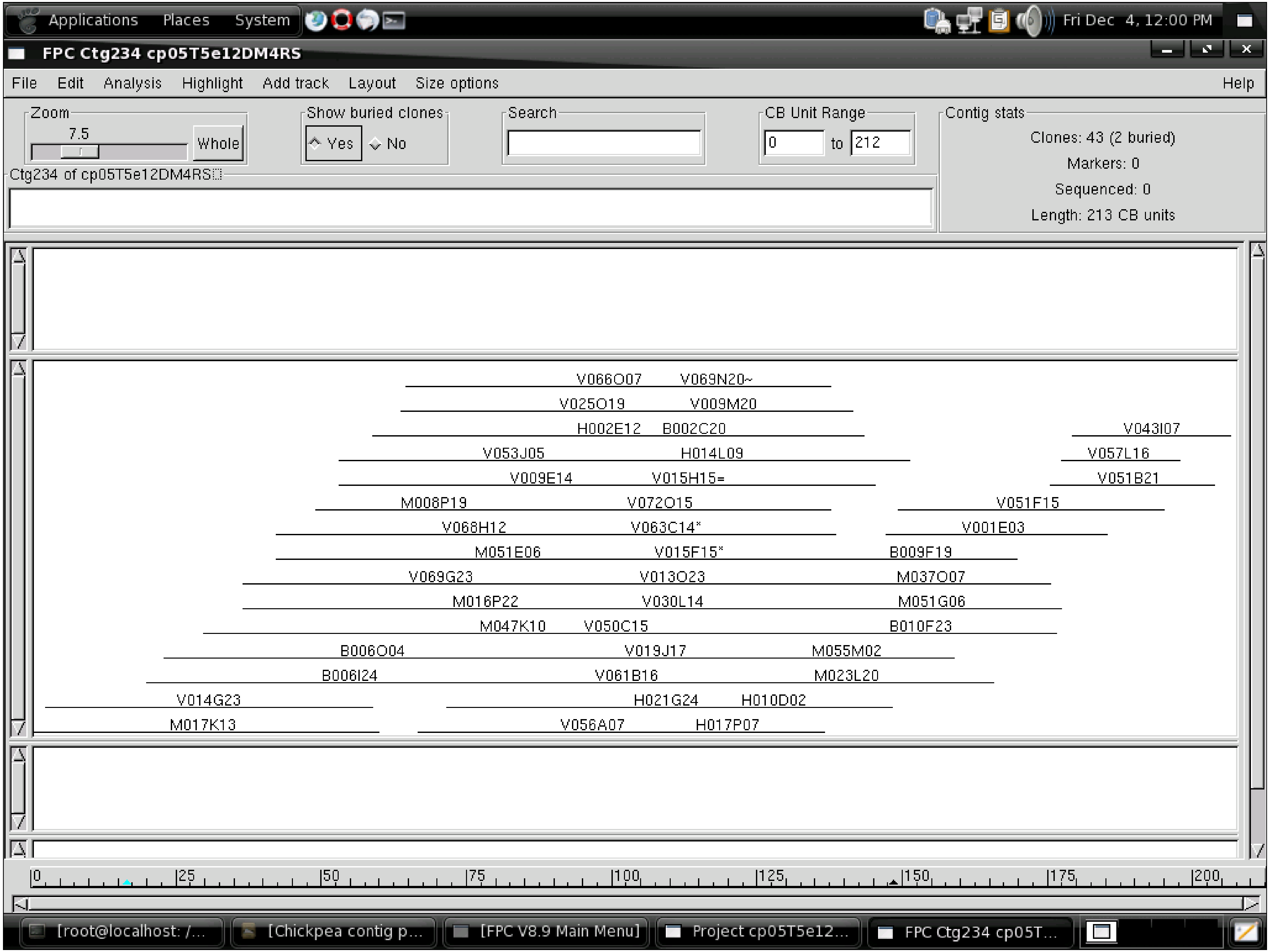
Applications Places System
Fri Dec 4, 12:00 PM
FPC Ctg234 cp05T5e12DM4RS
File Edit Analysis Highlight Add track Layout Size options Help
Zoom 7.5 Whole
Show buried clones Yes No
Search
CB Unit Range 0 to 212
Contig stats
Clones: 43 (2 buried)
Markers: 0
Sequenced: 0
Length: 213 CB units
Ctg234 of cp05T5e12DM4RS
V066O07 V069N20~
V025O19 V009M20
H002E12 B002C20 V043I07
V053J05 H014L09 V057L16
V009E14 V015H15= V051B21
M008P19 V072O15 V051F15
V068H12 V063C14* V001E03
M051E06 V015F15* B009F19
V069G23 V013O23 M037O07
M016P22 V030L14 M051G06
M047K10 V050C15 B010F23
B006O04 V019J17 M055M02
B006I24 V061B16 M023L20
V014G23 H021G24 H010D02
M017K13 V056A07 H017P07
0 25 50 75 100 125 150 175 200
[root@localhost: /... [Chickpea contig p... [FPC V8.9 Main Menu] Project cp05T5e12... FPC Ctg234 cp05T...

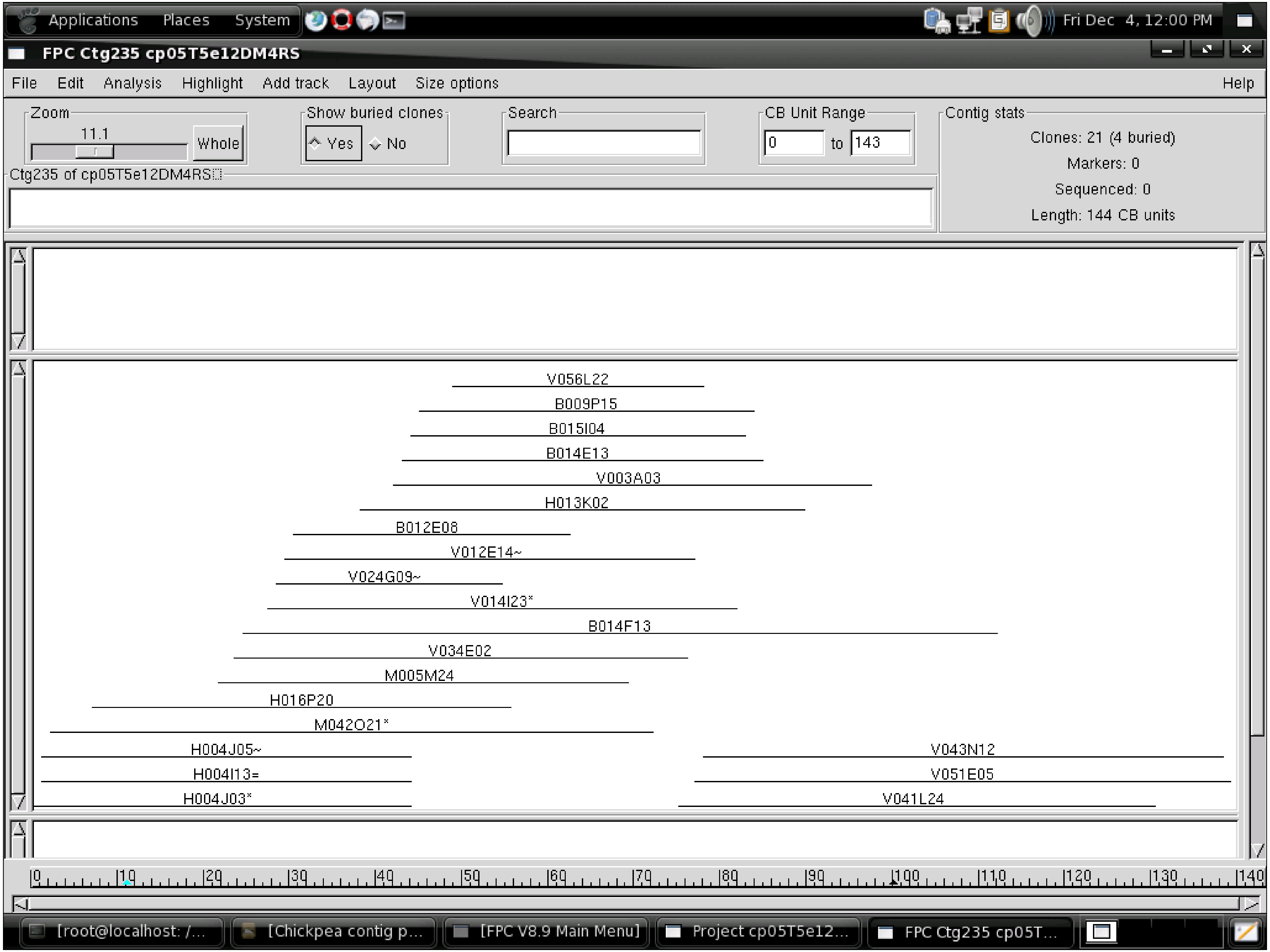
Applications Places System
Fri Dec 4, 12:00 PM
FPC Ctg235 cp05T5e12DM4RS
File Edit Analysis Highlight Add track Layout Size options Help
Zoom
11.1
Whole
Show buried clones
Yes No
Search
CB Unit Range
0 to 143
Contig stats
Clones: 21 (4 buried)
Markers: 0
Sequenced: 0
Length: 144 CB units
Ctg235 of cp05T5e12DM4RS
V056L22
B009P15
B015I04
B014E13
V003A03
H013K02
B012E08
V012E14~
V024G09~
V014I23*
B014F13
V034E02
M005M24
H016P20
M042O21*
H004J05~
V043N12
H004I13=
V051E05
H004J03*
V041L24
0 10 20 30 40 50 60 70 80 90 100 110 120 130 140
[root@localhost: /...
[Chickpea contig p...
[FPC V8.9 Main Menu]
Project cp05T5e12...
FPC Ctg235 cp05T...

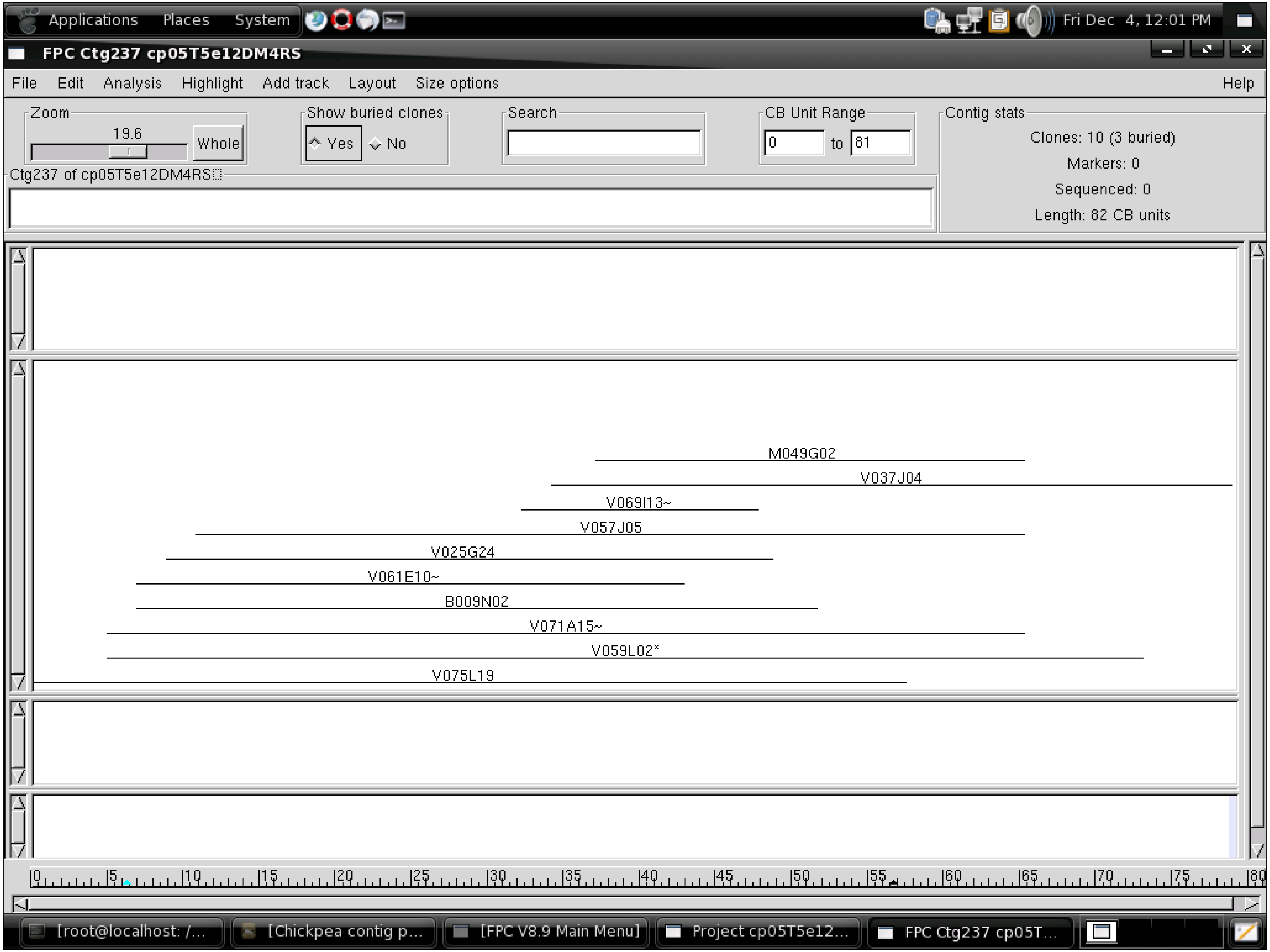
Applications Places System
Fri Dec 4, 12:01 PM
FPC Ctg237 cp05T5e12DM4RS
File Edit Analysis Highlight Add track Layout Size options Help
Zoom
19.6
Whole
Show buried clones
Yes No
Search
CB Unit Range
0 to 81
Contig stats
Clones: 10 (3 buried)
Markers: 0
Sequenced: 0
Length: 82 CB units
Ctg237 of cp05T5e12DM4RS
M049G02
V037J04
V069I13~
V057J05
V025G24
V061E10~
B009N02
V071A15~
V059L02*
V075L19
0 5 10 15 20 25 30 35 40 45 50 55 60 65 70 75 80
[root@localhost: /...
[Chickpea contig p...
[FPC V8.9 Main Menu]
Project cp05T5e12...
FPC Ctg237 cp05T...

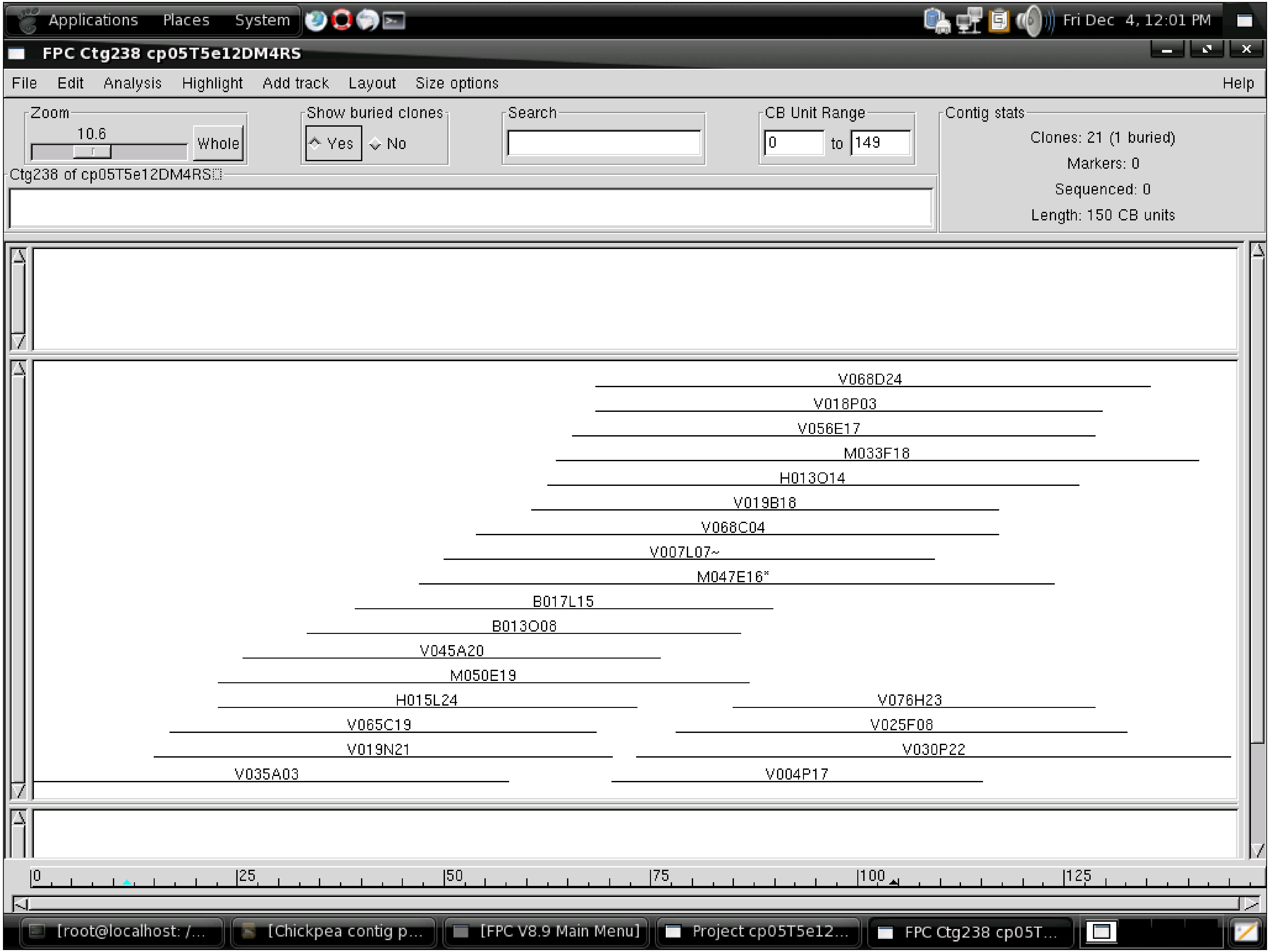

Applications Places System
Fri Dec 4, 12:01 PM
FPC Ctg238 cp05T5e12DM4RS
File Edit Analysis Highlight Add track Layout Size options Help
Zoom
10.6
Whole
Show buried clones
Yes No
Search
CB Unit Range
0 to 149
Contig stats
Clones: 21 (1 buried)
Markers: 0
Sequenced: 0
Length: 150 CB units
Ctg238 of cp05T5e12DM4RS
V068D24
V018P03
V056E17
M033F18
H013O14
V019B18
V068C04
V007L07~
M047E16*
B017L15
B013O08
V045A20
M050E19
H015L24
V076H23
V065C19
V025F08
V019N21
V030P22
V035A03
V004P17
0
25
50
75
100
125
[root@localhost: /...
[Chickpea contig p...
[FPC V8.9 Main Menu]
Project cp05T5e12...
FPC Ctg238 cp05T...

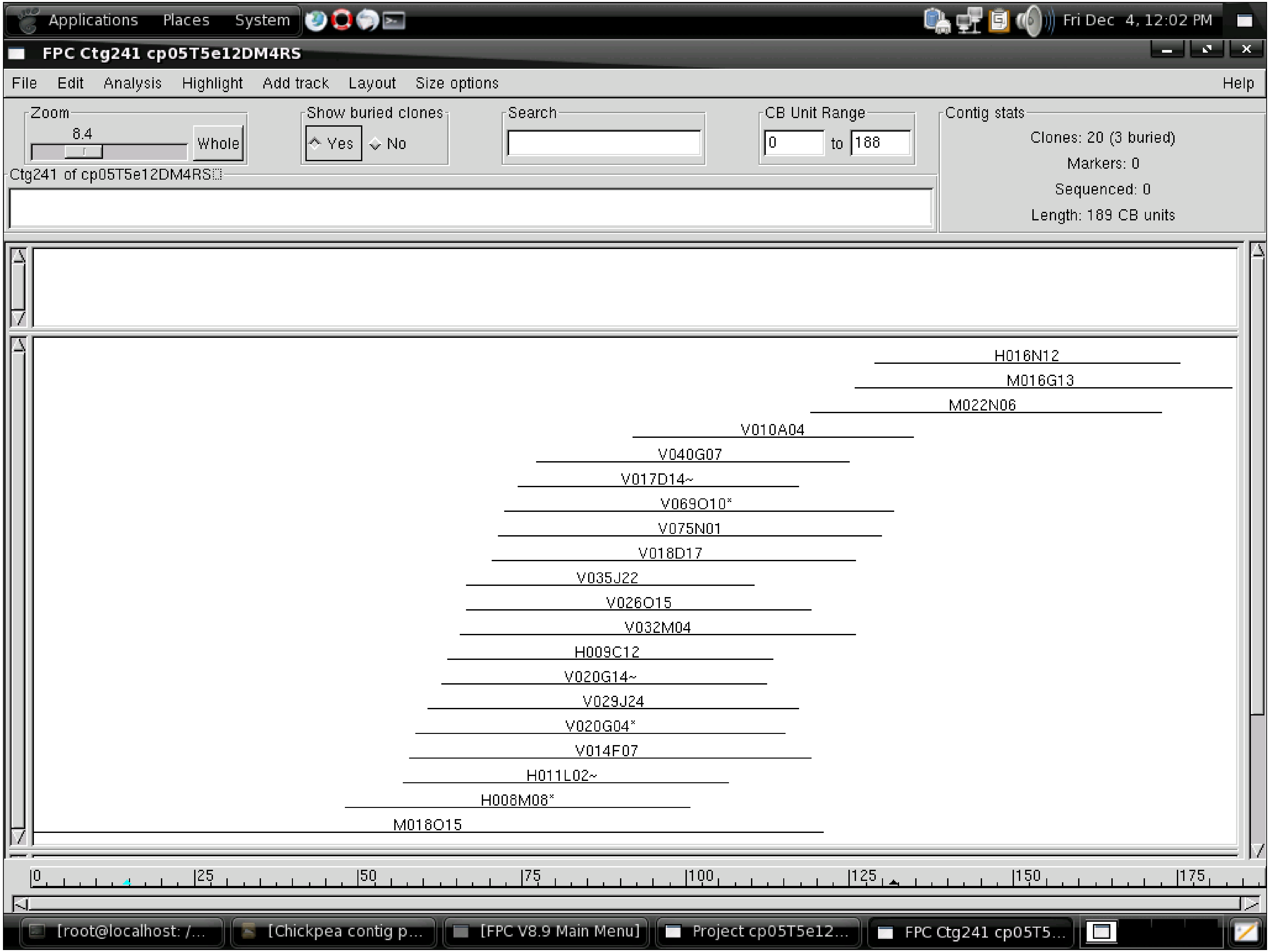
Applications Places System
Fri Dec 4, 12:02 PM
FPC Ctg241 cp05T5e12DM4RS
File Edit Analysis Highlight Add track Layout Size options Help
Zoom
8.4
Whole
Show buried clones
Yes No
Search
CB Unit Range
0 to 188
Contig stats
Clones: 20 (3 buried)
Markers: 0
Sequenced: 0
Length: 189 CB units
Ctg241 of cp05T5e12DM4RS
H016N12
M016G13
M022N06
V010A04
V040G07
V017D14~
V069O10*
V075N01
V018D17
V035J22
V026O15
V032M04
H009C12
V020G14~
V029J24
V020G04*
V014F07
H011L02~
H008M08*
M018O15
0 25 50 75 100 125 150 175
[root@localhost: /...
[Chickpea contig p...
[FPC V8.9 Main Menu]
Project cp05T5e12...
FPC Ctg241 cp05T5...

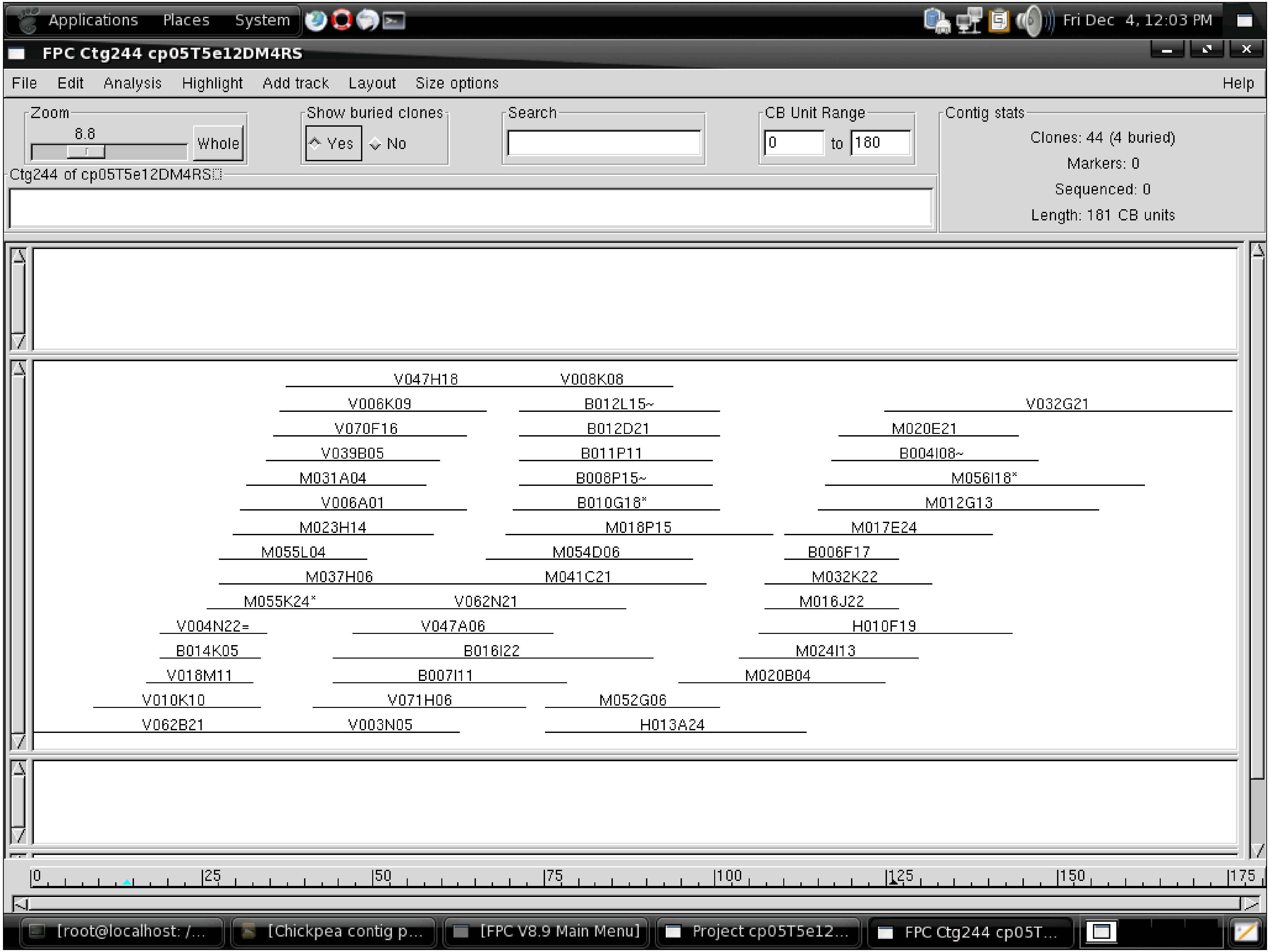
Applications Places System
Fri Dec 4, 12:03 PM
FPC Ctg244 cp05T5e12DM4RS
File Edit Analysis Highlight Add track Layout Size options Help
Zoom
8.8
Whole
Show buried clones
Yes
No
Search
CB Unit Range
0
to
180
Contig stats
Clones: 44 (4 buried)
Markers: 0
Sequenced: 0
Length: 181 CB units
Ctg244 of cp05T5e12DM4RS
V047H18
V008K08
V006K09
B012L15~
V032G21
V070F16
B012D21
M020E21
V039B05
B011P11
B004I08~
M031A04
B008P15~
M056I18*
V006A01
B010G18*
M012G13
M023H14
M018P15
M017E24
M055L04
M054D06
B006F17
M037H06
M041C21
M032K22
M055K24*
V062N21
M016J22
V004N22=
V047A06
H010F19
B014K05
B016I22
M024I13
V018M11
B007I11
M020B04
V010K10
V071H06
M052G06
V062B21
V003N05
H013A24
0
25
50
75
100
125
150
175
[root@localhost: /...
[Chickpea contig p...
[FPC V8.9 Main Menu]
Project cp05T5e12...
FPC Ctg244 cp05T...

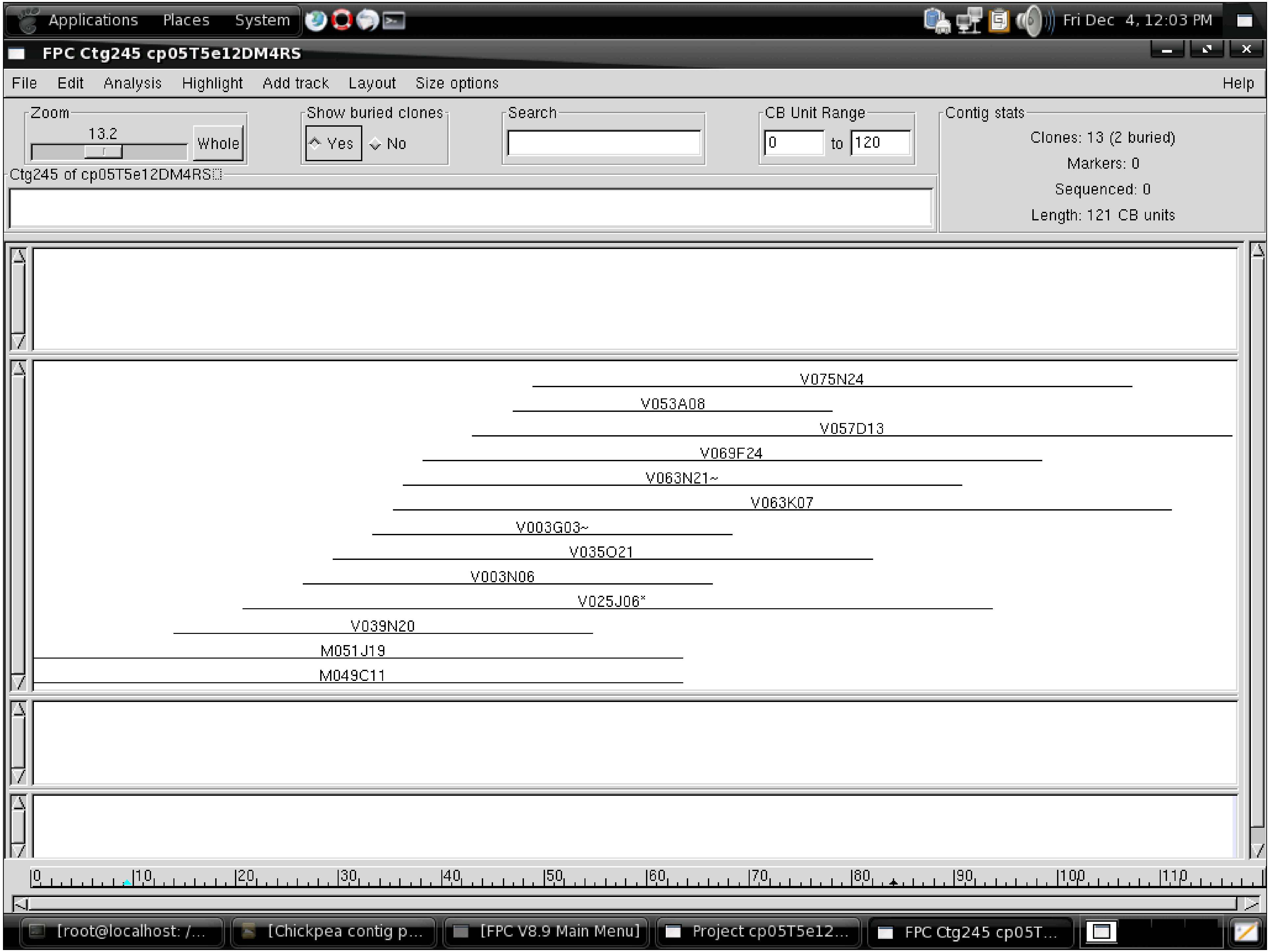
Applications
Places
System
Fri Dec 4, 12:03 PM
FPC Ctg245 cp05T5e12DM4RS
File
Edit
Analysis
Highlight
Add track
Layout
Size options
Help
Zoom
13.2
Whole
Show buried clones
Yes
No
Search
CB Unit Range
0
to
120
Contig stats
Clones: 13 (2 buried)
Markers: 0
Sequenced: 0
Length: 121 CB units
Ctg245 of cp05T5e12DM4RS
V075N24
V053A08
V057D13
V069F24
V063N21~
V063K07
V003G03~
V035O21
V003N06
V025J06*
V039N20
M051J19
M049C11
0
10
20
30
40
50
60
70
80
90
100
110
[root@localhost: /...
[Chickpea contig p...
[FPC V8.9 Main Menu]
Project cp05T5e12...
FPC Ctg245 cp05T...

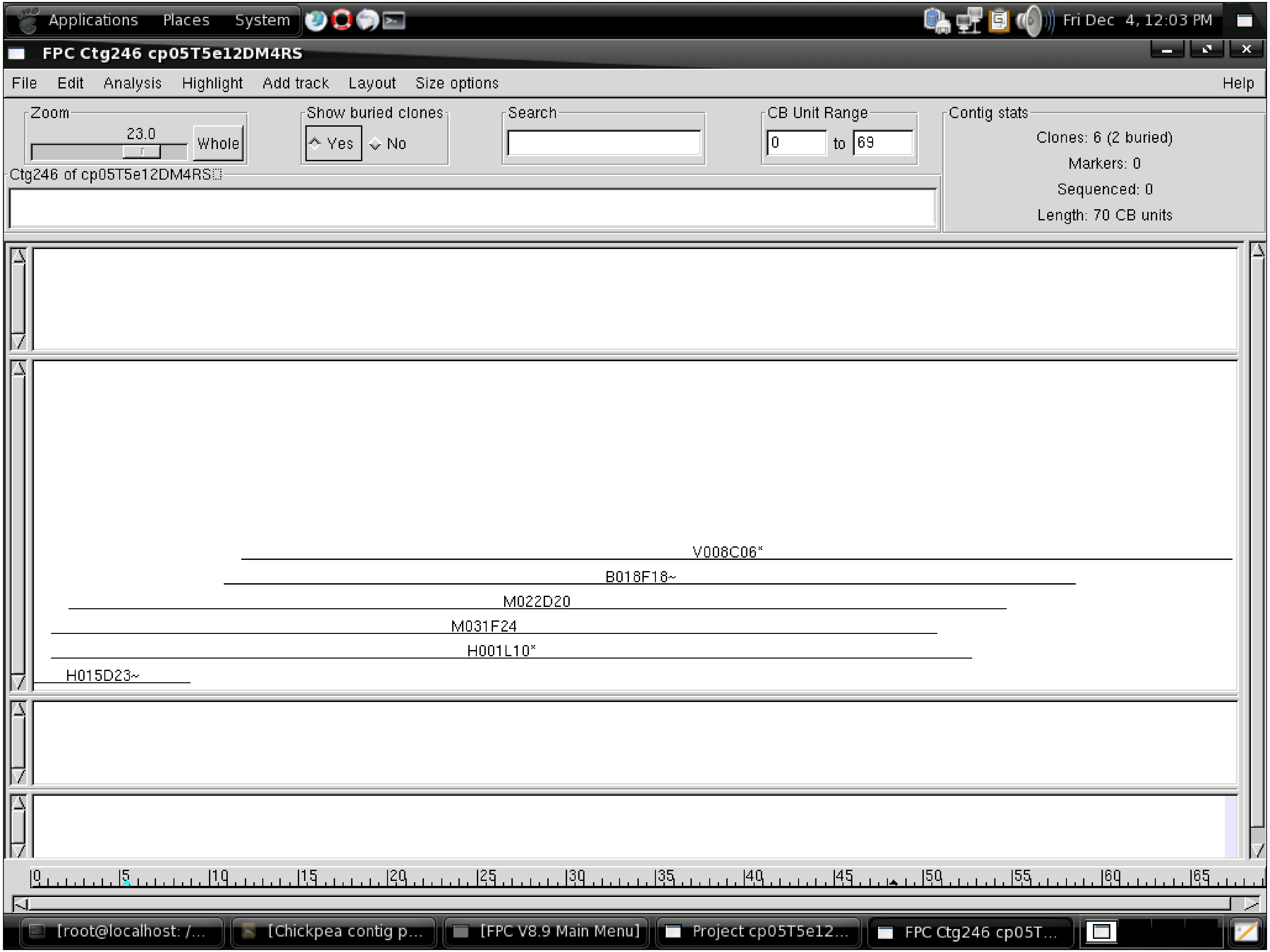
Applications Places System
Fri Dec 4, 12:03 PM
FPC Ctg246 cp05T5e12DM4RS
File Edit Analysis Highlight Add track Layout Size options Help
Zoom
23.0
Whole
Show buried clones
Yes
No
Search
CB Unit Range
0
to
69
Contig stats
Clones: 6 (2 buried)
Markers: 0
Sequenced: 0
Length: 70 CB units
Ctg246 of cp05T5e12DM4RS
V008C06*
B018F18~
M022D20
M031F24
H001L10*
H015D23~
0 5 10 15 20 25 30 35 40 45 50 55 60 65
[root@localhost: /...
[Chickpea contig p...
[FPC V8.9 Main Menu]
Project cp05T5e12...
FPC Ctg246 cp05T...

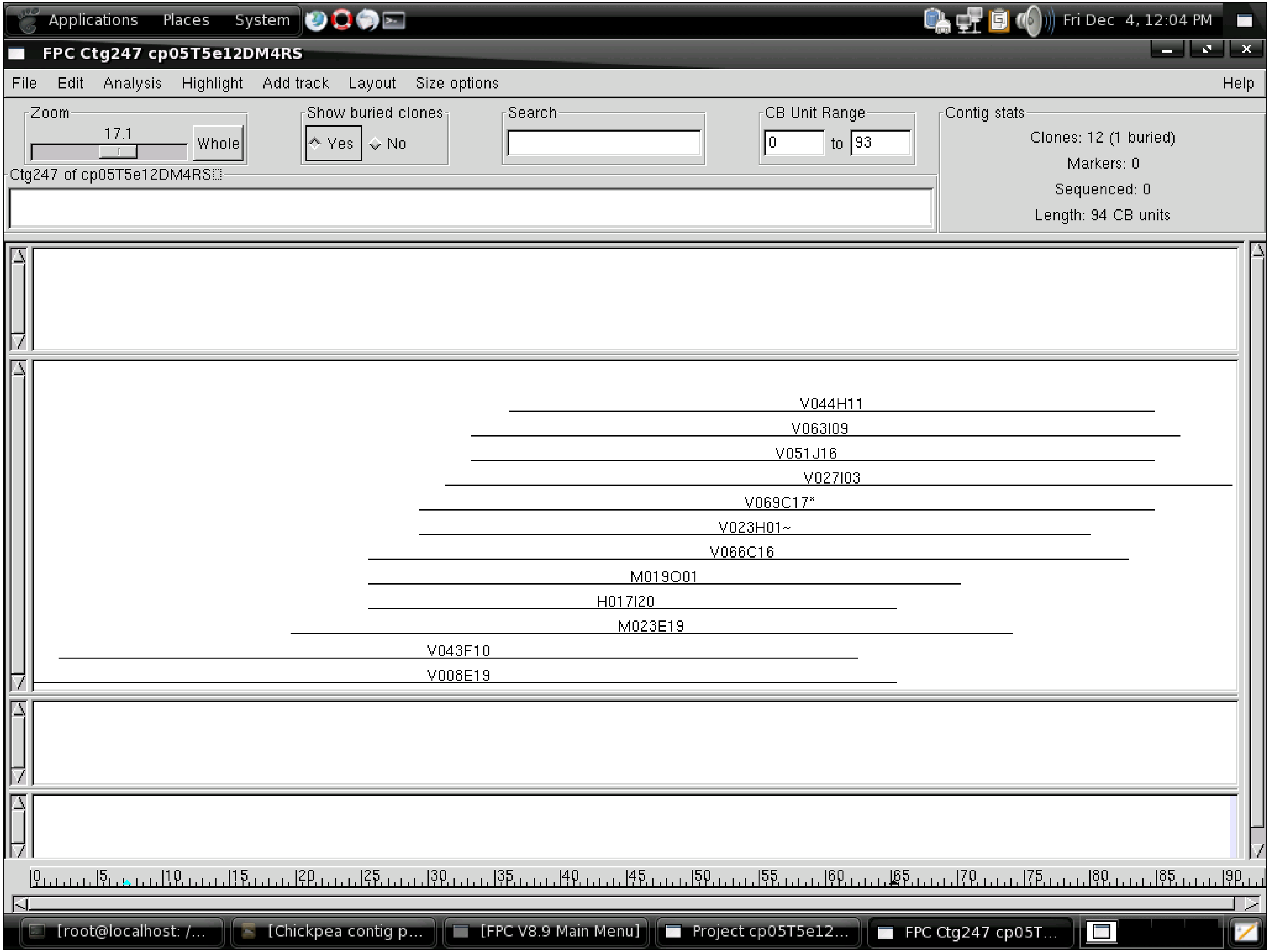
Applications Places System
Fri Dec 4, 12:04 PM
FPC Ctg247 cp05T5e12DM4RS
File Edit Analysis Highlight Add track Layout Size options Help
Zoom
17.1
Whole
Show buried clones
Yes No
Search
CB Unit Range
0 to 93
Contig stats
Clones: 12 (1 buried)
Markers: 0
Sequenced: 0
Length: 94 CB units
Ctg247 of cp05T5e12DM4RS
V044H11
V063I09
V051J16
V027I03
V069C17*
V023H01~
V066C16
M019O01
H017I20
M023E19
V043F10
V008E19
0 5 10 15 20 25 30 35 40 45 50 55 60 65 70 75 80 85 90
[root@localhost: /...
[Chickpea contig p...
[FPC V8.9 Main Menu]
Project cp05T5e12...
FPC Ctg247 cp05T...

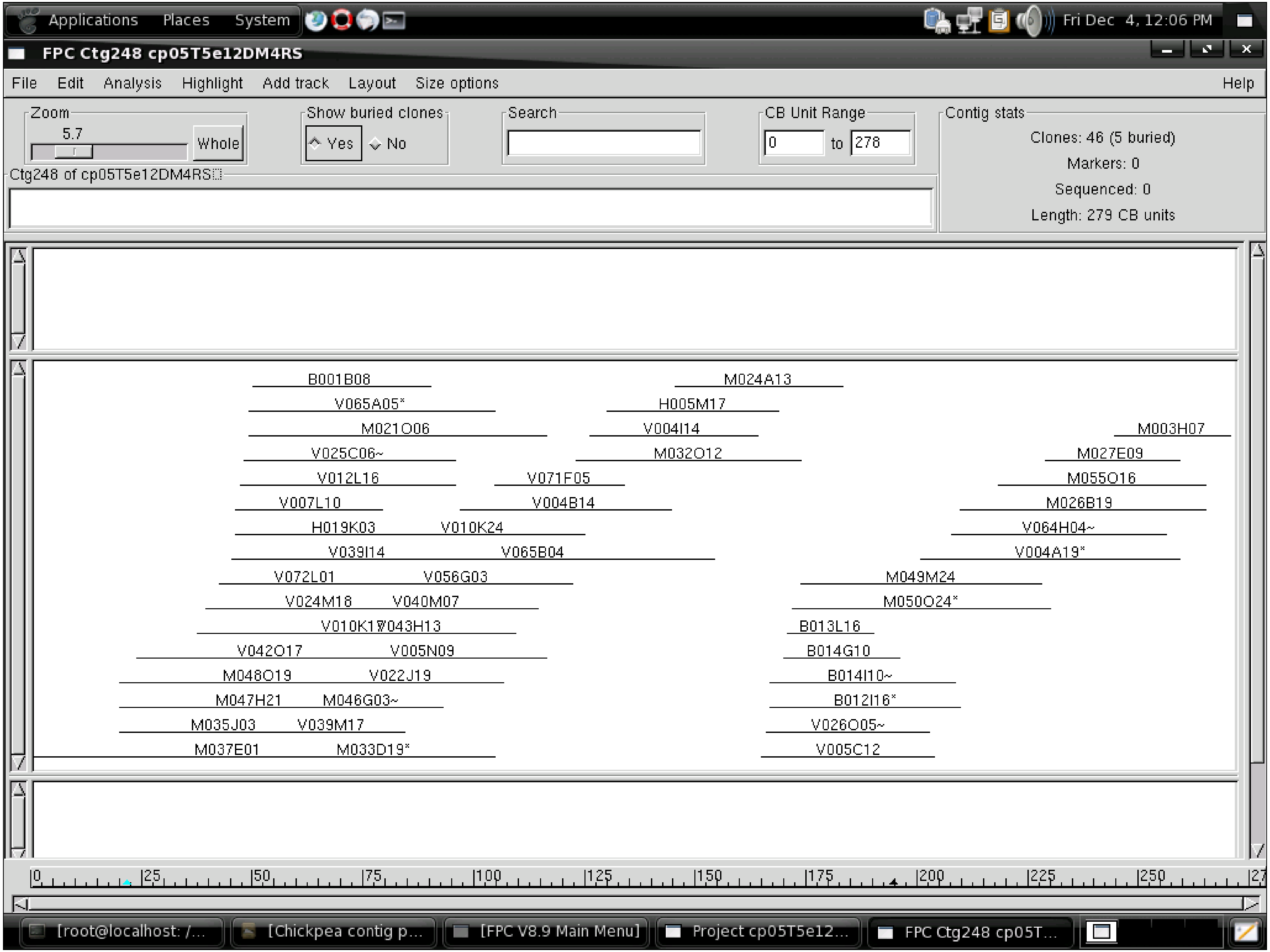
Applications Places System
Fri Dec 4, 12:06 PM
FPC Ctg248 cp05T5e12DM4RS
File Edit Analysis Highlight Add track Layout Size options Help
Zoom
5.7
Whole
Show buried clones
Yes
No
Search
CB Unit Range
0 to 278
Contig stats
Clones: 46 (5 buried)
Markers: 0
Sequenced: 0
Length: 279 CB units
Ctg248 of cp05T5e12DM4RS
B001B08
M024A13
V065A05*
H005M17
M021O06
V004I14
M003H07
V025C06~
M032O12
M027E09
V012L16
V071F05
M055O16
V007L10
V004B14
M026B19
H019K03
V010K24
V064H04~
V039I14
V065B04
V004A19*
V072L01
V056G03
M049M24
V024M18
V040M07
M050O24*
V010K1V043H13
B013L16
V042O17
V005N09
B014G10
M048O19
V022J19
B014I10~
M047H21
M046G03~
B012I16*
M035J03
V039M17
V026O05~
M037E01
M033D19*
V005C12
0 25 50 75 100 125 150 175 200 225 250
[root@localhost: /...
[Chickpea contig p...
[FPC V8.9 Main Menu]
Project cp05T5e12...
FPC Ctg248 cp05T...

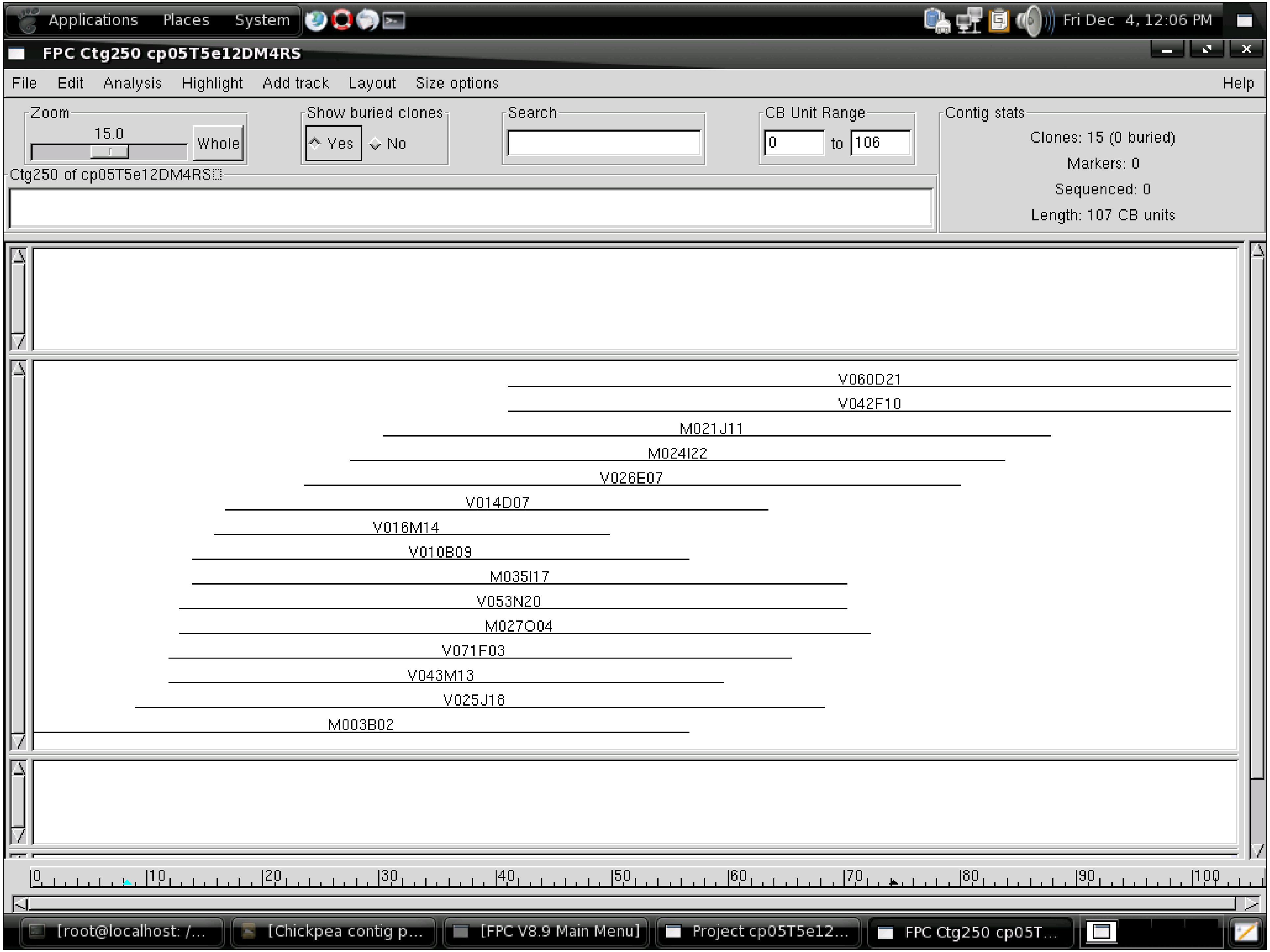
Applications Places System
Fri Dec 4, 12:06 PM
FPC Ctg250 cp05T5e12DM4RS
File Edit Analysis Highlight Add track Layout Size options Help
Zoom
15.0
Whole
Show buried clones
Yes No
Search
CB Unit Range
0 to 106
Contig stats
Clones: 15 (0 buried)
Markers: 0
Sequenced: 0
Length: 107 CB units
Ctg250 of cp05T5e12DM4RS
V060D21
V042F10
M021J11
M024I22
V026E07
V014D07
V016M14
V010B09
M035I17
V053N20
M027O04
V071F03
V043M13
V025J18
M003B02
0 10 20 30 40 50 60 70 80 90 100
[root@localhost: /...
[Chickpea contig p...
[FPC V8.9 Main Menu]
Project cp05T5e12...
FPC Ctg250 cp05T...

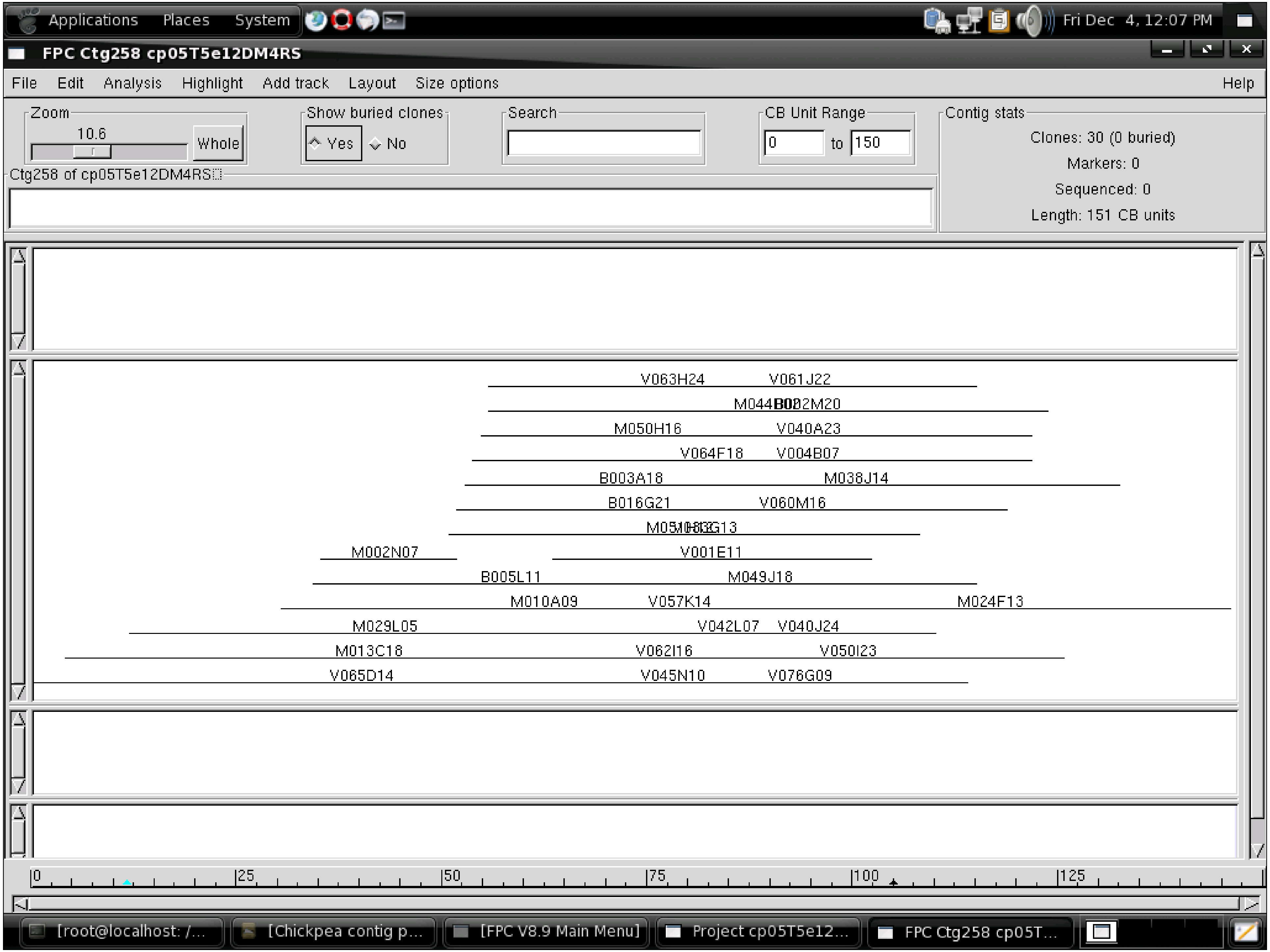
Applications Places System
Fri Dec 4, 12:07 PM
FPC Ctg258 cp05T5e12DM4RS
File Edit Analysis Highlight Add track Layout Size options Help
Zoom
10.6
Whole
Show buried clones
Yes
No
Search
CB Unit Range
0 to 150
Contig stats
Clones: 30 (0 buried)
Markers: 0
Sequenced: 0
Length: 151 CB units
Ctg258 of cp05T5e12DM4RS
V063H24 V061J22
M050H16 V040A23
V064F18 V004B07
B003A18 M038J14
B016G21 V060M16
M002N07 V001E11
B005L11 M049J18
M010A09 V057K14 M024F13
M029L05 V042L07 V040J24
M013C18 V062I16 V050I23
V065D14 V045N10 V076G09
0 25 50 75 100 125
[root@localhost: /...
[Chickpea contig p...
[FPC V8.9 Main Menu]
Project cp05T5e12...
FPC Ctg258 cp05T...

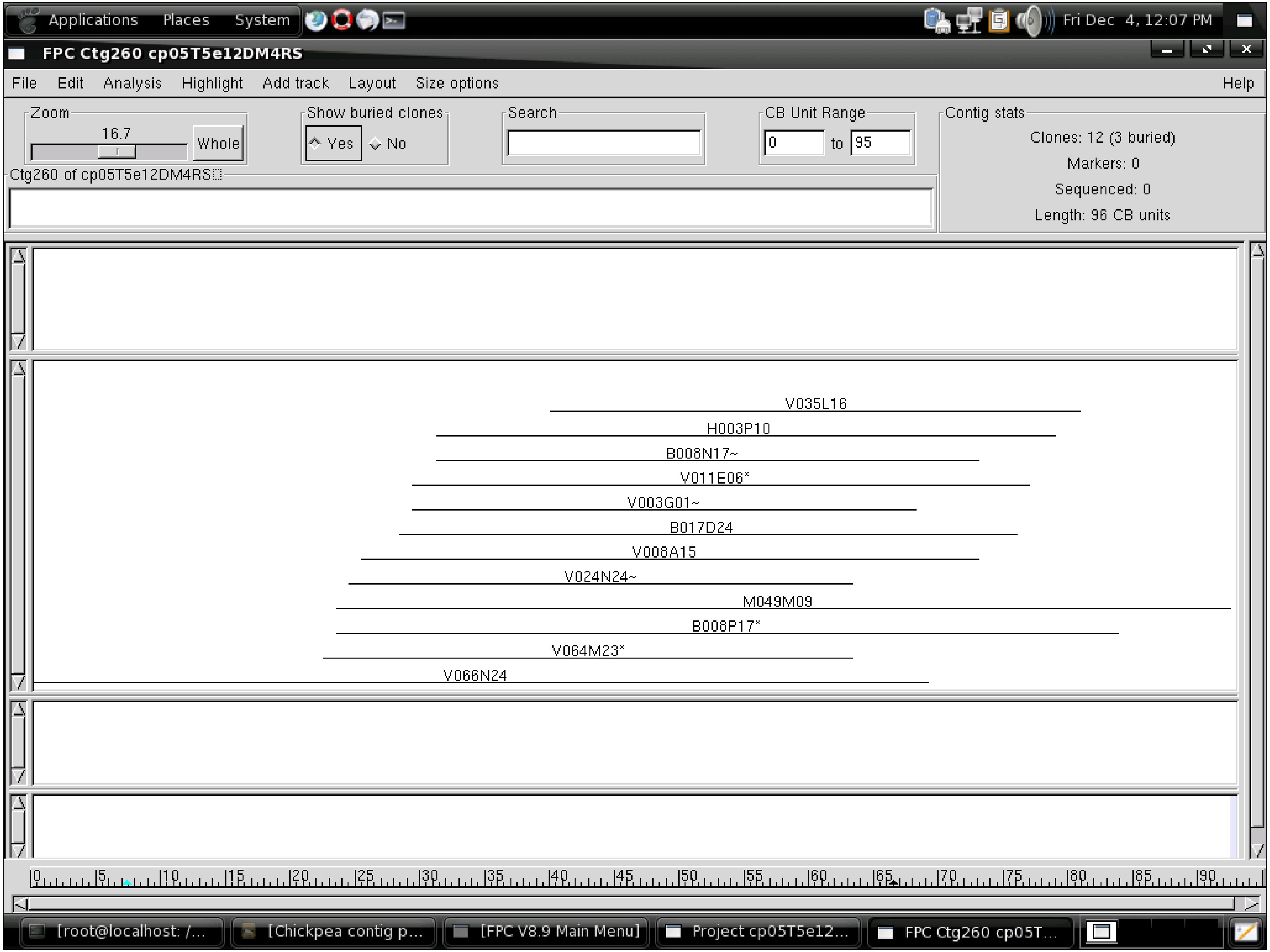
Applications Places System
Fri Dec 4, 12:07 PM
FPC Ctg260 cp05T5e12DM4RS
File Edit Analysis Highlight Add track Layout Size options Help
Zoom
16.7
Whole
Show buried clones
Yes No
Search
CB Unit Range
0 to 95
Contig stats
Clones: 12 (3 buried)
Markers: 0
Sequenced: 0
Length: 96 CB units
Ctg260 of cp05T5e12DM4RS
V035L16
H003P10
B008N17~
V011E06*
V003G01~
B017D24
V008A15
V024N24~
M049M09
B008P17*
V064M23*
V066N24
0 5 10 15 20 25 30 35 40 45 50 55 60 65 70 75 80 85 90
[root@localhost: /...
[Chickpea contig p...
[FPC V8.9 Main Menu]
Project cp05T5e12...
FPC Ctg260 cp05T...

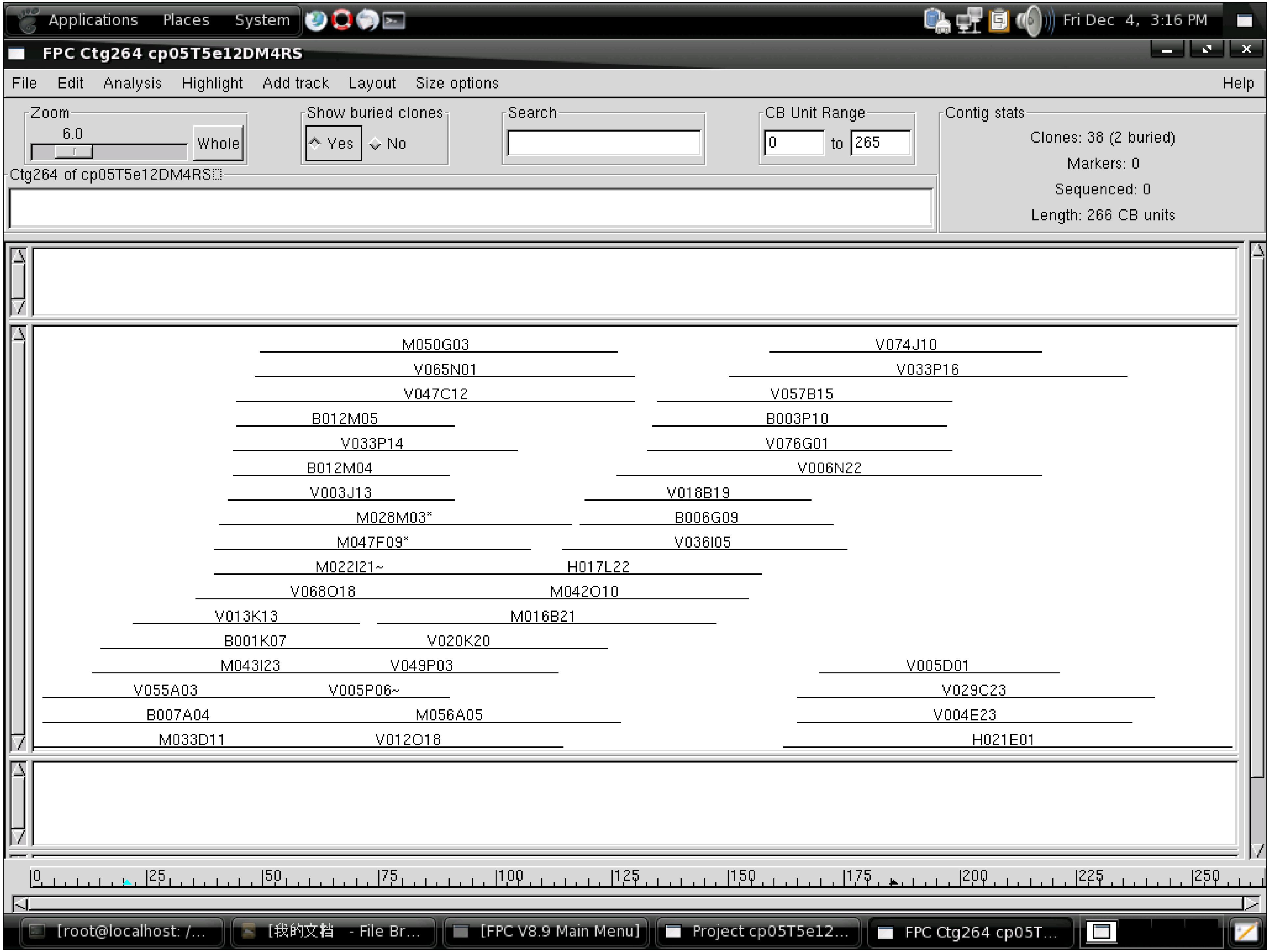
Applications Places System
Fri Dec 4, 3:16 PM
FPC Ctg264 cp05T5e12DM4RS
File Edit Analysis Highlight Add track Layout Size options Help
Zoom
6.0
Whole
Show buried clones
Yes
No
Search
CB Unit Range
0 to 265
Contig stats
Clones: 38 (2 buried)
Markers: 0
Sequenced: 0
Length: 266 CB units
Ctg264 of cp05T5e12DM4RS
M050G03
V074J10
V065N01
V033P16
V047C12
V057B15
B012M05
B003P10
V033P14
V076G01
B012M04
V006N22
V003J13
V018B19
M028M03*
B006G09
M047F09*
V036I05
M022I21~
H017L22
V068O18
M042O10
V013K13
M016B21
B001K07
V020K20
M043I23
V049P03
V005D01
V055A03
V005P06~
V029C23
B007A04
M056A05
V004E23
M033D11
V012O18
H021E01
0 25 50 75 100 125 150 175 200 225 250
[root@localhost: /...
[我的文档 - File Br...
[FPC V8.9 Main Menu]
Project cp05T5e12...
FPC Ctg264 cp05T...

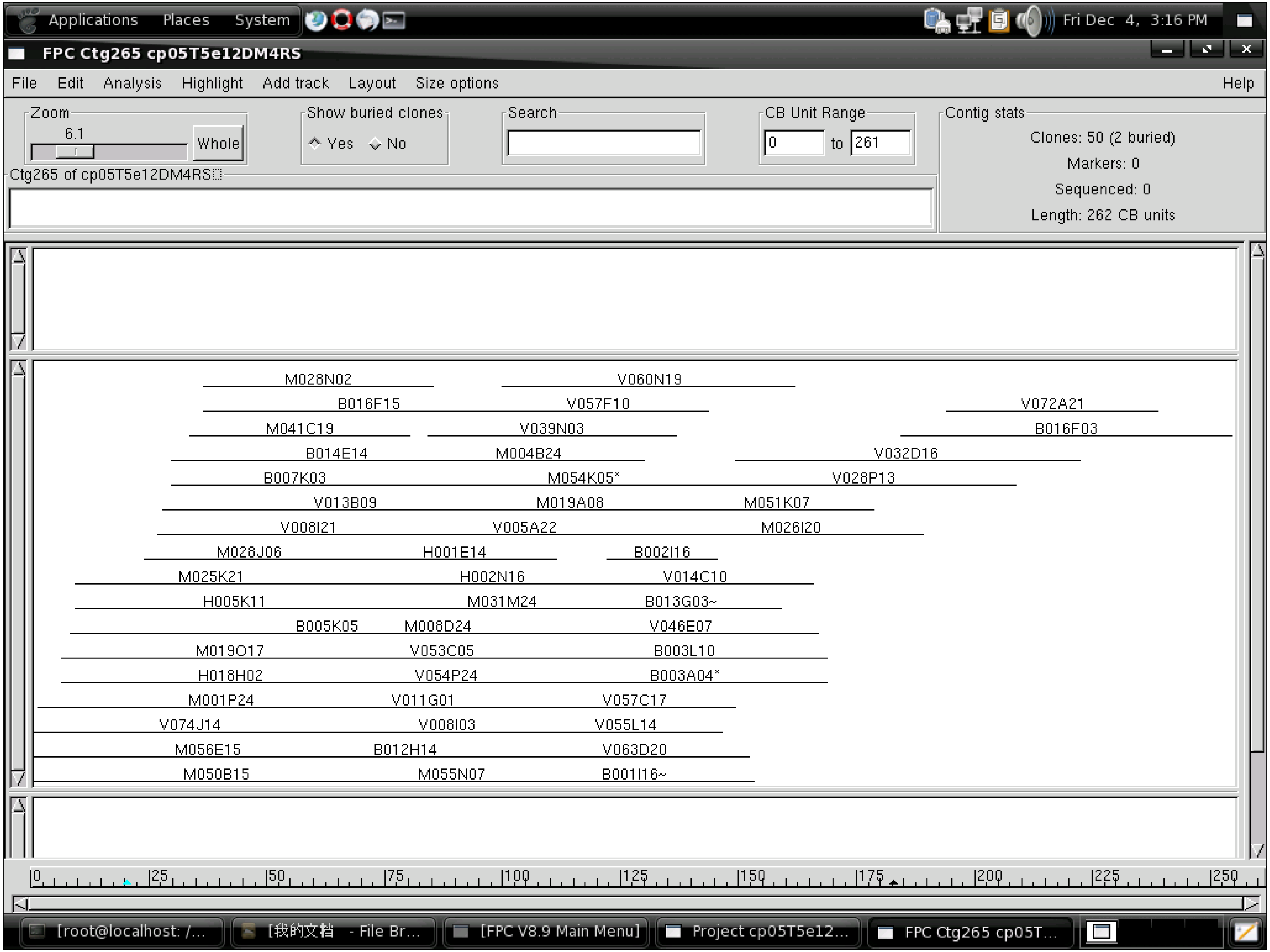
Applications Places System
Fri Dec 4, 3:16 PM
FPC Ctg265 cp05T5e12DM4RS
File Edit Analysis Highlight Add track Layout Size options Help
Zoom
6.1
Whole
Show buried clones
Yes No
Search
CB Unit Range
0 to 261
Contig stats
Clones: 50 (2 buried)
Markers: 0
Sequenced: 0
Length: 262 CB units
Ctg265 of cp05T5e12DM4RS
M028N02
V060N19
B016F15
V057F10
V072A21
M041C19
V039N03
B016F03
B014E14
M004B24
V032D16
B007K03
M054K05*
V028P13
V013B09
M019A08
M051K07
V008I21
V005A22
M026I20
M028J06
H001E14
B002I16
M025K21
H002N16
V014C10
H005K11
M031M24
B013G03~
B005K05
M008D24
V046E07
M019O17
V053C05
B003L10
H018H02
V054P24
B003A04*
M001P24
V011G01
V057C17
V074J14
V008I03
V055L14
M056E15
B012H14
V063D20
M050B15
M055N07
B001I16~
0 25 50 75 100 125 150 175 200 225 250
[root@localhost: /...
[我的文档 - File Br...
[FPC V8.9 Main Menu]
Project cp05T5e12...
FPC Ctg265 cp05T...

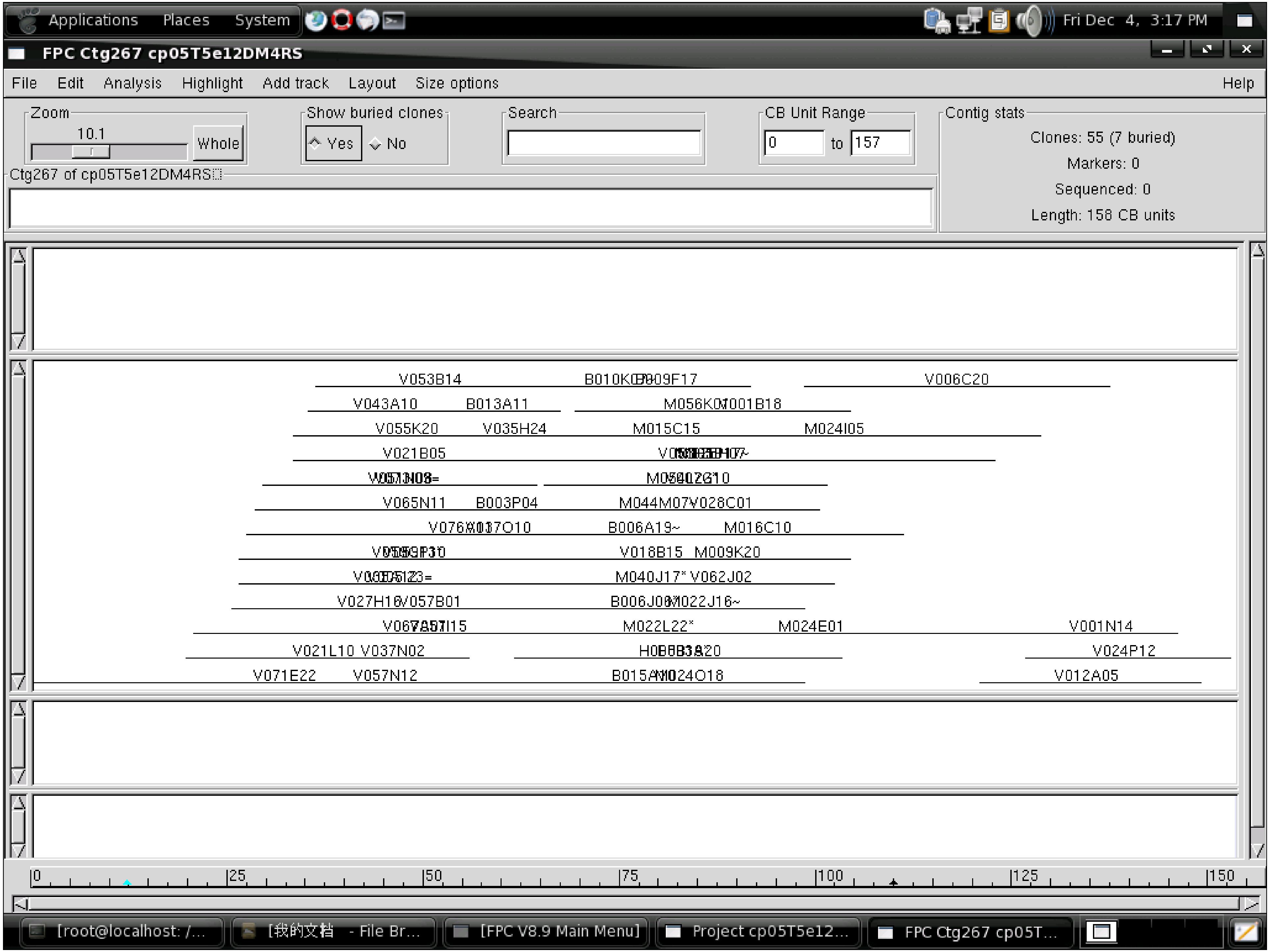

Applications Places System
Fri Dec 4, 3:17 PM
FPC Ctg267 cp05T5e12DM4RS
File Edit Analysis Highlight Add track Layout Size options Help
Zoom
10.1
Whole
Show buried clones
Yes
No
Search
CB Unit Range
0 to 157
Contig stats
Clones: 55 (7 buried)
Markers: 0
Sequenced: 0
Length: 158 CB units
Ctg267 of cp05T5e12DM4RS
V053B14
V006C20
V043A10
B013A11
V055K20
V035H24
M015C15
M024I05
V021B05
V065N11
B003P04
M016C10
V018B15
M009K20
V062J02
V027H16
M024E01
V001N14
V021L10
V037N02
V024P12
V071E22
V057N12
V012A05
0
25
50
75
100
125
150
[root@localhost: /...
[我的文档 - File Br...
[FPC V8.9 Main Menu]
Project cp05T5e12...
FPC Ctg267 cp05T...

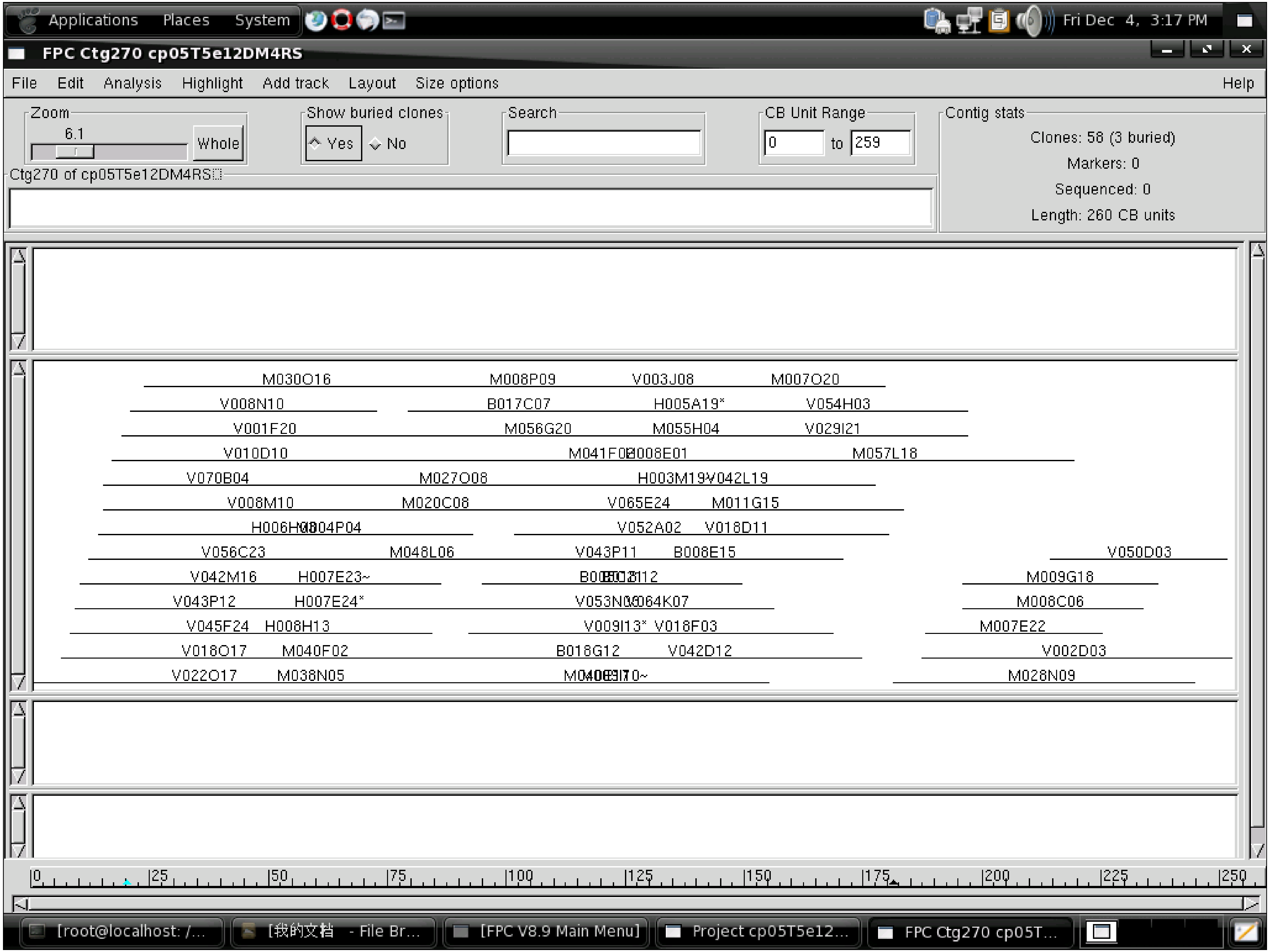
Applications
Places
System
Fri Dec 4, 3:17 PM
FPC Ctg270 cp05T5e12DM4RS
File
Edit
Analysis
Highlight
Add track
Layout
Size options
Help
Zoom
6.1
Whole
Show buried clones
Yes
No
Search
CB Unit Range
0
to
259
Contig stats
Clones: 58 (3 buried)
Markers: 0
Sequenced: 0
Length: 260 CB units
Ctg270 of cp05T5e12DM4RS
M030O16
M008P09
V003J08
M007O20
V008N10
B017C07
H005A19*
V054H03
V001F20
M056G20
M055H04
V029I21
V010D10
M057L18
V070B04
M027O08
V008M10
M020C08
V065E24
M011G15
V052A02
V018D11
V056C23
M048L06
V043P11
B008E15
V050D03
V042M16
H007E23~
M009G18
V043P12
H007E24*
M008C06
V045F24
H008H13
V018F03
M007E22
V018O17
M040F02
B018G12
V042D12
V002D03
V022O17
M038N05
M028N09
0
25
50
75
100
125
150
175
200
225
250
[root@localhost: /...
[我的文档 - File Br...
[FPC V8.9 Main Menu]
Project cp05T5e12...
FPC Ctg270 cp05T...

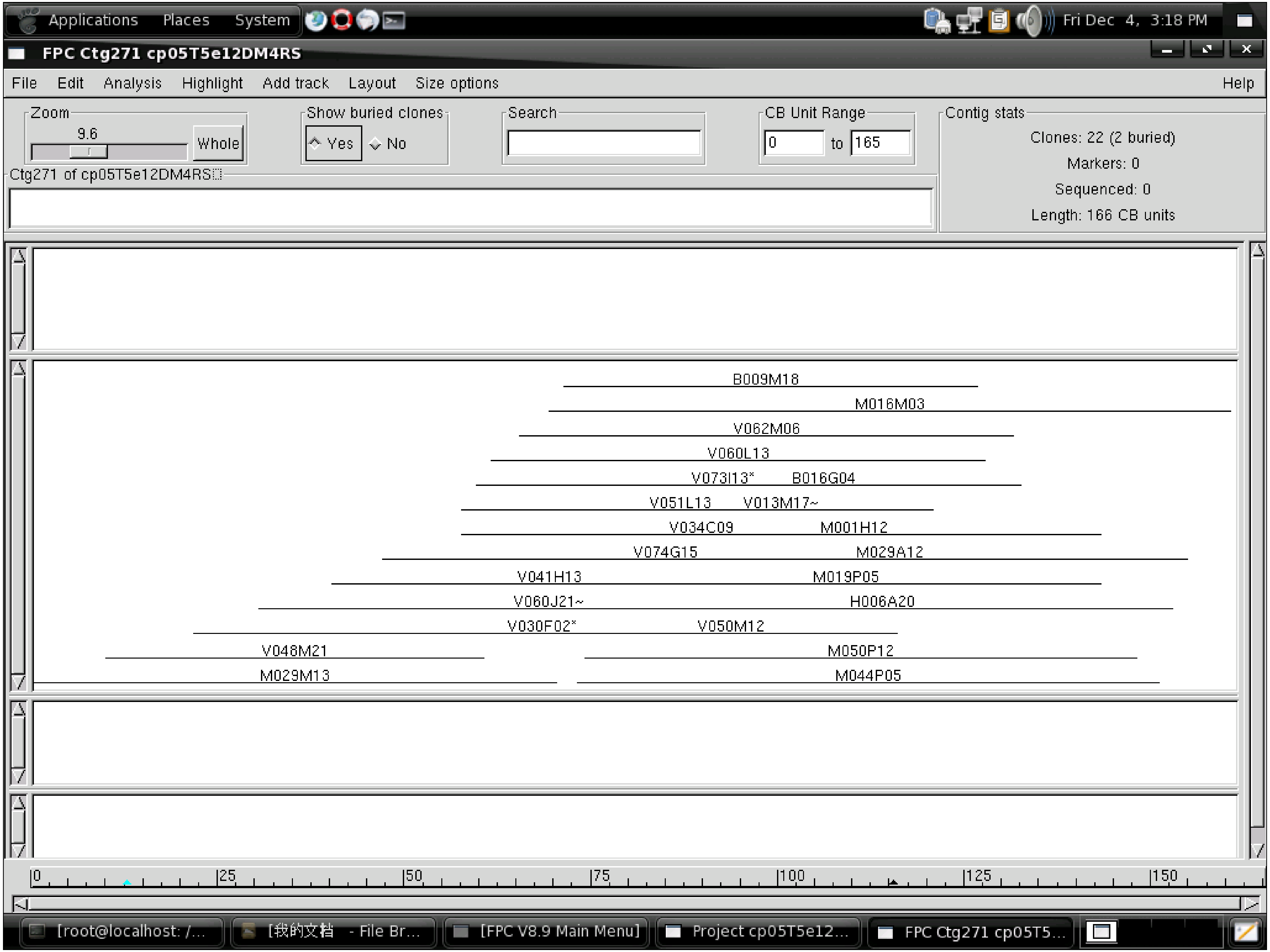
Applications Places System
Fri Dec 4, 3:18 PM
FPC Ctg271 cp05T5e12DM4RS
File Edit Analysis Highlight Add track Layout Size options Help
Zoom 9.6 Whole
Show buried clones Yes No
Search
CB Unit Range 0 to 165
Contig stats
Clones: 22 (2 buried)
Markers: 0
Sequenced: 0
Length: 166 CB units
Ctg271 of cp05T5e12DM4RS
B009M18
M016M03
V062M06
V060L13
V073I13* B016G04
V051L13 V013M17~
V034C09 M001H12
V074G15 M029A12
V041H13 M019P05
V060J21~ H006A20
V030F02* V050M12
V048M21 M050P12
M029M13 M044P05
0 25 50 75 100 125 150
[root@localhost: /... [我的文档 - File Br... [FPC V8.9 Main Menu] Project cp05T5e12... FPC Ctg271 cp05T5...

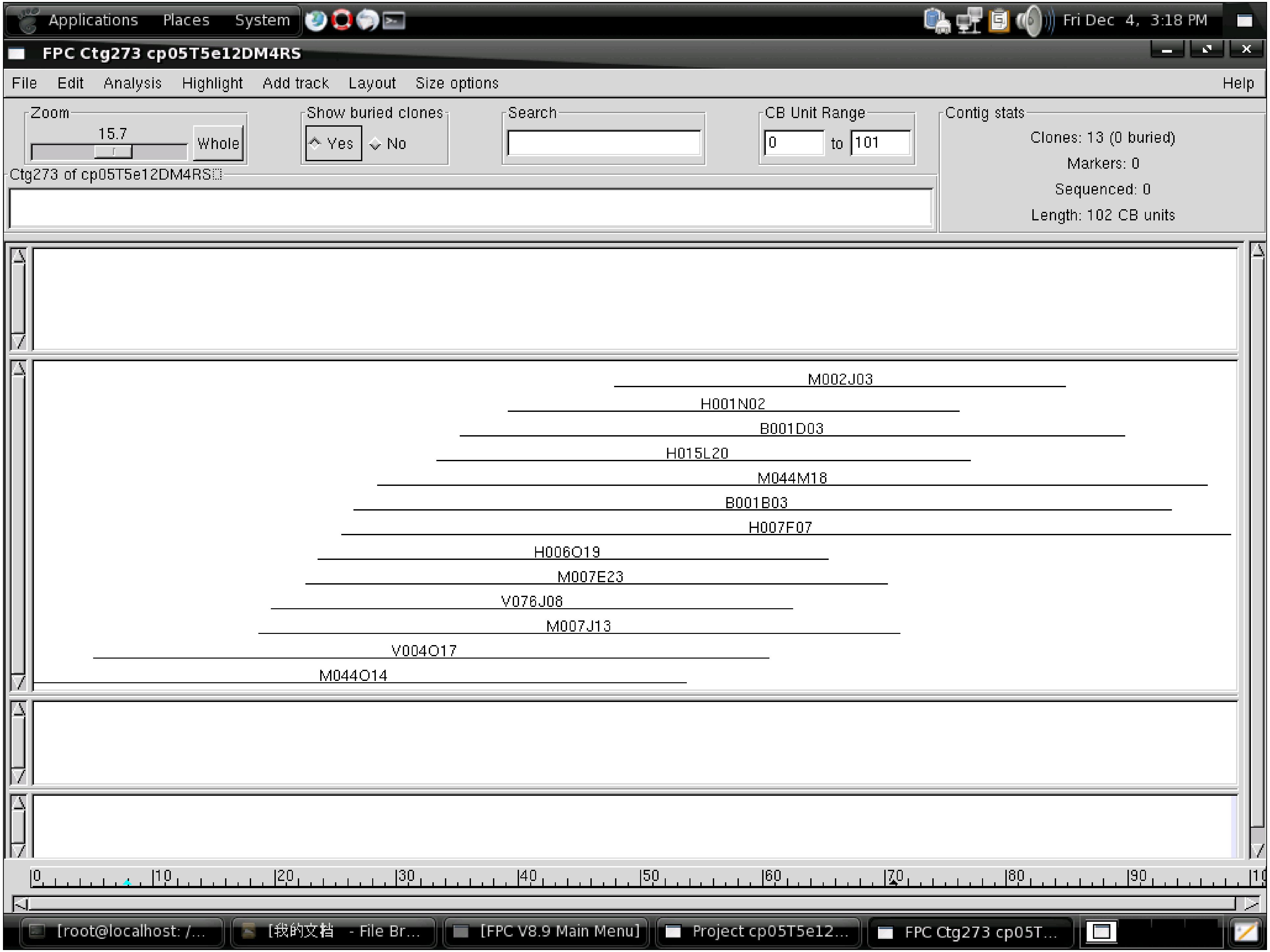
Applications Places System
Fri Dec 4, 3:18 PM
FPC Ctg273 cp05T5e12DM4RS
File Edit Analysis Highlight Add track Layout Size options Help
Zoom
15.7
Whole
Show buried clones
Yes
No
Search
CB Unit Range
0
to
101
Contig stats
Clones: 13 (0 buried)
Markers: 0
Sequenced: 0
Length: 102 CB units
Ctg273 of cp05T5e12DM4RS
M002J03
H001N02
B001D03
H015L20
M044M18
B001B03
H007F07
H006O19
M007E23
V076J08
M007J13
V004O17
M044O14
0 10 20 30 40 50 60 70 80 90
[root@localhost: /...
[我的文档 - File Br...
[FPC V8.9 Main Menu]
Project cp05T5e12...
FPC Ctg273 cp05T...

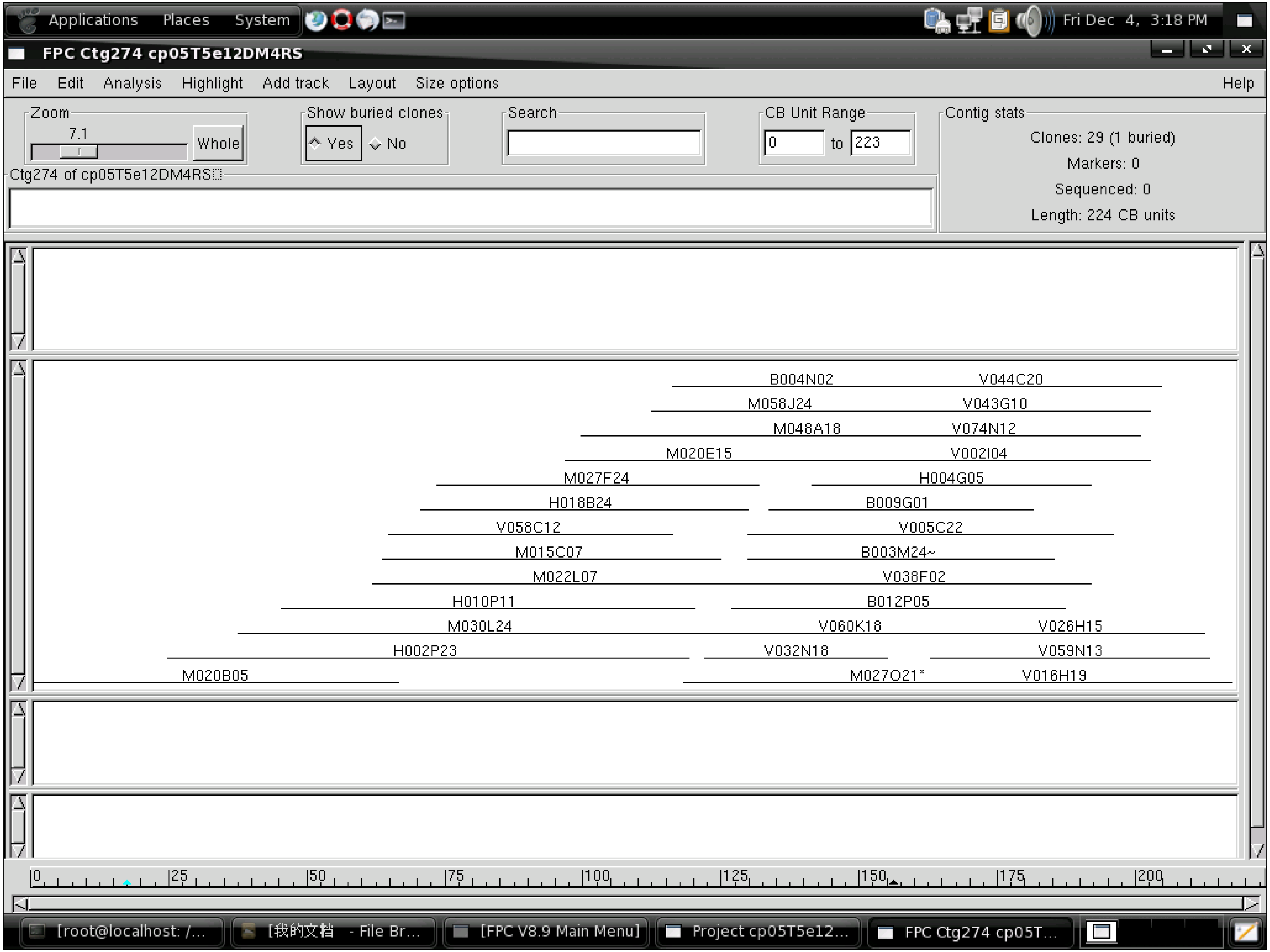
Applications Places System
Fri Dec 4, 3:18 PM
FPC Ctg274 cp05T5e12DM4RS
File Edit Analysis Highlight Add track Layout Size options Help
Zoom 7.1 Whole
Show buried clones Yes No
Search
CB Unit Range 0 to 223
Contig stats
Clones: 29 (1 buried)
Markers: 0
Sequenced: 0
Length: 224 CB units
Ctg274 of cp05T5e12DM4RS
B004N02
V044C20
M058J24
V043G10
M048A18
V074N12
M020E15
V002I04
M027F24
H004G05
H018B24
B009G01
V058C12
V005C22
M015C07
B003M24~
M022L07
V038F02
H010P11
B012P05
M030L24
V060K18
V026H15
H002P23
V032N18
V059N13
M020B05
M027O21*
V016H19
0 25 50 75 100 125 150 175 200
[root@localhost: /...
[我的文档 - File Br...
[FPC V8.9 Main Menu]
Project cp05T5e12...
FPC Ctg274 cp05T...

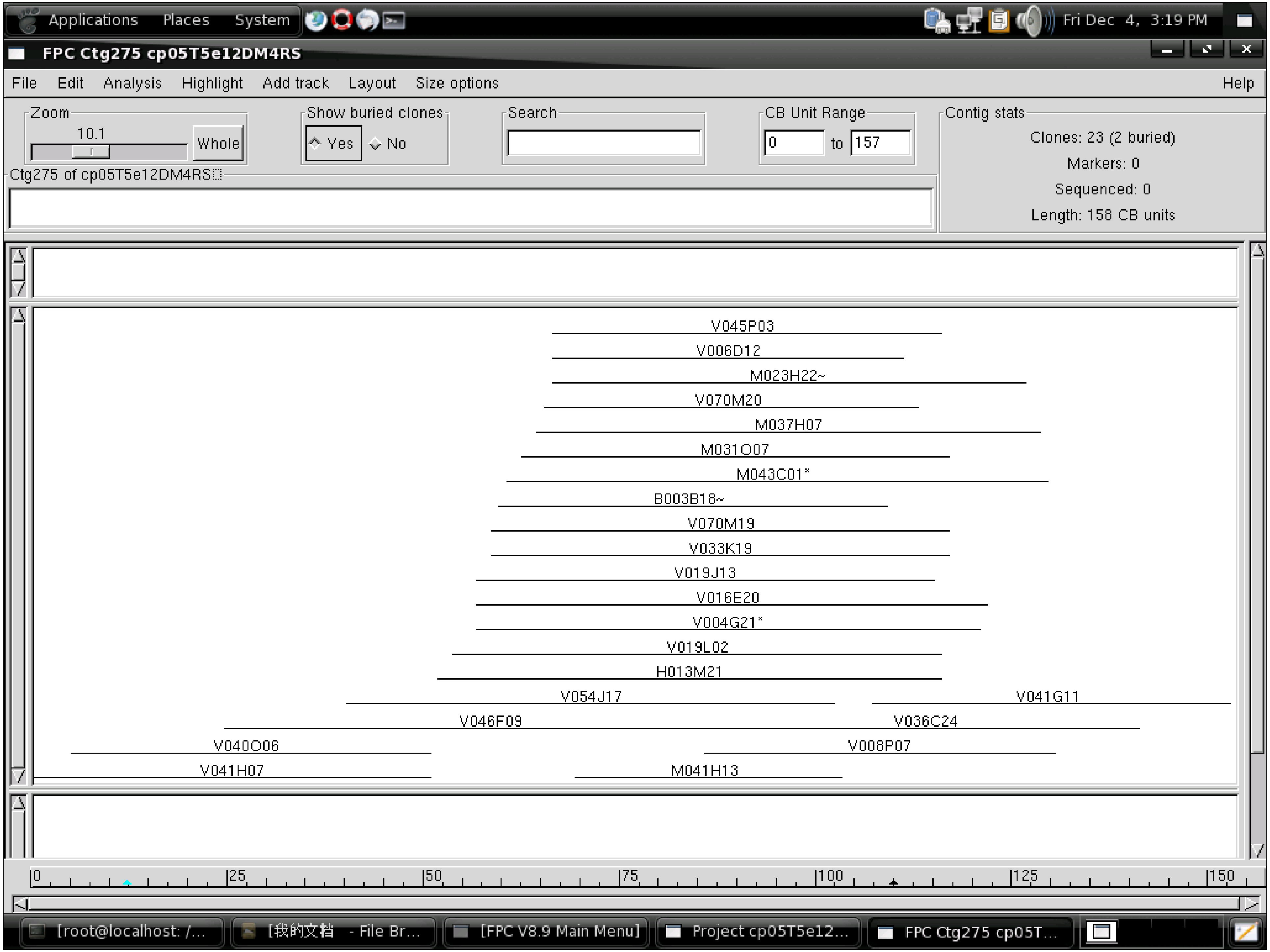
Applications Places System
Fri Dec 4, 3:19 PM
FPC Ctg275 cp05T5e12DM4RS
File Edit Analysis Highlight Add track Layout Size options Help
Zoom
10.1
Whole
Show buried clones
Yes No
Search
CB Unit Range
0 to 157
Contig stats
Clones: 23 (2 buried)
Markers: 0
Sequenced: 0
Length: 158 CB units
Ctg275 of cp05T5e12DM4RS
V045P03
V006D12
M023H22~
V070M20
M037H07
M031O07
M043C01*
B003B18~
V070M19
V033K19
V019J13
V016E20
V004G21*
V019L02
H013M21
V054J17
V041G11
V046F09
V036C24
V040O06
V008P07
V041H07
M041H13
0
25
50
75
100
125
150
[root@localhost: /...
[我的文档 - File Br...
[FPC V8.9 Main Menu]
Project cp05T5e12...
FPC Ctg275 cp05T...

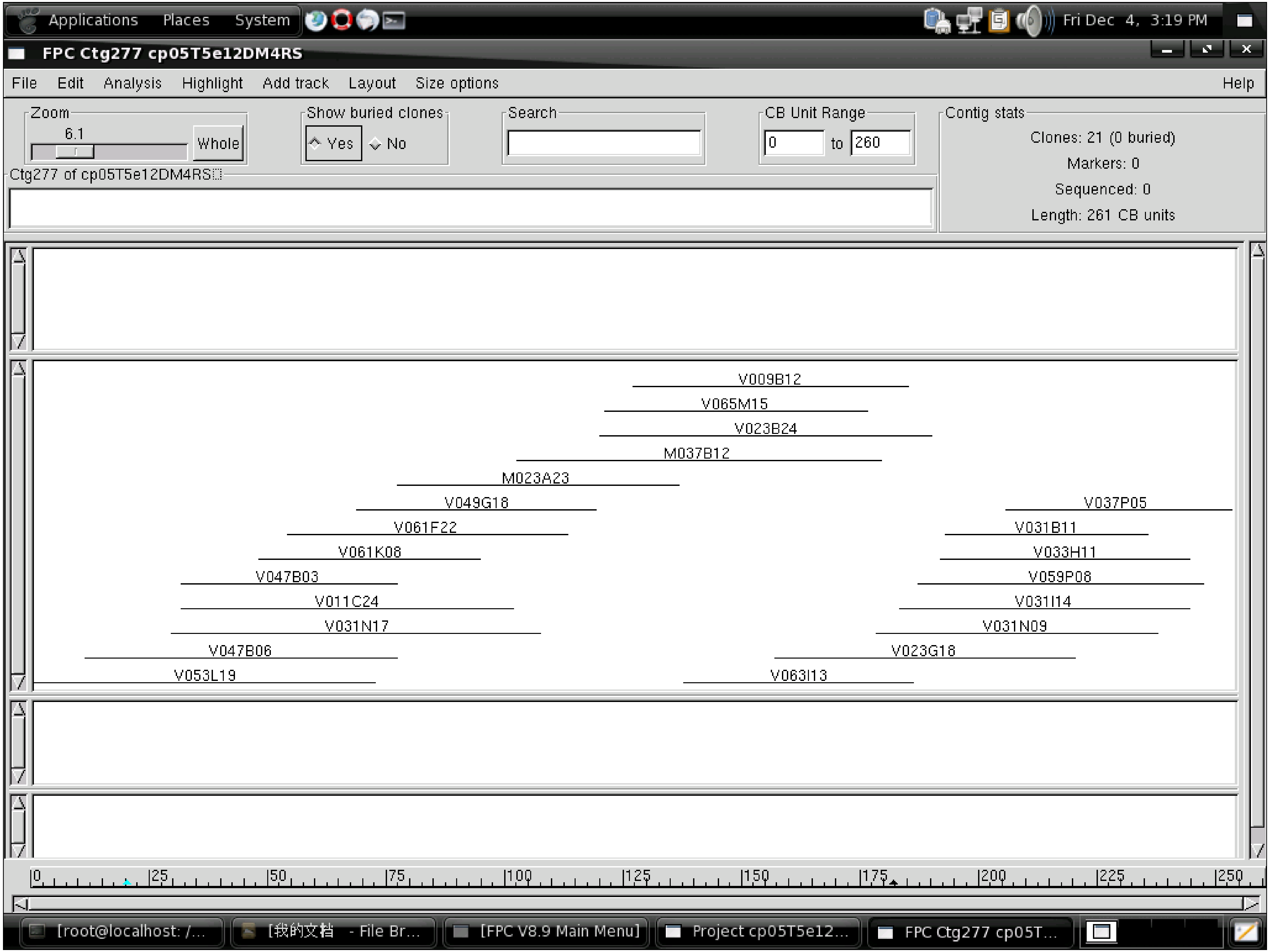
Applications Places System
Fri Dec 4, 3:19 PM
FPC Ctg277 cp05T5e12DM4RS
File Edit Analysis Highlight Add track Layout Size options Help
Zoom
6.1
Whole
Show buried clones
Yes
No
Search
CB Unit Range
0
to
260
Contig stats
Clones: 21 (0 buried)
Markers: 0
Sequenced: 0
Length: 261 CB units
Ctg277 of cp05T5e12DM4RS
V009B12
V065M15
V023B24
M037B12
M023A23
V049G18
V037P05
V061F22
V031B11
V061K08
V033H11
V047B03
V059P08
V011C24
V031I14
V031N17
V031N09
V047B06
V023G18
V053L19
V063I13
0
25
50
75
100
125
150
175
200
225
250
[root@localhost: /...
[我的文档 - File Br...
[FPC V8.9 Main Menu]
Project cp05T5e12...
FPC Ctg277 cp05T...

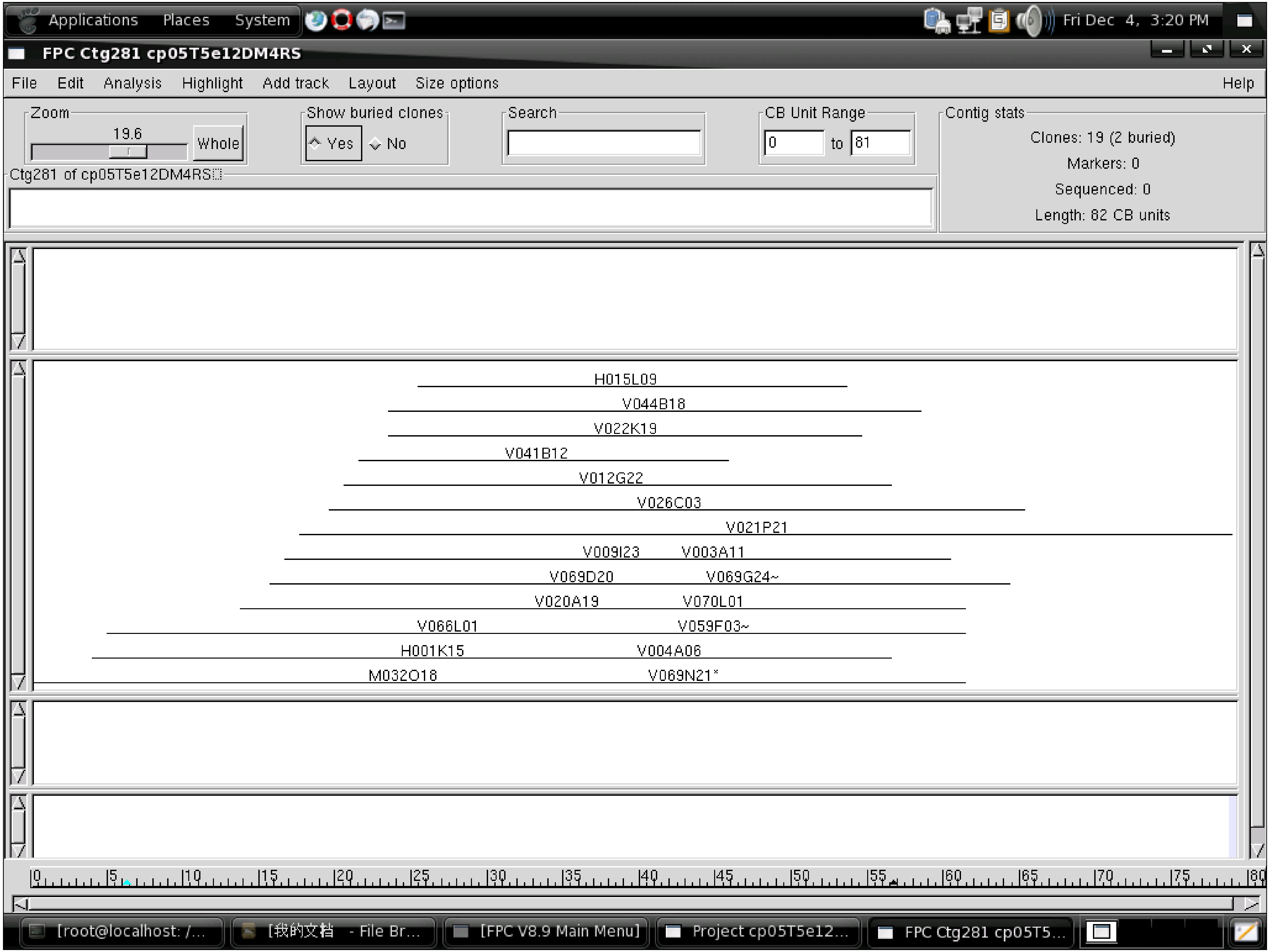
Applications Places System
Fri Dec 4, 3:20 PM
FPC Ctg281 cp05T5e12DM4RS
File Edit Analysis Highlight Add track Layout Size options
Help
Zoom
19.6
Whole
Show buried clones
Yes
No
Search
CB Unit Range
0
to
81
Contig stats
Clones: 19 (2 buried)
Markers: 0
Sequenced: 0
Length: 82 CB units
Ctg281 of cp05T5e12DM4RS
H015L09
V044B18
V022K19
V041B12
V012G22
V026C03
V021P21
V009I23
V003A11
V069D20
V069G24~
V020A19
V070L01
V066L01
V059F03~
H001K15
V004A06
M032O18
V069N21*
0 5 10 15 20 25 30 35 40 45 50 55 60 65 70 75 80
[root@localhost: /...
[我的文档 - File Br...
[FPC V8.9 Main Menu]
Project cp05T5e12...
FPC Ctg281 cp05T5...

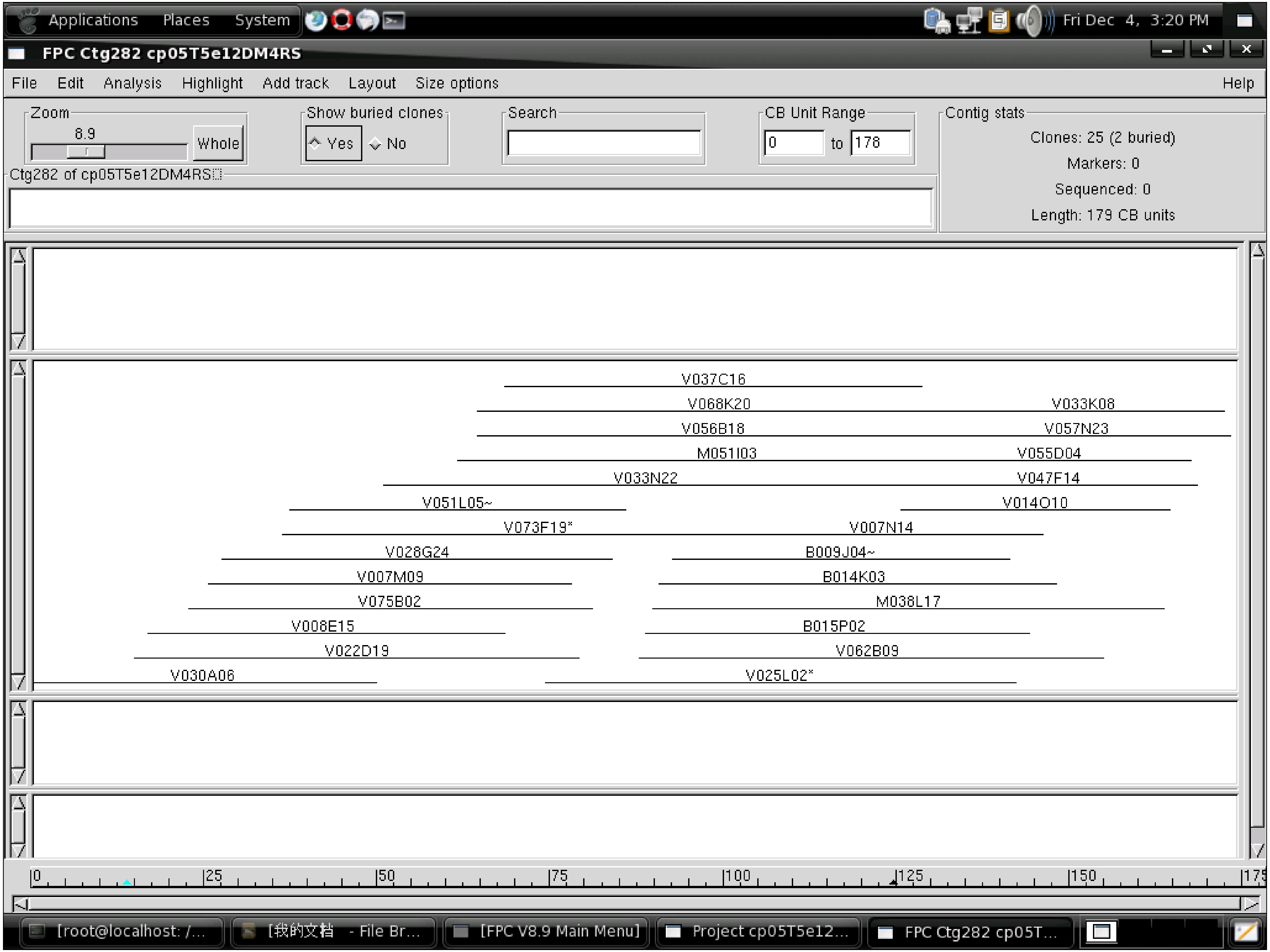
Applications Places System
Fri Dec 4, 3:20 PM
FPC Ctg282 cp05T5e12DM4RS
File Edit Analysis Highlight Add track Layout Size options Help
Zoom
8.9
Whole
Show buried clones
Yes
No
Search
CB Unit Range
0
to
178
Contig stats
Clones: 25 (2 buried)
Markers: 0
Sequenced: 0
Length: 179 CB units
Ctg282 of cp05T5e12DM4RS
V037C16
V068K20
V033K08
V056B18
V057N23
M051I03
V055D04
V033N22
V047F14
V051L05~
V014O10
V073F19*
V007N14
V028G24
B009J04~
V007M09
B014K03
V075B02
M038L17
V008E15
B015P02
V022D19
V062B09
V030A06
V025L02*
0
25
50
75
100
125
150
175
[root@localhost: /...
[我的文档 - File Br...
[FPC V8.9 Main Menu]
Project cp05T5e12...
FPC Ctg282 cp05T...

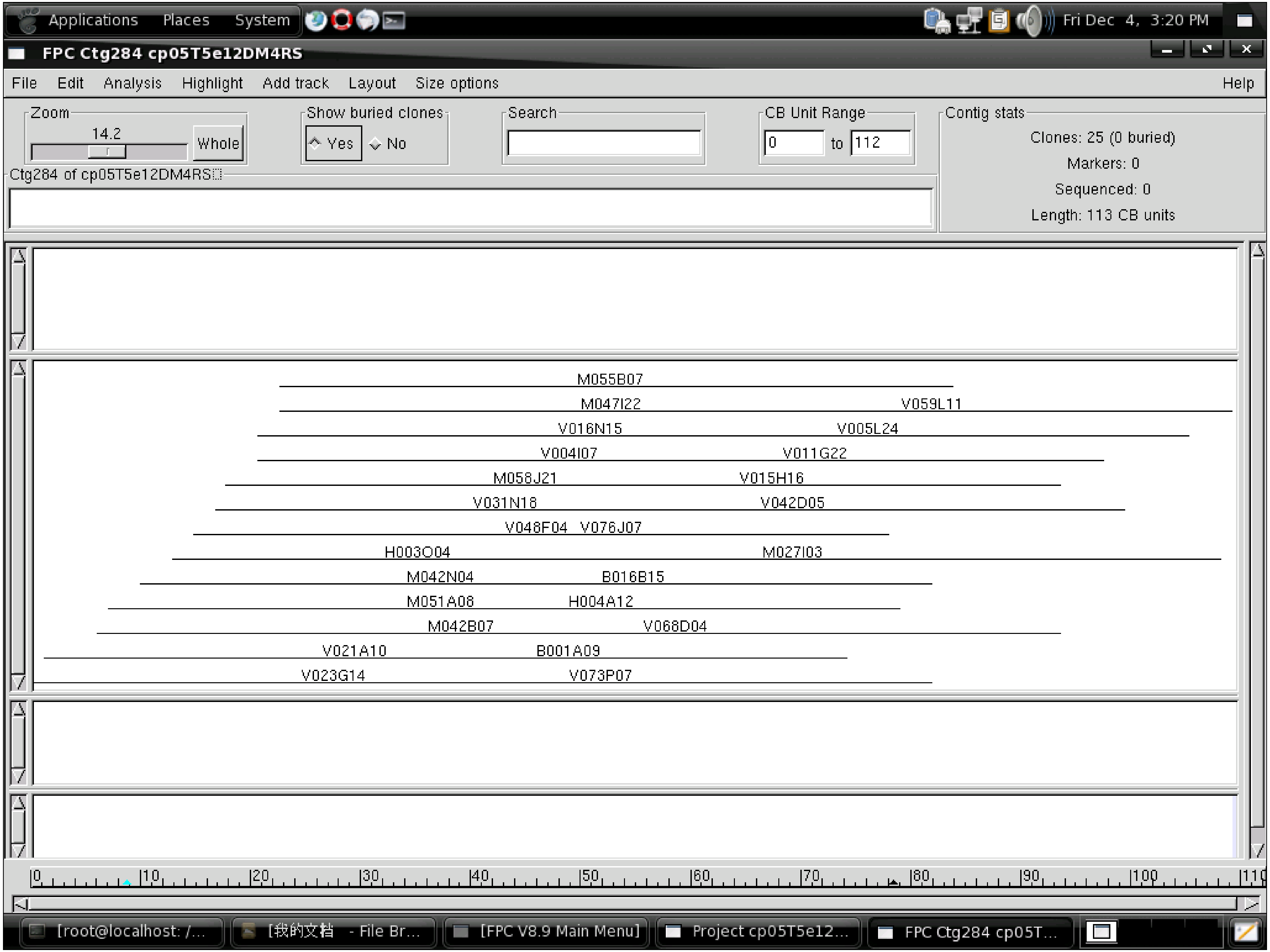
Applications Places System
Fri Dec 4, 3:20 PM
FPC Ctg284 cp05T5e12DM4RS
File Edit Analysis Highlight Add track Layout Size options Help
Zoom
14.2
Whole
Show buried clones
Yes
No
Search
CB Unit Range
0
to
112
Contig stats
Clones: 25 (0 buried)
Markers: 0
Sequenced: 0
Length: 113 CB units
Ctg284 of cp05T5e12DM4RS
M055B07
M047I22
V059L11
V016N15
V005L24
V004I07
V011G22
M058J21
V015H16
V031N18
V042D05
V048F04
V076J07
H003O04
M027I03
M042N04
B016B15
M051A08
H004A12
M042B07
V068D04
V021A10
B001A09
V023G14
V073P07
0
10
20
30
40
50
60
70
80
90
100
110
[root@localhost: /...
[我的文档 - File Br...
[FPC V8.9 Main Menu]
Project cp05T5e12...
FPC Ctg284 cp05T...

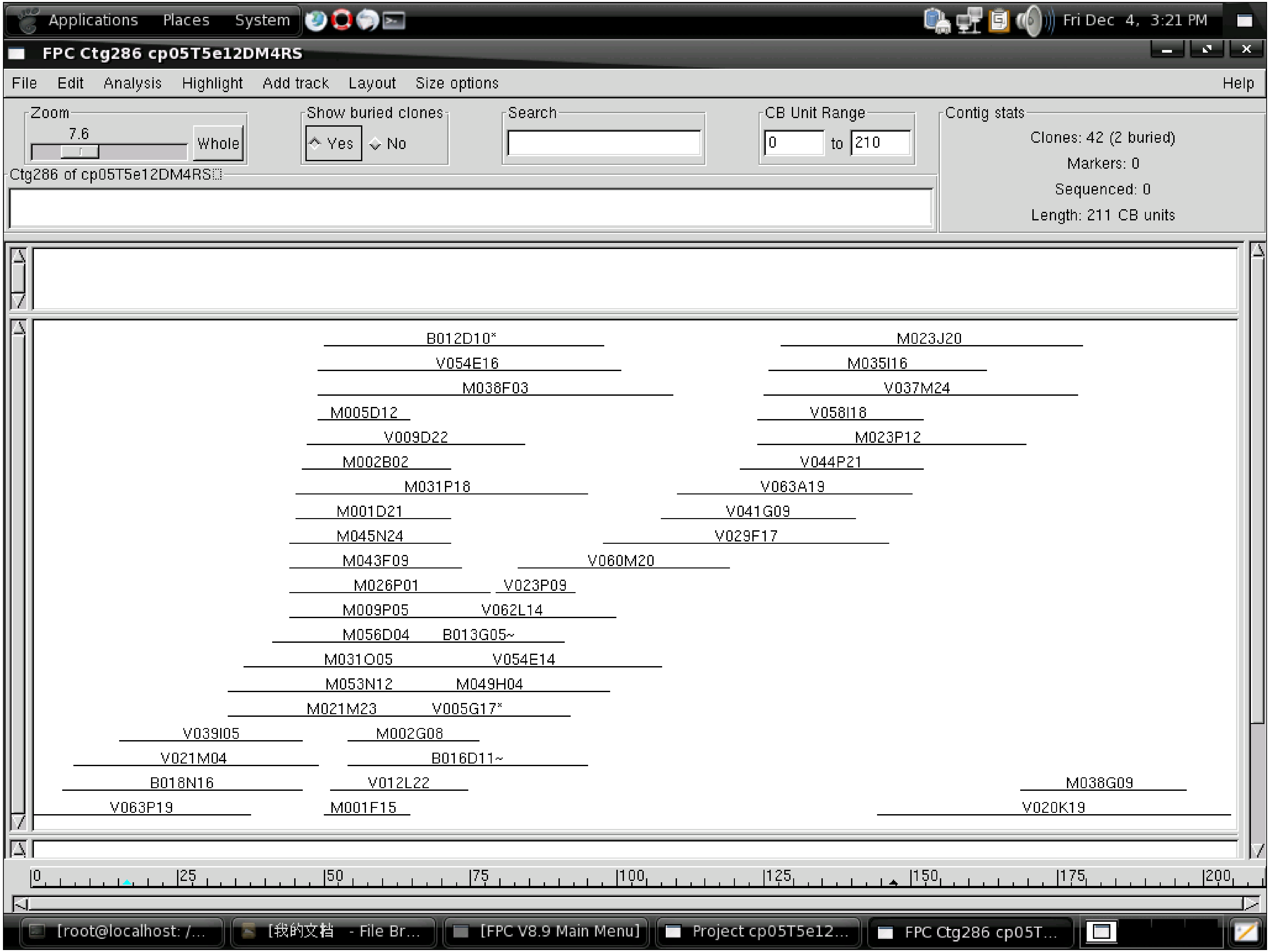
Applications Places System
Fri Dec 4, 3:21 PM
FPC Ctg286 cp05T5e12DM4RS
File Edit Analysis Highlight Add track Layout Size options Help
Zoom
7.6
Whole
Show buried clones
Yes
No
Search
CB Unit Range
0
to
210
Contig stats
Clones: 42 (2 buried)
Markers: 0
Sequenced: 0
Length: 211 CB units
Ctg286 of cp05T5e12DM4RS
B012D10*
M023J20
V054E16
M035I16
M038F03
V037M24
M005D12
V058I18
V009D22
M023P12
M002B02
V044P21
M031P18
V063A19
M001D21
V041G09
M045N24
V029F17
M043F09
V060M20
M026P01
V023P09
M009P05
V062L14
M056D04
B013G05~
M031O05
V054E14
M053N12
M049H04
M021M23
V005G17*
V039I05
M002G08
V021M04
B016D11~
B018N16
V012L22
M038G09
V063P19
M001F15
V020K19
0
25
50
75
100
125
150
175
200
[root@localhost: /...
[我的文档 - File Br...
[FPC V8.9 Main Menu]
Project cp05T5e12...
FPC Ctg286 cp05T...

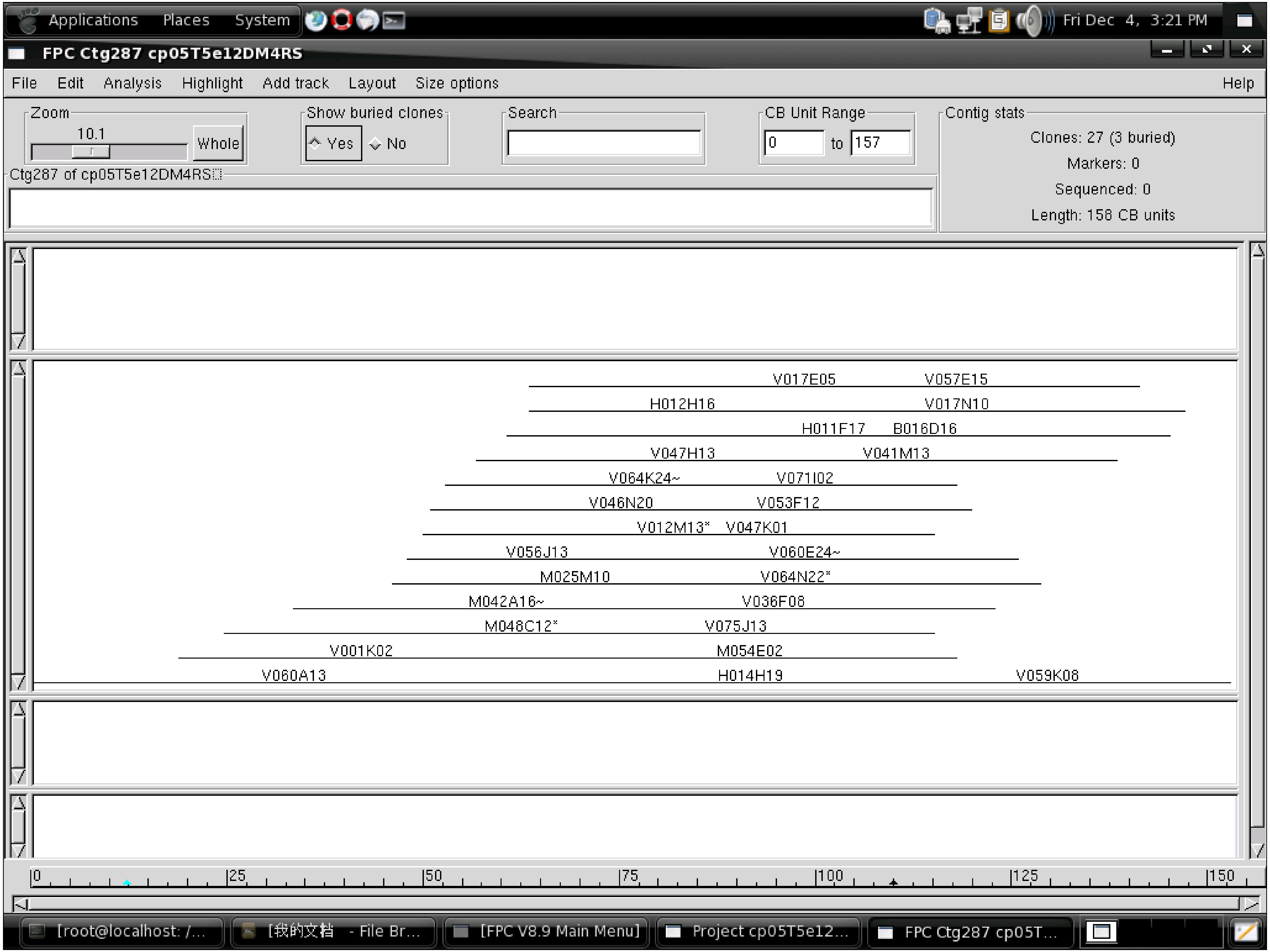
Applications Places System
Fri Dec 4, 3:21 PM
FPC Ctg287 cp05T5e12DM4RS
File Edit Analysis Highlight Add track Layout Size options Help
Zoom
10.1
Whole
Show buried clones
Yes No
Search
CB Unit Range
0 to 157
Contig stats
Clones: 27 (3 buried)
Markers: 0
Sequenced: 0
Length: 158 CB units
Ctg287 of cp05T5e12DM4RS
V017E05 V057E15
H012H16 V017N10
H011F17 B016D16
V047H13 V041M13
V064K24~ V071I02
V046N20 V053F12
V012M13* V047K01
V056J13 V060E24~
M025M10 V064N22*
M042A16~ V036F08
M048C12* V075J13
V001K02 M054E02
V060A13 H014H19 V059K08
0 25 50 75 100 125 150
[root@localhost: /...
[我的文档 - File Br...
[FPC V8.9 Main Menu]
Project cp05T5e12...
FPC Ctg287 cp05T...

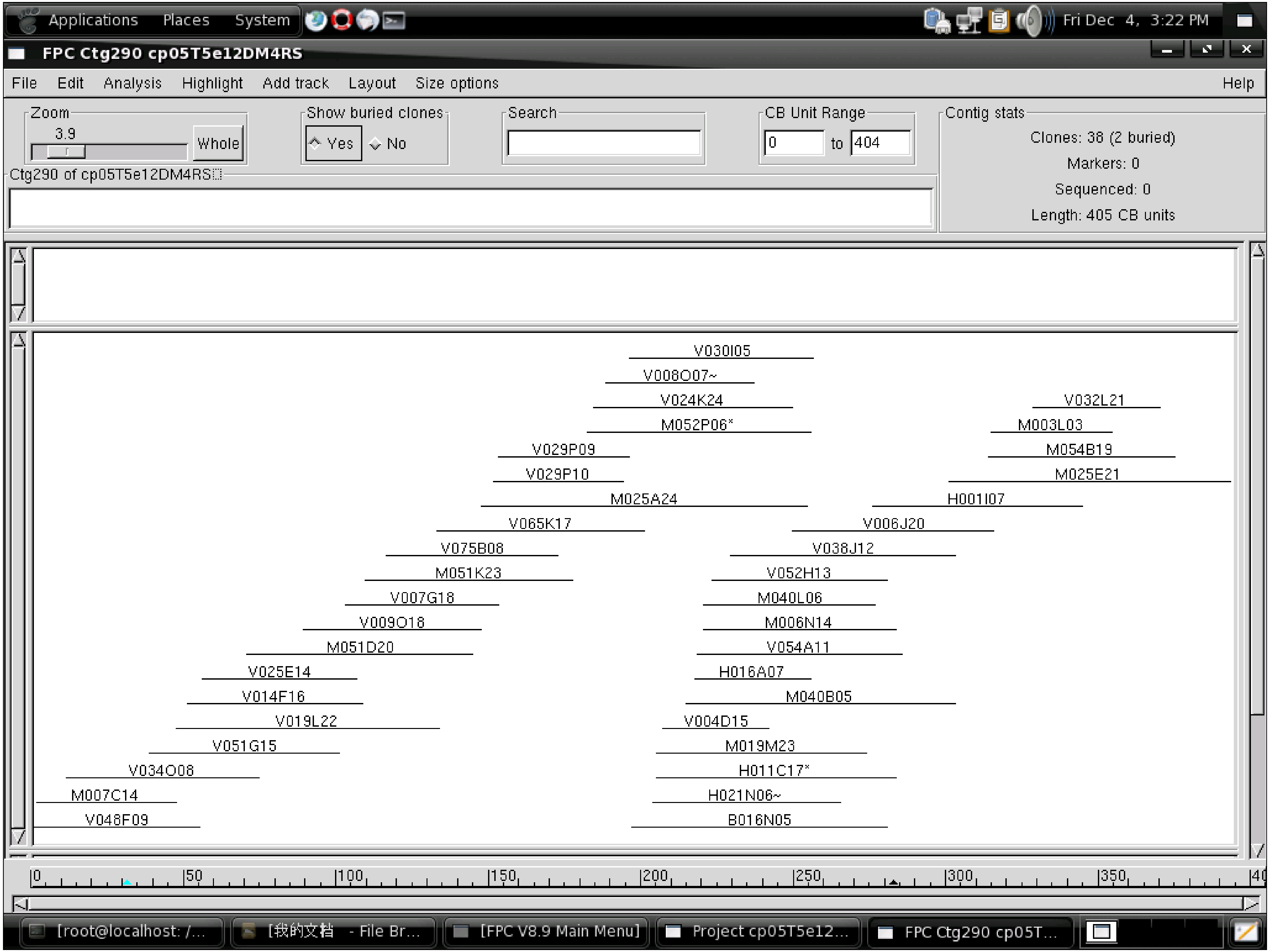
Applications Places System
Fri Dec 4, 3:22 PM
FPC Ctg290 cp05T5e12DM4RS
File Edit Analysis Highlight Add track Layout Size options Help
Zoom
3.9
Whole
Show buried clones
Yes No
Search
CB Unit Range
0 to 404
Contig stats
Clones: 38 (2 buried)
Markers: 0
Sequenced: 0
Length: 405 CB units
Ctg290 of cp05T5e12DM4RS
V030I05
V008O07~
V024K24
V032L21
M052P06*
M003L03
V029P09
M054B19
V029P10
M025E21
M025A24
H001I07
V065K17
V006J20
V075B08
V038J12
M051K23
V052H13
V007G18
M040L06
V009O18
M006N14
M051D20
V054A11
V025E14
H016A07
V014F16
M040B05
V019L22
V004D15
V051G15
M019M23
V034O08
H011C17*
M007C14
H021N06~
V048F09
B016N05
0 50 100 150 200 250 300 350 40
[root@localhost: /...
[我的文档 - File Br...
[FPC V8.9 Main Menu]
Project cp05T5e12...
FPC Ctg290 cp05T...

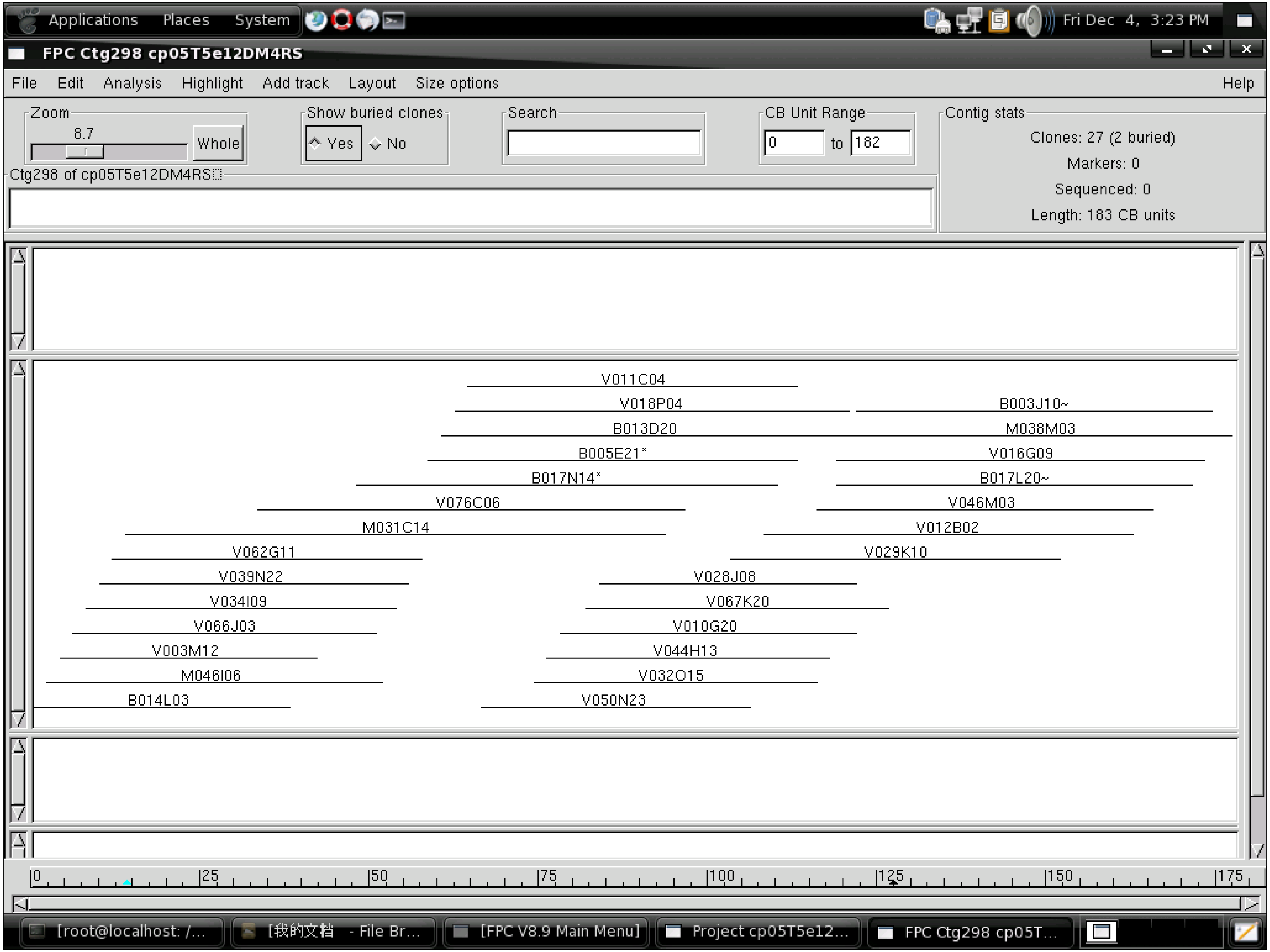
Applications Places System
Fri Dec 4, 3:23 PM
FPC Ctg298 cp05T5e12DM4RS
File Edit Analysis Highlight Add track Layout Size options Help
Zoom
8.7
Whole
Show buried clones
Yes No
Search
CB Unit Range
0 to 182
Contig stats
Clones: 27 (2 buried)
Markers: 0
Sequenced: 0
Length: 183 CB units
Ctg298 of cp05T5e12DM4RS
V011C04
V018P04
B003J10~
B013D20
M038M03
B005E21*
V016G09
B017N14*
B017L20~
V076C06
V046M03
M031C14
V012B02
V062G11
V029K10
V039N22
V028J08
V034I09
V067K20
V066J03
V010G20
V003M12
V044H13
M046I06
V032O15
B014L03
V050N23
0 25 50 75 100 125 150 175
[root@localhost: /...
[我的文档 - File Br...
[FPC V8.9 Main Menu]
Project cp05T5e12...
FPC Ctg298 cp05T...

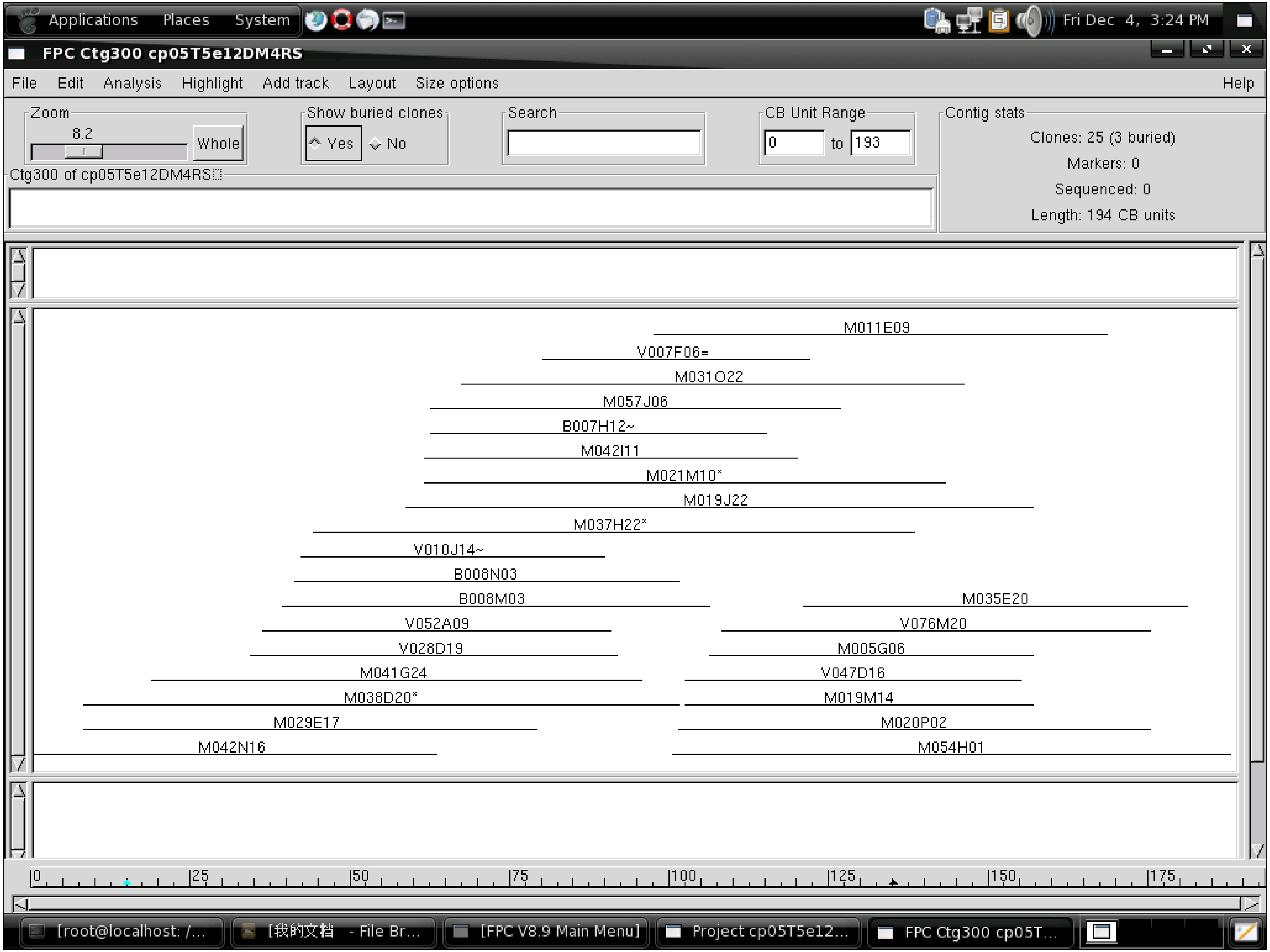
Applications Places System
Fri Dec 4, 3:24 PM
FPC Ctg300 cp05T5e12DM4RS
File Edit Analysis Highlight Add track Layout Size options Help
Zoom
8.2
Whole
Show buried clones
Yes No
Search
CB Unit Range
0 to 193
Contig stats
Clones: 25 (3 buried)
Markers: 0
Sequenced: 0
Length: 194 CB units
Ctg300 of cp05T5e12DM4RS
M011E09
V007F06=
M031O22
M057J06
B007H12~
M042I11
M021M10*
M019J22
M037H22*
V010J14~
B008N03
B008M03
M035E20
V052A09
V076M20
V028D19
M005G06
M041G24
V047D16
M038D20*
M019M14
M029E17
M020P02
M042N16
M054H01
0 25 50 75 100 125 150 175
[root@localhost: /...
[我的文档 - File Br...
[FPC V8.9 Main Menu]
Project cp05T5e12...
FPC Ctg300 cp05T...

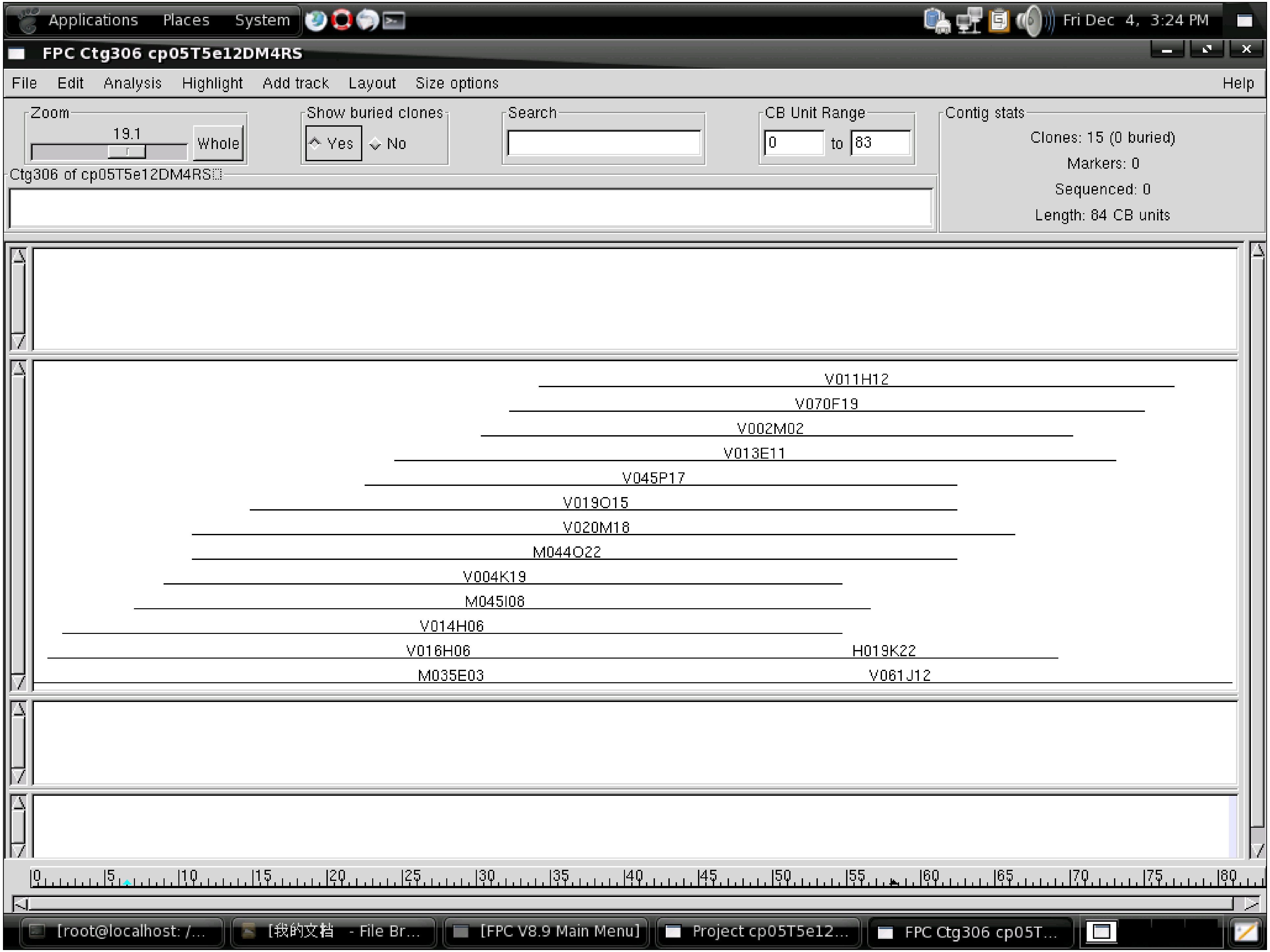
Applications Places System
Fri Dec 4, 3:24 PM
FPC Ctg306 cp05T5e12DM4RS
File Edit Analysis Highlight Add track Layout Size options
Help
Zoom
19.1
Whole
Show buried clones
Yes
No
Search
CB Unit Range
0
to
83
Contig stats
Clones: 15 (0 buried)
Markers: 0
Sequenced: 0
Length: 84 CB units
Ctg306 of cp05T5e12DM4RS
V011H12
V070F19
V002M02
V013E11
V045P17
V019O15
V020M18
M044O22
V004K19
M045I08
V014H06
V016H06
H019K22
M035E03
V061J12
0 5 10 15 20 25 30 35 40 45 50 55 60 65 70 75 80
[root@localhost: /...
[我的文档 - File Br...
[FPC V8.9 Main Menu]
Project cp05T5e12...
FPC Ctg306 cp05T...

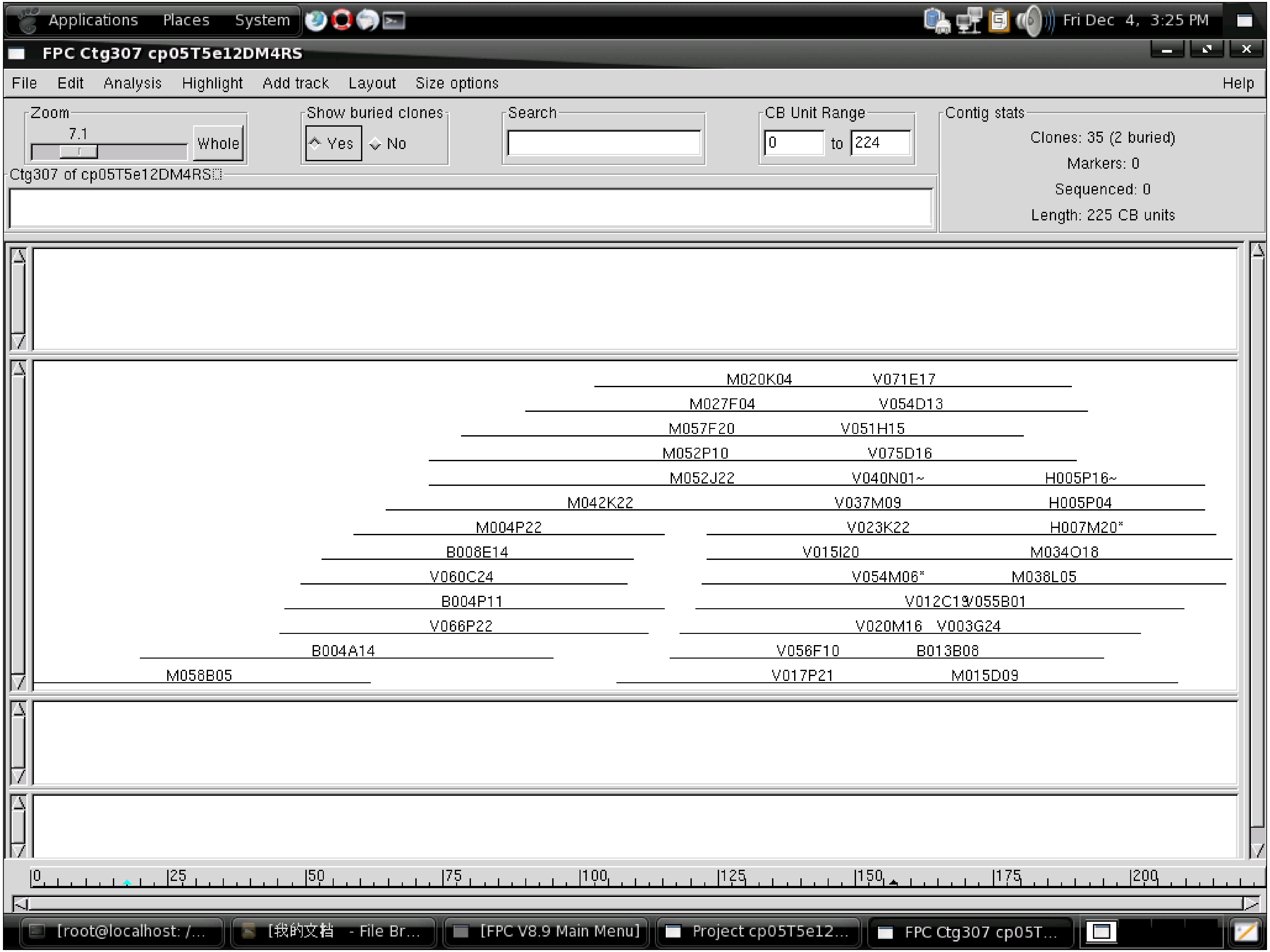
Applications Places System
Fri Dec 4, 3:25 PM
FPC Ctg307 cp05T5e12DM4RS
File Edit Analysis Highlight Add track Layout Size options Help
Zoom
7.1
Whole
Show buried clones
Yes No
Search
CB Unit Range
0 to 224
Contig stats
Clones: 35 (2 buried)
Markers: 0
Sequenced: 0
Length: 225 CB units
Ctg307 of cp05T5e12DM4RS
M020K04 V071E17
M027F04 V054D13
M057F20 V051H15
M052P10 V075D16
M052J22 V040N01~ H005P16~
M042K22 V037M09 H005P04
M004P22 V023K22 H007M20*
B008E14 V015I20 M034O18
V060C24 V054M06* M038L05
B004P11 V012C19 V055B01
V066P22 V020M16 V003G24
B004A14 V056F10 B013B08
M058B05 V017P21 M015D09
0 25 50 75 100 125 150 175 200
[root@localhost: /...
[我的文档 - File Br...
[FPC V8.9 Main Menu]
Project cp05T5e12...
FPC Ctg307 cp05T...

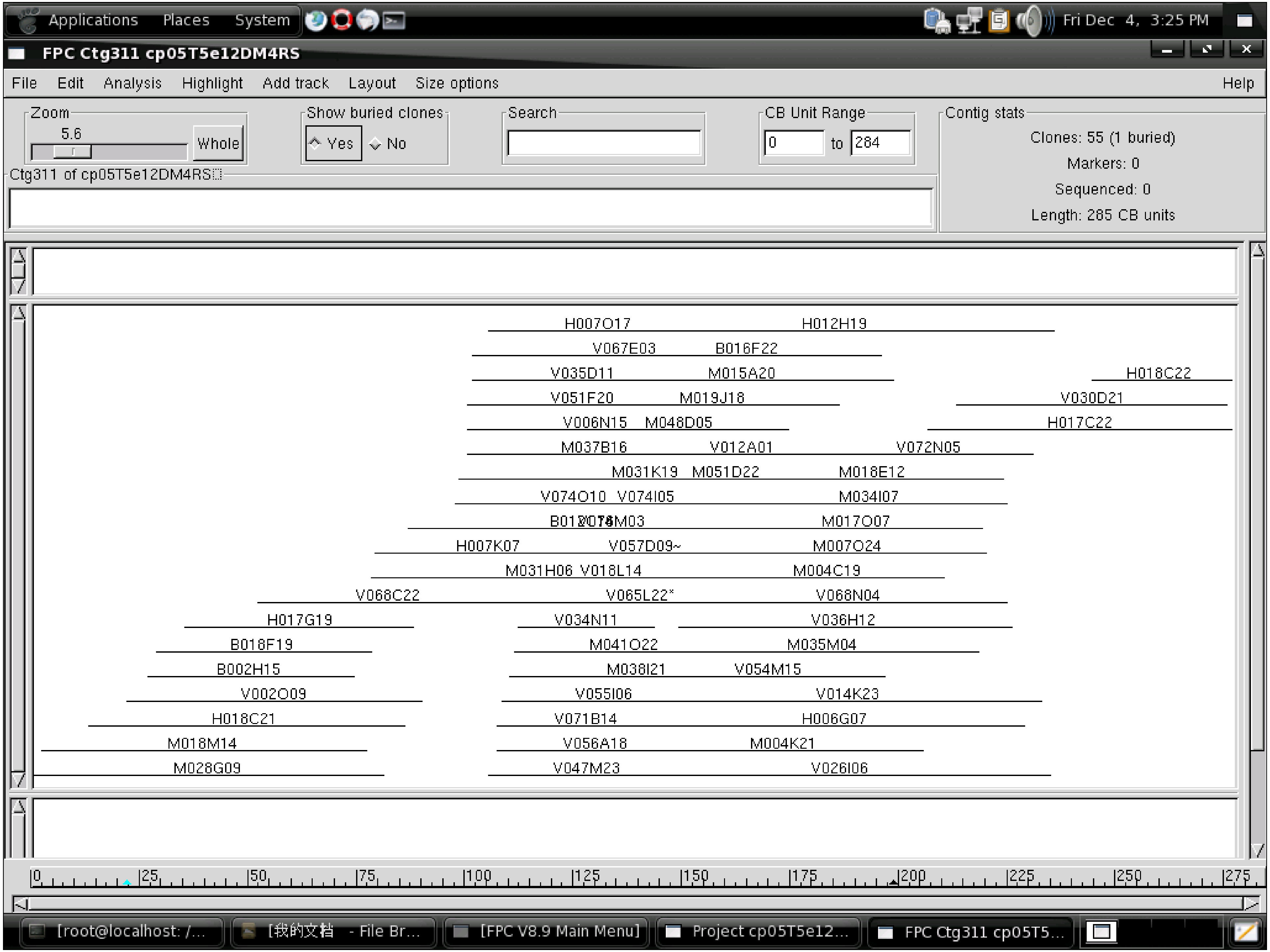
Applications Places System
Fri Dec 4, 3:25 PM
FPC Ctg311 cp05T5e12DM4RS
File Edit Analysis Highlight Add track Layout Size options Help
Zoom
5.6
Whole
Show buried clones
Yes
No
Search
CB Unit Range
0 to 284
Contig stats
Clones: 55 (1 buried)
Markers: 0
Sequenced: 0
Length: 285 CB units
Ctg311 of cp05T5e12DM4RS
H007O17 H012H19
V067E03 B016F22
V035D11 M015A20 H018C22
V051F20 M019J18 V030D21
V006N15 M048D05 H017C22
M037B16 V012A01 V072N05
M031K19 M051D22 M018E12
V074O10 V074I05 M034I07
M017O07
H007K07 V057D09~ M007O24
M031H06 V018L14 M004C19
V068C22 V065L22* V068N04
H017G19 V034N11 V036H12
B018F19 M041O22 M035M04
B002H15 M038I21 V054M15
V002O09 V055I06 V014K23
H018C21 V071B14 H006G07
M018M14 V056A18 M004K21
M028G09 V047M23 V026I06
0 25 50 75 100 125 150 175 200 225 250 275
[root@localhost: /...
[我的文档 - File Br...
[FPC V8.9 Main Menu]
Project cp05T5e12...
FPC Ctg311 cp05T5...

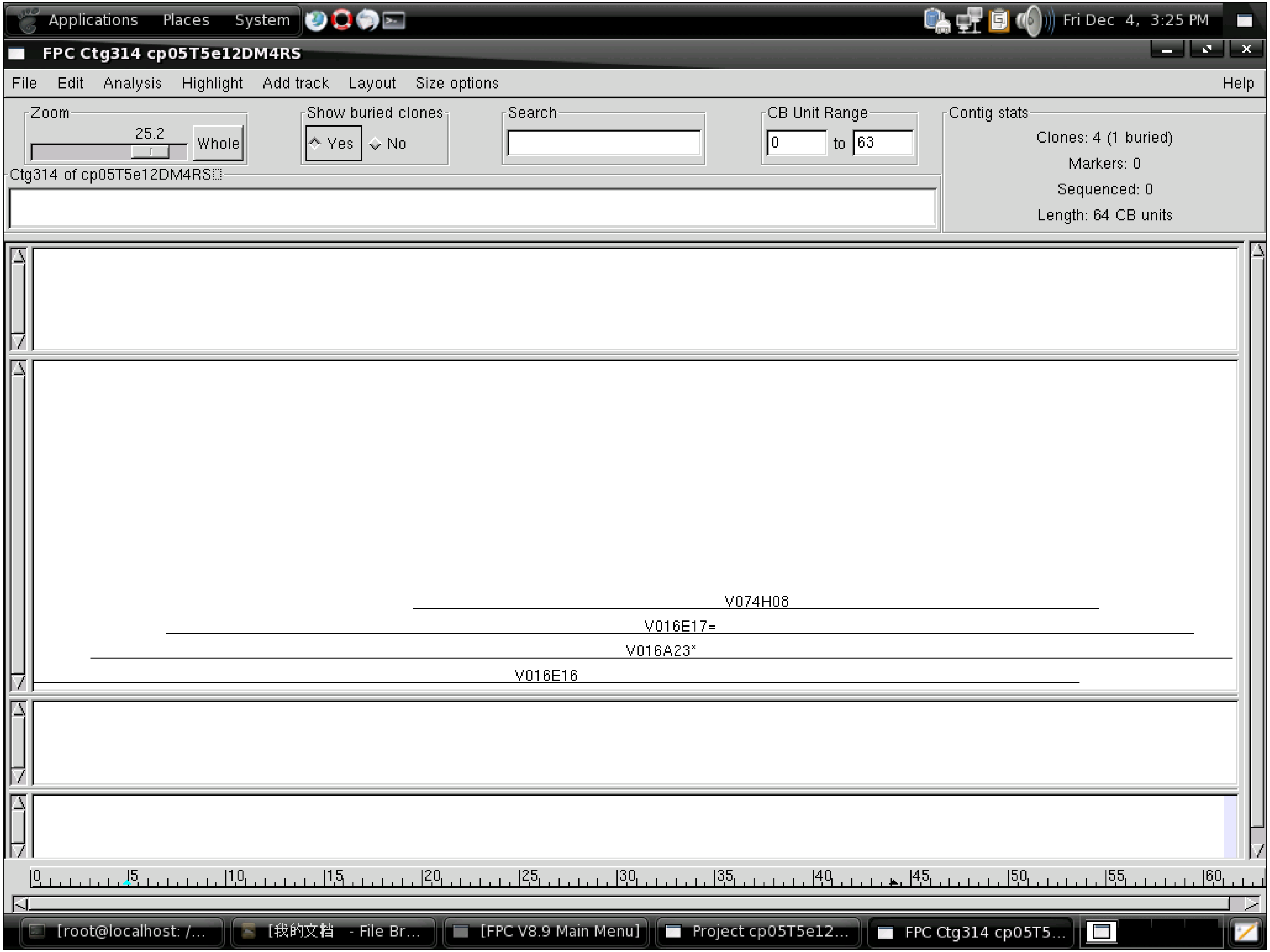
Applications Places System
Fri Dec 4, 3:25 PM
FPC Ctg314 cp05T5e12DM4RS
File Edit Analysis Highlight Add track Layout Size options Help
Zoom
25.2
Whole
Show buried clones
Yes No
Search
CB Unit Range
0 to 63
Contig stats
Clones: 4 (1 buried)
Markers: 0
Sequenced: 0
Length: 64 CB units
Ctg314 of cp05T5e12DM4RS
V074H08
V016E17=
V016A23*
V016E16
0 5 10 15 20 25 30 35 40 45 50 55 60
[root@localhost: /...
[我的文档 - File Br...
[FPC V8.9 Main Menu]
Project cp05T5e12...
FPC Ctg314 cp05T5...

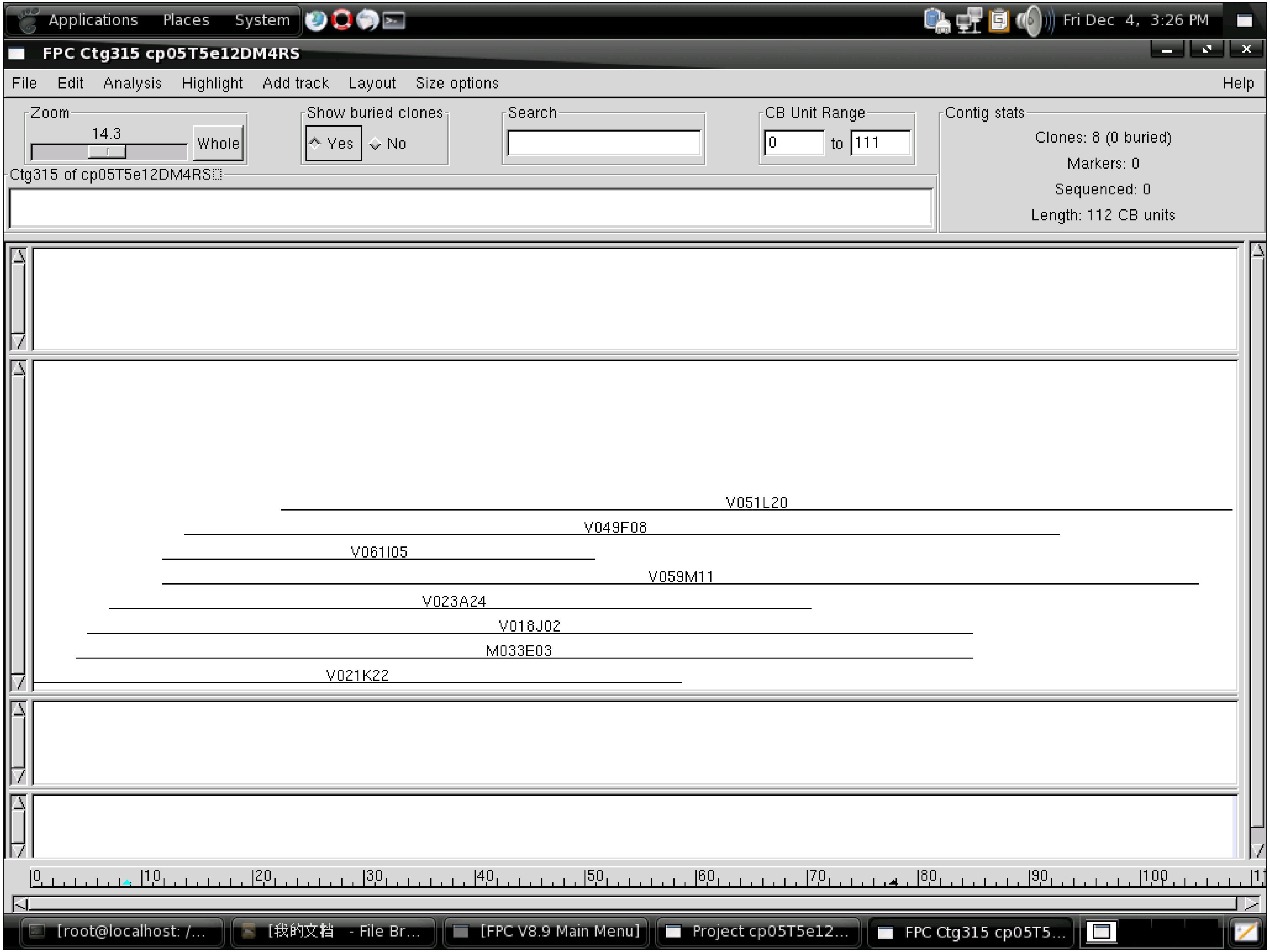

Applications Places System
Fri Dec 4, 3:26 PM
FPC Ctg315 cp05T5e12DM4RS
File Edit Analysis Highlight Add track Layout Size options Help
Zoom
14.3
Whole
Show buried clones
Yes No
Search
CB Unit Range
0 to 111
Contig stats
Clones: 8 (0 buried)
Markers: 0
Sequenced: 0
Length: 112 CB units
Ctg315 of cp05T5e12DM4RS
V051L20
V049F08
V061I05
V059M11
V023A24
V018J02
M033E03
V021K22
0 10 20 30 40 50 60 70 80 90 100
[root@localhost: /...
[我的文档 - File Br...
[FPC V8.9 Main Menu]
Project cp05T5e12...
FPC Ctg315 cp05T5...

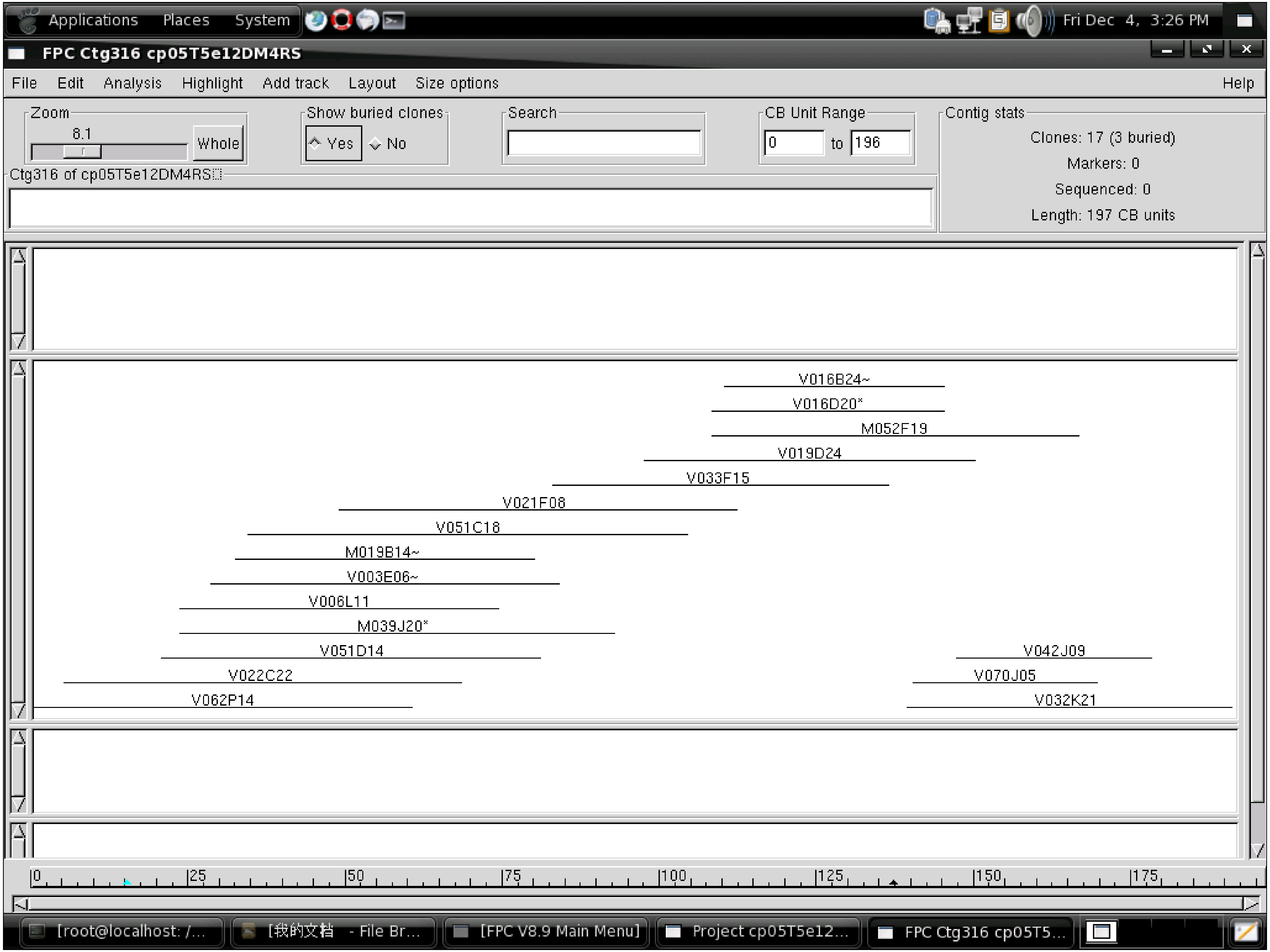

Applications
Places
System
Fri Dec 4, 3:26 PM
FPC Ctg316 cp05T5e12DM4RS
File
Edit
Analysis
Highlight
Add track
Layout
Size options
Help
Zoom
8.1
Whole
Show buried clones
Yes
No
Search
CB Unit Range
0
to
196
Contig stats
Clones: 17 (3 buried)
Markers: 0
Sequenced: 0
Length: 197 CB units
Ctg316 of cp05T5e12DM4RS
V016B24~
V016D20*
M052F19
V019D24
V033F15
V021F08
V051C18
M019B14~
V003E06~
V006L11
M039J20*
V051D14
V022C22
V062P14
V042J09
V070J05
V032K21
0
25
50
75
100
125
150
175
[root@localhost: /...
[我的文档 - File Br...
[FPC V8.9 Main Menu]
Project cp05T5e12...
FPC Ctg316 cp05T5...

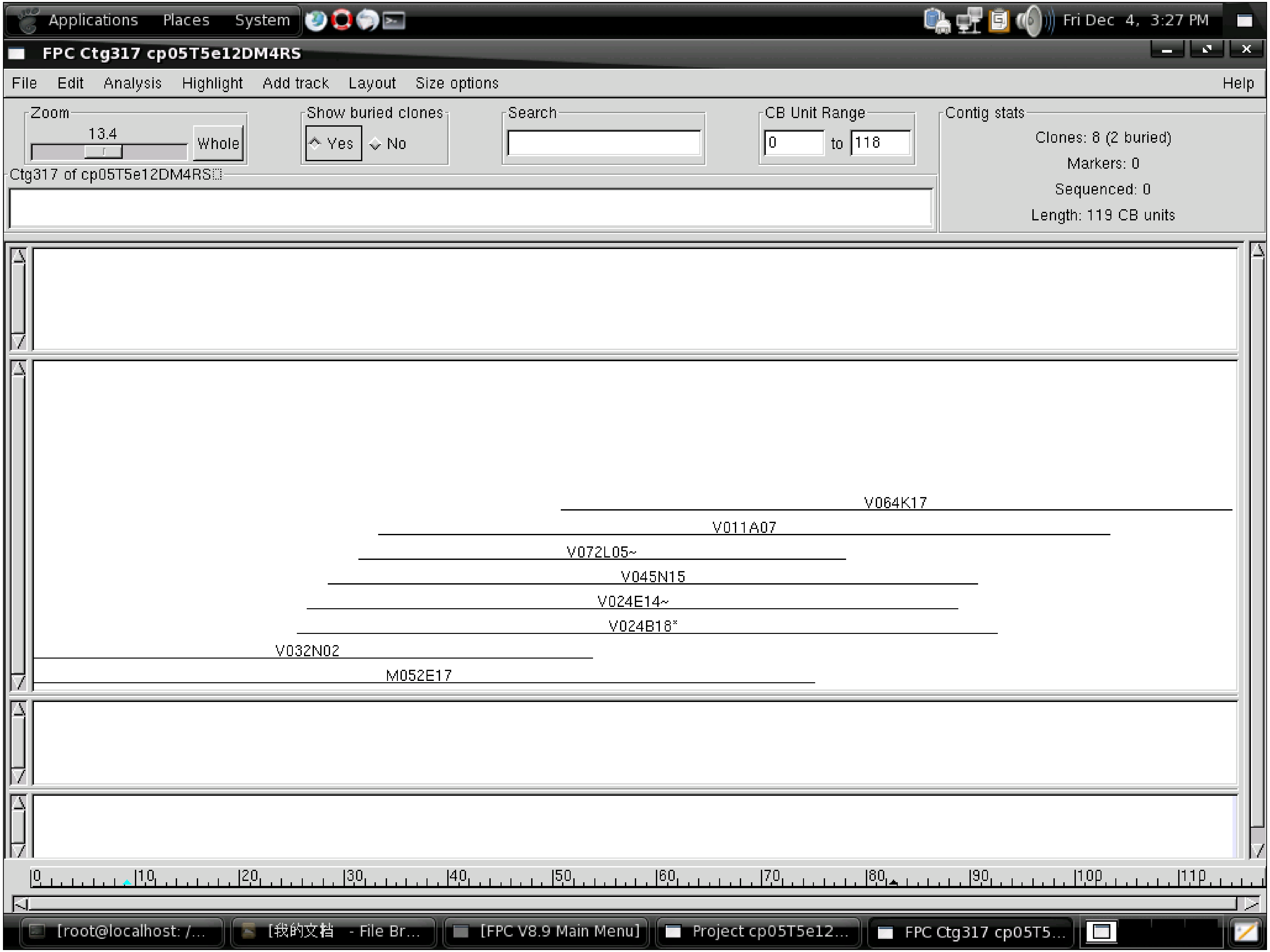
Applications Places System
Fri Dec 4, 3:27 PM
FPC Ctg317 cp05T5e12DM4RS
File Edit Analysis Highlight Add track Layout Size options Help
Zoom
13.4
Whole
Show buried clones
Yes No
Search
CB Unit Range
0 to 118
Contig stats
Clones: 8 (2 buried)
Markers: 0
Sequenced: 0
Length: 119 CB units
Ctg317 of cp05T5e12DM4RS
V064K17
V011A07
V072L05~
V045N15
V024E14~
V024B18*
V032N02
M052E17
0 10 20 30 40 50 60 70 80 90 100 110
[root@localhost: /...
[我的文档 - File Br...
[FPC V8.9 Main Menu]
Project cp05T5e12...
FPC Ctg317 cp05T5...

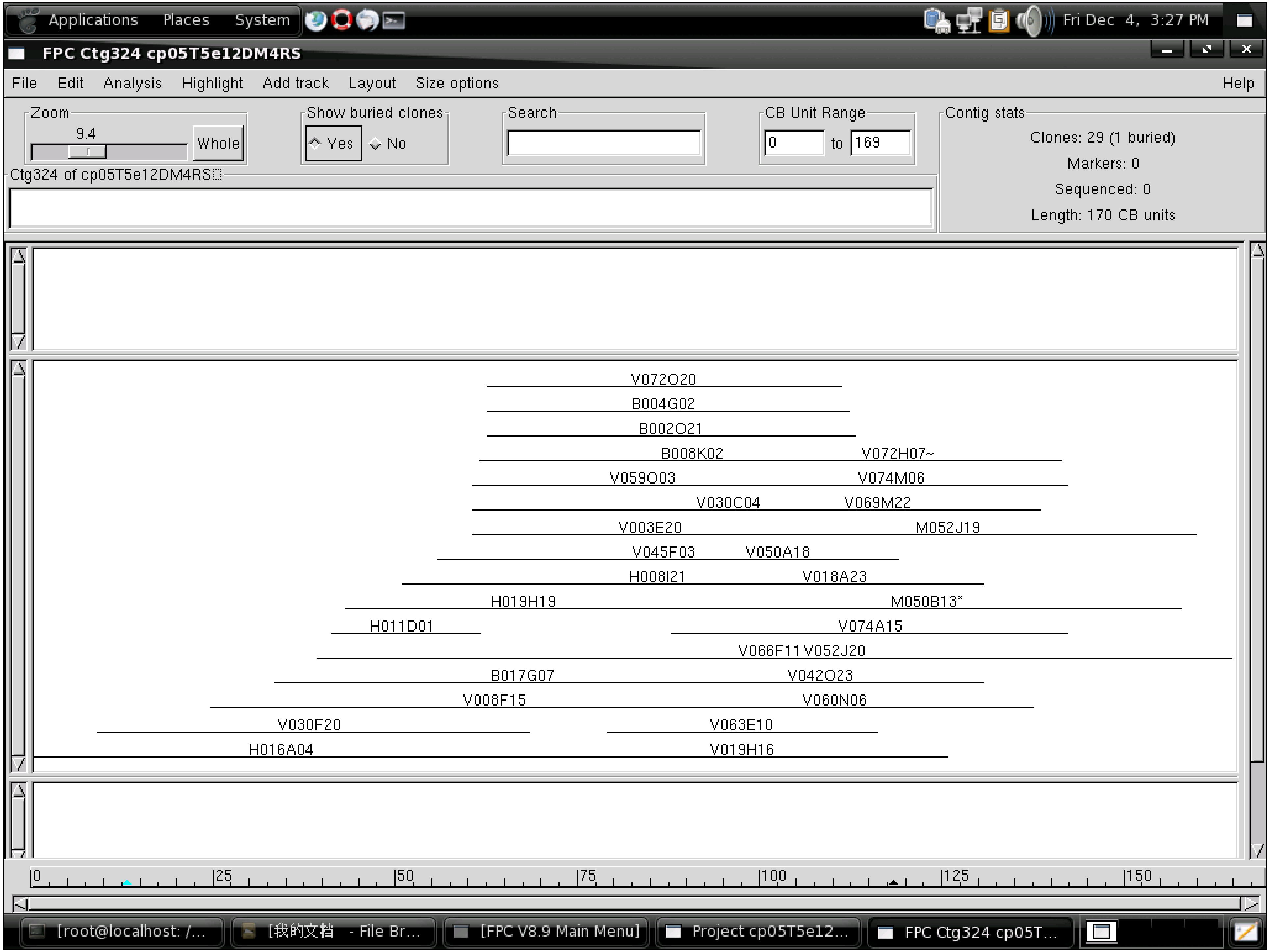
Applications Places System
Fri Dec 4, 3:27 PM
FPC Ctg324 cp05T5e12DM4RS
File Edit Analysis Highlight Add track Layout Size options Help
Zoom
9.4
Whole
Show buried clones
Yes No
Search
CB Unit Range
0 to 169
Contig stats
Clones: 29 (1 buried)
Markers: 0
Sequenced: 0
Length: 170 CB units
Ctg324 of cp05T5e12DM4RS
V072O20
B004G02
B002O21
B008K02 V072H07~
V059O03 V074M06
V030C04 V069M22
V003E20 M052J19
V045F03 V050A18
H008I21 V018A23
H019H19 M050B13*
H011D01 V074A15
V066F11 V052J20
B017G07 V042O23
V008F15 V060N06
V030F20 V063E10
H016A04 V019H16
0 25 50 75 100 125 150
[root@localhost: /...
[我的文档 - File Br...
[FPC V8.9 Main Menu]
Project cp05T5e12...
FPC Ctg324 cp05T...

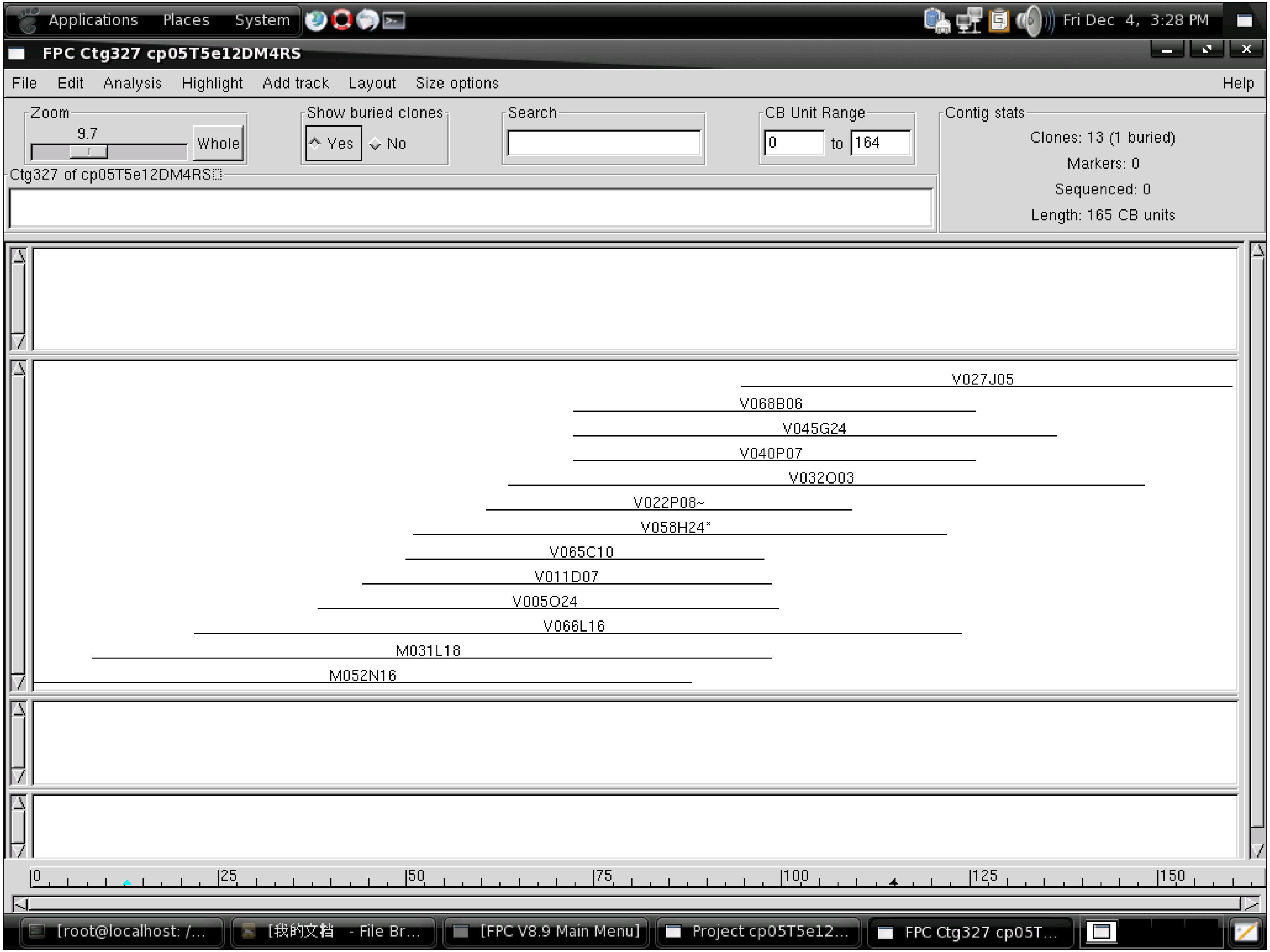
Applications Places System
Fri Dec 4, 3:28 PM
FPC Ctg327 cp05T5e12DM4RS
File Edit Analysis Highlight Add track Layout Size options Help
Zoom
9.7
Whole
Show buried clones
Yes No
Search
CB Unit Range
0 to 164
Contig stats
Clones: 13 (1 buried)
Markers: 0
Sequenced: 0
Length: 165 CB units
Ctg327 of cp05T5e12DM4RS
V027J05
V068B06
V045G24
V040P07
V032O03
V022P08~
V058H24*
V065C10
V011D07
V005O24
V066L16
M031L18
M052N16
0 25 50 75 100 125 150
[root@localhost: /...
[我的文档 - File Br...
[FPC V8.9 Main Menu]
Project cp05T5e12...
FPC Ctg327 cp05T...

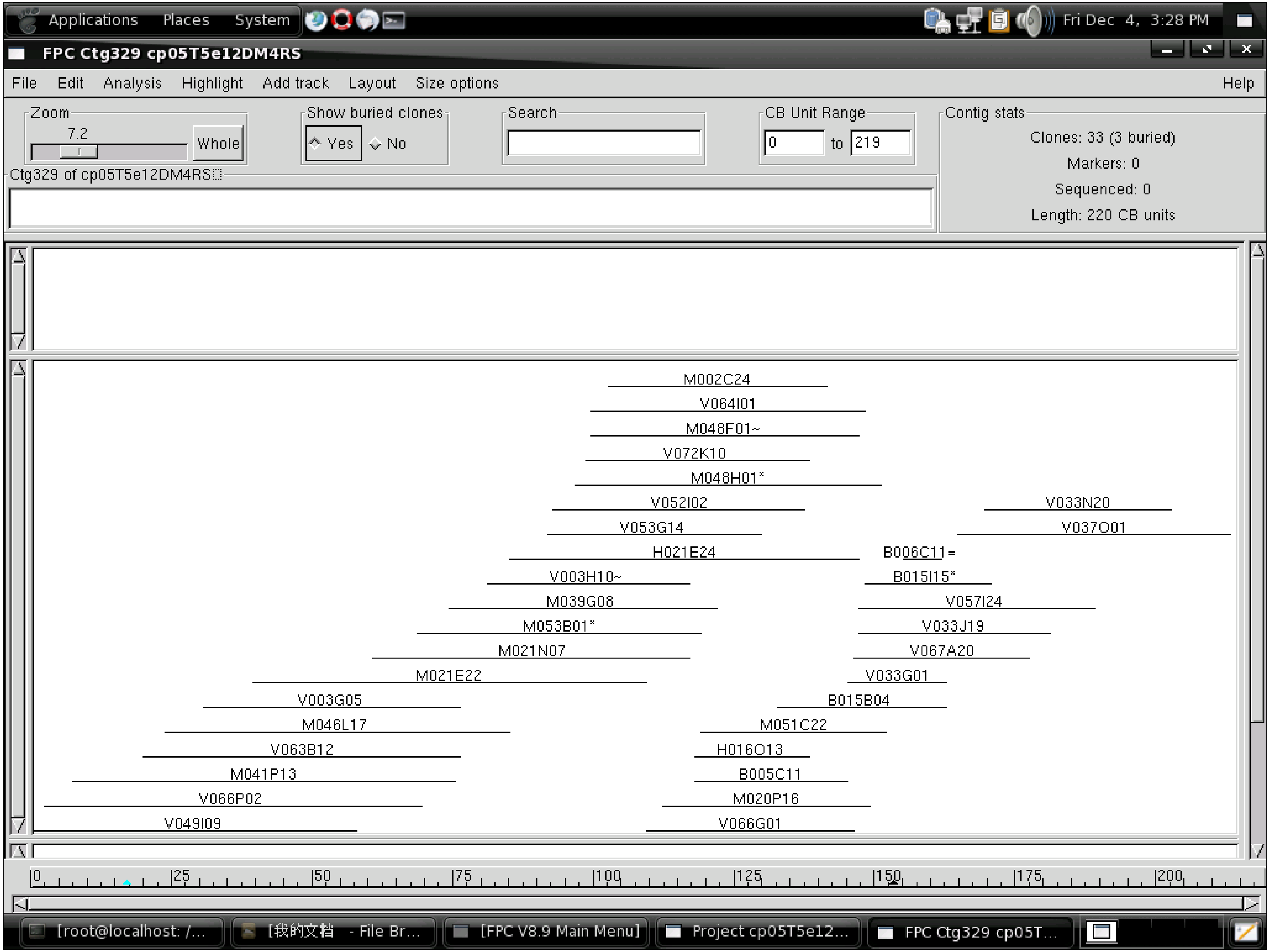
Applications Places System
Fri Dec 4, 3:28 PM
FPC Ctg329 cp05T5e12DM4RS
File Edit Analysis Highlight Add track Layout Size options Help
Zoom
7.2
Whole
Show buried clones
Yes No
Search
CB Unit Range
0 to 219
Contig stats
Clones: 33 (3 buried)
Markers: 0
Sequenced: 0
Length: 220 CB units
Ctg329 of cp05T5e12DM4RS
M002C24
V064I01
M048F01~
V072K10
M048H01*
V052I02
V033N20
V053G14
V037O01
H021E24
B006C11=
V003H10~
B015I15*
M039G08
V057I24
M053B01*
V033J19
M021N07
V067A20
M021E22
V033G01
V003G05
B015B04
M046L17
M051C22
V063B12
H016O13
M041P13
B005C11
V066P02
M020P16
V049I09
V066G01
0 25 50 75 100 125 150 175 200
[root@localhost: /...
[我的文档 - File Br...
[FPC V8.9 Main Menu]
Project cp05T5e12...
FPC Ctg329 cp05T...

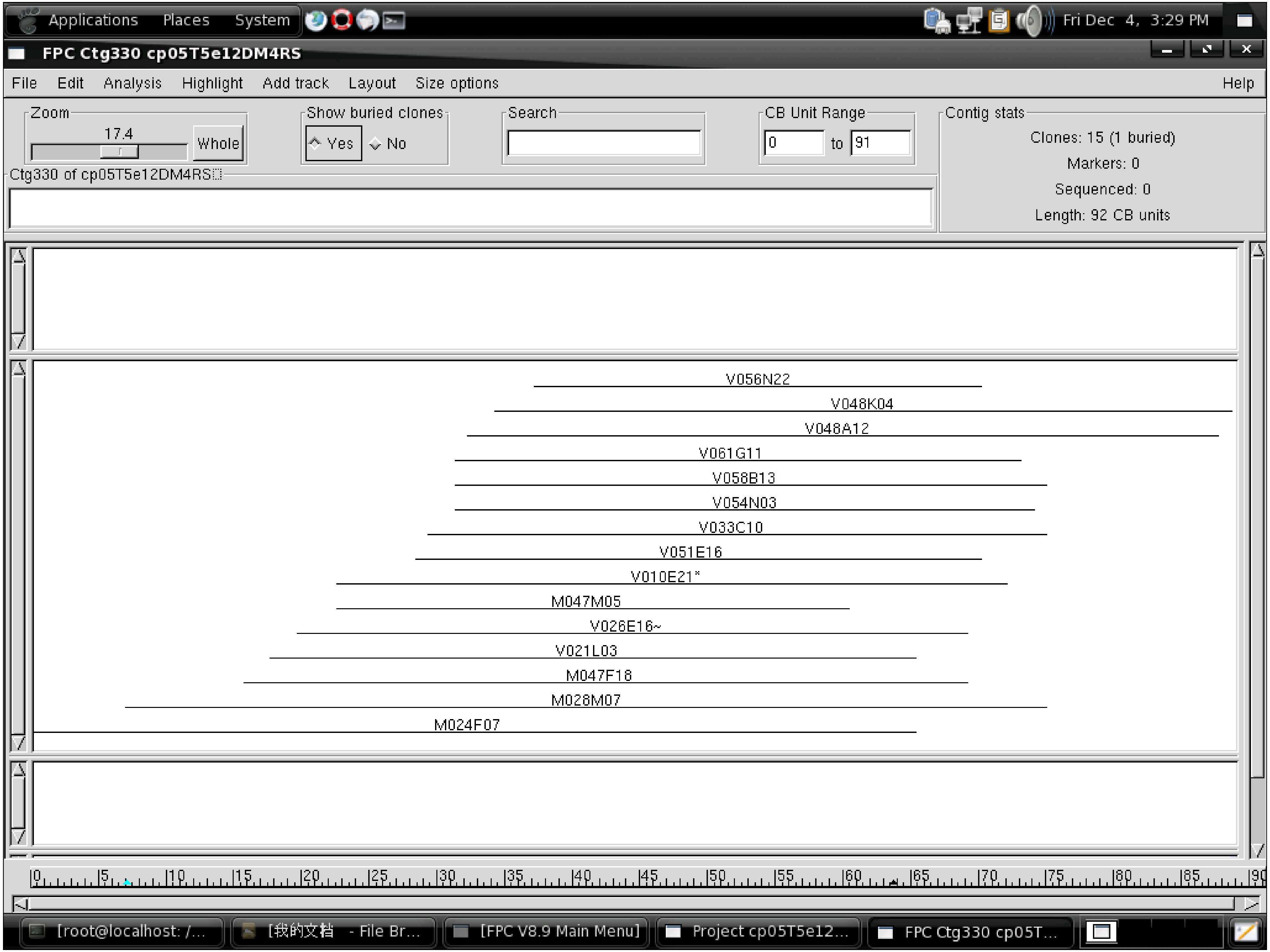
Applications Places System
Fri Dec 4, 3:29 PM
FPC Ctg330 cp05T5e12DM4RS
File Edit Analysis Highlight Add track Layout Size options Help
Zoom
17.4
Whole
Show buried clones
Yes
No
Search
CB Unit Range
0
to
91
Contig stats
Clones: 15 (1 buried)
Markers: 0
Sequenced: 0
Length: 92 CB units
Ctg330 of cp05T5e12DM4RS
V056N22
V048K04
V048A12
V061G11
V058B13
V054N03
V033C10
V051E16
V010E21*
M047M05
V026E16~
V021L03
M047F18
M028M07
M024F07
0 5 10 15 20 25 30 35 40 45 50 55 60 65 70 75 80 85 90
[root@localhost: /...
[我的文档 - File Br...
[FPC V8.9 Main Menu]
Project cp05T5e12...
FPC Ctg330 cp05T...

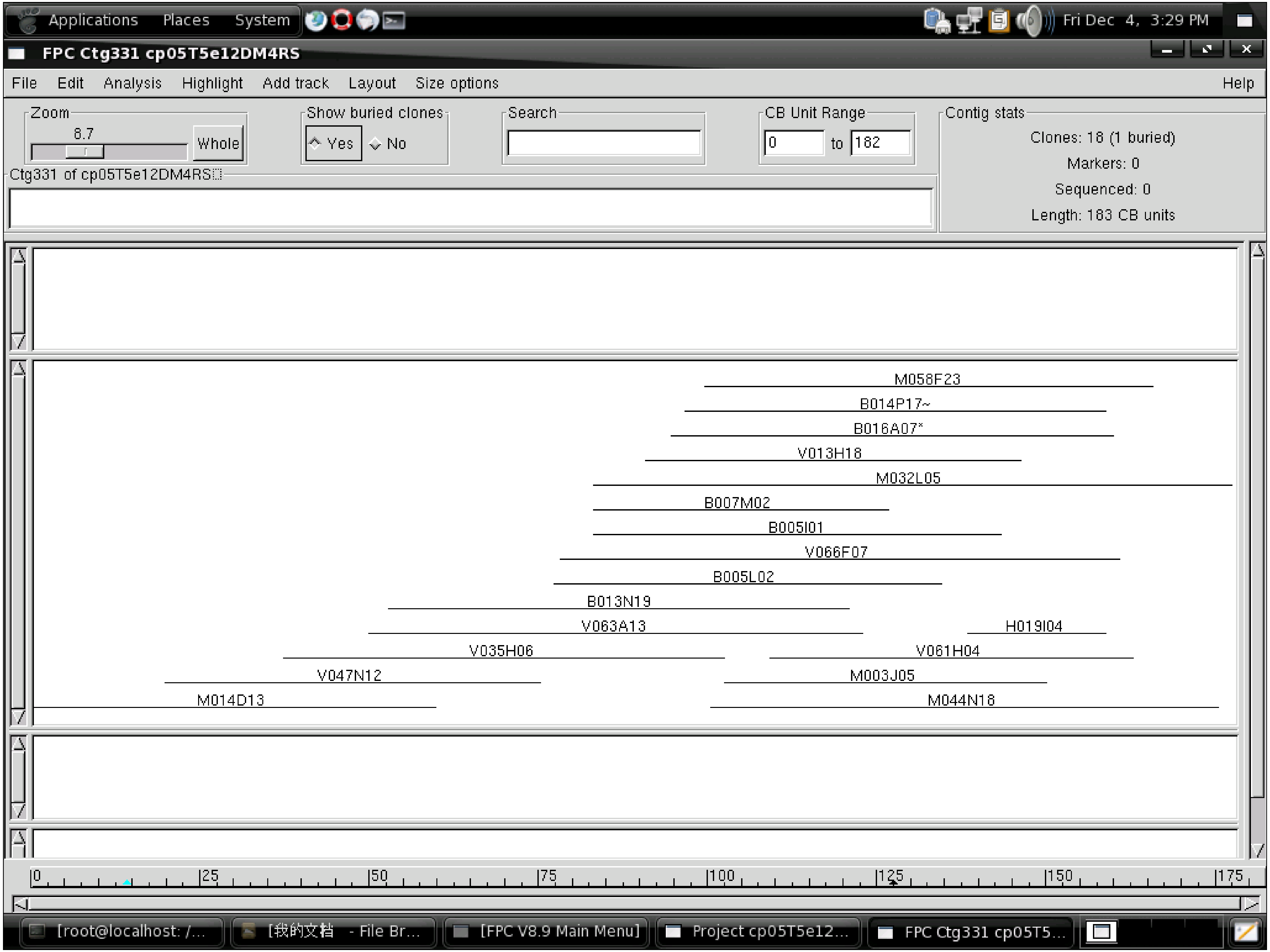
Applications
Places
System
Fri Dec 4, 3:29 PM
FPC Ctg331 cp05T5e12DM4RS
File
Edit
Analysis
Highlight
Add track
Layout
Size options
Help
Zoom
8.7
Whole
Show buried clones
Yes
No
Search
CB Unit Range
0
to
182
Contig stats
Clones: 18 (1 buried)
Markers: 0
Sequenced: 0
Length: 183 CB units
Ctg331 of cp05T5e12DM4RS
M058F23
B014P17~
B016A07*
V013H18
M032L05
B007M02
B005I01
V066F07
B005L02
B013N19
V063A13
H019I04
V035H06
V061H04
V047N12
M003J05
M014D13
M044N18
0
25
50
75
100
125
150
175
[root@localhost: /...
[我的文档 - File Br...
[FPC V8.9 Main Menu]
Project cp05T5e12...
FPC Ctg331 cp05T5...

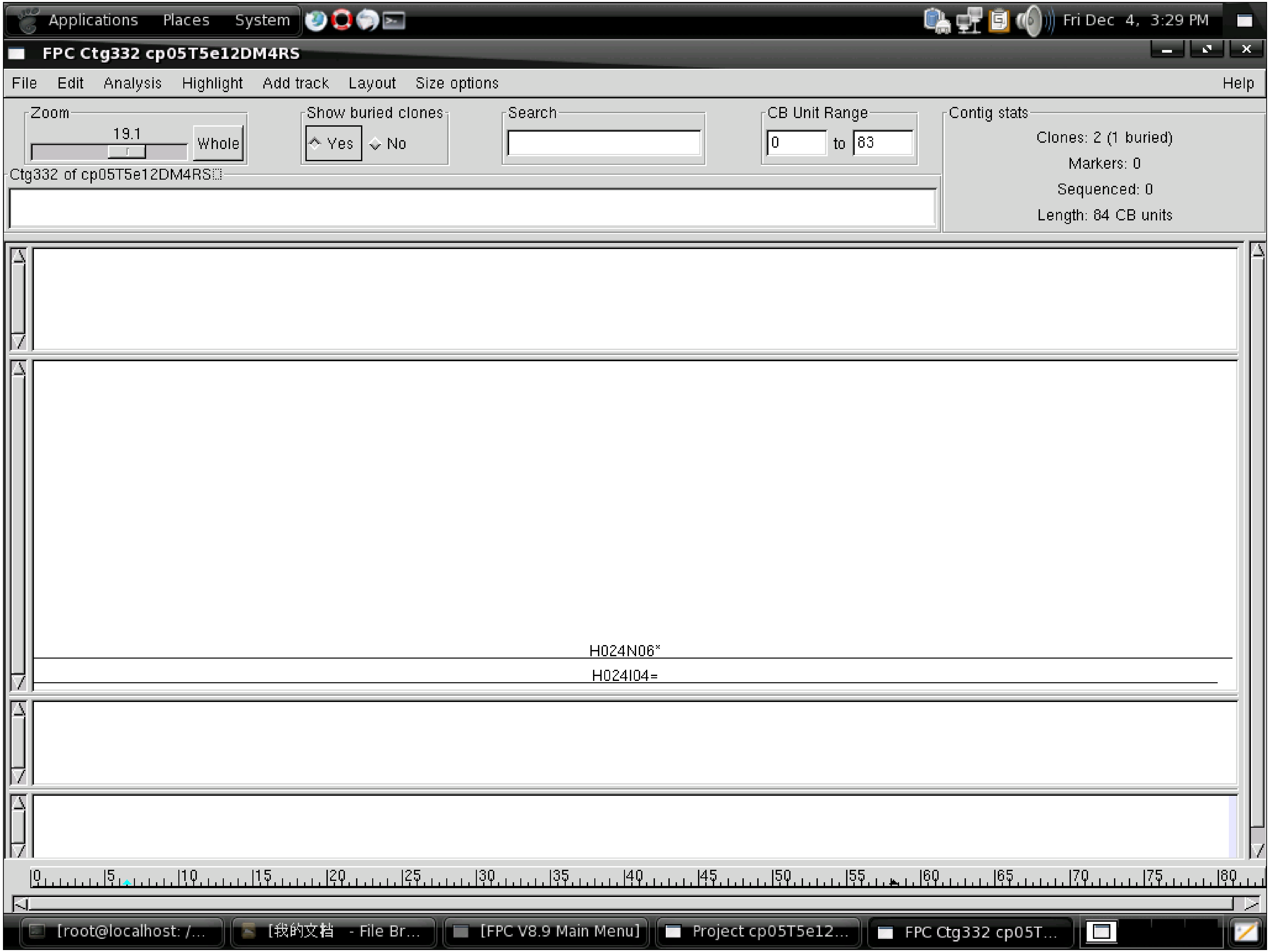
Applications
Places
System
Fri Dec 4, 3:29 PM
FPC Ctg332 cp05T5e12DM4RS
File
Edit
Analysis
Highlight
Add track
Layout
Size options
Help
Zoom
19.1
Whole
Show buried clones
Yes
No
Search
CB Unit Range
0
to
83
Contig stats
Clones: 2 (1 buried)
Markers: 0
Sequenced: 0
Length: 84 CB units
Ctg332 of cp05T5e12DM4RS
H024N06*
H024I04=
0 5 10 15 20 25 30 35 40 45 50 55 60 65 70 75 80
[root@localhost: /...
[我的文档 - File Br...
[FPC V8.9 Main Menu]
Project cp05T5e12...
FPC Ctg332 cp05T...

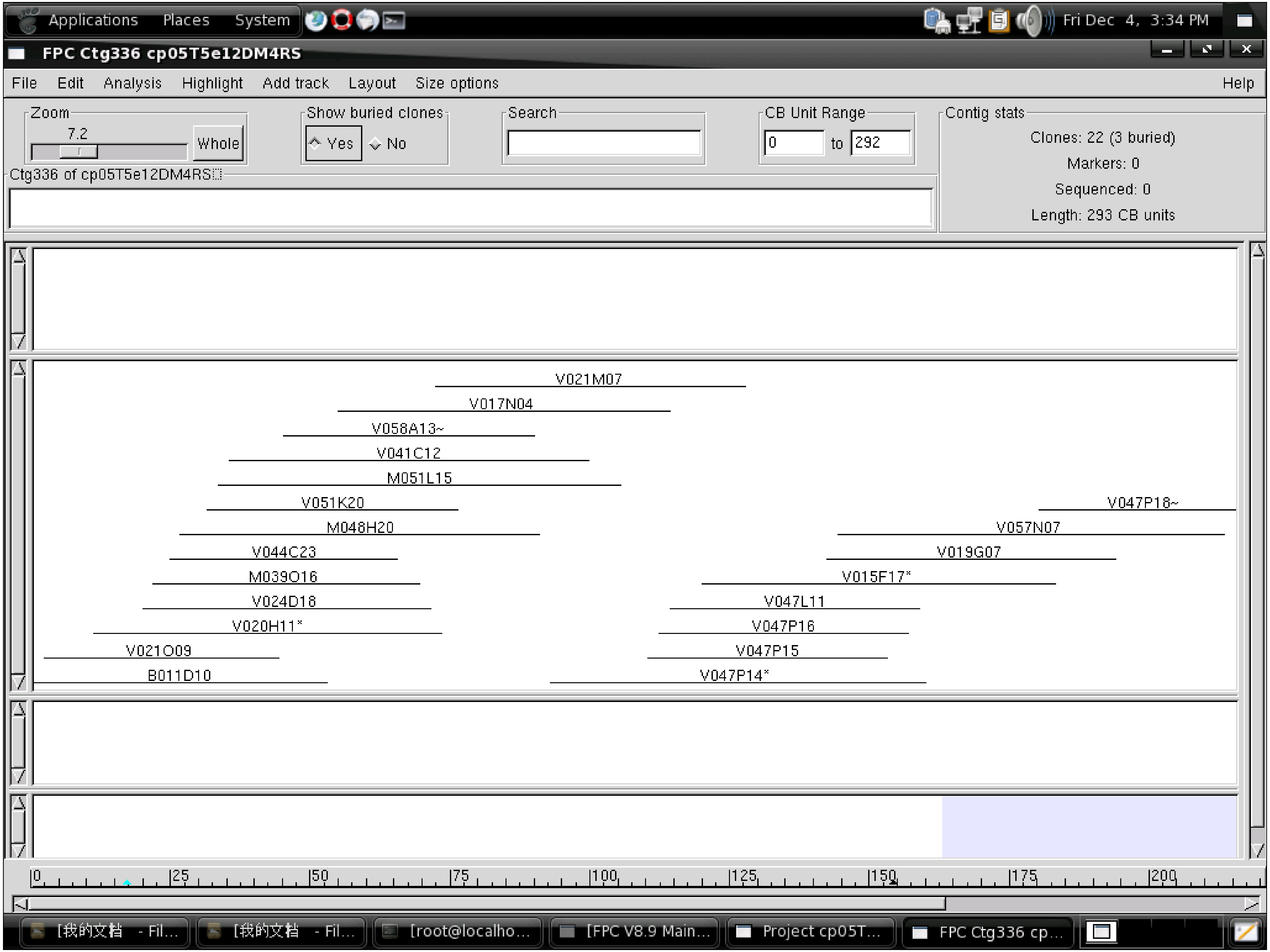
Applications Places System
Fri Dec 4, 3:34 PM
FPC Ctg336 cp05T5e12DM4RS
File Edit Analysis Highlight Add track Layout Size options Help
Zoom 7.2 Whole
Show buried clones Yes No
Search
CB Unit Range 0 to 292
Contig stats
Clones: 22 (3 buried)
Markers: 0
Sequenced: 0
Length: 293 CB units
Ctg336 of cp05T5e12DM4RS
V021M07
V017N04
V058A13~
V041C12
M051L15
V051K20
V047P18~
M048H20
V057N07
V044C23
V019G07
M039O16
V015F17*
V024D18
V047L11
V020H11*
V047P16
V021O09
V047P15
B011D10
V047P14*
0 25 50 75 100 125 150 175 200
[我的文档 - Fil...
[我的文档 - Fil...
[root@localho...
[FPC V8.9 Main...
Project cp05T...
FPC Ctg336 cp...

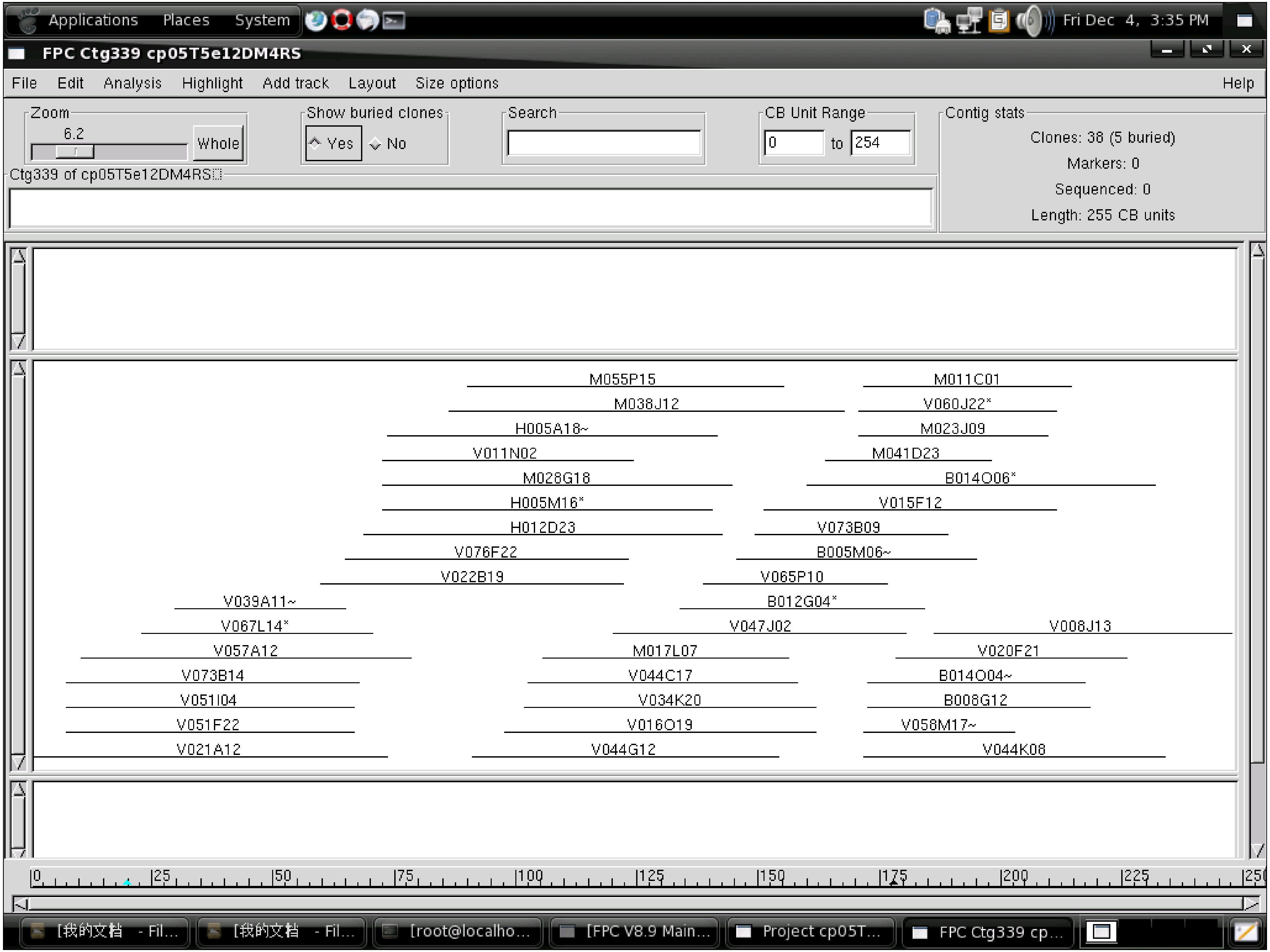
Applications Places System
Fri Dec 4, 3:35 PM
FPC Ctg339 cp05T5e12DM4RS
File Edit Analysis Highlight Add track Layout Size options Help
Zoom
6.2
Whole
Show buried clones
Yes No
Search
CB Unit Range
0 to 254
Contig stats
Clones: 38 (5 buried)
Markers: 0
Sequenced: 0
Length: 255 CB units
Ctg339 of cp05T5e12DM4RS
M055P15
M011C01
M038J12
V060J22*
H005A18~
M023J09
V011N02
M041D23
M028G18
B014O06*
H005M16*
V015F12
H012D23
V073B09
V076F22
B005M06~
V022B19
V065P10
V039A11~
B012G04*
V067L14*
V047J02
V008J13
V057A12
M017L07
V020F21
V073B14
V044C17
B014O04~
V051I04
V034K20
B008G12
V051F22
V016O19
V058M17~
V021A12
V044G12
V044K08
0 25 50 75 100 125 150 175 200 225 250
[我的文档 - Fil...
[我的文档 - Fil...
[root@localho...
[FPC V8.9 Main...
Project cp05T...
FPC Ctg339 cp...

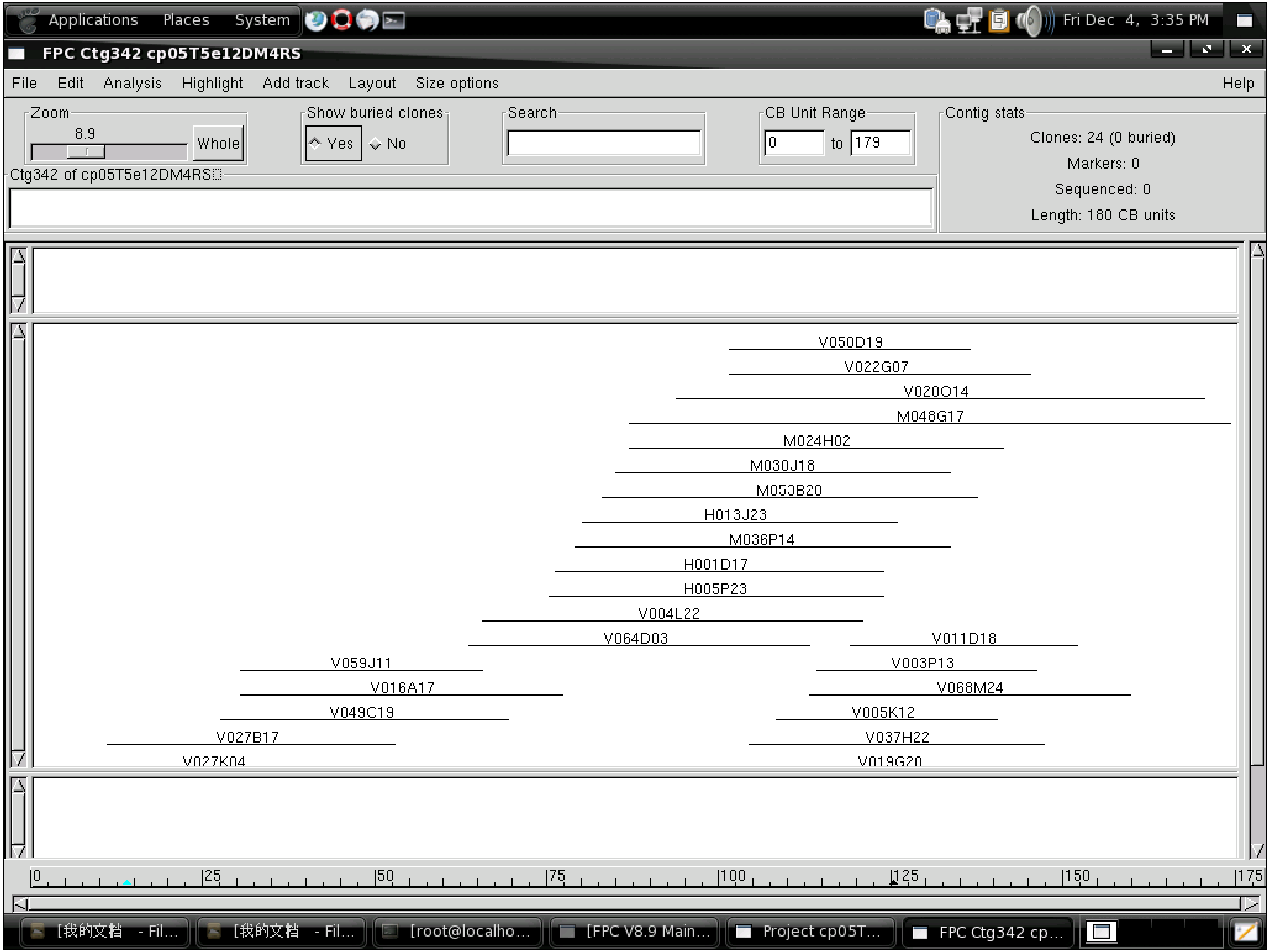
Applications Places System
Fri Dec 4, 3:35 PM
FPC Ctg342 cp05T5e12DM4RS
File Edit Analysis Highlight Add track Layout Size options Help
Zoom
8.9
Whole
Show buried clones
Yes No
Search
CB Unit Range
0 to 179
Contig stats
Clones: 24 (0 buried)
Markers: 0
Sequenced: 0
Length: 180 CB units
Ctg342 of cp05T5e12DM4RS
V050D19
V022G07
V020O14
M048G17
M024H02
M030J18
M053B20
H013J23
M036P14
H001D17
H005P23
V004L22
V064D03
V011D18
V059J11
V003P13
V016A17
V068M24
V049C19
V005K12
V027B17
V037H22
V027K04
V019G20
0 25 50 75 100 125 150 175
[我的文档 - Fil...
[我的文档 - Fil...
[root@localho...
[FPC V8.9 Main...
Project cp05T...
FPC Ctg342 cp...

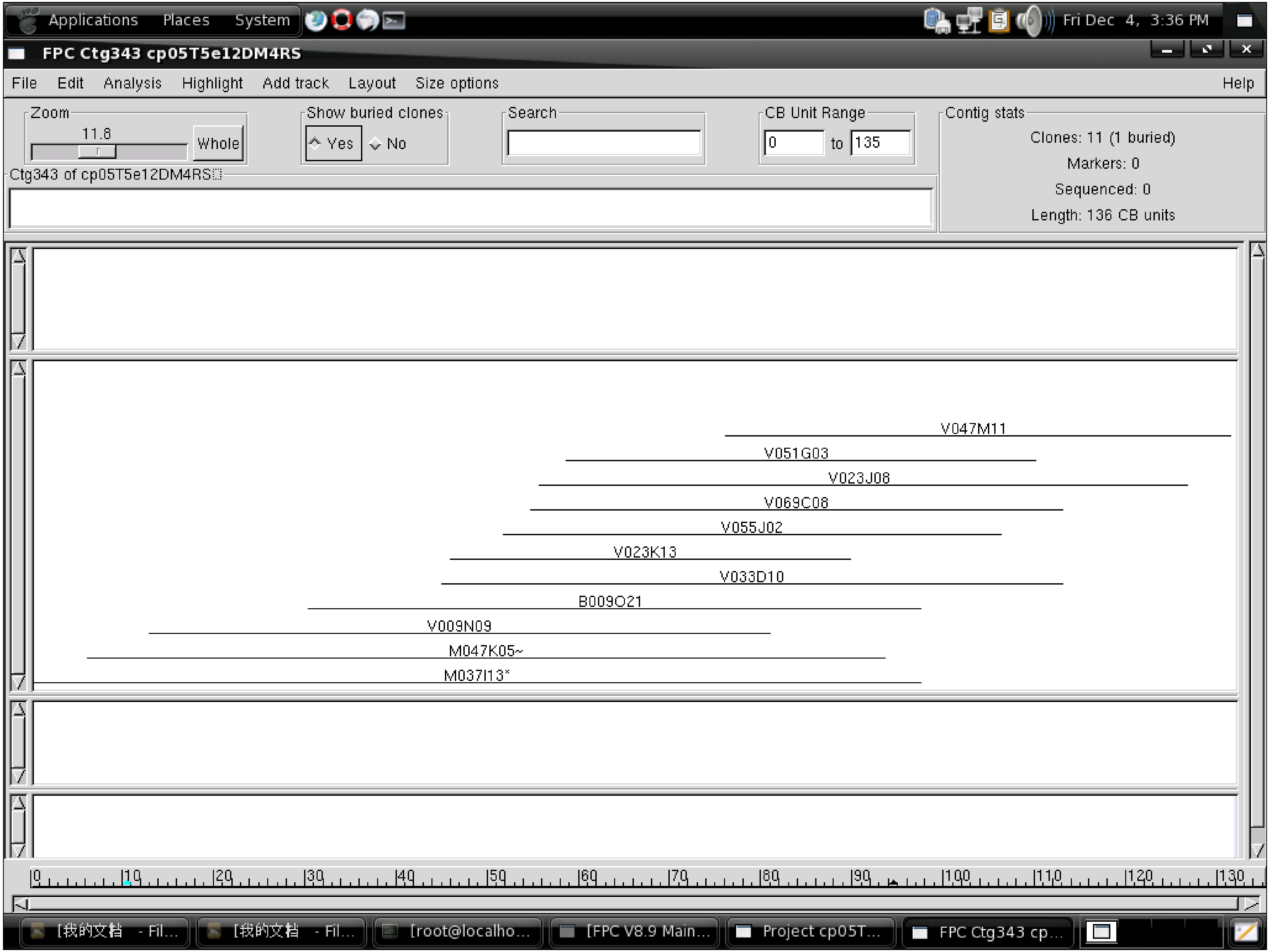
Applications Places System
Fri Dec 4, 3:36 PM
FPC Ctg343 cp05T5e12DM4RS
File Edit Analysis Highlight Add track Layout Size options Help
Zoom
11.8
Whole
Show buried clones
Yes
No
Search
CB Unit Range
0
to
135
Contig stats
Clones: 11 (1 buried)
Markers: 0
Sequenced: 0
Length: 136 CB units
Ctg343 of cp05T5e12DM4RS
V047M11
V051G03
V023J08
V069C08
V055J02
V023K13
V033D10
B009O21
V009N09
M047K05~
M037I13*
0 10 20 30 40 50 60 70 80 90 100 110 120 130
[我的文档 - Fil...
[我的文档 - Fil...
[root@localho...
[FPC V8.9 Main...
Project cp05T...
FPC Ctg343 cp...

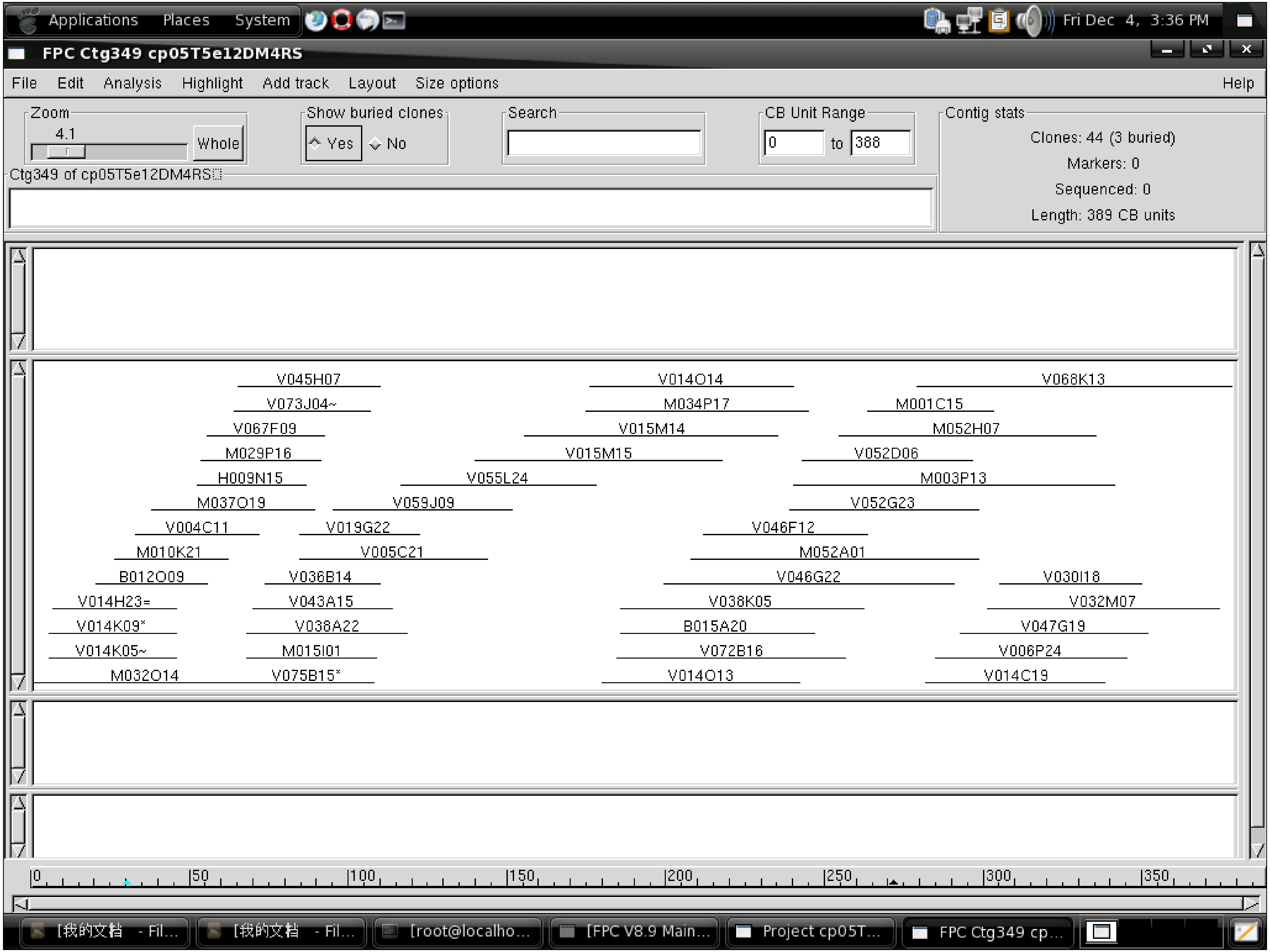
Applications Places System
Fri Dec 4, 3:36 PM
FPC Ctg349 cp05T5e12DM4RS
File Edit Analysis Highlight Add track Layout Size options Help
Zoom
4.1
Whole
Show buried clones
Yes No
Search
CB Unit Range
0 to 388
Contig stats
Clones: 44 (3 buried)
Markers: 0
Sequenced: 0
Length: 389 CB units
Ctg349 of cp05T5e12DM4RS
V045H07
V014O14
V068K13
V073J04~
M034P17
M001C15
V067F09
V015M14
M052H07
M029P16
V015M15
V052D06
H009N15
V055L24
M003P13
M037O19
V059J09
V052G23
V004C11
V019G22
V046F12
M010K21
V005C21
M052A01
B012O09
V036B14
V046G22
V030I18
V014H23=
V043A15
V038K05
V032M07
V014K09*
V038A22
B015A20
V047G19
V014K05~
M015I01
V072B16
V006P24
M032O14
V075B15*
V014O13
V014C19
0 50 100 150 200 250 300 350
[我的文档 - Fi...
[我的文档 - Fi...
[root@localho...
[FPC V8.9 Main...
Project cp05T...
FPC Ctg349 cp...

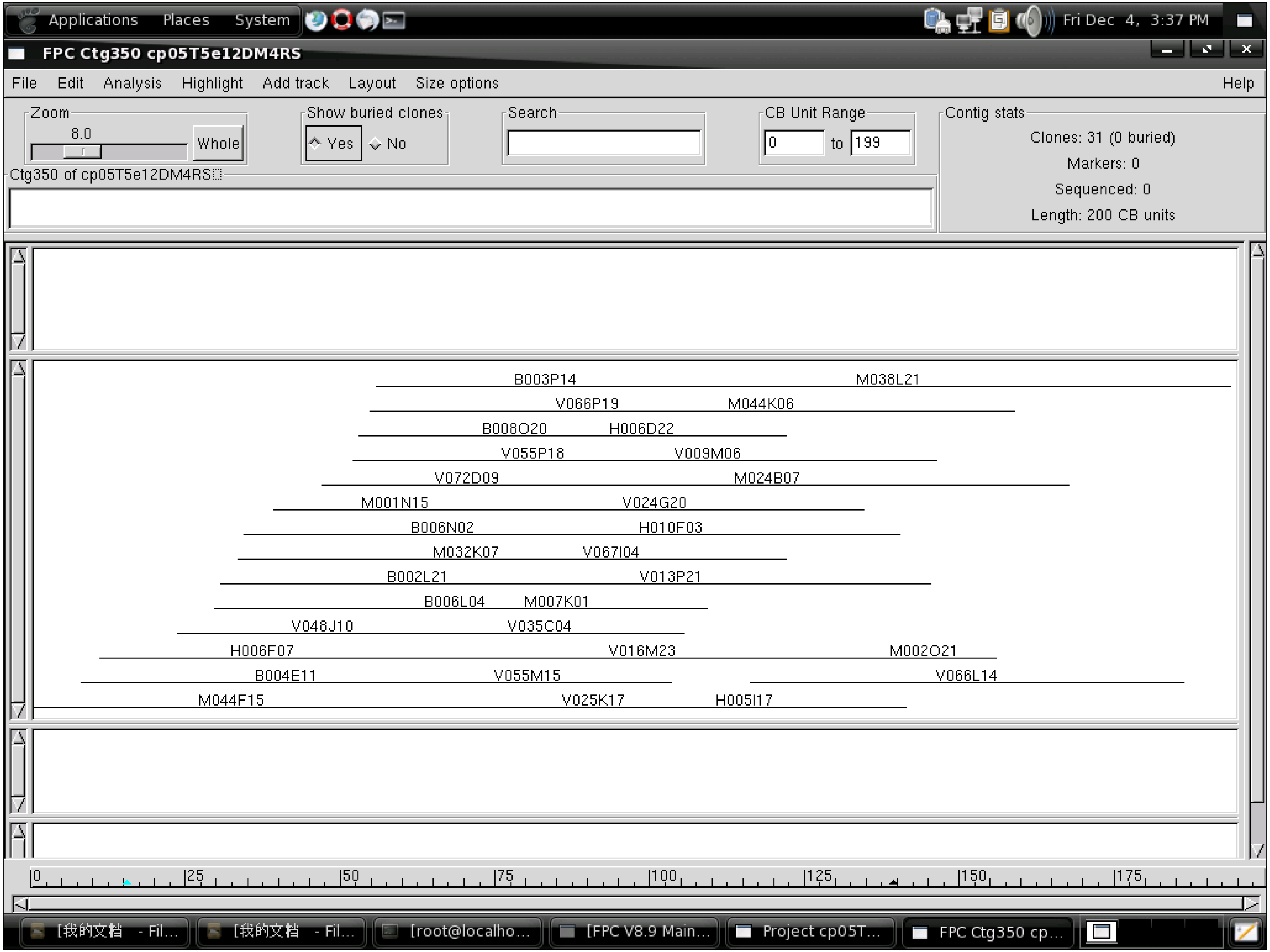
Applications Places System
Fri Dec 4, 3:37 PM
FPC Ctg350 cp05T5e12DM4RS
File Edit Analysis Highlight Add track Layout Size options Help
Zoom
8.0
Whole
Show buried clones
Yes
No
Search
CB Unit Range
0 to 199
Contig stats
Clones: 31 (0 buried)
Markers: 0
Sequenced: 0
Length: 200 CB units
Ctg350 of cp05T5e12DM4RS
B003P14
M038L21
V066P19
M044K06
B008O20
H006D22
V055P18
V009M06
V072D09
M024B07
M001N15
V024G20
B006N02
H010F03
M032K07
V067I04
B002L21
V013P21
B006L04
M007K01
V048J10
V035C04
H006F07
V016M23
M002O21
B004E11
V055M15
V066L14
M044F15
V025K17
H005I17
0
25
50
75
100
125
150
175
[我的文档 - Fil…
[我的文档 - Fil…
[root@localho…
[FPC V8.9 Main…
Project cp05T…
FPC Ctg350 cp…

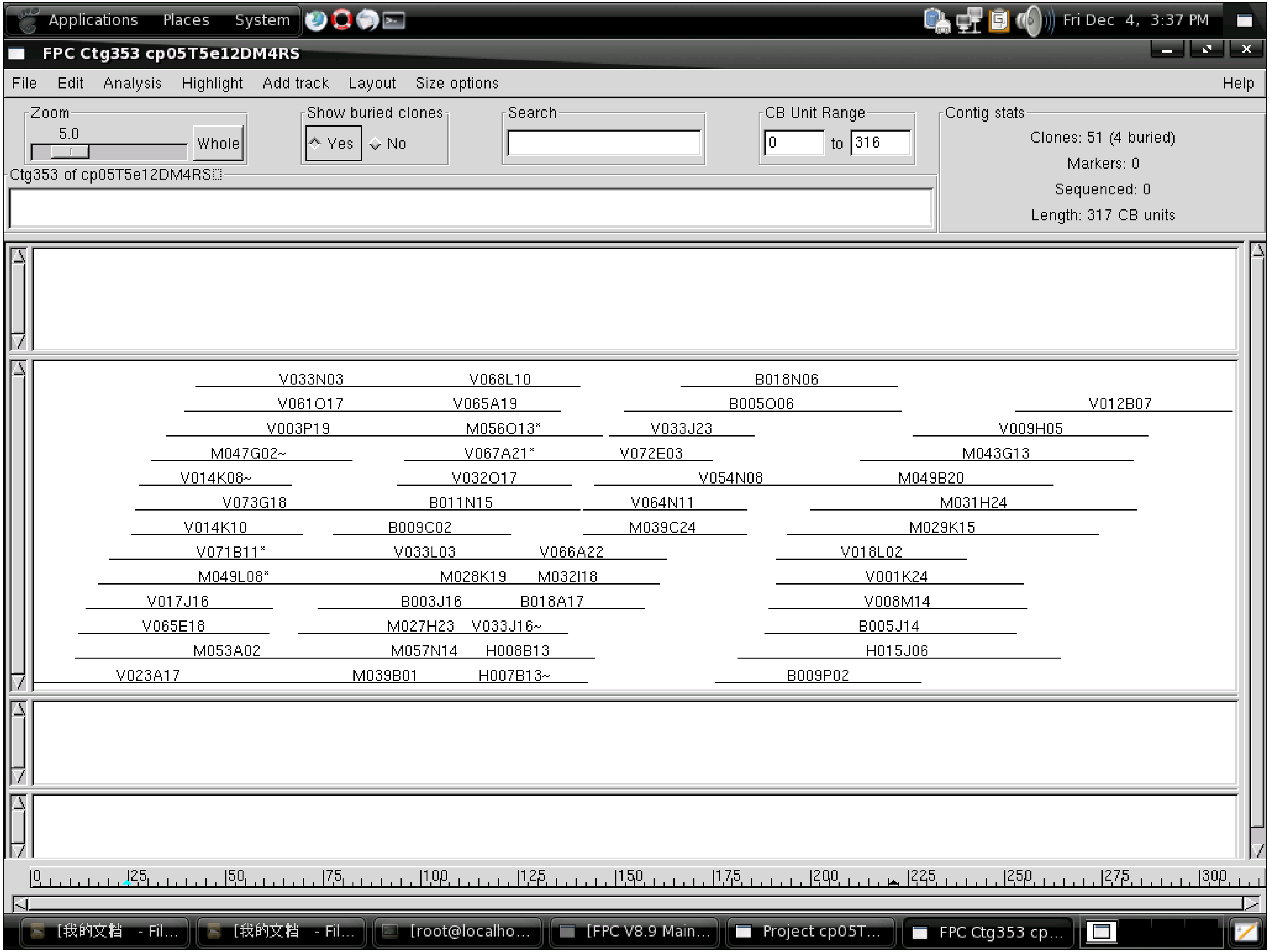
Applications Places System
Fri Dec 4, 3:37 PM
FPC Ctg353 cp05T5e12DM4RS
File Edit Analysis Highlight Add track Layout Size options Help
Zoom
5.0
Whole
Show buried clones
Yes
No
Search
CB Unit Range
0
to
316
Contig stats
Clones: 51 (4 buried)
Markers: 0
Sequenced: 0
Length: 317 CB units
Ctg353 of cp05T5e12DM4RS
V033N03
V068L10
B018N06
V061O17
V065A19
B005O06
V012B07
V003P19
M056O13*
V033J23
V009H05
M047G02~
V067A21*
V072E03
M043G13
V014K08~
V032O17
V054N08
M049B20
V073G18
B011N15
V064N11
M031H24
V014K10
B009C02
M039C24
M029K15
V071B11*
V033L03
V066A22
V018L02
M049L08*
M028K19
M032I18
V001K24
V017J16
B003J16
B018A17
V008M14
V065E18
M027H23
V033J16~
B005J14
M053A02
M057N14
H008B13
H015J06
V023A17
M039B01
H007B13~
B009P02
0
25
50
75
100
125
150
175
200
225
250
275
300
[我的文档 - Fil...
[我的文档 - Fil...
[root@localho...
[FPC V8.9 Main...
Project cp05T...
FPC Ctg353 cp...

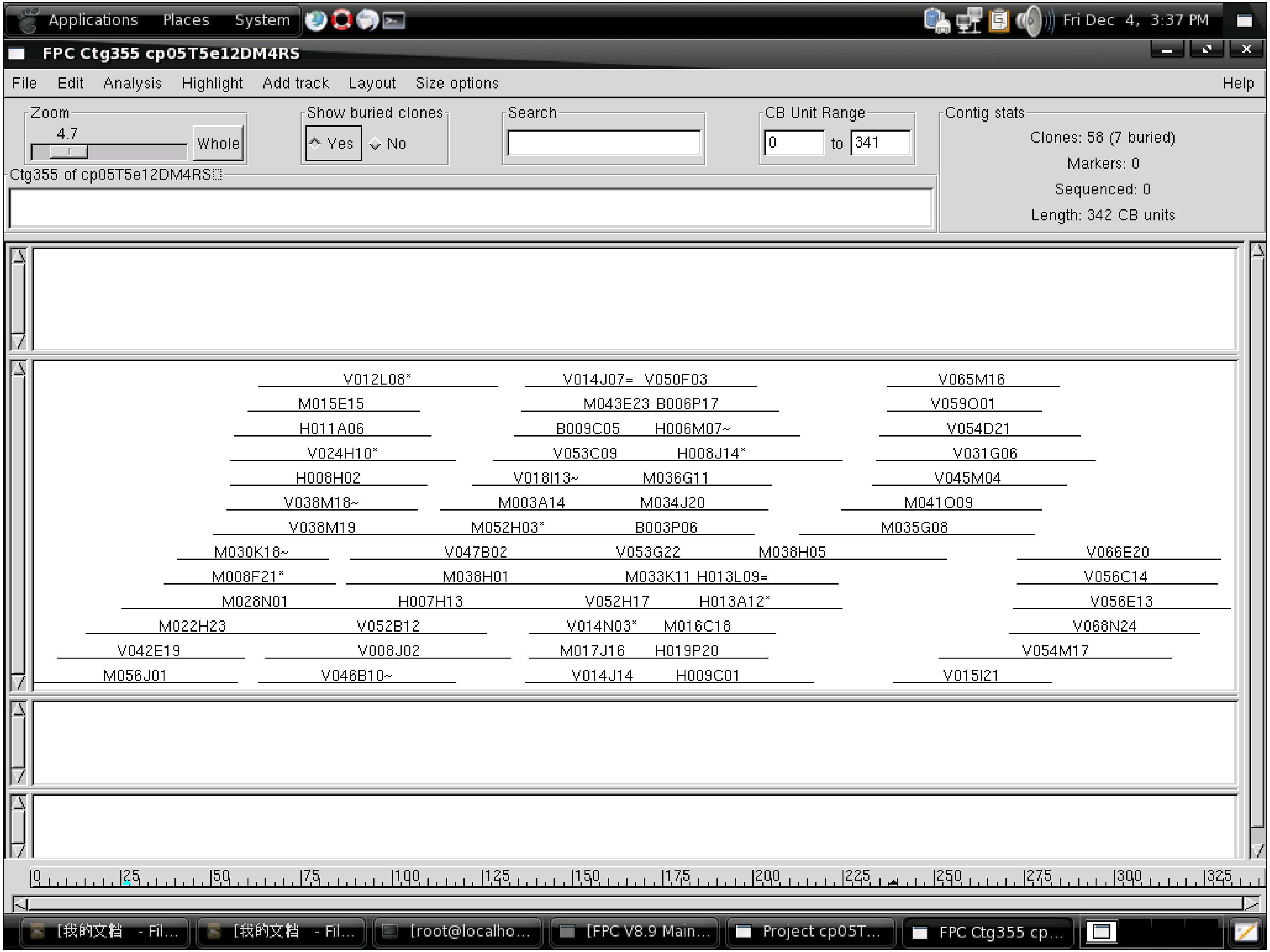
Applications Places System
Fri Dec 4, 3:37 PM
FPC Ctg355 cp05T5e12DM4RS
File Edit Analysis Highlight Add track Layout Size options Help
Zoom
4.7
Whole
Show buried clones
Yes
No
Search
CB Unit Range
0 to 341
Contig stats
Clones: 58 (7 buried)
Markers: 0
Sequenced: 0
Length: 342 CB units
Ctg355 of cp05T5e12DM4RS
V012L08*
V014J07= V050F03
V065M16
M015E15
M043E23 B006P17
V059O01
H011A06
B009C05 H006M07~
V054D21
V024H10*
V053C09 H008J14*
V031G06
H008H02
V018I13~ M036G11
V045M04
V038M18~
M003A14 M034J20
M041O09
V038M19
M052H03* B003P06
M035G08
M030K18~
V047B02 V053G22 M038H05
V066E20
M008F21*
M038H01 M033K11 H013L09=
V056C14
M028N01
H007H13 V052H17 H013A12*
V056E13
M022H23
V052B12
V014N03* M016C18
V068N24
V042E19
V008J02
M017J16 H019P20
V054M17
M056J01
V046B10~
V014J14 H009C01
V015I21
0 25 50 75 100 125 150 175 200 225 250 275 300 325
[我的文档 - Fil...
[我的文档 - Fil...
[root@localho...
[FPC V8.9 Main...
Project cp05T...
FPC Ctg355 cp...

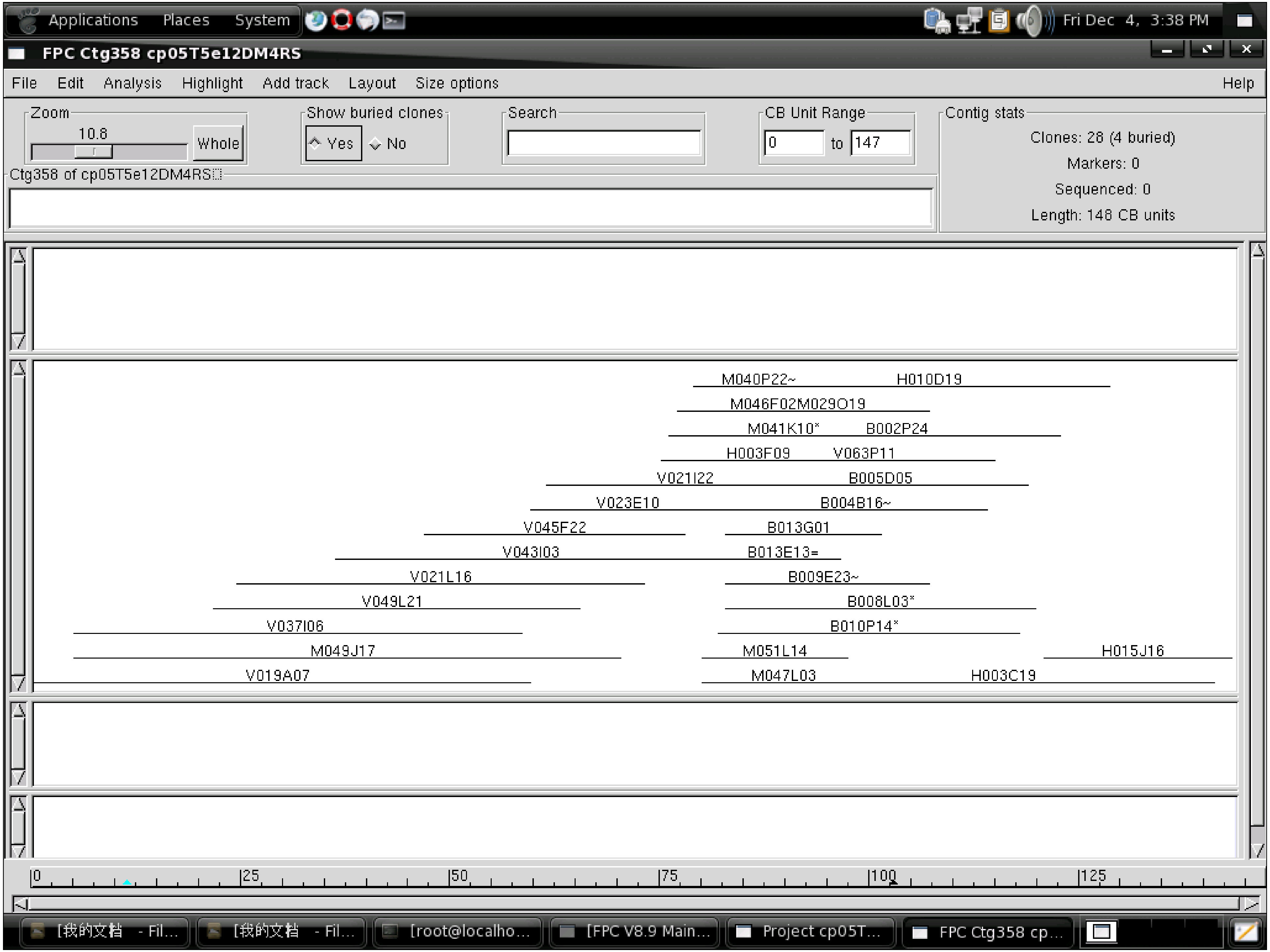

Applications Places System
Fri Dec 4, 3:38 PM
FPC Ctg358 cp05T5e12DM4RS
File Edit Analysis Highlight Add track Layout Size options Help
Zoom
10.8
Whole
Show buried clones
Yes No
Search
CB Unit Range
0 to 147
Contig stats
Clones: 28 (4 buried)
Markers: 0
Sequenced: 0
Length: 148 CB units
Ctg358 of cp05T5e12DM4RS
M040P22~
H010D19
M046F02M029O19
M041K10*
B002P24
H003F09
V063P11
V021I22
B005D05
V023E10
B004B16~
V045F22
B013G01
V043I03
B013E13=
V021L16
B009E23~
V049L21
B008L03*
V037I06
B010P14*
M049J17
M051L14
H015J16
V019A07
M047L03
H003C19
0 25 50 75 100 125
[我的文档 - Fil...
[我的文档 - Fil...
[root@localho...
[FPC V8.9 Main...
Project cp05T...
FPC Ctg358 cp...

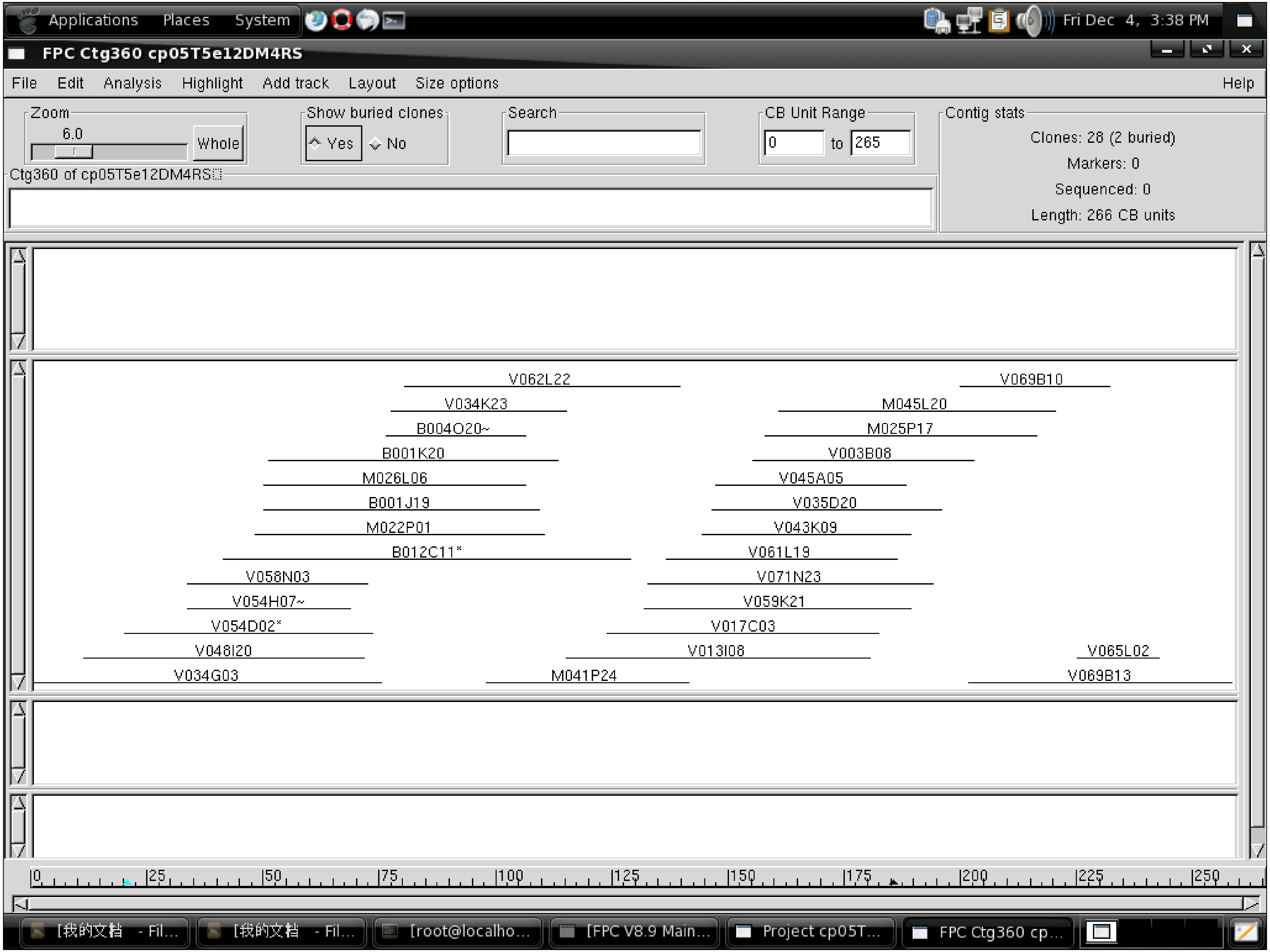

Applications
Places
System
Fri Dec 4, 3:38 PM
FPC Ctg360 cp05T5e12DM4RS
File
Edit
Analysis
Highlight
Add track
Layout
Size options
Help
Zoom
6.0
Whole
Show buried clones
Yes
No
Search
CB Unit Range
0
to
265
Contig stats
Clones: 28 (2 buried)
Markers: 0
Sequenced: 0
Length: 266 CB units
Ctg360 of cp05T5e12DM4RS
V062L22
V069B10
V034K23
M045L20
B004O20~
M025P17
B001K20
V003B08
M026L06
V045A05
B001J19
V035D20
M022P01
V043K09
B012C11*
V061L19
V058N03
V071N23
V054H07~
V059K21
V054D02*
V017C03
V048I20
V013I08
V065L02
V034G03
M041P24
V069B13
0
25
50
75
100
125
150
175
200
225
250
[我的文档 - Fil...
[我的文档 - Fil...
[root@localho...
[FPC V8.9 Main...
Project cp05T...
FPC Ctg360 cp...

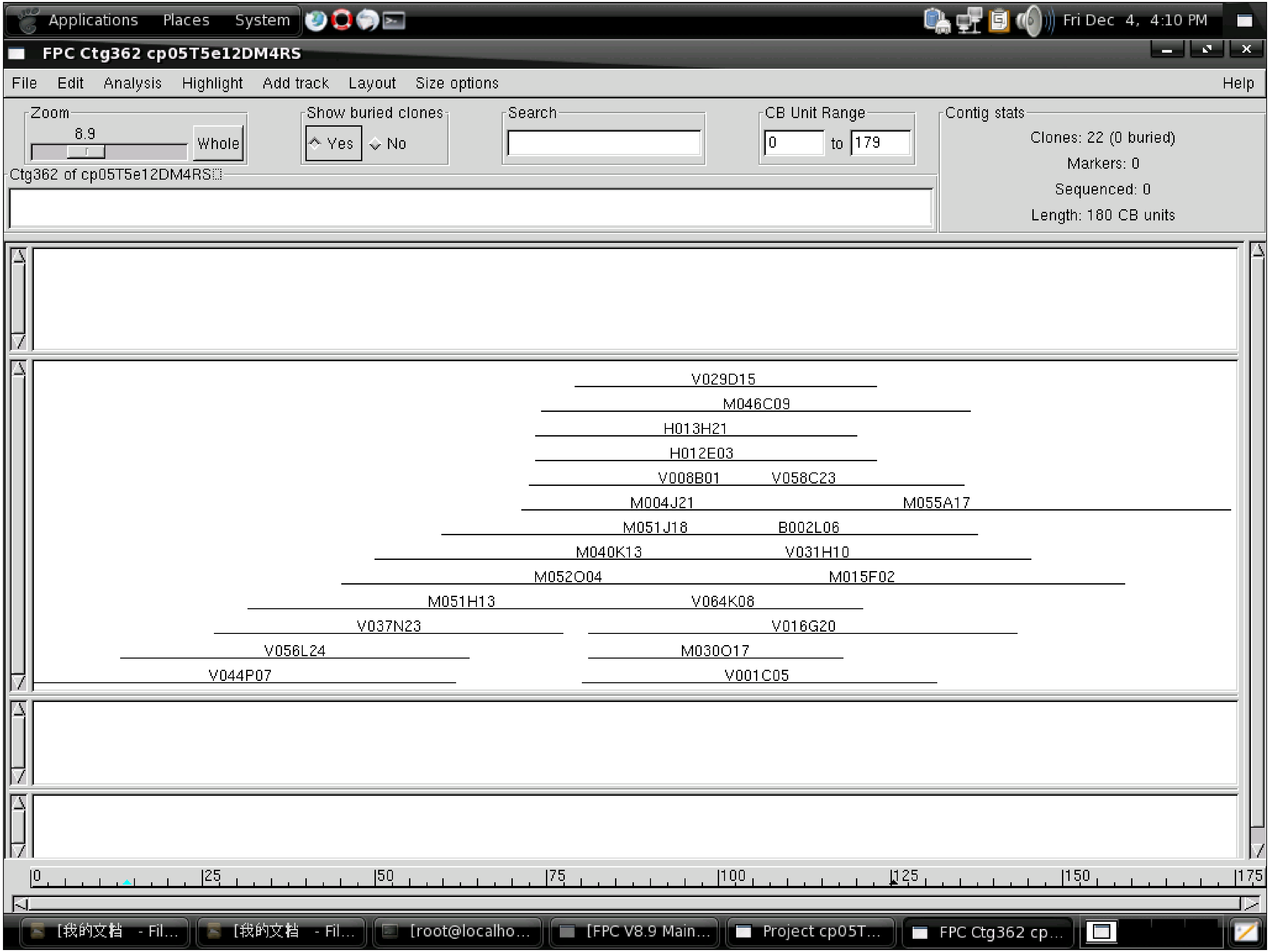
Applications Places System
Fri Dec 4, 4:10 PM
FPC Ctg362 cp05T5e12DM4RS
File Edit Analysis Highlight Add track Layout Size options Help
Zoom
8.9
Whole
Show buried clones
Yes No
Search
CB Unit Range
0 to 179
Contig stats
Clones: 22 (0 buried)
Markers: 0
Sequenced: 0
Length: 180 CB units
Ctg362 of cp05T5e12DM4RS
V029D15
M046C09
H013H21
H012E03
V008B01 V058C23
M004J21 M055A17
M051J18 B002L06
M040K13 V031H10
M052O04 M015F02
M051H13 V064K08
V037N23 V016G20
V056L24 M030O17
V044P07 V001C05
0 25 50 75 100 125 150 175
[我的文档 - Fil...
[我的文档 - Fil...
[root@localho...
[FPC V8.9 Main...
Project cp05T...
FPC Ctg362 cp...

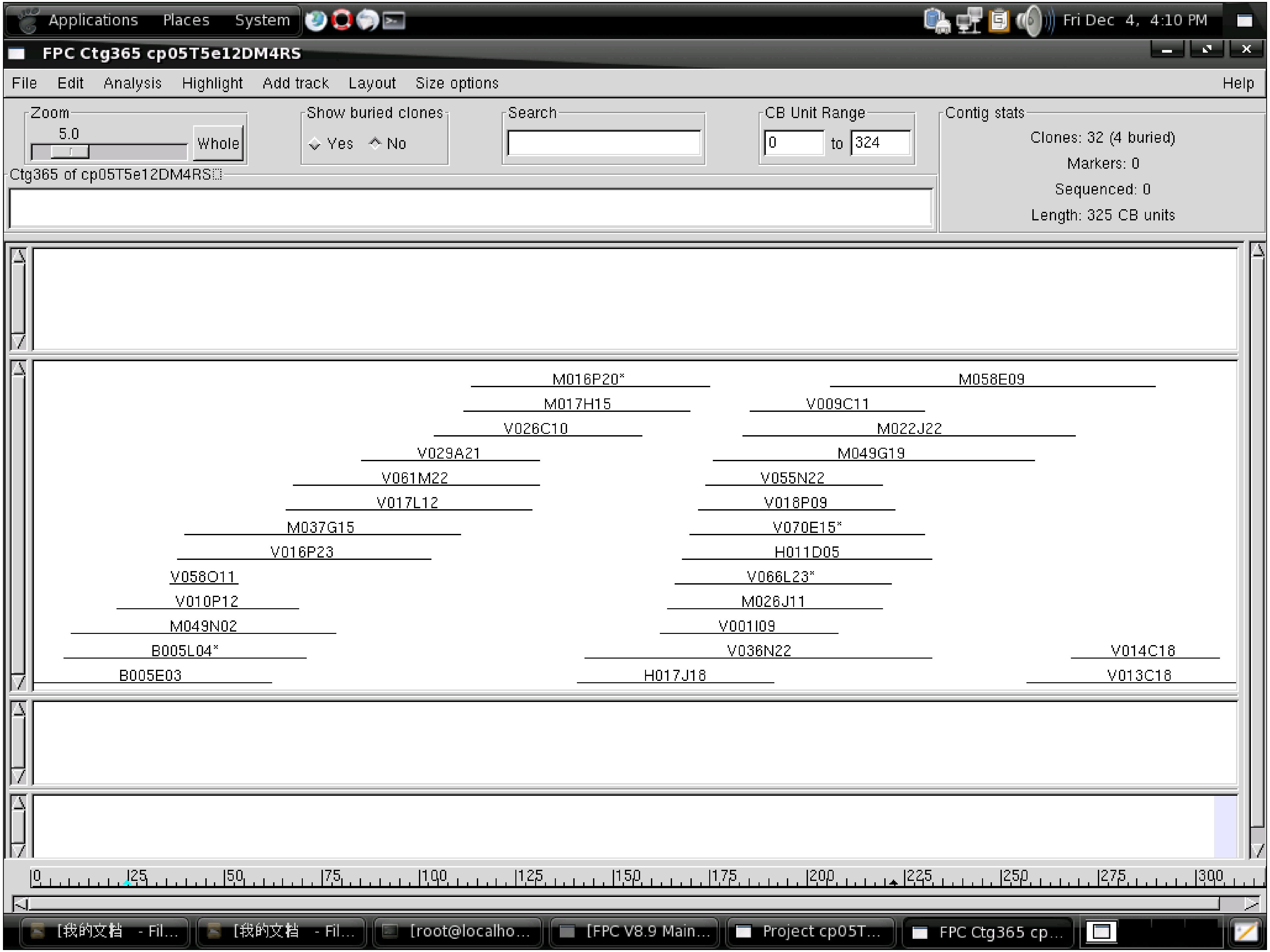
Applications Places System
Fri Dec 4, 4:10 PM
FPC Ctg365 cp05T5e12DM4RS
File Edit Analysis Highlight Add track Layout Size options Help
Zoom
5.0
Whole
Show buried clones
Yes No
Search
CB Unit Range
0 to 324
Contig stats
Clones: 32 (4 buried)
Markers: 0
Sequenced: 0
Length: 325 CB units
Ctg365 of cp05T5e12DM4RS
M016P20*
M058E09
M017H15
V009C11
V026C10
M022J22
V029A21
M049G19
V061M22
V055N22
V017L12
V018P09
M037G15
V070E15*
V016P23
H011D05
V058O11
V066L23*
V010P12
M026J11
M049N02
V001I09
B005L04*
V036N22
V014C18
B005E03
H017J18
V013C18
0 25 50 75 100 125 150 175 200 225 250 275 300
[我的文档 - Fil...
[我的文档 - Fil...
[root@localho...
[FPC V8.9 Main...
Project cp05T...
FPC Ctg365 cp...

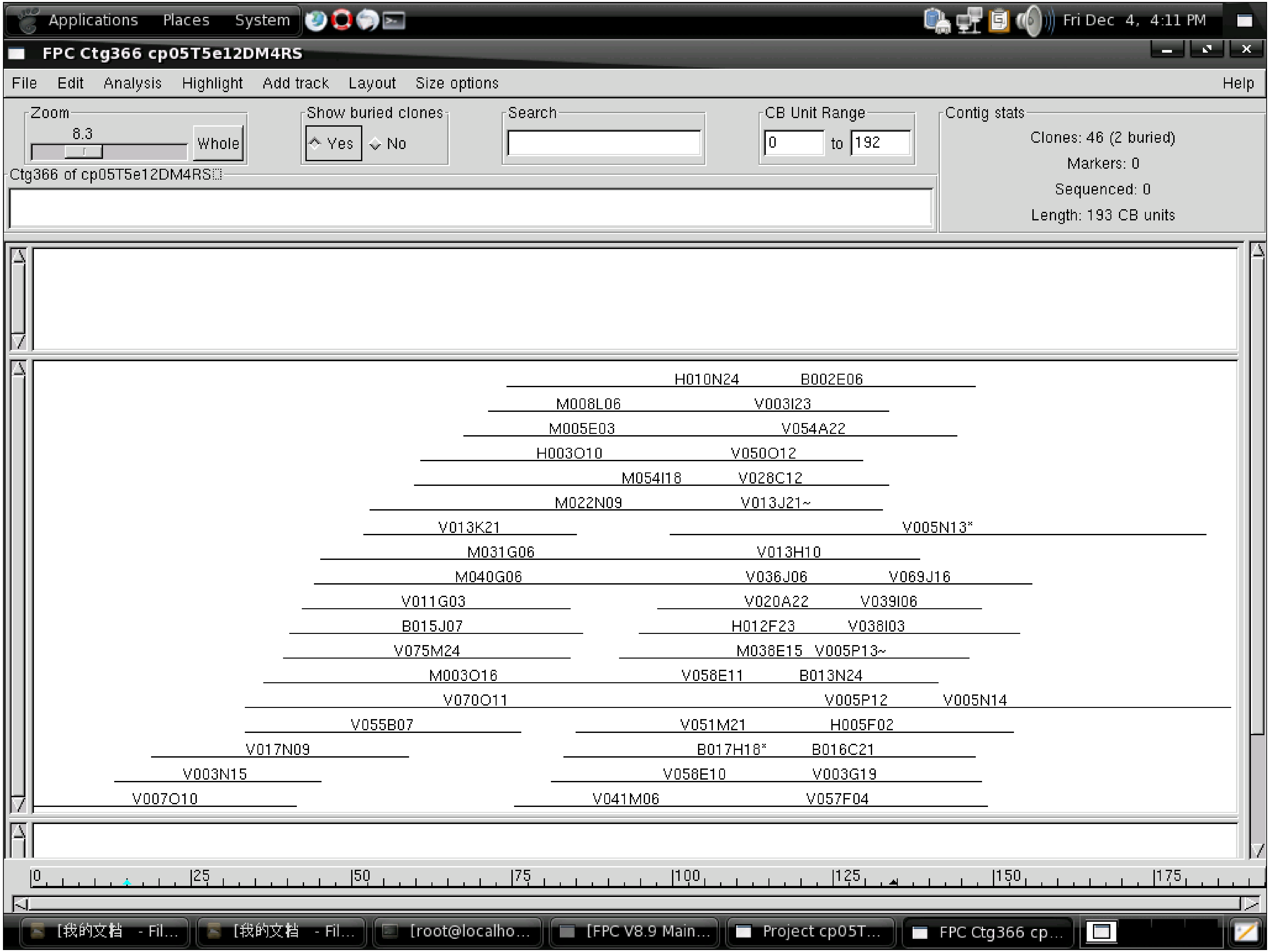
Applications Places System
Fri Dec 4, 4:11 PM
FPC Ctg366 cp05T5e12DM4RS
File Edit Analysis Highlight Add track Layout Size options Help
Zoom
8.3
Whole
Show buried clones
Yes
No
Search
CB Unit Range
0 to 192
Contig stats
Clones: 46 (2 buried)
Markers: 0
Sequenced: 0
Length: 193 CB units
Ctg366 of cp05T5e12DM4RS
H010N24 B002E06
M008L06 V003I23
M005E03 V054A22
H003O10 V050O12
M054I18 V028C12
M022N09 V013J21~
V013K21 V005N13*
M031G06 V013H10
M040G06 V036J06 V069J16
V011G03 V020A22 V039I06
B015J07 H012F23 V038I03
V075M24 M038E15 V005P13~
M003O16 V058E11 B013N24
V070O11 V005P12 V005N14
V055B07 V051M21 H005F02
V017N09 B017H18* B016C21
V003N15 V058E10 V003G19
V007O10 V041M06 V057F04
0 25 50 75 100 125 150 175
[我的文档 - Fil...
[我的文档 - Fil...
[root@localho...
[FPC V8.9 Main...
Project cp05T...
FPC Ctg366 cp...

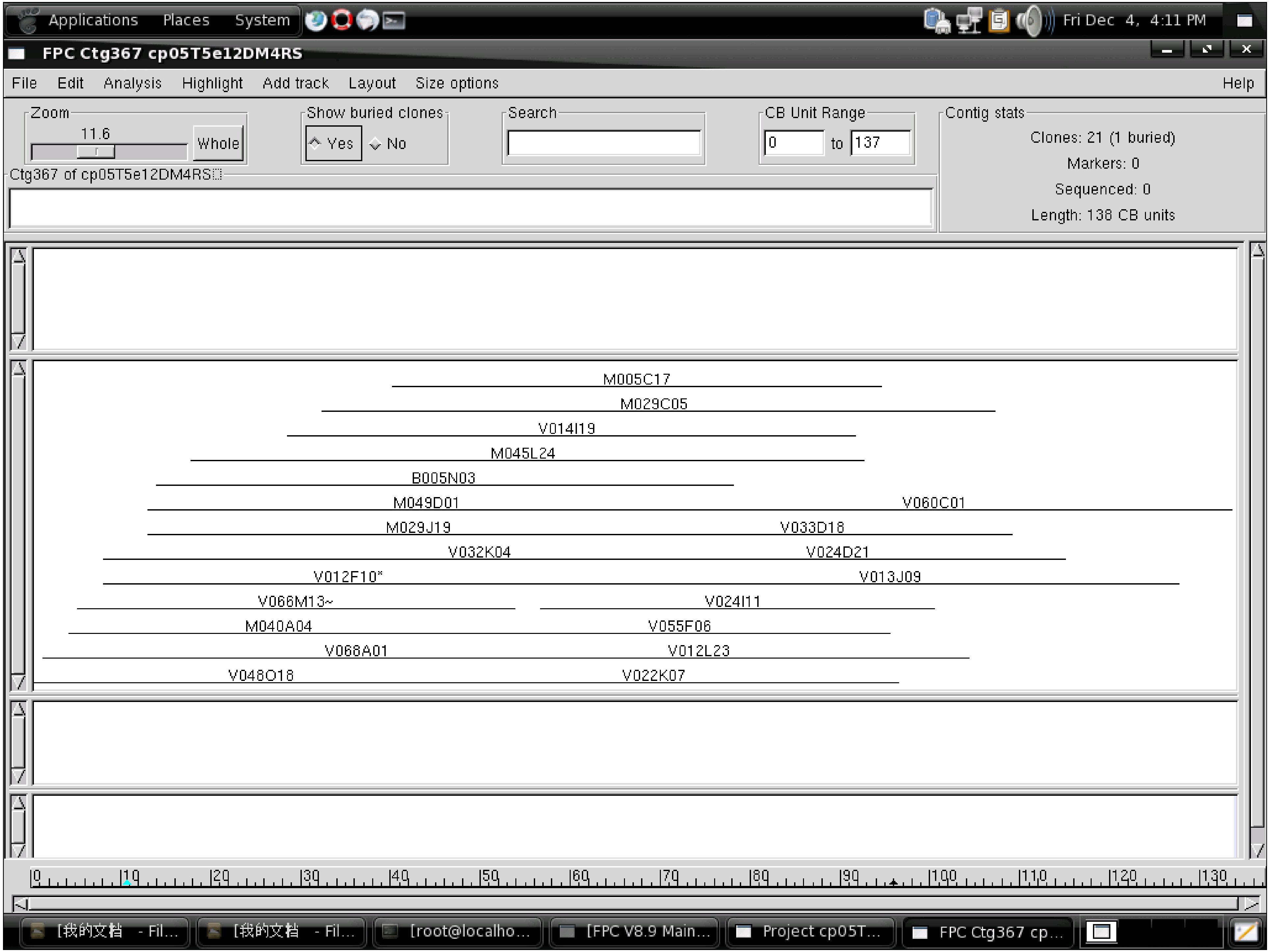
Applications Places System
Fri Dec 4, 4:11 PM
FPC Ctg367 cp05T5e12DM4RS
File Edit Analysis Highlight Add track Layout Size options Help
Zoom
11.6
Whole
Show buried clones
Yes
No
Search
CB Unit Range
0
to
137
Contig stats
Clones: 21 (1 buried)
Markers: 0
Sequenced: 0
Length: 138 CB units
Ctg367 of cp05T5e12DM4RS
M005C17
M029C05
V014I19
M045L24
B005N03
M049D01
V060C01
M029J19
V033D18
V032K04
V024D21
V012F10*
V013J09
V066M13~
V024I11
M040A04
V055F06
V068A01
V012L23
V048O18
V022K07
0 10 20 30 40 50 60 70 80 90 100 110 120 130
[我的文档 - Fil…
[我的文档 - Fil…
[root@localho…
[FPC V8.9 Main…
Project cp05T…
FPC Ctg367 cp…

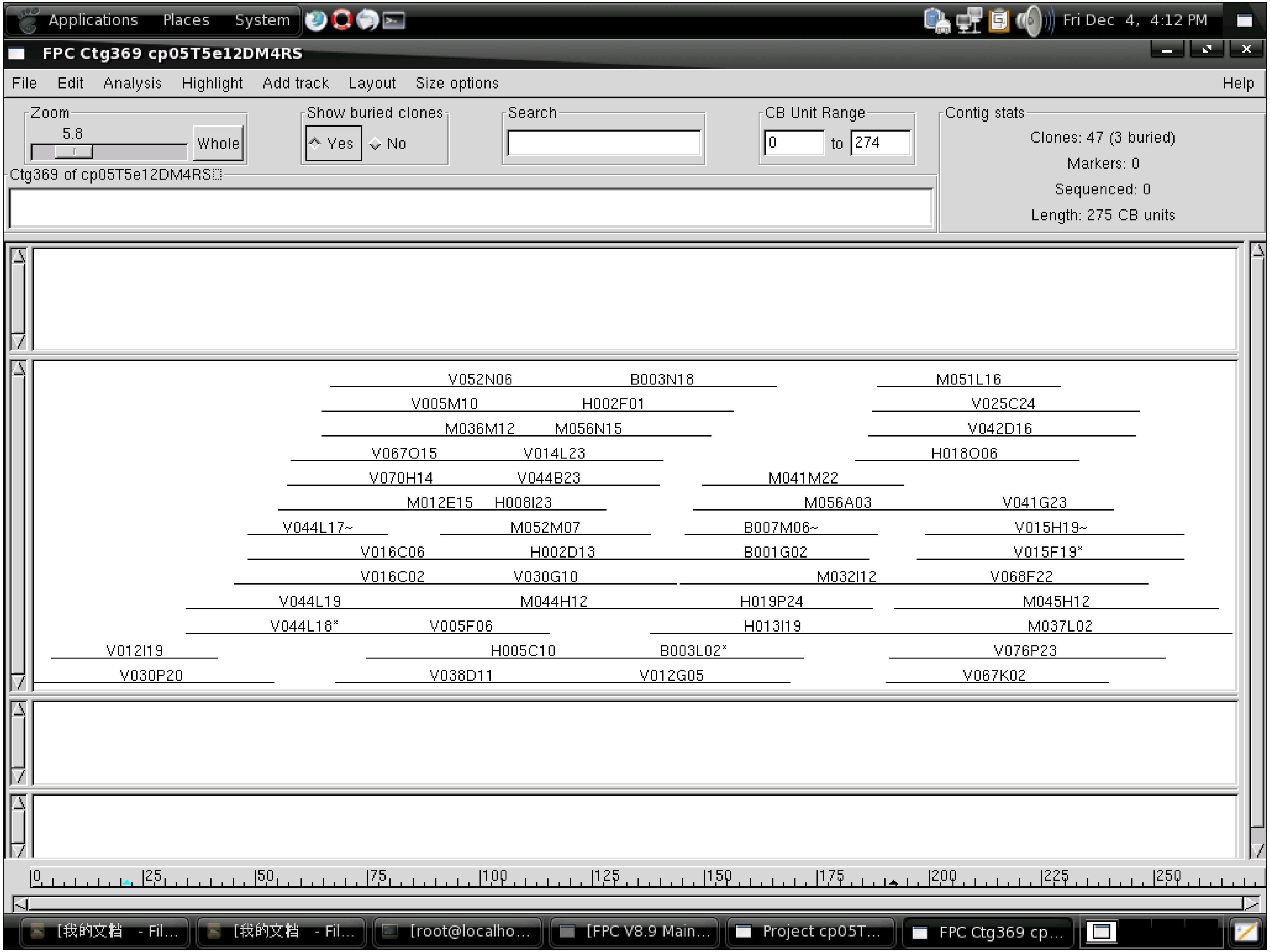
Applications Places System
Fri Dec 4, 4:12 PM
FPC Ctg369 cp05T5e12DM4RS
File Edit Analysis Highlight Add track Layout Size options Help
Zoom 5.8 Whole
Show buried clones Yes No
Search
CB Unit Range 0 to 274
Contig stats
Clones: 47 (3 buried)
Markers: 0
Sequenced: 0
Length: 275 CB units
Ctg369 of cp05T5e12DM4RS
V052N06 B003N18 M051L16
V005M10 H002F01 V025C24
M036M12 M056N15 V042D16
V067O15 V014L23 H018O06
V070H14 V044B23 M041M22
M012E15 H008I23 M056A03 V041G23
V044L17~ M052M07 B007M06~ V015H19~
V016C06 H002D13 B001G02 V015F19*
V016C02 V030G10 M032I12 V068F22
V044L19 M044H12 H019P24 M045H12
V044L18* V005F06 H013I19 M037L02
V012I19 H005C10 B003L02* V076P23
V030P20 V038D11 V012G05 V067K02
0 25 50 75 100 125 150 175 200 225 250
[我的文档 - Fil… [我的文档 - Fil… [root@localho… [FPC V8.9 Main… Project cp05T… FPC Ctg369 cp…

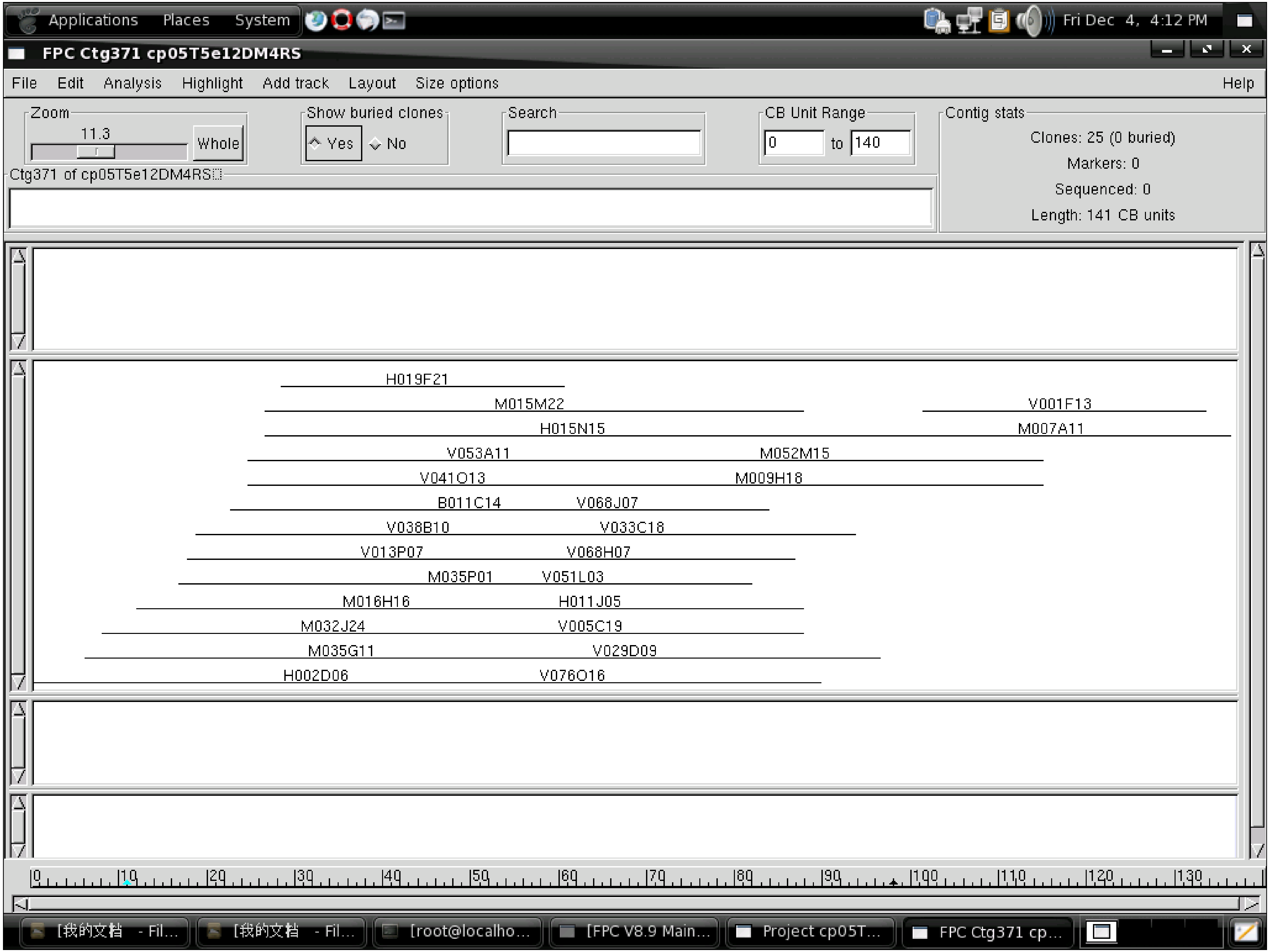

Applications Places System
Fri Dec 4, 4:12 PM
FPC Ctg371 cp05T5e12DM4RS
File Edit Analysis Highlight Add track Layout Size options Help
Zoom
11.3
Whole
Show buried clones
Yes No
Search
CB Unit Range
0 to 140
Contig stats
Clones: 25 (0 buried)
Markers: 0
Sequenced: 0
Length: 141 CB units
Ctg371 of cp05T5e12DM4RS
H019F21
M015M22
V001F13
H015N15
M007A11
V053A11
M052M15
V041O13
M009H18
B011C14
V068J07
V038B10
V033C18
V013P07
V068H07
M035P01
V051L03
M016H16
H011J05
M032J24
V005C19
M035G11
V029D09
H002D06
V076O16
0 10 20 30 40 50 60 70 80 90 100 110 120 130
[我的文档 - Fil...
[我的文档 - Fil...
[root@localho...
[FPC V8.9 Main...
Project cp05T...
FPC Ctg371 cp...

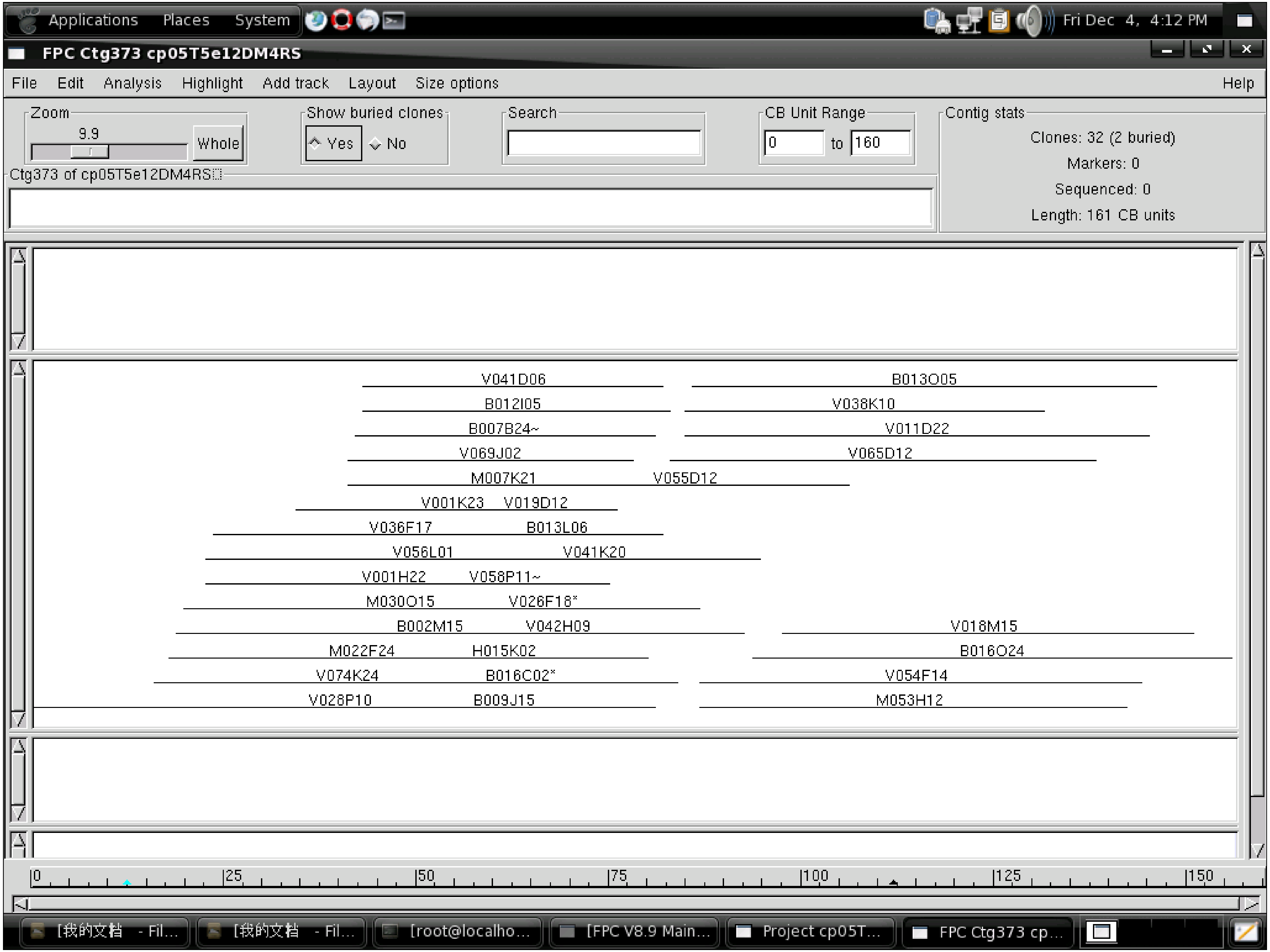
Applications Places System
Fri Dec 4, 4:12 PM
FPC Ctg373 cp05T5e12DM4RS
File Edit Analysis Highlight Add track Layout Size options Help
Zoom
9.9
Whole
Show buried clones
Yes No
Search
CB Unit Range
0 to 160
Contig stats
Clones: 32 (2 buried)
Markers: 0
Sequenced: 0
Length: 161 CB units
Ctg373 of cp05T5e12DM4RS
V041D06
B013O05
B012I05
V038K10
B007B24~
V011D22
V069J02
V065D12
M007K21
V055D12
V001K23
V019D12
V036F17
B013L06
V056L01
V041K20
V001H22
V058P11~
M030O15
V026F18*
B002M15
V042H09
V018M15
M022F24
H015K02
B016O24
V074K24
B016C02*
V054F14
V028P10
B009J15
M053H12
0 25 50 75 100 125 150
[我的文档 - Fil...
[我的文档 - Fil...
[root@localho...
[FPC V8.9 Main...
Project cp05T...
FPC Ctg373 cp...

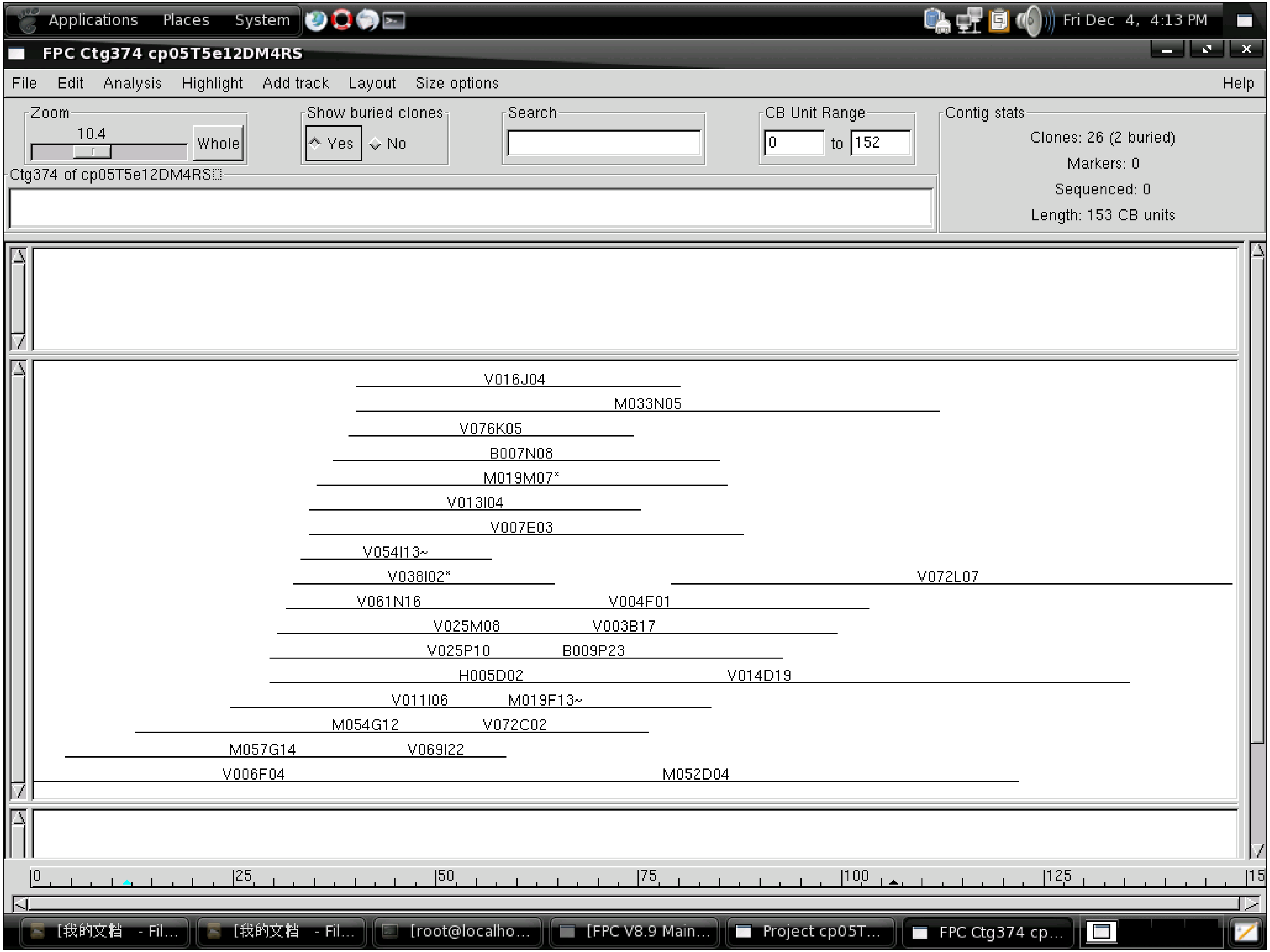
Applications Places System
Fri Dec 4, 4:13 PM
FPC Ctg374 cp05T5e12DM4RS
File Edit Analysis Highlight Add track Layout Size options Help
Zoom
10.4
Whole
Show buried clones
Yes
No
Search
CB Unit Range
0
to
152
Contig stats
Clones: 26 (2 buried)
Markers: 0
Sequenced: 0
Length: 153 CB units
Ctg374 of cp05T5e12DM4RS
V016J04
M033N05
V076K05
B007N08
M019M07*
V013I04
V007E03
V054I13~
V038I02*
V072L07
V061N16
V004F01
V025M08
V003B17
V025P10
B009P23
H005D02
V014D19
V011I06
M019F13~
M054G12
V072C02
M057G14
V069I22
V006F04
M052D04
0
25
50
75
100
125
[我的文档 - Fil...
[我的文档 - Fil...
[root@localho...
[FPC V8.9 Main...
Project cp05T...
FPC Ctg374 cp...

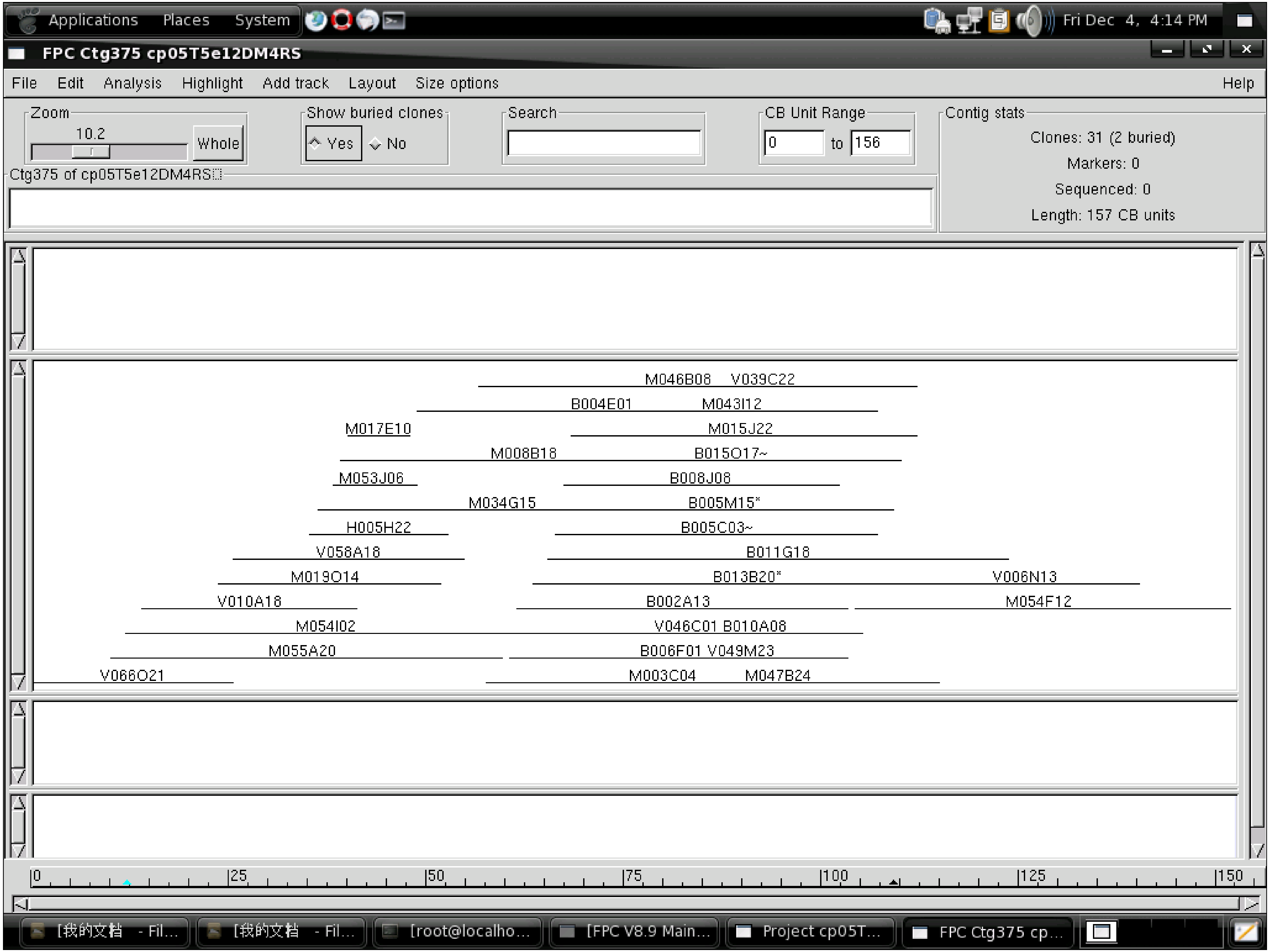
Applications Places System
Fri Dec 4, 4:14 PM
FPC Ctg375 cp05T5e12DM4RS
File Edit Analysis Highlight Add track Layout Size options Help
Zoom
10.2
Whole
Show buried clones
Yes No
Search
CB Unit Range
0 to 156
Contig stats
Clones: 31 (2 buried)
Markers: 0
Sequenced: 0
Length: 157 CB units
Ctg375 of cp05T5e12DM4RS
M046B08 V039C22
B004E01 M043I12
M017E10
M015J22
M008B18 B015O17~
M053J06
B008J08
M034G15 B005M15*
H005H22
B005C03~
V058A18
B011G18
M019O14
B013B20*
V006N13
V010A18
B002A13
M054F12
M054I02
V046C01 B010A08
M055A20
B006F01 V049M23
V066O21
M003C04 M047B24
0 25 50 75 100 125 150
[我的文档 - Fil...
[我的文档 - Fil...
[root@localho...
[FPC V8.9 Main...
Project cp05T...
FPC Ctg375 cp...

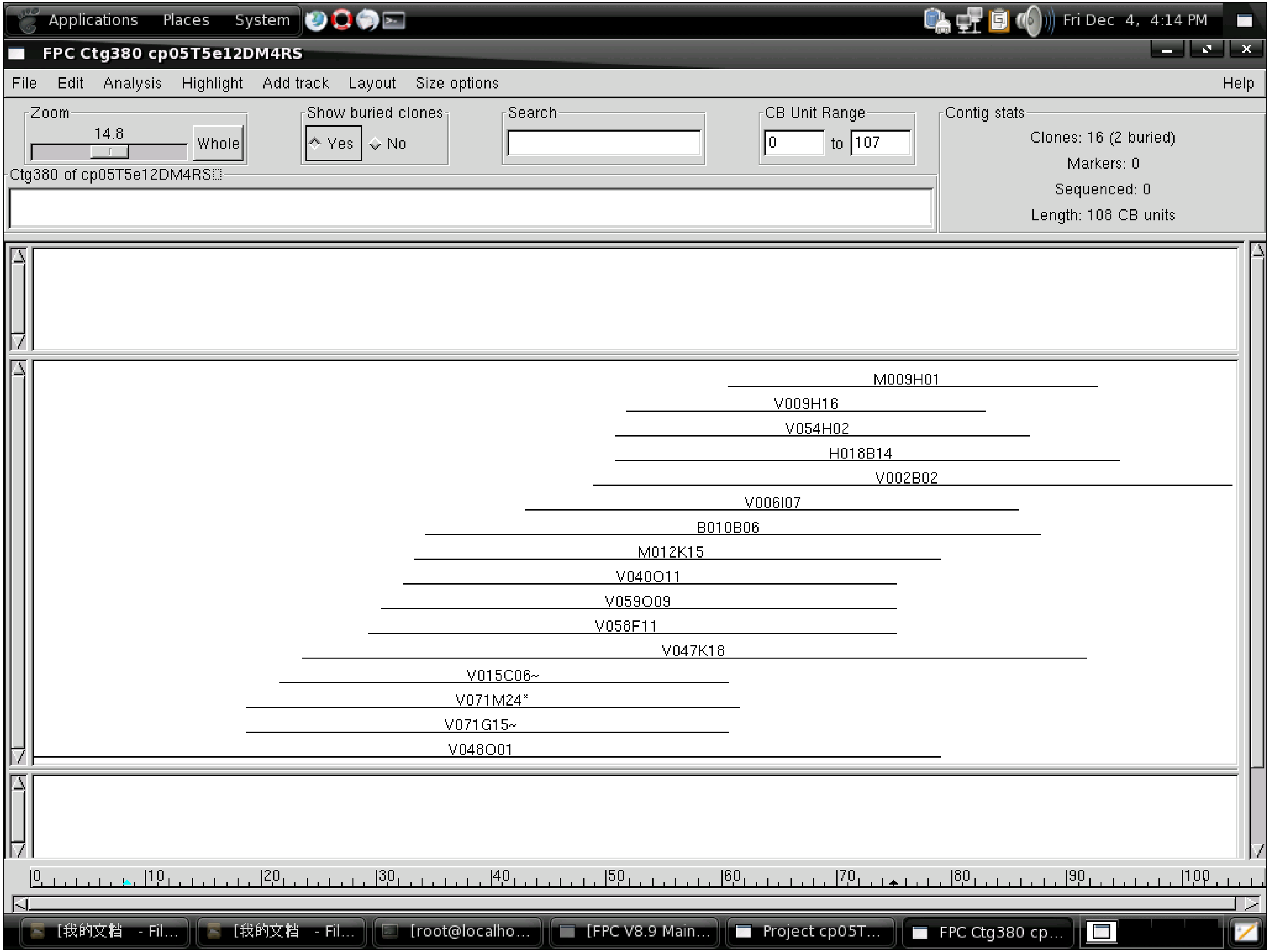
Applications Places System
Fri Dec 4, 4:14 PM
FPC Ctg380 cp05T5e12DM4RS
File Edit Analysis Highlight Add track Layout Size options Help
Zoom
14.8
Whole
Show buried clones
Yes No
Search
CB Unit Range
0 to 107
Contig stats
Clones: 16 (2 buried)
Markers: 0
Sequenced: 0
Length: 108 CB units
Ctg380 of cp05T5e12DM4RS
M009H01
V009H16
V054H02
H018B14
V002B02
V006I07
B010B06
M012K15
V040O11
V059O09
V058F11
V047K18
V015C06~
V071M24*
V071G15~
V048O01
0 10 20 30 40 50 60 70 80 90 100
[我的文档 - Fil…
[我的文档 - Fil…
[root@localho…
[FPC V8.9 Main…
Project cp05T…
FPC Ctg380 cp…

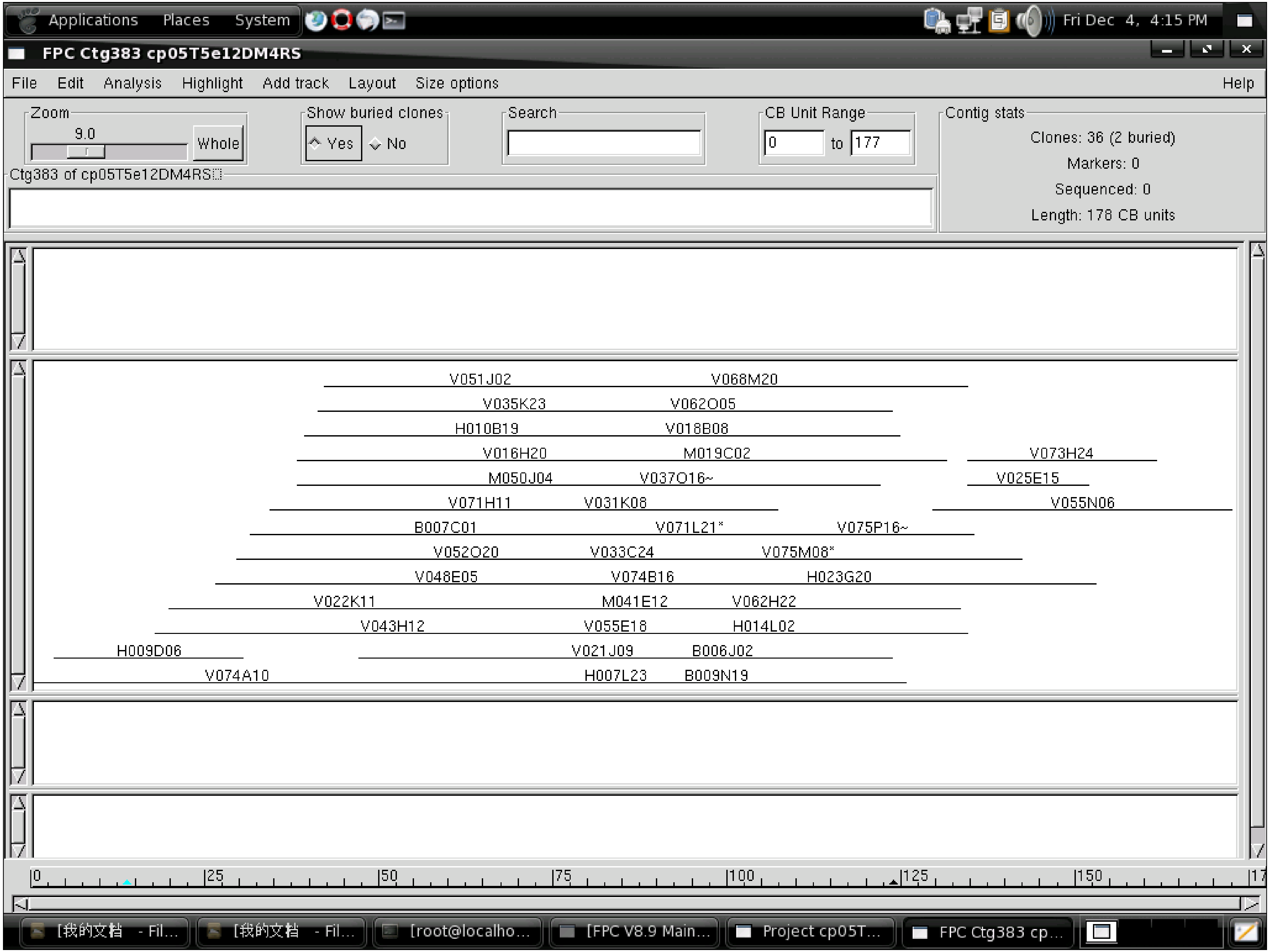
Applications Places System
Fri Dec 4, 4:15 PM
FPC Ctg383 cp05T5e12DM4RS
File Edit Analysis Highlight Add track Layout Size options Help
Zoom
9.0
Whole
Show buried clones
Yes No
Search
CB Unit Range
0 to 177
Contig stats
Clones: 36 (2 buried)
Markers: 0
Sequenced: 0
Length: 178 CB units
Ctg383 of cp05T5e12DM4RS
V051J02 V068M20
V035K23 V062O05
H010B19 V018B08
V016H20 M019C02 V073H24
M050J04 V037O16~ V025E15
V071H11 V031K08 V055N06
B007C01 V071L21* V075P16~
V052O20 V033C24 V075M08*
V048E05 V074B16 H023G20
V022K11 M041E12 V062H22
V043H12 V055E18 H014L02
H009D06 V021J09 B006J02
V074A10 H007L23 B009N19
0 25 50 75 100 125 150
[我的文档 - Fil...
[我的文档 - Fil...
[root@localho...
[FPC V8.9 Main...
Project cp05T...
FPC Ctg383 cp...

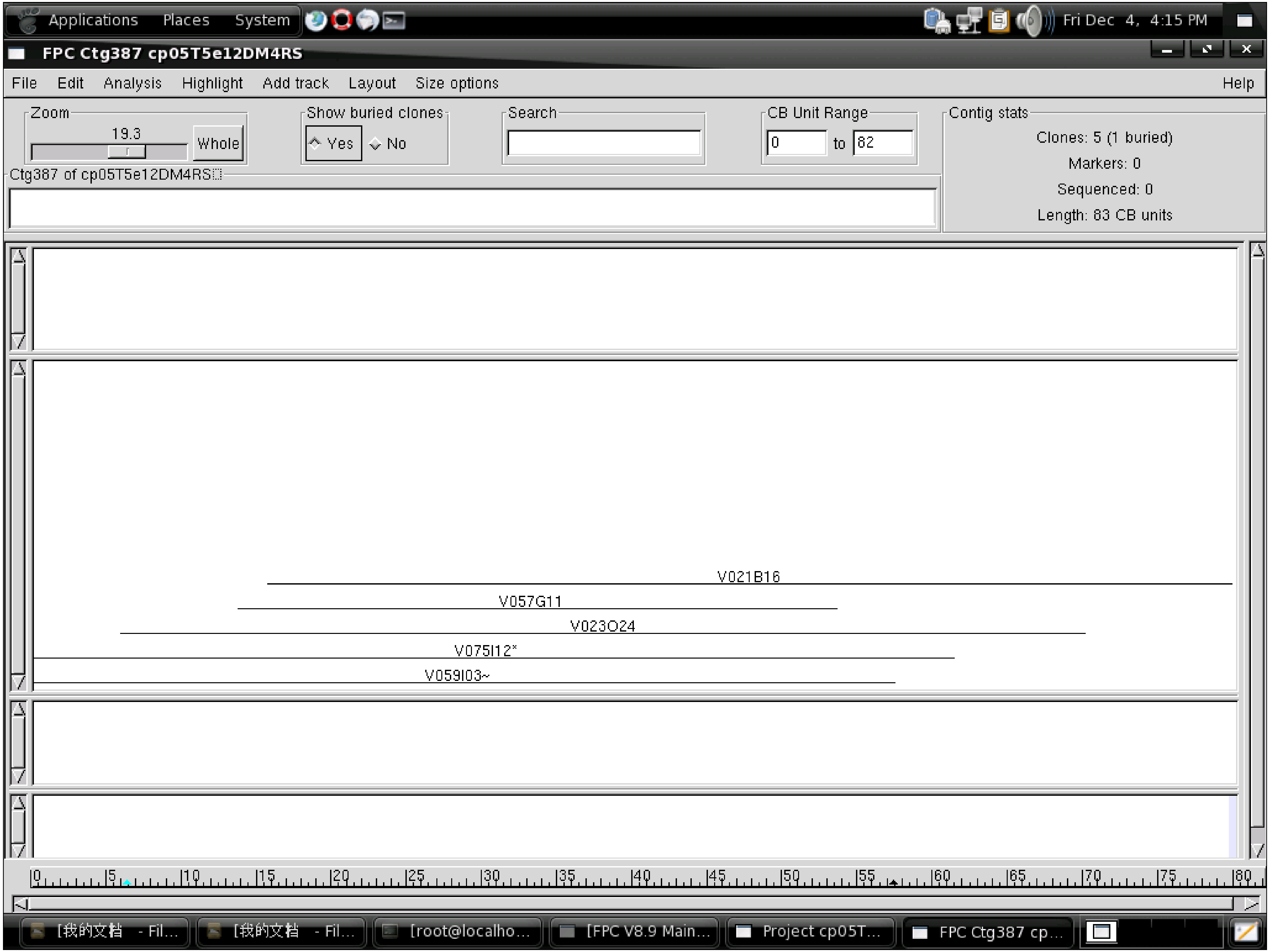
Applications Places System
Fri Dec 4, 4:15 PM
FPC Ctg387 cp05T5e12DM4RS
File Edit Analysis Highlight Add track Layout Size options Help
Zoom
19.3
Whole
Show buried clones
Yes
No
Search
CB Unit Range
0
to
82
Contig stats
Clones: 5 (1 buried)
Markers: 0
Sequenced: 0
Length: 83 CB units
Ctg387 of cp05T5e12DM4RS
V021B16
V057G11
V023O24
V075I12*
V059I03~
0 5 10 15 20 25 30 35 40 45 50 55 60 65 70 75 80
[我的文档 - Fil...
[我的文档 - Fil...
[root@localho...
[FPC V8.9 Main...
Project cp05T...
FPC Ctg387 cp...

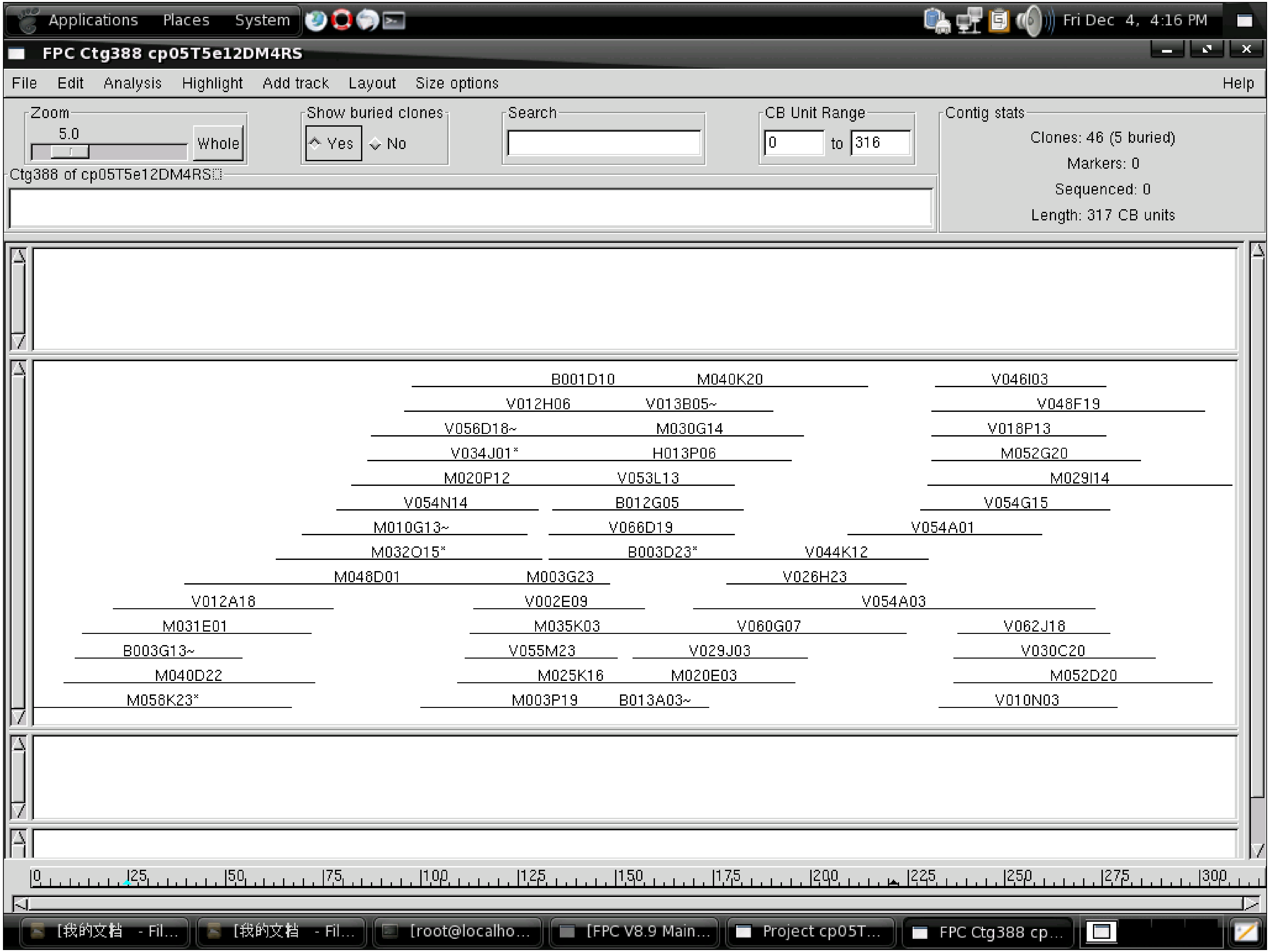
Applications Places System
Fri Dec 4, 4:16 PM
FPC Ctg388 cp05T5e12DM4RS
File Edit Analysis Highlight Add track Layout Size options Help
Zoom
5.0
Whole
Show buried clones
Yes
No
Search
CB Unit Range
0 to 316
Contig stats
Clones: 46 (5 buried)
Markers: 0
Sequenced: 0
Length: 317 CB units
Ctg388 of cp05T5e12DM4RS
B001D10
M040K20
V046I03
V012H06
V013B05~
V048F19
V056D18~
M030G14
V018P13
V034J01*
H013P06
M052G20
M020P12
V053L13
M029I14
V054N14
B012G05
V054G15
M010G13~
V066D19
V054A01
M032O15*
B003D23*
V044K12
M048D01
M003G23
V026H23
V012A18
V002E09
V054A03
M031E01
M035K03
V060G07
V062J18
B003G13~
V055M23
V029J03
V030C20
M040D22
M025K16
M020E03
M052D20
M058K23*
M003P19
B013A03~
V010N03
0 25 50 75 100 125 150 175 200 225 250 275 300
[我的文档 - Fil...
[我的文档 - Fil...
[root@localho...
[FPC V8.9 Main...
Project cp05T...
FPC Ctg388 cp...

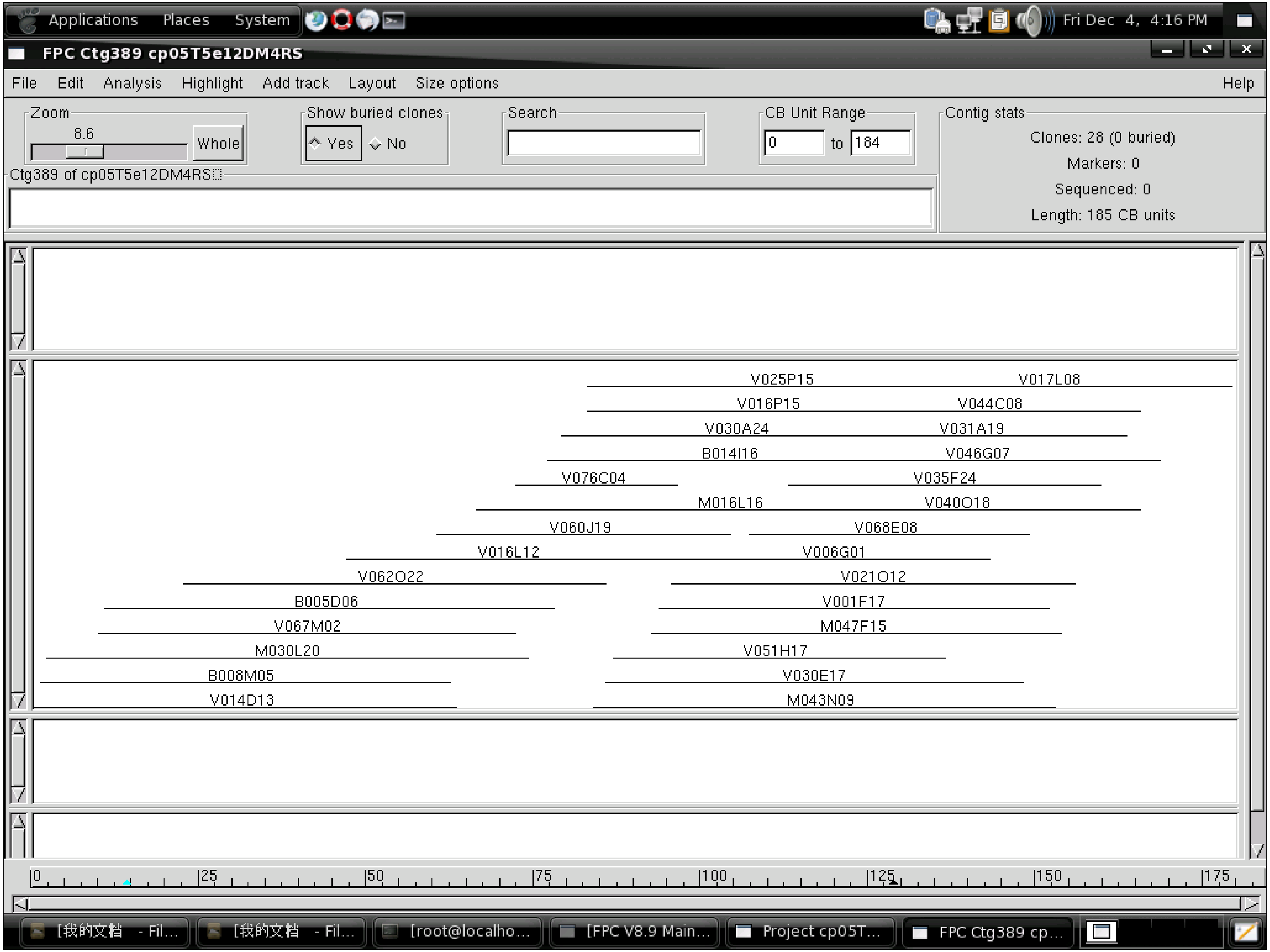
Applications Places System
Fri Dec 4, 4:16 PM
FPC Ctg389 cp05T5e12DM4RS
File Edit Analysis Highlight Add track Layout Size options Help
Zoom
8.6
Whole
Show buried clones
Yes
No
Search
CB Unit Range
0 to 184
Contig stats
Clones: 28 (0 buried)
Markers: 0
Sequenced: 0
Length: 185 CB units
Ctg389 of cp05T5e12DM4RS
V025P15
V017L08
V016P15
V044C08
V030A24
V031A19
B014I16
V046G07
V076C04
V035F24
M016L16
V040O18
V060J19
V068E08
V016L12
V006G01
V062O22
V021O12
B005D06
V001F17
V067M02
M047F15
M030L20
V051H17
B008M05
V030E17
V014D13
M043N09
0 25 50 75 100 125 150 175
[我的文档 - Fil...
[我的文档 - Fil...
[root@localho...
[FPC V8.9 Main...
Project cp05T...
FPC Ctg389 cp...

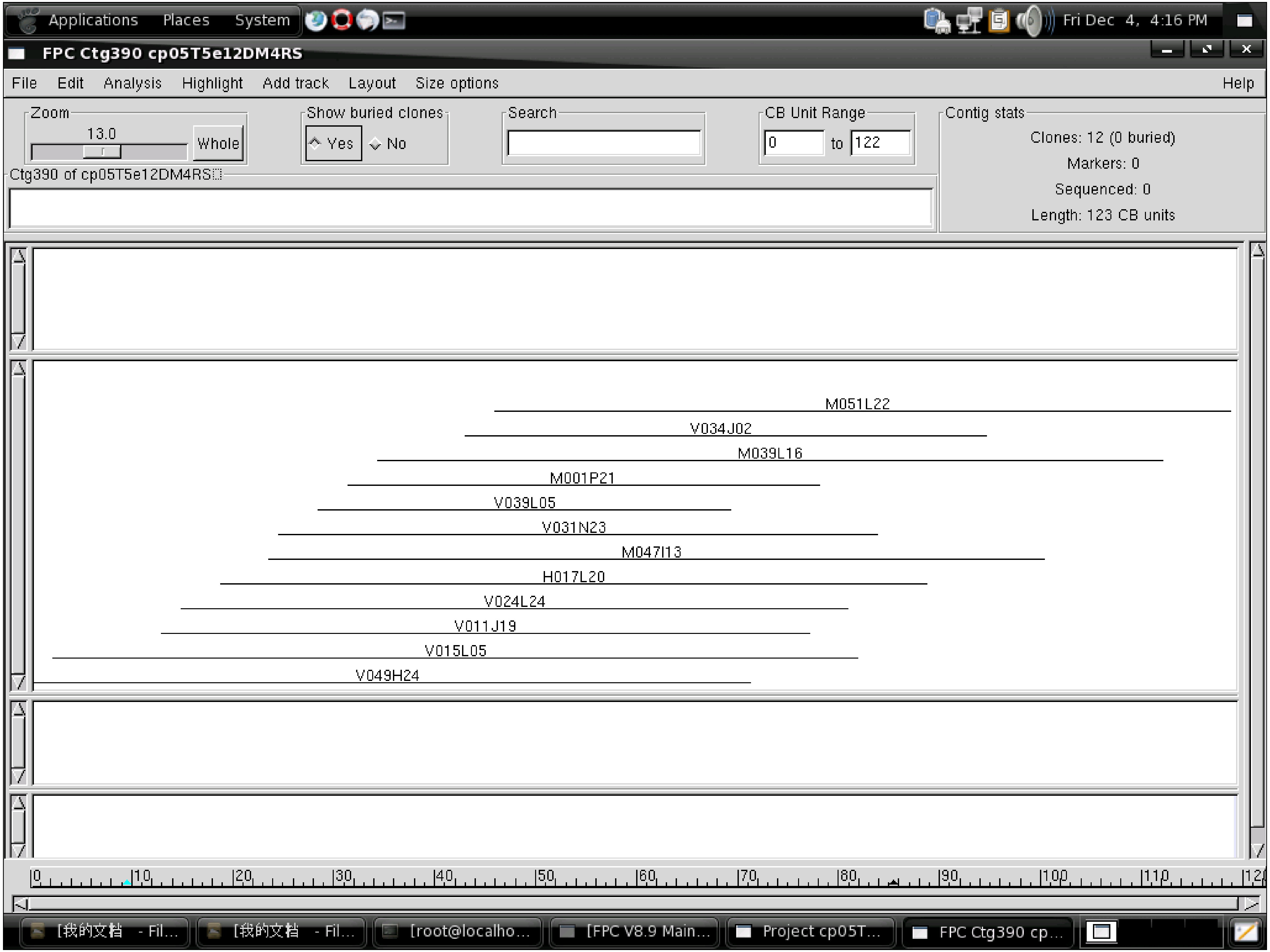
Applications Places System
Fri Dec 4, 4:16 PM
FPC Ctg390 cp05T5e12DM4RS
File Edit Analysis Highlight Add track Layout Size options Help
Zoom
13.0
Whole
Show buried clones
Yes
No
Search
CB Unit Range
0
to
122
Contig stats
Clones: 12 (0 buried)
Markers: 0
Sequenced: 0
Length: 123 CB units
Ctg390 of cp05T5e12DM4RS
M051L22
V034J02
M039L16
M001P21
V039L05
V031N23
M047I13
H017L20
V024L24
V011J19
V015L05
V049H24
0 10 20 30 40 50 60 70 80 90 100 110 12
[我的文档 - Fil...
[我的文档 - Fil...
[root@localho...
[FPC V8.9 Main...
Project cp05T...
FPC Ctg390 cp...

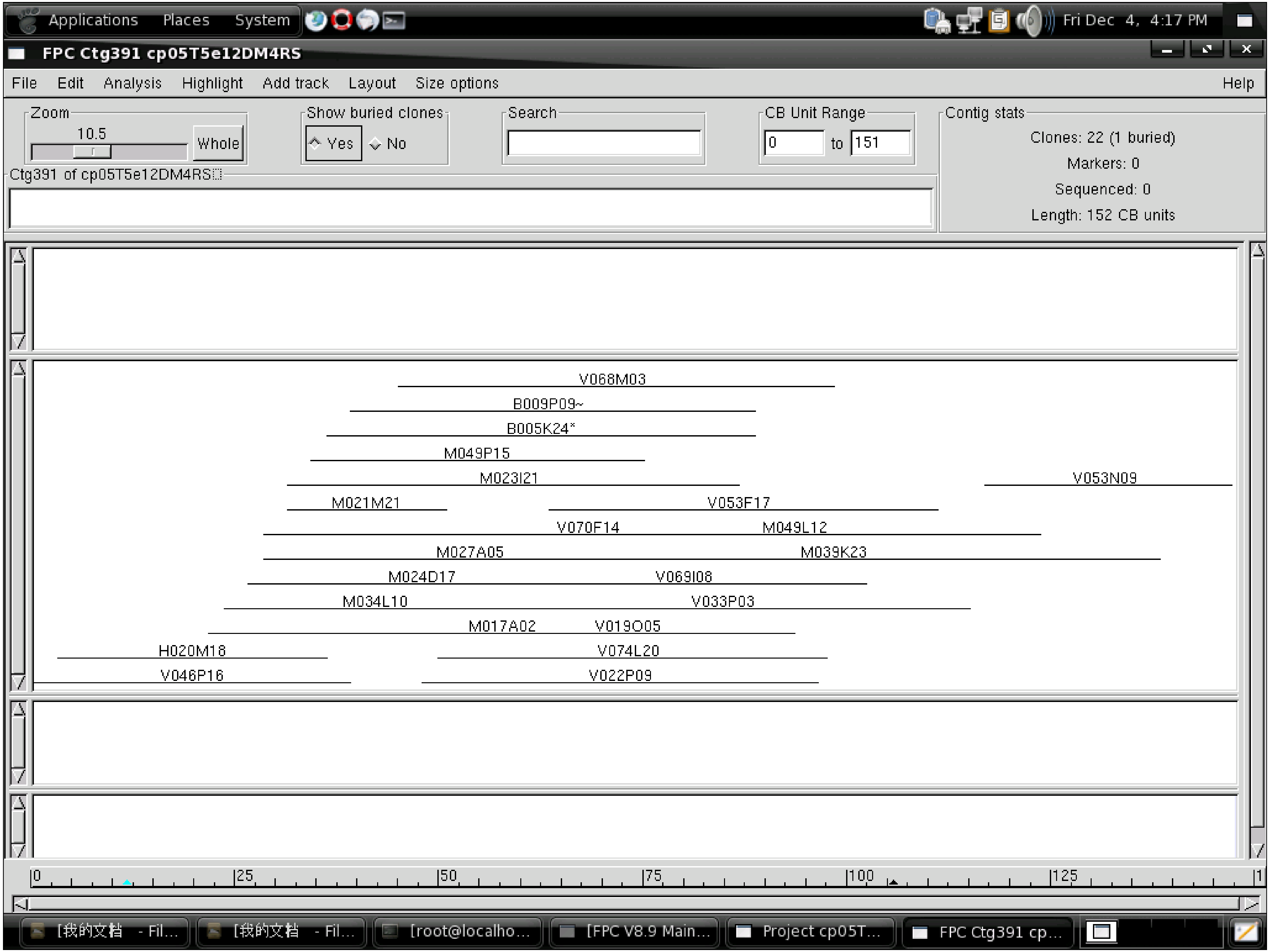
Applications Places System
Fri Dec 4, 4:17 PM
FPC Ctg391 cp05T5e12DM4RS
File Edit Analysis Highlight Add track Layout Size options Help
Zoom
10.5
Whole
Show buried clones
Yes No
Search
CB Unit Range
0 to 151
Contig stats
Clones: 22 (1 buried)
Markers: 0
Sequenced: 0
Length: 152 CB units
Ctg391 of cp05T5e12DM4RS
V068M03
B009P09~
B005K24*
M049P15
M023I21
V053N09
M021M21
V053F17
V070F14
M049L12
M027A05
M039K23
M024D17
V069I08
M034L10
V033P03
M017A02
V019O05
H020M18
V074L20
V046P16
V022P09
0 25 50 75 100 125
[我的文档 - Fil...
[我的文档 - Fil...
[root@localho...
[FPC V8.9 Main...
Project cp05T...
FPC Ctg391 cp...

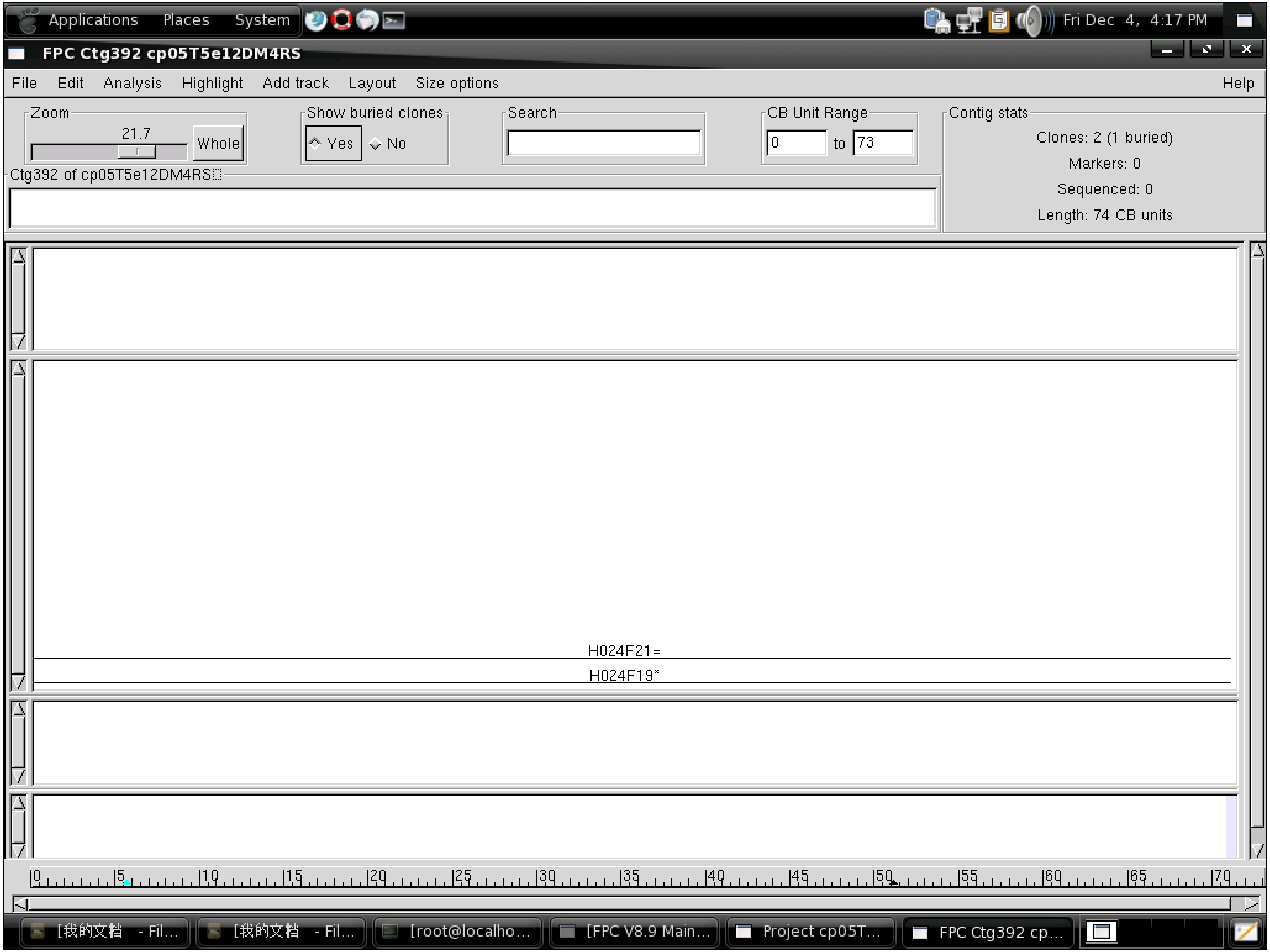
Applications Places System
Fri Dec 4, 4:17 PM
FPC Ctg392 cp05T5e12DM4RS
File Edit Analysis Highlight Add track Layout Size options Help
Zoom
21.7
Whole
Show buried clones
Yes
No
Search
CB Unit Range
0
to
73
Contig stats
Clones: 2 (1 buried)
Markers: 0
Sequenced: 0
Length: 74 CB units
Ctg392 of cp05T5e12DM4RS
H024F21=
H024F19*
0 5 10 15 20 25 30 35 40 45 50 55 60 65 70
[我的文档 - Fil...
[我的文档 - Fil...
[root@localho...
[FPC V8.9 Main...
Project cp05T...
FPC Ctg392 cp...

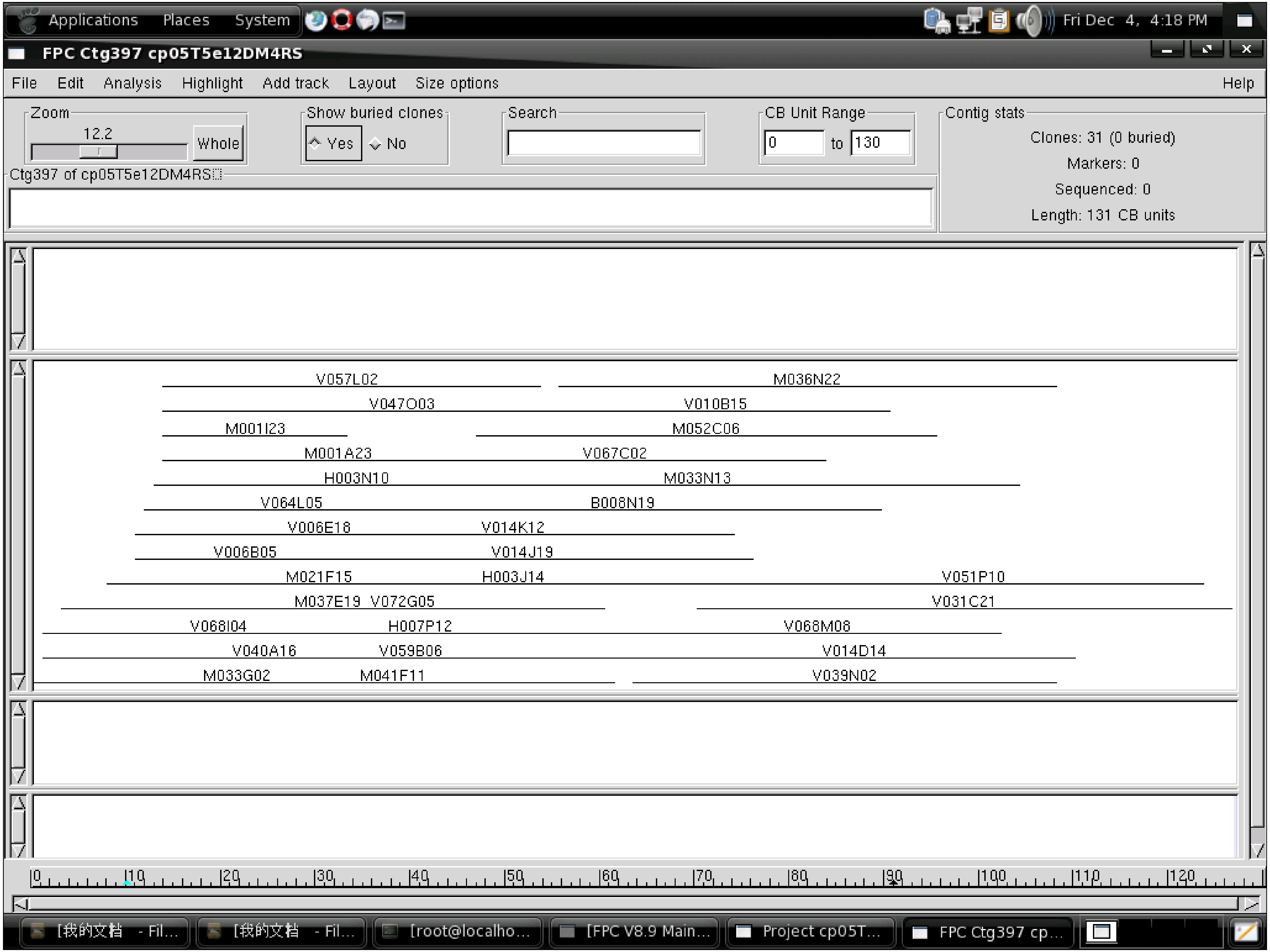
Applications Places System
Fri Dec 4, 4:18 PM
FPC Ctg397 cp05T5e12DM4RS
File Edit Analysis Highlight Add track Layout Size options Help
Zoom
12.2
Whole
Show buried clones
Yes No
Search
CB Unit Range
0 to 130
Contig stats
Clones: 31 (0 buried)
Markers: 0
Sequenced: 0
Length: 131 CB units
Ctg397 of cp05T5e12DM4RS
V057L02
M036N22
V047O03
V010B15
M001I23
M052C06
M001A23
V067C02
H003N10
M033N13
V064L05
B008N19
V006E18
V014K12
V006B05
V014J19
M021F15
H003J14
V051P10
M037E19 V072G05
V031C21
V068I04
H007P12
V068M08
V040A16
V059B06
V014D14
M033G02
M041F11
V039N02
0 10 20 30 40 50 60 70 80 90 100 110 120
[我的文档 - Fil...
[我的文档 - Fil...
[root@localho...
[FPC V8.9 Main...
Project cp05T...
FPC Ctg397 cp...

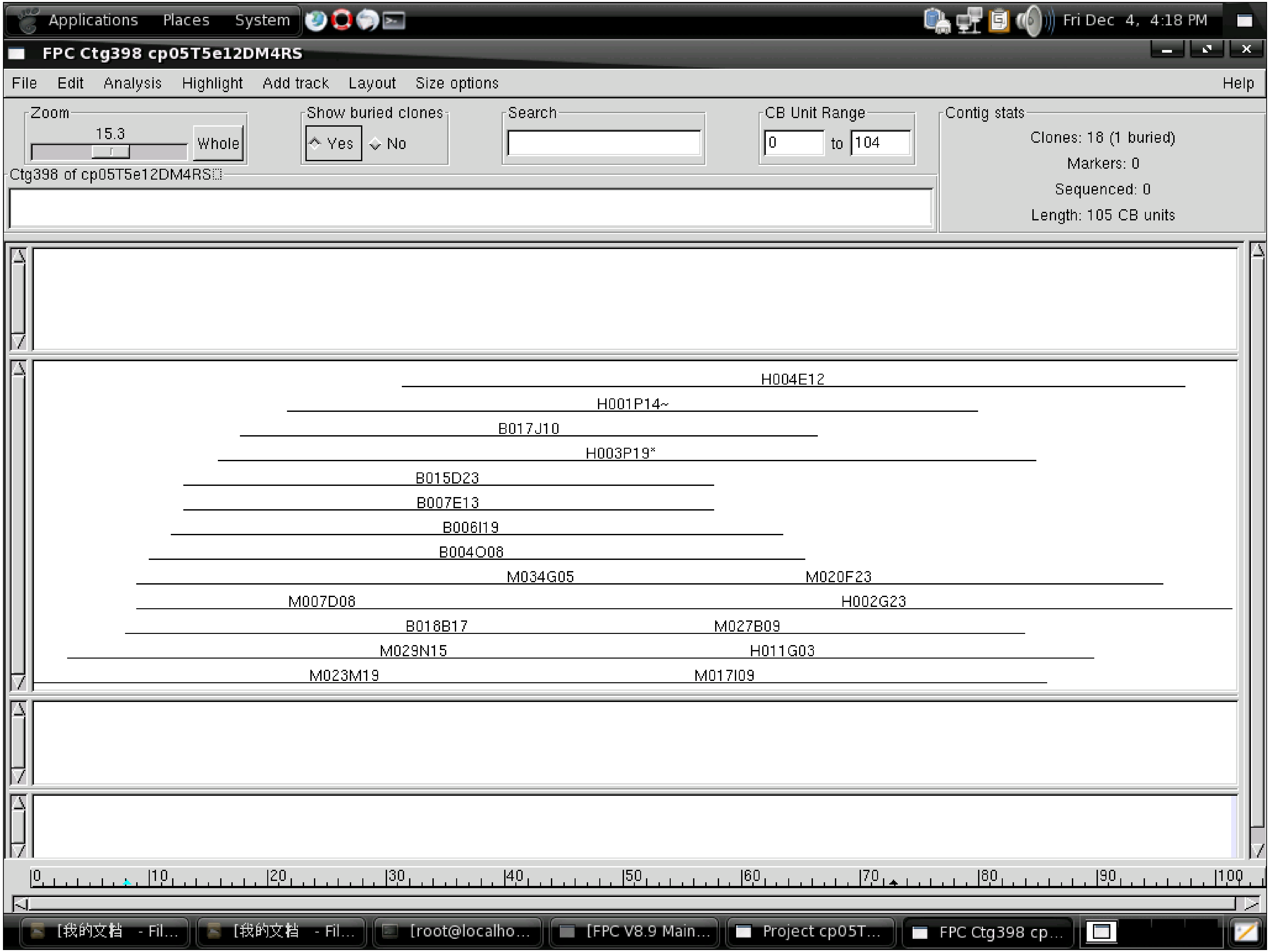

Applications
Places
System
Fri Dec 4, 4:18 PM
FPC Ctg398 cp05T5e12DM4RS
File
Edit
Analysis
Highlight
Add track
Layout
Size options
Help
Zoom
15.3
Whole
Show buried clones
Yes
No
Search
CB Unit Range
0
to
104
Contig stats
Clones: 18 (1 buried)
Markers: 0
Sequenced: 0
Length: 105 CB units
Ctg398 of cp05T5e12DM4RS
H004E12
H001P14~
B017J10
H003P19*
B015D23
B007E13
B006I19
B004O08
M034G05
M020F23
M007D08
H002G23
B018B17
M027B09
M029N15
H011G03
M023M19
M017I09
0
10
20
30
40
50
60
70
80
90
100
[我的文档 - Fil...
[我的文档 - Fil...
[root@localho...
[FPC V8.9 Main...
Project cp05T...
FPC Ctg398 cp...

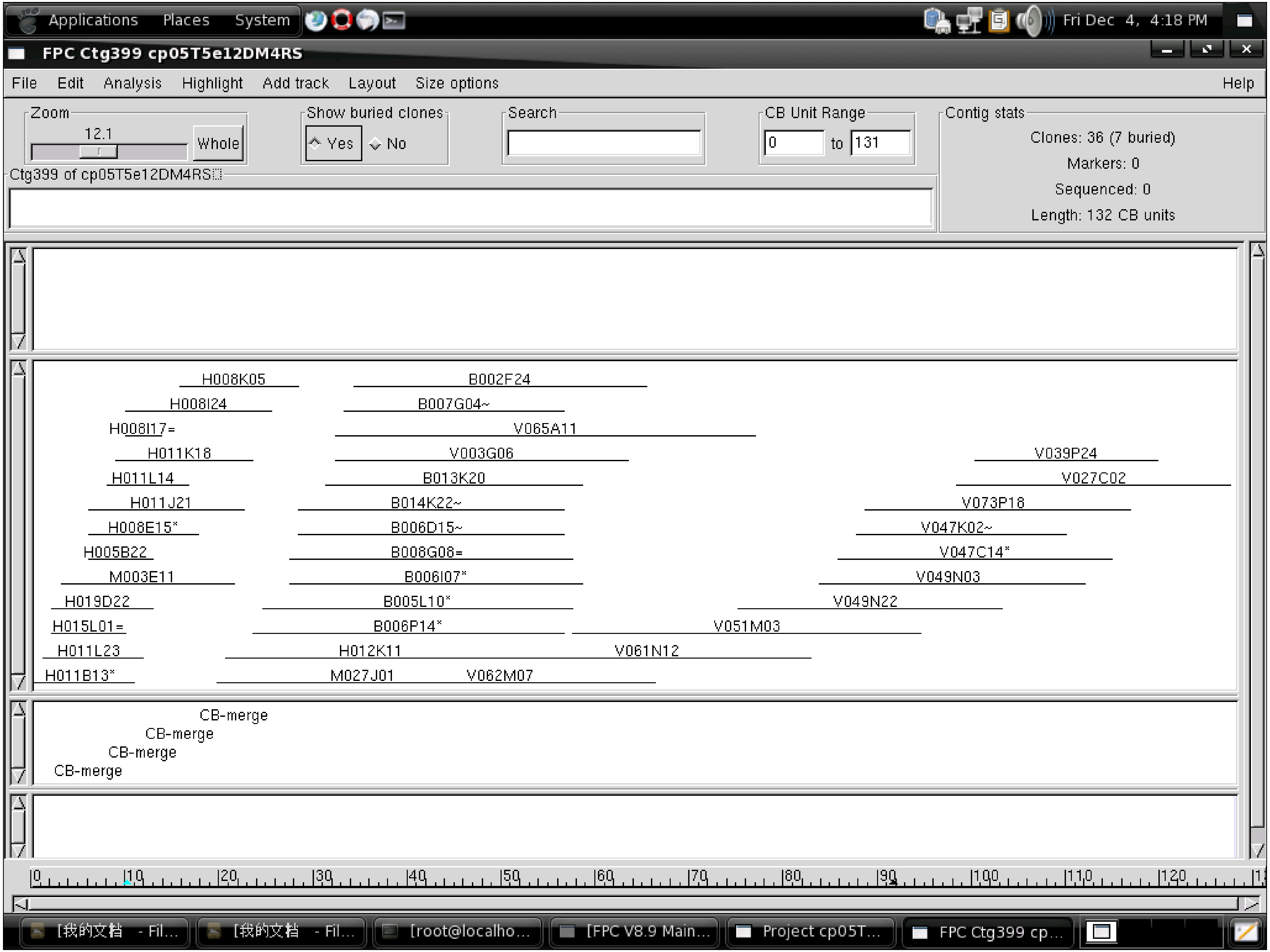
Applications Places System
Fri Dec 4, 4:18 PM
FPC Ctg399 cp05T5e12DM4RS
File Edit Analysis Highlight Add track Layout Size options Help
Zoom
12.1
Whole
Show buried clones
Yes
No
Search
CB Unit Range
0
to
131
Contig stats
Clones: 36 (7 buried)
Markers: 0
Sequenced: 0
Length: 132 CB units
Ctg399 of cp05T5e12DM4RS
H008K05
B002F24
H008I24
B007G04~
H008I17=
V065A11
H011K18
V003G06
V039P24
H011L14
B013K20
V027C02
H011J21
B014K22~
V073P18
H008E15*
B006D15~
V047K02~
H005B22
B008G08=
V047C14*
M003E11
B006I07*
V049N03
H019D22
B005L10*
V049N22
H015L01=
B006P14*
V051M03
H011L23
H012K11
V061N12
H011B13*
M027J01
V062M07
CB-merge
CB-merge
CB-merge
CB-merge
0 10 20 30 40 50 60 70 80 90 100 110 120
[我的文档 - Fil...
[我的文档 - Fil...
[root@localho...
[FPC V8.9 Main...
Project cp05T...
FPC Ctg399 cp...

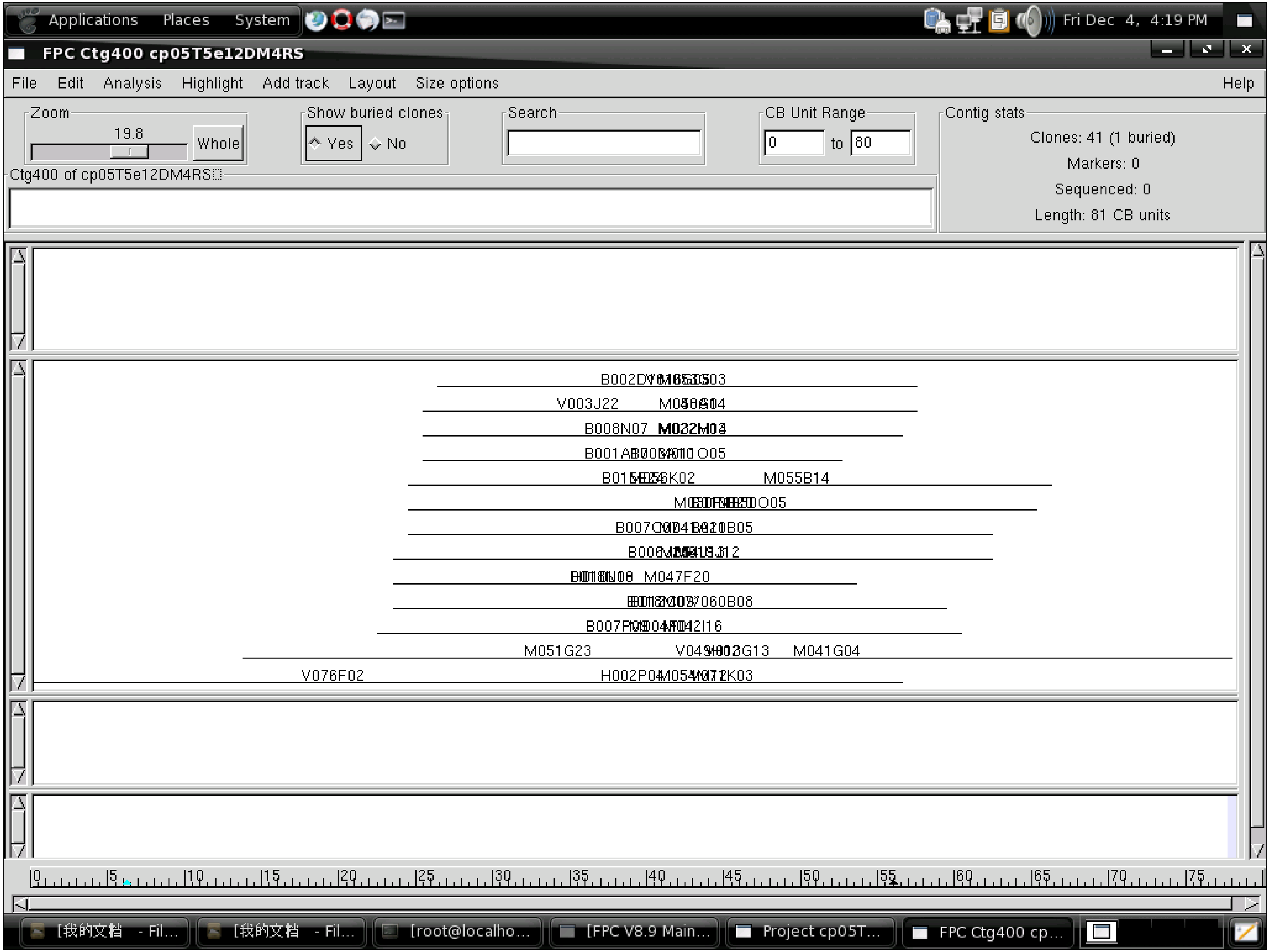
Applications Places System
Fri Dec 4, 4:19 PM
FPC Ctg400 cp05T5e12DM4RS
File Edit Analysis Highlight Add track Layout Size options
Help
Zoom
19.8
Whole
Show buried clones
Yes No
Search
CB Unit Range
0 to 80
Contig stats
Clones: 41 (1 buried)
Markers: 0
Sequenced: 0
Length: 81 CB units
Ctg400 of cp05T5e12DM4RS
V003J22
B008N07
M055B14
M047F20
M051G23
M041G04
V076F02
0 5 10 15 20 25 30 35 40 45 50 55 60 65 70 75
[root@localho…
[FPC V8.9 Main…
Project cp05T…
FPC Ctg400 cp…

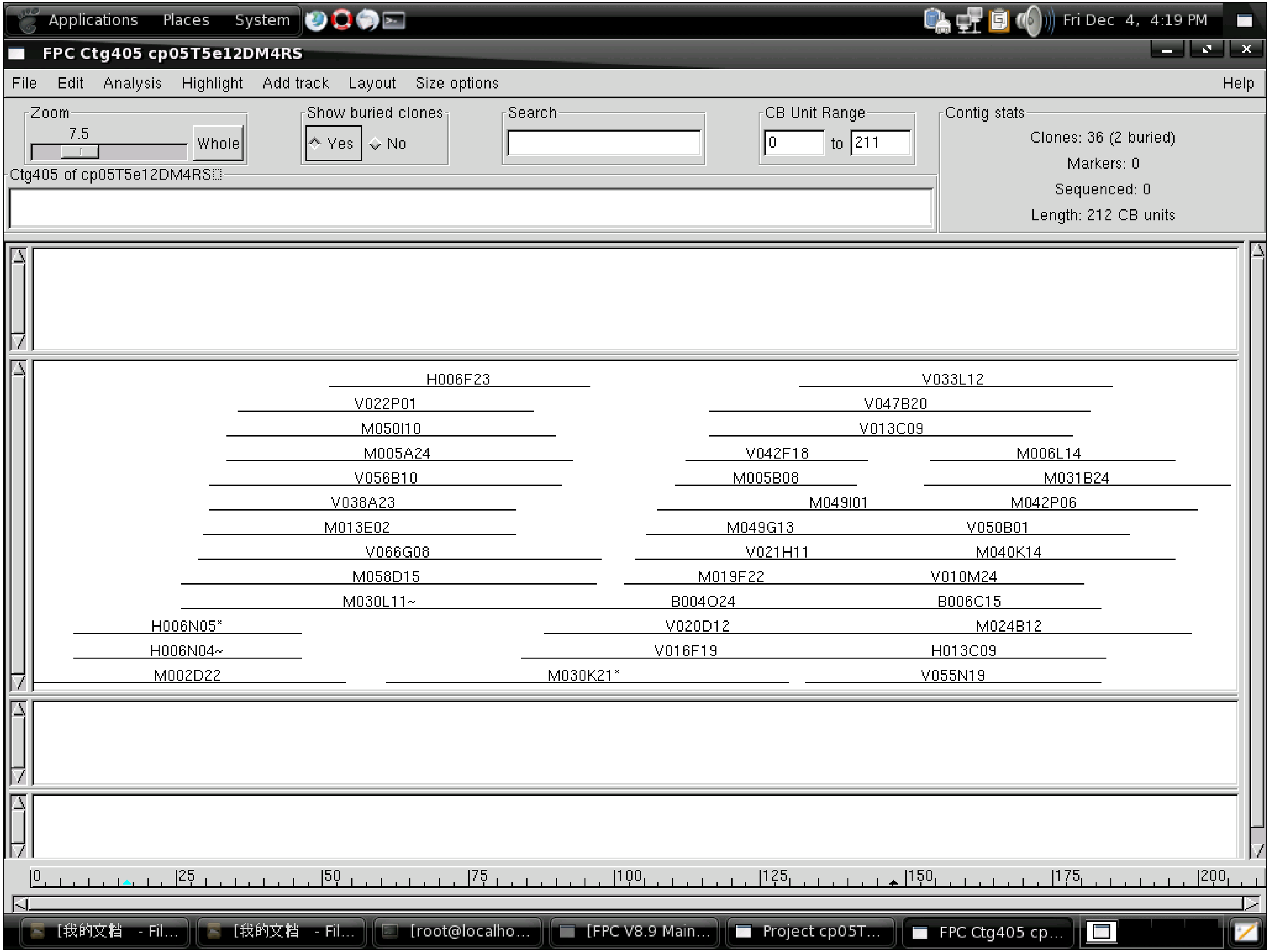
Applications Places System
Fri Dec 4, 4:19 PM
FPC Ctg405 cp05T5e12DM4RS
File Edit Analysis Highlight Add track Layout Size options Help
Zoom
7.5
Whole
Show buried clones
Yes
No
Search
CB Unit Range
0 to 211
Contig stats
Clones: 36 (2 buried)
Markers: 0
Sequenced: 0
Length: 212 CB units
Ctg405 of cp05T5e12DM4RS
H006F23
V033L12
V022P01
V047B20
M050I10
V013C09
M005A24
V042F18
M006L14
V056B10
M005B08
M031B24
V038A23
M049I01
M042P06
M013E02
M049G13
V050B01
V066G08
V021H11
M040K14
M058D15
M019F22
V010M24
M030L11~
B004O24
B006C15
H006N05*
V020D12
M024B12
H006N04~
V016F19
H013C09
M002D22
M030K21*
V055N19
0 25 50 75 100 125 150 175 200
[我的文档 - Fil...
[我的文档 - Fil...
[root@localho...
[FPC V8.9 Main...
Project cp05T...
FPC Ctg405 cp...

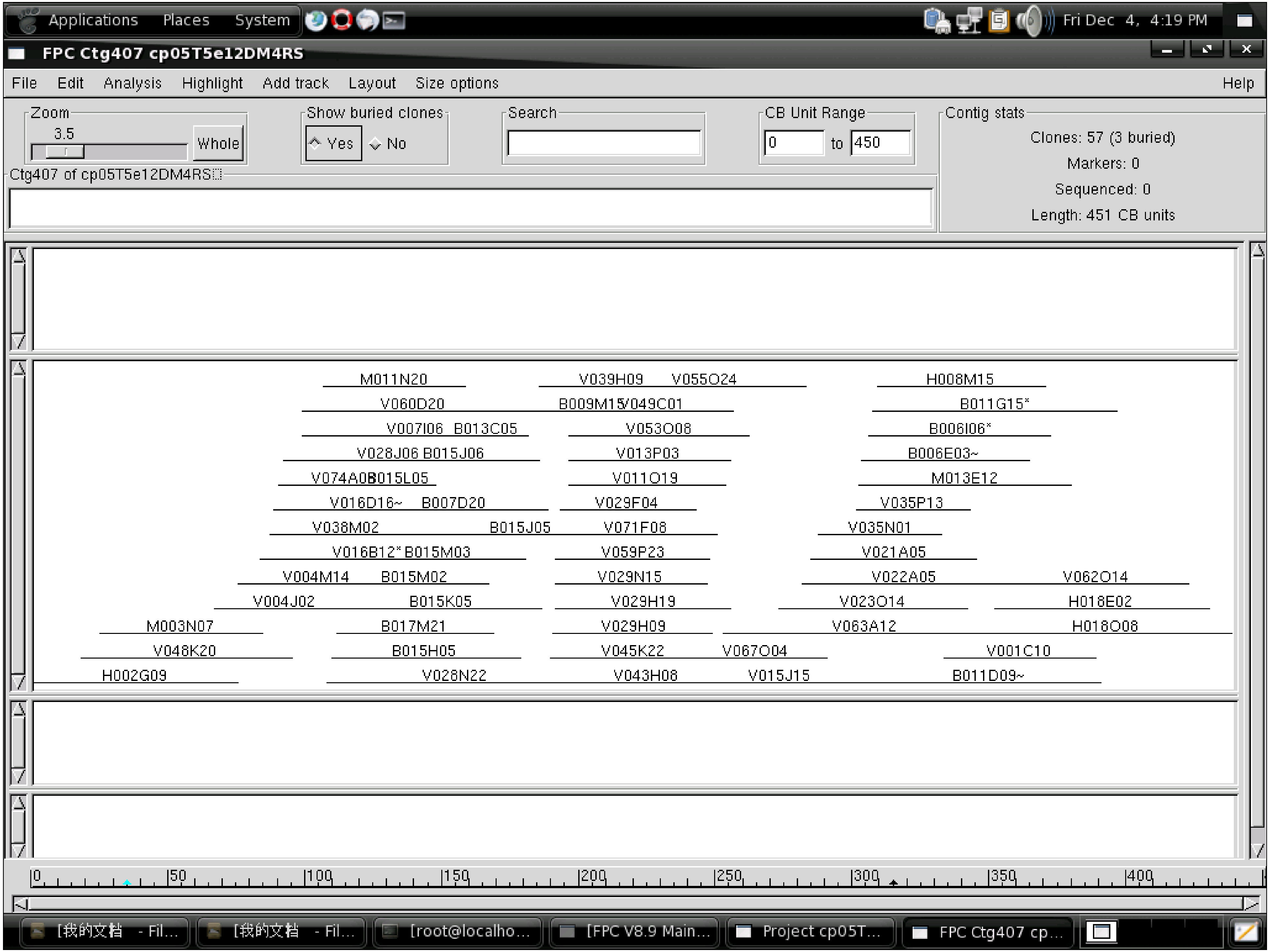
Applications Places System
Fri Dec 4, 4:19 PM
FPC Ctg407 cp05T5e12DM4RS
File Edit Analysis Highlight Add track Layout Size options Help
Zoom
3.5
Whole
Show buried clones
Yes
No
Search
CB Unit Range
0
to
450
Contig stats
Clones: 57 (3 buried)
Markers: 0
Sequenced: 0
Length: 451 CB units
Ctg407 of cp05T5e12DM4RS
M011N20
V039H09 V055O24
H008M15
V060D20
B009M15
V049C01
B011G15*
V007I06 B013C05
V053O08
B006I06*
V028J06 B015J06
V013P03
B006E03~
V074A08
B015L05
V011O19
M013E12
V016D16~ B007D20
V029F04
V035P13
V038M02
B015J05
V071F08
V035N01
V016B12* B015M03
V059P23
V021A05
V004M14 B015M02
V029N15
V022A05
V062O14
V004J02
B015K05
V029H19
V023O14
H018E02
M003N07
B017M21
V029H09
V063A12
H018O08
V048K20
B015H05
V045K22
V067O04
V001C10
H002G09
V028N22
V043H08
V015J15
B011D09~
0 50 100 150 200 250 300 350 400
[我的文档 - Fil...
[我的文档 - Fil...
[root@localho...
[FPC V8.9 Main...
Project cp05T...
FPC Ctg407 cp...

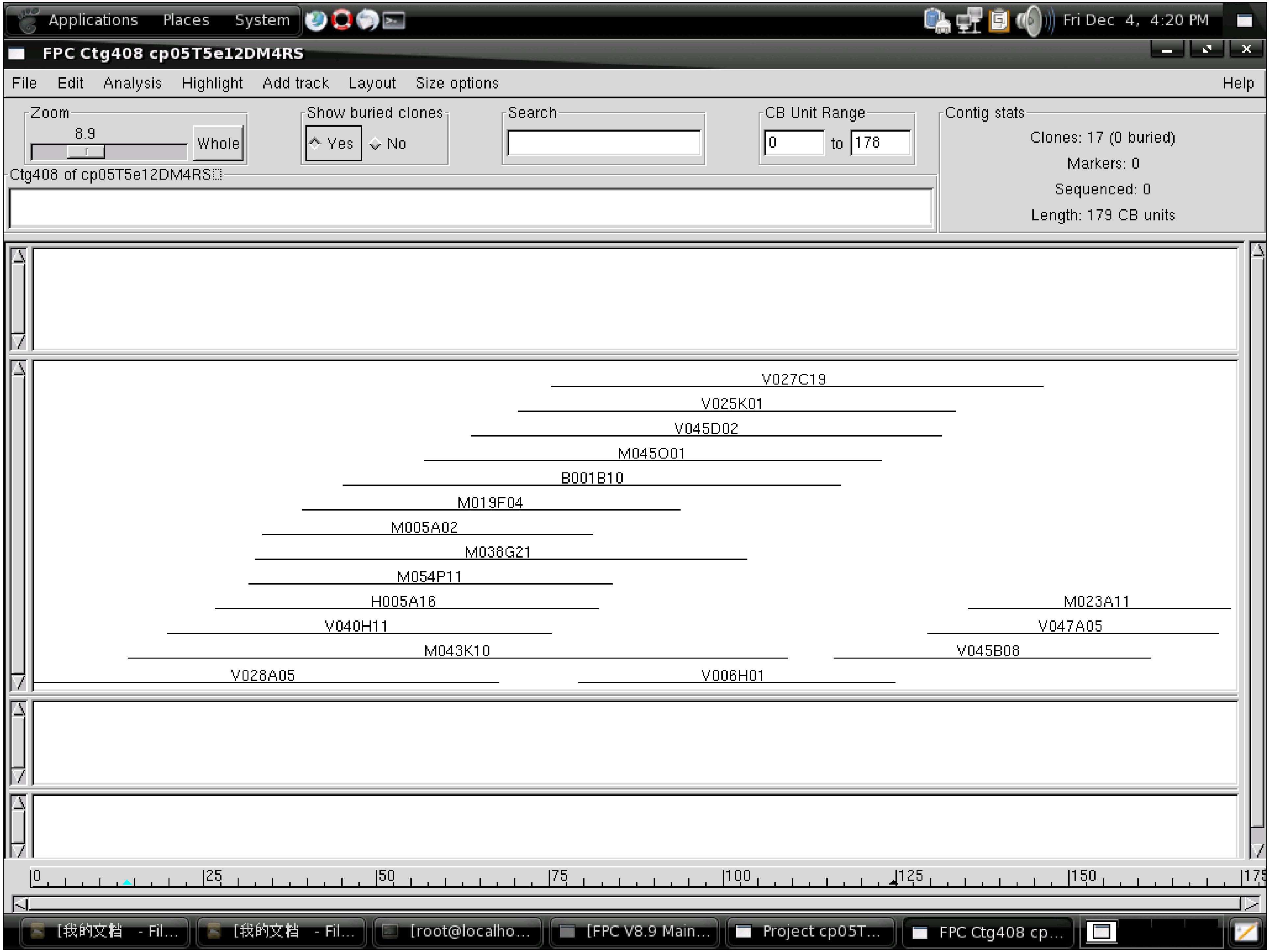

Applications Places System
Fri Dec 4, 4:20 PM
FPC Ctg408 cp05T5e12DM4RS
File Edit Analysis Highlight Add track Layout Size options Help
Zoom
8.9
Whole
Show buried clones
Yes
No
Search
CB Unit Range
0 to 178
Contig stats
Clones: 17 (0 buried)
Markers: 0
Sequenced: 0
Length: 179 CB units
Ctg408 of cp05T5e12DM4RS
V027C19
V025K01
V045D02
M045O01
B001B10
M019F04
M005A02
M038G21
M054P11
H005A16
M023A11
V040H11
V047A05
M043K10
V045B08
V028A05
V006H01
0
25
50
75
100
125
150
175
[我的文档 - Fil...
[我的文档 - Fil...
[root@localho...
[FPC V8.9 Main...
Project cp05T...
FPC Ctg408 cp...

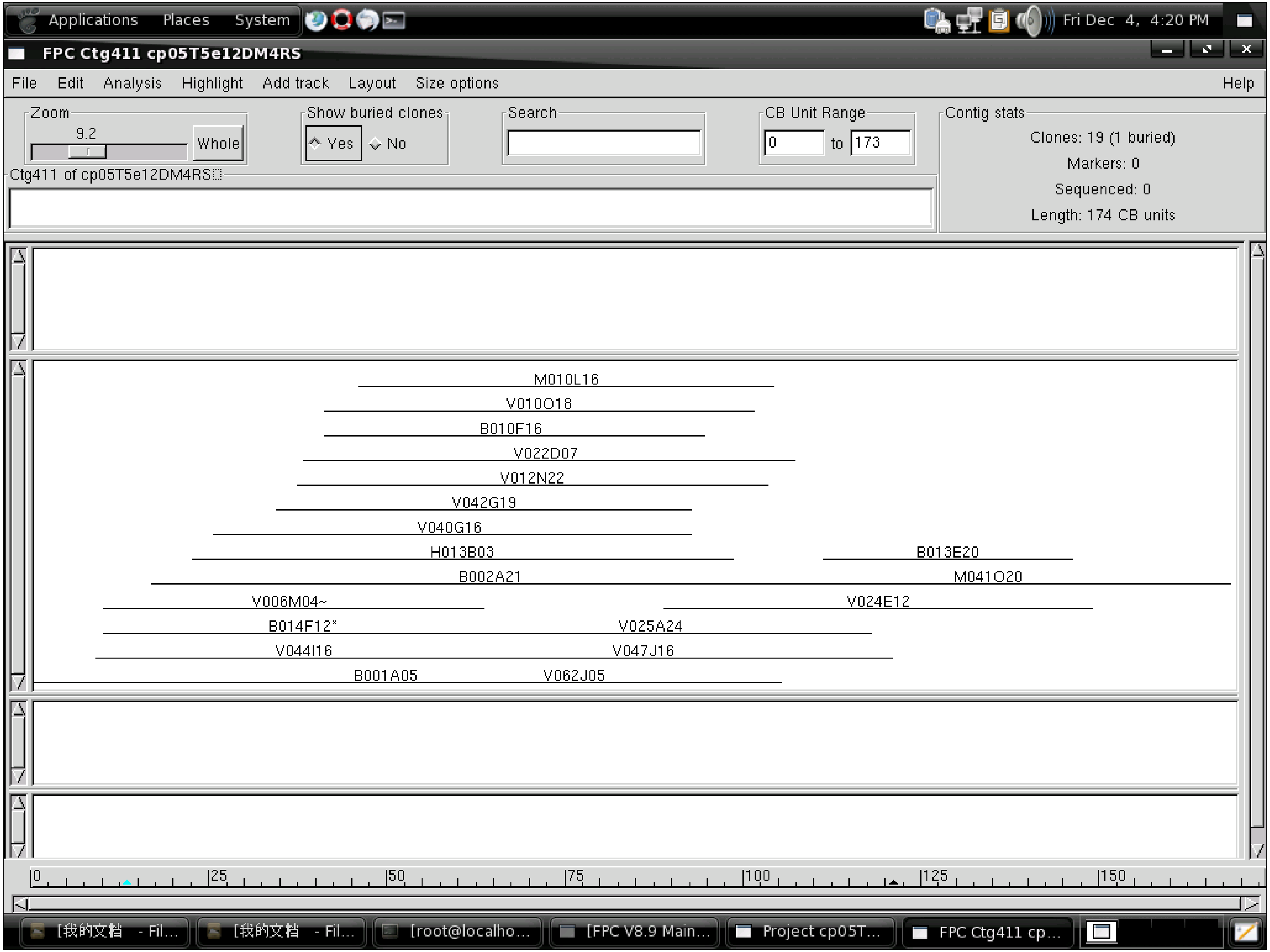
Applications Places System
Fri Dec 4, 4:20 PM
FPC Ctg411 cp05T5e12DM4RS
File Edit Analysis Highlight Add track Layout Size options Help
Zoom
9.2
Whole
Show buried clones
Yes No
Search
CB Unit Range
0 to 173
Contig stats
Clones: 19 (1 buried)
Markers: 0
Sequenced: 0
Length: 174 CB units
Ctg411 of cp05T5e12DM4RS
M010L16
V010O18
B010F16
V022D07
V012N22
V042G19
V040G16
H013B03
B013E20
B002A21
M041O20
V006M04~
V024E12
B014F12*
V025A24
V044I16
V047J16
B001A05
V062J05
0 25 50 75 100 125 150
[我的文档 - Fil...
[我的文档 - Fil...
[root@localho...
[FPC V8.9 Main...
Project cp05T...
FPC Ctg411 cp...

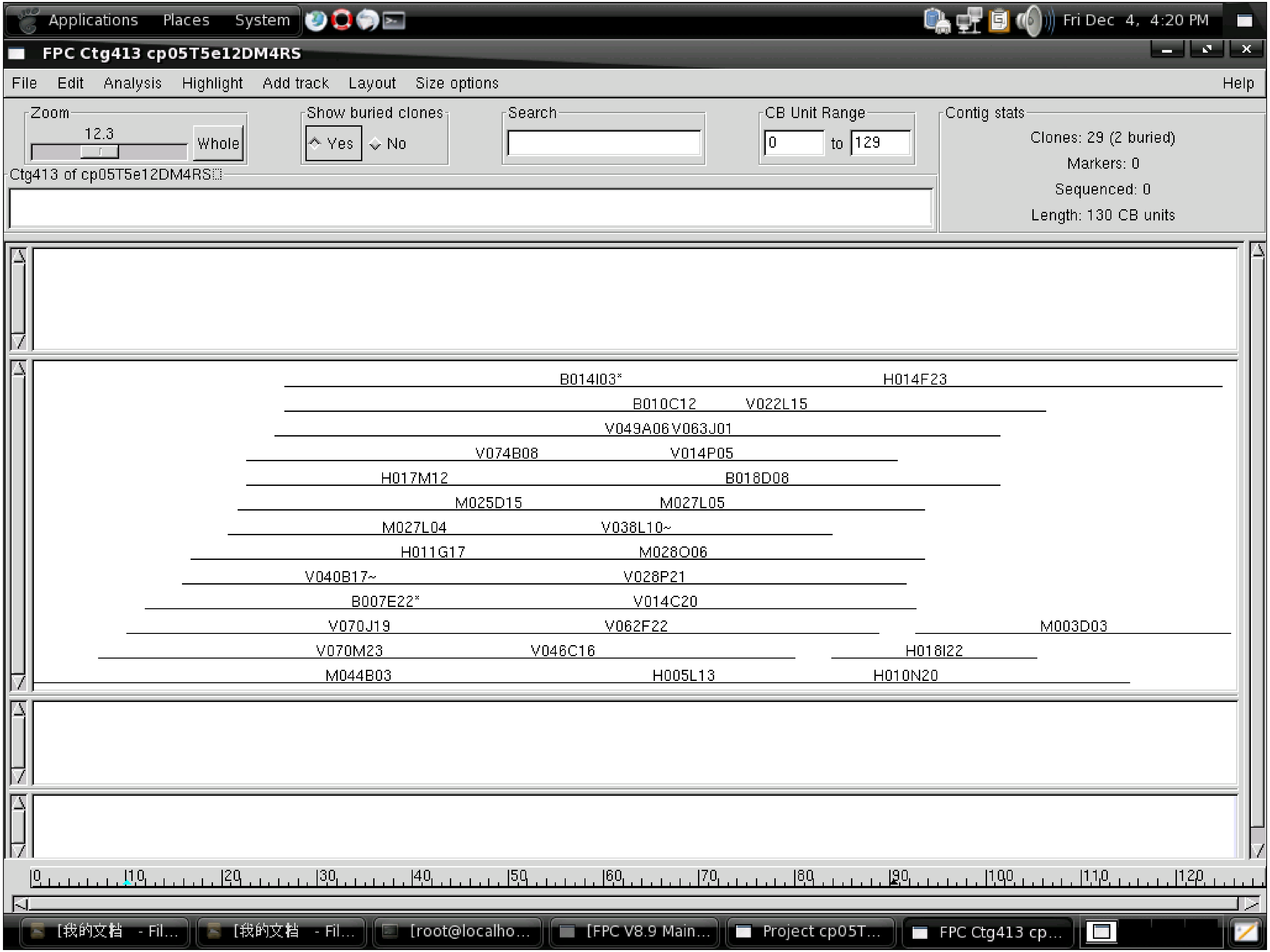
Applications Places System
Fri Dec 4, 4:20 PM
FPC Ctg413 cp05T5e12DM4RS
File Edit Analysis Highlight Add track Layout Size options Help
Zoom 12.3 Whole
Show buried clones Yes No
Search
CB Unit Range 0 to 129
Contig stats
Clones: 29 (2 buried)
Markers: 0
Sequenced: 0
Length: 130 CB units
Ctg413 of cp05T5e12DM4RS
B014I03* H014F23
B010C12 V022L15
V049A06 V063J01
V074B08 V014P05
H017M12 B018D08
M025D15 M027L05
M027L04 V038L10~
H011G17 M028O06
V040B17~ V028P21
B007E22* V014C20
V070J19 V062F22 M003D03
V070M23 V046C16 H018I22
M044B03 H005L13 H010N20
0 10 20 30 40 50 60 70 80 90 100 110 120
[我的文档 - Fil… [我的文档 - Fil… [root@localho… [FPC V8.9 Main… Project cp05T… FPC Ctg413 cp…

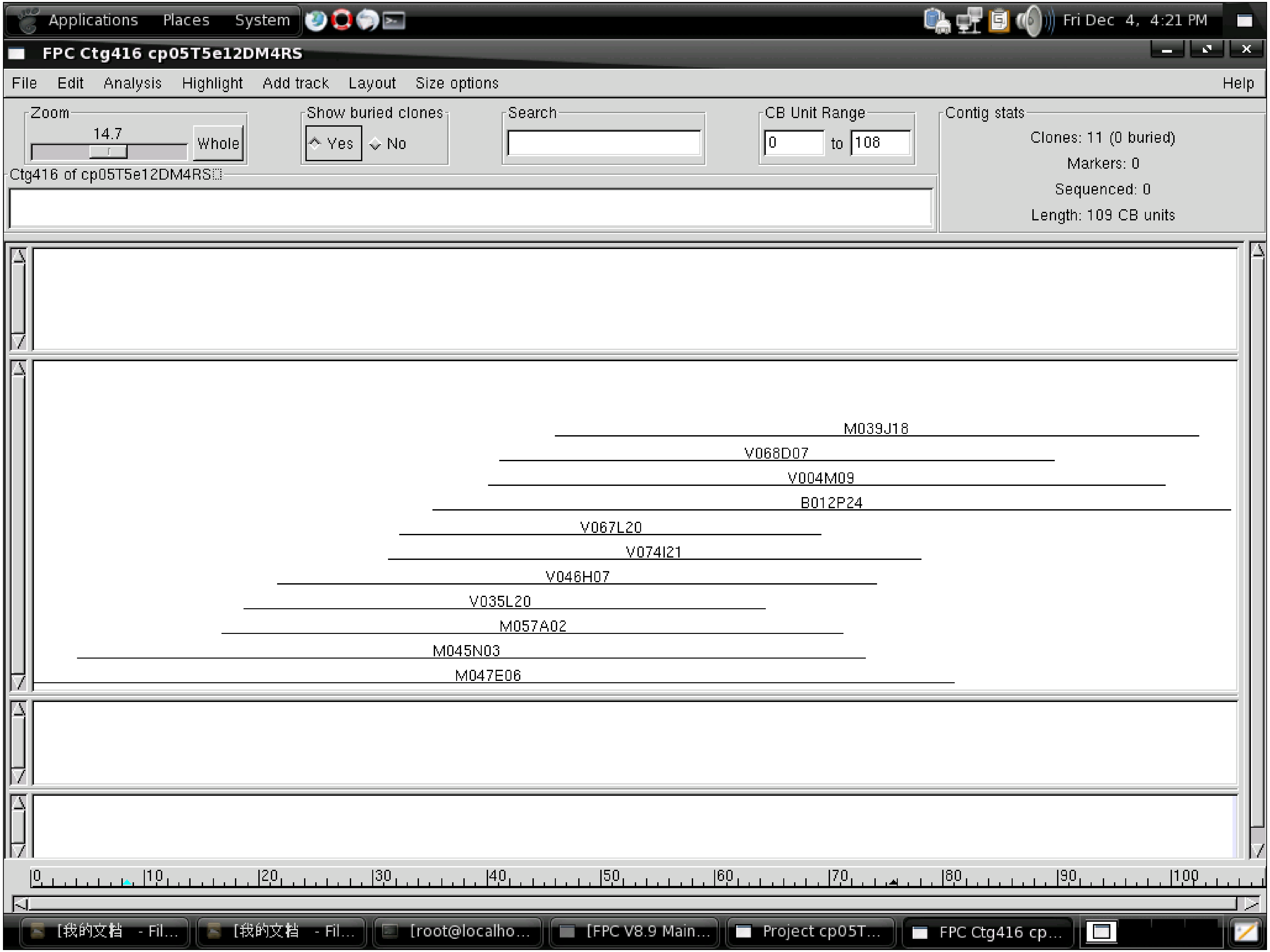
Applications Places System
Fri Dec 4, 4:21 PM
FPC Ctg416 cp05T5e12DM4RS
File Edit Analysis Highlight Add track Layout Size options Help
Zoom
14.7
Whole
Show buried clones
Yes No
Search
CB Unit Range
0 to 108
Contig stats
Clones: 11 (0 buried)
Markers: 0
Sequenced: 0
Length: 109 CB units
Ctg416 of cp05T5e12DM4RS
M039J18
V068D07
V004M09
B012P24
V067L20
V074I21
V046H07
V035L20
M057A02
M045N03
M047E06
0 10 20 30 40 50 60 70 80 90 100
[我的文档 - Fil...
[我的文档 - Fil...
[root@localho...
[FPC V8.9 Main...
Project cp05T...
FPC Ctg416 cp...

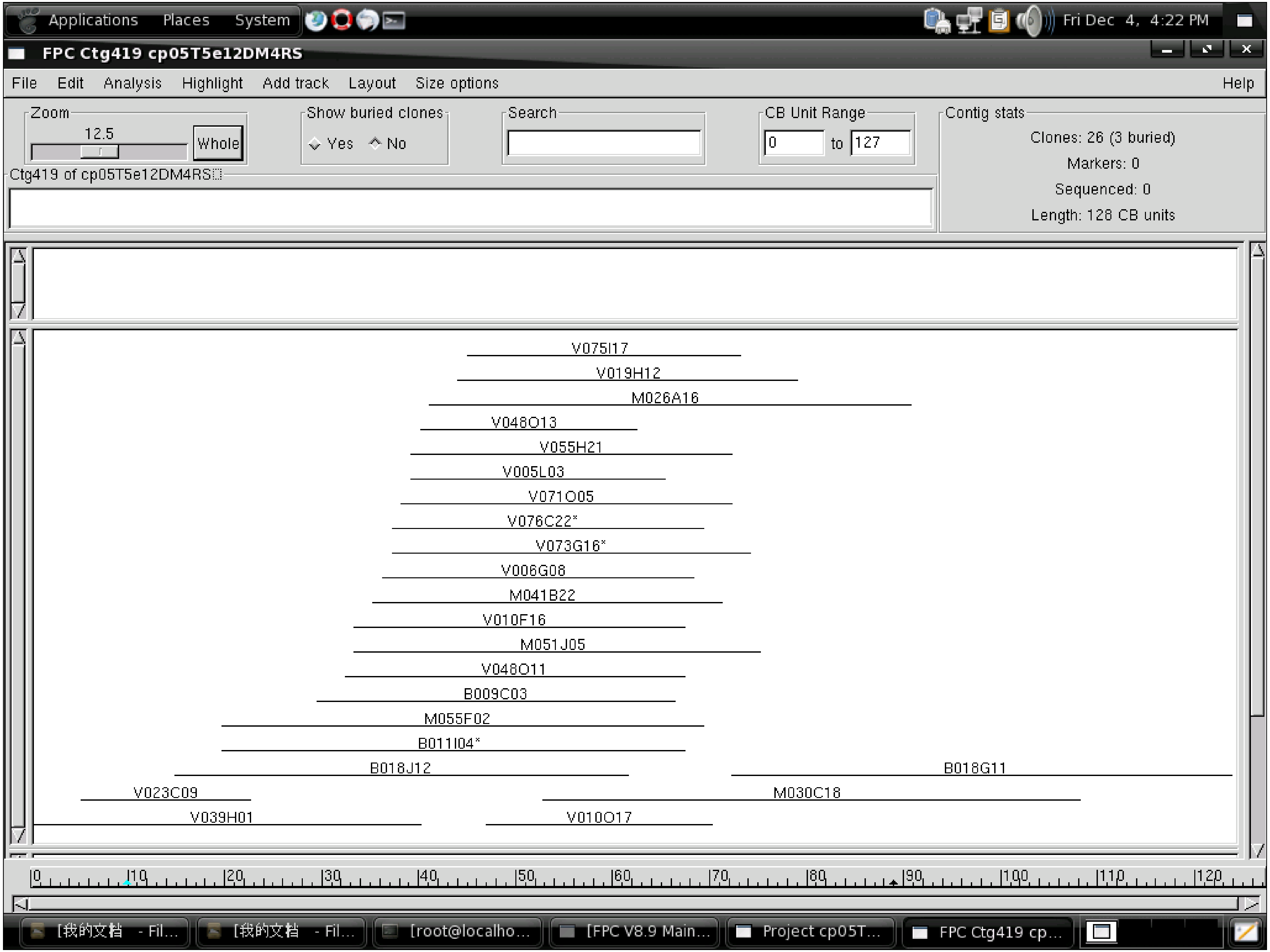
Applications Places System
Fri Dec 4, 4:22 PM
FPC Ctg419 cp05T5e12DM4RS
File Edit Analysis Highlight Add track Layout Size options Help
Zoom
12.5
Whole
Show buried clones
Yes No
Search
CB Unit Range
0 to 127
Contig stats
Clones: 26 (3 buried)
Markers: 0
Sequenced: 0
Length: 128 CB units
Ctg419 of cp05T5e12DM4RS
V075I17
V019H12
M026A16
V048O13
V055H21
V005L03
V071O05
V076C22*
V073G16*
V006G08
M041B22
V010F16
M051J05
V048O11
B009C03
M055F02
B011I04*
B018J12
B018G11
V023C09
M030C18
V039H01
V010O17
0 10 20 30 40 50 60 70 80 90 100 110 120
[我的文档 - Fil...
[我的文档 - Fil...
[root@localho...
[FPC V8.9 Main...
Project cp05T...
FPC Ctg419 cp...

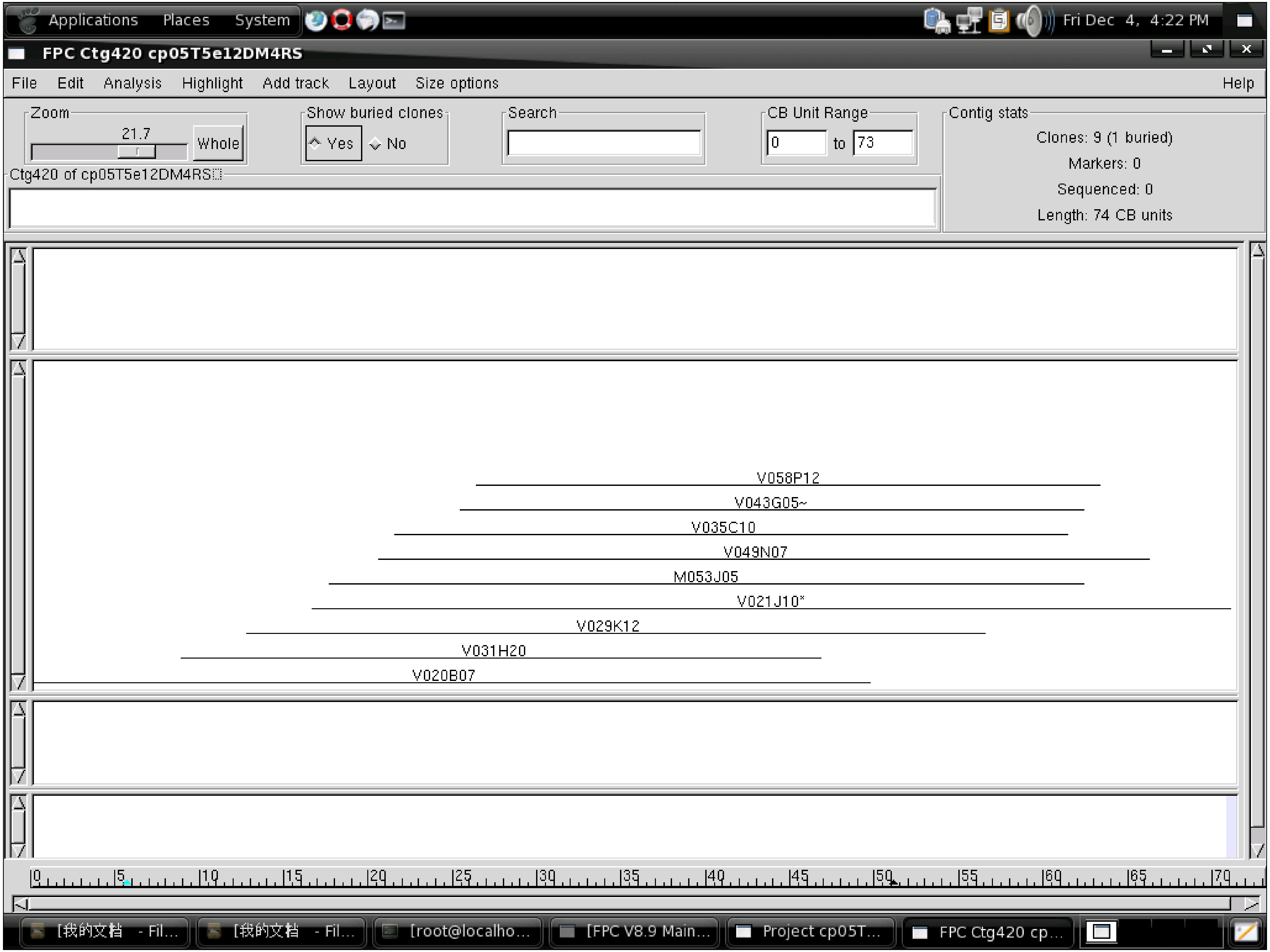
Applications Places System
Fri Dec 4, 4:22 PM
FPC Ctg420 cp05T5e12DM4RS
File Edit Analysis Highlight Add track Layout Size options Help
Zoom
21.7
Whole
Show buried clones
Yes
No
Search
CB Unit Range
0 to 73
Contig stats
Clones: 9 (1 buried)
Markers: 0
Sequenced: 0
Length: 74 CB units
Ctg420 of cp05T5e12DM4RS
V058P12
V043G05~
V035C10
V049N07
M053J05
V021J10*
V029K12
V031H20
V020B07
0 5 10 15 20 25 30 35 40 45 50 55 60 65 70
[我的文档 - Fil...
[我的文档 - Fil...
[root@localho...
[FPC V8.9 Main...
Project cp05T...
FPC Ctg420 cp...

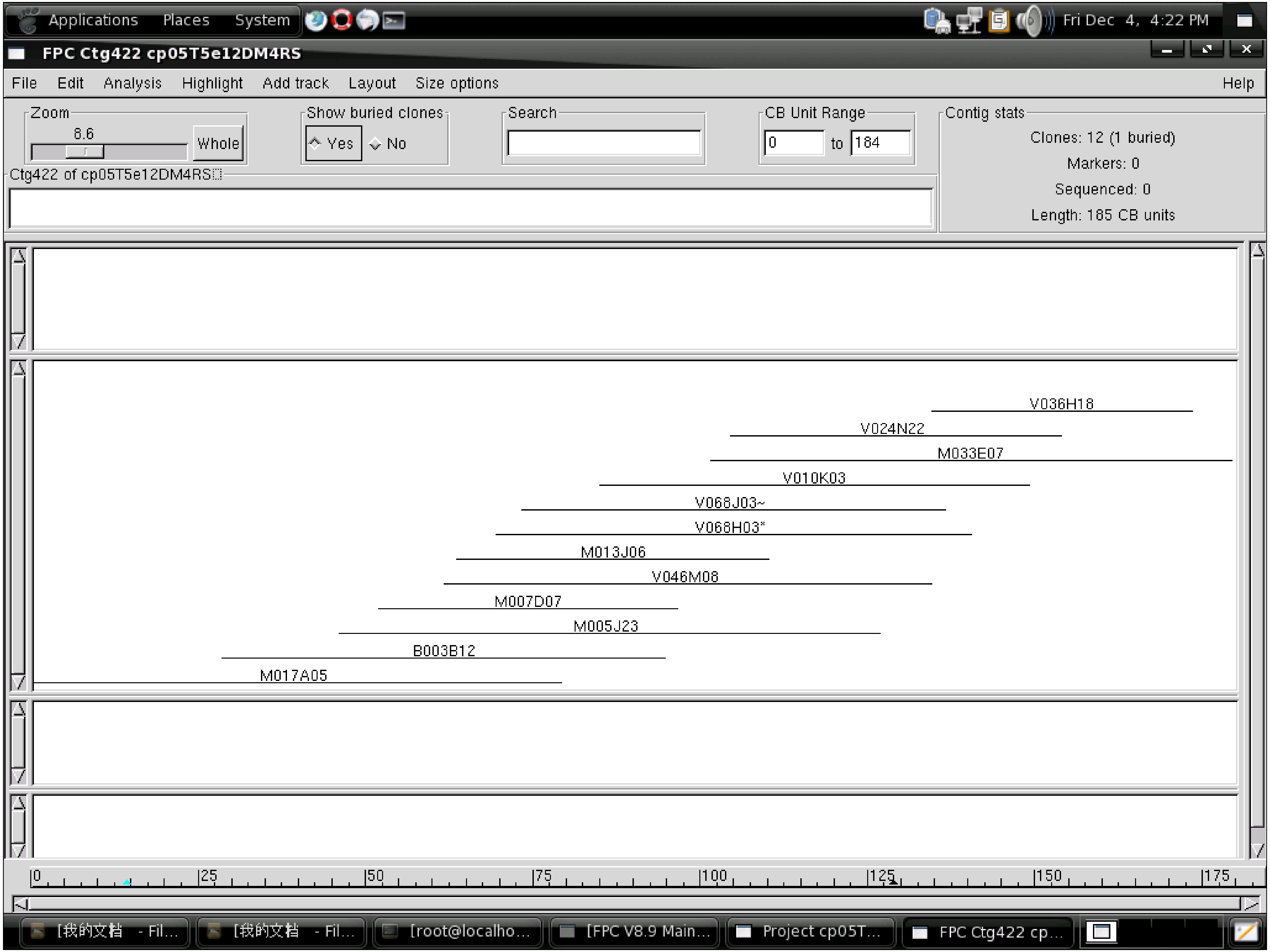
Applications Places System
Fri Dec 4, 4:22 PM
FPC Ctg422 cp05T5e12DM4RS
File Edit Analysis Highlight Add track Layout Size options Help
Zoom
8.6
Whole
Show buried clones
Yes
No
Search
CB Unit Range
0
to
184
Contig stats
Clones: 12 (1 buried)
Markers: 0
Sequenced: 0
Length: 185 CB units
Ctg422 of cp05T5e12DM4RS
V036H18
V024N22
M033E07
V010K03
V068J03~
V068H03*
M013J06
V046M08
M007D07
M005J23
B003B12
M017A05
0
25
50
75
100
125
150
175
[我的文档 - Fil...
[我的文档 - Fil...
[root@localho...
[FPC V8.9 Main...
Project cp05T...
FPC Ctg422 cp...

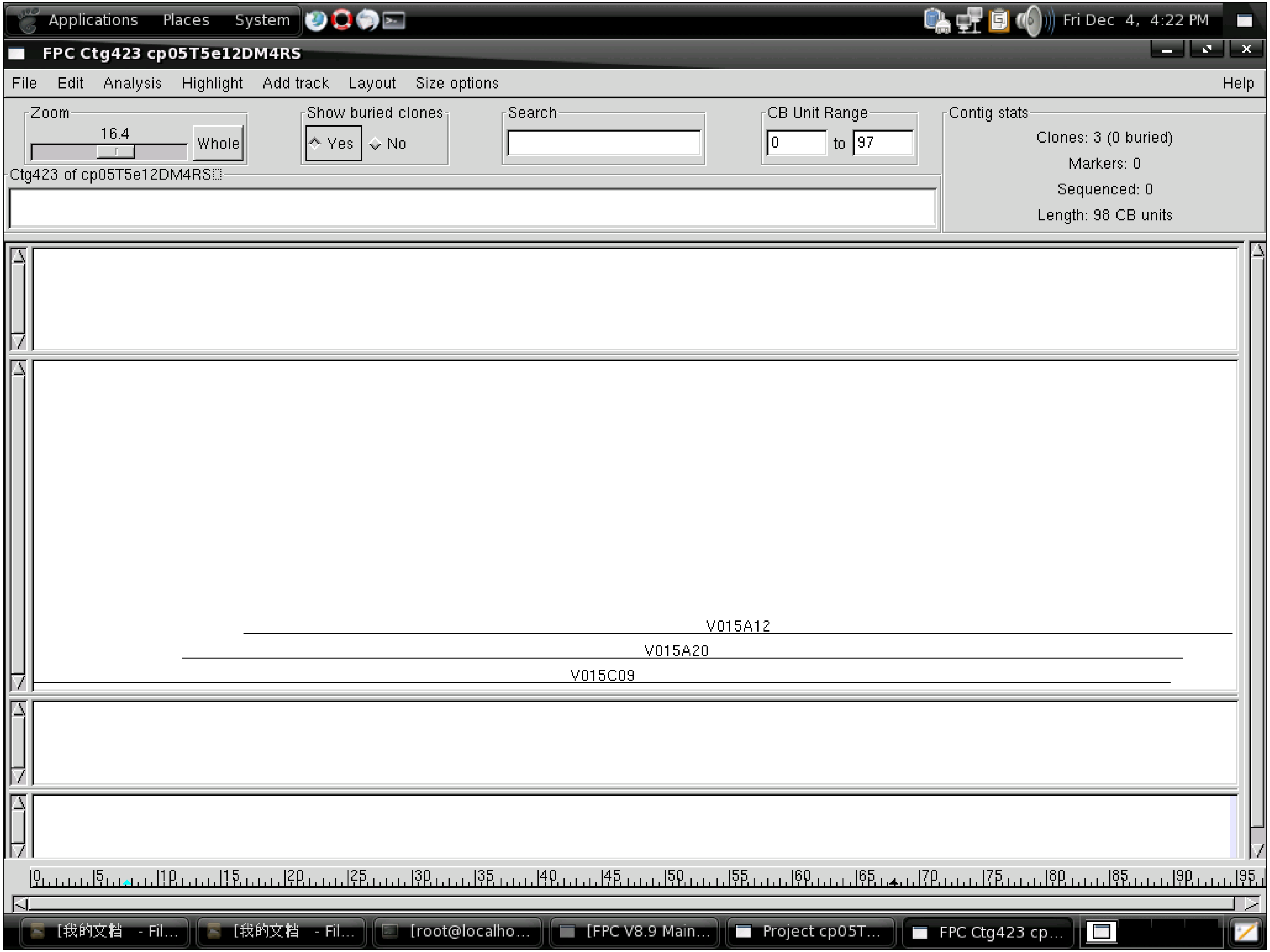
Applications Places System
Fri Dec 4, 4:22 PM
FPC Ctg423 cp05T5e12DM4RS
File Edit Analysis Highlight Add track Layout Size options Help
Zoom
16.4
Whole
Show buried clones
Yes
No
Search
CB Unit Range
0 to 97
Contig stats
Clones: 3 (0 buried)
Markers: 0
Sequenced: 0
Length: 98 CB units
Ctg423 of cp05T5e12DM4RS
V015A12
V015A20
V015C09
0 5 10 15 20 25 30 35 40 45 50 55 60 65 70 75 80 85 90 95
[我的文档 - Fil...
[我的文档 - Fil...
[root@localho...
[FPC V8.9 Main...
Project cp05T...
FPC Ctg423 cp...

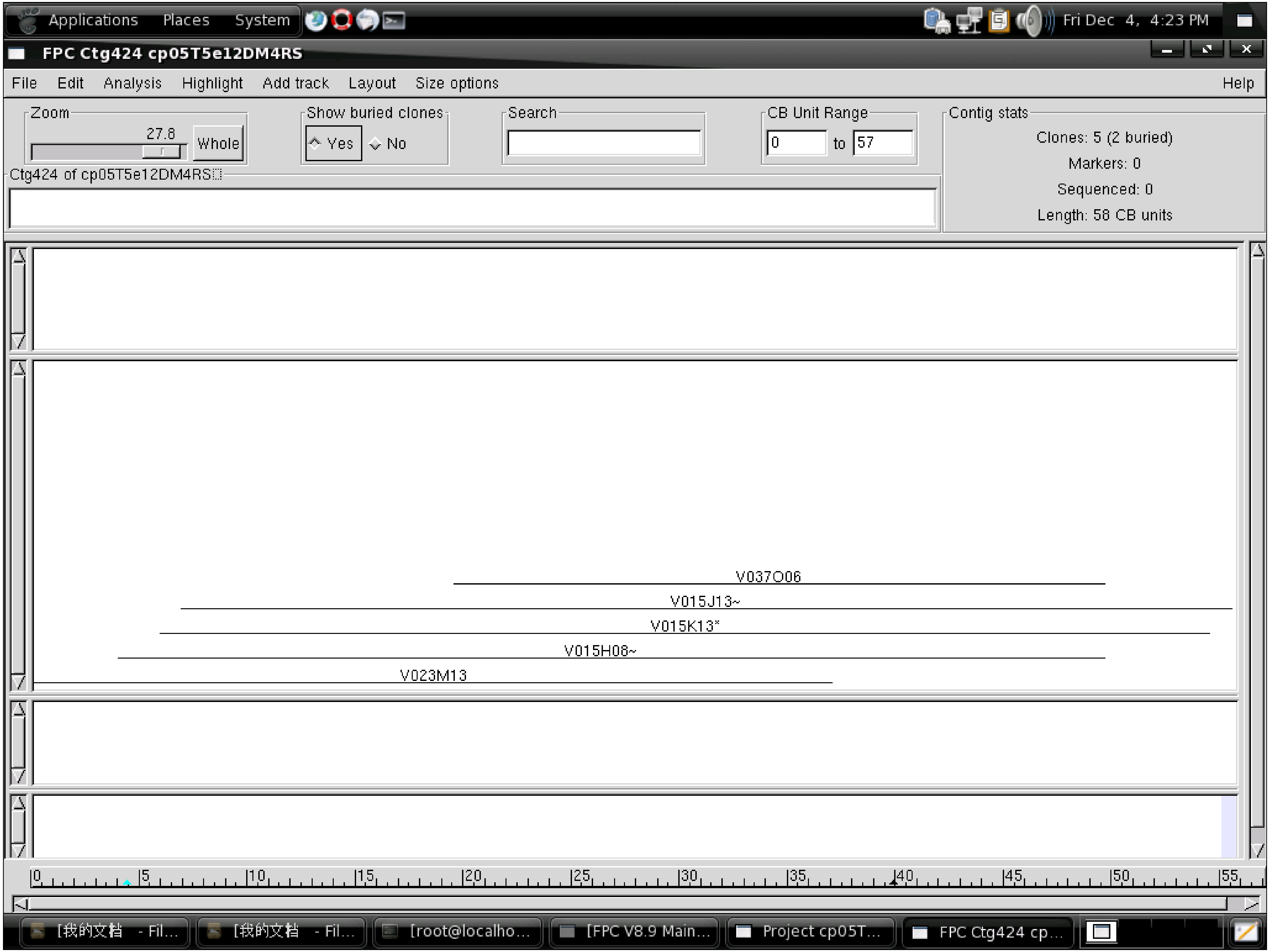
Applications
Places
System
Fri Dec 4, 4:23 PM
FPC Ctg424 cp05T5e12DM4RS
File
Edit
Analysis
Highlight
Add track
Layout
Size options
Help
Zoom
27.8
Whole
Show buried clones
Yes
No
Search
CB Unit Range
0
to
57
Contig stats
Clones: 5 (2 buried)
Markers: 0
Sequenced: 0
Length: 58 CB units
Ctg424 of cp05T5e12DM4RS
V037O06
V015J13~
V015K13*
V015H08~
V023M13
0
5
10
15
20
25
30
35
40
45
50
55
[我的文档 - Fil...
[我的文档 - Fil...
[root@localho...
[FPC V8.9 Main...
Project cp05T...
FPC Ctg424 cp...

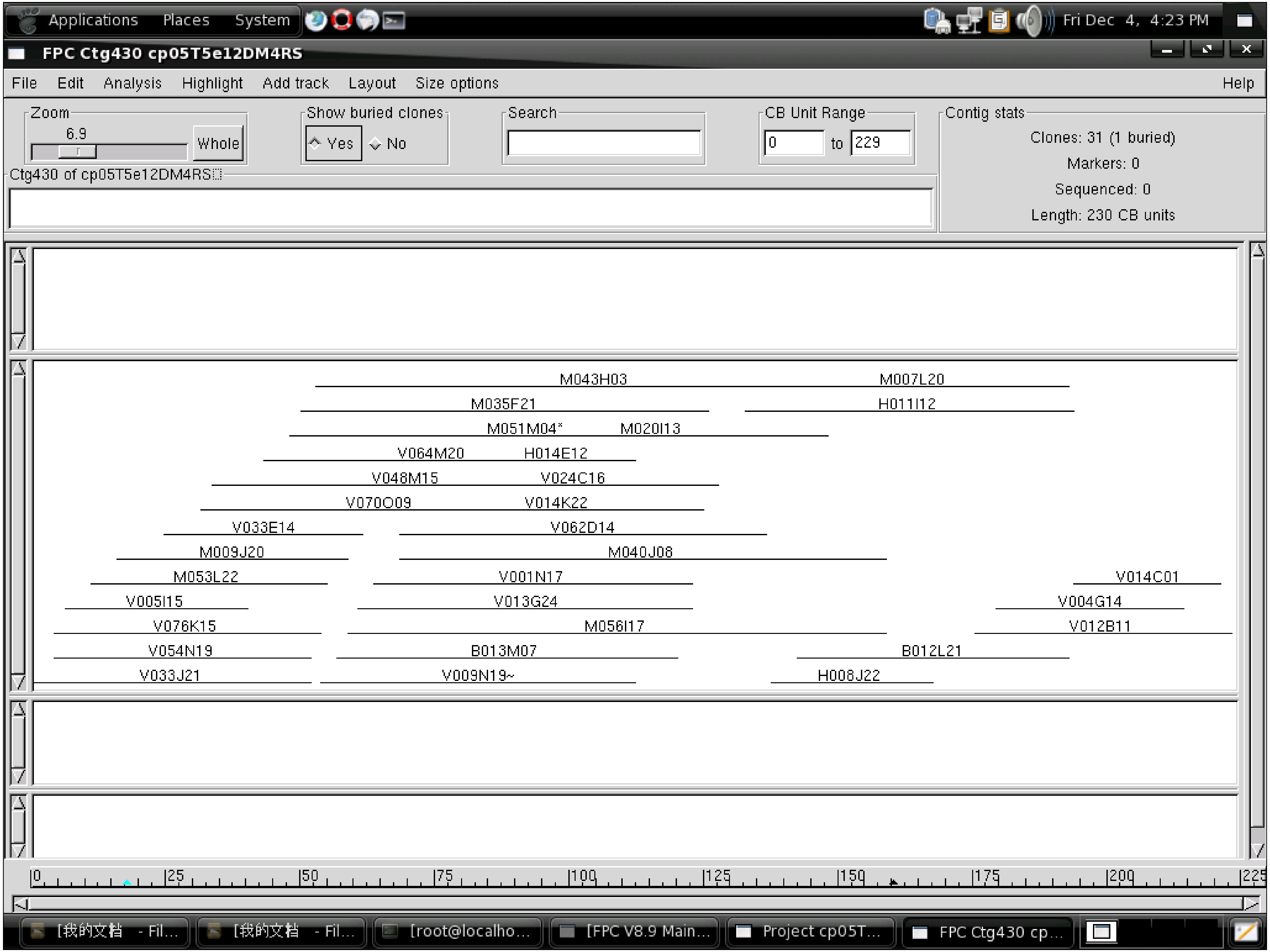
Applications Places System
Fri Dec 4, 4:23 PM
FPC Ctg430 cp05T5e12DM4RS
File Edit Analysis Highlight Add track Layout Size options Help
Zoom 6.9 Whole
Show buried clones Yes No
Search
CB Unit Range 0 to 229
Contig stats
Clones: 31 (1 buried)
Markers: 0
Sequenced: 0
Length: 230 CB units
Ctg430 of cp05T5e12DM4RS
M043H03
M007L20
M035F21
H011I12
M051M04*
M020I13
V064M20
H014E12
V048M15
V024C16
V070O09
V014K22
V033E14
V062D14
M009J20
M040J08
M053L22
V001N17
V014C01
V005I15
V013G24
V004G14
V076K15
M056I17
V012B11
V054N19
B013M07
B012L21
V033J21
V009N19~
H008J22
0 25 50 75 100 125 150 175 200 225
[root@localho...
[FPC V8.9 Main...
Project cp05T...
FPC Ctg430 cp...

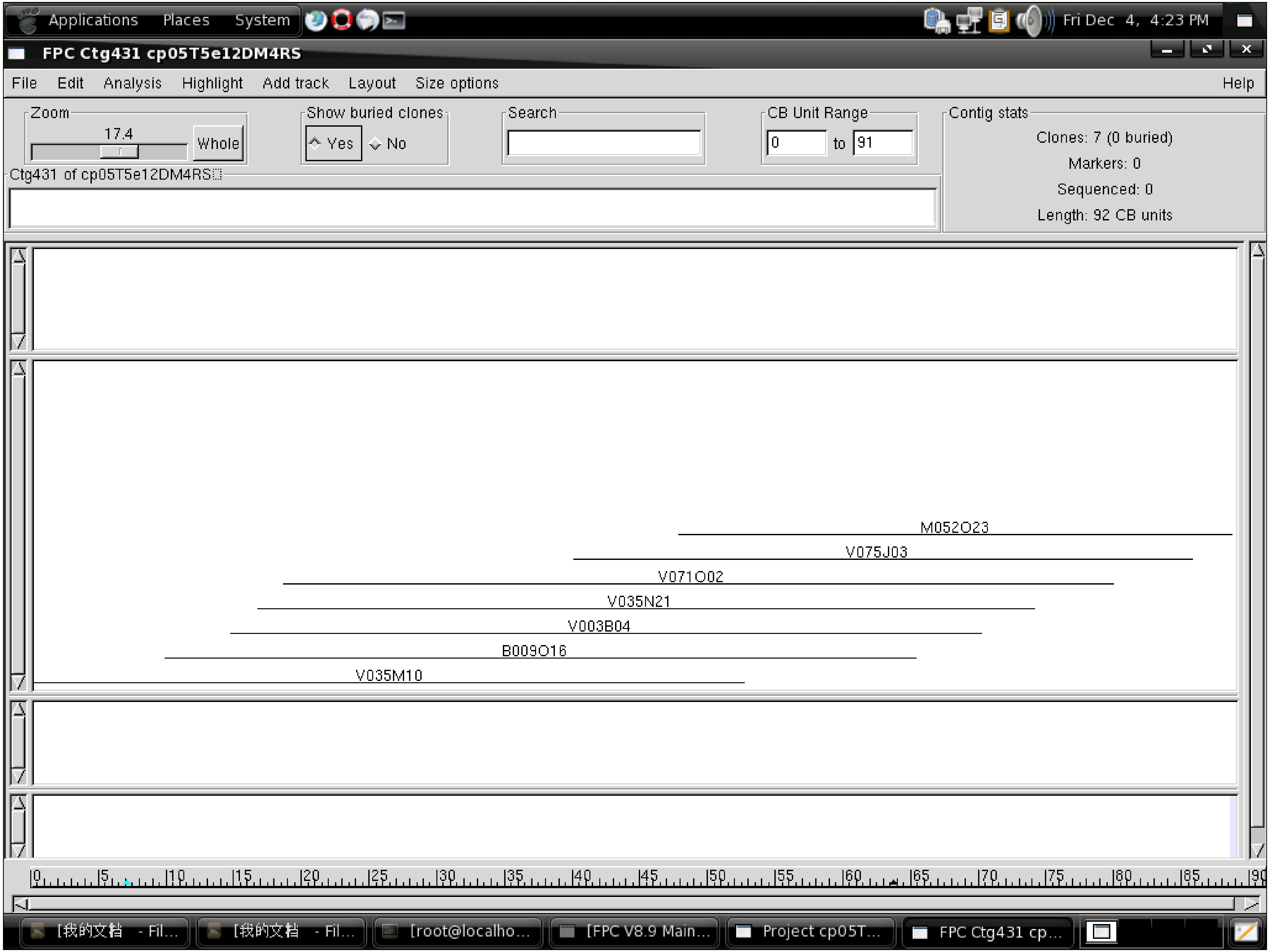
Applications Places System
Fri Dec 4, 4:23 PM
FPC Ctg431 cp05T5e12DM4RS
File Edit Analysis Highlight Add track Layout Size options Help
Zoom
17.4
Whole
Show buried clones
Yes
No
Search
CB Unit Range
0
to
91
Contig stats
Clones: 7 (0 buried)
Markers: 0
Sequenced: 0
Length: 92 CB units
Ctg431 of cp05T5e12DM4RS
M052O23
V075J03
V071O02
V035N21
V003B04
B009O16
V035M10
[root@localho...
[FPC V8.9 Main...
Project cp05T...
FPC Ctg431 cp...

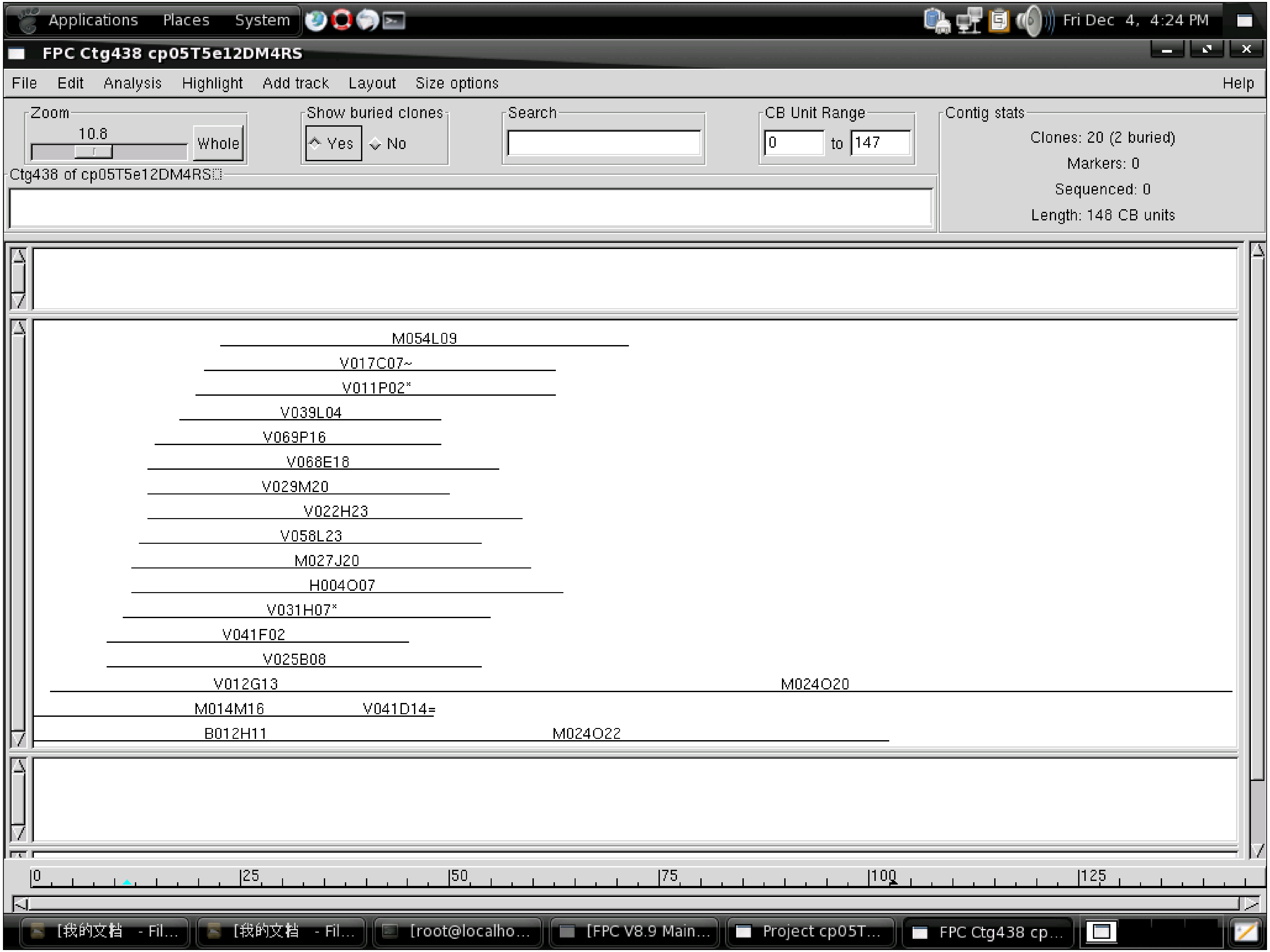

Applications Places System
Fri Dec 4, 4:24 PM
FPC Ctg438 cp05T5e12DM4RS
File Edit Analysis Highlight Add track Layout Size options Help
Zoom
10.8
Whole
Show buried clones
Yes
No
Search
CB Unit Range
0 to 147
Contig stats
Clones: 20 (2 buried)
Markers: 0
Sequenced: 0
Length: 148 CB units
Ctg438 of cp05T5e12DM4RS
M054L09
V017C07~
V011P02*
V039L04
V069P16
V068E18
V029M20
V022H23
V058L23
M027J20
H004O07
V031H07*
V041F02
V025B08
V012G13
M024O20
M014M16
V041D14=
B012H11
M024O22
0
25
50
75
100
125
[我的文档 - Fil...
[我的文档 - Fil...
[root@localho...
[FPC V8.9 Main...
Project cp05T...
FPC Ctg438 cp...

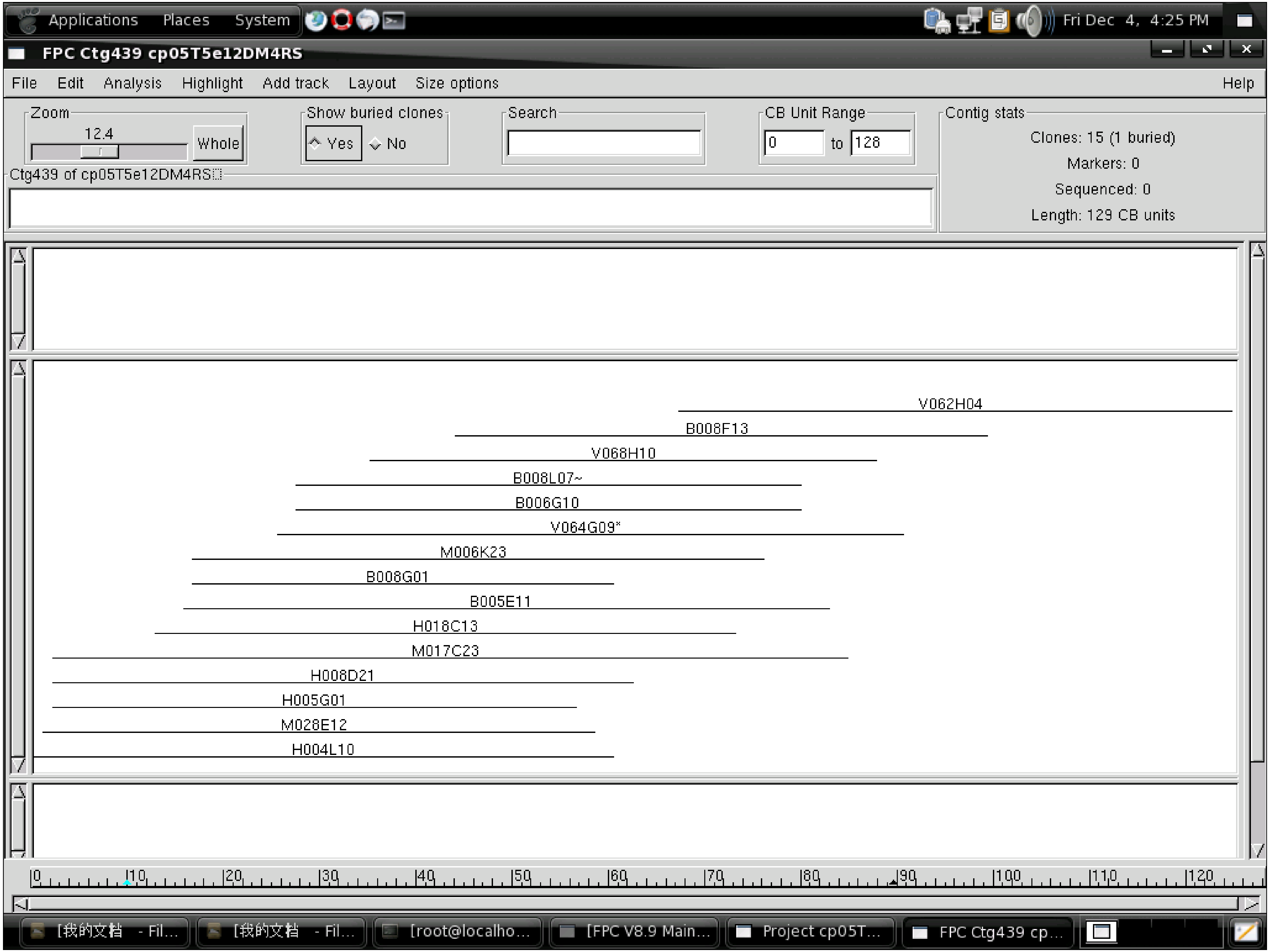

Applications Places System
Fri Dec 4, 4:25 PM
FPC Ctg439 cp05T5e12DM4RS
File Edit Analysis Highlight Add track Layout Size options Help
Zoom
12.4
Whole
Show buried clones
Yes
No
Search
CB Unit Range
0
to
128
Contig stats
Clones: 15 (1 buried)
Markers: 0
Sequenced: 0
Length: 129 CB units
Ctg439 of cp05T5e12DM4RS
V062H04
B008F13
V068H10
B008L07~
B006G10
V064G09*
M006K23
B008G01
B005E11
H018C13
M017C23
H008D21
H005G01
M028E12
H004L10
0 10 20 30 40 50 60 70 80 90 100 110 120
[我的文档 - Fil...
[我的文档 - Fil...
[root@localho...
[FPC V8.9 Main...
Project cp05T...
FPC Ctg439 cp...

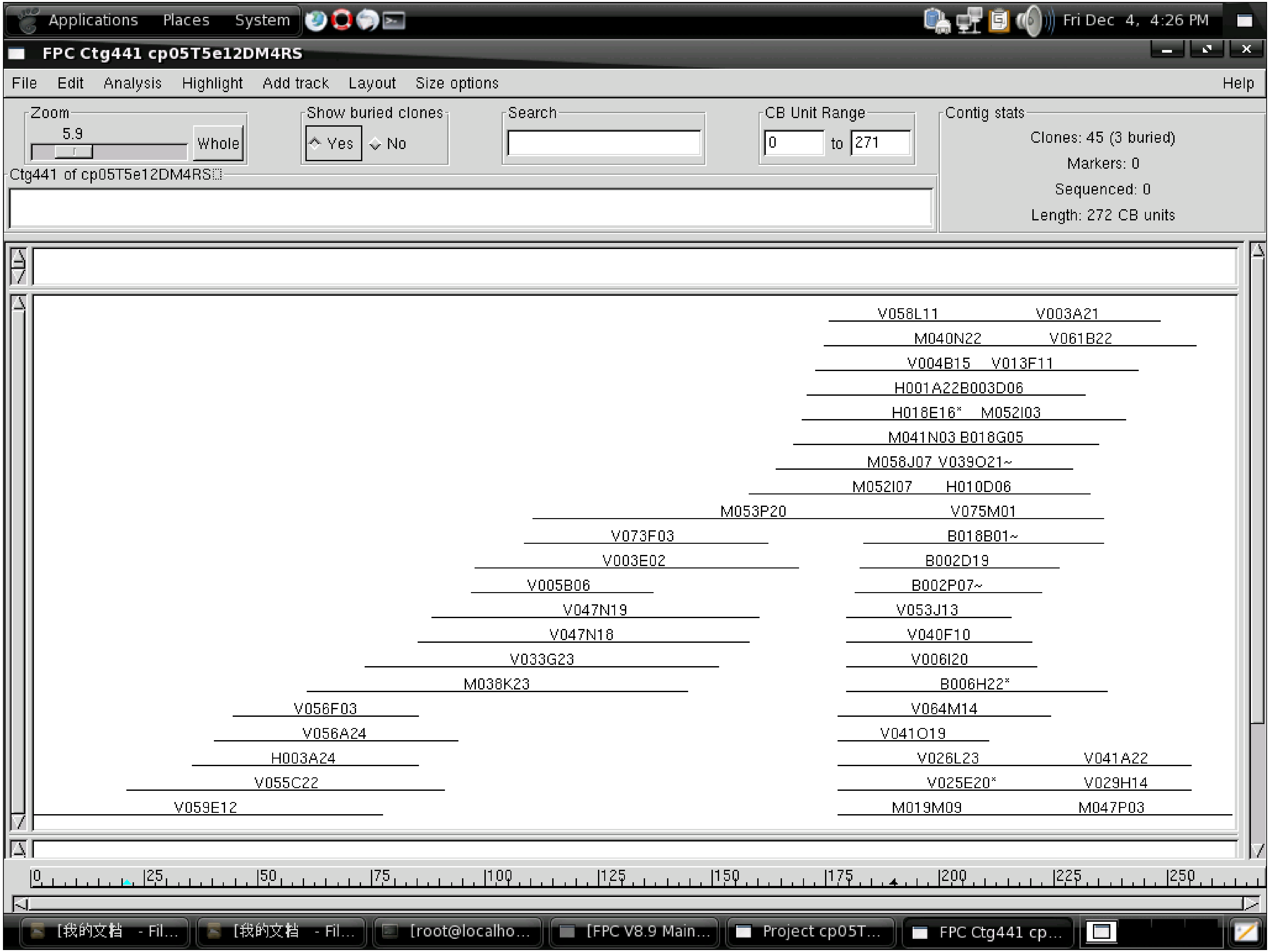
Applications Places System
Fri Dec 4, 4:26 PM
FPC Ctg441 cp05T5e12DM4RS
File Edit Analysis Highlight Add track Layout Size options Help
Zoom
5.9
Whole
Show buried clones
Yes No
Search
CB Unit Range
0 to 271
Contig stats
Clones: 45 (3 buried)
Markers: 0
Sequenced: 0
Length: 272 CB units
Ctg441 of cp05T5e12DM4RS
V058L11 V003A21
M040N22 V061B22
V004B15 V013F11
H001A22B003D06
H018E16* M052I03
M041N03 B018G05
M058J07 V039O21~
M052I07 H010D06
M053P20 V075M01
V073F03 B018B01~
V003E02 B002D19
V005B06 B002P07~
V047N19 V053J13
V047N18 V040F10
V033G23 V006I20
M038K23 B006H22*
V056F03 V064M14
V056A24 V041O19
H003A24 V026L23 V041A22
V055C22 V025E20* V029H14
V059E12 M019M09 M047P03
0 25 50 75 100 125 150 175 200 225 250
[我的文档 - Fil... [我的文档 - Fil... [root@localho... [FPC V8.9 Main... Project cp05T... FPC Ctg441 cp...

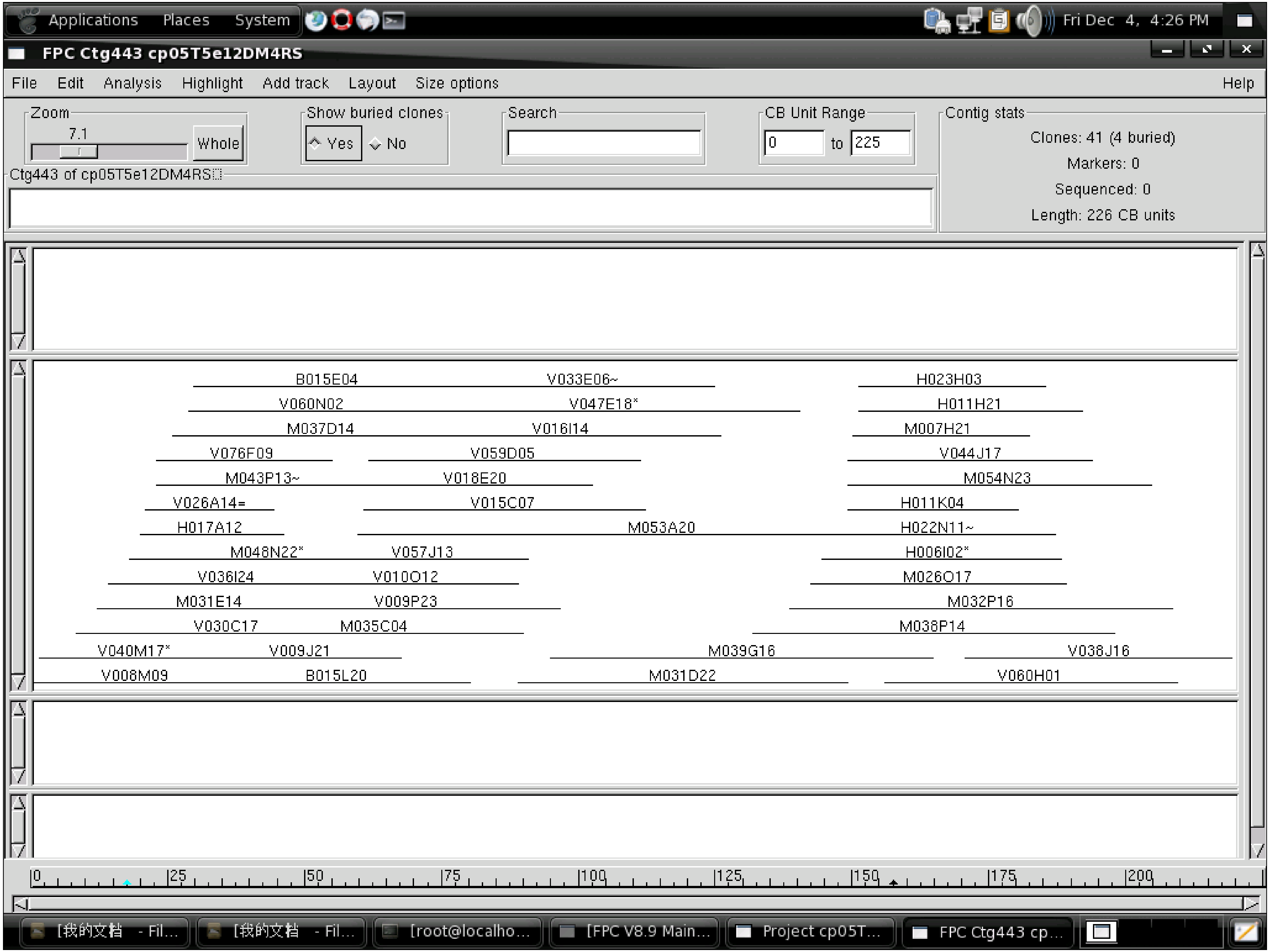
Applications Places System
Fri Dec 4, 4:26 PM
FPC Ctg443 cp05T5e12DM4RS
File Edit Analysis Highlight Add track Layout Size options Help
Zoom 7.1 Whole
Show buried clones Yes No
Search
CB Unit Range 0 to 225
Contig stats
Clones: 41 (4 buried)
Markers: 0
Sequenced: 0
Length: 226 CB units
Ctg443 of cp05T5e12DM4RS
B015E04
V033E06~
H023H03
V060N02
V047E18*
H011H21
M037D14
V016I14
M007H21
V076F09
V059D05
V044J17
M043P13~
V018E20
M054N23
V026A14=
V015C07
H011K04
H017A12
M053A20
H022N11~
M048N22*
V057J13
H006I02*
V036I24
V010O12
M026O17
M031E14
V009P23
M032P16
V030C17
M035C04
M038P14
V040M17*
V009J21
M039G16
V038J16
V008M09
B015L20
M031D22
V060H01
0 25 50 75 100 125 150 175 200
[我的文档 - Fil...
[我的文档 - Fil...
[root@localho...
[FPC V8.9 Main...
Project cp05T...
FPC Ctg443 cp...

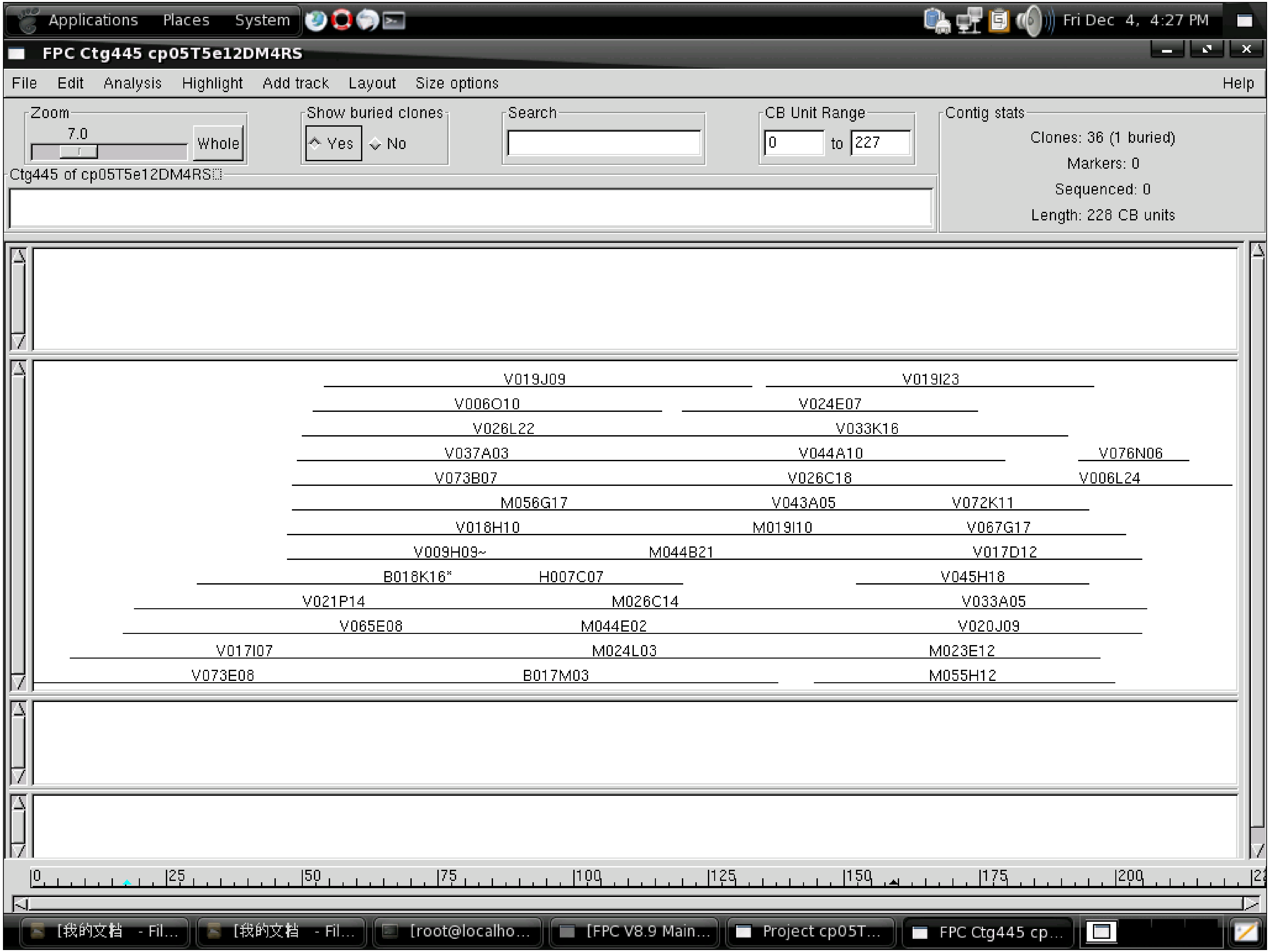
Applications Places System
Fri Dec 4, 4:27 PM
FPC Ctg445 cp05T5e12DM4RS
File Edit Analysis Highlight Add track Layout Size options Help
Zoom
7.0
Whole
Show buried clones
Yes No
Search
CB Unit Range
0 to 227
Contig stats
Clones: 36 (1 buried)
Markers: 0
Sequenced: 0
Length: 228 CB units
Ctg445 of cp05T5e12DM4RS
V019J09
V019I23
V006O10
V024E07
V026L22
V033K16
V037A03
V044A10
V076N06
V073B07
V026C18
V006L24
M056G17
V043A05
V072K11
V018H10
M019I10
V067G17
V009H09~
M044B21
V017D12
B018K16*
H007C07
V045H18
V021P14
M026C14
V033A05
V065E08
M044E02
V020J09
V017I07
M024L03
M023E12
V073E08
B017M03
M055H12
0 25 50 75 100 125 150 175 200
[我的文档 - Fil...
[我的文档 - Fil...
[root@localho...
[FPC V8.9 Main...
Project cp05T...
FPC Ctg445 cp...

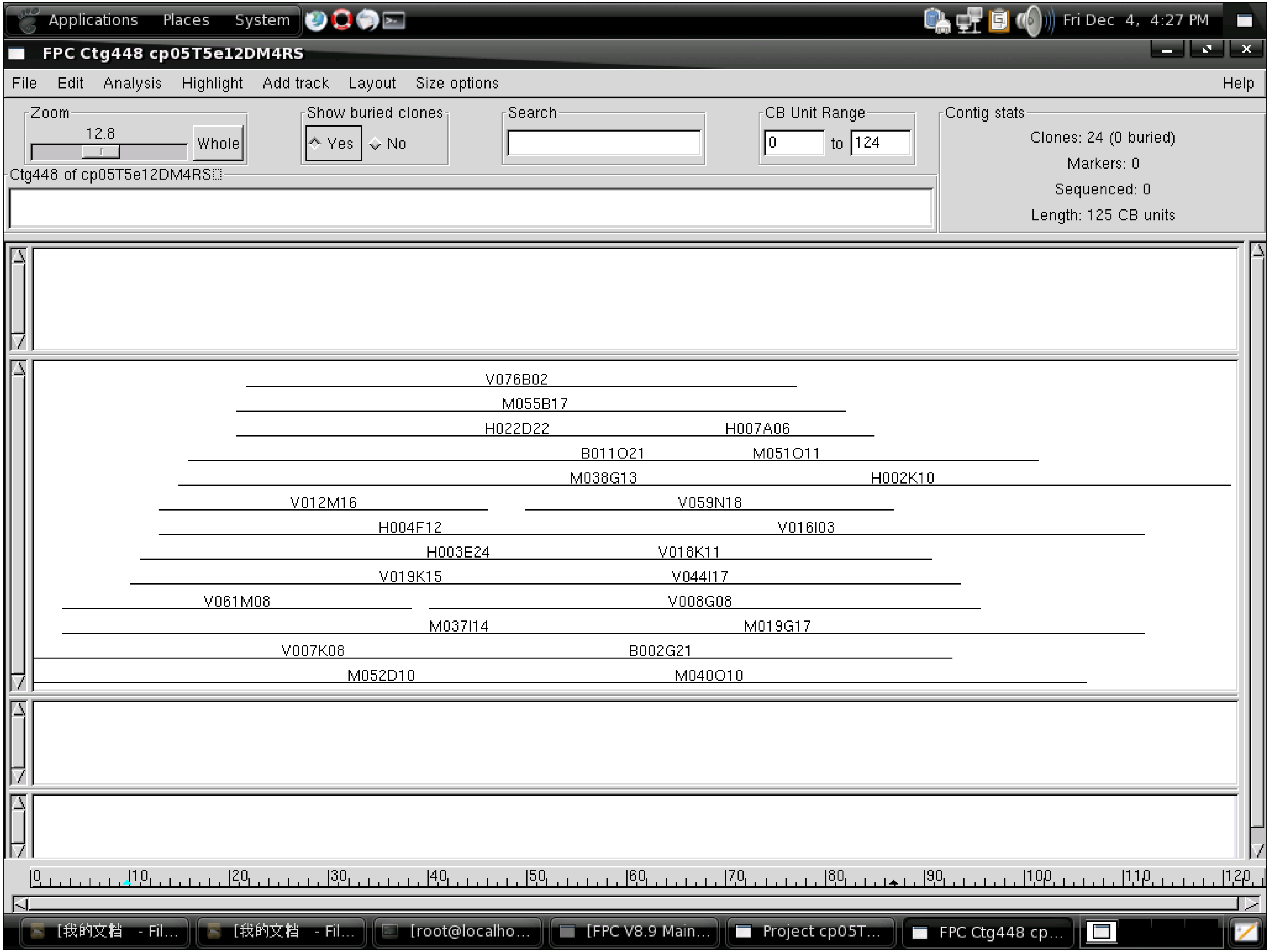
Applications Places System
Fri Dec 4, 4:27 PM
FPC Ctg448 cp05T5e12DM4RS
File Edit Analysis Highlight Add track Layout Size options Help
Zoom
12.8
Whole
Show buried clones
Yes No
Search
CB Unit Range
0 to 124
Contig stats
Clones: 24 (0 buried)
Markers: 0
Sequenced: 0
Length: 125 CB units
Ctg448 of cp05T5e12DM4RS
V076B02
M055B17
H022D22
H007A06
B011O21
M051O11
M038G13
H002K10
V012M16
V059N18
H004F12
V016I03
H003E24
V018K11
V019K15
V044I17
V061M08
V008G08
M037I14
M019G17
V007K08
B002G21
M052D10
M040O10
0 10 20 30 40 50 60 70 80 90 100 110 120
[我的文档 - Fil...
[我的文档 - Fil...
[root@localho...
[FPC V8.9 Main...
Project cp05T...
FPC Ctg448 cp...

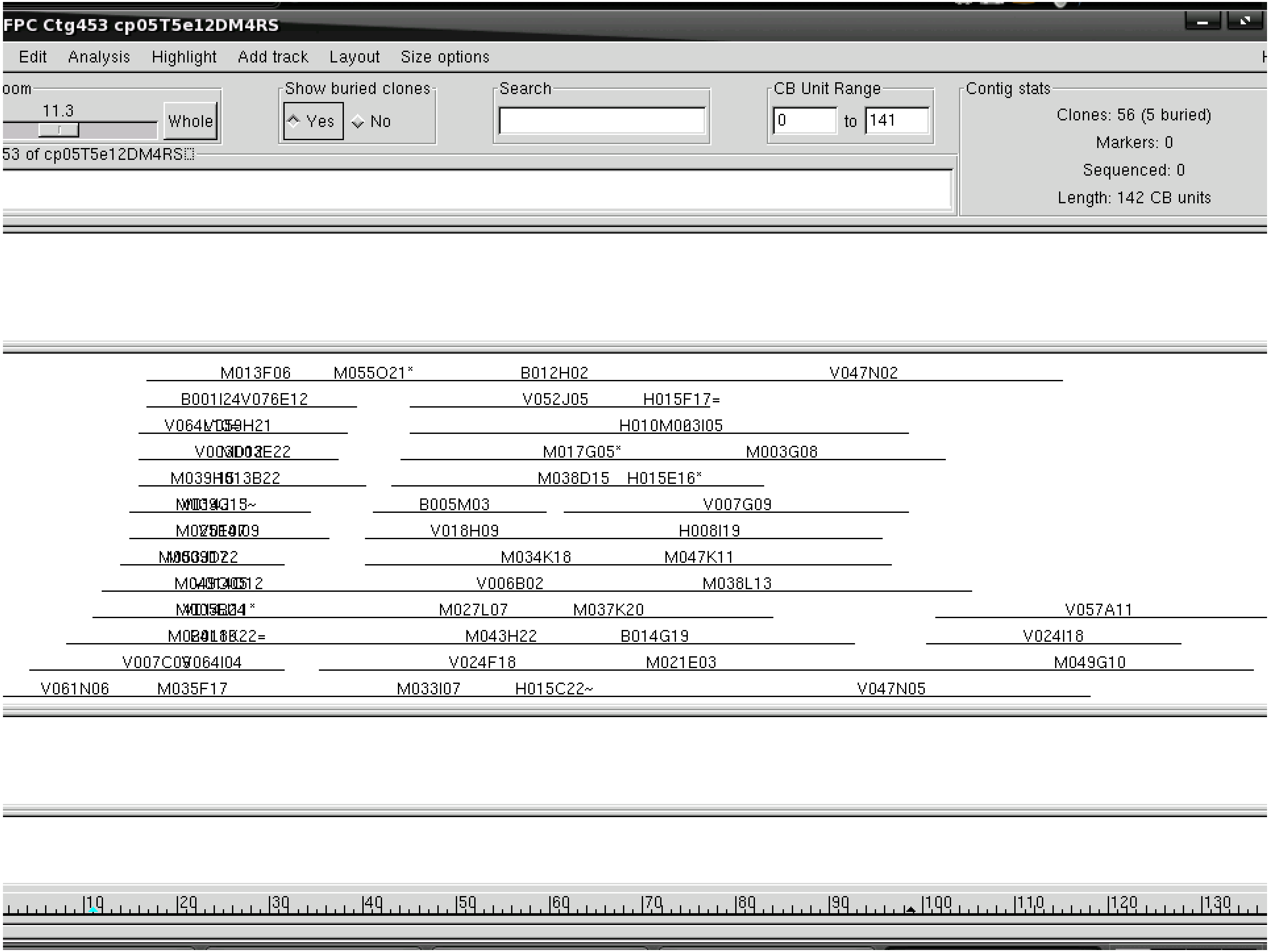

FPC Ctg453 cp05T5e12DM4RS
Edit
Analysis
Highlight
Add track
Layout
Size options
oom
11.3
Whole
Show buried clones
Yes
No
Search
CB Unit Range
0
to
141
Contig stats
Clones: 56 (5 buried)
Markers: 0
Sequenced: 0
Length: 142 CB units
53 of cp05T5e12DM4RS
M013F06
M055O21*
B012H02
V047N02
B001I24V076E12
V052J05
H015F17=
M017G05*
M003G08
M038D15
H015E16*
B005M03
V007G09
V018H09
H008I19
M034K18
M047K11
V006B02
M038L13
M027L07
M037K20
V057A11
M043H22
B014G19
V024I18
V024F18
M021E03
M049G10
V061N06
M035F17
M033I07
H015C22~
V047N05
10
20
30
40
50
60
70
80
90
100
110
120
130

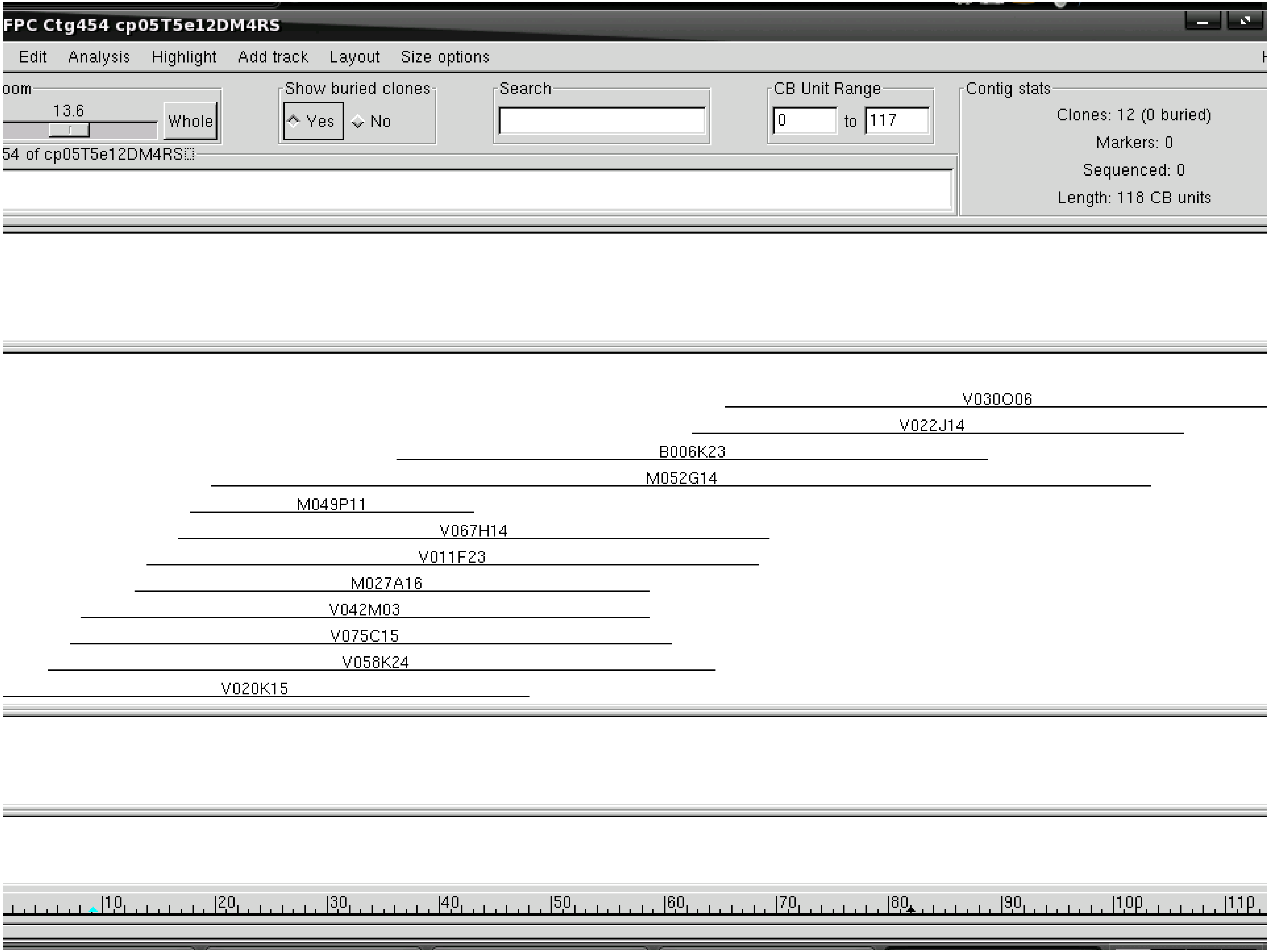
FPC Ctg454 cp05T5e12DM4RS
Edit
Analysis
Highlight
Add track
Layout
Size options
oom
13.6
Whole
Show buried clones
Yes
No
Search
CB Unit Range
0
to
117
Contig stats
Clones: 12 (0 buried)
Markers: 0
Sequenced: 0
Length: 118 CB units
54 of cp05T5e12DM4RS
V030O06
V022J14
B006K23
M052G14
M049P11
V067H14
V011F23
M027A16
V042M03
V075C15
V058K24
V020K15
10
20
30
40
50
60
70
80
90
100
110

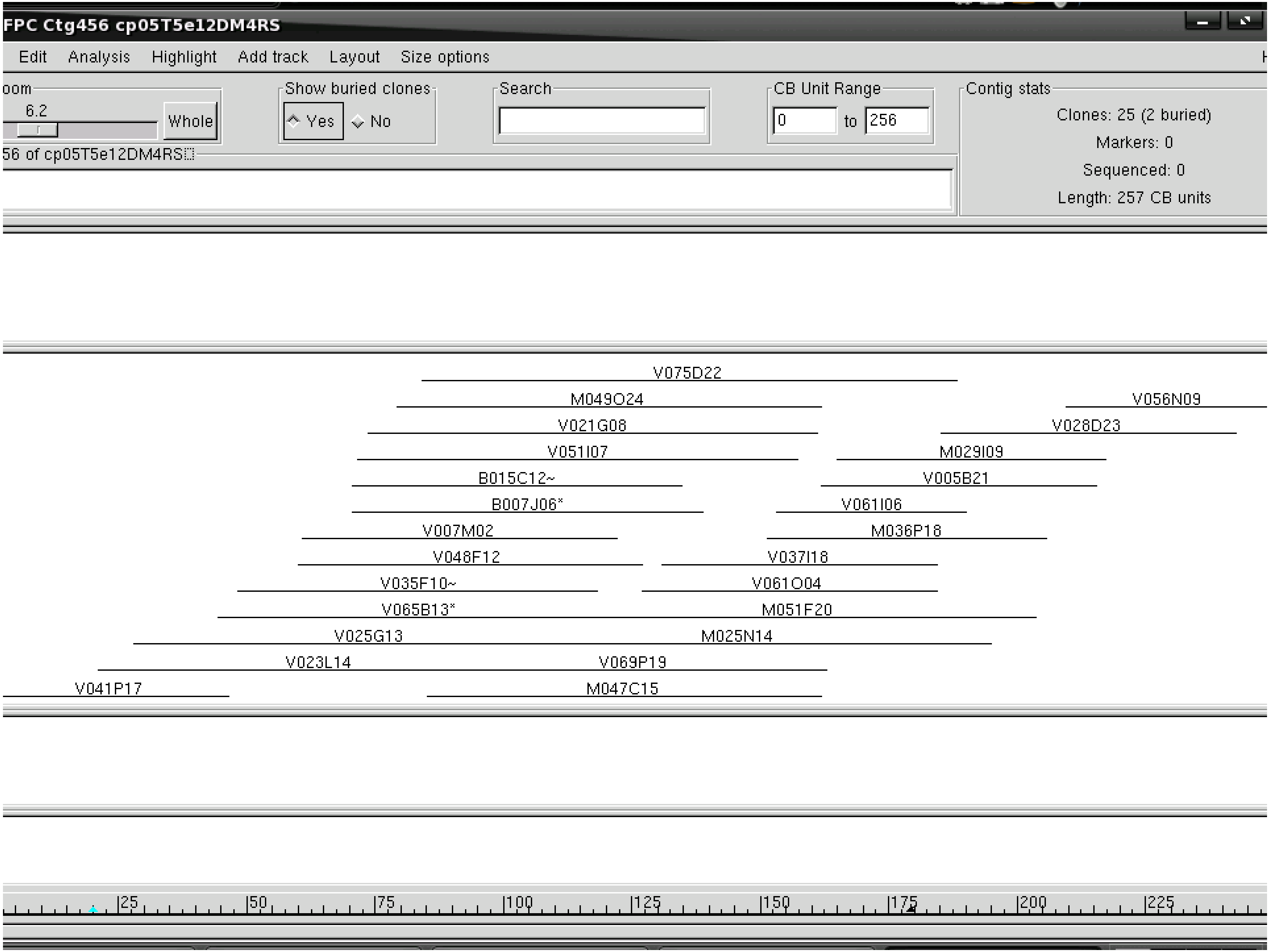
FPC Ctg456 cp05T5e12DM4RS
Edit Analysis Highlight Add track Layout Size options
oom
6.2
Whole
Show buried clones
Yes
No
Search
CB Unit Range
0
to
256
Contig stats
Clones: 25 (2 buried)
Markers: 0
Sequenced: 0
Length: 257 CB units
56 of cp05T5e12DM4RS
V075D22
M049O24
V056N09
V021G08
V028D23
V051I07
M029I09
B015C12~
V005B21
B007J06*
V061I06
V007M02
M036P18
V048F12
V037I18
V035F10~
V061O04
V065B13*
M051F20
V025G13
M025N14
V023L14
V069P19
V041P17
M047C15
25
50
75
100
125
150
175
200
225

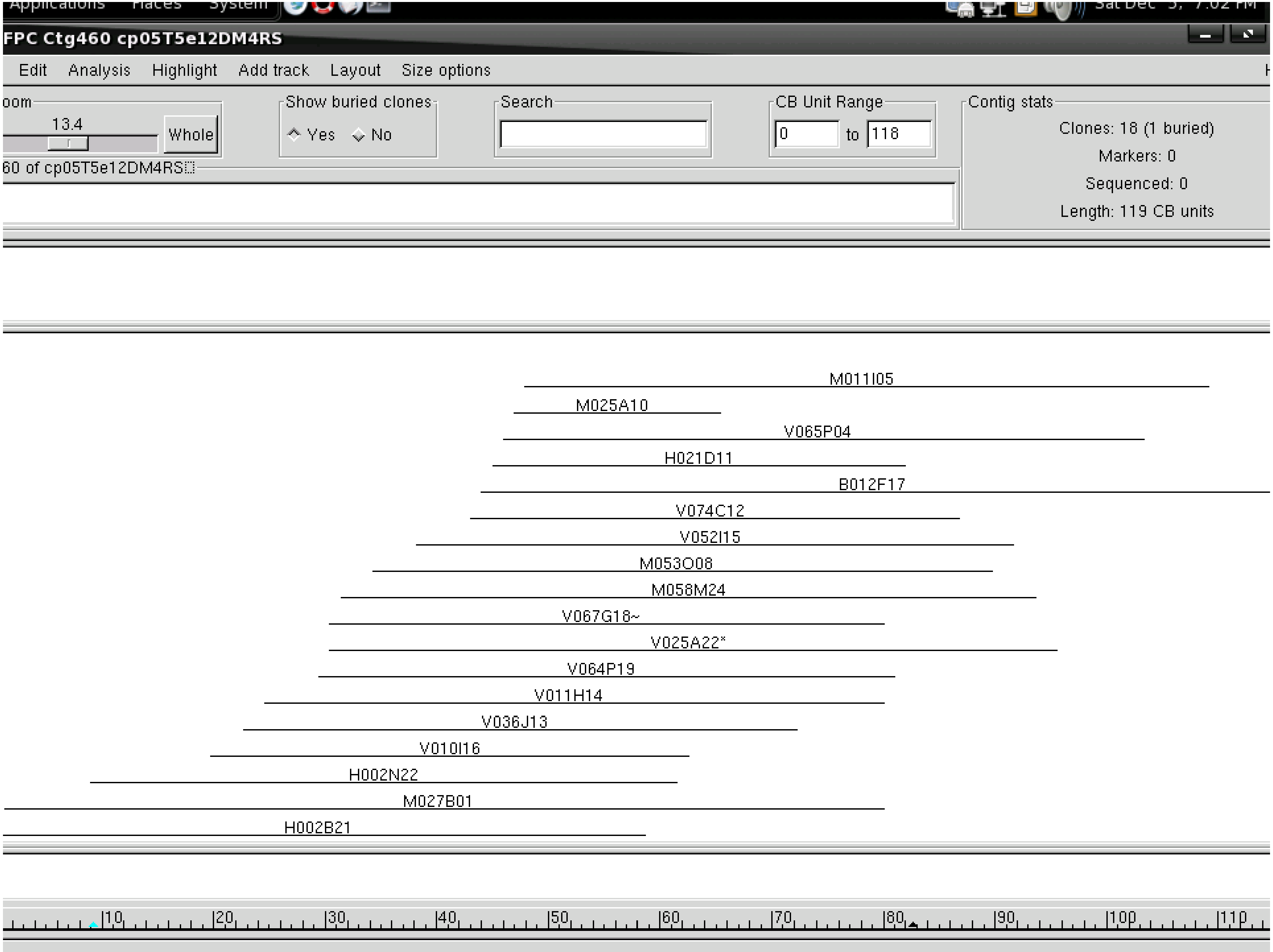
FPC Ctg460 cp05T5e12DM4RS
Edit
Analysis
Highlight
Add track
Layout
Size options
Show buried clones
Yes
No
Search
CB Unit Range
0
to
118
Contig stats
Clones: 18 (1 buried)
Markers: 0
Sequenced: 0
Length: 119 CB units
13.4
Whole
M011I05
M025A10
V065P04
H021D11
B012F17
V074C12
V052I15
M053O08
M058M24
V067G18~
V025A22*
V064P19
V011H14
V036J13
V010I16
H002N22
M027B01
H002B21
10
20
30
40
50
60
70
80
90
100
110

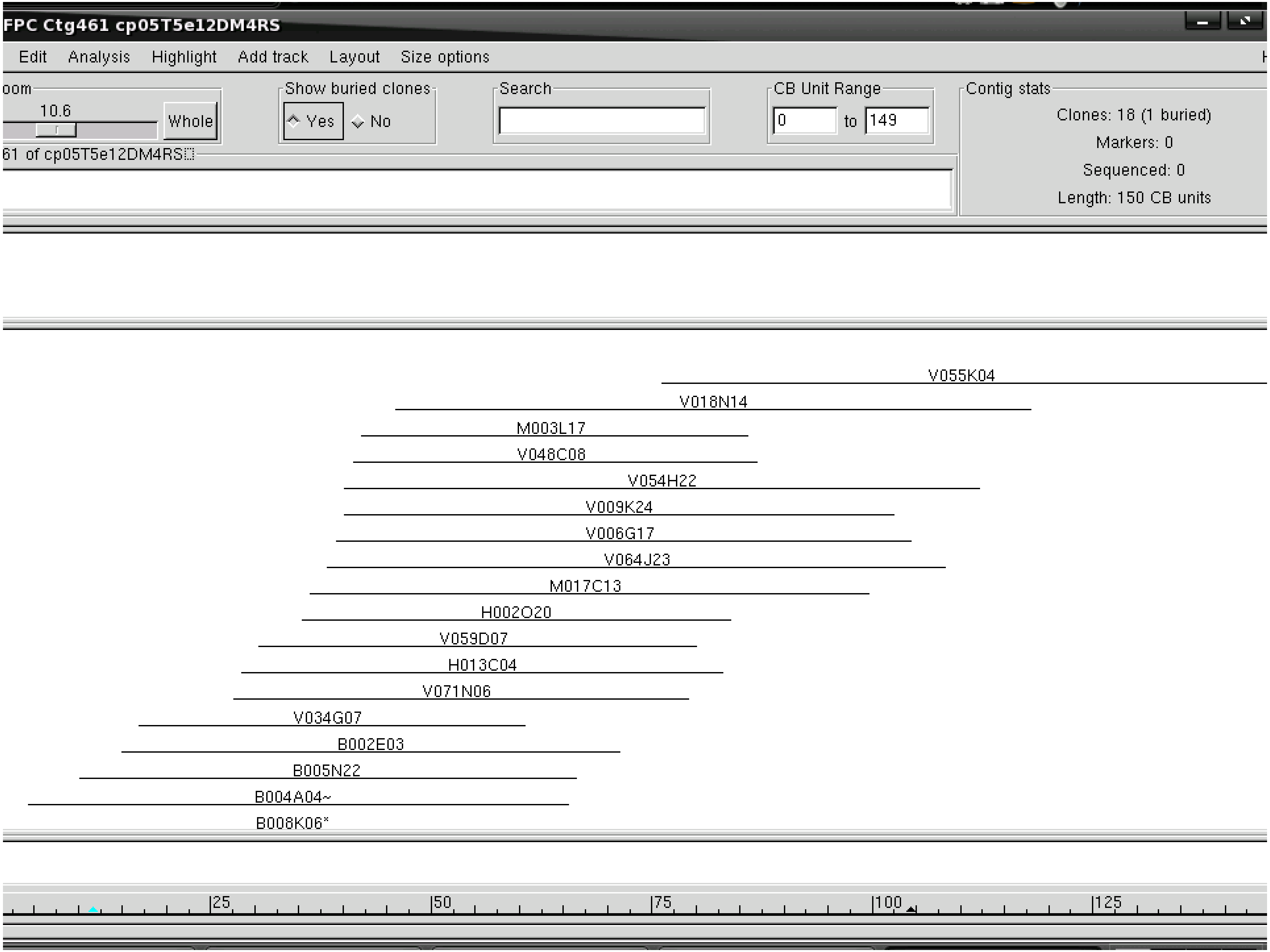
FPC Ctg461 cp05T5e12DM4RS
Edit Analysis Highlight Add track Layout Size options
oom
10.6
Whole
Show buried clones
Yes
No
Search
CB Unit Range
0
to
149
Contig stats
Clones: 18 (1 buried)
Markers: 0
Sequenced: 0
Length: 150 CB units
61 of cp05T5e12DM4RS
V055K04
V018N14
M003L17
V048C08
V054H22
V009K24
V006G17
V064J23
M017C13
H002O20
V059D07
H013C04
V071N06
V034G07
B002E03
B005N22
B004A04~
B008K06*
25
50
75
100
125

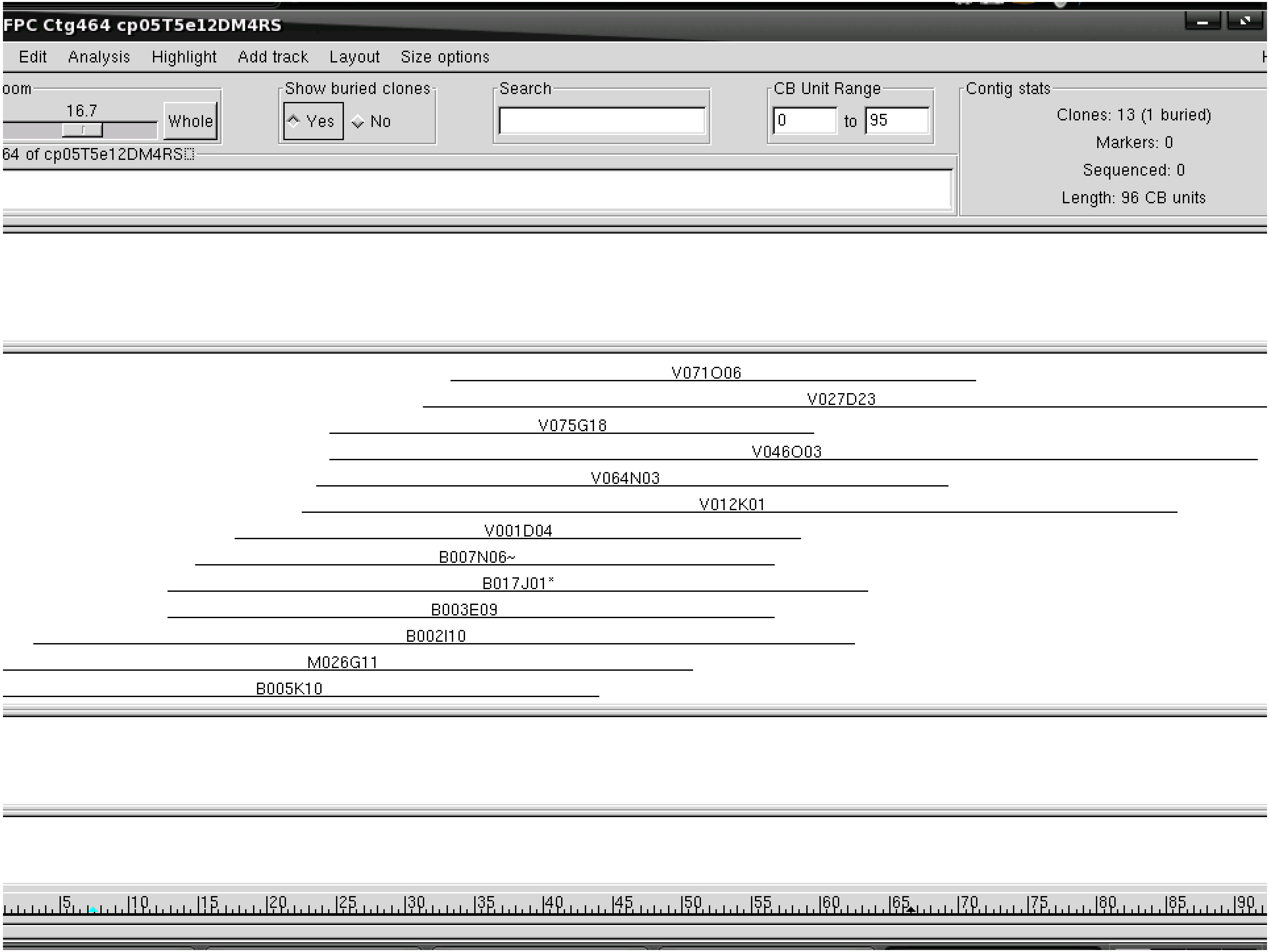
FPC Ctg464 cp05T5e12DM4RS
Edit
Analysis
Highlight
Add track
Layout
Size options
oom
16.7
Whole
Show buried clones
Yes
No
Search
CB Unit Range
0
to
95
Contig stats
Clones: 13 (1 buried)
Markers: 0
Sequenced: 0
Length: 96 CB units
64 of cp05T5e12DM4RS
V071O06
V027D23
V075G18
V046O03
V064N03
V012K01
V001D04
B007N06~
B017J01*
B003E09
B002I10
M026G11
B005K10
5 10 15 20 25 30 35 40 45 50 55 60 65 70 75 80 85 90

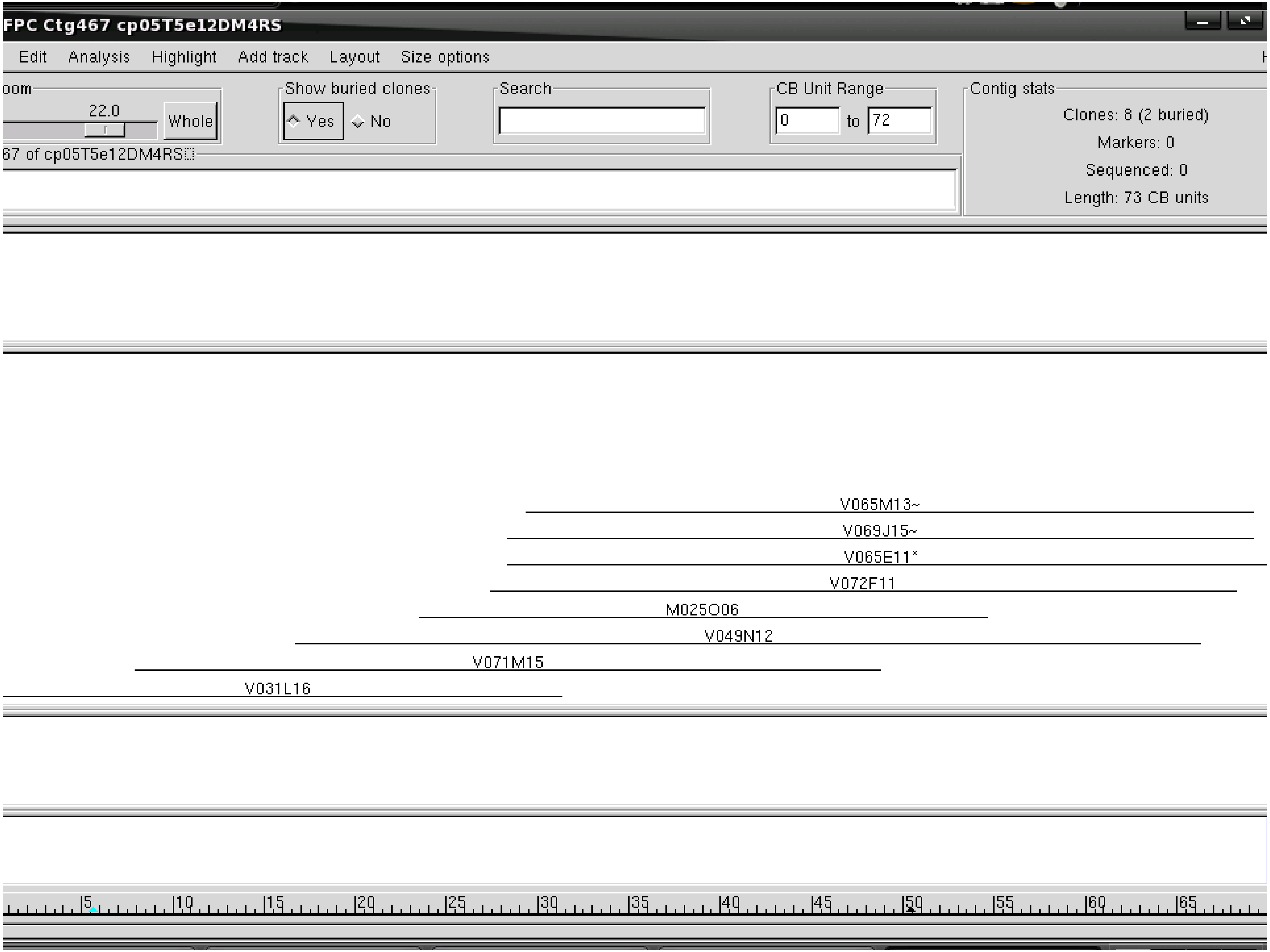
FPC Ctg467 cp05T5e12DM4RS
Edit
Analysis
Highlight
Add track
Layout
Size options
oom
22.0
Whole
Show buried clones
Yes
No
Search
CB Unit Range
0
to
72
Contig stats
Clones: 8 (2 buried)
Markers: 0
Sequenced: 0
Length: 73 CB units
67 of cp05T5e12DM4RS
V065M13~
V069J15~
V065E11*
V072F11
M025O06
V049N12
V071M15
V031L16
5
10
15
20
25
30
35
40
45
50
55
60
65

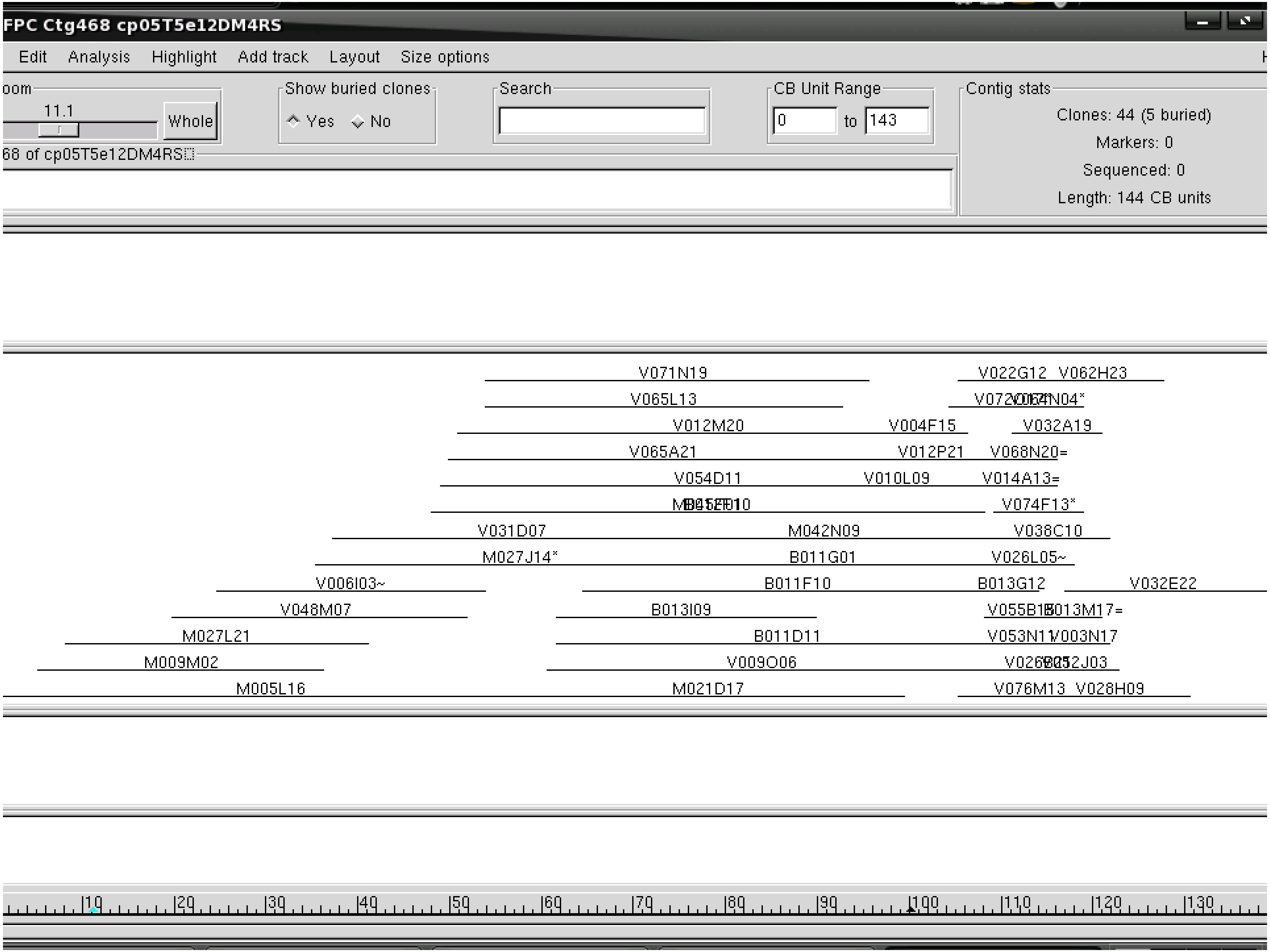

FPC Ctg468 cp05T5e12DM4RS
Edit Analysis Highlight Add track Layout Size options
Show buried clones
Yes No
Search
CB Unit Range
0 to 143
Contig stats
Clones: 44 (5 buried)
Markers: 0
Sequenced: 0
Length: 144 CB units
Whole
11.1
V071N19
V065L13
V012M20
V065A21
V054D11
V031D07
M027J14*
V006I03~
V048M07
M027L21
M009M02
M005L16
M042N09
B011G01
B011F10
B013I09
B011D11
V009O06
M021D17
V004F15
V012P21
V010L09
V022G12
V062H23
V032A19
V068N20=
V014A13=
V074F13*
V038C10
V026L05~
B013G12
V032E22
V076M13
V028H09
|10 |20 |30 |40 |50 |60 |70 |80 |90 |100 |110 |120 |130

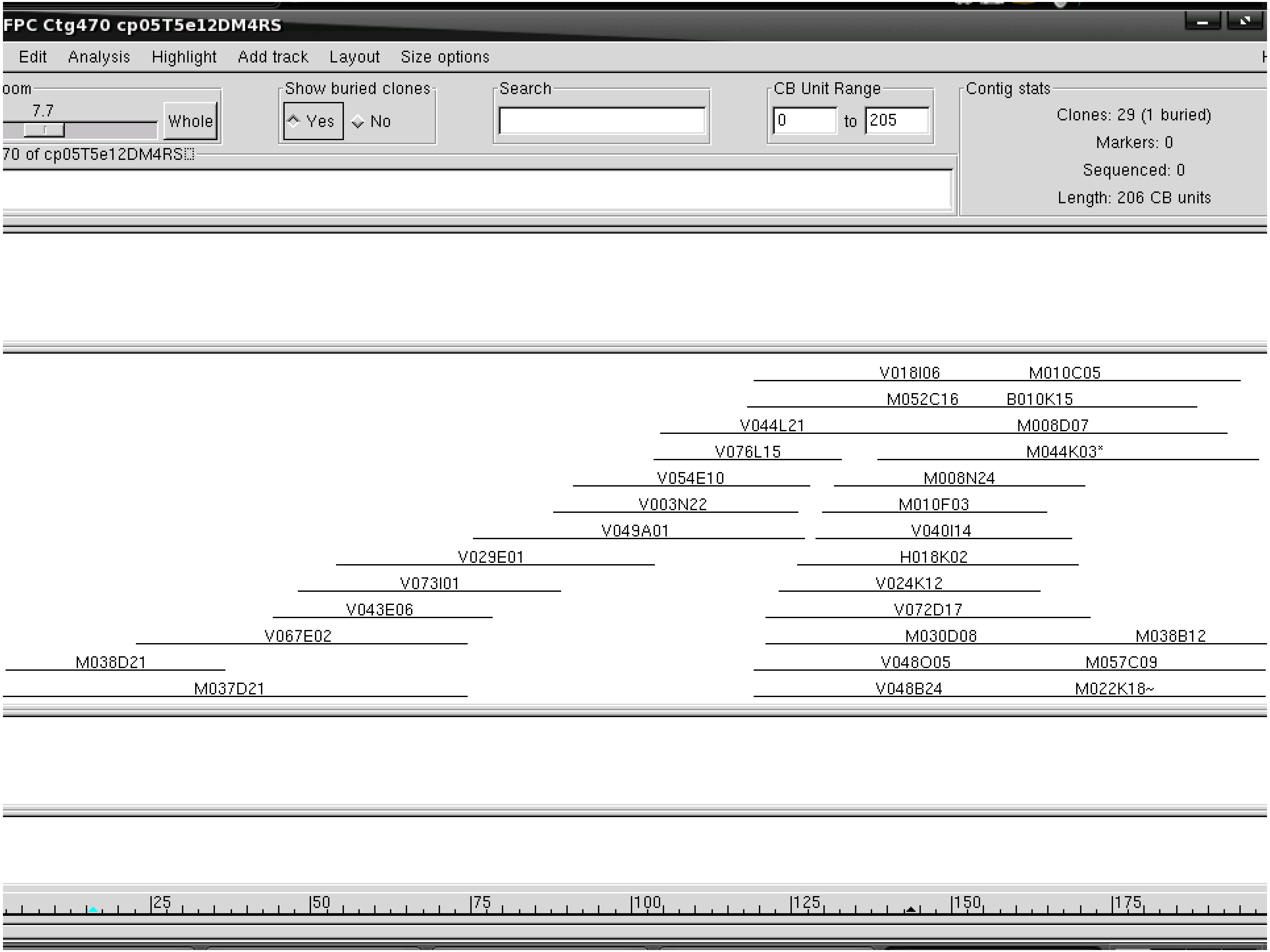
FPC Ctg470 cp05T5e12DM4RS
Edit Analysis Highlight Add track Layout Size options
oom
7.7
Whole
Show buried clones
Yes
No
Search
CB Unit Range
0
to
205
Contig stats
Clones: 29 (1 buried)
Markers: 0
Sequenced: 0
Length: 206 CB units
70 of cp05T5e12DM4RS
V018I06
M010C05
M052C16
B010K15
V044L21
M008D07
V076L15
M044K03*
V054E10
M008N24
V003N22
M010F03
V049A01
V040I14
V029E01
H018K02
V073I01
V024K12
V043E06
V072D17
V067E02
M030D08
M038B12
M038D21
V048O05
M057C09
M037D21
V048B24
M022K18~
25
50
75
100
125
150
175

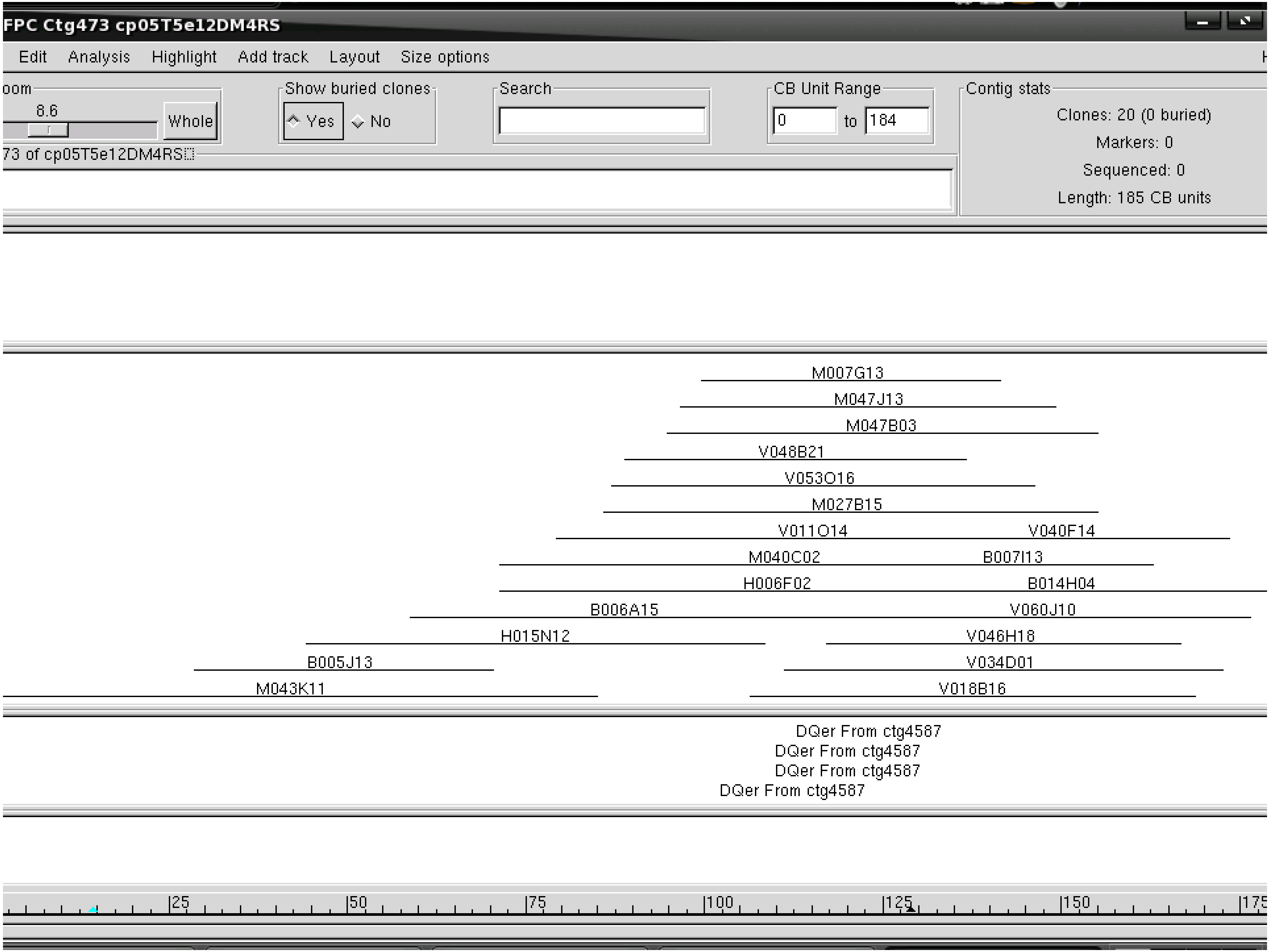
FPC Ctg473 cp05T5e12DM4RS
Edit Analysis Highlight Add track Layout Size options
oom
8.6
Whole
Show buried clones
Yes
No
Search
CB Unit Range
0
to
184
Contig stats
Clones: 20 (0 buried)
Markers: 0
Sequenced: 0
Length: 185 CB units
73 of cp05T5e12DM4RS
M007G13
M047J13
M047B03
V048B21
V053O16
M027B15
V011O14
V040F14
M040C02
B007I13
H006F02
B014H04
B006A15
V060J10
H015N12
V046H18
B005J13
V034D01
M043K11
V018B16
DQer From ctg4587
DQer From ctg4587
DQer From ctg4587
DQer From ctg4587
25
50
75
100
125
150
175

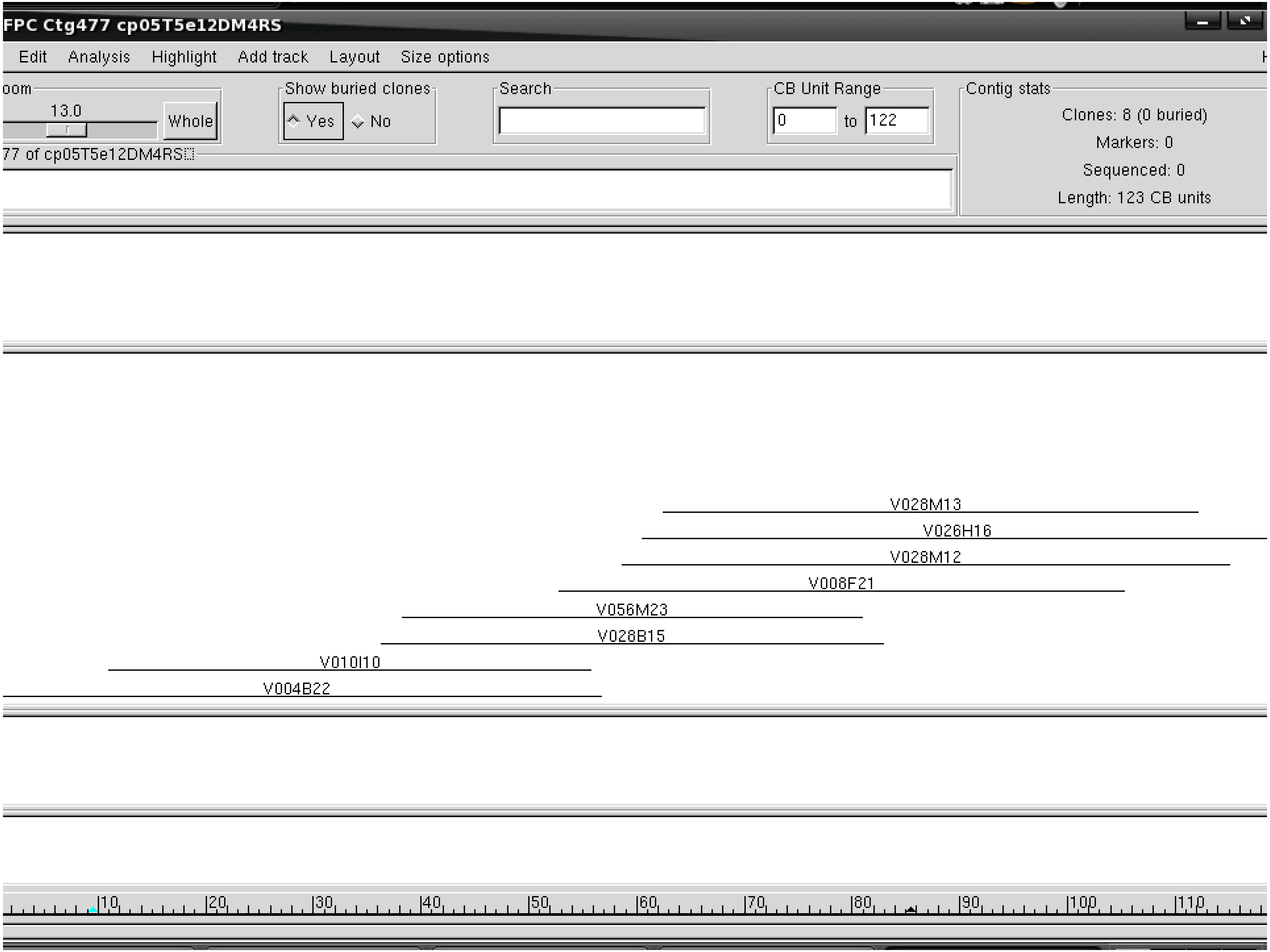
FPC Ctg477 cp05T5e12DM4RS
Edit
Analysis
Highlight
Add track
Layout
Size options
oom
13.0
Whole
Show buried clones
Yes
No
Search
CB Unit Range
0
to
122
Contig stats
Clones: 8 (0 buried)
Markers: 0
Sequenced: 0
Length: 123 CB units
77 of cp05T5e12DM4RS
V028M13
V026H16
V028M12
V008F21
V056M23
V028B15
V010I10
V004B22
10
20
30
40
50
60
70
80
90
100
110

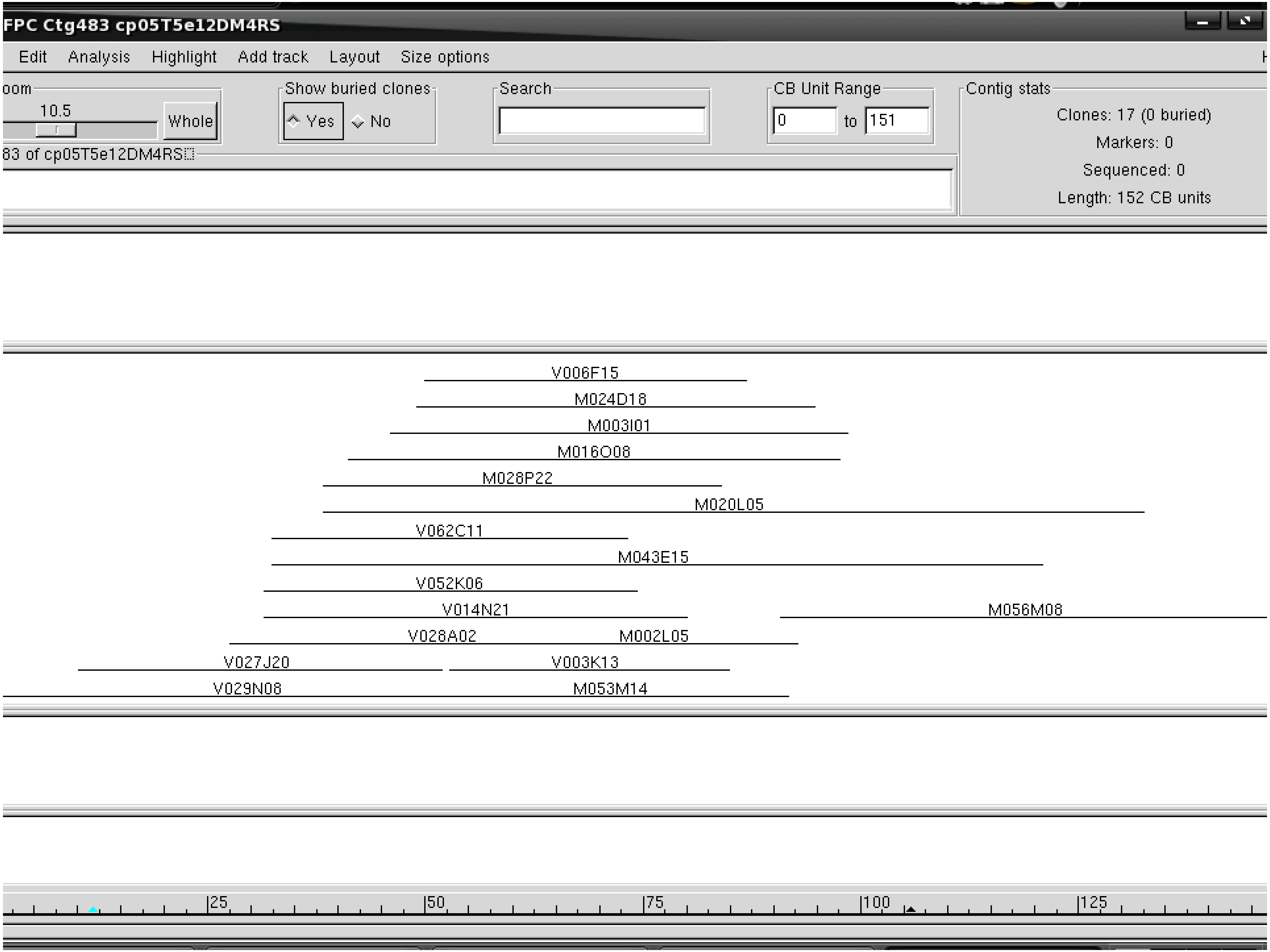
FPC Ctg483 cp05T5e12DM4RS
Edit Analysis Highlight Add track Layout Size options
oom
10.5
Whole
Show buried clones
Yes
No
Search
CB Unit Range
0
to
151
Contig stats
Clones: 17 (0 buried)
Markers: 0
Sequenced: 0
Length: 152 CB units
83 of cp05T5e12DM4RS
V006F15
M024D18
M003I01
M016O08
M028P22
M020L05
V062C11
M043E15
V052K06
V014N21
M056M08
V028A02
M002L05
V027J20
V003K13
V029N08
M053M14
25
50
75
100
125

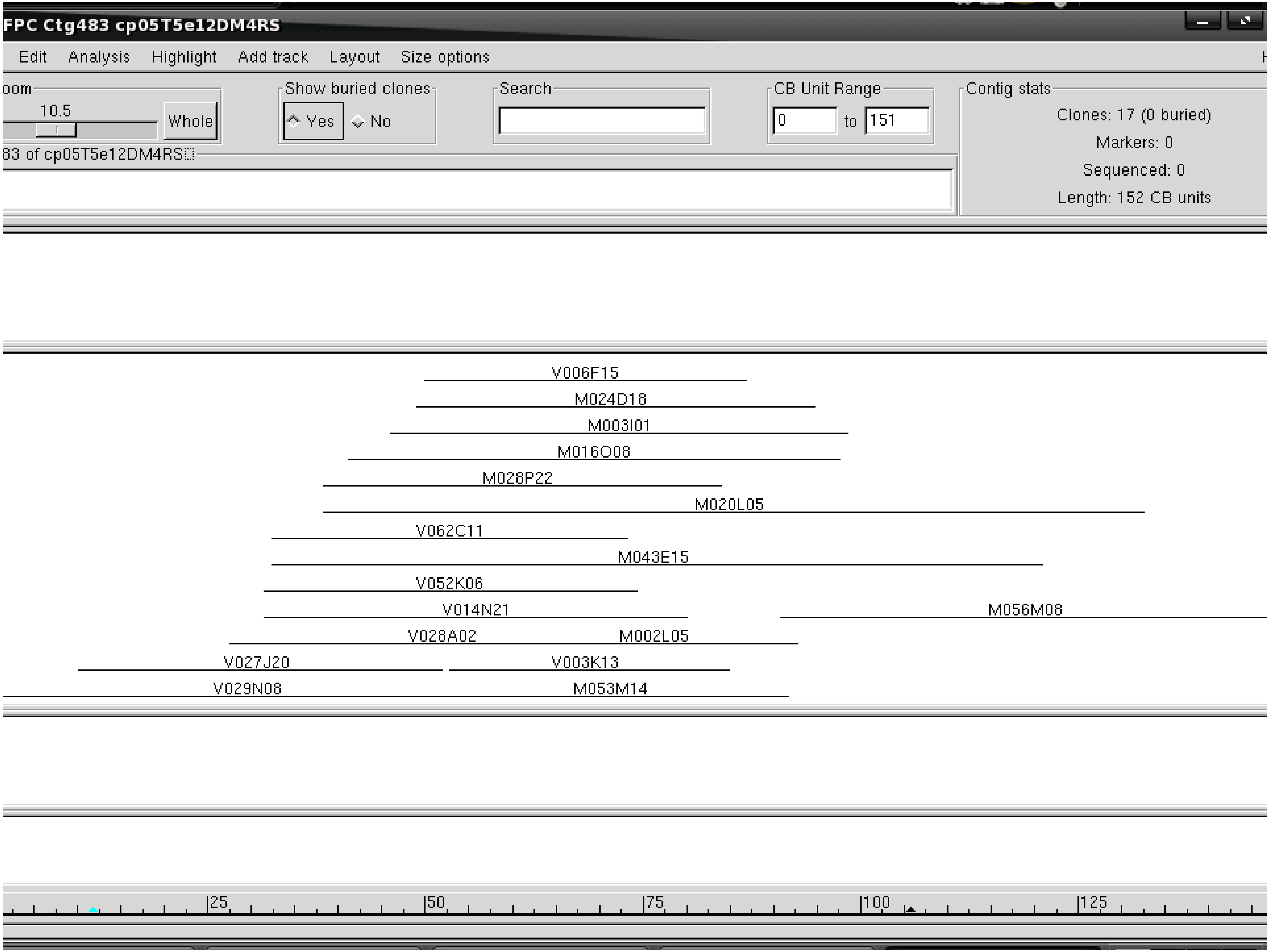
FPC Ctg483 cp05T5e12DM4RS
Edit Analysis Highlight Add track Layout Size options
oom
10.5
Whole
Show buried clones
Yes
No
Search
CB Unit Range
0
to
151
Contig stats
Clones: 17 (0 buried)
Markers: 0
Sequenced: 0
Length: 152 CB units
83 of cp05T5e12DM4RS
V006F15
M024D18
M003I01
M016O08
M028P22
M020L05
V062C11
M043E15
V052K06
V014N21
M056M08
V028A02
M002L05
V027J20
V003K13
V029N08
M053M14
25
50
75
100
125

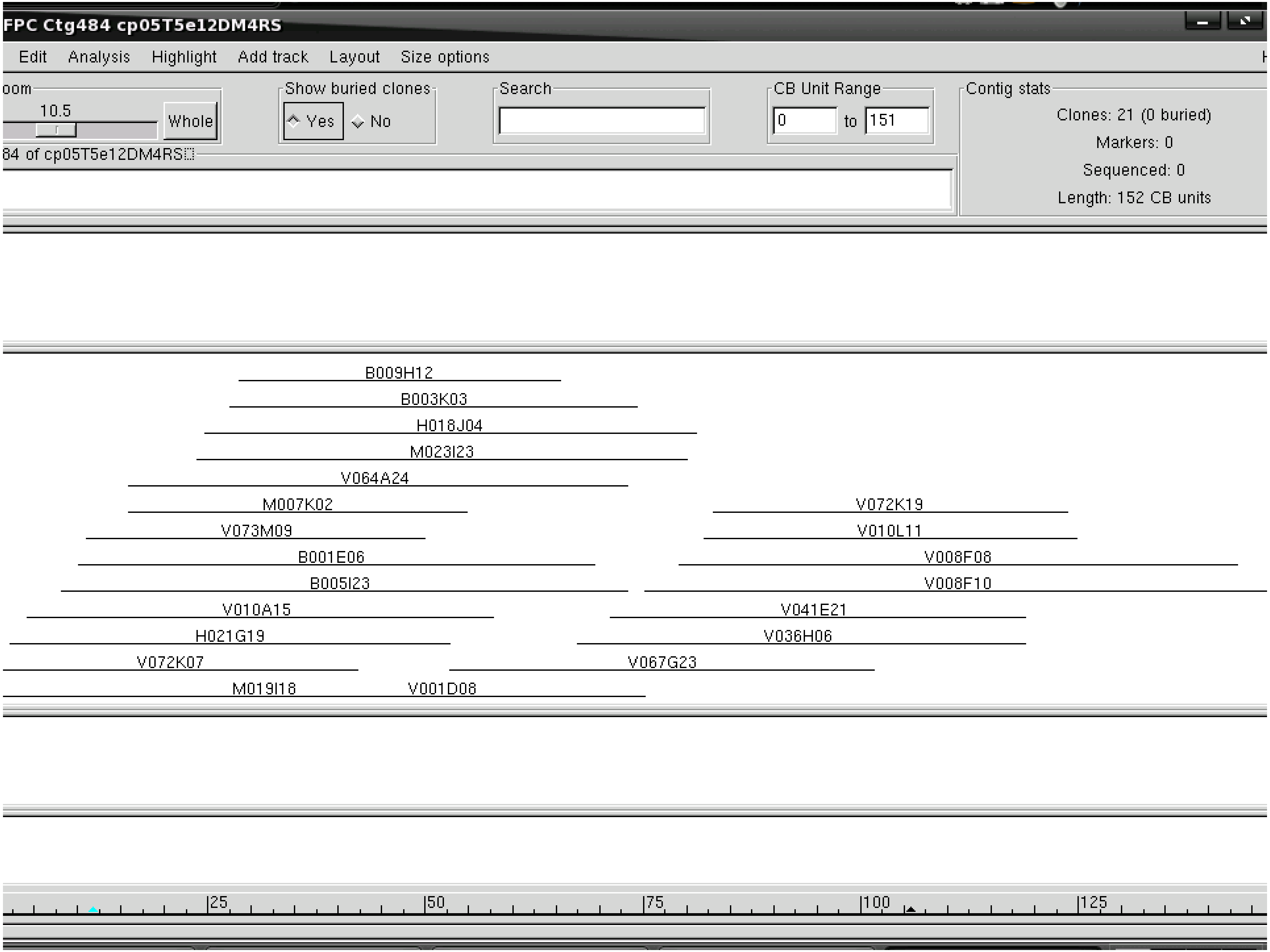
FPC Ctg484 cp05T5e12DM4RS
Edit
Analysis
Highlight
Add track
Layout
Size options
oom
10.5
Whole
Show buried clones
Yes
No
Search
CB Unit Range
0
to
151
Contig stats
Clones: 21 (0 buried)
Markers: 0
Sequenced: 0
Length: 152 CB units
84 of cp05T5e12DM4RS
B009H12
B003K03
H018J04
M023I23
V064A24
M007K02
V072K19
V073M09
V010L11
B001E06
V008F08
B005I23
V008F10
V010A15
V041E21
H021G19
V036H06
V072K07
V067G23
M019I18
V001D08
25
50
75
100
125

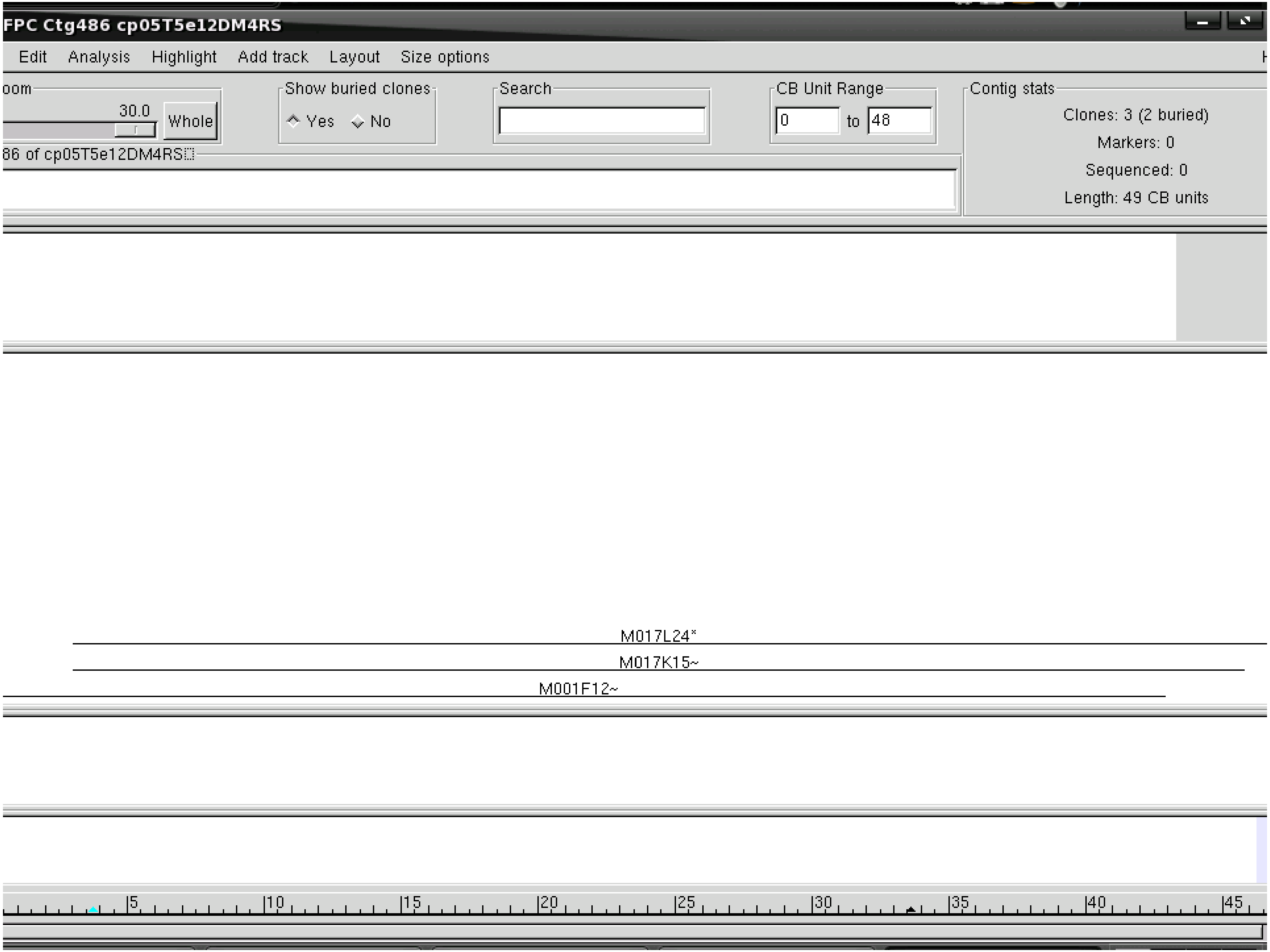
FPC Ctg486 cp05T5e12DM4RS
Edit Analysis Highlight Add track Layout Size options
oom
30.0
Whole
Show buried clones
Yes No
Search
CB Unit Range
0 to 48
Contig stats
Clones: 3 (2 buried)
Markers: 0
Sequenced: 0
Length: 49 CB units
86 of cp05T5e12DM4RS
M017L24*
M017K15~
M001F12~
5 10 15 20 25 30 35 40 45

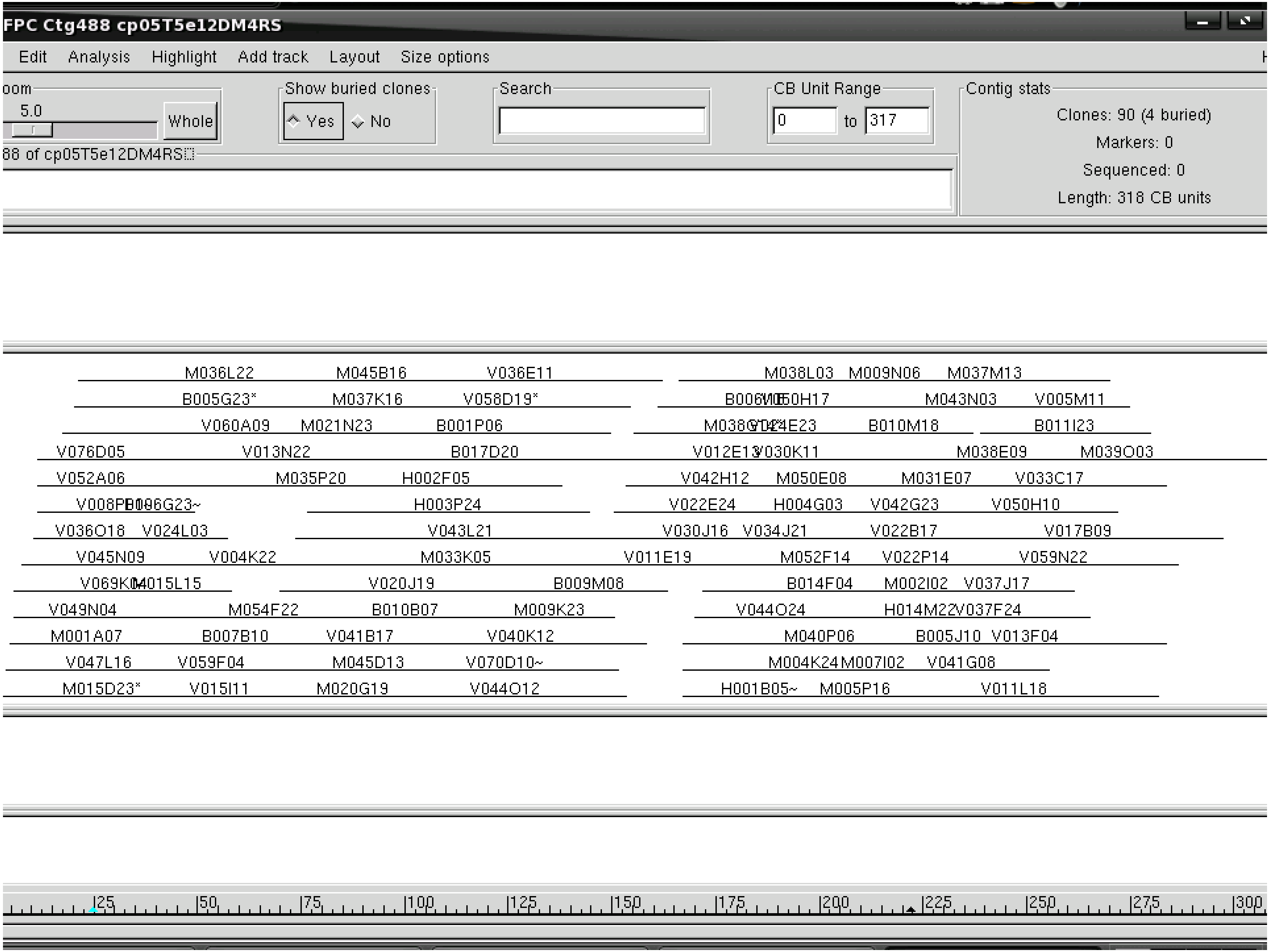
FPC Ctg488 cp05T5e12DM4RS
Edit Analysis Highlight Add track Layout Size options
Show buried clones
Yes No
Search
CB Unit Range
0 to 317
Contig stats
Clones: 90 (4 buried)
Markers: 0
Sequenced: 0
Length: 318 CB units
5.0
Whole
88 of cp05T5e12DM4RS
M036L22 M045B16 V036E11 M038L03 M009N06 M037M13
B005G23* M037K16 V058D19* M043N03 V005M11
V060A09 M021N23 B001P06 B010M18 B011I23
V076D05 V013N22 B017D20 M038E09 M039O03
V052A06 M035P20 H002F05 V042H12 M050E08 M031E07 V033C17
H003P24 V022E24 H004G03 V042G23 V050H10
V036O18 V024L03 V043L21 V030J16 V034J21 V022B17 V017B09
V045N09 V004K22 M033K05 V011E19 M052F14 V022P14 V059N22
V020J19 B009M08 B014F04 M002I02 V037J17
V049N04 M054F22 B010B07 M009K23 V044O24
M001A07 B007B10 V041B17 V040K12 M040P06 B005J10 V013F04
V047L16 V059F04 M045D13 V070D10~ V041G08
M015D23* V015I11 M020G19 V044O12 H001B05~ M005P16 V011L18
25 50 75 100 125 150 175 200 225 250 275 300

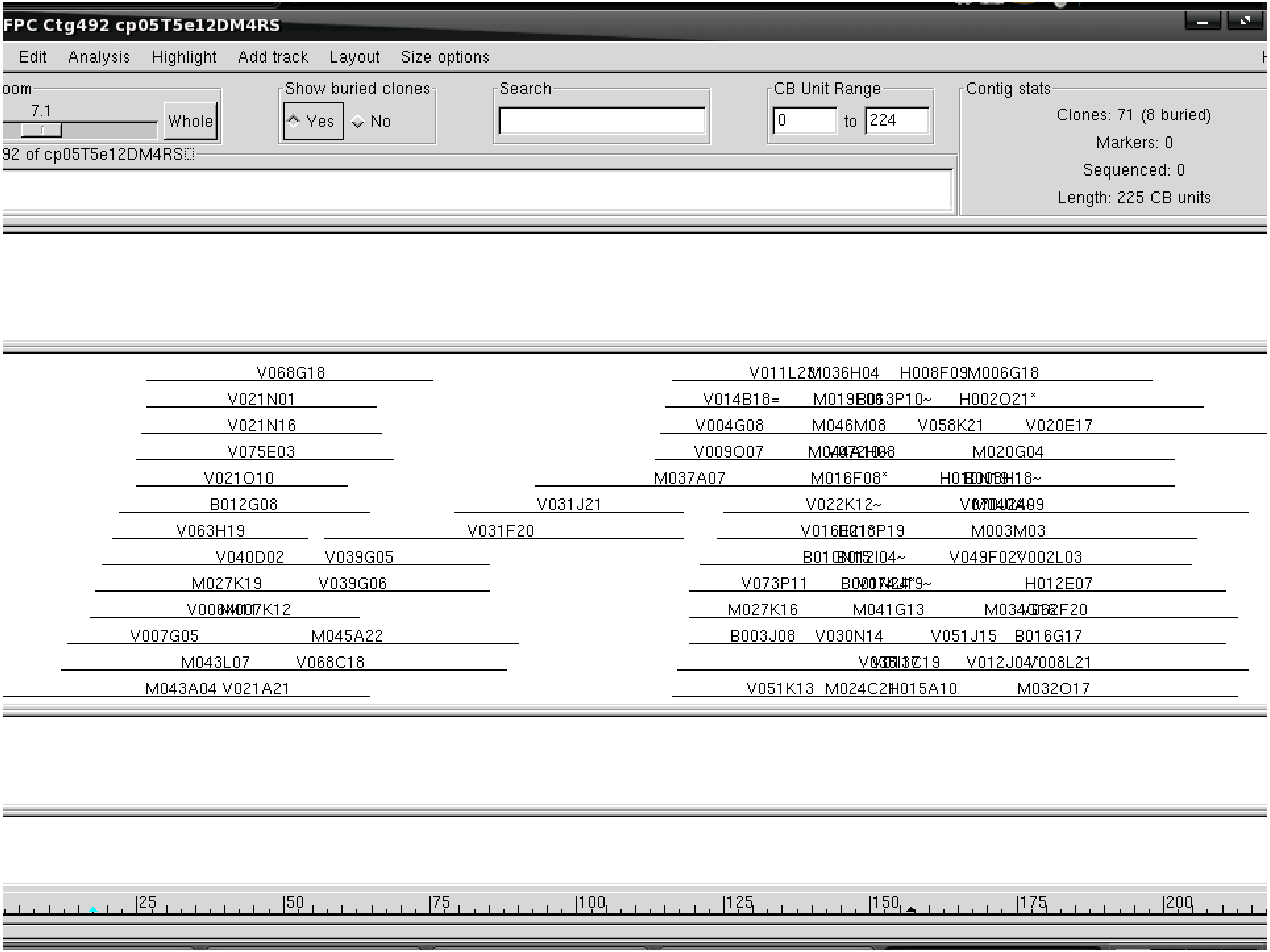
FPC Ctg492 cp05T5e12DM4RS
Edit
Analysis
Highlight
Add track
Layout
Size options
Show buried clones
Yes
No
Search
CB Unit Range
0
to
224
Contig stats
Clones: 71 (8 buried)
Markers: 0
Sequenced: 0
Length: 225 CB units
7.1
Whole
V068G18
V021N01
V021N16
V075E03
V021O10
B012G08
V063H19
V040D02
V039G05
M027K19
V039G06
V007G05
M045A22
M043L07
V068C18
M043A04
V021A21
V031J21
V031F20
M037A07
M036H04
H008F09
M006G18
V014B18=
H002O21*
V004G08
M046M08
V058K21
V020E17
V009O07
M020G04
M016F08*
V022K12~
M003M03
V049F02
V002L03
V073P11
H012E07
M027K16
M041G13
B003J08
V030N14
V051J15
B016G17
V012J04
V051K13
H015A10
M032O17
25
50
75
100
125
150
175
200

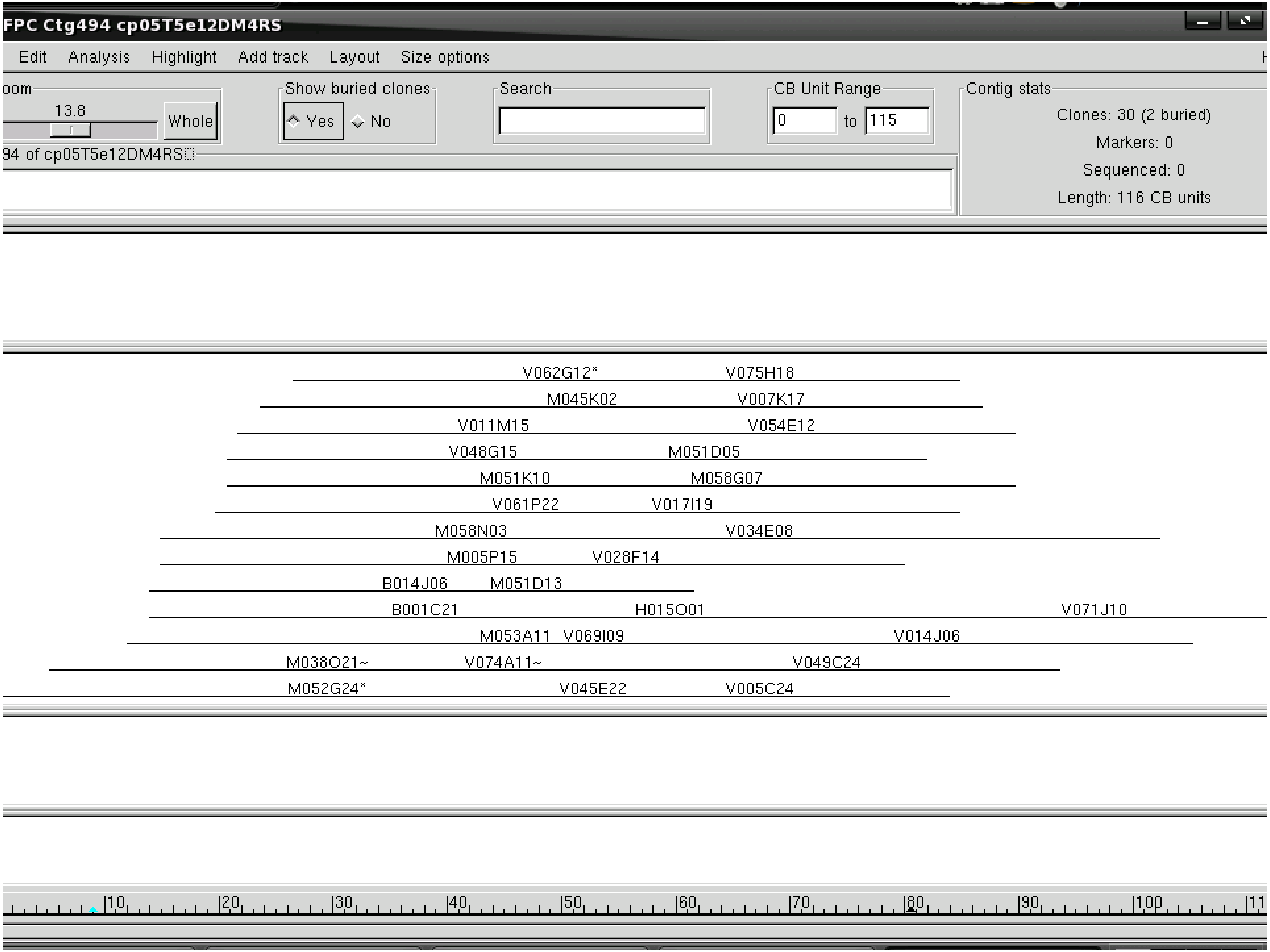
FPC Ctg494 cp05T5e12DM4RS
Edit
Analysis
Highlight
Add track
Layout
Size options
oom
13.8
Whole
Show buried clones
Yes
No
Search
CB Unit Range
0
to
115
Contig stats
Clones: 30 (2 buried)
Markers: 0
Sequenced: 0
Length: 116 CB units
94 of cp05T5e12DM4RS
V062G12*
V075H18
M045K02
V007K17
V011M15
V054E12
V048G15
M051D05
M051K10
M058G07
V061P22
V017I19
M058N03
V034E08
M005P15
V028F14
B014J06
M051D13
B001C21
H015O01
V071J10
M053A11
V069I09
V014J06
M038O21~
V074A11~
V049C24
M052G24*
V045E22
V005C24
10
20
30
40
50
60
70
80
90
100

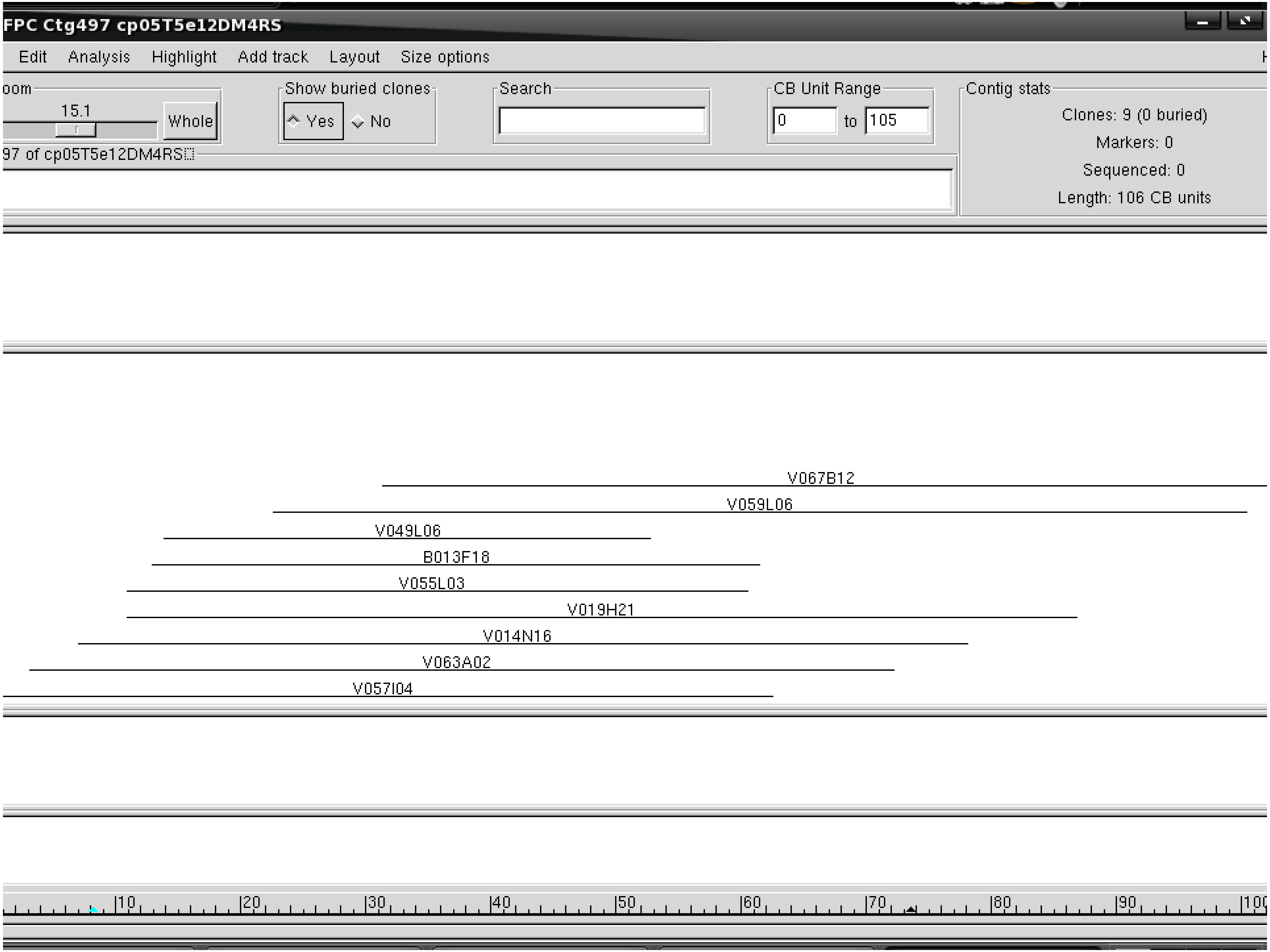
FPC Ctg497 cp05T5e12DM4RS
Edit Analysis Highlight Add track Layout Size options
oom
15.1
Whole
Show buried clones
Yes
No
Search
CB Unit Range
0
to
105
Contig stats
Clones: 9 (0 buried)
Markers: 0
Sequenced: 0
Length: 106 CB units
97 of cp05T5e12DM4RS
V067B12
V059L06
V049L06
B013F18
V055L03
V019H21
V014N16
V063A02
V057I04
10
20
30
40
50
60
70
80
90

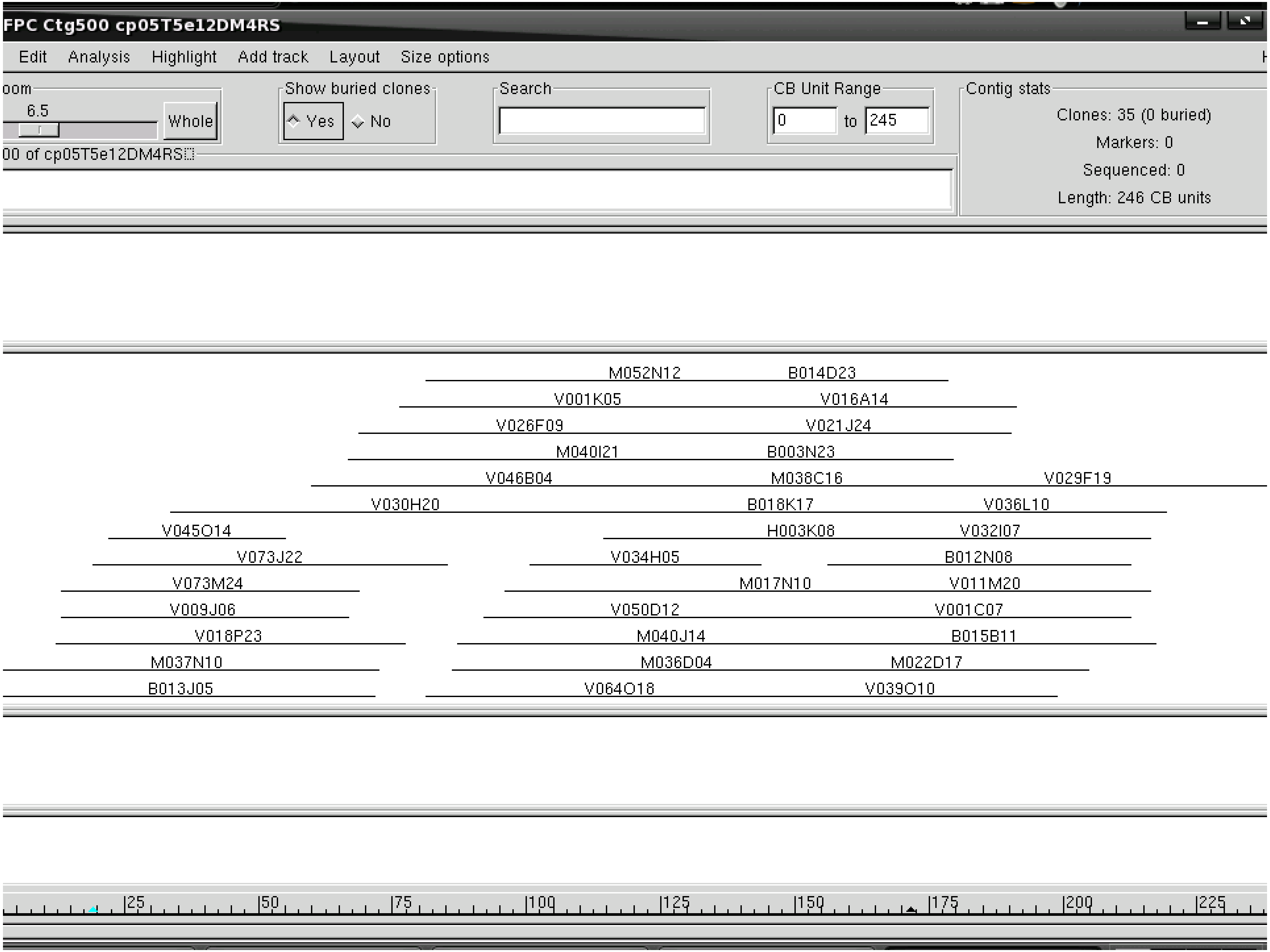
FPC Ctg500 cp05T5e12DM4RS
Edit
Analysis
Highlight
Add track
Layout
Size options
oom
6.5
Whole
Show buried clones
Yes
No
Search
CB Unit Range
0
to
245
Contig stats
Clones: 35 (0 buried)
Markers: 0
Sequenced: 0
Length: 246 CB units
00 of cp05T5e12DM4RS
M052N12
B014D23
V001K05
V016A14
V026F09
V021J24
M040I21
B003N23
V046B04
M038C16
V029F19
V030H20
B018K17
V036L10
V045O14
H003K08
V032I07
V073J22
V034H05
B012N08
V073M24
M017N10
V011M20
V009J06
V050D12
V001C07
V018P23
M040J14
B015B11
M037N10
M036D04
M022D17
B013J05
V064O18
V039O10
25
50
75
100
125
150
175
200
225

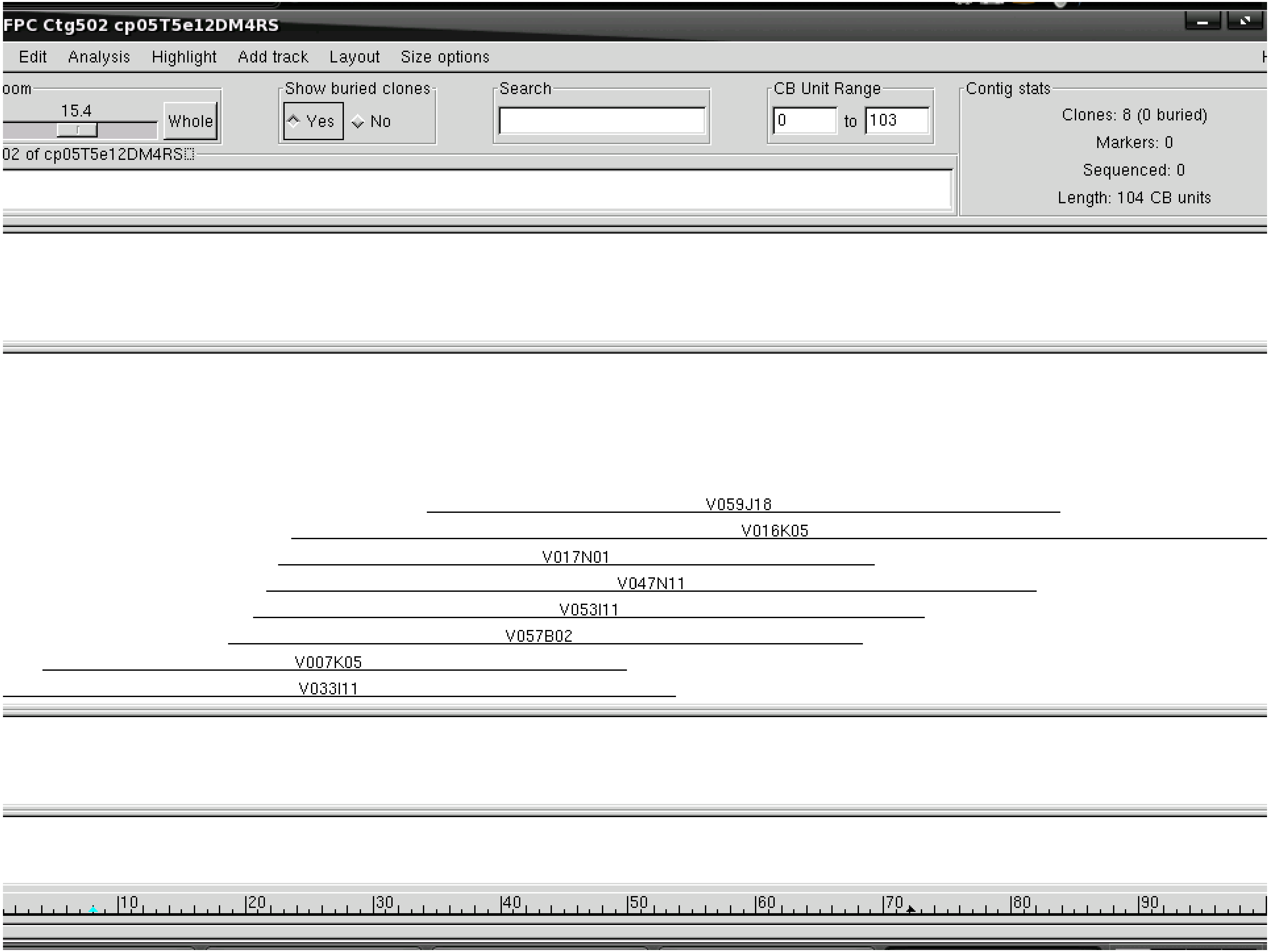
FPC Ctg502 cp05T5e12DM4RS
Edit Analysis Highlight Add track Layout Size options
oom
15.4
Whole
Show buried clones
Yes
No
Search
CB Unit Range
0
to
103
Contig stats
Clones: 8 (0 buried)
Markers: 0
Sequenced: 0
Length: 104 CB units
02 of cp05T5e12DM4RS
V059J18
V016K05
V017N01
V047N11
V053I11
V057B02
V007K05
V033I11
10
20
30
40
50
60
70
80
90

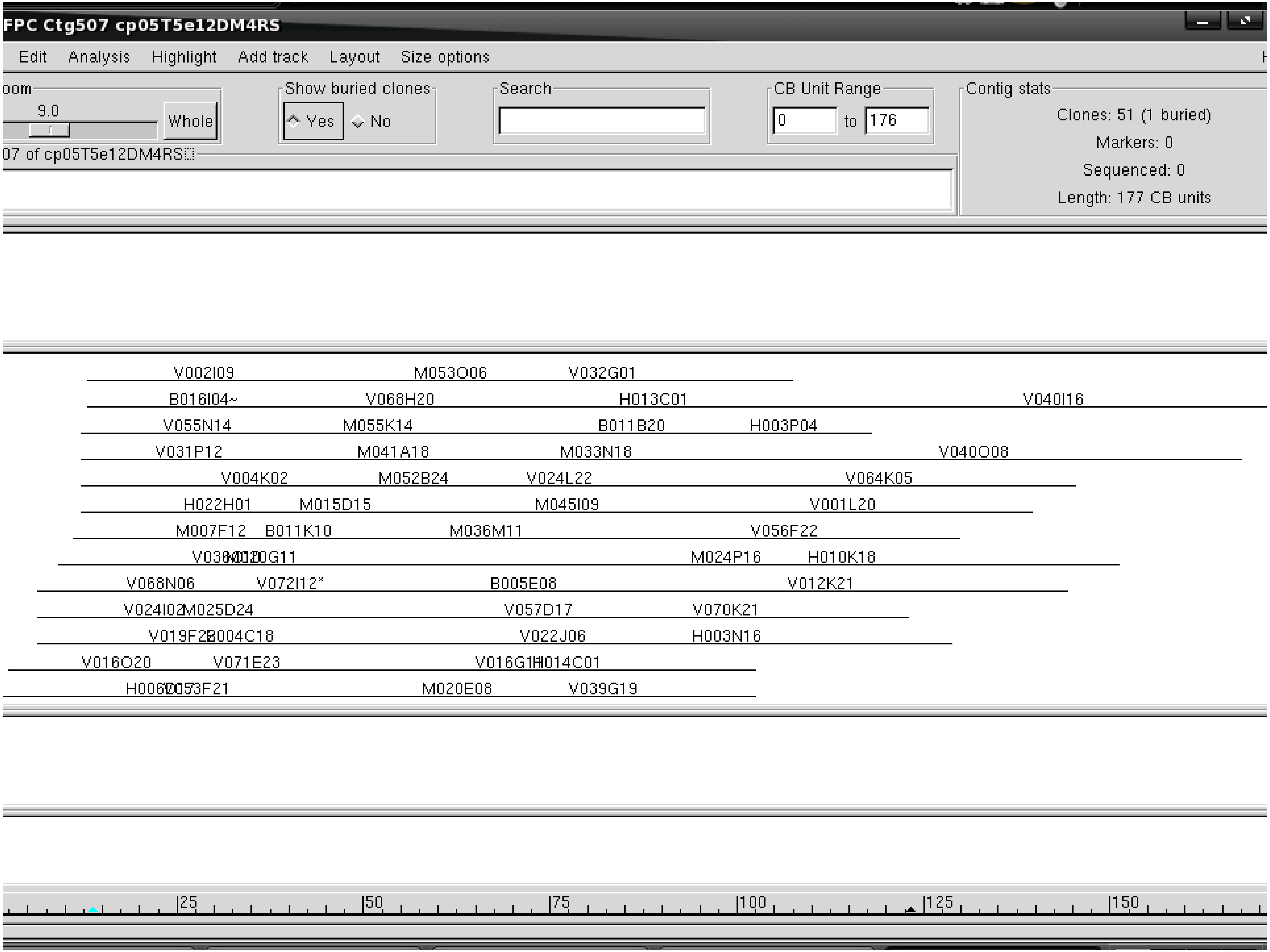

FPC Ctg507 cp05T5e12DM4RS
Edit Analysis Highlight Add track Layout Size options
oom 9.0 Whole
Show buried clones Yes No
Search
CB Unit Range 0 to 176
Contig stats
Clones: 51 (1 buried)
Markers: 0
Sequenced: 0
Length: 177 CB units
07 of cp05T5e12DM4RS
V002I09 M053O06 V032G01
B016I04~ V068H20 H013C01 V040I16
V055N14 M055K14 B011B20 H003P04
V031P12 M041A18 M033N18 V040O08
V004K02 M052B24 V024L22 V064K05
H022H01 M015D15 M045I09 V001L20
M007F12 B011K10 M036M11 V056F22
M024P16 H010K18
V068N06 V072I12* B005E08 V012K21
M025D24 V057D17 V070K21
B004C18 V022J06 H003N16
V016O20 V071E23
M020E08 V039G19
25 50 75 100 125 150

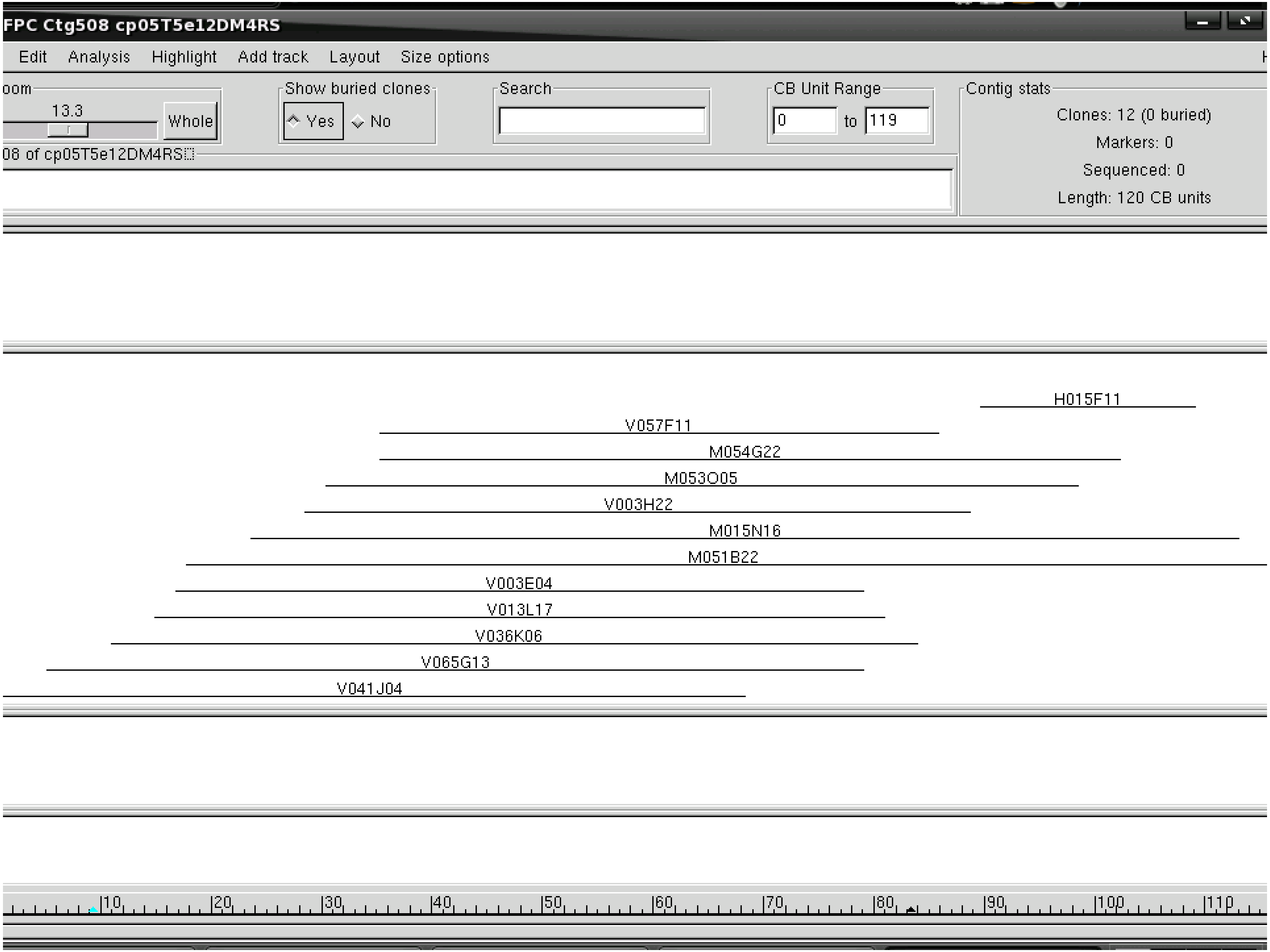
FPC Ctg508 cp05T5e12DM4RS
Edit
Analysis
Highlight
Add track
Layout
Size options
oom
13.3
Whole
Show buried clones
Yes
No
Search
CB Unit Range
0
to
119
Contig stats
Clones: 12 (0 buried)
Markers: 0
Sequenced: 0
Length: 120 CB units
08 of cp05T5e12DM4RS
H015F11
V057F11
M054G22
M053O05
V003H22
M015N16
M051B22
V003E04
V013L17
V036K06
V065G13
V041J04
10
20
30
40
50
60
70
80
90
100
110

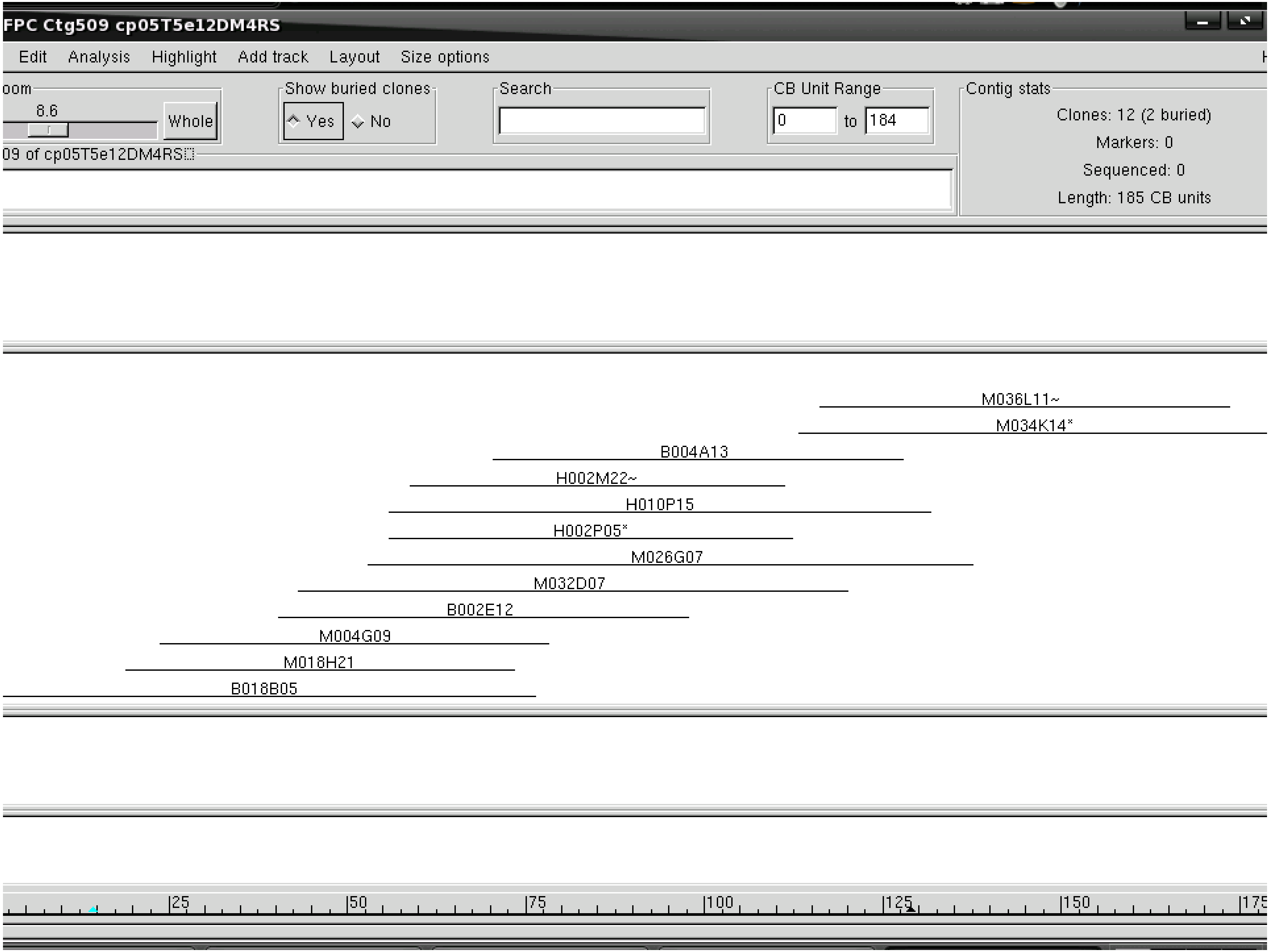
FPC Ctg509 cp05T5e12DM4RS
Edit
Analysis
Highlight
Add track
Layout
Size options
oom
8.6
Whole
Show buried clones
Yes
No
Search
CB Unit Range
0
to
184
Contig stats
Clones: 12 (2 buried)
Markers: 0
Sequenced: 0
Length: 185 CB units
09 of cp05T5e12DM4RS
M036L11~
M034K14*
B004A13
H002M22~
H010P15
H002P05*
M026G07
M032D07
B002E12
M004G09
M018H21
B018B05
25
50
75
100
125
150
175

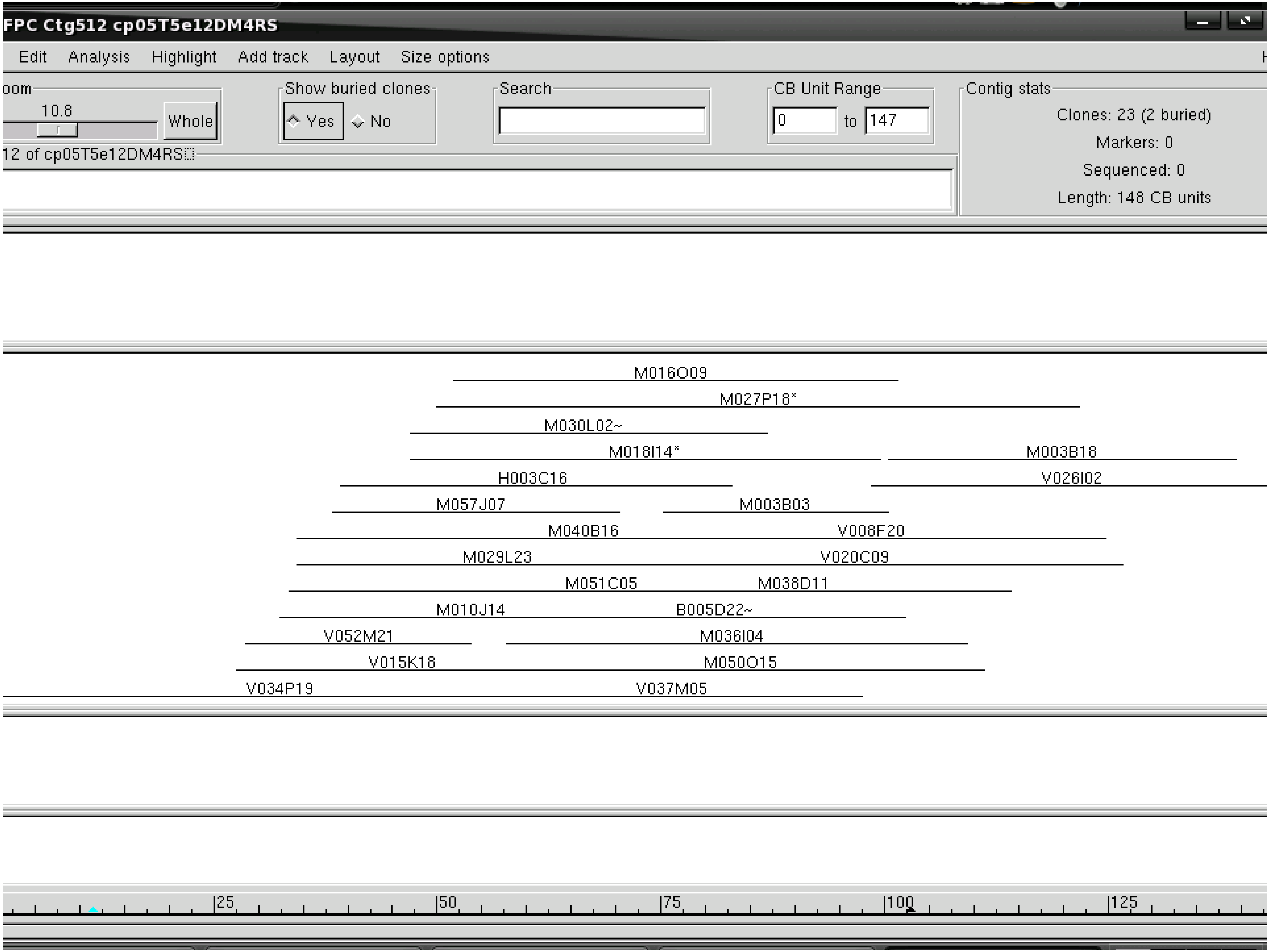

FPC Ctg512 cp05T5e12DM4RS
Edit Analysis Highlight Add track Layout Size options
oom
10.8
Whole
Show buried clones
Yes
No
Search
CB Unit Range
0
to
147
Contig stats
Clones: 23 (2 buried)
Markers: 0
Sequenced: 0
Length: 148 CB units
12 of cp05T5e12DM4RS
M016O09
M027P18*
M030L02~
M018I14*
M003B18
H003C16
V026I02
M057J07
M003B03
M040B16
V008F20
M029L23
V020C09
M051C05
M038D11
M010J14
B005D22~
V052M21
M036I04
V015K18
M050O15
V034P19
V037M05
25
50
75
100
125

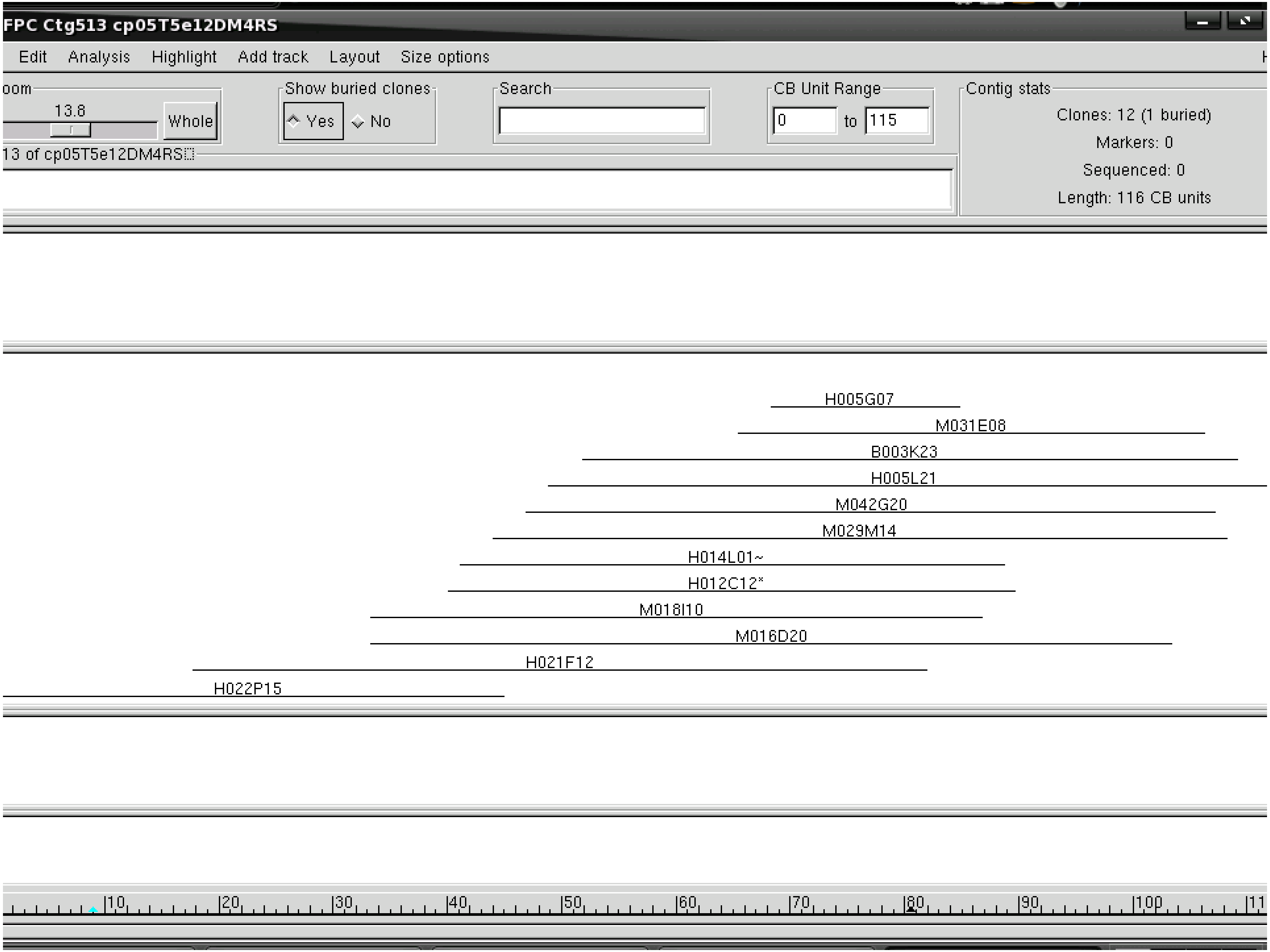
FPC Ctg513 cp05T5e12DM4RS
Edit Analysis Highlight Add track Layout Size options
oom
13.8
Whole
Show buried clones
Yes
No
Search
CB Unit Range
0
to
115
Contig stats
Clones: 12 (1 buried)
Markers: 0
Sequenced: 0
Length: 116 CB units
13 of cp05T5e12DM4RS
H005G07
M031E08
B003K23
H005L21
M042G20
M029M14
H014L01~
H012C12*
M018I10
M016D20
H021F12
H022P15
10
20
30
40
50
60
70
80
90
100

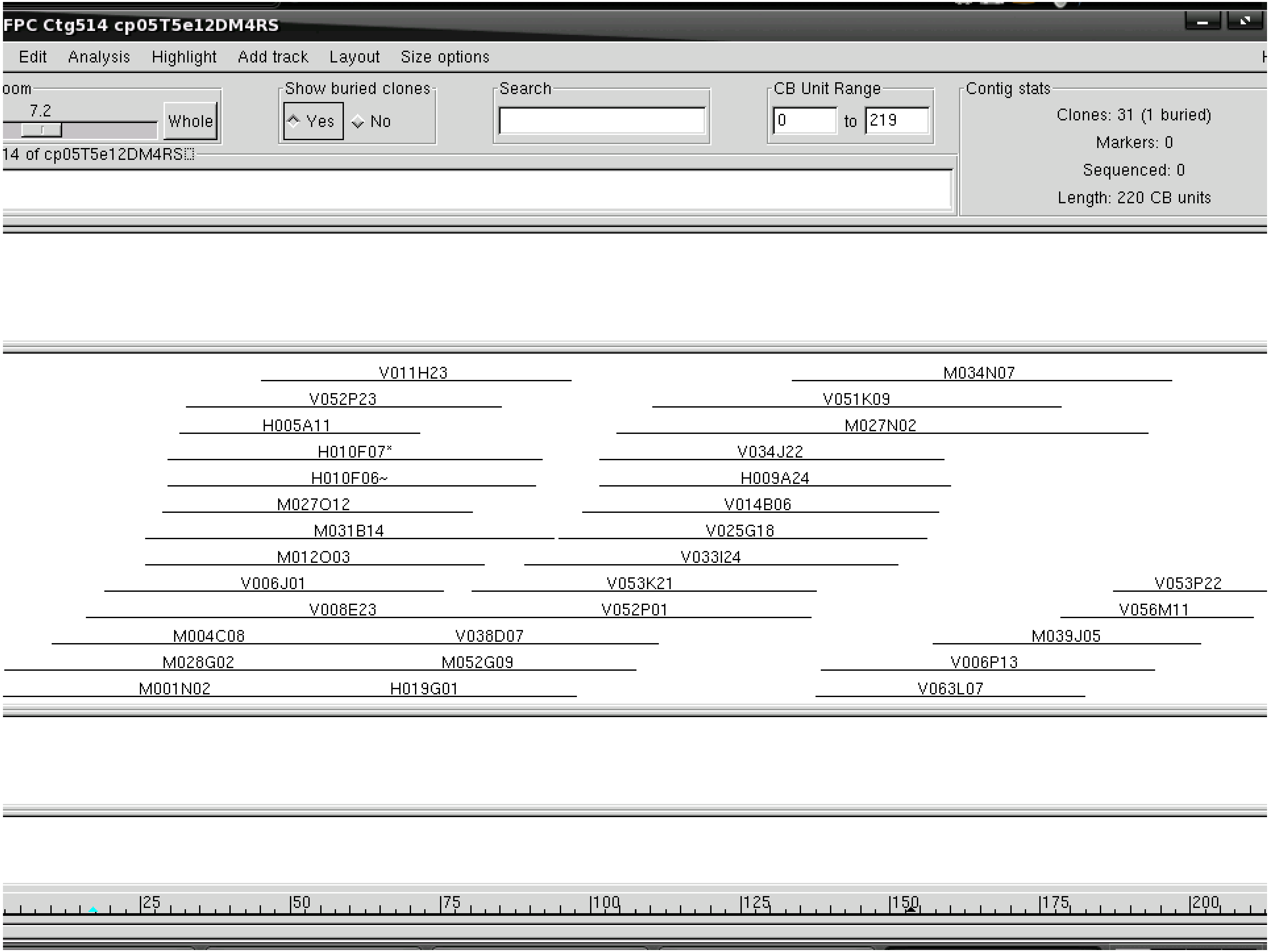
FPC Ctg514 cp05T5e12DM4RS
Edit
Analysis
Highlight
Add track
Layout
Size options
oom
7.2
Whole
Show buried clones
Yes
No
Search
CB Unit Range
0
to
219
Contig stats
Clones: 31 (1 buried)
Markers: 0
Sequenced: 0
Length: 220 CB units
14 of cp05T5e12DM4RS
V011H23
M034N07
V052P23
V051K09
H005A11
M027N02
H010F07*
V034J22
H010F06~
H009A24
M027O12
V014B06
M031B14
V025G18
M012O03
V033I24
V006J01
V053K21
V053P22
V008E23
V052P01
V056M11
M004C08
V038D07
M039J05
M028G02
M052G09
V006P13
M001N02
H019G01
V063L07
25
50
75
100
125
150
175
200

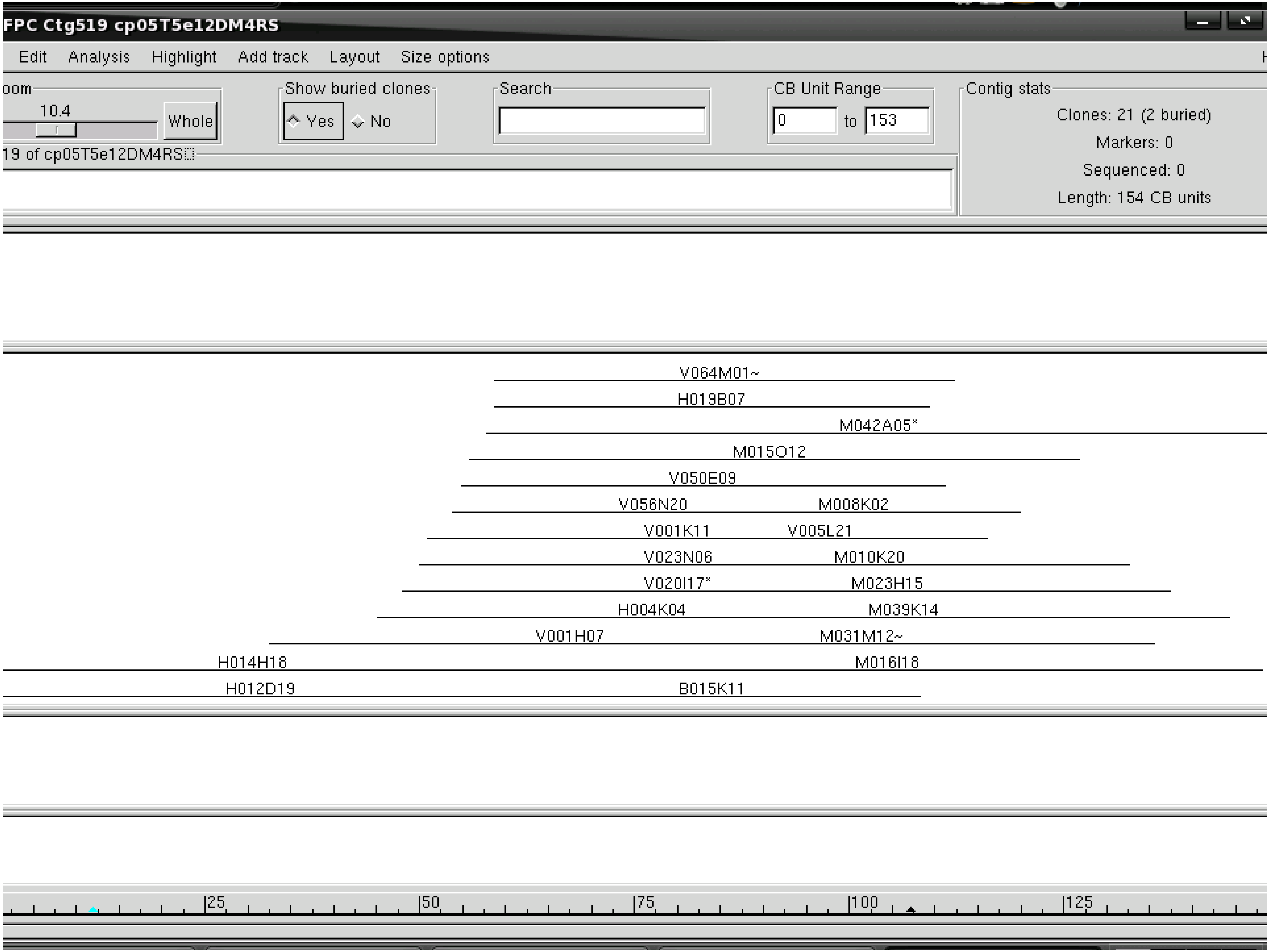
FPC Ctg519 cp05T5e12DM4RS
Edit Analysis Highlight Add track Layout Size options
oom
10.4
Whole
Show buried clones
Yes
No
Search
CB Unit Range
0 to 153
Contig stats
Clones: 21 (2 buried)
Markers: 0
Sequenced: 0
Length: 154 CB units
19 of cp05T5e12DM4RS
V064M01~
H019B07
M042A05*
M015O12
V050E09
V056N20
M008K02
V001K11
V005L21
V023N06
M010K20
V020I17*
M023H15
H004K04
M039K14
V001H07
M031M12~
H014H18
M016I18
H012D19
B015K11
25
50
75
100
125

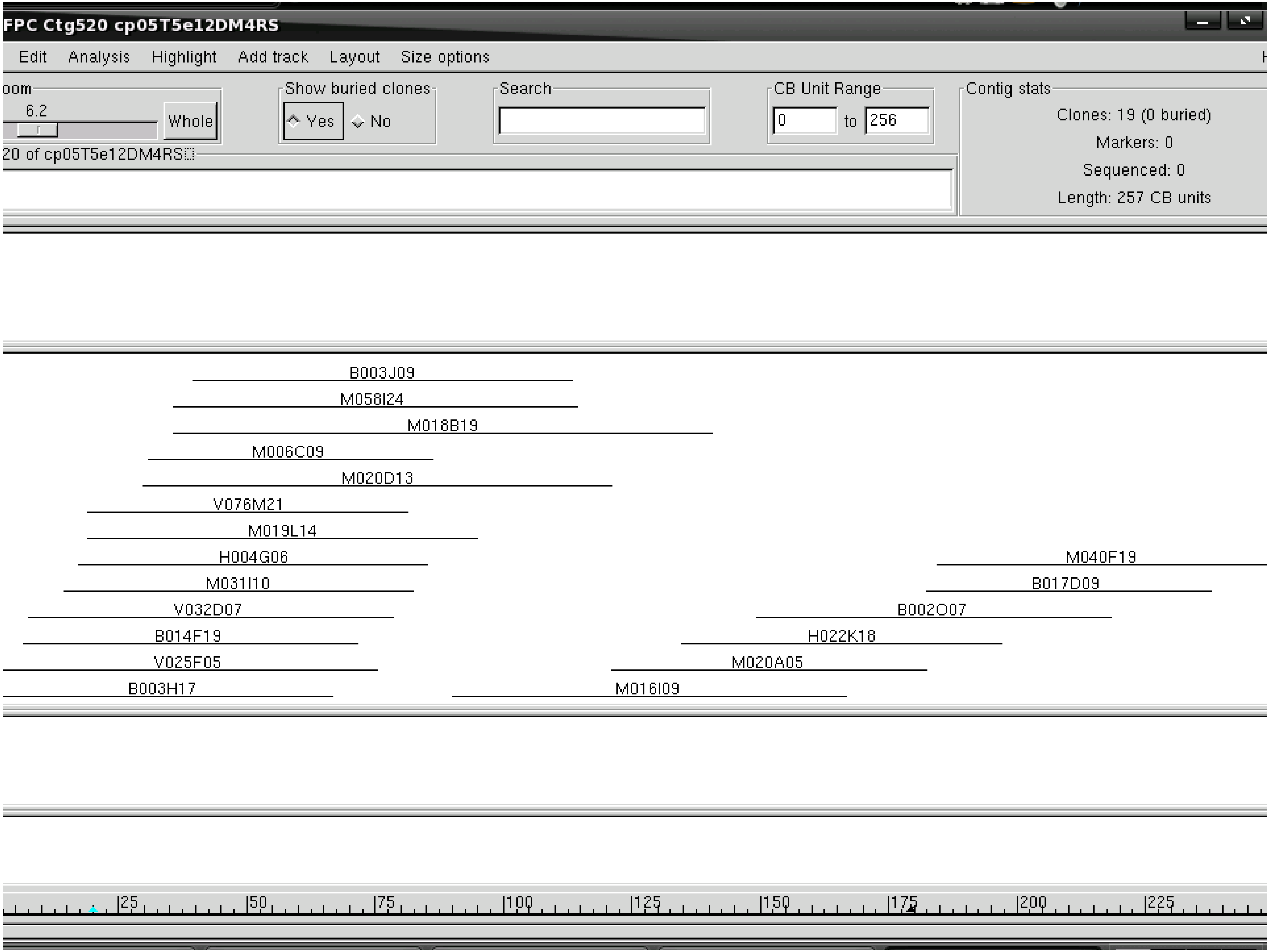
FPC Ctg520 cp05T5e12DM4RS
Edit
Analysis
Highlight
Add track
Layout
Size options
oom
6.2
Whole
Show buried clones
Yes
No
Search
CB Unit Range
0
to
256
Contig stats
Clones: 19 (0 buried)
Markers: 0
Sequenced: 0
Length: 257 CB units
20 of cp05T5e12DM4RS
B003J09
M058I24
M018B19
M006C09
M020D13
V076M21
M019L14
H004G06
M040F19
M031I10
B017D09
V032D07
B002O07
B014F19
H022K18
V025F05
M020A05
B003H17
M016I09
25
50
75
100
125
150
175
200
225

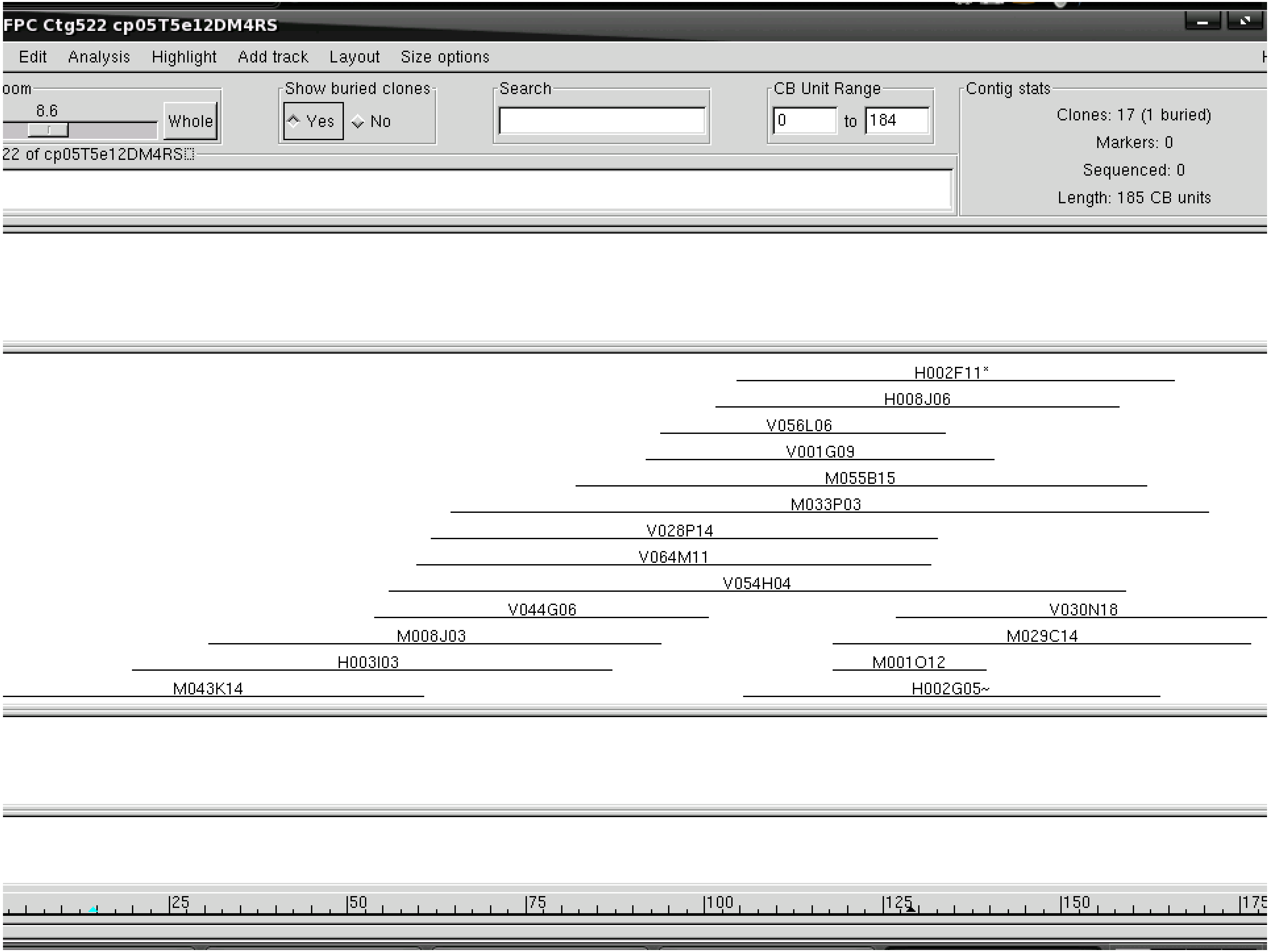
FPC Ctg522 cp05T5e12DM4RS
Edit
Analysis
Highlight
Add track
Layout
Size options
oom
8.6
Whole
Show buried clones
Yes
No
Search
CB Unit Range
0
to
184
Contig stats
Clones: 17 (1 buried)
Markers: 0
Sequenced: 0
Length: 185 CB units
22 of cp05T5e12DM4RS
H002F11*
H008J06
V056L06
V001G09
M055B15
M033P03
V028P14
V064M11
V054H04
V044G06
V030N18
M008J03
M029C14
H003I03
M001O12
M043K14
H002G05~
25
50
75
100
125
150
175

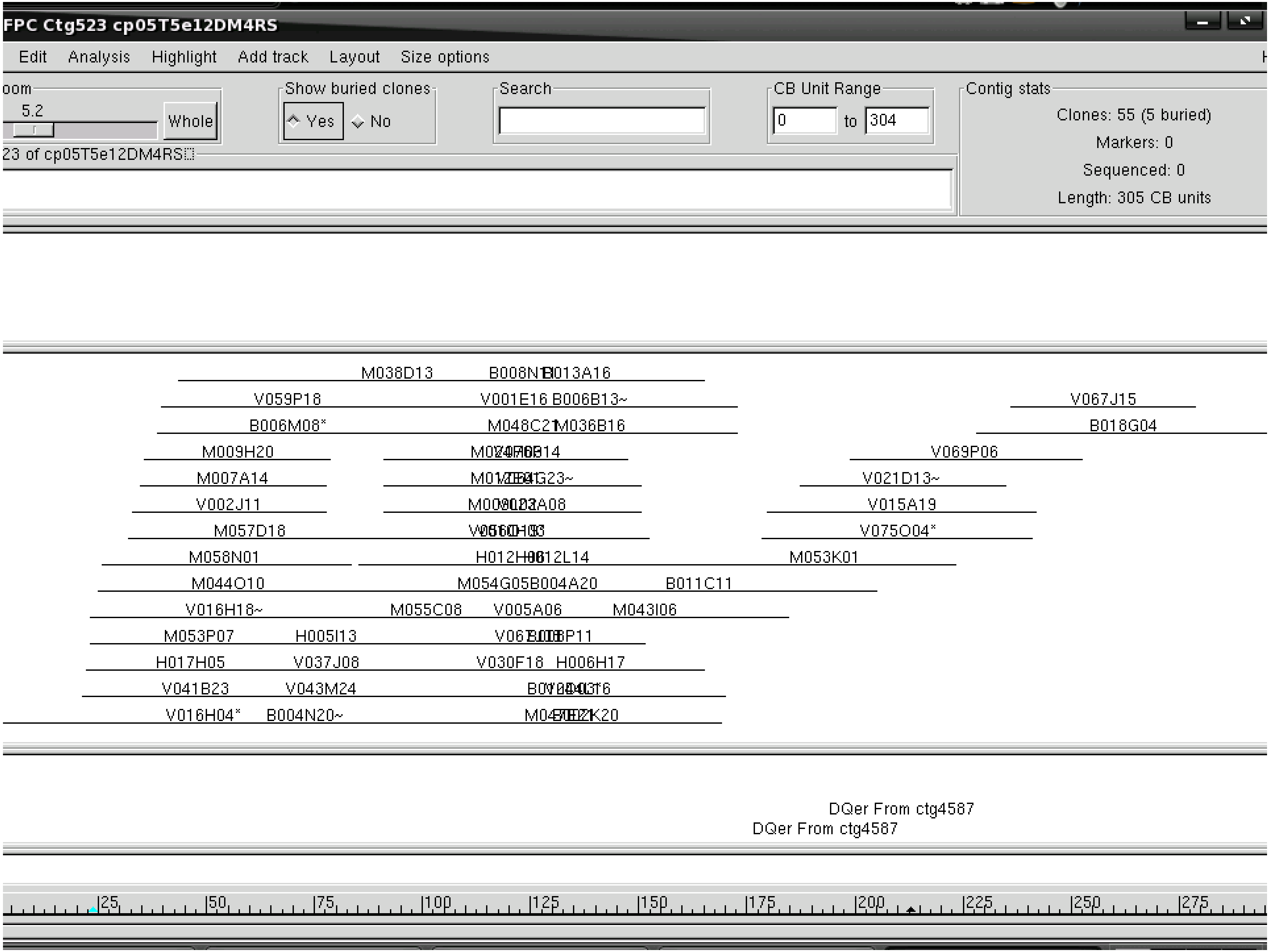
FPC Ctg523 cp05T5e12DM4RS
Edit Analysis Highlight Add track Layout Size options
oom
5.2
Whole
Show buried clones
Yes
No
Search
CB Unit Range
0
to
304
Contig stats
Clones: 55 (5 buried)
Markers: 0
Sequenced: 0
Length: 305 CB units
23 of cp05T5e12DM4RS
M038D13
V059P18
V001E16 B006B13~
V067J15
B006M08*
M048C21
M036B16
B018G04
M009H20
V069P06
M007A14
V021D13~
V002J11
V015A19
M057D18
V075O04*
M058N01
M053K01
M044O10
M054G05
B004A20
B011C11
V016H18~
M055C08
V005A06
M043I06
M053P07
H005I13
H017H05
V037J08
V030F18
H006H17
V041B23
V043M24
V016H04*
B004N20~
DQer From ctg4587
DQer From ctg4587
25
50
75
100
125
150
175
200
225
250
275

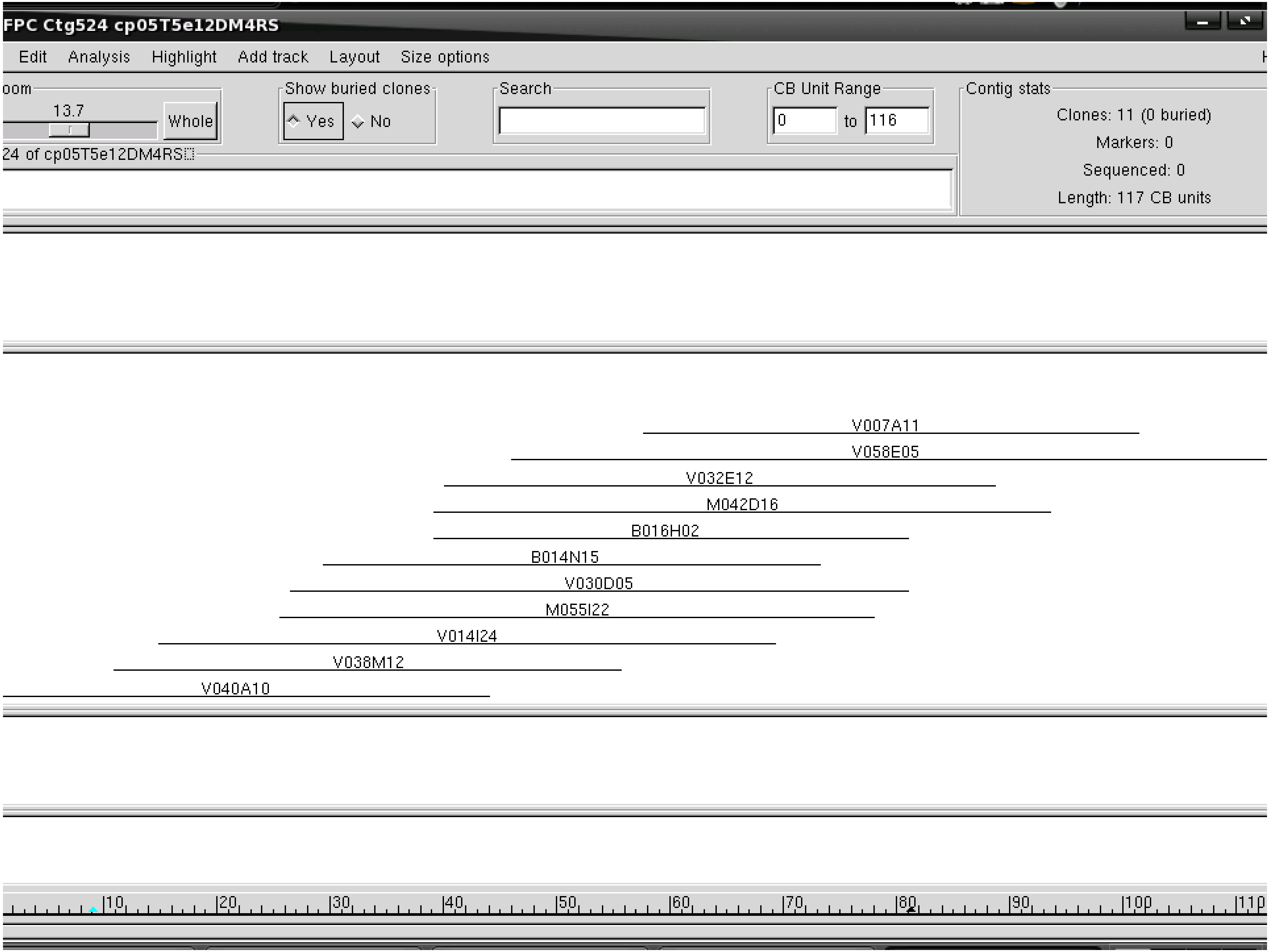
FPC Ctg524 cp05T5e12DM4RS
Edit Analysis Highlight Add track Layout Size options
oom
13.7
Whole
Show buried clones
Yes
No
Search
CB Unit Range
0
to
116
Contig stats
Clones: 11 (0 buried)
Markers: 0
Sequenced: 0
Length: 117 CB units
24 of cp05T5e12DM4RS
V007A11
V058E05
V032E12
M042D16
B016H02
B014N15
V030D05
M055I22
V014I24
V038M12
V040A10
10 20 30 40 50 60 70 80 90 100 110

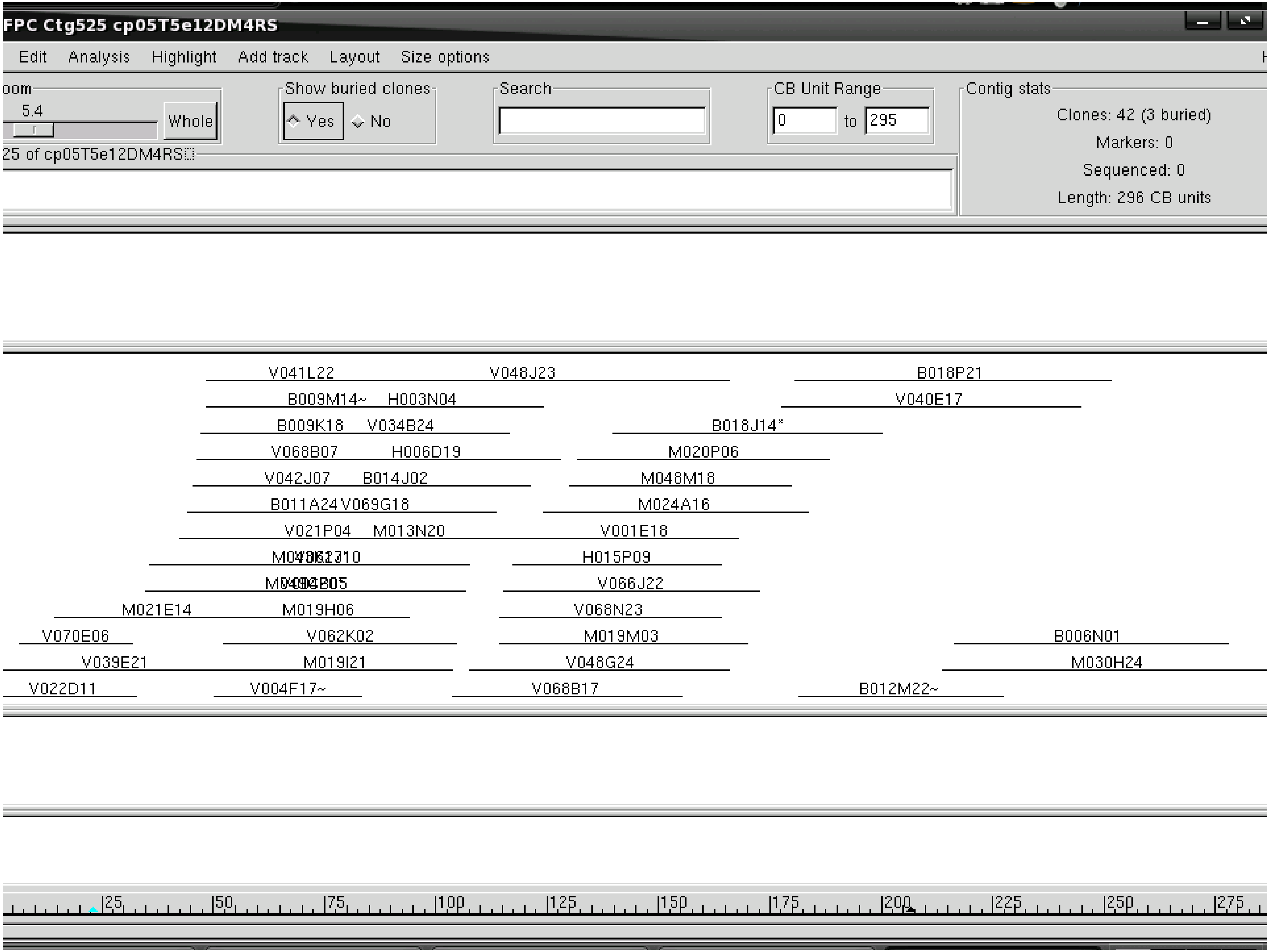

FPC Ctg525 cp05T5e12DM4RS
Edit
Analysis
Highlight
Add track
Layout
Size options
oom
5.4
Whole
Show buried clones
Yes
No
Search
CB Unit Range
0
to
295
Contig stats
Clones: 42 (3 buried)
Markers: 0
Sequenced: 0
Length: 296 CB units
25 of cp05T5e12DM4RS
V041L22
V048J23
B018P21
B009M14~
H003N04
V040E17
B009K18
V034B24
B018J14*
V068B07
H006D19
M020P06
V042J07
B014J02
M048M18
B011A24
V069G18
M024A16
V021P04
M013N20
V001E18
H015P09
V066J22
M021E14
M019H06
V068N23
V070E06
V062K02
M019M03
B006N01
V039E21
M019I21
V048G24
M030H24
V022D11
V004F17~
V068B17
B012M22~
25
50
75
100
125
150
175
200
225
250
275

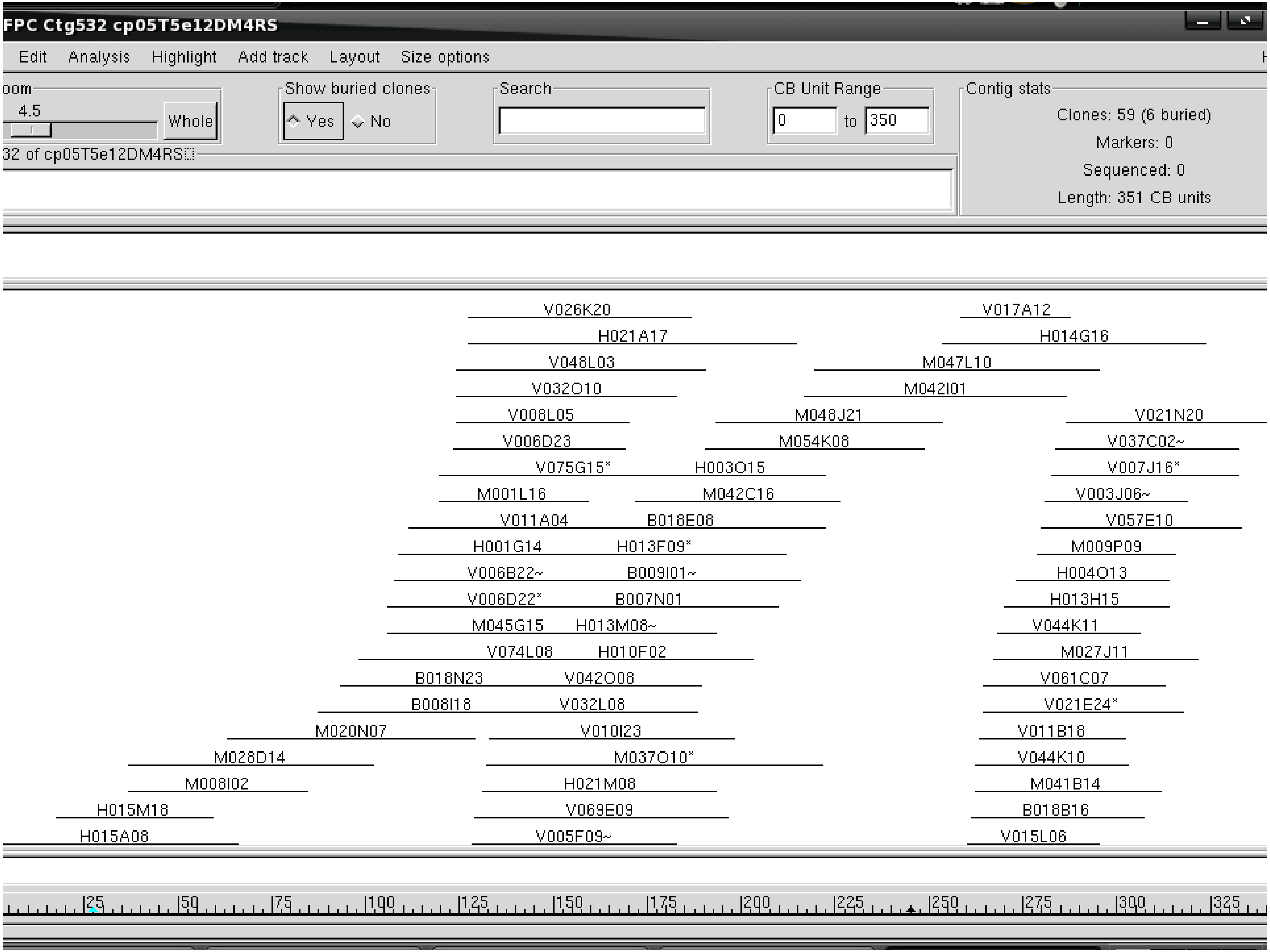
FPC Ctg532 cp05T5e12DM4RS
Edit Analysis Highlight Add track Layout Size options
oom
4.5
Whole
Show buried clones
Yes No
Search
CB Unit Range
0 to 350
Contig stats
Clones: 59 (6 buried)
Markers: 0
Sequenced: 0
Length: 351 CB units
32 of cp05T5e12DM4RS
V026K20
H021A17
V048L03
V032O10
V008L05
V006D23
V075G15*
M001L16
V011A04
H001G14
V006B22~
V006D22*
M045G15
V074L08
B018N23
B008I18
M020N07
M028D14
M008I02
H015M18
H015A08
H003O15
M042C16
B018E08
H013F09*
B009I01~
B007N01
H013M08~
H010F02
V042O08
V032L08
V010I23
M037O10*
H021M08
V069E09
V005F09~
M047L10
M042I01
M048J21
M054K08
V017A12
H014G16
V021N20
V037C02~
V007J16*
V003J06~
V057E10
M009P09
H004O13
H013H15
V044K11
M027J11
V061C07
V021E24*
V011B18
V044K10
M041B14
B018B16
V015L06
25 50 75 100 125 150 175 200 225 250 275 300 325

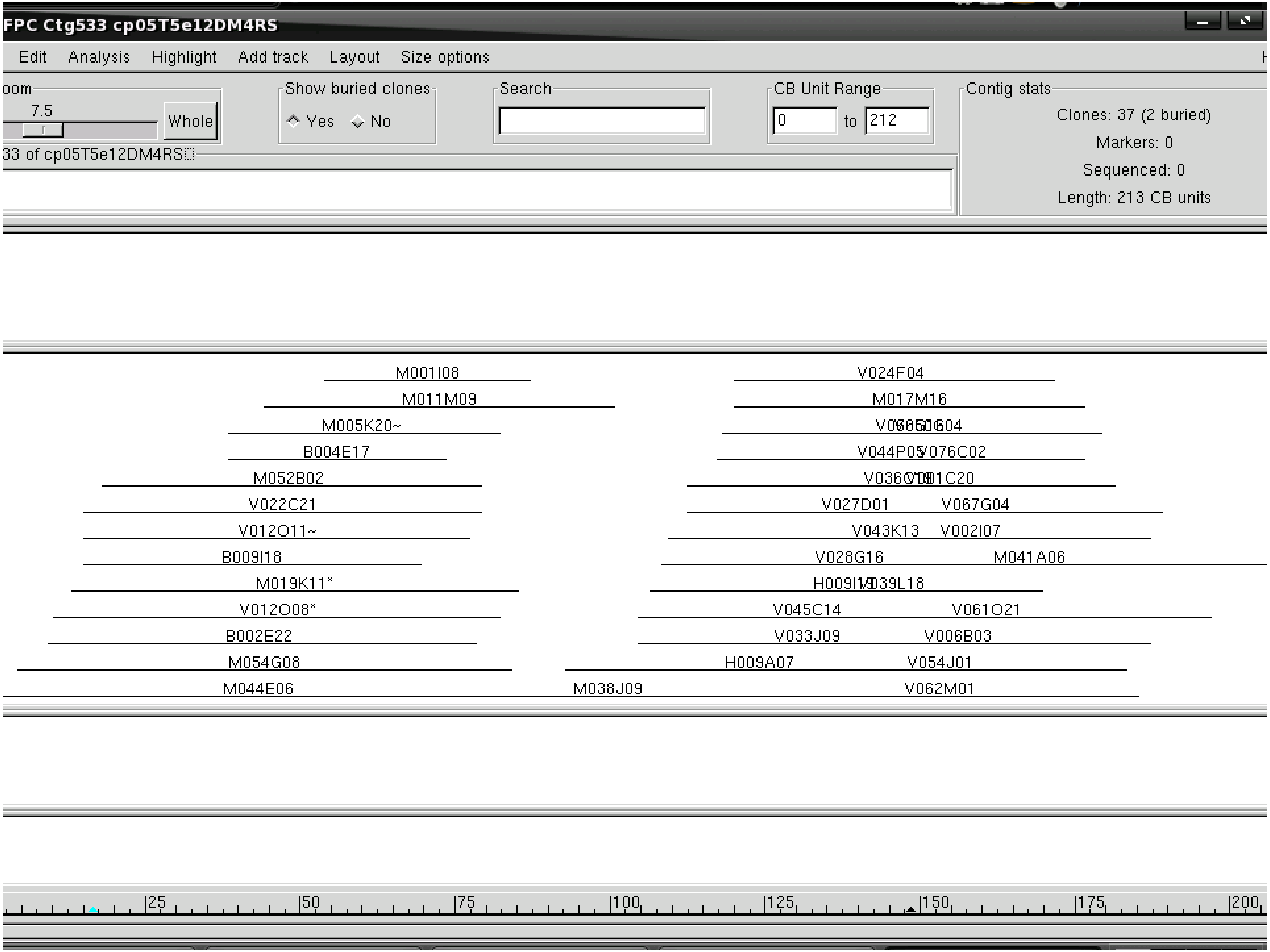
FPC Ctg533 cp05T5e12DM4RS
Edit Analysis Highlight Add track Layout Size options
oom
7.5
Whole
Show buried clones
Yes No
Search
CB Unit Range
0 to 212
Contig stats
Clones: 37 (2 buried)
Markers: 0
Sequenced: 0
Length: 213 CB units
33 of cp05T5e12DM4RS
M001I08
M011M09
M005K20~
B004E17
M052B02
V022C21
V012O11~
B009I18
M019K11*
V012O08*
B002E22
M054G08
M044E06
M038J09
V024F04
M017M16
V027D01
V067G04
V043K13
V002I07
V028G16
M041A06
V045C14
V061O21
V033J09
V006B03
H009A07
V054J01
V062M01
|25 |50 |75 |100 |125 |150 |175 |200

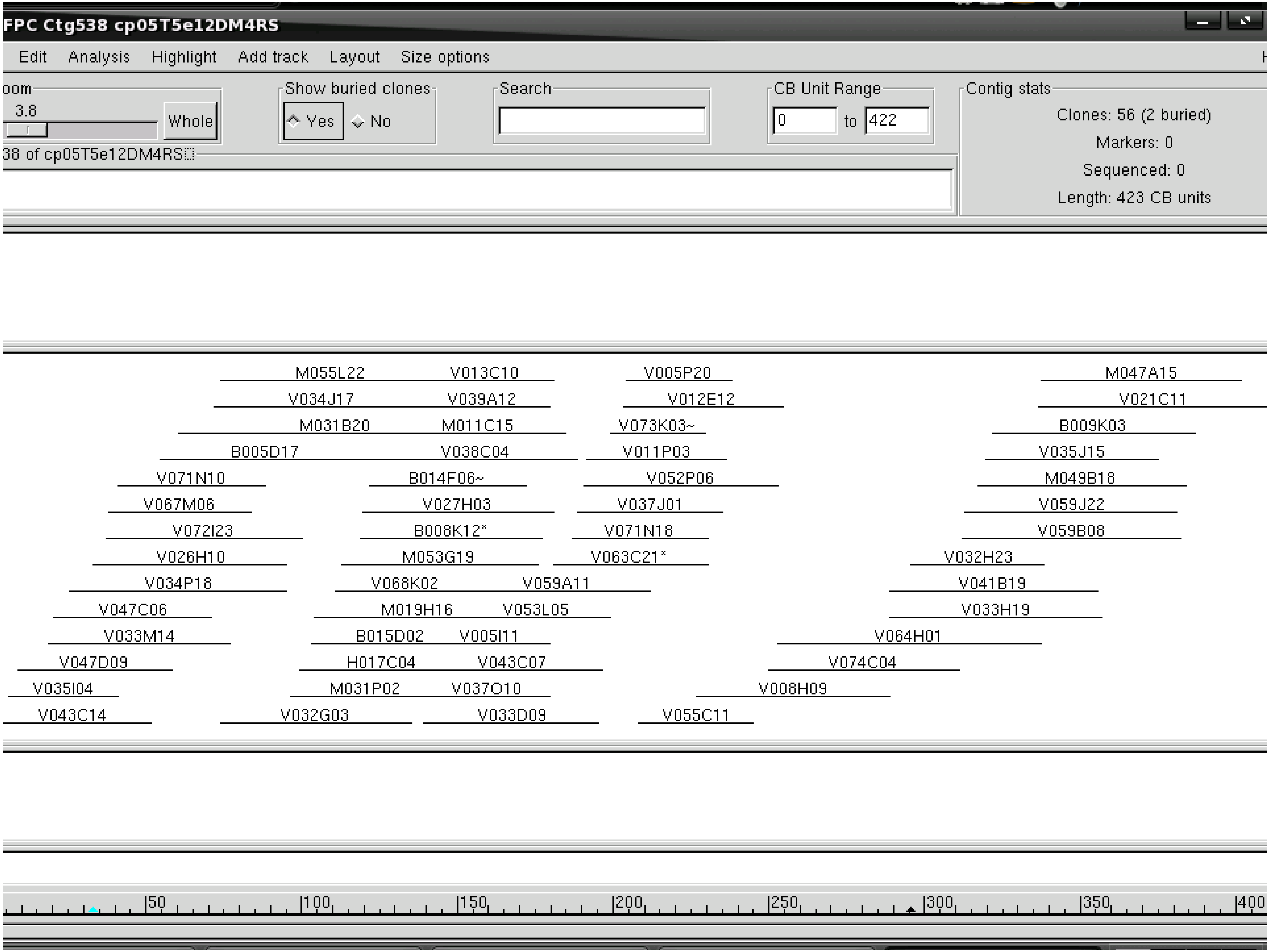
FPC Ctg538 cp05T5e12DM4RS
Edit Analysis Highlight Add track Layout Size options
oom
3.8
Whole
Show buried clones
Yes
No
Search
CB Unit Range
0
to
422
Contig stats
Clones: 56 (2 buried)
Markers: 0
Sequenced: 0
Length: 423 CB units
38 of cp05T5e12DM4RS
M055L22 V013C10 V005P20 M047A15
V034J17 V039A12 V012E12 V021C11
M031B20 M011C15 V073K03~ B009K03
B005D17 V038C04 V011P03 V035J15
V071N10 B014F06~ V052P06 M049B18
V067M06 V027H03 V037J01 V059J22
V072I23 B008K12* V071N18 V059B08
V026H10 M053G19 V063C21* V032H23
V034P18 V068K02 V059A11 V041B19
V047C06 M019H16 V053L05 V033H19
V033M14 B015D02 V005I11 V064H01
V047D09 H017C04 V043C07 V074C04
V035I04 M031P02 V037O10 V008H09
V043C14 V032G03 V033D09 V055C11
50 100 150 200 250 300 350 400

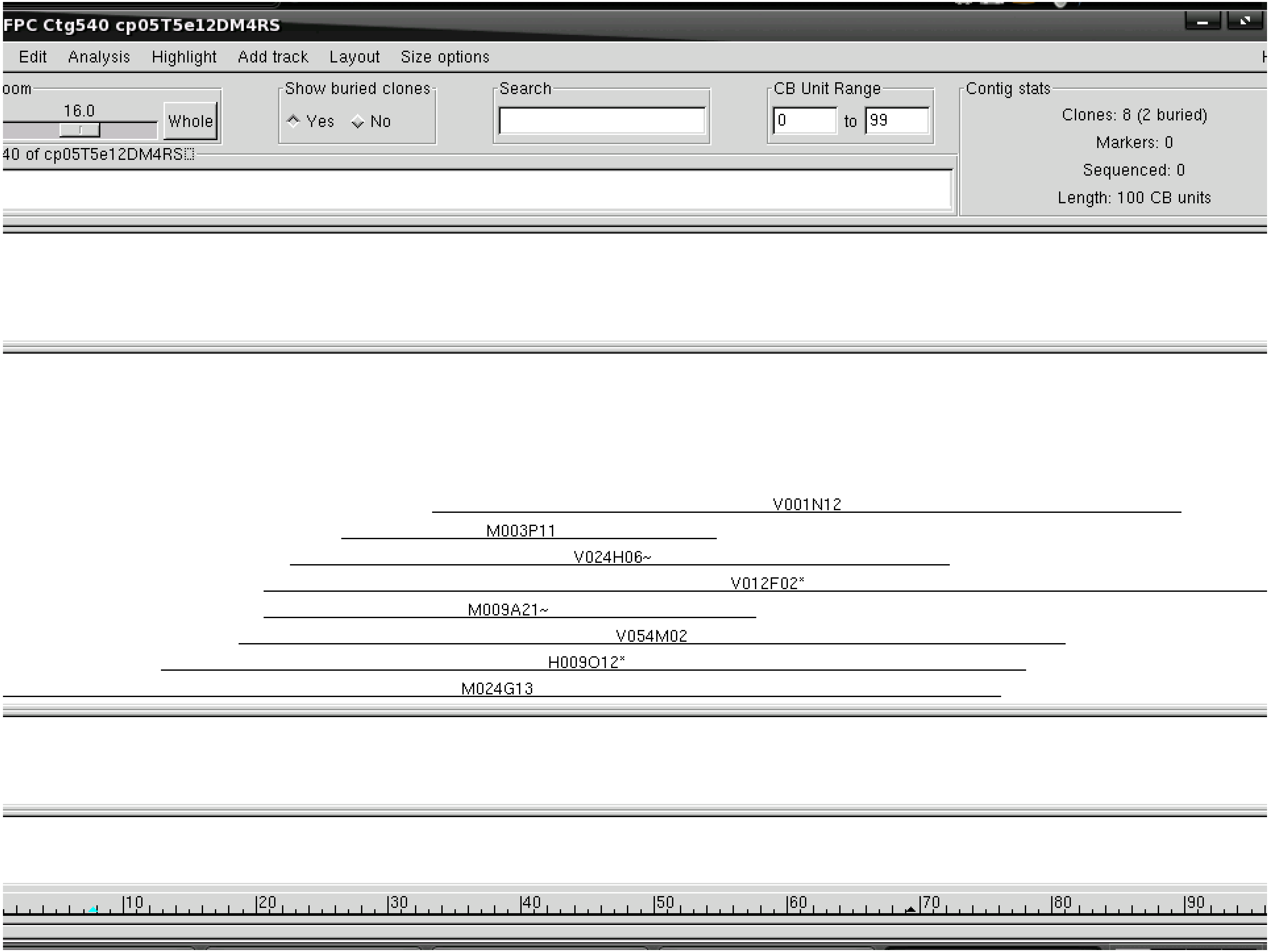
FPC Ctg540 cp05T5e12DM4RS
Edit
Analysis
Highlight
Add track
Layout
Size options
oom
16.0
Whole
Show buried clones
Yes
No
Search
CB Unit Range
0
to
99
Contig stats
Clones: 8 (2 buried)
Markers: 0
Sequenced: 0
Length: 100 CB units
40 of cp05T5e12DM4RS
V001N12
M003P11
V024H06~
V012F02*
M009A21~
V054M02
H009O12*
M024G13
10
20
30
40
50
60
70
80
90

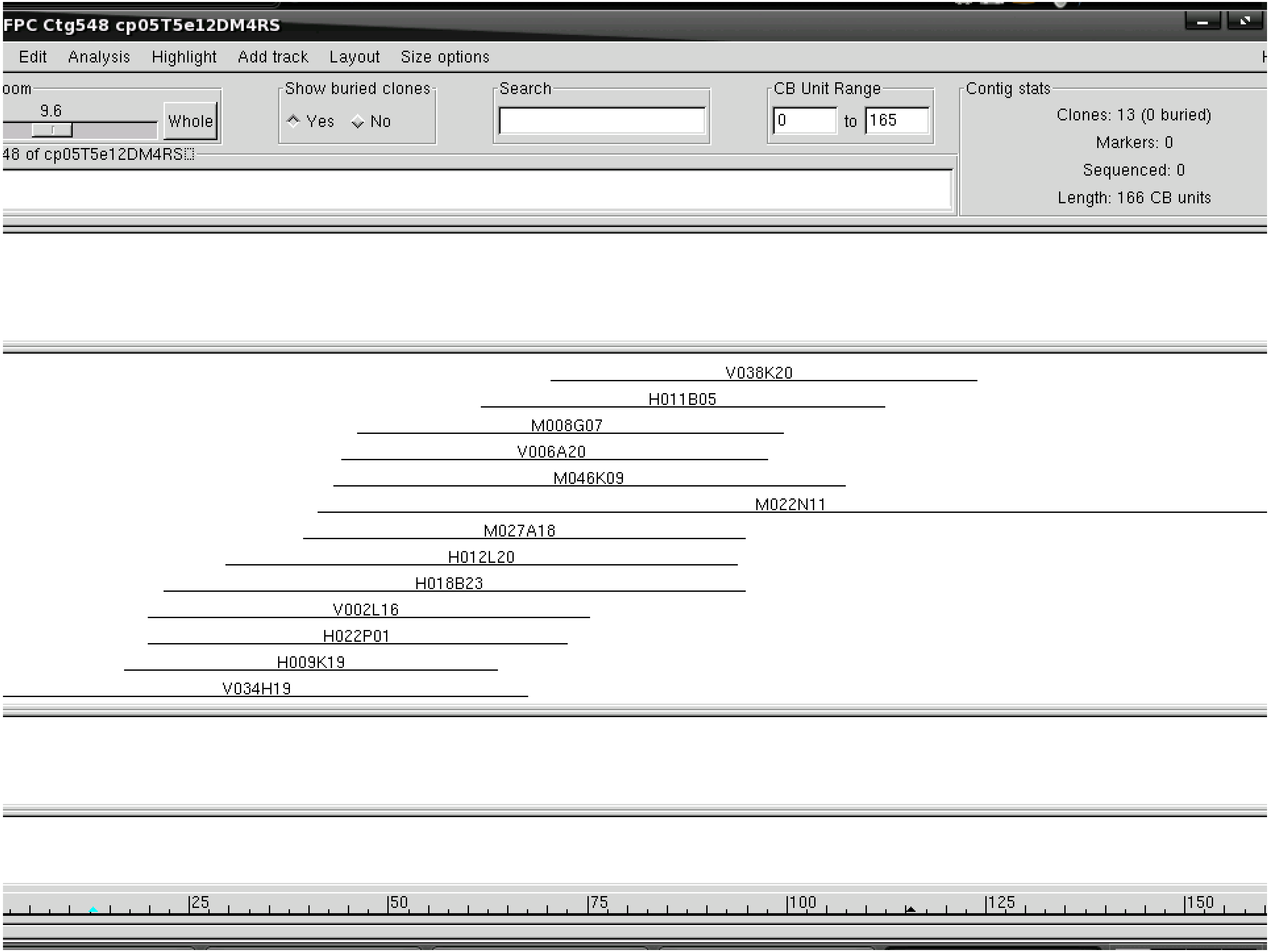
FPC Ctg548 cp05T5e12DM4RS
Edit
Analysis
Highlight
Add track
Layout
Size options
oom
9.6
Whole
Show buried clones
Yes
No
Search
CB Unit Range
0
to
165
Contig stats
Clones: 13 (0 buried)
Markers: 0
Sequenced: 0
Length: 166 CB units
48 of cp05T5e12DM4RS
V038K20
H011B05
M008G07
V006A20
M046K09
M022N11
M027A18
H012L20
H018B23
V002L16
H022P01
H009K19
V034H19
25
50
75
100
125
150

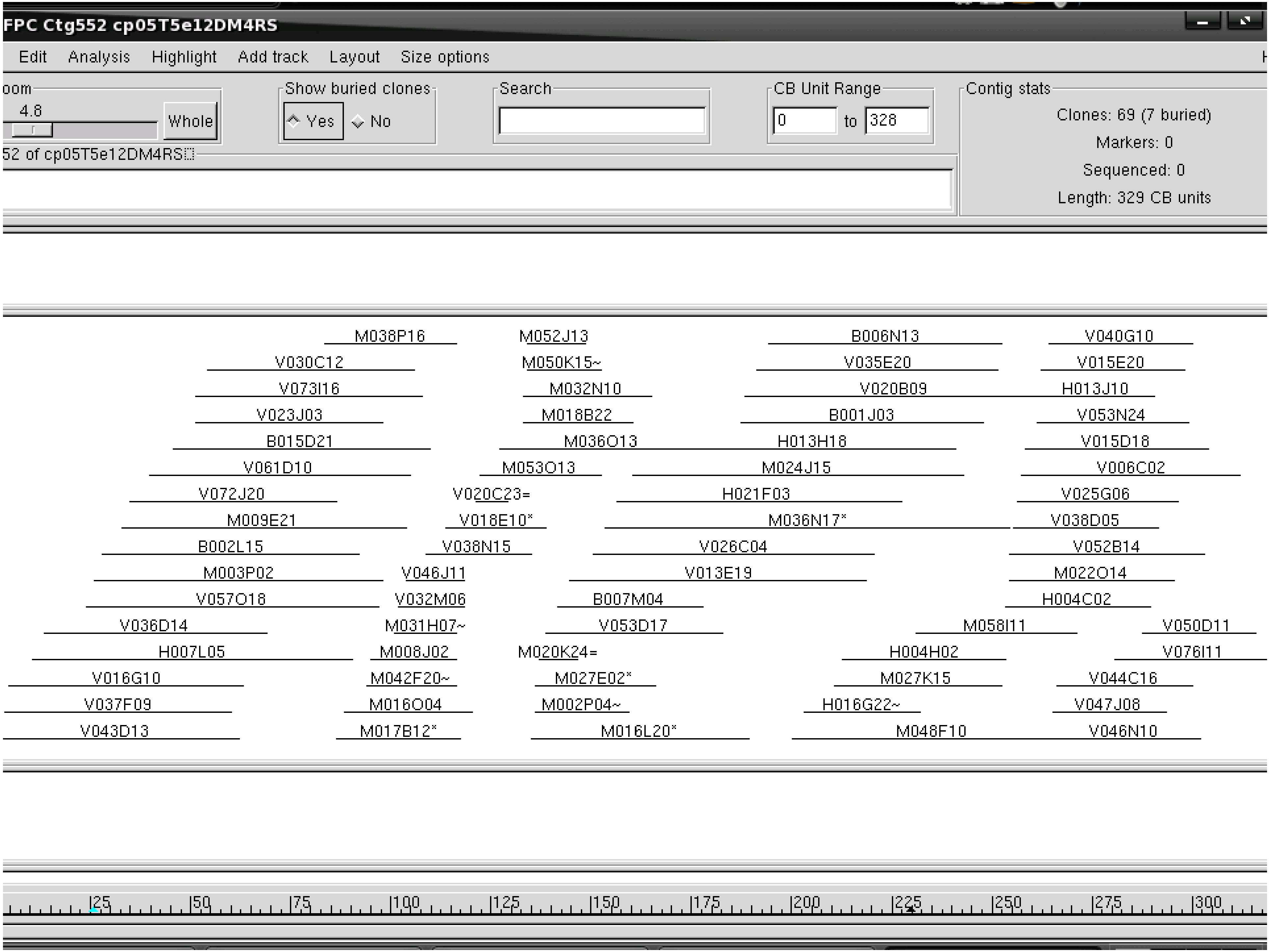
FPC Ctg552 cp05T5e12DM4RS
Edit Analysis Highlight Add track Layout Size options
oom
4.8
Whole
Show buried clones
Yes
No
Search
CB Unit Range
0
to
328
Contig stats
Clones: 69 (7 buried)
Markers: 0
Sequenced: 0
Length: 329 CB units
52 of cp05T5e12DM4RS
M038P16
M052J13
B006N13
V040G10
V030C12
M050K15~
V035E20
V015E20
V073I16
M032N10
V020B09
H013J10
V023J03
M018B22
B001J03
V053N24
B015D21
M036O13
H013H18
V015D18
V061D10
M053O13
M024J15
V006C02
V072J20
V020C23=
H021F03
V025G06
M009E21
V018E10*
M036N17*
V038D05
B002L15
V038N15
V026C04
V052B14
M003P02
V046J11
V013E19
M022O14
V057O18
V032M06
B007M04
H004C02
V036D14
M031H07~
V053D17
M058I11
V050D11
H007L05
M008J02
M020K24=
H004H02
V076I11
V016G10
M042F20~
M027E02*
M027K15
V044C16
V037F09
M016O04
M002P04~
H016G22~
V047J08
V043D13
M017B12*
M016L20*
M048F10
V046N10
25
50
75
100
125
150
175
200
225
250
275
300

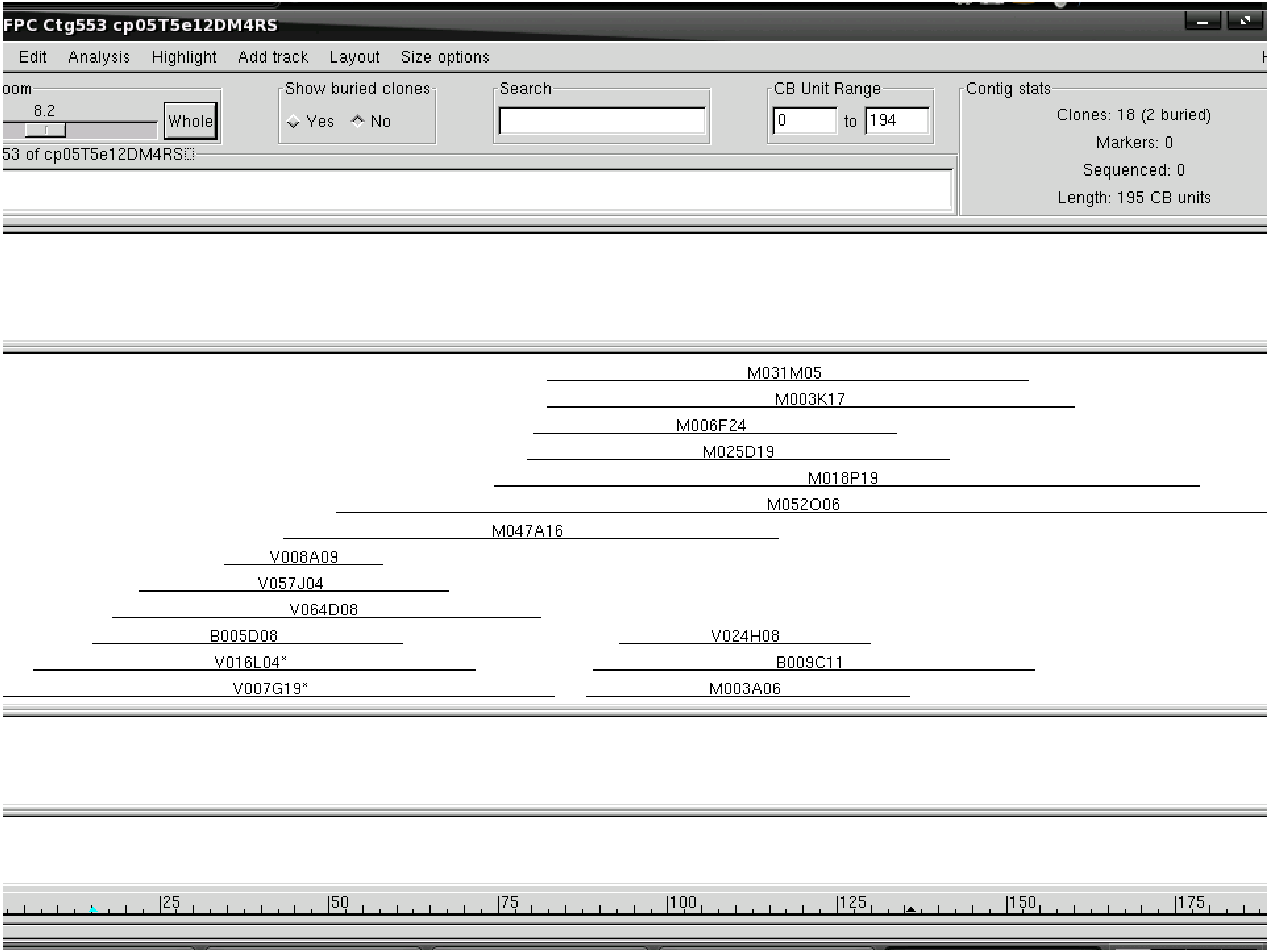
FPC Ctg553 cp05T5e12DM4RS
Edit
Analysis
Highlight
Add track
Layout
Size options
oom
8.2
Whole
Show buried clones
Yes
No
Search
CB Unit Range
0
to
194
Contig stats
Clones: 18 (2 buried)
Markers: 0
Sequenced: 0
Length: 195 CB units
53 of cp05T5e12DM4RS
M031M05
M003K17
M006F24
M025D19
M018P19
M052O06
M047A16
V008A09
V057J04
V064D08
B005D08
V024H08
V016L04*
B009C11
V007G19*
M003A06
25
50
75
100
125
150
175

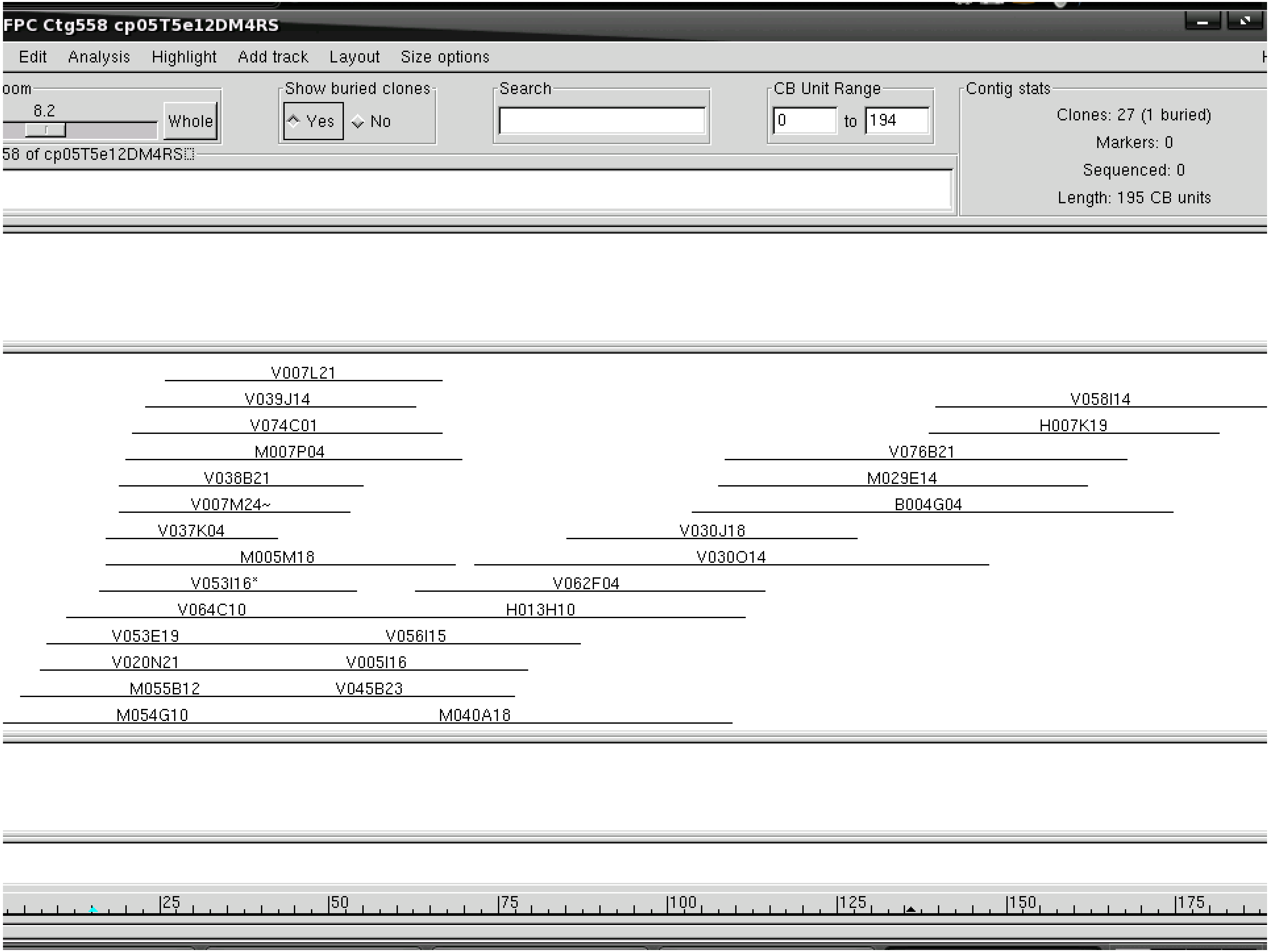

FPC Ctg558 cp05T5e12DM4RS
Edit
Analysis
Highlight
Add track
Layout
Size options
oom
8.2
Whole
Show buried clones
Yes
No
Search
CB Unit Range
0
to
194
Contig stats
Clones: 27 (1 buried)
Markers: 0
Sequenced: 0
Length: 195 CB units
58 of cp05T5e12DM4RS
V007L21
V039J14
V058I14
V074C01
H007K19
M007P04
V076B21
V038B21
M029E14
V007M24~
B004G04
V037K04
V030J18
M005M18
V030O14
V053I16*
V062F04
V064C10
H013H10
V053E19
V056I15
V020N21
V005I16
M055B12
V045B23
M054G10
M040A18
25
50
75
100
125
150
175

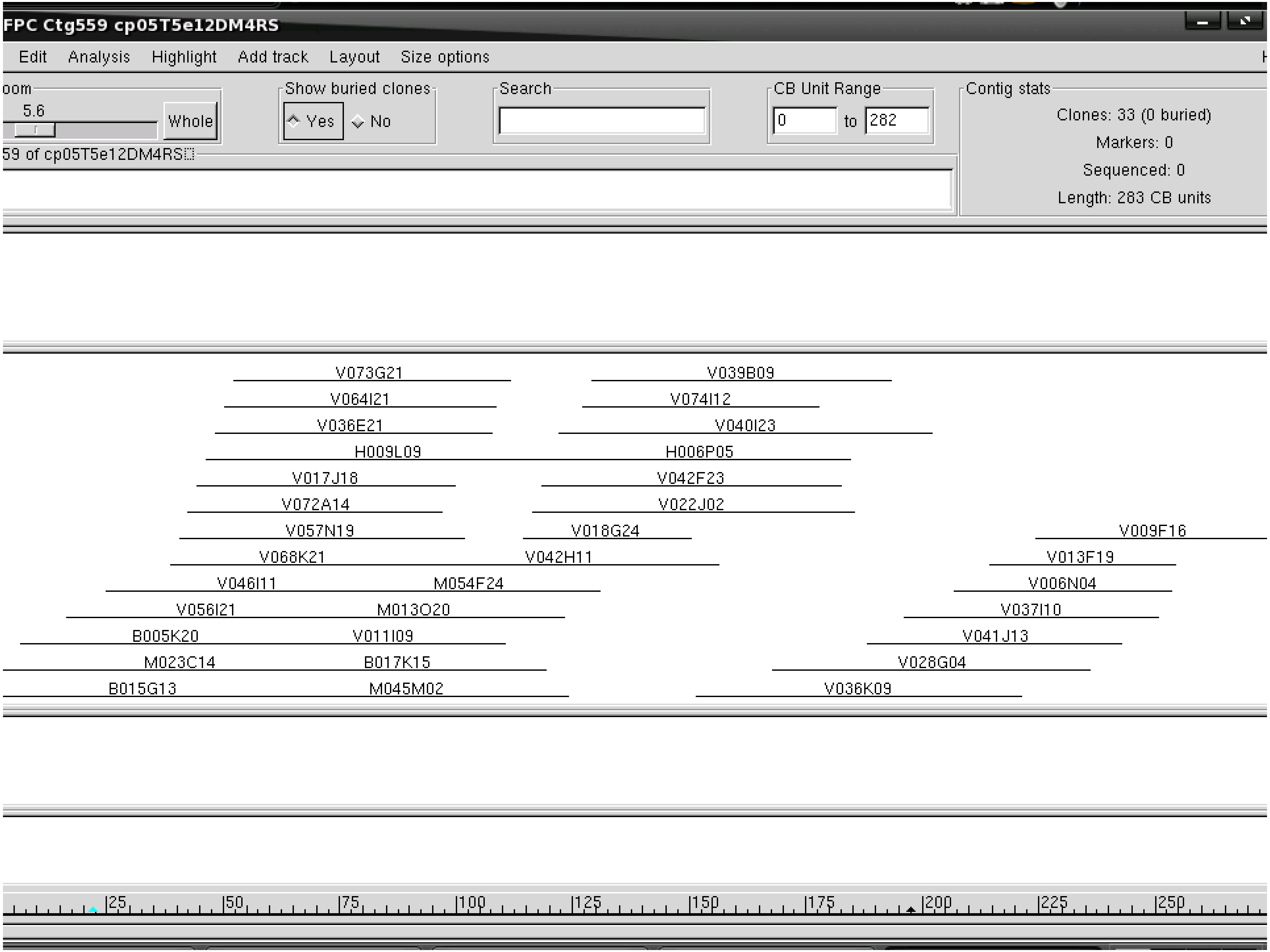
FPC Ctg559 cp05T5e12DM4RS
Edit
Analysis
Highlight
Add track
Layout
Size options
oom
5.6
Whole
Show buried clones
Yes
No
Search
CB Unit Range
0
to
282
Contig stats
Clones: 33 (0 buried)
Markers: 0
Sequenced: 0
Length: 283 CB units
59 of cp05T5e12DM4RS
V073G21
V039B09
V064I21
V074I12
V036E21
V040I23
H009L09
H006P05
V017J18
V042F23
V072A14
V022J02
V057N19
V018G24
V009F16
V068K21
V042H11
V013F19
V046I11
M054F24
V006N04
V056I21
M013O20
V037I10
B005K20
V011I09
V041J13
M023C14
B017K15
V028G04
B015G13
M045M02
V036K09
25
50
75
100
125
150
175
200
225
250

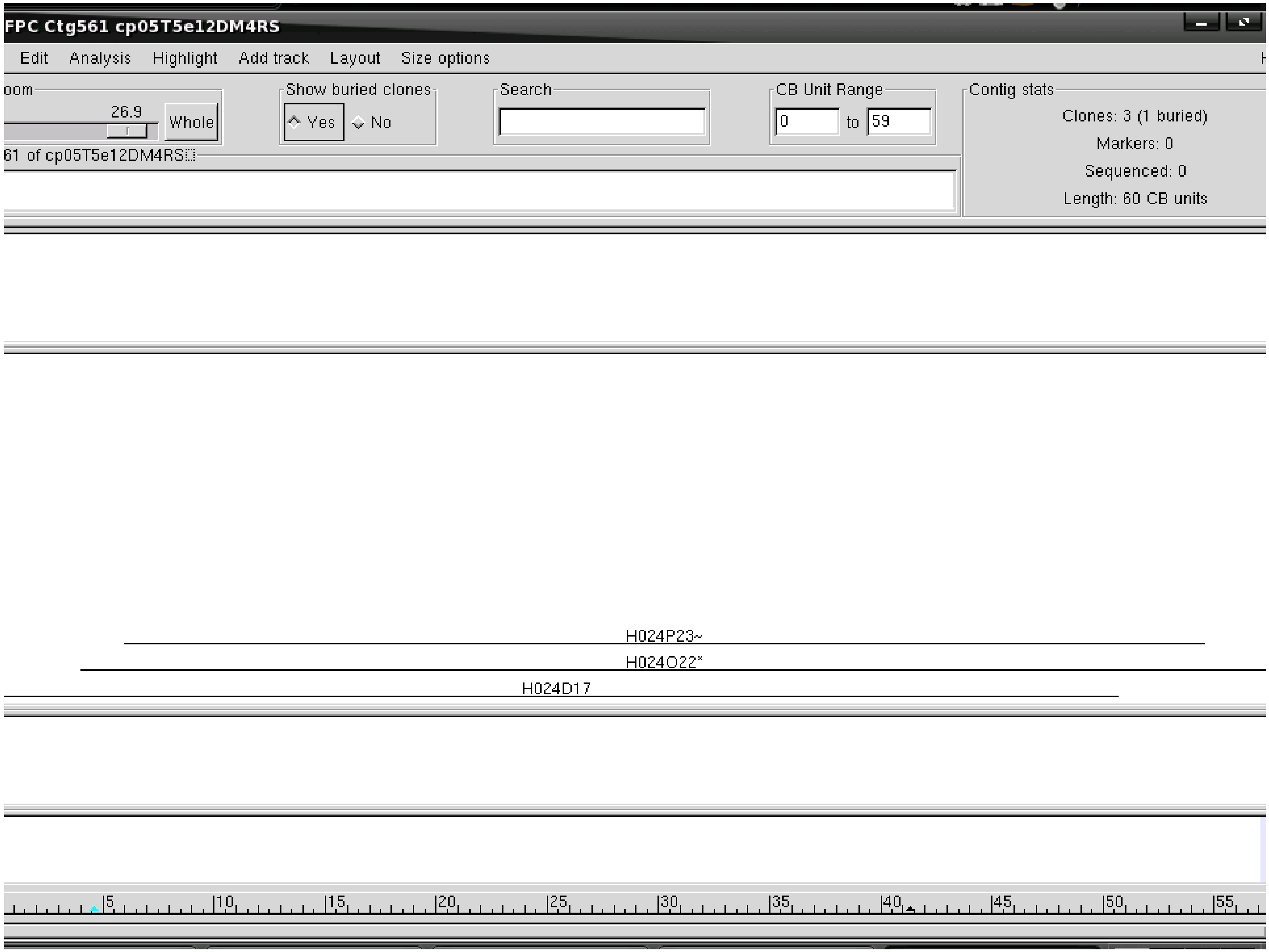
FPC Ctg561 cp05T5e12DM4RS
Edit Analysis Highlight Add track Layout Size options
oom
26.9
Whole
Show buried clones
Yes
No
Search
CB Unit Range
0
to
59
Contig stats
Clones: 3 (1 buried)
Markers: 0
Sequenced: 0
Length: 60 CB units
61 of cp05T5e12DM4RS
H024P23~
H024O22*
H024D17
5 10 15 20 25 30 35 40 45 50 55

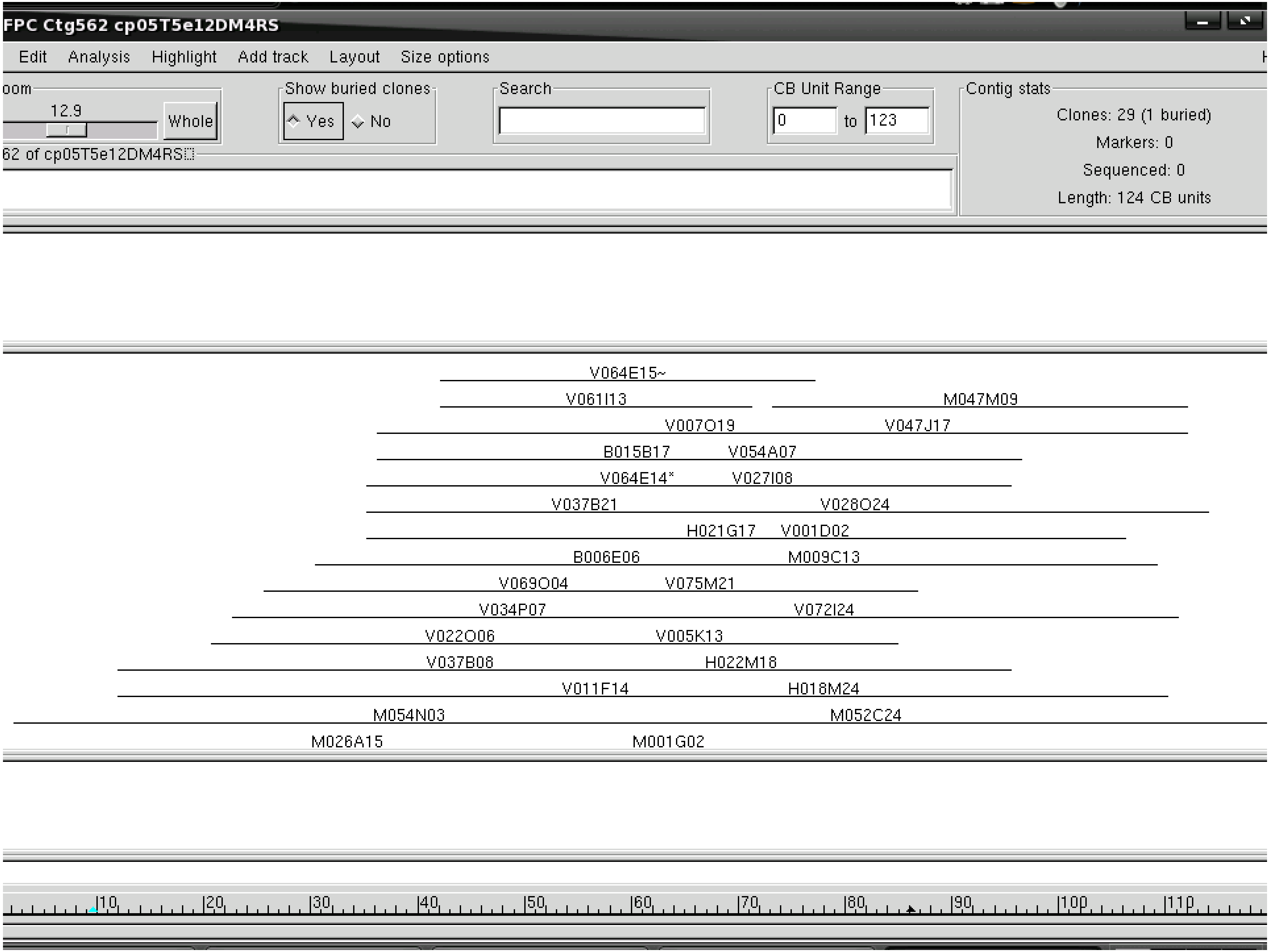
FPC Ctg562 cp05T5e12DM4RS
Edit
Analysis
Highlight
Add track
Layout
Size options
oom
12.9
Whole
Show buried clones
Yes
No
Search
CB Unit Range
0
to
123
Contig stats
Clones: 29 (1 buried)
Markers: 0
Sequenced: 0
Length: 124 CB units
62 of cp05T5e12DM4RS
V064E15~
V061I13
M047M09
V007O19
V047J17
B015B17
V054A07
V064E14*
V027I08
V037B21
V028O24
H021G17
V001D02
B006E06
M009C13
V069O04
V075M21
V034P07
V072I24
V022O06
V005K13
V037B08
H022M18
V011F14
H018M24
M054N03
M052C24
M026A15
M001G02
10
20
30
40
50
60
70
80
90
100
110

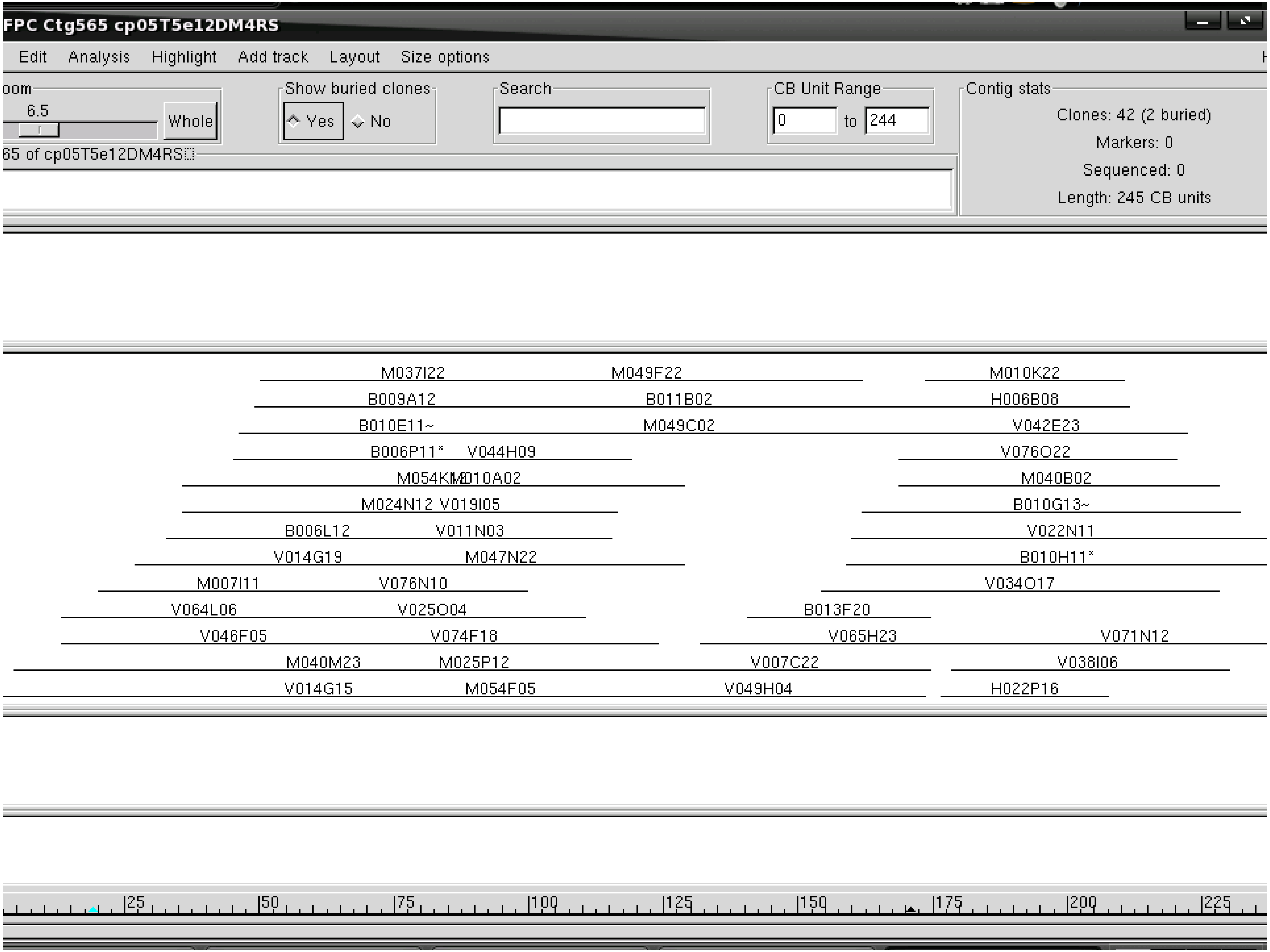
FPC Ctg565 cp05T5e12DM4RS
Edit
Analysis
Highlight
Add track
Layout
Size options
oom
6.5
Whole
65 of cp05T5e12DM4RS
Show buried clones
Yes
No
Search
CB Unit Range
0
to
244
Contig stats
Clones: 42 (2 buried)
Markers: 0
Sequenced: 0
Length: 245 CB units
M037I22
M049F22
M010K22
B009A12
B011B02
H006B08
B010E11~
M049C02
V042E23
B006P11*
V044H09
V076O22
M054K
M010A02
M040B02
M024N12
V019I05
B010G13~
B006L12
V011N03
V022N11
V014G19
M047N22
B010H11*
M007I11
V076N10
V034O17
V064L06
V025O04
B013F20
V046F05
V074F18
V065H23
V071N12
M040M23
M025P12
V007C22
V038I06
V014G15
M054F05
V049H04
H022P16
25
50
75
100
125
150
175
200
225

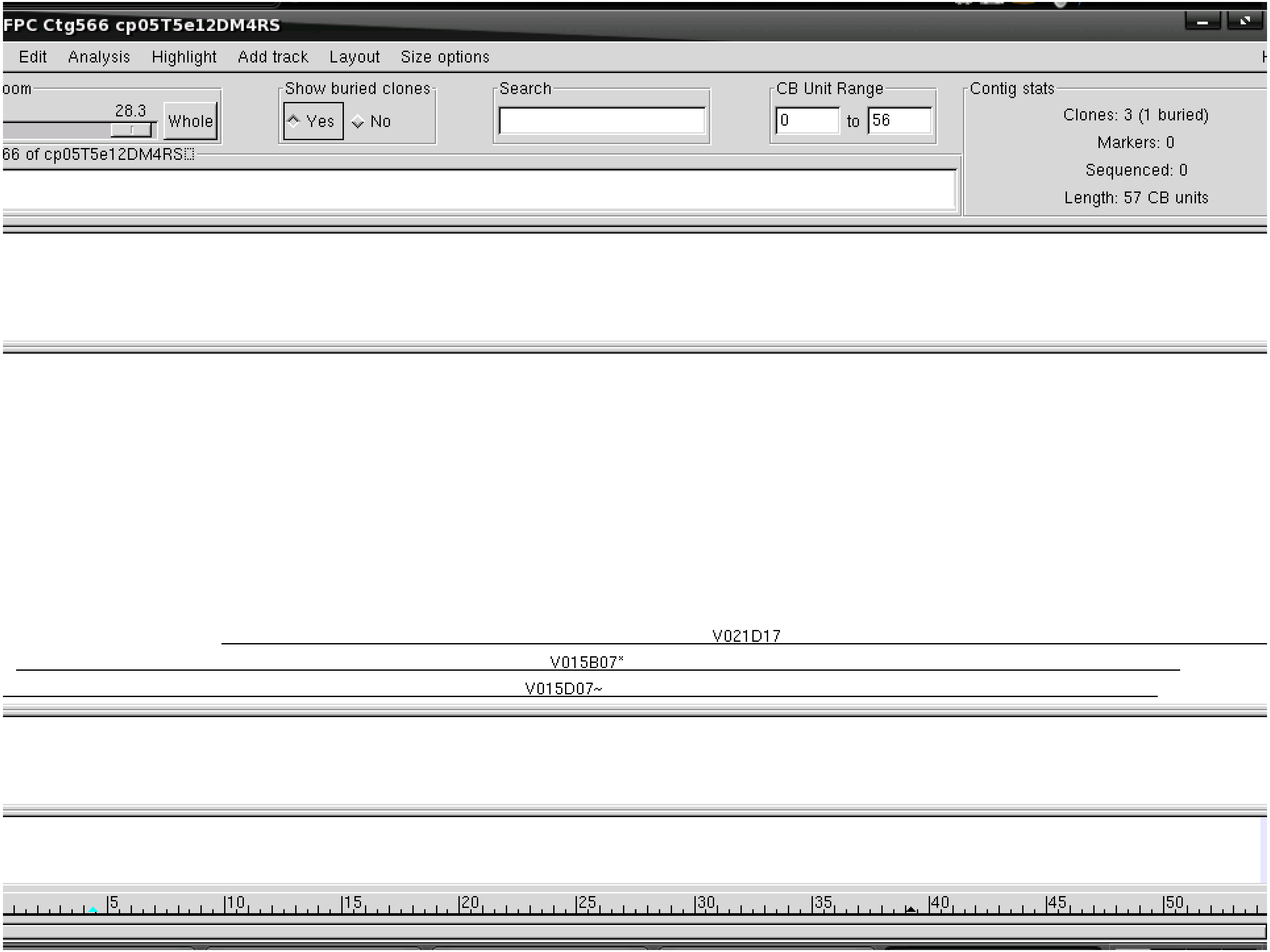
FPC Ctg566 cp05T5e12DM4RS
Edit
Analysis
Highlight
Add track
Layout
Size options
oom
28.3
Whole
Show buried clones
Yes
No
Search
CB Unit Range
0
to
56
Contig stats
Clones: 3 (1 buried)
Markers: 0
Sequenced: 0
Length: 57 CB units
66 of cp05T5e12DM4RS
V021D17
V015B07*
V015D07~
5
10
15
20
25
30
35
40
45
50

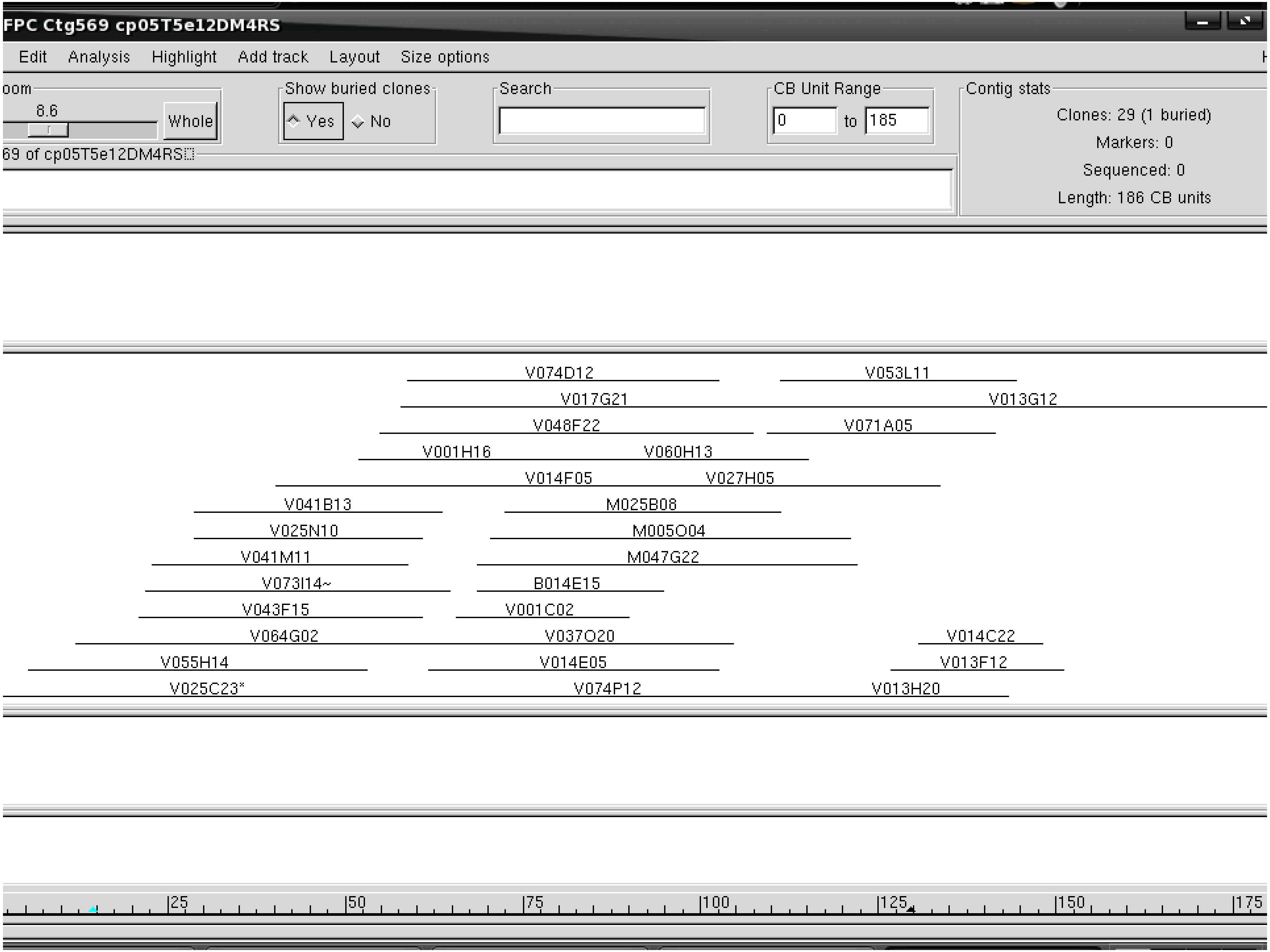
FPC Ctg569 cp05T5e12DM4RS
Edit
Analysis
Highlight
Add track
Layout
Size options
oom
8.6
Whole
Show buried clones
Yes
No
Search
CB Unit Range
0
to
185
Contig stats
Clones: 29 (1 buried)
Markers: 0
Sequenced: 0
Length: 186 CB units
69 of cp05T5e12DM4RS
V074D12
V053L11
V017G21
V013G12
V048F22
V071A05
V001H16
V060H13
V014F05
V027H05
V041B13
M025B08
V025N10
M005O04
V041M11
M047G22
V073I14~
B014E15
V043F15
V001C02
V064G02
V037O20
V014C22
V055H14
V014E05
V013F12
V025C23*
V074P12
V013H20
25
50
75
100
125
150
175

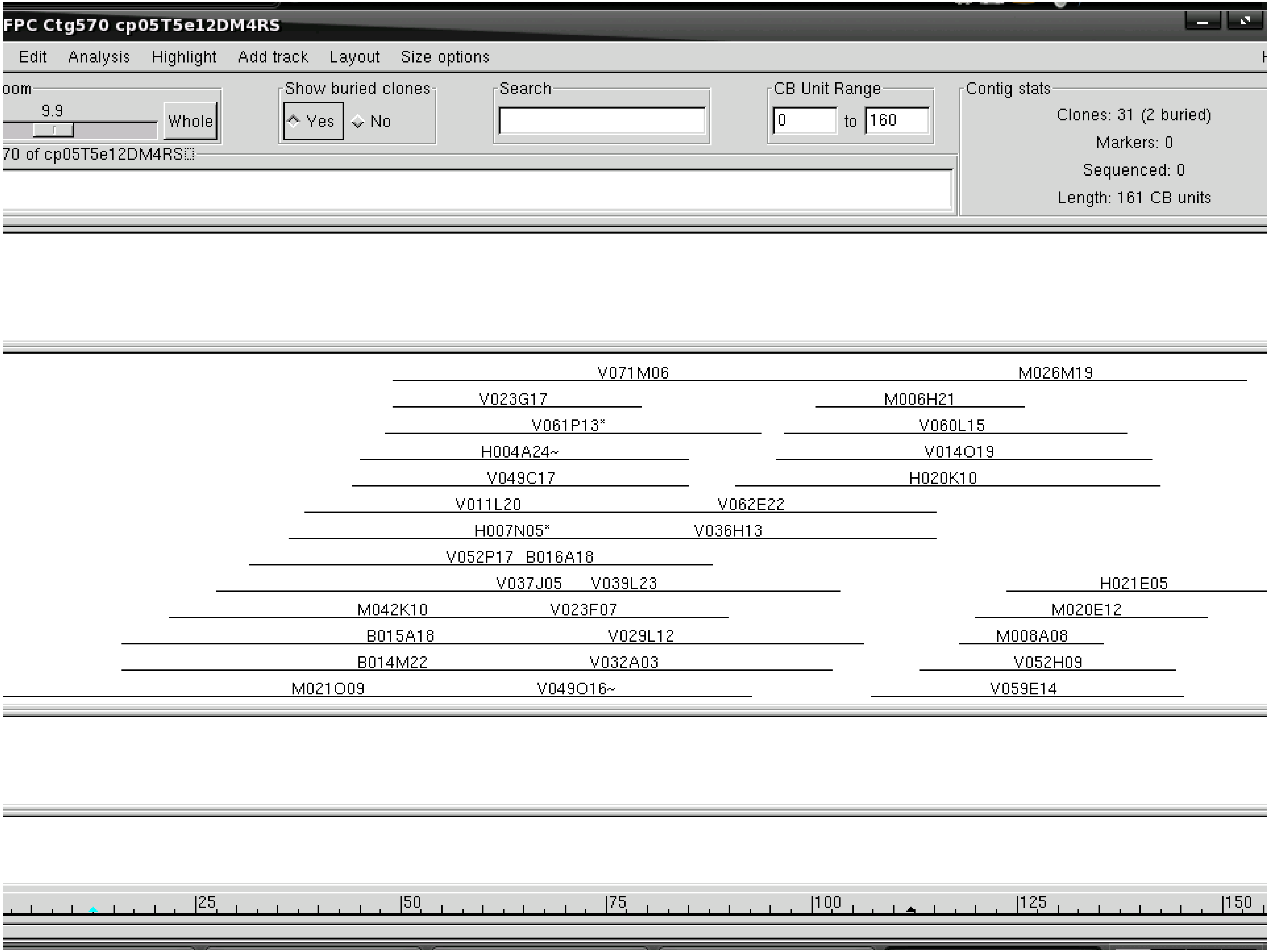
FPC Ctg570 cp05T5e12DM4RS
Edit Analysis Highlight Add track Layout Size options
oom
9.9
Whole
Show buried clones
Yes
No
Search
CB Unit Range
0 to 160
Contig stats
Clones: 31 (2 buried)
Markers: 0
Sequenced: 0
Length: 161 CB units
70 of cp05T5e12DM4RS
V071M06
M026M19
V023G17
M006H21
V061P13*
V060L15
H004A24~
V014O19
V049C17
H020K10
V011L20
V062E22
H007N05*
V036H13
V052P17
B016A18
V037J05
V039L23
H021E05
M042K10
V023F07
M020E12
B015A18
V029L12
M008A08
B014M22
V032A03
V052H09
M021O09
V049O16~
V059E14
|25
|50
|75
|100
|125
|150

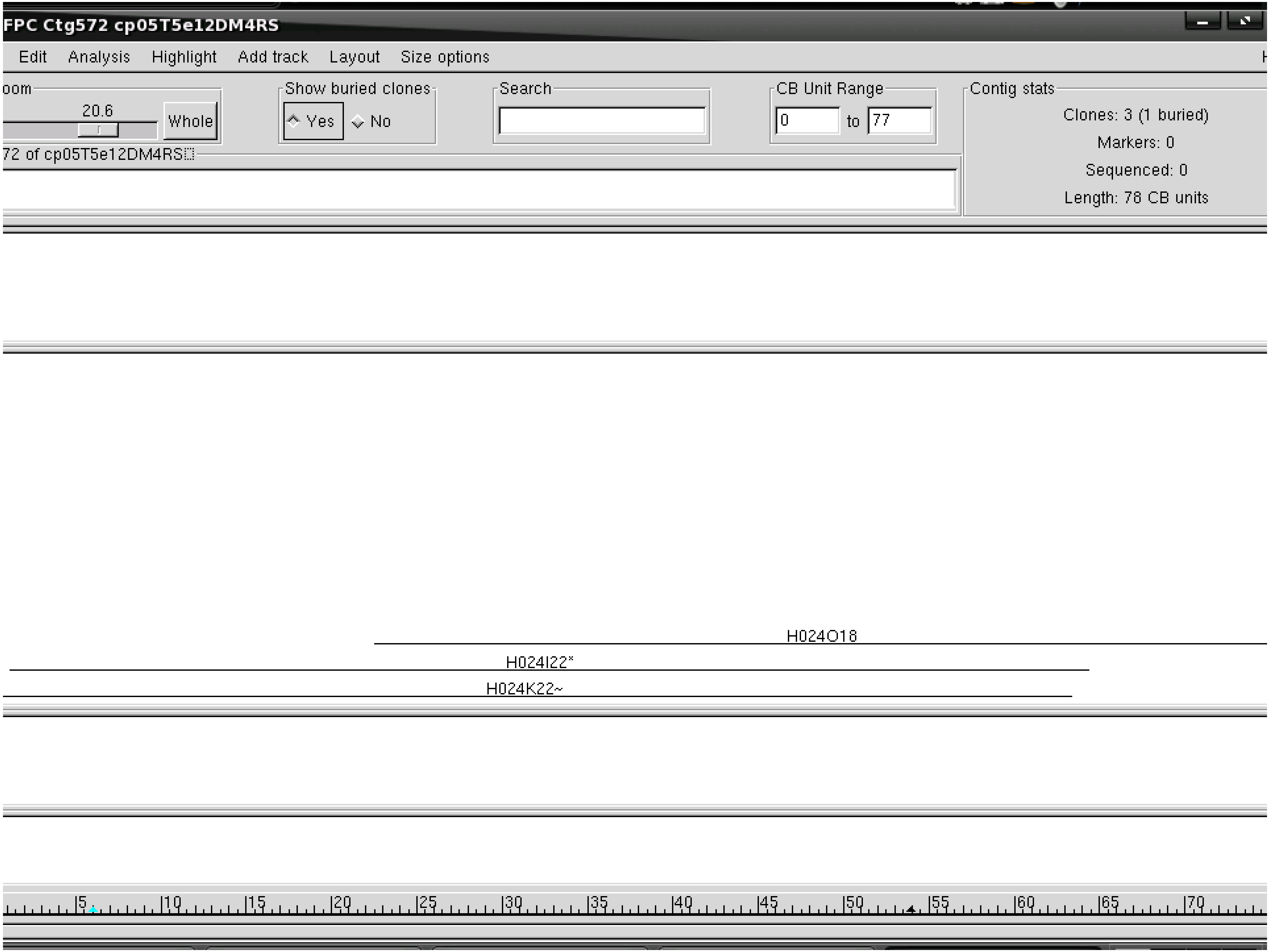
FPC Ctg572 cp05T5e12DM4RS
Edit
Analysis
Highlight
Add track
Layout
Size options
oom
20.6
Whole
Show buried clones
Yes
No
Search
CB Unit Range
0
to
77
Contig stats
Clones: 3 (1 buried)
Markers: 0
Sequenced: 0
Length: 78 CB units
72 of cp05T5e12DM4RS
H024O18
H024I22*
H024K22~
5
10
15
20
25
30
35
40
45
50
55
60
65
70

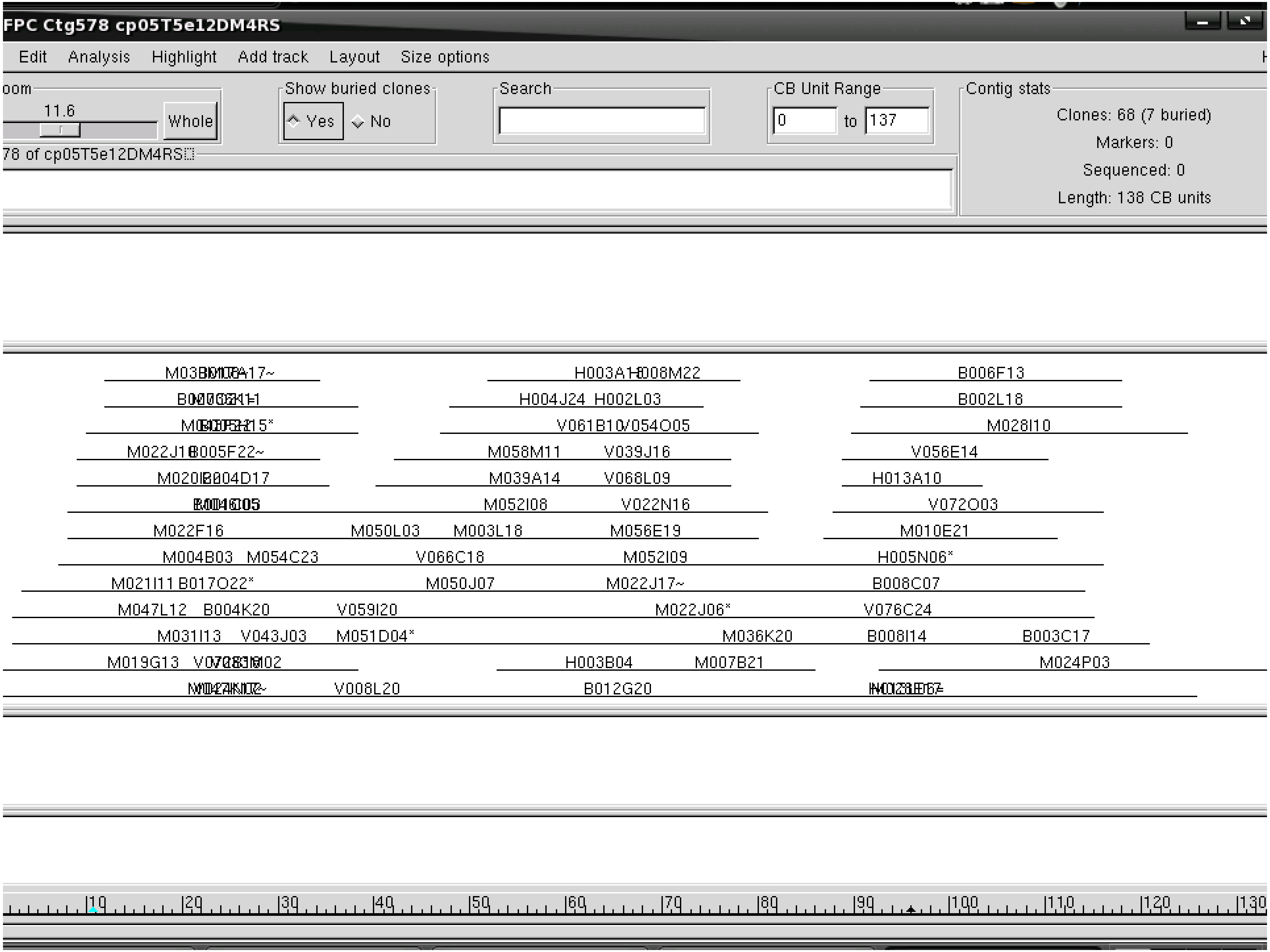
FPC Ctg578 cp05T5e12DM4RS
Edit
Analysis
Highlight
Add track
Layout
Size options
oom
11.6
Whole
Show buried clones
Yes
No
Search
CB Unit Range
0
to
137
Contig stats
Clones: 68 (7 buried)
Markers: 0
Sequenced: 0
Length: 138 CB units
78 of cp05T5e12DM4RS
H004J24
H002L03
B006F13
B002L18
M028I10
M058M11
V039J16
V056E14
M039A14
V068L09
H013A10
M052I08
V022N16
V072O03
M022F16
M050L03
M003L18
M056E19
M010E21
M004B03
M054C23
V066C18
M052I09
H005N06*
M021I11
B017O22*
M050J07
M022J17~
B008C07
M047L12
B004K20
V059I20
M022J06*
V076C24
M031I13
V043J03
M051D04*
M036K20
B008I14
B003C17
M019G13
H003B04
M007B21
M024P03
V008L20
B012G20
10
20
30
40
50
60
70
80
90
100
110
120
130

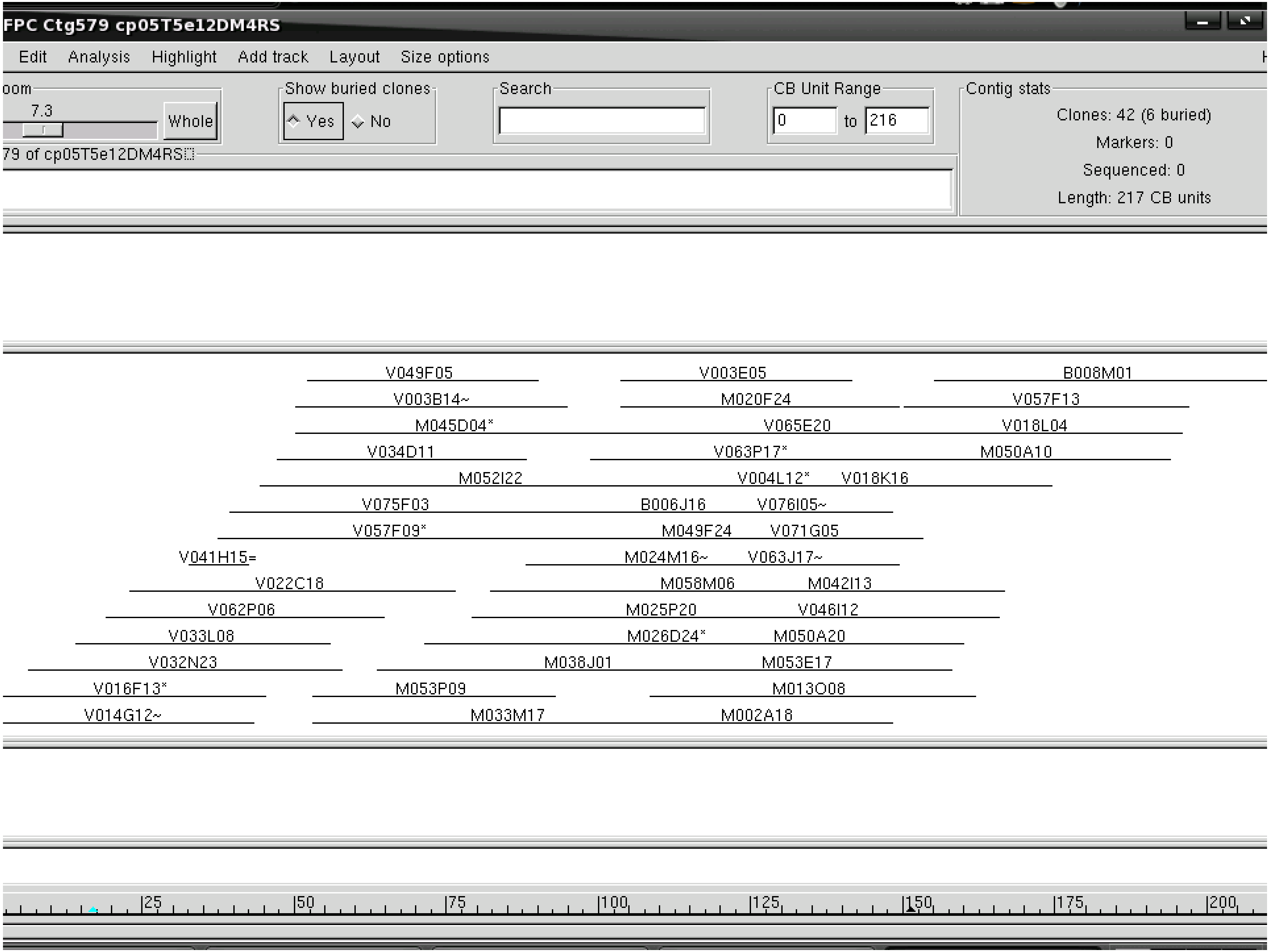
FPC Ctg579 cp05T5e12DM4RS
Edit Analysis Highlight Add track Layout Size options
oom 7.3 Whole
Show buried clones Yes No
Search
CB Unit Range 0 to 216
Contig stats
Clones: 42 (6 buried)
Markers: 0
Sequenced: 0
Length: 217 CB units
79 of cp05T5e12DM4RS
V049F05
V003E05
B008M01
V003B14~
M020F24
V057F13
M045D04*
V065E20
V018L04
V034D11
V063P17*
M050A10
M052I22
V004L12*
V018K16
V075F03
B006J16
V076I05~
V057F09*
M049F24
V071G05
V041H15=
M024M16~
V063J17~
V022C18
M058M06
M042I13
V062P06
M025P20
V046I12
V033L08
M026D24*
M050A20
V032N23
M038J01
M053E17
V016F13*
M053P09
M013O08
V014G12~
M033M17
M002A18
25 50 75 100 125 150 175 200

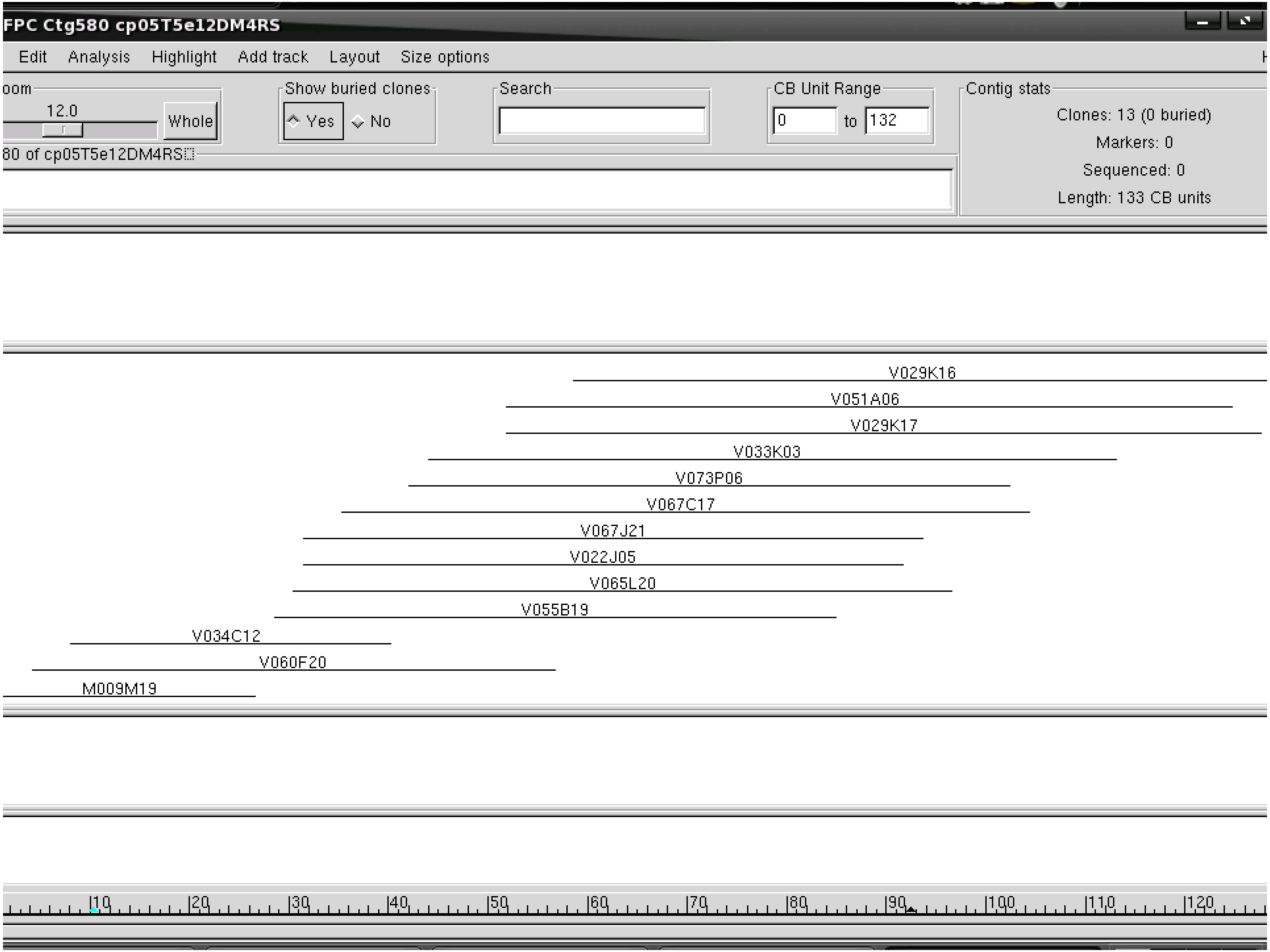
FPC Ctg580 cp05T5e12DM4RS
Edit
Analysis
Highlight
Add track
Layout
Size options
oom
12.0
Whole
Show buried clones
Yes
No
Search
CB Unit Range
0
to
132
Contig stats
Clones: 13 (0 buried)
Markers: 0
Sequenced: 0
Length: 133 CB units
80 of cp05T5e12DM4RS
V029K16
V051A06
V029K17
V033K03
V073P06
V067C17
V067J21
V022J05
V065L20
V055B19
V034C12
V060F20
M009M19
10
20
30
40
50
60
70
80
90
100
110
120

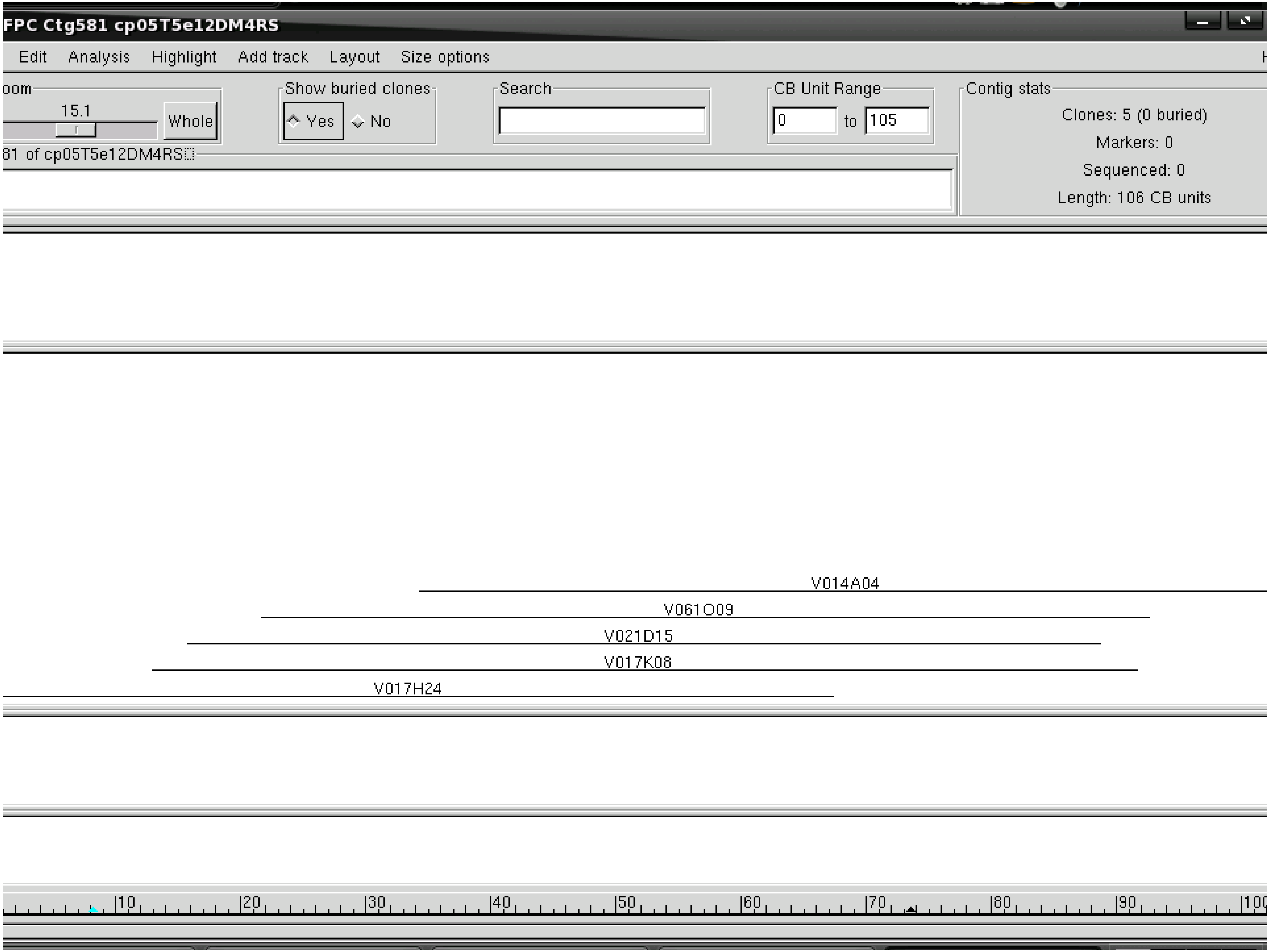
FPC Ctg581 cp05T5e12DM4RS
Edit
Analysis
Highlight
Add track
Layout
Size options
oom
15.1
Whole
Show buried clones
Yes
No
Search
CB Unit Range
0
to
105
Contig stats
Clones: 5 (0 buried)
Markers: 0
Sequenced: 0
Length: 106 CB units
81 of cp05T5e12DM4RS
V014A04
V061O09
V021D15
V017K08
V017H24
10
20
30
40
50
60
70
80
90

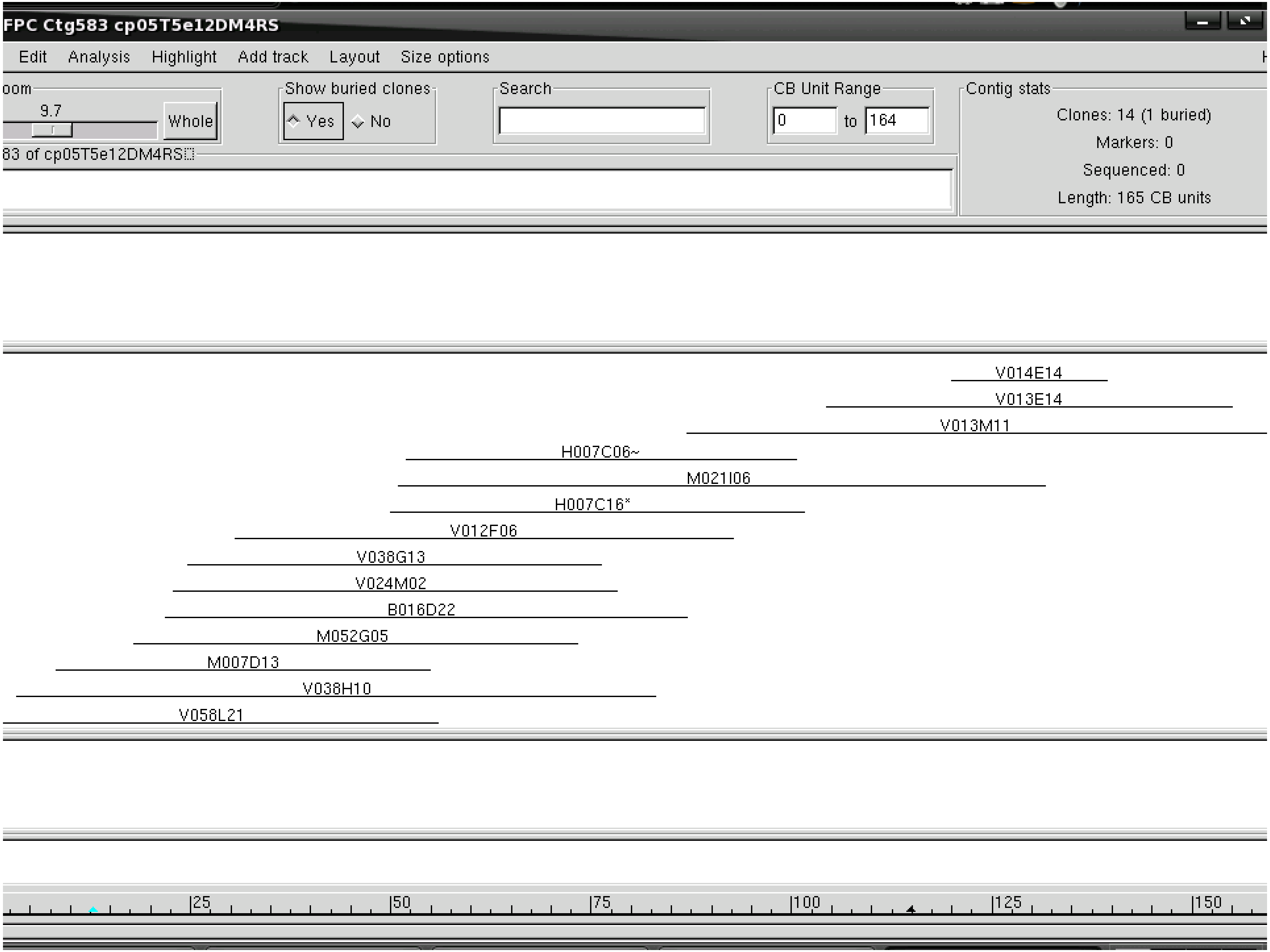
FPC Ctg583 cp05T5e12DM4RS
Edit
Analysis
Highlight
Add track
Layout
Size options
oom
9.7
Whole
Show buried clones
Yes
No
Search
CB Unit Range
0
to
164
Contig stats
Clones: 14 (1 buried)
Markers: 0
Sequenced: 0
Length: 165 CB units
83 of cp05T5e12DM4RS
V014E14
V013E14
V013M11
H007C06~
M021I06
H007C16*
V012F06
V038G13
V024M02
B016D22
M052G05
M007D13
V038H10
V058L21
25
50
75
100
125
150

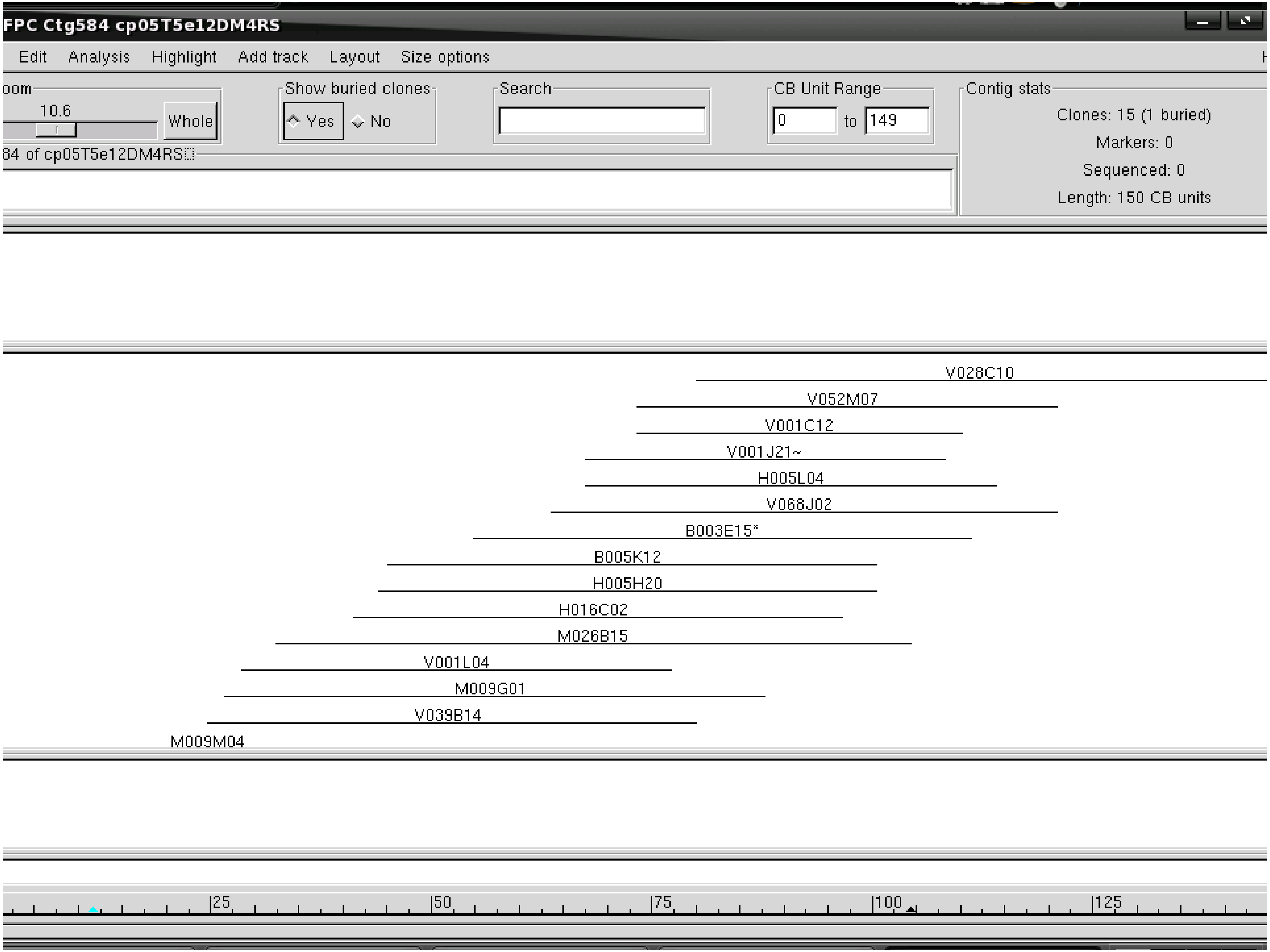
FPC Ctg584 cp05T5e12DM4RS
Edit Analysis Highlight Add track Layout Size options
oom
10.6
Whole
Show buried clones
Yes No
Search
CB Unit Range
0 to 149
Contig stats
Clones: 15 (1 buried)
Markers: 0
Sequenced: 0
Length: 150 CB units
84 of cp05T5e12DM4RS
V028C10
V052M07
V001C12
V001J21~
H005L04
V068J02
B003E15*
B005K12
H005H20
H016C02
M026B15
V001L04
M009G01
V039B14
M009M04
25
50
75
100
125

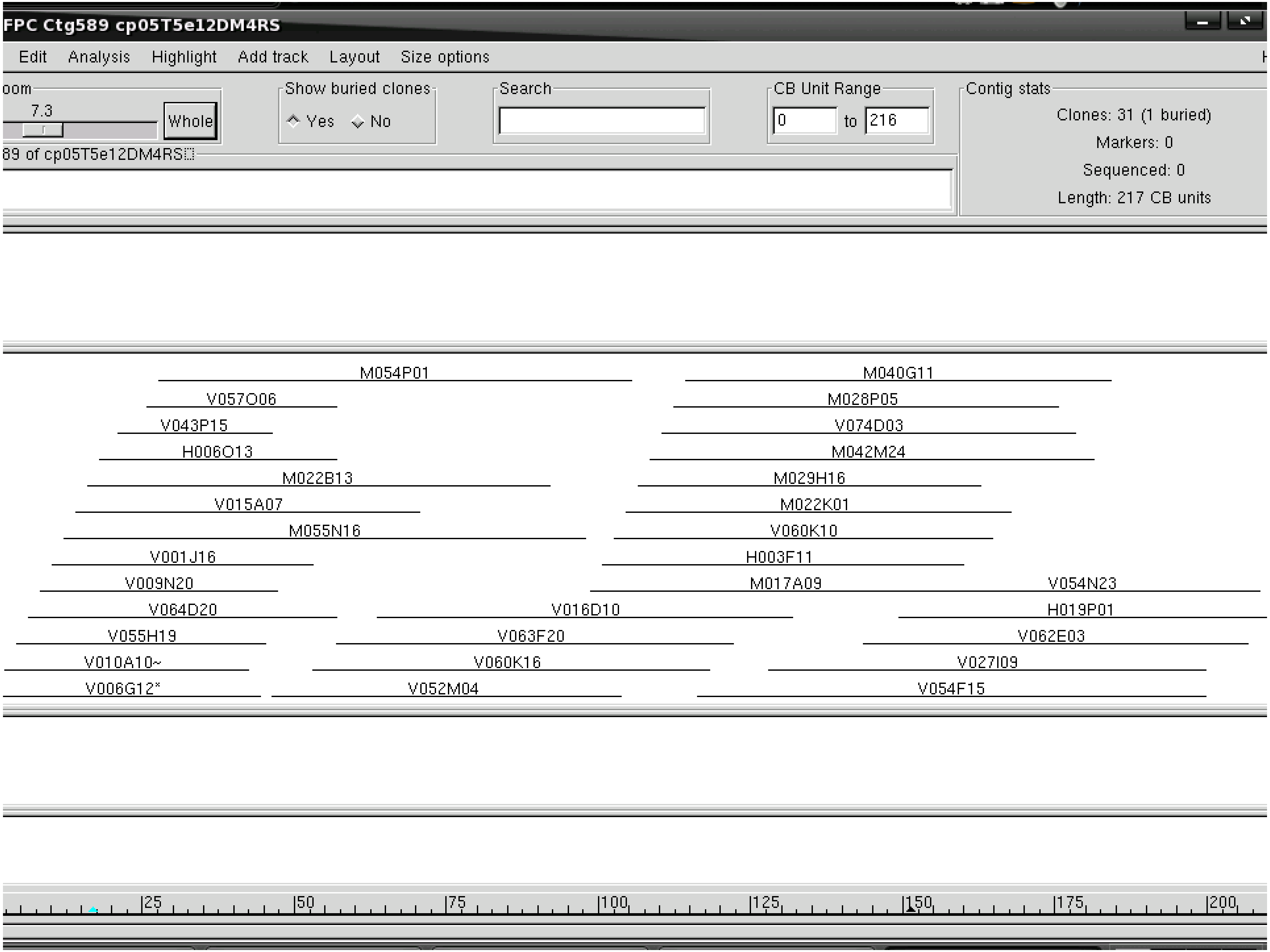
FPC Ctg589 cp05T5e12DM4RS
Edit
Analysis
Highlight
Add track
Layout
Size options
oom
7.3
Whole
Show buried clones
Yes
No
Search
CB Unit Range
0
to
216
Contig stats
Clones: 31 (1 buried)
Markers: 0
Sequenced: 0
Length: 217 CB units
89 of cp05T5e12DM4RS
M054P01
M040G11
V057O06
M028P05
V043P15
V074D03
H006O13
M042M24
M022B13
M029H16
V015A07
M022K01
M055N16
V060K10
V001J16
H003F11
V009N20
M017A09
V054N23
V064D20
V016D10
H019P01
V055H19
V063F20
V062E03
V010A10~
V060K16
V027I09
V006G12*
V052M04
V054F15
25
50
75
100
125
150
175
200

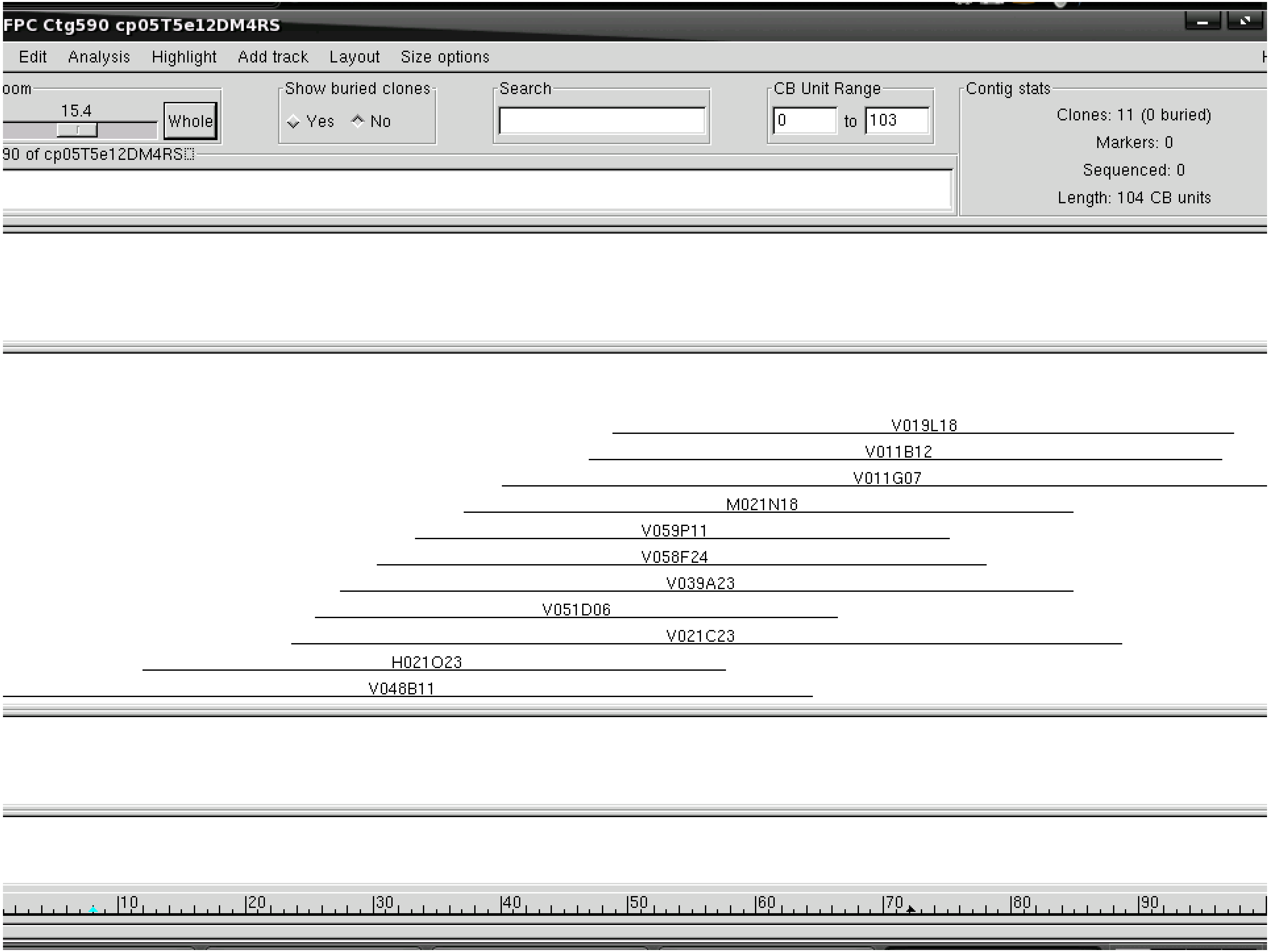
FPC Ctg590 cp05T5e12DM4RS
Edit
Analysis
Highlight
Add track
Layout
Size options
oom
15.4
Whole
Show buried clones
Yes
No
Search
CB Unit Range
0
to
103
Contig stats
Clones: 11 (0 buried)
Markers: 0
Sequenced: 0
Length: 104 CB units
90 of cp05T5e12DM4RS
V019L18
V011B12
V011G07
M021N18
V059P11
V058F24
V039A23
V051D06
V021C23
H021O23
V048B11
10
20
30
40
50
60
70
80
90

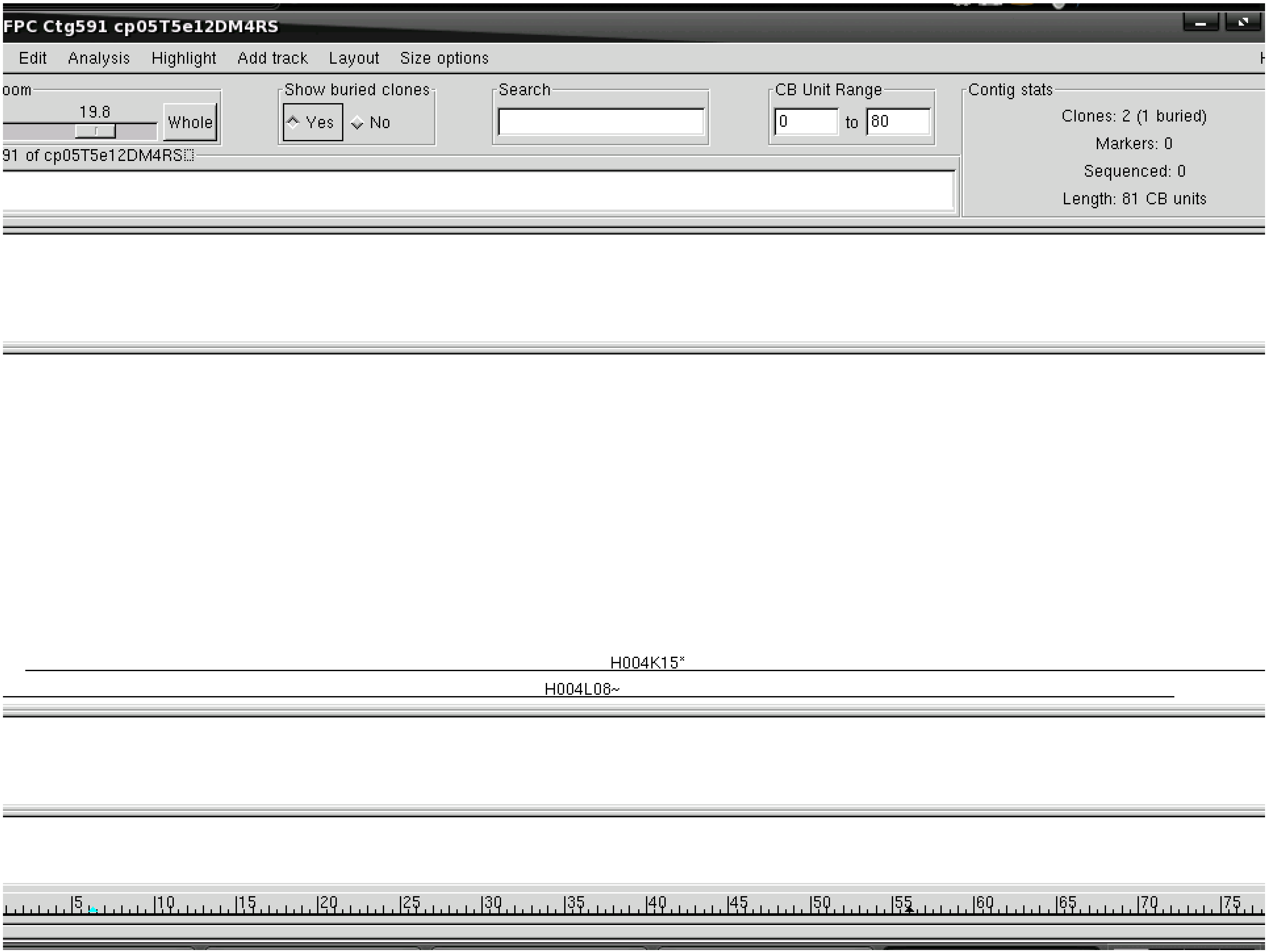
FPC Ctg591 cp05T5e12DM4RS
Edit
Analysis
Highlight
Add track
Layout
Size options
oom
19.8
Whole
Show buried clones
Yes
No
Search
CB Unit Range
0
to
80
Contig stats
Clones: 2 (1 buried)
Markers: 0
Sequenced: 0
Length: 81 CB units
91 of cp05T5e12DM4RS
H004K15*
H004L08~
5 10 15 20 25 30 35 40 45 50 55 60 65 70 75

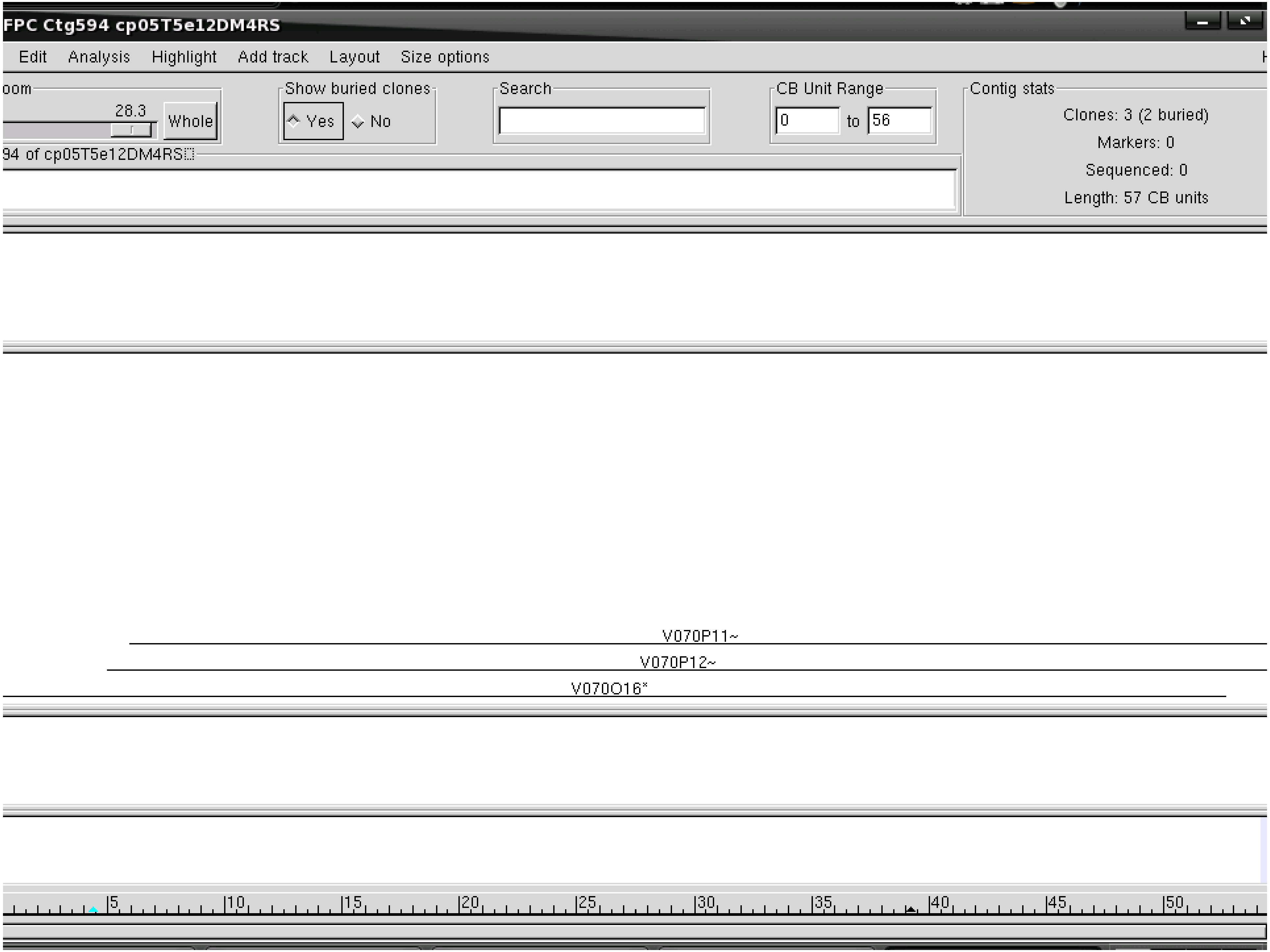
FPC Ctg594 cp05T5e12DM4RS
Edit
Analysis
Highlight
Add track
Layout
Size options
oom
28.3
Whole
Show buried clones
Yes
No
Search
CB Unit Range
0
to
56
Contig stats
Clones: 3 (2 buried)
Markers: 0
Sequenced: 0
Length: 57 CB units
94 of cp05T5e12DM4RS
V070P11~
V070P12~
V070O16*
5
10
15
20
25
30
35
40
45
50

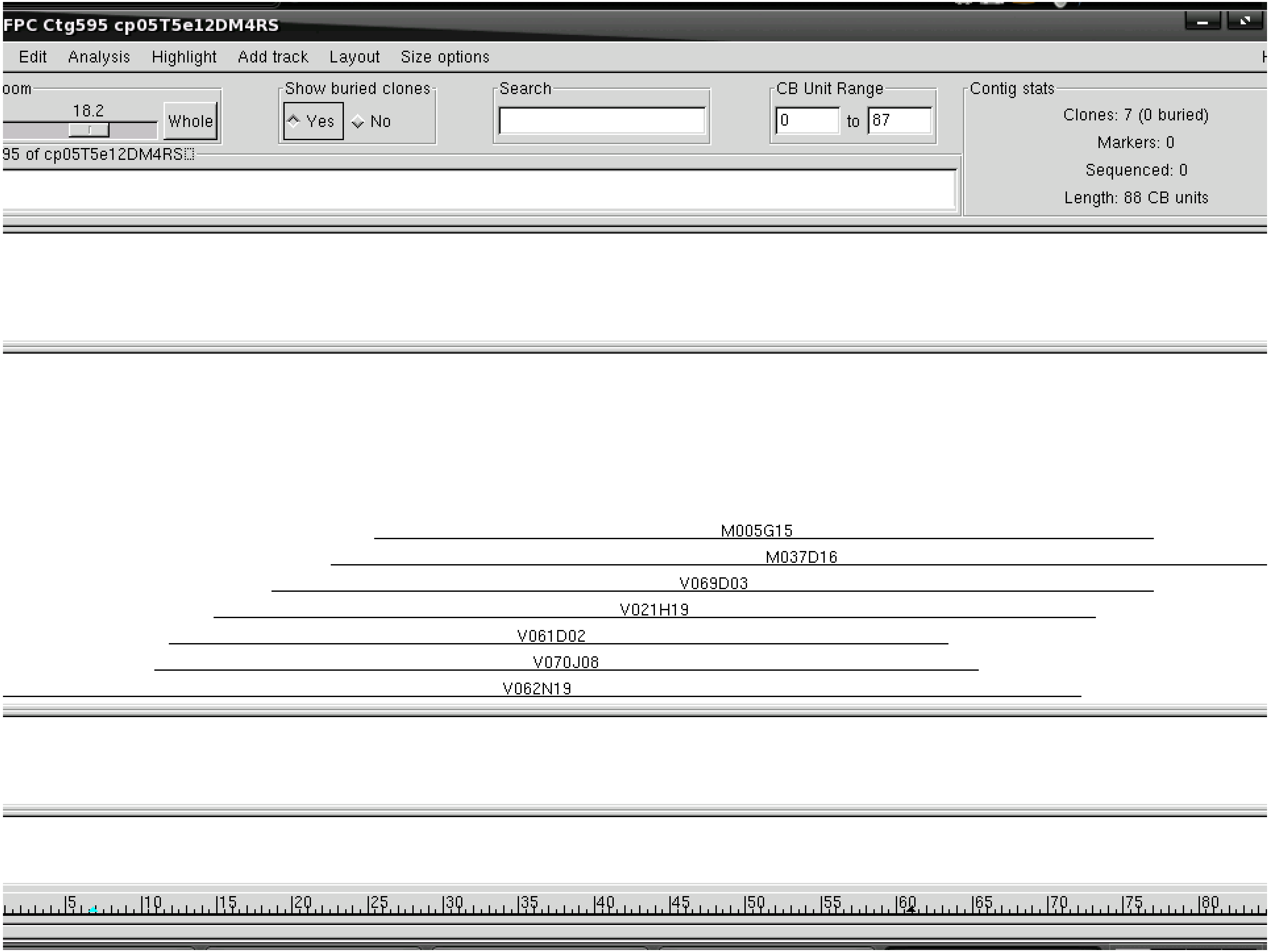
FPC Ctg595 cp05T5e12DM4RS
Edit
Analysis
Highlight
Add track
Layout
Size options
oom
18.2
Whole
Show buried clones
Yes
No
Search
CB Unit Range
0
to
87
Contig stats
Clones: 7 (0 buried)
Markers: 0
Sequenced: 0
Length: 88 CB units
95 of cp05T5e12DM4RS
M005G15
M037D16
V069D03
V021H19
V061D02
V070J08
V062N19
5
10
15
20
25
30
35
40
45
50
55
60
65
70
75
80

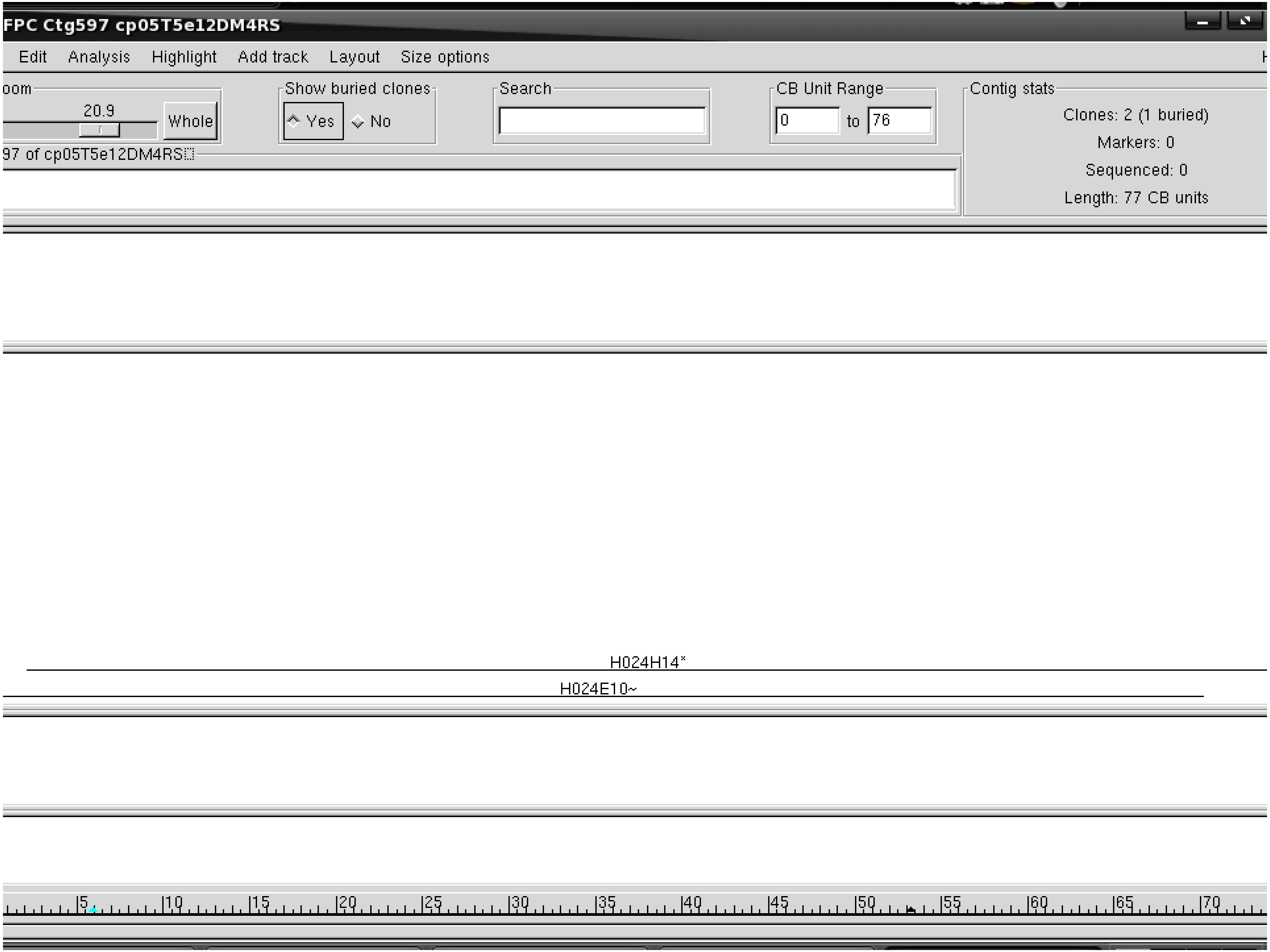
FPC Ctg597 cp05T5e12DM4RS
Edit Analysis Highlight Add track Layout Size options
oom
20.9
Whole
Show buried clones
Yes
No
Search
CB Unit Range
0
to
76
Contig stats
Clones: 2 (1 buried)
Markers: 0
Sequenced: 0
Length: 77 CB units
97 of cp05T5e12DM4RS
H024H14*
H024E10~
5 10 15 20 25 30 35 40 45 50 55 60 65 70

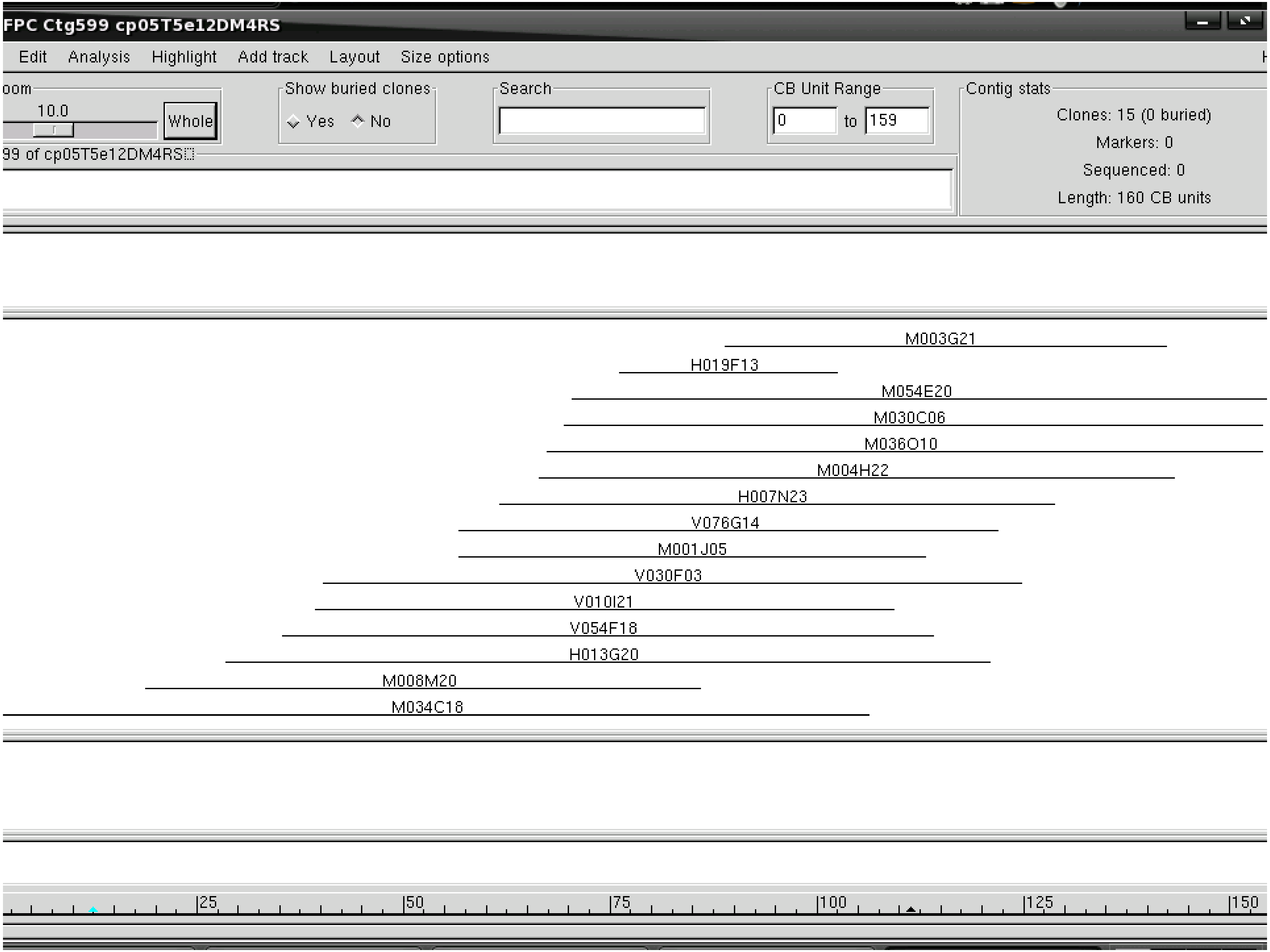
FPC Ctg599 cp05T5e12DM4RS
Edit
Analysis
Highlight
Add track
Layout
Size options
oom
10.0
Whole
Show buried clones
Yes
No
Search
CB Unit Range
0
to
159
Contig stats
Clones: 15 (0 buried)
Markers: 0
Sequenced: 0
Length: 160 CB units
99 of cp05T5e12DM4RS
M003G21
H019F13
M054E20
M030C06
M036O10
M004H22
H007N23
V076G14
M001J05
V030F03
V010I21
V054F18
H013G20
M008M20
M034C18
25
50
75
100
125
150

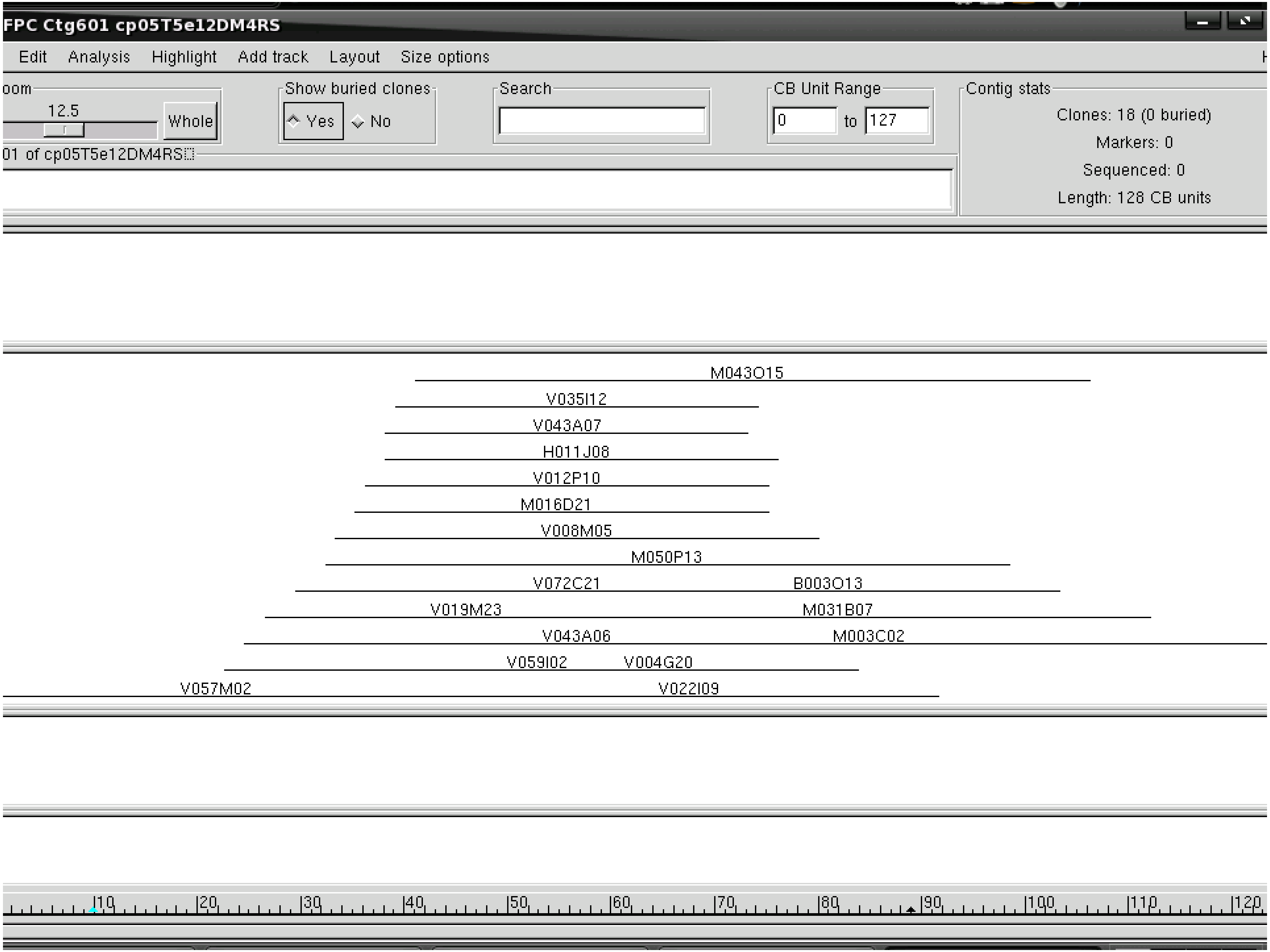
FPC Ctg601 cp05T5e12DM4RS
Edit
Analysis
Highlight
Add track
Layout
Size options
oom
12.5
Whole
Show buried clones
Yes
No
Search
CB Unit Range
0
to
127
Contig stats
Clones: 18 (0 buried)
Markers: 0
Sequenced: 0
Length: 128 CB units
01 of cp05T5e12DM4RS
M043O15
V035I12
V043A07
H011J08
V012P10
M016D21
V008M05
M050P13
V072C21
B003O13
V019M23
M031B07
V043A06
M003C02
V059I02
V004G20
V057M02
V022I09
10
20
30
40
50
60
70
80
90
100
110
120

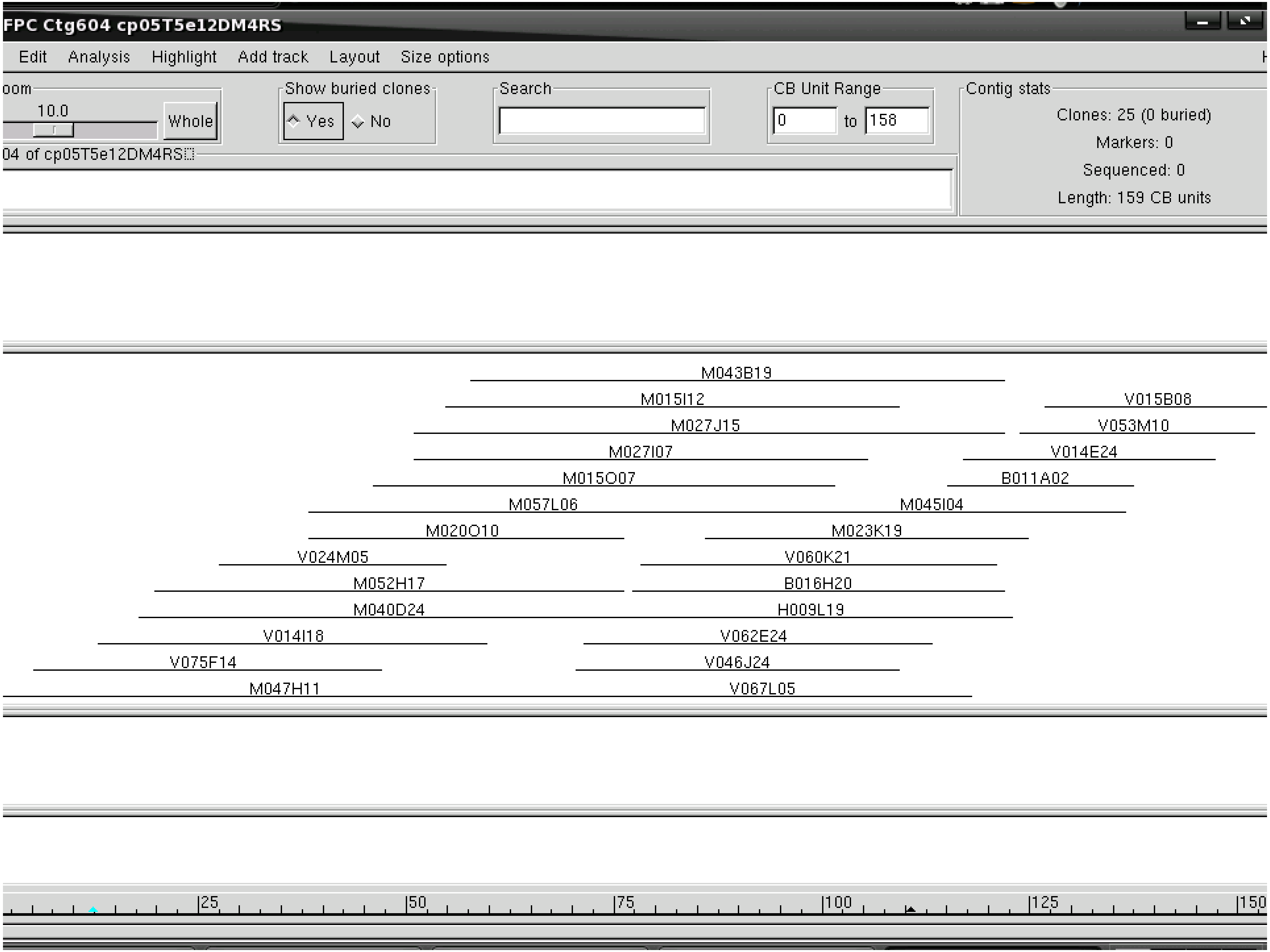
FPC Ctg604 cp05T5e12DM4RS
Edit
Analysis
Highlight
Add track
Layout
Size options
oom
10.0
Whole
Show buried clones
Yes
No
Search
CB Unit Range
0
to
158
Contig stats
Clones: 25 (0 buried)
Markers: 0
Sequenced: 0
Length: 159 CB units
04 of cp05T5e12DM4RS
M043B19
M015I12
V015B08
M027J15
V053M10
M027I07
V014E24
M015O07
B011A02
M057L06
M045I04
M020O10
M023K19
V024M05
V060K21
M052H17
B016H20
M040D24
H009L19
V014I18
V062E24
V075F14
V046J24
M047H11
V067L05
25
50
75
100
125
150

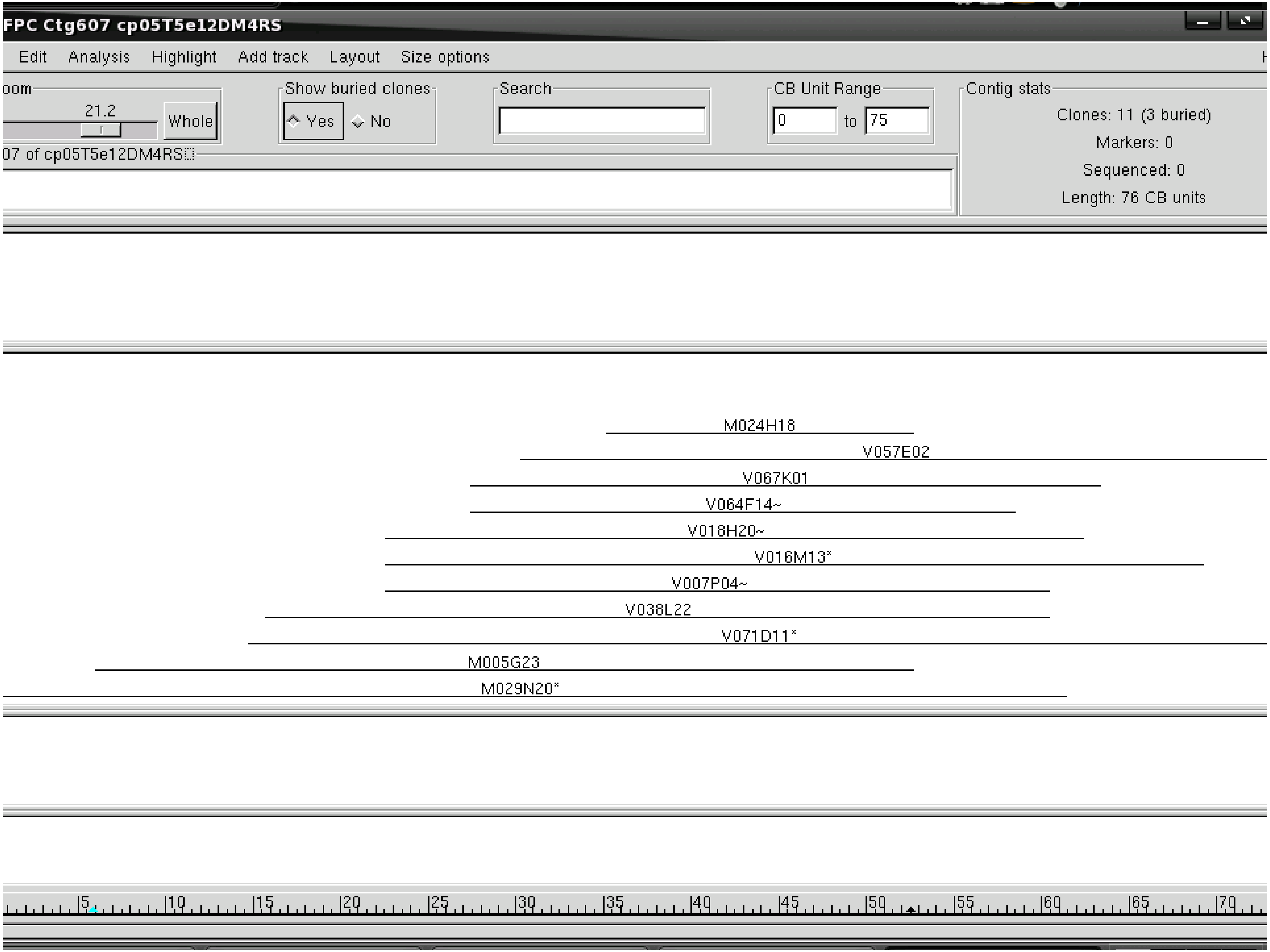
FPC Ctg607 cp05T5e12DM4RS
Edit Analysis Highlight Add track Layout Size options
oom
21.2
Whole
Show buried clones
Yes
No
Search
CB Unit Range
0
to
75
Contig stats
Clones: 11 (3 buried)
Markers: 0
Sequenced: 0
Length: 76 CB units
07 of cp05T5e12DM4RS
M024H18
V057E02
V067K01
V064F14~
V018H20~
V016M13*
V007P04~
V038L22
V071D11*
M005G23
M029N20*
5 10 15 20 25 30 35 40 45 50 55 60 65 70

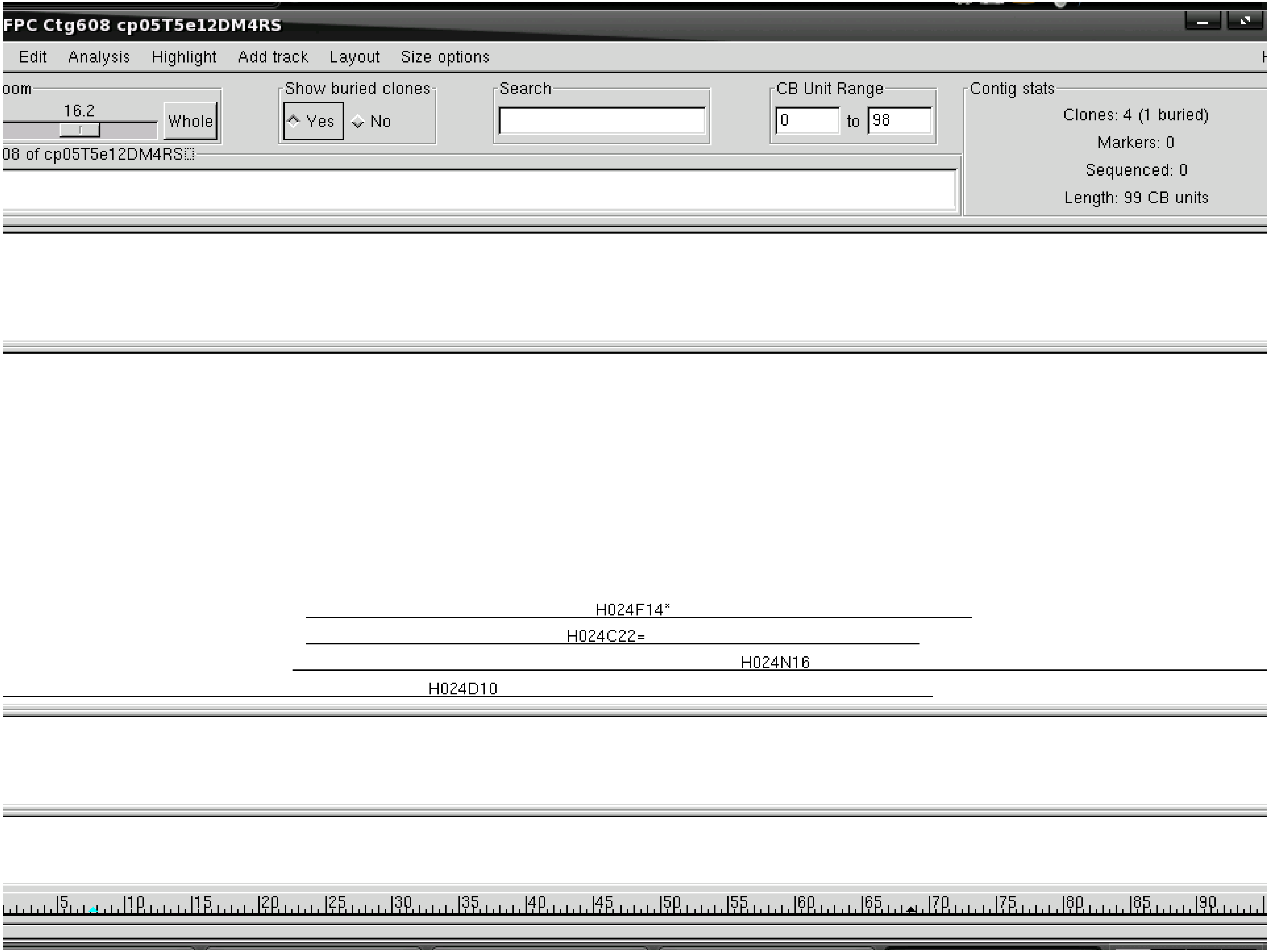
FPC Ctg608 cp05T5e12DM4RS
Edit
Analysis
Highlight
Add track
Layout
Size options
oom
16.2
Whole
Show buried clones
Yes
No
Search
CB Unit Range
0
to
98
Contig stats
Clones: 4 (1 buried)
Markers: 0
Sequenced: 0
Length: 99 CB units
08 of cp05T5e12DM4RS
H024F14*
H024C22=
H024N16
H024D10
5 10 15 20 25 30 35 40 45 50 55 60 65 70 75 80 85 90

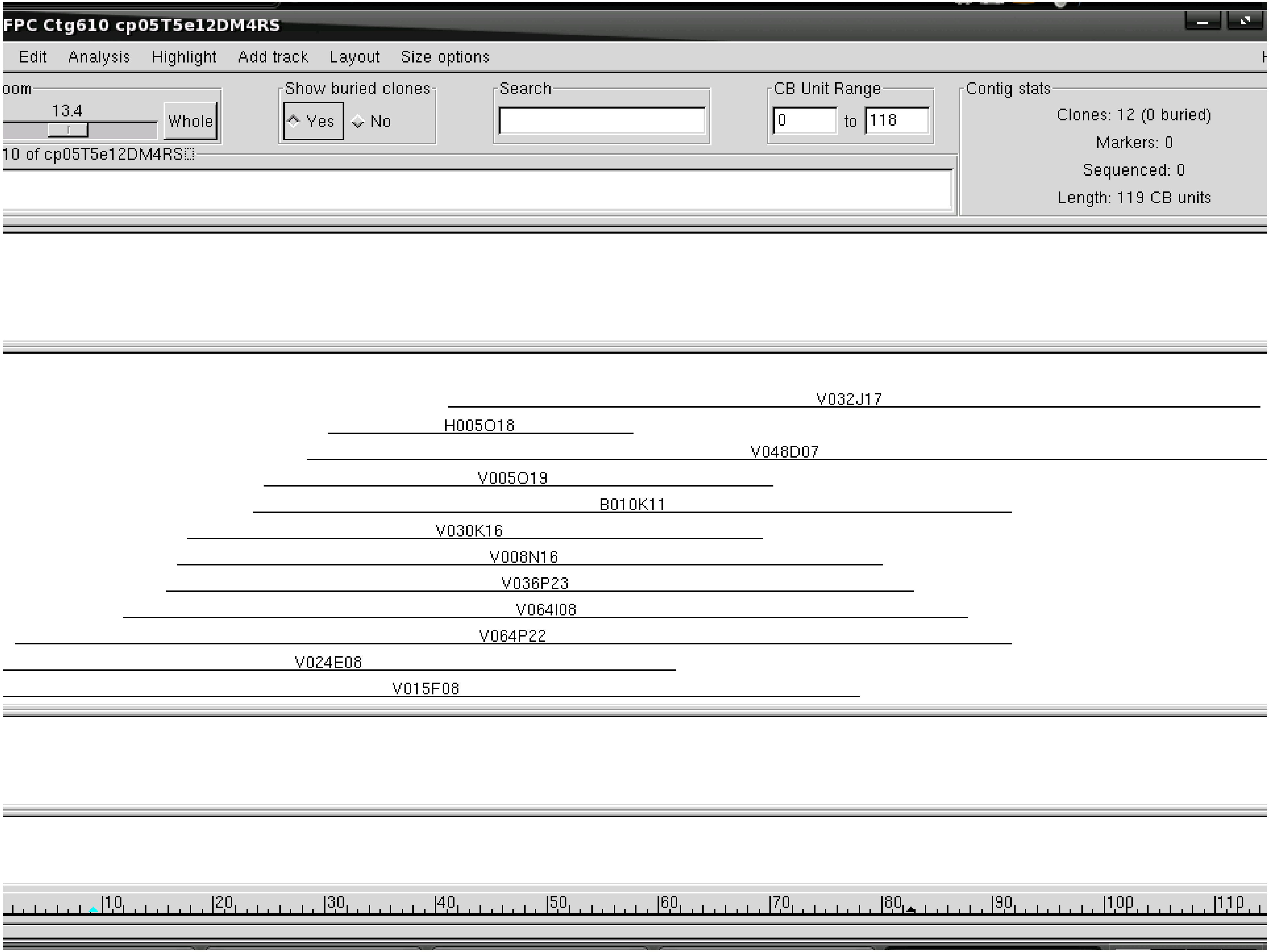
FPC Ctg610 cp05T5e12DM4RS
Edit
Analysis
Highlight
Add track
Layout
Size options
oom
13.4
Whole
Show buried clones
Yes
No
Search
CB Unit Range
0
to
118
Contig stats
Clones: 12 (0 buried)
Markers: 0
Sequenced: 0
Length: 119 CB units
10 of cp05T5e12DM4RS
V032J17
H005O18
V048D07
V005O19
B010K11
V030K16
V008N16
V036P23
V064I08
V064P22
V024E08
V015F08
10
20
30
40
50
60
70
80
90
100
110

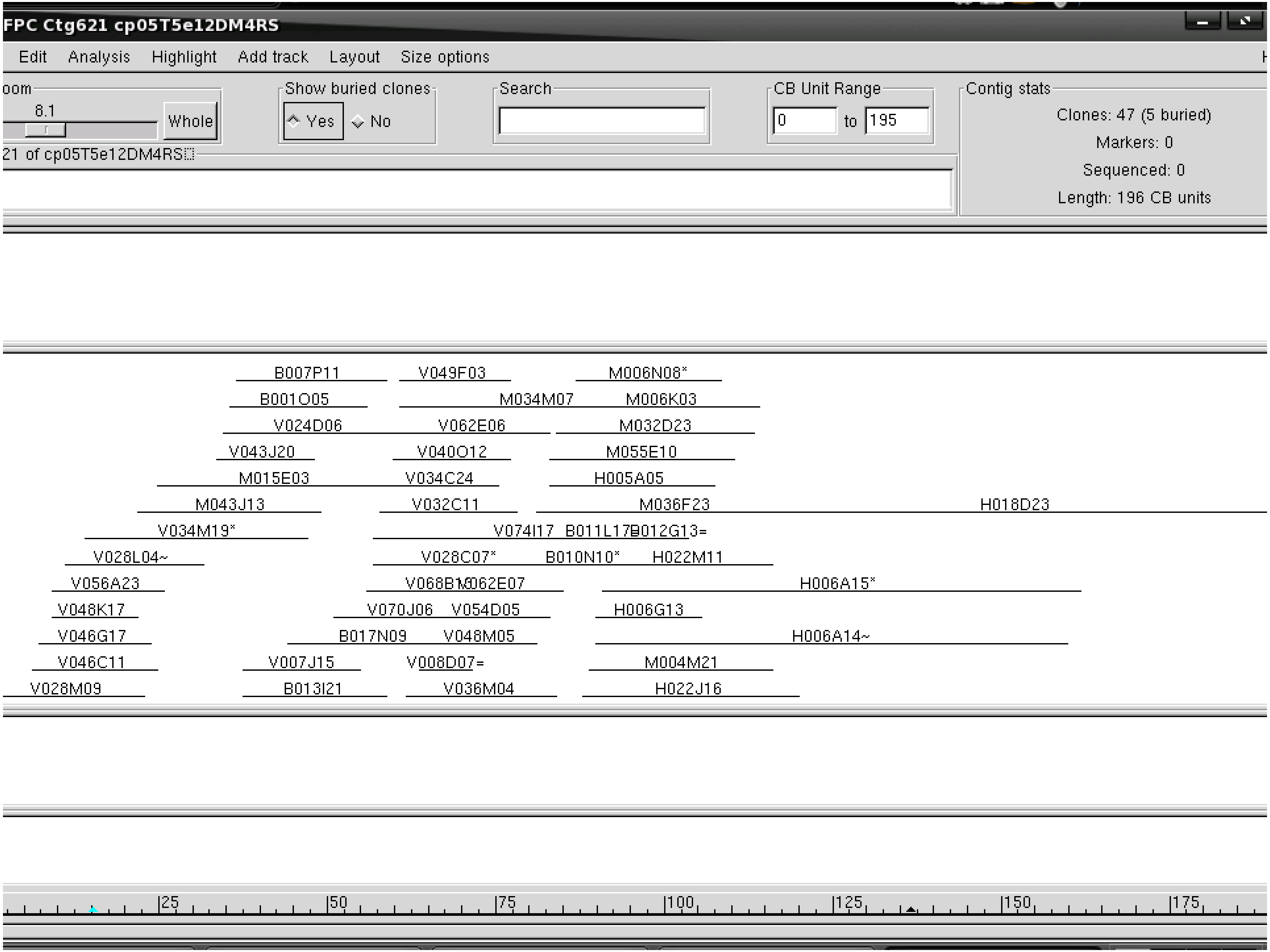
FPC Ctg621 cp05T5e12DM4RS
Edit
Analysis
Highlight
Add track
Layout
Size options
oom
8.1
Whole
Show buried clones
Yes
No
Search
CB Unit Range
0
to
195
Contig stats
Clones: 47 (5 buried)
Markers: 0
Sequenced: 0
Length: 196 CB units
21 of cp05T5e12DM4RS
B007P11
V049F03
M006N08*
B001O05
M034M07
M006K03
V024D06
V062E06
M032D23
V043J20
V040O12
M055E10
M015E03
V034C24
H005A05
M043J13
V032C11
M036F23
H018D23
V034M19*
V074I17
B011L17
B012G13=
V028L04~
V028C07*
B010N10*
H022M11
V056A23
V068B1
M062E07
H006A15*
V048K17
V070J06
V054D05
H006G13
V046G17
B017N09
V048M05
H006A14~
V046C11
V007J15
V008D07=
M004M21
V028M09
B013I21
V036M04
H022J16
25
50
75
100
125
150
175

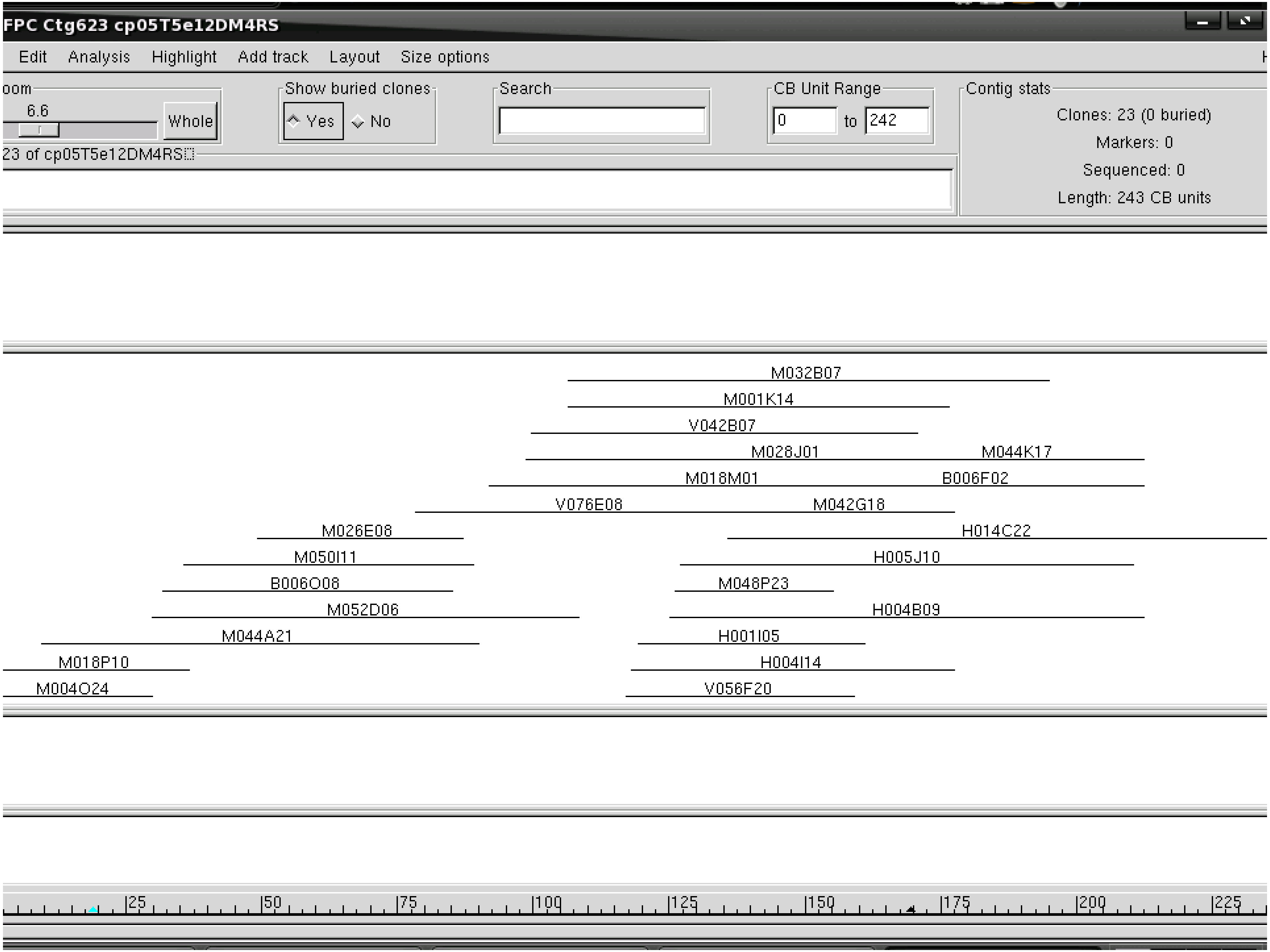

FPC Ctg623 cp05T5e12DM4RS
Edit
Analysis
Highlight
Add track
Layout
Size options
oom
6.6
Whole
Show buried clones
Yes
No
Search
CB Unit Range
0
to
242
Contig stats
Clones: 23 (0 buried)
Markers: 0
Sequenced: 0
Length: 243 CB units
23 of cp05T5e12DM4RS
M032B07
M001K14
V042B07
M028J01
M044K17
M018M01
B006F02
V076E08
M042G18
M026E08
H014C22
M050I11
H005J10
B006O08
M048P23
M052D06
H004B09
M044A21
H001I05
M018P10
H004I14
M004O24
V056F20
|25
|50
|75
|100
|125
|150
|175
|200
|225

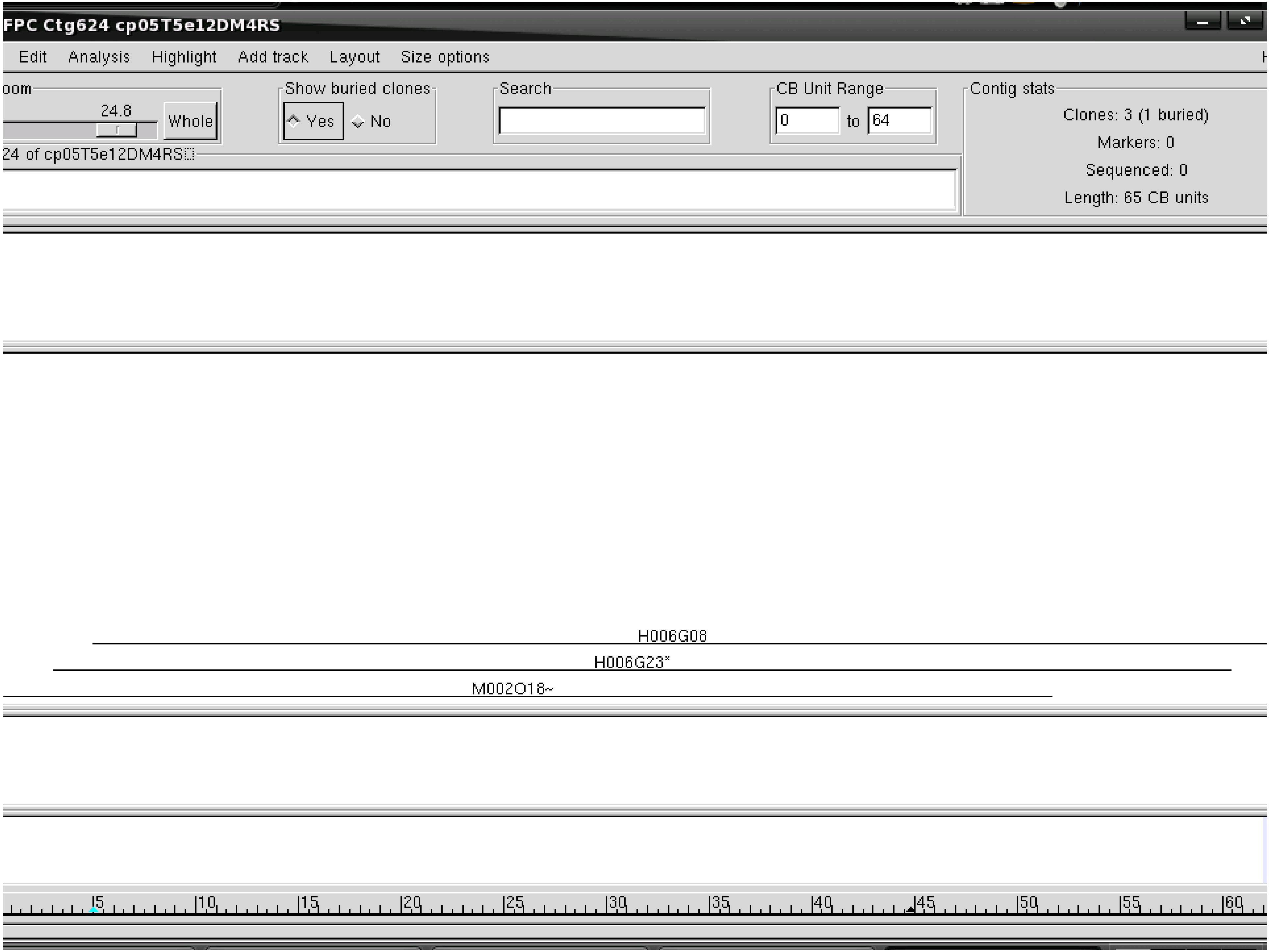
FPC Ctg624 cp05T5e12DM4RS
Edit Analysis Highlight Add track Layout Size options
oom
24.8
Whole
Show buried clones
Yes
No
Search
CB Unit Range
0
to
64
Contig stats
Clones: 3 (1 buried)
Markers: 0
Sequenced: 0
Length: 65 CB units
24 of cp05T5e12DM4RS
H006G08
H006G23*
M002O18~
5 10 15 20 25 30 35 40 45 50 55 60

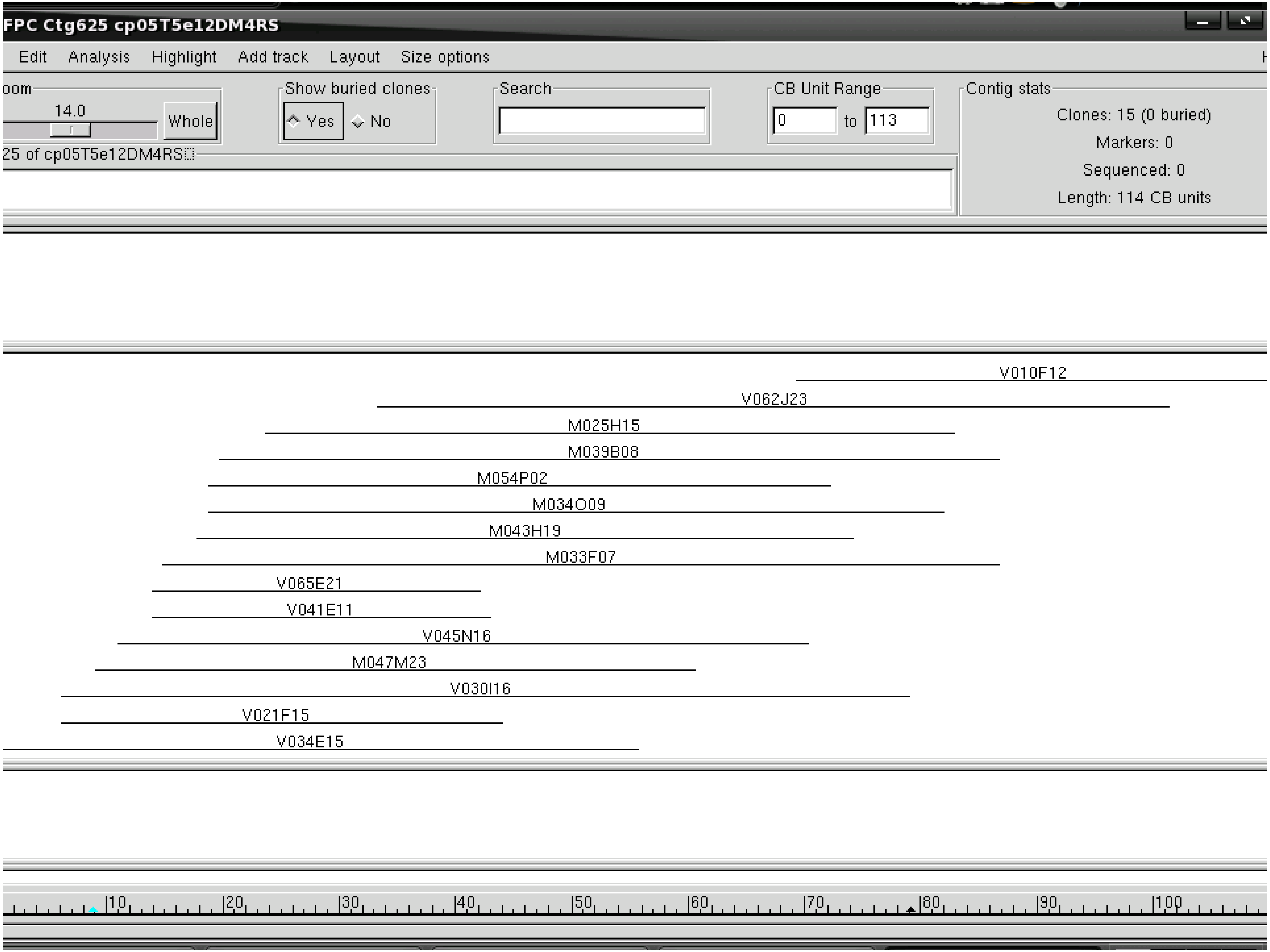
FPC Ctg625 cp05T5e12DM4RS
Edit
Analysis
Highlight
Add track
Layout
Size options
oom
14.0
Whole
Show buried clones
Yes
No
Search
CB Unit Range
0
to
113
Contig stats
Clones: 15 (0 buried)
Markers: 0
Sequenced: 0
Length: 114 CB units
25 of cp05T5e12DM4RS
V010F12
V062J23
M025H15
M039B08
M054P02
M034O09
M043H19
M033F07
V065E21
V041E11
V045N16
M047M23
V030I16
V021F15
V034E15
10
20
30
40
50
60
70
80
90
100

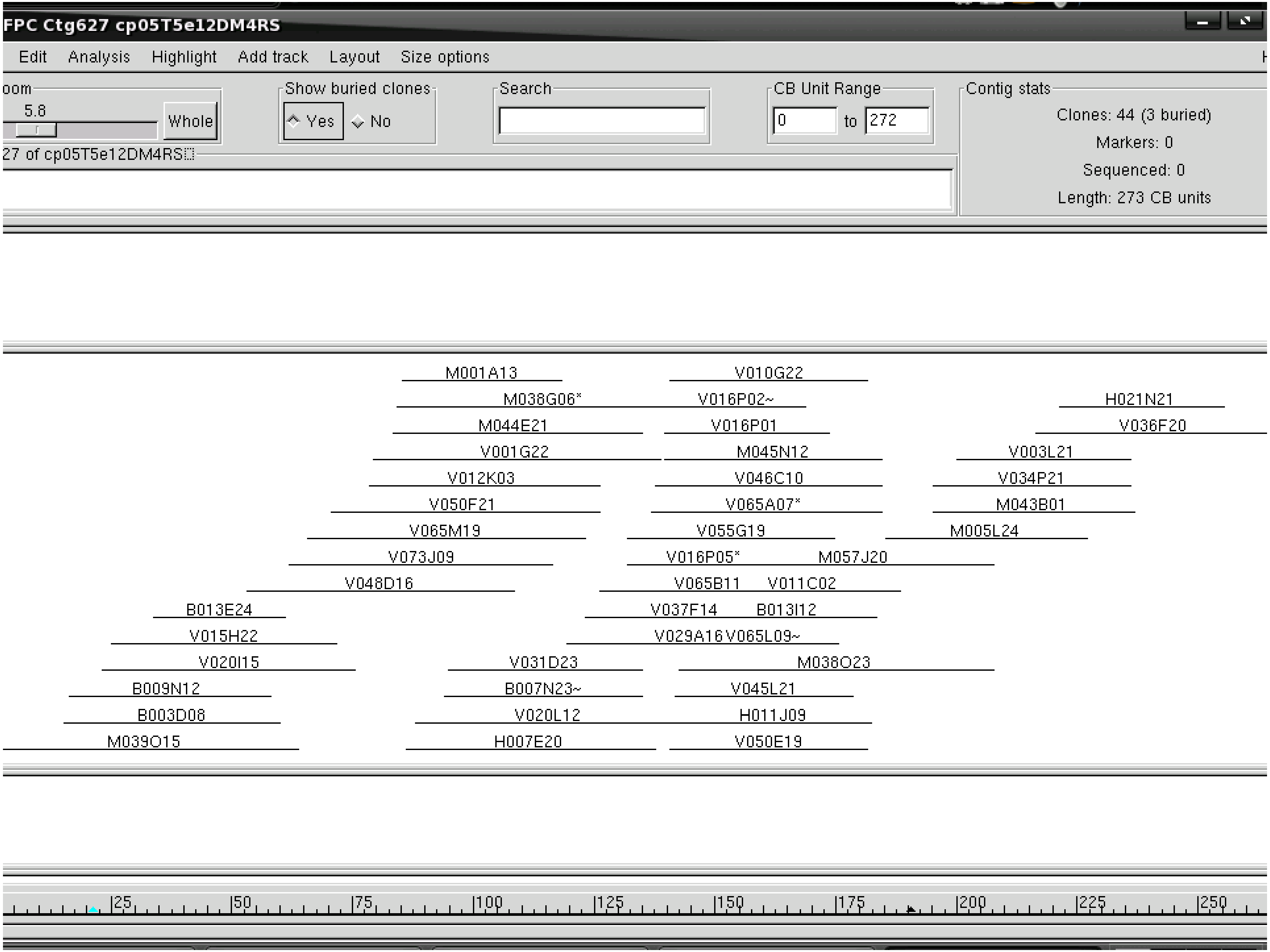
FPC Ctg627 cp05T5e12DM4RS
Edit
Analysis
Highlight
Add track
Layout
Size options
oom
5.8
Whole
Show buried clones
Yes
No
Search
CB Unit Range
0
to
272
Contig stats
Clones: 44 (3 buried)
Markers: 0
Sequenced: 0
Length: 273 CB units
27 of cp05T5e12DM4RS
M001A13
V010G22
M038G06*
V016P02~
H021N21
M044E21
V016P01
V036F20
V001G22
M045N12
V003L21
V012K03
V046C10
V034P21
V050F21
V065A07*
M043B01
V065M19
V055G19
M005L24
V073J09
V016P05*
M057J20
V048D16
V065B11
V011C02
B013E24
V037F14
B013I12
V015H22
V029A16
V065L09~
V020I15
V031D23
M038O23
B009N12
B007N23~
V045L21
B003D08
V020L12
H011J09
M039O15
H007E20
V050E19
25
50
75
100
125
150
175
200
225
250

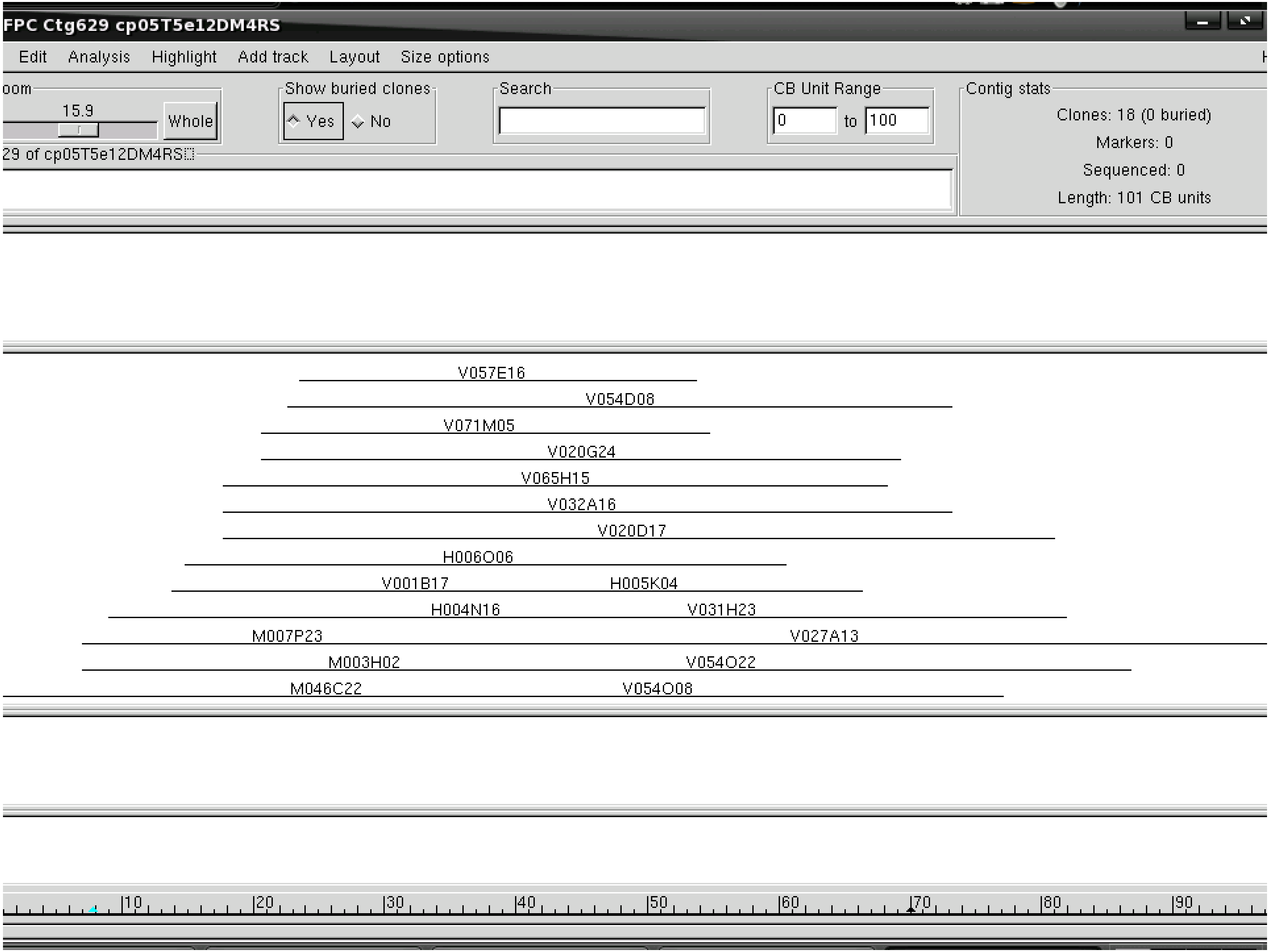
FPC Ctg629 cp05T5e12DM4RS
Edit
Analysis
Highlight
Add track
Layout
Size options
oom
15.9
Whole
Show buried clones
Yes
No
Search
CB Unit Range
0
to
100
Contig stats
Clones: 18 (0 buried)
Markers: 0
Sequenced: 0
Length: 101 CB units
29 of cp05T5e12DM4RS
V057E16
V054D08
V071M05
V020G24
V065H15
V032A16
V020D17
H006O06
V001B17
H005K04
H004N16
V031H23
M007P23
V027A13
M003H02
V054O22
M046C22
V054O08
10
20
30
40
50
60
70
80
90

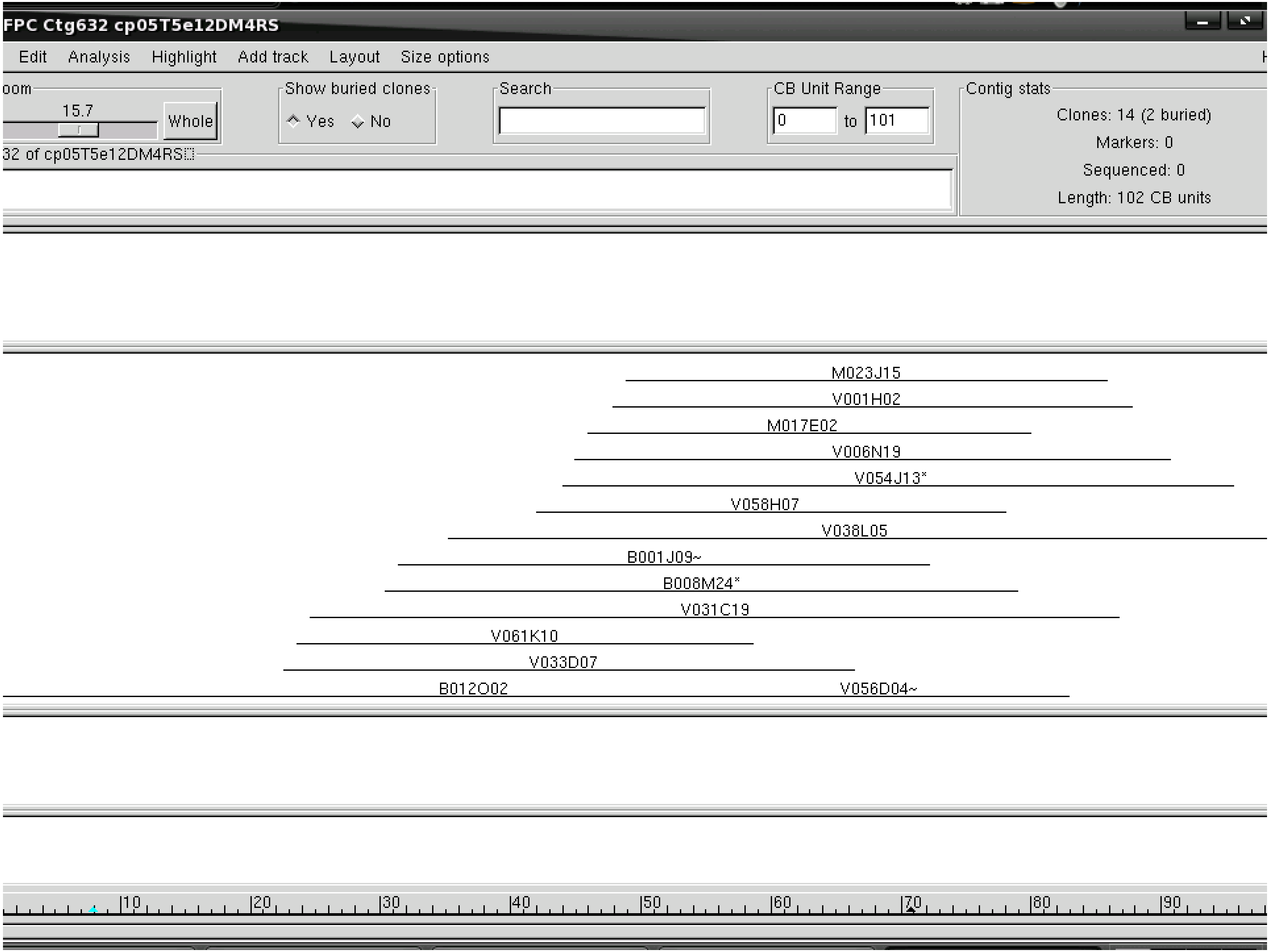
FPC Ctg632 cp05T5e12DM4RS
Edit Analysis Highlight Add track Layout Size options
oom
15.7
Whole
Show buried clones
Yes No
Search
CB Unit Range
0 to 101
Contig stats
Clones: 14 (2 buried)
Markers: 0
Sequenced: 0
Length: 102 CB units
32 of cp05T5e12DM4RS
M023J15
V001H02
M017E02
V006N19
V054J13*
V058H07
V038L05
B001J09~
B008M24*
V031C19
V061K10
V033D07
B012O02
V056D04~
10 20 30 40 50 60 70 80 90

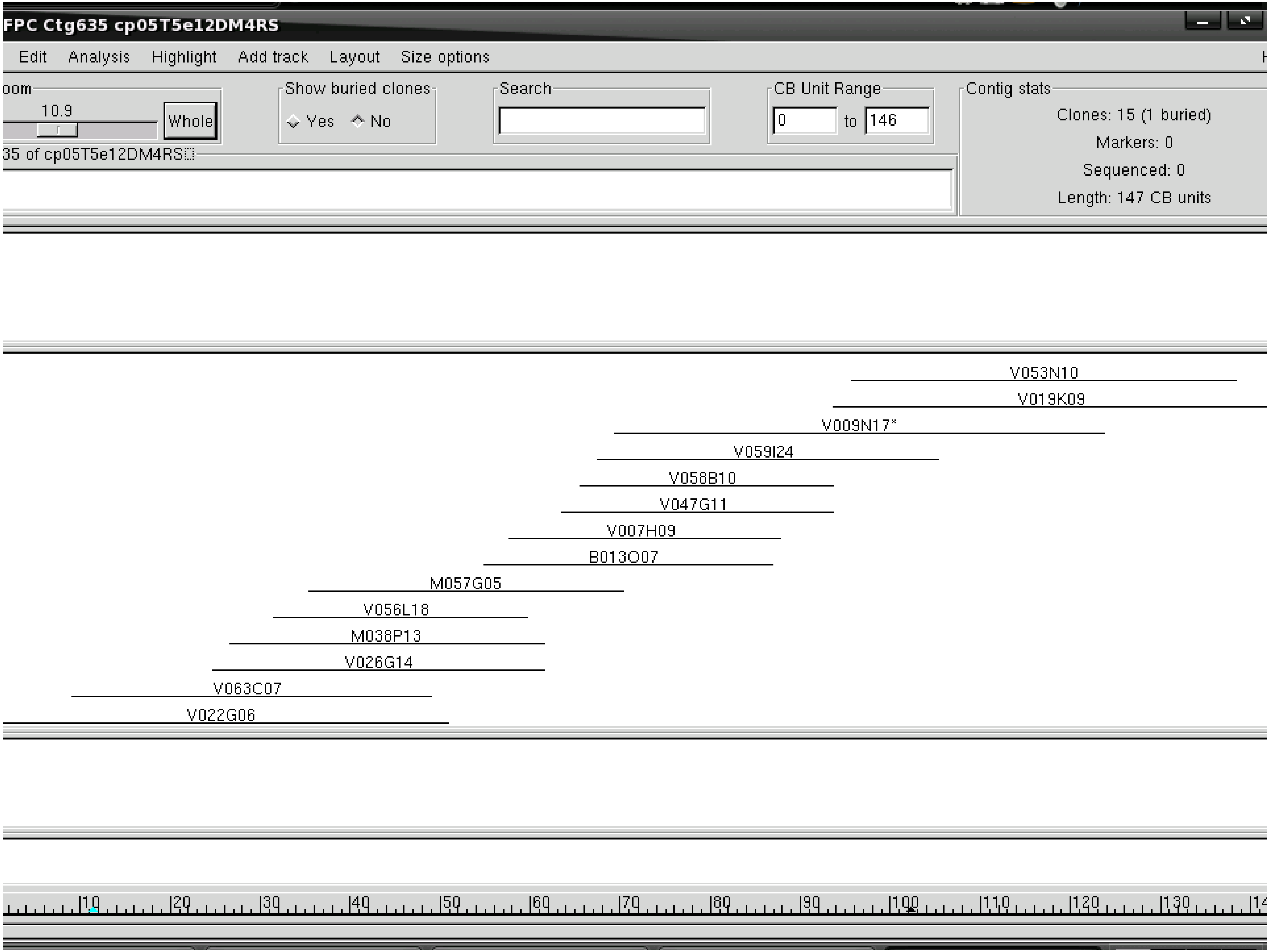
FPC Ctg635 cp05T5e12DM4RS
Edit
Analysis
Highlight
Add track
Layout
Size options
oom
10.9
Whole
Show buried clones
Yes
No
Search
CB Unit Range
0
to
146
Contig stats
Clones: 15 (1 buried)
Markers: 0
Sequenced: 0
Length: 147 CB units
35 of cp05T5e12DM4RS
V053N10
V019K09
V009N17*
V059I24
V058B10
V047G11
V007H09
B013O07
M057G05
V056L18
M038P13
V026G14
V063C07
V022G06
10
20
30
40
50
60
70
80
90
100
110
120
130

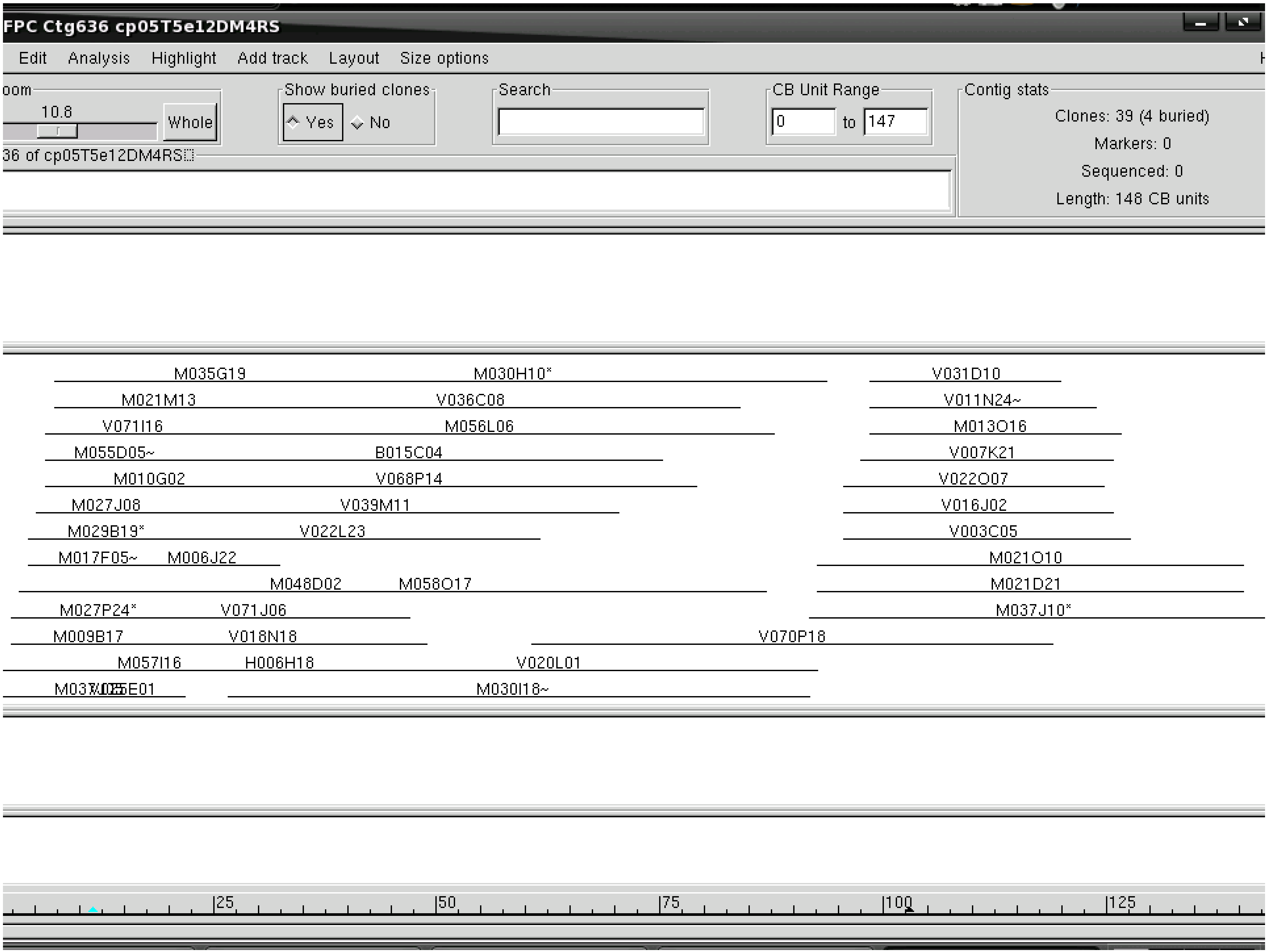
FPC Ctg636 cp05T5e12DM4RS
Edit Analysis Highlight Add track Layout Size options
oom
10.8
Whole
Show buried clones
Yes
No
Search
CB Unit Range
0
to
147
Contig stats
Clones: 39 (4 buried)
Markers: 0
Sequenced: 0
Length: 148 CB units
36 of cp05T5e12DM4RS
M035G19
M030H10*
V031D10
M021M13
V036C08
V011N24~
V071I16
M056L06
M013O16
M055D05~
B015C04
V007K21
M010G02
V068P14
V022O07
M027J08
V039M11
V016J02
M029B19*
V022L23
V003C05
M017F05~
M006J22
M021O10
M048D02
M058O17
M021D21
M027P24*
V071J06
M037J10*
M009B17
V018N18
V070P18
M057I16
H006H18
V020L01
M030I18~
25
50
75
100
125

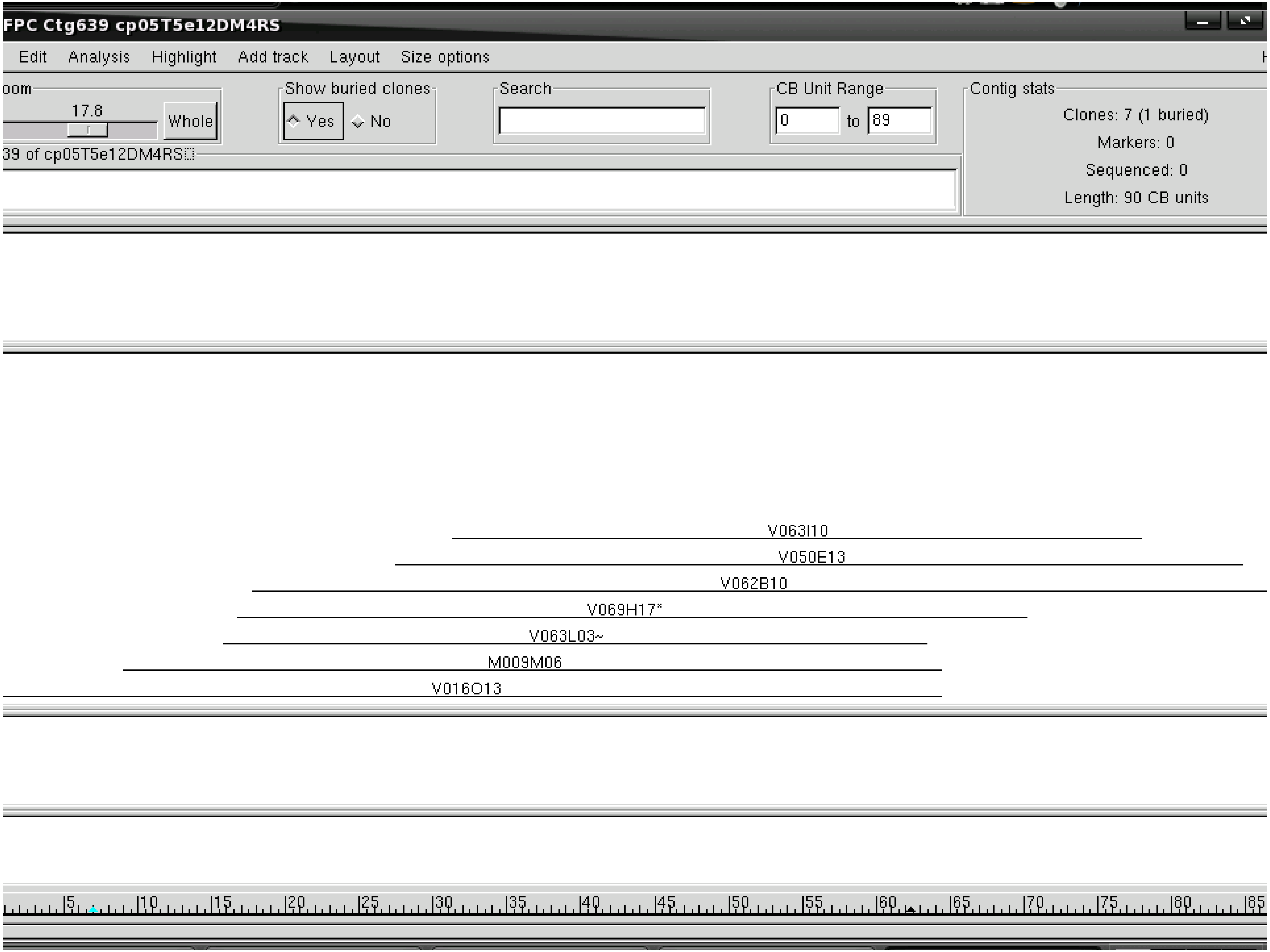
FPC Ctg639 cp05T5e12DM4RS
Edit
Analysis
Highlight
Add track
Layout
Size options
oom
17.8
Whole
Show buried clones
Yes
No
Search
CB Unit Range
0
to
89
Contig stats
Clones: 7 (1 buried)
Markers: 0
Sequenced: 0
Length: 90 CB units
39 of cp05T5e12DM4RS
V063I10
V050E13
V062B10
V069H17*
V063L03~
M009M06
V016O13
5
10
15
20
25
30
35
40
45
50
55
60
65
70
75
80
85

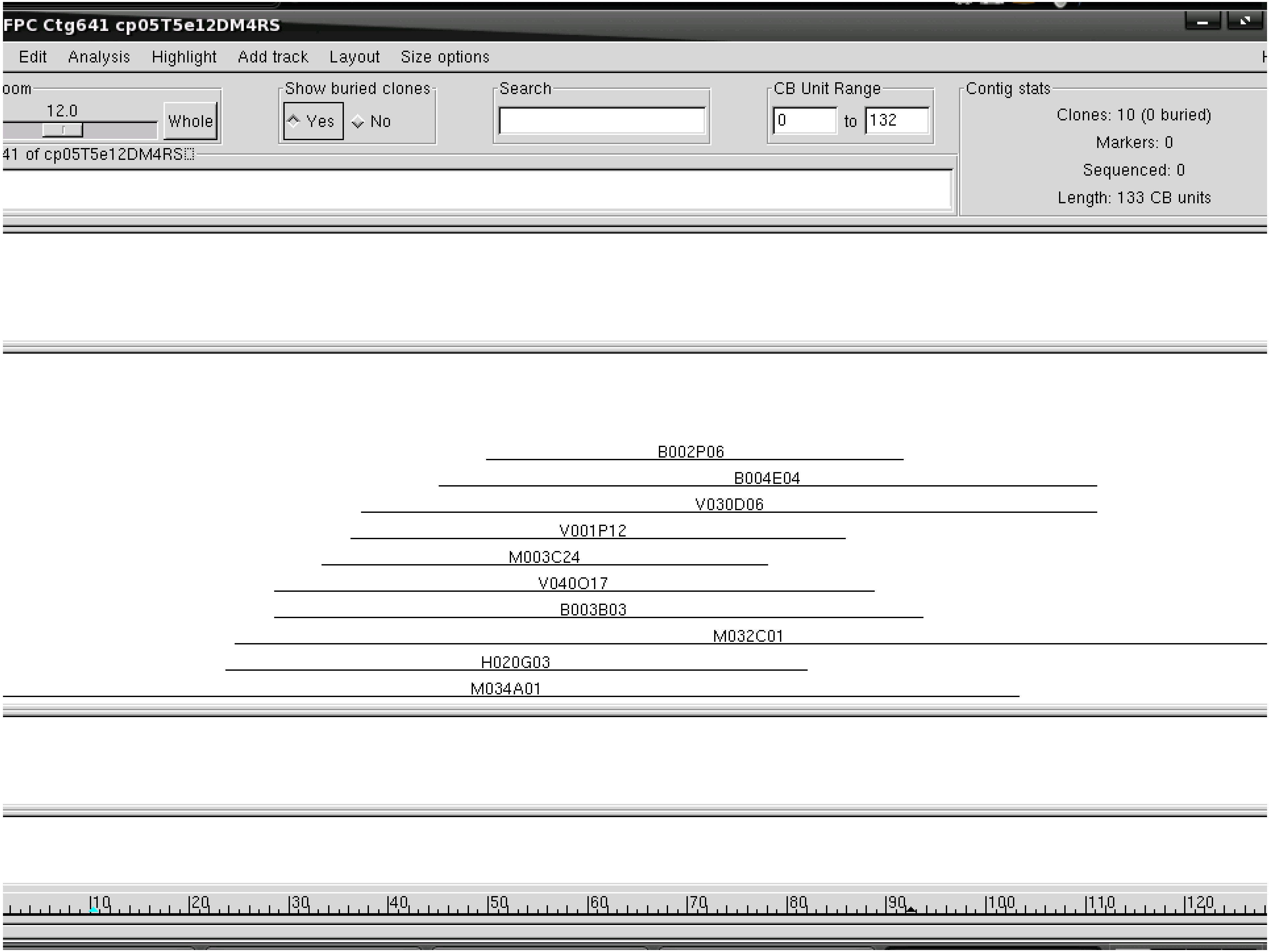
FPC Ctg641 cp05T5e12DM4RS
Edit
Analysis
Highlight
Add track
Layout
Size options
oom
12.0
Whole
Show buried clones
Yes
No
Search
CB Unit Range
0
to
132
Contig stats
Clones: 10 (0 buried)
Markers: 0
Sequenced: 0
Length: 133 CB units
41 of cp05T5e12DM4RS
B002P06
B004E04
V030D06
V001P12
M003C24
V040O17
B003B03
M032C01
H020G03
M034A01
10
20
30
40
50
60
70
80
90
100
110
120

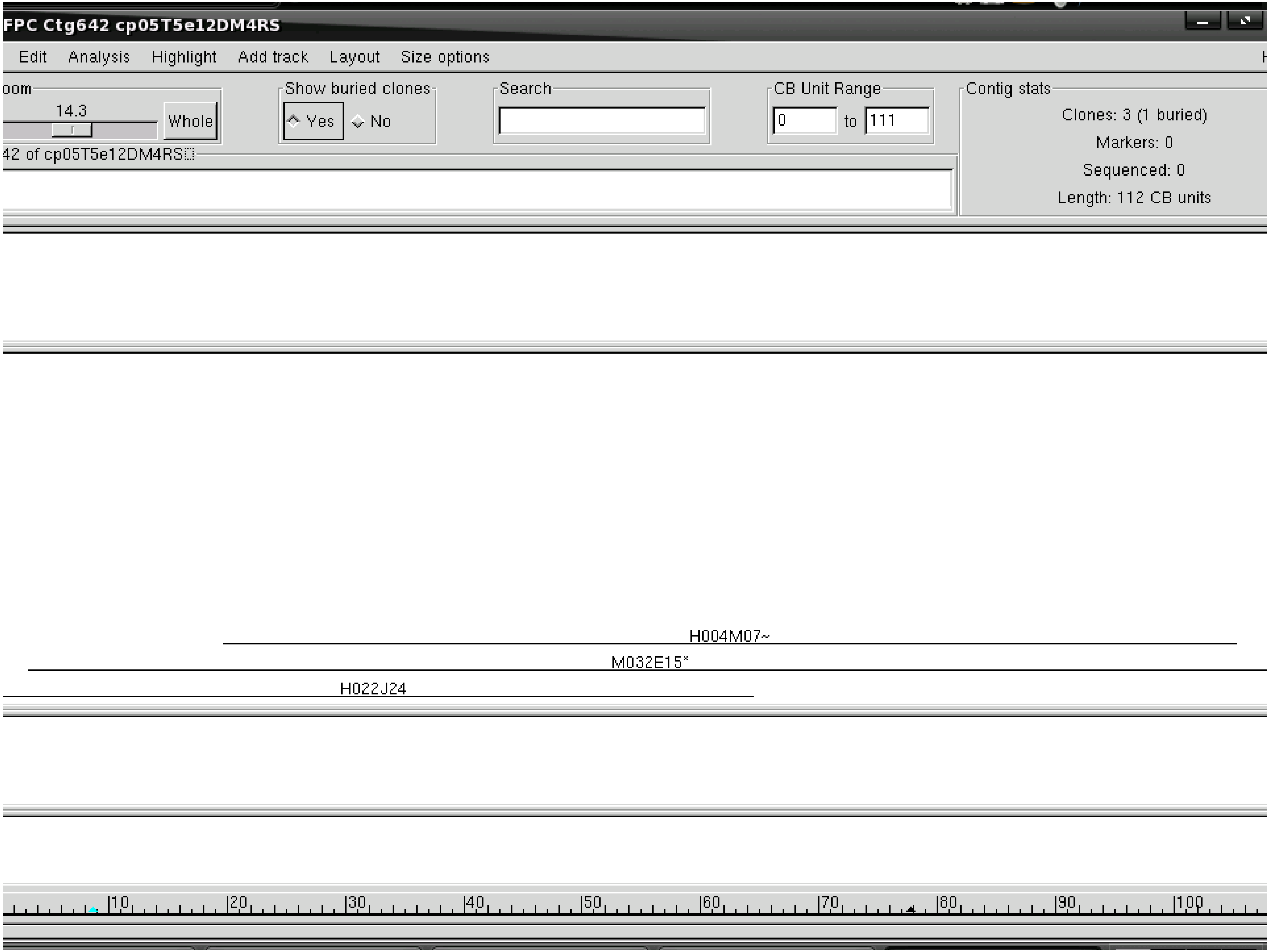
FPC Ctg642 cp05T5e12DM4RS
Edit
Analysis
Highlight
Add track
Layout
Size options
oom
14.3
Whole
Show buried clones
Yes
No
Search
CB Unit Range
0
to
111
Contig stats
Clones: 3 (1 buried)
Markers: 0
Sequenced: 0
Length: 112 CB units
42 of cp05T5e12DM4RS
H004M07~
M032E15*
H022J24
10
20
30
40
50
60
70
80
90
100

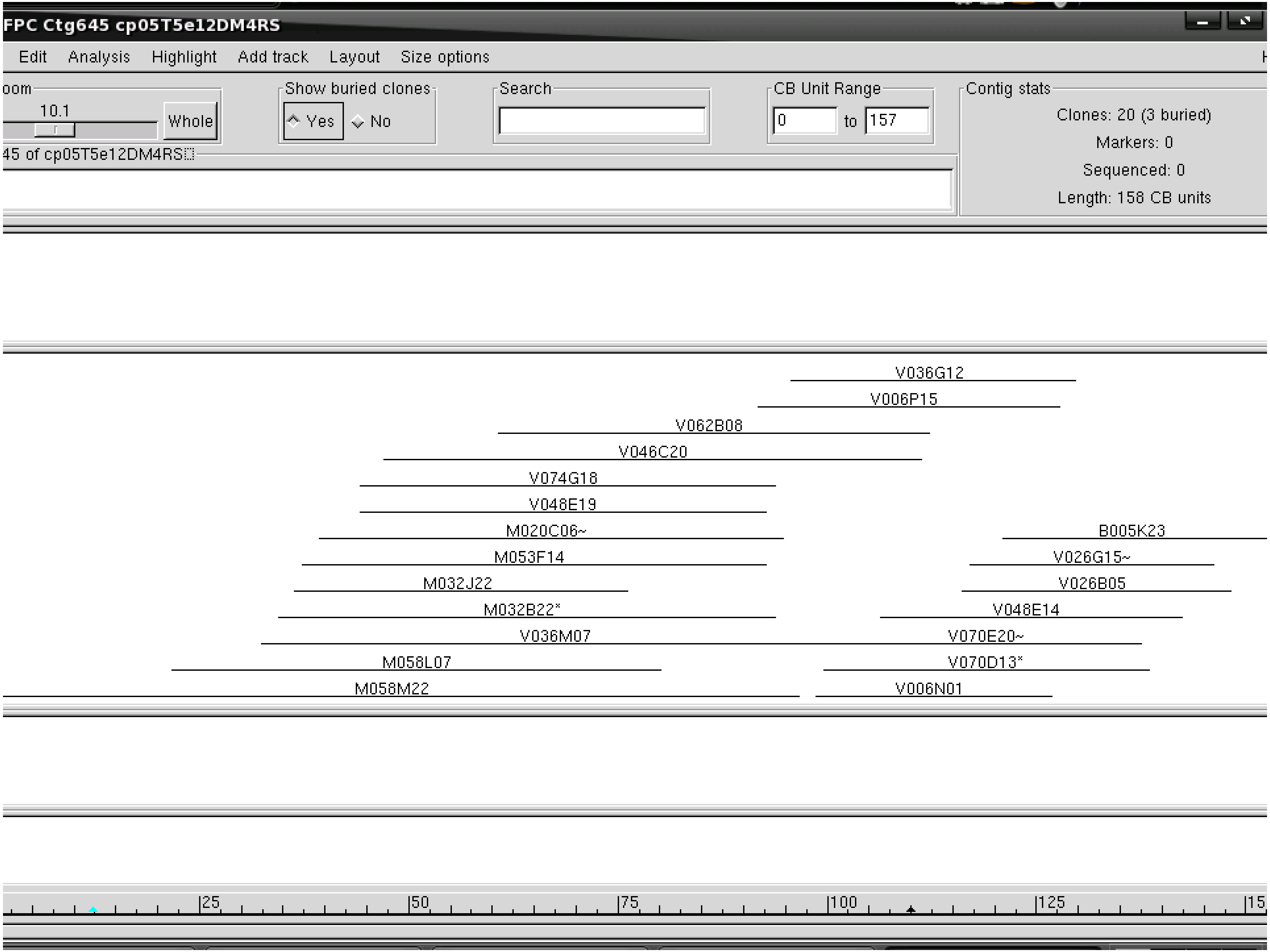
FPC Ctg645 cp05T5e12DM4RS
Edit
Analysis
Highlight
Add track
Layout
Size options
oom
10.1
Whole
Show buried clones
Yes
No
Search
CB Unit Range
0
to
157
Contig stats
Clones: 20 (3 buried)
Markers: 0
Sequenced: 0
Length: 158 CB units
45 of cp05T5e12DM4RS
V036G12
V006P15
V062B08
V046C20
V074G18
V048E19
M020C06~
B005K23
M053F14
V026G15~
M032J22
V026B05
M032B22*
V048E14
V036M07
V070E20~
M058L07
V070D13*
M058M22
V006N01
25
50
75
100
125
15

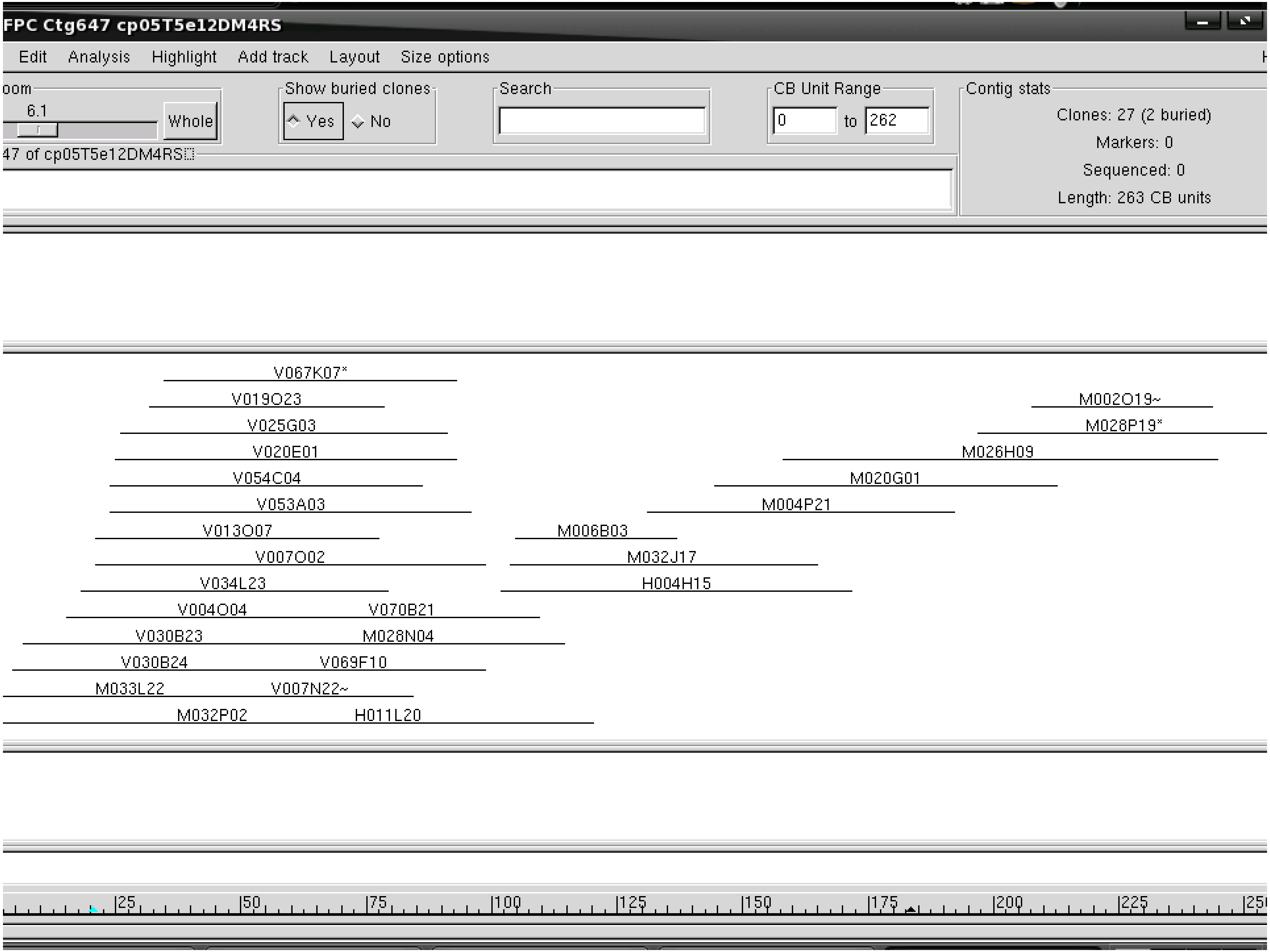
FPC Ctg647 cp05T5e12DM4RS
Edit
Analysis
Highlight
Add track
Layout
Size options
oom
6.1
Whole
Show buried clones
Yes
No
Search
CB Unit Range
0
to
262
Contig stats
Clones: 27 (2 buried)
Markers: 0
Sequenced: 0
Length: 263 CB units
47 of cp05T5e12DM4RS
V067K07*
V019O23
M002O19~
V025G03
M028P19*
V020E01
M026H09
V054C04
M020G01
V053A03
M004P21
V013O07
M006B03
V007O02
M032J17
V034L23
H004H15
V004O04
V070B21
V030B23
M028N04
V030B24
V069F10
M033L22
V007N22~
M032P02
H011L20
25
50
75
100
125
150
175
200
225
25

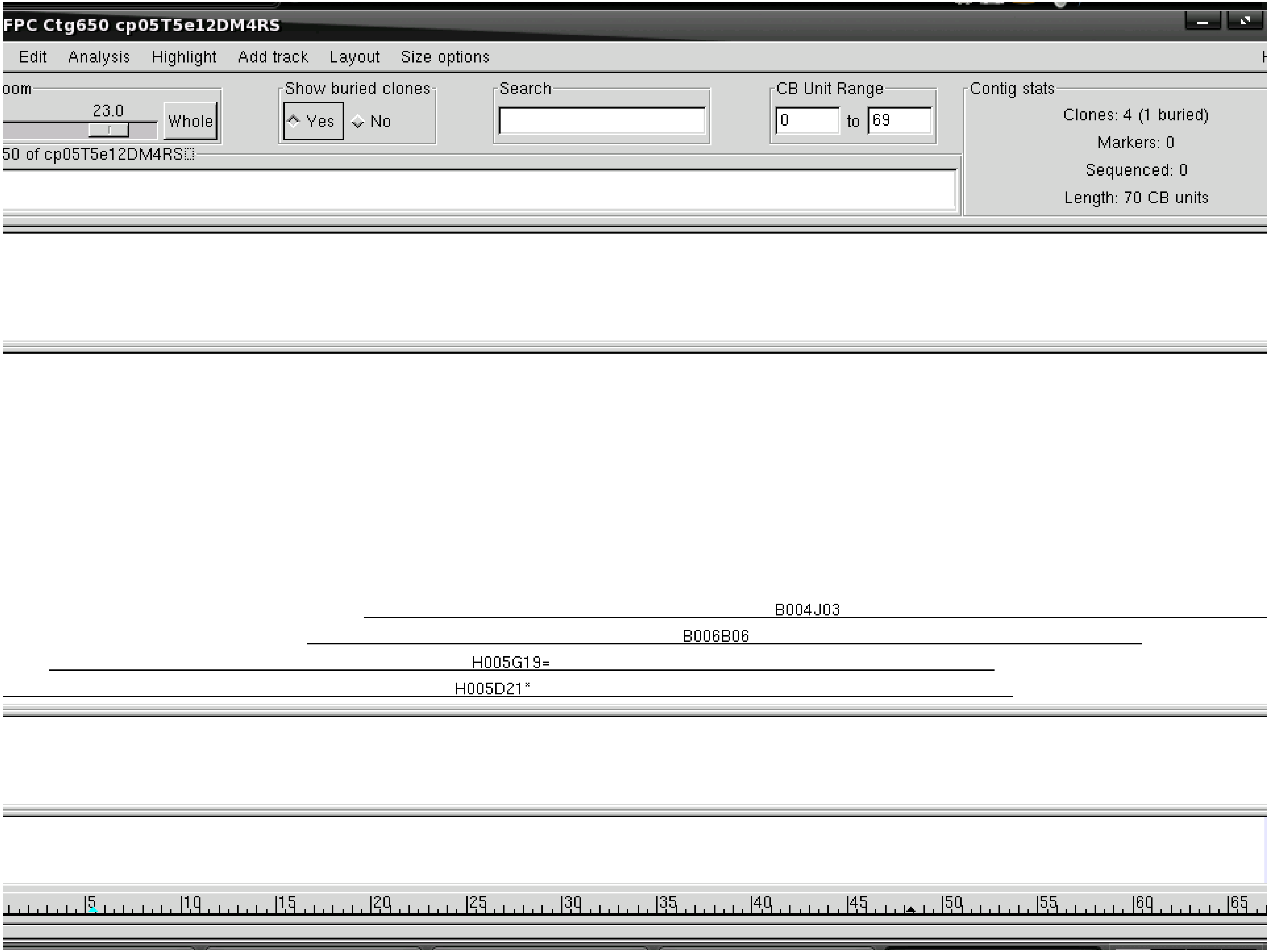
FPC Ctg650 cp05T5e12DM4RS
Edit
Analysis
Highlight
Add track
Layout
Size options
oom
23.0
Whole
Show buried clones
Yes
No
Search
CB Unit Range
0
to
69
Contig stats
Clones: 4 (1 buried)
Markers: 0
Sequenced: 0
Length: 70 CB units
50 of cp05T5e12DM4RS
B004J03
B006B06
H005G19=
H005D21*
5
10
15
20
25
30
35
40
45
50
55
60
65

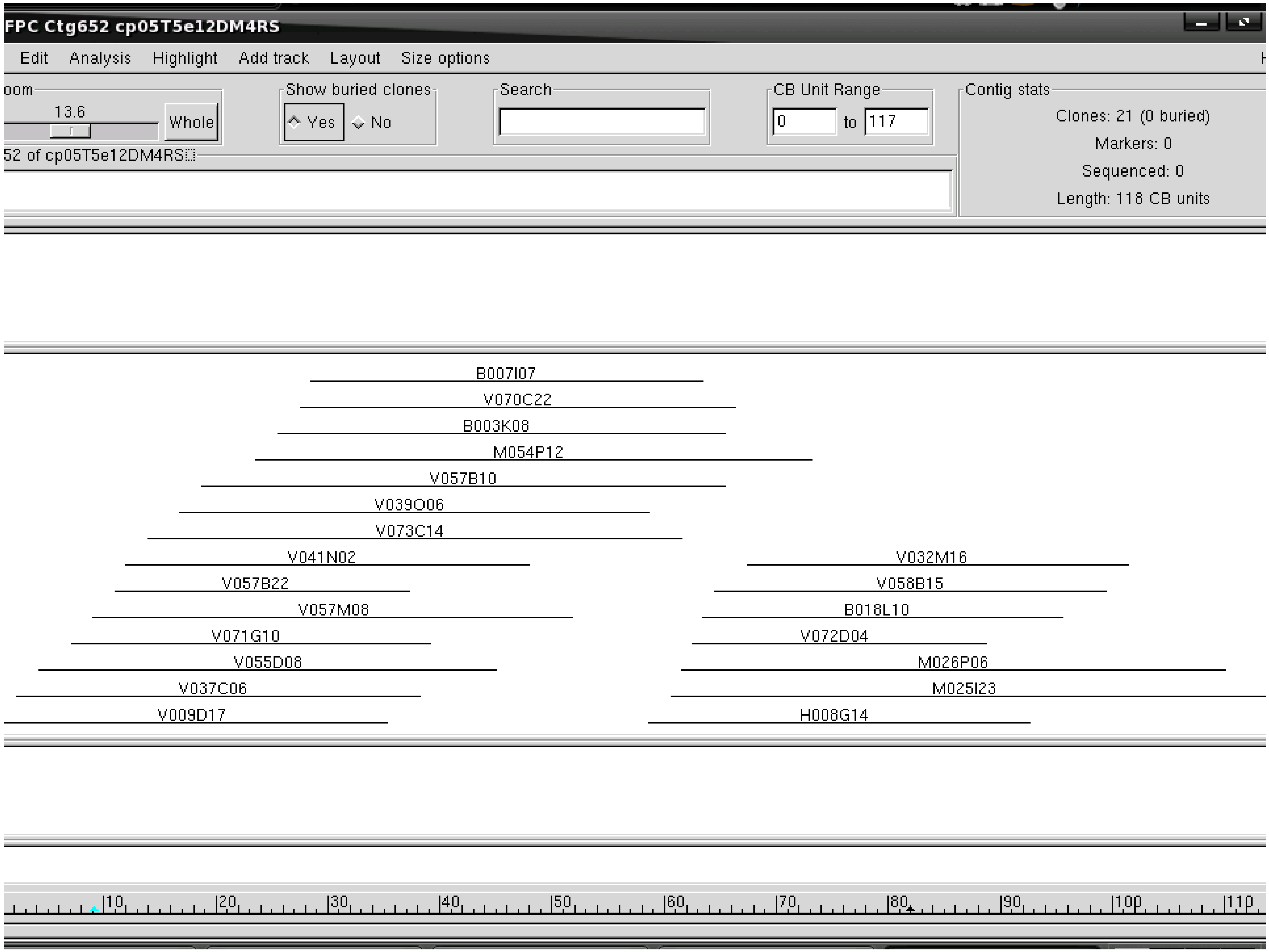
FPC Ctg652 cp05T5e12DM4RS
Edit
Analysis
Highlight
Add track
Layout
Size options
oom
13.6
Whole
Show buried clones
Yes
No
Search
CB Unit Range
0
to
117
Contig stats
Clones: 21 (0 buried)
Markers: 0
Sequenced: 0
Length: 118 CB units
52 of cp05T5e12DM4RS
B007I07
V070C22
B003K08
M054P12
V057B10
V039O06
V073C14
V041N02
V032M16
V057B22
V058B15
V057M08
B018L10
V071G10
V072D04
V055D08
M026P06
V037C06
M025I23
V009D17
H008G14
10
20
30
40
50
60
70
80
90
100
110

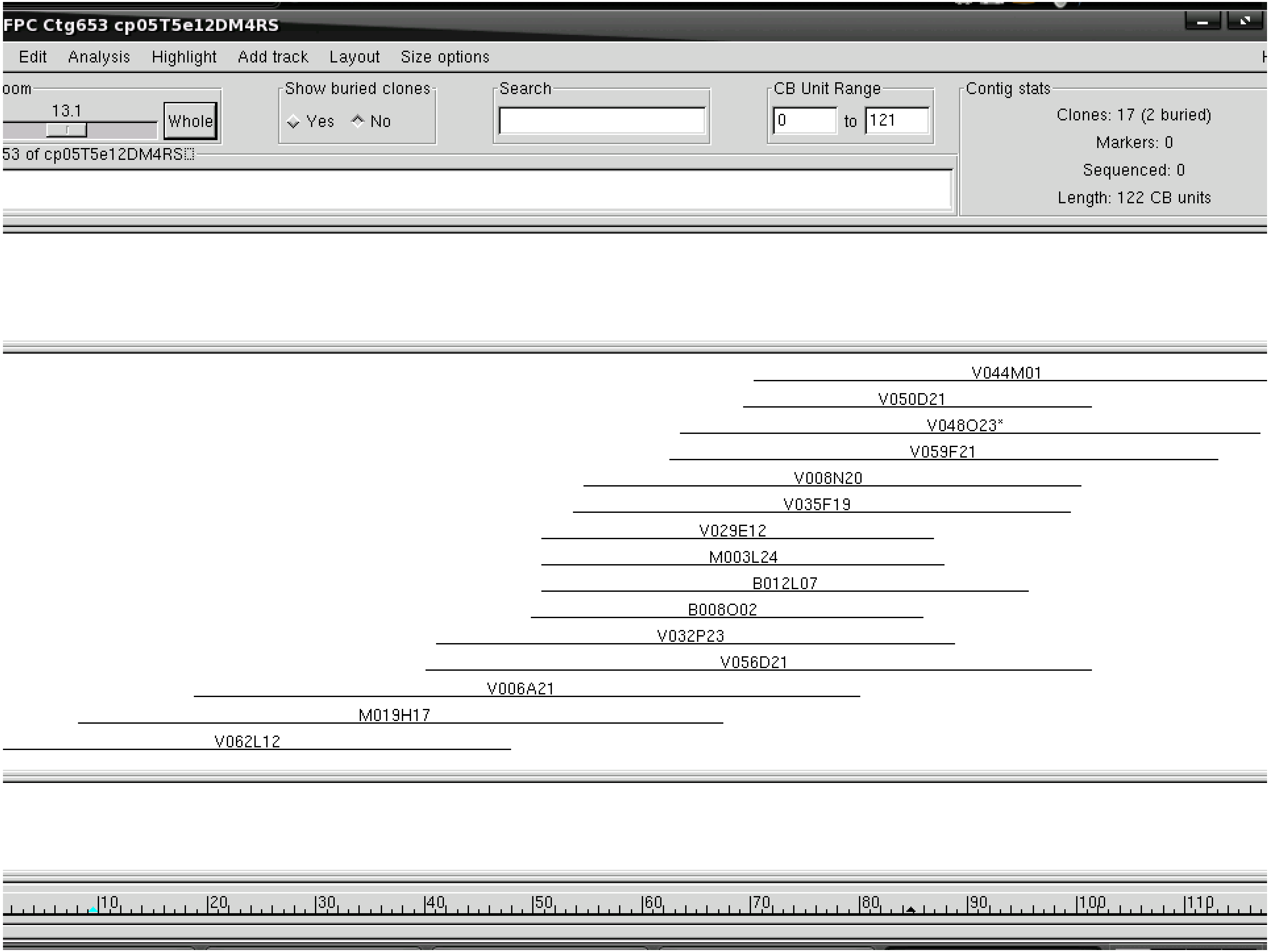
FPC Ctg653 cp05T5e12DM4RS
Edit
Analysis
Highlight
Add track
Layout
Size options
oom
13.1
Whole
Show buried clones
Yes
No
Search
CB Unit Range
0
to
121
Contig stats
Clones: 17 (2 buried)
Markers: 0
Sequenced: 0
Length: 122 CB units
53 of cp05T5e12DM4RS
V044M01
V050D21
V048O23*
V059F21
V008N20
V035F19
V029E12
M003L24
B012L07
B008O02
V032P23
V056D21
V006A21
M019H17
V062L12
10
20
30
40
50
60
70
80
90
100
110

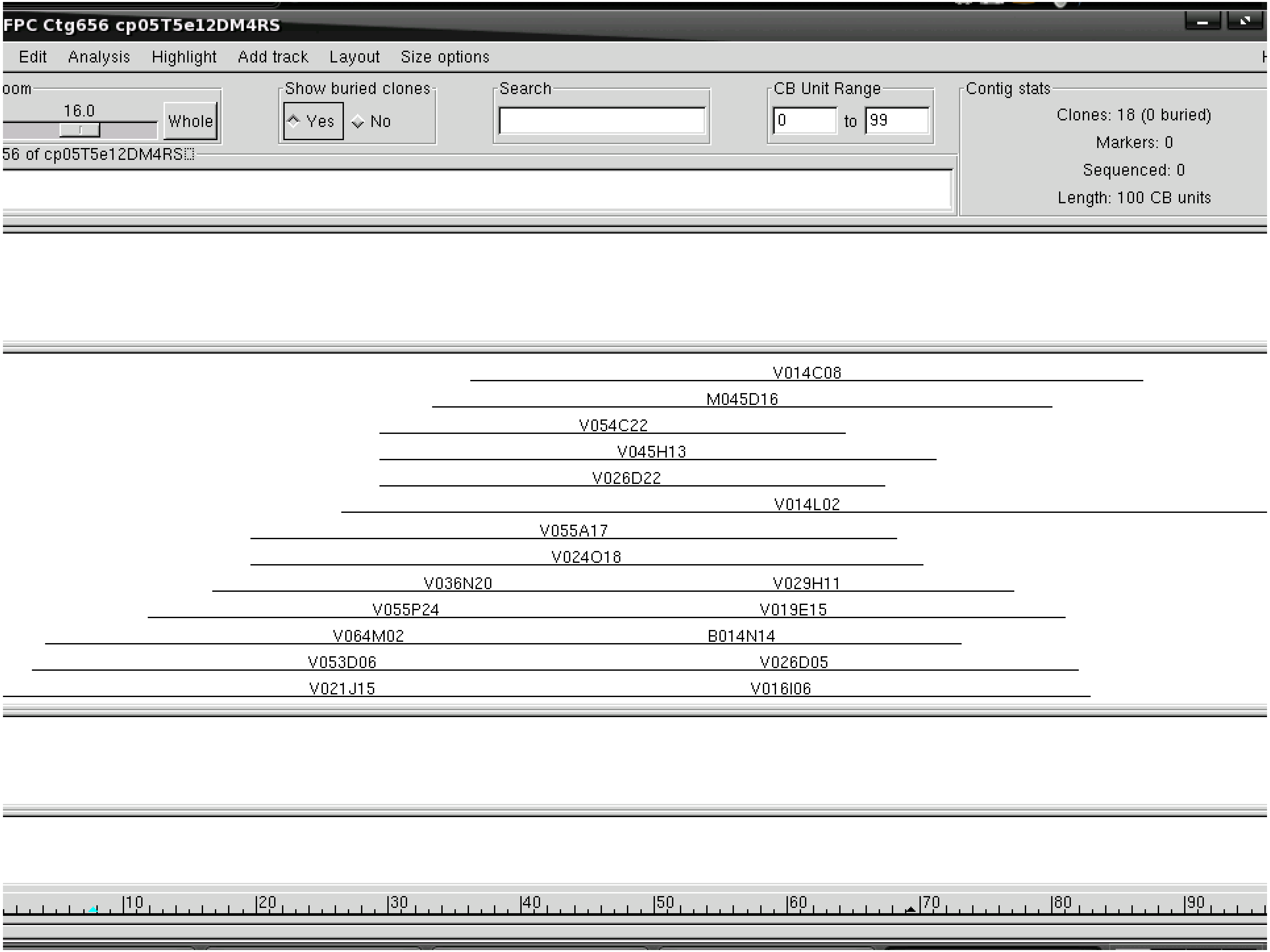
FPC Ctg656 cp05T5e12DM4RS
Edit
Analysis
Highlight
Add track
Layout
Size options
oom
16.0
Whole
Show buried clones
Yes
No
Search
CB Unit Range
0
to
99
Contig stats
Clones: 18 (0 buried)
Markers: 0
Sequenced: 0
Length: 100 CB units
56 of cp05T5e12DM4RS
V014C08
M045D16
V054C22
V045H13
V026D22
V014L02
V055A17
V024O18
V036N20
V029H11
V055P24
V019E15
V064M02
B014N14
V053D06
V026D05
V021J15
V016I06
10
20
30
40
50
60
70
80
90

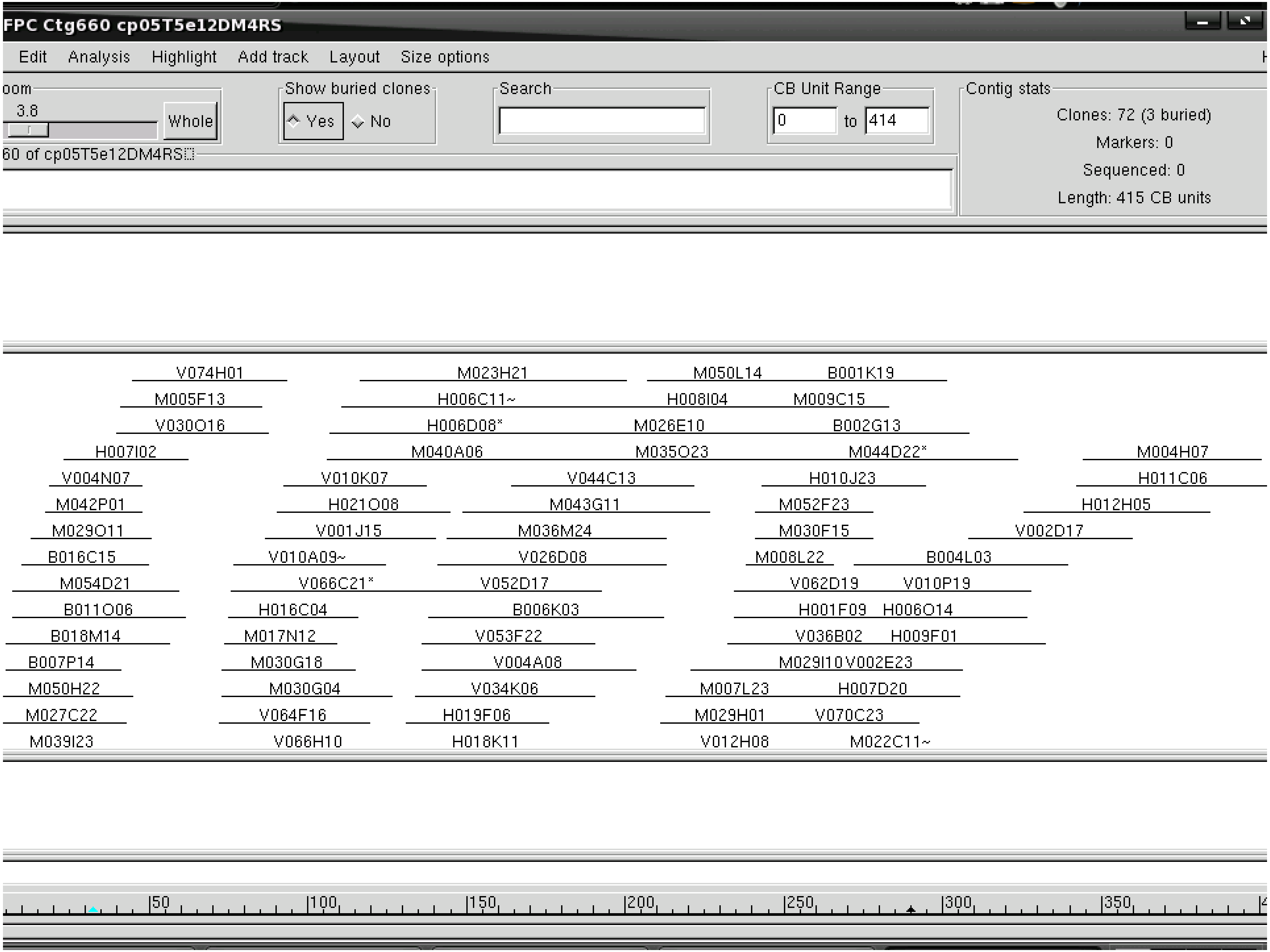
FPC Ctg660 cp05T5e12DM4RS
Edit Analysis Highlight Add track Layout Size options
oom
3.8
Whole
Show buried clones
Yes No
Search
CB Unit Range
0 to 414
Contig stats
Clones: 72 (3 buried)
Markers: 0
Sequenced: 0
Length: 415 CB units
60 of cp05T5e12DM4RS
V074H01
M023H21
M050L14
B001K19
M005F13
H006C11~
H008I04
M009C15
V030O16
H006D08*
M026E10
B002G13
H007I02
M040A06
M035O23
M044D22*
M004H07
V004N07
V010K07
V044C13
H010J23
H011C06
M042P01
H021O08
M043G11
M052F23
H012H05
M029O11
V001J15
M036M24
M030F15
V002D17
B016C15
V010A09~
V026D08
M008L22
B004L03
M054D21
V066C21*
V052D17
V062D19
V010P19
B011O06
H016C04
B006K03
H001F09
H006O14
B018M14
M017N12
V053F22
V036B02
H009F01
B007P14
M030G18
V004A08
M029I10
V002E23
M050H22
M030G04
V034K06
M007L23
H007D20
M027C22
V064F16
H019F06
M029H01
V070C23
M039I23
V066H10
H018K11
V012H08
M022C11~
50
100
150
200
250
300
350

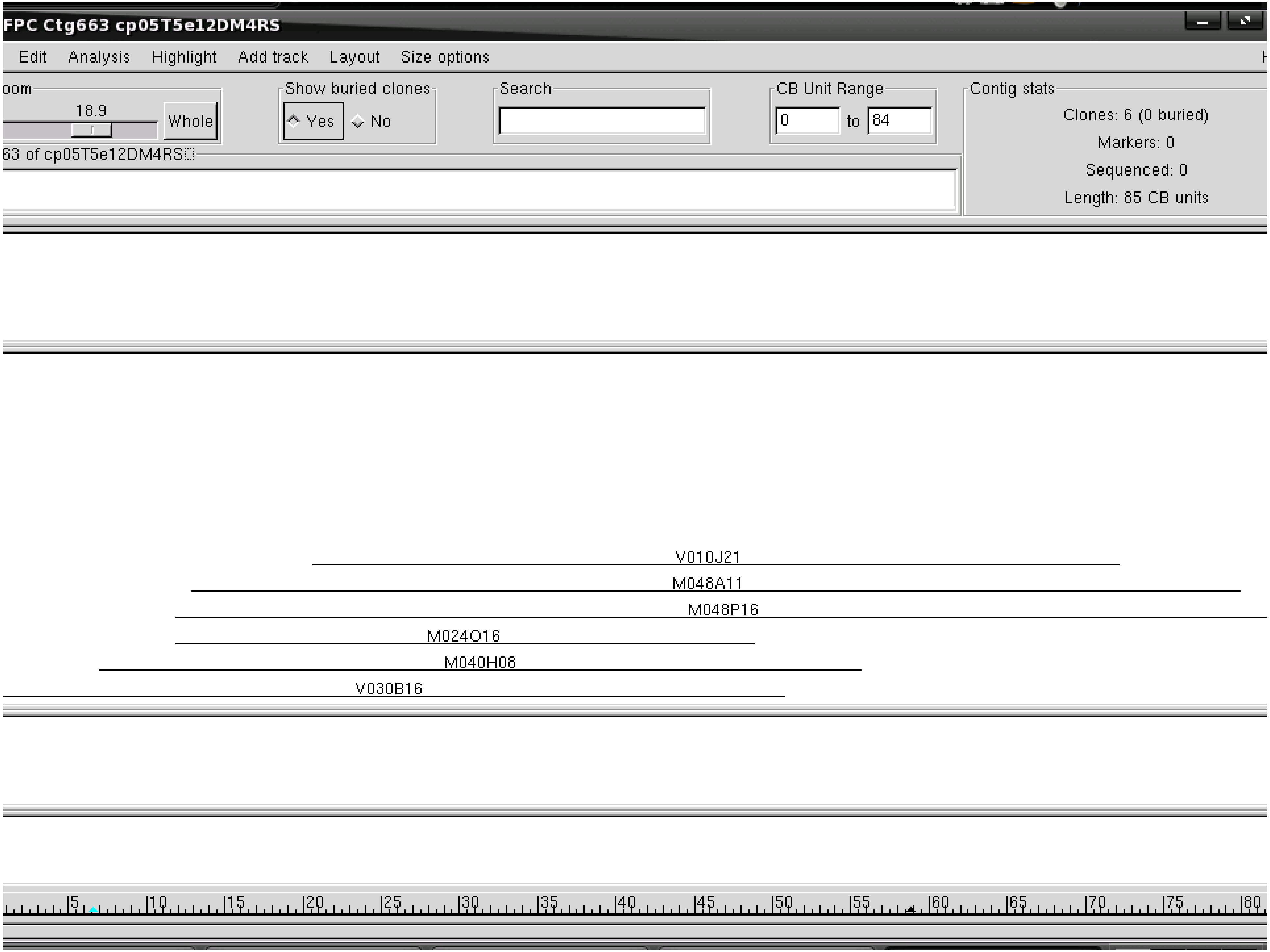
FPC Ctg663 cp05T5e12DM4RS
Edit
Analysis
Highlight
Add track
Layout
Size options
oom
18.9
Whole
Show buried clones
Yes
No
Search
CB Unit Range
0
to
84
Contig stats
Clones: 6 (0 buried)
Markers: 0
Sequenced: 0
Length: 85 CB units
63 of cp05T5e12DM4RS
V010J21
M048A11
M048P16
M024O16
M040H08
V030B16
5
10
15
20
25
30
35
40
45
50
55
60
65
70
75
80

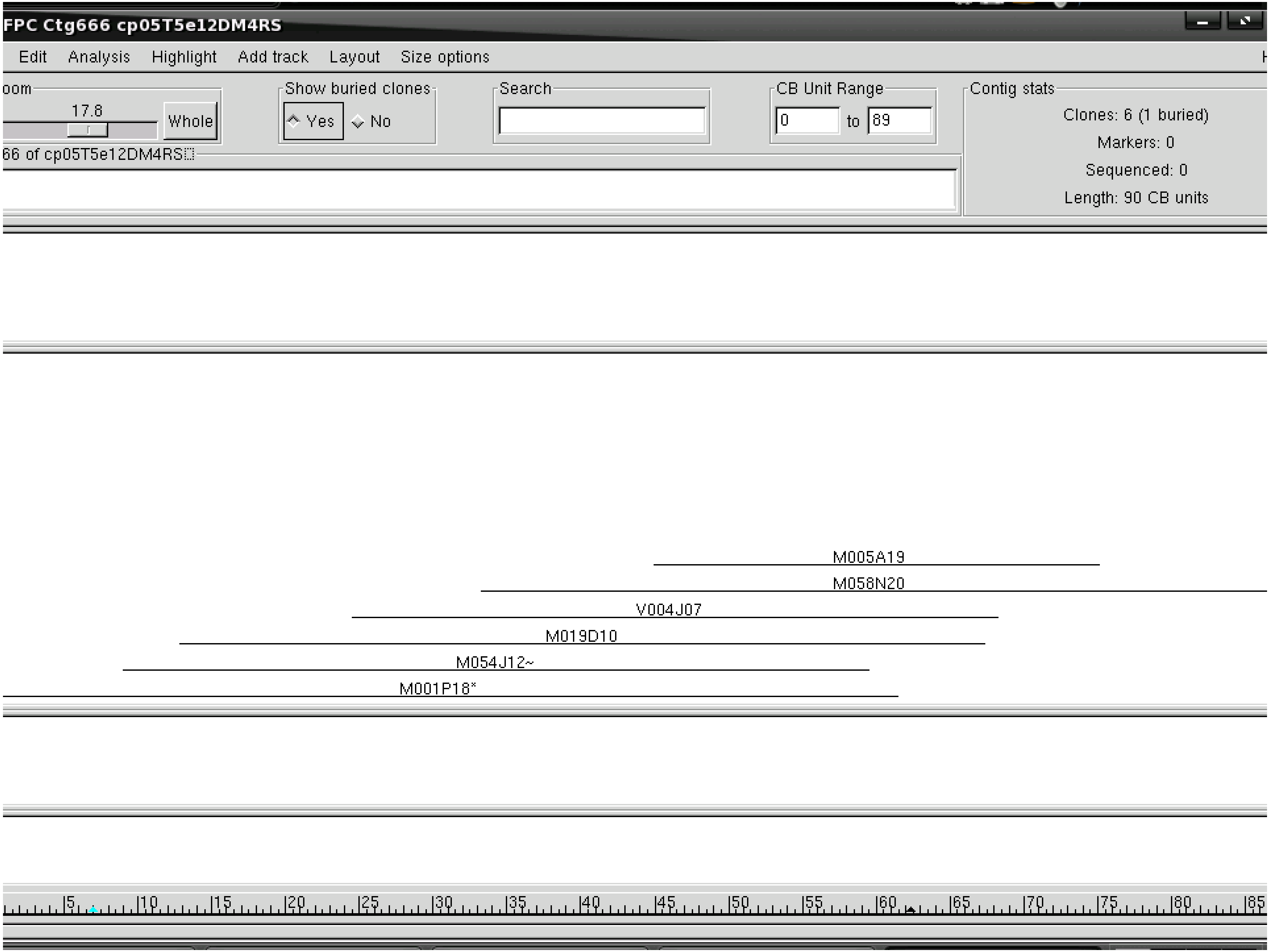
FPC Ctg666 cp05T5e12DM4RS
Edit
Analysis
Highlight
Add track
Layout
Size options
oom
17.8
Whole
Show buried clones
Yes
No
Search
CB Unit Range
0
to
89
Contig stats
Clones: 6 (1 buried)
Markers: 0
Sequenced: 0
Length: 90 CB units
66 of cp05T5e12DM4RS
M005A19
M058N20
V004J07
M019D10
M054J12~
M001P18*
5
10
15
20
25
30
35
40
45
50
55
60
65
70
75
80
85

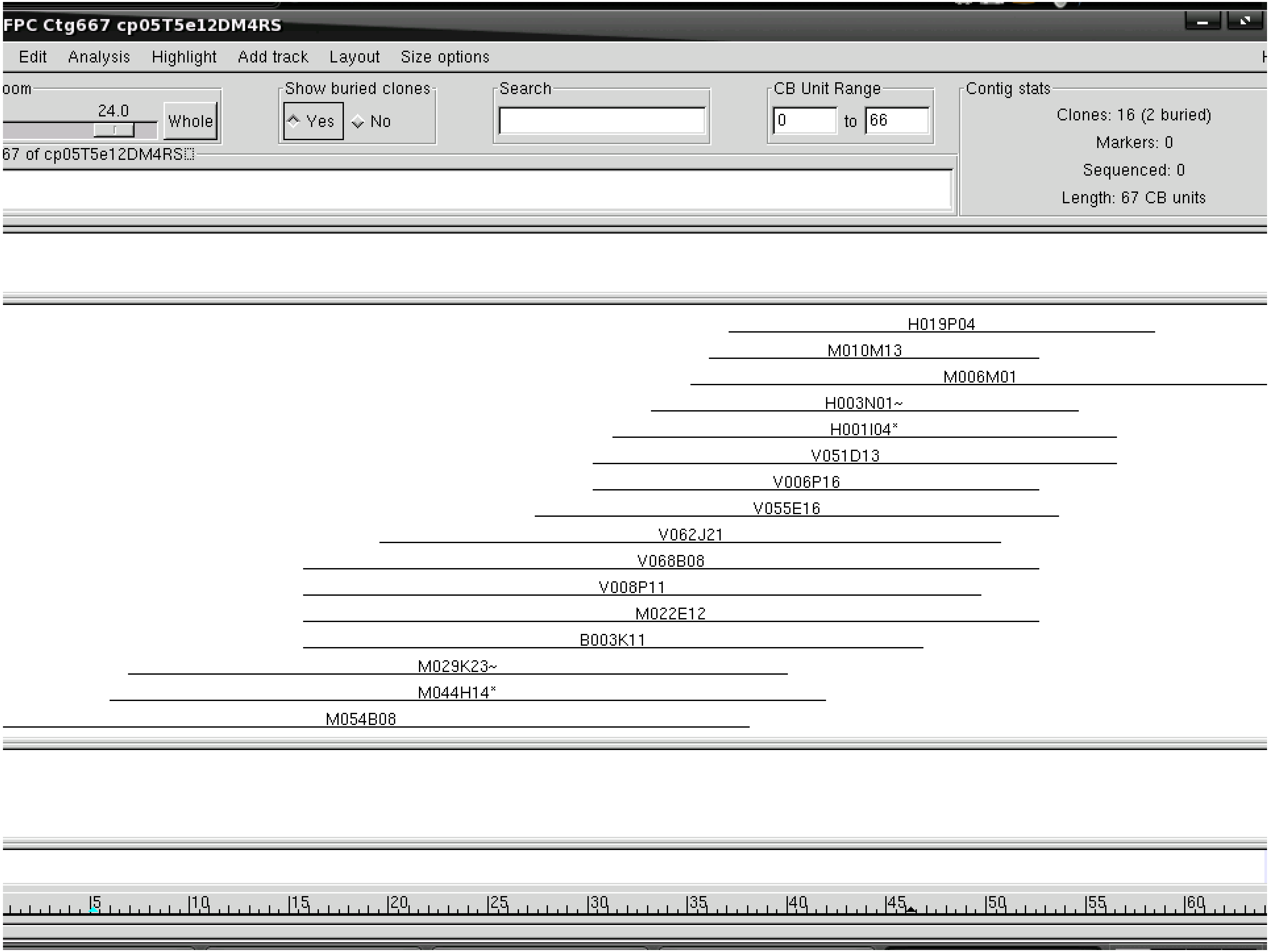
FPC Ctg667 cp05T5e12DM4RS
Edit
Analysis
Highlight
Add track
Layout
Size options
oom
24.0
Whole
Show buried clones
Yes
No
Search
CB Unit Range
0
to
66
Contig stats
Clones: 16 (2 buried)
Markers: 0
Sequenced: 0
Length: 67 CB units
67 of cp05T5e12DM4RS
H019P04
M010M13
M006M01
H003N01~
H001I04*
V051D13
V006P16
V055E16
V062J21
V068B08
V008P11
M022E12
B003K11
M029K23~
M044H14*
M054B08
5
10
15
20
25
30
35
40
45
50
55
60

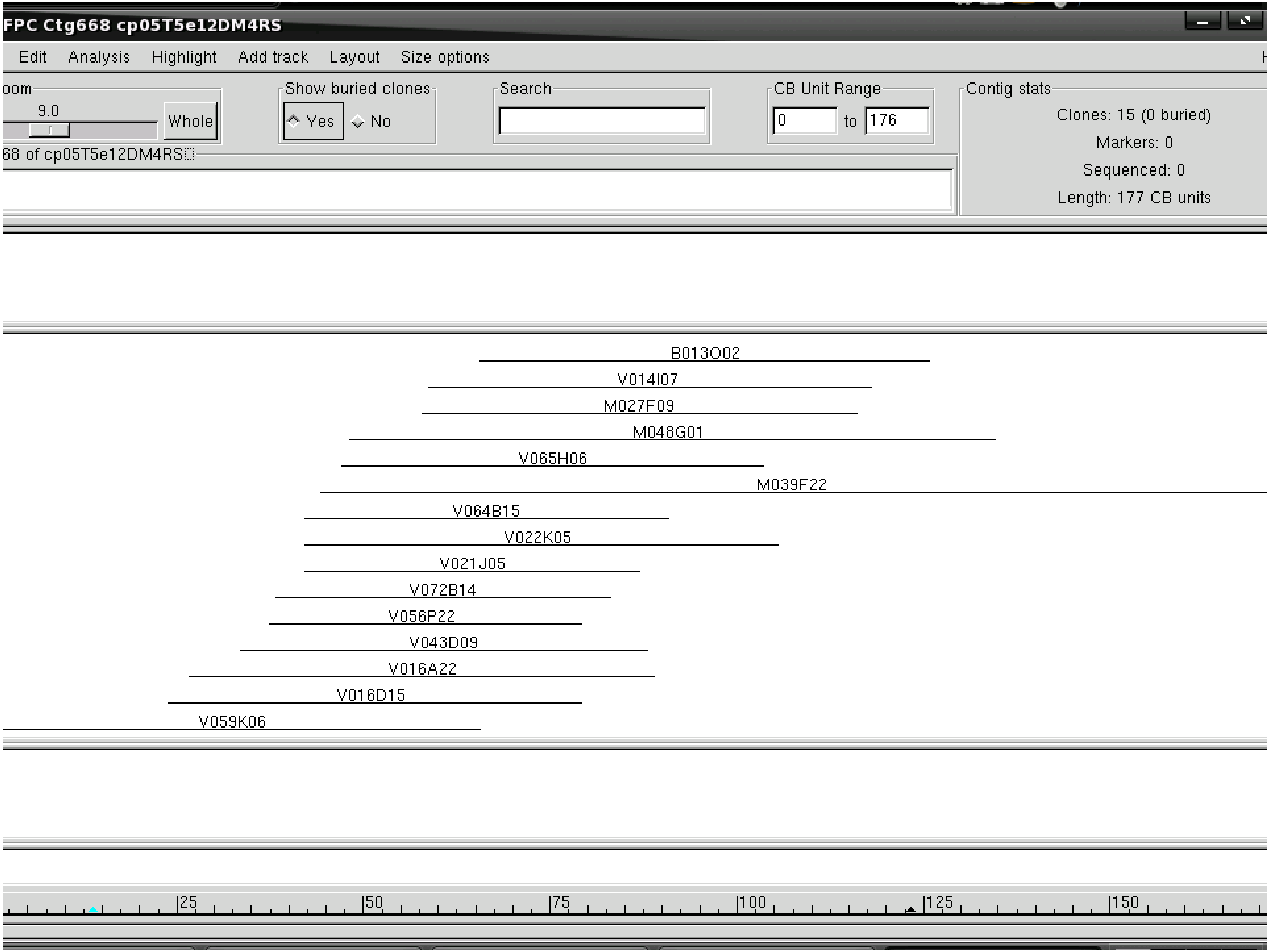
FPC Ctg668 cp05T5e12DM4RS
Edit
Analysis
Highlight
Add track
Layout
Size options
oom
9.0
Whole
Show buried clones
Yes
No
Search
CB Unit Range
0
to
176
Contig stats
Clones: 15 (0 buried)
Markers: 0
Sequenced: 0
Length: 177 CB units
68 of cp05T5e12DM4RS
B013O02
V014I07
M027F09
M048G01
V065H06
M039F22
V064B15
V022K05
V021J05
V072B14
V056P22
V043D09
V016A22
V016D15
V059K06
25
50
75
100
125
150

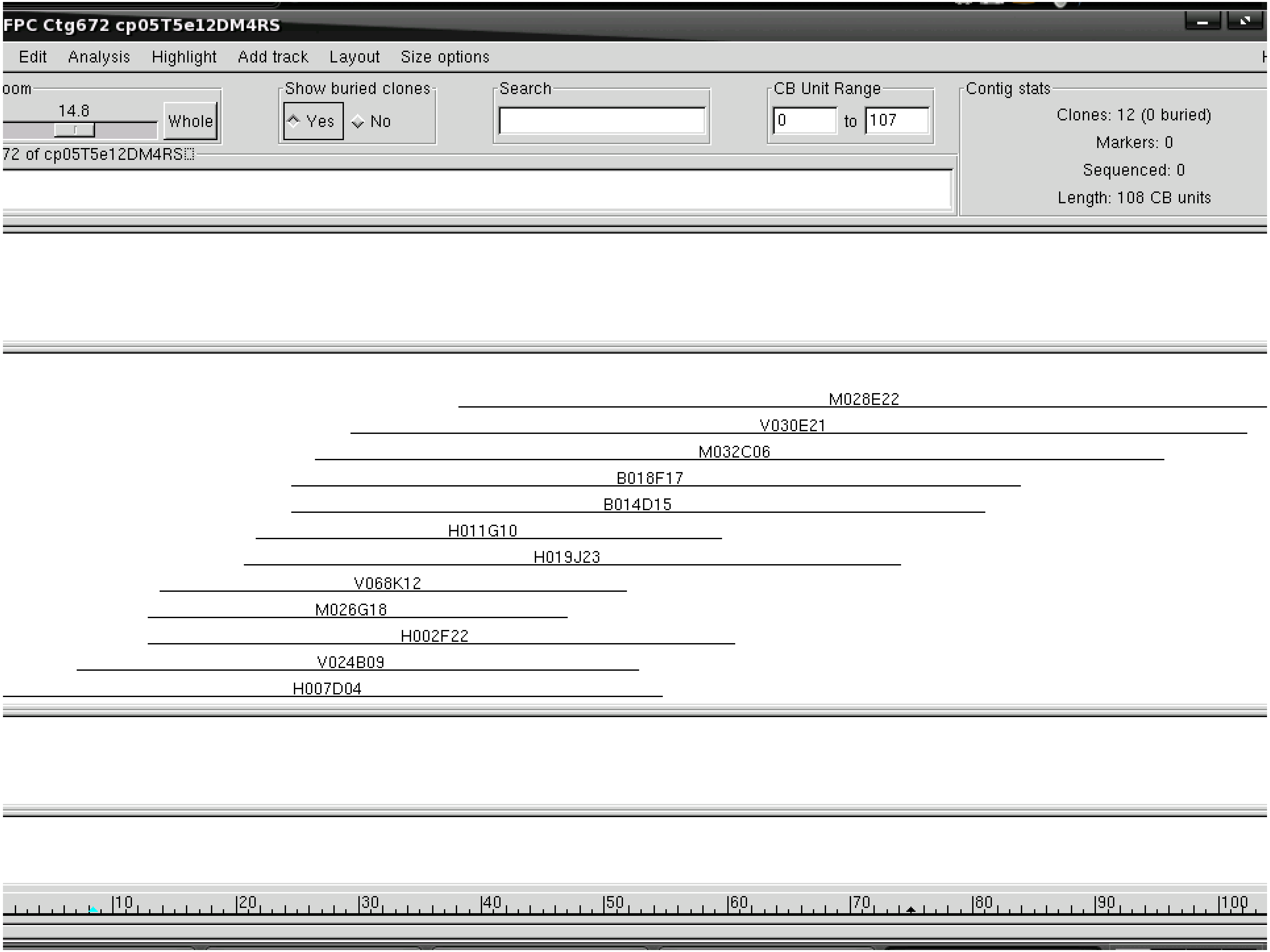
FPC Ctg672 cp05T5e12DM4RS
Edit
Analysis
Highlight
Add track
Layout
Size options
oom
14.8
Whole
Show buried clones
Yes
No
Search
CB Unit Range
0
to
107
Contig stats
Clones: 12 (0 buried)
Markers: 0
Sequenced: 0
Length: 108 CB units
72 of cp05T5e12DM4RS
M028E22
V030E21
M032C06
B018F17
B014D15
H011G10
H019J23
V068K12
M026G18
H002F22
V024B09
H007D04
10
20
30
40
50
60
70
80
90
100

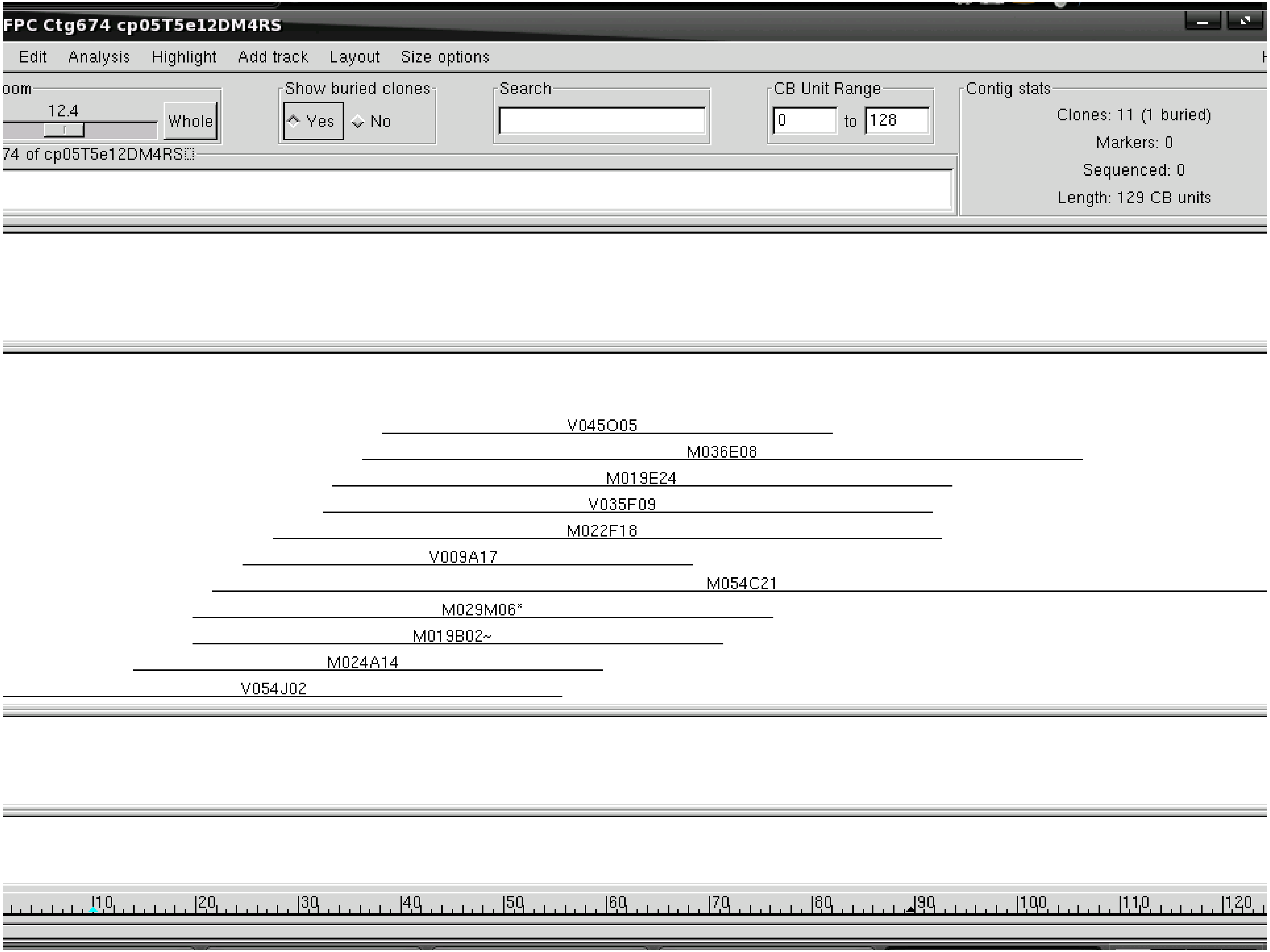
FPC Ctg674 cp05T5e12DM4RS
Edit
Analysis
Highlight
Add track
Layout
Size options
oom
12.4
Whole
Show buried clones
Yes
No
Search
CB Unit Range
0
to
128
Contig stats
Clones: 11 (1 buried)
Markers: 0
Sequenced: 0
Length: 129 CB units
74 of cp05T5e12DM4RS
V045O05
M036E08
M019E24
V035F09
M022F18
V009A17
M054C21
M029M06*
M019B02~
M024A14
V054J02
10
20
30
40
50
60
70
80
90
100
110
120

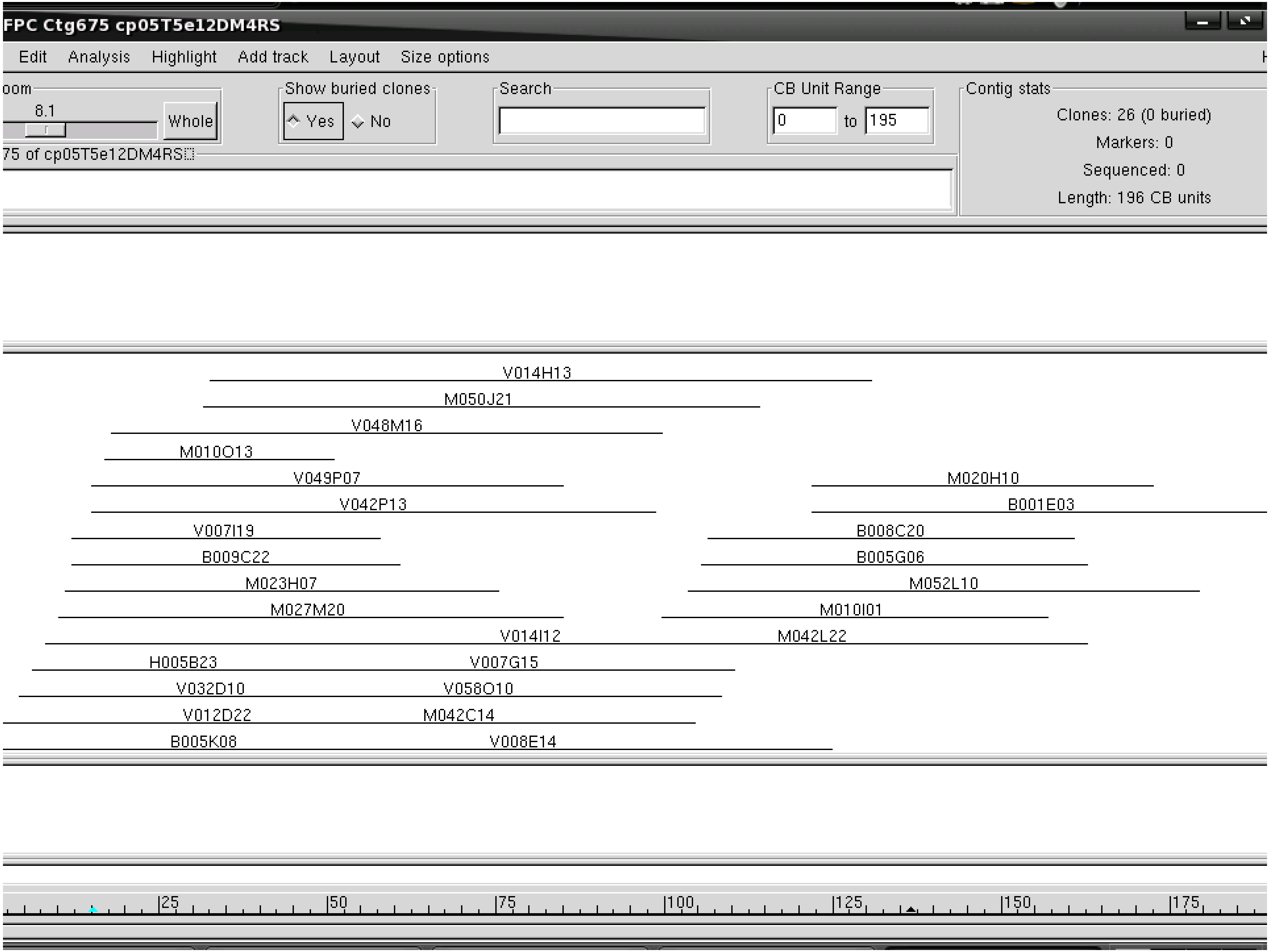
FPC Ctg675 cp05T5e12DM4RS
Edit
Analysis
Highlight
Add track
Layout
Size options
oom
8.1
Whole
Show buried clones
Yes
No
Search
CB Unit Range
0
to
195
Contig stats
Clones: 26 (0 buried)
Markers: 0
Sequenced: 0
Length: 196 CB units
75 of cp05T5e12DM4RS
V014H13
M050J21
V048M16
M010O13
V049P07
M020H10
V042P13
B001E03
V007I19
B008C20
B009C22
B005G06
M023H07
M052L10
M027M20
M010I01
V014I12
M042L22
H005B23
V007G15
V032D10
V058O10
V012D22
M042C14
B005K08
V008E14
25
50
75
100
125
150
175

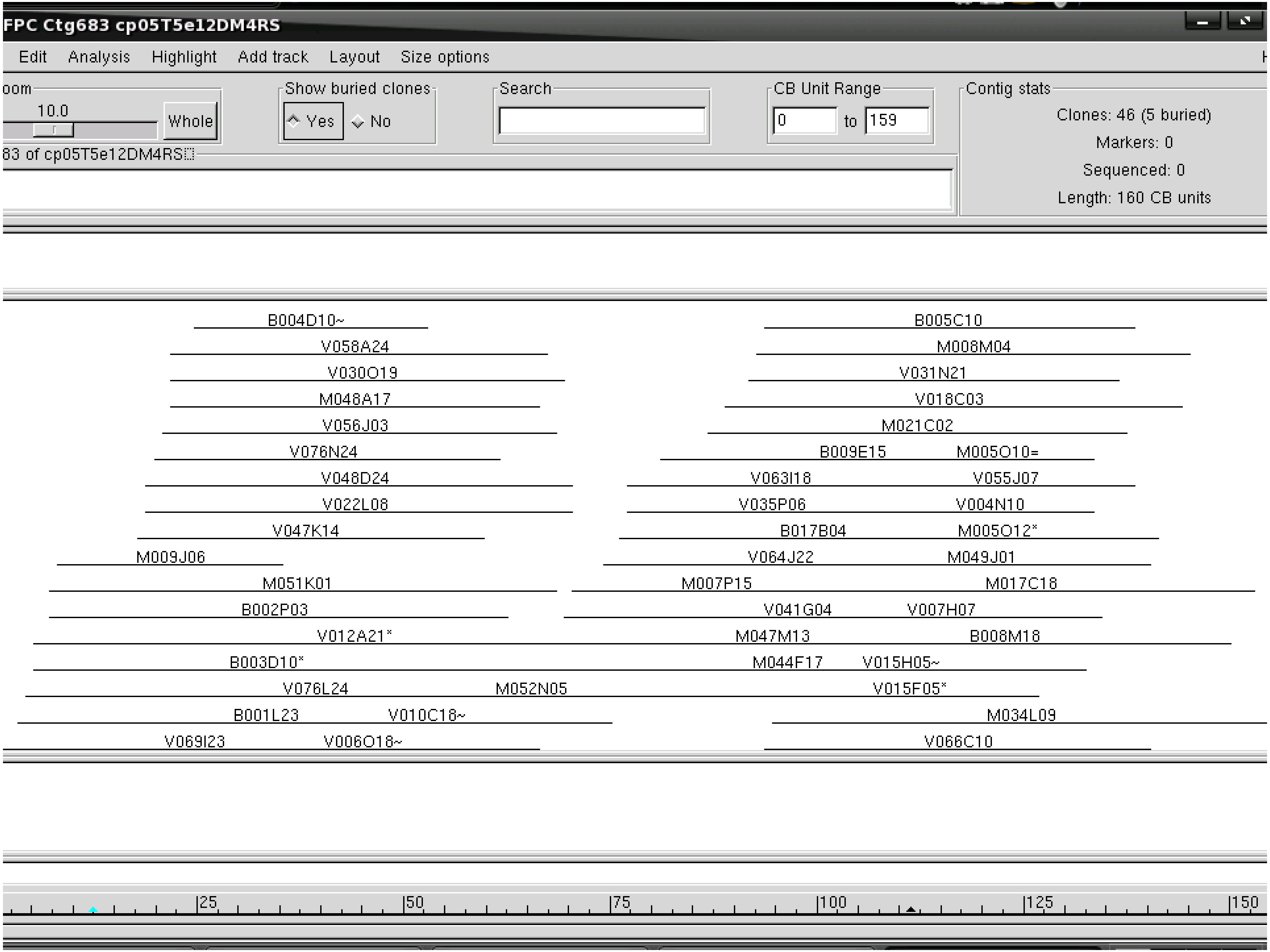
FPC Ctg683 cp05T5e12DM4RS
Edit Analysis Highlight Add track Layout Size options
oom
10.0
Whole
Show buried clones
Yes
No
Search
CB Unit Range
0
to
159
Contig stats
Clones: 46 (5 buried)
Markers: 0
Sequenced: 0
Length: 160 CB units
83 of cp05T5e12DM4RS
B004D10~
V058A24
V030O19
M048A17
V056J03
V076N24
V048D24
V022L08
V047K14
M009J06
M051K01
B002P03
V012A21*
B003D10*
V076L24
M052N05
B001L23
V010C18~
V069I23
V006O18~
B005C10
M008M04
V031N21
V018C03
M021C02
B009E15
M005O10=
V063I18
V055J07
V035P06
V004N10
B017B04
M005O12*
V064J22
M049J01
M007P15
M017C18
V041G04
V007H07
M047M13
B008M18
M044F17
V015H05~
V015F05*
M034L09
V066C10
25
50
75
100
125
150

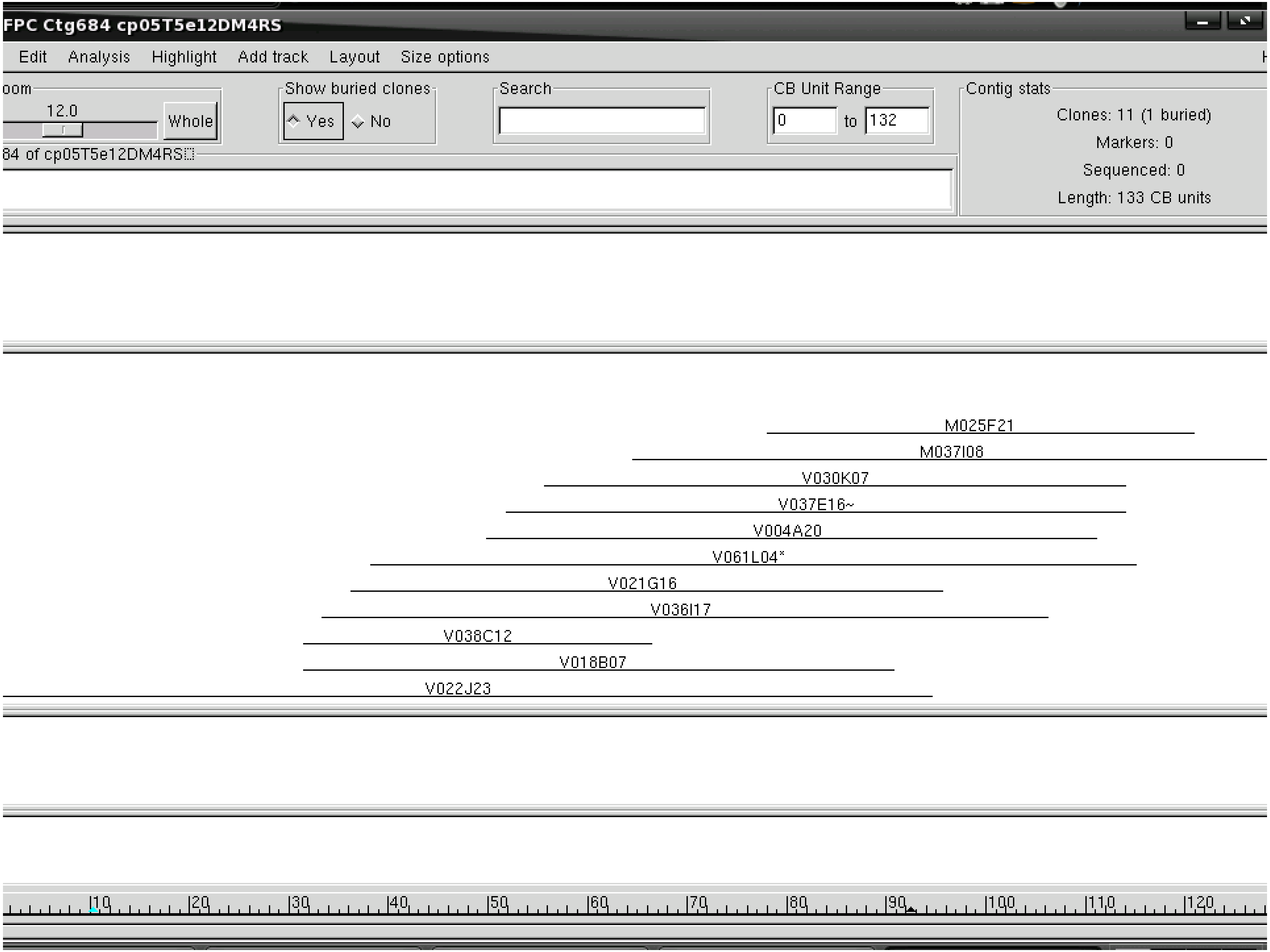

FPC Ctg684 cp05T5e12DM4RS
Edit
Analysis
Highlight
Add track
Layout
Size options
oom
12.0
Whole
Show buried clones
Yes
No
Search
CB Unit Range
0
to
132
Contig stats
Clones: 11 (1 buried)
Markers: 0
Sequenced: 0
Length: 133 CB units
84 of cp05T5e12DM4RS
M025F21
M037I08
V030K07
V037E16~
V004A20
V061L04*
V021G16
V036I17
V038C12
V018B07
V022J23
10
20
30
40
50
60
70
80
90
100
110
120

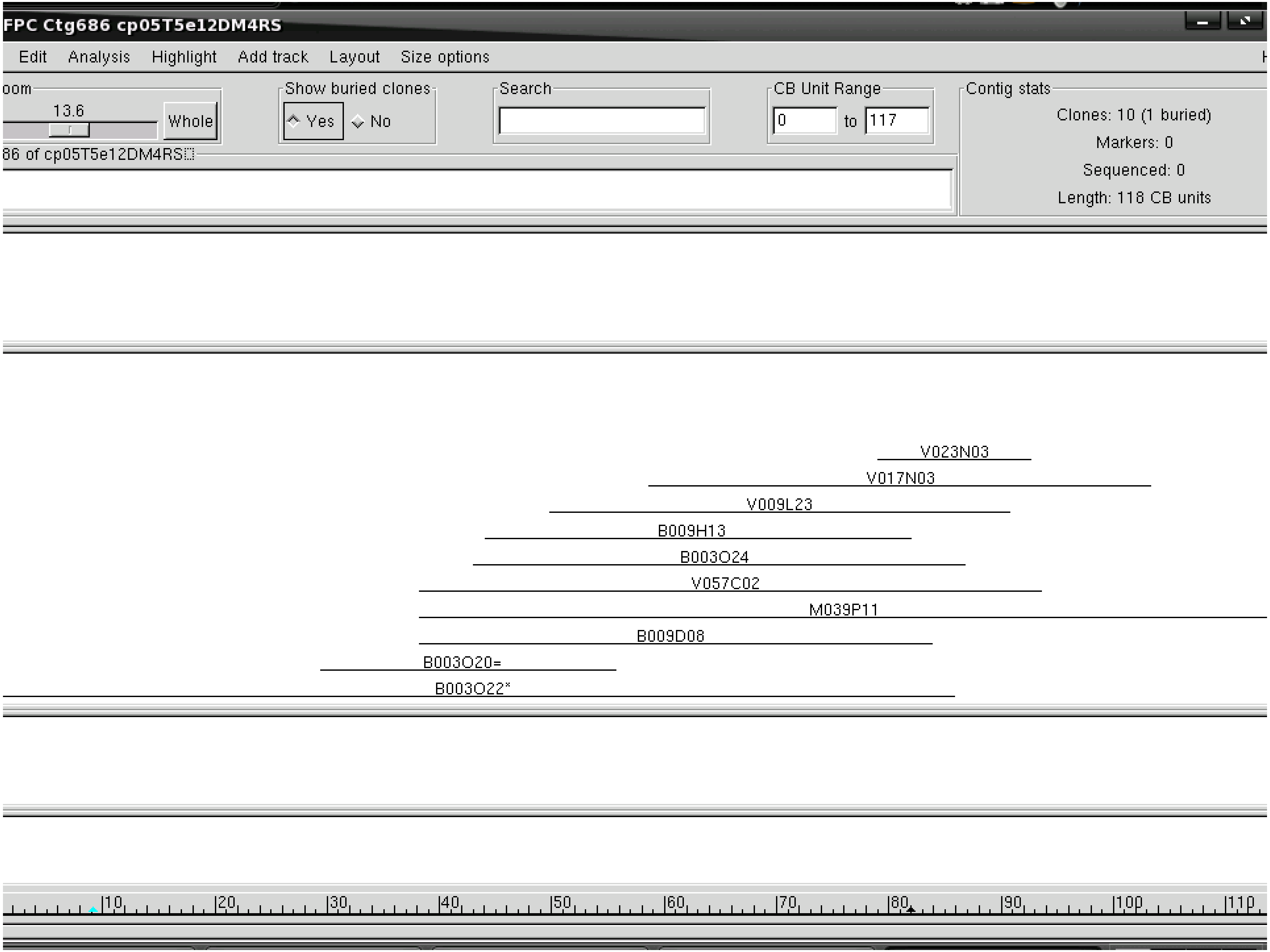
FPC Ctg686 cp05T5e12DM4RS
Edit
Analysis
Highlight
Add track
Layout
Size options
oom
13.6
Whole
Show buried clones
Yes
No
Search
CB Unit Range
0
to
117
Contig stats
Clones: 10 (1 buried)
Markers: 0
Sequenced: 0
Length: 118 CB units
86 of cp05T5e12DM4RS
V023N03
V017N03
V009L23
B009H13
B003O24
V057C02
M039P11
B009D08
B003O20=
B003O22*
10
20
30
40
50
60
70
80
90
100
110

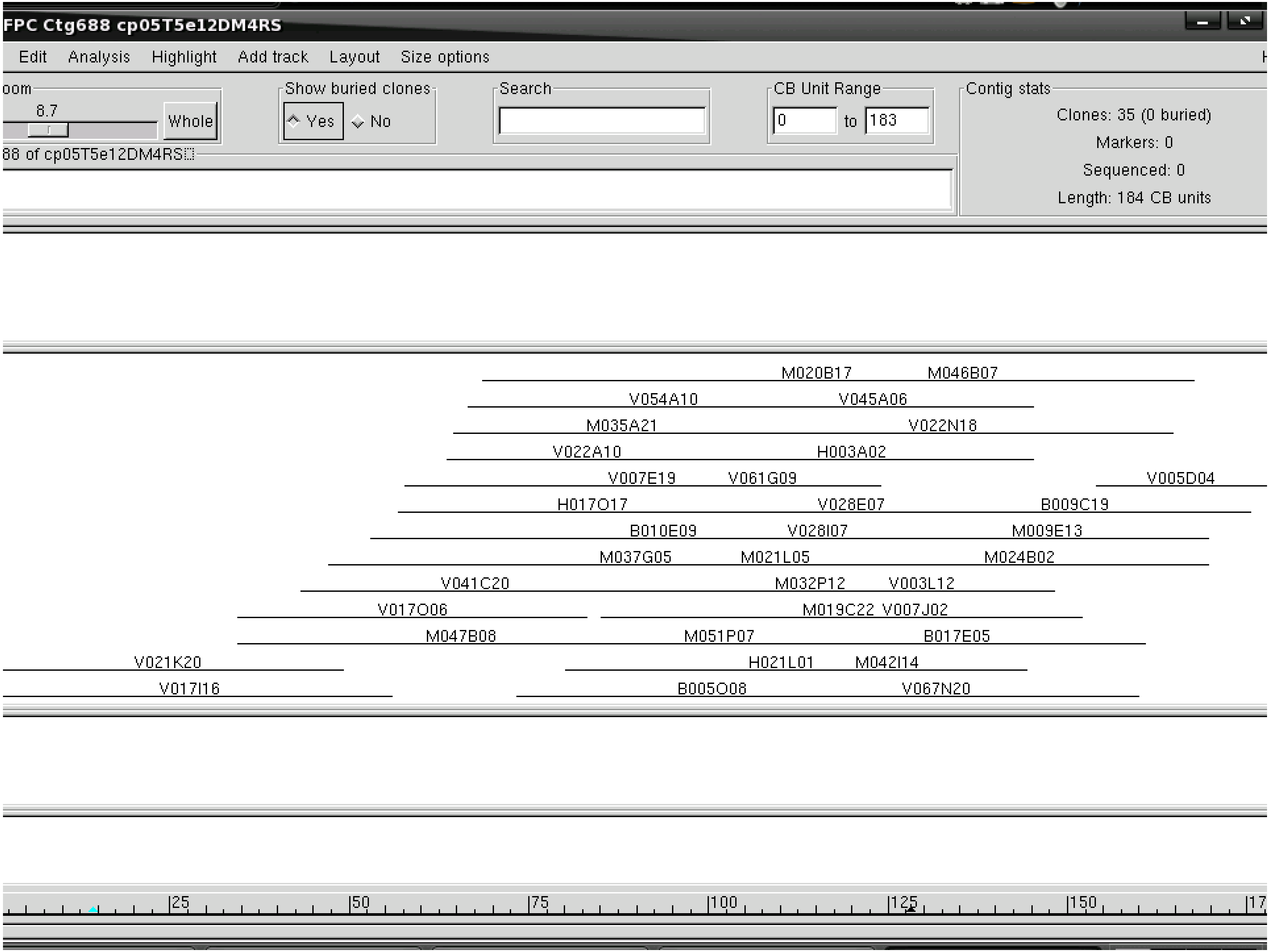
FPC Ctg688 cp05T5e12DM4RS
Edit
Analysis
Highlight
Add track
Layout
Size options
oom
8.7
Whole
Show buried clones
Yes
No
Search
CB Unit Range
0
to
183
Contig stats
Clones: 35 (0 buried)
Markers: 0
Sequenced: 0
Length: 184 CB units
88 of cp05T5e12DM4RS
M020B17
M046B07
V054A10
V045A06
M035A21
V022N18
V022A10
H003A02
V007E19
V061G09
V005D04
H017O17
V028E07
B009C19
B010E09
V028I07
M009E13
M037G05
M021L05
M024B02
V041C20
M032P12
V003L12
V017O06
M019C22
V007J02
M047B08
M051P07
B017E05
V021K20
H021L01
M042I14
V017I16
B005O08
V067N20
25
50
75
100
125
150
17

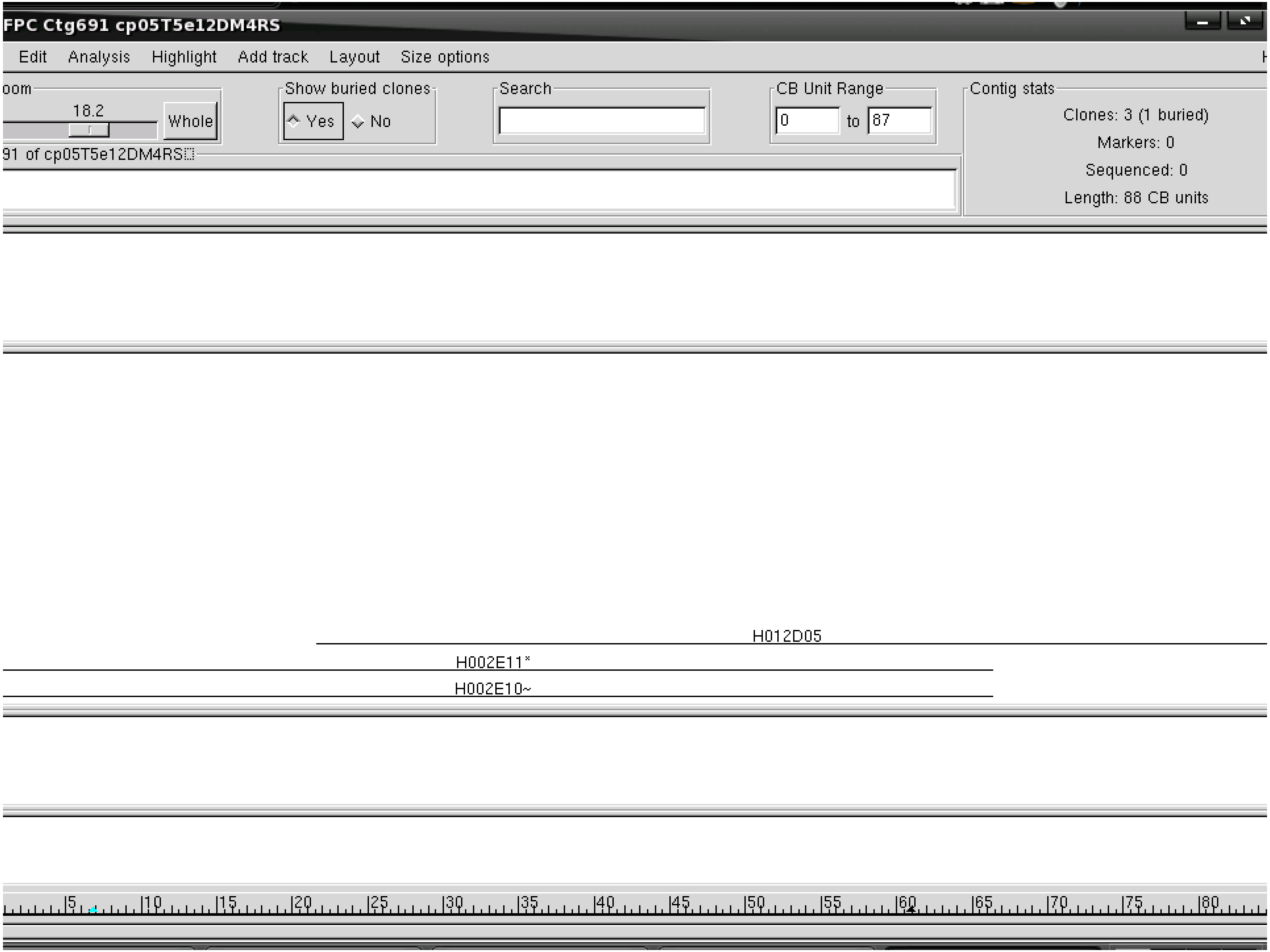
FPC Ctg691 cp05T5e12DM4RS
Edit
Analysis
Highlight
Add track
Layout
Size options
oom
18.2
Whole
Show buried clones
Yes
No
Search
CB Unit Range
0
to
87
Contig stats
Clones: 3 (1 buried)
Markers: 0
Sequenced: 0
Length: 88 CB units
91 of cp05T5e12DM4RS
H012D05
H002E11*
H002E10~
5 10 15 20 25 30 35 40 45 50 55 60 65 70 75 80

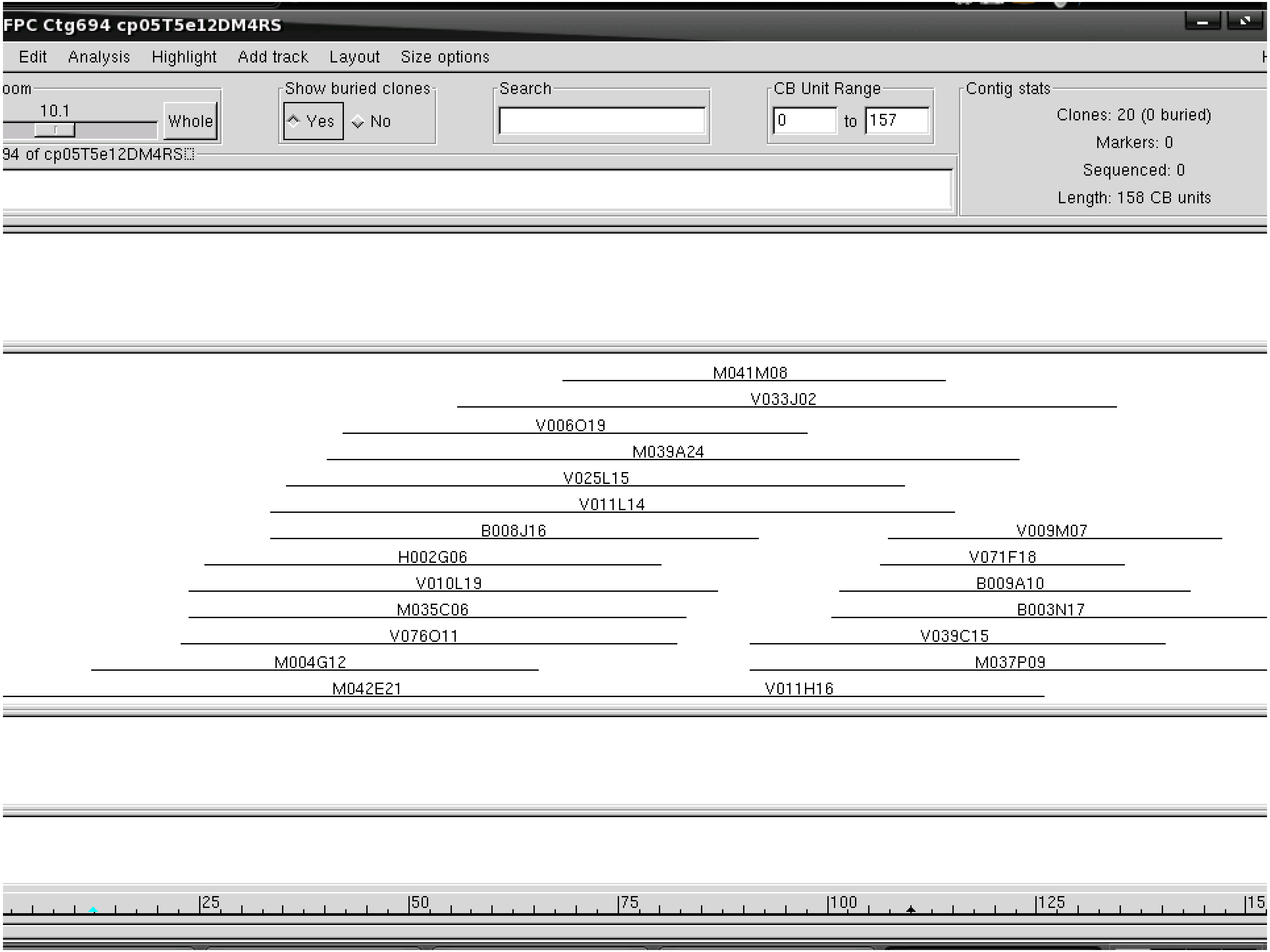
FPC Ctg694 cp05T5e12DM4RS
Edit
Analysis
Highlight
Add track
Layout
Size options
oom
10.1
Whole
Show buried clones
Yes
No
Search
CB Unit Range
0
to
157
Contig stats
Clones: 20 (0 buried)
Markers: 0
Sequenced: 0
Length: 158 CB units
94 of cp05T5e12DM4RS
M041M08
V033J02
V006O19
M039A24
V025L15
V011L14
B008J16
V009M07
H002G06
V071F18
V010L19
B009A10
M035C06
B003N17
V076O11
V039C15
M004G12
M037P09
M042E21
V011H16
25
50
75
100
125
15

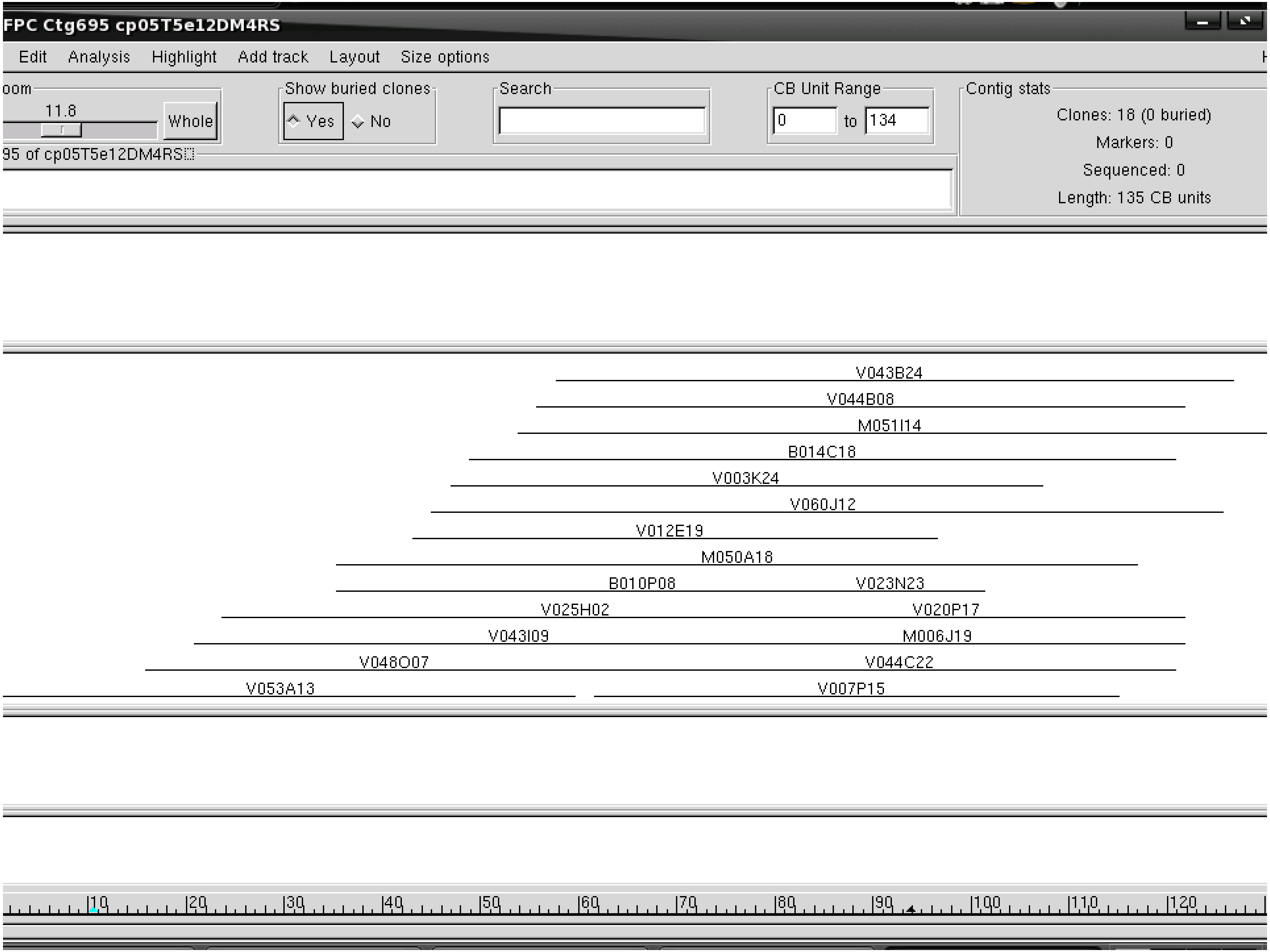
FPC Ctg695 cp05T5e12DM4RS
Edit
Analysis
Highlight
Add track
Layout
Size options
oom
11.8
Whole
Show buried clones
Yes
No
Search
CB Unit Range
0
to
134
Contig stats
Clones: 18 (0 buried)
Markers: 0
Sequenced: 0
Length: 135 CB units
95 of cp05T5e12DM4RS
V043B24
V044B08
M051I14
B014C18
V003K24
V060J12
V012E19
M050A18
B010P08
V023N23
V025H02
V020P17
V043I09
M006J19
V048O07
V044C22
V053A13
V007P15
10
20
30
40
50
60
70
80
90
100
110
120

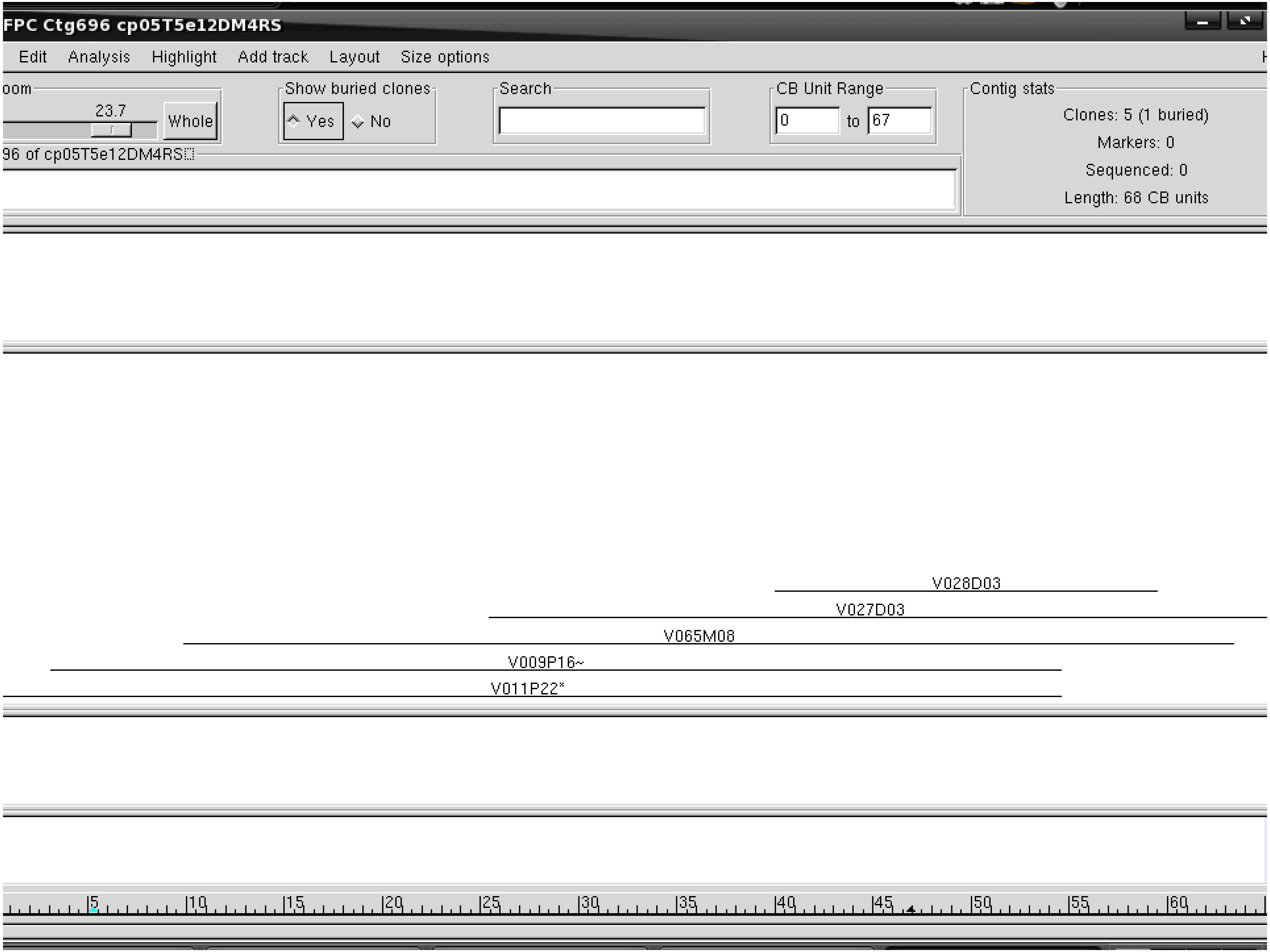
FPC Ctg696 cp05T5e12DM4RS
Edit
Analysis
Highlight
Add track
Layout
Size options
oom
23.7
Whole
Show buried clones
Yes
No
Search
CB Unit Range
0
to
67
Contig stats
Clones: 5 (1 buried)
Markers: 0
Sequenced: 0
Length: 68 CB units
96 of cp05T5e12DM4RS
V028D03
V027D03
V065M08
V009P16~
V011P22*
5
10
15
20
25
30
35
40
45
50
55
60

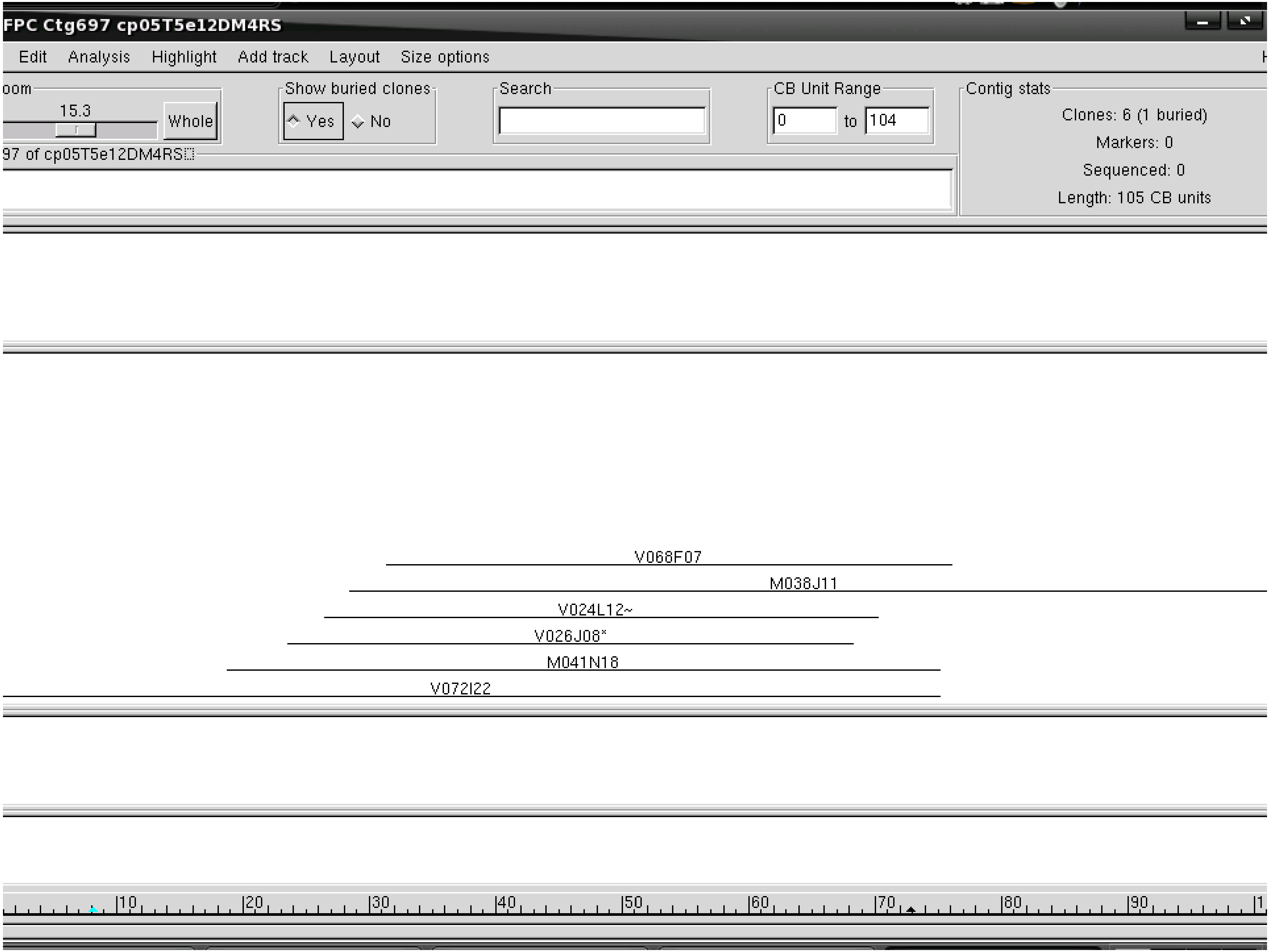
FPC Ctg697 cp05T5e12DM4RS
Edit Analysis Highlight Add track Layout Size options
oom
15.3
Whole
Show buried clones
Yes
No
Search
CB Unit Range
0
to
104
Contig stats
Clones: 6 (1 buried)
Markers: 0
Sequenced: 0
Length: 105 CB units
97 of cp05T5e12DM4RS
V068F07
M038J11
V024L12~
V026J08*
M041N18
V072I22
10
20
30
40
50
60
70
80
90

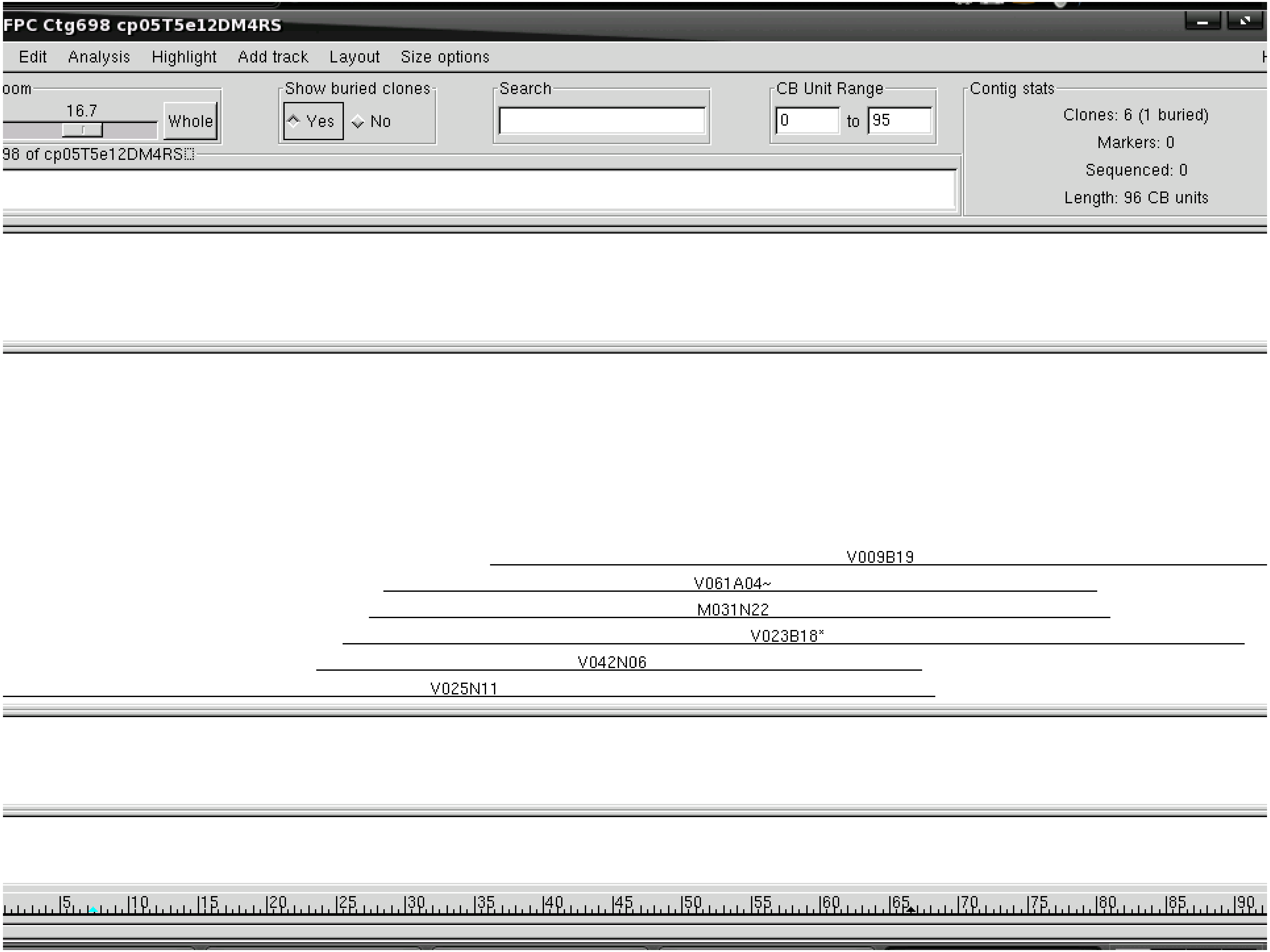
FPC Ctg698 cp05T5e12DM4RS
Edit
Analysis
Highlight
Add track
Layout
Size options
oom
16.7
Whole
Show buried clones
Yes
No
Search
CB Unit Range
0
to
95
Contig stats
Clones: 6 (1 buried)
Markers: 0
Sequenced: 0
Length: 96 CB units
98 of cp05T5e12DM4RS
V009B19
V061A04~
M031N22
V023B18*
V042N06
V025N11
5
10
15
20
25
30
35
40
45
50
55
60
65
70
75
80
85
90

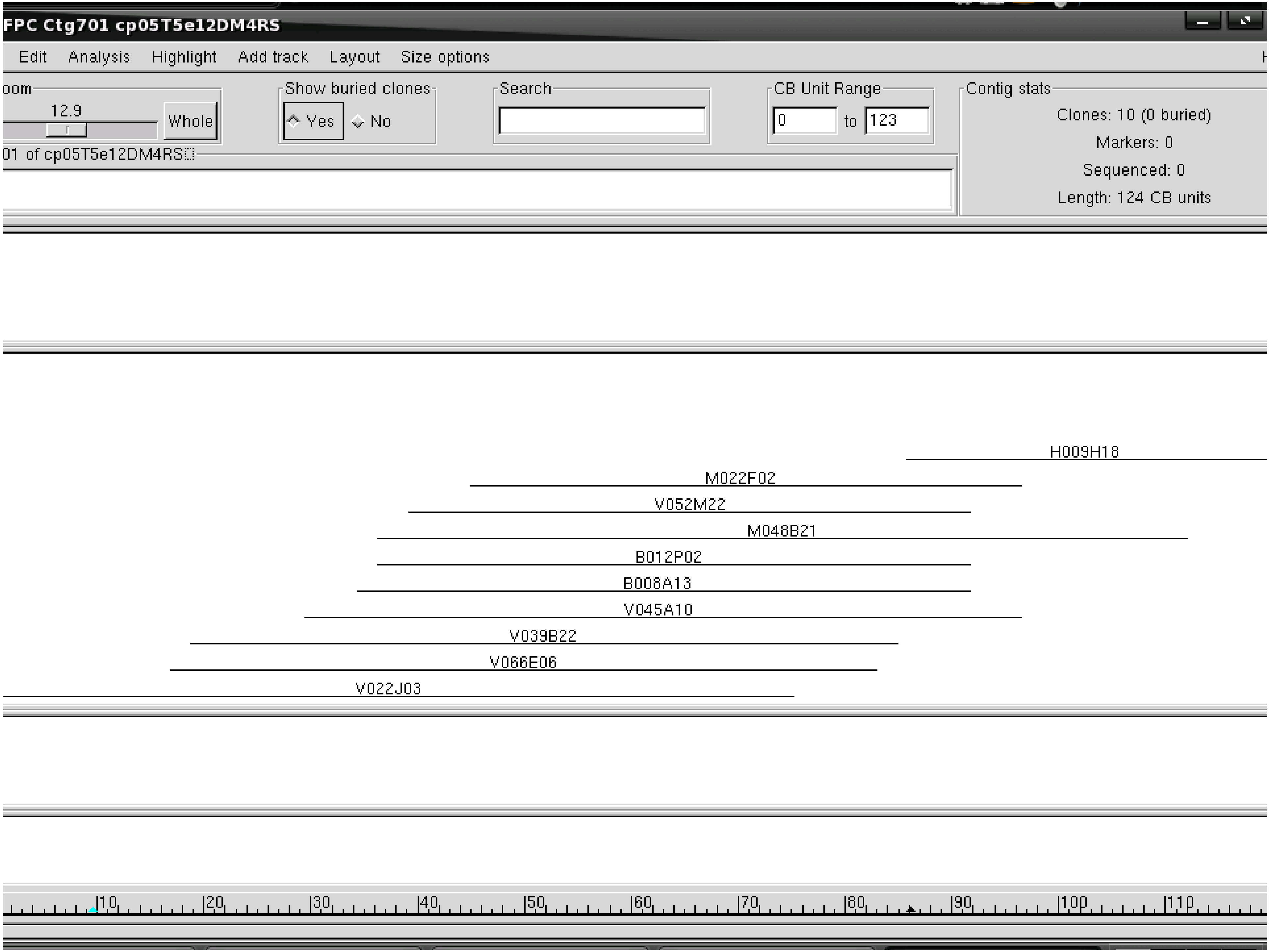
FPC Ctg701 cp05T5e12DM4RS
Edit
Analysis
Highlight
Add track
Layout
Size options
Show buried clones
Yes
No
Search
CB Unit Range
0
to
123
Contig stats
Clones: 10 (0 buried)
Markers: 0
Sequenced: 0
Length: 124 CB units
12.9
Whole
H009H18
M022F02
V052M22
M048B21
B012P02
B008A13
V045A10
V039B22
V066E06
V022J03
10
20
30
40
50
60
70
80
90
100
110

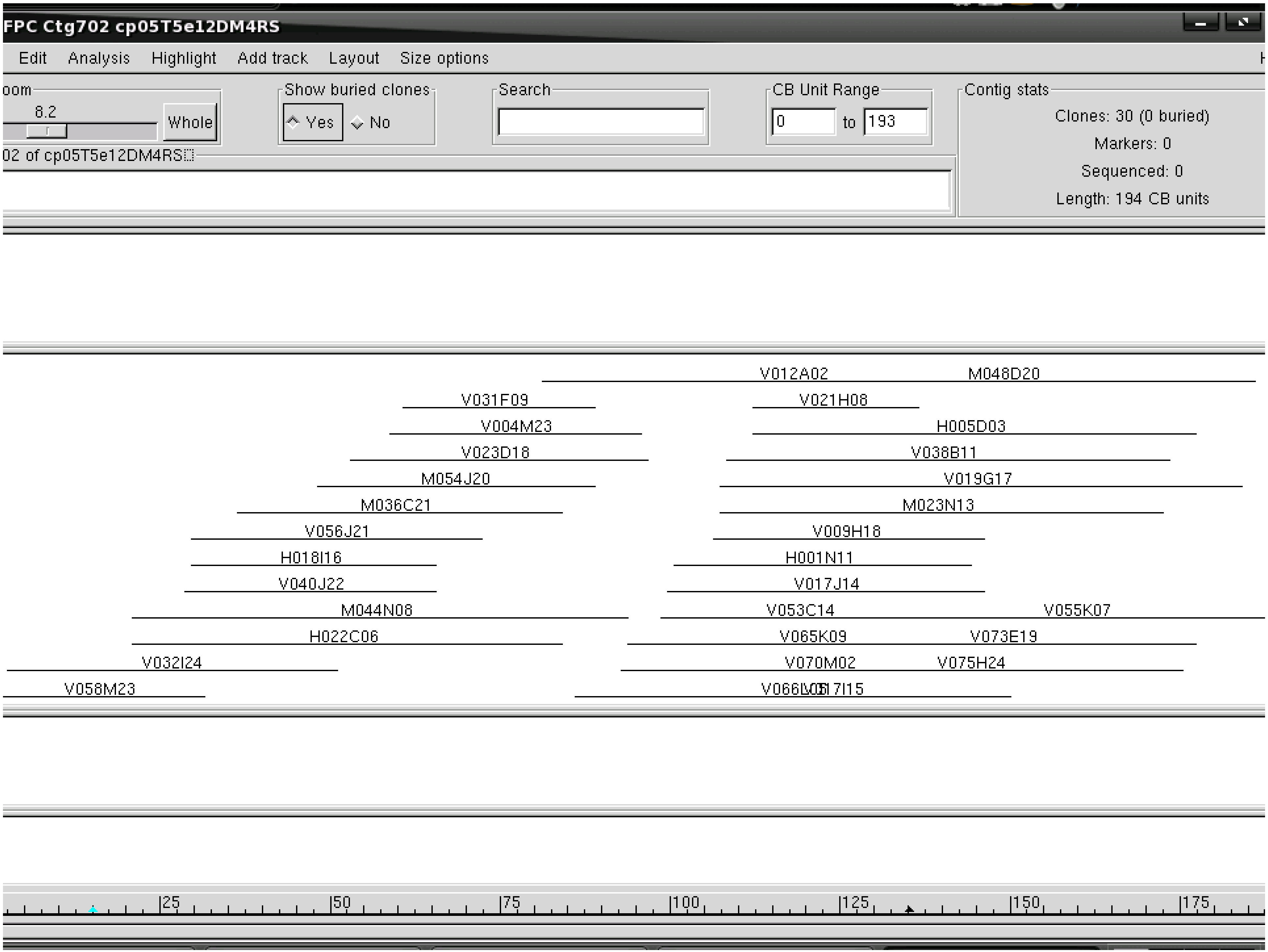
FPC Ctg702 cp05T5e12DM4RS
Edit Analysis Highlight Add track Layout Size options
oom
8.2
Whole
Show buried clones
Yes
No
Search
CB Unit Range
0
to
193
Contig stats
Clones: 30 (0 buried)
Markers: 0
Sequenced: 0
Length: 194 CB units
02 of cp05T5e12DM4RS
V012A02
M048D20
V031F09
V021H08
V004M23
H005D03
V023D18
V038B11
M054J20
V019G17
M036C21
M023N13
V056J21
V009H18
H018I16
H001N11
V040J22
V017J14
M044N08
V053C14
V055K07
H022C06
V065K09
V073E19
V032I24
V070M02
V075H24
V058M23
V066L05
V017I15
25
50
75
100
125
150
175

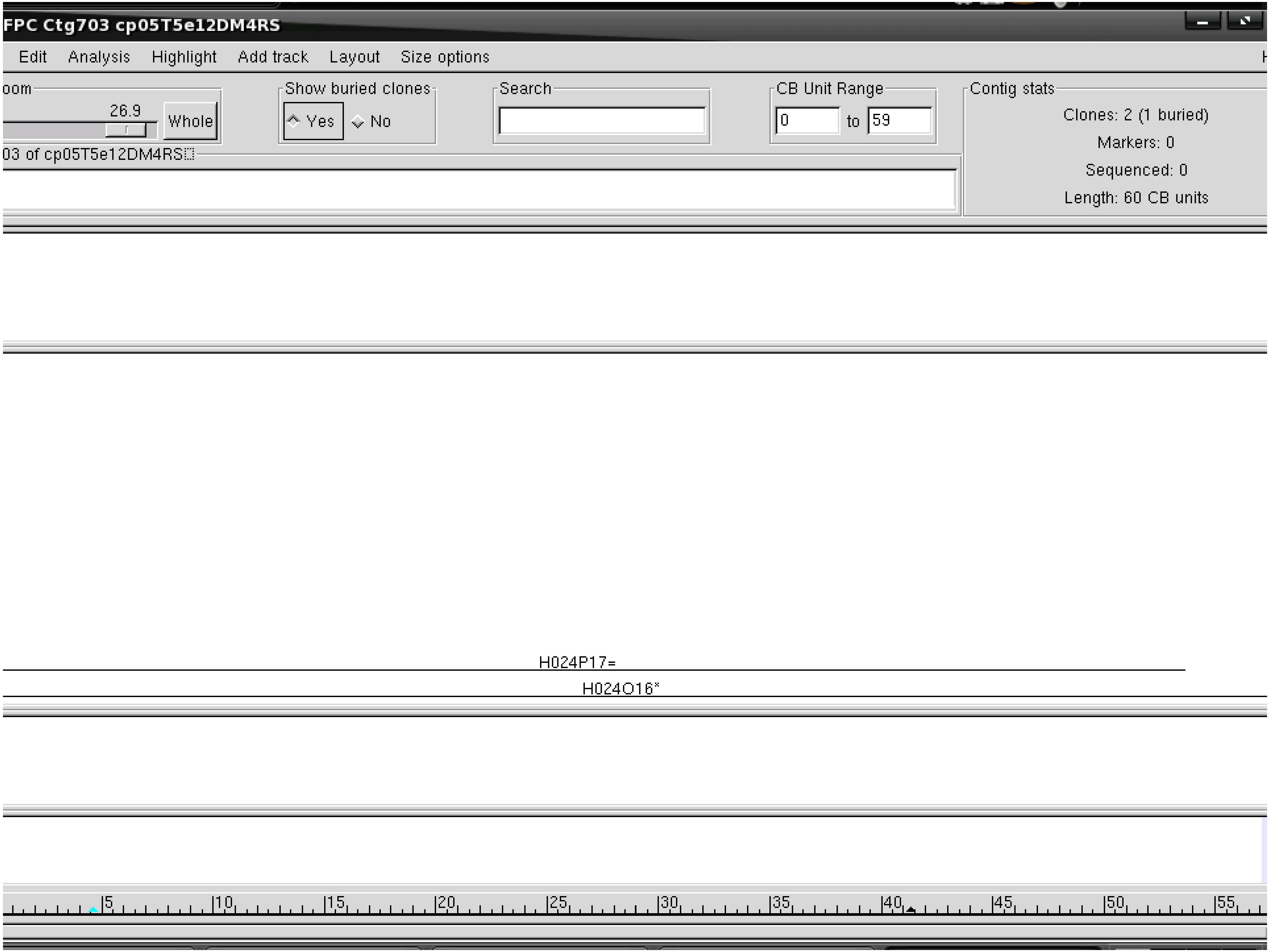
FPC Ctg703 cp05T5e12DM4RS
Edit Analysis Highlight Add track Layout Size options
oom
26.9
Whole
Show buried clones
Yes
No
Search
CB Unit Range
0
to
59
Contig stats
Clones: 2 (1 buried)
Markers: 0
Sequenced: 0
Length: 60 CB units
03 of cp05T5e12DM4RS
H024P17=
H024O16*
5 10 15 20 25 30 35 40 45 50 55

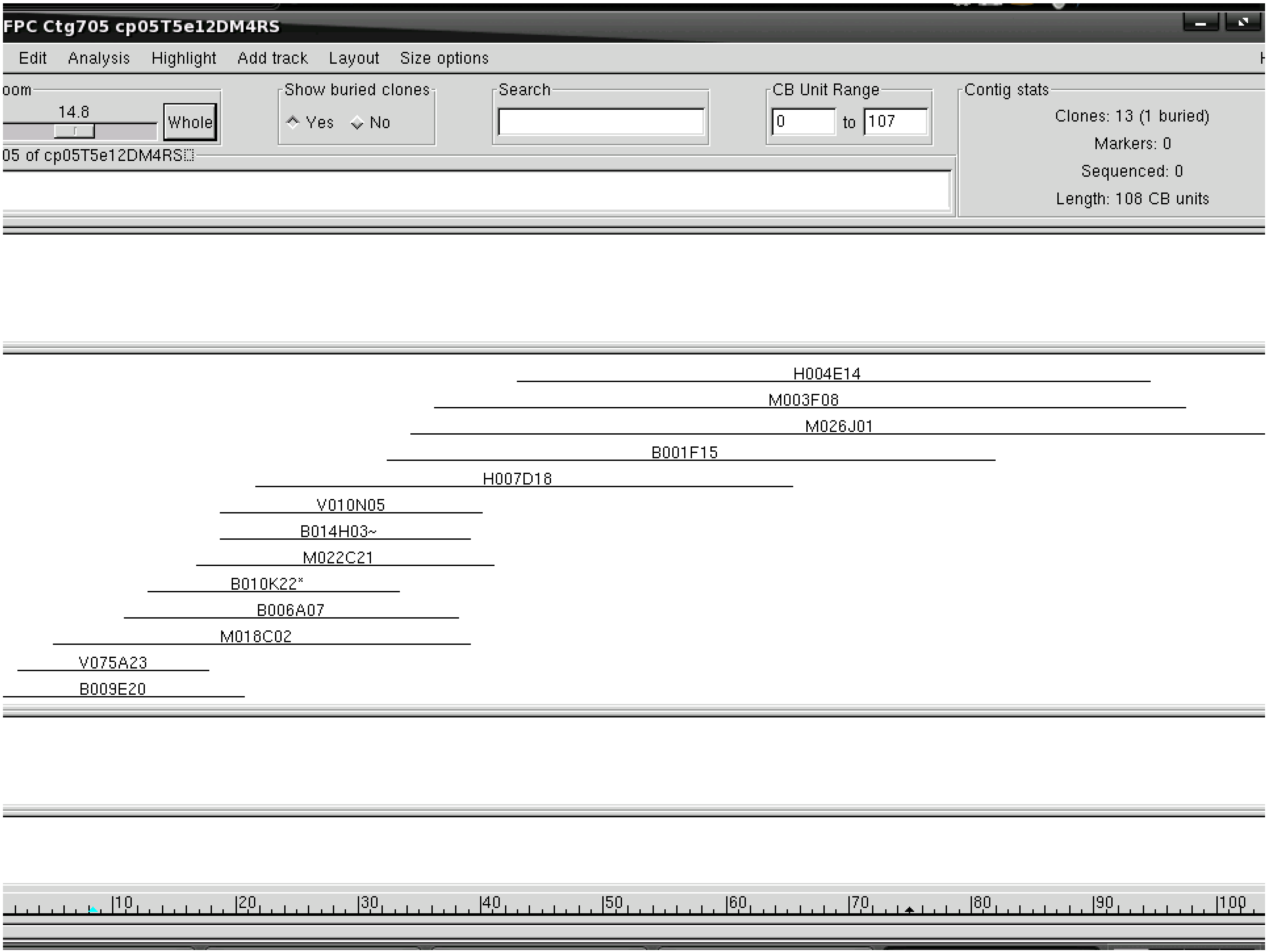

FPC Ctg705 cp05T5e12DM4RS
Edit
Analysis
Highlight
Add track
Layout
Size options
oom
14.8
Whole
Show buried clones
Yes
No
Search
CB Unit Range
0
to
107
Contig stats
Clones: 13 (1 buried)
Markers: 0
Sequenced: 0
Length: 108 CB units
05 of cp05T5e12DM4RS
H004E14
M003F08
M026J01
B001F15
H007D18
V010N05
B014H03~
M022C21
B010K22*
B006A07
M018C02
V075A23
B009E20
10
20
30
40
50
60
70
80
90
100

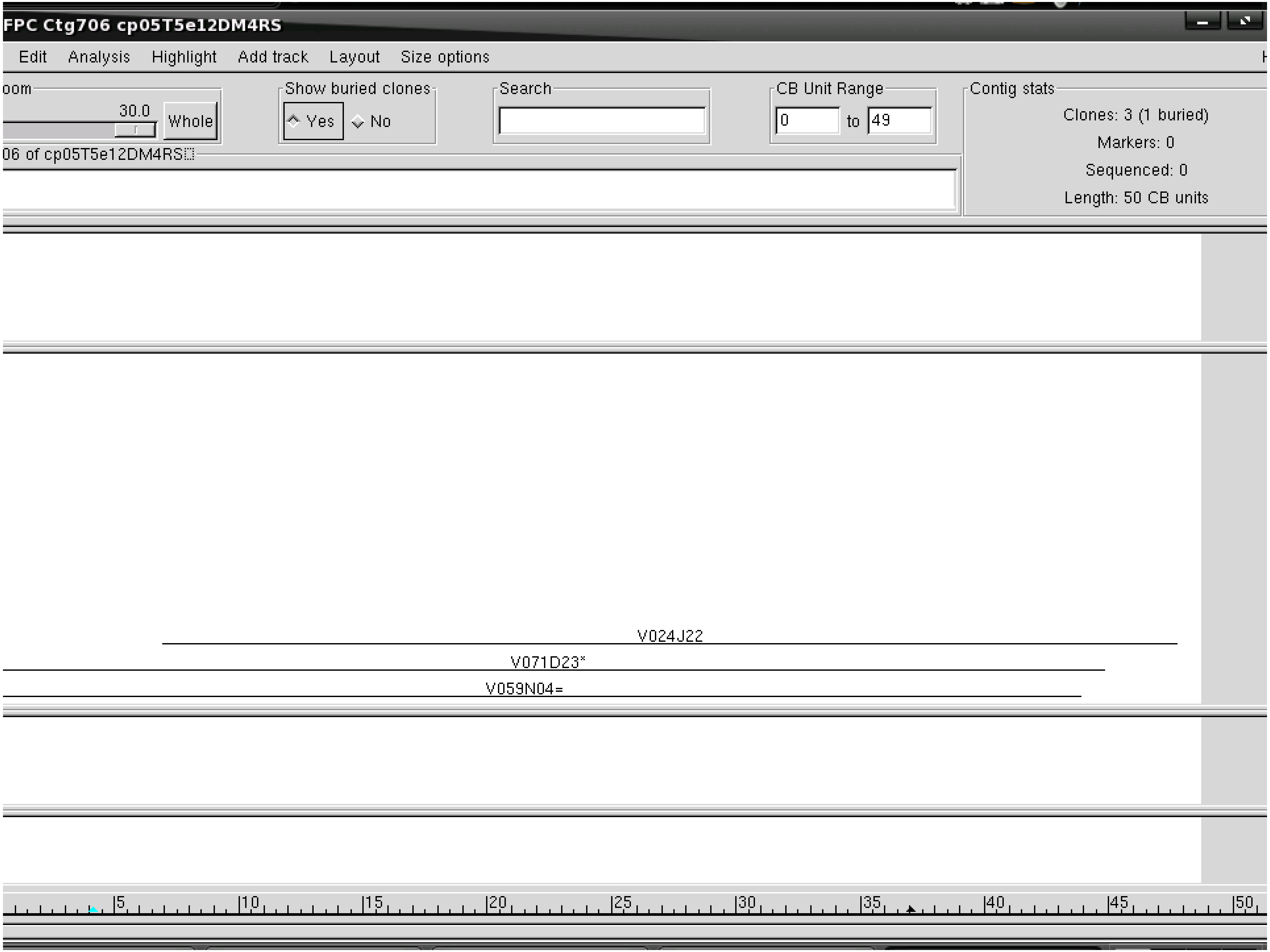
FPC Ctg706 cp05T5e12DM4RS
Edit
Analysis
Highlight
Add track
Layout
Size options
oom
30.0
Whole
Show buried clones
Yes
No
Search
CB Unit Range
0
to
49
Contig stats
Clones: 3 (1 buried)
Markers: 0
Sequenced: 0
Length: 50 CB units
06 of cp05T5e12DM4RS
V024J22
V071D23*
V059N04=
5
10
15
20
25
30
35
40
45
50

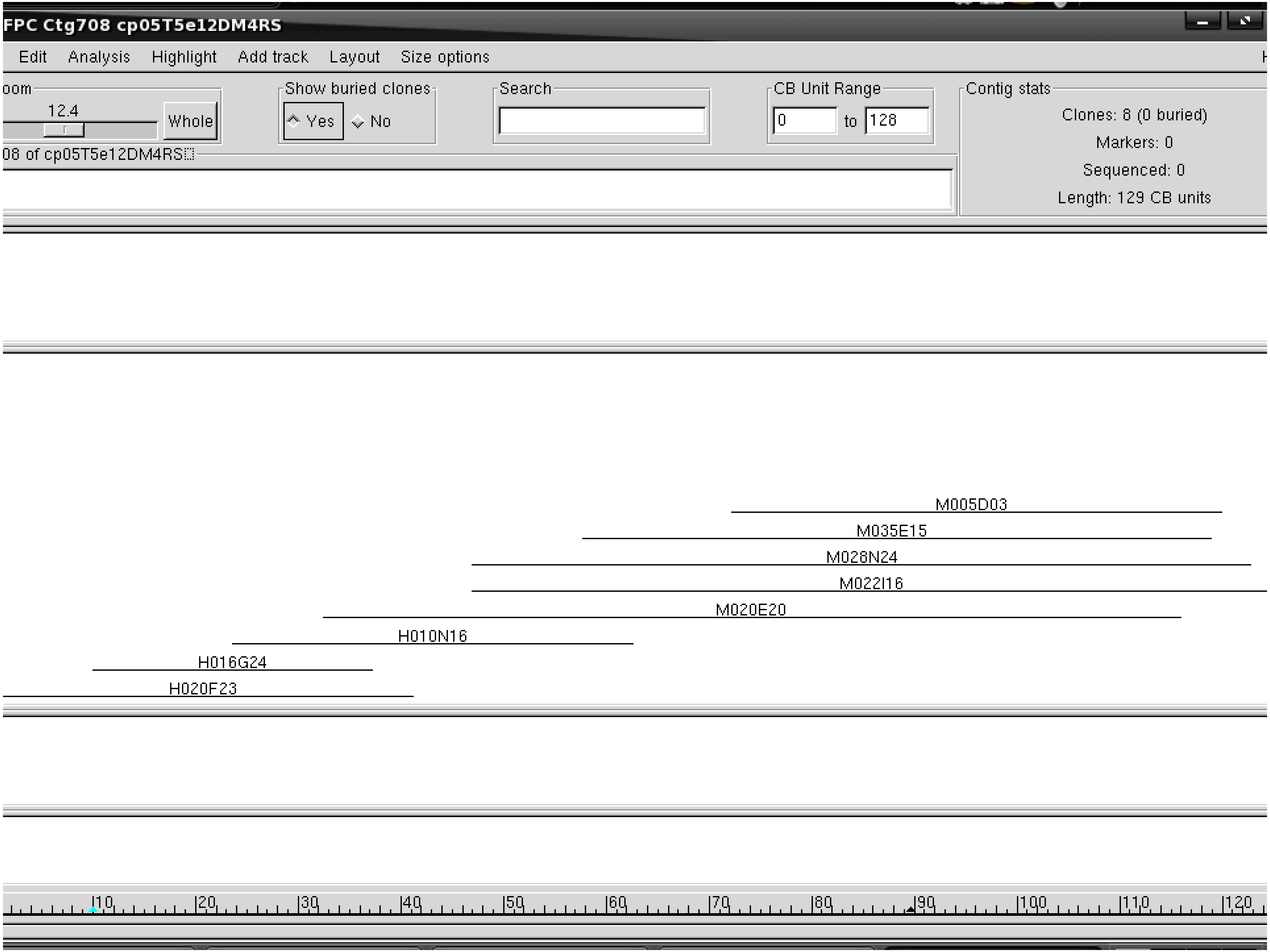
FPC Ctg708 cp05T5e12DM4RS
Edit
Analysis
Highlight
Add track
Layout
Size options
oom
12.4
Whole
Show buried clones
Yes
No
Search
CB Unit Range
0
to
128
Contig stats
Clones: 8 (0 buried)
Markers: 0
Sequenced: 0
Length: 129 CB units
08 of cp05T5e12DM4RS
M005D03
M035E15
M028N24
M022I16
M020E20
H010N16
H016G24
H020F23
10
20
30
40
50
60
70
80
90
100
110
120

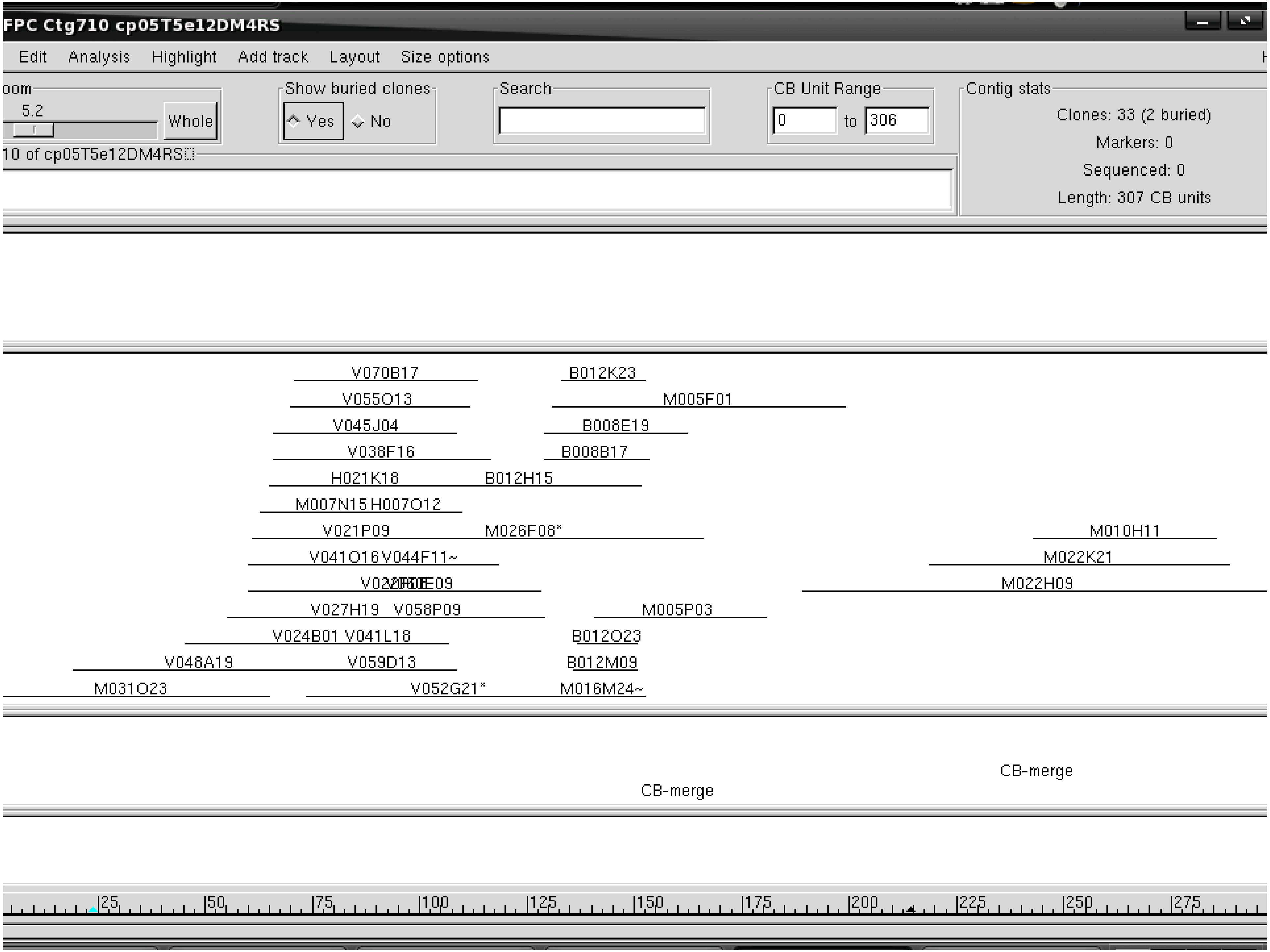
FPC Ctg710 cp05T5e12DM4RS
Edit Analysis Highlight Add track Layout Size options
oom
5.2
Whole
Show buried clones
Yes
No
Search
CB Unit Range
0 to 306
Contig stats
Clones: 33 (2 buried)
Markers: 0
Sequenced: 0
Length: 307 CB units
10 of cp05T5e12DM4RS
V070B17
B012K23
V055O13
M005F01
V045J04
B008E19
V038F16
B008B17
H021K18
B012H15
M007N15
H007O12
V021P09
M026F08*
M010H11
V041O16
V044F11~
M022K21
M022H09
V027H19
V058P09
M005P03
V024B01
V041L18
B012O23
V048A19
V059D13
B012M09
M031O23
V052G21*
M016M24~
CB-merge
CB-merge
25 50 75 100 125 150 175 200 225 250 275

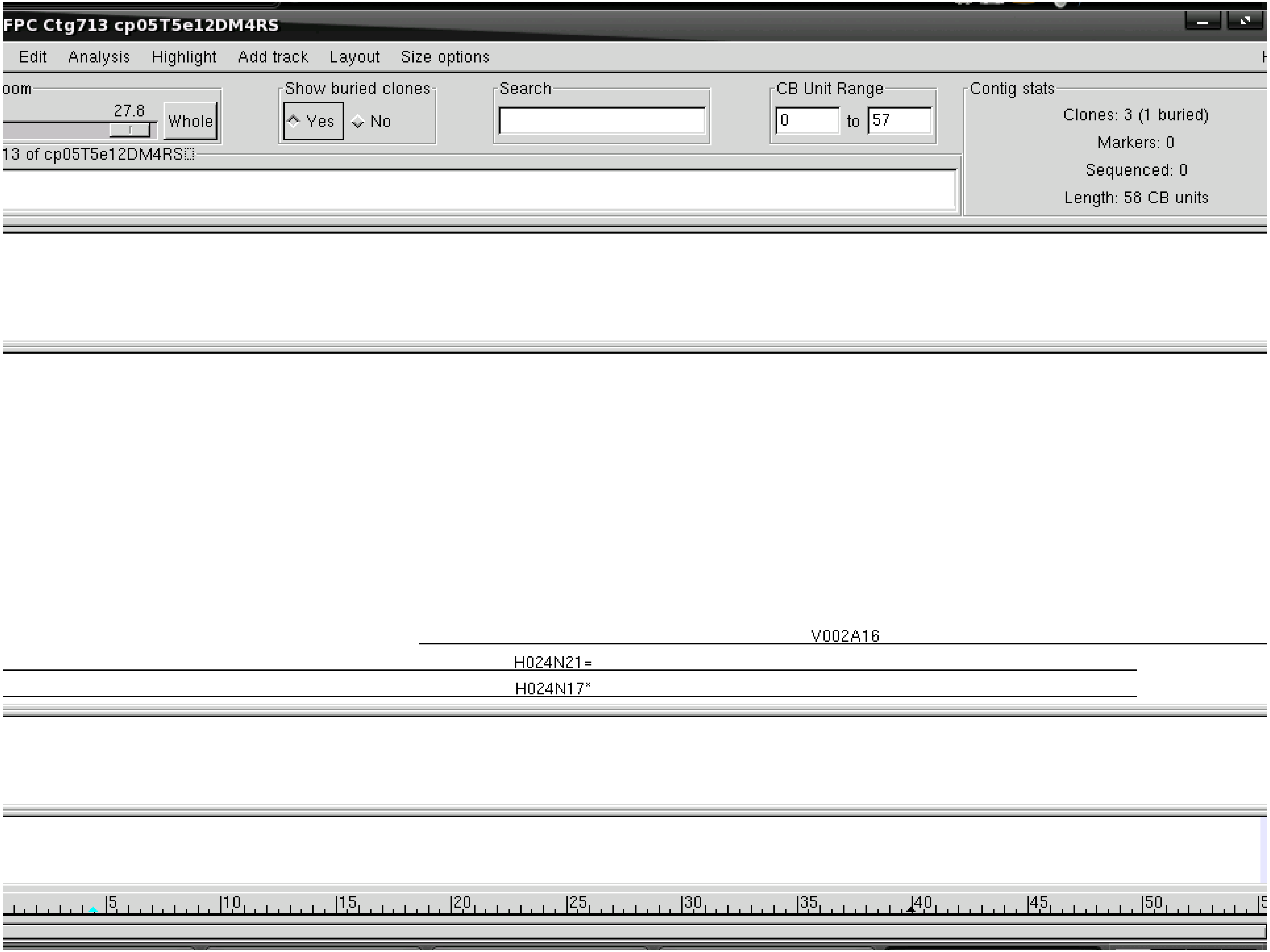
FPC Ctg713 cp05T5e12DM4RS
Edit Analysis Highlight Add track Layout Size options
oom
27.8
Whole
Show buried clones
Yes
No
Search
CB Unit Range
0
to
57
Contig stats
Clones: 3 (1 buried)
Markers: 0
Sequenced: 0
Length: 58 CB units
13 of cp05T5e12DM4RS
V002A16
H024N21=
H024N17*
5 10 15 20 25 30 35 40 45 50

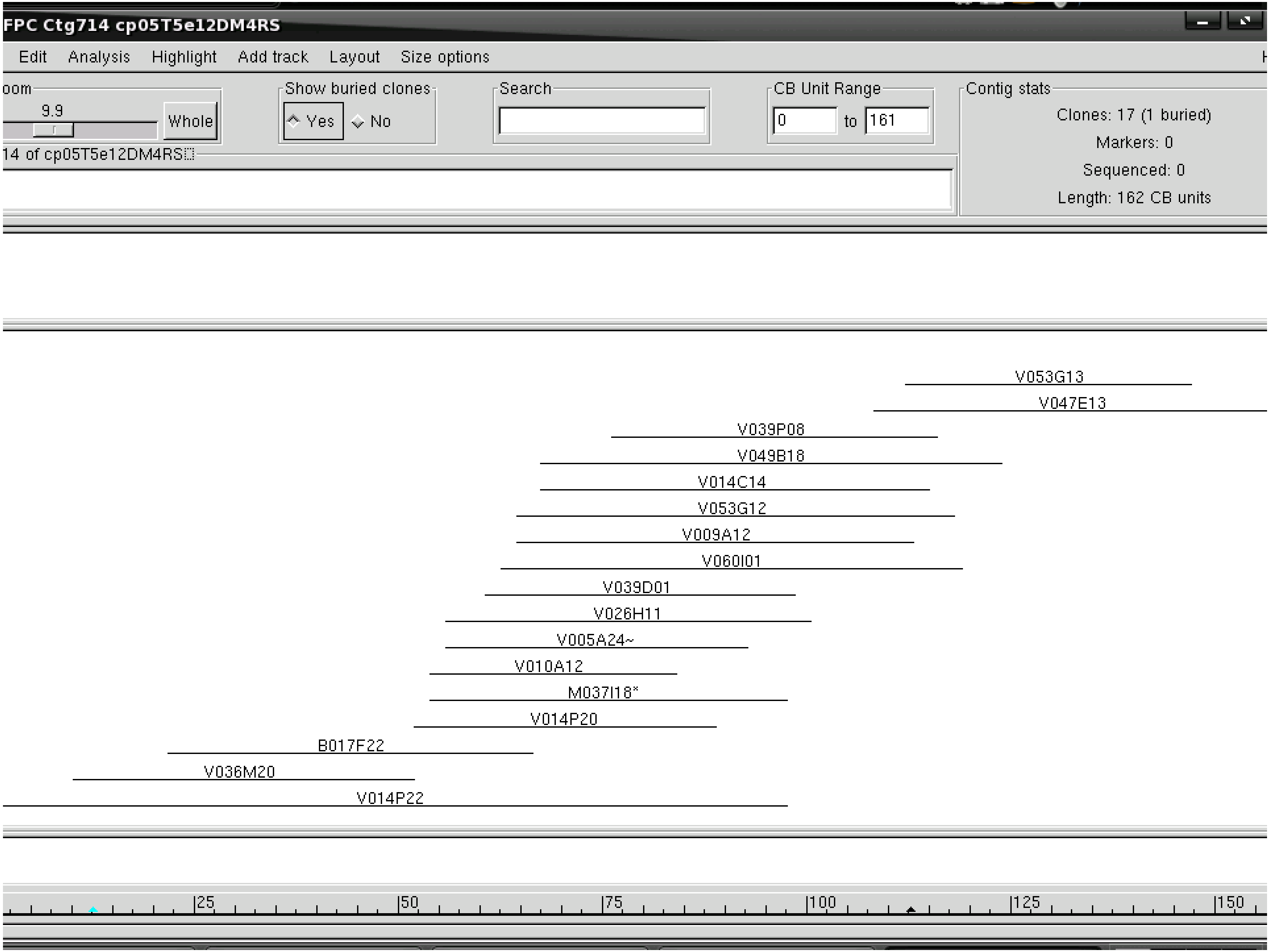
FPC Ctg714 cp05T5e12DM4RS
Edit
Analysis
Highlight
Add track
Layout
Size options
oom
9.9
Whole
Show buried clones
Yes
No
Search
CB Unit Range
0
to
161
Contig stats
Clones: 17 (1 buried)
Markers: 0
Sequenced: 0
Length: 162 CB units
14 of cp05T5e12DM4RS
V053G13
V047E13
V039P08
V049B18
V014C14
V053G12
V009A12
V060I01
V039D01
V026H11
V005A24~
V010A12
M037I18*
V014P20
B017F22
V036M20
V014P22
25
50
75
100
125
150

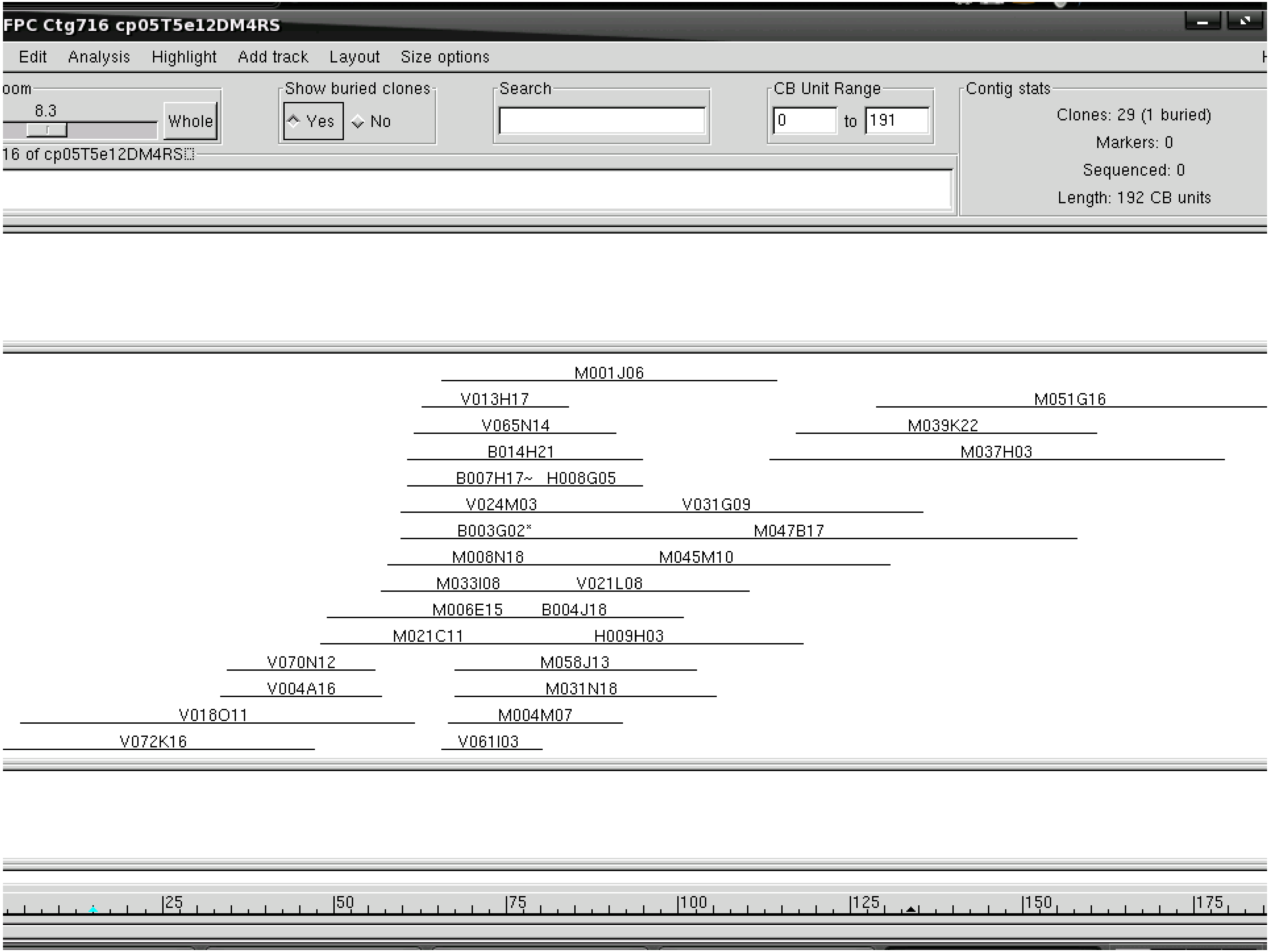
FPC Ctg716 cp05T5e12DM4RS
Edit
Analysis
Highlight
Add track
Layout
Size options
oom
8.3
Whole
Show buried clones
Yes
No
Search
CB Unit Range
0
to
191
Contig stats
Clones: 29 (1 buried)
Markers: 0
Sequenced: 0
Length: 192 CB units
16 of cp05T5e12DM4RS
M001J06
V013H17
M051G16
V065N14
M039K22
B014H21
M037H03
B007H17~
H008G05
V024M03
V031G09
B003G02*
M047B17
M008N18
M045M10
M033I08
V021L08
M006E15
B004J18
M021C11
H009H03
V070N12
M058J13
V004A16
M031N18
V018O11
M004M07
V072K16
V061I03
25
50
75
100
125
150
175

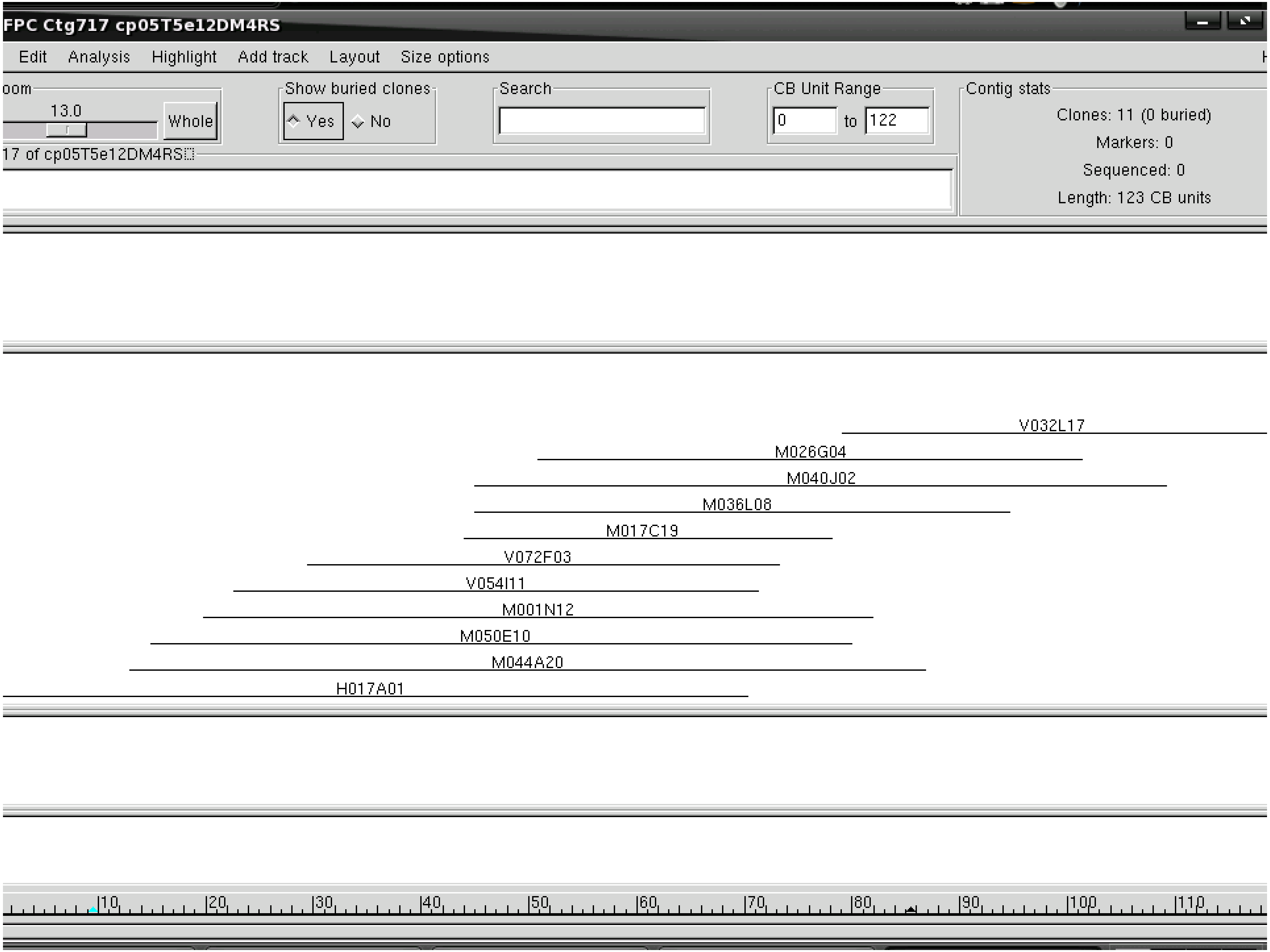
FPC Ctg717 cp05T5e12DM4RS
Edit
Analysis
Highlight
Add track
Layout
Size options
oom
13.0
Whole
Show buried clones
Yes
No
Search
CB Unit Range
0
to
122
Contig stats
Clones: 11 (0 buried)
Markers: 0
Sequenced: 0
Length: 123 CB units
17 of cp05T5e12DM4RS
V032L17
M026G04
M040J02
M036L08
M017C19
V072F03
V054I11
M001N12
M050E10
M044A20
H017A01
10
20
30
40
50
60
70
80
90
100
110

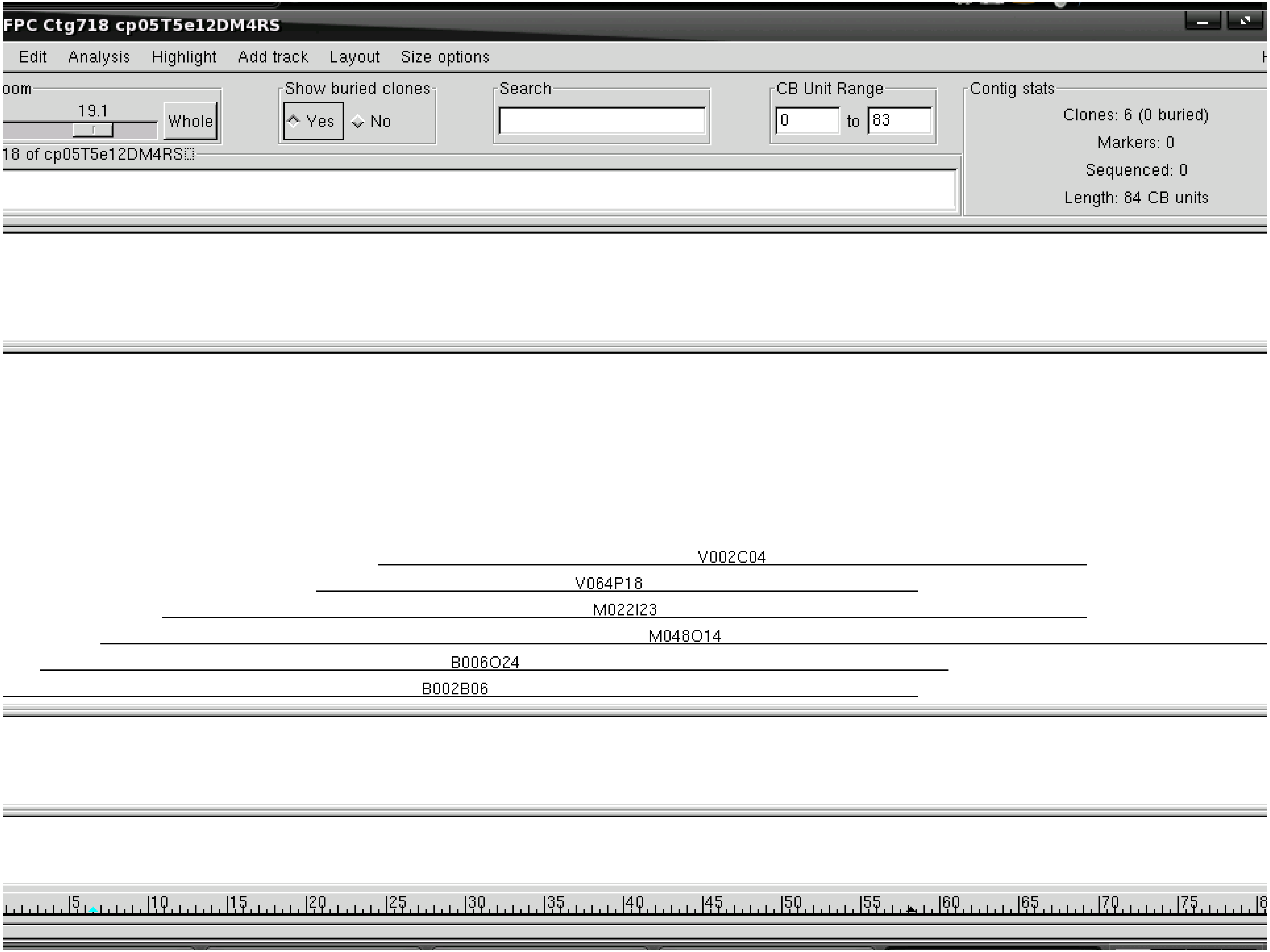
FPC Ctg718 cp05T5e12DM4RS
Edit
Analysis
Highlight
Add track
Layout
Size options
oom
19.1
Whole
Show buried clones
Yes
No
Search
CB Unit Range
0
to
83
Contig stats
Clones: 6 (0 buried)
Markers: 0
Sequenced: 0
Length: 84 CB units
18 of cp05T5e12DM4RS
V002C04
V064P18
M022I23
M048O14
B006O24
B002B06
5
10
15
20
25
30
35
40
45
50
55
60
65
70
75

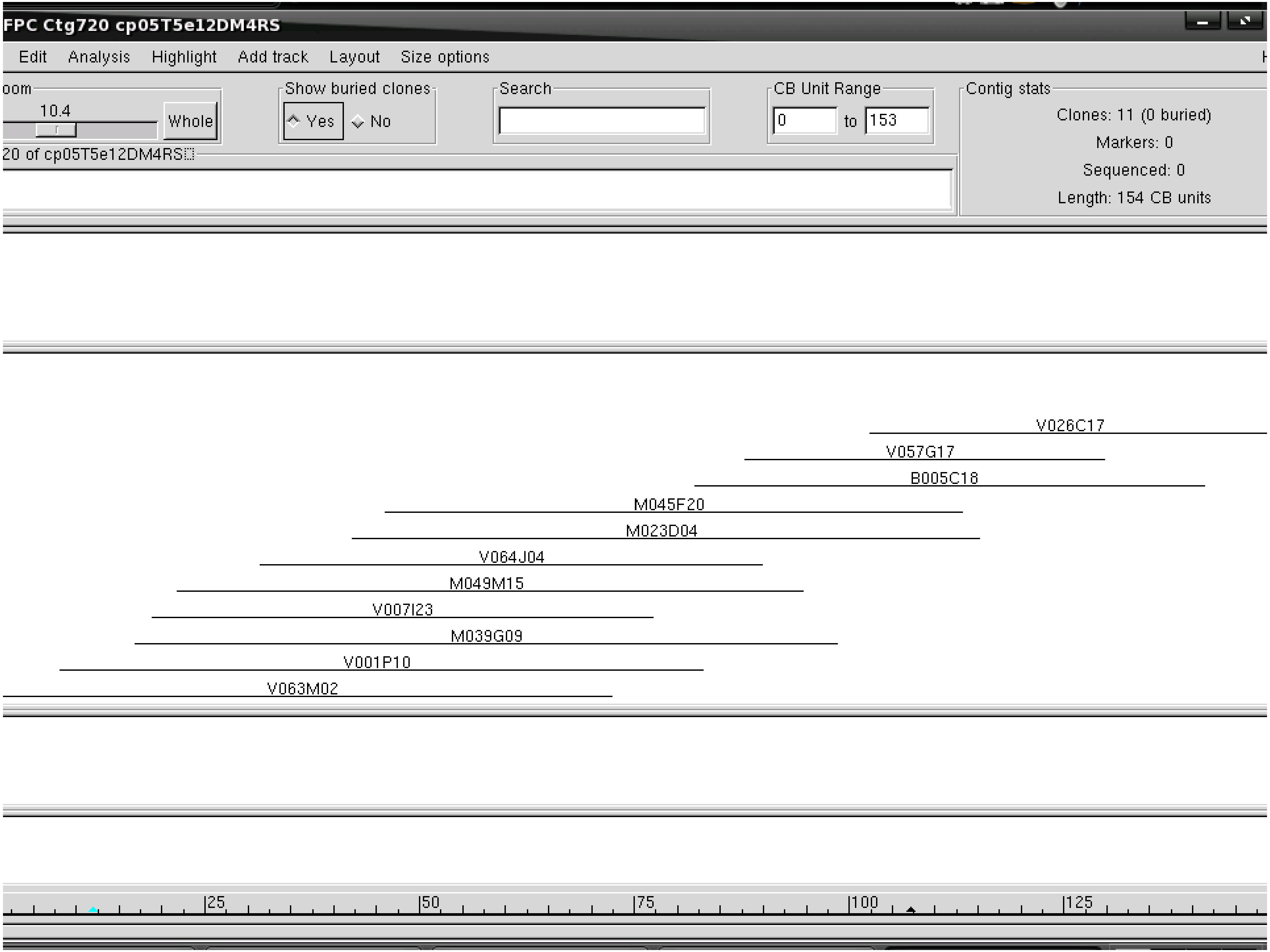
FPC Ctg720 cp05T5e12DM4RS
Edit
Analysis
Highlight
Add track
Layout
Size options
oom
10.4
Whole
Show buried clones
Yes
No
Search
CB Unit Range
0
to
153
Contig stats
Clones: 11 (0 buried)
Markers: 0
Sequenced: 0
Length: 154 CB units
20 of cp05T5e12DM4RS
V026C17
V057G17
B005C18
M045F20
M023D04
V064J04
M049M15
V007I23
M039G09
V001P10
V063M02
25
50
75
100
125

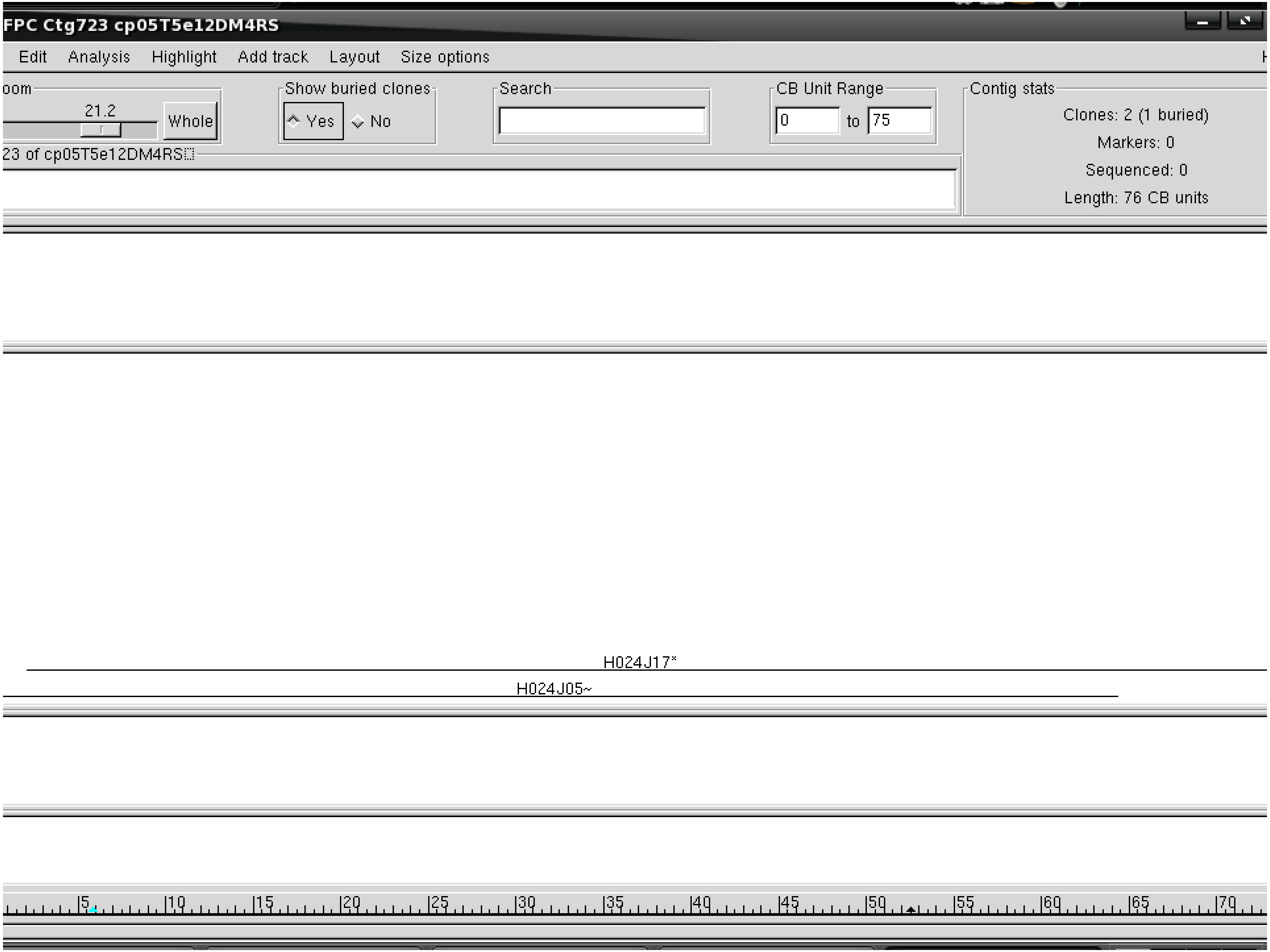
FPC Ctg723 cp05T5e12DM4RS
Edit
Analysis
Highlight
Add track
Layout
Size options
oom
21.2
Whole
Show buried clones
Yes
No
Search
CB Unit Range
0
to
75
Contig stats
Clones: 2 (1 buried)
Markers: 0
Sequenced: 0
Length: 76 CB units
23 of cp05T5e12DM4RS
H024J17*
H024J05~
5 10 15 20 25 30 35 40 45 50 55 60 65 70

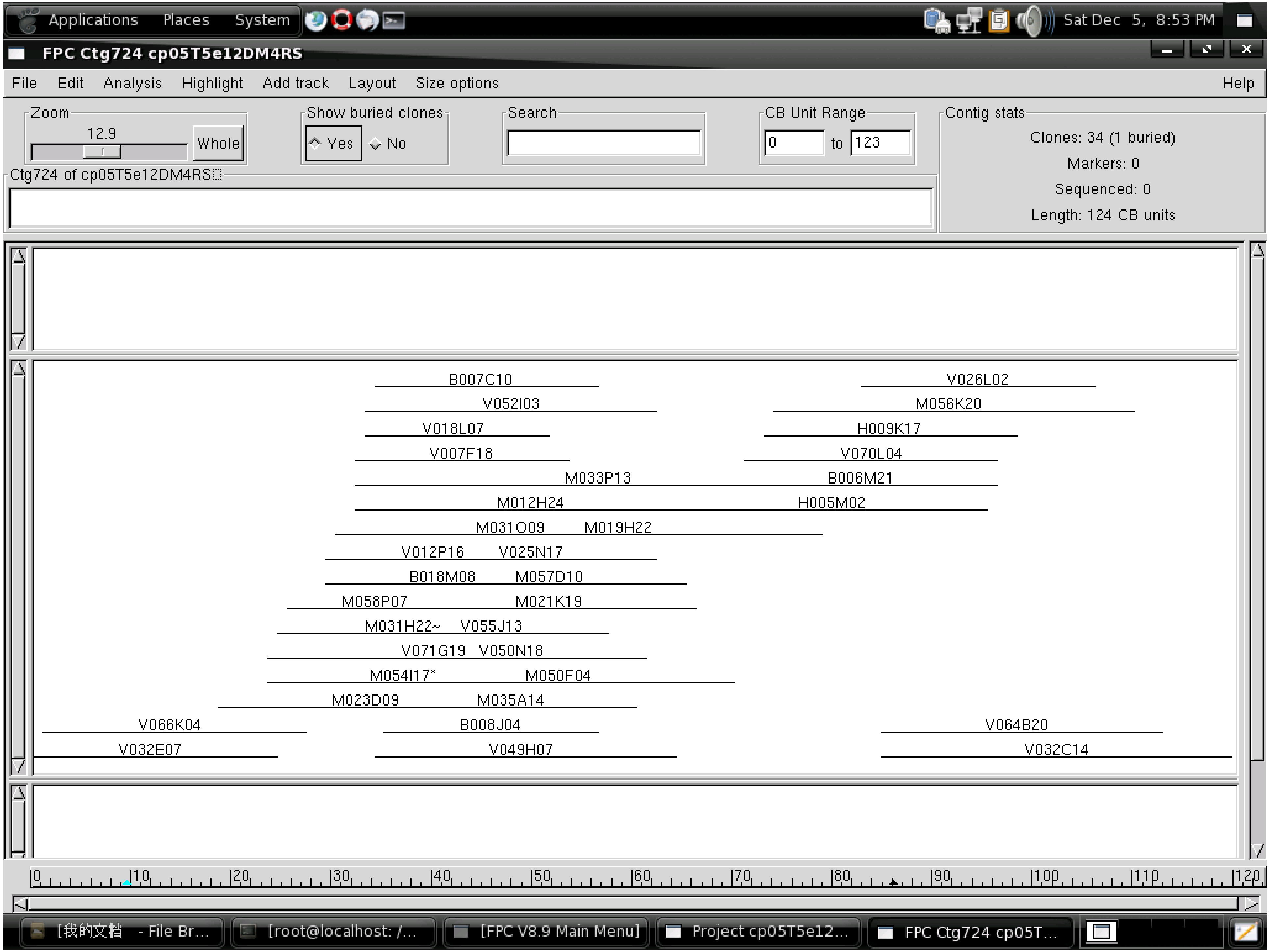
Applications Places System
Sat Dec 5, 8:53 PM
FPC Ctg724 cp05T5e12DM4RS
File Edit Analysis Highlight Add track Layout Size options Help
Zoom
12.9
Whole
Show buried clones
Yes
No
Search
CB Unit Range
0
to
123
Contig stats
Clones: 34 (1 buried)
Markers: 0
Sequenced: 0
Length: 124 CB units
Ctg724 of cp05T5e12DM4RS
B007C10
V026L02
V052I03
M056K20
V018L07
H009K17
V007F18
V070L04
M033P13
B006M21
M012H24
H005M02
M031O09
M019H22
V012P16
V025N17
B018M08
M057D10
M058P07
M021K19
M031H22~
V055J13
V071G19
V050N18
M054I17*
M050F04
M023D09
M035A14
V066K04
B008J04
V064B20
V032E07
V049H07
V032C14
0 10 20 30 40 50 60 70 80 90 100 110 120
[我的文档 - File Br...
[root@localhost: /...
[FPC V8.9 Main Menu]
Project cp05T5e12...
FPC Ctg724 cp05T...

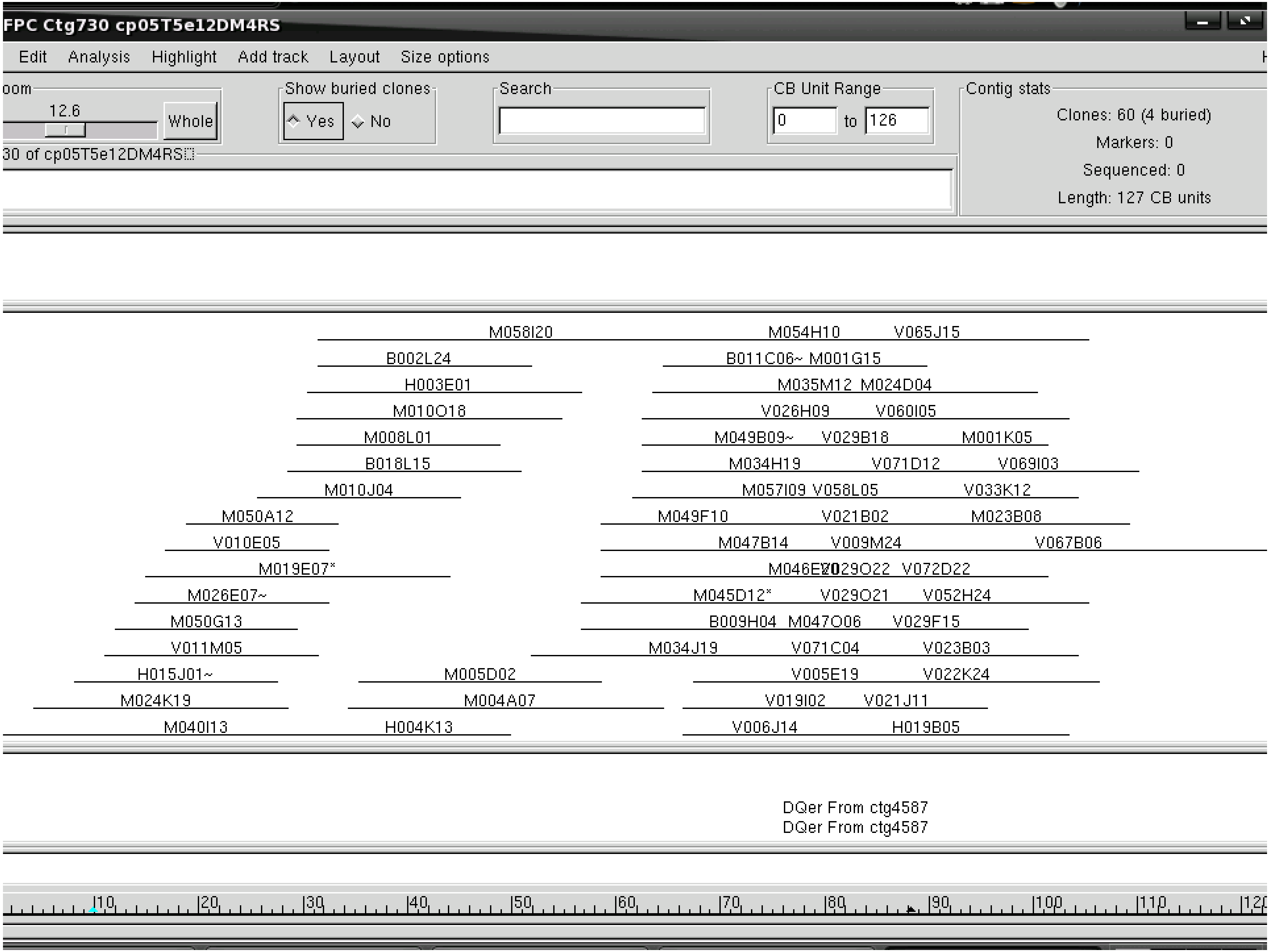
FPC Ctg730 cp05T5e12DM4RS
Edit Analysis Highlight Add track Layout Size options
oom
12.6
Whole
Show buried clones
Yes No
Search
CB Unit Range
0 to 126
Contig stats
Clones: 60 (4 buried)
Markers: 0
Sequenced: 0
Length: 127 CB units
30 of cp05T5e12DM4RS
M058I20 M054H10 V065J15
B002L24 B011C06~ M001G15
H003E01 M035M12 M024D04
M010O18 V026H09 V060I05
M008L01 M049B09~ V029B18 M001K05
B018L15 M034H19 V071D12 V069I03
M010J04 M057I09 V058L05 V033K12
M050A12 M049F10 V021B02 M023B08
V010E05 M047B14 V009M24 V067B06
M019E07* V029O22 V072D22
M026E07~ M045D12* V029O21 V052H24
M050G13 B009H04 M047O06 V029F15
V011M05 M034J19 V071C04 V023B03
H015J01~ M005D02 V005E19 V022K24
M024K19 M004A07 V019I02 V021J11
M040I13 H004K13 V006J14 H019B05
DQer From ctg4587
DQer From ctg4587
10 20 30 40 50 60 70 80 90 100 110

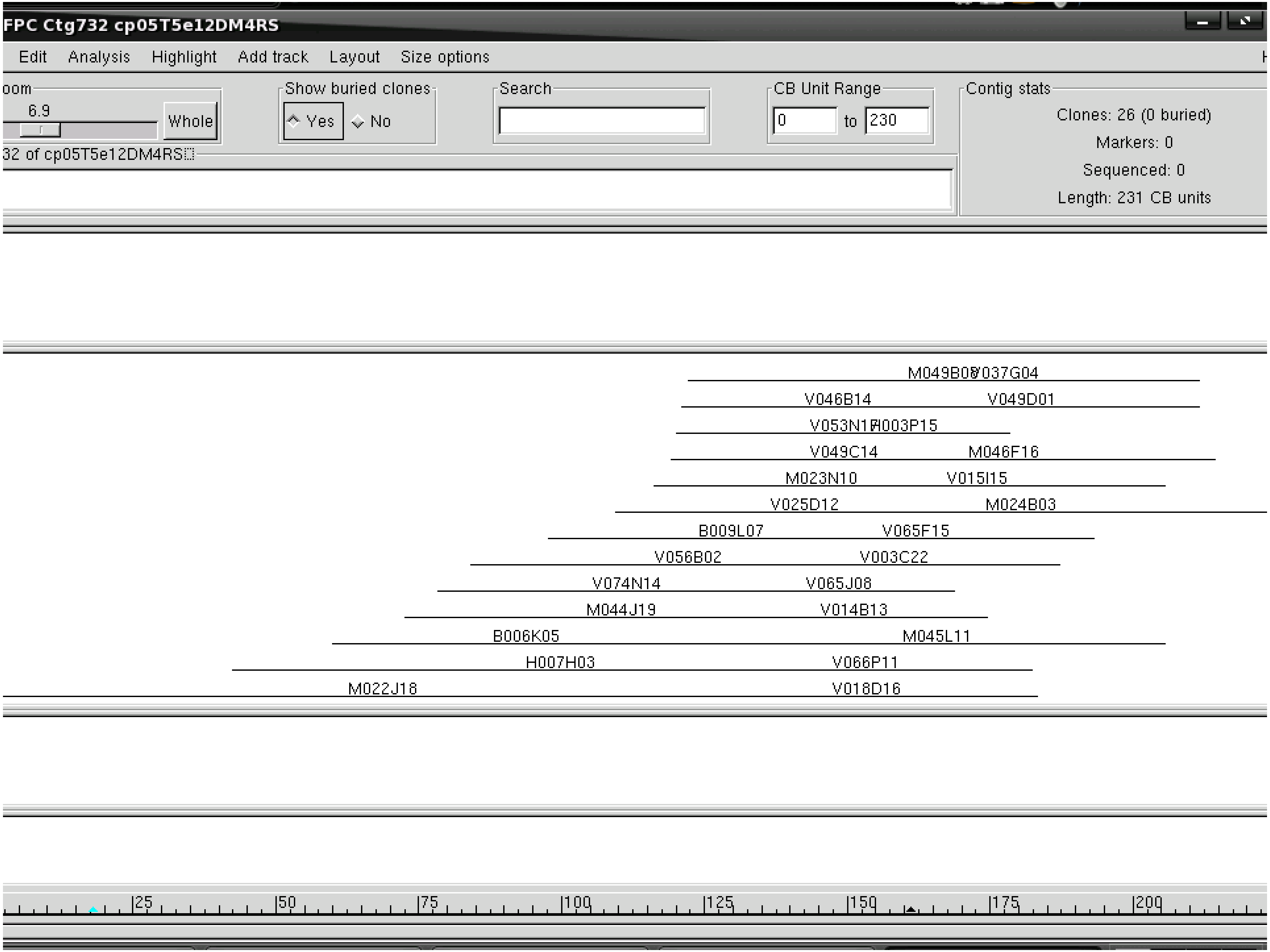
FPC Ctg732 cp05T5e12DM4RS
Edit
Analysis
Highlight
Add track
Layout
Size options
oom
6.9
Whole
Show buried clones
Yes
No
Search
CB Unit Range
0
to
230
Contig stats
Clones: 26 (0 buried)
Markers: 0
Sequenced: 0
Length: 231 CB units
32 of cp05T5e12DM4RS
V037G04
V046B14
V049D01
H003P15
V049C14
M046F16
M023N10
V015I15
V025D12
M024B03
B009L07
V065F15
V056B02
V003C22
V074N14
V065J08
M044J19
V014B13
B006K05
M045L11
H007H03
V066P11
M022J18
V018D16
25
50
75
100
125
150
175
200

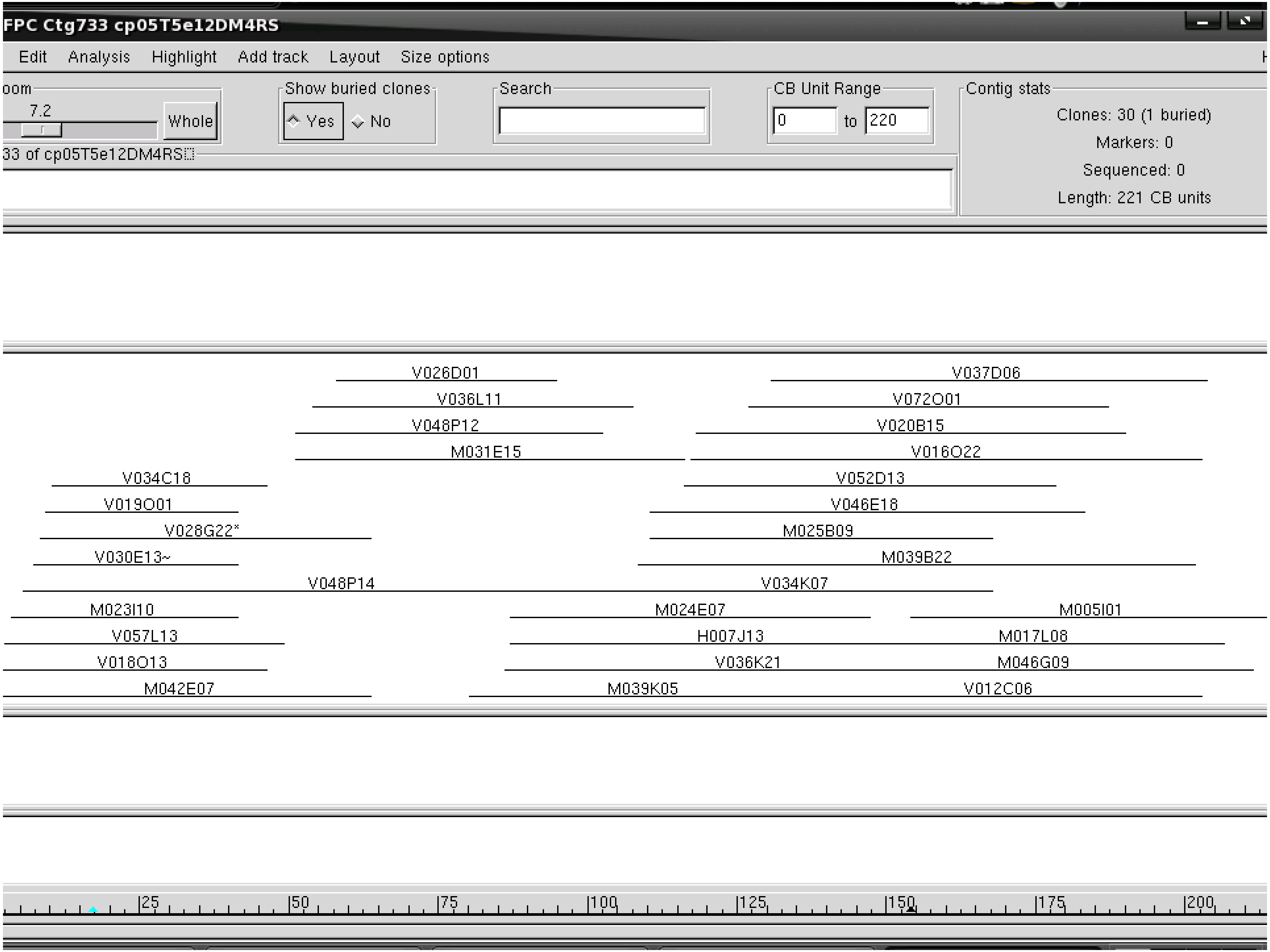
FPC Ctg733 cp05T5e12DM4RS
Edit
Analysis
Highlight
Add track
Layout
Size options
oom
7.2
Whole
Show buried clones
Yes
No
Search
CB Unit Range
0
to
220
Contig stats
Clones: 30 (1 buried)
Markers: 0
Sequenced: 0
Length: 221 CB units
33 of cp05T5e12DM4RS
V026D01
V037D06
V036L11
V072O01
V048P12
V020B15
M031E15
V016O22
V034C18
V052D13
V019O01
V046E18
V028G22*
M025B09
V030E13~
M039B22
V048P14
V034K07
M023I10
M024E07
M005I01
V057L13
H007J13
M017L08
V018O13
V036K21
M046G09
M042E07
M039K05
V012C06
25
50
75
100
125
150
175
200

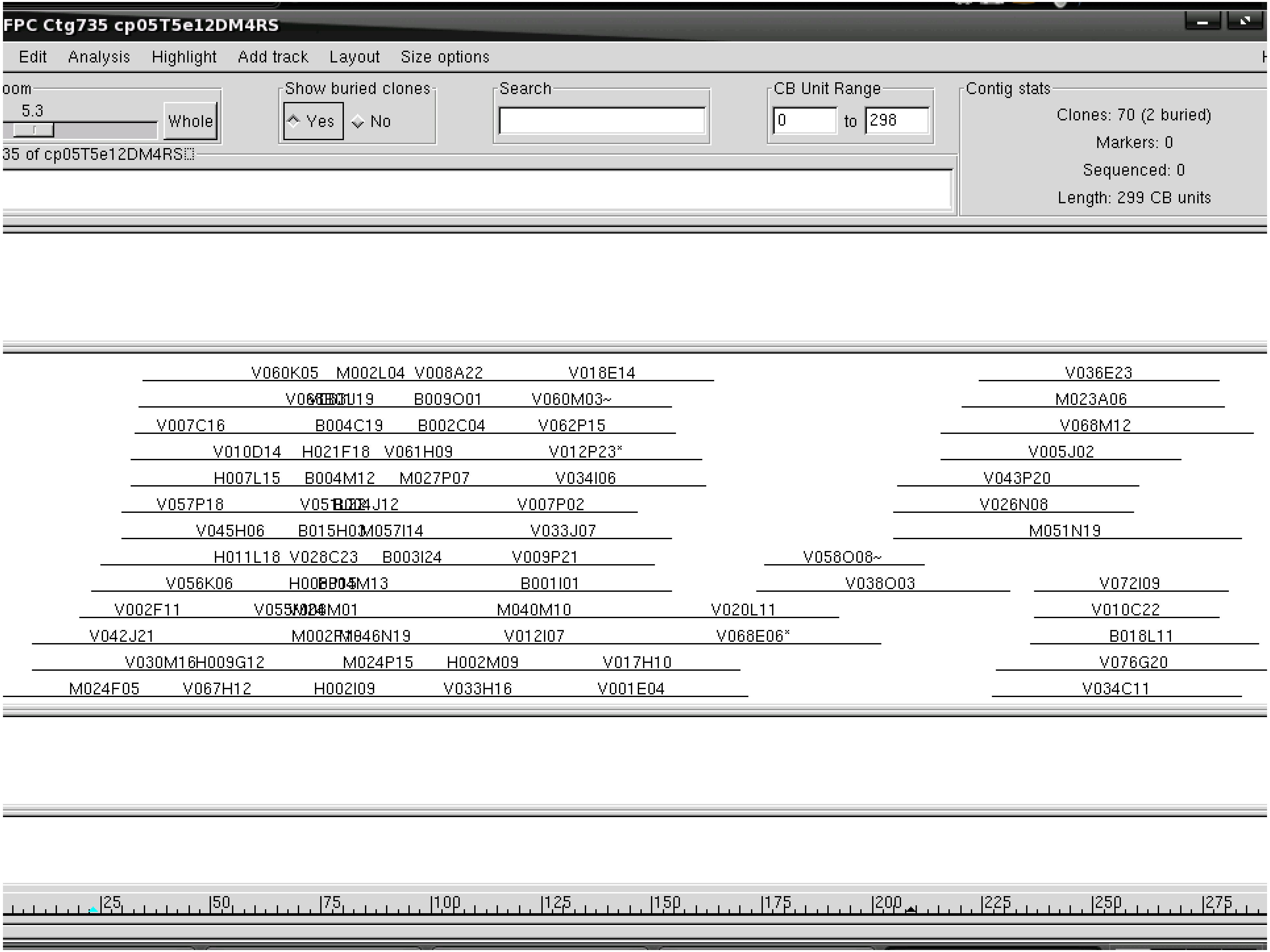

FPC Ctg735 cp05T5e12DM4RS
Edit Analysis Highlight Add track Layout Size options
5.3
Whole
Show buried clones
Yes
No
Search
CB Unit Range
0 to 298
Contig stats
Clones: 70 (2 buried)
Markers: 0
Sequenced: 0
Length: 299 CB units
V060K05 M002L04 V008A22 V018E14 V036E23
B009O01 V060M03~ M023A06
V007C16 B004C19 B002C04 V062P15 V068M12
V010D14 H021F18 V061H09 V012P23* V005J02
H007L15 B004M12 M027P07 V034I06 V043P20
V057P18 V007P02 V026N08
V045H06 V033J07 M051N19
H011L18 V028C23 B003I24 V009P21 V058O08~
V056K06 B001I01 V038O03 V072I09
V002F11 M040M10 V020L11 V010C22
V042J21 V012I07 V068E06* B018L11
M024P15 H002M09 V017H10 V076G20
M024F05 V067H12 H002I09 V033H16 V001E04 V034C11
25 50 75 100 125 150 175 200 225 250 275

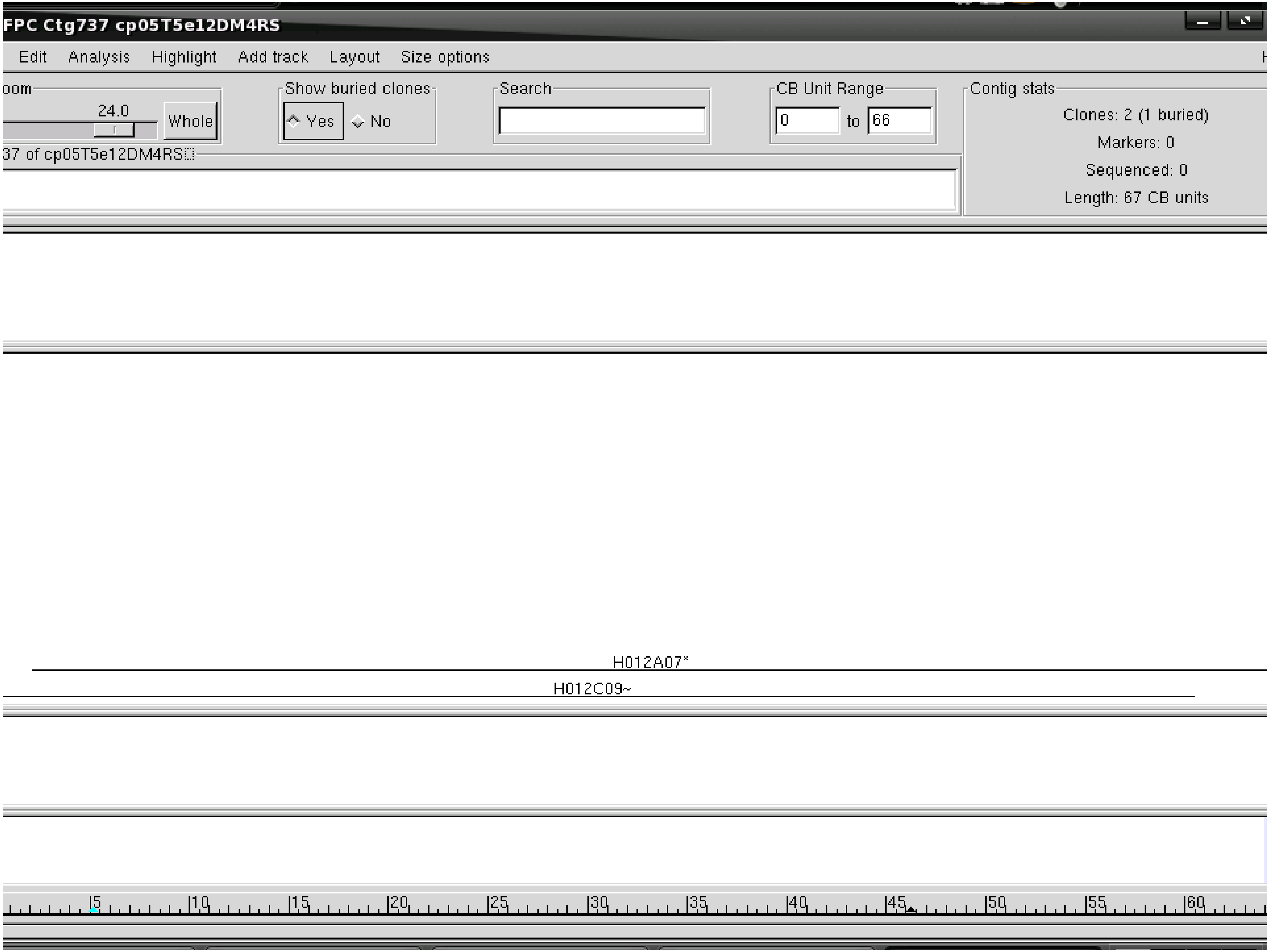
FPC Ctg737 cp05T5e12DM4RS
Edit
Analysis
Highlight
Add track
Layout
Size options
oom
24.0
Whole
Show buried clones
Yes
No
Search
CB Unit Range
0
to
66
Contig stats
Clones: 2 (1 buried)
Markers: 0
Sequenced: 0
Length: 67 CB units
37 of cp05T5e12DM4RS
H012A07*
H012C09~
5
10
15
20
25
30
35
40
45
50
55
60

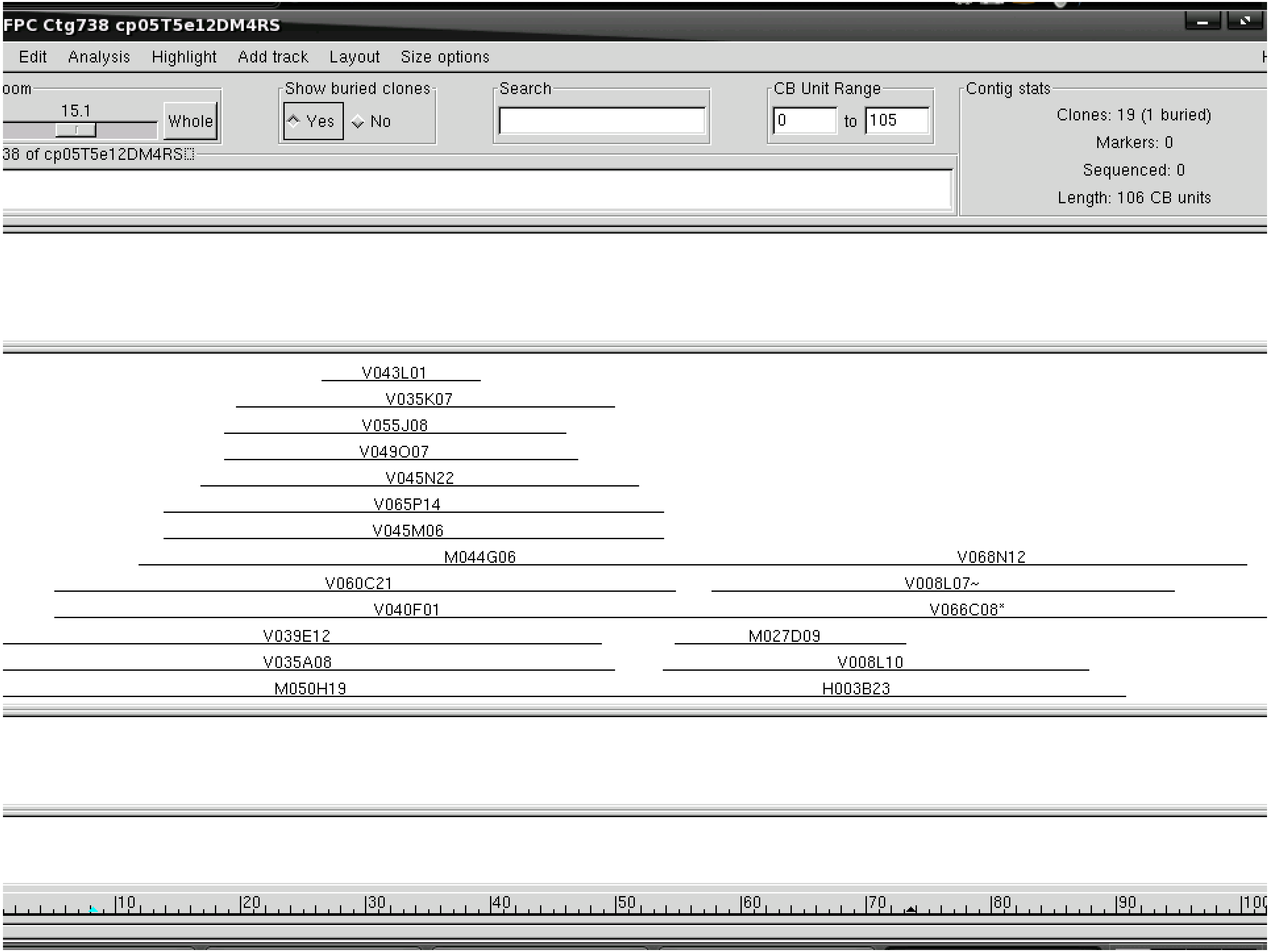
FPC Ctg738 cp05T5e12DM4RS
Edit Analysis Highlight Add track Layout Size options
oom
15.1
Whole
Show buried clones
Yes
No
Search
CB Unit Range
0
to
105
Contig stats
Clones: 19 (1 buried)
Markers: 0
Sequenced: 0
Length: 106 CB units
38 of cp05T5e12DM4RS
V043L01
V035K07
V055J08
V049O07
V045N22
V065P14
V045M06
M044G06
V068N12
V060C21
V008L07~
V040F01
V066C08*
V039E12
M027D09
V035A08
V008L10
M050H19
H003B23
10
20
30
40
50
60
70
80
90
100

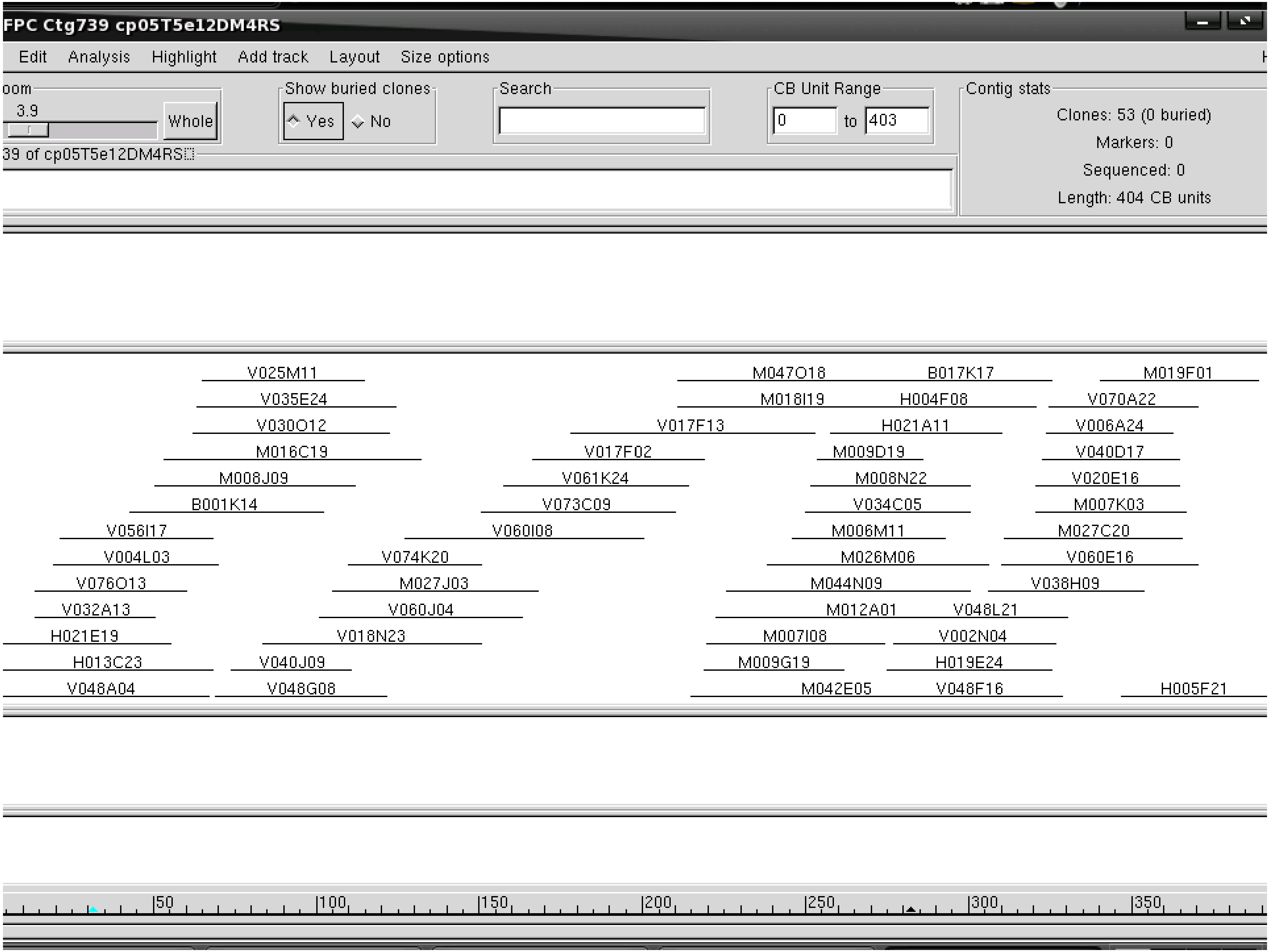
FPC Ctg739 cp05T5e12DM4RS
Edit Analysis Highlight Add track Layout Size options
oom
3.9
Whole
Show buried clones
Yes
No
Search
CB Unit Range
0 to 403
Contig stats
Clones: 53 (0 buried)
Markers: 0
Sequenced: 0
Length: 404 CB units
39 of cp05T5e12DM4RS
V025M11
V035E24
V030O12
M016C19
M008J09
B001K14
V056I17
V004L03
V076O13
V032A13
H021E19
H013C23
V048A04
V048G08
V040J09
V018N23
V060J04
M027J03
V074K20
V060I08
V073C09
V061K24
V017F02
V017F13
M047O18
M018I19
B017K17
H004F08
H021A11
M009D19
M008N22
V034C05
M006M11
M026M06
M044N09
M012A01
M007I08
M009G19
M042E05
V048L21
V002N04
H019E24
V048F16
M019F01
V070A22
V006A24
V040D17
V020E16
M007K03
M027C20
V060E16
V038H09
H005F21
50
100
150
200
250
300
350

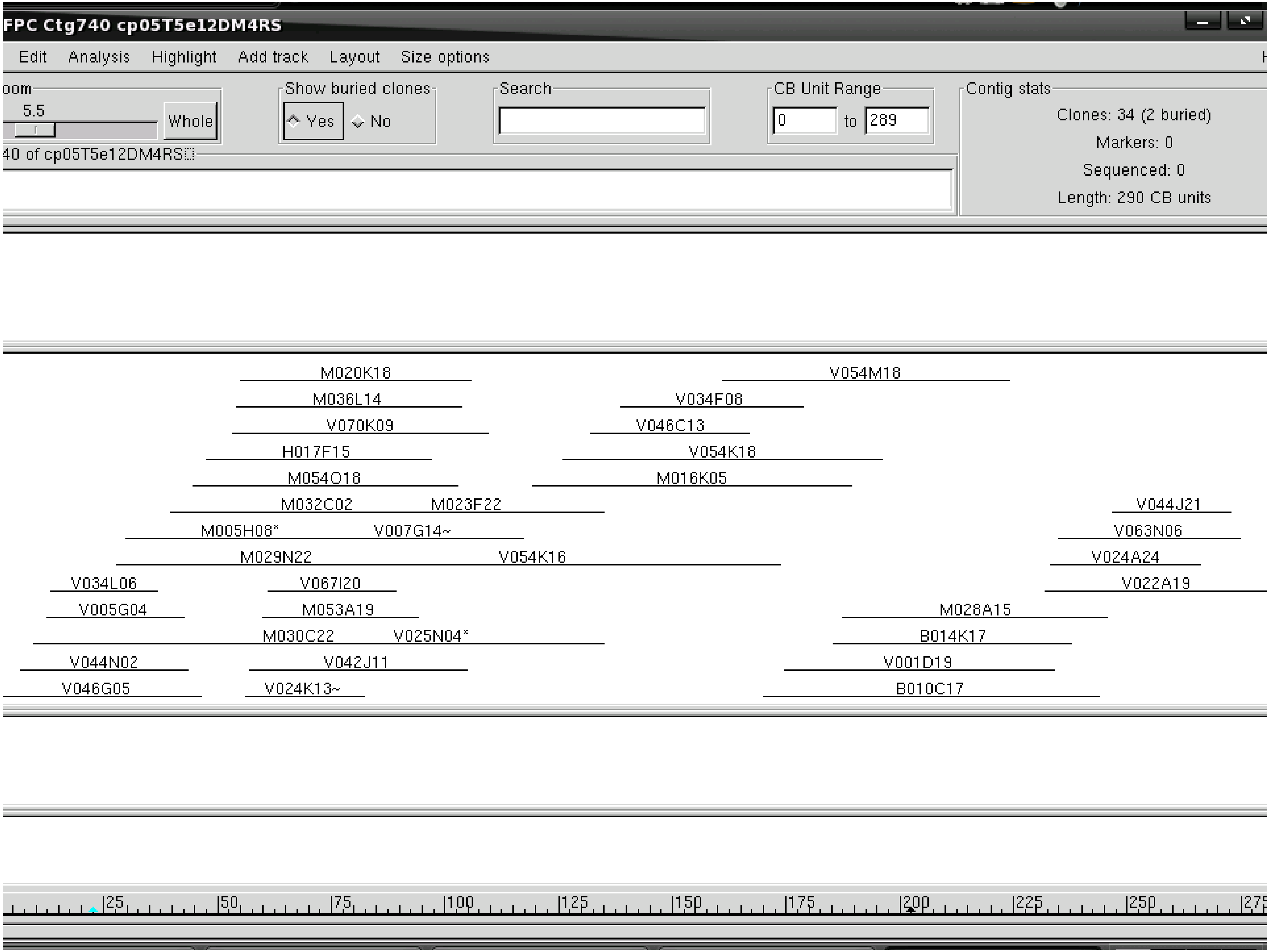
FPC Ctg740 cp05T5e12DM4RS
Edit
Analysis
Highlight
Add track
Layout
Size options
oom
5.5
Whole
Show buried clones
Yes
No
Search
CB Unit Range
0
to
289
Contig stats
Clones: 34 (2 buried)
Markers: 0
Sequenced: 0
Length: 290 CB units
40 of cp05T5e12DM4RS
M020K18
V054M18
M036L14
V034F08
V070K09
V046C13
H017F15
V054K18
M054O18
M016K05
M032C02
M023F22
V044J21
M005H08*
V007G14~
V063N06
M029N22
V054K16
V024A24
V034L06
V067I20
V022A19
V005G04
M053A19
M028A15
M030C22
V025N04*
B014K17
V044N02
V042J11
V001D19
V046G05
V024K13~
B010C17
25
50
75
100
125
150
175
200
225
250

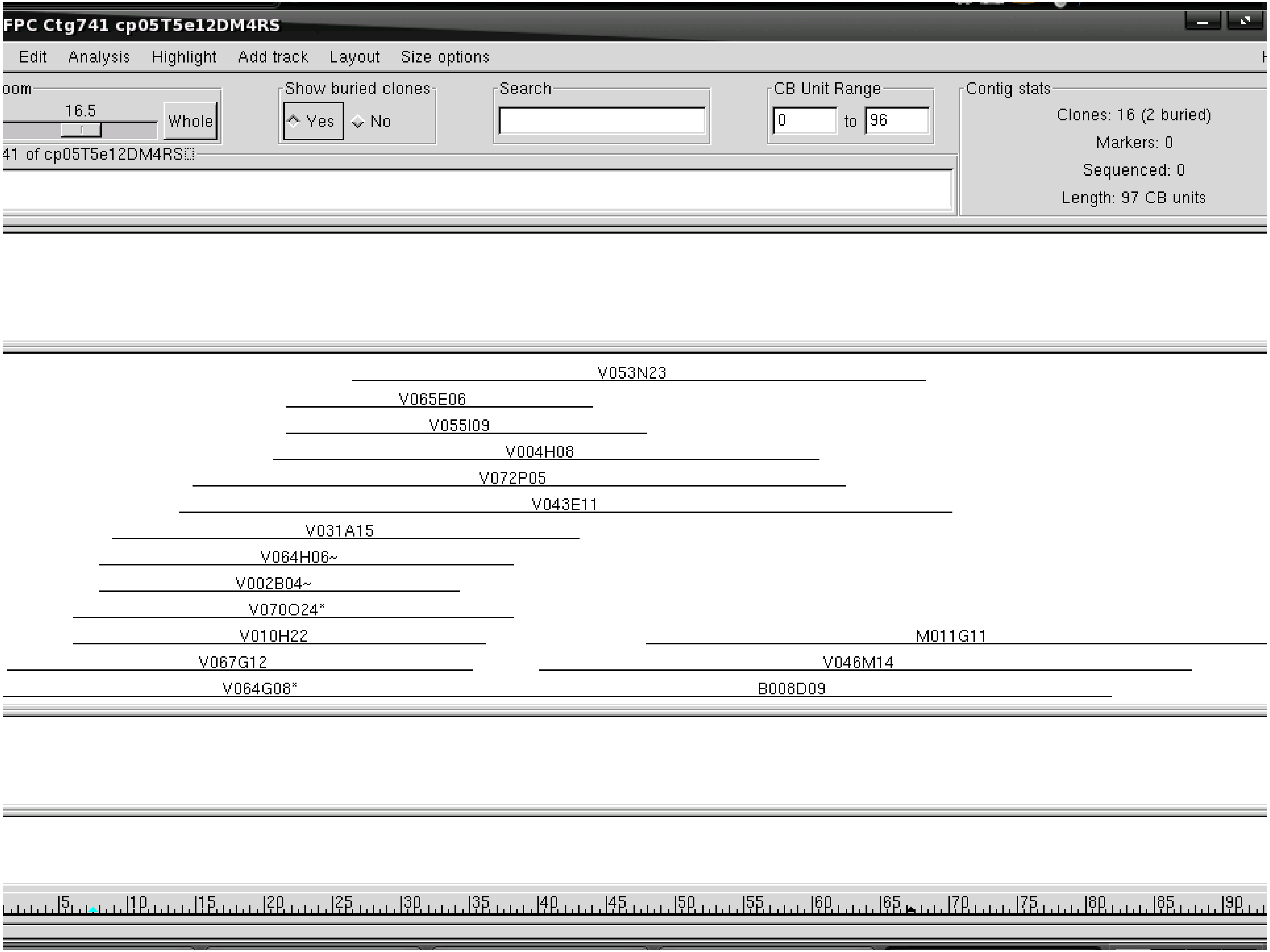
FPC Ctg741 cp05T5e12DM4RS
Edit
Analysis
Highlight
Add track
Layout
Size options
oom
16.5
Whole
Show buried clones
Yes
No
Search
CB Unit Range
0
to
96
Contig stats
Clones: 16 (2 buried)
Markers: 0
Sequenced: 0
Length: 97 CB units
41 of cp05T5e12DM4RS
V053N23
V065E06
V055I09
V004H08
V072P05
V043E11
V031A15
V064H06~
V002B04~
V070O24*
V010H22
M011G11
V067G12
V046M14
V064G08*
B008D09
5
10
15
20
25
30
35
40
45
50
55
60
65
70
75
80
85
90

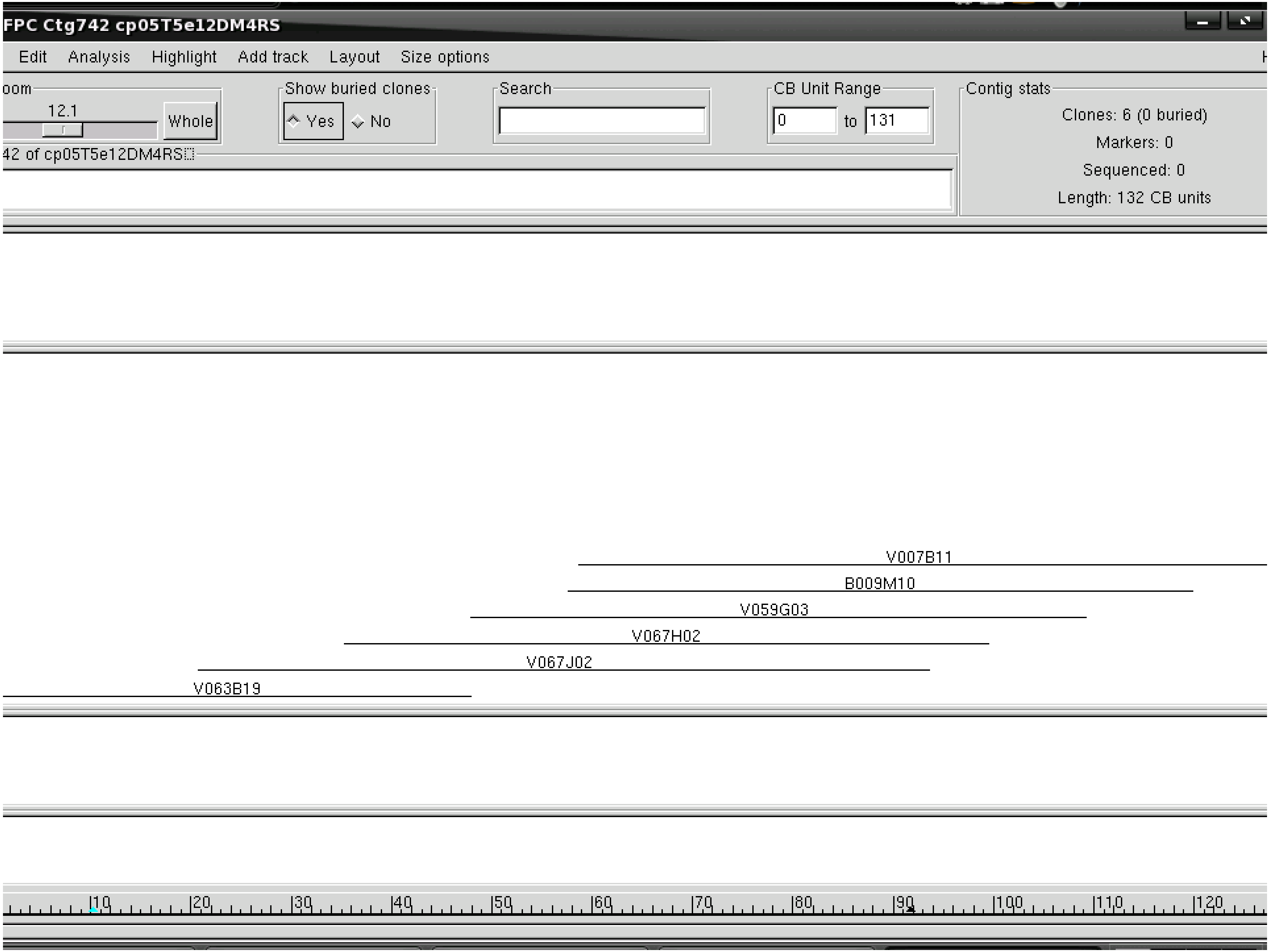
FPC Ctg742 cp05T5e12DM4RS
Edit
Analysis
Highlight
Add track
Layout
Size options
oom
12.1
Whole
Show buried clones
Yes
No
Search
CB Unit Range
0
to
131
Contig stats
Clones: 6 (0 buried)
Markers: 0
Sequenced: 0
Length: 132 CB units
42 of cp05T5e12DM4RS
V007B11
B009M10
V059G03
V067H02
V067J02
V063B19
10
20
30
40
50
60
70
80
90
100
110
120

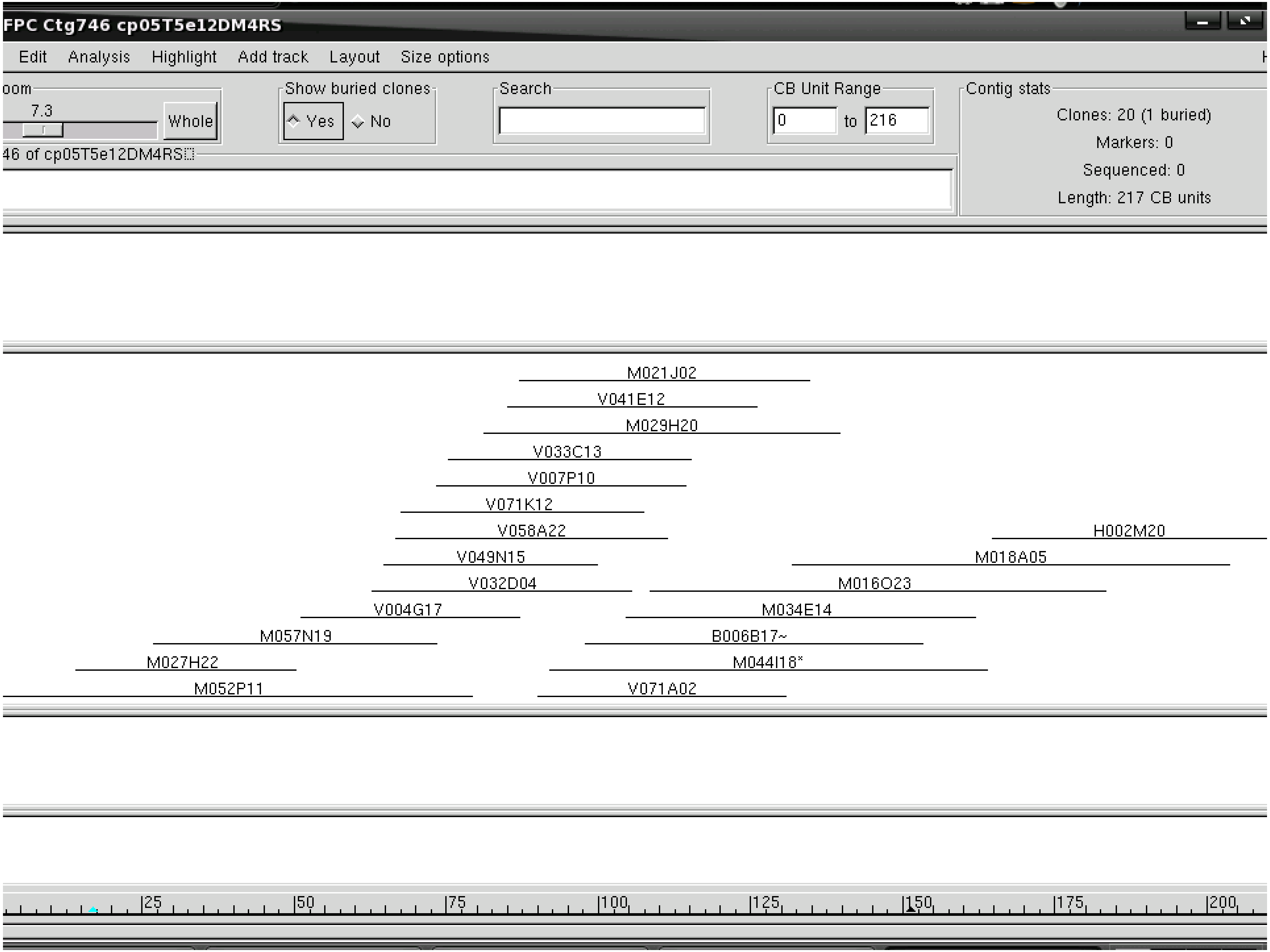
FPC Ctg746 cp05T5e12DM4RS
Edit Analysis Highlight Add track Layout Size options
oom
7.3
Whole
Show buried clones
Yes
No
Search
CB Unit Range
0 to 216
Contig stats
Clones: 20 (1 buried)
Markers: 0
Sequenced: 0
Length: 217 CB units
46 of cp05T5e12DM4RS
M021J02
V041E12
M029H20
V033C13
V007P10
V071K12
V058A22
H002M20
V049N15
M018A05
V032D04
M016O23
V004G17
M034E14
M057N19
B006B17~
M027H22
M044I18*
M052P11
V071A02
25 50 75 100 125 150 175 200

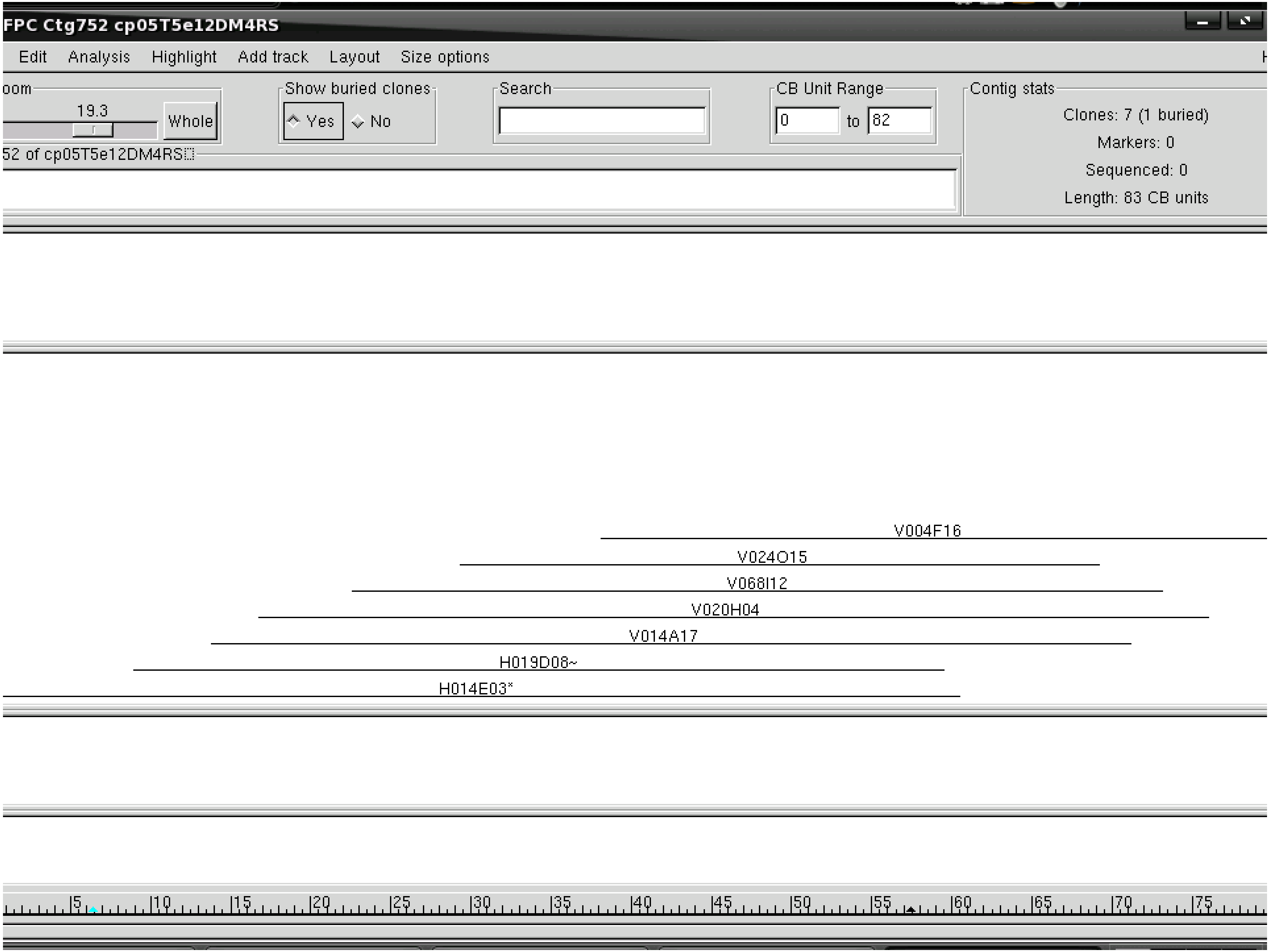
FPC Ctg752 cp05T5e12DM4RS
Edit
Analysis
Highlight
Add track
Layout
Size options
Show buried clones
Yes
No
Search
CB Unit Range
0
to
82
Contig stats
Clones: 7 (1 buried)
Markers: 0
Sequenced: 0
Length: 83 CB units
19.3
Whole
V004F16
V024O15
V068I12
V020H04
V014A17
H019D08~
H014E03*
5
10
15
20
25
30
35
40
45
50
55
60
65
70
75

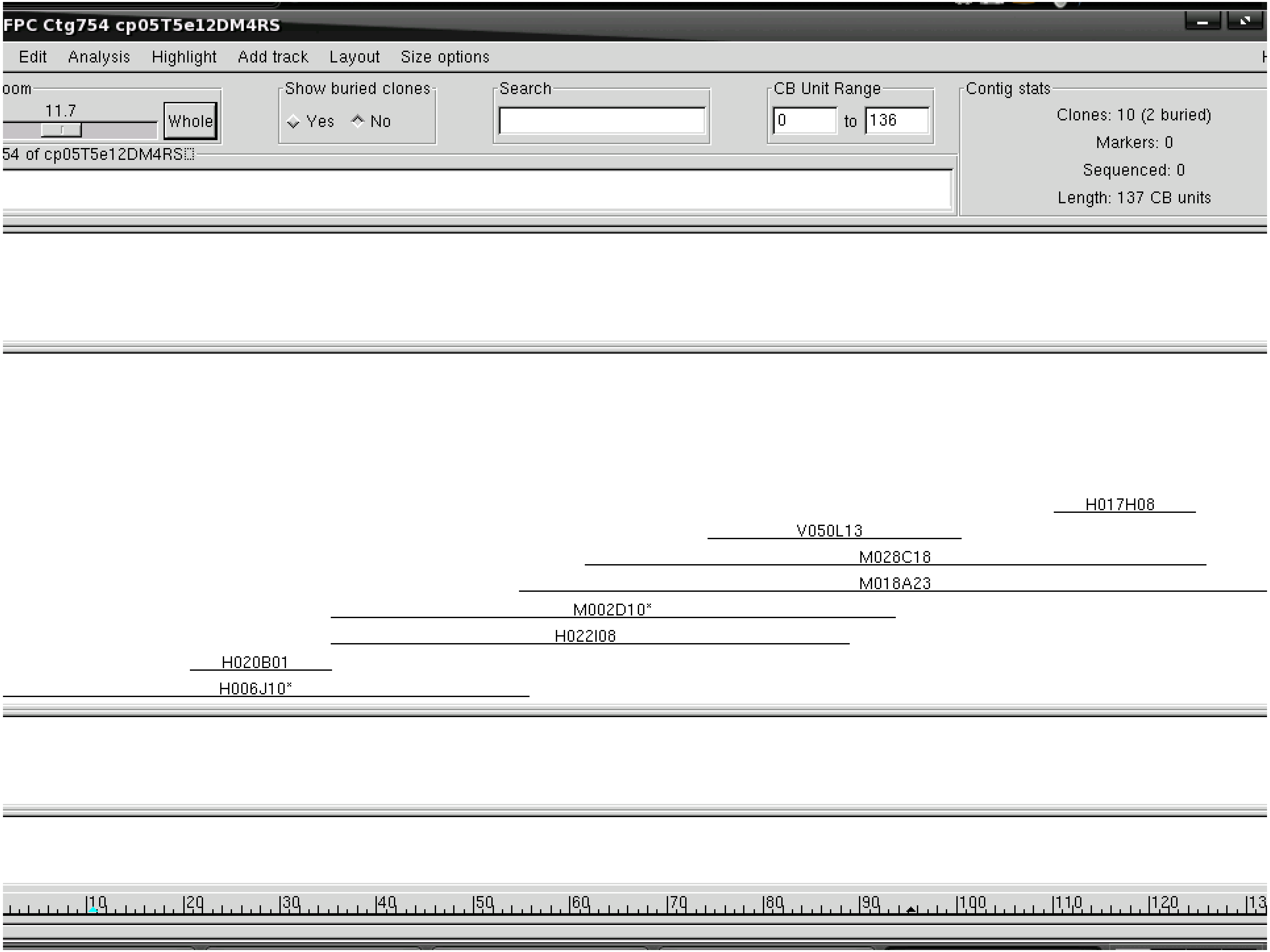
FPC Ctg754 cp05T5e12DM4RS
Edit
Analysis
Highlight
Add track
Layout
Size options
oom
11.7
Whole
Show buried clones
Yes
No
Search
CB Unit Range
0
to
136
Contig stats
Clones: 10 (2 buried)
Markers: 0
Sequenced: 0
Length: 137 CB units
54 of cp05T5e12DM4RS
H017H08
V050L13
M028C18
M018A23
M002D10*
H022I08
H020B01
H006J10*
10
20
30
40
50
60
70
80
90
100
110
120

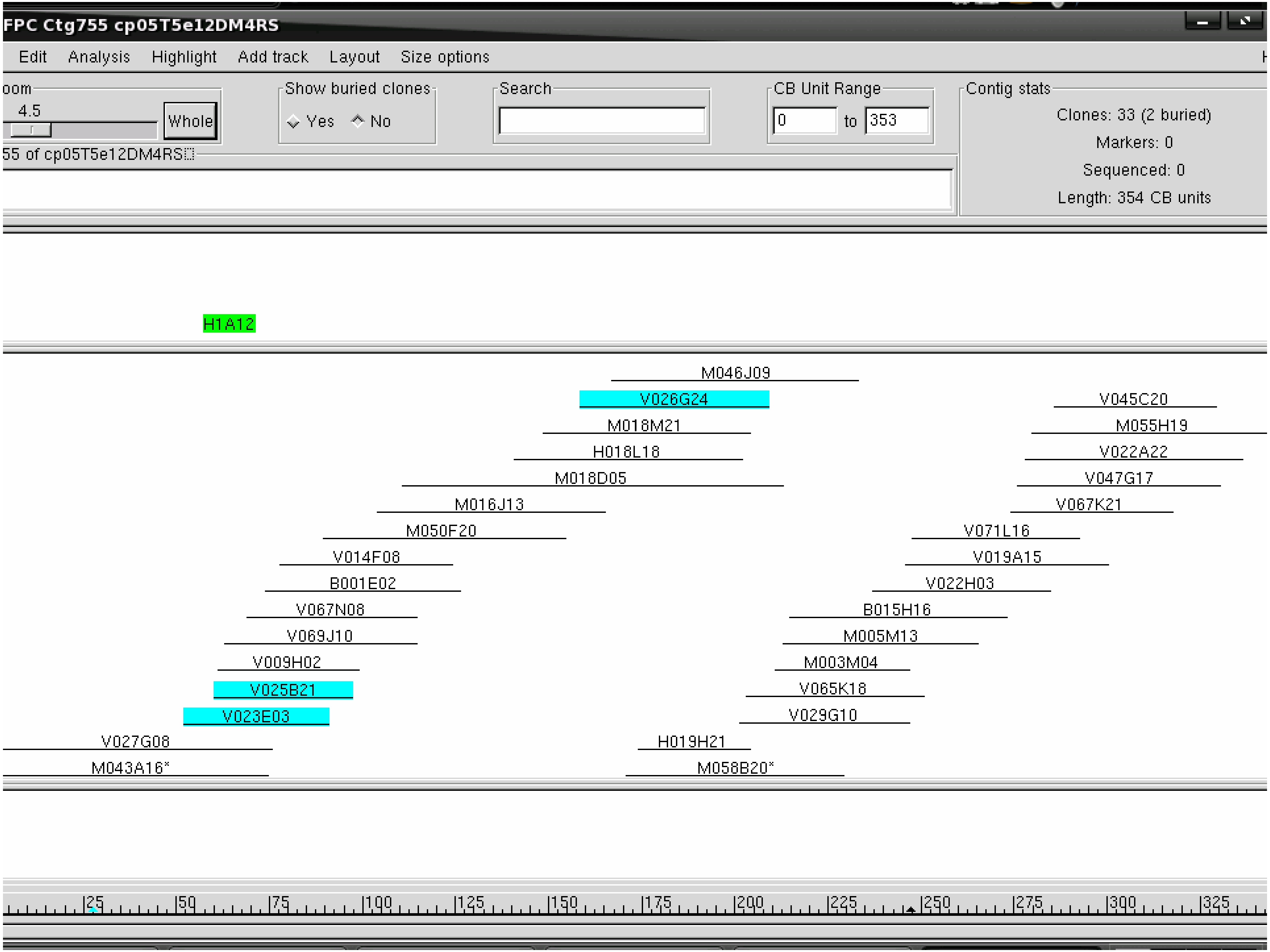
FPC Ctg755 cp05T5e12DM4RS
Edit
Analysis
Highlight
Add track
Layout
Size options
oom
4.5
Whole
Show buried clones
Yes
No
Search
CB Unit Range
0
to
353
Contig stats
Clones: 33 (2 buried)
Markers: 0
Sequenced: 0
Length: 354 CB units
55 of cp05T5e12DM4RS
H1A12
M046J09
V026G24
V045C20
M018M21
M055H19
H018L18
V022A22
M018D05
V047G17
M016J13
V067K21
M050F20
V071L16
V014F08
V019A15
B001E02
V022H03
V067N08
B015H16
V069J10
M005M13
V009H02
M003M04
V025B21
V065K18
V023E03
V029G10
V027G08
H019H21
M043A16*
M058B20*
25
50
75
100
125
150
175
200
225
250
275
300
325

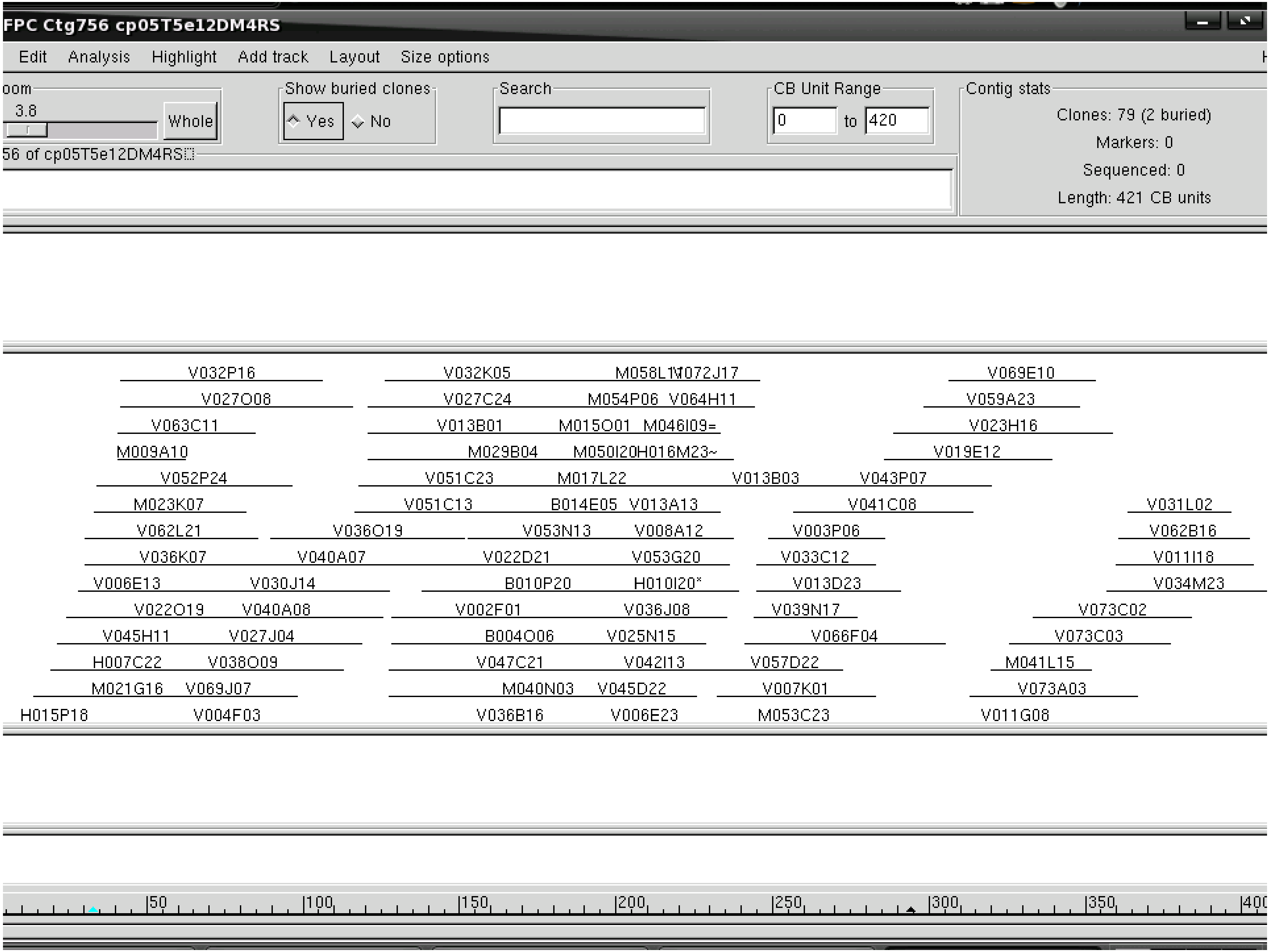
FPC Ctg756 cp05T5e12DM4RS
Edit
Analysis
Highlight
Add track
Layout
Size options
Show buried clones
Yes
No
Search
CB Unit Range
0
to
420
Contig stats
Clones: 79 (2 buried)
Markers: 0
Sequenced: 0
Length: 421 CB units
3.8
Whole
V032P16
V032K05
V069E10
V027O08
V027C24
M054P06
V064H11
V059A23
V063C11
V013B01
M015O01
V023H16
M009A10
M029B04
V019E12
V052P24
V051C23
M017L22
V013B03
V043P07
M023K07
V051C13
B014E05
V013A13
V041C08
V031L02
V062L21
V036O19
V053N13
V008A12
V003P06
V062B16
V036K07
V040A07
V022D21
V053G20
V033C12
V006E13
V030J14
B010P20
V013D23
V034M23
V022O19
V040A08
V002F01
V036J08
V039N17
V073C02
V045H11
V027J04
B004O06
V025N15
V066F04
V073C03
H007C22
V038O09
V047C21
V042I13
V057D22
M041L15
M021G16
V069J07
M040N03
V045D22
V007K01
V073A03
H015P18
V004F03
V036B16
V006E23
M053C23
V011G08
50
100
150
200
250
300
350

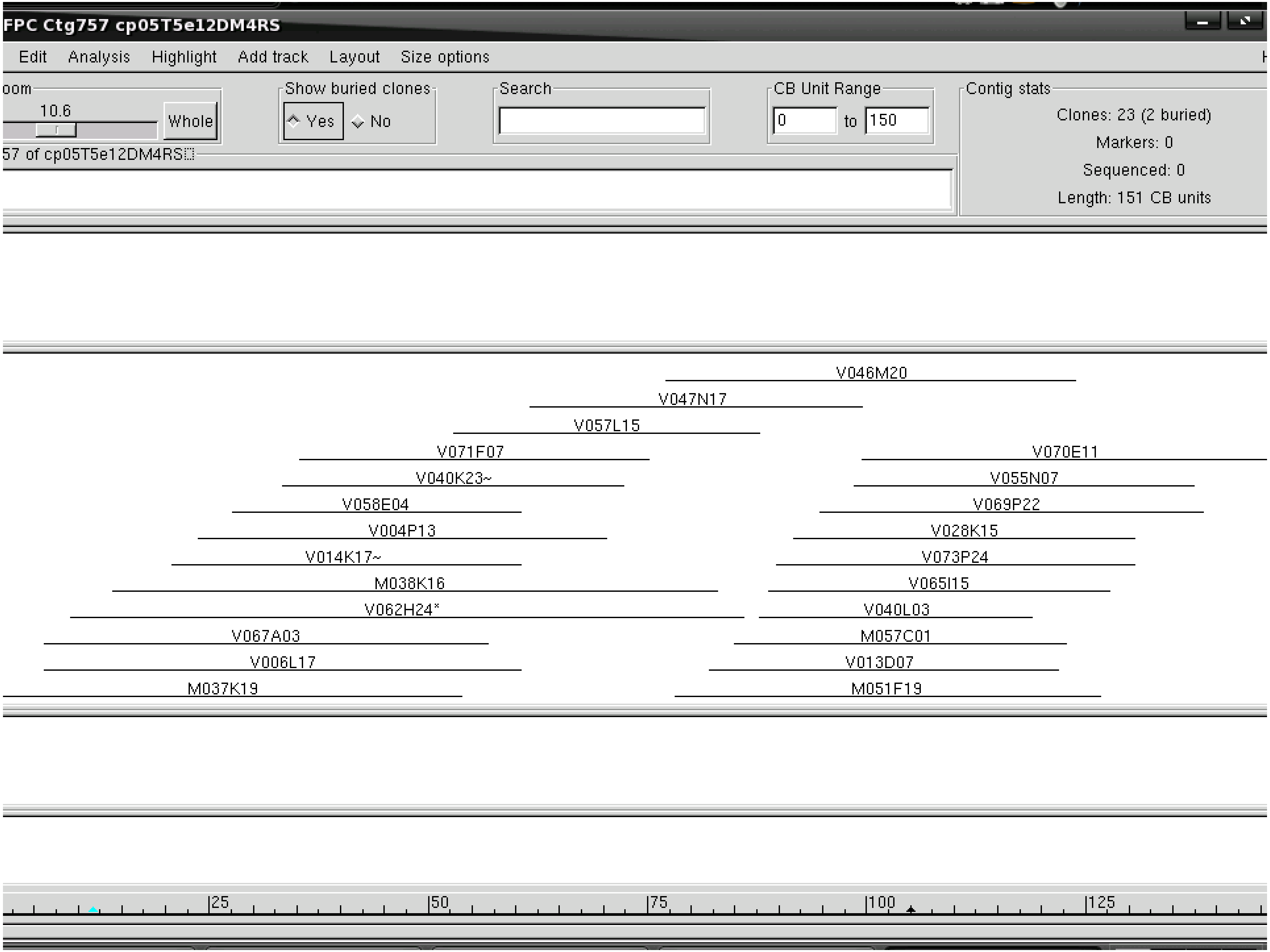
FPC Ctg757 cp05T5e12DM4RS
Edit
Analysis
Highlight
Add track
Layout
Size options
oom
10.6
Whole
Show buried clones
Yes
No
Search
CB Unit Range
0
to
150
Contig stats
Clones: 23 (2 buried)
Markers: 0
Sequenced: 0
Length: 151 CB units
57 of cp05T5e12DM4RS
V046M20
V047N17
V057L15
V071F07
V070E11
V040K23~
V055N07
V058E04
V069P22
V004P13
V028K15
V014K17~
V073P24
M038K16
V065I15
V062H24*
V040L03
V067A03
M057C01
V006L17
V013D07
M037K19
M051F19
25
50
75
100
125

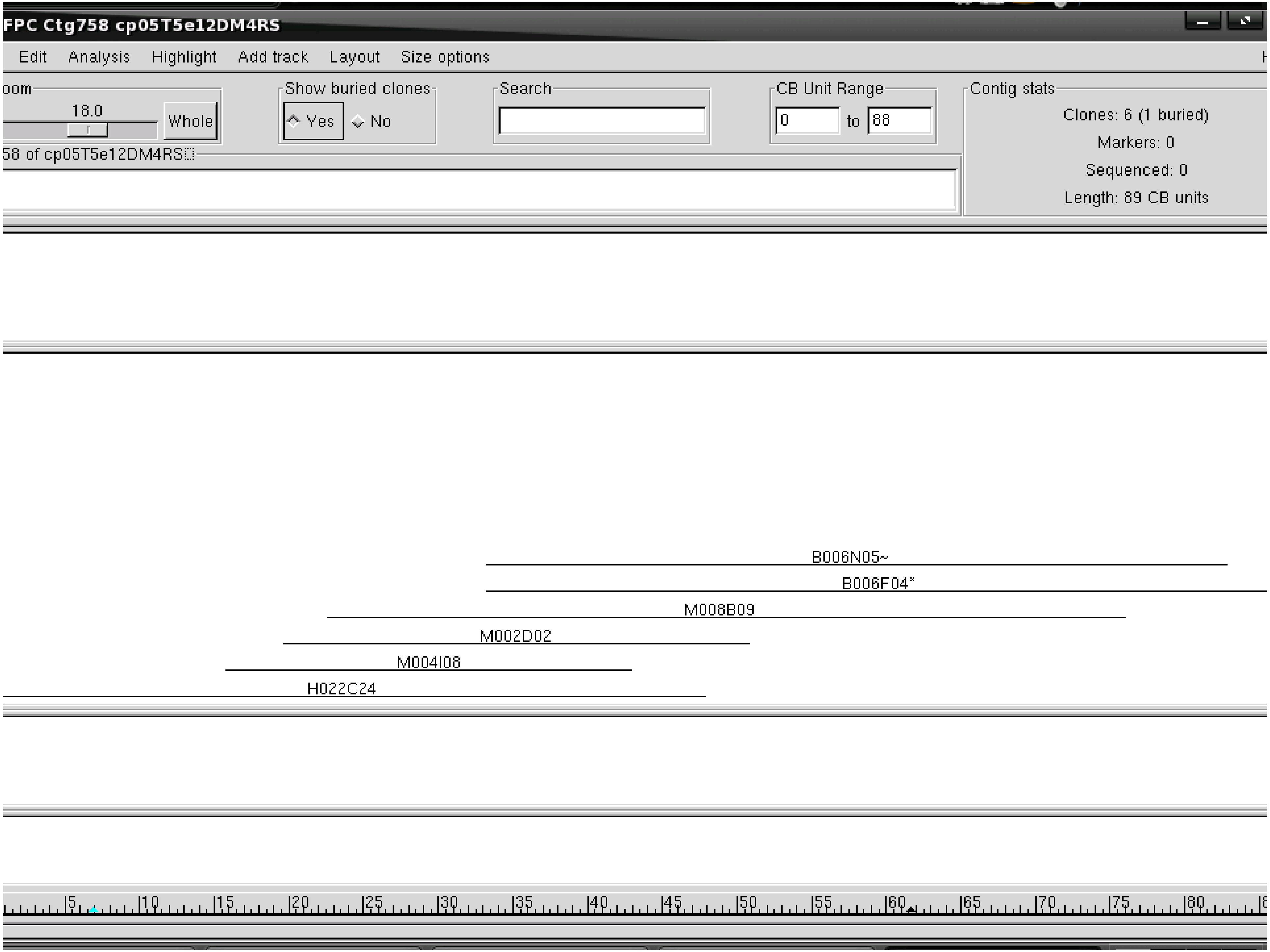
FPC Ctg758 cp05T5e12DM4RS
Edit
Analysis
Highlight
Add track
Layout
Size options
oom
18.0
Whole
Show buried clones
Yes
No
Search
CB Unit Range
0
to
88
Contig stats
Clones: 6 (1 buried)
Markers: 0
Sequenced: 0
Length: 89 CB units
58 of cp05T5e12DM4RS
B006N05~
B006F04*
M008B09
M002D02
M004I08
H022C24
5 10 15 20 25 30 35 40 45 50 55 60 65 70 75 80

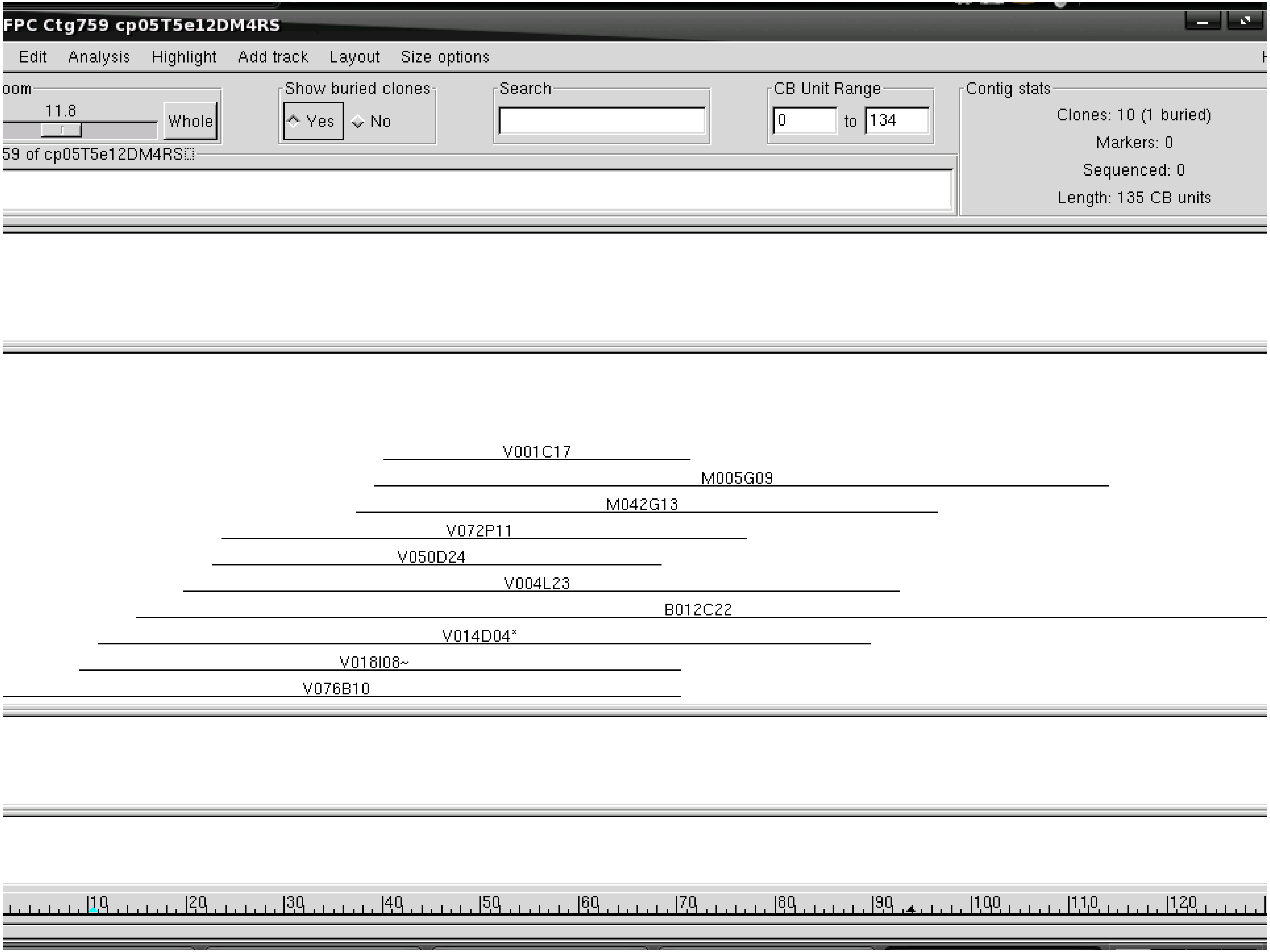
FPC Ctg759 cp05T5e12DM4RS
Edit
Analysis
Highlight
Add track
Layout
Size options
oom
11.8
Whole
Show buried clones
Yes
No
Search
CB Unit Range
0
to
134
Contig stats
Clones: 10 (1 buried)
Markers: 0
Sequenced: 0
Length: 135 CB units
59 of cp05T5e12DM4RS
V001C17
M005G09
M042G13
V072P11
V050D24
V004L23
B012C22
V014D04*
V018I08~
V076B10
10
20
30
40
50
60
70
80
90
100
110
120

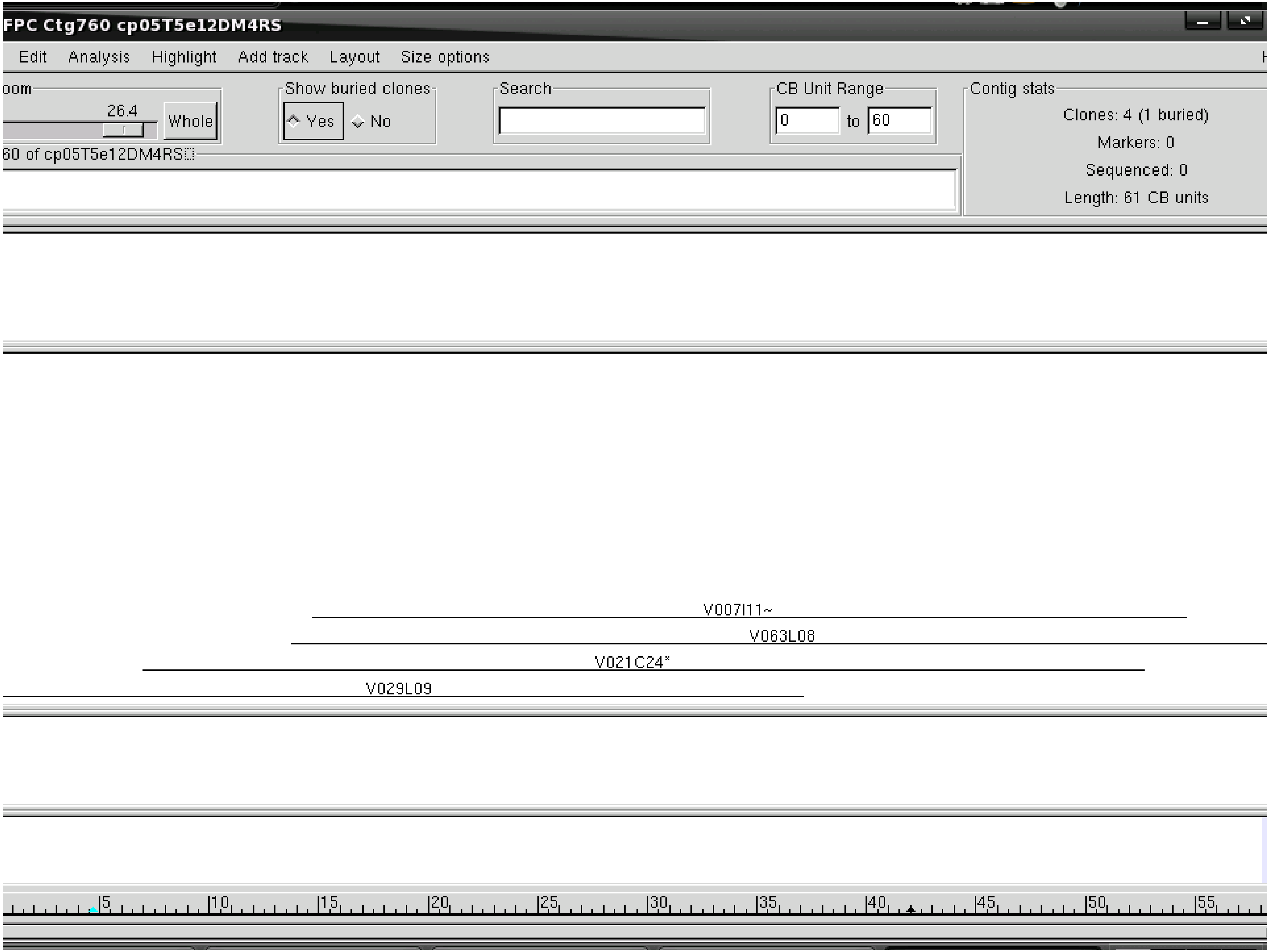
FPC Ctg760 cp05T5e12DM4RS
Edit Analysis Highlight Add track Layout Size options
oom
26.4
Whole
Show buried clones
Yes
No
Search
CB Unit Range
0
to
60
Contig stats
Clones: 4 (1 buried)
Markers: 0
Sequenced: 0
Length: 61 CB units
60 of cp05T5e12DM4RS
V007I11~
V063L08
V021C24*
V029L09
5
10
15
20
25
30
35
40
45
50
55

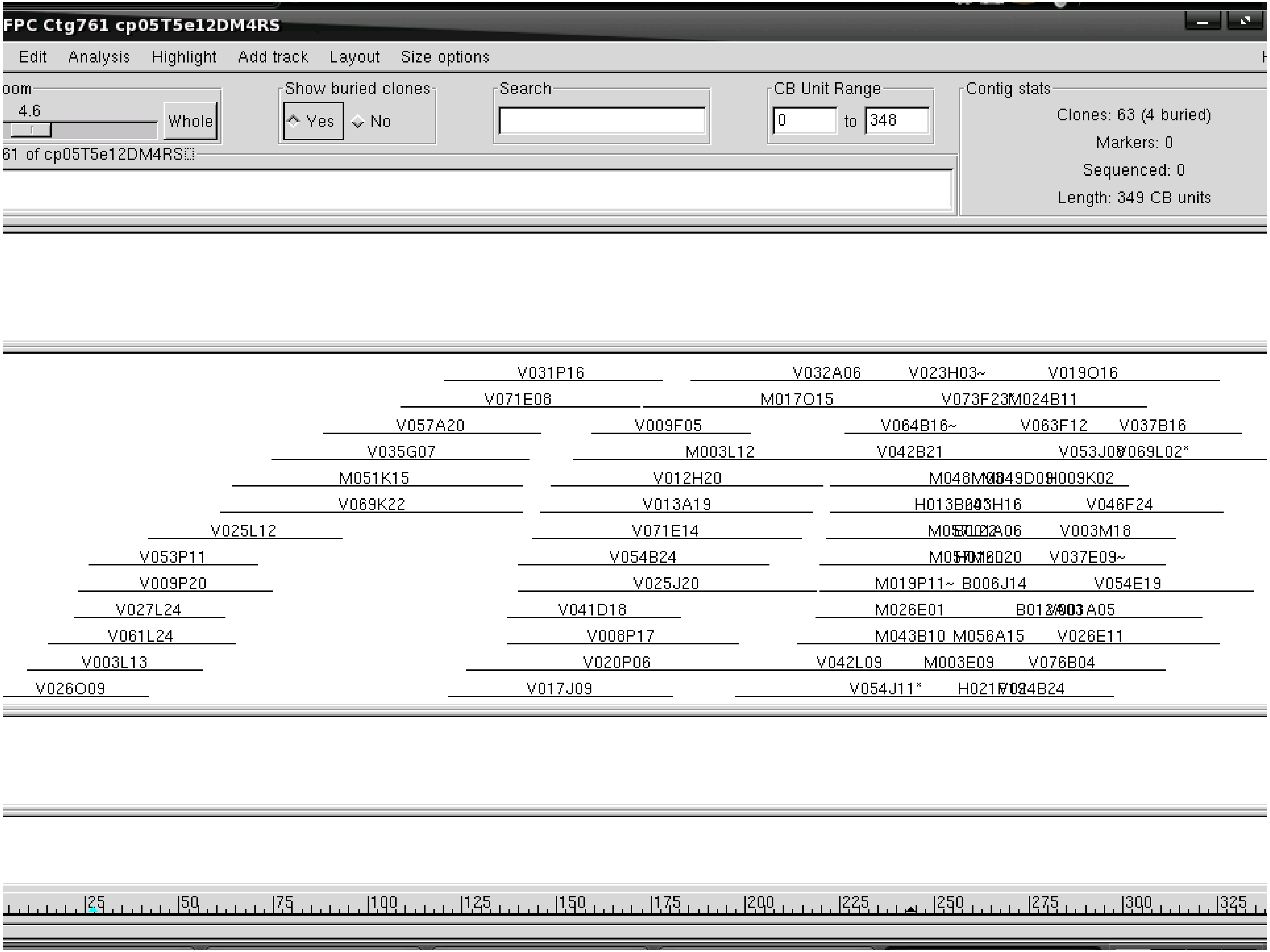
FPC Ctg761 cp05T5e12DM4RS
Edit Analysis Highlight Add track Layout Size options
Show buried clones
Yes No
Whole
4.6
Search
CB Unit Range
0 to 348
Contig stats
Clones: 63 (4 buried)
Markers: 0
Sequenced: 0
Length: 349 CB units
61 of cp05T5e12DM4RS
V031P16
V032A06
V023H03~
V019O16
V071E08
M017O15
V057A20
V009F05
V064B16~
V063F12
V037B16
V035G07
M003L12
V042B21
M051K15
V012H20
V069K22
V013A19
V046F24
V025L12
V071E14
V003M18
V053P11
V054B24
V037E09~
V009P20
V025J20
M019P11~
B006J14
V054E19
V027L24
V041D18
M026E01
V061L24
V008P17
M043B10
M056A15
V026E11
V003L13
V020P06
V042L09
M003E09
V076B04
V026O09
V017J09
V054J11*
25 50 75 100 125 150 175 200 225 250 275 300 325

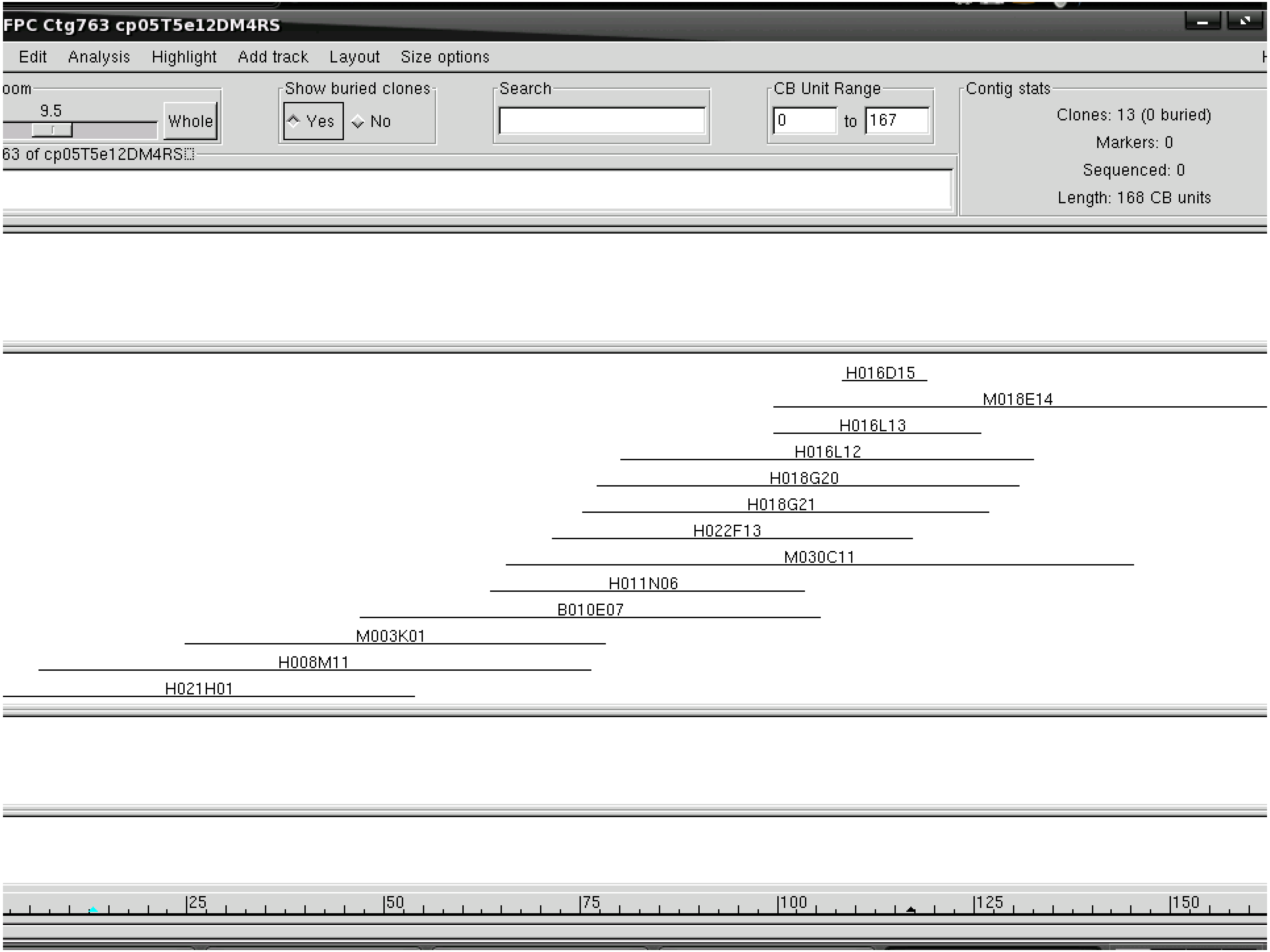
FPC Ctg763 cp05T5e12DM4RS
Edit Analysis Highlight Add track Layout Size options
oom
9.5
Whole
Show buried clones
Yes
No
Search
CB Unit Range
0
to
167
Contig stats
Clones: 13 (0 buried)
Markers: 0
Sequenced: 0
Length: 168 CB units
63 of cp05T5e12DM4RS
H016D15
M018E14
H016L13
H016L12
H018G20
H018G21
H022F13
M030C11
H011N06
B010E07
M003K01
H008M11
H021H01
25
50
75
100
125
150

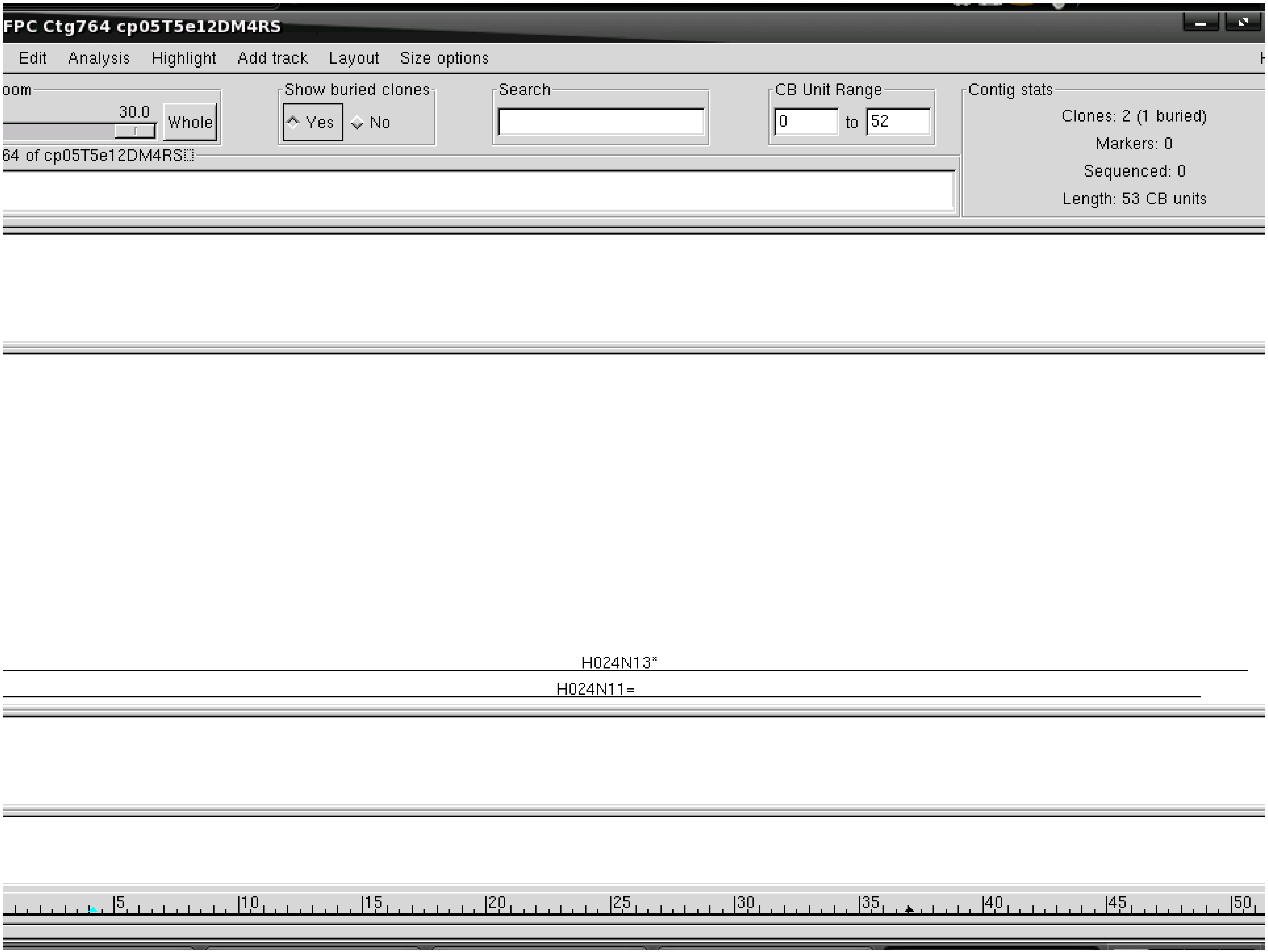
FPC Ctg764 cp05T5e12DM4RS
Edit Analysis Highlight Add track Layout Size options
oom
30.0
Whole
Show buried clones
Yes
No
Search
CB Unit Range
0
to
52
Contig stats
Clones: 2 (1 buried)
Markers: 0
Sequenced: 0
Length: 53 CB units
64 of cp05T5e12DM4RS
H024N13*
H024N11=
5 10 15 20 25 30 35 40 45 50

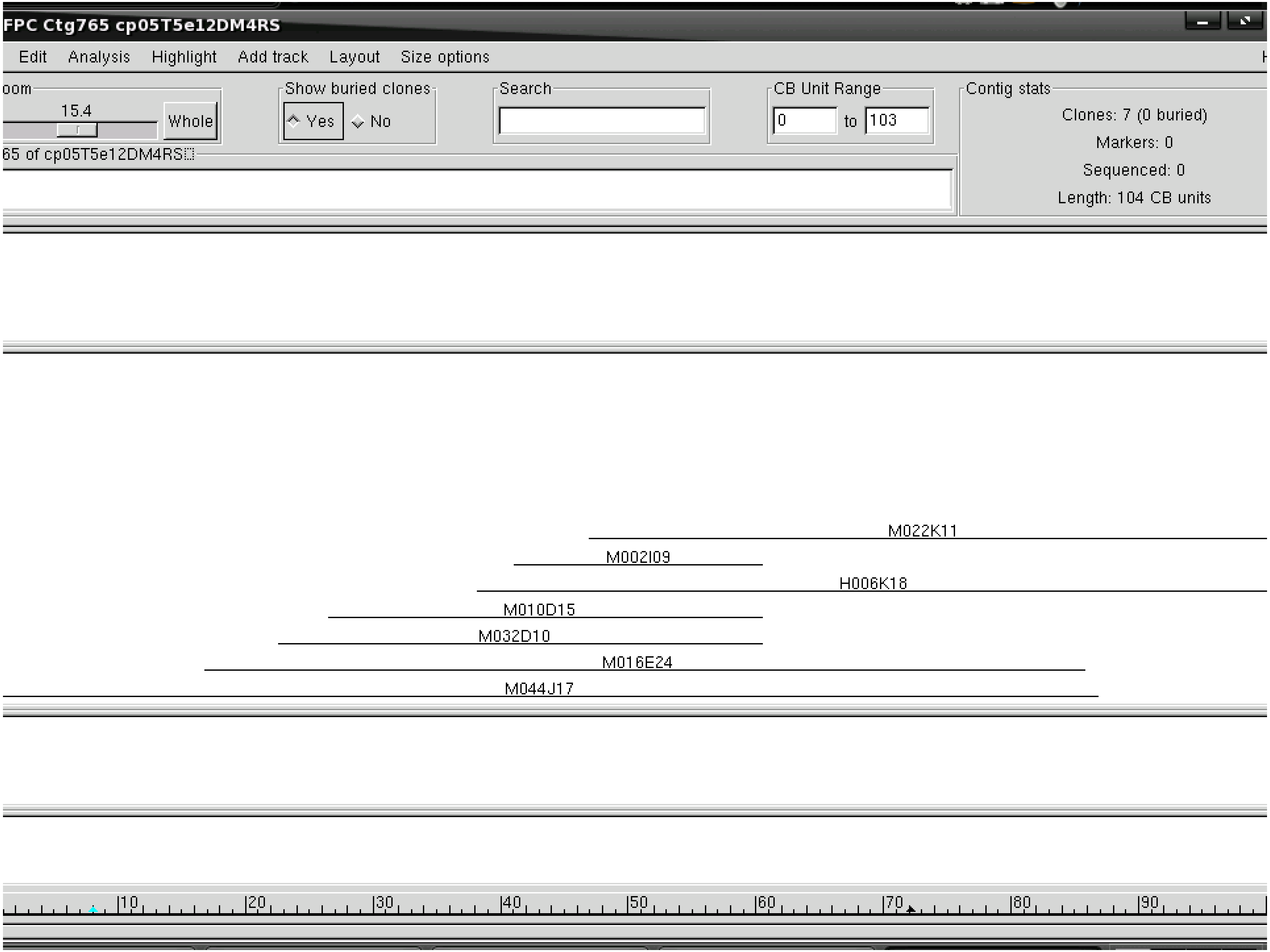
FPC Ctg765 cp05T5e12DM4RS
Edit
Analysis
Highlight
Add track
Layout
Size options
oom
15.4
Whole
Show buried clones
Yes
No
Search
CB Unit Range
0
to
103
Contig stats
Clones: 7 (0 buried)
Markers: 0
Sequenced: 0
Length: 104 CB units
65 of cp05T5e12DM4RS
M022K11
M002I09
H006K18
M010D15
M032D10
M016E24
M044J17
10
20
30
40
50
60
70
80
90

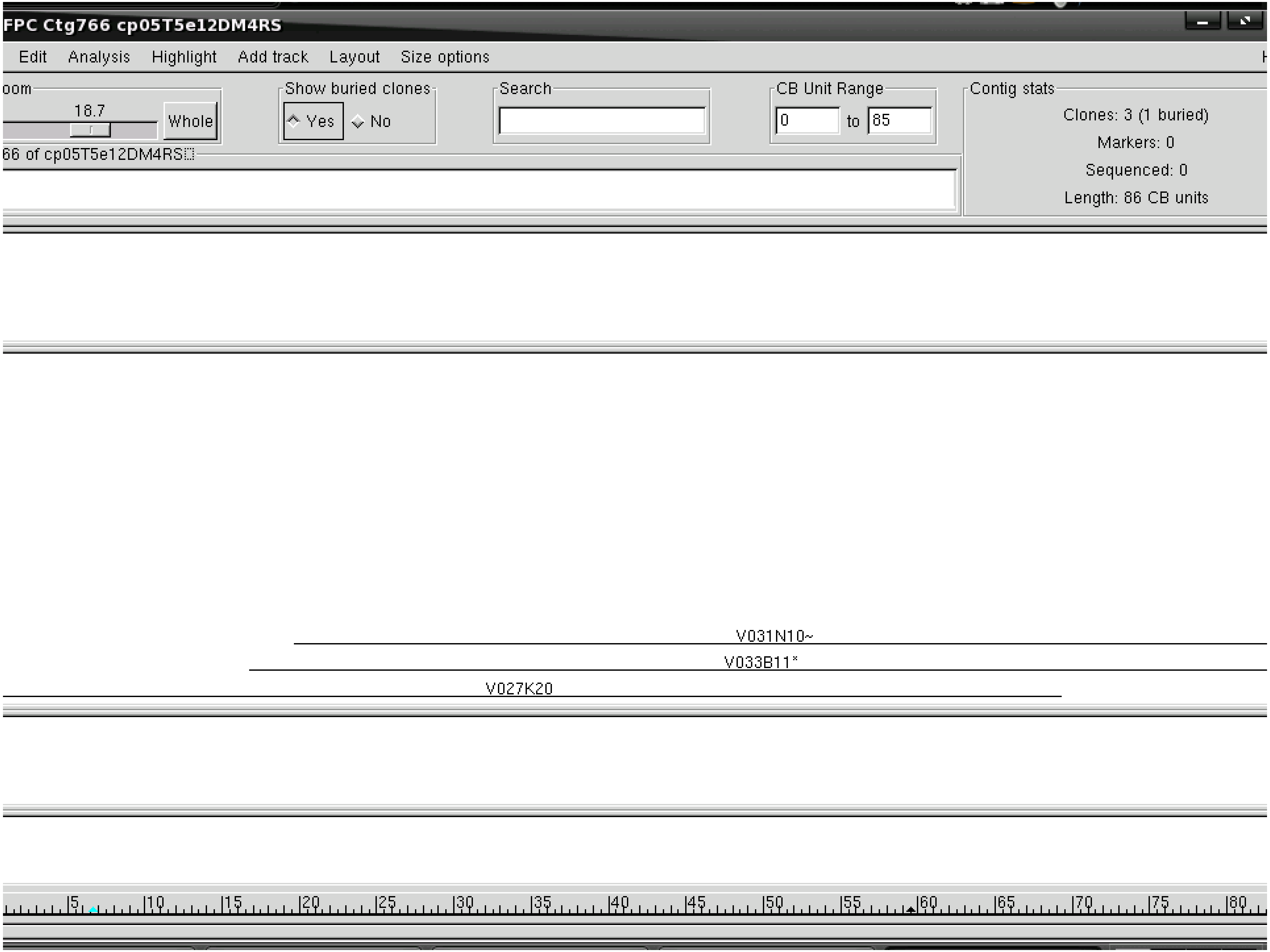
FPC Ctg766 cp05T5e12DM4RS
Edit
Analysis
Highlight
Add track
Layout
Size options
oom
18.7
Whole
Show buried clones
Yes
No
Search
CB Unit Range
0
to
85
Contig stats
Clones: 3 (1 buried)
Markers: 0
Sequenced: 0
Length: 86 CB units
66 of cp05T5e12DM4RS
V031N10~
V033B11*
V027K20
5 10 15 20 25 30 35 40 45 50 55 60 65 70 75 80

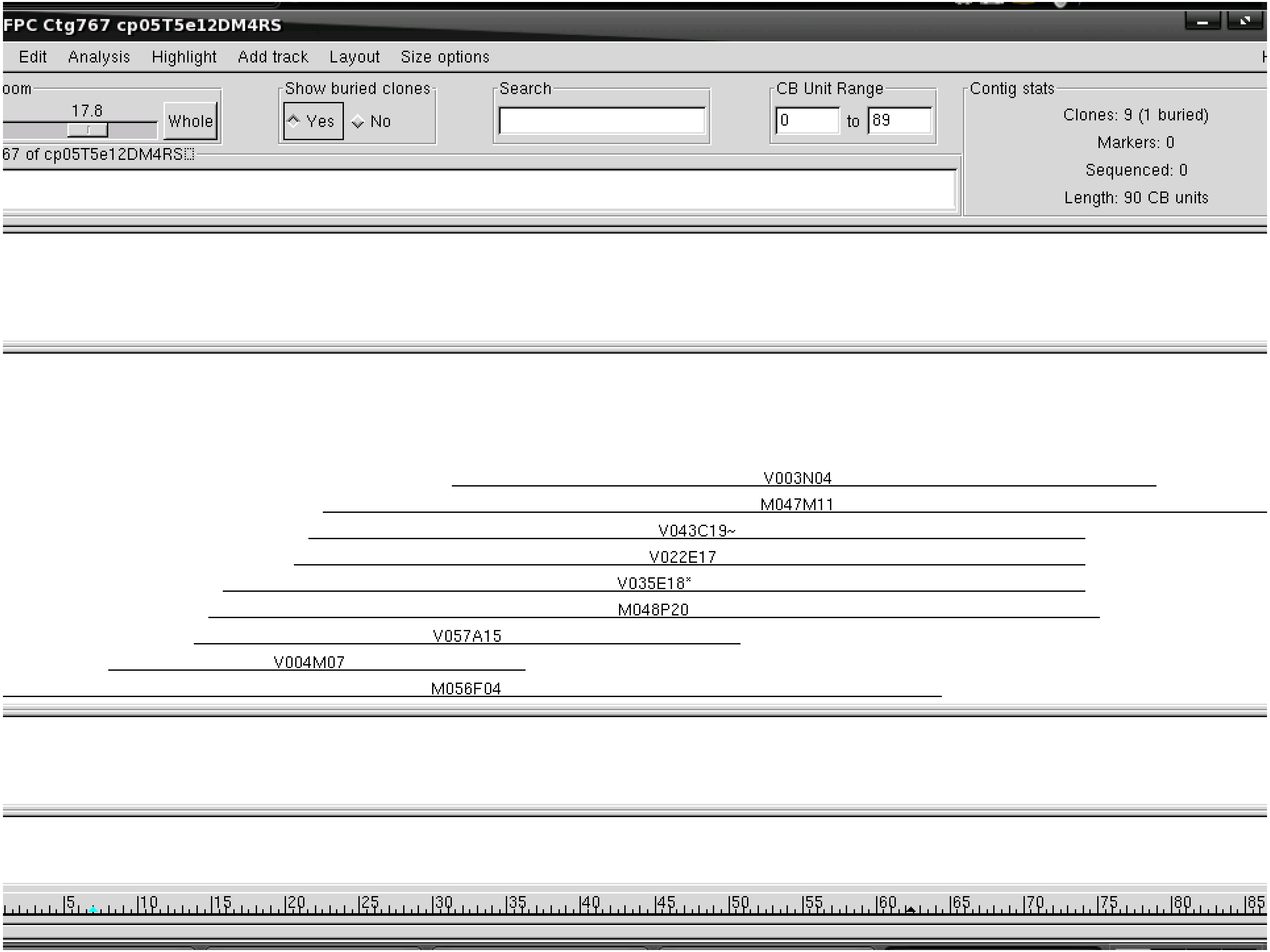
FPC Ctg767 cp05T5e12DM4RS
Edit
Analysis
Highlight
Add track
Layout
Size options
oom
17.8
Whole
Show buried clones
Yes
No
Search
CB Unit Range
0
to
89
Contig stats
Clones: 9 (1 buried)
Markers: 0
Sequenced: 0
Length: 90 CB units
67 of cp05T5e12DM4RS
V003N04
M047M11
V043C19~
V022E17
V035E18*
M048P20
V057A15
V004M07
M056F04
5
10
15
20
25
30
35
40
45
50
55
60
65
70
75
80
85

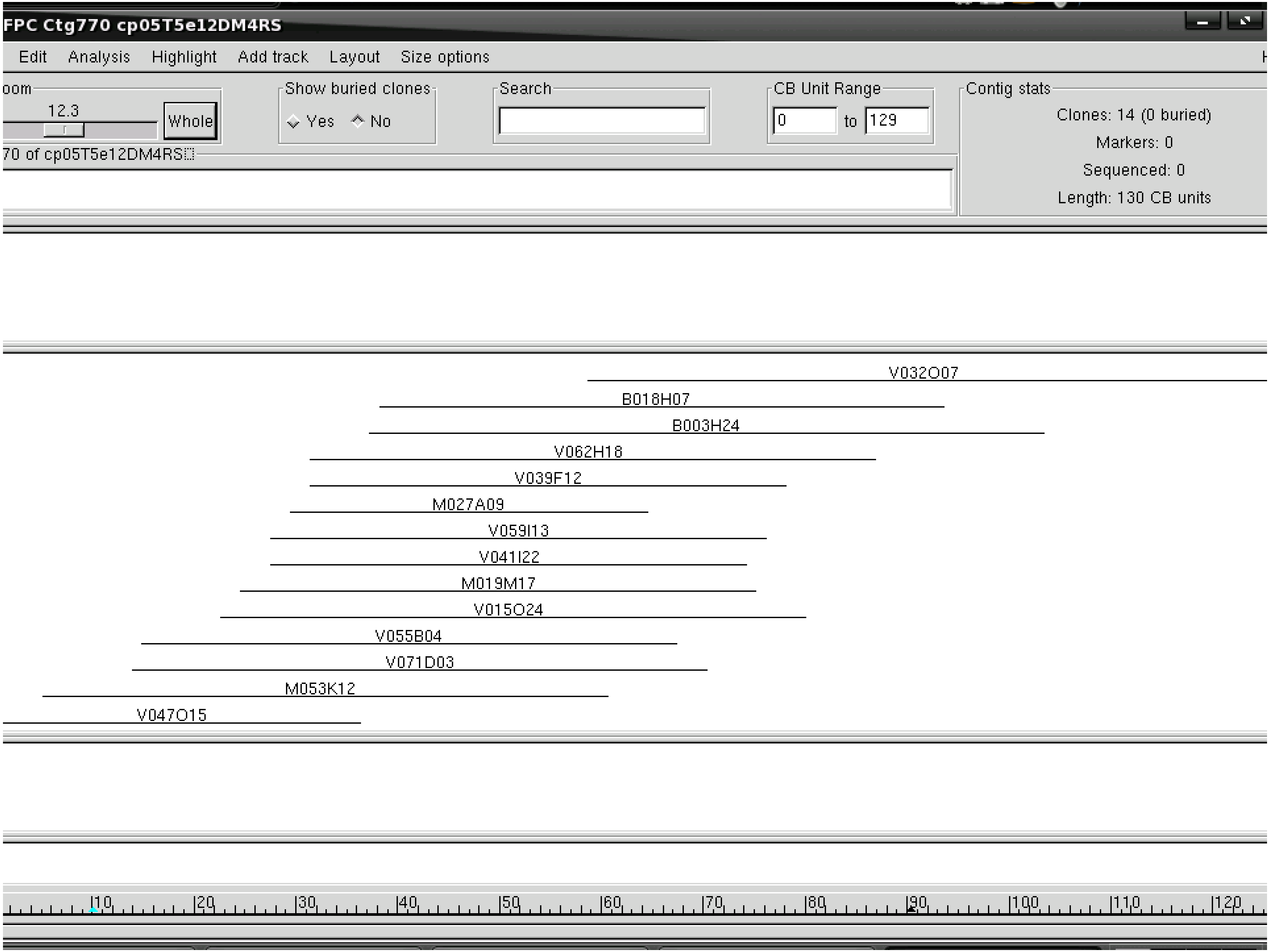
FPC Ctg770 cp05T5e12DM4RS
Edit
Analysis
Highlight
Add track
Layout
Size options
oom
12.3
Whole
Show buried clones
Yes
No
Search
CB Unit Range
0
to
129
Contig stats
Clones: 14 (0 buried)
Markers: 0
Sequenced: 0
Length: 130 CB units
70 of cp05T5e12DM4RS
V032O07
B018H07
B003H24
V062H18
V039F12
M027A09
V059I13
V041I22
M019M17
V015O24
V055B04
V071D03
M053K12
V047O15
10
20
30
40
50
60
70
80
90
100
110
120

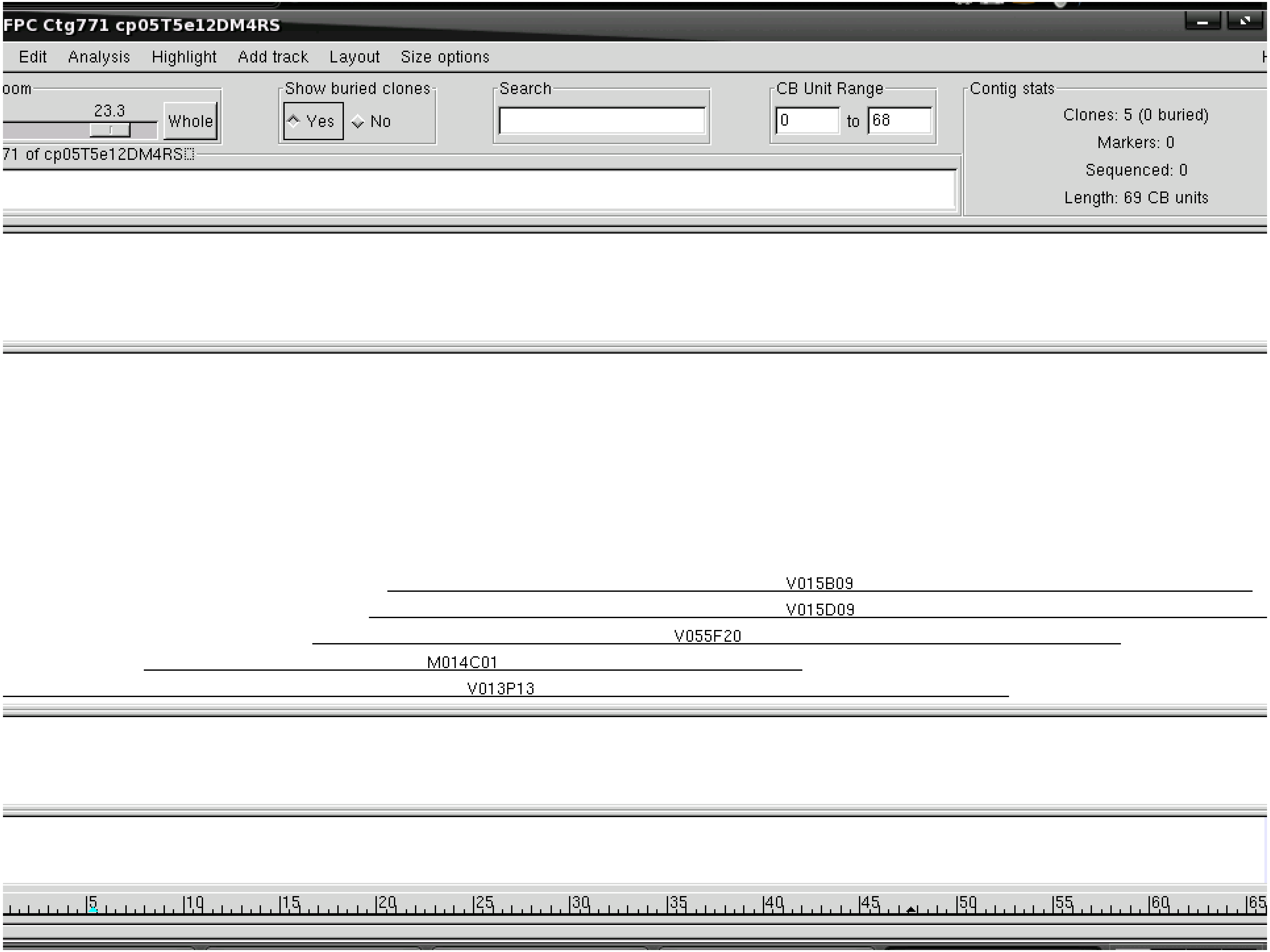
FPC Ctg771 cp05T5e12DM4RS
Edit
Analysis
Highlight
Add track
Layout
Size options
oom
23.3
Whole
Show buried clones
Yes
No
Search
CB Unit Range
0
to
68
Contig stats
Clones: 5 (0 buried)
Markers: 0
Sequenced: 0
Length: 69 CB units
71 of cp05T5e12DM4RS
V015B09
V015D09
V055F20
M014C01
V013P13
5
10
15
20
25
30
35
40
45
50
55
60
65

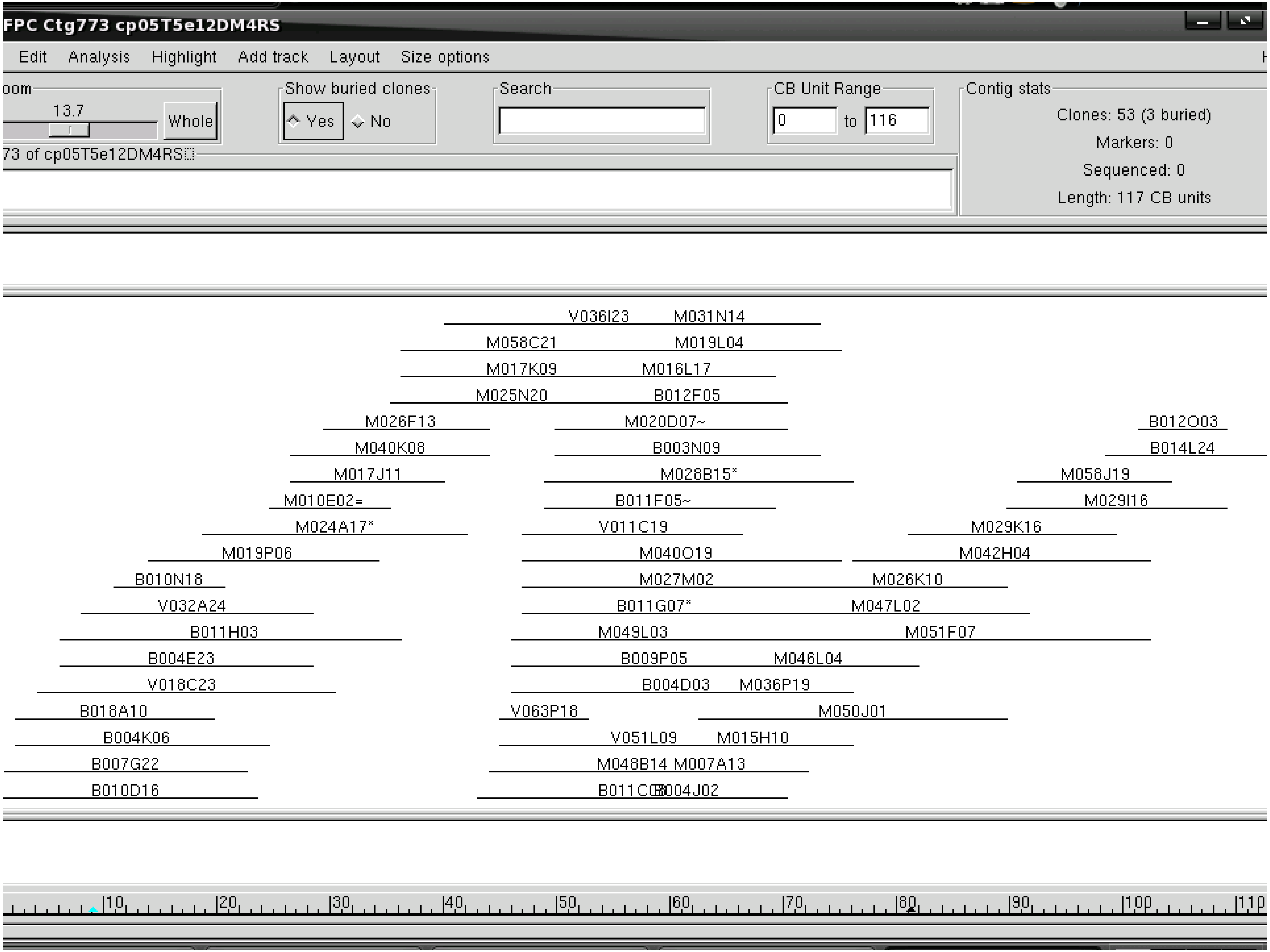
FPC Ctg773 cp05T5e12DM4RS
Edit Analysis Highlight Add track Layout Size options
oom
13.7
Whole
Show buried clones
Yes
No
Search
CB Unit Range
0
to
116
Contig stats
Clones: 53 (3 buried)
Markers: 0
Sequenced: 0
Length: 117 CB units
73 of cp05T5e12DM4RS
V036I23
M031N14
M058C21
M019L04
M017K09
M016L17
M025N20
B012F05
M026F13
M020D07~
B012O03
M040K08
B003N09
B014L24
M017J11
M028B15*
M058J19
M010E02=
B011F05~
M029I16
M024A17*
V011C19
M029K16
M019P06
M040O19
M042H04
B010N18
M027M02
M026K10
V032A24
B011G07*
M047L02
B011H03
M049L03
M051F07
B004E23
B009P05
M046L04
V018C23
B004D03
M036P19
B018A10
V063P18
M050J01
B004K06
V051L09
M015H10
B007G22
M048B14 M007A13
B010D16
|10
|20
|30
|40
|50
|60
|70
|80
|90
|100
|110

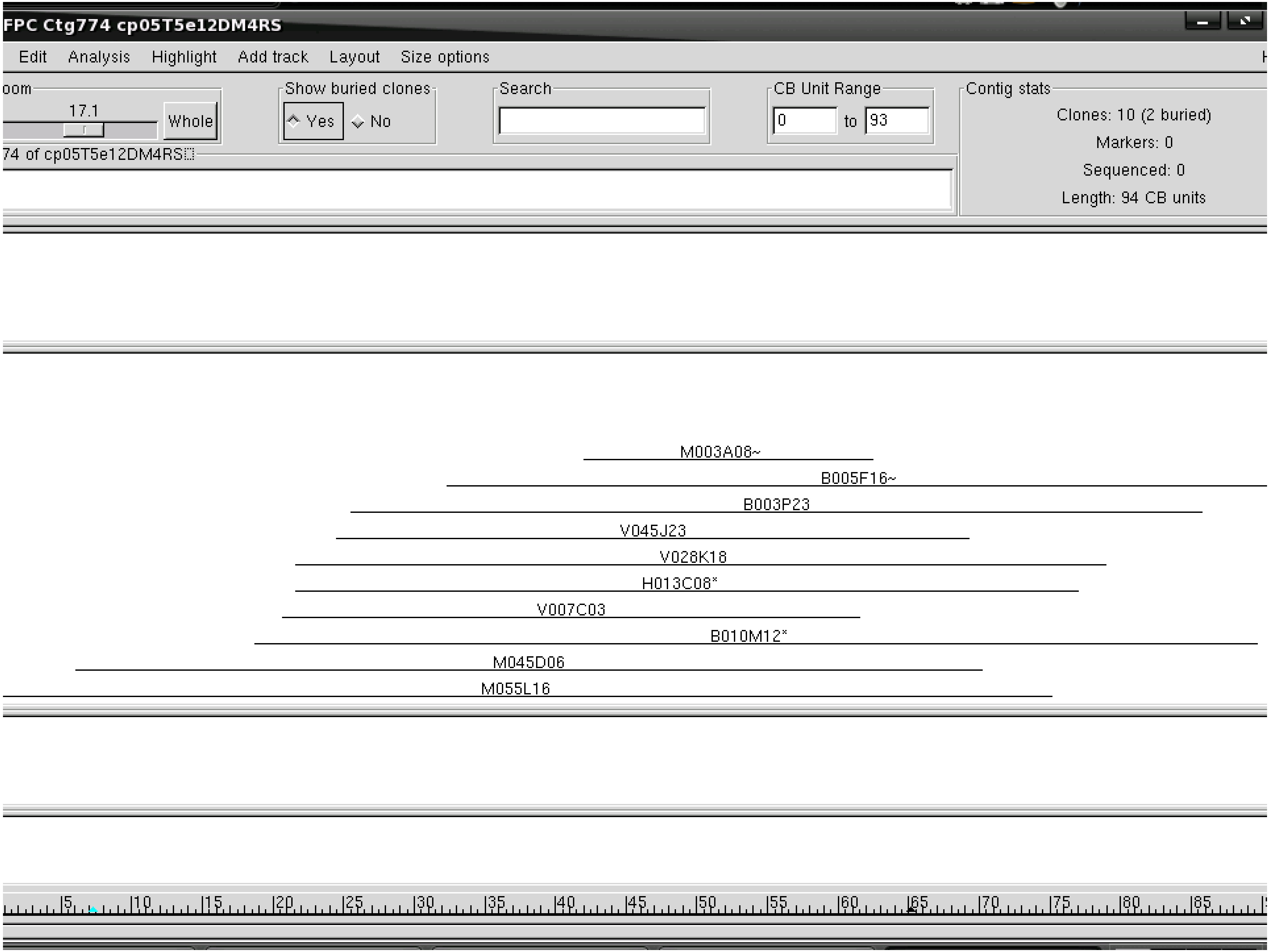
FPC Ctg774 cp05T5e12DM4RS
Edit Analysis Highlight Add track Layout Size options
oom
17.1
Whole
Show buried clones
Yes
No
Search
CB Unit Range
0 to 93
Contig stats
Clones: 10 (2 buried)
Markers: 0
Sequenced: 0
Length: 94 CB units
74 of cp05T5e12DM4RS
M003A08~
B005F16~
B003P23
V045J23
V028K18
H013C08*
V007C03
B010M12*
M045D06
M055L16
5 10 15 20 25 30 35 40 45 50 55 60 65 70 75 80 85

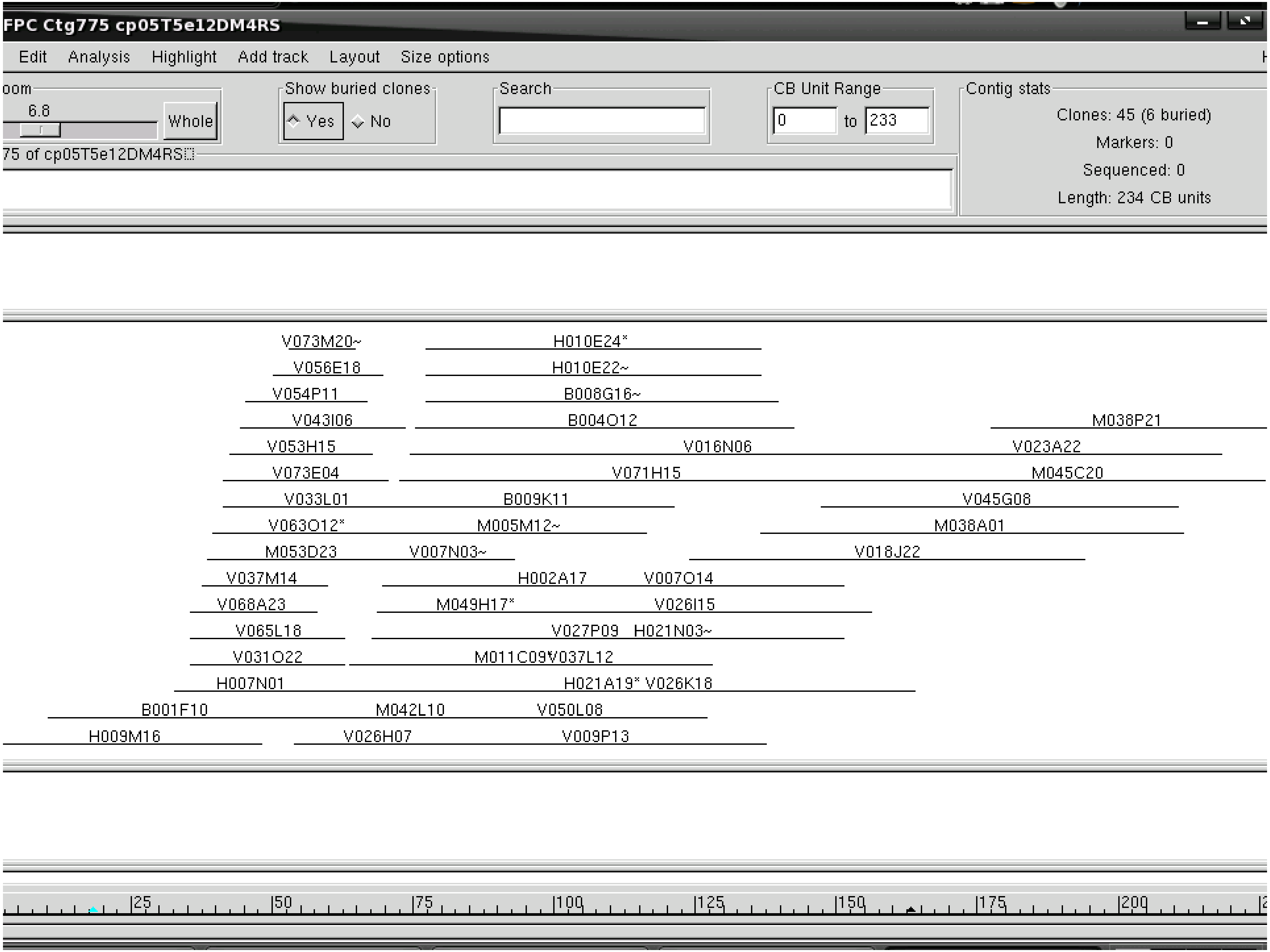
FPC Ctg775 cp05T5e12DM4RS
Edit Analysis Highlight Add track Layout Size options
oom
6.8
Whole
Show buried clones
Yes
No
Search
CB Unit Range
0
to
233
Contig stats
Clones: 45 (6 buried)
Markers: 0
Sequenced: 0
Length: 234 CB units
75 of cp05T5e12DM4RS
V073M20~
H010E24*
V056E18
H010E22~
V054P11
B008G16~
V043I06
B004O12
M038P21
V053H15
V016N06
V023A22
V073E04
V071H15
M045C20
V033L01
B009K11
V045G08
V063O12*
M005M12~
M038A01
M053D23
V007N03~
V018J22
V037M14
H002A17
V007O14
V068A23
M049H17*
V026I15
V065L18
V027P09
H021N03~
V031O22
M011C09
V037L12
H007N01
H021A19*
V026K18
B001F10
M042L10
V050L08
H009M16
V026H07
V009P13
25
50
75
100
125
150
175
200

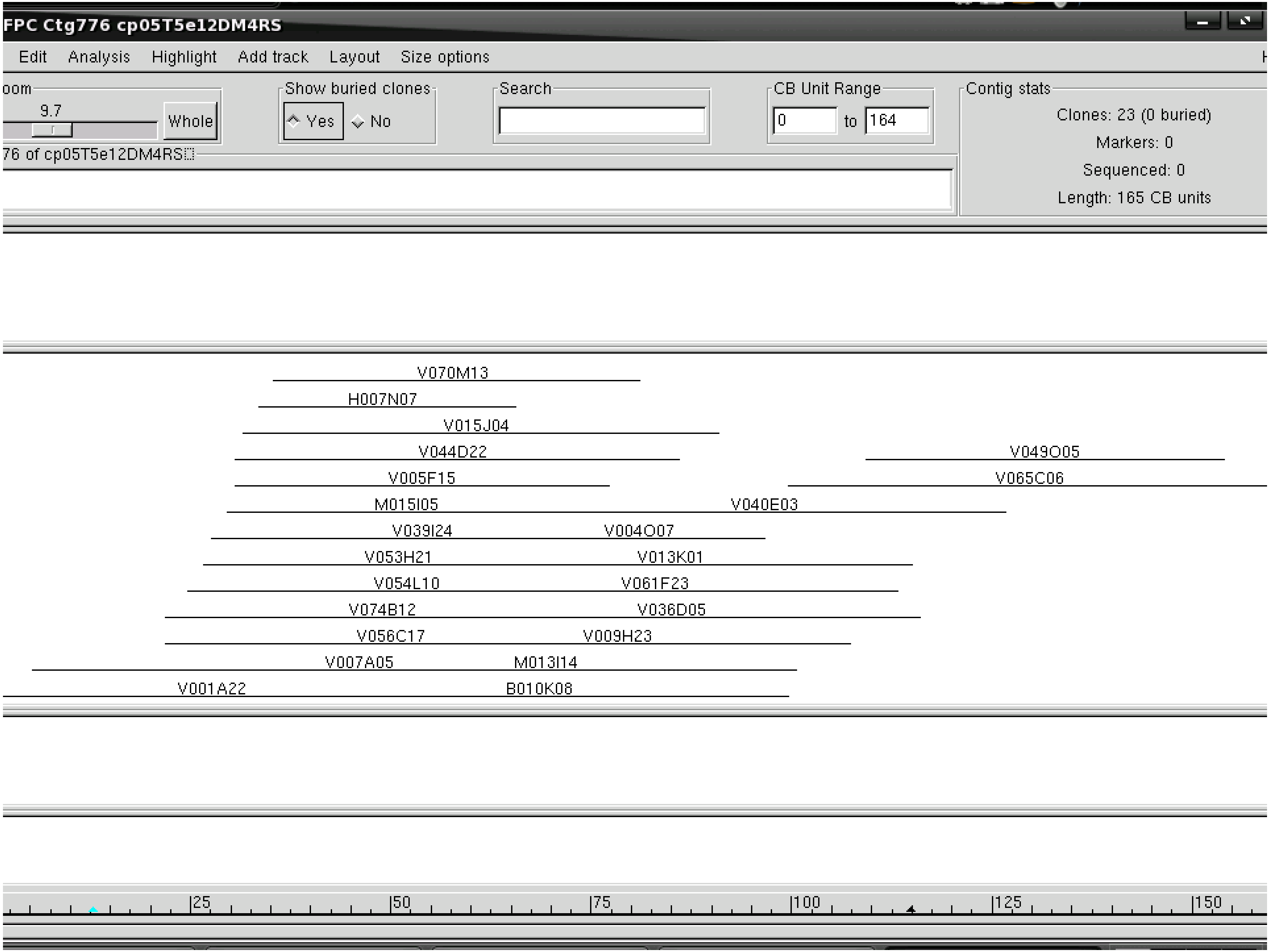
FPC Ctg776 cp05T5e12DM4RS
Edit
Analysis
Highlight
Add track
Layout
Size options
oom
9.7
Whole
Show buried clones
Yes
No
Search
CB Unit Range
0
to
164
Contig stats
Clones: 23 (0 buried)
Markers: 0
Sequenced: 0
Length: 165 CB units
76 of cp05T5e12DM4RS
V070M13
H007N07
V015J04
V044D22
V049O05
V005F15
V065C06
M015I05
V040E03
V039I24
V004O07
V053H21
V013K01
V054L10
V061F23
V074B12
V036D05
V056C17
V009H23
V007A05
M013I14
V001A22
B010K08
25
50
75
100
125
150

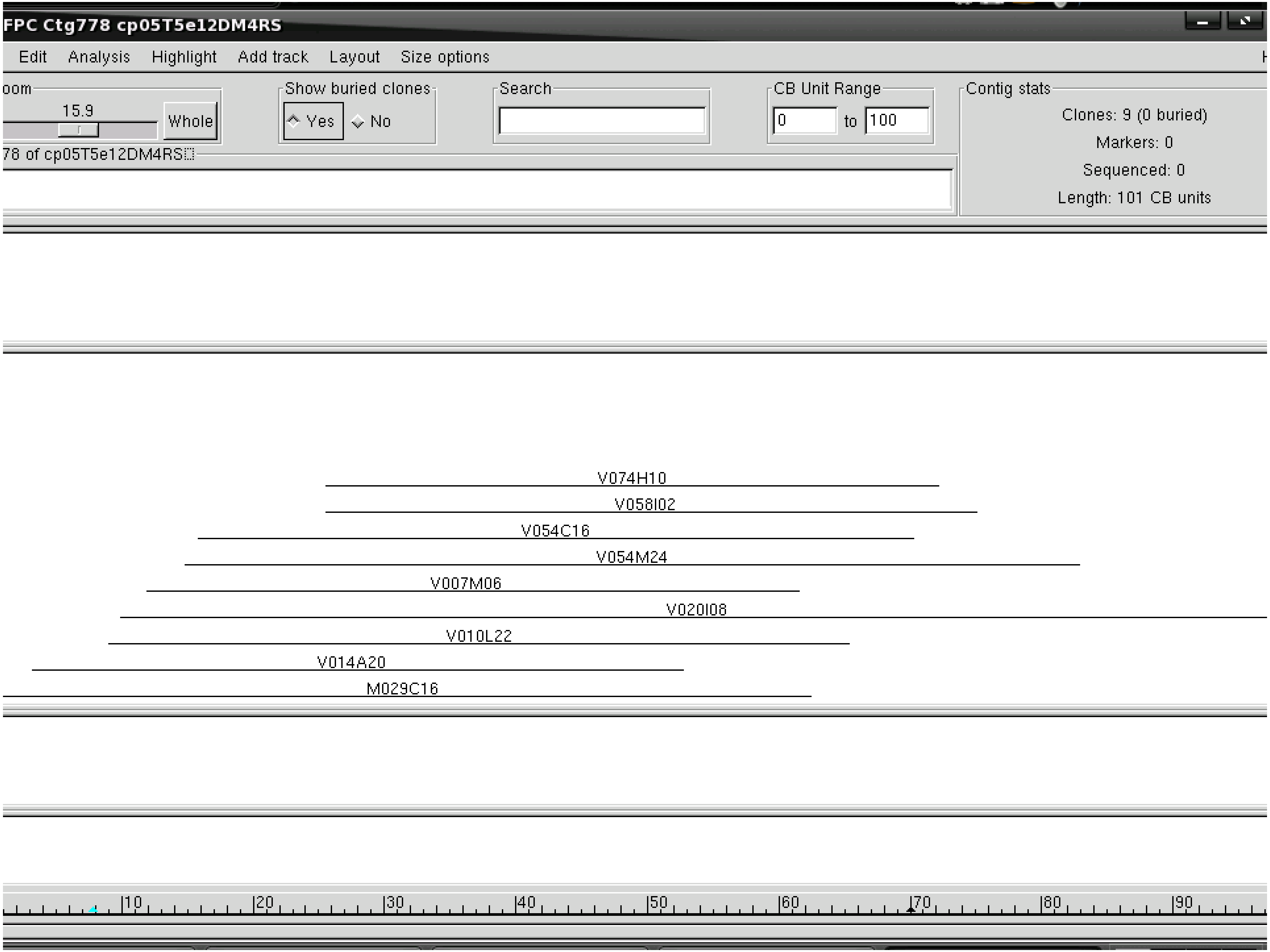
FPC Ctg778 cp05T5e12DM4RS
Edit
Analysis
Highlight
Add track
Layout
Size options
oom
15.9
Whole
Show buried clones
Yes
No
Search
CB Unit Range
0
to
100
Contig stats
Clones: 9 (0 buried)
Markers: 0
Sequenced: 0
Length: 101 CB units
78 of cp05T5e12DM4RS
V074H10
V058I02
V054C16
V054M24
V007M06
V020I08
V010L22
V014A20
M029C16
10
20
30
40
50
60
70
80
90

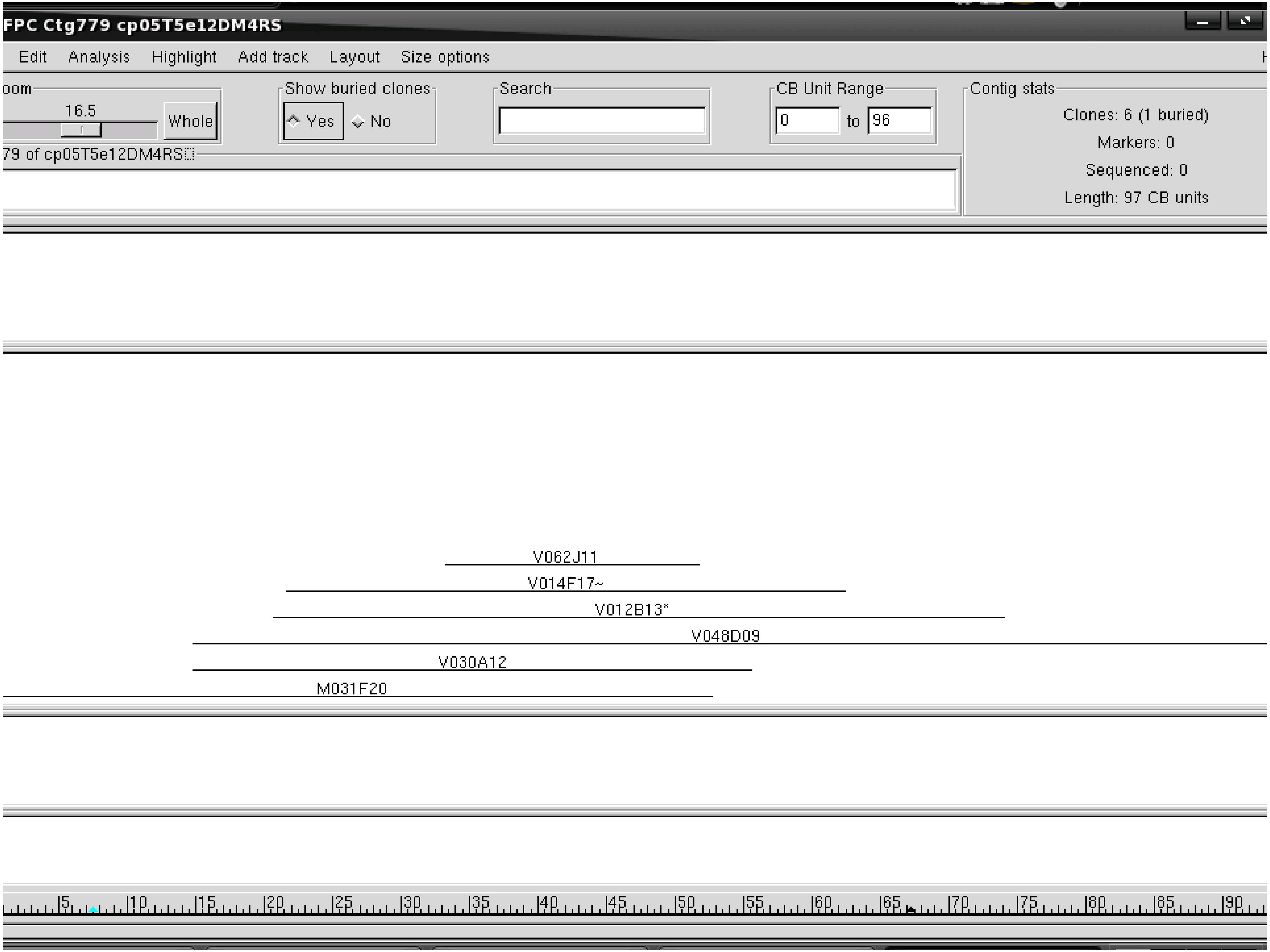
FPC Ctg779 cp05T5e12DM4RS
Edit
Analysis
Highlight
Add track
Layout
Size options
oom
16.5
Whole
Show buried clones
Yes
No
Search
CB Unit Range
0
to
96
Contig stats
Clones: 6 (1 buried)
Markers: 0
Sequenced: 0
Length: 97 CB units
79 of cp05T5e12DM4RS
V062J11
V014F17~
V012B13*
V048D09
V030A12
M031F20
5 10 15 20 25 30 35 40 45 50 55 60 65 70 75 80 85 90

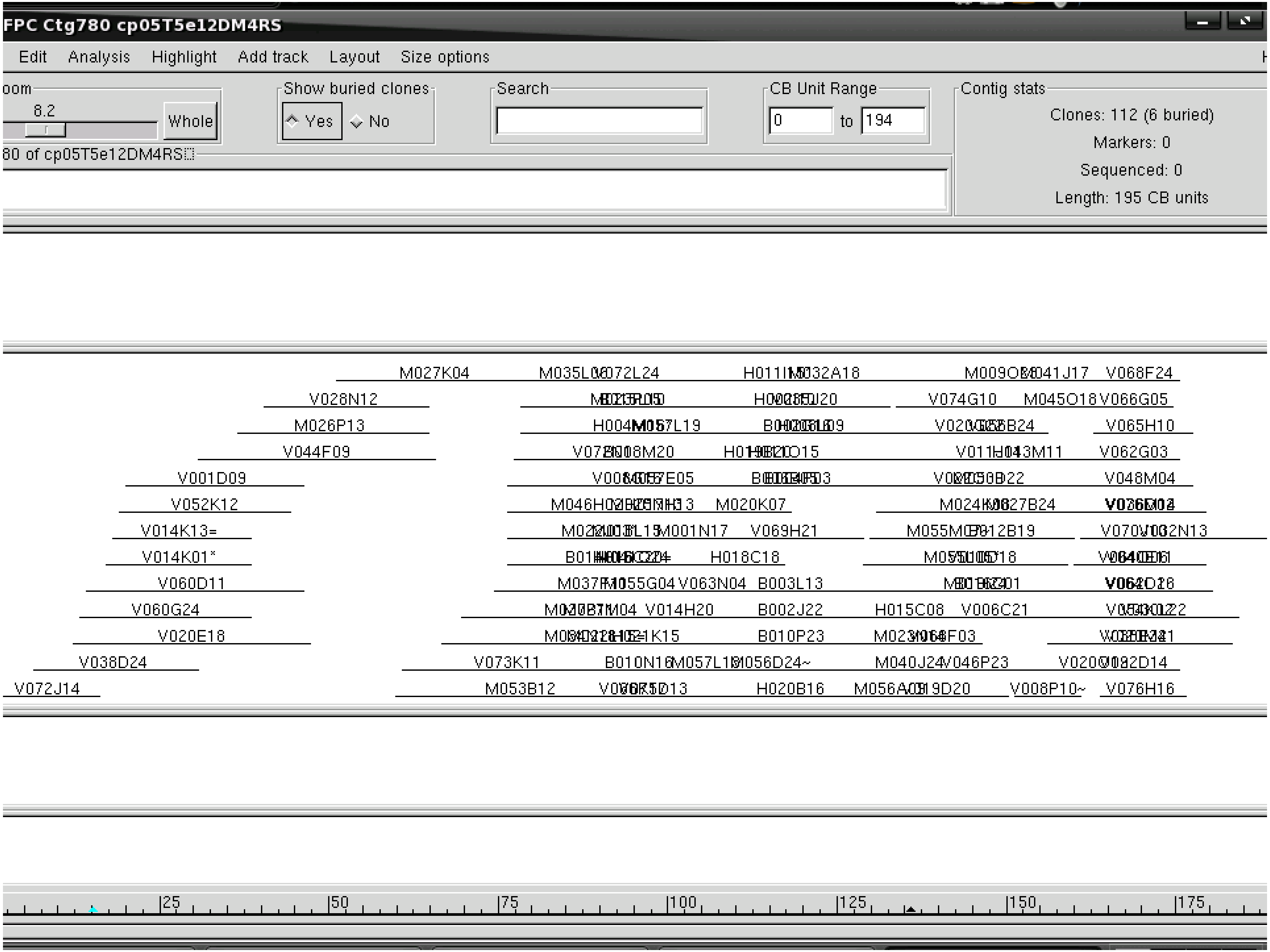
FPC Ctg780 cp05T5e12DM4RS
Edit
Analysis
Highlight
Add track
Layout
Size options
Show buried clones
Yes
No
Search
CB Unit Range
0
to
194
Contig stats
Clones: 112 (6 buried)
Markers: 0
Sequenced: 0
Length: 195 CB units
8.2
Whole
80 of cp05T5e12DM4RS
M027K04
V068F24
V028N12
M045O18
V066G05
M026P13
V074G10
V065H10
V044F09
V062G03
V001D09
V048M04
V052K12
M020K07
V014K13=
V069H21
V014K01*
H018C18
V060D11
V063N04
B003L13
V060G24
V014H20
B002J22
H015C08
V006C21
V020E18
B010P23
V038D24
V073K11
M040J24
V046P23
V072J14
M053B12
H020B16
V008P10~
V076H16
|25
|50
|75
|100
|125
|150
|175

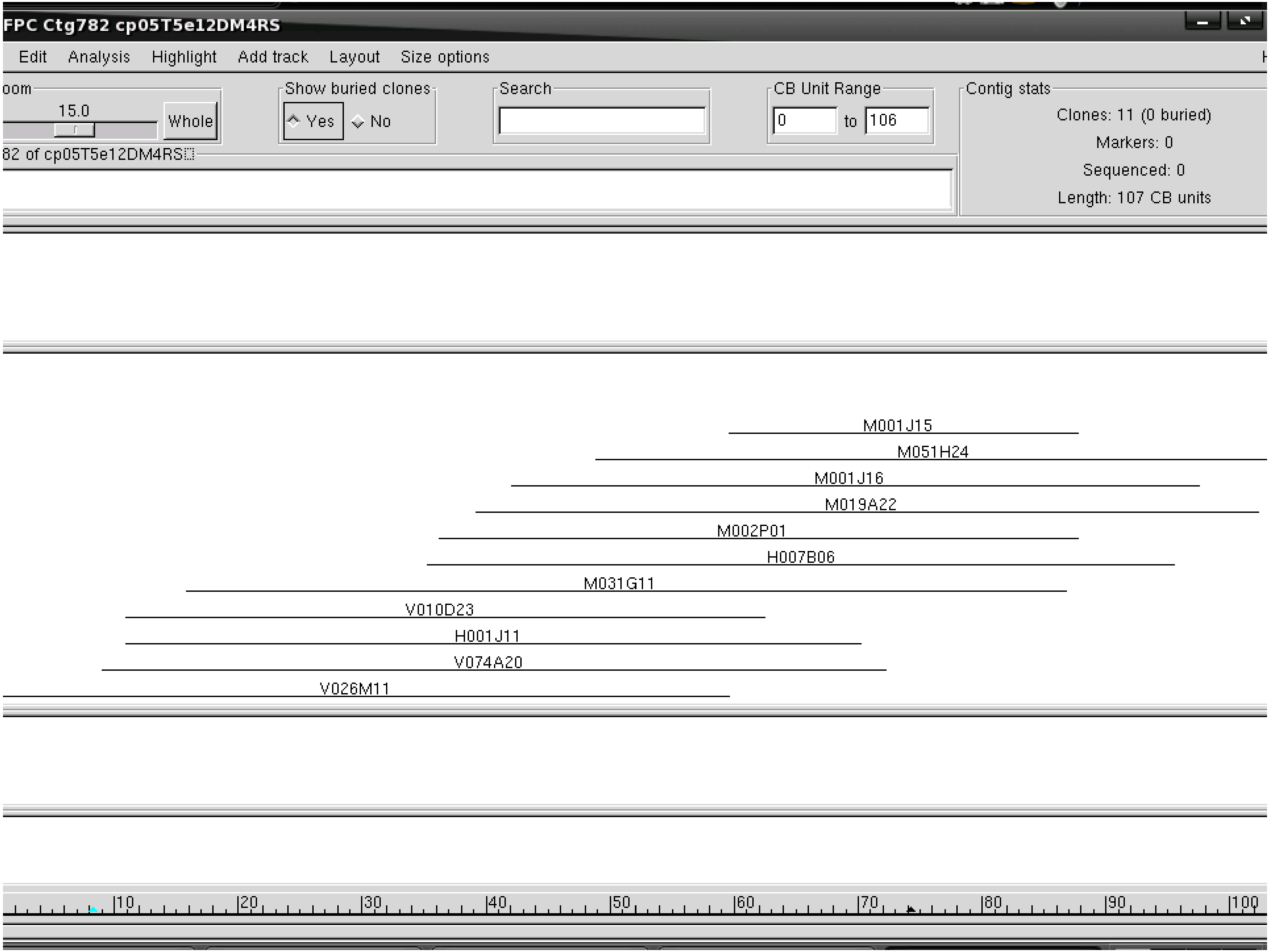
FPC Ctg782 cp05T5e12DM4RS
Edit
Analysis
Highlight
Add track
Layout
Size options
oom
15.0
Whole
Show buried clones
Yes
No
Search
CB Unit Range
0
to
106
Contig stats
Clones: 11 (0 buried)
Markers: 0
Sequenced: 0
Length: 107 CB units
82 of cp05T5e12DM4RS
M001J15
M051H24
M001J16
M019A22
M002P01
H007B06
M031G11
V010D23
H001J11
V074A20
V026M11
10
20
30
40
50
60
70
80
90
100

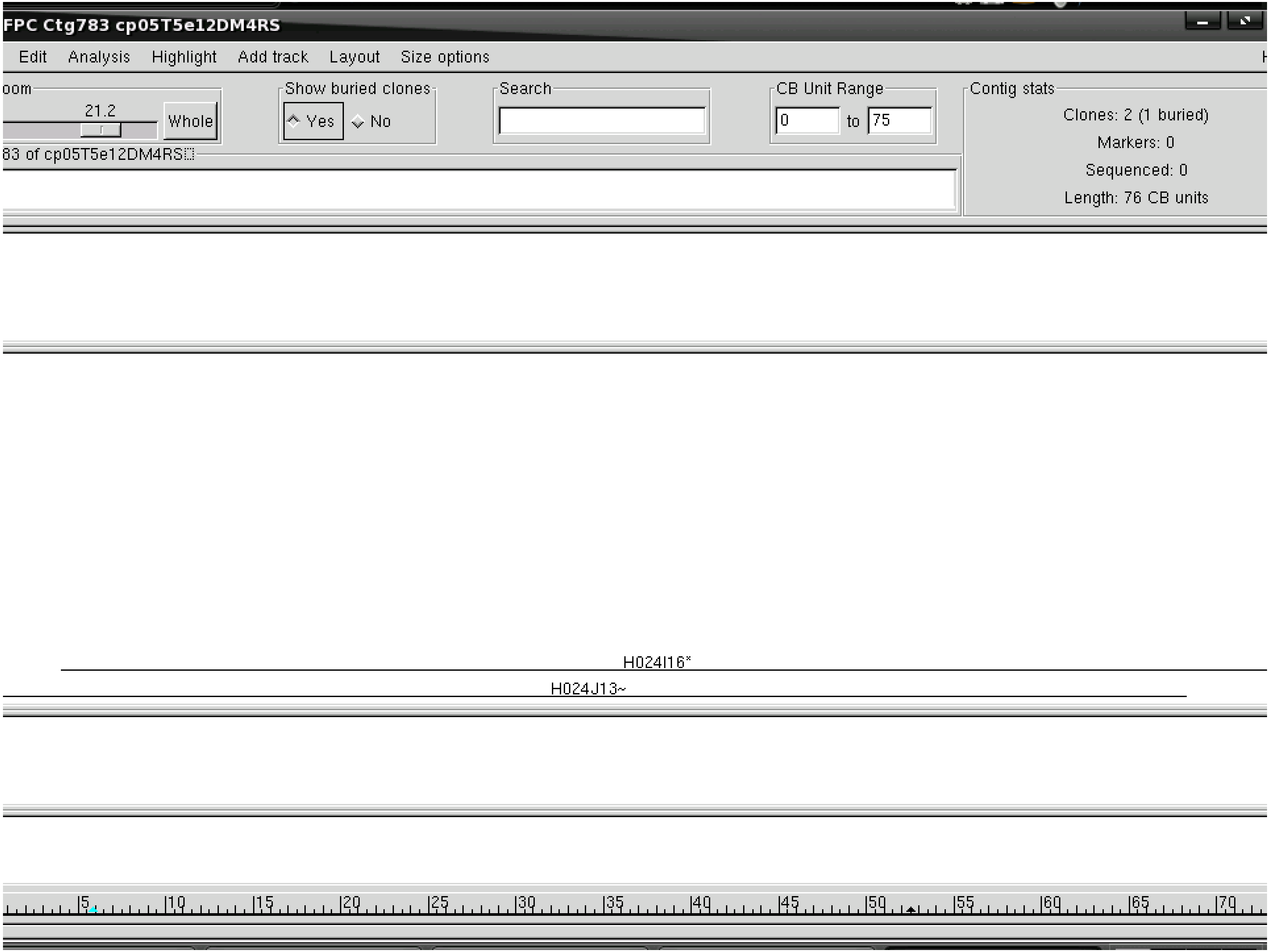
FPC Ctg783 cp05T5e12DM4RS
Edit
Analysis
Highlight
Add track
Layout
Size options
oom
21.2
Whole
Show buried clones
Yes
No
Search
CB Unit Range
0
to
75
Contig stats
Clones: 2 (1 buried)
Markers: 0
Sequenced: 0
Length: 76 CB units
83 of cp05T5e12DM4RS
H024I16*
H024J13~
5 10 15 20 25 30 35 40 45 50 55 60 65 70

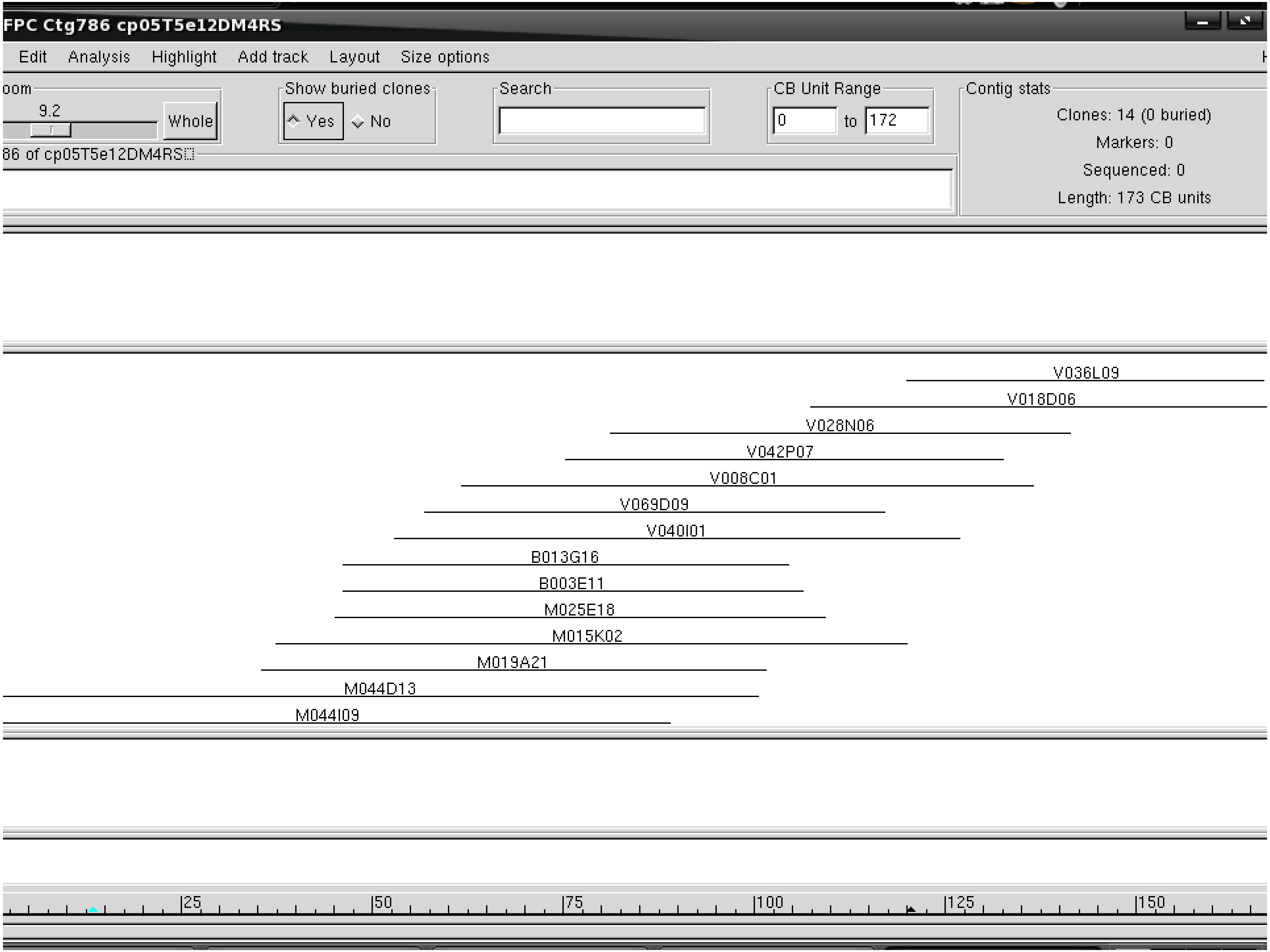
FPC Ctg786 cp05T5e12DM4RS
Edit
Analysis
Highlight
Add track
Layout
Size options
oom
9.2
Whole
Show buried clones
Yes
No
Search
CB Unit Range
0
to
172
Contig stats
Clones: 14 (0 buried)
Markers: 0
Sequenced: 0
Length: 173 CB units
86 of cp05T5e12DM4RS
V036L09
V018D06
V028N06
V042P07
V008C01
V069D09
V040I01
B013G16
B003E11
M025E18
M015K02
M019A21
M044D13
M044I09
25
50
75
100
125
150

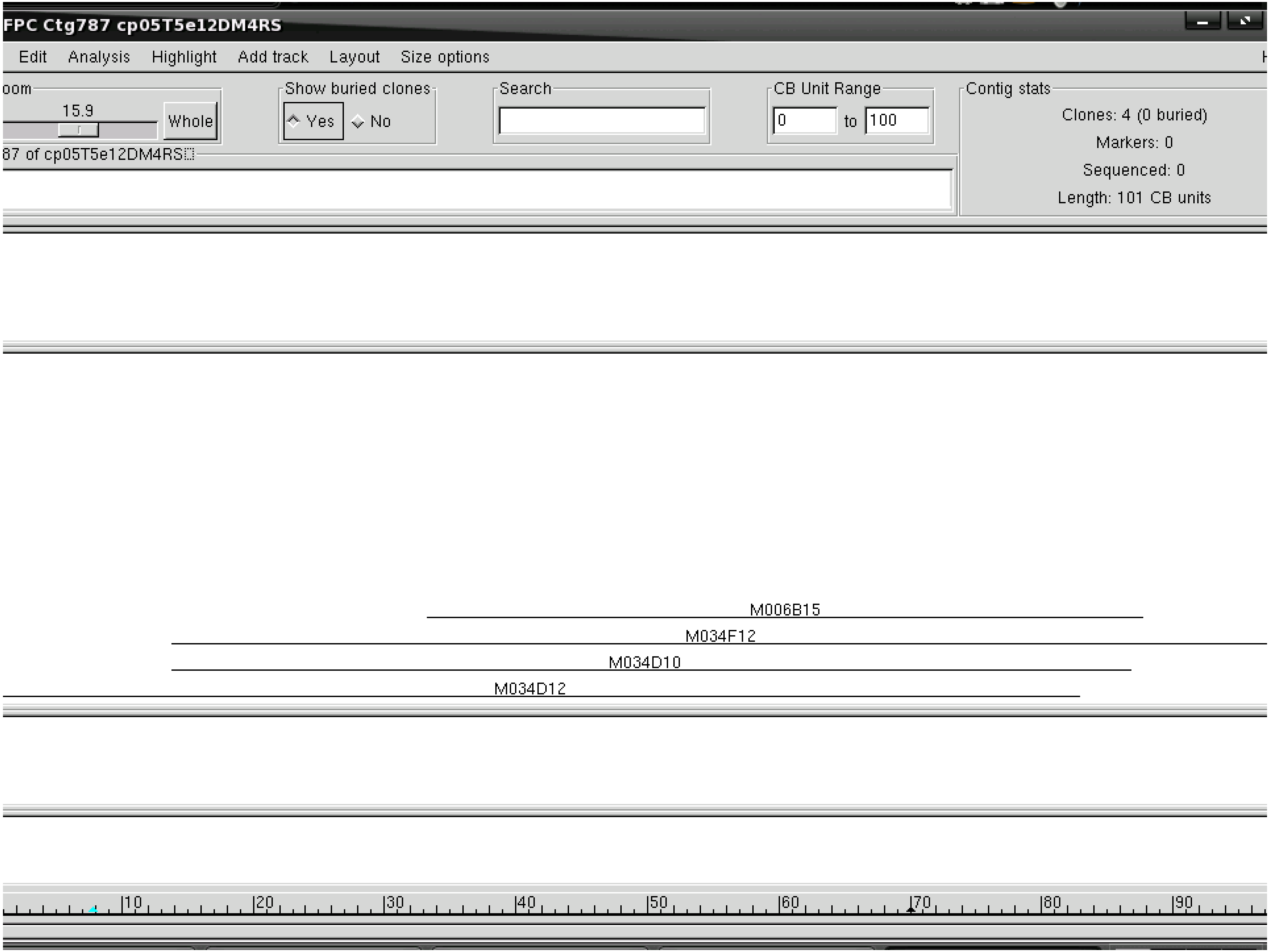
FPC Ctg787 cp05T5e12DM4RS
Edit
Analysis
Highlight
Add track
Layout
Size options
oom
15.9
Whole
Show buried clones
Yes
No
Search
CB Unit Range
0
to
100
Contig stats
Clones: 4 (0 buried)
Markers: 0
Sequenced: 0
Length: 101 CB units
87 of cp05T5e12DM4RS
M006B15
M034F12
M034D10
M034D12
10
20
30
40
50
60
70
80
90

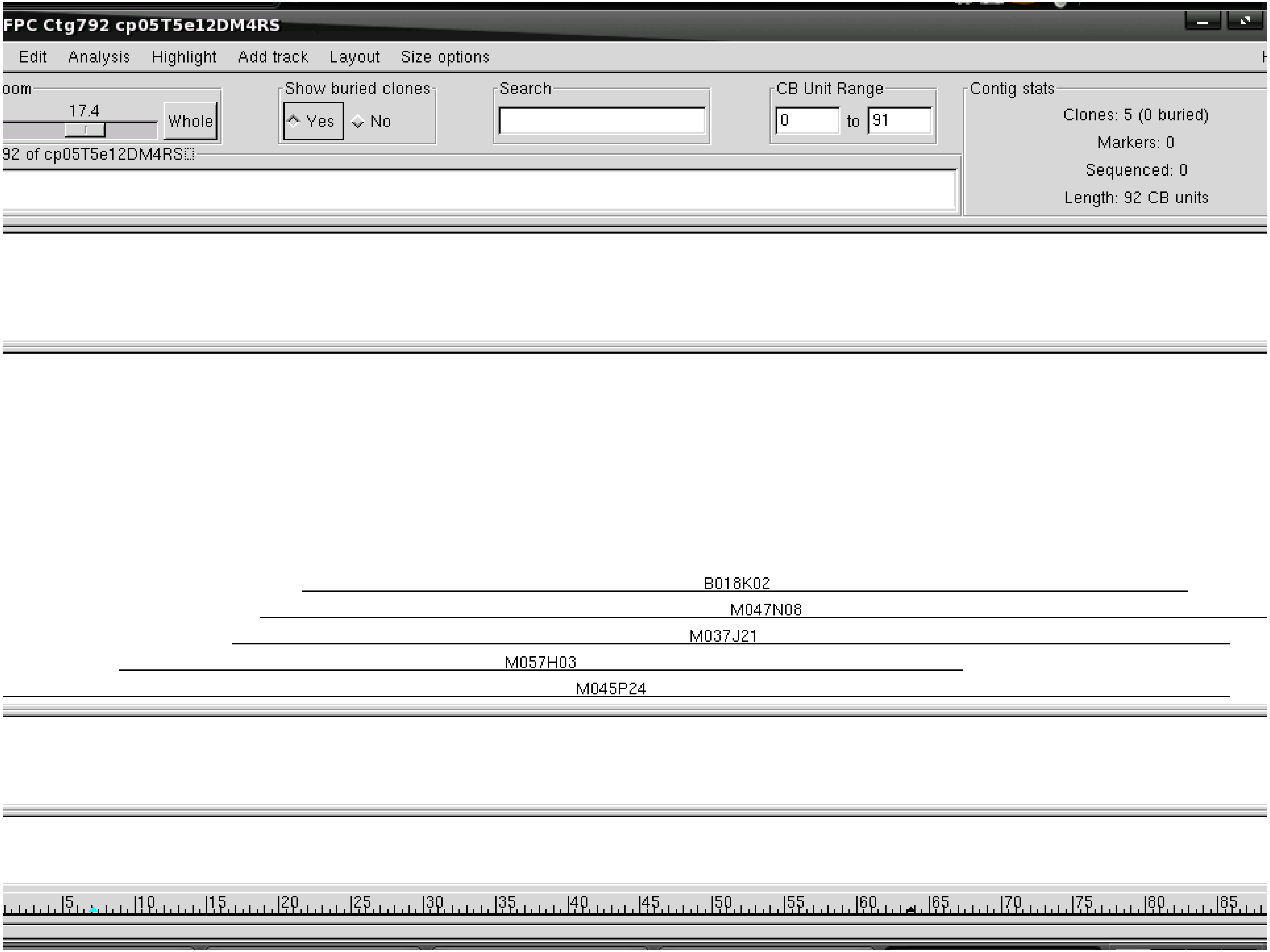
FPC Ctg792 cp05T5e12DM4RS
Edit
Analysis
Highlight
Add track
Layout
Size options
oom
17.4
Whole
Show buried clones
Yes
No
Search
CB Unit Range
0
to
91
Contig stats
Clones: 5 (0 buried)
Markers: 0
Sequenced: 0
Length: 92 CB units
92 of cp05T5e12DM4RS
B018K02
M047N08
M037J21
M057H03
M045P24
5 10 15 20 25 30 35 40 45 50 55 60 65 70 75 80 85

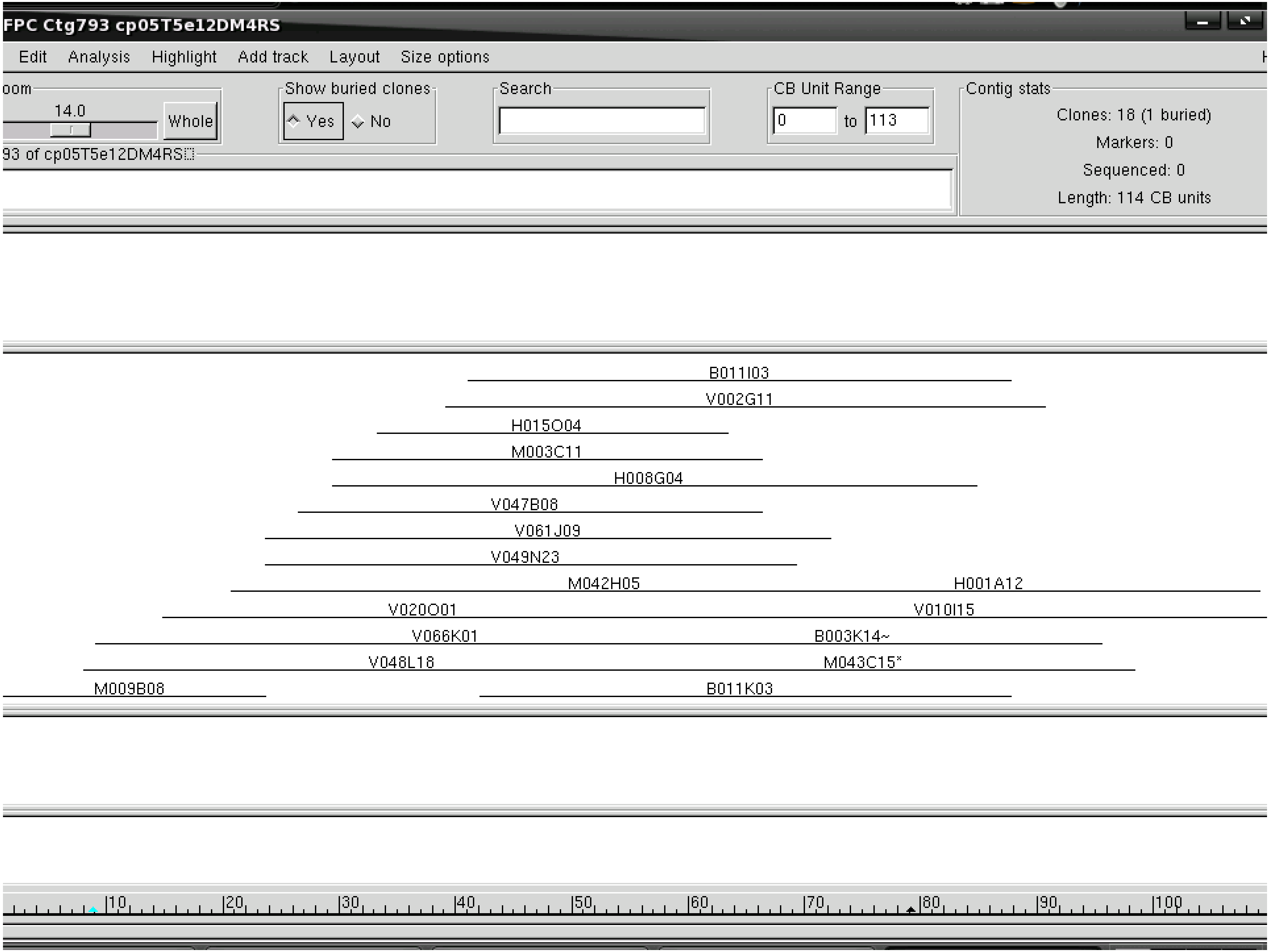
FPC Ctg793 cp05T5e12DM4RS
Edit Analysis Highlight Add track Layout Size options
oom
14.0
Whole
Show buried clones
Yes
No
Search
CB Unit Range
0
to
113
Contig stats
Clones: 18 (1 buried)
Markers: 0
Sequenced: 0
Length: 114 CB units
93 of cp05T5e12DM4RS
B011I03
V002G11
H015O04
M003C11
H008G04
V047B08
V061J09
V049N23
M042H05
H001A12
V020O01
V010I15
V066K01
B003K14~
V048L18
M043C15*
M009B08
B011K03
10 20 30 40 50 60 70 80 90 100

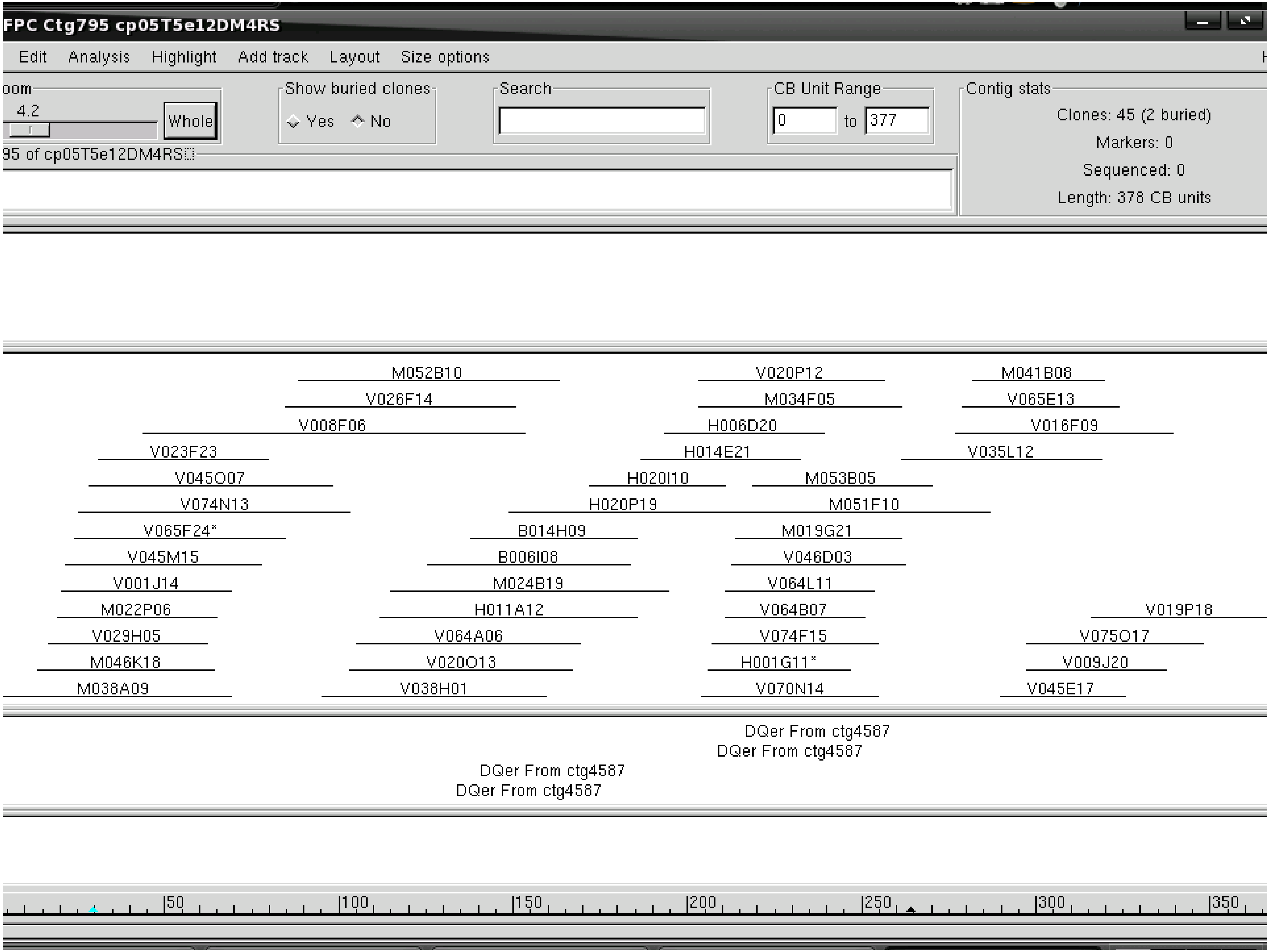
FPC Ctg795 cp05T5e12DM4RS
Edit
Analysis
Highlight
Add track
Layout
Size options
oom
4.2
Whole
Show buried clones
Yes
No
Search
CB Unit Range
0
to
377
Contig stats
Clones: 45 (2 buried)
Markers: 0
Sequenced: 0
Length: 378 CB units
95 of cp05T5e12DM4RS
M052B10
V020P12
M041B08
V026F14
M034F05
V065E13
V008F06
H006D20
V016F09
V023F23
H014E21
V035L12
V045O07
H020I10
M053B05
V074N13
H020P19
M051F10
V065F24*
B014H09
M019G21
V045M15
B006I08
V046D03
V001J14
M024B19
V064L11
M022P06
H011A12
V064B07
V019P18
V029H05
V064A06
V074F15
V075O17
M046K18
V020O13
H001G11*
V009J20
M038A09
V038H01
V070N14
V045E17
DQer From ctg4587
DQer From ctg4587
DQer From ctg4587
DQer From ctg4587
50
100
150
200
250
300
350

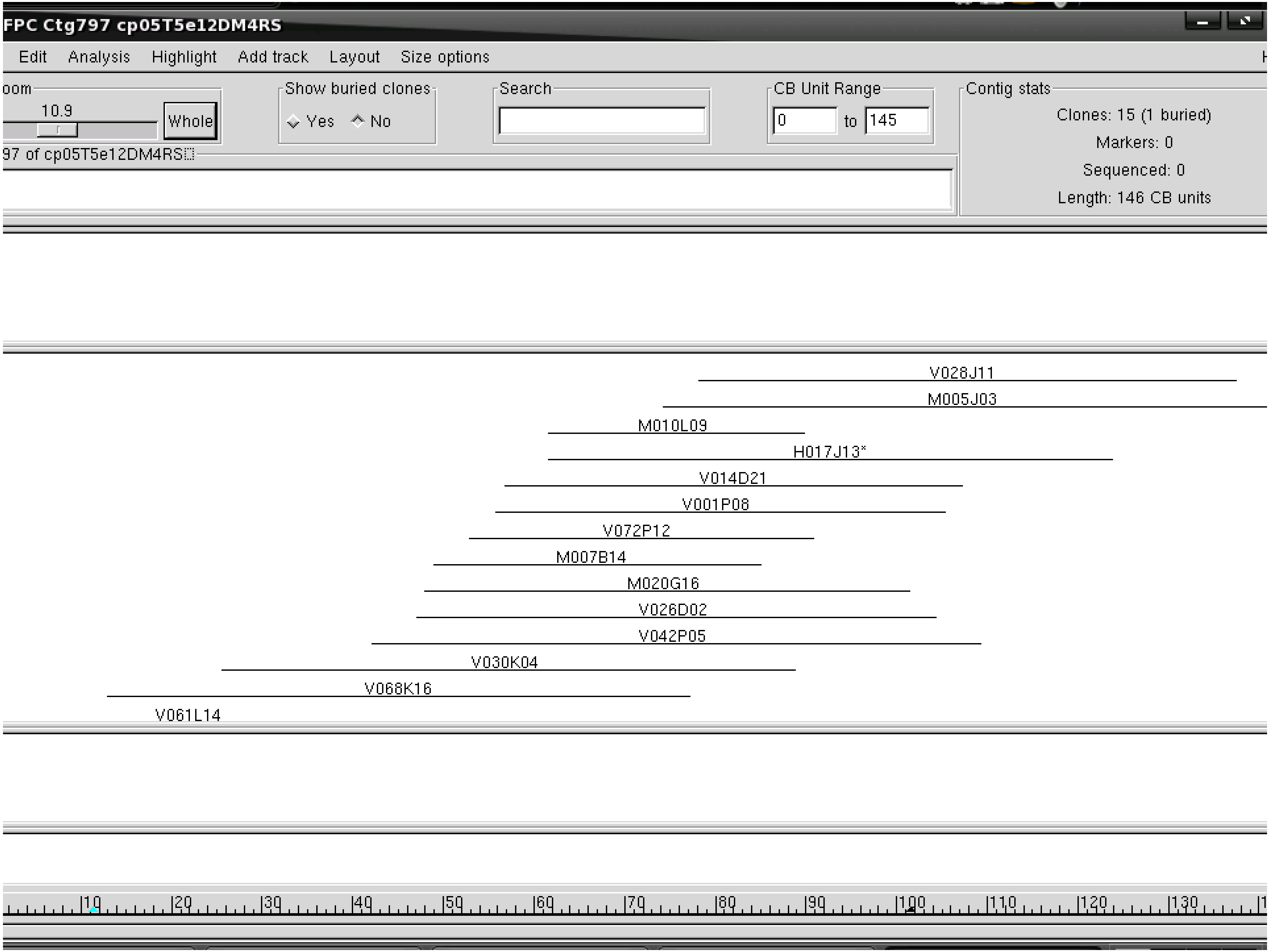
FPC Ctg797 cp05T5e12DM4RS
Edit Analysis Highlight Add track Layout Size options
oom
10.9
Whole
Show buried clones
Yes
No
Search
CB Unit Range
0
to
145
Contig stats
Clones: 15 (1 buried)
Markers: 0
Sequenced: 0
Length: 146 CB units
97 of cp05T5e12DM4RS
V028J11
M005J03
M010L09
H017J13*
V014D21
V001P08
V072P12
M007B14
M020G16
V026D02
V042P05
V030K04
V068K16
V061L14
10 20 30 40 50 60 70 80 90 100 110 120 130

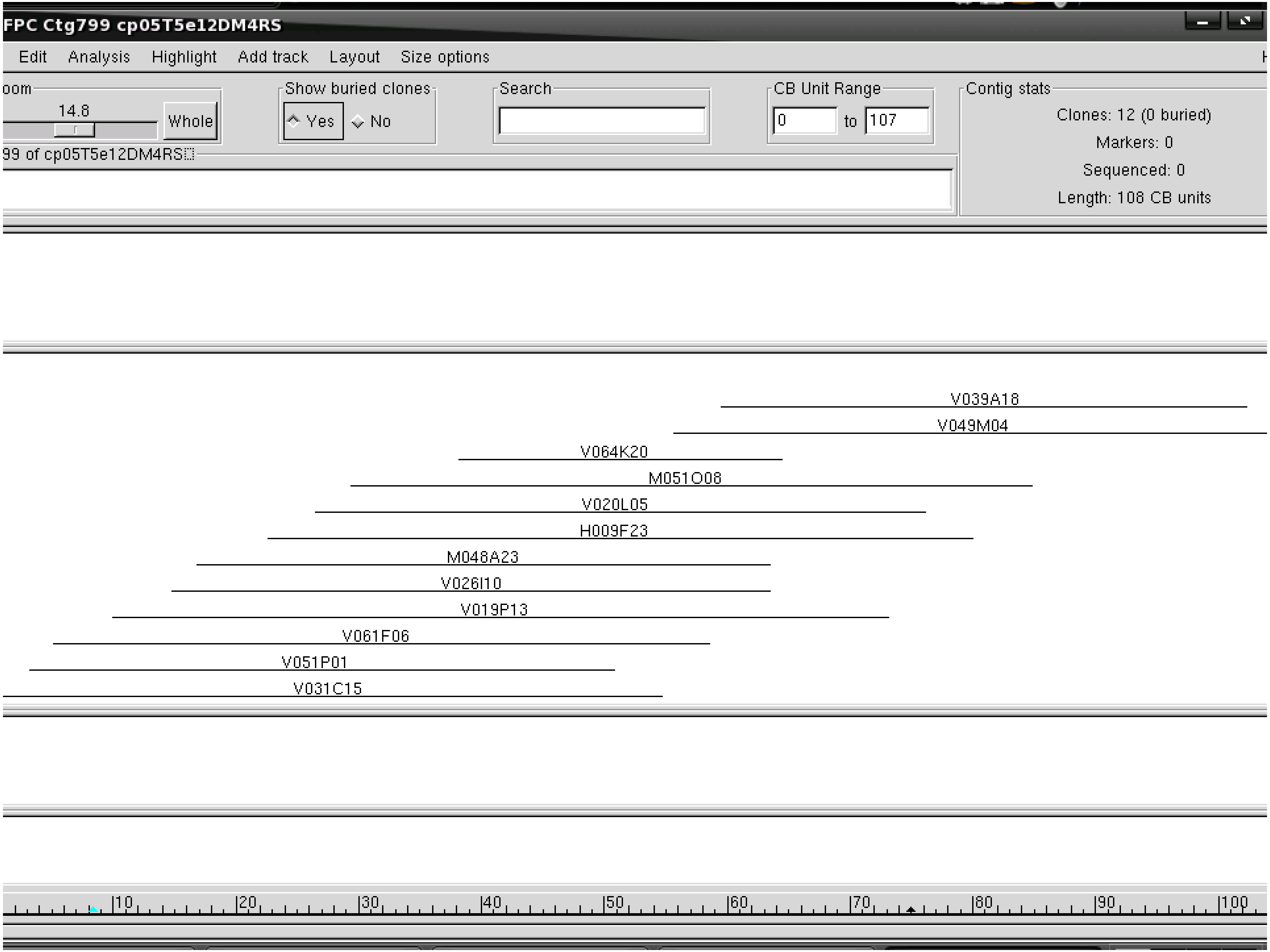
FPC Ctg799 cp05T5e12DM4RS
Edit
Analysis
Highlight
Add track
Layout
Size options
oom
14.8
Whole
Show buried clones
Yes
No
Search
CB Unit Range
0
to
107
Contig stats
Clones: 12 (0 buried)
Markers: 0
Sequenced: 0
Length: 108 CB units
99 of cp05T5e12DM4RS
V039A18
V049M04
V064K20
M051O08
V020L05
H009F23
M048A23
V026I10
V019P13
V061F06
V051P01
V031C15
10
20
30
40
50
60
70
80
90
100

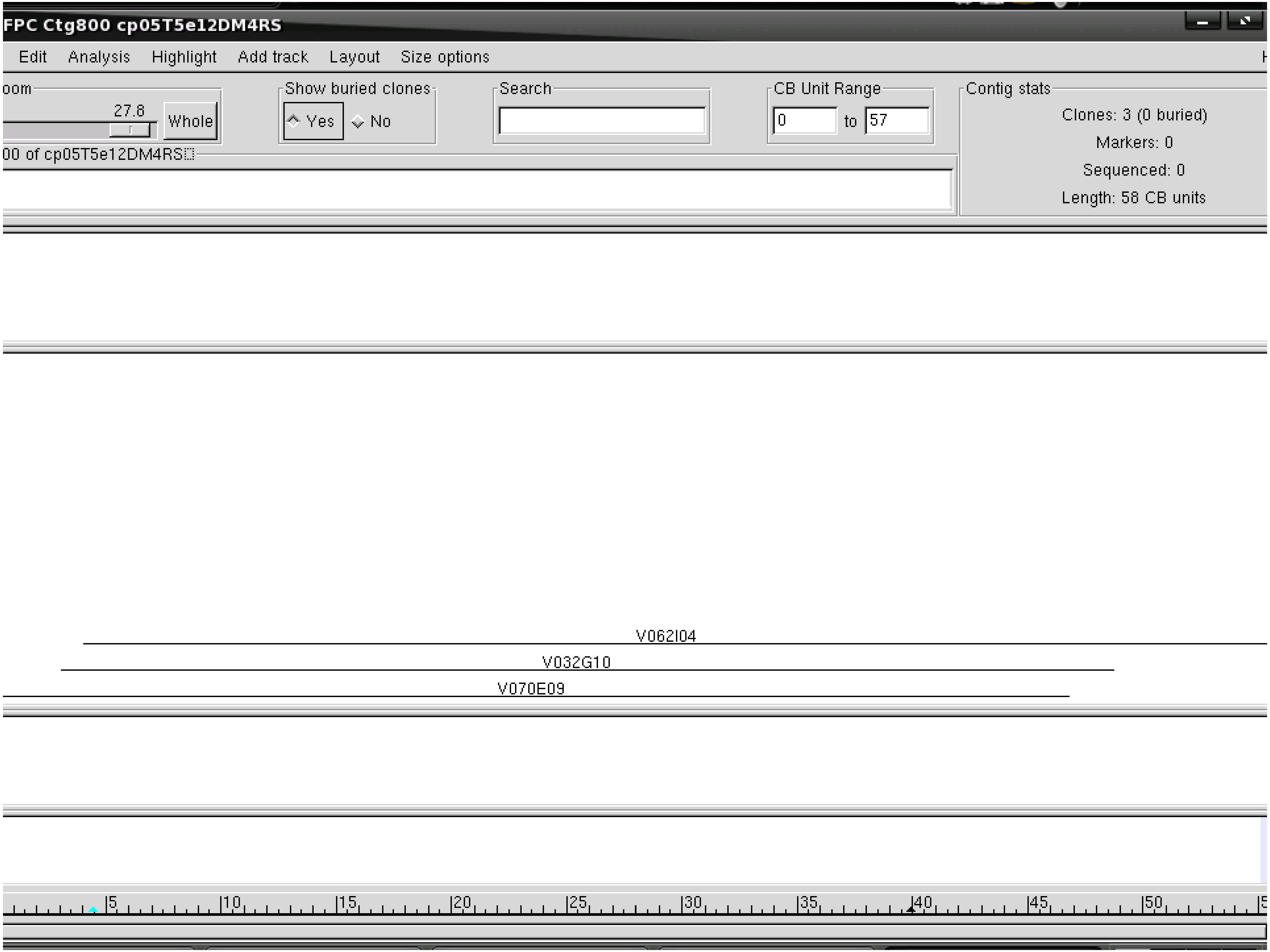
FPC Ctg800 cp05T5e12DM4RS
Edit
Analysis
Highlight
Add track
Layout
Size options
oom
27.8
Whole
Show buried clones
Yes
No
Search
CB Unit Range
0
to
57
Contig stats
Clones: 3 (0 buried)
Markers: 0
Sequenced: 0
Length: 58 CB units
00 of cp05T5e12DM4RS
V062I04
V032G10
V070E09
5
10
15
20
25
30
35
40
45
50

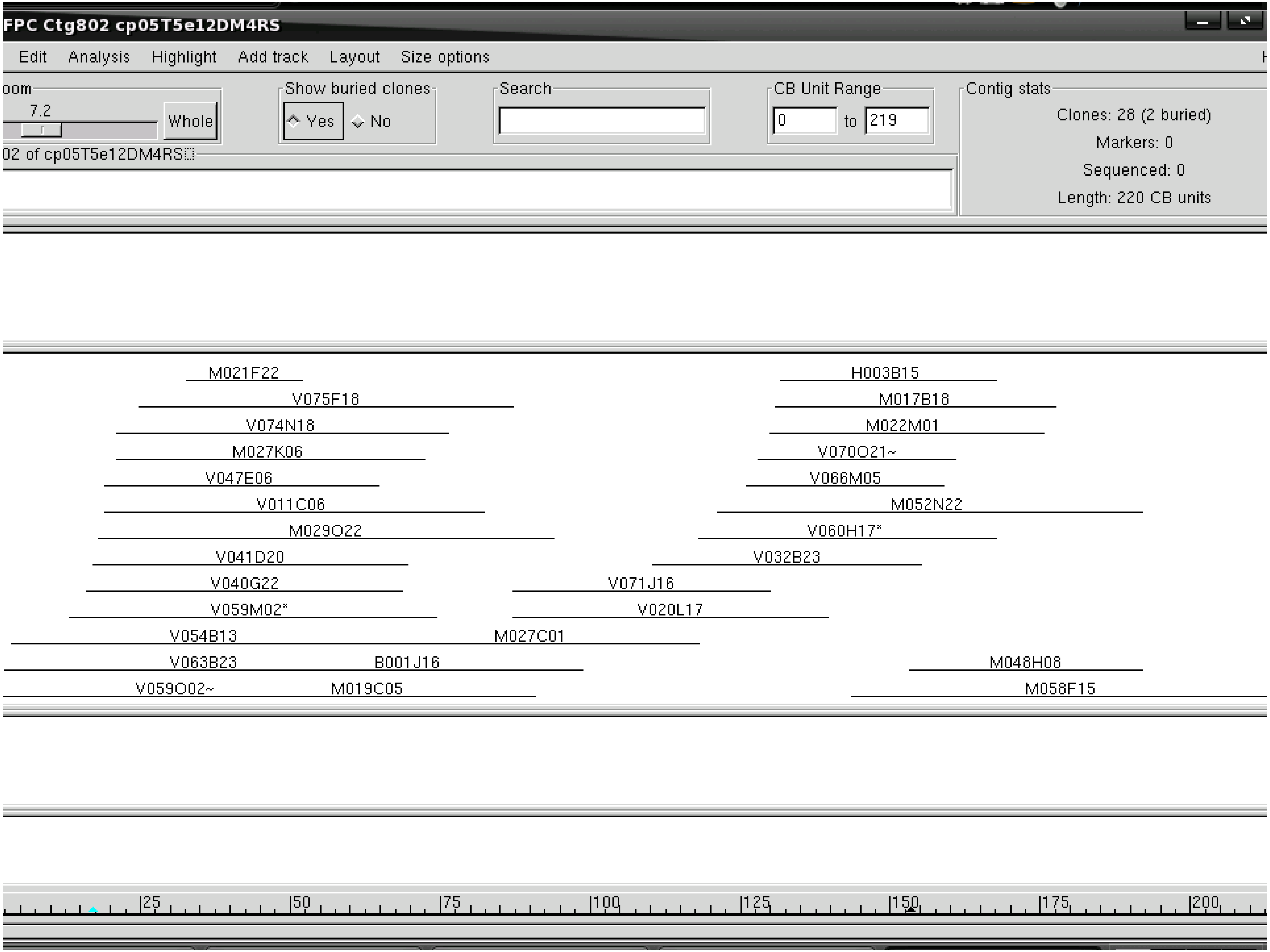
FPC Ctg802 cp05T5e12DM4RS
Edit
Analysis
Highlight
Add track
Layout
Size options
oom
7.2
Whole
Show buried clones
Yes
No
Search
CB Unit Range
0
to
219
Contig stats
Clones: 28 (2 buried)
Markers: 0
Sequenced: 0
Length: 220 CB units
02 of cp05T5e12DM4RS
M021F22
V075F18
V074N18
M027K06
V047E06
V011C06
M029O22
V041D20
V040G22
V059M02*
V054B13
M027C01
V063B23
B001J16
V059O02~
M019C05
H003B15
M017B18
M022M01
V070O21~
V066M05
M052N22
V060H17*
V032B23
V071J16
V020L17
M048H08
M058F15
25
50
75
100
125
150
175
200

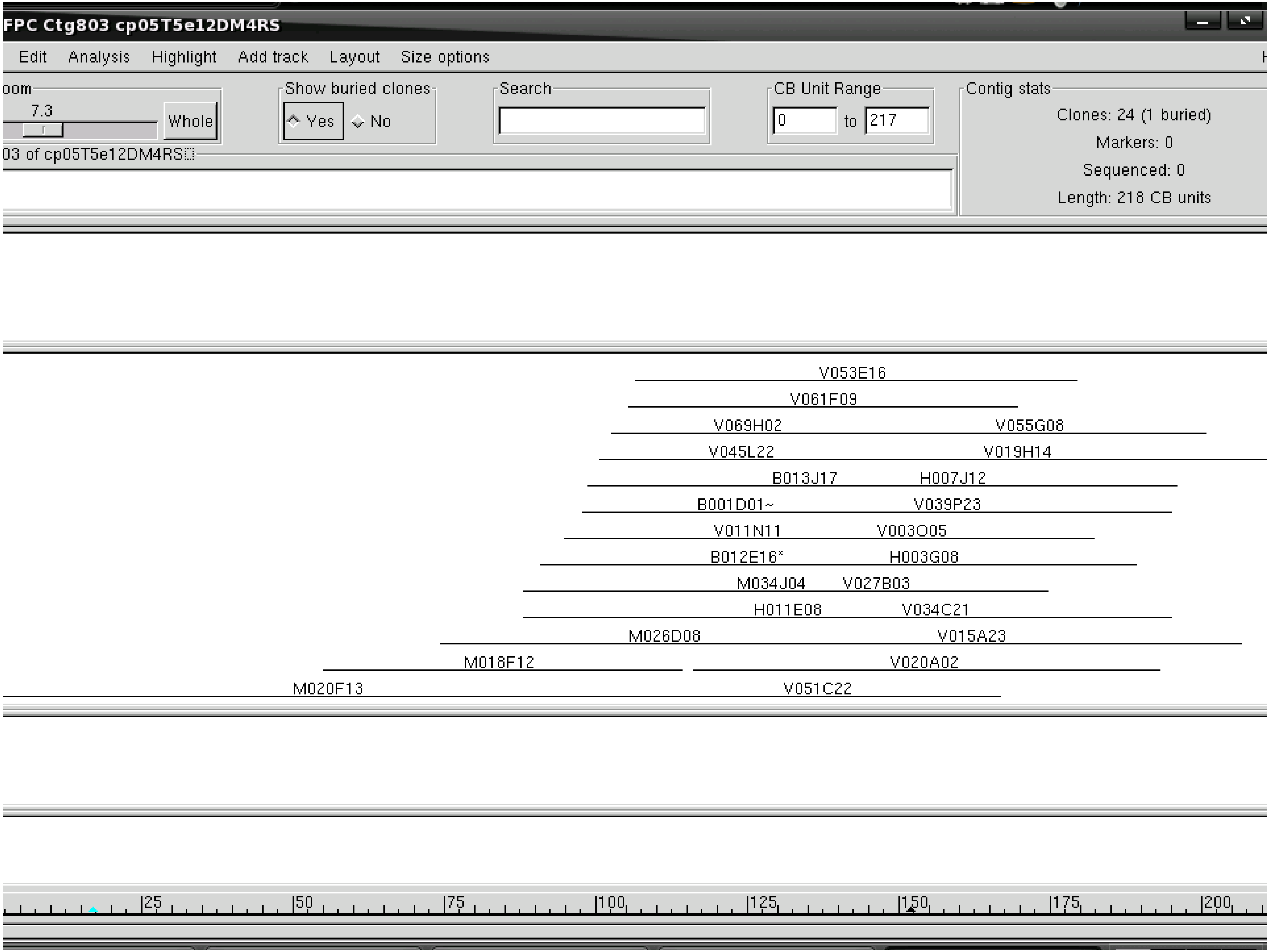
FPC Ctg803 cp05T5e12DM4RS
Edit
Analysis
Highlight
Add track
Layout
Size options
oom
7.3
Whole
Show buried clones
Yes
No
Search
CB Unit Range
0
to
217
Contig stats
Clones: 24 (1 buried)
Markers: 0
Sequenced: 0
Length: 218 CB units
03 of cp05T5e12DM4RS
V053E16
V061F09
V069H02
V055G08
V045L22
V019H14
B013J17
H007J12
B001D01~
V039P23
V011N11
V003O05
B012E16*
H003G08
M034J04
V027B03
H011E08
V034C21
M026D08
V015A23
M018F12
V020A02
M020F13
V051C22
25
50
75
100
125
150
175
200

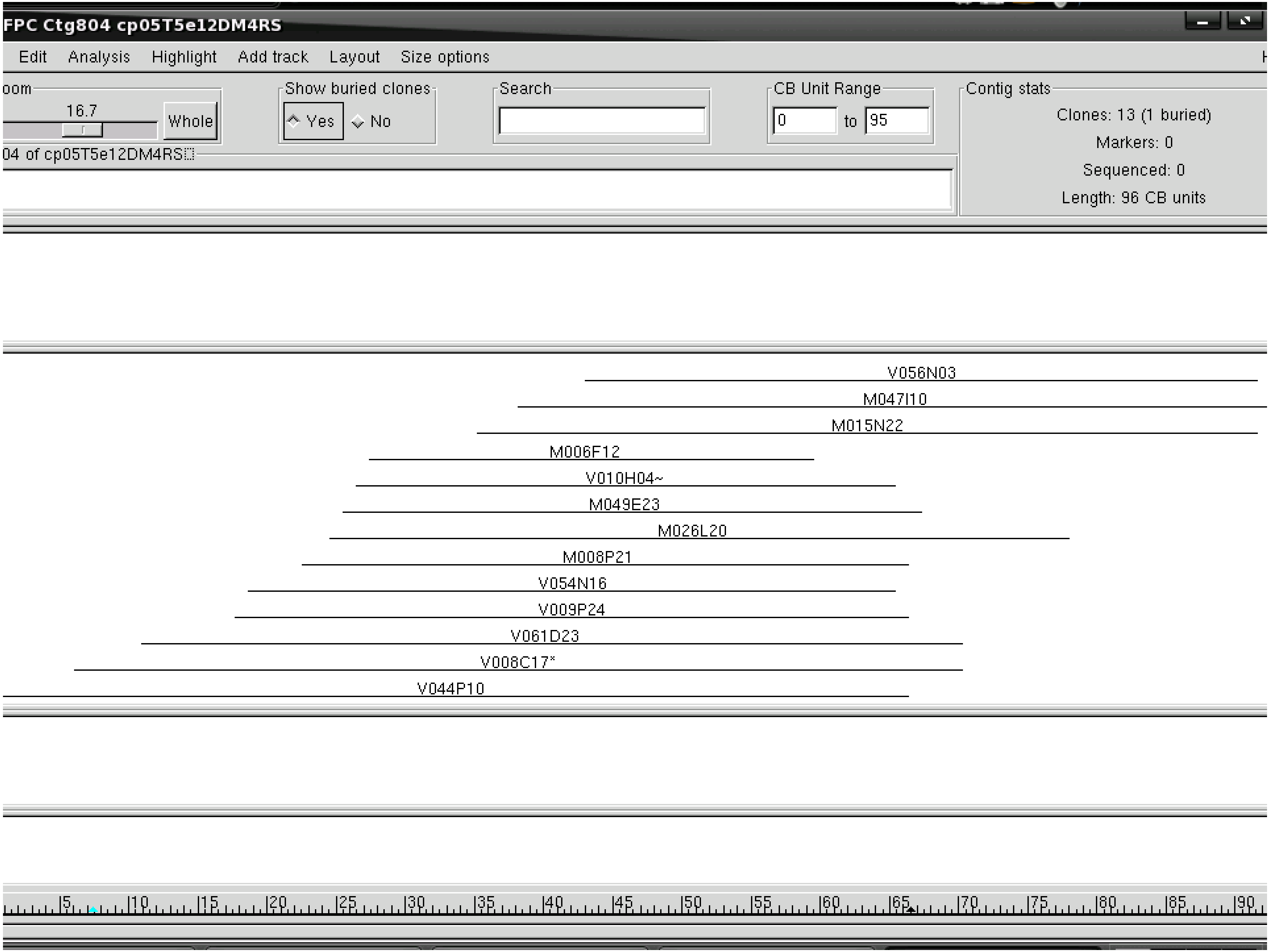
FPC Ctg804 cp05T5e12DM4RS
Edit
Analysis
Highlight
Add track
Layout
Size options
oom
16.7
Whole
Show buried clones
Yes
No
Search
CB Unit Range
0
to
95
Contig stats
Clones: 13 (1 buried)
Markers: 0
Sequenced: 0
Length: 96 CB units
04 of cp05T5e12DM4RS
V056N03
M047I10
M015N22
M006F12
V010H04~
M049E23
M026L20
M008P21
V054N16
V009P24
V061D23
V008C17*
V044P10
5
10
15
20
25
30
35
40
45
50
55
60
65
70
75
80
85
90

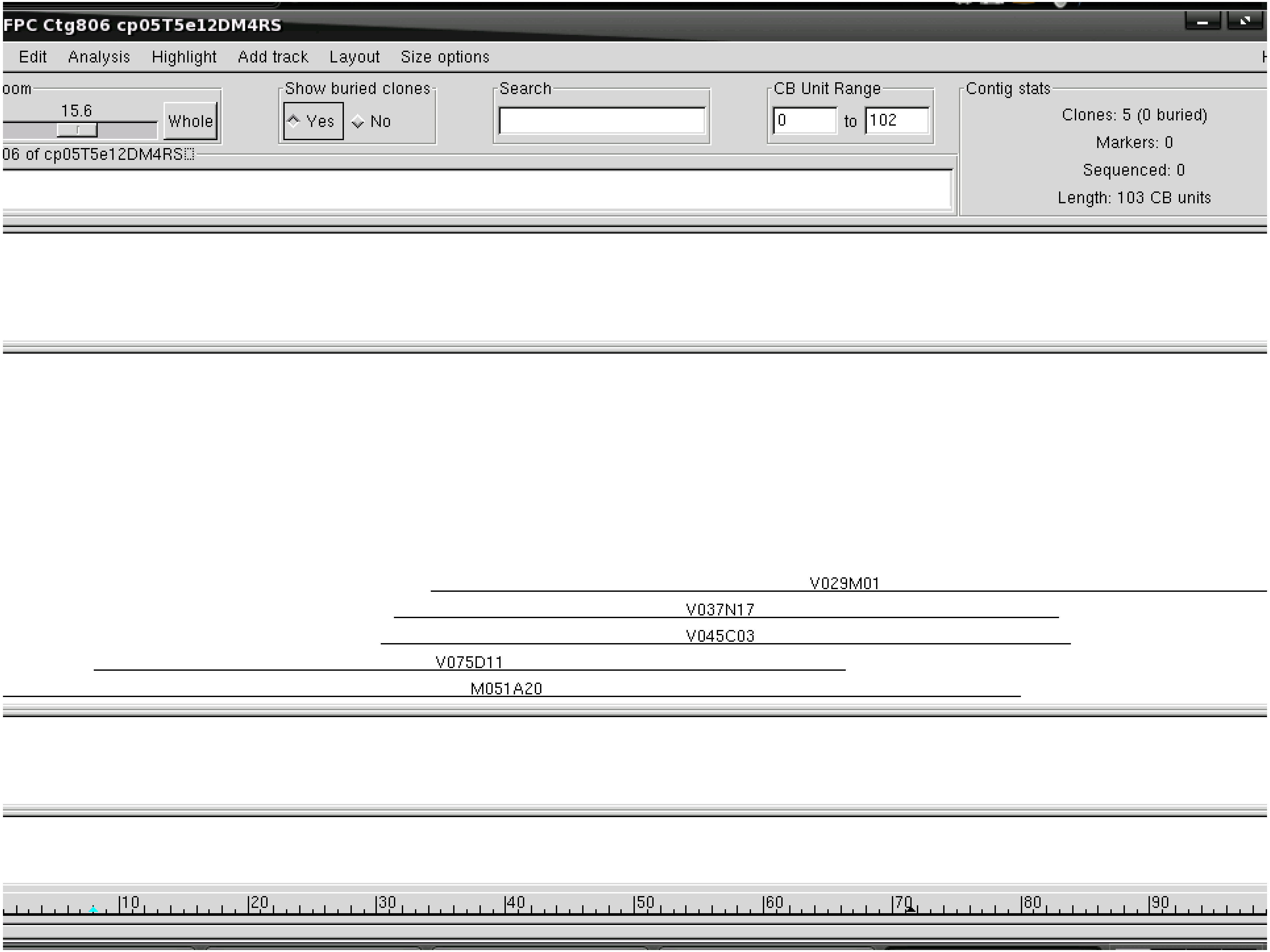
FPC Ctg806 cp05T5e12DM4RS
Edit
Analysis
Highlight
Add track
Layout
Size options
oom
15.6
Whole
Show buried clones
Yes
No
Search
CB Unit Range
0
to
102
Contig stats
Clones: 5 (0 buried)
Markers: 0
Sequenced: 0
Length: 103 CB units
06 of cp05T5e12DM4RS
V029M01
V037N17
V045C03
V075D11
M051A20
10
20
30
40
50
60
70
80
90

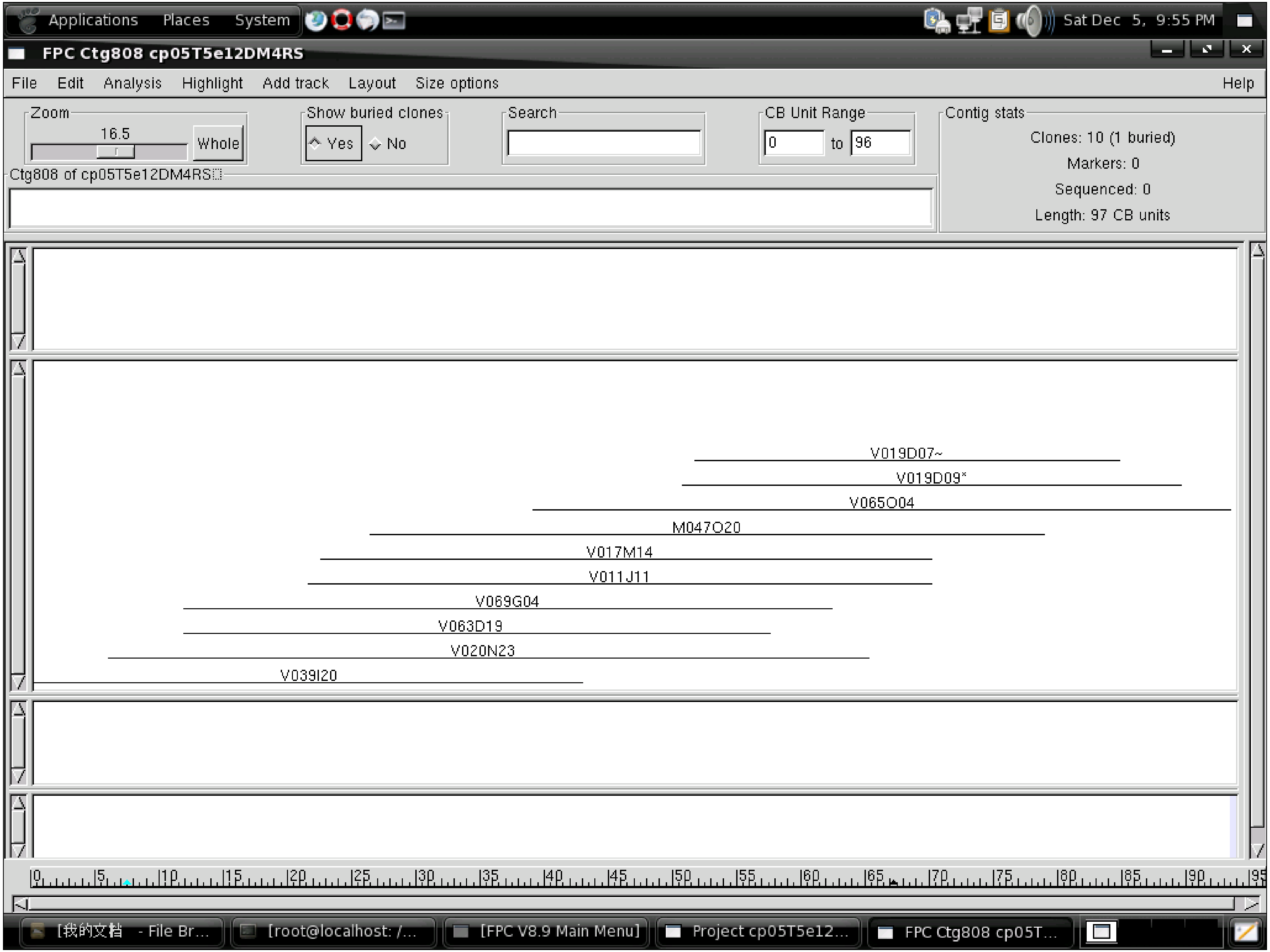
Applications Places System
Sat Dec 5, 9:55 PM
FPC Ctg808 cp05T5e12DM4RS
File Edit Analysis Highlight Add track Layout Size options Help
Zoom
16.5
Whole
Show buried clones
Yes No
Search
CB Unit Range
0 to 96
Contig stats
Clones: 10 (1 buried)
Markers: 0
Sequenced: 0
Length: 97 CB units
Ctg808 of cp05T5e12DM4RS
V019D07~
V019D09*
V065O04
M047O20
V017M14
V011J11
V069G04
V063D19
V020N23
V039I20
0 5 10 15 20 25 30 35 40 45 50 55 60 65 70 75 80 85 90 95
[我的文档 - File Br...
[root@localhost: /...
[FPC V8.9 Main Menu]
Project cp05T5e12...
FPC Ctg808 cp05T...

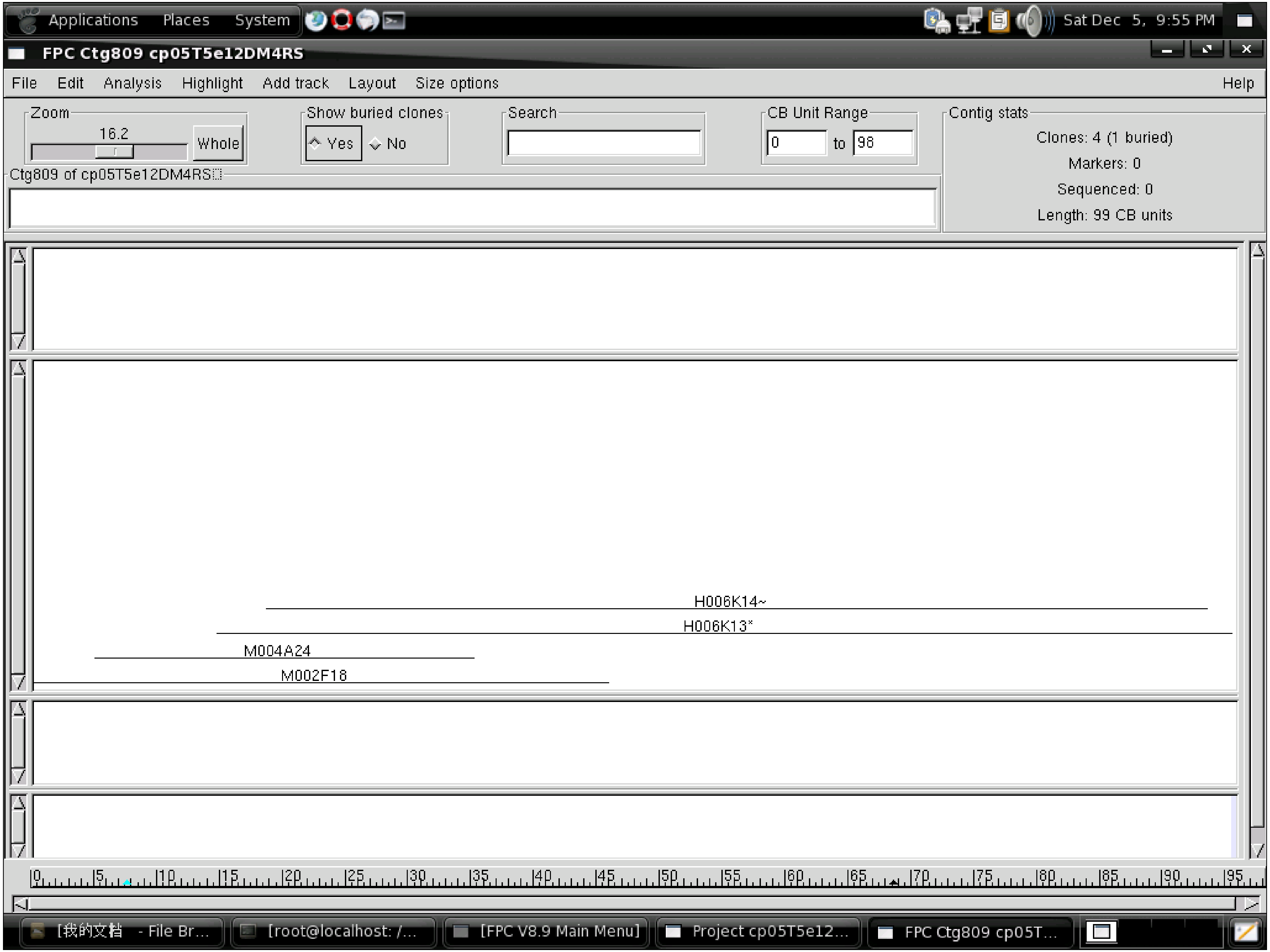
Applications Places System
Sat Dec 5, 9:55 PM
FPC Ctg809 cp05T5e12DM4RS
File Edit Analysis Highlight Add track Layout Size options Help
Zoom
16.2
Whole
Show buried clones
Yes
No
Search
CB Unit Range
0 to 98
Contig stats
Clones: 4 (1 buried)
Markers: 0
Sequenced: 0
Length: 99 CB units
Ctg809 of cp05T5e12DM4RS
H006K14~
H006K13*
M004A24
M002F18
0 5 10 15 20 25 30 35 40 45 50 55 60 65 70 75 80 85 90 95
[我的文档 - File Br...
[root@localhost: /...
[FPC V8.9 Main Menu]
Project cp05T5e12...
FPC Ctg809 cp05T...

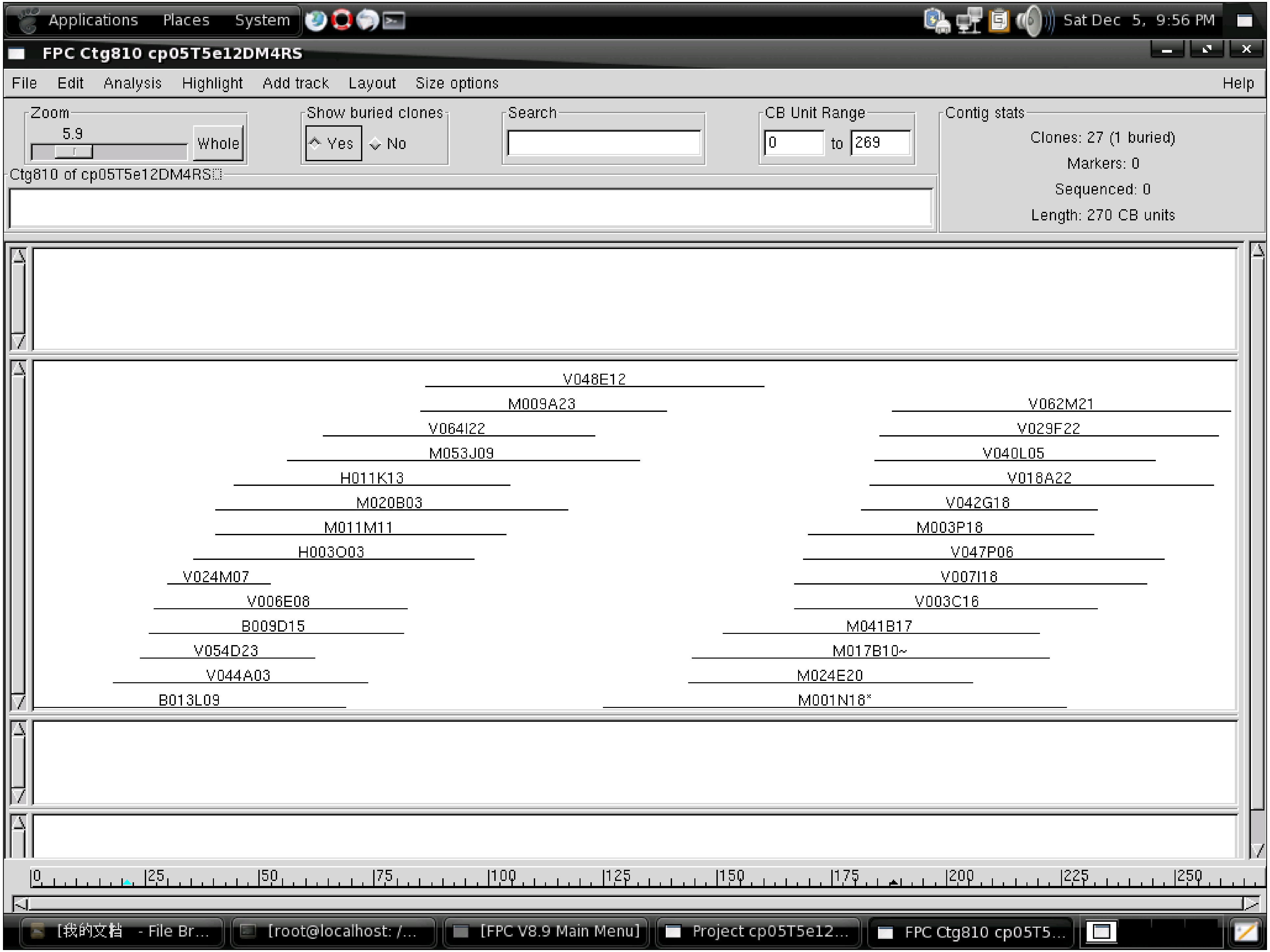
Applications
Places
System
Sat Dec 5, 9:56 PM
FPC Ctg810 cp05T5e12DM4RS
File
Edit
Analysis
Highlight
Add track
Layout
Size options
Help
Zoom
5.9
Whole
Show buried clones
Yes
No
Search
CB Unit Range
0
to
269
Contig stats
Clones: 27 (1 buried)
Markers: 0
Sequenced: 0
Length: 270 CB units
Ctg810 of cp05T5e12DM4RS
V048E12
M009A23
V062M21
V064I22
V029F22
M053J09
V040L05
H011K13
V018A22
M020B03
V042G18
M011M11
M003P18
H003O03
V047P06
V024M07
V007I18
V006E08
V003C16
B009D15
M041B17
V054D23
M017B10~
V044A03
M024E20
B013L09
M001N18*
0
25
50
75
100
125
150
175
200
225
250
[我的文档 - File Br...
[root@localhost: /...
[FPC V8.9 Main Menu]
Project cp05T5e12...
FPC Ctg810 cp05T5...

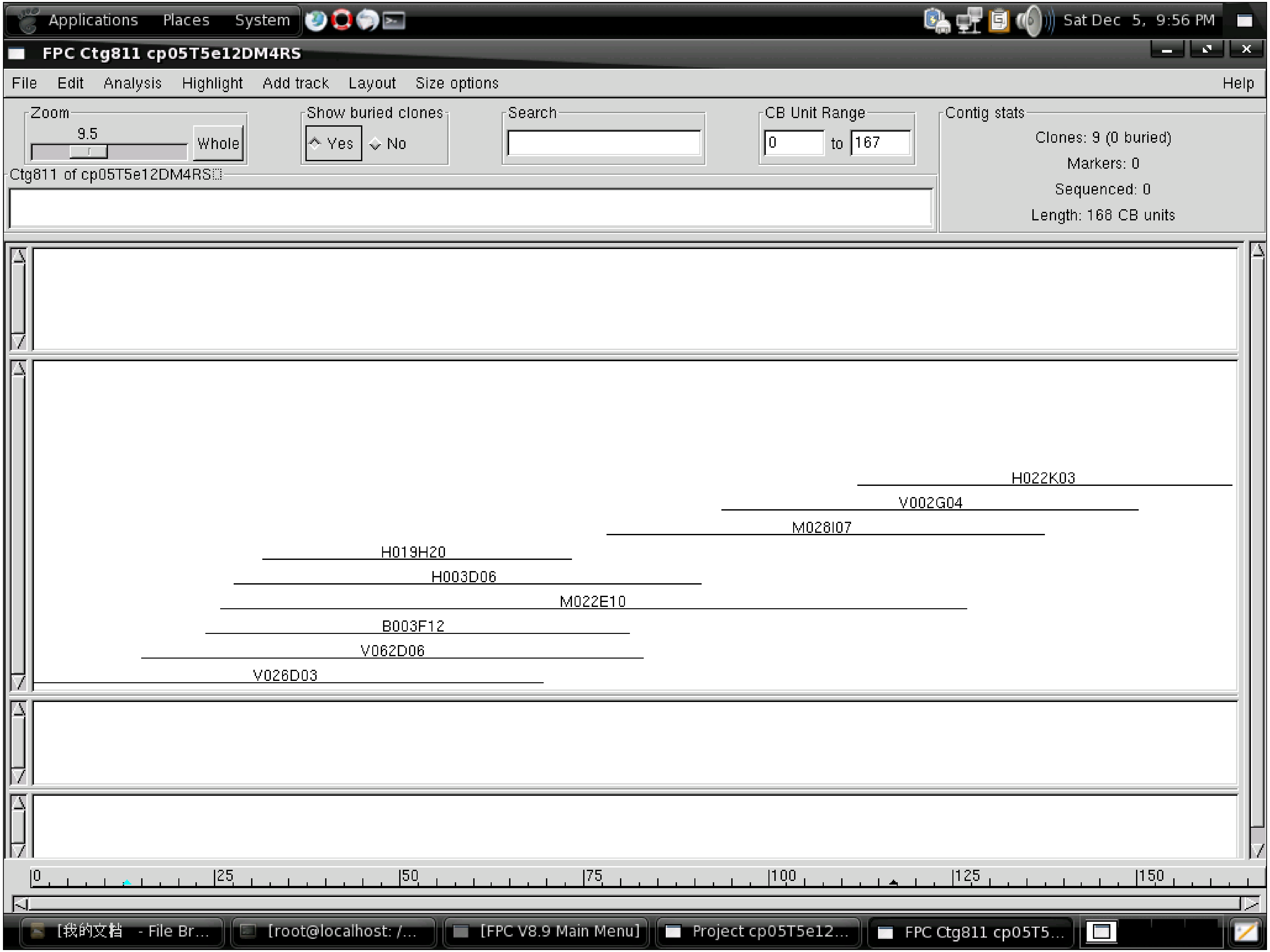
Applications Places System
Sat Dec 5, 9:56 PM
FPC Ctg811 cp05T5e12DM4RS
File Edit Analysis Highlight Add track Layout Size options Help
Zoom
9.5
Whole
Show buried clones
Yes No
Search
CB Unit Range
0 to 167
Contig stats
Clones: 9 (0 buried)
Markers: 0
Sequenced: 0
Length: 168 CB units
Ctg811 of cp05T5e12DM4RS
H022K03
V002G04
M028I07
H019H20
H003D06
M022E10
B003F12
V062D06
V026D03
0 25 50 75 100 125 150
[我的文档 - File Br...
[root@localhost: /...
[FPC V8.9 Main Menu]
Project cp05T5e12...
FPC Ctg811 cp05T5...

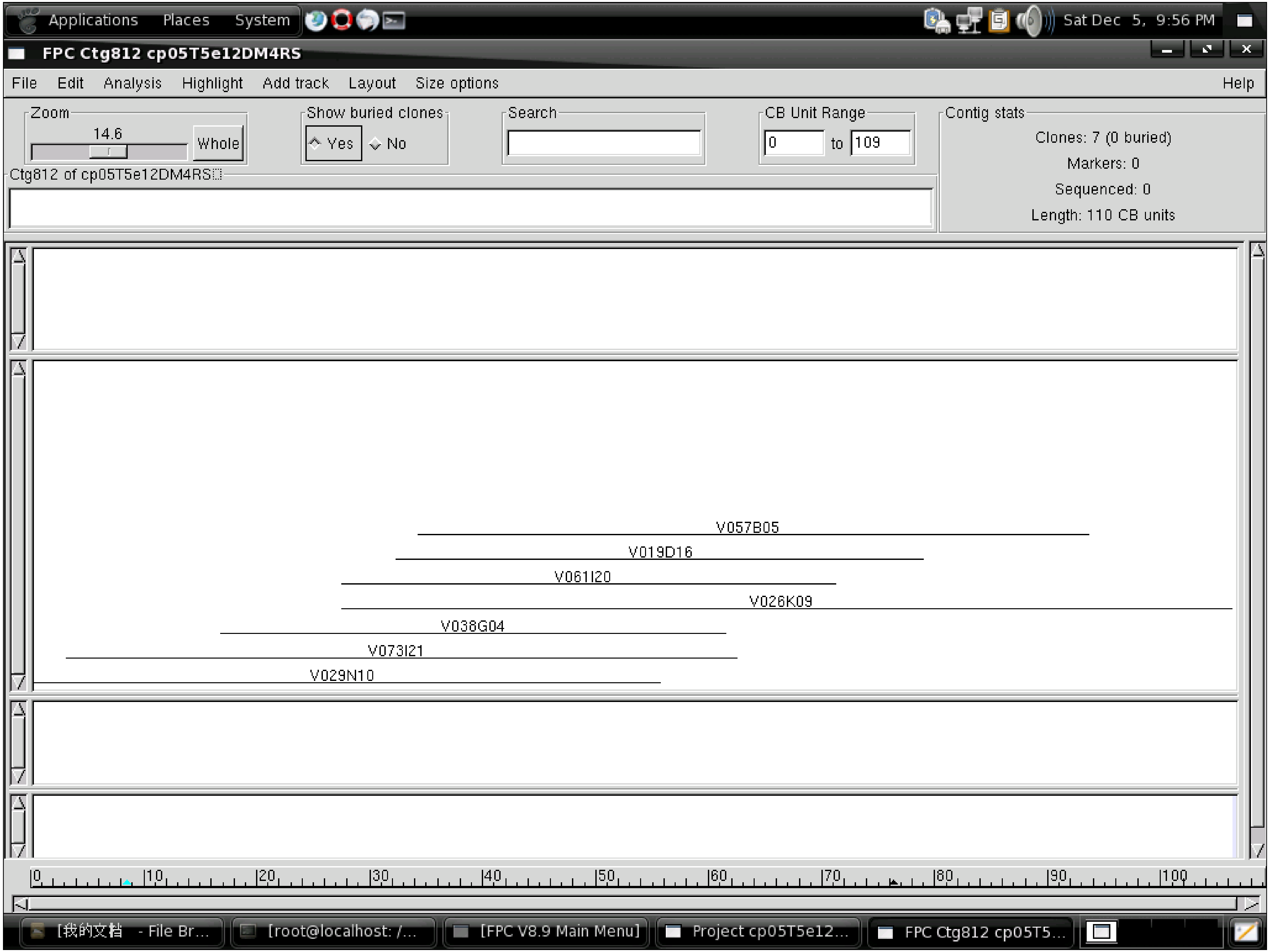
Applications Places System
Sat Dec 5, 9:56 PM
FPC Ctg812 cp05T5e12DM4RS
File Edit Analysis Highlight Add track Layout Size options Help
Zoom
14.6
Whole
Show buried clones
Yes
No
Search
CB Unit Range
0
to
109
Contig stats
Clones: 7 (0 buried)
Markers: 0
Sequenced: 0
Length: 110 CB units
Ctg812 of cp05T5e12DM4RS
V057B05
V019D16
V061I20
V026K09
V038G04
V073I21
V029N10
0 10 20 30 40 50 60 70 80 90 100
[我的文档 - File Br...
[root@localhost: /...
[FPC V8.9 Main Menu]
Project cp05T5e12...
FPC Ctg812 cp05T5...

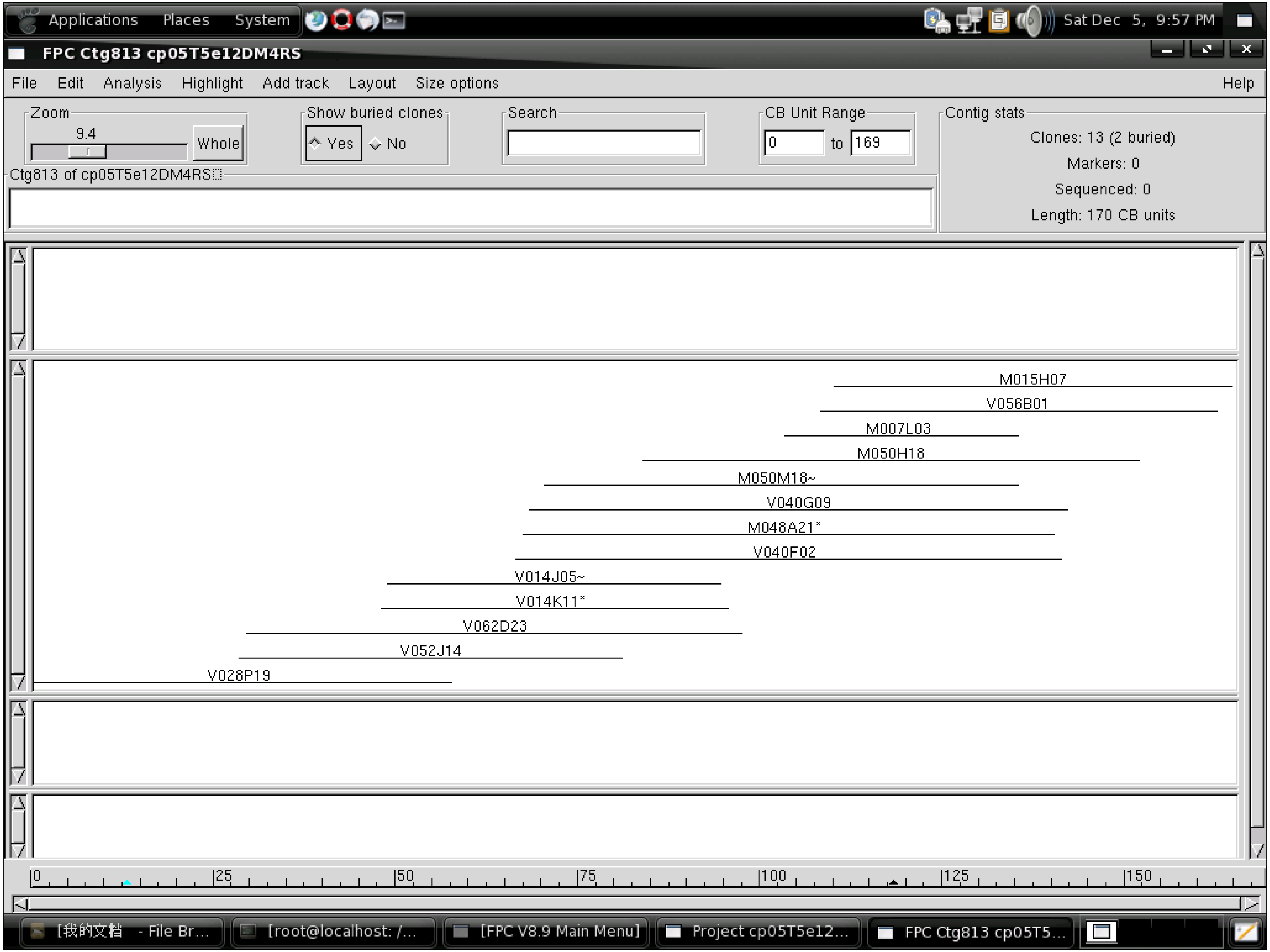
Applications Places System
Sat Dec 5, 9:57 PM
FPC Ctg813 cp05T5e12DM4RS
File Edit Analysis Highlight Add track Layout Size options Help
Zoom
9.4
Whole
Show buried clones
Yes
No
Search
CB Unit Range
0
to
169
Contig stats
Clones: 13 (2 buried)
Markers: 0
Sequenced: 0
Length: 170 CB units
Ctg813 of cp05T5e12DM4RS
M015H07
V056B01
M007L03
M050H18
M050M18~
V040G09
M048A21*
V040F02
V014J05~
V014K11*
V062D23
V052J14
V028P19
0
25
50
75
100
125
150
[root@localhost: /...
[FPC V8.9 Main Menu]
Project cp05T5e12...
FPC Ctg813 cp05T5...

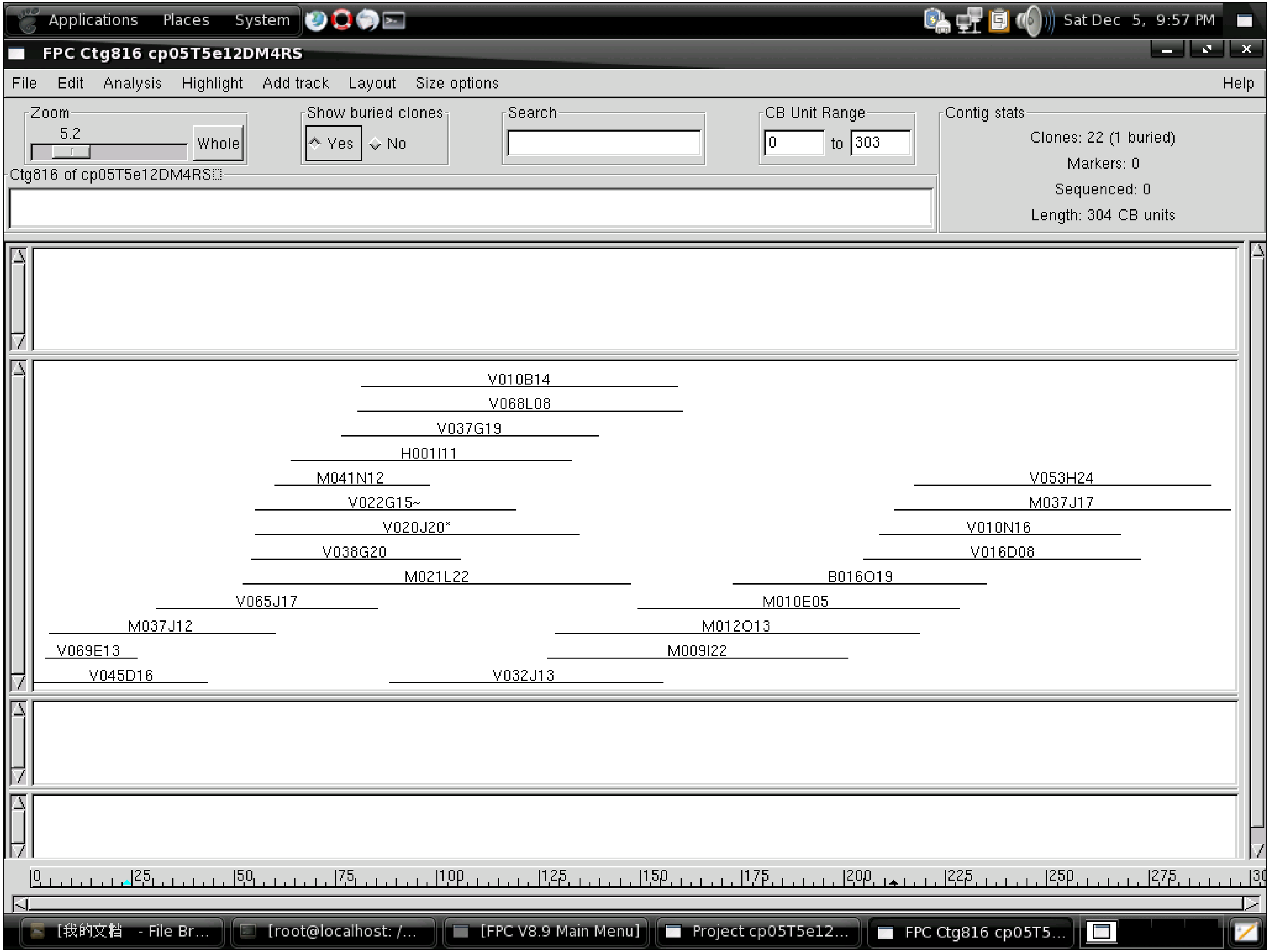
Applications Places System
Sat Dec 5, 9:57 PM
FPC Ctg816 cp05T5e12DM4RS
File Edit Analysis Highlight Add track Layout Size options Help
Zoom
5.2
Whole
Show buried clones
Yes
No
Search
CB Unit Range
0
to
303
Contig stats
Clones: 22 (1 buried)
Markers: 0
Sequenced: 0
Length: 304 CB units
Ctg816 of cp05T5e12DM4RS
V010B14
V068L08
V037G19
H001I11
M041N12
V053H24
V022G15~
M037J17
V020J20*
V010N16
V038G20
V016D08
M021L22
B016O19
V065J17
M010E05
M037J12
M012O13
V069E13
M009I22
V045D16
V032J13
0 25 50 75 100 125 150 175 200 225 250 275
[我的文档 - File Br...
[root@localhost: /...
[FPC V8.9 Main Menu]
Project cp05T5e12...
FPC Ctg816 cp05T5...

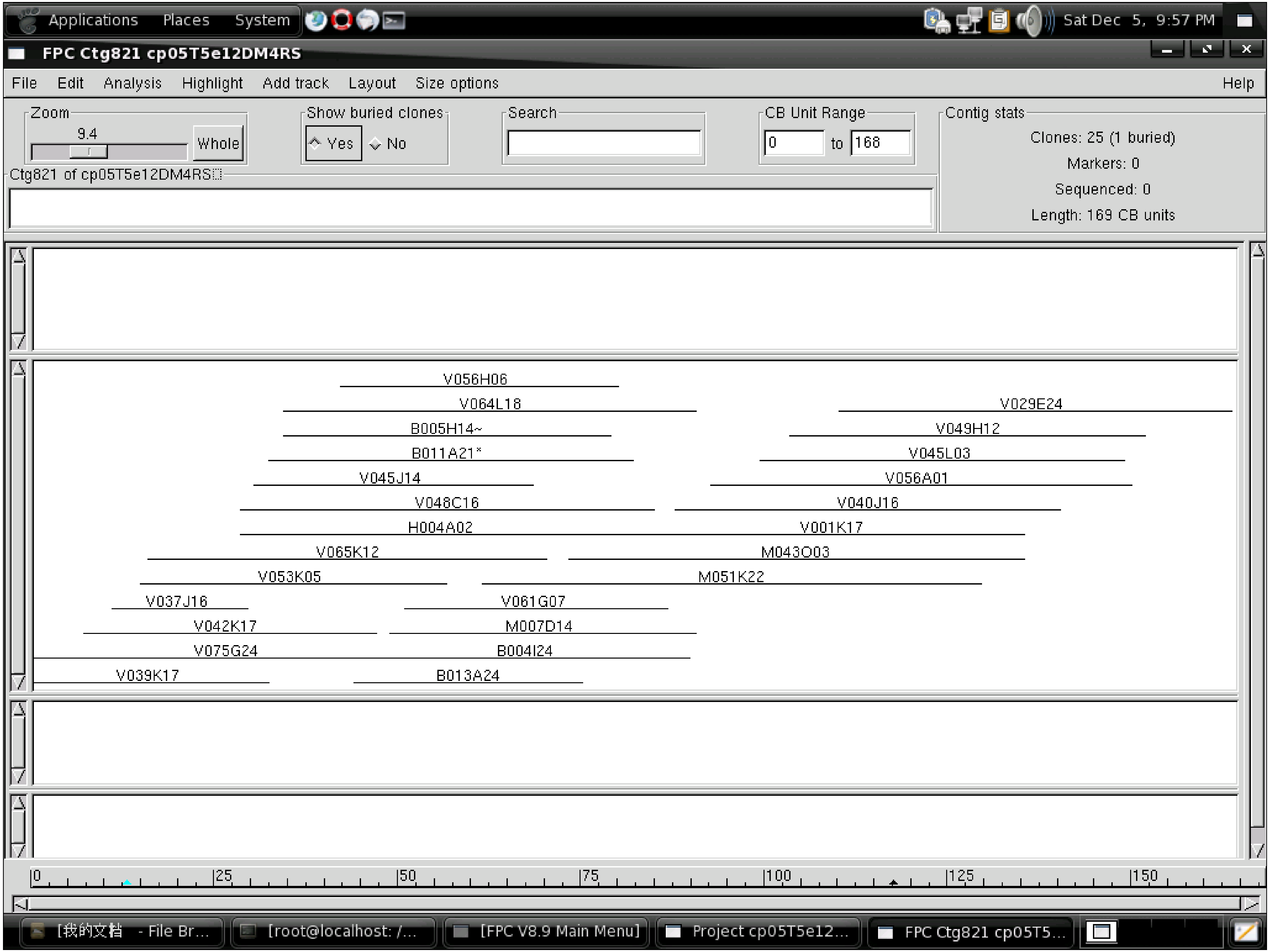
Applications Places System
Sat Dec 5, 9:57 PM
FPC Ctg821 cp05T5e12DM4RS
File Edit Analysis Highlight Add track Layout Size options
Help
Zoom
9.4
Whole
Show buried clones
Yes No
Search
CB Unit Range
0 to 168
Contig stats
Clones: 25 (1 buried)
Markers: 0
Sequenced: 0
Length: 169 CB units
Ctg821 of cp05T5e12DM4RS
V056H06
V064L18
V029E24
B005H14~
V049H12
B011A21*
V045L03
V045J14
V056A01
V048C16
V040J16
H004A02
V001K17
V065K12
M043O03
V053K05
M051K22
V037J16
V061G07
V042K17
M007D14
V075G24
B004I24
V039K17
B013A24
0
25
50
75
100
125
150
[我的文档 - File Br...
[root@localhost: /...
[FPC V8.9 Main Menu]
Project cp05T5e12...
FPC Ctg821 cp05T5...

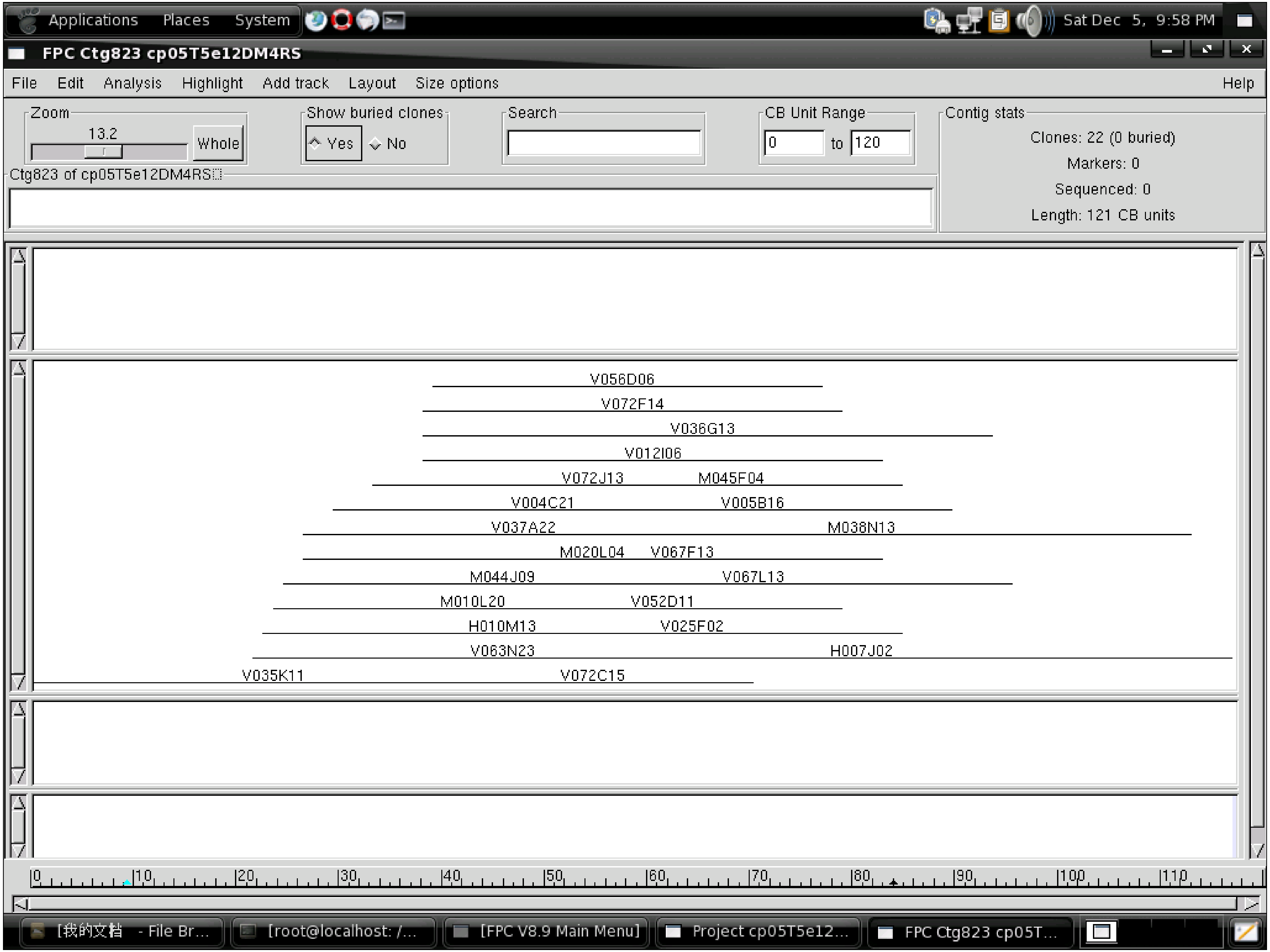
Applications Places System
Sat Dec 5, 9:58 PM
FPC Ctg823 cp05T5e12DM4RS
File Edit Analysis Highlight Add track Layout Size options Help
Zoom
13.2
Whole
Show buried clones
Yes No
Search
CB Unit Range
0 to 120
Contig stats
Clones: 22 (0 buried)
Markers: 0
Sequenced: 0
Length: 121 CB units
Ctg823 of cp05T5e12DM4RS
V056D06
V072F14
V036G13
V012I06
V072J13 M045F04
V004C21 V005B16
V037A22 M038N13
M020L04 V067F13
M044J09 V067L13
M010L20 V052D11
H010M13 V025F02
V063N23 H007J02
V035K11 V072C15
0 10 20 30 40 50 60 70 80 90 100 110
[我的文档 - File Br...
[root@localhost: /...
[FPC V8.9 Main Menu]
Project cp05T5e12...
FPC Ctg823 cp05T...

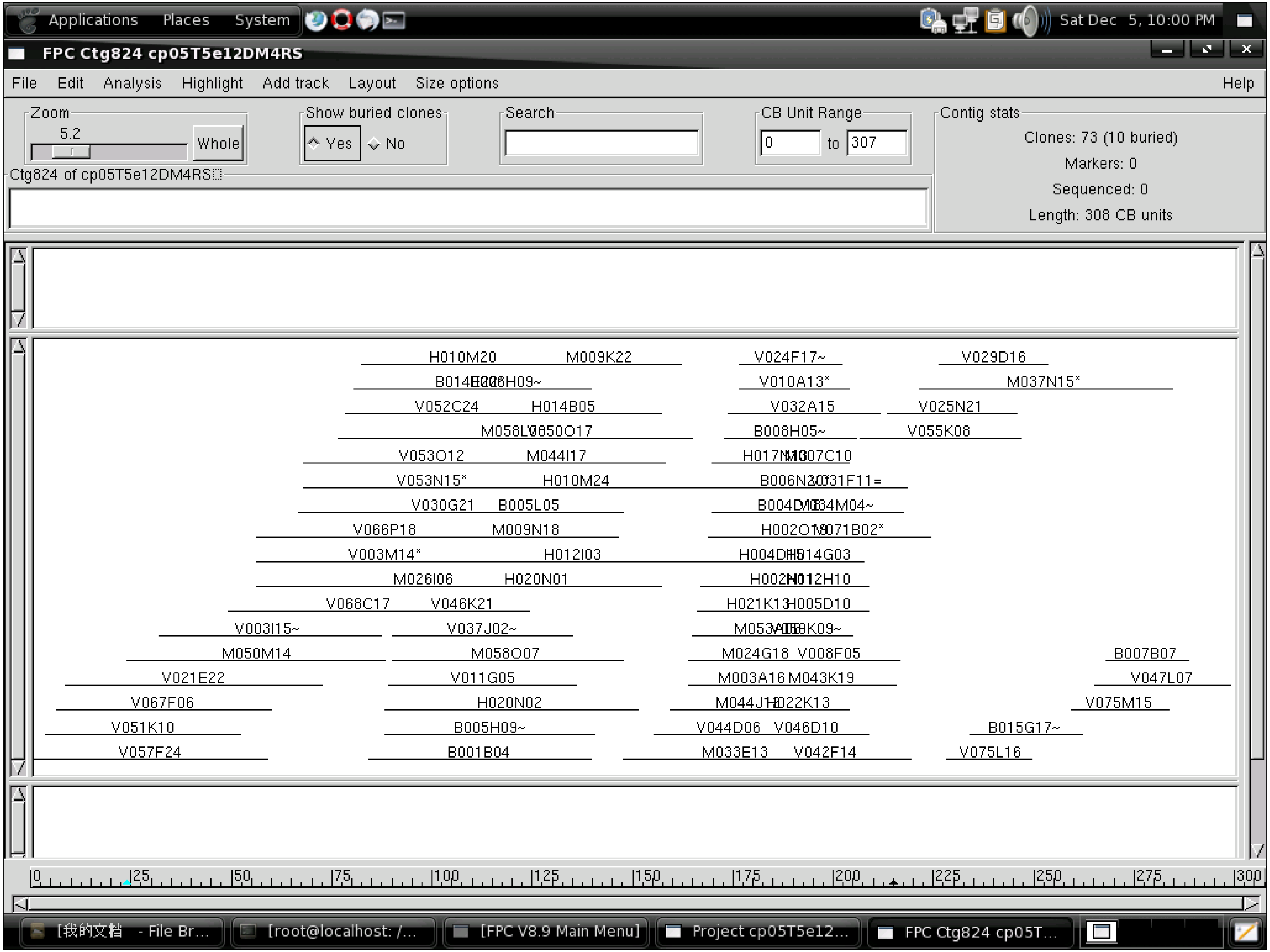
Applications Places System
Sat Dec 5, 10:00 PM
FPC Ctg824 cp05T5e12DM4RS
File Edit Analysis Highlight Add track Layout Size options Help
Zoom
5.2
Whole
Show buried clones
Yes
No
Search
CB Unit Range
0
to
307
Contig stats
Clones: 73 (10 buried)
Markers: 0
Sequenced: 0
Length: 308 CB units
Ctg824 of cp05T5e12DM4RS
H010M20
M009K22
V024F17~
V029D16
V010A13*
M037N15*
V052C24
H014B05
V032A15
V025N21
B008H05~
V055K08
V053O12
M044I17
V053N15*
H010M24
V030G21
B005L05
V066P18
M009N18
V003M14*
H012I03
M026I06
H020N01
V068C17
V046K21
H021K13
H005D10
V003I15~
V037J02~
M050M14
M058O07
M024G18
V008F05
B007B07
V021E22
V011G05
M003A16
M043K19
V047L07
V067F06
H020N02
V075M15
V051K10
B005H09~
V044D06
V046D10
B015G17~
V057F24
B001B04
M033E13
V042F14
V075L16
0 25 50 75 100 125 150 175 200 225 250 275 300
[我的文档 - File Br...
[root@localhost: /...
[FPC V8.9 Main Menu]
Project cp05T5e12...
FPC Ctg824 cp05T...

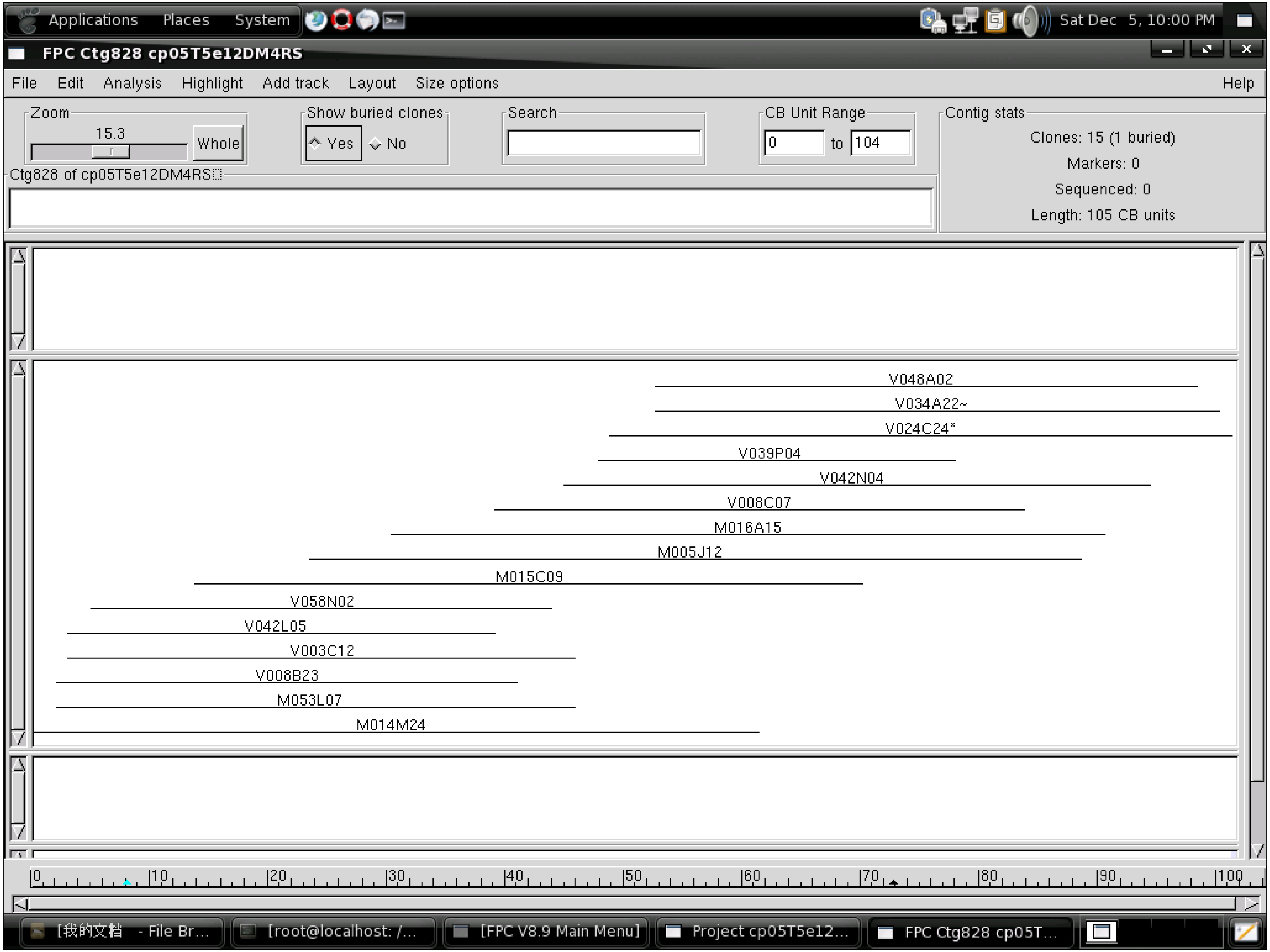
Applications Places System
Sat Dec 5, 10:00 PM
FPC Ctg828 cp05T5e12DM4RS
File Edit Analysis Highlight Add track Layout Size options Help
Zoom
15.3
Whole
Show buried clones
Yes No
Search
CB Unit Range
0 to 104
Contig stats
Clones: 15 (1 buried)
Markers: 0
Sequenced: 0
Length: 105 CB units
Ctg828 of cp05T5e12DM4RS
V048A02
V034A22~
V024C24*
V039P04
V042N04
V008C07
M016A15
M005J12
M015C09
V058N02
V042L05
V003C12
V008B23
M053L07
M014M24
0 10 20 30 40 50 60 70 80 90 100
[我的文档 - File Br...
[root@localhost: /...
[FPC V8.9 Main Menu]
Project cp05T5e12...
FPC Ctg828 cp05T...

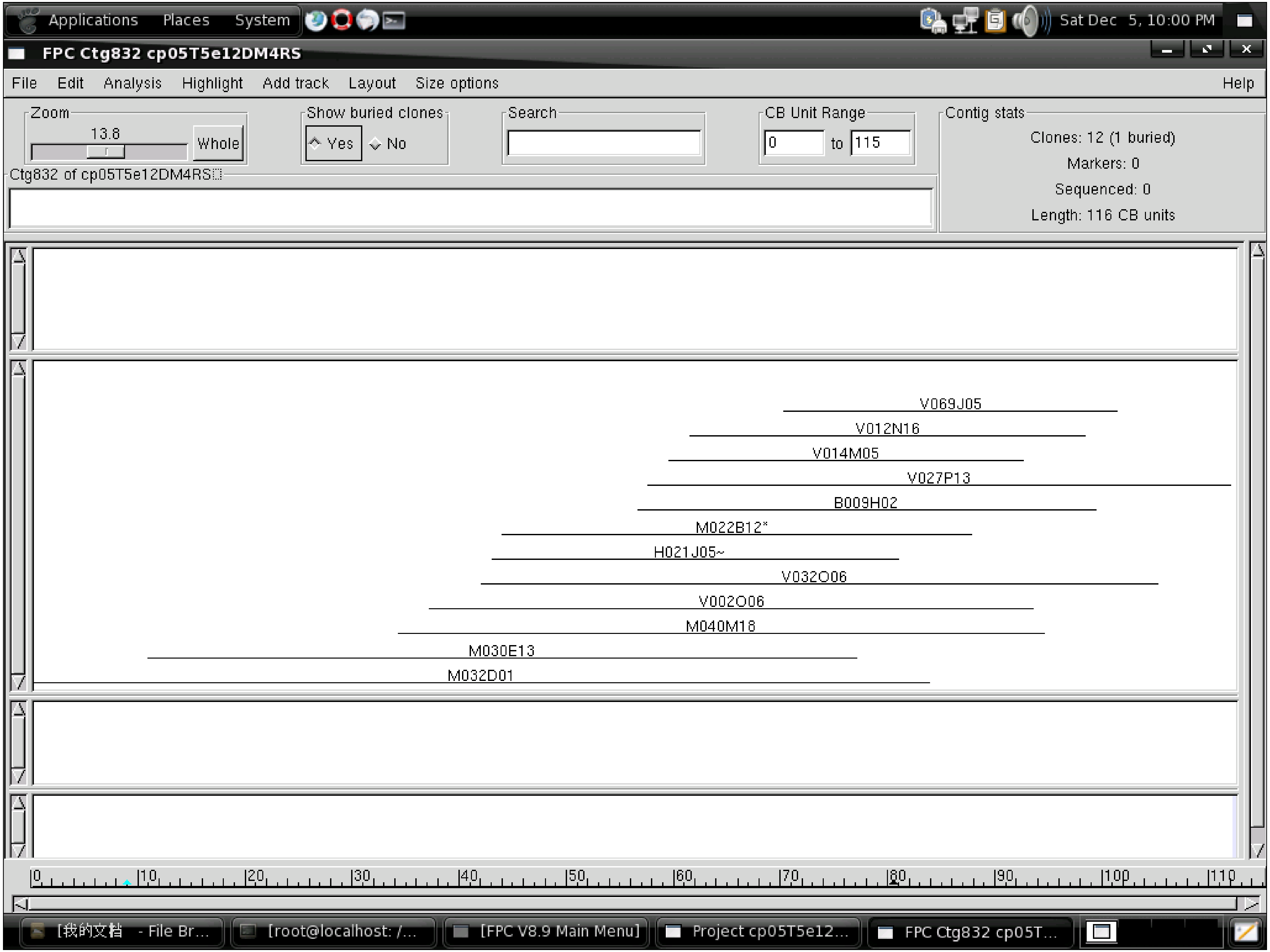
Applications Places System
Sat Dec 5, 10:00 PM
FPC Ctg832 cp05T5e12DM4RS
File Edit Analysis Highlight Add track Layout Size options Help
Zoom
13.8
Whole
Show buried clones
Yes
No
Search
CB Unit Range
0 to 115
Contig stats
Clones: 12 (1 buried)
Markers: 0
Sequenced: 0
Length: 116 CB units
Ctg832 of cp05T5e12DM4RS
V069J05
V012N16
V014M05
V027P13
B009H02
M022B12*
H021J05~
V032O06
V002O06
M040M18
M030E13
M032D01
0 10 20 30 40 50 60 70 80 90 100 110
[我的文档 - File Br...
[root@localhost: /...
[FPC V8.9 Main Menu]
Project cp05T5e12...
FPC Ctg832 cp05T...

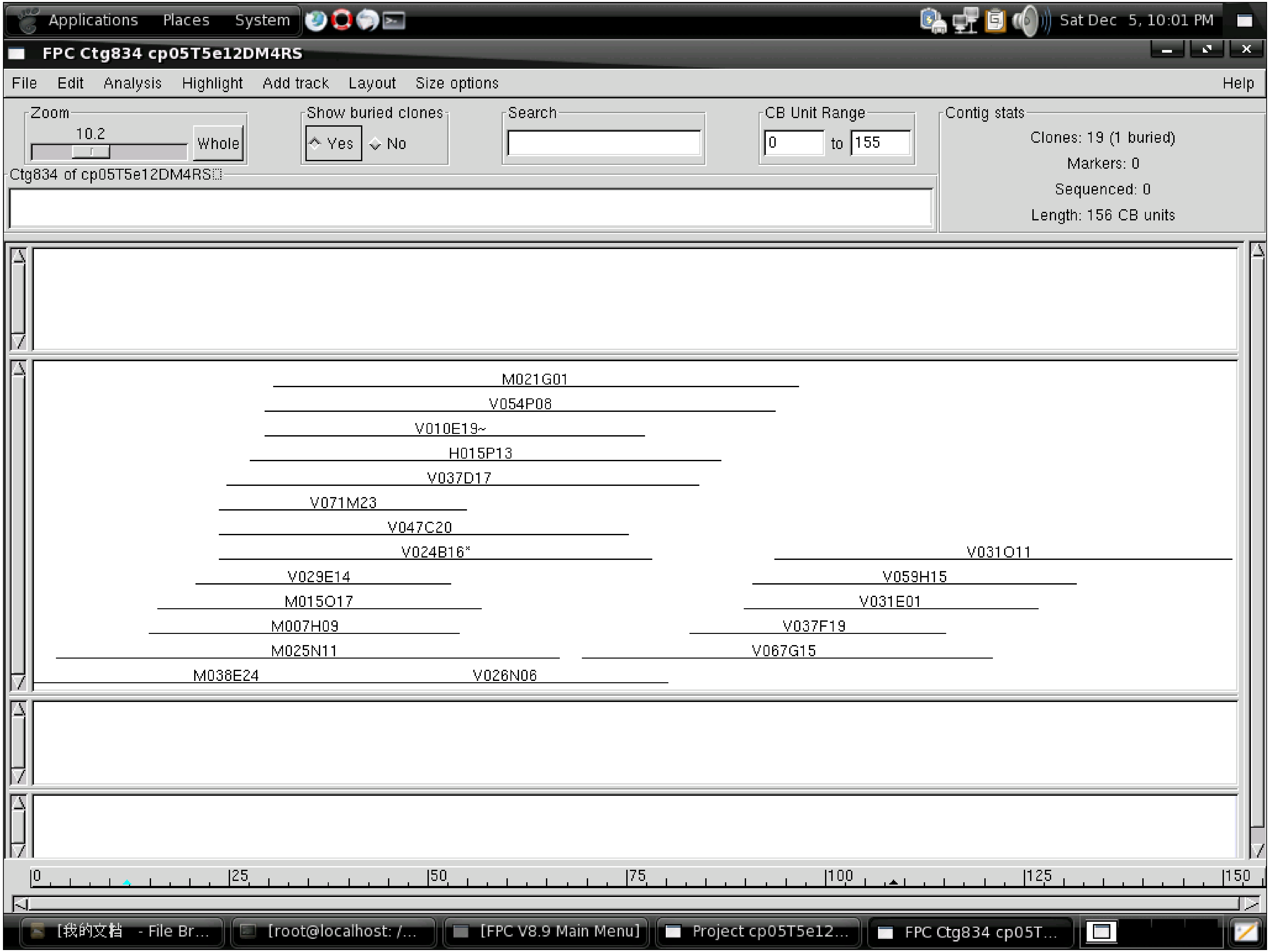

Applications
Places
System
Sat Dec 5, 10:01 PM
FPC Ctg834 cp05T5e12DM4RS
File
Edit
Analysis
Highlight
Add track
Layout
Size options
Help
Zoom
10.2
Whole
Show buried clones
Yes
No
Search
CB Unit Range
0
to
155
Contig stats
Clones: 19 (1 buried)
Markers: 0
Sequenced: 0
Length: 156 CB units
Ctg834 of cp05T5e12DM4RS
M021G01
V054P08
V010E19~
H015P13
V037D17
V071M23
V047C20
V024B16*
V031O11
V029E14
V059H15
M015O17
V031E01
M007H09
V037F19
M025N11
V067G15
M038E24
V026N06
0
25
50
75
100
125
150
[我的文档 - File Br...
[root@localhost: /...
[FPC V8.9 Main Menu]
Project cp05T5e12...
FPC Ctg834 cp05T...

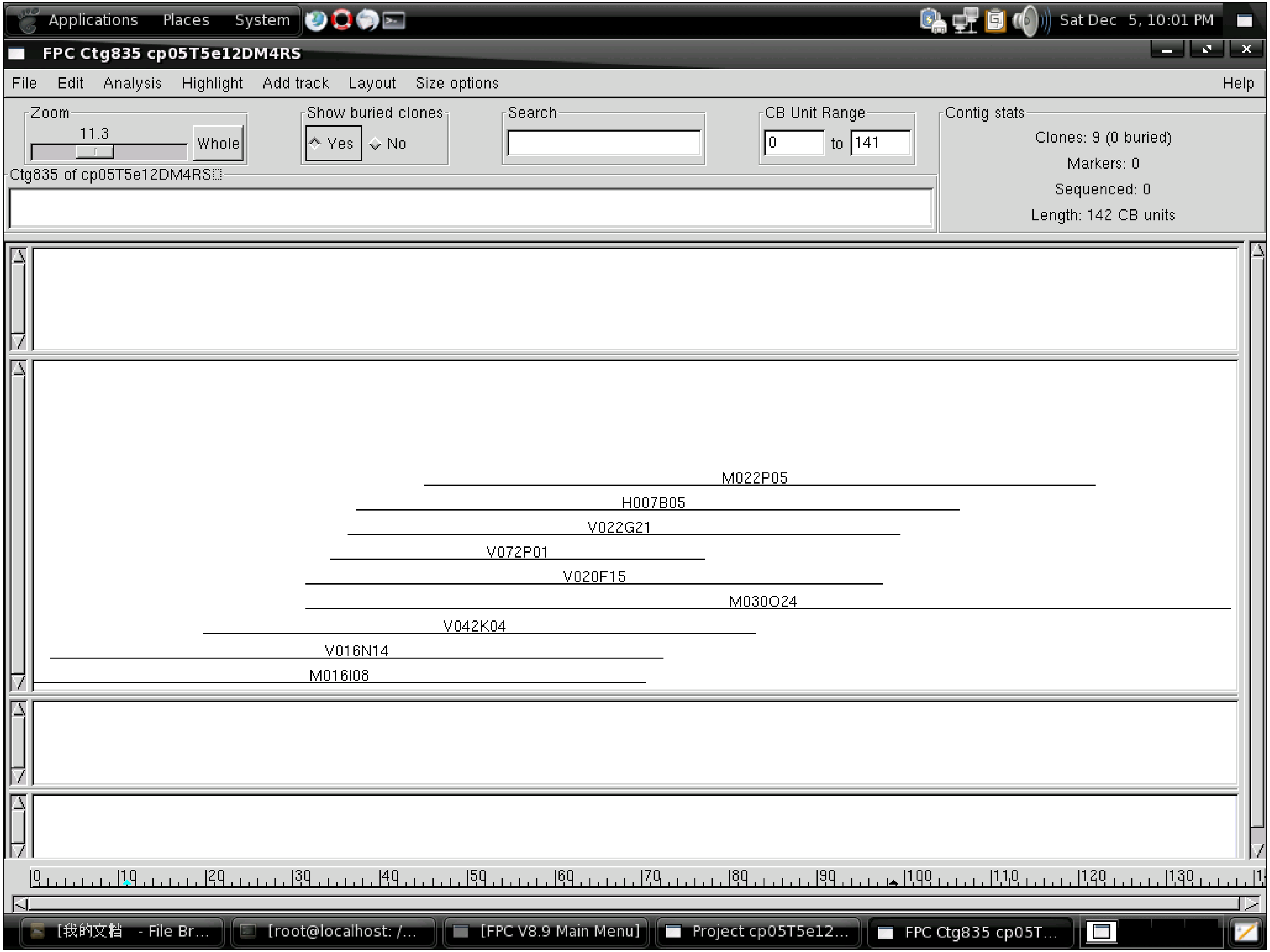
Applications
Places
System
Sat Dec 5, 10:01 PM
FPC Ctg835 cp05T5e12DM4RS
File
Edit
Analysis
Highlight
Add track
Layout
Size options
Help
Zoom
11.3
Whole
Show buried clones
Yes
No
Search
CB Unit Range
0
to
141
Contig stats
Clones: 9 (0 buried)
Markers: 0
Sequenced: 0
Length: 142 CB units
Ctg835 of cp05T5e12DM4RS
M022P05
H007B05
V022G21
V072P01
V020F15
M030O24
V042K04
V016N14
M016I08
0
10
20
30
40
50
60
70
80
90
100
110
120
130
[我的文档 - File Br...
[root@localhost: /...
[FPC V8.9 Main Menu]
Project cp05T5e12...
FPC Ctg835 cp05T...

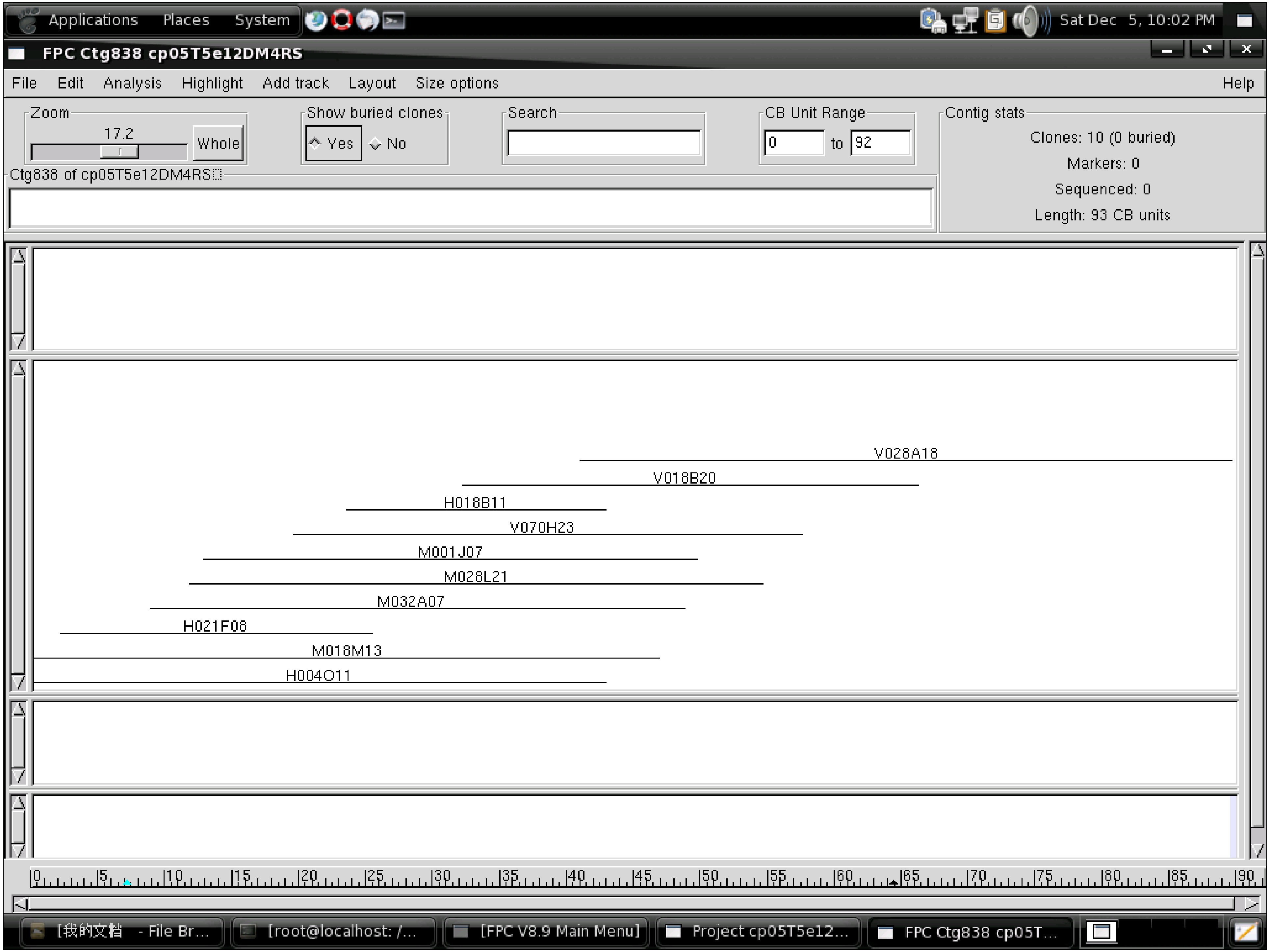
Applications Places System
Sat Dec 5, 10:02 PM
FPC Ctg838 cp05T5e12DM4RS
File Edit Analysis Highlight Add track Layout Size options Help
Zoom
17.2
Whole
Show buried clones
Yes
No
Search
CB Unit Range
0
to
92
Contig stats
Clones: 10 (0 buried)
Markers: 0
Sequenced: 0
Length: 93 CB units
Ctg838 of cp05T5e12DM4RS
V028A18
V018B20
H018B11
V070H23
M001J07
M028L21
M032A07
H021F08
M018M13
H004O11
0 5 10 15 20 25 30 35 40 45 50 55 60 65 70 75 80 85 90
[我的文档 - File Br...
[root@localhost: /...
[FPC V8.9 Main Menu]
Project cp05T5e12...
FPC Ctg838 cp05T...

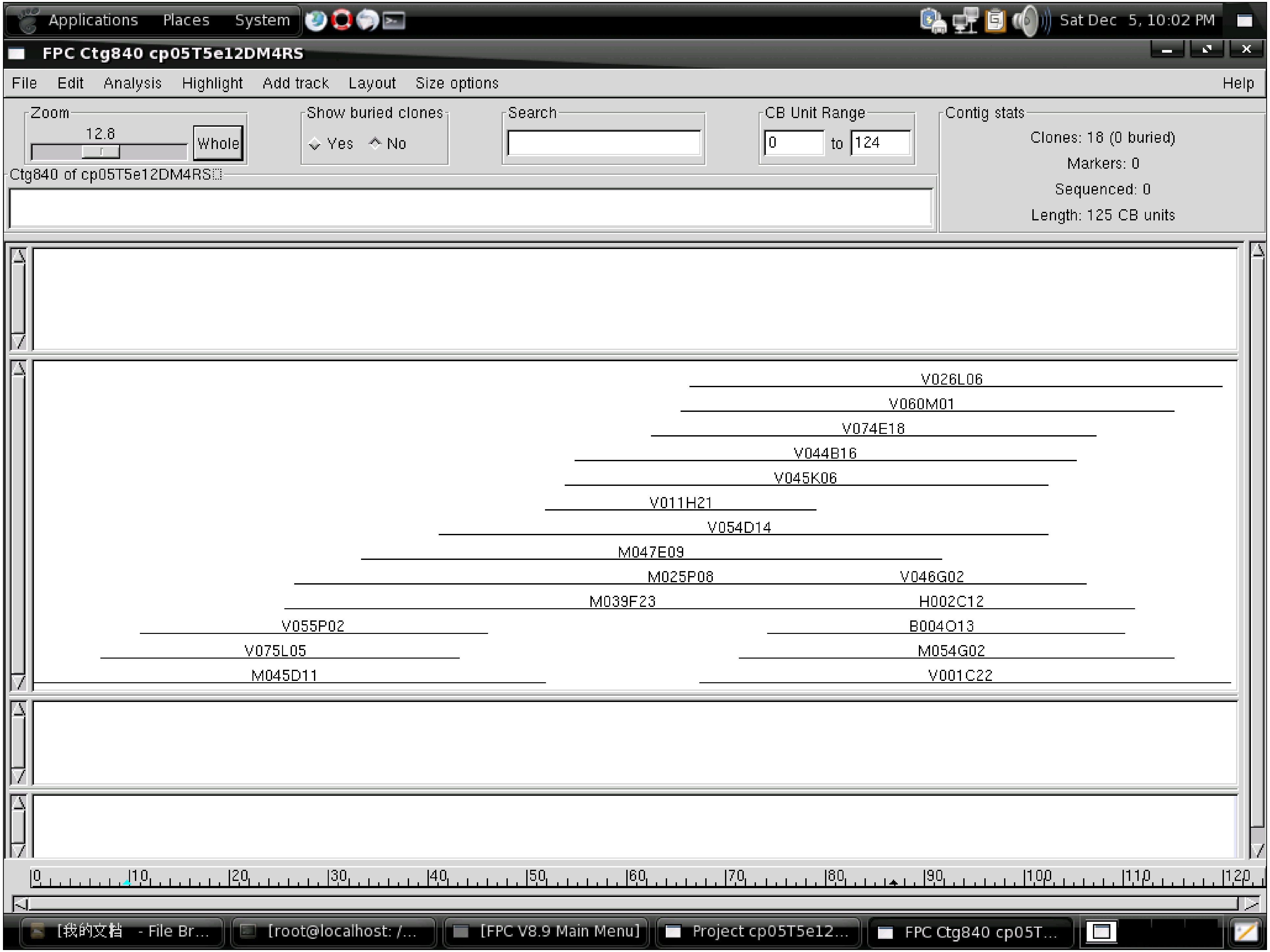
Applications Places System
Sat Dec 5, 10:02 PM
FPC Ctg840 cp05T5e12DM4RS
File Edit Analysis Highlight Add track Layout Size options Help
Zoom
12.8
Whole
Show buried clones
Yes No
Search
CB Unit Range
0 to 124
Contig stats
Clones: 18 (0 buried)
Markers: 0
Sequenced: 0
Length: 125 CB units
Ctg840 of cp05T5e12DM4RS
V026L06
V060M01
V074E18
V044B16
V045K06
V011H21
V054D14
M047E09
M025P08
V046G02
M039F23
H002C12
V055P02
B004O13
V075L05
M054G02
M045D11
V001C22
0 10 20 30 40 50 60 70 80 90 100 110 120
[我的文档 - File Br...
[root@localhost: /...
[FPC V8.9 Main Menu]
Project cp05T5e12...
FPC Ctg840 cp05T...

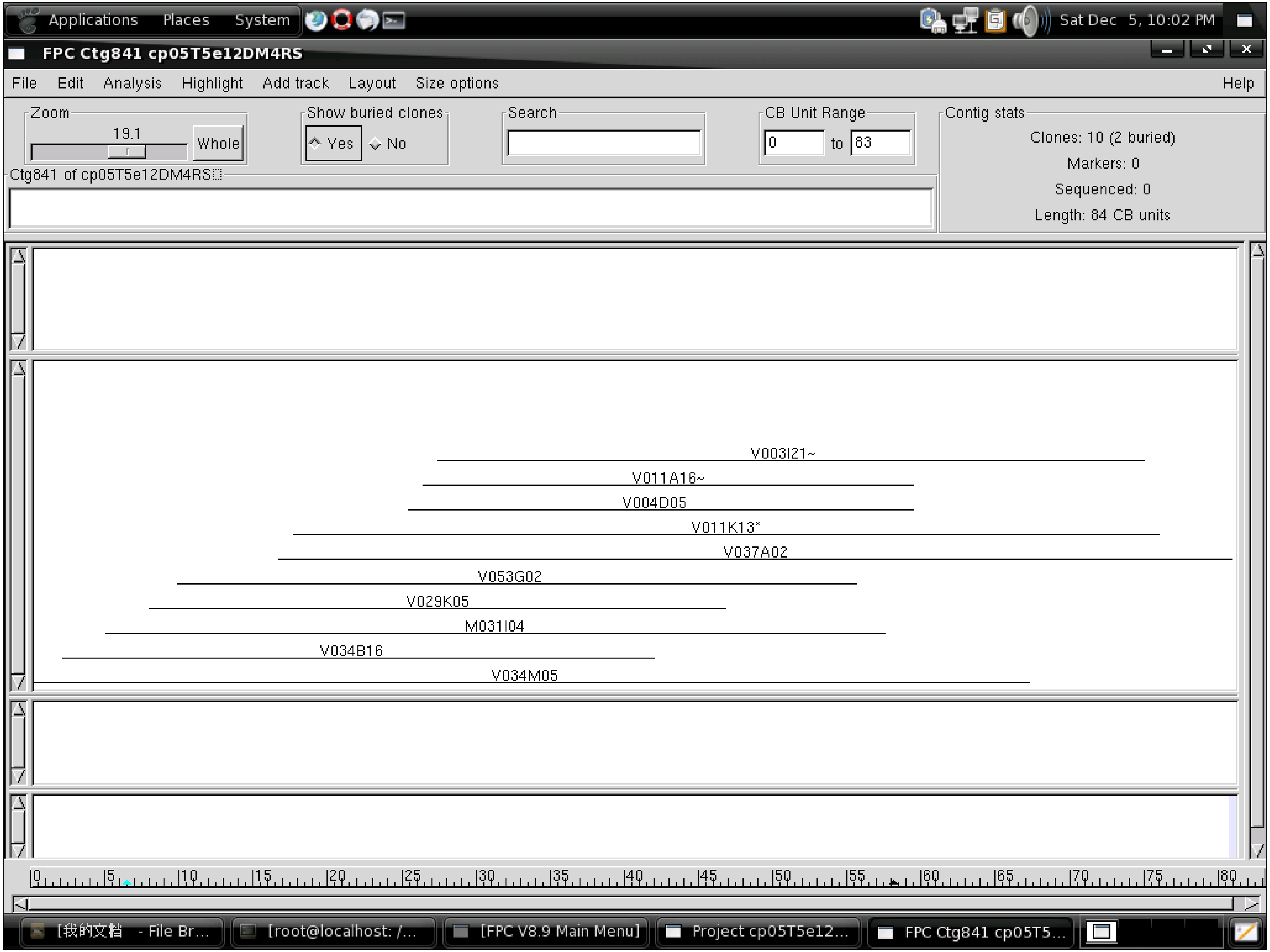
Applications Places System
Sat Dec 5, 10:02 PM
FPC Ctg841 cp05T5e12DM4RS
File Edit Analysis Highlight Add track Layout Size options Help
Zoom
19.1
Whole
Show buried clones
Yes
No
Search
CB Unit Range
0 to 83
Contig stats
Clones: 10 (2 buried)
Markers: 0
Sequenced: 0
Length: 84 CB units
Ctg841 of cp05T5e12DM4RS
V003I21~
V011A16~
V004D05
V011K13*
V037A02
V053G02
V029K05
M031I04
V034B16
V034M05
0 5 10 15 20 25 30 35 40 45 50 55 60 65 70 75 80
[我的文档 - File Br...
[root@localhost: /...
[FPC V8.9 Main Menu]
Project cp05T5e12...
FPC Ctg841 cp05T5...

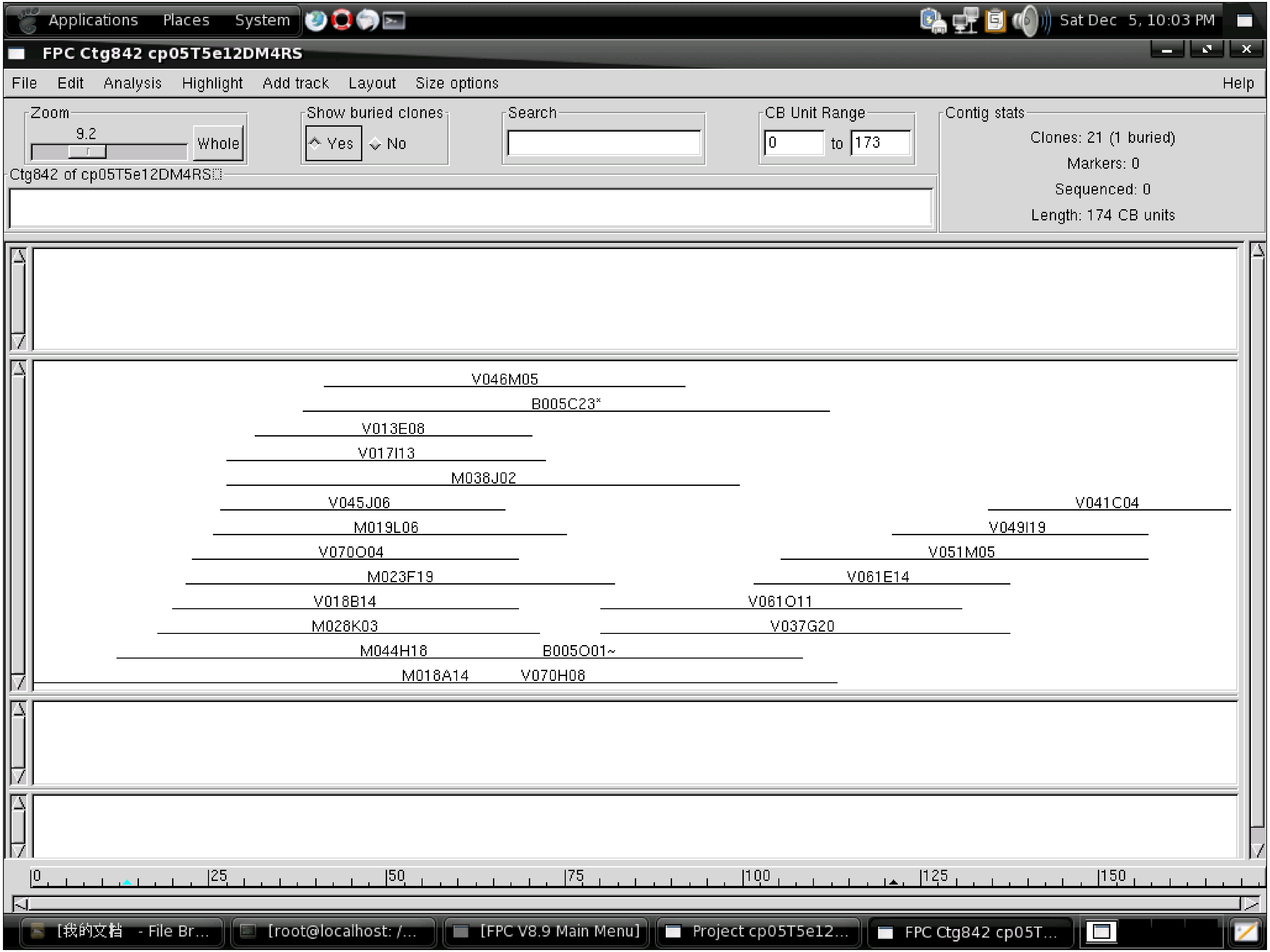
Applications Places System
Sat Dec 5, 10:03 PM
FPC Ctg842 cp05T5e12DM4RS
File Edit Analysis Highlight Add track Layout Size options Help
Zoom
9.2
Whole
Show buried clones
Yes
No
Search
CB Unit Range
0 to 173
Contig stats
Clones: 21 (1 buried)
Markers: 0
Sequenced: 0
Length: 174 CB units
Ctg842 of cp05T5e12DM4RS
V046M05
B005C23*
V013E08
V017I13
M038J02
V045J06
V041C04
M019L06
V049I19
V070O04
V051M05
M023F19
V061E14
V018B14
V061O11
M028K03
V037G20
M044H18
B005O01~
M018A14
V070H08
0 25 50 75 100 125 150
[我的文档 - File Br...
[root@localhost: /...
[FPC V8.9 Main Menu]
Project cp05T5e12...
FPC Ctg842 cp05T...

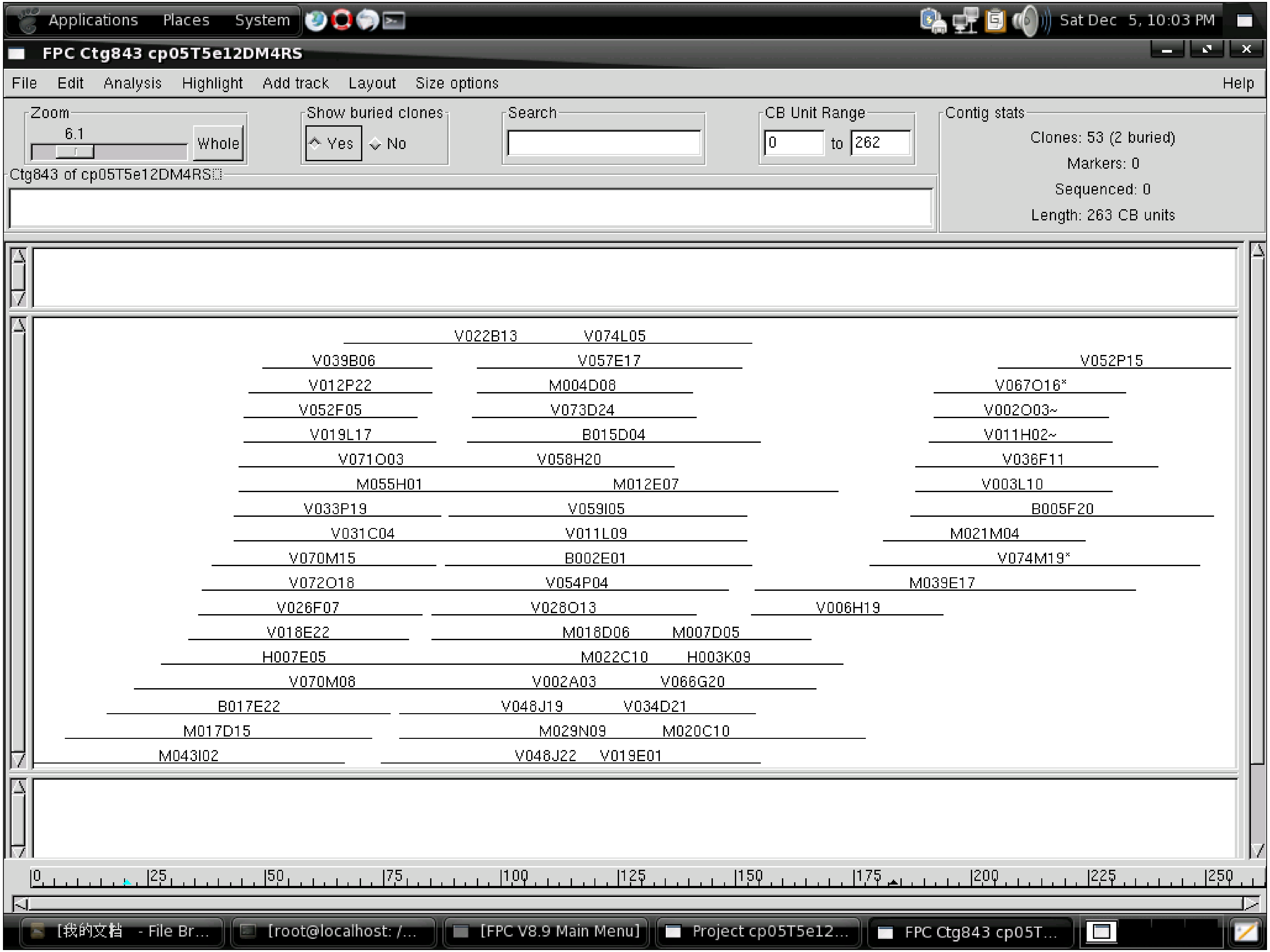
Applications Places System
Sat Dec 5, 10:03 PM
FPC Ctg843 cp05T5e12DM4RS
File Edit Analysis Highlight Add track Layout Size options Help
Zoom
6.1
Whole
Show buried clones
Yes
No
Search
CB Unit Range
0
to
262
Contig stats
Clones: 53 (2 buried)
Markers: 0
Sequenced: 0
Length: 263 CB units
Ctg843 of cp05T5e12DM4RS
V022B13
V074L05
V039B06
V057E17
V052P15
V012P22
M004D08
V067O16*
V052F05
V073D24
V002O03~
V019L17
B015D04
V011H02~
V071O03
V058H20
V036F11
M055H01
M012E07
V003L10
V033P19
V059I05
B005F20
V031C04
V011L09
M021M04
V070M15
B002E01
V074M19*
V072O18
V054P04
M039E17
V026F07
V028O13
V006H19
V018E22
M018D06
M007D05
H007E05
M022C10
H003K09
V070M08
V002A03
V066G20
B017E22
V048J19
V034D21
M017D15
M029N09
M020C10
M043I02
V048J22
V019E01
0
25
50
75
100
125
150
175
200
225
250
[我的文档 - File Br...
[root@localhost: /...
[FPC V8.9 Main Menu]
Project cp05T5e12...
FPC Ctg843 cp05T...

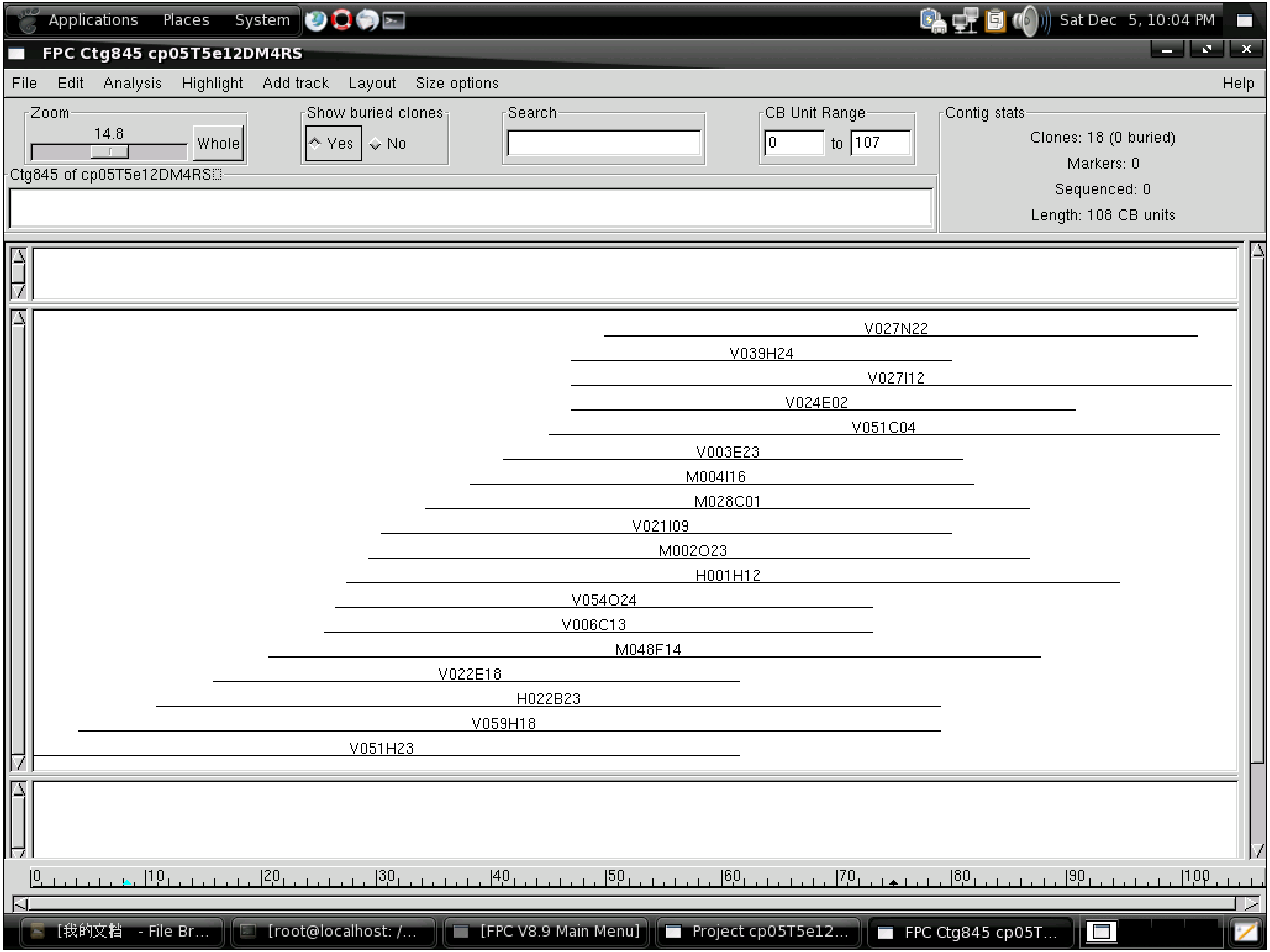
Applications Places System
Sat Dec 5, 10:04 PM
FPC Ctg845 cp05T5e12DM4RS
File Edit Analysis Highlight Add track Layout Size options Help
Zoom
14.8
Whole
Show buried clones
Yes
No
Search
CB Unit Range
0 to 107
Contig stats
Clones: 18 (0 buried)
Markers: 0
Sequenced: 0
Length: 108 CB units
Ctg845 of cp05T5e12DM4RS
V027N22
V039H24
V027I12
V024E02
V051C04
V003E23
M004I16
M028C01
V021I09
M002O23
H001H12
V054O24
V006C13
M048F14
V022E18
H022B23
V059H18
V051H23
0 10 20 30 40 50 60 70 80 90 100
[我的文档 - File Br...
[root@localhost: /...
[FPC V8.9 Main Menu]
Project cp05T5e12...
FPC Ctg845 cp05T...

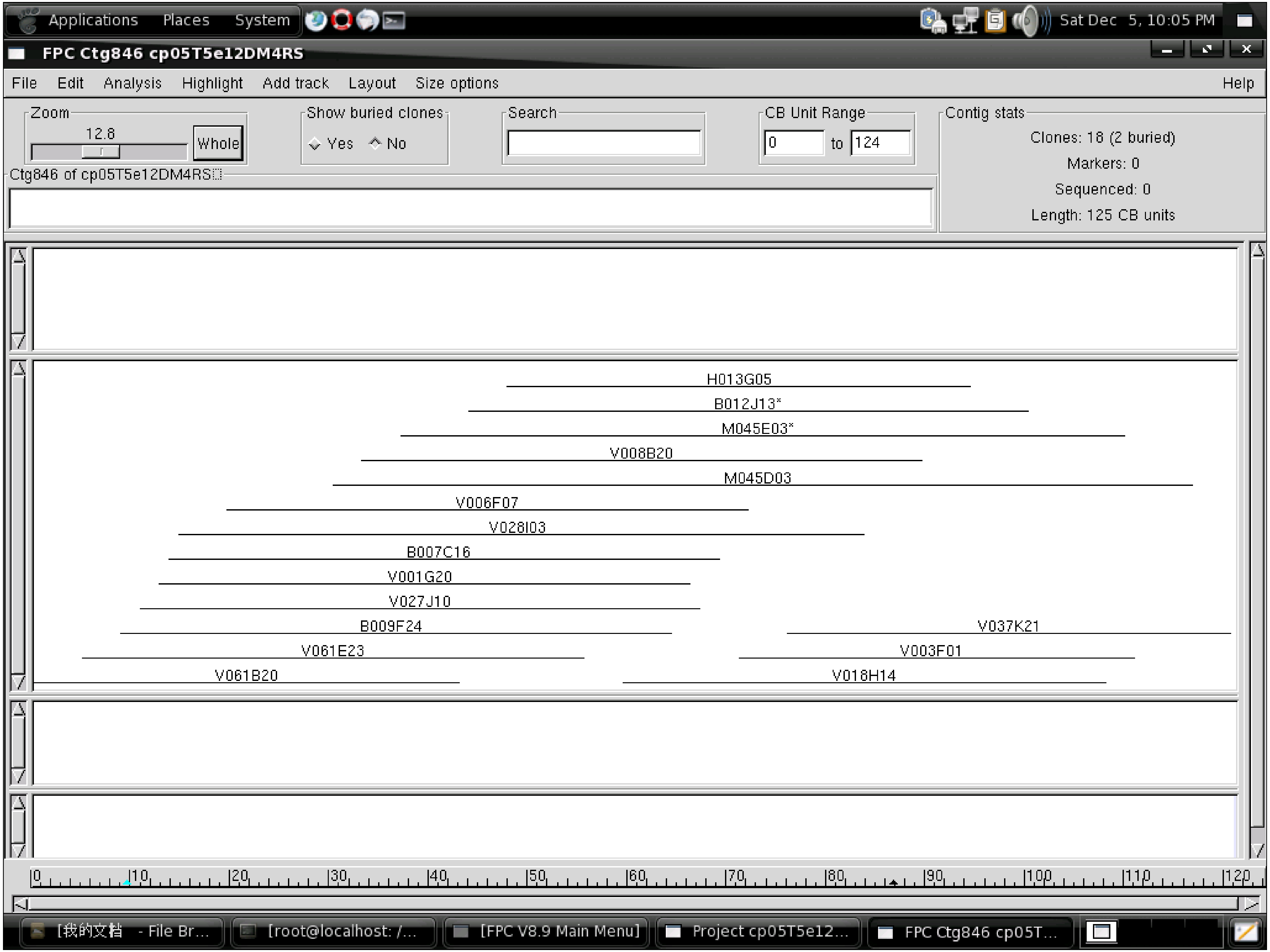

Applications
Places
System
Sat Dec 5, 10:05 PM
FPC Ctg846 cp05T5e12DM4RS
File
Edit
Analysis
Highlight
Add track
Layout
Size options
Help
Zoom
12.8
Whole
Show buried clones
Yes
No
Search
CB Unit Range
0
to
124
Contig stats
Clones: 18 (2 buried)
Markers: 0
Sequenced: 0
Length: 125 CB units
Ctg846 of cp05T5e12DM4RS
H013G05
B012J13*
M045E03*
V008B20
M045D03
V006F07
V028I03
B007C16
V001G20
V027J10
B009F24
V037K21
V061E23
V003F01
V061B20
V018H14
0
10
20
30
40
50
60
70
80
90
100
110
120
[我的文档 - File Br...
[root@localhost: /...
[FPC V8.9 Main Menu]
Project cp05T5e12...
FPC Ctg846 cp05T...

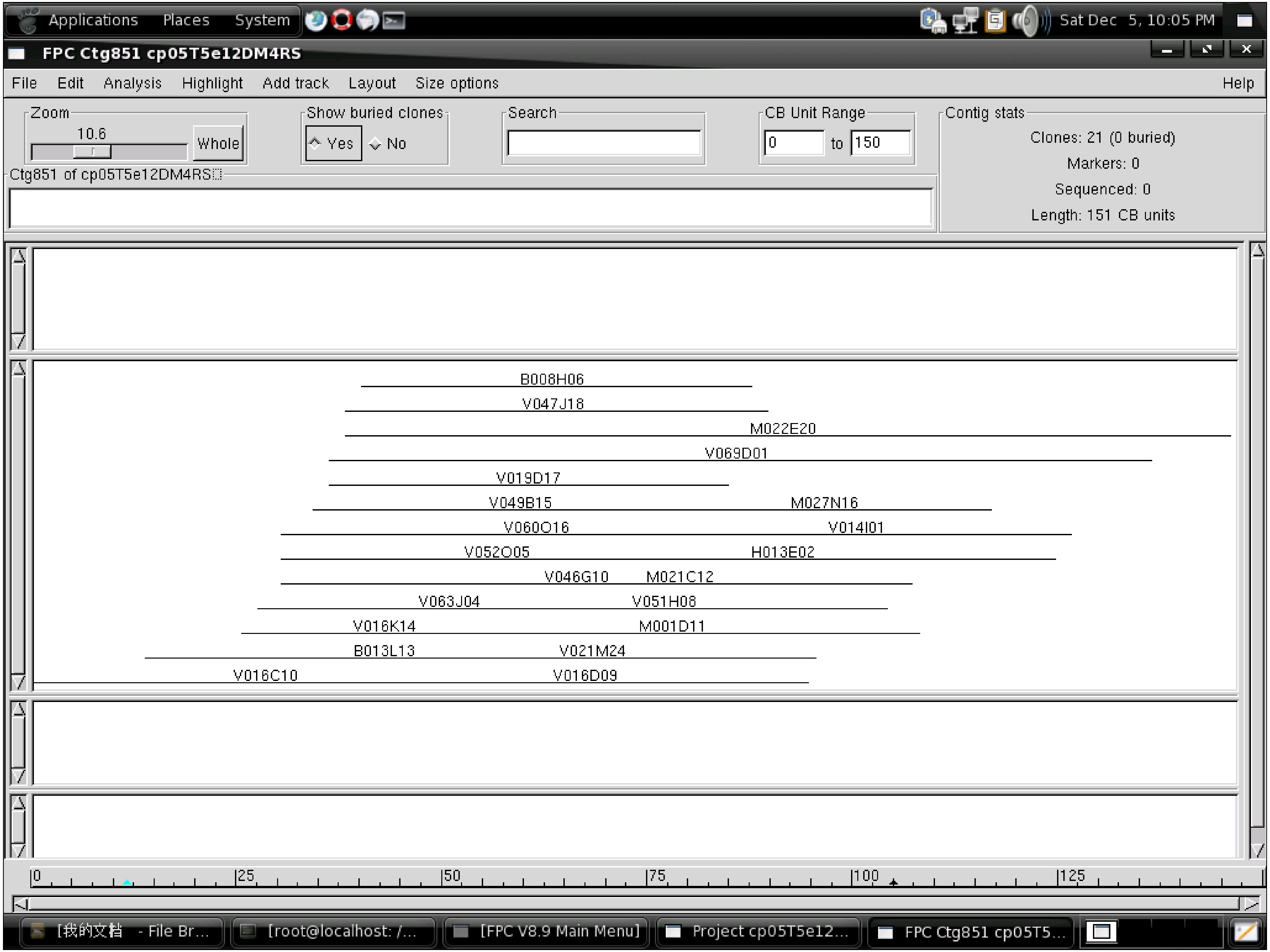
Applications Places System
Sat Dec 5, 10:05 PM
FPC Ctg851 cp05T5e12DM4RS
File Edit Analysis Highlight Add track Layout Size options Help
Zoom
10.6
Whole
Show buried clones
Yes No
Search
CB Unit Range
0 to 150
Contig stats
Clones: 21 (0 buried)
Markers: 0
Sequenced: 0
Length: 151 CB units
Ctg851 of cp05T5e12DM4RS
B008H06
V047J18
M022E20
V069D01
V019D17
V049B15
M027N16
V060O16
V014I01
V052O05
H013E02
V046G10
M021C12
V063J04
V051H08
V016K14
M001D11
B013L13
V021M24
V016C10
V016D09
0
25
50
75
100
125
[我的文档 - File Br...
[root@localhost: /...
[FPC V8.9 Main Menu]
Project cp05T5e12...
FPC Ctg851 cp05T5...

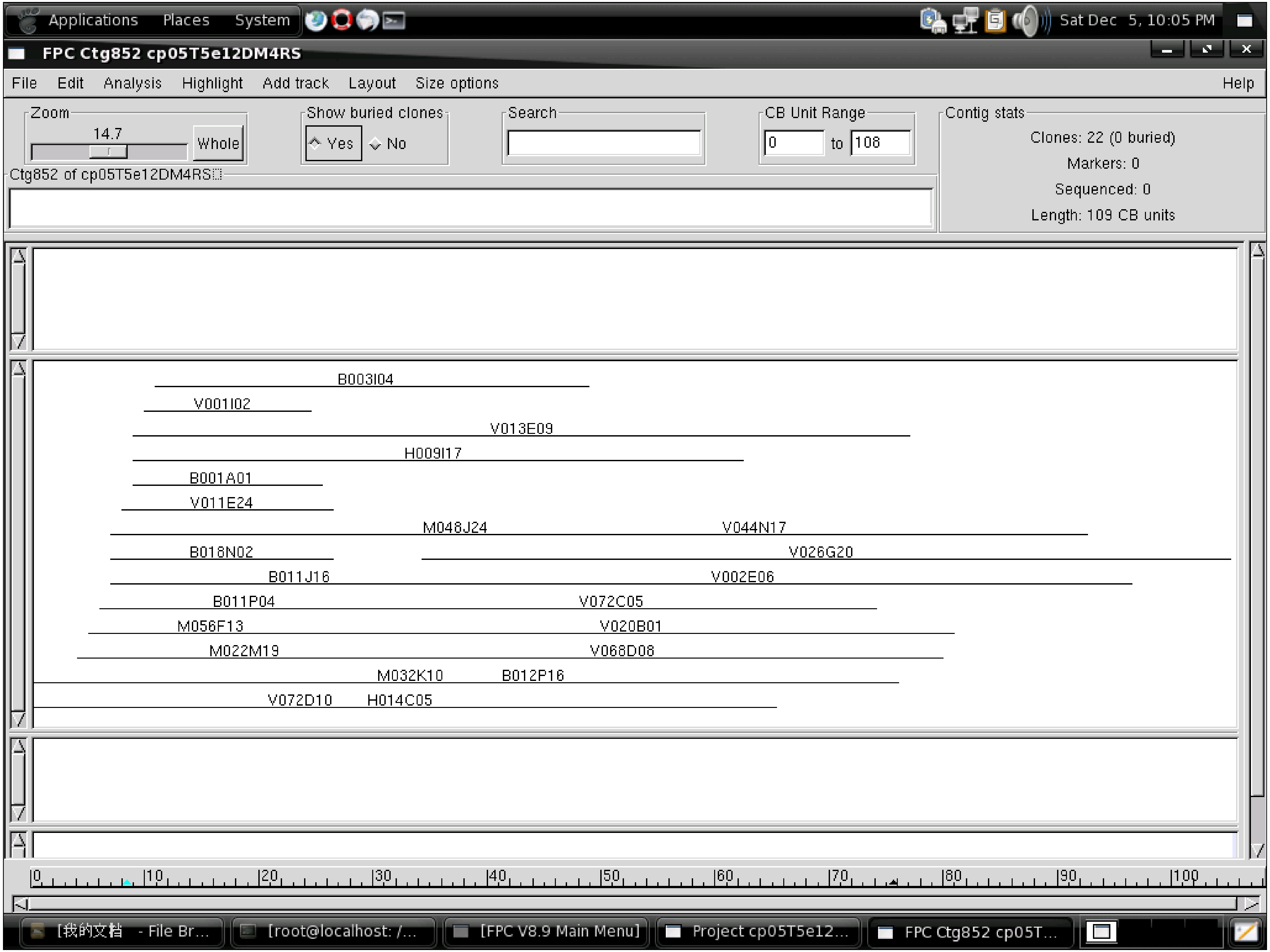

Applications
Places
System
Sat Dec 5, 10:05 PM
FPC Ctg852 cp05T5e12DM4RS
File
Edit
Analysis
Highlight
Add track
Layout
Size options
Help
Zoom
14.7
Whole
Show buried clones
Yes
No
Search
CB Unit Range
0
to
108
Contig stats
Clones: 22 (0 buried)
Markers: 0
Sequenced: 0
Length: 109 CB units
Ctg852 of cp05T5e12DM4RS
B003I04
V001I02
V013E09
H009I17
B001A01
V011E24
M048J24
V044N17
B018N02
V026G20
B011J16
V002E06
B011P04
V072C05
M056F13
V020B01
M022M19
V068D08
M032K10
B012P16
V072D10
H014C05
0
10
20
30
40
50
60
70
80
90
100
[我的文档 - File Br...
[root@localhost: /...
[FPC V8.9 Main Menu]
Project cp05T5e12...
FPC Ctg852 cp05T...

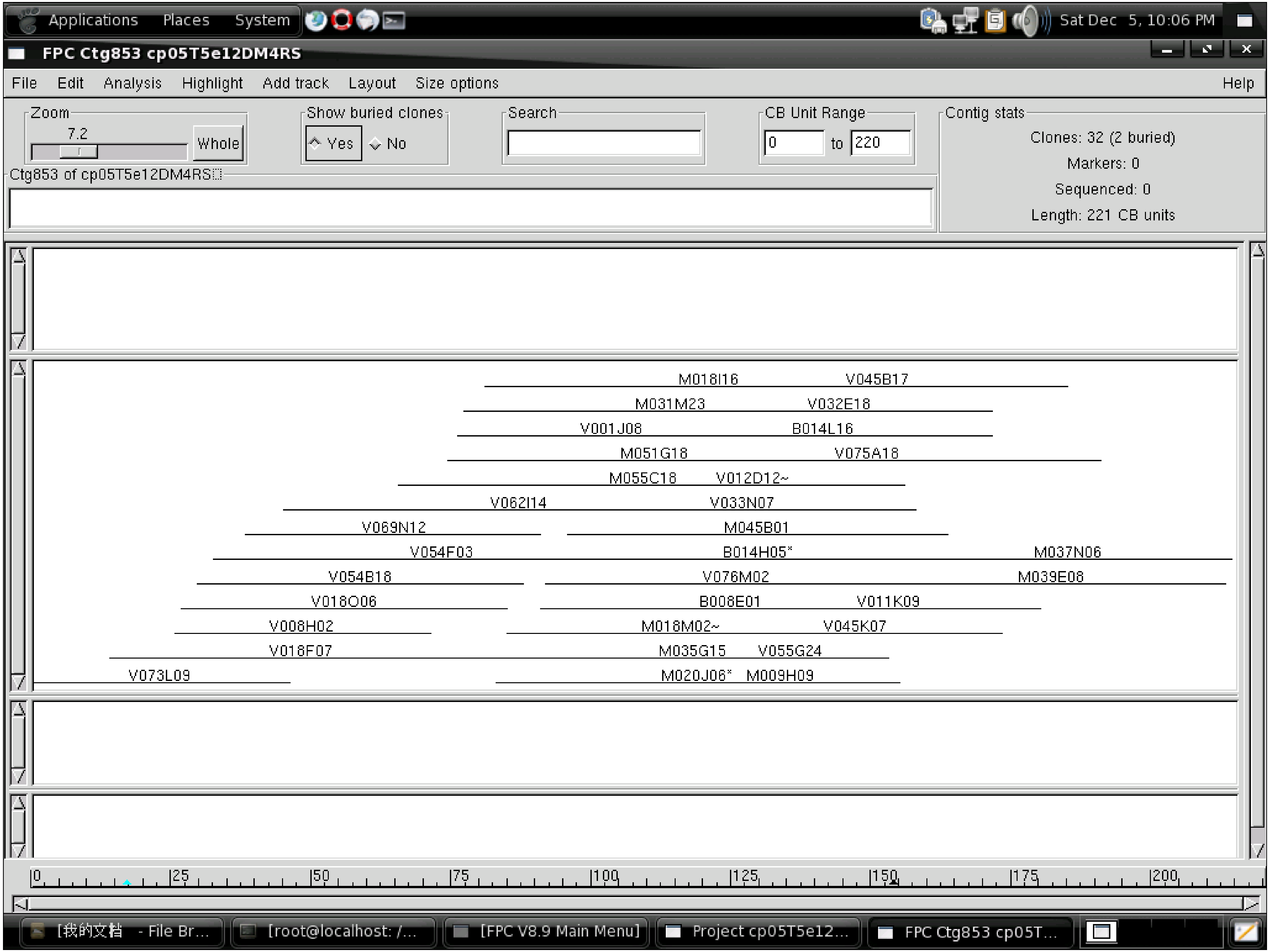
Applications Places System
Sat Dec 5, 10:06 PM
FPC Ctg853 cp05T5e12DM4RS
File Edit Analysis Highlight Add track Layout Size options Help
Zoom
7.2
Whole
Show buried clones
Yes
No
Search
CB Unit Range
0 to 220
Contig stats
Clones: 32 (2 buried)
Markers: 0
Sequenced: 0
Length: 221 CB units
Ctg853 of cp05T5e12DM4RS
M018I16 V045B17
M031M23 V032E18
V001J08 B014L16
M051G18 V075A18
M055C18 V012D12~
V062I14 V033N07
V069N12 M045B01
V054F03 B014H05* M037N06
V054B18 V076M02 M039E08
V018O06 B008E01 V011K09
V008H02 M018M02~ V045K07
V018F07 M035G15 V055G24
V073L09 M020J06* M009H09
0 25 50 75 100 125 150 175 200
[我的文档 - File Br...
[root@localhost: /...
[FPC V8.9 Main Menu]
Project cp05T5e12...
FPC Ctg853 cp05T...

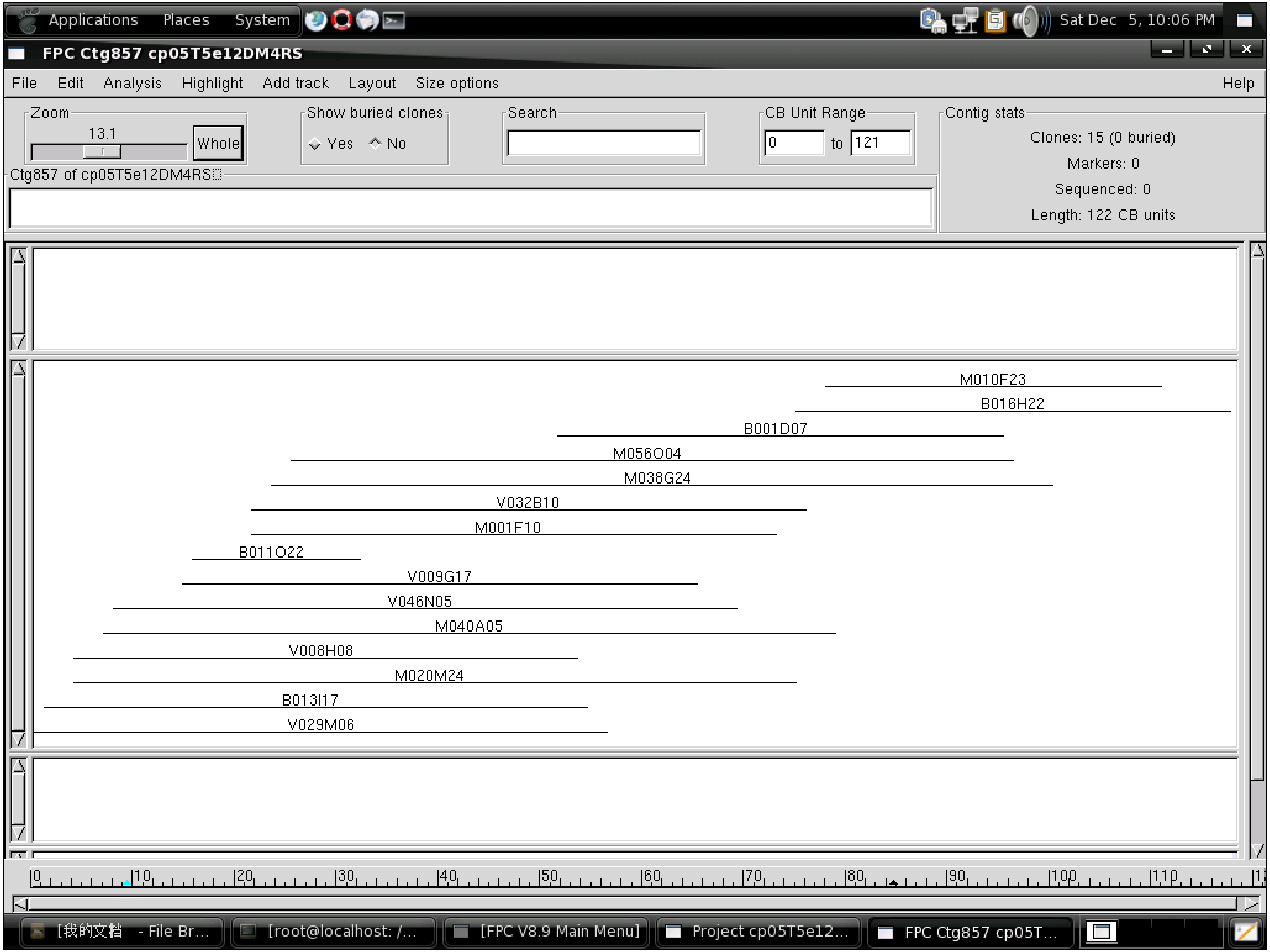
Applications Places System
Sat Dec 5, 10:06 PM
FPC Ctg857 cp05T5e12DM4RS
File Edit Analysis Highlight Add track Layout Size options Help
Zoom
13.1
Whole
Show buried clones
Yes No
Search
CB Unit Range
0 to 121
Contig stats
Clones: 15 (0 buried)
Markers: 0
Sequenced: 0
Length: 122 CB units
Ctg857 of cp05T5e12DM4RS
M010F23
B016H22
B001D07
M056O04
M038G24
V032B10
M001F10
B011O22
V009G17
V046N05
M040A05
V008H08
M020M24
B013I17
V029M06
0 10 20 30 40 50 60 70 80 90 100 110
[我的文档 - File Br...
[root@localhost: /...
[FPC V8.9 Main Menu]
Project cp05T5e12...
FPC Ctg857 cp05T...

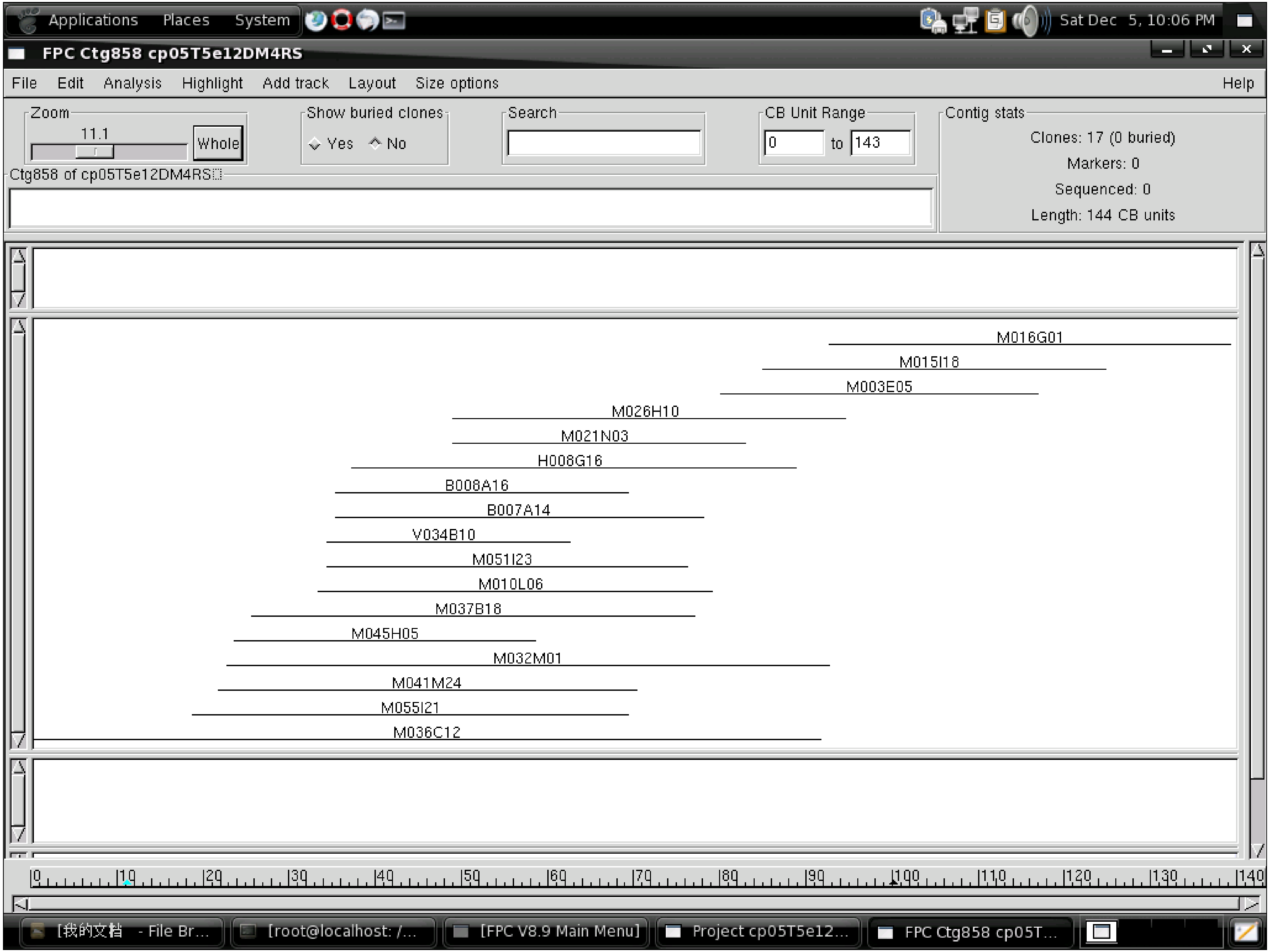
Applications
Places
System
Sat Dec 5, 10:06 PM
FPC Ctg858 cp05T5e12DM4RS
File
Edit
Analysis
Highlight
Add track
Layout
Size options
Help
Zoom
11.1
Whole
Show buried clones
Yes
No
Search
CB Unit Range
0
to
143
Contig stats
Clones: 17 (0 buried)
Markers: 0
Sequenced: 0
Length: 144 CB units
Ctg858 of cp05T5e12DM4RS
M016G01
M015I18
M003E05
M026H10
M021N03
H008G16
B008A16
B007A14
V034B10
M051I23
M010L06
M037B18
M045H05
M032M01
M041M24
M055I21
M036C12
0
10
20
30
40
50
60
70
80
90
100
110
120
130
140
[我的文档 - File Br...
[root@localhost: /...
[FPC V8.9 Main Menu]
Project cp05T5e12...
FPC Ctg858 cp05T...

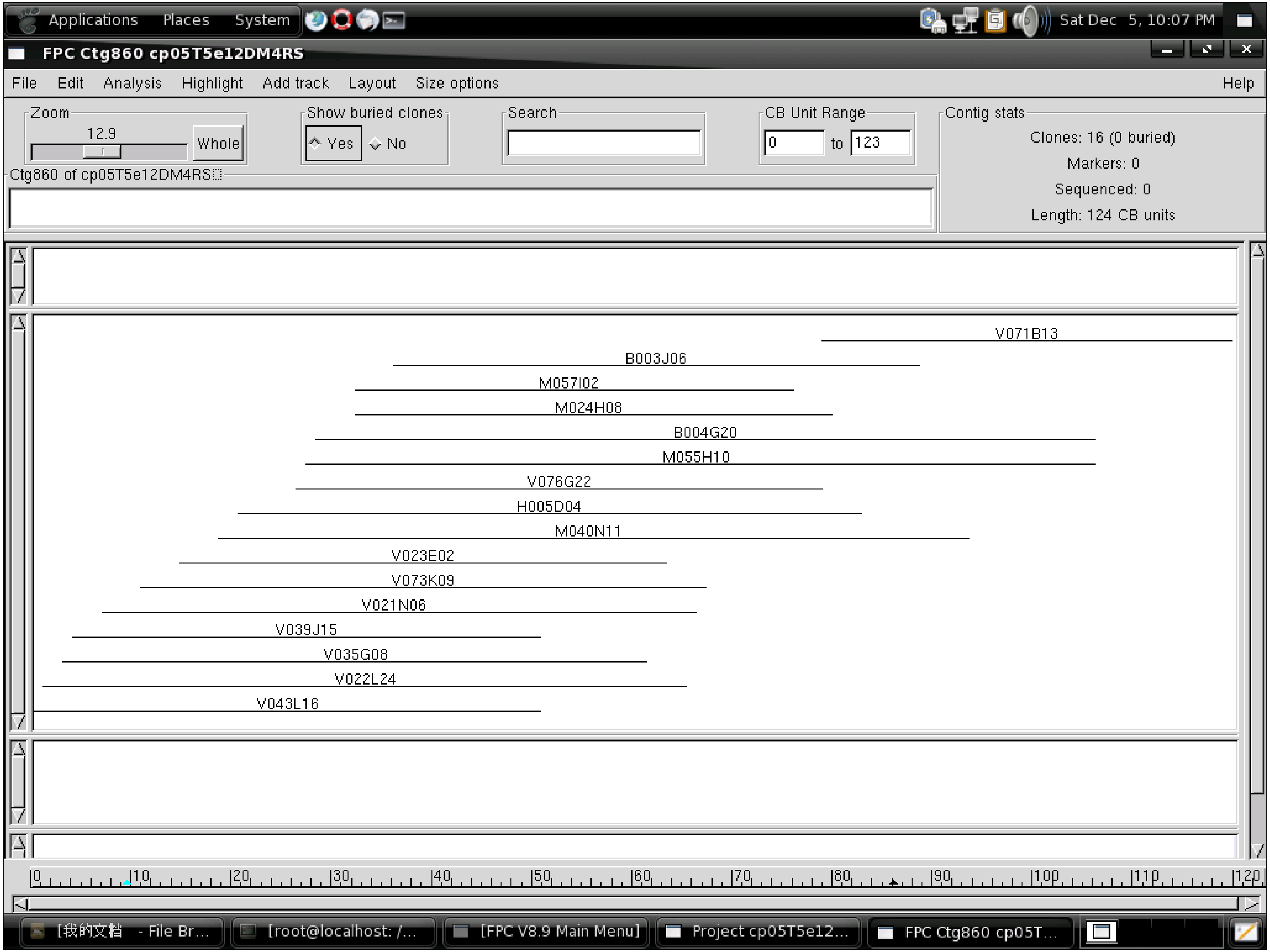
Applications
Places
System
Sat Dec 5, 10:07 PM
FPC Ctg860 cp05T5e12DM4RS
File
Edit
Analysis
Highlight
Add track
Layout
Size options
Help
Zoom
12.9
Whole
Show buried clones
Yes
No
Search
CB Unit Range
0
to
123
Contig stats
Clones: 16 (0 buried)
Markers: 0
Sequenced: 0
Length: 124 CB units
Ctg860 of cp05T5e12DM4RS
V071B13
B003J06
M057I02
M024H08
B004G20
M055H10
V076G22
H005D04
M040N11
V023E02
V073K09
V021N06
V039J15
V035G08
V022L24
V043L16
0
10
20
30
40
50
60
70
80
90
100
110
120
[我的文档 - File Br...
[root@localhost: /...
[FPC V8.9 Main Menu]
Project cp05T5e12...
FPC Ctg860 cp05T...

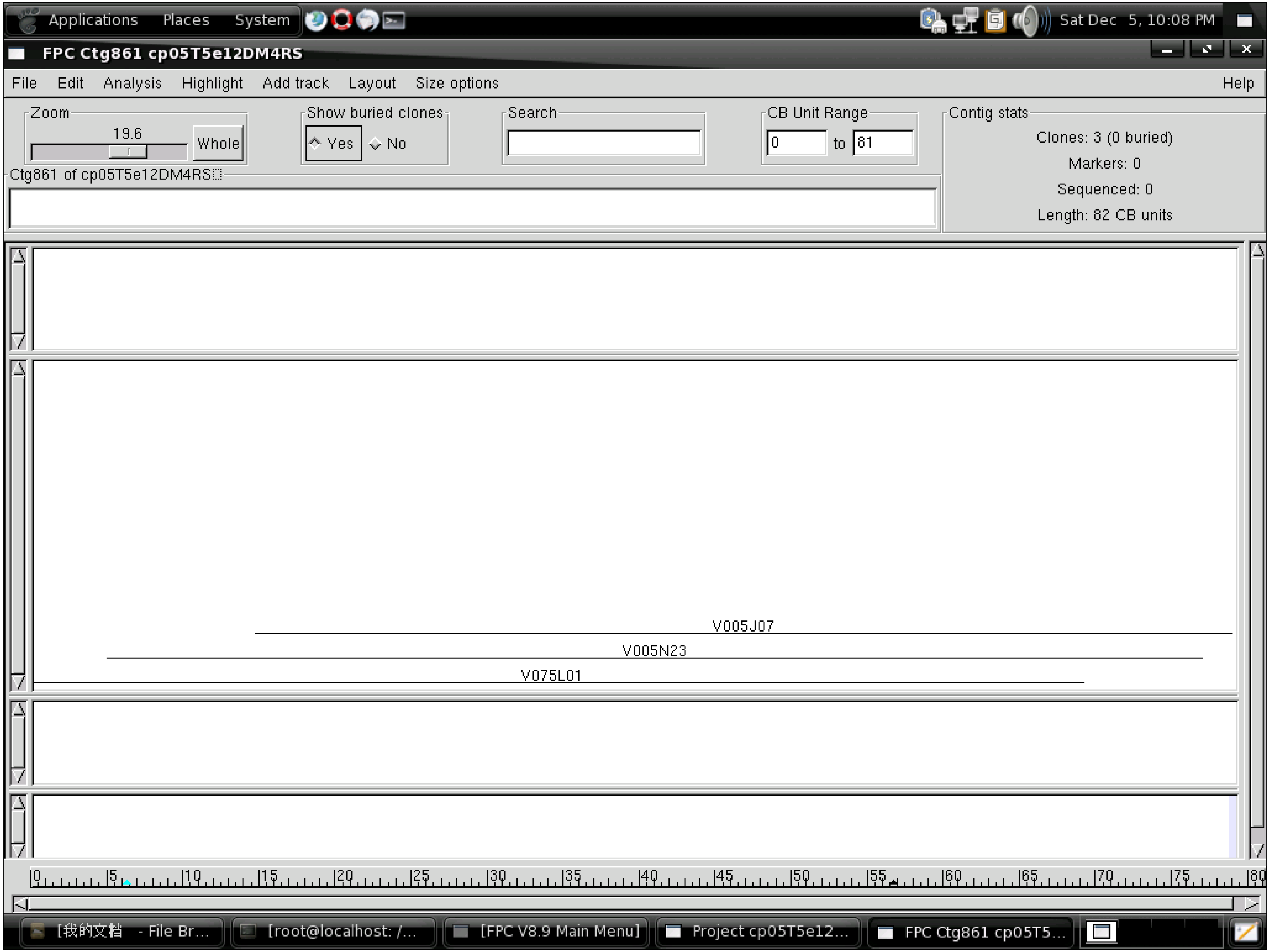
Applications
Places
System
Sat Dec 5, 10:08 PM
FPC Ctg861 cp05T5e12DM4RS
File
Edit
Analysis
Highlight
Add track
Layout
Size options
Help
Zoom
19.6
Whole
Show buried clones
Yes
No
Search
CB Unit Range
0
to
81
Contig stats
Clones: 3 (0 buried)
Markers: 0
Sequenced: 0
Length: 82 CB units
Ctg861 of cp05T5e12DM4RS
V005J07
V005N23
V075L01
0 5 10 15 20 25 30 35 40 45 50 55 60 65 70 75 80
[我的文档 - File Br...
[root@localhost: /...
[FPC V8.9 Main Menu]
Project cp05T5e12...
FPC Ctg861 cp05T5...

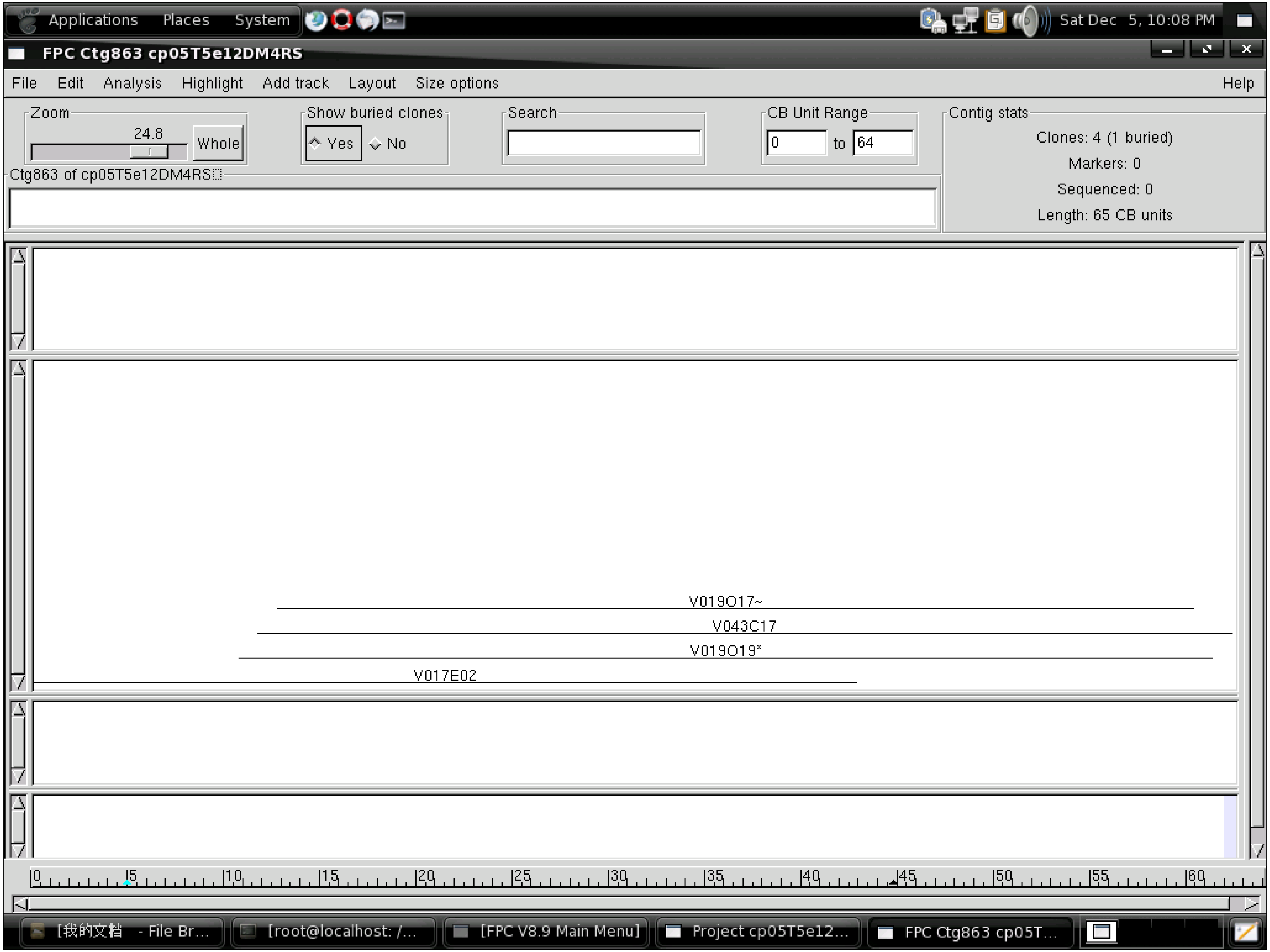
Applications Places System
Sat Dec 5, 10:08 PM
FPC Ctg863 cp05T5e12DM4RS
File Edit Analysis Highlight Add track Layout Size options Help
Zoom
24.8
Whole
Show buried clones
Yes No
Search
CB Unit Range
0 to 64
Contig stats
Clones: 4 (1 buried)
Markers: 0
Sequenced: 0
Length: 65 CB units
Ctg863 of cp05T5e12DM4RS
V019O17~
V043C17
V019O19*
V017E02
0 5 10 15 20 25 30 35 40 45 50 55 60
[我的文档 - File Br...
[root@localhost: /...
[FPC V8.9 Main Menu]
Project cp05T5e12...
FPC Ctg863 cp05T...

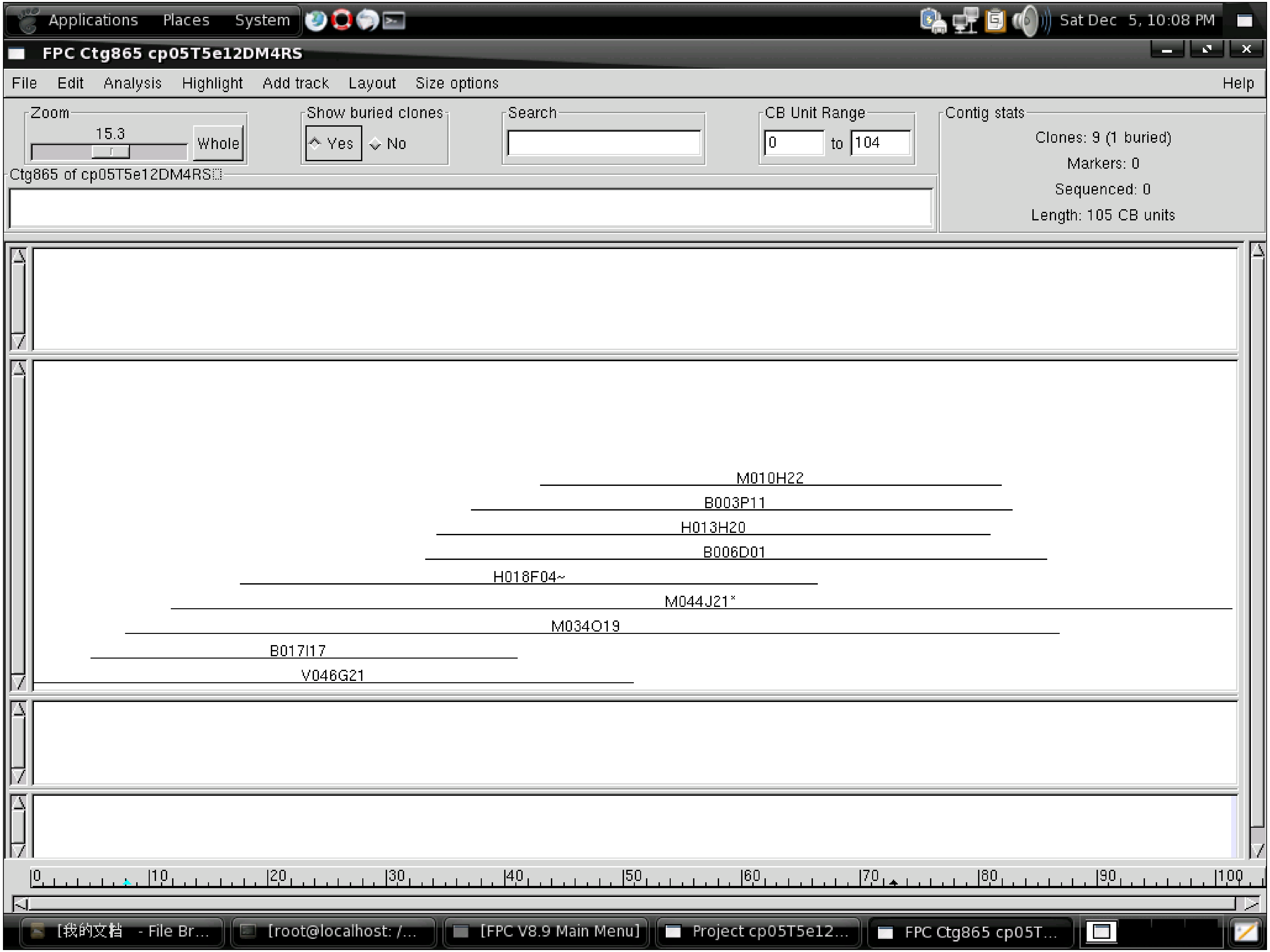
Applications Places System
Sat Dec 5, 10:08 PM
FPC Ctg865 cp05T5e12DM4RS
File Edit Analysis Highlight Add track Layout Size options Help
Zoom
15.3
Whole
Show buried clones
Yes No
Search
CB Unit Range
0 to 104
Contig stats
Clones: 9 (1 buried)
Markers: 0
Sequenced: 0
Length: 105 CB units
Ctg865 of cp05T5e12DM4RS
M010H22
B003P11
H013H20
B006D01
H018F04~
M044J21*
M034O19
B017I17
V046G21
0 10 20 30 40 50 60 70 80 90 100
[我的文档 - File Br...
[root@localhost: /...
[FPC V8.9 Main Menu]
Project cp05T5e12...
FPC Ctg865 cp05T...

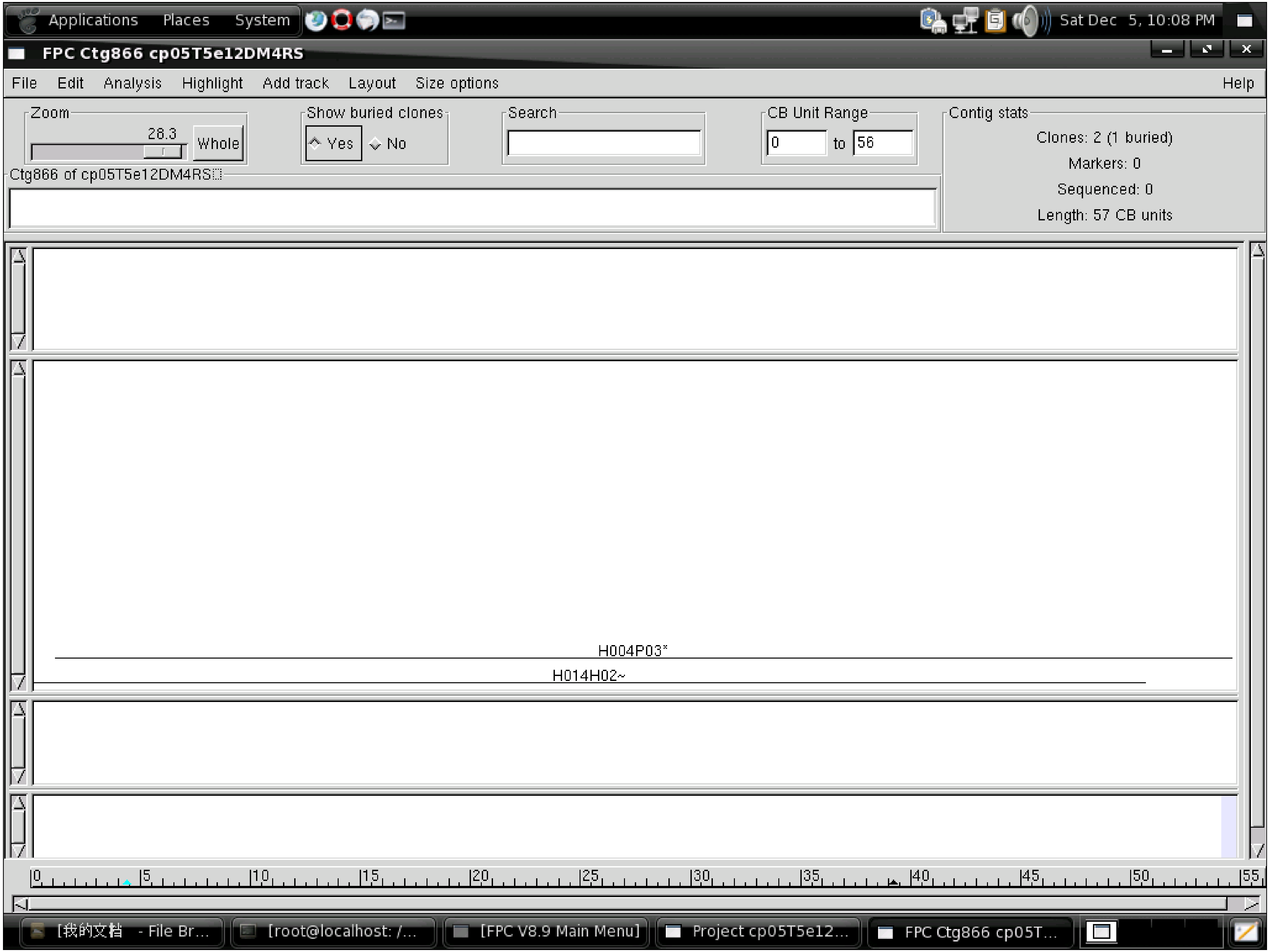
Applications Places System
Sat Dec 5, 10:08 PM
FPC Ctg866 cp05T5e12DM4RS
File Edit Analysis Highlight Add track Layout Size options Help
Zoom
28.3
Whole
Show buried clones
Yes
No
Search
CB Unit Range
0 to 56
Contig stats
Clones: 2 (1 buried)
Markers: 0
Sequenced: 0
Length: 57 CB units
Ctg866 of cp05T5e12DM4RS
H004P03*
H014H02~
0 5 10 15 20 25 30 35 40 45 50 55
[我的文档 - File Br...
[root@localhost: /...
[FPC V8.9 Main Menu]
Project cp05T5e12...
FPC Ctg866 cp05T...

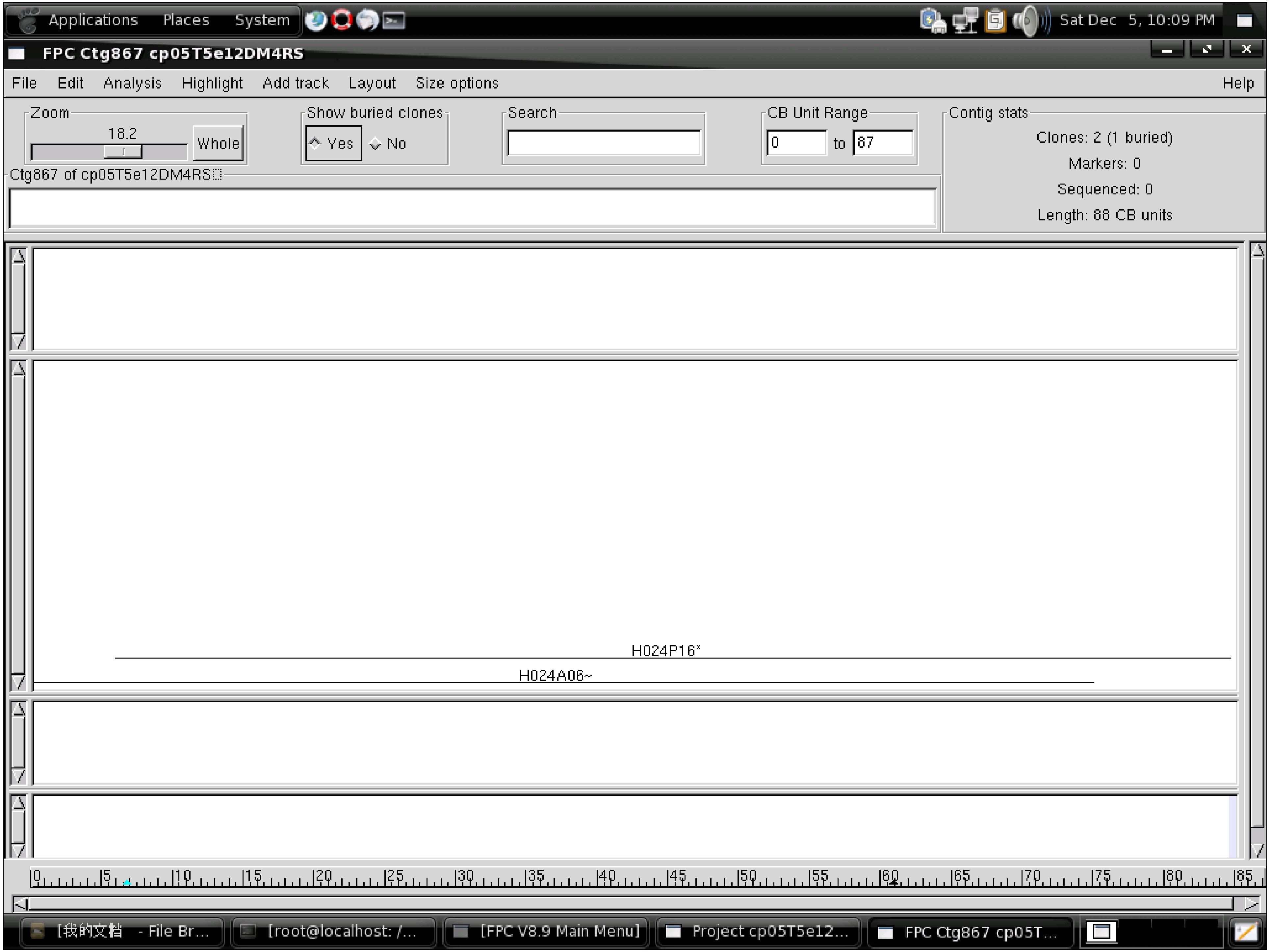

Applications Places System
Sat Dec 5, 10:09 PM
FPC Ctg867 cp05T5e12DM4RS
File Edit Analysis Highlight Add track Layout Size options Help
Zoom
18.2
Whole
Show buried clones
Yes No
Search
CB Unit Range
0 to 87
Contig stats
Clones: 2 (1 buried)
Markers: 0
Sequenced: 0
Length: 88 CB units
Ctg867 of cp05T5e12DM4RS
H024P16*
H024A06~
0 5 10 15 20 25 30 35 40 45 50 55 60 65 70 75 80 85
[我的文档 - File Br...
[root@localhost: /...
[FPC V8.9 Main Menu]
Project cp05T5e12...
FPC Ctg867 cp05T...

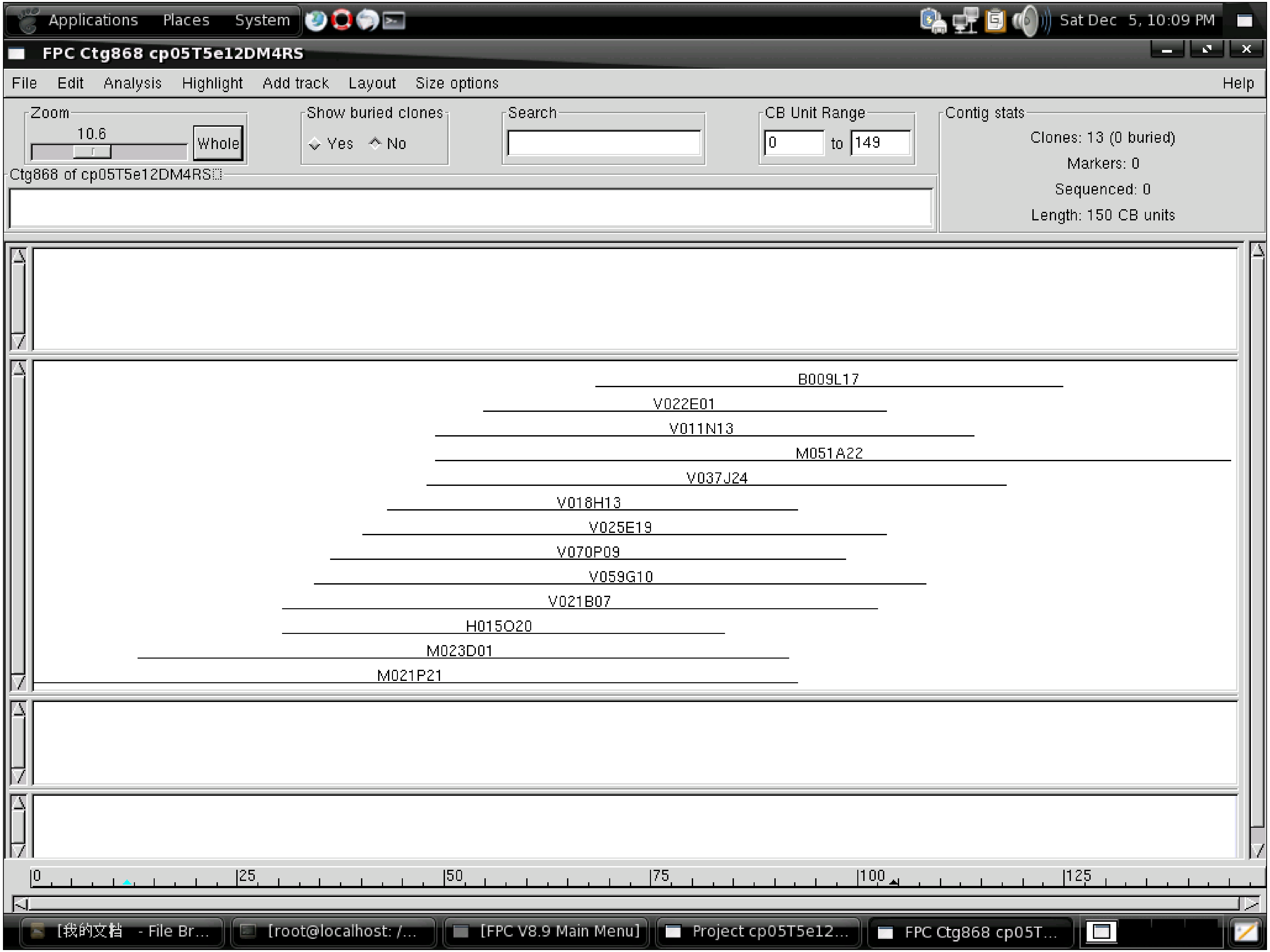
Applications
Places
System
Sat Dec 5, 10:09 PM
FPC Ctg868 cp05T5e12DM4RS
File
Edit
Analysis
Highlight
Add track
Layout
Size options
Help
Zoom
10.6
Whole
Show buried clones
Yes
No
Search
CB Unit Range
0
to
149
Contig stats
Clones: 13 (0 buried)
Markers: 0
Sequenced: 0
Length: 150 CB units
Ctg868 of cp05T5e12DM4RS
B009L17
V022E01
V011N13
M051A22
V037J24
V018H13
V025E19
V070P09
V059G10
V021B07
H015O20
M023D01
M021P21
0
25
50
75
100
125
[我的文档 - File Br...
[root@localhost: /...
[FPC V8.9 Main Menu]
Project cp05T5e12...
FPC Ctg868 cp05T...

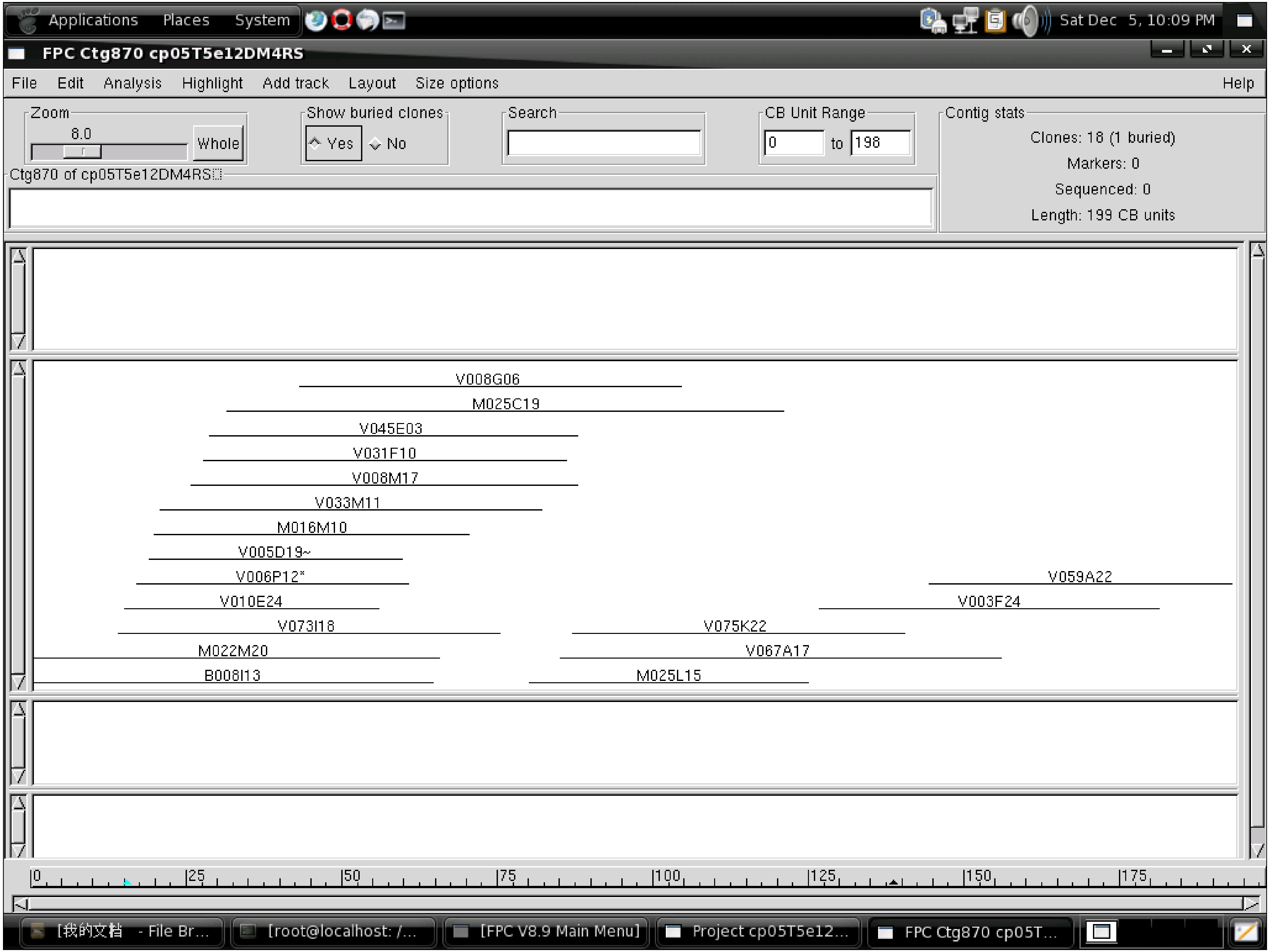
Applications Places System
Sat Dec 5, 10:09 PM
FPC Ctg870 cp05T5e12DM4RS
File Edit Analysis Highlight Add track Layout Size options Help
Zoom
8.0
Whole
Show buried clones
Yes No
Search
CB Unit Range
0 to 198
Contig stats
Clones: 18 (1 buried)
Markers: 0
Sequenced: 0
Length: 199 CB units
Ctg870 of cp05T5e12DM4RS
V008G06
M025C19
V045E03
V031F10
V008M17
V033M11
M016M10
V005D19~
V006P12*
V010E24
V073I18
M022M20
B008I13
V059A22
V003F24
V075K22
V067A17
M025L15
0 25 50 75 100 125 150 175
[我的文档 - File Br...
[root@localhost: /...
[FPC V8.9 Main Menu]
Project cp05T5e12...
FPC Ctg870 cp05T...

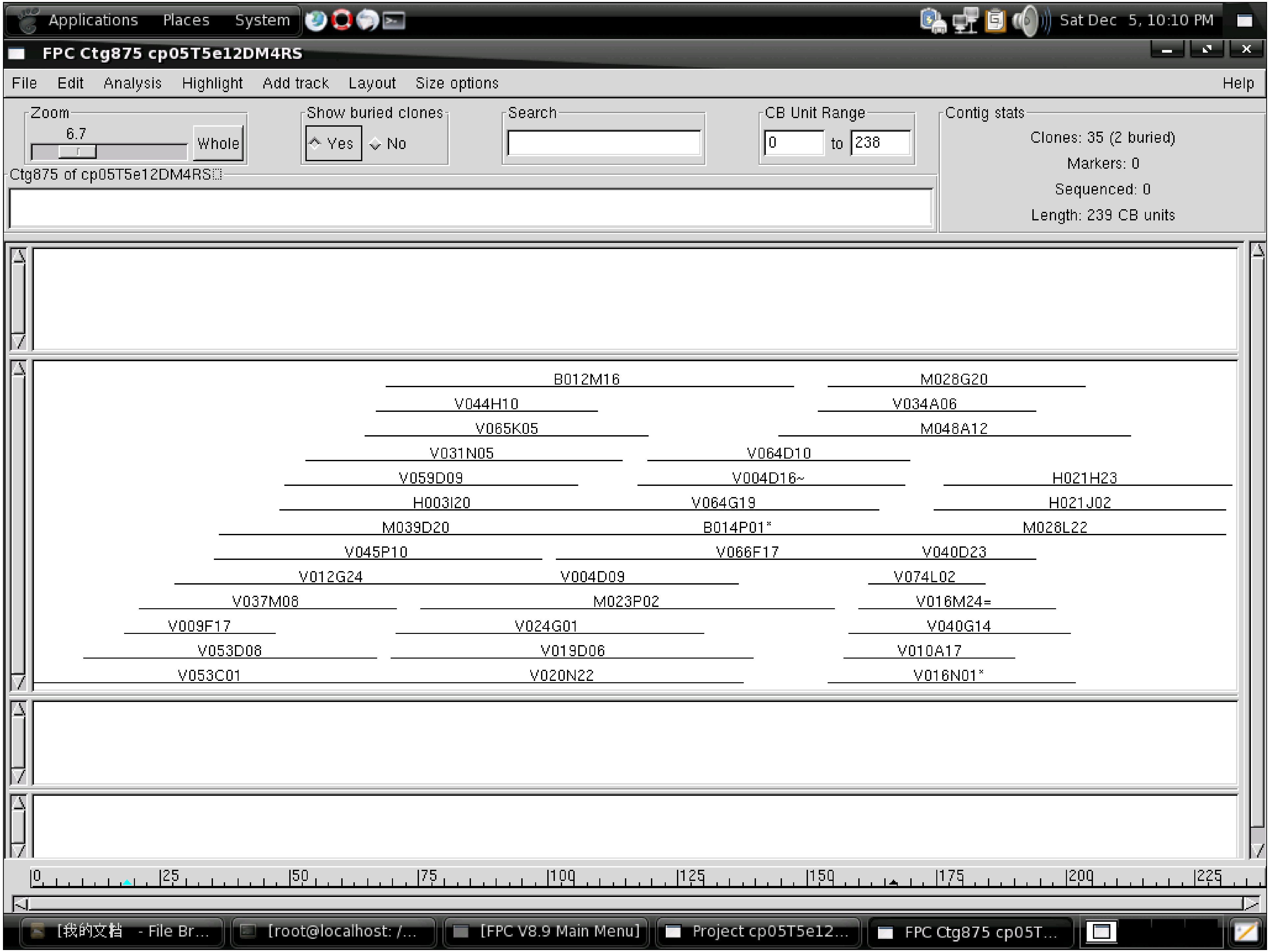
Applications Places System
Sat Dec 5, 10:10 PM
FPC Ctg875 cp05T5e12DM4RS
File Edit Analysis Highlight Add track Layout Size options Help
Zoom
6.7
Whole
Show buried clones
Yes No
Search
CB Unit Range
0 to 238
Contig stats
Clones: 35 (2 buried)
Markers: 0
Sequenced: 0
Length: 239 CB units
Ctg875 of cp05T5e12DM4RS
B012M16
M028G20
V044H10
V034A06
V065K05
M048A12
V031N05
V064D10
V059D09
V004D16~
H021H23
H003I20
V064G19
H021J02
M039D20
B014P01*
M028L22
V045P10
V066F17
V040D23
V012G24
V004D09
V074L02
V037M08
M023P02
V016M24=
V009F17
V024G01
V040G14
V053D08
V019D06
V010A17
V053C01
V020N22
V016N01*
0 25 50 75 100 125 150 175 200 225
[我的文档 - File Br...
[root@localhost: /...
[FPC V8.9 Main Menu]
Project cp05T5e12...
FPC Ctg875 cp05T...

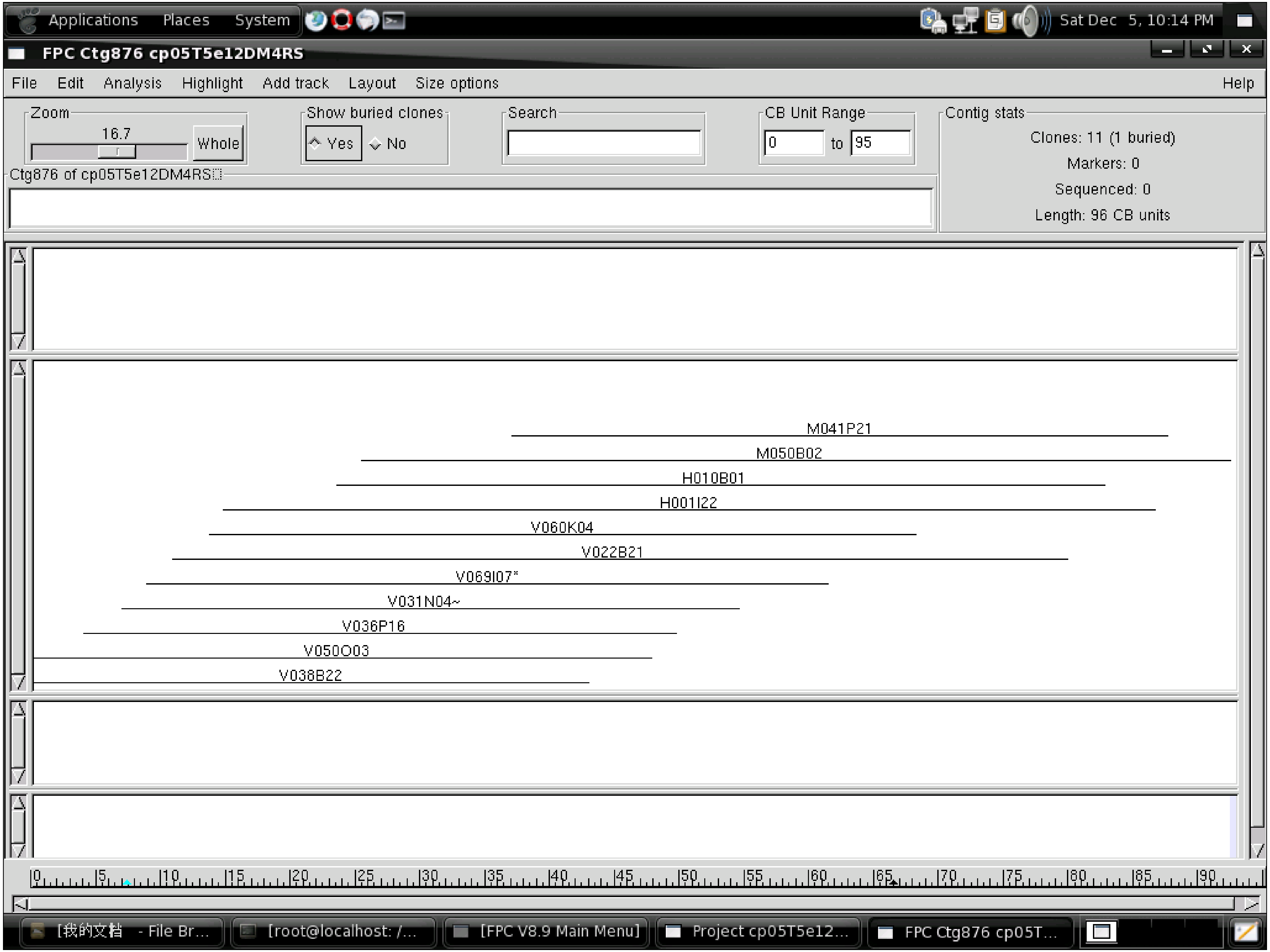
Applications Places System
Sat Dec 5, 10:14 PM
FPC Ctg876 cp05T5e12DM4RS
File Edit Analysis Highlight Add track Layout Size options Help
Zoom
16.7
Whole
Show buried clones
Yes No
Search
CB Unit Range
0 to 95
Contig stats
Clones: 11 (1 buried)
Markers: 0
Sequenced: 0
Length: 96 CB units
Ctg876 of cp05T5e12DM4RS
M041P21
M050B02
H010B01
H001I22
V060K04
V022B21
V069I07*
V031N04~
V036P16
V050O03
V038B22
0 5 10 15 20 25 30 35 40 45 50 55 60 65 70 75 80 85 90
[我的文档 - File Br...
[root@localhost: /...
[FPC V8.9 Main Menu]
Project cp05T5e12...
FPC Ctg876 cp05T...

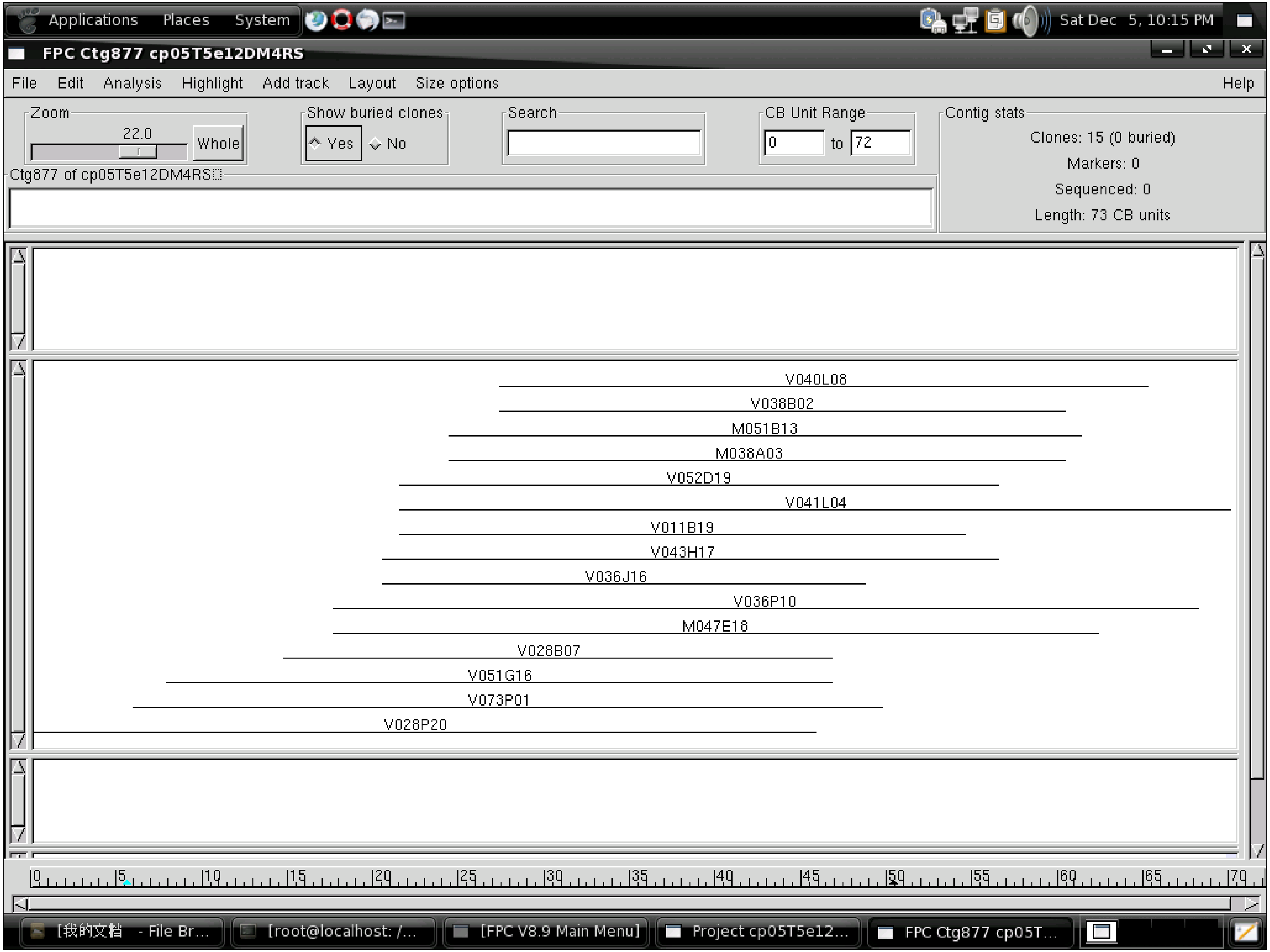
Applications
Places
System
Sat Dec 5, 10:15 PM
FPC Ctg877 cp05T5e12DM4RS
File
Edit
Analysis
Highlight
Add track
Layout
Size options
Help
Zoom
22.0
Whole
Show buried clones
Yes
No
Search
CB Unit Range
0
to
72
Contig stats
Clones: 15 (0 buried)
Markers: 0
Sequenced: 0
Length: 73 CB units
Ctg877 of cp05T5e12DM4RS
V040L08
V038B02
M051B13
M038A03
V052D19
V041L04
V011B19
V043H17
V036J16
V036P10
M047E18
V028B07
V051G16
V073P01
V028P20
0
5
10
15
20
25
30
35
40
45
50
55
60
65
70
[我的文档 - File Br...
[root@localhost: /...
[FPC V8.9 Main Menu]
Project cp05T5e12...
FPC Ctg877 cp05T...

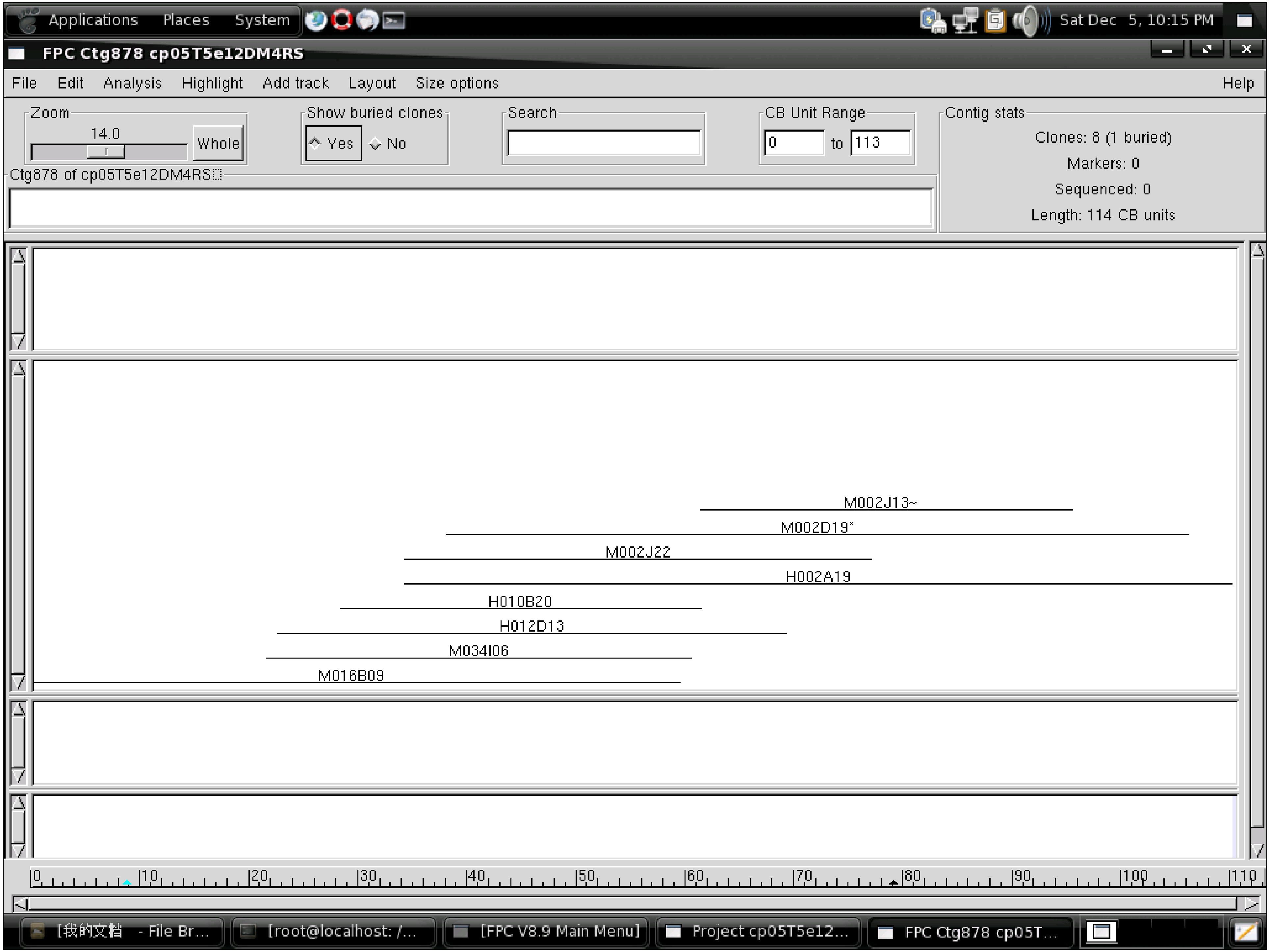

Applications
Places
System
Sat Dec 5, 10:15 PM
FPC Ctg878 cp05T5e12DM4RS
File
Edit
Analysis
Highlight
Add track
Layout
Size options
Help
Zoom
14.0
Whole
Show buried clones
Yes
No
Search
CB Unit Range
0
to
113
Contig stats
Clones: 8 (1 buried)
Markers: 0
Sequenced: 0
Length: 114 CB units
Ctg878 of cp05T5e12DM4RS
M002J13~
M002D19*
M002J22
H002A19
H010B20
H012D13
M034I06
M016B09
0
10
20
30
40
50
60
70
80
90
100
110
[FPC V8.9 Main Menu]

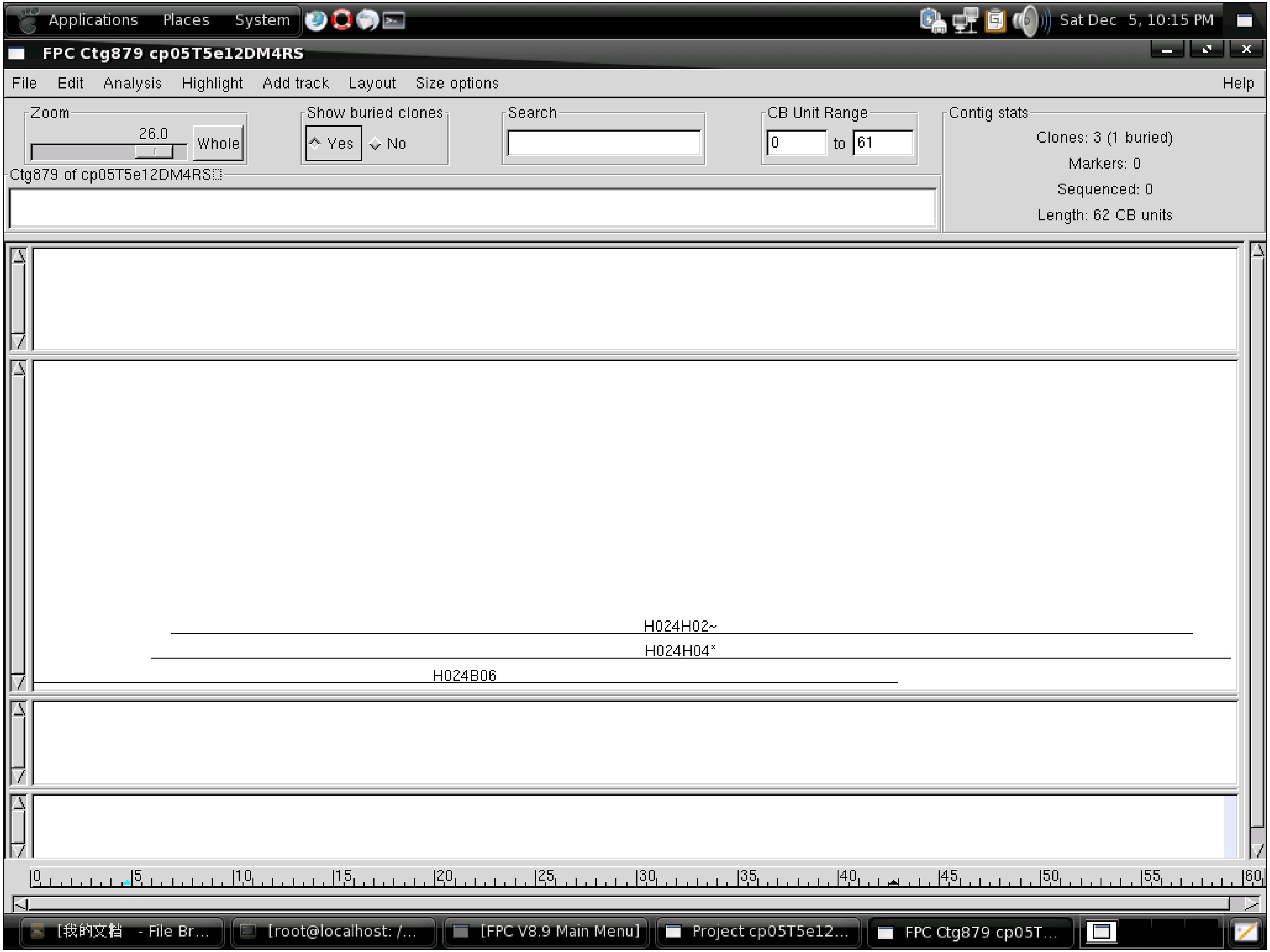
Applications Places System
Sat Dec 5, 10:15 PM
FPC Ctg879 cp05T5e12DM4RS
File Edit Analysis Highlight Add track Layout Size options Help
Zoom
26.0
Whole
Show buried clones
Yes
No
Search
CB Unit Range
0 to 61
Contig stats
Clones: 3 (1 buried)
Markers: 0
Sequenced: 0
Length: 62 CB units
Ctg879 of cp05T5e12DM4RS
H024H02~
H024H04*
H024B06
0 5 10 15 20 25 30 35 40 45 50 55 60
[我的文档 - File Br...
[root@localhost: /...
[FPC V8.9 Main Menu]
Project cp05T5e12...
FPC Ctg879 cp05T...

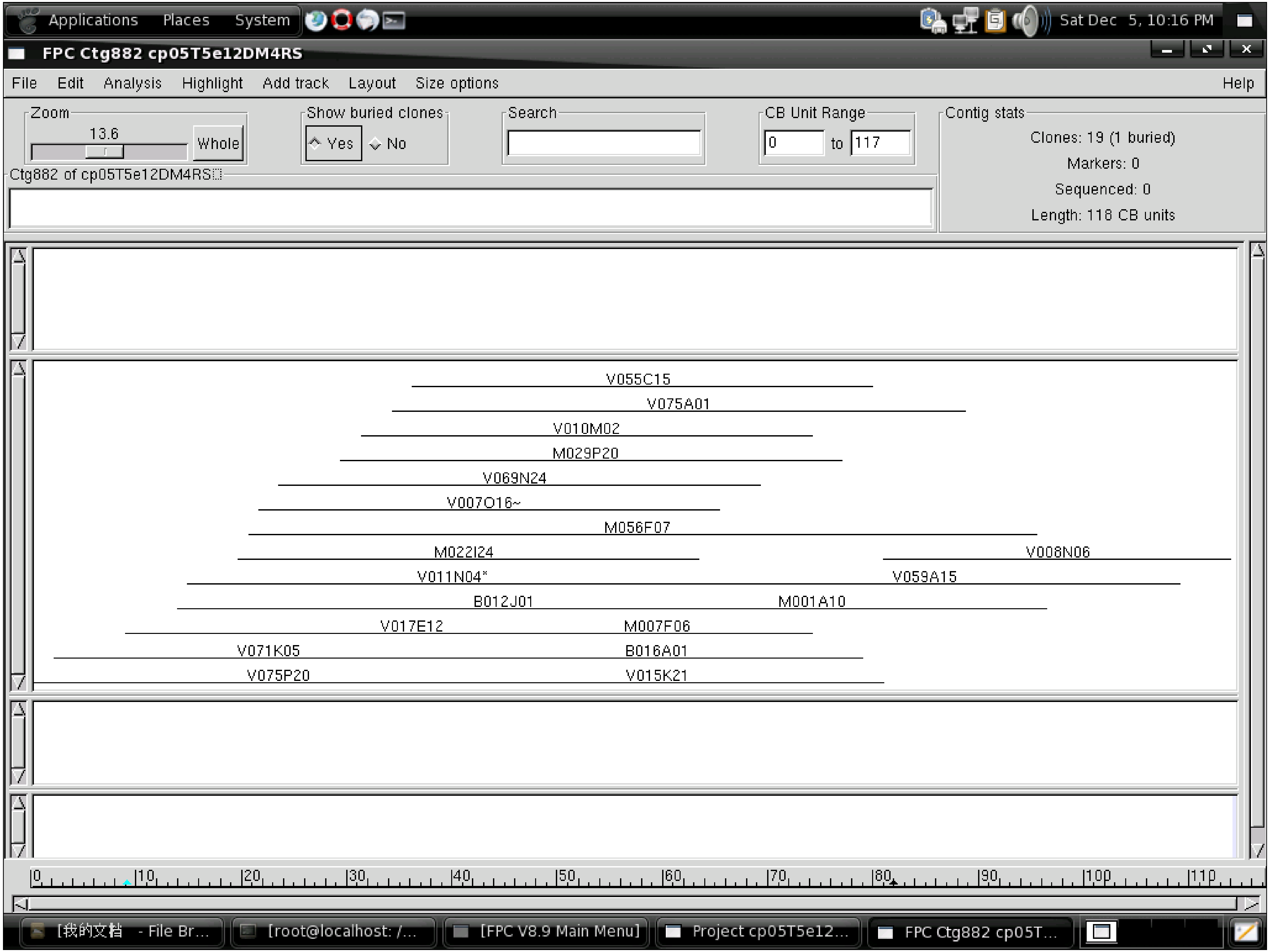

Applications Places System
Sat Dec 5, 10:16 PM
FPC Ctg882 cp05T5e12DM4RS
File Edit Analysis Highlight Add track Layout Size options Help
Zoom
13.6
Whole
Show buried clones
Yes No
Search
CB Unit Range
0 to 117
Contig stats
Clones: 19 (1 buried)
Markers: 0
Sequenced: 0
Length: 118 CB units
Ctg882 of cp05T5e12DM4RS
V055C15
V075A01
V010M02
M029P20
V069N24
V007O16~
M056F07
M022I24
V008N06
V011N04*
V059A15
B012J01
M001A10
V017E12
M007F06
V071K05
B016A01
V075P20
V015K21
0 10 20 30 40 50 60 70 80 90 100 110
[root@localhost: /...
[FPC V8.9 Main Menu]
Project cp05T5e12...
FPC Ctg882 cp05T...

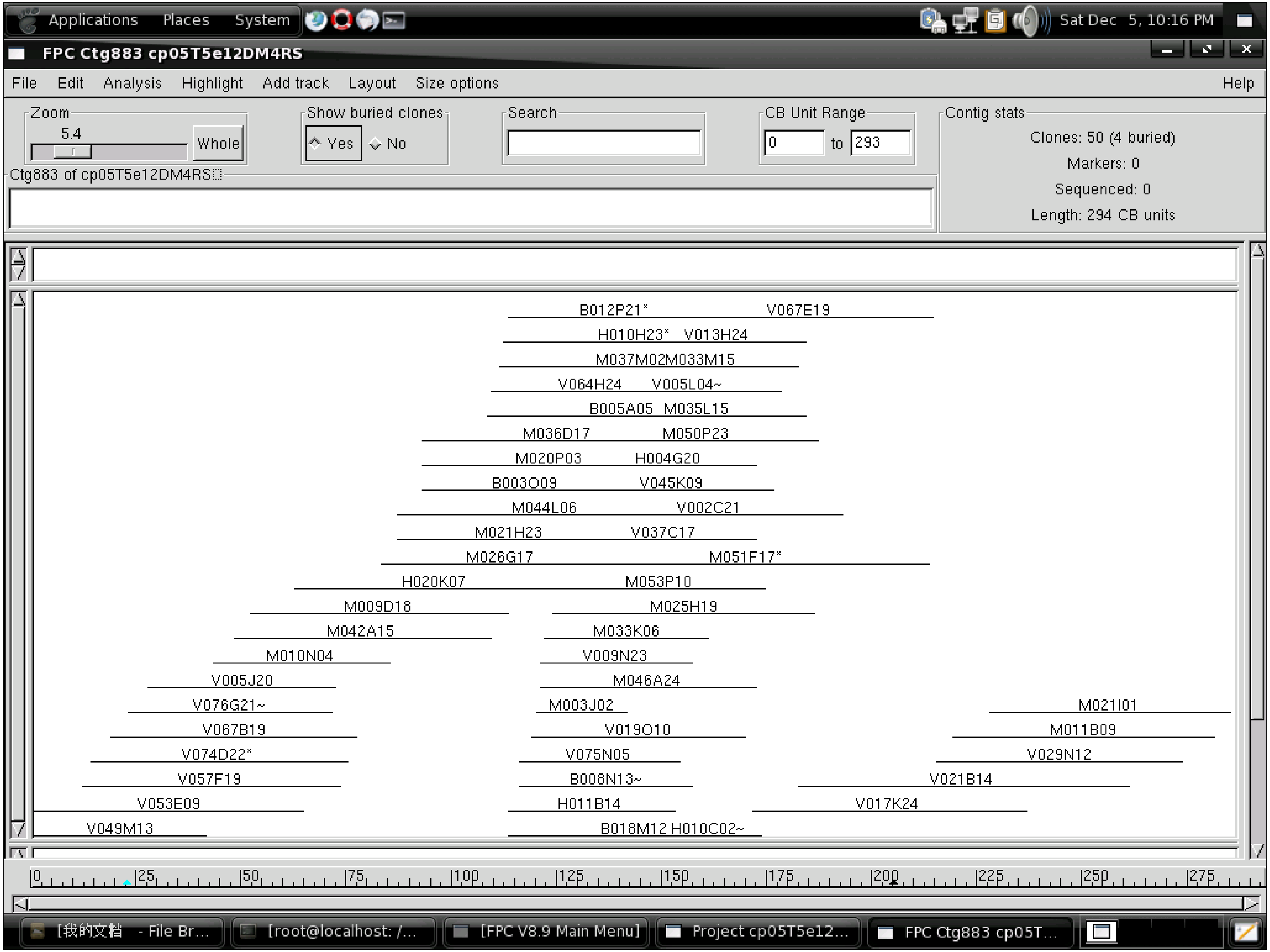
Applications Places System
Sat Dec 5, 10:16 PM
FPC Ctg883 cp05T5e12DM4RS
File Edit Analysis Highlight Add track Layout Size options Help
Zoom
5.4
Whole
Show buried clones
Yes
No
Search
CB Unit Range
0 to 293
Contig stats
Clones: 50 (4 buried)
Markers: 0
Sequenced: 0
Length: 294 CB units
Ctg883 of cp05T5e12DM4RS
B012P21* V067E19
H010H23* V013H24
M037M02M033M15
V064H24 V005L04~
B005A05 M035L15
M036D17 M050P23
M020P03 H004G20
B003O09 V045K09
M044L06 V002C21
M021H23 V037C17
M026G17 M051F17*
H020K07 M053P10
M009D18 M025H19
M042A15 M033K06
M010N04 V009N23
V005J20 M046A24
V076G21~ M003J02 M021I01
V067B19 V019O10 M011B09
V074D22* V075N05 V029N12
V057F19 B008N13~ V021B14
V053E09 H011B14 V017K24
V049M13 B018M12 H010C02~
0 25 50 75 100 125 150 175 200 225 250 275
[我的文档 - File Br...
[root@localhost: /...
[FPC V8.9 Main Menu]
Project cp05T5e12...
FPC Ctg883 cp05T...

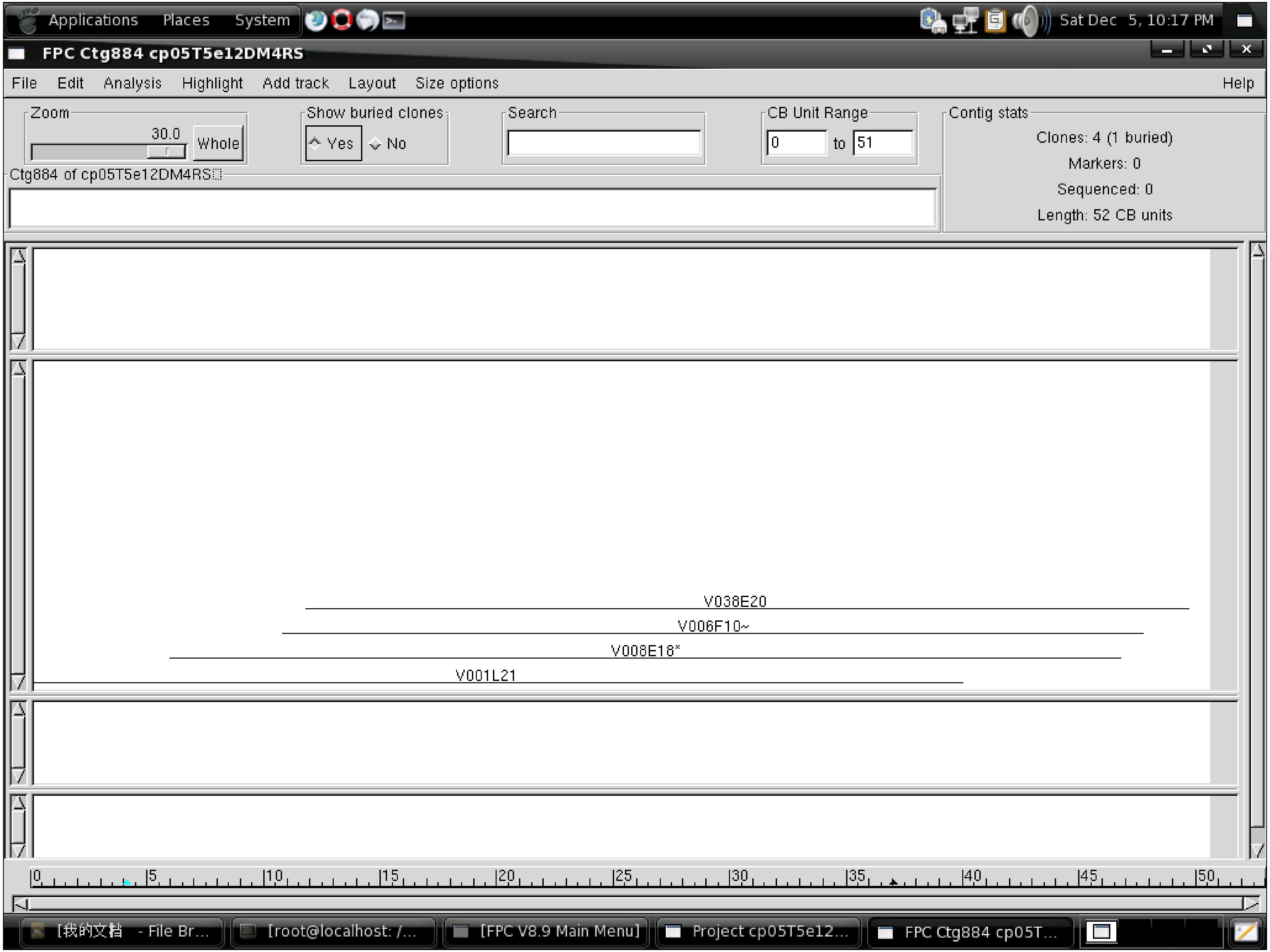
Applications
Places
System
Sat Dec 5, 10:17 PM
FPC Ctg884 cp05T5e12DM4RS
File
Edit
Analysis
Highlight
Add track
Layout
Size options
Help
Zoom
30.0
Whole
Show buried clones
Yes
No
Search
CB Unit Range
0
to
51
Contig stats
Clones: 4 (1 buried)
Markers: 0
Sequenced: 0
Length: 52 CB units
Ctg884 of cp05T5e12DM4RS
V038E20
V006F10~
V008E18*
V001L21
0 5 10 15 20 25 30 35 40 45 50
[我的文档 - File Br...
[root@localhost: /...
[FPC V8.9 Main Menu]
Project cp05T5e12...
FPC Ctg884 cp05T...

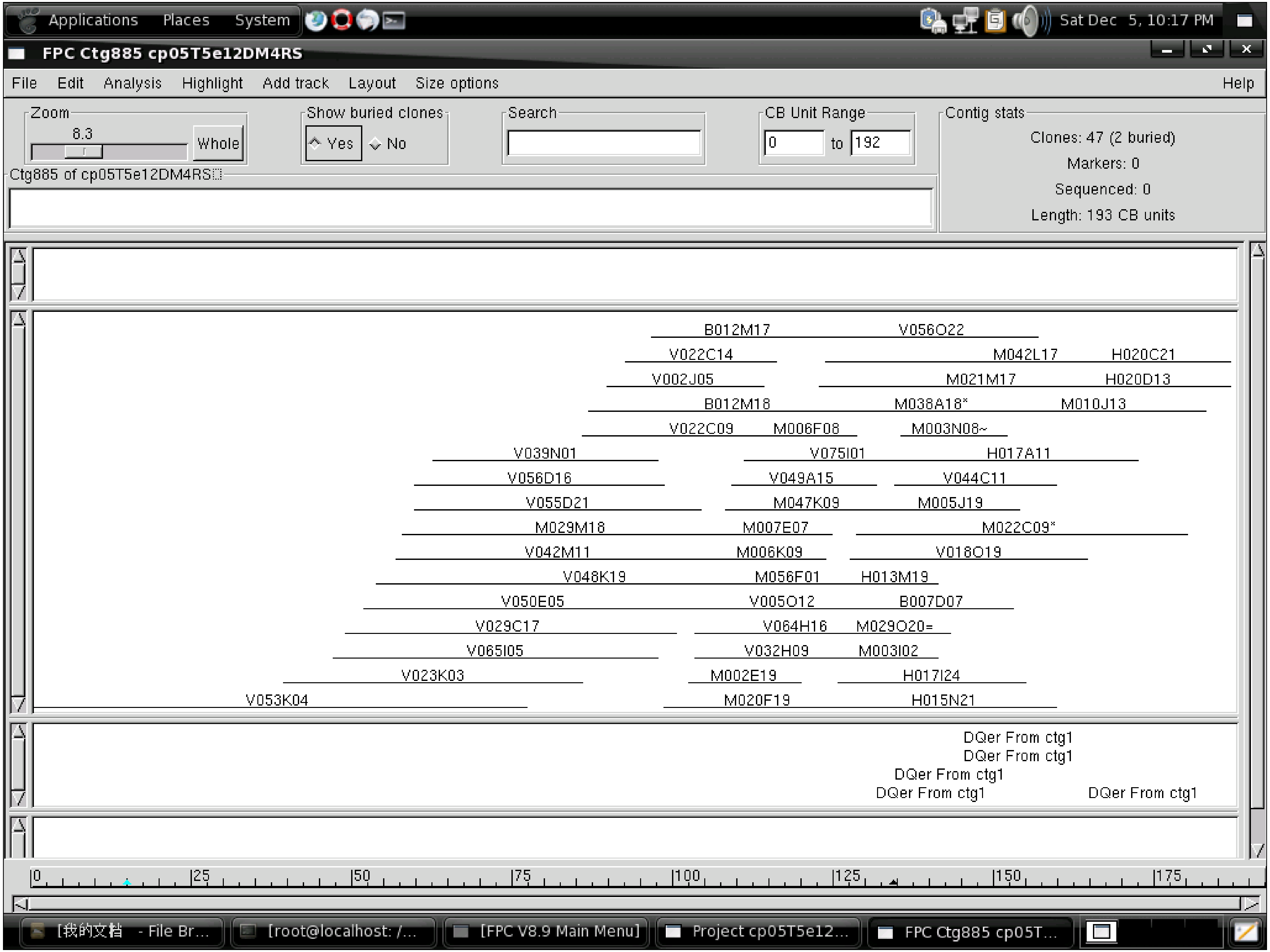
Applications Places System
Sat Dec 5, 10:17 PM
FPC Ctg885 cp05T5e12DM4RS
File Edit Analysis Highlight Add track Layout Size options Help
Zoom
8.3
Whole
Show buried clones
Yes No
Search
CB Unit Range
0 to 192
Contig stats
Clones: 47 (2 buried)
Markers: 0
Sequenced: 0
Length: 193 CB units
Ctg885 of cp05T5e12DM4RS
B012M17 V056O22
V022C14 M042L17 H020C21
V002J05 M021M17 H020D13
B012M18 M038A18* M010J13
V022C09 M006F08 M003N08~
V039N01 V075I01 H017A11
V056D16 V049A15 V044C11
V055D21 M047K09 M005J19
M029M18 M007E07 M022C09*
V042M11 M006K09 V018O19
V048K19 M056F01 H013M19
V050E05 V005O12 B007D07
V029C17 V064H16 M029O20=
V065I05 V032H09 M003I02
V023K03 M002E19 H017I24
V053K04 M020F19 H015N21
DQer From ctg1
DQer From ctg1
DQer From ctg1
DQer From ctg1
DQer From ctg1
0 25 50 75 100 125 150 175
[我的文档 - File Br...
[root@localhost: /...
[FPC V8.9 Main Menu]
Project cp05T5e12...
FPC Ctg885 cp05T...

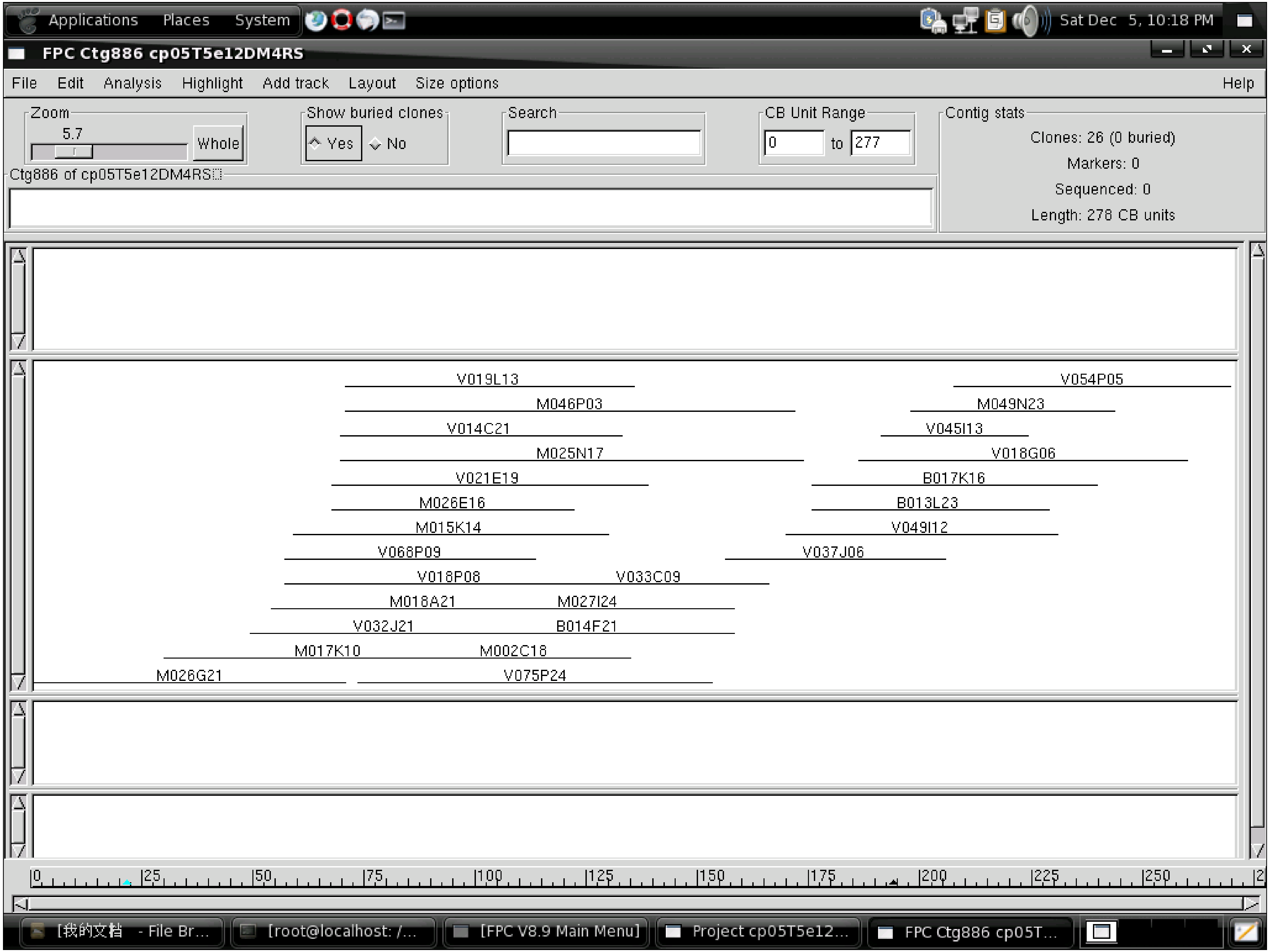
Applications Places System
Sat Dec 5, 10:18 PM
FPC Ctg886 cp05T5e12DM4RS
File Edit Analysis Highlight Add track Layout Size options Help
Zoom
5.7
Whole
Show buried clones
Yes No
Search
CB Unit Range
0 to 277
Contig stats
Clones: 26 (0 buried)
Markers: 0
Sequenced: 0
Length: 278 CB units
Ctg886 of cp05T5e12DM4RS
V019L13
V054P05
M046P03
M049N23
V014C21
V045I13
M025N17
V018G06
V021E19
B017K16
M026E16
B013L23
M015K14
V049I12
V068P09
V037J06
V018P08
V033C09
M018A21
M027I24
V032J21
B014F21
M017K10
M002C18
M026G21
V075P24
0 25 50 75 100 125 150 175 200 225 250
[我的文档 - File Br...
[root@localhost: /...
[FPC V8.9 Main Menu]
Project cp05T5e12...
FPC Ctg886 cp05T...

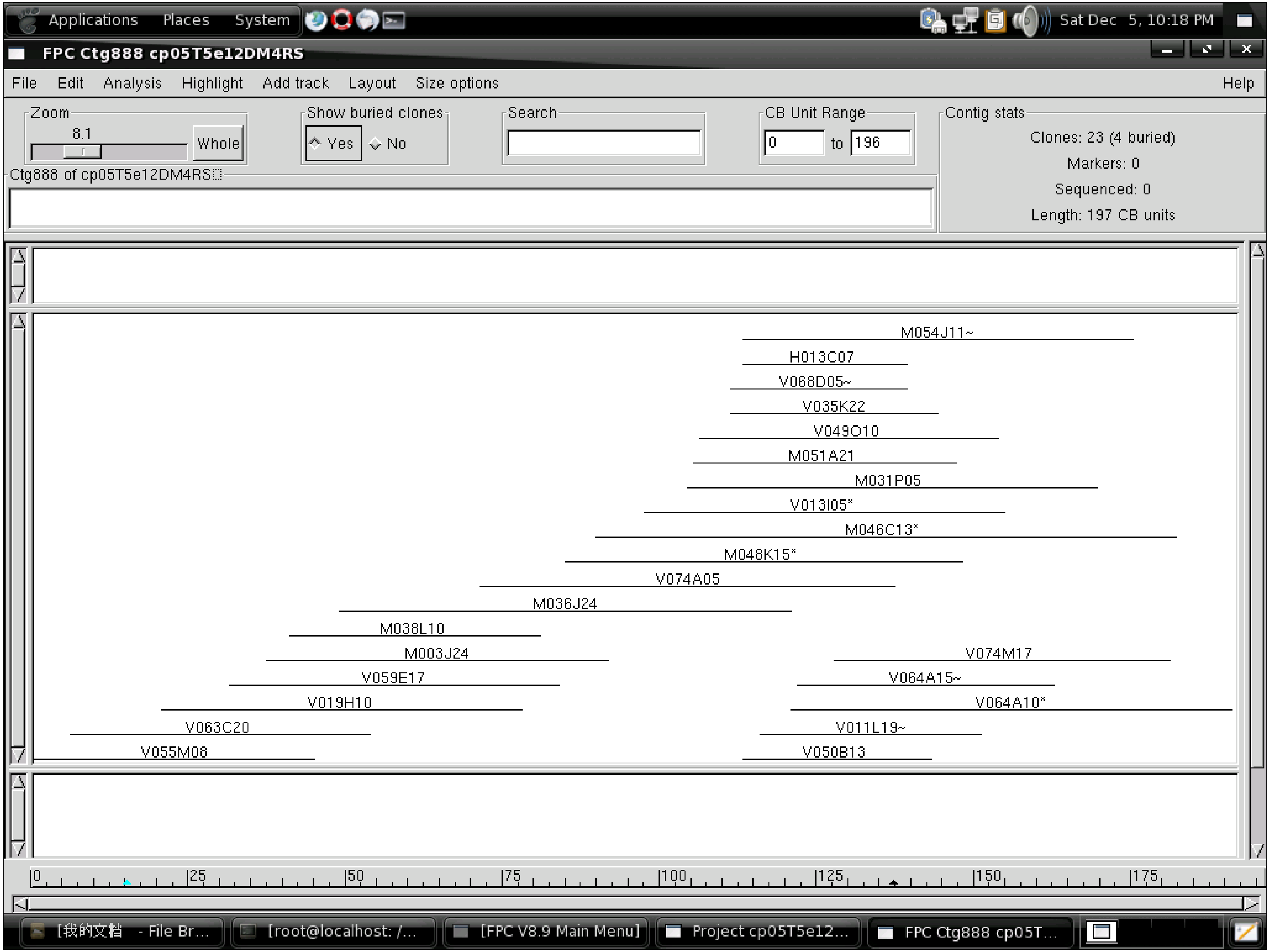
Applications Places System
Sat Dec 5, 10:18 PM
FPC Ctg888 cp05T5e12DM4RS
File Edit Analysis Highlight Add track Layout Size options Help
Zoom
8.1
Whole
Show buried clones
Yes
No
Search
CB Unit Range
0
to
196
Contig stats
Clones: 23 (4 buried)
Markers: 0
Sequenced: 0
Length: 197 CB units
Ctg888 of cp05T5e12DM4RS
M054J11~
H013C07
V068D05~
V035K22
V049O10
M051A21
M031P05
V013I05*
M046C13*
M048K15*
V074A05
M036J24
M038L10
M003J24
V074M17
V059E17
V064A15~
V019H10
V064A10*
V063C20
V011L19~
V055M08
V050B13
0
25
50
75
100
125
150
175
[我的文档 - File Br...
[root@localhost: /...
[FPC V8.9 Main Menu]
Project cp05T5e12...
FPC Ctg888 cp05T...

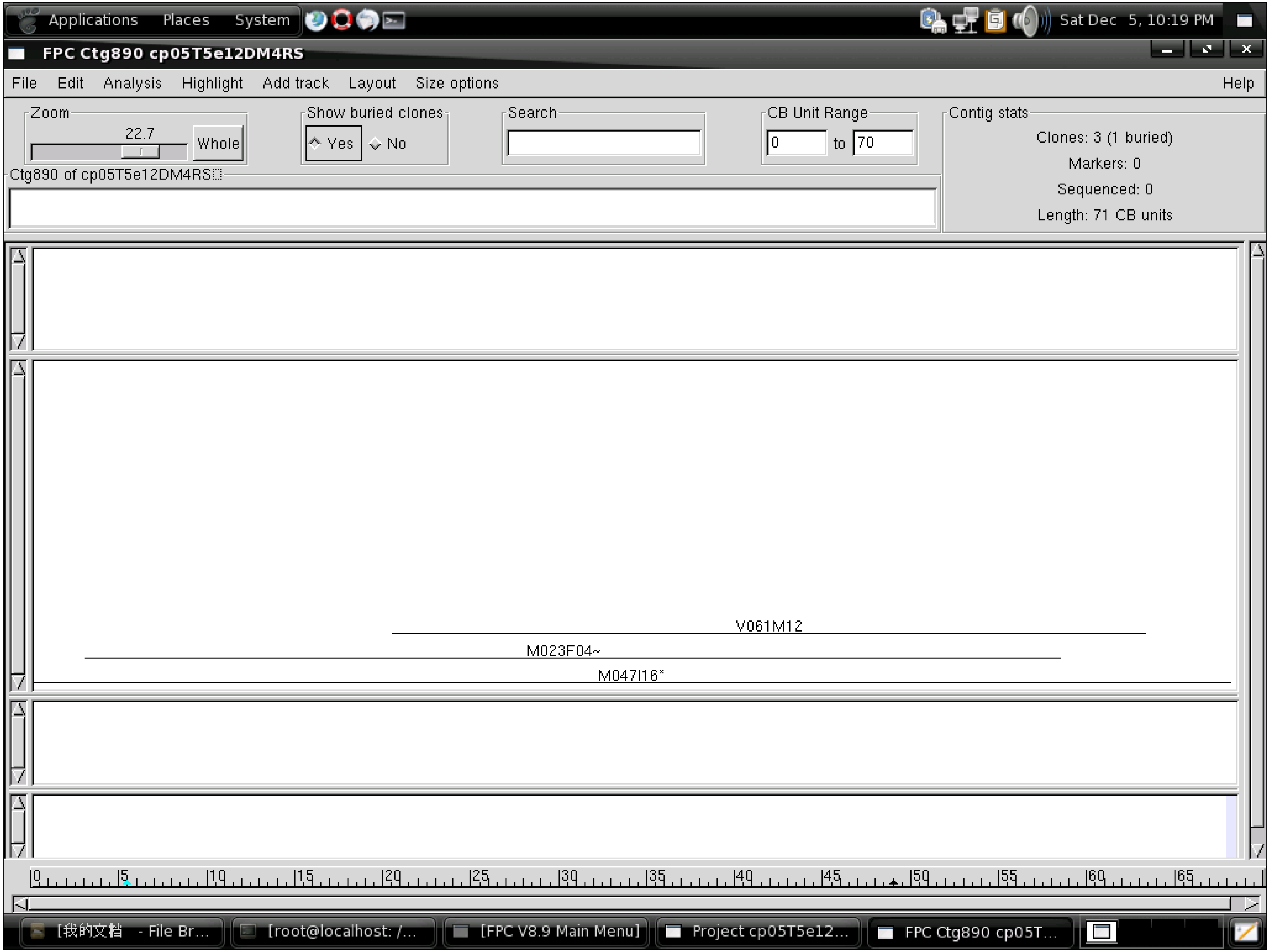

Applications Places System
Sat Dec 5, 10:19 PM
FPC Ctg890 cp05T5e12DM4RS
File Edit Analysis Highlight Add track Layout Size options Help
Zoom
22.7
Whole
Show buried clones
Yes No
Search
CB Unit Range
0 to 70
Contig stats
Clones: 3 (1 buried)
Markers: 0
Sequenced: 0
Length: 71 CB units
Ctg890 of cp05T5e12DM4RS
V061M12
M023F04~
M047I16*
0 5 10 15 20 25 30 35 40 45 50 55 60 65
[我的文档 - File Br...
[root@localhost: /...
[FPC V8.9 Main Menu]
Project cp05T5e12...
FPC Ctg890 cp05T...

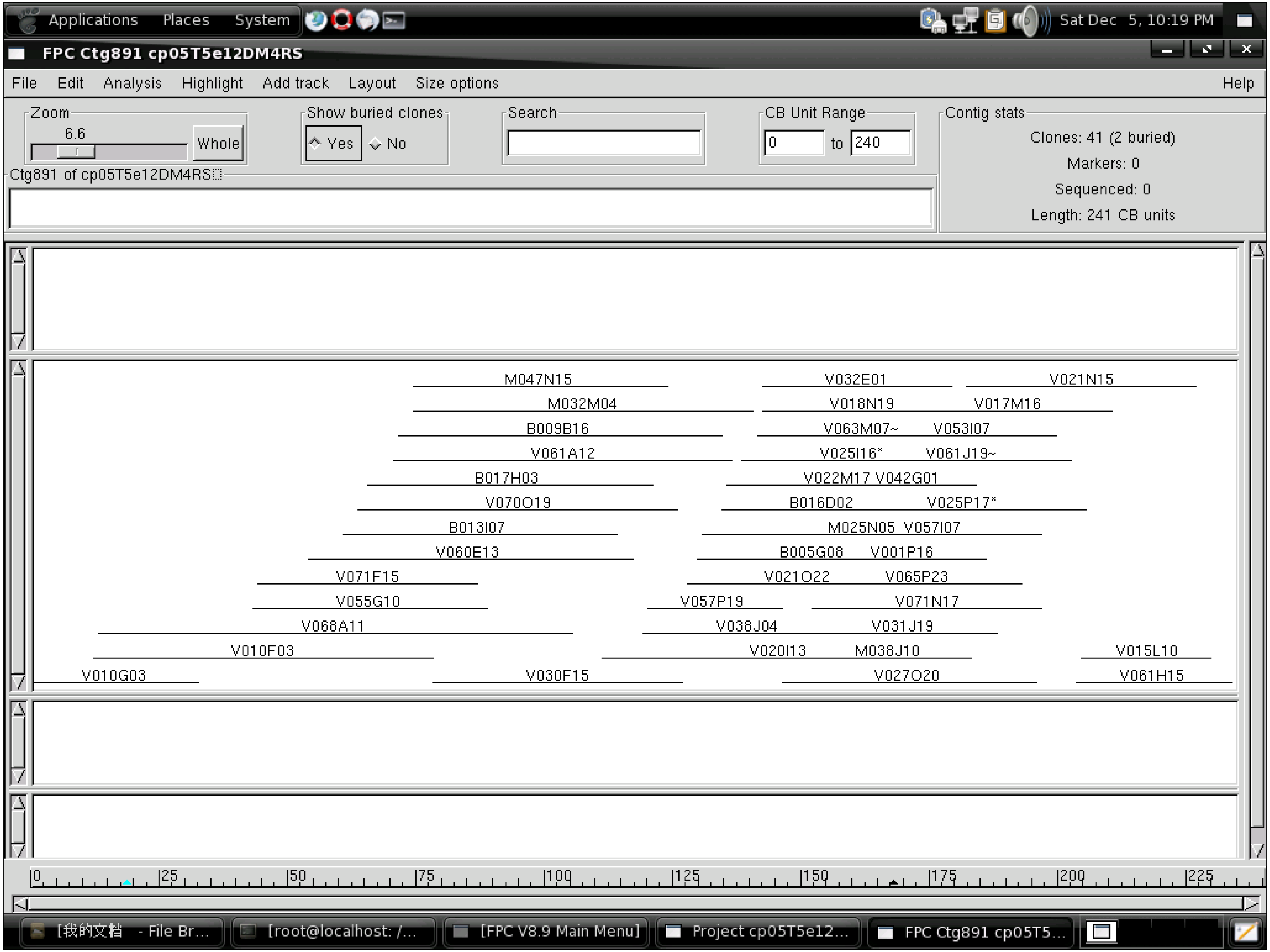
Applications Places System
Sat Dec 5, 10:19 PM
FPC Ctg891 cp05T5e12DM4RS
File Edit Analysis Highlight Add track Layout Size options Help
Zoom
6.6
Whole
Show buried clones
Yes No
Search
CB Unit Range
0 to 240
Contig stats
Clones: 41 (2 buried)
Markers: 0
Sequenced: 0
Length: 241 CB units
Ctg891 of cp05T5e12DM4RS
M047N15
V032E01
V021N15
M032M04
V018N19
V017M16
B009B16
V063M07~
V053I07
V061A12
V025I16*
V061J19~
B017H03
V022M17 V042G01
V070O19
B016D02
V025P17*
B013I07
M025N05 V057I07
V060E13
B005G08
V001P16
V071F15
V021O22
V065P23
V055G10
V057P19
V071N17
V068A11
V038J04
V031J19
V010F03
V020I13
M038J10
V015L10
V010G03
V030F15
V027O20
V061H15
0 25 50 75 100 125 150 175 200 225
[我的文档 - File Br...
[root@localhost: /...
[FPC V8.9 Main Menu]
Project cp05T5e12...
FPC Ctg891 cp05T5...

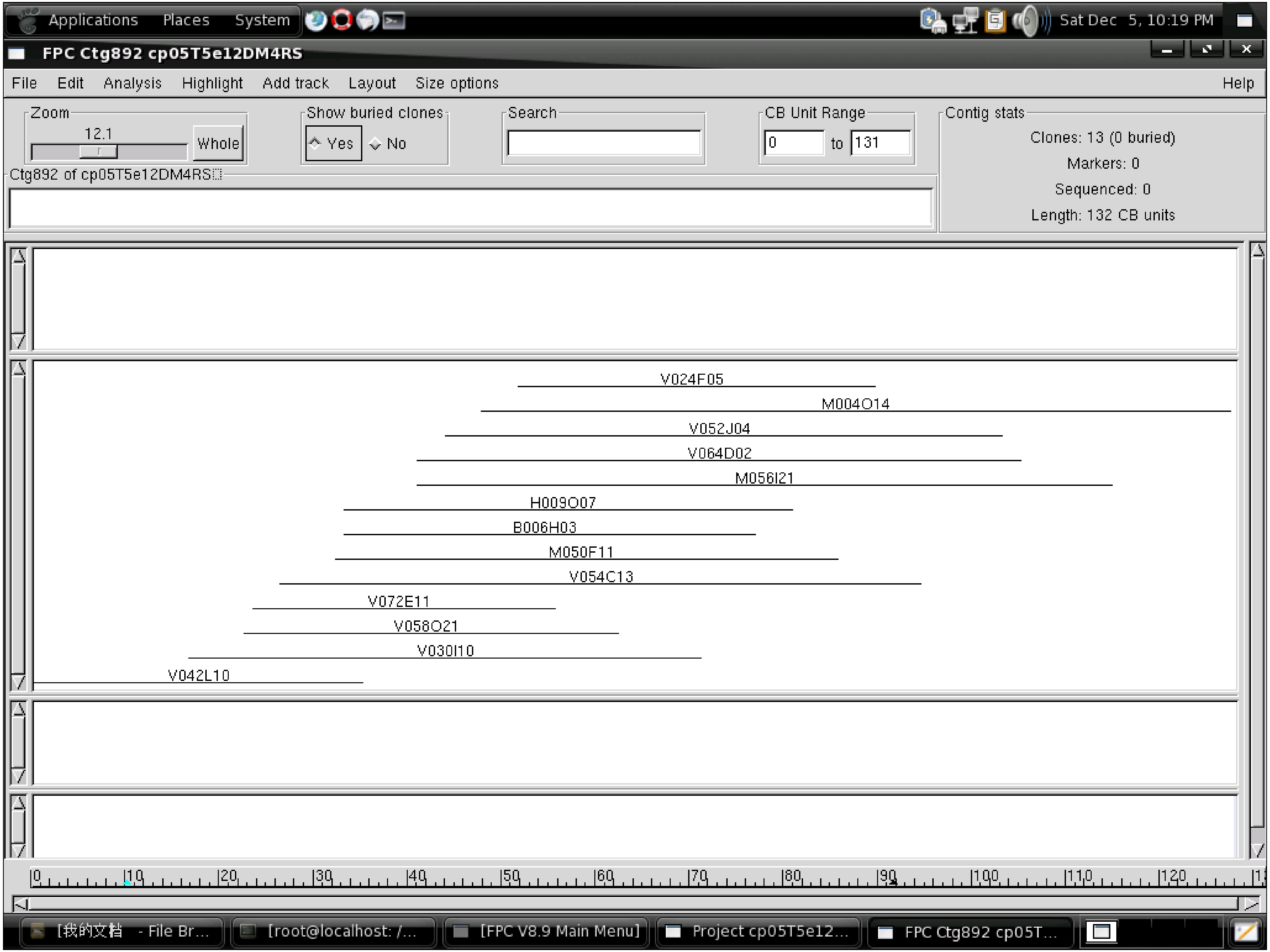
Applications
Places
System
Sat Dec 5, 10:19 PM
FPC Ctg892 cp05T5e12DM4RS
File
Edit
Analysis
Highlight
Add track
Layout
Size options
Help
Zoom
12.1
Whole
Show buried clones
Yes
No
Search
CB Unit Range
0
to
131
Contig stats
Clones: 13 (0 buried)
Markers: 0
Sequenced: 0
Length: 132 CB units
Ctg892 of cp05T5e12DM4RS
V024F05
M004O14
V052J04
V064D02
M056I21
H009O07
B006H03
M050F11
V054C13
V072E11
V058O21
V030I10
V042L10
0
10
20
30
40
50
60
70
80
90
100
110
120
[我的文档 - File Br...
[root@localhost: /...
[FPC V8.9 Main Menu]
Project cp05T5e12...
FPC Ctg892 cp05T...

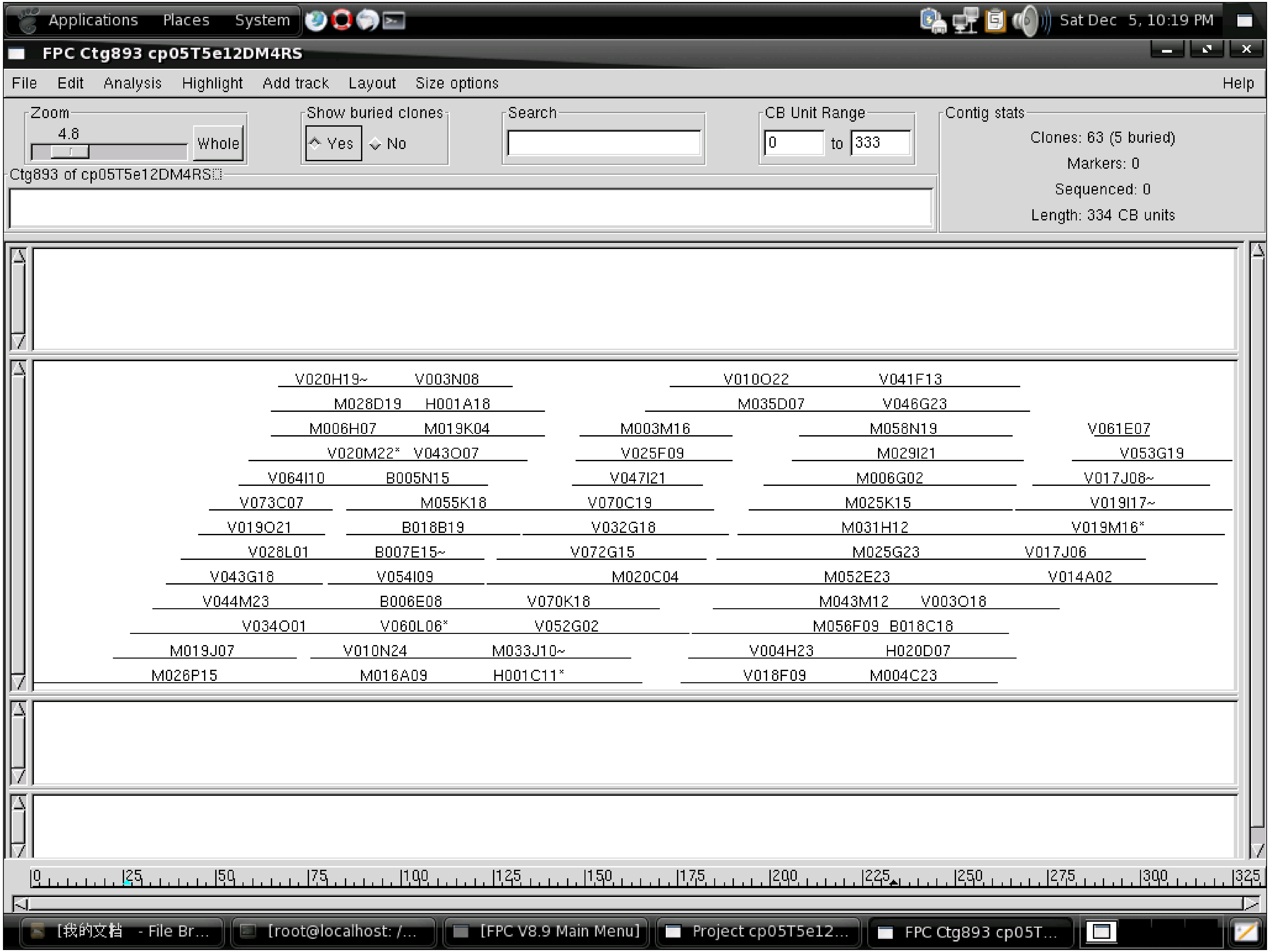
Applications Places System
Sat Dec 5, 10:19 PM
FPC Ctg893 cp05T5e12DM4RS
File Edit Analysis Highlight Add track Layout Size options Help
Zoom
4.8
Whole
Show buried clones
Yes No
Search
CB Unit Range
0 to 333
Contig stats
Clones: 63 (5 buried)
Markers: 0
Sequenced: 0
Length: 334 CB units
Ctg893 of cp05T5e12DM4RS
V020H19~ V003N08 V010O22 V041F13
M028D19 H001A18 M035D07 V046G23
M006H07 M019K04 M003M16 M058N19 V061E07
V020M22* V043O07 V025F09 M029I21 V053G19
V064I10 B005N15 V047I21 M006G02 V017J08~
V073C07 M055K18 V070C19 M025K15 V019I17~
V019O21 B018B19 V032G18 M031H12 V019M16*
V028L01 B007E15~ V072G15 M025G23 V017J06
V043G18 V054I09 M020C04 M052E23 V014A02
V044M23 B006E08 V070K18 M043M12 V003O18
V034O01 V060L06* V052G02 M056F09 B018C18
M019J07 V010N24 M033J10~ V004H23 H020D07
M026P15 M016A09 H001C11* V018F09 M004C23
0 25 50 75 100 125 150 175 200 225 250 275 300 325
[我的文档 - File Br...
[root@localhost: /...
[FPC V8.9 Main Menu]
Project cp05T5e12...
FPC Ctg893 cp05T...

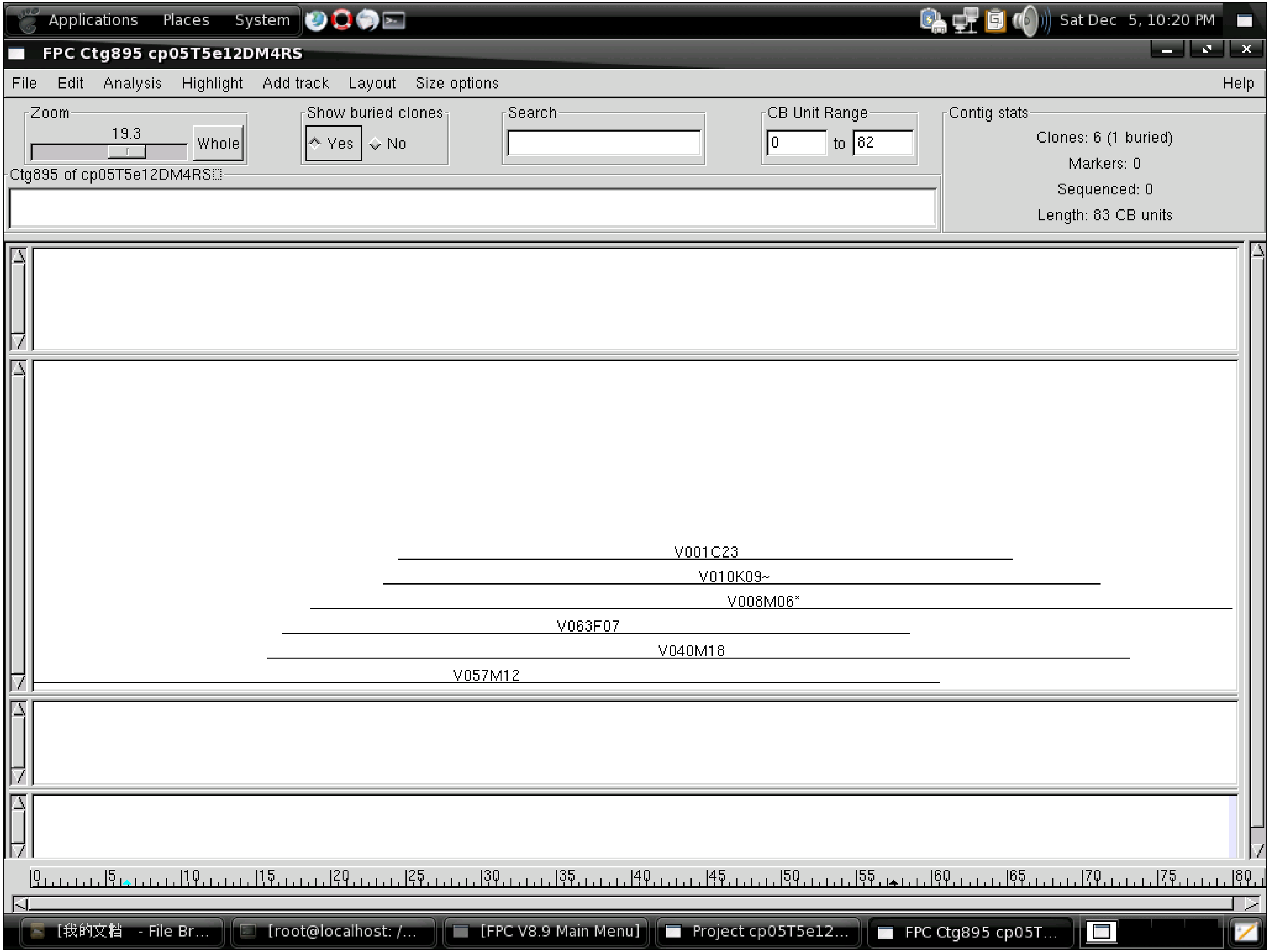
Applications Places System
Sat Dec 5, 10:20 PM
FPC Ctg895 cp05T5e12DM4RS
File Edit Analysis Highlight Add track Layout Size options
Help
Zoom
19.3
Whole
Show buried clones
Yes
No
Search
CB Unit Range
0
to
82
Contig stats
Clones: 6 (1 buried)
Markers: 0
Sequenced: 0
Length: 83 CB units
Ctg895 of cp05T5e12DM4RS
V001C23
V010K09~
V008M06*
V063F07
V040M18
V057M12
0 5 10 15 20 25 30 35 40 45 50 55 60 65 70 75 80
[我的文档 - File Br...
[root@localhost: /...
[FPC V8.9 Main Menu]
Project cp05T5e12...
FPC Ctg895 cp05T...

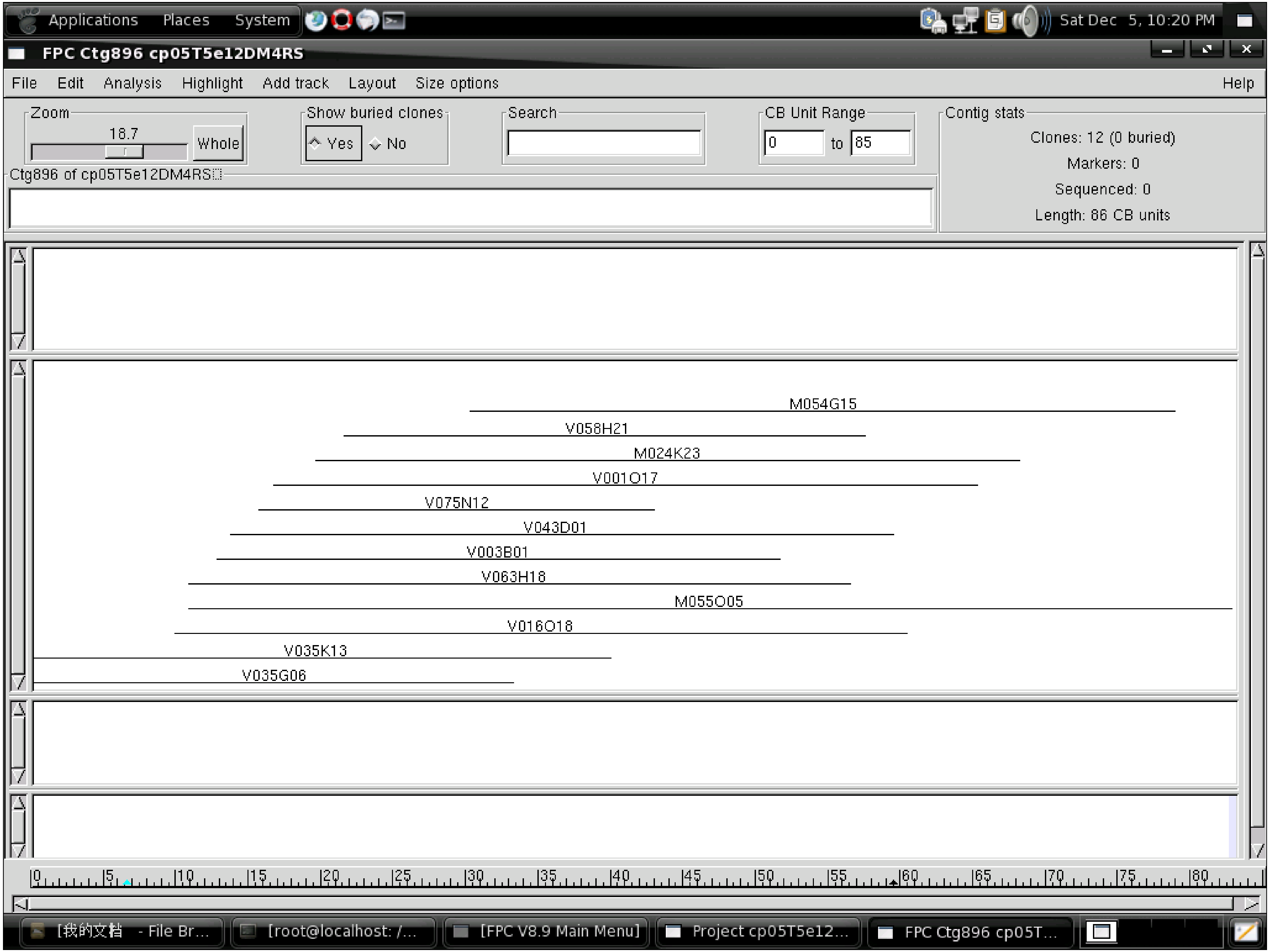
Applications Places System
Sat Dec 5, 10:20 PM
FPC Ctg896 cp05T5e12DM4RS
File Edit Analysis Highlight Add track Layout Size options Help
Zoom
18.7
Whole
Show buried clones
Yes No
Search
CB Unit Range
0 to 85
Contig stats
Clones: 12 (0 buried)
Markers: 0
Sequenced: 0
Length: 86 CB units
Ctg896 of cp05T5e12DM4RS
M054G15
V058H21
M024K23
V001O17
V075N12
V043D01
V003B01
V063H18
M055O05
V016O18
V035K13
V035G06
0 5 10 15 20 25 30 35 40 45 50 55 60 65 70 75 80
[我的文档 - File Br...
[root@localhost: /...
[FPC V8.9 Main Menu]
Project cp05T5e12...
FPC Ctg896 cp05T...

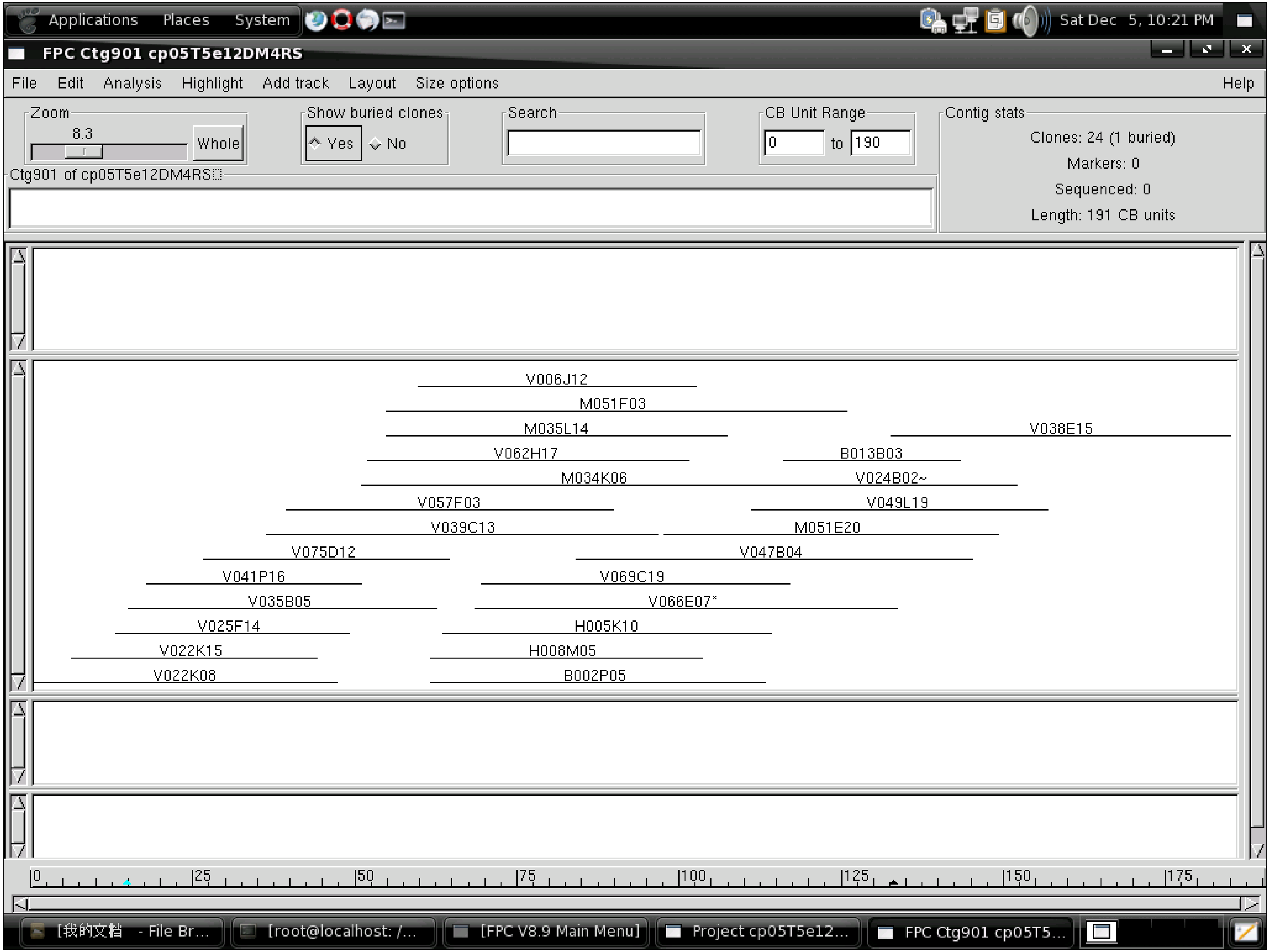
Applications
Places
System
Sat Dec 5, 10:21 PM
FPC Ctg901 cp05T5e12DM4RS
File
Edit
Analysis
Highlight
Add track
Layout
Size options
Help
Zoom
8.3
Whole
Show buried clones
Yes
No
Search
CB Unit Range
0
to
190
Contig stats
Clones: 24 (1 buried)
Markers: 0
Sequenced: 0
Length: 191 CB units
Ctg901 of cp05T5e12DM4RS
V006J12
M051F03
M035L14
V038E15
V062H17
B013B03
M034K06
V024B02~
V057F03
V049L19
V039C13
M051E20
V075D12
V047B04
V041P16
V069C19
V035B05
V066E07*
V025F14
H005K10
V022K15
H008M05
V022K08
B002P05
0
25
50
75
100
125
150
175
[我的文档 - File Br...
[root@localhost: /...
[FPC V8.9 Main Menu]
Project cp05T5e12...
FPC Ctg901 cp05T5...

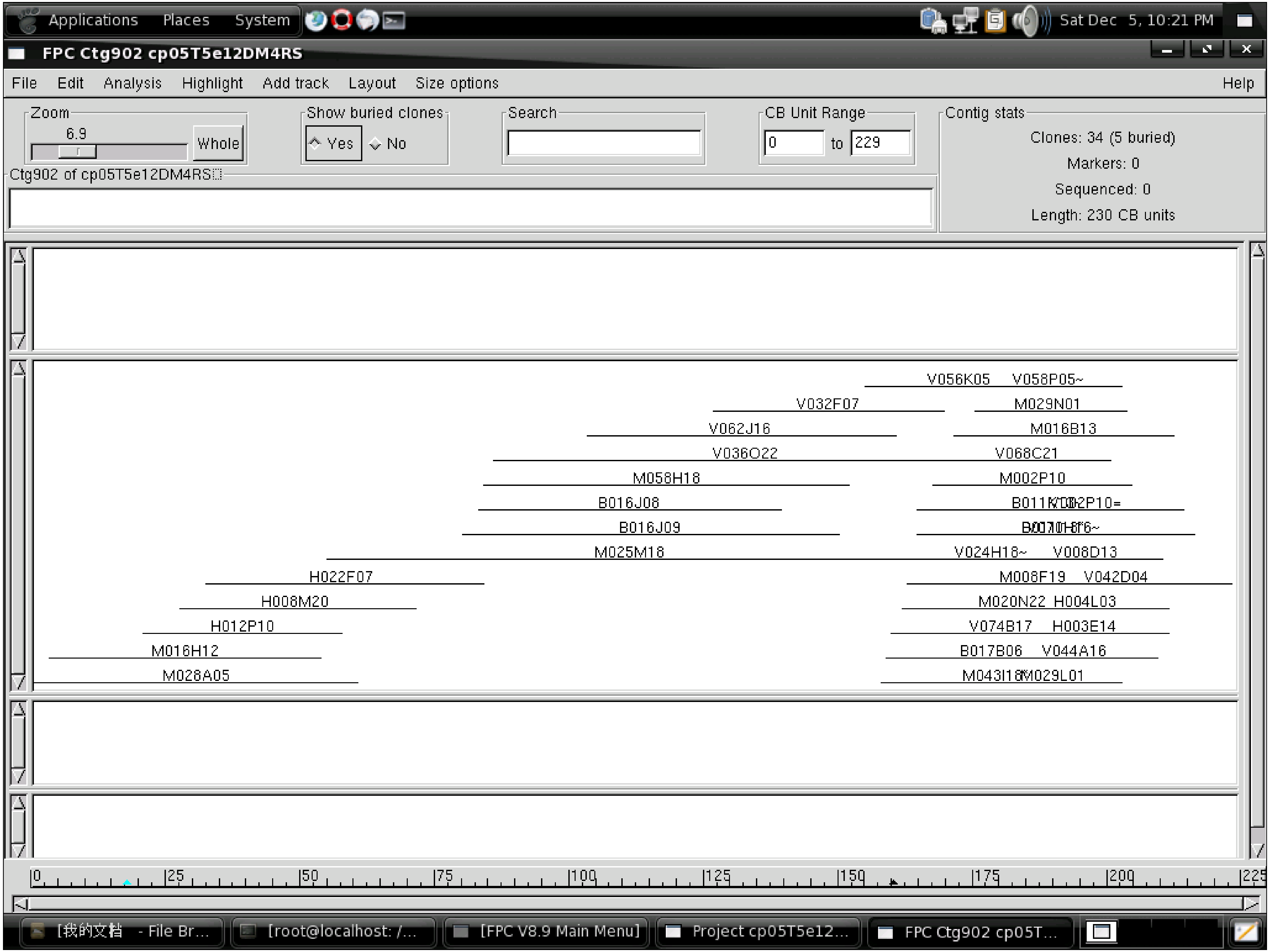
Applications Places System
Sat Dec 5, 10:21 PM
FPC Ctg902 cp05T5e12DM4RS
File Edit Analysis Highlight Add track Layout Size options Help
Zoom
6.9
Whole
Show buried clones
Yes
No
Search
CB Unit Range
0 to 229
Contig stats
Clones: 34 (5 buried)
Markers: 0
Sequenced: 0
Length: 230 CB units
Ctg902 of cp05T5e12DM4RS
V056K05
V058P05~
V032F07
M029N01
V062J16
M016B13
V036O22
V068C21
M058H18
M002P10
B016J08
B016J09
M025M18
V024H18~
V008D13
H022F07
M008F19
V042D04
H008M20
M020N22
H004L03
H012P10
V074B17
H003E14
M016H12
B017B06
V044A16
M028A05
0 25 50 75 100 125 150 175 200 225
[我的文档 - File Br...
[root@localhost: /...
[FPC V8.9 Main Menu]
Project cp05T5e12...
FPC Ctg902 cp05T...

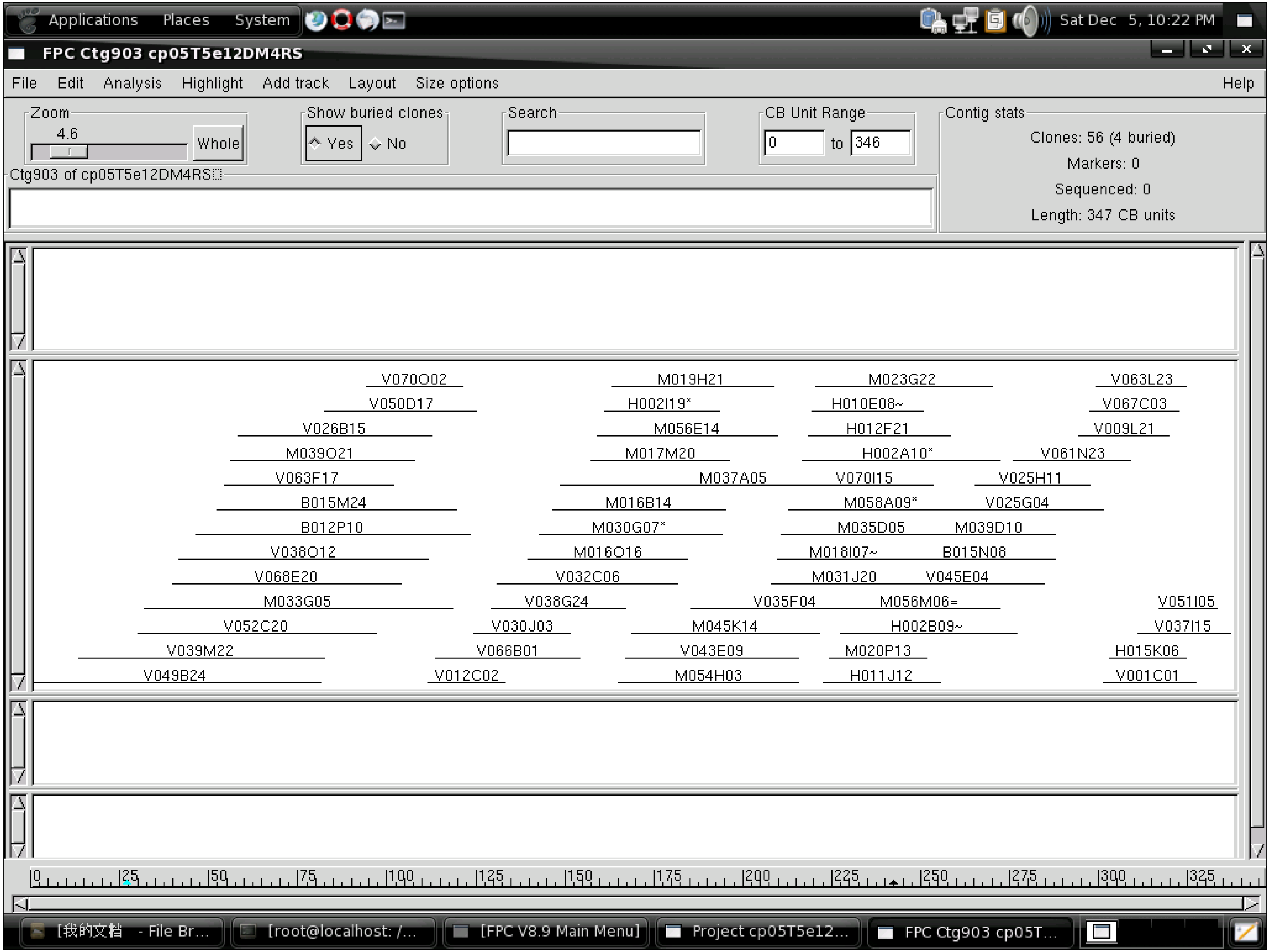
Applications Places System
Sat Dec 5, 10:22 PM
FPC Ctg903 cp05T5e12DM4RS
File Edit Analysis Highlight Add track Layout Size options Help
Zoom
4.6
Whole
Show buried clones
Yes No
Search
CB Unit Range
0 to 346
Contig stats
Clones: 56 (4 buried)
Markers: 0
Sequenced: 0
Length: 347 CB units
Ctg903 of cp05T5e12DM4RS
V070O02
M019H21
M023G22
V063L23
V050D17
H002I19*
H010E08~
V067C03
V026B15
M056E14
H012F21
V009L21
M039O21
M017M20
H002A10*
V061N23
V063F17
M037A05
V070I15
V025H11
B015M24
M016B14
M058A09*
V025G04
B012P10
M030G07*
M035D05
M039D10
V038O12
M016O16
M018I07~
B015N08
V068E20
V032C06
M031J20
V045E04
M033G05
V038G24
V035F04
M056M06=
V051I05
V052C20
V030J03
M045K14
H002B09~
V037I15
V039M22
V066B01
V043E09
M020P13
H015K06
V049B24
V012C02
M054H03
H011J12
V001C01
0 25 50 75 100 125 150 175 200 225 250 275 300 325
[我的文档 - File Br...
[root@localhost: /...
[FPC V8.9 Main Menu]
Project cp05T5e12...
FPC Ctg903 cp05T...

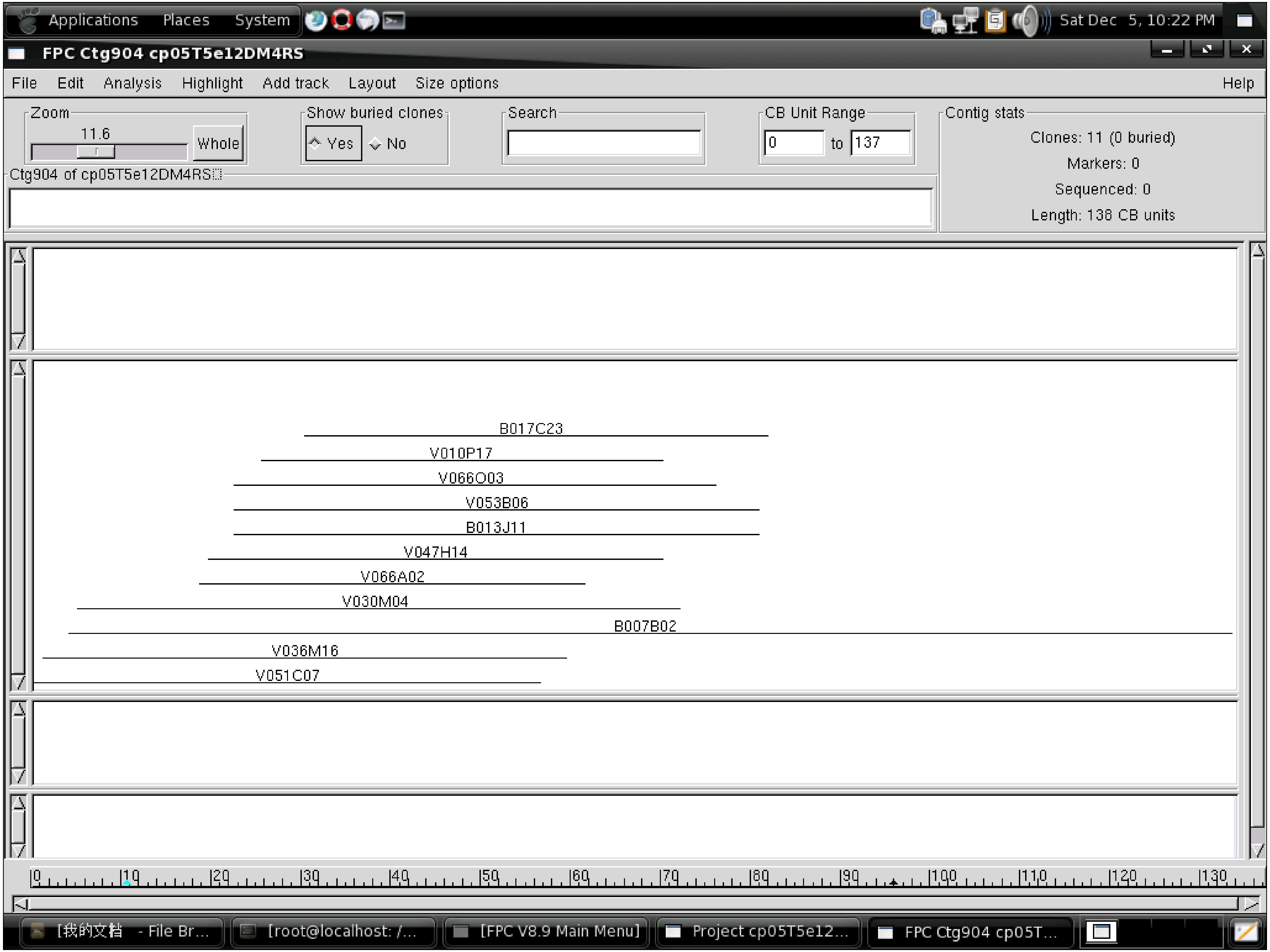
Applications Places System
Sat Dec 5, 10:22 PM
FPC Ctg904 cp05T5e12DM4RS
File Edit Analysis Highlight Add track Layout Size options Help
Zoom
11.6
Whole
Show buried clones
Yes No
Search
CB Unit Range
0 to 137
Contig stats
Clones: 11 (0 buried)
Markers: 0
Sequenced: 0
Length: 138 CB units
Ctg904 of cp05T5e12DM4RS
B017C23
V010P17
V066O03
V053B06
B013J11
V047H14
V066A02
V030M04
B007B02
V036M16
V051C07
0 10 20 30 40 50 60 70 80 90 100 110 120 130
[我的文档 - File Br...
[root@localhost: /...
[FPC V8.9 Main Menu]
Project cp05T5e12...
FPC Ctg904 cp05T...
