# Supplementary material for: A BAC/BIBAC-based physical map of chickpea, Cicer arietinum L
Source: BMC Genomics. 2010 Sep 17;11:501. doi: 10.1186/1471-2164-11-501 (PMC2996997; doi:10.1186/1471-2164-11-501)
Supplement: Additional file 6 — Shows the positive clones and associated contigs of SSR primers identified from 36,864 clones of the chickpea-CHV BIBAC library (384-well microplates 1 - 96). [file 1471-2164-11-501-S6.PDF]

Additional file 6. Positive clones and associated contigs of SSR primers identified from 36,864 clones of the chickpea-CHV BIBAC library (384-well microplates 1 - 96). The clones highlighted in bold face were fingerprinted and used in the physical map construction (microplates 1-76) whereas the clones not highlighted were not used for the map construction (microplates 77 - 96). For the markers giving a large number of positive clones, only examples of positive clones and their associated contigs are listed. ctg, contig; FP, fingerprinting.

SSR marker	Positive clone	Contig	
		Name	Size (kb)
H3C041	**V058I22**	ctg2831	2,036
	V095I18		
TA2	**V053O04**	ctg3270	955
	**V066I24**	ctg3270	
H1A12	**V025B21**	ctg755	1,149
	**V026G24**	ctg755	
	**V023E03**	ctg755	
H1C92	**V016G24**	ctg87	458
	**V025E24**	ctg87	
	**V066C22**	ctg87	
	**V009N04**	singleton	
H1G20	**V037I10**	ctg559	1,159
	**V073G21**	ctg559	
	**V022A06**	ctg42	737
	V078L12		
	V090L12		
	…		
	672 positive clones		
H1C092	**V053P03**	ctg3579	1,253
	**V065C07**	ctg1843	692
	V077A04		
	…		
	101 positive clones		
H3C11a	**V027D09**	ctg71	2,939
	**V039D20**	ctg71	
	**V038N18**	ctg71	
	**V029O02**	ctg71	
	V080F17		
	V090C16		
	V093O21		
	V094C23		
H1B17	**V019A15**	ctg755	1,149
	**V022H03**	ctg755	
	**V045C20**	ctg755	
	**V047G17**	ctg755	
	**V067K21**	ctg755	
	**V035J07**	singleton	
TA3	**V057B04**	ctg89	1,208
	**V063P17**	ctg89	
	**V022F04**	ctg111	1,069
	**V036A03**	singleton	
	...		
	59 positive clones		

H1A19	**V074C07**	ctg61	1,032
	**V074C18**	ctg61	
	**V051P08**	ctg61	
	**V066P07**	ctg61	
	**V058K10**	ctg87	458
	**V075K18**	ctg87	
	**V037M10**	ctg2849	
	**V051P02**	singleton	
	V079A12		
	...		
	104 positive clones		
H1H22	**V036A06**	ctg3579	1,253
	**V033A03**	ctg3579	
	**V033J06**	ctg3579	
	**V055H13**	ctg3579	
	**V072E10**	ctg3579	
	**V062A16**	ctg3579	
	**V062B18**	ctg3579	
	**V019M06**	ctg993	782
	**V034A07**	ctg993	
	**V031K21**	ctg993	
	**V025K04**	ctg993	
	**V028J12**	ctg993	
	**V066I04**	ctg993	
	**V037D06**	ctg733	905
	**V016O22**	ctg733	
	**V052D13**	ctg733	
	V086I19		
	V096H12		
	…		
	47 positive clones		
H1C9-2	**V004E03**	ctg906	1,400
	**V017B07**	ctg4013	200
	**V019L02**	ctg275	647
	**V019K04**	ctg157	946
	**...**		
	216 positive clones		
H2F24	**V008A01**	ctg3165	925
	**V039D10**	singleton	
	**V054J03**	ctg3165	
H1A18	**V023C02**	singleton	
	**V017N03**	ctg686	483
	**V020M06**	singleton	
	**V027G01**	failed in FP	
	**V057C02**	ctg686	
H1B6	**V049N08**	failed in FP	
	**V049N09**	ctg3481	622
	**V035A06**	ctg3481	
H2J2	**V017C08**	ctg3585	700
	**V022C06**	ctg1751	897
	**V020C07**	ctg2436	1,310
	…		
	248 positive clones		
