# Supplementary material for: A BAC/BIBAC-based physical map of chickpea, Cicer arietinum L
Source: BMC Genomics. 2010 Sep 17;11:501. doi: 10.1186/1471-2164-11-501 (PMC2996997; doi:10.1186/1471-2164-11-501)
Supplement: Additional file 2 — Shows the contigs constituting the physical map of the chickpea genome 2 (ctg904-1902). [file 1471-2164-11-501-S2.PDF]

A BAC/BIBAC-based physical map of chickpea, *Cicer arietinum* L.

Xiaojun Zhang[1,6], Chantel F Scheuring[1], Meiping Zhang[1,2], Jennifer J Dong[1], Yang Zhang[1], James J Huang[1], Mi-Kyung Lee[1], Shahal Abbo[3], Amir Sherman[4], Dani Shtienberg[4], Weidong Chen[5], Fred Muehlbauer[5], Hong-Bin Zhang[1,*]

[1] Department of Soil and Crop Sciences, Texas A&M University, College Station, Texas 77843-2474, USA
[2] College of Life Science, Jilin Agricultural University, Changchun, Jilin 130118, China
[3] Institute of Plant Science and Genetics in Agriculture, The Hebrew University of Jerusalem, Rehovot, 76100, Israel
[4] The Volcani Center, P.O. Box 6, Bet-Dagan, 50250, Israel
[5] USDA-ARS and Department of Crop and Soil Sciences, Washington State University, Pullman, WA 99164-6434, USA
[6] The Key Laboratory of Experimental Marine Biology, Institute of Oceanology, Chinese Academy of Sciences, Qingdao 266071, China

***Corresponding author:** H.-B. Zhang, Tel.: +1-979-862-2244; Fax: +1-979-845-0456; E-mail: hbz7049@tamu.edu

Additional file 2. The contigs constituting the physical map of the chickpea genome 2(ctg905-1902)

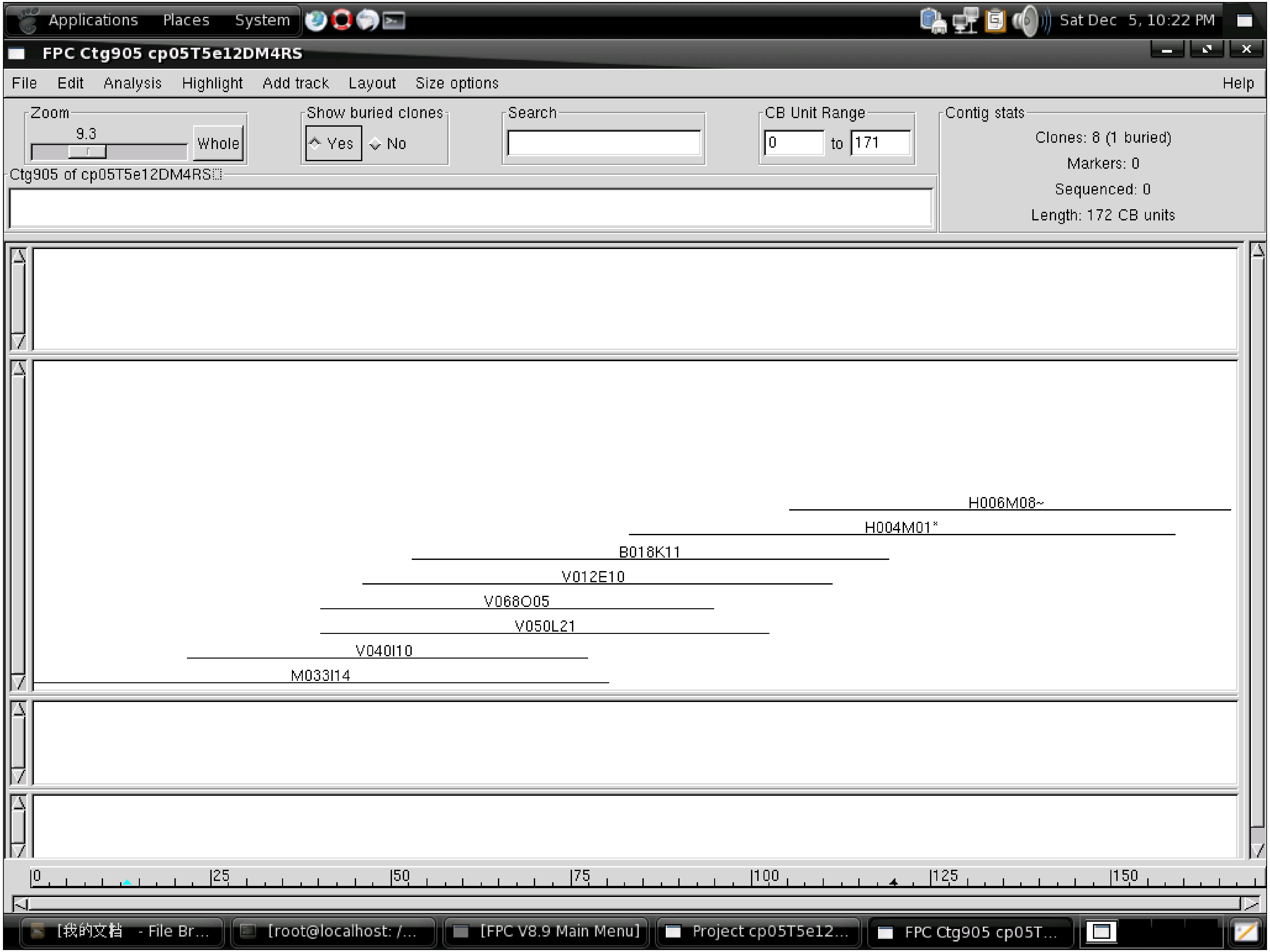
Applications Places System
Sat Dec 5, 10:22 PM
FPC Ctg905 cp05T5e12DM4RS
File Edit Analysis Highlight Add track Layout Size options Help
Zoom
9.3
Whole
Show buried clones
Yes
No
Search
CB Unit Range
0
to
171
Contig stats
Clones: 8 (1 buried)
Markers: 0
Sequenced: 0
Length: 172 CB units
Ctg905 of cp05T5e12DM4RS
H006M08~
H004M01*
B018K11
V012E10
V068O05
V050L21
V040I10
M033I14
0
25
50
75
100
125
150
[我的文档 - File Br...
[root@localhost: /...
[FPC V8.9 Main Menu]
Project cp05T5e12...
FPC Ctg905 cp05T...

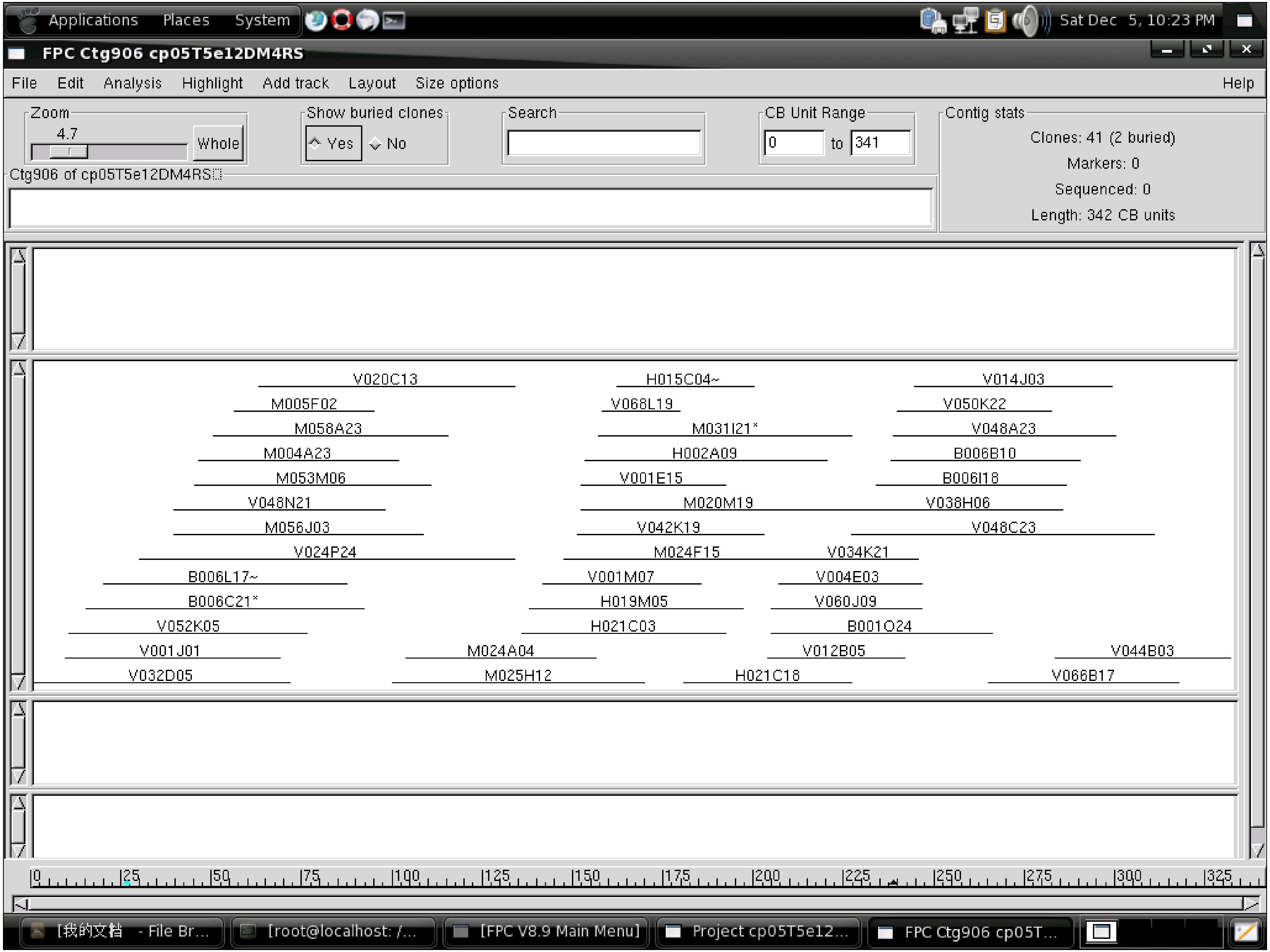

Applications Places System
Sat Dec 5, 10:23 PM
FPC Ctg906 cp05T5e12DM4RS
File Edit Analysis Highlight Add track Layout Size options Help
Zoom
4.7
Whole
Show buried clones
Yes No
Search
CB Unit Range
0 to 341
Contig stats
Clones: 41 (2 buried)
Markers: 0
Sequenced: 0
Length: 342 CB units
Ctg906 of cp05T5e12DM4RS
V020C13
H015C04~
V014J03
M005F02
V068L19
V050K22
M058A23
M031I21*
V048A23
M004A23
H002A09
B006B10
M053M06
V001E15
B006I18
M020M19
V048N21
V038H06
M056J03
V042K19
V048C23
V024P24
M024F15
V034K21
B006L17~
V001M07
V004E03
B006C21*
H019M05
V060J09
V052K05
H021C03
B001O24
V001J01
M024A04
V012B05
V044B03
V032D05
M025H12
H021C18
V066B17
0 25 50 75 100 125 150 175 200 225 250 275 300 325
[我的文档 - File Br...
[root@localhost: /...
[FPC V8.9 Main Menu]
Project cp05T5e12...
FPC Ctg906 cp05T...

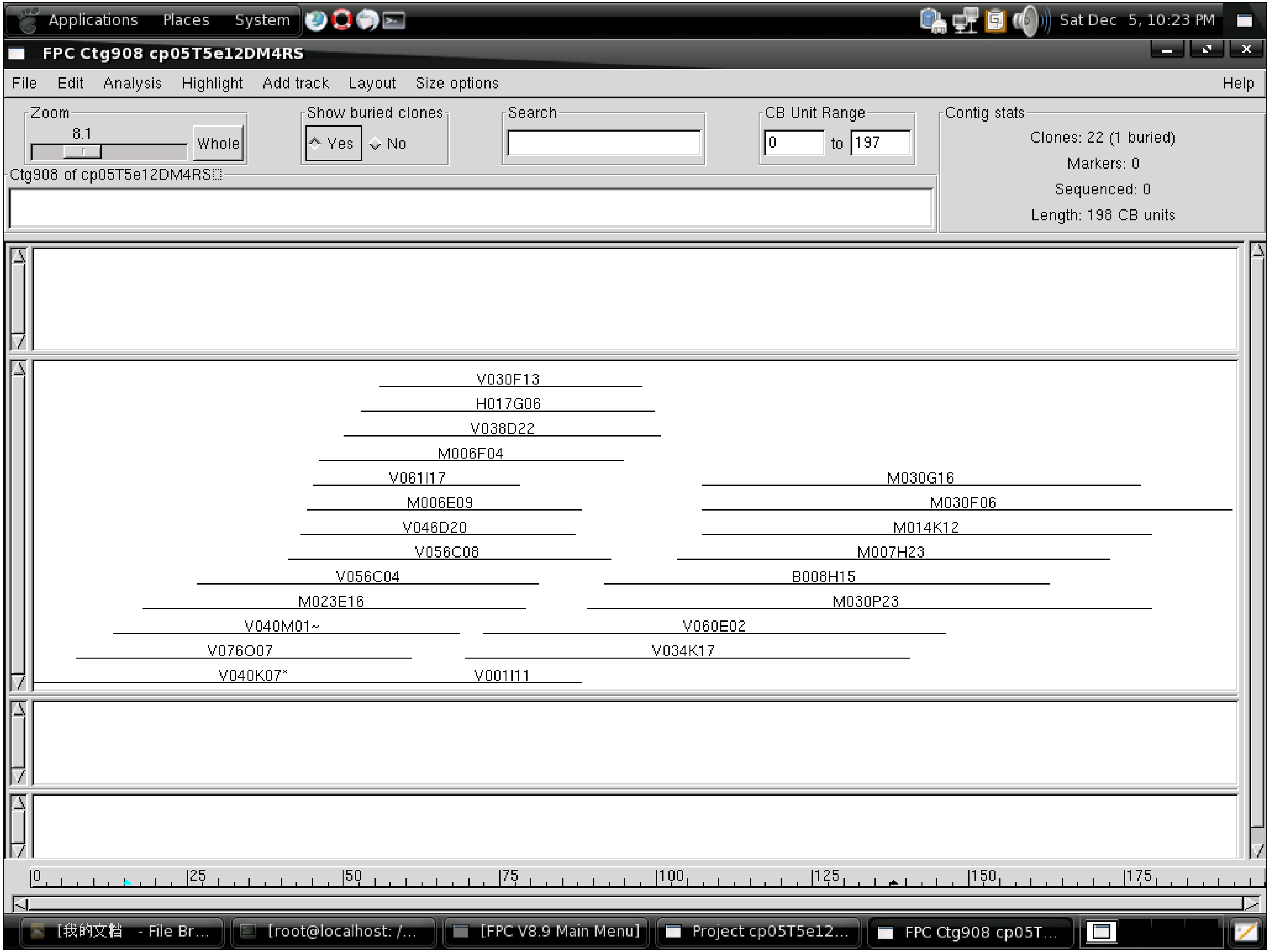
Applications Places System
Sat Dec 5, 10:23 PM
FPC Ctg908 cp05T5e12DM4RS
File Edit Analysis Highlight Add track Layout Size options Help
Zoom
8.1
Whole
Show buried clones
Yes No
Search
CB Unit Range
0 to 197
Contig stats
Clones: 22 (1 buried)
Markers: 0
Sequenced: 0
Length: 198 CB units
Ctg908 of cp05T5e12DM4RS
V030F13
H017G06
V038D22
M006F04
V061I17
M030G16
M006E09
M030F06
V046D20
M014K12
V056C08
M007H23
V056C04
B008H15
M023E16
M030P23
V040M01~
V060E02
V076O07
V034K17
V040K07*
V001I11
0 25 50 75 100 125 150 175
[我的文档 - File Br...
[root@localhost: /...
[FPC V8.9 Main Menu]
Project cp05T5e12...
FPC Ctg908 cp05T...

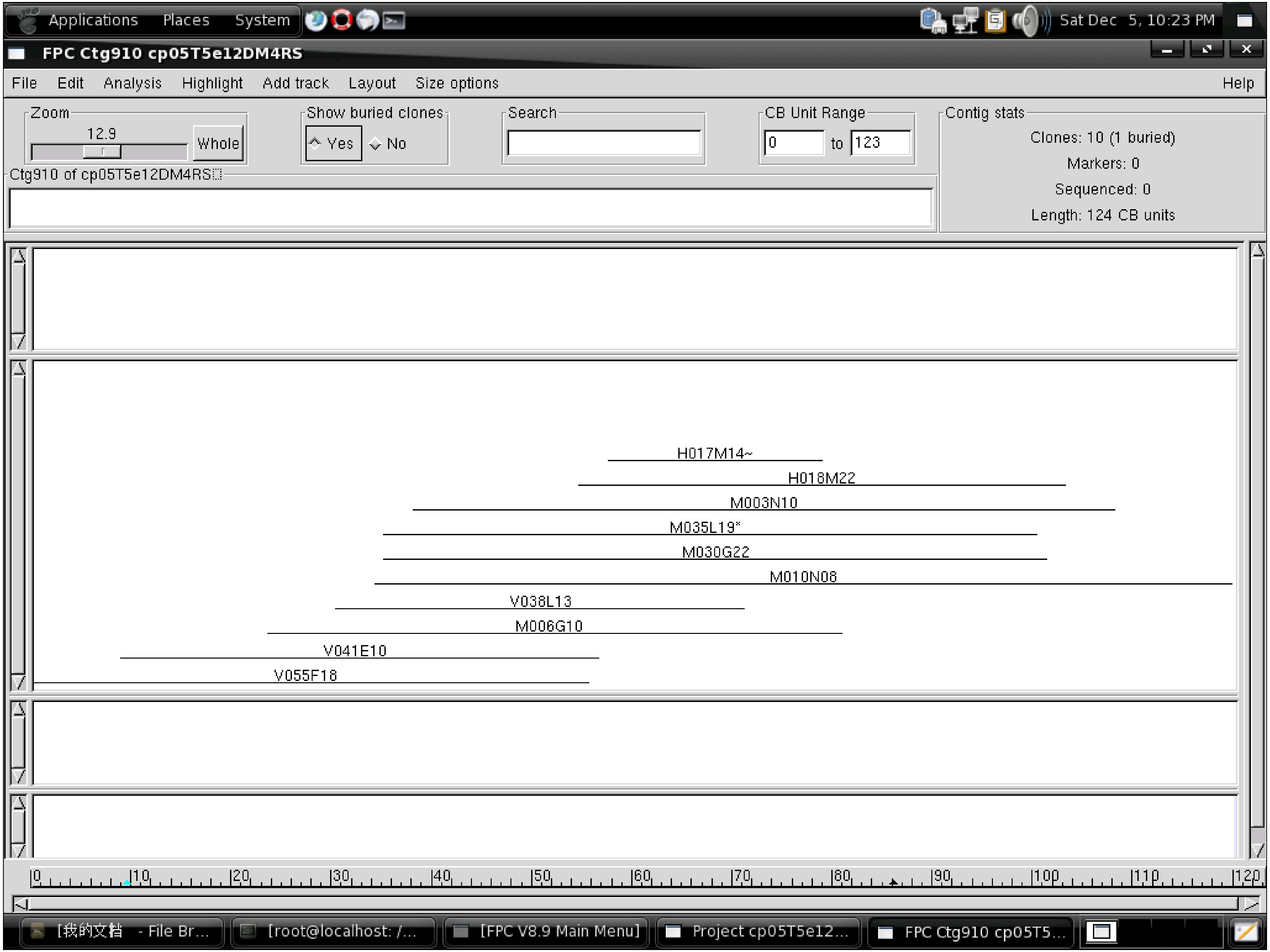
Applications Places System
Sat Dec 5, 10:23 PM
FPC Ctg910 cp05T5e12DM4RS
File Edit Analysis Highlight Add track Layout Size options Help
Zoom
12.9
Whole
Show buried clones
Yes No
Search
CB Unit Range
0 to 123
Contig stats
Clones: 10 (1 buried)
Markers: 0
Sequenced: 0
Length: 124 CB units
Ctg910 of cp05T5e12DM4RS
H017M14~
H018M22
M003N10
M035L19*
M030G22
M010N08
V038L13
M006G10
V041E10
V055F18
0 10 20 30 40 50 60 70 80 90 100 110 120
[我的文档 - File Br...
[root@localhost: /...
[FPC V8.9 Main Menu]
Project cp05T5e12...
FPC Ctg910 cp05T5...

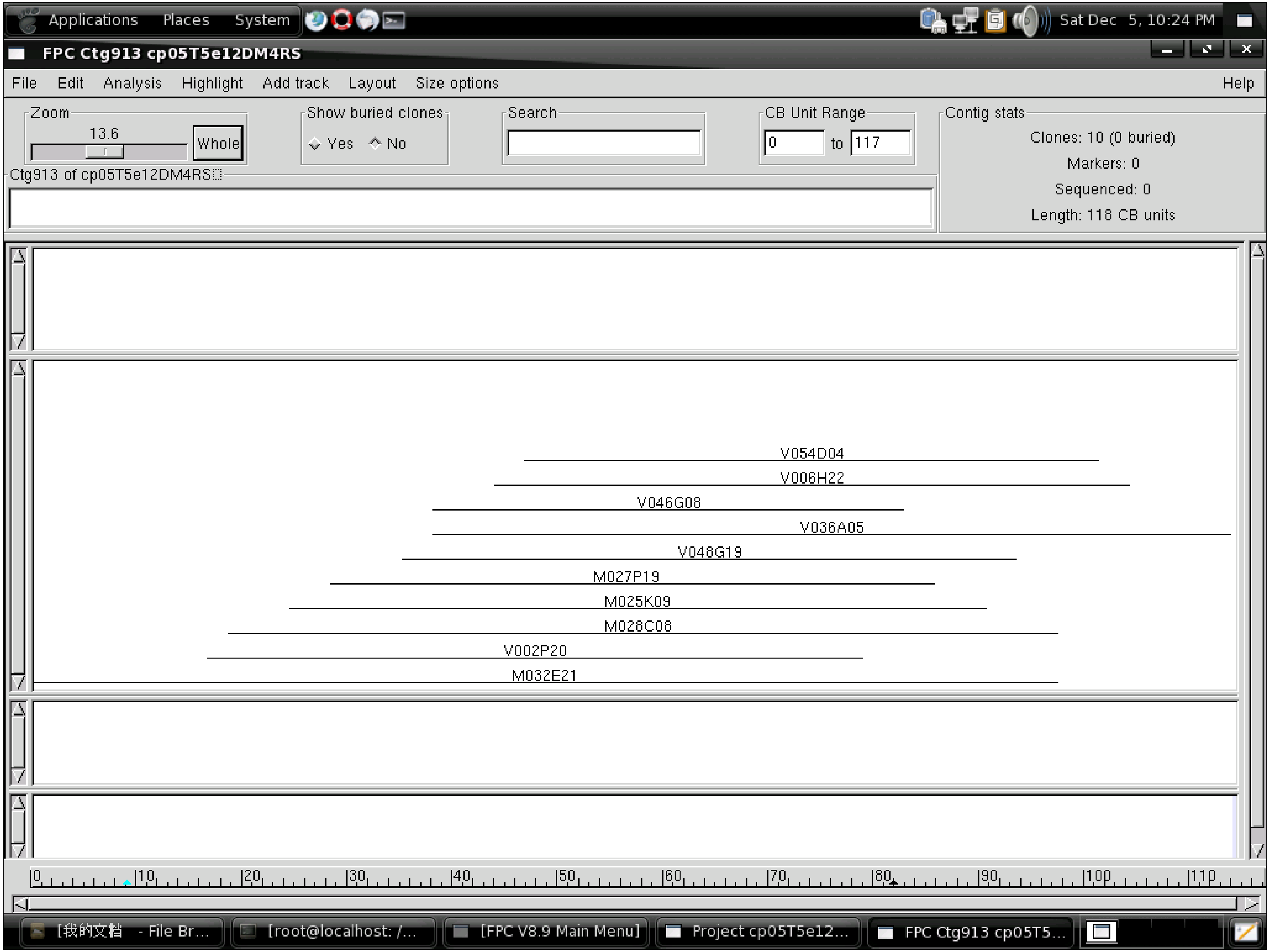
Applications Places System
Sat Dec 5, 10:24 PM
FPC Ctg913 cp05T5e12DM4RS
File Edit Analysis Highlight Add track Layout Size options Help
Zoom
13.6
Whole
Show buried clones
Yes No
Search
CB Unit Range
0 to 117
Contig stats
Clones: 10 (0 buried)
Markers: 0
Sequenced: 0
Length: 118 CB units
Ctg913 of cp05T5e12DM4RS
V054D04
V006H22
V046G08
V036A05
V048G19
M027P19
M025K09
M028C08
V002P20
M032E21
0 10 20 30 40 50 60 70 80 90 100 110
[我的文档 - File Br...
[root@localhost: /...
[FPC V8.9 Main Menu]
Project cp05T5e12...
FPC Ctg913 cp05T5...

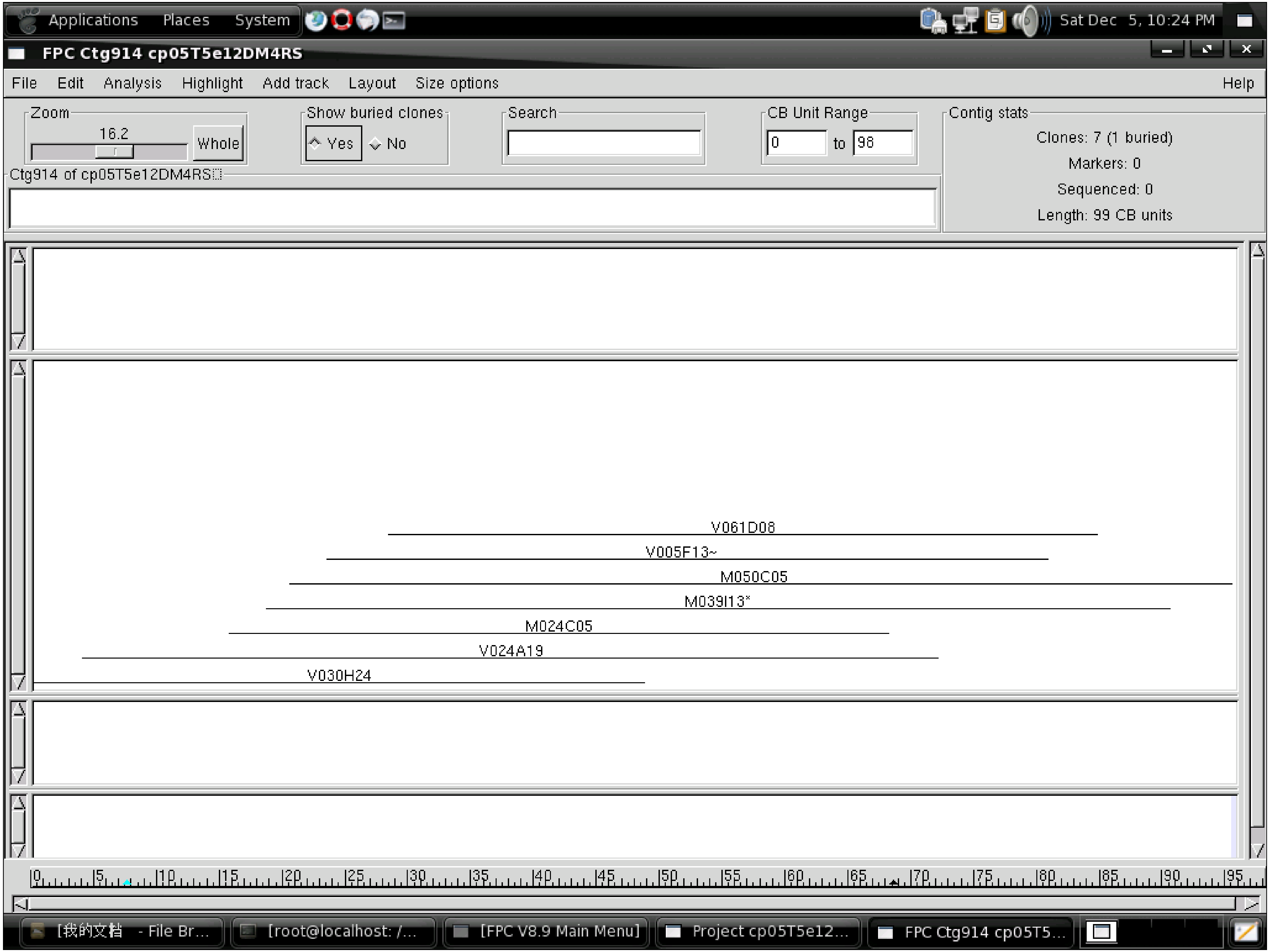
Applications Places System
Sat Dec 5, 10:24 PM
FPC Ctg914 cp05T5e12DM4RS
File Edit Analysis Highlight Add track Layout Size options Help
Zoom
16.2
Whole
Show buried clones
Yes
No
Search
CB Unit Range
0
to
98
Contig stats
Clones: 7 (1 buried)
Markers: 0
Sequenced: 0
Length: 99 CB units
Ctg914 of cp05T5e12DM4RS
V061D08
V005F13~
M050C05
M039I13*
M024C05
V024A19
V030H24
0 5 10 15 20 25 30 35 40 45 50 55 60 65 70 75 80 85 90 95
[我的文档 - File Br...
[root@localhost: /...
[FPC V8.9 Main Menu]
Project cp05T5e12...
FPC Ctg914 cp05T5...

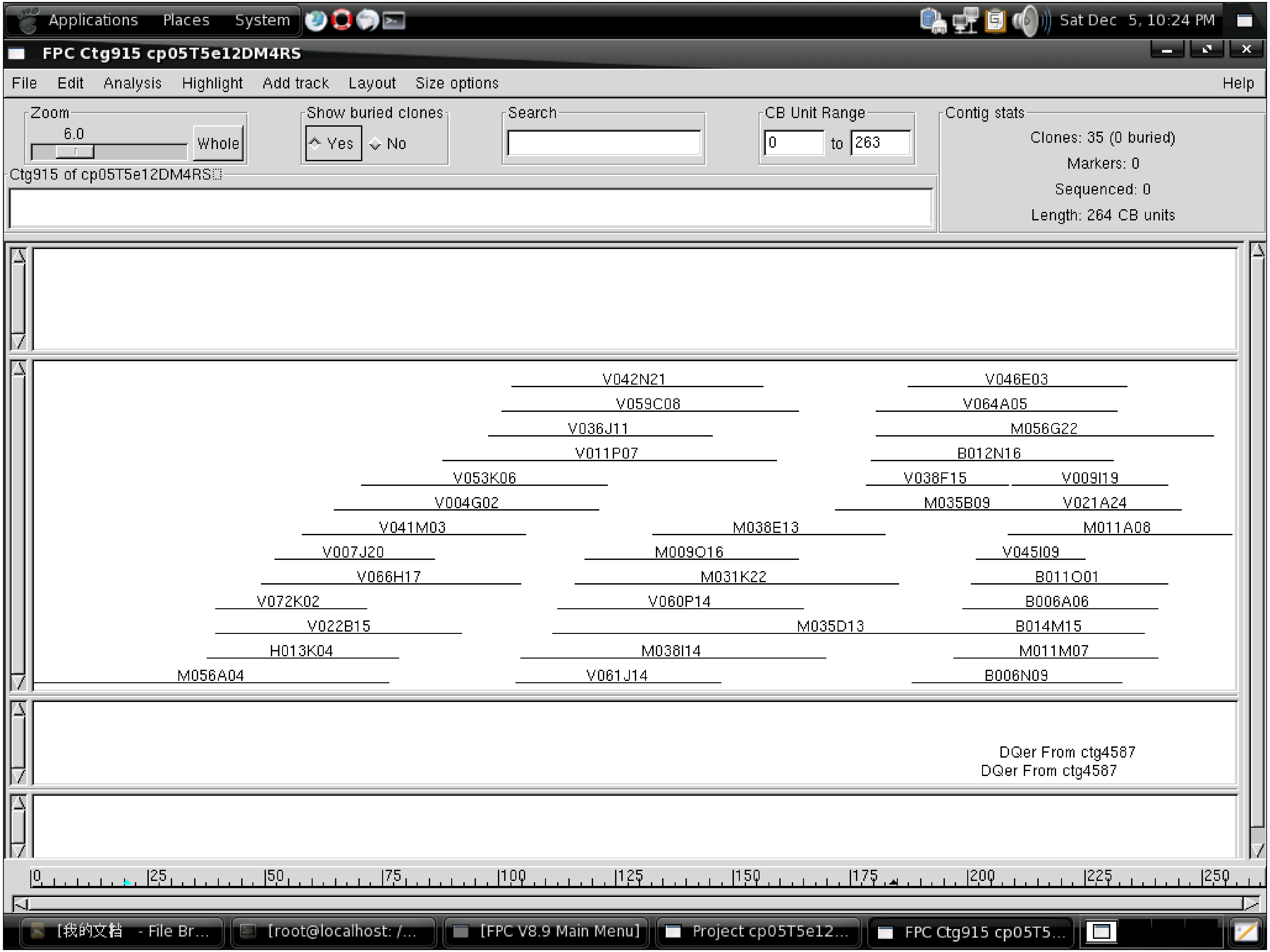
Applications Places System
Sat Dec 5, 10:24 PM
FPC Ctg915 cp05T5e12DM4RS
File Edit Analysis Highlight Add track Layout Size options Help
Zoom
6.0
Whole
Show buried clones
Yes No
Search
CB Unit Range
0 to 263
Contig stats
Clones: 35 (0 buried)
Markers: 0
Sequenced: 0
Length: 264 CB units
Ctg915 of cp05T5e12DM4RS
V042N21
V046E03
V059C08
V064A05
V036J11
M056G22
V011P07
B012N16
V053K06
V038F15
V009I19
V004G02
M035B09
V021A24
V041M03
M038E13
M011A08
V007J20
M009O16
V045I09
V066H17
M031K22
B011O01
V072K02
V060P14
B006A06
V022B15
M035D13
B014M15
H013K04
M038I14
M011M07
M056A04
V061J14
B006N09
DQer From ctg4587
DQer From ctg4587
0 25 50 75 100 125 150 175 200 225 250
[我的文档 - File Br...
[root@localhost: /...
[FPC V8.9 Main Menu]
Project cp05T5e12...
FPC Ctg915 cp05T5...

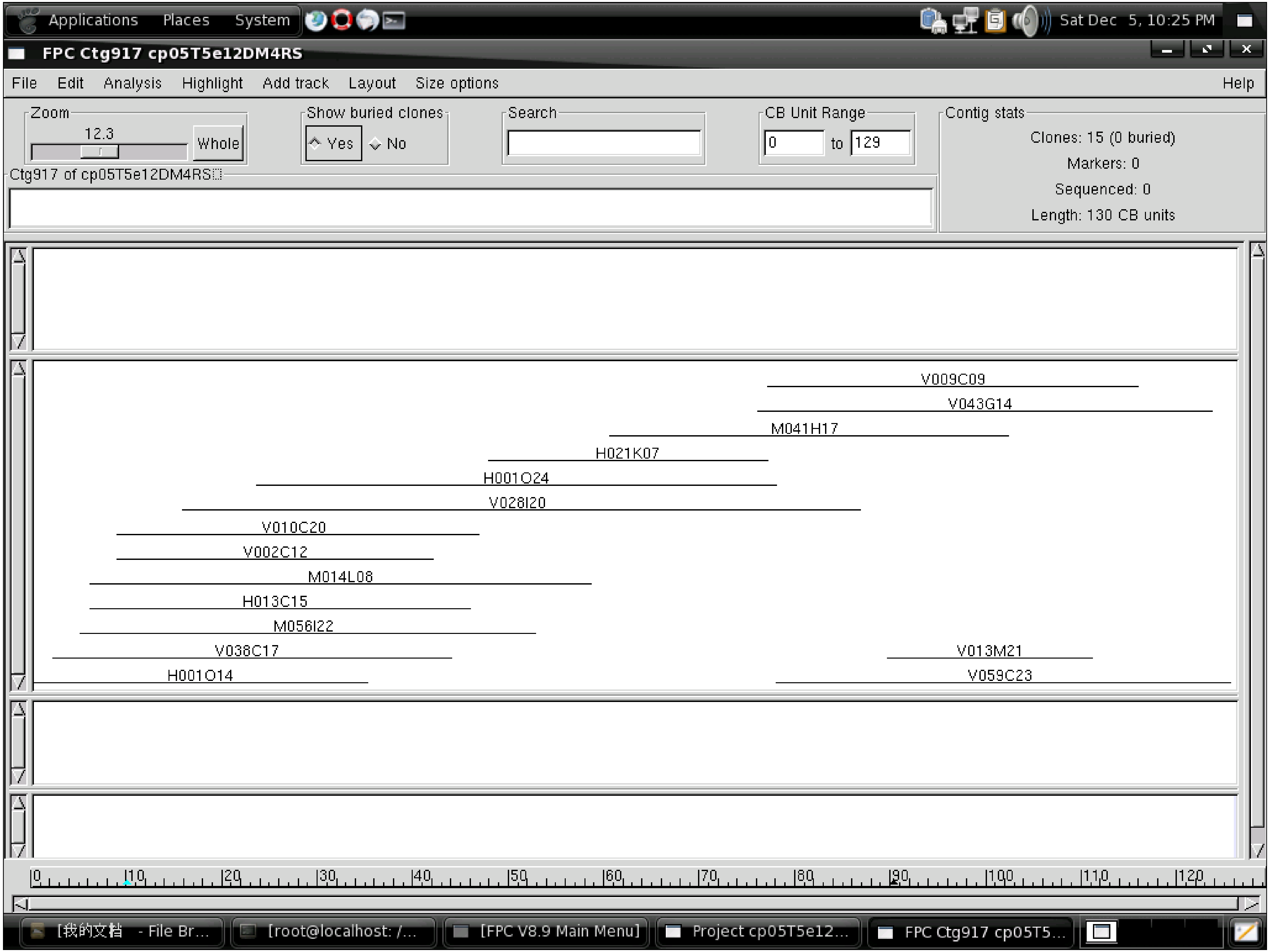
Applications Places System
Sat Dec 5, 10:25 PM
FPC Ctg917 cp05T5e12DM4RS
File Edit Analysis Highlight Add track Layout Size options Help
Zoom
12.3
Whole
Show buried clones
Yes No
Search
CB Unit Range
0 to 129
Contig stats
Clones: 15 (0 buried)
Markers: 0
Sequenced: 0
Length: 130 CB units
Ctg917 of cp05T5e12DM4RS
V009C09
V043G14
M041H17
H021K07
H001O24
V028I20
V010C20
V002C12
M014L08
H013C15
M056I22
V038C17
V013M21
H001O14
V059C23
0 10 20 30 40 50 60 70 80 90 100 110 120
[我的文档 - File Br...
[root@localhost: /...
[FPC V8.9 Main Menu]
Project cp05T5e12...
FPC Ctg917 cp05T5...

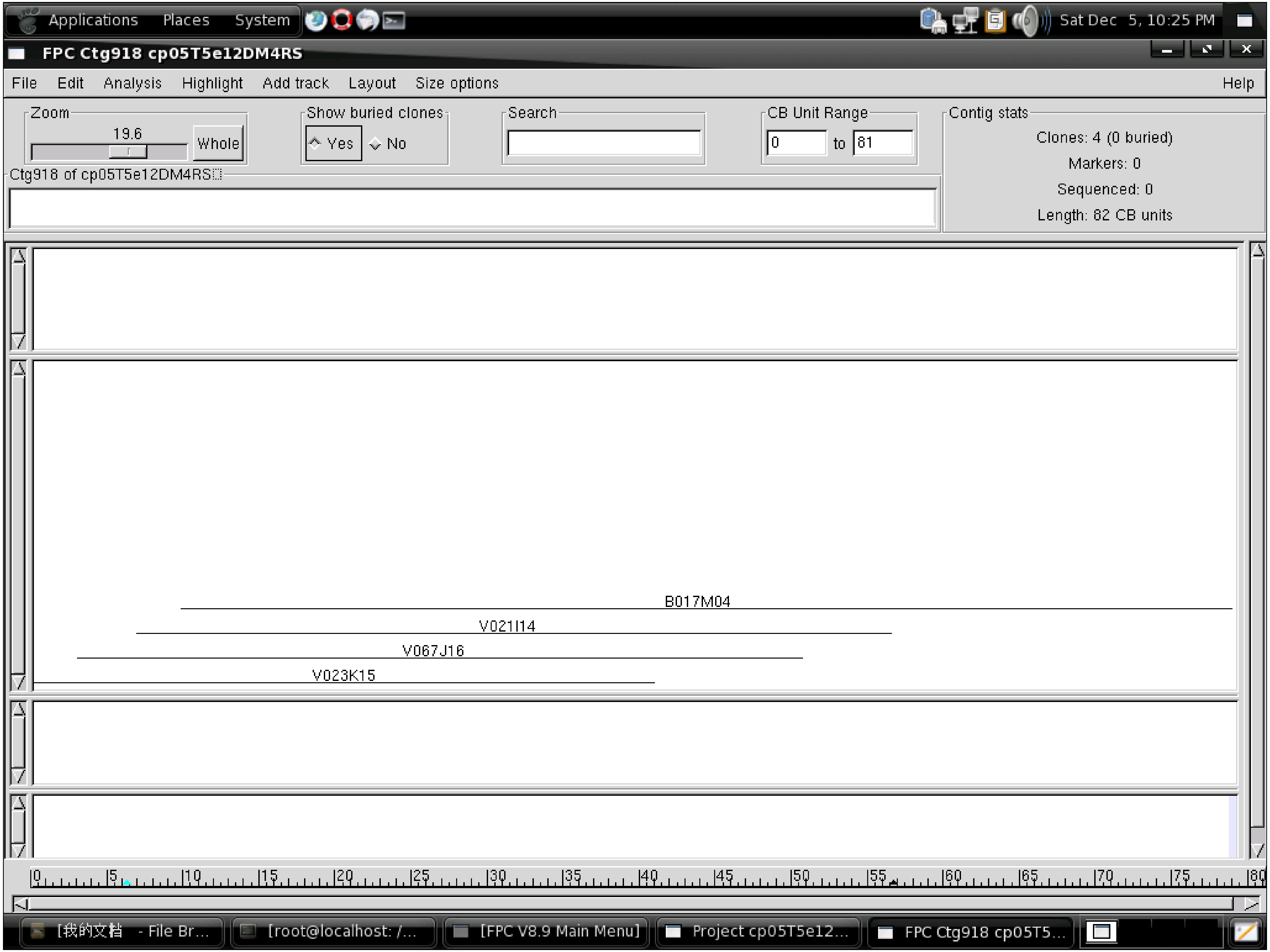
Applications
Places
System
Sat Dec 5, 10:25 PM
FPC Ctg918 cp05T5e12DM4RS
File
Edit
Analysis
Highlight
Add track
Layout
Size options
Help
Zoom
19.6
Whole
Show buried clones
Yes
No
Search
CB Unit Range
0
to
81
Contig stats
Clones: 4 (0 buried)
Markers: 0
Sequenced: 0
Length: 82 CB units
Ctg918 of cp05T5e12DM4RS
B017M04
V021I14
V067J16
V023K15
0 5 10 15 20 25 30 35 40 45 50 55 60 65 70 75 80
[root@localhost: /...
[FPC V8.9 Main Menu]
Project cp05T5e12...
FPC Ctg918 cp05T5...

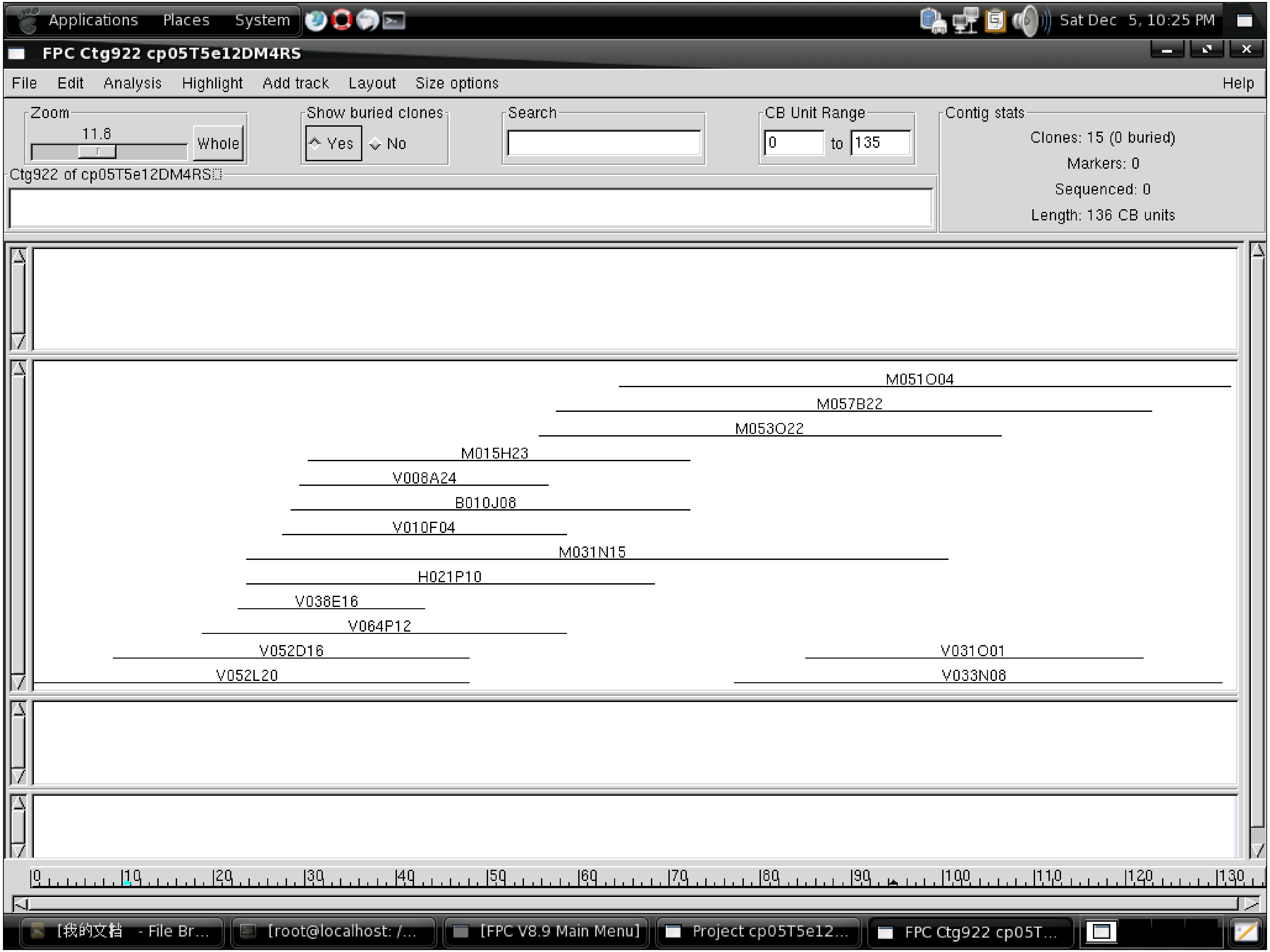
Applications Places System
Sat Dec 5, 10:25 PM
FPC Ctg922 cp05T5e12DM4RS
File Edit Analysis Highlight Add track Layout Size options Help
Zoom
11.8
Whole
Show buried clones
Yes No
Search
CB Unit Range
0 to 135
Contig stats
Clones: 15 (0 buried)
Markers: 0
Sequenced: 0
Length: 136 CB units
Ctg922 of cp05T5e12DM4RS
M051O04
M057B22
M053O22
M015H23
V008A24
B010J08
V010F04
M031N15
H021P10
V038E16
V064P12
V052D16
V031O01
V052L20
V033N08
0 10 20 30 40 50 60 70 80 90 100 110 120 130
[我的文档 - File Br...
[root@localhost: /...
[FPC V8.9 Main Menu]
Project cp05T5e12...
FPC Ctg922 cp05T...

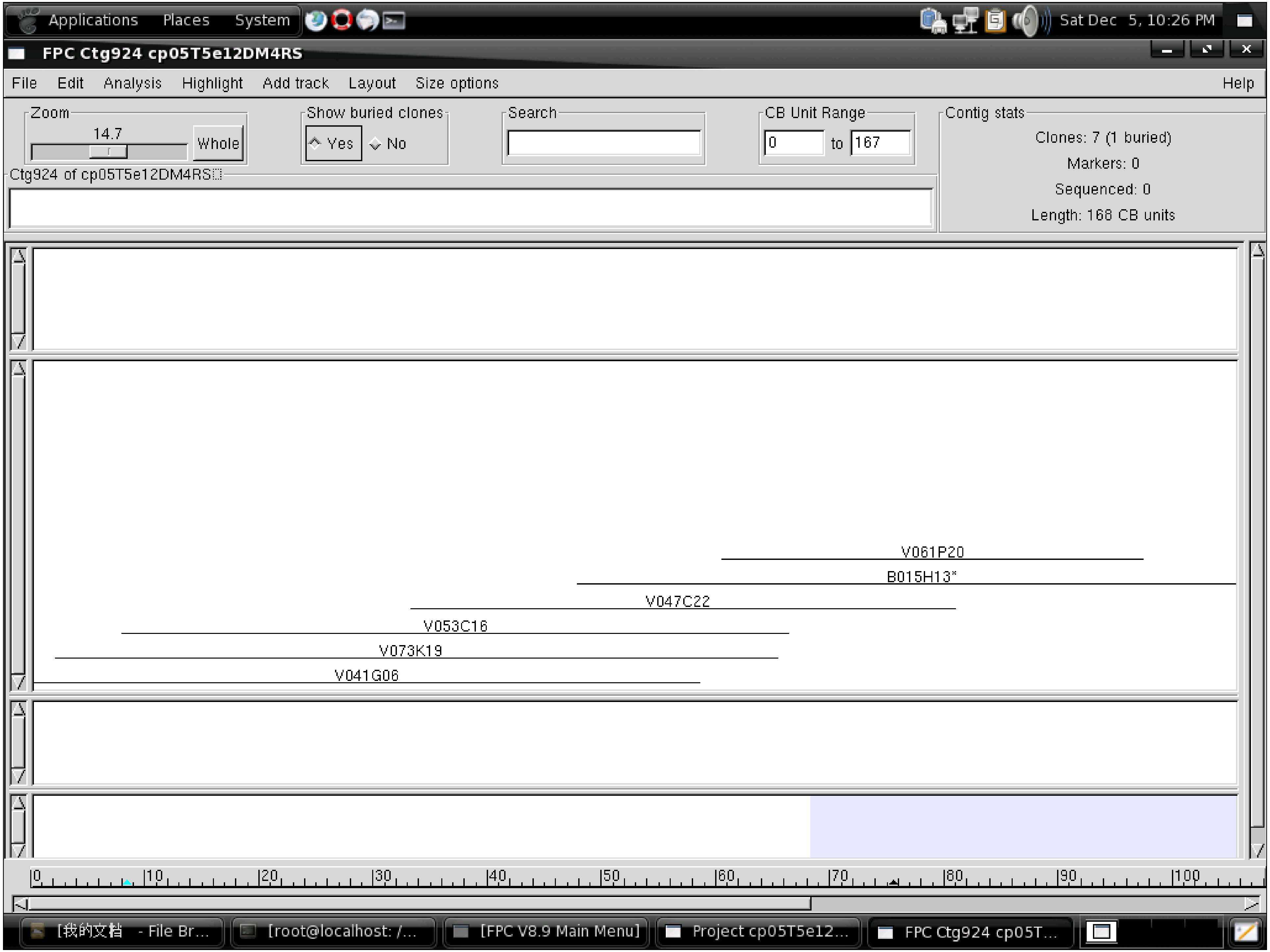
Applications Places System
Sat Dec 5, 10:26 PM
FPC Ctg924 cp05T5e12DM4RS
File Edit Analysis Highlight Add track Layout Size options Help
Zoom
14.7
Whole
Show buried clones
Yes No
Search
CB Unit Range
0 to 167
Contig stats
Clones: 7 (1 buried)
Markers: 0
Sequenced: 0
Length: 168 CB units
Ctg924 of cp05T5e12DM4RS
V061P20
B015H13*
V047C22
V053C16
V073K19
V041G06
0 10 20 30 40 50 60 70 80 90 100
[我的文档 - File Br...
[root@localhost: /...
[FPC V8.9 Main Menu]
Project cp05T5e12...
FPC Ctg924 cp05T...

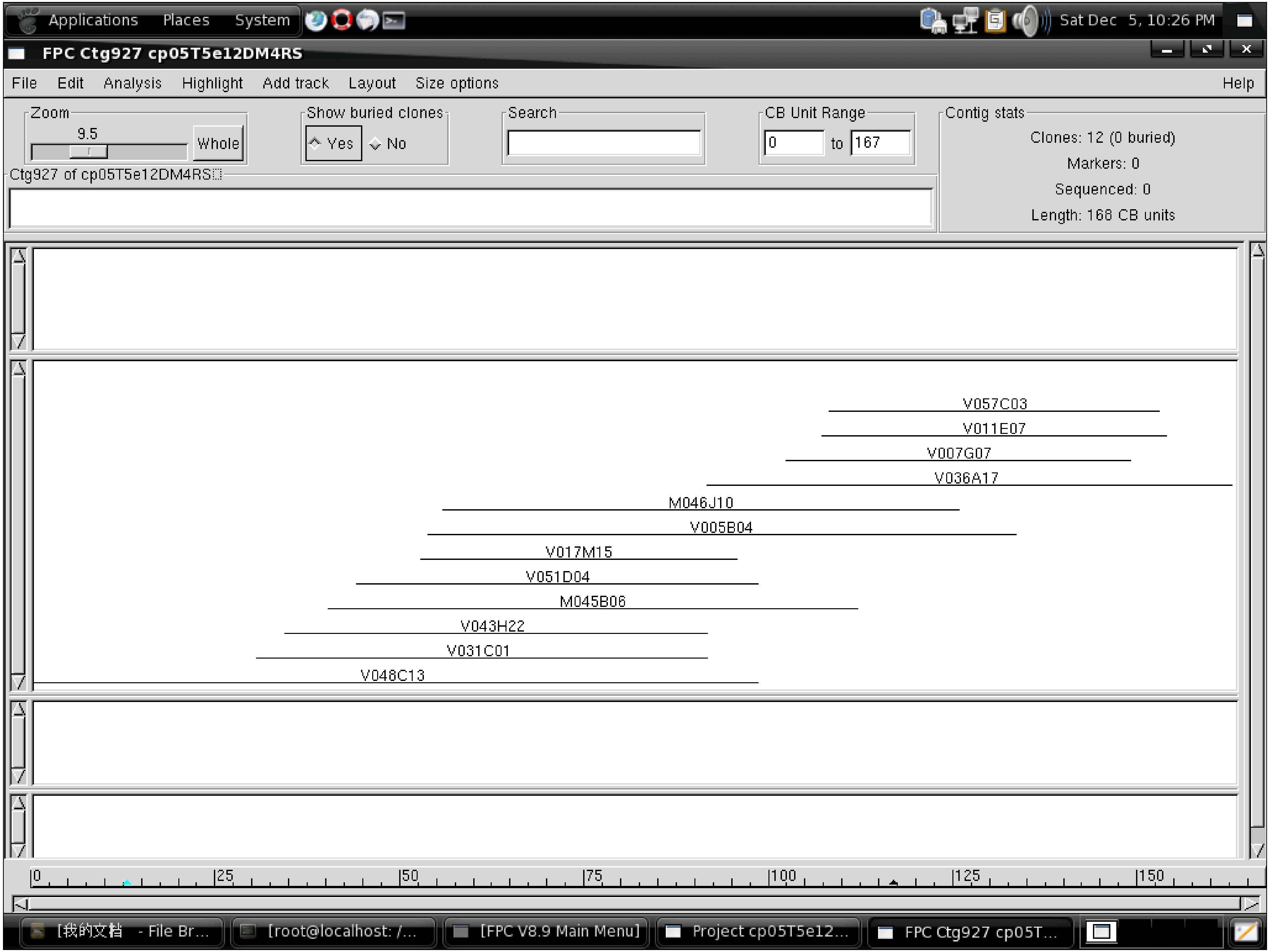
Applications Places System
Sat Dec 5, 10:26 PM
FPC Ctg927 cp05T5e12DM4RS
File Edit Analysis Highlight Add track Layout Size options Help
Zoom
9.5
Whole
Show buried clones
Yes No
Search
CB Unit Range
0 to 167
Contig stats
Clones: 12 (0 buried)
Markers: 0
Sequenced: 0
Length: 168 CB units
Ctg927 of cp05T5e12DM4RS
V057C03
V011E07
V007G07
V036A17
M046J10
V005B04
V017M15
V051D04
M045B06
V043H22
V031C01
V048C13
0 25 50 75 100 125 150
[我的文档 - File Br...
[root@localhost: /...
[FPC V8.9 Main Menu]
Project cp05T5e12...
FPC Ctg927 cp05T...

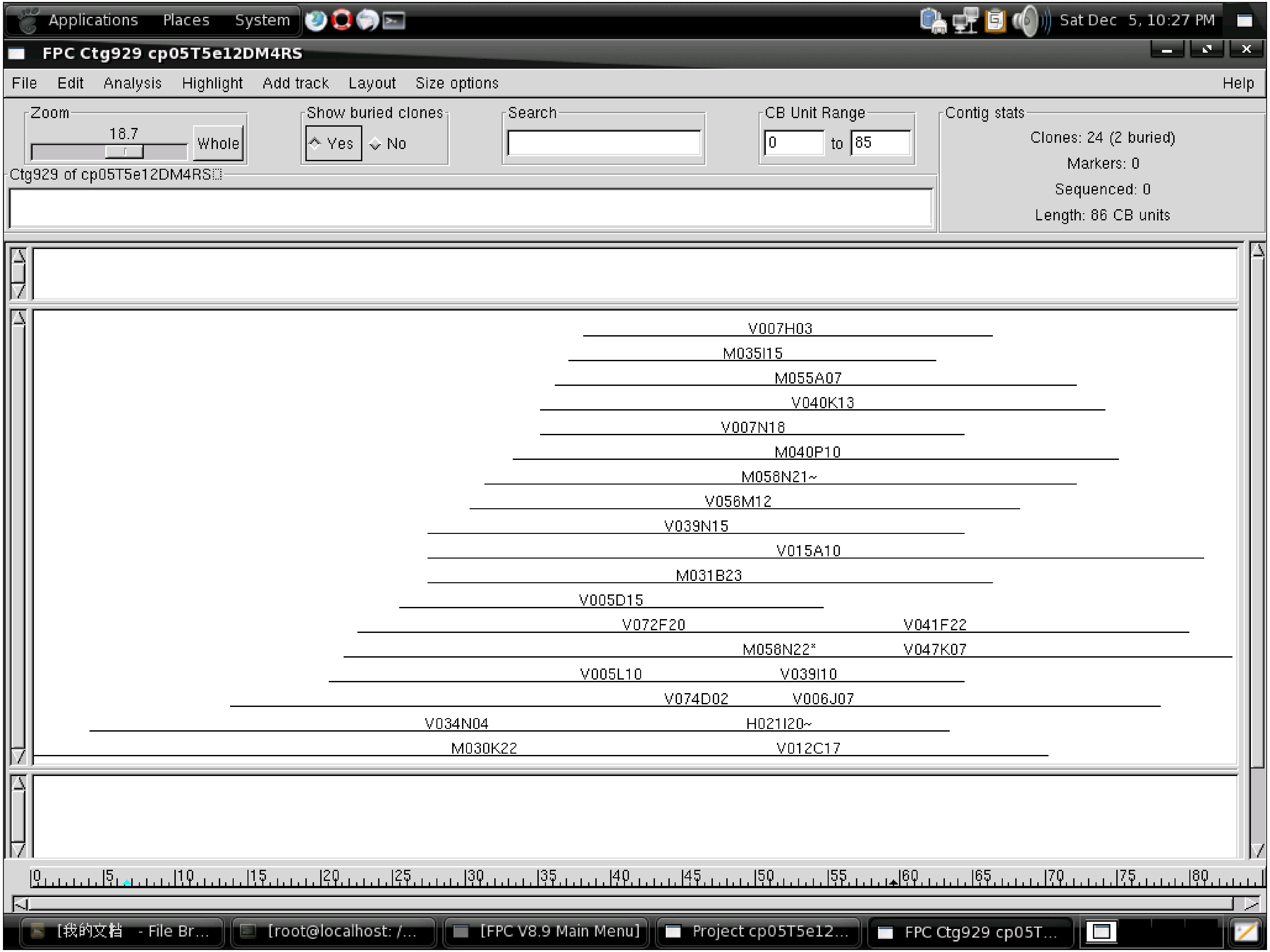
Applications Places System
Sat Dec 5, 10:27 PM
FPC Ctg929 cp05T5e12DM4RS
File Edit Analysis Highlight Add track Layout Size options Help
Zoom
18.7
Whole
Show buried clones
Yes No
Search
CB Unit Range
0 to 85
Contig stats
Clones: 24 (2 buried)
Markers: 0
Sequenced: 0
Length: 86 CB units
Ctg929 of cp05T5e12DM4RS
V007H03
M035I15
M055A07
V040K13
V007N18
M040P10
M058N21~
V056M12
V039N15
V015A10
M031B23
V005D15
V072F20
V041F22
M058N22*
V047K07
V005L10
V039I10
V074D02
V006J07
V034N04
H021I20~
M030K22
V012C17
0 5 10 15 20 25 30 35 40 45 50 55 60 65 70 75 80
[我的文档 - File Br...
[root@localhost: /...
[FPC V8.9 Main Menu]
Project cp05T5e12...
FPC Ctg929 cp05T...

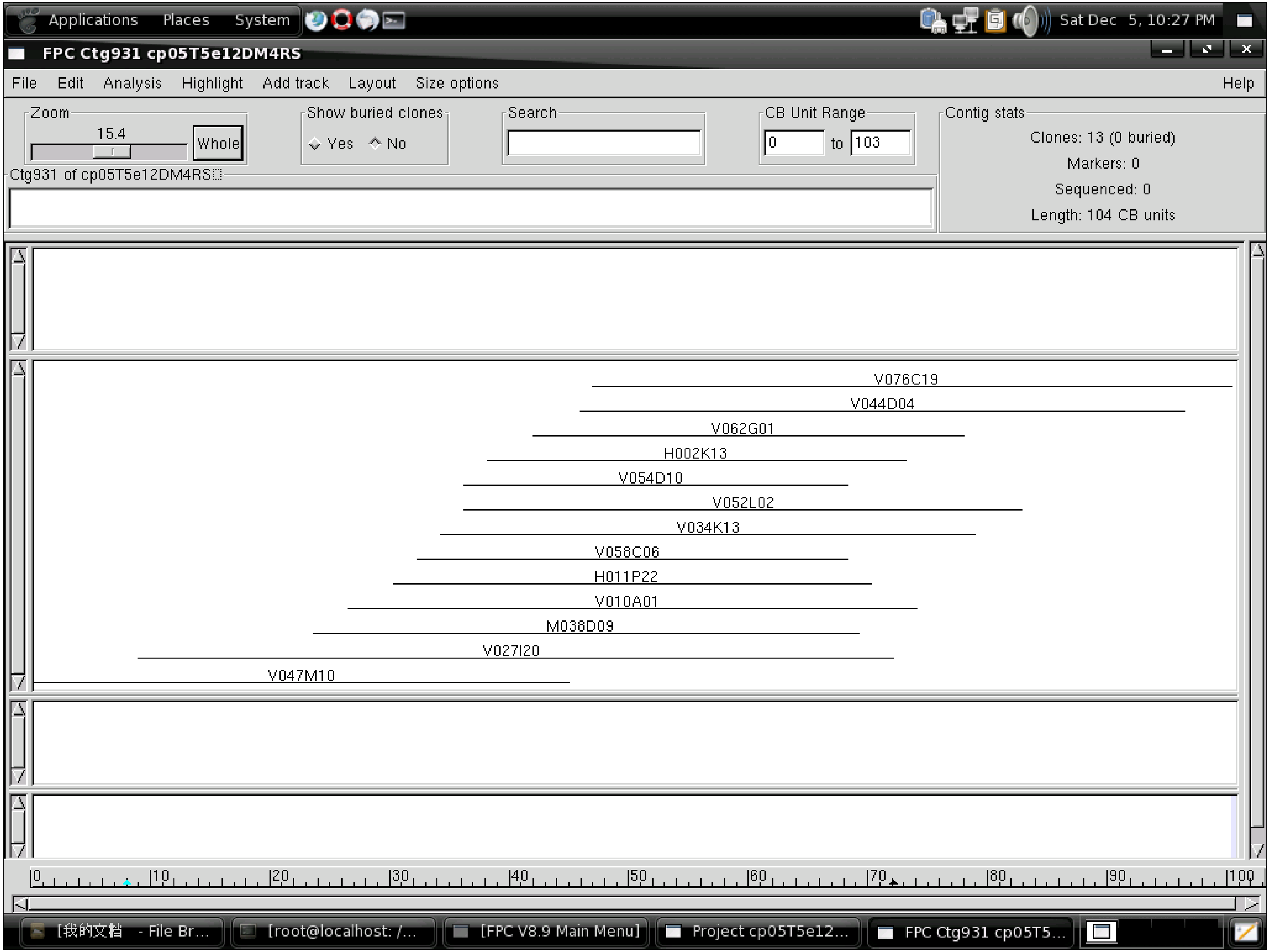
Applications Places System
Sat Dec 5, 10:27 PM
FPC Ctg931 cp05T5e12DM4RS
File Edit Analysis Highlight Add track Layout Size options Help
Zoom
15.4
Whole
Show buried clones
Yes No
Search
CB Unit Range
0 to 103
Contig stats
Clones: 13 (0 buried)
Markers: 0
Sequenced: 0
Length: 104 CB units
Ctg931 of cp05T5e12DM4RS
V076C19
V044D04
V062G01
H002K13
V054D10
V052L02
V034K13
V058C06
H011P22
V010A01
M038D09
V027I20
V047M10
0 10 20 30 40 50 60 70 80 90 100
[我的文档 - File Br...
[root@localhost: /...
[FPC V8.9 Main Menu]
Project cp05T5e12...
FPC Ctg931 cp05T5...

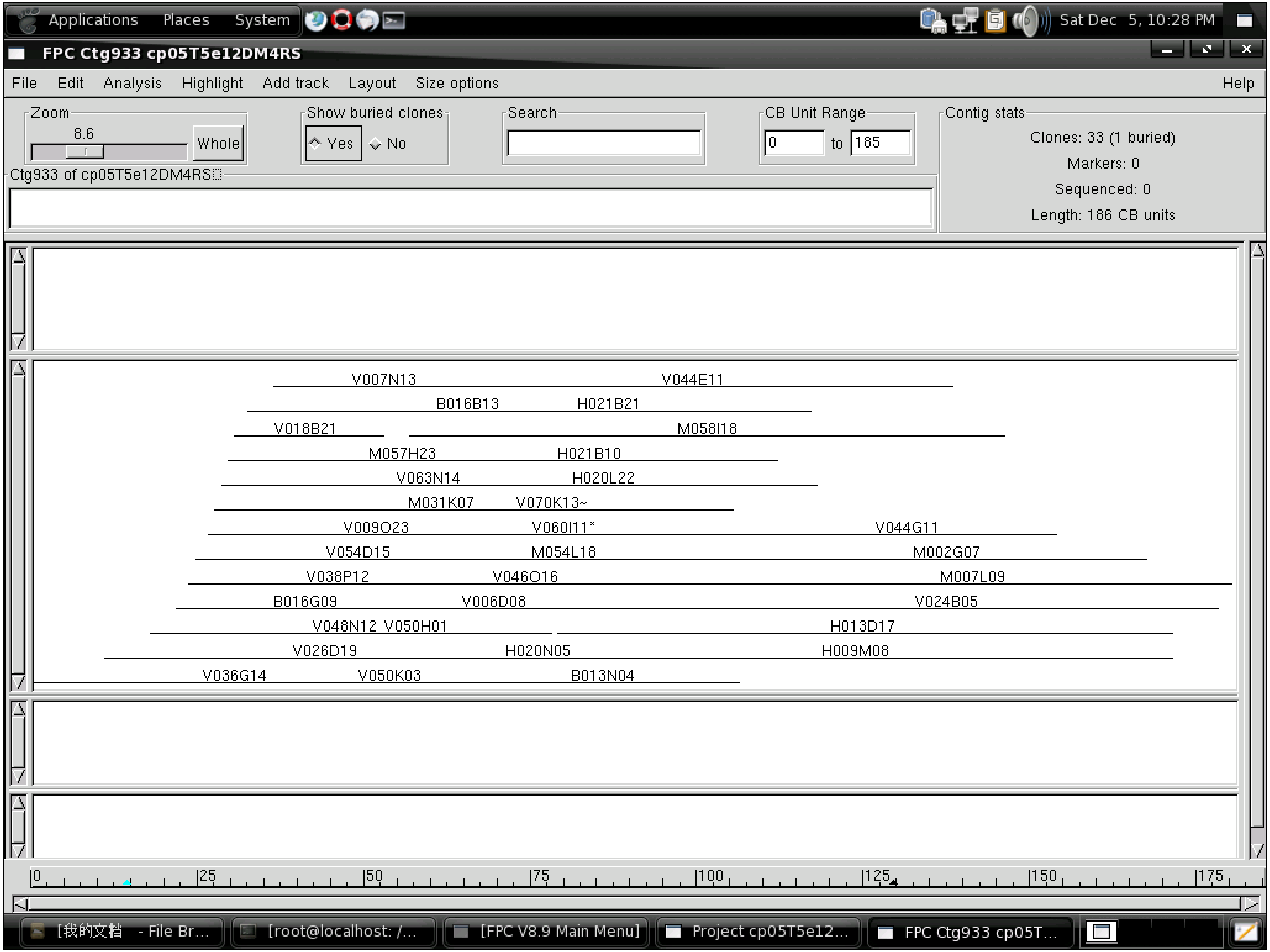

Applications Places System
Sat Dec 5, 10:28 PM
FPC Ctg933 cp05T5e12DM4RS
File Edit Analysis Highlight Add track Layout Size options Help
Zoom
8.6
Whole
Show buried clones
Yes No
Search
CB Unit Range
0 to 185
Contig stats
Clones: 33 (1 buried)
Markers: 0
Sequenced: 0
Length: 186 CB units
Ctg933 of cp05T5e12DM4RS
V007N13
V044E11
B016B13
H021B21
V018B21
M058I18
M057H23
H021B10
V063N14
H020L22
M031K07
V070K13~
V009O23
V060I11*
V044G11
V054D15
M054L18
M002G07
V038P12
V046O16
M007L09
B016G09
V006D08
V024B05
V048N12 V050H01
H013D17
V026D19
H020N05
H009M08
V036G14
V050K03
B013N04
0 25 50 75 100 125 150 175
[我的文档 - File Br...
[root@localhost: /...
[FPC V8.9 Main Menu]
Project cp05T5e12...
FPC Ctg933 cp05T...

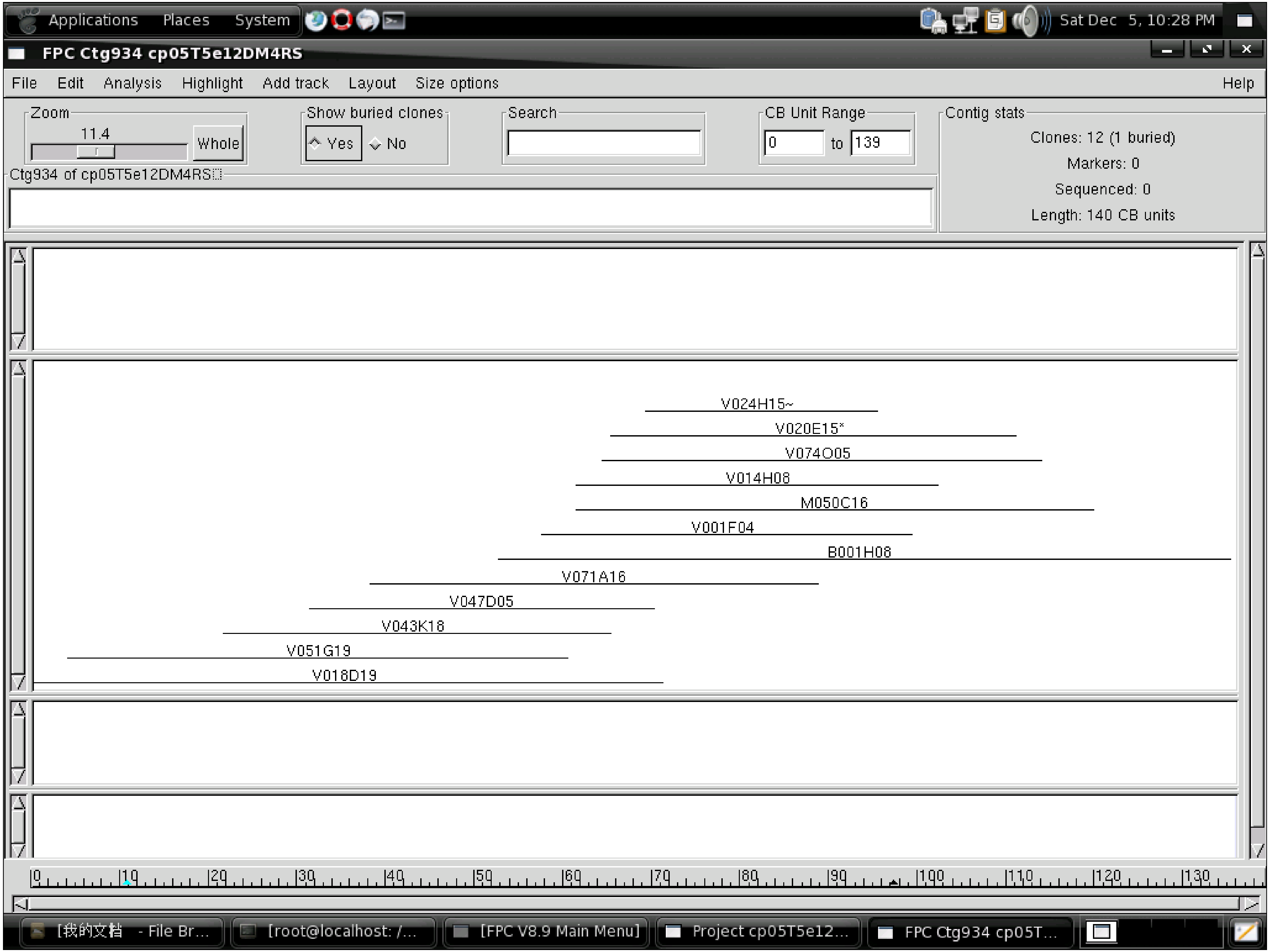
Applications Places System
Sat Dec 5, 10:28 PM
FPC Ctg934 cp05T5e12DM4RS
File Edit Analysis Highlight Add track Layout Size options Help
Zoom
11.4
Whole
Show buried clones
Yes No
Search
CB Unit Range
0 to 139
Contig stats
Clones: 12 (1 buried)
Markers: 0
Sequenced: 0
Length: 140 CB units
Ctg934 of cp05T5e12DM4RS
V024H15~
V020E15*
V074O05
V014H08
M050C16
V001F04
B001H08
V071A16
V047D05
V043K18
V051G19
V018D19
0 10 20 30 40 50 60 70 80 90 100 110 120 130
[我的文档 - File Br...
[root@localhost: /...
[FPC V8.9 Main Menu]
Project cp05T5e12...
FPC Ctg934 cp05T...

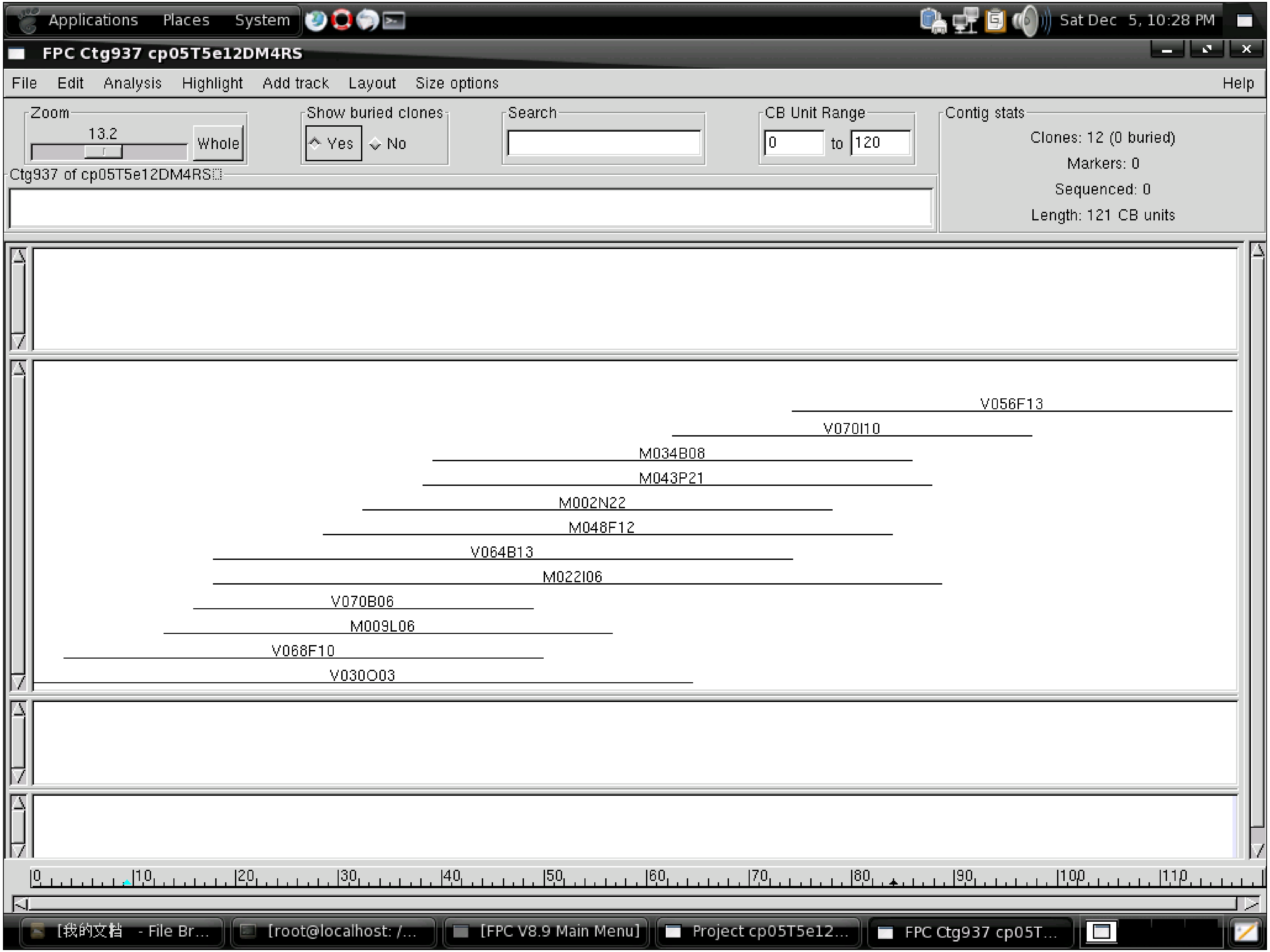
Applications Places System
Sat Dec 5, 10:28 PM
FPC Ctg937 cp05T5e12DM4RS
File Edit Analysis Highlight Add track Layout Size options Help
Zoom
13.2
Whole
Show buried clones
Yes
No
Search
CB Unit Range
0
to
120
Contig stats
Clones: 12 (0 buried)
Markers: 0
Sequenced: 0
Length: 121 CB units
Ctg937 of cp05T5e12DM4RS
V056F13
V070I10
M034B08
M043P21
M002N22
M048F12
V064B13
M022I06
V070B06
M009L06
V068F10
V030O03
0 10 20 30 40 50 60 70 80 90 100 110
[我的文档 - File Br...
[root@localhost: /...
[FPC V8.9 Main Menu]
Project cp05T5e12...
FPC Ctg937 cp05T...

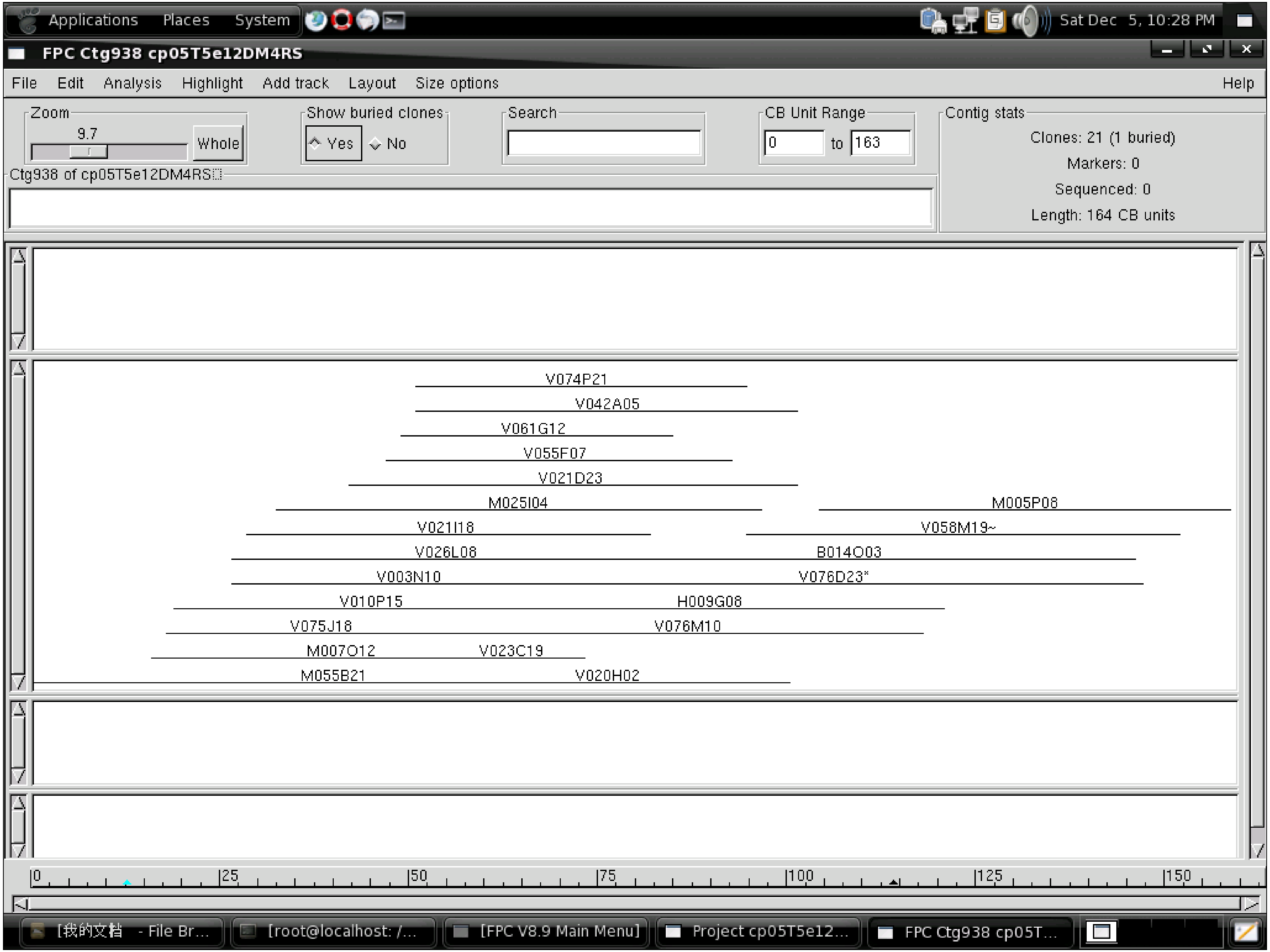
Applications Places System
Sat Dec 5, 10:28 PM
FPC Ctg938 cp05T5e12DM4RS
File Edit Analysis Highlight Add track Layout Size options Help
Zoom
9.7
Whole
Show buried clones
Yes
No
Search
CB Unit Range
0 to 163
Contig stats
Clones: 21 (1 buried)
Markers: 0
Sequenced: 0
Length: 164 CB units
Ctg938 of cp05T5e12DM4RS
V074P21
V042A05
V061G12
V055F07
V021D23
M025I04
M005P08
V021I18
V058M19~
V026L08
B014O03
V003N10
V076D23*
V010P15
H009G08
V075J18
V076M10
M007O12
V023C19
M055B21
V020H02
0
25
50
75
100
125
150
[我的文档 - File Br...
[root@localhost: /...
[FPC V8.9 Main Menu]
Project cp05T5e12...
FPC Ctg938 cp05T...

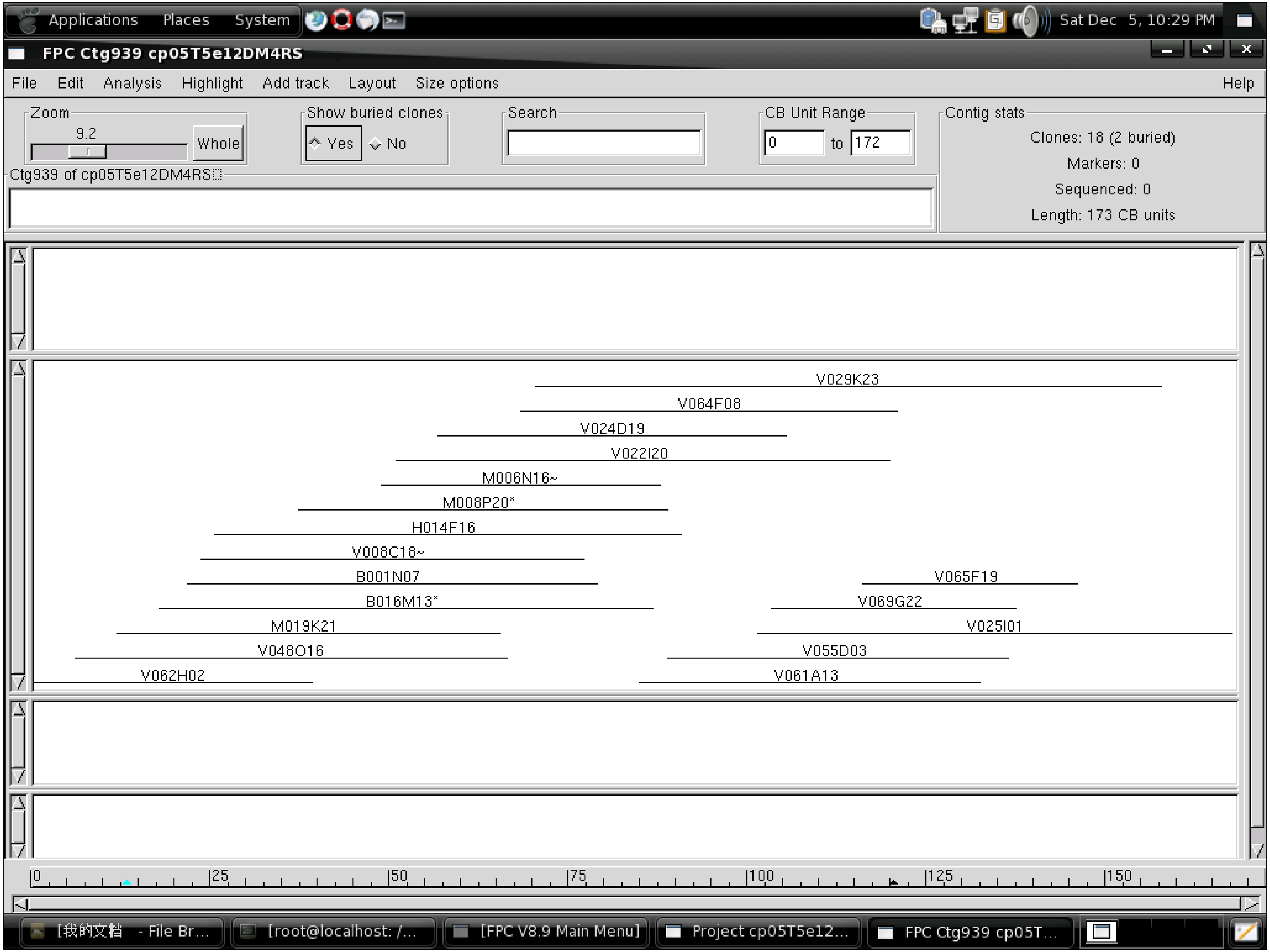
Applications Places System
Sat Dec 5, 10:29 PM
FPC Ctg939 cp05T5e12DM4RS
File Edit Analysis Highlight Add track Layout Size options Help
Zoom
9.2
Whole
Show buried clones
Yes No
Search
CB Unit Range
0 to 172
Contig stats
Clones: 18 (2 buried)
Markers: 0
Sequenced: 0
Length: 173 CB units
Ctg939 of cp05T5e12DM4RS
V029K23
V064F08
V024D19
V022I20
M006N16~
M008P20*
H014F16
V008C18~
B001N07
V065F19
B016M13*
V069G22
M019K21
V025I01
V048O16
V055D03
V062H02
V061A13
0 25 50 75 100 125 150
[我的文档 - File Br...
[root@localhost: /...
[FPC V8.9 Main Menu]
Project cp05T5e12...
FPC Ctg939 cp05T...

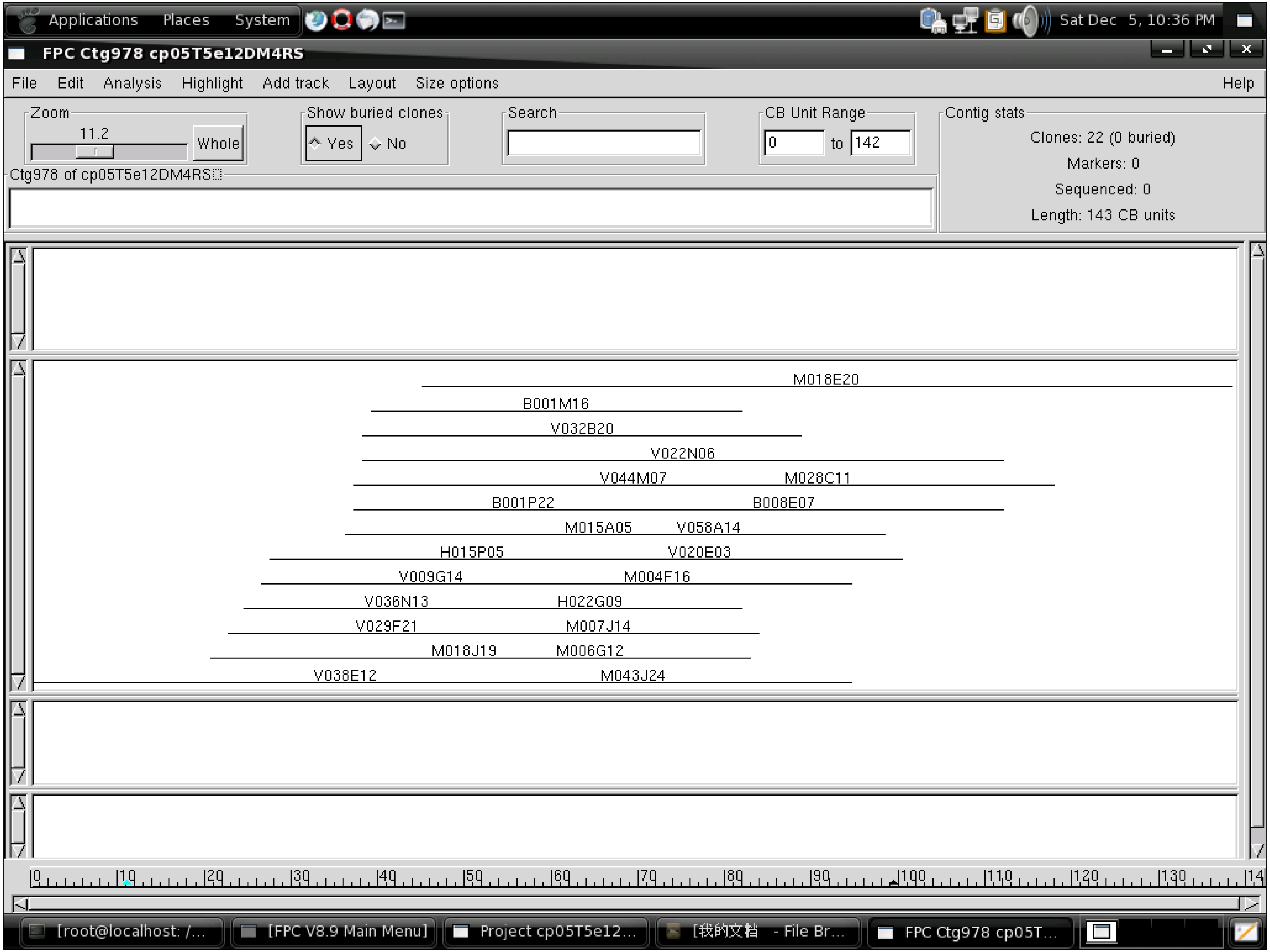
Applications Places System
Sat Dec 5, 10:36 PM
FPC Ctg978 cp05T5e12DM4RS
File Edit Analysis Highlight Add track Layout Size options Help
Zoom
11.2
Whole
Show buried clones
Yes No
Search
CB Unit Range
0 to 142
Contig stats
Clones: 22 (0 buried)
Markers: 0
Sequenced: 0
Length: 143 CB units
Ctg978 of cp05T5e12DM4RS
M018E20
B001M16
V032B20
V022N06
V044M07 M028C11
B001P22 B008E07
M015A05 V058A14
H015P05 V020E03
V009G14 M004F16
V036N13 H022G09
V029F21 M007J14
M018J19 M006G12
V038E12 M043J24
0 10 20 30 40 50 60 70 80 90 100 110 120 130
[root@localhost: /...
[FPC V8.9 Main Menu]
Project cp05T5e12...
[我的文档 - File Br...
FPC Ctg978 cp05T...

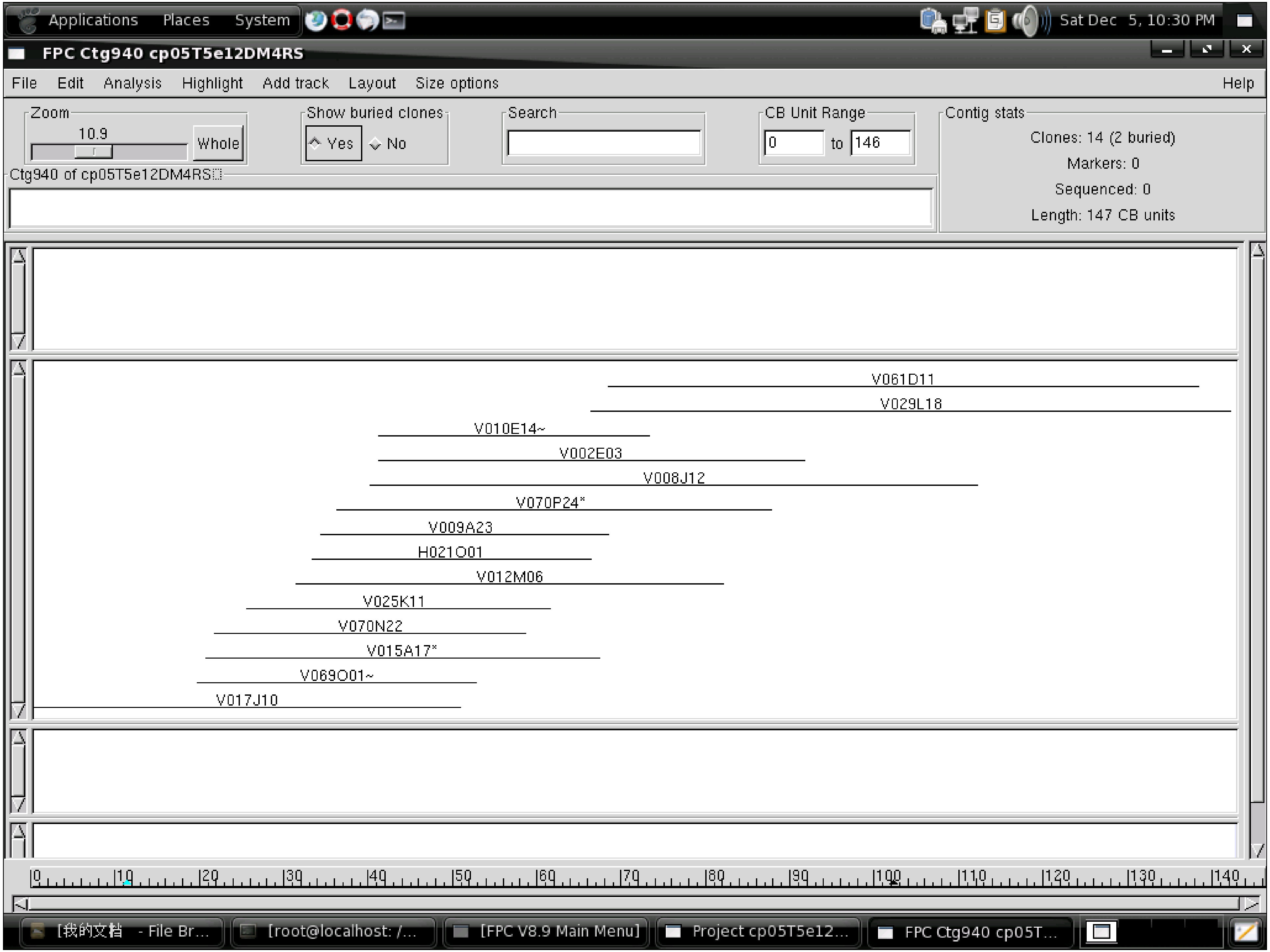
Applications Places System
Sat Dec 5, 10:30 PM
FPC Ctg940 cp05T5e12DM4RS
File Edit Analysis Highlight Add track Layout Size options Help
Zoom
10.9
Whole
Show buried clones
Yes No
Search
CB Unit Range
0 to 146
Contig stats
Clones: 14 (2 buried)
Markers: 0
Sequenced: 0
Length: 147 CB units
Ctg940 of cp05T5e12DM4RS
V061D11
V029L18
V010E14~
V002E03
V008J12
V070P24*
V009A23
H021O01
V012M06
V025K11
V070N22
V015A17*
V069O01~
V017J10
0 10 20 30 40 50 60 70 80 90 100 110 120 130 140
[我的文档 - File Br...
[root@localhost: /...
[FPC V8.9 Main Menu]
Project cp05T5e12...
FPC Ctg940 cp05T...

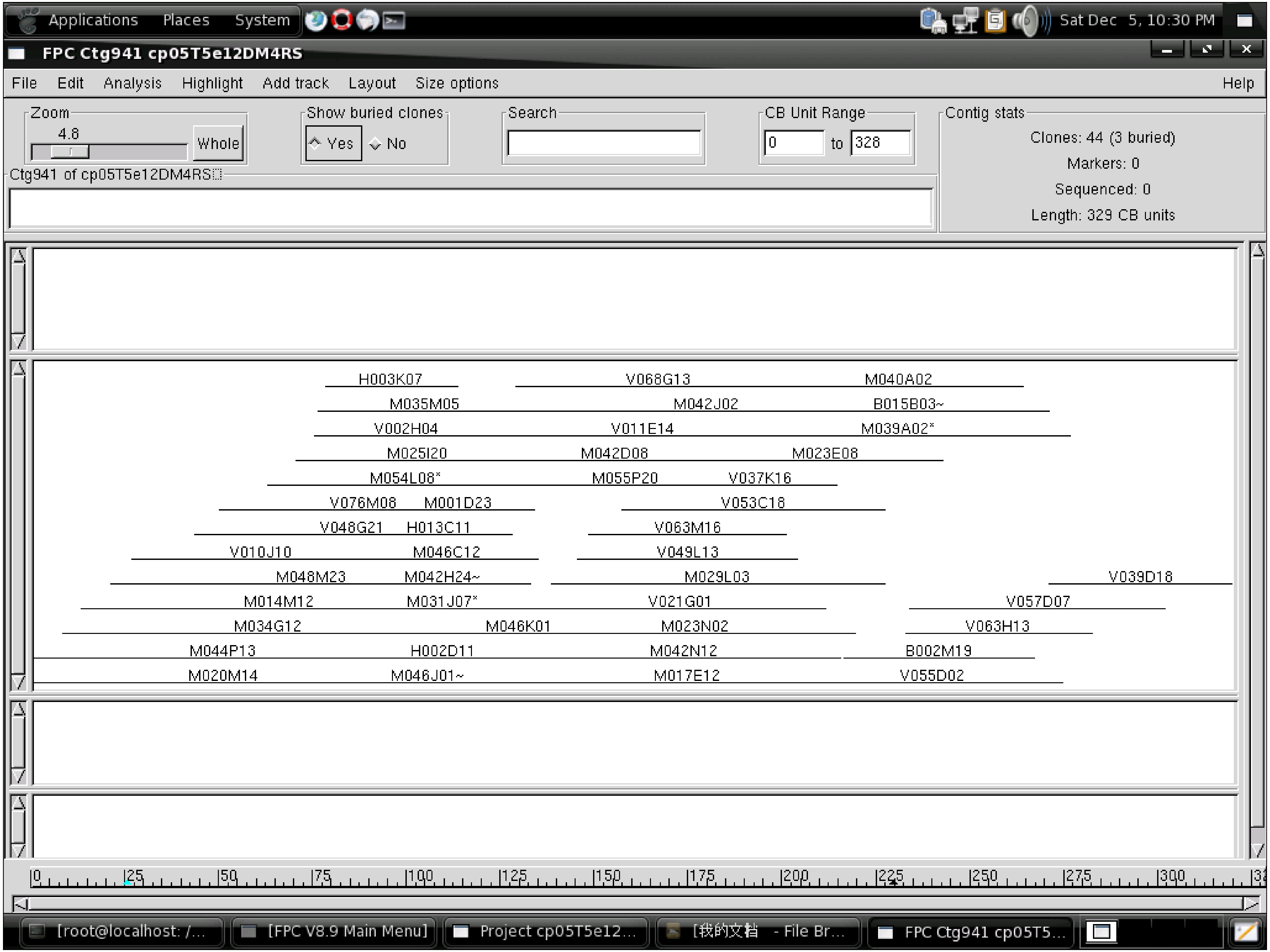
Applications
Places
System
Sat Dec 5, 10:30 PM
FPC Ctg941 cp05T5e12DM4RS
File
Edit
Analysis
Highlight
Add track
Layout
Size options
Help
Zoom
4.8
Whole
Show buried clones
Yes
No
Search
CB Unit Range
0
to
328
Contig stats
Clones: 44 (3 buried)
Markers: 0
Sequenced: 0
Length: 329 CB units
Ctg941 of cp05T5e12DM4RS
H003K07
V068G13
M040A02
M035M05
M042J02
B015B03~
V002H04
V011E14
M039A02*
M025I20
M042D08
M023E08
M054L08*
M055P20
V037K16
V076M08
M001D23
V053C18
V048G21
H013C11
V063M16
V010J10
M046C12
V049L13
M048M23
M042H24~
M029L03
V039D18
M014M12
M031J07*
V021G01
V057D07
M034G12
M046K01
M023N02
V063H13
M044P13
H002D11
M042N12
B002M19
M020M14
M046J01~
M017E12
V055D02
0
25
50
75
100
125
150
175
200
225
250
275
300
[root@localhost: /...
[FPC V8.9 Main Menu]
Project cp05T5e12...
[我的文档 - File Br...
FPC Ctg941 cp05T5...

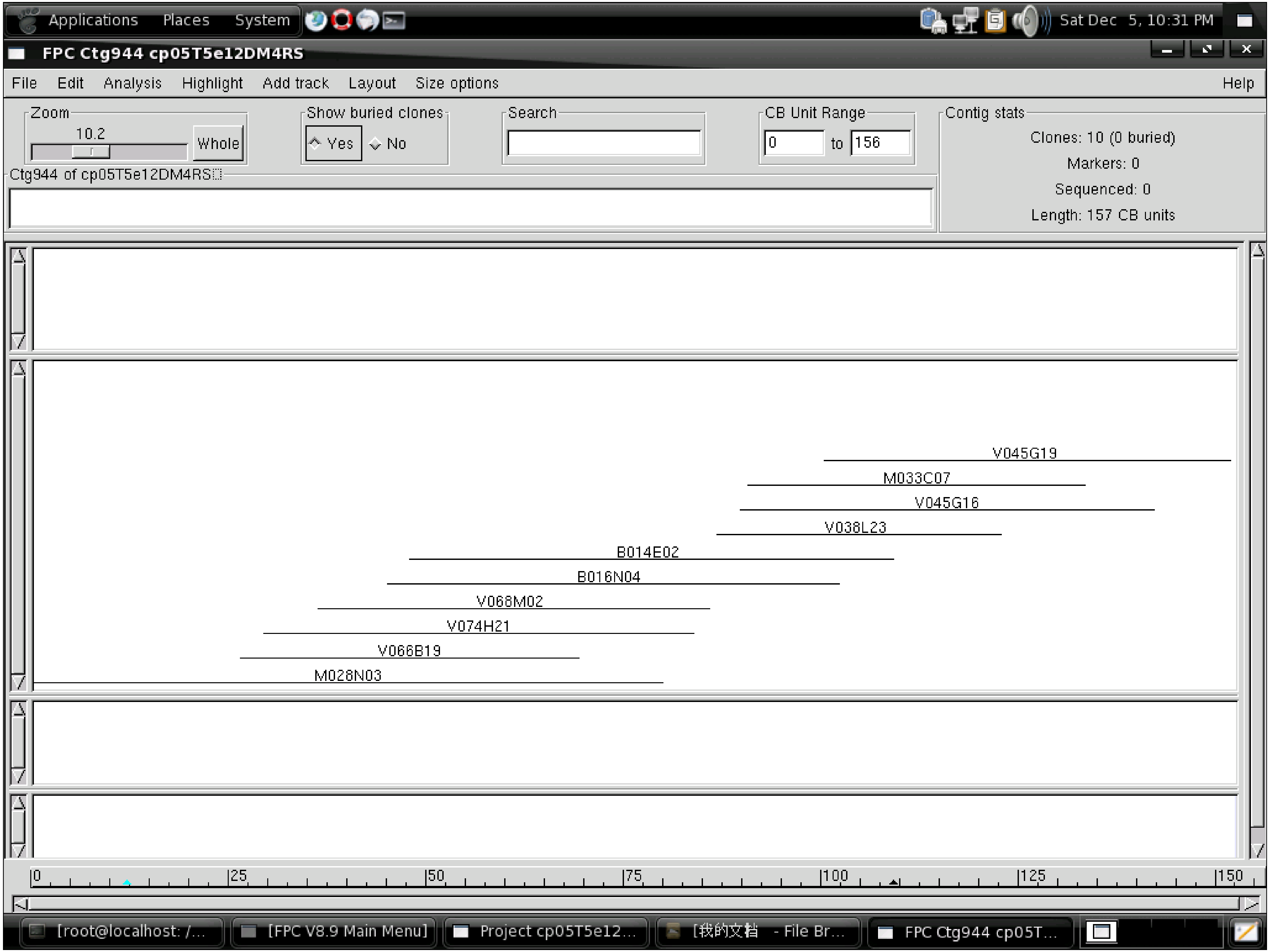
Applications Places System
Sat Dec 5, 10:31 PM
FPC Ctg944 cp05T5e12DM4RS
File Edit Analysis Highlight Add track Layout Size options Help
Zoom
10.2
Whole
Show buried clones
Yes
No
Search
CB Unit Range
0 to 156
Contig stats
Clones: 10 (0 buried)
Markers: 0
Sequenced: 0
Length: 157 CB units
Ctg944 of cp05T5e12DM4RS
V045G19
M033C07
V045G16
V038L23
B014E02
B016N04
V068M02
V074H21
V066B19
M028N03
0 25 50 75 100 125 150
[root@localhost: /...
[FPC V8.9 Main Menu]
Project cp05T5e12...
[我的文档 - File Br...
FPC Ctg944 cp05T...

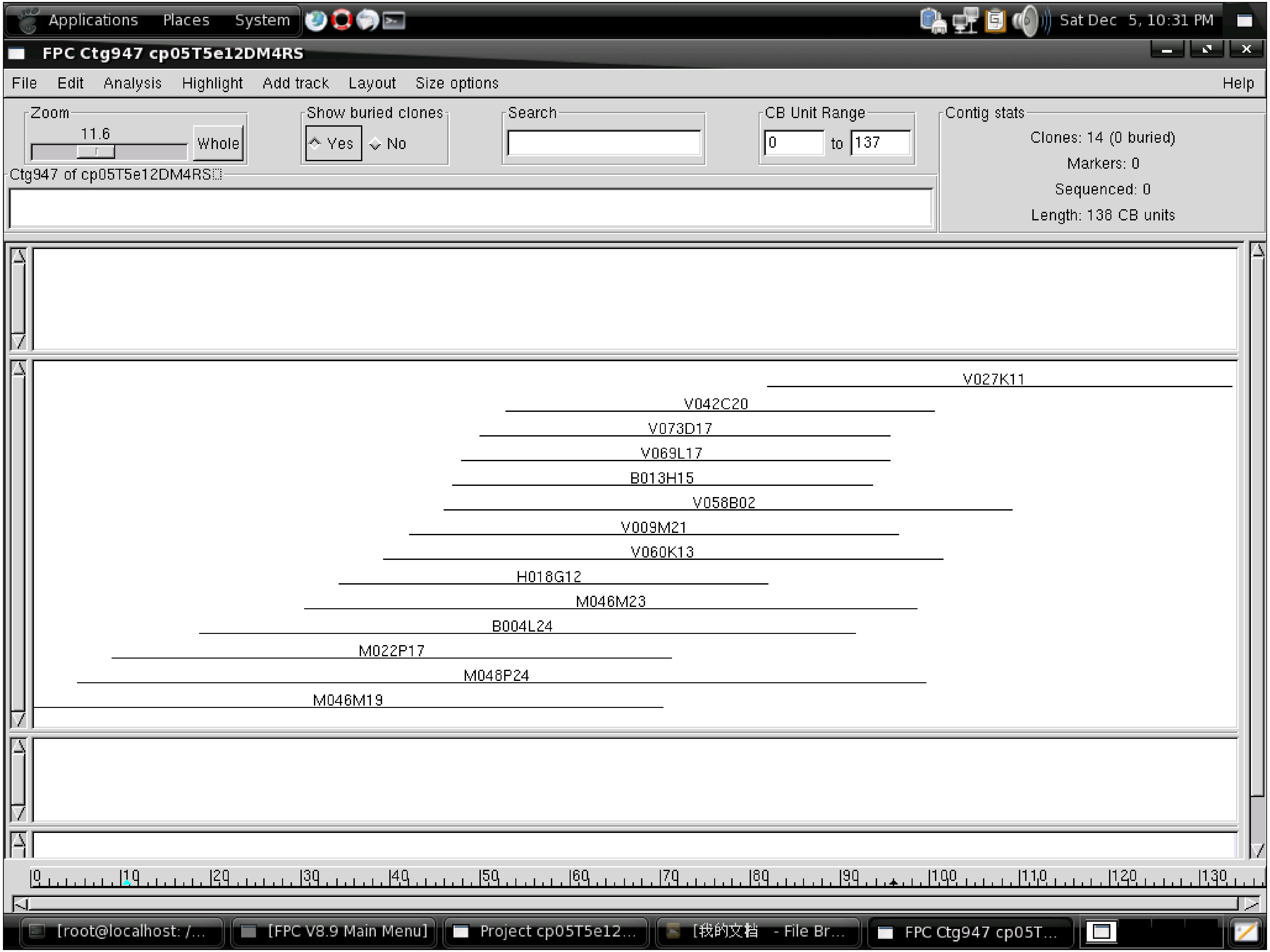

Applications Places System
Sat Dec 5, 10:31 PM
FPC Ctg947 cp05T5e12DM4RS
File Edit Analysis Highlight Add track Layout Size options Help
Zoom
11.6
Whole
Show buried clones
Yes
No
Search
CB Unit Range
0 to 137
Contig stats
Clones: 14 (0 buried)
Markers: 0
Sequenced: 0
Length: 138 CB units
Ctg947 of cp05T5e12DM4RS
V027K11
V042C20
V073D17
V069L17
B013H15
V058B02
V009M21
V060K13
H018G12
M046M23
B004L24
M022P17
M048P24
M046M19
0 10 20 30 40 50 60 70 80 90 100 110 120 130
[root@localhost: /...
[FPC V8.9 Main Menu]
Project cp05T5e12...
[我的文档 - File Br...
FPC Ctg947 cp05T...

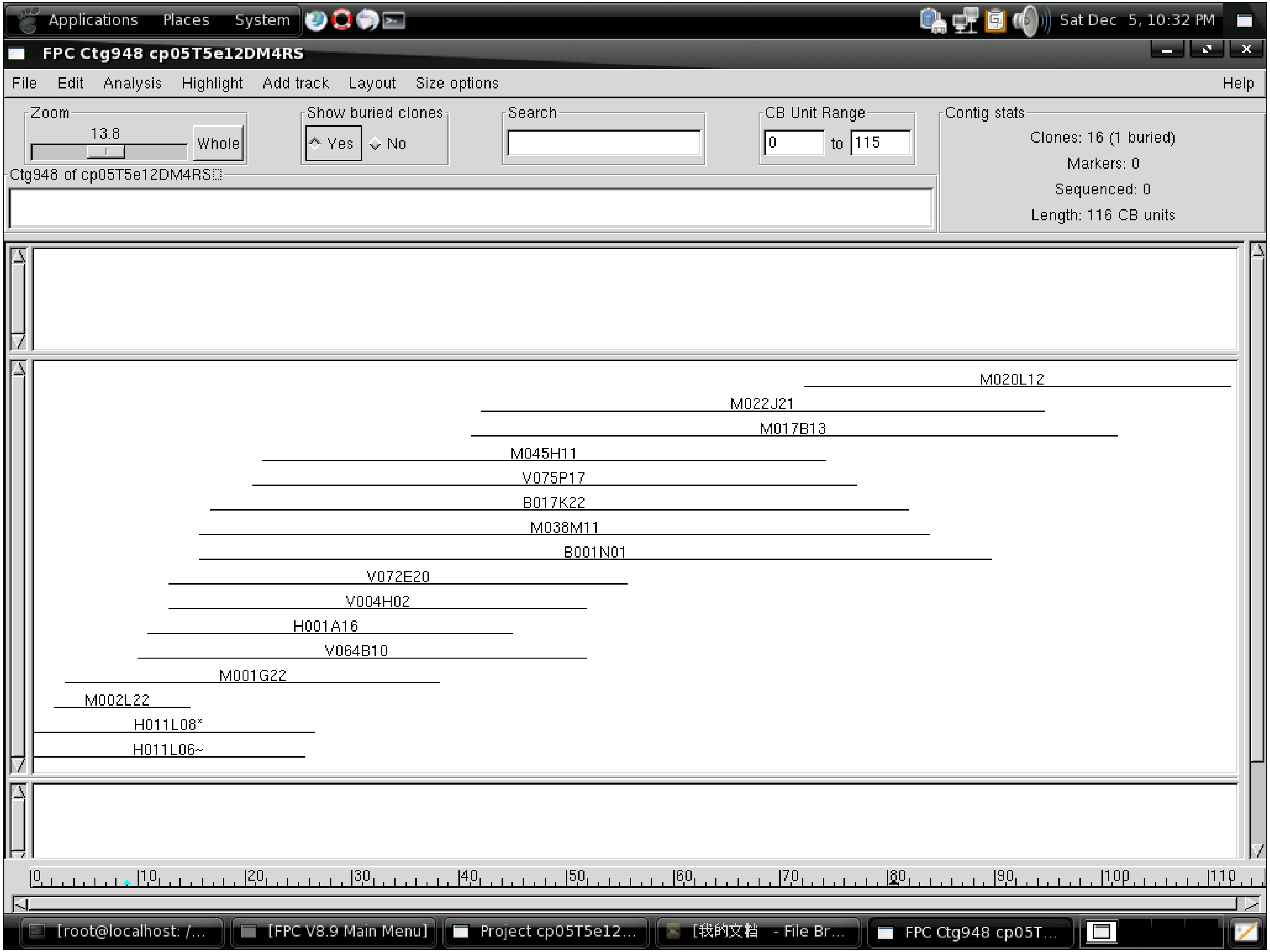

Applications Places System
Sat Dec 5, 10:32 PM
FPC Ctg948 cp05T5e12DM4RS
File Edit Analysis Highlight Add track Layout Size options Help
Zoom
13.8
Whole
Show buried clones
Yes No
Search
CB Unit Range
0 to 115
Contig stats
Clones: 16 (1 buried)
Markers: 0
Sequenced: 0
Length: 116 CB units
Ctg948 of cp05T5e12DM4RS
M020L12
M022J21
M017B13
M045H11
V075P17
B017K22
M038M11
B001N01
V072E20
V004H02
H001A16
V064B10
M001G22
M002L22
H011L08*
H011L06~
0 10 20 30 40 50 60 70 80 90 100 110
[root@localhost: /...
[FPC V8.9 Main Menu]
Project cp05T5e12...
[我的文档 - File Br...
FPC Ctg948 cp05T...

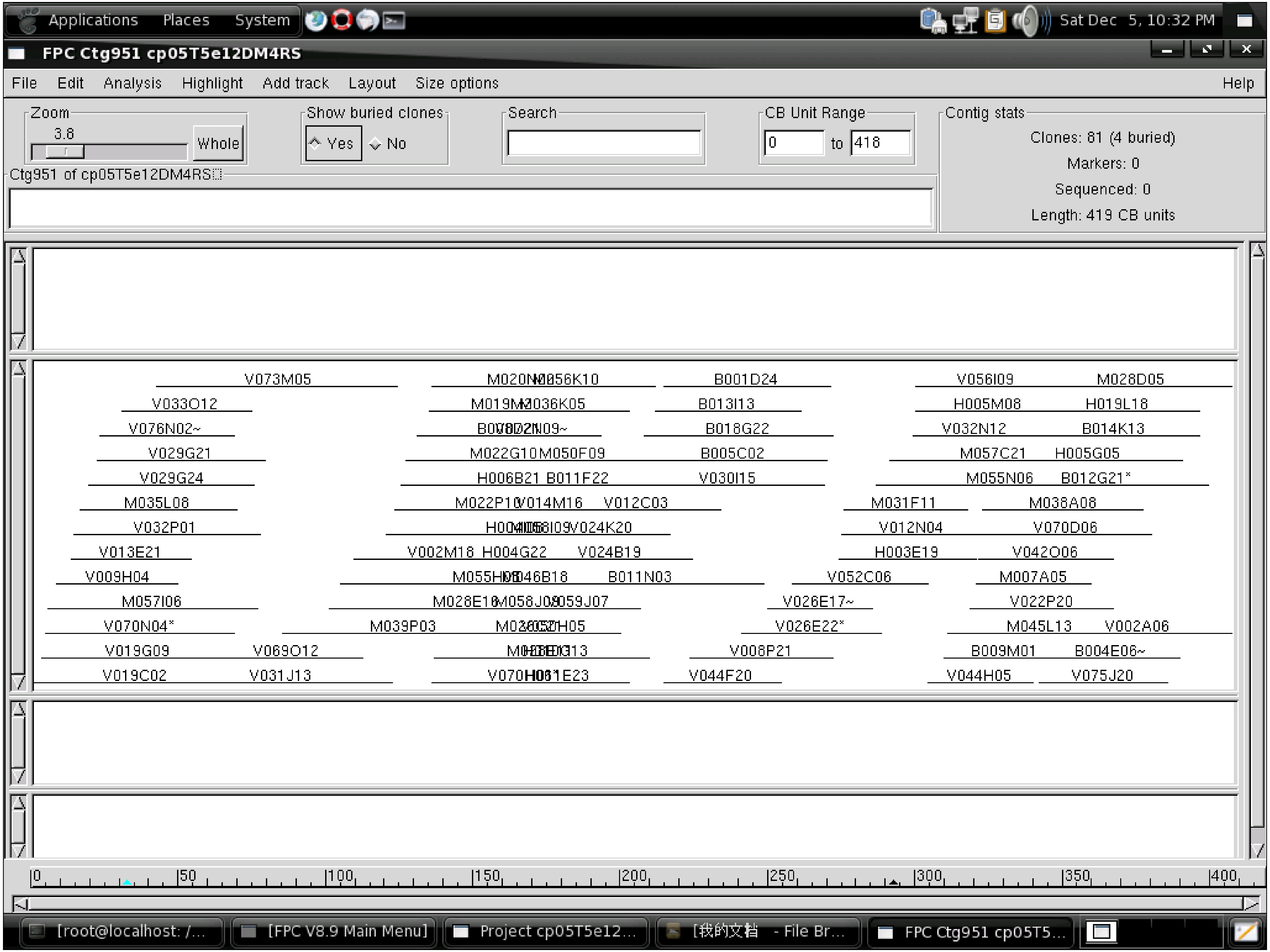
Applications Places System
Sat Dec 5, 10:32 PM
FPC Ctg951 cp05T5e12DM4RS
File Edit Analysis Highlight Add track Layout Size options Help
Zoom
3.8
Whole
Show buried clones
Yes
No
Search
CB Unit Range
0
to
418
Contig stats
Clones: 81 (4 buried)
Markers: 0
Sequenced: 0
Length: 419 CB units
Ctg951 of cp05T5e12DM4RS
V073M05
V033O12
V076N02~
V029G21
V029G24
M035L08
V032P01
V013E21
V009H04
M057I06
V070N04*
V019G09
V069O12
V019C02
V031J13
M039P03
M022G10
M050F09
H006B21
B011F22
V002M18
H004G22
V024B19
V012C03
V024K20
B011N03
B001D24
B013I13
B018G22
B005C02
V030I15
V044F20
V008P21
V026E22*
V026E17~
V052C06
V056I09
M028D05
H005M08
H019L18
V032N12
B014K13
M057C21
H005G05
M055N06
B012G21*
M031F11
M038A08
V012N04
V070D06
H003E19
V042O06
M007A05
V022P20
M045L13
V002A06
B009M01
B004E06~
V044H05
V075J20
0 50 100 150 200 250 300 350 400
[root@localhost: /...
[FPC V8.9 Main Menu]
Project cp05T5e12...
[我的文档 - File Br...
FPC Ctg951 cp05T5...

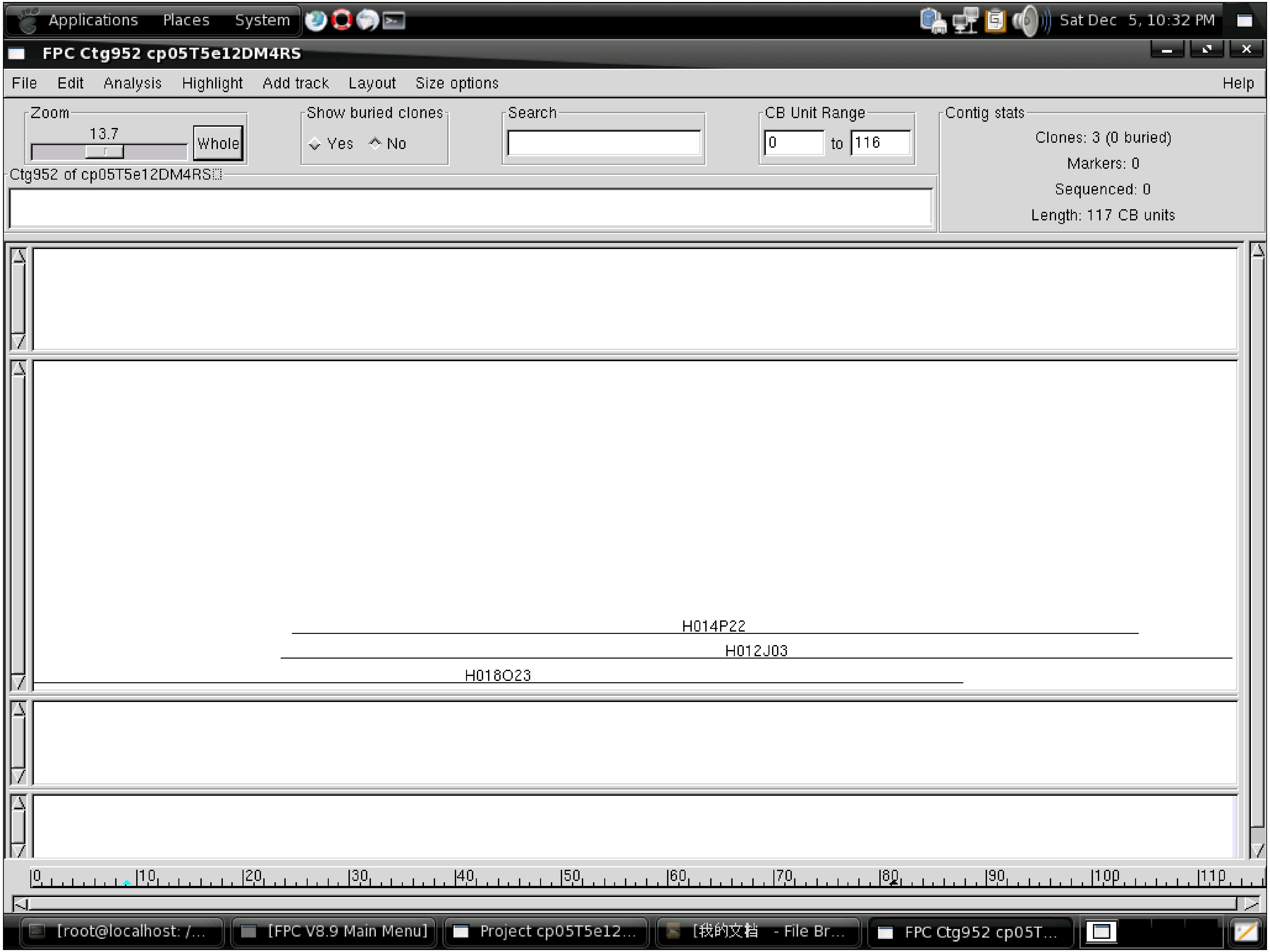

Applications Places System
Sat Dec 5, 10:32 PM
FPC Ctg952 cp05T5e12DM4RS
File Edit Analysis Highlight Add track Layout Size options Help
Zoom
13.7
Whole
Show buried clones
Yes No
Search
CB Unit Range
0 to 116
Contig stats
Clones: 3 (0 buried)
Markers: 0
Sequenced: 0
Length: 117 CB units
Ctg952 of cp05T5e12DM4RS
H014P22
H012J03
H018O23
0 10 20 30 40 50 60 70 80 90 100 110
[root@localhost: /...
[FPC V8.9 Main Menu]
Project cp05T5e12...
[我的文档 - File Br...
FPC Ctg952 cp05T...

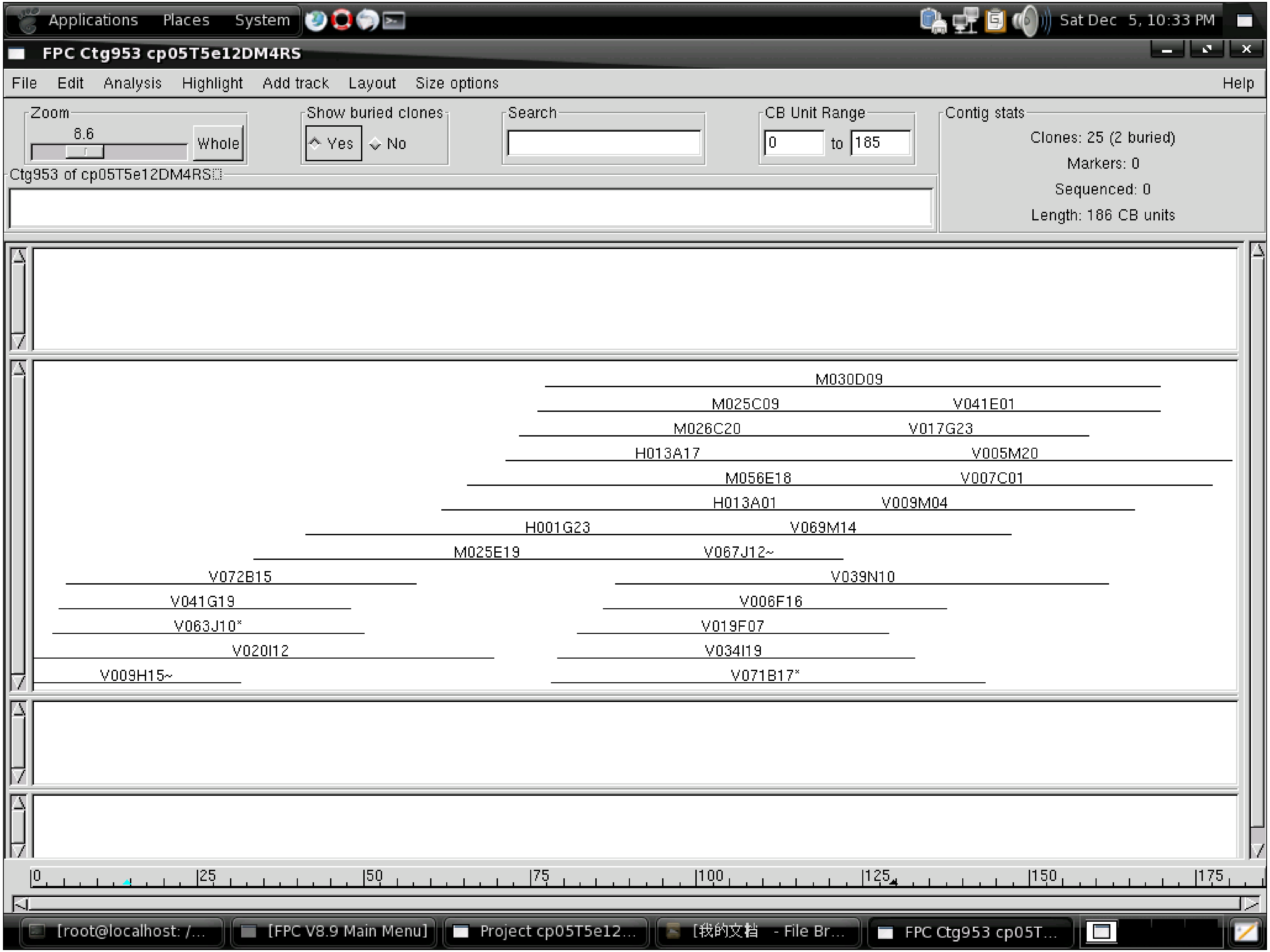
Applications Places System
Sat Dec 5, 10:33 PM
FPC Ctg953 cp05T5e12DM4RS
File Edit Analysis Highlight Add track Layout Size options Help
Zoom
8.6
Whole
Show buried clones
Yes No
Search
CB Unit Range
0 to 185
Contig stats
Clones: 25 (2 buried)
Markers: 0
Sequenced: 0
Length: 186 CB units
Ctg953 of cp05T5e12DM4RS
M030D09
M025C09
V041E01
M026C20
V017G23
H013A17
V005M20
M056E18
V007C01
H013A01
V009M04
H001G23
V069M14
M025E19
V067J12~
V072B15
V039N10
V041G19
V006F16
V063J10*
V019F07
V020I12
V034I19
V009H15~
V071B17*
0 25 50 75 100 125 150 175
[root@localhost: /...
[FPC V8.9 Main Menu]
Project cp05T5e12...
[我的文档 - File Br...
FPC Ctg953 cp05T...

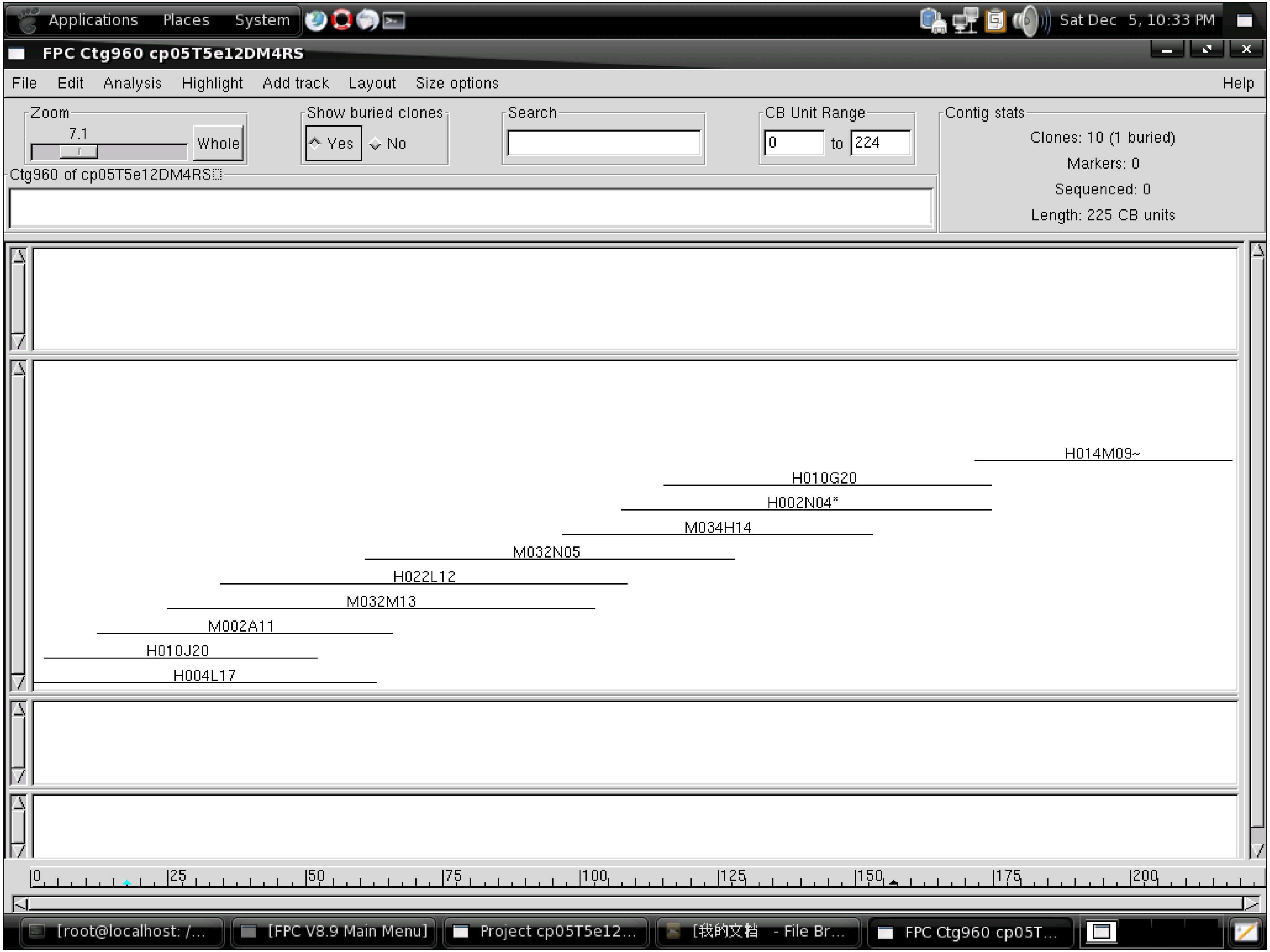
Applications
Places
System
Sat Dec 5, 10:33 PM
FPC Ctg960 cp05T5e12DM4RS
File
Edit
Analysis
Highlight
Add track
Layout
Size options
Help
Zoom
7.1
Whole
Show buried clones
Yes
No
Search
CB Unit Range
0
to
224
Contig stats
Clones: 10 (1 buried)
Markers: 0
Sequenced: 0
Length: 225 CB units
Ctg960 of cp05T5e12DM4RS
H014M09~
H010G20
H002N04*
M034H14
M032N05
H022L12
M032M13
M002A11
H010J20
H004L17
0
25
50
75
100
125
150
175
200
[root@localhost: /...
[FPC V8.9 Main Menu]
Project cp05T5e12...
[我的文档 - File Br...
FPC Ctg960 cp05T...

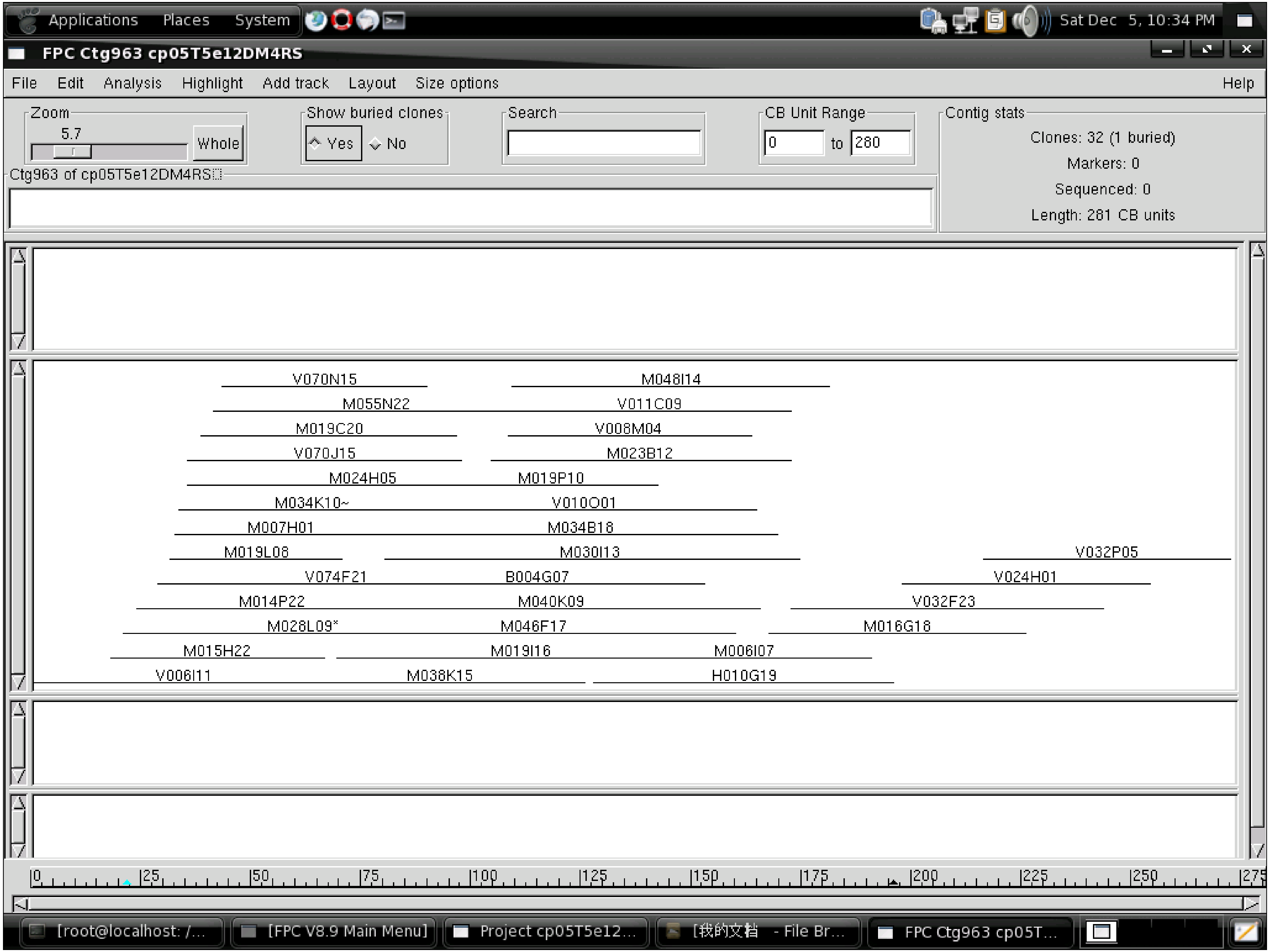

Applications Places System
Sat Dec 5, 10:34 PM
FPC Ctg963 cp05T5e12DM4RS
File Edit Analysis Highlight Add track Layout Size options Help
Zoom
5.7
Whole
Show buried clones
Yes
No
Search
CB Unit Range
0
to
280
Contig stats
Clones: 32 (1 buried)
Markers: 0
Sequenced: 0
Length: 281 CB units
Ctg963 of cp05T5e12DM4RS
V070N15
M048I14
M055N22
V011C09
M019C20
V008M04
V070J15
M023B12
M024H05
M019P10
M034K10~
V010O01
M007H01
M034B18
M019L08
M030I13
V032P05
V074F21
B004G07
V024H01
M014P22
M040K09
V032F23
M028L09*
M046F17
M016G18
M015H22
M019I16
M006I07
V006I11
M038K15
H010G19
0 25 50 75 100 125 150 175 200 225 250 275
[root@localhost: /...
[FPC V8.9 Main Menu]
Project cp05T5e12...
[我的文档 - File Br...
FPC Ctg963 cp05T...

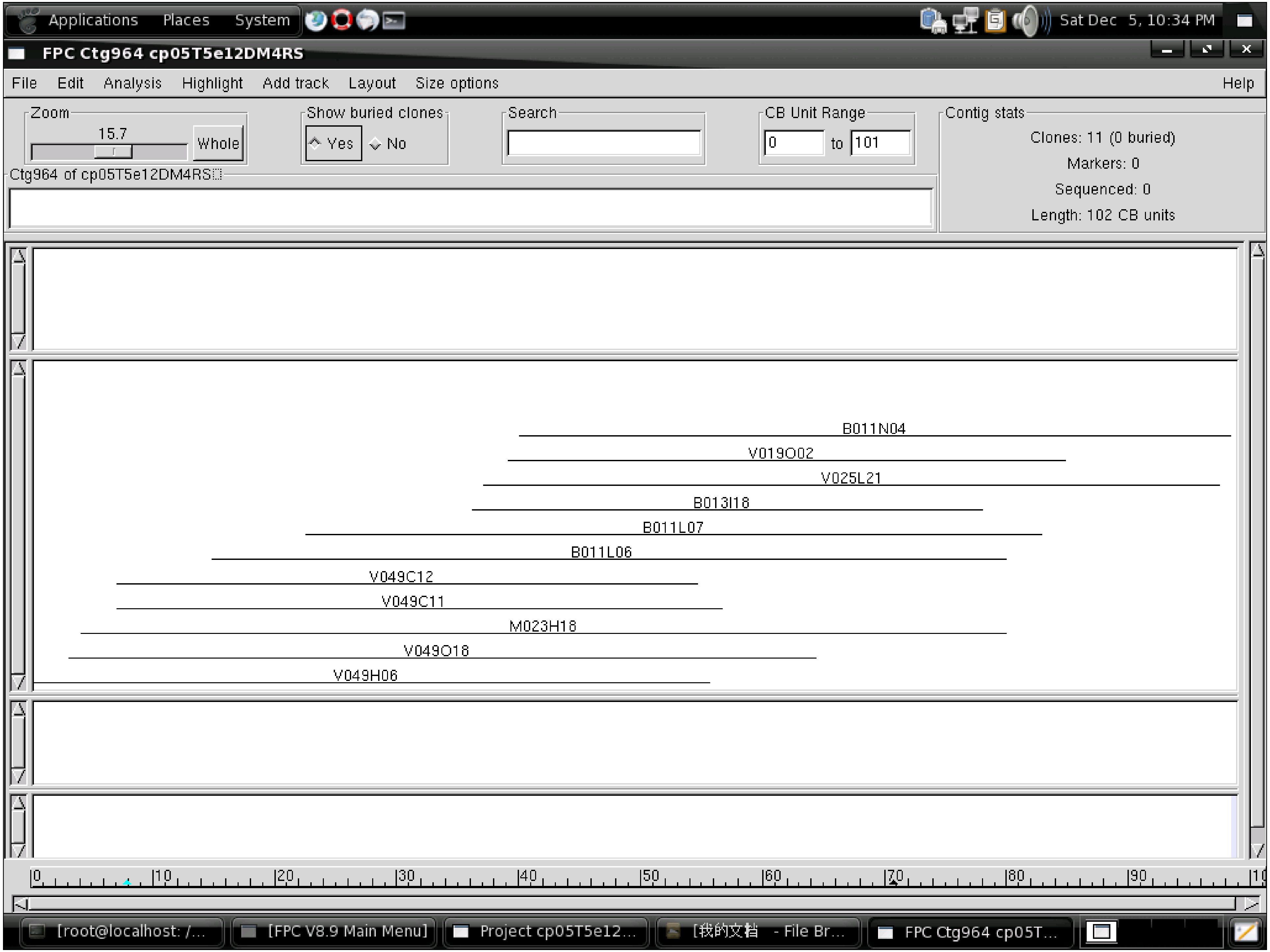
Applications Places System
Sat Dec 5, 10:34 PM
FPC Ctg964 cp05T5e12DM4RS
File Edit Analysis Highlight Add track Layout Size options Help
Zoom
15.7
Whole
Show buried clones
Yes No
Search
CB Unit Range
0 to 101
Contig stats
Clones: 11 (0 buried)
Markers: 0
Sequenced: 0
Length: 102 CB units
Ctg964 of cp05T5e12DM4RS
B011N04
V019O02
V025L21
B013I18
B011L07
B011L06
V049C12
V049C11
M023H18
V049O18
V049H06
0 10 20 30 40 50 60 70 80 90
[root@localhost: /...
[FPC V8.9 Main Menu]
Project cp05T5e12...
[我的文档 - File Br...
FPC Ctg964 cp05T...

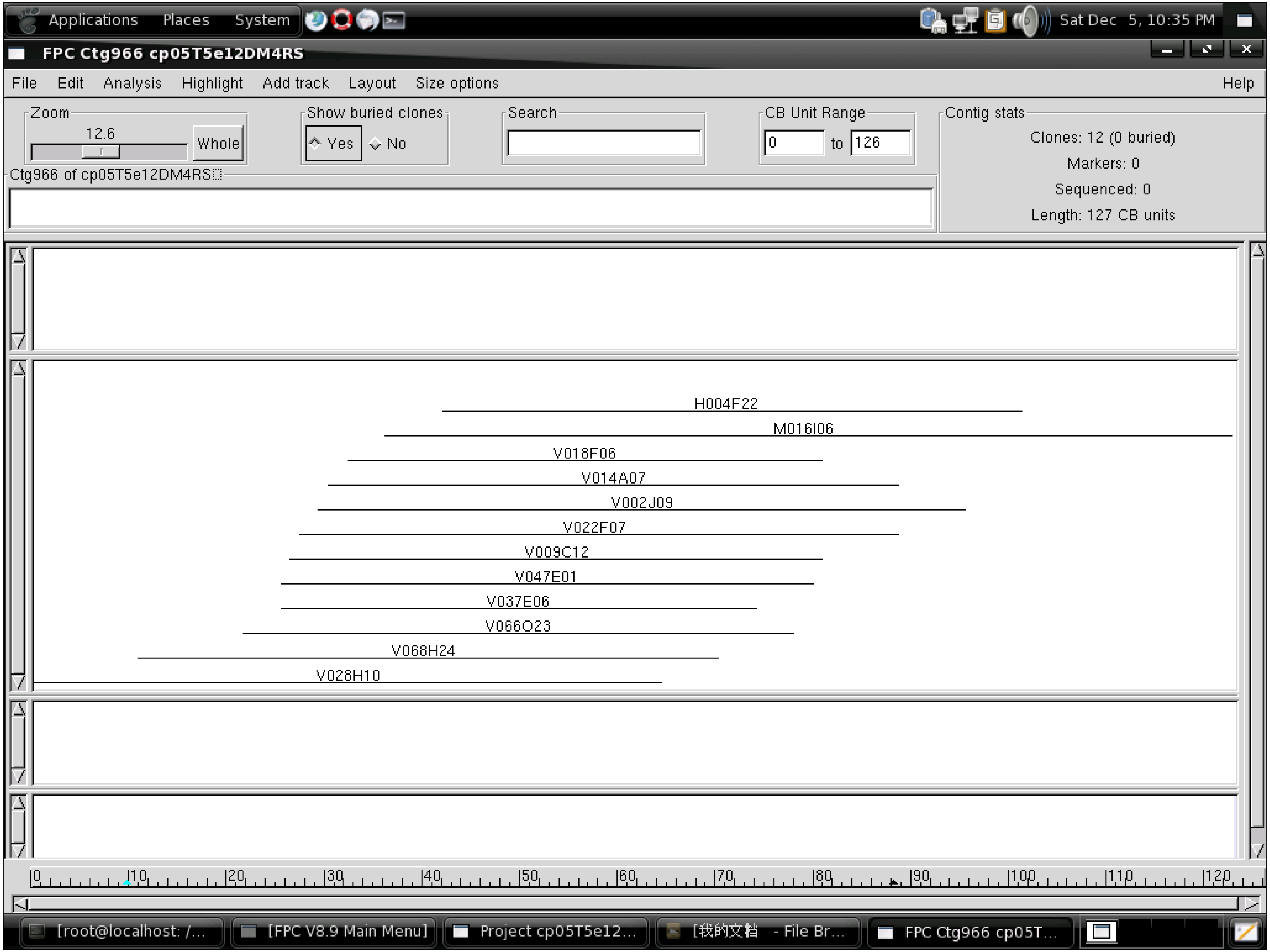
Applications Places System
Sat Dec 5, 10:35 PM
FPC Ctg966 cp05T5e12DM4RS
File Edit Analysis Highlight Add track Layout Size options Help
Zoom
12.6
Whole
Show buried clones
Yes No
Search
CB Unit Range
0 to 126
Contig stats
Clones: 12 (0 buried)
Markers: 0
Sequenced: 0
Length: 127 CB units
Ctg966 of cp05T5e12DM4RS
H004F22
M016I06
V018F06
V014A07
V002J09
V022F07
V009C12
V047E01
V037E06
V066O23
V068H24
V028H10
0 10 20 30 40 50 60 70 80 90 100 110 120
[root@localhost: /...
[FPC V8.9 Main Menu]
Project cp05T5e12...
[我的文档 - File Br...
FPC Ctg966 cp05T...

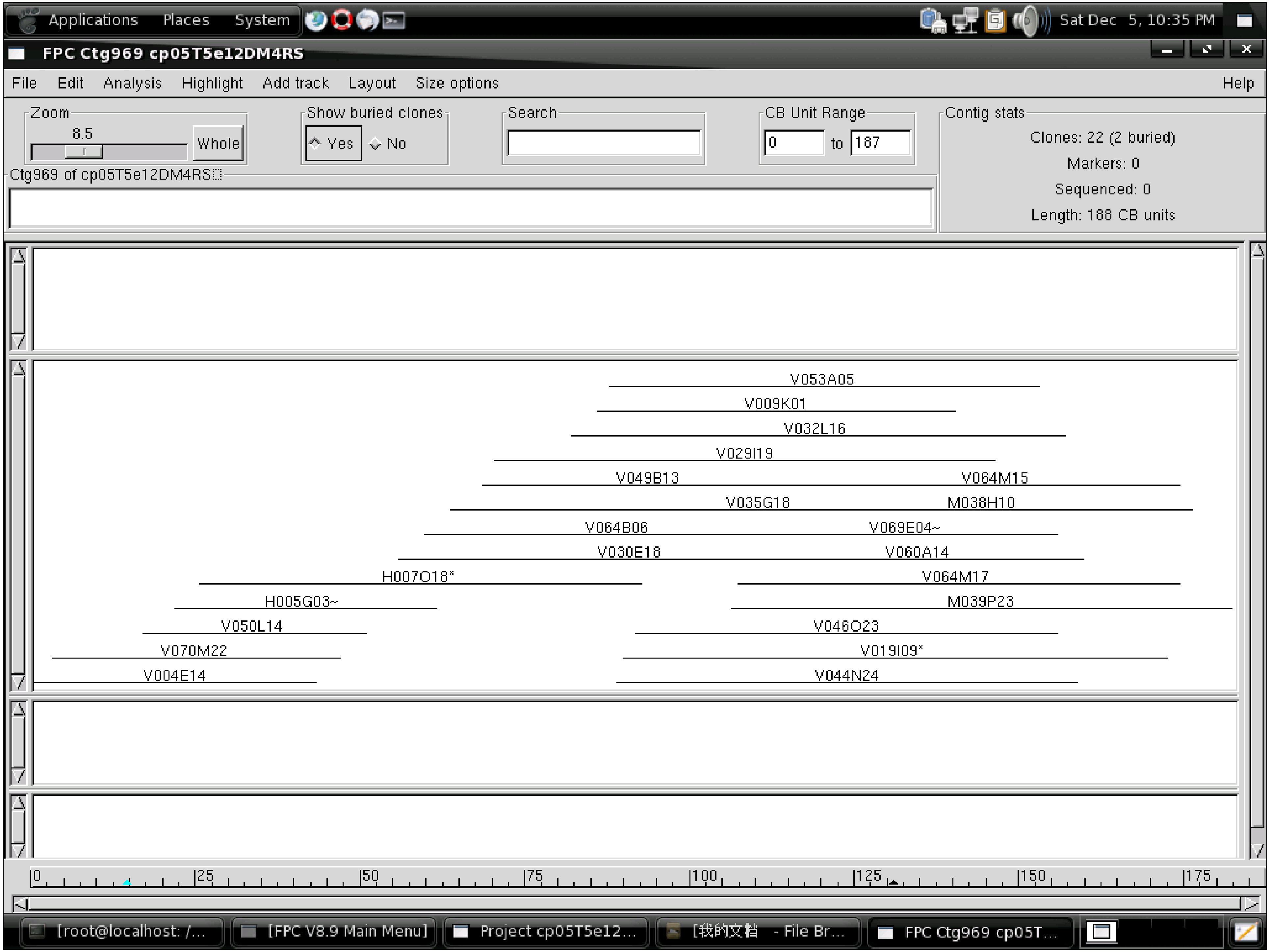
Applications Places System
Sat Dec 5, 10:35 PM
FPC Ctg969 cp05T5e12DM4RS
File Edit Analysis Highlight Add track Layout Size options Help
Zoom
8.5
Whole
Show buried clones
Yes No
Search
CB Unit Range
0 to 187
Contig stats
Clones: 22 (2 buried)
Markers: 0
Sequenced: 0
Length: 188 CB units
Ctg969 of cp05T5e12DM4RS
V053A05
V009K01
V032L16
V029I19
V049B13
V064M15
V035G18
M038H10
V064B06
V069E04~
V030E18
V060A14
H007O18*
V064M17
H005G03~
M039P23
V050L14
V046O23
V070M22
V019I09*
V004E14
V044N24
0 25 50 75 100 125 150 175
[root@localhost: /...
[FPC V8.9 Main Menu]
Project cp05T5e12...
[我的文档 - File Br...
FPC Ctg969 cp05T...

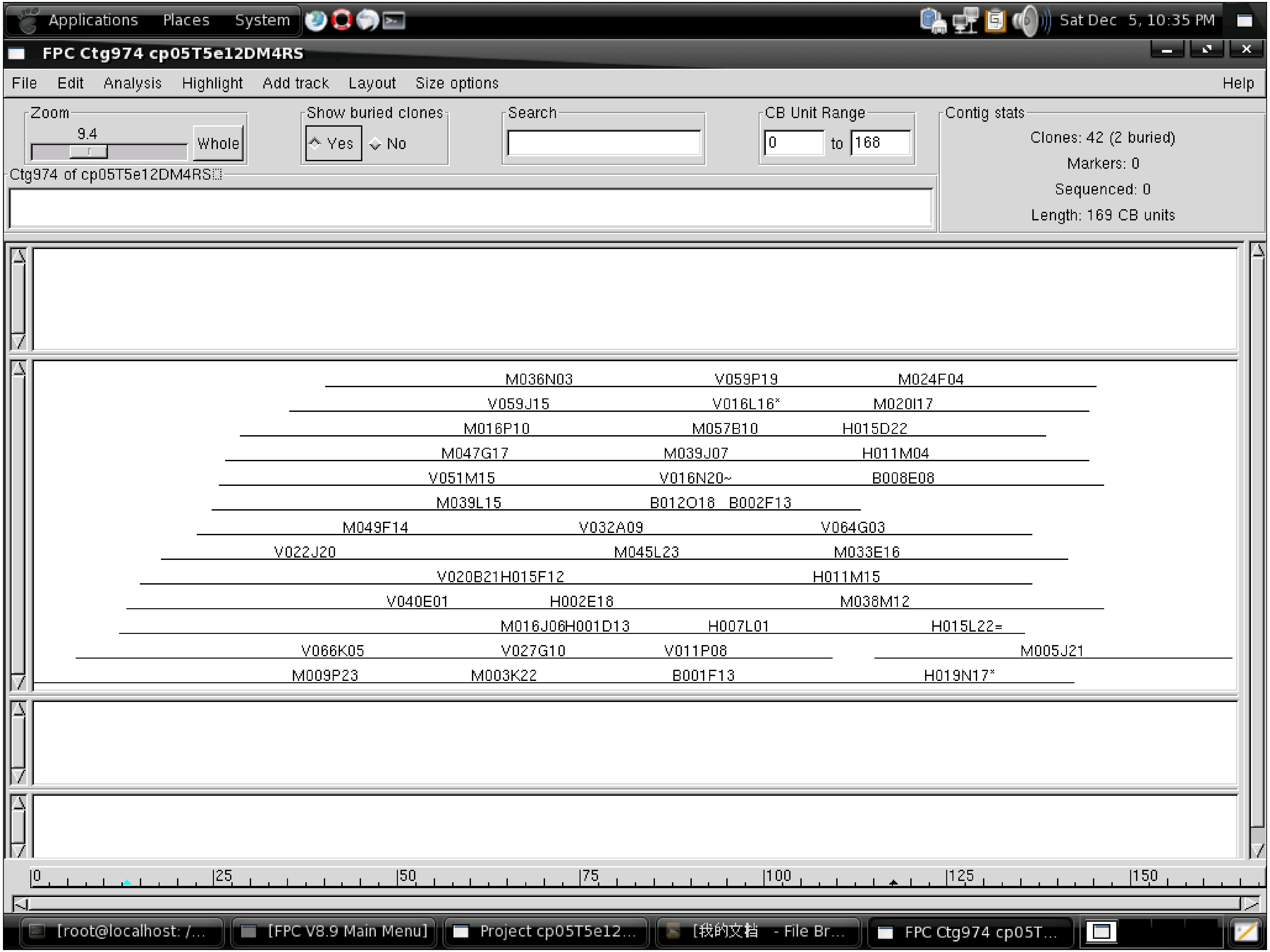
Applications Places System
Sat Dec 5, 10:35 PM
FPC Ctg974 cp05T5e12DM4RS
File Edit Analysis Highlight Add track Layout Size options Help
Zoom
9.4
Whole
Show buried clones
Yes
No
Search
CB Unit Range
0
to
168
Contig stats
Clones: 42 (2 buried)
Markers: 0
Sequenced: 0
Length: 169 CB units
Ctg974 of cp05T5e12DM4RS
M036N03 V059P19 M024F04
V059J15 V016L16* M020I17
M016P10 M057B10 H015D22
M047G17 M039J07 H011M04
V051M15 V016N20~ B008E08
M039L15 B012O18 B002F13
M049F14 V032A09 V064G03
V022J20 M045L23 M033E16
V020B21H015F12 H011M15
V040E01 H002E18 M038M12
M016J06H001D13 H007L01 H015L22=
V066K05 V027G10 V011P08 M005J21
M009P23 M003K22 B001F13 H019N17*
0 25 50 75 100 125 150
[root@localhost: /...
[FPC V8.9 Main Menu]
Project cp05T5e12...
[我的文档 - File Br...
FPC Ctg974 cp05T...

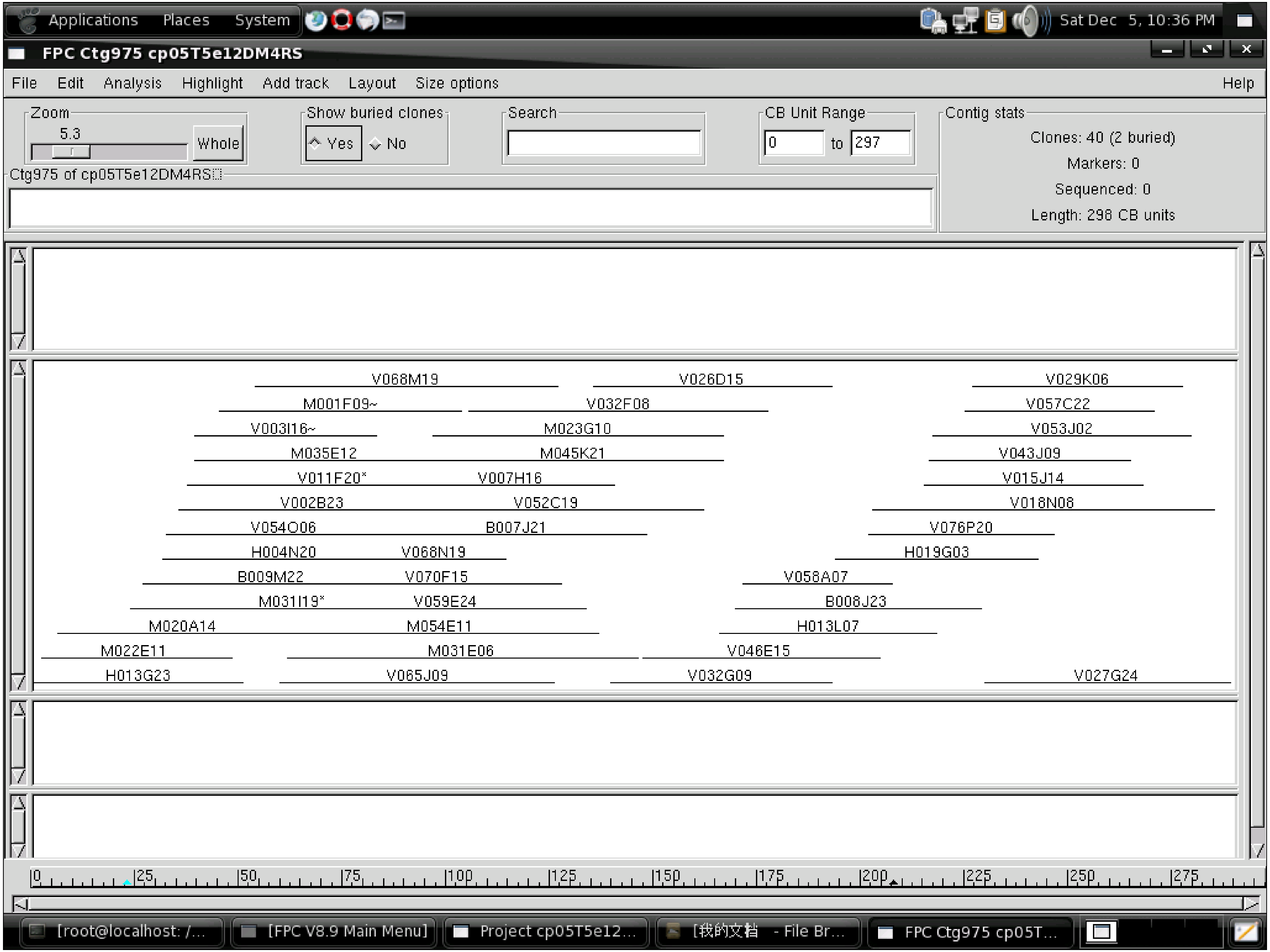
Applications Places System
Sat Dec 5, 10:36 PM
FPC Ctg975 cp05T5e12DM4RS
File Edit Analysis Highlight Add track Layout Size options Help
Zoom
5.3
Whole
Show buried clones
Yes No
Search
CB Unit Range
0 to 297
Contig stats
Clones: 40 (2 buried)
Markers: 0
Sequenced: 0
Length: 298 CB units
Ctg975 of cp05T5e12DM4RS
V068M19
V026D15
V029K06
M001F09~
V032F08
V057C22
V003I16~
M023G10
V053J02
M035E12
M045K21
V043J09
V011F20*
V007H16
V015J14
V002B23
V052C19
V018N08
V054O06
B007J21
V076P20
H004N20
V068N19
H019G03
B009M22
V070F15
V058A07
M031I19*
V059E24
B008J23
M020A14
M054E11
H013L07
M022E11
M031E06
V046E15
H013G23
V065J09
V032G09
V027G24
0 25 50 75 100 125 150 175 200 225 250 275
[root@localhost: /...
[FPC V8.9 Main Menu]
Project cp05T5e12...
[我的文档 - File Br...
FPC Ctg975 cp05T...

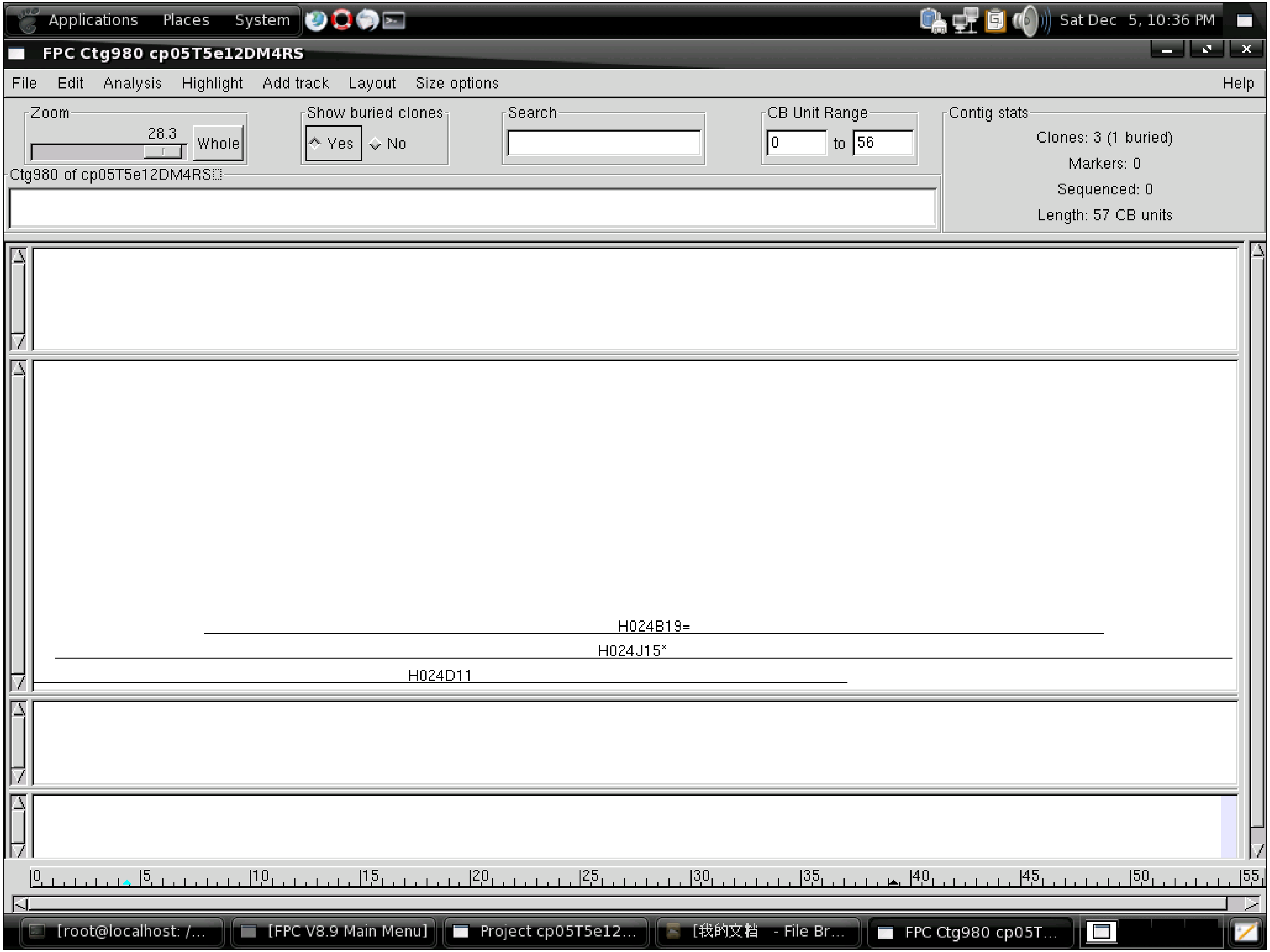
Applications
Places
System
Sat Dec 5, 10:36 PM
FPC Ctg980 cp05T5e12DM4RS
File
Edit
Analysis
Highlight
Add track
Layout
Size options
Help
Zoom
28.3
Whole
Show buried clones
Yes
No
Search
CB Unit Range
0
to
56
Contig stats
Clones: 3 (1 buried)
Markers: 0
Sequenced: 0
Length: 57 CB units
Ctg980 of cp05T5e12DM4RS
H024B19=
H024J15*
H024D11
0
5
10
15
20
25
30
35
40
45
50
55
[root@localhost: /...
[FPC V8.9 Main Menu]
Project cp05T5e12...
[我的文档 - File Br...
FPC Ctg980 cp05T...

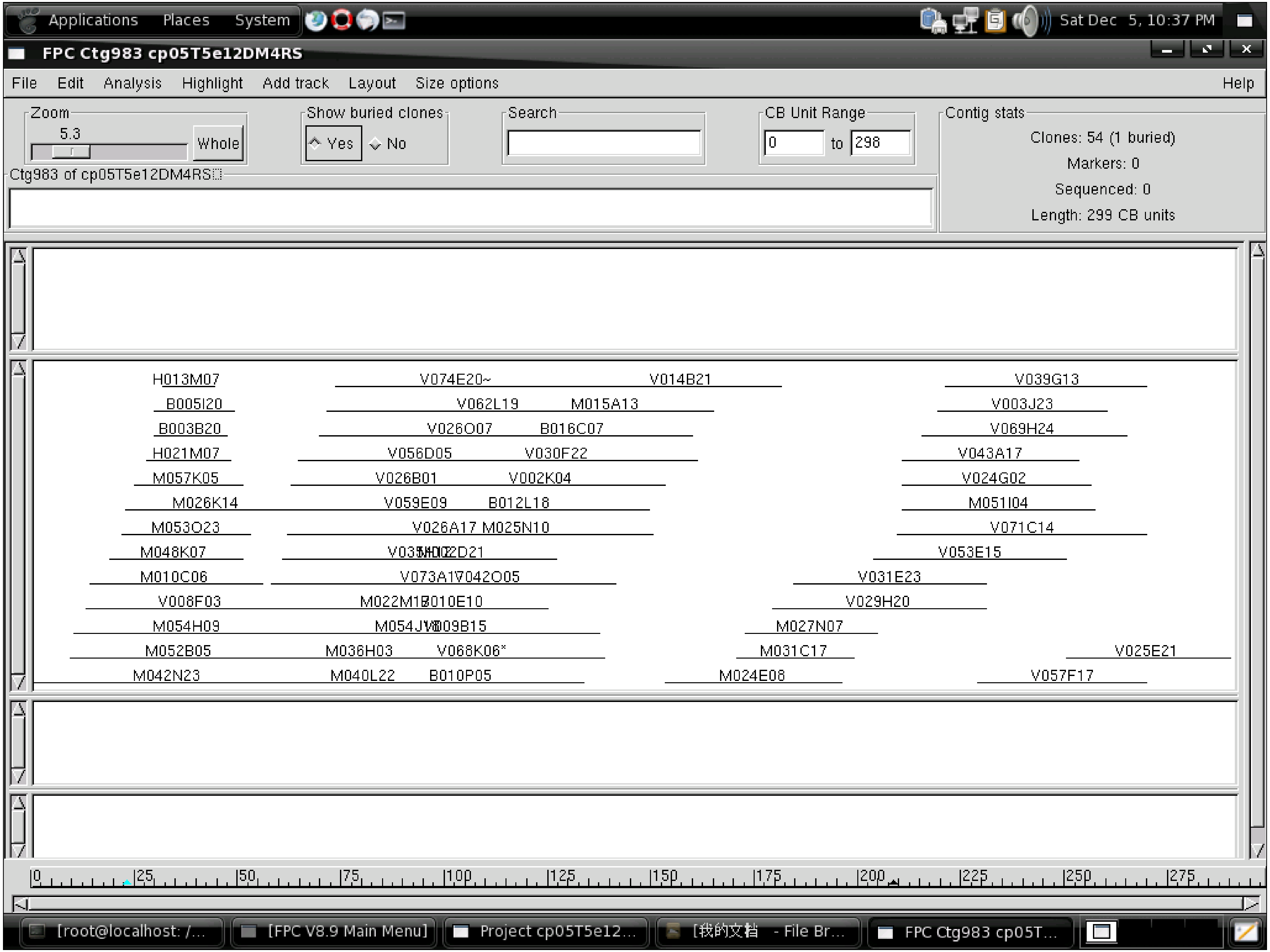
Applications Places System
Sat Dec 5, 10:37 PM
FPC Ctg983 cp05T5e12DM4RS
File Edit Analysis Highlight Add track Layout Size options Help
Zoom
5.3
Whole
Show buried clones
Yes No
Search
CB Unit Range
0 to 298
Contig stats
Clones: 54 (1 buried)
Markers: 0
Sequenced: 0
Length: 299 CB units
Ctg983 of cp05T5e12DM4RS
H013M07
V074E20~
V014B21
V039G13
B005I20
V062L19
M015A13
V003J23
B003B20
V026O07
B016C07
V069H24
H021M07
V056D05
V030F22
V043A17
M057K05
V026B01
V002K04
V024G02
M026K14
V059E09
B012L18
M051I04
M053O23
V026A17 M025N10
V071C14
M048K07
V053E15
M010C06
V031E23
V008F03
V029H20
M054H09
M027N07
M052B05
M036H03
V068K06*
M031C17
V025E21
M042N23
M040L22
B010P05
M024E08
V057F17
0 25 50 75 100 125 150 175 200 225 250 275
[root@localhost: /...
[FPC V8.9 Main Menu]
Project cp05T5e12...
[我的文档 - File Br...
FPC Ctg983 cp05T...

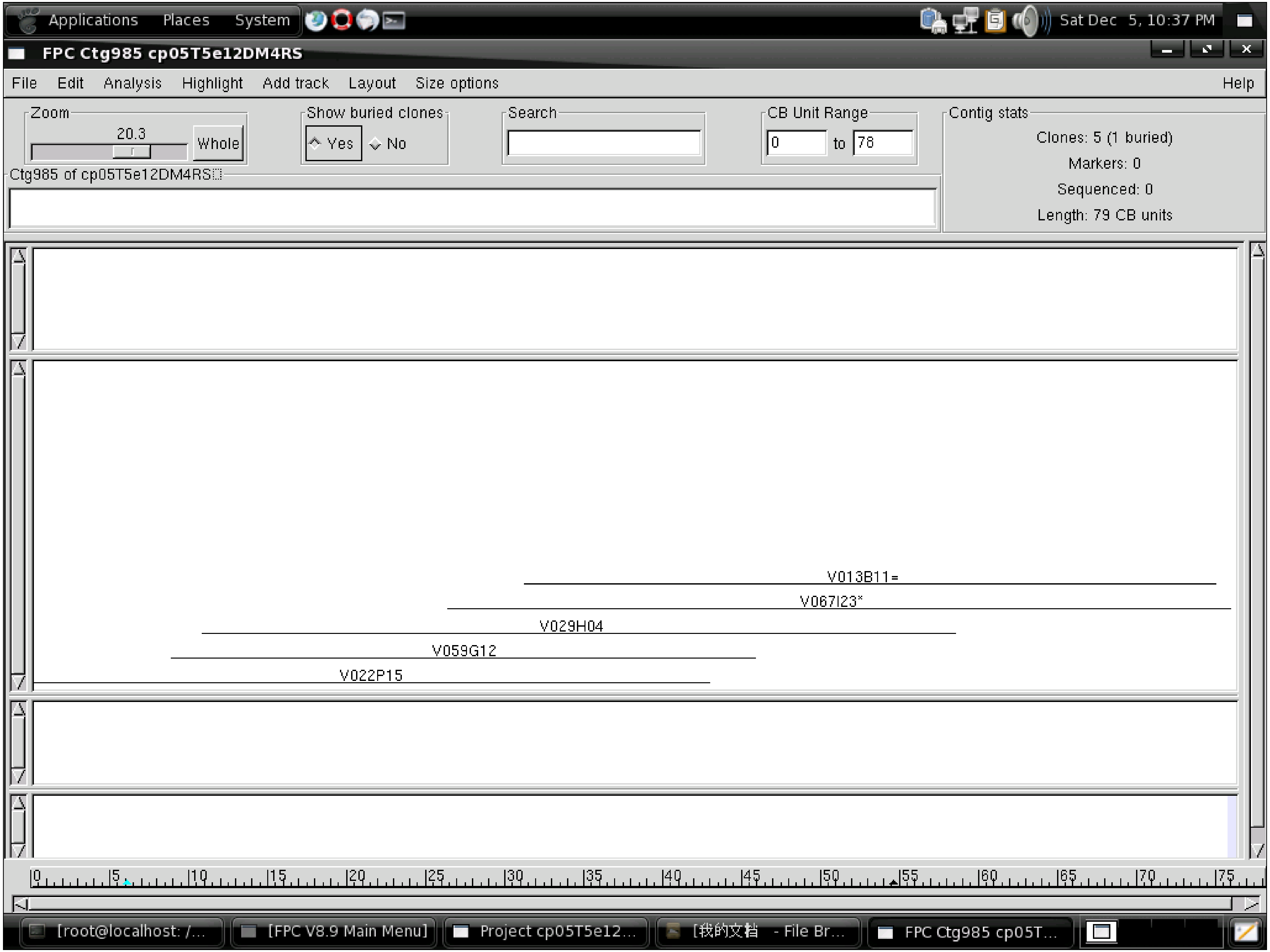

Applications Places System
Sat Dec 5, 10:37 PM
FPC Ctg985 cp05T5e12DM4RS
File Edit Analysis Highlight Add track Layout Size options Help
Zoom
20.3
Whole
Show buried clones
Yes No
Search
CB Unit Range
0 to 78
Contig stats
Clones: 5 (1 buried)
Markers: 0
Sequenced: 0
Length: 79 CB units
Ctg985 of cp05T5e12DM4RS
V013B11=
V067I23*
V029H04
V059G12
V022P15
0 5 10 15 20 25 30 35 40 45 50 55 60 65 70 75
[root@localhost: /...
[FPC V8.9 Main Menu]
Project cp05T5e12...
[我的文档 - File Br...
FPC Ctg985 cp05T...

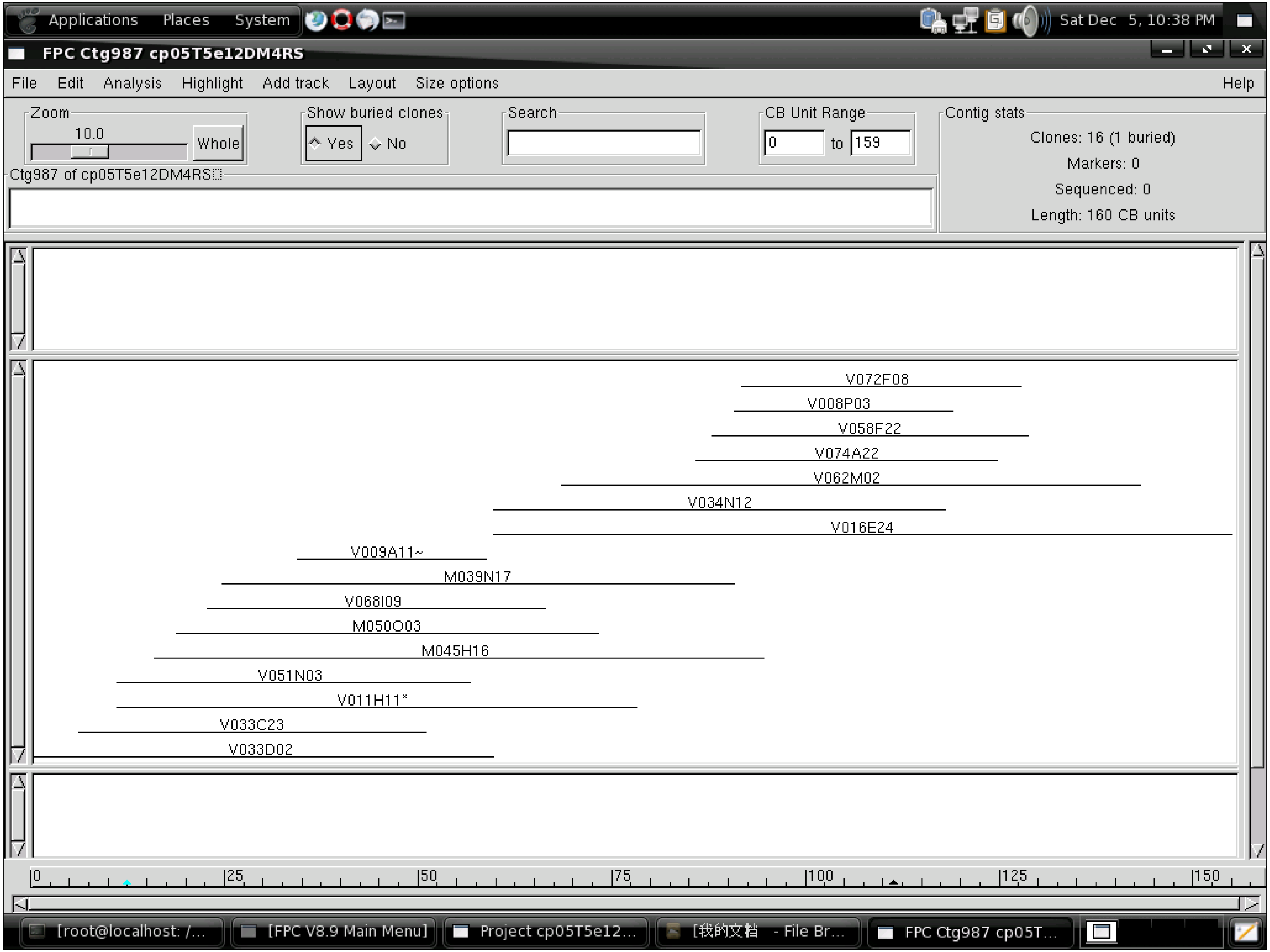
Applications Places System
Sat Dec 5, 10:38 PM
FPC Ctg987 cp05T5e12DM4RS
File Edit Analysis Highlight Add track Layout Size options Help
Zoom
10.0
Whole
Show buried clones
Yes
No
Search
CB Unit Range
0 to 159
Contig stats
Clones: 16 (1 buried)
Markers: 0
Sequenced: 0
Length: 160 CB units
Ctg987 of cp05T5e12DM4RS
V072F08
V008P03
V058F22
V074A22
V062M02
V034N12
V016E24
V009A11~
M039N17
V068I09
M050O03
M045H16
V051N03
V011H11*
V033C23
V033D02
0
25
50
75
100
125
150
[root@localhost: /...
[FPC V8.9 Main Menu]
Project cp05T5e12...
[我的文档 - File Br...
FPC Ctg987 cp05T...

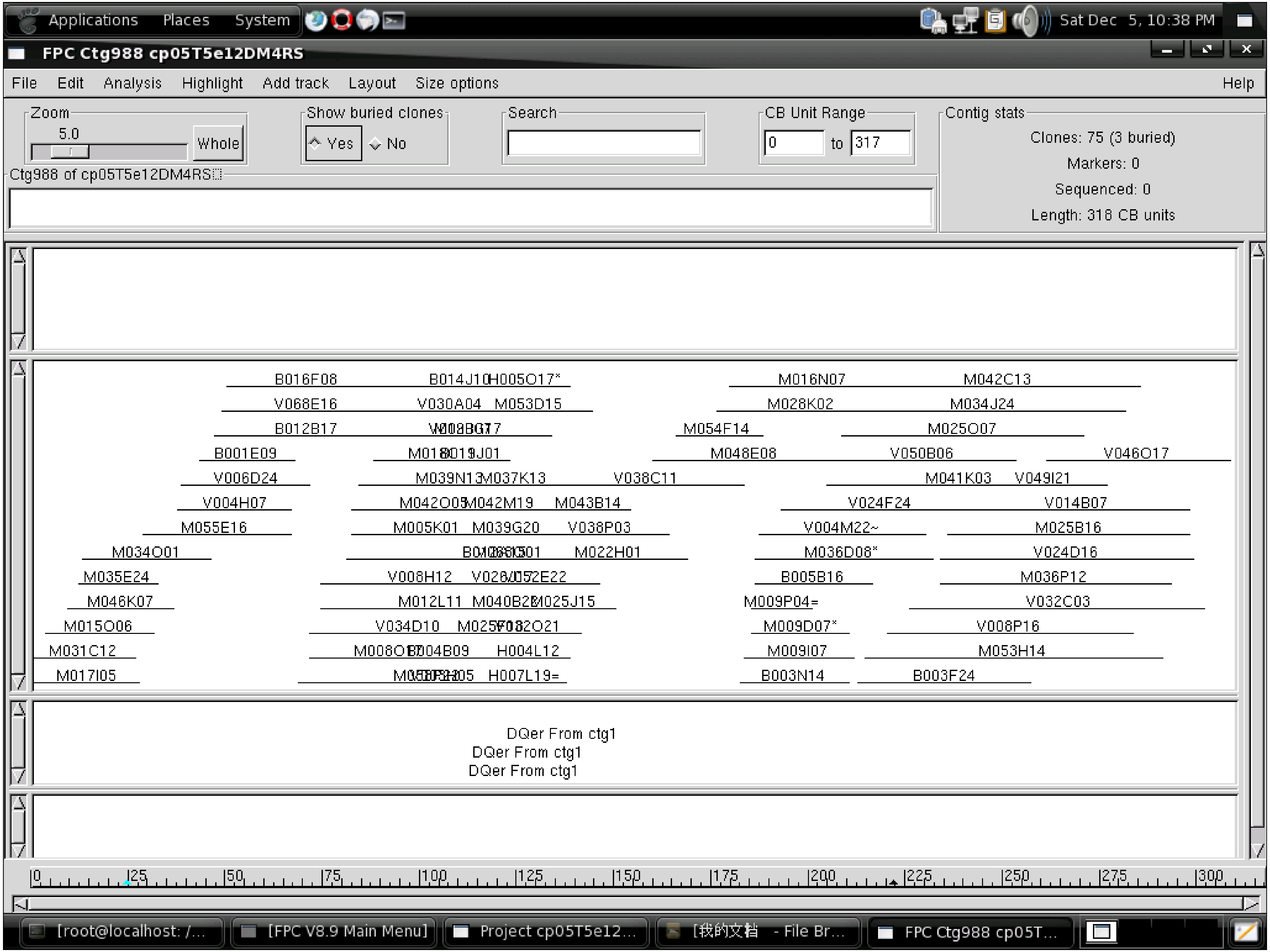
Applications Places System
Sat Dec 5, 10:38 PM
FPC Ctg988 cp05T5e12DM4RS
File Edit Analysis Highlight Add track Layout Size options Help
Zoom
5.0
Whole
Show buried clones
Yes No
Search
CB Unit Range
0 to 317
Contig stats
Clones: 75 (3 buried)
Markers: 0
Sequenced: 0
Length: 318 CB units
Ctg988 of cp05T5e12DM4RS
B016F08
M016N07
M042C13
V068E16
V030A04
M053D15
M028K02
M034J24
B012B17
M054F14
M025O07
B001E09
M048E08
V050B06
V046O17
V006D24
V038C11
M041K03
V049I21
V004H07
M043B14
V024F24
V014B07
M055E16
M005K01
M039G20
V038P03
V004M22~
M025B16
M034O01
M022H01
M036D08*
V024D16
M035E24
V008H12
B005B16
M036P12
M046K07
M012L11
M009P04=
V032C03
M015O06
V034D10
M009D07*
V008P16
M031C12
H004L12
M009I07
M053H14
M017I05
H007L19=
B003N14
B003F24
DQer From ctg1
DQer From ctg1
DQer From ctg1
0
25
50
75
100
125
150
175
200
225
250
275
300
[root@localhost: /...
[FPC V8.9 Main Menu]
Project cp05T5e12...
[我的文档 - File Br...
FPC Ctg988 cp05T...

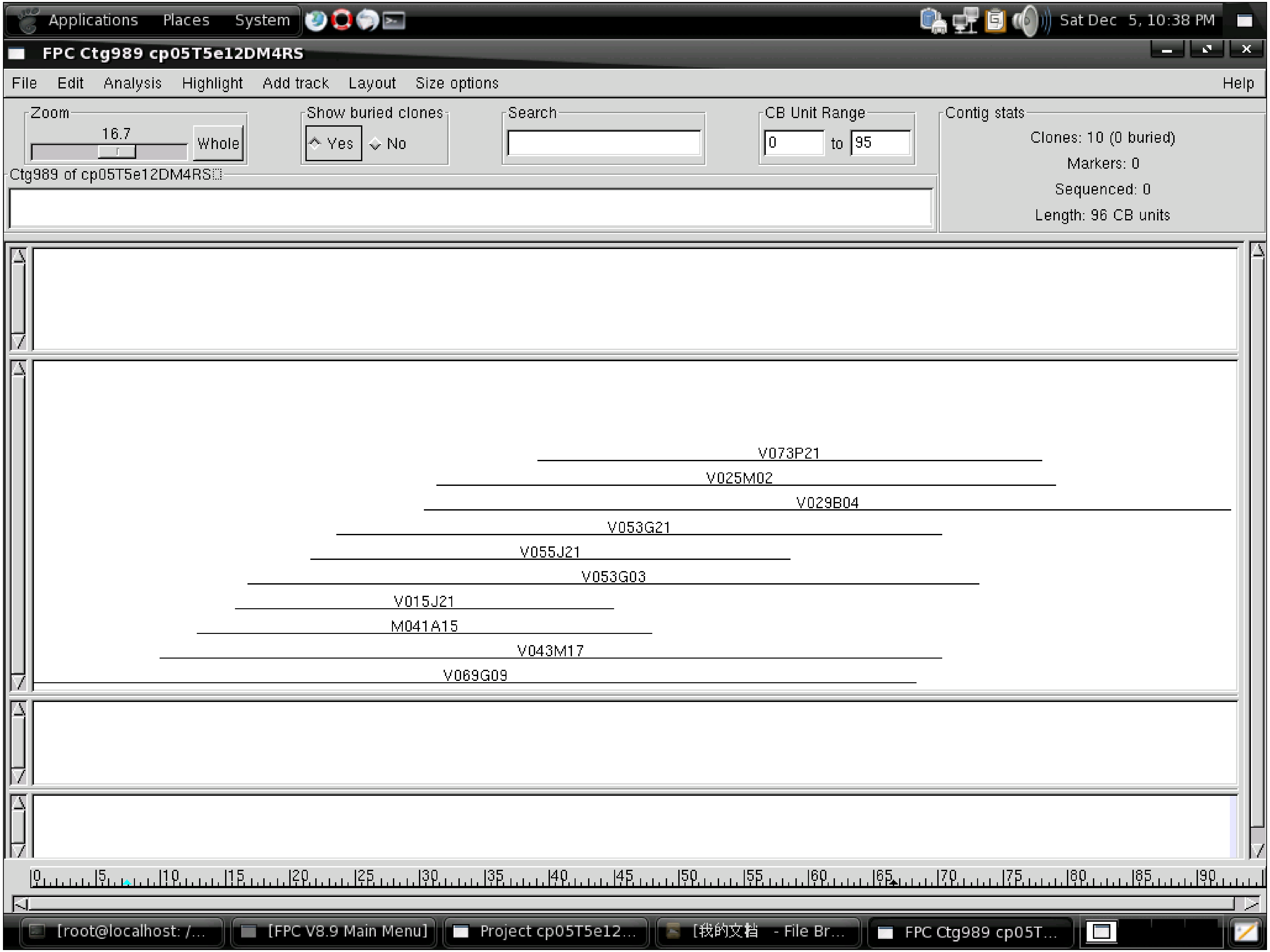
Applications Places System
Sat Dec 5, 10:38 PM
FPC Ctg989 cp05T5e12DM4RS
File Edit Analysis Highlight Add track Layout Size options Help
Zoom
16.7
Whole
Show buried clones
Yes
No
Search
CB Unit Range
0 to 95
Contig stats
Clones: 10 (0 buried)
Markers: 0
Sequenced: 0
Length: 96 CB units
Ctg989 of cp05T5e12DM4RS
V073P21
V025M02
V029B04
V053G21
V055J21
V053G03
V015J21
M041A15
V043M17
V069G09
0 5 10 15 20 25 30 35 40 45 50 55 60 65 70 75 80 85 90
[root@localhost: /...
[FPC V8.9 Main Menu]
Project cp05T5e12...
[我的文档 - File Br...
FPC Ctg989 cp05T...

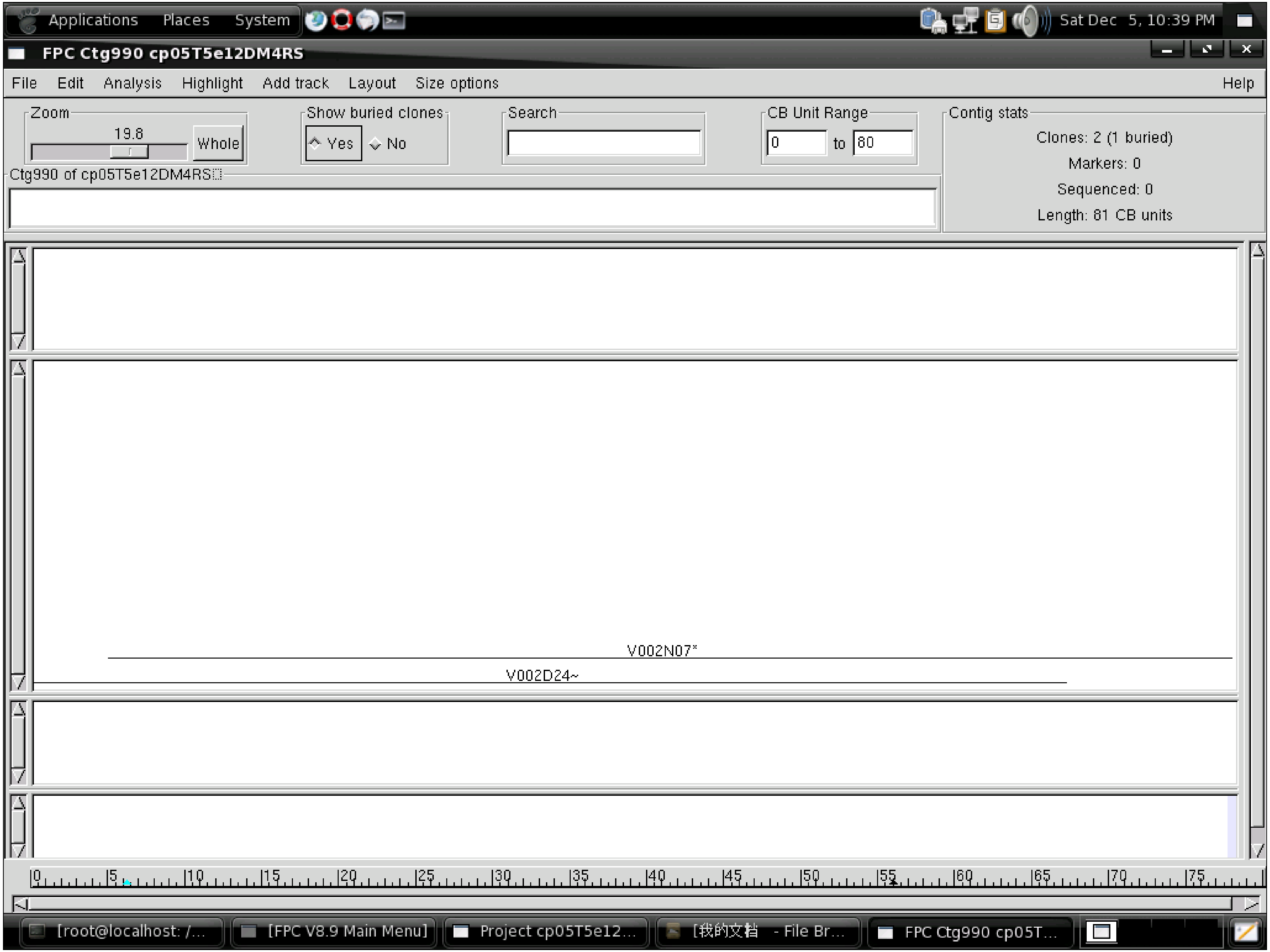
Applications Places System
Sat Dec 5, 10:39 PM
FPC Ctg990 cp05T5e12DM4RS
File Edit Analysis Highlight Add track Layout Size options Help
Zoom
19.8
Whole
Show buried clones
Yes No
Search
CB Unit Range
0 to 80
Contig stats
Clones: 2 (1 buried)
Markers: 0
Sequenced: 0
Length: 81 CB units
Ctg990 of cp05T5e12DM4RS
V002N07*
V002D24~
0 5 10 15 20 25 30 35 40 45 50 55 60 65 70 75
[root@localhost: /...
[FPC V8.9 Main Menu]
Project cp05T5e12...
[我的文档 - File Br...
FPC Ctg990 cp05T...

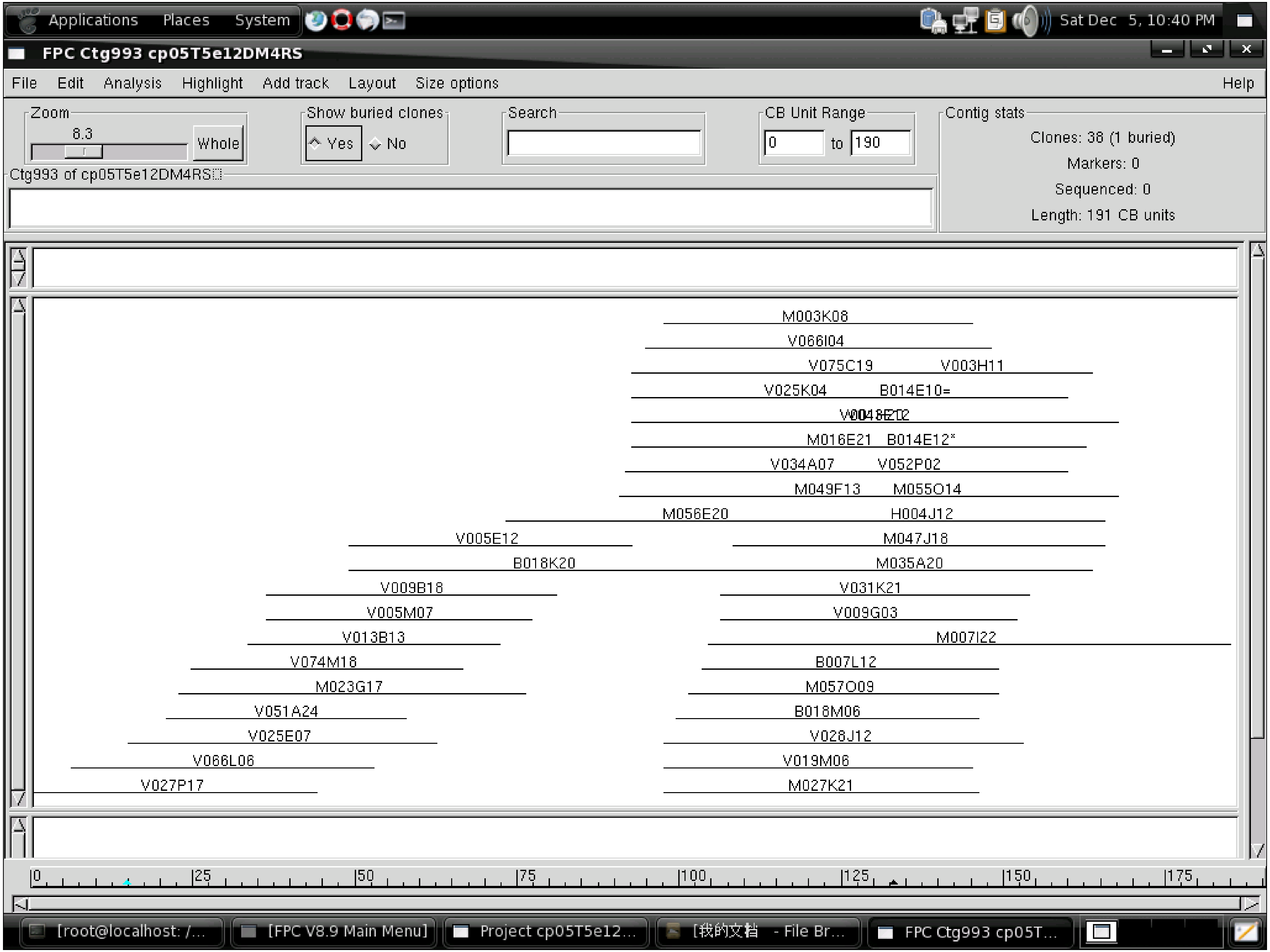

Applications Places System
Sat Dec 5, 10:40 PM
FPC Ctg993 cp05T5e12DM4RS
File Edit Analysis Highlight Add track Layout Size options Help
Zoom
8.3
Whole
Show buried clones
Yes
No
Search
CB Unit Range
0
to
190
Contig stats
Clones: 38 (1 buried)
Markers: 0
Sequenced: 0
Length: 191 CB units
Ctg993 of cp05T5e12DM4RS
M003K08
V066I04
V075C19
V003H11
V025K04
B014E10=
M016E21
B014E12*
V034A07
V052P02
M049F13
M055O14
M056E20
H004J12
V005E12
M047J18
B018K20
M035A20
V009B18
V031K21
V005M07
V009G03
V013B13
M007I22
V074M18
B007L12
M023G17
M057O09
V051A24
B018M06
V025E07
V028J12
V066L06
V019M06
V027P17
M027K21
0 25 50 75 100 125 150 175
[root@localhost: /...
[FPC V8.9 Main Menu]
Project cp05T5e12...
[我的文档 - File Br...
FPC Ctg993 cp05T...

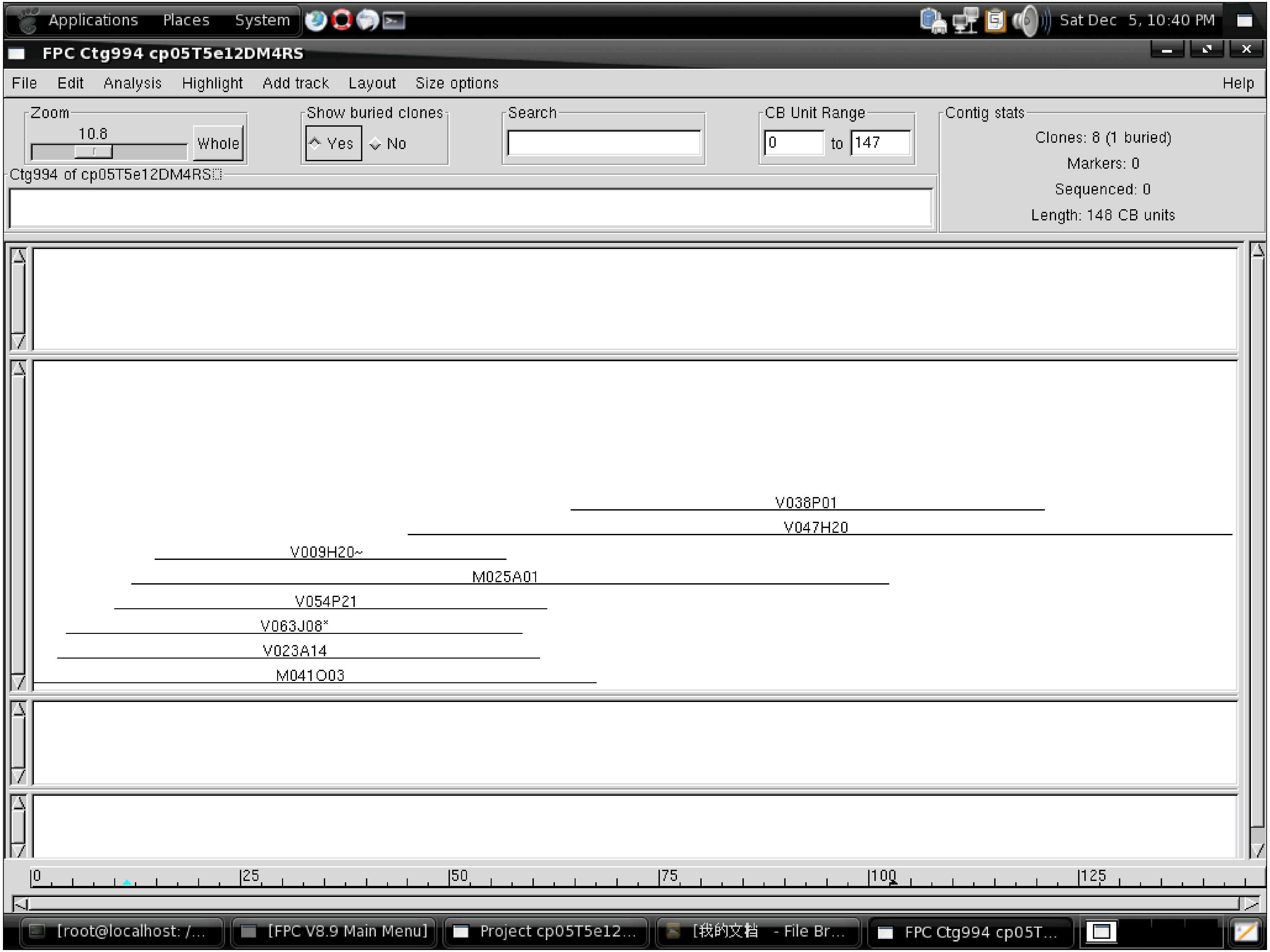
Applications
Places
System
Sat Dec 5, 10:40 PM
FPC Ctg994 cp05T5e12DM4RS
File
Edit
Analysis
Highlight
Add track
Layout
Size options
Help
Zoom
10.8
Whole
Show buried clones
Yes
No
Search
CB Unit Range
0
to
147
Contig stats
Clones: 8 (1 buried)
Markers: 0
Sequenced: 0
Length: 148 CB units
Ctg994 of cp05T5e12DM4RS
V038P01
V047H20
V009H20~
M025A01
V054P21
V063J08*
V023A14
M041O03
0
25
50
75
100
125
[root@localhost: /...
[FPC V8.9 Main Menu]
Project cp05T5e12...
[我的文档 - File Br...
FPC Ctg994 cp05T...

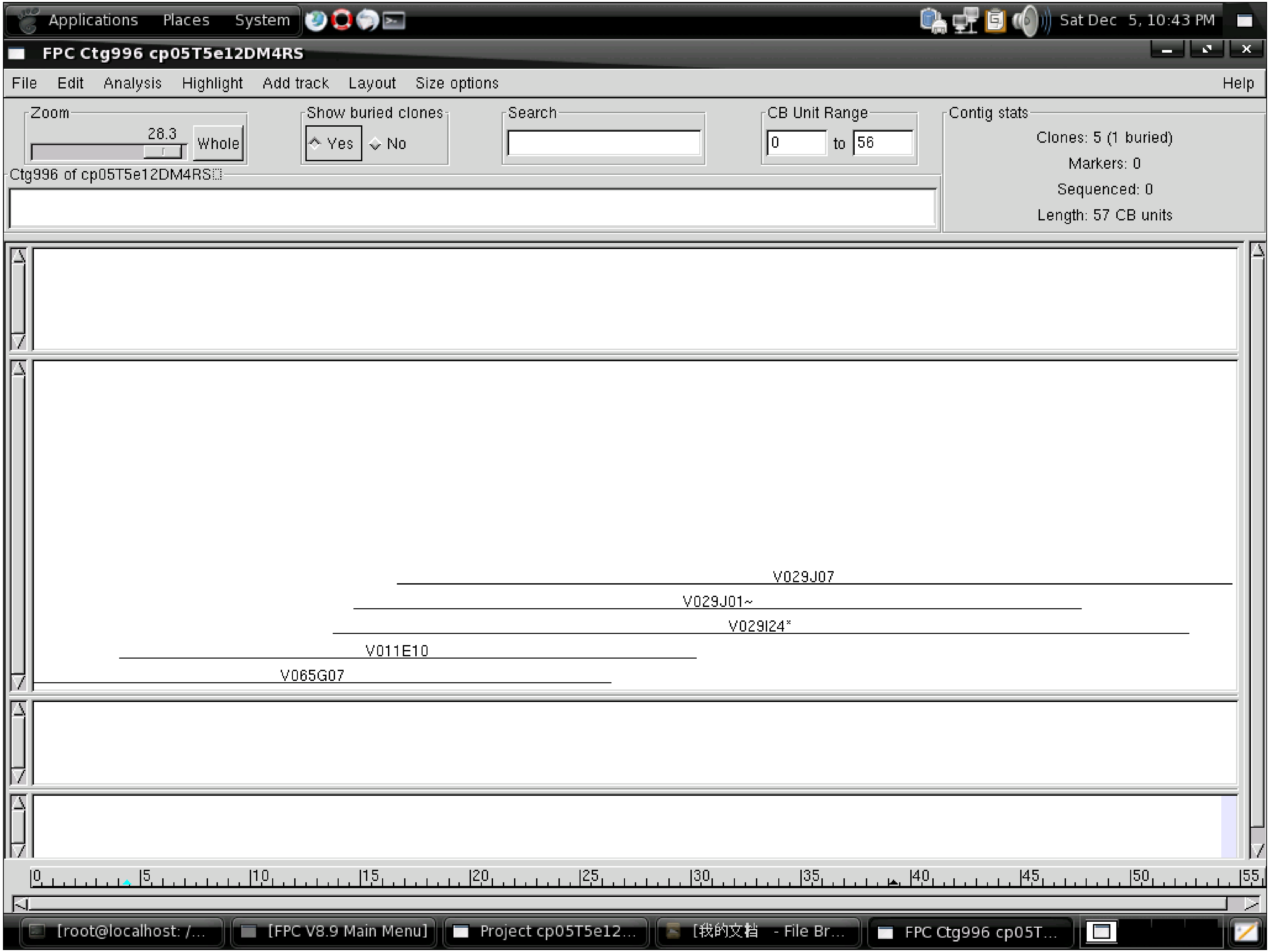
Applications Places System
Sat Dec 5, 10:43 PM
FPC Ctg996 cp05T5e12DM4RS
File Edit Analysis Highlight Add track Layout Size options Help
Zoom
28.3
Whole
Show buried clones
Yes
No
Search
CB Unit Range
0
to
56
Contig stats
Clones: 5 (1 buried)
Markers: 0
Sequenced: 0
Length: 57 CB units
Ctg996 of cp05T5e12DM4RS
V029J07
V029J01~
V029I24*
V011E10
V065G07
0 5 10 15 20 25 30 35 40 45 50 55
[root@localhost: /...
[FPC V8.9 Main Menu]
Project cp05T5e12...
[我的文档 - File Br...
FPC Ctg996 cp05T...

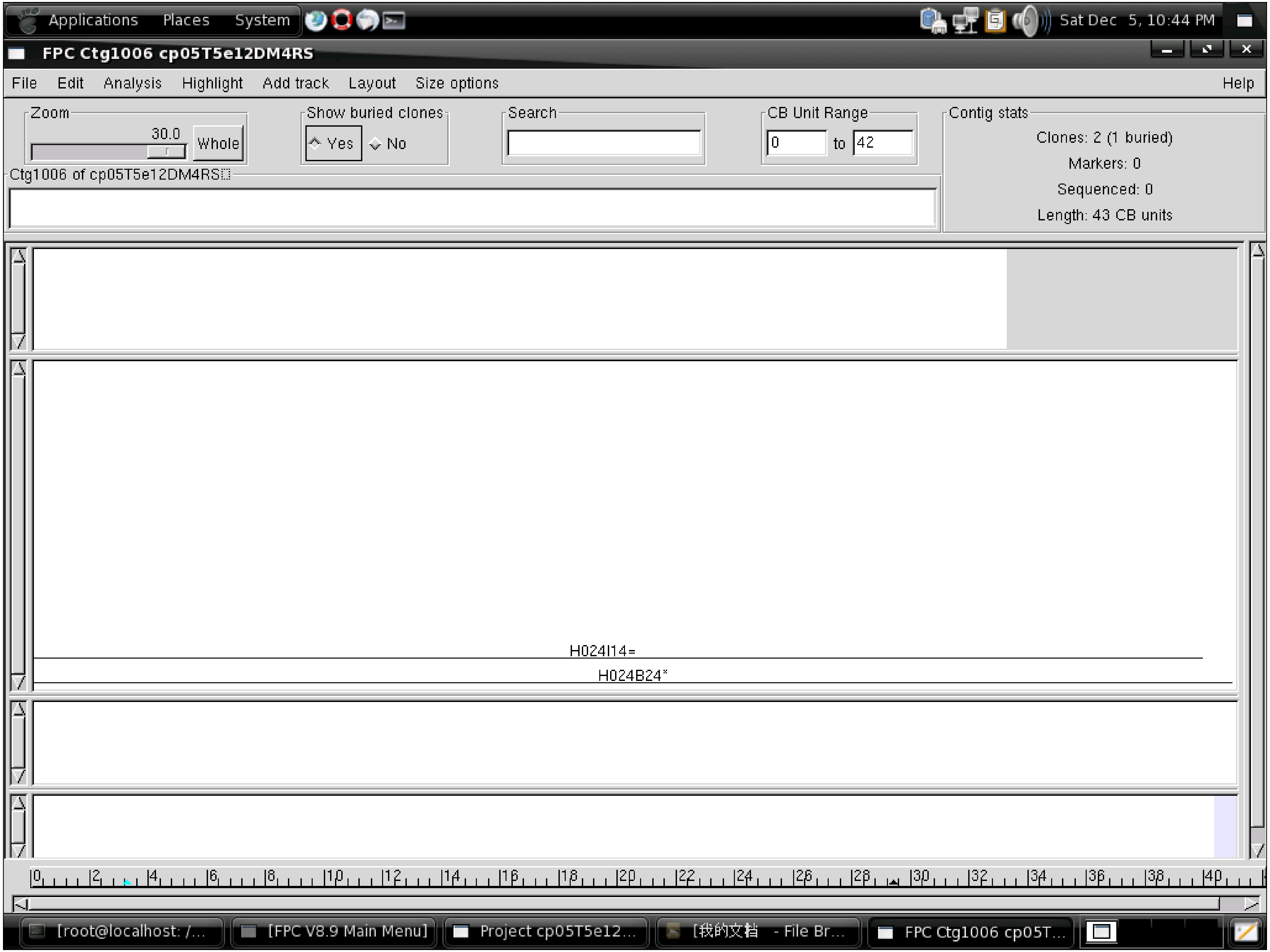
Applications Places System
Sat Dec 5, 10:44 PM
FPC Ctg1006 cp05T5e12DM4RS
File Edit Analysis Highlight Add track Layout Size options Help
Zoom
30.0
Whole
Show buried clones
Yes No
Search
CB Unit Range
0 to 42
Contig stats
Clones: 2 (1 buried)
Markers: 0
Sequenced: 0
Length: 43 CB units
Ctg1006 of cp05T5e12DM4RS
H024I14=
H024B24*
0 2 4 6 8 10 12 14 16 18 20 22 24 26 28 30 32 34 36 38 40
[root@localhost: /...
[FPC V8.9 Main Menu]
Project cp05T5e12...
[我的文档 - File Br...
FPC Ctg1006 cp05T...

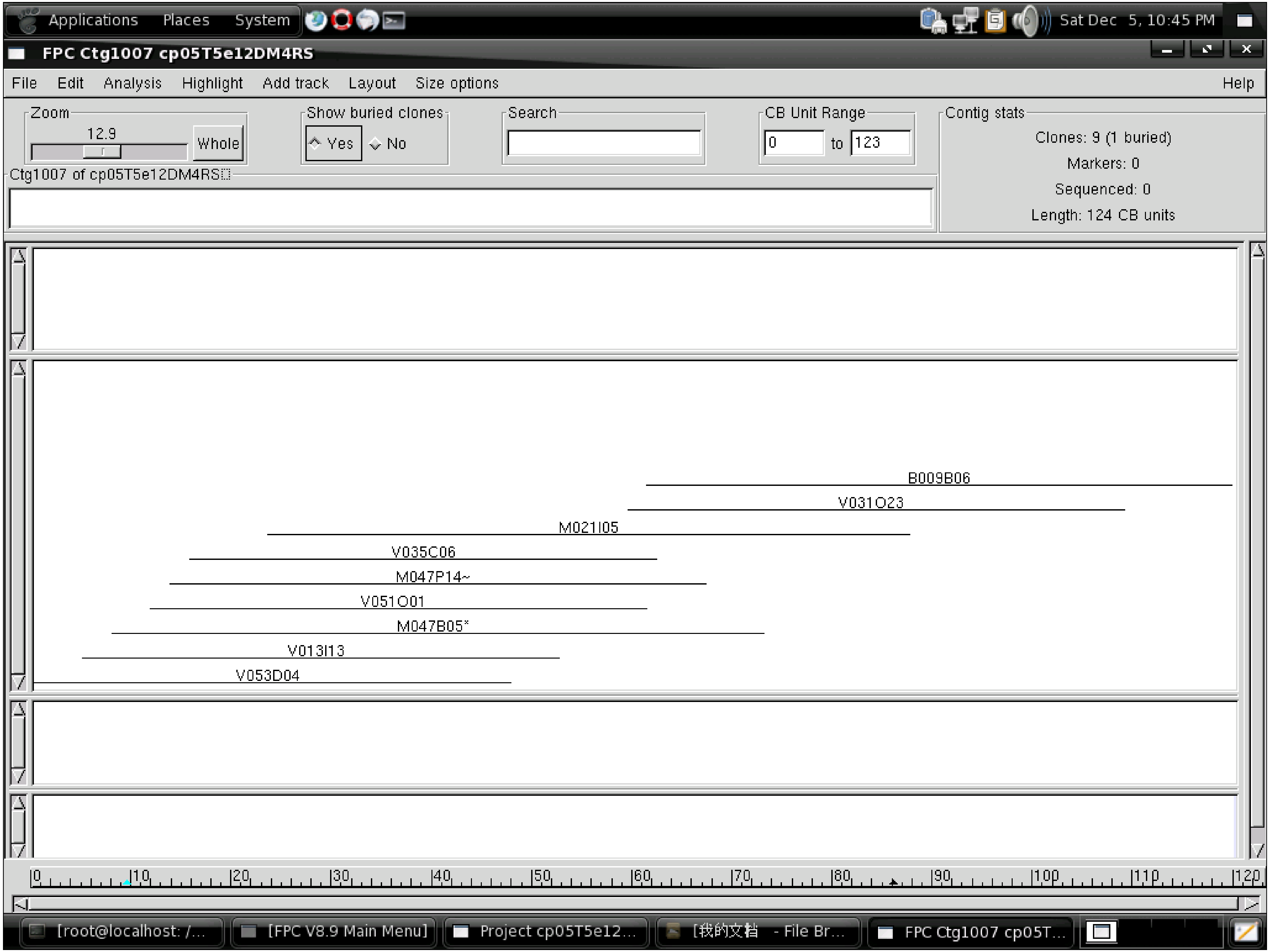
Applications Places System
Sat Dec 5, 10:45 PM
FPC Ctg1007 cp05T5e12DM4RS
File Edit Analysis Highlight Add track Layout Size options Help
Zoom
12.9
Whole
Show buried clones
Yes
No
Search
CB Unit Range
0
to
123
Contig stats
Clones: 9 (1 buried)
Markers: 0
Sequenced: 0
Length: 124 CB units
Ctg1007 of cp05T5e12DM4RS
B009B06
V031O23
M021I05
V035C06
M047P14~
V051O01
M047B05*
V013I13
V053D04
0 10 20 30 40 50 60 70 80 90 100 110 120
[root@localhost: /...
[FPC V8.9 Main Menu]
Project cp05T5e12...
[我的文档 - File Br...
FPC Ctg1007 cp05T...

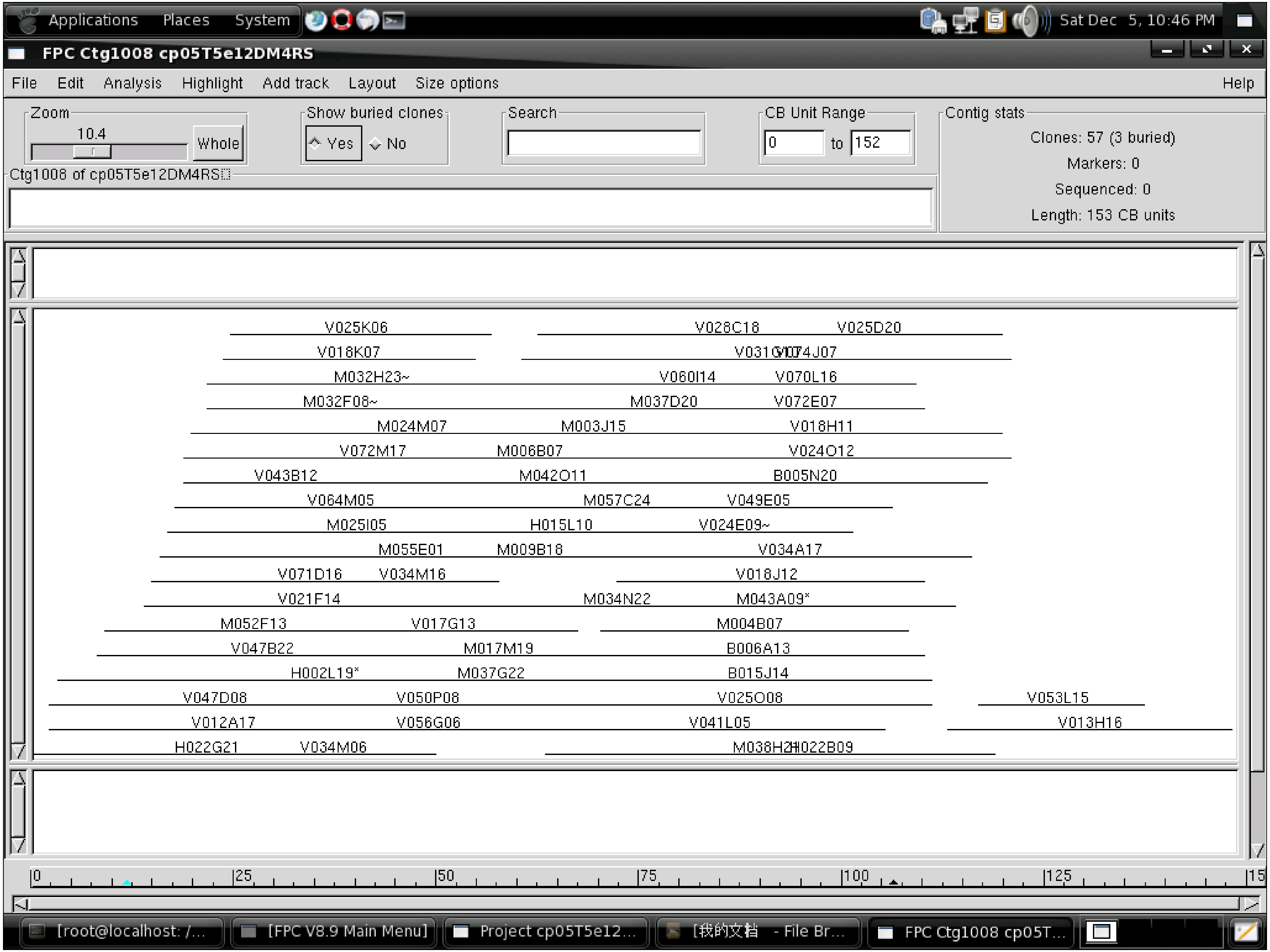
Applications Places System
Sat Dec 5, 10:46 PM
FPC Ctg1008 cp05T5e12DM4RS
File Edit Analysis Highlight Add track Layout Size options Help
Zoom
10.4
Whole
Show buried clones
Yes
No
Search
CB Unit Range
0
to
152
Contig stats
Clones: 57 (3 buried)
Markers: 0
Sequenced: 0
Length: 153 CB units
Ctg1008 of cp05T5e12DM4RS
V025K06
V028C18
V025D20
V018K07
V074J07
M032H23~
V060I14
V070L16
M032F08~
M037D20
V072E07
M024M07
M003J15
V018H11
V072M17
M006B07
V024O12
V043B12
M042O11
B005N20
V064M05
M057C24
V049E05
M025I05
H015L10
V024E09~
M055E01
M009B18
V034A17
V071D16
V034M16
V018J12
V021F14
M034N22
M043A09*
M052F13
V017G13
M004B07
V047B22
M017M19
B006A13
H002L19*
M037G22
B015J14
V047D08
V050P08
V025O08
V053L15
V012A17
V056G06
V041L05
V013H16
H022G21
V034M06
H022B09
0
25
50
75
100
125
[root@localhost: /...
[FPC V8.9 Main Menu]
Project cp05T5e12...
[我的文档 - File Br...
FPC Ctg1008 cp05T...

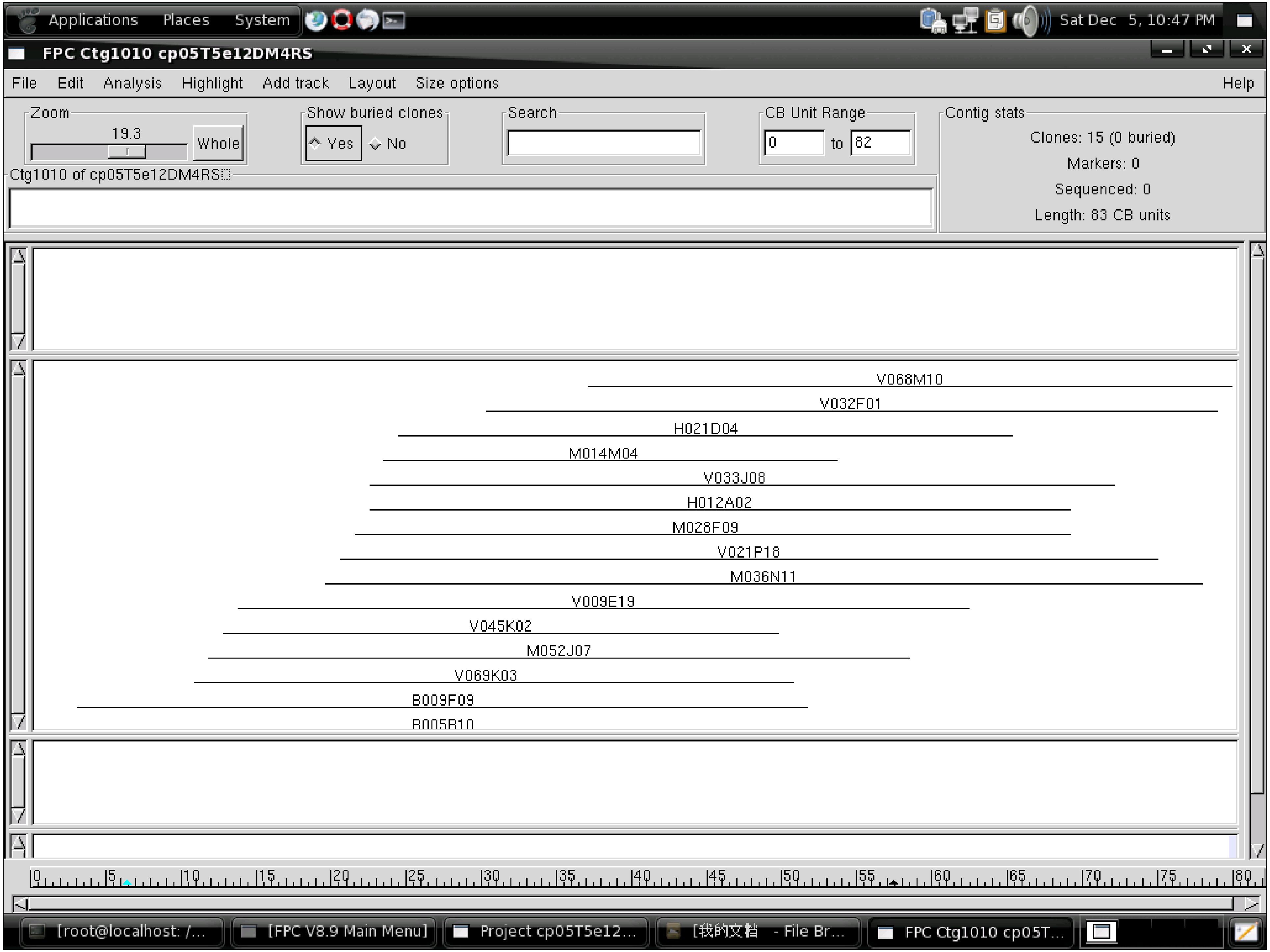
Applications Places System
Sat Dec 5, 10:47 PM
FPC Ctg1010 cp05T5e12DM4RS
File Edit Analysis Highlight Add track Layout Size options Help
Zoom
19.3
Whole
Show buried clones
Yes No
Search
CB Unit Range
0 to 82
Contig stats
Clones: 15 (0 buried)
Markers: 0
Sequenced: 0
Length: 83 CB units
Ctg1010 of cp05T5e12DM4RS
V068M10
V032F01
H021D04
M014M04
V033J08
H012A02
M028F09
V021P18
M036N11
V009E19
V045K02
M052J07
V069K03
B009F09
B005B10
0 5 10 15 20 25 30 35 40 45 50 55 60 65 70 75 80
[root@localhost: /...
[FPC V8.9 Main Menu]
Project cp05T5e12...
[我的文档 - File Br...
FPC Ctg1010 cp05T...

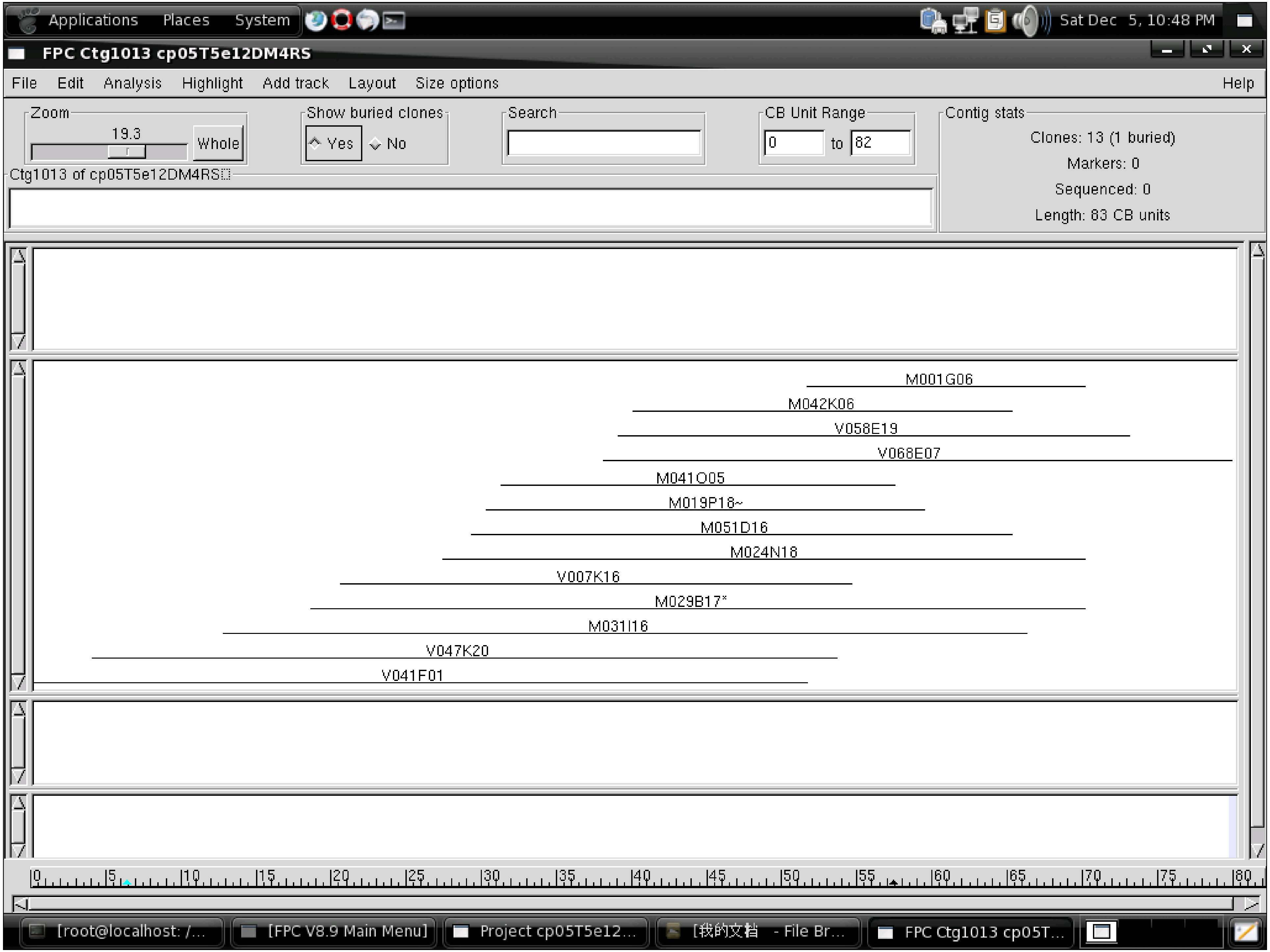
Applications
Places
System
Sat Dec 5, 10:48 PM
FPC Ctg1013 cp05T5e12DM4RS
File
Edit
Analysis
Highlight
Add track
Layout
Size options
Help
Zoom
19.3
Whole
Show buried clones
Yes
No
Search
CB Unit Range
0
to
82
Contig stats
Clones: 13 (1 buried)
Markers: 0
Sequenced: 0
Length: 83 CB units
Ctg1013 of cp05T5e12DM4RS
M001G06
M042K06
V058E19
V068E07
M041O05
M019P18~
M051D16
M024N18
V007K16
M029B17*
M031I16
V047K20
V041F01
0
5
10
15
20
25
30
35
40
45
50
55
60
65
70
75
80
[root@localhost: /...
[FPC V8.9 Main Menu]
Project cp05T5e12...
[我的文档 - File Br...
FPC Ctg1013 cp05T...

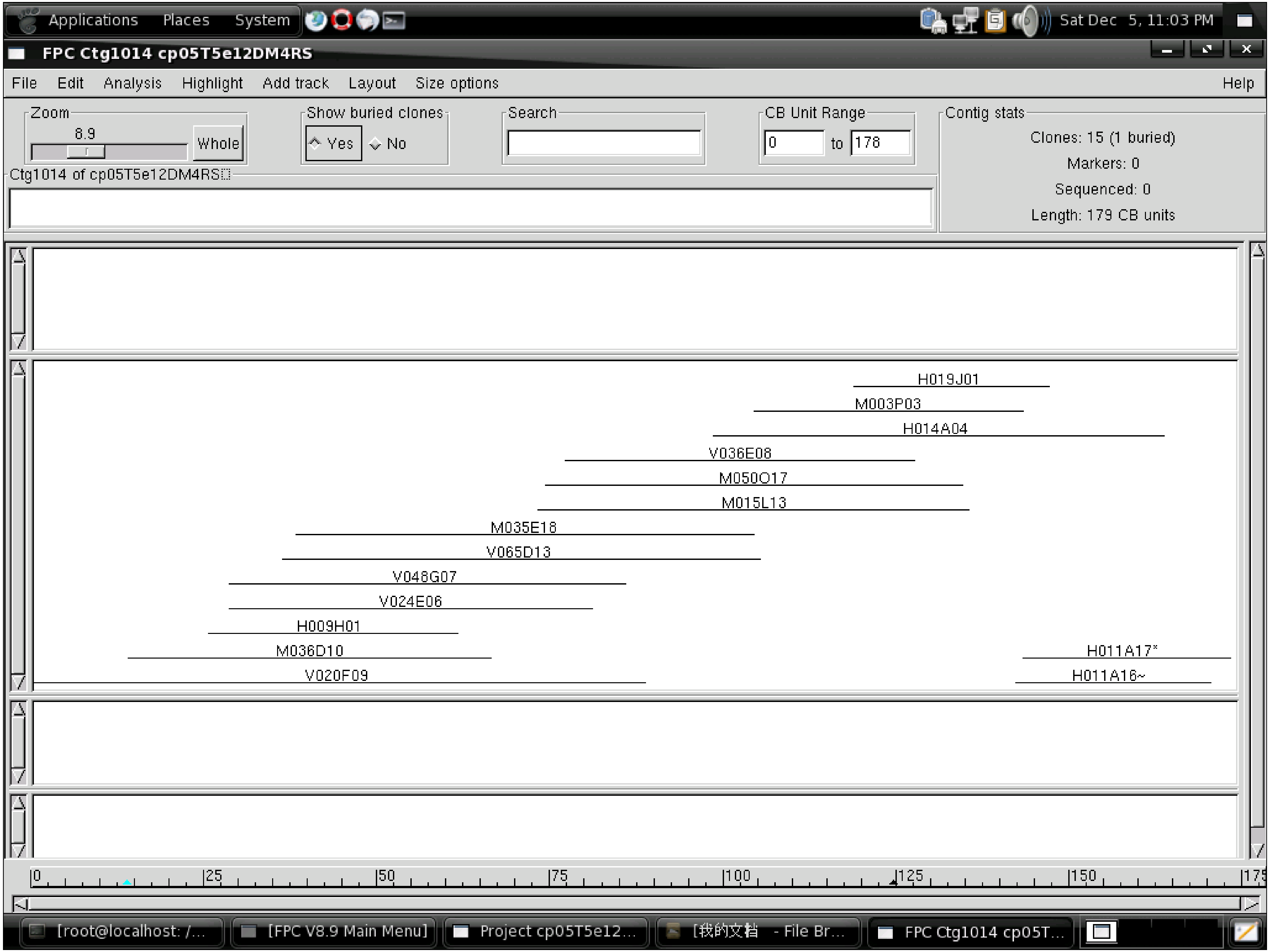
Applications Places System
Sat Dec 5, 11:03 PM
FPC Ctg1014 cp05T5e12DM4RS
File Edit Analysis Highlight Add track Layout Size options Help
Zoom
8.9
Whole
Show buried clones
Yes
No
Search
CB Unit Range
0
to
178
Contig stats
Clones: 15 (1 buried)
Markers: 0
Sequenced: 0
Length: 179 CB units
Ctg1014 of cp05T5e12DM4RS
H019J01
M003P03
H014A04
V036E08
M050O17
M015L13
M035E18
V065D13
V048G07
V024E06
H009H01
M036D10
H011A17*
V020F09
H011A16~
0
25
50
75
100
125
150
175
[root@localhost: /...
[FPC V8.9 Main Menu]
Project cp05T5e12...
[我的文档 - File Br...
FPC Ctg1014 cp05T...

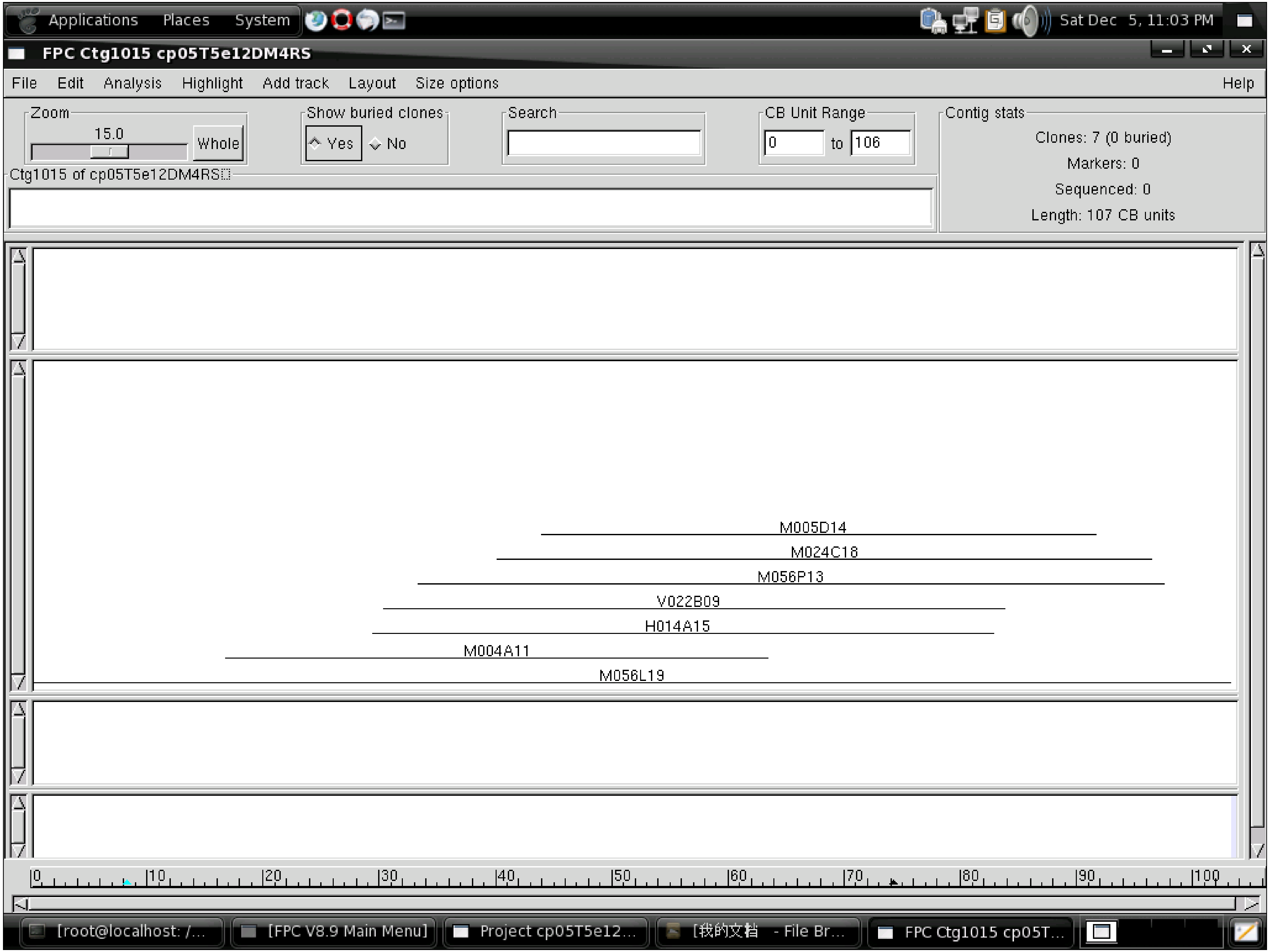
Applications Places System
Sat Dec 5, 11:03 PM
FPC Ctg1015 cp05T5e12DM4RS
File Edit Analysis Highlight Add track Layout Size options Help
Zoom
15.0
Whole
Show buried clones
Yes No
Search
CB Unit Range
0 to 106
Contig stats
Clones: 7 (0 buried)
Markers: 0
Sequenced: 0
Length: 107 CB units
Ctg1015 of cp05T5e12DM4RS
M005D14
M024C18
M056P13
V022B09
H014A15
M004A11
M056L19
0 10 20 30 40 50 60 70 80 90 100
[root@localhost: /...
[FPC V8.9 Main Menu]
Project cp05T5e12...
[我的文档 - File Br...
FPC Ctg1015 cp05T...

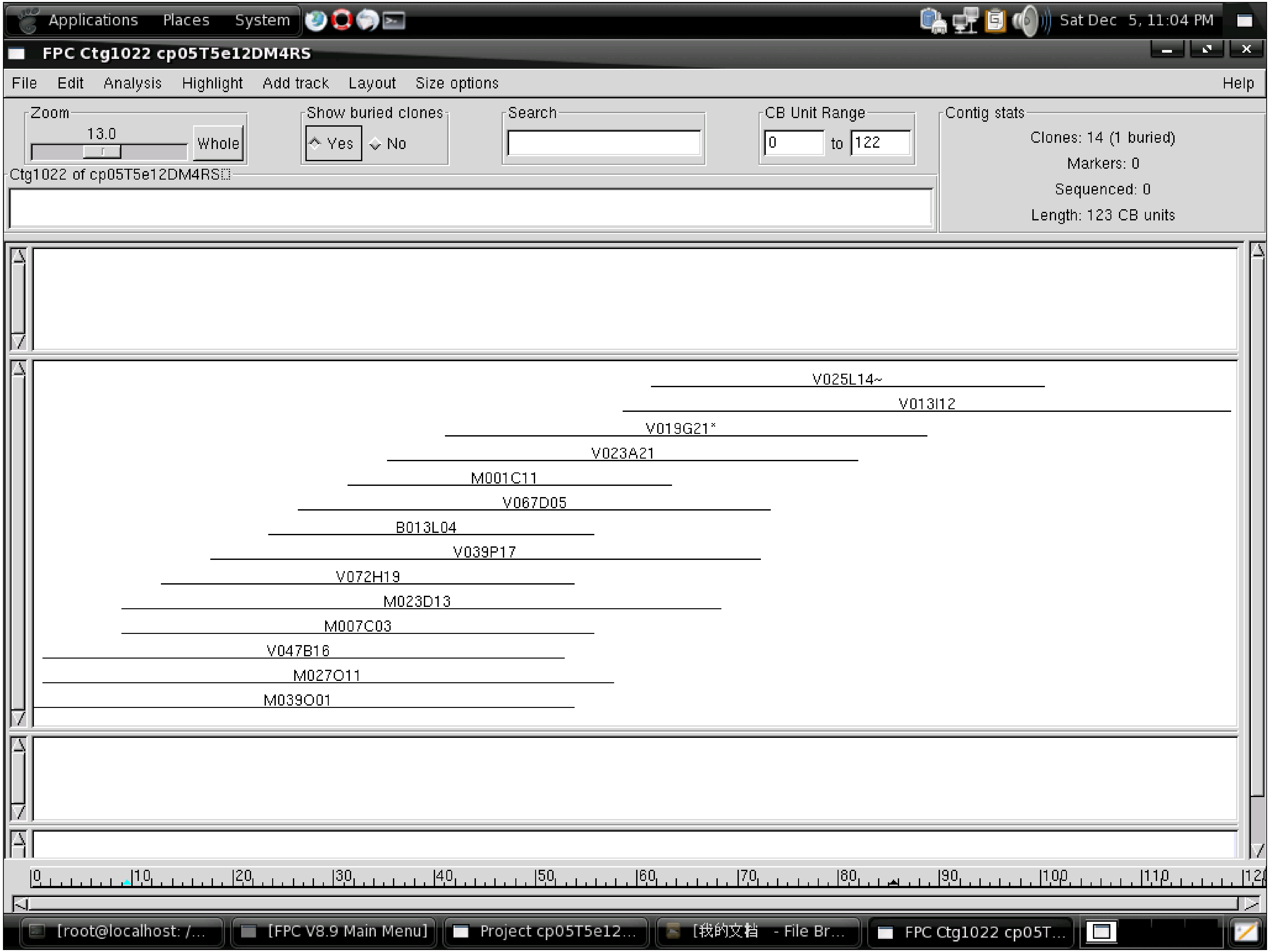
Applications Places System
Sat Dec 5, 11:04 PM
FPC Ctg1022 cp05T5e12DM4RS
File Edit Analysis Highlight Add track Layout Size options Help
Zoom
13.0
Whole
Show buried clones
Yes No
Search
CB Unit Range
0 to 122
Contig stats
Clones: 14 (1 buried)
Markers: 0
Sequenced: 0
Length: 123 CB units
Ctg1022 of cp05T5e12DM4RS
V025L14~
V013I12
V019G21*
V023A21
M001C11
V067D05
B013L04
V039P17
V072H19
M023D13
M007C03
V047B16
M027O11
M039O01
0 10 20 30 40 50 60 70 80 90 100 110 12
[root@localhost: /...
[FPC V8.9 Main Menu]
Project cp05T5e12...
[我的文档 - File Br...
FPC Ctg1022 cp05T...

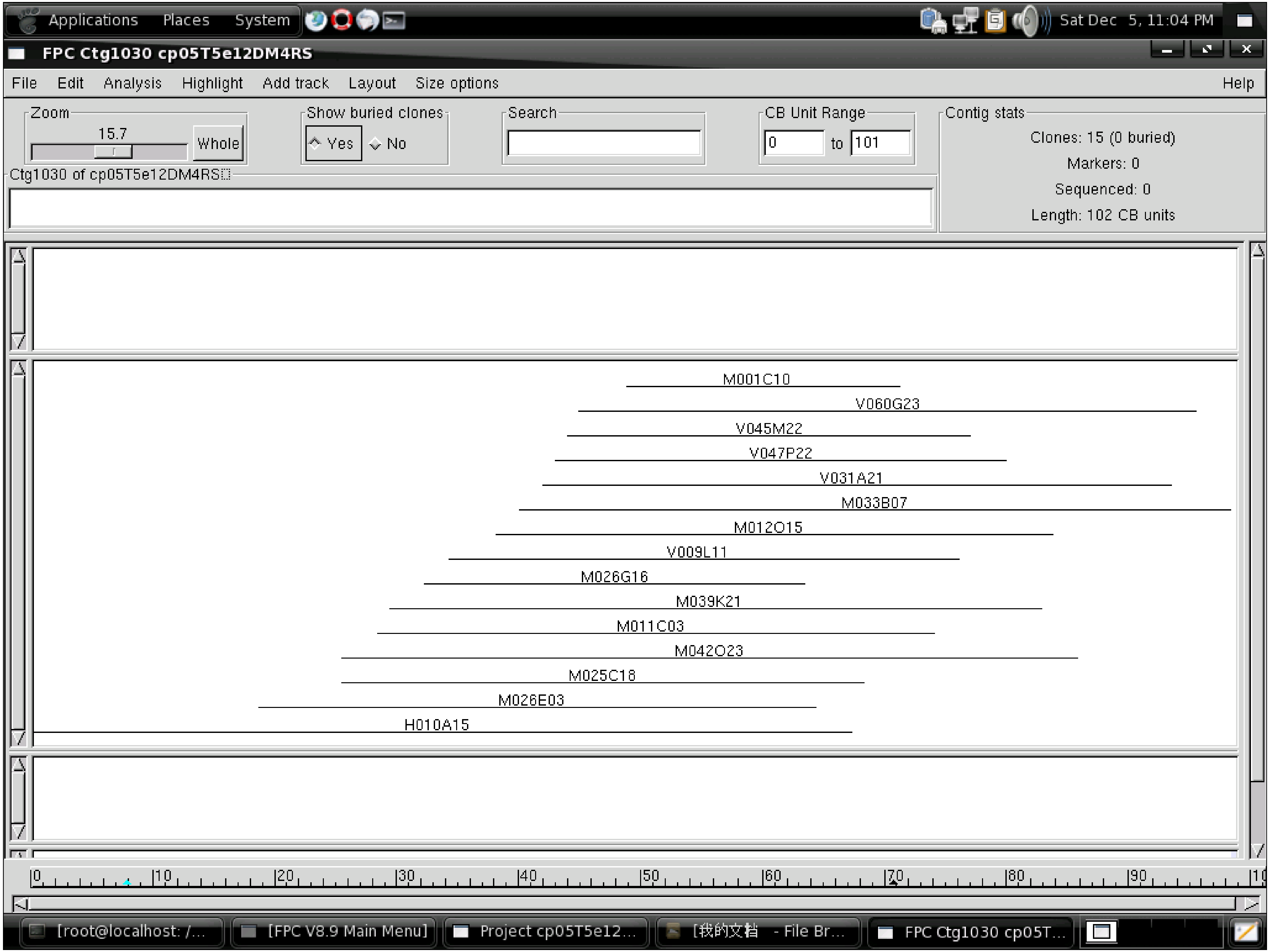
Applications
Places
System
Sat Dec 5, 11:04 PM
FPC Ctg1030 cp05T5e12DM4RS
File
Edit
Analysis
Highlight
Add track
Layout
Size options
Help
Zoom
15.7
Whole
Show buried clones
Yes
No
Search
CB Unit Range
0
to
101
Contig stats
Clones: 15 (0 buried)
Markers: 0
Sequenced: 0
Length: 102 CB units
Ctg1030 of cp05T5e12DM4RS
M001C10
V060G23
V045M22
V047P22
V031A21
M033B07
M012O15
V009L11
M026G16
M039K21
M011C03
M042O23
M025C18
M026E03
H010A15
0
10
20
30
40
50
60
70
80
90
[root@localhost: /...
[FPC V8.9 Main Menu]
Project cp05T5e12...
[我的文档 - File Br...
FPC Ctg1030 cp05T...

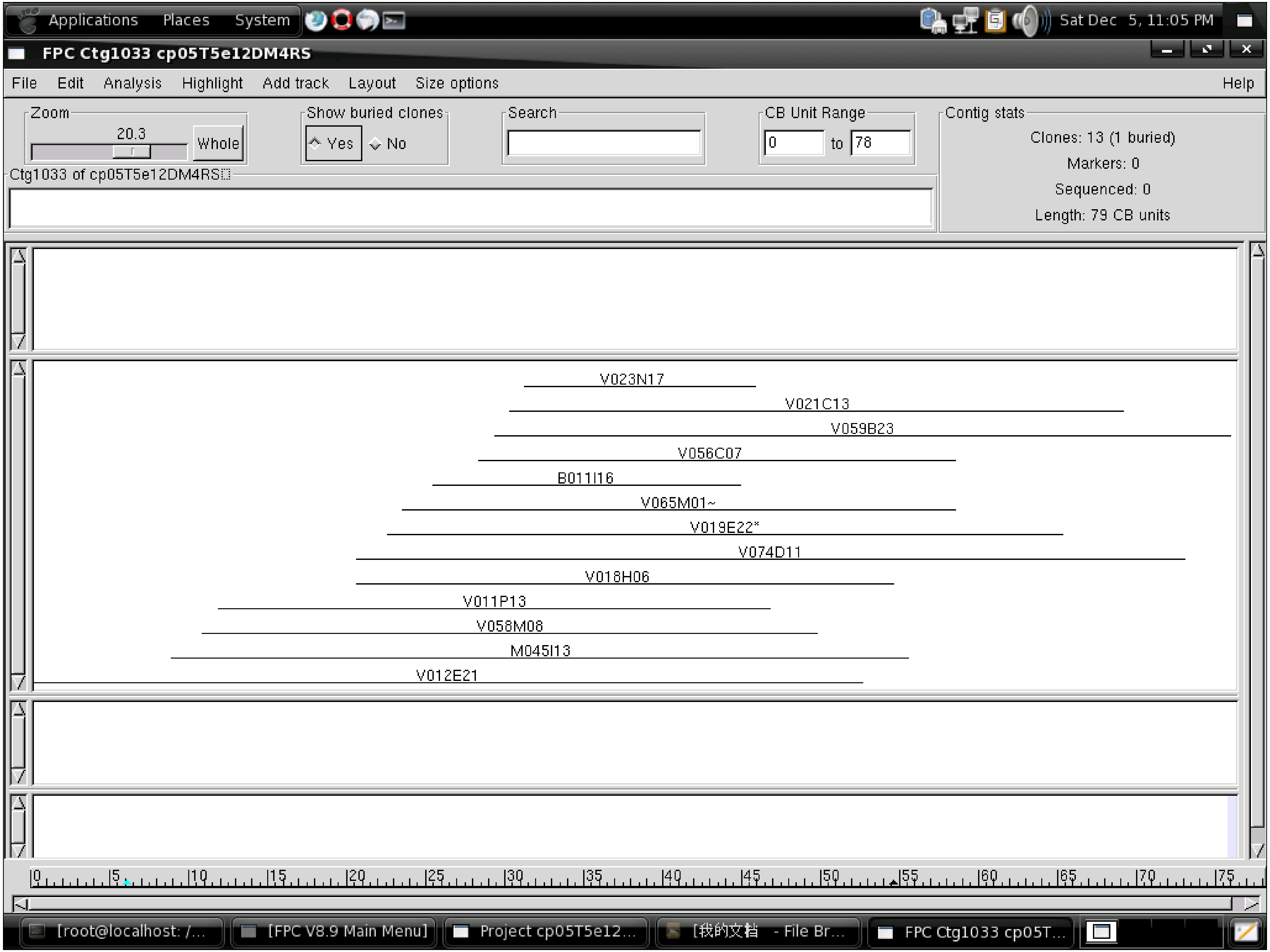
Applications Places System
Sat Dec 5, 11:05 PM
FPC Ctg1033 cp05T5e12DM4RS
File Edit Analysis Highlight Add track Layout Size options Help
Zoom
20.3
Whole
Show buried clones
Yes
No
Search
CB Unit Range
0 to 78
Contig stats
Clones: 13 (1 buried)
Markers: 0
Sequenced: 0
Length: 79 CB units
Ctg1033 of cp05T5e12DM4RS
V023N17
V021C13
V059B23
V056C07
B011I16
V065M01~
V019E22*
V074D11
V018H06
V011P13
V058M08
M045I13
V012E21
0 5 10 15 20 25 30 35 40 45 50 55 60 65 70 75
[root@localhost: /...
[FPC V8.9 Main Menu]
Project cp05T5e12...
FPC Ctg1033 cp05T...

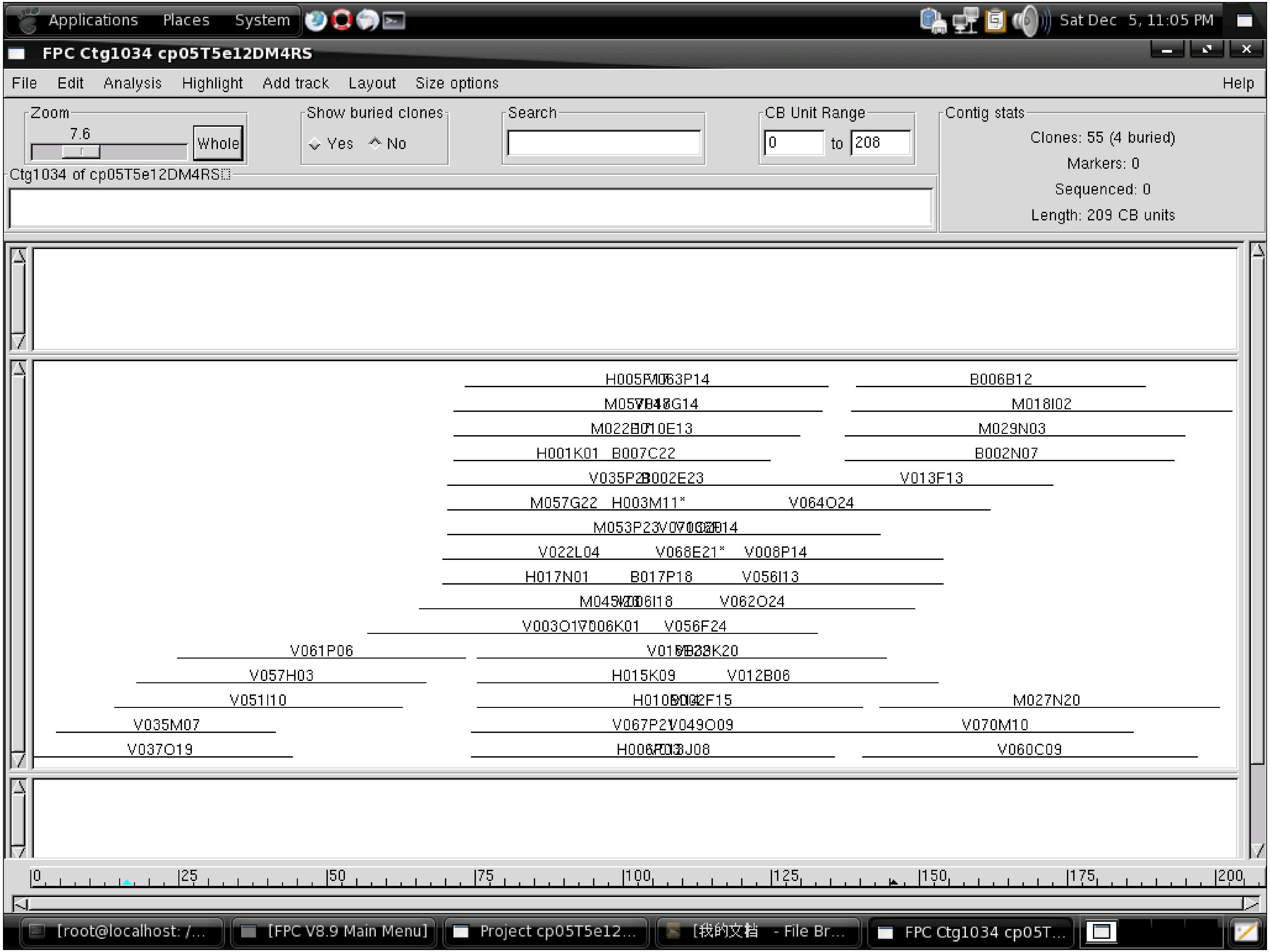
Applications Places System
Sat Dec 5, 11:05 PM
FPC Ctg1034 cp05T5e12DM4RS
File Edit Analysis Highlight Add track Layout Size options Help
Zoom 7.6 Whole
Show buried clones Yes No
Search
CB Unit Range 0 to 208
Contig stats
Clones: 55 (4 buried)
Markers: 0
Sequenced: 0
Length: 209 CB units
Ctg1034 of cp05T5e12DM4RS
B006B12
M018I02
M029N03
B002N07
V013F13
V064O24
V008P14
V056I13
V062O24
V056F24
V061P06
V057H03
H015K09
V012B06
V051I10
M027N20
V035M07
V070M10
V037O19
V060C09
H001K01
B007C22
M057G22
H003M11*
V022L04
V068E21*
H017N01
B017P18
0 25 50 75 100 125 150 175 200
[root@localhost: /...
[FPC V8.9 Main Menu]
Project cp05T5e12...
FPC Ctg1034 cp05T...

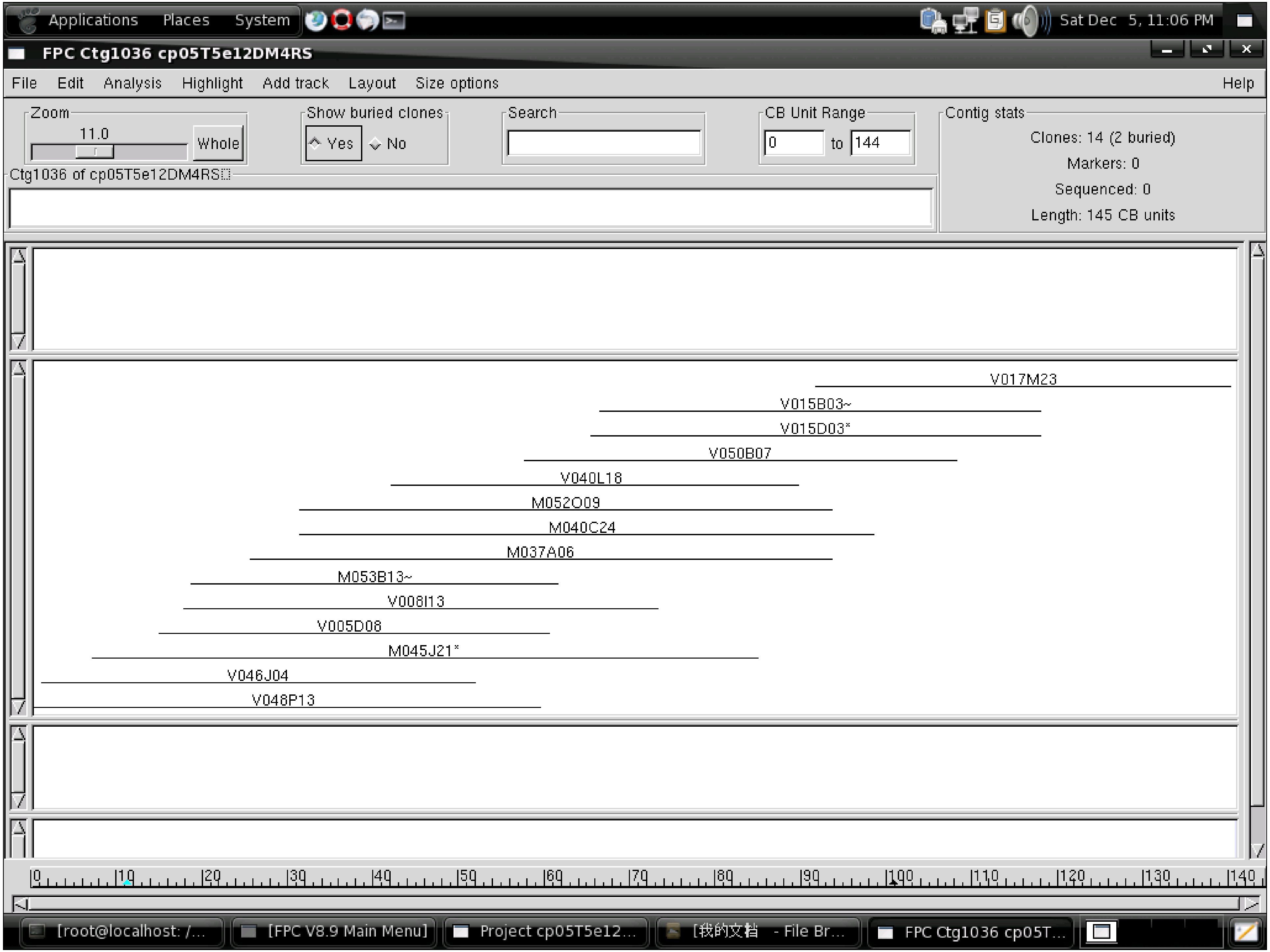
Applications Places System
Sat Dec 5, 11:06 PM
FPC Ctg1036 cp05T5e12DM4RS
File Edit Analysis Highlight Add track Layout Size options Help
Zoom
11.0
Whole
Show buried clones
Yes
No
Search
CB Unit Range
0
to
144
Contig stats
Clones: 14 (2 buried)
Markers: 0
Sequenced: 0
Length: 145 CB units
Ctg1036 of cp05T5e12DM4RS
V017M23
V015B03~
V015D03*
V050B07
V040L18
M052O09
M040C24
M037A06
M053B13~
V008I13
V005D08
M045J21*
V046J04
V048P13
0 10 20 30 40 50 60 70 80 90 100 110 120 130 140
[root@localhost: /...
[FPC V8.9 Main Menu]
Project cp05T5e12...
[我的文档 - File Br...
FPC Ctg1036 cp05T...

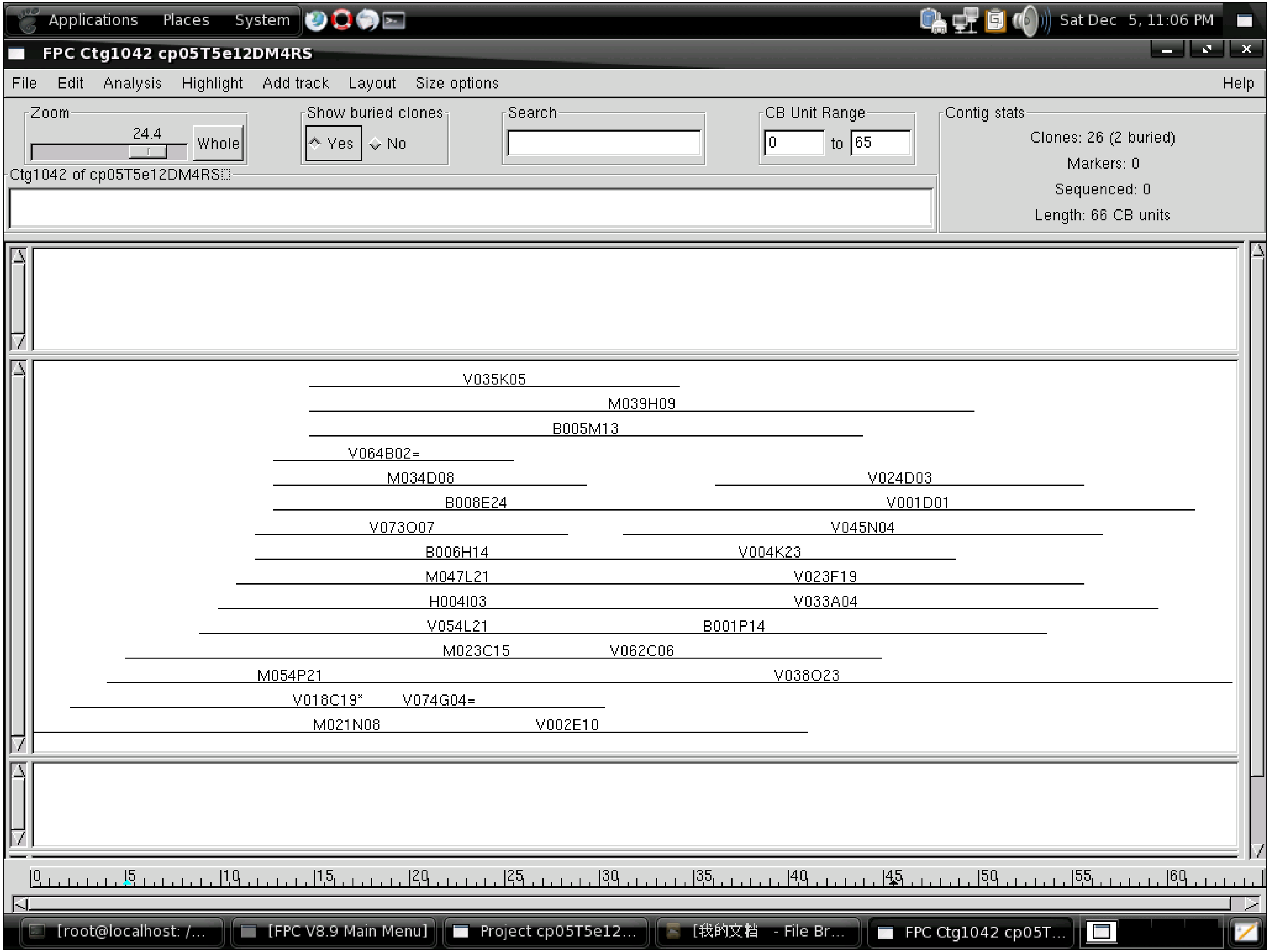
Applications Places System
Sat Dec 5, 11:06 PM
FPC Ctg1042 cp05T5e12DM4RS
File Edit Analysis Highlight Add track Layout Size options Help
Zoom
24.4
Whole
Show buried clones
Yes No
Search
CB Unit Range
0 to 65
Contig stats
Clones: 26 (2 buried)
Markers: 0
Sequenced: 0
Length: 66 CB units
Ctg1042 of cp05T5e12DM4RS
V035K05
M039H09
B005M13
V064B02=
M034D08
V024D03
B008E24
V001D01
V073O07
V045N04
B006H14
V004K23
M047L21
V023F19
H004I03
V033A04
V054L21
B001P14
M023C15
V062C06
M054P21
V038O23
V018C19*
V074G04=
M021N08
V002E10
0 5 10 15 20 25 30 35 40 45 50 55 60
[root@localhost: /...
[FPC V8.9 Main Menu]
Project cp05T5e12...
[我的文档 - File Br...
FPC Ctg1042 cp05T...

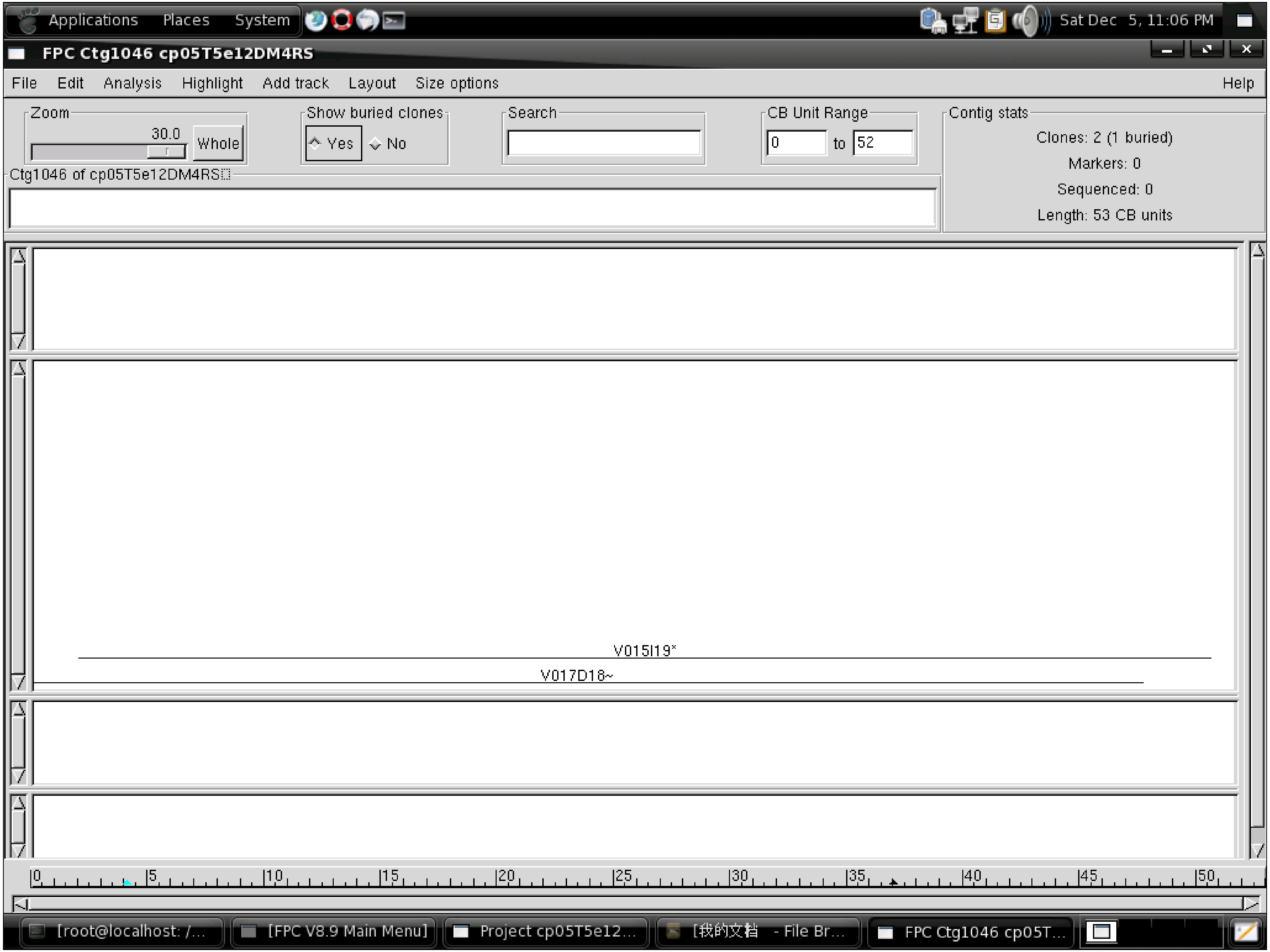

Applications Places System
Sat Dec 5, 11:06 PM
FPC Ctg1046 cp05T5e12DM4RS
File Edit Analysis Highlight Add track Layout Size options Help
Zoom
30.0
Whole
Show buried clones
Yes
No
Search
CB Unit Range
0 to 52
Contig stats
Clones: 2 (1 buried)
Markers: 0
Sequenced: 0
Length: 53 CB units
Ctg1046 of cp05T5e12DM4RS
V015I19*
V017D18~
0 5 10 15 20 25 30 35 40 45 50
[root@localhost: /...
[FPC V8.9 Main Menu]
Project cp05T5e12...
[我的文档 - File Br...
FPC Ctg1046 cp05T...

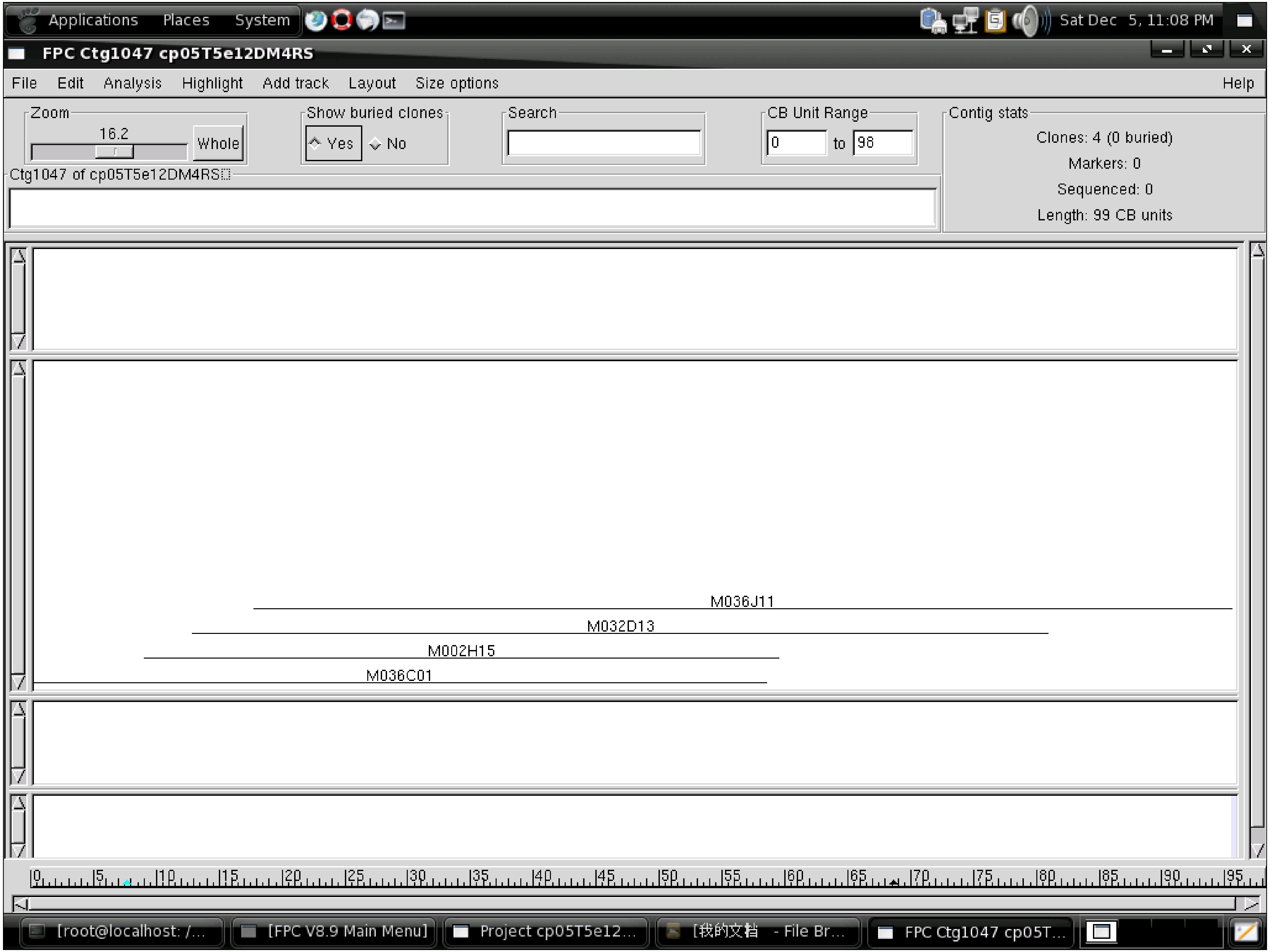

Applications
Places
System
Sat Dec 5, 11:08 PM
FPC Ctg1047 cp05T5e12DM4RS
File
Edit
Analysis
Highlight
Add track
Layout
Size options
Help
Zoom
16.2
Whole
Show buried clones
Yes
No
Search
CB Unit Range
0
to
98
Contig stats
Clones: 4 (0 buried)
Markers: 0
Sequenced: 0
Length: 99 CB units
Ctg1047 of cp05T5e12DM4RS
M036J11
M032D13
M002H15
M036C01
0 5 10 15 20 25 30 35 40 45 50 55 60 65 70 75 80 85 90 95
[root@localhost: /...
[FPC V8.9 Main Menu]
Project cp05T5e12...
[我的文档 - File Br...
FPC Ctg1047 cp05T...

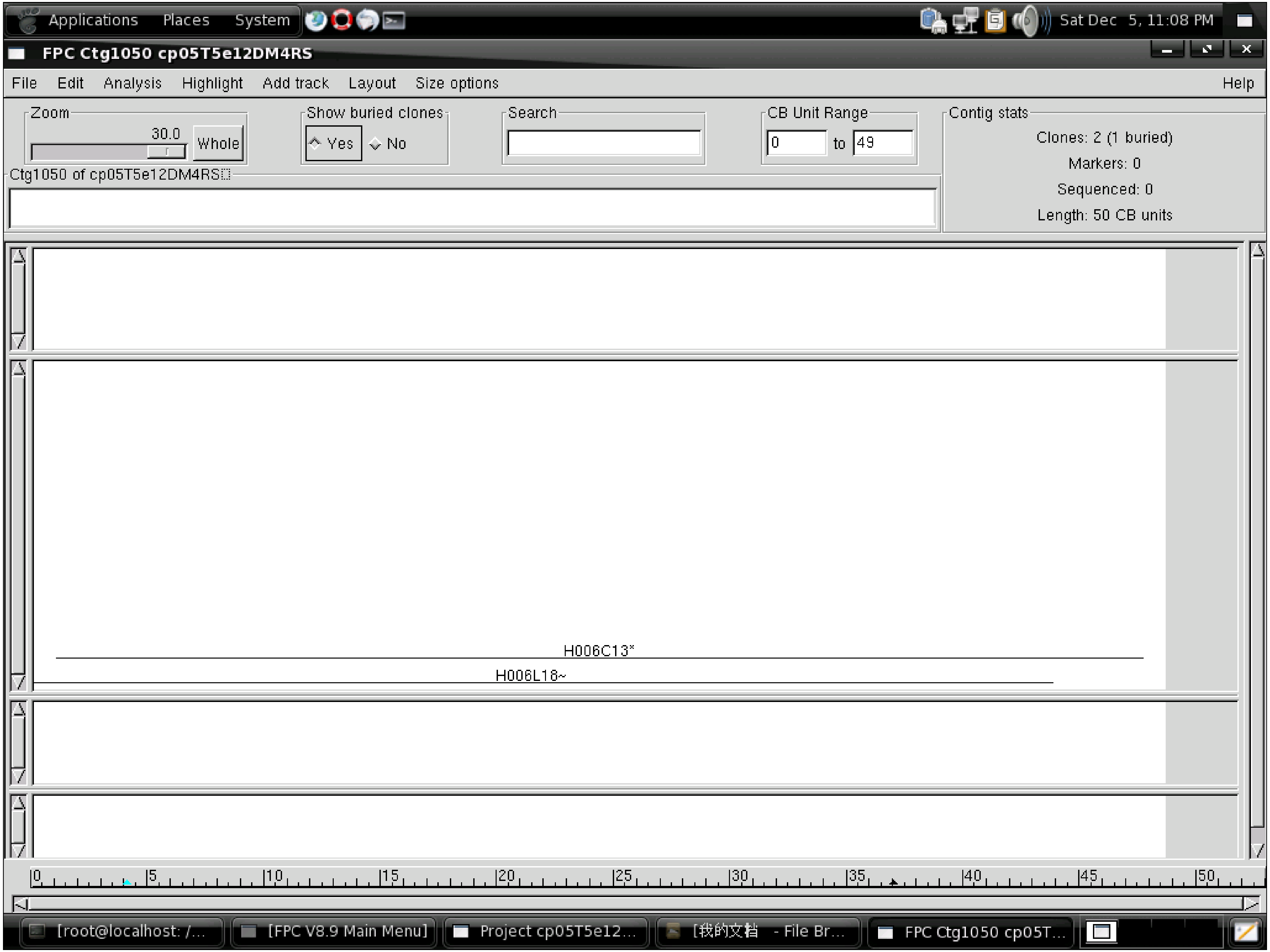
Applications Places System
Sat Dec 5, 11:08 PM
FPC Ctg1050 cp05T5e12DM4RS
File Edit Analysis Highlight Add track Layout Size options Help
Zoom
30.0
Whole
Show buried clones
Yes No
Search
CB Unit Range
0 to 49
Contig stats
Clones: 2 (1 buried)
Markers: 0
Sequenced: 0
Length: 50 CB units
Ctg1050 of cp05T5e12DM4RS
H006C13*
H006L18~
0 5 10 15 20 25 30 35 40 45 50
[root@localhost: /...
[FPC V8.9 Main Menu]
Project cp05T5e12...
[我的文档 - File Br...
FPC Ctg1050 cp05T...

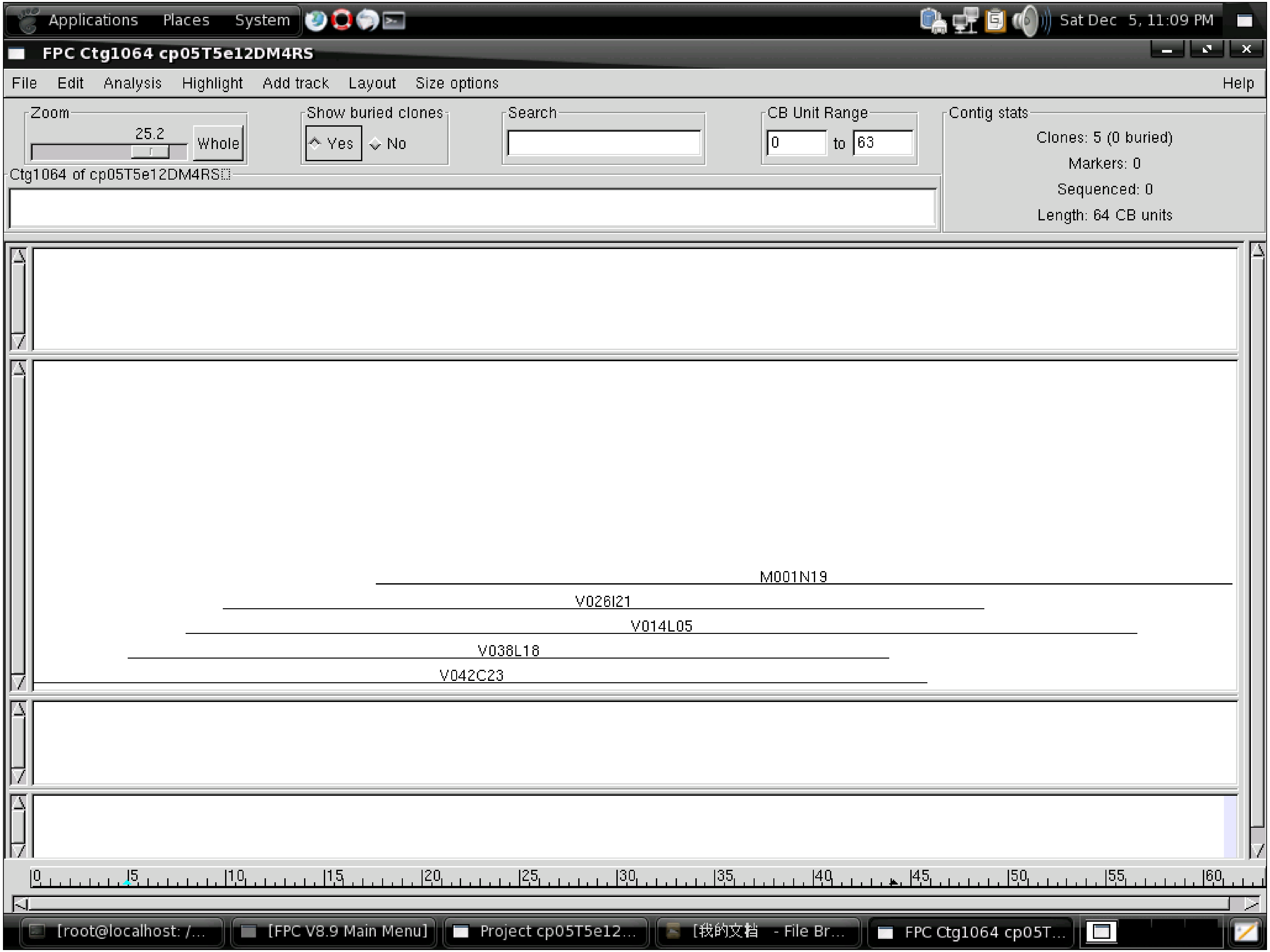
Applications Places System
Sat Dec 5, 11:09 PM
FPC Ctg1064 cp05T5e12DM4RS
File Edit Analysis Highlight Add track Layout Size options Help
Zoom
25.2
Whole
Show buried clones
Yes No
Search
CB Unit Range
0 to 63
Contig stats
Clones: 5 (0 buried)
Markers: 0
Sequenced: 0
Length: 64 CB units
Ctg1064 of cp05T5e12DM4RS
M001N19
V026I21
V014L05
V038L18
V042C23
0 5 10 15 20 25 30 35 40 45 50 55 60
[root@localhost: /...
[FPC V8.9 Main Menu]
Project cp05T5e12...
[我的文档 - File Br...
FPC Ctg1064 cp05T...

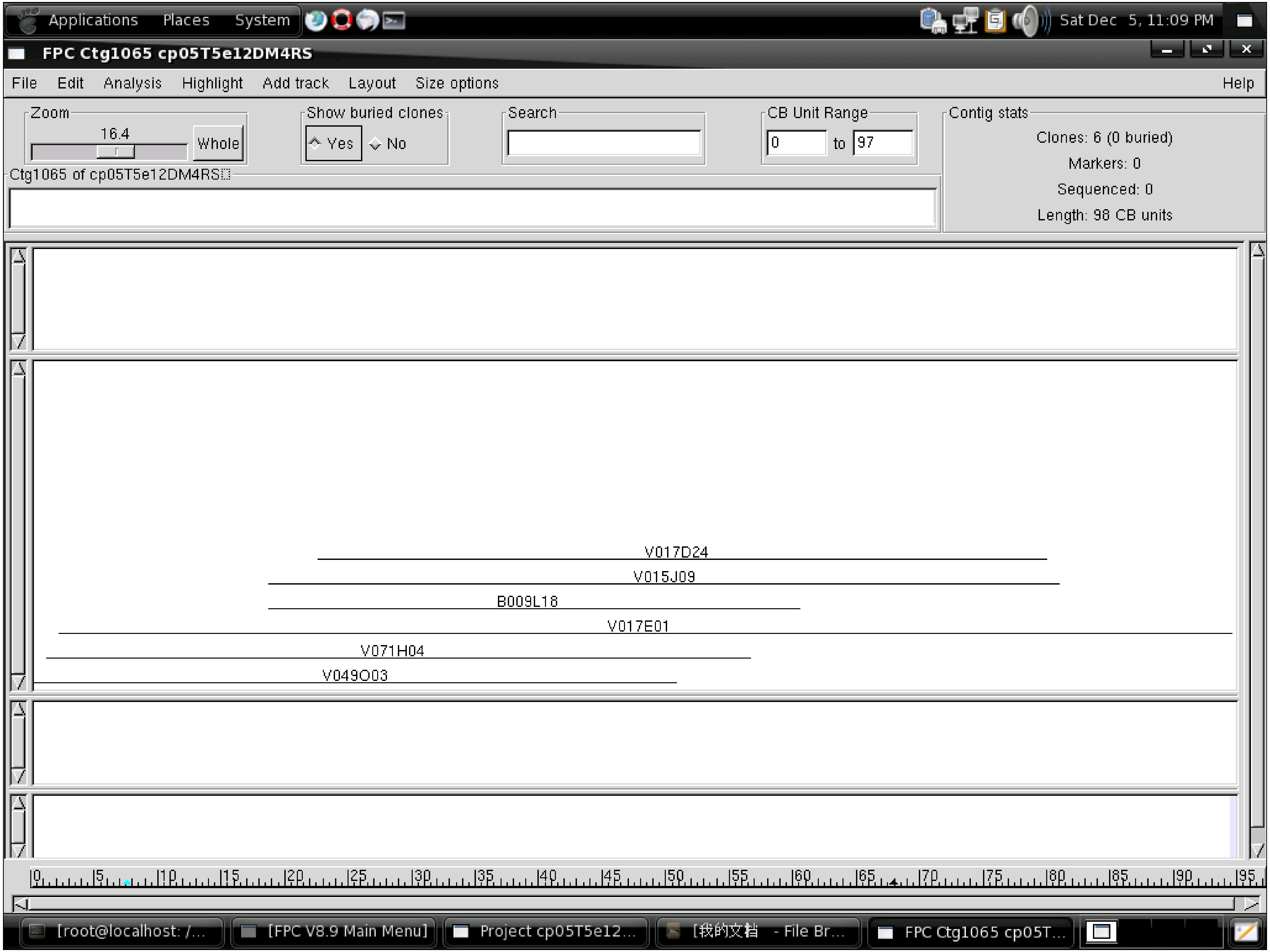
Applications Places System
Sat Dec 5, 11:09 PM
FPC Ctg1065 cp05T5e12DM4RS
File Edit Analysis Highlight Add track Layout Size options Help
Zoom
16.4
Whole
Show buried clones
Yes No
Search
CB Unit Range
0 to 97
Contig stats
Clones: 6 (0 buried)
Markers: 0
Sequenced: 0
Length: 98 CB units
Ctg1065 of cp05T5e12DM4RS
V017D24
V015J09
B009L18
V017E01
V071H04
V049O03
0 5 10 15 20 25 30 35 40 45 50 55 60 65 70 75 80 85 90 95
[root@localhost: /...
[FPC V8.9 Main Menu]
Project cp05T5e12...
[我的文档 - File Br...
FPC Ctg1065 cp05T...

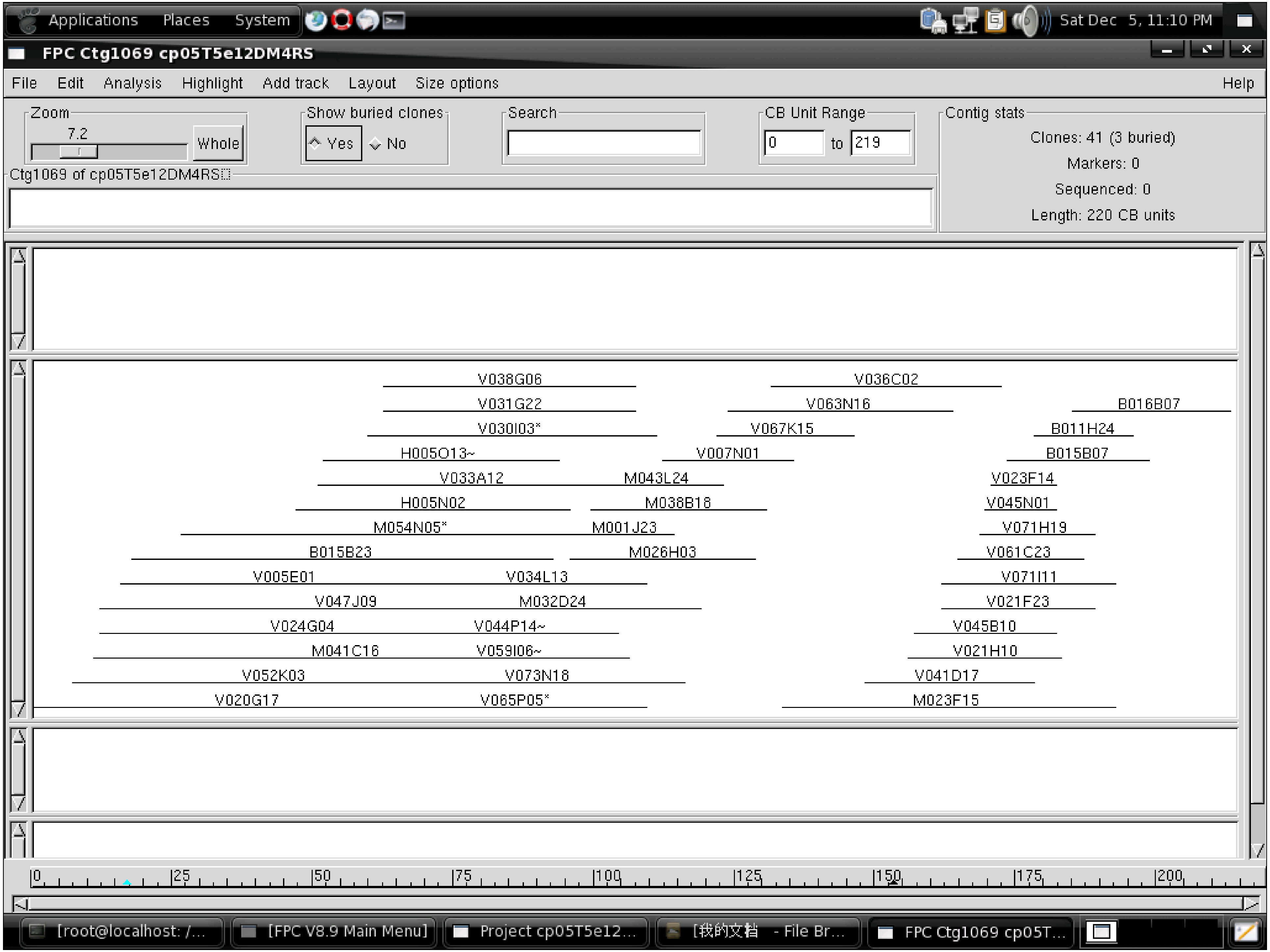

Applications Places System
Sat Dec 5, 11:10 PM
FPC Ctg1069 cp05T5e12DM4RS
File Edit Analysis Highlight Add track Layout Size options Help
Zoom
7.2
Whole
Show buried clones
Yes
No
Search
CB Unit Range
0
to
219
Contig stats
Clones: 41 (3 buried)
Markers: 0
Sequenced: 0
Length: 220 CB units
Ctg1069 of cp05T5e12DM4RS
V038G06
V036C02
V031G22
V063N16
B016B07
V030I03*
V067K15
B011H24
H005O13~
V007N01
B015B07
V033A12
M043L24
V023F14
H005N02
M038B18
V045N01
M054N05*
M001J23
V071H19
B015B23
M026H03
V061C23
V005E01
V034L13
V071I11
V047J09
M032D24
V021F23
V024G04
V044P14~
V045B10
M041C16
V059I06~
V021H10
V052K03
V073N18
V041D17
V020G17
V065P05*
M023F15
0
25
50
75
100
125
150
175
200
[root@localhost: /...
[FPC V8.9 Main Menu]
Project cp05T5e12...
[我的文档 - File Br...
FPC Ctg1069 cp05T...

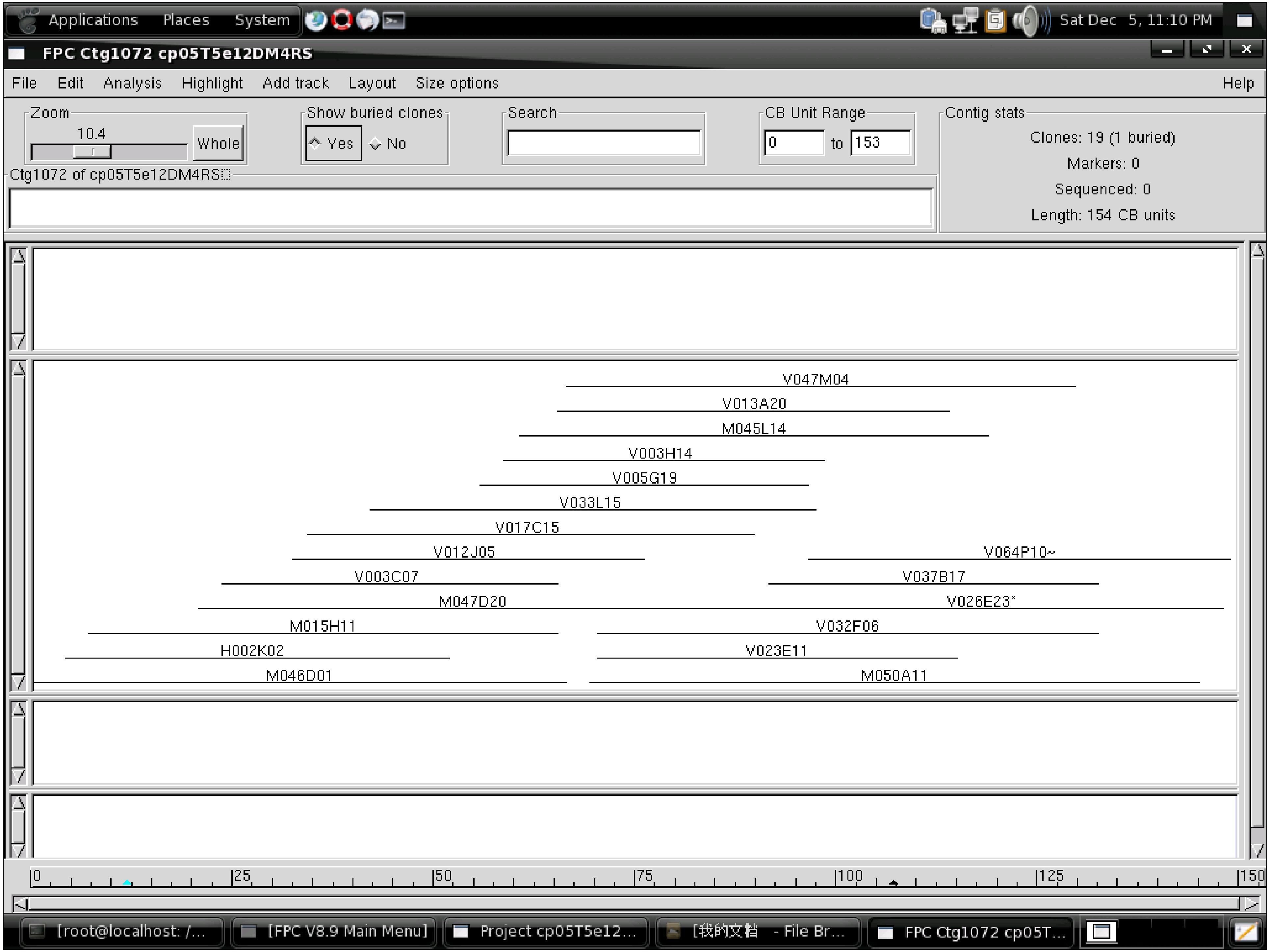
Applications Places System
Sat Dec 5, 11:10 PM
FPC Ctg1072 cp05T5e12DM4RS
File Edit Analysis Highlight Add track Layout Size options Help
Zoom
10.4
Whole
Show buried clones
Yes No
Search
CB Unit Range
0 to 153
Contig stats
Clones: 19 (1 buried)
Markers: 0
Sequenced: 0
Length: 154 CB units
Ctg1072 of cp05T5e12DM4RS
V047M04
V013A20
M045L14
V003H14
V005G19
V033L15
V017C15
V012J05
V064P10~
V003C07
V037B17
M047D20
V026E23*
M015H11
V032F06
H002K02
V023E11
M046D01
M050A11
0 25 50 75 100 125 150
[root@localhost: /...
[FPC V8.9 Main Menu]
Project cp05T5e12...
[我的文档 - File Br...
FPC Ctg1072 cp05T...

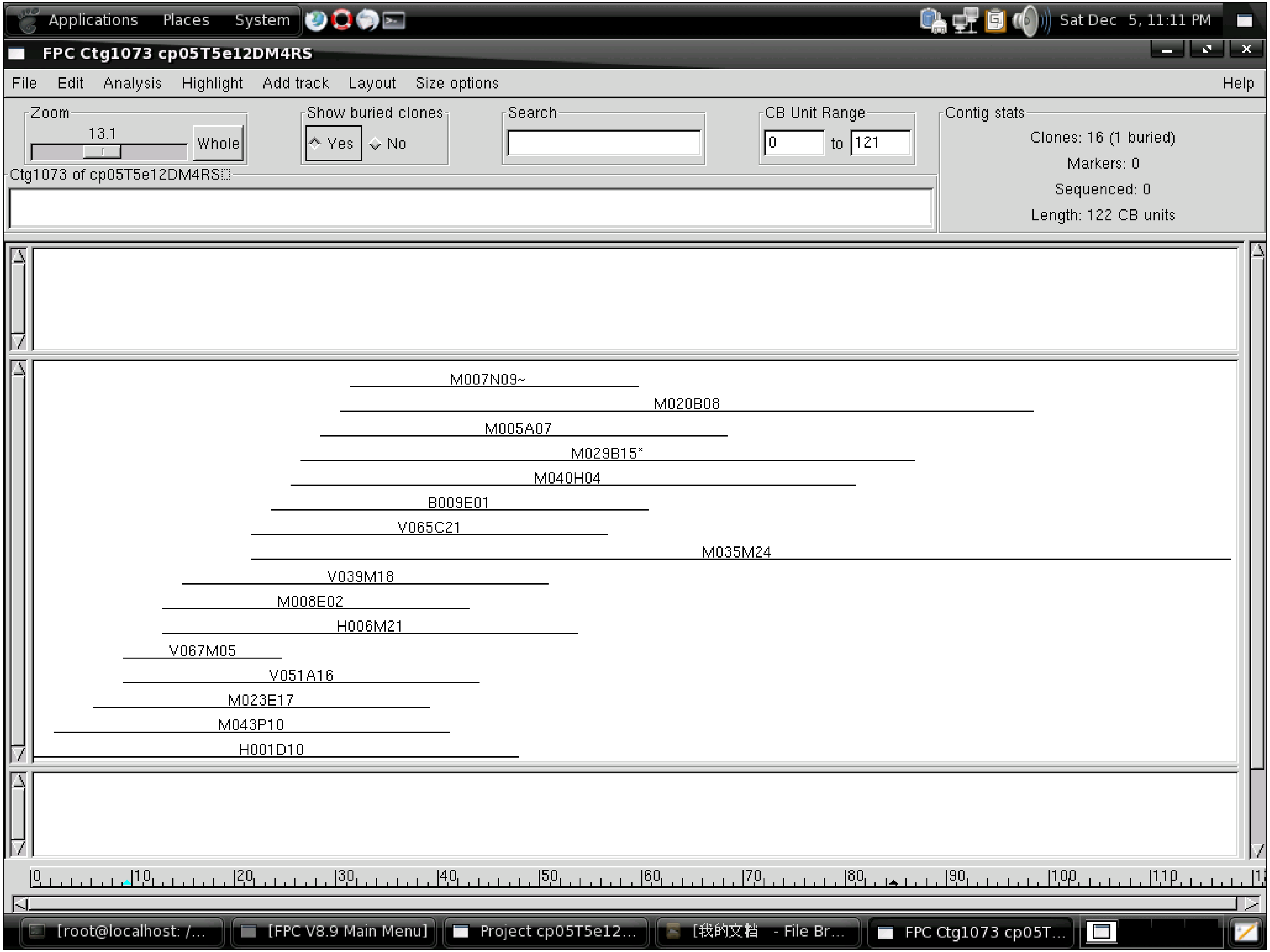
Applications Places System
Sat Dec 5, 11:11 PM
FPC Ctg1073 cp05T5e12DM4RS
File Edit Analysis Highlight Add track Layout Size options Help
Zoom
13.1
Whole
Show buried clones
Yes
No
Search
CB Unit Range
0
to
121
Contig stats
Clones: 16 (1 buried)
Markers: 0
Sequenced: 0
Length: 122 CB units
Ctg1073 of cp05T5e12DM4RS
M007N09~
M020B08
M005A07
M029B15*
M040H04
B009E01
V065C21
M035M24
V039M18
M008E02
H006M21
V067M05
V051A16
M023E17
M043P10
H001D10
0 10 20 30 40 50 60 70 80 90 100 110
[root@localhost: /...
[FPC V8.9 Main Menu]
Project cp05T5e12...
[我的文档 - File Br...
FPC Ctg1073 cp05T...

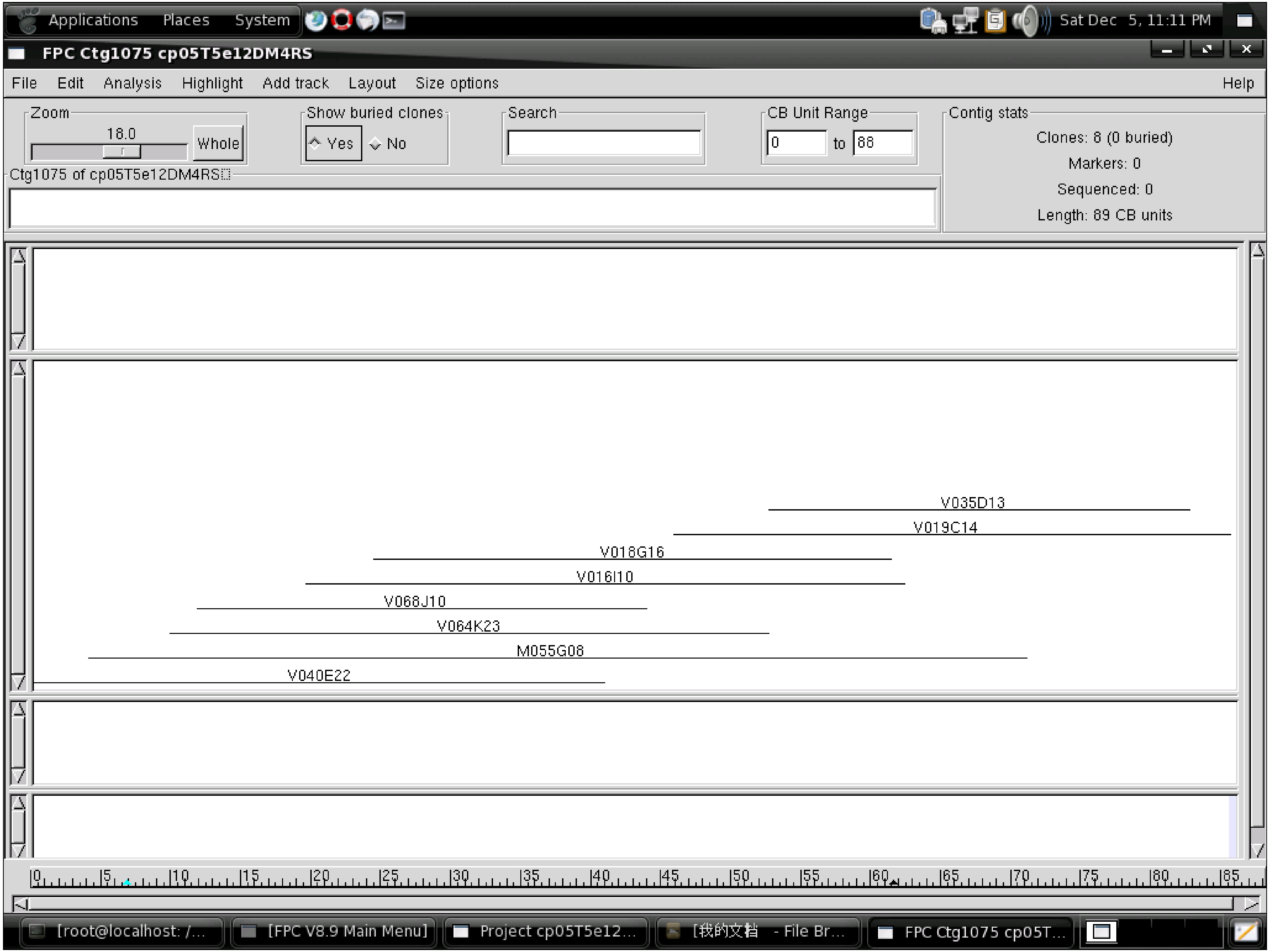
Applications Places System
Sat Dec 5, 11:11 PM
FPC Ctg1075 cp05T5e12DM4RS
File Edit Analysis Highlight Add track Layout Size options Help
Zoom
18.0
Whole
Show buried clones
Yes
No
Search
CB Unit Range
0
to
88
Contig stats
Clones: 8 (0 buried)
Markers: 0
Sequenced: 0
Length: 89 CB units
Ctg1075 of cp05T5e12DM4RS
V035D13
V019C14
V018G16
V016I10
V068J10
V064K23
M055G08
V040E22
0 5 10 15 20 25 30 35 40 45 50 55 60 65 70 75 80 85
[root@localhost: /...
[FPC V8.9 Main Menu]
Project cp05T5e12...
[我的文档 - File Br...
FPC Ctg1075 cp05T...

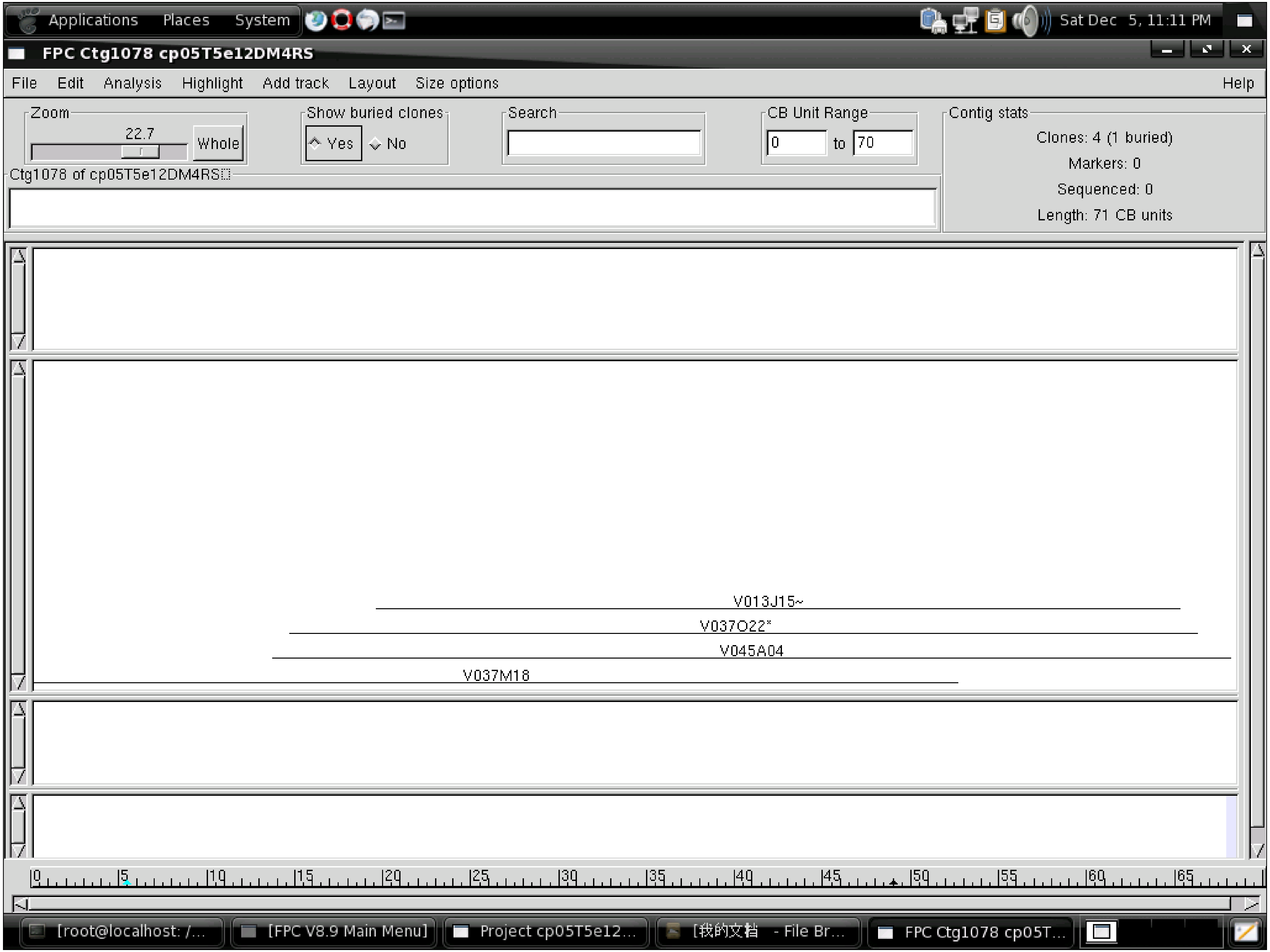
Applications Places System
Sat Dec 5, 11:11 PM
FPC Ctg1078 cp05T5e12DM4RS
File Edit Analysis Highlight Add track Layout Size options Help
Zoom
22.7
Whole
Show buried clones
Yes No
Search
CB Unit Range
0 to 70
Contig stats
Clones: 4 (1 buried)
Markers: 0
Sequenced: 0
Length: 71 CB units
Ctg1078 of cp05T5e12DM4RS
V013J15~
V037O22*
V045A04
V037M18
0 5 10 15 20 25 30 35 40 45 50 55 60 65
[root@localhost: /...
[FPC V8.9 Main Menu]
Project cp05T5e12...
[我的文档 - File Br...
FPC Ctg1078 cp05T...

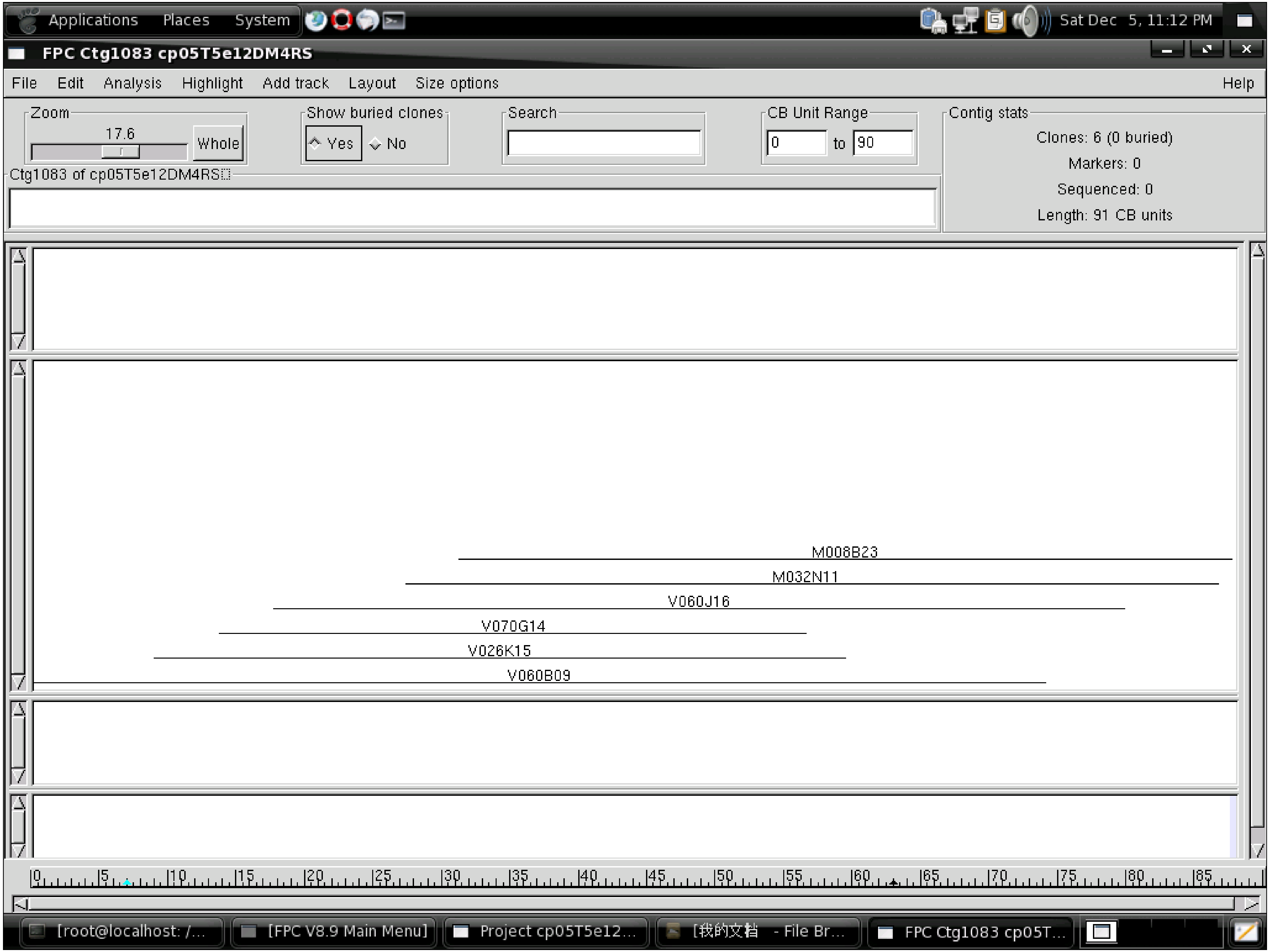
Applications Places System
Sat Dec 5, 11:12 PM
FPC Ctg1083 cp05T5e12DM4RS
File Edit Analysis Highlight Add track Layout Size options Help
Zoom
17.6
Whole
Show buried clones
Yes
No
Search
CB Unit Range
0 to 90
Contig stats
Clones: 6 (0 buried)
Markers: 0
Sequenced: 0
Length: 91 CB units
Ctg1083 of cp05T5e12DM4RS
M008B23
M032N11
V060J16
V070G14
V026K15
V060B09
0 5 10 15 20 25 30 35 40 45 50 55 60 65 70 75 80 85
[root@localhost: /...
[FPC V8.9 Main Menu]
Project cp05T5e12...
[我的文档 - File Br...
FPC Ctg1083 cp05T...

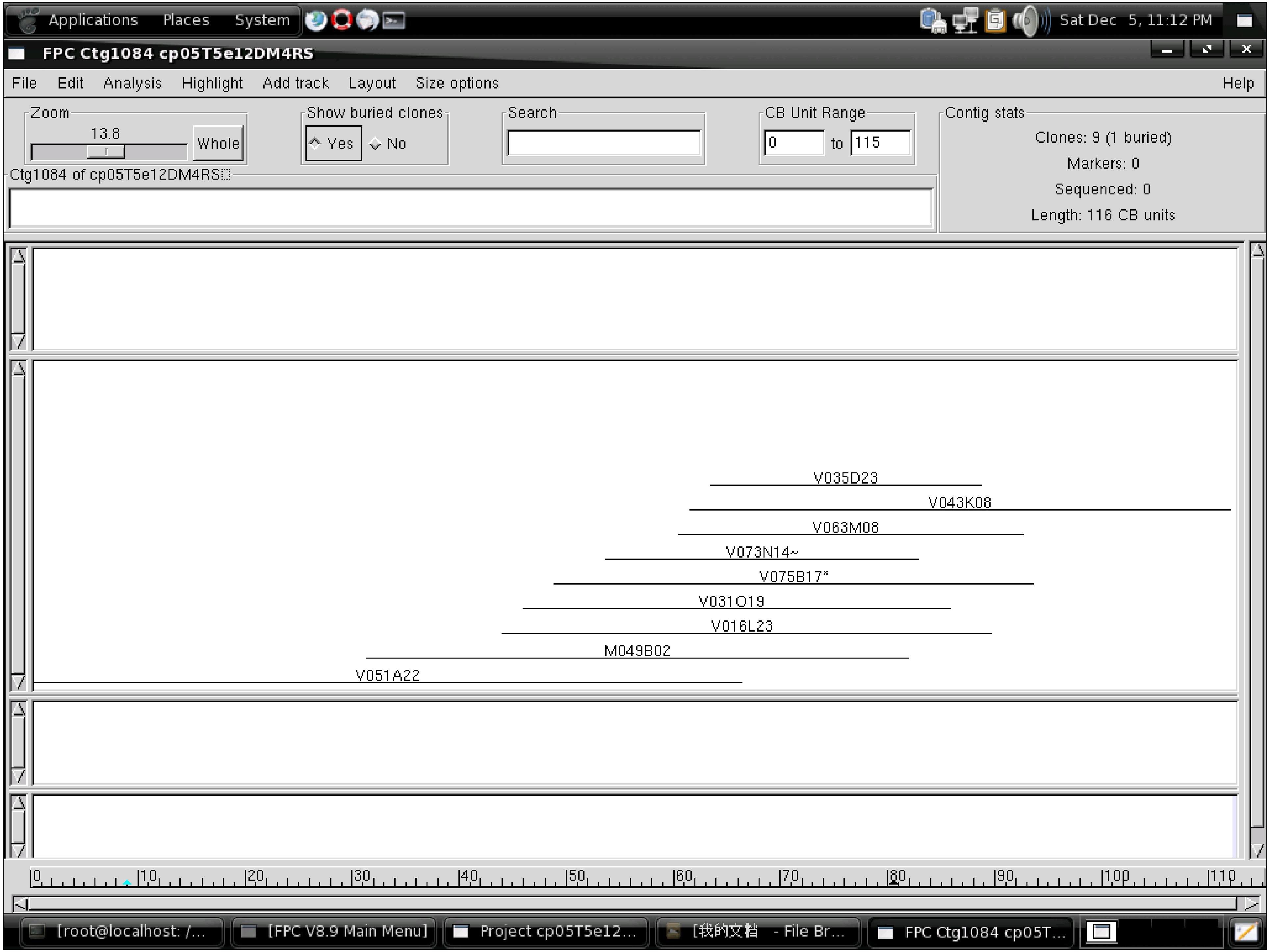
Applications Places System
Sat Dec 5, 11:12 PM
FPC Ctg1084 cp05T5e12DM4RS
File Edit Analysis Highlight Add track Layout Size options Help
Zoom
13.8
Whole
Show buried clones
Yes
No
Search
CB Unit Range
0 to 115
Contig stats
Clones: 9 (1 buried)
Markers: 0
Sequenced: 0
Length: 116 CB units
Ctg1084 of cp05T5e12DM4RS
V035D23
V043K08
V063M08
V073N14~
V075B17*
V031O19
V016L23
M049B02
V051A22
0 10 20 30 40 50 60 70 80 90 100 110
[root@localhost: /...
[FPC V8.9 Main Menu]
Project cp05T5e12...
[我的文档 - File Br...
FPC Ctg1084 cp05T...

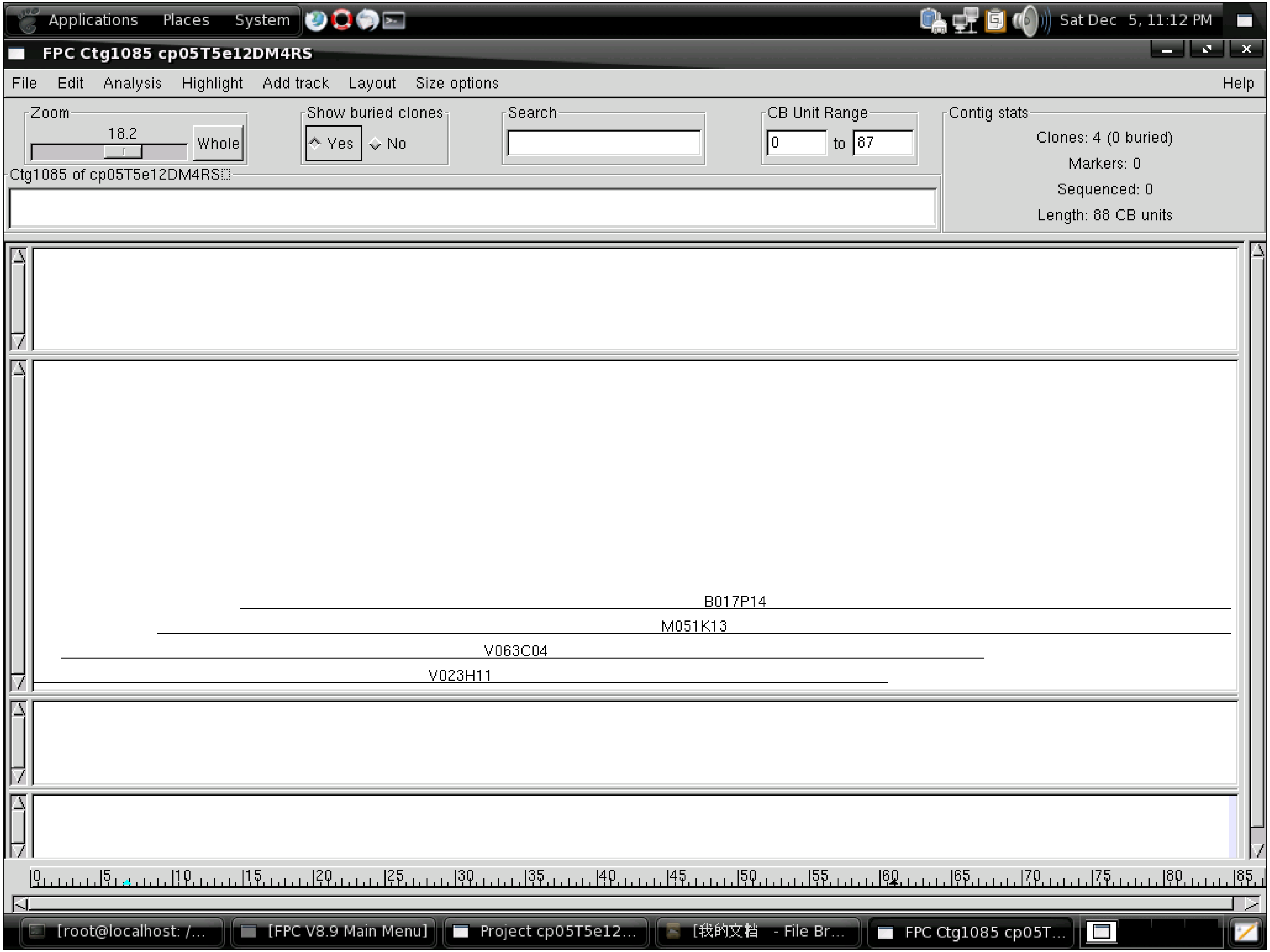
Applications
Places
System
Sat Dec 5, 11:12 PM
FPC Ctg1085 cp05T5e12DM4RS
File
Edit
Analysis
Highlight
Add track
Layout
Size options
Help
Zoom
18.2
Whole
Show buried clones
Yes
No
Search
CB Unit Range
0
to
87
Contig stats
Clones: 4 (0 buried)
Markers: 0
Sequenced: 0
Length: 88 CB units
Ctg1085 of cp05T5e12DM4RS
B017P14
M051K13
V063C04
V023H11
[root@localhost: /...
[FPC V8.9 Main Menu]
Project cp05T5e12...
[我的文档 - File Br...
FPC Ctg1085 cp05T...

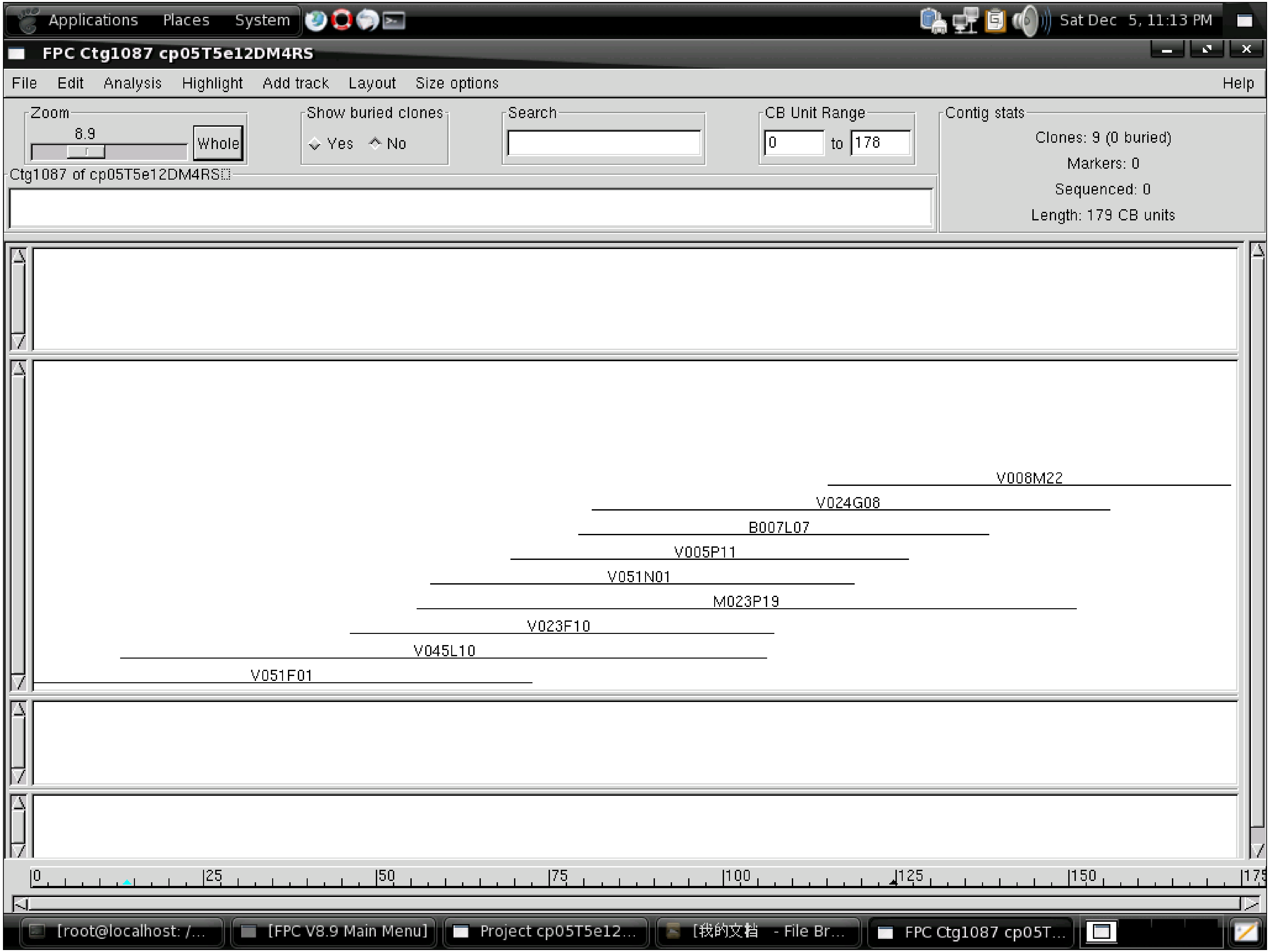
Applications Places System
Sat Dec 5, 11:13 PM
FPC Ctg1087 cp05T5e12DM4RS
File Edit Analysis Highlight Add track Layout Size options Help
Zoom
8.9
Whole
Show buried clones
Yes No
Search
CB Unit Range
0 to 178
Contig stats
Clones: 9 (0 buried)
Markers: 0
Sequenced: 0
Length: 179 CB units
Ctg1087 of cp05T5e12DM4RS
V008M22
V024G08
B007L07
V005P11
V051N01
M023P19
V023F10
V045L10
V051F01
0 25 50 75 100 125 150 175
[root@localhost: /...
[FPC V8.9 Main Menu]
Project cp05T5e12...
[我的文档 - File Br...
FPC Ctg1087 cp05T...

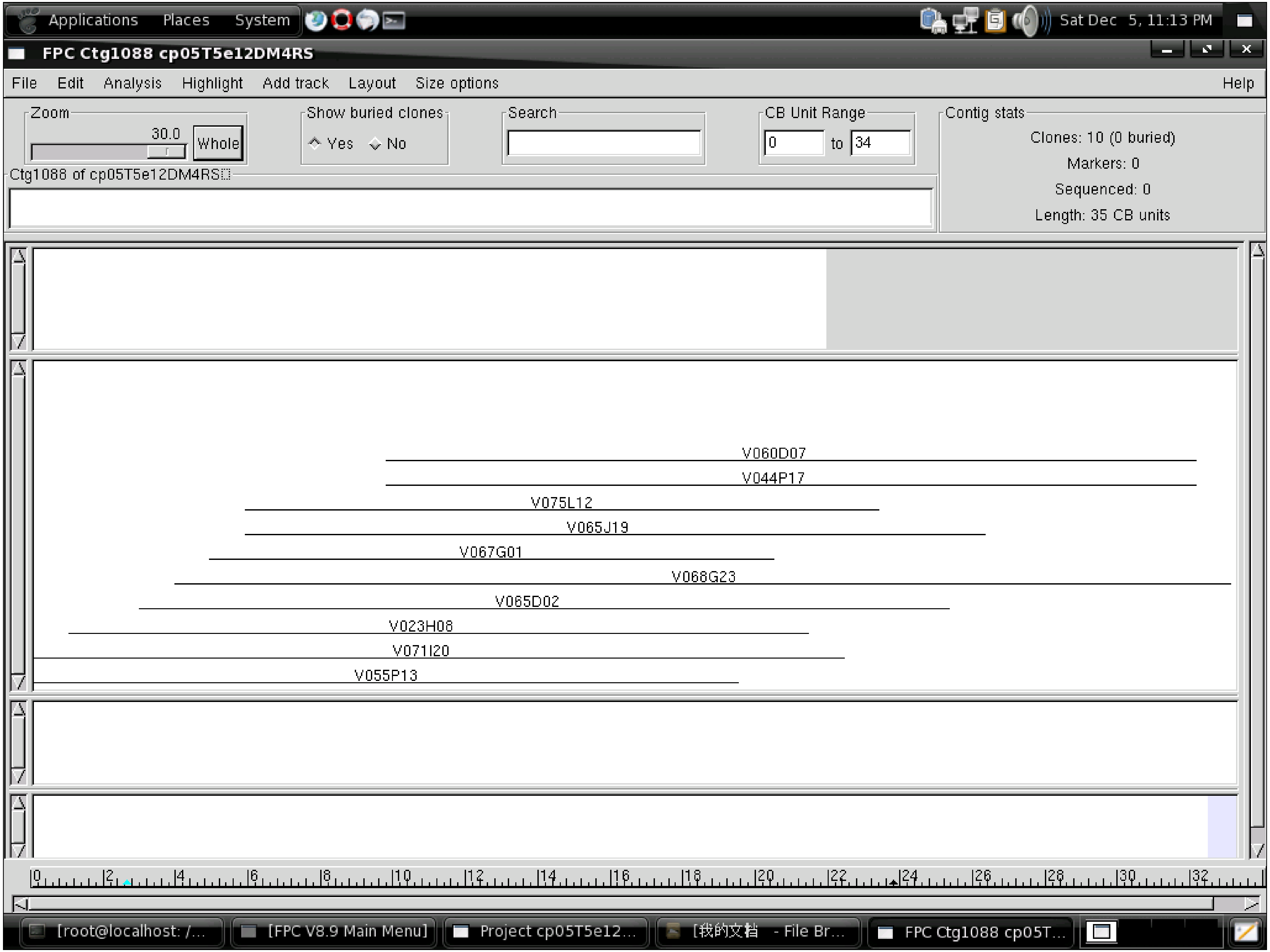
Applications Places System
Sat Dec 5, 11:13 PM
FPC Ctg1088 cp05T5e12DM4RS
File Edit Analysis Highlight Add track Layout Size options Help
Zoom
30.0
Whole
Show buried clones
Yes No
Search
CB Unit Range
0 to 34
Contig stats
Clones: 10 (0 buried)
Markers: 0
Sequenced: 0
Length: 35 CB units
Ctg1088 of cp05T5e12DM4RS
V060D07
V044P17
V075L12
V065J19
V067G01
V068G23
V065D02
V023H08
V071I20
V055P13
0 2 4 6 8 10 12 14 16 18 20 22 24 26 28 30 32
[root@localhost: /...
[FPC V8.9 Main Menu]
Project cp05T5e12...
[我的文档 - File Br...
FPC Ctg1088 cp05T...

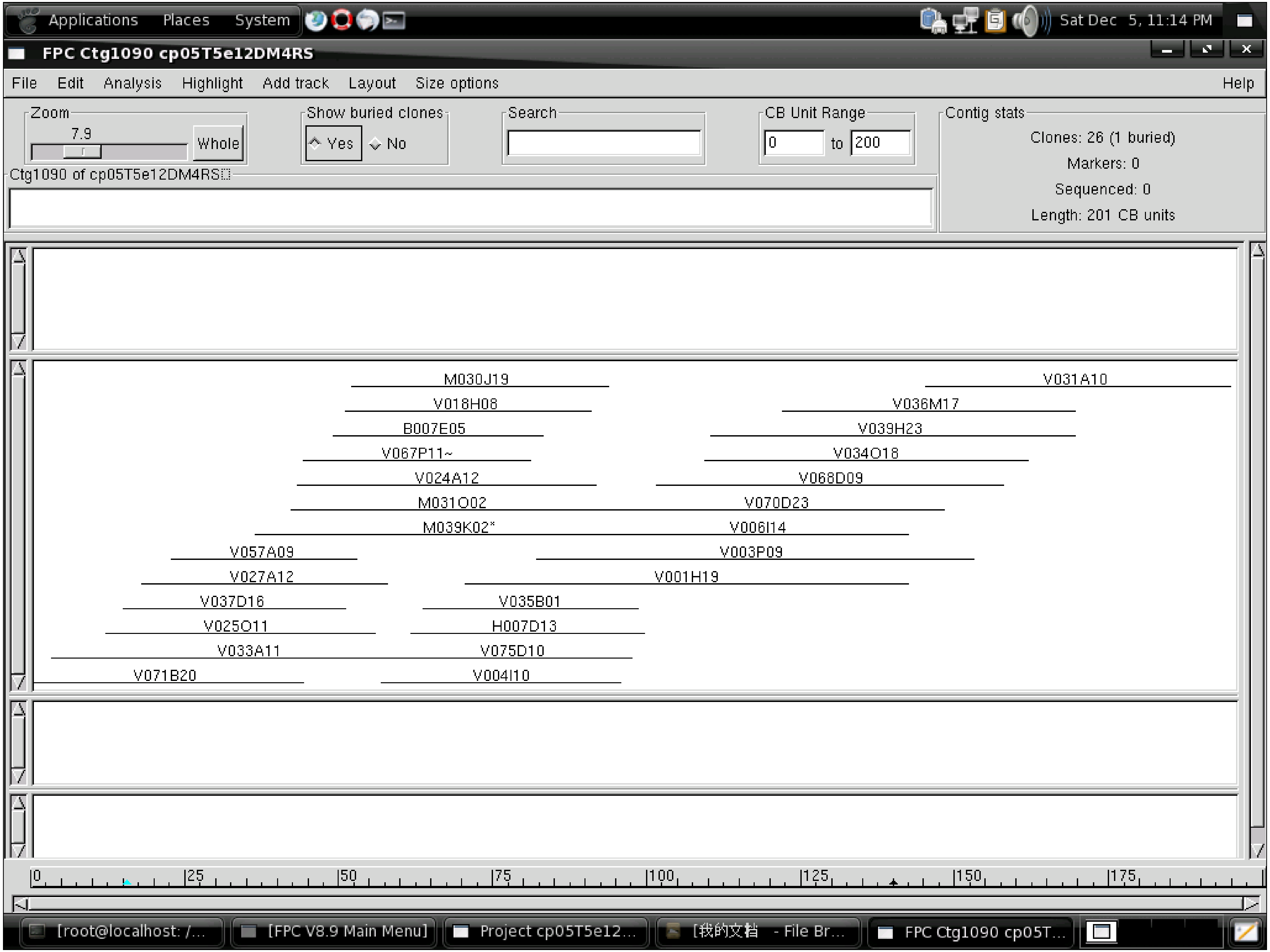
Applications
Places
System
Sat Dec 5, 11:14 PM
FPC Ctg1090 cp05T5e12DM4RS
File
Edit
Analysis
Highlight
Add track
Layout
Size options
Help
Zoom
7.9
Whole
Show buried clones
Yes
No
Search
CB Unit Range
0
to
200
Contig stats
Clones: 26 (1 buried)
Markers: 0
Sequenced: 0
Length: 201 CB units
Ctg1090 of cp05T5e12DM4RS
M030J19
V031A10
V018H08
V036M17
B007E05
V039H23
V067P11~
V034O18
V024A12
V068D09
M031O02
V070D23
M039K02*
V006I14
V057A09
V003P09
V027A12
V001H19
V037D16
V035B01
V025O11
H007D13
V033A11
V075D10
V071B20
V004I10
0
25
50
75
100
125
150
175
[root@localhost: /...
[FPC V8.9 Main Menu]
Project cp05T5e12...
[我的文档 - File Br...
FPC Ctg1090 cp05T...

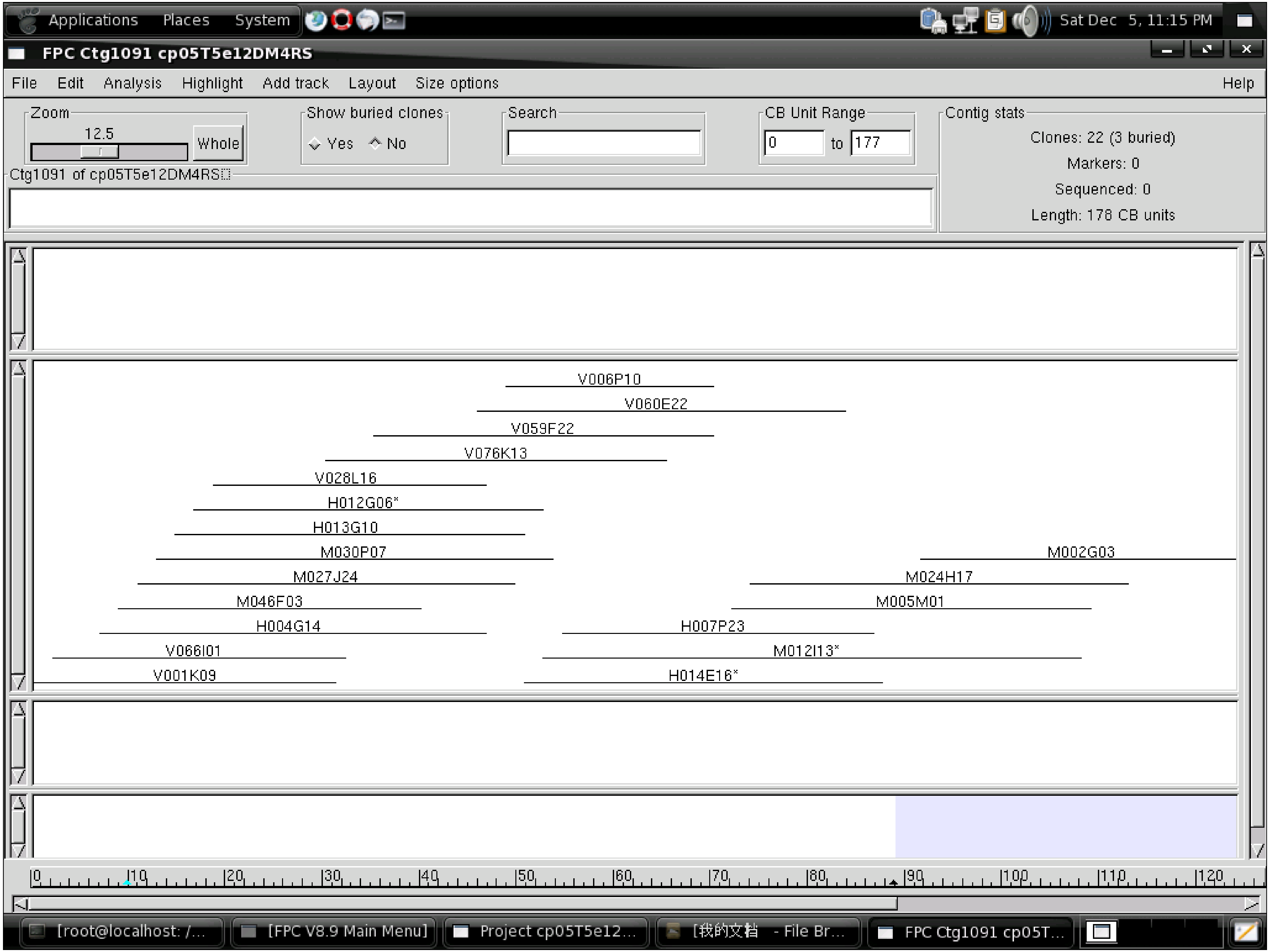

Applications Places System
Sat Dec 5, 11:15 PM
FPC Ctg1091 cp05T5e12DM4RS
File Edit Analysis Highlight Add track Layout Size options Help
Zoom
12.5
Whole
Show buried clones
Yes No
Search
CB Unit Range
0 to 177
Contig stats
Clones: 22 (3 buried)
Markers: 0
Sequenced: 0
Length: 178 CB units
Ctg1091 of cp05T5e12DM4RS
V006P10
V060E22
V059F22
V076K13
V028L16
H012G06*
H013G10
M030P07
M002G03
M027J24
M024H17
M046F03
M005M01
H004G14
H007P23
V066I01
M012I13*
V001K09
H014E16*
0 10 20 30 40 50 60 70 80 90 100 110 120
[root@localhost: /...
[FPC V8.9 Main Menu]
Project cp05T5e12...
[我的文档 - File Br...
FPC Ctg1091 cp05T...

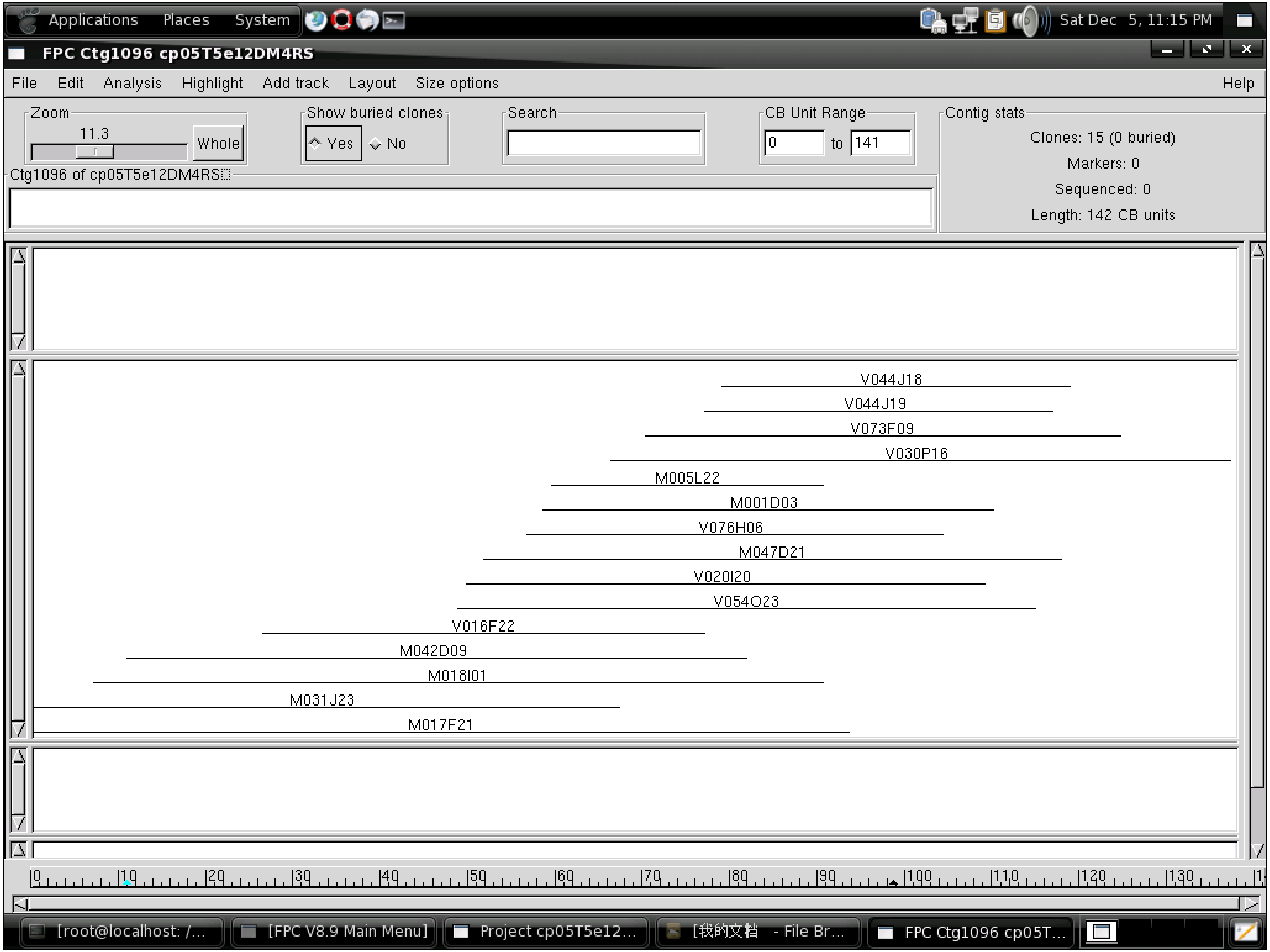
Applications
Places
System
Sat Dec 5, 11:15 PM
FPC Ctg1096 cp05T5e12DM4RS
File
Edit
Analysis
Highlight
Add track
Layout
Size options
Help
Zoom
11.3
Whole
Show buried clones
Yes
No
Search
CB Unit Range
0
to
141
Contig stats
Clones: 15 (0 buried)
Markers: 0
Sequenced: 0
Length: 142 CB units
Ctg1096 of cp05T5e12DM4RS
V044J18
V044J19
V073F09
V030P16
M005L22
M001D03
V076H06
M047D21
V020I20
V054O23
V016F22
M042D09
M018I01
M031J23
M017F21
0
10
20
30
40
50
60
70
80
90
100
110
120
130
[root@localhost: /...
[FPC V8.9 Main Menu]
Project cp05T5e12...
[我的文档 - File Br...
FPC Ctg1096 cp05T...

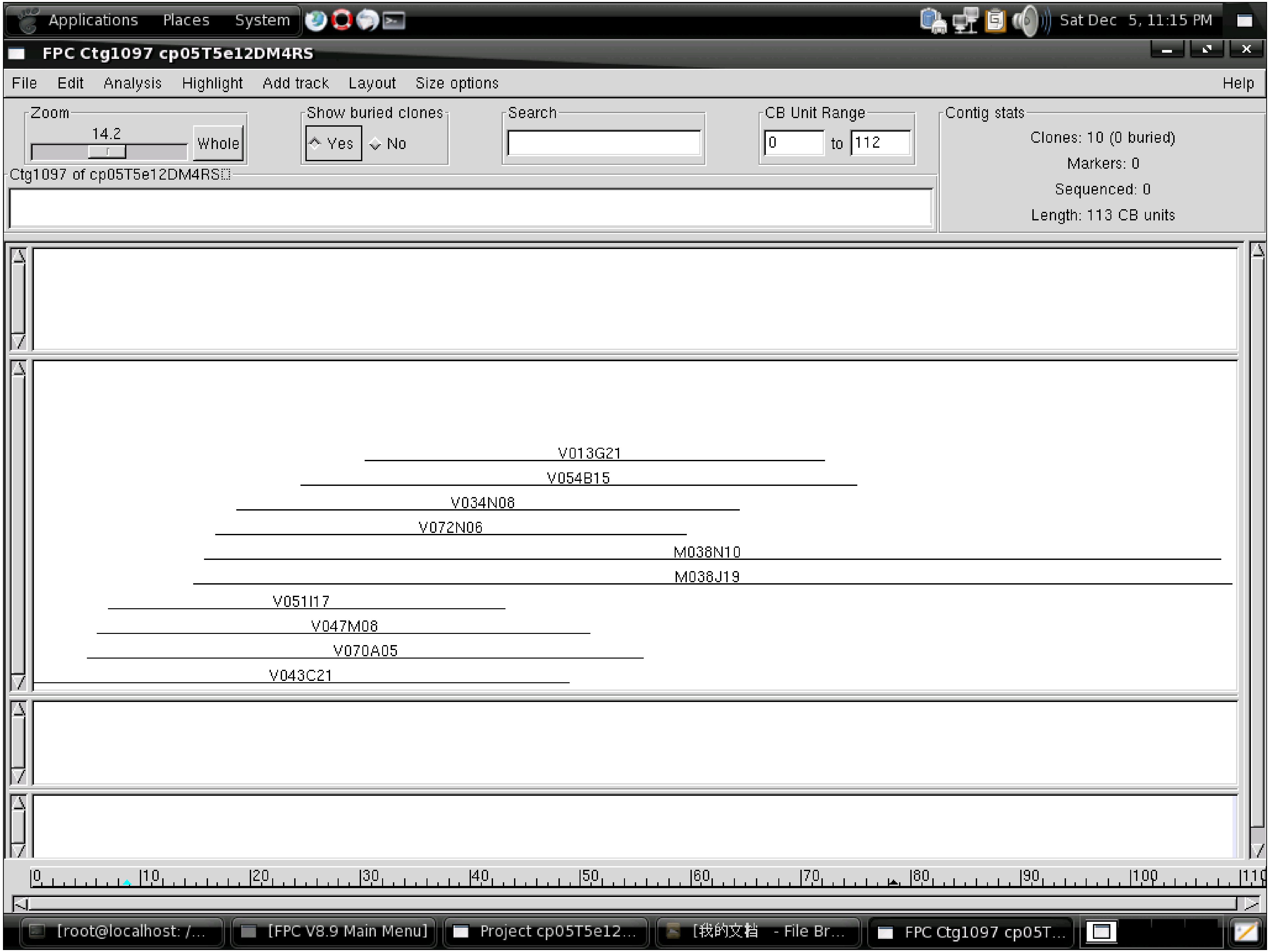
Applications Places System
Sat Dec 5, 11:15 PM
FPC Ctg1097 cp05T5e12DM4RS
File Edit Analysis Highlight Add track Layout Size options Help
Zoom
14.2
Whole
Show buried clones
Yes No
Search
CB Unit Range
0 to 112
Contig stats
Clones: 10 (0 buried)
Markers: 0
Sequenced: 0
Length: 113 CB units
Ctg1097 of cp05T5e12DM4RS
V013G21
V054B15
V034N08
V072N06
M038N10
M038J19
V051I17
V047M08
V070A05
V043C21
0 10 20 30 40 50 60 70 80 90 100 110
[root@localhost: /...
[FPC V8.9 Main Menu]
Project cp05T5e12...
[我的文档 - File Br...
FPC Ctg1097 cp05T...

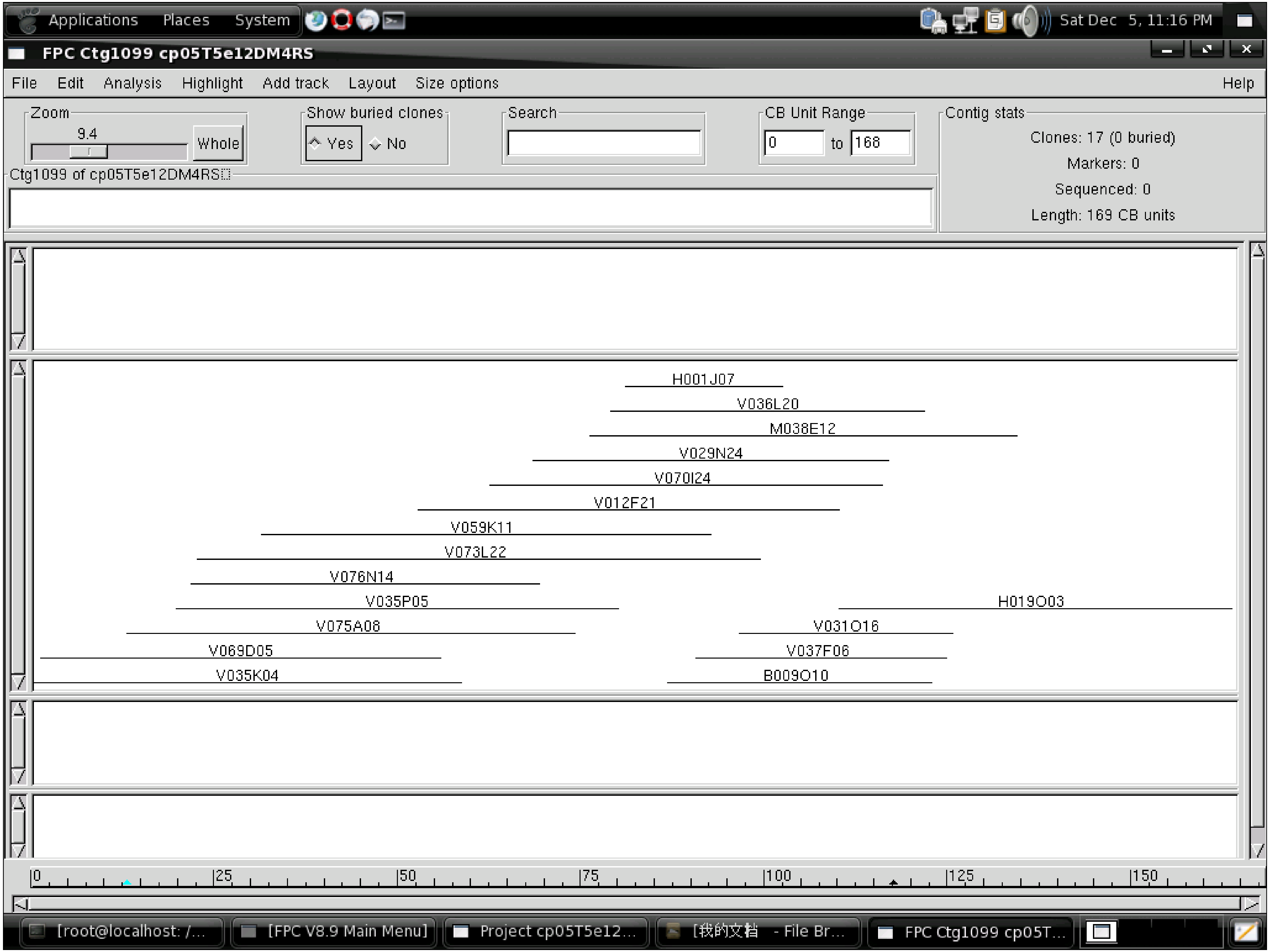
Applications Places System
Sat Dec 5, 11:16 PM
FPC Ctg1099 cp05T5e12DM4RS
File Edit Analysis Highlight Add track Layout Size options Help
Zoom
9.4
Whole
Show buried clones
Yes No
Search
CB Unit Range
0 to 168
Contig stats
Clones: 17 (0 buried)
Markers: 0
Sequenced: 0
Length: 169 CB units
Ctg1099 of cp05T5e12DM4RS
H001J07
V036L20
M038E12
V029N24
V070I24
V012F21
V059K11
V073L22
V076N14
V035P05
H019O03
V075A08
V031O16
V069D05
V037F06
V035K04
B009O10
0 25 50 75 100 125 150
[root@localhost: /...
[FPC V8.9 Main Menu]
Project cp05T5e12...
[我的文档 - File Br...
FPC Ctg1099 cp05T...

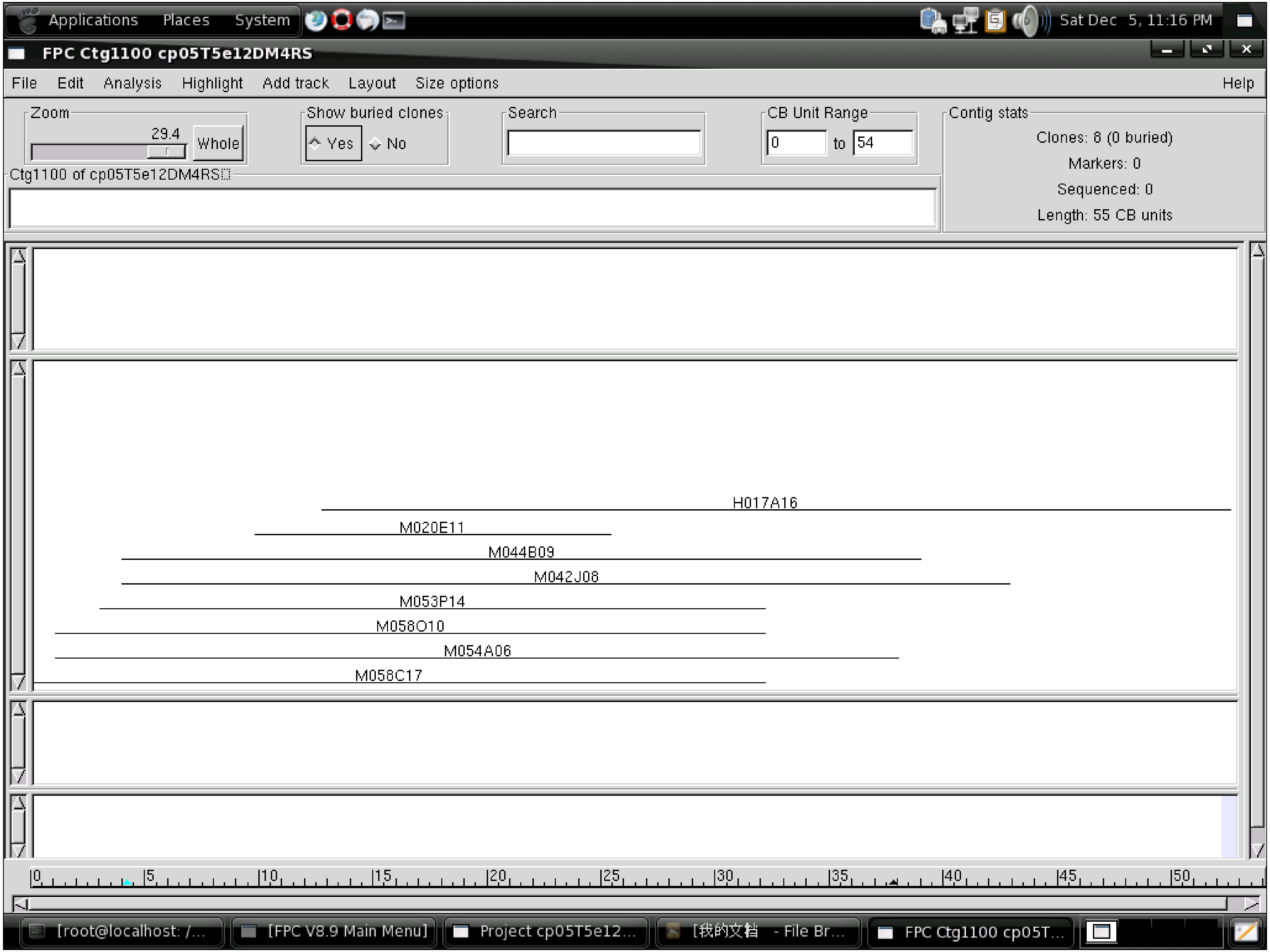
Applications Places System
Sat Dec 5, 11:16 PM
FPC Ctg1100 cp05T5e12DM4RS
File Edit Analysis Highlight Add track Layout Size options Help
Zoom
29.4
Whole
Show buried clones
Yes No
Search
CB Unit Range
0 to 54
Contig stats
Clones: 8 (0 buried)
Markers: 0
Sequenced: 0
Length: 55 CB units
Ctg1100 of cp05T5e12DM4RS
H017A16
M020E11
M044B09
M042J08
M053P14
M058O10
M054A06
M058C17
0 5 10 15 20 25 30 35 40 45 50
[root@localhost: /...
[FPC V8.9 Main Menu]
Project cp05T5e12...
[我的文档 - File Br...
FPC Ctg1100 cp05T...

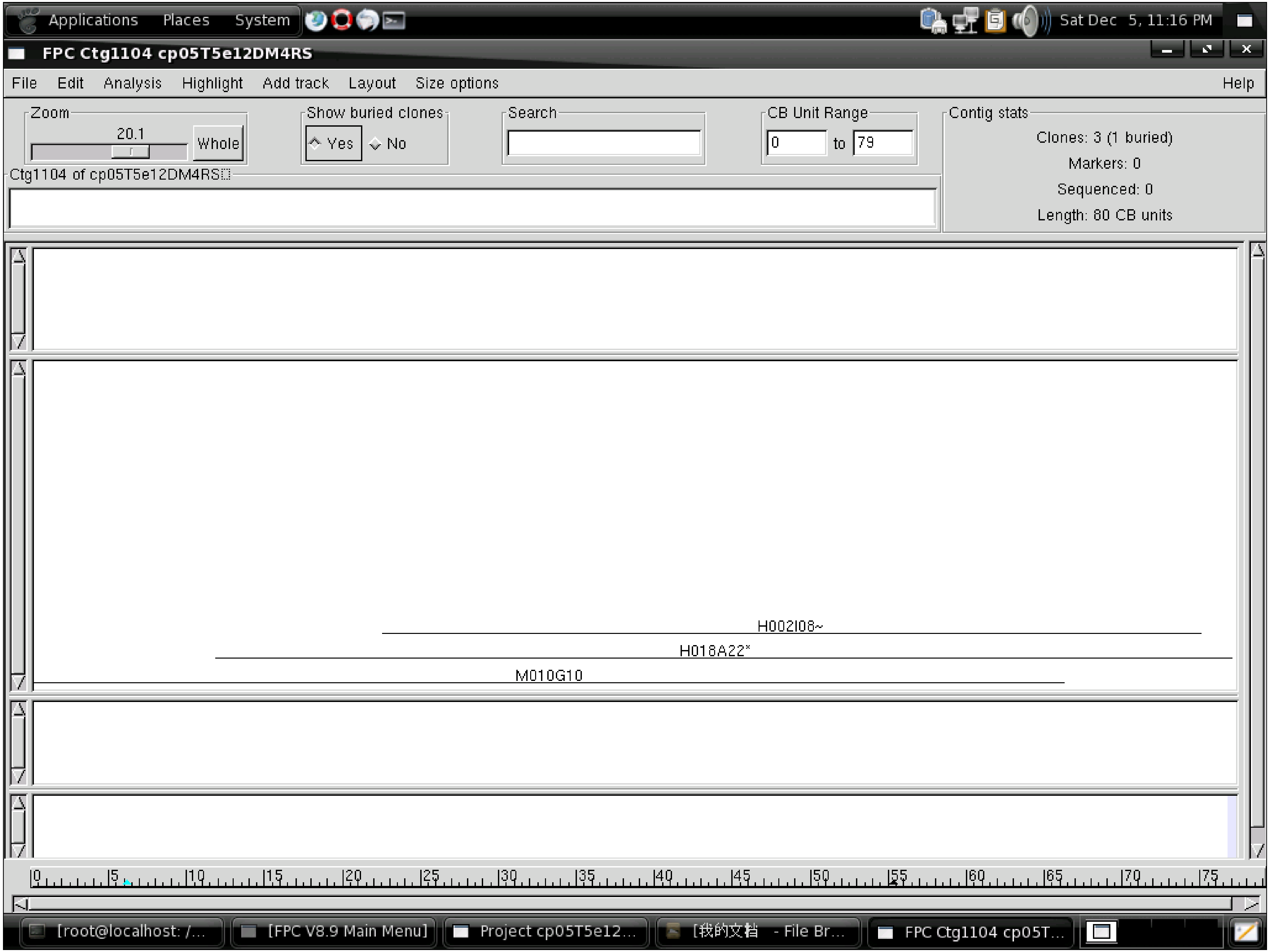

Applications Places System
Sat Dec 5, 11:16 PM
FPC Ctg1104 cp05T5e12DM4RS
File Edit Analysis Highlight Add track Layout Size options Help
Zoom
20.1
Whole
Show buried clones
Yes No
Search
CB Unit Range
0 to 79
Contig stats
Clones: 3 (1 buried)
Markers: 0
Sequenced: 0
Length: 80 CB units
Ctg1104 of cp05T5e12DM4RS
H002I08~
H018A22*
M010G10
0 5 10 15 20 25 30 35 40 45 50 55 60 65 70 75
[root@localhost: /...
[FPC V8.9 Main Menu]
Project cp05T5e12...
[我的文档 - File Br...
FPC Ctg1104 cp05T...

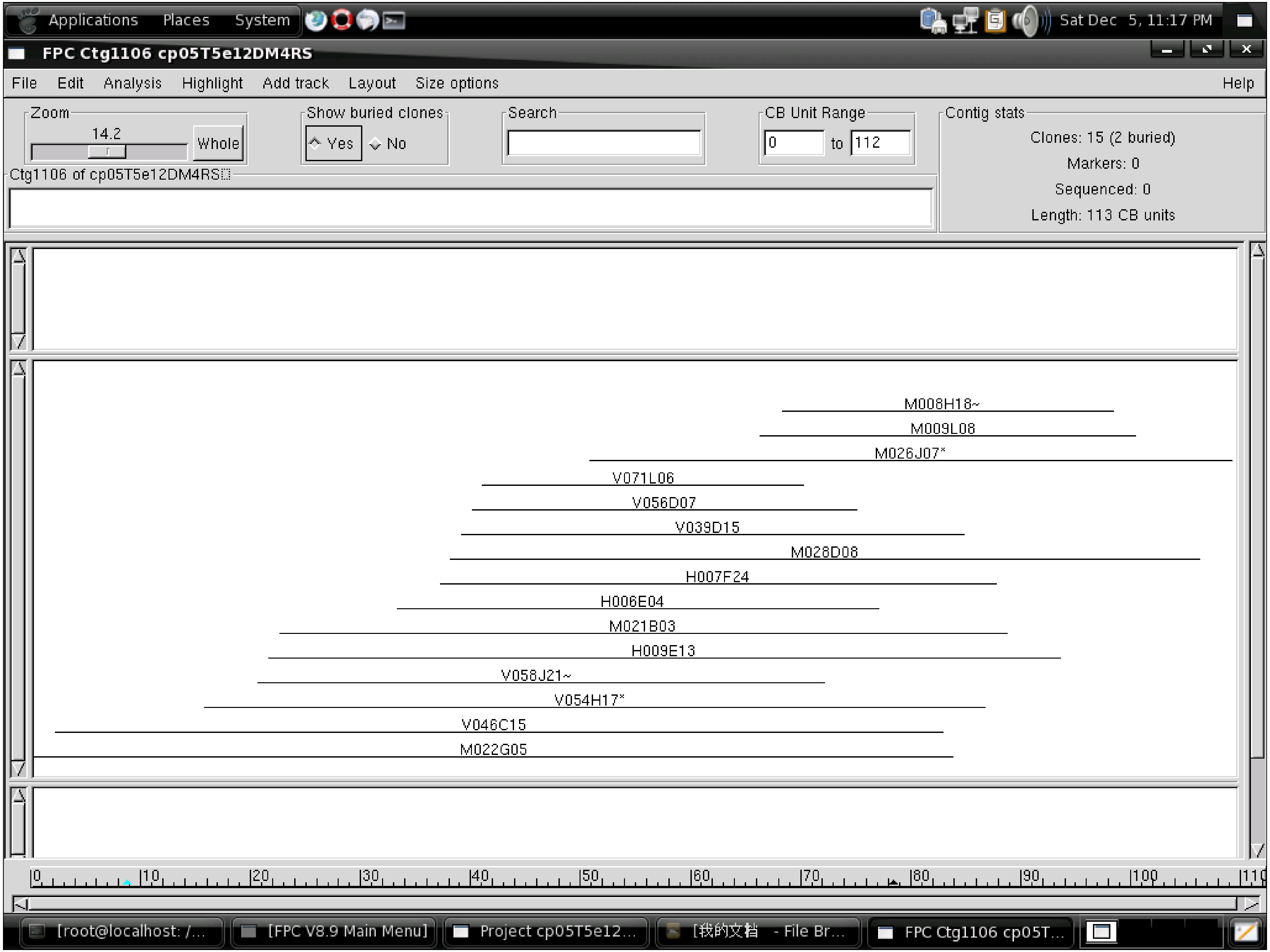
Applications Places System
Sat Dec 5, 11:17 PM
FPC Ctg1106 cp05T5e12DM4RS
File Edit Analysis Highlight Add track Layout Size options Help
Zoom
14.2
Whole
Show buried clones
Yes No
Search
CB Unit Range
0 to 112
Contig stats
Clones: 15 (2 buried)
Markers: 0
Sequenced: 0
Length: 113 CB units
Ctg1106 of cp05T5e12DM4RS
M008H18~
M009L08
M026J07*
V071L06
V056D07
V039D15
M028D08
H007F24
H006E04
M021B03
H009E13
V058J21~
V054H17*
V046C15
M022G05
0 10 20 30 40 50 60 70 80 90 100 110
[root@localhost: /...
[FPC V8.9 Main Menu]
Project cp05T5e12...
[我的文档 - File Br...
FPC Ctg1106 cp05T...

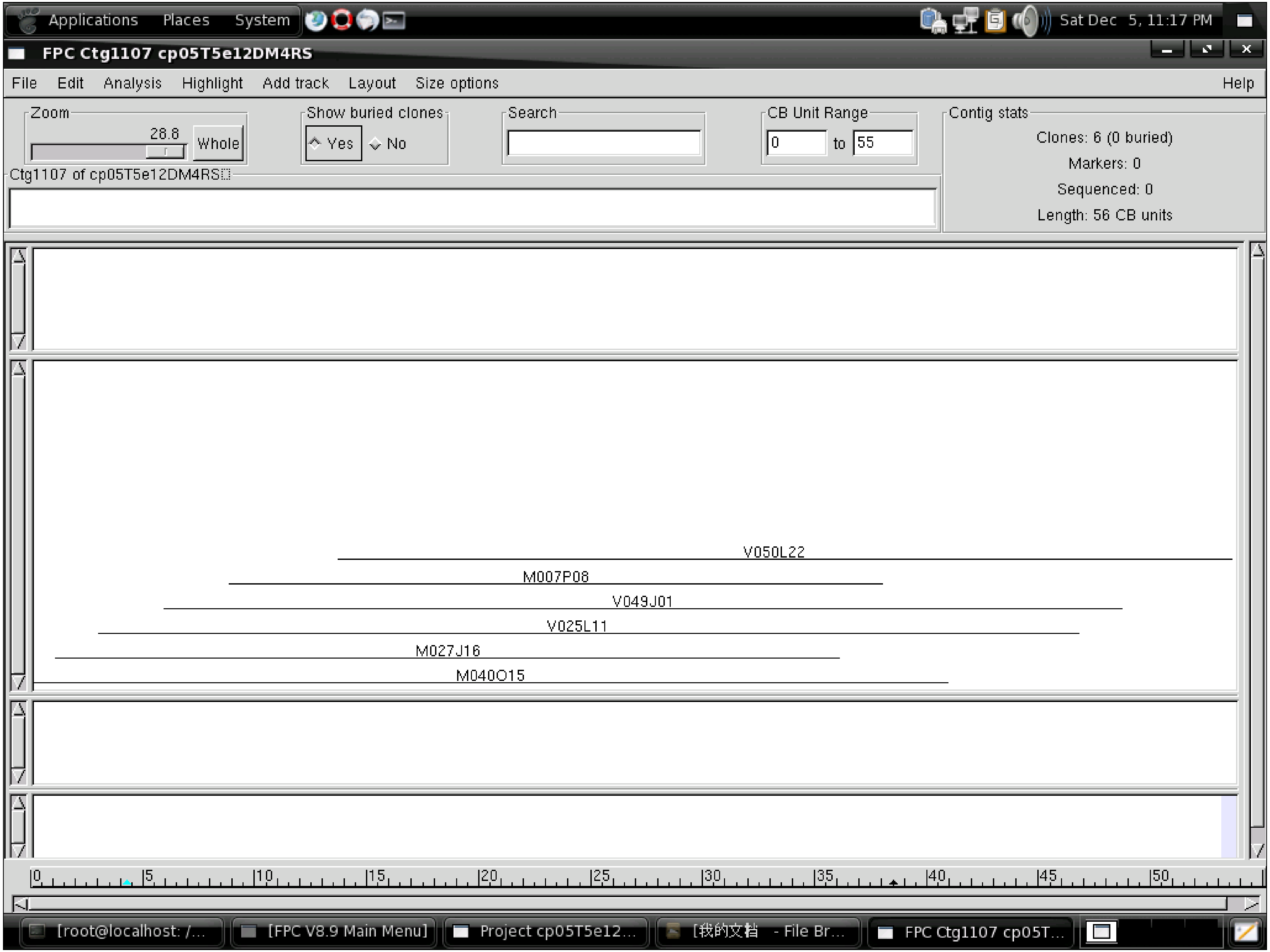
Applications
Places
System
Sat Dec 5, 11:17 PM
FPC Ctg1107 cp05T5e12DM4RS
File
Edit
Analysis
Highlight
Add track
Layout
Size options
Help
Zoom
28.8
Whole
Show buried clones
Yes
No
Search
CB Unit Range
0
to
55
Contig stats
Clones: 6 (0 buried)
Markers: 0
Sequenced: 0
Length: 56 CB units
Ctg1107 of cp05T5e12DM4RS
V050L22
M007P08
V049J01
V025L11
M027J16
M040O15
0
5
10
15
20
25
30
35
40
45
50
[root@localhost: /...
[FPC V8.9 Main Menu]
Project cp05T5e12...
[我的文档 - File Br...
FPC Ctg1107 cp05T...

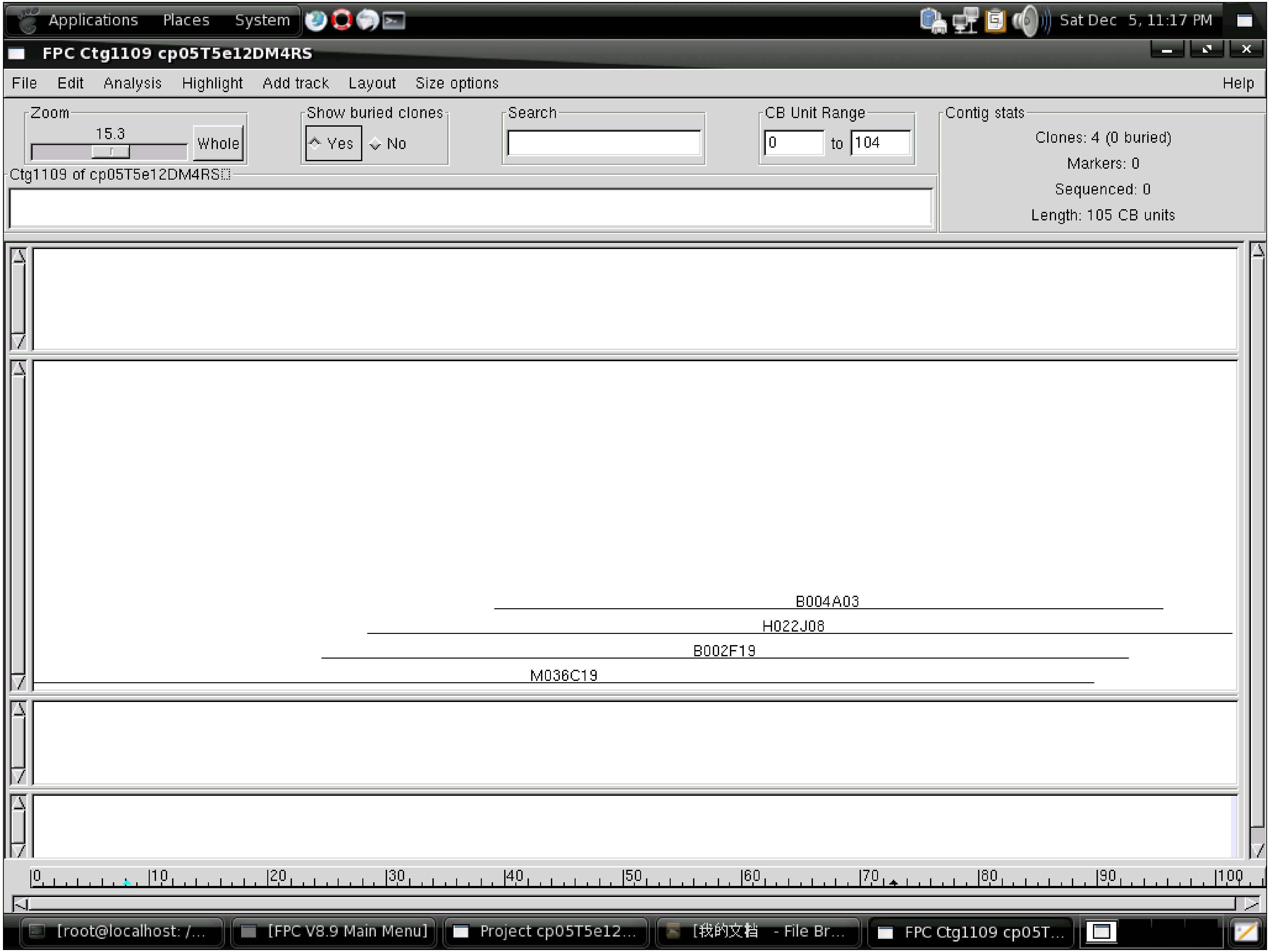
Applications
Places
System
Sat Dec 5, 11:17 PM
FPC Ctg1109 cp05T5e12DM4RS
File
Edit
Analysis
Highlight
Add track
Layout
Size options
Help
Zoom
15.3
Whole
Show buried clones
Yes
No
Search
CB Unit Range
0
to
104
Contig stats
Clones: 4 (0 buried)
Markers: 0
Sequenced: 0
Length: 105 CB units
Ctg1109 of cp05T5e12DM4RS
B004A03
H022J08
B002F19
M036C19
0
10
20
30
40
50
60
70
80
90
100
[root@localhost: /...
[FPC V8.9 Main Menu]
Project cp05T5e12...
[我的文档 - File Br...
FPC Ctg1109 cp05T...

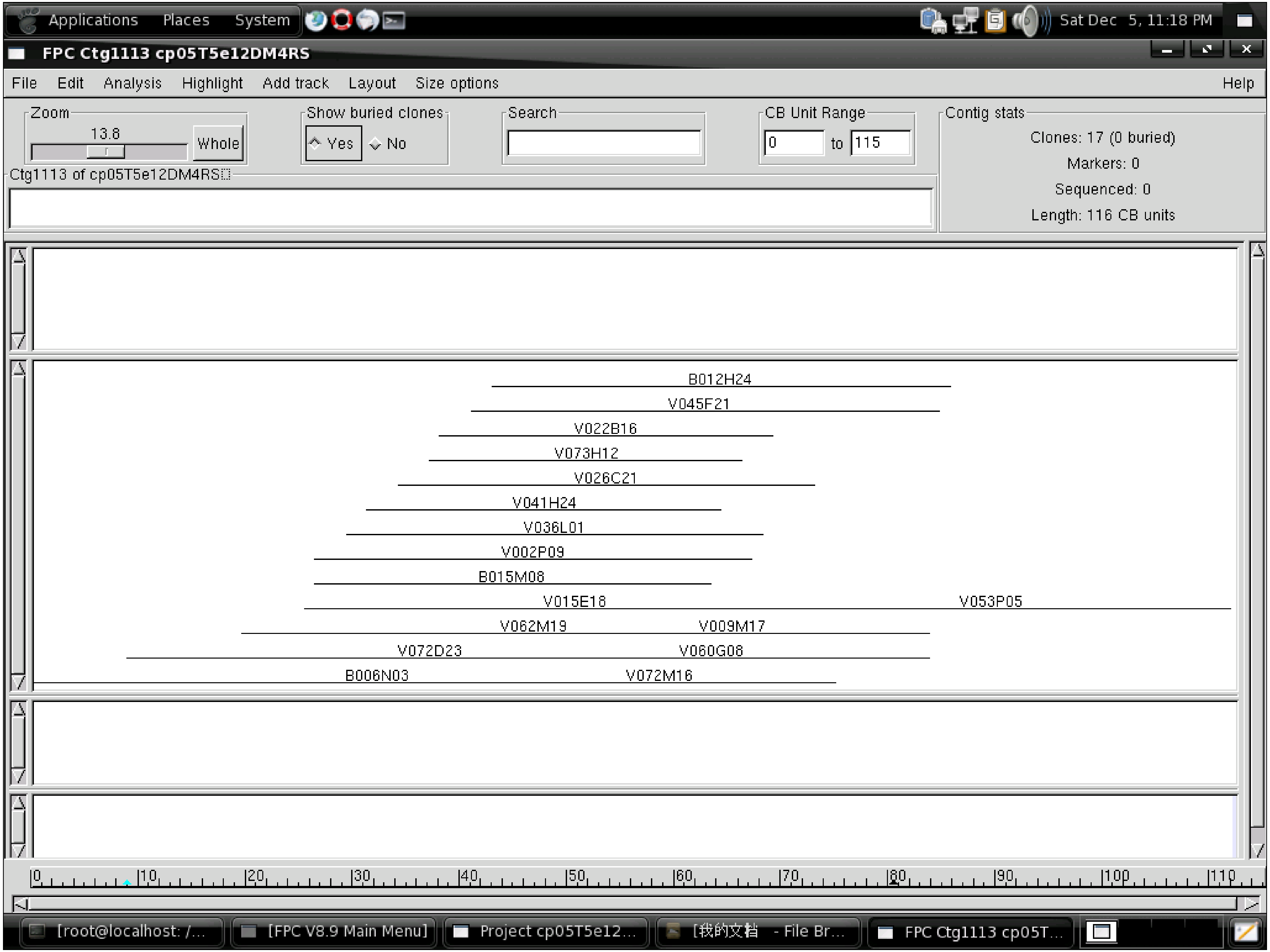
Applications Places System
Sat Dec 5, 11:18 PM
FPC Ctg1113 cp05T5e12DM4RS
File Edit Analysis Highlight Add track Layout Size options Help
Zoom
13.8
Whole
Show buried clones
Yes No
Search
CB Unit Range
0 to 115
Contig stats
Clones: 17 (0 buried)
Markers: 0
Sequenced: 0
Length: 116 CB units
Ctg1113 of cp05T5e12DM4RS
B012H24
V045F21
V022B16
V073H12
V026C21
V041H24
V036L01
V002P09
B015M08
V015E18
V053P05
V062M19
V009M17
V072D23
V060G08
B006N03
V072M16
0 10 20 30 40 50 60 70 80 90 100 110
[root@localhost: /...
[FPC V8.9 Main Menu]
Project cp05T5e12...
[我的文档 - File Br...
FPC Ctg1113 cp05T...

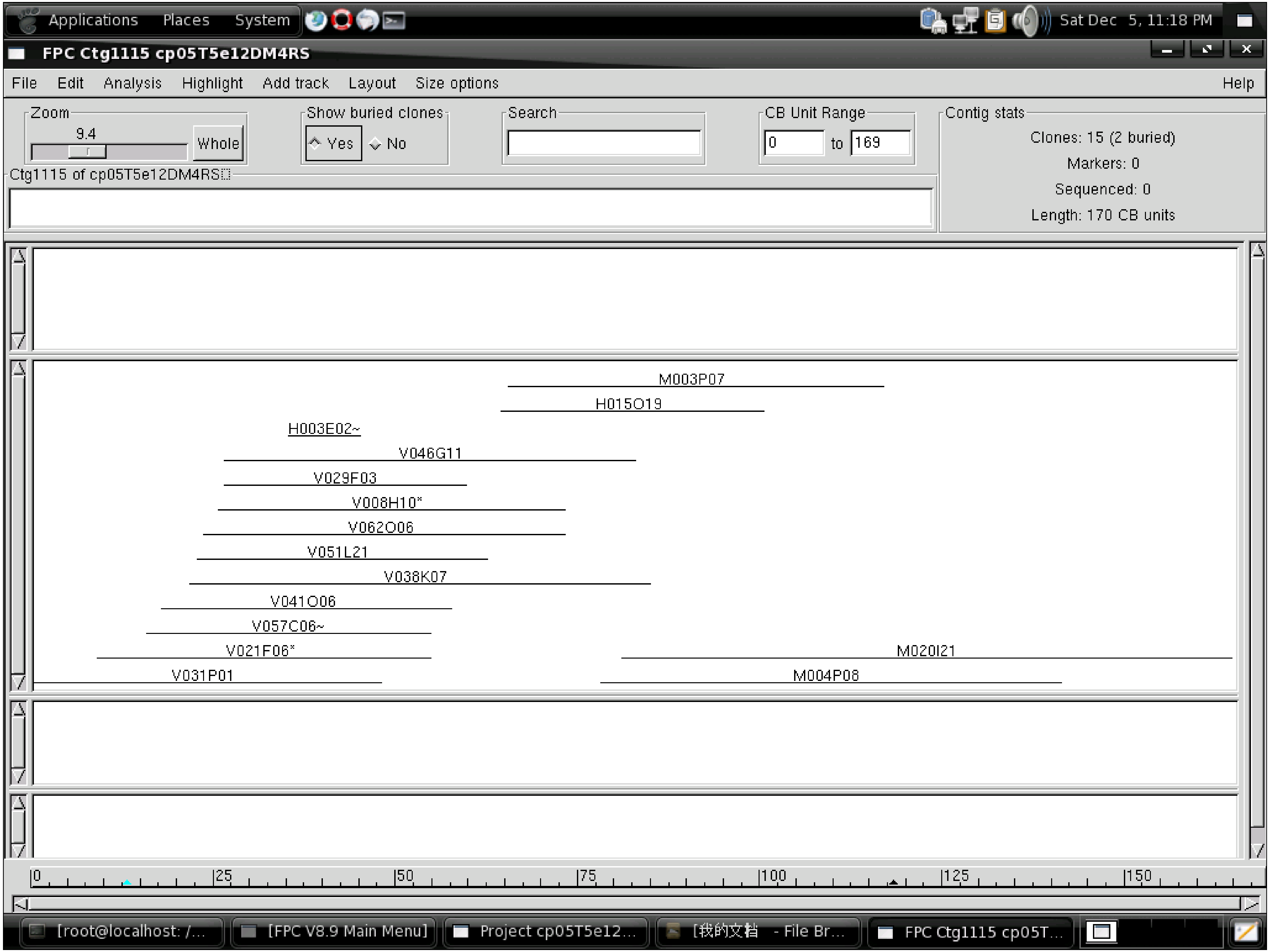
Applications Places System
Sat Dec 5, 11:18 PM
FPC Ctg1115 cp05T5e12DM4RS
File Edit Analysis Highlight Add track Layout Size options Help
Zoom
9.4
Whole
Show buried clones
Yes No
Search
CB Unit Range
0 to 169
Contig stats
Clones: 15 (2 buried)
Markers: 0
Sequenced: 0
Length: 170 CB units
Ctg1115 of cp05T5e12DM4RS
M003P07
H015O19
H003E02~
V046G11
V029F03
V008H10*
V062O06
V051L21
V038K07
V041O06
V057C06~
V021F06*
M020I21
V031P01
M004P08
0 25 50 75 100 125 150
[root@localhost: /...
[FPC V8.9 Main Menu]
Project cp05T5e12...
[我的文档 - File Br...
FPC Ctg1115 cp05T...

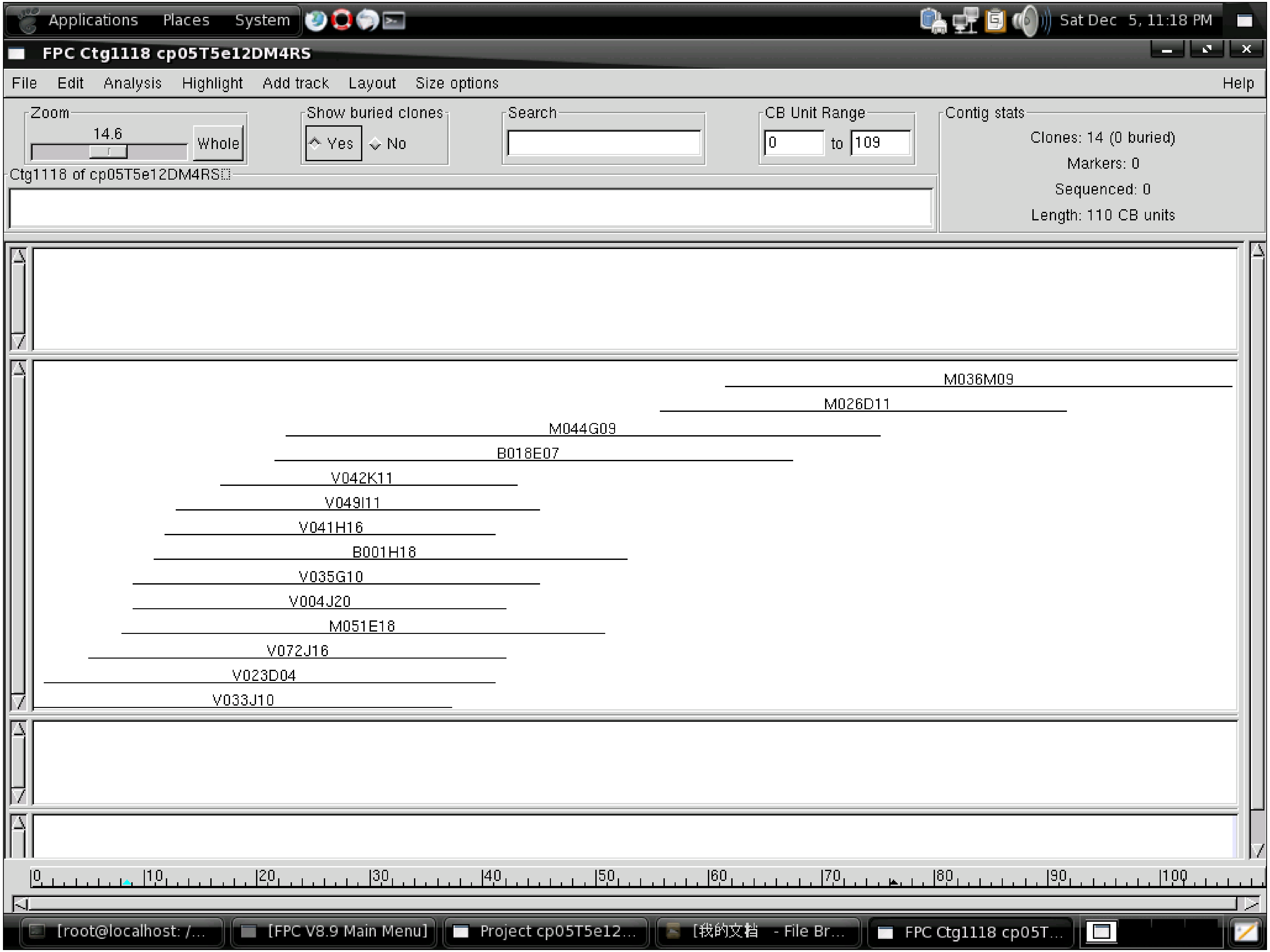
Applications Places System
Sat Dec 5, 11:18 PM
FPC Ctg1118 cp05T5e12DM4RS
File Edit Analysis Highlight Add track Layout Size options Help
Zoom
14.6
Whole
Show buried clones
Yes No
Search
CB Unit Range
0 to 109
Contig stats
Clones: 14 (0 buried)
Markers: 0
Sequenced: 0
Length: 110 CB units
Ctg1118 of cp05T5e12DM4RS
M036M09
M026D11
M044G09
B018E07
V042K11
V049I11
V041H16
B001H18
V035G10
V004J20
M051E18
V072J16
V023D04
V033J10
0 10 20 30 40 50 60 70 80 90 100
[root@localhost: /...
[FPC V8.9 Main Menu]
Project cp05T5e12...
[我的文档 - File Br...
FPC Ctg1118 cp05T...

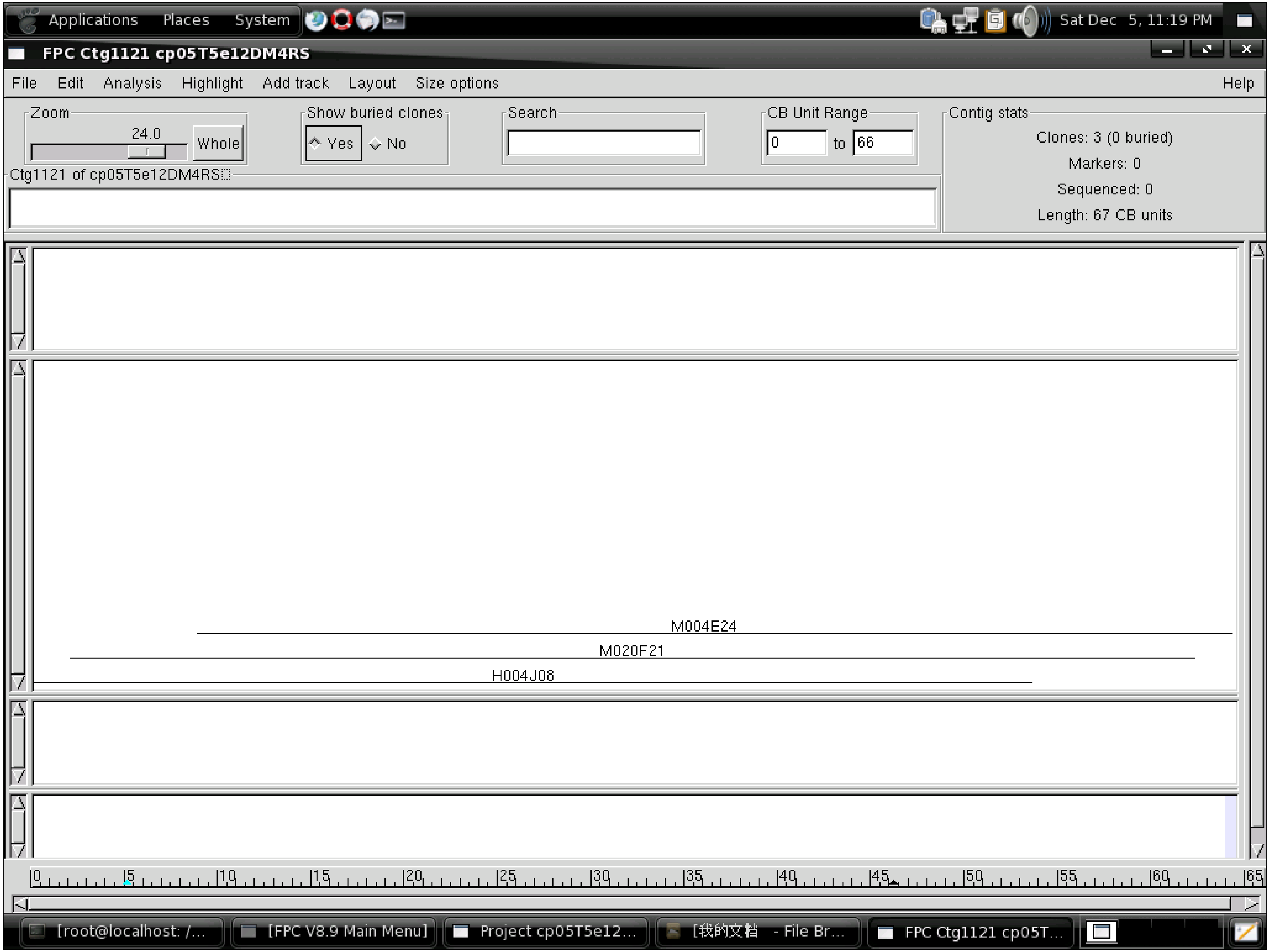
Applications Places System
Sat Dec 5, 11:19 PM
FPC Ctg1121 cp05T5e12DM4RS
File Edit Analysis Highlight Add track Layout Size options Help
Zoom
24.0
Whole
Show buried clones
Yes
No
Search
CB Unit Range
0
to
66
Contig stats
Clones: 3 (0 buried)
Markers: 0
Sequenced: 0
Length: 67 CB units
Ctg1121 of cp05T5e12DM4RS
M004E24
M020F21
H004J08
0 5 10 15 20 25 30 35 40 45 50 55 60 65
[root@localhost: /...
[FPC V8.9 Main Menu]
Project cp05T5e12...
[我的文档 - File Br...
FPC Ctg1121 cp05T...

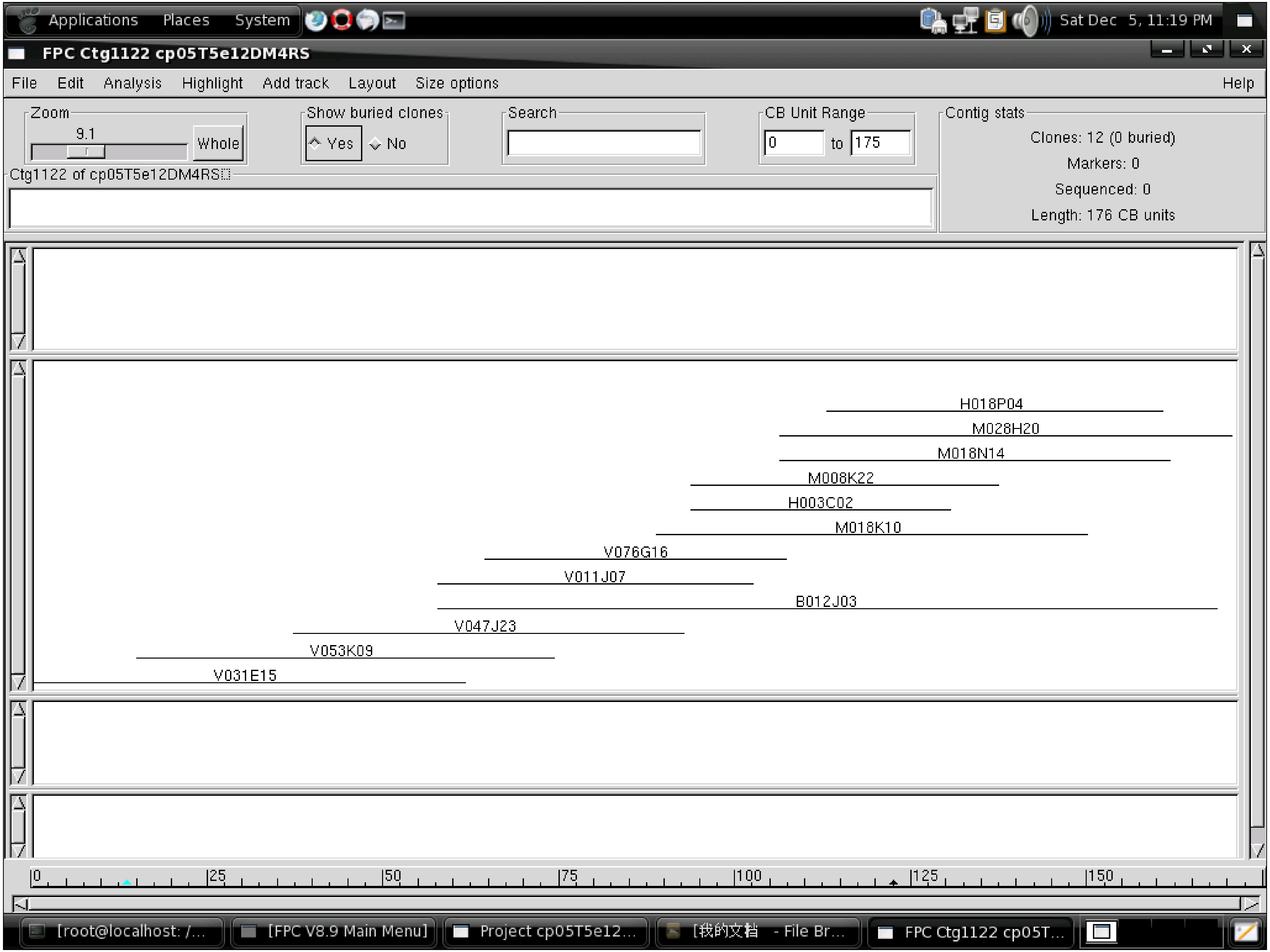
Applications
Places
System
Sat Dec 5, 11:19 PM
FPC Ctg1122 cp05T5e12DM4RS
File
Edit
Analysis
Highlight
Add track
Layout
Size options
Help
Zoom
9.1
Whole
Show buried clones
Yes
No
Search
CB Unit Range
0
to
175
Contig stats
Clones: 12 (0 buried)
Markers: 0
Sequenced: 0
Length: 176 CB units
Ctg1122 of cp05T5e12DM4RS
H018P04
M028H20
M018N14
M008K22
H003C02
M018K10
V076G16
V011J07
B012J03
V047J23
V053K09
V031E15
0
25
50
75
100
125
150
[root@localhost: /...
[FPC V8.9 Main Menu]
Project cp05T5e12...
[我的文档 - File Br...
FPC Ctg1122 cp05T...

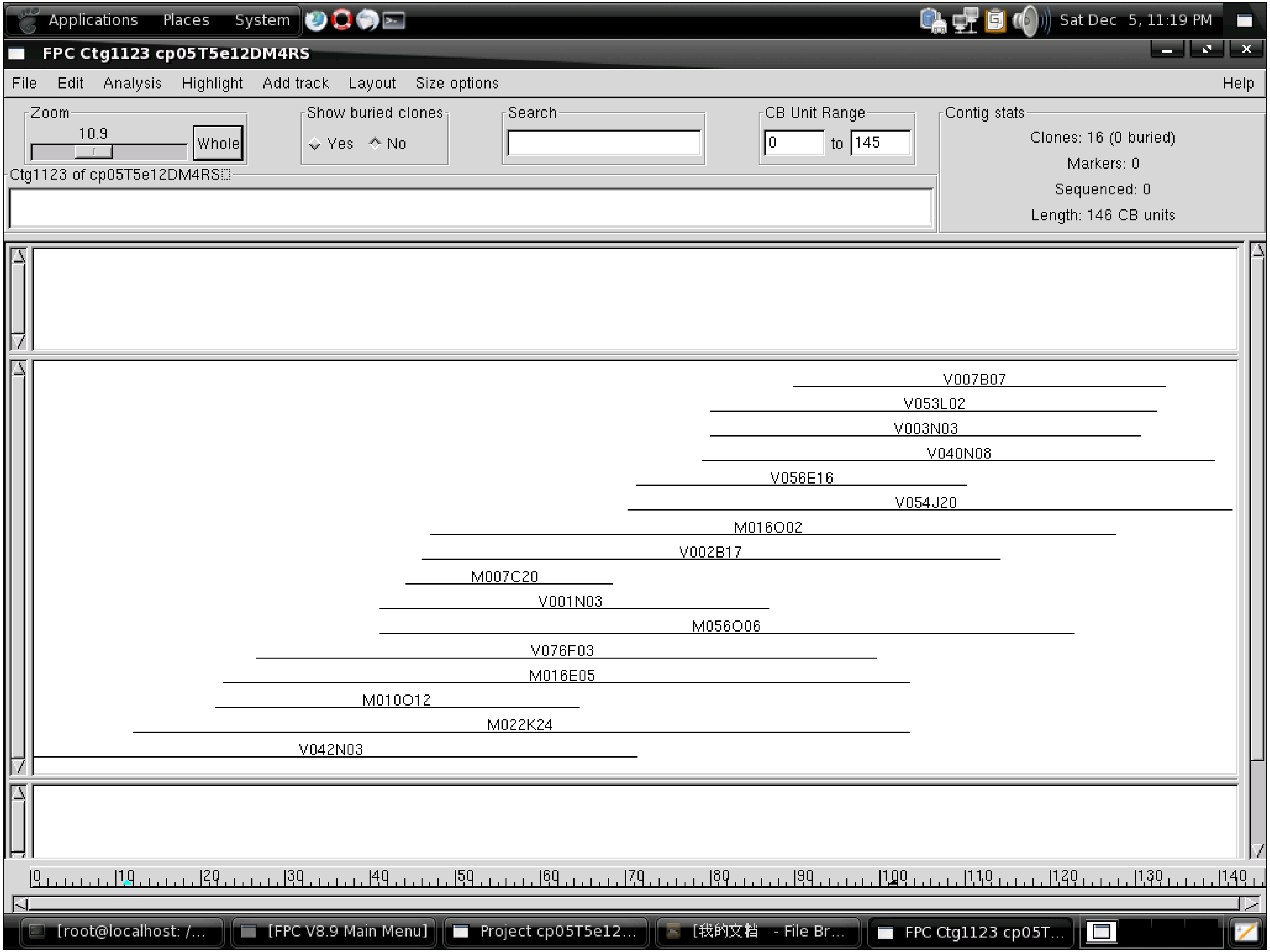
Applications Places System
Sat Dec 5, 11:19 PM
FPC Ctg1123 cp05T5e12DM4RS
File Edit Analysis Highlight Add track Layout Size options Help
Zoom
10.9
Whole
Show buried clones
Yes No
Search
CB Unit Range
0 to 145
Contig stats
Clones: 16 (0 buried)
Markers: 0
Sequenced: 0
Length: 146 CB units
Ctg1123 of cp05T5e12DM4RS
V007B07
V053L02
V003N03
V040N08
V056E16
V054J20
M016O02
V002B17
M007C20
V001N03
M056O06
V076F03
M016E05
M010O12
M022K24
V042N03
0 10 20 30 40 50 60 70 80 90 100 110 120 130 140
[root@localhost: /...
[FPC V8.9 Main Menu]
Project cp05T5e12...
[我的文档 - File Br...
FPC Ctg1123 cp05T...

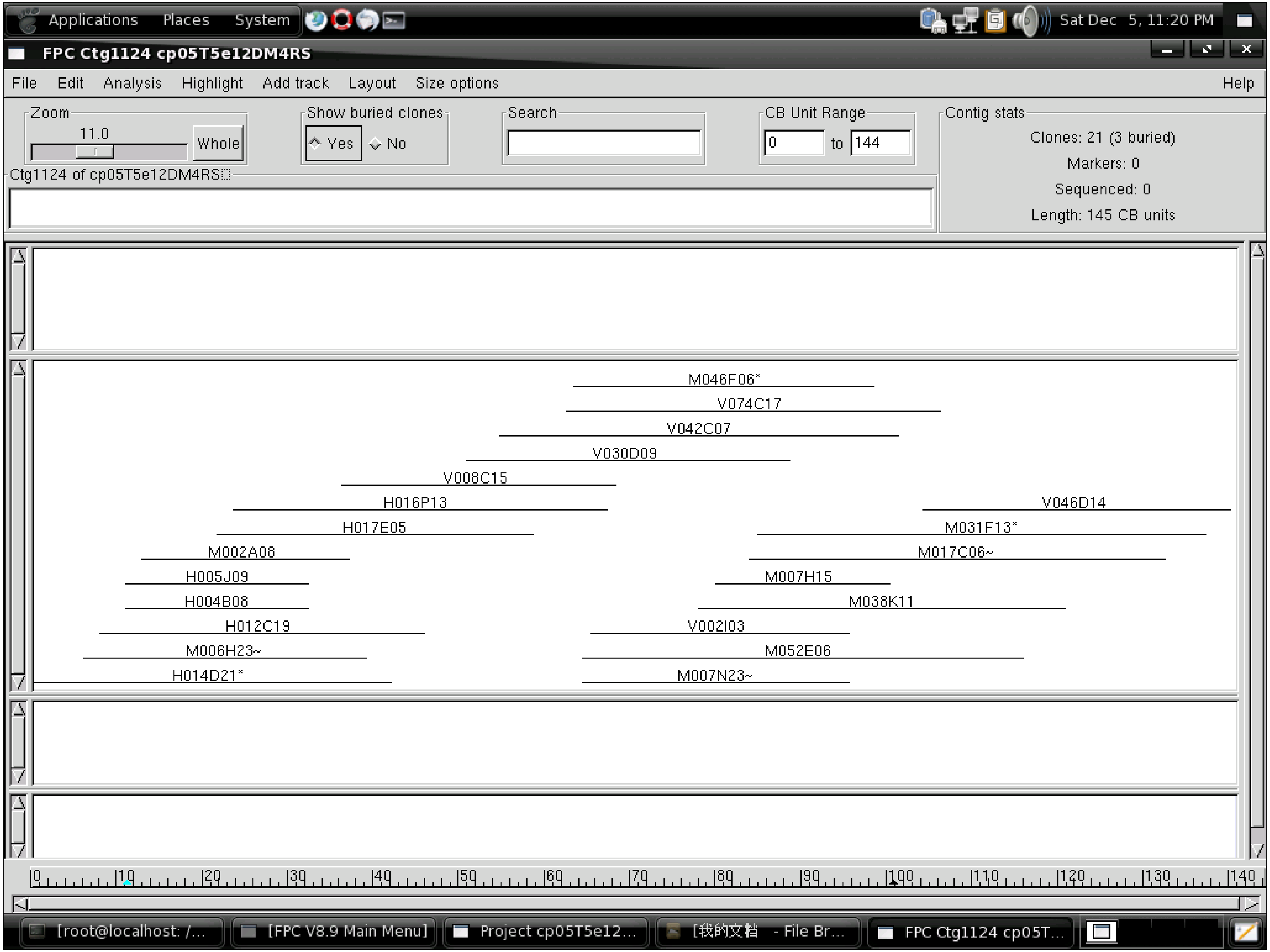

Applications Places System
Sat Dec 5, 11:20 PM
FPC Ctg1124 cp05T5e12DM4RS
File Edit Analysis Highlight Add track Layout Size options Help
Zoom
11.0
Whole
Show buried clones
Yes
No
Search
CB Unit Range
0
to
144
Contig stats
Clones: 21 (3 buried)
Markers: 0
Sequenced: 0
Length: 145 CB units
Ctg1124 of cp05T5e12DM4RS
M046F06*
V074C17
V042C07
V030D09
V008C15
H016P13
V046D14
H017E05
M031F13*
M002A08
M017C06~
H005J09
M007H15
H004B08
M038K11
H012C19
V002I03
M006H23~
M052E06
H014D21*
M007N23~
0 10 20 30 40 50 60 70 80 90 100 110 120 130 140
[root@localhost: /...
[FPC V8.9 Main Menu]
Project cp05T5e12...
[我的文档 - File Br...
FPC Ctg1124 cp05T...

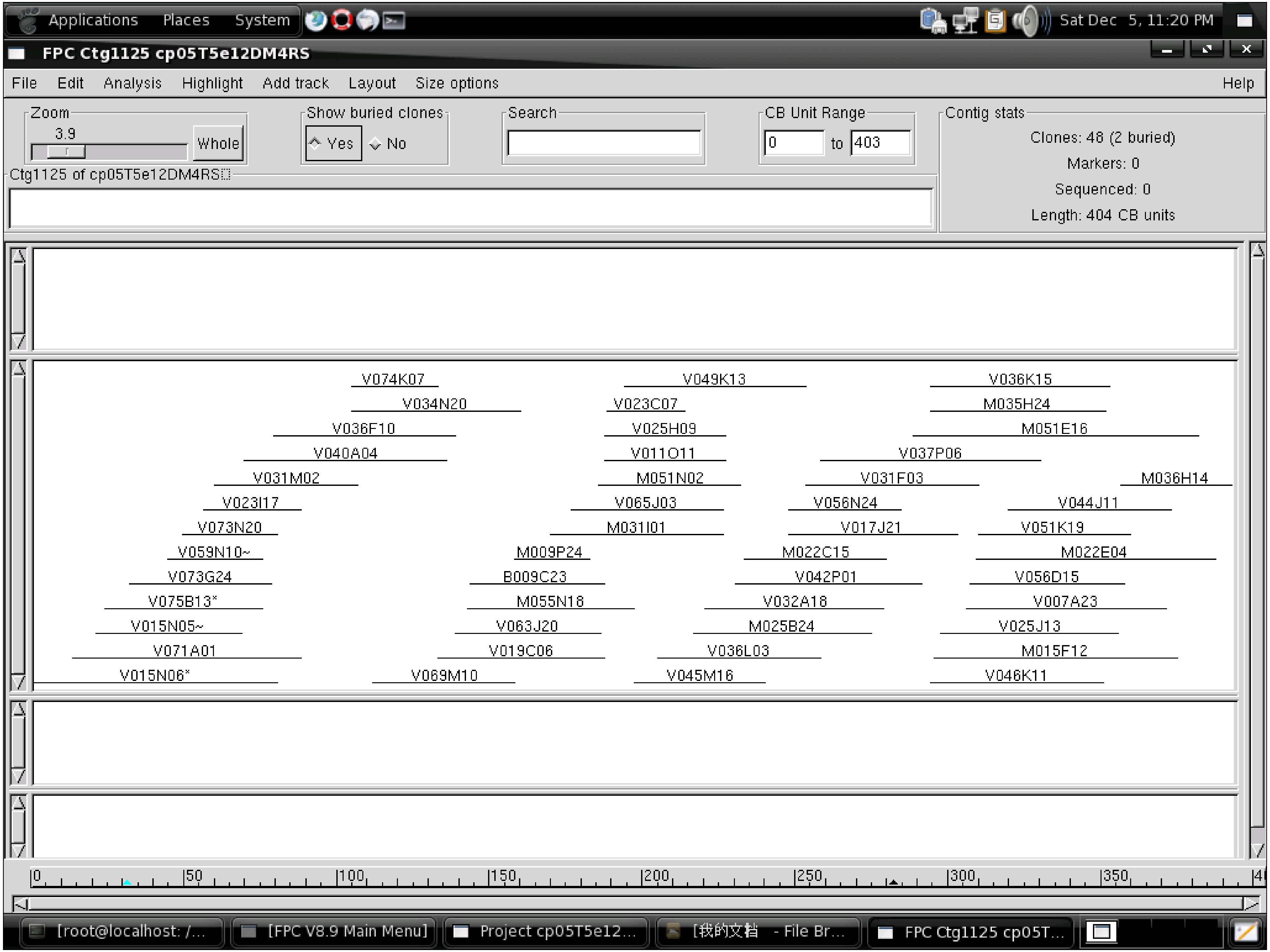
Applications Places System
Sat Dec 5, 11:20 PM
FPC Ctg1125 cp05T5e12DM4RS
File Edit Analysis Highlight Add track Layout Size options Help
Zoom
3.9
Whole
Show buried clones
Yes No
Search
CB Unit Range
0 to 403
Contig stats
Clones: 48 (2 buried)
Markers: 0
Sequenced: 0
Length: 404 CB units
Ctg1125 of cp05T5e12DM4RS
V074K07
V049K13
V036K15
V034N20
V023C07
M035H24
V036F10
V025H09
M051E16
V040A04
V011O11
V037P06
V031M02
M051N02
V031F03
M036H14
V023I17
V065J03
V056N24
V044J11
V073N20
M031I01
V017J21
V051K19
V059N10~
M009P24
M022C15
M022E04
V073G24
B009C23
V042P01
V056D15
V075B13*
M055N18
V032A18
V007A23
V015N05~
V063J20
M025B24
V025J13
V071A01
V019C06
V036L03
M015F12
V015N06*
V069M10
V045M16
V046K11
0 50 100 150 200 250 300 350
[root@localhost: /...
[FPC V8.9 Main Menu]
Project cp05T5e12...
[我的文档 - File Br...
FPC Ctg1125 cp05T...

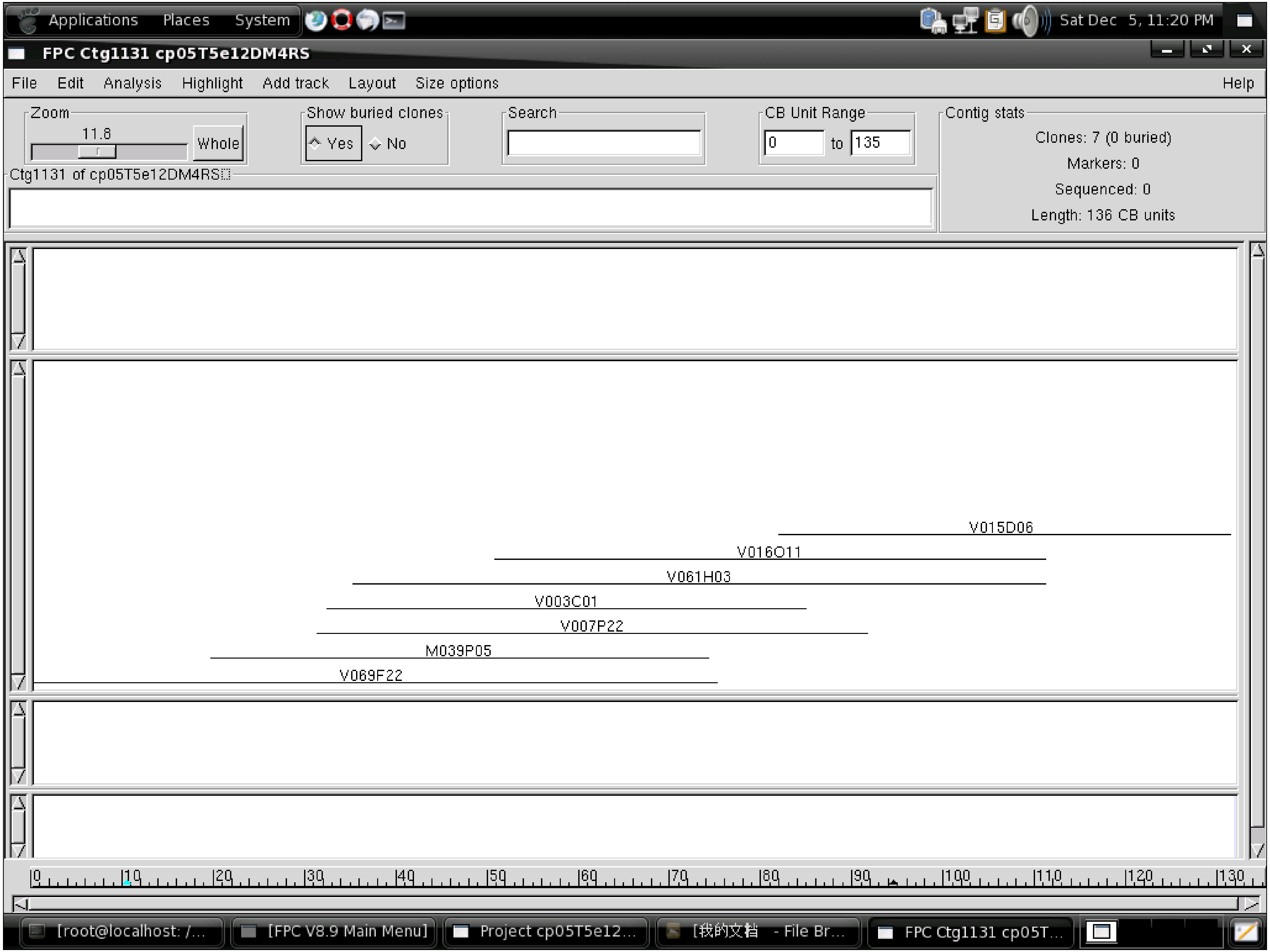
Applications
Places
System
Sat Dec 5, 11:20 PM
FPC Ctg1131 cp05T5e12DM4RS
File
Edit
Analysis
Highlight
Add track
Layout
Size options
Help
Zoom
11.8
Whole
Show buried clones
Yes
No
Search
CB Unit Range
0
to
135
Contig stats
Clones: 7 (0 buried)
Markers: 0
Sequenced: 0
Length: 136 CB units
Ctg1131 of cp05T5e12DM4RS
V015D06
V016O11
V061H03
V003C01
V007P22
M039P05
V069F22
0
10
20
30
40
50
60
70
80
90
100
110
120
130
[root@localhost: /...
[FPC V8.9 Main Menu]
Project cp05T5e12...
FPC Ctg1131 cp05T...

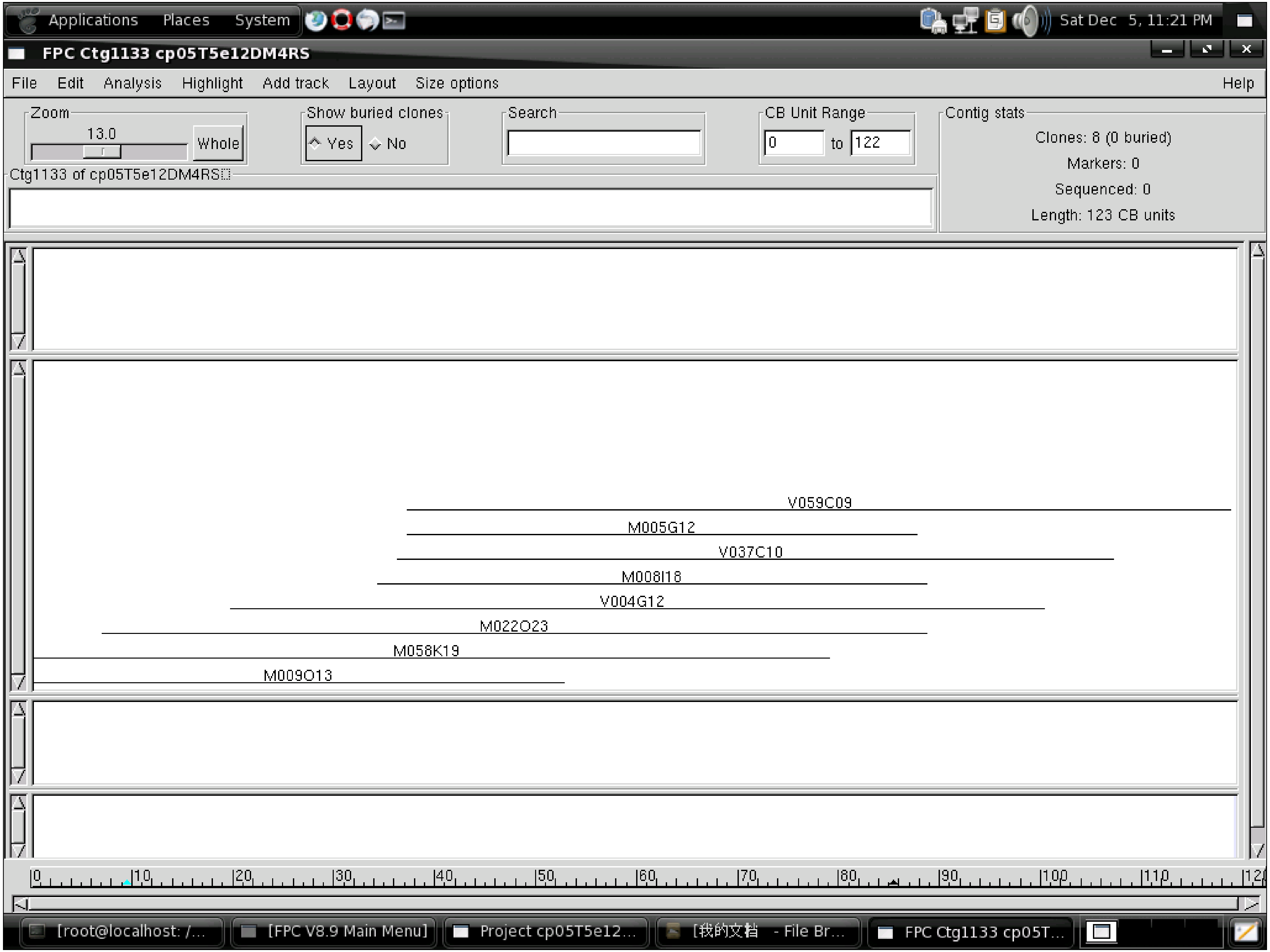
Applications Places System
Sat Dec 5, 11:21 PM
FPC Ctg1133 cp05T5e12DM4RS
File Edit Analysis Highlight Add track Layout Size options
Help
Zoom
13.0
Whole
Show buried clones
Yes
No
Search
CB Unit Range
0
to
122
Contig stats
Clones: 8 (0 buried)
Markers: 0
Sequenced: 0
Length: 123 CB units
Ctg1133 of cp05T5e12DM4RS
V059C09
M005G12
V037C10
M008I18
V004G12
M022O23
M058K19
M009O13
0 10 20 30 40 50 60 70 80 90 100 110
[root@localhost: /...
[FPC V8.9 Main Menu]
Project cp05T5e12...
[我的文档 - File Br...
FPC Ctg1133 cp05T...

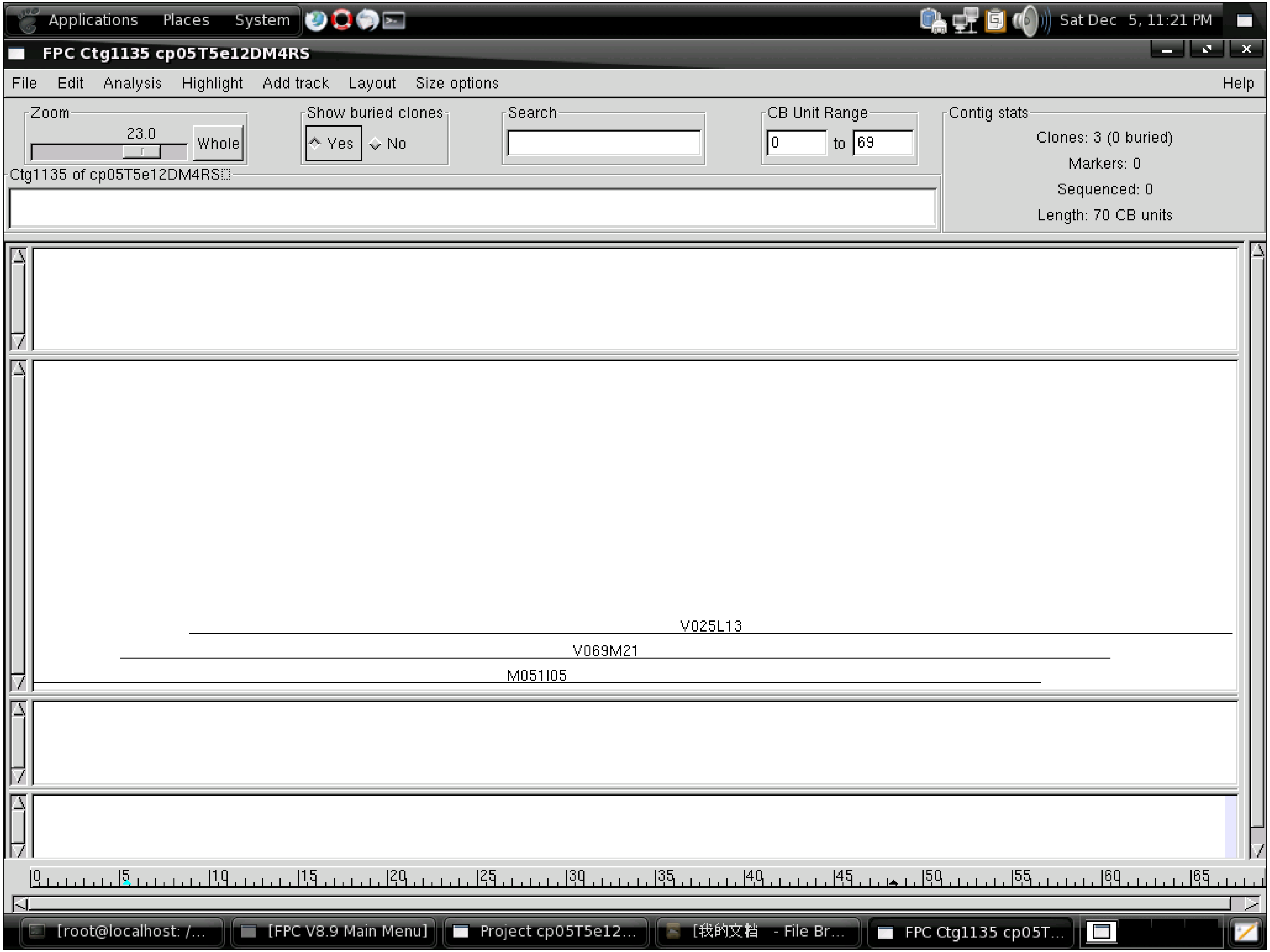
Applications Places System
Sat Dec 5, 11:21 PM
FPC Ctg1135 cp05T5e12DM4RS
File Edit Analysis Highlight Add track Layout Size options Help
Zoom
23.0
Whole
Show buried clones
Yes
No
Search
CB Unit Range
0 to 69
Contig stats
Clones: 3 (0 buried)
Markers: 0
Sequenced: 0
Length: 70 CB units
Ctg1135 of cp05T5e12DM4RS
V025L13
V069M21
M051I05
0 5 10 15 20 25 30 35 40 45 50 55 60 65
[root@localhost: /...
[FPC V8.9 Main Menu]
Project cp05T5e12...
[我的文档 - File Br...
FPC Ctg1135 cp05T...

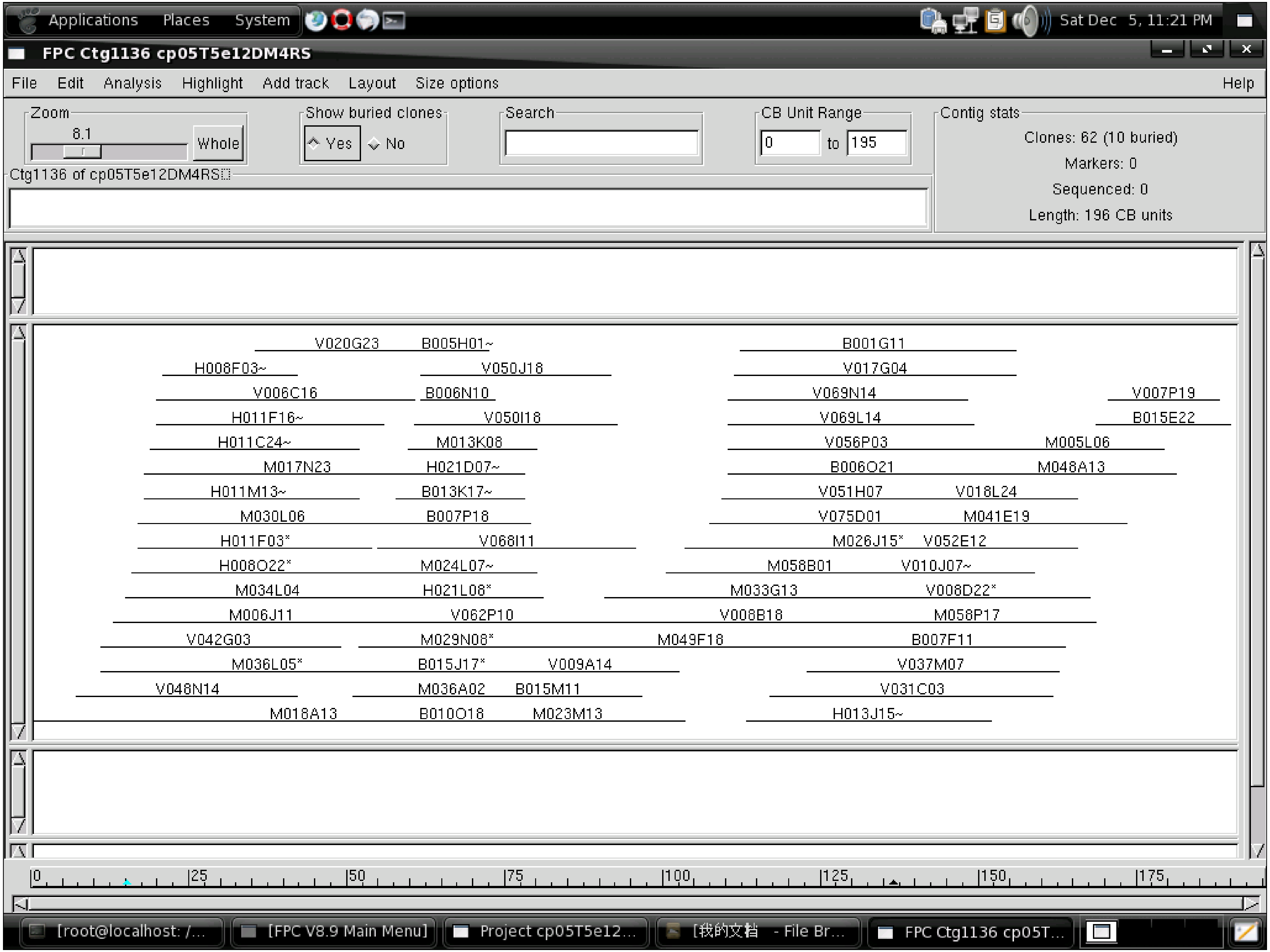
Applications Places System
Sat Dec 5, 11:21 PM
FPC Ctg1136 cp05T5e12DM4RS
File Edit Analysis Highlight Add track Layout Size options Help
Zoom
8.1
Whole
Show buried clones
Yes No
Search
CB Unit Range
0 to 195
Contig stats
Clones: 62 (10 buried)
Markers: 0
Sequenced: 0
Length: 196 CB units
Ctg1136 of cp05T5e12DM4RS
V020G23 B005H01~ B001G11
H008F03~ V050J18 V017G04
V006C16 B006N10 V069N14 V007P19
H011F16~ V050I18 V069L14 B015E22
H011C24~ M013K08 V056P03 M005L06
M017N23 H021D07~ B006O21 M048A13
H011M13~ B013K17~ V051H07 V018L24
M030L06 B007P18 V075D01 M041E19
H011F03* V068I11 M026J15* V052E12
H008O22* M024L07~ M058B01 V010J07~
M034L04 H021L08* M033G13 V008D22*
M006J11 V062P10 V008B18 M058P17
V042G03 M029N08* M049F18 B007F11
M036L05* B015J17* V009A14 V037M07
V048N14 M036A02 B015M11 V031C03
M018A13 B010O18 M023M13 H013J15~
0 25 50 75 100 125 150 175
[root@localhost: /... [FPC V8.9 Main Menu] Project cp05T5e12... [我的文档 - File Br... FPC Ctg1136 cp05T...

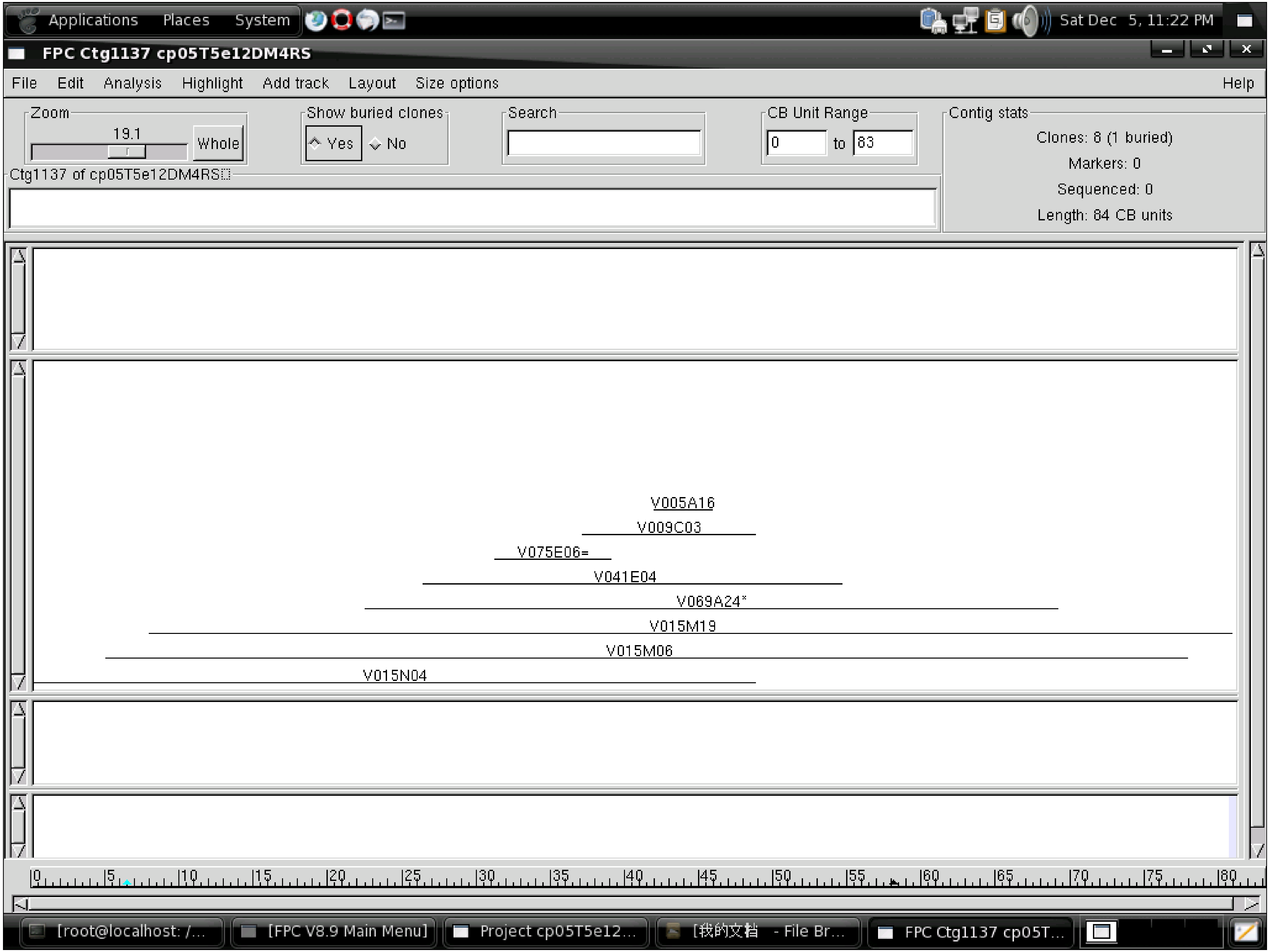
Applications Places System
Sat Dec 5, 11:22 PM
FPC Ctg1137 cp05T5e12DM4RS
File Edit Analysis Highlight Add track Layout Size options Help
Zoom
19.1
Whole
Show buried clones
Yes No
Search
CB Unit Range
0 to 83
Contig stats
Clones: 8 (1 buried)
Markers: 0
Sequenced: 0
Length: 84 CB units
Ctg1137 of cp05T5e12DM4RS
V005A16
V009C03
V075E06=
V041E04
V069A24*
V015M19
V015M06
V015N04
0 5 10 15 20 25 30 35 40 45 50 55 60 65 70 75 80
[root@localhost: /...
[FPC V8.9 Main Menu]
Project cp05T5e12...
[我的文档 - File Br...
FPC Ctg1137 cp05T...

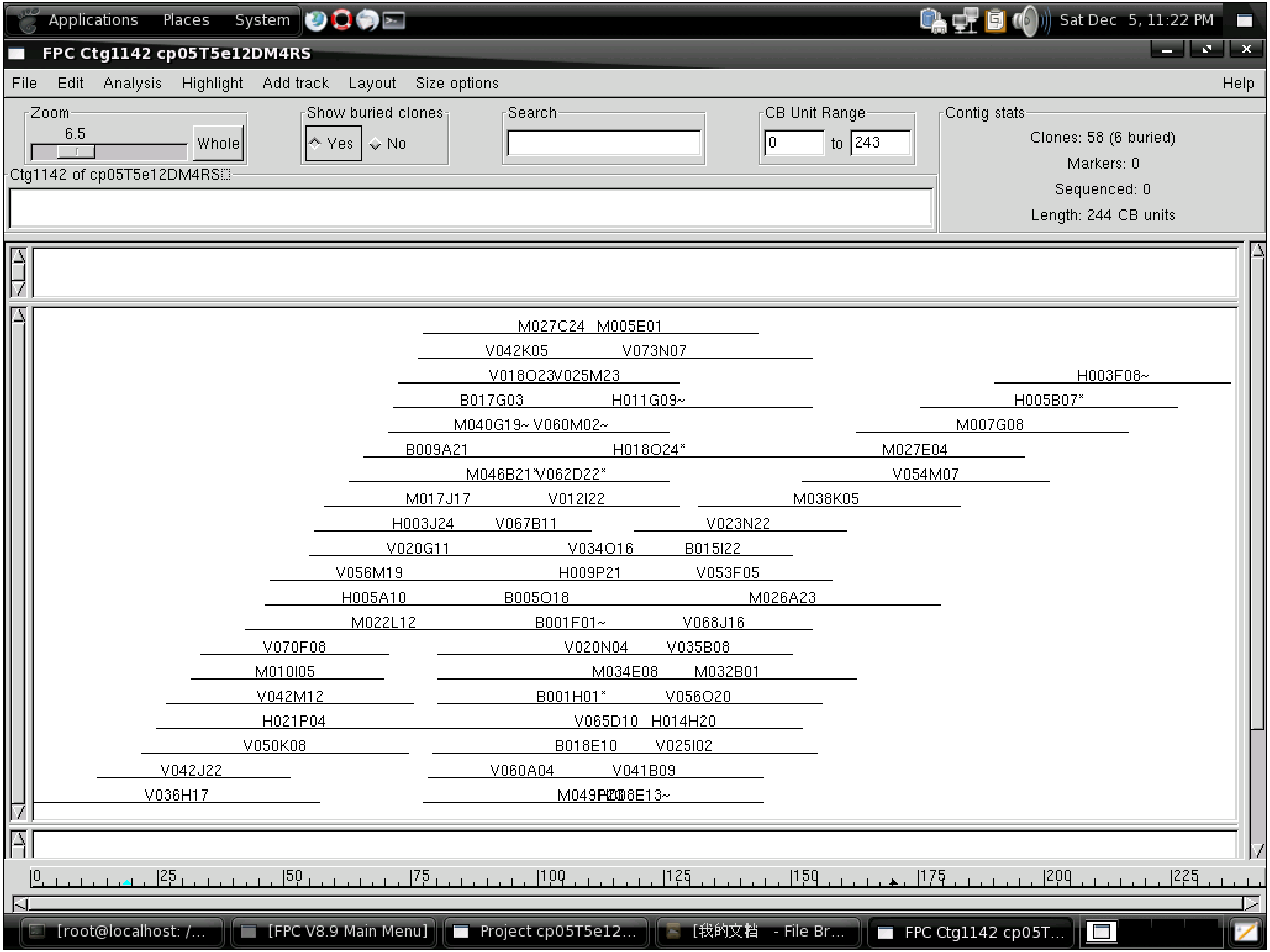
Applications Places System
Sat Dec 5, 11:22 PM
FPC Ctg1142 cp05T5e12DM4RS
File Edit Analysis Highlight Add track Layout Size options Help
Zoom
6.5
Whole
Show buried clones
Yes
No
Search
CB Unit Range
0 to 243
Contig stats
Clones: 58 (6 buried)
Markers: 0
Sequenced: 0
Length: 244 CB units
Ctg1142 of cp05T5e12DM4RS
M027C24 M005E01
V042K05 V073N07
V018O23V025M23
H003F08~
B017G03 H011G09~
H005B07*
M040G19~ V060M02~
M007G08
B009A21 H018O24*
M027E04
M046B21*V062D22*
V054M07
M017J17 V012I22
M038K05
H003J24 V067B11
V023N22
V020G11 V034O16 B015I22
V056M19 H009P21 V053F05
H005A10 B005O18 M026A23
M022L12 B001F01~ V068J16
V070F08 V020N04 V035B08
M010I05 M034E08 M032B01
V042M12 B001H01* V056O20
H021P04 V065D10 H014H20
V050K08 B018E10 V025I02
V042J22 V060A04 V041B09
V036H17 M049P20 B08E13~
0 25 50 75 100 125 150 175 200 225
[root@localhost: /...
[FPC V8.9 Main Menu]
Project cp05T5e12...
[我的文档 - File Br...
FPC Ctg1142 cp05T...

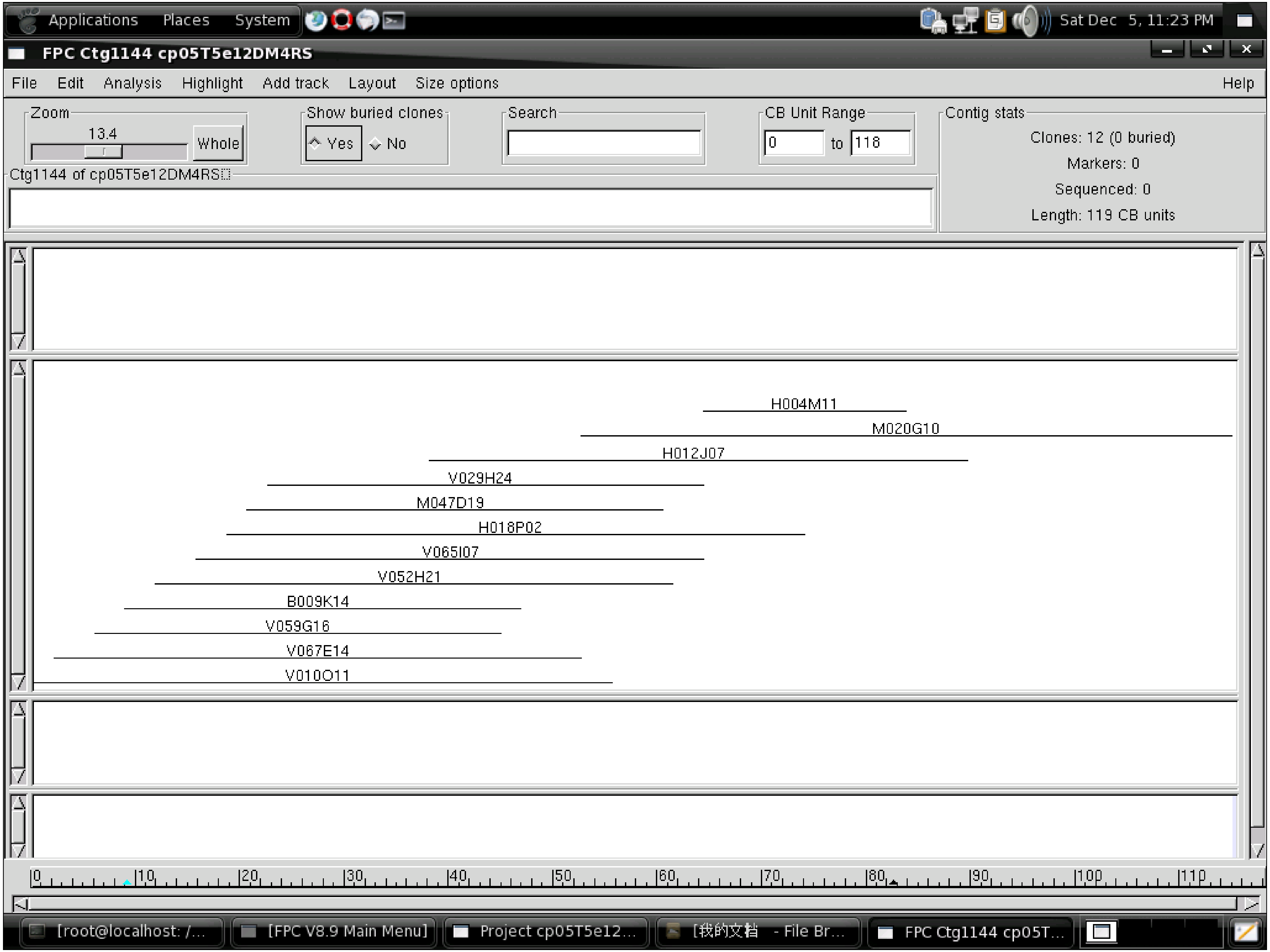
Applications Places System
Sat Dec 5, 11:23 PM
FPC Ctg1144 cp05T5e12DM4RS
File Edit Analysis Highlight Add track Layout Size options Help
Zoom
13.4
Whole
Show buried clones
Yes No
Search
CB Unit Range
0 to 118
Contig stats
Clones: 12 (0 buried)
Markers: 0
Sequenced: 0
Length: 119 CB units
Ctg1144 of cp05T5e12DM4RS
H004M11
M020G10
H012J07
V029H24
M047D19
H018P02
V065I07
V052H21
B009K14
V059G16
V067E14
V010O11
0 10 20 30 40 50 60 70 80 90 100 110
[root@localhost: /...
[FPC V8.9 Main Menu]
Project cp05T5e12...
FPC Ctg1144 cp05T...

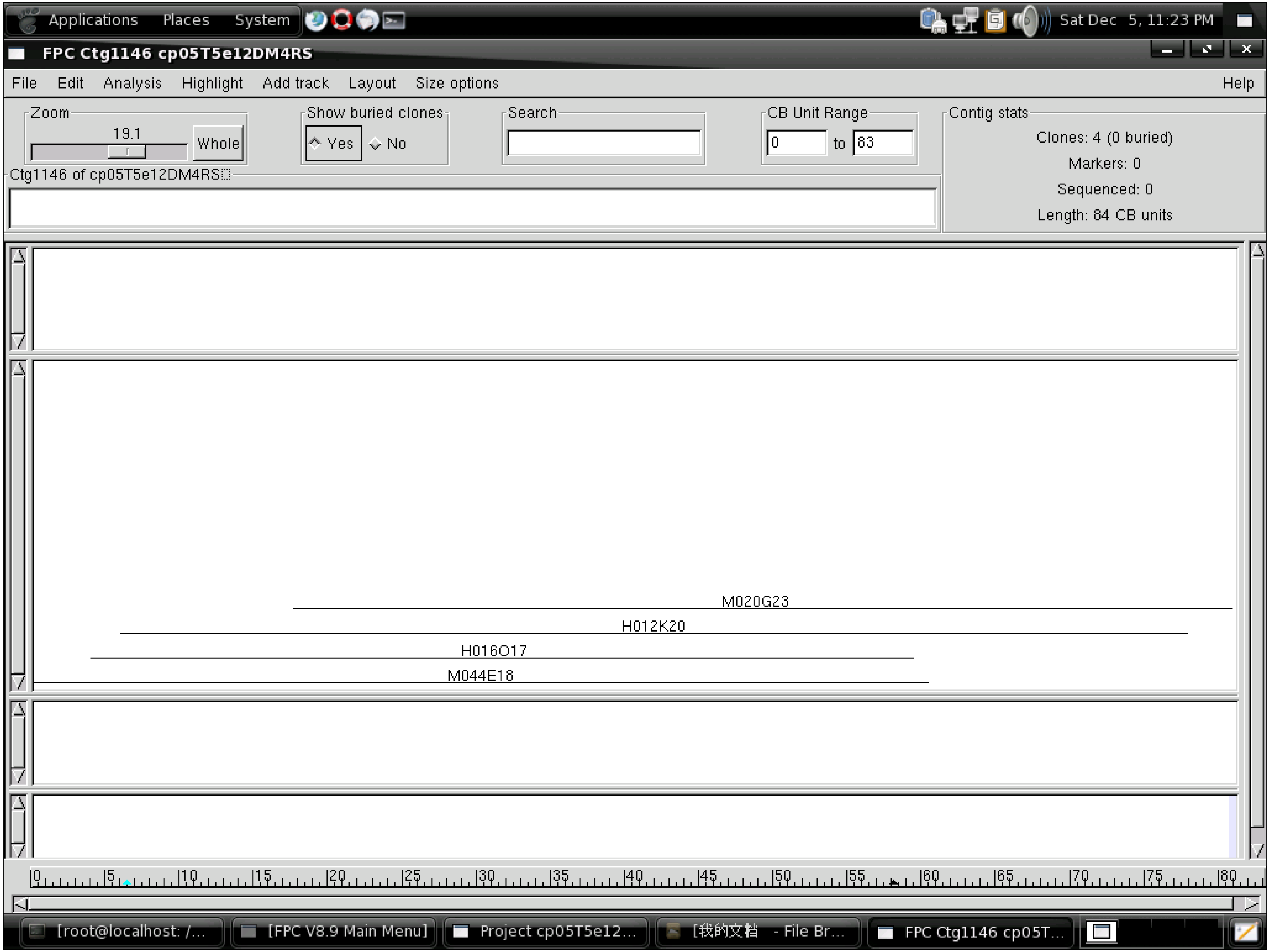
Applications
Places
System
Sat Dec 5, 11:23 PM
FPC Ctg1146 cp05T5e12DM4RS
File
Edit
Analysis
Highlight
Add track
Layout
Size options
Help
Zoom
19.1
Whole
Show buried clones
Yes
No
Search
CB Unit Range
0
to
83
Contig stats
Clones: 4 (0 buried)
Markers: 0
Sequenced: 0
Length: 84 CB units
Ctg1146 of cp05T5e12DM4RS
M020G23
H012K20
H016O17
M044E18
0 5 10 15 20 25 30 35 40 45 50 55 60 65 70 75 80
[root@localhost: /...
[FPC V8.9 Main Menu]
Project cp05T5e12...
[我的文档 - File Br...
FPC Ctg1146 cp05T...

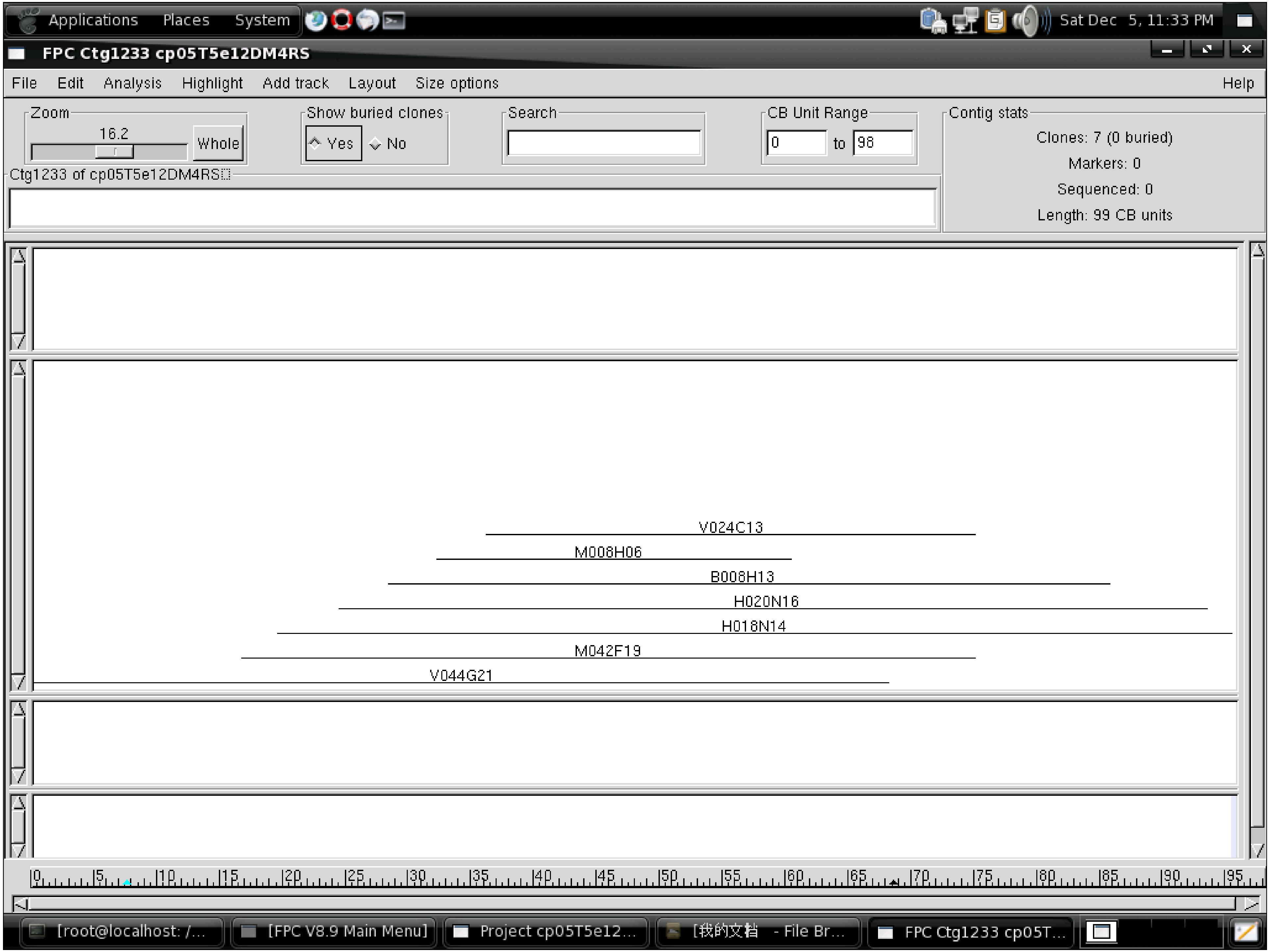

Applications
Places
System
Sat Dec 5, 11:33 PM
FPC Ctg1233 cp05T5e12DM4RS
File
Edit
Analysis
Highlight
Add track
Layout
Size options
Help
Zoom
16.2
Whole
Show buried clones
Yes
No
Search
CB Unit Range
0
to
98
Contig stats
Clones: 7 (0 buried)
Markers: 0
Sequenced: 0
Length: 99 CB units
Ctg1233 of cp05T5e12DM4RS
V024C13
M008H06
B008H13
H020N16
H018N14
M042F19
V044G21
0
5
10
15
20
25
30
35
40
45
50
55
60
65
70
75
80
85
90
95
[root@localhost: /...
[FPC V8.9 Main Menu]
Project cp05T5e12...
[我的文档 - File Br...
FPC Ctg1233 cp05T...

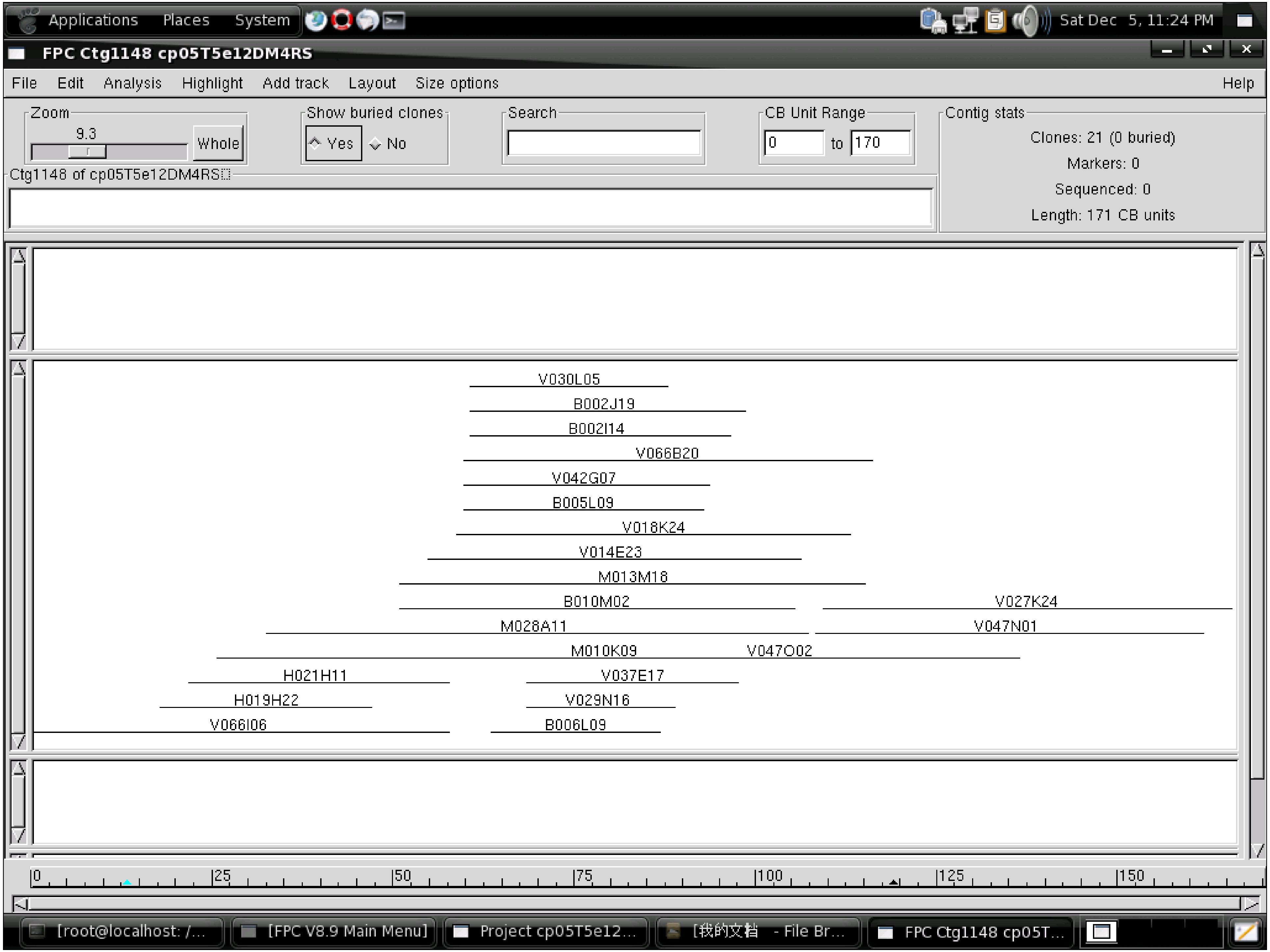
Applications Places System
Sat Dec 5, 11:24 PM
FPC Ctg1148 cp05T5e12DM4RS
File Edit Analysis Highlight Add track Layout Size options Help
Zoom
9.3
Whole
Show buried clones
Yes No
Search
CB Unit Range
0 to 170
Contig stats
Clones: 21 (0 buried)
Markers: 0
Sequenced: 0
Length: 171 CB units
Ctg1148 of cp05T5e12DM4RS
V030L05
B002J19
B002I14
V066B20
V042G07
B005L09
V018K24
V014E23
M013M18
B010M02
V027K24
M028A11
V047N01
M010K09
V047O02
H021H11
V037E17
H019H22
V029N16
V066I06
B006L09
0 25 50 75 100 125 150
[root@localhost: /...
[FPC V8.9 Main Menu]
Project cp05T5e12...
[我的文档 - File Br...
FPC Ctg1148 cp05T...

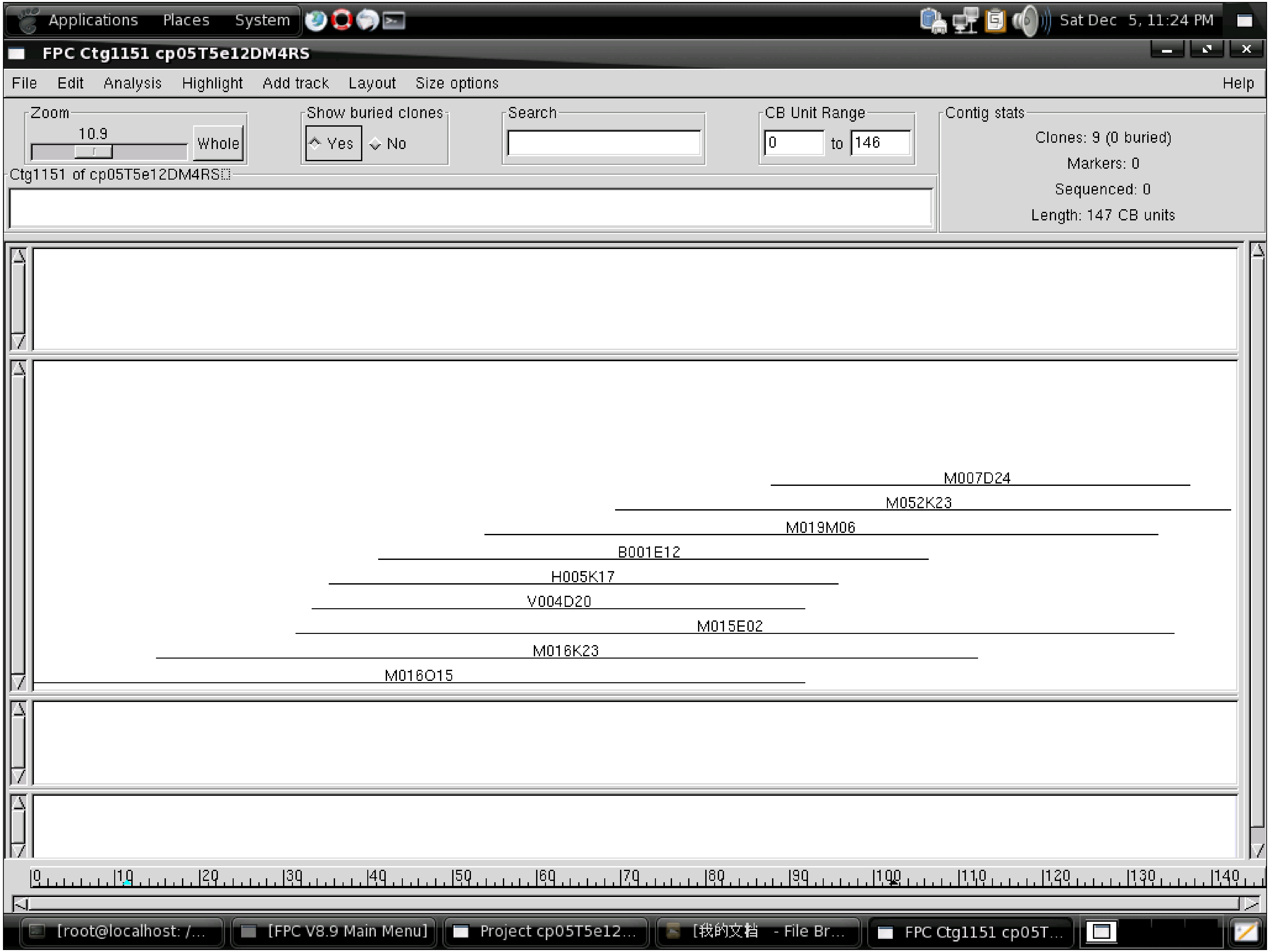
Applications Places System
Sat Dec 5, 11:24 PM
FPC Ctg1151 cp05T5e12DM4RS
File Edit Analysis Highlight Add track Layout Size options Help
Zoom
10.9
Whole
Show buried clones
Yes
No
Search
CB Unit Range
0 to 146
Contig stats
Clones: 9 (0 buried)
Markers: 0
Sequenced: 0
Length: 147 CB units
Ctg1151 of cp05T5e12DM4RS
M007D24
M052K23
M019M06
B001E12
H005K17
V004D20
M015E02
M016K23
M016O15
0 10 20 30 40 50 60 70 80 90 100 110 120 130 140
[root@localhost: /...
[FPC V8.9 Main Menu]
Project cp05T5e12...
[我的文档 - File Br...
FPC Ctg1151 cp05T...

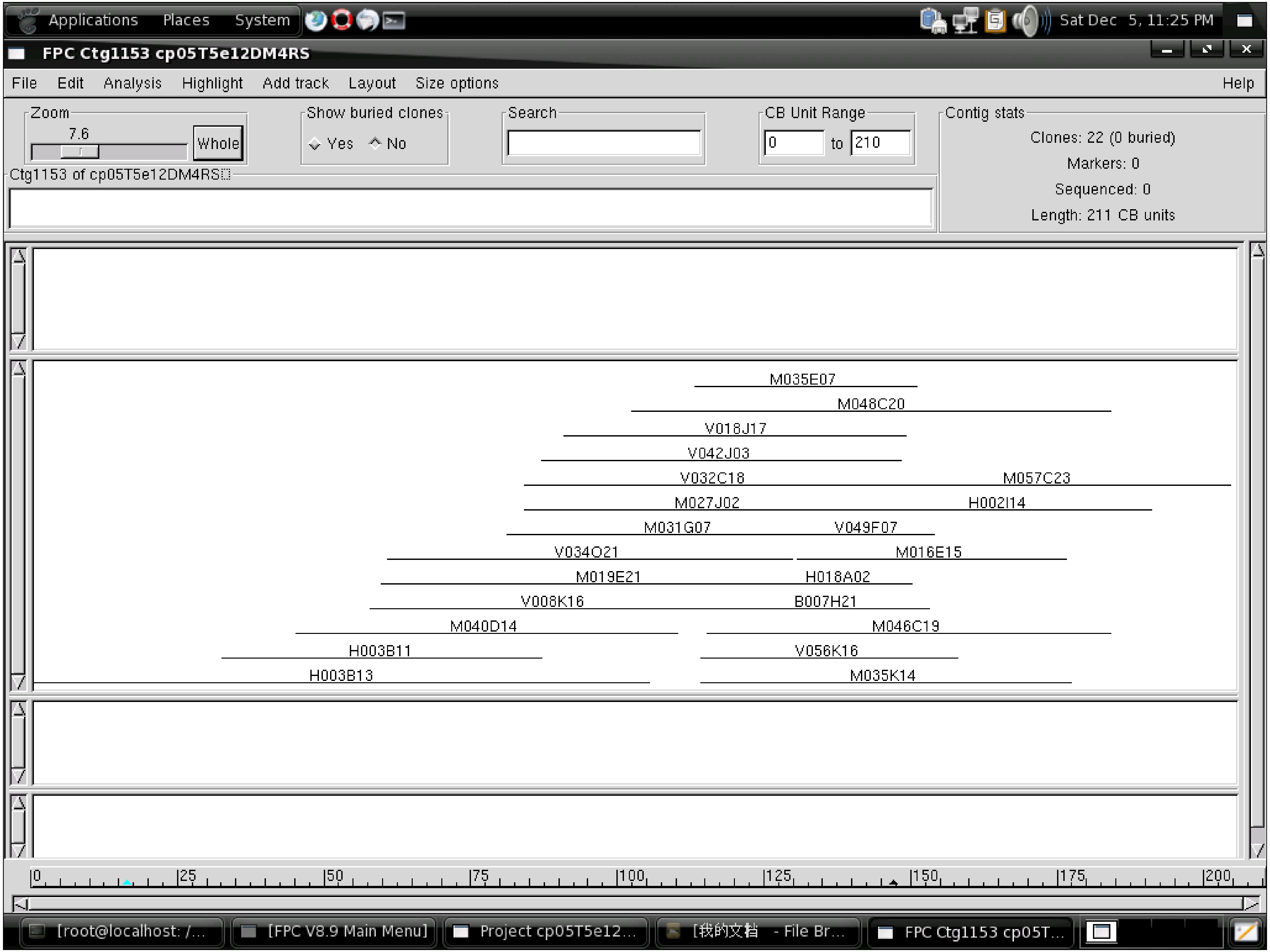
Applications Places System
Sat Dec 5, 11:25 PM
FPC Ctg1153 cp05T5e12DM4RS
File Edit Analysis Highlight Add track Layout Size options Help
Zoom
7.6
Whole
Show buried clones
Yes No
Search
CB Unit Range
0 to 210
Contig stats
Clones: 22 (0 buried)
Markers: 0
Sequenced: 0
Length: 211 CB units
Ctg1153 of cp05T5e12DM4RS
M035E07
M048C20
V018J17
V042J03
V032C18
M057C23
M027J02
H002I14
M031G07
V049F07
V034O21
M016E15
M019E21
H018A02
V008K16
B007H21
M040D14
M046C19
H003B11
V056K16
H003B13
M035K14
0 25 50 75 100 125 150 175 200
[root@localhost: /...
[FPC V8.9 Main Menu]
Project cp05T5e12...
FPC Ctg1153 cp05T...

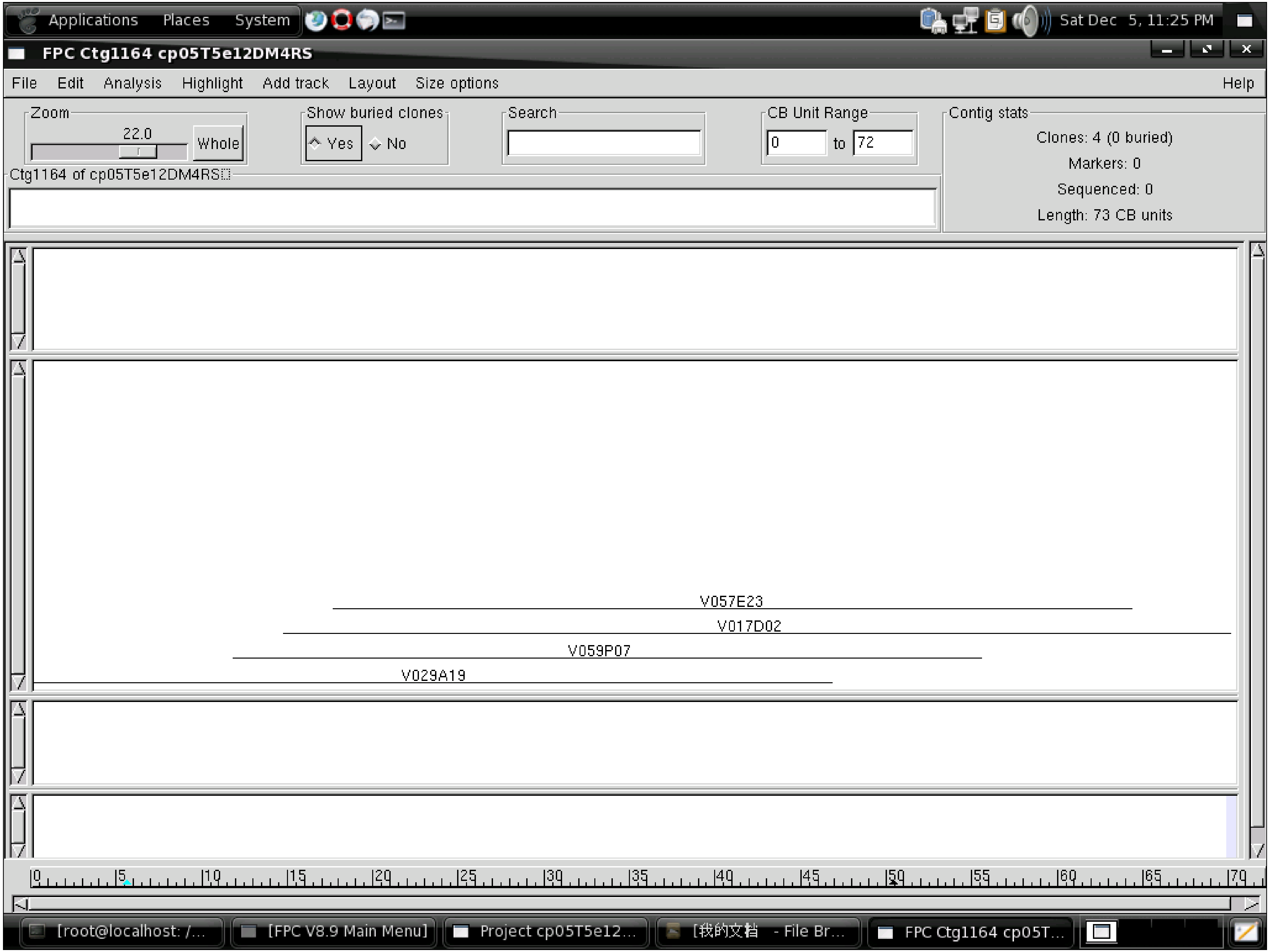
Applications Places System
Sat Dec 5, 11:25 PM
FPC Ctg1164 cp05T5e12DM4RS
File Edit Analysis Highlight Add track Layout Size options Help
Zoom
22.0
Whole
Show buried clones
Yes No
Search
CB Unit Range
0 to 72
Contig stats
Clones: 4 (0 buried)
Markers: 0
Sequenced: 0
Length: 73 CB units
Ctg1164 of cp05T5e12DM4RS
V057E23
V017D02
V059P07
V029A19
0 5 10 15 20 25 30 35 40 45 50 55 60 65 70
[root@localhost: /...
[FPC V8.9 Main Menu]
Project cp05T5e12...
[我的文档 - File Br...
FPC Ctg1164 cp05T...

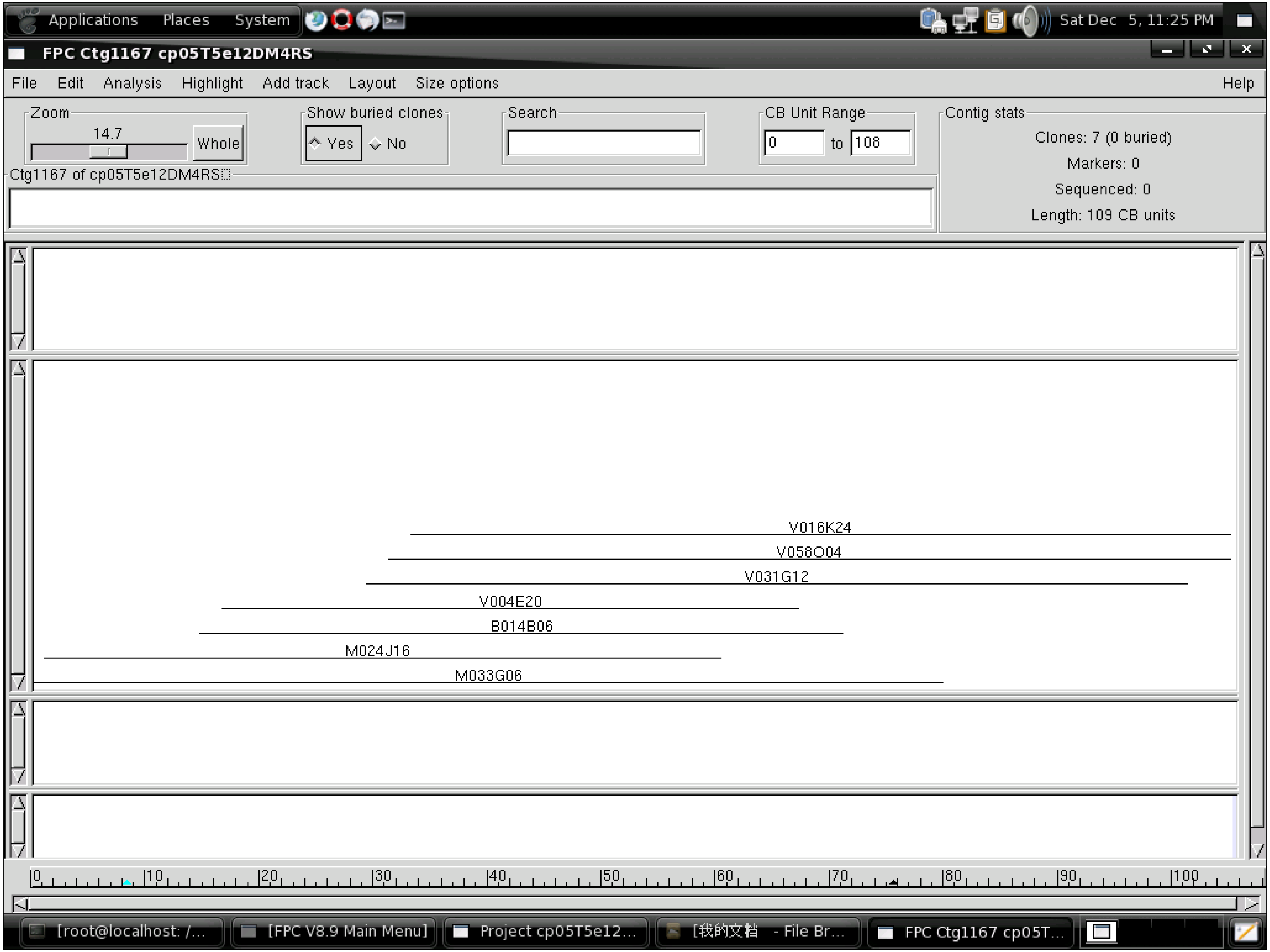
Applications Places System
Sat Dec 5, 11:25 PM
FPC Ctg1167 cp05T5e12DM4RS
File Edit Analysis Highlight Add track Layout Size options Help
Zoom
14.7
Whole
Show buried clones
Yes
No
Search
CB Unit Range
0
to
108
Contig stats
Clones: 7 (0 buried)
Markers: 0
Sequenced: 0
Length: 109 CB units
Ctg1167 of cp05T5e12DM4RS
V016K24
V058O04
V031G12
V004E20
B014B06
M024J16
M033G06
0 10 20 30 40 50 60 70 80 90 100
[root@localhost: /...
[FPC V8.9 Main Menu]
Project cp05T5e12...
[我的文档 - File Br...
FPC Ctg1167 cp05T...

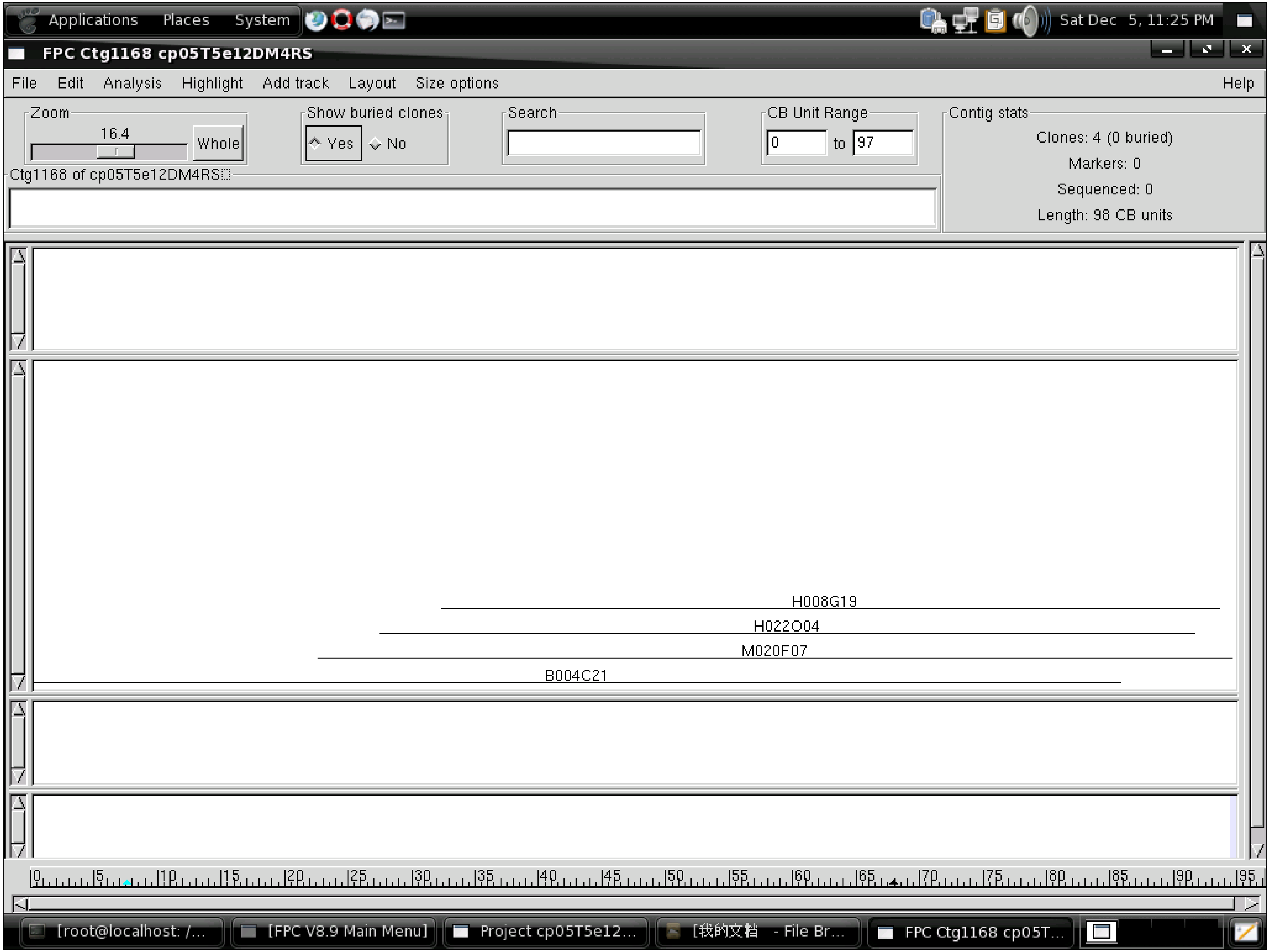
Applications Places System
Sat Dec 5, 11:25 PM
FPC Ctg1168 cp05T5e12DM4RS
File Edit Analysis Highlight Add track Layout Size options Help
Zoom
16.4
Whole
Show buried clones
Yes
No
Search
CB Unit Range
0 to 97
Contig stats
Clones: 4 (0 buried)
Markers: 0
Sequenced: 0
Length: 98 CB units
Ctg1168 of cp05T5e12DM4RS
H008G19
H022O04
M020F07
B004C21
0 5 10 15 20 25 30 35 40 45 50 55 60 65 70 75 80 85 90 95
[root@localhost: /...
[FPC V8.9 Main Menu]
Project cp05T5e12...
[我的文档 - File Br...
FPC Ctg1168 cp05T...

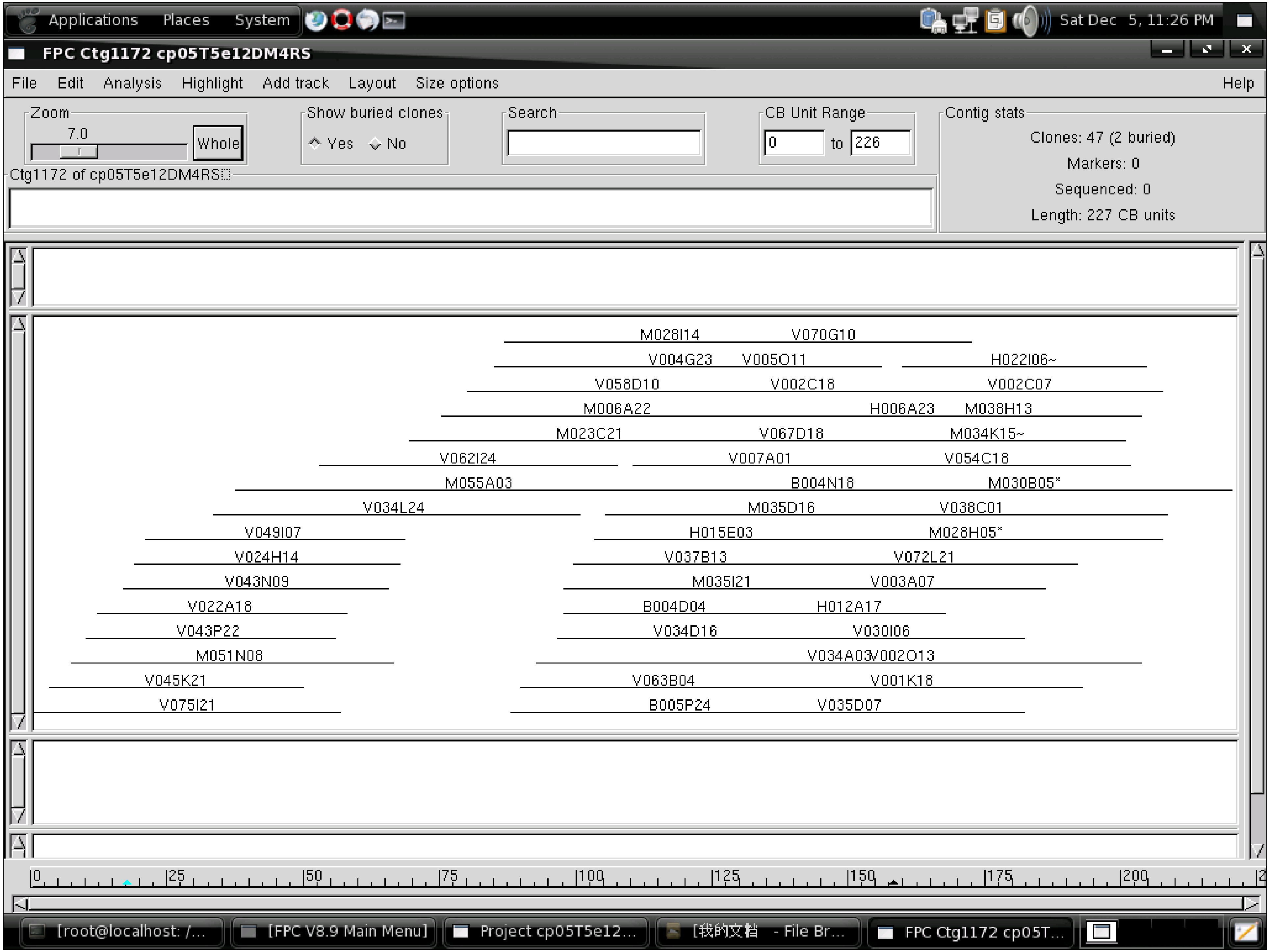
Applications Places System
Sat Dec 5, 11:26 PM
FPC Ctg1172 cp05T5e12DM4RS
File Edit Analysis Highlight Add track Layout Size options Help
Zoom
7.0
Whole
Show buried clones
Yes No
Search
CB Unit Range
0 to 226
Contig stats
Clones: 47 (2 buried)
Markers: 0
Sequenced: 0
Length: 227 CB units
Ctg1172 of cp05T5e12DM4RS
M028I14 V070G10
V004G23 V005O11 H022I06~
V058D10 V002C18 V002C07
M006A22 H006A23 M038H13
M023C21 V067D18 M034K15~
V062I24 V007A01 V054C18
M055A03 B004N18 M030B05*
V034L24 M035D16 V038C01
V049I07 H015E03 M028H05*
V024H14 V037B13 V072L21
V043N09 M035I21 V003A07
V022A18 B004D04 H012A17
V043P22 V034D16 V030I06
M051N08 V034A03V002O13
V045K21 V063B04 V001K18
V075I21 B005P24 V035D07
0 25 50 75 100 125 150 175 200
[root@localhost: /...
[FPC V8.9 Main Menu]
Project cp05T5e12...
[我的文档 - File Br...
FPC Ctg1172 cp05T...

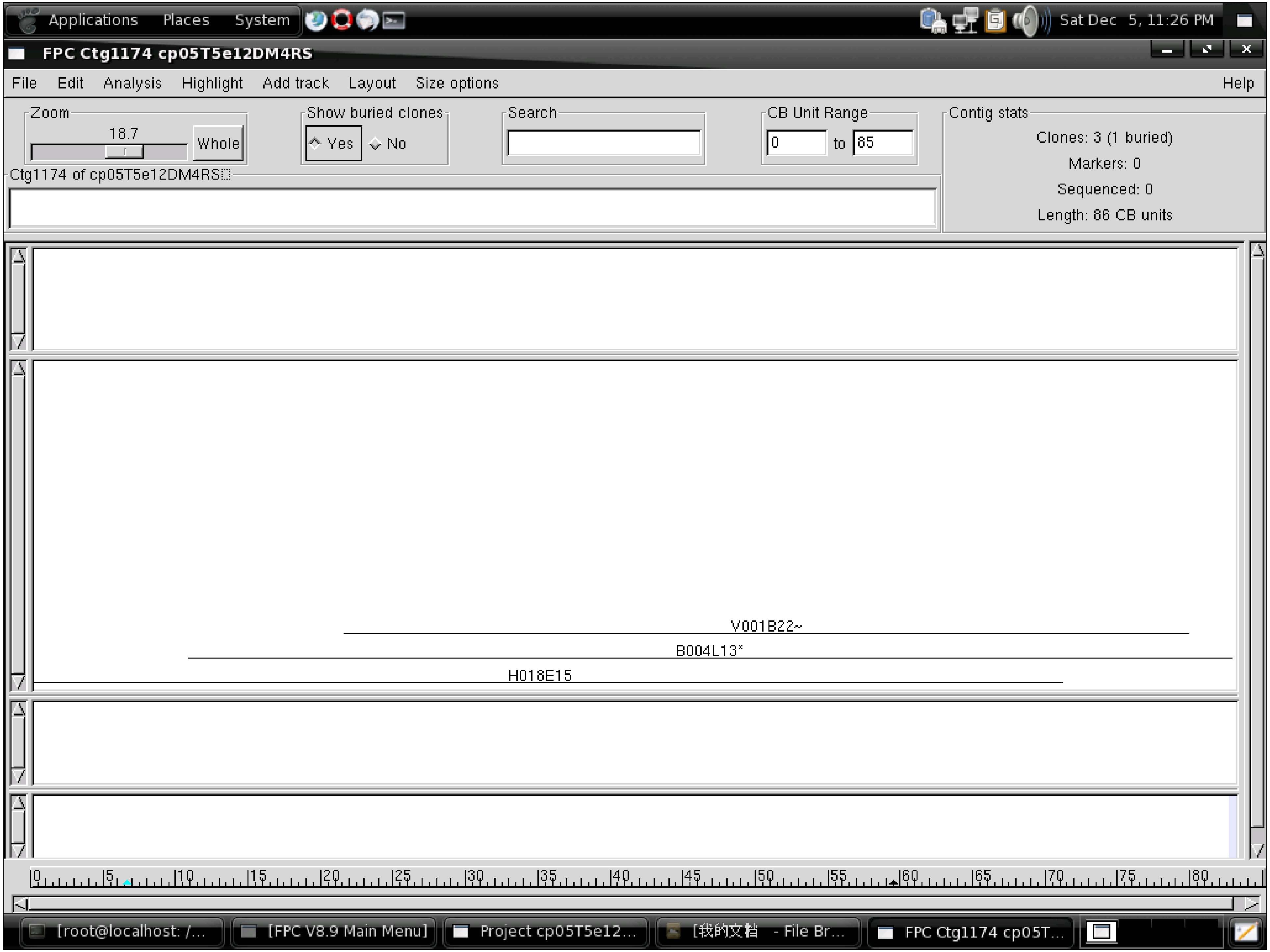
Applications Places System
Sat Dec 5, 11:26 PM
FPC Ctg1174 cp05T5e12DM4RS
File Edit Analysis Highlight Add track Layout Size options Help
Zoom
18.7
Whole
Show buried clones
Yes No
Search
CB Unit Range
0 to 85
Contig stats
Clones: 3 (1 buried)
Markers: 0
Sequenced: 0
Length: 86 CB units
Ctg1174 of cp05T5e12DM4RS
V001B22~
B004L13*
H018E15
0 5 10 15 20 25 30 35 40 45 50 55 60 65 70 75 80
[root@localhost: /...
[FPC V8.9 Main Menu]
Project cp05T5e12...
[我的文档 - File Br...
FPC Ctg1174 cp05T...

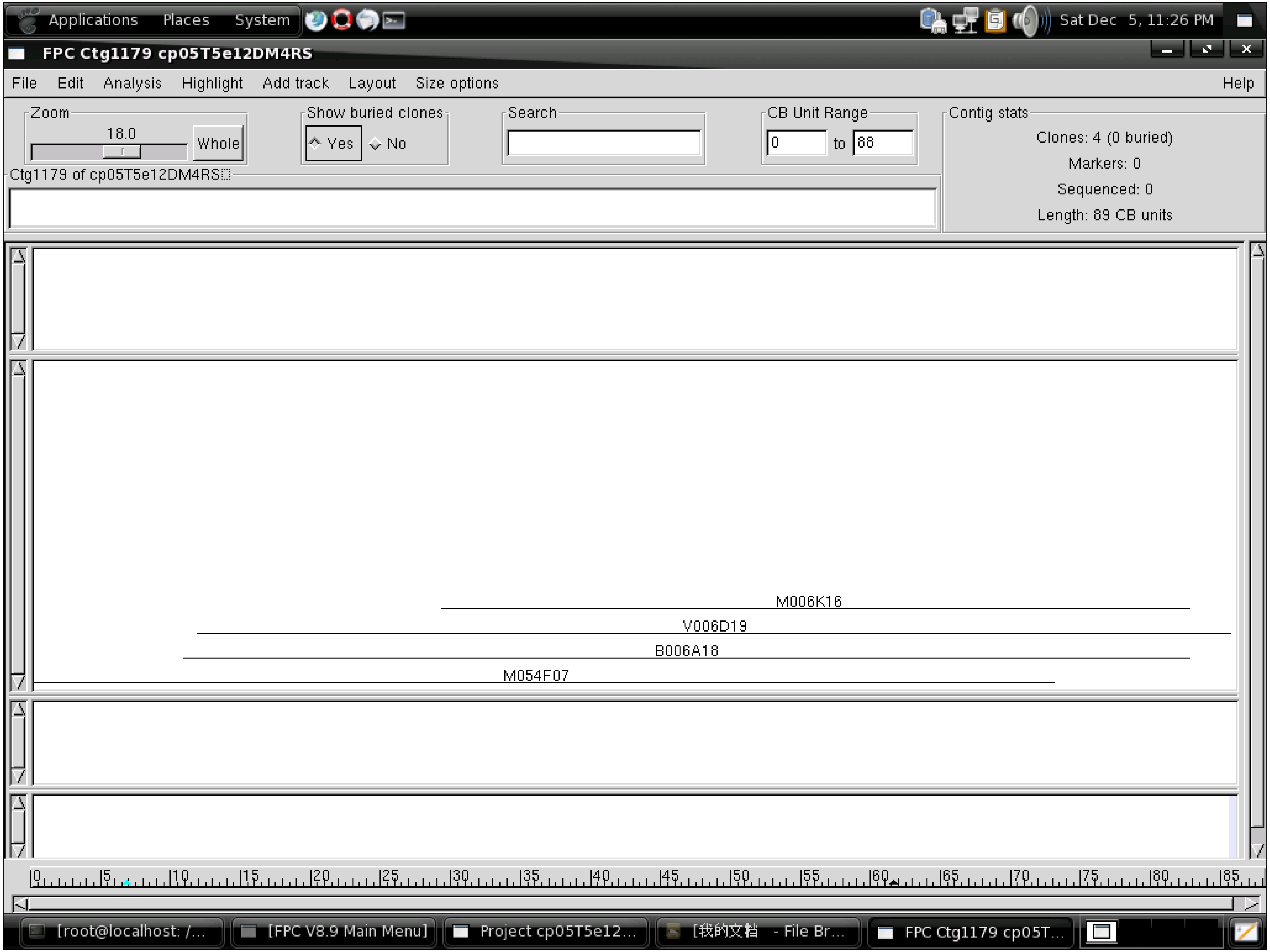
Applications
Places
System
Sat Dec 5, 11:26 PM
FPC Ctg1179 cp05T5e12DM4RS
File
Edit
Analysis
Highlight
Add track
Layout
Size options
Help
Zoom
18.0
Whole
Show buried clones
Yes
No
Search
CB Unit Range
0
to
88
Contig stats
Clones: 4 (0 buried)
Markers: 0
Sequenced: 0
Length: 89 CB units
Ctg1179 of cp05T5e12DM4RS
M006K16
V006D19
B006A18
M054F07
0 5 10 15 20 25 30 35 40 45 50 55 60 65 70 75 80 85
[root@localhost: /...
[FPC V8.9 Main Menu]
Project cp05T5e12...
[我的文档 - File Br...
FPC Ctg1179 cp05T...

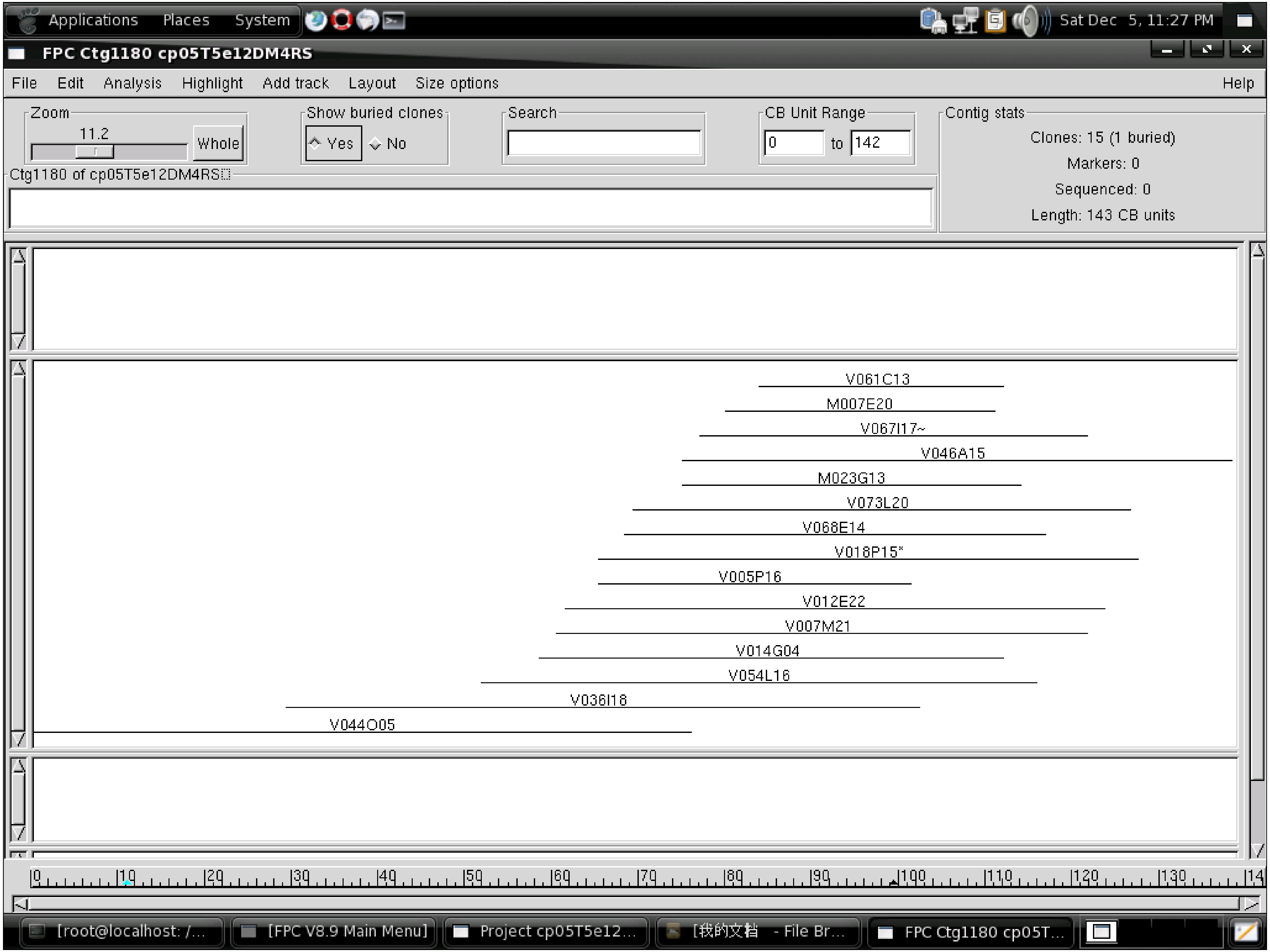
Applications Places System
Sat Dec 5, 11:27 PM
FPC Ctg1180 cp05T5e12DM4RS
File Edit Analysis Highlight Add track Layout Size options Help
Zoom
11.2
Whole
Show buried clones
Yes
No
Search
CB Unit Range
0
to
142
Contig stats
Clones: 15 (1 buried)
Markers: 0
Sequenced: 0
Length: 143 CB units
Ctg1180 of cp05T5e12DM4RS
V061C13
M007E20
V067I17~
V046A15
M023G13
V073L20
V068E14
V018P15*
V005P16
V012E22
V007M21
V014G04
V054L16
V036I18
V044O05
0 10 20 30 40 50 60 70 80 90 100 110 120 130
[root@localhost: /...
[FPC V8.9 Main Menu]
Project cp05T5e12...
[我的文档 - File Br...
FPC Ctg1180 cp05T...

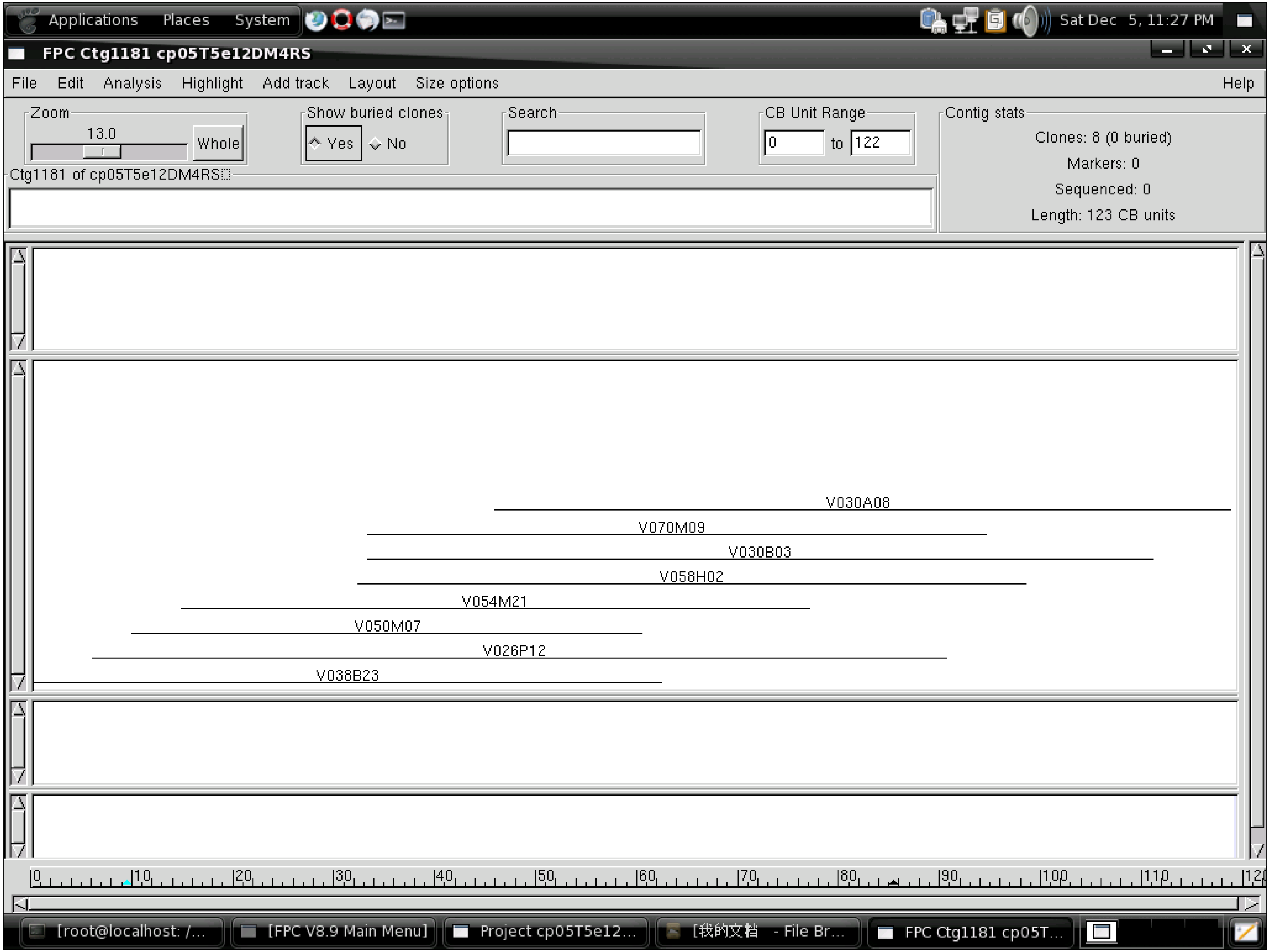
Applications Places System
Sat Dec 5, 11:27 PM
FPC Ctg1181 cp05T5e12DM4RS
File Edit Analysis Highlight Add track Layout Size options Help
Zoom
13.0
Whole
Show buried clones
Yes No
Search
CB Unit Range
0 to 122
Contig stats
Clones: 8 (0 buried)
Markers: 0
Sequenced: 0
Length: 123 CB units
Ctg1181 of cp05T5e12DM4RS
V030A08
V070M09
V030B03
V058H02
V054M21
V050M07
V026P12
V038B23
0 10 20 30 40 50 60 70 80 90 100 110
[root@localhost: /...
[FPC V8.9 Main Menu]
Project cp05T5e12...
[我的文档 - File Br...
FPC Ctg1181 cp05T...

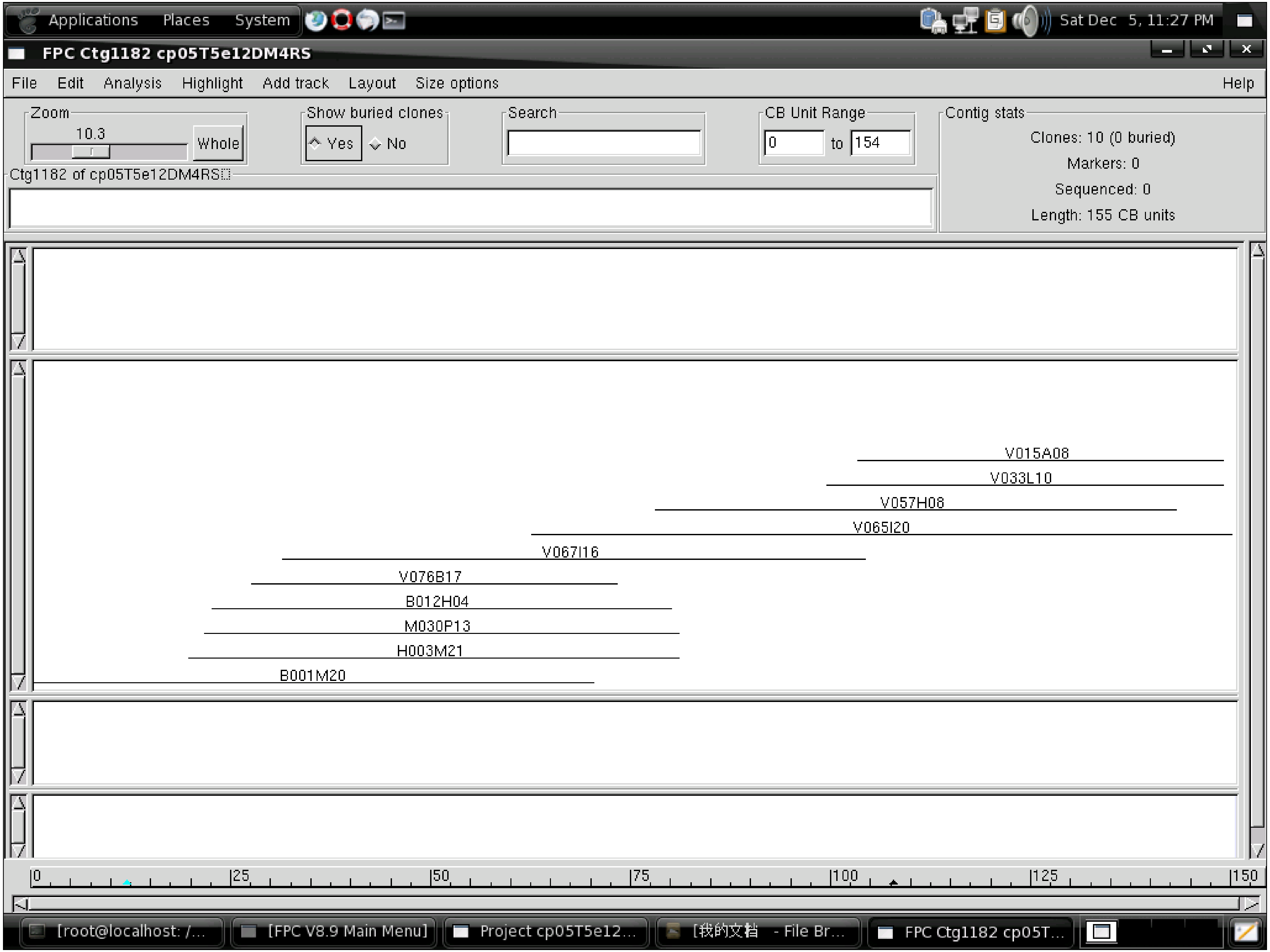
Applications Places System
Sat Dec 5, 11:27 PM
FPC Ctg1182 cp05T5e12DM4RS
File Edit Analysis Highlight Add track Layout Size options Help
Zoom
10.3
Whole
Show buried clones
Yes
No
Search
CB Unit Range
0 to 154
Contig stats
Clones: 10 (0 buried)
Markers: 0
Sequenced: 0
Length: 155 CB units
Ctg1182 of cp05T5e12DM4RS
V015A08
V033L10
V057H08
V065I20
V067I16
V076B17
B012H04
M030P13
H003M21
B001M20
0 25 50 75 100 125 150
[root@localhost: /...
[FPC V8.9 Main Menu]
Project cp05T5e12...
[我的文档 - File Br...
FPC Ctg1182 cp05T...

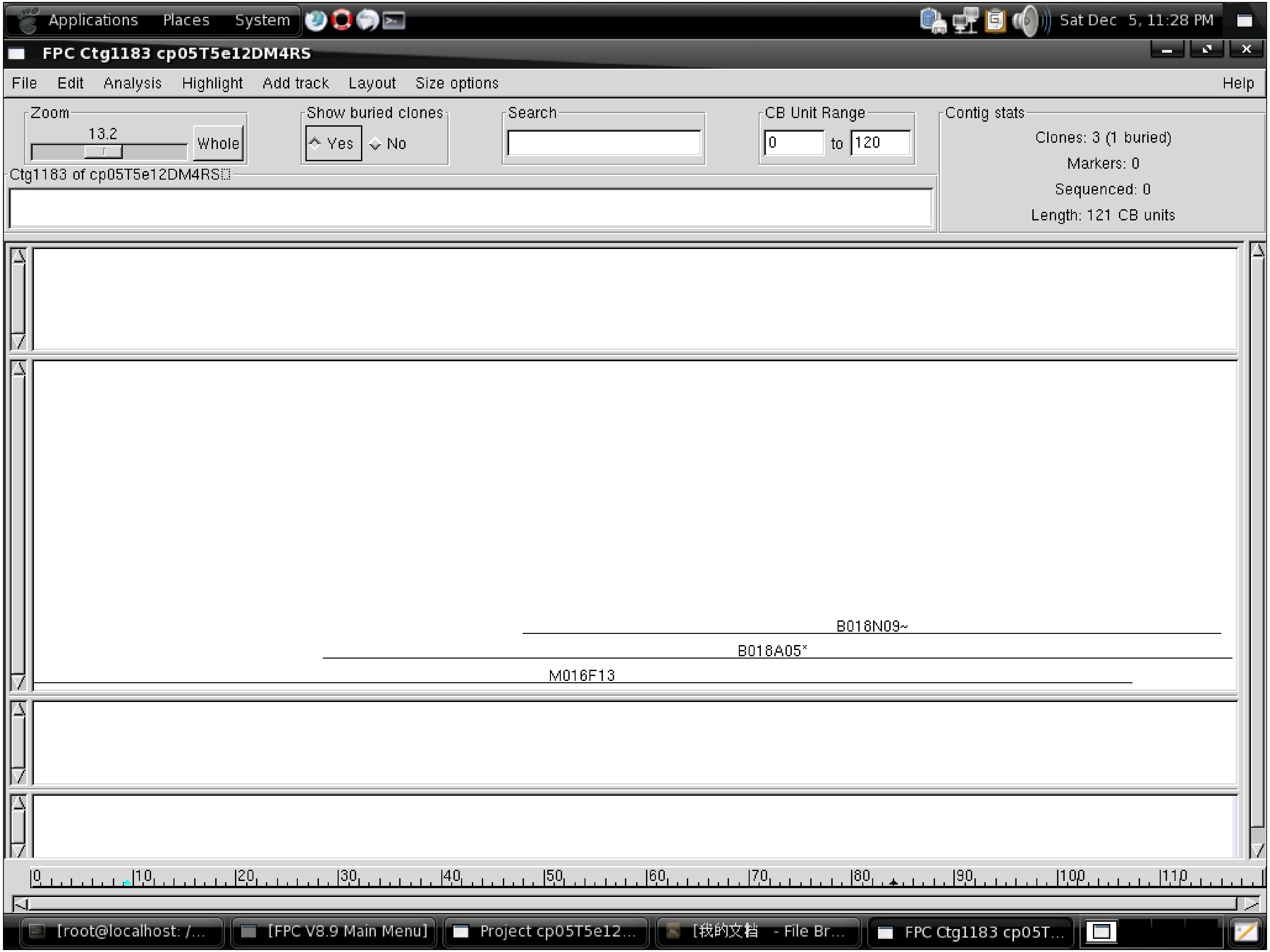
Applications
Places
System
Sat Dec 5, 11:28 PM
FPC Ctg1183 cp05T5e12DM4RS
File
Edit
Analysis
Highlight
Add track
Layout
Size options
Help
Zoom
13.2
Whole
Show buried clones
Yes
No
Search
CB Unit Range
0
to
120
Contig stats
Clones: 3 (1 buried)
Markers: 0
Sequenced: 0
Length: 121 CB units
Ctg1183 of cp05T5e12DM4RS
B018N09~
B018A05*
M016F13
0
10
20
30
40
50
60
70
80
90
100
110
[root@localhost: /...
[FPC V8.9 Main Menu]
Project cp05T5e12...
[我的文档 - File Br...
FPC Ctg1183 cp05T...

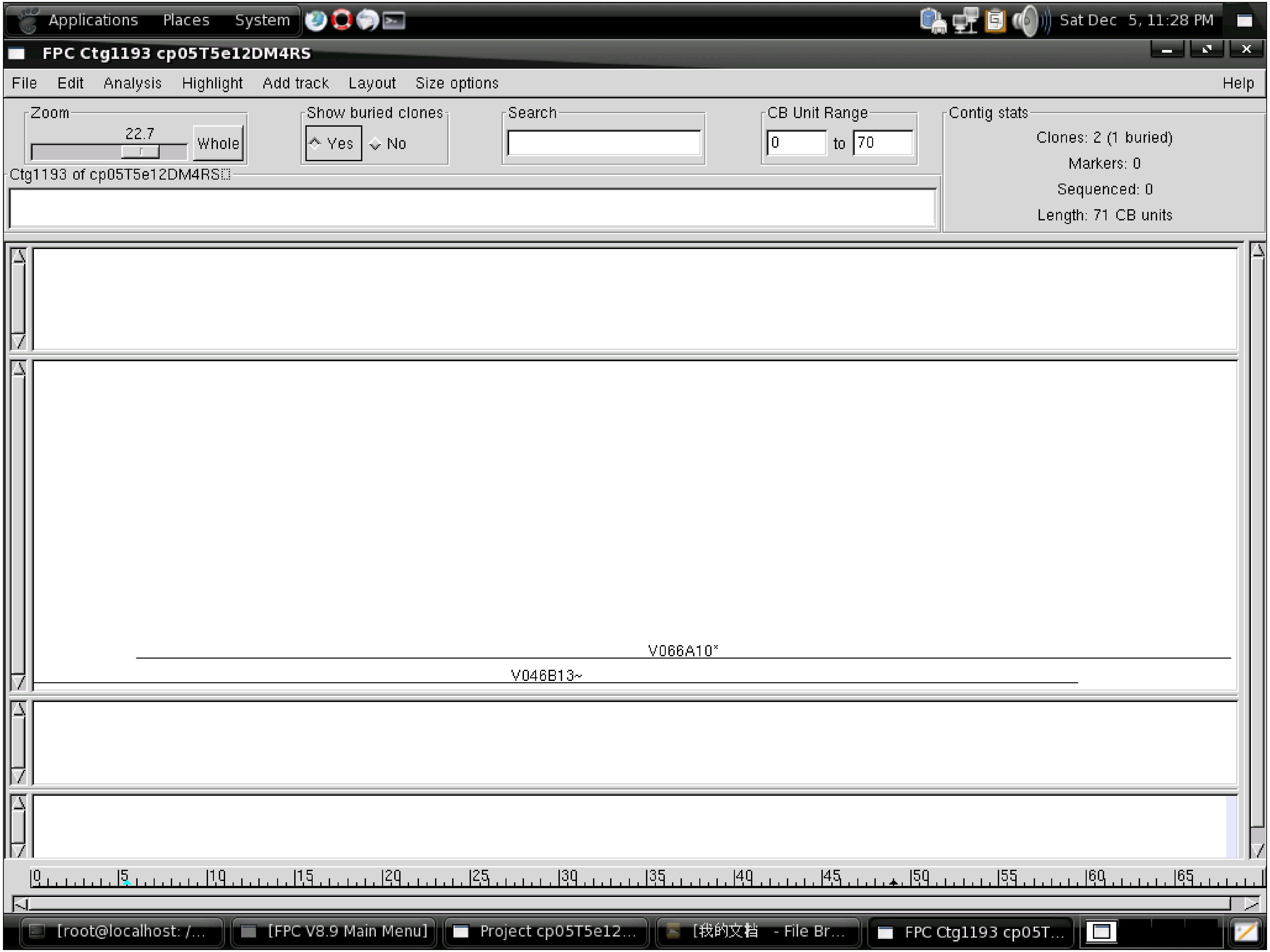
Applications Places System
Sat Dec 5, 11:28 PM
FPC Ctg1193 cp05T5e12DM4RS
File Edit Analysis Highlight Add track Layout Size options Help
Zoom
22.7
Whole
Show buried clones
Yes
No
Search
CB Unit Range
0
to
70
Contig stats
Clones: 2 (1 buried)
Markers: 0
Sequenced: 0
Length: 71 CB units
Ctg1193 of cp05T5e12DM4RS
V066A10*
V046B13~
0 5 10 15 20 25 30 35 40 45 50 55 60 65
[root@localhost: /...
[FPC V8.9 Main Menu]
Project cp05T5e12...
[我的文档 - File Br...
FPC Ctg1193 cp05T...

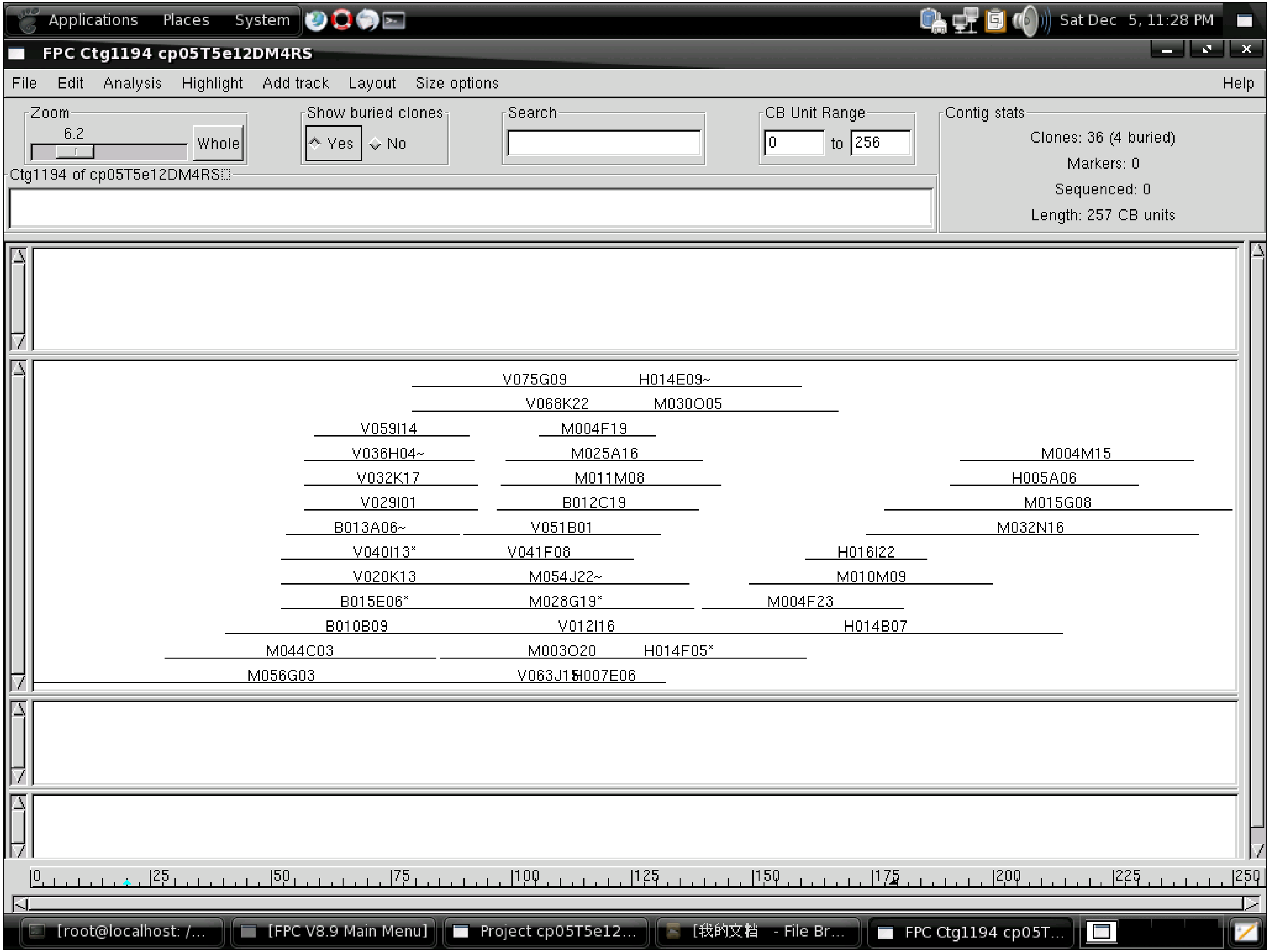
Applications Places System
Sat Dec 5, 11:28 PM
FPC Ctg1194 cp05T5e12DM4RS
File Edit Analysis Highlight Add track Layout Size options Help
Zoom
6.2
Whole
Show buried clones
Yes No
Search
CB Unit Range
0 to 256
Contig stats
Clones: 36 (4 buried)
Markers: 0
Sequenced: 0
Length: 257 CB units
Ctg1194 of cp05T5e12DM4RS
V075G09 H014E09~
V068K22 M030O05
V059I14 M004F19
V036H04~ M025A16 M004M15
V032K17 M011M08 H005A06
V029I01 B012C19 M015G08
B013A06~ V051B01 M032N16
V040I13* V041F08 H016I22
V020K13 M054J22~ M010M09
B015E06* M028G19* M004F23
B010B09 V012I16 H014B07
M044C03 M003O20 H014F05*
M056G03 V063J15 H007E06
0 25 50 75 100 125 150 175 200 225 250
[root@localhost: /...
[FPC V8.9 Main Menu]
Project cp05T5e12...
[我的文档 - File Br...
FPC Ctg1194 cp05T...

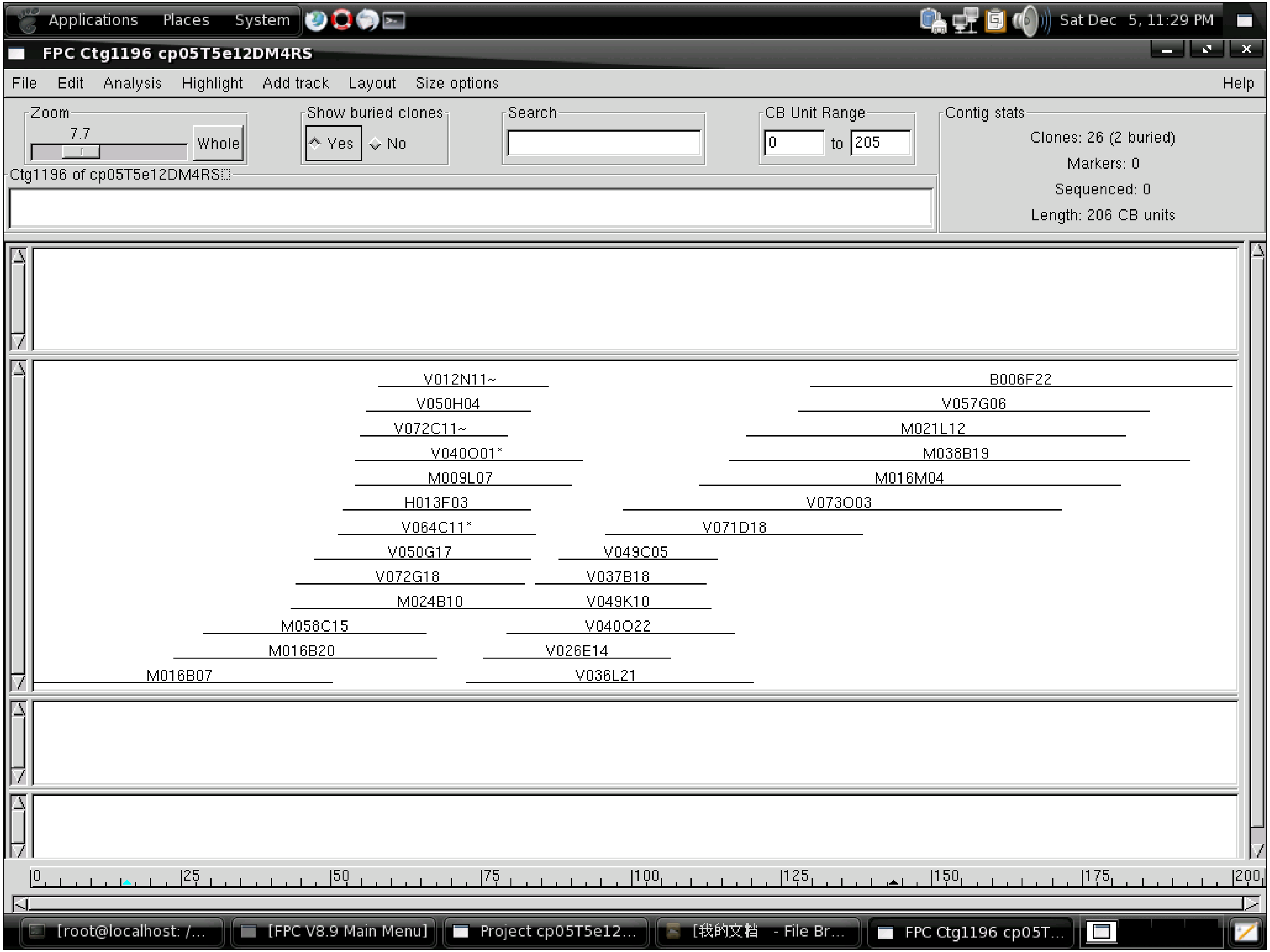
Applications Places System
Sat Dec 5, 11:29 PM
FPC Ctg1196 cp05T5e12DM4RS
File Edit Analysis Highlight Add track Layout Size options Help
Zoom
7.7
Whole
Show buried clones
Yes No
Search
CB Unit Range
0 to 205
Contig stats
Clones: 26 (2 buried)
Markers: 0
Sequenced: 0
Length: 206 CB units
Ctg1196 of cp05T5e12DM4RS
V012N11~
B006F22
V050H04
V057G06
V072C11~
M021L12
V040O01*
M038B19
M009L07
M016M04
H013F03
V073O03
V064C11*
V071D18
V050G17
V049C05
V072G18
V037B18
M024B10
V049K10
M058C15
V040O22
M016B20
V026E14
M016B07
V036L21
0 25 50 75 100 125 150 175 200
[root@localhost: /...
[FPC V8.9 Main Menu]
Project cp05T5e12...
[我的文档 - File Br...
FPC Ctg1196 cp05T...

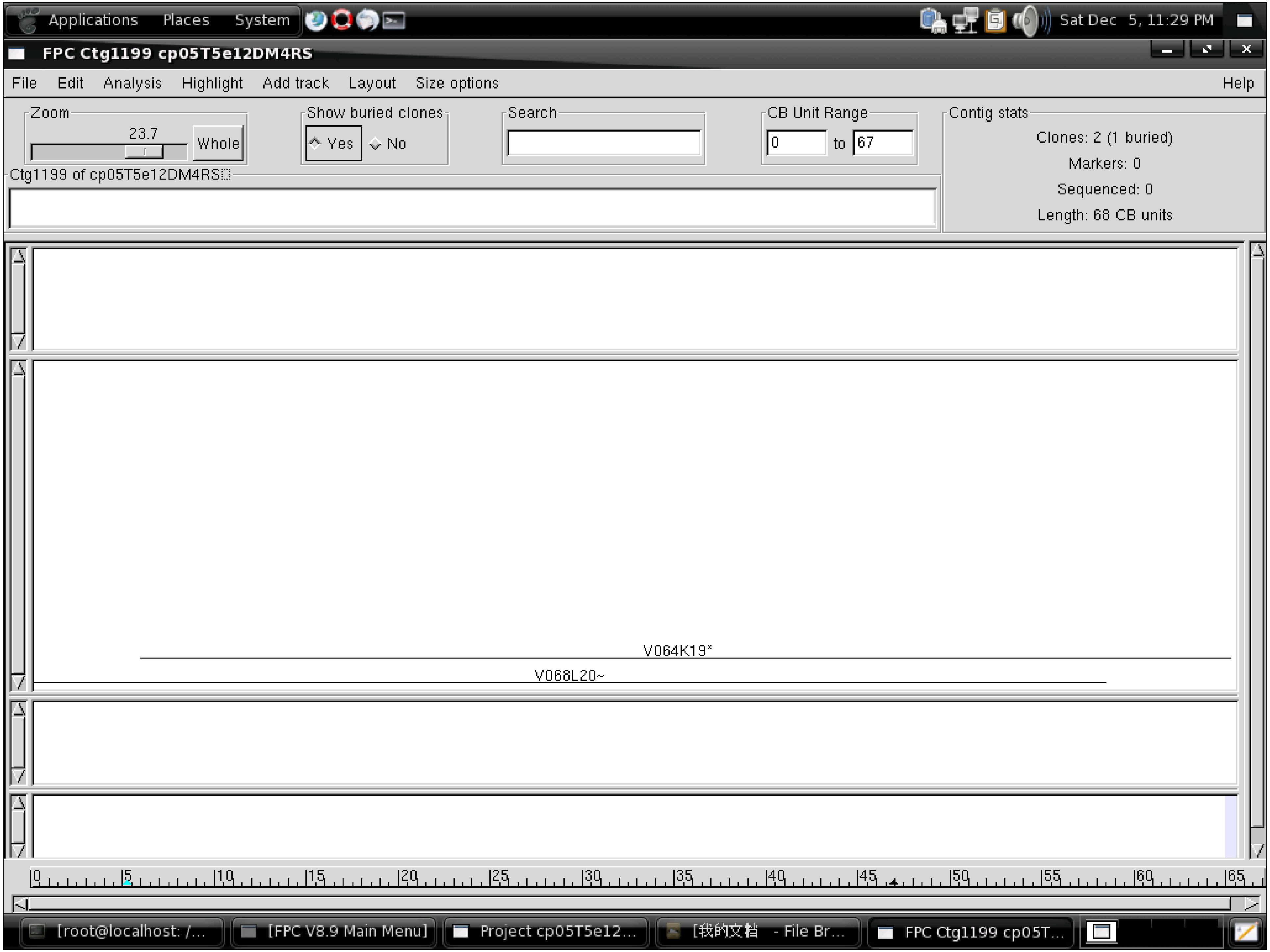
Applications Places System
Sat Dec 5, 11:29 PM
FPC Ctg1199 cp05T5e12DM4RS
File Edit Analysis Highlight Add track Layout Size options Help
Zoom
23.7
Whole
Show buried clones
Yes
No
Search
CB Unit Range
0
to
67
Contig stats
Clones: 2 (1 buried)
Markers: 0
Sequenced: 0
Length: 68 CB units
Ctg1199 of cp05T5e12DM4RS
V064K19*
V068L20~
0 5 10 15 20 25 30 35 40 45 50 55 60 65
[root@localhost: /...
[FPC V8.9 Main Menu]
Project cp05T5e12...
[我的文档 - File Br...
FPC Ctg1199 cp05T...

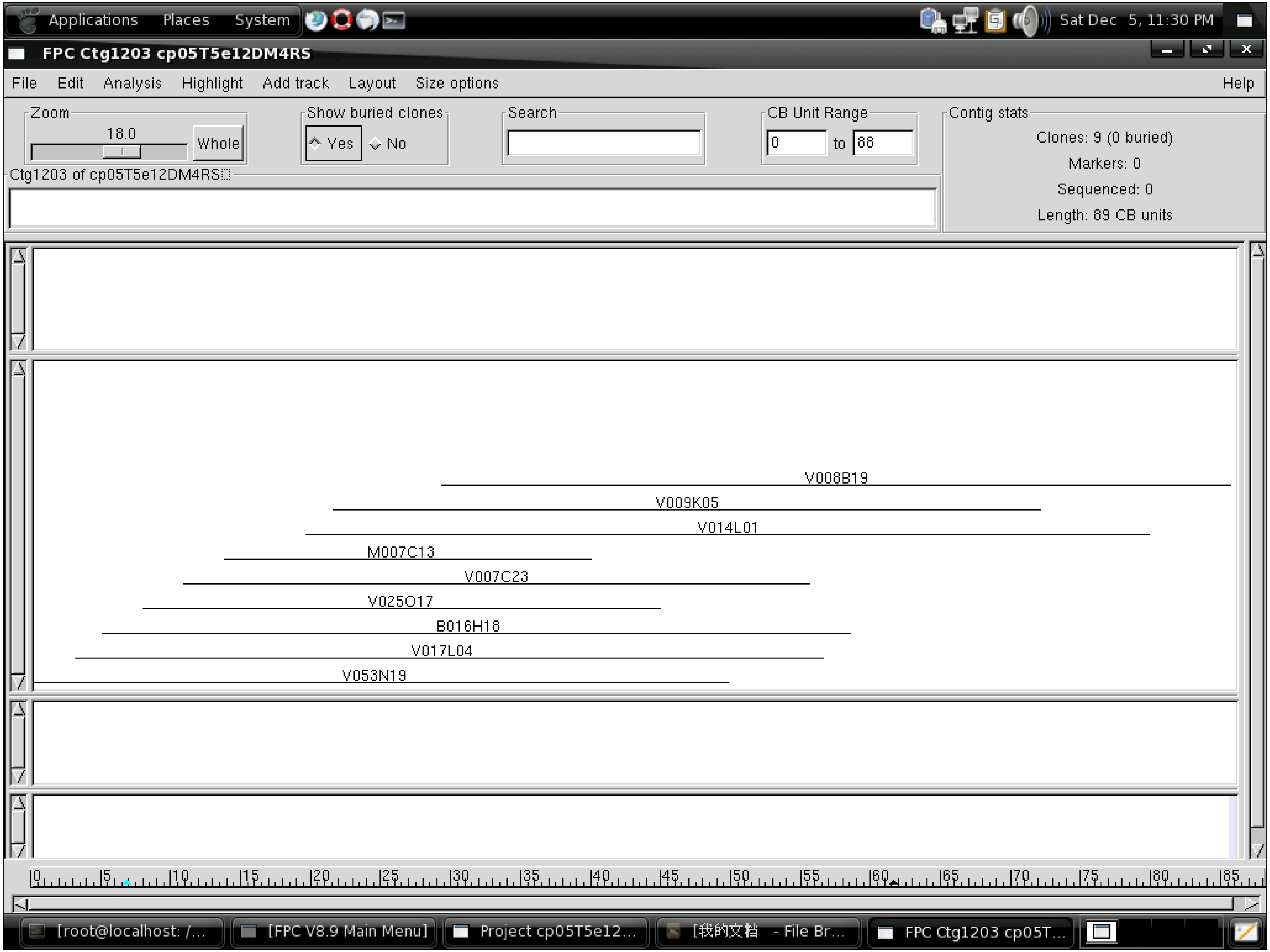
Applications Places System
Sat Dec 5, 11:30 PM
FPC Ctg1203 cp05T5e12DM4RS
File Edit Analysis Highlight Add track Layout Size options Help
Zoom
18.0
Whole
Show buried clones
Yes No
Search
CB Unit Range
0 to 88
Contig stats
Clones: 9 (0 buried)
Markers: 0
Sequenced: 0
Length: 89 CB units
Ctg1203 of cp05T5e12DM4RS
V008B19
V009K05
V014L01
M007C13
V007C23
V025O17
B016H18
V017L04
V053N19
0 5 10 15 20 25 30 35 40 45 50 55 60 65 70 75 80 85
[root@localhost: /...
[FPC V8.9 Main Menu]
Project cp05T5e12...
[我的文档 - File Br...
FPC Ctg1203 cp05T...

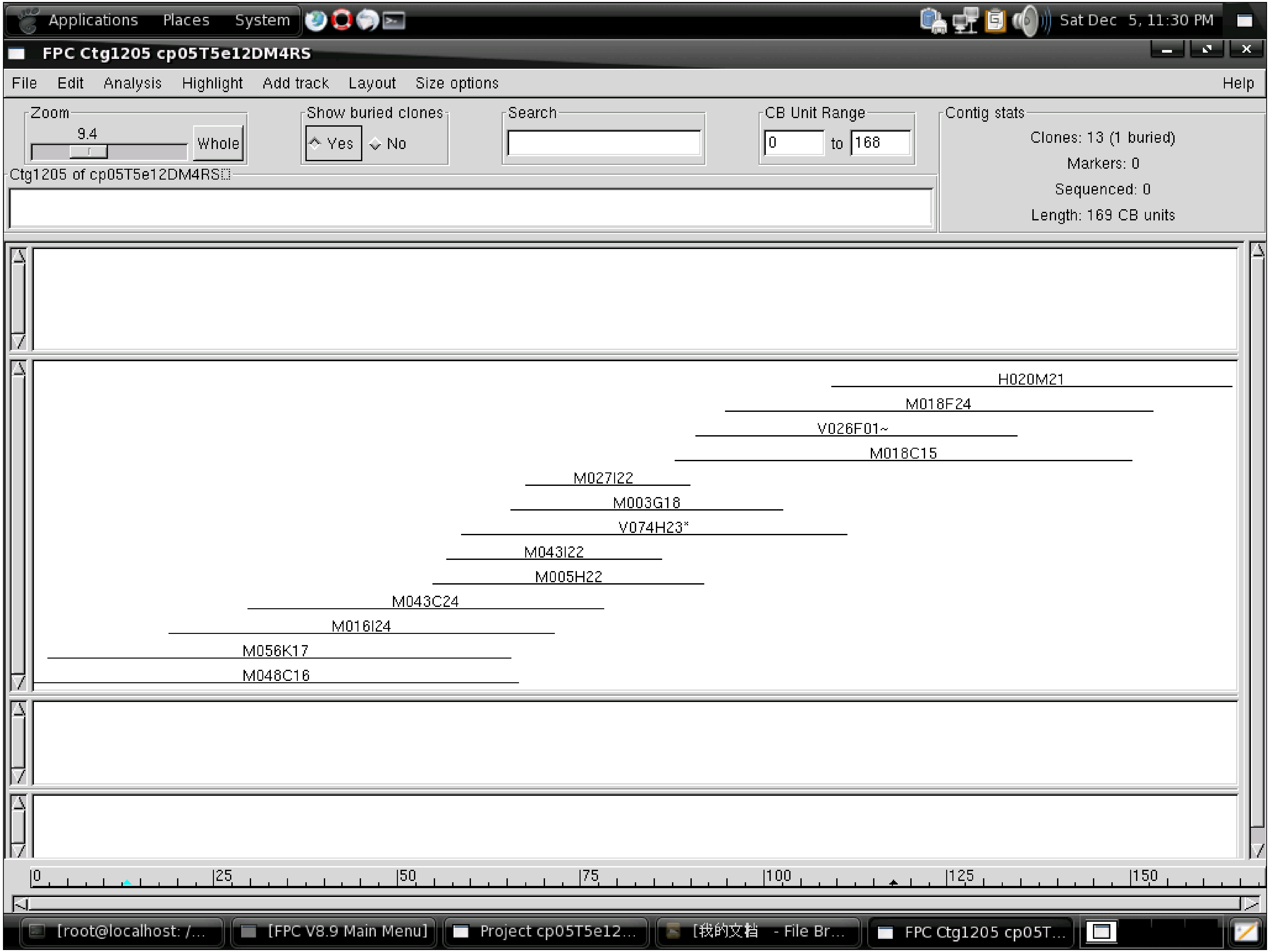
Applications Places System
Sat Dec 5, 11:30 PM
FPC Ctg1205 cp05T5e12DM4RS
File Edit Analysis Highlight Add track Layout Size options Help
Zoom
9.4
Whole
Show buried clones
Yes
No
Search
CB Unit Range
0 to 168
Contig stats
Clones: 13 (1 buried)
Markers: 0
Sequenced: 0
Length: 169 CB units
Ctg1205 of cp05T5e12DM4RS
H020M21
M018F24
V026F01~
M018C15
M027I22
M003G18
V074H23*
M043I22
M005H22
M043C24
M016I24
M056K17
M048C16
0 25 50 75 100 125 150
[root@localhost: /...
[FPC V8.9 Main Menu]
Project cp05T5e12...
[我的文档 - File Br...
FPC Ctg1205 cp05T...

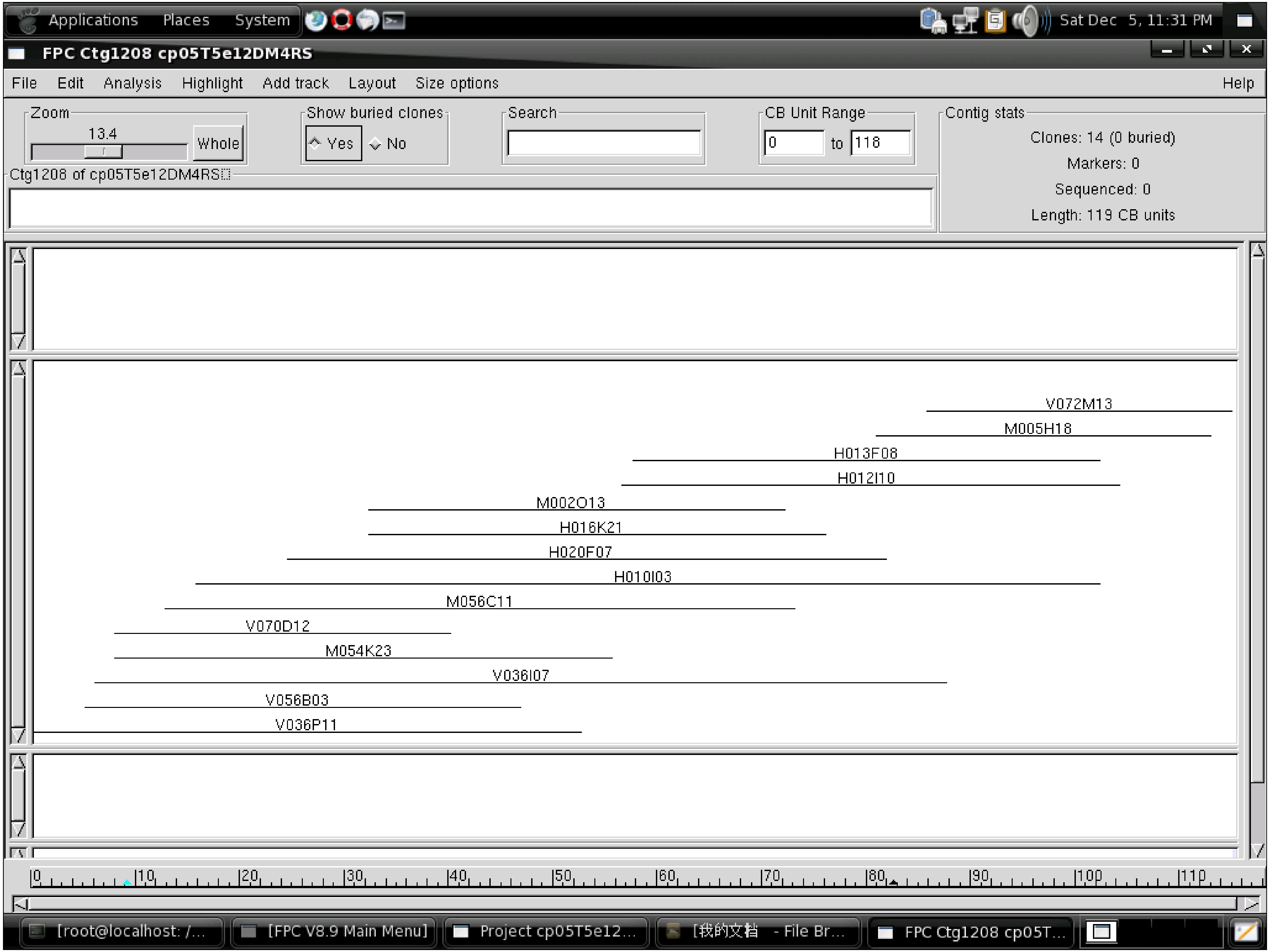
Applications Places System
Sat Dec 5, 11:31 PM
FPC Ctg1208 cp05T5e12DM4RS
File Edit Analysis Highlight Add track Layout Size options Help
Zoom
13.4
Whole
Show buried clones
Yes
No
Search
CB Unit Range
0
to
118
Contig stats
Clones: 14 (0 buried)
Markers: 0
Sequenced: 0
Length: 119 CB units
Ctg1208 of cp05T5e12DM4RS
V072M13
M005H18
H013F08
H012I10
M002O13
H016K21
H020F07
H010I03
M056C11
V070D12
M054K23
V036I07
V056B03
V036P11
0 10 20 30 40 50 60 70 80 90 100 110
[root@localhost: /...
[FPC V8.9 Main Menu]
Project cp05T5e12...
[我的文档 - File Br...
FPC Ctg1208 cp05T...

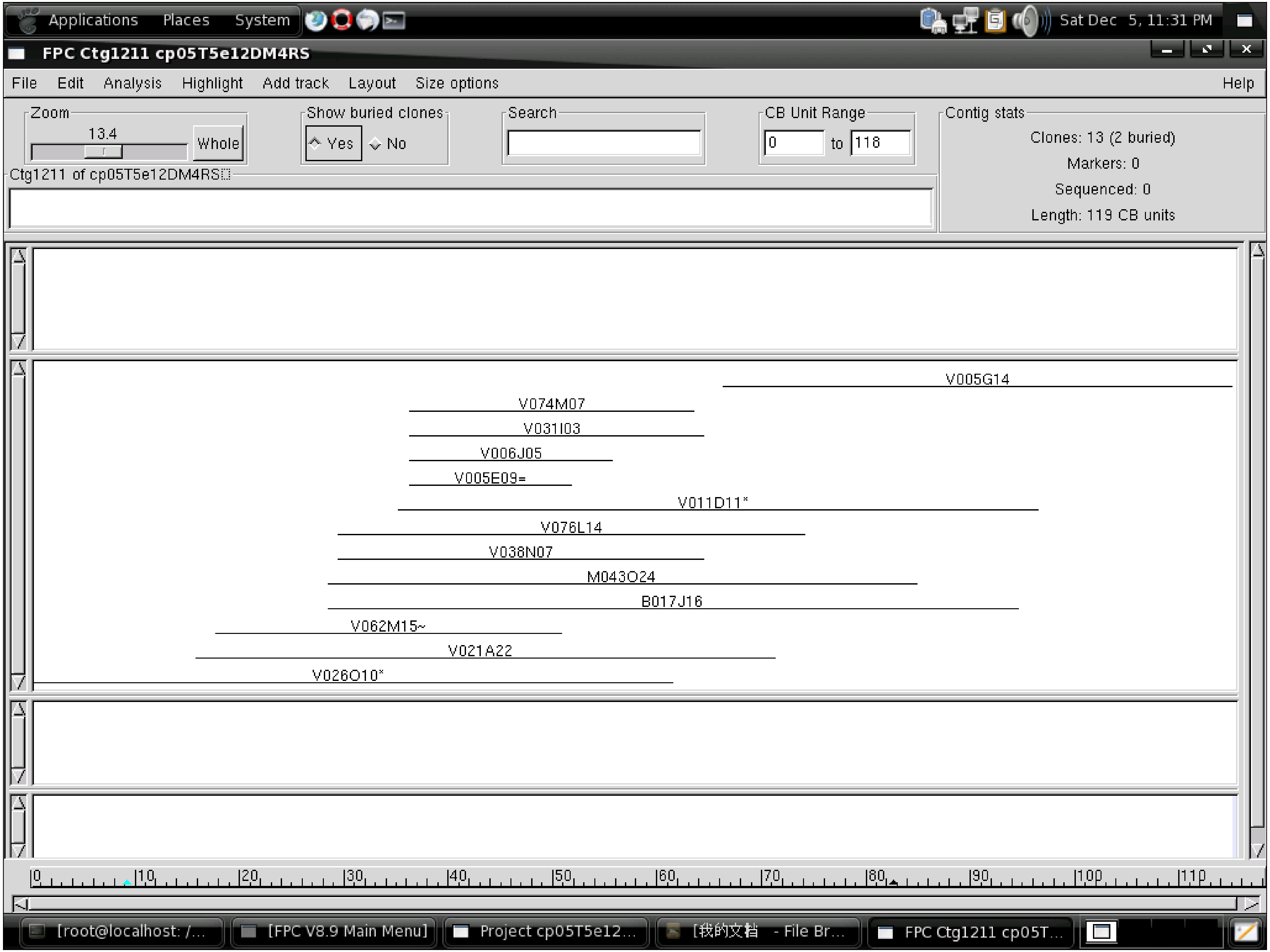
Applications Places System
Sat Dec 5, 11:31 PM
FPC Ctg1211 cp05T5e12DM4RS
File Edit Analysis Highlight Add track Layout Size options Help
Zoom
13.4
Whole
Show buried clones
Yes
No
Search
CB Unit Range
0 to 118
Contig stats
Clones: 13 (2 buried)
Markers: 0
Sequenced: 0
Length: 119 CB units
Ctg1211 of cp05T5e12DM4RS
V005G14
V074M07
V031I03
V006J05
V005E09=
V011D11*
V076L14
V038N07
M043O24
B017J16
V062M15~
V021A22
V026O10*
0 10 20 30 40 50 60 70 80 90 100 110
[root@localhost: /...
[FPC V8.9 Main Menu]
Project cp05T5e12...
[我的文档 - File Br...
FPC Ctg1211 cp05T...

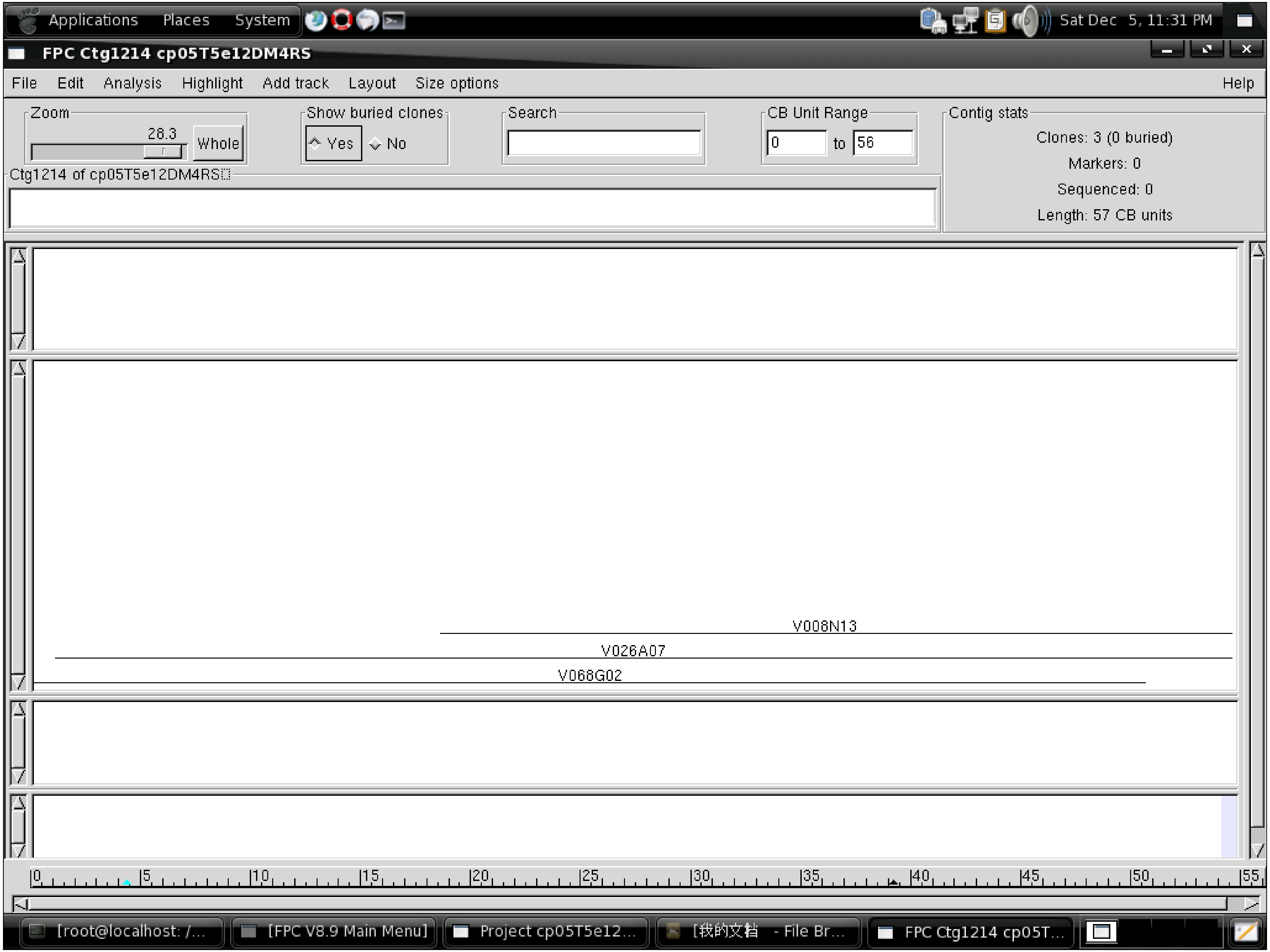
Applications Places System
Sat Dec 5, 11:31 PM
FPC Ctg1214 cp05T5e12DM4RS
File Edit Analysis Highlight Add track Layout Size options Help
Zoom
28.3
Whole
Show buried clones
Yes No
Search
CB Unit Range
0 to 56
Contig stats
Clones: 3 (0 buried)
Markers: 0
Sequenced: 0
Length: 57 CB units
Ctg1214 of cp05T5e12DM4RS
V008N13
V026A07
V068G02
0 5 10 15 20 25 30 35 40 45 50 55
[root@localhost: /...
[FPC V8.9 Main Menu]
Project cp05T5e12...
[我的文档 - File Br...
FPC Ctg1214 cp05T...

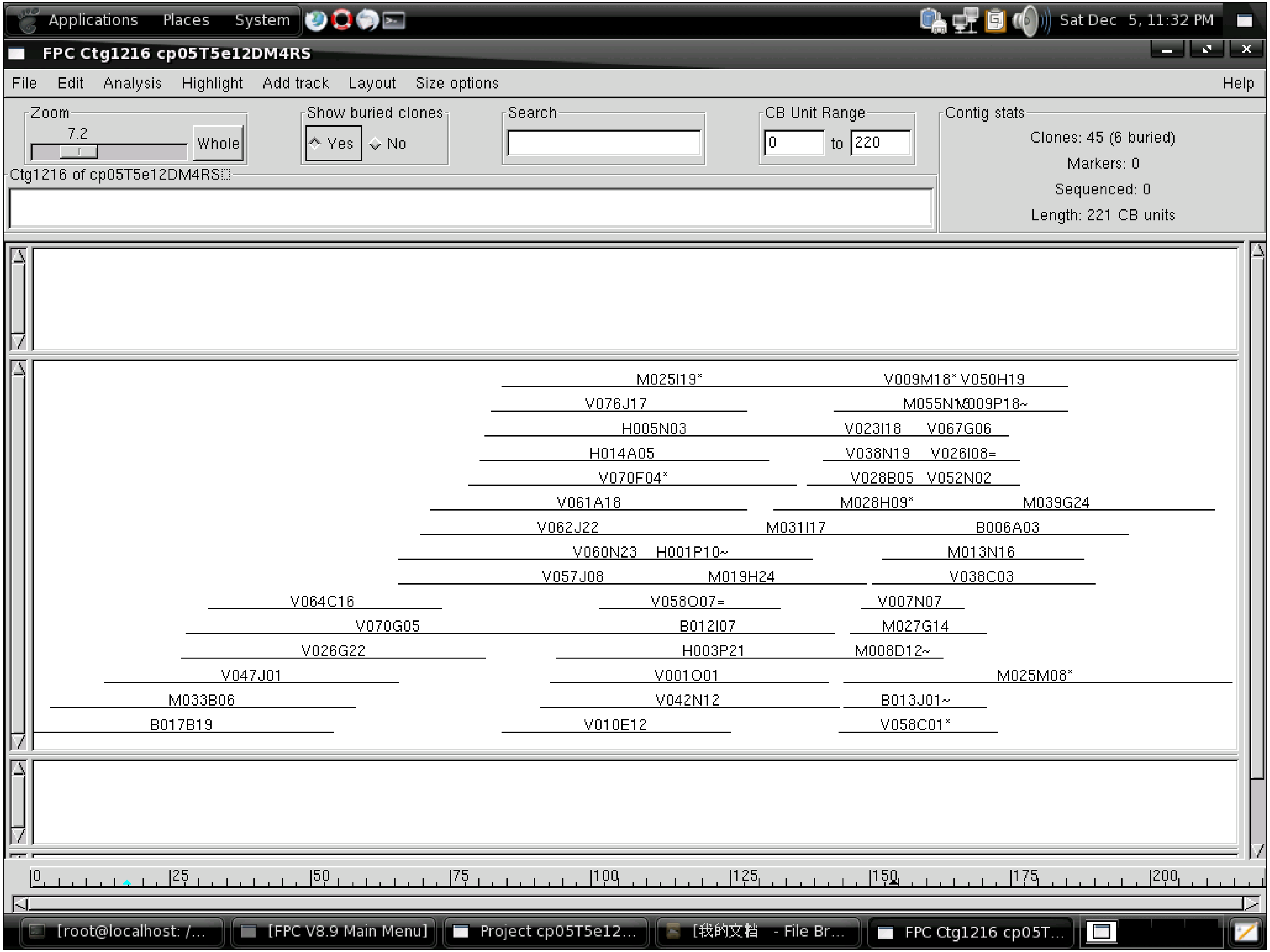
Applications Places System
Sat Dec 5, 11:32 PM
FPC Ctg1216 cp05T5e12DM4RS
File Edit Analysis Highlight Add track Layout Size options Help
Zoom 7.2 Whole
Show buried clones Yes No
Search
CB Unit Range 0 to 220
Contig stats
Clones: 45 (6 buried)
Markers: 0
Sequenced: 0
Length: 221 CB units
Ctg1216 of cp05T5e12DM4RS
M025I19*
V009M18* V050H19
V076J17
M055N1M009P18~
H005N03
V023I18 V067G06
H014A05
V038N19 V026I08=
V070F04*
V028B05 V052N02
V061A18
M028H09*
M039G24
V062J22
M031I17
B006A03
V060N23 H001P10~
M013N16
V057J08
M019H24
V038C03
V064C16
V058O07=
V007N07
V070G05
B012I07
M027G14
V026G22
H003P21
M008D12~
V047J01
V001O01
M025M08*
M033B06
V042N12
B013J01~
B017B19
V010E12
V058C01*
0 25 50 75 100 125 150 175 200
[root@localhost: /...
[FPC V8.9 Main Menu]
Project cp05T5e12...
[我的文档 - File Br...
FPC Ctg1216 cp05T...

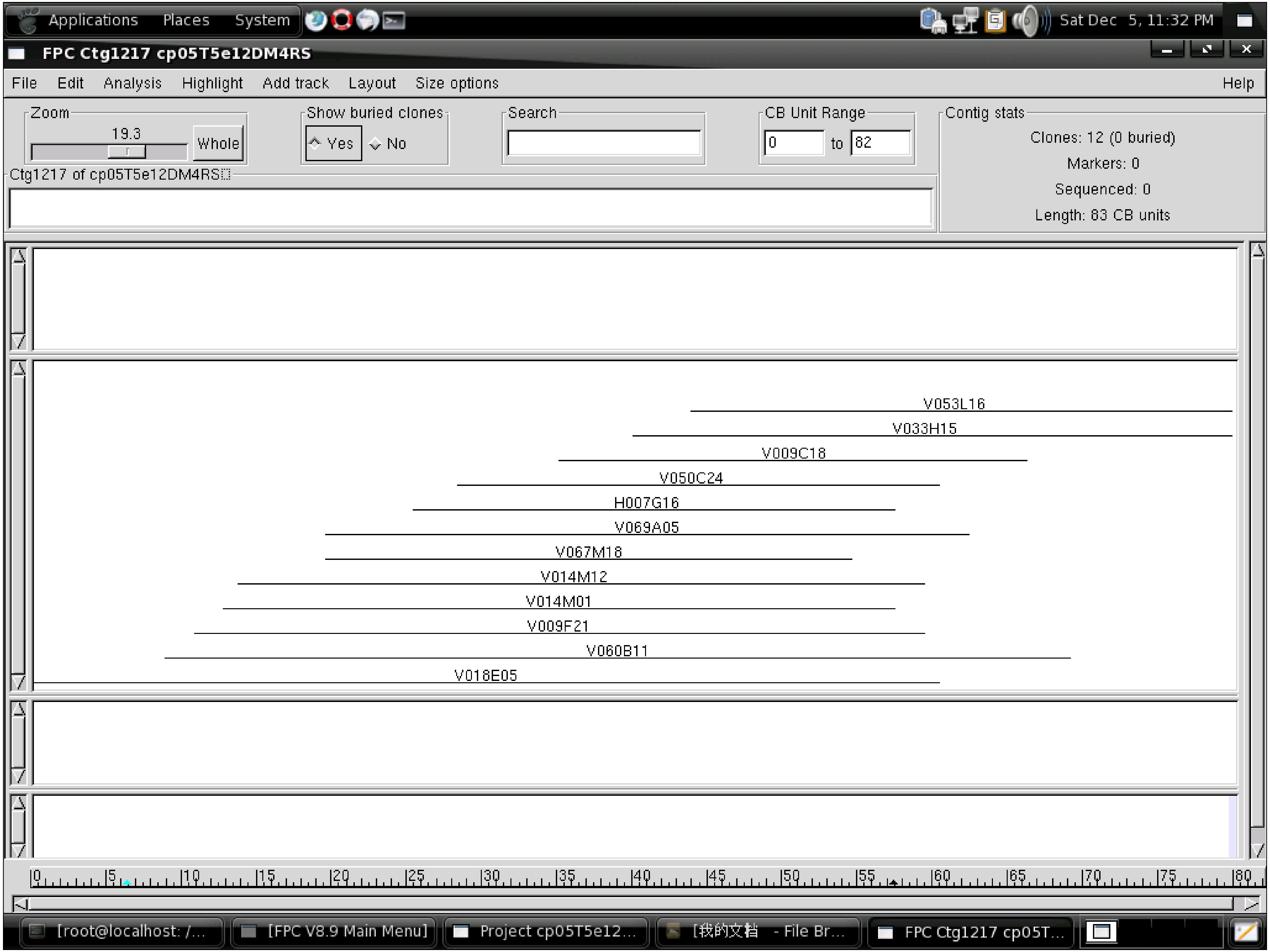
Applications Places System
Sat Dec 5, 11:32 PM
FPC Ctg1217 cp05T5e12DM4RS
File Edit Analysis Highlight Add track Layout Size options Help
Zoom
19.3
Whole
Show buried clones
Yes
No
Search
CB Unit Range
0
to
82
Contig stats
Clones: 12 (0 buried)
Markers: 0
Sequenced: 0
Length: 83 CB units
Ctg1217 of cp05T5e12DM4RS
V053L16
V033H15
V009C18
V050C24
H007G16
V069A05
V067M18
V014M12
V014M01
V009F21
V060B11
V018E05
0 5 10 15 20 25 30 35 40 45 50 55 60 65 70 75 80
[root@localhost: /...
[FPC V8.9 Main Menu]
Project cp05T5e12...
[我的文档 - File Br...
FPC Ctg1217 cp05T...

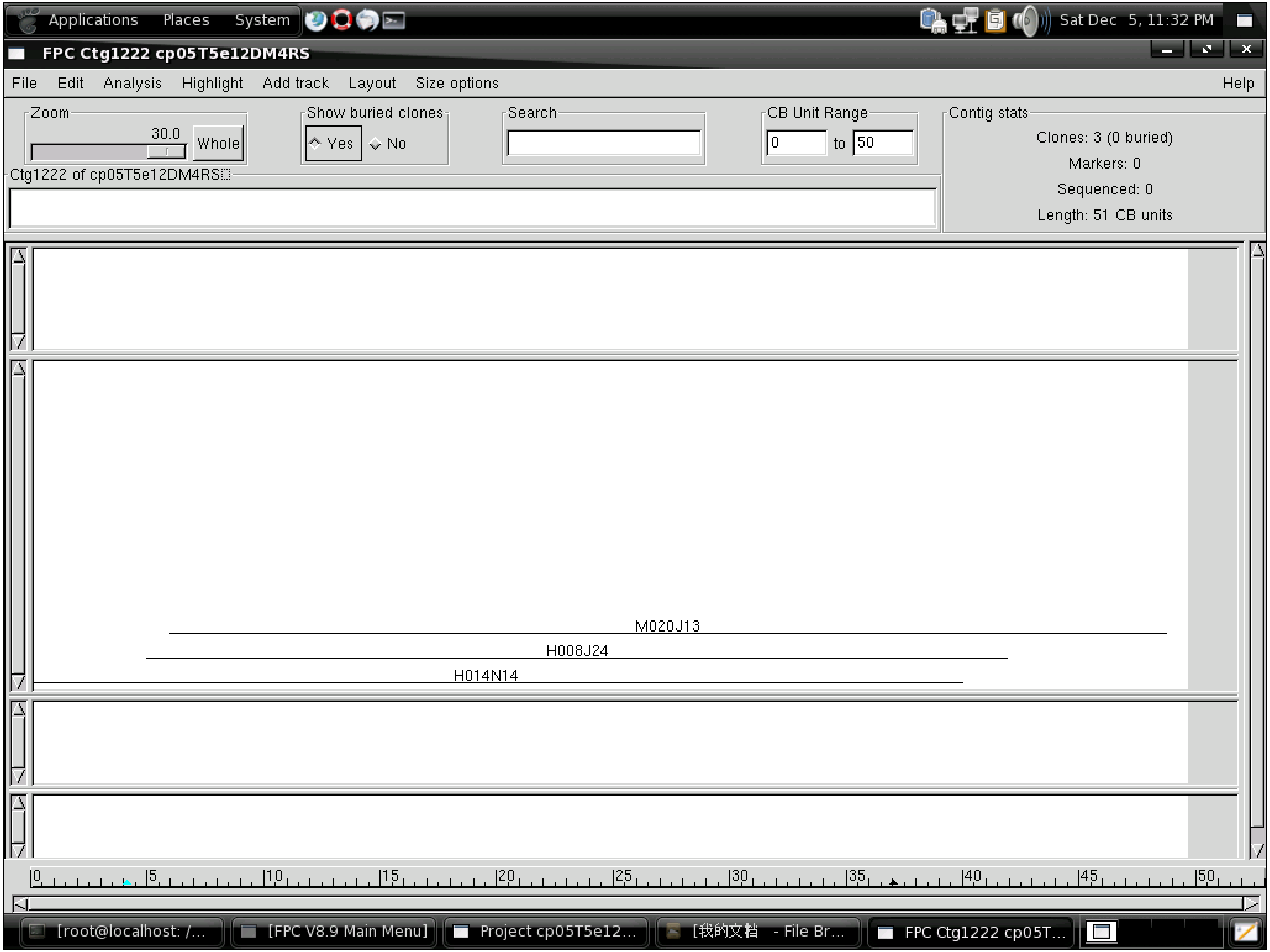
Applications
Places
System
Sat Dec 5, 11:32 PM
FPC Ctg1222 cp05T5e12DM4RS
File
Edit
Analysis
Highlight
Add track
Layout
Size options
Help
Zoom
30.0
Whole
Show buried clones
Yes
No
Search
CB Unit Range
0
to
50
Contig stats
Clones: 3 (0 buried)
Markers: 0
Sequenced: 0
Length: 51 CB units
Ctg1222 of cp05T5e12DM4RS
M020J13
H008J24
H014N14
0
5
10
15
20
25
30
35
40
45
50
[root@localhost: /...
[FPC V8.9 Main Menu]
Project cp05T5e12...
[我的文档 - File Br...
FPC Ctg1222 cp05T...

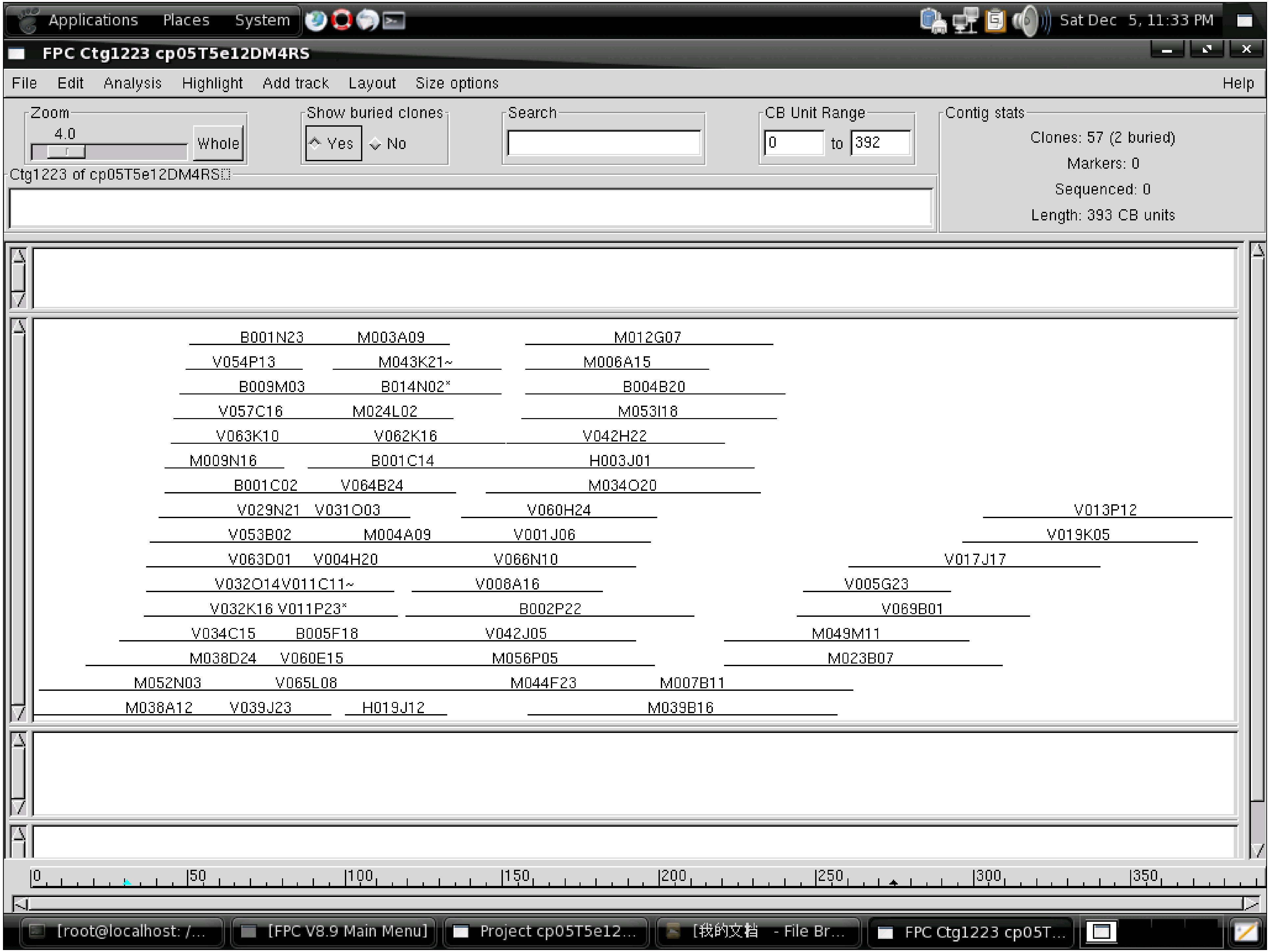
Applications Places System
Sat Dec 5, 11:33 PM
FPC Ctg1223 cp05T5e12DM4RS
File Edit Analysis Highlight Add track Layout Size options Help
Zoom
4.0
Whole
Show buried clones
Yes
No
Search
CB Unit Range
0 to 392
Contig stats
Clones: 57 (2 buried)
Markers: 0
Sequenced: 0
Length: 393 CB units
Ctg1223 of cp05T5e12DM4RS
B001N23
M003A09
M012G07
V054P13
M043K21~
M006A15
B009M03
B014N02*
B004B20
V057C16
M024L02
M053I18
V063K10
V062K16
V042H22
M009N16
B001C14
H003J01
B001C02
V064B24
M034O20
V029N21
V031O03
V060H24
V013P12
V053B02
M004A09
V001J06
V019K05
V063D01
V004H20
V066N10
V017J17
V032O14V011C11~
V008A16
V005G23
V032K16 V011P23*
B002P22
V069B01
V034C15
B005F18
V042J05
M049M11
M038D24
V060E15
M056P05
M023B07
M052N03
V065L08
M044F23
M007B11
M038A12
V039J23
H019J12
M039B16
0
50
100
150
200
250
300
350
[root@localhost: /...
[FPC V8.9 Main Menu]
Project cp05T5e12...
[我的文档 - File Br...
FPC Ctg1223 cp05T...

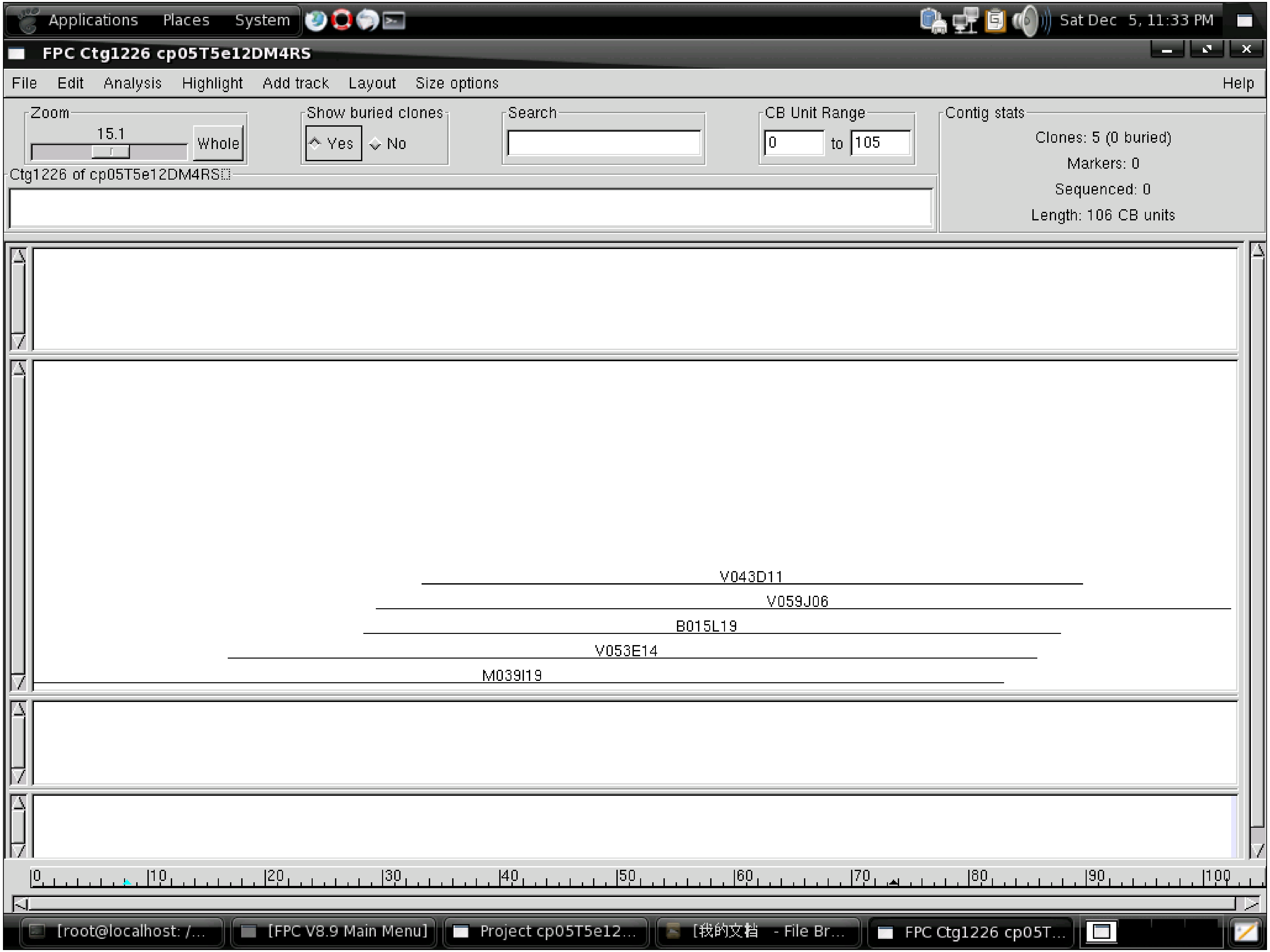
Applications Places System
Sat Dec 5, 11:33 PM
FPC Ctg1226 cp05T5e12DM4RS
File Edit Analysis Highlight Add track Layout Size options Help
Zoom
15.1
Whole
Show buried clones
Yes
No
Search
CB Unit Range
0
to
105
Contig stats
Clones: 5 (0 buried)
Markers: 0
Sequenced: 0
Length: 106 CB units
Ctg1226 of cp05T5e12DM4RS
V043D11
V059J06
B015L19
V053E14
M039I19
0 10 20 30 40 50 60 70 80 90 100
[root@localhost: /...
[FPC V8.9 Main Menu]
Project cp05T5e12...
[我的文档 - File Br...
FPC Ctg1226 cp05T...

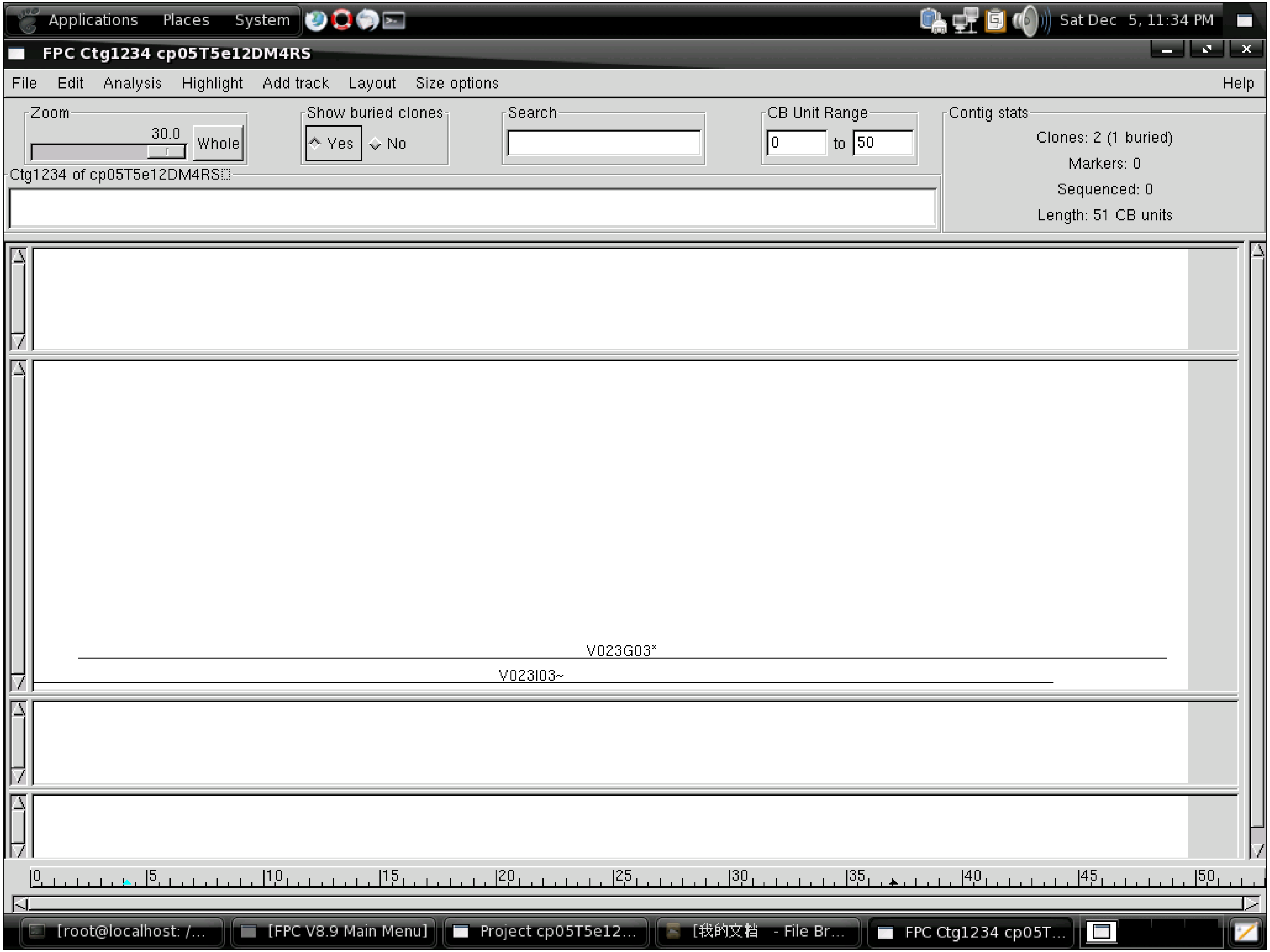
Applications Places System
Sat Dec 5, 11:34 PM
FPC Ctg1234 cp05T5e12DM4RS
File Edit Analysis Highlight Add track Layout Size options Help
Zoom
30.0
Whole
Show buried clones
Yes No
Search
CB Unit Range
0 to 50
Contig stats
Clones: 2 (1 buried)
Markers: 0
Sequenced: 0
Length: 51 CB units
Ctg1234 of cp05T5e12DM4RS
V023G03*
V023I03~
0 5 10 15 20 25 30 35 40 45 50
[root@localhost: /...
[FPC V8.9 Main Menu]
Project cp05T5e12...
[我的文档 - File Br...
FPC Ctg1234 cp05T...

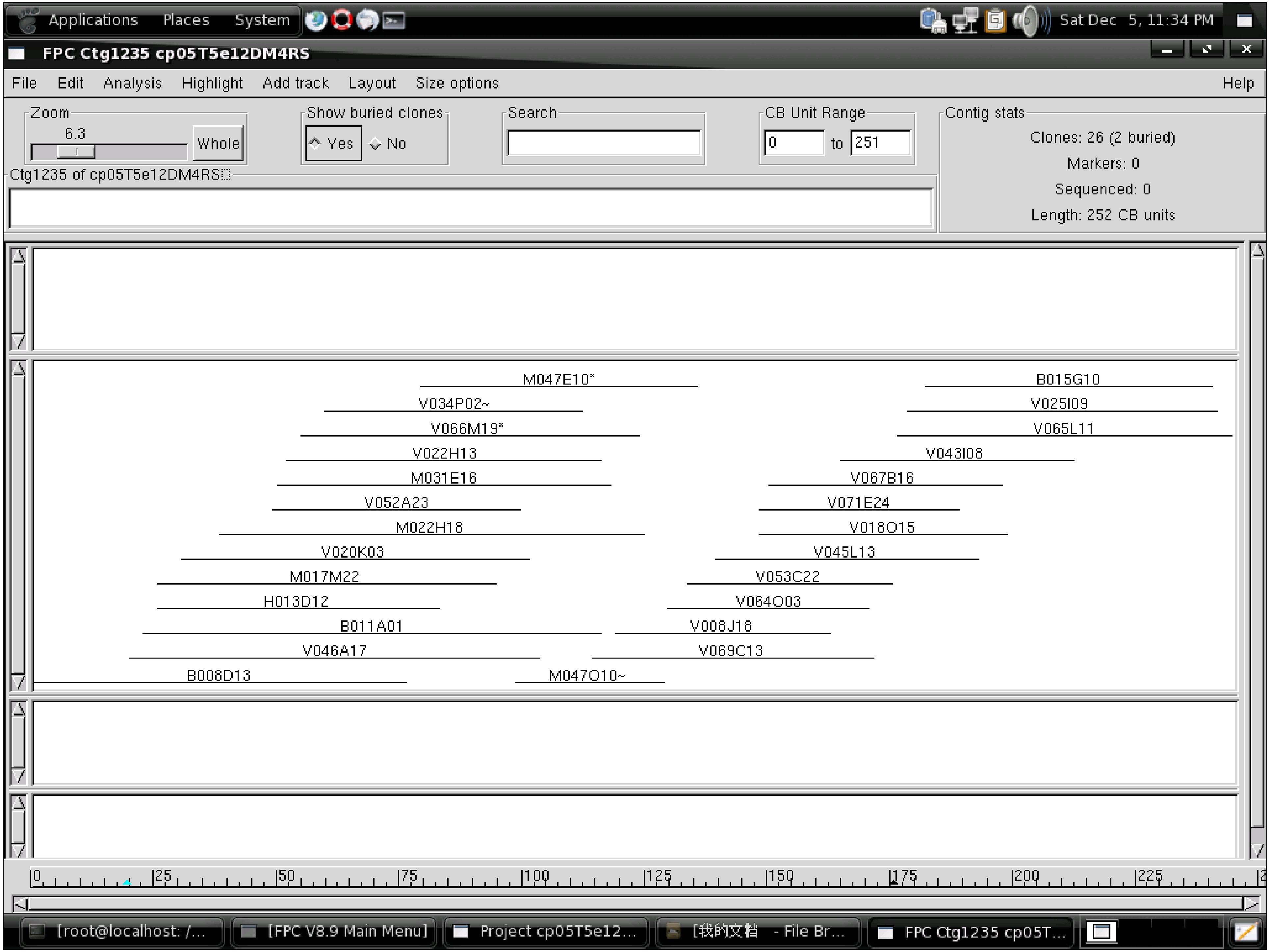
Applications Places System
Sat Dec 5, 11:34 PM
FPC Ctg1235 cp05T5e12DM4RS
File Edit Analysis Highlight Add track Layout Size options Help
Zoom
6.3
Whole
Show buried clones
Yes No
Search
CB Unit Range
0 to 251
Contig stats
Clones: 26 (2 buried)
Markers: 0
Sequenced: 0
Length: 252 CB units
Ctg1235 of cp05T5e12DM4RS
M047E10*
B015G10
V034P02~
V025I09
V066M19*
V065L11
V022H13
V043I08
M031E16
V067B16
V052A23
V071E24
M022H18
V018O15
V020K03
V045L13
M017M22
V053C22
H013D12
V064O03
B011A01
V008J18
V046A17
V069C13
B008D13
M047O10~
0 25 50 75 100 125 150 175 200 225
[root@localhost: /...
[FPC V8.9 Main Menu]
Project cp05T5e12...
[我的文档 - File Br...
FPC Ctg1235 cp05T...

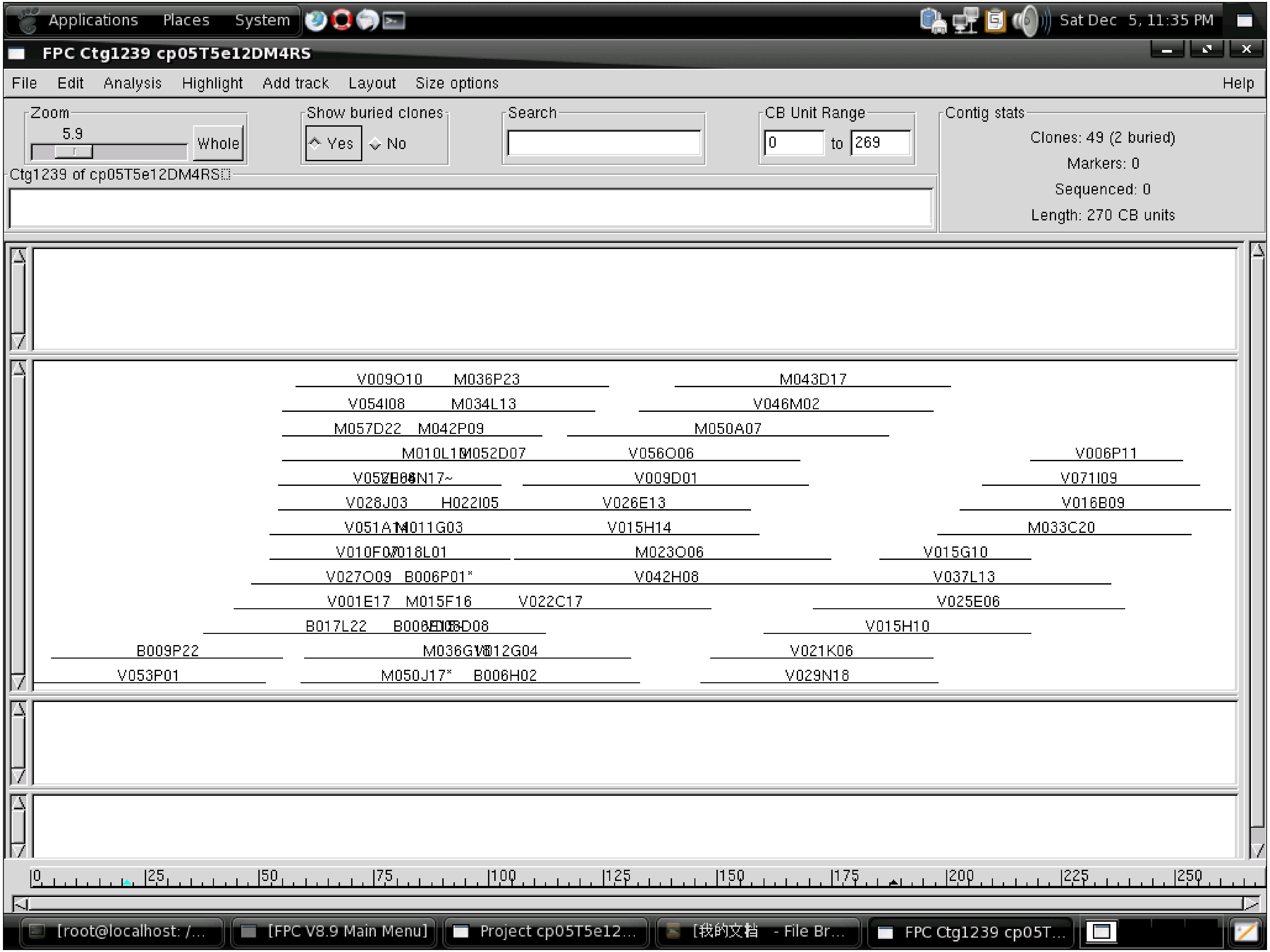

Applications Places System
Sat Dec 5, 11:35 PM
FPC Ctg1239 cp05T5e12DM4RS
File Edit Analysis Highlight Add track Layout Size options Help
Zoom
5.9
Whole
Show buried clones
Yes
No
Search
CB Unit Range
0 to 269
Contig stats
Clones: 49 (2 buried)
Markers: 0
Sequenced: 0
Length: 270 CB units
Ctg1239 of cp05T5e12DM4RS
V009O10
M036P23
M043D17
V054I08
M034L13
V046M02
M057D22
M042P09
M050A07
V056O06
V006P11
V009D01
V071I09
V028J03
H022I05
V026E13
V016B09
M011G03
V015H14
M033C20
M023O06
V015G10
V027O09
B006P01*
V042H08
V037L13
V001E17
M015F16
V022C17
V025E06
B017L22
V015H10
B009P22
V021K06
V053P01
M050J17*
B006H02
V029N18
0 25 50 75 100 125 150 175 200 225 250
[root@localhost: /...
[FPC V8.9 Main Menu]
Project cp05T5e12...
FPC Ctg1239 cp05T...

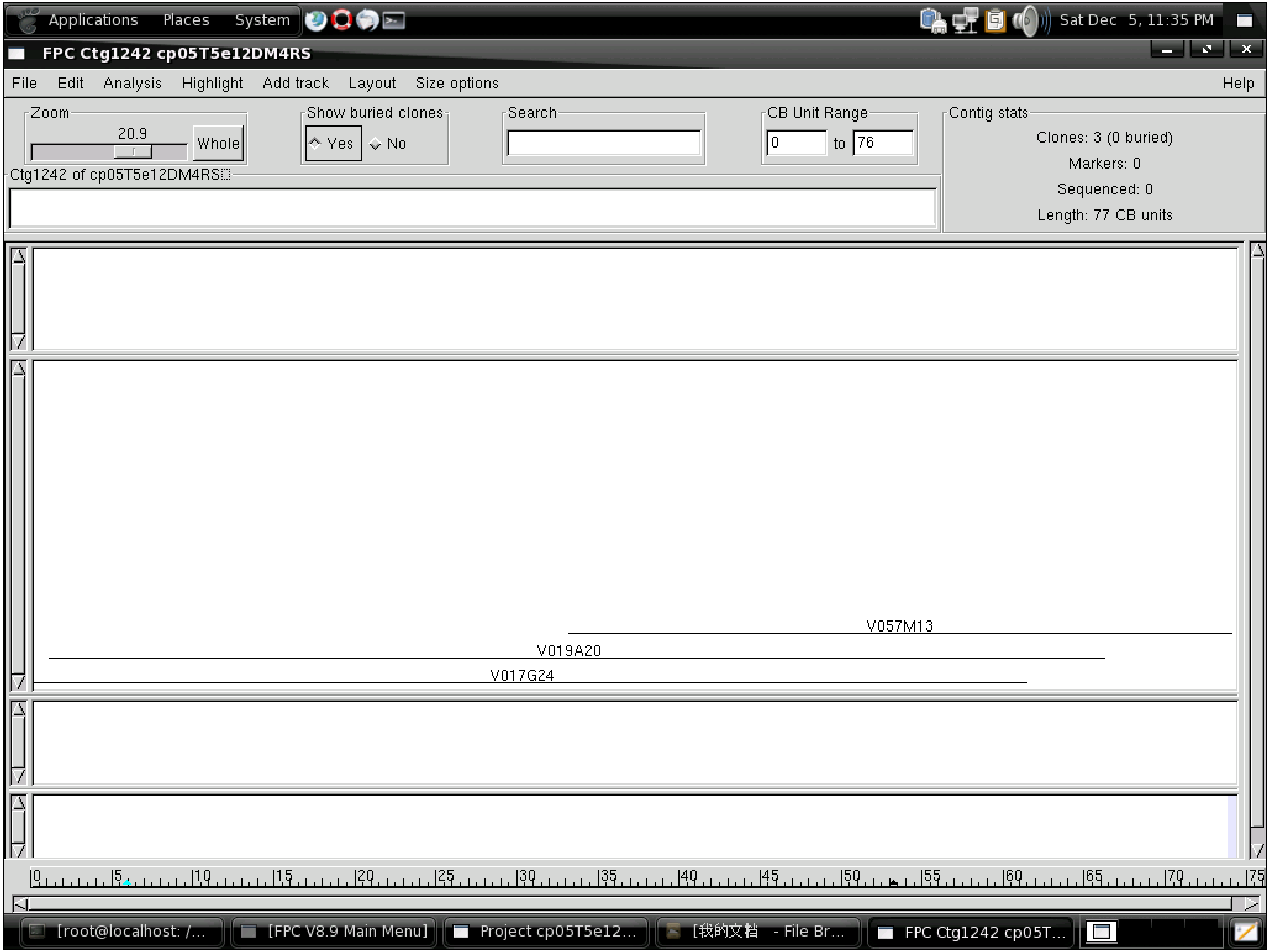
Applications Places System
Sat Dec 5, 11:35 PM
FPC Ctg1242 cp05T5e12DM4RS
File Edit Analysis Highlight Add track Layout Size options Help
Zoom
20.9
Whole
Show buried clones
Yes No
Search
CB Unit Range
0 to 76
Contig stats
Clones: 3 (0 buried)
Markers: 0
Sequenced: 0
Length: 77 CB units
Ctg1242 of cp05T5e12DM4RS
V057M13
V019A20
V017G24
0 5 10 15 20 25 30 35 40 45 50 55 60 65 70 75
[root@localhost: /...
[FPC V8.9 Main Menu]
Project cp05T5e12...
[我的文档 - File Br...
FPC Ctg1242 cp05T...

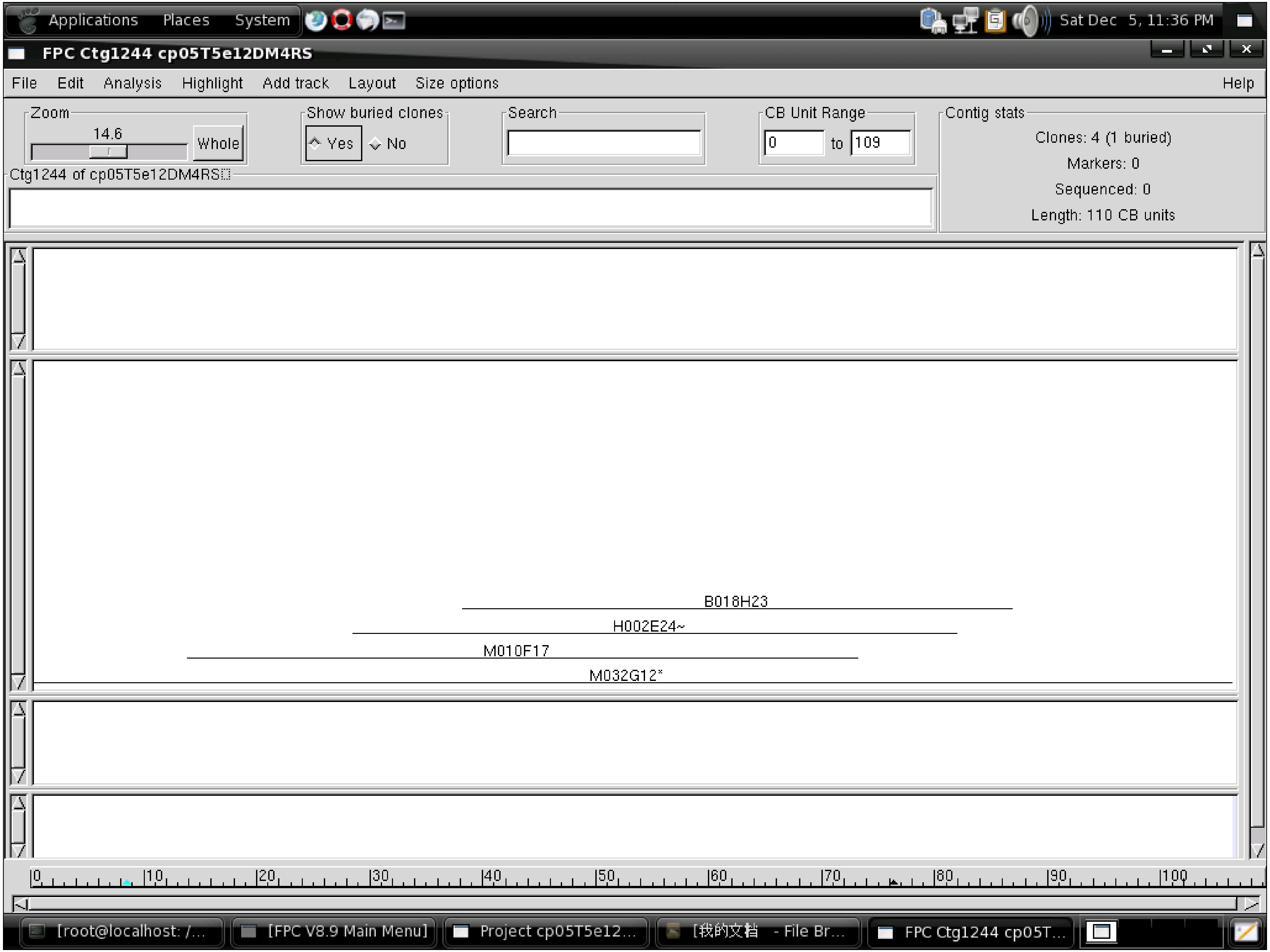
Applications Places System
Sat Dec 5, 11:36 PM
FPC Ctg1244 cp05T5e12DM4RS
File Edit Analysis Highlight Add track Layout Size options
Help
Zoom
14.6
Whole
Show buried clones
Yes
No
Search
CB Unit Range
0
to
109
Contig stats
Clones: 4 (1 buried)
Markers: 0
Sequenced: 0
Length: 110 CB units
Ctg1244 of cp05T5e12DM4RS
B018H23
H002E24~
M010F17
M032G12*
0 10 20 30 40 50 60 70 80 90 100
[root@localhost: /...
[FPC V8.9 Main Menu]
Project cp05T5e12...
[我的文档 - File Br...
FPC Ctg1244 cp05T...

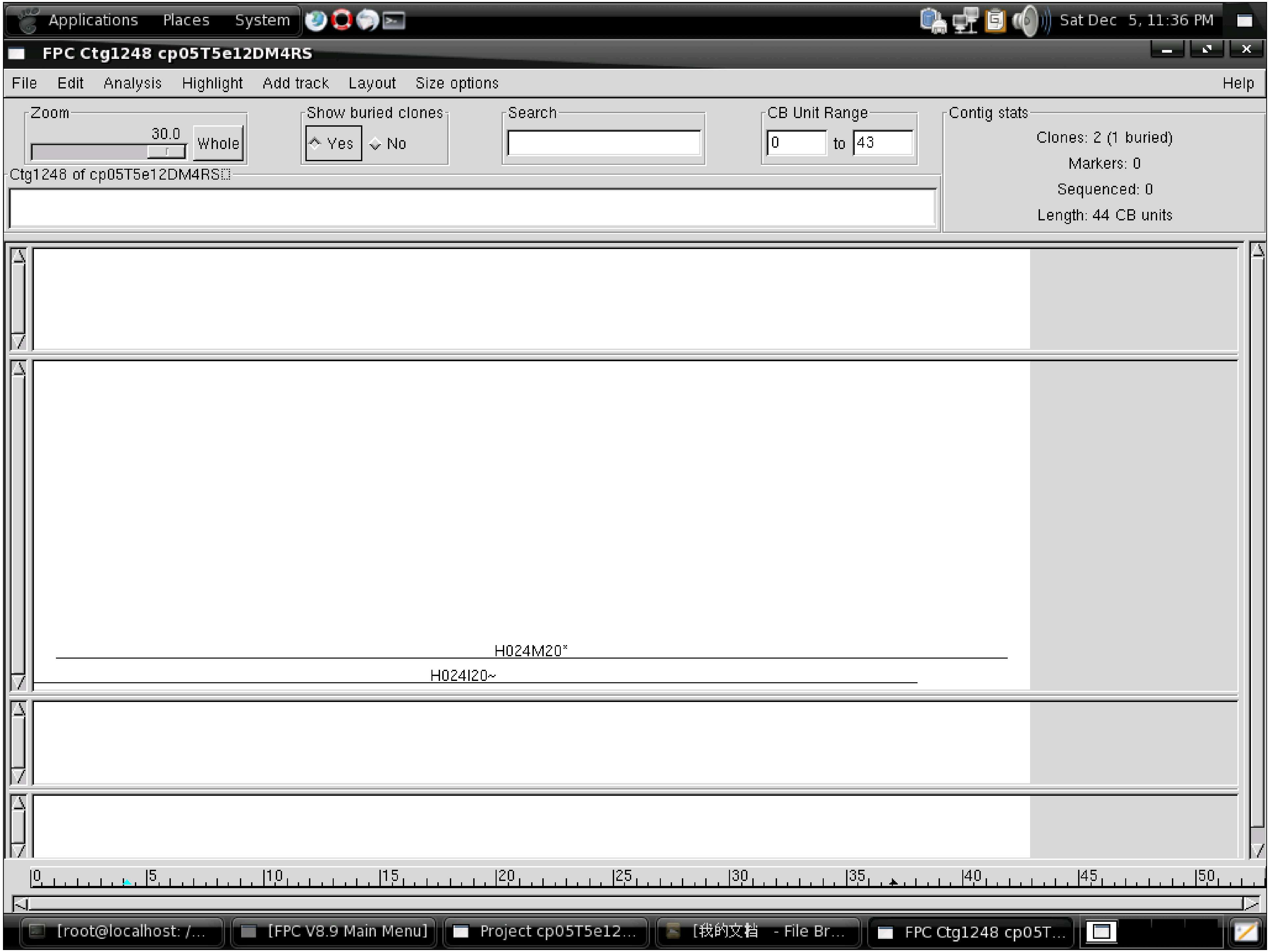
Applications
Places
System
Sat Dec 5, 11:36 PM
FPC Ctg1248 cp05T5e12DM4RS
File
Edit
Analysis
Highlight
Add track
Layout
Size options
Help
Zoom
30.0
Whole
Show buried clones
Yes
No
Search
CB Unit Range
0
to
43
Contig stats
Clones: 2 (1 buried)
Markers: 0
Sequenced: 0
Length: 44 CB units
Ctg1248 of cp05T5e12DM4RS
H024M20*
H024I20~
0
5
10
15
20
25
30
35
40
45
50
[root@localhost: /...
[FPC V8.9 Main Menu]
Project cp05T5e12...
[我的文档 - File Br...
FPC Ctg1248 cp05T...

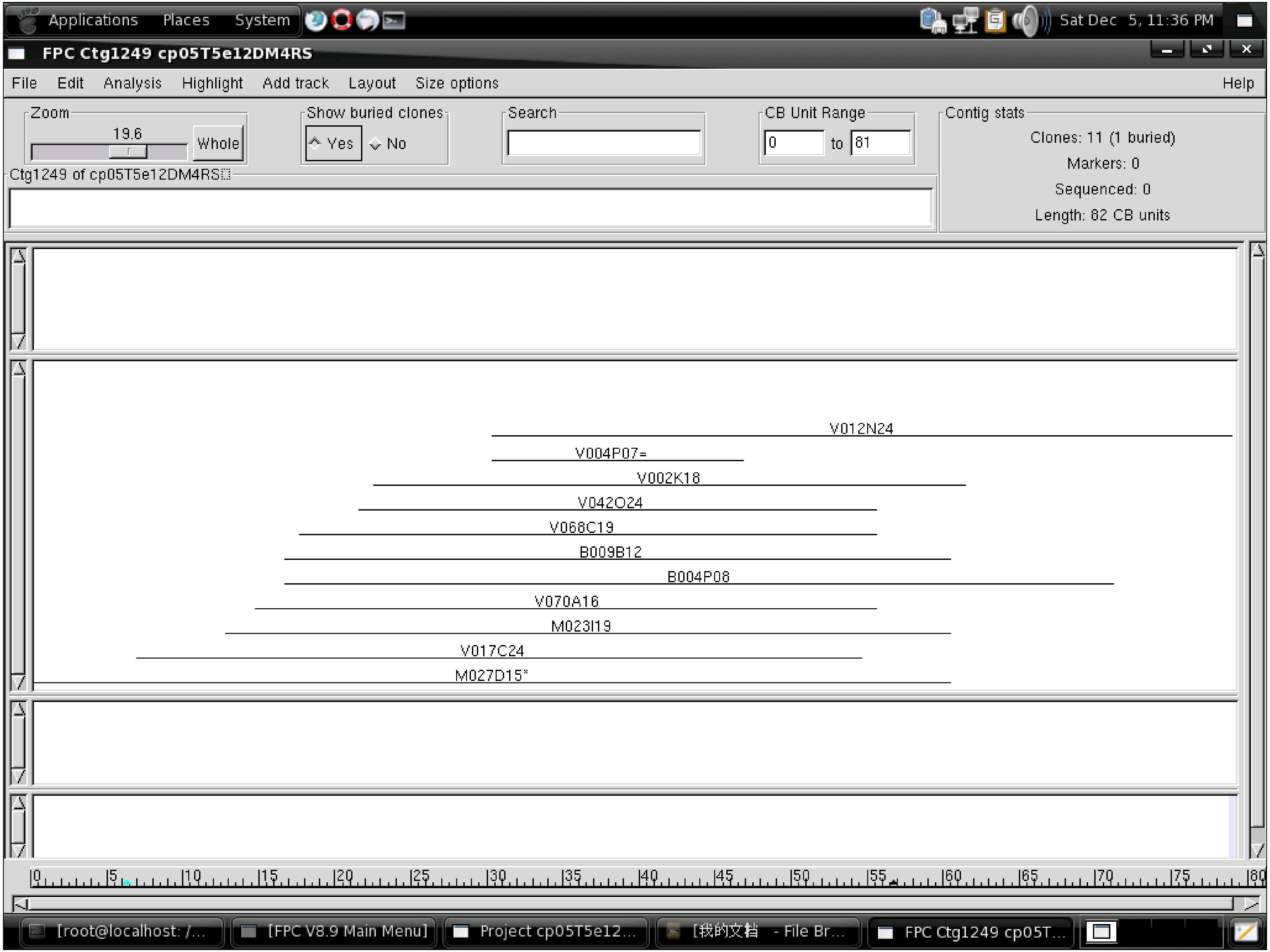
Applications Places System
Sat Dec 5, 11:36 PM
FPC Ctg1249 cp05T5e12DM4RS
File Edit Analysis Highlight Add track Layout Size options Help
Zoom
19.6
Whole
Show buried clones
Yes
No
Search
CB Unit Range
0
to
81
Contig stats
Clones: 11 (1 buried)
Markers: 0
Sequenced: 0
Length: 82 CB units
Ctg1249 of cp05T5e12DM4RS
V012N24
V004P07=
V002K18
V042O24
V068C19
B009B12
B004P08
V070A16
M023I19
V017C24
M027D15*
0 5 10 15 20 25 30 35 40 45 50 55 60 65 70 75 80
[root@localhost: /...
[FPC V8.9 Main Menu]
Project cp05T5e12...
[我的文档 - File Br...
FPC Ctg1249 cp05T...

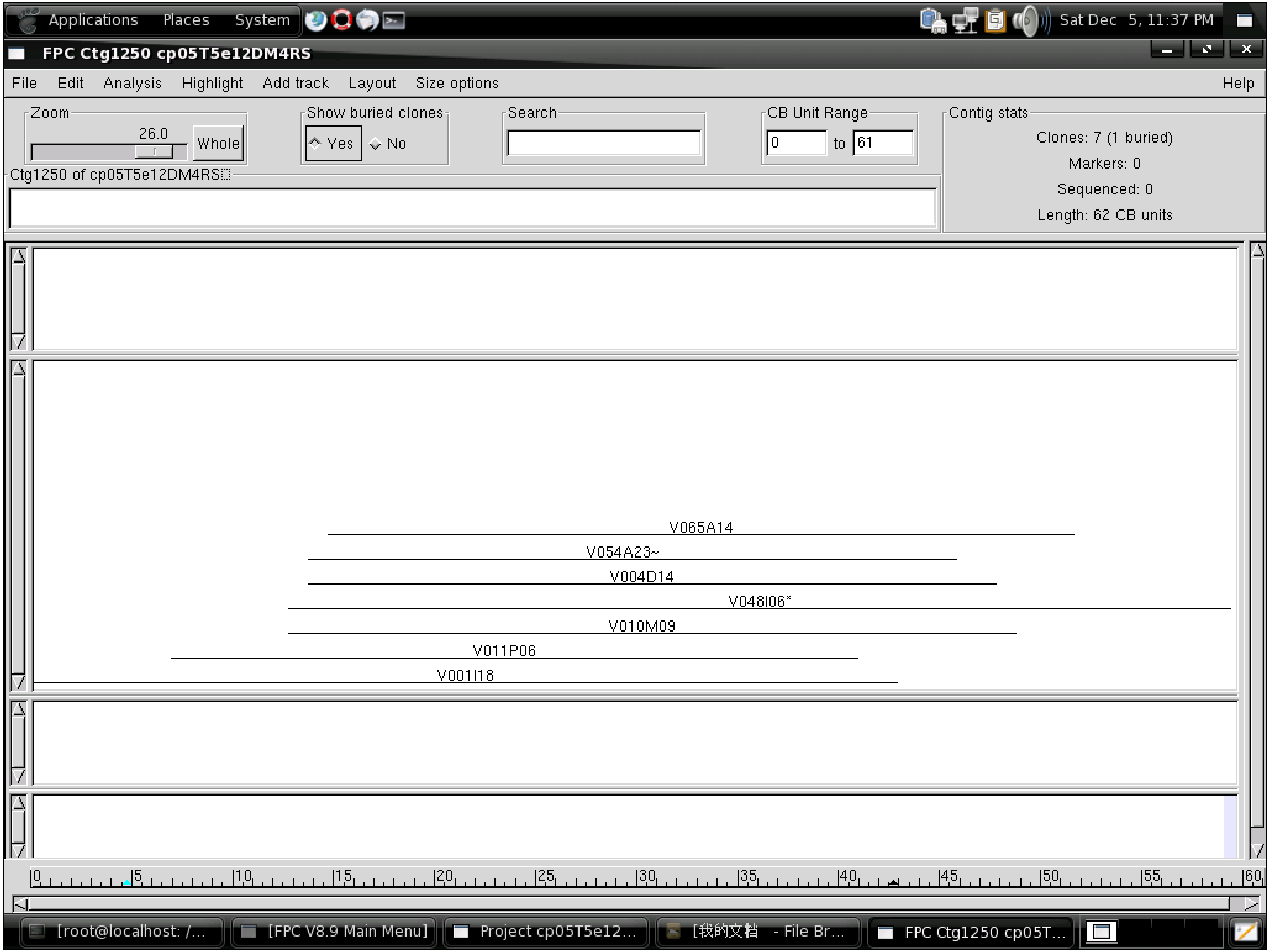
Applications Places System
Sat Dec 5, 11:37 PM
FPC Ctg1250 cp05T5e12DM4RS
File Edit Analysis Highlight Add track Layout Size options Help
Zoom
26.0
Whole
Show buried clones
Yes No
Search
CB Unit Range
0 to 61
Contig stats
Clones: 7 (1 buried)
Markers: 0
Sequenced: 0
Length: 62 CB units
Ctg1250 of cp05T5e12DM4RS
V065A14
V054A23~
V004D14
V048I06*
V010M09
V011P06
V001I18
0 5 10 15 20 25 30 35 40 45 50 55 60
[root@localhost: /...
[FPC V8.9 Main Menu]
Project cp05T5e12...
[我的文档 - File Br...
FPC Ctg1250 cp05T...

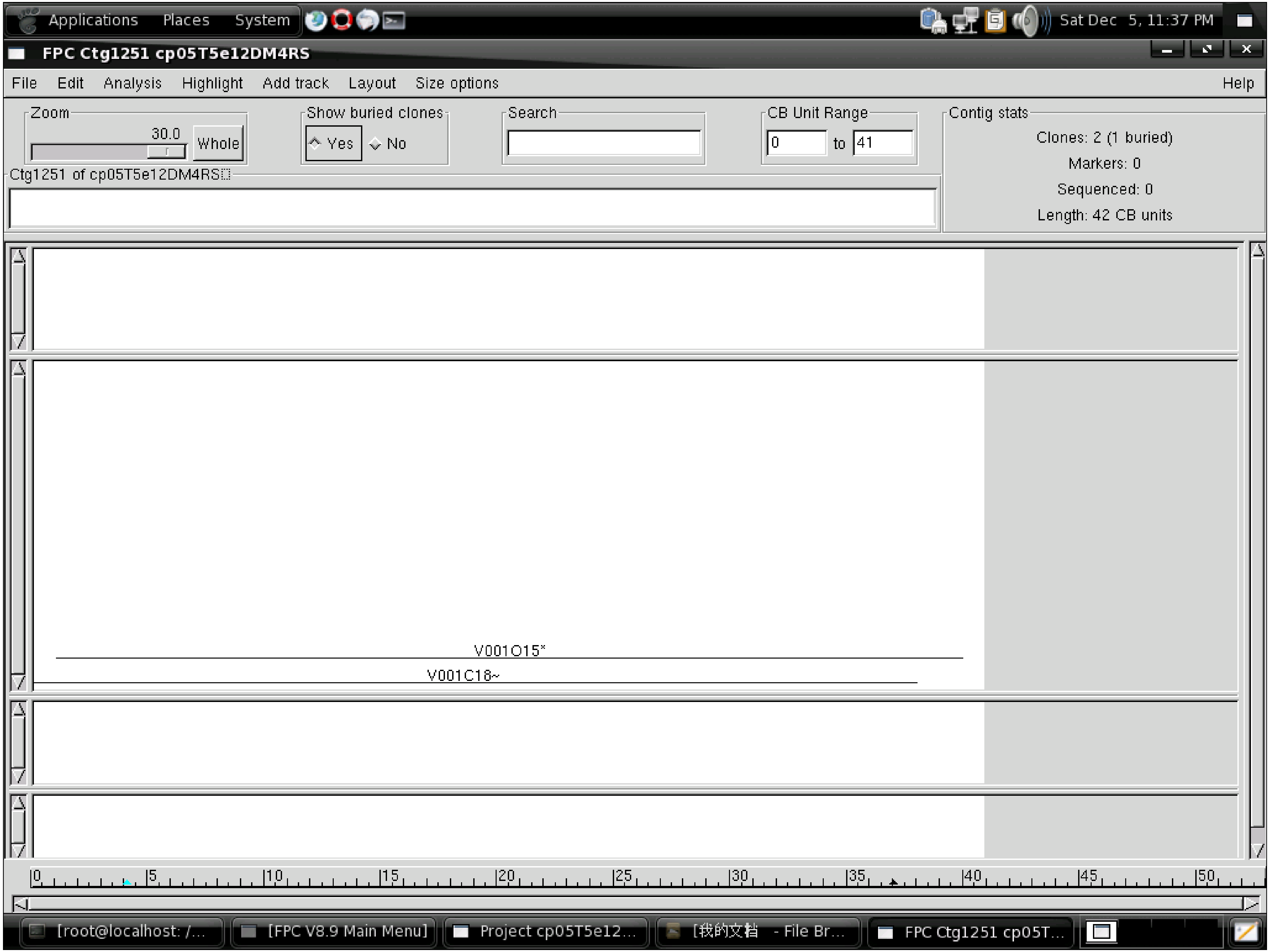
Applications
Places
System
Sat Dec 5, 11:37 PM
FPC Ctg1251 cp05T5e12DM4RS
File
Edit
Analysis
Highlight
Add track
Layout
Size options
Help
Zoom
30.0
Whole
Show buried clones
Yes
No
Search
CB Unit Range
0
to
41
Contig stats
Clones: 2 (1 buried)
Markers: 0
Sequenced: 0
Length: 42 CB units
Ctg1251 of cp05T5e12DM4RS
V001O15*
V001C18~
0
5
10
15
20
25
30
35
40
45
50
[root@localhost: /...
[FPC V8.9 Main Menu]
Project cp05T5e12...
[我的文档 - File Br...
FPC Ctg1251 cp05T...

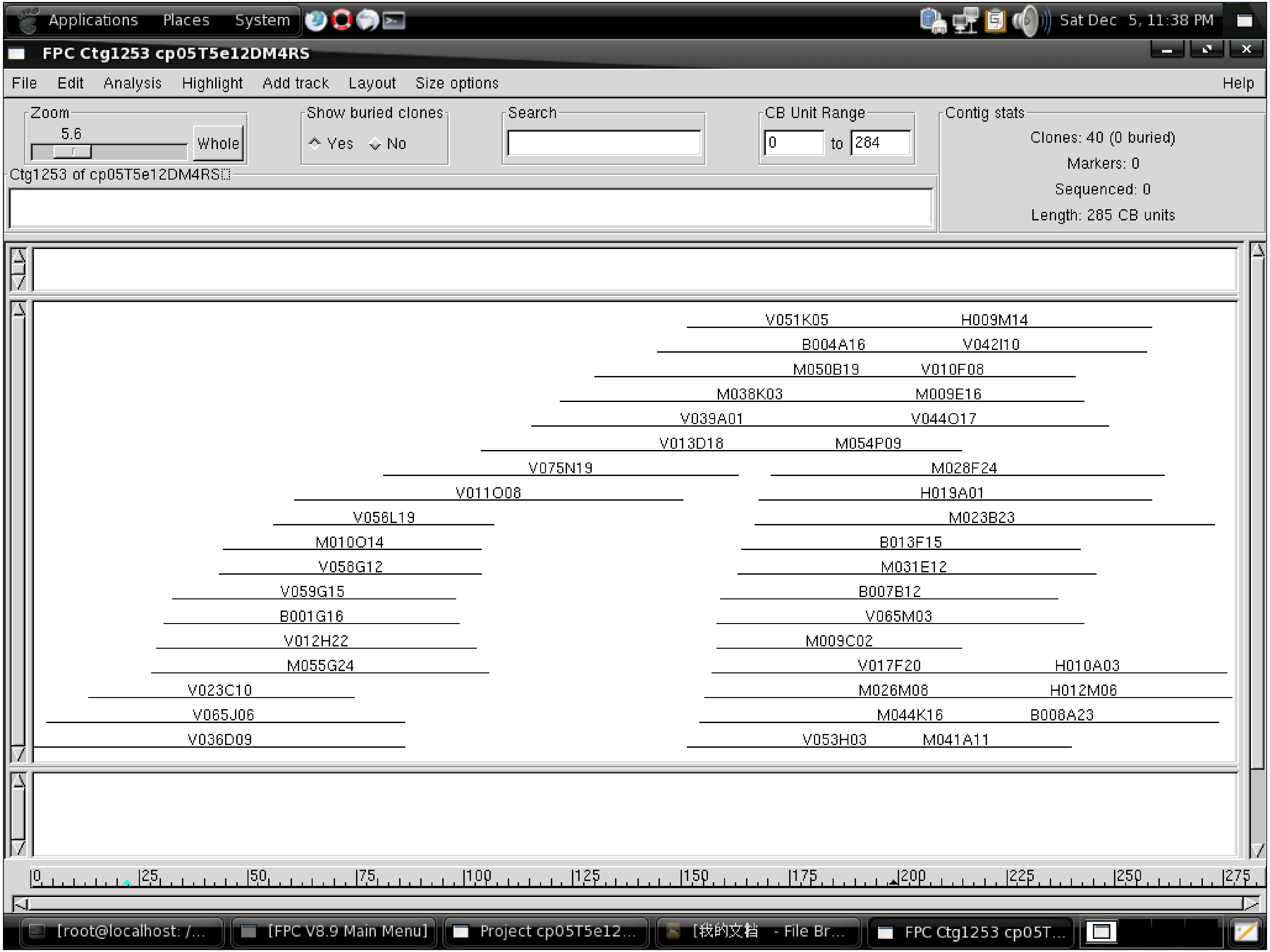
Applications
Places
System
Sat Dec 5, 11:38 PM
FPC Ctg1253 cp05T5e12DM4RS
File
Edit
Analysis
Highlight
Add track
Layout
Size options
Help
Zoom
5.6
Whole
Show buried clones
Yes
No
Search
CB Unit Range
0
to
284
Contig stats
Clones: 40 (0 buried)
Markers: 0
Sequenced: 0
Length: 285 CB units
Ctg1253 of cp05T5e12DM4RS
V051K05
H009M14
B004A16
V042I10
M050B19
V010F08
M038K03
M009E16
V039A01
V044O17
V013D18
M054P09
V075N19
M028F24
V011O08
H019A01
V056L19
M023B23
M010O14
B013F15
V058G12
M031E12
V059G15
B007B12
B001G16
V065M03
V012H22
M009C02
M055G24
V017F20
H010A03
V023C10
M026M08
H012M06
V065J06
M044K16
B008A23
V036D09
V053H03
M041A11
0
25
50
75
100
125
150
175
200
225
250
275
[root@localhost: /...
[FPC V8.9 Main Menu]
Project cp05T5e12...
[我的文档 - File Br...
FPC Ctg1253 cp05T...

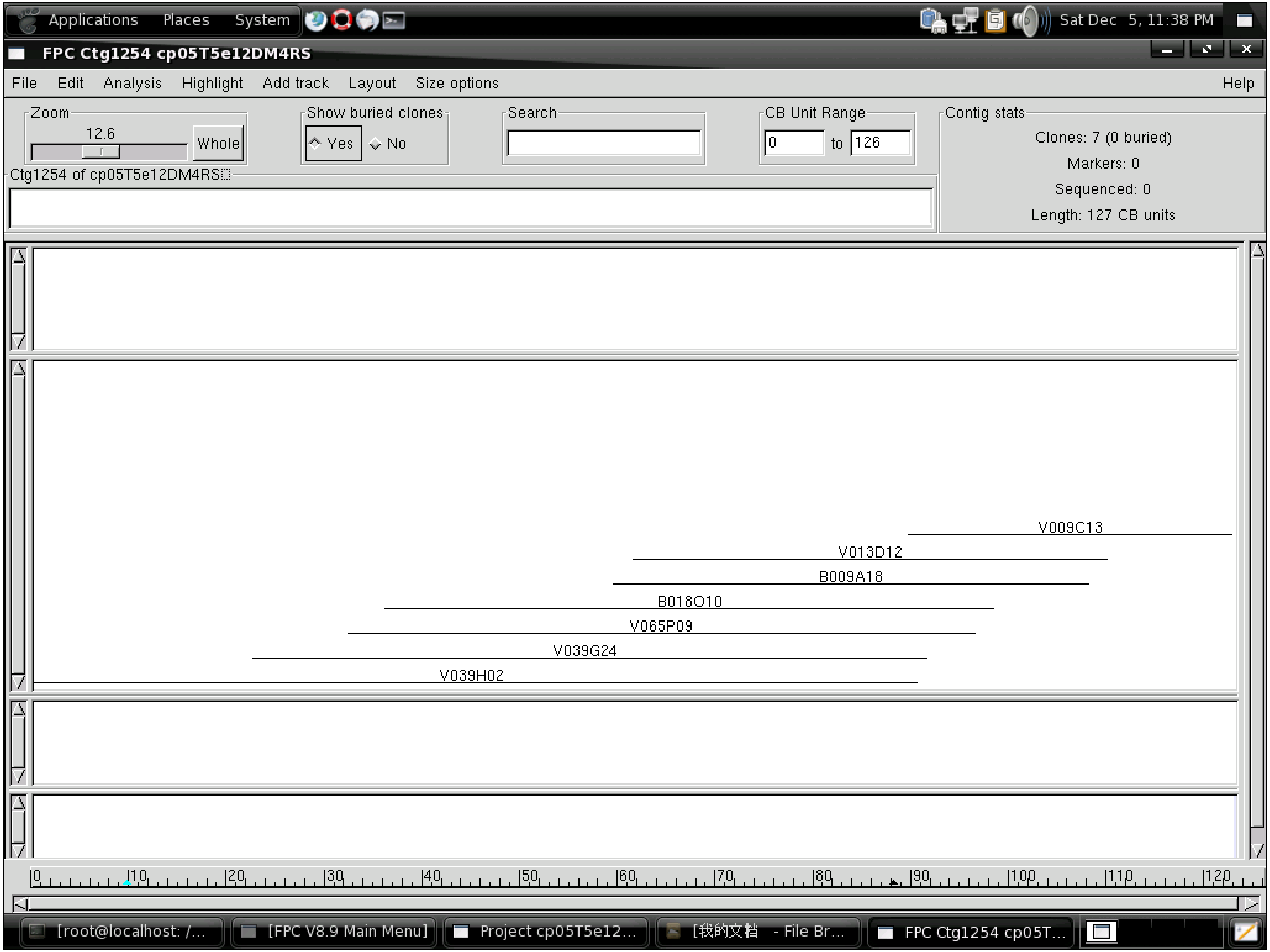
Applications Places System
Sat Dec 5, 11:38 PM
FPC Ctg1254 cp05T5e12DM4RS
File Edit Analysis Highlight Add track Layout Size options Help
Zoom
12.6
Whole
Show buried clones
Yes
No
Search
CB Unit Range
0 to 126
Contig stats
Clones: 7 (0 buried)
Markers: 0
Sequenced: 0
Length: 127 CB units
Ctg1254 of cp05T5e12DM4RS
V009C13
V013D12
B009A18
B018O10
V065P09
V039G24
V039H02
0 10 20 30 40 50 60 70 80 90 100 110 120
[root@localhost: /...
[FPC V8.9 Main Menu]
Project cp05T5e12...
[我的文档 - File Br...
FPC Ctg1254 cp05T...

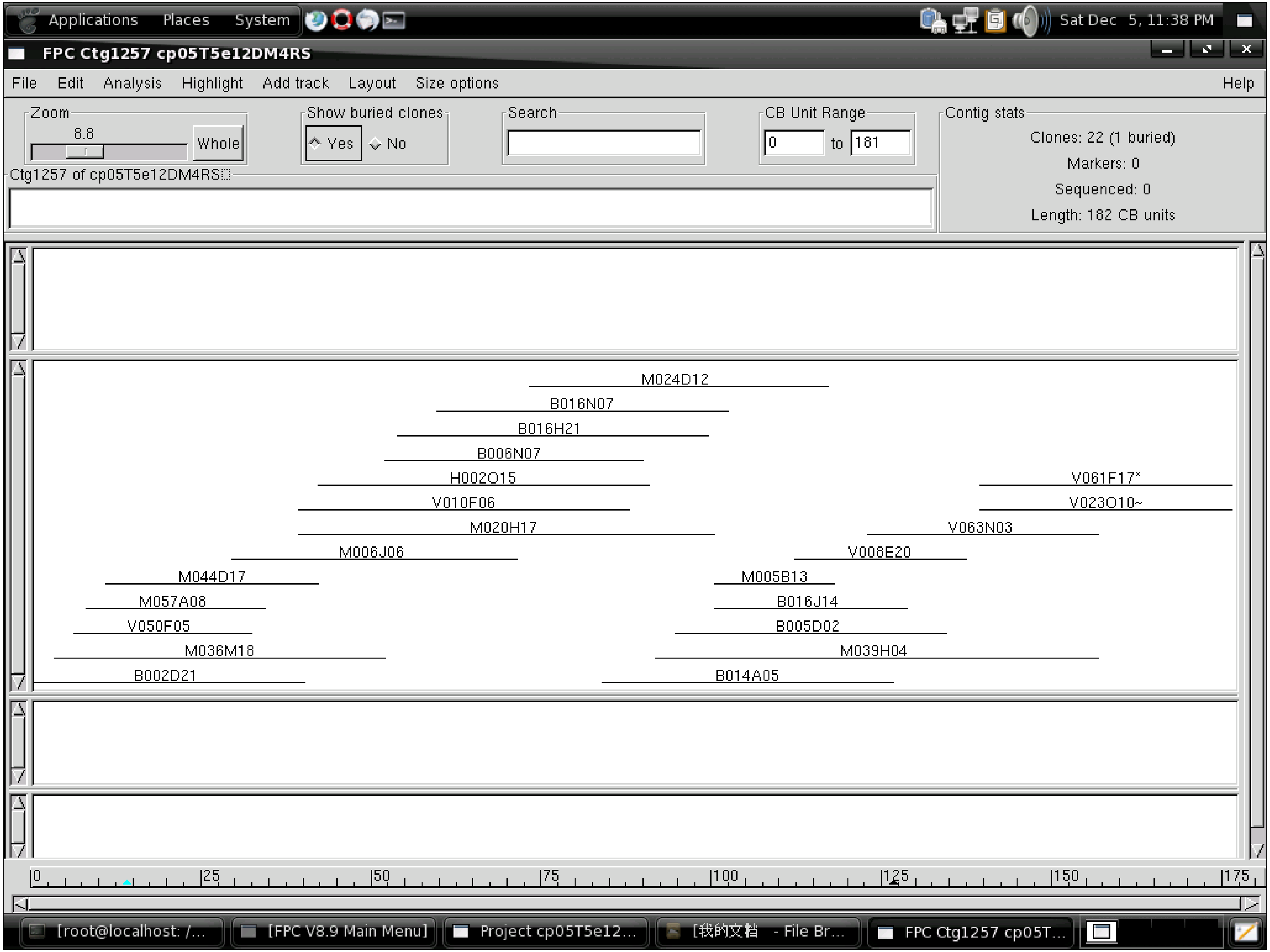
Applications Places System
Sat Dec 5, 11:38 PM
FPC Ctg1257 cp05T5e12DM4RS
File Edit Analysis Highlight Add track Layout Size options Help
Zoom
8.8
Whole
Show buried clones
Yes No
Search
CB Unit Range
0 to 181
Contig stats
Clones: 22 (1 buried)
Markers: 0
Sequenced: 0
Length: 182 CB units
Ctg1257 of cp05T5e12DM4RS
M024D12
B016N07
B016H21
B006N07
H002O15
V061F17*
V010F06
V023O10~
M020H17
V063N03
M006J06
V008E20
M044D17
M005B13
M057A08
B016J14
V050F05
B005D02
M036M18
M039H04
B002D21
B014A05
0 25 50 75 100 125 150 175
[root@localhost: /...
[FPC V8.9 Main Menu]
Project cp05T5e12...
[我的文档 - File Br...
FPC Ctg1257 cp05T...

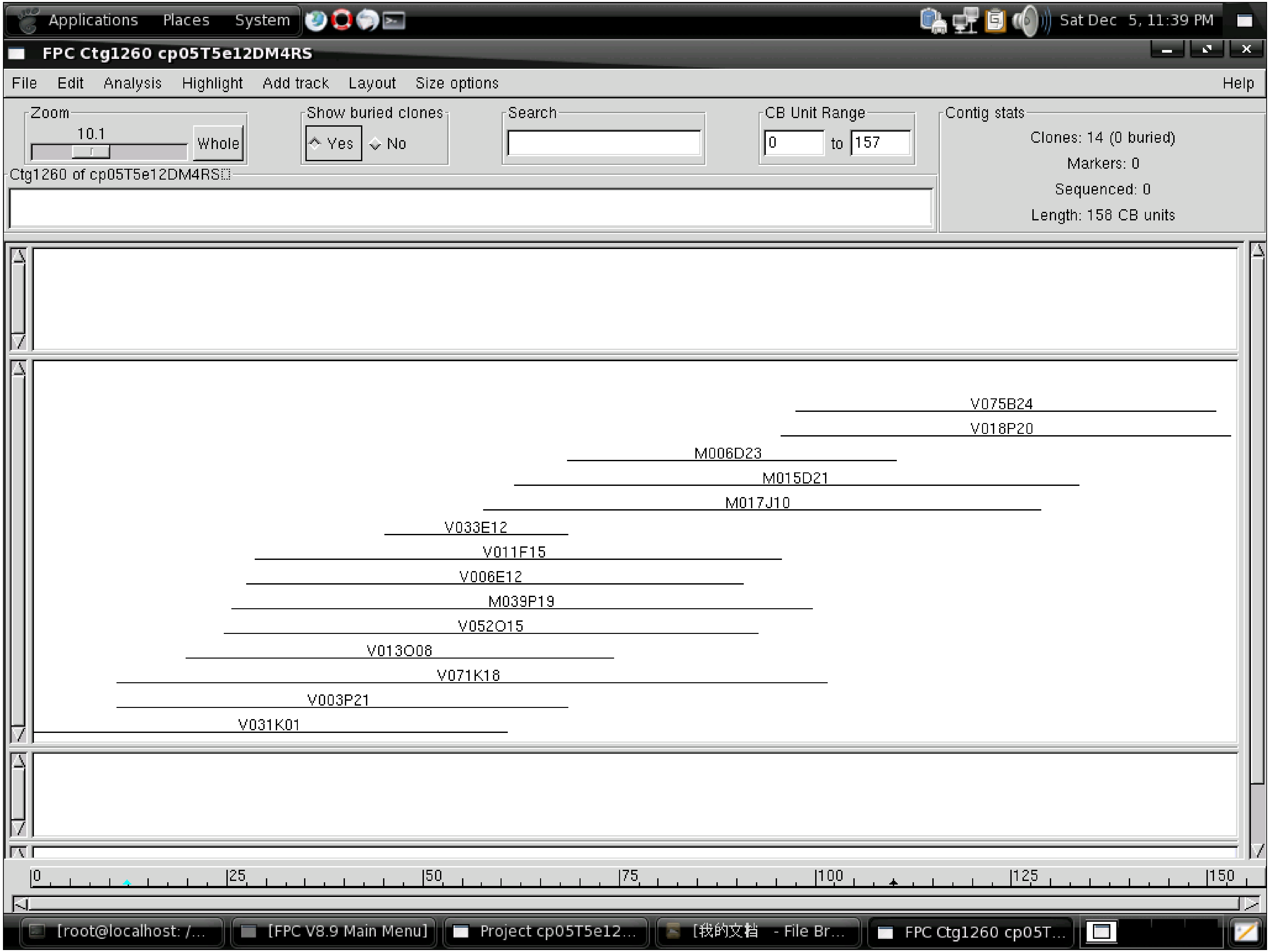
Applications Places System
Sat Dec 5, 11:39 PM
FPC Ctg1260 cp05T5e12DM4RS
File Edit Analysis Highlight Add track Layout Size options Help
Zoom
10.1
Whole
Show buried clones
Yes
No
Search
CB Unit Range
0 to 157
Contig stats
Clones: 14 (0 buried)
Markers: 0
Sequenced: 0
Length: 158 CB units
Ctg1260 of cp05T5e12DM4RS
V075B24
V018P20
M006D23
M015D21
M017J10
V033E12
V011F15
V006E12
M039P19
V052O15
V013O08
V071K18
V003P21
V031K01
0
25
50
75
100
125
150
[root@localhost: /...
[FPC V8.9 Main Menu]
Project cp05T5e12...
[我的文档 - File Br...
FPC Ctg1260 cp05T...

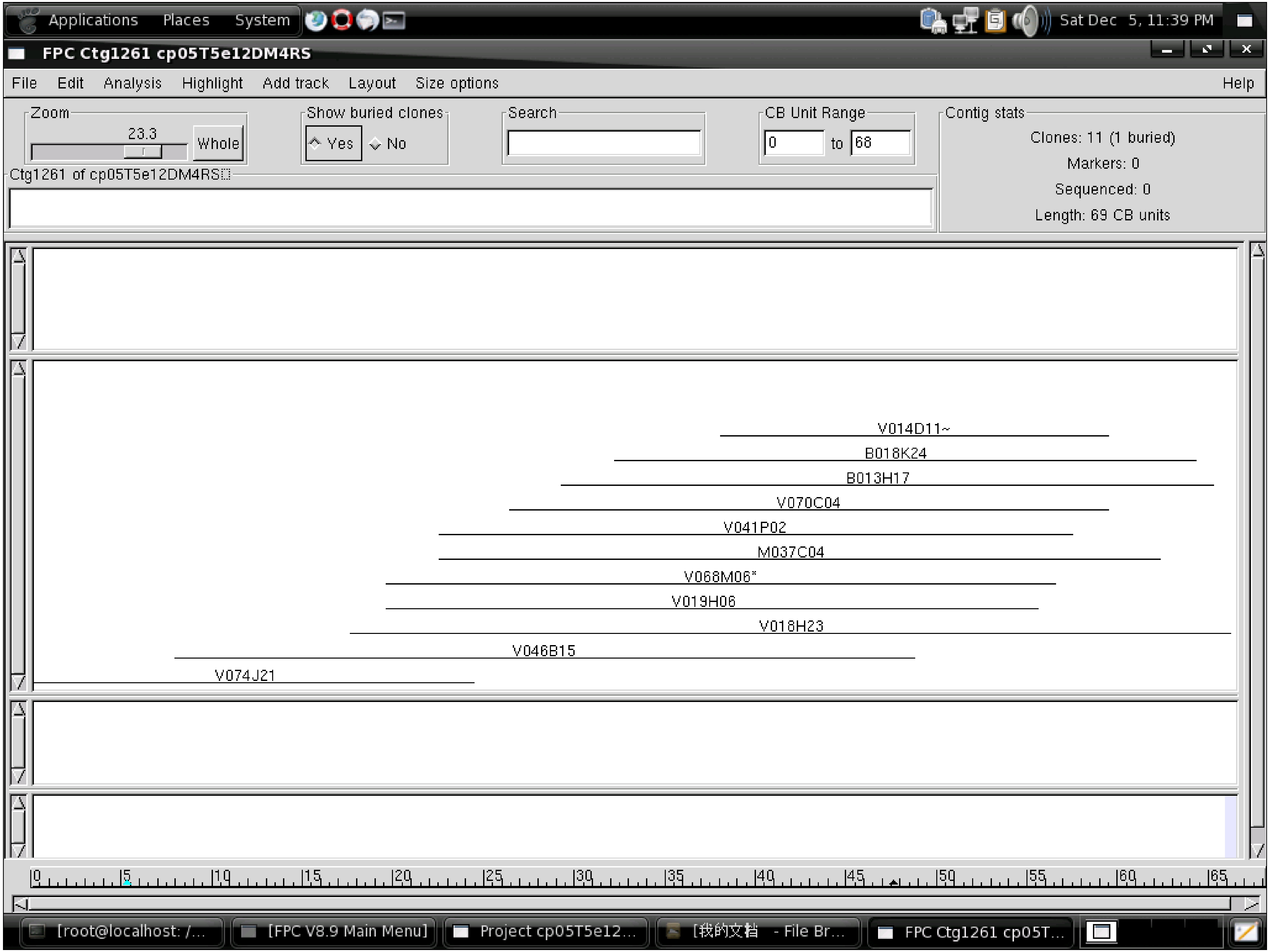
Applications Places System
Sat Dec 5, 11:39 PM
FPC Ctg1261 cp05T5e12DM4RS
File Edit Analysis Highlight Add track Layout Size options Help
Zoom
23.3
Whole
Show buried clones
Yes
No
Search
CB Unit Range
0
to
68
Contig stats
Clones: 11 (1 buried)
Markers: 0
Sequenced: 0
Length: 69 CB units
Ctg1261 of cp05T5e12DM4RS
V014D11~
B018K24
B013H17
V070C04
V041P02
M037C04
V068M06*
V019H06
V018H23
V046B15
V074J21
0 5 10 15 20 25 30 35 40 45 50 55 60 65
[root@localhost: /...
[FPC V8.9 Main Menu]
Project cp05T5e12...
[我的文档 - File Br...
FPC Ctg1261 cp05T...

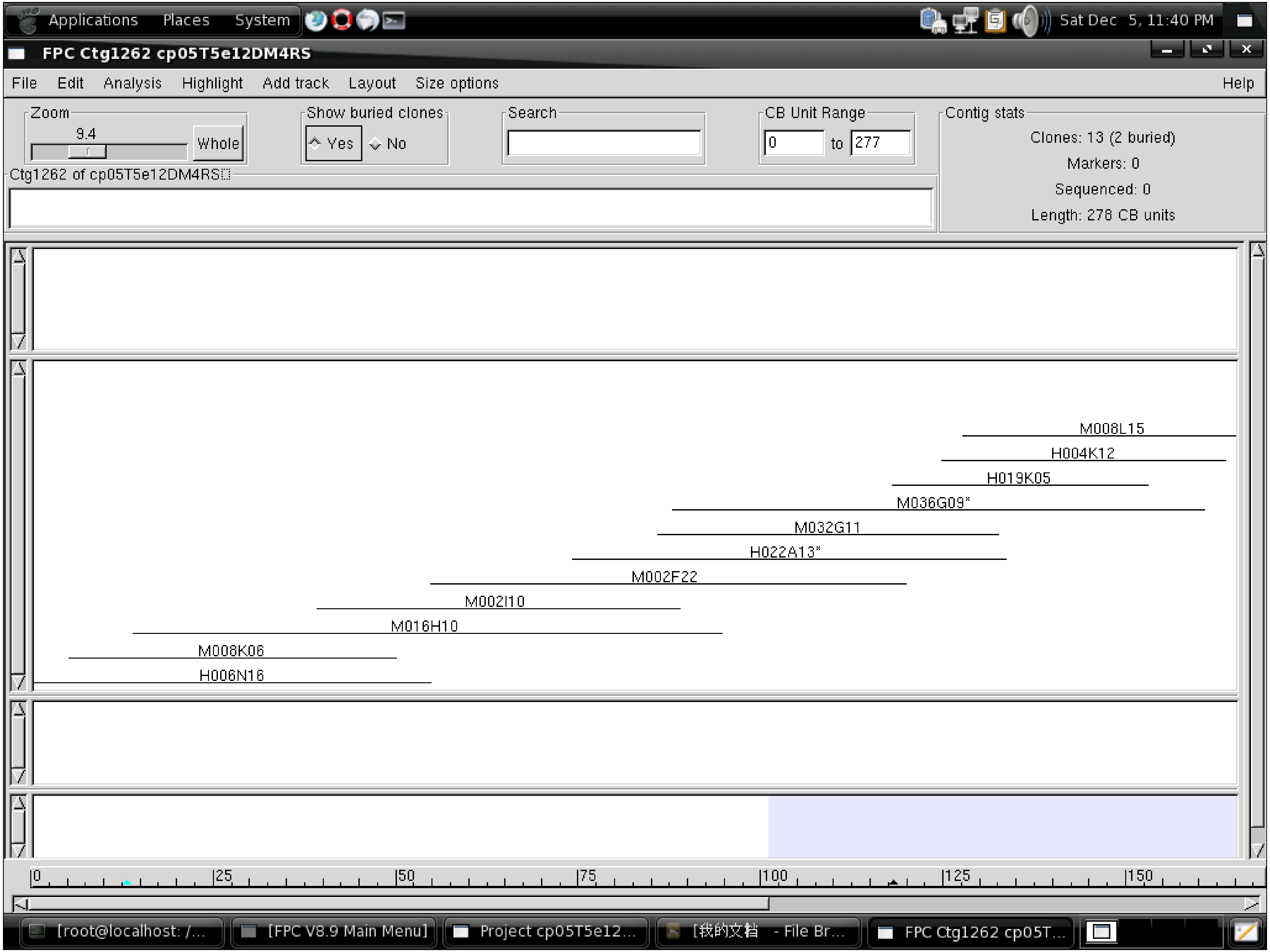
Applications Places System
Sat Dec 5, 11:40 PM
FPC Ctg1262 cp05T5e12DM4RS
File Edit Analysis Highlight Add track Layout Size options Help
Zoom
9.4
Whole
Show buried clones
Yes
No
Search
CB Unit Range
0
to
277
Contig stats
Clones: 13 (2 buried)
Markers: 0
Sequenced: 0
Length: 278 CB units
Ctg1262 of cp05T5e12DM4RS
M008L15
H004K12
H019K05
M036G09*
M032G11
H022A13*
M002F22
M002I10
M016H10
M008K06
H006N16
0
25
50
75
100
125
150
[root@localhost: /...
[FPC V8.9 Main Menu]
Project cp05T5e12...
[我的文档 - File Br...
FPC Ctg1262 cp05T...

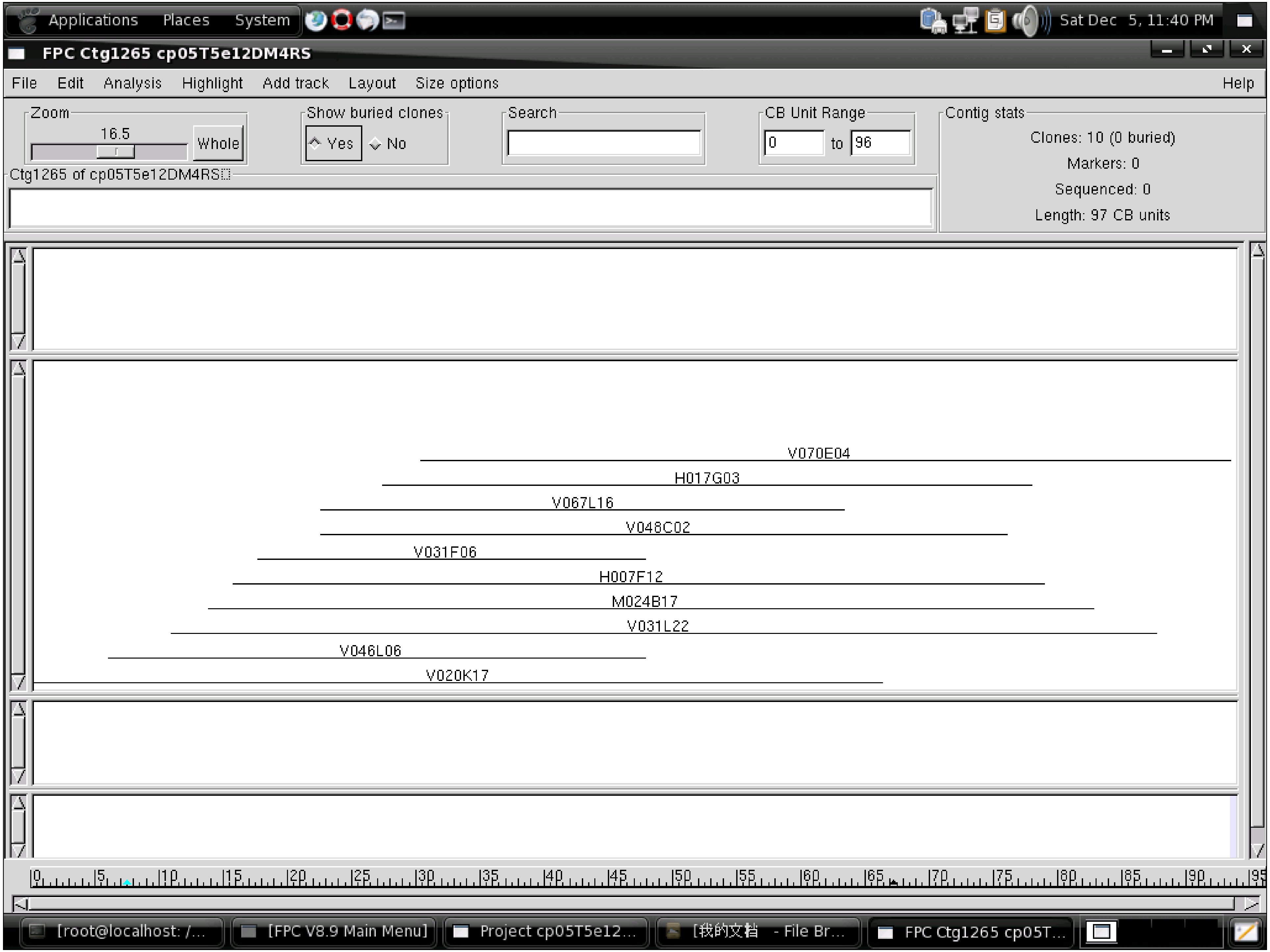
Applications Places System
Sat Dec 5, 11:40 PM
FPC Ctg1265 cp05T5e12DM4RS
File Edit Analysis Highlight Add track Layout Size options Help
Zoom
16.5
Whole
Show buried clones
Yes
No
Search
CB Unit Range
0 to 96
Contig stats
Clones: 10 (0 buried)
Markers: 0
Sequenced: 0
Length: 97 CB units
Ctg1265 of cp05T5e12DM4RS
V070E04
H017G03
V067L16
V048C02
V031F06
H007F12
M024B17
V031L22
V046L06
V020K17
0 5 10 15 20 25 30 35 40 45 50 55 60 65 70 75 80 85 90 95
[root@localhost: /...
[FPC V8.9 Main Menu]
Project cp05T5e12...
[我的文档 - File Br...
FPC Ctg1265 cp05T...

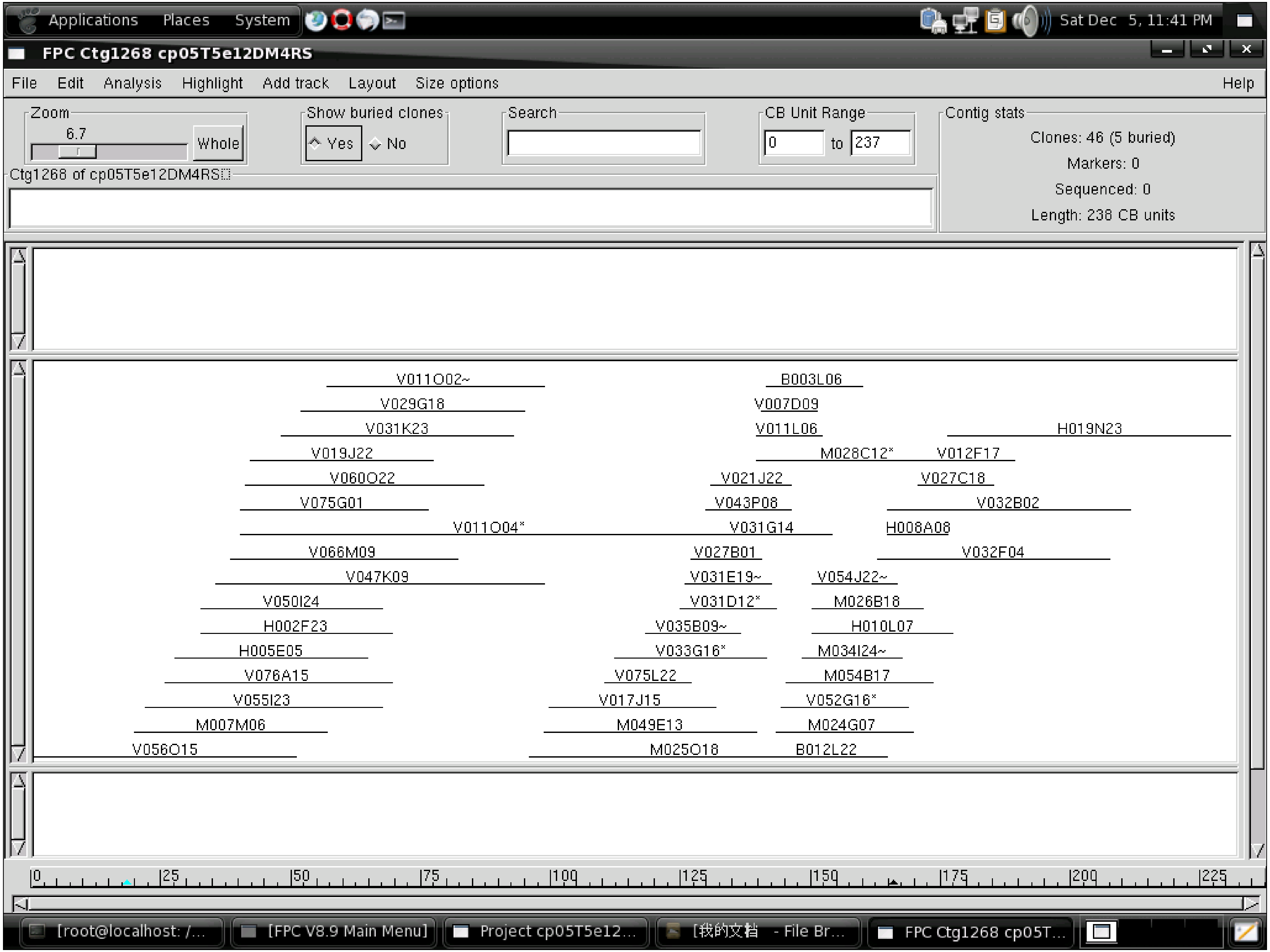
Applications Places System
Sat Dec 5, 11:41 PM
FPC Ctg1268 cp05T5e12DM4RS
File Edit Analysis Highlight Add track Layout Size options Help
Zoom 6.7 Whole
Show buried clones Yes No
Search
CB Unit Range 0 to 237
Contig stats
Clones: 46 (5 buried)
Markers: 0
Sequenced: 0
Length: 238 CB units
Ctg1268 of cp05T5e12DM4RS
V011O02~
V029G18
V031K23
V019J22
V060O22
V075G01
V011O04*
V066M09
V047K09
V050I24
H002F23
H005E05
V076A15
V055I23
M007M06
V056O15
B003L06
V007D09
V011L06
H019N23
M028C12*
V012F17
V021J22
V027C18
V043P08
V032B02
V031G14
H008A08
V027B01
V032F04
V031E19~
V054J22~
V031D12*
M026B18
V035B09~
H010L07
V033G16*
M034I24~
V075L22
M054B17
V017J15
V052G16*
M049E13
M024G07
M025O18
B012L22
0 25 50 75 100 125 150 175 200 225
[root@localhost: /...
[FPC V8.9 Main Menu]
Project cp05T5e12...
[我的文档 - File Br...
FPC Ctg1268 cp05T...

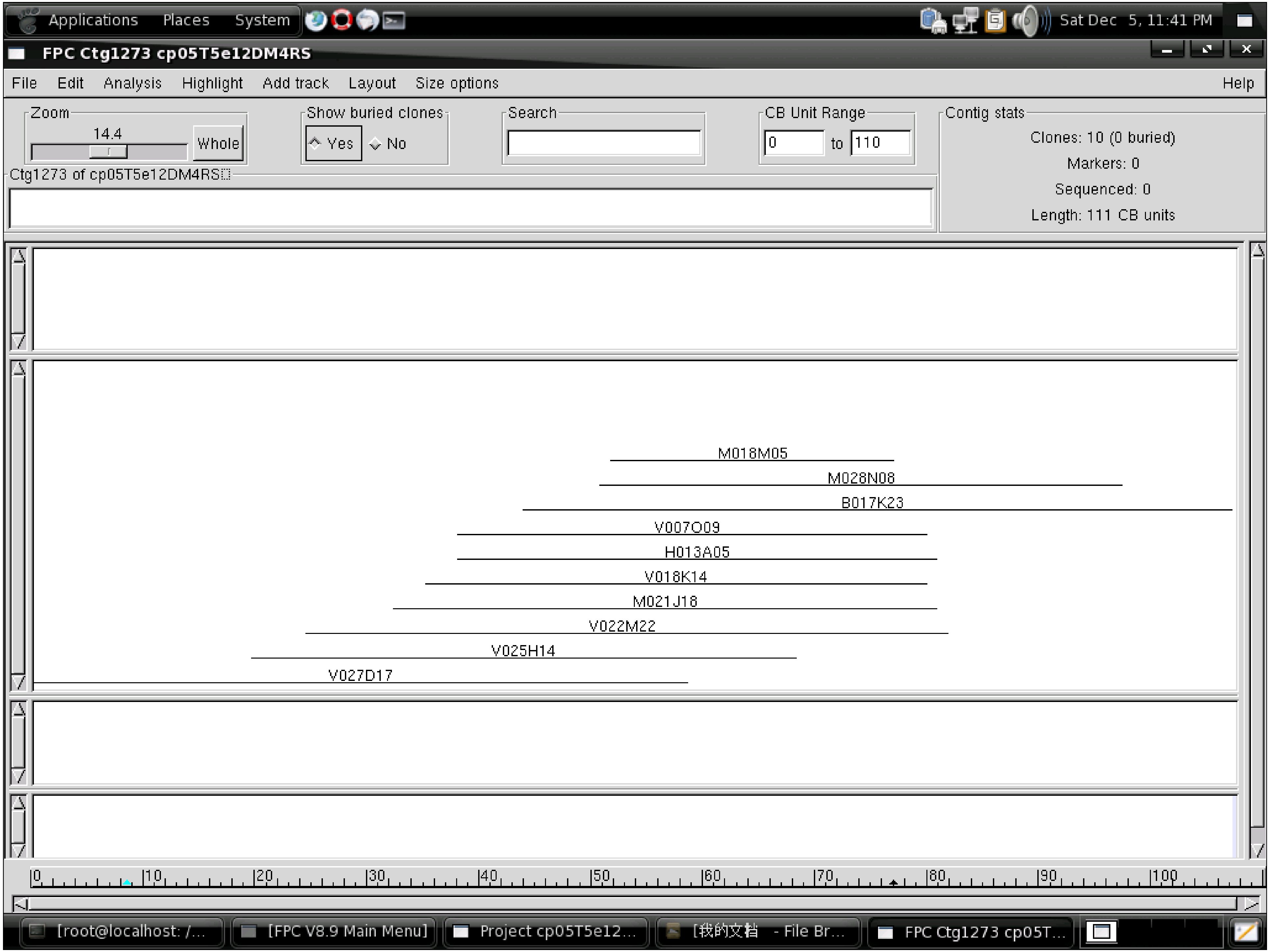

Applications Places System
Sat Dec 5, 11:41 PM
FPC Ctg1273 cp05T5e12DM4RS
File Edit Analysis Highlight Add track Layout Size options Help
Zoom
14.4
Whole
Show buried clones
Yes No
Search
CB Unit Range
0 to 110
Contig stats
Clones: 10 (0 buried)
Markers: 0
Sequenced: 0
Length: 111 CB units
Ctg1273 of cp05T5e12DM4RS
M018M05
M028N08
B017K23
V007O09
H013A05
V018K14
M021J18
V022M22
V025H14
V027D17
0 10 20 30 40 50 60 70 80 90 100
[root@localhost: /...
[FPC V8.9 Main Menu]
Project cp05T5e12...
[我的文档 - File Br...
FPC Ctg1273 cp05T...

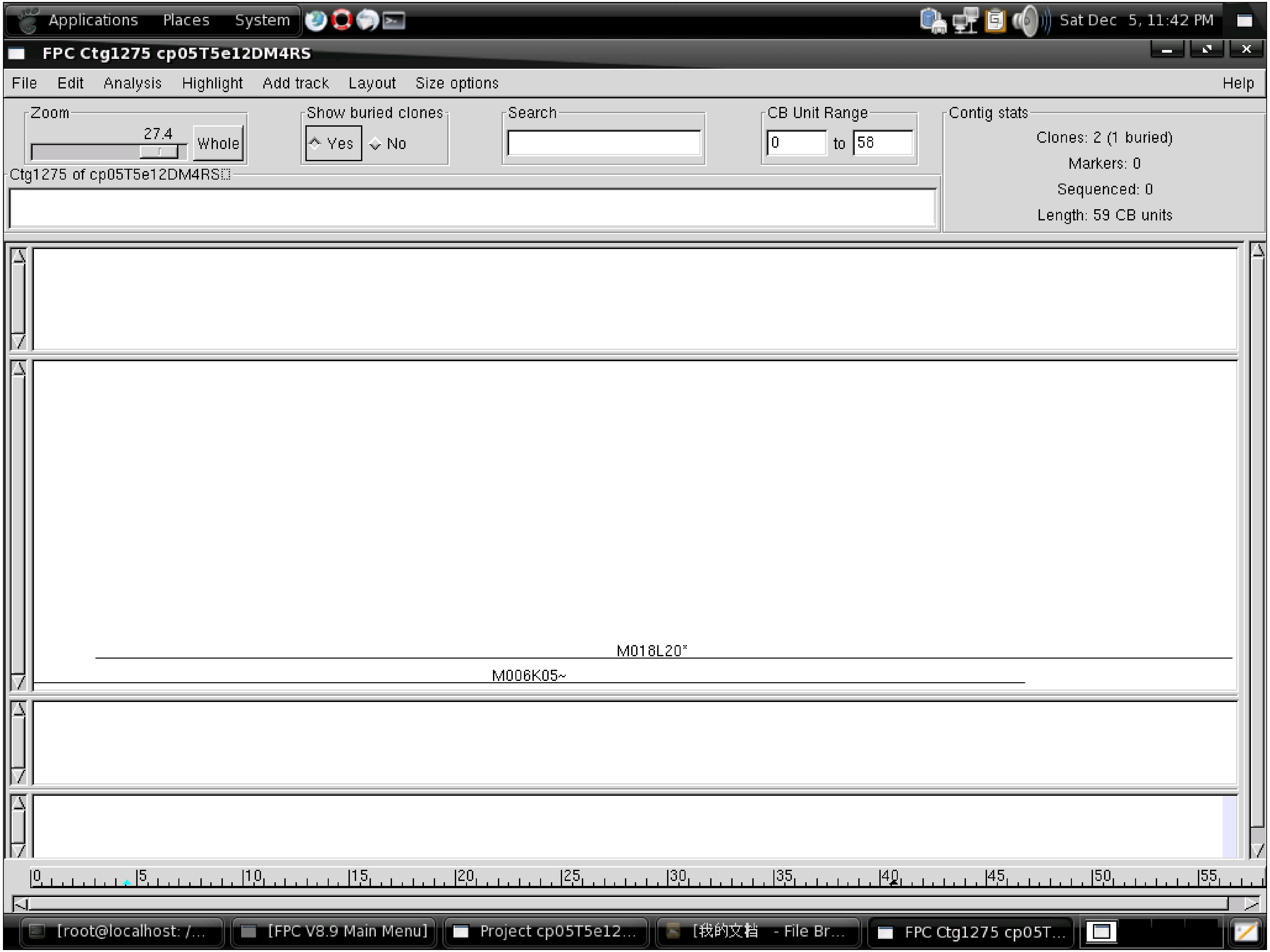
Applications Places System
Sat Dec 5, 11:42 PM
FPC Ctg1275 cp05T5e12DM4RS
File Edit Analysis Highlight Add track Layout Size options Help
Zoom
27.4
Whole
Show buried clones
Yes No
Search
CB Unit Range
0 to 58
Contig stats
Clones: 2 (1 buried)
Markers: 0
Sequenced: 0
Length: 59 CB units
Ctg1275 of cp05T5e12DM4RS
M018L20*
M006K05~
0 5 10 15 20 25 30 35 40 45 50 55
[root@localhost: /...
[FPC V8.9 Main Menu]
Project cp05T5e12...
[我的文档 - File Br...
FPC Ctg1275 cp05T...

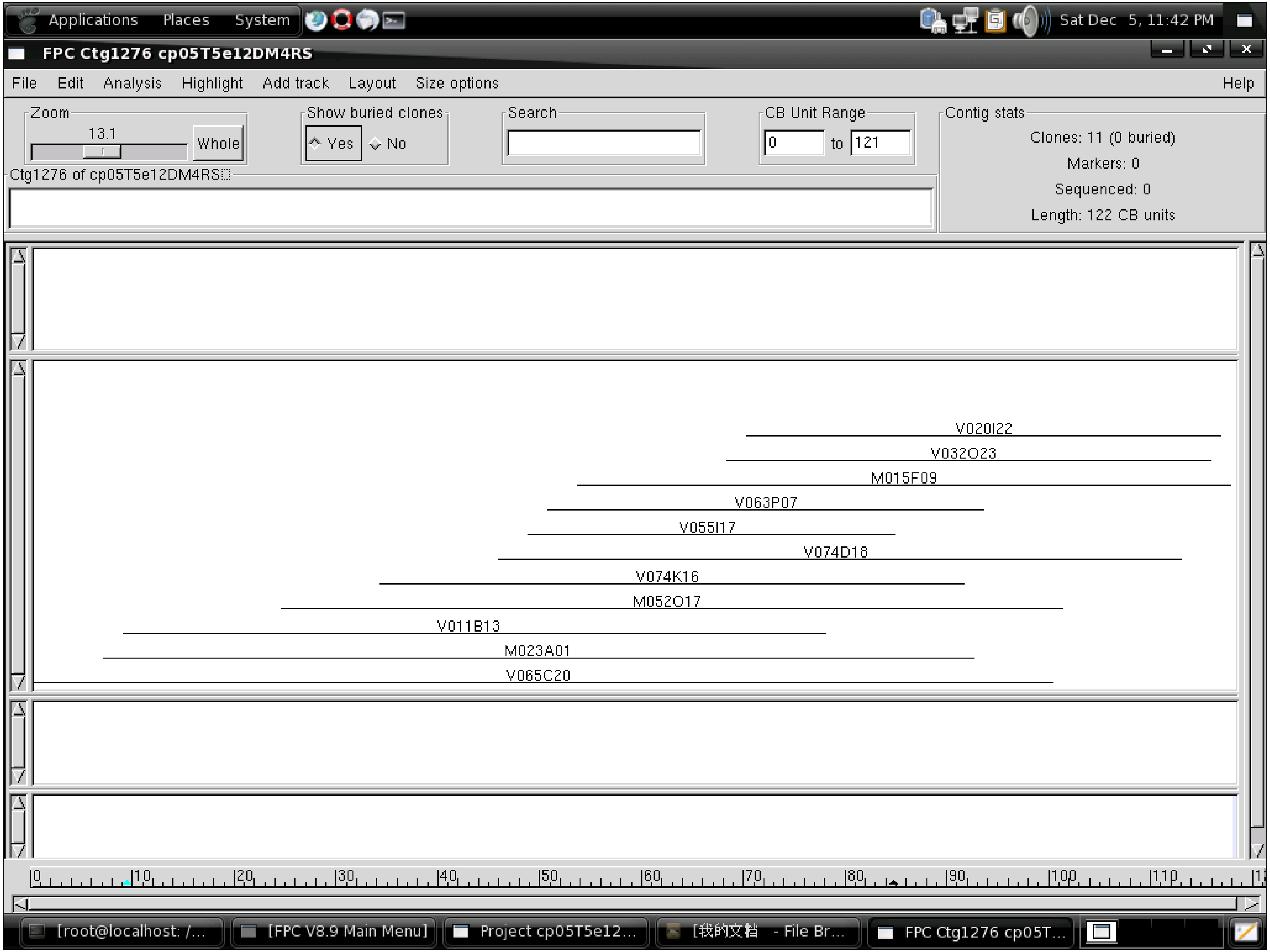
Applications Places System
Sat Dec 5, 11:42 PM
FPC Ctg1276 cp05T5e12DM4RS
File Edit Analysis Highlight Add track Layout Size options Help
Zoom
13.1
Whole
Show buried clones
Yes No
Search
CB Unit Range
0 to 121
Contig stats
Clones: 11 (0 buried)
Markers: 0
Sequenced: 0
Length: 122 CB units
Ctg1276 of cp05T5e12DM4RS
V020I22
V032O23
M015F09
V063P07
V055I17
V074D18
V074K16
M052O17
V011B13
M023A01
V065C20
0 10 20 30 40 50 60 70 80 90 100 110
[root@localhost: /...
[FPC V8.9 Main Menu]
Project cp05T5e12...
[我的文档 - File Br...
FPC Ctg1276 cp05T...

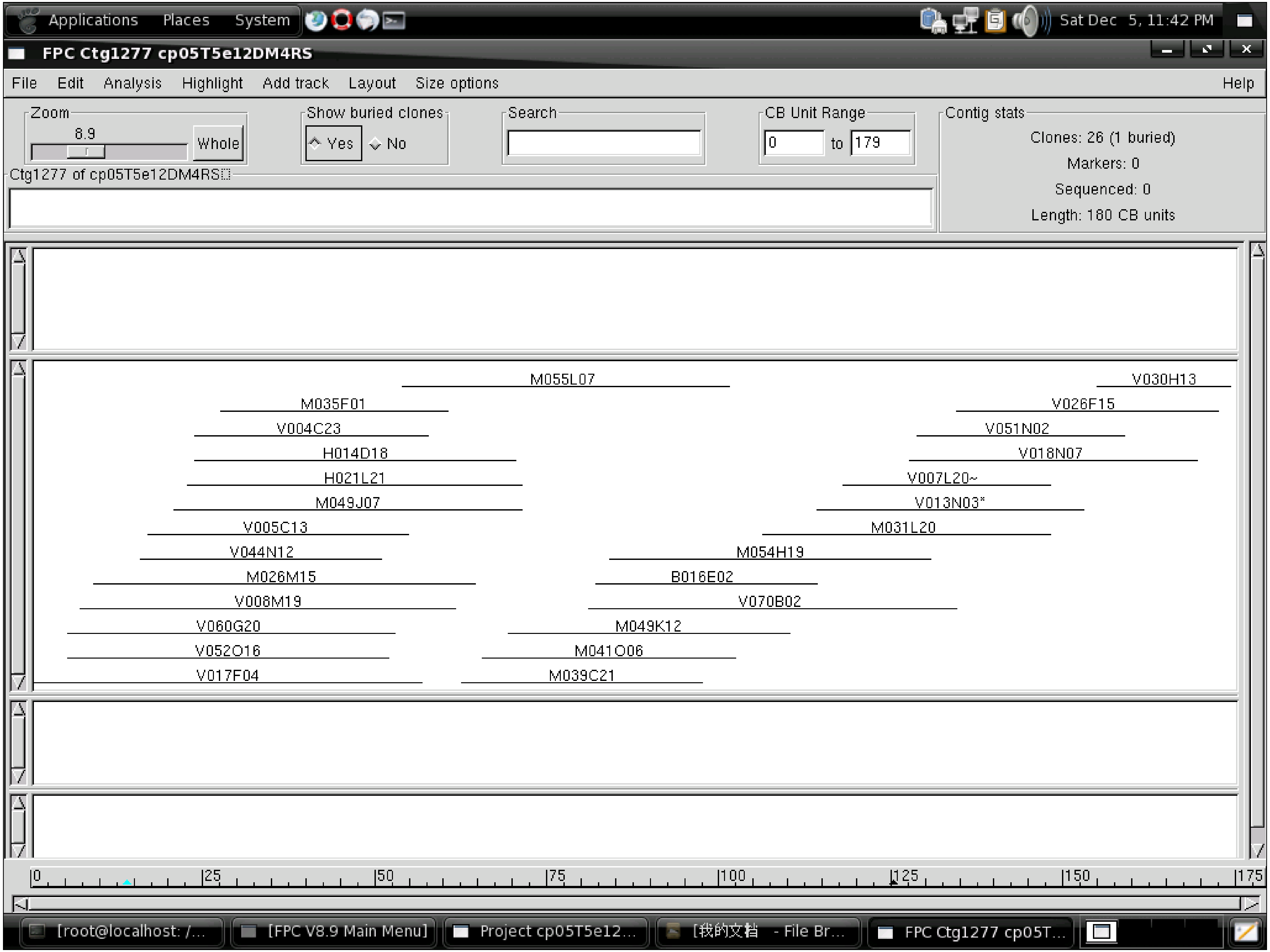
Applications Places System
Sat Dec 5, 11:42 PM
FPC Ctg1277 cp05T5e12DM4RS
File Edit Analysis Highlight Add track Layout Size options Help
Zoom
8.9
Whole
Show buried clones
Yes No
Search
CB Unit Range
0 to 179
Contig stats
Clones: 26 (1 buried)
Markers: 0
Sequenced: 0
Length: 180 CB units
Ctg1277 of cp05T5e12DM4RS
M055L07
V030H13
M035F01
V026F15
V004C23
V051N02
H014D18
V018N07
H021L21
V007L20~
M049J07
V013N03*
V005C13
M031L20
V044N12
M054H19
M026M15
B016E02
V008M19
V070B02
V060G20
M049K12
V052O16
M041O06
V017F04
M039C21
0 25 50 75 100 125 150 175
[root@localhost: /...
[FPC V8.9 Main Menu]
Project cp05T5e12...
[我的文档 - File Br...
FPC Ctg1277 cp05T...

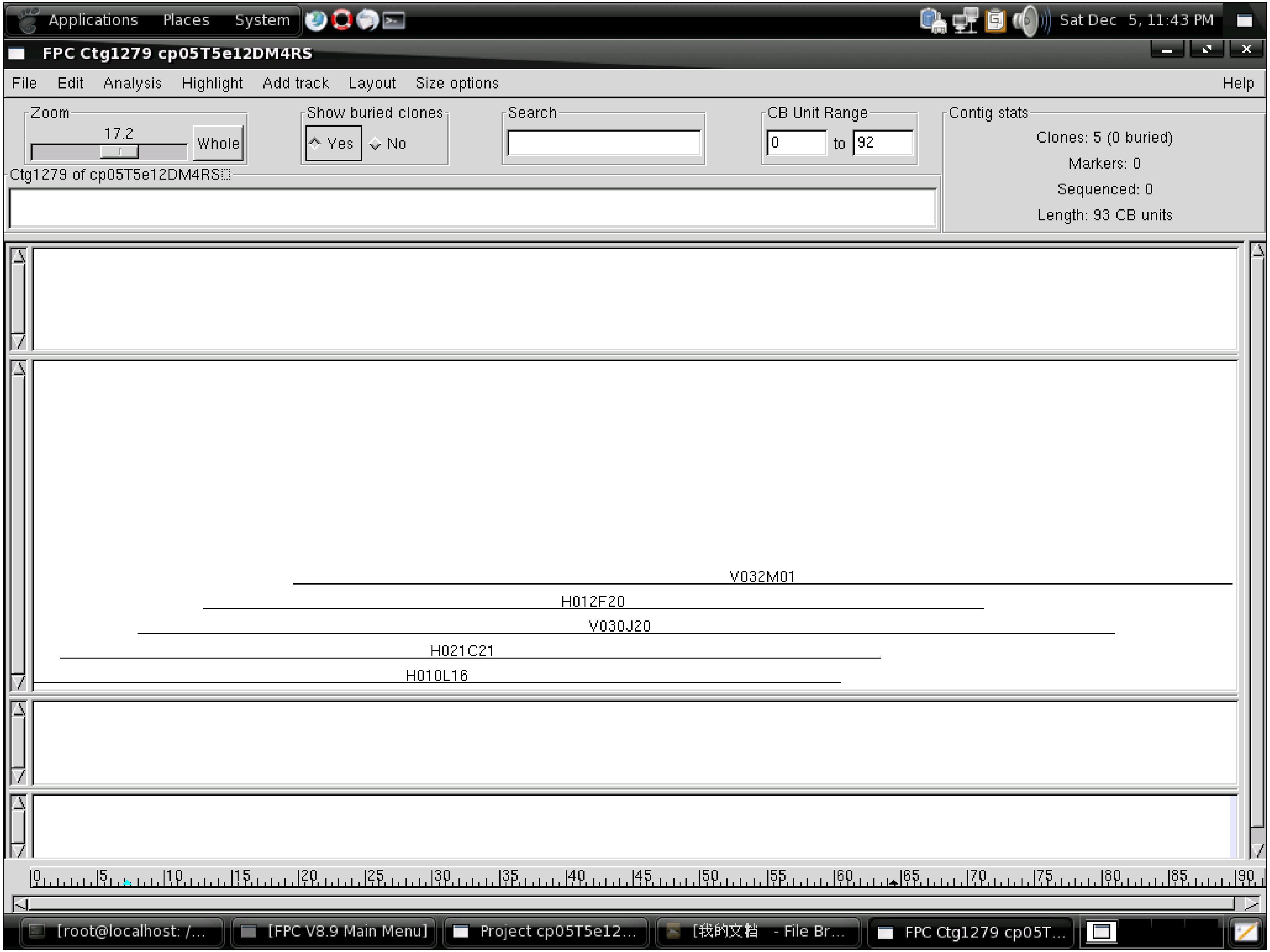

Applications Places System
Sat Dec 5, 11:43 PM
FPC Ctg1279 cp05T5e12DM4RS
File Edit Analysis Highlight Add track Layout Size options Help
Zoom
17.2
Whole
Show buried clones
Yes No
Search
CB Unit Range
0 to 92
Contig stats
Clones: 5 (0 buried)
Markers: 0
Sequenced: 0
Length: 93 CB units
Ctg1279 of cp05T5e12DM4RS
V032M01
H012F20
V030J20
H021C21
H010L16
0 5 10 15 20 25 30 35 40 45 50 55 60 65 70 75 80 85 90
[root@localhost: /...
[FPC V8.9 Main Menu]
Project cp05T5e12...
[我的文档 - File Br...
FPC Ctg1279 cp05T...

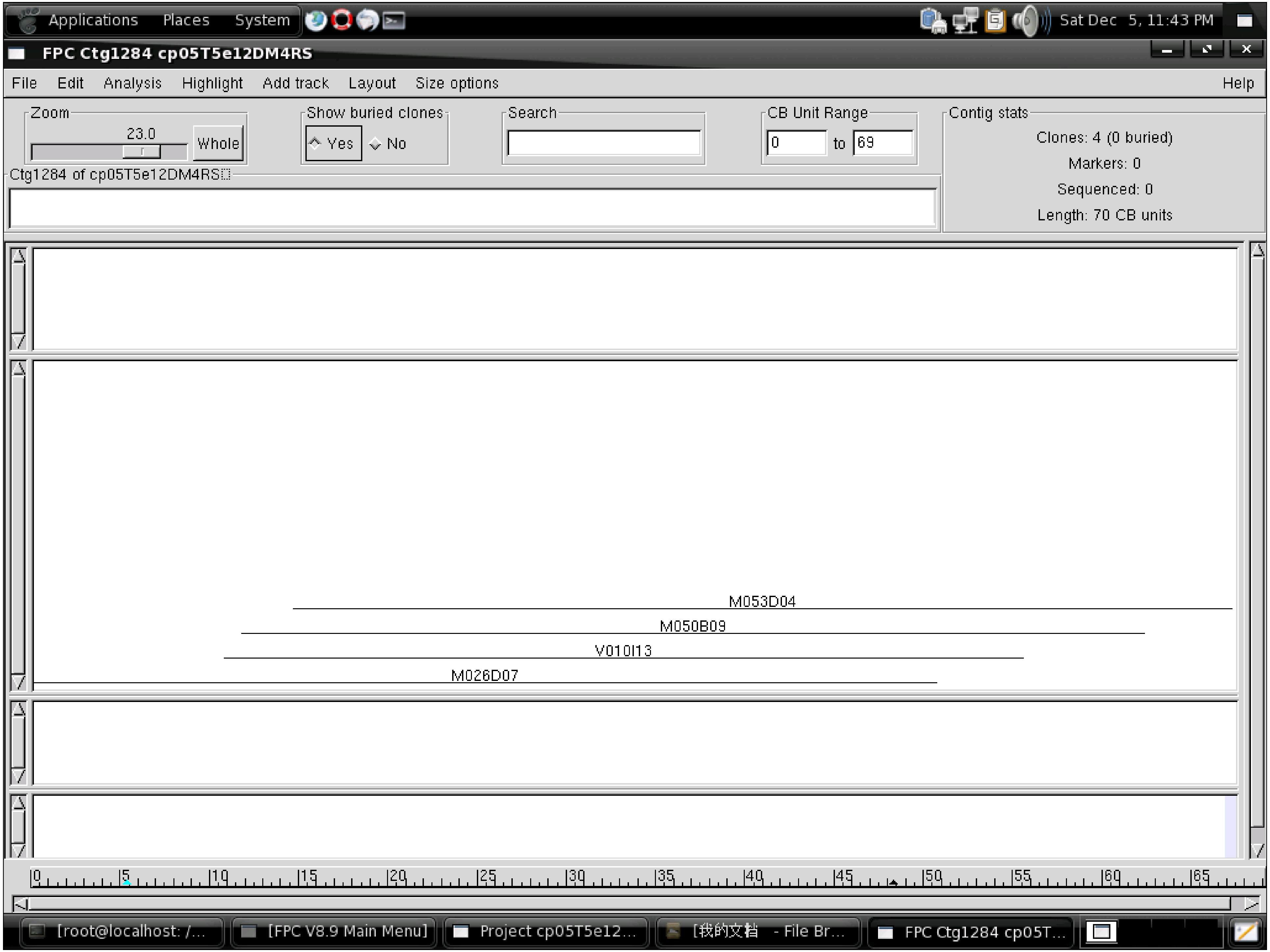
Applications Places System
Sat Dec 5, 11:43 PM
FPC Ctg1284 cp05T5e12DM4RS
File Edit Analysis Highlight Add track Layout Size options Help
Zoom
23.0
Whole
Show buried clones
Yes
No
Search
CB Unit Range
0 to 69
Contig stats
Clones: 4 (0 buried)
Markers: 0
Sequenced: 0
Length: 70 CB units
Ctg1284 of cp05T5e12DM4RS
M053D04
M050B09
V010I13
M026D07
0 5 10 15 20 25 30 35 40 45 50 55 60 65
[root@localhost: /...
[FPC V8.9 Main Menu]
Project cp05T5e12...
[我的文档 - File Br...
FPC Ctg1284 cp05T...

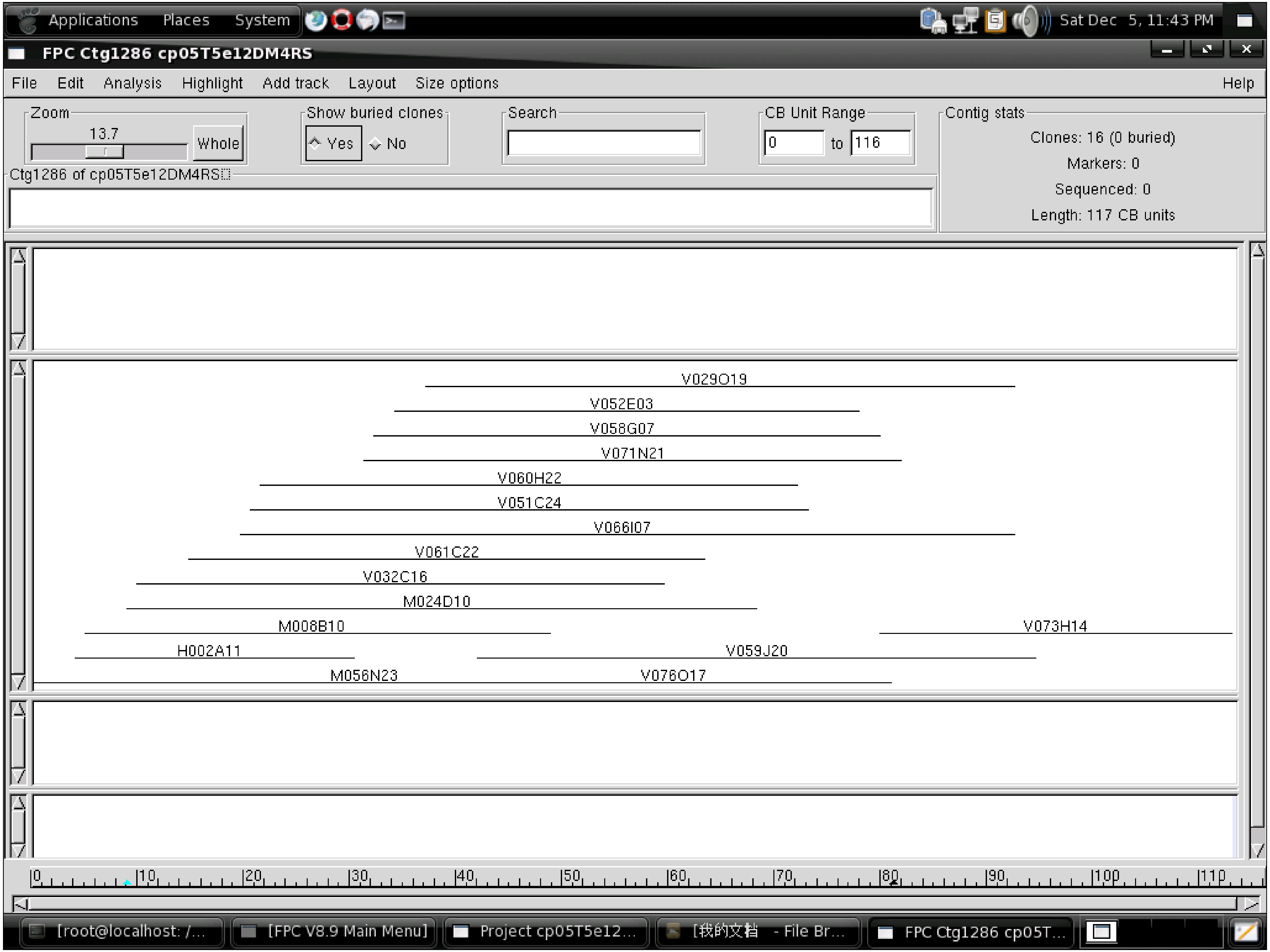
Applications Places System
Sat Dec 5, 11:43 PM
FPC Ctg1286 cp05T5e12DM4RS
File Edit Analysis Highlight Add track Layout Size options Help
Zoom
13.7
Whole
Show buried clones
Yes
No
Search
CB Unit Range
0
to
116
Contig stats
Clones: 16 (0 buried)
Markers: 0
Sequenced: 0
Length: 117 CB units
Ctg1286 of cp05T5e12DM4RS
V029O19
V052E03
V058G07
V071N21
V060H22
V051C24
V066I07
V061C22
V032C16
M024D10
M008B10
V073H14
H002A11
V059J20
M056N23
V076O17
0 10 20 30 40 50 60 70 80 90 100 110
[root@localhost: /...
[FPC V8.9 Main Menu]
Project cp05T5e12...
[我的文档 - File Br...
FPC Ctg1286 cp05T...

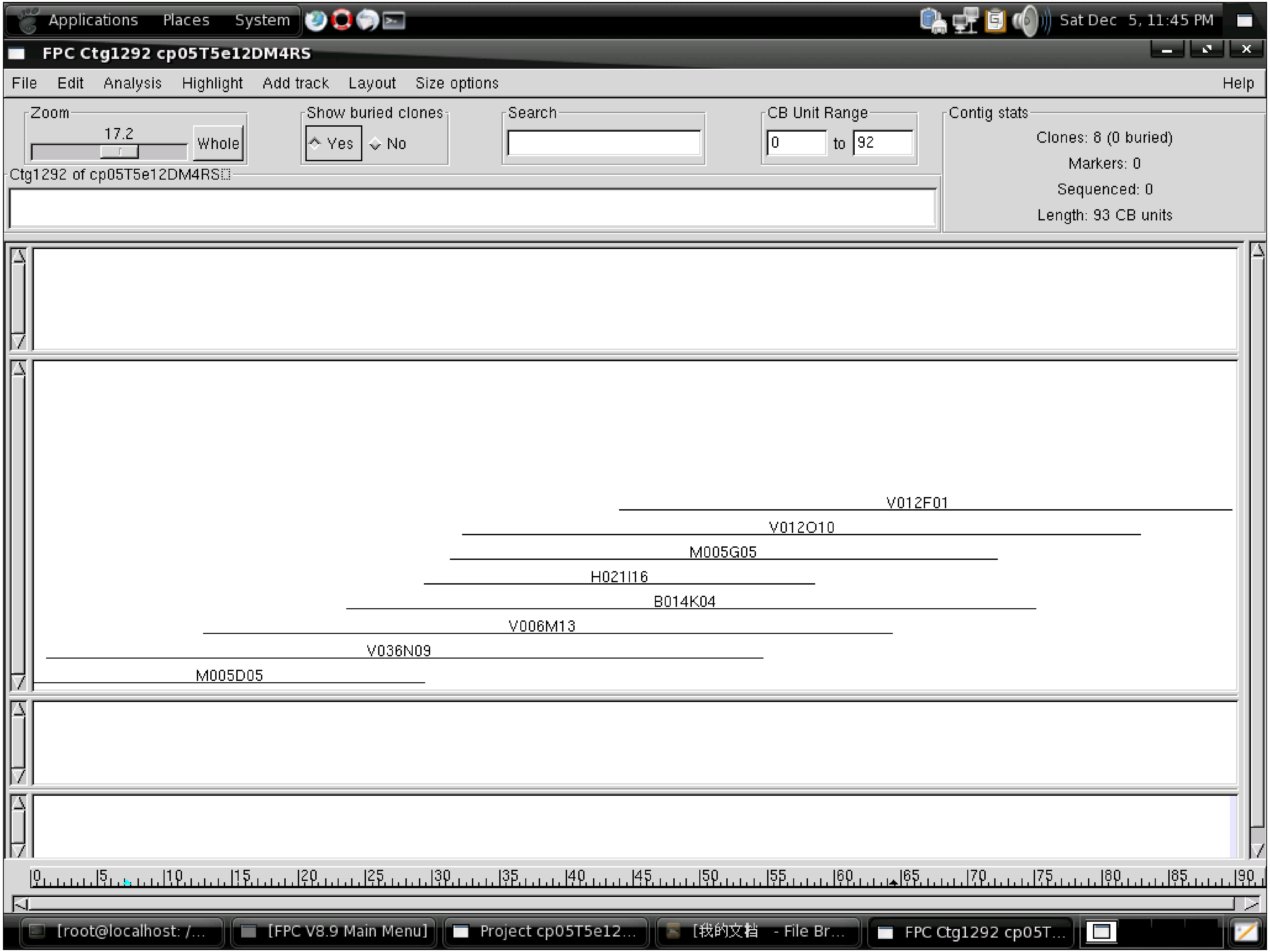
Applications Places System
Sat Dec 5, 11:45 PM
FPC Ctg1292 cp05T5e12DM4RS
File Edit Analysis Highlight Add track Layout Size options Help
Zoom
17.2
Whole
Show buried clones
Yes
No
Search
CB Unit Range
0 to 92
Contig stats
Clones: 8 (0 buried)
Markers: 0
Sequenced: 0
Length: 93 CB units
Ctg1292 of cp05T5e12DM4RS
V012F01
V012O10
M005G05
H021I16
B014K04
V006M13
V036N09
M005D05
0 5 10 15 20 25 30 35 40 45 50 55 60 65 70 75 80 85 90
[root@localhost: /...
[FPC V8.9 Main Menu]
Project cp05T5e12...
[我的文档 - File Br...
FPC Ctg1292 cp05T...

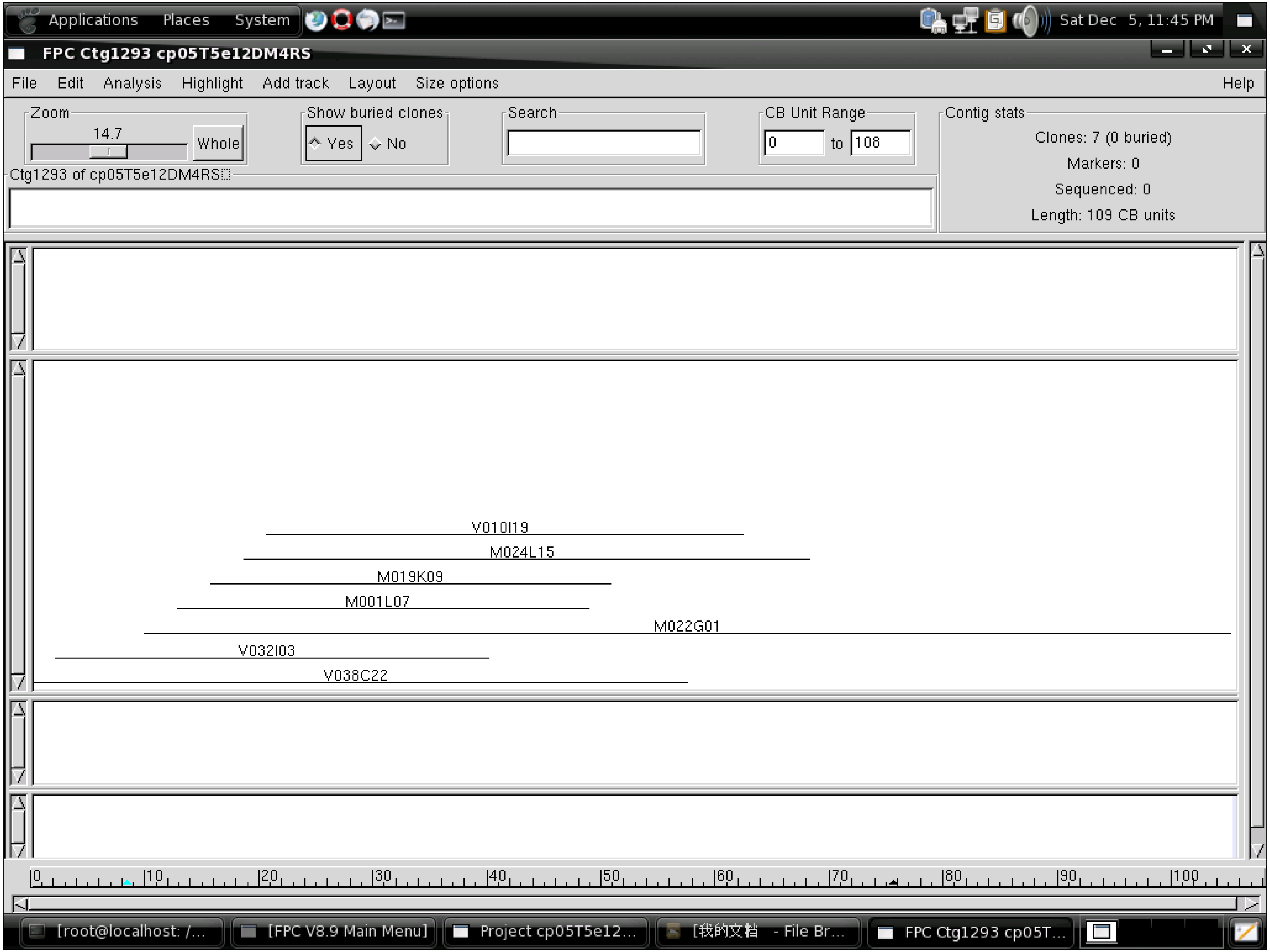
Applications Places System
Sat Dec 5, 11:45 PM
FPC Ctg1293 cp05T5e12DM4RS
File Edit Analysis Highlight Add track Layout Size options Help
Zoom
14.7
Whole
Show buried clones
Yes No
Search
CB Unit Range
0 to 108
Contig stats
Clones: 7 (0 buried)
Markers: 0
Sequenced: 0
Length: 109 CB units
Ctg1293 of cp05T5e12DM4RS
V010I19
M024L15
M019K09
M001L07
M022G01
V032I03
V038C22
0 10 20 30 40 50 60 70 80 90 100
[root@localhost: /...
[FPC V8.9 Main Menu]
Project cp05T5e12...
FPC Ctg1293 cp05T...

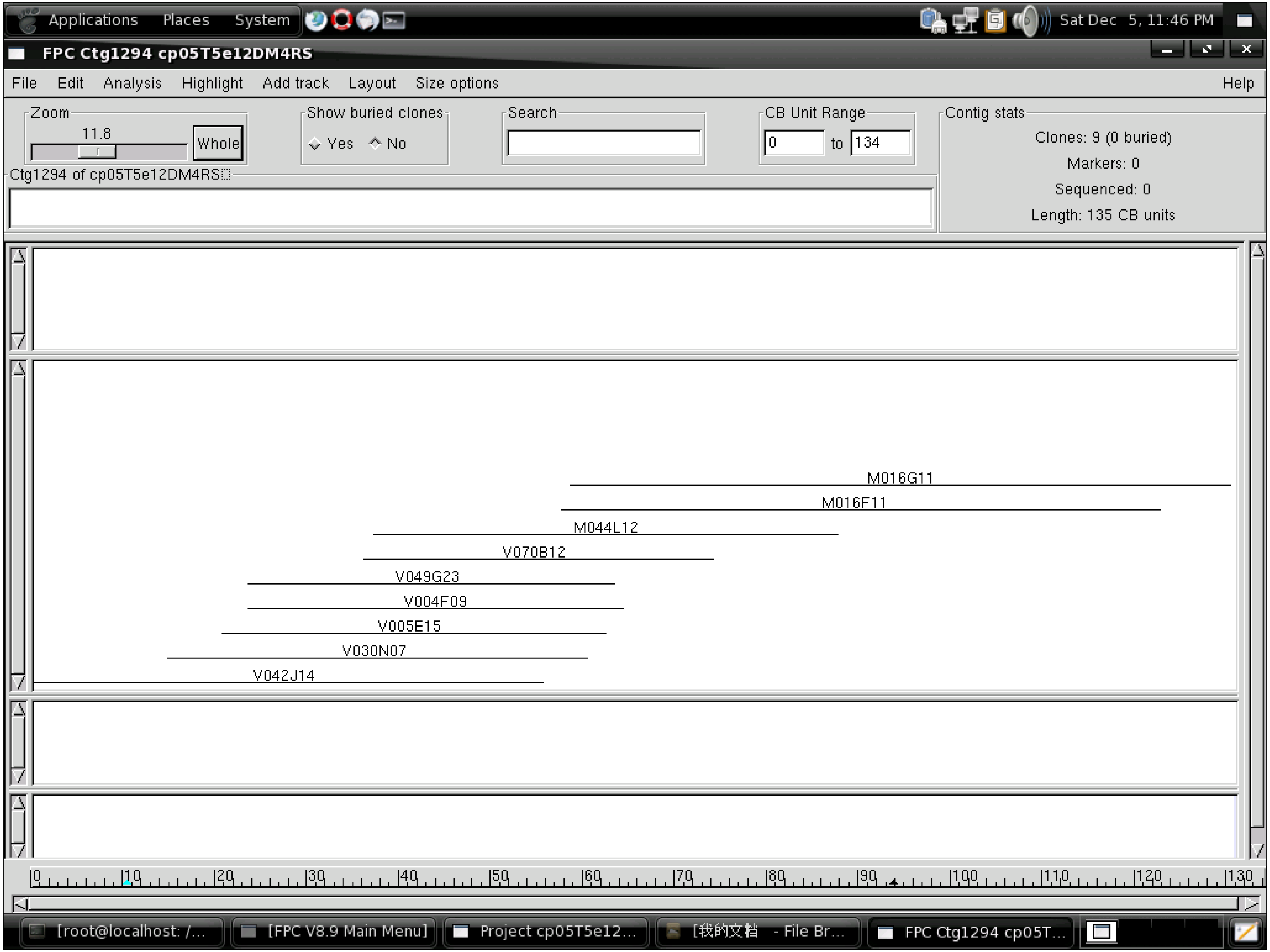
Applications Places System
Sat Dec 5, 11:46 PM
FPC Ctg1294 cp05T5e12DM4RS
File Edit Analysis Highlight Add track Layout Size options Help
Zoom
11.8
Whole
Show buried clones
Yes No
Search
CB Unit Range
0 to 134
Contig stats
Clones: 9 (0 buried)
Markers: 0
Sequenced: 0
Length: 135 CB units
Ctg1294 of cp05T5e12DM4RS
M016G11
M016F11
M044L12
V070B12
V049G23
V004F09
V005E15
V030N07
V042J14
0 10 20 30 40 50 60 70 80 90 100 110 120 130
[root@localhost: /...
[FPC V8.9 Main Menu]
Project cp05T5e12...
[我的文档 - File Br...
FPC Ctg1294 cp05T...

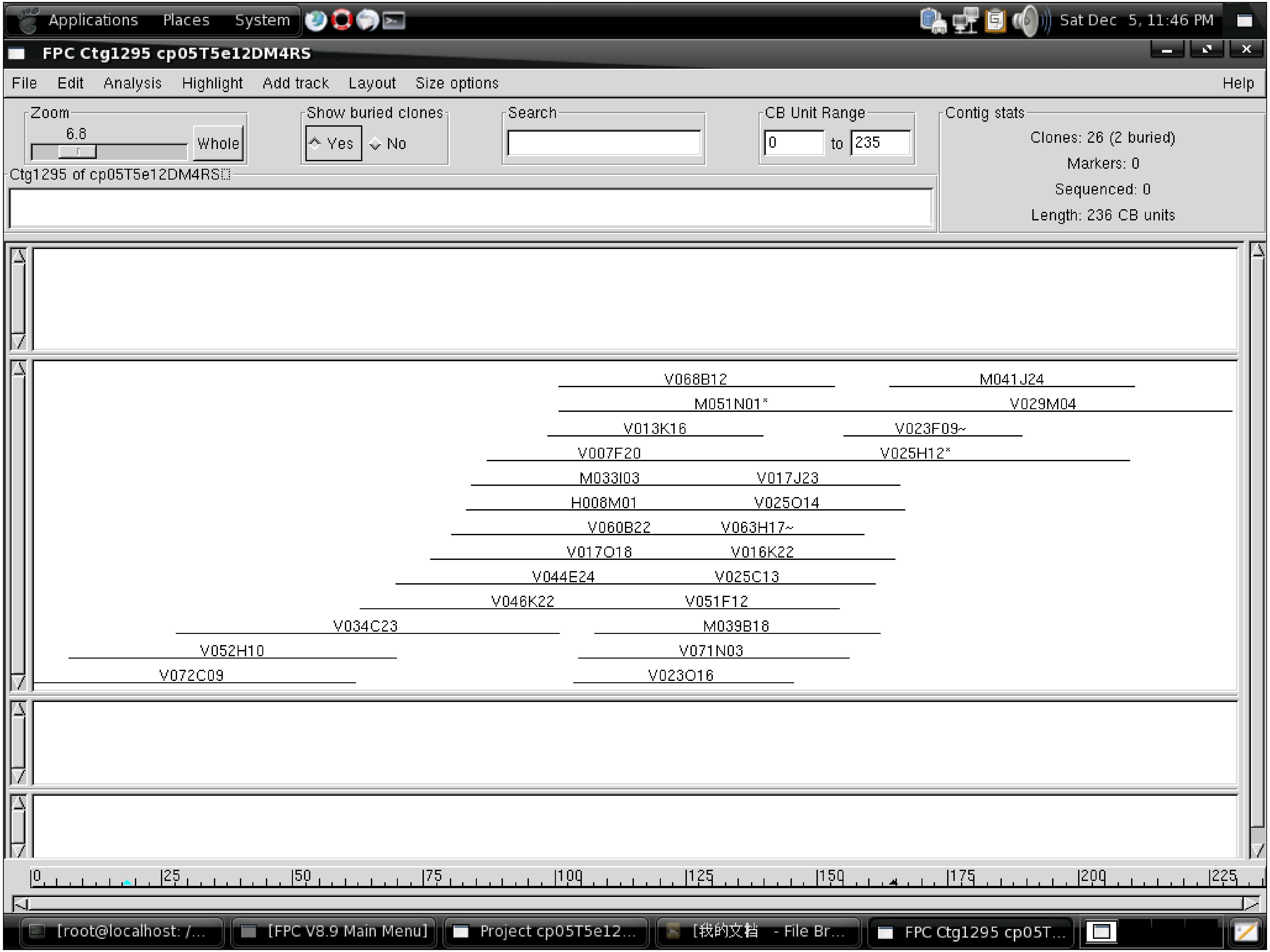
Applications Places System
Sat Dec 5, 11:46 PM
FPC Ctg1295 cp05T5e12DM4RS
File Edit Analysis Highlight Add track Layout Size options Help
Zoom
6.8
Whole
Show buried clones
Yes No
Search
CB Unit Range
0 to 235
Contig stats
Clones: 26 (2 buried)
Markers: 0
Sequenced: 0
Length: 236 CB units
Ctg1295 of cp05T5e12DM4RS
V068B12
M041J24
M051N01*
V029M04
V013K16
V023F09~
V007F20
V025H12*
M033I03
V017J23
H008M01
V025O14
V060B22
V063H17~
V017O18
V016K22
V044E24
V025C13
V046K22
V051F12
V034C23
M039B18
V052H10
V071N03
V072C09
V023O16
0 25 50 75 100 125 150 175 200 225
[root@localhost: /...
[FPC V8.9 Main Menu]
Project cp05T5e12...
[我的文档 - File Br...
FPC Ctg1295 cp05T...

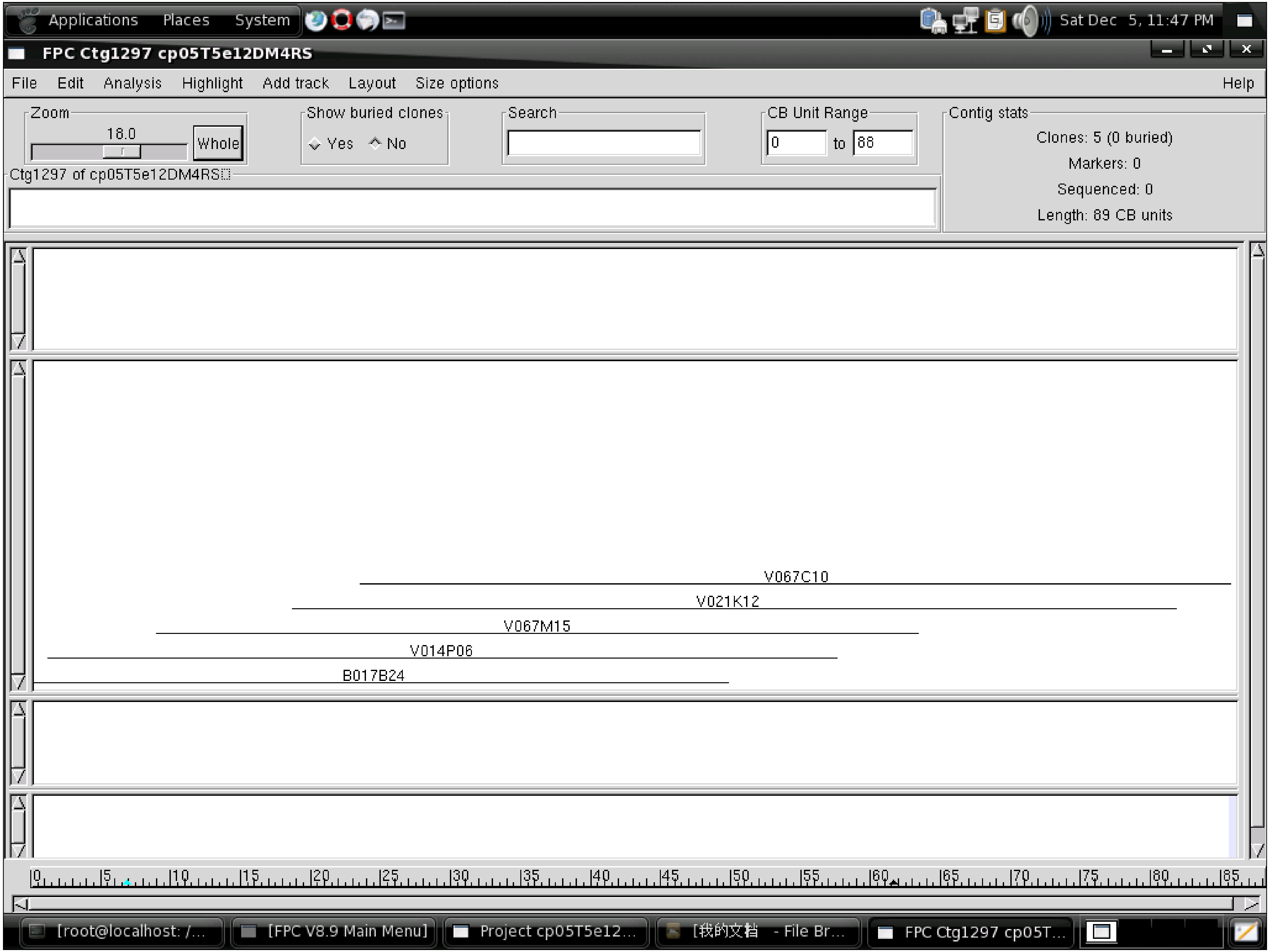

Applications Places System
Sat Dec 5, 11:47 PM
FPC Ctg1297 cp05T5e12DM4RS
File Edit Analysis Highlight Add track Layout Size options Help
Zoom
18.0
Whole
Show buried clones
Yes No
Search
CB Unit Range
0 to 88
Contig stats
Clones: 5 (0 buried)
Markers: 0
Sequenced: 0
Length: 89 CB units
Ctg1297 of cp05T5e12DM4RS
V067C10
V021K12
V067M15
V014P06
B017B24
0 5 10 15 20 25 30 35 40 45 50 55 60 65 70 75 80 85
[root@localhost: /...
[FPC V8.9 Main Menu]
Project cp05T5e12...
[我的文档 - File Br...
FPC Ctg1297 cp05T...

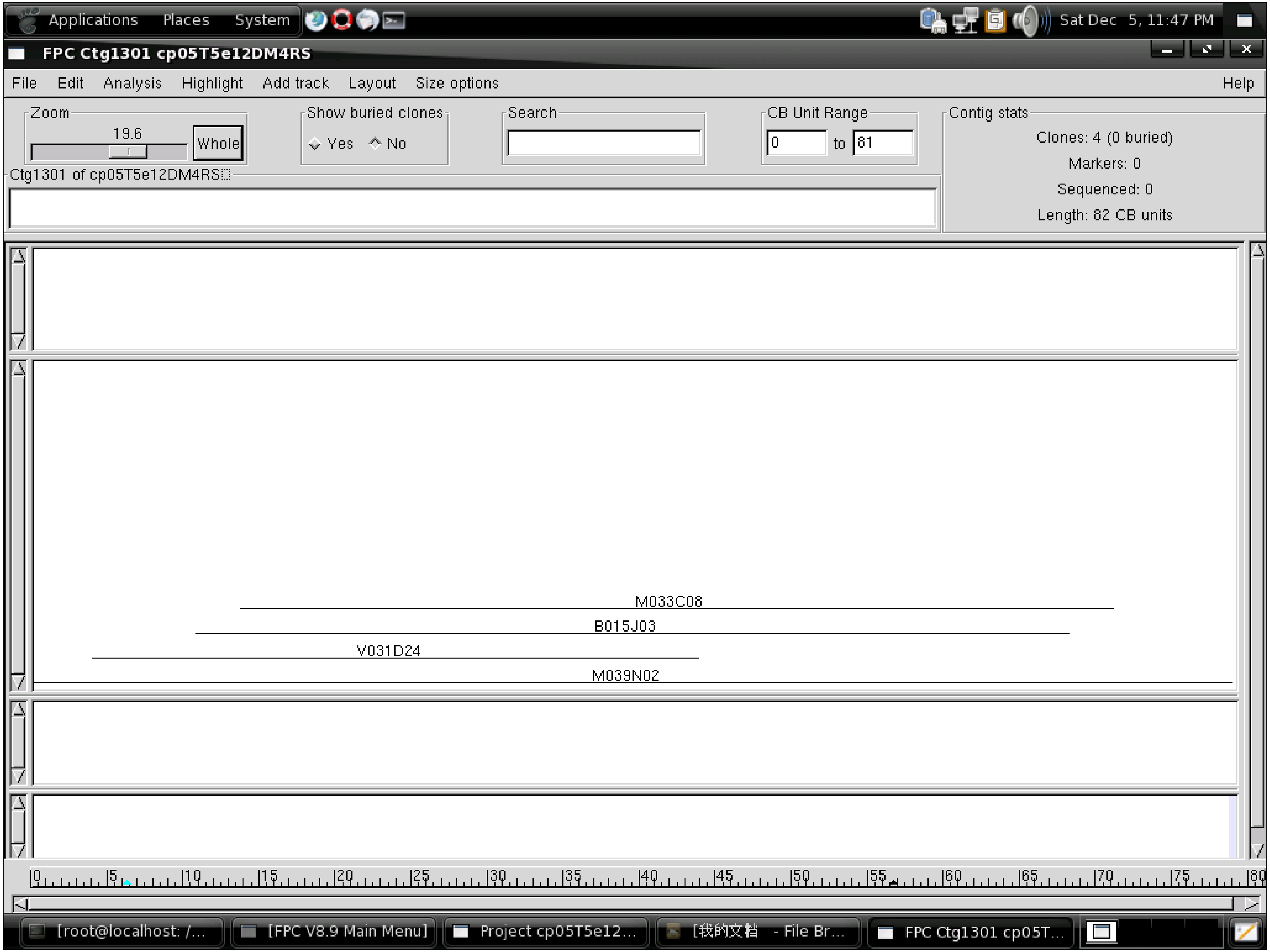
Applications Places System
Sat Dec 5, 11:47 PM
FPC Ctg1301 cp05T5e12DM4RS
File Edit Analysis Highlight Add track Layout Size options Help
Zoom
19.6
Whole
Show buried clones
Yes No
Search
CB Unit Range
0 to 81
Contig stats
Clones: 4 (0 buried)
Markers: 0
Sequenced: 0
Length: 82 CB units
Ctg1301 of cp05T5e12DM4RS
M033C08
B015J03
V031D24
M039N02
0 5 10 15 20 25 30 35 40 45 50 55 60 65 70 75 80
[root@localhost: /...
[FPC V8.9 Main Menu]
Project cp05T5e12...
[我的文档 - File Br...
FPC Ctg1301 cp05T...

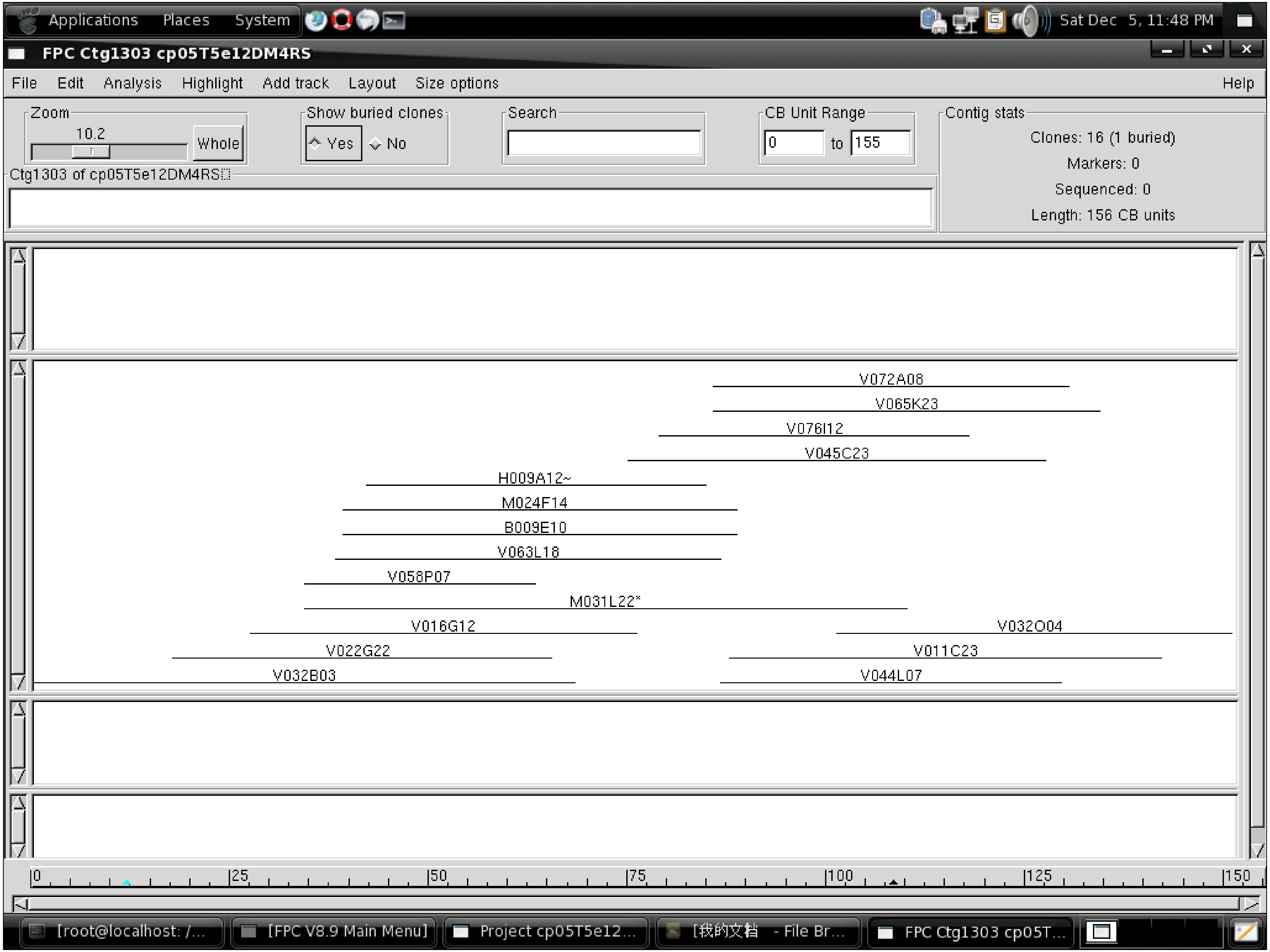
Applications Places System
Sat Dec 5, 11:48 PM
FPC Ctg1303 cp05T5e12DM4RS
File Edit Analysis Highlight Add track Layout Size options Help
Zoom
10.2
Whole
Show buried clones
Yes No
Search
CB Unit Range
0 to 155
Contig stats
Clones: 16 (1 buried)
Markers: 0
Sequenced: 0
Length: 156 CB units
Ctg1303 of cp05T5e12DM4RS
V072A08
V065K23
V076I12
V045C23
H009A12~
M024F14
B009E10
V063L18
V058P07
M031L22*
V016G12
V032O04
V022G22
V011C23
V032B03
V044L07
0 25 50 75 100 125 150
[root@localhost: /...
[FPC V8.9 Main Menu]
Project cp05T5e12...
[我的文档 - File Br...
FPC Ctg1303 cp05T...

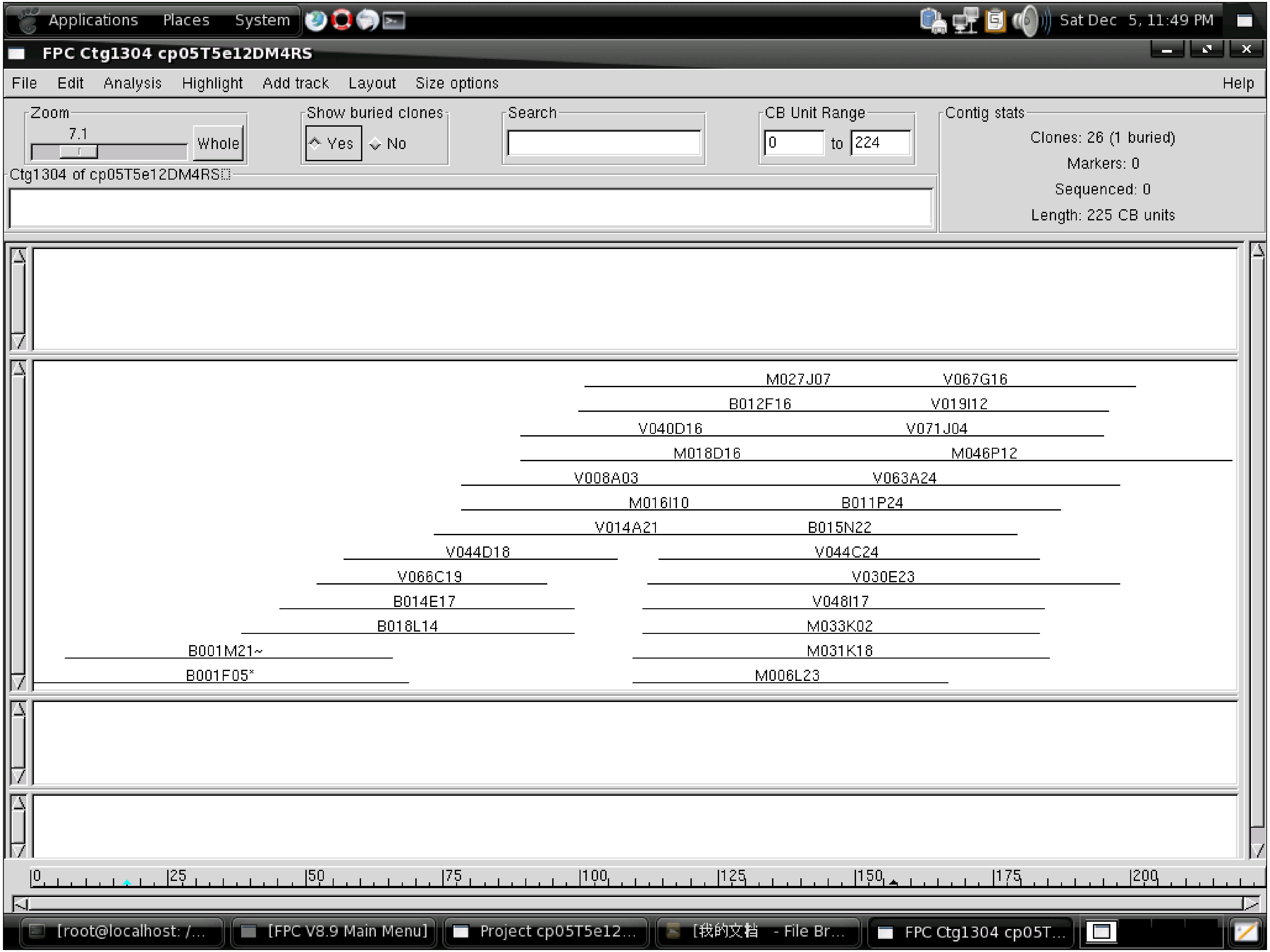
Applications Places System
Sat Dec 5, 11:49 PM
FPC Ctg1304 cp05T5e12DM4RS
File Edit Analysis Highlight Add track Layout Size options Help
Zoom
7.1
Whole
Show buried clones
Yes
No
Search
CB Unit Range
0
to
224
Contig stats
Clones: 26 (1 buried)
Markers: 0
Sequenced: 0
Length: 225 CB units
Ctg1304 of cp05T5e12DM4RS
M027J07
V067G16
B012F16
V019I12
V040D16
V071J04
M018D16
M046P12
V008A03
V063A24
M016I10
B011P24
V014A21
B015N22
V044D18
V044C24
V066C19
V030E23
B014E17
V048I17
B018L14
M033K02
B001M21~
M031K18
B001F05*
M006L23
0
25
50
75
100
125
150
175
200
[root@localhost: /...
[FPC V8.9 Main Menu]
Project cp05T5e12...
[我的文档 - File Br...
FPC Ctg1304 cp05T...

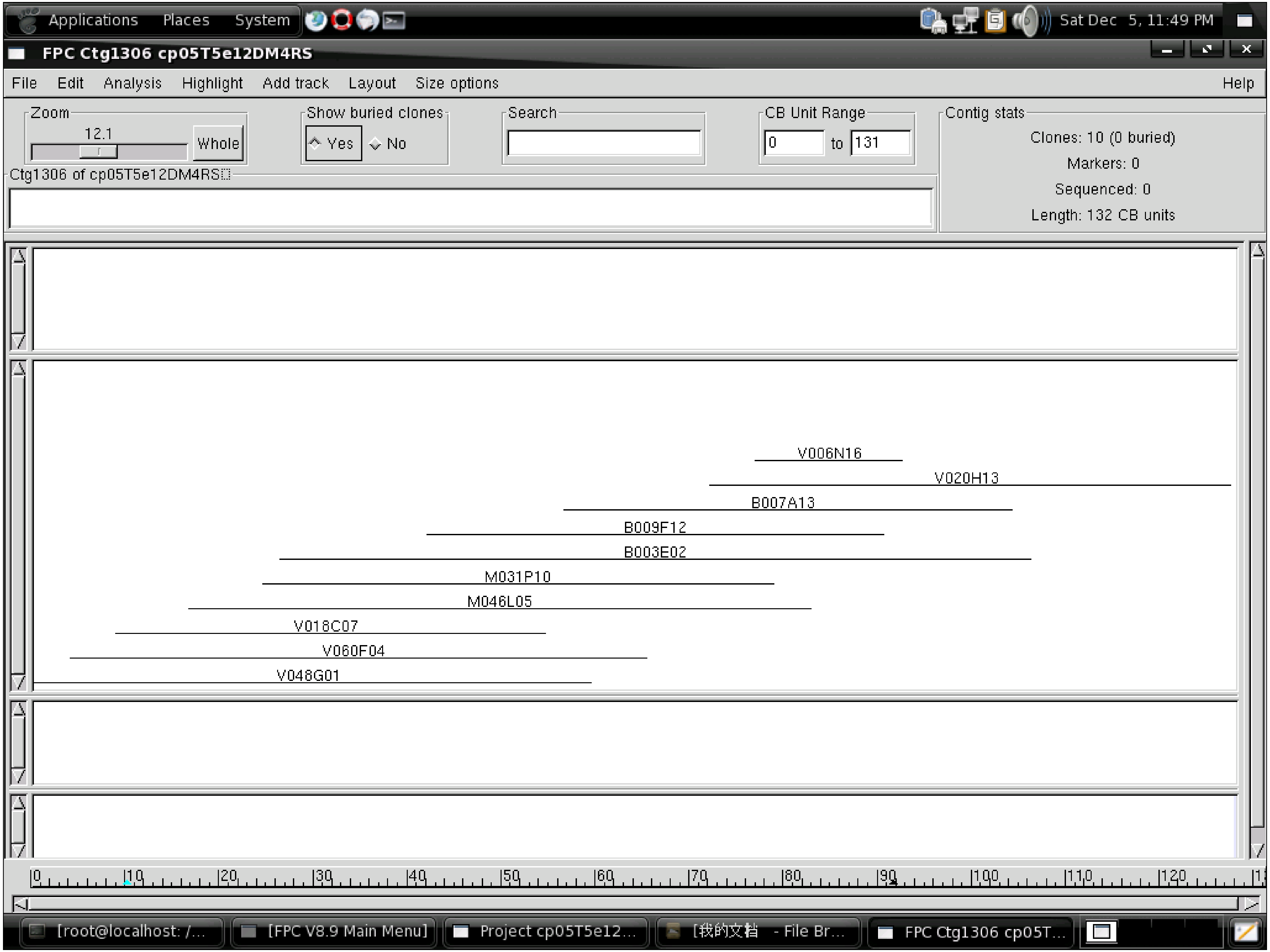
Applications Places System
Sat Dec 5, 11:49 PM
FPC Ctg1306 cp05T5e12DM4RS
File Edit Analysis Highlight Add track Layout Size options Help
Zoom
12.1
Whole
Show buried clones
Yes No
Search
CB Unit Range
0 to 131
Contig stats
Clones: 10 (0 buried)
Markers: 0
Sequenced: 0
Length: 132 CB units
Ctg1306 of cp05T5e12DM4RS
V006N16
V020H13
B007A13
B009F12
B003E02
M031P10
M046L05
V018C07
V060F04
V048G01
0 10 20 30 40 50 60 70 80 90 100 110 120
[root@localhost: /...
[FPC V8.9 Main Menu]
Project cp05T5e12...
[我的文档 - File Br...
FPC Ctg1306 cp05T...

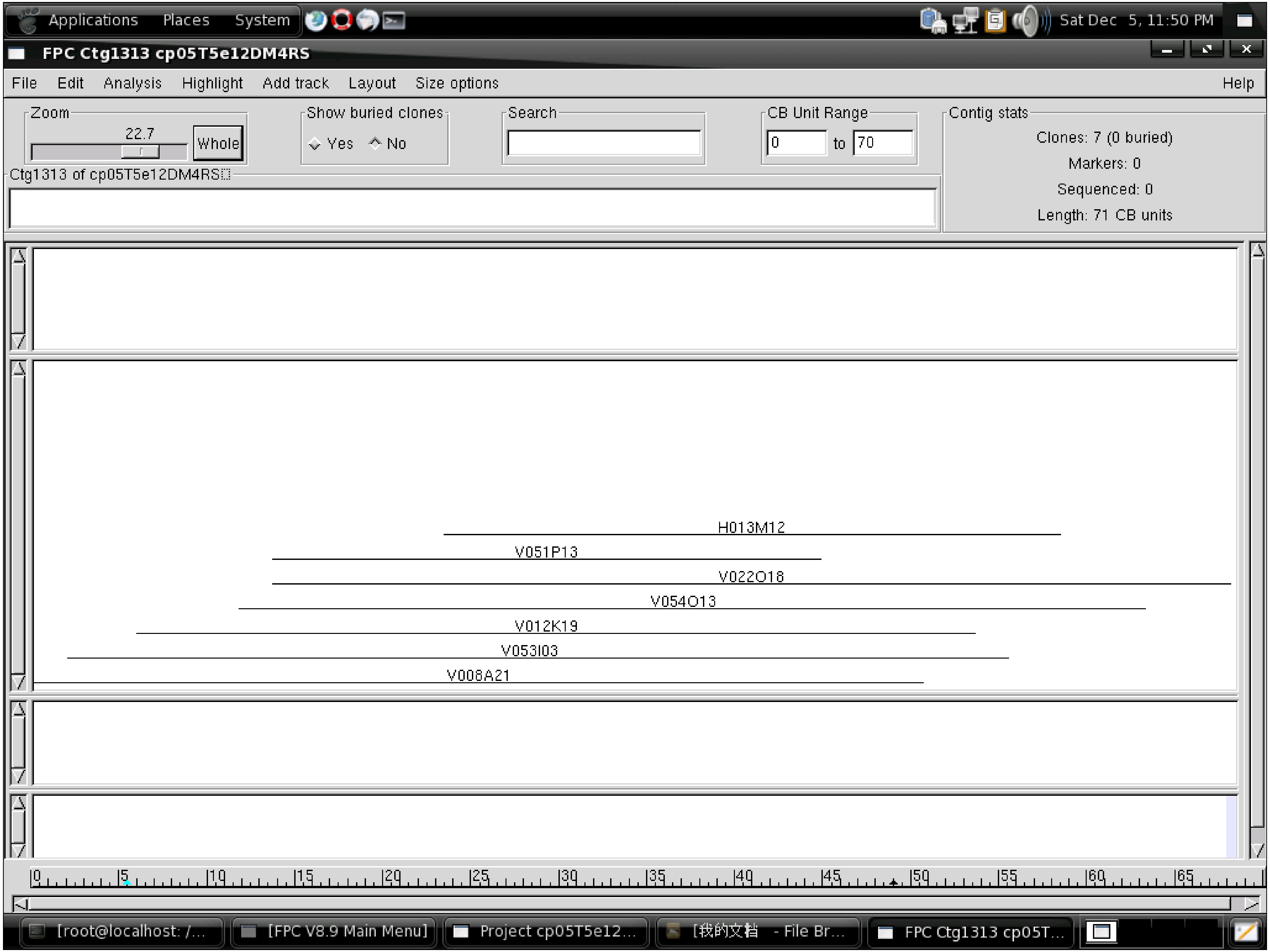
Applications Places System
Sat Dec 5, 11:50 PM
FPC Ctg1313 cp05T5e12DM4RS
File Edit Analysis Highlight Add track Layout Size options Help
Zoom
22.7
Whole
Show buried clones
Yes No
Search
CB Unit Range
0 to 70
Contig stats
Clones: 7 (0 buried)
Markers: 0
Sequenced: 0
Length: 71 CB units
Ctg1313 of cp05T5e12DM4RS
H013M12
V051P13
V022O18
V054O13
V012K19
V053I03
V008A21
0 5 10 15 20 25 30 35 40 45 50 55 60 65
[root@localhost: /...
[FPC V8.9 Main Menu]
Project cp05T5e12...
[我的文档 - File Br...
FPC Ctg1313 cp05T...

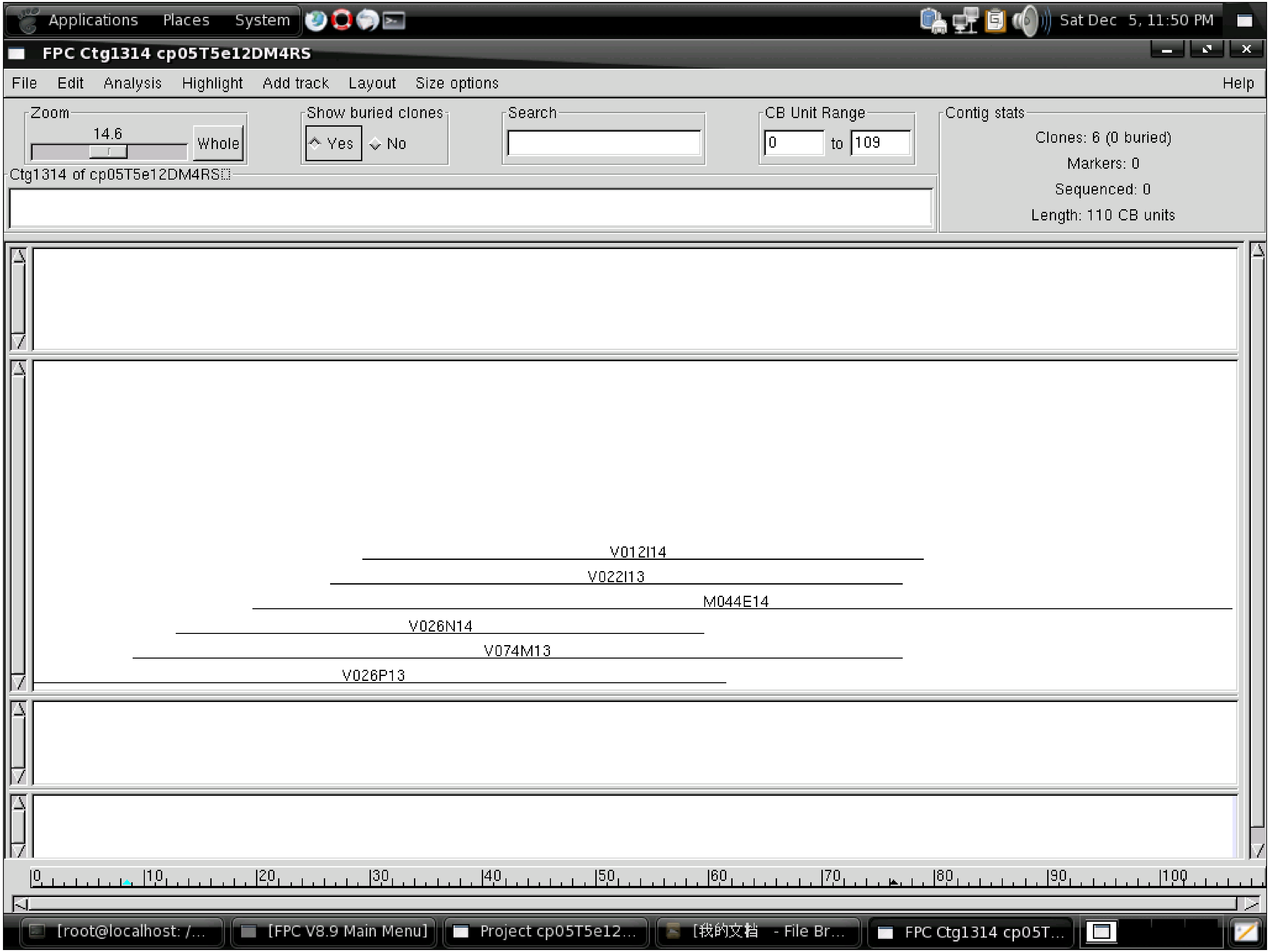
Applications Places System
Sat Dec 5, 11:50 PM
FPC Ctg1314 cp05T5e12DM4RS
File Edit Analysis Highlight Add track Layout Size options Help
Zoom
14.6
Whole
Show buried clones
Yes
No
Search
CB Unit Range
0 to 109
Contig stats
Clones: 6 (0 buried)
Markers: 0
Sequenced: 0
Length: 110 CB units
Ctg1314 of cp05T5e12DM4RS
V012I14
V022I13
M044E14
V026N14
V074M13
V026P13
0 10 20 30 40 50 60 70 80 90 100
[root@localhost: /...
[FPC V8.9 Main Menu]
Project cp05T5e12...
[我的文档 - File Br...
FPC Ctg1314 cp05T...

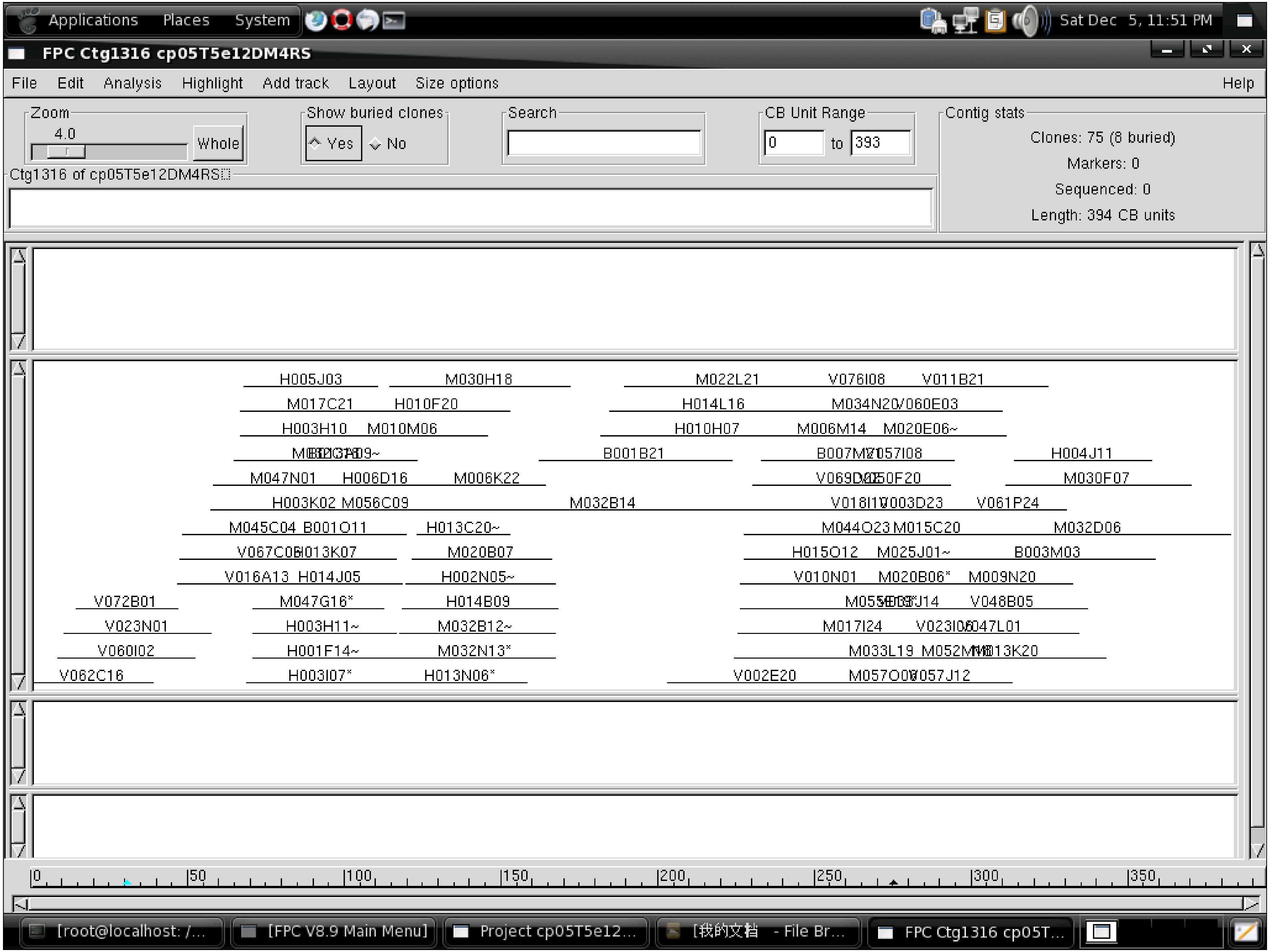
Applications Places System
Sat Dec 5, 11:51 PM
FPC Ctg1316 cp05T5e12DM4RS
File Edit Analysis Highlight Add track Layout Size options Help
Zoom
4.0
Whole
Show buried clones
Yes No
Search
CB Unit Range
0 to 393
Contig stats
Clones: 75 (8 buried)
Markers: 0
Sequenced: 0
Length: 394 CB units
Ctg1316 of cp05T5e12DM4RS
H005J03 M030H18 M022L21 V076I08 V011B21
M017C21 H010F20 H014L16
H003H10 M010M06 H010H07 M006M14 M020E06~
B001B21 H004J11
M047N01 H006D16 M006K22 M030F07
H003K02 M056C09 M032B14 V061P24
M045C04 B001O11 H013C20~ M015C20 M032D06
M020B07 H015O12 M025J01~ B003M03
V016A13 H014J05 H002N05~ V010N01 M020B06* M009N20
V072B01 M047G16* H014B09 V048B05
V023N01 H003H11~ M032B12~ M017I24
V060I02 H001F14~ M032N13* M033L19
V062C16 H003I07* H013N06* V002E20
0 50 100 150 200 250 300 350
[root@localhost: /... [FPC V8.9 Main Menu] Project cp05T5e12... [我的文档 - File Br... FPC Ctg1316 cp05T...

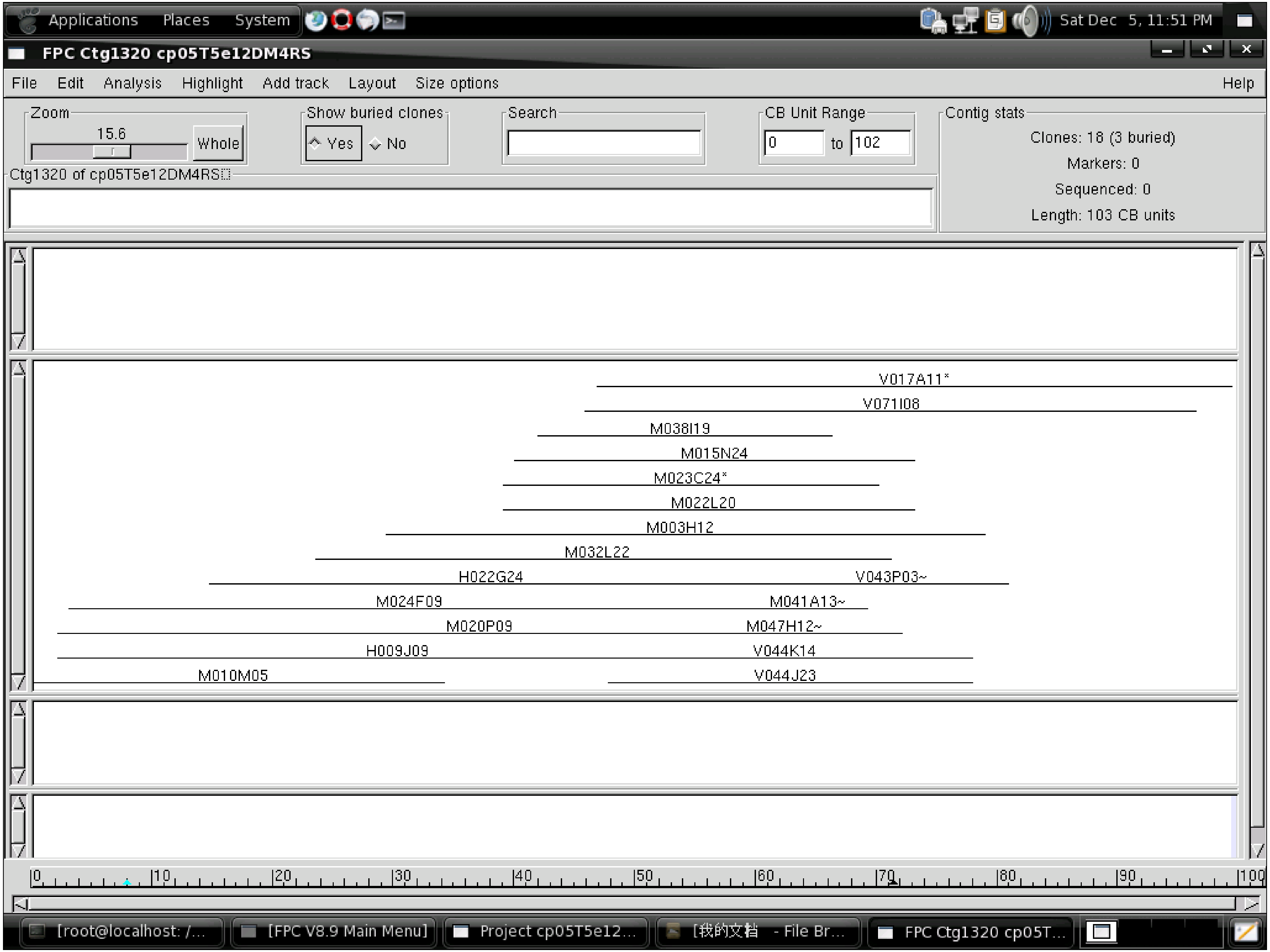
Applications Places System
Sat Dec 5, 11:51 PM
FPC Ctg1320 cp05T5e12DM4RS
File Edit Analysis Highlight Add track Layout Size options Help
Zoom
15.6
Whole
Show buried clones
Yes No
Search
CB Unit Range
0 to 102
Contig stats
Clones: 18 (3 buried)
Markers: 0
Sequenced: 0
Length: 103 CB units
Ctg1320 of cp05T5e12DM4RS
V017A11*
V071I08
M038I19
M015N24
M023C24*
M022L20
M003H12
M032L22
H022G24
V043P03~
M024F09
M041A13~
M020P09
M047H12~
H009J09
V044K14
M010M05
V044J23
0 10 20 30 40 50 60 70 80 90 100
[root@localhost: /...
[FPC V8.9 Main Menu]
Project cp05T5e12...
[我的文档 - File Br...
FPC Ctg1320 cp05T...

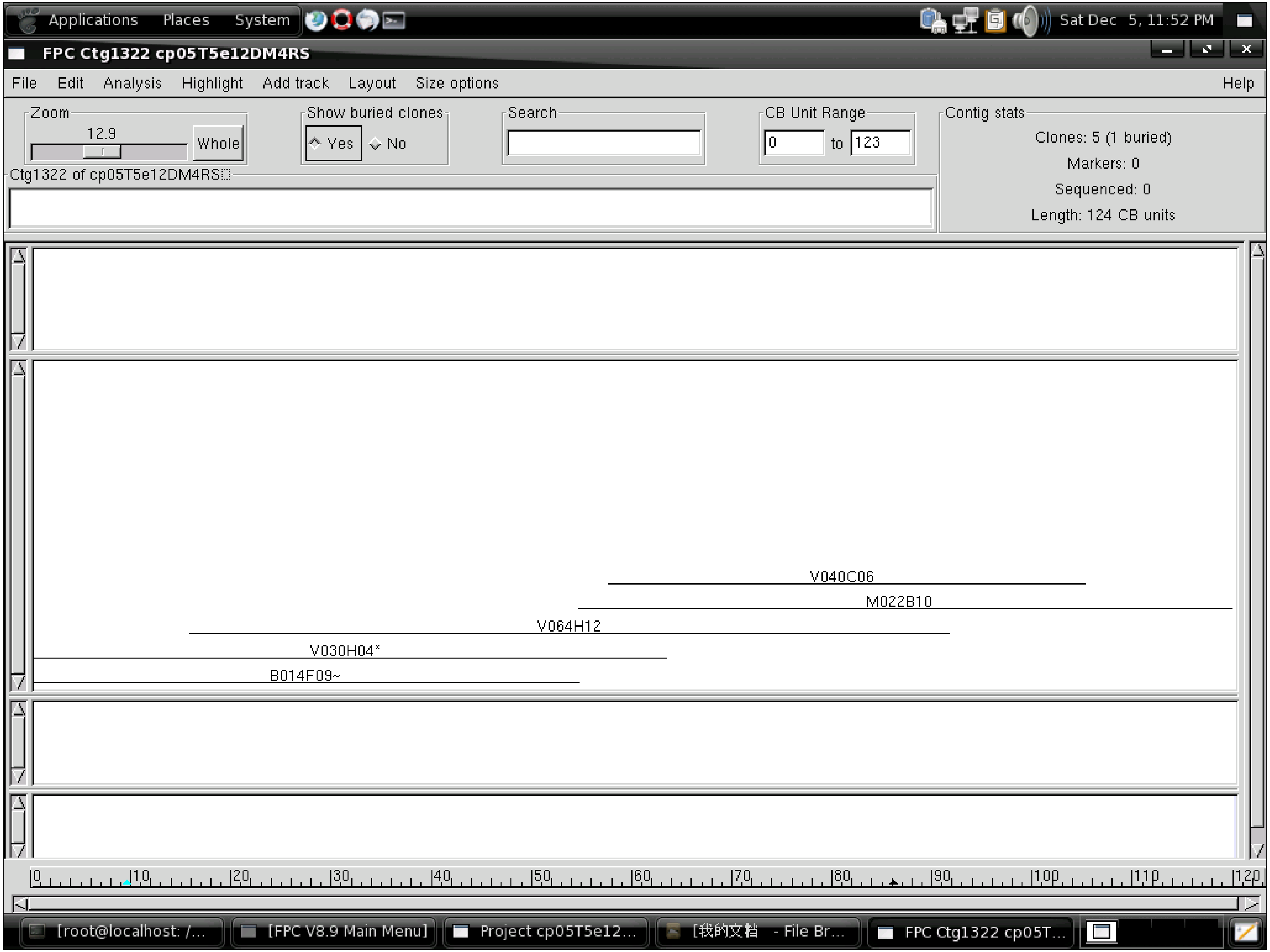
Applications Places System
Sat Dec 5, 11:52 PM
FPC Ctg1322 cp05T5e12DM4RS
File Edit Analysis Highlight Add track Layout Size options Help
Zoom
12.9
Whole
Show buried clones
Yes No
Search
CB Unit Range
0 to 123
Contig stats
Clones: 5 (1 buried)
Markers: 0
Sequenced: 0
Length: 124 CB units
Ctg1322 of cp05T5e12DM4RS
V040C06
M022B10
V064H12
V030H04*
B014F09~
0 10 20 30 40 50 60 70 80 90 100 110 120
[root@localhost: /...
[FPC V8.9 Main Menu]
Project cp05T5e12...
[我的文档 - File Br...
FPC Ctg1322 cp05T...

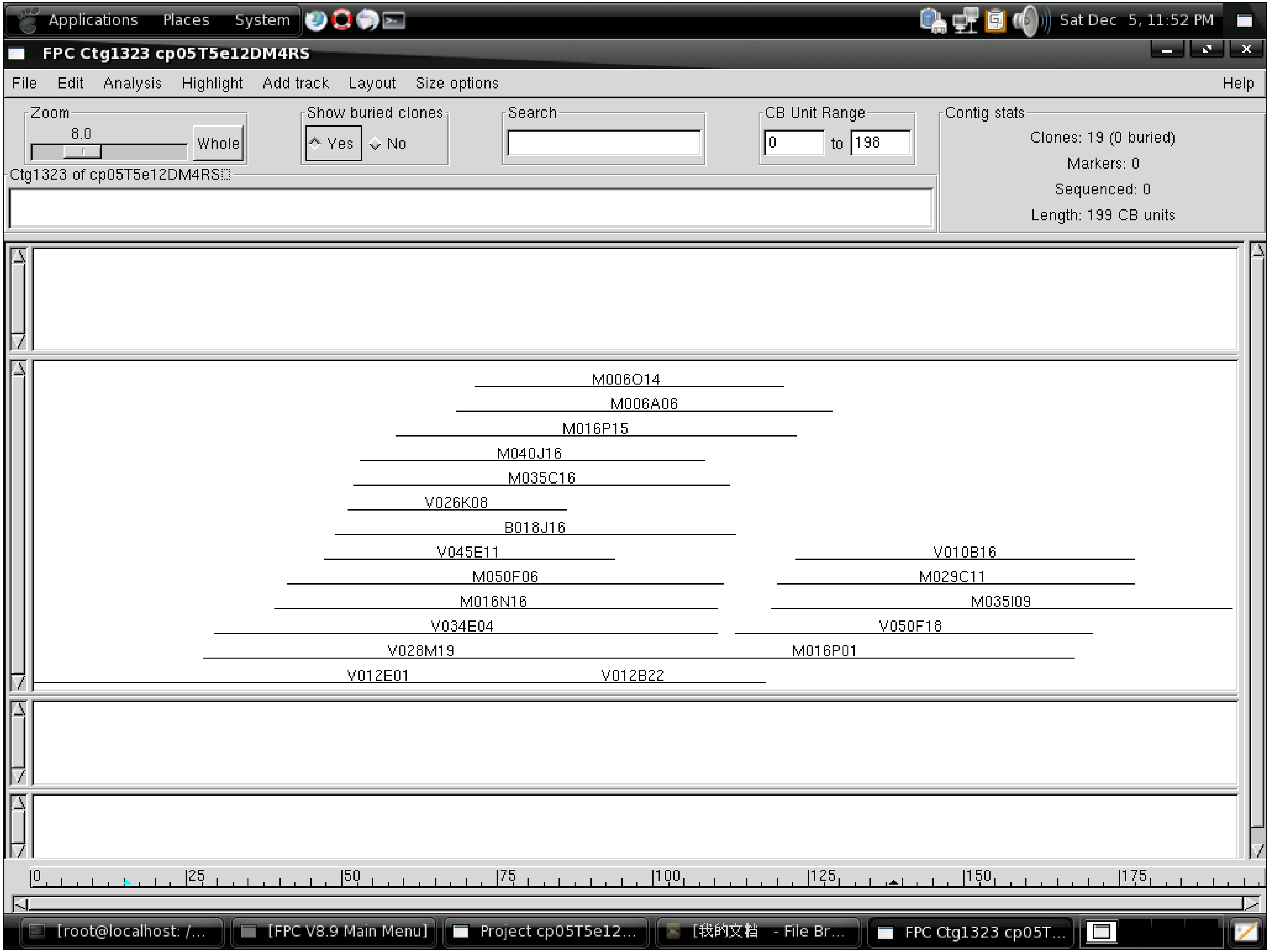
Applications Places System
Sat Dec 5, 11:52 PM
FPC Ctg1323 cp05T5e12DM4RS
File Edit Analysis Highlight Add track Layout Size options Help
Zoom
8.0
Whole
Show buried clones
Yes
No
Search
CB Unit Range
0
to
198
Contig stats
Clones: 19 (0 buried)
Markers: 0
Sequenced: 0
Length: 199 CB units
Ctg1323 of cp05T5e12DM4RS
M006O14
M006A06
M016P15
M040J16
M035C16
V026K08
B018J16
V045E11
V010B16
M050F06
M029C11
M016N16
M035I09
V034E04
V050F18
V028M19
M016P01
V012E01
V012B22
0
25
50
75
100
125
150
175
[root@localhost: /...
[FPC V8.9 Main Menu]
Project cp05T5e12...
[我的文档 - File Br...
FPC Ctg1323 cp05T...

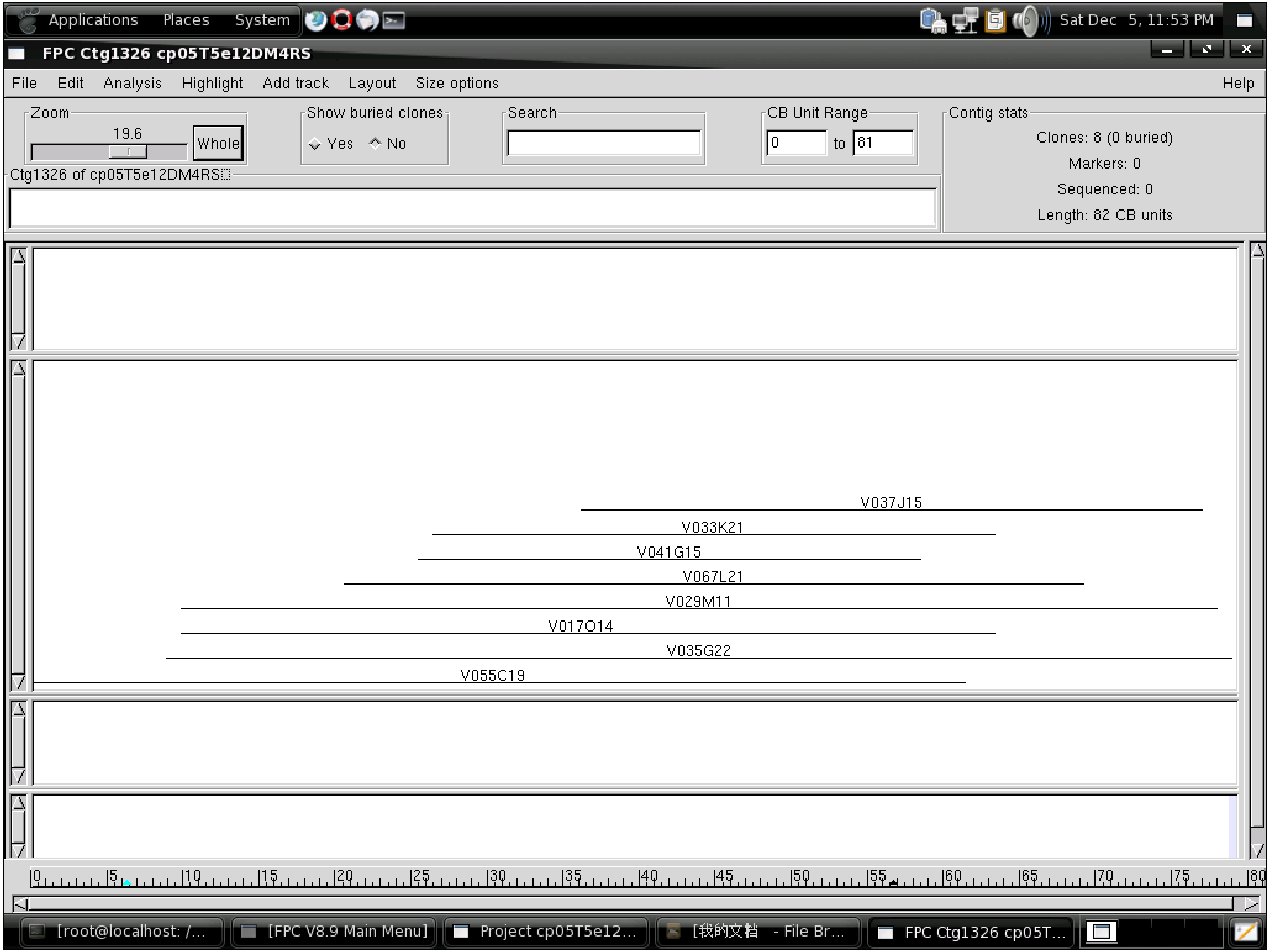
Applications Places System
Sat Dec 5, 11:53 PM
FPC Ctg1326 cp05T5e12DM4RS
File Edit Analysis Highlight Add track Layout Size options Help
Zoom
19.6
Whole
Show buried clones
Yes No
Search
CB Unit Range
0 to 81
Contig stats
Clones: 8 (0 buried)
Markers: 0
Sequenced: 0
Length: 82 CB units
Ctg1326 of cp05T5e12DM4RS
V037J15
V033K21
V041G15
V067L21
V029M11
V017O14
V035G22
V055C19
0 5 10 15 20 25 30 35 40 45 50 55 60 65 70 75 80
[root@localhost: /...
[FPC V8.9 Main Menu]
Project cp05T5e12...
[我的文档 - File Br...
FPC Ctg1326 cp05T...

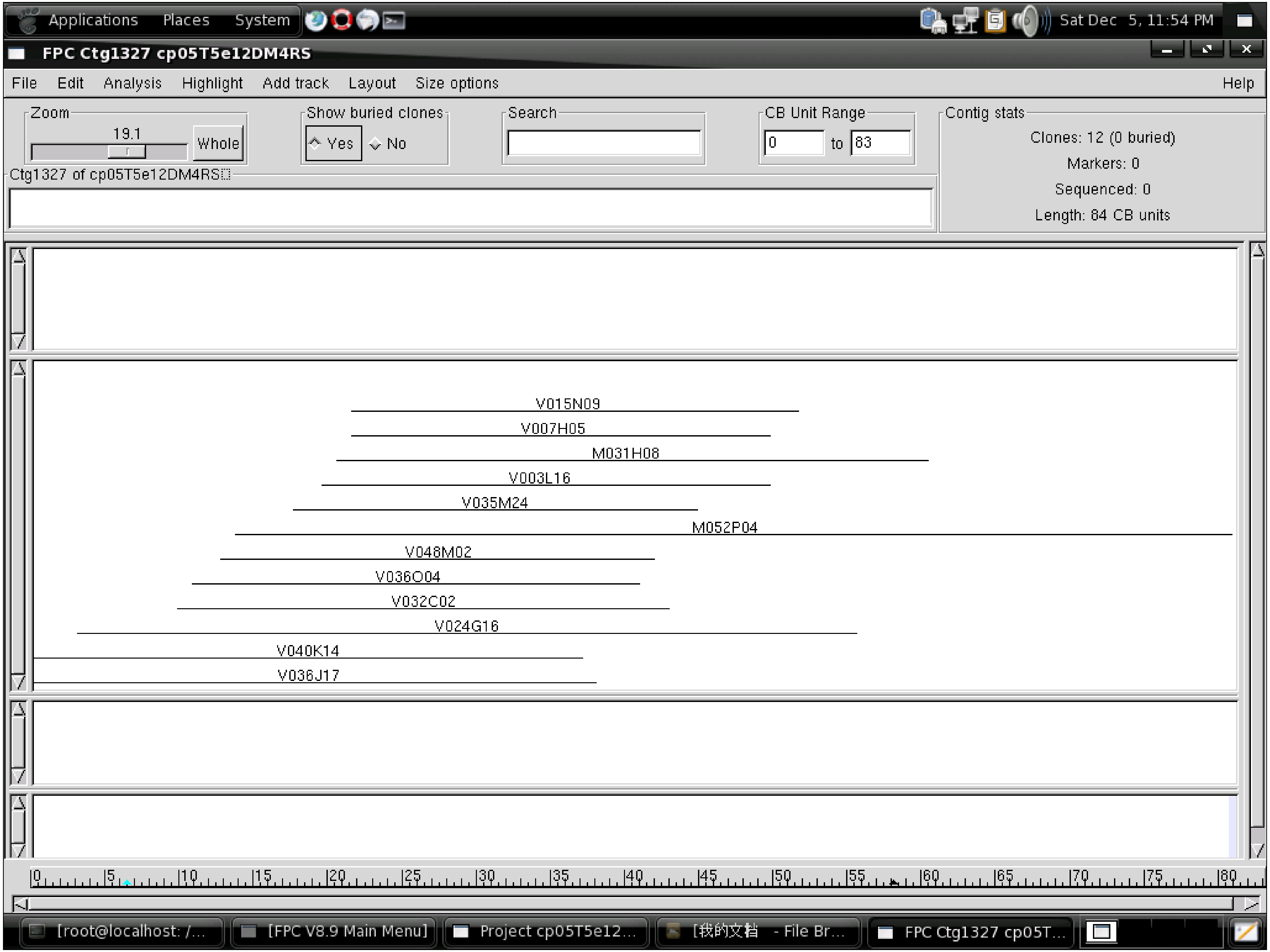
Applications Places System
Sat Dec 5, 11:54 PM
FPC Ctg1327 cp05T5e12DM4RS
File Edit Analysis Highlight Add track Layout Size options Help
Zoom
19.1
Whole
Show buried clones
Yes No
Search
CB Unit Range
0 to 83
Contig stats
Clones: 12 (0 buried)
Markers: 0
Sequenced: 0
Length: 84 CB units
Ctg1327 of cp05T5e12DM4RS
V015N09
V007H05
M031H08
V003L16
V035M24
M052P04
V048M02
V036O04
V032C02
V024G16
V040K14
V036J17
0 5 10 15 20 25 30 35 40 45 50 55 60 65 70 75 80
[root@localhost: /...
[FPC V8.9 Main Menu]
Project cp05T5e12...
[我的文档 - File Br...
FPC Ctg1327 cp05T...

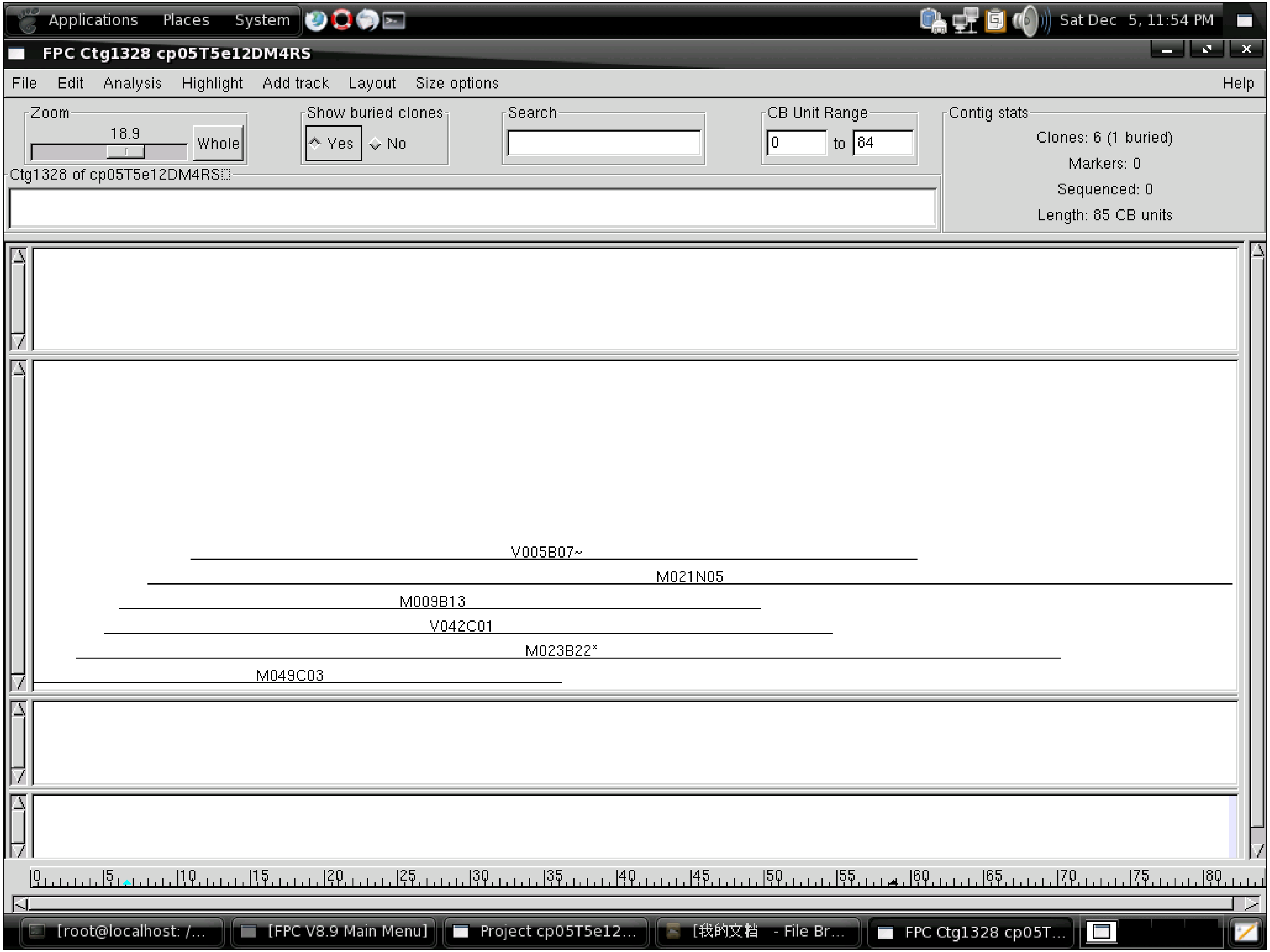
Applications Places System
Sat Dec 5, 11:54 PM
FPC Ctg1328 cp05T5e12DM4RS
File Edit Analysis Highlight Add track Layout Size options Help
Zoom
18.9
Whole
Show buried clones
Yes No
Search
CB Unit Range
0 to 84
Contig stats
Clones: 6 (1 buried)
Markers: 0
Sequenced: 0
Length: 85 CB units
Ctg1328 of cp05T5e12DM4RS
V005B07~
M021N05
M009B13
V042C01
M023B22*
M049C03
0 5 10 15 20 25 30 35 40 45 50 55 60 65 70 75 80
[root@localhost: /...
[FPC V8.9 Main Menu]
Project cp05T5e12...
[我的文档 - File Br...
FPC Ctg1328 cp05T...

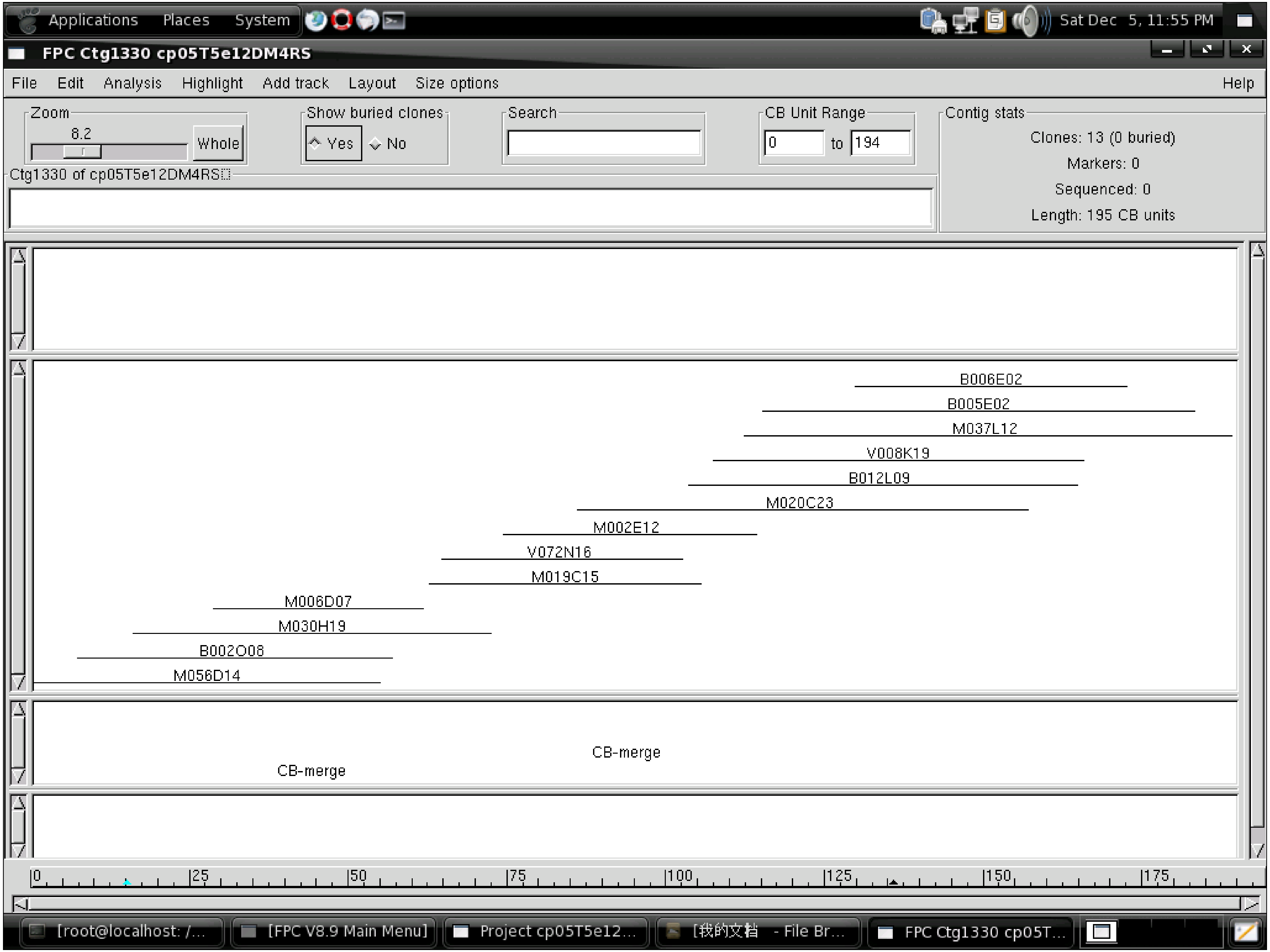

Applications Places System
Sat Dec 5, 11:55 PM
FPC Ctg1330 cp05T5e12DM4RS
File Edit Analysis Highlight Add track Layout Size options Help
Zoom
8.2
Whole
Show buried clones
Yes No
Search
CB Unit Range
0 to 194
Contig stats
Clones: 13 (0 buried)
Markers: 0
Sequenced: 0
Length: 195 CB units
Ctg1330 of cp05T5e12DM4RS
B006E02
B005E02
M037L12
V008K19
B012L09
M020C23
M002E12
V072N16
M019C15
M006D07
M030H19
B002O08
M056D14
CB-merge
CB-merge
0 25 50 75 100 125 150 175
[root@localhost: /...
[FPC V8.9 Main Menu]
Project cp05T5e12...
[我的文档 - File Br...
FPC Ctg1330 cp05T...

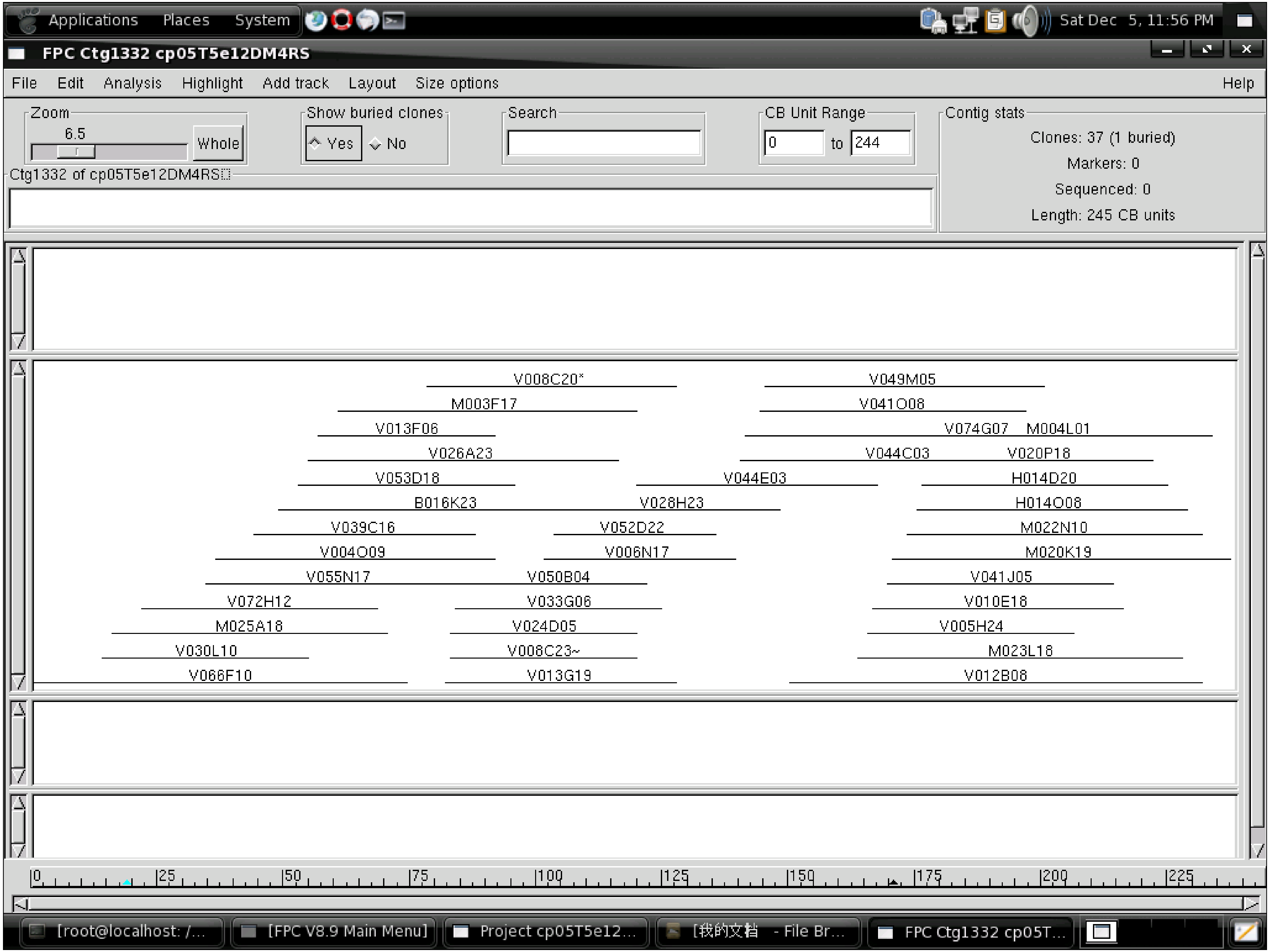
Applications Places System
Sat Dec 5, 11:56 PM
FPC Ctg1332 cp05T5e12DM4RS
File Edit Analysis Highlight Add track Layout Size options Help
Zoom
6.5
Whole
Show buried clones
Yes No
Search
CB Unit Range
0 to 244
Contig stats
Clones: 37 (1 buried)
Markers: 0
Sequenced: 0
Length: 245 CB units
Ctg1332 of cp05T5e12DM4RS
V008C20*
V049M05
M003F17
V041O08
V013F06
V074G07
M004L01
V026A23
V044C03
V020P18
V053D18
V044E03
H014D20
B016K23
V028H23
H014O08
V039C16
V052D22
M022N10
V004O09
V006N17
M020K19
V055N17
V050B04
V041J05
V072H12
V033G06
V010E18
M025A18
V024D05
V005H24
V030L10
V008C23~
M023L18
V066F10
V013G19
V012B08
0 25 50 75 100 125 150 175 200 225
[root@localhost: /...
[FPC V8.9 Main Menu]
Project cp05T5e12...
[我的文档 - File Br...
FPC Ctg1332 cp05T...

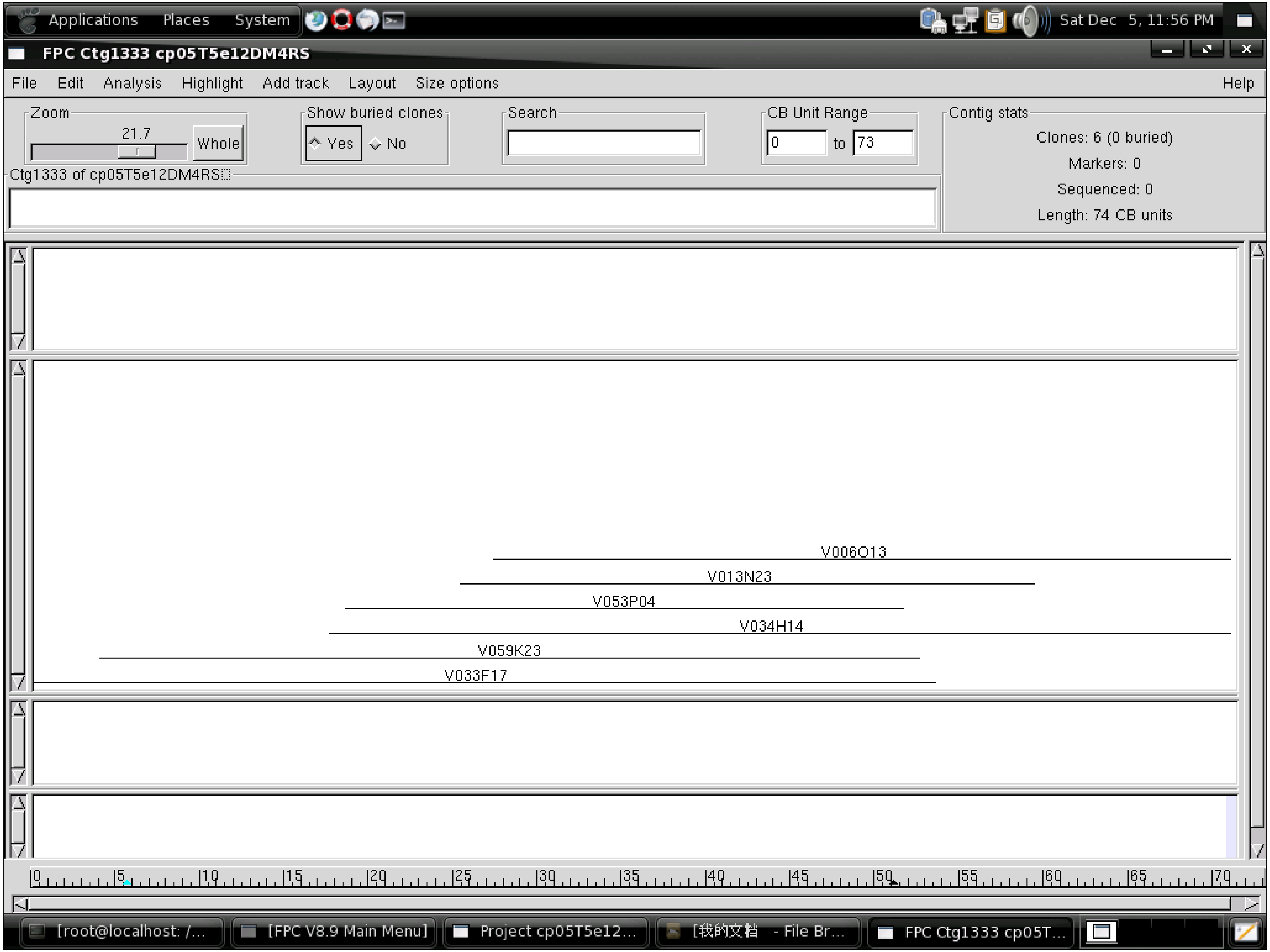
Applications
Places
System
Sat Dec 5, 11:56 PM
FPC Ctg1333 cp05T5e12DM4RS
File
Edit
Analysis
Highlight
Add track
Layout
Size options
Help
Zoom
21.7
Whole
Show buried clones
Yes
No
Search
CB Unit Range
0
to
73
Contig stats
Clones: 6 (0 buried)
Markers: 0
Sequenced: 0
Length: 74 CB units
Ctg1333 of cp05T5e12DM4RS
V006O13
V013N23
V053P04
V034H14
V059K23
V033F17
0 5 10 15 20 25 30 35 40 45 50 55 60 65 70
[root@localhost: /...
[FPC V8.9 Main Menu]
Project cp05T5e12...
[我的文档 - File Br...
FPC Ctg1333 cp05T...

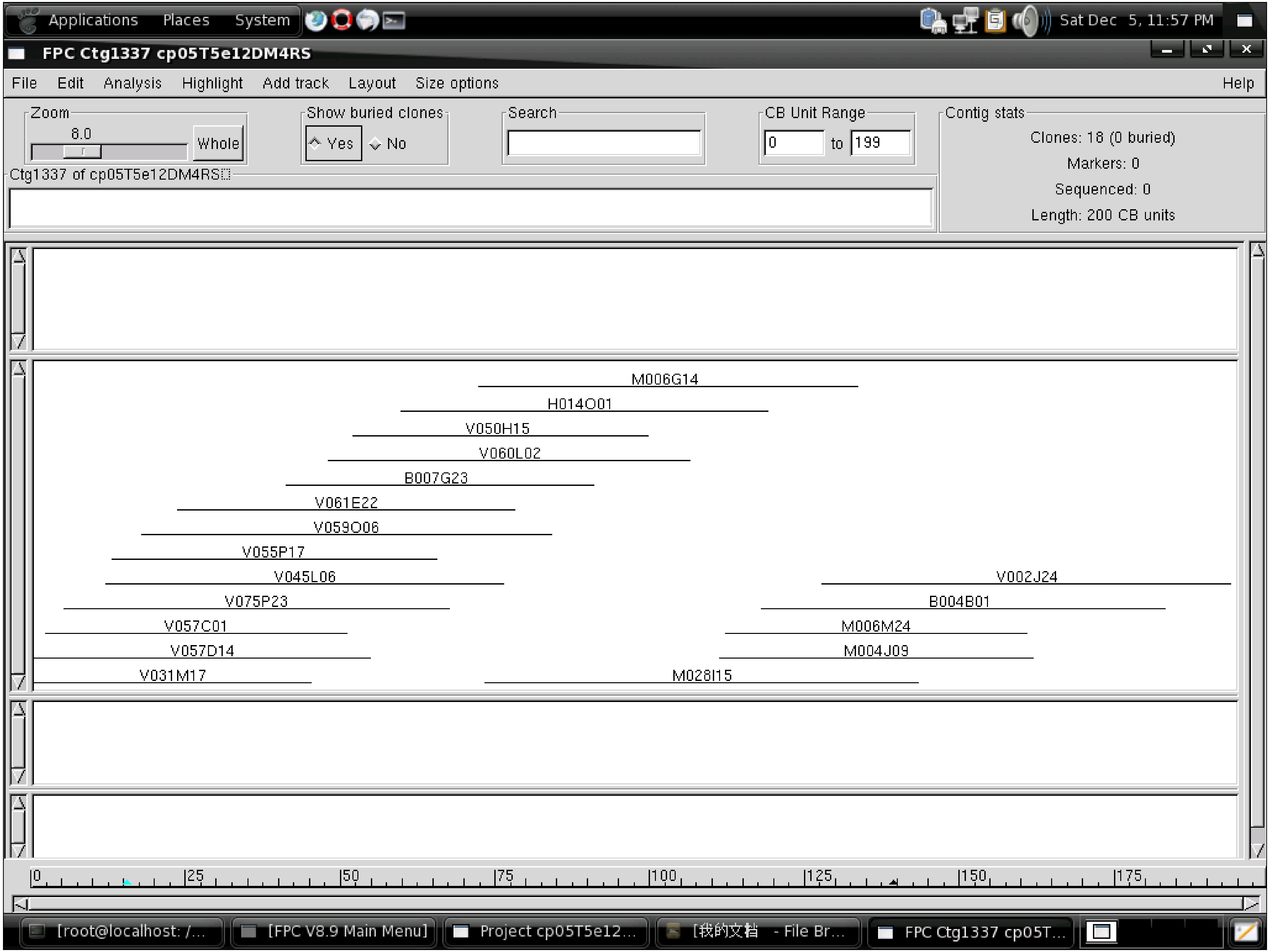
Applications Places System
Sat Dec 5, 11:57 PM
FPC Ctg1337 cp05T5e12DM4RS
File Edit Analysis Highlight Add track Layout Size options Help
Zoom
8.0
Whole
Show buried clones
Yes No
Search
CB Unit Range
0 to 199
Contig stats
Clones: 18 (0 buried)
Markers: 0
Sequenced: 0
Length: 200 CB units
Ctg1337 of cp05T5e12DM4RS
M006G14
H014O01
V050H15
V060L02
B007G23
V061E22
V059O06
V055P17
V045L06
V002J24
V075P23
B004B01
V057C01
M006M24
V057D14
M004J09
V031M17
M028I15
0 25 50 75 100 125 150 175
[root@localhost: /...
[FPC V8.9 Main Menu]
Project cp05T5e12...
[我的文档 - File Br...
FPC Ctg1337 cp05T...

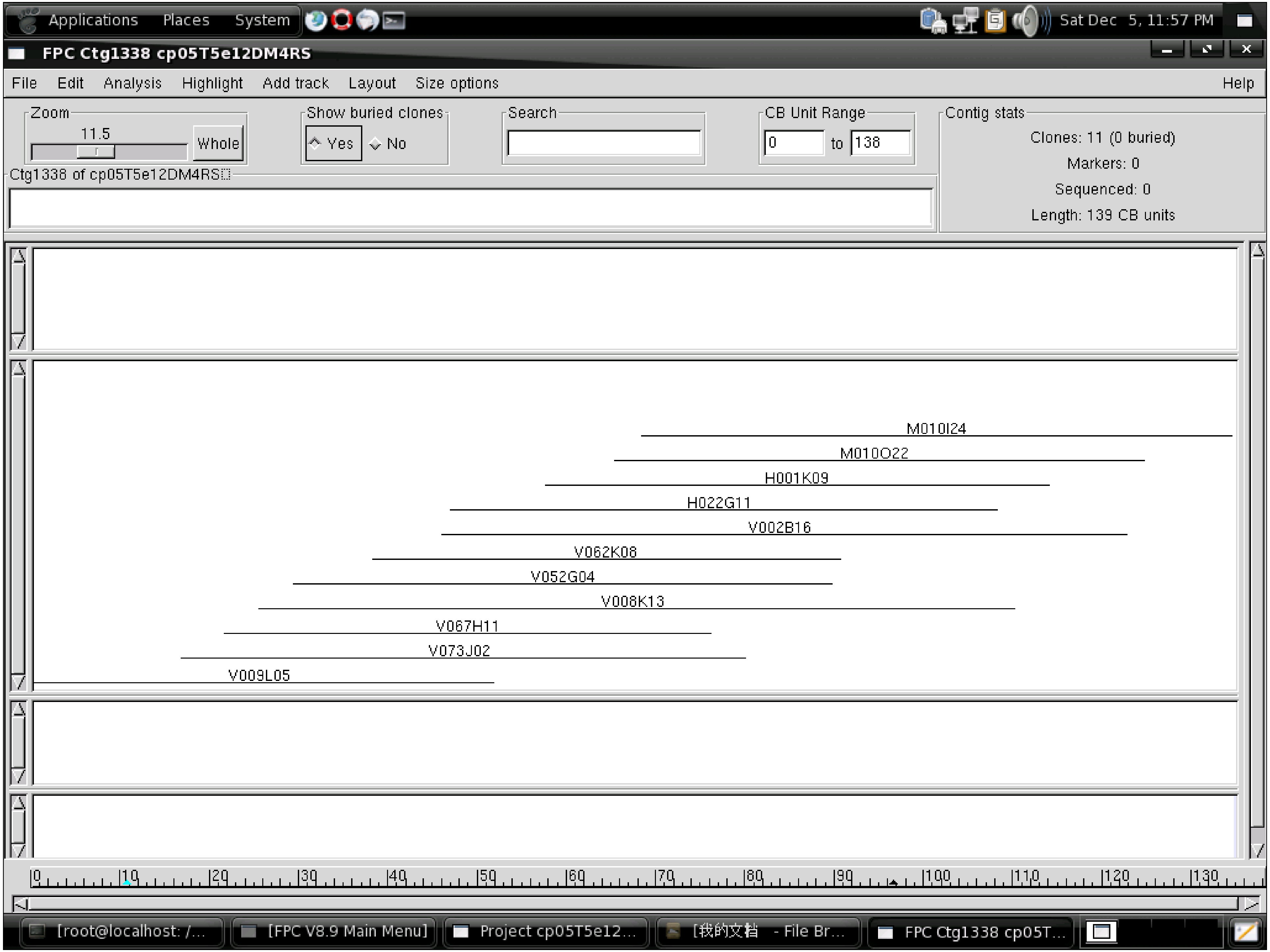
Applications Places System
Sat Dec 5, 11:57 PM
FPC Ctg1338 cp05T5e12DM4RS
File Edit Analysis Highlight Add track Layout Size options Help
Zoom
11.5
Whole
Show buried clones
Yes
No
Search
CB Unit Range
0 to 138
Contig stats
Clones: 11 (0 buried)
Markers: 0
Sequenced: 0
Length: 139 CB units
Ctg1338 of cp05T5e12DM4RS
M010I24
M010O22
H001K09
H022G11
V002B16
V062K08
V052G04
V008K13
V067H11
V073J02
V009L05
0 10 20 30 40 50 60 70 80 90 100 110 120 130
[root@localhost: /...
[FPC V8.9 Main Menu]
Project cp05T5e12...
[我的文档 - File Br...
FPC Ctg1338 cp05T...

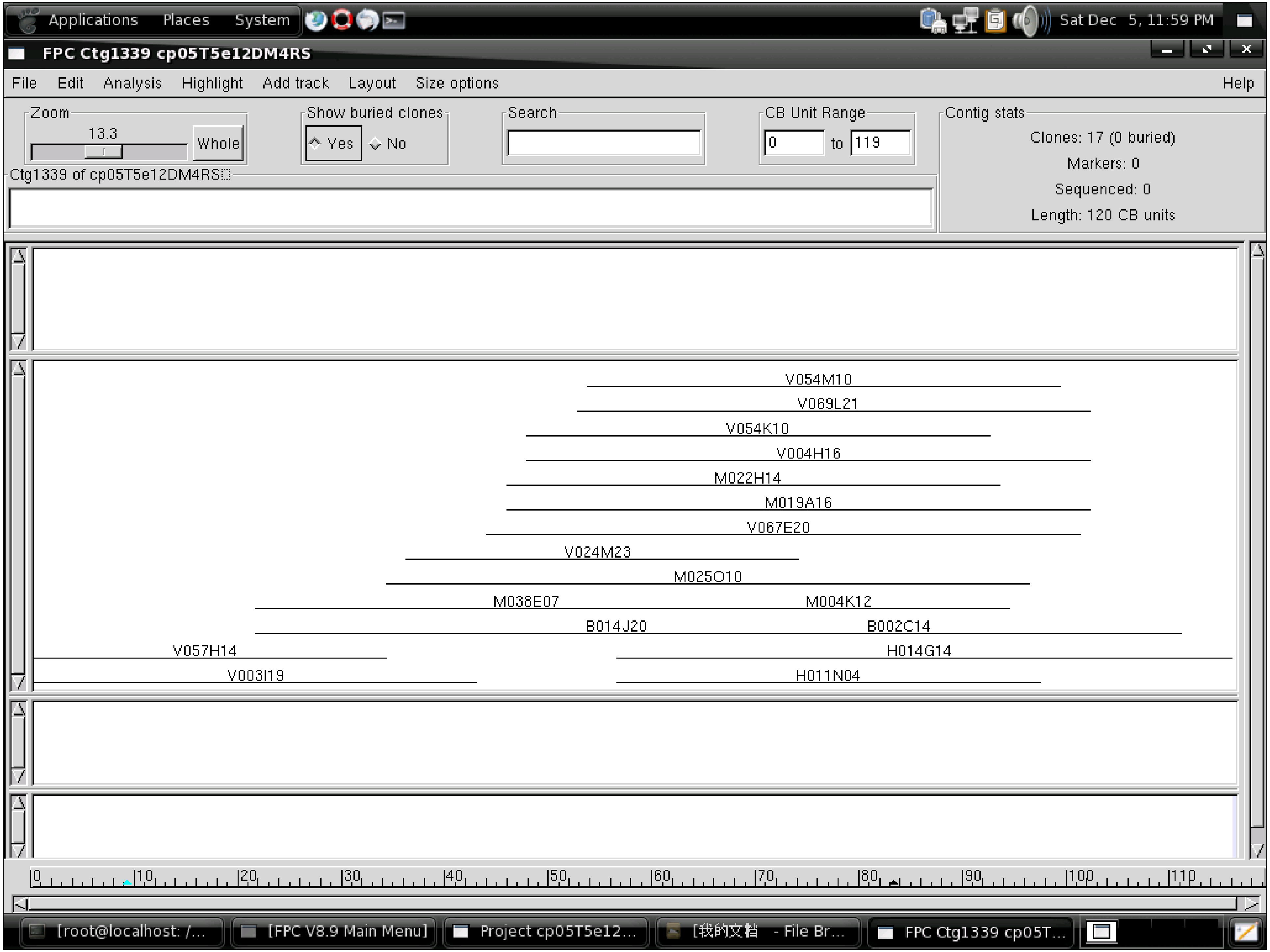
Applications Places System
Sat Dec 5, 11:59 PM
FPC Ctg1339 cp05T5e12DM4RS
File Edit Analysis Highlight Add track Layout Size options Help
Zoom
13.3
Whole
Show buried clones
Yes No
Search
CB Unit Range
0 to 119
Contig stats
Clones: 17 (0 buried)
Markers: 0
Sequenced: 0
Length: 120 CB units
Ctg1339 of cp05T5e12DM4RS
V054M10
V069L21
V054K10
V004H16
M022H14
M019A16
V067E20
V024M23
M025O10
M038E07
M004K12
B014J20
B002C14
V057H14
H014G14
V003I19
H011N04
0 10 20 30 40 50 60 70 80 90 100 110
[root@localhost: /...
[FPC V8.9 Main Menu]
Project cp05T5e12...
[我的文档 - File Br...
FPC Ctg1339 cp05T...

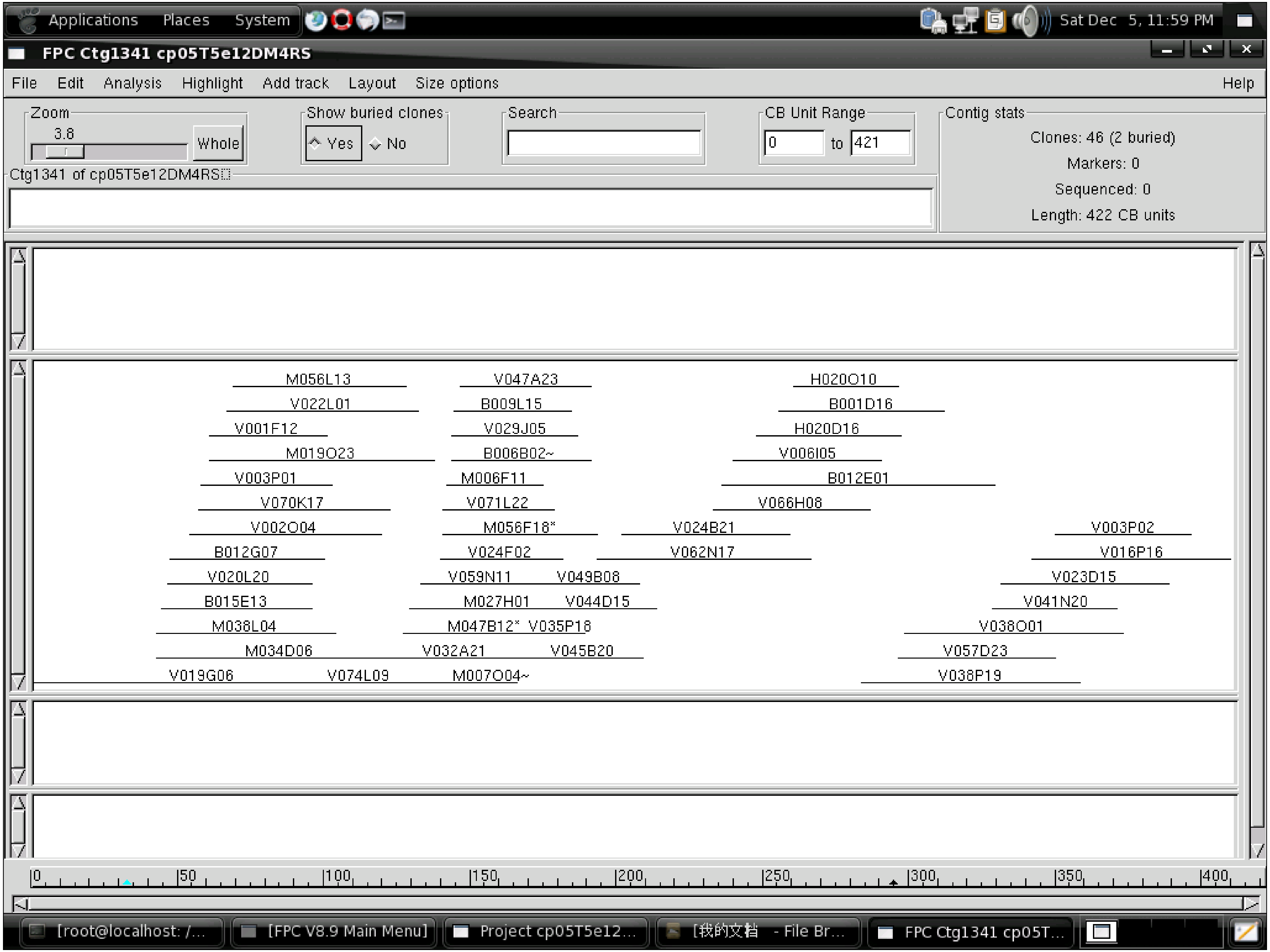

Applications Places System
Sat Dec 5, 11:59 PM
FPC Ctg1341 cp05T5e12DM4RS
File Edit Analysis Highlight Add track Layout Size options Help
Zoom 3.8 Whole
Show buried clones Yes No
Search
CB Unit Range 0 to 421
Contig stats
Clones: 46 (2 buried)
Markers: 0
Sequenced: 0
Length: 422 CB units
Ctg1341 of cp05T5e12DM4RS
M056L13
V047A23
H020O10
V022L01
B009L15
B001D16
V001F12
V029J05
H020D16
M019O23
B006B02~
V006I05
V003P01
M006F11
B012E01
V070K17
V071L22
V066H08
V002O04
M056F18*
V024B21
V003P02
B012G07
V024F02
V062N17
V016P16
V020L20
V059N11
V049B08
V023D15
B015E13
M027H01
V044D15
V041N20
M038L04
M047B12*
V035P18
V038O01
M034D06
V032A21
V045B20
V057D23
V019G06
V074L09
M007O04~
V038P19
0 50 100 150 200 250 300 350 400
[root@localhost: /...
[FPC V8.9 Main Menu]
Project cp05T5e12...
[我的文档 - File Br...
FPC Ctg1341 cp05T...

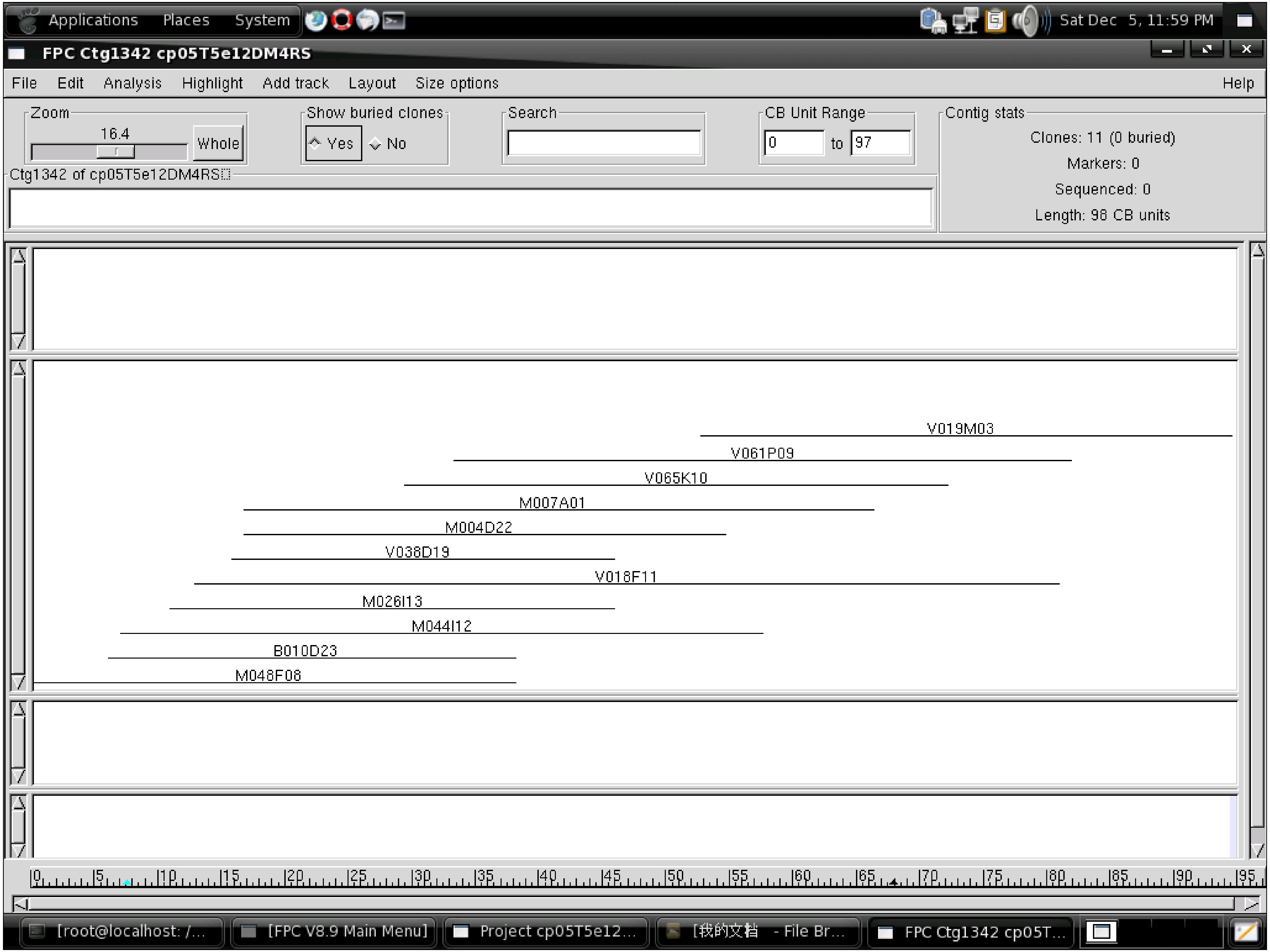
Applications
Places
System
Sat Dec 5, 11:59 PM
FPC Ctg1342 cp05T5e12DM4RS
File
Edit
Analysis
Highlight
Add track
Layout
Size options
Help
Zoom
16.4
Whole
Show buried clones
Yes
No
Search
CB Unit Range
0
to
97
Contig stats
Clones: 11 (0 buried)
Markers: 0
Sequenced: 0
Length: 98 CB units
Ctg1342 of cp05T5e12DM4RS
V019M03
V061P09
V065K10
M007A01
M004D22
V038D19
V018F11
M026I13
M044I12
B010D23
M048F08
0 5 10 15 20 25 30 35 40 45 50 55 60 65 70 75 80 85 90 95
[root@localhost: /...
[FPC V8.9 Main Menu]
Project cp05T5e12...
[我的文档 - File Br...
FPC Ctg1342 cp05T...

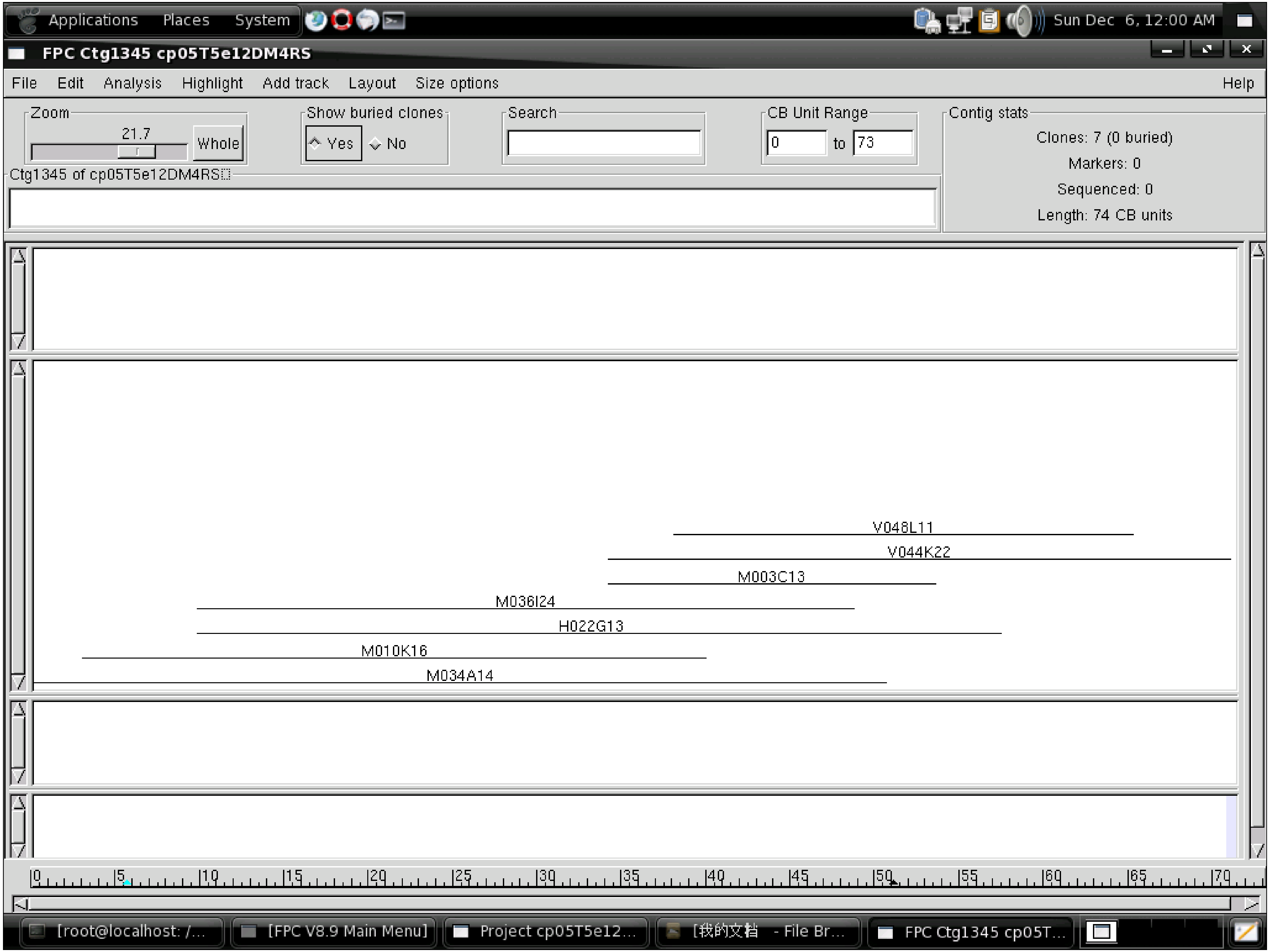
Applications Places System
Sun Dec 6, 12:00 AM
FPC Ctg1345 cp05T5e12DM4RS
File Edit Analysis Highlight Add track Layout Size options Help
Zoom
21.7
Whole
Show buried clones
Yes
No
Search
CB Unit Range
0
to
73
Contig stats
Clones: 7 (0 buried)
Markers: 0
Sequenced: 0
Length: 74 CB units
Ctg1345 of cp05T5e12DM4RS
V048L11
V044K22
M003C13
M036I24
H022G13
M010K16
M034A14
0 5 10 15 20 25 30 35 40 45 50 55 60 65 70
[root@localhost: /...
[FPC V8.9 Main Menu]
Project cp05T5e12...
[我的文档 - File Br...
FPC Ctg1345 cp05T...

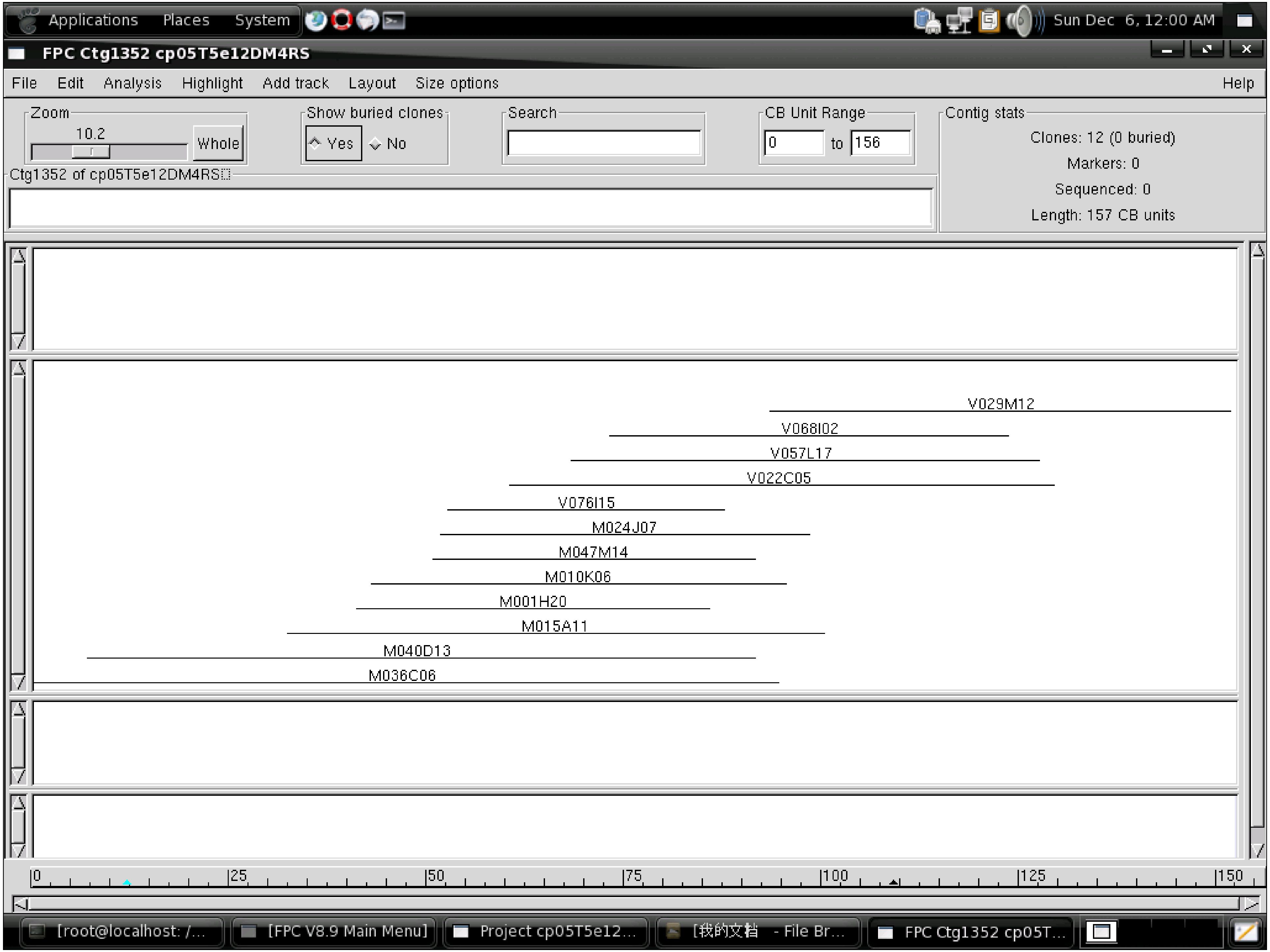
Applications Places System
Sun Dec 6, 12:00 AM
FPC Ctg1352 cp05T5e12DM4RS
File Edit Analysis Highlight Add track Layout Size options Help
Zoom
10.2
Whole
Show buried clones
Yes
No
Search
CB Unit Range
0 to 156
Contig stats
Clones: 12 (0 buried)
Markers: 0
Sequenced: 0
Length: 157 CB units
Ctg1352 of cp05T5e12DM4RS
V029M12
V068I02
V057L17
V022C05
V076I15
M024J07
M047M14
M010K06
M001H20
M015A11
M040D13
M036C06
0 25 50 75 100 125 150
[root@localhost: /...
[FPC V8.9 Main Menu]
Project cp05T5e12...
[我的文档 - File Br...
FPC Ctg1352 cp05T...

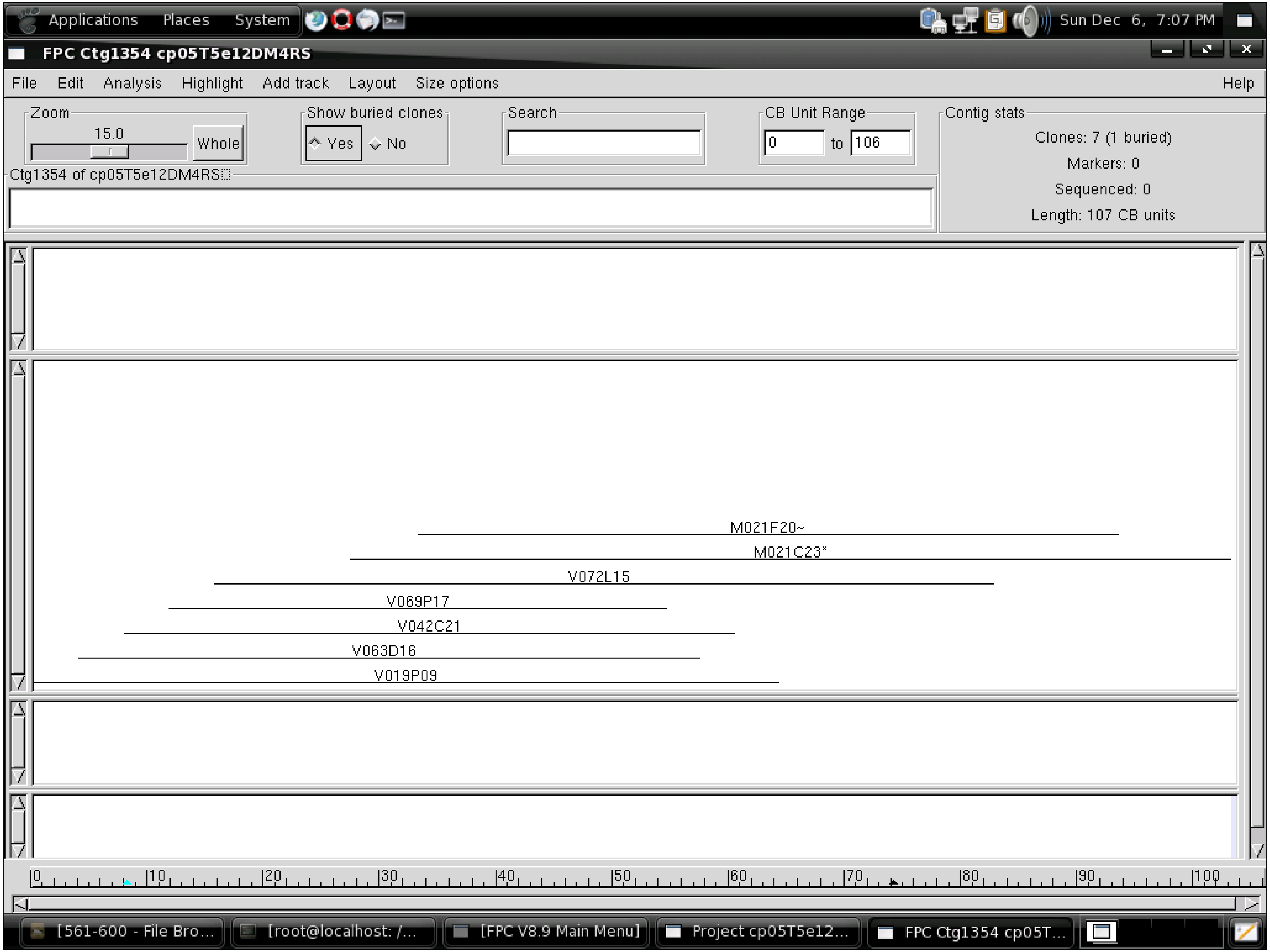
Applications Places System
Sun Dec 6, 7:07 PM
FPC Ctg1354 cp05T5e12DM4RS
File Edit Analysis Highlight Add track Layout Size options Help
Zoom
15.0
Whole
Show buried clones
Yes
No
Search
CB Unit Range
0
to
106
Contig stats
Clones: 7 (1 buried)
Markers: 0
Sequenced: 0
Length: 107 CB units
Ctg1354 of cp05T5e12DM4RS
M021F20~
M021C23*
V072L15
V069P17
V042C21
V063D16
V019P09
0 10 20 30 40 50 60 70 80 90 100
[561-600 - File Bro...
[root@localhost: /...
[FPC V8.9 Main Menu]
Project cp05T5e12...
FPC Ctg1354 cp05T...

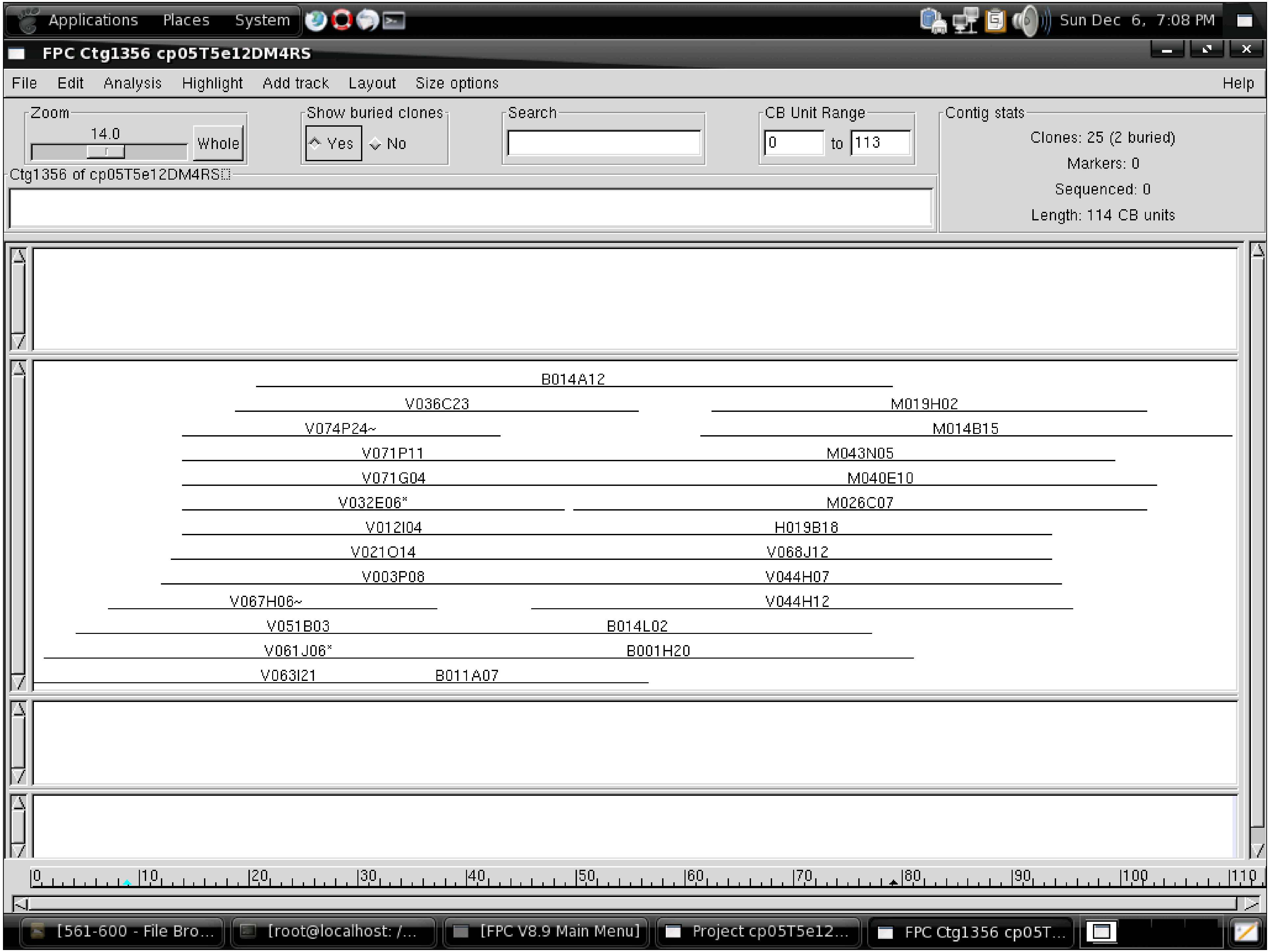
Applications Places System
Sun Dec 6, 7:08 PM
FPC Ctg1356 cp05T5e12DM4RS
File Edit Analysis Highlight Add track Layout Size options Help
Zoom
14.0
Whole
Show buried clones
Yes No
Search
CB Unit Range
0 to 113
Contig stats
Clones: 25 (2 buried)
Markers: 0
Sequenced: 0
Length: 114 CB units
Ctg1356 of cp05T5e12DM4RS
B014A12
V036C23
M019H02
V074P24~
M014B15
V071P11
M043N05
V071G04
M040E10
V032E06*
M026C07
V012I04
H019B18
V021O14
V068J12
V003P08
V044H07
V067H06~
V044H12
V051B03
B014L02
V061J06*
B001H20
V063I21
B011A07
0 10 20 30 40 50 60 70 80 90 100 110
[561-600 - File Bro...
[root@localhost: /...
[FPC V8.9 Main Menu]
Project cp05T5e12...
FPC Ctg1356 cp05T...

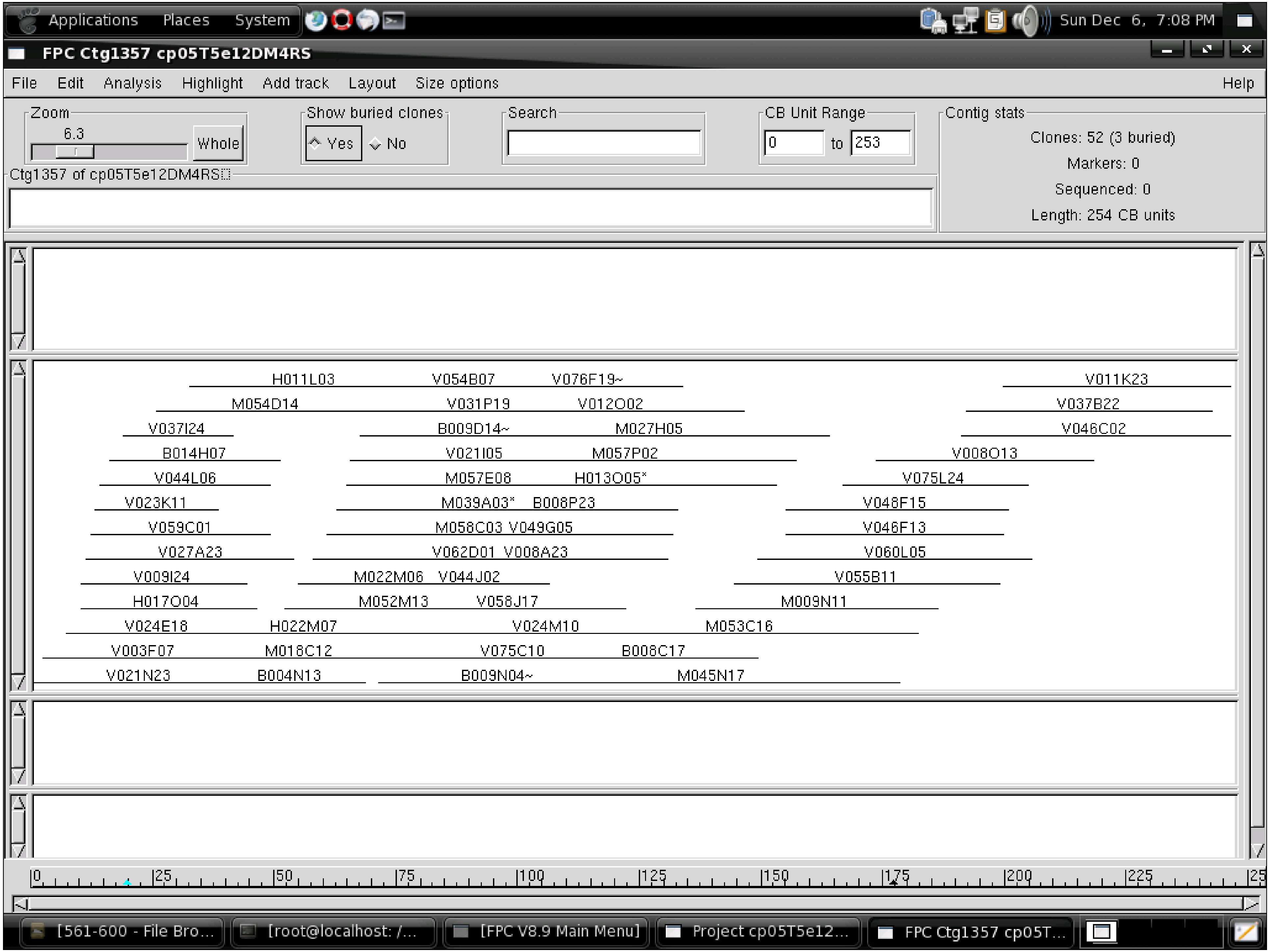
Applications Places System
Sun Dec 6, 7:08 PM
FPC Ctg1357 cp05T5e12DM4RS
File Edit Analysis Highlight Add track Layout Size options Help
Zoom
6.3
Whole
Show buried clones
Yes No
Search
CB Unit Range
0 to 253
Contig stats
Clones: 52 (3 buried)
Markers: 0
Sequenced: 0
Length: 254 CB units
Ctg1357 of cp05T5e12DM4RS
H011L03 V054B07 V076F19~ V011K23
M054D14 V031P19 V012O02 V037B22
V037I24 B009D14~ M027H05 V046C02
B014H07 V021I05 M057P02 V008O13
V044L06 M057E08 H013O05* V075L24
V023K11 M039A03* B008P23 V048F15
V059C01 M058C03 V049G05 V046F13
V027A23 V062D01 V008A23 V060L05
V009I24 M022M06 V044J02 V055B11
H017O04 M052M13 V058J17 M009N11
V024E18 H022M07 V024M10 M053C16
V003F07 M018C12 V075C10 B008C17
V021N23 B004N13 B009N04~ M045N17
0 25 50 75 100 125 150 175 200 225
[561-600 - File Bro...
[root@localhost: /...
[FPC V8.9 Main Menu]
Project cp05T5e12...
FPC Ctg1357 cp05T...

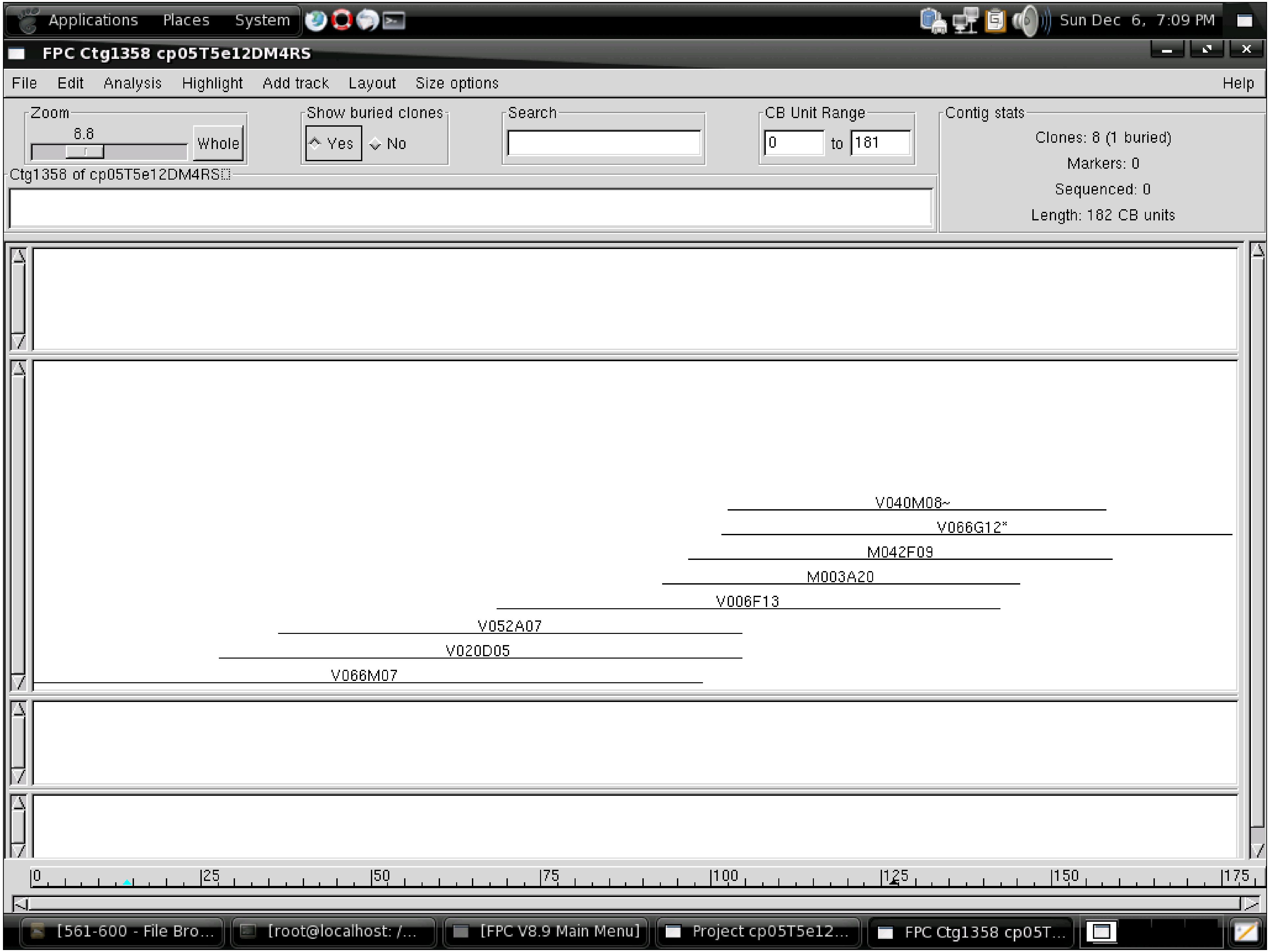
Applications Places System
Sun Dec 6, 7:09 PM
FPC Ctg1358 cp05T5e12DM4RS
File Edit Analysis Highlight Add track Layout Size options Help
Zoom
8.8
Whole
Show buried clones
Yes No
Search
CB Unit Range
0 to 181
Contig stats
Clones: 8 (1 buried)
Markers: 0
Sequenced: 0
Length: 182 CB units
Ctg1358 of cp05T5e12DM4RS
V040M08~
V066G12*
M042F09
M003A20
V006F13
V052A07
V020D05
V066M07
0 25 50 75 100 125 150 175
[561-600 - File Bro...
[root@localhost: /...
[FPC V8.9 Main Menu]
Project cp05T5e12...
FPC Ctg1358 cp05T...

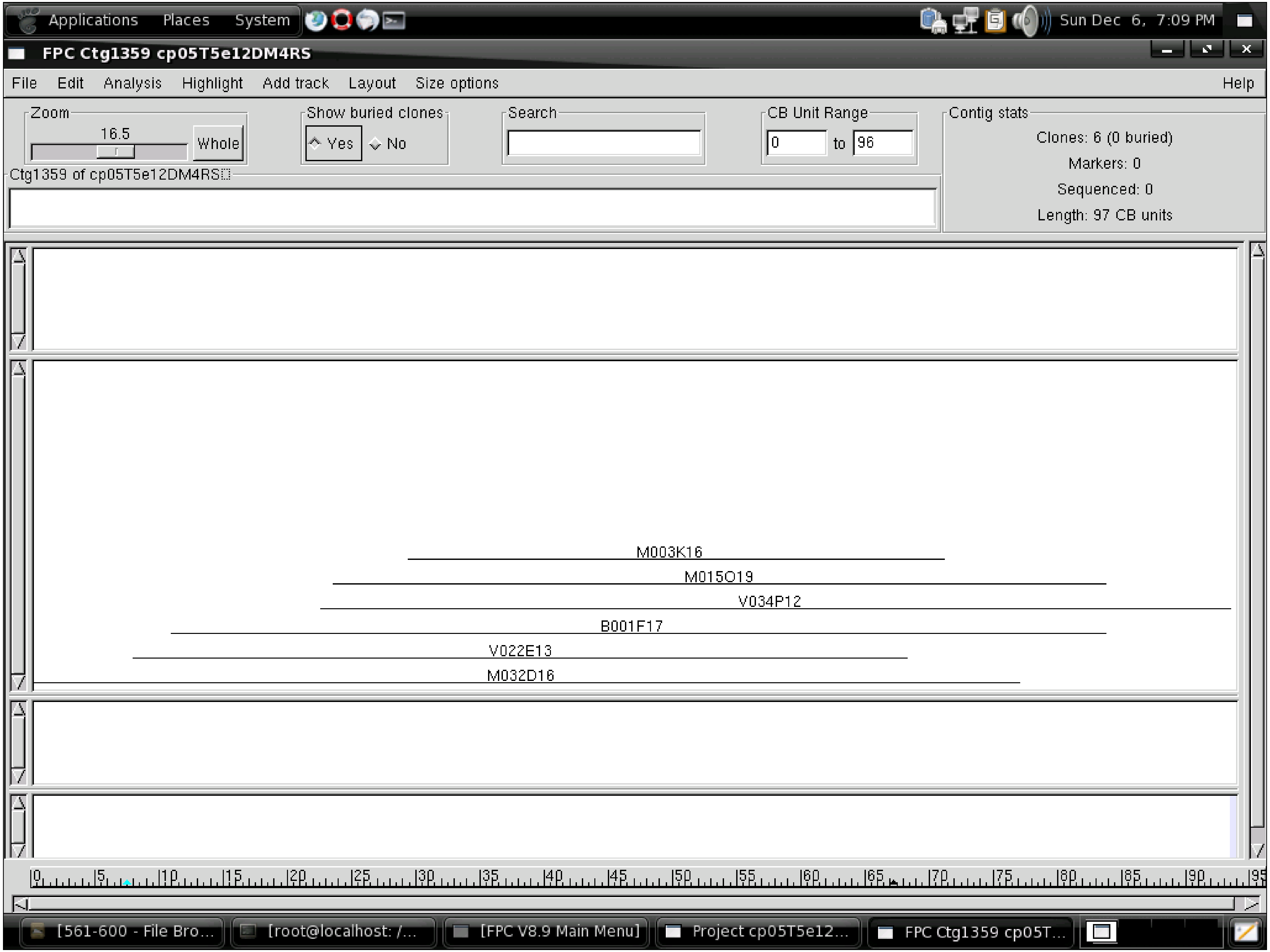
Applications Places System
Sun Dec 6, 7:09 PM
FPC Ctg1359 cp05T5e12DM4RS
File Edit Analysis Highlight Add track Layout Size options Help
Zoom
16.5
Whole
Show buried clones
Yes No
Search
CB Unit Range
0 to 96
Contig stats
Clones: 6 (0 buried)
Markers: 0
Sequenced: 0
Length: 97 CB units
Ctg1359 of cp05T5e12DM4RS
M003K16
M015O19
V034P12
B001F17
V022E13
M032D16
0 5 10 15 20 25 30 35 40 45 50 55 60 65 70 75 80 85 90 95
[561-600 - File Bro...
[root@localhost: /...
[FPC V8.9 Main Menu]
Project cp05T5e12...
FPC Ctg1359 cp05T...

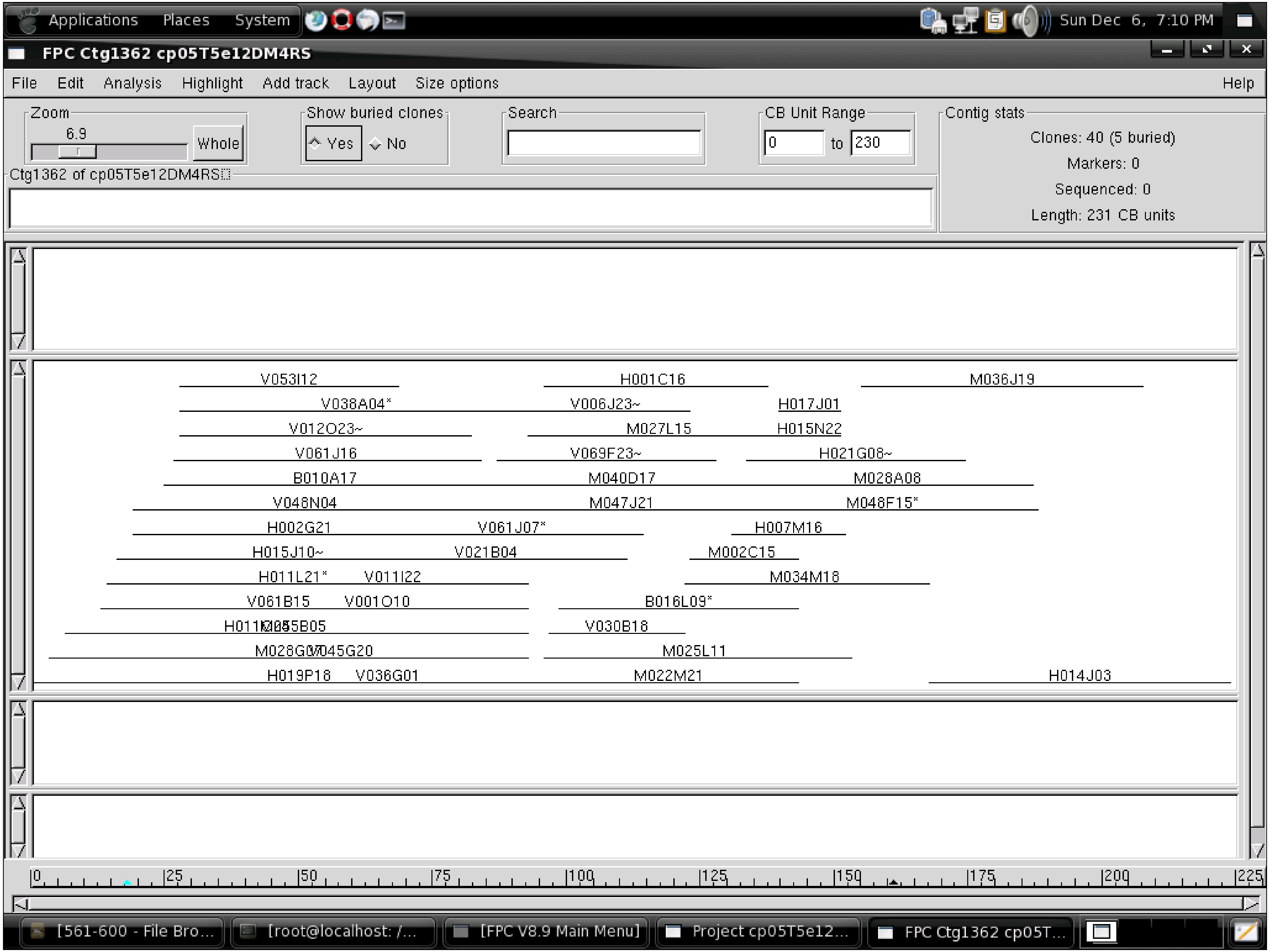

Applications Places System
Sun Dec 6, 7:10 PM
FPC Ctg1362 cp05T5e12DM4RS
File Edit Analysis Highlight Add track Layout Size options Help
Zoom
6.9
Whole
Show buried clones
Yes
No
Search
CB Unit Range
0
to
230
Contig stats
Clones: 40 (5 buried)
Markers: 0
Sequenced: 0
Length: 231 CB units
Ctg1362 of cp05T5e12DM4RS
V053I12
H001C16
M036J19
V038A04*
V006J23~
H017J01
V012O23~
M027L15
H015N22
V061J16
V069F23~
H021G08~
B010A17
M040D17
M028A08
V048N04
M047J21
M048F15*
H002G21
V061J07*
H007M16
H015J10~
V021B04
M002C15
H011L21*
V011I22
M034M18
V061B15
V001O10
B016L09*
V030B18
M025L11
H019P18
V036G01
M022M21
H014J03
0 25 50 75 100 125 150 175 200 225
[561-600 - File Bro...
[root@localhost: /...
[FPC V8.9 Main Menu]
Project cp05T5e12...
FPC Ctg1362 cp05T...

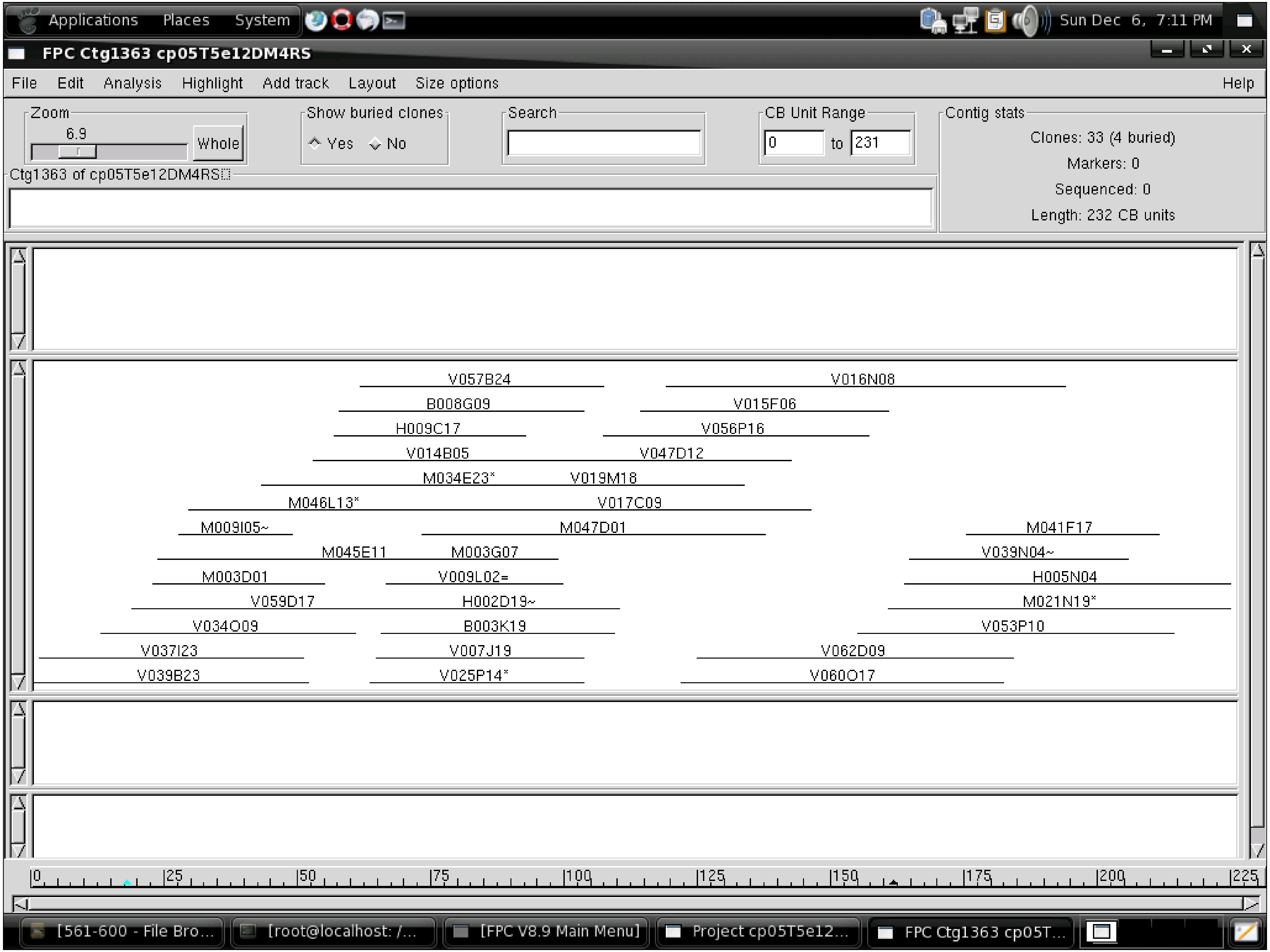
Applications Places System
Sun Dec 6, 7:11 PM
FPC Ctg1363 cp05T5e12DM4RS
File Edit Analysis Highlight Add track Layout Size options Help
Zoom
6.9
Whole
Show buried clones
Yes No
Search
CB Unit Range
0 to 231
Contig stats
Clones: 33 (4 buried)
Markers: 0
Sequenced: 0
Length: 232 CB units
Ctg1363 of cp05T5e12DM4RS
V057B24
V016N08
B008G09
V015F06
H009C17
V056P16
V014B05
V047D12
M034E23*
V019M18
M046L13*
V017C09
M009I05~
M047D01
M041F17
M045E11
M003G07
V039N04~
M003D01
V009L02=
H005N04
V059D17
H002D19~
M021N19*
V034O09
B003K19
V053P10
V037I23
V007J19
V062D09
V039B23
V025P14*
V060O17
0 25 50 75 100 125 150 175 200 225
[561-600 - File Bro...
[root@localhost: /...
[FPC V8.9 Main Menu]
Project cp05T5e12...
FPC Ctg1363 cp05T...

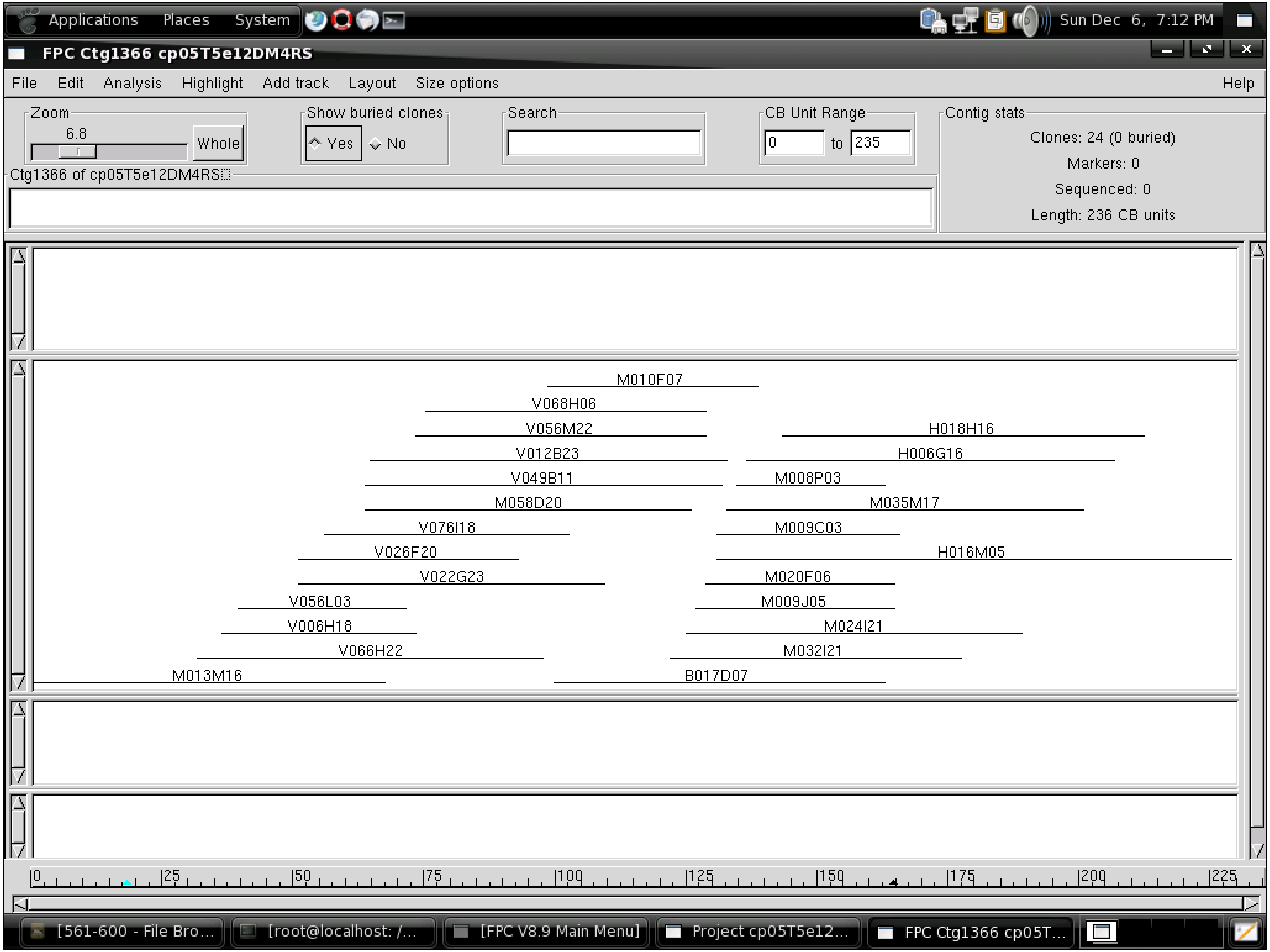

Applications
Places
System
Sun Dec 6, 7:12 PM
FPC Ctg1366 cp05T5e12DM4RS
File
Edit
Analysis
Highlight
Add track
Layout
Size options
Help
Zoom
6.8
Whole
Show buried clones
Yes
No
Search
CB Unit Range
0
to
235
Contig stats
Clones: 24 (0 buried)
Markers: 0
Sequenced: 0
Length: 236 CB units
Ctg1366 of cp05T5e12DM4RS
M010F07
V068H06
V056M22
H018H16
V012B23
H006G16
V049B11
M008P03
M058D20
M035M17
V076I18
M009C03
V026F20
H016M05
V022G23
M020F06
V056L03
M009J05
V006H18
M024I21
V066H22
M032I21
M013M16
B017D07
0
25
50
75
100
125
150
175
200
225
[561-600 - File Bro...
[root@localhost: /...
[FPC V8.9 Main Menu]
Project cp05T5e12...
FPC Ctg1366 cp05T...

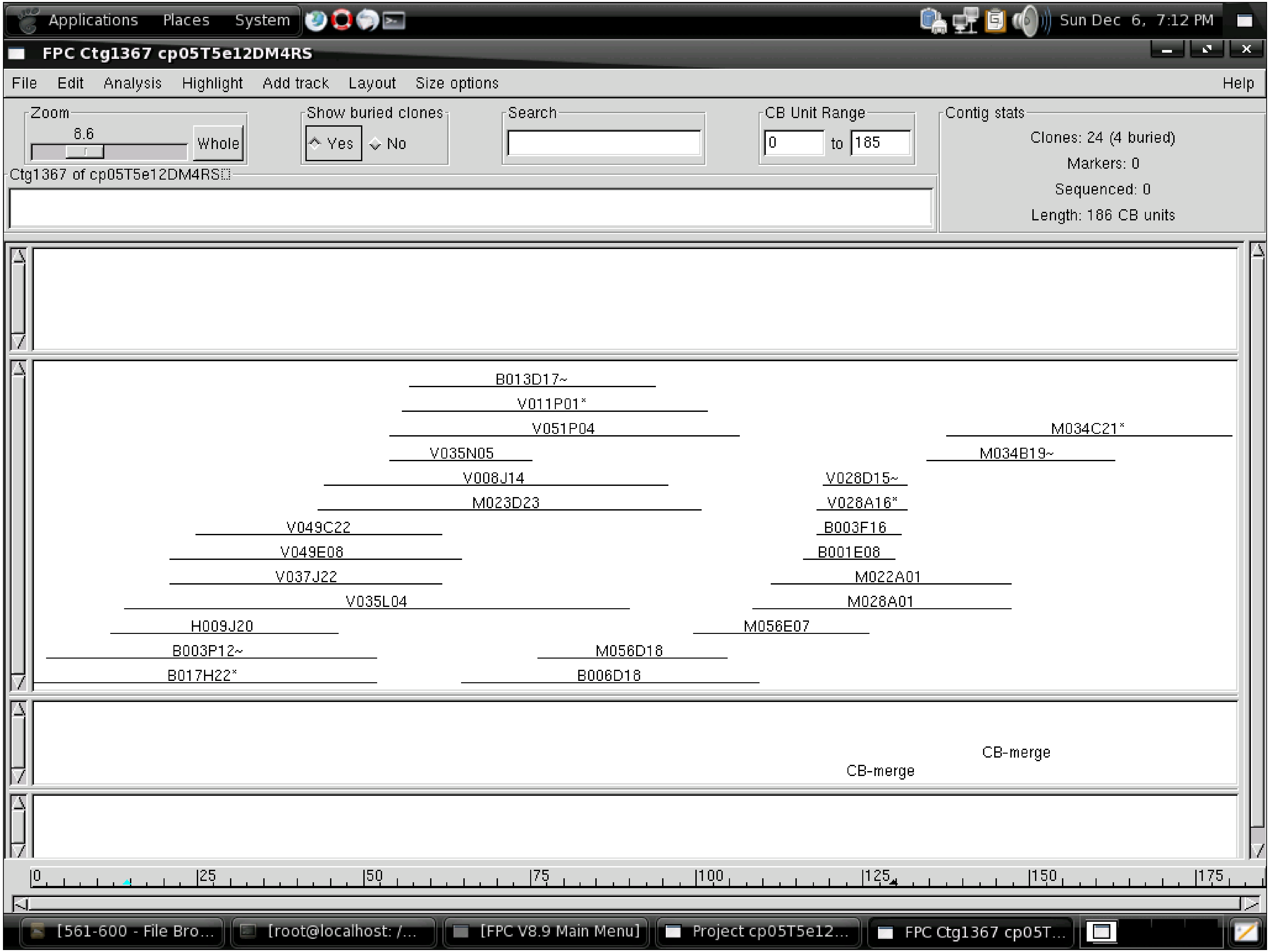
Applications Places System
Sun Dec 6, 7:12 PM
FPC Ctg1367 cp05T5e12DM4RS
File Edit Analysis Highlight Add track Layout Size options Help
Zoom
8.6
Whole
Show buried clones
Yes
No
Search
CB Unit Range
0
to
185
Contig stats
Clones: 24 (4 buried)
Markers: 0
Sequenced: 0
Length: 186 CB units
Ctg1367 of cp05T5e12DM4RS
B013D17~
V011P01*
V051P04
M034C21*
V035N05
M034B19~
V008J14
V028D15~
M023D23
V028A16*
V049C22
B003F16
V049E08
B001E08
V037J22
M022A01
V035L04
M028A01
H009J20
M056E07
B003P12~
M056D18
B017H22*
B006D18
CB-merge
CB-merge
0
25
50
75
100
125
150
175
[561-600 - File Bro...
[root@localhost: /...
[FPC V8.9 Main Menu]
Project cp05T5e12...
FPC Ctg1367 cp05T...

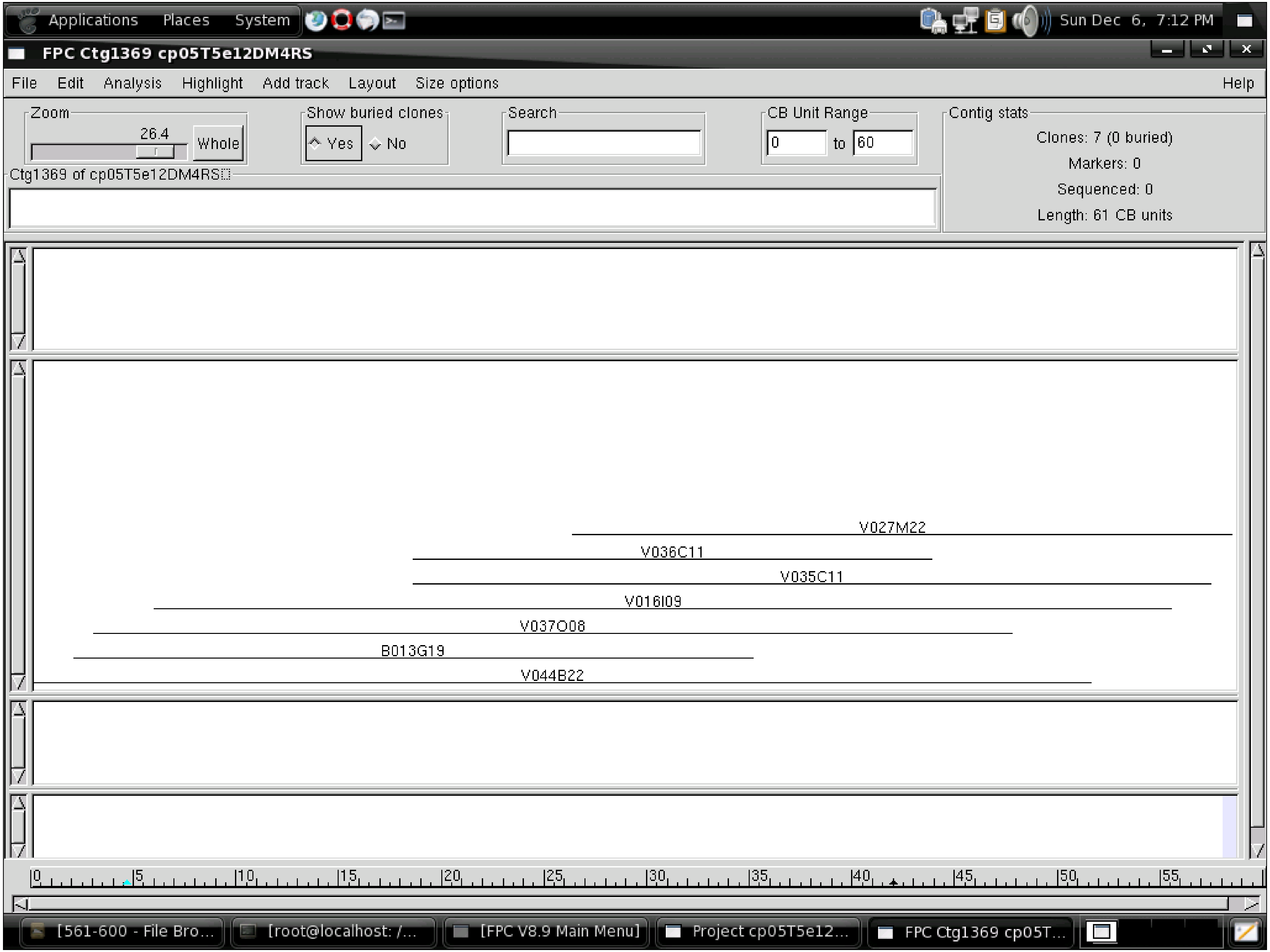

Applications Places System
Sun Dec 6, 7:12 PM
FPC Ctg1369 cp05T5e12DM4RS
File Edit Analysis Highlight Add track Layout Size options Help
Zoom
26.4
Whole
Show buried clones
Yes
No
Search
CB Unit Range
0 to 60
Contig stats
Clones: 7 (0 buried)
Markers: 0
Sequenced: 0
Length: 61 CB units
Ctg1369 of cp05T5e12DM4RS
V027M22
V036C11
V035C11
V016I09
V037O08
B013G19
V044B22
0 5 10 15 20 25 30 35 40 45 50 55
[561-600 - File Bro...
[root@localhost: /...
[FPC V8.9 Main Menu]
Project cp05T5e12...
FPC Ctg1369 cp05T...

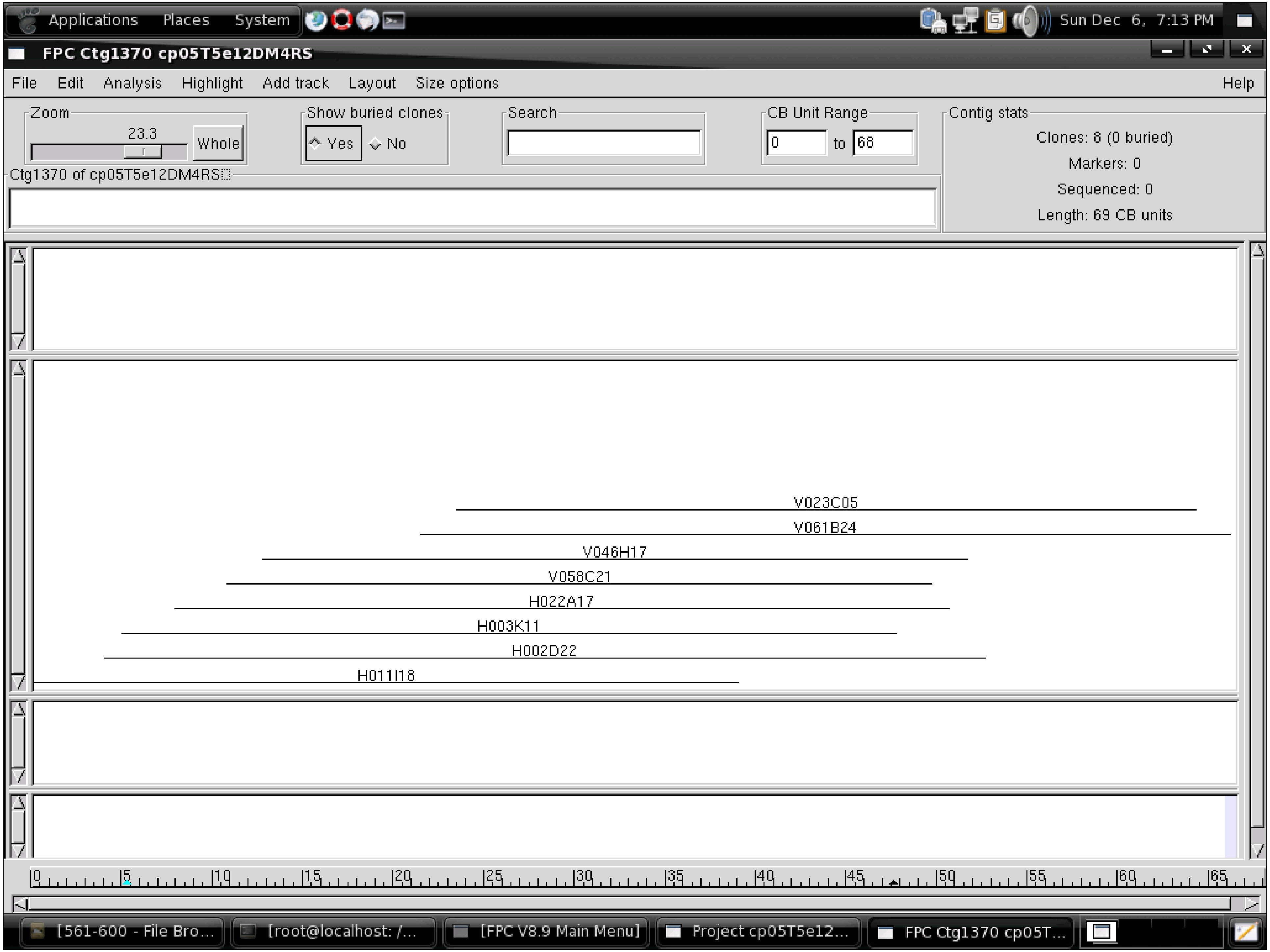
Applications Places System
Sun Dec 6, 7:13 PM
FPC Ctg1370 cp05T5e12DM4RS
File Edit Analysis Highlight Add track Layout Size options Help
Zoom
23.3
Whole
Show buried clones
Yes
No
Search
CB Unit Range
0
to
68
Contig stats
Clones: 8 (0 buried)
Markers: 0
Sequenced: 0
Length: 69 CB units
Ctg1370 of cp05T5e12DM4RS
V023C05
V061B24
V046H17
V058C21
H022A17
H003K11
H002D22
H011I18
0 5 10 15 20 25 30 35 40 45 50 55 60 65
[561-600 - File Bro...
[root@localhost: /...
[FPC V8.9 Main Menu]
Project cp05T5e12...
FPC Ctg1370 cp05T...

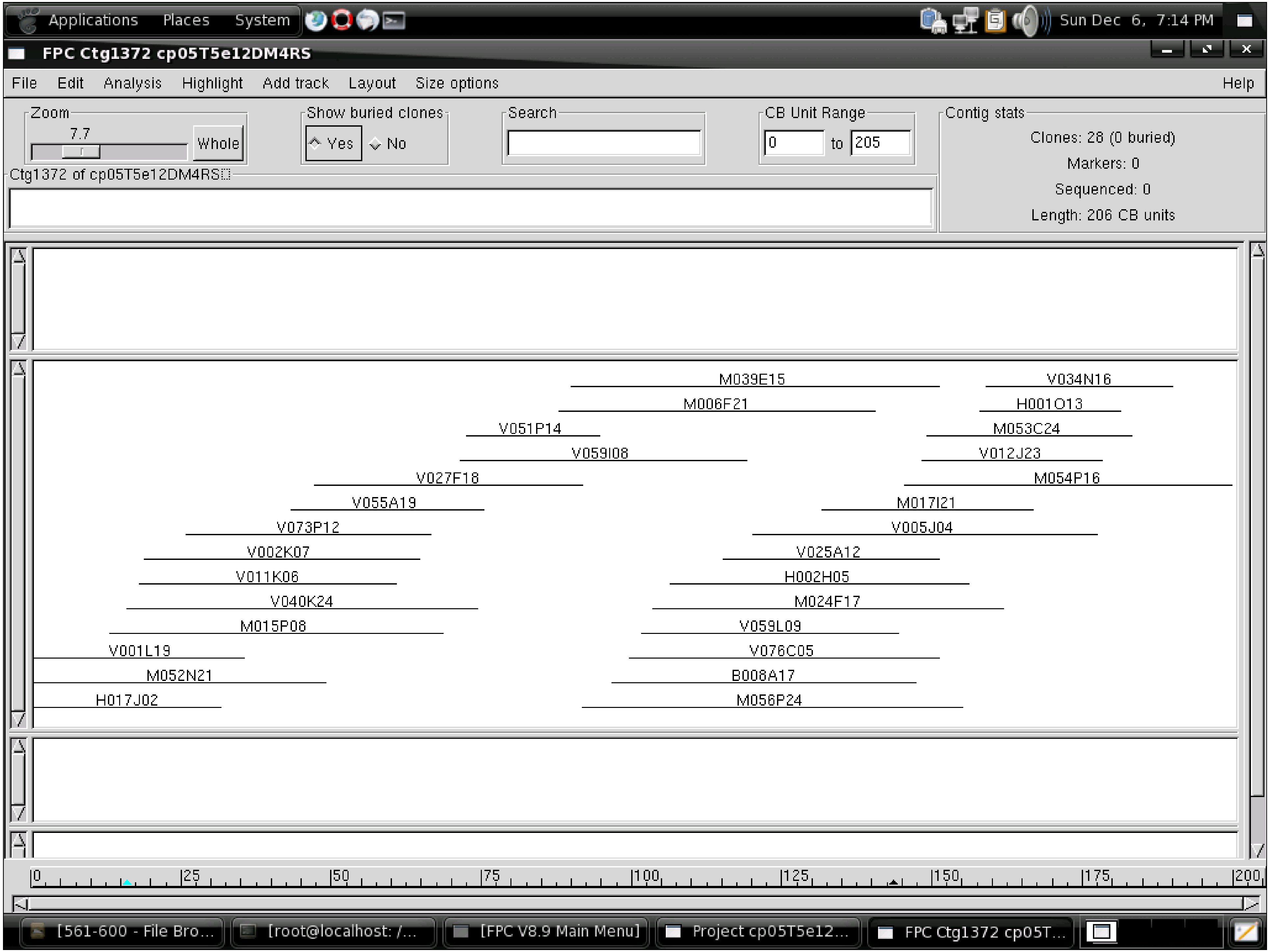
Applications Places System
Sun Dec 6, 7:14 PM
FPC Ctg1372 cp05T5e12DM4RS
File Edit Analysis Highlight Add track Layout Size options Help
Zoom
7.7
Whole
Show buried clones
Yes
No
Search
CB Unit Range
0 to 205
Contig stats
Clones: 28 (0 buried)
Markers: 0
Sequenced: 0
Length: 206 CB units
Ctg1372 of cp05T5e12DM4RS
M039E15
V034N16
M006F21
H001O13
V051P14
M053C24
V059I08
V012J23
V027F18
M054P16
V055A19
M017I21
V073P12
V005J04
V002K07
V025A12
V011K06
H002H05
V040K24
M024F17
M015P08
V059L09
V001L19
V076C05
M052N21
B008A17
H017J02
M056P24
0 25 50 75 100 125 150 175 200
[561-600 - File Bro...
[root@localhost: /...
[FPC V8.9 Main Menu]
Project cp05T5e12...
FPC Ctg1372 cp05T...

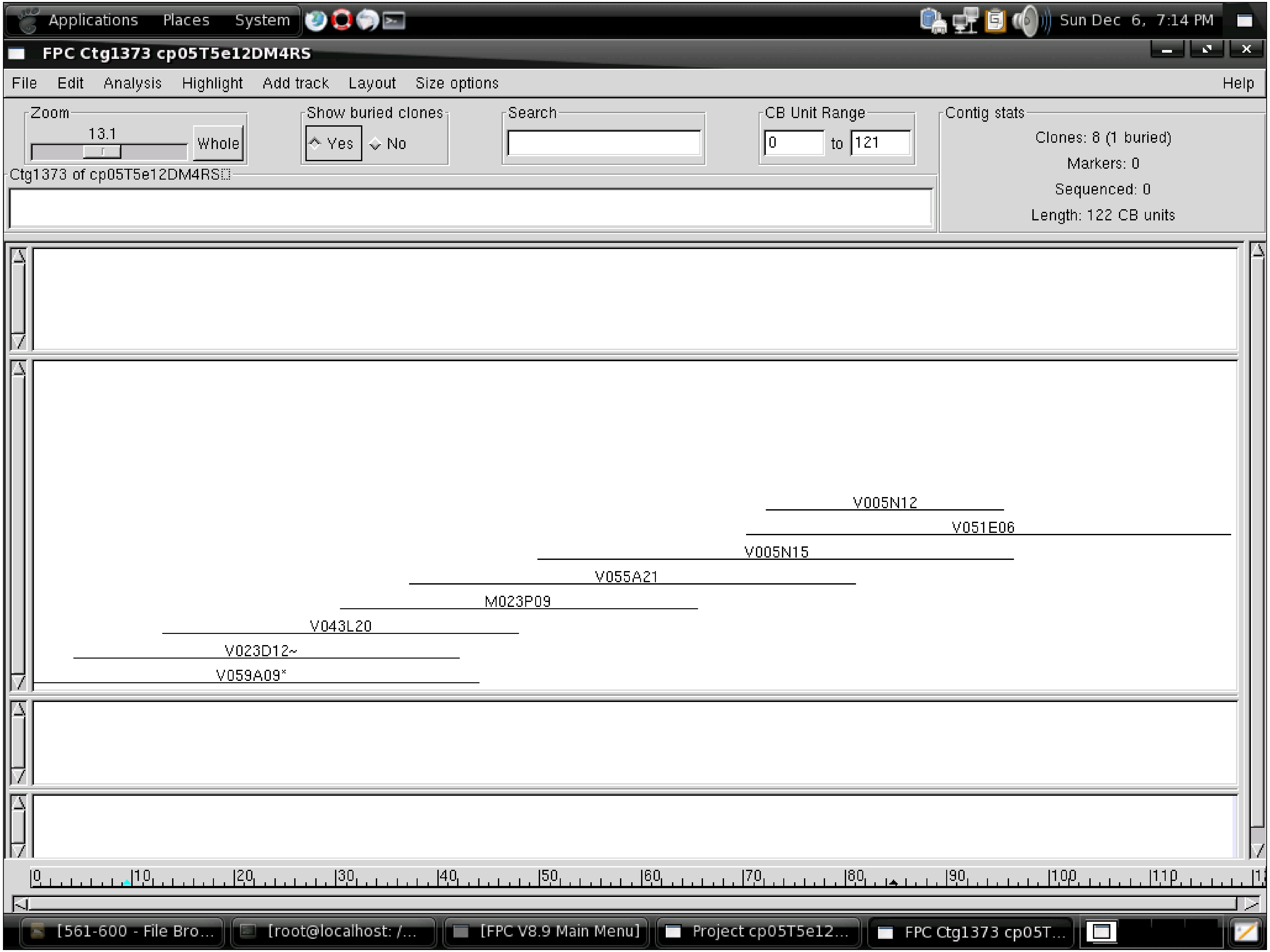
Applications Places System
Sun Dec 6, 7:14 PM
FPC Ctg1373 cp05T5e12DM4RS
File Edit Analysis Highlight Add track Layout Size options Help
Zoom
13.1
Whole
Show buried clones
Yes No
Search
CB Unit Range
0 to 121
Contig stats
Clones: 8 (1 buried)
Markers: 0
Sequenced: 0
Length: 122 CB units
Ctg1373 of cp05T5e12DM4RS
V005N12
V051E06
V005N15
V055A21
M023P09
V043L20
V023D12~
V059A09*
0 10 20 30 40 50 60 70 80 90 100 110
[561-600 - File Bro...
[root@localhost: /...
[FPC V8.9 Main Menu]
Project cp05T5e12...
FPC Ctg1373 cp05T...

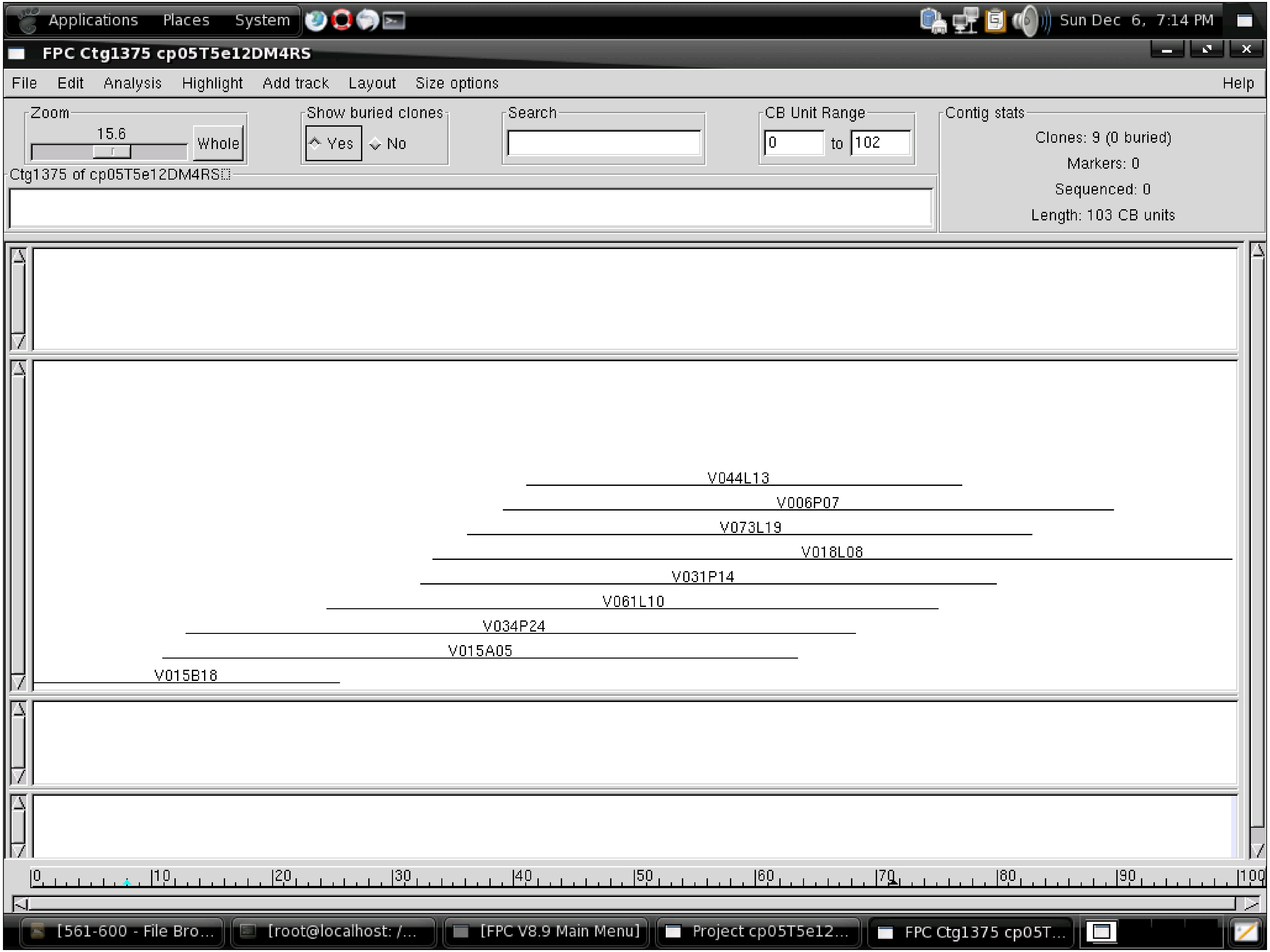
Applications Places System
Sun Dec 6, 7:14 PM
FPC Ctg1375 cp05T5e12DM4RS
File Edit Analysis Highlight Add track Layout Size options Help
Zoom
15.6
Whole
Show buried clones
Yes No
Search
CB Unit Range
0 to 102
Contig stats
Clones: 9 (0 buried)
Markers: 0
Sequenced: 0
Length: 103 CB units
Ctg1375 of cp05T5e12DM4RS
V044L13
V006P07
V073L19
V018L08
V031P14
V061L10
V034P24
V015A05
V015B18
0 10 20 30 40 50 60 70 80 90 100
[561-600 - File Bro...
[root@localhost: /...
[FPC V8.9 Main Menu]
Project cp05T5e12...
FPC Ctg1375 cp05T...

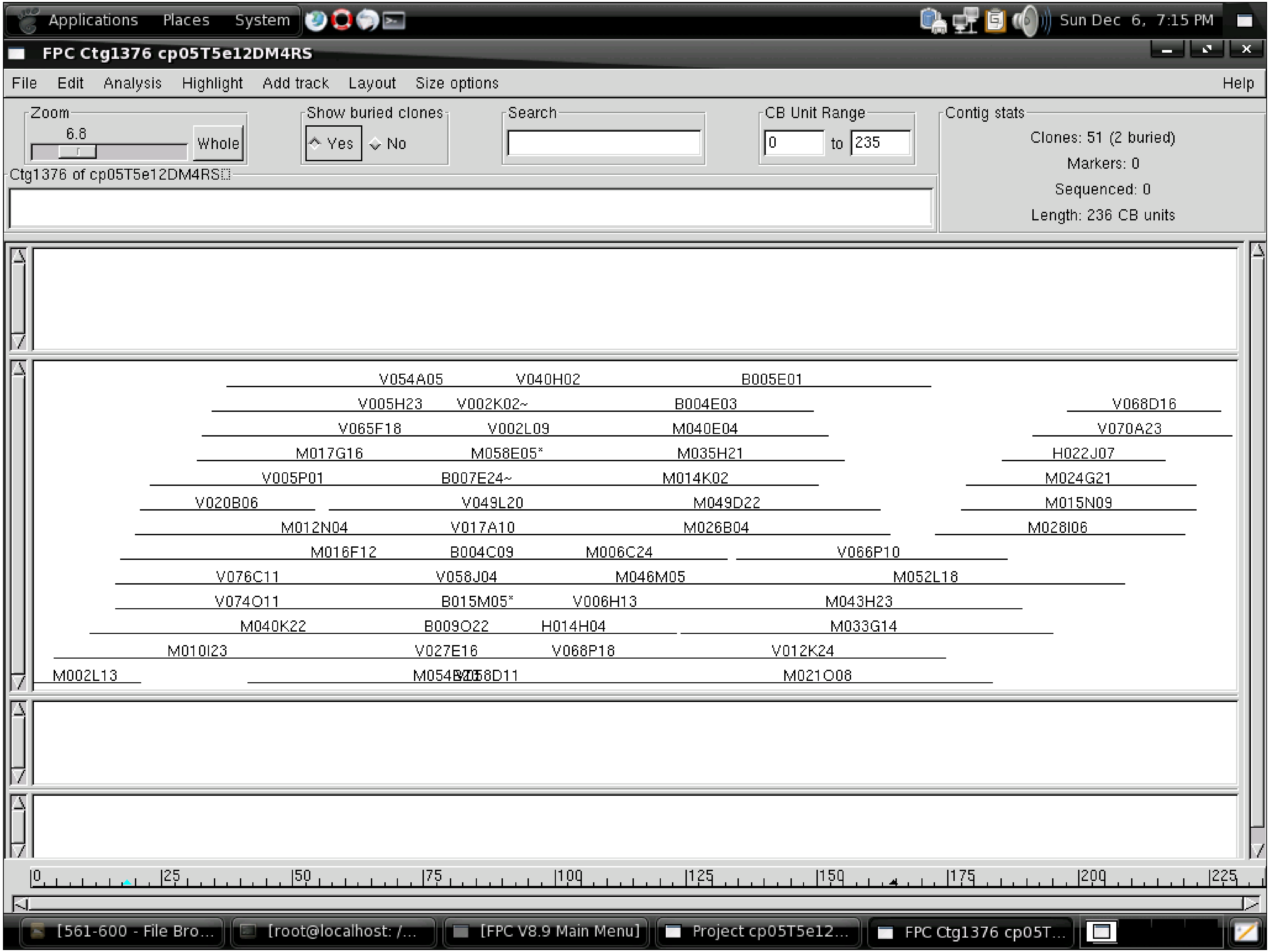
Applications Places System
Sun Dec 6, 7:15 PM
FPC Ctg1376 cp05T5e12DM4RS
File Edit Analysis Highlight Add track Layout Size options Help
Zoom
6.8
Whole
Show buried clones
Yes
No
Search
CB Unit Range
0
to
235
Contig stats
Clones: 51 (2 buried)
Markers: 0
Sequenced: 0
Length: 236 CB units
Ctg1376 of cp05T5e12DM4RS
V054A05 V040H02 B005E01
V005H23 V002K02~ B004E03 V068D16
V065F18 V002L09 M040E04 V070A23
M017G16 M058E05* M035H21 H022J07
V005P01 B007E24~ M014K02 M024G21
V020B06 V049L20 M049D22 M015N09
M012N04 V017A10 M026B04 M028I06
M016F12 B004C09 M006C24 V066P10
V076C11 V058J04 M046M05 M052L18
V074O11 B015M05* V006H13 M043H23
M040K22 B009O22 H014H04 M033G14
M010I23 V027E16 V068P18 V012K24
M002L13 M054B20 B058D11 M021O08
0 25 50 75 100 125 150 175 200 225
[561-600 - File Bro...
[root@localhost: /...
[FPC V8.9 Main Menu]
Project cp05T5e12...
FPC Ctg1376 cp05T...

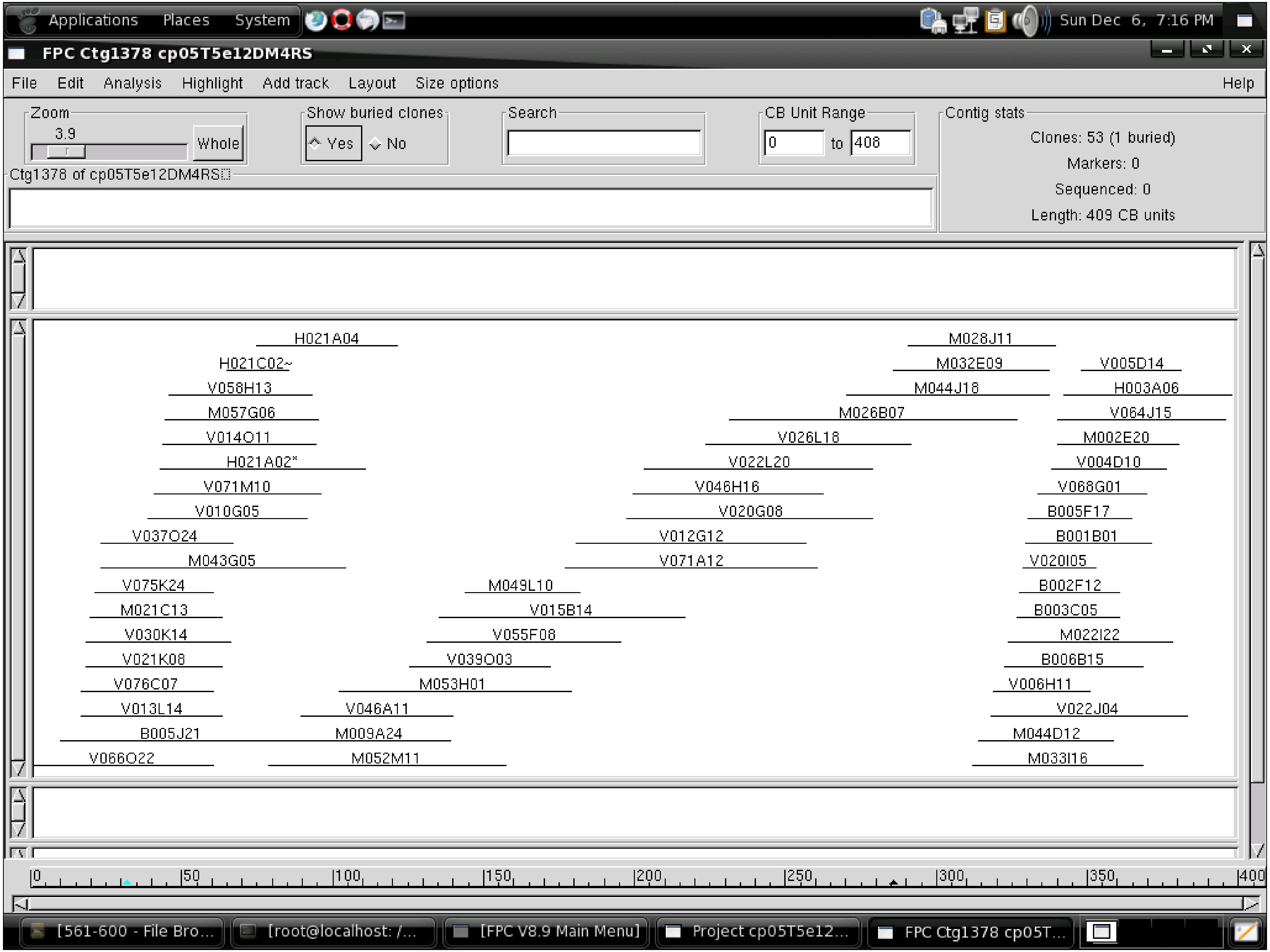
Applications Places System
Sun Dec 6, 7:16 PM
FPC Ctg1378 cp05T5e12DM4RS
File Edit Analysis Highlight Add track Layout Size options Help
Zoom
3.9
Whole
Show buried clones
Yes
No
Search
CB Unit Range
0
to
408
Contig stats
Clones: 53 (1 buried)
Markers: 0
Sequenced: 0
Length: 409 CB units
Ctg1378 of cp05T5e12DM4RS
H021A04
H021C02~
V058H13
M057G06
V014O11
H021A02*
V071M10
V010G05
V037O24
M043G05
V075K24
M021C13
V030K14
V021K08
V076C07
V013L14
B005J21
V066O22
M049L10
V015B14
V055F08
V039O03
M053H01
V046A11
M009A24
M052M11
M026B07
V026L18
V022L20
V046H16
V020G08
V012G12
V071A12
M028J11
M032E09
M044J18
V005D14
H003A06
V064J15
M002E20
V004D10
V068G01
B005F17
B001B01
V020I05
B002F12
B003C05
M022I22
B006B15
V006H11
V022J04
M044D12
M033I16
0 50 100 150 200 250 300 350 400
[561-600 - File Bro...
[root@localhost: /...
[FPC V8.9 Main Menu]
Project cp05T5e12...
FPC Ctg1378 cp05T...

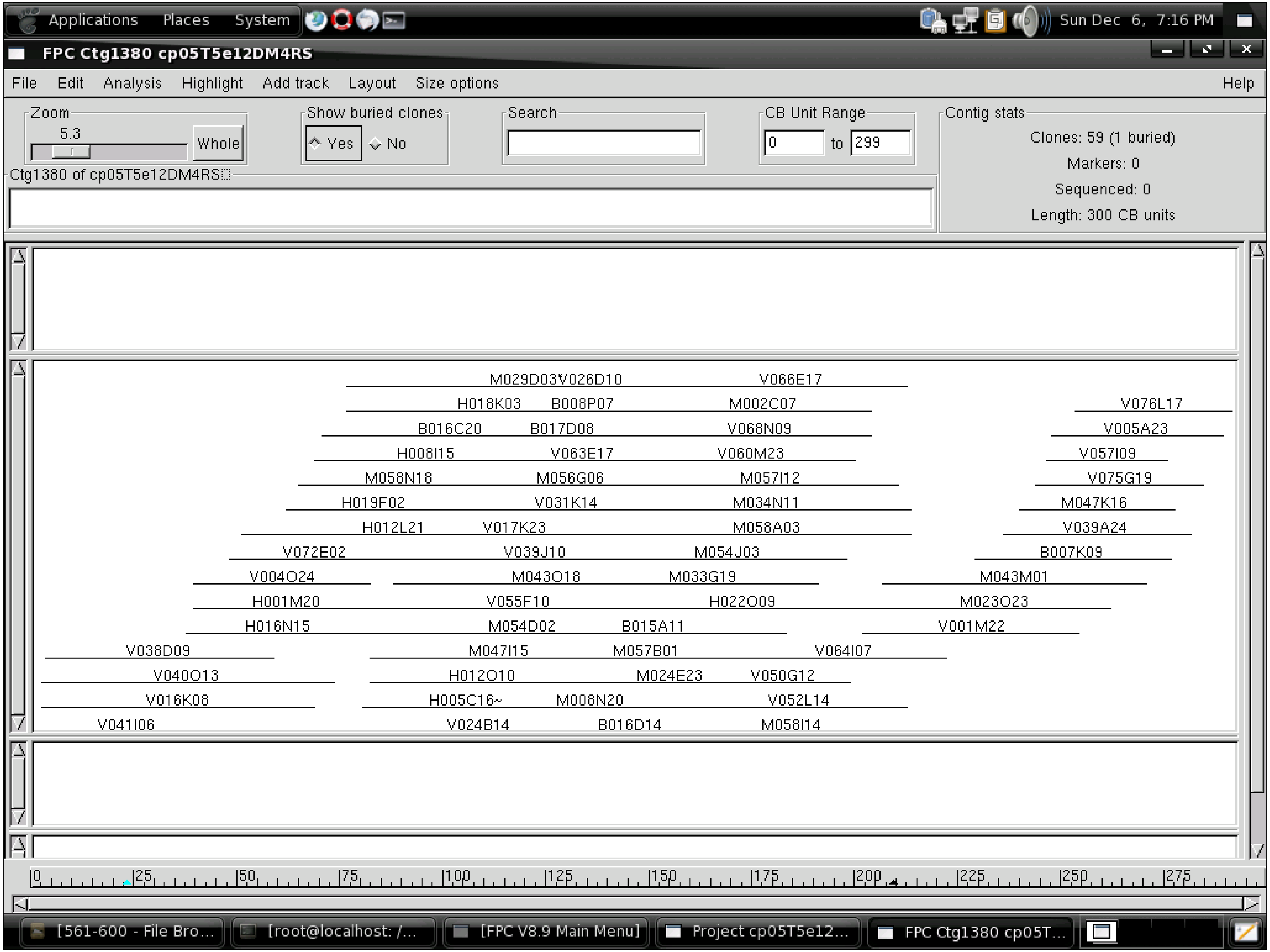
Applications Places System
Sun Dec 6, 7:16 PM
FPC Ctg1380 cp05T5e12DM4RS
File Edit Analysis Highlight Add track Layout Size options Help
Zoom
5.3
Whole
Show buried clones
Yes
No
Search
CB Unit Range
0
to
299
Contig stats
Clones: 59 (1 buried)
Markers: 0
Sequenced: 0
Length: 300 CB units
Ctg1380 of cp05T5e12DM4RS
M029D03V026D10
V066E17
H018K03
B008P07
M002C07
V076L17
B016C20
B017D08
V068N09
V005A23
H008I15
V063E17
V060M23
V057I09
M058N18
M056G06
M057I12
V075G19
H019F02
V031K14
M034N11
M047K16
H012L21
V017K23
M058A03
V039A24
V072E02
V039J10
M054J03
B007K09
V004O24
M043O18
M033G19
M043M01
H001M20
V055F10
H022O09
M023O23
H016N15
M054D02
B015A11
V001M22
V038D09
M047I15
M057B01
V064I07
V040O13
H012O10
M024E23
V050G12
V016K08
H005C16~
M008N20
V052L14
V041I06
V024B14
B016D14
M058I14
0
25
50
75
100
125
150
175
200
225
250
275
[561-600 - File Bro...
[root@localhost: /...
[FPC V8.9 Main Menu]
Project cp05T5e12...
FPC Ctg1380 cp05T...

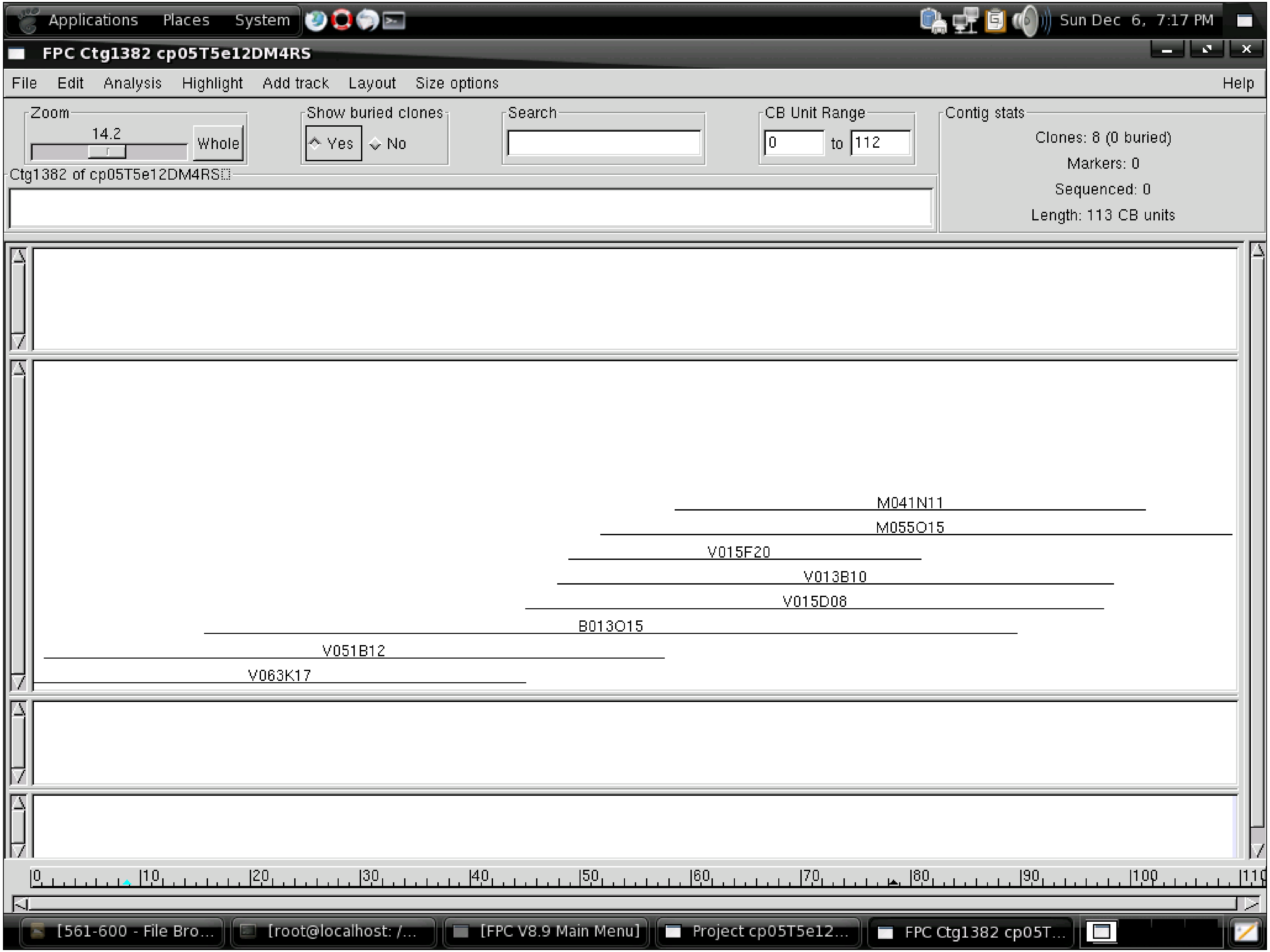
Applications Places System
Sun Dec 6, 7:17 PM
FPC Ctg1382 cp05T5e12DM4RS
File Edit Analysis Highlight Add track Layout Size options Help
Zoom
14.2
Whole
Show buried clones
Yes No
Search
CB Unit Range
0 to 112
Contig stats
Clones: 8 (0 buried)
Markers: 0
Sequenced: 0
Length: 113 CB units
Ctg1382 of cp05T5e12DM4RS
M041N11
M055O15
V015F20
V013B10
V015D08
B013O15
V051B12
V063K17
0 10 20 30 40 50 60 70 80 90 100 110
[561-600 - File Bro...
[root@localhost: /...
[FPC V8.9 Main Menu]
Project cp05T5e12...
FPC Ctg1382 cp05T...

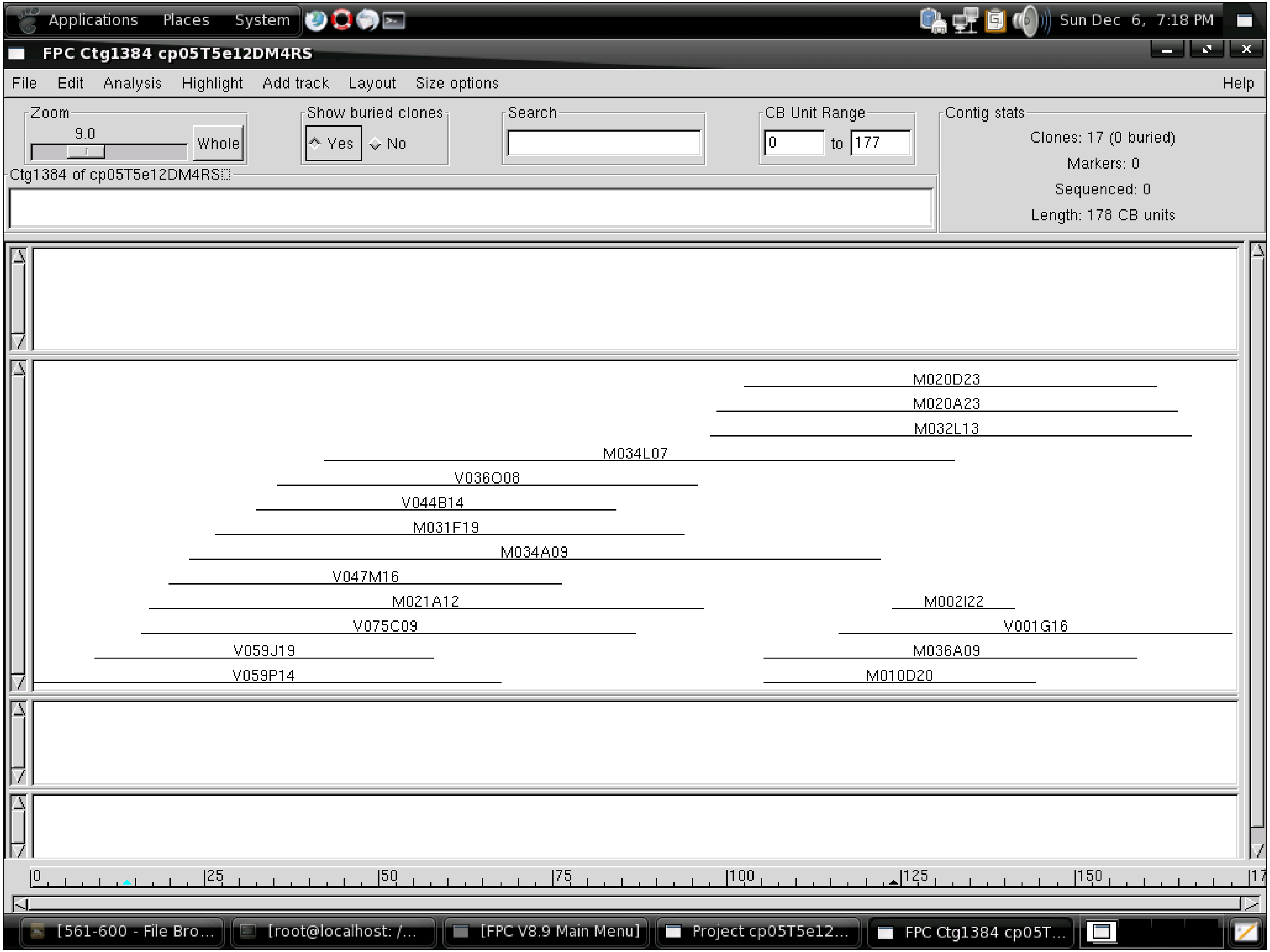
Applications Places System
Sun Dec 6, 7:18 PM
FPC Ctg1384 cp05T5e12DM4RS
File Edit Analysis Highlight Add track Layout Size options Help
Zoom
9.0
Whole
Show buried clones
Yes
No
Search
CB Unit Range
0 to 177
Contig stats
Clones: 17 (0 buried)
Markers: 0
Sequenced: 0
Length: 178 CB units
Ctg1384 of cp05T5e12DM4RS
M020D23
M020A23
M032L13
M034L07
V036O08
V044B14
M031F19
M034A09
V047M16
M021A12
M002I22
V075C09
V001G16
V059J19
M036A09
V059P14
M010D20
0
25
50
75
100
125
150
[561-600 - File Bro...
[root@localhost: /...
[FPC V8.9 Main Menu]
Project cp05T5e12...
FPC Ctg1384 cp05T...

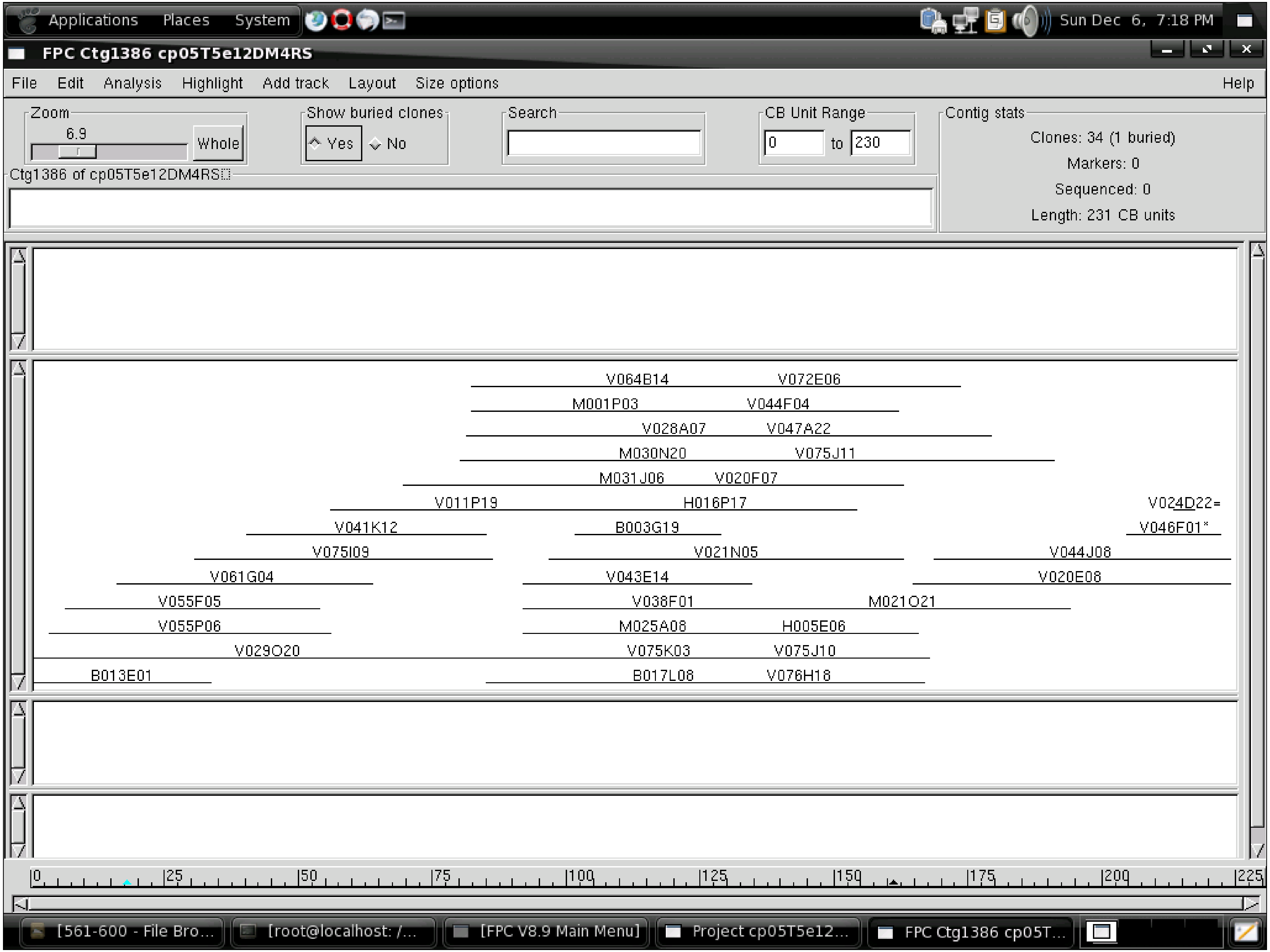
Applications Places System
Sun Dec 6, 7:18 PM
FPC Ctg1386 cp05T5e12DM4RS
File Edit Analysis Highlight Add track Layout Size options Help
Zoom
6.9
Whole
Show buried clones
Yes No
Search
CB Unit Range
0 to 230
Contig stats
Clones: 34 (1 buried)
Markers: 0
Sequenced: 0
Length: 231 CB units
Ctg1386 of cp05T5e12DM4RS
V064B14
V072E06
M001P03
V044F04
V028A07
V047A22
M030N20
V075J11
M031J06
V020F07
V011P19
H016P17
V024D22=
V041K12
B003G19
V046F01*
V075I09
V021N05
V044J08
V061G04
V043E14
V020E08
V055F05
V038F01
M021O21
V055P06
M025A08
H005E06
V029O20
V075K03
V075J10
B013E01
B017L08
V076H18
0 25 50 75 100 125 150 175 200 225
[561-600 - File Bro...
[root@localhost: /...
[FPC V8.9 Main Menu]
Project cp05T5e12...
FPC Ctg1386 cp05T...

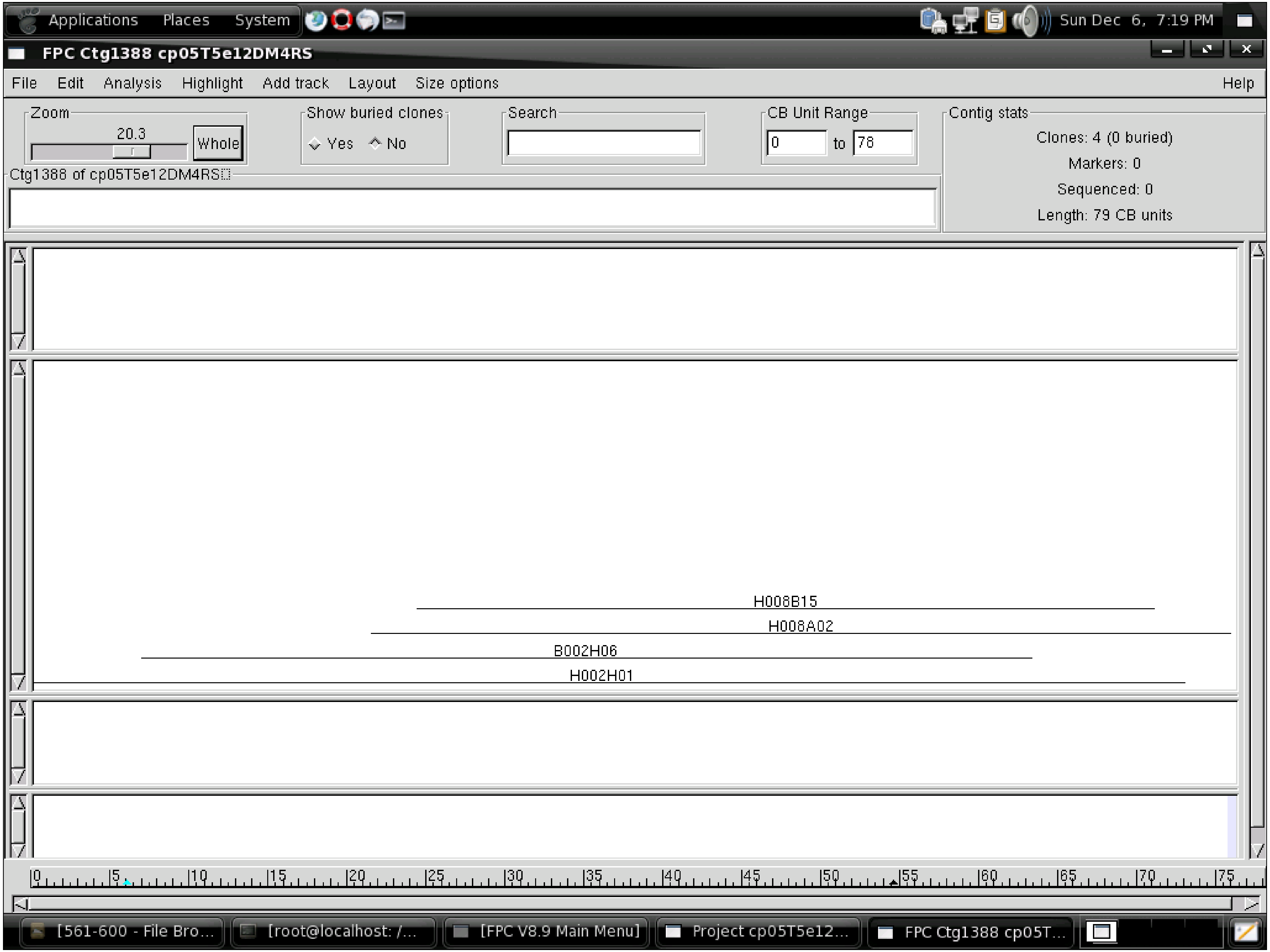
Applications
Places
System
Sun Dec 6, 7:19 PM
FPC Ctg1388 cp05T5e12DM4RS
File
Edit
Analysis
Highlight
Add track
Layout
Size options
Help
Zoom
20.3
Whole
Show buried clones
Yes
No
Search
CB Unit Range
0
to
78
Contig stats
Clones: 4 (0 buried)
Markers: 0
Sequenced: 0
Length: 79 CB units
Ctg1388 of cp05T5e12DM4RS
H008B15
H008A02
B002H06
H002H01
0
5
10
15
20
25
30
35
40
45
50
55
60
65
70
75
[561-600 - File Bro...
[root@localhost: /...
[FPC V8.9 Main Menu]
Project cp05T5e12...
FPC Ctg1388 cp05T...

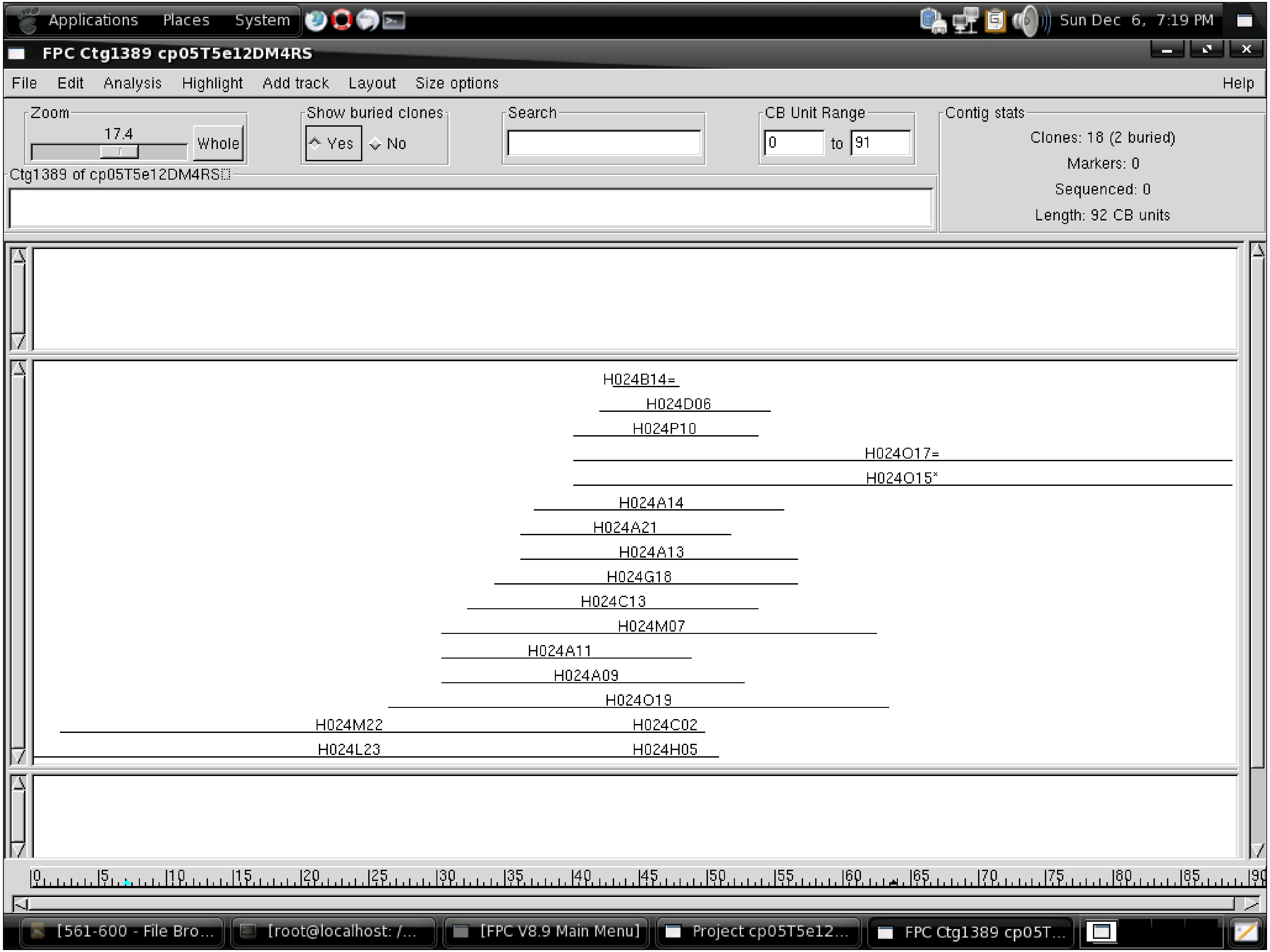
Applications Places System
Sun Dec 6, 7:19 PM
FPC Ctg1389 cp05T5e12DM4RS
File Edit Analysis Highlight Add track Layout Size options Help
Zoom
17.4
Whole
Show buried clones
Yes No
Search
CB Unit Range
0 to 91
Contig stats
Clones: 18 (2 buried)
Markers: 0
Sequenced: 0
Length: 92 CB units
Ctg1389 of cp05T5e12DM4RS
H024B14=
H024D06
H024P10
H024O17=
H024O15*
H024A14
H024A21
H024A13
H024G18
H024C13
H024M07
H024A11
H024A09
H024O19
H024M22
H024C02
H024L23
H024H05
0 5 10 15 20 25 30 35 40 45 50 55 60 65 70 75 80 85 90
[561-600 - File Bro...
[root@localhost: /...
[FPC V8.9 Main Menu]
Project cp05T5e12...
FPC Ctg1389 cp05T...

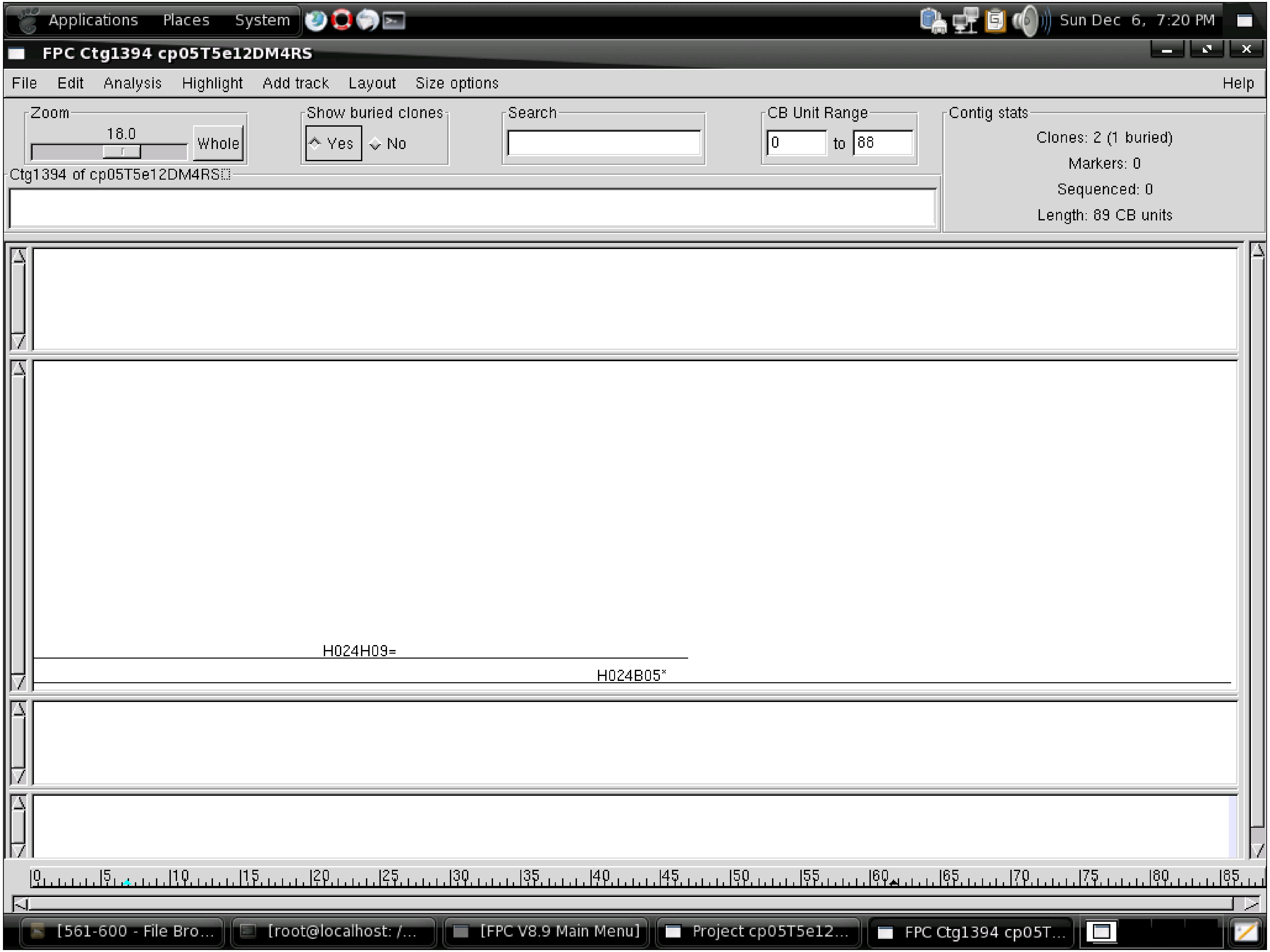
Applications Places System
Sun Dec 6, 7:20 PM
FPC Ctg1394 cp05T5e12DM4RS
File Edit Analysis Highlight Add track Layout Size options Help
Zoom
18.0
Whole
Show buried clones
Yes No
Search
CB Unit Range
0 to 88
Contig stats
Clones: 2 (1 buried)
Markers: 0
Sequenced: 0
Length: 89 CB units
Ctg1394 of cp05T5e12DM4RS
H024H09=
H024B05*
0 5 10 15 20 25 30 35 40 45 50 55 60 65 70 75 80 85
[561-600 - File Bro...
[root@localhost: /...
[FPC V8.9 Main Menu]
Project cp05T5e12...
FPC Ctg1394 cp05T...

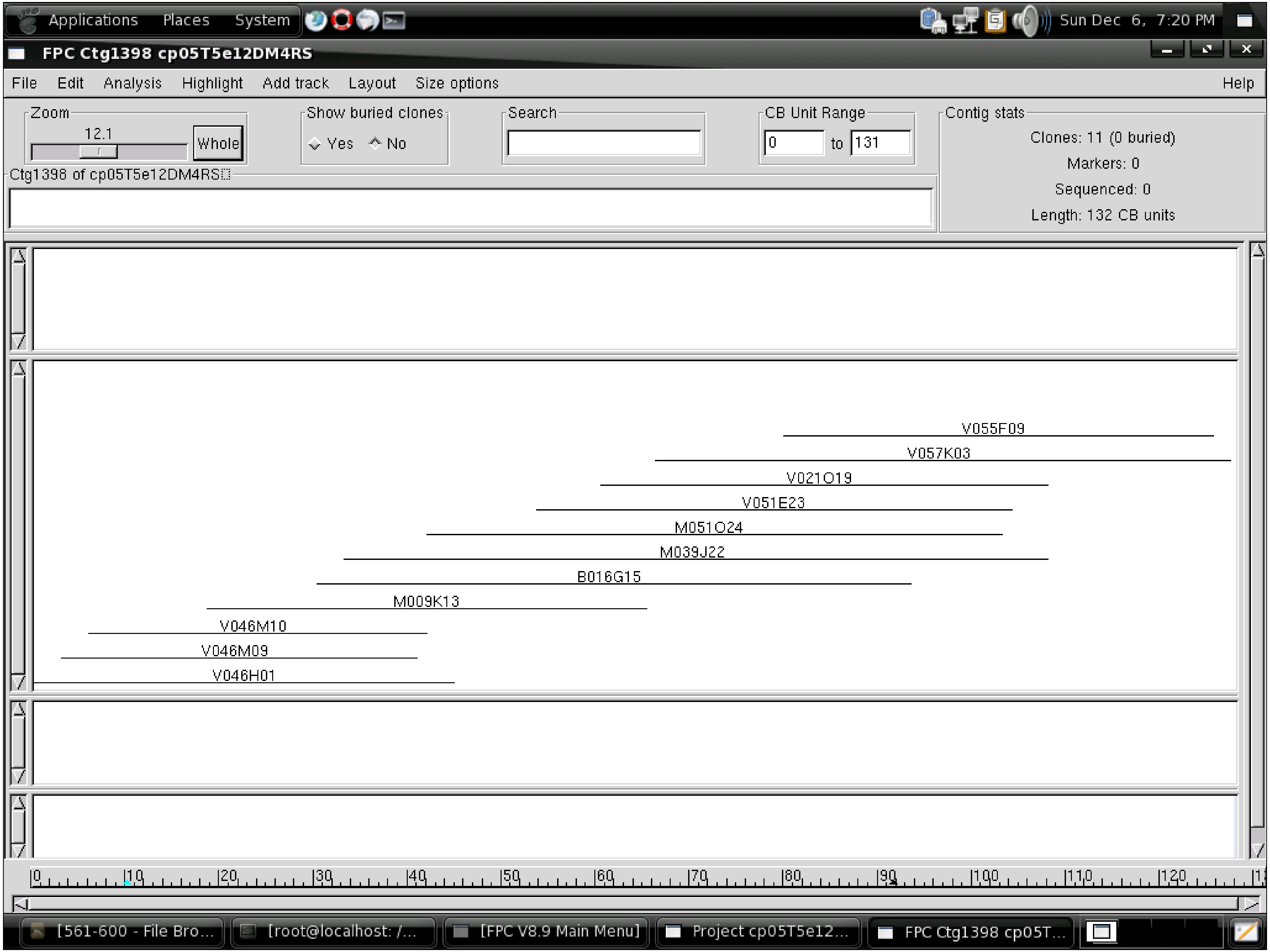
Applications Places System
Sun Dec 6, 7:20 PM
FPC Ctg1398 cp05T5e12DM4RS
File Edit Analysis Highlight Add track Layout Size options Help
Zoom
12.1
Whole
Show buried clones
Yes No
Search
CB Unit Range
0 to 131
Contig stats
Clones: 11 (0 buried)
Markers: 0
Sequenced: 0
Length: 132 CB units
Ctg1398 of cp05T5e12DM4RS
V055F09
V057K03
V021O19
V051E23
M051O24
M039J22
B016G15
M009K13
V046M10
V046M09
V046H01
0 10 20 30 40 50 60 70 80 90 100 110 120
[561-600 - File Bro...
[root@localhost: /...
[FPC V8.9 Main Menu]
Project cp05T5e12...
FPC Ctg1398 cp05T...

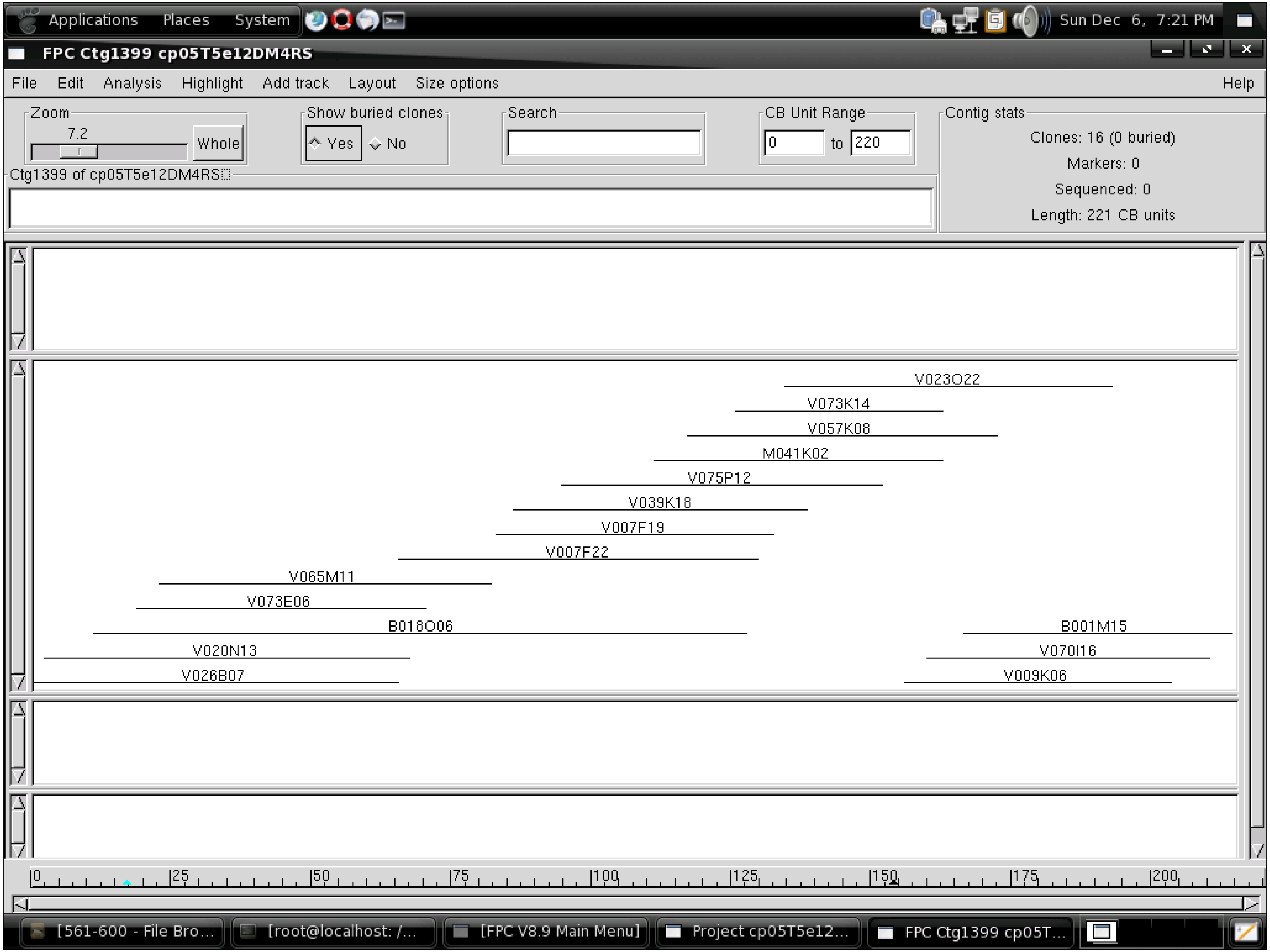
Applications Places System
Sun Dec 6, 7:21 PM
FPC Ctg1399 cp05T5e12DM4RS
File Edit Analysis Highlight Add track Layout Size options Help
Zoom
7.2
Whole
Show buried clones
Yes No
Search
CB Unit Range
0 to 220
Contig stats
Clones: 16 (0 buried)
Markers: 0
Sequenced: 0
Length: 221 CB units
Ctg1399 of cp05T5e12DM4RS
V023O22
V073K14
V057K08
M041K02
V075P12
V039K18
V007F19
V007F22
V065M11
V073E06
B018O06
B001M15
V020N13
V070I16
V026B07
V009K06
0 25 50 75 100 125 150 175 200
[561-600 - File Bro...
[root@localhost: /...
[FPC V8.9 Main Menu]
Project cp05T5e12...
FPC Ctg1399 cp05T...

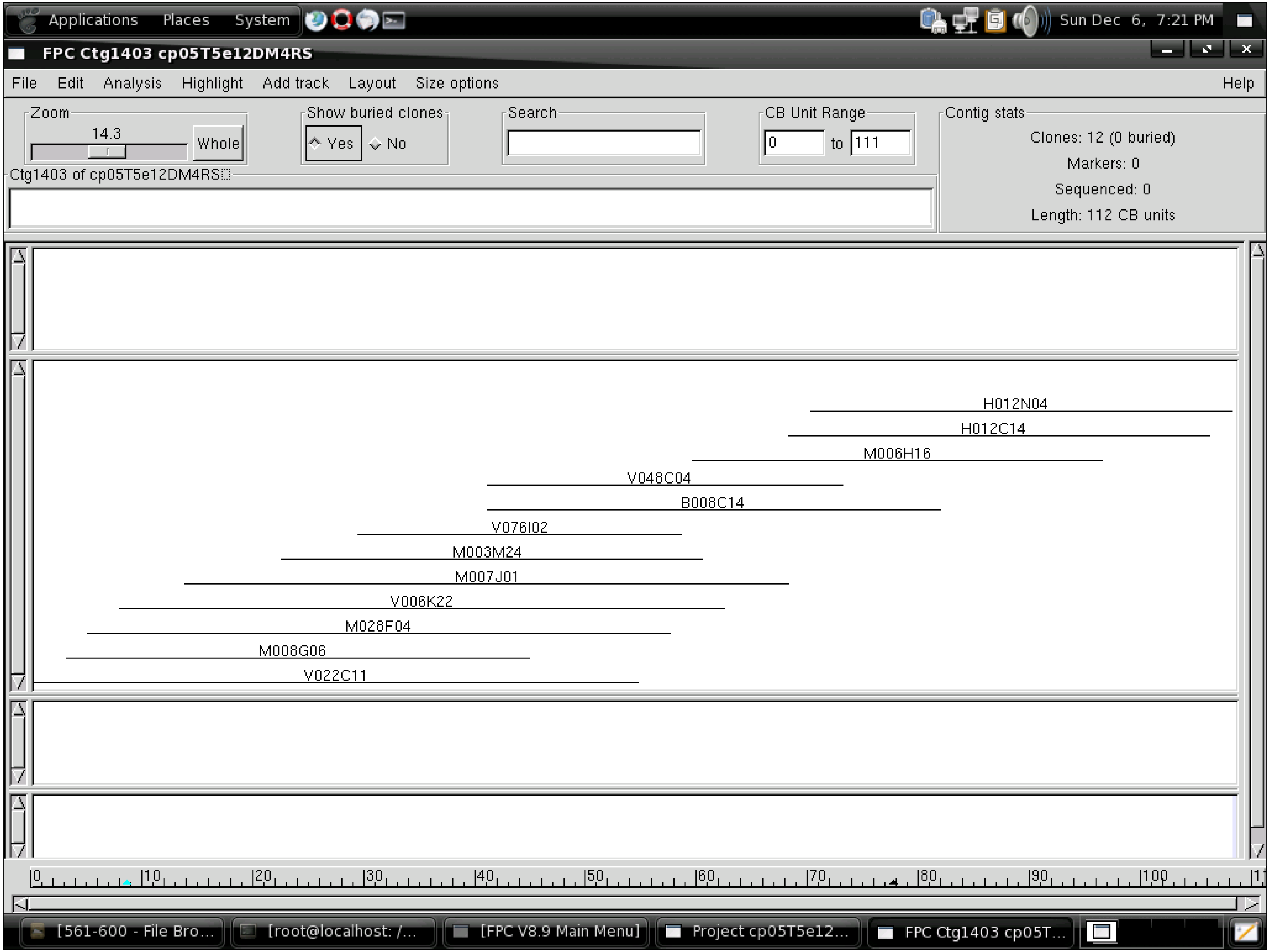
Applications Places System
Sun Dec 6, 7:21 PM
FPC Ctg1403 cp05T5e12DM4RS
File Edit Analysis Highlight Add track Layout Size options Help
Zoom
14.3
Whole
Show buried clones
Yes No
Search
CB Unit Range
0 to 111
Contig stats
Clones: 12 (0 buried)
Markers: 0
Sequenced: 0
Length: 112 CB units
Ctg1403 of cp05T5e12DM4RS
H012N04
H012C14
M006H16
V048C04
B008C14
V076I02
M003M24
M007J01
V006K22
M028F04
M008G06
V022C11
0 10 20 30 40 50 60 70 80 90 100
[561-600 - File Bro...
[root@localhost: /...
[FPC V8.9 Main Menu]
Project cp05T5e12...
FPC Ctg1403 cp05T...

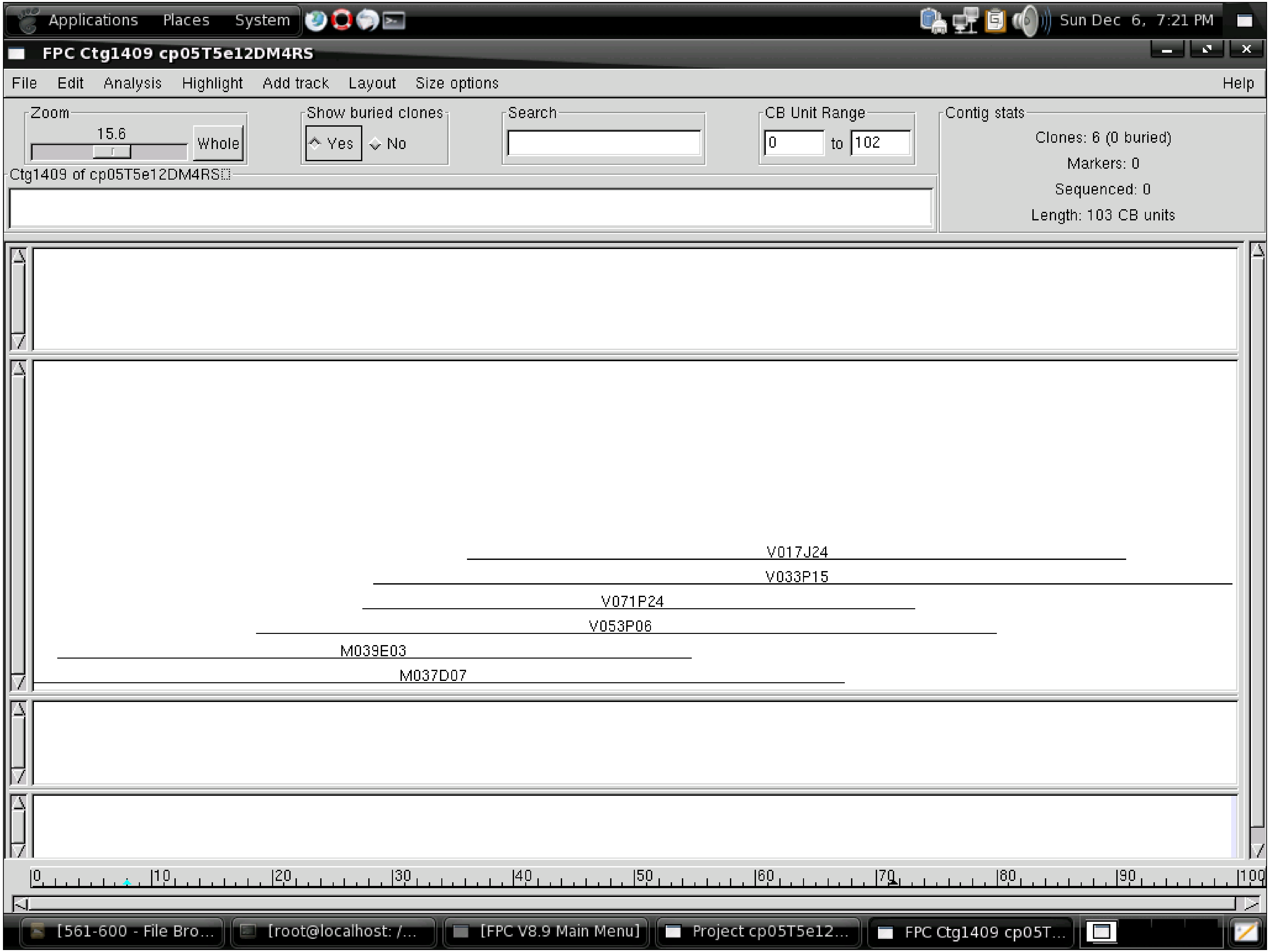
Applications Places System
Sun Dec 6, 7:21 PM
FPC Ctg1409 cp05T5e12DM4RS
File Edit Analysis Highlight Add track Layout Size options Help
Zoom
15.6
Whole
Show buried clones
Yes No
Search
CB Unit Range
0 to 102
Contig stats
Clones: 6 (0 buried)
Markers: 0
Sequenced: 0
Length: 103 CB units
Ctg1409 of cp05T5e12DM4RS
V017J24
V033P15
V071P24
V053P06
M039E03
M037D07
0 10 20 30 40 50 60 70 80 90 100
[561-600 - File Bro...
[root@localhost: /...
[FPC V8.9 Main Menu]
Project cp05T5e12...
FPC Ctg1409 cp05T...

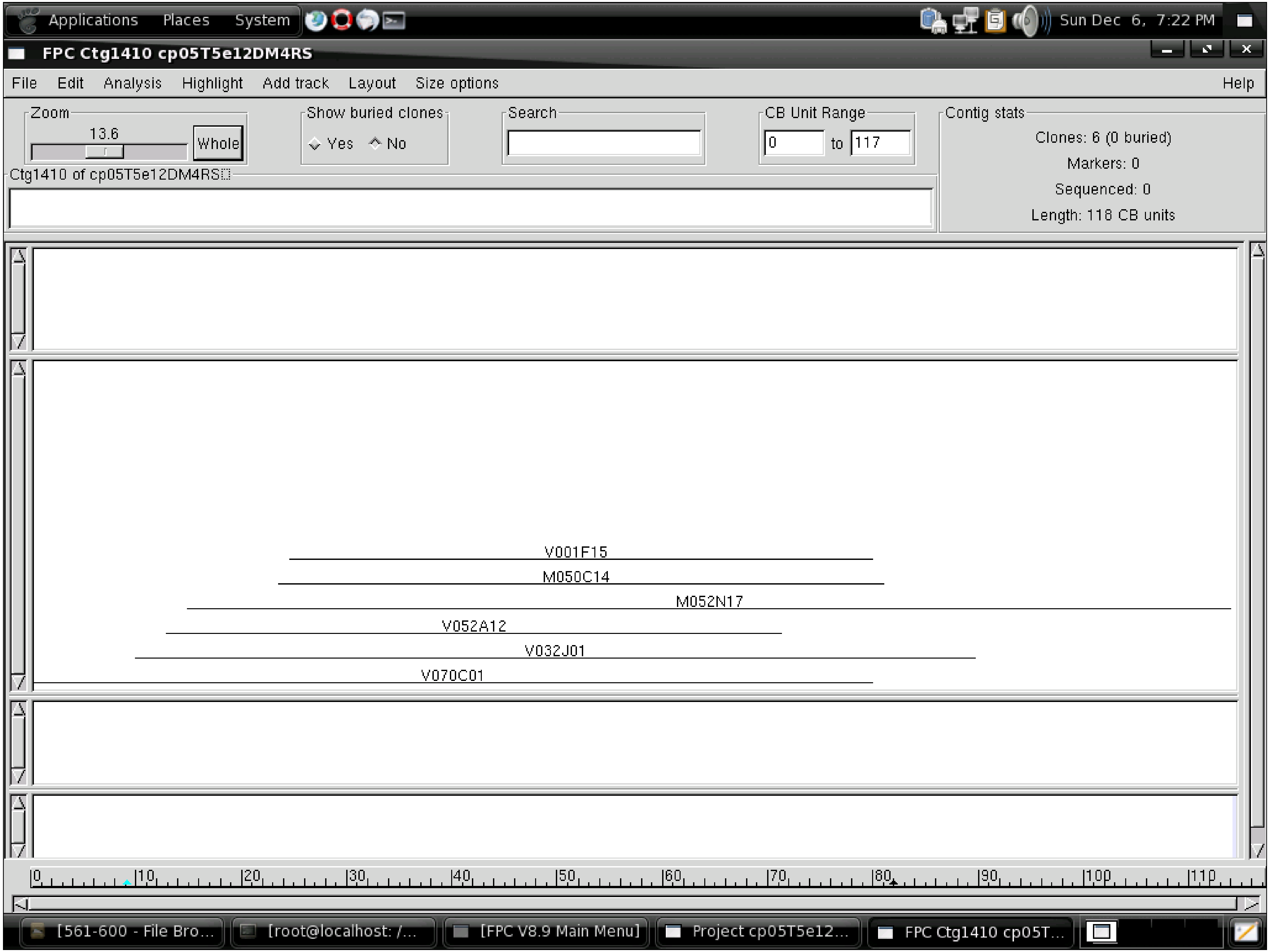
Applications
Places
System
Sun Dec 6, 7:22 PM
FPC Ctg1410 cp05T5e12DM4RS
File
Edit
Analysis
Highlight
Add track
Layout
Size options
Help
Zoom
13.6
Whole
Show buried clones
Yes
No
Search
CB Unit Range
0
to
117
Contig stats
Clones: 6 (0 buried)
Markers: 0
Sequenced: 0
Length: 118 CB units
Ctg1410 of cp05T5e12DM4RS
V001F15
M050C14
M052N17
V052A12
V032J01
V070C01
0
10
20
30
40
50
60
70
80
90
100
110
[561-600 - File Bro...
[root@localhost: /...
[FPC V8.9 Main Menu]
Project cp05T5e12...
FPC Ctg1410 cp05T...

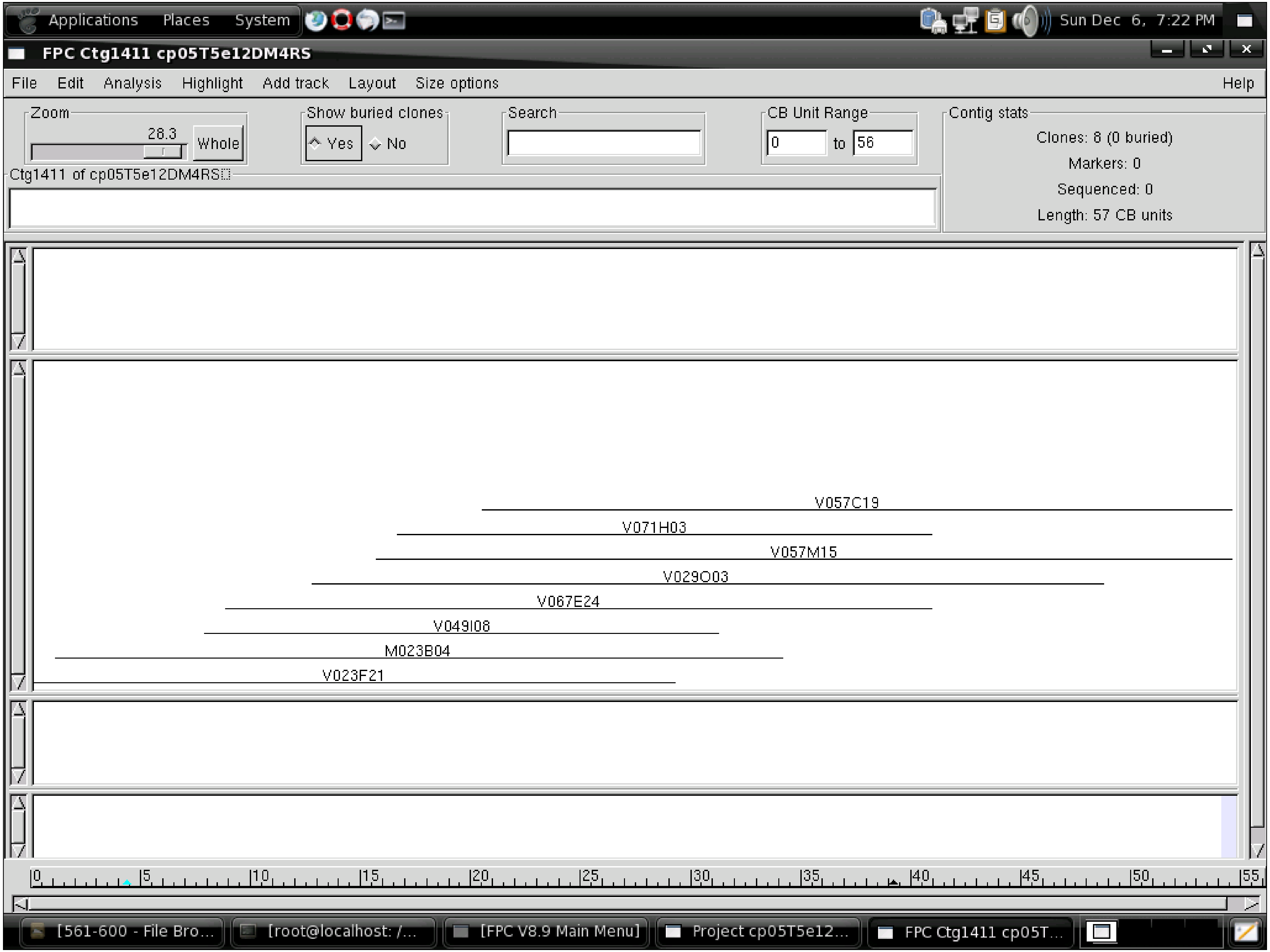
Applications Places System
Sun Dec 6, 7:22 PM
FPC Ctg1411 cp05T5e12DM4RS
File Edit Analysis Highlight Add track Layout Size options Help
Zoom
28.3
Whole
Show buried clones
Yes
No
Search
CB Unit Range
0 to 56
Contig stats
Clones: 8 (0 buried)
Markers: 0
Sequenced: 0
Length: 57 CB units
Ctg1411 of cp05T5e12DM4RS
V057C19
V071H03
V057M15
V029O03
V067E24
V049I08
M023B04
V023F21
0 5 10 15 20 25 30 35 40 45 50 55
[561-600 - File Bro...
[root@localhost: /...
[FPC V8.9 Main Menu]
Project cp05T5e12...
FPC Ctg1411 cp05T...

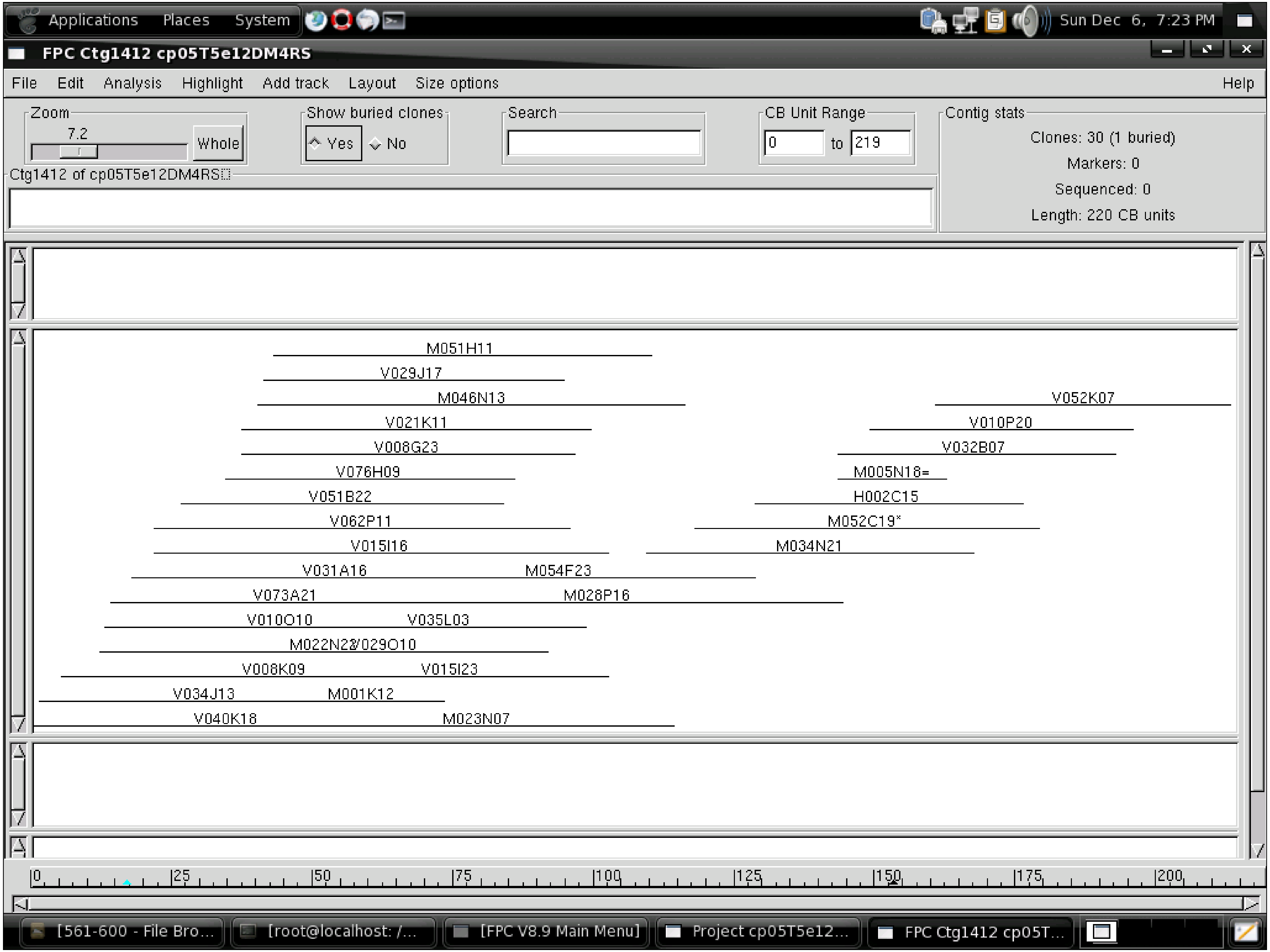
Applications Places System
Sun Dec 6, 7:23 PM
FPC Ctg1412 cp05T5e12DM4RS
File Edit Analysis Highlight Add track Layout Size options Help
Zoom
7.2
Whole
Show buried clones
Yes No
Search
CB Unit Range
0 to 219
Contig stats
Clones: 30 (1 buried)
Markers: 0
Sequenced: 0
Length: 220 CB units
Ctg1412 of cp05T5e12DM4RS
M051H11
V029J17
M046N13
V052K07
V021K11
V010P20
V008G23
V032B07
V076H09
M005N18=
V051B22
H002C15
V062P11
M052C19*
V015I16
M034N21
V031A16
M054F23
V073A21
M028P16
V010O10
V035L03
M022N22
V029O10
V008K09
V015I23
V034J13
M001K12
V040K18
M023N07
0 25 50 75 100 125 150 175 200
[561-600 - File Bro...
[root@localhost: /...
[FPC V8.9 Main Menu]
Project cp05T5e12...
FPC Ctg1412 cp05T...

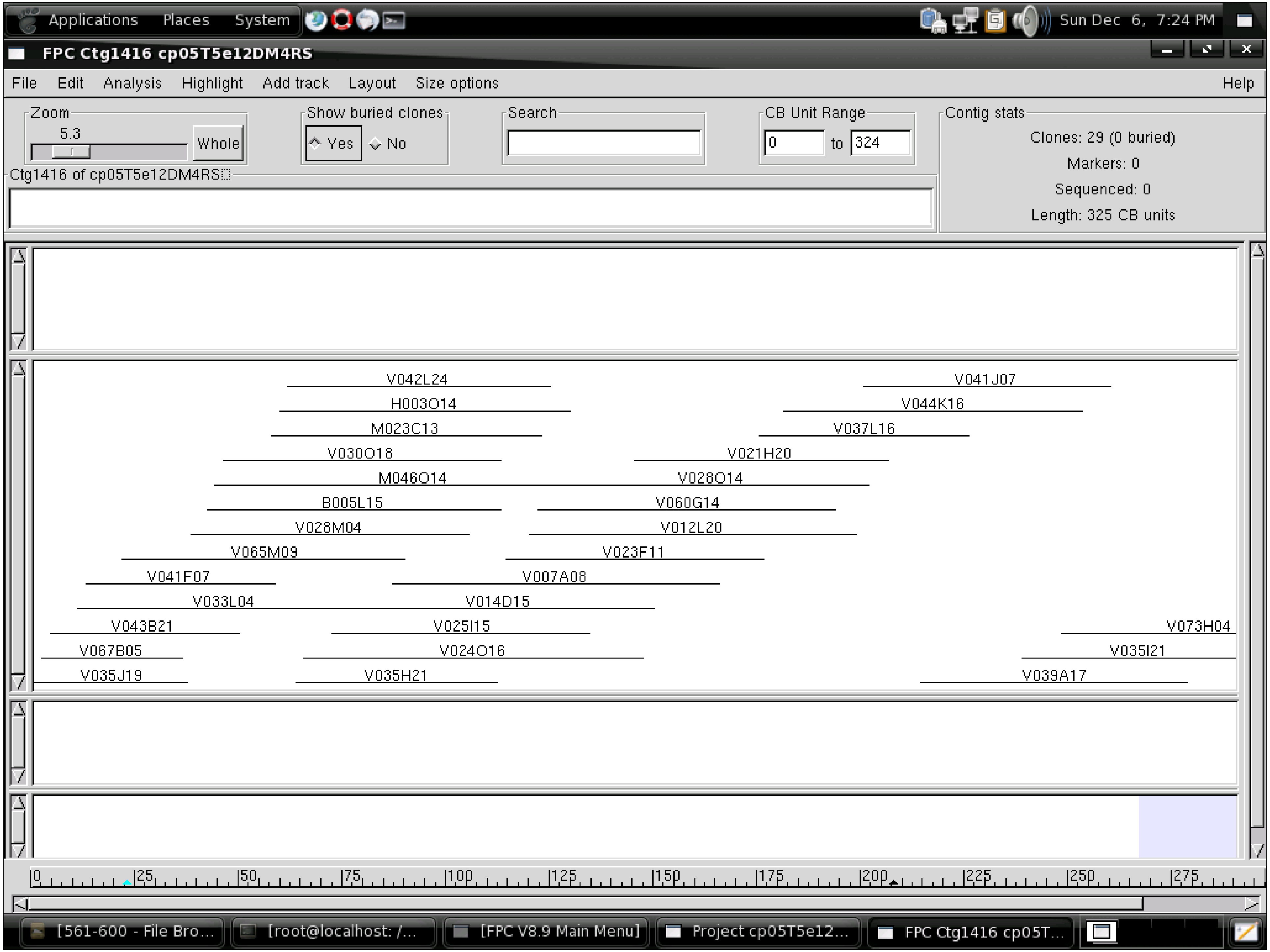
Applications Places System
Sun Dec 6, 7:24 PM
FPC Ctg1416 cp05T5e12DM4RS
File Edit Analysis Highlight Add track Layout Size options Help
Zoom
5.3
Whole
Show buried clones
Yes No
Search
CB Unit Range
0 to 324
Contig stats
Clones: 29 (0 buried)
Markers: 0
Sequenced: 0
Length: 325 CB units
Ctg1416 of cp05T5e12DM4RS
V042L24
V041J07
H003O14
V044K16
M023C13
V037L16
V030O18
V021H20
M046O14
V028O14
B005L15
V060G14
V028M04
V012L20
V065M09
V023F11
V041F07
V007A08
V033L04
V014D15
V043B21
V025I15
V073H04
V067B05
V024O16
V035I21
V035J19
V035H21
V039A17
0 25 50 75 100 125 150 175 200 225 250 275
[561-600 - File Bro...
[root@localhost: /...
[FPC V8.9 Main Menu]
Project cp05T5e12...
FPC Ctg1416 cp05T...

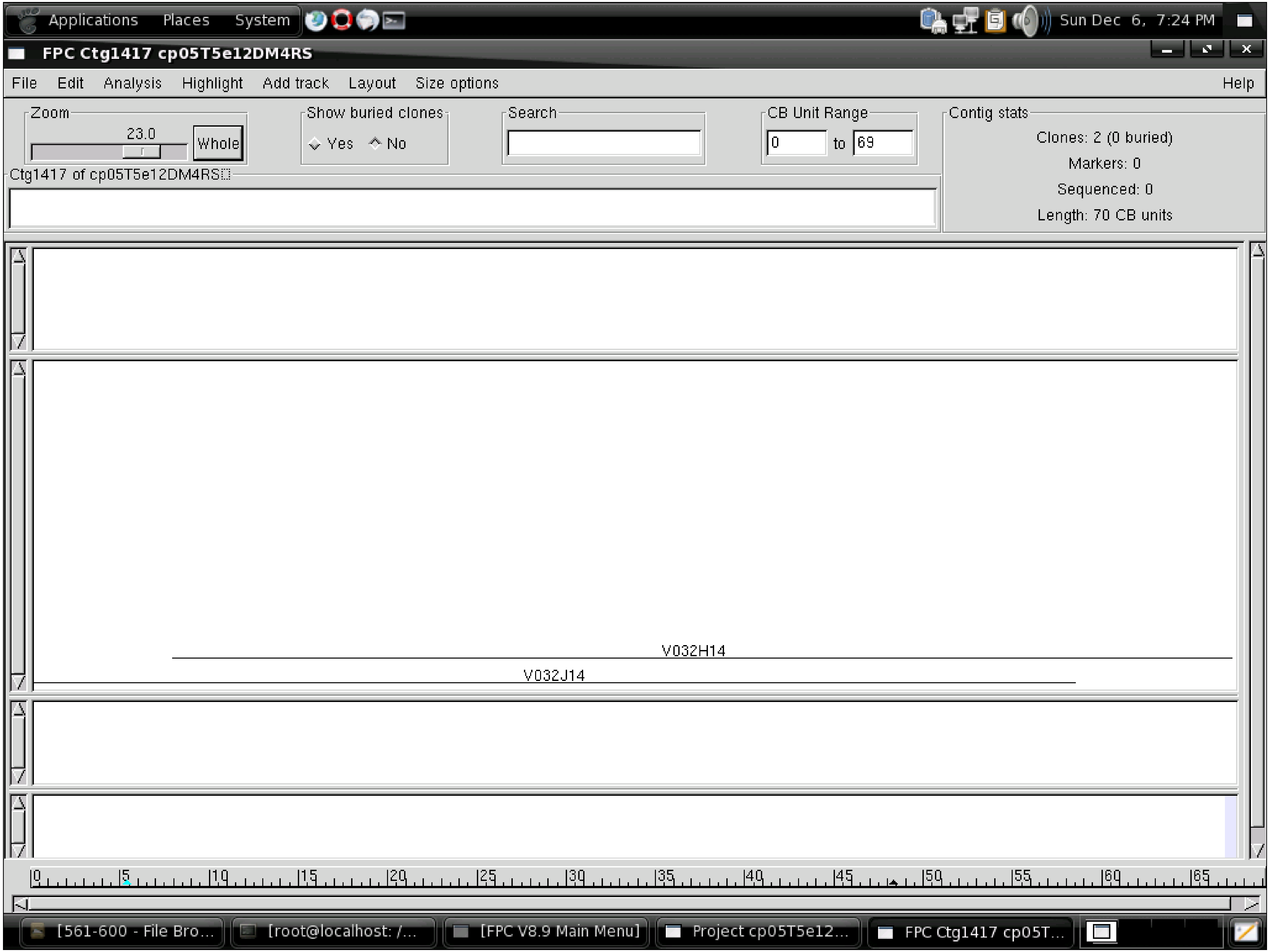

Applications Places System
Sun Dec 6, 7:24 PM
FPC Ctg1417 cp05T5e12DM4RS
File Edit Analysis Highlight Add track Layout Size options Help
Zoom
23.0
Whole
Show buried clones
Yes No
Search
CB Unit Range
0 to 69
Contig stats
Clones: 2 (0 buried)
Markers: 0
Sequenced: 0
Length: 70 CB units
Ctg1417 of cp05T5e12DM4RS
V032H14
V032J14
0 5 10 15 20 25 30 35 40 45 50 55 60 65
[561-600 - File Bro...
[root@localhost: /...
[FPC V8.9 Main Menu]
Project cp05T5e12...
FPC Ctg1417 cp05T...

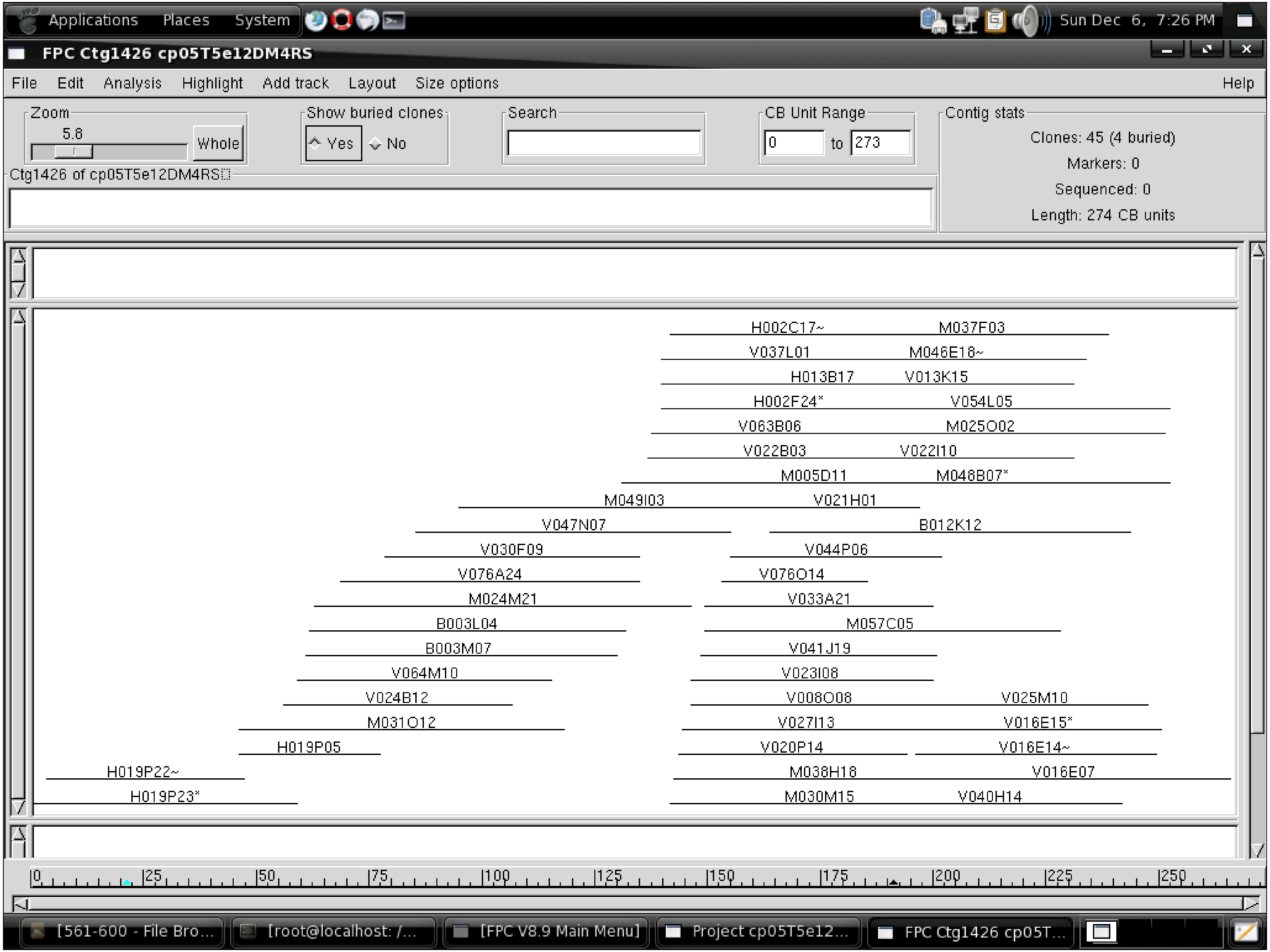
Applications Places System
Sun Dec 6, 7:26 PM
FPC Ctg1426 cp05T5e12DM4RS
File Edit Analysis Highlight Add track Layout Size options Help
Zoom
5.8
Whole
Show buried clones
Yes
No
Search
CB Unit Range
0 to 273
Contig stats
Clones: 45 (4 buried)
Markers: 0
Sequenced: 0
Length: 274 CB units
Ctg1426 of cp05T5e12DM4RS
H002C17~
M037F03
V037L01
M046E18~
H013B17
V013K15
H002F24*
V054L05
V063B06
M025O02
V022B03
V022I10
M005D11
M048B07*
M049I03
V021H01
V047N07
B012K12
V030F09
V044P06
V076A24
V076O14
M024M21
V033A21
B003L04
M057C05
B003M07
V041J19
V064M10
V023I08
V024B12
V008O08
V025M10
M031O12
V027I13
V016E15*
H019P05
V020P14
V016E14~
H019P22~
M038H18
V016E07
H019P23*
M030M15
V040H14
0 25 50 75 100 125 150 175 200 225 250
[561-600 - File Bro...
[root@localhost: /...
[FPC V8.9 Main Menu]
Project cp05T5e12...
FPC Ctg1426 cp05T...

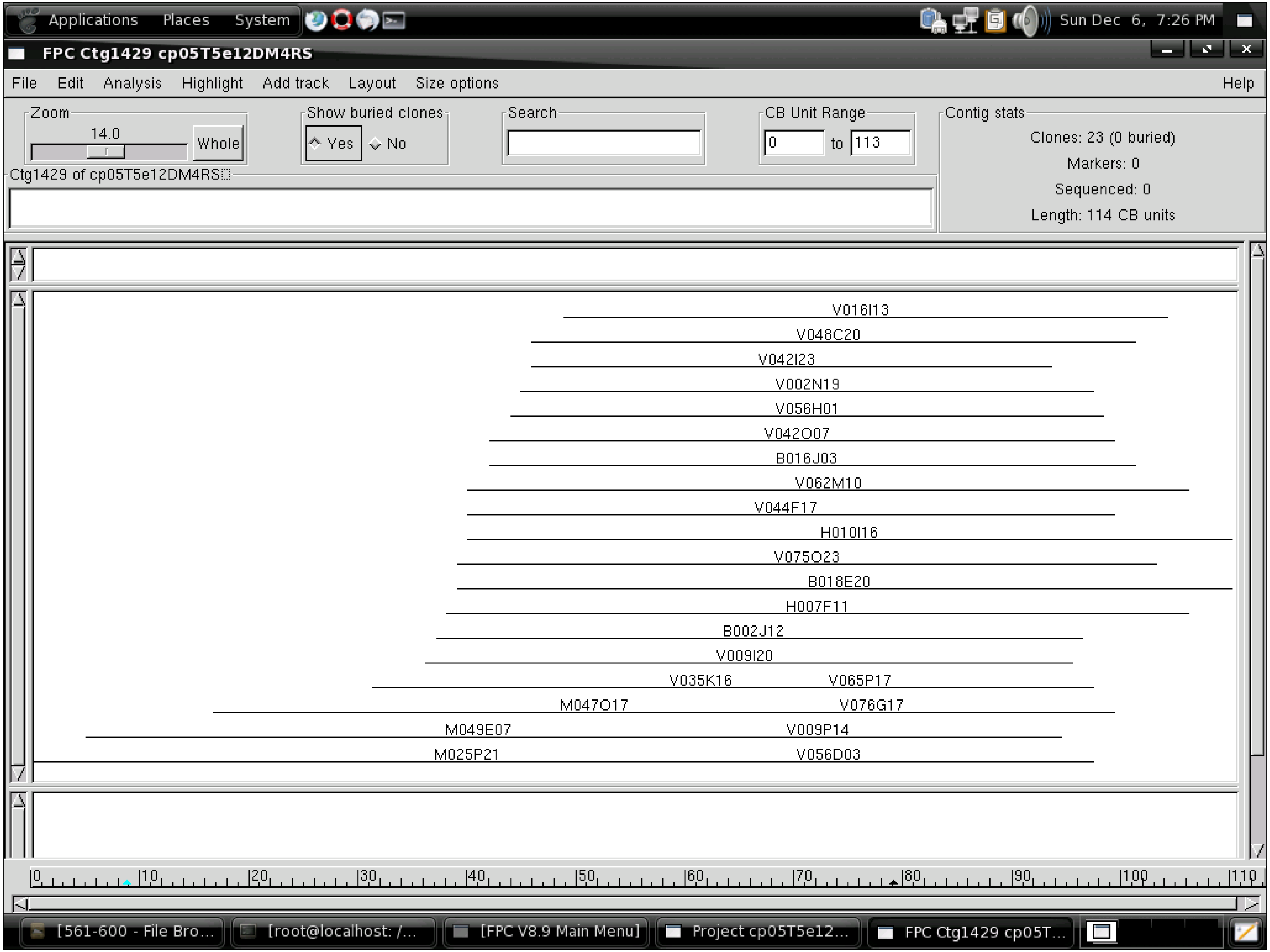
Applications Places System
Sun Dec 6, 7:26 PM
FPC Ctg1429 cp05T5e12DM4RS
File Edit Analysis Highlight Add track Layout Size options Help
Zoom
14.0
Whole
Show buried clones
Yes No
Search
CB Unit Range
0 to 113
Contig stats
Clones: 23 (0 buried)
Markers: 0
Sequenced: 0
Length: 114 CB units
Ctg1429 of cp05T5e12DM4RS
V016I13
V048C20
V042I23
V002N19
V056H01
V042O07
B016J03
V062M10
V044F17
H010I16
V075O23
B018E20
H007F11
B002J12
V009I20
V035K16
V065P17
M047O17
V076G17
M049E07
V009P14
M025P21
V056D03
0 10 20 30 40 50 60 70 80 90 100 110
[561-600 - File Bro...
[root@localhost: /...
[FPC V8.9 Main Menu]
Project cp05T5e12...
FPC Ctg1429 cp05T...

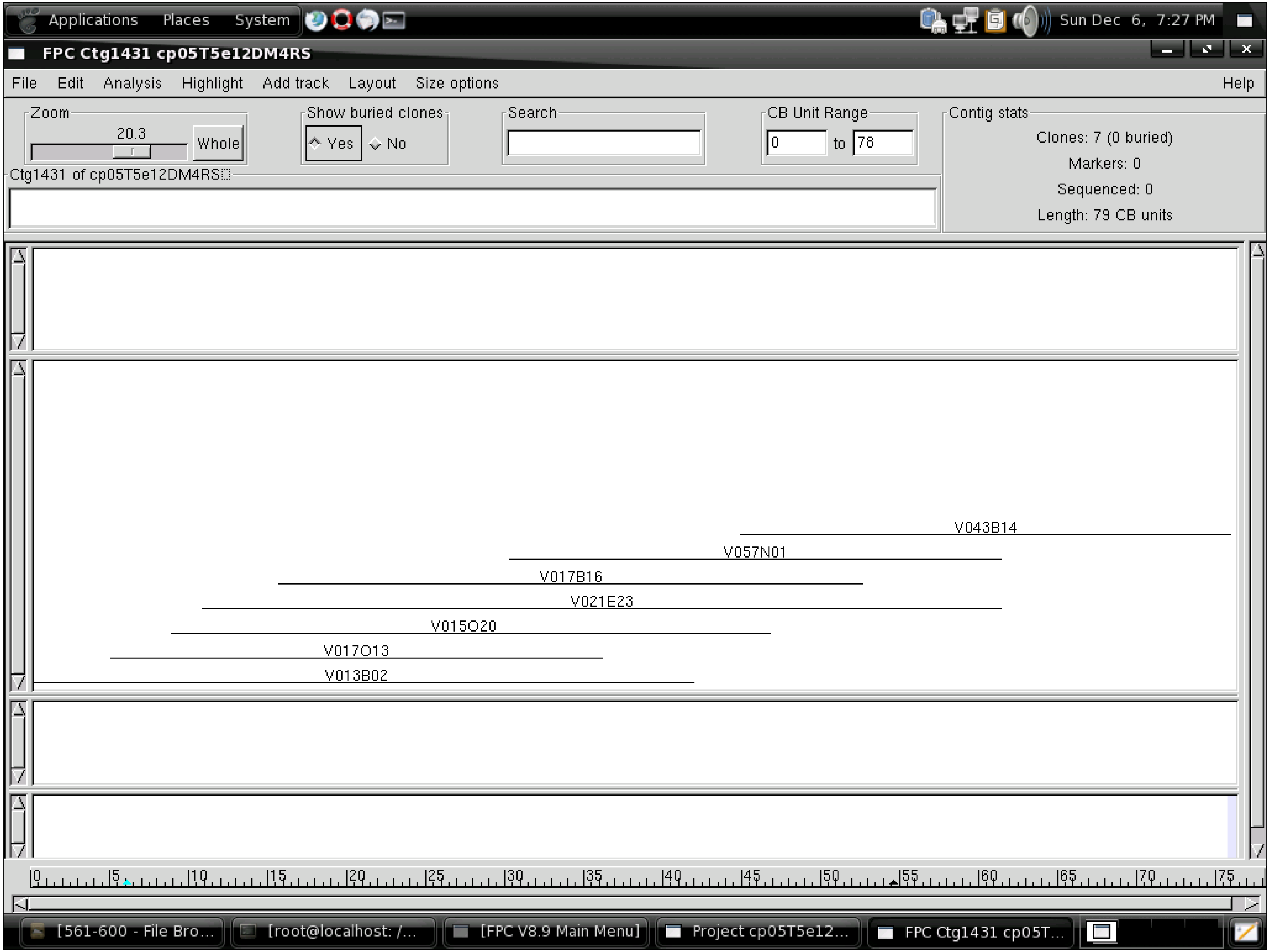
Applications
Places
System
Sun Dec 6, 7:27 PM
FPC Ctg1431 cp05T5e12DM4RS
File
Edit
Analysis
Highlight
Add track
Layout
Size options
Help
Zoom
20.3
Whole
Show buried clones
Yes
No
Search
CB Unit Range
0
to
78
Contig stats
Clones: 7 (0 buried)
Markers: 0
Sequenced: 0
Length: 79 CB units
Ctg1431 of cp05T5e12DM4RS
V043B14
V057N01
V017B16
V021E23
V015O20
V017O13
V013B02
0
5
10
15
20
25
30
35
40
45
50
55
60
65
70
75
[561-600 - File Bro...
[root@localhost: /...
[FPC V8.9 Main Menu]
Project cp05T5e12...
FPC Ctg1431 cp05T...

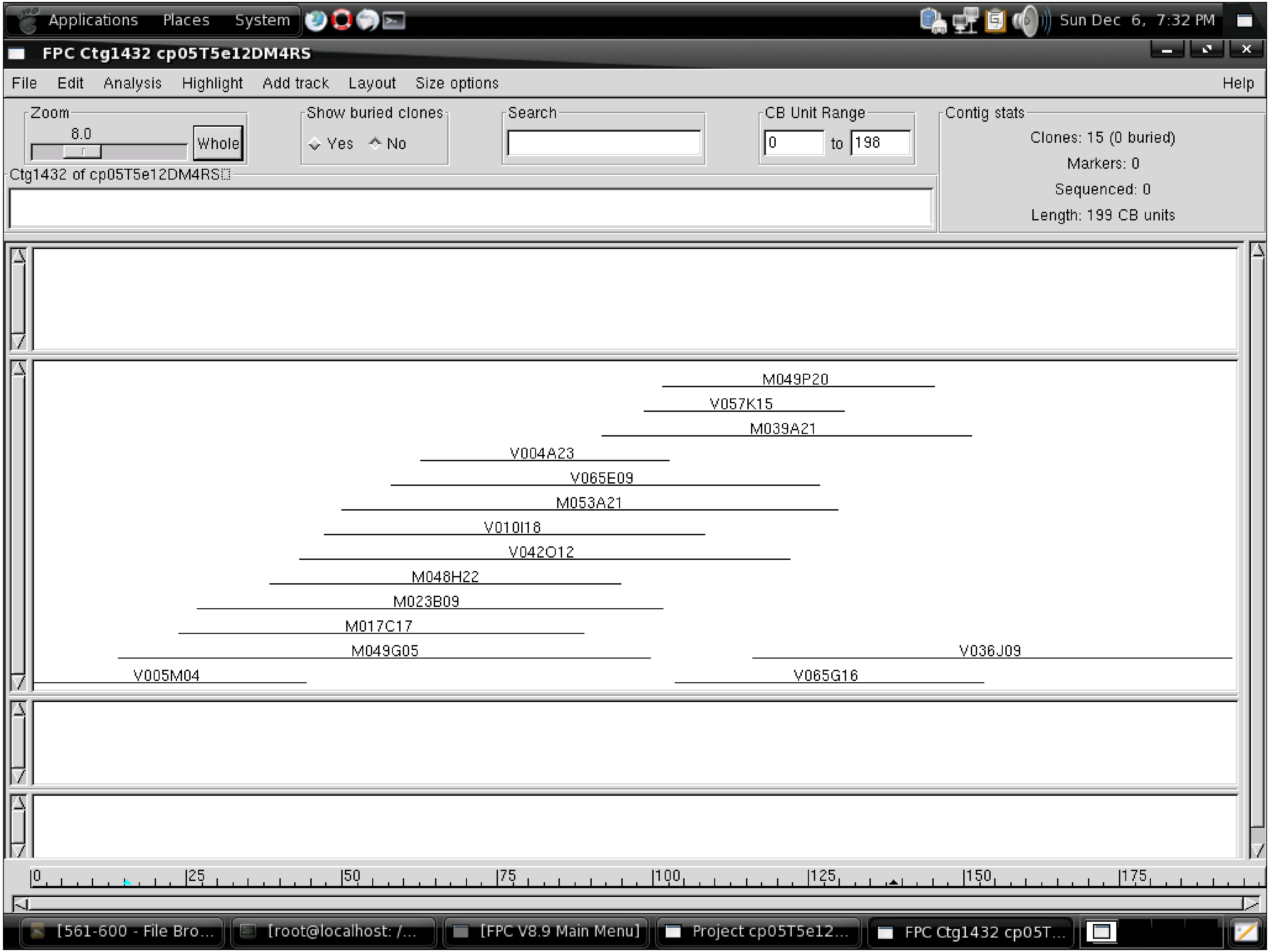
Applications Places System
Sun Dec 6, 7:32 PM
FPC Ctg1432 cp05T5e12DM4RS
File Edit Analysis Highlight Add track Layout Size options Help
Zoom
8.0
Whole
Show buried clones
Yes No
Search
CB Unit Range
0 to 198
Contig stats
Clones: 15 (0 buried)
Markers: 0
Sequenced: 0
Length: 199 CB units
Ctg1432 of cp05T5e12DM4RS
M049P20
V057K15
M039A21
V004A23
V065E09
M053A21
V010I18
V042O12
M048H22
M023B09
M017C17
M049G05
V036J09
V005M04
V065G16
0 25 50 75 100 125 150 175
[561-600 - File Bro...
[root@localhost: /...
[FPC V8.9 Main Menu]
Project cp05T5e12...
FPC Ctg1432 cp05T...

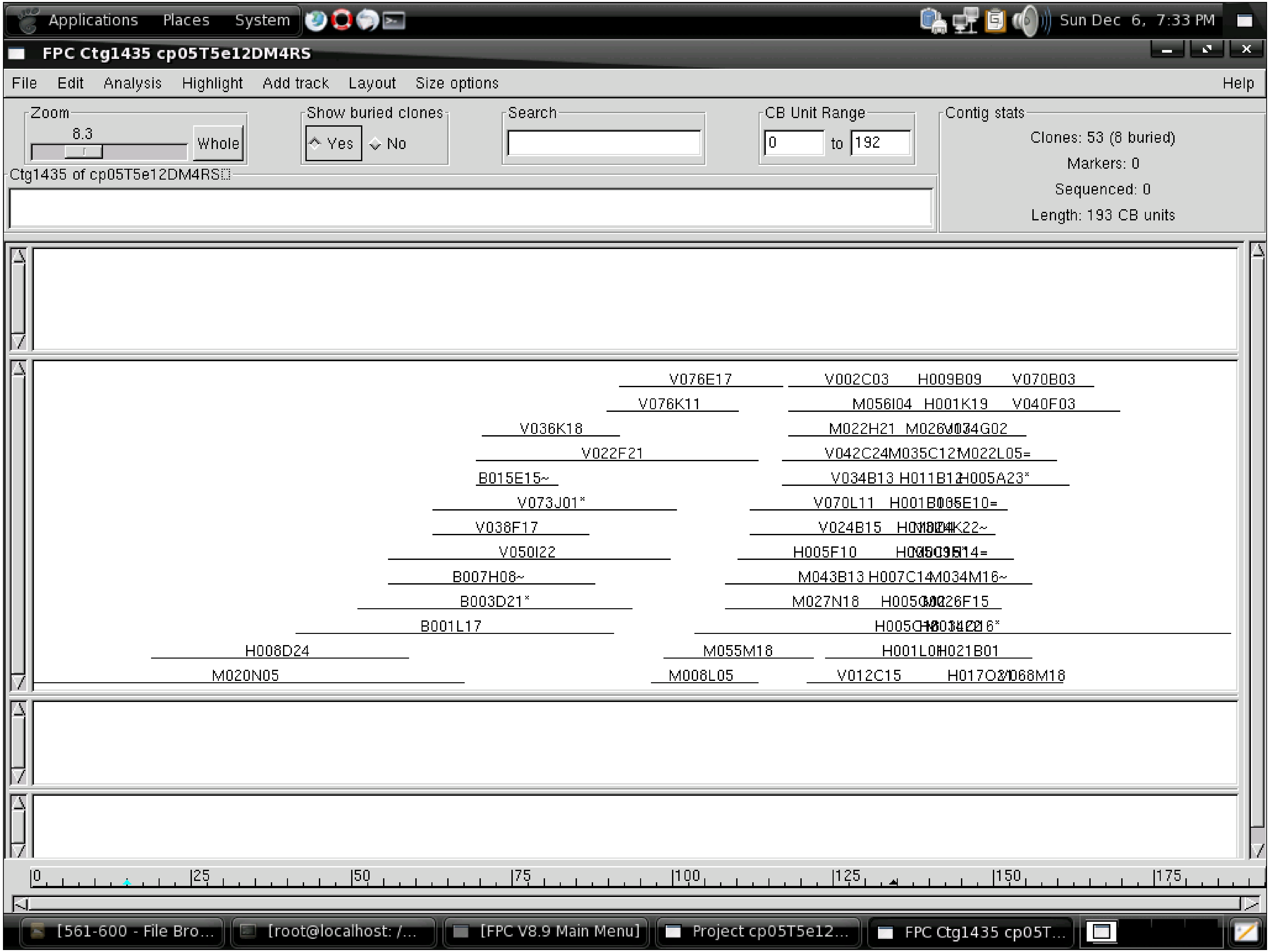
Applications Places System
Sun Dec 6, 7:33 PM
FPC Ctg1435 cp05T5e12DM4RS
File Edit Analysis Highlight Add track Layout Size options Help
Zoom
8.3
Whole
Show buried clones
Yes
No
Search
CB Unit Range
0
to
192
Contig stats
Clones: 53 (8 buried)
Markers: 0
Sequenced: 0
Length: 193 CB units
Ctg1435 of cp05T5e12DM4RS
V076E17
V002C03
H009B09
V070B03
V076K11
M056I04
H001K19
V040F03
V036K18
M022H21
V022F21
B015E15~
V073J01*
V038F17
V050I22
B007H08~
B003D21*
B001L17
H008D24
M020N05
V042C24
V034B13
V070L11
V024B15
H005F10
M043B13
H007C14
M034M16~
M027N18
M026F15
H001L01
H021B01
M055M18
M008L05
V012C15
0
25
50
75
100
125
150
175
[561-600 - File Bro...
[root@localhost: /...
[FPC V8.9 Main Menu]
Project cp05T5e12...
FPC Ctg1435 cp05T...

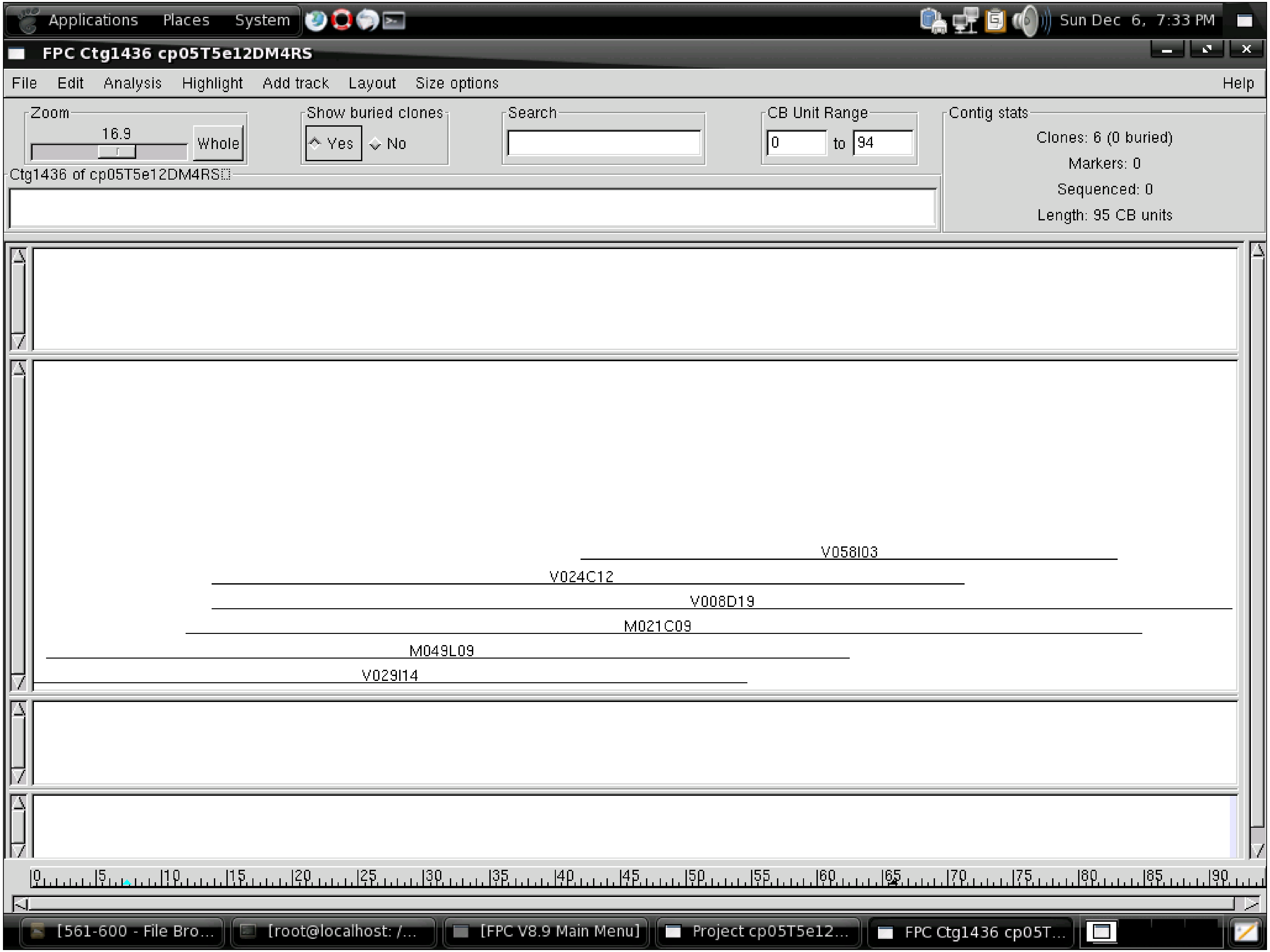
Applications
Places
System
Sun Dec 6, 7:33 PM
FPC Ctg1436 cp05T5e12DM4RS
File
Edit
Analysis
Highlight
Add track
Layout
Size options
Help
Zoom
16.9
Whole
Show buried clones
Yes
No
Search
CB Unit Range
0
to
94
Contig stats
Clones: 6 (0 buried)
Markers: 0
Sequenced: 0
Length: 95 CB units
Ctg1436 of cp05T5e12DM4RS
V058I03
V024C12
V008D19
M021C09
M049L09
V029I14
0 5 10 15 20 25 30 35 40 45 50 55 60 65 70 75 80 85 90
[561-600 - File Bro...
[root@localhost: /...
[FPC V8.9 Main Menu]
Project cp05T5e12...
FPC Ctg1436 cp05T...

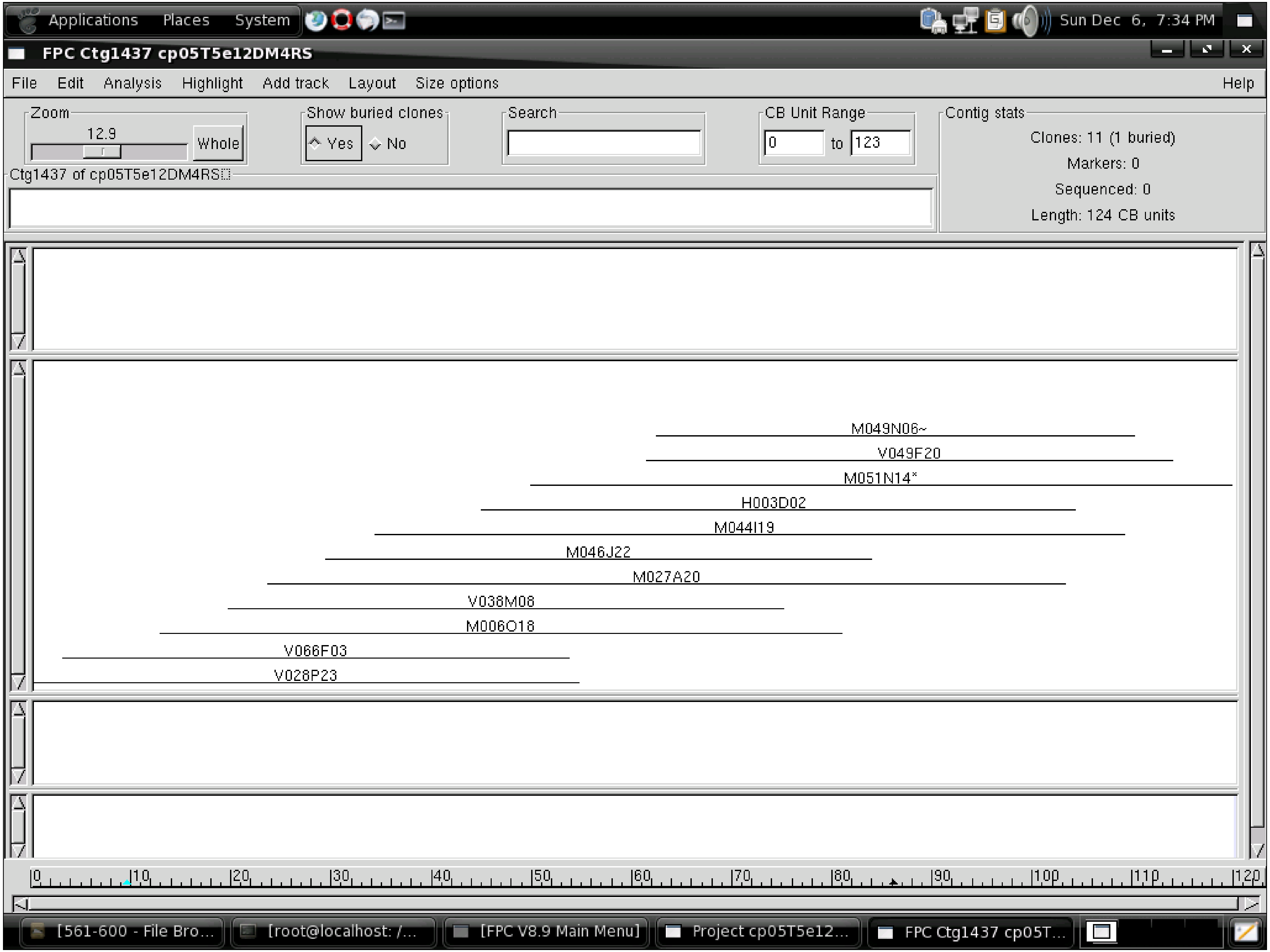
Applications Places System
Sun Dec 6, 7:34 PM
FPC Ctg1437 cp05T5e12DM4RS
File Edit Analysis Highlight Add track Layout Size options Help
Zoom
12.9
Whole
Show buried clones
Yes No
Search
CB Unit Range
0 to 123
Contig stats
Clones: 11 (1 buried)
Markers: 0
Sequenced: 0
Length: 124 CB units
Ctg1437 of cp05T5e12DM4RS
M049N06~
V049F20
M051N14*
H003D02
M044I19
M046J22
M027A20
V038M08
M006O18
V066F03
V028P23
0 10 20 30 40 50 60 70 80 90 100 110 120
[561-600 - File Bro...
[root@localhost: /...
[FPC V8.9 Main Menu]
Project cp05T5e12...
FPC Ctg1437 cp05T...

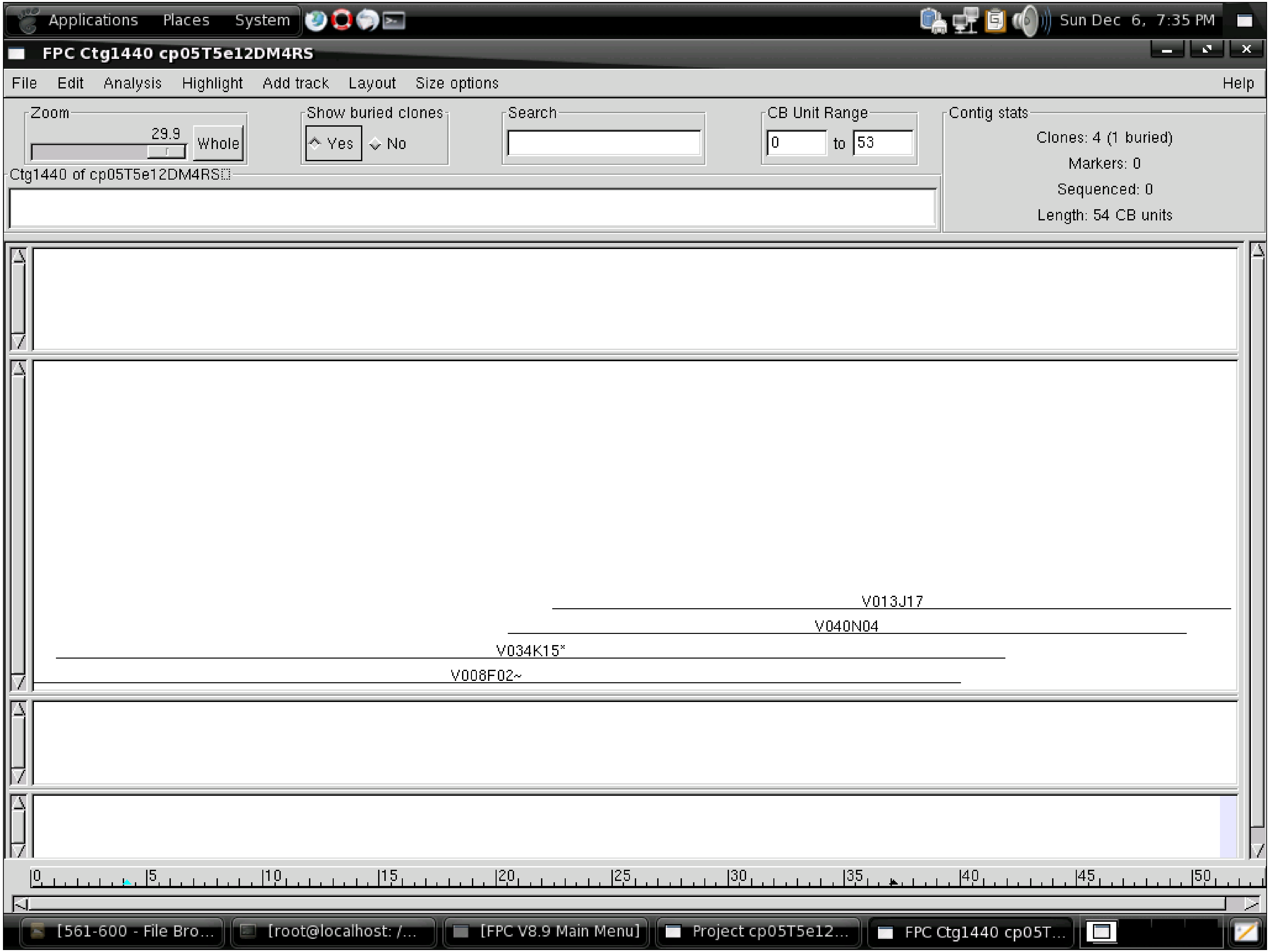
Applications Places System
Sun Dec 6, 7:35 PM
FPC Ctg1440 cp05T5e12DM4RS
File Edit Analysis Highlight Add track Layout Size options
Help
Zoom
29.9
Whole
Show buried clones
Yes
No
Search
CB Unit Range
0
to
53
Contig stats
Clones: 4 (1 buried)
Markers: 0
Sequenced: 0
Length: 54 CB units
Ctg1440 of cp05T5e12DM4RS
V013J17
V040N04
V034K15*
V008F02~
0 5 10 15 20 25 30 35 40 45 50
[561-600 - File Bro...
[root@localhost: /...
[FPC V8.9 Main Menu]
Project cp05T5e12...
FPC Ctg1440 cp05T...

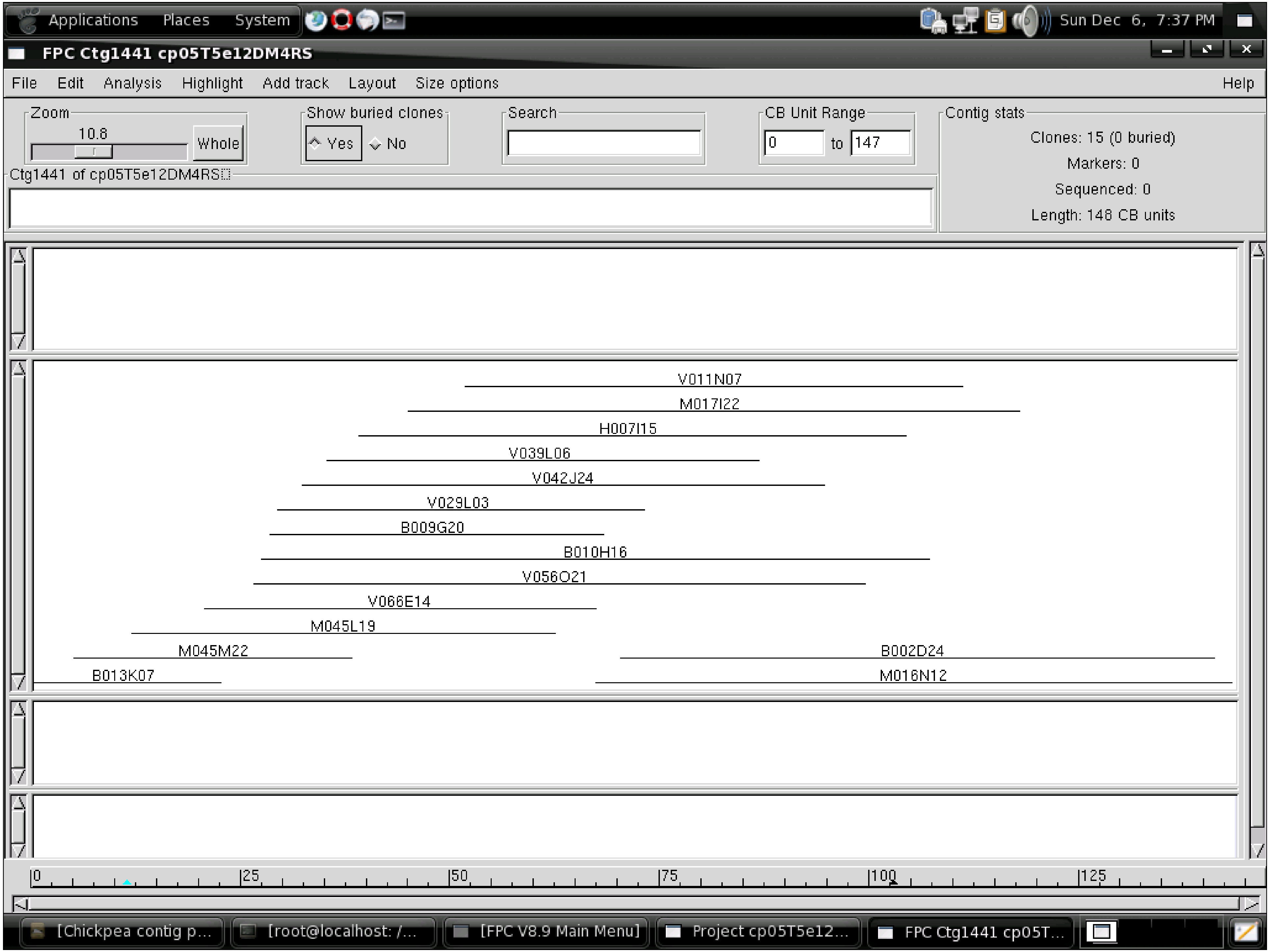
Applications Places System
Sun Dec 6, 7:37 PM
FPC Ctg1441 cp05T5e12DM4RS
File Edit Analysis Highlight Add track Layout Size options Help
Zoom
10.8
Whole
Show buried clones
Yes No
Search
CB Unit Range
0 to 147
Contig stats
Clones: 15 (0 buried)
Markers: 0
Sequenced: 0
Length: 148 CB units
Ctg1441 of cp05T5e12DM4RS
V011N07
M017I22
H007I15
V039L06
V042J24
V029L03
B009G20
B010H16
V056O21
V066E14
M045L19
M045M22
B002D24
B013K07
M016N12
0
25
50
75
100
125
[Chickpea contig p...
[root@localhost: /...
[FPC V8.9 Main Menu]
Project cp05T5e12...
FPC Ctg1441 cp05T...

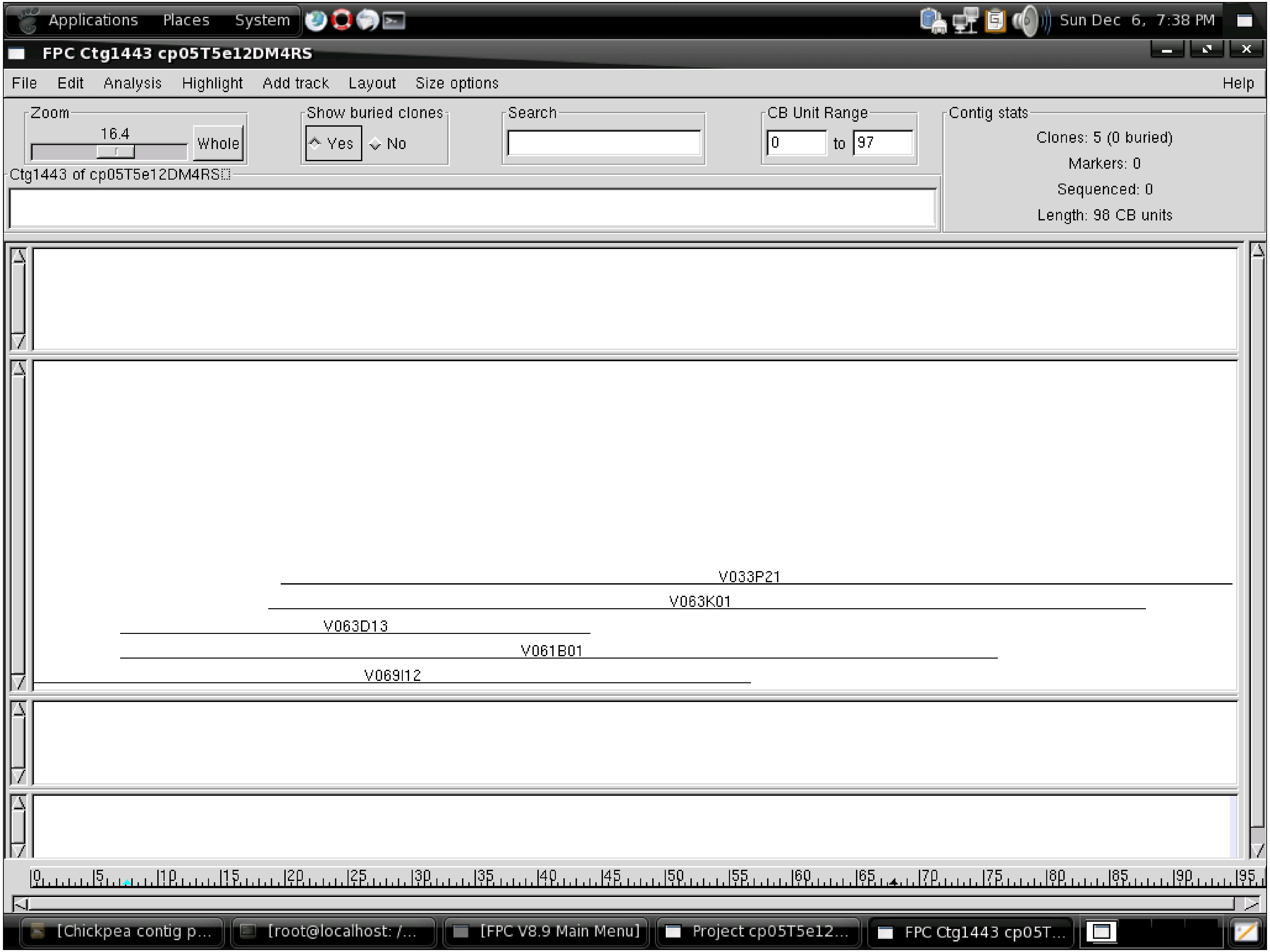
Applications Places System
Sun Dec 6, 7:38 PM
FPC Ctg1443 cp05T5e12DM4RS
File Edit Analysis Highlight Add track Layout Size options Help
Zoom
16.4
Whole
Show buried clones
Yes No
Search
CB Unit Range
0 to 97
Contig stats
Clones: 5 (0 buried)
Markers: 0
Sequenced: 0
Length: 98 CB units
Ctg1443 of cp05T5e12DM4RS
V033P21
V063K01
V063D13
V061B01
V069I12
0 5 10 15 20 25 30 35 40 45 50 55 60 65 70 75 80 85 90 95
[Chickpea contig p...
[root@localhost: /...
[FPC V8.9 Main Menu]
Project cp05T5e12...
FPC Ctg1443 cp05T...

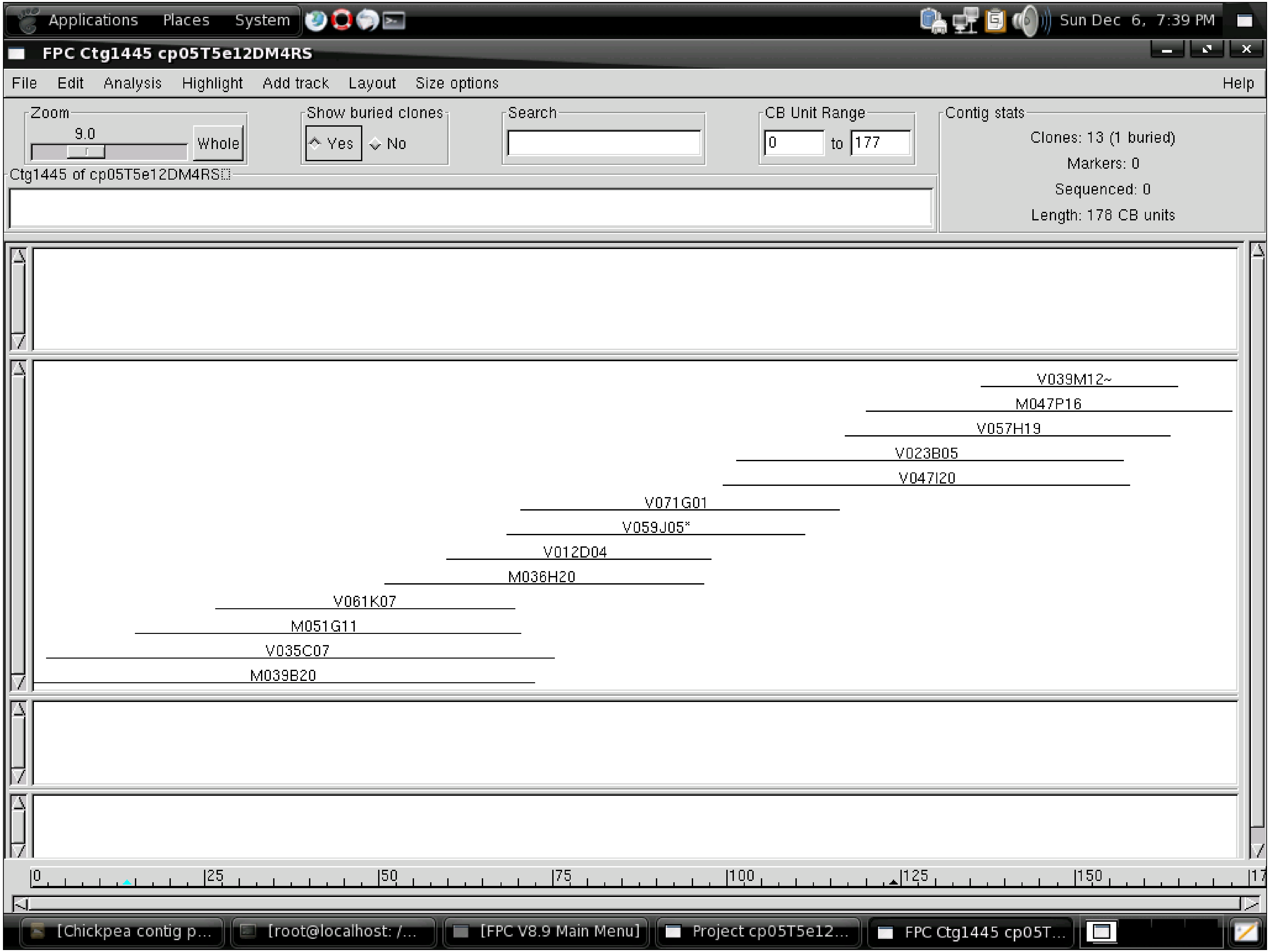
Applications Places System
Sun Dec 6, 7:39 PM
FPC Ctg1445 cp05T5e12DM4RS
File Edit Analysis Highlight Add track Layout Size options Help
Zoom
9.0
Whole
Show buried clones
Yes
No
Search
CB Unit Range
0 to 177
Contig stats
Clones: 13 (1 buried)
Markers: 0
Sequenced: 0
Length: 178 CB units
Ctg1445 of cp05T5e12DM4RS
V039M12~
M047P16
V057H19
V023B05
V047I20
V071G01
V059J05*
V012D04
M036H20
V061K07
M051G11
V035C07
M039B20
0
25
50
75
100
125
150
[Chickpea contig p...
[root@localhost: /...
[FPC V8.9 Main Menu]
Project cp05T5e12...
FPC Ctg1445 cp05T...

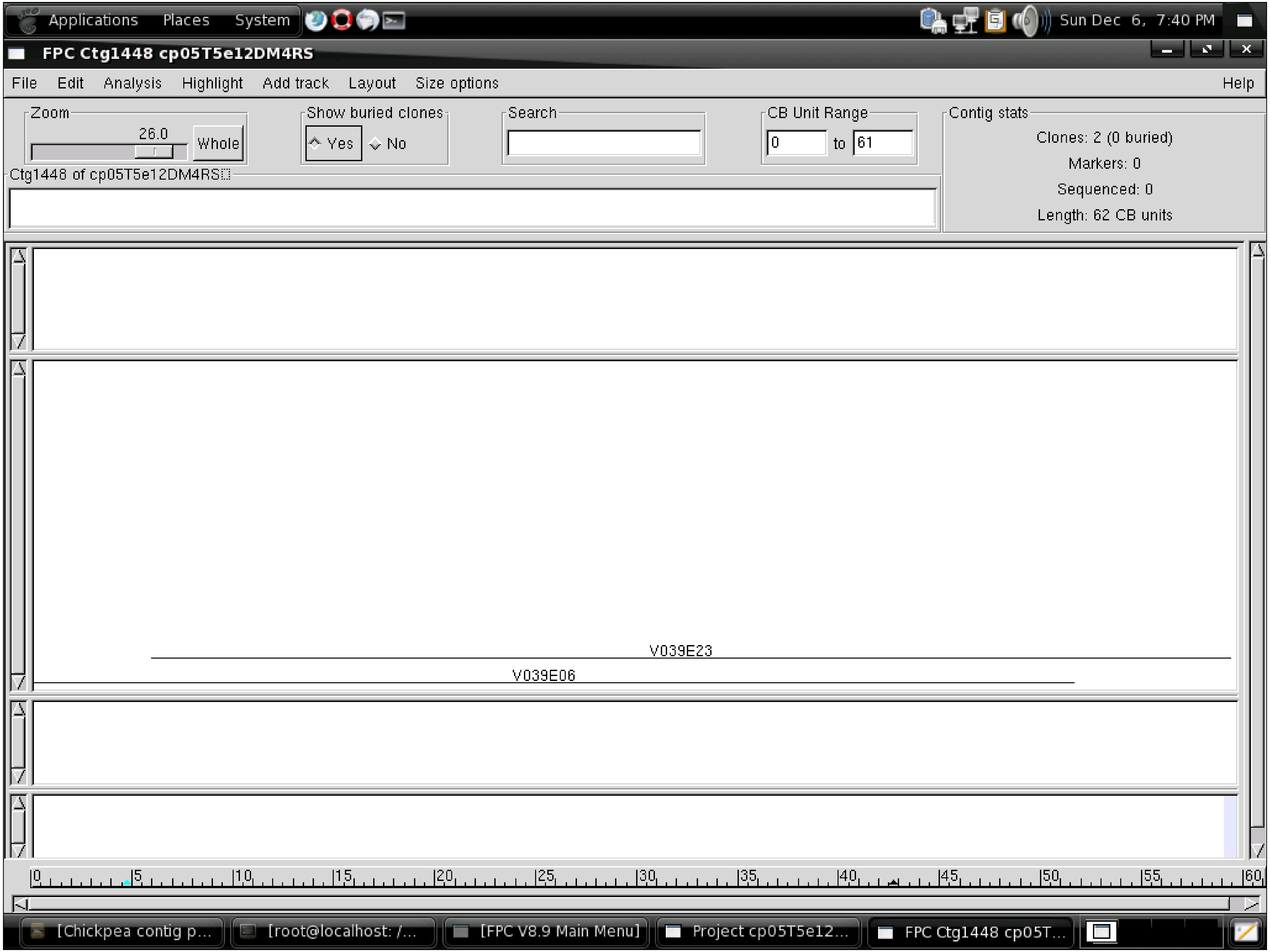
Applications Places System
Sun Dec 6, 7:40 PM
FPC Ctg1448 cp05T5e12DM4RS
File Edit Analysis Highlight Add track Layout Size options Help
Zoom
26.0
Whole
Show buried clones
Yes
No
Search
CB Unit Range
0 to 61
Contig stats
Clones: 2 (0 buried)
Markers: 0
Sequenced: 0
Length: 62 CB units
Ctg1448 of cp05T5e12DM4RS
V039E23
V039E06
0 5 10 15 20 25 30 35 40 45 50 55 60
[Chickpea contig p...
[root@localhost: /...
[FPC V8.9 Main Menu]
Project cp05T5e12...
FPC Ctg1448 cp05T...

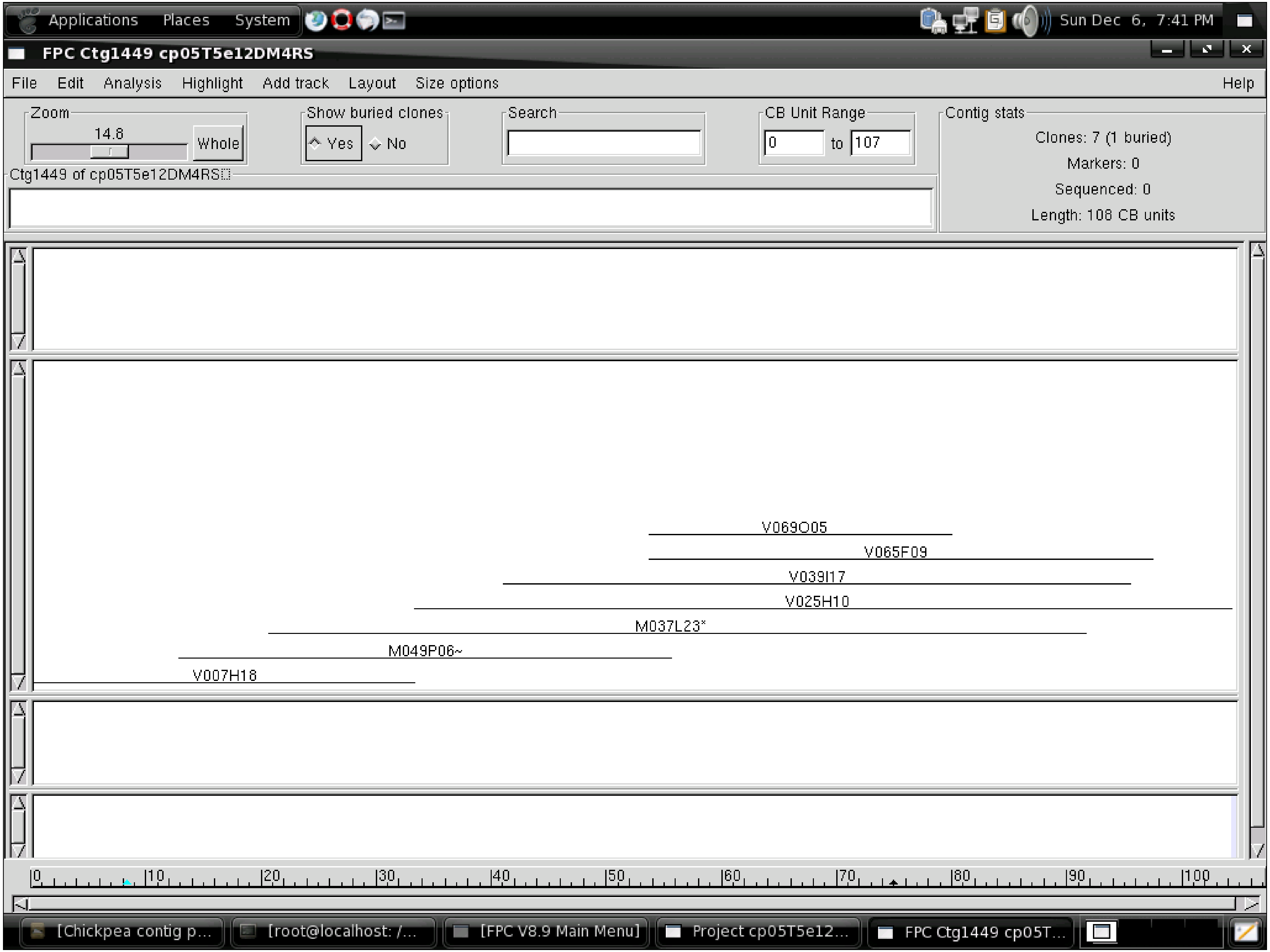
Applications Places System
Sun Dec 6, 7:41 PM
FPC Ctg1449 cp05T5e12DM4RS
File Edit Analysis Highlight Add track Layout Size options Help
Zoom 14.8 Whole
Show buried clones Yes No
Search
CB Unit Range 0 to 107
Contig stats
Clones: 7 (1 buried)
Markers: 0
Sequenced: 0
Length: 108 CB units
Ctg1449 of cp05T5e12DM4RS
V069O05
V065F09
V039I17
V025H10
M037L23*
M049P06~
V007H18
0 10 20 30 40 50 60 70 80 90 100
[Chickpea contig p...
[root@localhost: /...
[FPC V8.9 Main Menu]
Project cp05T5e12...
FPC Ctg1449 cp05T...

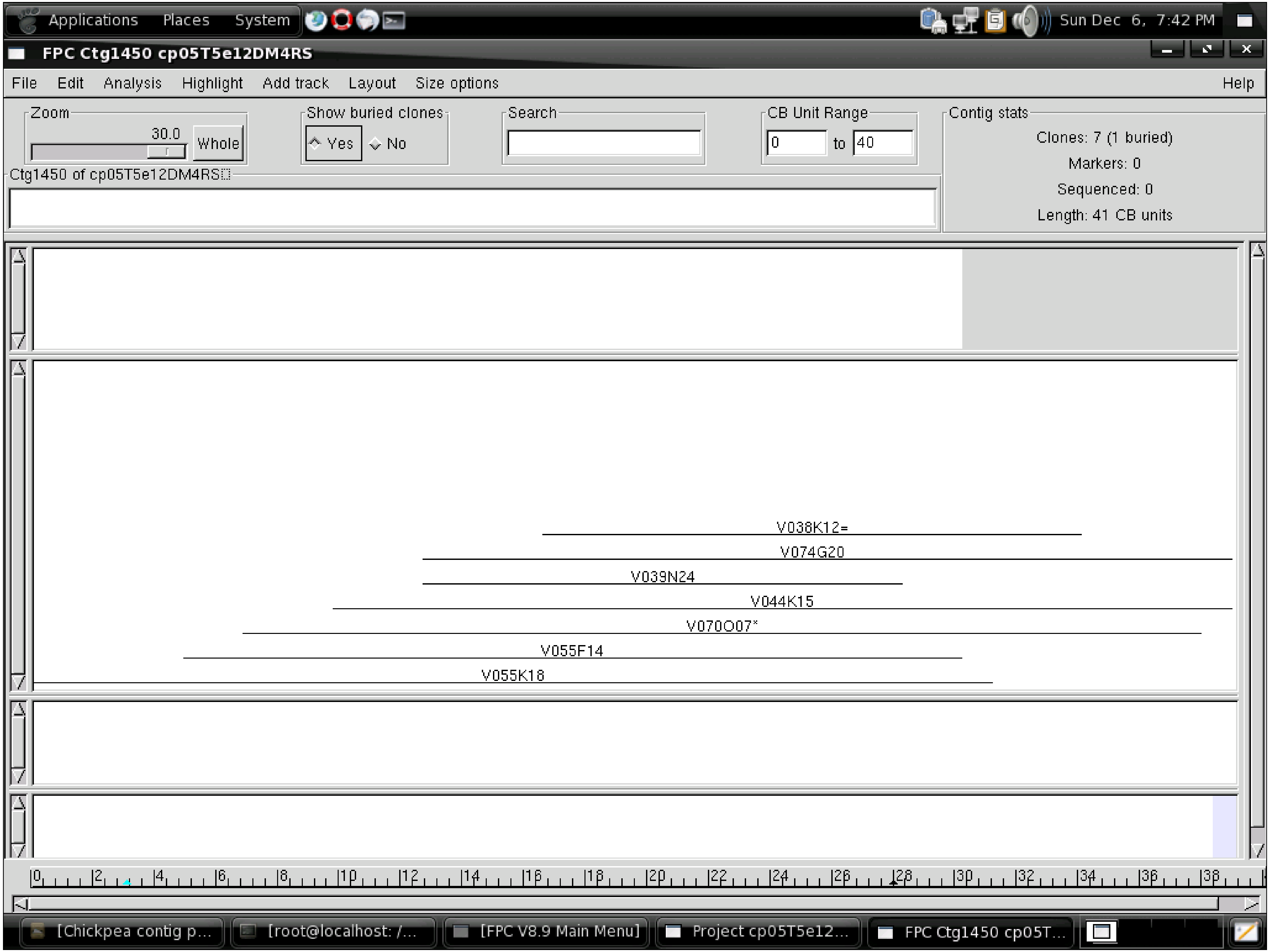

Applications
Places
System
Sun Dec 6, 7:42 PM
FPC Ctg1450 cp05T5e12DM4RS
File
Edit
Analysis
Highlight
Add track
Layout
Size options
Help
Zoom
30.0
Whole
Show buried clones
Yes
No
Search
CB Unit Range
0
to
40
Contig stats
Clones: 7 (1 buried)
Markers: 0
Sequenced: 0
Length: 41 CB units
Ctg1450 of cp05T5e12DM4RS
V038K12=
V074G20
V039N24
V044K15
V070O07*
V055F14
V055K18
0 2 4 6 8 10 12 14 16 18 20 22 24 26 28 30 32 34 36 38
[Chickpea contig p...
[root@localhost: /...
[FPC V8.9 Main Menu]
Project cp05T5e12...
FPC Ctg1450 cp05T...

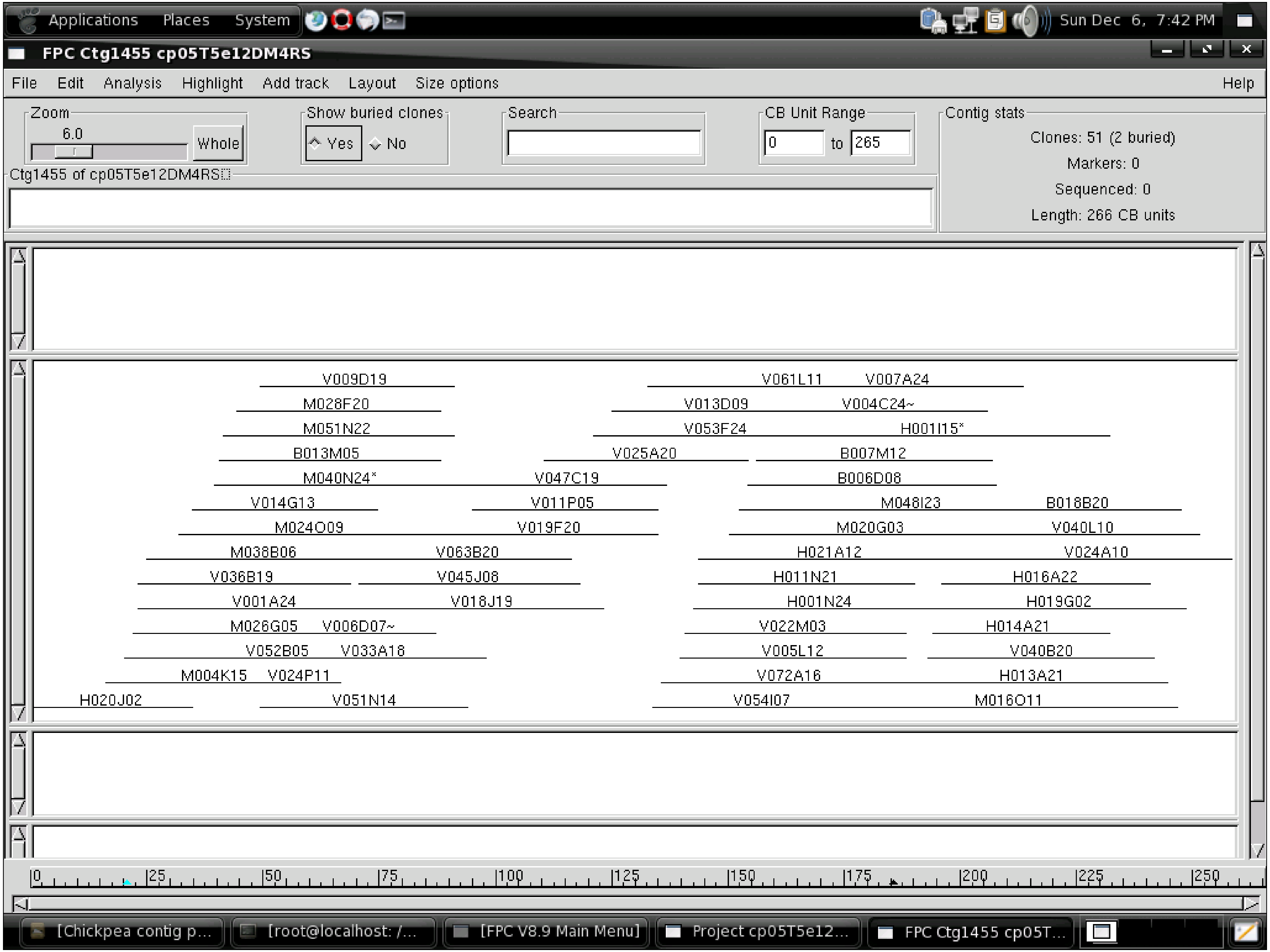

Applications Places System
Sun Dec 6, 7:42 PM
FPC Ctg1455 cp05T5e12DM4RS
File Edit Analysis Highlight Add track Layout Size options Help
Zoom
6.0
Whole
Show buried clones
Yes
No
Search
CB Unit Range
0 to 265
Contig stats
Clones: 51 (2 buried)
Markers: 0
Sequenced: 0
Length: 266 CB units
Ctg1455 of cp05T5e12DM4RS
V009D19
V061L11
V007A24
M028F20
V013D09
V004C24~
M051N22
V053F24
H001I15*
B013M05
V025A20
B007M12
M040N24*
V047C19
B006D08
V014G13
V011P05
M048I23
B018B20
M024O09
V019F20
M020G03
V040L10
M038B06
V063B20
H021A12
V024A10
V036B19
V045J08
H011N21
H016A22
V001A24
V018J19
H001N24
H019G02
M026G05
V006D07~
V022M03
H014A21
V052B05
V033A18
V005L12
V040B20
M004K15
V024P11
V072A16
H013A21
H020J02
V051N14
V054I07
M016O11
0 25 50 75 100 125 150 175 200 225 250
[Chickpea contig p...
[root@localhost: /...
[FPC V8.9 Main Menu]
Project cp05T5e12...
FPC Ctg1455 cp05T...

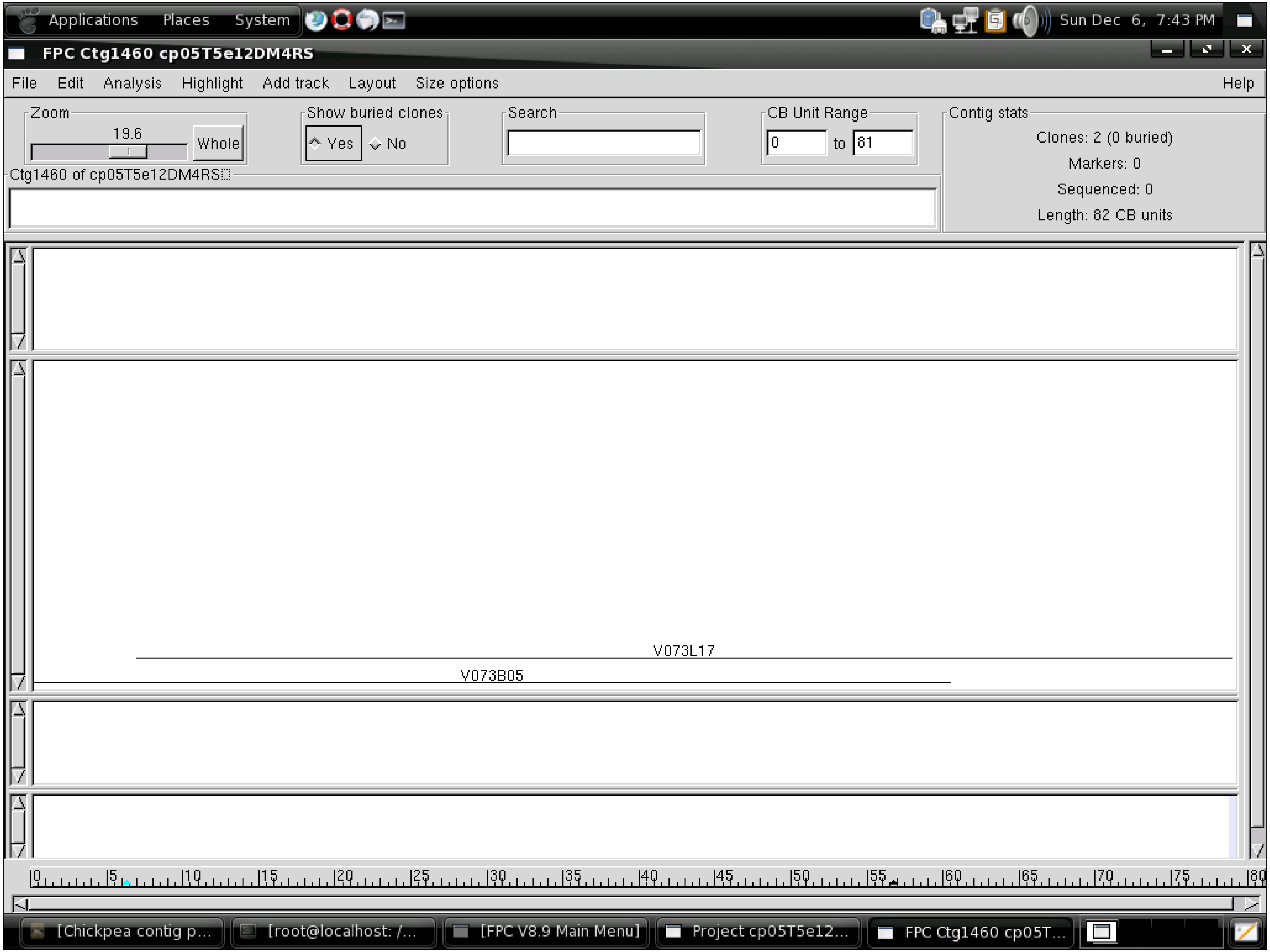
Applications
Places
System
Sun Dec 6, 7:43 PM
FPC Ctg1460 cp05T5e12DM4RS
File
Edit
Analysis
Highlight
Add track
Layout
Size options
Help
Zoom
19.6
Whole
Show buried clones
Yes
No
Search
CB Unit Range
0
to
81
Contig stats
Clones: 2 (0 buried)
Markers: 0
Sequenced: 0
Length: 82 CB units
Ctg1460 of cp05T5e12DM4RS
V073L17
V073B05
0 5 10 15 20 25 30 35 40 45 50 55 60 65 70 75 80
[Chickpea contig p...
[root@localhost: /...
[FPC V8.9 Main Menu]
Project cp05T5e12...
FPC Ctg1460 cp05T...

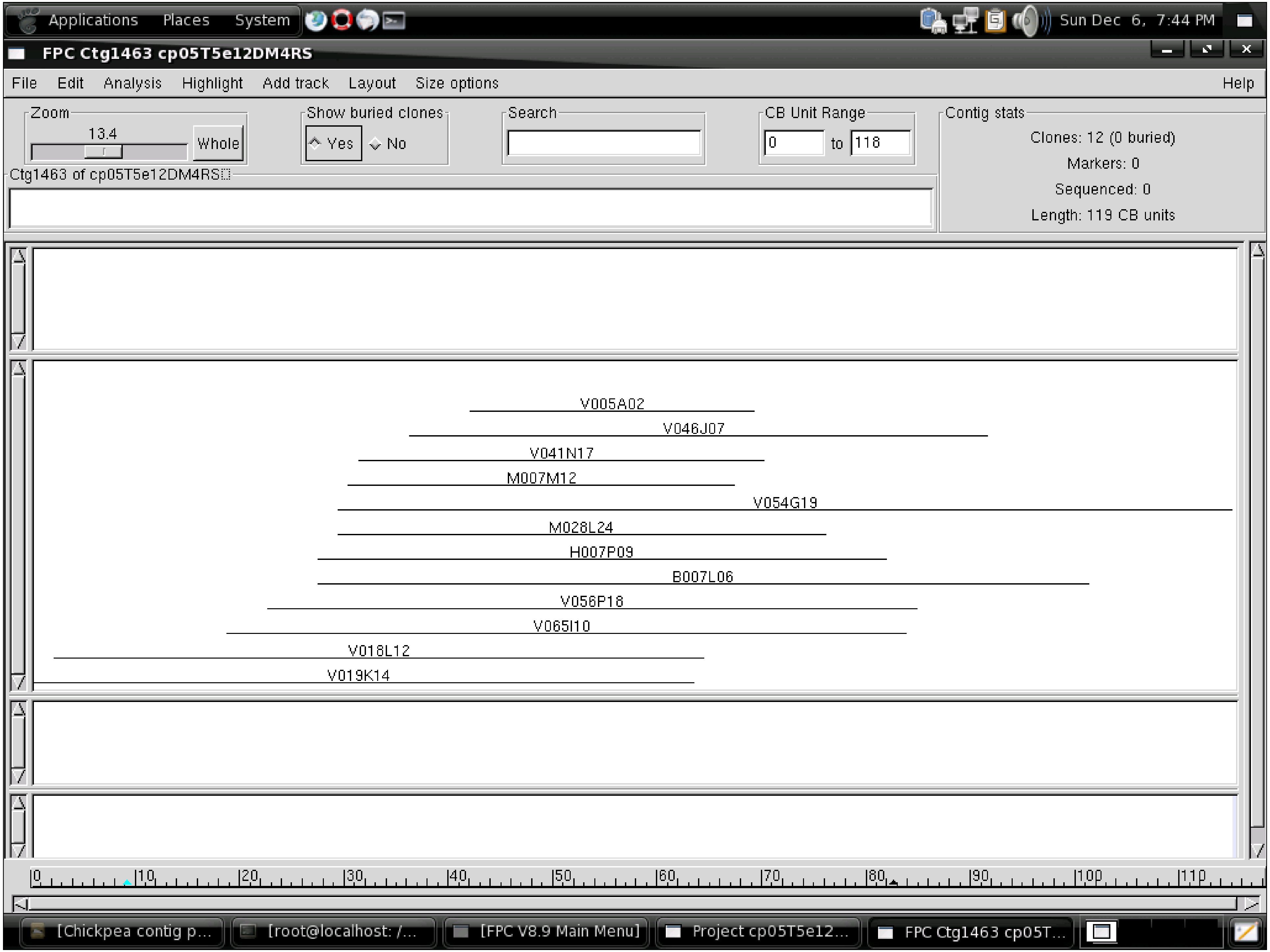
Applications Places System
Sun Dec 6, 7:44 PM
FPC Ctg1463 cp05T5e12DM4RS
File Edit Analysis Highlight Add track Layout Size options Help
Zoom
13.4
Whole
Show buried clones
Yes No
Search
CB Unit Range
0 to 118
Contig stats
Clones: 12 (0 buried)
Markers: 0
Sequenced: 0
Length: 119 CB units
Ctg1463 of cp05T5e12DM4RS
V005A02
V046J07
V041N17
M007M12
V054G19
M028L24
H007P09
B007L06
V056P18
V065I10
V018L12
V019K14
0 10 20 30 40 50 60 70 80 90 100 110
[Chickpea contig p...
[root@localhost: /...
[FPC V8.9 Main Menu]
Project cp05T5e12...
FPC Ctg1463 cp05T...

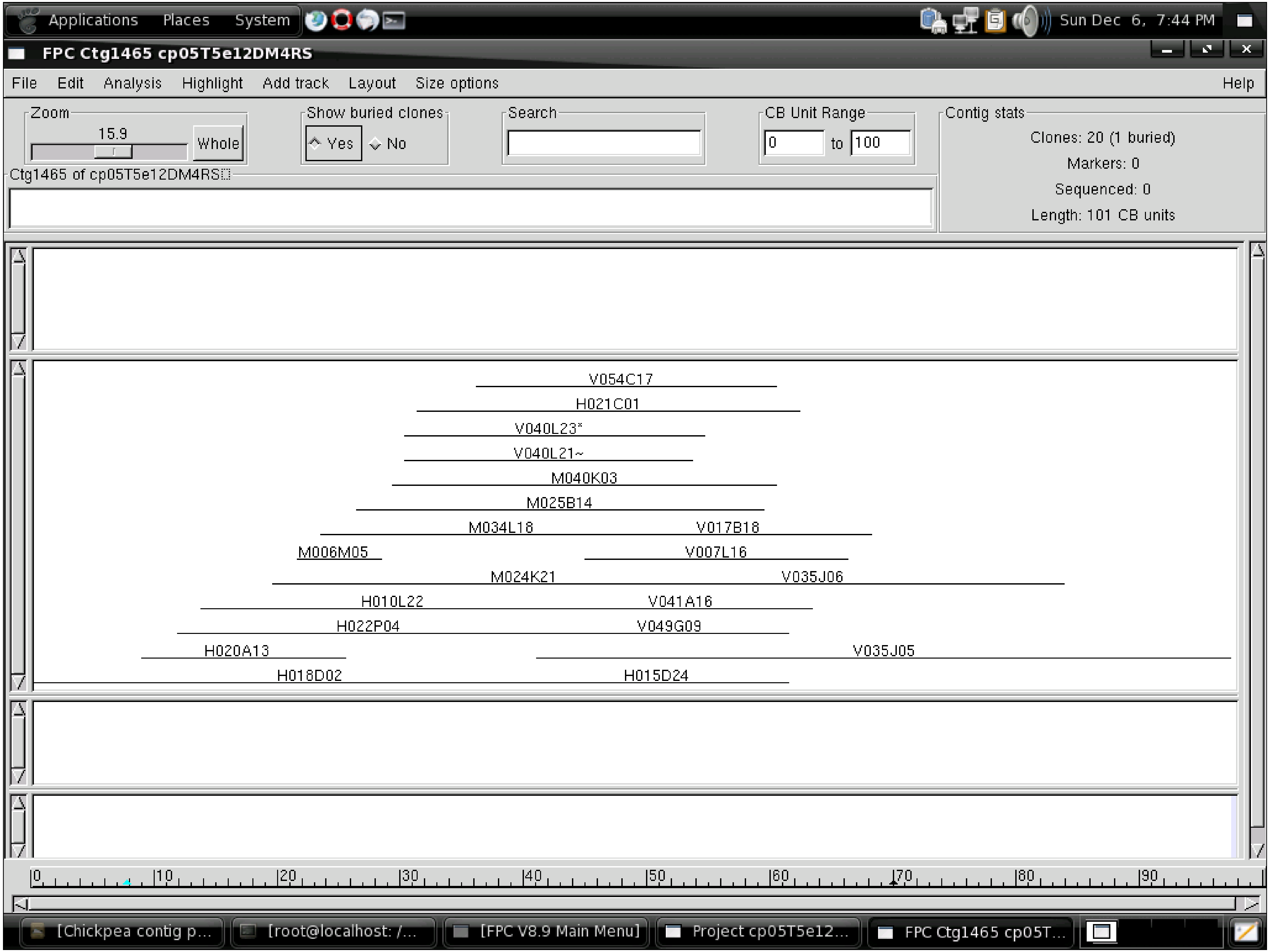
Applications Places System
Sun Dec 6, 7:44 PM
FPC Ctg1465 cp05T5e12DM4RS
File Edit Analysis Highlight Add track Layout Size options Help
Zoom
15.9
Whole
Show buried clones
Yes
No
Search
CB Unit Range
0 to 100
Contig stats
Clones: 20 (1 buried)
Markers: 0
Sequenced: 0
Length: 101 CB units
Ctg1465 of cp05T5e12DM4RS
V054C17
H021C01
V040L23*
V040L21~
M040K03
M025B14
M034L18
V017B18
M006M05
V007L16
M024K21
V035J06
H010L22
V041A16
H022P04
V049G09
H020A13
V035J05
H018D02
H015D24
0 10 20 30 40 50 60 70 80 90
[Chickpea contig p...
[root@localhost: /...
[FPC V8.9 Main Menu]
Project cp05T5e12...
FPC Ctg1465 cp05T...

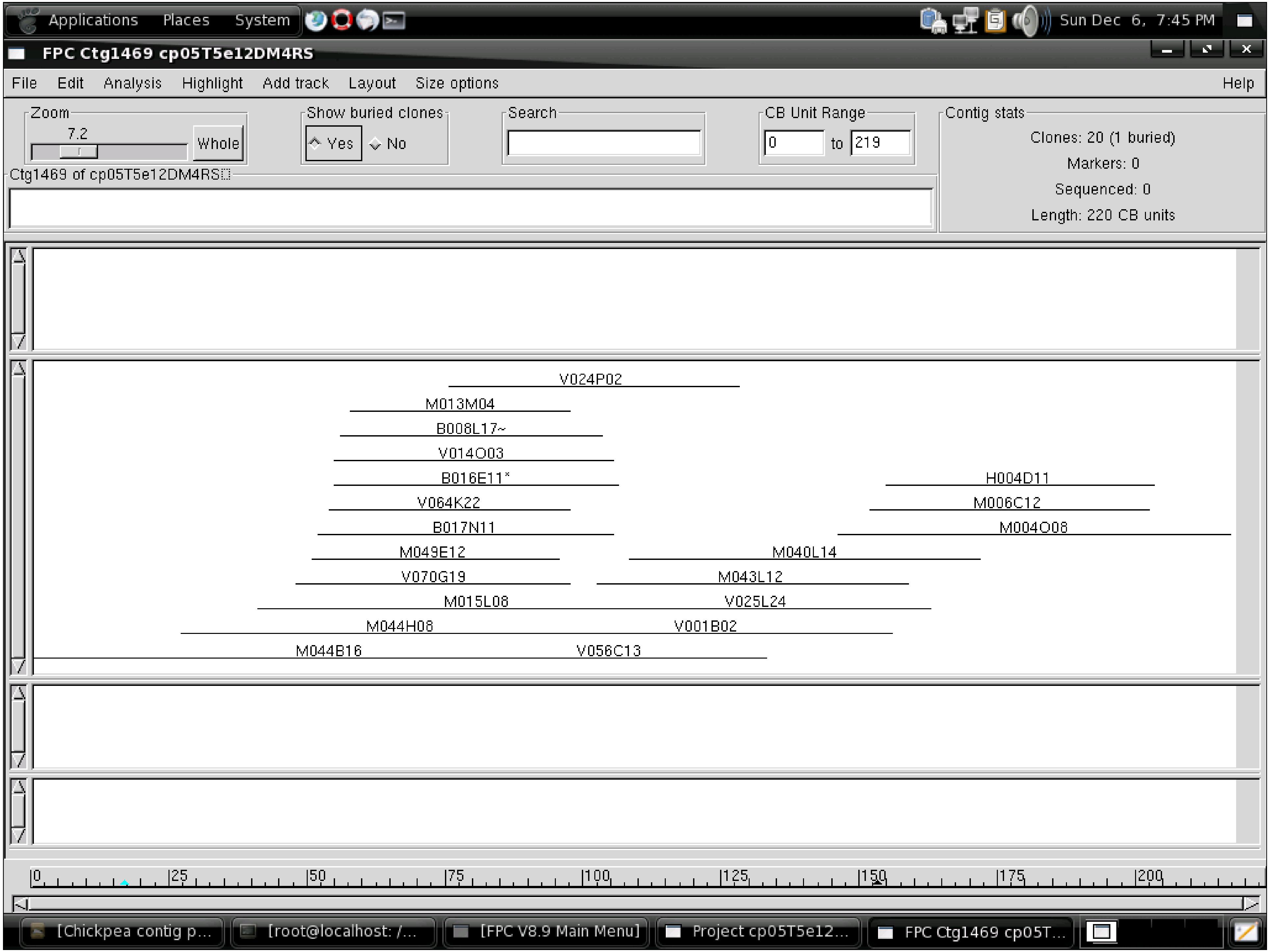
Applications
Places
System
Sun Dec 6, 7:45 PM
FPC Ctg1469 cp05T5e12DM4RS
File
Edit
Analysis
Highlight
Add track
Layout
Size options
Help
Zoom
7.2
Whole
Show buried clones
Yes
No
Search
CB Unit Range
0
to
219
Contig stats
Clones: 20 (1 buried)
Markers: 0
Sequenced: 0
Length: 220 CB units
Ctg1469 of cp05T5e12DM4RS
V024P02
M013M04
B008L17~
V014O03
B016E11*
H004D11
V064K22
M006C12
B017N11
M004O08
M049E12
M040L14
V070G19
M043L12
M015L08
V025L24
M044H08
V001B02
M044B16
V056C13
0
25
50
75
100
125
150
175
200
[Chickpea contig p...
[root@localhost: /...
[FPC V8.9 Main Menu]
Project cp05T5e12...
FPC Ctg1469 cp05T...

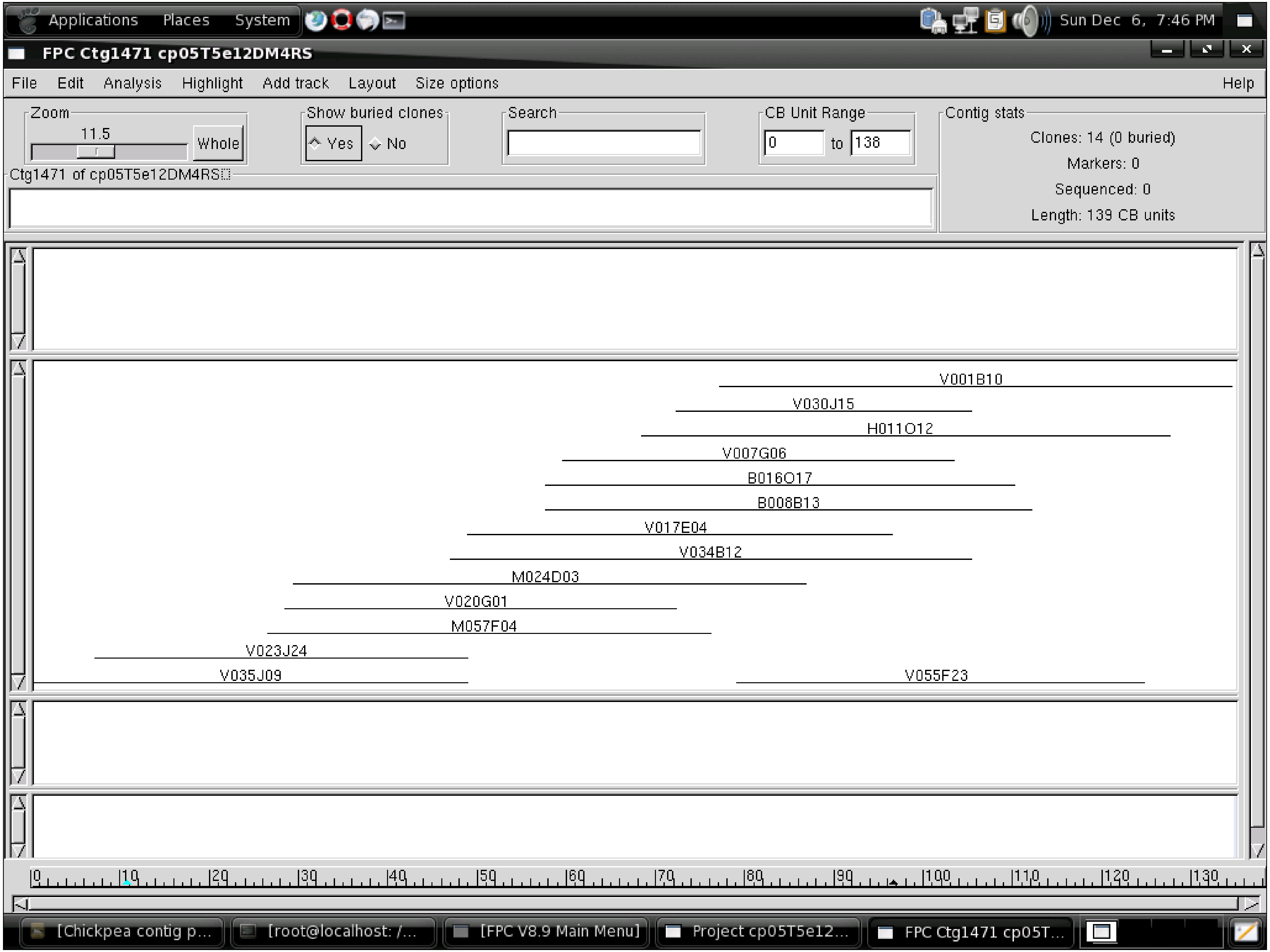
Applications Places System
Sun Dec 6, 7:46 PM
FPC Ctg1471 cp05T5e12DM4RS
File Edit Analysis Highlight Add track Layout Size options Help
Zoom
11.5
Whole
Show buried clones
Yes
No
Search
CB Unit Range
0
to
138
Contig stats
Clones: 14 (0 buried)
Markers: 0
Sequenced: 0
Length: 139 CB units
Ctg1471 of cp05T5e12DM4RS
V001B10
V030J15
H011O12
V007G06
B016O17
B008B13
V017E04
V034B12
M024D03
V020G01
M057F04
V023J24
V035J09
V055F23
0 10 20 30 40 50 60 70 80 90 100 110 120 130
[Chickpea contig p...
[root@localhost: /...
[FPC V8.9 Main Menu]
Project cp05T5e12...
FPC Ctg1471 cp05T...

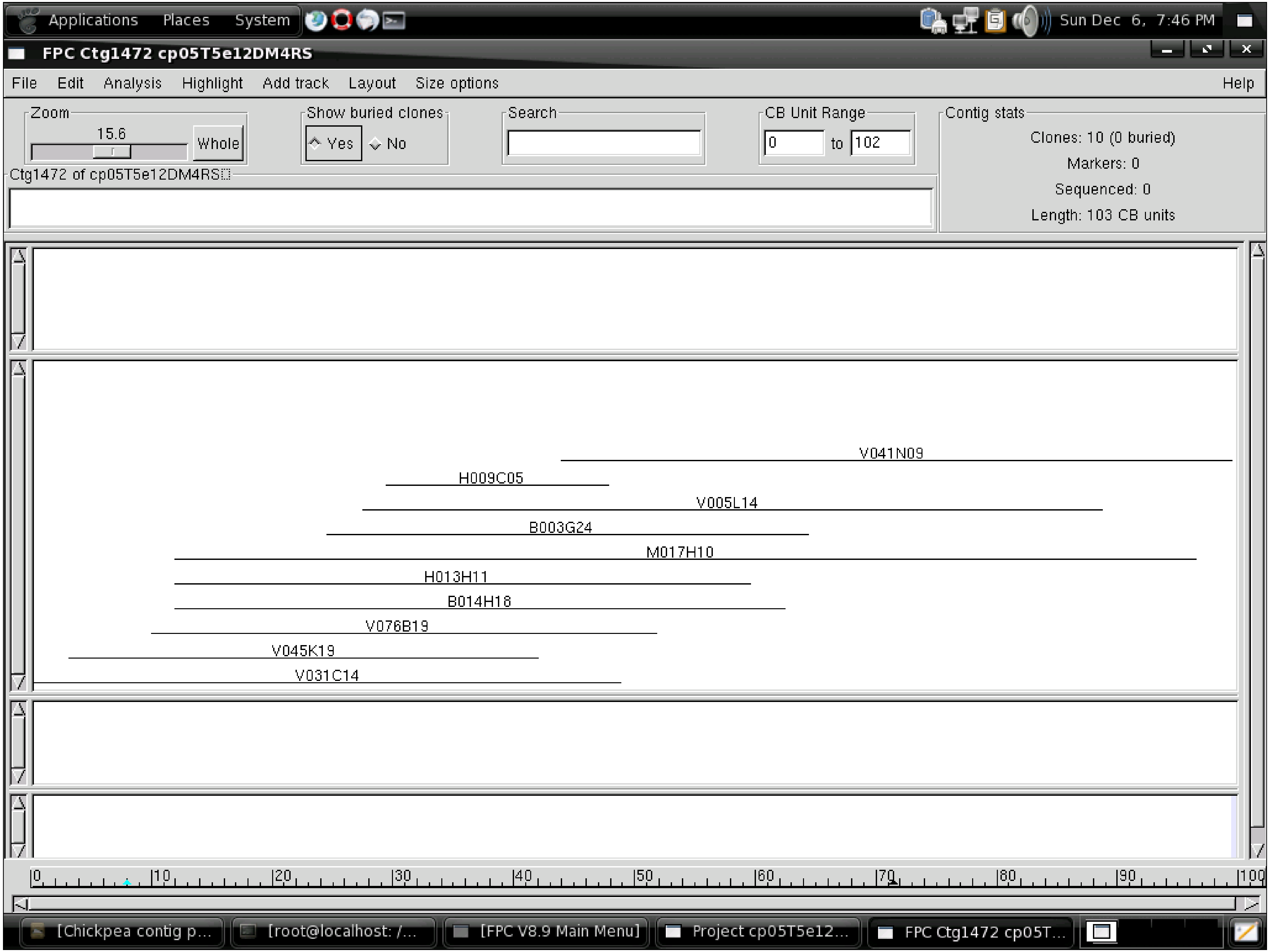
Applications Places System
Sun Dec 6, 7:46 PM
FPC Ctg1472 cp05T5e12DM4RS
File Edit Analysis Highlight Add track Layout Size options Help
Zoom
15.6
Whole
Show buried clones
Yes No
Search
CB Unit Range
0 to 102
Contig stats
Clones: 10 (0 buried)
Markers: 0
Sequenced: 0
Length: 103 CB units
Ctg1472 of cp05T5e12DM4RS
V041N09
H009C05
V005L14
B003G24
M017H10
H013H11
B014H18
V076B19
V045K19
V031C14
0 10 20 30 40 50 60 70 80 90 100
[Chickpea contig p...
[root@localhost: /...
[FPC V8.9 Main Menu]
Project cp05T5e12...
FPC Ctg1472 cp05T...

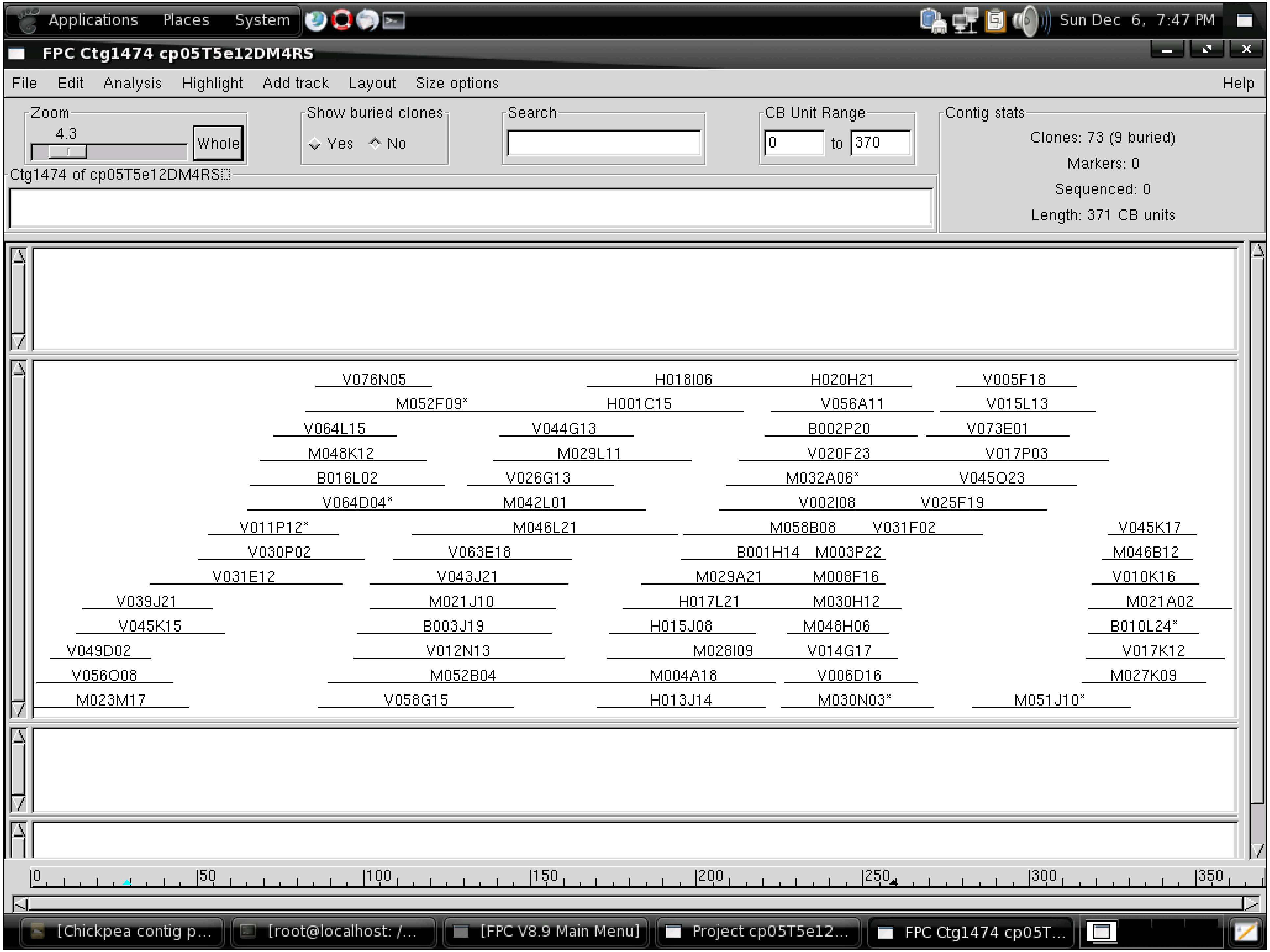
Applications Places System
Sun Dec 6, 7:47 PM
FPC Ctg1474 cp05T5e12DM4RS
File Edit Analysis Highlight Add track Layout Size options Help
Zoom
4.3
Whole
Show buried clones
Yes No
Search
CB Unit Range
0 to 370
Contig stats
Clones: 73 (9 buried)
Markers: 0
Sequenced: 0
Length: 371 CB units
Ctg1474 of cp05T5e12DM4RS
V076N05 H018I06 H020H21 V005F18
M052F09* H001C15 V056A11 V015L13
V064L15 V044G13 B002P20 V073E01
M048K12 M029L11 V020F23 V017P03
B016L02 V026G13 M032A06* V045O23
V064D04* M042L01 V002I08 V025F19
V011P12* M046L21 M058B08 V031F02 V045K17
V030P02 V063E18 B001H14 M003P22 M046B12
V031E12 V043J21 M029A21 M008F16 V010K16
V039J21 M021J10 H017L21 M030H12 M021A02
V045K15 B003J19 H015J08 M048H06 B010L24*
V049D02 V012N13 M028I09 V014G17 V017K12
V056O08 M052B04 M004A18 V006D16 M027K09
M023M17 V058G15 H013J14 M030N03* M051J10*
0 50 100 150 200 250 300 350
[Chickpea contig p...
[root@localhost: /...
[FPC V8.9 Main Menu]
Project cp05T5e12...
FPC Ctg1474 cp05T...

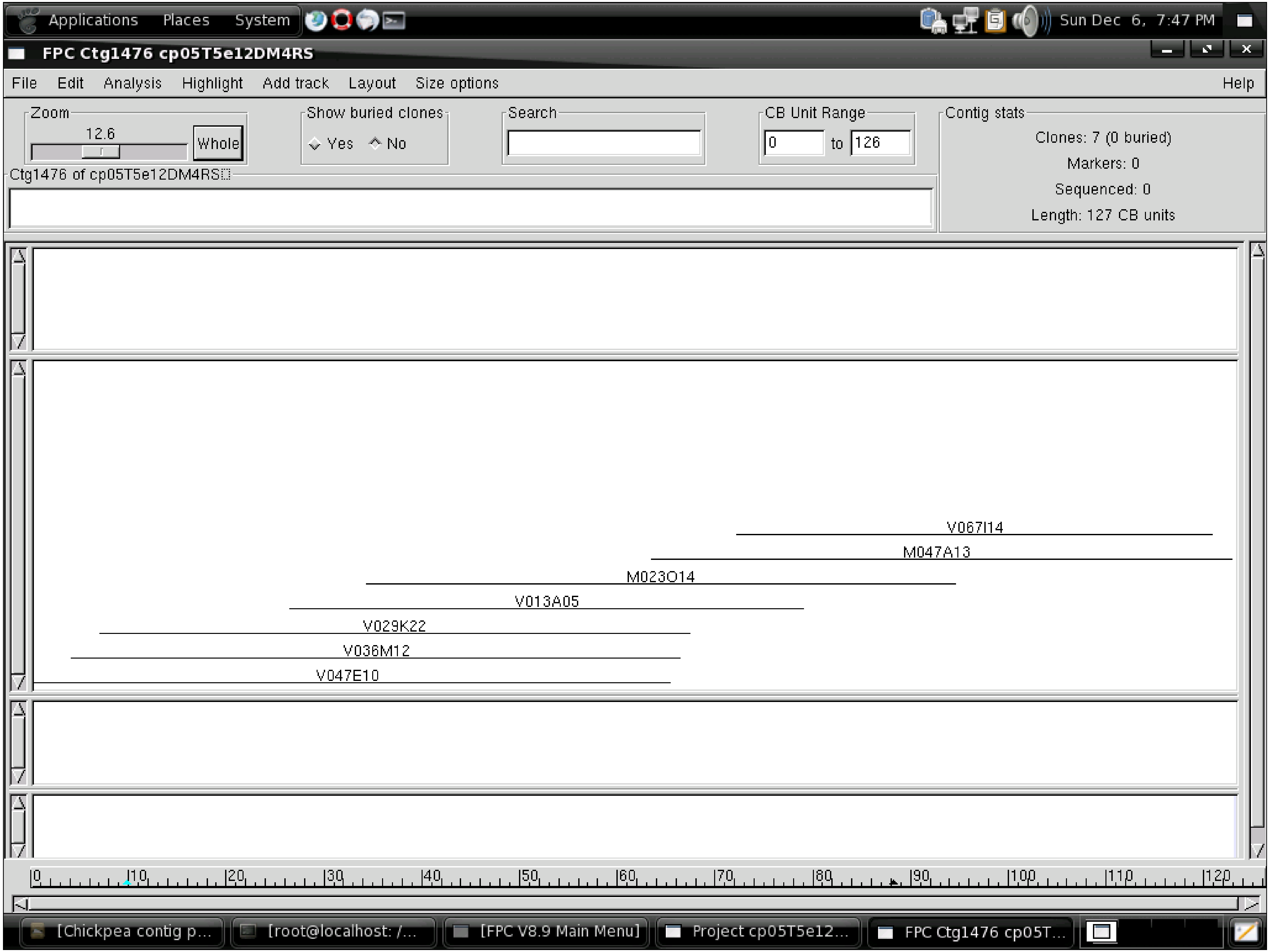
Applications Places System
Sun Dec 6, 7:47 PM
FPC Ctg1476 cp05T5e12DM4RS
File Edit Analysis Highlight Add track Layout Size options Help
Zoom
12.6
Whole
Show buried clones
Yes No
Search
CB Unit Range
0 to 126
Contig stats
Clones: 7 (0 buried)
Markers: 0
Sequenced: 0
Length: 127 CB units
Ctg1476 of cp05T5e12DM4RS
V067I14
M047A13
M023O14
V013A05
V029K22
V036M12
V047E10
0 10 20 30 40 50 60 70 80 90 100 110 120
[Chickpea contig p...
[root@localhost: /...
[FPC V8.9 Main Menu]
Project cp05T5e12...
FPC Ctg1476 cp05T...

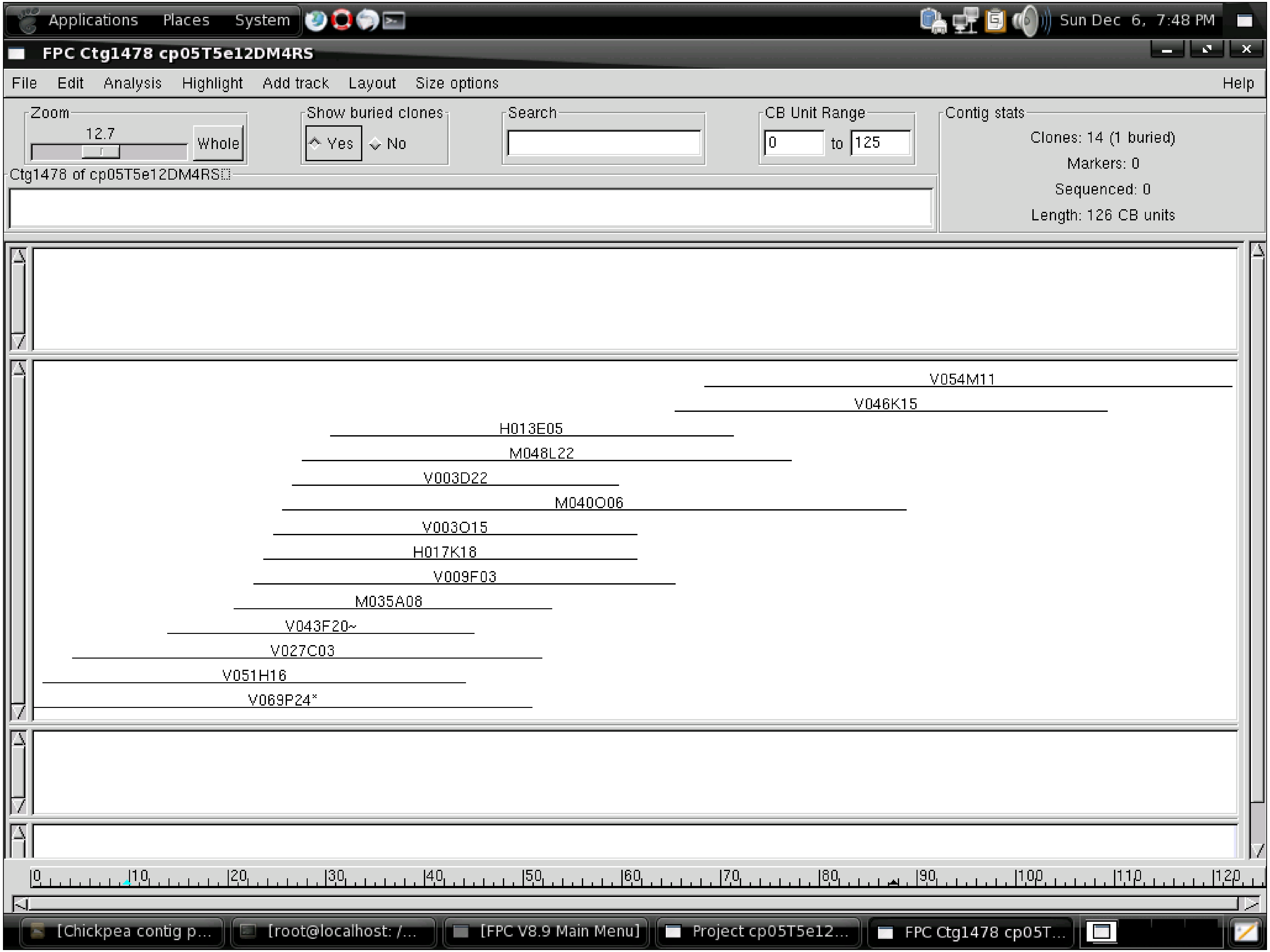
Applications Places System
Sun Dec 6, 7:48 PM
FPC Ctg1478 cp05T5e12DM4RS
File Edit Analysis Highlight Add track Layout Size options Help
Zoom
12.7
Whole
Show buried clones
Yes
No
Search
CB Unit Range
0
to
125
Contig stats
Clones: 14 (1 buried)
Markers: 0
Sequenced: 0
Length: 126 CB units
Ctg1478 of cp05T5e12DM4RS
V054M11
V046K15
H013E05
M048L22
V003D22
M040O06
V003O15
H017K18
V009F03
M035A08
V043F20~
V027C03
V051H16
V069P24*
0 10 20 30 40 50 60 70 80 90 100 110 120
[Chickpea contig p...
[root@localhost: /...
[FPC V8.9 Main Menu]
Project cp05T5e12...
FPC Ctg1478 cp05T...

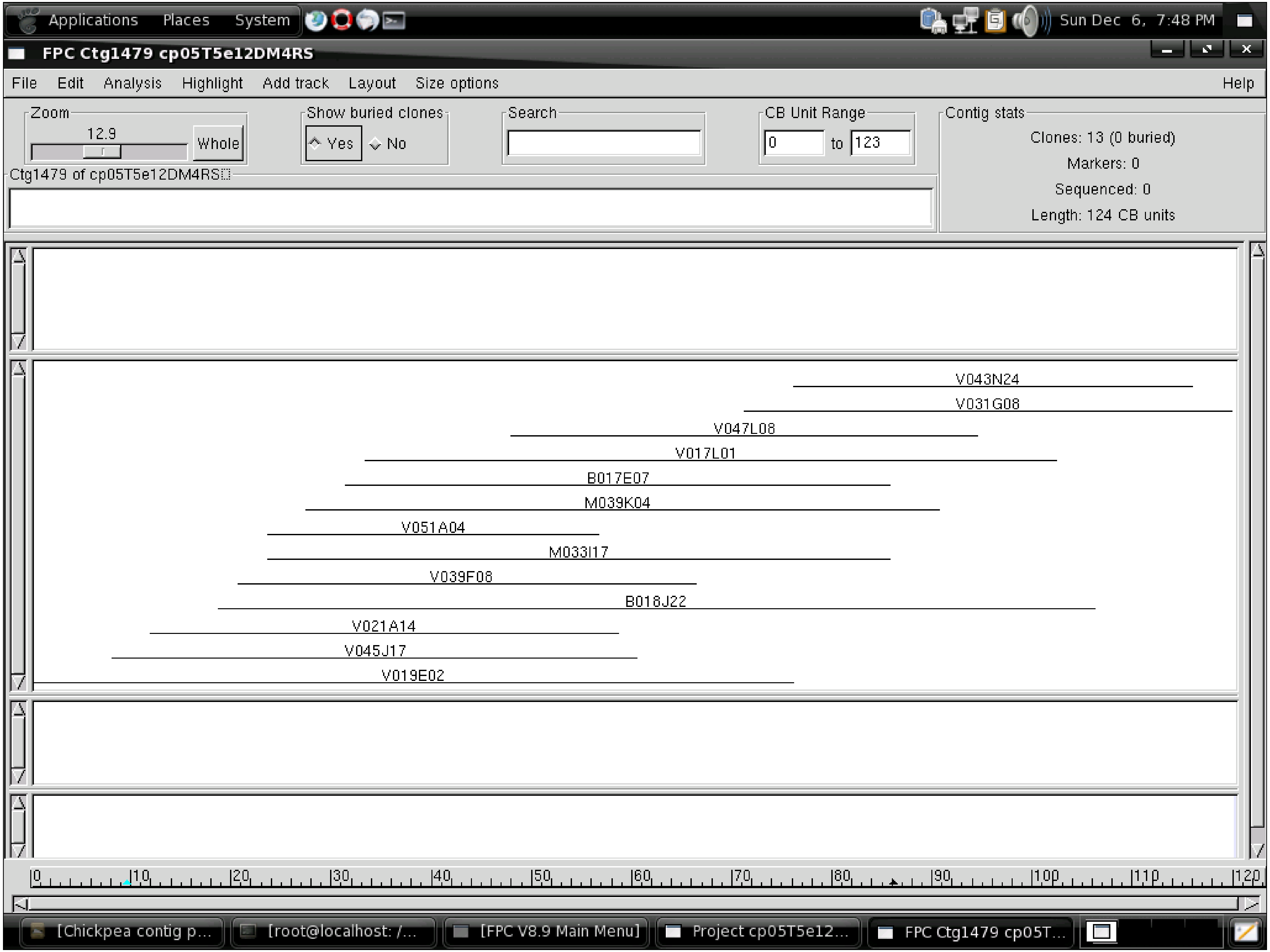
Applications Places System
Sun Dec 6, 7:48 PM
FPC Ctg1479 cp05T5e12DM4RS
File Edit Analysis Highlight Add track Layout Size options Help
Zoom
12.9
Whole
Show buried clones
Yes
No
Search
CB Unit Range
0
to
123
Contig stats
Clones: 13 (0 buried)
Markers: 0
Sequenced: 0
Length: 124 CB units
Ctg1479 of cp05T5e12DM4RS
V043N24
V031G08
V047L08
V017L01
B017E07
M039K04
V051A04
M033I17
V039F08
B018J22
V021A14
V045J17
V019E02
0 10 20 30 40 50 60 70 80 90 100 110 120
[Chickpea contig p...
[root@localhost: /...
[FPC V8.9 Main Menu]
Project cp05T5e12...
FPC Ctg1479 cp05T...

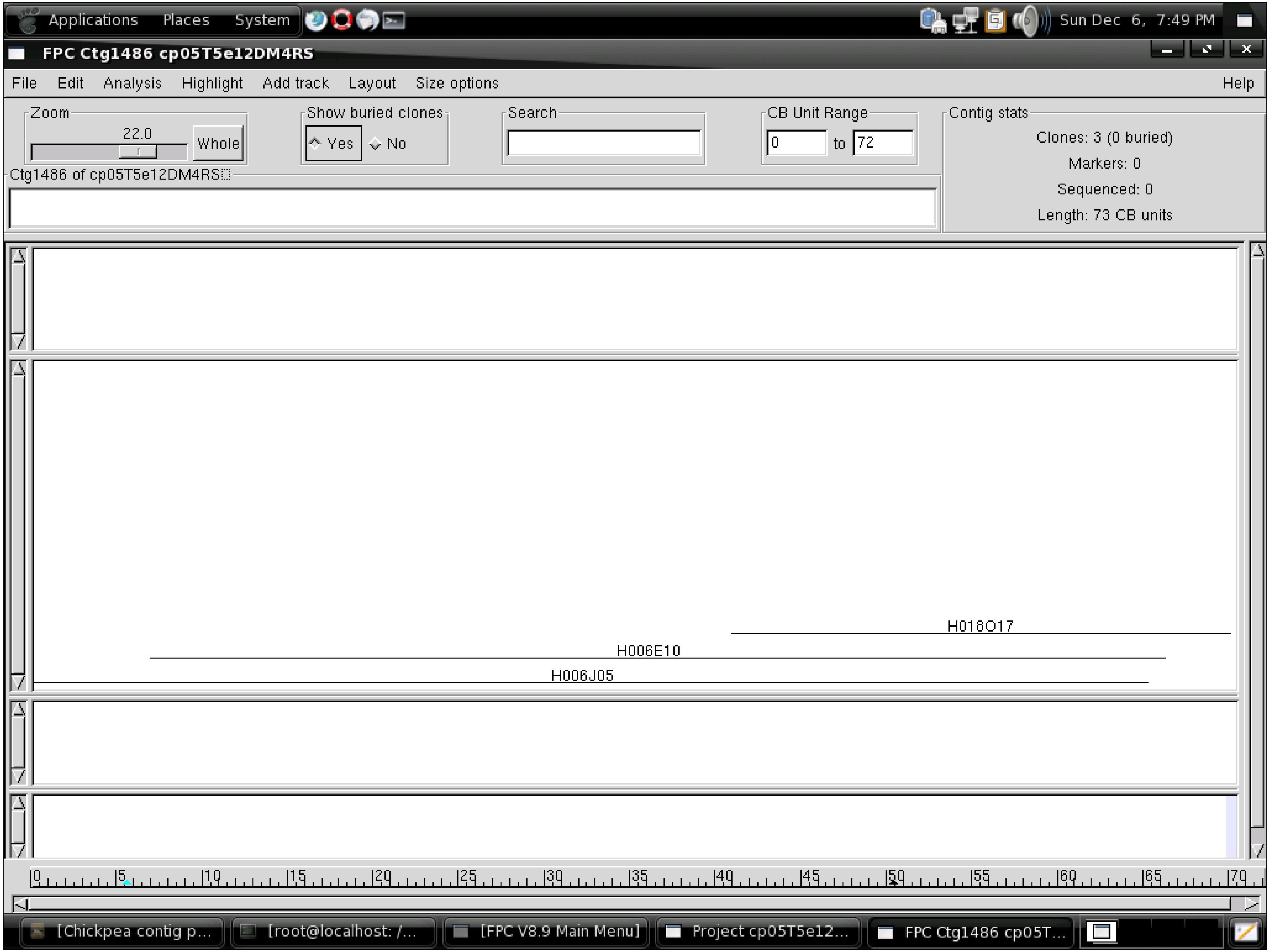
Applications Places System
Sun Dec 6, 7:49 PM
FPC Ctg1486 cp05T5e12DM4RS
File Edit Analysis Highlight Add track Layout Size options Help
Zoom
22.0
Whole
Show buried clones
Yes No
Search
CB Unit Range
0 to 72
Contig stats
Clones: 3 (0 buried)
Markers: 0
Sequenced: 0
Length: 73 CB units
Ctg1486 of cp05T5e12DM4RS
H018O17
H006E10
H006J05
0 5 10 15 20 25 30 35 40 45 50 55 60 65 70
[Chickpea contig p...
[root@localhost: /...
[FPC V8.9 Main Menu]
Project cp05T5e12...
FPC Ctg1486 cp05T...

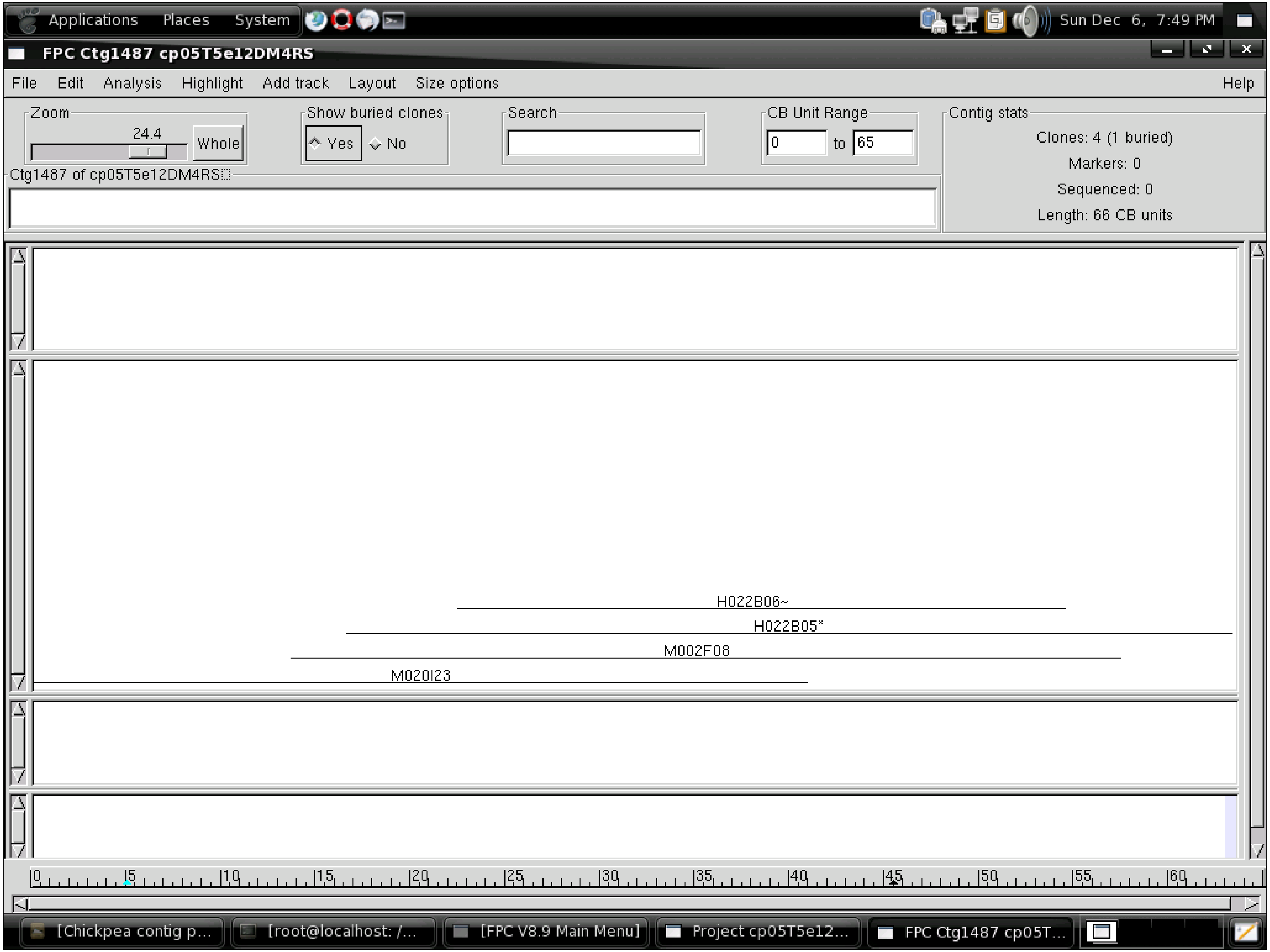
Applications Places System
Sun Dec 6, 7:49 PM
FPC Ctg1487 cp05T5e12DM4RS
File Edit Analysis Highlight Add track Layout Size options Help
Zoom
24.4
Whole
Show buried clones
Yes No
Search
CB Unit Range
0 to 65
Contig stats
Clones: 4 (1 buried)
Markers: 0
Sequenced: 0
Length: 66 CB units
Ctg1487 of cp05T5e12DM4RS
H022B06~
H022B05*
M002F08
M020I23
0 5 10 15 20 25 30 35 40 45 50 55 60
[Chickpea contig p...
[root@localhost: /...
[FPC V8.9 Main Menu]
Project cp05T5e12...
FPC Ctg1487 cp05T...

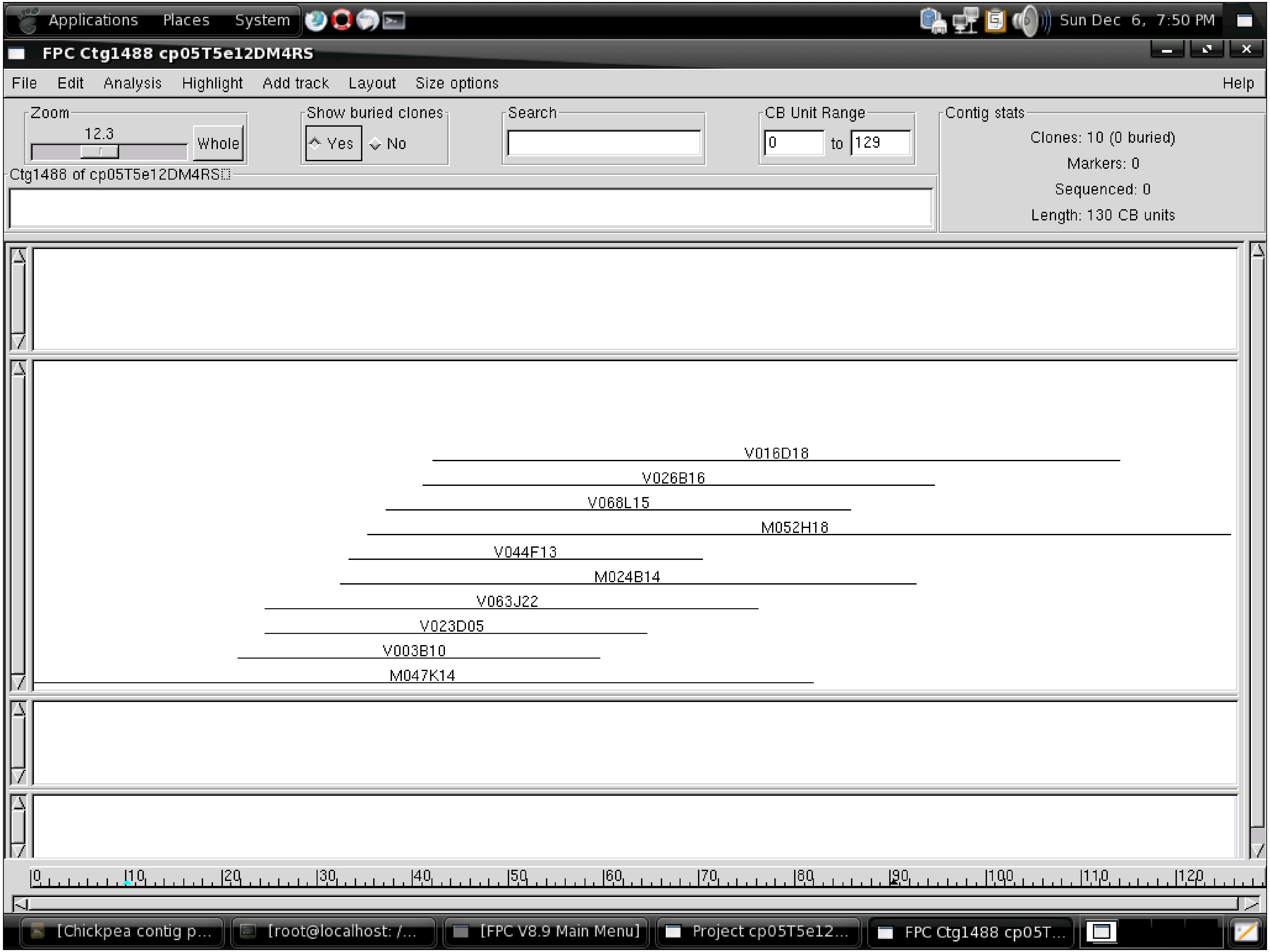
Applications
Places
System
Sun Dec 6, 7:50 PM
FPC Ctg1488 cp05T5e12DM4RS
File
Edit
Analysis
Highlight
Add track
Layout
Size options
Help
Zoom
12.3
Whole
Show buried clones
Yes
No
Search
CB Unit Range
0
to
129
Contig stats
Clones: 10 (0 buried)
Markers: 0
Sequenced: 0
Length: 130 CB units
Ctg1488 of cp05T5e12DM4RS
V016D18
V026B16
V068L15
M052H18
V044F13
M024B14
V063J22
V023D05
V003B10
M047K14
0
10
20
30
40
50
60
70
80
90
100
110
120
[Chickpea contig p...
[root@localhost: /...
[FPC V8.9 Main Menu]
Project cp05T5e12...
FPC Ctg1488 cp05T...

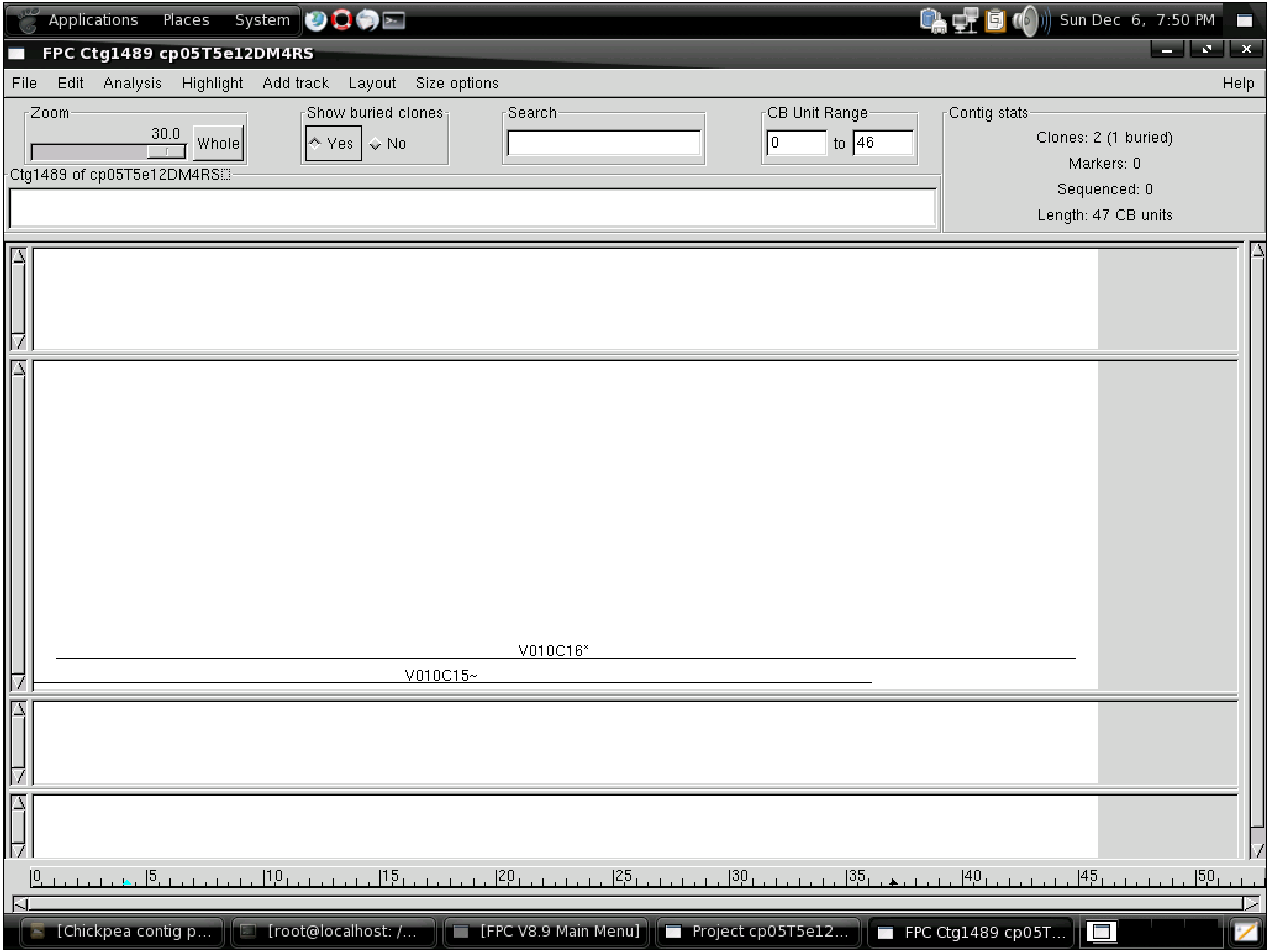

Applications Places System
Sun Dec 6, 7:50 PM
FPC Ctg1489 cp05T5e12DM4RS
File Edit Analysis Highlight Add track Layout Size options Help
Zoom
30.0
Whole
Show buried clones
Yes No
Search
CB Unit Range
0 to 46
Contig stats
Clones: 2 (1 buried)
Markers: 0
Sequenced: 0
Length: 47 CB units
Ctg1489 of cp05T5e12DM4RS
V010C16*
V010C15~
0 5 10 15 20 25 30 35 40 45 50
[Chickpea contig p...
[root@localhost: /...
[FPC V8.9 Main Menu]
Project cp05T5e12...
FPC Ctg1489 cp05T...

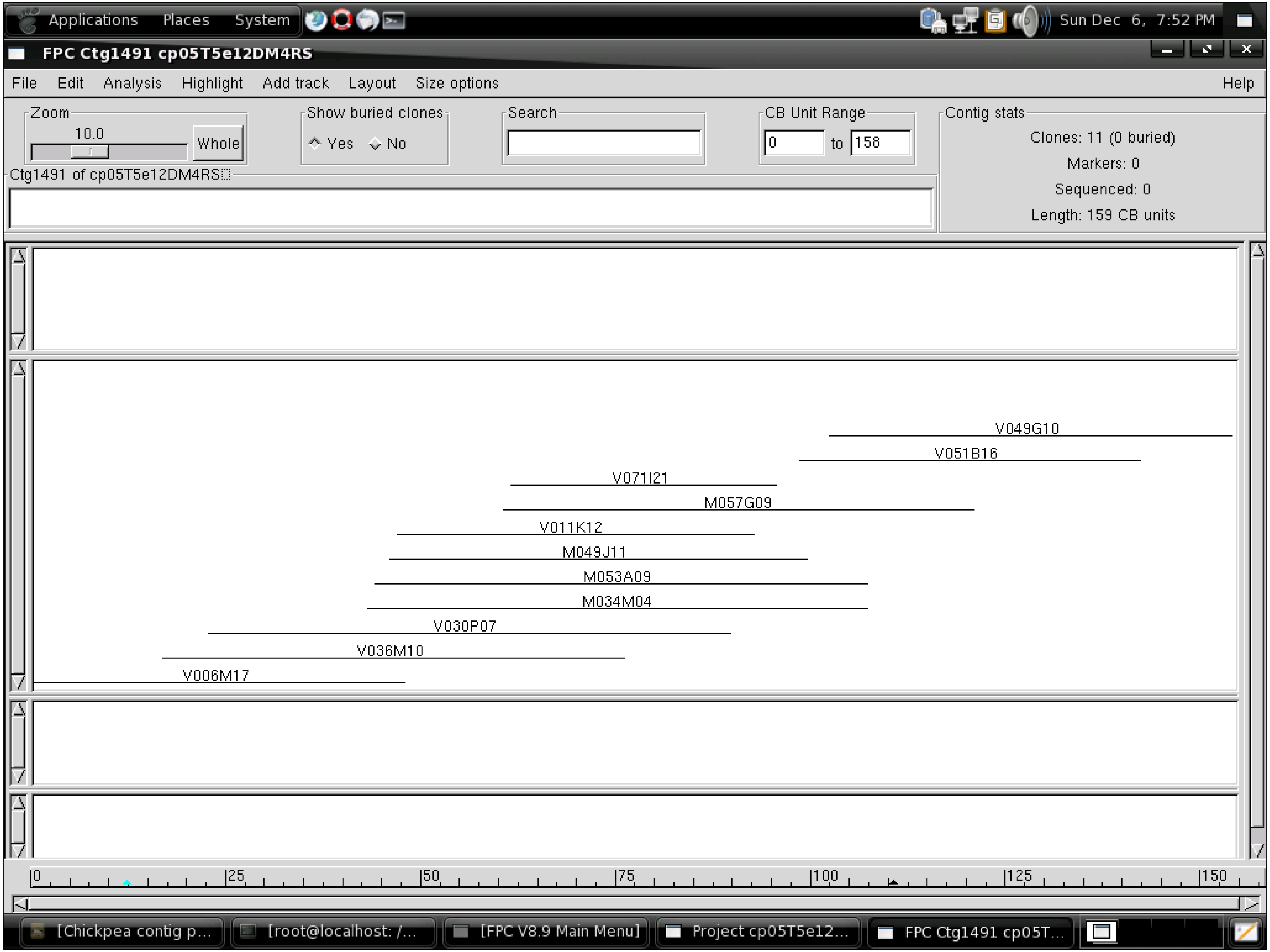
Applications Places System
Sun Dec 6, 7:52 PM
FPC Ctg1491 cp05T5e12DM4RS
File Edit Analysis Highlight Add track Layout Size options Help
Zoom
10.0
Whole
Show buried clones
Yes No
Search
CB Unit Range
0 to 158
Contig stats
Clones: 11 (0 buried)
Markers: 0
Sequenced: 0
Length: 159 CB units
Ctg1491 of cp05T5e12DM4RS
V049G10
V051B16
V071I21
M057G09
V011K12
M049J11
M053A09
M034M04
V030P07
V036M10
V006M17
0 25 50 75 100 125 150
[Chickpea contig p...
[root@localhost: /...
[FPC V8.9 Main Menu]
Project cp05T5e12...
FPC Ctg1491 cp05T...

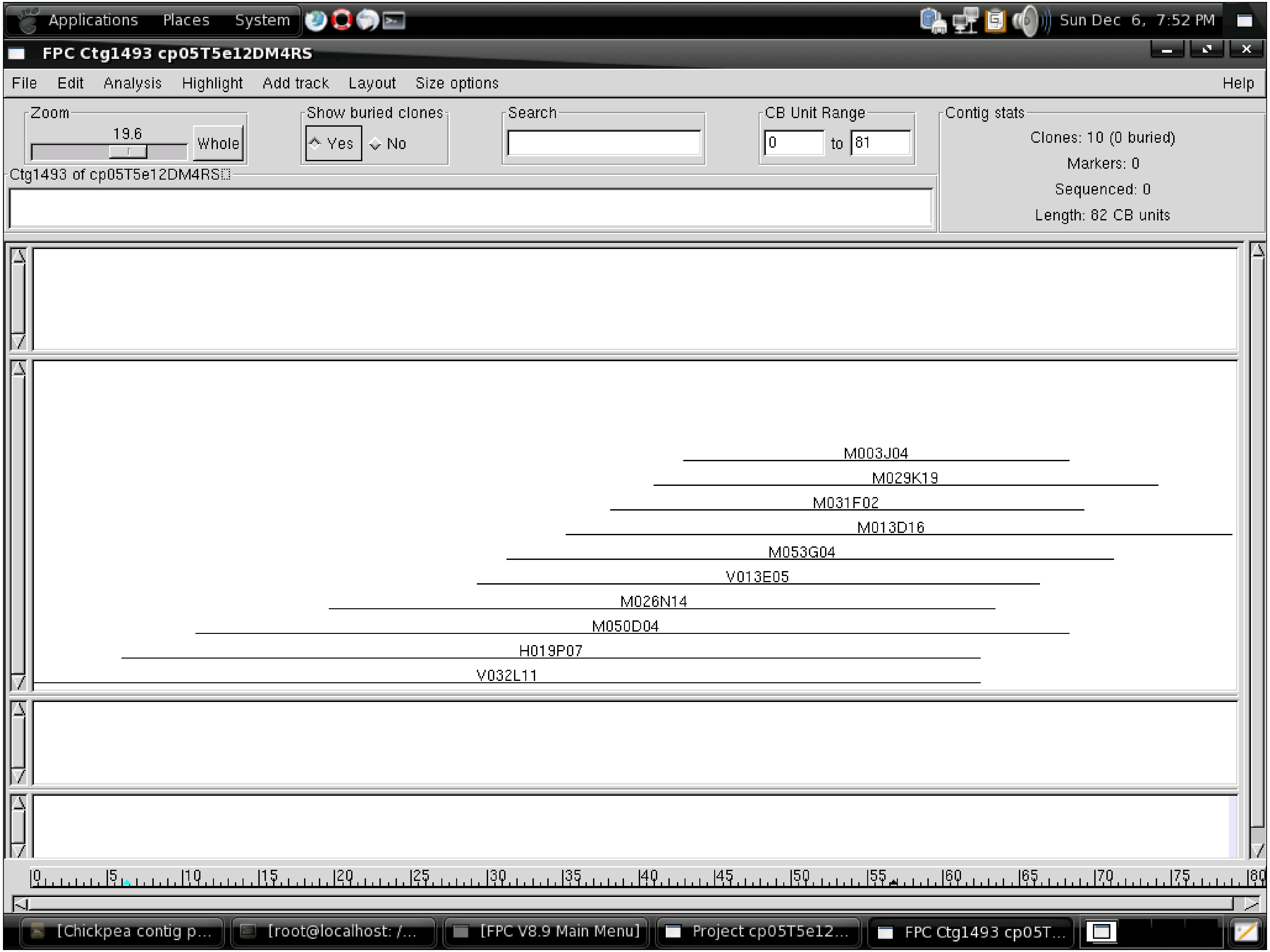
Applications Places System
Sun Dec 6, 7:52 PM
FPC Ctg1493 cp05T5e12DM4RS
File Edit Analysis Highlight Add track Layout Size options Help
Zoom
19.6
Whole
Show buried clones
Yes No
Search
CB Unit Range
0 to 81
Contig stats
Clones: 10 (0 buried)
Markers: 0
Sequenced: 0
Length: 82 CB units
Ctg1493 of cp05T5e12DM4RS
M003J04
M029K19
M031F02
M013D16
M053G04
V013E05
M026N14
M050D04
H019P07
V032L11
0 5 10 15 20 25 30 35 40 45 50 55 60 65 70 75 80
[Chickpea contig p...
[root@localhost: /...
[FPC V8.9 Main Menu]
Project cp05T5e12...
FPC Ctg1493 cp05T...

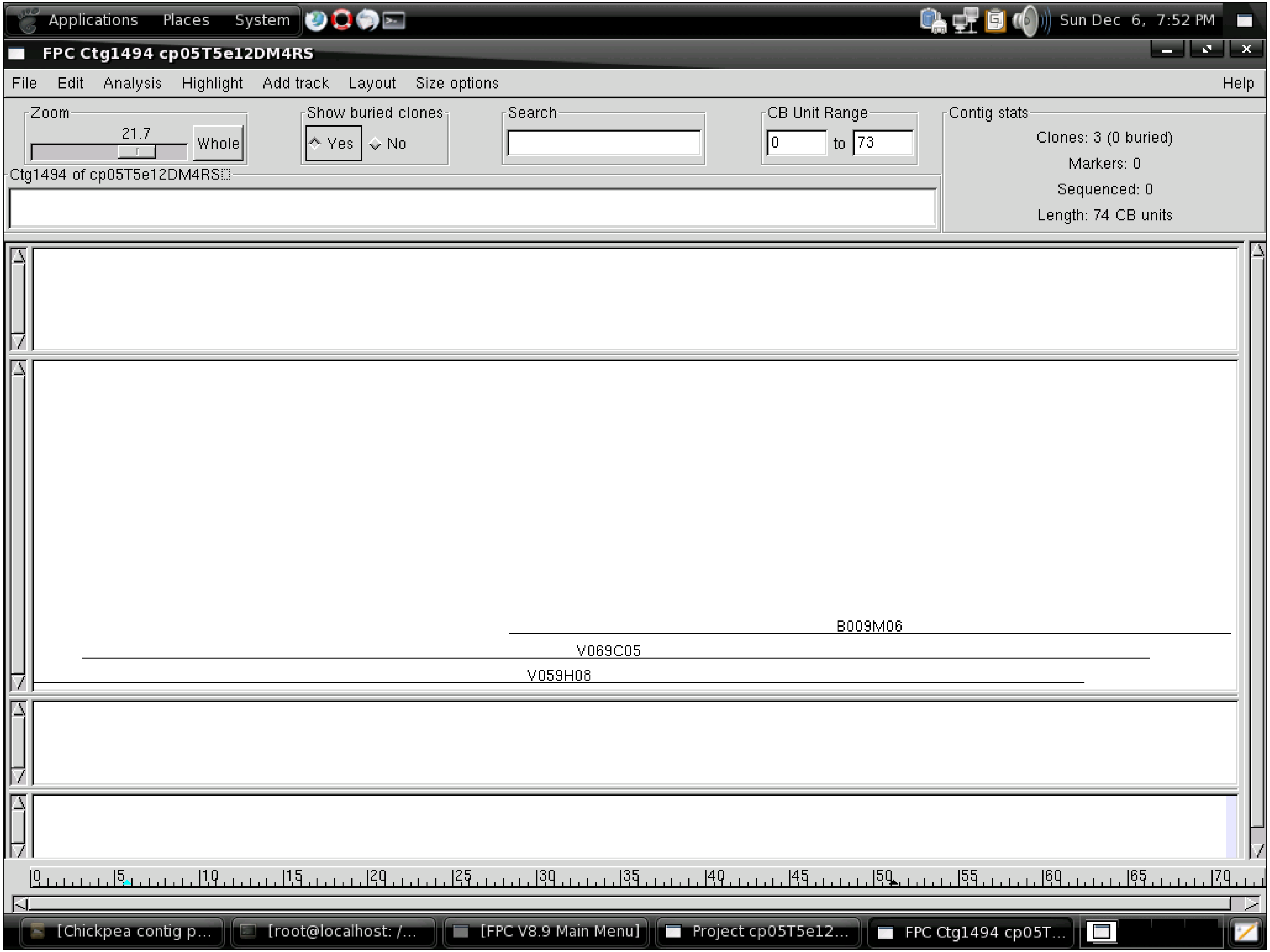
Applications Places System
Sun Dec 6, 7:52 PM
FPC Ctg1494 cp05T5e12DM4RS
File Edit Analysis Highlight Add track Layout Size options Help
Zoom
21.7
Whole
Show buried clones
Yes No
Search
CB Unit Range
0 to 73
Contig stats
Clones: 3 (0 buried)
Markers: 0
Sequenced: 0
Length: 74 CB units
Ctg1494 of cp05T5e12DM4RS
B009M06
V069C05
V059H08
0 5 10 15 20 25 30 35 40 45 50 55 60 65 70
[Chickpea contig p...
[root@localhost: /...
[FPC V8.9 Main Menu]
Project cp05T5e12...
FPC Ctg1494 cp05T...

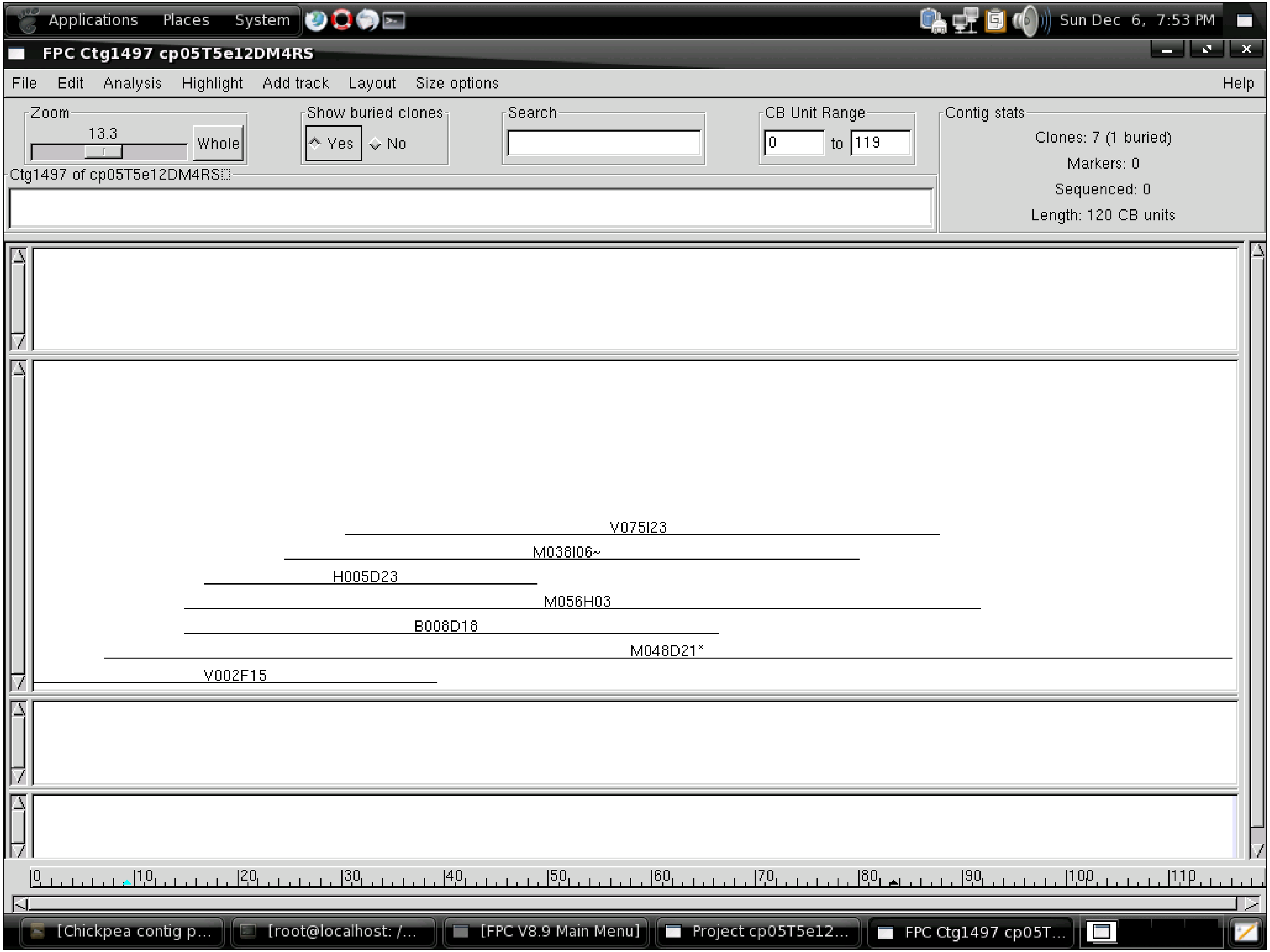
Applications Places System
Sun Dec 6, 7:53 PM
FPC Ctg1497 cp05T5e12DM4RS
File Edit Analysis Highlight Add track Layout Size options Help
Zoom
13.3
Whole
Show buried clones
Yes
No
Search
CB Unit Range
0
to
119
Contig stats
Clones: 7 (1 buried)
Markers: 0
Sequenced: 0
Length: 120 CB units
Ctg1497 of cp05T5e12DM4RS
V075I23
M038I06~
H005D23
M056H03
B008D18
M048D21*
V002F15
0 10 20 30 40 50 60 70 80 90 100 110
[Chickpea contig p...
[root@localhost: /...
[FPC V8.9 Main Menu]
Project cp05T5e12...
FPC Ctg1497 cp05T...

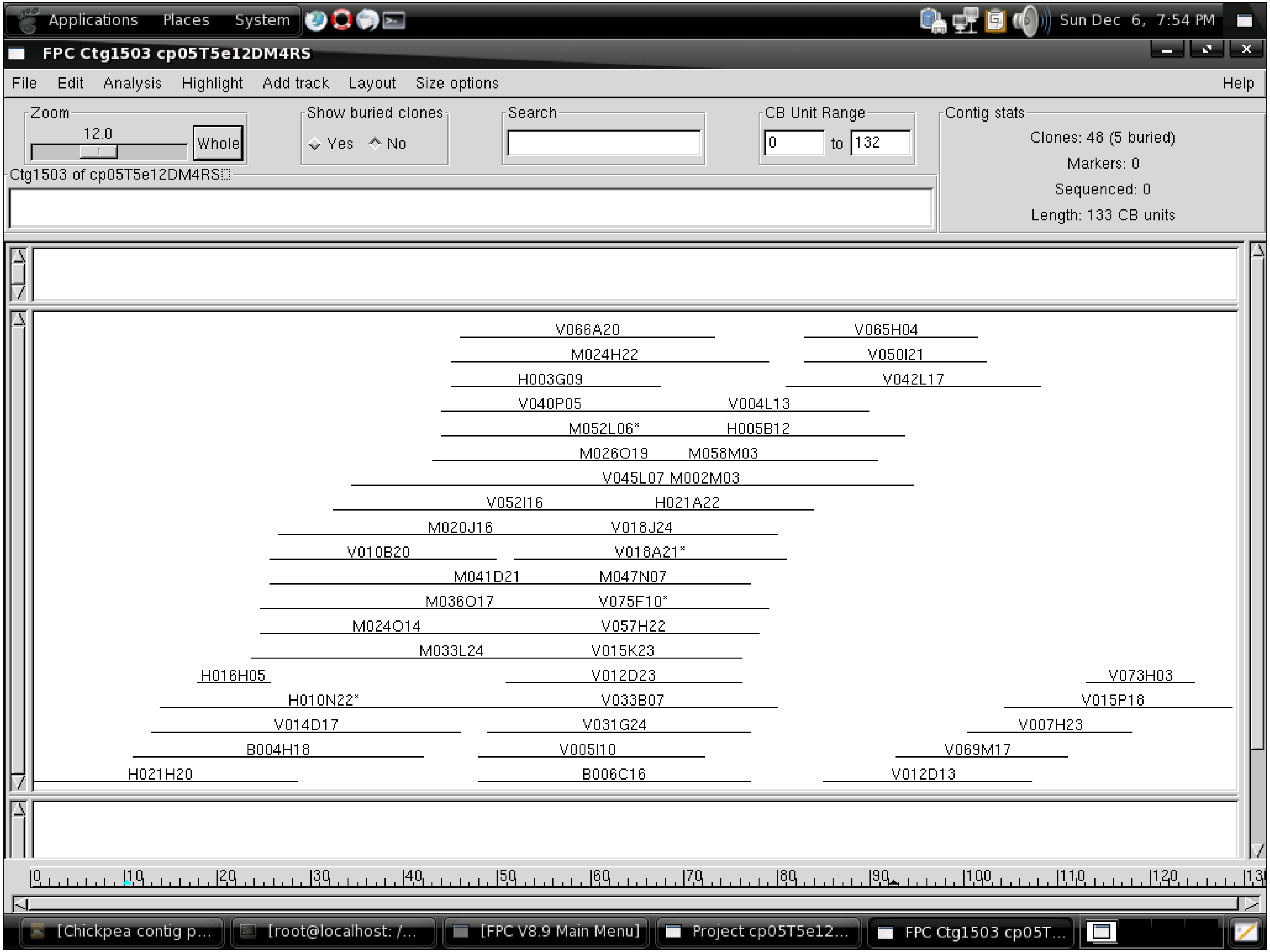
Applications Places System
Sun Dec 6, 7:54 PM
FPC Ctg1503 cp05T5e12DM4RS
File Edit Analysis Highlight Add track Layout Size options Help
Zoom
12.0
Whole
Show buried clones
Yes No
Search
CB Unit Range
0 to 132
Contig stats
Clones: 48 (5 buried)
Markers: 0
Sequenced: 0
Length: 133 CB units
Ctg1503 of cp05T5e12DM4RS
V066A20
V065H04
M024H22
V050I21
H003G09
V042L17
V040P05
V004L13
M052L06*
H005B12
M026O19
M058M03
V045L07 M002M03
V052I16
H021A22
M020J16
V018J24
V010B20
V018A21*
M041D21
M047N07
M036O17
V075F10*
M024O14
V057H22
M033L24
V015K23
H016H05
V012D23
V073H03
H010N22*
V033B07
V015P18
V014D17
V031G24
V007H23
B004H18
V005I10
V069M17
H021H20
B006C16
V012D13
0 10 20 30 40 50 60 70 80 90 100 110 120 13
[Chickpea contig p...
[root@localhost: /...
[FPC V8.9 Main Menu]
Project cp05T5e12...
FPC Ctg1503 cp05T...

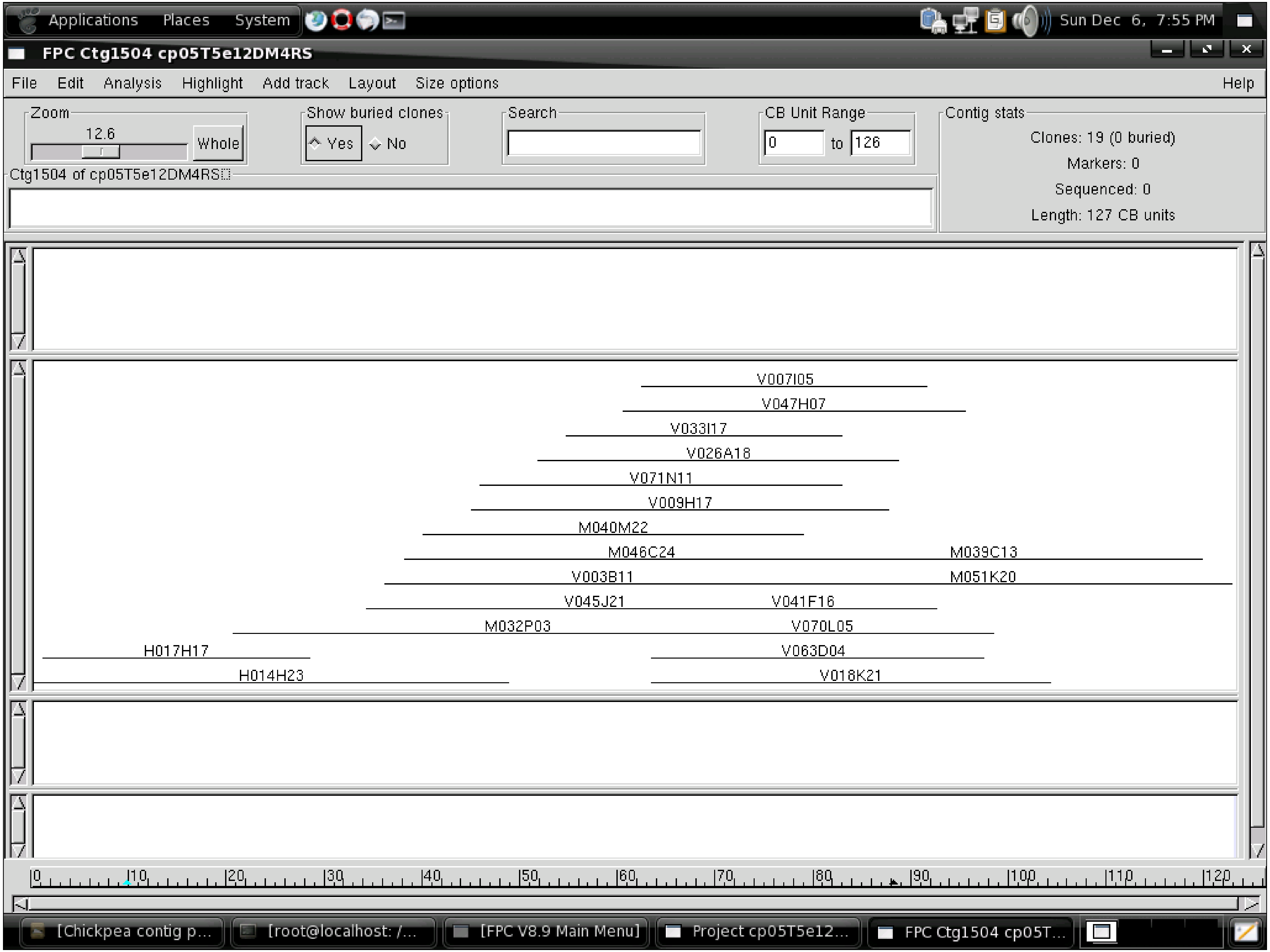
Applications
Places
System
Sun Dec 6, 7:55 PM
FPC Ctg1504 cp05T5e12DM4RS
File
Edit
Analysis
Highlight
Add track
Layout
Size options
Help
Zoom
12.6
Whole
Show buried clones
Yes
No
Search
CB Unit Range
0
to
126
Contig stats
Clones: 19 (0 buried)
Markers: 0
Sequenced: 0
Length: 127 CB units
Ctg1504 of cp05T5e12DM4RS
V007I05
V047H07
V033I17
V026A18
V071N11
V009H17
M040M22
M046C24
M039C13
V003B11
M051K20
V045J21
V041F16
M032P03
V070L05
H017H17
V063D04
H014H23
V018K21
0
10
20
30
40
50
60
70
80
90
100
110
120
[Chickpea contig p...
[root@localhost: /...
[FPC V8.9 Main Menu]
Project cp05T5e12...
FPC Ctg1504 cp05T...

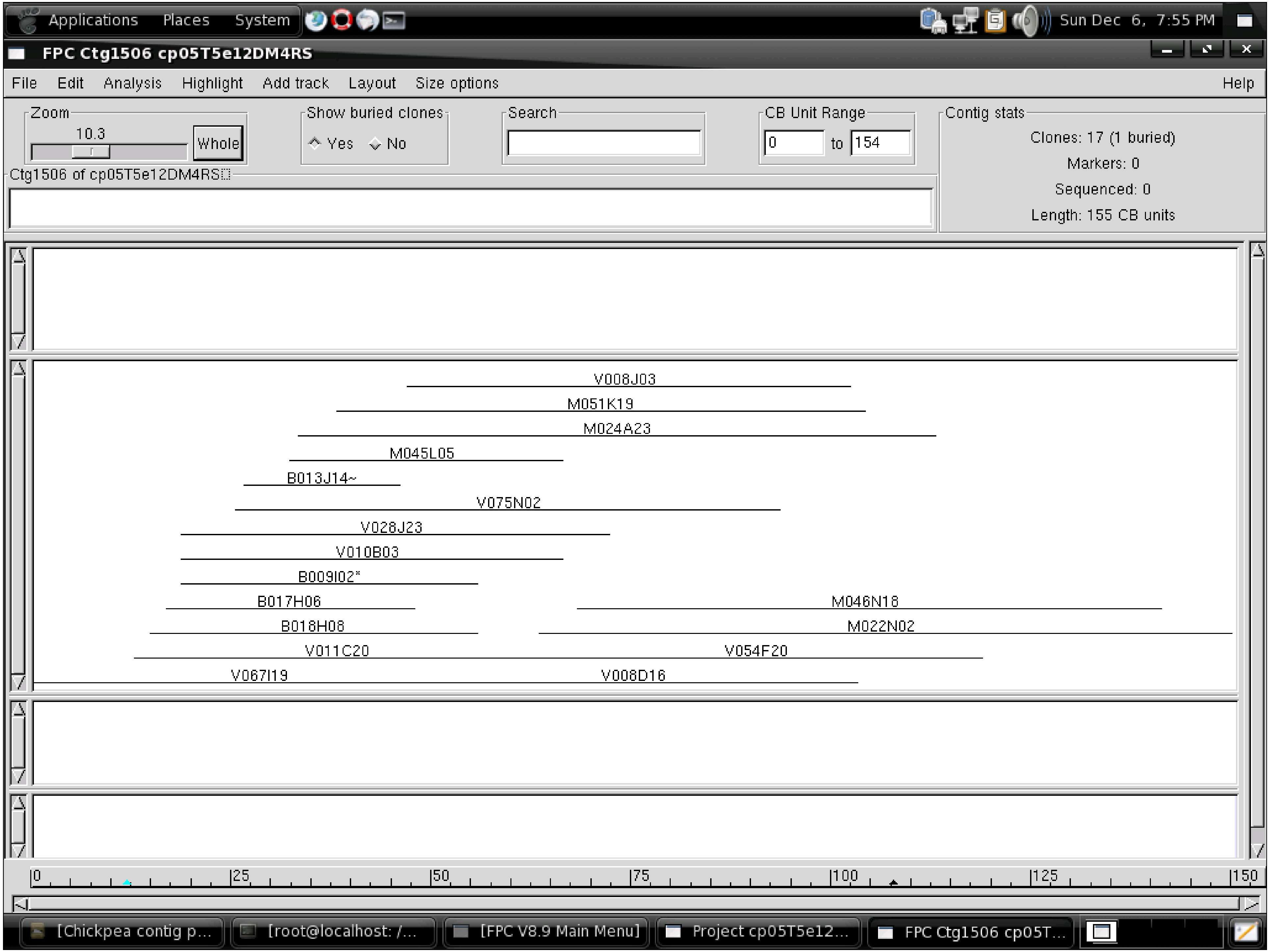
Applications
Places
System
Sun Dec 6, 7:55 PM
FPC Ctg1506 cp05T5e12DM4RS
File
Edit
Analysis
Highlight
Add track
Layout
Size options
Help
Zoom
10.3
Whole
Show buried clones
Yes
No
Search
CB Unit Range
0
to
154
Contig stats
Clones: 17 (1 buried)
Markers: 0
Sequenced: 0
Length: 155 CB units
Ctg1506 of cp05T5e12DM4RS
V008J03
M051K19
M024A23
M045L05
B013J14~
V075N02
V028J23
V010B03
B009I02*
B017H06
M046N18
B018H08
M022N02
V011C20
V054F20
V067I19
V008D16
0
25
50
75
100
125
150
[Chickpea contig p...
[root@localhost: /...
[FPC V8.9 Main Menu]
Project cp05T5e12...
FPC Ctg1506 cp05T...

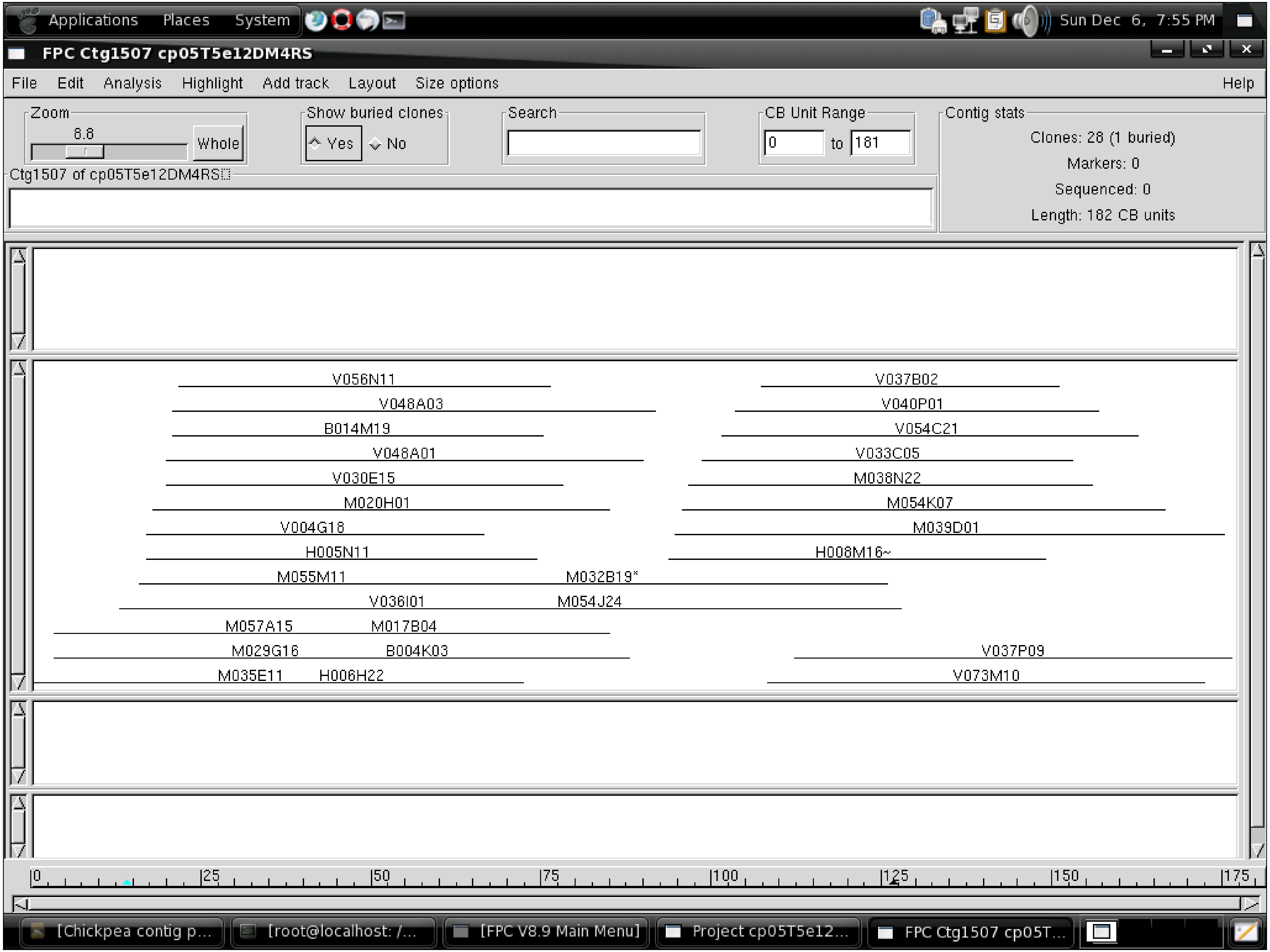

Applications Places System
Sun Dec 6, 7:55 PM
FPC Ctg1507 cp05T5e12DM4RS
File Edit Analysis Highlight Add track Layout Size options Help
Zoom
8.8
Whole
Show buried clones
Yes No
Search
CB Unit Range
0 to 181
Contig stats
Clones: 28 (1 buried)
Markers: 0
Sequenced: 0
Length: 182 CB units
Ctg1507 of cp05T5e12DM4RS
V056N11
V037B02
V048A03
V040P01
B014M19
V054C21
V048A01
V033C05
V030E15
M038N22
M020H01
M054K07
V004G18
M039D01
H005N11
H008M16~
M055M11
M032B19*
V036I01
M054J24
M057A15
M017B04
M029G16
B004K03
V037P09
M035E11
H006H22
V073M10
0 25 50 75 100 125 150 175
[Chickpea contig p...
[root@localhost: /...
[FPC V8.9 Main Menu]
Project cp05T5e12...
FPC Ctg1507 cp05T...

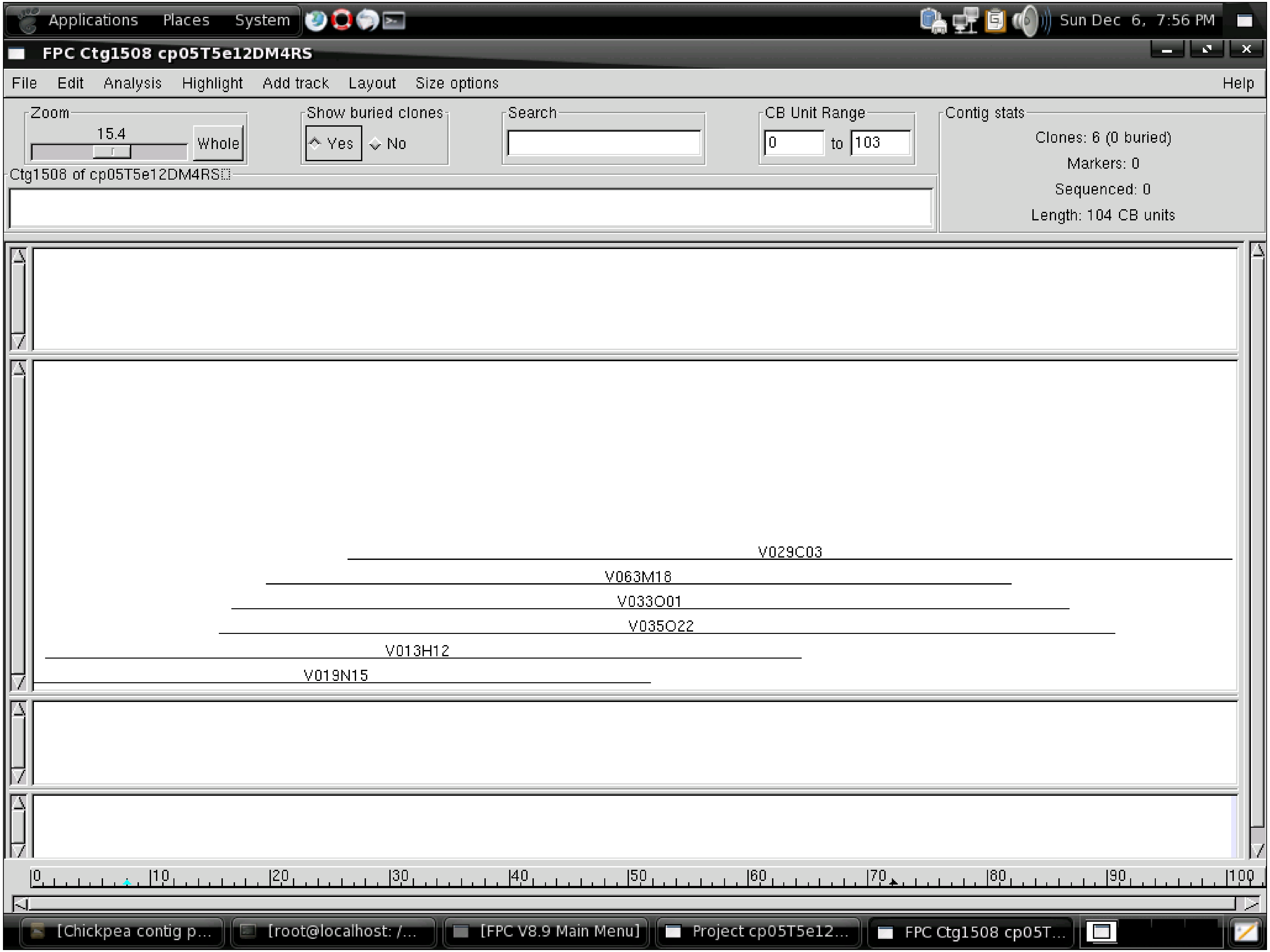
Applications Places System
Sun Dec 6, 7:56 PM
FPC Ctg1508 cp05T5e12DM4RS
File Edit Analysis Highlight Add track Layout Size options Help
Zoom
15.4
Whole
Show buried clones
Yes
No
Search
CB Unit Range
0
to
103
Contig stats
Clones: 6 (0 buried)
Markers: 0
Sequenced: 0
Length: 104 CB units
Ctg1508 of cp05T5e12DM4RS
V029C03
V063M18
V033O01
V035O22
V013H12
V019N15
0 10 20 30 40 50 60 70 80 90 100
[Chickpea contig p...
[root@localhost: /...
[FPC V8.9 Main Menu]
Project cp05T5e12...
FPC Ctg1508 cp05T...

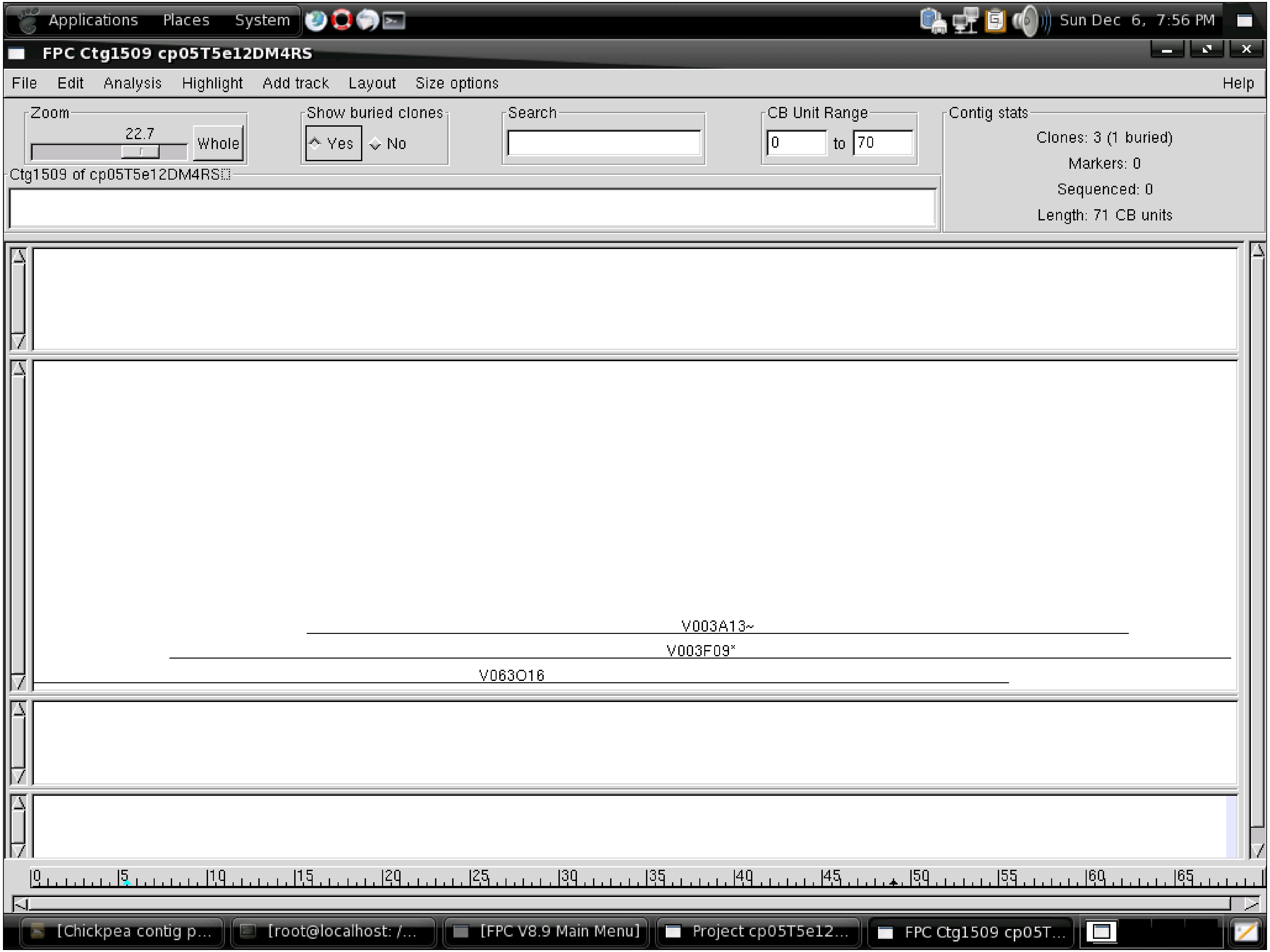

Applications Places System
Sun Dec 6, 7:56 PM
FPC Ctg1509 cp05T5e12DM4RS
File Edit Analysis Highlight Add track Layout Size options Help
Zoom
22.7
Whole
Show buried clones
Yes
No
Search
CB Unit Range
0
to
70
Contig stats
Clones: 3 (1 buried)
Markers: 0
Sequenced: 0
Length: 71 CB units
Ctg1509 of cp05T5e12DM4RS
V003A13~
V003F09*
V063O16
0 5 10 15 20 25 30 35 40 45 50 55 60 65
[Chickpea contig p...
[root@localhost: /...
[FPC V8.9 Main Menu]
Project cp05T5e12...
FPC Ctg1509 cp05T...

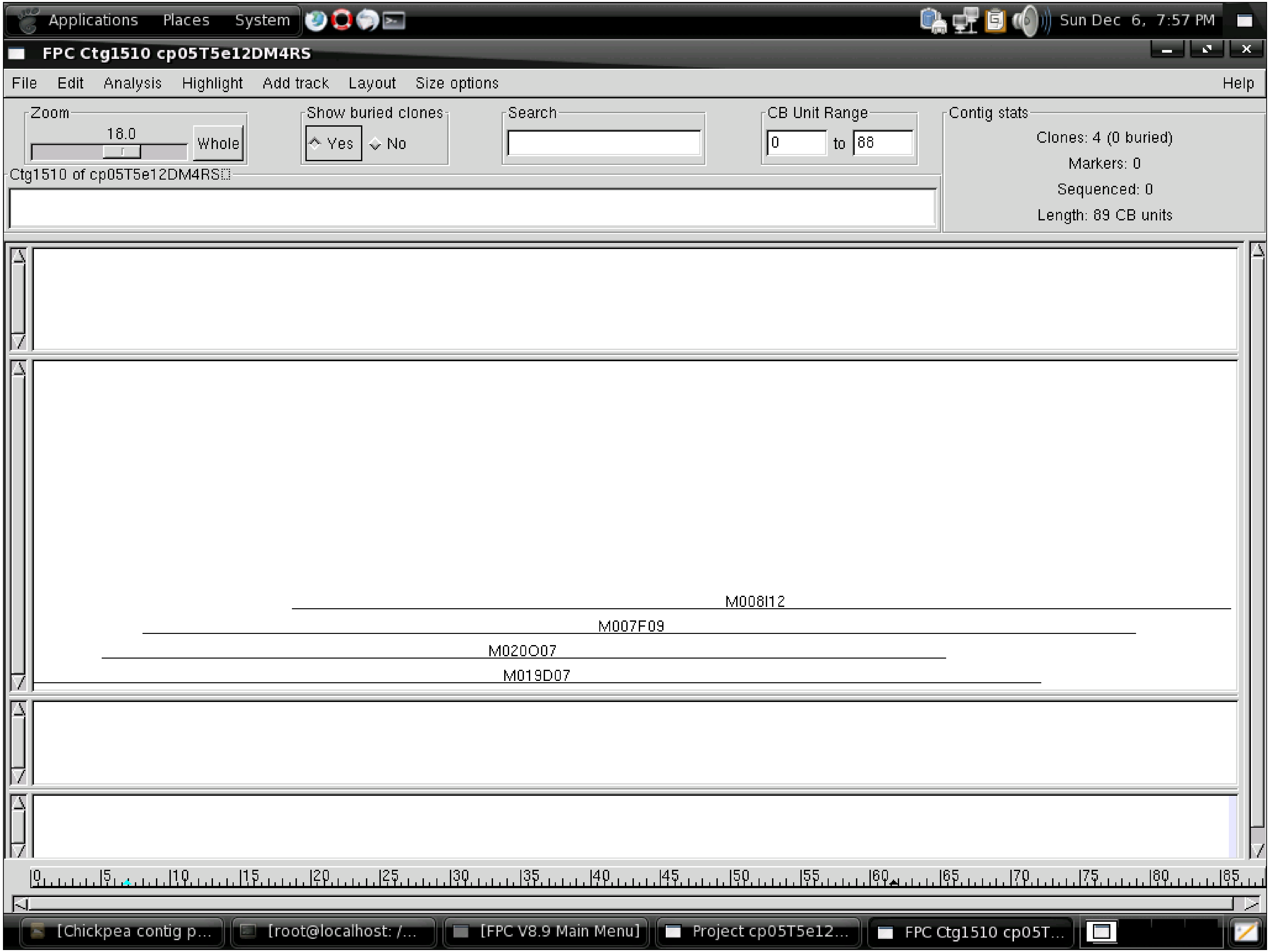
Applications Places System
Sun Dec 6, 7:57 PM
FPC Ctg1510 cp05T5e12DM4RS
File Edit Analysis Highlight Add track Layout Size options Help
Zoom
18.0
Whole
Show buried clones
Yes
No
Search
CB Unit Range
0
to
88
Contig stats
Clones: 4 (0 buried)
Markers: 0
Sequenced: 0
Length: 89 CB units
Ctg1510 of cp05T5e12DM4RS
M008I12
M007F09
M020O07
M019D07
0 5 10 15 20 25 30 35 40 45 50 55 60 65 70 75 80 85
[Chickpea contig p...
[root@localhost: /...
[FPC V8.9 Main Menu]
Project cp05T5e12...
FPC Ctg1510 cp05T...

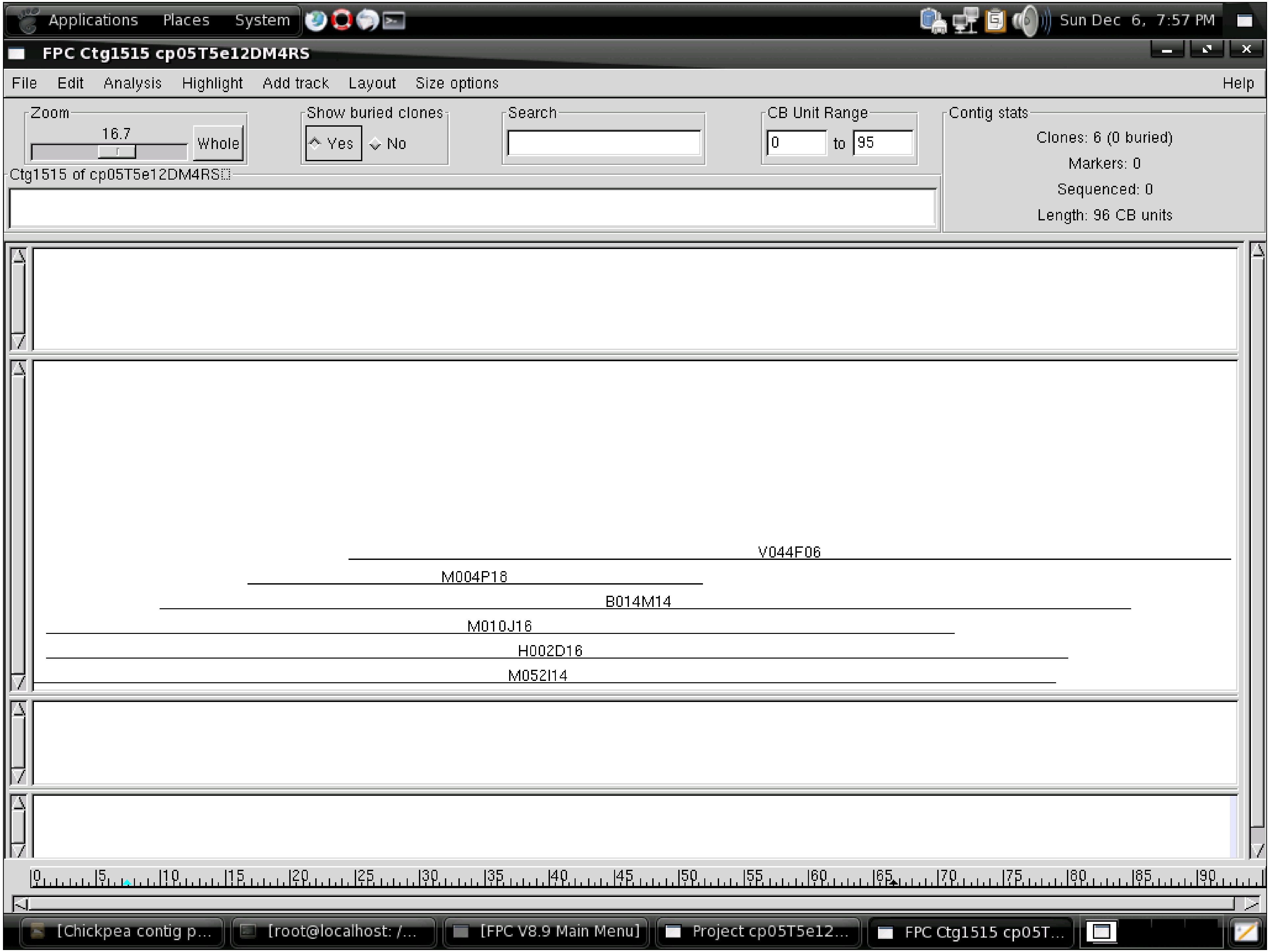
Applications Places System
Sun Dec 6, 7:57 PM
FPC Ctg1515 cp05T5e12DM4RS
File Edit Analysis Highlight Add track Layout Size options Help
Zoom
16.7
Whole
Show buried clones
Yes
No
Search
CB Unit Range
0 to 95
Contig stats
Clones: 6 (0 buried)
Markers: 0
Sequenced: 0
Length: 96 CB units
Ctg1515 of cp05T5e12DM4RS
V044F06
M004P18
B014M14
M010J16
H002D16
M052I14
0 5 10 15 20 25 30 35 40 45 50 55 60 65 70 75 80 85 90
[Chickpea contig p...
[root@localhost: /...
[FPC V8.9 Main Menu]
Project cp05T5e12...
FPC Ctg1515 cp05T...

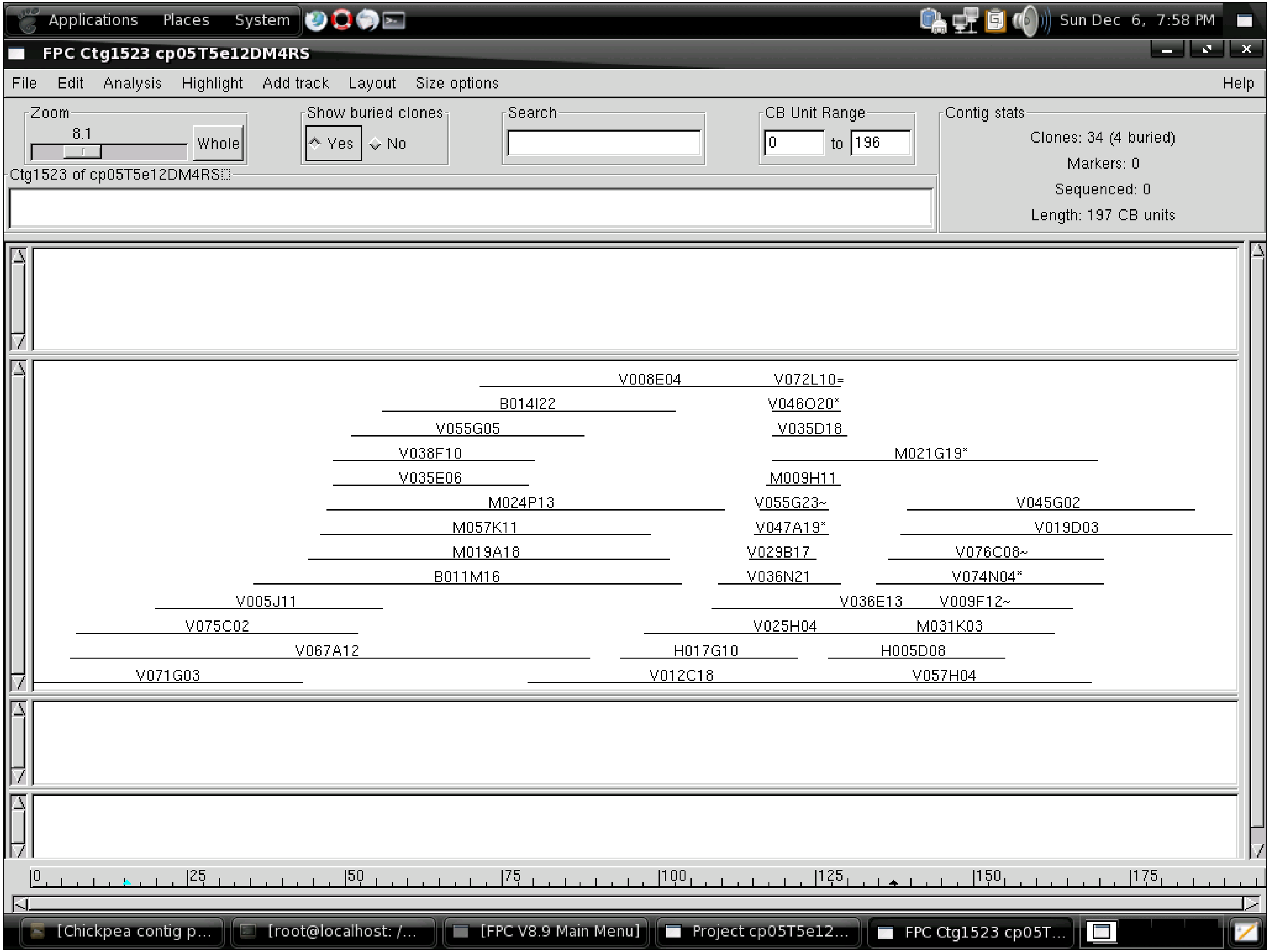
Applications Places System
Sun Dec 6, 7:58 PM
FPC Ctg1523 cp05T5e12DM4RS
File Edit Analysis Highlight Add track Layout Size options Help
Zoom
8.1
Whole
Show buried clones
Yes
No
Search
CB Unit Range
0 to 196
Contig stats
Clones: 34 (4 buried)
Markers: 0
Sequenced: 0
Length: 197 CB units
Ctg1523 of cp05T5e12DM4RS
V008E04
V072L10=
B014I22
V046O20*
V055G05
V035D18
V038F10
M021G19*
V035E06
M009H11
M024P13
V055G23~
V045G02
M057K11
V047A19*
V019D03
M019A18
V029B17
V076C08~
B011M16
V036N21
V074N04*
V005J11
V036E13
V009F12~
V075C02
V025H04
M031K03
V067A12
H017G10
H005D08
V071G03
V012C18
V057H04
0 25 50 75 100 125 150 175
[Chickpea contig p...
[root@localhost: /...
[FPC V8.9 Main Menu]
Project cp05T5e12...
FPC Ctg1523 cp05T...

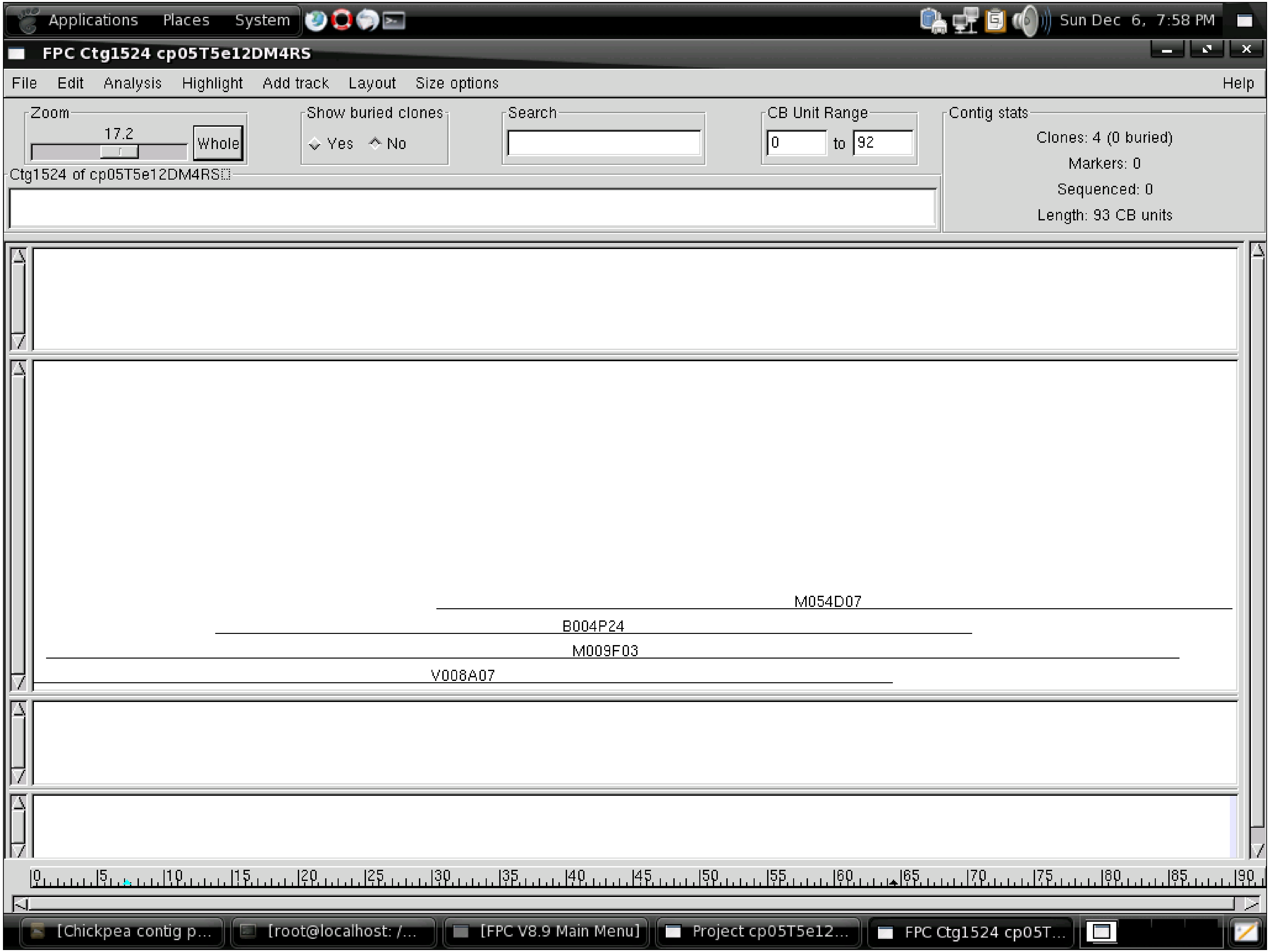

Applications Places System
Sun Dec 6, 7:58 PM
FPC Ctg1524 cp05T5e12DM4RS
File Edit Analysis Highlight Add track Layout Size options Help
Zoom
17.2
Whole
Show buried clones
Yes No
Search
CB Unit Range
0 to 92
Contig stats
Clones: 4 (0 buried)
Markers: 0
Sequenced: 0
Length: 93 CB units
Ctg1524 of cp05T5e12DM4RS
M054D07
B004P24
M009F03
V008A07
0 5 10 15 20 25 30 35 40 45 50 55 60 65 70 75 80 85 90
[Chickpea contig p...
[root@localhost: /...
[FPC V8.9 Main Menu]
Project cp05T5e12...
FPC Ctg1524 cp05T...

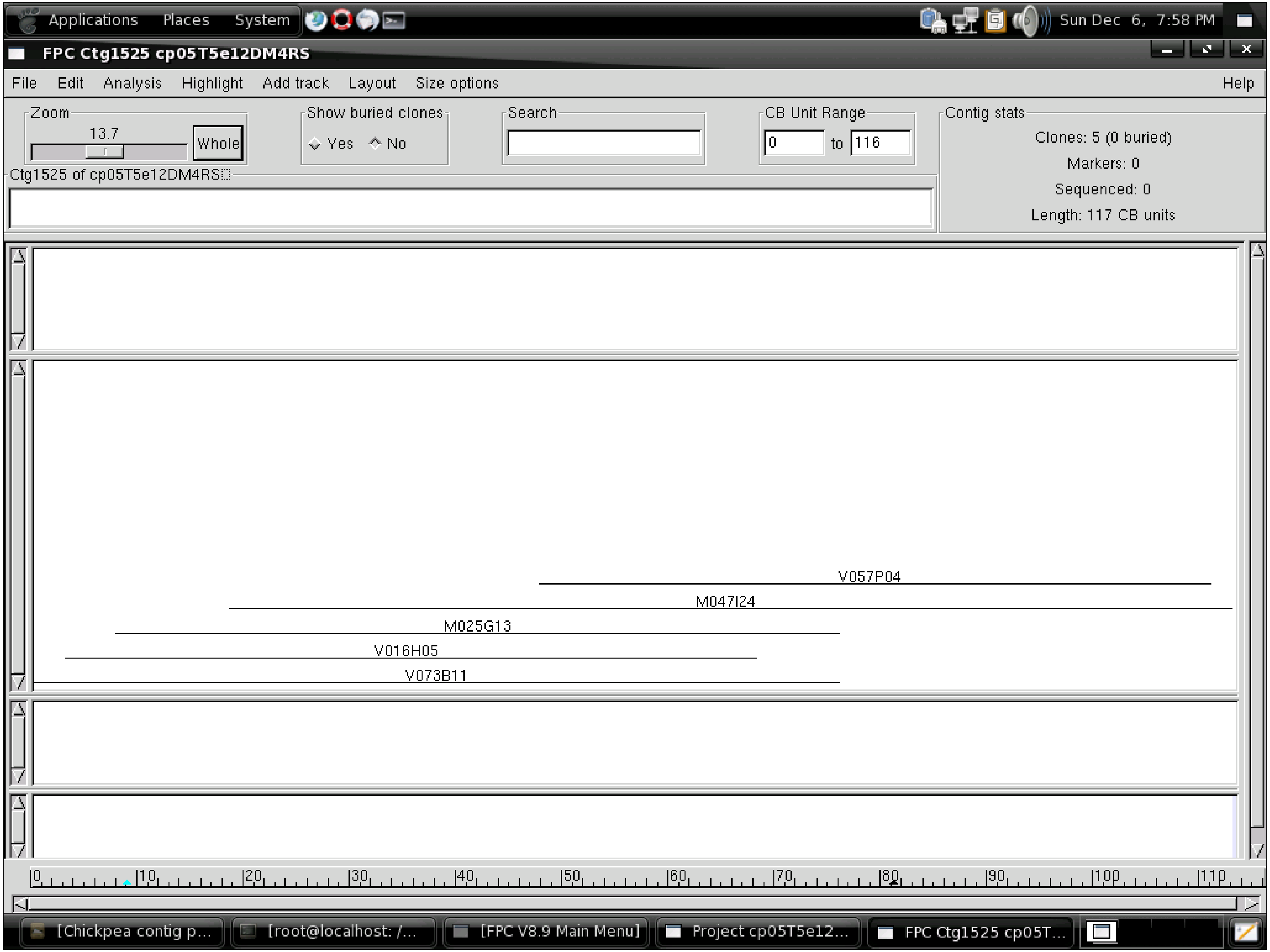
Applications Places System
Sun Dec 6, 7:58 PM
FPC Ctg1525 cp05T5e12DM4RS
File Edit Analysis Highlight Add track Layout Size options Help
Zoom
13.7
Whole
Show buried clones
Yes No
Search
CB Unit Range
0 to 116
Contig stats
Clones: 5 (0 buried)
Markers: 0
Sequenced: 0
Length: 117 CB units
Ctg1525 of cp05T5e12DM4RS
V057P04
M047I24
M025G13
V016H05
V073B11
0 10 20 30 40 50 60 70 80 90 100 110
[Chickpea contig p...
[root@localhost: /...
[FPC V8.9 Main Menu]
Project cp05T5e12...
FPC Ctg1525 cp05T...

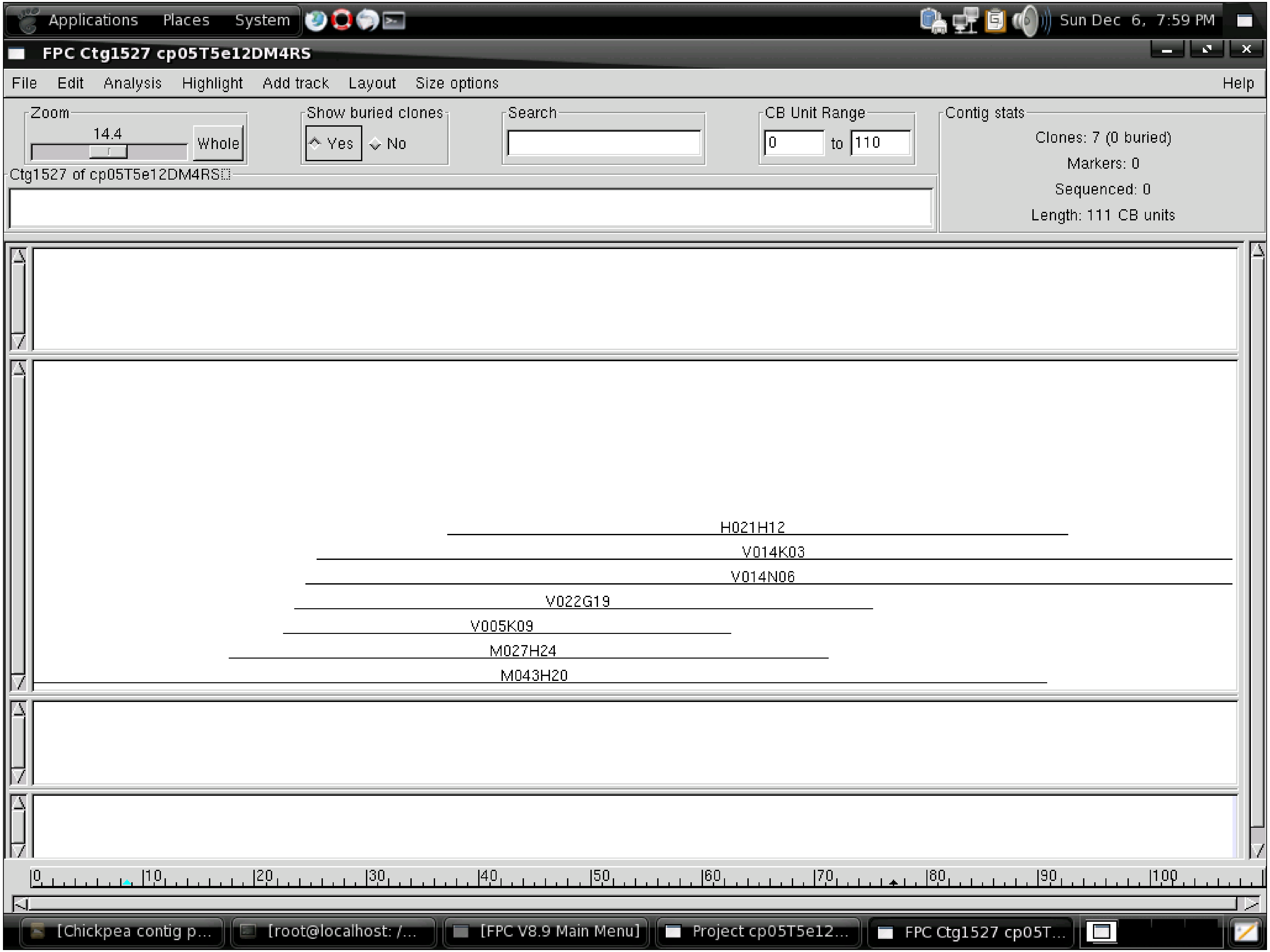

Applications Places System
Sun Dec 6, 7:59 PM
FPC Ctg1527 cp05T5e12DM4RS
File Edit Analysis Highlight Add track Layout Size options Help
Zoom
14.4
Whole
Show buried clones
Yes No
Search
CB Unit Range
0 to 110
Contig stats
Clones: 7 (0 buried)
Markers: 0
Sequenced: 0
Length: 111 CB units
Ctg1527 of cp05T5e12DM4RS
H021H12
V014K03
V014N06
V022G19
V005K09
M027H24
M043H20
0 10 20 30 40 50 60 70 80 90 100
[Chickpea contig p...
[root@localhost: /...
[FPC V8.9 Main Menu]
Project cp05T5e12...
FPC Ctg1527 cp05T...

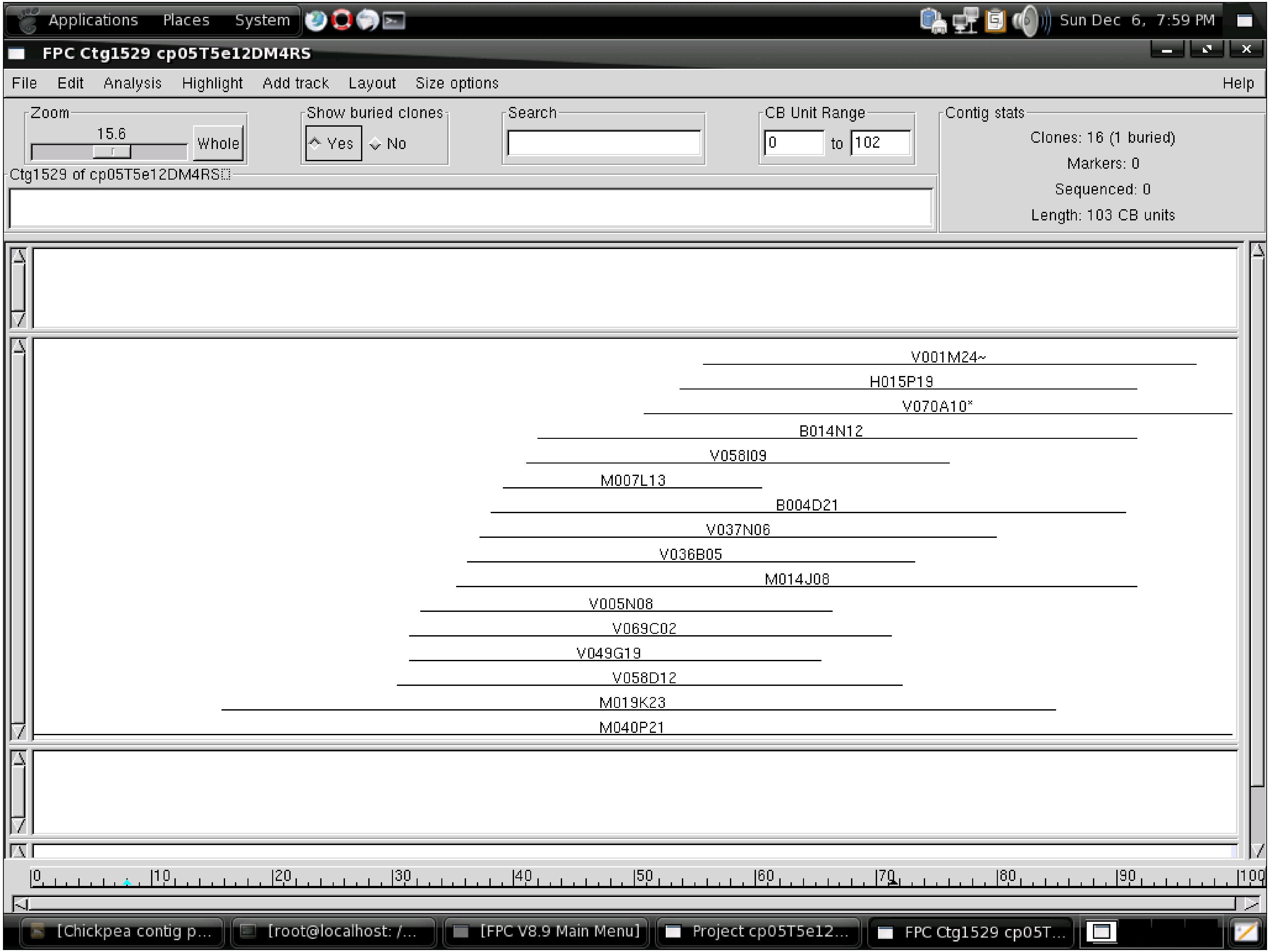
Applications Places System
Sun Dec 6, 7:59 PM
FPC Ctg1529 cp05T5e12DM4RS
File Edit Analysis Highlight Add track Layout Size options Help
Zoom
15.6
Whole
Show buried clones
Yes No
Search
CB Unit Range
0 to 102
Contig stats
Clones: 16 (1 buried)
Markers: 0
Sequenced: 0
Length: 103 CB units
Ctg1529 of cp05T5e12DM4RS
V001M24~
H015P19
V070A10*
B014N12
V058I09
M007L13
B004D21
V037N06
V036B05
M014J08
V005N08
V069C02
V049G19
V058D12
M019K23
M040P21
0 10 20 30 40 50 60 70 80 90 100
[Chickpea contig p...
[root@localhost: /...
[FPC V8.9 Main Menu]
Project cp05T5e12...
FPC Ctg1529 cp05T...

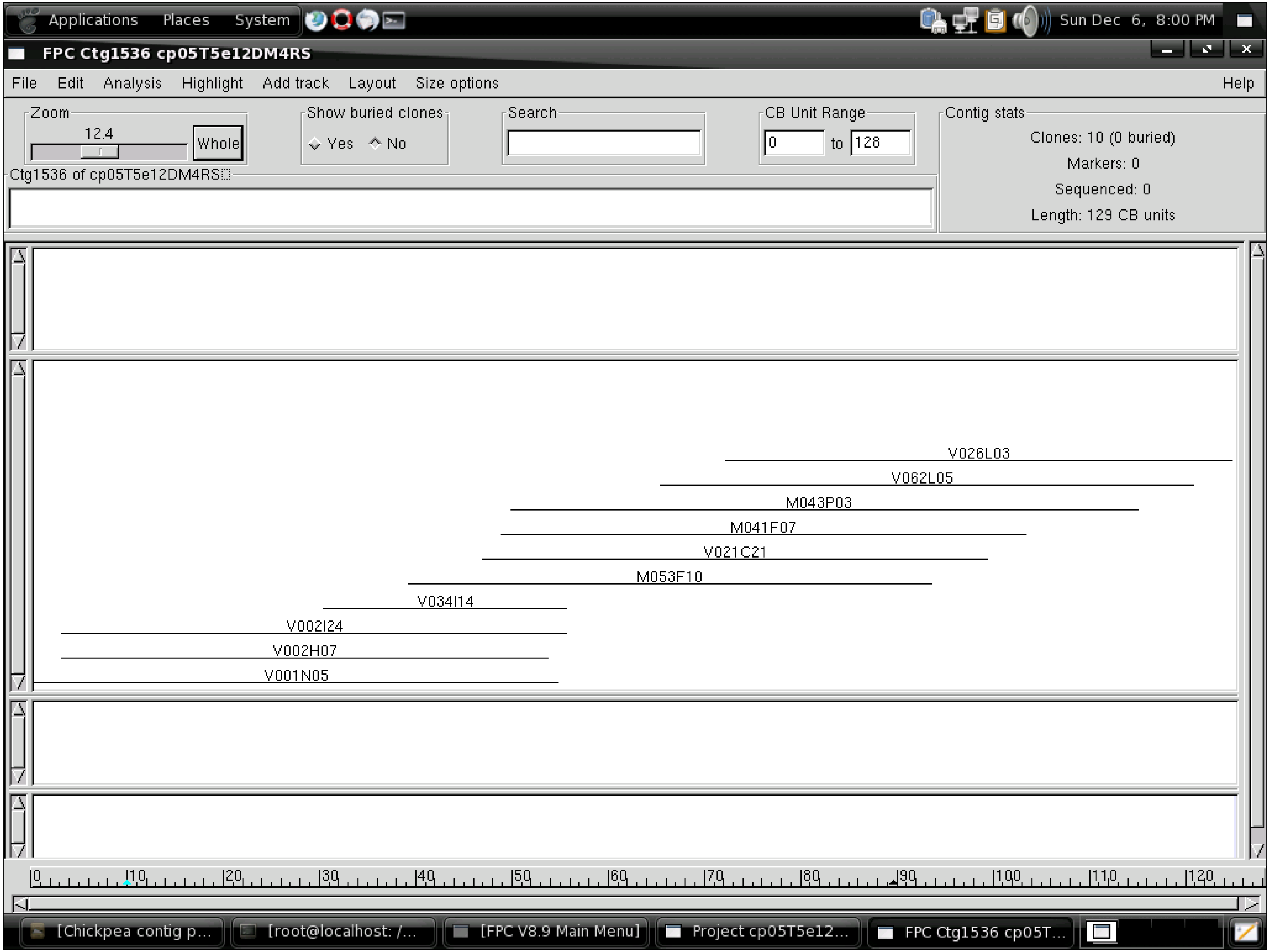
Applications
Places
System
Sun Dec 6, 8:00 PM
FPC Ctg1536 cp05T5e12DM4RS
File
Edit
Analysis
Highlight
Add track
Layout
Size options
Help
Zoom
12.4
Whole
Show buried clones
Yes
No
Search
CB Unit Range
0
to
128
Contig stats
Clones: 10 (0 buried)
Markers: 0
Sequenced: 0
Length: 129 CB units
Ctg1536 of cp05T5e12DM4RS
V026L03
V062L05
M043P03
M041F07
V021C21
M053F10
V034I14
V002I24
V002H07
V001N05
0
10
20
30
40
50
60
70
80
90
100
110
120
[Chickpea contig p...
[root@localhost: /...
[FPC V8.9 Main Menu]
Project cp05T5e12...
FPC Ctg1536 cp05T...

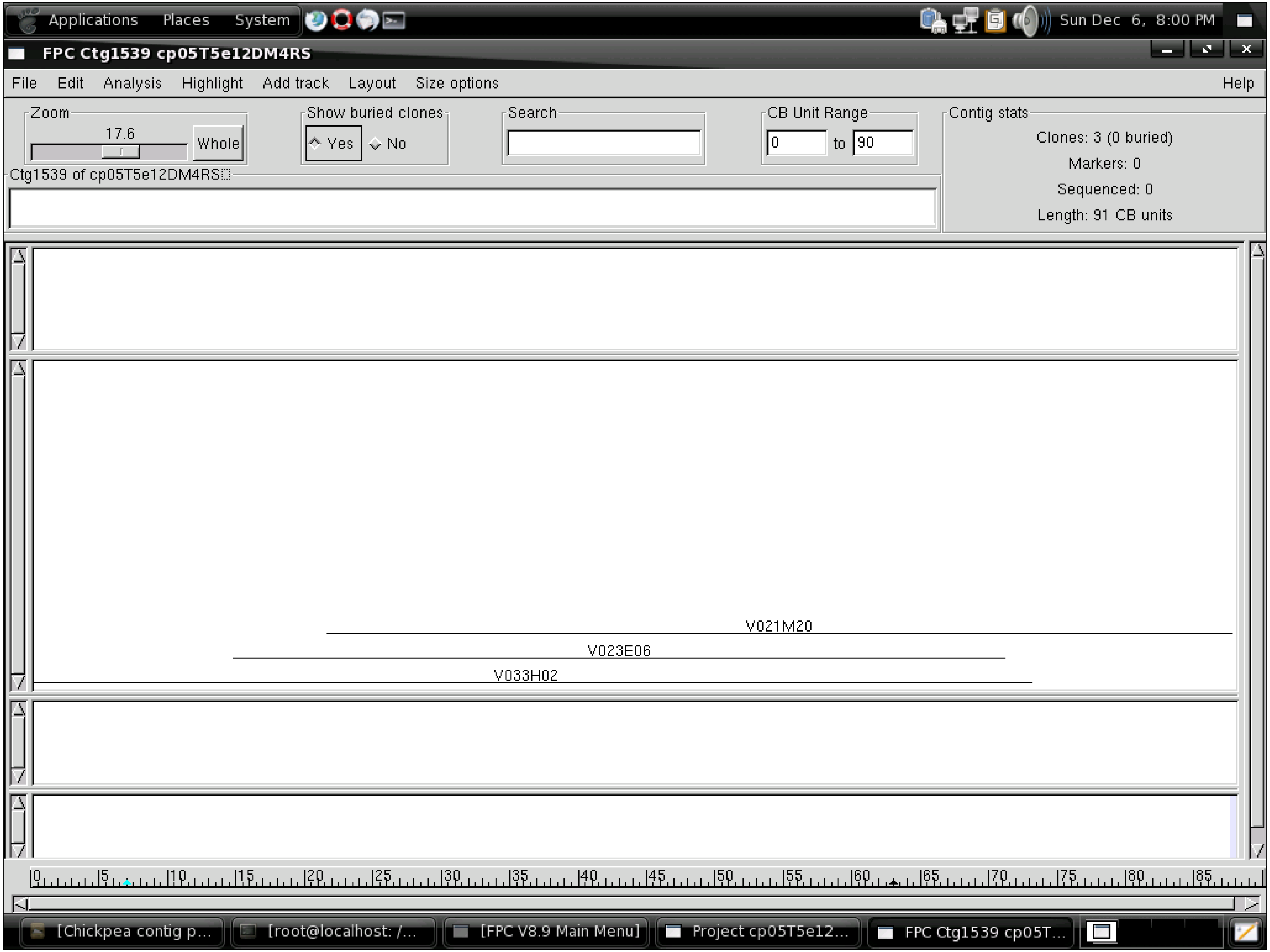
Applications Places System
Sun Dec 6, 8:00 PM
FPC Ctg1539 cp05T5e12DM4RS
File Edit Analysis Highlight Add track Layout Size options Help
Zoom
17.6
Whole
Show buried clones
Yes
No
Search
CB Unit Range
0
to
90
Contig stats
Clones: 3 (0 buried)
Markers: 0
Sequenced: 0
Length: 91 CB units
Ctg1539 of cp05T5e12DM4RS
V021M20
V023E06
V033H02
0 5 10 15 20 25 30 35 40 45 50 55 60 65 70 75 80 85
[Chickpea contig p...
[root@localhost: /...
[FPC V8.9 Main Menu]
Project cp05T5e12...
FPC Ctg1539 cp05T...

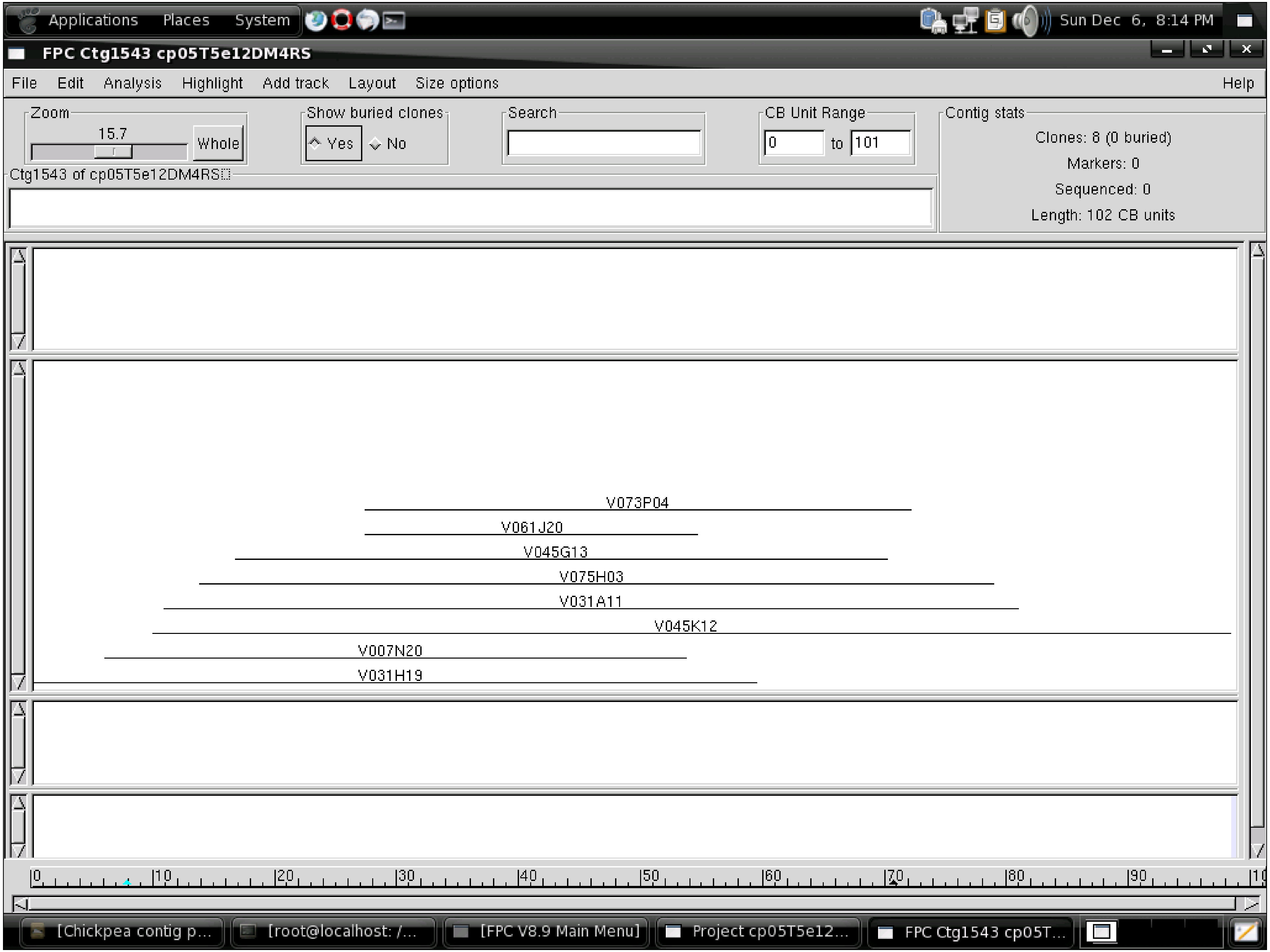
Applications
Places
System
Sun Dec 6, 8:14 PM
FPC Ctg1543 cp05T5e12DM4RS
File
Edit
Analysis
Highlight
Add track
Layout
Size options
Help
Zoom
15.7
Whole
Show buried clones
Yes
No
Search
CB Unit Range
0
to
101
Contig stats
Clones: 8 (0 buried)
Markers: 0
Sequenced: 0
Length: 102 CB units
Ctg1543 of cp05T5e12DM4RS
V073P04
V061J20
V045G13
V075H03
V031A11
V045K12
V007N20
V031H19
0
10
20
30
40
50
60
70
80
90
[Chickpea contig p...
[root@localhost: /...
[FPC V8.9 Main Menu]
Project cp05T5e12...
FPC Ctg1543 cp05T...

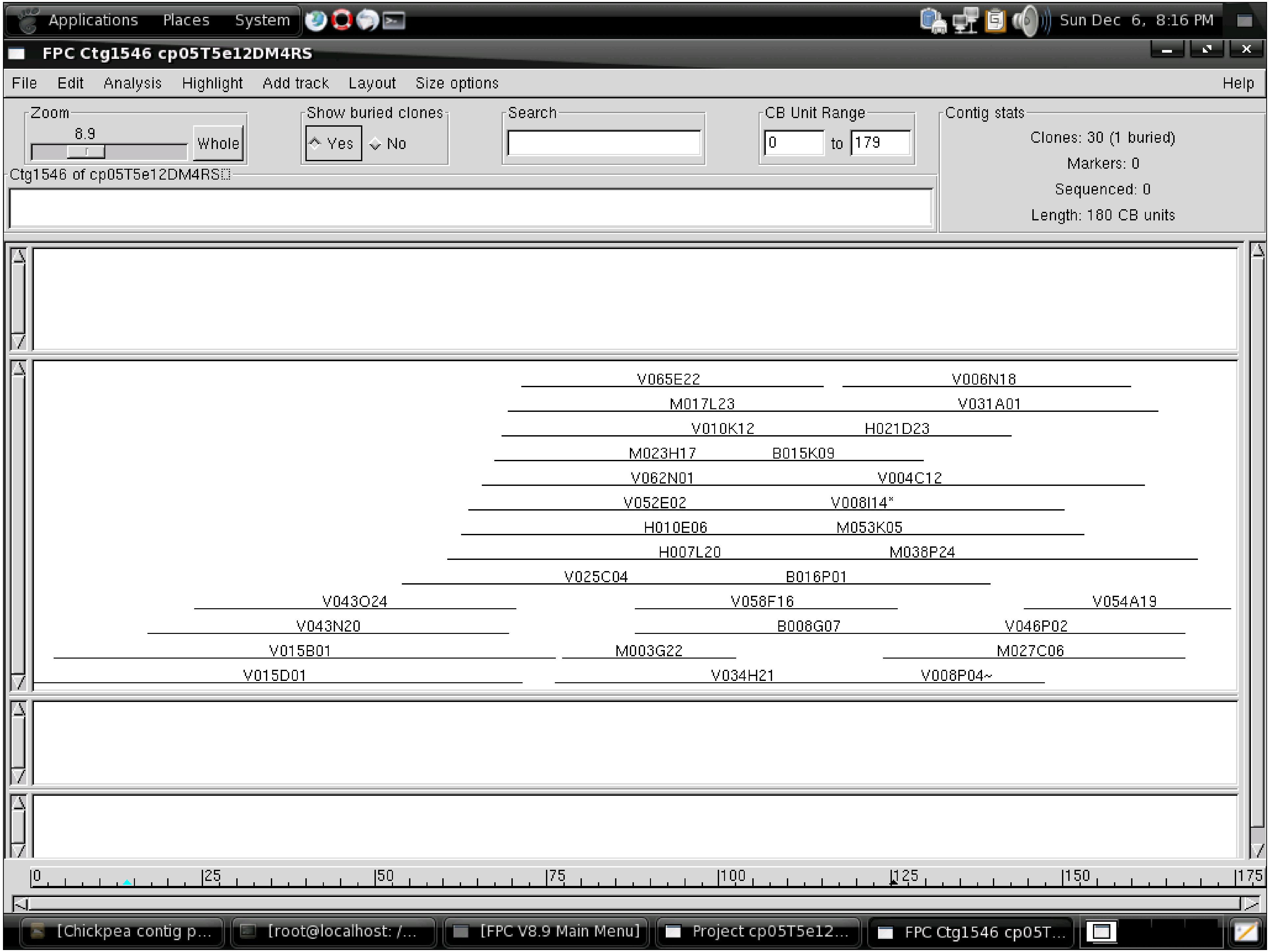
Applications Places System
Sun Dec 6, 8:16 PM
FPC Ctg1546 cp05T5e12DM4RS
File Edit Analysis Highlight Add track Layout Size options Help
Zoom
8.9
Whole
Show buried clones
Yes
No
Search
CB Unit Range
0 to 179
Contig stats
Clones: 30 (1 buried)
Markers: 0
Sequenced: 0
Length: 180 CB units
Ctg1546 of cp05T5e12DM4RS
V065E22
V006N18
M017L23
V031A01
V010K12
H021D23
M023H17
B015K09
V062N01
V004C12
V052E02
V008I14*
H010E06
M053K05
H007L20
M038P24
V025C04
B016P01
V043O24
V058F16
V054A19
V043N20
B008G07
V046P02
V015B01
M003G22
M027C06
V015D01
V034H21
V008P04~
0
25
50
75
100
125
150
175
[Chickpea contig p...
[root@localhost: /...
[FPC V8.9 Main Menu]
Project cp05T5e12...
FPC Ctg1546 cp05T...

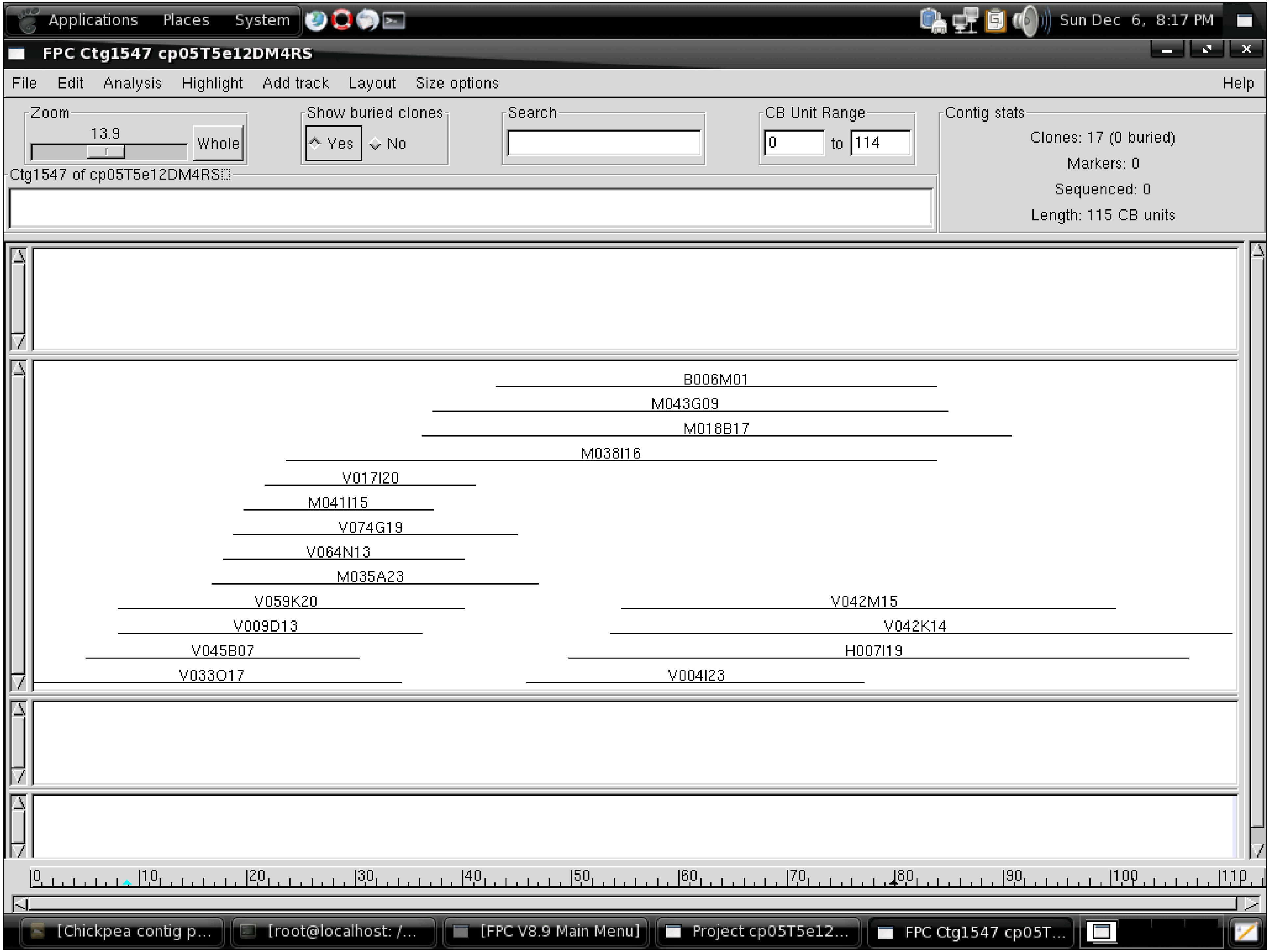
Applications Places System
Sun Dec 6, 8:17 PM
FPC Ctg1547 cp05T5e12DM4RS
File Edit Analysis Highlight Add track Layout Size options Help
Zoom
13.9
Whole
Show buried clones
Yes
No
Search
CB Unit Range
0 to 114
Contig stats
Clones: 17 (0 buried)
Markers: 0
Sequenced: 0
Length: 115 CB units
Ctg1547 of cp05T5e12DM4RS
B006M01
M043G09
M018B17
M038I16
V017I20
M041I15
V074G19
V064N13
M035A23
V059K20
V042M15
V009D13
V042K14
V045B07
H007I19
V033O17
V004I23
0 10 20 30 40 50 60 70 80 90 100 110
[Chickpea contig p...
[root@localhost: /...
[FPC V8.9 Main Menu]
Project cp05T5e12...
FPC Ctg1547 cp05T...

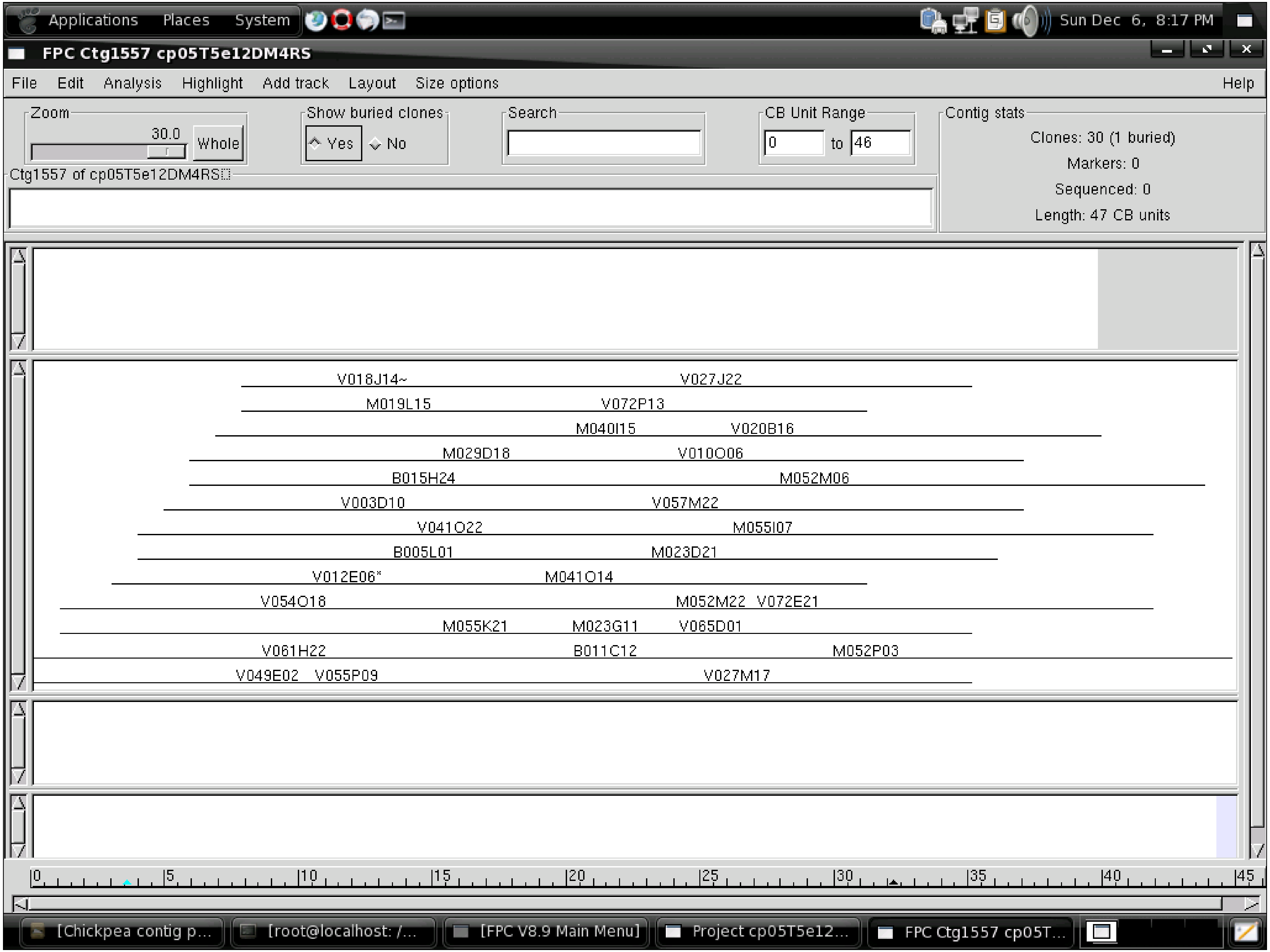
Applications Places System
Sun Dec 6, 8:17 PM
FPC Ctg1557 cp05T5e12DM4RS
File Edit Analysis Highlight Add track Layout Size options Help
Zoom
30.0
Whole
Show buried clones
Yes
No
Search
CB Unit Range
0 to 46
Contig stats
Clones: 30 (1 buried)
Markers: 0
Sequenced: 0
Length: 47 CB units
Ctg1557 of cp05T5e12DM4RS
V018J14~
V027J22
M019L15
V072P13
M040I15
V020B16
M029D18
V010O06
B015H24
M052M06
V003D10
V057M22
V041O22
M055I07
B005L01
M023D21
V012E06*
M041O14
V054O18
M052M22 V072E21
M055K21
M023G11
V065D01
V061H22
B011C12
M052P03
V049E02 V055P09
V027M17
0 5 10 15 20 25 30 35 40 45
[Chickpea contig p...
[root@localhost: /...
[FPC V8.9 Main Menu]
Project cp05T5e12...
FPC Ctg1557 cp05T...

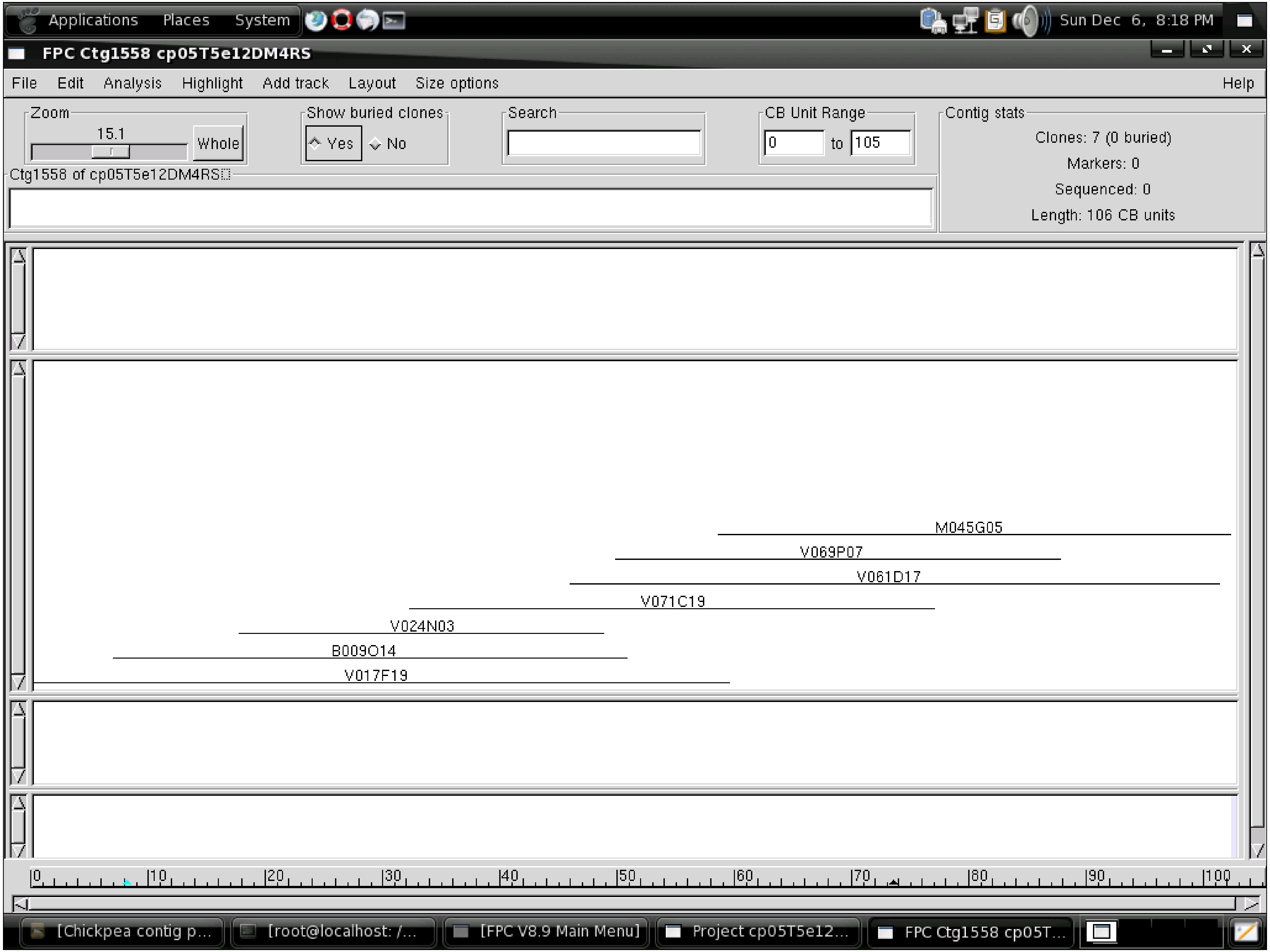
Applications Places System
Sun Dec 6, 8:18 PM
FPC Ctg1558 cp05T5e12DM4RS
File Edit Analysis Highlight Add track Layout Size options Help
Zoom
15.1
Whole
Show buried clones
Yes No
Search
CB Unit Range
0 to 105
Contig stats
Clones: 7 (0 buried)
Markers: 0
Sequenced: 0
Length: 106 CB units
Ctg1558 of cp05T5e12DM4RS
M045G05
V069P07
V061D17
V071C19
V024N03
B009O14
V017F19
0 10 20 30 40 50 60 70 80 90 100
[Chickpea contig p...
[root@localhost: /...
[FPC V8.9 Main Menu]
Project cp05T5e12...
FPC Ctg1558 cp05T...

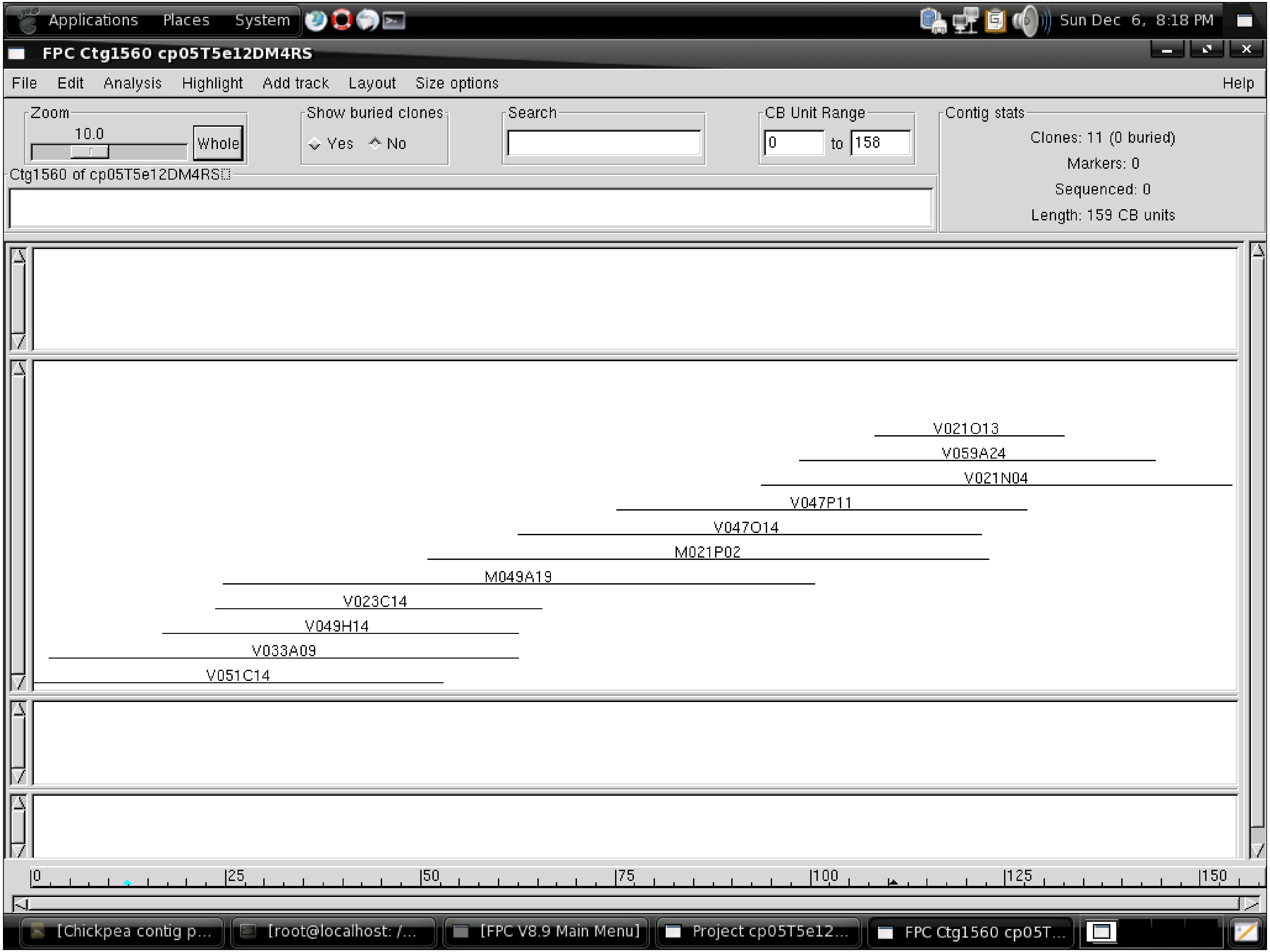
Applications Places System
Sun Dec 6, 8:18 PM
FPC Ctg1560 cp05T5e12DM4RS
File Edit Analysis Highlight Add track Layout Size options Help
Zoom
10.0
Whole
Show buried clones
Yes No
Search
CB Unit Range
0 to 158
Contig stats
Clones: 11 (0 buried)
Markers: 0
Sequenced: 0
Length: 159 CB units
Ctg1560 of cp05T5e12DM4RS
V021O13
V059A24
V021N04
V047P11
V047O14
M021P02
M049A19
V023C14
V049H14
V033A09
V051C14
0 25 50 75 100 125 150
[Chickpea contig p...
[root@localhost: /...
[FPC V8.9 Main Menu]
Project cp05T5e12...
FPC Ctg1560 cp05T...

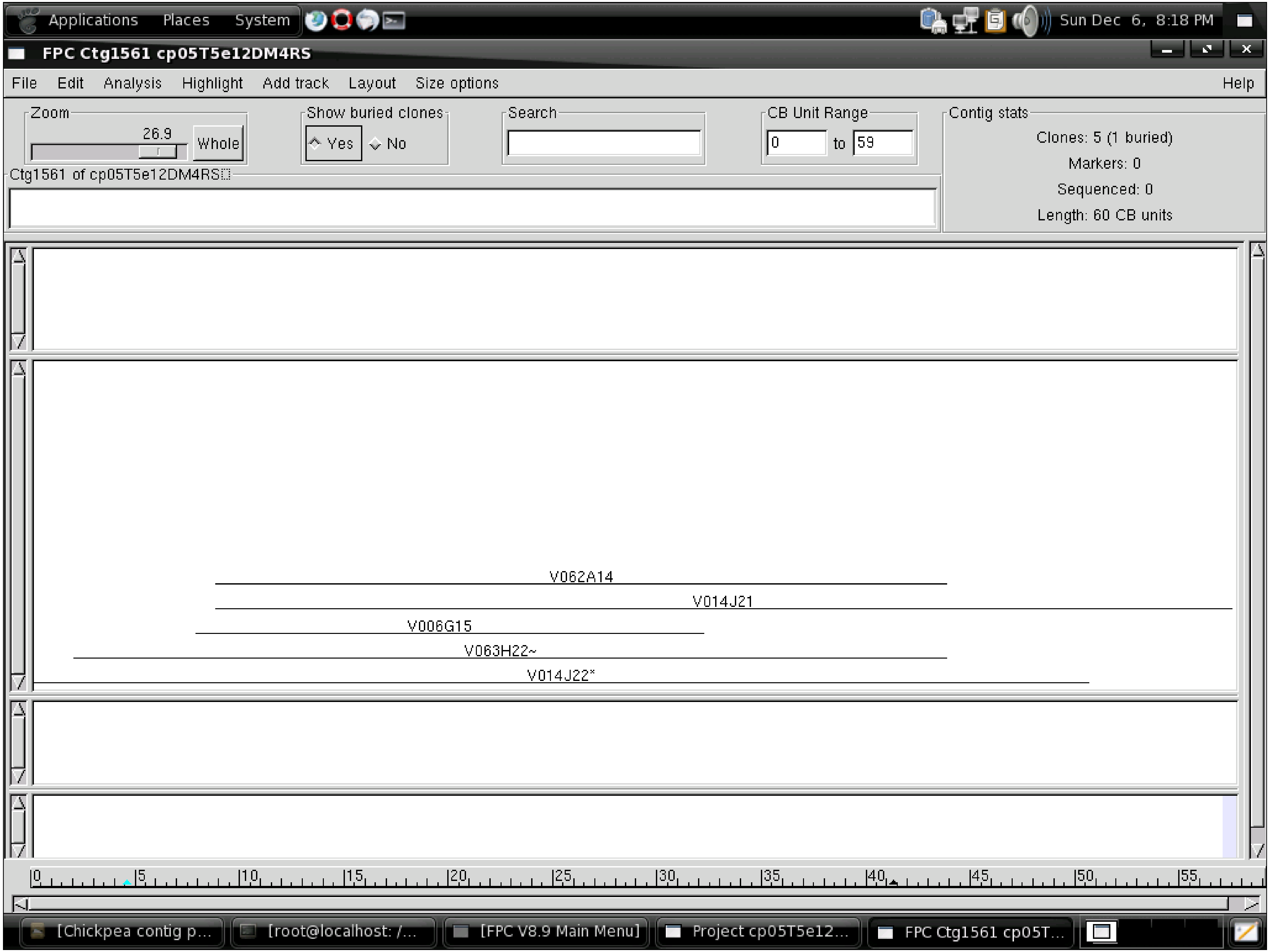
Applications Places System
Sun Dec 6, 8:18 PM
FPC Ctg1561 cp05T5e12DM4RS
File Edit Analysis Highlight Add track Layout Size options Help
Zoom
26.9
Whole
Show buried clones
Yes
No
Search
CB Unit Range
0 to 59
Contig stats
Clones: 5 (1 buried)
Markers: 0
Sequenced: 0
Length: 60 CB units
Ctg1561 of cp05T5e12DM4RS
V062A14
V014J21
V006G15
V063H22~
V014J22*
0 5 10 15 20 25 30 35 40 45 50 55
[Chickpea contig p...
[root@localhost: /...
[FPC V8.9 Main Menu]
Project cp05T5e12...
FPC Ctg1561 cp05T...

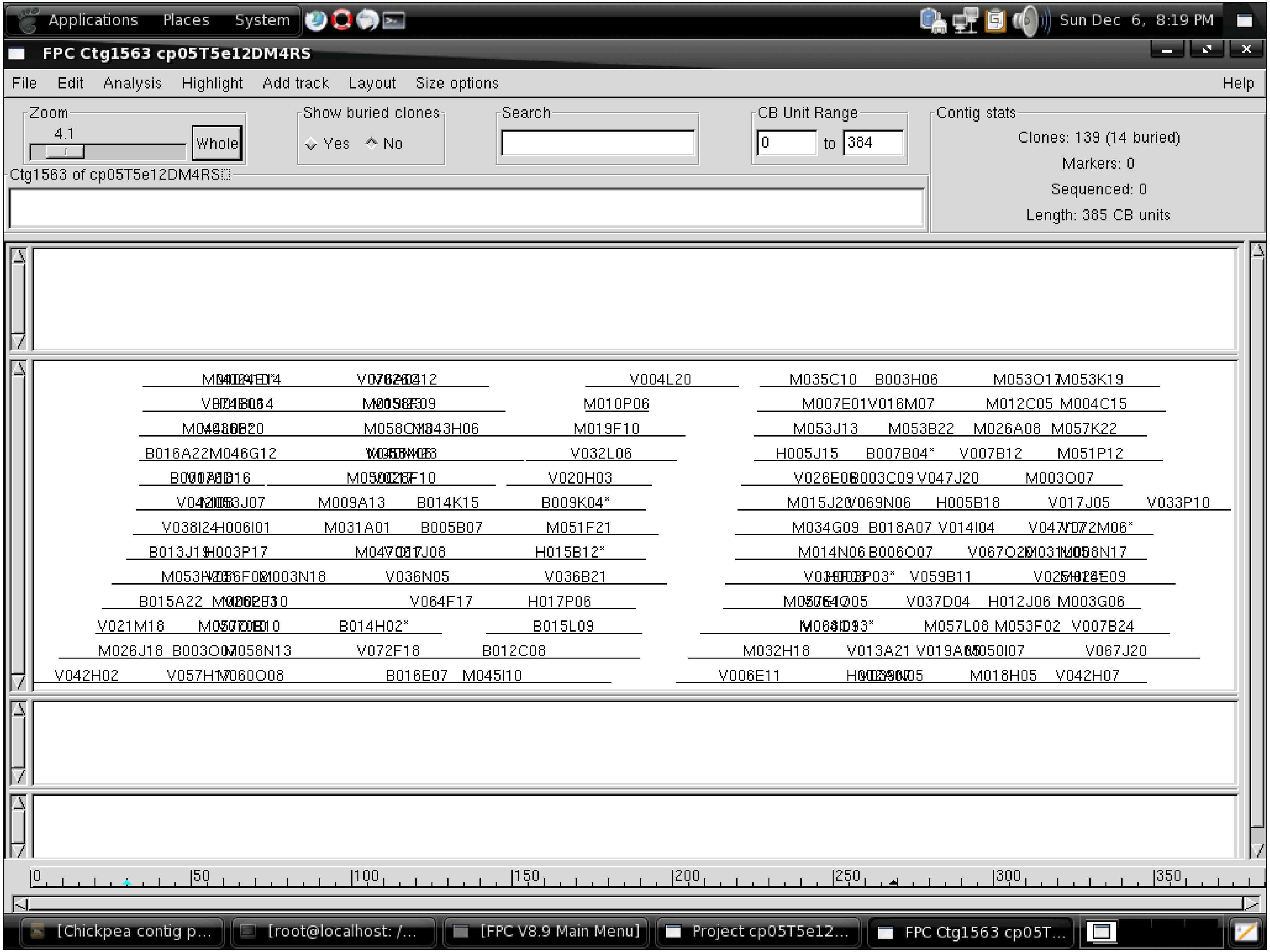
Applications Places System
Sun Dec 6, 8:19 PM
FPC Ctg1563 cp05T5e12DM4RS
File Edit Analysis Highlight Add track Layout Size options Help
Zoom
4.1
Whole
Show buried clones
Yes No
Search
CB Unit Range
0 to 384
Contig stats
Clones: 139 (14 buried)
Markers: 0
Sequenced: 0
Length: 385 CB units
Ctg1563 of cp05T5e12DM4RS
V004L20
M035C10 B003H06
M010P06
M019F10
M053J13 M053B22 M026A08 M057K22
V032L06
H005J15 B007B04* V007B12 M051P12
V020H03
M003O07
M009A13 B014K15 B009K04*
V033P10
M031A01 B005B07 M051F21
H015B12*
V036N05 V036B21 V059B11
V064F17 H017P06
V037D04 H012J06 M003G06
B014H02* B015L09
M057L08 M053F02 V007B24
M026J18 V072F18 B012C08
M032H18 V067J20
V042H02 B016E07 M045I10
V006E11 M018H05 V042H07
0 50 100 150 200 250 300 350
[Chickpea contig p...
[root@localhost: /...
[FPC V8.9 Main Menu]
Project cp05T5e12...
FPC Ctg1563 cp05T...

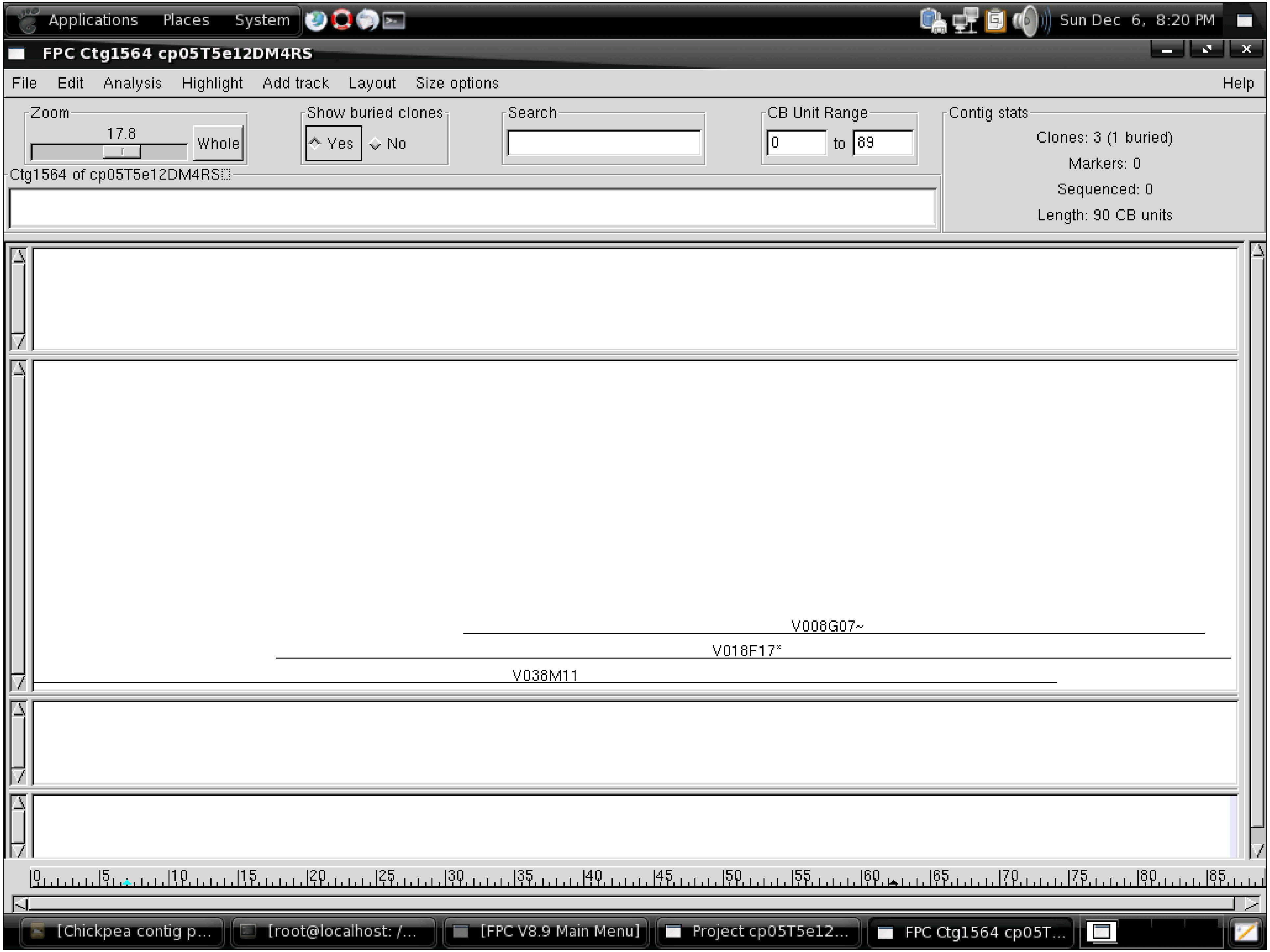

Applications Places System
Sun Dec 6, 8:20 PM
FPC Ctg1564 cp05T5e12DM4RS
File Edit Analysis Highlight Add track Layout Size options Help
Zoom
17.8
Whole
Show buried clones
Yes No
Search
CB Unit Range
0 to 89
Contig stats
Clones: 3 (1 buried)
Markers: 0
Sequenced: 0
Length: 90 CB units
Ctg1564 of cp05T5e12DM4RS
V008G07~
V018F17*
V038M11
0 5 10 15 20 25 30 35 40 45 50 55 60 65 70 75 80 85
[Chickpea contig p...
[root@localhost: /...
[FPC V8.9 Main Menu]
Project cp05T5e12...
FPC Ctg1564 cp05T...

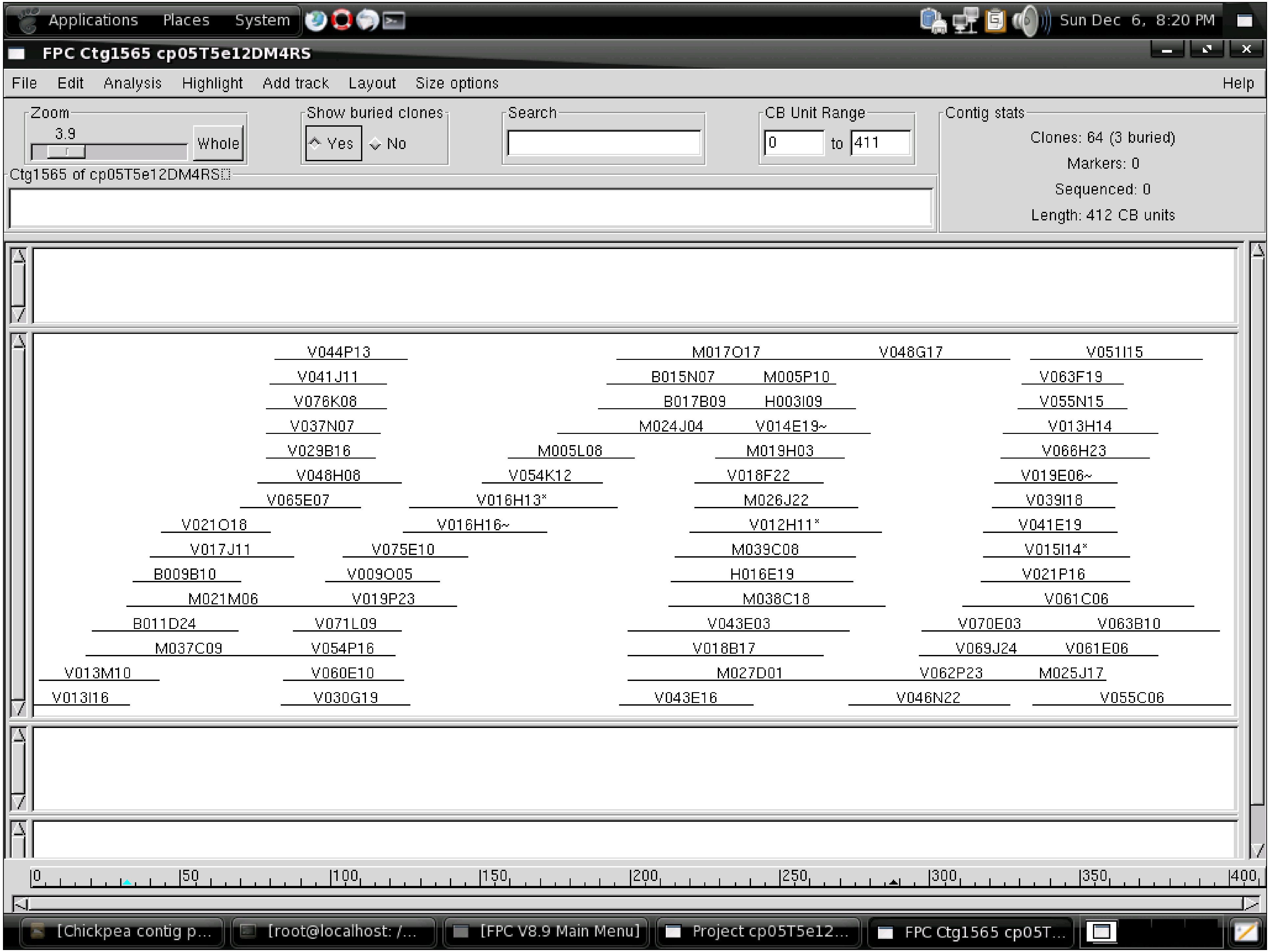
Applications Places System
Sun Dec 6, 8:20 PM
FPC Ctg1565 cp05T5e12DM4RS
File Edit Analysis Highlight Add track Layout Size options Help
Zoom
3.9
Whole
Show buried clones
Yes No
Search
CB Unit Range
0 to 411
Contig stats
Clones: 64 (3 buried)
Markers: 0
Sequenced: 0
Length: 412 CB units
Ctg1565 of cp05T5e12DM4RS
V044P13
V041J11
V076K08
V037N07
V029B16
V048H08
V065E07
V021O18
V017J11
B009B10
M021M06
B011D24
M037C09
V013M10
V013I16
M005L08
V054K12
V016H13*
V016H16~
V075E10
V009O05
V019P23
V071L09
V054P16
V060E10
V030G19
M017O17
B015N07
M005P10
B017B09
H003I09
M024J04
V014E19~
M019H03
V018F22
M026J22
V012H11*
M039C08
H016E19
M038C18
V043E03
V018B17
M027D01
V043E16
V048G17
V051I15
V063F19
V055N15
V013H14
V066H23
V019E06~
V039I18
V041E19
V015I14*
V021P16
V061C06
V070E03
V063B10
V069J24
V061E06
V062P23
M025J17
V046N22
V055C06
0 50 100 150 200 250 300 350 400
[Chickpea contig p...
[root@localhost: /...
[FPC V8.9 Main Menu]
Project cp05T5e12...
FPC Ctg1565 cp05T...

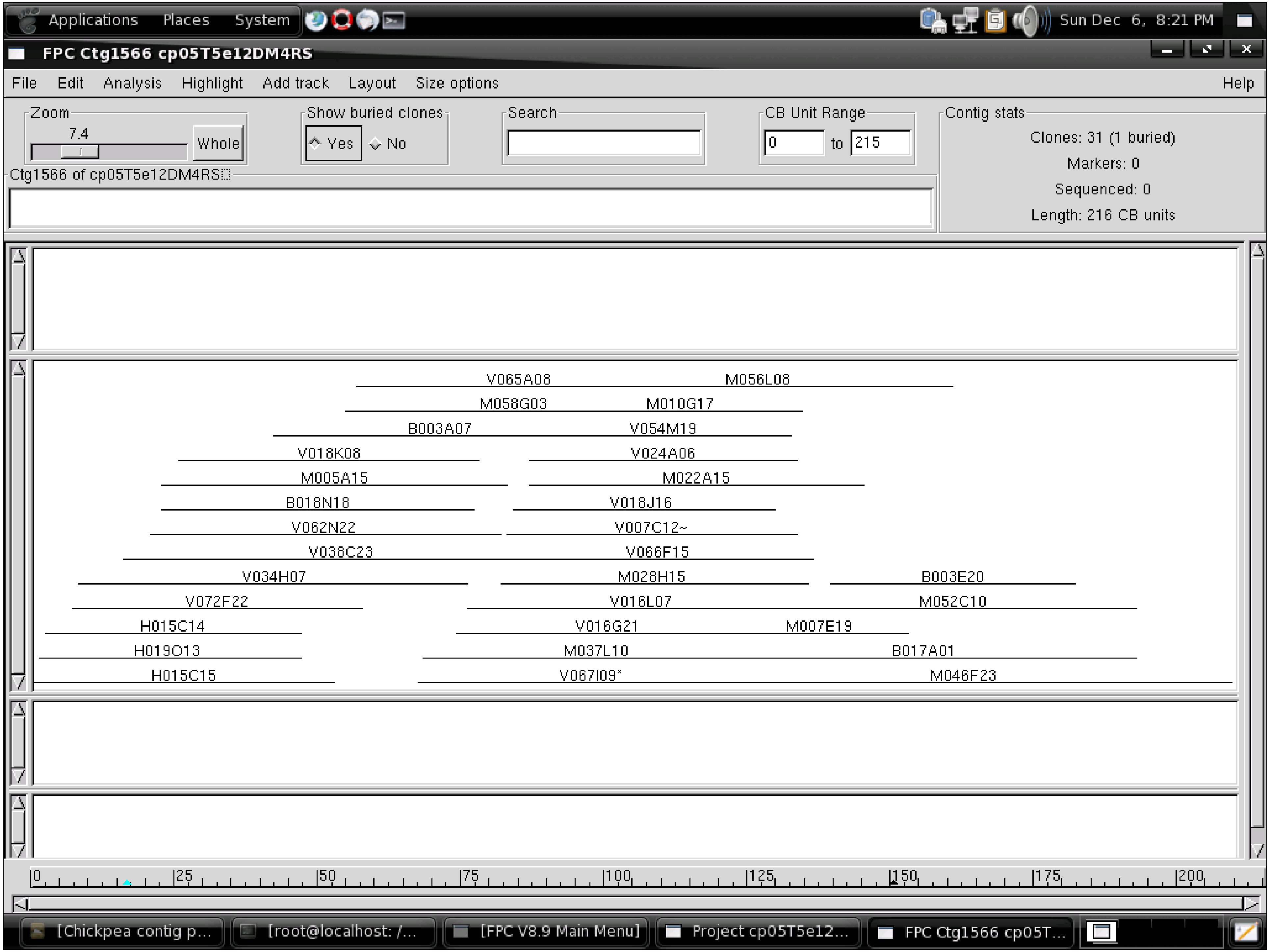
Applications Places System
Sun Dec 6, 8:21 PM
FPC Ctg1566 cp05T5e12DM4RS
File Edit Analysis Highlight Add track Layout Size options Help
Zoom
7.4
Whole
Show buried clones
Yes No
Search
CB Unit Range
0 to 215
Contig stats
Clones: 31 (1 buried)
Markers: 0
Sequenced: 0
Length: 216 CB units
Ctg1566 of cp05T5e12DM4RS
V065A08
M056L08
M058G03
M010G17
B003A07
V054M19
V018K08
V024A06
M005A15
M022A15
B018N18
V018J16
V062N22
V007C12~
V038C23
V066F15
V034H07
M028H15
B003E20
V072F22
V016L07
M052C10
H015C14
V016G21
M007E19
H019O13
M037L10
B017A01
H015C15
V067I09*
M046F23
0 25 50 75 100 125 150 175 200
[Chickpea contig p...
[root@localhost: /...
[FPC V8.9 Main Menu]
Project cp05T5e12...
FPC Ctg1566 cp05T...

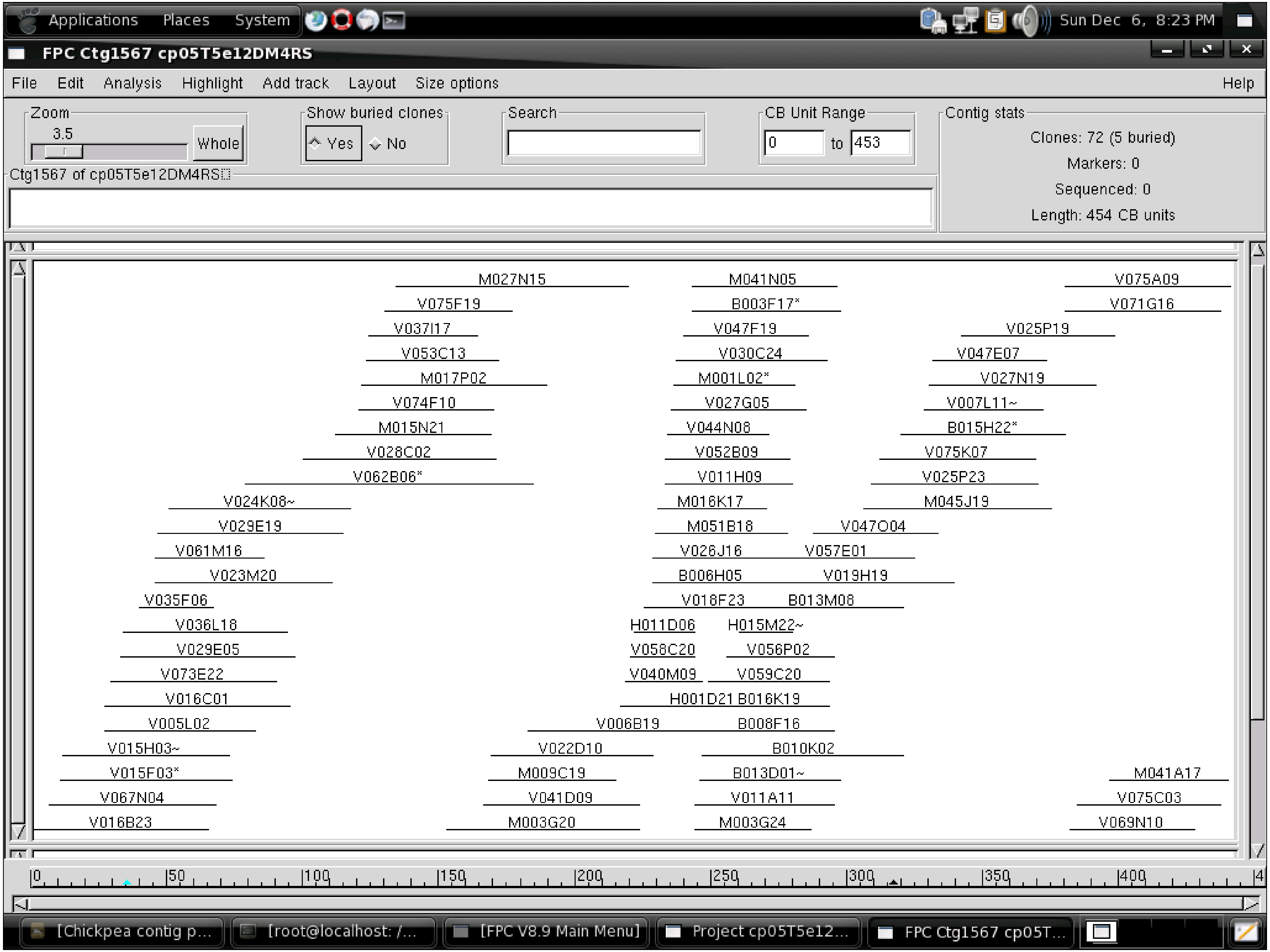
Applications Places System
Sun Dec 6, 8:23 PM
FPC Ctg1567 cp05T5e12DM4RS
File Edit Analysis Highlight Add track Layout Size options Help
Zoom 3.5 Whole
Show buried clones Yes No
Search
CB Unit Range 0 to 453
Contig stats
Clones: 72 (5 buried)
Markers: 0
Sequenced: 0
Length: 454 CB units
Ctg1567 of cp05T5e12DM4RS
M027N15
M041N05
V075A09
V075F19
B003F17*
V071G16
V037I17
V047F19
V025P19
V053C13
V030C24
V047E07
M017P02
M001L02*
V027N19
V074F10
V027G05
V007L11~
M015N21
V044N08
B015H22*
V028C02
V052B09
V075K07
V062B06*
V011H09
V025P23
V024K08~
M016K17
M045J19
V029E19
M051B18
V047O04
V061M16
V026J16
V057E01
V023M20
B006H05
V019H19
V035F06
V018F23
B013M08
V036L18
H011D06
H015M22~
V029E05
V058C20
V056P02
V073E22
V040M09
V059C20
V016C01
H001D21 B016K19
V005L02
V006B19
B008F16
V015H03~
V022D10
B010K02
V015F03*
M009C19
B013D01~
M041A17
V067N04
V041D09
V011A11
V075C03
V016B23
M003G20
M003G24
V069N10
0 50 100 150 200 250 300 350 400
[Chickpea contig p...
[root@localhost: /...
[FPC V8.9 Main Menu]
Project cp05T5e12...
FPC Ctg1567 cp05T...

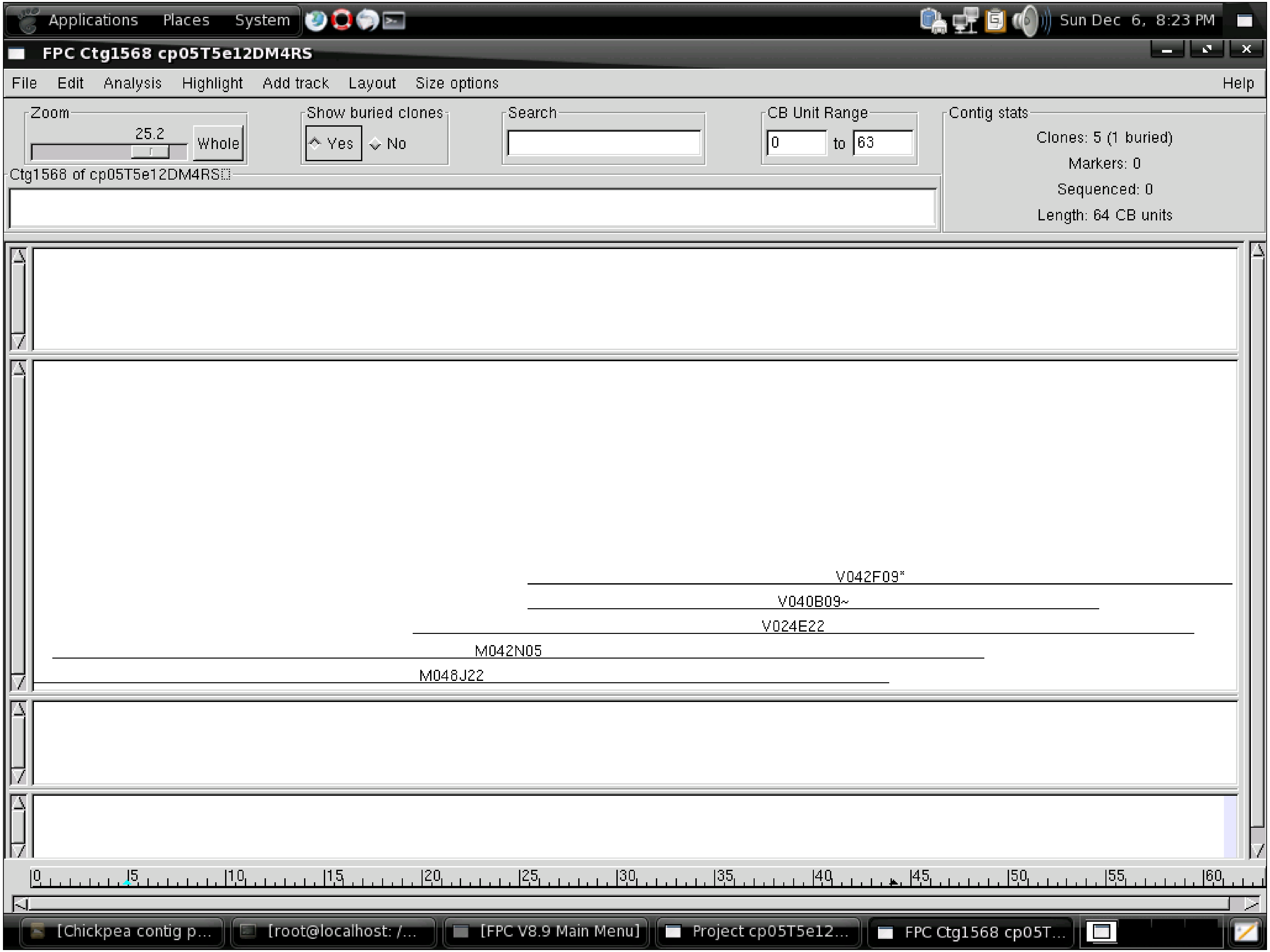
Applications Places System
Sun Dec 6, 8:23 PM
FPC Ctg1568 cp05T5e12DM4RS
File Edit Analysis Highlight Add track Layout Size options Help
Zoom
25.2
Whole
Show buried clones
Yes
No
Search
CB Unit Range
0 to 63
Contig stats
Clones: 5 (1 buried)
Markers: 0
Sequenced: 0
Length: 64 CB units
Ctg1568 of cp05T5e12DM4RS
V042F09*
V040B09~
V024E22
M042N05
M048J22
0 5 10 15 20 25 30 35 40 45 50 55 60
[Chickpea contig p...
[root@localhost: /...
[FPC V8.9 Main Menu]
Project cp05T5e12...
FPC Ctg1568 cp05T...

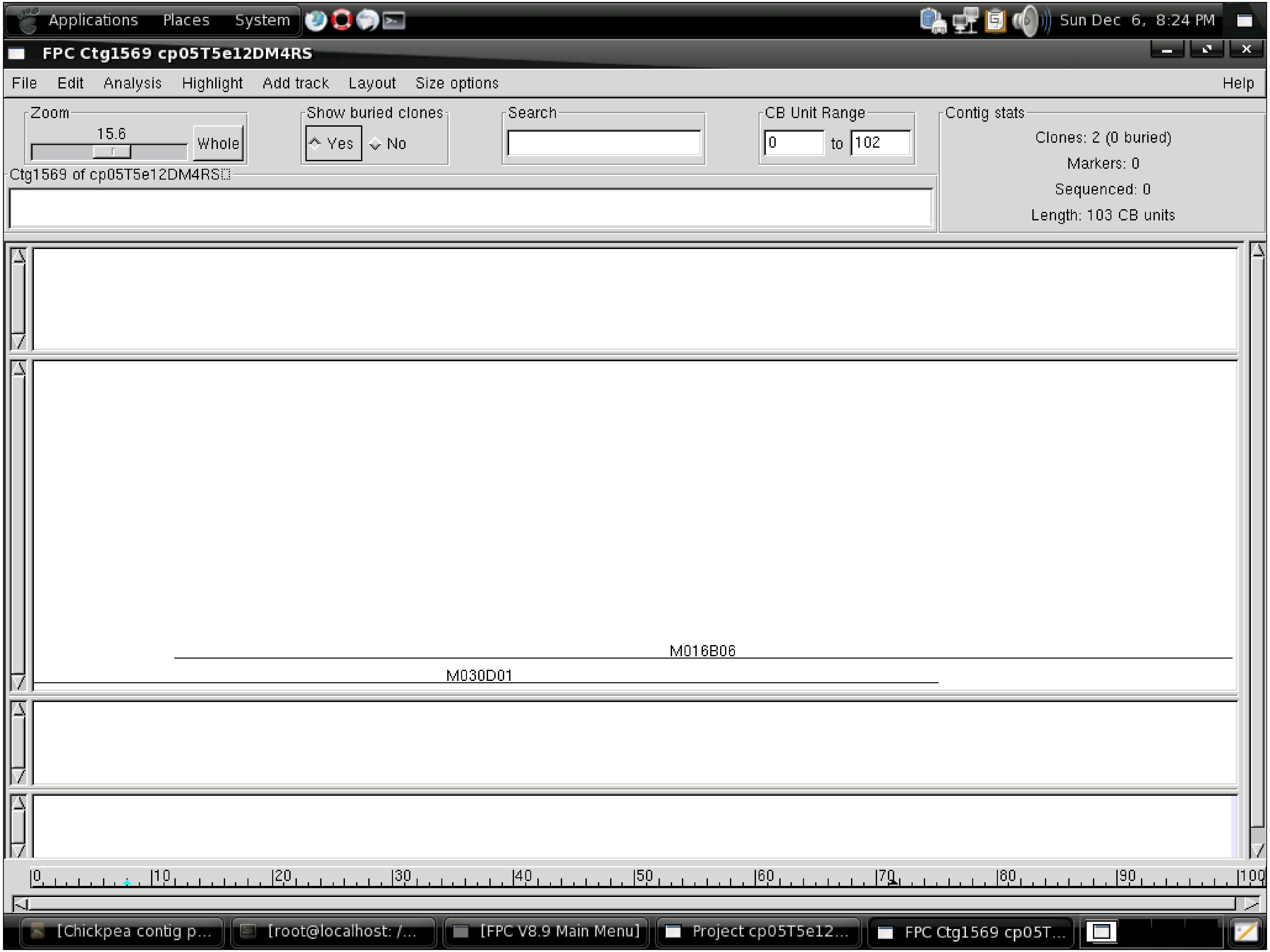
Applications
Places
System
Sun Dec 6, 8:24 PM
FPC Ctg1569 cp05T5e12DM4RS
File
Edit
Analysis
Highlight
Add track
Layout
Size options
Help
Zoom
15.6
Whole
Show buried clones
Yes
No
Search
CB Unit Range
0
to
102
Contig stats
Clones: 2 (0 buried)
Markers: 0
Sequenced: 0
Length: 103 CB units
Ctg1569 of cp05T5e12DM4RS
M016B06
M030D01
0
10
20
30
40
50
60
70
80
90
100
[Chickpea contig p...
[root@localhost: /...
[FPC V8.9 Main Menu]
Project cp05T5e12...
FPC Ctg1569 cp05T...

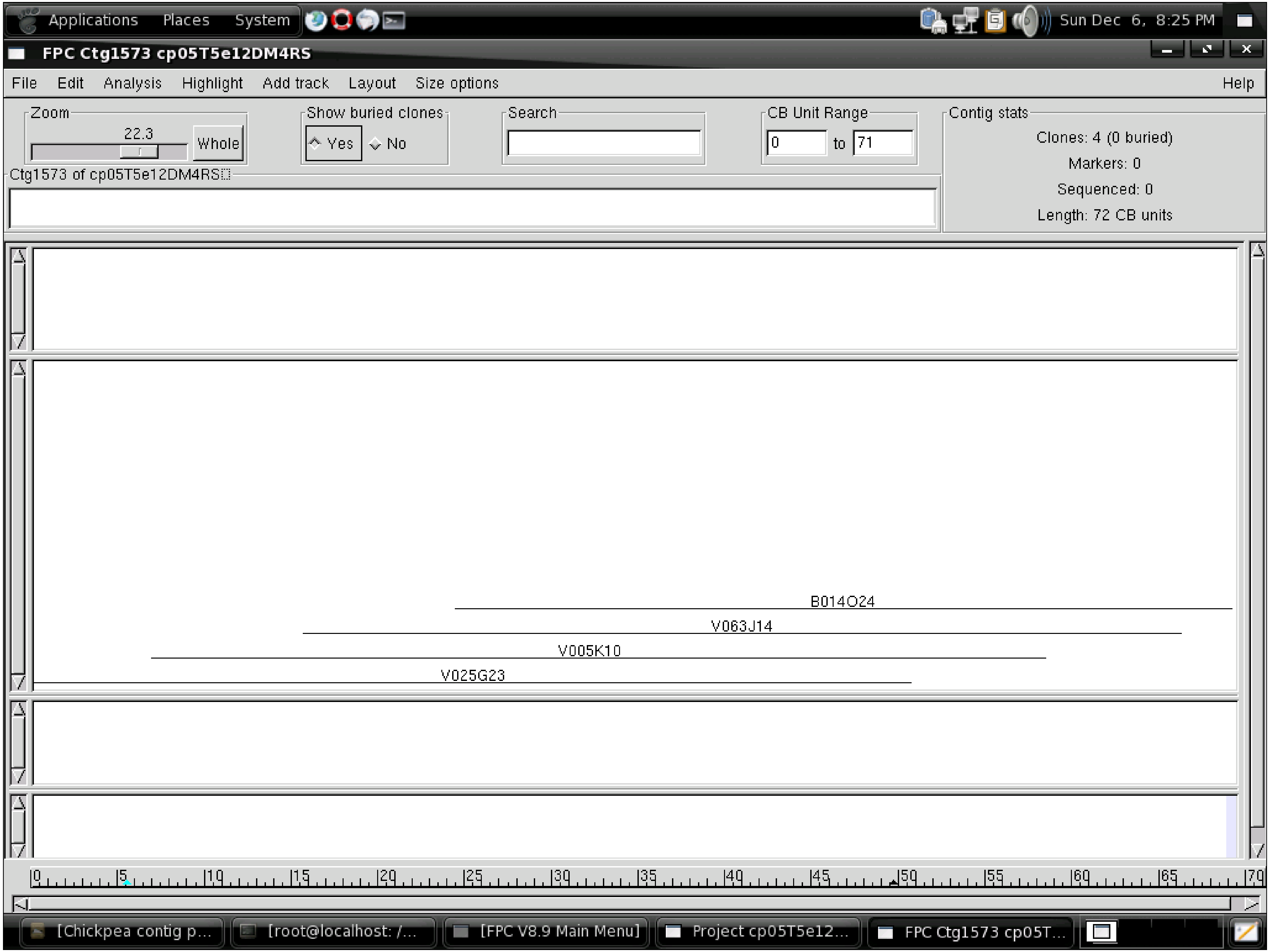
Applications Places System
Sun Dec 6, 8:25 PM
FPC Ctg1573 cp05T5e12DM4RS
File Edit Analysis Highlight Add track Layout Size options Help
Zoom
22.3
Whole
Show buried clones
Yes No
Search
CB Unit Range
0 to 71
Contig stats
Clones: 4 (0 buried)
Markers: 0
Sequenced: 0
Length: 72 CB units
Ctg1573 of cp05T5e12DM4RS
B014O24
V063J14
V005K10
V025G23
0 5 10 15 20 25 30 35 40 45 50 55 60 65 70
[Chickpea contig p...
[root@localhost: /...
[FPC V8.9 Main Menu]
Project cp05T5e12...
FPC Ctg1573 cp05T...

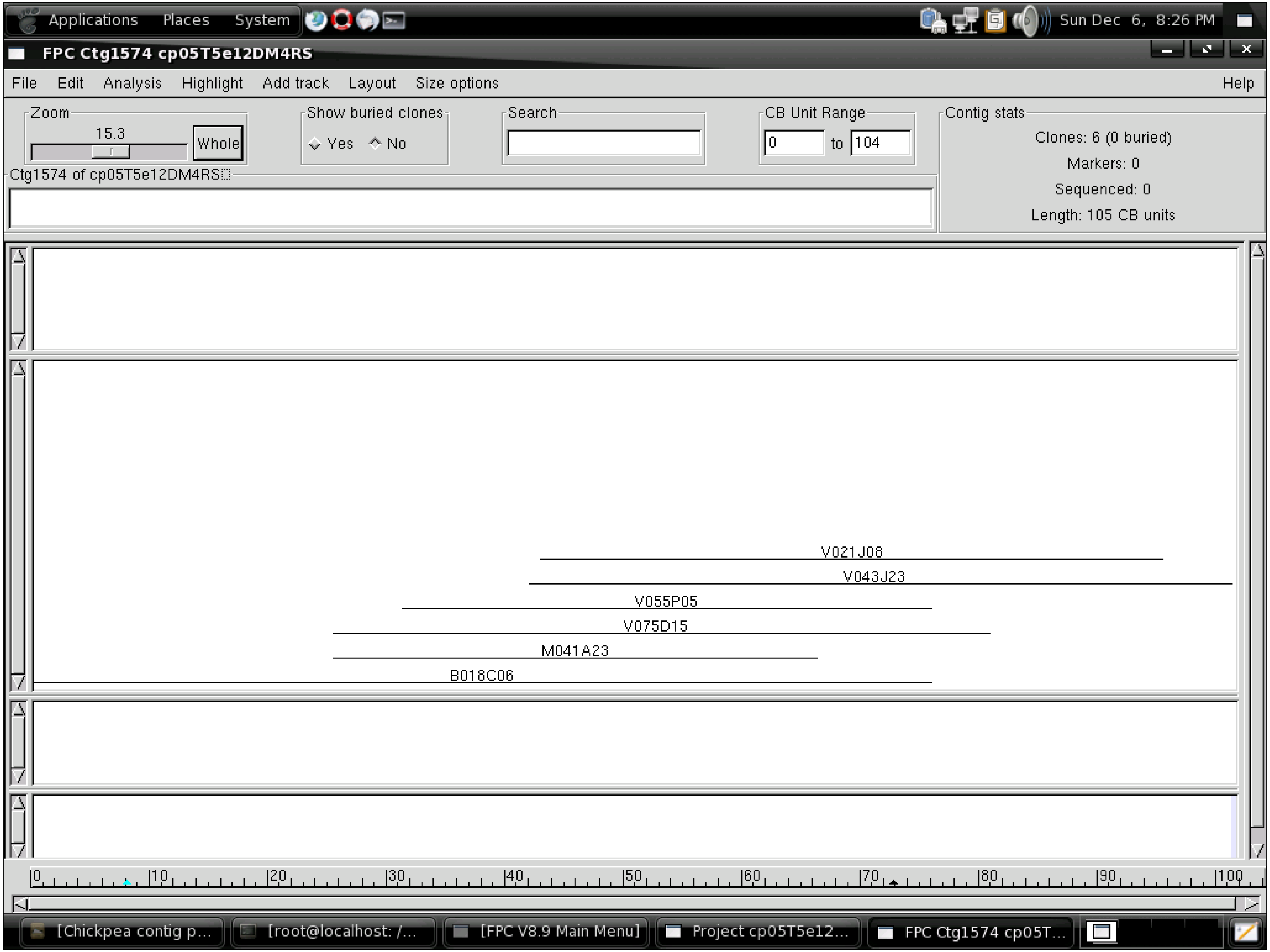
Applications Places System
Sun Dec 6, 8:26 PM
FPC Ctg1574 cp05T5e12DM4RS
File Edit Analysis Highlight Add track Layout Size options Help
Zoom
15.3
Whole
Show buried clones
Yes No
Search
CB Unit Range
0 to 104
Contig stats
Clones: 6 (0 buried)
Markers: 0
Sequenced: 0
Length: 105 CB units
Ctg1574 of cp05T5e12DM4RS
V021J08
V043J23
V055P05
V075D15
M041A23
B018C06
0 10 20 30 40 50 60 70 80 90 100
[Chickpea contig p...
[root@localhost: /...
[FPC V8.9 Main Menu]
Project cp05T5e12...
FPC Ctg1574 cp05T...

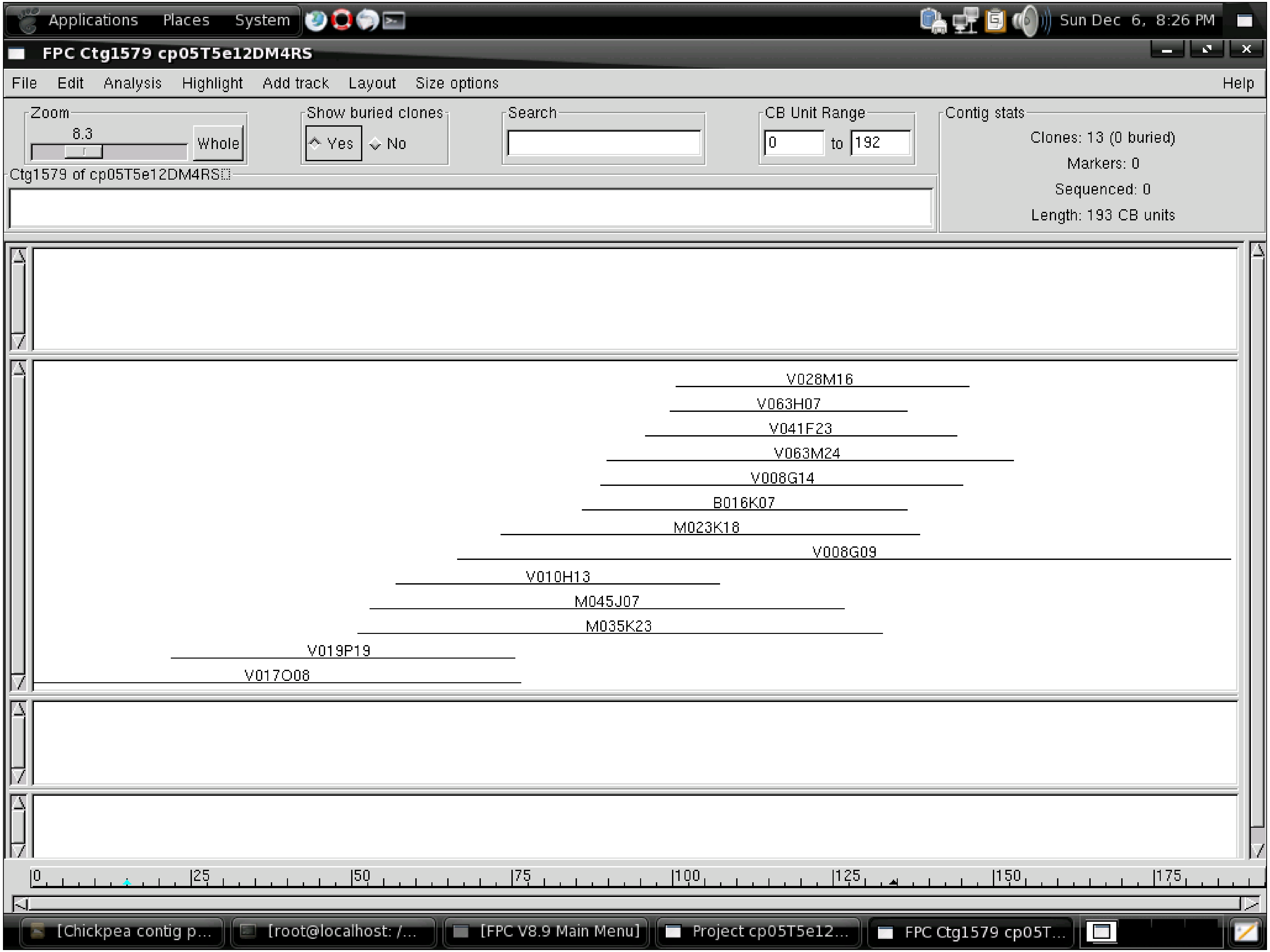
Applications Places System
Sun Dec 6, 8:26 PM
FPC Ctg1579 cp05T5e12DM4RS
File Edit Analysis Highlight Add track Layout Size options Help
Zoom
8.3
Whole
Show buried clones
Yes
No
Search
CB Unit Range
0
to
192
Contig stats
Clones: 13 (0 buried)
Markers: 0
Sequenced: 0
Length: 193 CB units
Ctg1579 of cp05T5e12DM4RS
V028M16
V063H07
V041F23
V063M24
V008G14
B016K07
M023K18
V008G09
V010H13
M045J07
M035K23
V019P19
V017O08
0
25
50
75
100
125
150
175
[Chickpea contig p...
[root@localhost: /...
[FPC V8.9 Main Menu]
Project cp05T5e12...
FPC Ctg1579 cp05T...

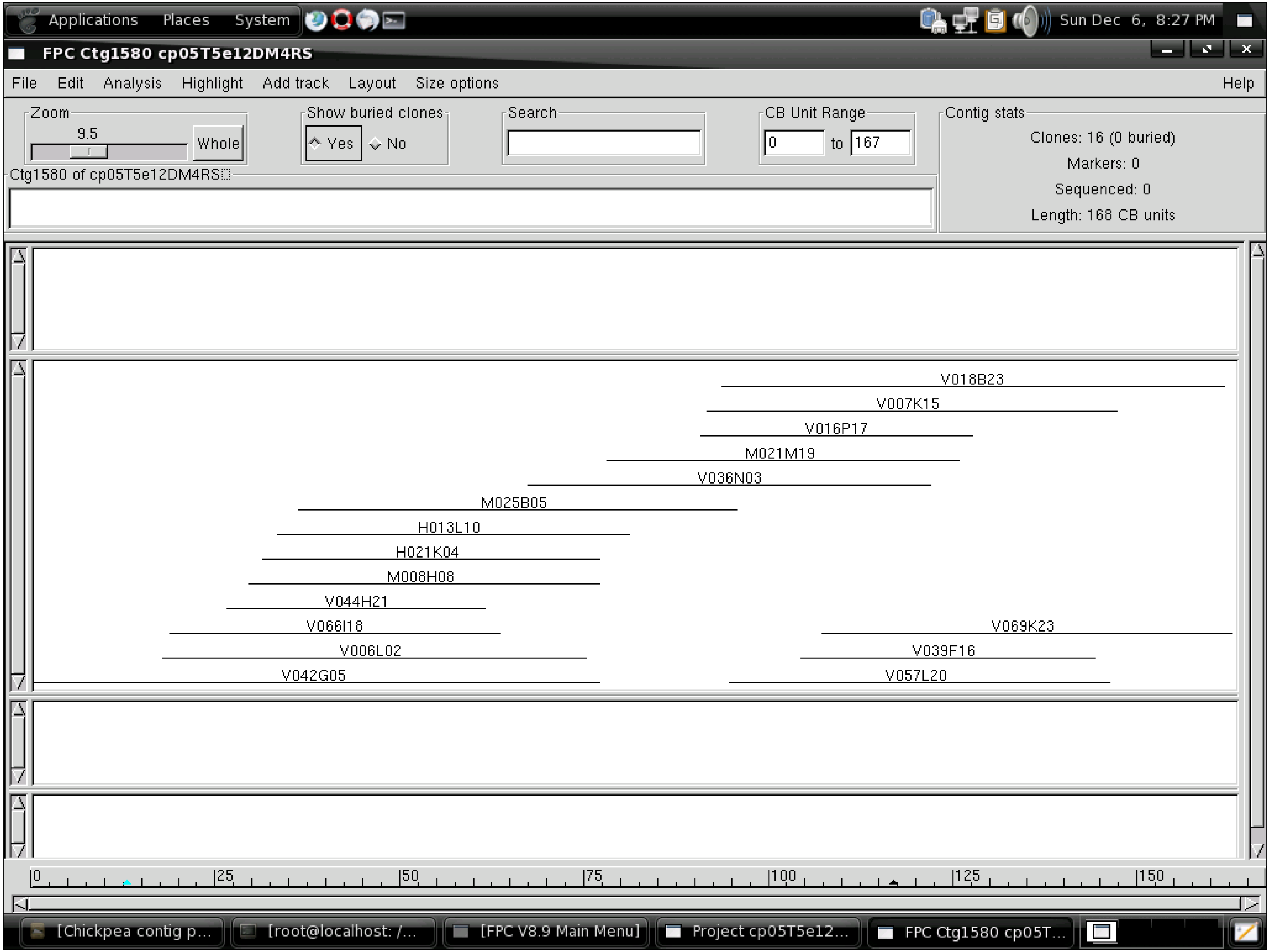

Applications Places System
Sun Dec 6, 8:27 PM
FPC Ctg1580 cp05T5e12DM4RS
File Edit Analysis Highlight Add track Layout Size options Help
Zoom 9.5 Whole
Show buried clones Yes No
Search
CB Unit Range 0 to 167
Contig stats
Clones: 16 (0 buried)
Markers: 0
Sequenced: 0
Length: 168 CB units
Ctg1580 of cp05T5e12DM4RS
V018B23
V007K15
V016P17
M021M19
V036N03
M025B05
H013L10
H021K04
M008H08
V044H21
V066I18
V069K23
V006L02
V039F16
V042G05
V057L20
0 25 50 75 100 125 150
[Chickpea contig p...
[root@localhost: /...
[FPC V8.9 Main Menu]
Project cp05T5e12...
FPC Ctg1580 cp05T...

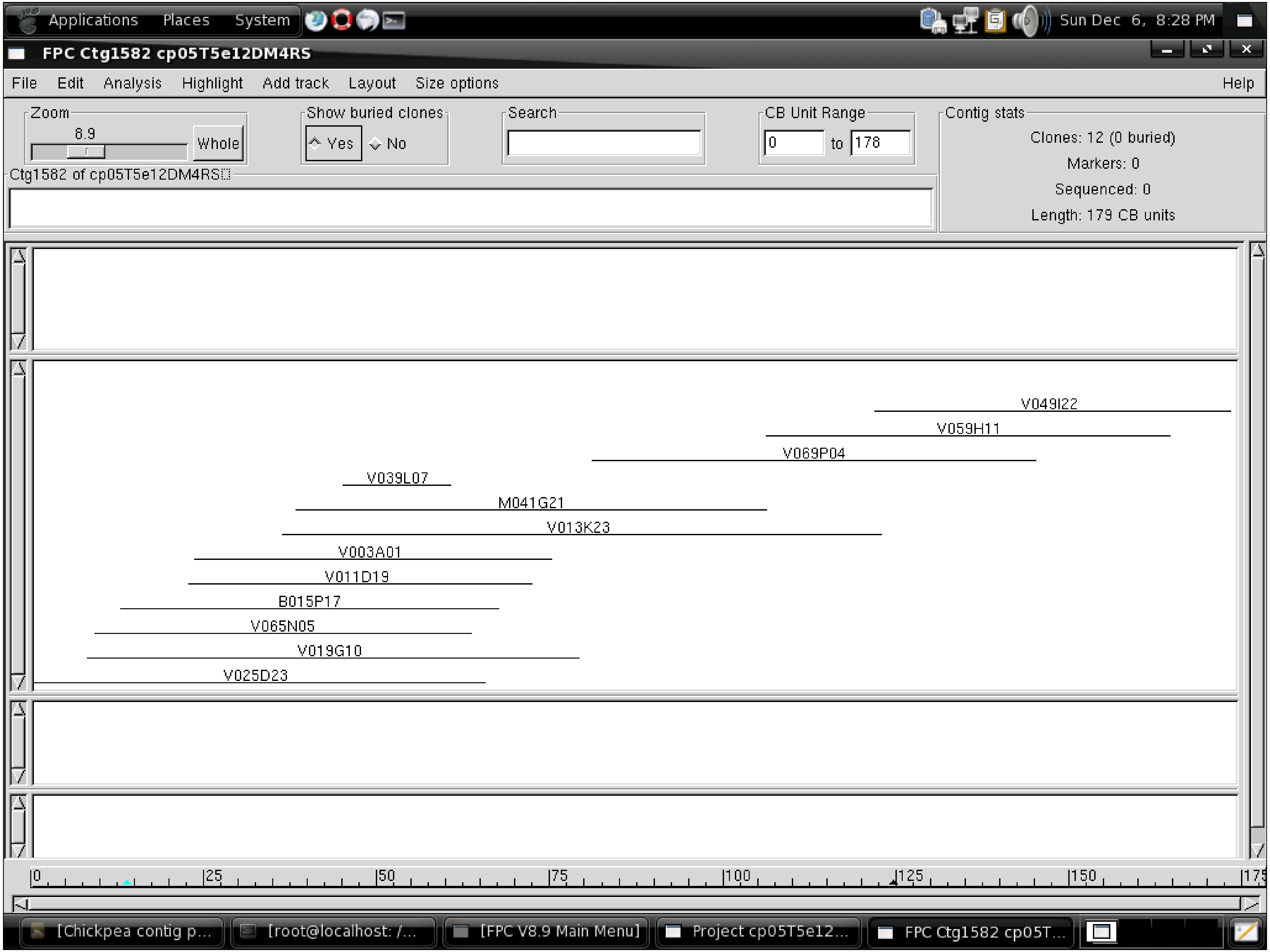
Applications Places System
Sun Dec 6, 8:28 PM
FPC Ctg1582 cp05T5e12DM4RS
File Edit Analysis Highlight Add track Layout Size options Help
Zoom
8.9
Whole
Show buried clones
Yes No
Search
CB Unit Range
0 to 178
Contig stats
Clones: 12 (0 buried)
Markers: 0
Sequenced: 0
Length: 179 CB units
Ctg1582 of cp05T5e12DM4RS
V049I22
V059H11
V069P04
V039L07
M041G21
V013K23
V003A01
V011D19
B015P17
V065N05
V019G10
V025D23
0 25 50 75 100 125 150 175
[Chickpea contig p...
[root@localhost: /...
[FPC V8.9 Main Menu]
Project cp05T5e12...
FPC Ctg1582 cp05T...

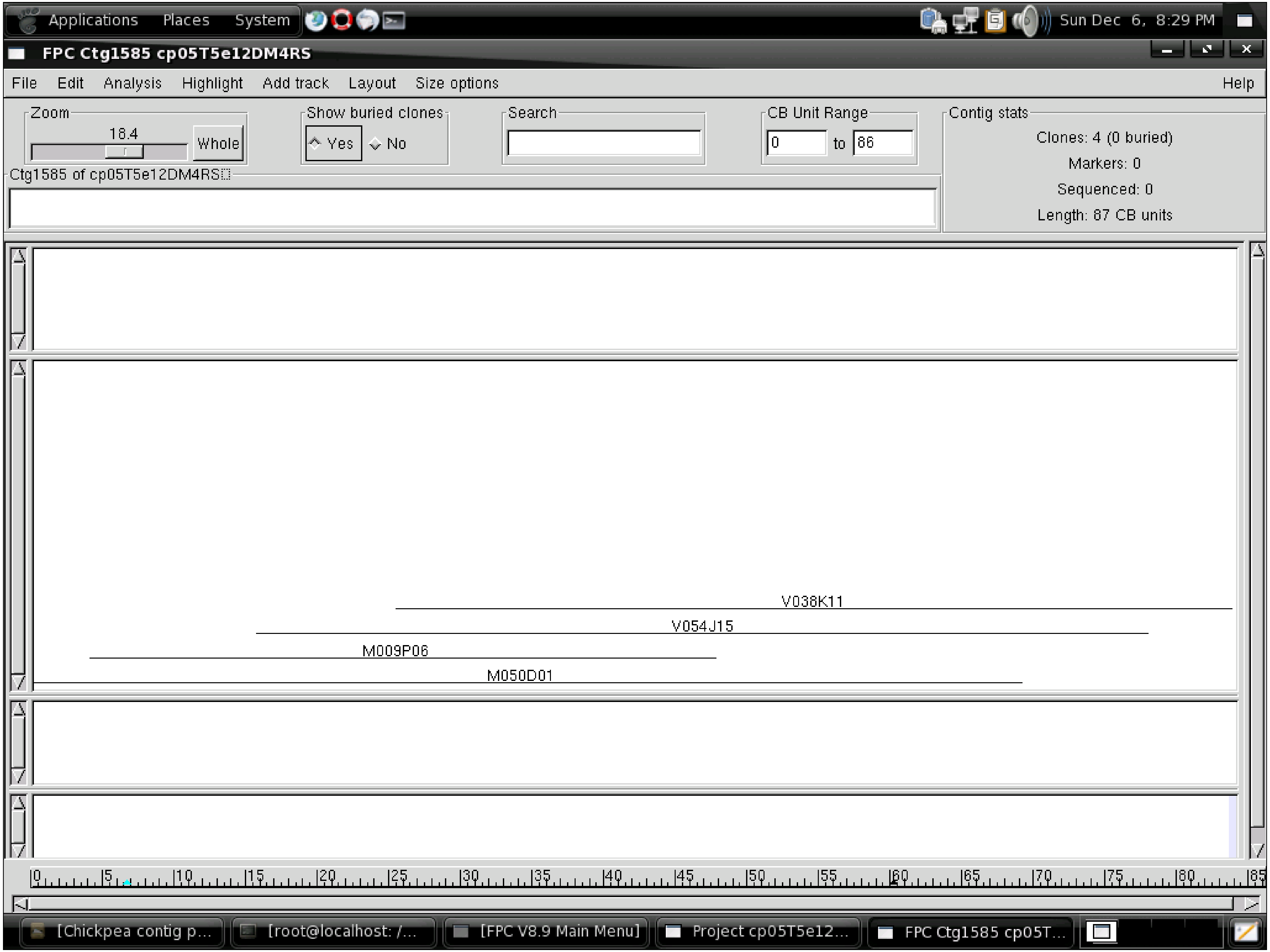
Applications Places System
Sun Dec 6, 8:29 PM
FPC Ctg1585 cp05T5e12DM4RS
File Edit Analysis Highlight Add track Layout Size options Help
Zoom
18.4
Whole
Show buried clones
Yes
No
Search
CB Unit Range
0
to
86
Contig stats
Clones: 4 (0 buried)
Markers: 0
Sequenced: 0
Length: 87 CB units
Ctg1585 of cp05T5e12DM4RS
V038K11
V054J15
M009P06
M050D01
[Chickpea contig p...
[root@localhost: /...
[FPC V8.9 Main Menu]
Project cp05T5e12...
FPC Ctg1585 cp05T...

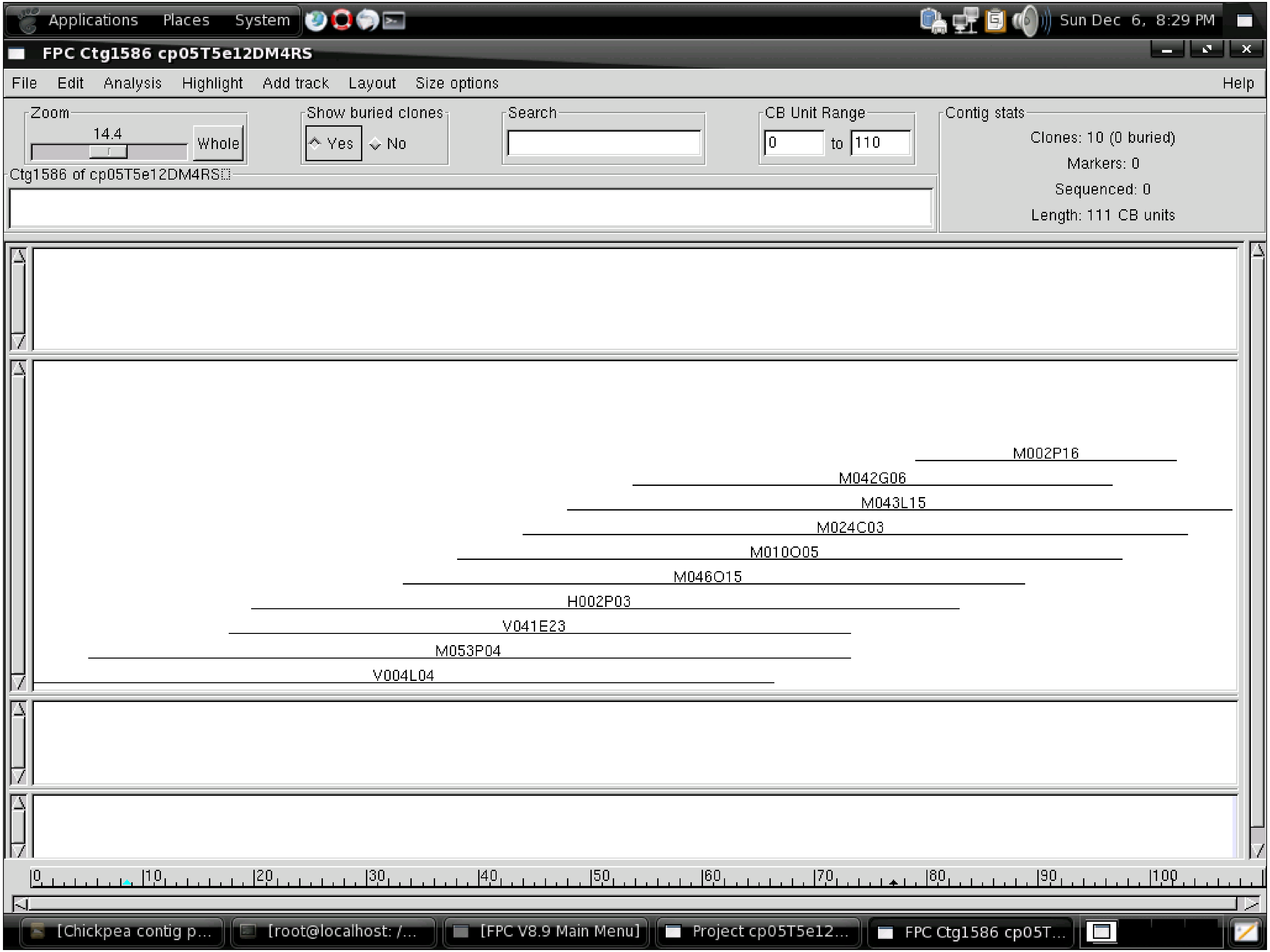
Applications Places System
Sun Dec 6, 8:29 PM
FPC Ctg1586 cp05T5e12DM4RS
File Edit Analysis Highlight Add track Layout Size options Help
Zoom
14.4
Whole
Show buried clones
Yes No
Search
CB Unit Range
0 to 110
Contig stats
Clones: 10 (0 buried)
Markers: 0
Sequenced: 0
Length: 111 CB units
Ctg1586 of cp05T5e12DM4RS
M002P16
M042G06
M043L15
M024C03
M010O05
M046O15
H002P03
V041E23
M053P04
V004L04
0 10 20 30 40 50 60 70 80 90 100
[Chickpea contig p...
[root@localhost: /...
[FPC V8.9 Main Menu]
Project cp05T5e12...
FPC Ctg1586 cp05T...

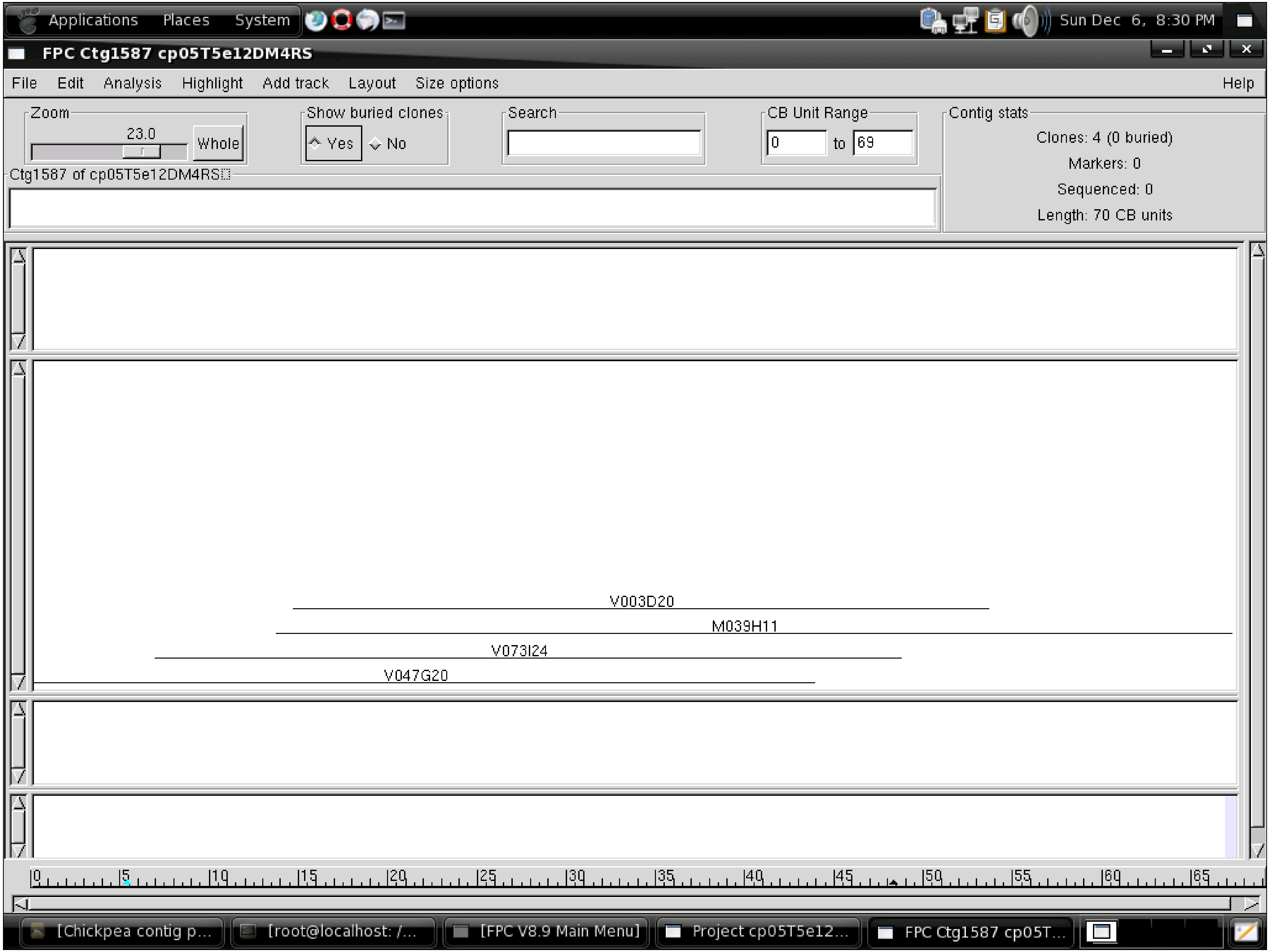
Applications Places System
Sun Dec 6, 8:30 PM
FPC Ctg1587 cp05T5e12DM4RS
File Edit Analysis Highlight Add track Layout Size options Help
Zoom
23.0
Whole
Show buried clones
Yes No
Search
CB Unit Range
0 to 69
Contig stats
Clones: 4 (0 buried)
Markers: 0
Sequenced: 0
Length: 70 CB units
Ctg1587 of cp05T5e12DM4RS
V003D20
M039H11
V073I24
V047G20
0 5 10 15 20 25 30 35 40 45 50 55 60 65
[Chickpea contig p...
[root@localhost: /...
[FPC V8.9 Main Menu]
Project cp05T5e12...
FPC Ctg1587 cp05T...

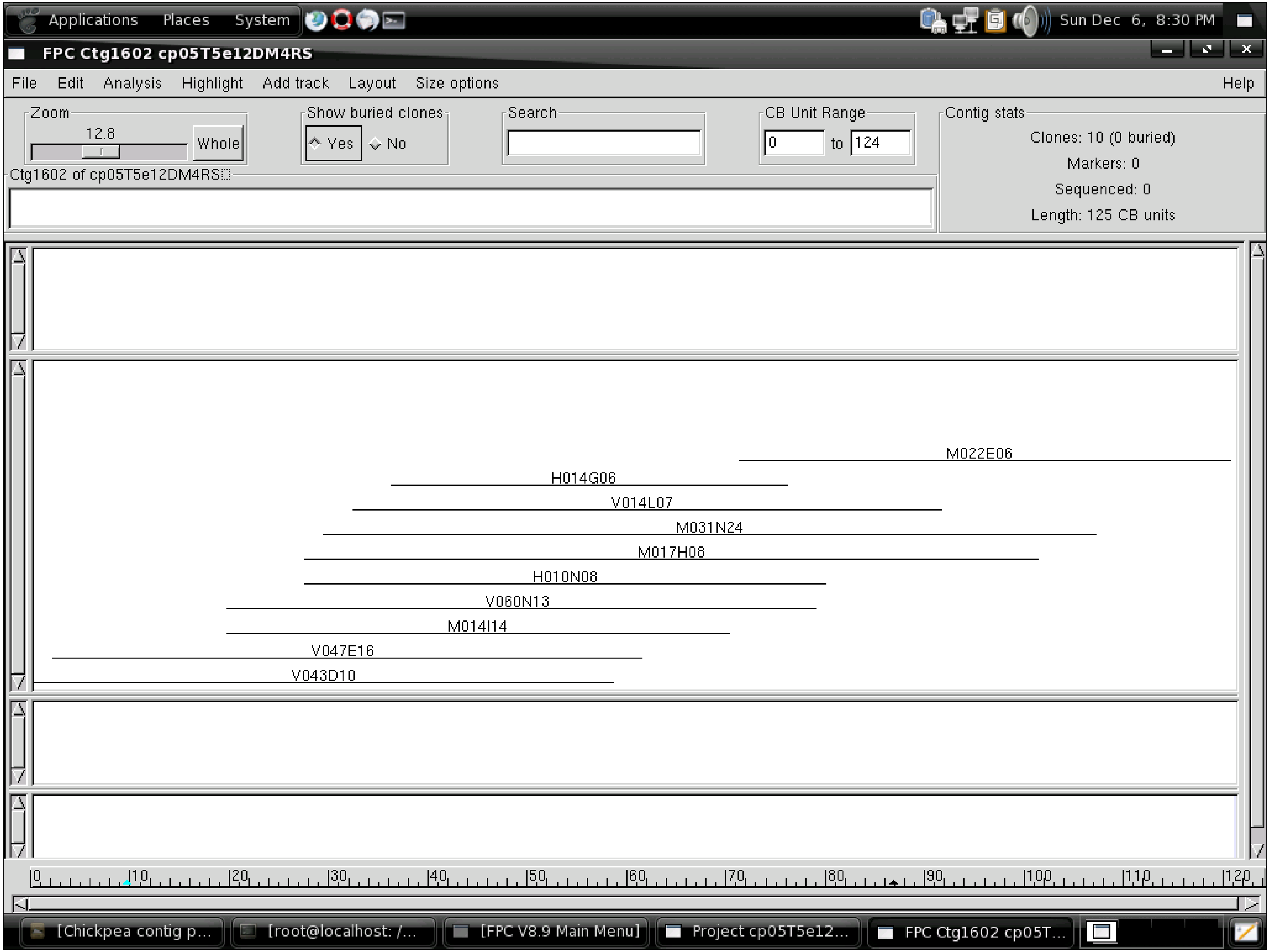
Applications Places System
Sun Dec 6, 8:30 PM
FPC Ctg1602 cp05T5e12DM4RS
File Edit Analysis Highlight Add track Layout Size options Help
Zoom
12.8
Whole
Show buried clones
Yes No
Search
CB Unit Range
0 to 124
Contig stats
Clones: 10 (0 buried)
Markers: 0
Sequenced: 0
Length: 125 CB units
Ctg1602 of cp05T5e12DM4RS
M022E06
H014G06
V014L07
M031N24
M017H08
H010N08
V060N13
M014I14
V047E16
V043D10
0 10 20 30 40 50 60 70 80 90 100 110 120
[Chickpea contig p...
[root@localhost: /...
[FPC V8.9 Main Menu]
Project cp05T5e12...
FPC Ctg1602 cp05T...

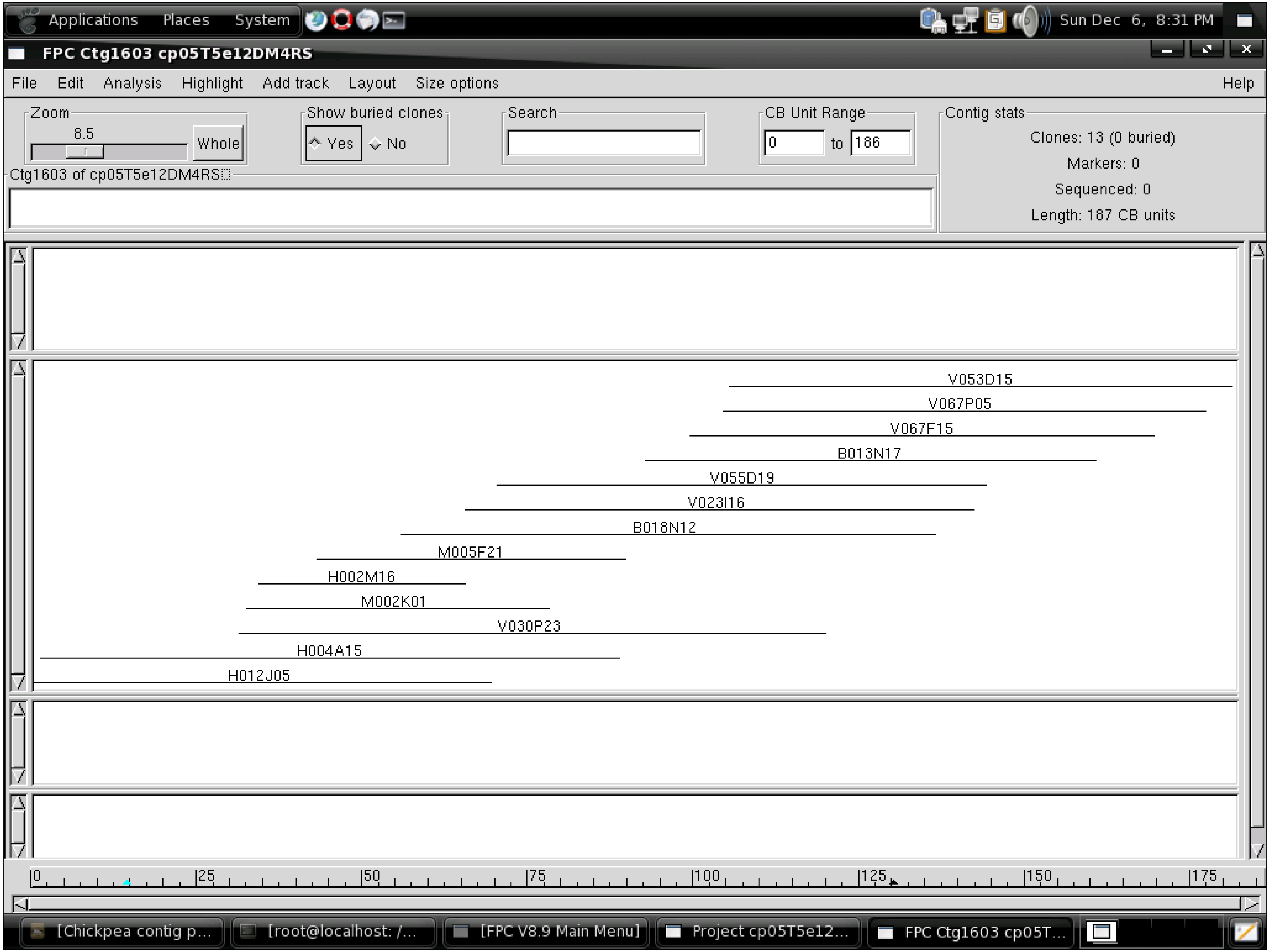
Applications Places System
Sun Dec 6, 8:31 PM
FPC Ctg1603 cp05T5e12DM4RS
File Edit Analysis Highlight Add track Layout Size options Help
Zoom
8.5
Whole
Show buried clones
Yes
No
Search
CB Unit Range
0 to 186
Contig stats
Clones: 13 (0 buried)
Markers: 0
Sequenced: 0
Length: 187 CB units
Ctg1603 of cp05T5e12DM4RS
V053D15
V067P05
V067F15
B013N17
V055D19
V023I16
B018N12
M005F21
H002M16
M002K01
V030P23
H004A15
H012J05
0
25
50
75
100
125
150
175
[Chickpea contig p...
[root@localhost: /...
[FPC V8.9 Main Menu]
Project cp05T5e12...
FPC Ctg1603 cp05T...

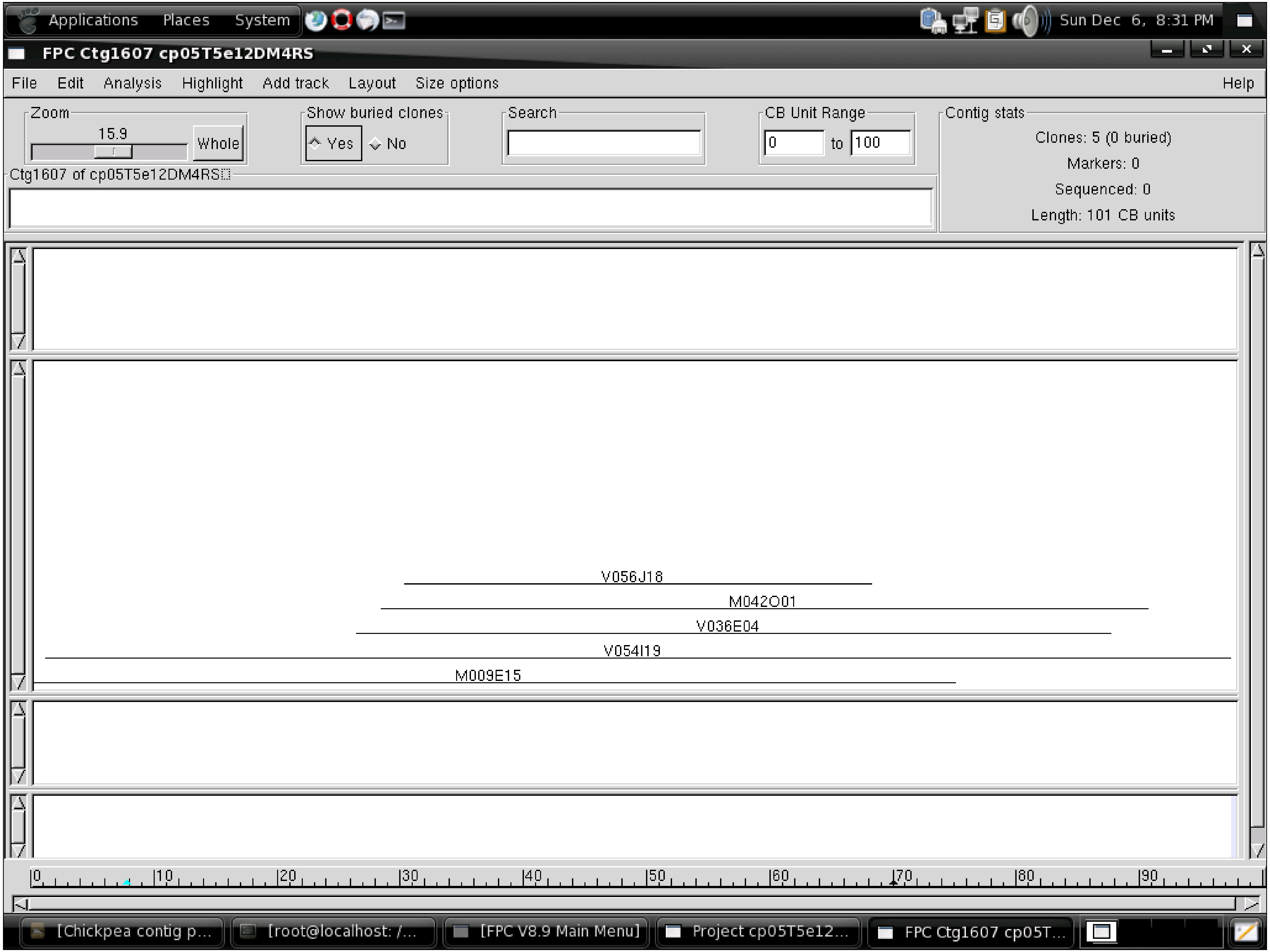

Applications Places System
Sun Dec 6, 8:31 PM
FPC Ctg1607 cp05T5e12DM4RS
File Edit Analysis Highlight Add track Layout Size options Help
Zoom
15.9
Whole
Show buried clones
Yes
No
Search
CB Unit Range
0
to
100
Contig stats
Clones: 5 (0 buried)
Markers: 0
Sequenced: 0
Length: 101 CB units
Ctg1607 of cp05T5e12DM4RS
V056J18
M042O01
V036E04
V054I19
M009E15
0 10 20 30 40 50 60 70 80 90
[Chickpea contig p...
[root@localhost: /...
[FPC V8.9 Main Menu]
Project cp05T5e12...
FPC Ctg1607 cp05T...

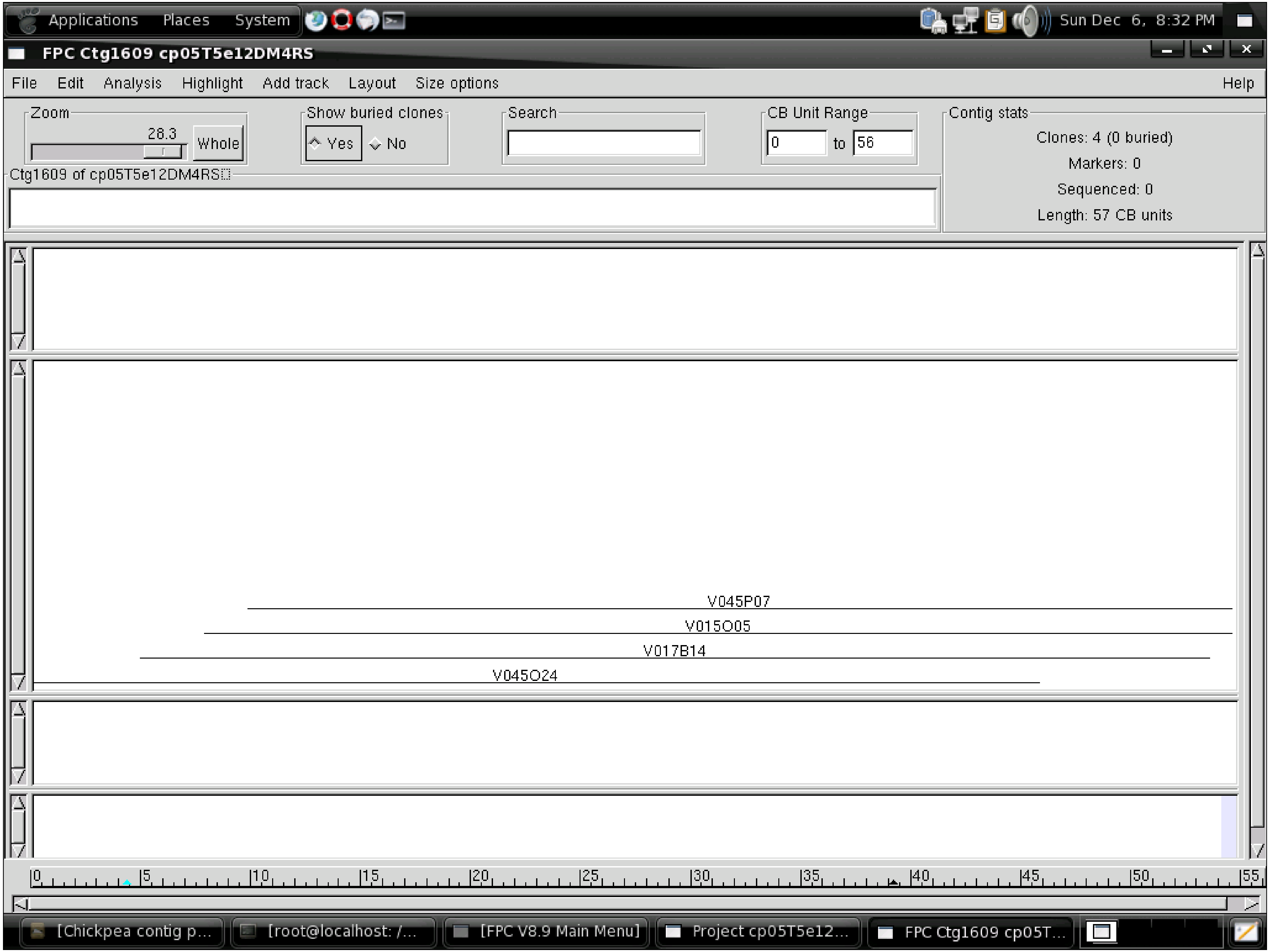
Applications Places System
Sun Dec 6, 8:32 PM
FPC Ctg1609 cp05T5e12DM4RS
File Edit Analysis Highlight Add track Layout Size options Help
Zoom
28.3
Whole
Show buried clones
Yes No
Search
CB Unit Range
0 to 56
Contig stats
Clones: 4 (0 buried)
Markers: 0
Sequenced: 0
Length: 57 CB units
Ctg1609 of cp05T5e12DM4RS
V045P07
V015O05
V017B14
V045O24
0 5 10 15 20 25 30 35 40 45 50 55
[Chickpea contig p...
[root@localhost: /...
[FPC V8.9 Main Menu]
Project cp05T5e12...
FPC Ctg1609 cp05T...

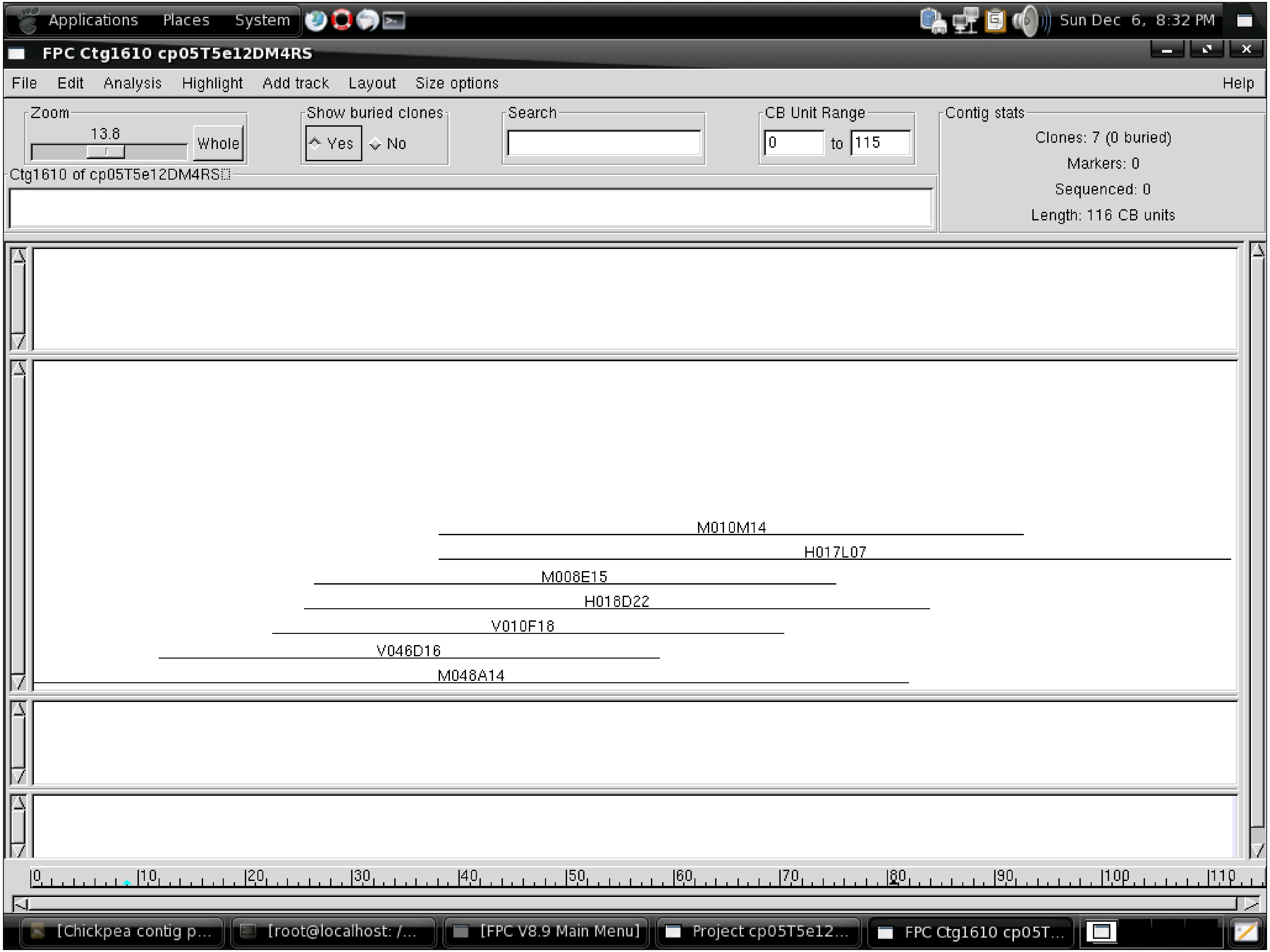

Applications
Places
System
Sun Dec 6, 8:32 PM
FPC Ctg1610 cp05T5e12DM4RS
File
Edit
Analysis
Highlight
Add track
Layout
Size options
Help
Zoom
13.8
Whole
Show buried clones
Yes
No
Search
CB Unit Range
0
to
115
Contig stats
Clones: 7 (0 buried)
Markers: 0
Sequenced: 0
Length: 116 CB units
Ctg1610 of cp05T5e12DM4RS
M010M14
H017L07
M008E15
H018D22
V010F18
V046D16
M048A14
0
10
20
30
40
50
60
70
80
90
100
110
[Chickpea contig p...
[root@localhost: /...
[FPC V8.9 Main Menu]
Project cp05T5e12...
FPC Ctg1610 cp05T...

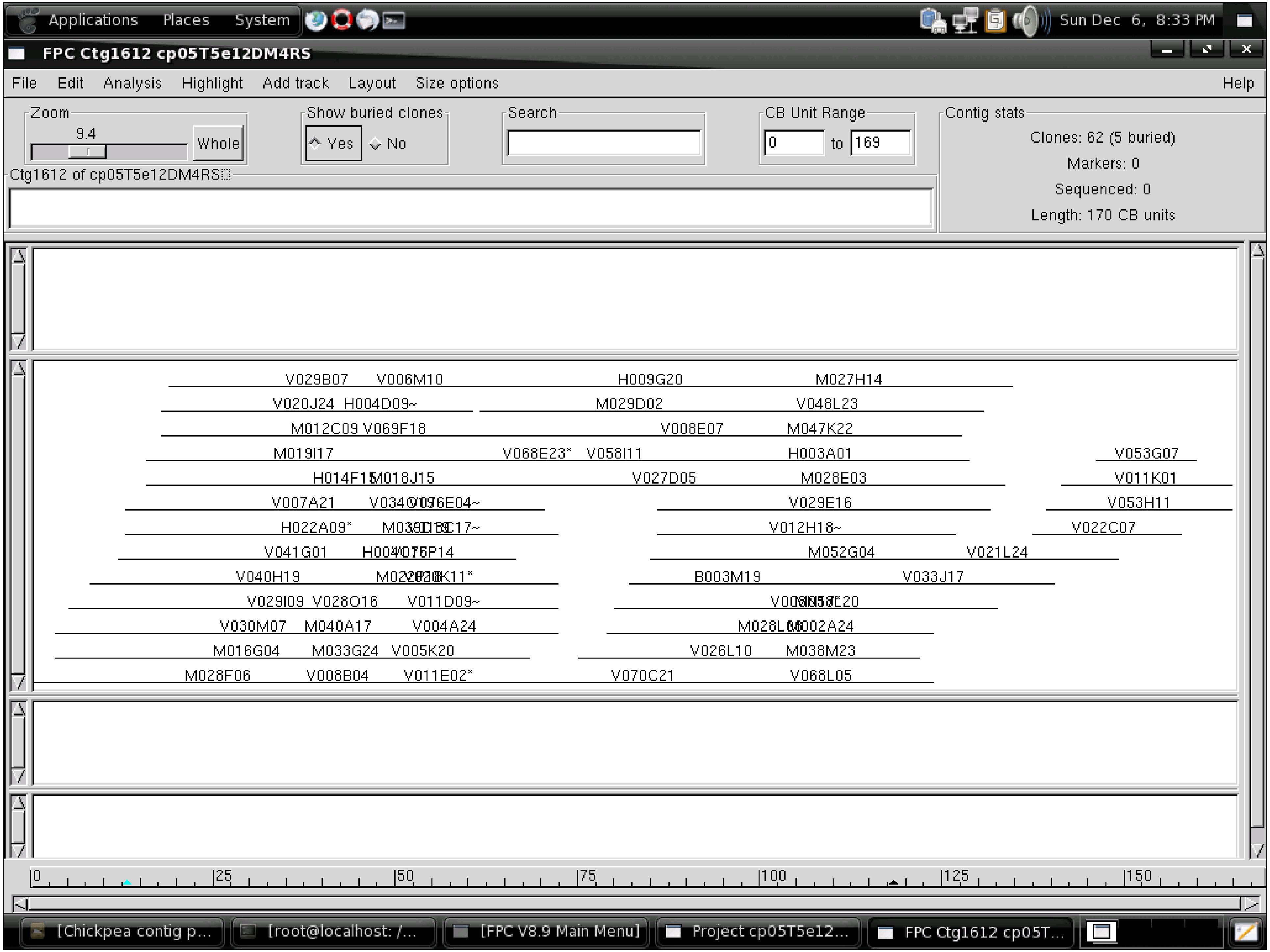
Applications Places System
Sun Dec 6, 8:33 PM
FPC Ctg1612 cp05T5e12DM4RS
File Edit Analysis Highlight Add track Layout Size options Help
Zoom
9.4
Whole
Show buried clones
Yes
No
Search
CB Unit Range
0 to 169
Contig stats
Clones: 62 (5 buried)
Markers: 0
Sequenced: 0
Length: 170 CB units
Ctg1612 of cp05T5e12DM4RS
V029B07 V006M10 H009G20 M027H14
V020J24 H004D09~ M029D02 V048L23
M012C09 V069F18 V008E07 M047K22
M019I17 V068E23* V058I11 H003A01 V053G07
V027D05 M028E03 V011K01
V007A21 V029E16 V053H11
H022A09* V012H18~ V022C07
V041G01 M052G04 V021L24
V040H19 B003M19 V033J17
V029I09 V028O16 V011D09~
V030M07 M040A17 V004A24
M016G04 M033G24 V005K20 V026L10 M038M23
M028F06 V008B04 V011E02* V070C21 V068L05
0 25 50 75 100 125 150
[Chickpea contig p...
[root@localhost: /...
[FPC V8.9 Main Menu]
Project cp05T5e12...
FPC Ctg1612 cp05T...

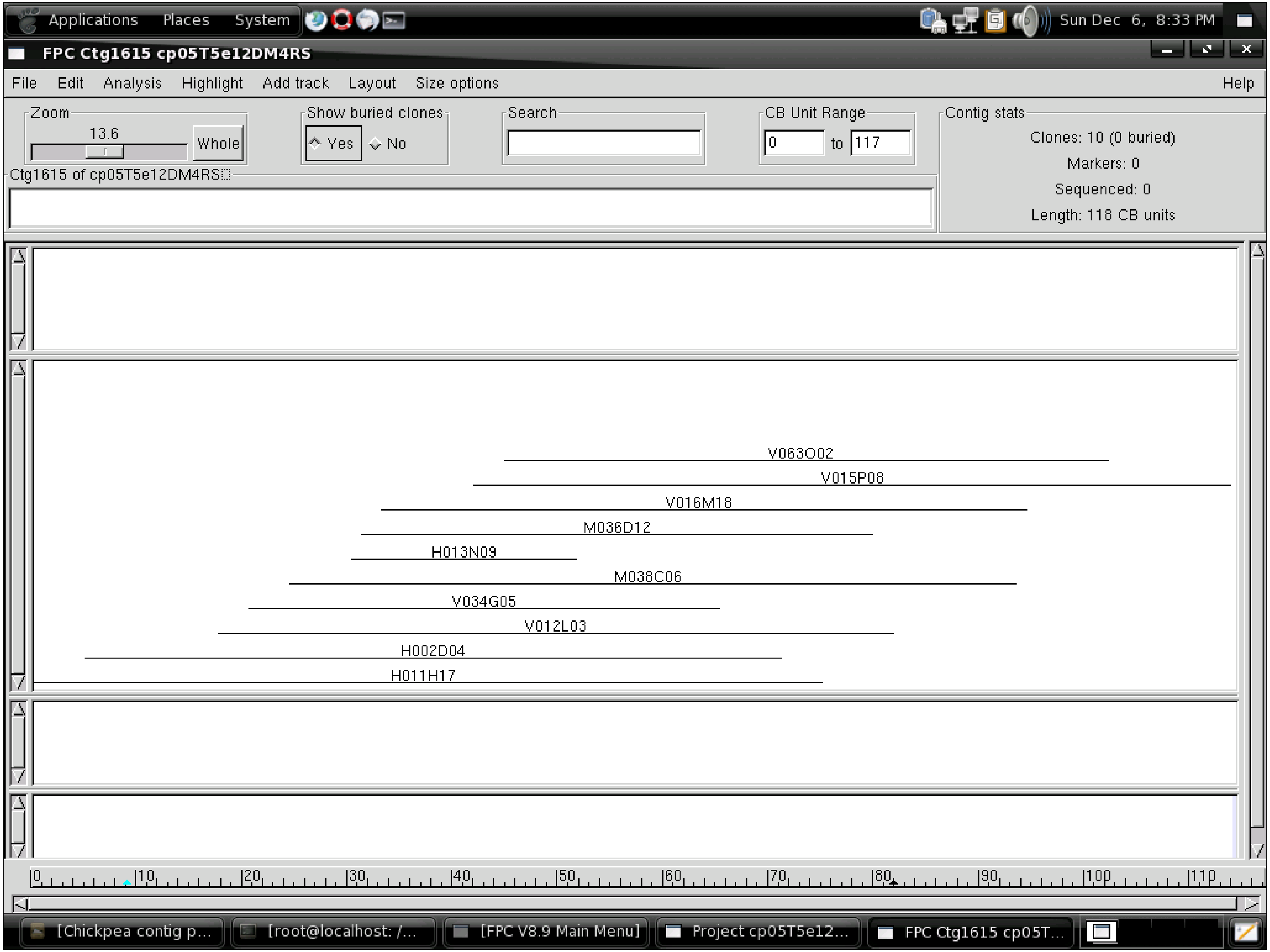
Applications Places System
Sun Dec 6, 8:33 PM
FPC Ctg1615 cp05T5e12DM4RS
File Edit Analysis Highlight Add track Layout Size options Help
Zoom
13.6
Whole
Show buried clones
Yes No
Search
CB Unit Range
0 to 117
Contig stats
Clones: 10 (0 buried)
Markers: 0
Sequenced: 0
Length: 118 CB units
Ctg1615 of cp05T5e12DM4RS
V063O02
V015P08
V016M18
M036D12
H013N09
M038C06
V034G05
V012L03
H002D04
H011H17
0 10 20 30 40 50 60 70 80 90 100 110
[Chickpea contig p...
[root@localhost: /...
[FPC V8.9 Main Menu]
Project cp05T5e12...
FPC Ctg1615 cp05T...

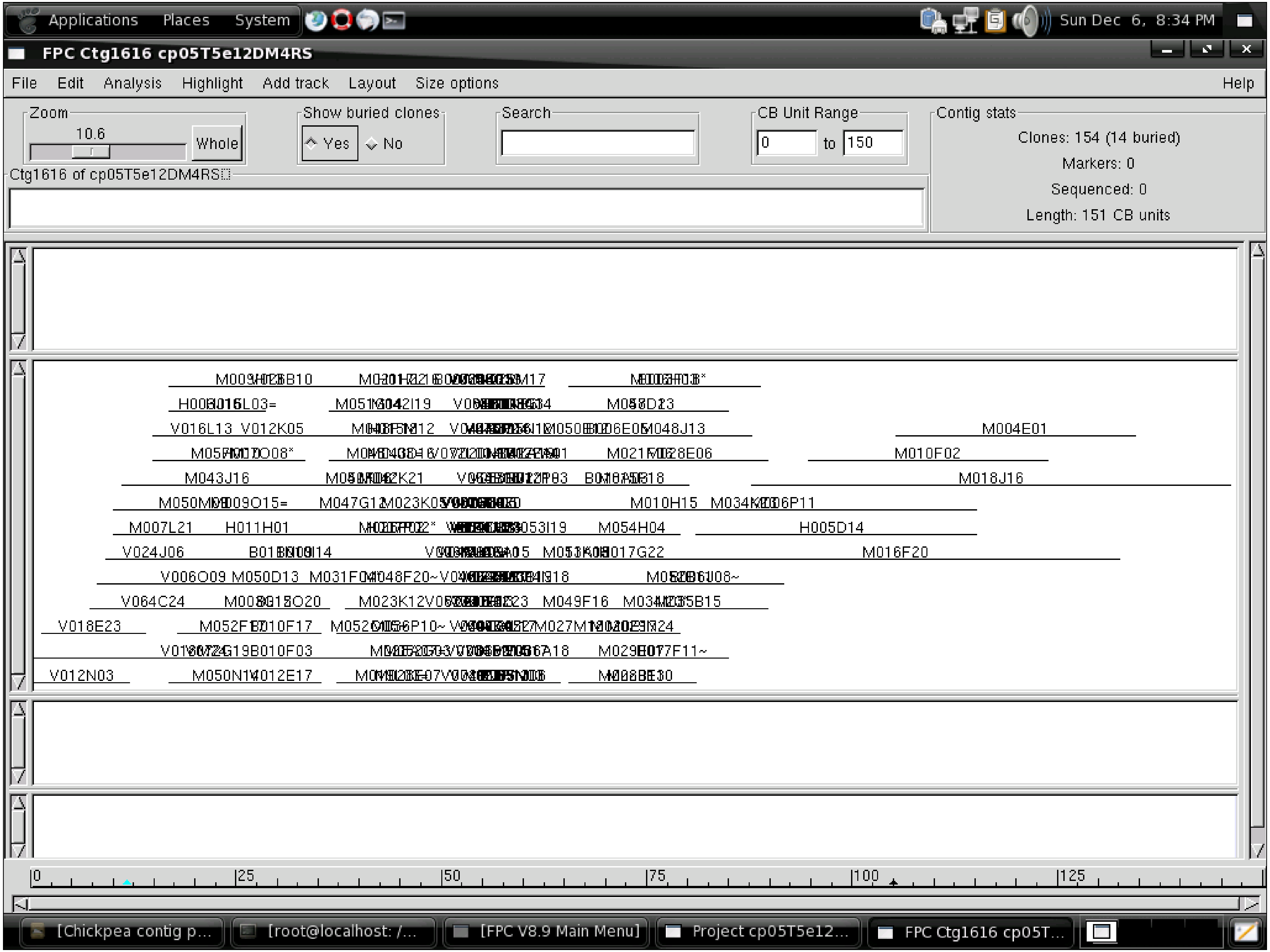

Applications Places System
Sun Dec 6, 8:34 PM
FPC Ctg1616 cp05T5e12DM4RS
File Edit Analysis Highlight Add track Layout Size options Help
Zoom
10.6
Whole
Show buried clones
Yes No
Search
CB Unit Range
0 to 150
Contig stats
Clones: 154 (14 buried)
Markers: 0
Sequenced: 0
Length: 151 CB units
Ctg1616 of cp05T5e12DM4RS
M004E01
M010F02
M018J16
H005D14
M016F20
V016L13 V012K05
M043J16
M007L21 H011H01
V024J06
V064C24
V018E23
V012N03
M054H04
M049F16
0 25 50 75 100 125
[Chickpea contig p...
[root@localhost: /...
[FPC V8.9 Main Menu]
Project cp05T5e12...
FPC Ctg1616 cp05T...

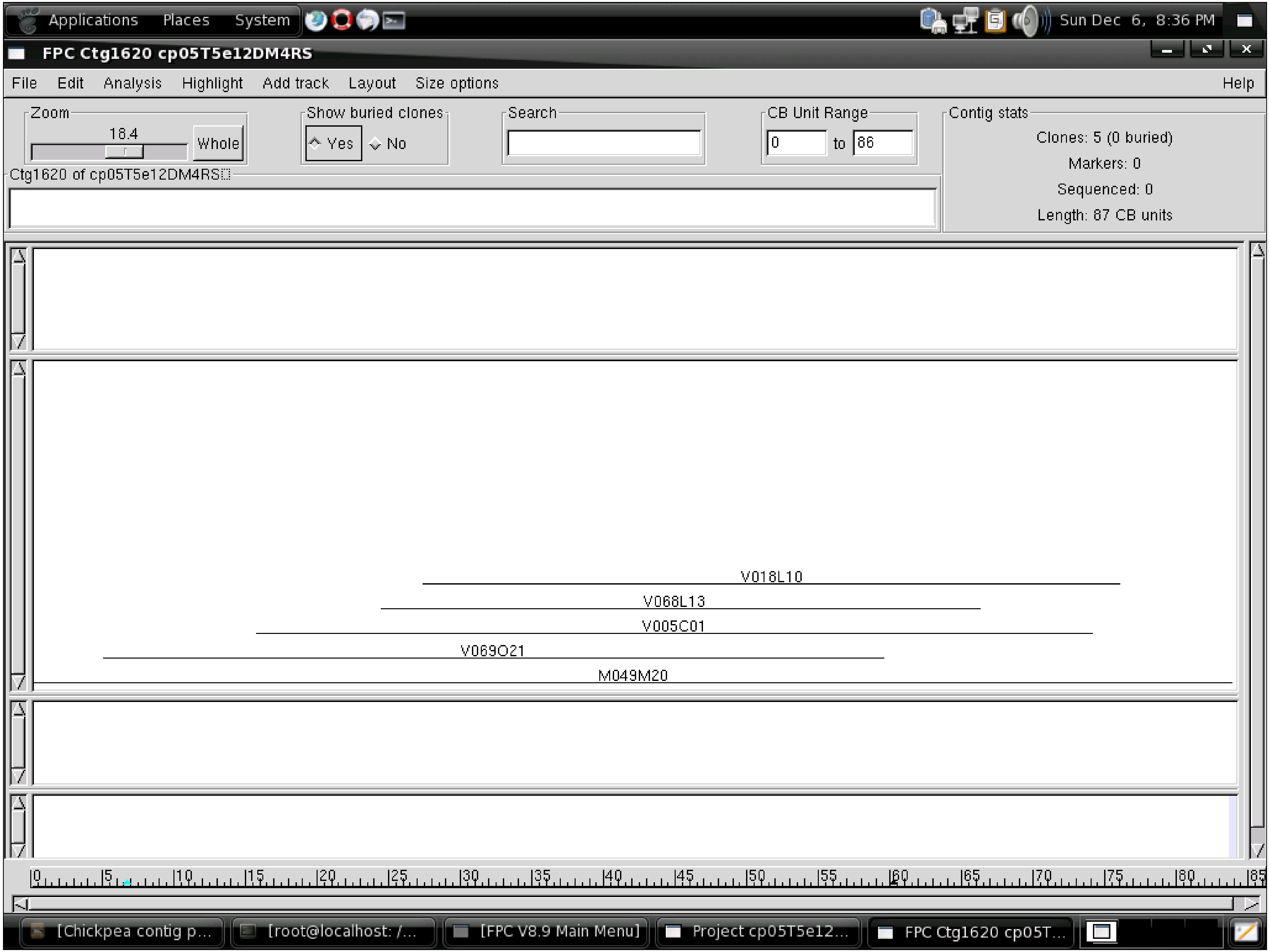

Applications Places System
Sun Dec 6, 8:36 PM
FPC Ctg1620 cp05T5e12DM4RS
File Edit Analysis Highlight Add track Layout Size options Help
Zoom
18.4
Whole
Show buried clones
Yes
No
Search
CB Unit Range
0
to
86
Contig stats
Clones: 5 (0 buried)
Markers: 0
Sequenced: 0
Length: 87 CB units
Ctg1620 of cp05T5e12DM4RS
V018L10
V068L13
V005C01
V069O21
M049M20
0 5 10 15 20 25 30 35 40 45 50 55 60 65 70 75 80 85
[Chickpea contig p...
[root@localhost: /...
[FPC V8.9 Main Menu]
Project cp05T5e12...
FPC Ctg1620 cp05T...

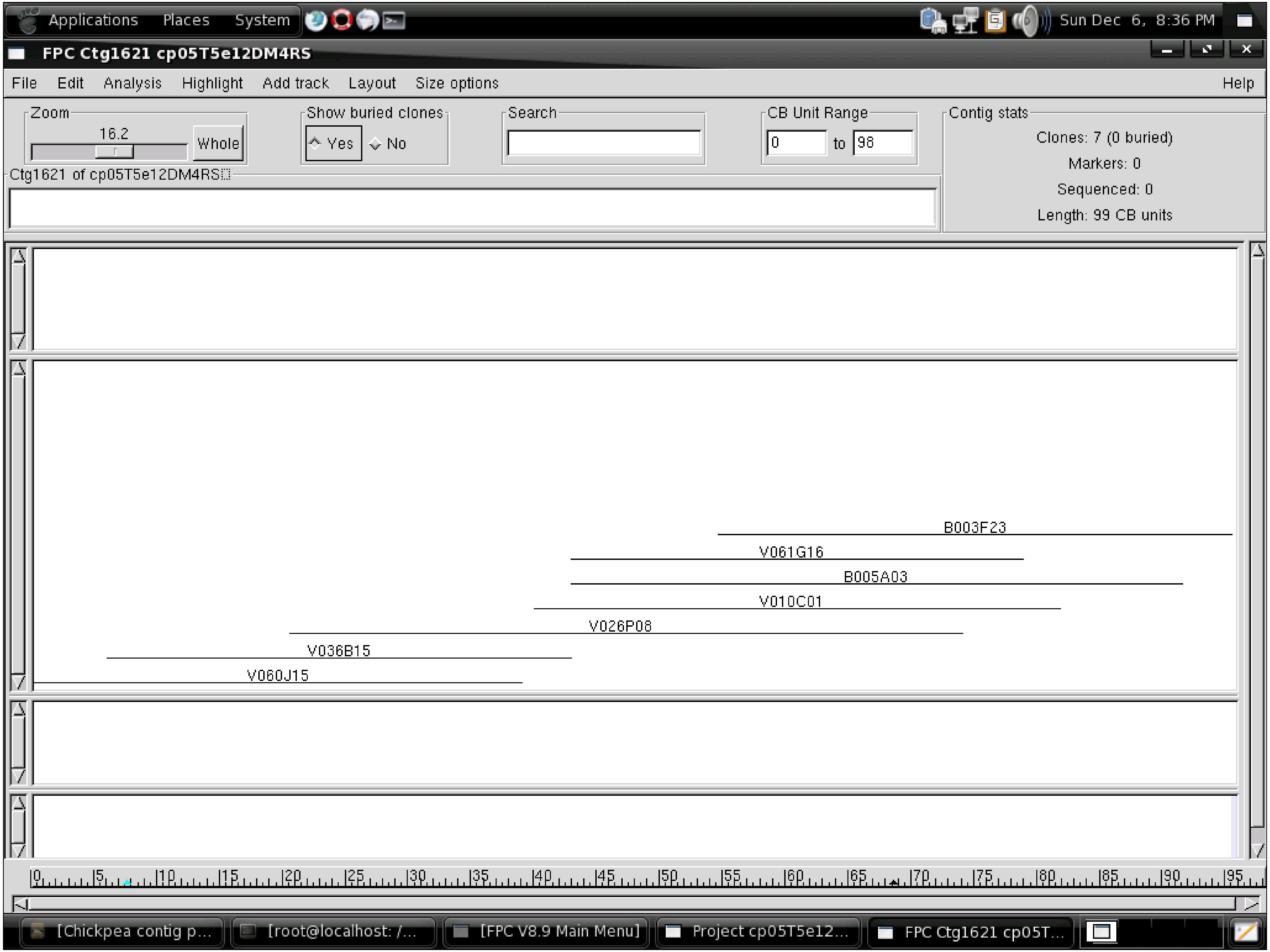

Applications Places System
Sun Dec 6, 8:36 PM
FPC Ctg1621 cp05T5e12DM4RS
File Edit Analysis Highlight Add track Layout Size options Help
Zoom
16.2
Whole
Show buried clones
Yes
No
Search
CB Unit Range
0 to 98
Contig stats
Clones: 7 (0 buried)
Markers: 0
Sequenced: 0
Length: 99 CB units
Ctg1621 of cp05T5e12DM4RS
B003F23
V061G16
B005A03
V010C01
V026P08
V036B15
V060J15
0 5 10 15 20 25 30 35 40 45 50 55 60 65 70 75 80 85 90 95
[Chickpea contig p...
[root@localhost: /...
[FPC V8.9 Main Menu]
Project cp05T5e12...
FPC Ctg1621 cp05T...

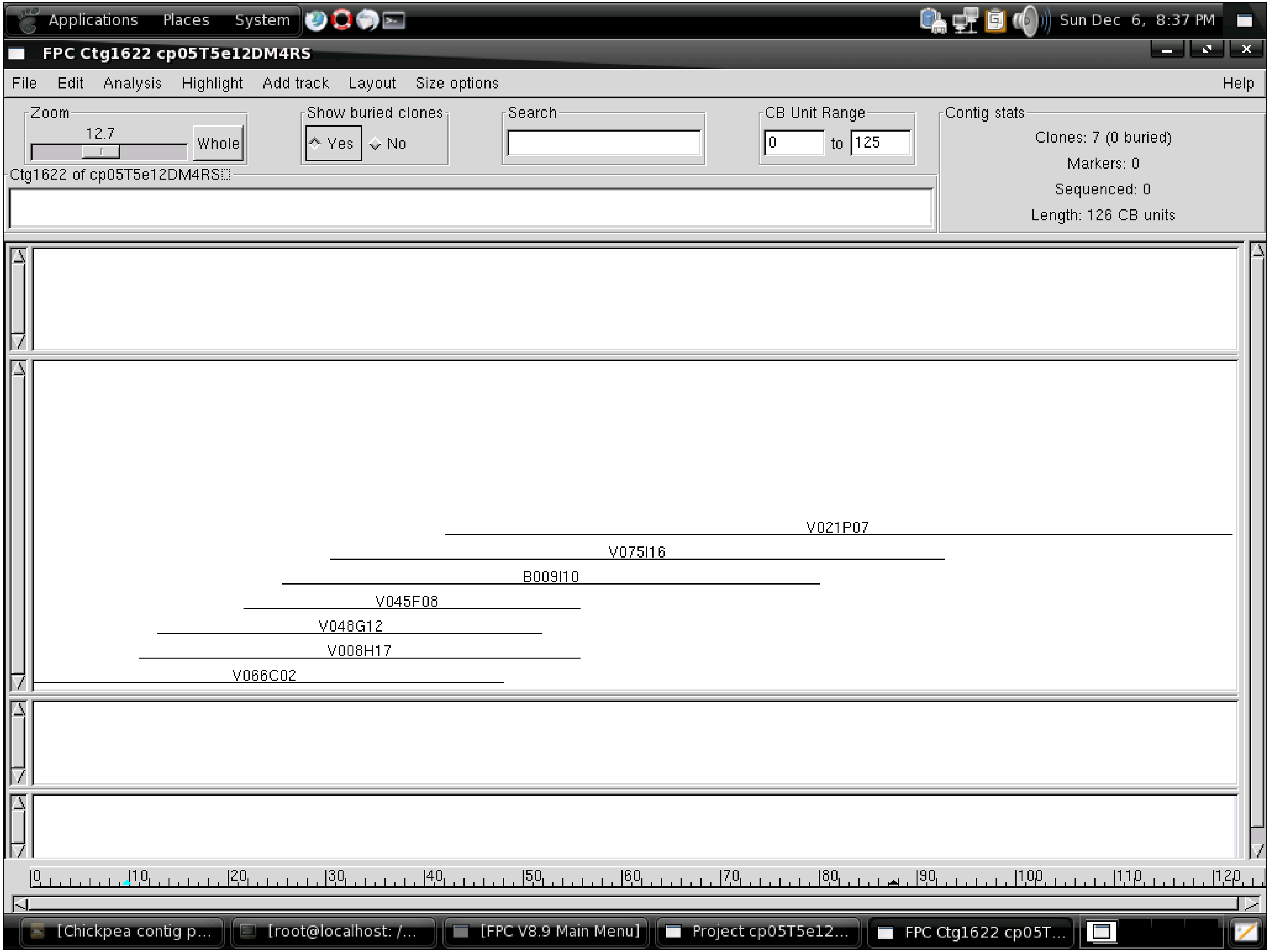
Applications Places System
Sun Dec 6, 8:37 PM
FPC Ctg1622 cp05T5e12DM4RS
File Edit Analysis Highlight Add track Layout Size options Help
Zoom
12.7
Whole
Show buried clones
Yes
No
Search
CB Unit Range
0 to 125
Contig stats
Clones: 7 (0 buried)
Markers: 0
Sequenced: 0
Length: 126 CB units
Ctg1622 of cp05T5e12DM4RS
V021P07
V075I16
B009I10
V045F08
V048G12
V008H17
V066C02
0 10 20 30 40 50 60 70 80 90 100 110 120
[Chickpea contig p...
[root@localhost: /...
[FPC V8.9 Main Menu]
Project cp05T5e12...
FPC Ctg1622 cp05T...

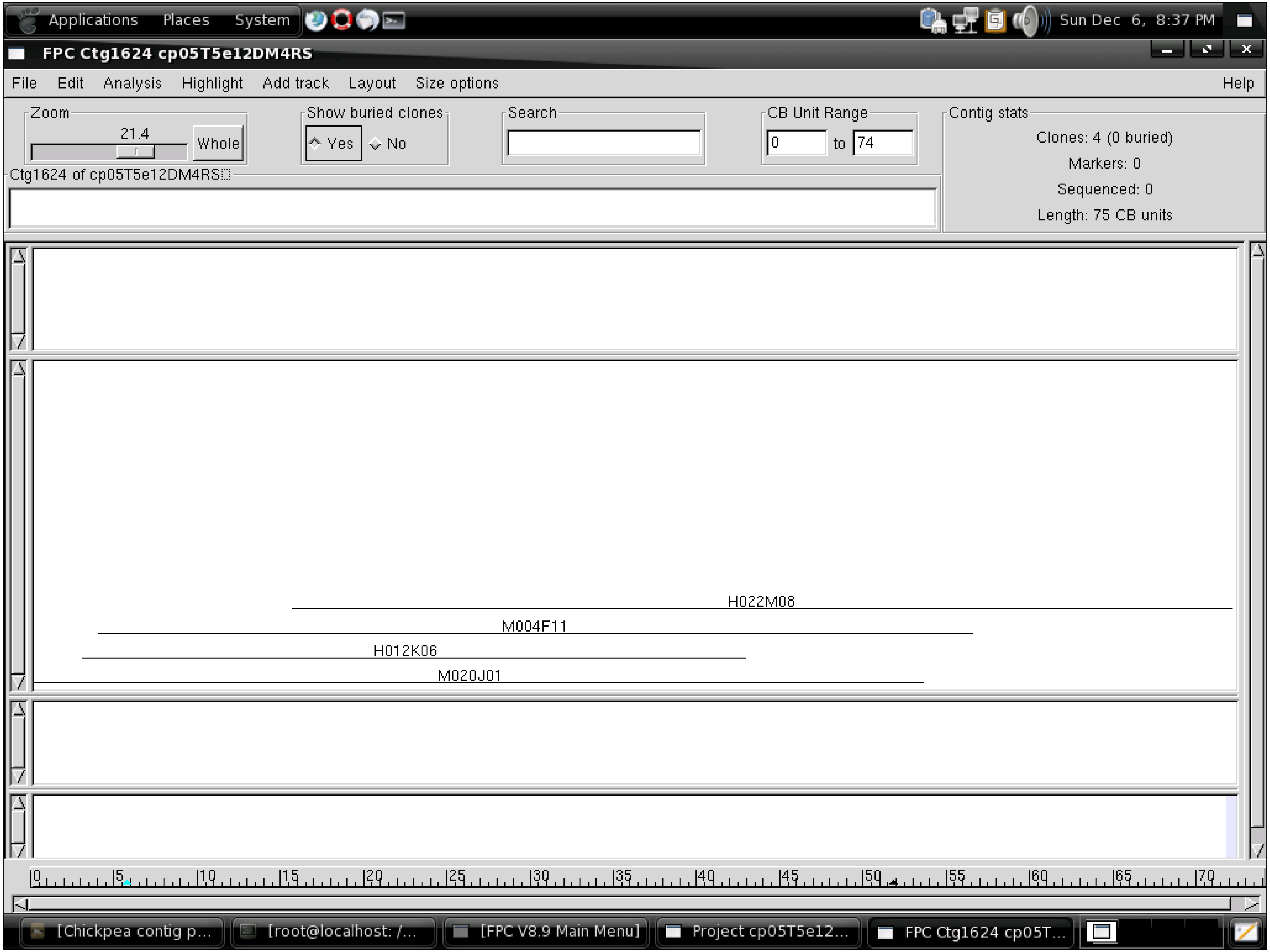
Applications Places System
Sun Dec 6, 8:37 PM
FPC Ctg1624 cp05T5e12DM4RS
File Edit Analysis Highlight Add track Layout Size options Help
Zoom
21.4
Whole
Show buried clones
Yes
No
Search
CB Unit Range
0
to
74
Contig stats
Clones: 4 (0 buried)
Markers: 0
Sequenced: 0
Length: 75 CB units
Ctg1624 of cp05T5e12DM4RS
H022M08
M004F11
H012K06
M020J01
0 5 10 15 20 25 30 35 40 45 50 55 60 65 70
[Chickpea contig p...
[root@localhost: /...
[FPC V8.9 Main Menu]
Project cp05T5e12...
FPC Ctg1624 cp05T...

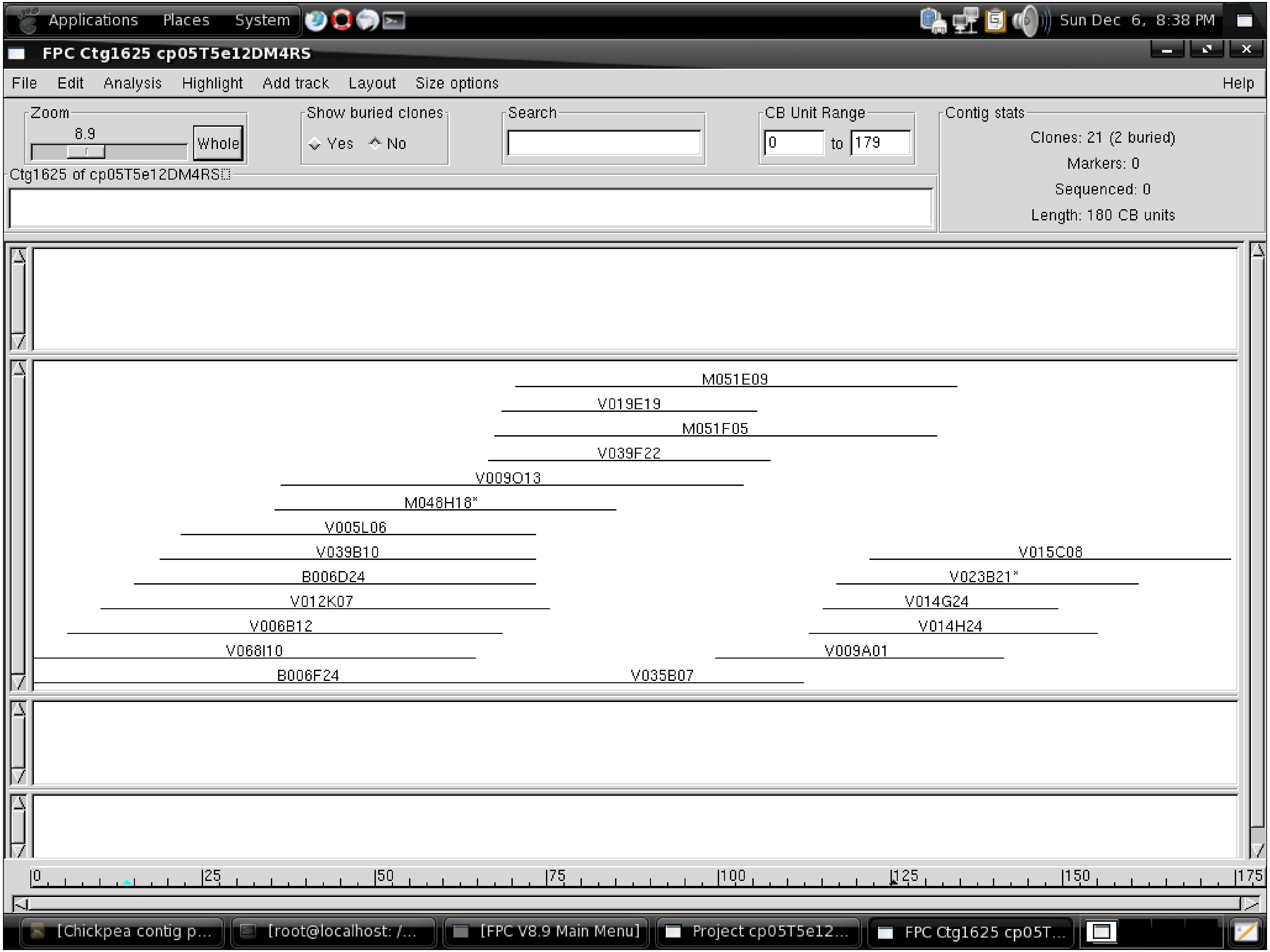
Applications Places System
Sun Dec 6, 8:38 PM
FPC Ctg1625 cp05T5e12DM4RS
File Edit Analysis Highlight Add track Layout Size options Help
Zoom
8.9
Whole
Show buried clones
Yes No
Search
CB Unit Range
0 to 179
Contig stats
Clones: 21 (2 buried)
Markers: 0
Sequenced: 0
Length: 180 CB units
Ctg1625 of cp05T5e12DM4RS
M051E09
V019E19
M051F05
V039F22
V009O13
M048H18*
V005L06
V039B10
V015C08
B006D24
V023B21*
V012K07
V014G24
V006B12
V014H24
V068I10
V009A01
B006F24
V035B07
0 25 50 75 100 125 150 175
[Chickpea contig p...
[root@localhost: /...
[FPC V8.9 Main Menu]
Project cp05T5e12...
FPC Ctg1625 cp05T...

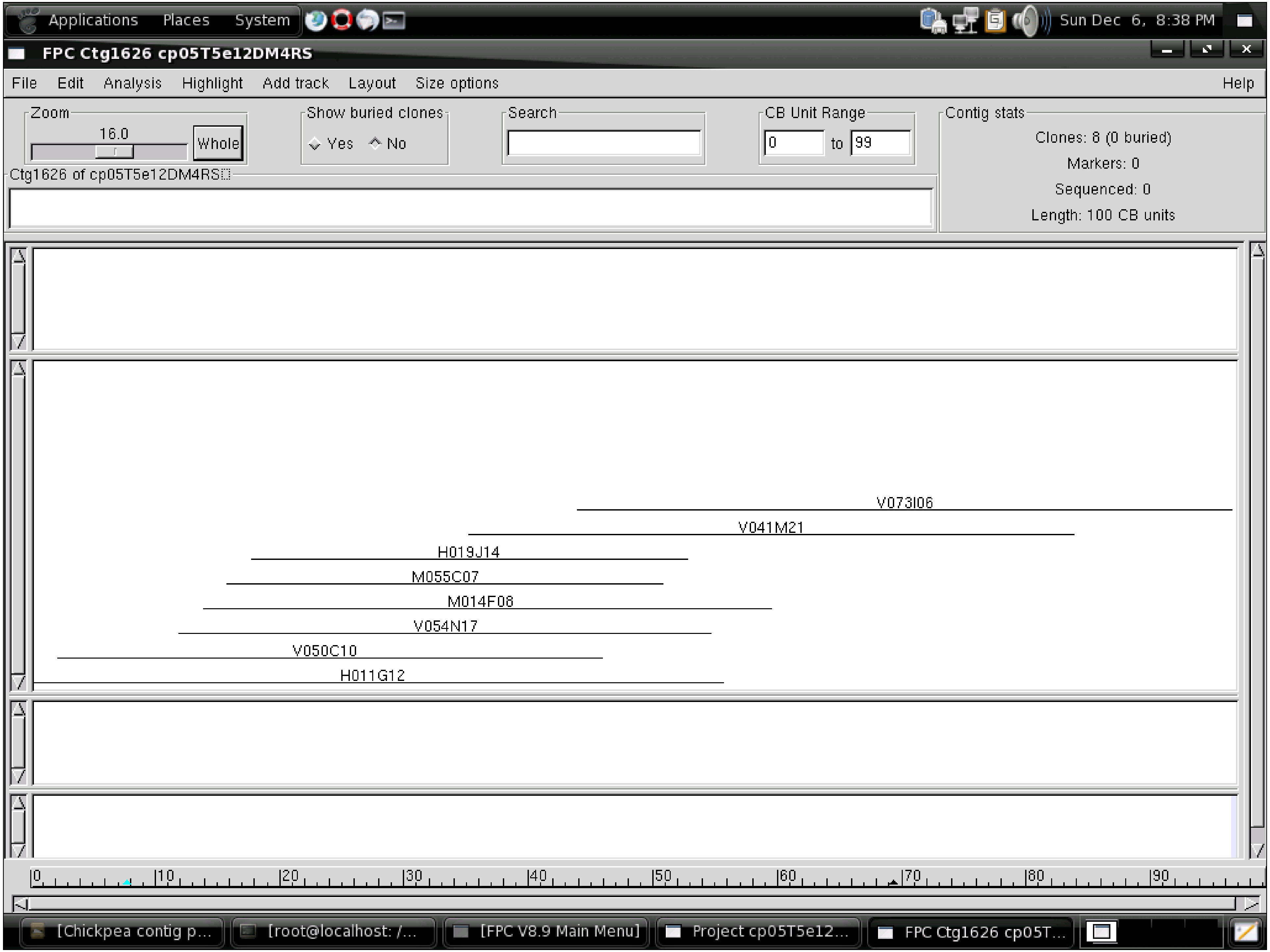
Applications Places System
Sun Dec 6, 8:38 PM
FPC Ctg1626 cp05T5e12DM4RS
File Edit Analysis Highlight Add track Layout Size options Help
Zoom
16.0
Whole
Show buried clones
Yes No
Search
CB Unit Range
0 to 99
Contig stats
Clones: 8 (0 buried)
Markers: 0
Sequenced: 0
Length: 100 CB units
Ctg1626 of cp05T5e12DM4RS
V073I06
V041M21
H019J14
M055C07
M014F08
V054N17
V050C10
H011G12
0 10 20 30 40 50 60 70 80 90
[Chickpea contig p...
[root@localhost: /...
[FPC V8.9 Main Menu]
Project cp05T5e12...
FPC Ctg1626 cp05T...

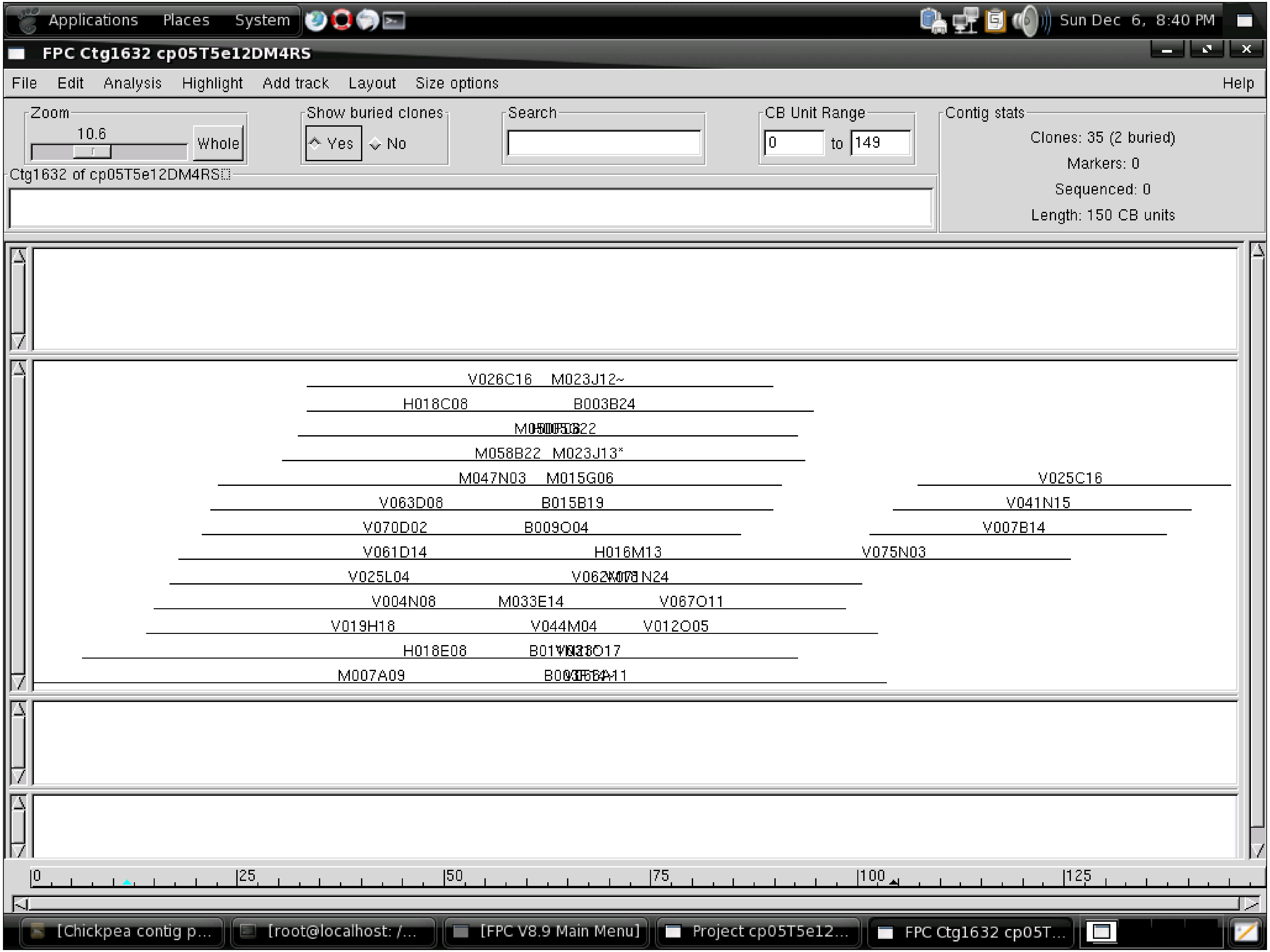

Applications Places System
Sun Dec 6, 8:40 PM
FPC Ctg1632 cp05T5e12DM4RS
File Edit Analysis Highlight Add track Layout Size options Help
Zoom
10.6
Whole
Show buried clones
Yes
No
Search
CB Unit Range
0 to 149
Contig stats
Clones: 35 (2 buried)
Markers: 0
Sequenced: 0
Length: 150 CB units
Ctg1632 of cp05T5e12DM4RS
V026C16
M023J12~
H018C08
B003B24
M058B22
M023J13*
M047N03
M015G06
V025C16
V063D08
B015B19
V041N15
V070D02
B009O04
V007B14
V061D14
H016M13
V075N03
V025L04
V004N08
M033E14
V067O11
V019H18
V044M04
V012O05
H018E08
M007A09
0
25
50
75
100
125
[Chickpea contig p...
[root@localhost: /...
[FPC V8.9 Main Menu]
Project cp05T5e12...
FPC Ctg1632 cp05T...

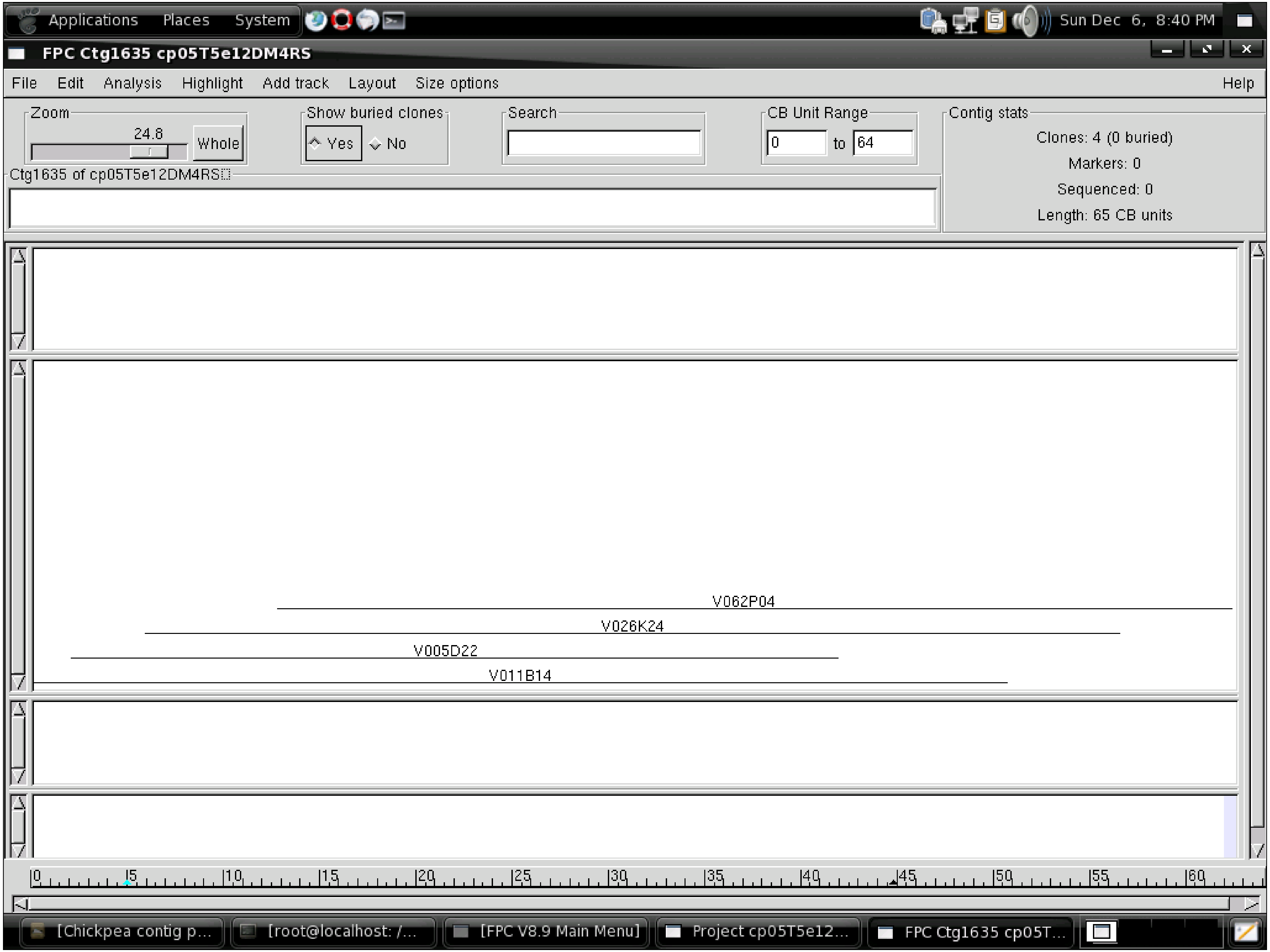
Applications
Places
System
Sun Dec 6, 8:40 PM
FPC Ctg1635 cp05T5e12DM4RS
File
Edit
Analysis
Highlight
Add track
Layout
Size options
Help
Zoom
24.8
Whole
Show buried clones
Yes
No
Search
CB Unit Range
0
to
64
Contig stats
Clones: 4 (0 buried)
Markers: 0
Sequenced: 0
Length: 65 CB units
Ctg1635 of cp05T5e12DM4RS
V062P04
V026K24
V005D22
V011B14
0
5
10
15
20
25
30
35
40
45
50
55
60
[Chickpea contig p...
[root@localhost: /...
[FPC V8.9 Main Menu]
Project cp05T5e12...
FPC Ctg1635 cp05T...

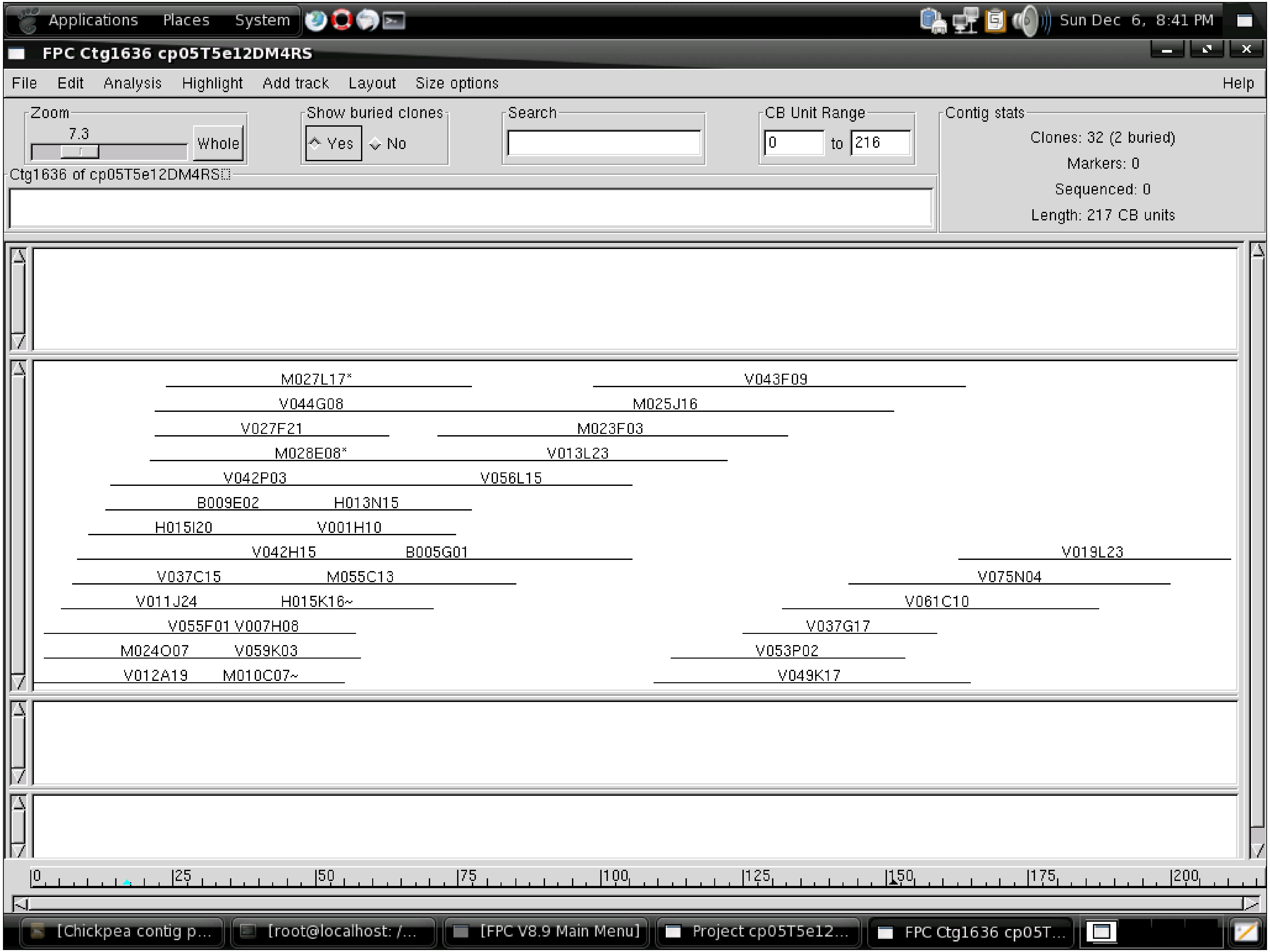
Applications Places System
Sun Dec 6, 8:41 PM
FPC Ctg1636 cp05T5e12DM4RS
File Edit Analysis Highlight Add track Layout Size options Help
Zoom 7.3 Whole
Show buried clones Yes No
Search
CB Unit Range 0 to 216
Contig stats
Clones: 32 (2 buried)
Markers: 0
Sequenced: 0
Length: 217 CB units
Ctg1636 of cp05T5e12DM4RS
M027L17*
V043F09
V044G08
M025J16
V027F21
M023F03
M028E08*
V013L23
V042P03
V056L15
B009E02
H013N15
H015I20
V001H10
V042H15
B005G01
V019L23
V037C15
M055C13
V075N04
V011J24
H015K16~
V061C10
V055F01 V007H08
V037G17
M024O07
V059K03
V053P02
V012A19
M010C07~
V049K17
0 25 50 75 100 125 150 175 200
[Chickpea contig p...
[root@localhost: /...
[FPC V8.9 Main Menu]
Project cp05T5e12...
FPC Ctg1636 cp05T...

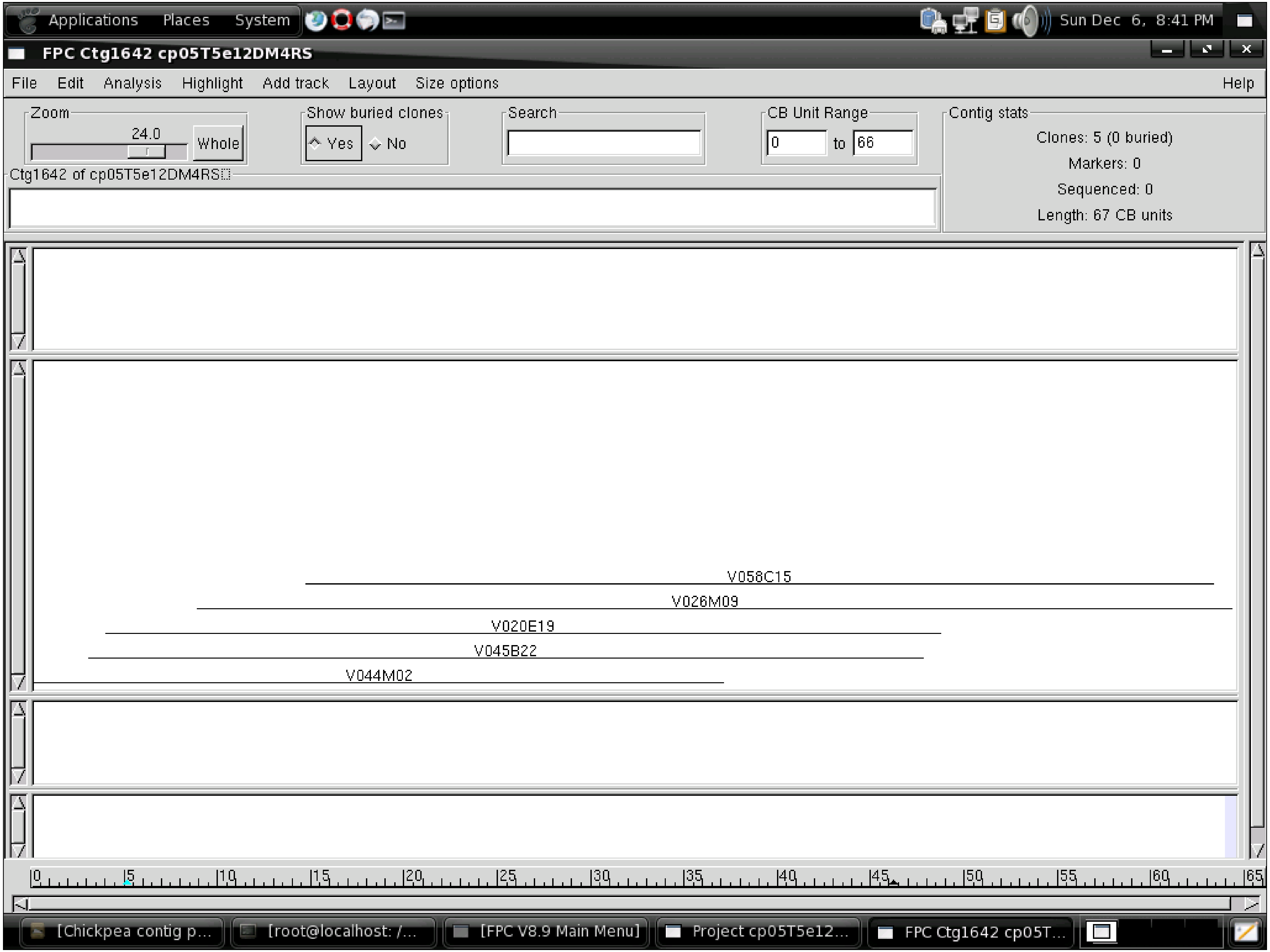
Applications Places System
Sun Dec 6, 8:41 PM
FPC Ctg1642 cp05T5e12DM4RS
File Edit Analysis Highlight Add track Layout Size options Help
Zoom
24.0
Whole
Show buried clones
Yes No
Search
CB Unit Range
0 to 66
Contig stats
Clones: 5 (0 buried)
Markers: 0
Sequenced: 0
Length: 67 CB units
Ctg1642 of cp05T5e12DM4RS
V058C15
V026M09
V020E19
V045B22
V044M02
0 5 10 15 20 25 30 35 40 45 50 55 60 65
[Chickpea contig p...
[root@localhost: /...
[FPC V8.9 Main Menu]
Project cp05T5e12...
FPC Ctg1642 cp05T...

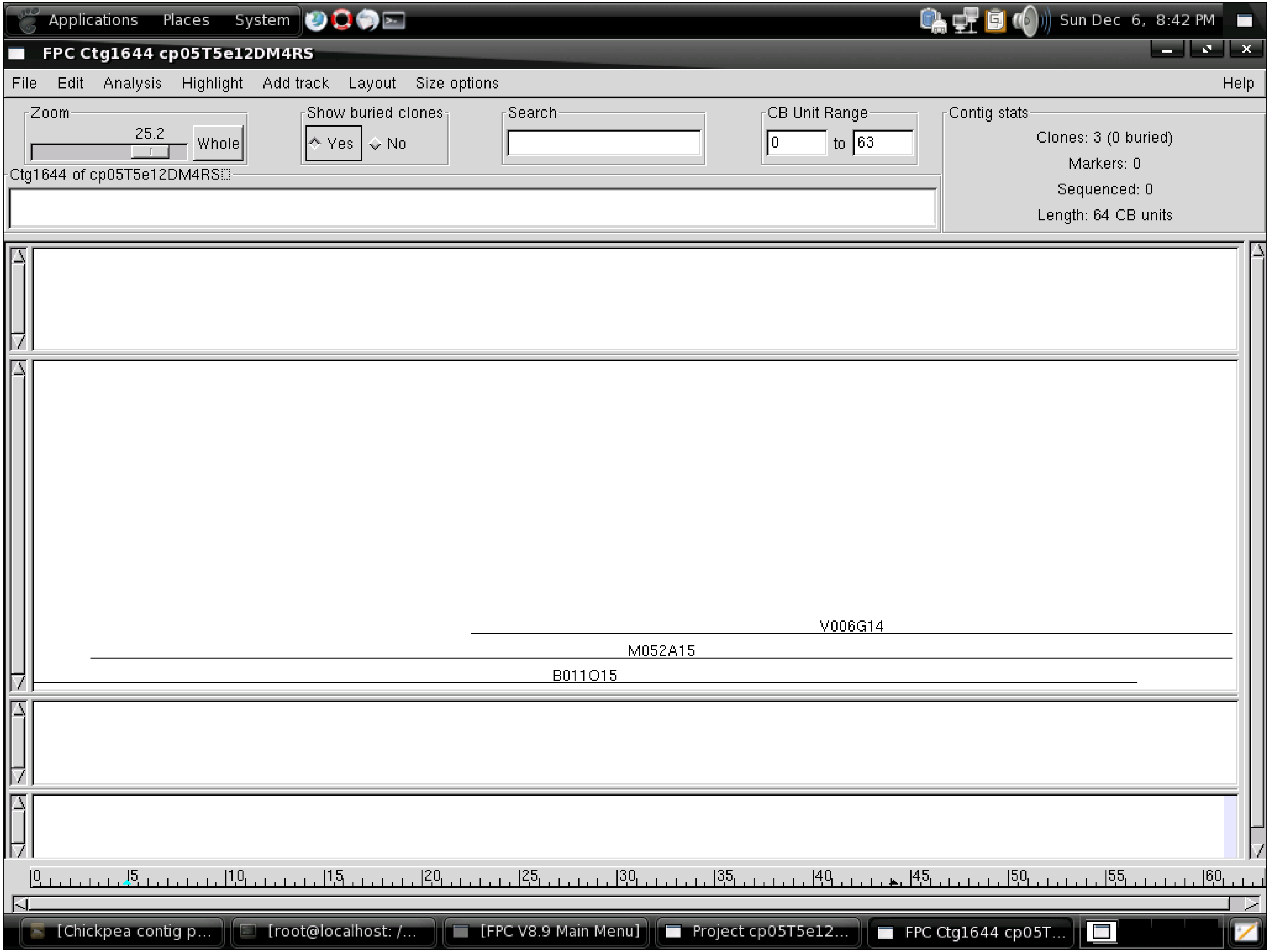
Applications Places System
Sun Dec 6, 8:42 PM
FPC Ctg1644 cp05T5e12DM4RS
File Edit Analysis Highlight Add track Layout Size options Help
Zoom
25.2
Whole
Show buried clones
Yes No
Search
CB Unit Range
0 to 63
Contig stats
Clones: 3 (0 buried)
Markers: 0
Sequenced: 0
Length: 64 CB units
Ctg1644 of cp05T5e12DM4RS
V006G14
M052A15
B011O15
0 5 10 15 20 25 30 35 40 45 50 55 60
[Chickpea contig p...
[root@localhost: /...
[FPC V8.9 Main Menu]
Project cp05T5e12...
FPC Ctg1644 cp05T...

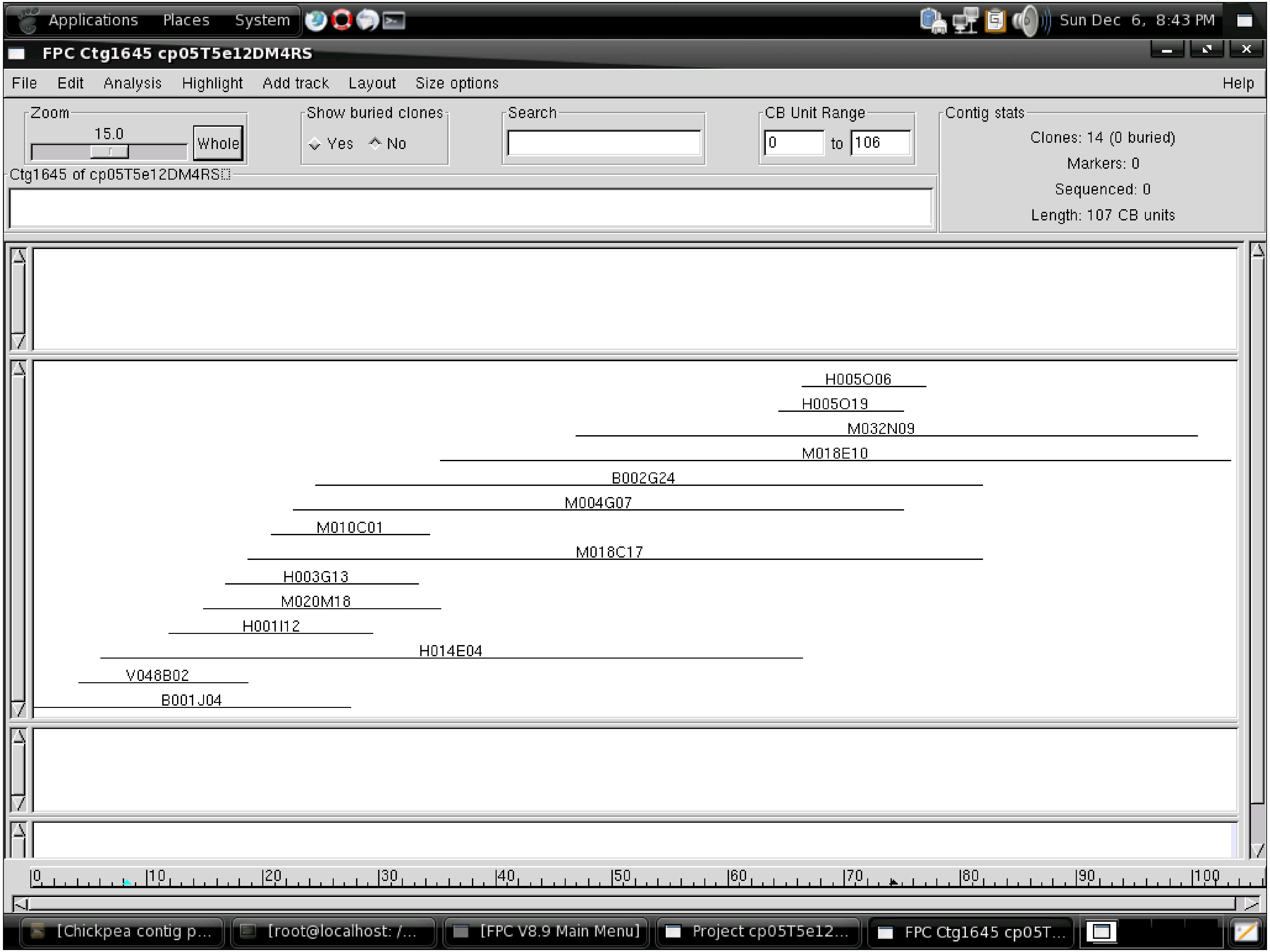

Applications Places System
Sun Dec 6, 8:43 PM
FPC Ctg1645 cp05T5e12DM4RS
File Edit Analysis Highlight Add track Layout Size options Help
Zoom
15.0
Whole
Show buried clones
Yes No
Search
CB Unit Range
0 to 106
Contig stats
Clones: 14 (0 buried)
Markers: 0
Sequenced: 0
Length: 107 CB units
Ctg1645 of cp05T5e12DM4RS
H005O06
H005O19
M032N09
M018E10
B002G24
M004G07
M010C01
M018C17
H003G13
M020M18
H001I12
H014E04
V048B02
B001J04
0 10 20 30 40 50 60 70 80 90 100
[Chickpea contig p...
[root@localhost: /...
[FPC V8.9 Main Menu]
Project cp05T5e12...
FPC Ctg1645 cp05T...

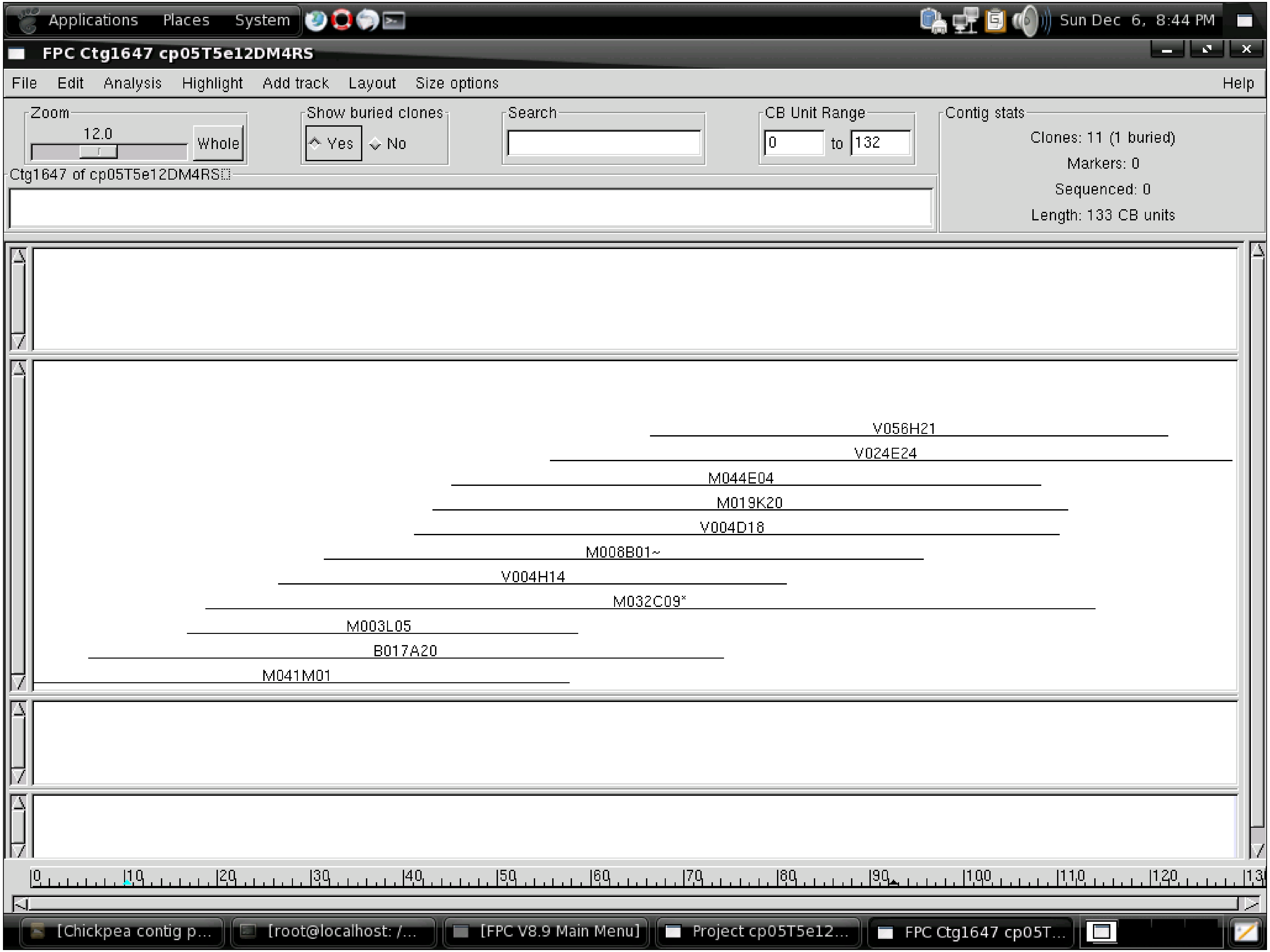
Applications
Places
System
Sun Dec 6, 8:44 PM
FPC Ctg1647 cp05T5e12DM4RS
File
Edit
Analysis
Highlight
Add track
Layout
Size options
Help
Zoom
12.0
Whole
Show buried clones
Yes
No
Search
CB Unit Range
0
to
132
Contig stats
Clones: 11 (1 buried)
Markers: 0
Sequenced: 0
Length: 133 CB units
Ctg1647 of cp05T5e12DM4RS
V056H21
V024E24
M044E04
M019K20
V004D18
M008B01~
V004H14
M032C09*
M003L05
B017A20
M041M01
0
10
20
30
40
50
60
70
80
90
100
110
120
[Chickpea contig p...
[root@localhost: /...
[FPC V8.9 Main Menu]
Project cp05T5e12...
FPC Ctg1647 cp05T...

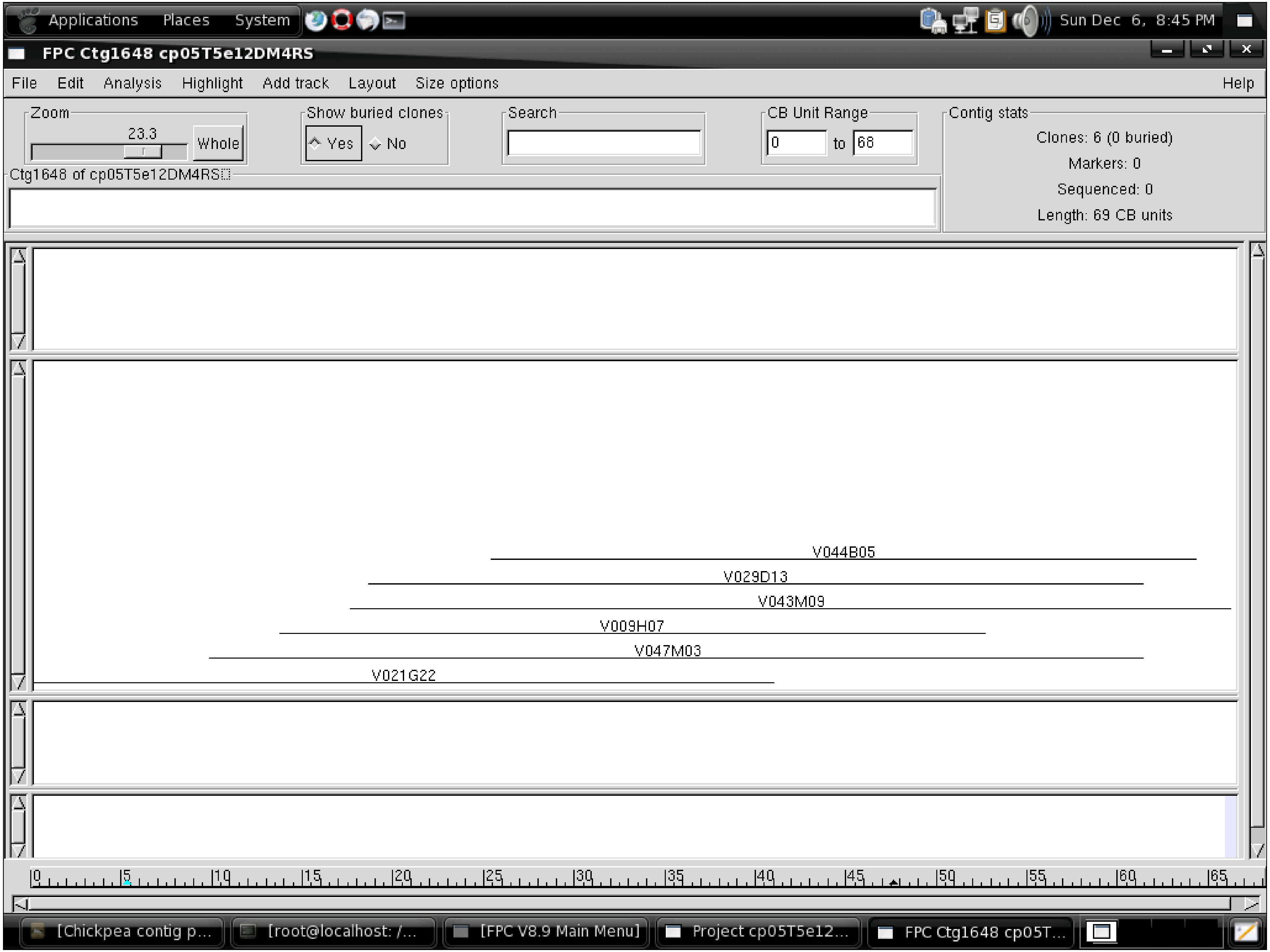
Applications Places System
Sun Dec 6, 8:45 PM
FPC Ctg1648 cp05T5e12DM4RS
File Edit Analysis Highlight Add track Layout Size options Help
Zoom
23.3
Whole
Show buried clones
Yes
No
Search
CB Unit Range
0
to
68
Contig stats
Clones: 6 (0 buried)
Markers: 0
Sequenced: 0
Length: 69 CB units
Ctg1648 of cp05T5e12DM4RS
V044B05
V029D13
V043M09
V009H07
V047M03
V021G22
0 5 10 15 20 25 30 35 40 45 50 55 60 65
[Chickpea contig p...
[root@localhost: /...
[FPC V8.9 Main Menu]
Project cp05T5e12...
FPC Ctg1648 cp05T...

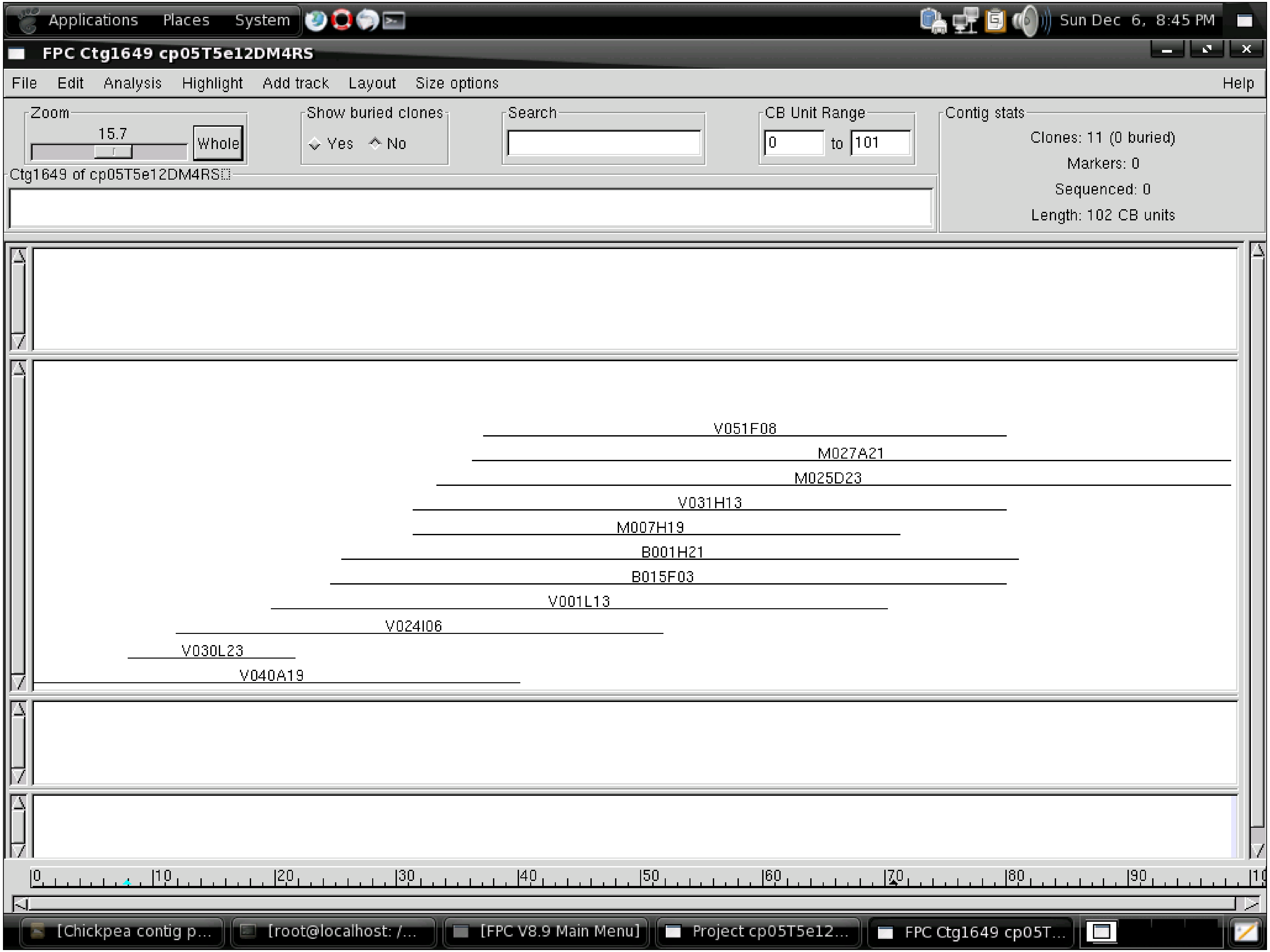
Applications Places System
Sun Dec 6, 8:45 PM
FPC Ctg1649 cp05T5e12DM4RS
File Edit Analysis Highlight Add track Layout Size options Help
Zoom
15.7
Whole
Show buried clones
Yes No
Search
CB Unit Range
0 to 101
Contig stats
Clones: 11 (0 buried)
Markers: 0
Sequenced: 0
Length: 102 CB units
Ctg1649 of cp05T5e12DM4RS
V051F08
M027A21
M025D23
V031H13
M007H19
B001H21
B015F03
V001L13
V024I06
V030L23
V040A19
0 10 20 30 40 50 60 70 80 90
[Chickpea contig p...
[root@localhost: /...
[FPC V8.9 Main Menu]
Project cp05T5e12...
FPC Ctg1649 cp05T...

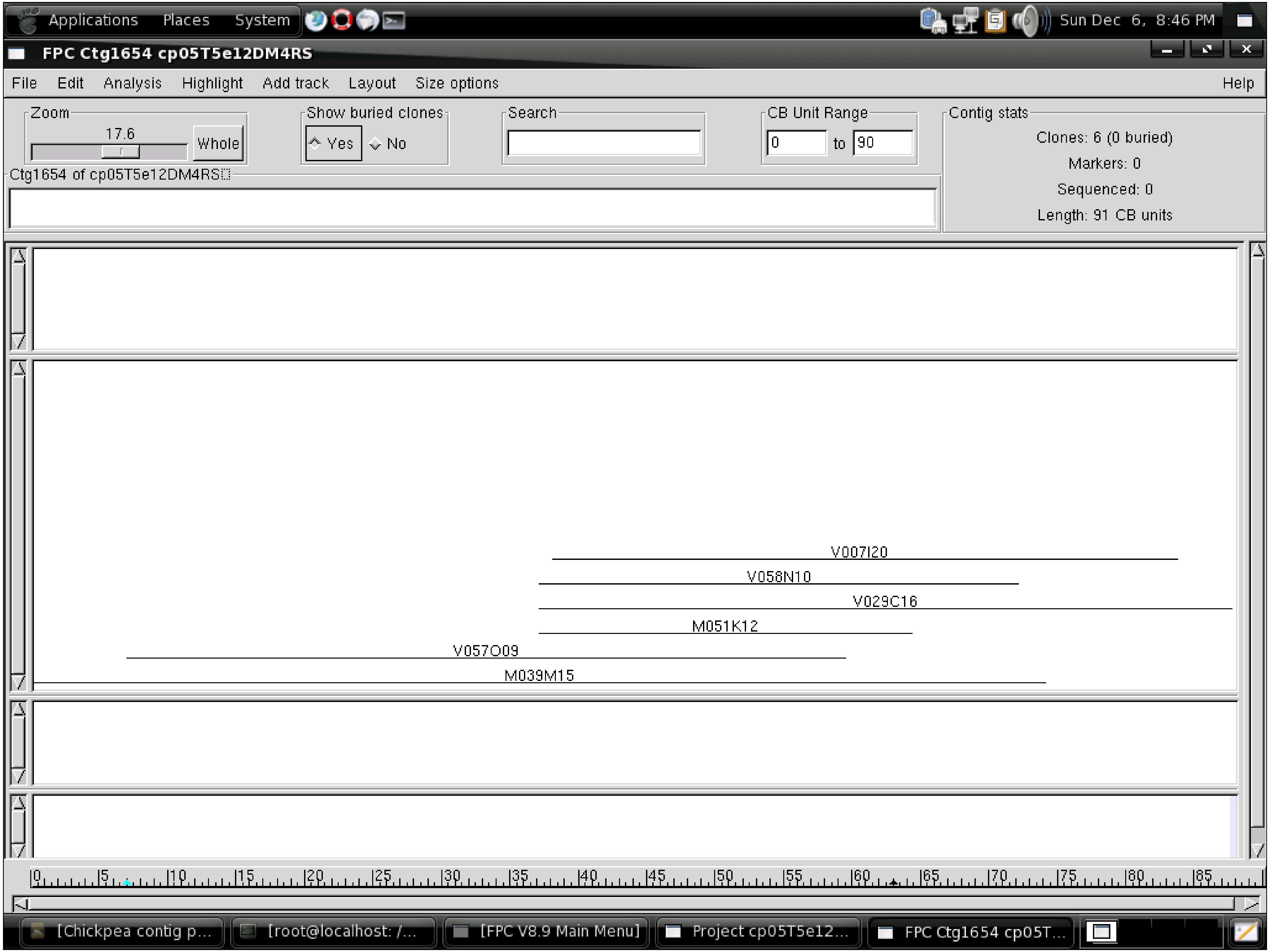
Applications
Places
System
Sun Dec 6, 8:46 PM
FPC Ctg1654 cp05T5e12DM4RS
File
Edit
Analysis
Highlight
Add track
Layout
Size options
Help
Zoom
17.6
Whole
Show buried clones
Yes
No
Search
CB Unit Range
0
to
90
Contig stats
Clones: 6 (0 buried)
Markers: 0
Sequenced: 0
Length: 91 CB units
Ctg1654 of cp05T5e12DM4RS
V007I20
V058N10
V029C16
M051K12
V057O09
M039M15
0 5 10 15 20 25 30 35 40 45 50 55 60 65 70 75 80 85
[Chickpea contig p...
[root@localhost: /...
[FPC V8.9 Main Menu]
Project cp05T5e12...
FPC Ctg1654 cp05T...

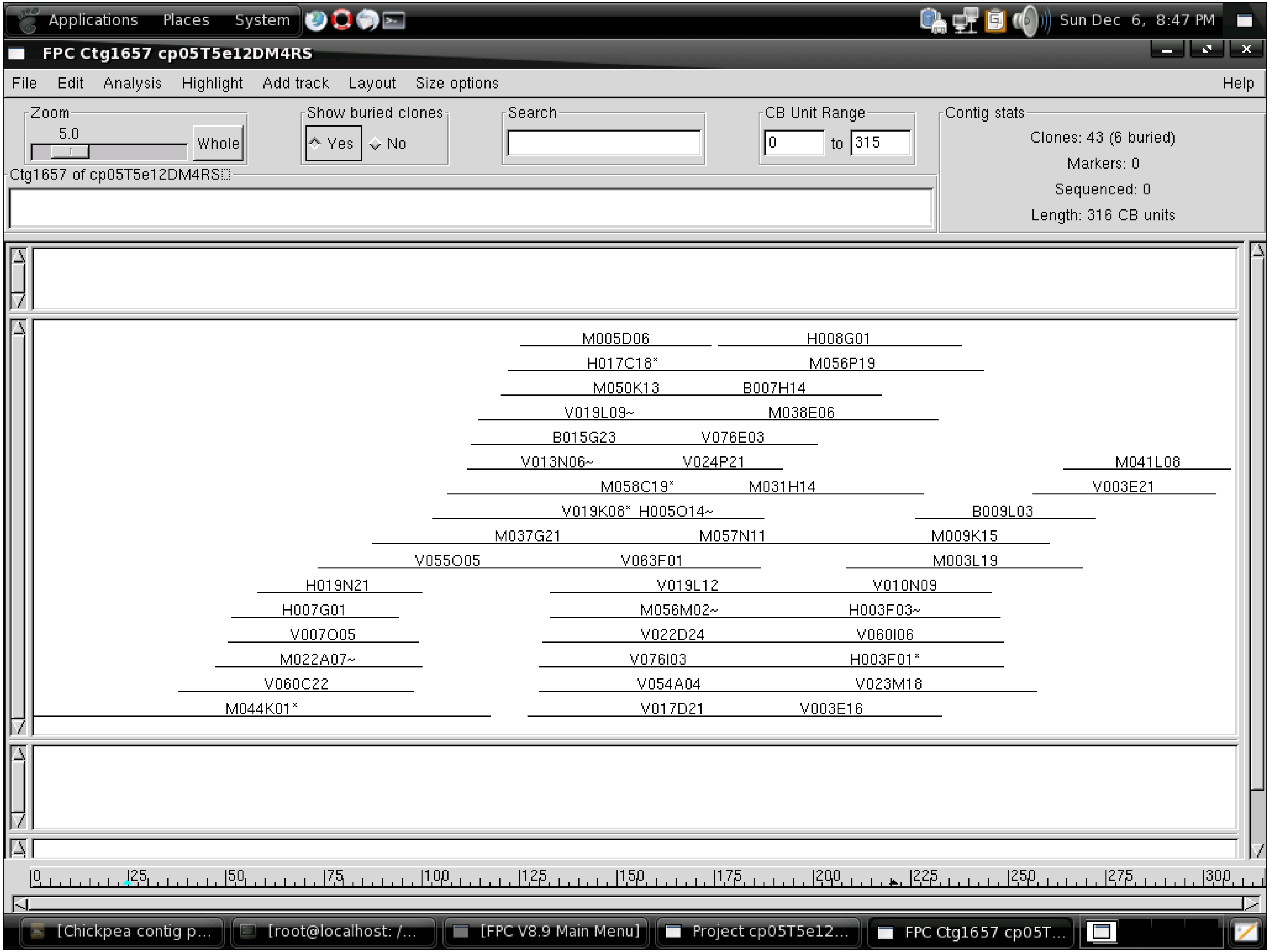
Applications Places System
Sun Dec 6, 8:47 PM
FPC Ctg1657 cp05T5e12DM4RS
File Edit Analysis Highlight Add track Layout Size options Help
Zoom
5.0
Whole
Show buried clones
Yes
No
Search
CB Unit Range
0
to
315
Contig stats
Clones: 43 (6 buried)
Markers: 0
Sequenced: 0
Length: 316 CB units
Ctg1657 of cp05T5e12DM4RS
M005D06
H008G01
H017C18*
M056P19
M050K13
B007H14
V019L09~
M038E06
B015G23
V076E03
V013N06~
V024P21
M041L08
M058C19*
M031H14
V003E21
V019K08* H005O14~
B009L03
M037G21
M057N11
M009K15
V055O05
V063F01
M003L19
H019N21
V019L12
V010N09
H007G01
M056M02~
H003F03~
V007O05
V022D24
V060I06
M022A07~
V076I03
H003F01*
V060C22
V054A04
V023M18
M044K01*
V017D21
V003E16
0 25 50 75 100 125 150 175 200 225 250 275 300
[Chickpea contig p...
[root@localhost: /...
[FPC V8.9 Main Menu]
Project cp05T5e12...
FPC Ctg1657 cp05T...

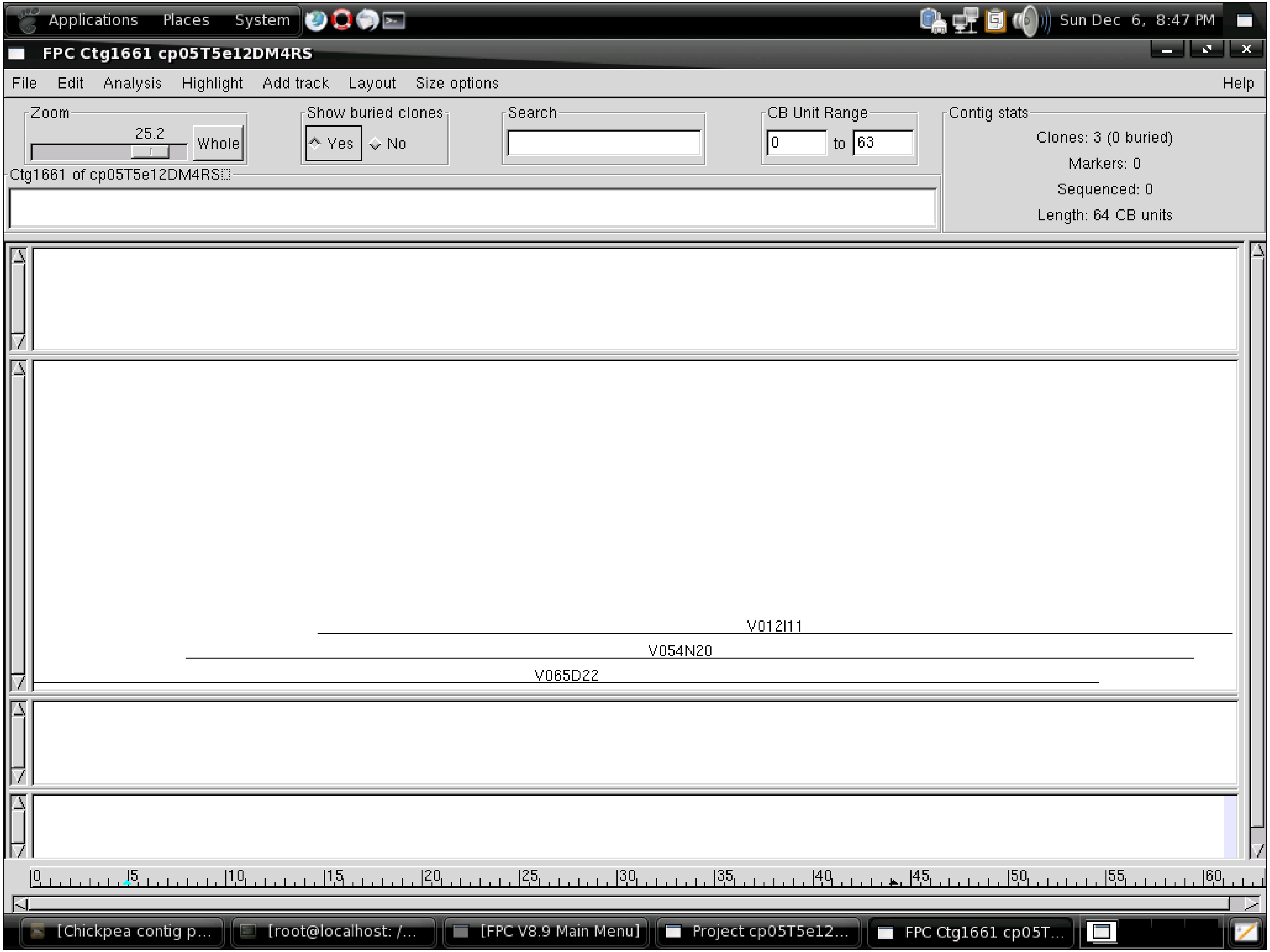

Applications Places System
Sun Dec 6, 8:47 PM
FPC Ctg1661 cp05T5e12DM4RS
File Edit Analysis Highlight Add track Layout Size options Help
Zoom
25.2
Whole
Show buried clones
Yes No
Search
CB Unit Range
0 to 63
Contig stats
Clones: 3 (0 buried)
Markers: 0
Sequenced: 0
Length: 64 CB units
Ctg1661 of cp05T5e12DM4RS
V012I11
V054N20
V065D22
0 5 10 15 20 25 30 35 40 45 50 55 60
[Chickpea contig p...
[root@localhost: /...
[FPC V8.9 Main Menu]
Project cp05T5e12...
FPC Ctg1661 cp05T...

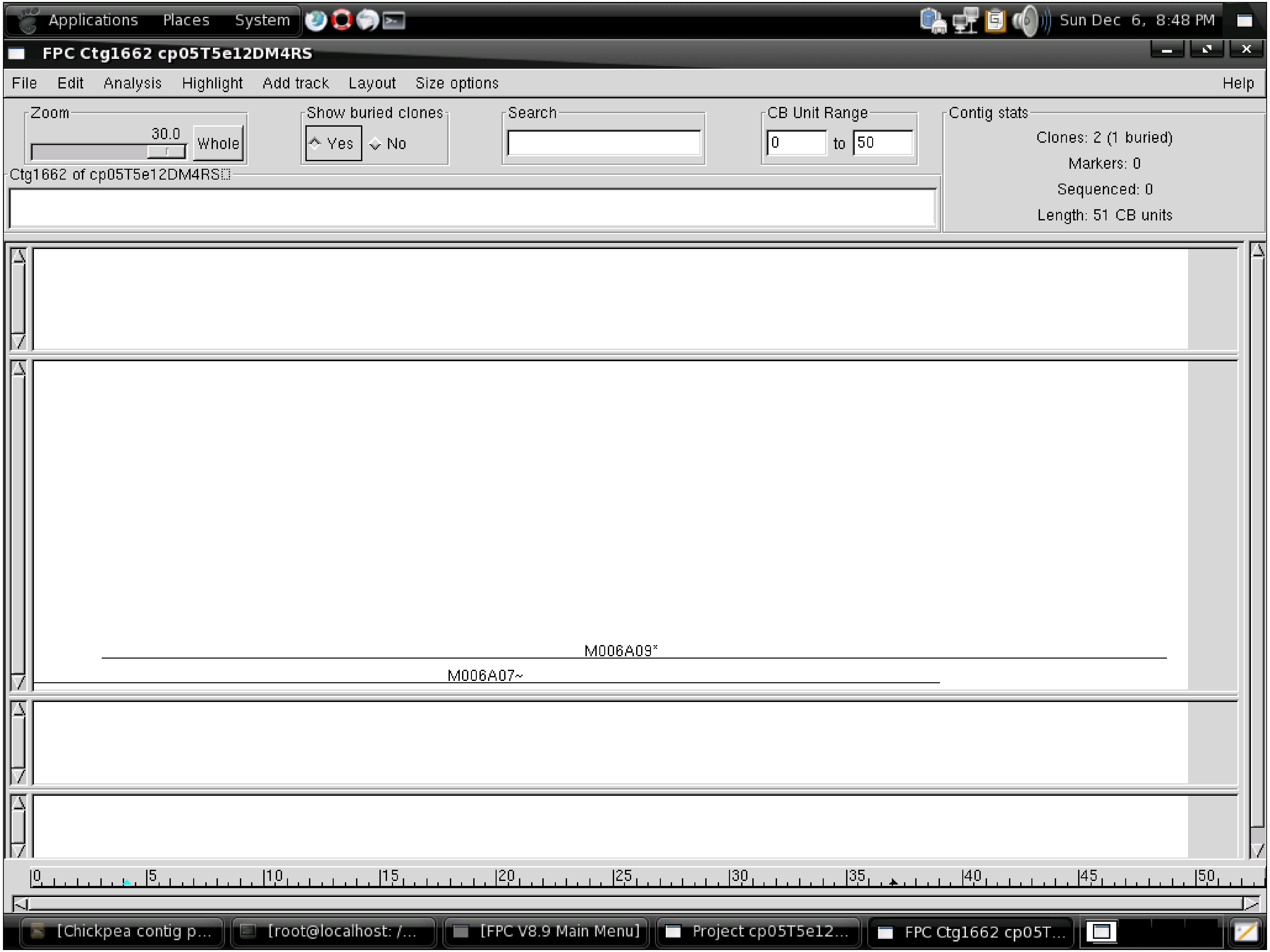
Applications
Places
System
Sun Dec 6, 8:48 PM
FPC Ctg1662 cp05T5e12DM4RS
File
Edit
Analysis
Highlight
Add track
Layout
Size options
Help
Zoom
30.0
Whole
Show buried clones
Yes
No
Search
CB Unit Range
0
to
50
Contig stats
Clones: 2 (1 buried)
Markers: 0
Sequenced: 0
Length: 51 CB units
Ctg1662 of cp05T5e12DM4RS
M006A09*
M006A07~
0
5
10
15
20
25
30
35
40
45
50
[Chickpea contig p...
[root@localhost: /...
[FPC V8.9 Main Menu]
Project cp05T5e12...
FPC Ctg1662 cp05T...

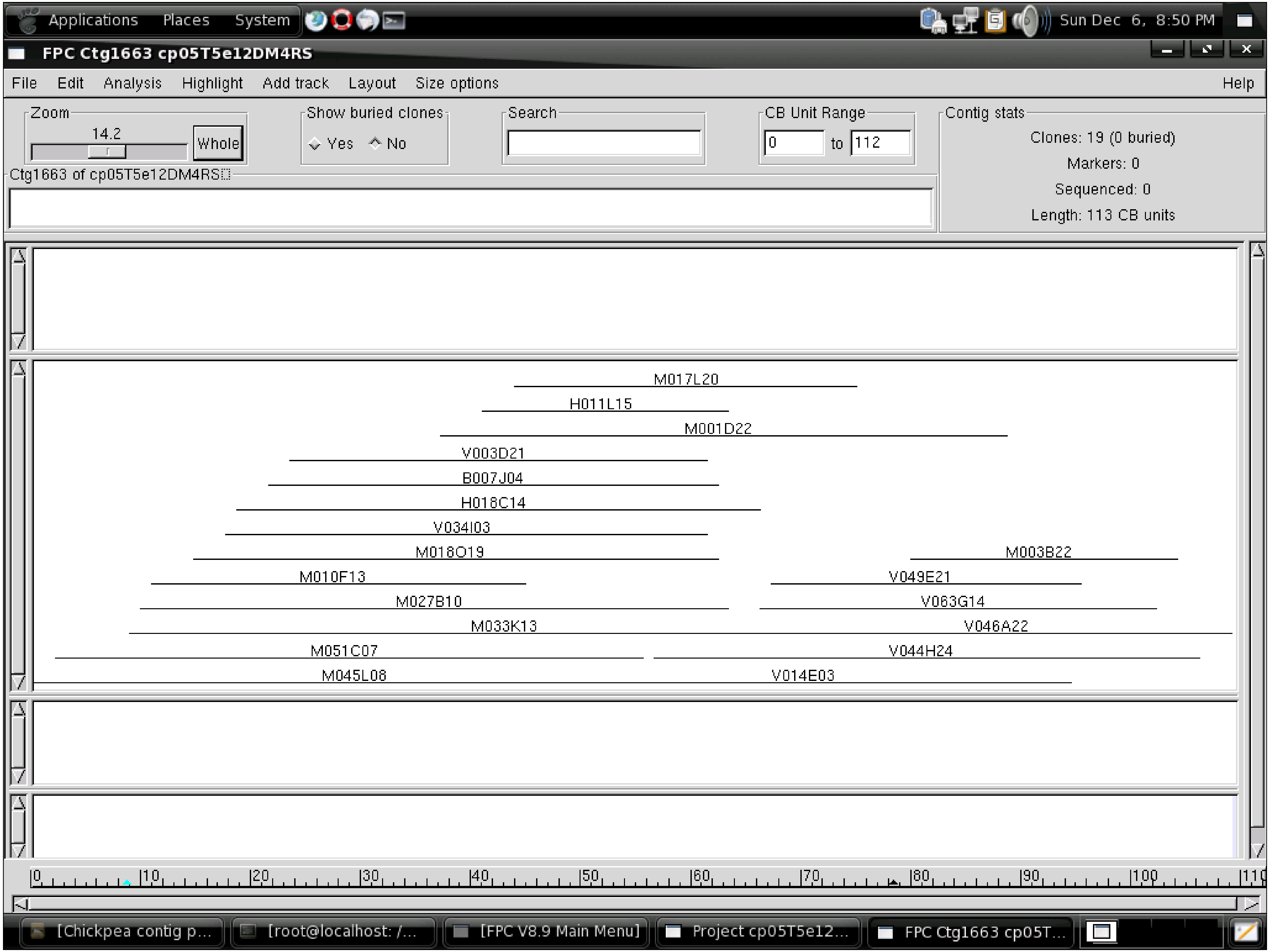
Applications Places System
Sun Dec 6, 8:50 PM
FPC Ctg1663 cp05T5e12DM4RS
File Edit Analysis Highlight Add track Layout Size options Help
Zoom
14.2
Whole
Show buried clones
Yes No
Search
CB Unit Range
0 to 112
Contig stats
Clones: 19 (0 buried)
Markers: 0
Sequenced: 0
Length: 113 CB units
Ctg1663 of cp05T5e12DM4RS
M017L20
H011L15
M001D22
V003D21
B007J04
H018C14
V034I03
M018O19
M003B22
M010F13
V049E21
M027B10
V063G14
M033K13
V046A22
M051C07
V044H24
M045L08
V014E03
0 10 20 30 40 50 60 70 80 90 100 110
[Chickpea contig p...
[root@localhost: /...
[FPC V8.9 Main Menu]
Project cp05T5e12...
FPC Ctg1663 cp05T...

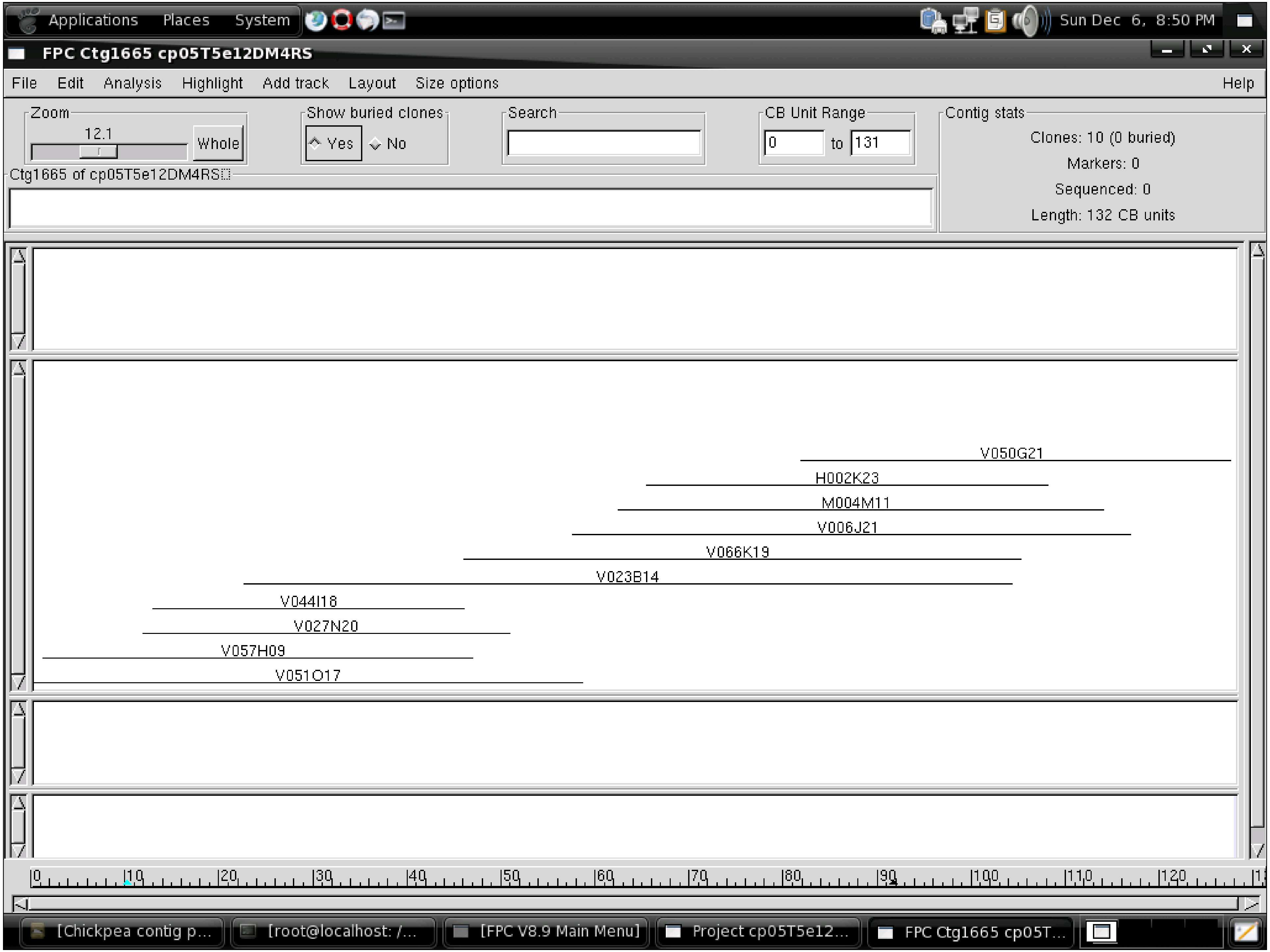
Applications
Places
System
Sun Dec 6, 8:50 PM
FPC Ctg1665 cp05T5e12DM4RS
File
Edit
Analysis
Highlight
Add track
Layout
Size options
Help
Zoom
12.1
Whole
Show buried clones
Yes
No
Search
CB Unit Range
0
to
131
Contig stats
Clones: 10 (0 buried)
Markers: 0
Sequenced: 0
Length: 132 CB units
Ctg1665 of cp05T5e12DM4RS
V050G21
H002K23
M004M11
V006J21
V066K19
V023B14
V044I18
V027N20
V057H09
V051O17
0
10
20
30
40
50
60
70
80
90
100
110
120
[Chickpea contig p...
[root@localhost: /...
[FPC V8.9 Main Menu]
Project cp05T5e12...
FPC Ctg1665 cp05T...

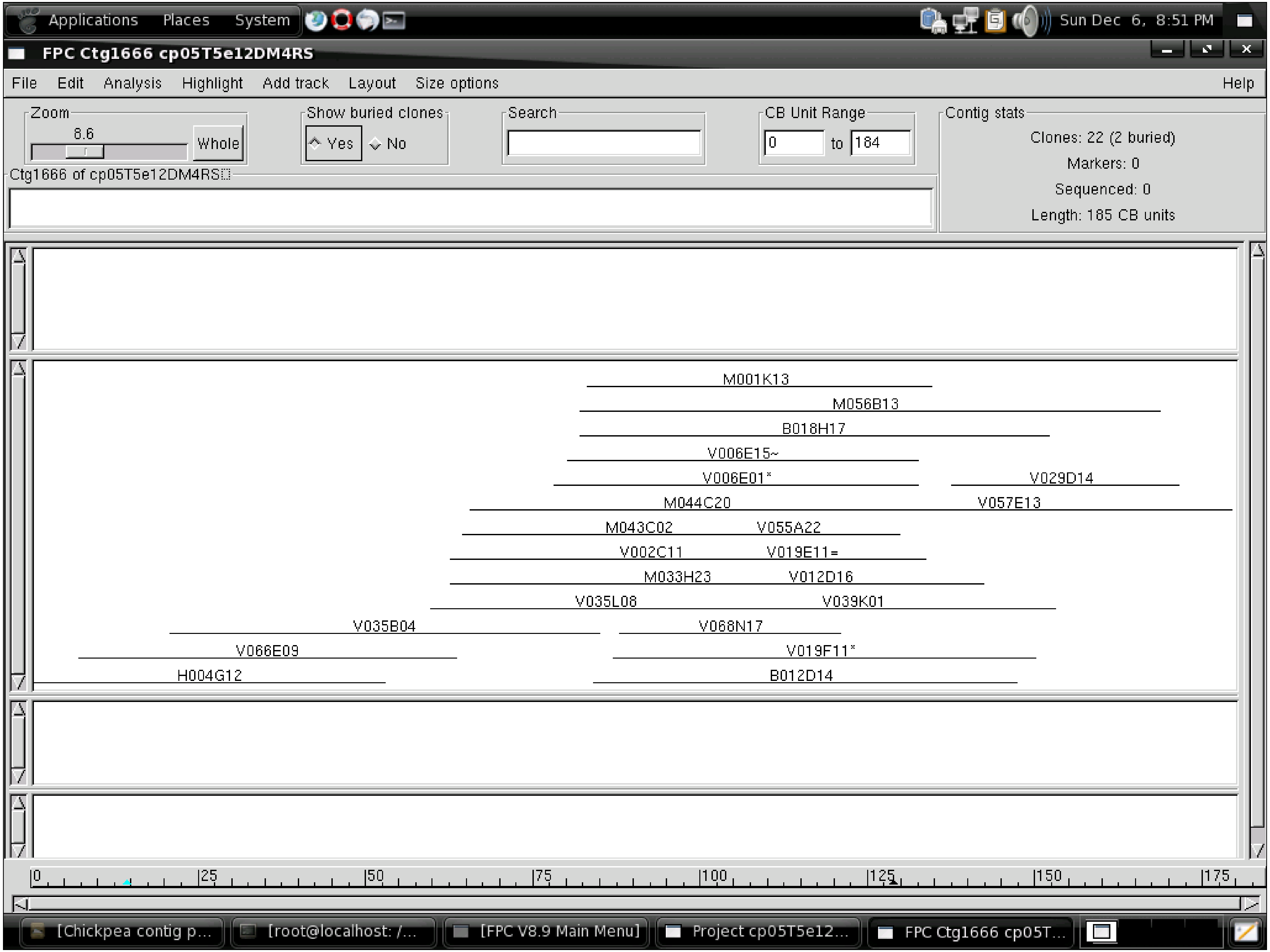
Applications Places System
Sun Dec 6, 8:51 PM
FPC Ctg1666 cp05T5e12DM4RS
File Edit Analysis Highlight Add track Layout Size options Help
Zoom
8.6
Whole
Show buried clones
Yes
No
Search
CB Unit Range
0 to 184
Contig stats
Clones: 22 (2 buried)
Markers: 0
Sequenced: 0
Length: 185 CB units
Ctg1666 of cp05T5e12DM4RS
M001K13
M056B13
B018H17
V006E15~
V006E01*
V029D14
M044C20
V057E13
M043C02
V055A22
V002C11
V019E11=
M033H23
V012D16
V035L08
V039K01
V035B04
V068N17
V066E09
V019F11*
H004G12
B012D14
0 25 50 75 100 125 150 175
[Chickpea contig p...
[root@localhost: /...
[FPC V8.9 Main Menu]
Project cp05T5e12...
FPC Ctg1666 cp05T...

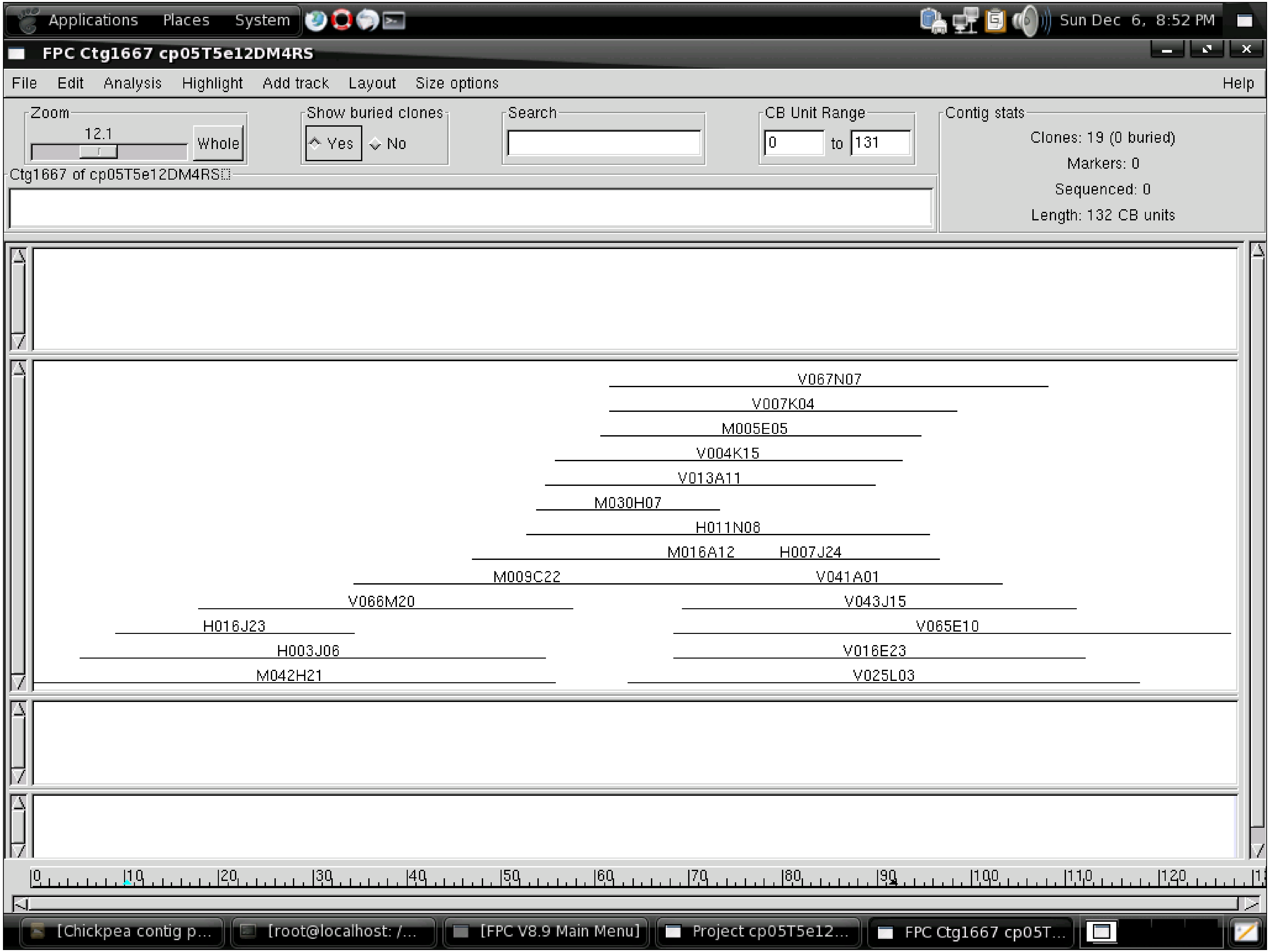
Applications Places System
Sun Dec 6, 8:52 PM
FPC Ctg1667 cp05T5e12DM4RS
File Edit Analysis Highlight Add track Layout Size options Help
Zoom
12.1
Whole
Show buried clones
Yes No
Search
CB Unit Range
0 to 131
Contig stats
Clones: 19 (0 buried)
Markers: 0
Sequenced: 0
Length: 132 CB units
Ctg1667 of cp05T5e12DM4RS
V067N07
V007K04
M005E05
V004K15
V013A11
M030H07
H011N08
M016A12
H007J24
M009C22
V041A01
V066M20
V043J15
H016J23
V065E10
H003J06
V016E23
M042H21
V025L03
0 10 20 30 40 50 60 70 80 90 100 110 120
[Chickpea contig p...
[root@localhost: /...
[FPC V8.9 Main Menu]
Project cp05T5e12...
FPC Ctg1667 cp05T...

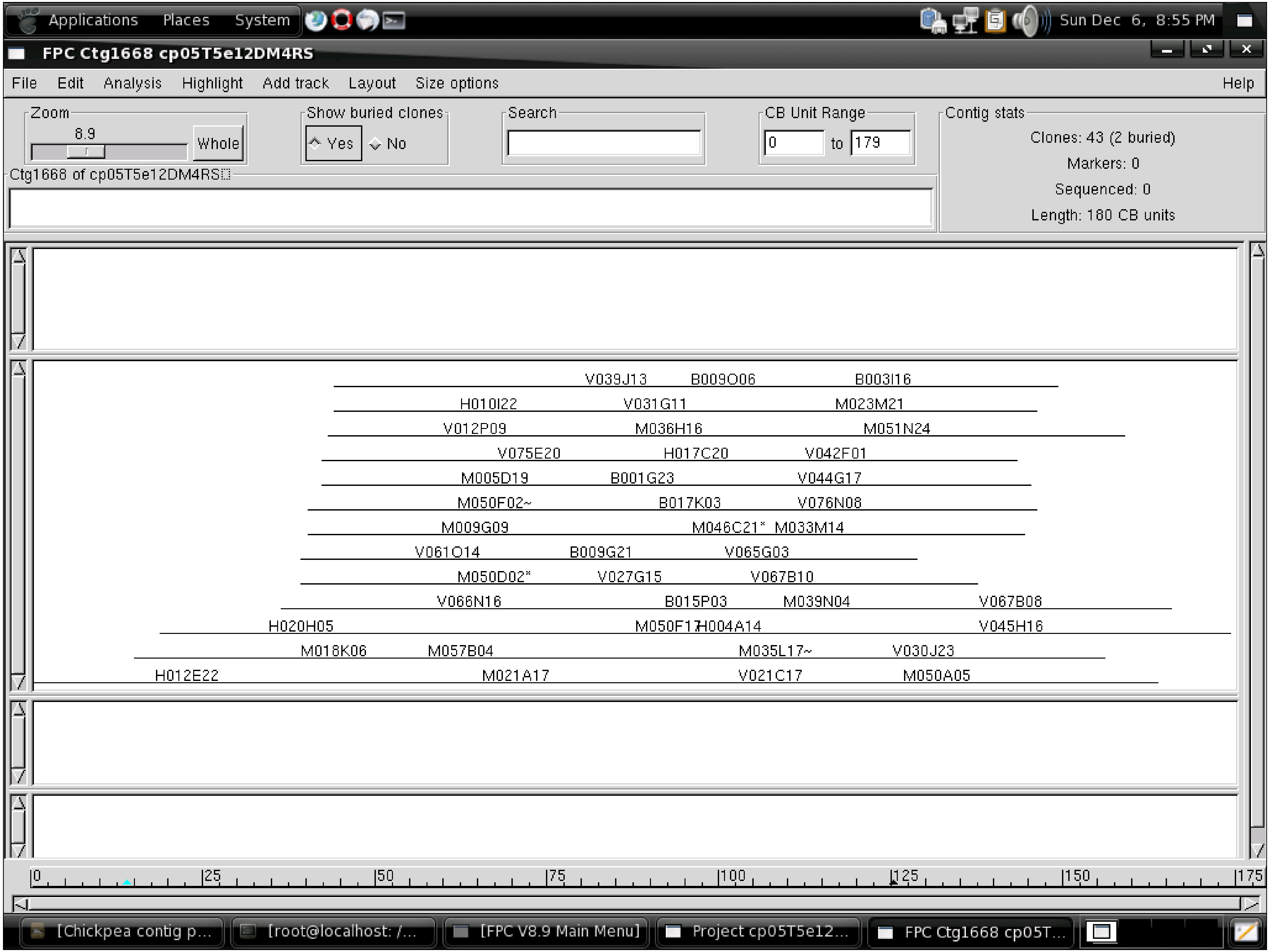
Applications Places System
Sun Dec 6, 8:55 PM
FPC Ctg1668 cp05T5e12DM4RS
File Edit Analysis Highlight Add track Layout Size options Help
Zoom
8.9
Whole
Show buried clones
Yes
No
Search
CB Unit Range
0
to
179
Contig stats
Clones: 43 (2 buried)
Markers: 0
Sequenced: 0
Length: 180 CB units
Ctg1668 of cp05T5e12DM4RS
V039J13 B009O06 B003I16
H010I22 V031G11 M023M21
V012P09 M036H16 M051N24
V075E20 H017C20 V042F01
M005D19 B001G23 V044G17
M050F02~ B017K03 V076N08
M009G09 M046C21* M033M14
V061O14 B009G21 V065G03
M050D02* V027G15 V067B10
V066N16 B015P03 M039N04 V067B08
H020H05 M050F17 H004A14 V045H16
M018K06 M057B04 M035L17~ V030J23
H012E22 M021A17 V021C17 M050A05
0 25 50 75 100 125 150 175
[Chickpea contig p...
[root@localhost: /...
[FPC V8.9 Main Menu]
Project cp05T5e12...
FPC Ctg1668 cp05T...

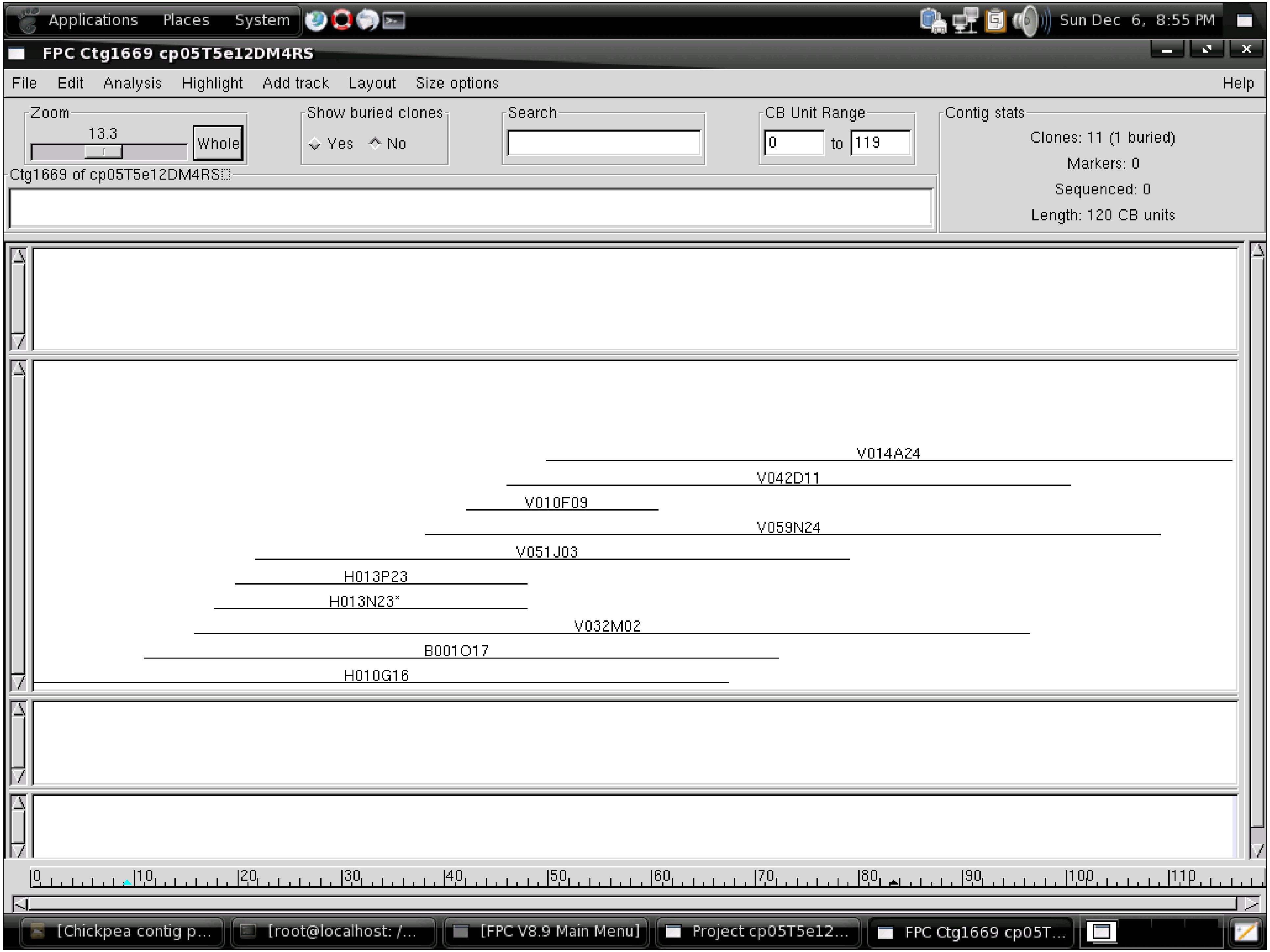

Applications Places System
Sun Dec 6, 8:55 PM
FPC Ctg1669 cp05T5e12DM4RS
File Edit Analysis Highlight Add track Layout Size options Help
Zoom
13.3
Whole
Show buried clones
Yes No
Search
CB Unit Range
0 to 119
Contig stats
Clones: 11 (1 buried)
Markers: 0
Sequenced: 0
Length: 120 CB units
Ctg1669 of cp05T5e12DM4RS
V014A24
V042D11
V010F09
V059N24
V051J03
H013P23
H013N23*
V032M02
B001O17
H010G16
0 10 20 30 40 50 60 70 80 90 100 110
[Chickpea contig p...
[root@localhost: /...
[FPC V8.9 Main Menu]
Project cp05T5e12...
FPC Ctg1669 cp05T...

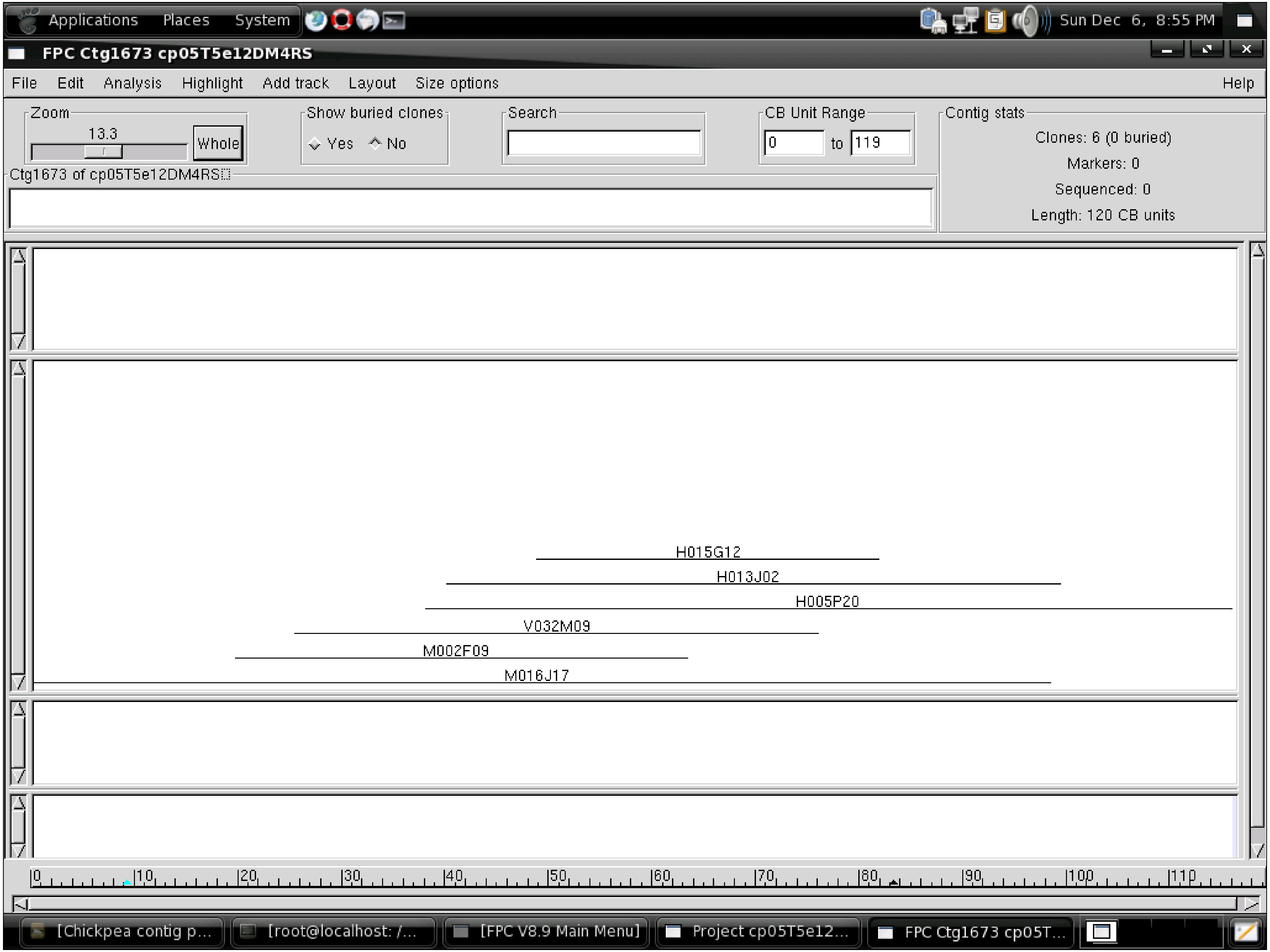
Applications Places System
Sun Dec 6, 8:55 PM
FPC Ctg1673 cp05T5e12DM4RS
File Edit Analysis Highlight Add track Layout Size options Help
Zoom
13.3
Whole
Show buried clones
Yes No
Search
CB Unit Range
0 to 119
Contig stats
Clones: 6 (0 buried)
Markers: 0
Sequenced: 0
Length: 120 CB units
Ctg1673 of cp05T5e12DM4RS
H015G12
H013J02
H005P20
V032M09
M002F09
M016J17
0 10 20 30 40 50 60 70 80 90 100 110
[Chickpea contig p...
[root@localhost: /...
[FPC V8.9 Main Menu]
Project cp05T5e12...
FPC Ctg1673 cp05T...

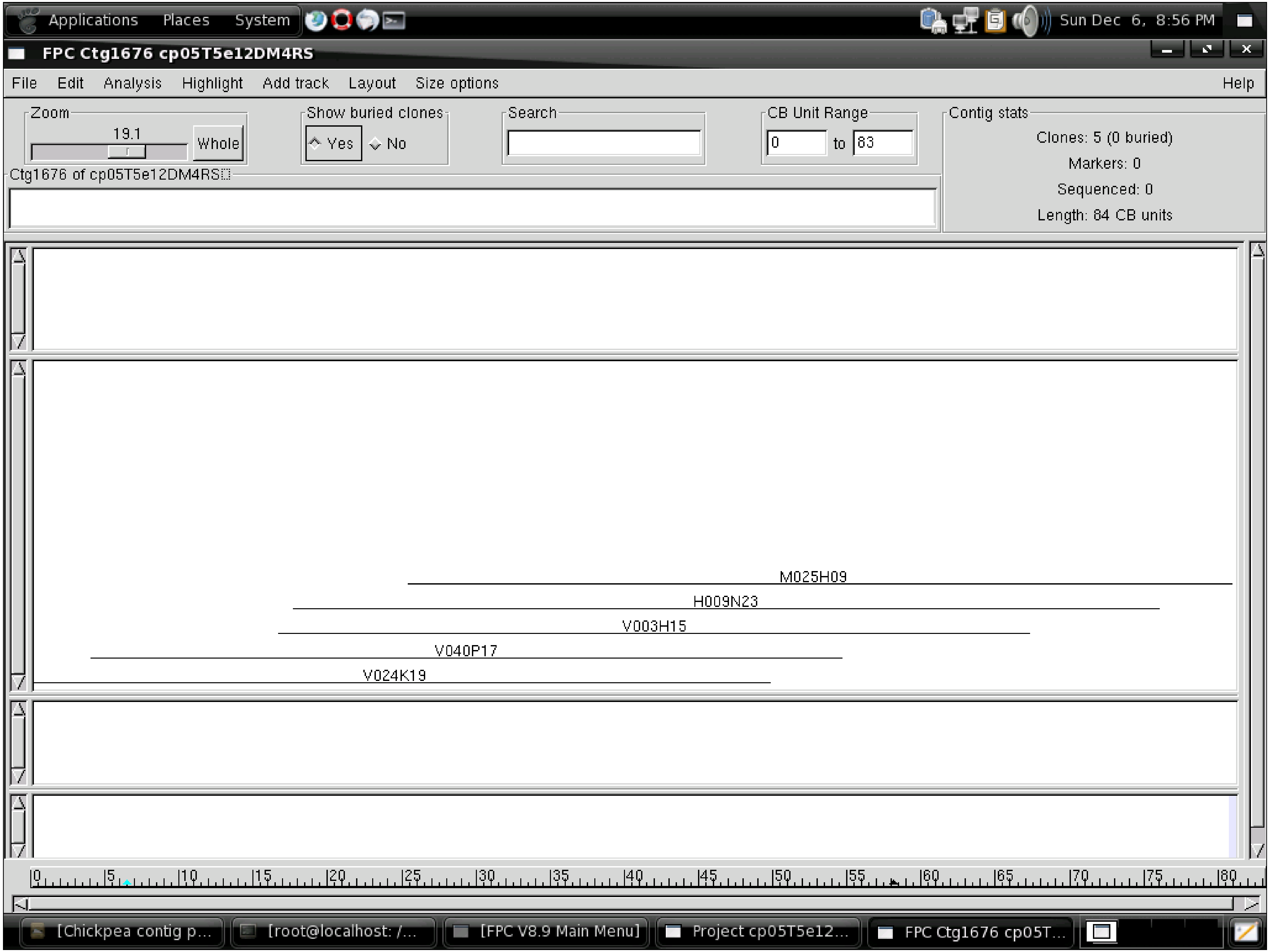
Applications Places System
Sun Dec 6, 8:56 PM
FPC Ctg1676 cp05T5e12DM4RS
File Edit Analysis Highlight Add track Layout Size options Help
Zoom
19.1
Whole
Show buried clones
Yes
No
Search
CB Unit Range
0
to
83
Contig stats
Clones: 5 (0 buried)
Markers: 0
Sequenced: 0
Length: 84 CB units
Ctg1676 of cp05T5e12DM4RS
M025H09
H009N23
V003H15
V040P17
V024K19
0 5 10 15 20 25 30 35 40 45 50 55 60 65 70 75 80
[Chickpea contig p...
[root@localhost: /...
[FPC V8.9 Main Menu]
Project cp05T5e12...
FPC Ctg1676 cp05T...

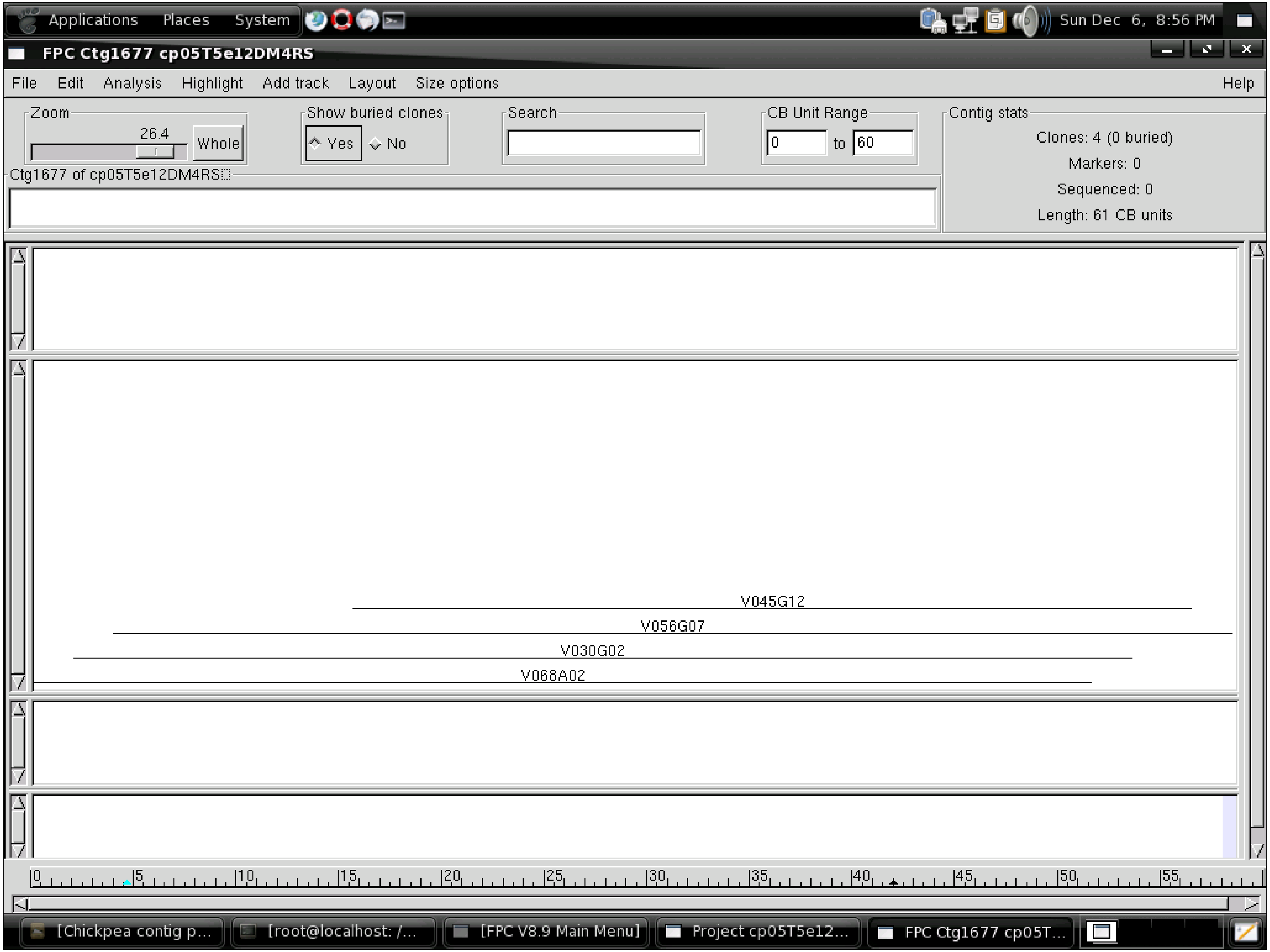
Applications Places System
Sun Dec 6, 8:56 PM
FPC Ctg1677 cp05T5e12DM4RS
File Edit Analysis Highlight Add track Layout Size options Help
Zoom
26.4
Whole
Show buried clones
Yes
No
Search
CB Unit Range
0 to 60
Contig stats
Clones: 4 (0 buried)
Markers: 0
Sequenced: 0
Length: 61 CB units
Ctg1677 of cp05T5e12DM4RS
V045G12
V056G07
V030G02
V068A02
0 5 10 15 20 25 30 35 40 45 50 55
[Chickpea contig p...
[root@localhost: /...
[FPC V8.9 Main Menu]
Project cp05T5e12...
FPC Ctg1677 cp05T...

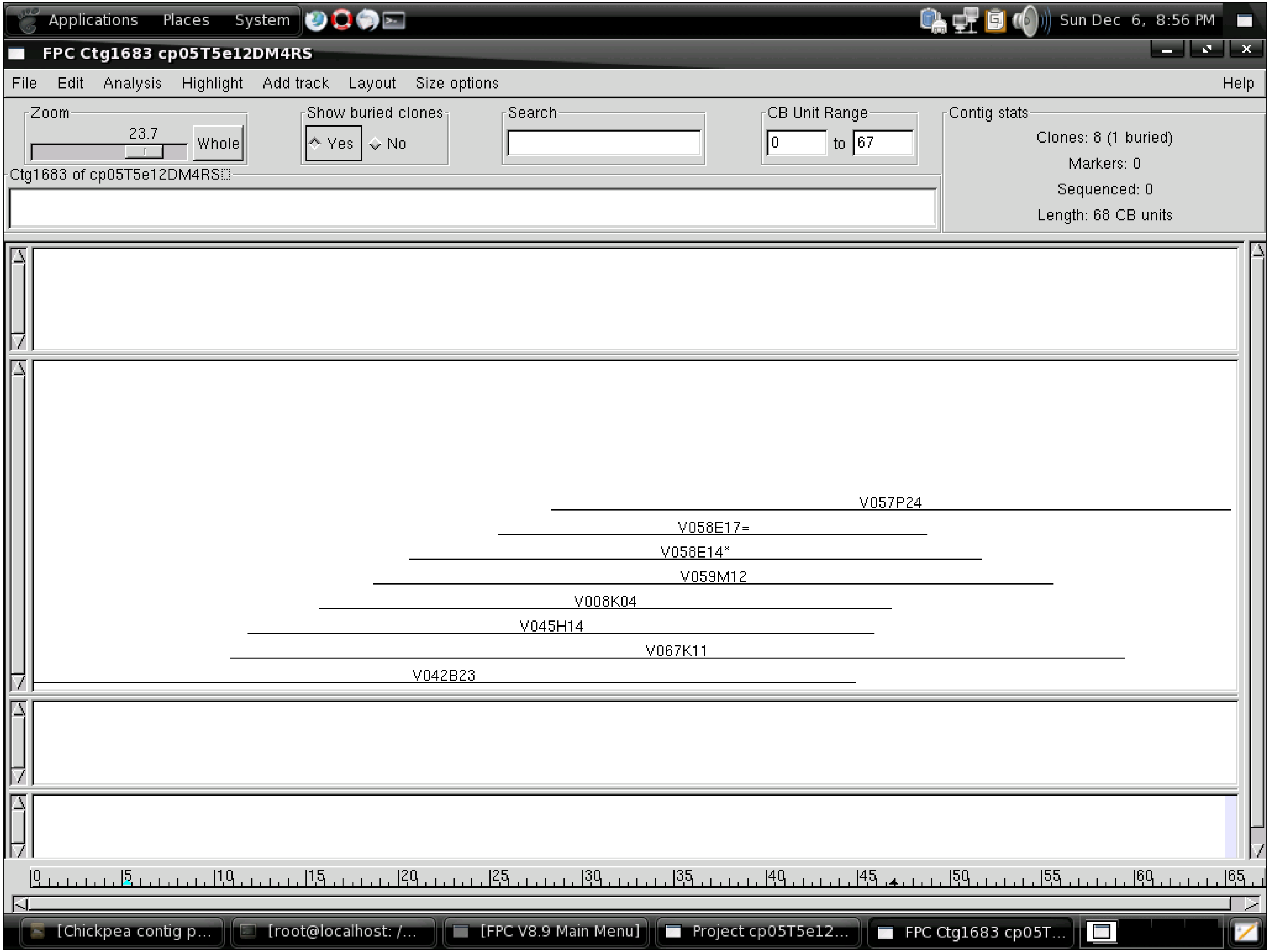
Applications Places System
Sun Dec 6, 8:56 PM
FPC Ctg1683 cp05T5e12DM4RS
File Edit Analysis Highlight Add track Layout Size options Help
Zoom
23.7
Whole
Show buried clones
Yes No
Search
CB Unit Range
0 to 67
Contig stats
Clones: 8 (1 buried)
Markers: 0
Sequenced: 0
Length: 68 CB units
Ctg1683 of cp05T5e12DM4RS
V057P24
V058E17=
V058E14*
V059M12
V008K04
V045H14
V067K11
V042B23
0 5 10 15 20 25 30 35 40 45 50 55 60 65
[Chickpea contig p...
[root@localhost: /...
[FPC V8.9 Main Menu]
Project cp05T5e12...
FPC Ctg1683 cp05T...

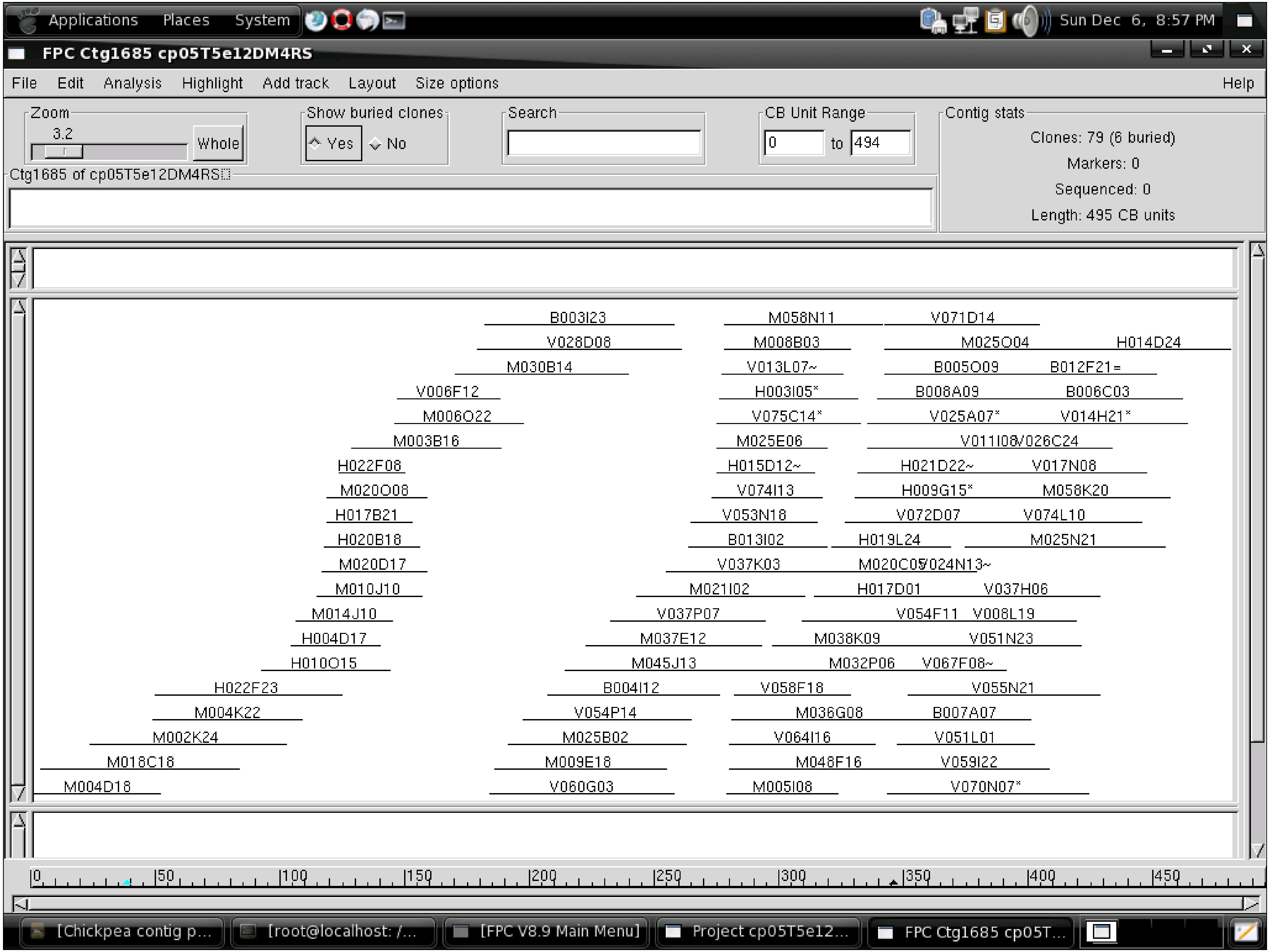
Applications Places System
Sun Dec 6, 8:57 PM
FPC Ctg1685 cp05T5e12DM4RS
File Edit Analysis Highlight Add track Layout Size options Help
Zoom
3.2
Whole
Show buried clones
Yes
No
Search
CB Unit Range
0
to
494
Contig stats
Clones: 79 (6 buried)
Markers: 0
Sequenced: 0
Length: 495 CB units
Ctg1685 of cp05T5e12DM4RS
B003I23
M058N11
V071D14
V028D08
M008B03
M025O04
H014D24
M030B14
V013L07~
B005O09
B012F21=
V006F12
H003I05*
B008A09
B006C03
M006O22
V075C14*
V025A07*
V014H21*
M003B16
M025E06
V011I08
V026C24
H022F08
H015D12~
H021D22~
V017N08
M020O08
V074I13
H009G15*
M058K20
H017B21
V053N18
V072D07
V074L10
H020B18
B013I02
H019L24
M025N21
M020D17
V037K03
M020C05
V024N13~
M010J10
M021I02
H017D01
V037H06
M014J10
V037P07
V054F11
V008L19
H004D17
M037E12
M038K09
V051N23
H010O15
M045J13
M032P06
V067F08~
H022F23
B004I12
V058F18
V055N21
M004K22
V054P14
M036G08
B007A07
M002K24
M025B02
V064I16
V051L01
M018C18
M009E18
M048F16
V059I22
M004D18
V060G03
M005I08
V070N07*
0
50
100
150
200
250
300
350
400
450
[Chickpea contig p...
[root@localhost: /...
[FPC V8.9 Main Menu]
Project cp05T5e12...
FPC Ctg1685 cp05T...

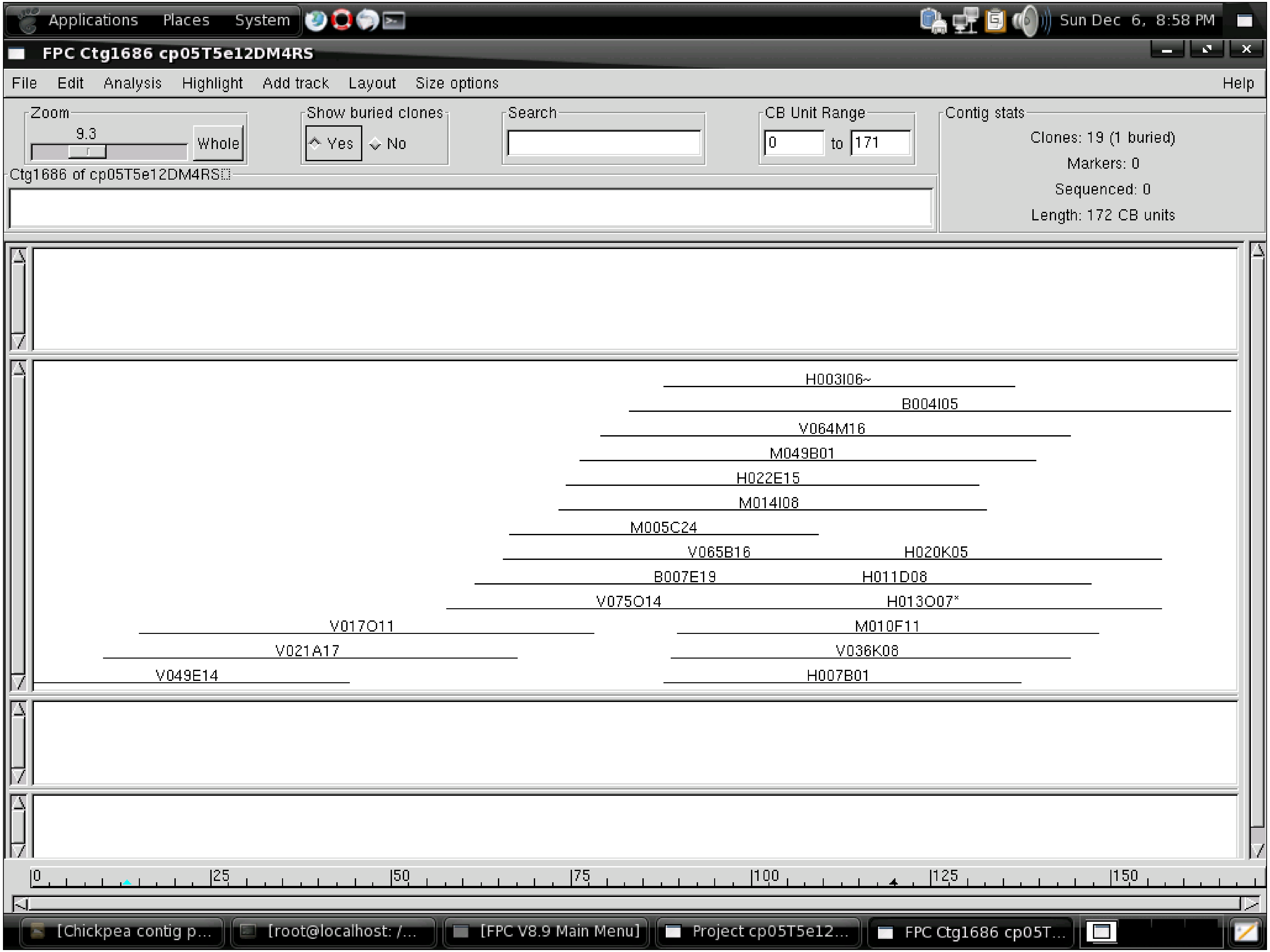
Applications Places System
Sun Dec 6, 8:58 PM
FPC Ctg1686 cp05T5e12DM4RS
File Edit Analysis Highlight Add track Layout Size options Help
Zoom
9.3
Whole
Show buried clones
Yes
No
Search
CB Unit Range
0
to
171
Contig stats
Clones: 19 (1 buried)
Markers: 0
Sequenced: 0
Length: 172 CB units
Ctg1686 of cp05T5e12DM4RS
H003I06~
B004I05
V064M16
M049B01
H022E15
M014I08
M005C24
V065B16
H020K05
B007E19
H011D08
V075O14
H013O07*
V017O11
M010F11
V021A17
V036K08
V049E14
H007B01
0
25
50
75
100
125
150
[Chickpea contig p...
[root@localhost: /...
[FPC V8.9 Main Menu]
Project cp05T5e12...
FPC Ctg1686 cp05T...

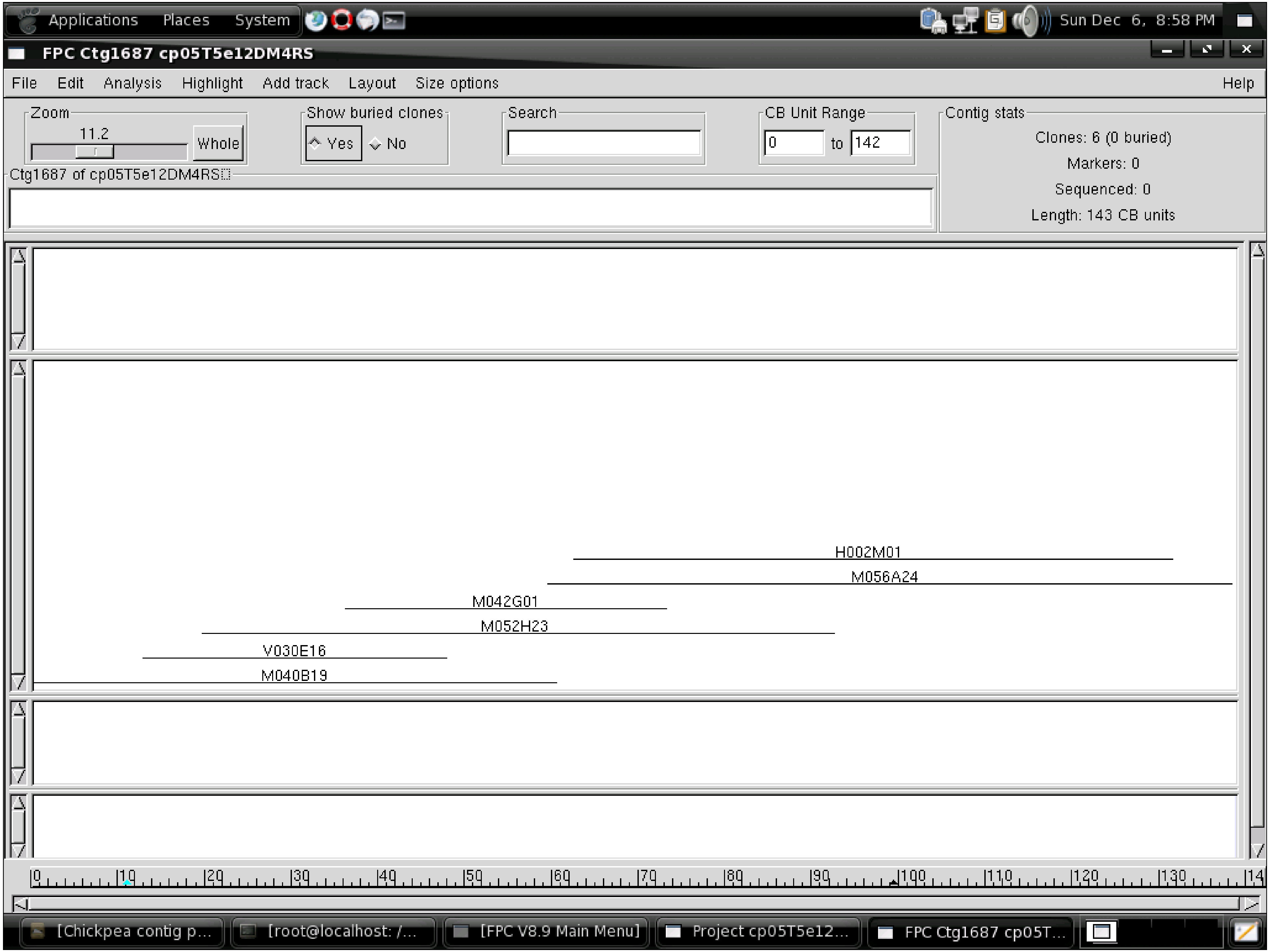
Applications
Places
System
Sun Dec 6, 8:58 PM
FPC Ctg1687 cp05T5e12DM4RS
File
Edit
Analysis
Highlight
Add track
Layout
Size options
Help
Zoom
11.2
Whole
Show buried clones
Yes
No
Search
CB Unit Range
0
to
142
Contig stats
Clones: 6 (0 buried)
Markers: 0
Sequenced: 0
Length: 143 CB units
Ctg1687 of cp05T5e12DM4RS
H002M01
M056A24
M042G01
M052H23
V030E16
M040B19
0
10
20
30
40
50
60
70
80
90
100
110
120
130
[Chickpea contig p...
[root@localhost: /...
[FPC V8.9 Main Menu]
Project cp05T5e12...
FPC Ctg1687 cp05T...

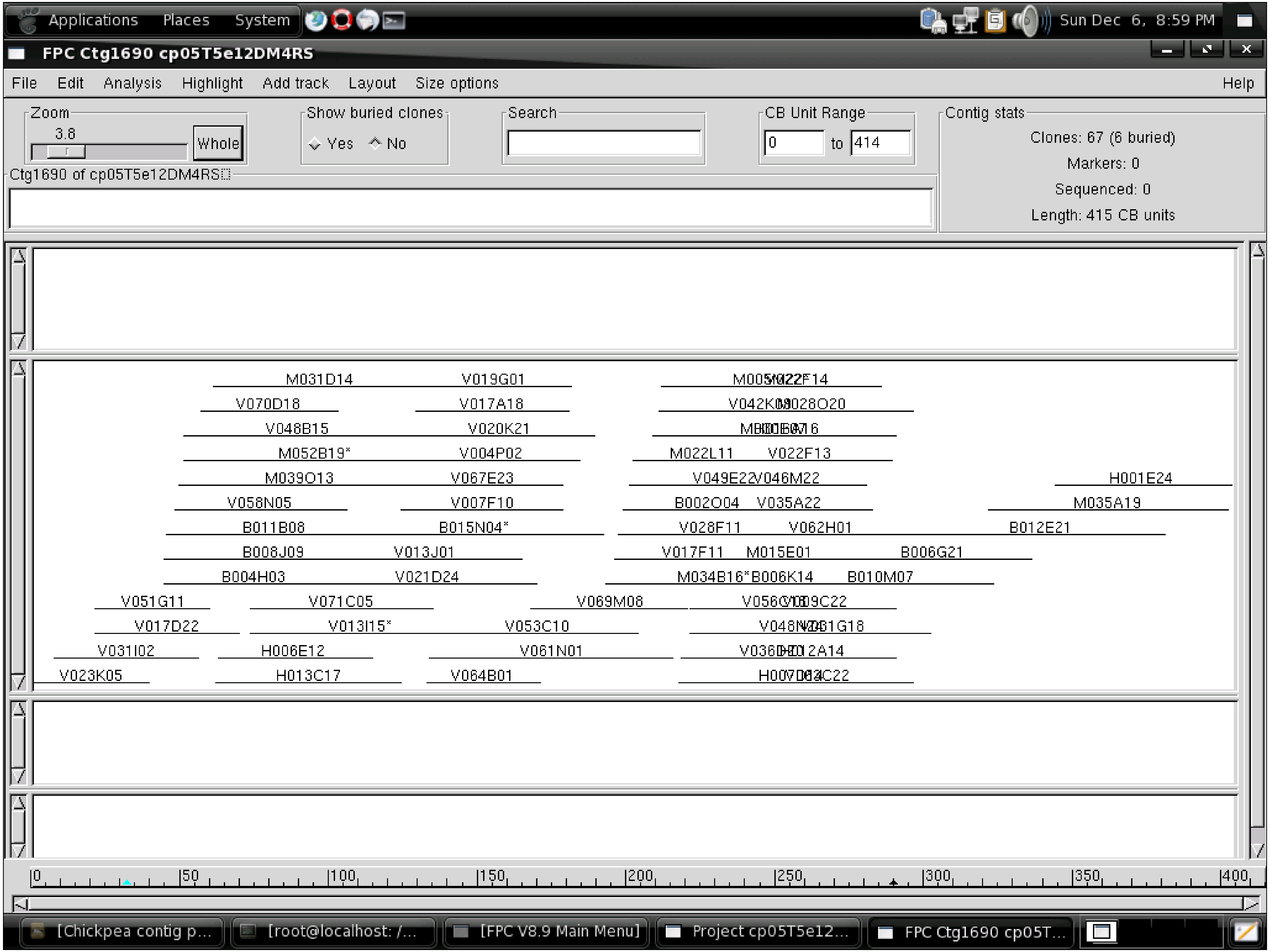
Applications
Places
System
Sun Dec 6, 8:59 PM
FPC Ctg1690 cp05T5e12DM4RS
File
Edit
Analysis
Highlight
Add track
Layout
Size options
Help
Zoom
3.8
Whole
Show buried clones
Yes
No
Search
CB Unit Range
0
to
414
Contig stats
Clones: 67 (6 buried)
Markers: 0
Sequenced: 0
Length: 415 CB units
Ctg1690 of cp05T5e12DM4RS
M031D14
V019G01
V070D18
V017A18
V048B15
V020K21
M052B19*
V004P02
M039O13
V067E23
V058N05
V007F10
B011B08
B015N04*
B008J09
V013J01
B004H03
V021D24
V051G11
V071C05
V069M08
V017D22
V013I15*
V053C10
V031I02
H006E12
V061N01
V023K05
H013C17
V064B01
M022L11
V022F13
B002O04
V035A22
V028F11
V062H01
V017F11
M015E01
B006G21
M034B16*
B006K14
B010M07
H001E24
M035A19
B012E21
0
50
100
150
200
250
300
350
400
[Chickpea contig p...
[root@localhost: /...
[FPC V8.9 Main Menu]
Project cp05T5e12...
FPC Ctg1690 cp05T...

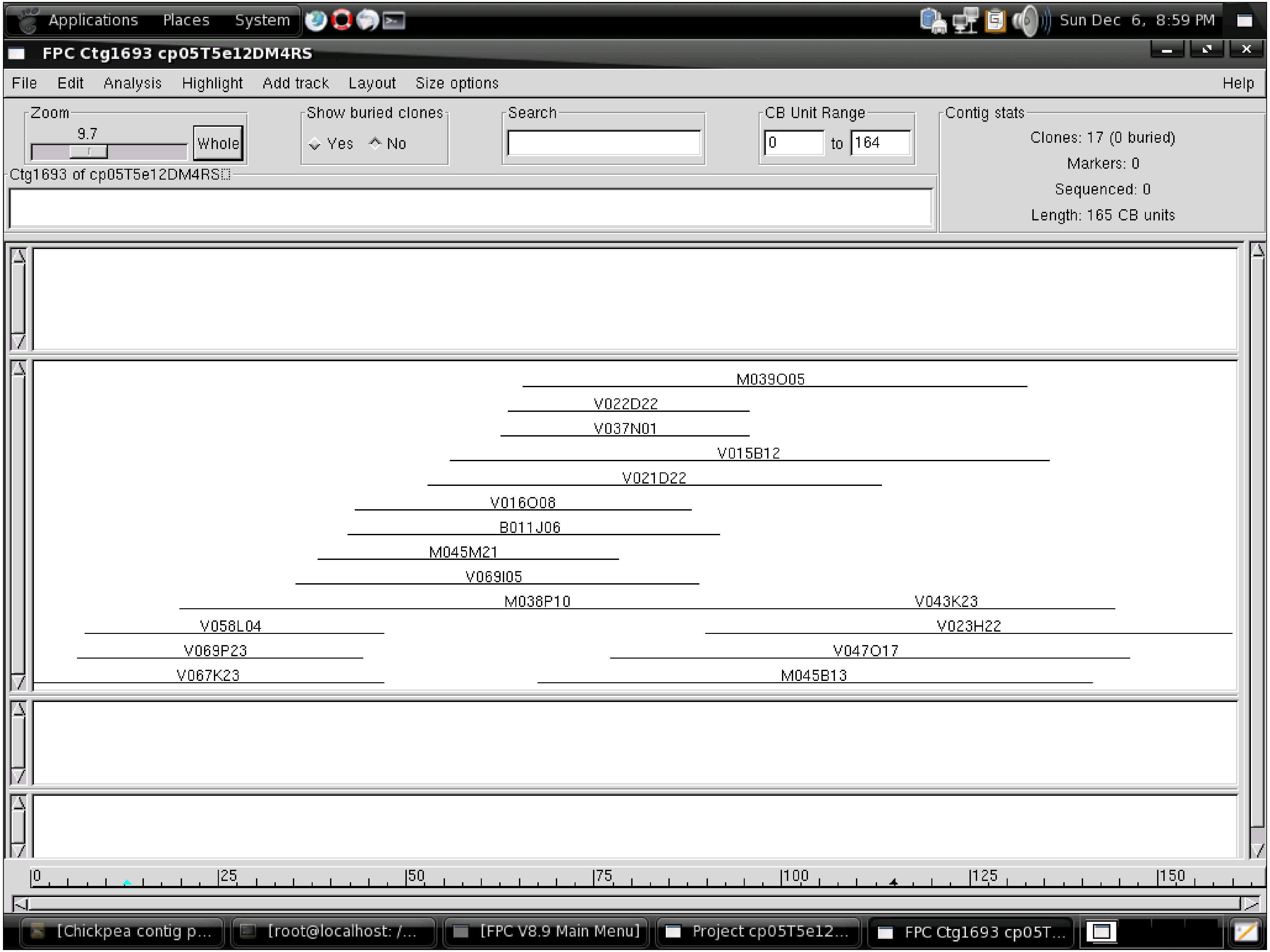
Applications Places System
Sun Dec 6, 8:59 PM
FPC Ctg1693 cp05T5e12DM4RS
File Edit Analysis Highlight Add track Layout Size options Help
Zoom
9.7
Whole
Show buried clones
Yes No
Search
CB Unit Range
0 to 164
Contig stats
Clones: 17 (0 buried)
Markers: 0
Sequenced: 0
Length: 165 CB units
Ctg1693 of cp05T5e12DM4RS
M039O05
V022D22
V037N01
V015B12
V021D22
V016O08
B011J06
M045M21
V069I05
M038P10
V043K23
V058L04
V023H22
V069P23
V047O17
V067K23
M045B13
0 25 50 75 100 125 150
[Chickpea contig p...
[root@localhost: /...
[FPC V8.9 Main Menu]
Project cp05T5e12...
FPC Ctg1693 cp05T...

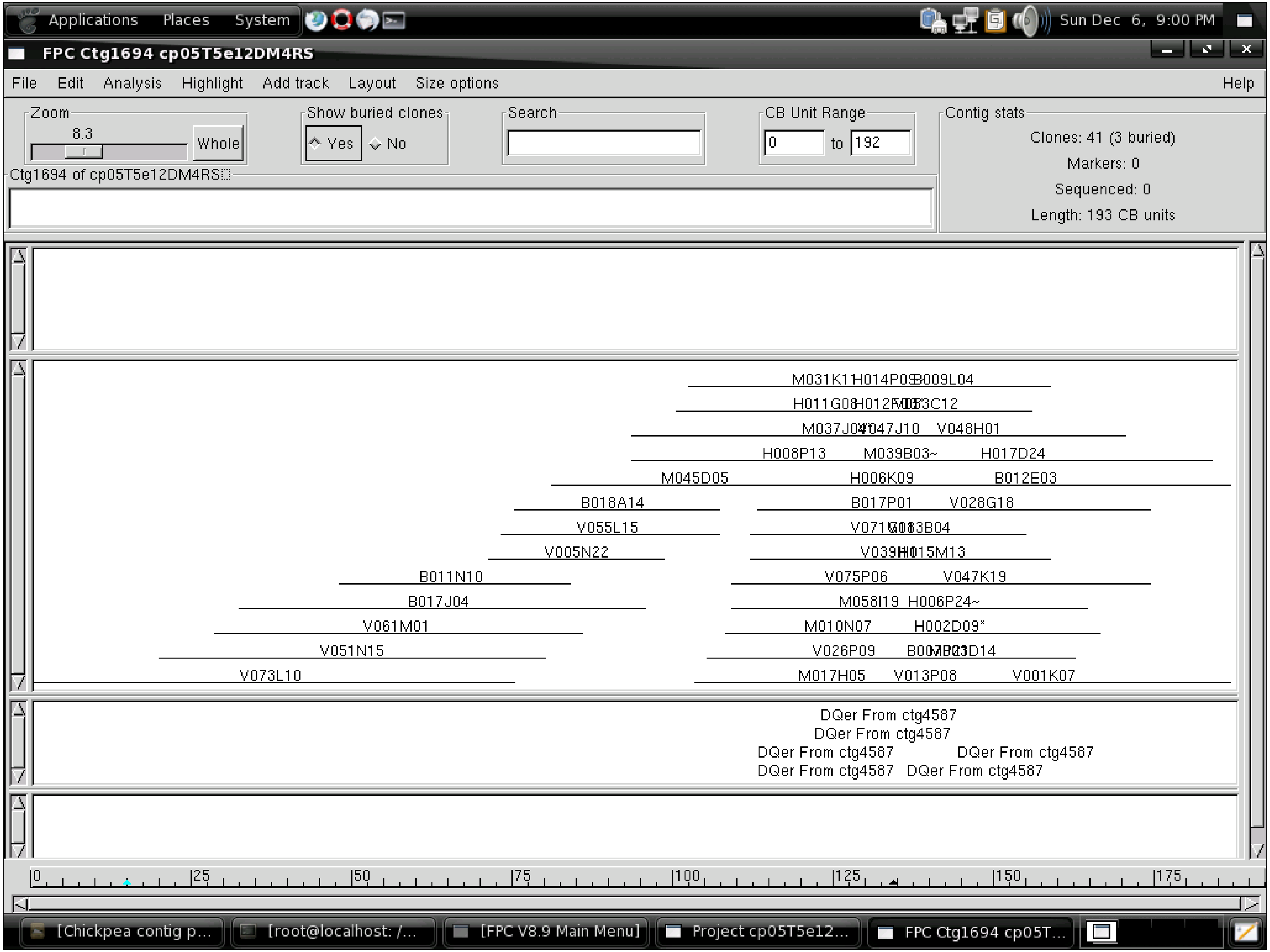
Applications Places System
Sun Dec 6, 9:00 PM
FPC Ctg1694 cp05T5e12DM4RS
File Edit Analysis Highlight Add track Layout Size options Help
Zoom
8.3
Whole
Show buried clones
Yes No
Search
CB Unit Range
0 to 192
Contig stats
Clones: 41 (3 buried)
Markers: 0
Sequenced: 0
Length: 193 CB units
Ctg1694 of cp05T5e12DM4RS
H008P13 M039B03~ H017D24
M045D05 H006K09 B012E03
B018A14 B017P01 V028G18
V055L15
V005N22
B011N10 V075P06 V047K19
B017J04 M058I19 H006P24~
V061M01 M010N07 H002D09*
V051N15 V026P09
V073L10 M017H05 V013P08 V001K07
DQer From ctg4587
DQer From ctg4587
DQer From ctg4587 DQer From ctg4587
DQer From ctg4587 DQer From ctg4587
0 25 50 75 100 125 150 175
[Chickpea contig p...
[root@localhost: /...
[FPC V8.9 Main Menu]
Project cp05T5e12...
FPC Ctg1694 cp05T...

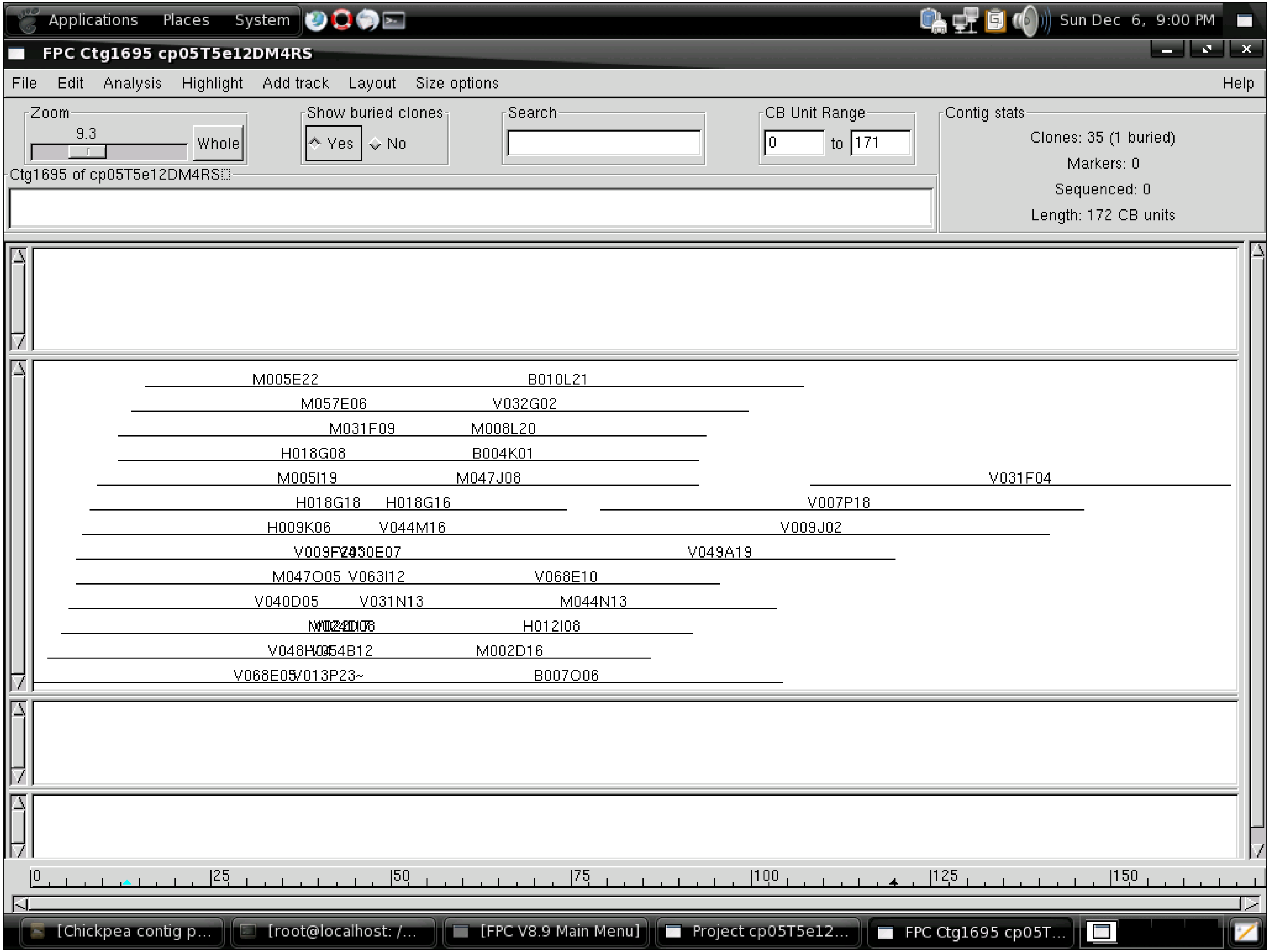
Applications Places System
Sun Dec 6, 9:00 PM
FPC Ctg1695 cp05T5e12DM4RS
File Edit Analysis Highlight Add track Layout Size options Help
Zoom
9.3
Whole
Show buried clones
Yes
No
Search
CB Unit Range
0 to 171
Contig stats
Clones: 35 (1 buried)
Markers: 0
Sequenced: 0
Length: 172 CB units
Ctg1695 of cp05T5e12DM4RS
M005E22
B010L21
M057E06
V032G02
M031F09
M008L20
H018G08
B004K01
M005I19
M047J08
V031F04
H018G16
V007P18
H009K06
V044M16
V009J02
V049A19
M047O05
V063I12
V068E10
V040D05
V031N13
M044N13
H012I08
M002D16
B007O06
0 25 50 75 100 125 150
[Chickpea contig p...
[root@localhost: /...
[FPC V8.9 Main Menu]
Project cp05T5e12...
FPC Ctg1695 cp05T...

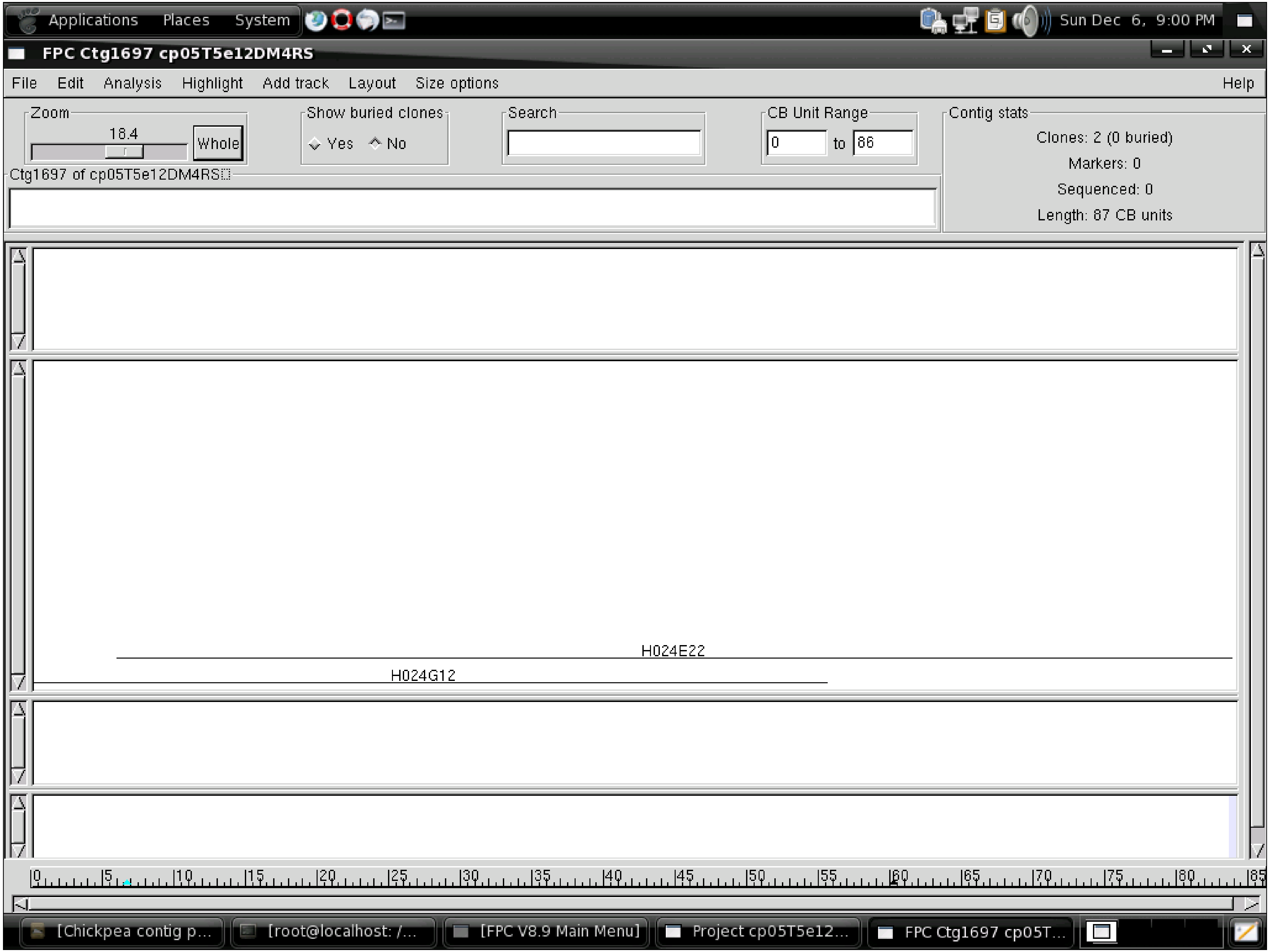
Applications Places System
Sun Dec 6, 9:00 PM
FPC Ctg1697 cp05T5e12DM4RS
File Edit Analysis Highlight Add track Layout Size options Help
Zoom
18.4
Whole
Show buried clones
Yes No
Search
CB Unit Range
0 to 86
Contig stats
Clones: 2 (0 buried)
Markers: 0
Sequenced: 0
Length: 87 CB units
Ctg1697 of cp05T5e12DM4RS
H024E22
H024G12
0 5 10 15 20 25 30 35 40 45 50 55 60 65 70 75 80 85
[Chickpea contig p...
[root@localhost: /...
[FPC V8.9 Main Menu]
Project cp05T5e12...
FPC Ctg1697 cp05T...

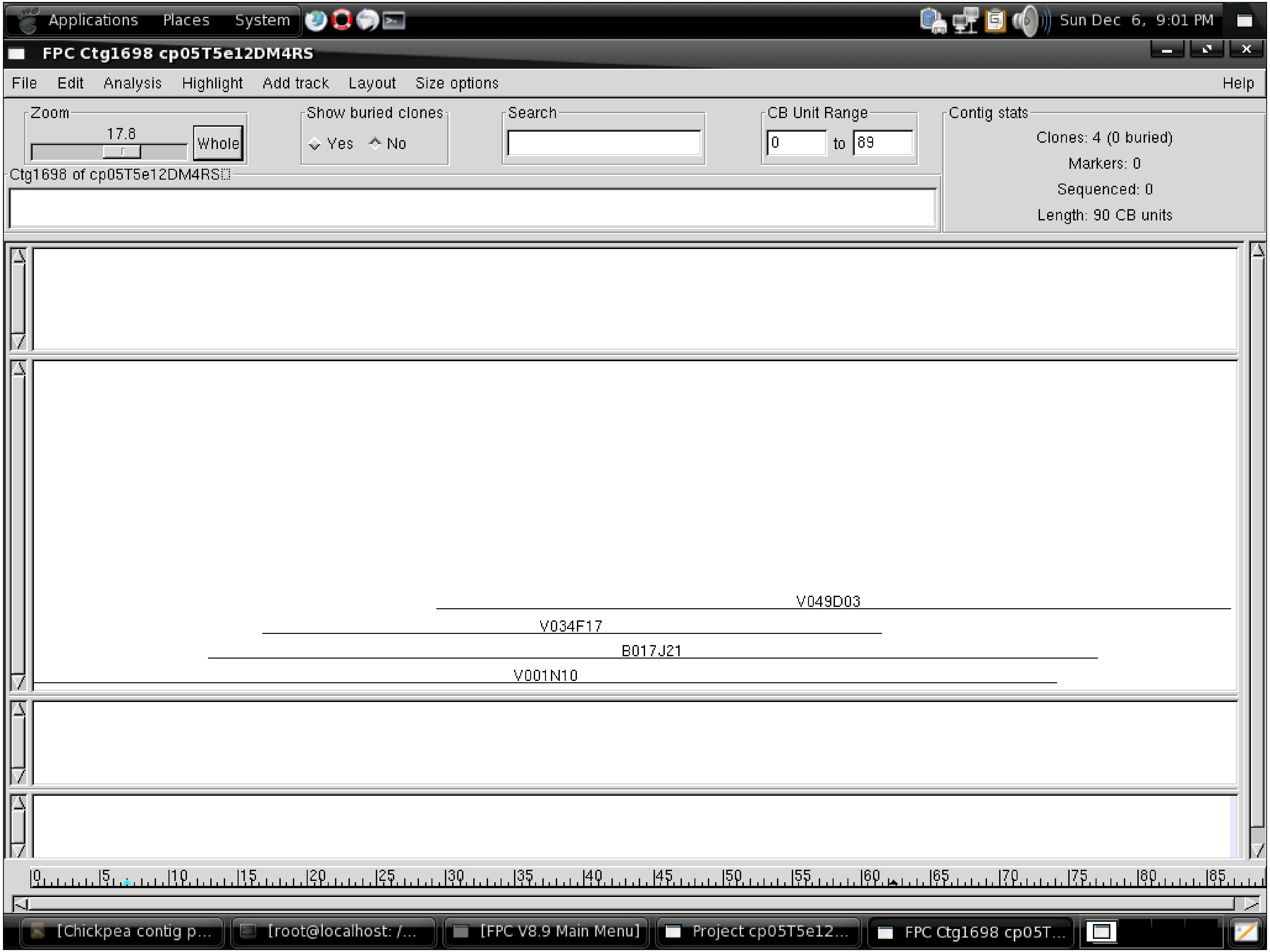
Applications Places System
Sun Dec 6, 9:01 PM
FPC Ctg1698 cp05T5e12DM4RS
File Edit Analysis Highlight Add track Layout Size options Help
Zoom
17.8
Whole
Show buried clones
Yes No
Search
CB Unit Range
0 to 89
Contig stats
Clones: 4 (0 buried)
Markers: 0
Sequenced: 0
Length: 90 CB units
Ctg1698 of cp05T5e12DM4RS
V049D03
V034F17
B017J21
V001N10
0 5 10 15 20 25 30 35 40 45 50 55 60 65 70 75 80 85
[Chickpea contig p...
[root@localhost: /...
[FPC V8.9 Main Menu]
Project cp05T5e12...
FPC Ctg1698 cp05T...

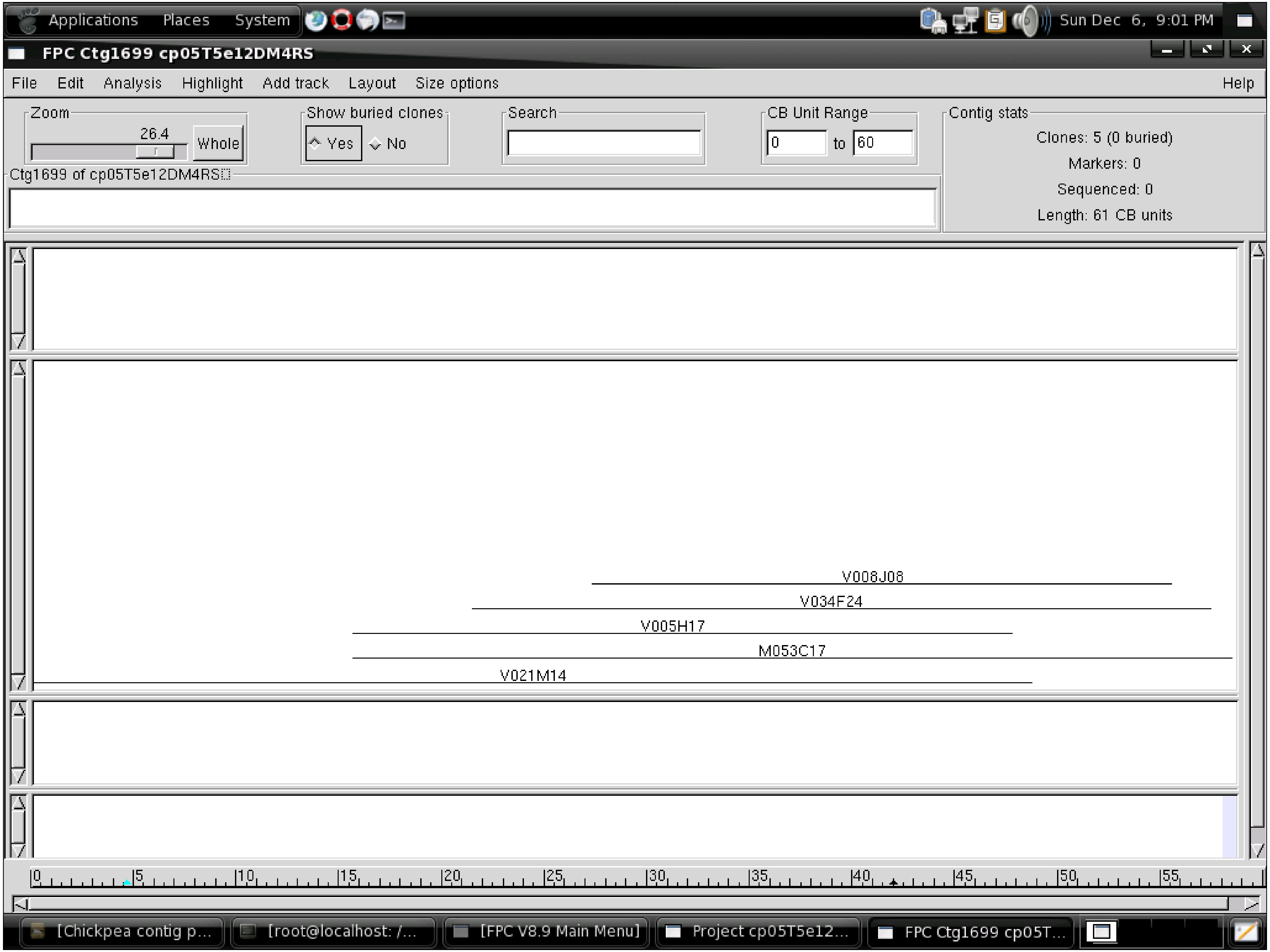
Applications Places System
Sun Dec 6, 9:01 PM
FPC Ctg1699 cp05T5e12DM4RS
File Edit Analysis Highlight Add track Layout Size options Help
Zoom
26.4
Whole
Show buried clones
Yes No
Search
CB Unit Range
0 to 60
Contig stats
Clones: 5 (0 buried)
Markers: 0
Sequenced: 0
Length: 61 CB units
Ctg1699 of cp05T5e12DM4RS
V008J08
V034F24
V005H17
M053C17
V021M14
0 5 10 15 20 25 30 35 40 45 50 55
[Chickpea contig p...
[root@localhost: /...
[FPC V8.9 Main Menu]
Project cp05T5e12...
FPC Ctg1699 cp05T...

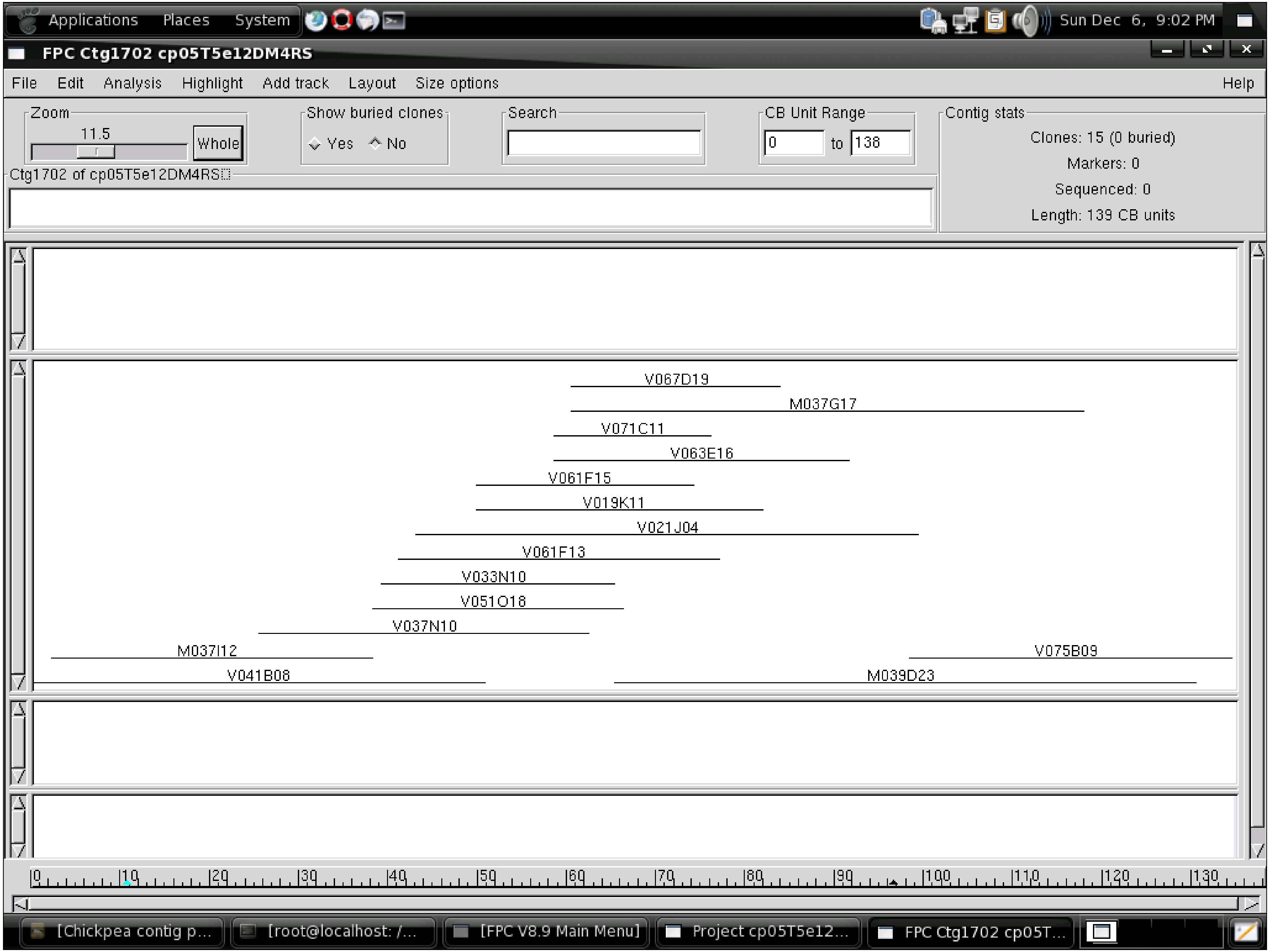
Applications Places System
Sun Dec 6, 9:02 PM
FPC Ctg1702 cp05T5e12DM4RS
File Edit Analysis Highlight Add track Layout Size options Help
Zoom
11.5
Whole
Show buried clones
Yes No
Search
CB Unit Range
0 to 138
Contig stats
Clones: 15 (0 buried)
Markers: 0
Sequenced: 0
Length: 139 CB units
Ctg1702 of cp05T5e12DM4RS
V067D19
M037G17
V071C11
V063E16
V061F15
V019K11
V021J04
V061F13
V033N10
V051O18
V037N10
M037I12
V075B09
V041B08
M039D23
0 10 20 30 40 50 60 70 80 90 100 110 120 130
[Chickpea contig p...
[root@localhost: /...
[FPC V8.9 Main Menu]
Project cp05T5e12...
FPC Ctg1702 cp05T...

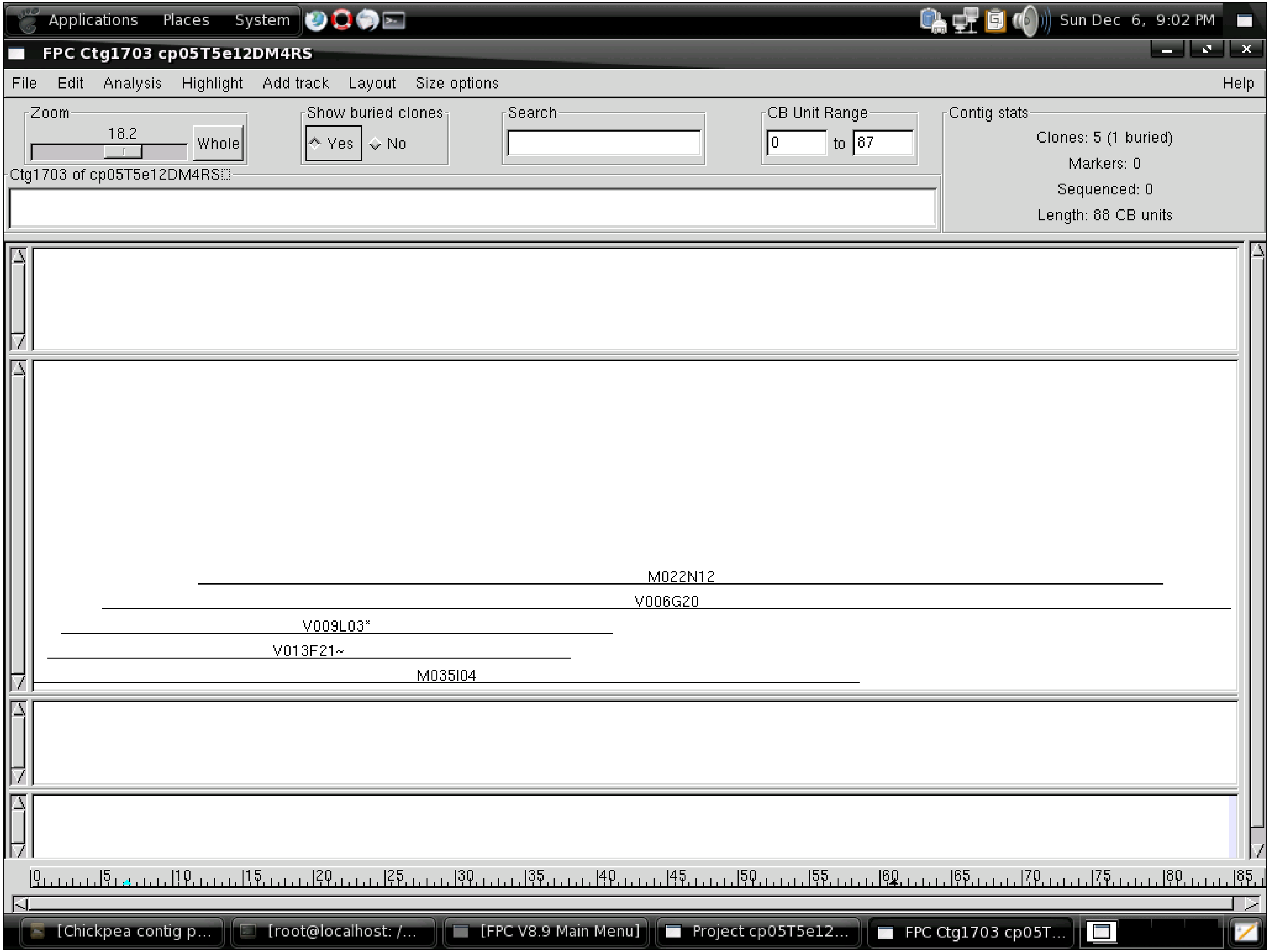
Applications Places System
Sun Dec 6, 9:02 PM
FPC Ctg1703 cp05T5e12DM4RS
File Edit Analysis Highlight Add track Layout Size options Help
Zoom
18.2
Whole
Show buried clones
Yes
No
Search
CB Unit Range
0
to
87
Contig stats
Clones: 5 (1 buried)
Markers: 0
Sequenced: 0
Length: 88 CB units
Ctg1703 of cp05T5e12DM4RS
M022N12
V006G20
V009L03*
V013F21~
M035I04
0 5 10 15 20 25 30 35 40 45 50 55 60 65 70 75 80 85
[Chickpea contig p...
[root@localhost: /...
[FPC V8.9 Main Menu]
Project cp05T5e12...
FPC Ctg1703 cp05T...

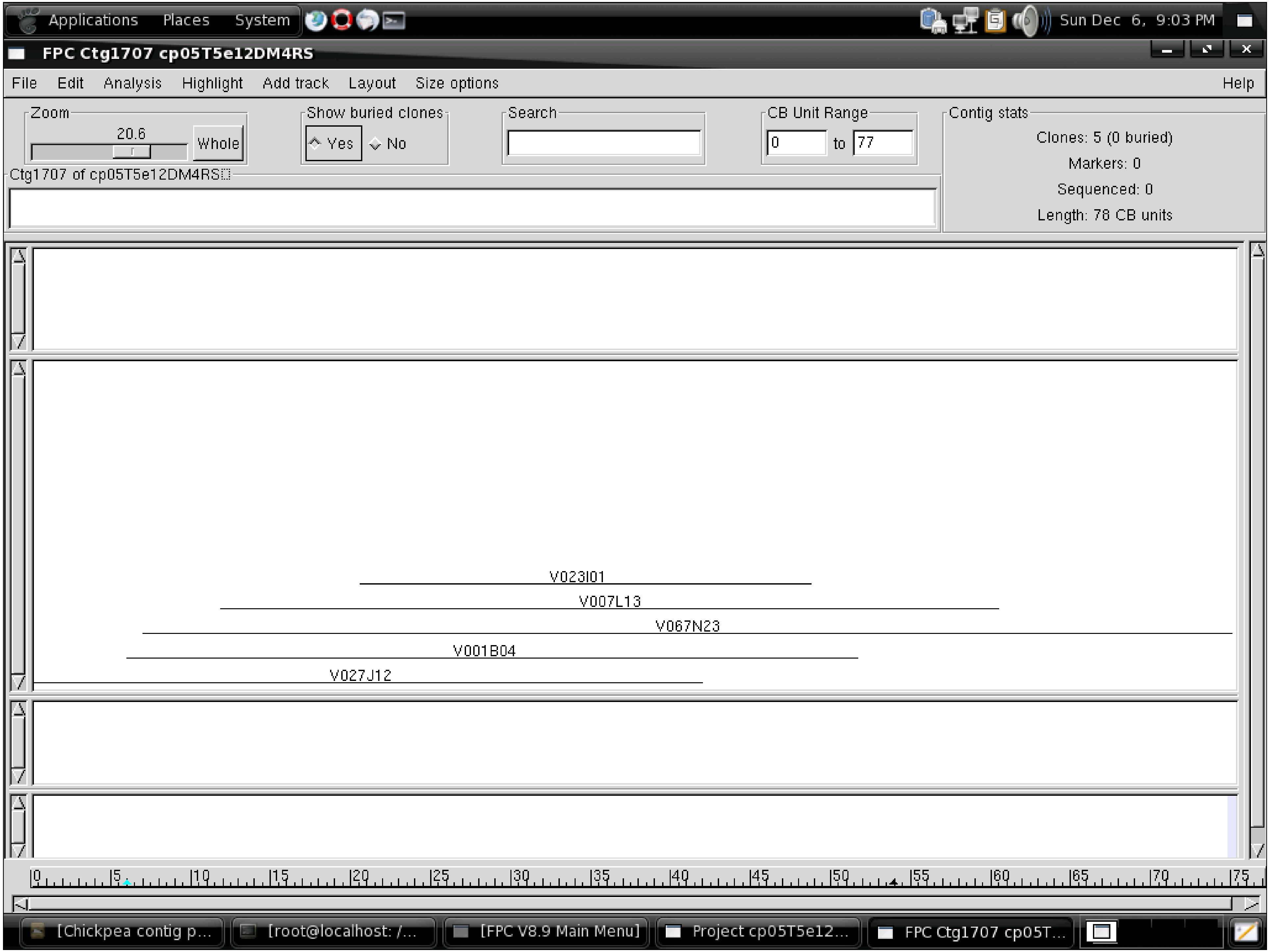
Applications Places System
Sun Dec 6, 9:03 PM
FPC Ctg1707 cp05T5e12DM4RS
File Edit Analysis Highlight Add track Layout Size options Help
Zoom
20.6
Whole
Show buried clones
Yes
No
Search
CB Unit Range
0
to
77
Contig stats
Clones: 5 (0 buried)
Markers: 0
Sequenced: 0
Length: 78 CB units
Ctg1707 of cp05T5e12DM4RS
V023I01
V007L13
V067N23
V001B04
V027J12
0 5 10 15 20 25 30 35 40 45 50 55 60 65 70 75
[Chickpea contig p...
[root@localhost: /...
[FPC V8.9 Main Menu]
Project cp05T5e12...
FPC Ctg1707 cp05T...

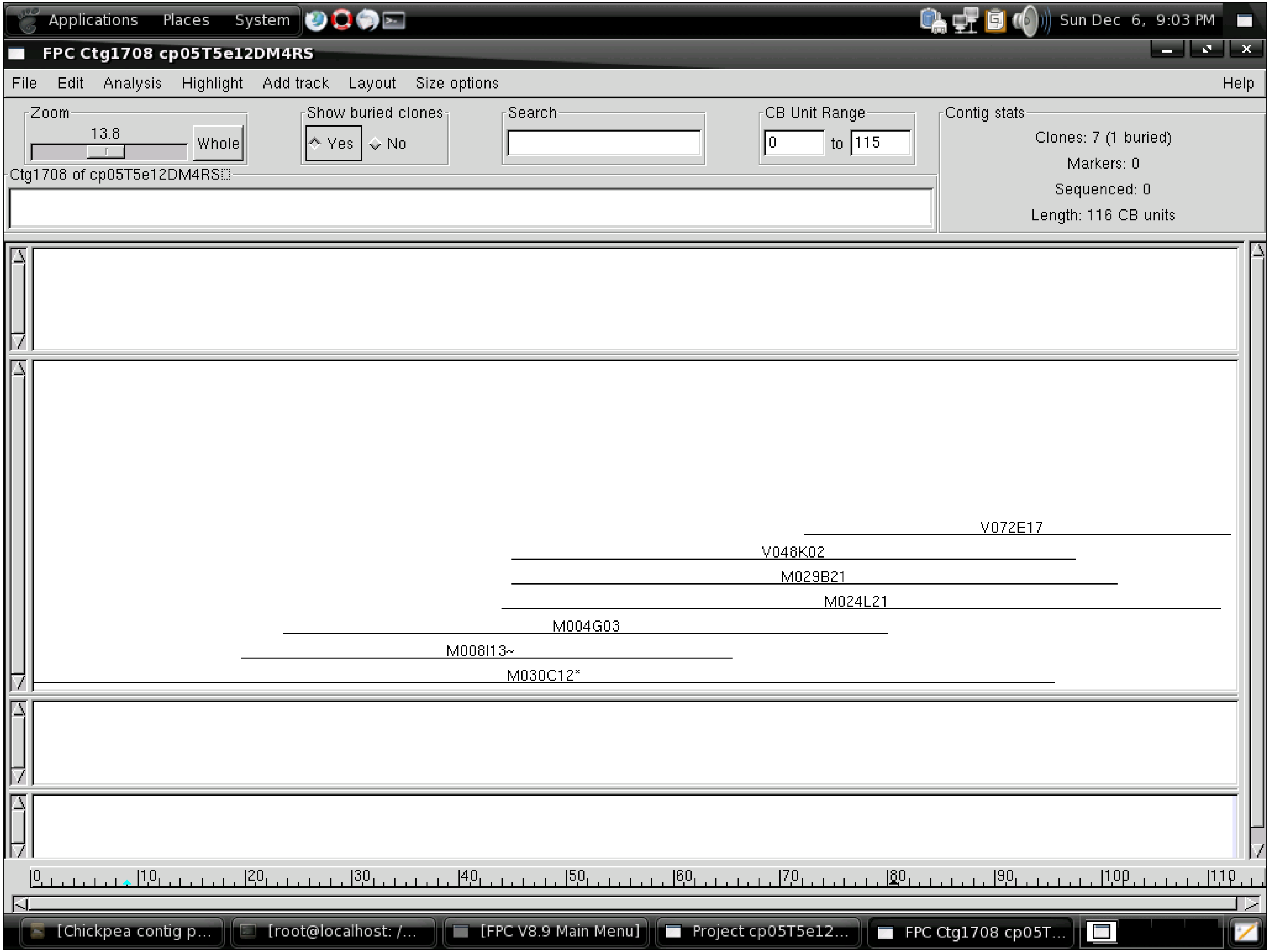
Applications Places System
Sun Dec 6, 9:03 PM
FPC Ctg1708 cp05T5e12DM4RS
File Edit Analysis Highlight Add track Layout Size options Help
Zoom
13.8
Whole
Show buried clones
Yes
No
Search
CB Unit Range
0 to 115
Contig stats
Clones: 7 (1 buried)
Markers: 0
Sequenced: 0
Length: 116 CB units
Ctg1708 of cp05T5e12DM4RS
V072E17
V048K02
M029B21
M024L21
M004G03
M008I13~
M030C12*
0 10 20 30 40 50 60 70 80 90 100 110
[Chickpea contig p...
[root@localhost: /...
[FPC V8.9 Main Menu]
Project cp05T5e12...
FPC Ctg1708 cp05T...

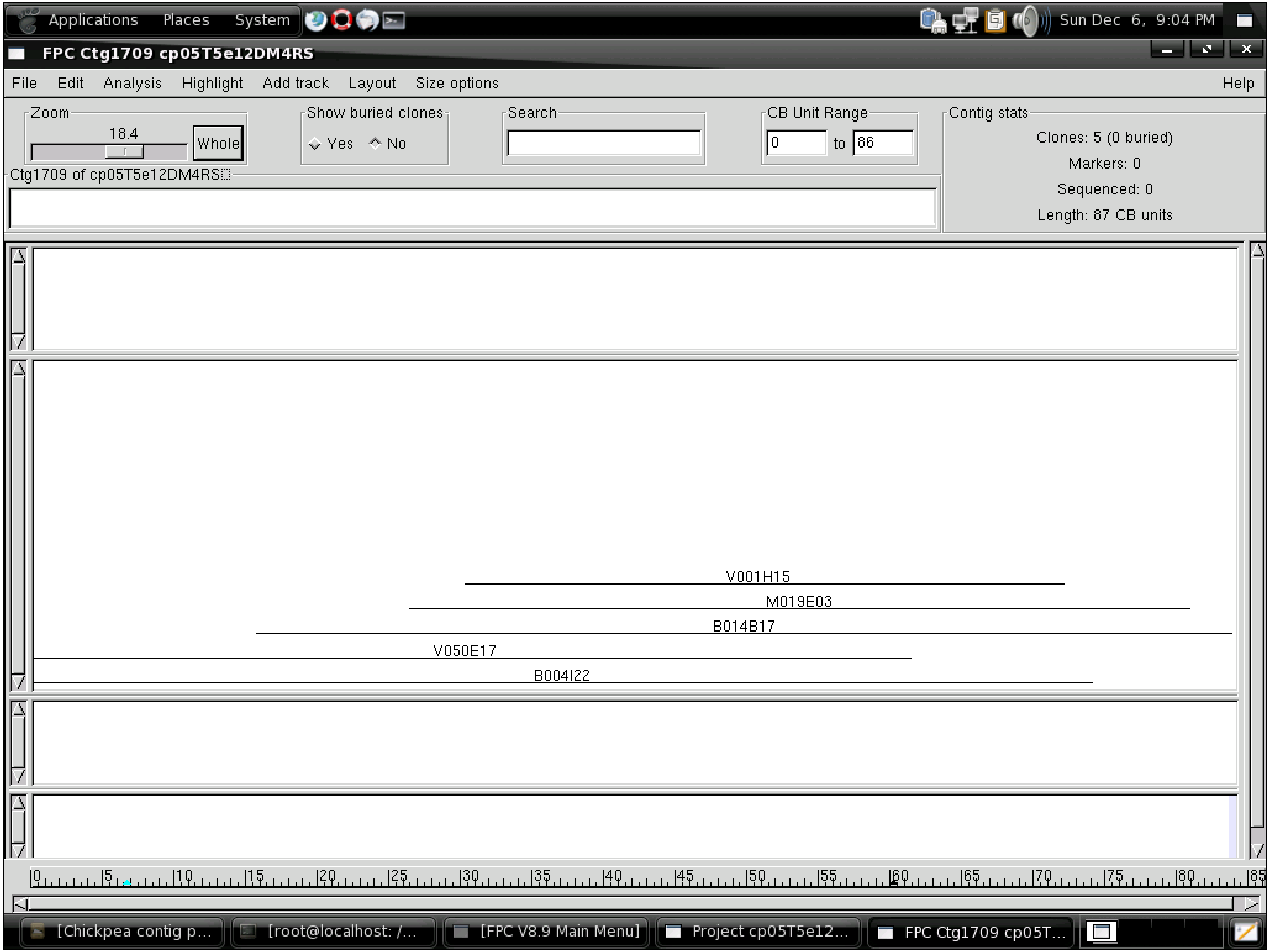
Applications Places System
Sun Dec 6, 9:04 PM
FPC Ctg1709 cp05T5e12DM4RS
File Edit Analysis Highlight Add track Layout Size options Help
Zoom
18.4
Whole
Show buried clones
Yes No
Search
CB Unit Range
0 to 86
Contig stats
Clones: 5 (0 buried)
Markers: 0
Sequenced: 0
Length: 87 CB units
Ctg1709 of cp05T5e12DM4RS
V001H15
M019E03
B014B17
V050E17
B004I22
0 5 10 15 20 25 30 35 40 45 50 55 60 65 70 75 80 85
[Chickpea contig p...
[root@localhost: /...
[FPC V8.9 Main Menu]
Project cp05T5e12...
FPC Ctg1709 cp05T...

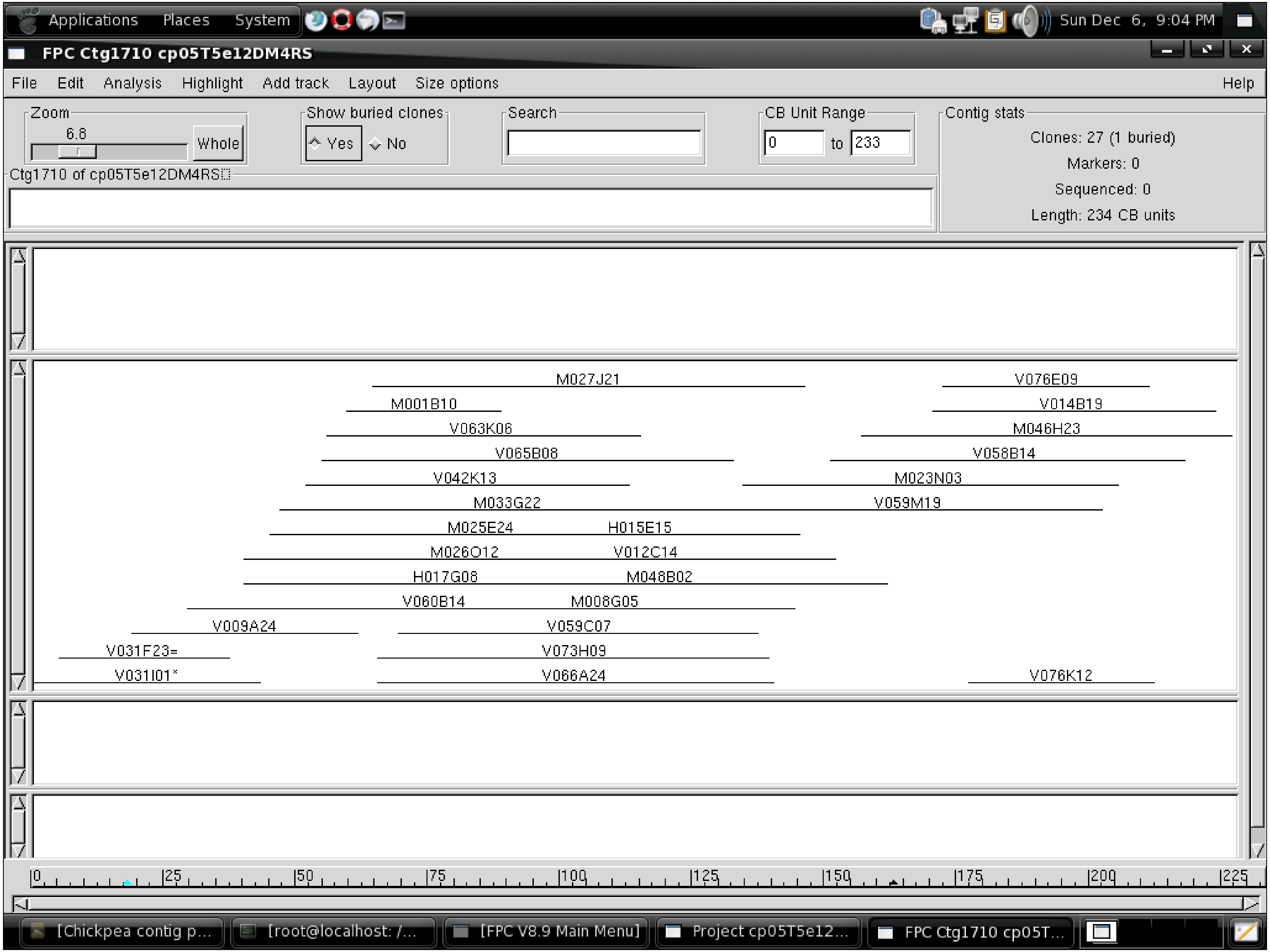
Applications Places System
Sun Dec 6, 9:04 PM
FPC Ctg1710 cp05T5e12DM4RS
File Edit Analysis Highlight Add track Layout Size options Help
Zoom
6.8
Whole
Show buried clones
Yes No
Search
CB Unit Range
0 to 233
Contig stats
Clones: 27 (1 buried)
Markers: 0
Sequenced: 0
Length: 234 CB units
Ctg1710 of cp05T5e12DM4RS
M027J21
V076E09
M001B10
V014B19
V063K06
M046H23
V065B08
V058B14
V042K13
M023N03
M033G22
V059M19
M025E24
H015E15
M026O12
V012C14
H017G08
M048B02
V060B14
M008G05
V009A24
V059C07
V031F23=
V073H09
V031I01*
V066A24
V076K12
0 25 50 75 100 125 150 175 200 225
[Chickpea contig p...
[root@localhost: /...
[FPC V8.9 Main Menu]
Project cp05T5e12...
FPC Ctg1710 cp05T...

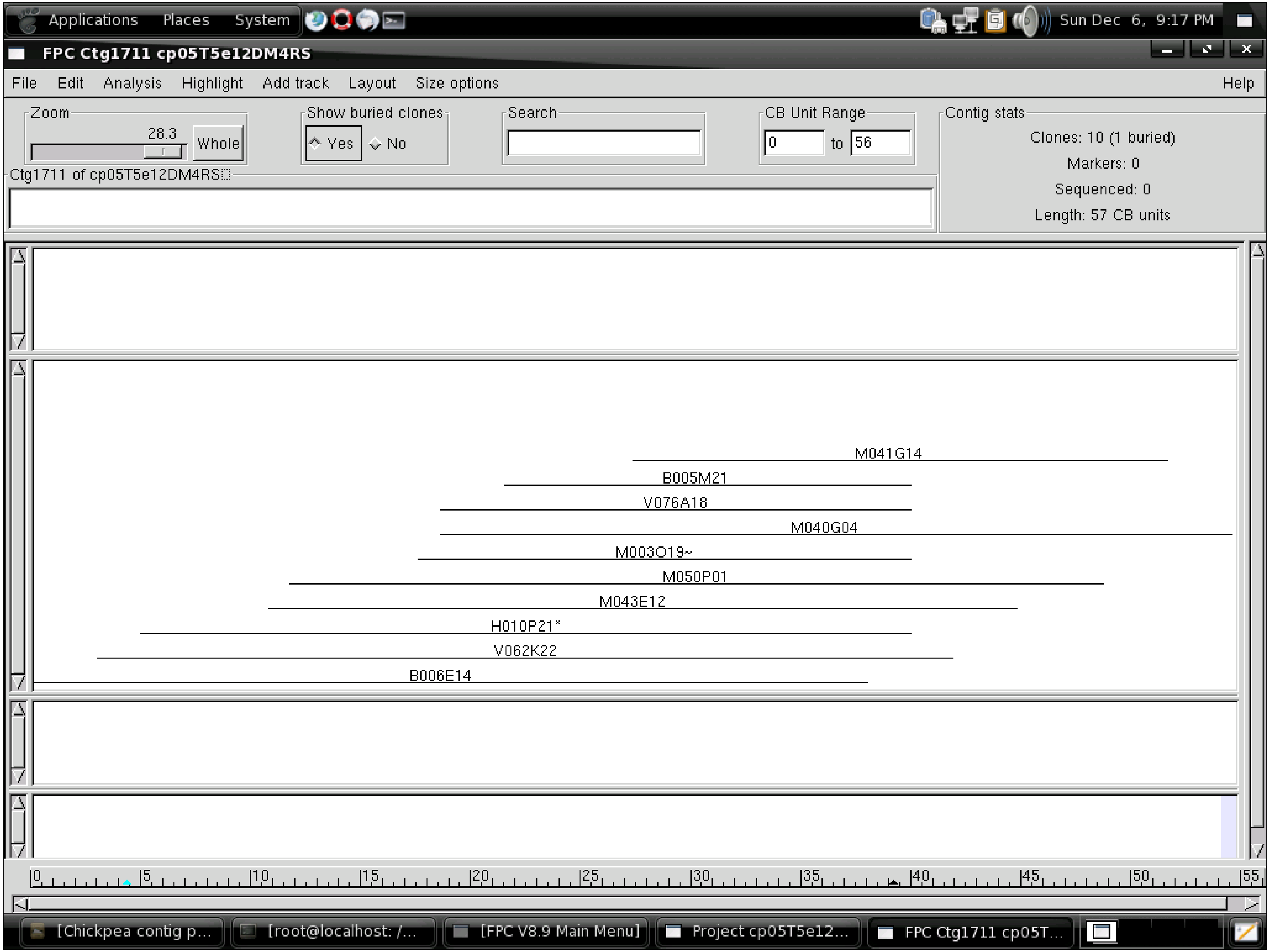
Applications Places System
Sun Dec 6, 9:17 PM
FPC Ctg1711 cp05T5e12DM4RS
File Edit Analysis Highlight Add track Layout Size options Help
Zoom
28.3
Whole
Show buried clones
Yes
No
Search
CB Unit Range
0 to 56
Contig stats
Clones: 10 (1 buried)
Markers: 0
Sequenced: 0
Length: 57 CB units
Ctg1711 of cp05T5e12DM4RS
M041G14
B005M21
V076A18
M040G04
M003O19~
M050P01
M043E12
H010P21*
V062K22
B006E14
0 5 10 15 20 25 30 35 40 45 50 55
[Chickpea contig p...
[root@localhost: /...
[FPC V8.9 Main Menu]
Project cp05T5e12...
FPC Ctg1711 cp05T...

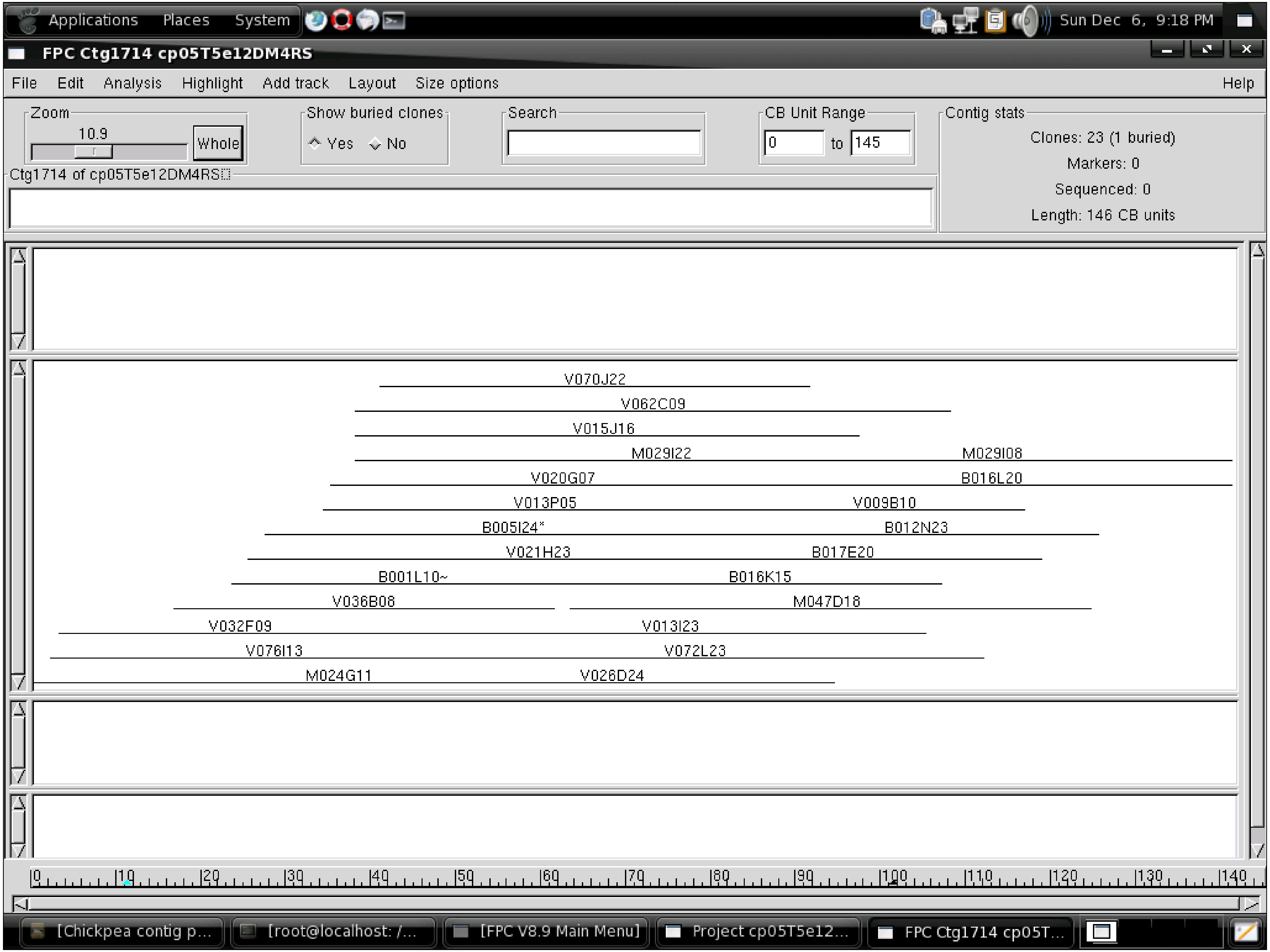
Applications Places System
Sun Dec 6, 9:18 PM
FPC Ctg1714 cp05T5e12DM4RS
File Edit Analysis Highlight Add track Layout Size options Help
Zoom
10.9
Whole
Show buried clones
Yes No
Search
CB Unit Range
0 to 145
Contig stats
Clones: 23 (1 buried)
Markers: 0
Sequenced: 0
Length: 146 CB units
Ctg1714 of cp05T5e12DM4RS
V070J22
V062C09
V015J16
M029I22
M029I08
V020G07
B016L20
V013P05
V009B10
B005I24*
B012N23
V021H23
B017E20
B001L10~
B016K15
V036B08
M047D18
V032F09
V013I23
V076I13
V072L23
M024G11
V026D24
0 10 20 30 40 50 60 70 80 90 100 110 120 130 140
[Chickpea contig p...
[root@localhost: /...
[FPC V8.9 Main Menu]
Project cp05T5e12...
FPC Ctg1714 cp05T...

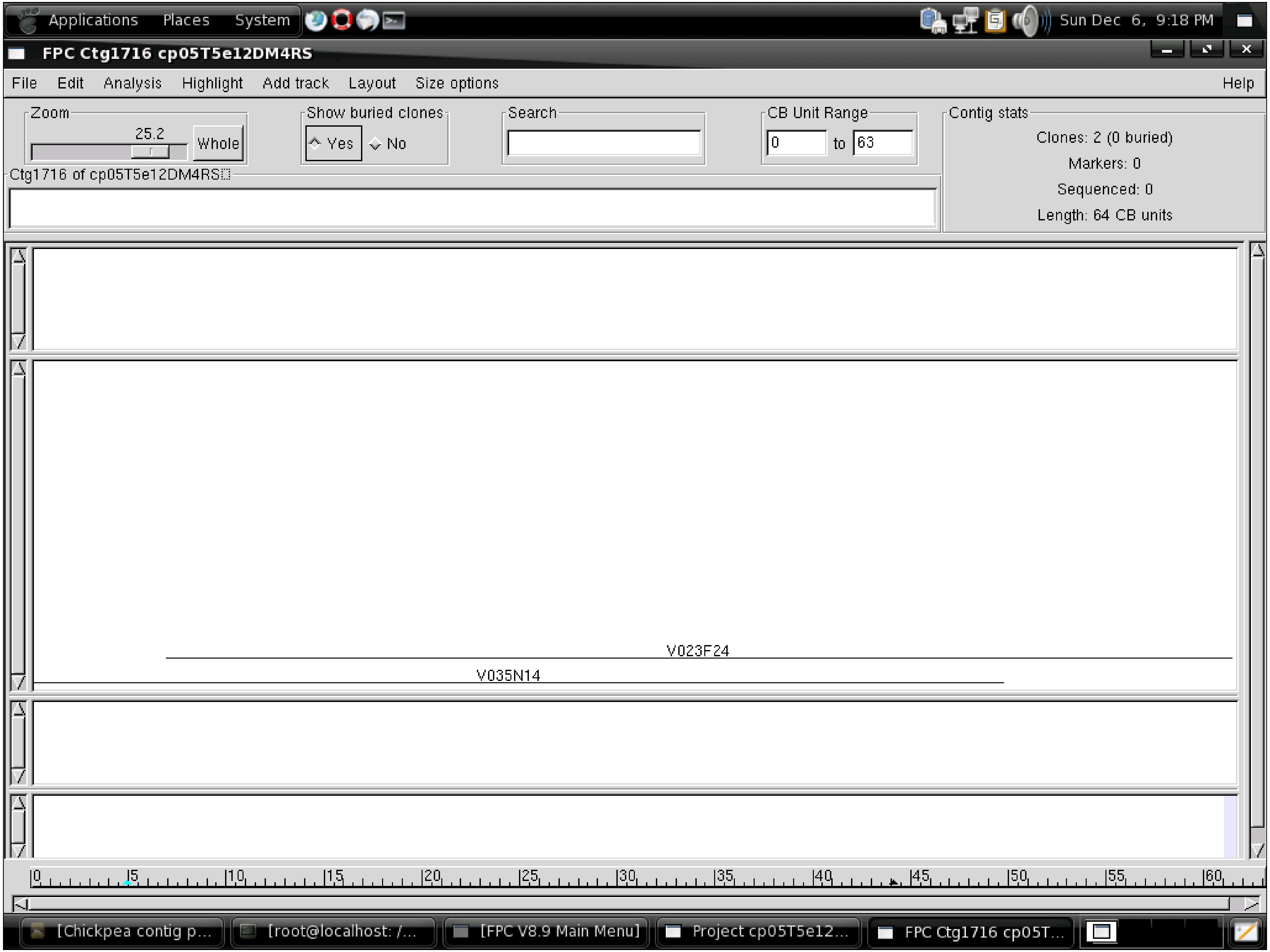
Applications Places System
Sun Dec 6, 9:18 PM
FPC Ctg1716 cp05T5e12DM4RS
File Edit Analysis Highlight Add track Layout Size options Help
Zoom
25.2
Whole
Show buried clones
Yes
No
Search
CB Unit Range
0 to 63
Contig stats
Clones: 2 (0 buried)
Markers: 0
Sequenced: 0
Length: 64 CB units
Ctg1716 of cp05T5e12DM4RS
V023F24
V035N14
0 5 10 15 20 25 30 35 40 45 50 55 60
[Chickpea contig p...
[root@localhost: /...
[FPC V8.9 Main Menu]
Project cp05T5e12...
FPC Ctg1716 cp05T...

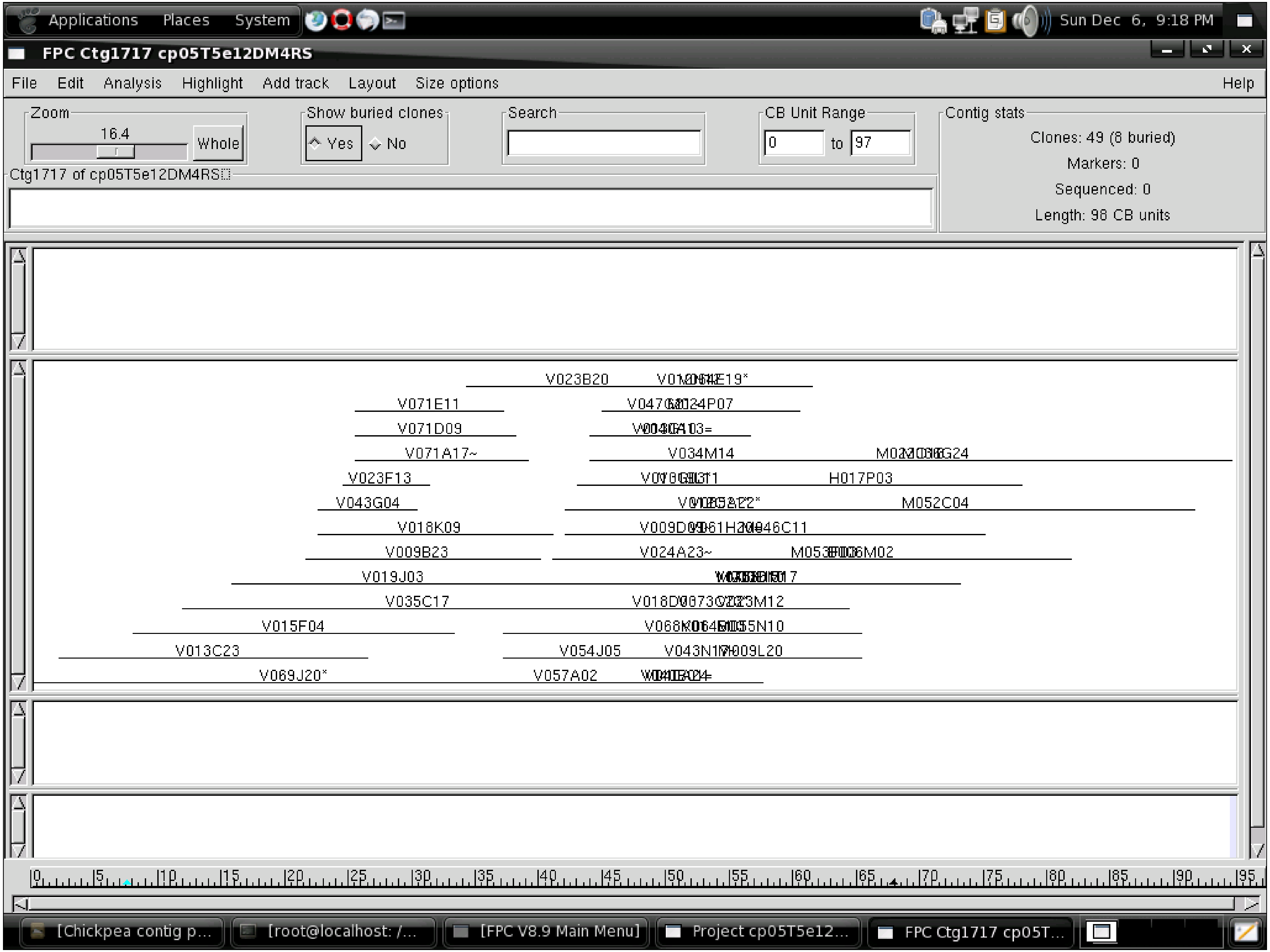
Applications Places System
Sun Dec 6, 9:18 PM
FPC Ctg1717 cp05T5e12DM4RS
File Edit Analysis Highlight Add track Layout Size options Help
Zoom
16.4
Whole
Show buried clones
Yes No
Search
CB Unit Range
0 to 97
Contig stats
Clones: 49 (8 buried)
Markers: 0
Sequenced: 0
Length: 98 CB units
Ctg1717 of cp05T5e12DM4RS
V023B20
V071E11
V071D09
V071A17~
V034M14
V023F13
H017P03
V043G04
M052C04
V018K09
V009B23
V024A23~
V019J03
V035C17
V015F04
V013C23
V054J05
V069J20*
V057A02
[Chickpea contig p...
[root@localhost: /...
[FPC V8.9 Main Menu]
Project cp05T5e12...
FPC Ctg1717 cp05T...

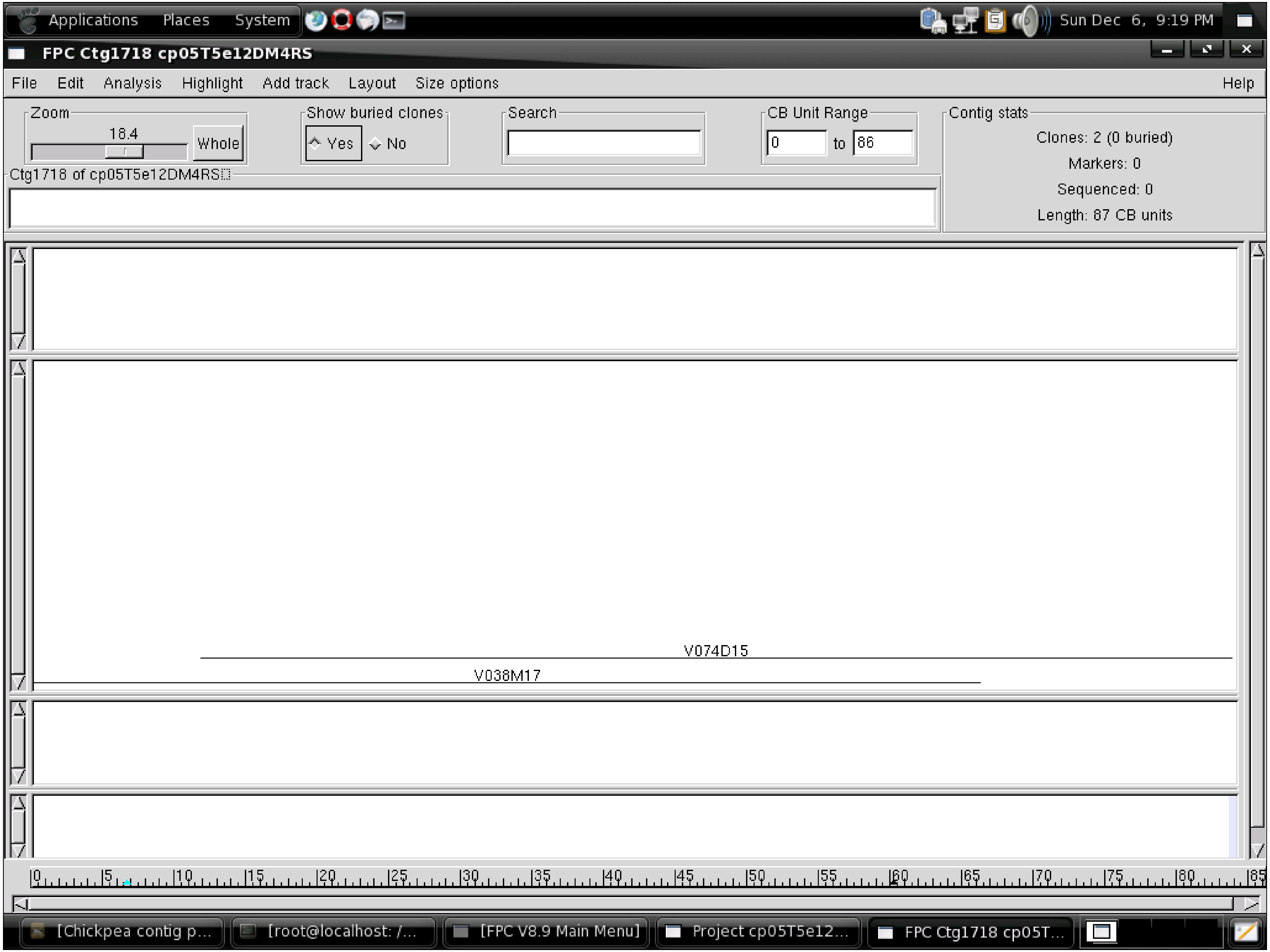
Applications Places System
Sun Dec 6, 9:19 PM
FPC Ctg1718 cp05T5e12DM4RS
File Edit Analysis Highlight Add track Layout Size options Help
Zoom
18.4
Whole
Show buried clones
Yes No
Search
CB Unit Range
0 to 86
Contig stats
Clones: 2 (0 buried)
Markers: 0
Sequenced: 0
Length: 87 CB units
Ctg1718 of cp05T5e12DM4RS
V074D15
V038M17
0 5 10 15 20 25 30 35 40 45 50 55 60 65 70 75 80 85
[Chickpea contig p...
[root@localhost: /...
[FPC V8.9 Main Menu]
Project cp05T5e12...
FPC Ctg1718 cp05T...

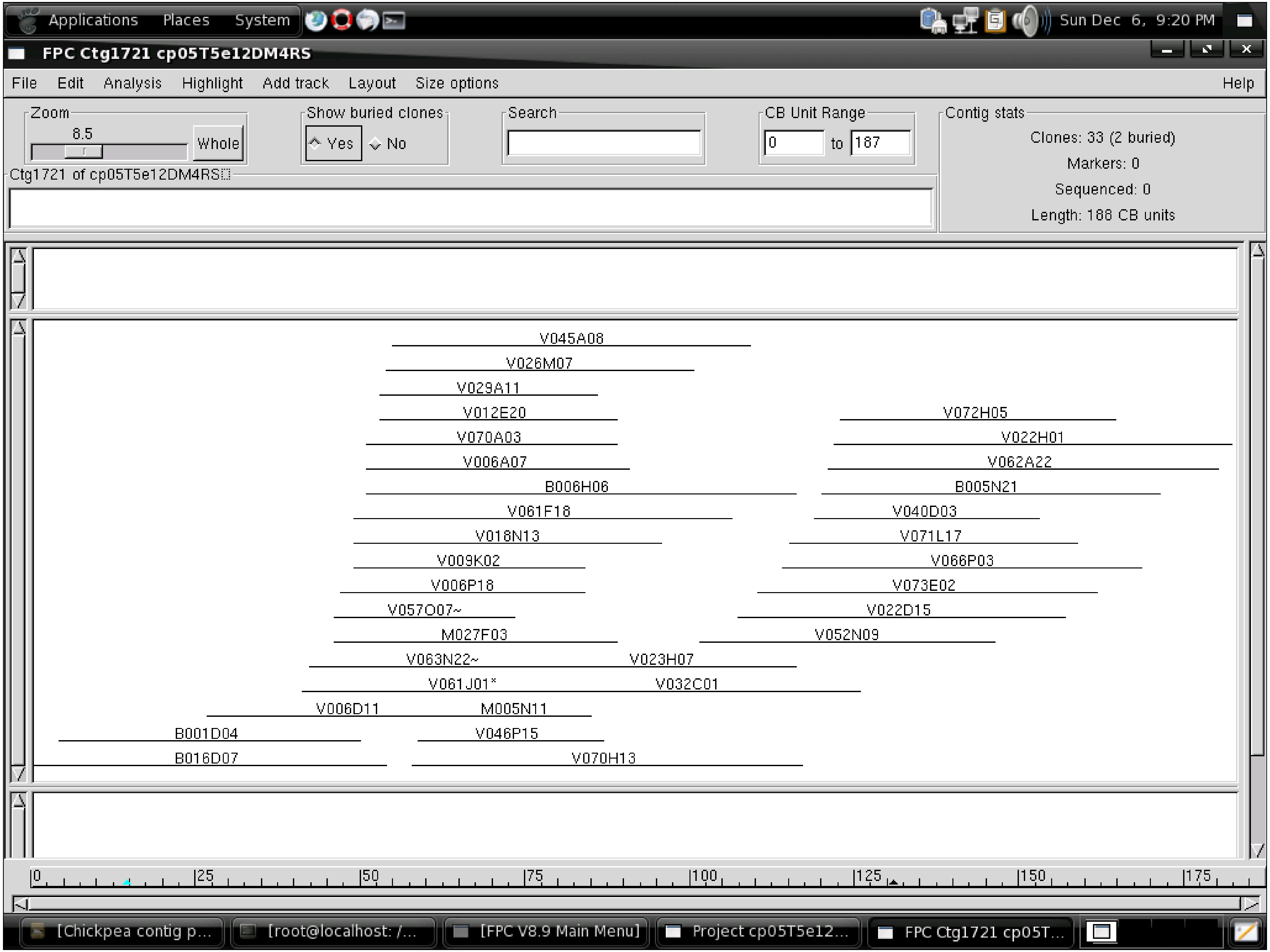
Applications Places System
Sun Dec 6, 9:20 PM
FPC Ctg1721 cp05T5e12DM4RS
File Edit Analysis Highlight Add track Layout Size options Help
Zoom
8.5
Whole
Show buried clones
Yes
No
Search
CB Unit Range
0
to
187
Contig stats
Clones: 33 (2 buried)
Markers: 0
Sequenced: 0
Length: 188 CB units
Ctg1721 of cp05T5e12DM4RS
V045A08
V026M07
V029A11
V012E20
V072H05
V070A03
V022H01
V006A07
V062A22
B006H06
B005N21
V061F18
V040D03
V018N13
V071L17
V009K02
V066P03
V006P18
V073E02
V057O07~
V022D15
M027F03
V052N09
V063N22~
V023H07
V061J01*
V032C01
V006D11
M005N11
B001D04
V046P15
B016D07
V070H13
0 25 50 75 100 125 150 175
[Chickpea contig p...
[root@localhost: /...
[FPC V8.9 Main Menu]
Project cp05T5e12...
FPC Ctg1721 cp05T...

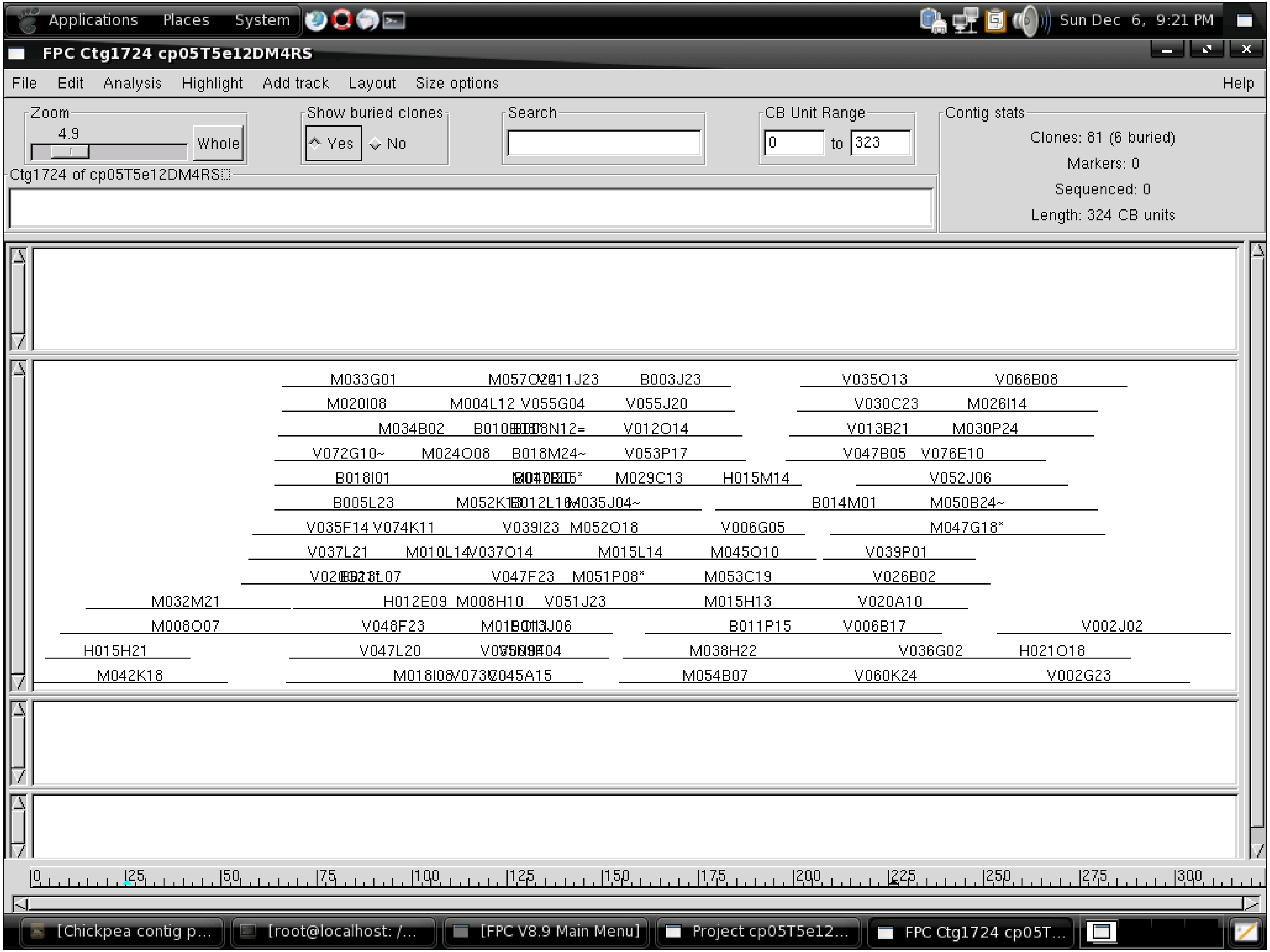
Applications Places System
Sun Dec 6, 9:21 PM
FPC Ctg1724 cp05T5e12DM4RS
File Edit Analysis Highlight Add track Layout Size options Help
Zoom
4.9
Whole
Show buried clones
Yes No
Search
CB Unit Range
0 to 323
Contig stats
Clones: 81 (6 buried)
Markers: 0
Sequenced: 0
Length: 324 CB units
Ctg1724 of cp05T5e12DM4RS
M033G01 B003J23 V035O13 V066B08
M020I08 M004L12 V055G04 V055J20 V030C23 M026I14
M034B02 V012O14 V013B21 M030P24
V072G10~ M024O08 B018M24~ V053P17 V047B05 V076E10
B018I01 M029C13 H015M14 V052J06
B005L23 B014M01 M050B24~
V035F14 V074K11 V039I23 M052O18 V006G05 M047G18*
V037L21 M015L14 M045O10 V039P01
V047F23 M051P08* M053C19 V026B02
M032M21 H012E09 M008H10 V051J23 M015H13 V020A10
M008O07 V048F23 B011P15 V006B17 V002J02
H015H21 V047L20 M038H22 V036G02 H021O18
M042K18 M054B07 V060K24 V002G23
0 25 50 75 100 125 150 175 200 225 250 275 300
[Chickpea contig p... [root@localhost: /... [FPC V8.9 Main Menu] Project cp05T5e12... FPC Ctg1724 cp05T...

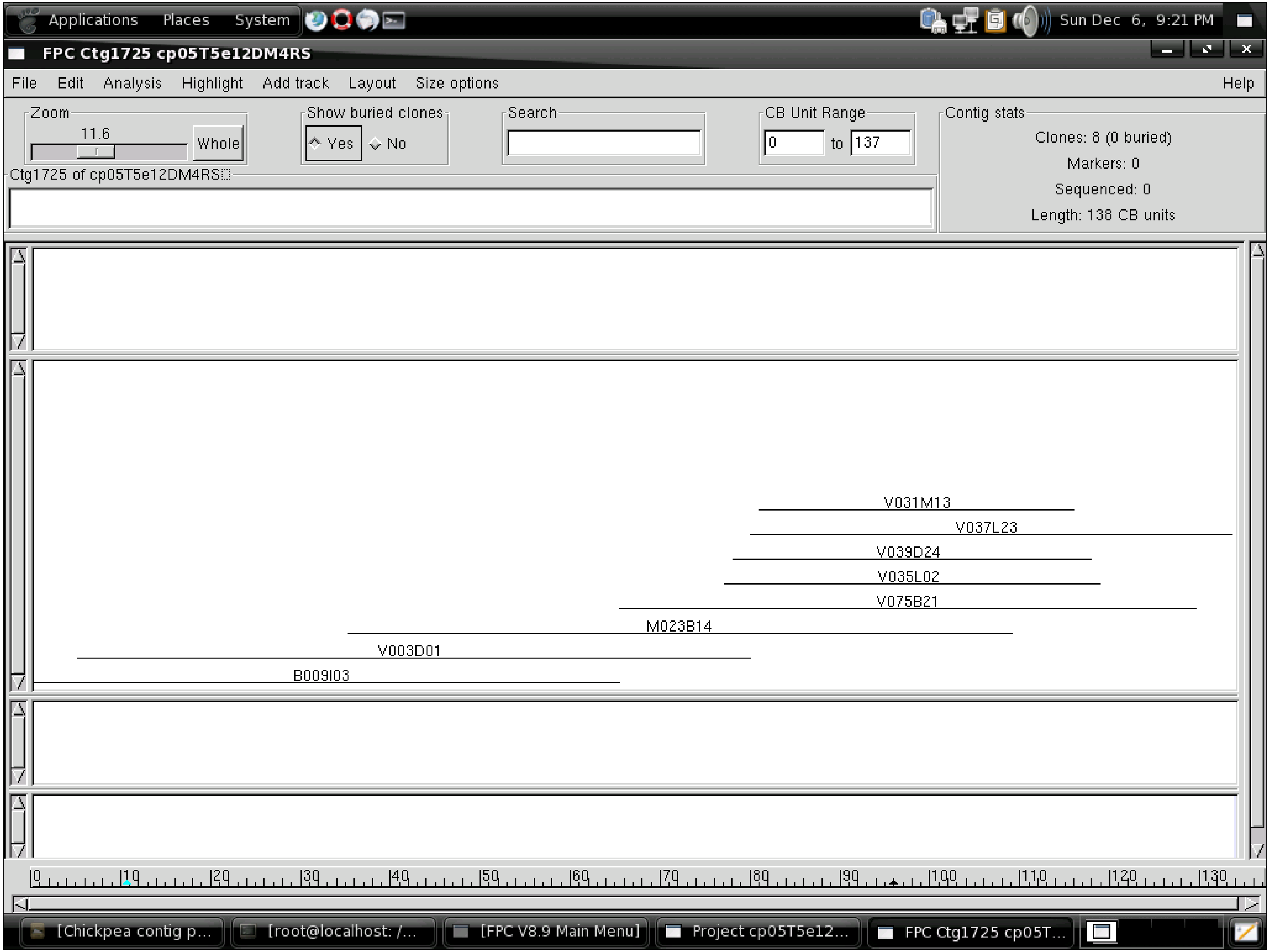
Applications Places System
Sun Dec 6, 9:21 PM
FPC Ctg1725 cp05T5e12DM4RS
File Edit Analysis Highlight Add track Layout Size options Help
Zoom
11.6
Whole
Show buried clones
Yes
No
Search
CB Unit Range
0 to 137
Contig stats
Clones: 8 (0 buried)
Markers: 0
Sequenced: 0
Length: 138 CB units
Ctg1725 of cp05T5e12DM4RS
V031M13
V037L23
V039D24
V035L02
V075B21
M023B14
V003D01
B009I03
0 10 20 30 40 50 60 70 80 90 100 110 120 130
[Chickpea contig p...
[root@localhost: /...
[FPC V8.9 Main Menu]
Project cp05T5e12...
FPC Ctg1725 cp05T...

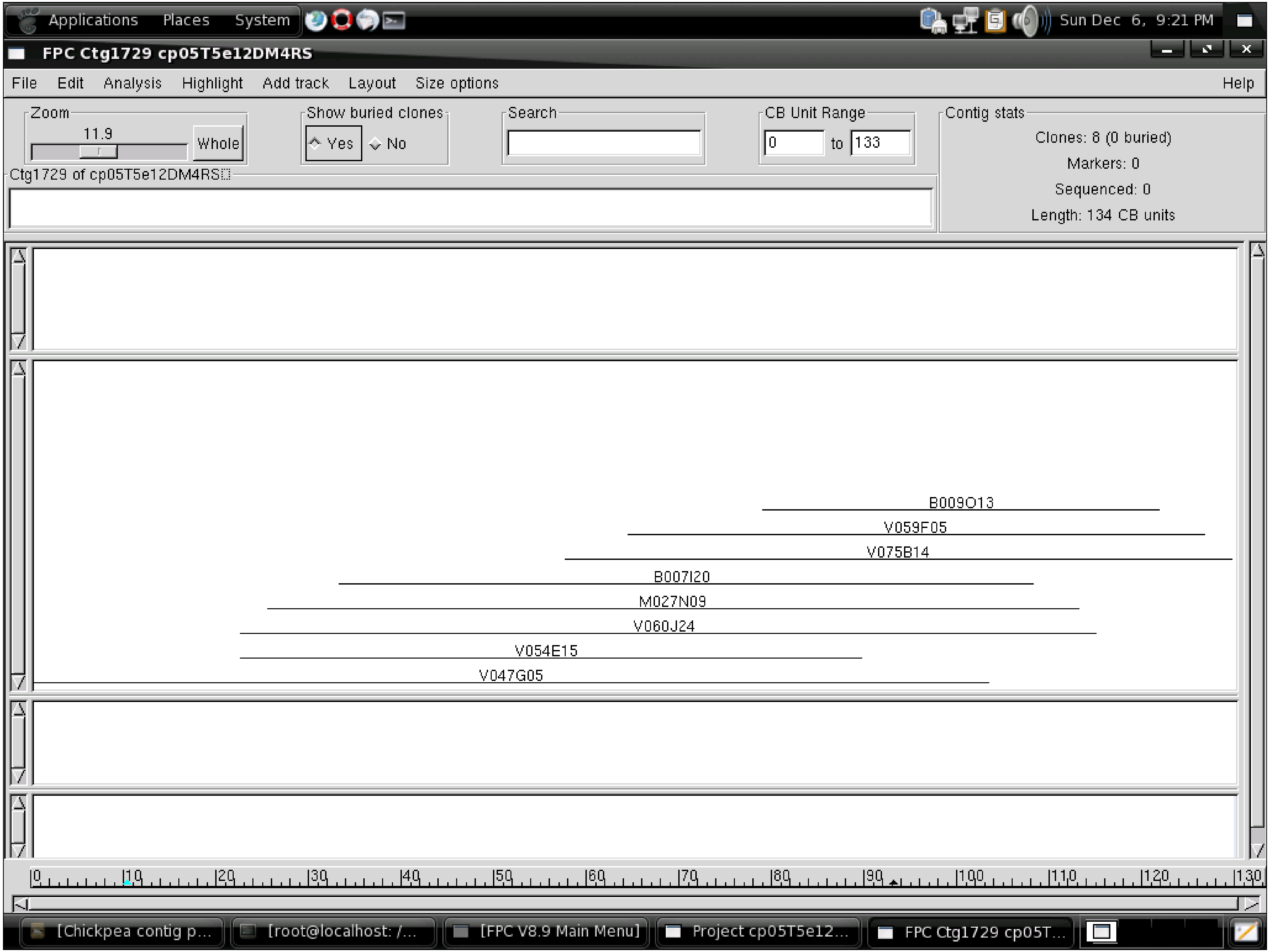
Applications Places System
Sun Dec 6, 9:21 PM
FPC Ctg1729 cp05T5e12DM4RS
File Edit Analysis Highlight Add track Layout Size options Help
Zoom
11.9
Whole
Show buried clones
Yes
No
Search
CB Unit Range
0
to
133
Contig stats
Clones: 8 (0 buried)
Markers: 0
Sequenced: 0
Length: 134 CB units
Ctg1729 of cp05T5e12DM4RS
B009O13
V059F05
V075B14
B007I20
M027N09
V060J24
V054E15
V047G05
0 10 20 30 40 50 60 70 80 90 100 110 120 130
[Chickpea contig p...
[root@localhost: /...
[FPC V8.9 Main Menu]
Project cp05T5e12...
FPC Ctg1729 cp05T...

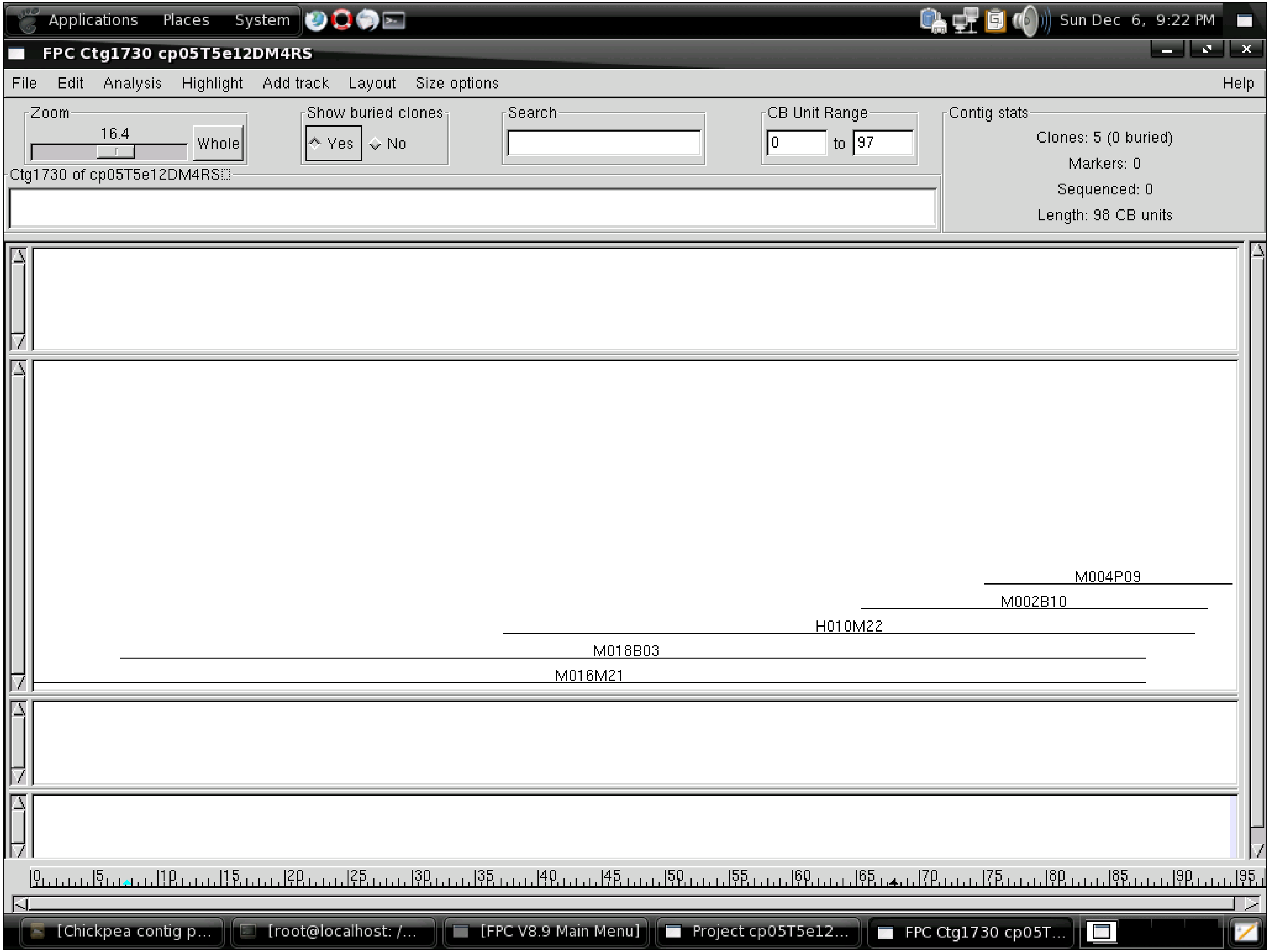
Applications Places System
Sun Dec 6, 9:22 PM
FPC Ctg1730 cp05T5e12DM4RS
File Edit Analysis Highlight Add track Layout Size options Help
Zoom
16.4
Whole
Show buried clones
Yes No
Search
CB Unit Range
0 to 97
Contig stats
Clones: 5 (0 buried)
Markers: 0
Sequenced: 0
Length: 98 CB units
Ctg1730 of cp05T5e12DM4RS
M004P09
M002B10
H010M22
M018B03
M016M21
0 5 10 15 20 25 30 35 40 45 50 55 60 65 70 75 80 85 90 95
[Chickpea contig p...
[root@localhost: /...
[FPC V8.9 Main Menu]
Project cp05T5e12...
FPC Ctg1730 cp05T...

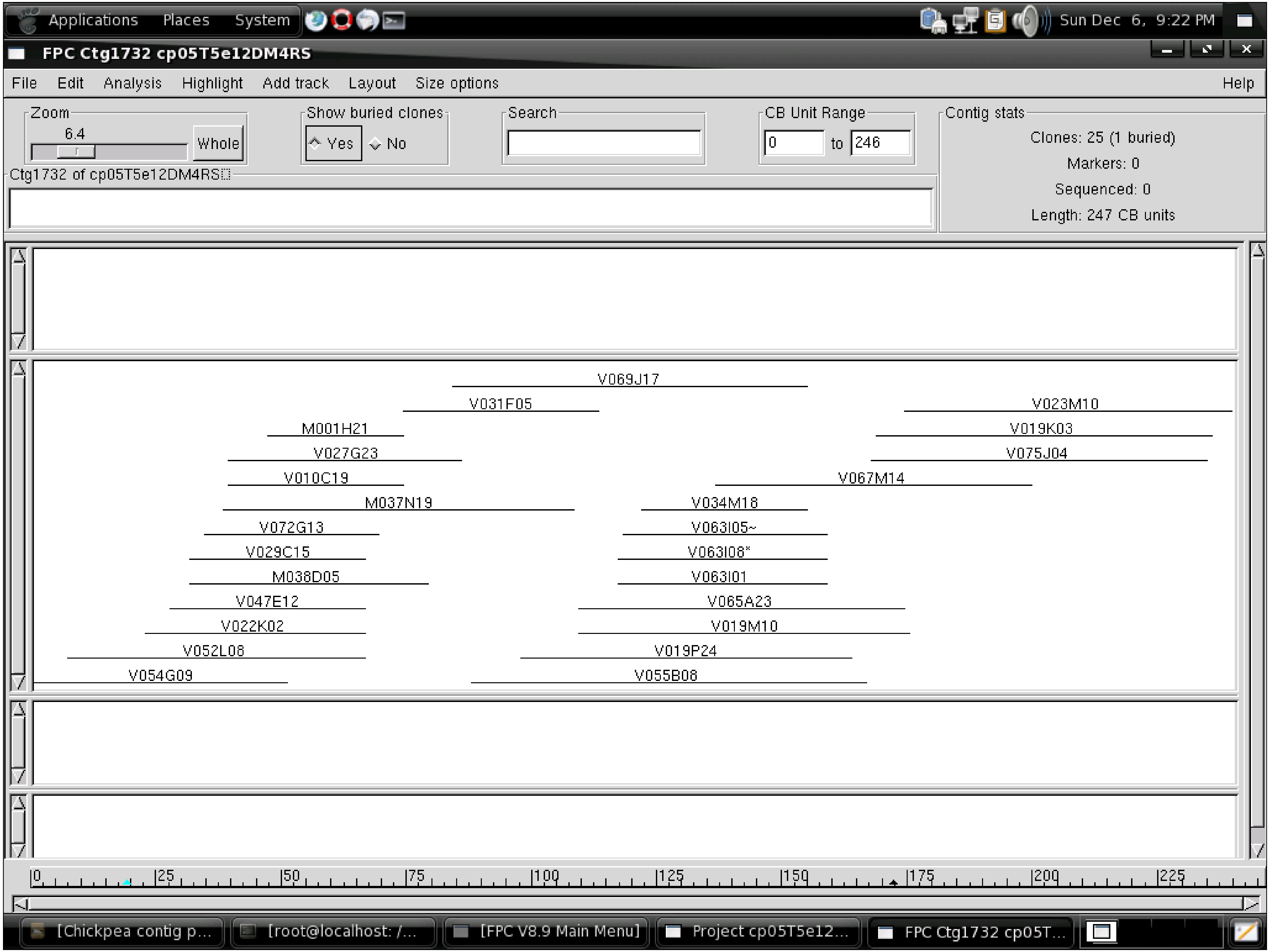
Applications Places System
Sun Dec 6, 9:22 PM
FPC Ctg1732 cp05T5e12DM4RS
File Edit Analysis Highlight Add track Layout Size options Help
Zoom
6.4
Whole
Show buried clones
Yes
No
Search
CB Unit Range
0 to 246
Contig stats
Clones: 25 (1 buried)
Markers: 0
Sequenced: 0
Length: 247 CB units
Ctg1732 of cp05T5e12DM4RS
V069J17
V031F05
V023M10
M001H21
V019K03
V027G23
V075J04
V010C19
V067M14
M037N19
V034M18
V072G13
V063I05~
V029C15
V063I08*
M038D05
V063I01
V047E12
V065A23
V022K02
V019M10
V052L08
V019P24
V054G09
V055B08
0 25 50 75 100 125 150 175 200 225
[Chickpea contig p...
[root@localhost: /...
[FPC V8.9 Main Menu]
Project cp05T5e12...
FPC Ctg1732 cp05T...

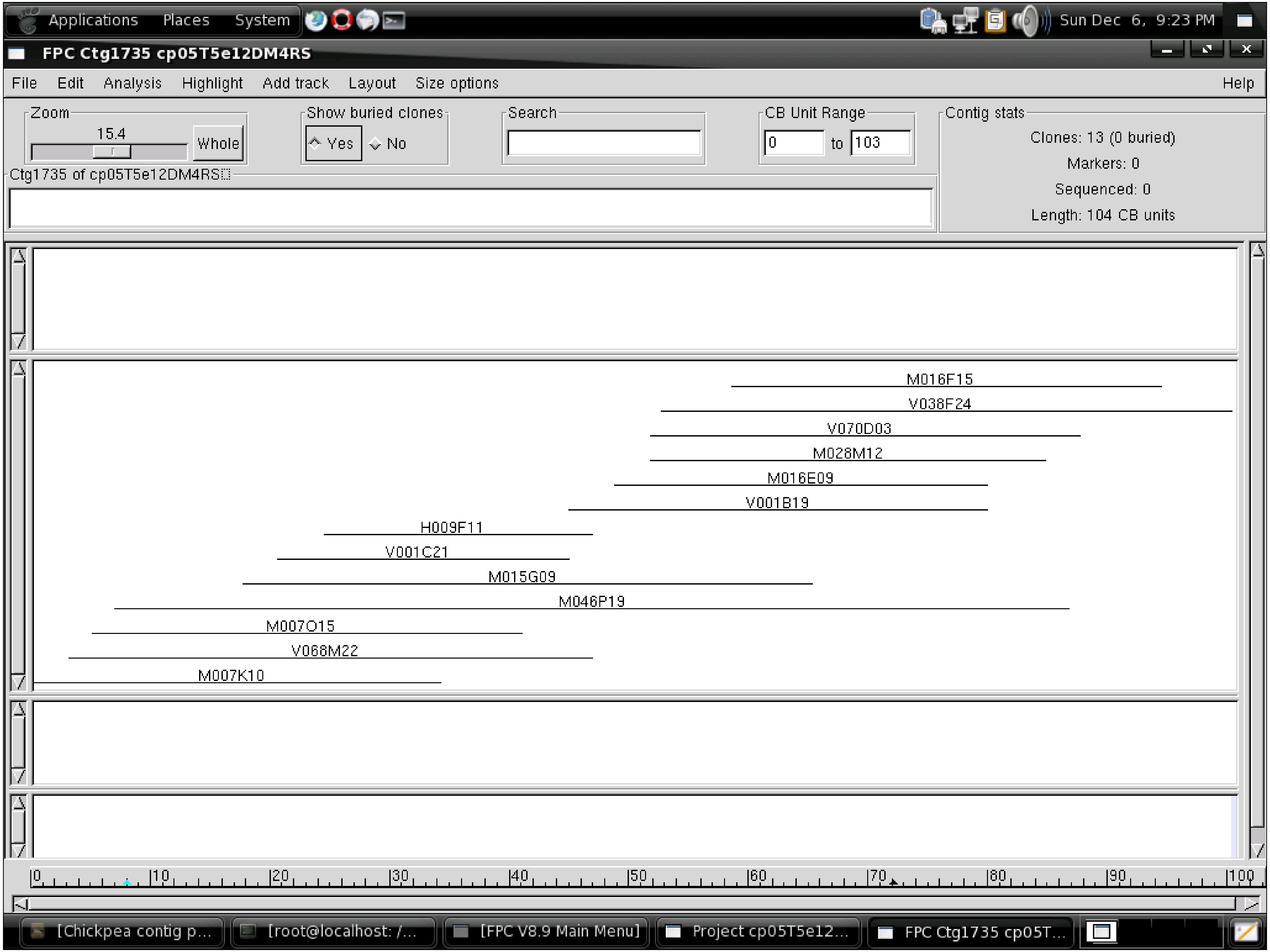
Applications Places System
Sun Dec 6, 9:23 PM
FPC Ctg1735 cp05T5e12DM4RS
File Edit Analysis Highlight Add track Layout Size options Help
Zoom
15.4
Whole
Show buried clones
Yes
No
Search
CB Unit Range
0
to
103
Contig stats
Clones: 13 (0 buried)
Markers: 0
Sequenced: 0
Length: 104 CB units
Ctg1735 of cp05T5e12DM4RS
M016F15
V038F24
V070D03
M028M12
M016E09
V001B19
H009F11
V001C21
M015G09
M046P19
M007O15
V068M22
M007K10
0 10 20 30 40 50 60 70 80 90 100
[Chickpea contig p...
[root@localhost: /...
[FPC V8.9 Main Menu]
Project cp05T5e12...
FPC Ctg1735 cp05T...

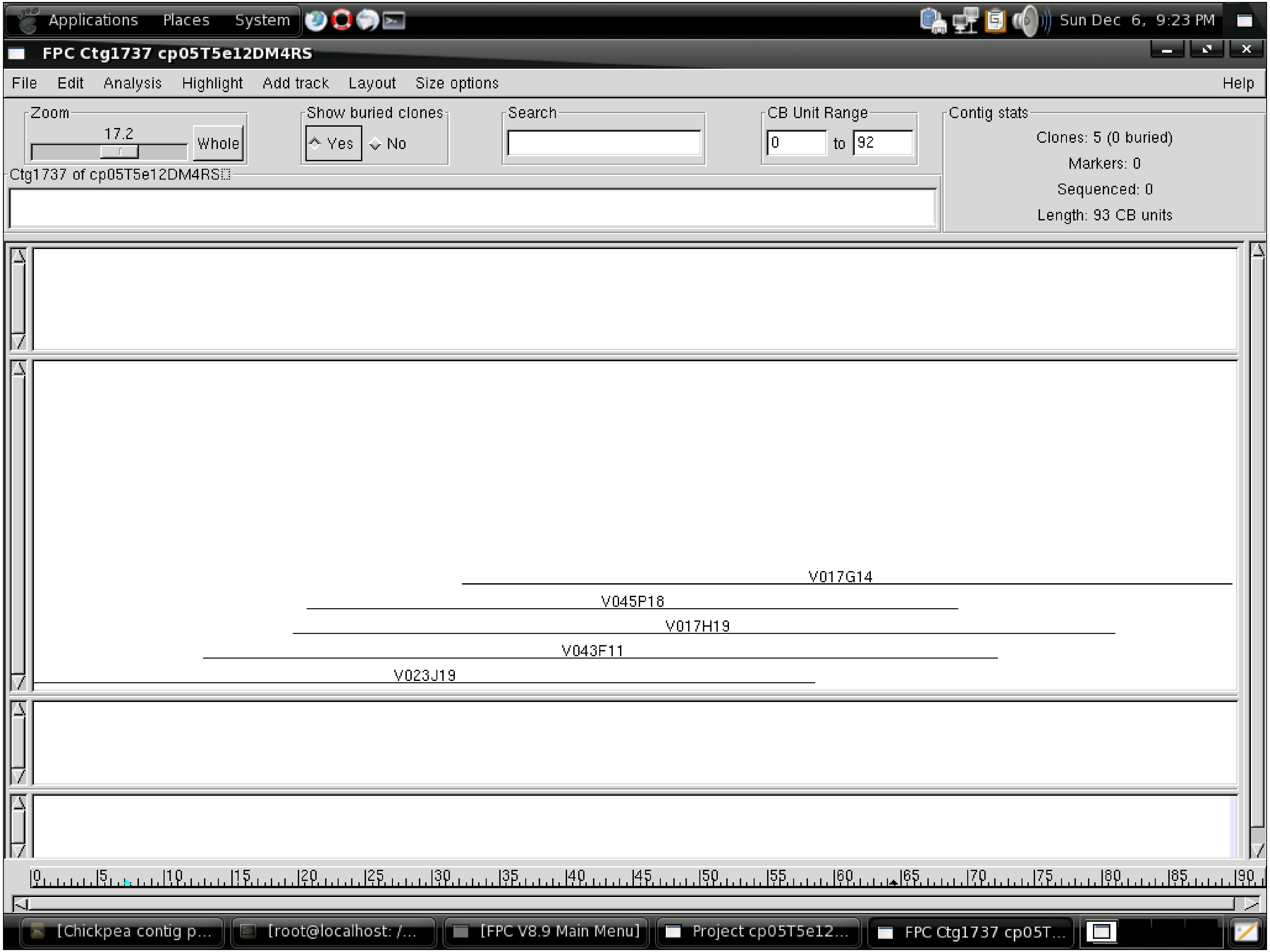

Applications Places System
Sun Dec 6, 9:23 PM
FPC Ctg1737 cp05T5e12DM4RS
File Edit Analysis Highlight Add track Layout Size options Help
Zoom
17.2
Whole
Show buried clones
Yes
No
Search
CB Unit Range
0 to 92
Contig stats
Clones: 5 (0 buried)
Markers: 0
Sequenced: 0
Length: 93 CB units
Ctg1737 of cp05T5e12DM4RS
V017G14
V045P18
V017H19
V043F11
V023J19
0 5 10 15 20 25 30 35 40 45 50 55 60 65 70 75 80 85 90
[Chickpea contig p...
[root@localhost: /...
[FPC V8.9 Main Menu]
Project cp05T5e12...
FPC Ctg1737 cp05T...

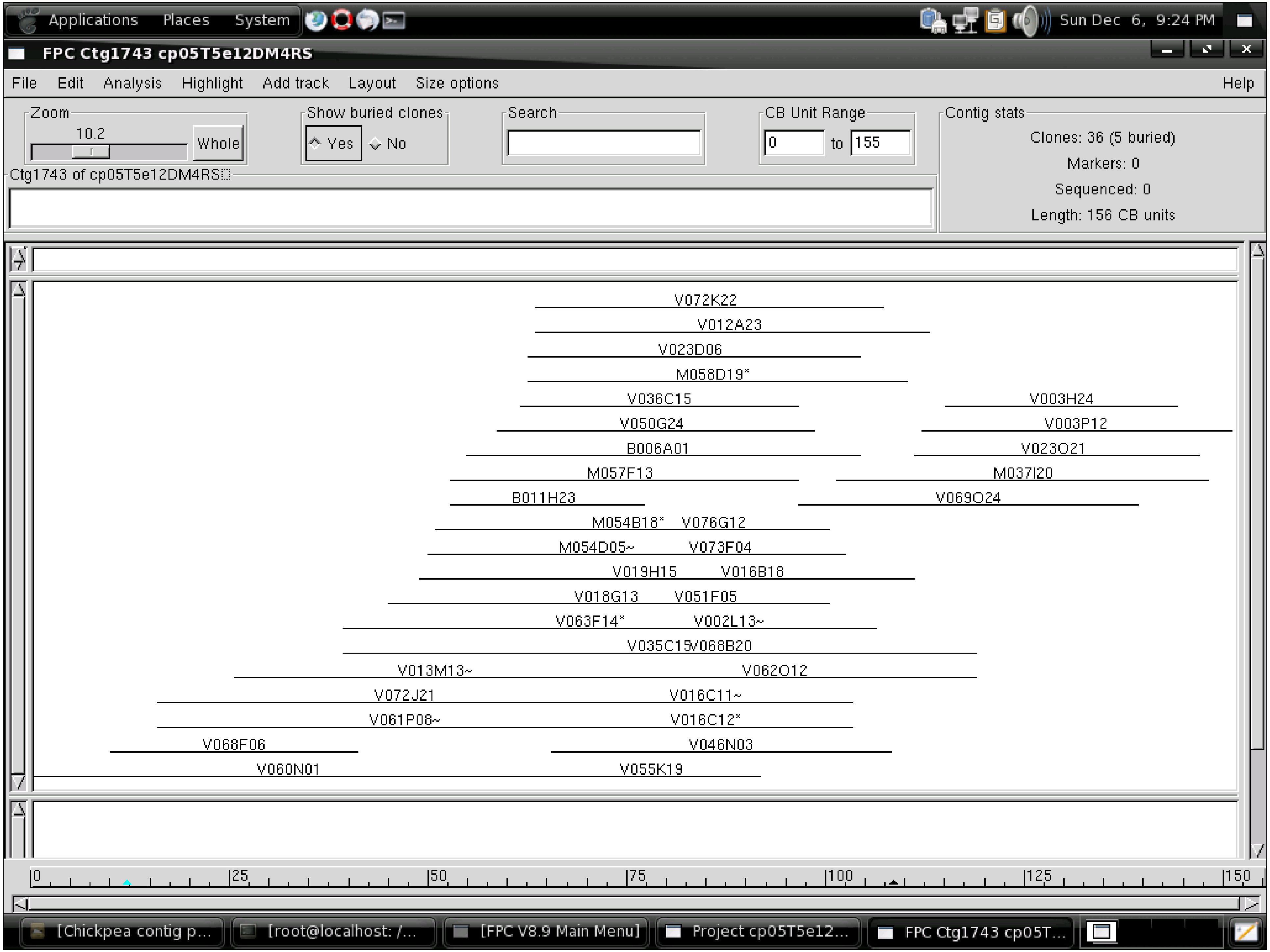
Applications Places System
Sun Dec 6, 9:24 PM
FPC Ctg1743 cp05T5e12DM4RS
File Edit Analysis Highlight Add track Layout Size options Help
Zoom
10.2
Whole
Show buried clones
Yes
No
Search
CB Unit Range
0 to 155
Contig stats
Clones: 36 (5 buried)
Markers: 0
Sequenced: 0
Length: 156 CB units
Ctg1743 of cp05T5e12DM4RS
V072K22
V012A23
V023D06
M058D19*
V036C15
V003H24
V050G24
V003P12
B006A01
V023O21
M057F13
M037I20
B011H23
V069O24
M054B18*
V076G12
M054D05~
V073F04
V019H15
V016B18
V018G13
V051F05
V063F14*
V002L13~
V035C15
V068B20
V013M13~
V062O12
V072J21
V016C11~
V061P08~
V016C12*
V068F06
V046N03
V060N01
V055K19
0 25 50 75 100 125 150
[Chickpea contig p...
[root@localhost: /...
[FPC V8.9 Main Menu]
Project cp05T5e12...
FPC Ctg1743 cp05T...

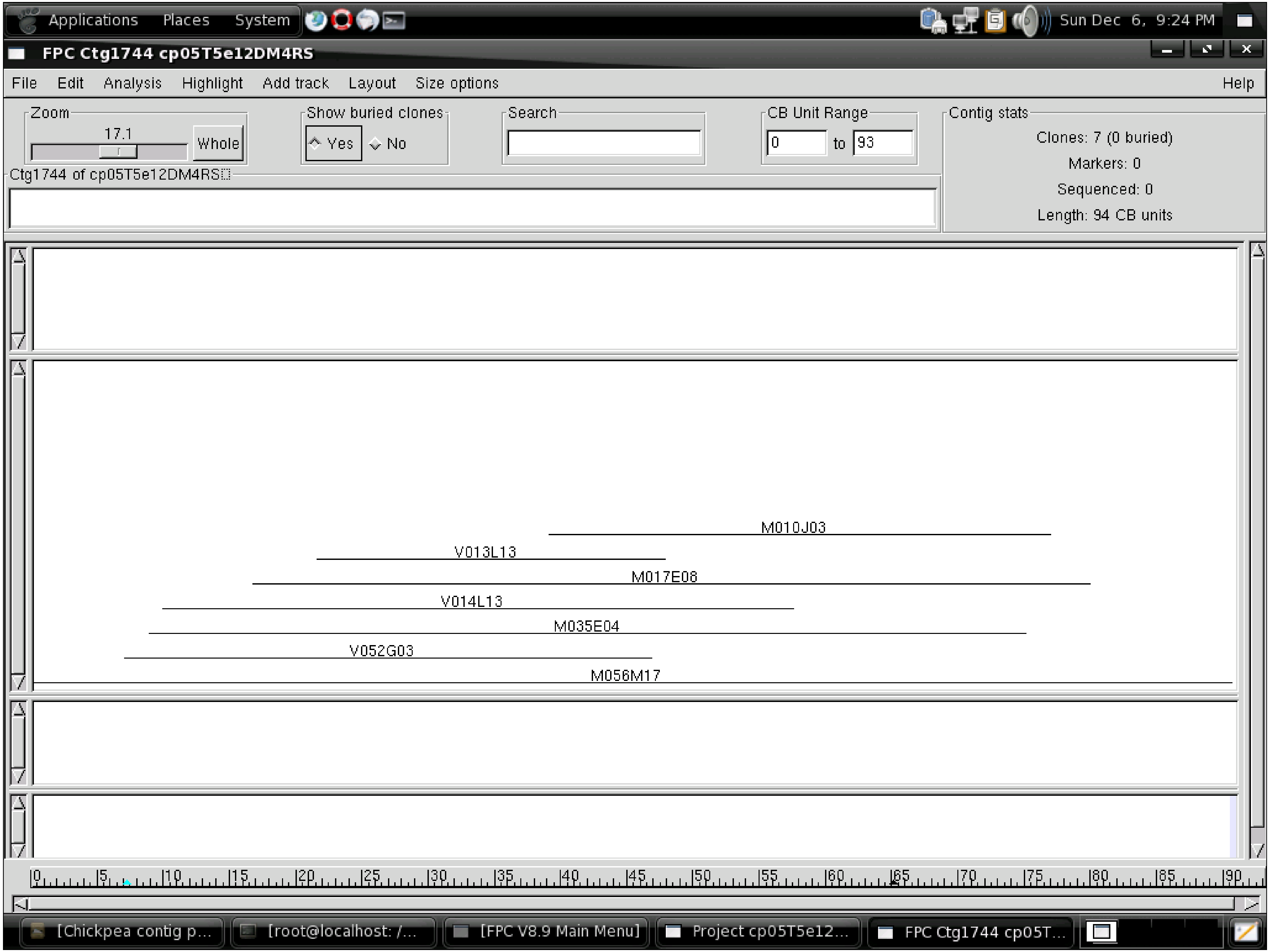
Applications Places System
Sun Dec 6, 9:24 PM
FPC Ctg1744 cp05T5e12DM4RS
File Edit Analysis Highlight Add track Layout Size options Help
Zoom
17.1
Whole
Show buried clones
Yes
No
Search
CB Unit Range
0 to 93
Contig stats
Clones: 7 (0 buried)
Markers: 0
Sequenced: 0
Length: 94 CB units
Ctg1744 of cp05T5e12DM4RS
M010J03
V013L13
M017E08
V014L13
M035E04
V052G03
M056M17
0 5 10 15 20 25 30 35 40 45 50 55 60 65 70 75 80 85 90
[Chickpea contig p...
[root@localhost: /...
[FPC V8.9 Main Menu]
Project cp05T5e12...
FPC Ctg1744 cp05T...

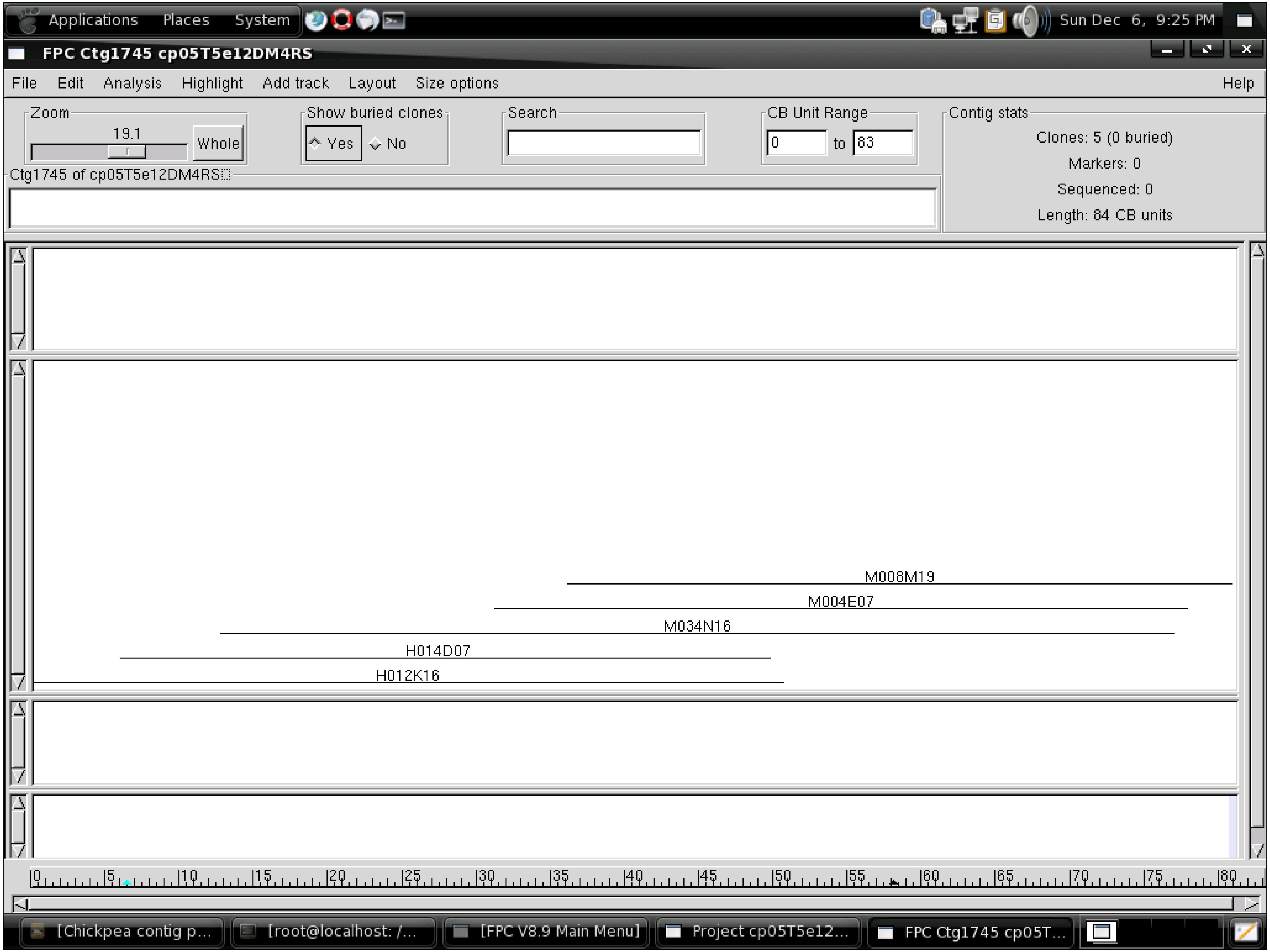
Applications Places System
Sun Dec 6, 9:25 PM
FPC Ctg1745 cp05T5e12DM4RS
File Edit Analysis Highlight Add track Layout Size options Help
Zoom
19.1
Whole
Show buried clones
Yes
No
Search
CB Unit Range
0 to 83
Contig stats
Clones: 5 (0 buried)
Markers: 0
Sequenced: 0
Length: 84 CB units
Ctg1745 of cp05T5e12DM4RS
M008M19
M004E07
M034N16
H014D07
H012K16
0 5 10 15 20 25 30 35 40 45 50 55 60 65 70 75 80
[Chickpea contig p...
[root@localhost: /...
[FPC V8.9 Main Menu]
Project cp05T5e12...
FPC Ctg1745 cp05T...

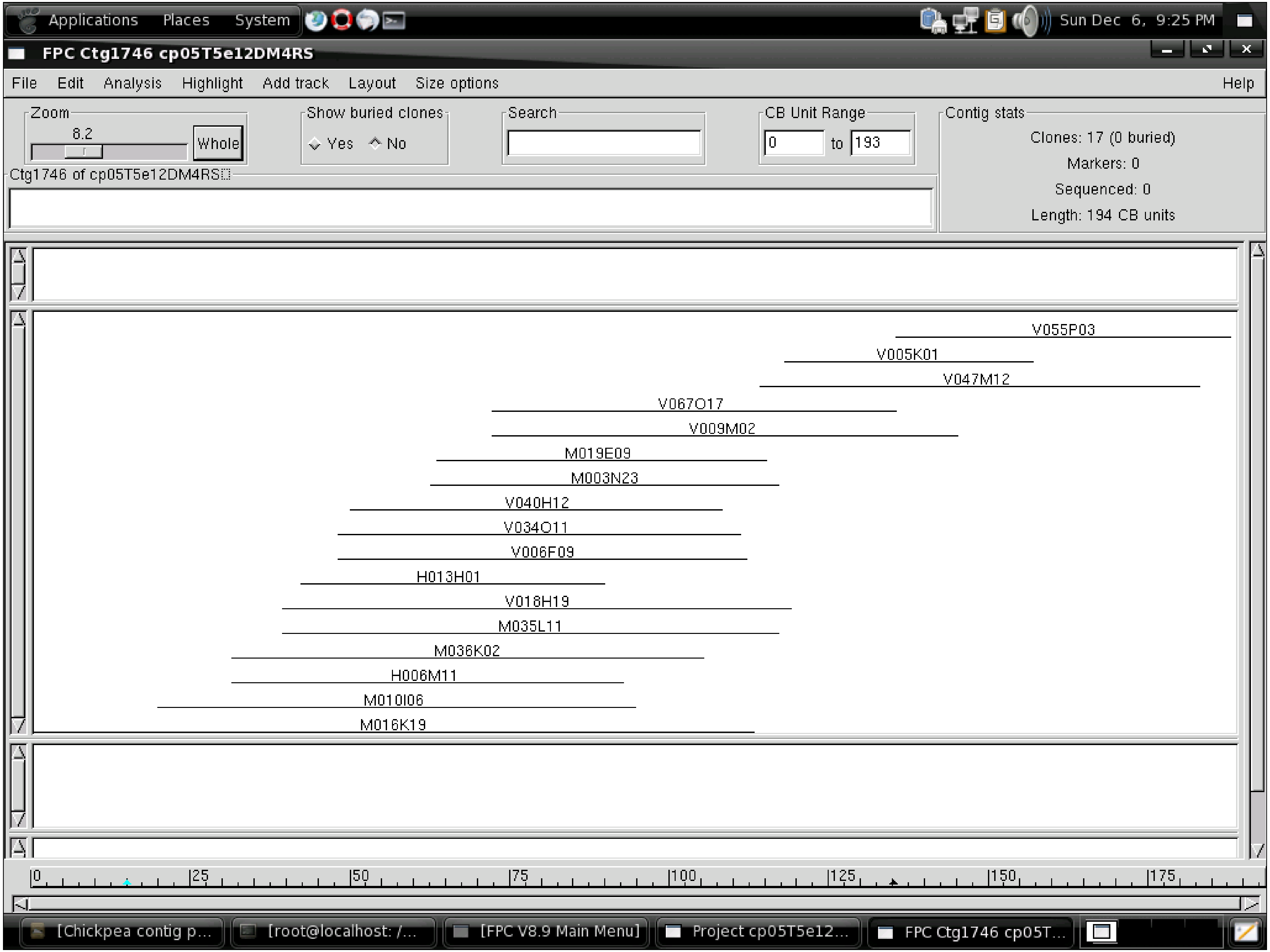
Applications Places System
Sun Dec 6, 9:25 PM
FPC Ctg1746 cp05T5e12DM4RS
File Edit Analysis Highlight Add track Layout Size options Help
Zoom
8.2
Whole
Show buried clones
Yes No
Search
CB Unit Range
0 to 193
Contig stats
Clones: 17 (0 buried)
Markers: 0
Sequenced: 0
Length: 194 CB units
Ctg1746 of cp05T5e12DM4RS
V055P03
V005K01
V047M12
V067O17
V009M02
M019E09
M003N23
V040H12
V034O11
V006F09
H013H01
V018H19
M035L11
M036K02
H006M11
M010I06
M016K19
0 25 50 75 100 125 150 175
[Chickpea contig p...
[root@localhost: /...
[FPC V8.9 Main Menu]
Project cp05T5e12...
FPC Ctg1746 cp05T...

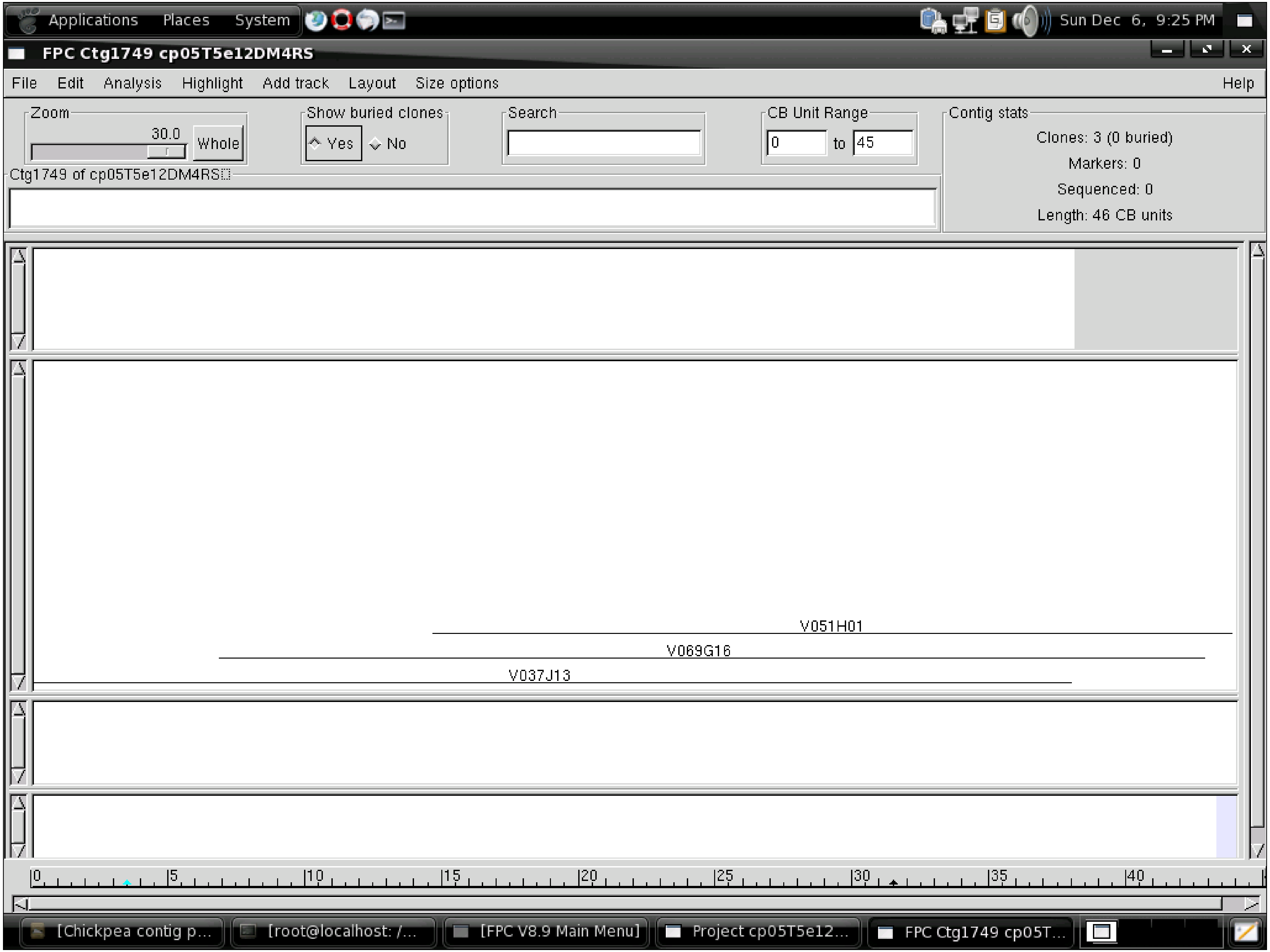
Applications Places System
Sun Dec 6, 9:25 PM
FPC Ctg1749 cp05T5e12DM4RS
File Edit Analysis Highlight Add track Layout Size options Help
Zoom
30.0
Whole
Show buried clones
Yes No
Search
CB Unit Range
0 to 45
Contig stats
Clones: 3 (0 buried)
Markers: 0
Sequenced: 0
Length: 46 CB units
Ctg1749 of cp05T5e12DM4RS
V051H01
V069G16
V037J13
0 5 10 15 20 25 30 35 40
[Chickpea contig p...
[root@localhost: /...
[FPC V8.9 Main Menu]
Project cp05T5e12...
FPC Ctg1749 cp05T...

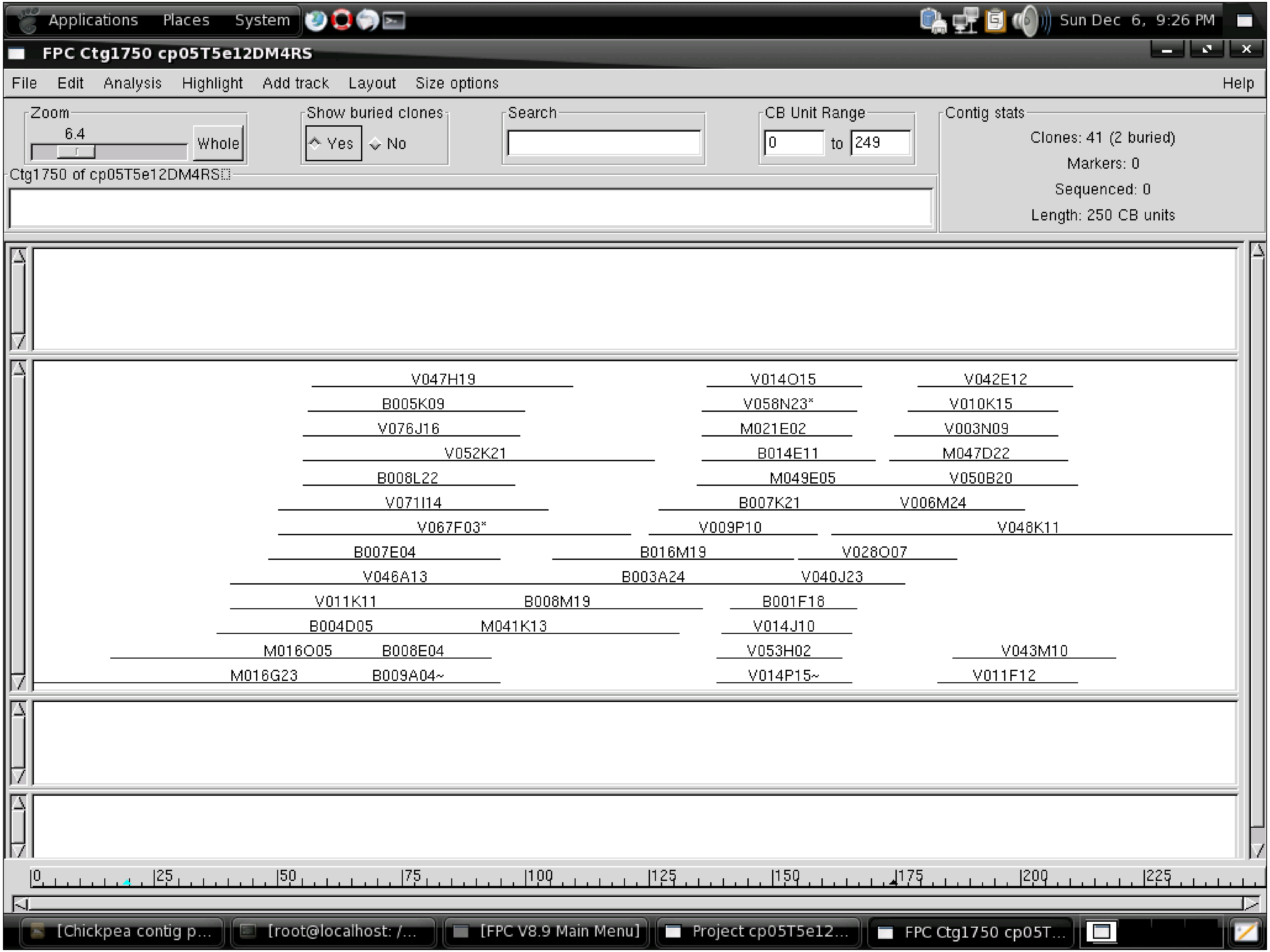
Applications
Places
System
Sun Dec 6, 9:26 PM
FPC Ctg1750 cp05T5e12DM4RS
File
Edit
Analysis
Highlight
Add track
Layout
Size options
Help
Zoom
6.4
Whole
Show buried clones
Yes
No
Search
CB Unit Range
0
to
249
Contig stats
Clones: 41 (2 buried)
Markers: 0
Sequenced: 0
Length: 250 CB units
Ctg1750 of cp05T5e12DM4RS
V047H19
V014O15
V042E12
B005K09
V058N23*
V010K15
V076J16
M021E02
V003N09
V052K21
B014E11
M047D22
B008L22
M049E05
V050B20
V071I14
B007K21
V006M24
V067F03*
V009P10
V048K11
B007E04
B016M19
V028O07
V046A13
B003A24
V040J23
V011K11
B008M19
B001F18
B004D05
M041K13
V014J10
M016O05
B008E04
V053H02
V043M10
M016G23
B009A04~
V014P15~
V011F12
0
25
50
75
100
125
150
175
200
225
[Chickpea contig p...
[root@localhost: /...
[FPC V8.9 Main Menu]
Project cp05T5e12...
FPC Ctg1750 cp05T...

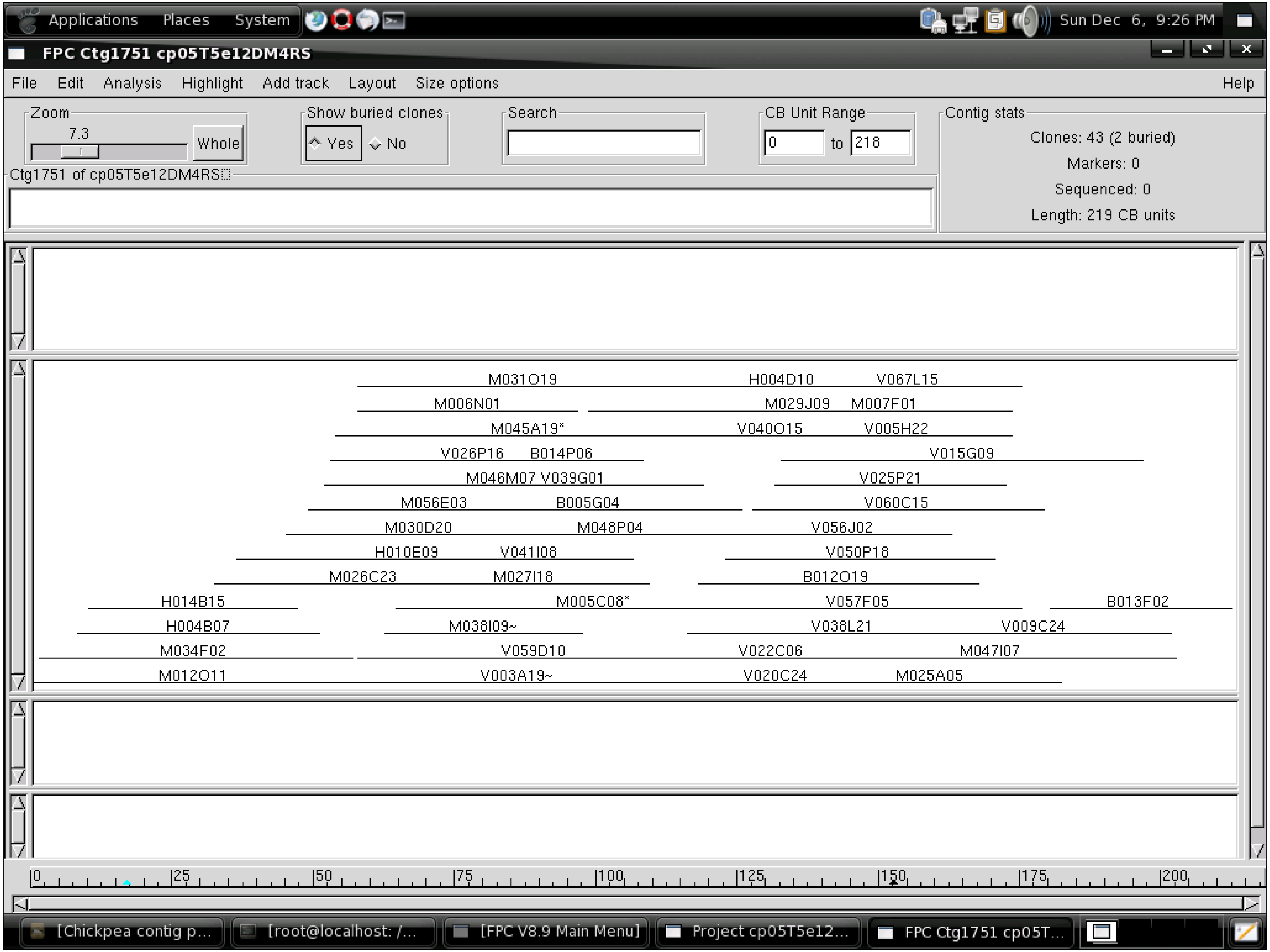
Applications Places System
Sun Dec 6, 9:26 PM
FPC Ctg1751 cp05T5e12DM4RS
File Edit Analysis Highlight Add track Layout Size options Help
Zoom
7.3
Whole
Show buried clones
Yes
No
Search
CB Unit Range
0 to 218
Contig stats
Clones: 43 (2 buried)
Markers: 0
Sequenced: 0
Length: 219 CB units
Ctg1751 of cp05T5e12DM4RS
M031O19
H004D10
V067L15
M006N01
M029J09
M007F01
M045A19*
V040O15
V005H22
V026P16
B014P06
V015G09
M046M07 V039G01
V025P21
M056E03
B005G04
V060C15
M030D20
M048P04
V056J02
H010E09
V041I08
V050P18
M026C23
M027I18
B012O19
H014B15
M005C08*
V057F05
B013F02
H004B07
M038I09~
V038L21
V009C24
M034F02
V059D10
V022C06
M047I07
M012O11
V003A19~
V020C24
M025A05
0 25 50 75 100 125 150 175 200
[Chickpea contig p...
[root@localhost: /...
[FPC V8.9 Main Menu]
Project cp05T5e12...
FPC Ctg1751 cp05T...

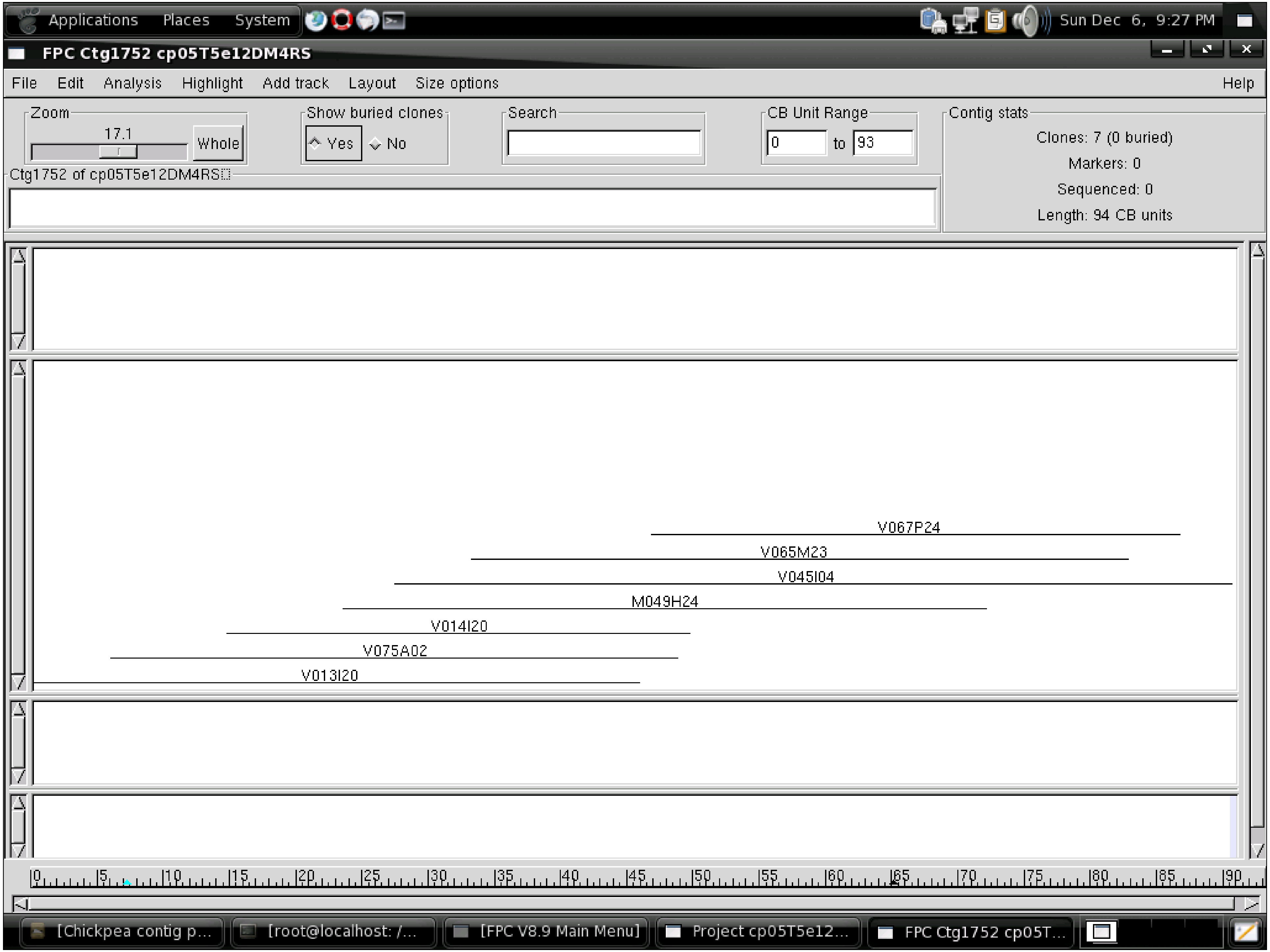
Applications Places System
Sun Dec 6, 9:27 PM
FPC Ctg1752 cp05T5e12DM4RS
File Edit Analysis Highlight Add track Layout Size options Help
Zoom
17.1
Whole
Show buried clones
Yes
No
Search
CB Unit Range
0 to 93
Contig stats
Clones: 7 (0 buried)
Markers: 0
Sequenced: 0
Length: 94 CB units
Ctg1752 of cp05T5e12DM4RS
V067P24
V065M23
V045I04
M049H24
V014I20
V075A02
V013I20
0 5 10 15 20 25 30 35 40 45 50 55 60 65 70 75 80 85 90
[Chickpea contig p...
[root@localhost: /...
[FPC V8.9 Main Menu]
Project cp05T5e12...
FPC Ctg1752 cp05T...

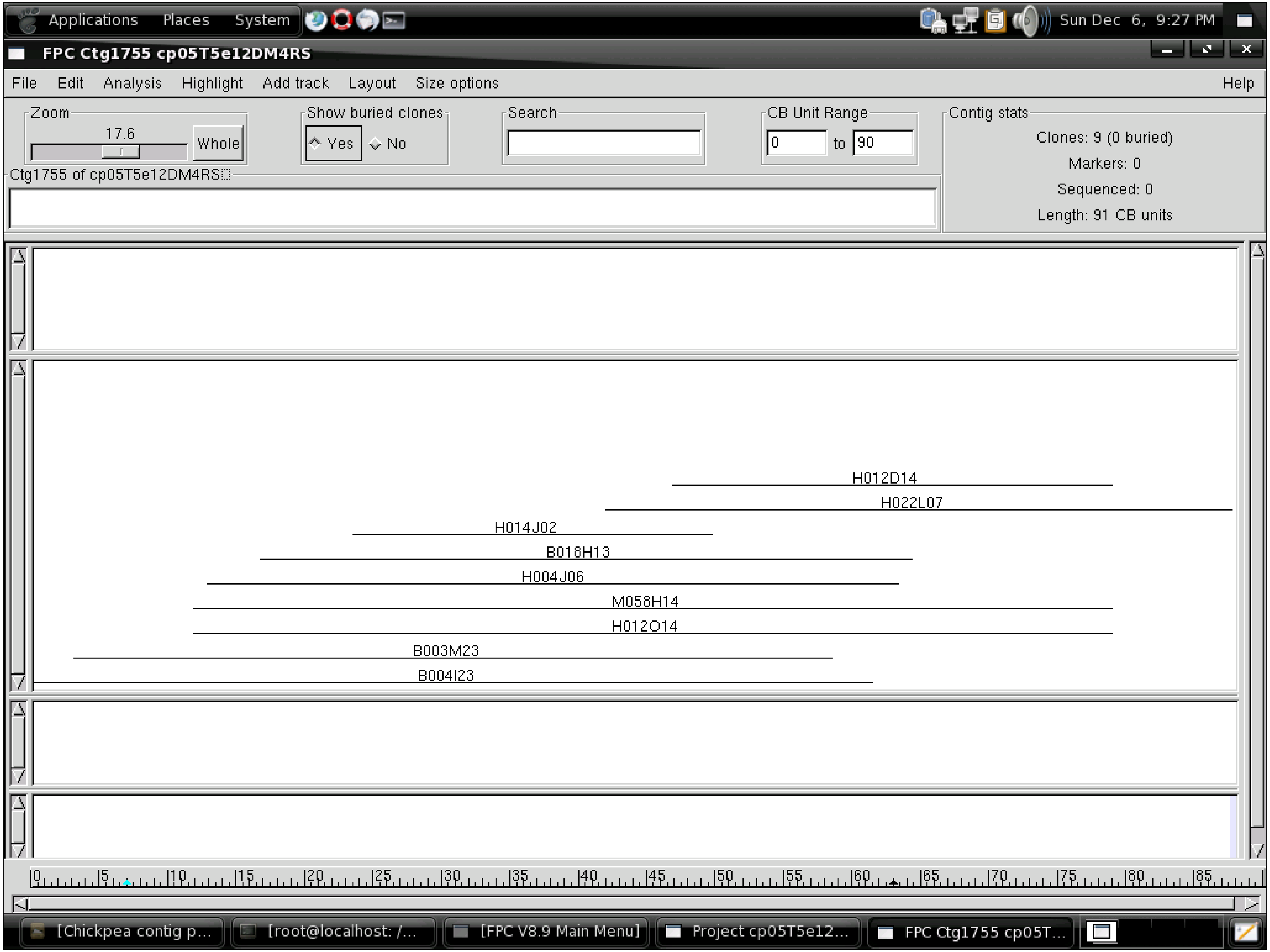
Applications Places System
Sun Dec 6, 9:27 PM
FPC Ctg1755 cp05T5e12DM4RS
File Edit Analysis Highlight Add track Layout Size options
Help
Zoom
17.6
Whole
Show buried clones
Yes
No
Search
CB Unit Range
0
to
90
Contig stats
Clones: 9 (0 buried)
Markers: 0
Sequenced: 0
Length: 91 CB units
Ctg1755 of cp05T5e12DM4RS
H012D14
H022L07
H014J02
B018H13
H004J06
M058H14
H012O14
B003M23
B004I23
0 5 10 15 20 25 30 35 40 45 50 55 60 65 70 75 80 85
[Chickpea contig p...
[root@localhost: /...
[FPC V8.9 Main Menu]
Project cp05T5e12...
FPC Ctg1755 cp05T...

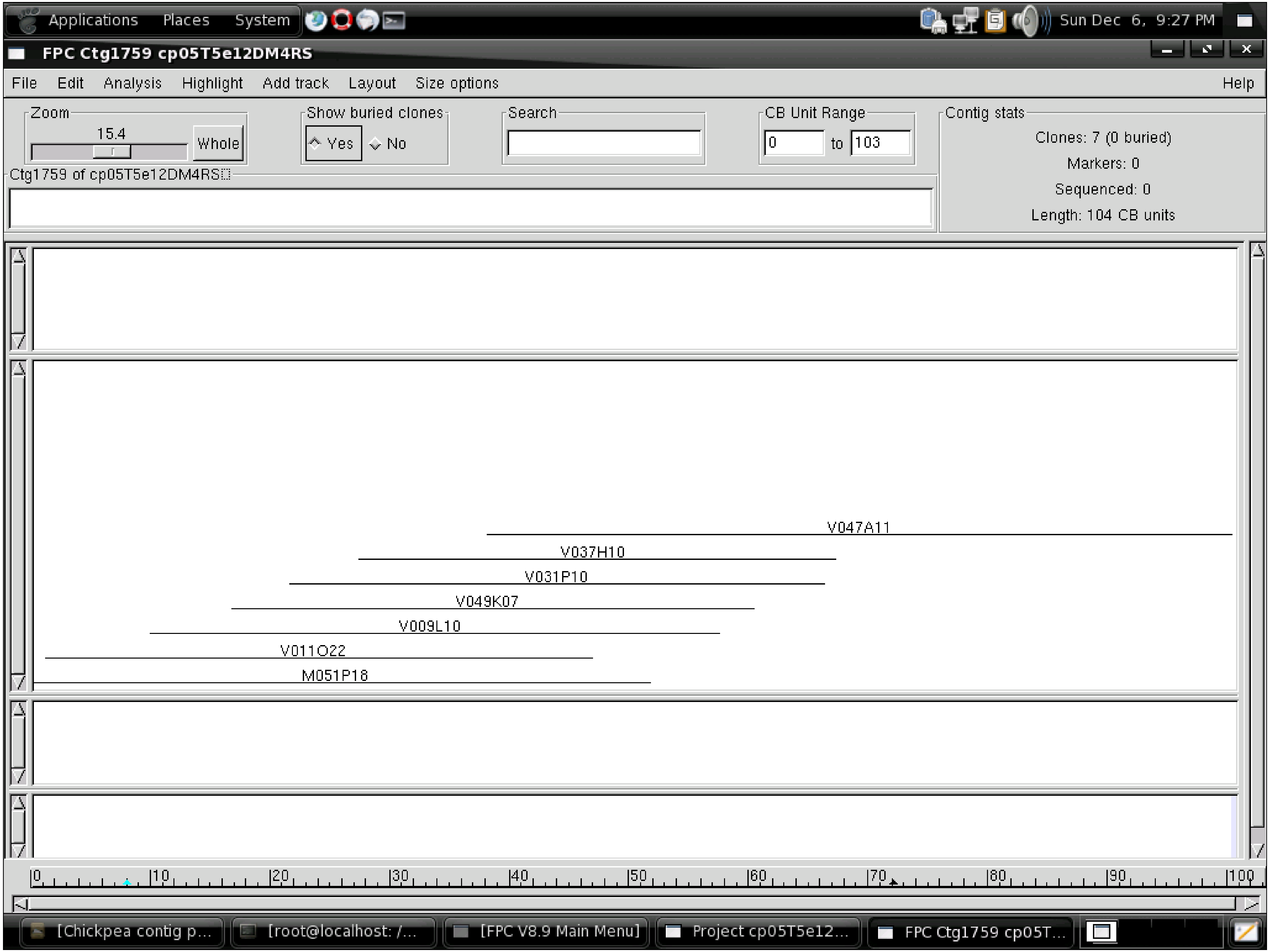
Applications Places System
Sun Dec 6, 9:27 PM
FPC Ctg1759 cp05T5e12DM4RS
File Edit Analysis Highlight Add track Layout Size options Help
Zoom
15.4
Whole
Show buried clones
Yes
No
Search
CB Unit Range
0
to
103
Contig stats
Clones: 7 (0 buried)
Markers: 0
Sequenced: 0
Length: 104 CB units
Ctg1759 of cp05T5e12DM4RS
V047A11
V037H10
V031P10
V049K07
V009L10
V011O22
M051P18
0 10 20 30 40 50 60 70 80 90 100
[Chickpea contig p...
[root@localhost: /...
[FPC V8.9 Main Menu]
Project cp05T5e12...
FPC Ctg1759 cp05T...

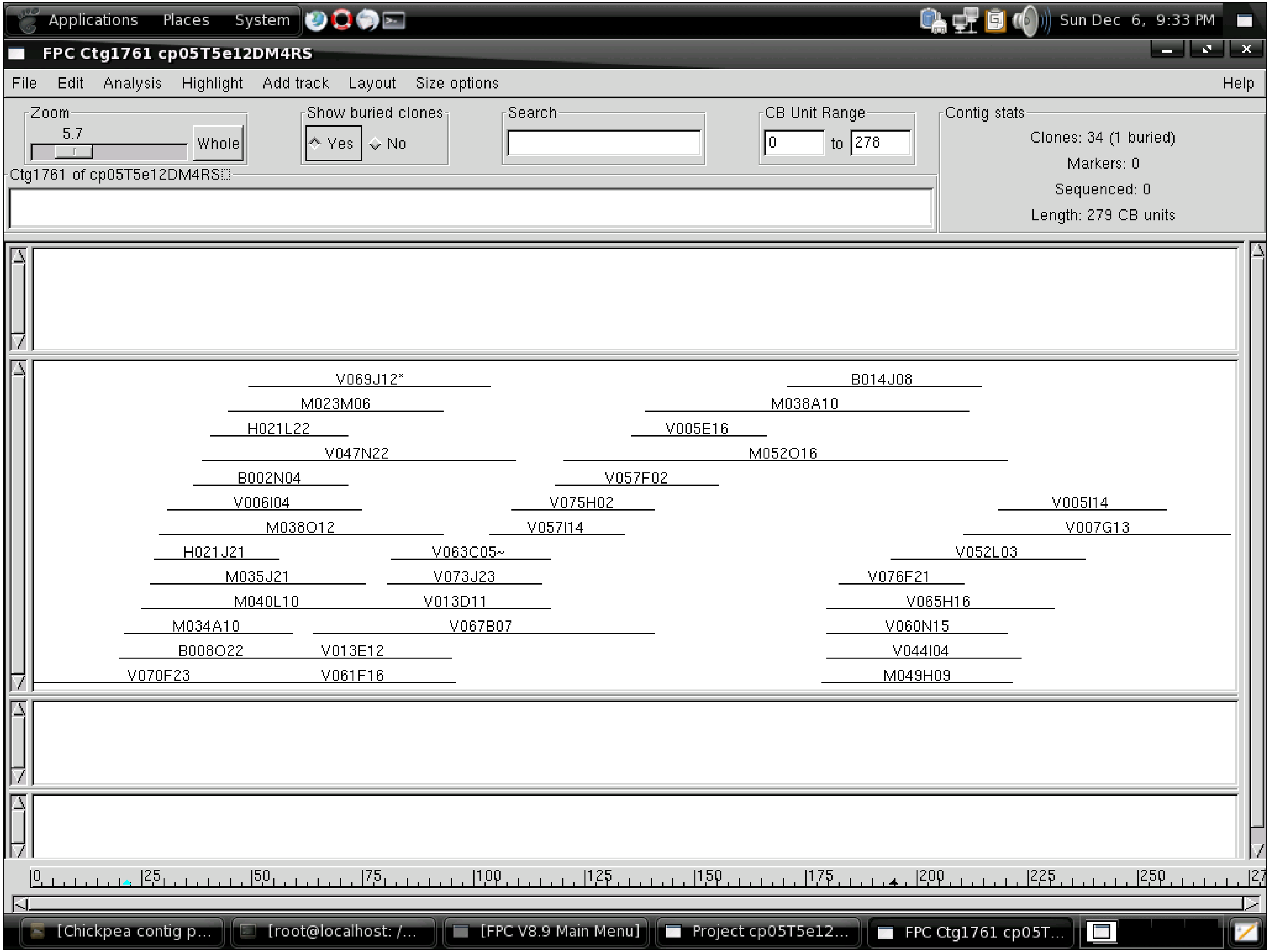
Applications Places System
Sun Dec 6, 9:33 PM
FPC Ctg1761 cp05T5e12DM4RS
File Edit Analysis Highlight Add track Layout Size options Help
Zoom
5.7
Whole
Show buried clones
Yes No
Search
CB Unit Range
0 to 278
Contig stats
Clones: 34 (1 buried)
Markers: 0
Sequenced: 0
Length: 279 CB units
Ctg1761 of cp05T5e12DM4RS
V069J12*
B014J08
M023M06
M038A10
H021L22
V005E16
V047N22
M052O16
B002N04
V057F02
V006I04
V075H02
V005I14
M038O12
V057I14
V007G13
H021J21
V063C05~
V052L03
M035J21
V073J23
V076F21
M040L10
V013D11
V065H16
M034A10
V067B07
V060N15
B008O22
V013E12
V044I04
V070F23
V061F16
M049H09
0 25 50 75 100 125 150 175 200 225 250
[Chickpea contig p...
[root@localhost: /...
[FPC V8.9 Main Menu]
Project cp05T5e12...
FPC Ctg1761 cp05T...

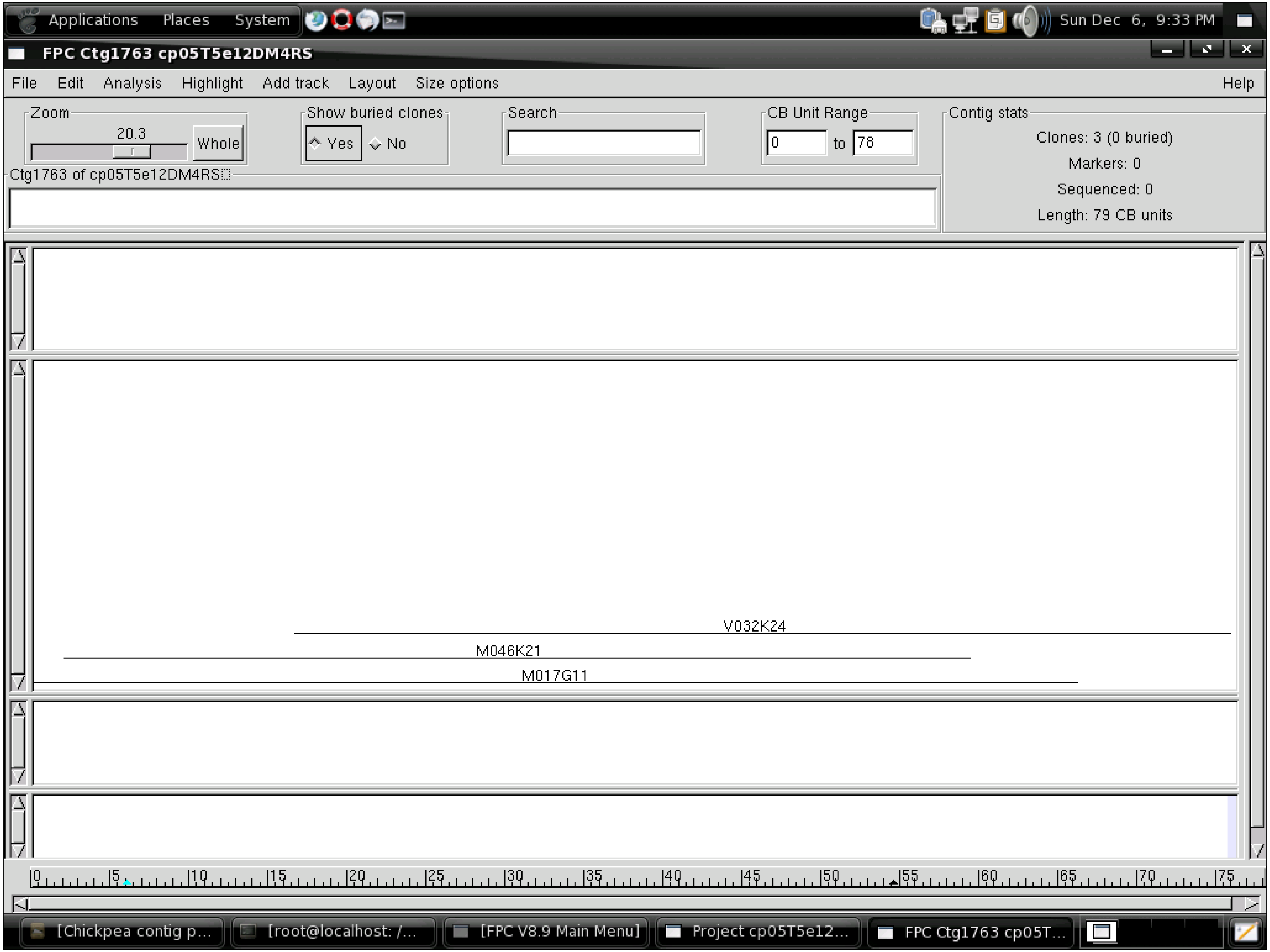
Applications Places System
Sun Dec 6, 9:33 PM
FPC Ctg1763 cp05T5e12DM4RS
File Edit Analysis Highlight Add track Layout Size options Help
Zoom
20.3
Whole
Show buried clones
Yes No
Search
CB Unit Range
0 to 78
Contig stats
Clones: 3 (0 buried)
Markers: 0
Sequenced: 0
Length: 79 CB units
Ctg1763 of cp05T5e12DM4RS
V032K24
M046K21
M017G11
0 5 10 15 20 25 30 35 40 45 50 55 60 65 70 75
[Chickpea contig p...
[root@localhost: /...
[FPC V8.9 Main Menu]
Project cp05T5e12...
FPC Ctg1763 cp05T...

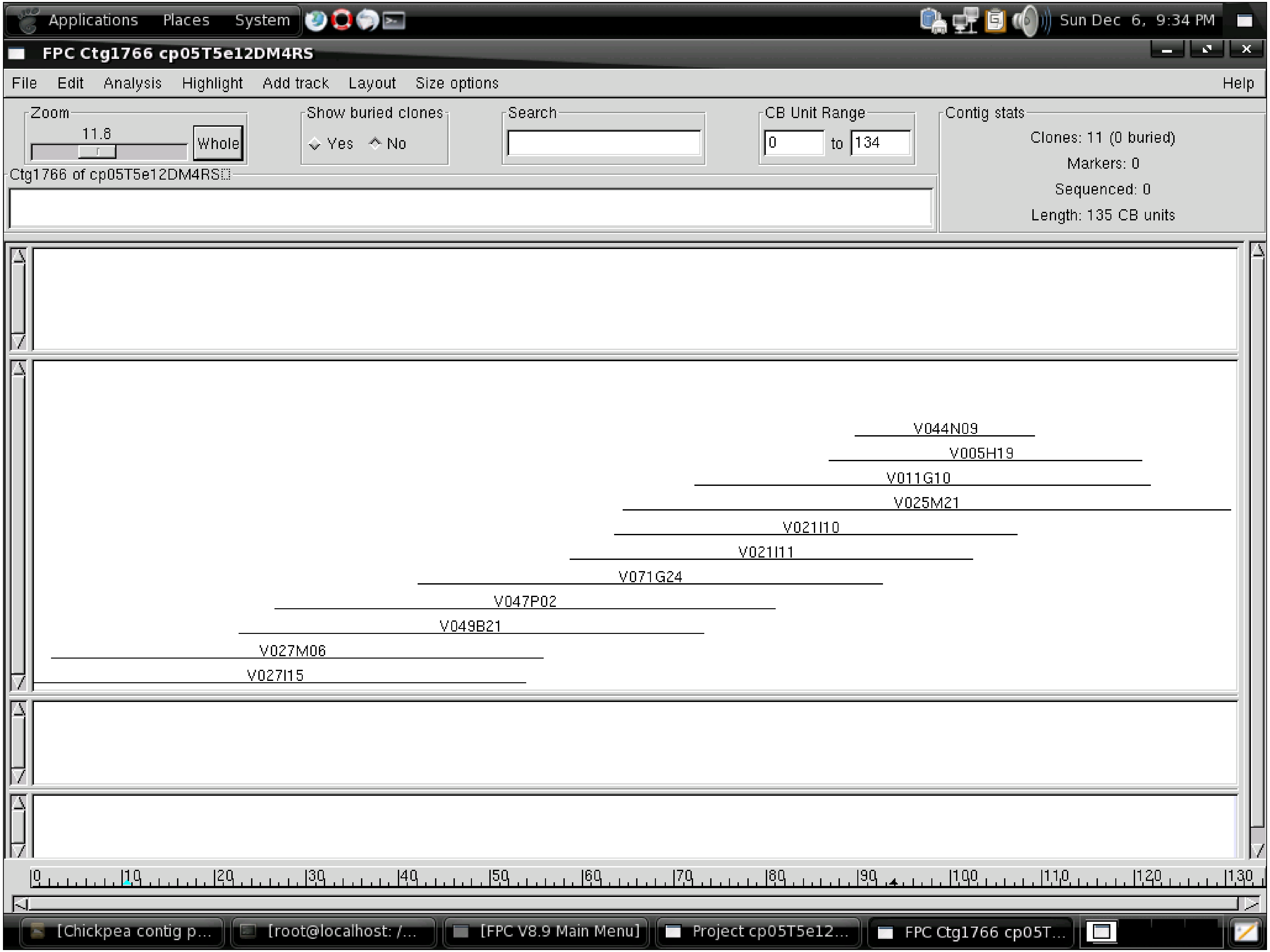
Applications Places System
Sun Dec 6, 9:34 PM
FPC Ctg1766 cp05T5e12DM4RS
File Edit Analysis Highlight Add track Layout Size options Help
Zoom
11.8
Whole
Show buried clones
Yes No
Search
CB Unit Range
0 to 134
Contig stats
Clones: 11 (0 buried)
Markers: 0
Sequenced: 0
Length: 135 CB units
Ctg1766 of cp05T5e12DM4RS
V044N09
V005H19
V011G10
V025M21
V021I10
V021I11
V071G24
V047P02
V049B21
V027M06
V027I15
0 10 20 30 40 50 60 70 80 90 100 110 120 130
[Chickpea contig p...
[root@localhost: /...
[FPC V8.9 Main Menu]
Project cp05T5e12...
FPC Ctg1766 cp05T...

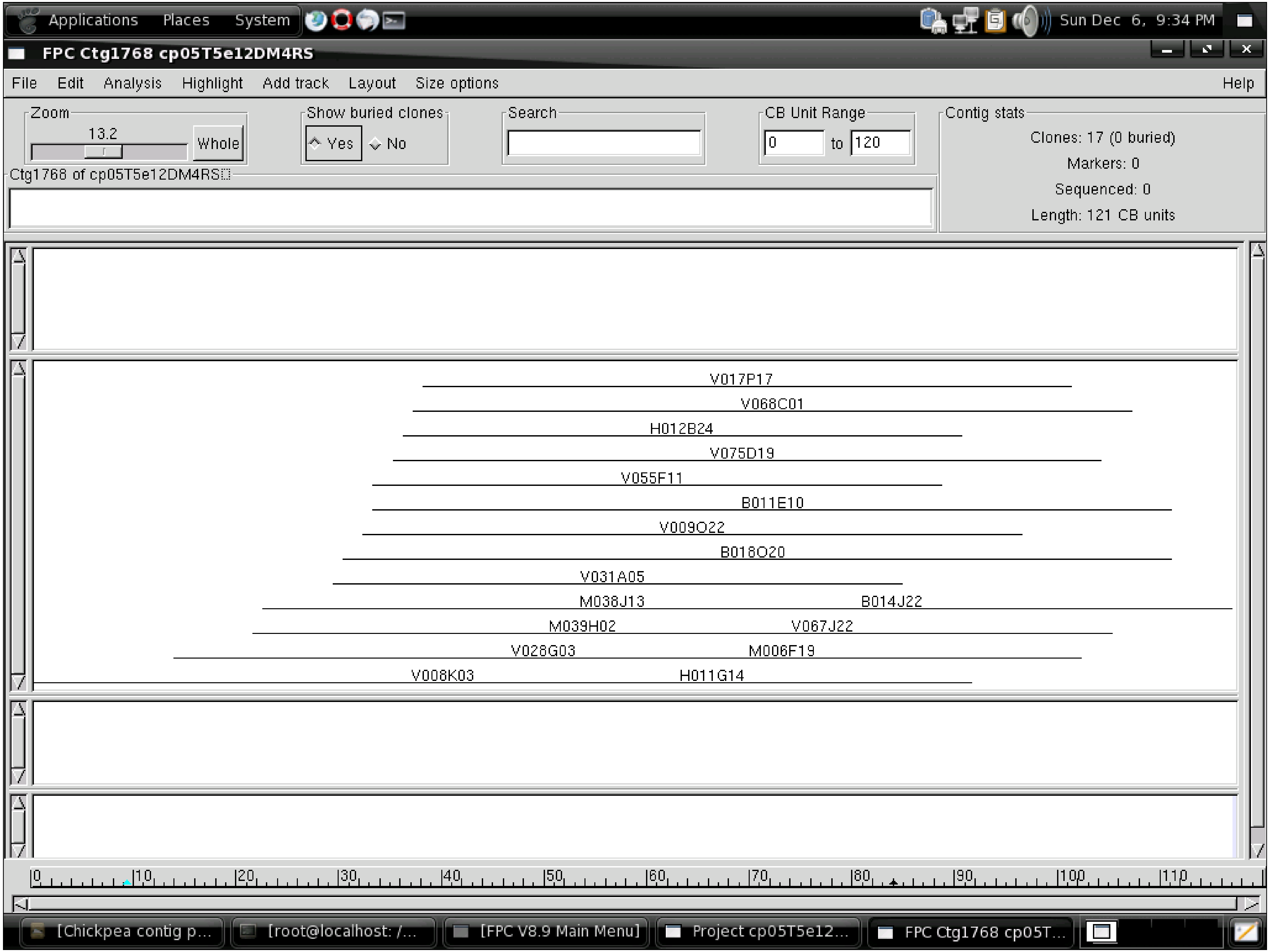
Applications Places System
Sun Dec 6, 9:34 PM
FPC Ctg1768 cp05T5e12DM4RS
File Edit Analysis Highlight Add track Layout Size options Help
Zoom
13.2
Whole
Show buried clones
Yes No
Search
CB Unit Range
0 to 120
Contig stats
Clones: 17 (0 buried)
Markers: 0
Sequenced: 0
Length: 121 CB units
Ctg1768 of cp05T5e12DM4RS
V017P17
V068C01
H012B24
V075D19
V055F11
B011E10
V009O22
B018O20
V031A05
M038J13
B014J22
M039H02
V067J22
V028G03
M006F19
V008K03
H011G14
0 10 20 30 40 50 60 70 80 90 100 110
[Chickpea contig p...
[root@localhost: /...
[FPC V8.9 Main Menu]
Project cp05T5e12...
FPC Ctg1768 cp05T...

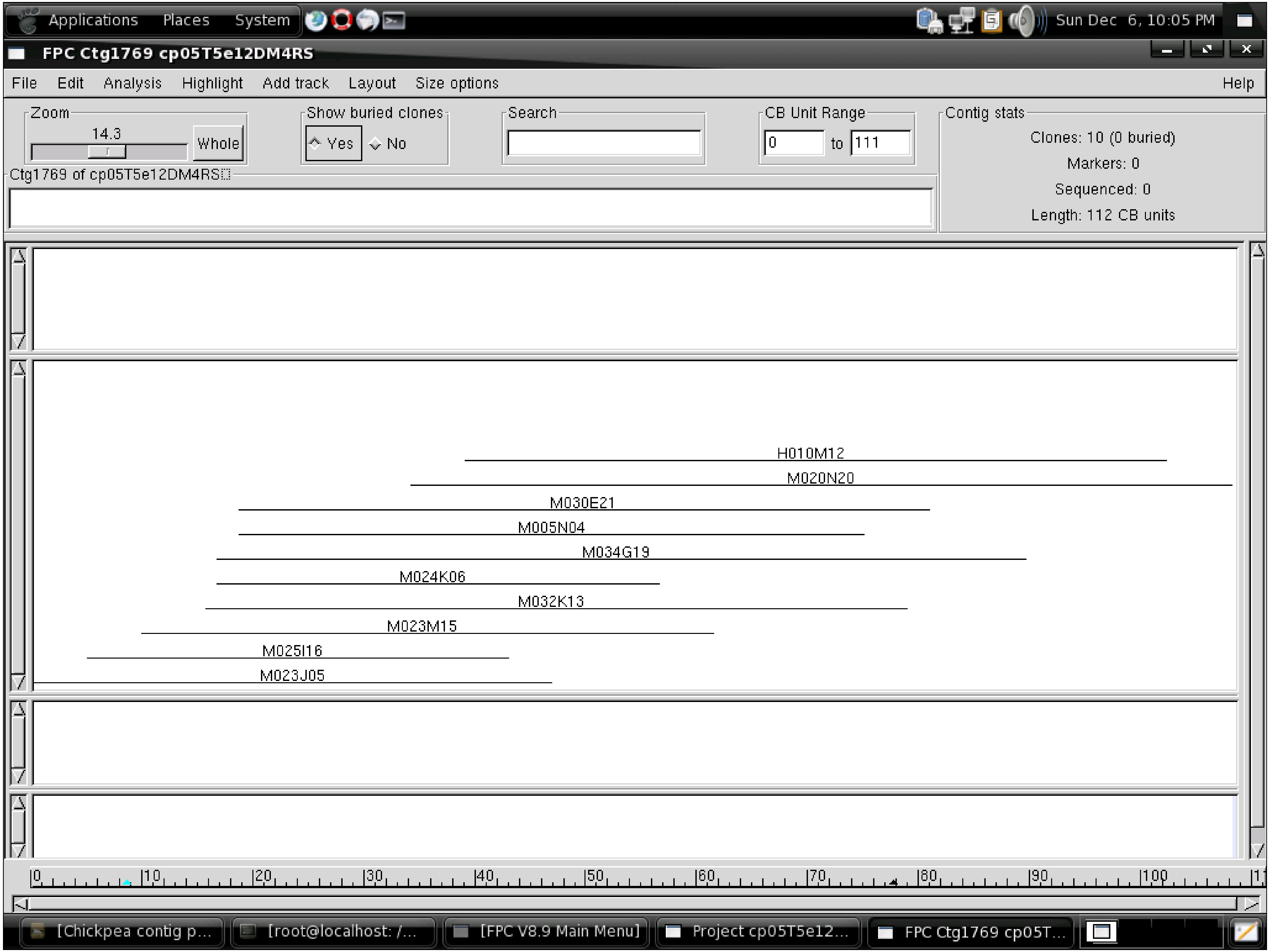
Applications Places System
Sun Dec 6, 10:05 PM
FPC Ctg1769 cp05T5e12DM4RS
File Edit Analysis Highlight Add track Layout Size options Help
Zoom
14.3
Whole
Show buried clones
Yes
No
Search
CB Unit Range
0
to
111
Contig stats
Clones: 10 (0 buried)
Markers: 0
Sequenced: 0
Length: 112 CB units
Ctg1769 of cp05T5e12DM4RS
H010M12
M020N20
M030E21
M005N04
M034G19
M024K06
M032K13
M023M15
M025I16
M023J05
0 10 20 30 40 50 60 70 80 90 100
[Chickpea contig p...
[root@localhost: /...
[FPC V8.9 Main Menu]
Project cp05T5e12...
FPC Ctg1769 cp05T...

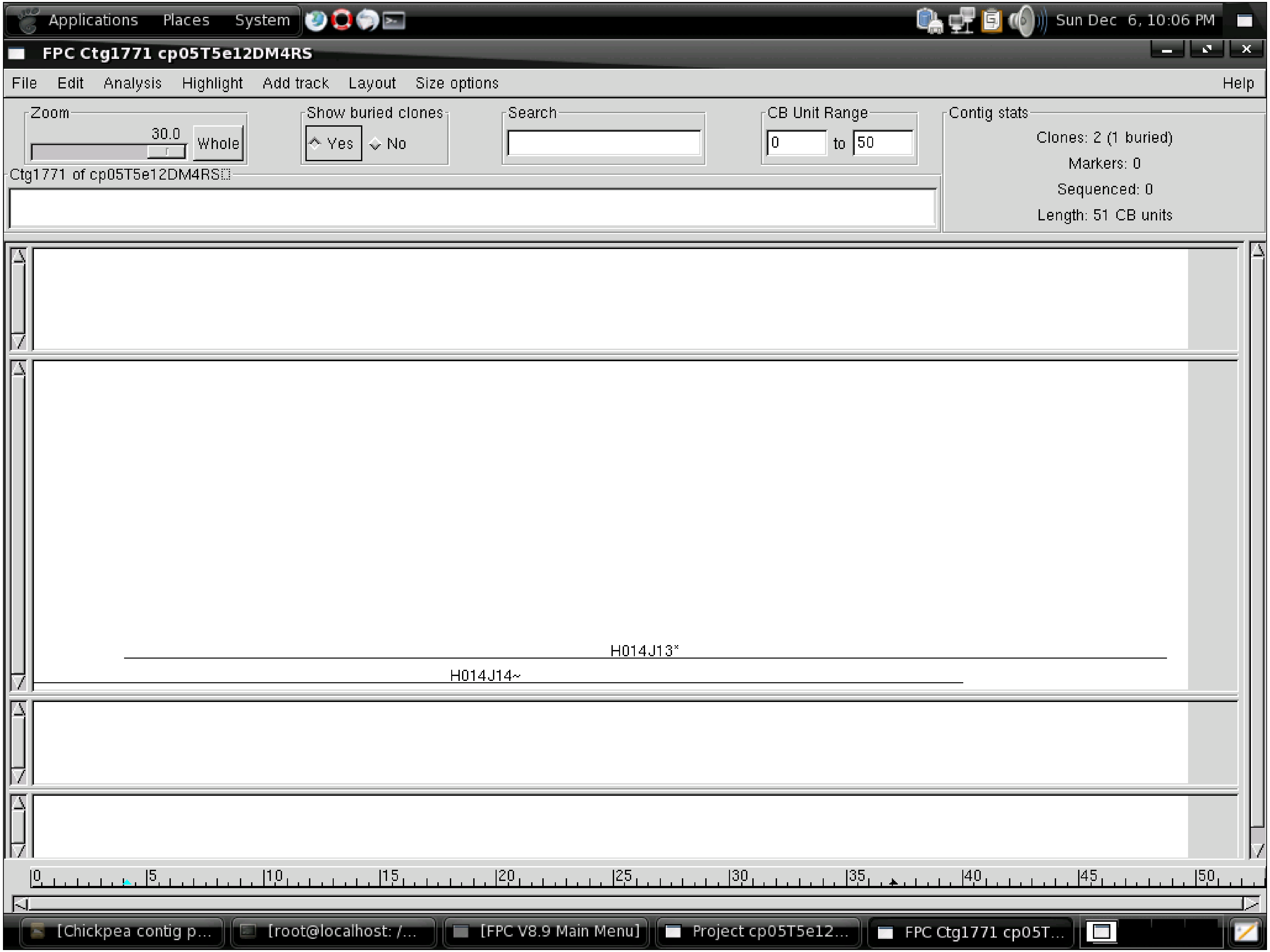

Applications Places System
Sun Dec 6, 10:06 PM
FPC Ctg1771 cp05T5e12DM4RS
File Edit Analysis Highlight Add track Layout Size options Help
Zoom
30.0
Whole
Show buried clones
Yes No
Search
CB Unit Range
0 to 50
Contig stats
Clones: 2 (1 buried)
Markers: 0
Sequenced: 0
Length: 51 CB units
Ctg1771 of cp05T5e12DM4RS
H014J13*
H014J14~
0 5 10 15 20 25 30 35 40 45 50
[Chickpea contig p...
[root@localhost: /...
[FPC V8.9 Main Menu]
Project cp05T5e12...
FPC Ctg1771 cp05T...

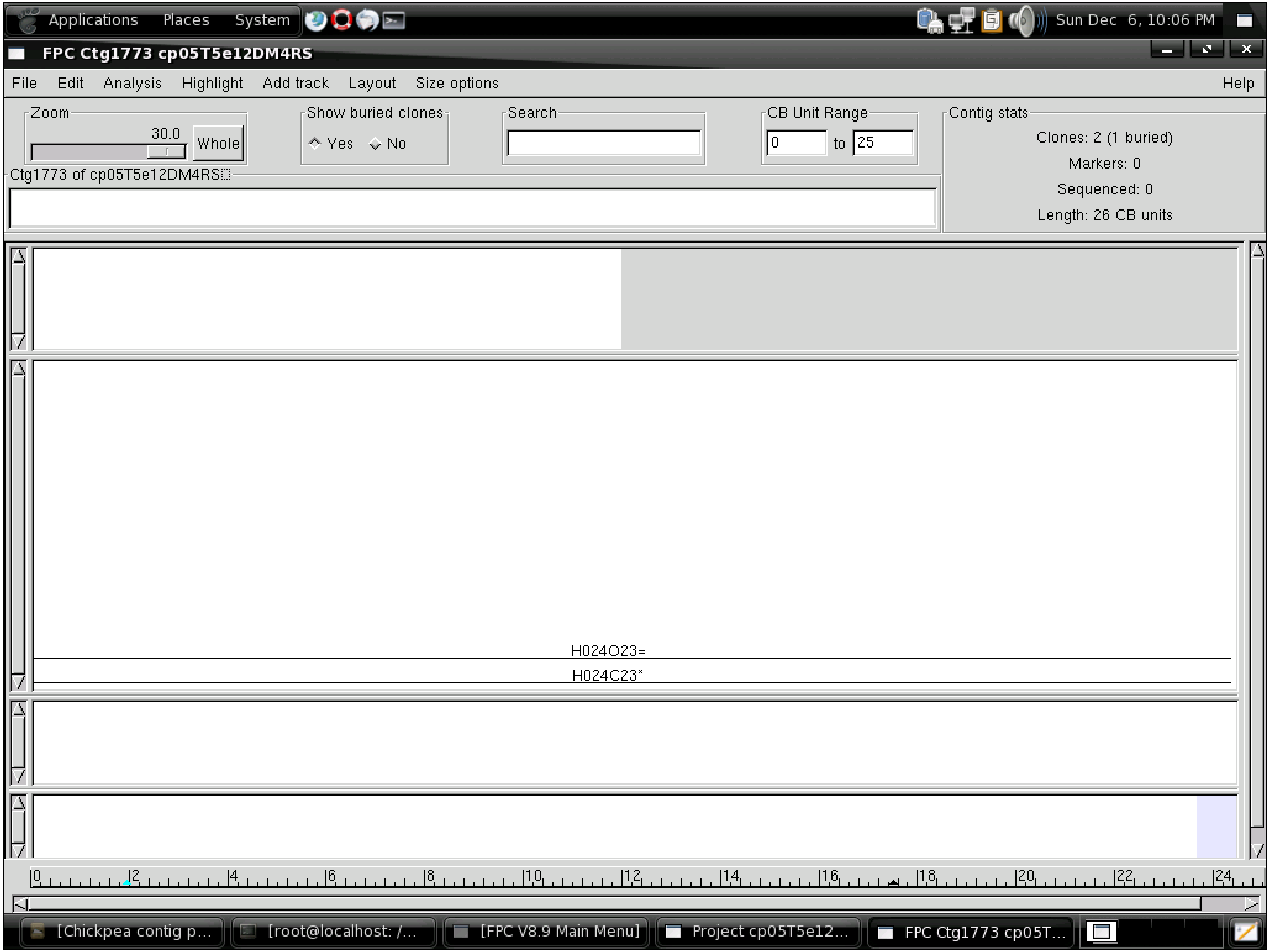
Applications Places System
Sun Dec 6, 10:06 PM
FPC Ctg1773 cp05T5e12DM4RS
File Edit Analysis Highlight Add track Layout Size options Help
Zoom
30.0
Whole
Show buried clones
Yes No
Search
CB Unit Range
0 to 25
Contig stats
Clones: 2 (1 buried)
Markers: 0
Sequenced: 0
Length: 26 CB units
Ctg1773 of cp05T5e12DM4RS
H024O23=
H024C23*
0 2 4 6 8 10 12 14 16 18 20 22 24
[Chickpea contig p...
[root@localhost: /...
[FPC V8.9 Main Menu]
Project cp05T5e12...
FPC Ctg1773 cp05T...

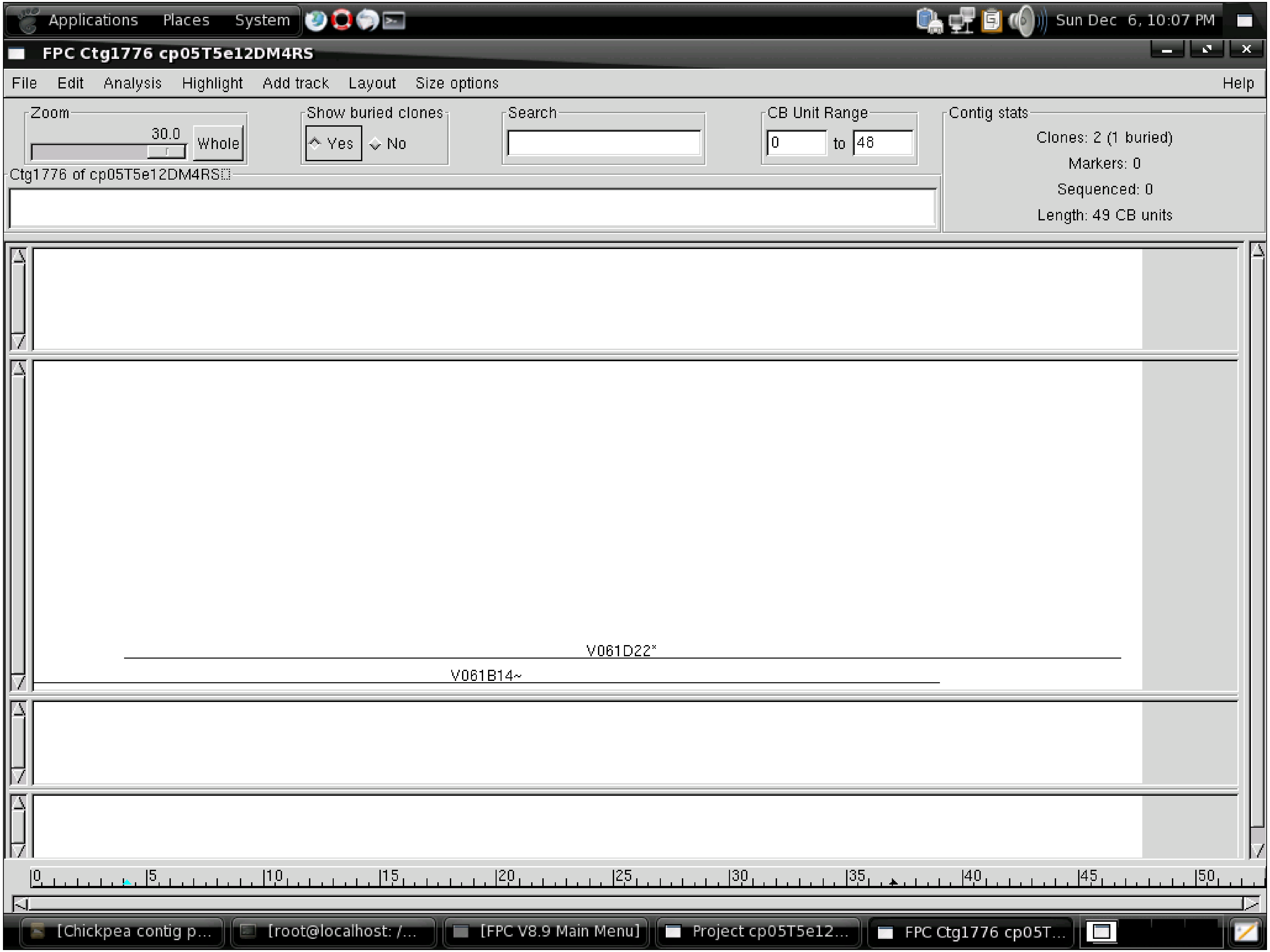
Applications Places System
Sun Dec 6, 10:07 PM
FPC Ctg1776 cp05T5e12DM4RS
File Edit Analysis Highlight Add track Layout Size options Help
Zoom
30.0
Whole
Show buried clones
Yes
No
Search
CB Unit Range
0
to
48
Contig stats
Clones: 2 (1 buried)
Markers: 0
Sequenced: 0
Length: 49 CB units
Ctg1776 of cp05T5e12DM4RS
V061D22*
V061B14~
0 5 10 15 20 25 30 35 40 45 50
[Chickpea contig p...
[root@localhost: /...
[FPC V8.9 Main Menu]
Project cp05T5e12...
FPC Ctg1776 cp05T...

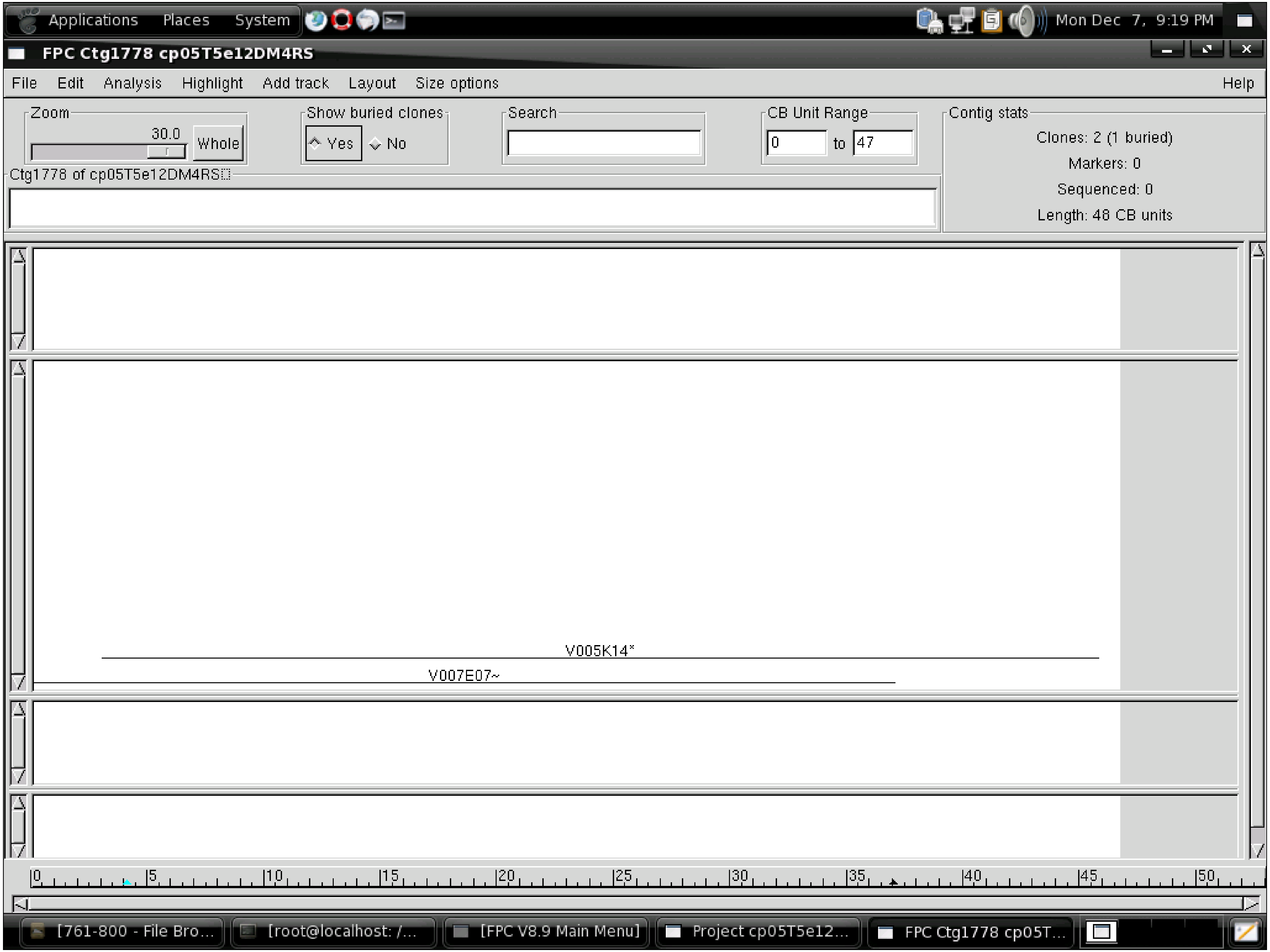
Applications Places System
Mon Dec 7, 9:19 PM
FPC Ctg1778 cp05T5e12DM4RS
File Edit Analysis Highlight Add track Layout Size options Help
Zoom
30.0
Whole
Show buried clones
Yes
No
Search
CB Unit Range
0
to
47
Contig stats
Clones: 2 (1 buried)
Markers: 0
Sequenced: 0
Length: 48 CB units
Ctg1778 of cp05T5e12DM4RS
V005K14*
V007E07~
0 5 10 15 20 25 30 35 40 45 50
[761-800 - File Bro...
[root@localhost: /...
[FPC V8.9 Main Menu]
Project cp05T5e12...
FPC Ctg1778 cp05T...

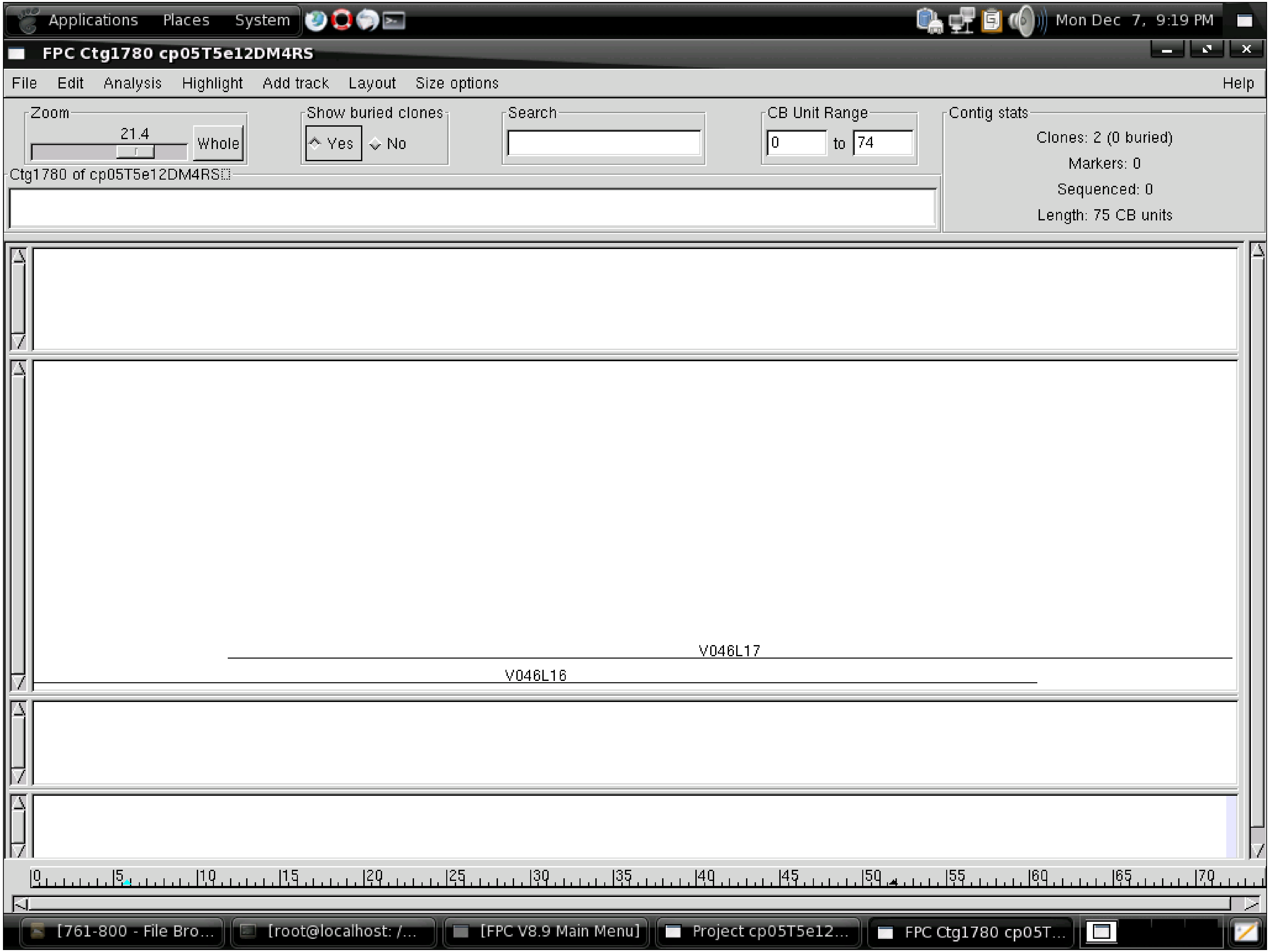
Applications Places System
Mon Dec 7, 9:19 PM
FPC Ctg1780 cp05T5e12DM4RS
File Edit Analysis Highlight Add track Layout Size options Help
Zoom
21.4
Whole
Show buried clones
Yes
No
Search
CB Unit Range
0 to 74
Contig stats
Clones: 2 (0 buried)
Markers: 0
Sequenced: 0
Length: 75 CB units
Ctg1780 of cp05T5e12DM4RS
V046L17
V046L16
0 5 10 15 20 25 30 35 40 45 50 55 60 65 70
[761-800 - File Bro...
[root@localhost: /...
[FPC V8.9 Main Menu]
Project cp05T5e12...
FPC Ctg1780 cp05T...

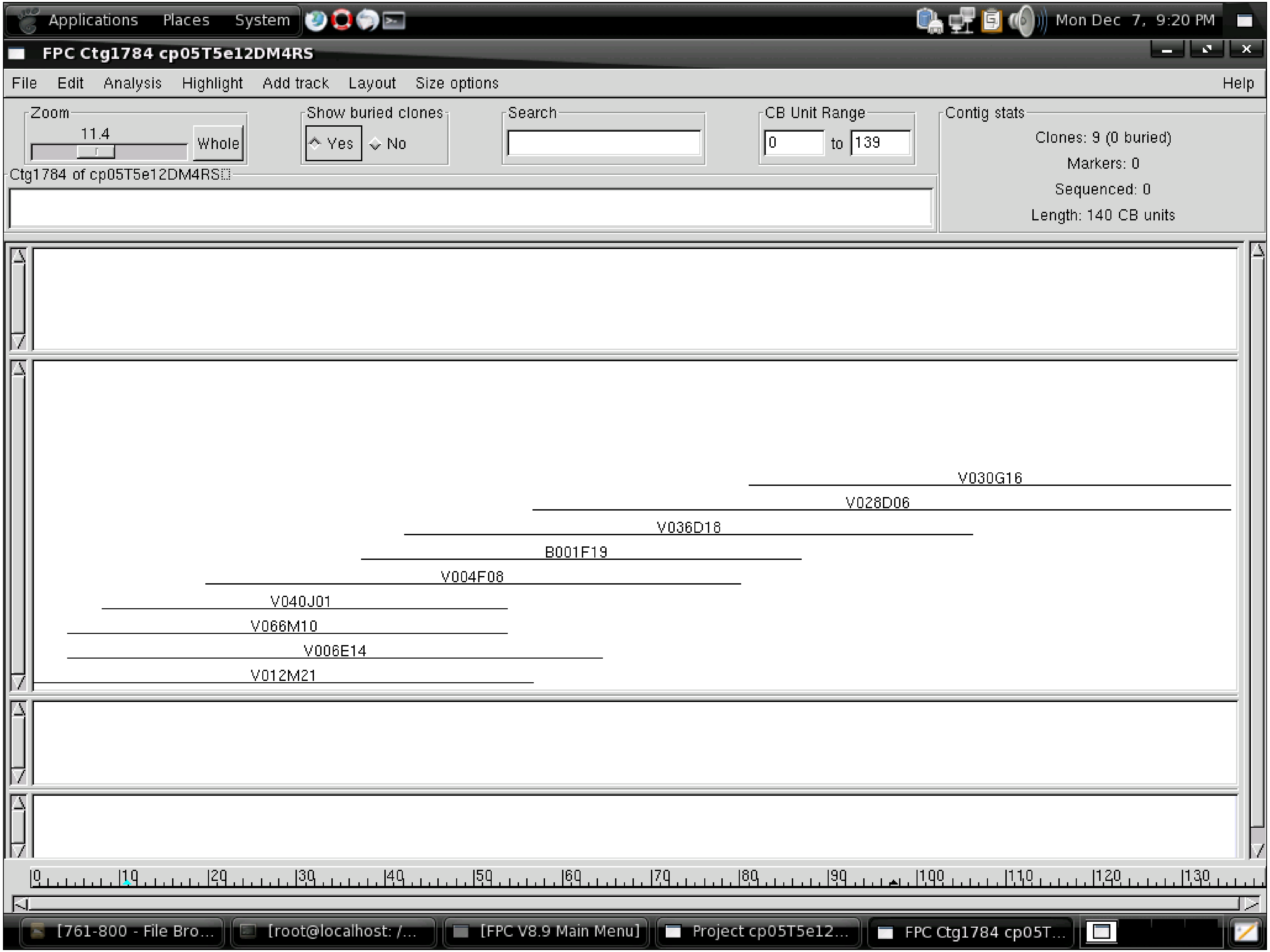
Applications Places System
Mon Dec 7, 9:20 PM
FPC Ctg1784 cp05T5e12DM4RS
File Edit Analysis Highlight Add track Layout Size options Help
Zoom
11.4
Whole
Show buried clones
Yes No
Search
CB Unit Range
0 to 139
Contig stats
Clones: 9 (0 buried)
Markers: 0
Sequenced: 0
Length: 140 CB units
Ctg1784 of cp05T5e12DM4RS
V030G16
V028D06
V036D18
B001F19
V004F08
V040J01
V066M10
V006E14
V012M21
0 10 20 30 40 50 60 70 80 90 100 110 120 130
[761-800 - File Bro...
[root@localhost: /...
[FPC V8.9 Main Menu]
Project cp05T5e12...
FPC Ctg1784 cp05T...

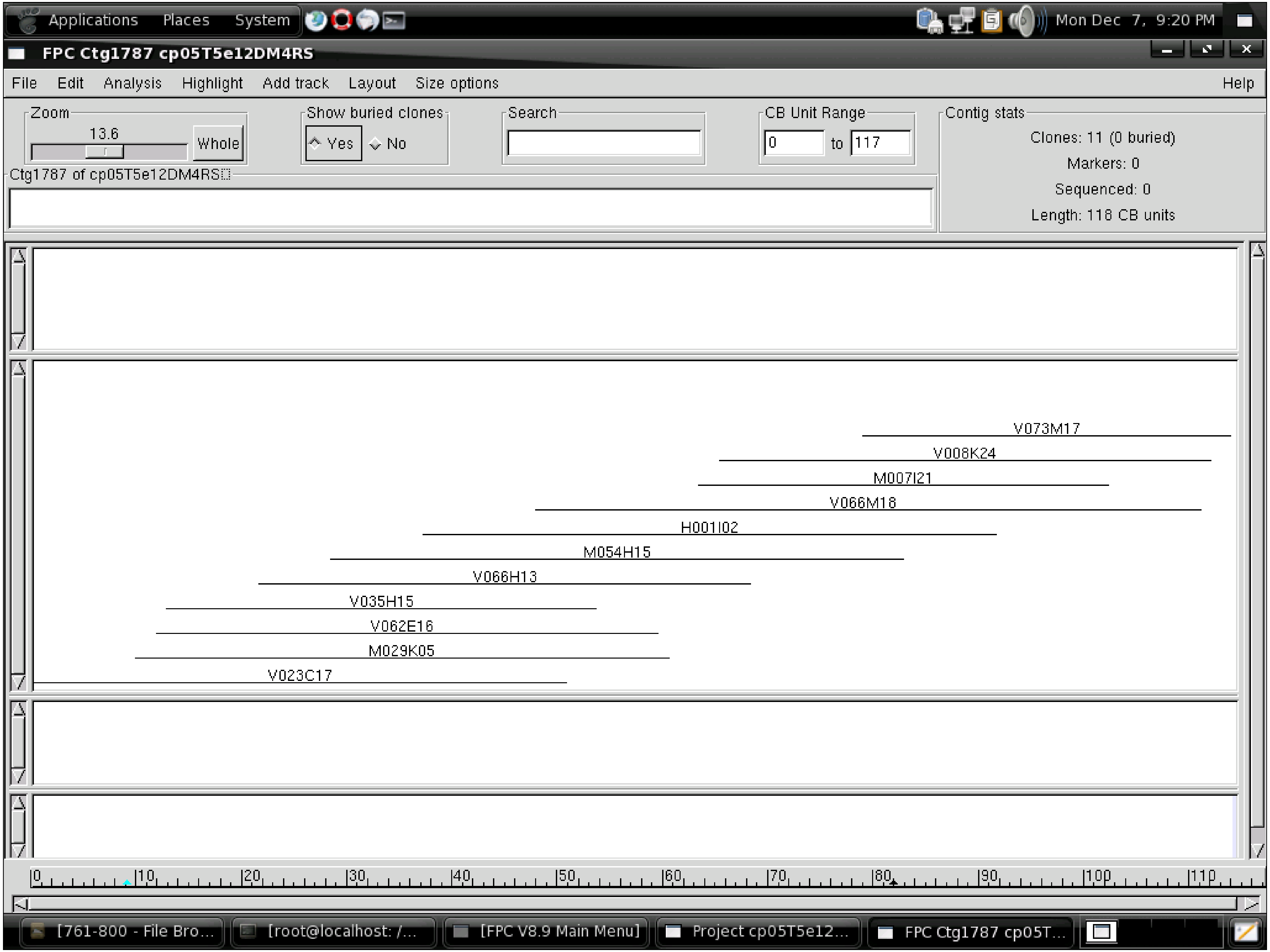

Applications Places System
Mon Dec 7, 9:20 PM
FPC Ctg1787 cp05T5e12DM4RS
File Edit Analysis Highlight Add track Layout Size options
Help
Zoom
13.6
Whole
Show buried clones
Yes
No
Search
CB Unit Range
0
to
117
Contig stats
Clones: 11 (0 buried)
Markers: 0
Sequenced: 0
Length: 118 CB units
Ctg1787 of cp05T5e12DM4RS
V073M17
V008K24
M007I21
V066M18
H001I02
M054H15
V066H13
V035H15
V062E16
M029K05
V023C17
0 10 20 30 40 50 60 70 80 90 100 110
[761-800 - File Bro...
[root@localhost: /...
[FPC V8.9 Main Menu]
Project cp05T5e12...
FPC Ctg1787 cp05T...

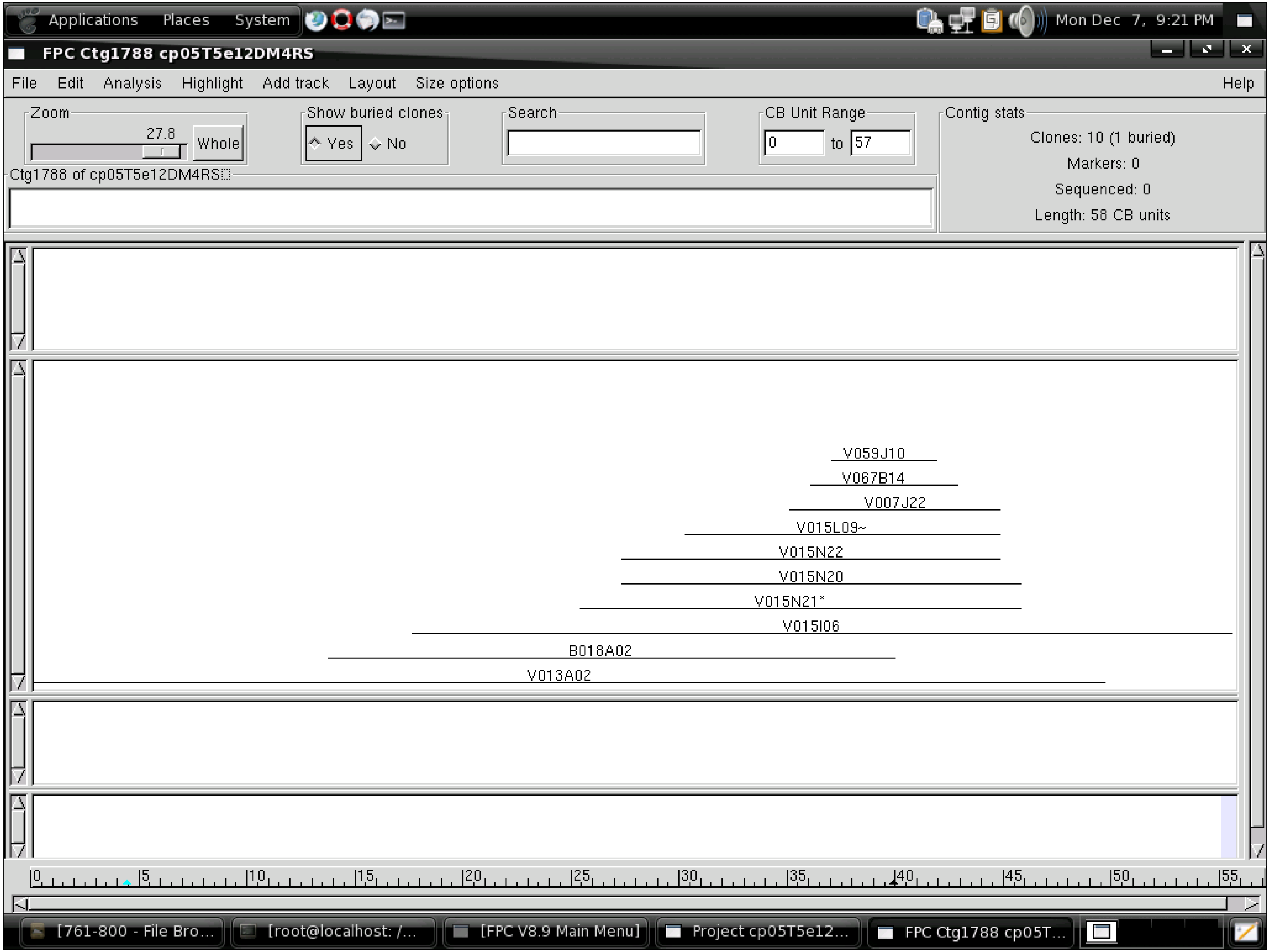
Applications
Places
System
Mon Dec 7, 9:21 PM
FPC Ctg1788 cp05T5e12DM4RS
File
Edit
Analysis
Highlight
Add track
Layout
Size options
Help
Zoom
27.8
Whole
Show buried clones
Yes
No
Search
CB Unit Range
0
to
57
Contig stats
Clones: 10 (1 buried)
Markers: 0
Sequenced: 0
Length: 58 CB units
Ctg1788 of cp05T5e12DM4RS
V059J10
V067B14
V007J22
V015L09~
V015N22
V015N20
V015N21*
V015I06
B018A02
V013A02
0
5
10
15
20
25
30
35
40
45
50
55
[761-800 - File Bro...
[root@localhost: /...
[FPC V8.9 Main Menu]
Project cp05T5e12...
FPC Ctg1788 cp05T...

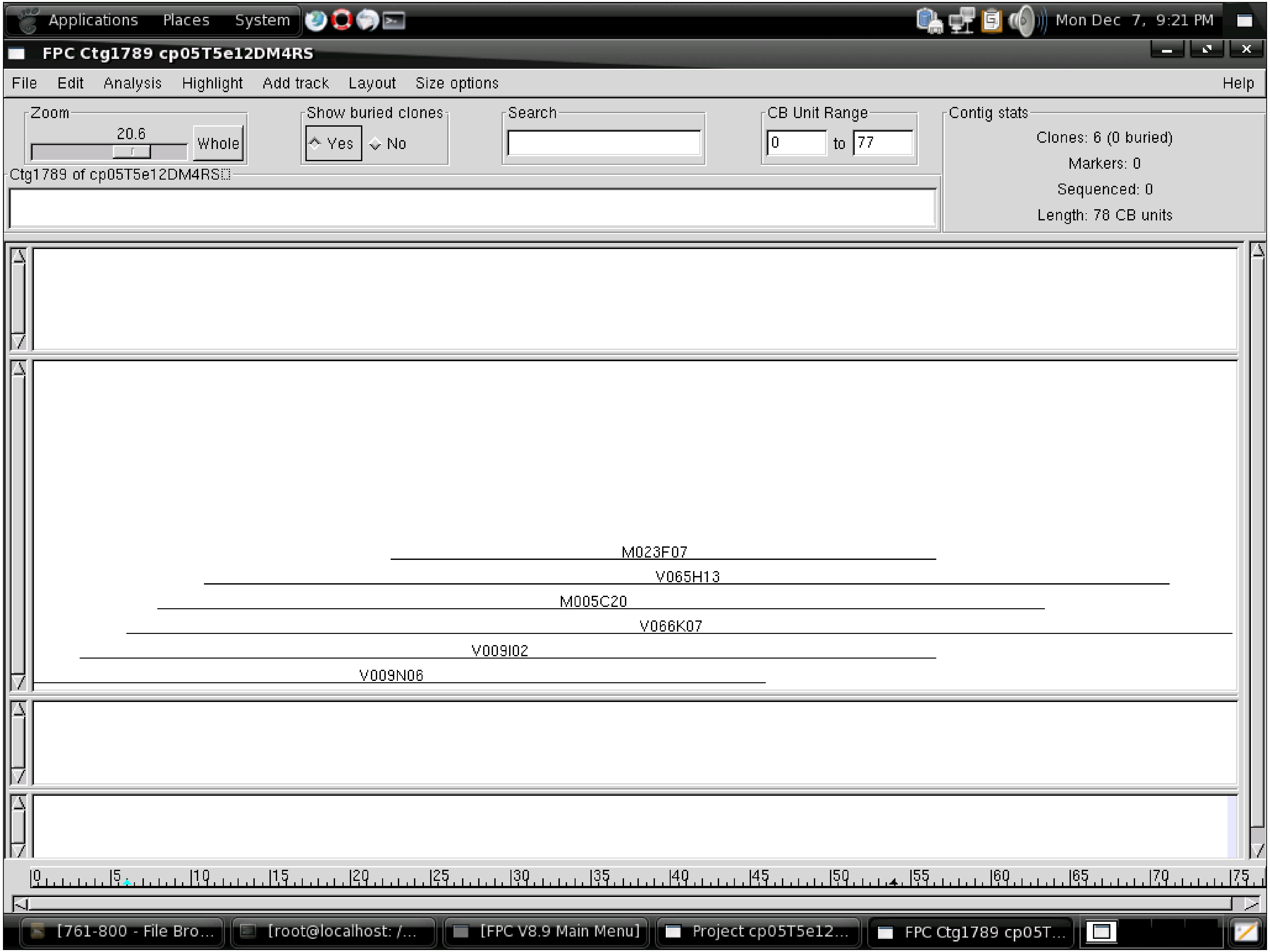
Applications Places System
Mon Dec 7, 9:21 PM
FPC Ctg1789 cp05T5e12DM4RS
File Edit Analysis Highlight Add track Layout Size options Help
Zoom
20.6
Whole
Show buried clones
Yes No
Search
CB Unit Range
0 to 77
Contig stats
Clones: 6 (0 buried)
Markers: 0
Sequenced: 0
Length: 78 CB units
Ctg1789 of cp05T5e12DM4RS
M023F07
V065H13
M005C20
V066K07
V009I02
V009N06
0 5 10 15 20 25 30 35 40 45 50 55 60 65 70 75
[761-800 - File Bro...
[root@localhost: /...
[FPC V8.9 Main Menu]
Project cp05T5e12...
FPC Ctg1789 cp05T...

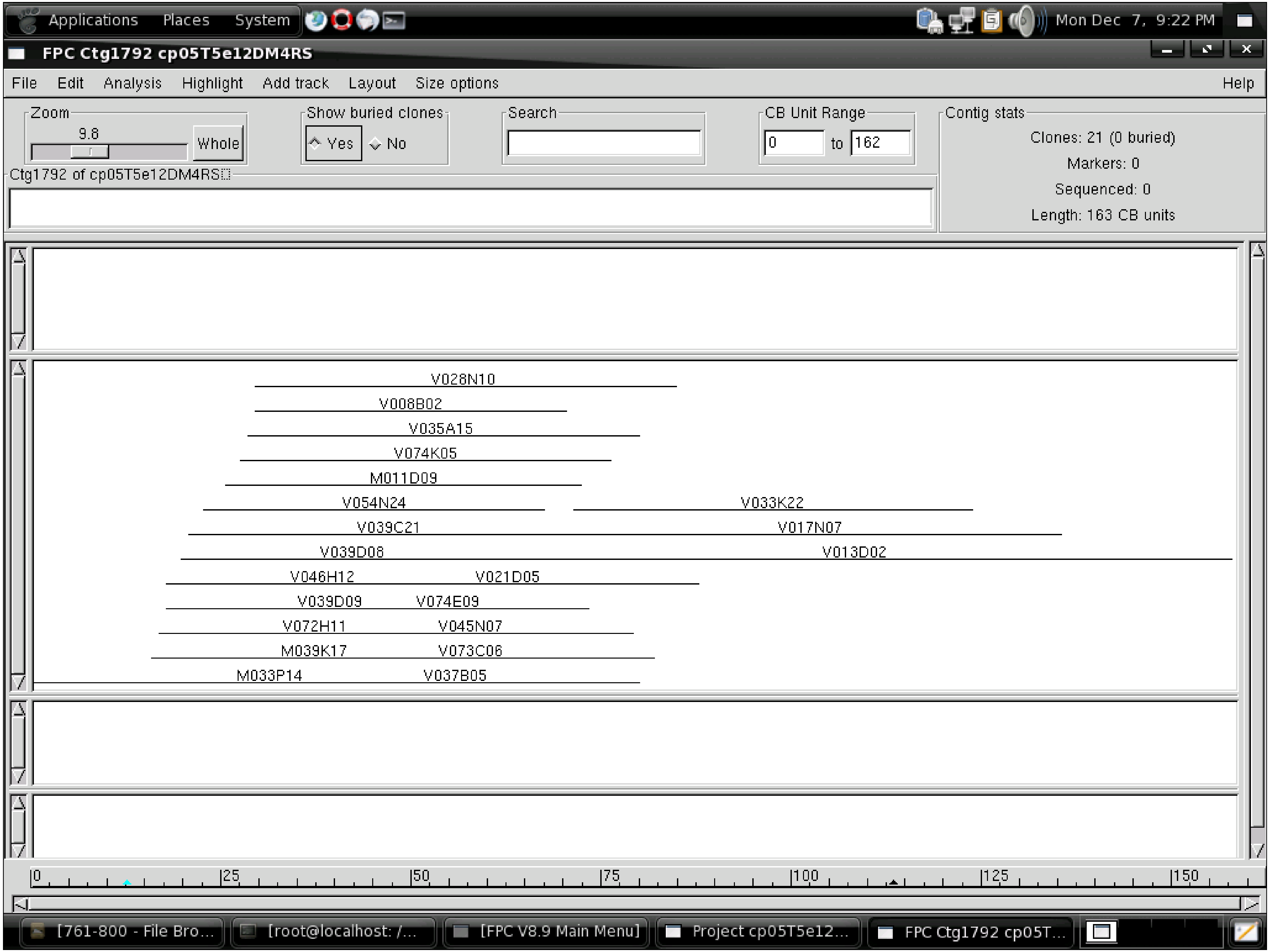
Applications Places System
Mon Dec 7, 9:22 PM
FPC Ctg1792 cp05T5e12DM4RS
File Edit Analysis Highlight Add track Layout Size options Help
Zoom
9.8
Whole
Show buried clones
Yes No
Search
CB Unit Range
0 to 162
Contig stats
Clones: 21 (0 buried)
Markers: 0
Sequenced: 0
Length: 163 CB units
Ctg1792 of cp05T5e12DM4RS
V028N10
V008B02
V035A15
V074K05
M011D09
V054N24
V033K22
V039C21
V017N07
V039D08
V013D02
V046H12
V021D05
V039D09
V074E09
V072H11
V045N07
M039K17
V073C06
M033P14
V037B05
0 25 50 75 100 125 150
[761-800 - File Bro...
[root@localhost: /...
[FPC V8.9 Main Menu]
Project cp05T5e12...
FPC Ctg1792 cp05T...

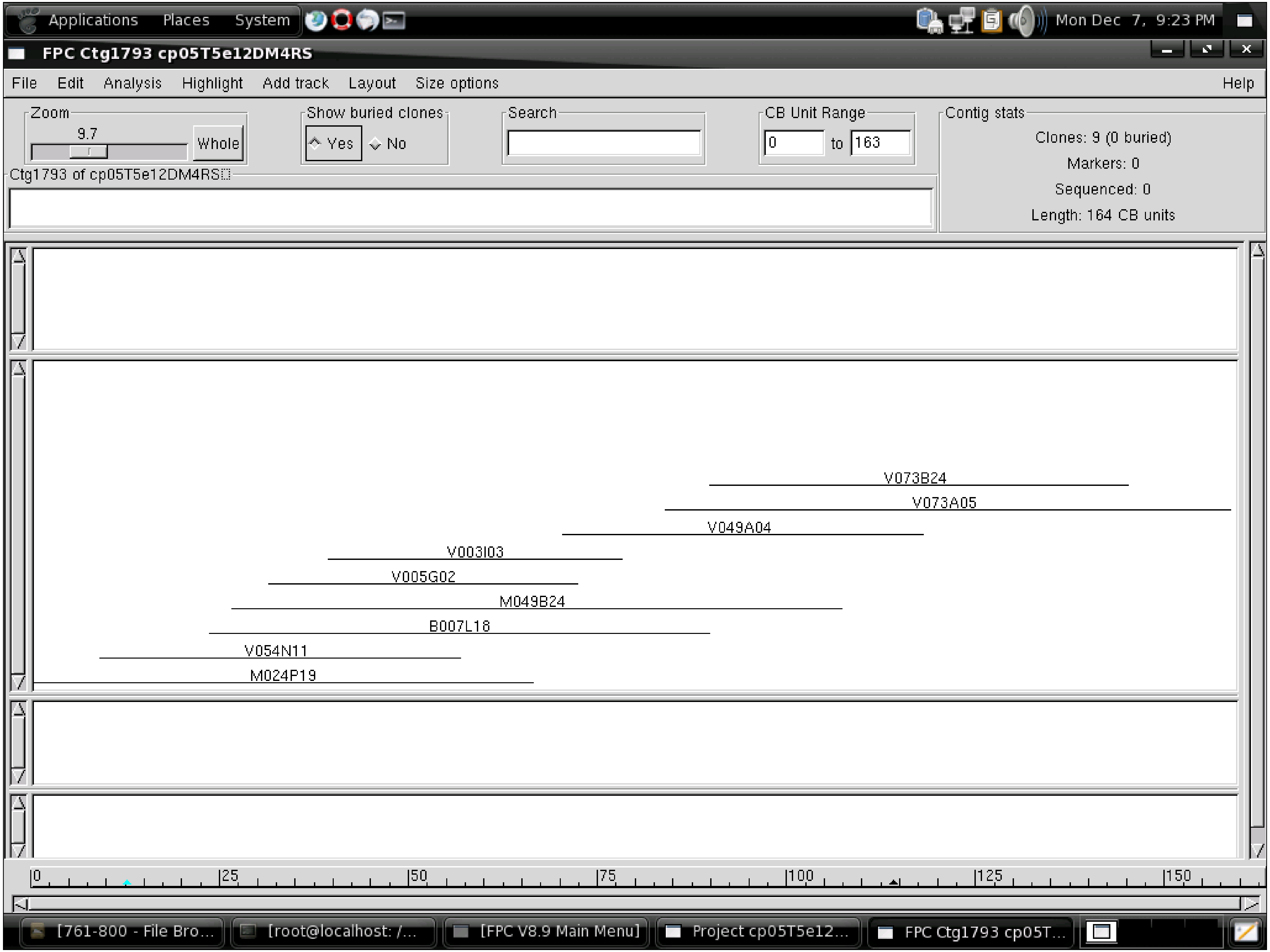
Applications
Places
System
Mon Dec 7, 9:23 PM
FPC Ctg1793 cp05T5e12DM4RS
File
Edit
Analysis
Highlight
Add track
Layout
Size options
Help
Zoom
9.7
Whole
Show buried clones
Yes
No
Search
CB Unit Range
0
to
163
Contig stats
Clones: 9 (0 buried)
Markers: 0
Sequenced: 0
Length: 164 CB units
Ctg1793 of cp05T5e12DM4RS
V073B24
V073A05
V049A04
V003I03
V005G02
M049B24
B007L18
V054N11
M024P19
0
25
50
75
100
125
150
[761-800 - File Bro...
[root@localhost: /...
[FPC V8.9 Main Menu]
Project cp05T5e12...
FPC Ctg1793 cp05T...

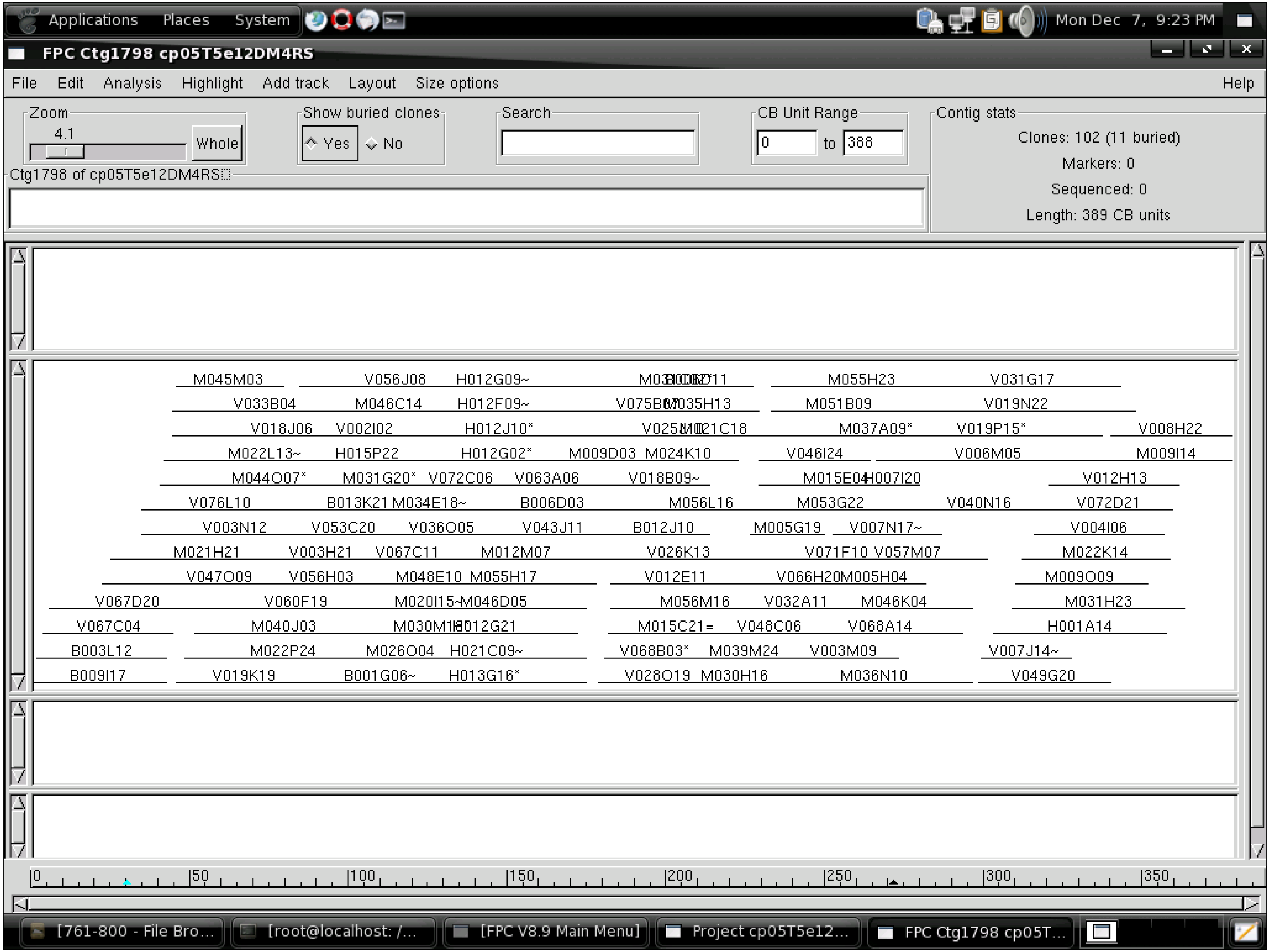
Applications Places System
Mon Dec 7, 9:23 PM
FPC Ctg1798 cp05T5e12DM4RS
File Edit Analysis Highlight Add track Layout Size options Help
Zoom
4.1
Whole
Show buried clones
Yes No
Search
CB Unit Range
0 to 388
Contig stats
Clones: 102 (11 buried)
Markers: 0
Sequenced: 0
Length: 389 CB units
Ctg1798 of cp05T5e12DM4RS
M045M03 V056J08 H012G09~ M055H23 V031G17
V033B04 M046C14 H012F09~ M051B09 V019N22
V018J06 V002I02 H012J10* M037A09* V019P15* V008H22
M022L13~ H015P22 H012G02* M009D03 M024K10 V046I24 V006M05 M009I14
M044O07* M031G20* V072C06 V063A06 V018B09~ V012H13
V076L10 B013K21 M034E18~ B006D03 M056L16 M053G22 V040N16 V072D21
V003N12 V053C20 V036O05 V043J11 B012J10 M005G19 V007N17~ V004I06
M021H21 V003H21 V067C11 M012M07 V026K13 V071F10 V057M07 M022K14
V047O09 V056H03 M048E10 M055H17 V012E11 M009O09
V067D20 V060F19 M056M16 V032A11 M046K04 M031H23
V067C04 M040J03 M015C21= V048C06 V068A14 H001A14
B003L12 M022P24 M026O04 H021C09~ V068B03* M039M24 V003M09 V007J14~
B009I17 V019K19 B001G06~ H013G16* V028O19 M030H16 M036N10 V049G20
0 50 100 150 200 250 300 350
[761-800 - File Bro... [root@localhost: /... [FPC V8.9 Main Menu] Project cp05T5e12... FPC Ctg1798 cp05T...

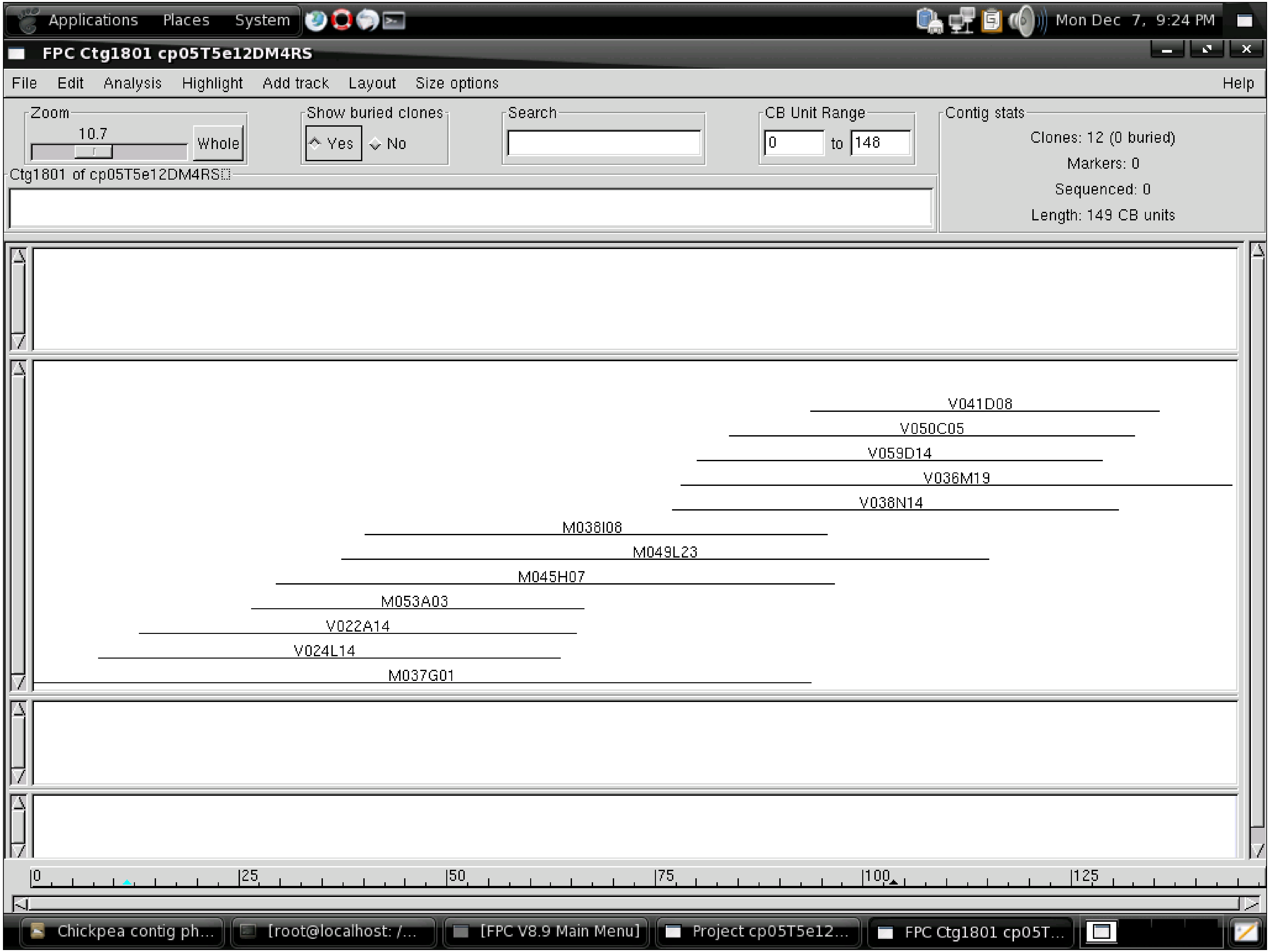
Applications
Places
System
Mon Dec 7, 9:24 PM
FPC Ctg1801 cp05T5e12DM4RS
File
Edit
Analysis
Highlight
Add track
Layout
Size options
Help
Zoom
10.7
Whole
Show buried clones
Yes
No
Search
CB Unit Range
0
to
148
Contig stats
Clones: 12 (0 buried)
Markers: 0
Sequenced: 0
Length: 149 CB units
Ctg1801 of cp05T5e12DM4RS
V041D08
V050C05
V059D14
V036M19
V038N14
M038I08
M049L23
M045H07
M053A03
V022A14
V024L14
M037G01
0
25
50
75
100
125
Chickpea contig ph...
[root@localhost: /...
[FPC V8.9 Main Menu]
Project cp05T5e12...
FPC Ctg1801 cp05T...

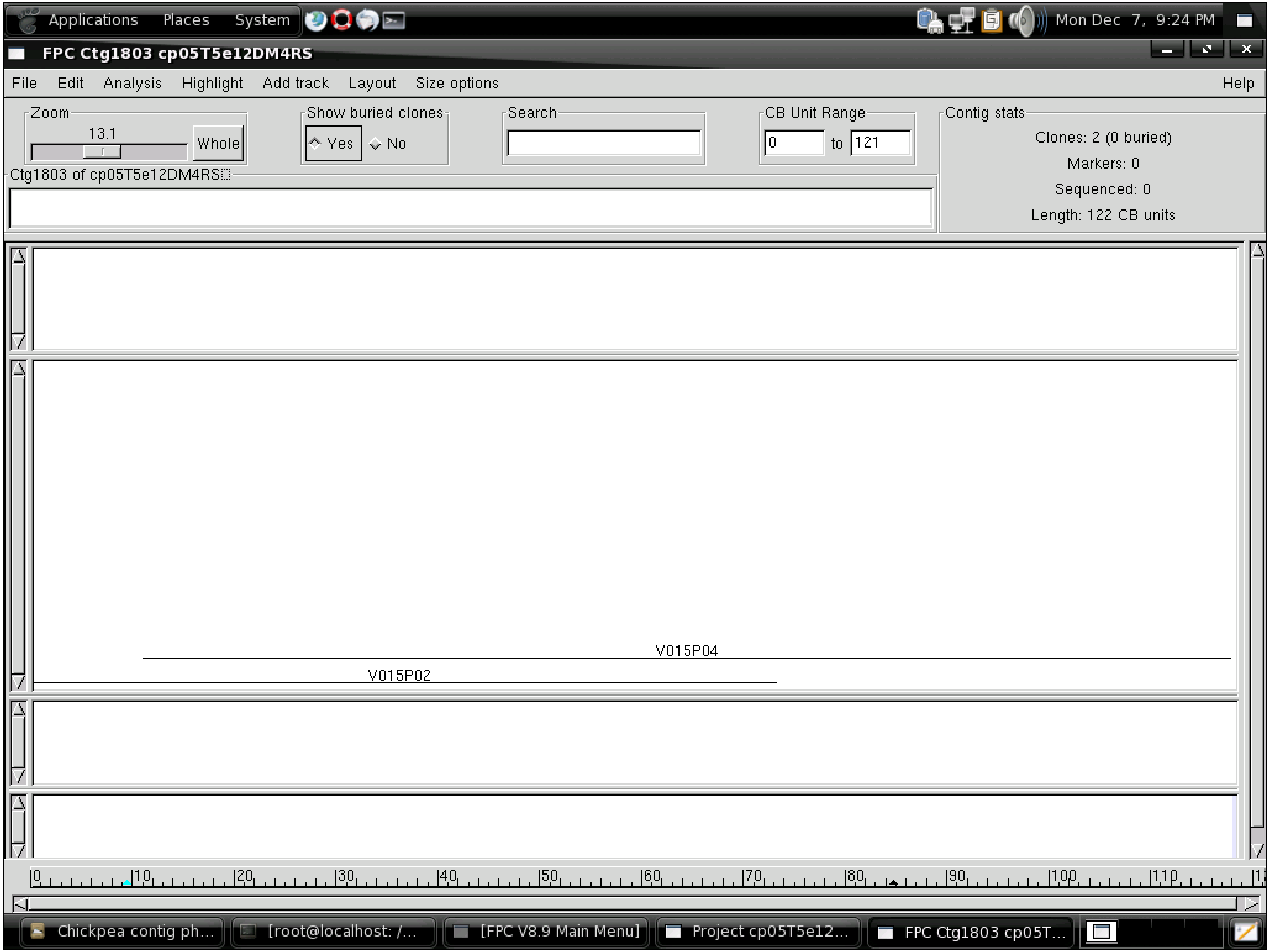
Applications Places System
Mon Dec 7, 9:24 PM
FPC Ctg1803 cp05T5e12DM4RS
File Edit Analysis Highlight Add track Layout Size options Help
Zoom
13.1
Whole
Show buried clones
Yes No
Search
CB Unit Range
0 to 121
Contig stats
Clones: 2 (0 buried)
Markers: 0
Sequenced: 0
Length: 122 CB units
Ctg1803 of cp05T5e12DM4RS
V015P04
V015P02
0 10 20 30 40 50 60 70 80 90 100 110
Chickpea contig ph...
[root@localhost: /...
[FPC V8.9 Main Menu]
Project cp05T5e12...
FPC Ctg1803 cp05T...

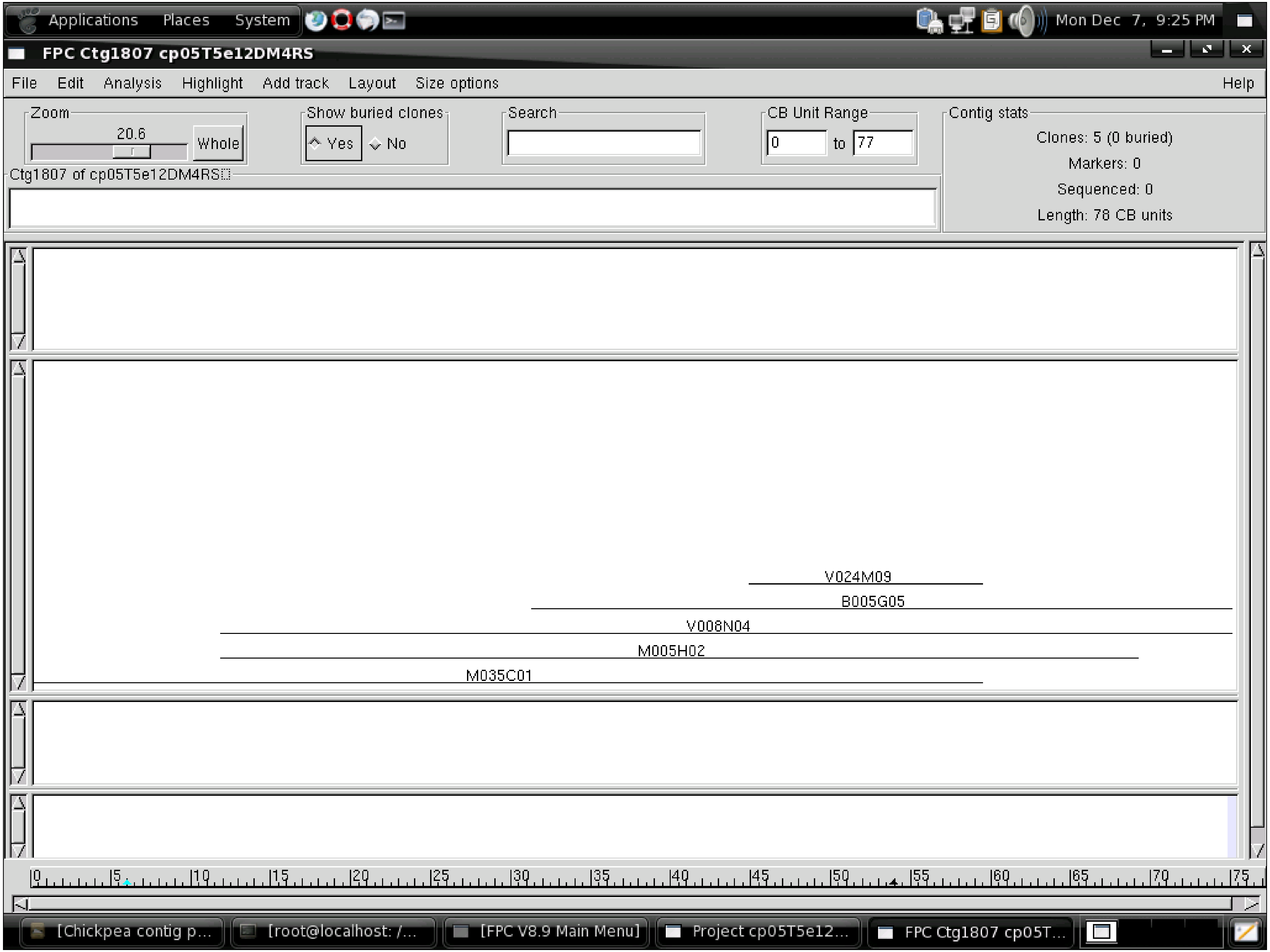
Applications
Places
System
Mon Dec 7, 9:25 PM
FPC Ctg1807 cp05T5e12DM4RS
File
Edit
Analysis
Highlight
Add track
Layout
Size options
Help
Zoom
20.6
Whole
Show buried clones
Yes
No
Search
CB Unit Range
0
to
77
Contig stats
Clones: 5 (0 buried)
Markers: 0
Sequenced: 0
Length: 78 CB units
Ctg1807 of cp05T5e12DM4RS
V024M09
B005G05
V008N04
M005H02
M035C01
0
5
10
15
20
25
30
35
40
45
50
55
60
65
70
75
[Chickpea contig p...
[root@localhost: /...
[FPC V8.9 Main Menu]
Project cp05T5e12...
FPC Ctg1807 cp05T...

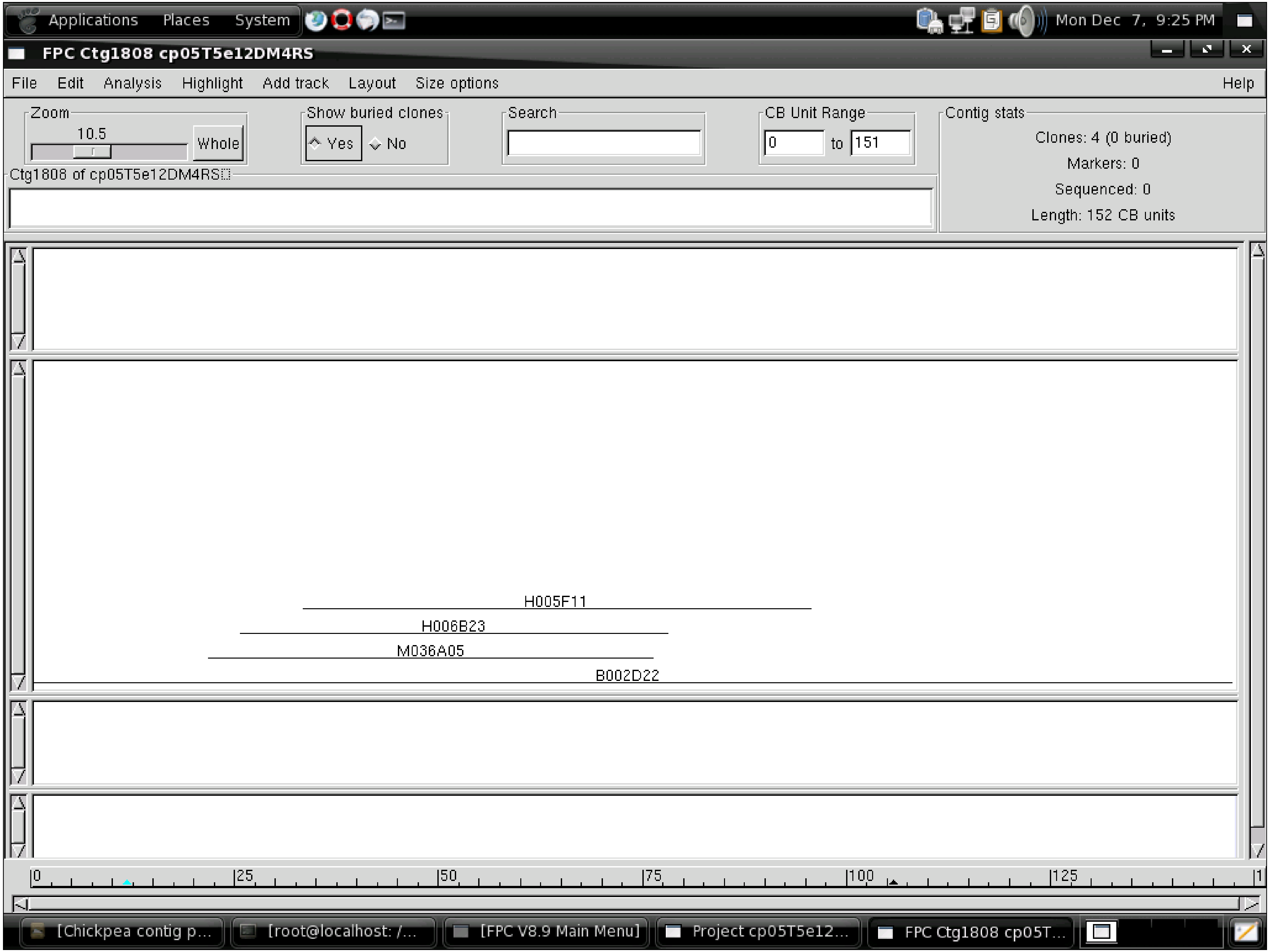
Applications
Places
System
Mon Dec 7, 9:25 PM
FPC Ctg1808 cp05T5e12DM4RS
File
Edit
Analysis
Highlight
Add track
Layout
Size options
Help
Zoom
10.5
Whole
Show buried clones
Yes
No
Search
CB Unit Range
0
to
151
Contig stats
Clones: 4 (0 buried)
Markers: 0
Sequenced: 0
Length: 152 CB units
Ctg1808 of cp05T5e12DM4RS
H005F11
H006B23
M036A05
B002D22
0
25
50
75
100
125
[Chickpea contig p...
[root@localhost: /...
[FPC V8.9 Main Menu]
Project cp05T5e12...
FPC Ctg1808 cp05T...

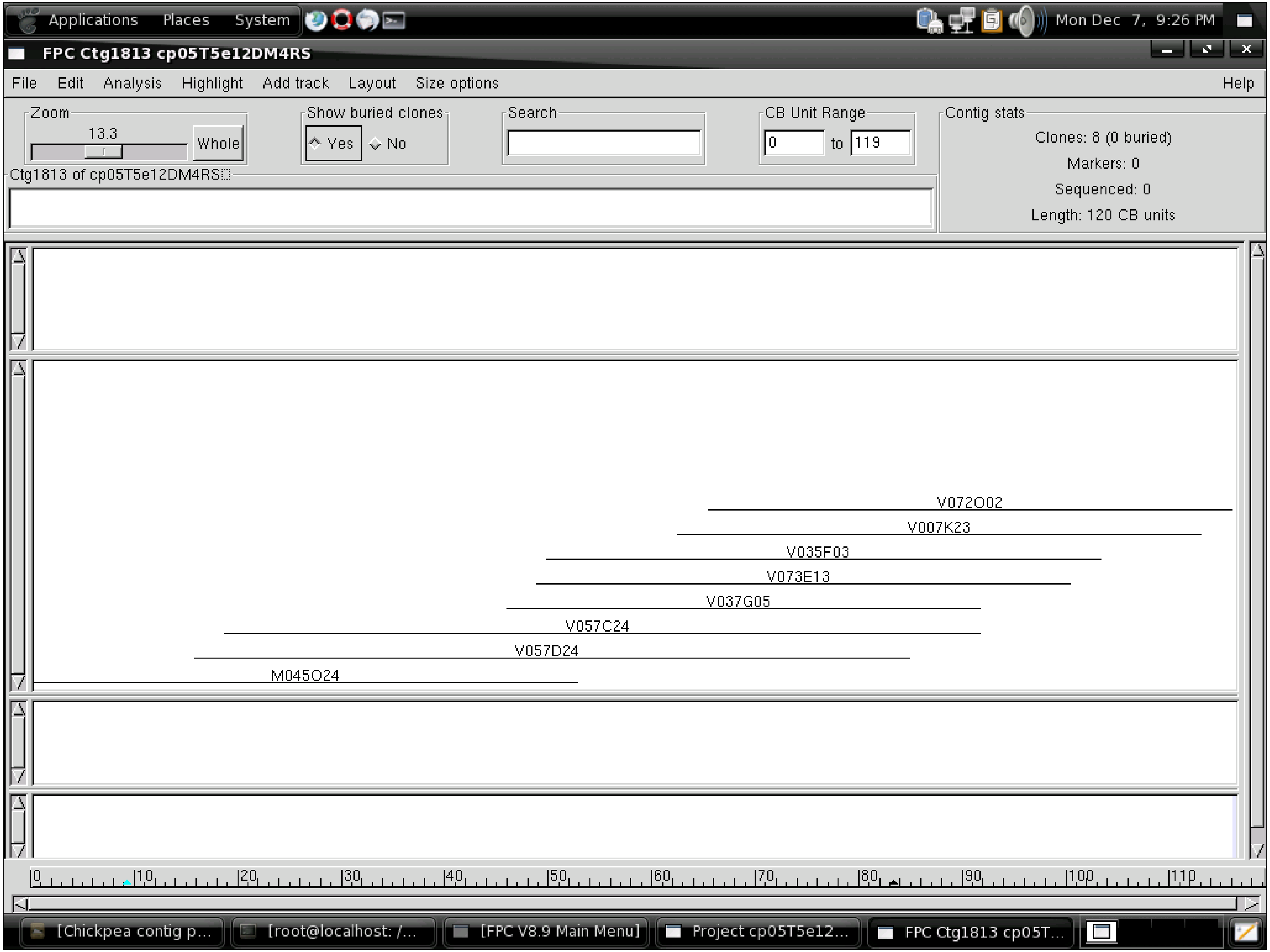
Applications Places System
Mon Dec 7, 9:26 PM
FPC Ctg1813 cp05T5e12DM4RS
File Edit Analysis Highlight Add track Layout Size options Help
Zoom
13.3
Whole
Show buried clones
Yes
No
Search
CB Unit Range
0 to 119
Contig stats
Clones: 8 (0 buried)
Markers: 0
Sequenced: 0
Length: 120 CB units
Ctg1813 of cp05T5e12DM4RS
V072O02
V007K23
V035F03
V073E13
V037G05
V057C24
V057D24
M045O24
0 10 20 30 40 50 60 70 80 90 100 110
[Chickpea contig p...
[root@localhost: /...
[FPC V8.9 Main Menu]
Project cp05T5e12...
FPC Ctg1813 cp05T...

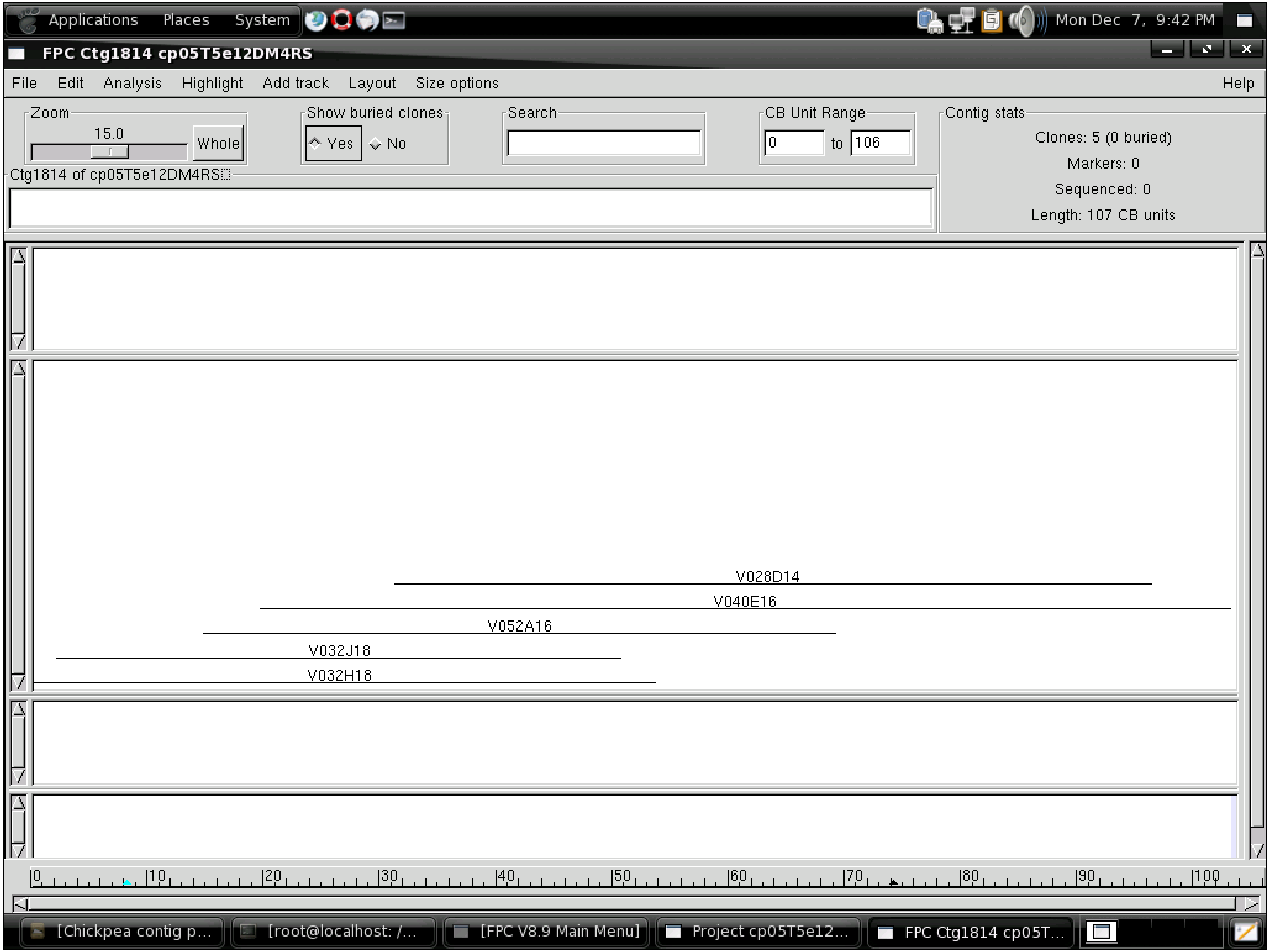
Applications Places System
Mon Dec 7, 9:42 PM
FPC Ctg1814 cp05T5e12DM4RS
File Edit Analysis Highlight Add track Layout Size options Help
Zoom
15.0
Whole
Show buried clones
Yes No
Search
CB Unit Range
0 to 106
Contig stats
Clones: 5 (0 buried)
Markers: 0
Sequenced: 0
Length: 107 CB units
Ctg1814 of cp05T5e12DM4RS
V028D14
V040E16
V052A16
V032J18
V032H18
0 10 20 30 40 50 60 70 80 90 100
[Chickpea contig p...
[root@localhost: /...
[FPC V8.9 Main Menu]
Project cp05T5e12...
FPC Ctg1814 cp05T...

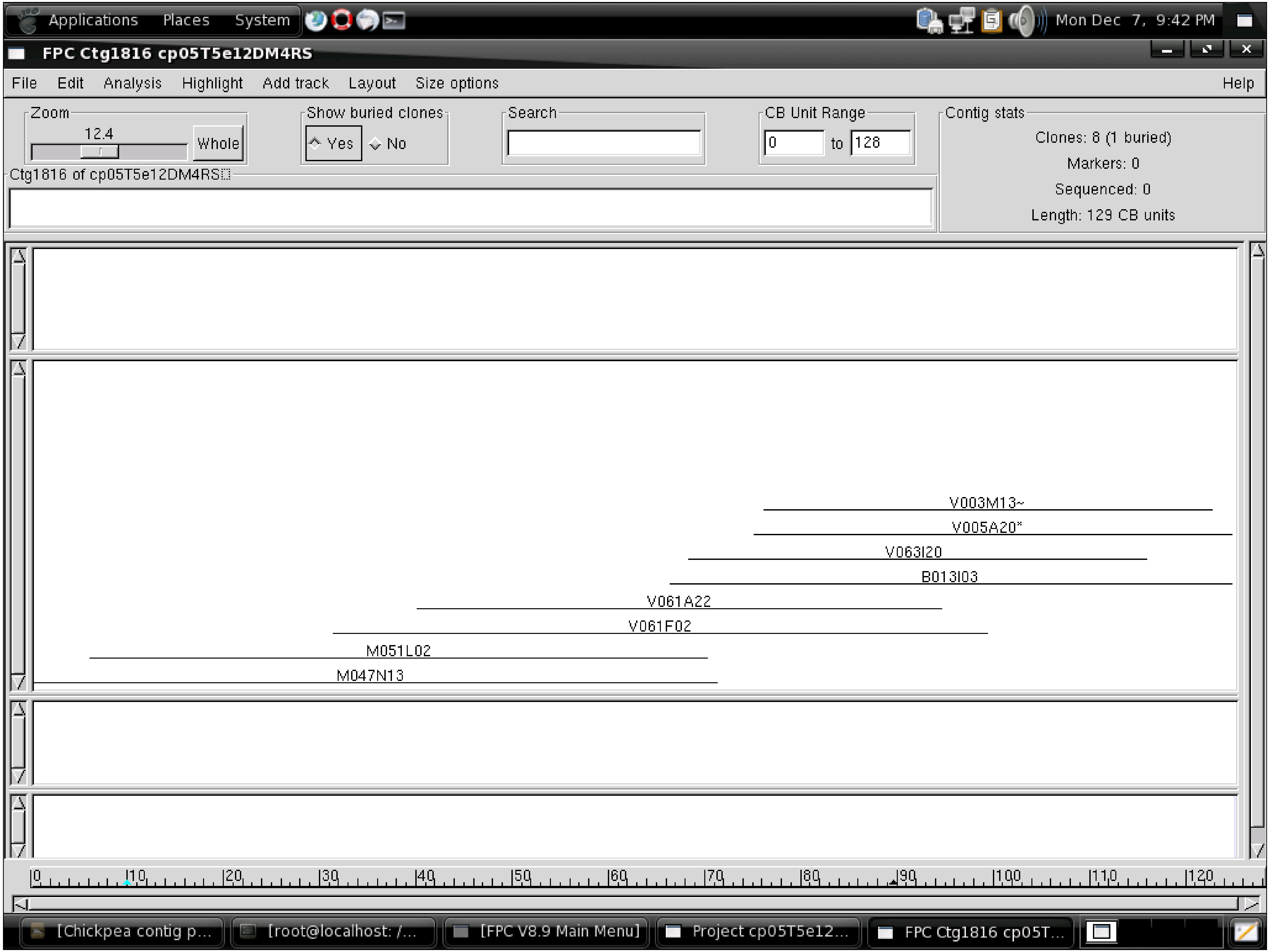
Applications
Places
System
Mon Dec 7, 9:42 PM
FPC Ctg1816 cp05T5e12DM4RS
File
Edit
Analysis
Highlight
Add track
Layout
Size options
Help
Zoom
12.4
Whole
Show buried clones
Yes
No
Search
CB Unit Range
0
to
128
Contig stats
Clones: 8 (1 buried)
Markers: 0
Sequenced: 0
Length: 129 CB units
Ctg1816 of cp05T5e12DM4RS
V003M13~
V005A20*
V063I20
B013I03
V061A22
V061F02
M051L02
M047N13
0 10 20 30 40 50 60 70 80 90 100 110 120
[Chickpea contig p...
[root@localhost: /...
[FPC V8.9 Main Menu]
Project cp05T5e12...
FPC Ctg1816 cp05T...

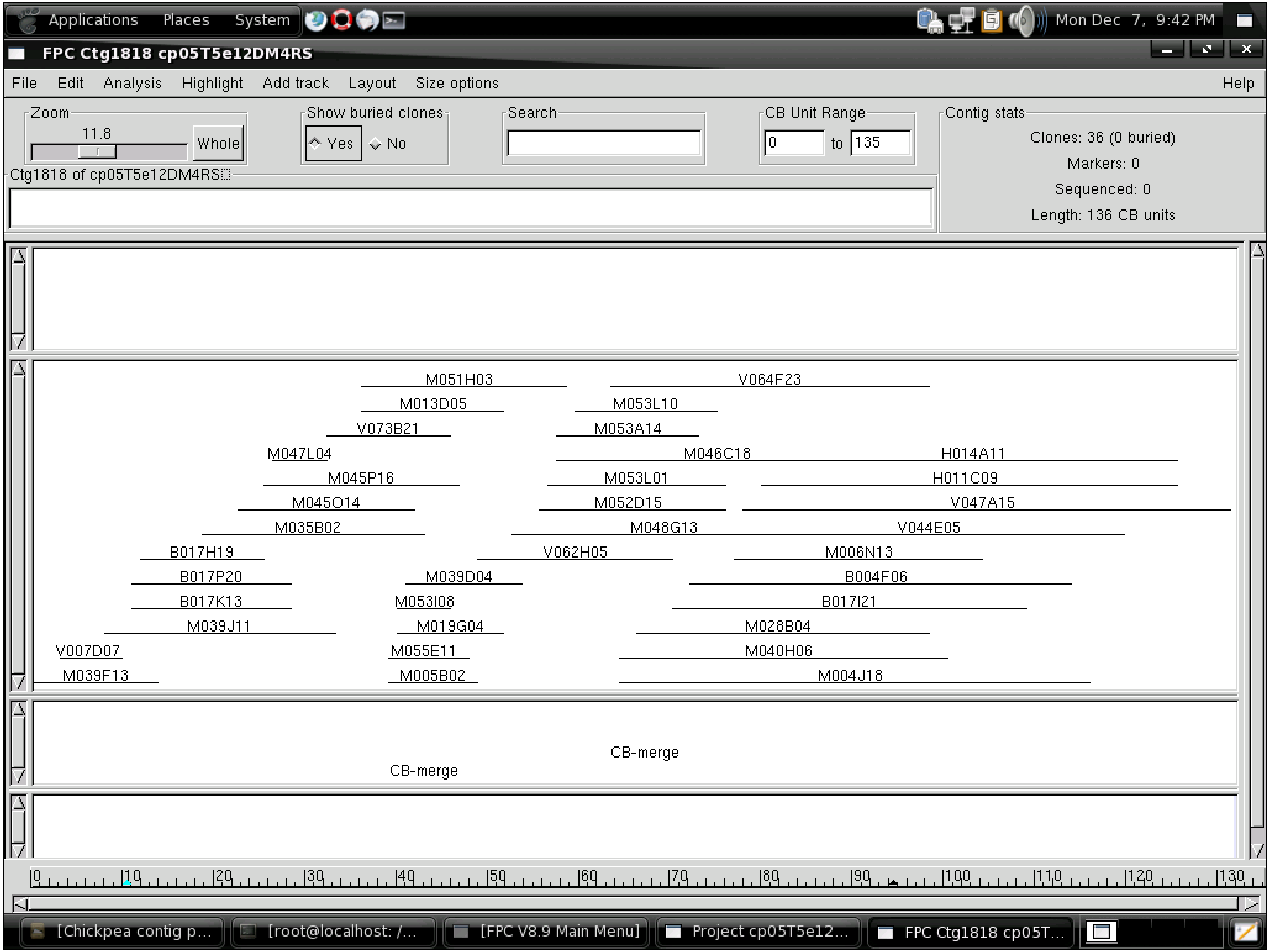
Applications Places System
Mon Dec 7, 9:42 PM
FPC Ctg1818 cp05T5e12DM4RS
File Edit Analysis Highlight Add track Layout Size options Help
Zoom
11.8
Whole
Show buried clones
Yes No
Search
CB Unit Range
0 to 135
Contig stats
Clones: 36 (0 buried)
Markers: 0
Sequenced: 0
Length: 136 CB units
Ctg1818 of cp05T5e12DM4RS
M051H03
V064F23
M013D05
M053L10
V073B21
M053A14
M047L04
M046C18
H014A11
M045P16
M053L01
H011C09
M045O14
M052D15
V047A15
M035B02
M048G13
V044E05
B017H19
V062H05
M006N13
B017P20
M039D04
B004F06
B017K13
M053I08
B017I21
M039J11
M019G04
M028B04
V007D07
M055E11
M040H06
M039F13
M005B02
M004J18
CB-merge
CB-merge
0 10 20 30 40 50 60 70 80 90 100 110 120 130
[Chickpea contig p...
[root@localhost: /...
[FPC V8.9 Main Menu]
Project cp05T5e12...
FPC Ctg1818 cp05T...

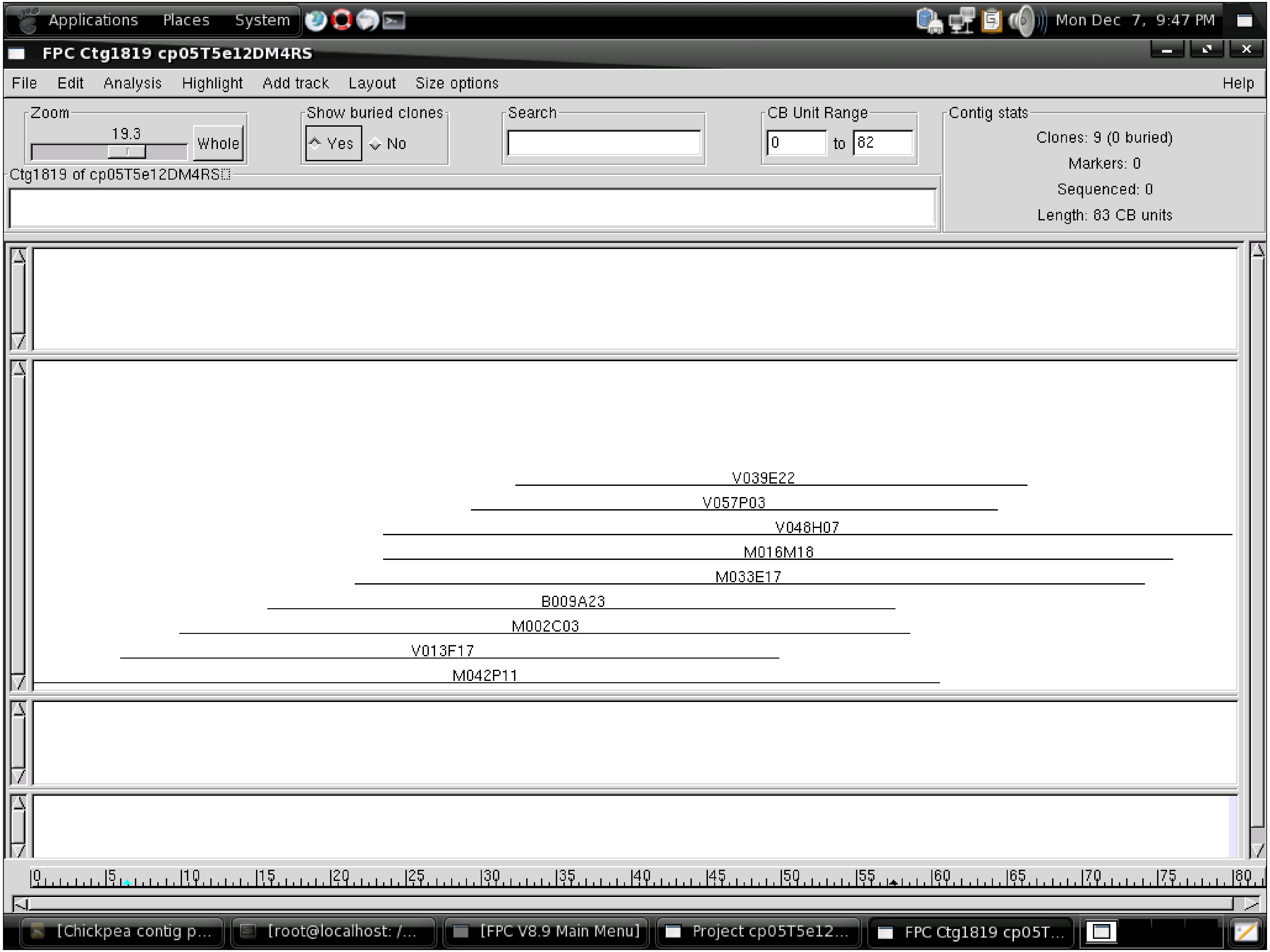

Applications Places System
Mon Dec 7, 9:47 PM
FPC Ctg1819 cp05T5e12DM4RS
File Edit Analysis Highlight Add track Layout Size options Help
Zoom
19.3
Whole
Show buried clones
Yes No
Search
CB Unit Range
0 to 82
Contig stats
Clones: 9 (0 buried)
Markers: 0
Sequenced: 0
Length: 83 CB units
Ctg1819 of cp05T5e12DM4RS
V039E22
V057P03
V048H07
M016M18
M033E17
B009A23
M002C03
V013F17
M042P11
0 5 10 15 20 25 30 35 40 45 50 55 60 65 70 75 80
[Chickpea contig p...
[root@localhost: /...
[FPC V8.9 Main Menu]
Project cp05T5e12...
FPC Ctg1819 cp05T...

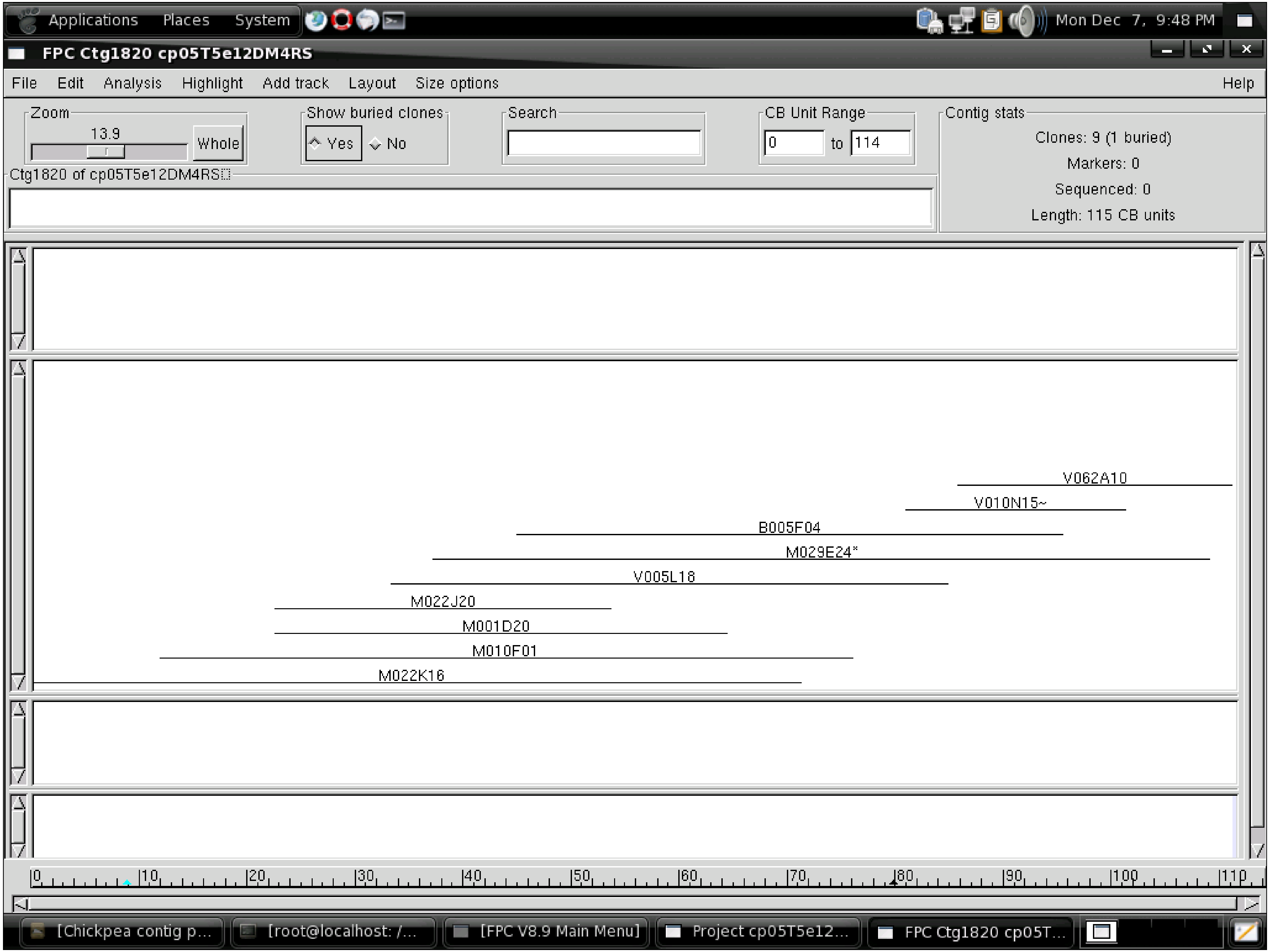
Applications Places System
Mon Dec 7, 9:48 PM
FPC Ctg1820 cp05T5e12DM4RS
File Edit Analysis Highlight Add track Layout Size options Help
Zoom
13.9
Whole
Show buried clones
Yes No
Search
CB Unit Range
0 to 114
Contig stats
Clones: 9 (1 buried)
Markers: 0
Sequenced: 0
Length: 115 CB units
Ctg1820 of cp05T5e12DM4RS
V062A10
V010N15~
B005F04
M029E24*
V005L18
M022J20
M001D20
M010F01
M022K16
0 10 20 30 40 50 60 70 80 90 100 110
[Chickpea contig p...
[root@localhost: /...
[FPC V8.9 Main Menu]
Project cp05T5e12...
FPC Ctg1820 cp05T...

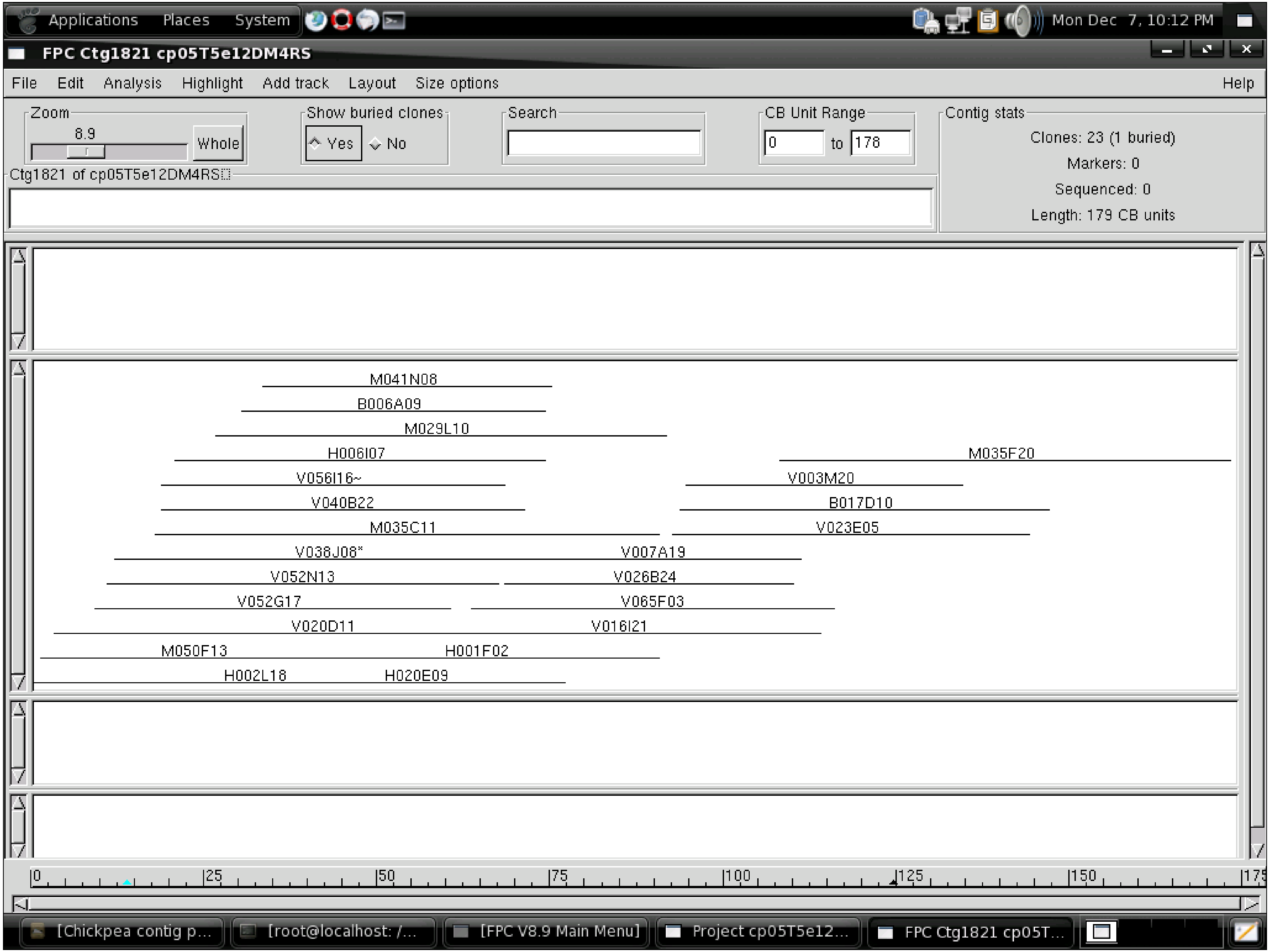
Applications Places System
Mon Dec 7, 10:12 PM
FPC Ctg1821 cp05T5e12DM4RS
File Edit Analysis Highlight Add track Layout Size options Help
Zoom
8.9
Whole
Show buried clones
Yes No
Search
CB Unit Range
0 to 178
Contig stats
Clones: 23 (1 buried)
Markers: 0
Sequenced: 0
Length: 179 CB units
Ctg1821 of cp05T5e12DM4RS
M041N08
B006A09
M029L10
H006I07
M035F20
V056I16~
V003M20
V040B22
B017D10
M035C11
V023E05
V038J08*
V007A19
V052N13
V026B24
V052G17
V065F03
V020D11
V016I21
M050F13
H001F02
H002L18
H020E09
0 25 50 75 100 125 150 175
[Chickpea contig p...
[root@localhost: /...
[FPC V8.9 Main Menu]
Project cp05T5e12...
FPC Ctg1821 cp05T...

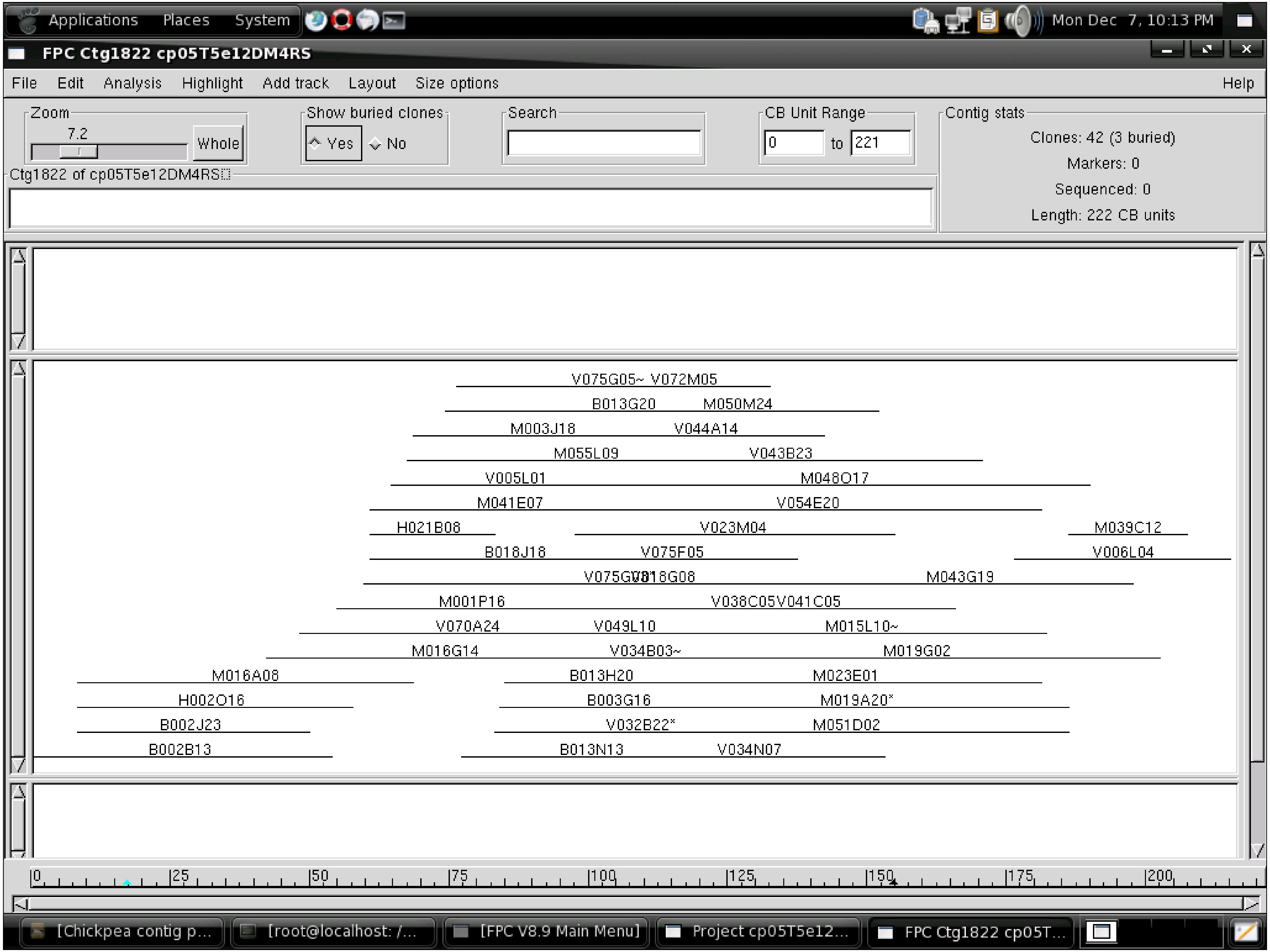

Applications Places System
Mon Dec 7, 10:13 PM
FPC Ctg1822 cp05T5e12DM4RS
File Edit Analysis Highlight Add track Layout Size options Help
Zoom 7.2 Whole
Show buried clones Yes No
Search
CB Unit Range 0 to 221
Contig stats
Clones: 42 (3 buried)
Markers: 0
Sequenced: 0
Length: 222 CB units
Ctg1822 of cp05T5e12DM4RS
V075G05~ V072M05
B013G20 M050M24
M003J18 V044A14
M055L09 V043B23
V005L01 M048O17
M041E07 V054E20
H021B08 V023M04 M039C12
B018J18 V075F05 V006L04
M043G19
M001P16 V038C05V041C05
V070A24 V049L10 M015L10~
M016G14 V034B03~ M019G02
M016A08 B013H20 M023E01
H002O16 B003G16 M019A20*
B002J23 V032B22* M051D02
B002B13 B013N13 V034N07
0 25 50 75 100 125 150 175 200
[Chickpea contig p... [root@localhost: /... [FPC V8.9 Main Menu] Project cp05T5e12... FPC Ctg1822 cp05T...

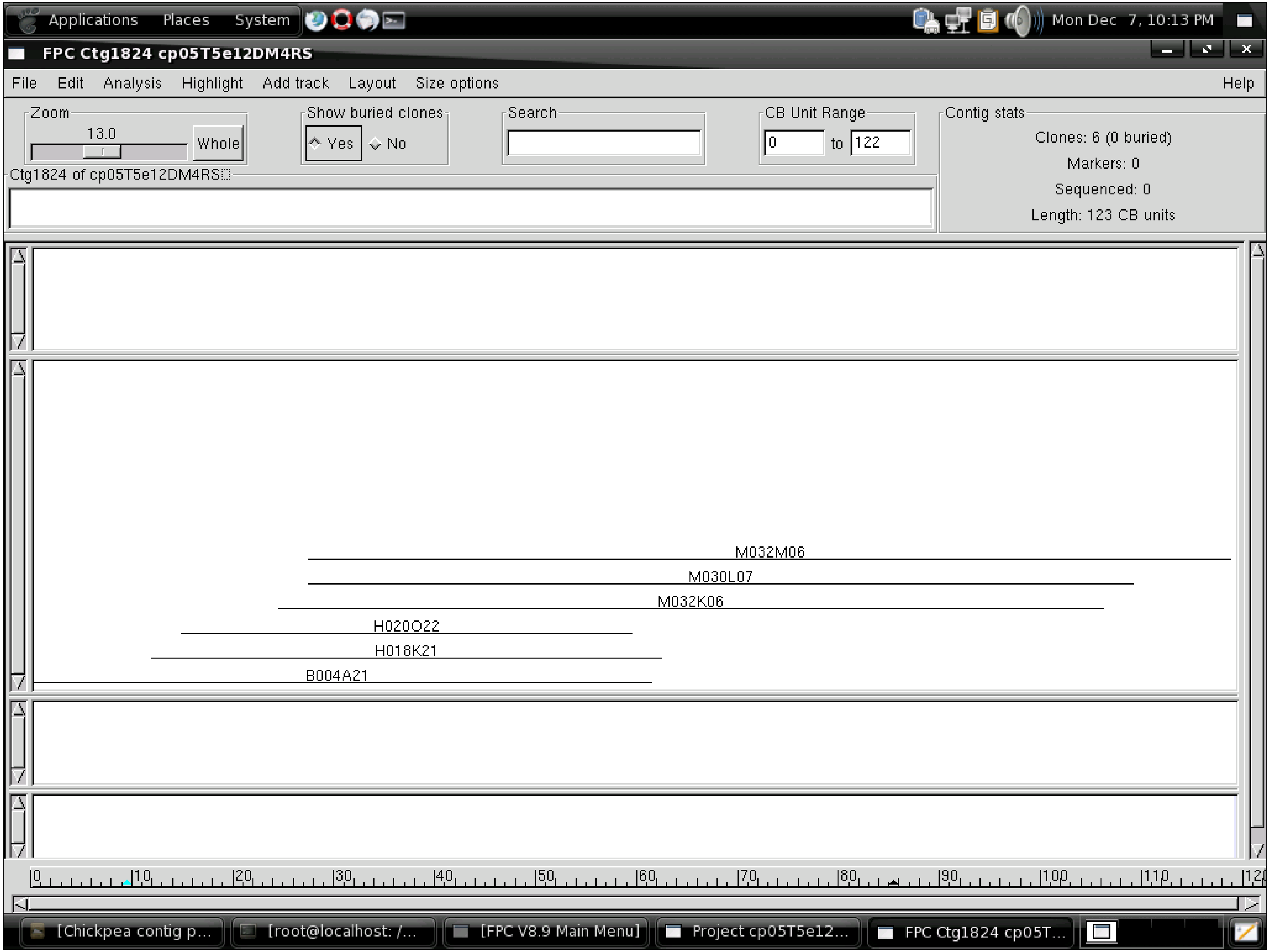
Applications Places System
Mon Dec 7, 10:13 PM
FPC Ctg1824 cp05T5e12DM4RS
File Edit Analysis Highlight Add track Layout Size options Help
Zoom
13.0
Whole
Show buried clones
Yes No
Search
CB Unit Range
0 to 122
Contig stats
Clones: 6 (0 buried)
Markers: 0
Sequenced: 0
Length: 123 CB units
Ctg1824 of cp05T5e12DM4RS
M032M06
M030L07
M032K06
H020O22
H018K21
B004A21
0 10 20 30 40 50 60 70 80 90 100 110
[Chickpea contig p...
[root@localhost: /...
[FPC V8.9 Main Menu]
Project cp05T5e12...
FPC Ctg1824 cp05T...

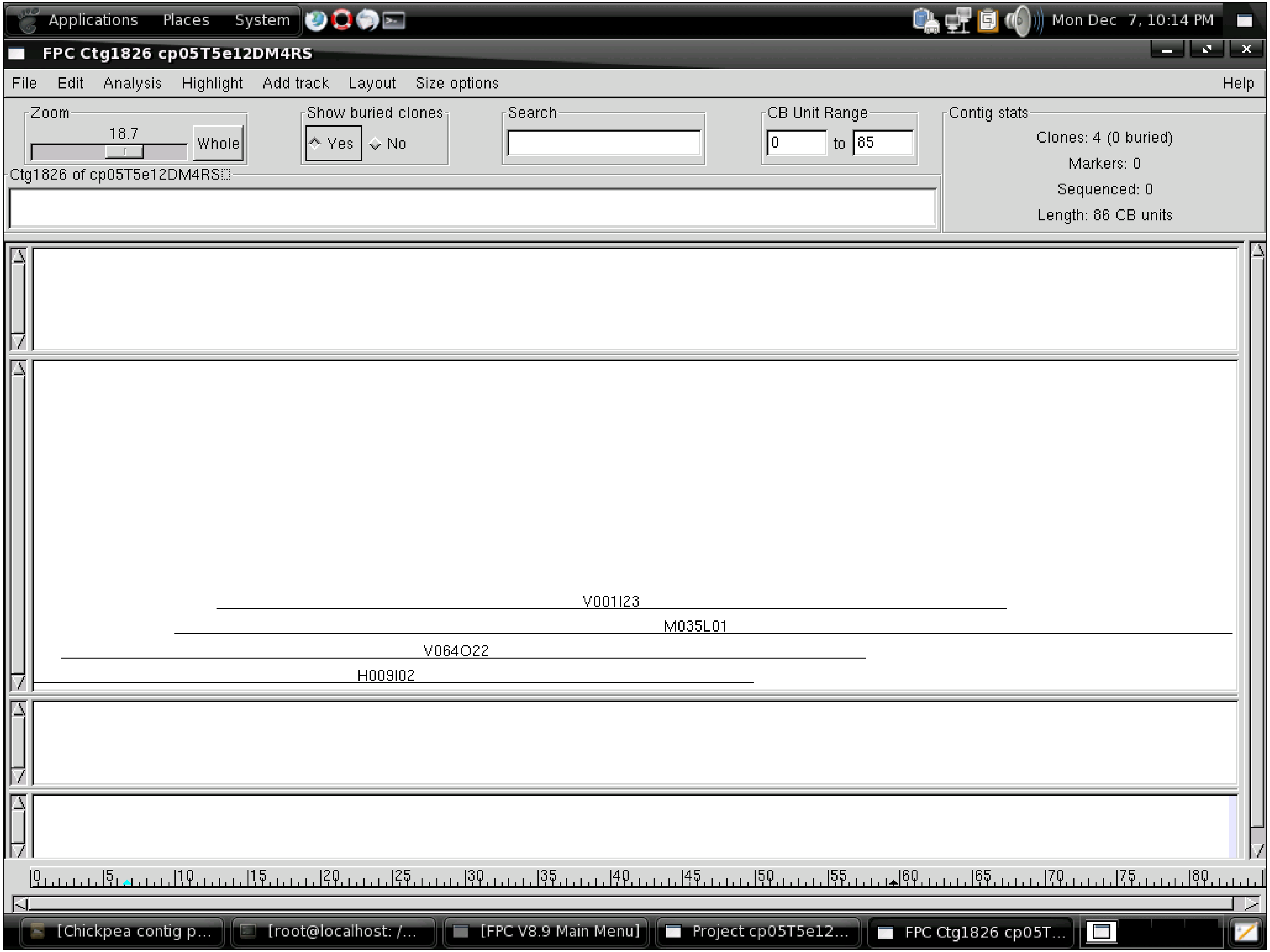

Applications Places System
Mon Dec 7, 10:14 PM
FPC Ctg1826 cp05T5e12DM4RS
File Edit Analysis Highlight Add track Layout Size options Help
Zoom
18.7
Whole
Show buried clones
Yes
No
Search
CB Unit Range
0
to
85
Contig stats
Clones: 4 (0 buried)
Markers: 0
Sequenced: 0
Length: 86 CB units
Ctg1826 of cp05T5e12DM4RS
V001I23
M035L01
V064O22
H009I02
0 5 10 15 20 25 30 35 40 45 50 55 60 65 70 75 80
[Chickpea contig p...
[root@localhost: /...
[FPC V8.9 Main Menu]
Project cp05T5e12...
FPC Ctg1826 cp05T...

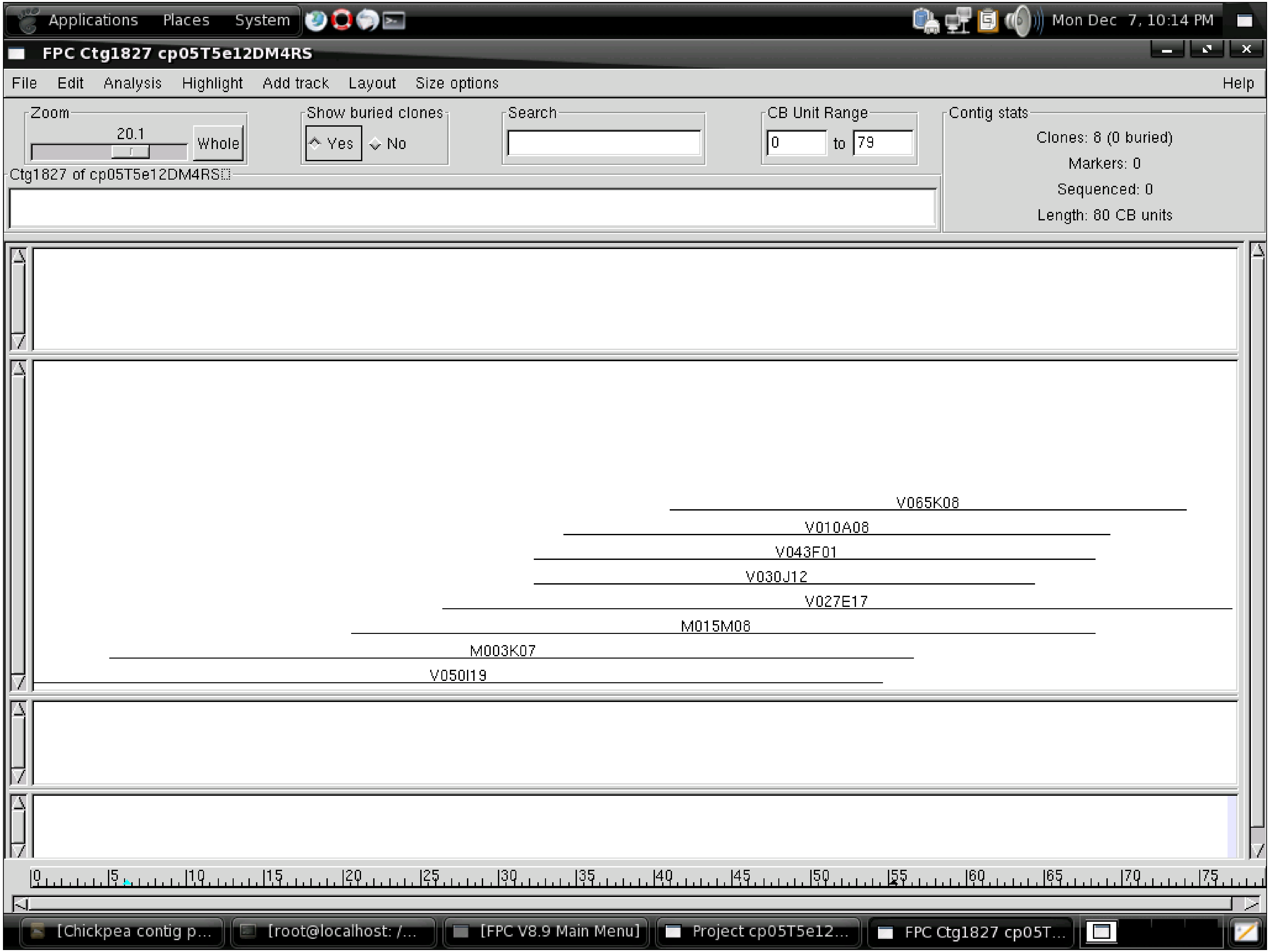
Applications Places System
Mon Dec 7, 10:14 PM
FPC Ctg1827 cp05T5e12DM4RS
File Edit Analysis Highlight Add track Layout Size options Help
Zoom
20.1
Whole
Show buried clones
Yes No
Search
CB Unit Range
0 to 79
Contig stats
Clones: 8 (0 buried)
Markers: 0
Sequenced: 0
Length: 80 CB units
Ctg1827 of cp05T5e12DM4RS
V065K08
V010A08
V043F01
V030J12
V027E17
M015M08
M003K07
V050I19
0 5 10 15 20 25 30 35 40 45 50 55 60 65 70 75
[Chickpea contig p...
[root@localhost: /...
[FPC V8.9 Main Menu]
Project cp05T5e12...
FPC Ctg1827 cp05T...

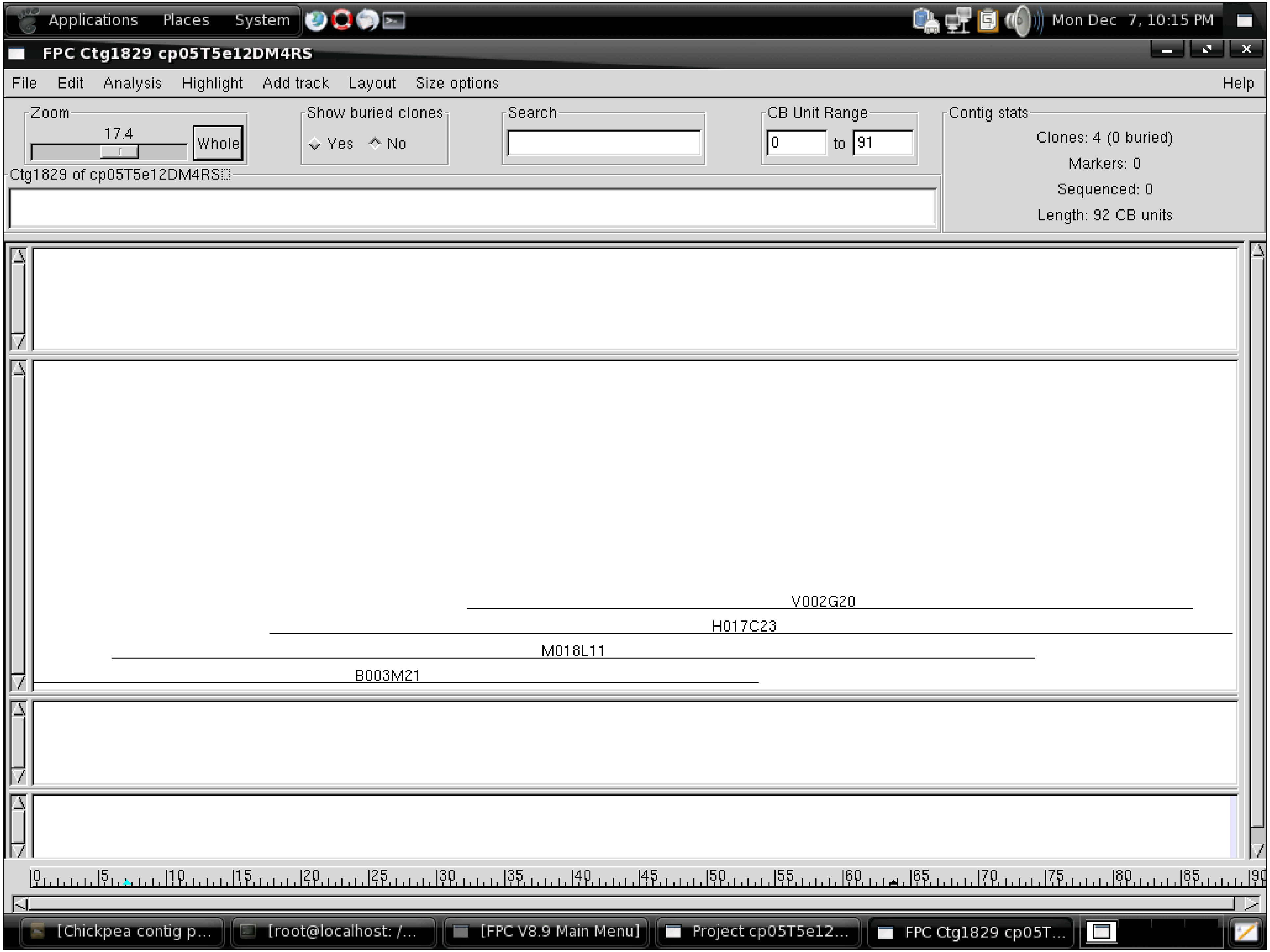
Applications Places System
Mon Dec 7, 10:15 PM
FPC Ctg1829 cp05T5e12DM4RS
File Edit Analysis Highlight Add track Layout Size options
Help
Zoom
17.4
Whole
Show buried clones
Yes No
Search
CB Unit Range
0 to 91
Contig stats
Clones: 4 (0 buried)
Markers: 0
Sequenced: 0
Length: 92 CB units
Ctg1829 of cp05T5e12DM4RS
V002G20
H017C23
M018L11
B003M21
0 5 10 15 20 25 30 35 40 45 50 55 60 65 70 75 80 85 90
[Chickpea contig p...
[root@localhost: /...
[FPC V8.9 Main Menu]
Project cp05T5e12...
FPC Ctg1829 cp05T...

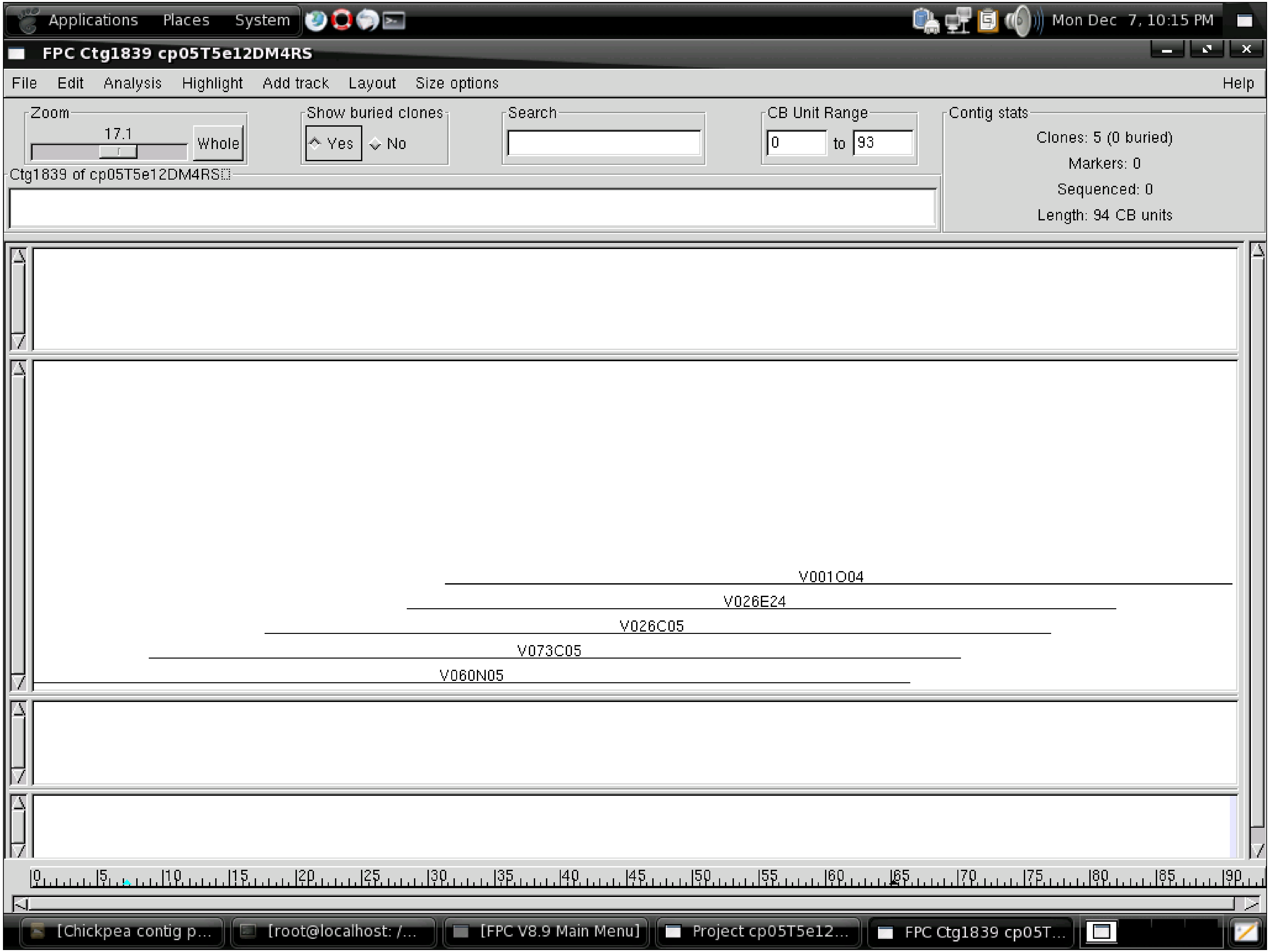
Applications Places System
Mon Dec 7, 10:15 PM
FPC Ctg1839 cp05T5e12DM4RS
File Edit Analysis Highlight Add track Layout Size options Help
Zoom
17.1
Whole
Show buried clones
Yes No
Search
CB Unit Range
0 to 93
Contig stats
Clones: 5 (0 buried)
Markers: 0
Sequenced: 0
Length: 94 CB units
Ctg1839 of cp05T5e12DM4RS
V001O04
V026E24
V026C05
V073C05
V060N05
0 5 10 15 20 25 30 35 40 45 50 55 60 65 70 75 80 85 90
[Chickpea contig p...
[root@localhost: /...
[FPC V8.9 Main Menu]
Project cp05T5e12...
FPC Ctg1839 cp05T...

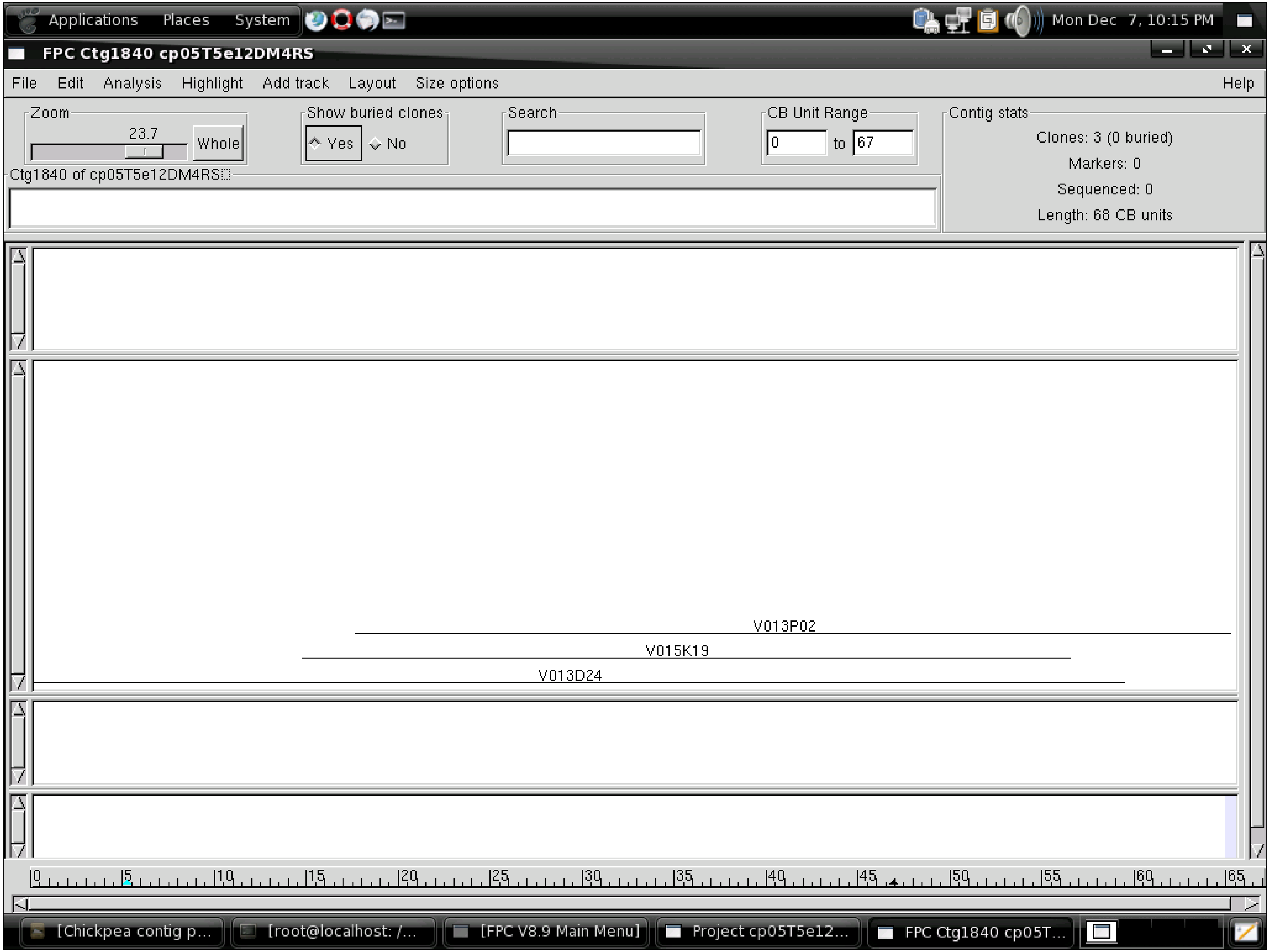
Applications Places System
Mon Dec 7, 10:15 PM
FPC Ctg1840 cp05T5e12DM4RS
File Edit Analysis Highlight Add track Layout Size options Help
Zoom
23.7
Whole
Show buried clones
Yes No
Search
CB Unit Range
0 to 67
Contig stats
Clones: 3 (0 buried)
Markers: 0
Sequenced: 0
Length: 68 CB units
Ctg1840 of cp05T5e12DM4RS
V013P02
V015K19
V013D24
0 5 10 15 20 25 30 35 40 45 50 55 60 65
[Chickpea contig p...
[root@localhost: /...
[FPC V8.9 Main Menu]
Project cp05T5e12...
FPC Ctg1840 cp05T...

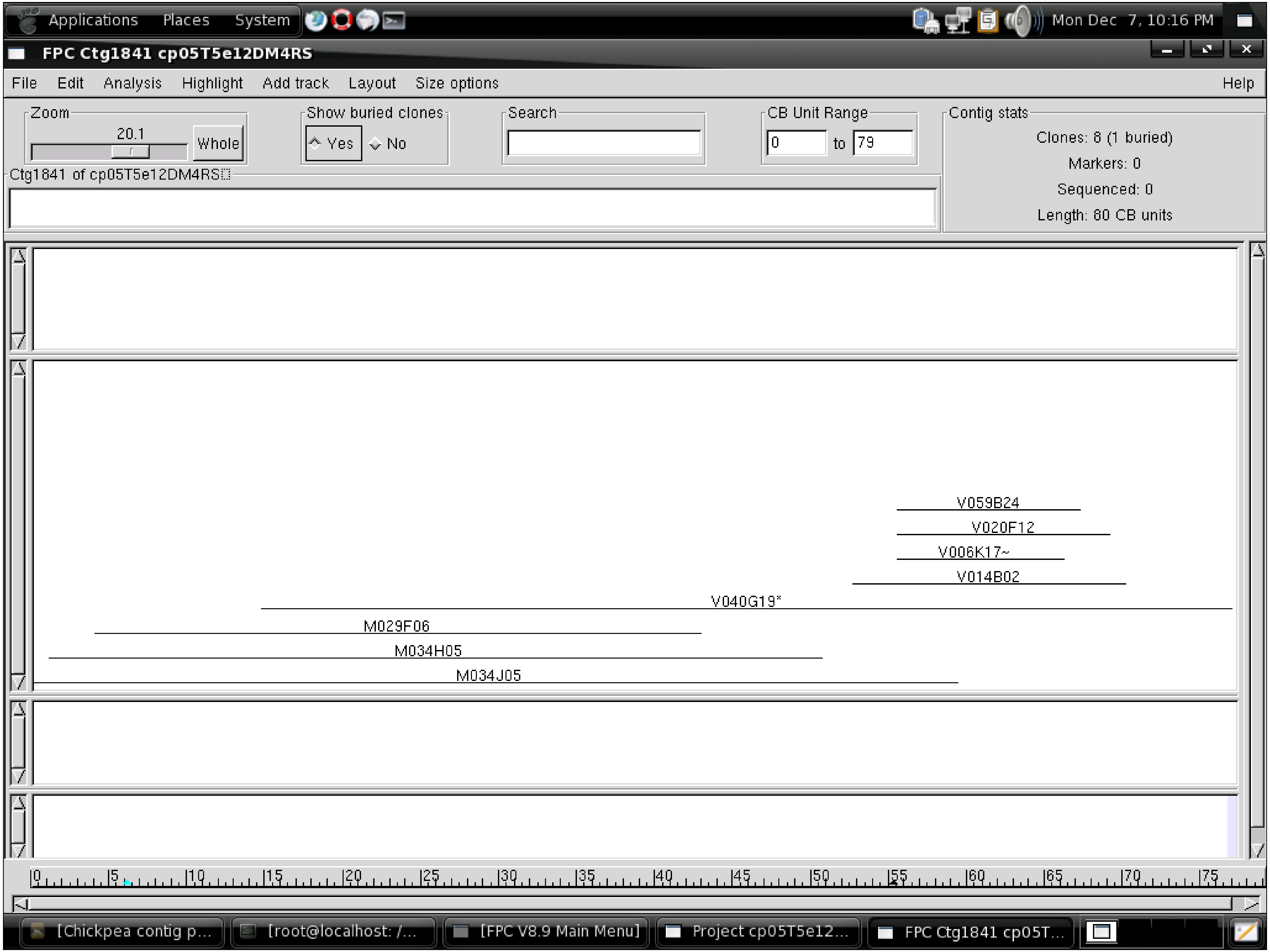

Applications Places System
Mon Dec 7, 10:16 PM
FPC Ctg1841 cp05T5e12DM4RS
File Edit Analysis Highlight Add track Layout Size options Help
Zoom 20.1 Whole
Show buried clones Yes No
Search
CB Unit Range 0 to 79
Contig stats
Clones: 8 (1 buried)
Markers: 0
Sequenced: 0
Length: 80 CB units
Ctg1841 of cp05T5e12DM4RS
V059B24
V020F12
V006K17~
V014B02
V040G19*
M029F06
M034H05
M034J05
0 5 10 15 20 25 30 35 40 45 50 55 60 65 70 75
[Chickpea contig p...
[root@localhost: /...
[FPC V8.9 Main Menu]
Project cp05T5e12...
FPC Ctg1841 cp05T...

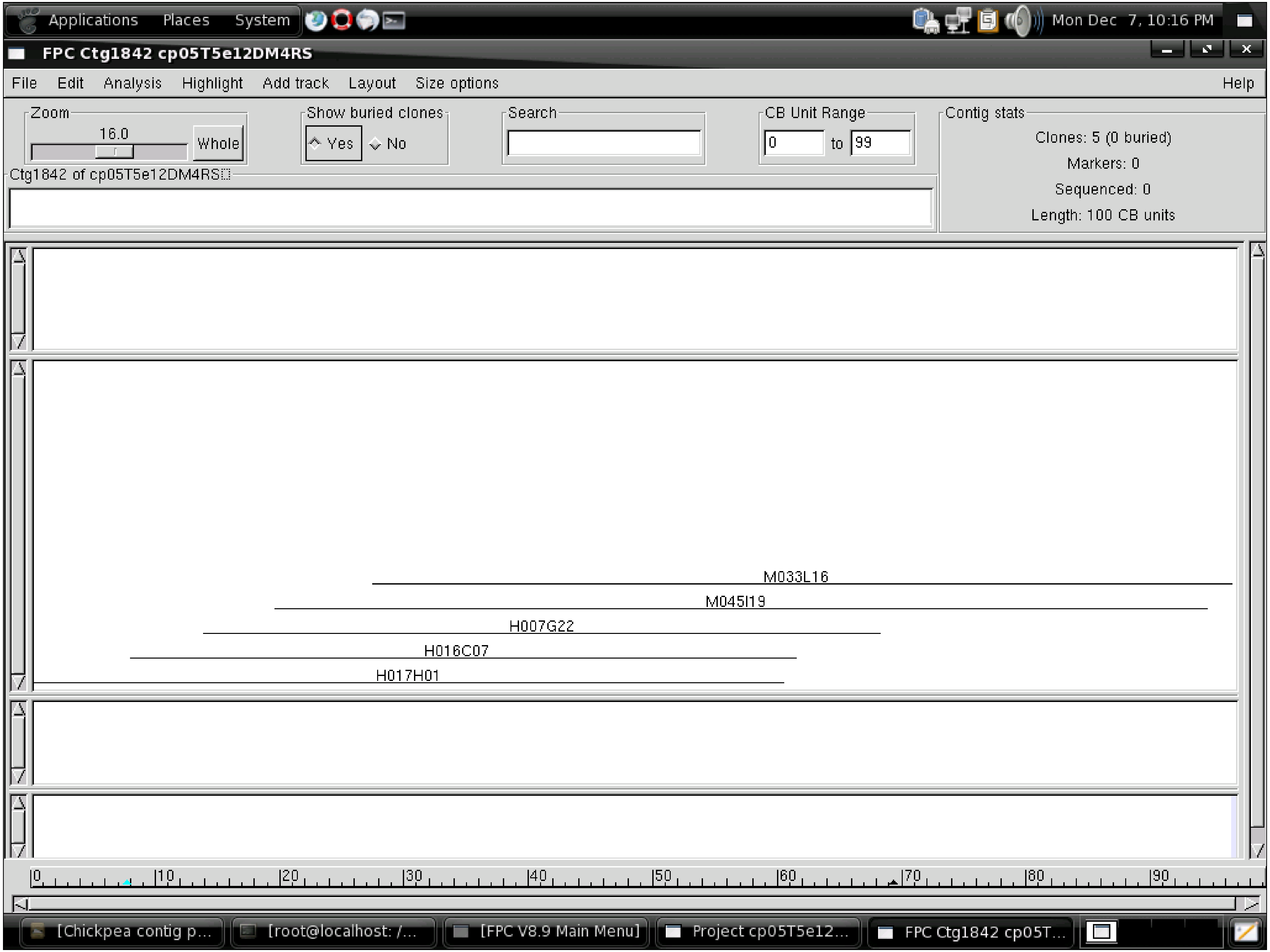
Applications Places System
Mon Dec 7, 10:16 PM
FPC Ctg1842 cp05T5e12DM4RS
File Edit Analysis Highlight Add track Layout Size options Help
Zoom
16.0
Whole
Show buried clones
Yes No
Search
CB Unit Range
0 to 99
Contig stats
Clones: 5 (0 buried)
Markers: 0
Sequenced: 0
Length: 100 CB units
Ctg1842 of cp05T5e12DM4RS
M033L16
M045I19
H007G22
H016C07
H017H01
0 10 20 30 40 50 60 70 80 90
[Chickpea contig p...
[root@localhost: /...
[FPC V8.9 Main Menu]
Project cp05T5e12...
FPC Ctg1842 cp05T...

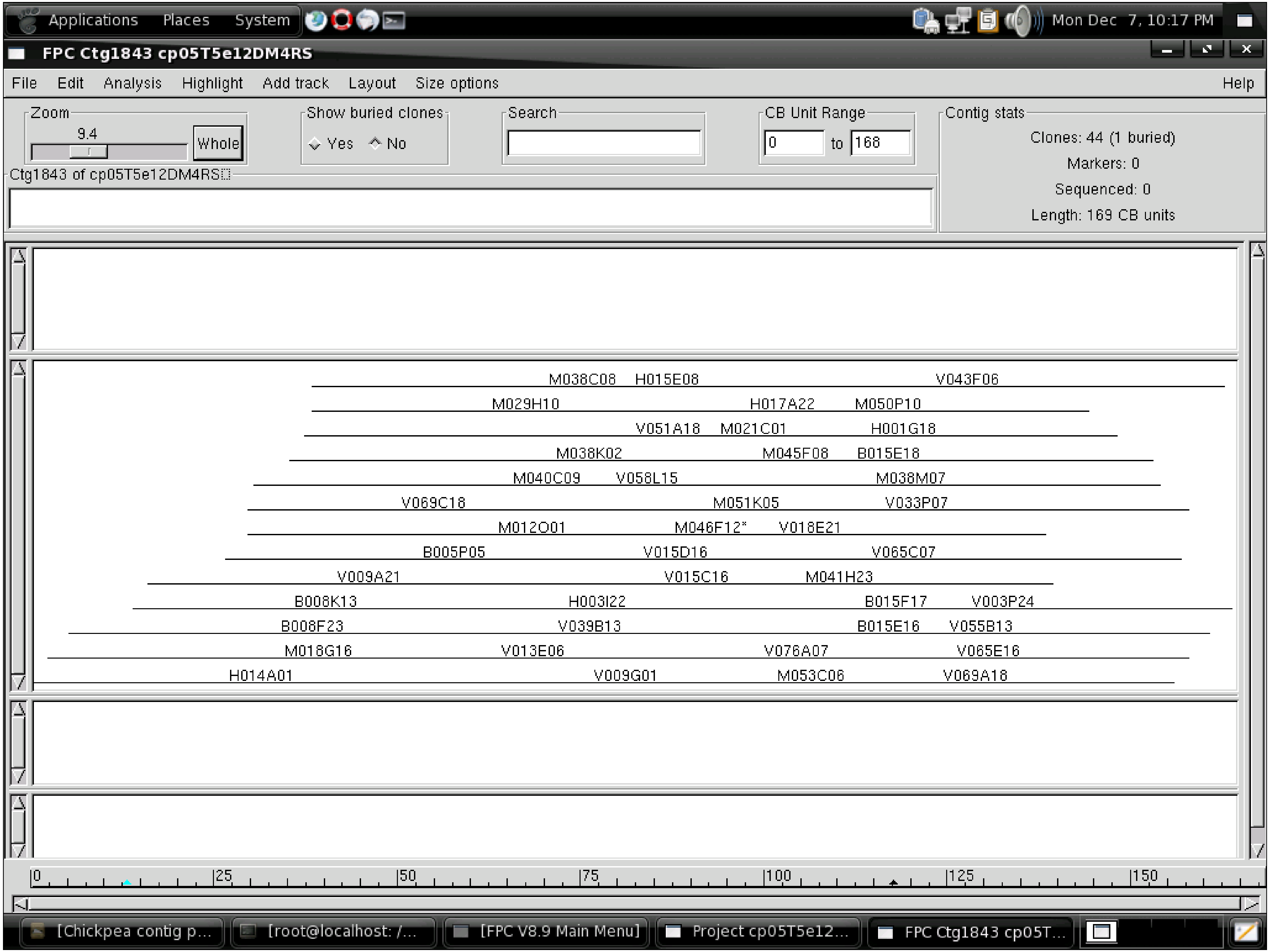
Applications Places System
Mon Dec 7, 10:17 PM
FPC Ctg1843 cp05T5e12DM4RS
File Edit Analysis Highlight Add track Layout Size options Help
Zoom
9.4
Whole
Show buried clones
Yes No
Search
CB Unit Range
0 to 168
Contig stats
Clones: 44 (1 buried)
Markers: 0
Sequenced: 0
Length: 169 CB units
Ctg1843 of cp05T5e12DM4RS
M038C08 H015E08 V043F06
M029H10 H017A22 M050P10
V051A18 M021C01 H001G18
M038K02 M045F08 B015E18
M040C09 V058L15 M038M07
V069C18 M051K05 V033P07
M012O01 M046F12* V018E21
B005P05 V015D16 V065C07
V009A21 V015C16 M041H23
B008K13 H003I22 B015F17 V003P24
B008F23 V039B13 B015E16 V055B13
M018G16 V013E06 V076A07 V065E16
H014A01 V009G01 M053C06 V069A18
0 25 50 75 100 125 150
[Chickpea contig p... [root@localhost: /... [FPC V8.9 Main Menu] Project cp05T5e12... FPC Ctg1843 cp05T...

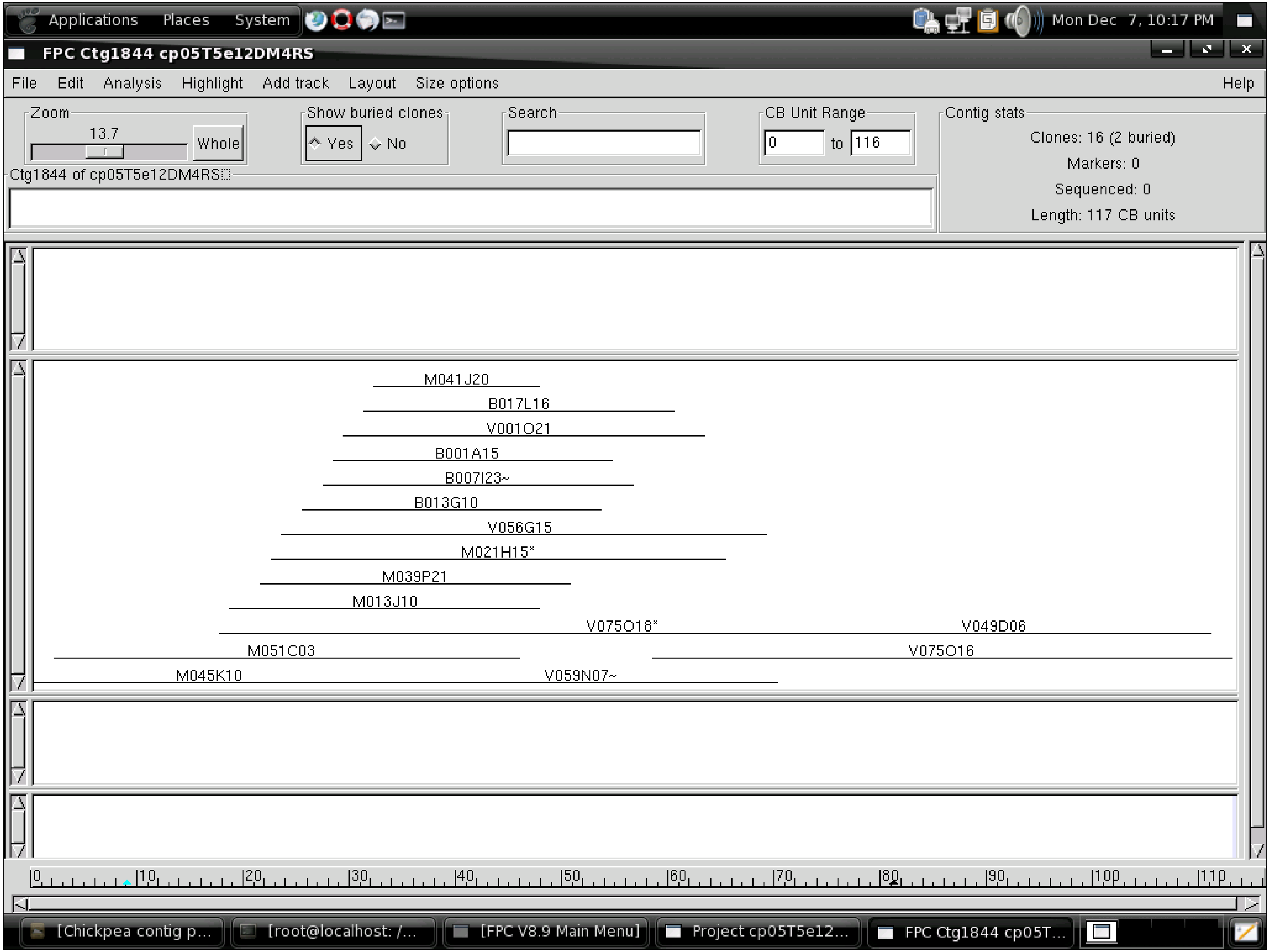

Applications Places System
Mon Dec 7, 10:17 PM
FPC Ctg1844 cp05T5e12DM4RS
File Edit Analysis Highlight Add track Layout Size options Help
Zoom
13.7
Whole
Show buried clones
Yes No
Search
CB Unit Range
0 to 116
Contig stats
Clones: 16 (2 buried)
Markers: 0
Sequenced: 0
Length: 117 CB units
Ctg1844 of cp05T5e12DM4RS
M041J20
B017L16
V001O21
B001A15
B007I23~
B013G10
V056G15
M021H15*
M039P21
M013J10
V075O18*
V049D06
M051C03
V075O16
M045K10
V059N07~
0 10 20 30 40 50 60 70 80 90 100 110
[Chickpea contig p...
[root@localhost: /...
[FPC V8.9 Main Menu]
Project cp05T5e12...
FPC Ctg1844 cp05T...

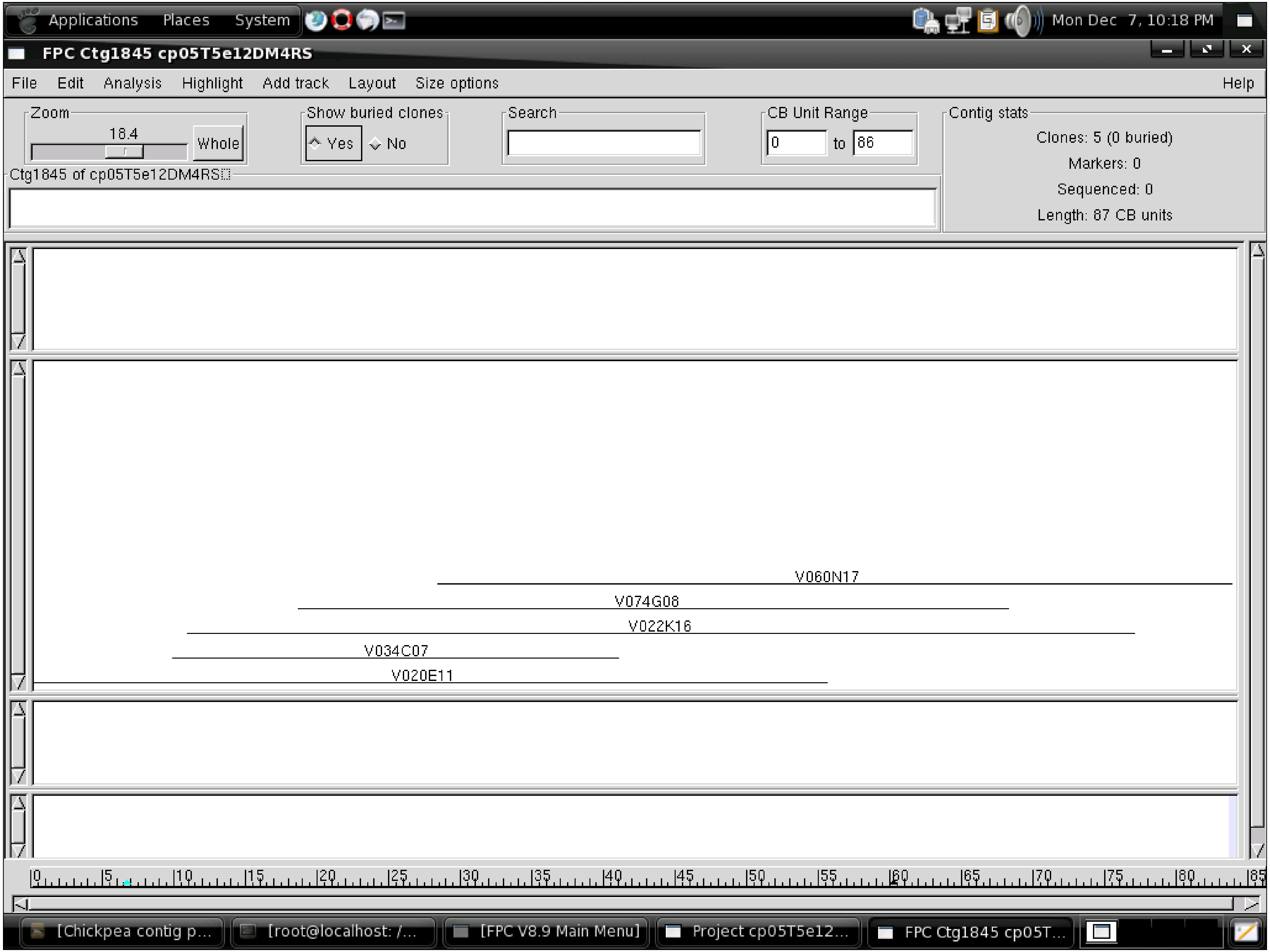
Applications Places System
Mon Dec 7, 10:18 PM
FPC Ctg1845 cp05T5e12DM4RS
File Edit Analysis Highlight Add track Layout Size options Help
Zoom
18.4
Whole
Show buried clones
Yes No
Search
CB Unit Range
0 to 86
Contig stats
Clones: 5 (0 buried)
Markers: 0
Sequenced: 0
Length: 87 CB units
Ctg1845 of cp05T5e12DM4RS
V060N17
V074G08
V022K16
V034C07
V020E11
0 5 10 15 20 25 30 35 40 45 50 55 60 65 70 75 80 85
[Chickpea contig p...
[root@localhost: /...
[FPC V8.9 Main Menu]
Project cp05T5e12...
FPC Ctg1845 cp05T...

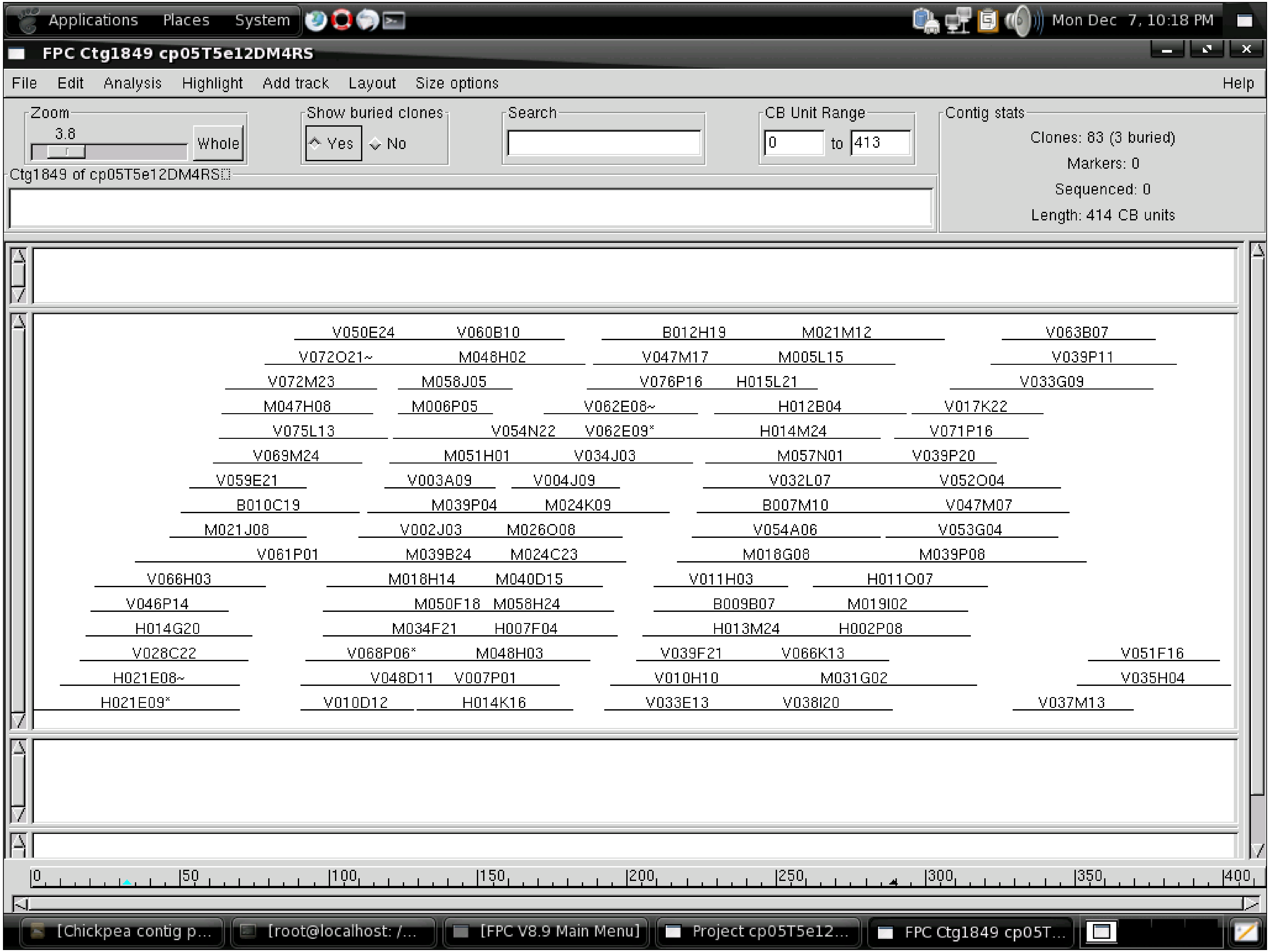
Applications Places System
Mon Dec 7, 10:18 PM
FPC Ctg1849 cp05T5e12DM4RS
File Edit Analysis Highlight Add track Layout Size options Help
Zoom
3.8
Whole
Show buried clones
Yes
No
Search
CB Unit Range
0
to
413
Contig stats
Clones: 83 (3 buried)
Markers: 0
Sequenced: 0
Length: 414 CB units
Ctg1849 of cp05T5e12DM4RS
V050E24 V060B10 B012H19 M021M12 V063B07
V072O21~ M048H02 V047M17 M005L15 V039P11
V072M23 M058J05 V076P16 H015L21 V033G09
M047H08 M006P05 V062E08~ H012B04 V017K22
V075L13 V054N22 V062E09* H014M24 V071P16
V069M24 M051H01 V034J03 M057N01 V039P20
V059E21 V003A09 V004J09 V032L07 V052O04
B010C19 M039P04 M024K09 B007M10 V047M07
M021J08 V002J03 M026O08 V054A06 V053G04
V061P01 M039B24 M024C23 M018G08 M039P08
V066H03 M018H14 M040D15 V011H03 H011O07
V046P14 M050F18 M058H24 B009B07 M019I02
H014G20 M034F21 H007F04 H013M24 H002P08
V028C22 V068P06* M048H03 V039F21 V066K13 V051F16
H021E08~ V048D11 V007P01 V010H10 M031G02 V035H04
H021E09* V010D12 H014K16 V033E13 V038I20 V037M13
0 50 100 150 200 250 300 350 400
[Chickpea contig p...
[root@localhost: /...
[FPC V8.9 Main Menu]
Project cp05T5e12...
FPC Ctg1849 cp05T...

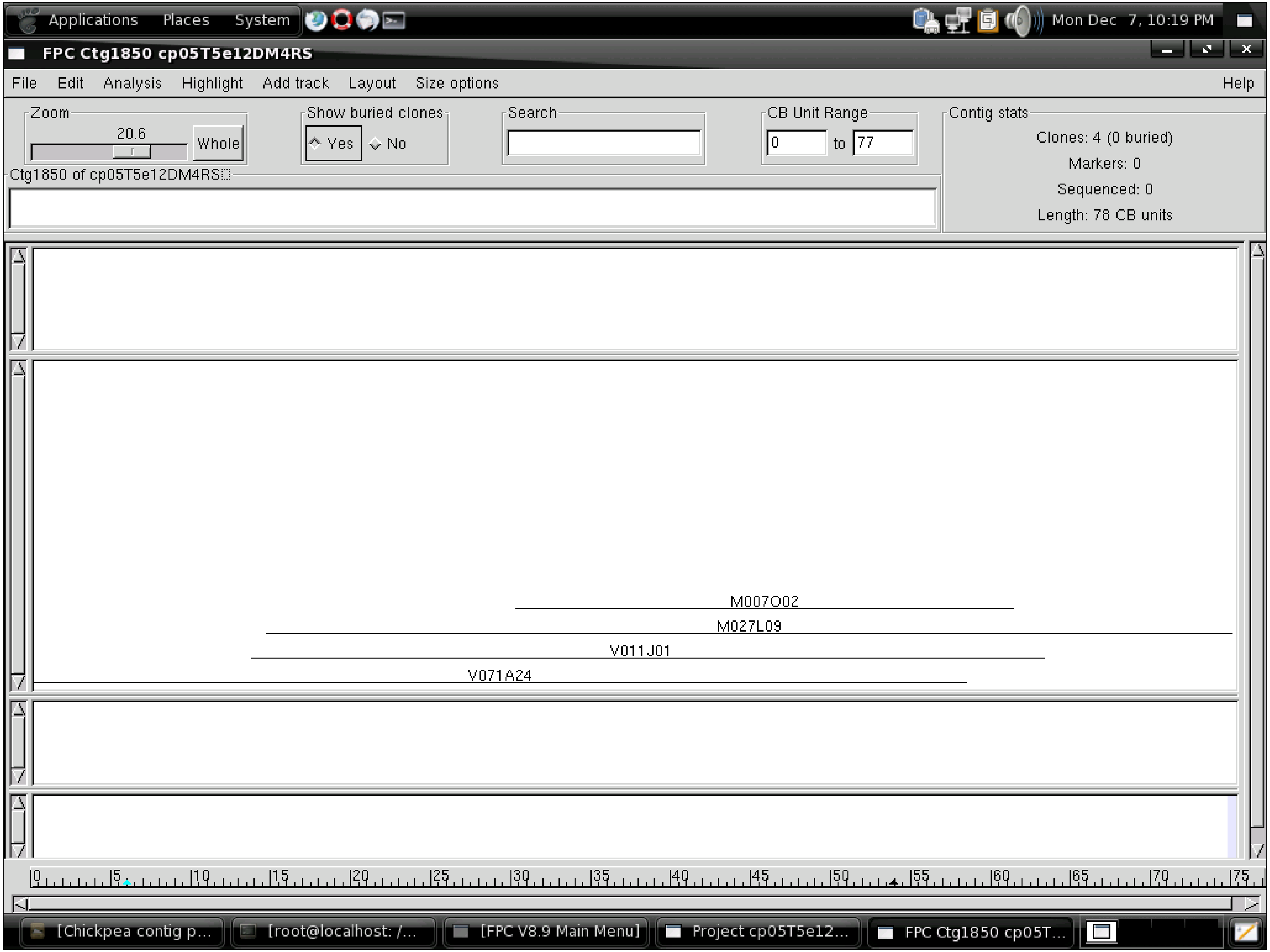
Applications Places System
Mon Dec 7, 10:19 PM
FPC Ctg1850 cp05T5e12DM4RS
File Edit Analysis Highlight Add track Layout Size options Help
Zoom
20.6
Whole
Show buried clones
Yes No
Search
CB Unit Range
0 to 77
Contig stats
Clones: 4 (0 buried)
Markers: 0
Sequenced: 0
Length: 78 CB units
Ctg1850 of cp05T5e12DM4RS
M007O02
M027L09
V011J01
V071A24
0 5 10 15 20 25 30 35 40 45 50 55 60 65 70 75
[Chickpea contig p...
[root@localhost: /...
[FPC V8.9 Main Menu]
Project cp05T5e12...
FPC Ctg1850 cp05T...

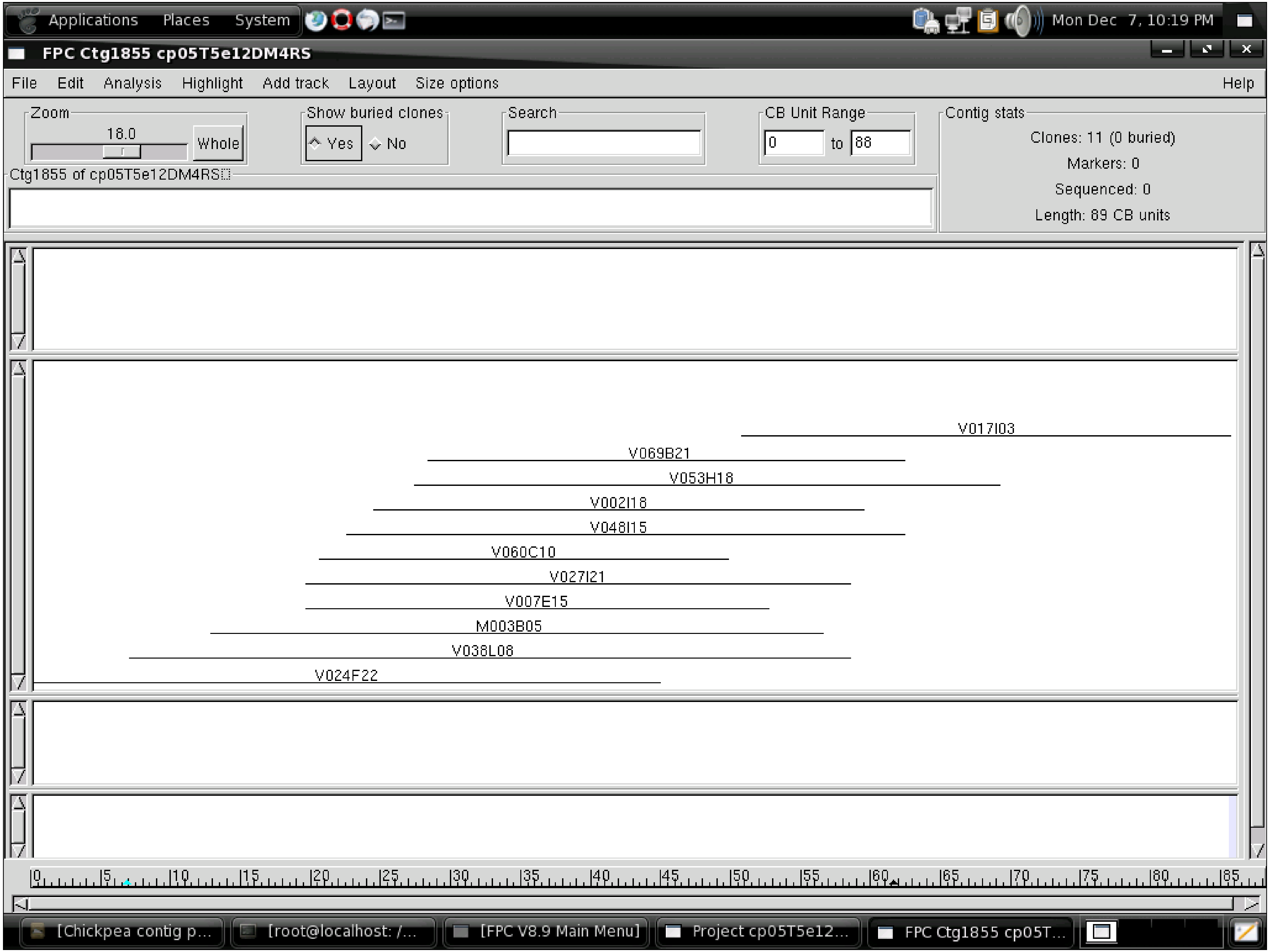
Applications Places System
Mon Dec 7, 10:19 PM
FPC Ctg1855 cp05T5e12DM4RS
File Edit Analysis Highlight Add track Layout Size options Help
Zoom
18.0
Whole
Show buried clones
Yes No
Search
CB Unit Range
0 to 88
Contig stats
Clones: 11 (0 buried)
Markers: 0
Sequenced: 0
Length: 89 CB units
Ctg1855 of cp05T5e12DM4RS
V017I03
V069B21
V053H18
V002I18
V048I15
V060C10
V027I21
V007E15
M003B05
V038L08
V024F22
0 5 10 15 20 25 30 35 40 45 50 55 60 65 70 75 80 85
[Chickpea contig p...
[root@localhost: /...
[FPC V8.9 Main Menu]
Project cp05T5e12...
FPC Ctg1855 cp05T...

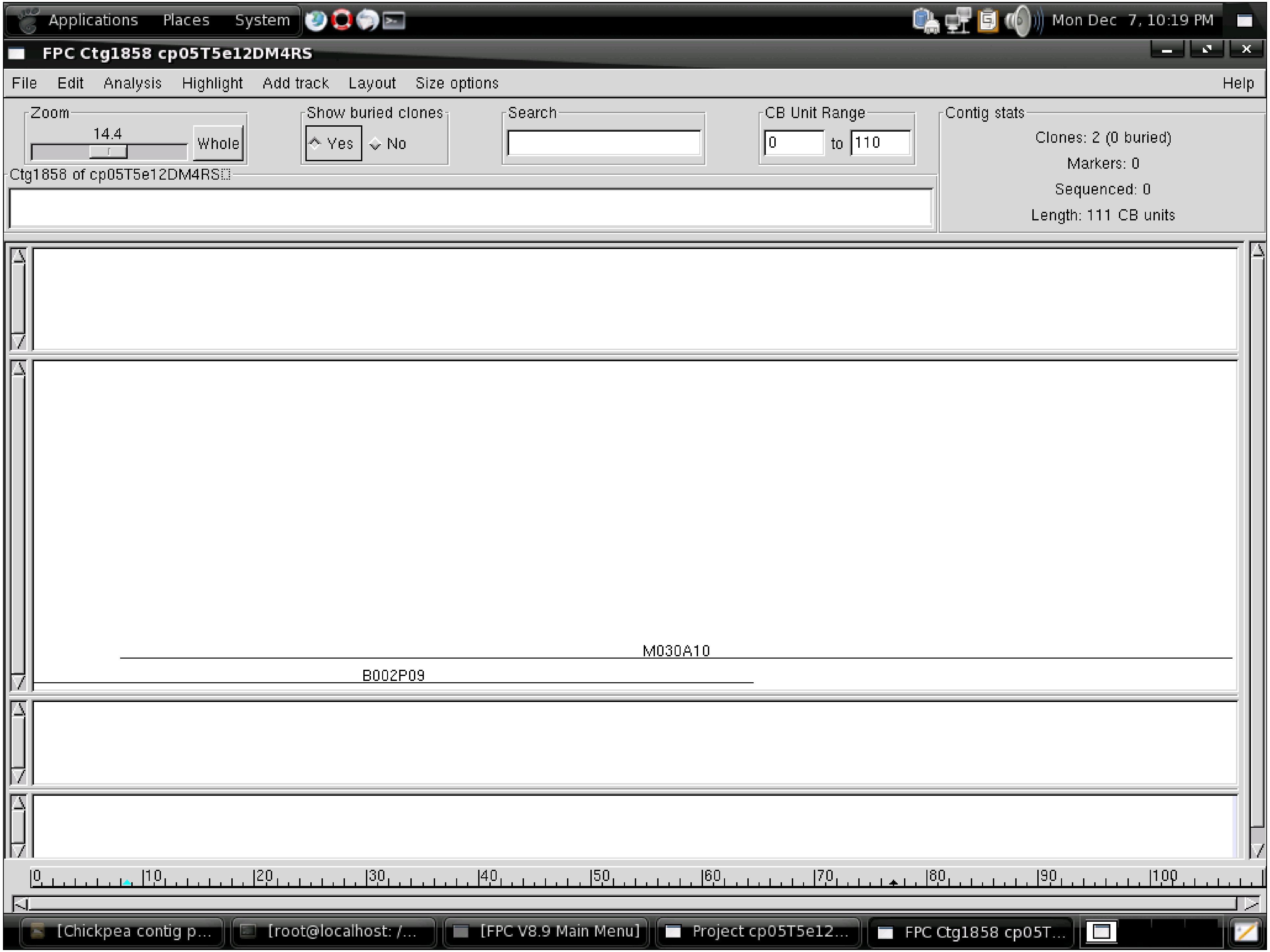

Applications Places System
Mon Dec 7, 10:19 PM
FPC Ctg1858 cp05T5e12DM4RS
File Edit Analysis Highlight Add track Layout Size options Help
Zoom
14.4
Whole
Show buried clones
Yes
No
Search
CB Unit Range
0 to 110
Contig stats
Clones: 2 (0 buried)
Markers: 0
Sequenced: 0
Length: 111 CB units
Ctg1858 of cp05T5e12DM4RS
M030A10
B002P09
0 10 20 30 40 50 60 70 80 90 100
[Chickpea contig p...
[root@localhost: /...
[FPC V8.9 Main Menu]
Project cp05T5e12...
FPC Ctg1858 cp05T...

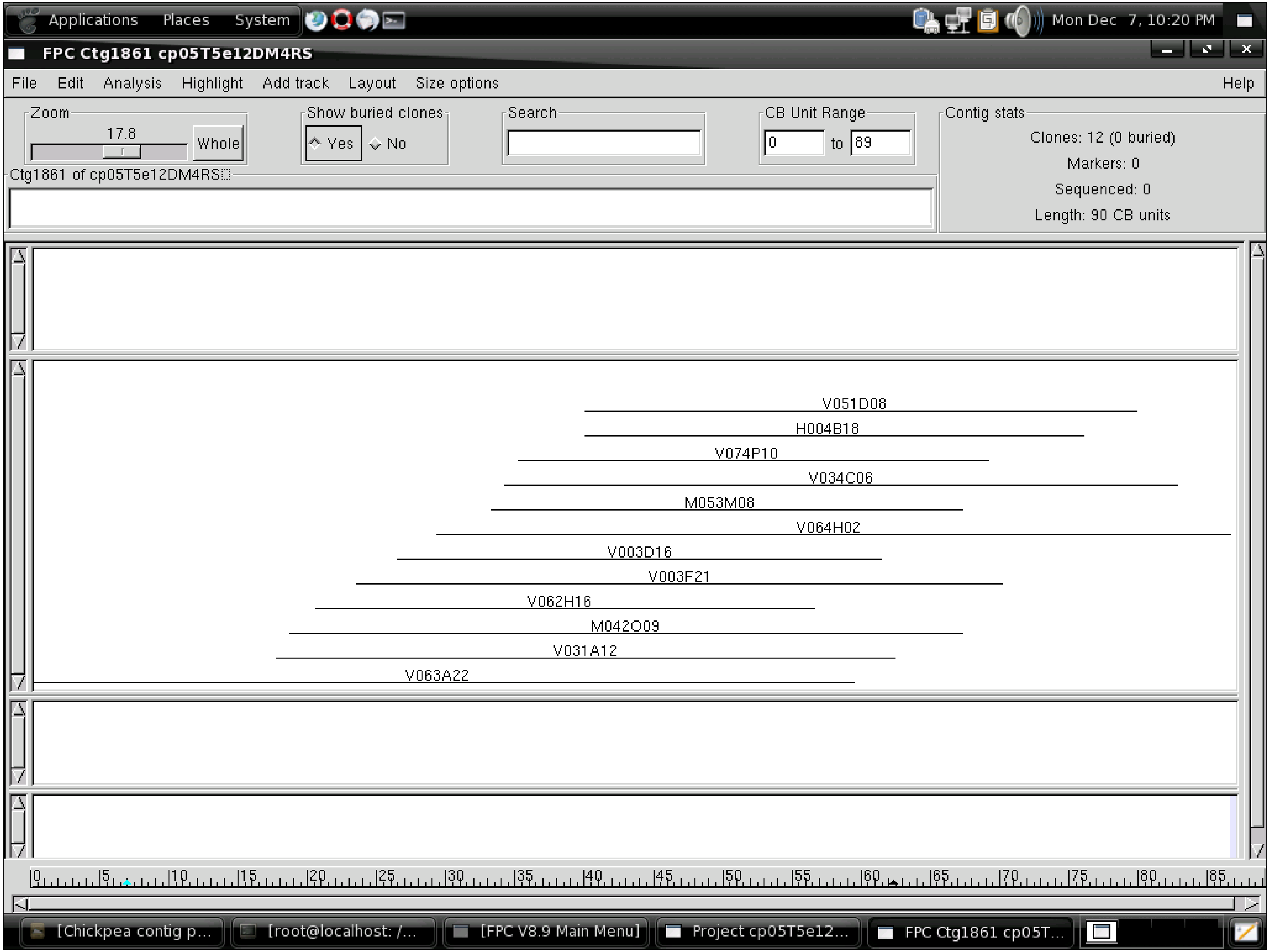
Applications Places System
Mon Dec 7, 10:20 PM
FPC Ctg1861 cp05T5e12DM4RS
File Edit Analysis Highlight Add track Layout Size options Help
Zoom
17.8
Whole
Show buried clones
Yes
No
Search
CB Unit Range
0
to
89
Contig stats
Clones: 12 (0 buried)
Markers: 0
Sequenced: 0
Length: 90 CB units
Ctg1861 of cp05T5e12DM4RS
V051D08
H004B18
V074P10
V034C06
M053M08
V064H02
V003D16
V003F21
V062H16
M042O09
V031A12
V063A22
0 5 10 15 20 25 30 35 40 45 50 55 60 65 70 75 80 85
[Chickpea contig p...
[root@localhost: /...
[FPC V8.9 Main Menu]
Project cp05T5e12...
FPC Ctg1861 cp05T...

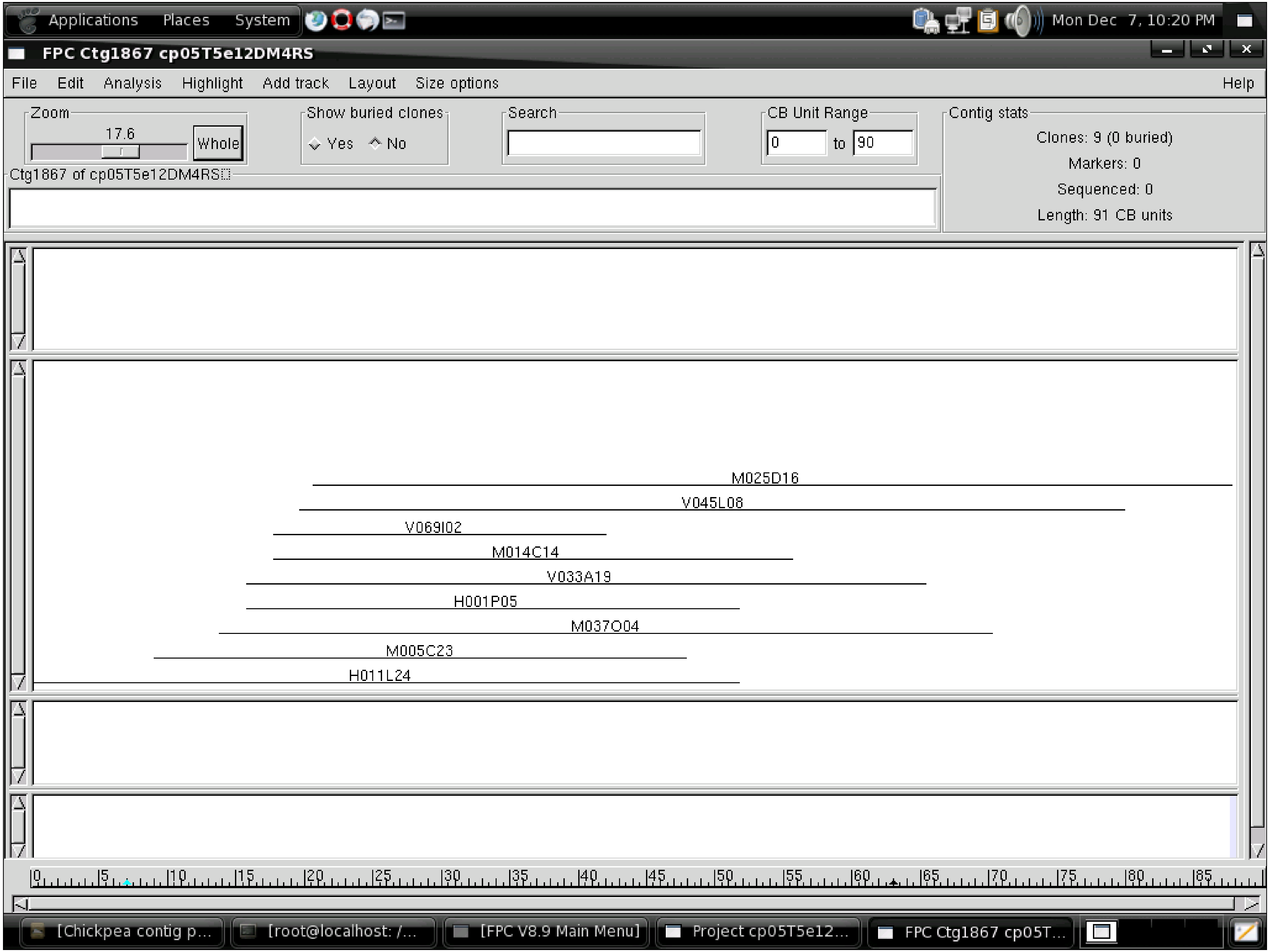
Applications Places System
Mon Dec 7, 10:20 PM
FPC Ctg1867 cp05T5e12DM4RS
File Edit Analysis Highlight Add track Layout Size options Help
Zoom
17.6
Whole
Show buried clones
Yes No
Search
CB Unit Range
0 to 90
Contig stats
Clones: 9 (0 buried)
Markers: 0
Sequenced: 0
Length: 91 CB units
Ctg1867 of cp05T5e12DM4RS
M025D16
V045L08
V069I02
M014C14
V033A19
H001P05
M037O04
M005C23
H011L24
0 5 10 15 20 25 30 35 40 45 50 55 60 65 70 75 80 85
[Chickpea contig p...
[root@localhost: /...
[FPC V8.9 Main Menu]
Project cp05T5e12...
FPC Ctg1867 cp05T...

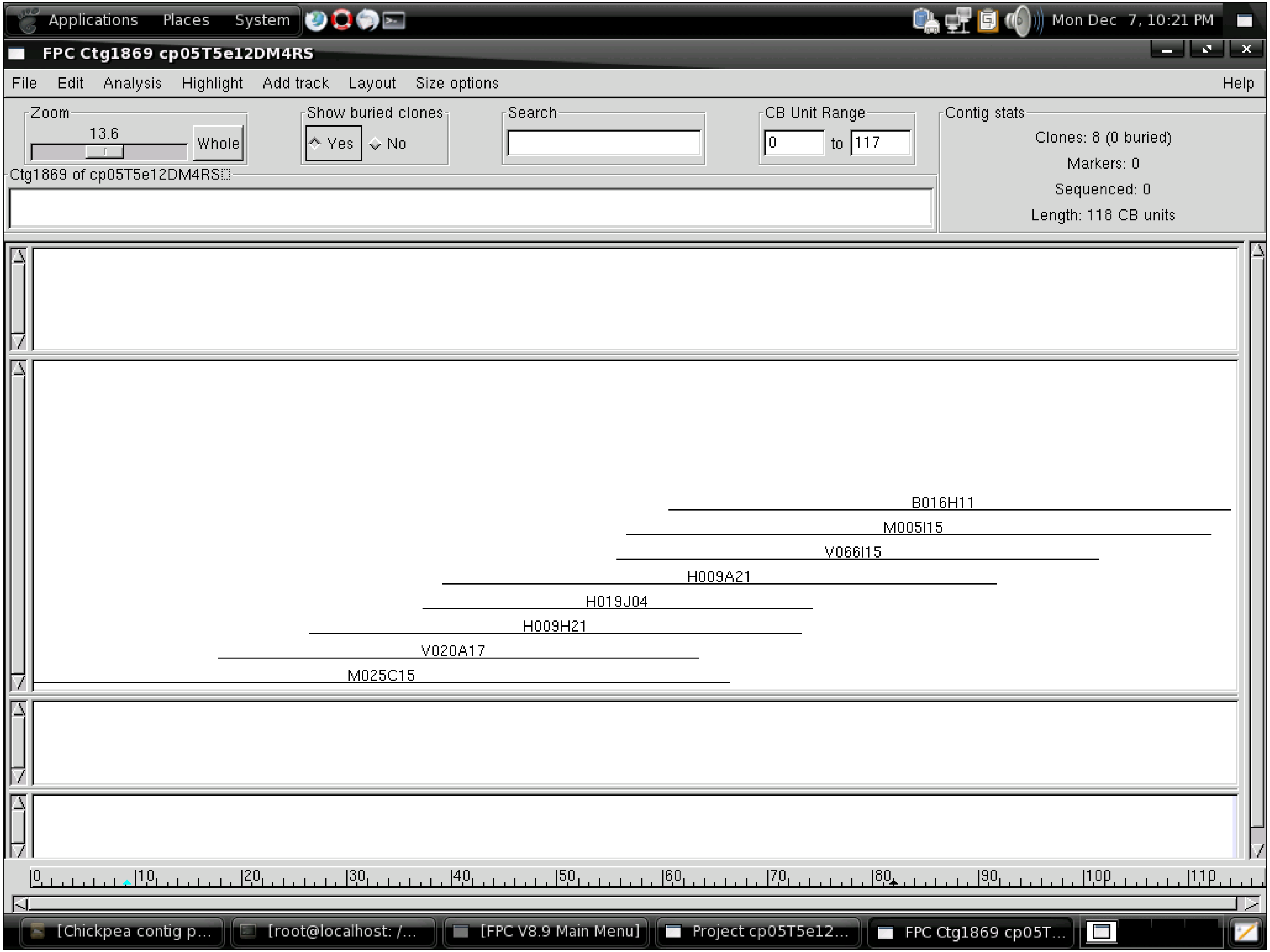
Applications Places System
Mon Dec 7, 10:21 PM
FPC Ctg1869 cp05T5e12DM4RS
File Edit Analysis Highlight Add track Layout Size options Help
Zoom
13.6
Whole
Show buried clones
Yes No
Search
CB Unit Range
0 to 117
Contig stats
Clones: 8 (0 buried)
Markers: 0
Sequenced: 0
Length: 118 CB units
Ctg1869 of cp05T5e12DM4RS
B016H11
M005I15
V066I15
H009A21
H019J04
H009H21
V020A17
M025C15
0 10 20 30 40 50 60 70 80 90 100 110
[Chickpea contig p...
[root@localhost: /...
[FPC V8.9 Main Menu]
Project cp05T5e12...
FPC Ctg1869 cp05T...

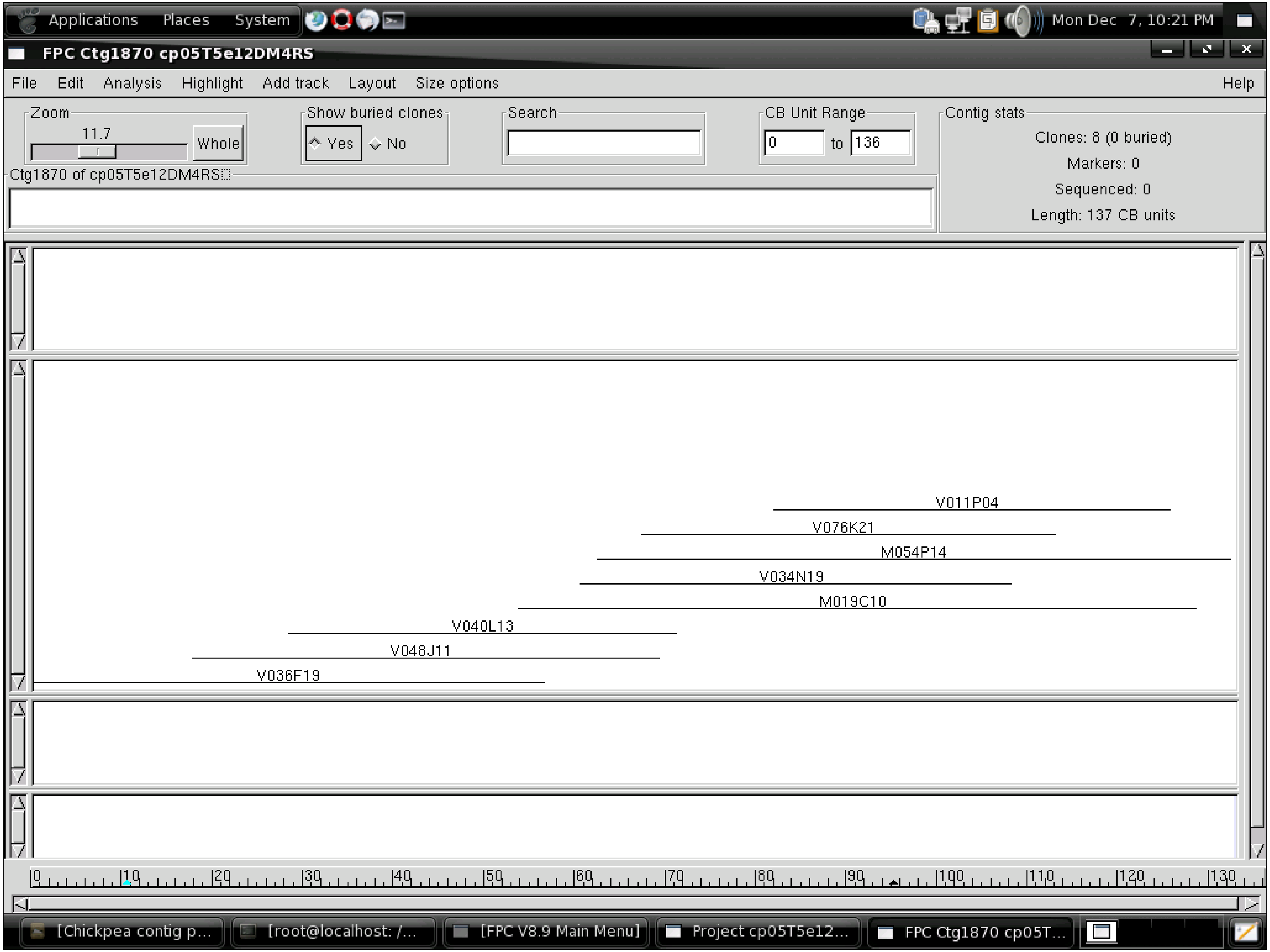
Applications Places System
Mon Dec 7, 10:21 PM
FPC Ctg1870 cp05T5e12DM4RS
File Edit Analysis Highlight Add track Layout Size options Help
Zoom
11.7
Whole
Show buried clones
Yes
No
Search
CB Unit Range
0
to
136
Contig stats
Clones: 8 (0 buried)
Markers: 0
Sequenced: 0
Length: 137 CB units
Ctg1870 of cp05T5e12DM4RS
V011P04
V076K21
M054P14
V034N19
M019C10
V040L13
V048J11
V036F19
0 10 20 30 40 50 60 70 80 90 100 110 120 130
[Chickpea contig p...
[root@localhost: /...
[FPC V8.9 Main Menu]
Project cp05T5e12...
FPC Ctg1870 cp05T...

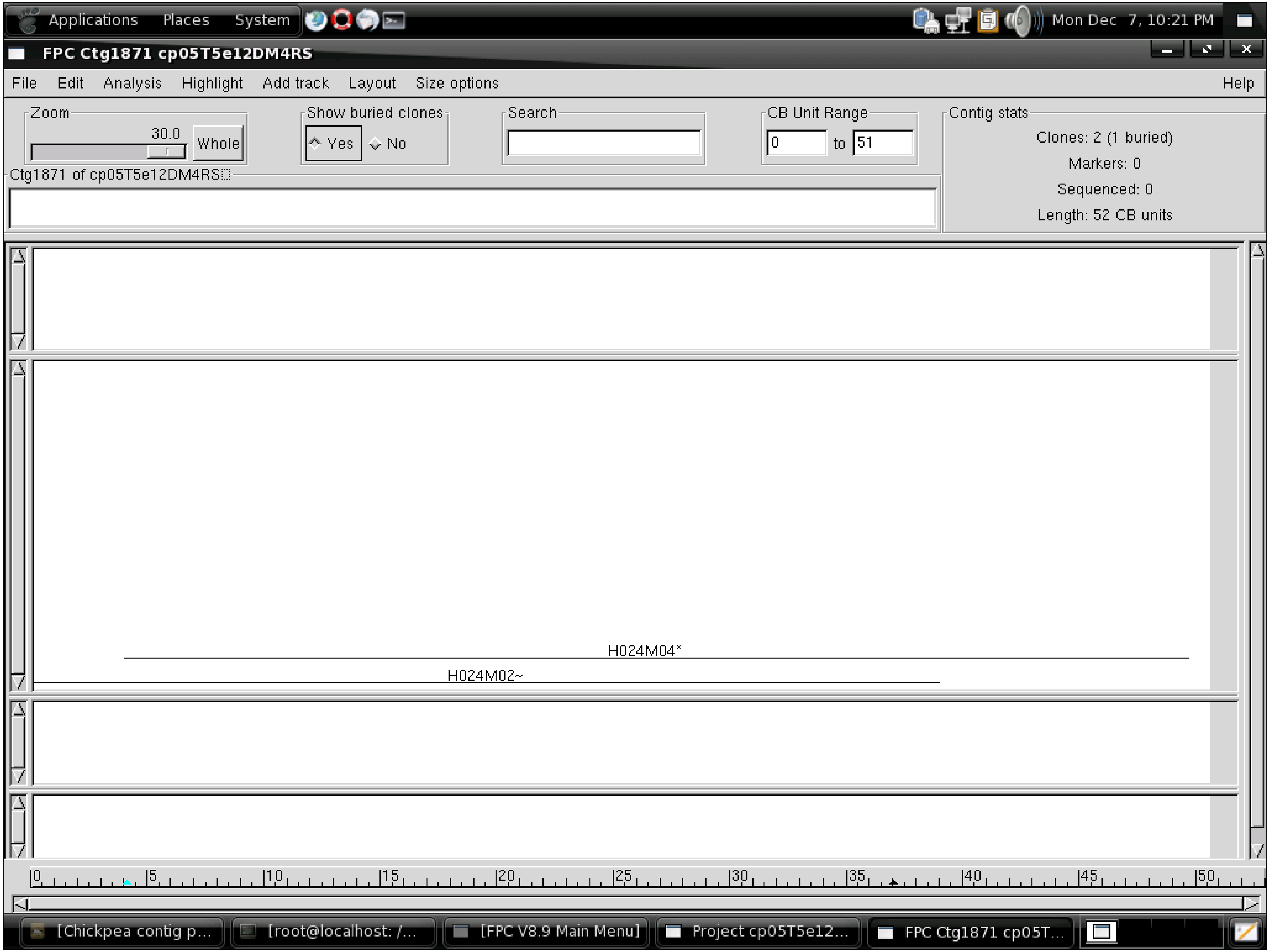
Applications Places System
Mon Dec 7, 10:21 PM
FPC Ctg1871 cp05T5e12DM4RS
File Edit Analysis Highlight Add track Layout Size options Help
Zoom
30.0
Whole
Show buried clones
Yes No
Search
CB Unit Range
0 to 51
Contig stats
Clones: 2 (1 buried)
Markers: 0
Sequenced: 0
Length: 52 CB units
Ctg1871 of cp05T5e12DM4RS
H024M04*
H024M02~
0 5 10 15 20 25 30 35 40 45 50
[Chickpea contig p...
[root@localhost: /...
[FPC V8.9 Main Menu]
Project cp05T5e12...
FPC Ctg1871 cp05T...

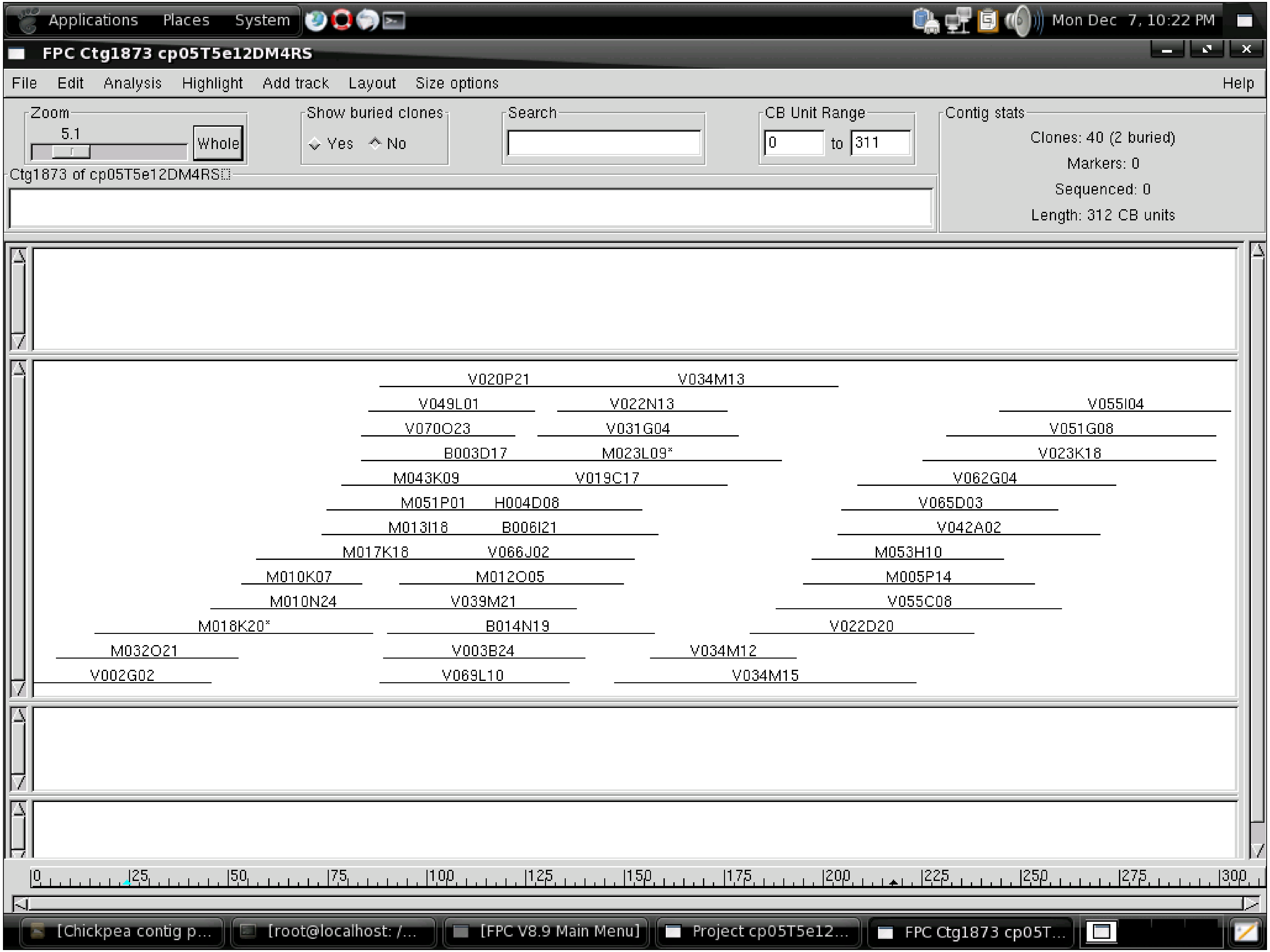
Applications Places System
Mon Dec 7, 10:22 PM
FPC Ctg1873 cp05T5e12DM4RS
File Edit Analysis Highlight Add track Layout Size options Help
Zoom
5.1
Whole
Show buried clones
Yes No
Search
CB Unit Range
0 to 311
Contig stats
Clones: 40 (2 buried)
Markers: 0
Sequenced: 0
Length: 312 CB units
Ctg1873 of cp05T5e12DM4RS
V020P21
V034M13
V049L01
V022N13
V055I04
V070O23
V031G04
V051G08
B003D17
M023L09*
V023K18
M043K09
V019C17
V062G04
M051P01
H004D08
V065D03
M013I18
B006I21
V042A02
M017K18
V066J02
M053H10
M010K07
M012O05
M005P14
M010N24
V039M21
V055C08
M018K20*
B014N19
V022D20
M032O21
V003B24
V034M12
V002G02
V069L10
V034M15
0 25 50 75 100 125 150 175 200 225 250 275 300
[Chickpea contig p...
[root@localhost: /...
[FPC V8.9 Main Menu]
Project cp05T5e12...
FPC Ctg1873 cp05T...

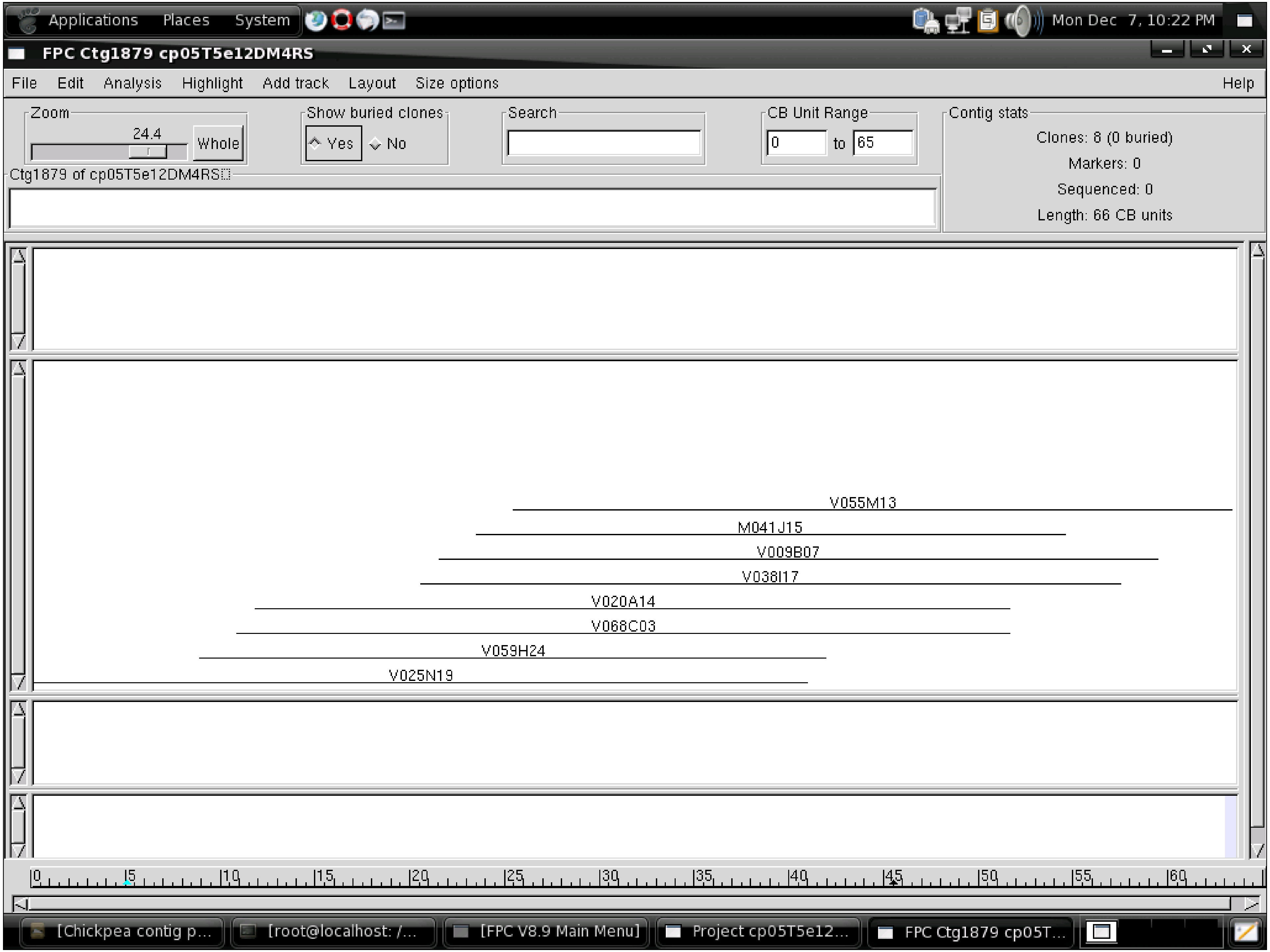
Applications
Places
System
Mon Dec 7, 10:22 PM
FPC Ctg1879 cp05T5e12DM4RS
File
Edit
Analysis
Highlight
Add track
Layout
Size options
Help
Zoom
24.4
Whole
Show buried clones
Yes
No
Search
CB Unit Range
0
to
65
Contig stats
Clones: 8 (0 buried)
Markers: 0
Sequenced: 0
Length: 66 CB units
Ctg1879 of cp05T5e12DM4RS
V055M13
M041J15
V009B07
V038I17
V020A14
V068C03
V059H24
V025N19
0
5
10
15
20
25
30
35
40
45
50
55
60
[Chickpea contig p...
[root@localhost: /...
[FPC V8.9 Main Menu]
Project cp05T5e12...
FPC Ctg1879 cp05T...

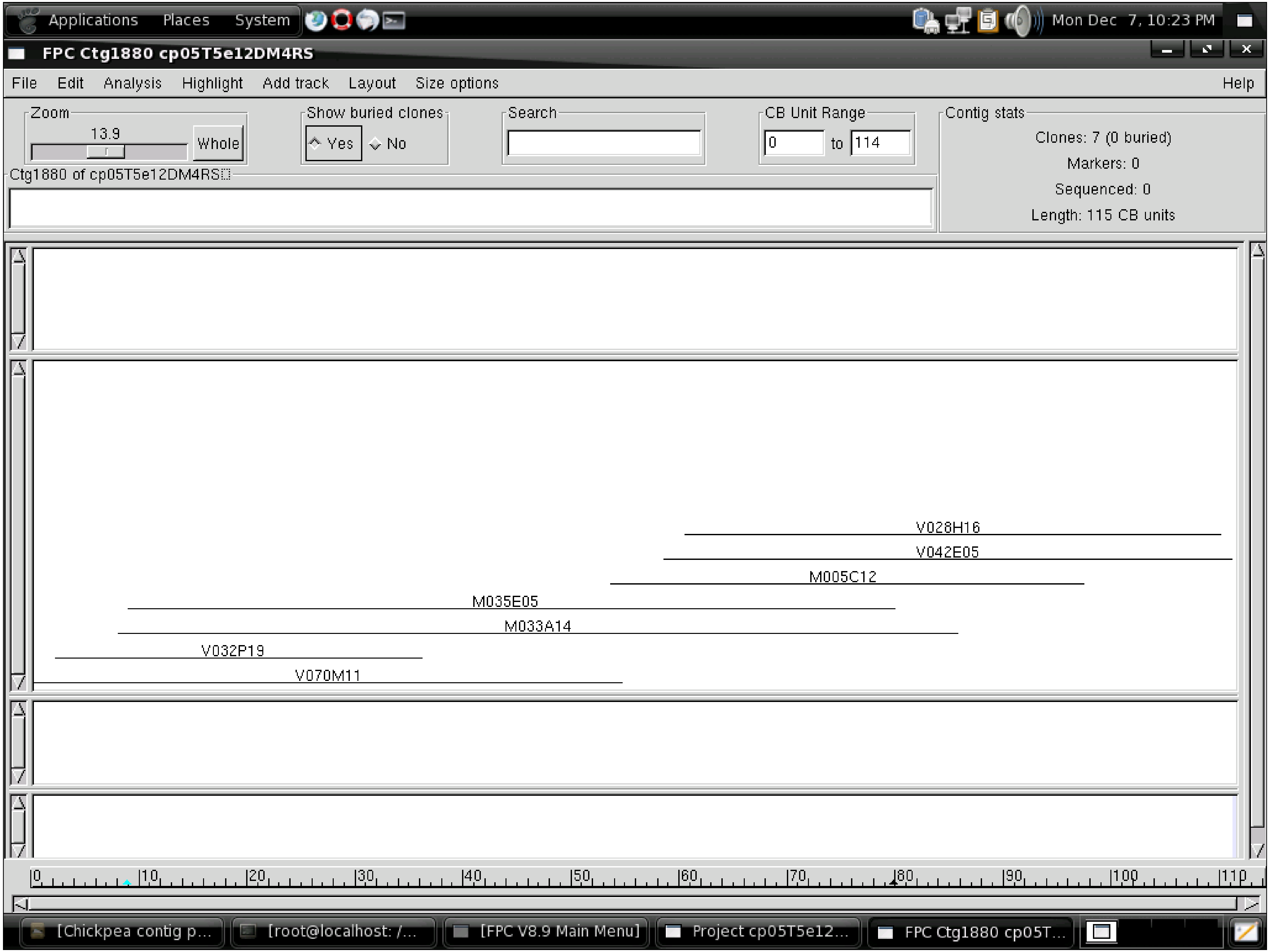
Applications
Places
System
Mon Dec 7, 10:23 PM
FPC Ctg1880 cp05T5e12DM4RS
File
Edit
Analysis
Highlight
Add track
Layout
Size options
Help
Zoom
13.9
Whole
Show buried clones
Yes
No
Search
CB Unit Range
0
to
114
Contig stats
Clones: 7 (0 buried)
Markers: 0
Sequenced: 0
Length: 115 CB units
Ctg1880 of cp05T5e12DM4RS
V028H16
V042E05
M005C12
M035E05
M033A14
V032P19
V070M11
0
10
20
30
40
50
60
70
80
90
100
110
[Chickpea contig p...
[root@localhost: /...
[FPC V8.9 Main Menu]
Project cp05T5e12...
FPC Ctg1880 cp05T...

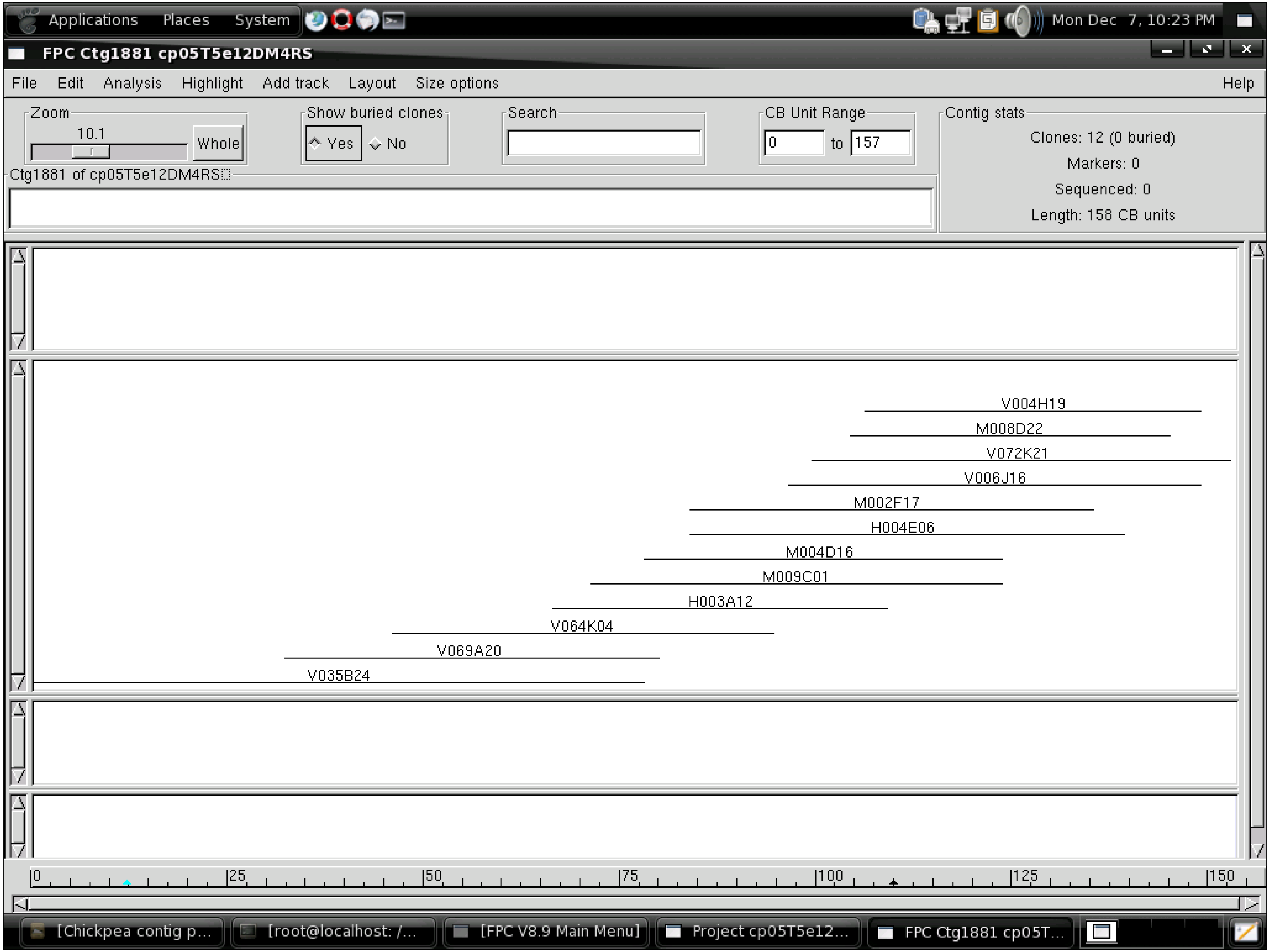

Applications Places System
Mon Dec 7, 10:23 PM
FPC Ctg1881 cp05T5e12DM4RS
File Edit Analysis Highlight Add track Layout Size options Help
Zoom
10.1
Whole
Show buried clones
Yes
No
Search
CB Unit Range
0
to
157
Contig stats
Clones: 12 (0 buried)
Markers: 0
Sequenced: 0
Length: 158 CB units
Ctg1881 of cp05T5e12DM4RS
V004H19
M008D22
V072K21
V006J16
M002F17
H004E06
M004D16
M009C01
H003A12
V064K04
V069A20
V035B24
0
25
50
75
100
125
150
[Chickpea contig p...
[root@localhost: /...
[FPC V8.9 Main Menu]
Project cp05T5e12...
FPC Ctg1881 cp05T...

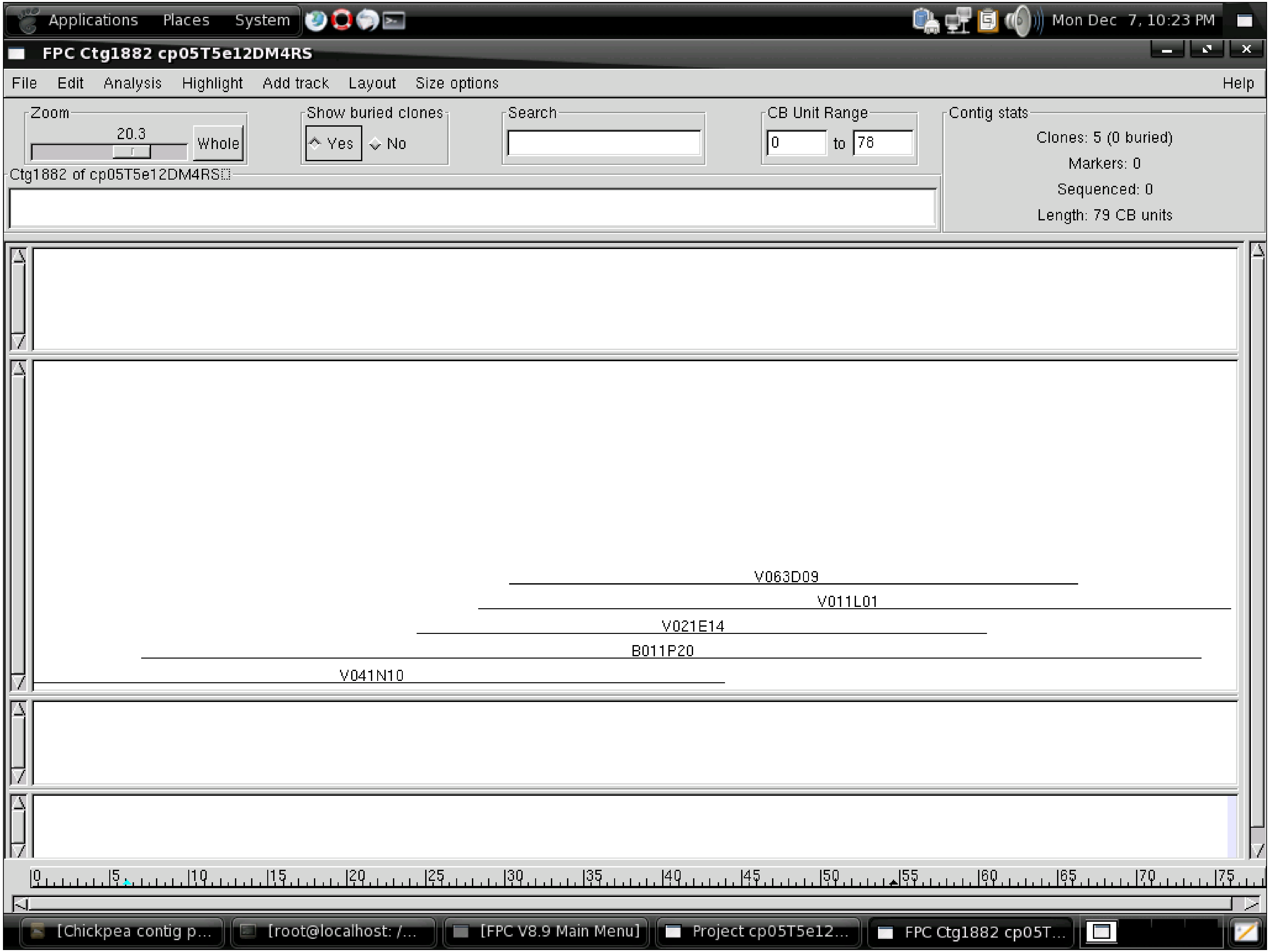

Applications
Places
System
Mon Dec 7, 10:23 PM
FPC Ctg1882 cp05T5e12DM4RS
File
Edit
Analysis
Highlight
Add track
Layout
Size options
Help
Zoom
20.3
Whole
Show buried clones
Yes
No
Search
CB Unit Range
0
to
78
Contig stats
Clones: 5 (0 buried)
Markers: 0
Sequenced: 0
Length: 79 CB units
Ctg1882 of cp05T5e12DM4RS
V063D09
V011L01
V021E14
B011P20
V041N10
0 5 10 15 20 25 30 35 40 45 50 55 60 65 70 75
[Chickpea contig p...
[root@localhost: /...
[FPC V8.9 Main Menu]
Project cp05T5e12...
FPC Ctg1882 cp05T...

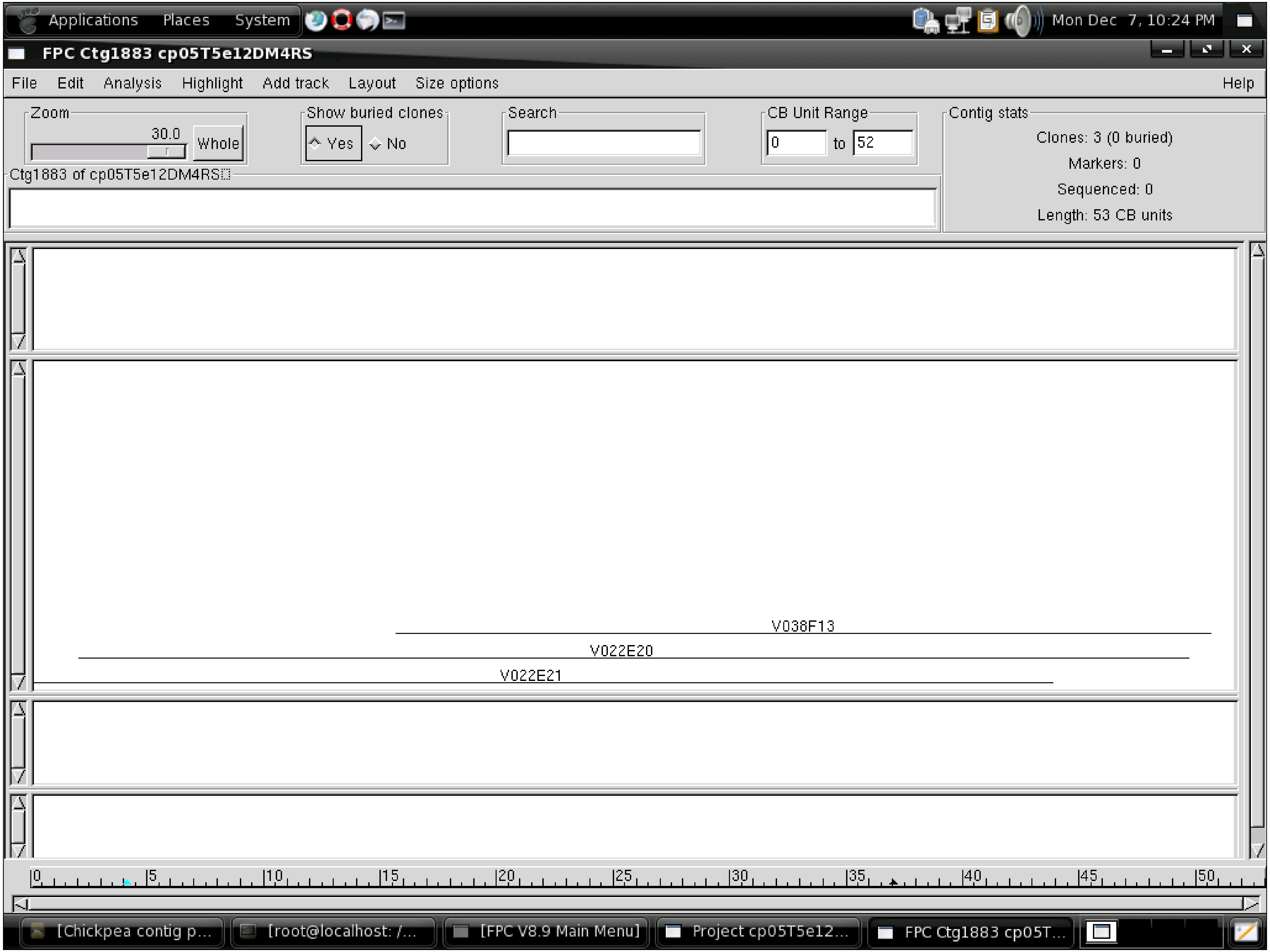
Applications
Places
System
Mon Dec 7, 10:24 PM
FPC Ctg1883 cp05T5e12DM4RS
File
Edit
Analysis
Highlight
Add track
Layout
Size options
Help
Zoom
30.0
Whole
Show buried clones
Yes
No
Search
CB Unit Range
0
to
52
Contig stats
Clones: 3 (0 buried)
Markers: 0
Sequenced: 0
Length: 53 CB units
Ctg1883 of cp05T5e12DM4RS
V038F13
V022E20
V022E21
0
5
10
15
20
25
30
35
40
45
50
[Chickpea contig p...
[root@localhost: /...
[FPC V8.9 Main Menu]
Project cp05T5e12...
FPC Ctg1883 cp05T...

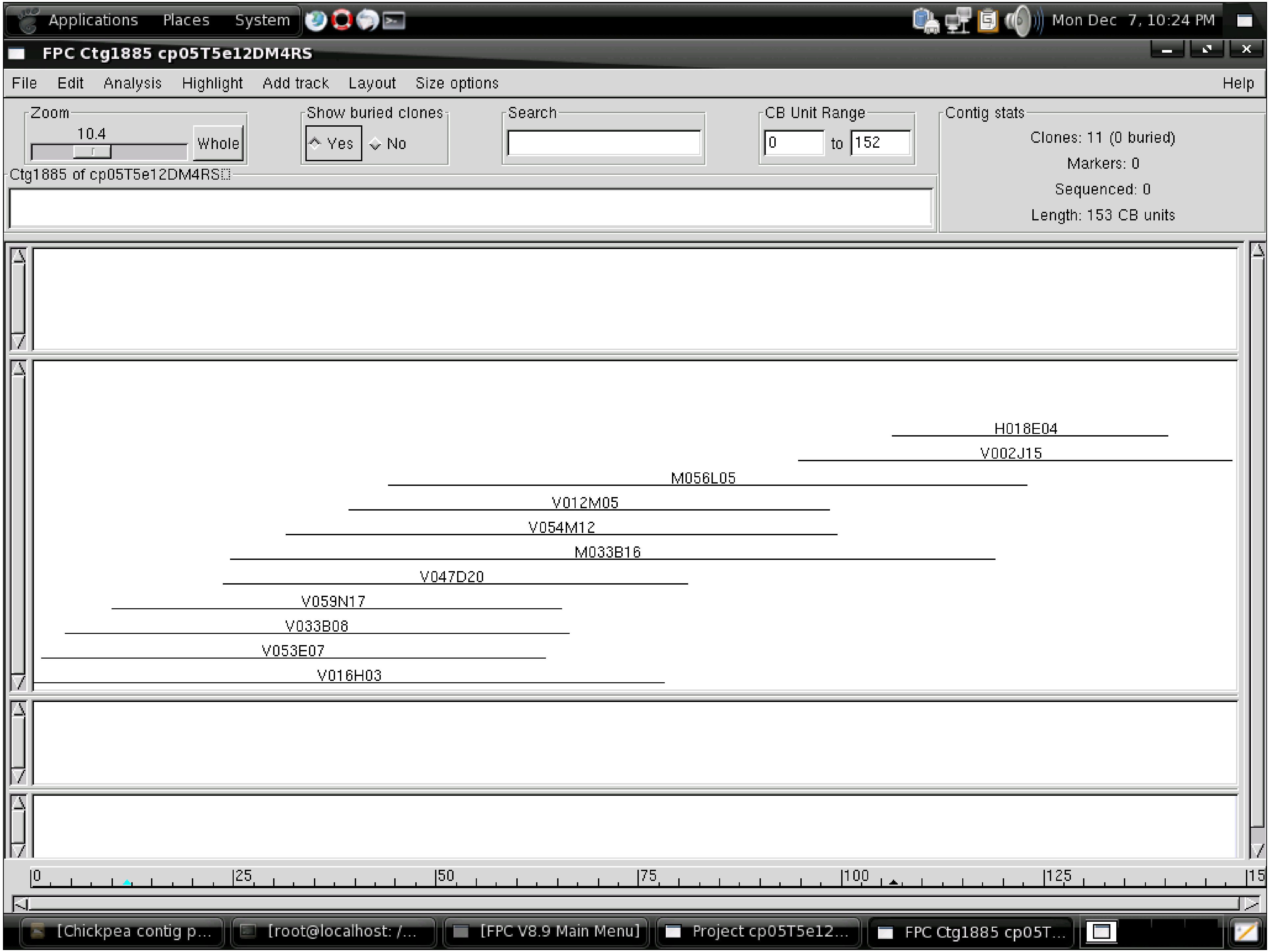
Applications Places System
Mon Dec 7, 10:24 PM
FPC Ctg1885 cp05T5e12DM4RS
File Edit Analysis Highlight Add track Layout Size options Help
Zoom
10.4
Whole
Show buried clones
Yes
No
Search
CB Unit Range
0 to 152
Contig stats
Clones: 11 (0 buried)
Markers: 0
Sequenced: 0
Length: 153 CB units
Ctg1885 of cp05T5e12DM4RS
H018E04
V002J15
M056L05
V012M05
V054M12
M033B16
V047D20
V059N17
V033B08
V053E07
V016H03
0
25
50
75
100
125
[Chickpea contig p...
[root@localhost: /...
[FPC V8.9 Main Menu]
Project cp05T5e12...
FPC Ctg1885 cp05T...

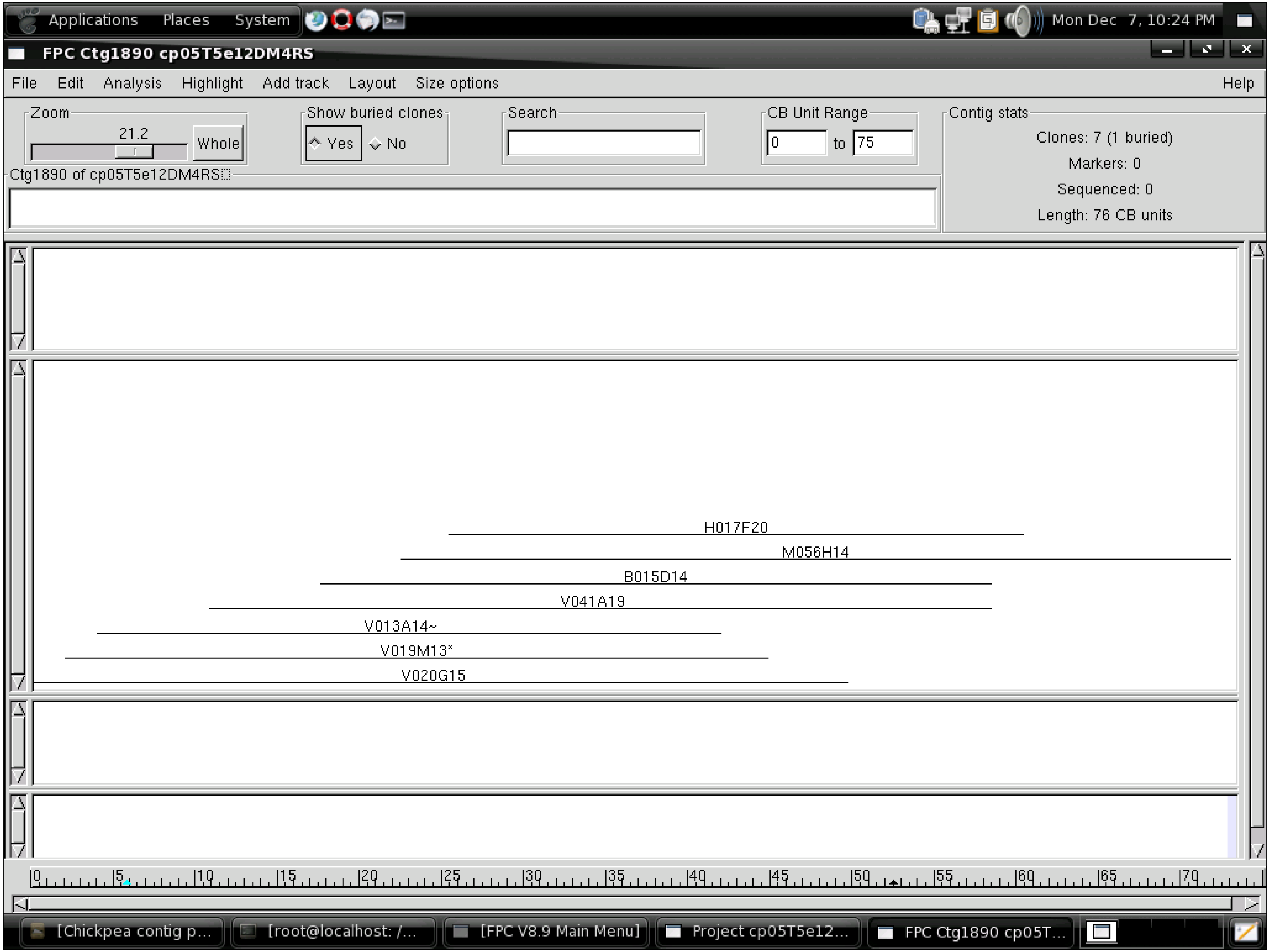
Applications Places System
Mon Dec 7, 10:24 PM
FPC Ctg1890 cp05T5e12DM4RS
File Edit Analysis Highlight Add track Layout Size options Help
Zoom
21.2
Whole
Show buried clones
Yes
No
Search
CB Unit Range
0
to
75
Contig stats
Clones: 7 (1 buried)
Markers: 0
Sequenced: 0
Length: 76 CB units
Ctg1890 of cp05T5e12DM4RS
H017F20
M056H14
B015D14
V041A19
V013A14~
V019M13*
V020G15
0 5 10 15 20 25 30 35 40 45 50 55 60 65 70
[Chickpea contig p...
[root@localhost: /...
[FPC V8.9 Main Menu]
Project cp05T5e12...
FPC Ctg1890 cp05T...

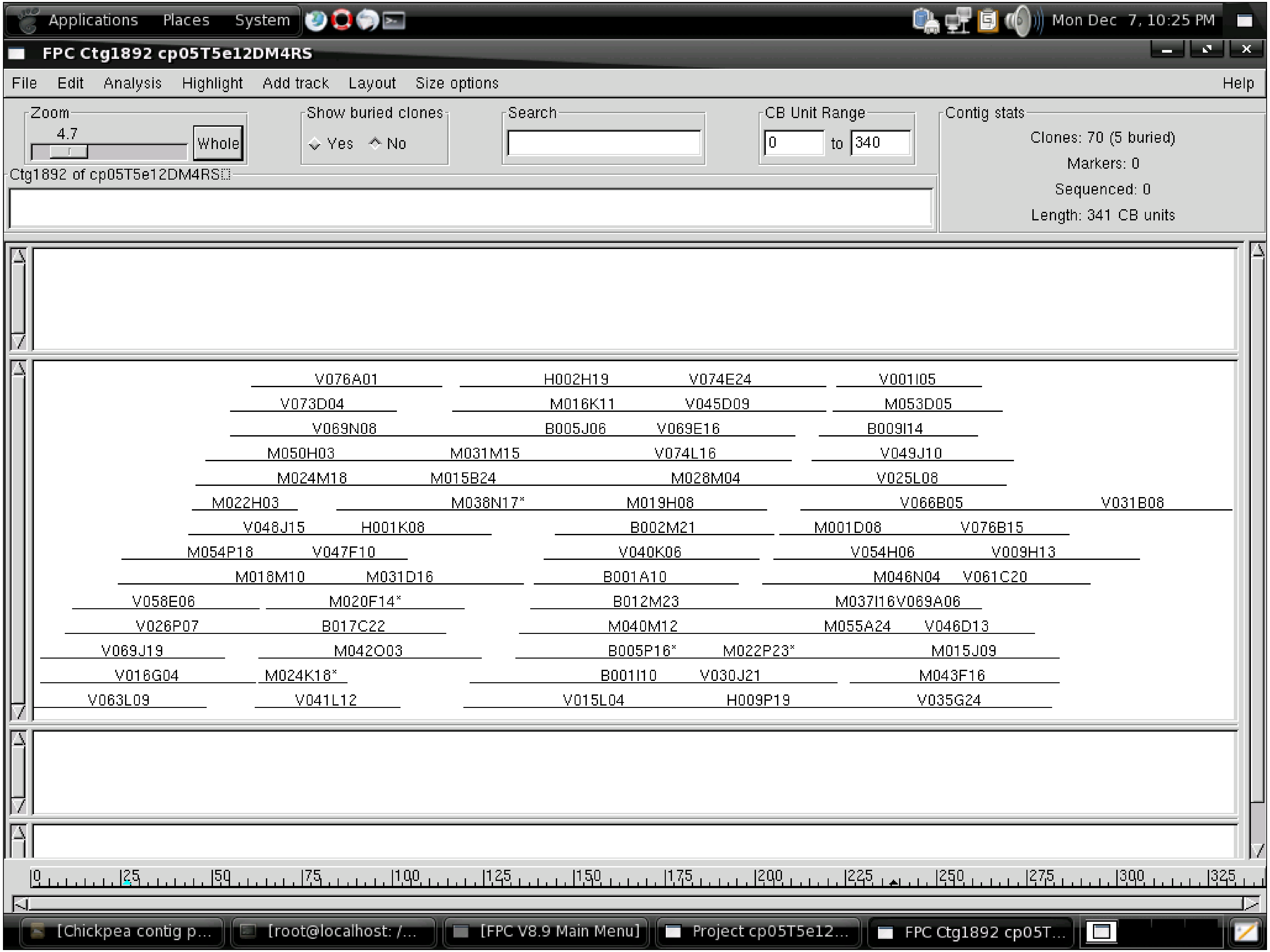
Applications Places System
Mon Dec 7, 10:25 PM
FPC Ctg1892 cp05T5e12DM4RS
File Edit Analysis Highlight Add track Layout Size options Help
Zoom
4.7
Whole
Show buried clones
Yes No
Search
CB Unit Range
0 to 340
Contig stats
Clones: 70 (5 buried)
Markers: 0
Sequenced: 0
Length: 341 CB units
Ctg1892 of cp05T5e12DM4RS
V076A01 H002H19 V074E24 V001I05
V073D04 M016K11 V045D09 M053D05
V069N08 B005J06 V069E16 B009I14
M050H03 M031M15 V074L16 V049J10
M024M18 M015B24 M028M04 V025L08
M022H03 M038N17* M019H08 V066B05 V031B08
V048J15 H001K08 B002M21 M001D08 V076B15
M054P18 V047F10 V040K06 V054H06 V009H13
M018M10 M031D16 B001A10 M046N04 V061C20
V058E06 M020F14* B012M23 M037I16V069A06
V026P07 B017C22 M040M12 M055A24 V046D13
V069J19 M042O03 B005P16* M022P23* M015J09
V016G04 M024K18* B001I10 V030J21 M043F16
V063L09 V041L12 V015L04 H009P19 V035G24
0 25 50 75 100 125 150 175 200 225 250 275 300 325
[Chickpea contig p...
[root@localhost: /...
[FPC V8.9 Main Menu]
Project cp05T5e12...
FPC Ctg1892 cp05T...

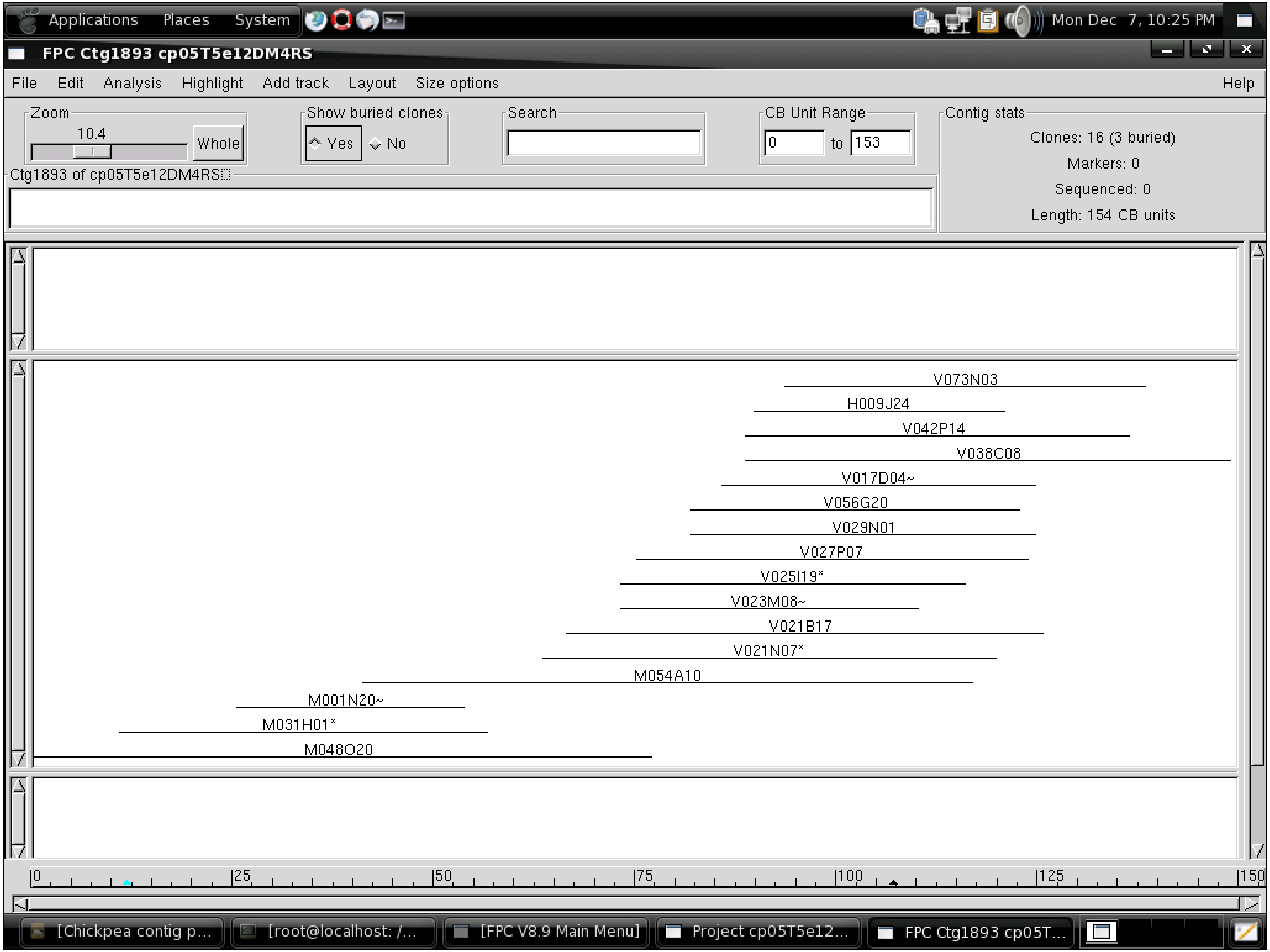

Applications Places System
Mon Dec 7, 10:25 PM
FPC Ctg1893 cp05T5e12DM4RS
File Edit Analysis Highlight Add track Layout Size options Help
Zoom
10.4
Whole
Show buried clones
Yes
No
Search
CB Unit Range
0
to
153
Contig stats
Clones: 16 (3 buried)
Markers: 0
Sequenced: 0
Length: 154 CB units
Ctg1893 of cp05T5e12DM4RS
V073N03
H009J24
V042P14
V038C08
V017D04~
V056G20
V029N01
V027P07
V025I19*
V023M08~
V021B17
V021N07*
M054A10
M001N20~
M031H01*
M048O20
0
25
50
75
100
125
150
[Chickpea contig p...
[root@localhost: /...
[FPC V8.9 Main Menu]
Project cp05T5e12...
FPC Ctg1893 cp05T...

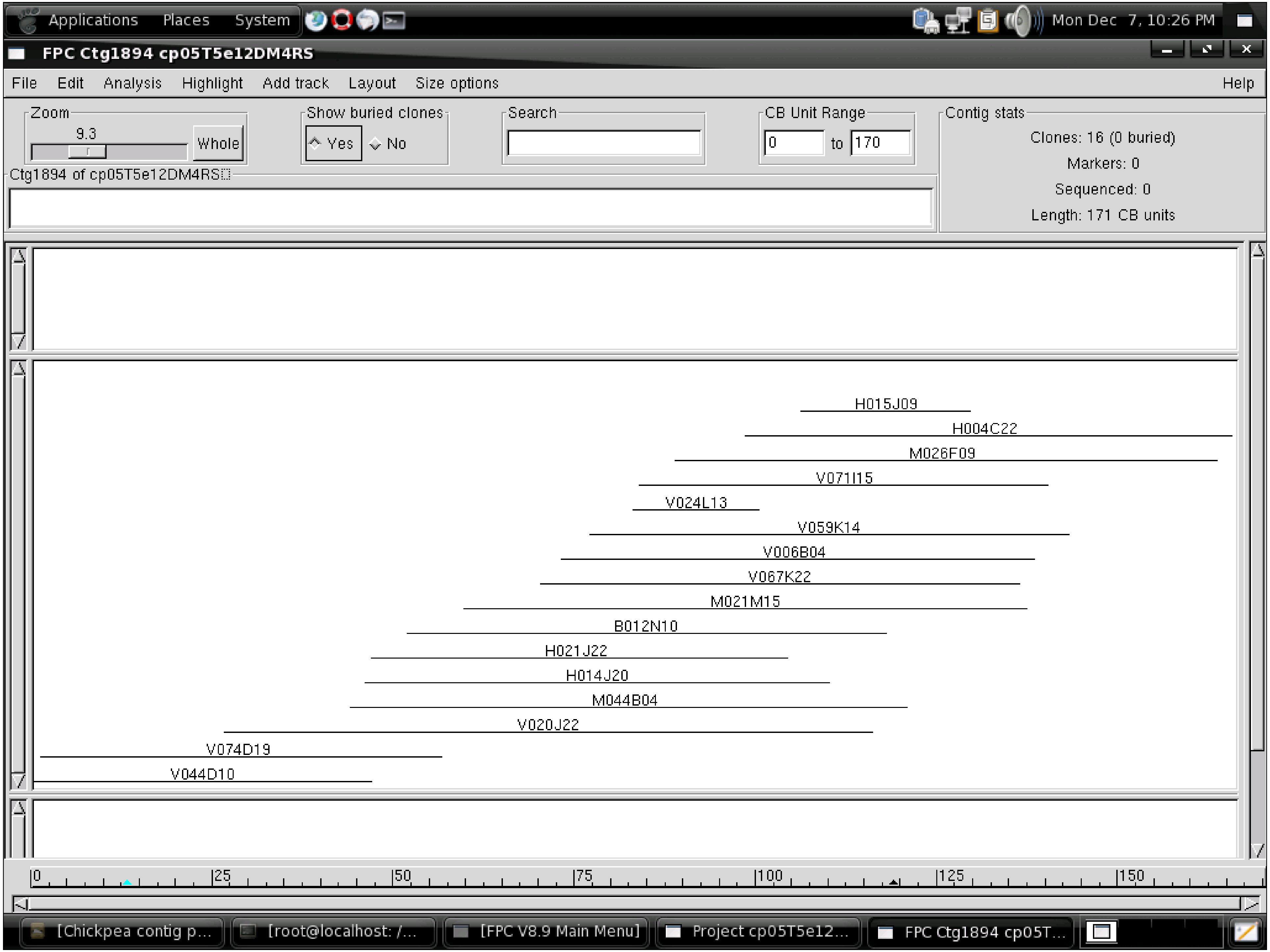
Applications Places System
Mon Dec 7, 10:26 PM
FPC Ctg1894 cp05T5e12DM4RS
File Edit Analysis Highlight Add track Layout Size options Help
Zoom
9.3
Whole
Show buried clones
Yes No
Search
CB Unit Range
0 to 170
Contig stats
Clones: 16 (0 buried)
Markers: 0
Sequenced: 0
Length: 171 CB units
Ctg1894 of cp05T5e12DM4RS
H015J09
H004C22
M026F09
V071I15
V024L13
V059K14
V006B04
V067K22
M021M15
B012N10
H021J22
H014J20
M044B04
V020J22
V074D19
V044D10
0 25 50 75 100 125 150
[Chickpea contig p...
[root@localhost: /...
[FPC V8.9 Main Menu]
Project cp05T5e12...
FPC Ctg1894 cp05T...

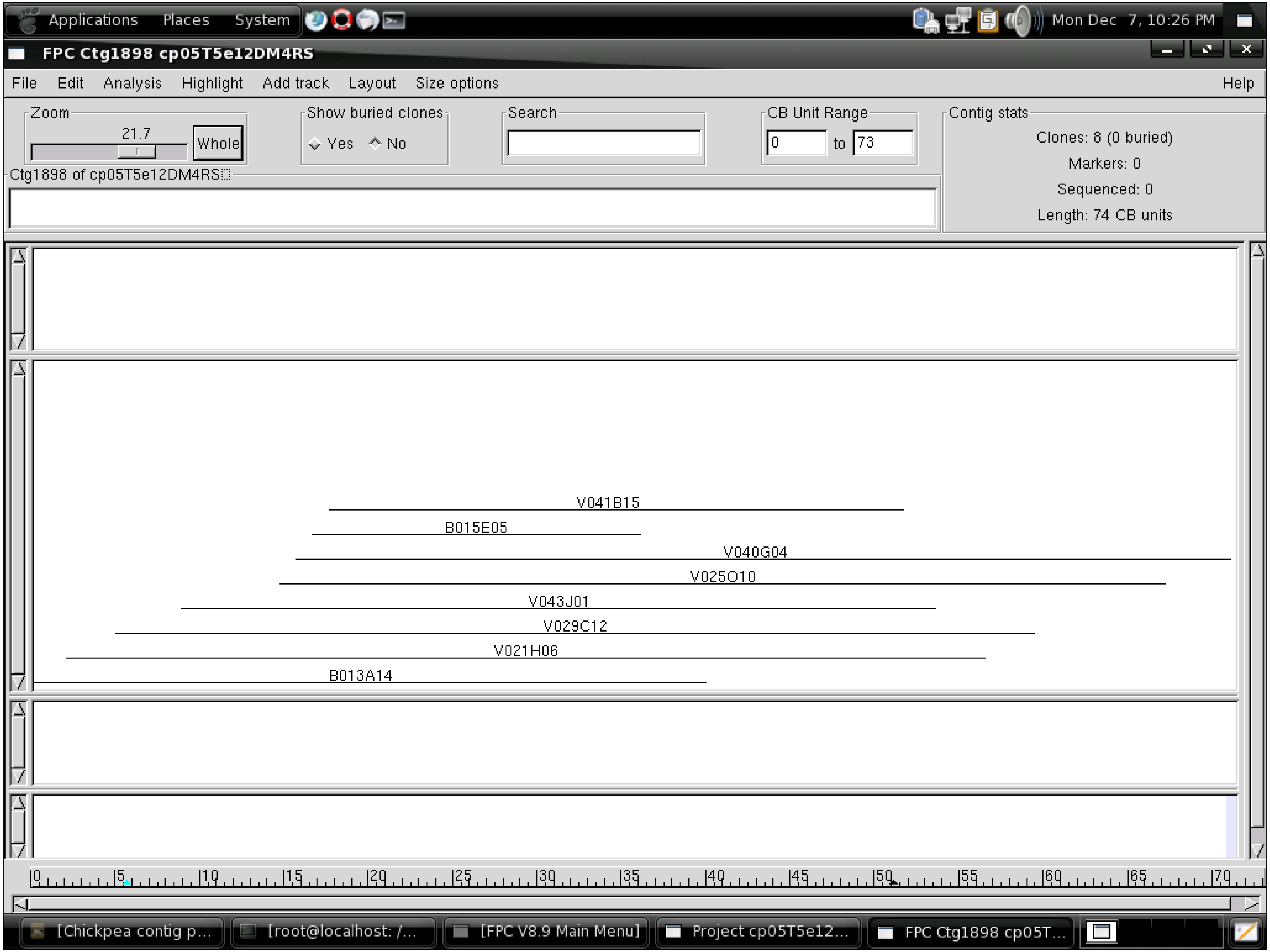
Applications Places System
Mon Dec 7, 10:26 PM
FPC Ctg1898 cp05T5e12DM4RS
File Edit Analysis Highlight Add track Layout Size options Help
Zoom
21.7
Whole
Show buried clones
Yes No
Search
CB Unit Range
0 to 73
Contig stats
Clones: 8 (0 buried)
Markers: 0
Sequenced: 0
Length: 74 CB units
Ctg1898 of cp05T5e12DM4RS
V041B15
B015E05
V040G04
V025O10
V043J01
V029C12
V021H06
B013A14
0 5 10 15 20 25 30 35 40 45 50 55 60 65 70
[Chickpea contig p...
[root@localhost: /...
[FPC V8.9 Main Menu]
Project cp05T5e12...
FPC Ctg1898 cp05T...

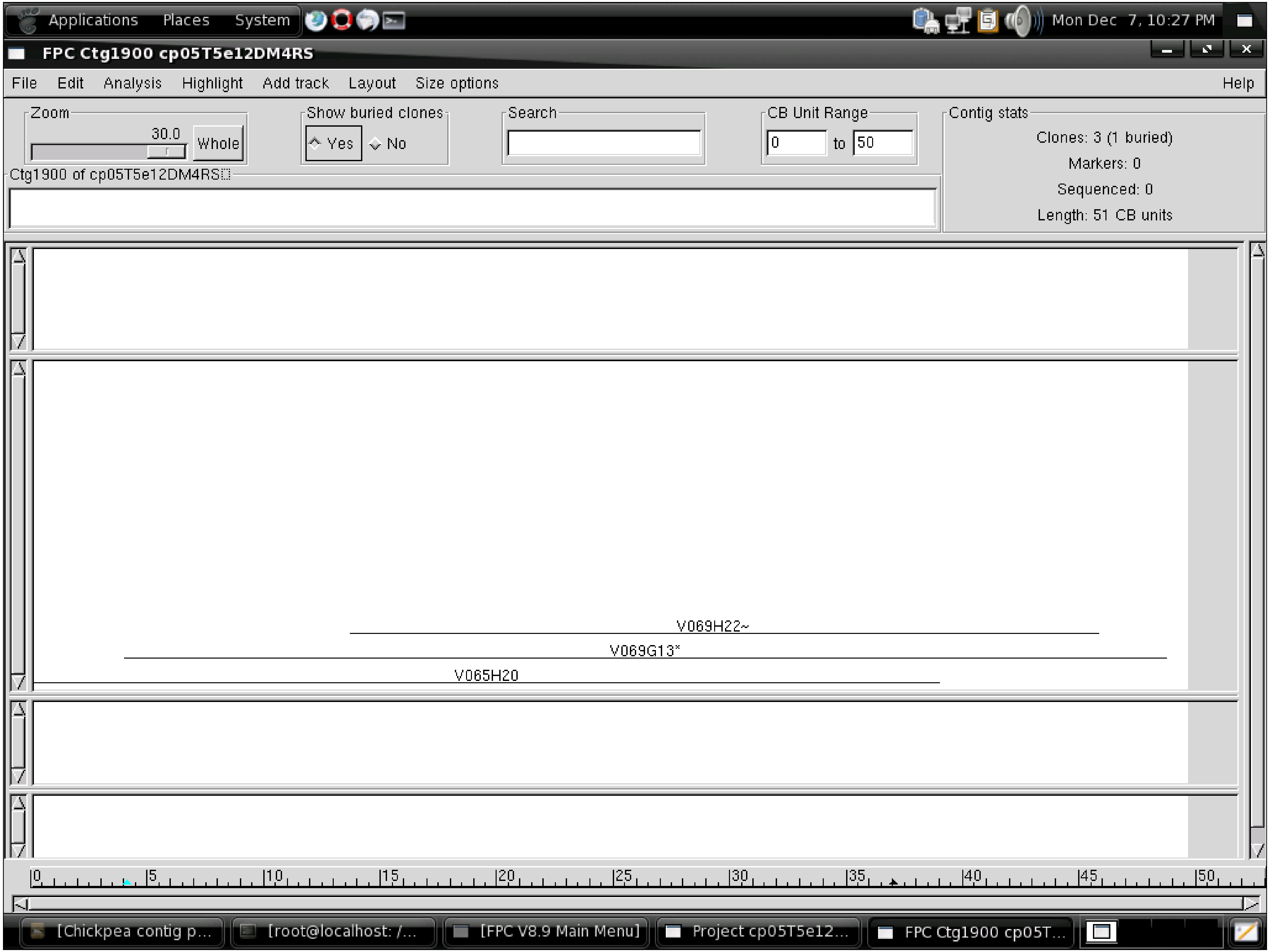
Applications
Places
System
Mon Dec 7, 10:27 PM
FPC Ctg1900 cp05T5e12DM4RS
File
Edit
Analysis
Highlight
Add track
Layout
Size options
Help
Zoom
30.0
Whole
Show buried clones
Yes
No
Search
CB Unit Range
0
to
50
Contig stats
Clones: 3 (1 buried)
Markers: 0
Sequenced: 0
Length: 51 CB units
Ctg1900 of cp05T5e12DM4RS
V069H22~
V069G13*
V065H20
0 5 10 15 20 25 30 35 40 45 50
[Chickpea contig p...
[root@localhost: /...
[FPC V8.9 Main Menu]
Project cp05T5e12...
FPC Ctg1900 cp05T...

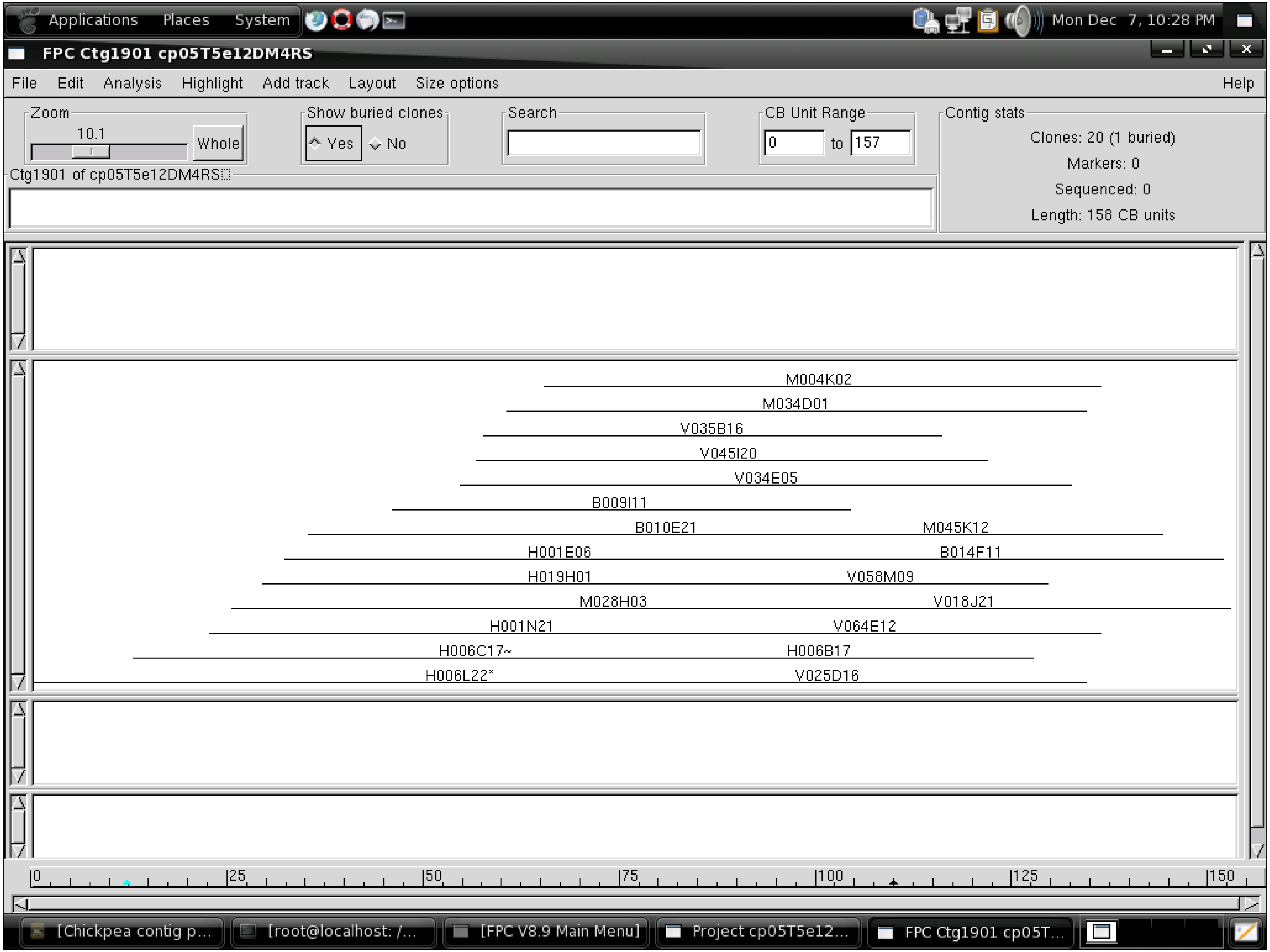
Applications Places System
Mon Dec 7, 10:28 PM
FPC Ctg1901 cp05T5e12DM4RS
File Edit Analysis Highlight Add track Layout Size options Help
Zoom
10.1
Whole
Show buried clones
Yes No
Search
CB Unit Range
0 to 157
Contig stats
Clones: 20 (1 buried)
Markers: 0
Sequenced: 0
Length: 158 CB units
Ctg1901 of cp05T5e12DM4RS
M004K02
M034D01
V035B16
V045I20
V034E05
B009I11
B010E21
M045K12
H001E06
B014F11
H019H01
V058M09
M028H03
V018J21
H001N21
V064E12
H006C17~
H006B17
H006L22*
V025D16
0
25
50
75
100
125
150
[Chickpea contig p...
[root@localhost: /...
[FPC V8.9 Main Menu]
Project cp05T5e12...
FPC Ctg1901 cp05T...

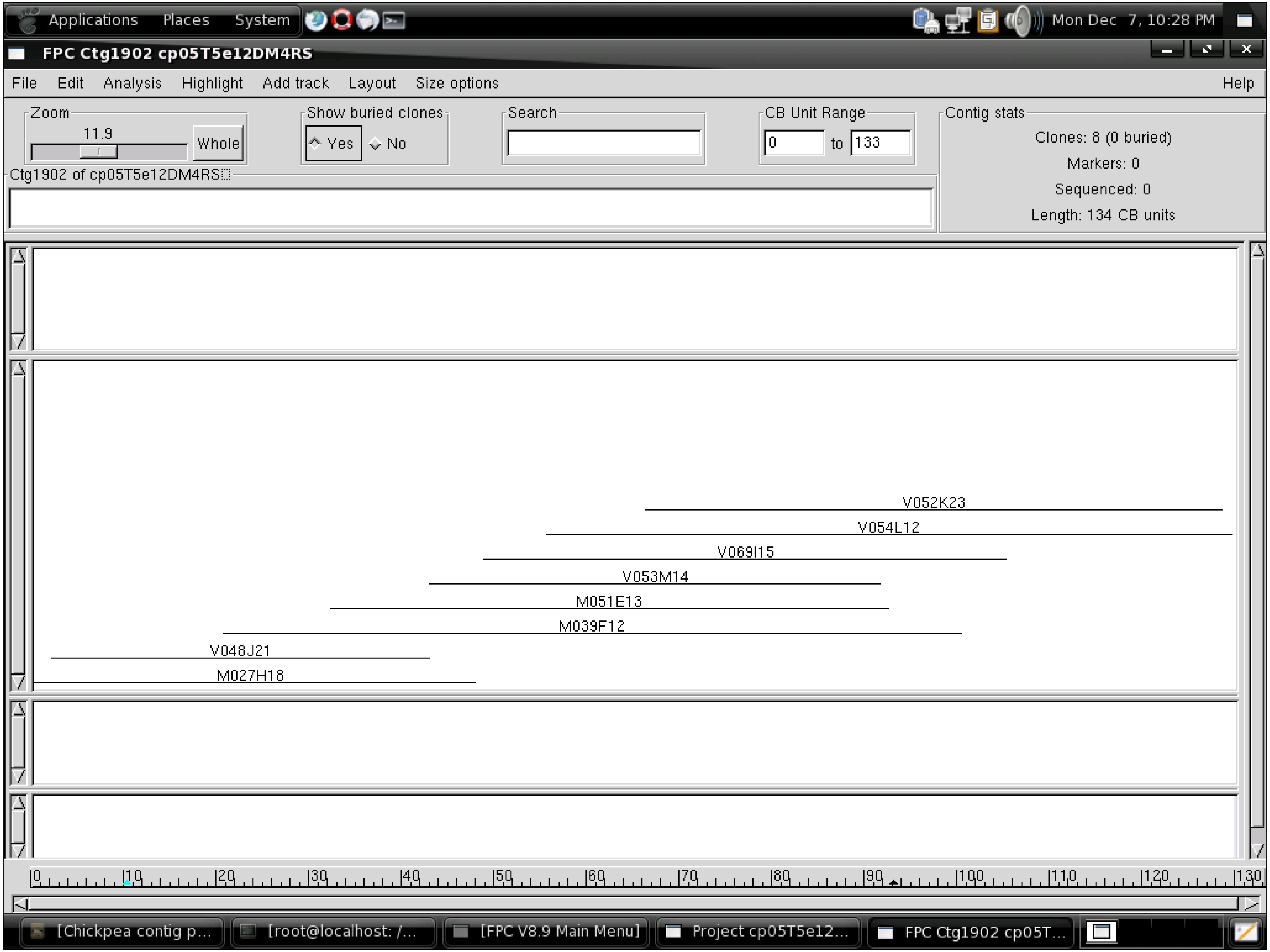
Applications
Places
System
Mon Dec 7, 10:28 PM
FPC Ctg1902 cp05T5e12DM4RS
File
Edit
Analysis
Highlight
Add track
Layout
Size options
Help
Zoom
11.9
Whole
Show buried clones
Yes
No
Search
CB Unit Range
0
to
133
Contig stats
Clones: 8 (0 buried)
Markers: 0
Sequenced: 0
Length: 134 CB units
Ctg1902 of cp05T5e12DM4RS
V052K23
V054L12
V069I15
V053M14
M051E13
M039F12
V048J21
M027H18
0
10
20
30
40
50
60
70
80
90
100
110
120
130
[Chickpea contig p...
[root@localhost: /...
[FPC V8.9 Main Menu]
Project cp05T5e12...
FPC Ctg1902 cp05T...
