# Supplementary material for: A BAC/BIBAC-based physical map of chickpea, Cicer arietinum L
Source: BMC Genomics. 2010 Sep 17;11:501. doi: 10.1186/1471-2164-11-501 (PMC2996997; doi:10.1186/1471-2164-11-501)
Supplement: Additional file 3 — Shows the contigs constituting the physical map of the chickpea genome 3 (ctg1903-2845). [file 1471-2164-11-501-S3.PDF]

A BAC/BIBAC-based physical map of chickpea, *Cicer arietinum* L.

Xiaojun Zhang[1,6], Chantel F Scheuring[1], Meiping Zhang[1,2], Jennifer J Dong[1], Yang Zhang[1], James J Huang[1], Mi-Kyung Lee[1], Shahal Abbo[3], Amir Sherman[4], Dani Shtienberg[4], Weidong Chen[5], Fred Muehlbauer[5], Hong-Bin Zhang[1,]*

[1] Department of Soil and Crop Sciences, Texas A&M University, College Station, Texas 77843-2474, USA
[2] College of Life Science, Jilin Agricultural University, Changchun, Jilin 130118, China
[3] Institute of Plant Science and Genetics in Agriculture, The Hebrew University of Jerusalem, Rehovot, 76100, Israel
[4] The Volcani Center, P.O. Box 6, Bet-Dagan, 50250, Israel
[5] USDA-ARS and Department of Crop and Soil Sciences, Washington State University, Pullman, WA 99164-6434, USA
[6] The Key Laboratory of Experimental Marine Biology, Institute of Oceanology, Chinese Academy of Sciences, Qingdao 266071, China

***Corresponding author:** H.-B. Zhang, Tel.: +1-979-862-2244; Fax: +1-979-845-0456; E-mail: hbz7049@tamu.edu

Additional file 3. The contigs constituting the physical map of the chickpea genome 3(ctg1903-2845)

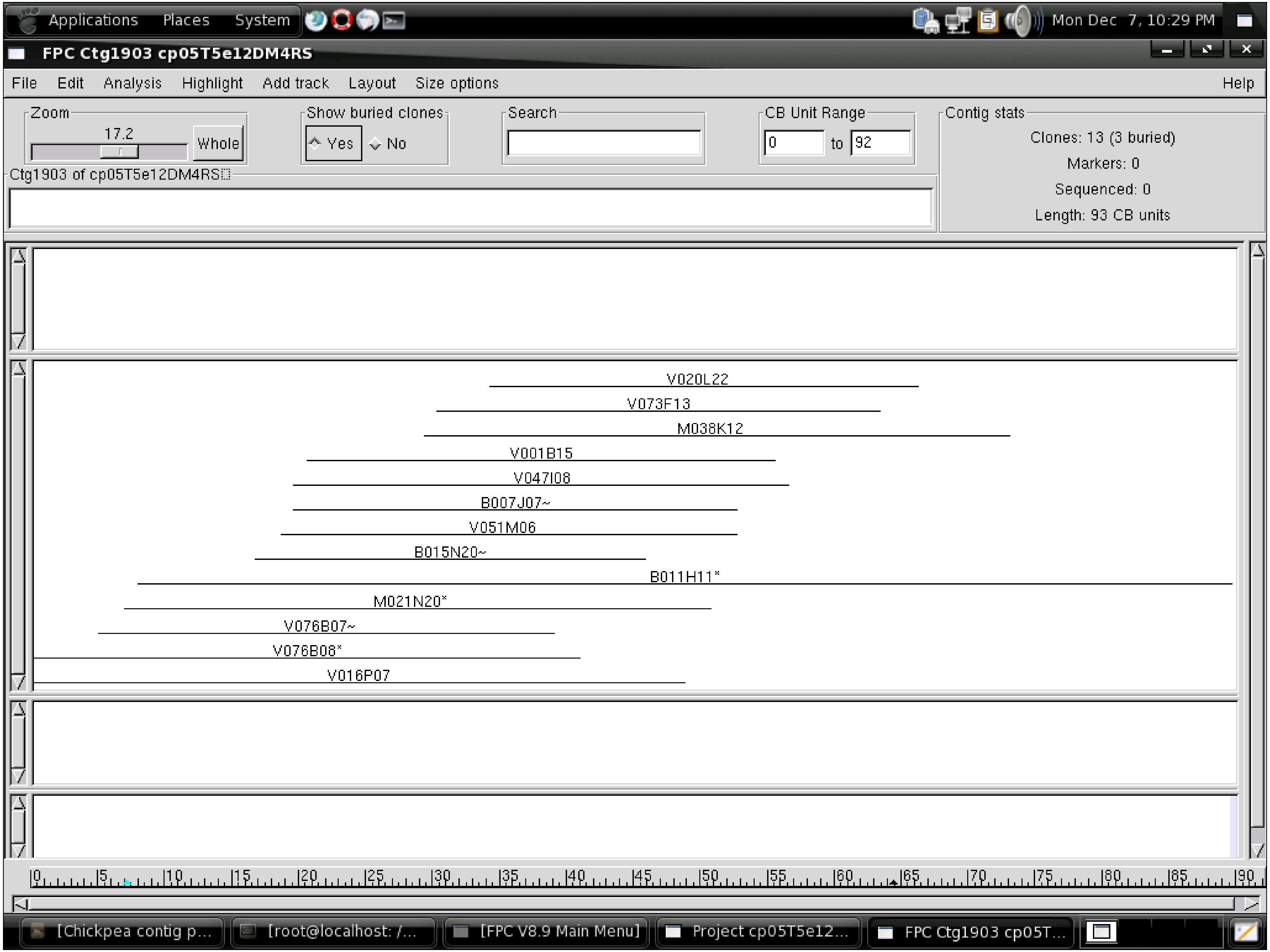
Applications Places System
Mon Dec 7, 10:29 PM
FPC Ctg1903 cp05T5e12DM4RS
File Edit Analysis Highlight Add track Layout Size options Help
Zoom
17.2
Whole
Show buried clones
Yes No
Search
CB Unit Range
0 to 92
Contig stats
Clones: 13 (3 buried)
Markers: 0
Sequenced: 0
Length: 93 CB units
Ctg1903 of cp05T5e12DM4RS
V020L22
V073F13
M038K12
V001B15
V047I08
B007J07~
V051M06
B015N20~
B011H11*
M021N20*
V076B07~
V076B08*
V016P07
0 5 10 15 20 25 30 35 40 45 50 55 60 65 70 75 80 85 90
[Chickpea contig p...
[root@localhost: /...
[FPC V8.9 Main Menu]
Project cp05T5e12...
FPC Ctg1903 cp05T...

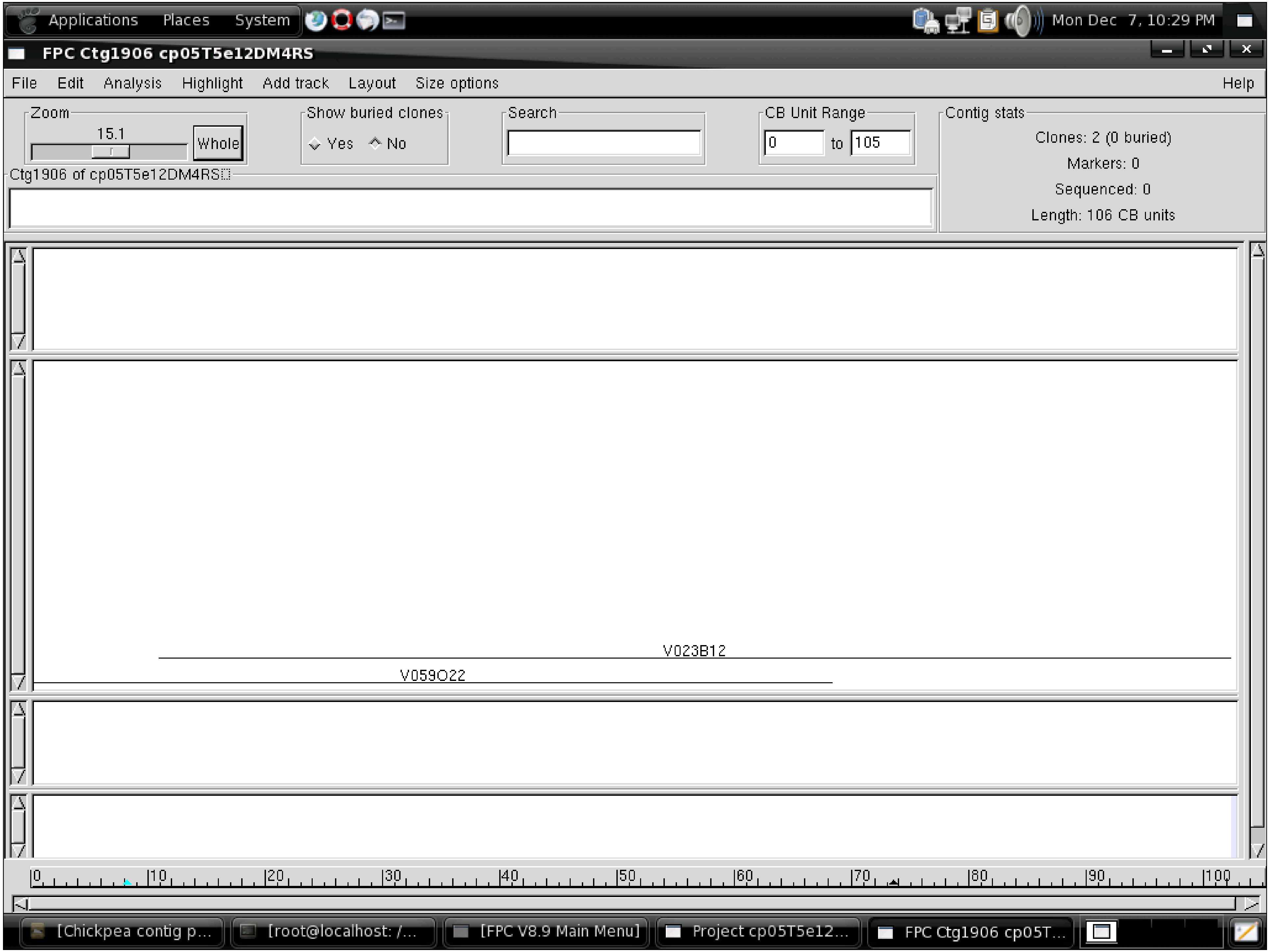
Applications
Places
System
Mon Dec 7, 10:29 PM
FPC Ctg1906 cp05T5e12DM4RS
File
Edit
Analysis
Highlight
Add track
Layout
Size options
Help
Zoom
15.1
Whole
Show buried clones
Yes
No
Search
CB Unit Range
0
to
105
Contig stats
Clones: 2 (0 buried)
Markers: 0
Sequenced: 0
Length: 106 CB units
Ctg1906 of cp05T5e12DM4RS
V023B12
V059O22
0
10
20
30
40
50
60
70
80
90
100
[Chickpea contig p...
[root@localhost: /...
[FPC V8.9 Main Menu]
Project cp05T5e12...
FPC Ctg1906 cp05T...

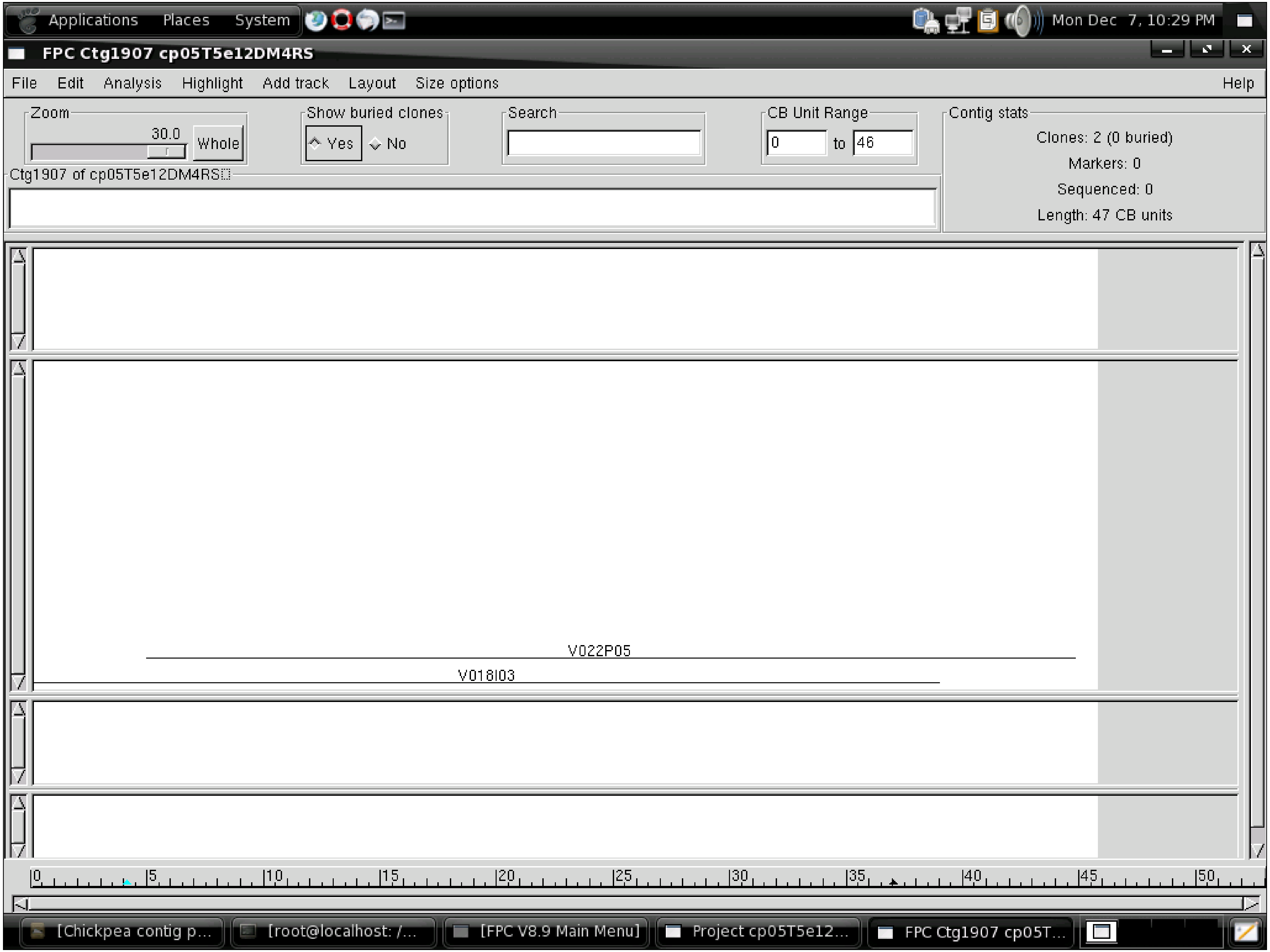
Applications Places System
Mon Dec 7, 10:29 PM
FPC Ctg1907 cp05T5e12DM4RS
File Edit Analysis Highlight Add track Layout Size options Help
Zoom
30.0
Whole
Show buried clones
Yes
No
Search
CB Unit Range
0
to
46
Contig stats
Clones: 2 (0 buried)
Markers: 0
Sequenced: 0
Length: 47 CB units
Ctg1907 of cp05T5e12DM4RS
V022P05
V018I03
0 5 10 15 20 25 30 35 40 45 50
[Chickpea contig p...
[root@localhost: /...
[FPC V8.9 Main Menu]
Project cp05T5e12...
FPC Ctg1907 cp05T...

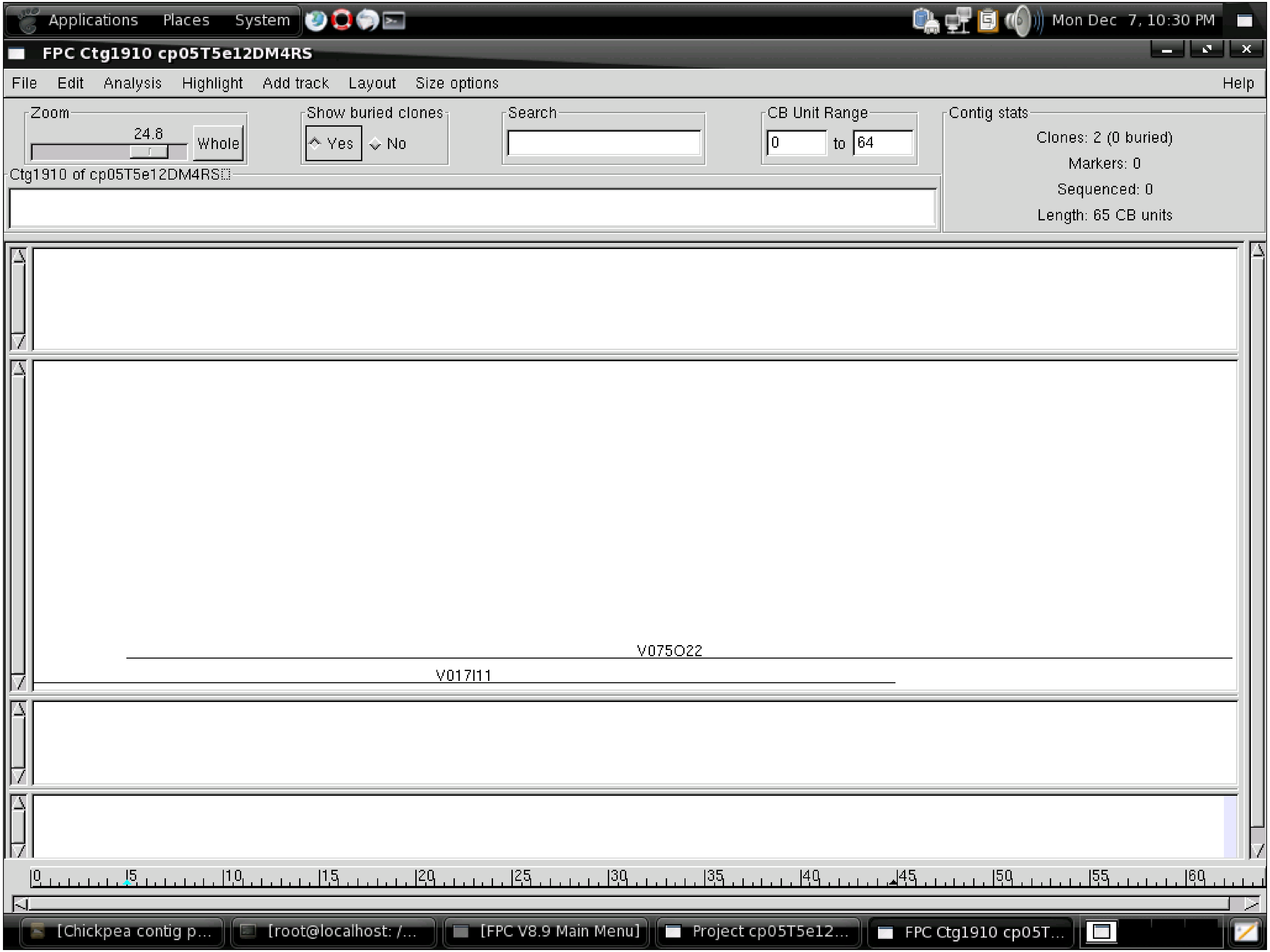
Applications
Places
System
Mon Dec 7, 10:30 PM
FPC Ctg1910 cp05T5e12DM4RS
File
Edit
Analysis
Highlight
Add track
Layout
Size options
Help
Zoom
24.8
Whole
Show buried clones
Yes
No
Search
CB Unit Range
0
to
64
Contig stats
Clones: 2 (0 buried)
Markers: 0
Sequenced: 0
Length: 65 CB units
Ctg1910 of cp05T5e12DM4RS
V075O22
V017I11
0 5 10 15 20 25 30 35 40 45 50 55 60
[Chickpea contig p...
[root@localhost: /...
[FPC V8.9 Main Menu]
Project cp05T5e12...
FPC Ctg1910 cp05T...

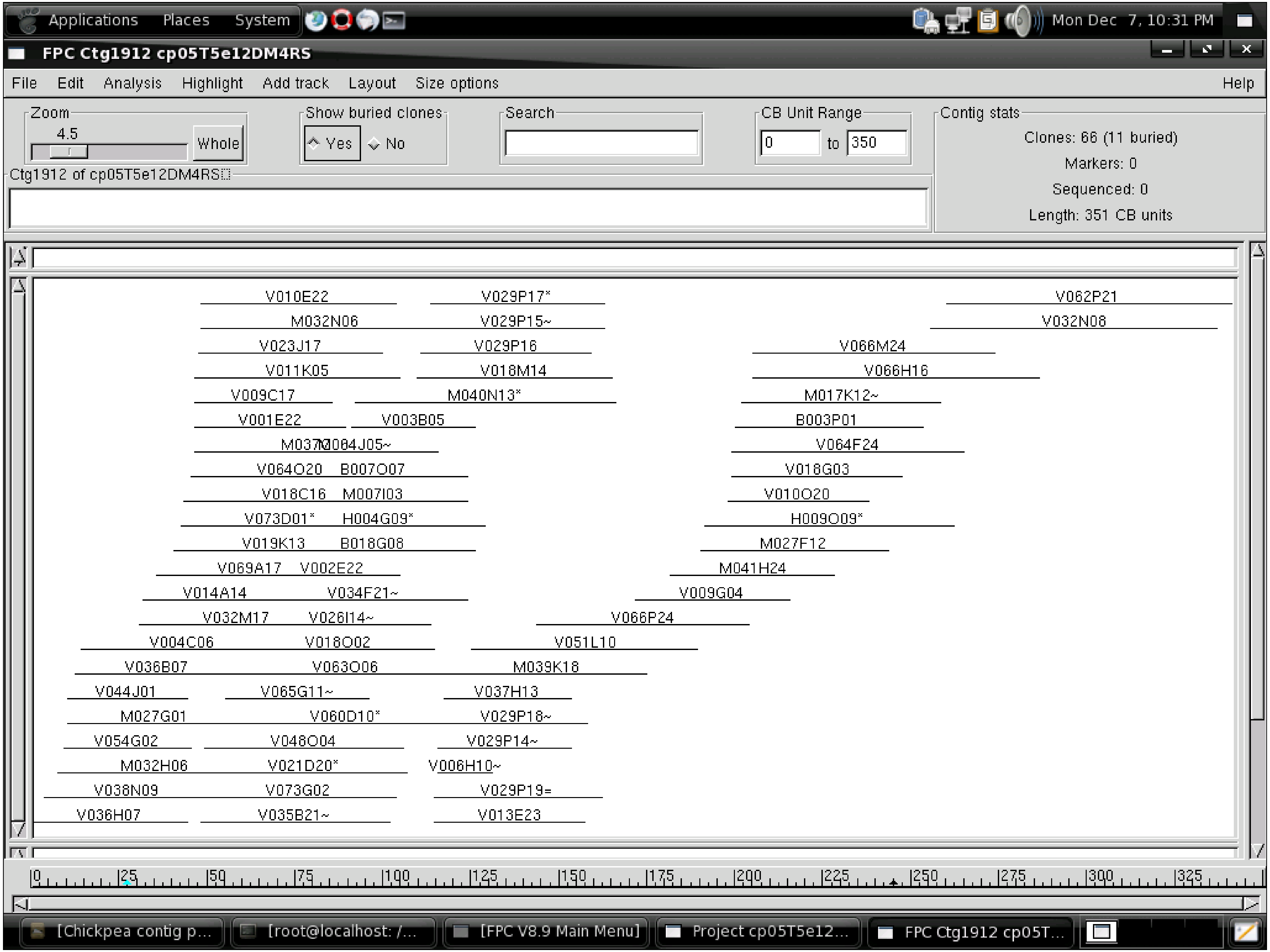
Applications Places System
Mon Dec 7, 10:31 PM
FPC Ctg1912 cp05T5e12DM4RS
File Edit Analysis Highlight Add track Layout Size options Help
Zoom
4.5
Whole
Show buried clones
Yes
No
Search
CB Unit Range
0
to
350
Contig stats
Clones: 66 (11 buried)
Markers: 0
Sequenced: 0
Length: 351 CB units
Ctg1912 of cp05T5e12DM4RS
V010E22
V029P17*
V062P21
M032N06
V029P15~
V032N08
V023J17
V029P16
V066M24
V011K05
V018M14
V066H16
V009C17
M040N13*
M017K12~
V001E22
V003B05
B003P01
V064F24
V064O20
B007O07
V018G03
V018C16
M007I03
V010O20
V073D01*
H004G09*
H009O09*
V019K13
B018G08
M027F12
V069A17
V002E22
M041H24
V014A14
V034F21~
V009G04
V032M17
V026I14~
V066P24
V004C06
V018O02
V051L10
V036B07
V063O06
M039K18
V044J01
V065G11~
V037H13
M027G01
V060D10*
V029P18~
V054G02
V048O04
V029P14~
M032H06
V021D20*
V006H10~
V038N09
V073G02
V029P19=
V036H07
V035B21~
V013E23
0
25
50
75
100
125
150
175
200
225
250
275
300
325
[Chickpea contig p...
[root@localhost: /...
[FPC V8.9 Main Menu]
Project cp05T5e12...
FPC Ctg1912 cp05T...

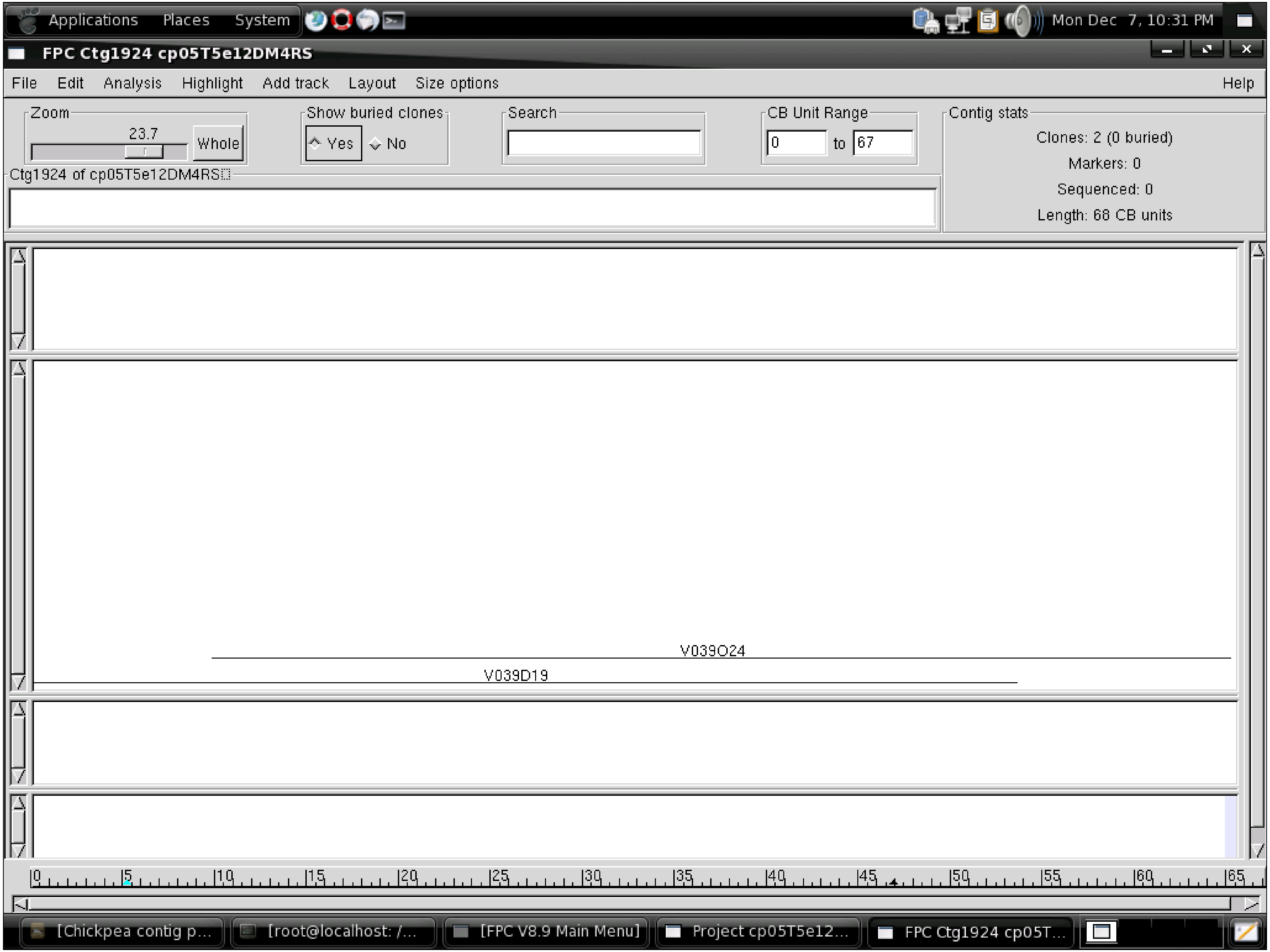
Applications Places System
Mon Dec 7, 10:31 PM
FPC Ctg1924 cp05T5e12DM4RS
File Edit Analysis Highlight Add track Layout Size options Help
Zoom
23.7
Whole
Show buried clones
Yes
No
Search
CB Unit Range
0
to
67
Contig stats
Clones: 2 (0 buried)
Markers: 0
Sequenced: 0
Length: 68 CB units
Ctg1924 of cp05T5e12DM4RS
V039O24
V039D19
0 5 10 15 20 25 30 35 40 45 50 55 60 65
[Chickpea contig p...
[root@localhost: /...
[FPC V8.9 Main Menu]
Project cp05T5e12...
FPC Ctg1924 cp05T...

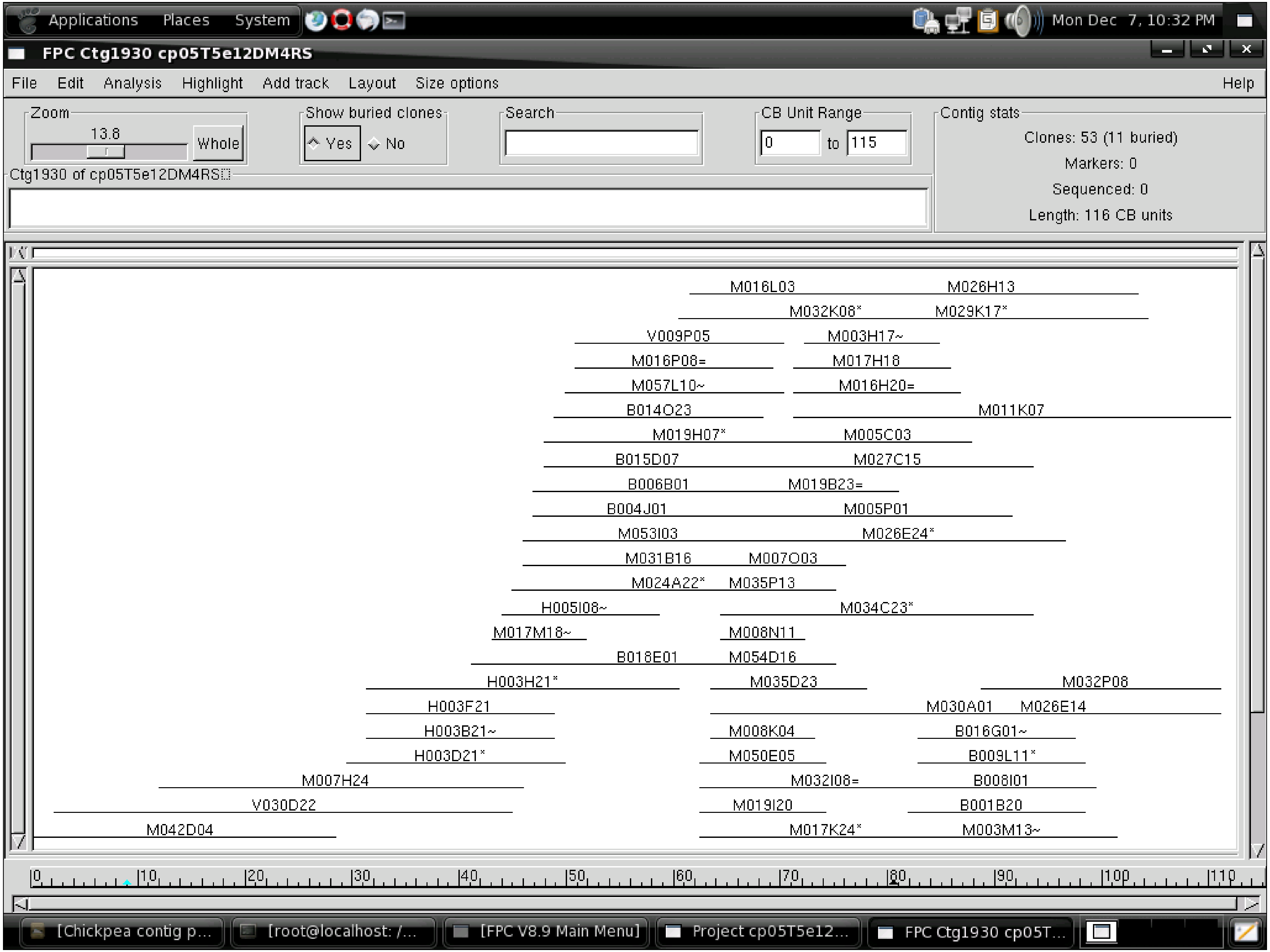
Applications Places System
Mon Dec 7, 10:32 PM
FPC Ctg1930 cp05T5e12DM4RS
File Edit Analysis Highlight Add track Layout Size options Help
Zoom
13.8
Whole
Show buried clones
Yes
No
Search
CB Unit Range
0 to 115
Contig stats
Clones: 53 (11 buried)
Markers: 0
Sequenced: 0
Length: 116 CB units
Ctg1930 of cp05T5e12DM4RS
M016L03
M026H13
M032K08*
M029K17*
V009P05
M003H17~
M016P08=
M017H18
M057L10~
M016H20=
B014O23
M011K07
M019H07*
M005C03
B015D07
M027C15
B006B01
M019B23=
B004J01
M005P01
M053I03
M026E24*
M031B16
M007O03
M024A22*
M035P13
H005I08~
M034C23*
M017M18~
M008N11
B018E01
M054D16
H003H21*
M035D23
M032P08
H003F21
M030A01
M026E14
H003B21~
M008K04
B016G01~
H003D21*
M050E05
B009L11*
M007H24
M032I08=
B008I01
V030D22
M019I20
B001B20
M042D04
M017K24*
M003M13~
0 10 20 30 40 50 60 70 80 90 100 110
[Chickpea contig p...
[root@localhost: /...
[FPC V8.9 Main Menu]
Project cp05T5e12...
FPC Ctg1930 cp05T...

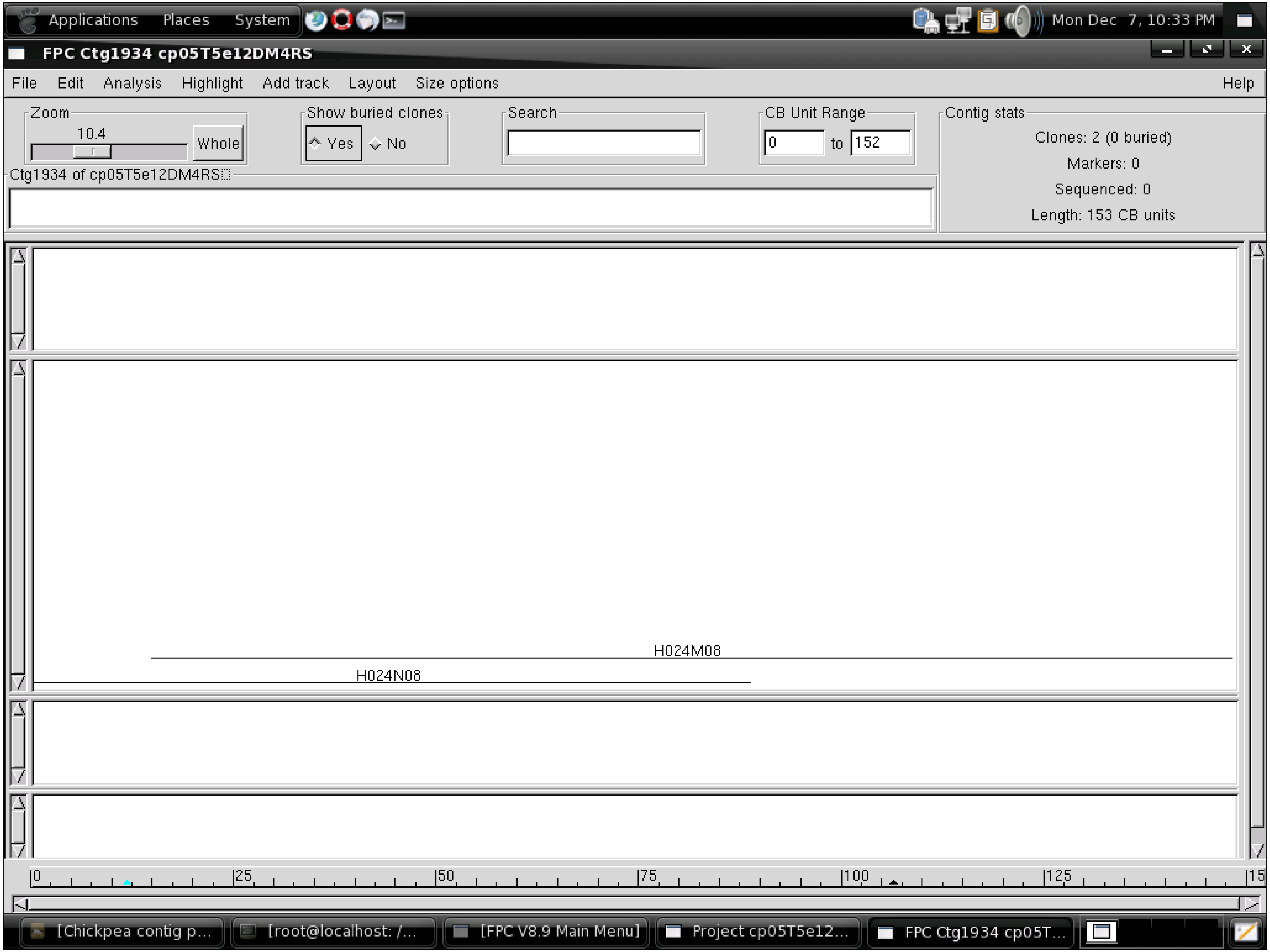
Applications Places System
Mon Dec 7, 10:33 PM
FPC Ctg1934 cp05T5e12DM4RS
File Edit Analysis Highlight Add track Layout Size options Help
Zoom
10.4
Whole
Show buried clones
Yes
No
Search
CB Unit Range
0
to
152
Contig stats
Clones: 2 (0 buried)
Markers: 0
Sequenced: 0
Length: 153 CB units
Ctg1934 of cp05T5e12DM4RS
H024M08
H024N08
0
25
50
75
100
125
[Chickpea contig p...
[root@localhost: /...
[FPC V8.9 Main Menu]
Project cp05T5e12...
FPC Ctg1934 cp05T...

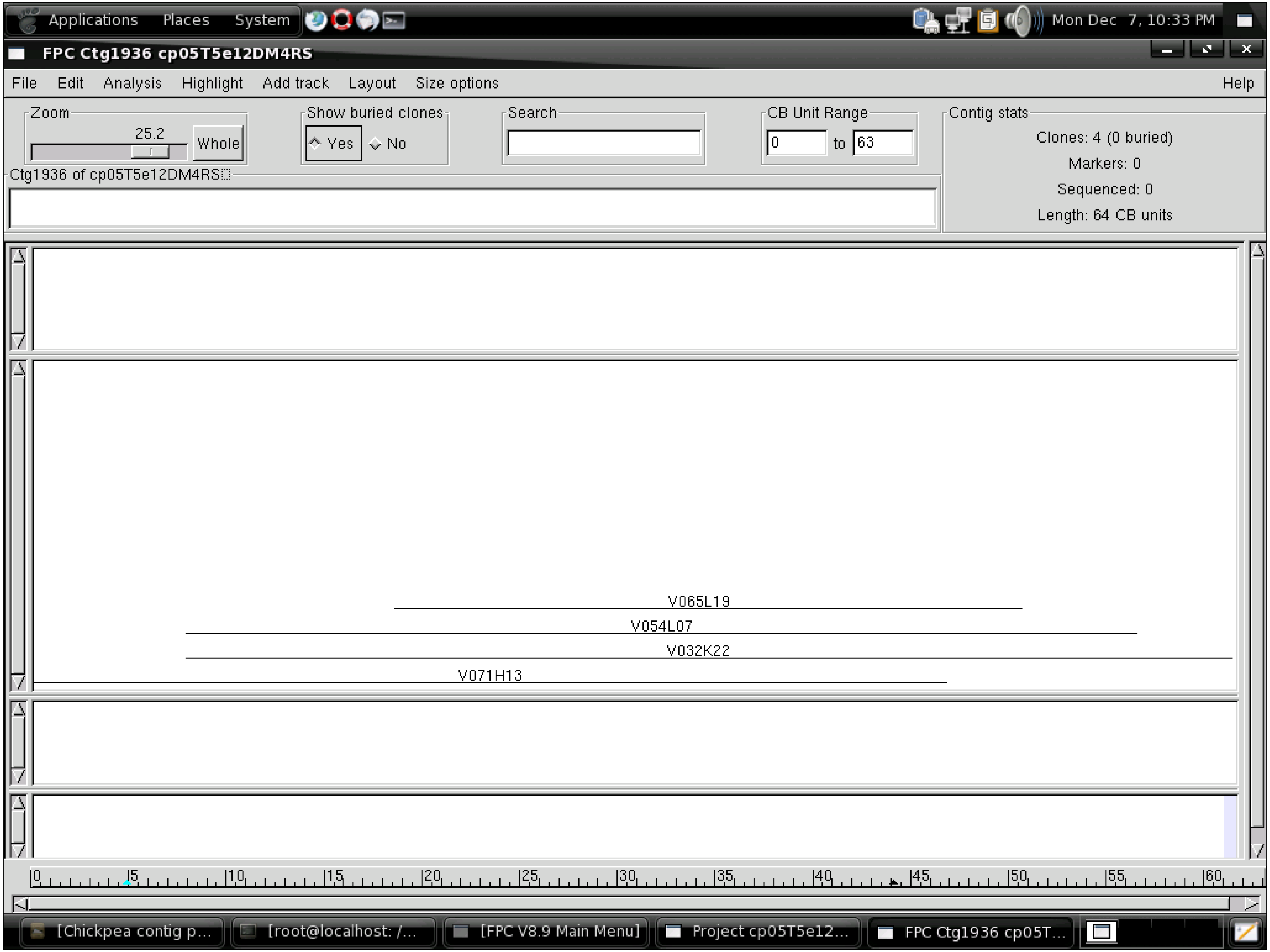
Applications Places System
Mon Dec 7, 10:33 PM
FPC Ctg1936 cp05T5e12DM4RS
File Edit Analysis Highlight Add track Layout Size options Help
Zoom
25.2
Whole
Show buried clones
Yes
No
Search
CB Unit Range
0 to 63
Contig stats
Clones: 4 (0 buried)
Markers: 0
Sequenced: 0
Length: 64 CB units
Ctg1936 of cp05T5e12DM4RS
V065L19
V054L07
V032K22
V071H13
0 5 10 15 20 25 30 35 40 45 50 55 60
[Chickpea contig p...
[root@localhost: /...
[FPC V8.9 Main Menu]
Project cp05T5e12...
FPC Ctg1936 cp05T...

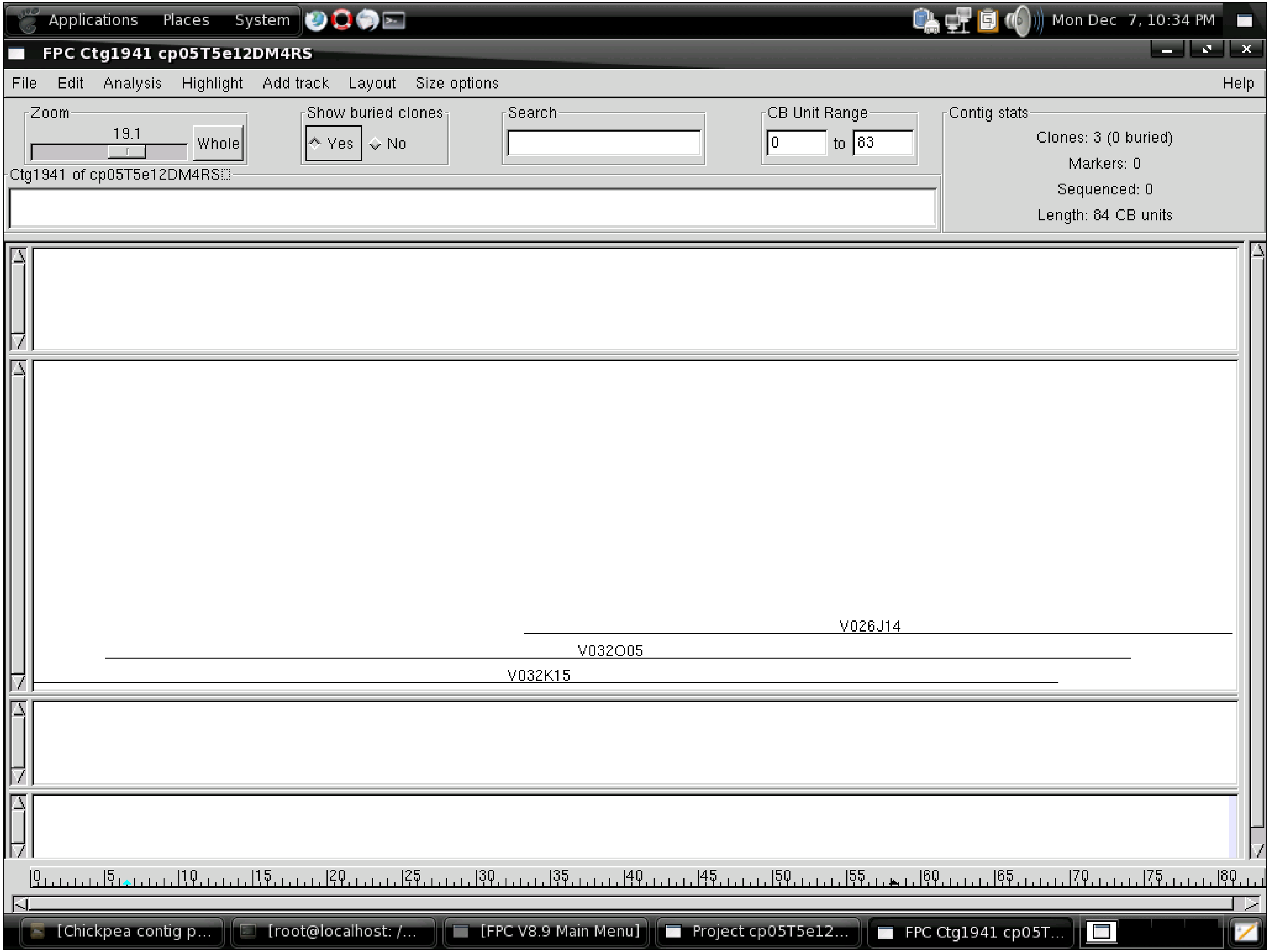
Applications
Places
System
Mon Dec 7, 10:34 PM
FPC Ctg1941 cp05T5e12DM4RS
File
Edit
Analysis
Highlight
Add track
Layout
Size options
Help
Zoom
19.1
Whole
Show buried clones
Yes
No
Search
CB Unit Range
0
to
83
Contig stats
Clones: 3 (0 buried)
Markers: 0
Sequenced: 0
Length: 84 CB units
Ctg1941 of cp05T5e12DM4RS
V026J14
V032O05
V032K15
0
5
10
15
20
25
30
35
40
45
50
55
60
65
70
75
80
[Chickpea contig p...
[root@localhost: /...
[FPC V8.9 Main Menu]
Project cp05T5e12...
FPC Ctg1941 cp05T...

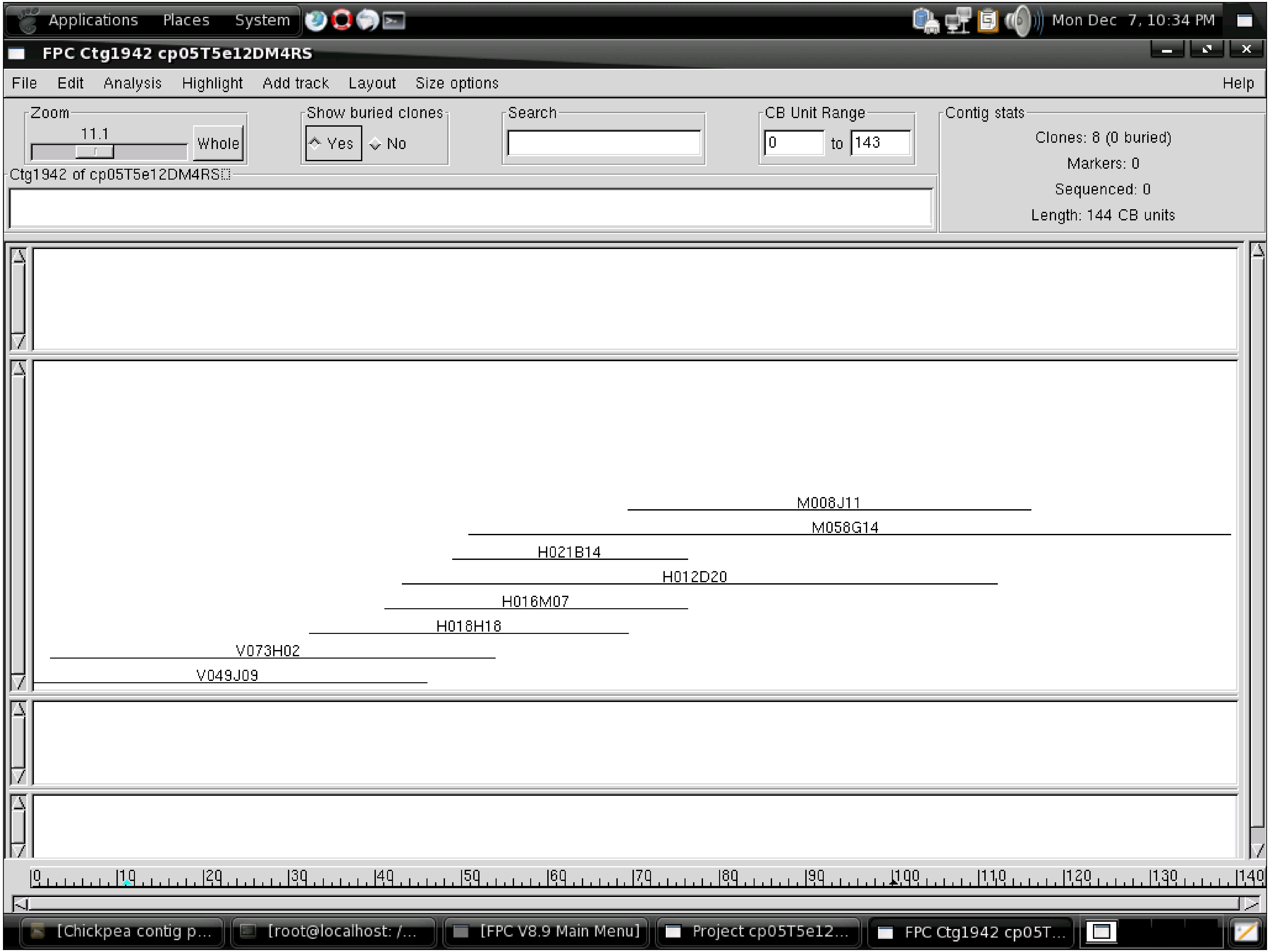
Applications Places System
Mon Dec 7, 10:34 PM
FPC Ctg1942 cp05T5e12DM4RS
File Edit Analysis Highlight Add track Layout Size options Help
Zoom
11.1
Whole
Show buried clones
Yes No
Search
CB Unit Range
0 to 143
Contig stats
Clones: 8 (0 buried)
Markers: 0
Sequenced: 0
Length: 144 CB units
Ctg1942 of cp05T5e12DM4RS
M008J11
M058G14
H021B14
H012D20
H016M07
H018H18
V073H02
V049J09
0 10 20 30 40 50 60 70 80 90 100 110 120 130 140
[Chickpea contig p...
[root@localhost: /...
[FPC V8.9 Main Menu]
Project cp05T5e12...
FPC Ctg1942 cp05T...

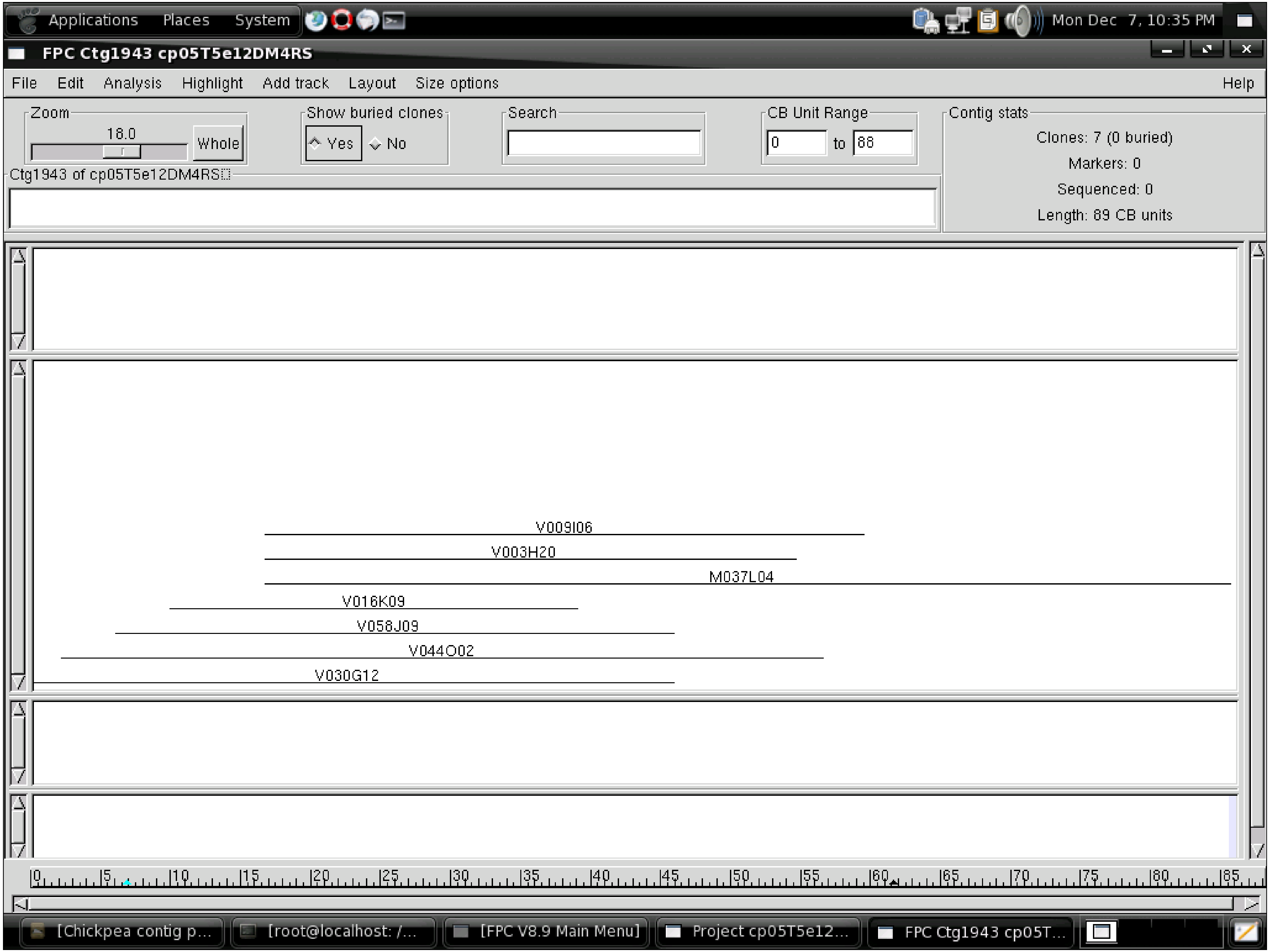
Applications Places System
Mon Dec 7, 10:35 PM
FPC Ctg1943 cp05T5e12DM4RS
File Edit Analysis Highlight Add track Layout Size options Help
Zoom
18.0
Whole
Show buried clones
Yes No
Search
CB Unit Range
0 to 88
Contig stats
Clones: 7 (0 buried)
Markers: 0
Sequenced: 0
Length: 89 CB units
Ctg1943 of cp05T5e12DM4RS
V009I06
V003H20
M037L04
V016K09
V058J09
V044O02
V030G12
0 5 10 15 20 25 30 35 40 45 50 55 60 65 70 75 80 85
[Chickpea contig p...
[root@localhost: /...
[FPC V8.9 Main Menu]
Project cp05T5e12...
FPC Ctg1943 cp05T...

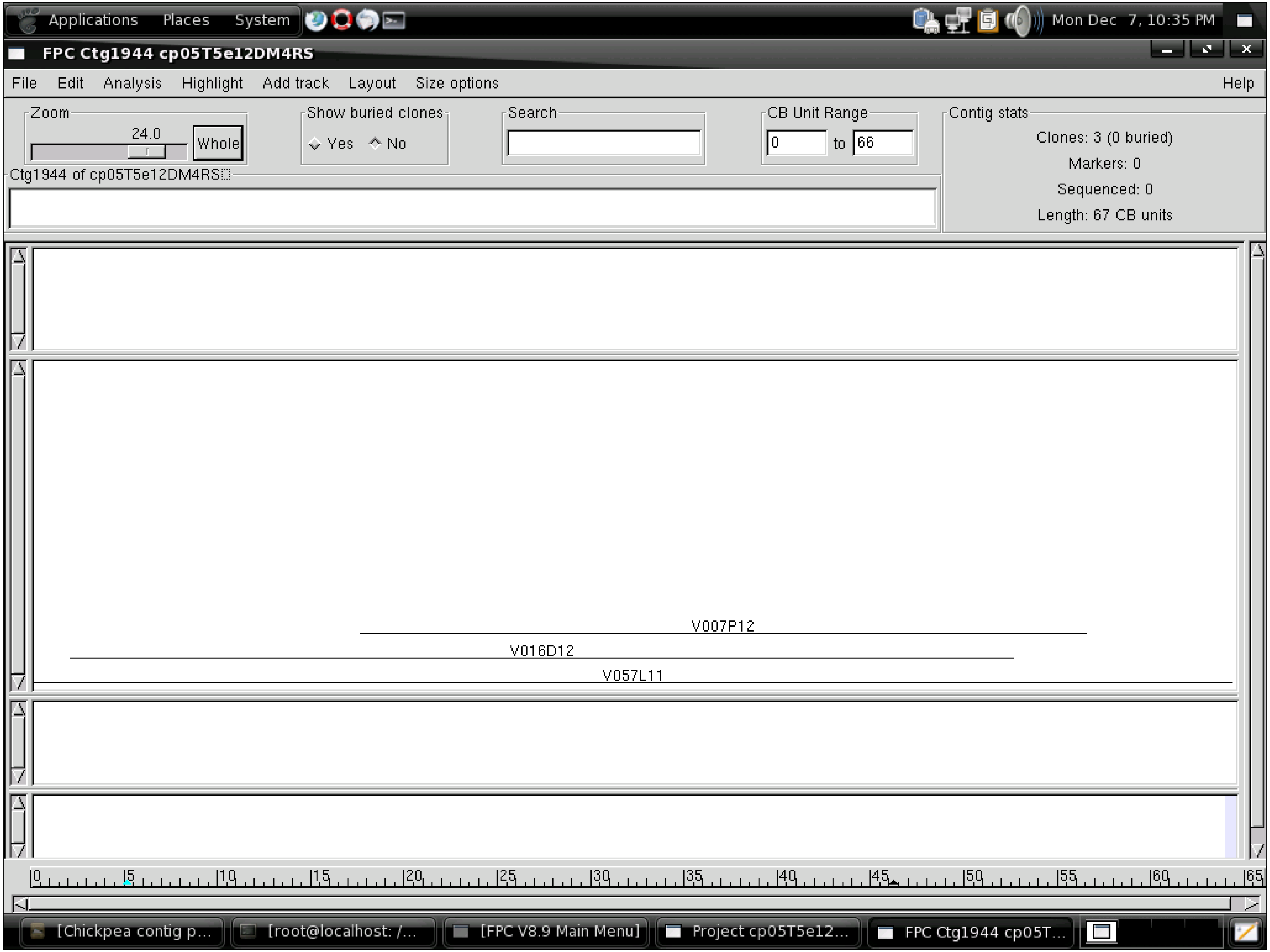
Applications Places System
Mon Dec 7, 10:35 PM
FPC Ctg1944 cp05T5e12DM4RS
File Edit Analysis Highlight Add track Layout Size options Help
Zoom
24.0
Whole
Show buried clones
Yes No
Search
CB Unit Range
0 to 66
Contig stats
Clones: 3 (0 buried)
Markers: 0
Sequenced: 0
Length: 67 CB units
Ctg1944 of cp05T5e12DM4RS
V007P12
V016D12
V057L11
0 5 10 15 20 25 30 35 40 45 50 55 60 65
[Chickpea contig p...
[root@localhost: /...
[FPC V8.9 Main Menu]
Project cp05T5e12...
FPC Ctg1944 cp05T...

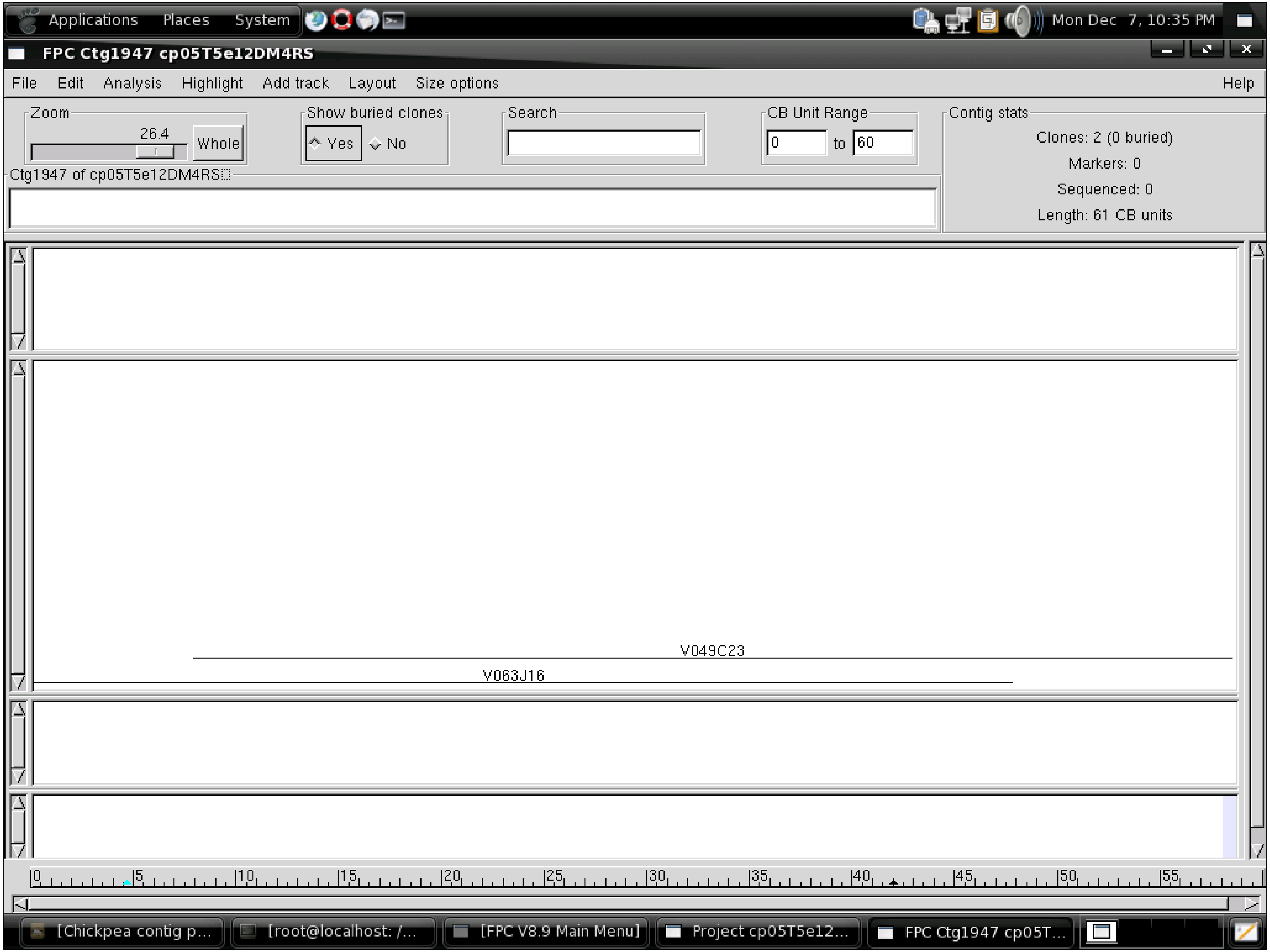
Applications Places System
Mon Dec 7, 10:35 PM
FPC Ctg1947 cp05T5e12DM4RS
File Edit Analysis Highlight Add track Layout Size options Help
Zoom
26.4
Whole
Show buried clones
Yes
No
Search
CB Unit Range
0 to 60
Contig stats
Clones: 2 (0 buried)
Markers: 0
Sequenced: 0
Length: 61 CB units
Ctg1947 of cp05T5e12DM4RS
V049C23
V063J16
0 5 10 15 20 25 30 35 40 45 50 55
[Chickpea contig p...
[root@localhost: /...
[FPC V8.9 Main Menu]
Project cp05T5e12...
FPC Ctg1947 cp05T...

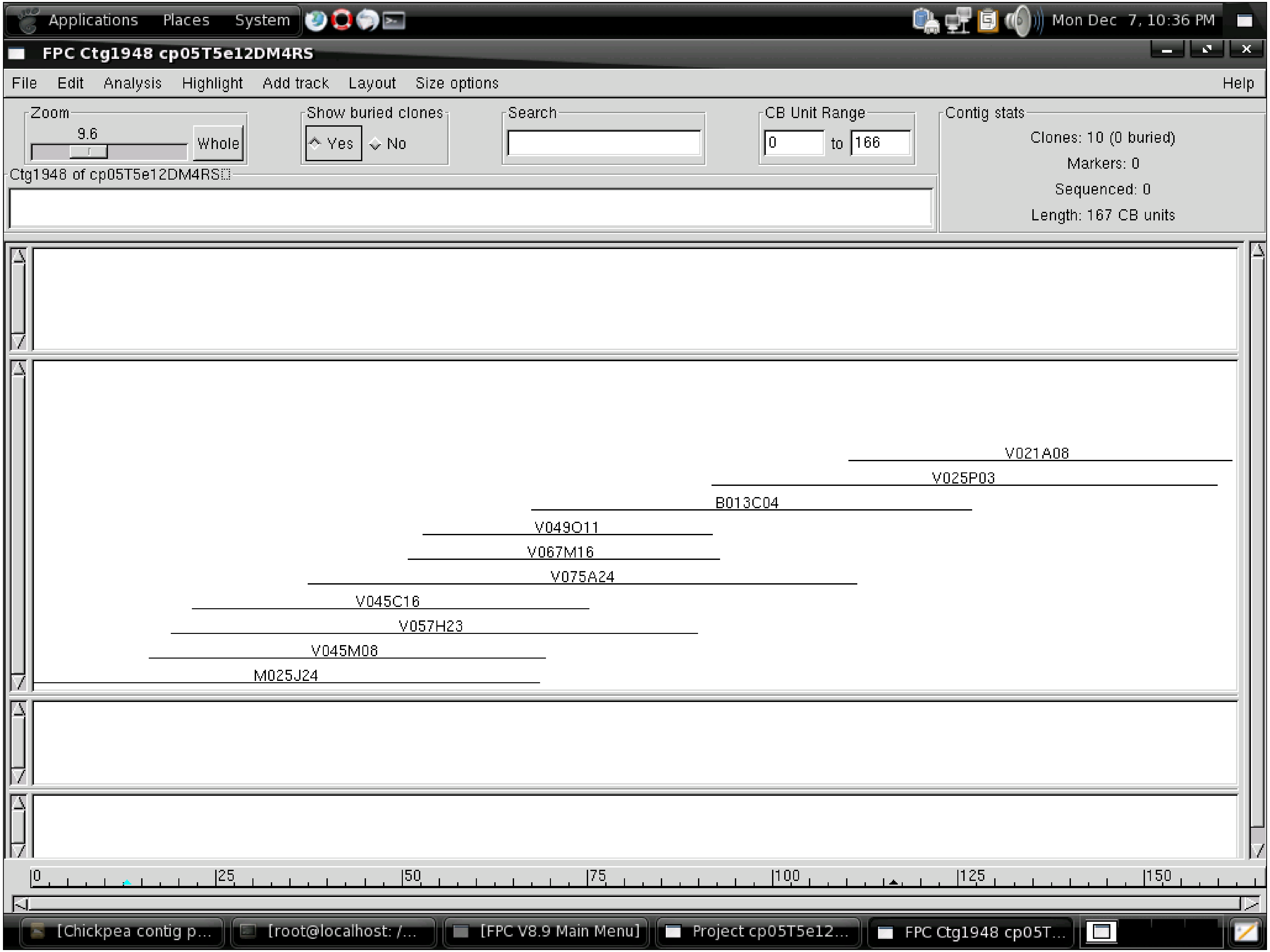
Applications Places System
Mon Dec 7, 10:36 PM
FPC Ctg1948 cp05T5e12DM4RS
File Edit Analysis Highlight Add track Layout Size options Help
Zoom
9.6
Whole
Show buried clones
Yes
No
Search
CB Unit Range
0 to 166
Contig stats
Clones: 10 (0 buried)
Markers: 0
Sequenced: 0
Length: 167 CB units
Ctg1948 of cp05T5e12DM4RS
V021A08
V025P03
B013C04
V049O11
V067M16
V075A24
V045C16
V057H23
V045M08
M025J24
0
25
50
75
100
125
150
[Chickpea contig p...
[root@localhost: /...
[FPC V8.9 Main Menu]
Project cp05T5e12...
FPC Ctg1948 cp05T...

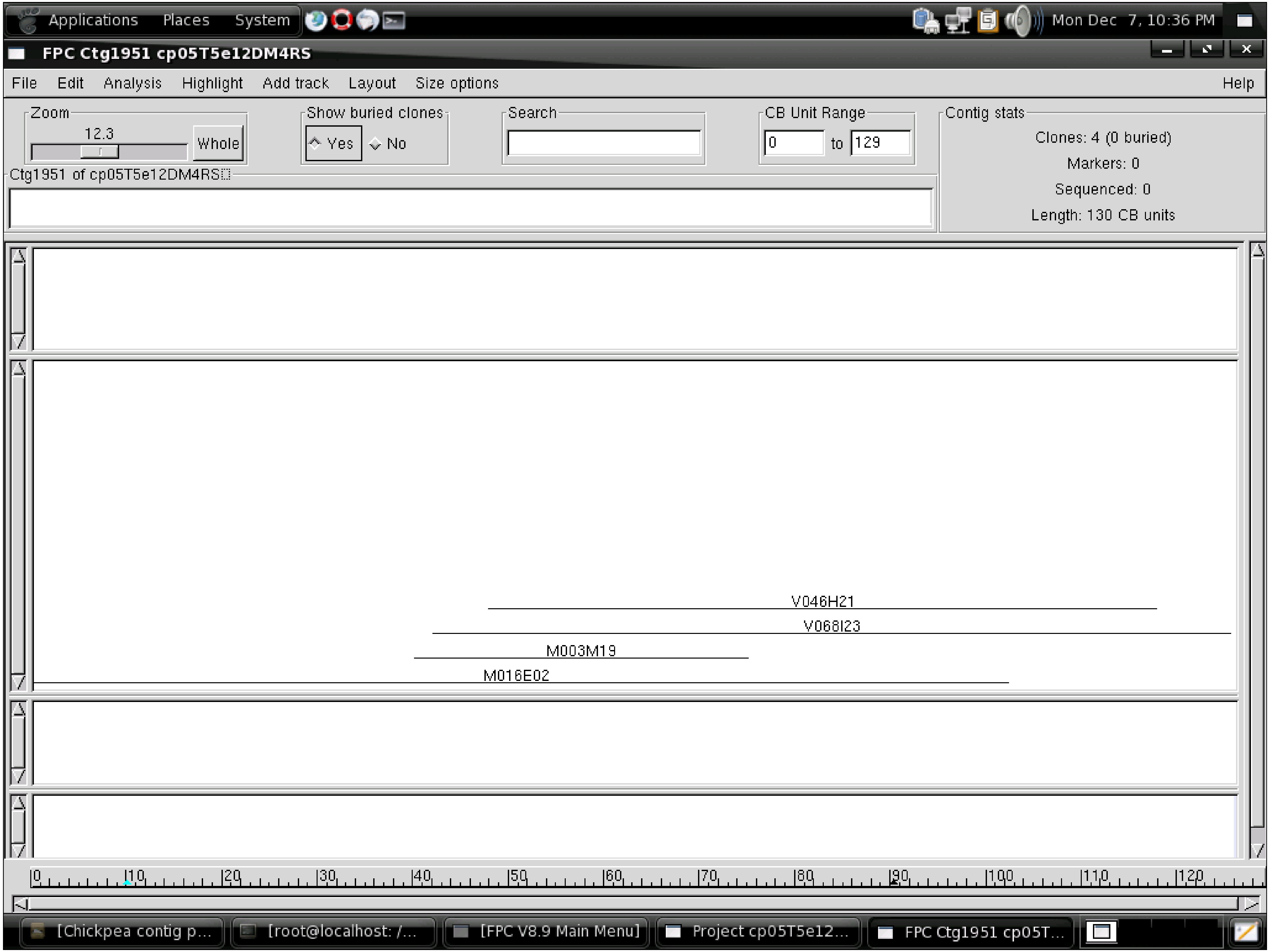
Applications Places System
Mon Dec 7, 10:36 PM
FPC Ctg1951 cp05T5e12DM4RS
File Edit Analysis Highlight Add track Layout Size options Help
Zoom
12.3
Whole
Show buried clones
Yes No
Search
CB Unit Range
0 to 129
Contig stats
Clones: 4 (0 buried)
Markers: 0
Sequenced: 0
Length: 130 CB units
Ctg1951 of cp05T5e12DM4RS
V046H21
V068I23
M003M19
M016E02
0 10 20 30 40 50 60 70 80 90 100 110 120
[Chickpea contig p...
[root@localhost: /...
[FPC V8.9 Main Menu]
Project cp05T5e12...
FPC Ctg1951 cp05T...

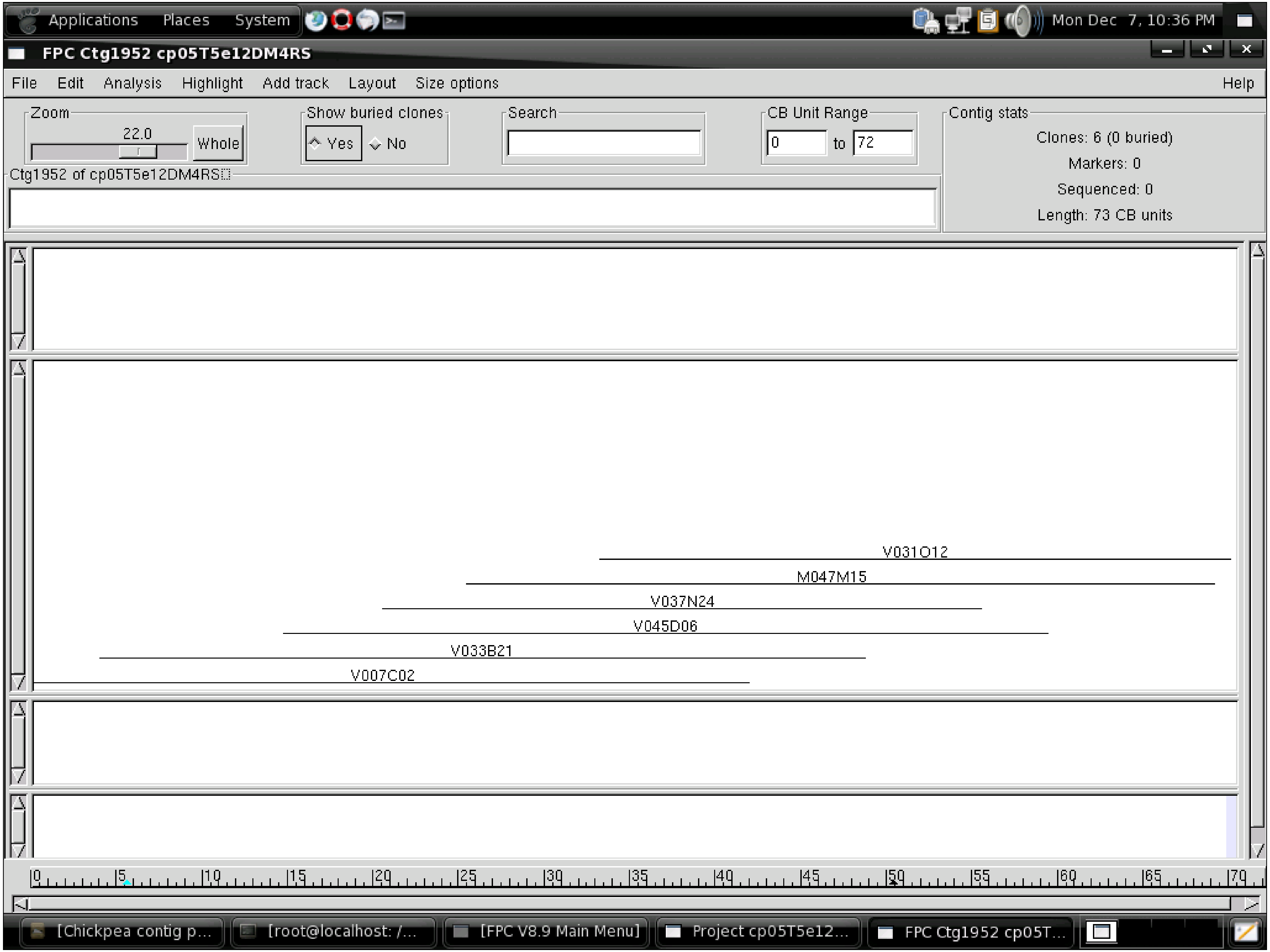
Applications Places System
Mon Dec 7, 10:36 PM
FPC Ctg1952 cp05T5e12DM4RS
File Edit Analysis Highlight Add track Layout Size options Help
Zoom
22.0
Whole
Show buried clones
Yes
No
Search
CB Unit Range
0 to 72
Contig stats
Clones: 6 (0 buried)
Markers: 0
Sequenced: 0
Length: 73 CB units
Ctg1952 of cp05T5e12DM4RS
V031O12
M047M15
V037N24
V045D06
V033B21
V007C02
0 5 10 15 20 25 30 35 40 45 50 55 60 65 70
[Chickpea contig p...
[root@localhost: /...
[FPC V8.9 Main Menu]
Project cp05T5e12...
FPC Ctg1952 cp05T...

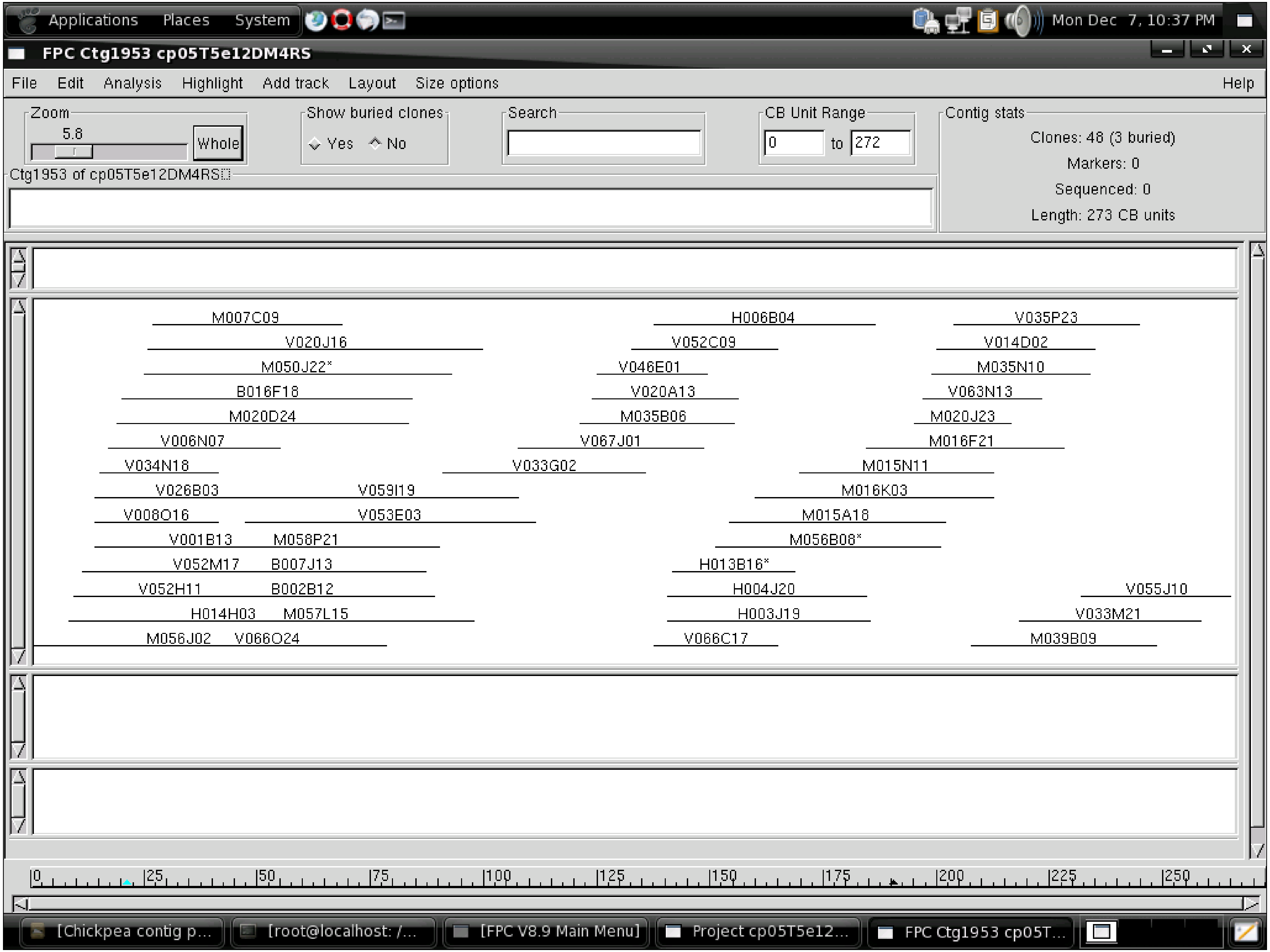
Applications Places System
Mon Dec 7, 10:37 PM
FPC Ctg1953 cp05T5e12DM4RS
File Edit Analysis Highlight Add track Layout Size options Help
Zoom 5.8 Whole
Show buried clones Yes No
Search
CB Unit Range 0 to 272
Contig stats
Clones: 48 (3 buried)
Markers: 0
Sequenced: 0
Length: 273 CB units
Ctg1953 of cp05T5e12DM4RS
M007C09
V020J16
M050J22*
B016F18
M020D24
V006N07
V034N18
V026B03
V059I19
V008O16
V053E03
V001B13
M058P21
V052M17
B007J13
V052H11
B002B12
H014H03
M057L15
M056J02
V066O24
H006B04
V052C09
V046E01
V020A13
M035B06
V067J01
V033G02
M015N11
M016K03
M015A18
M056B08*
H013B16*
H004J20
H003J19
V066C17
V035P23
V014D02
M035N10
V063N13
M020J23
M016F21
V055J10
V033M21
M039B09
0 25 50 75 100 125 150 175 200 225 250
[Chickpea contig p...
[root@localhost: /...
[FPC V8.9 Main Menu]
Project cp05T5e12...
FPC Ctg1953 cp05T...

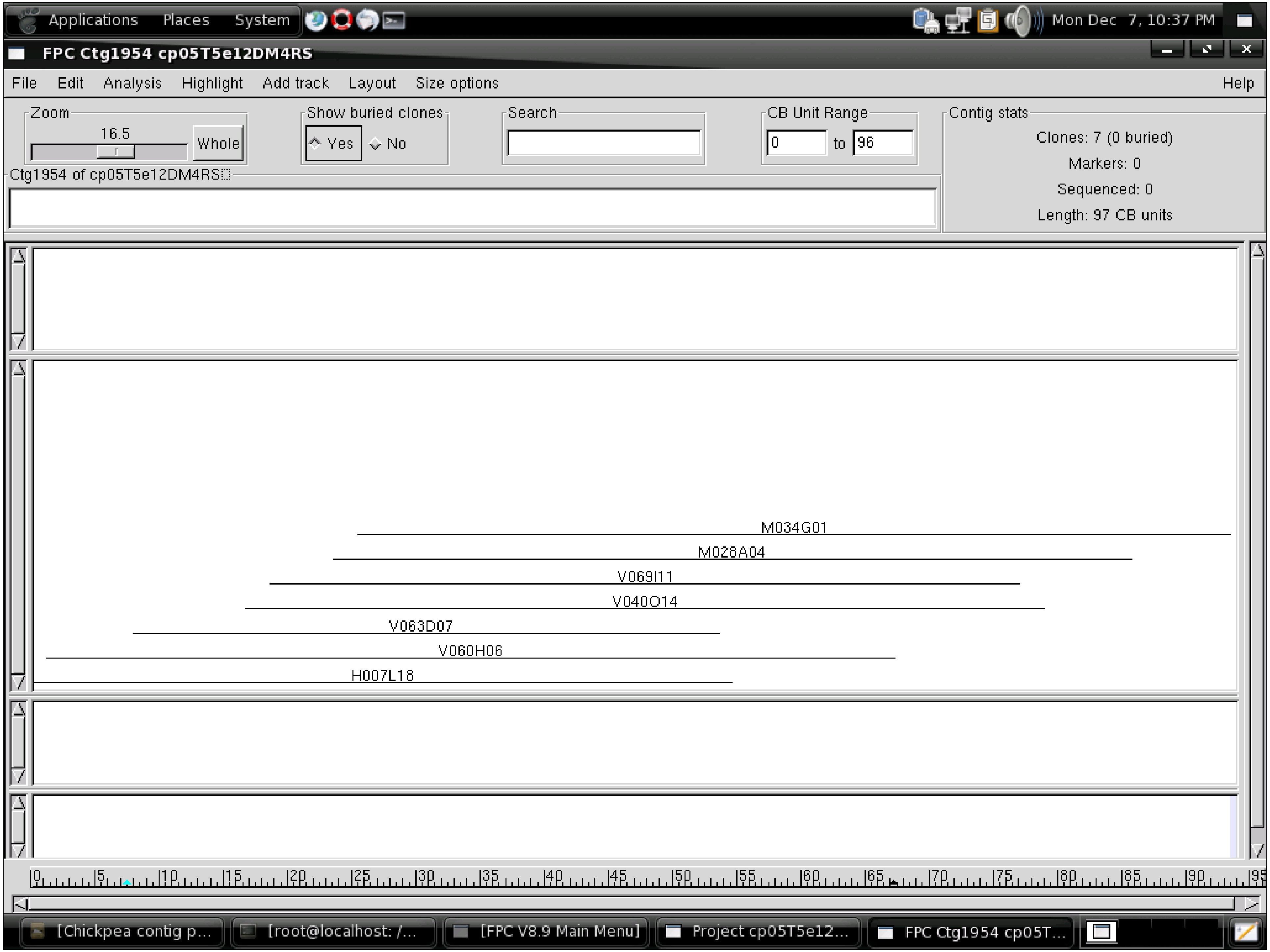
Applications Places System
Mon Dec 7, 10:37 PM
FPC Ctg1954 cp05T5e12DM4RS
File Edit Analysis Highlight Add track Layout Size options Help
Zoom
16.5
Whole
Show buried clones
Yes No
Search
CB Unit Range
0 to 96
Contig stats
Clones: 7 (0 buried)
Markers: 0
Sequenced: 0
Length: 97 CB units
Ctg1954 of cp05T5e12DM4RS
M034G01
M028A04
V069I11
V040O14
V063D07
V060H06
H007L18
0 5 10 15 20 25 30 35 40 45 50 55 60 65 70 75 80 85 90 95
[Chickpea contig p...
[root@localhost: /...
[FPC V8.9 Main Menu]
Project cp05T5e12...
FPC Ctg1954 cp05T...

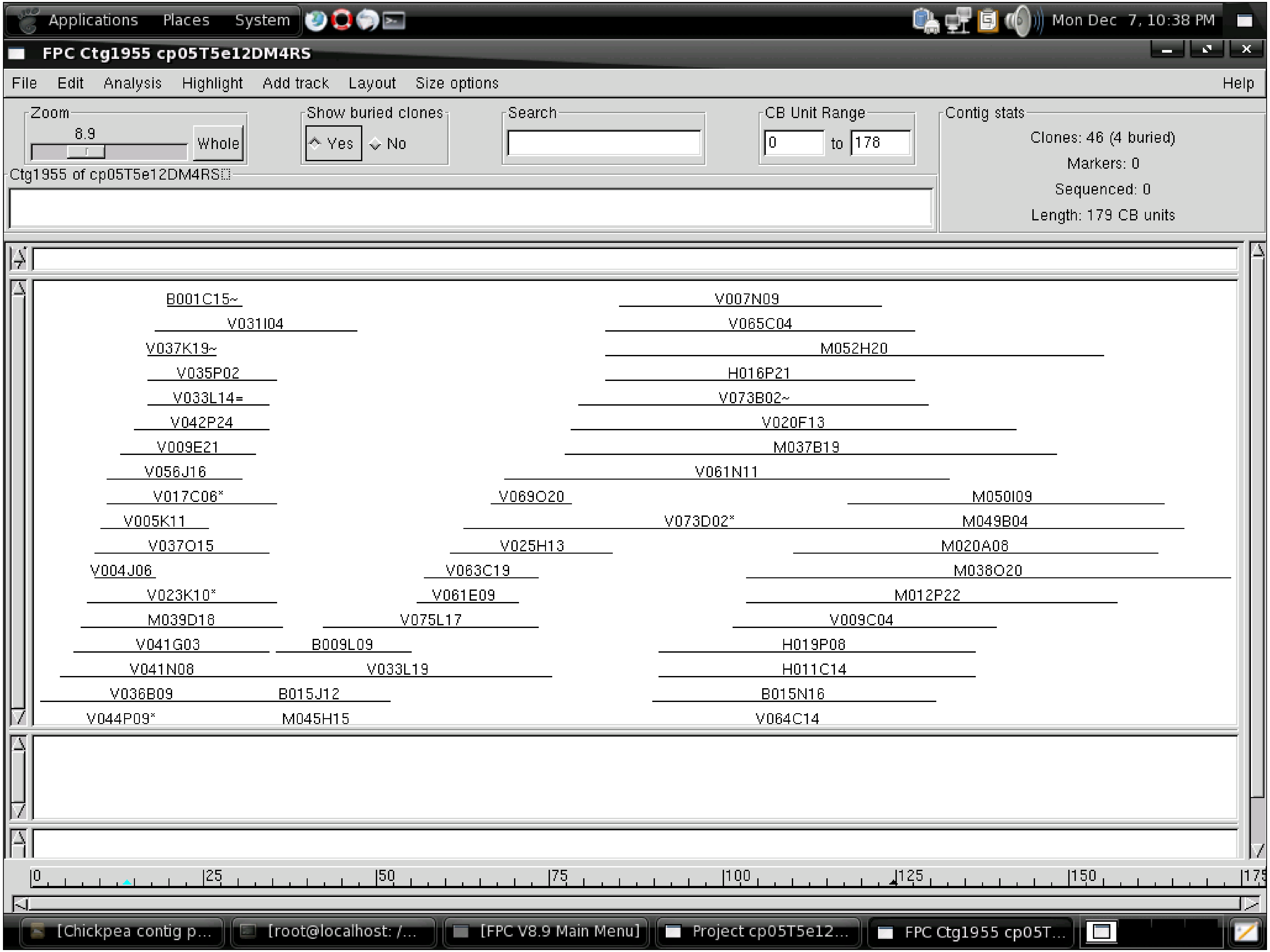
Applications Places System
Mon Dec 7, 10:38 PM
FPC Ctg1955 cp05T5e12DM4RS
File Edit Analysis Highlight Add track Layout Size options Help
Zoom
8.9
Whole
Show buried clones
Yes
No
Search
CB Unit Range
0 to 178
Contig stats
Clones: 46 (4 buried)
Markers: 0
Sequenced: 0
Length: 179 CB units
Ctg1955 of cp05T5e12DM4RS
B001C15~
V031I04
V037K19~
V035P02
V033L14=
V042P24
V009E21
V056J16
V017C06*
V005K11
V037O15
V004J06
V023K10*
M039D18
V041G03
V041N08
V036B09
V044P09*
V007N09
V065C04
M052H20
H016P21
V073B02~
V020F13
M037B19
V061N11
V069O20
M050I09
V073D02*
M049B04
V025H13
M020A08
V063C19
M038O20
V061E09
M012P22
V075L17
V009C04
B009L09
H019P08
V033L19
H011C14
B015J12
B015N16
M045H15
V064C14
0
25
50
75
100
125
150
175
[Chickpea contig p...
[root@localhost: /...
[FPC V8.9 Main Menu]
Project cp05T5e12...
FPC Ctg1955 cp05T...

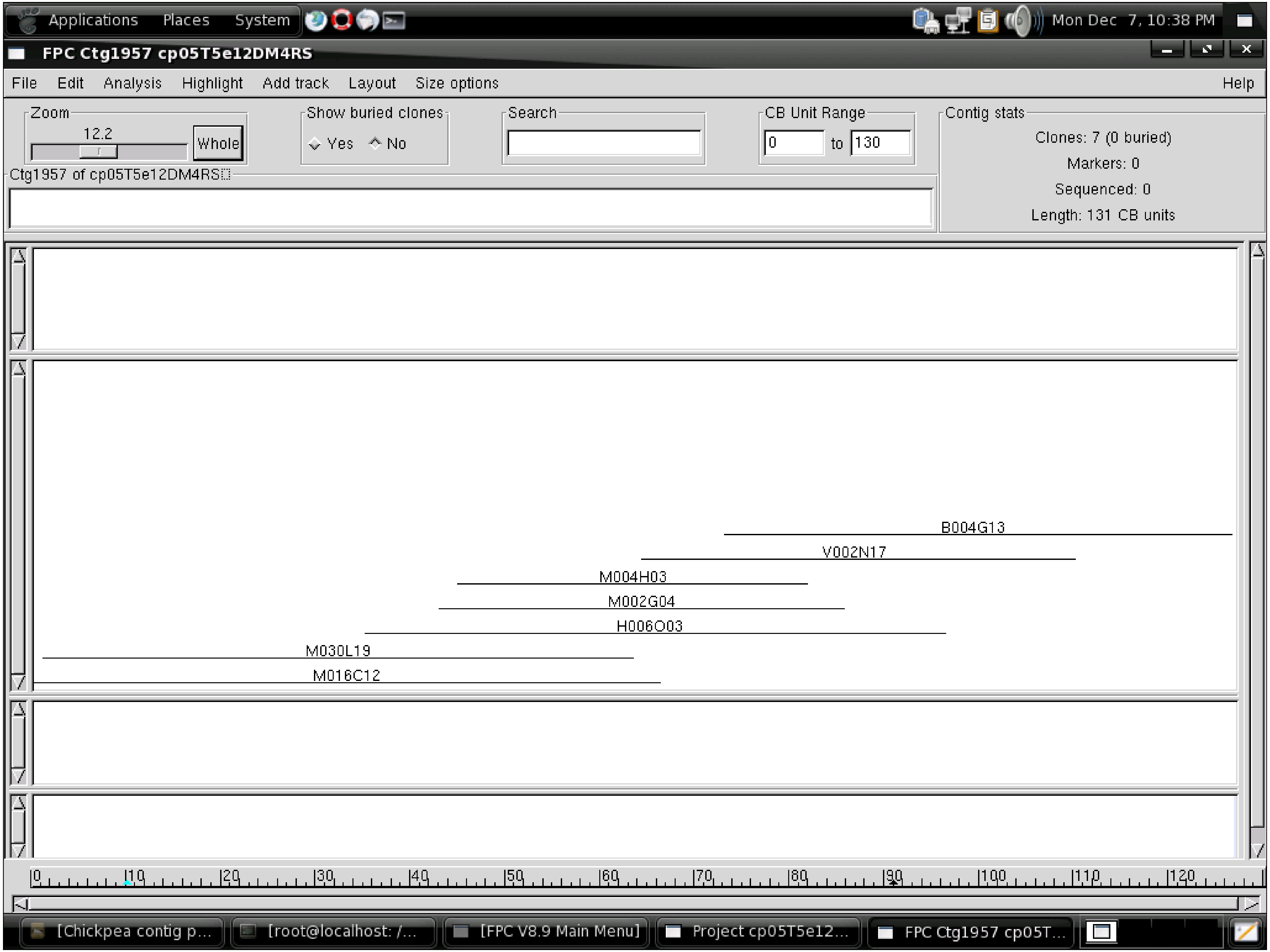
Applications Places System
Mon Dec 7, 10:38 PM
FPC Ctg1957 cp05T5e12DM4RS
File Edit Analysis Highlight Add track Layout Size options Help
Zoom
12.2
Whole
Show buried clones
Yes No
Search
CB Unit Range
0 to 130
Contig stats
Clones: 7 (0 buried)
Markers: 0
Sequenced: 0
Length: 131 CB units
Ctg1957 of cp05T5e12DM4RS
B004G13
V002N17
M004H03
M002G04
H006O03
M030L19
M016C12
0 10 20 30 40 50 60 70 80 90 100 110 120
[Chickpea contig p...
[root@localhost: /...
[FPC V8.9 Main Menu]
Project cp05T5e12...
FPC Ctg1957 cp05T...

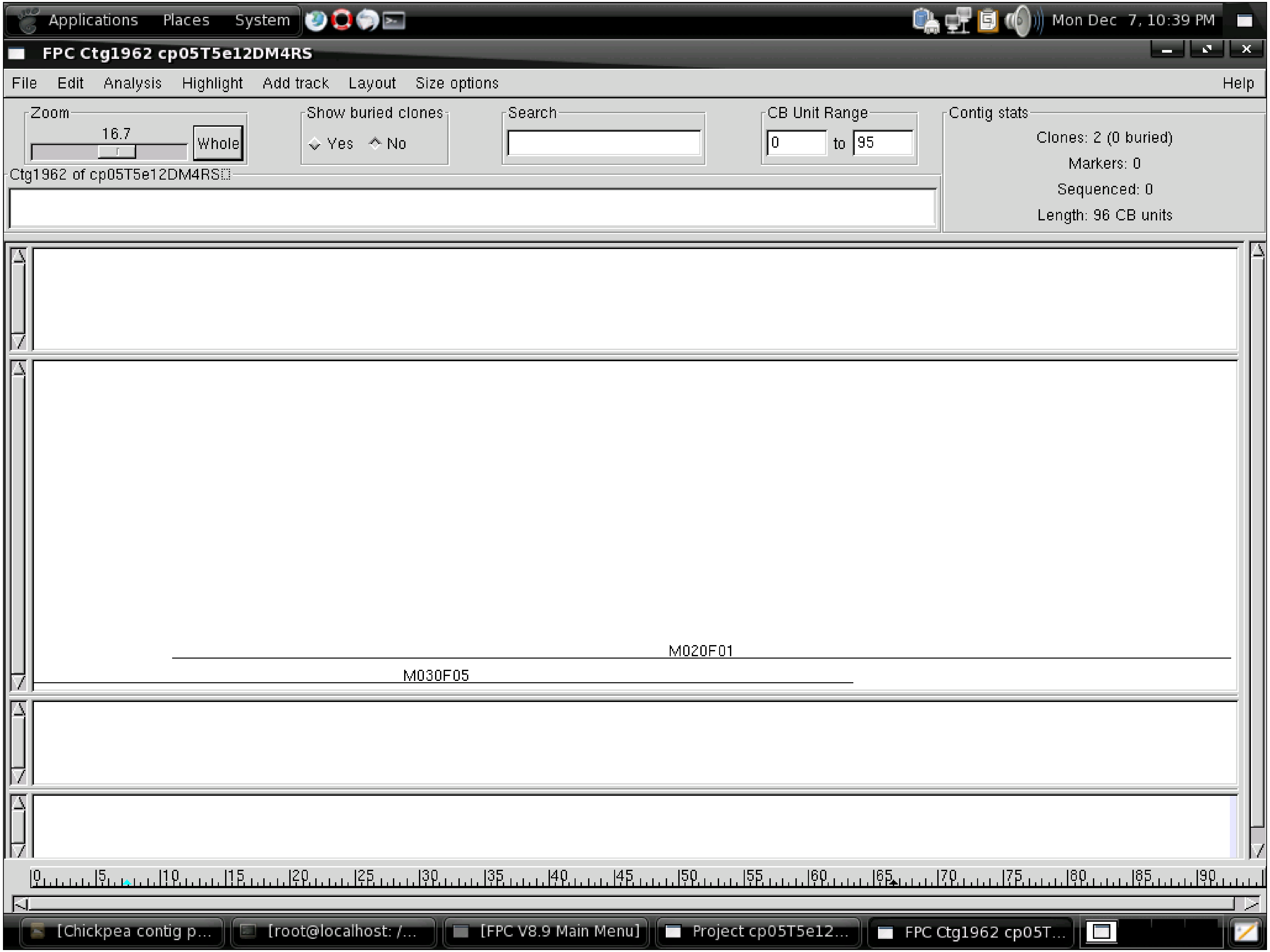

Applications Places System
Mon Dec 7, 10:39 PM
FPC Ctg1962 cp05T5e12DM4RS
File Edit Analysis Highlight Add track Layout Size options Help
Zoom
16.7
Whole
Show buried clones
Yes No
Search
CB Unit Range
0 to 95
Contig stats
Clones: 2 (0 buried)
Markers: 0
Sequenced: 0
Length: 96 CB units
Ctg1962 of cp05T5e12DM4RS
M020F01
M030F05
0 5 10 15 20 25 30 35 40 45 50 55 60 65 70 75 80 85 90
[Chickpea contig p...
[root@localhost: /...
[FPC V8.9 Main Menu]
Project cp05T5e12...
FPC Ctg1962 cp05T...

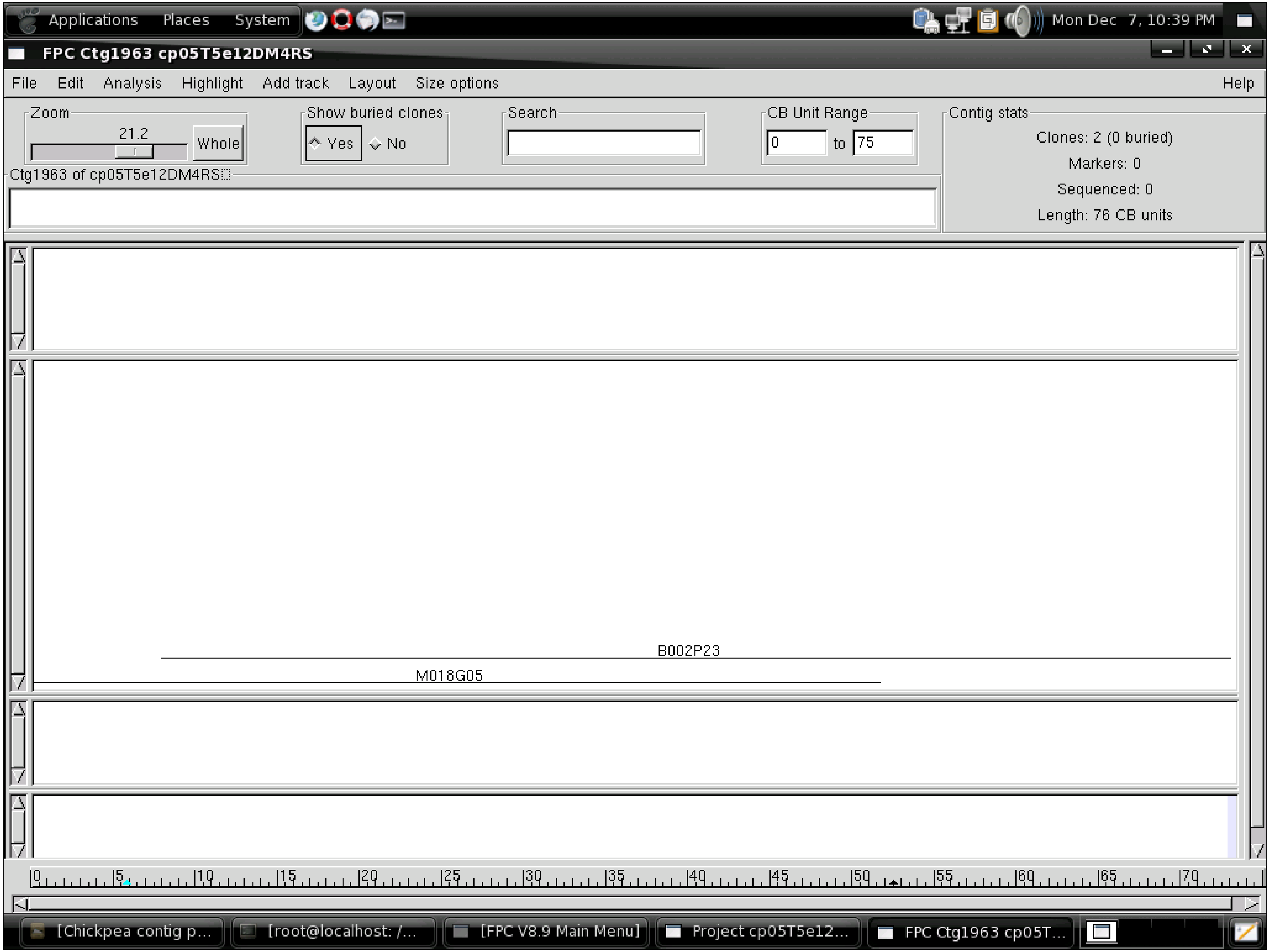

Applications
Places
System
Mon Dec 7, 10:39 PM
FPC Ctg1963 cp05T5e12DM4RS
File
Edit
Analysis
Highlight
Add track
Layout
Size options
Help
Zoom
21.2
Whole
Show buried clones
Yes
No
Search
CB Unit Range
0
to
75
Contig stats
Clones: 2 (0 buried)
Markers: 0
Sequenced: 0
Length: 76 CB units
Ctg1963 of cp05T5e12DM4RS
B002P23
M018G05
0
5
10
15
20
25
30
35
40
45
50
55
60
65
70
[Chickpea contig p...
[root@localhost: /...
[FPC V8.9 Main Menu]
Project cp05T5e12...
FPC Ctg1963 cp05T...

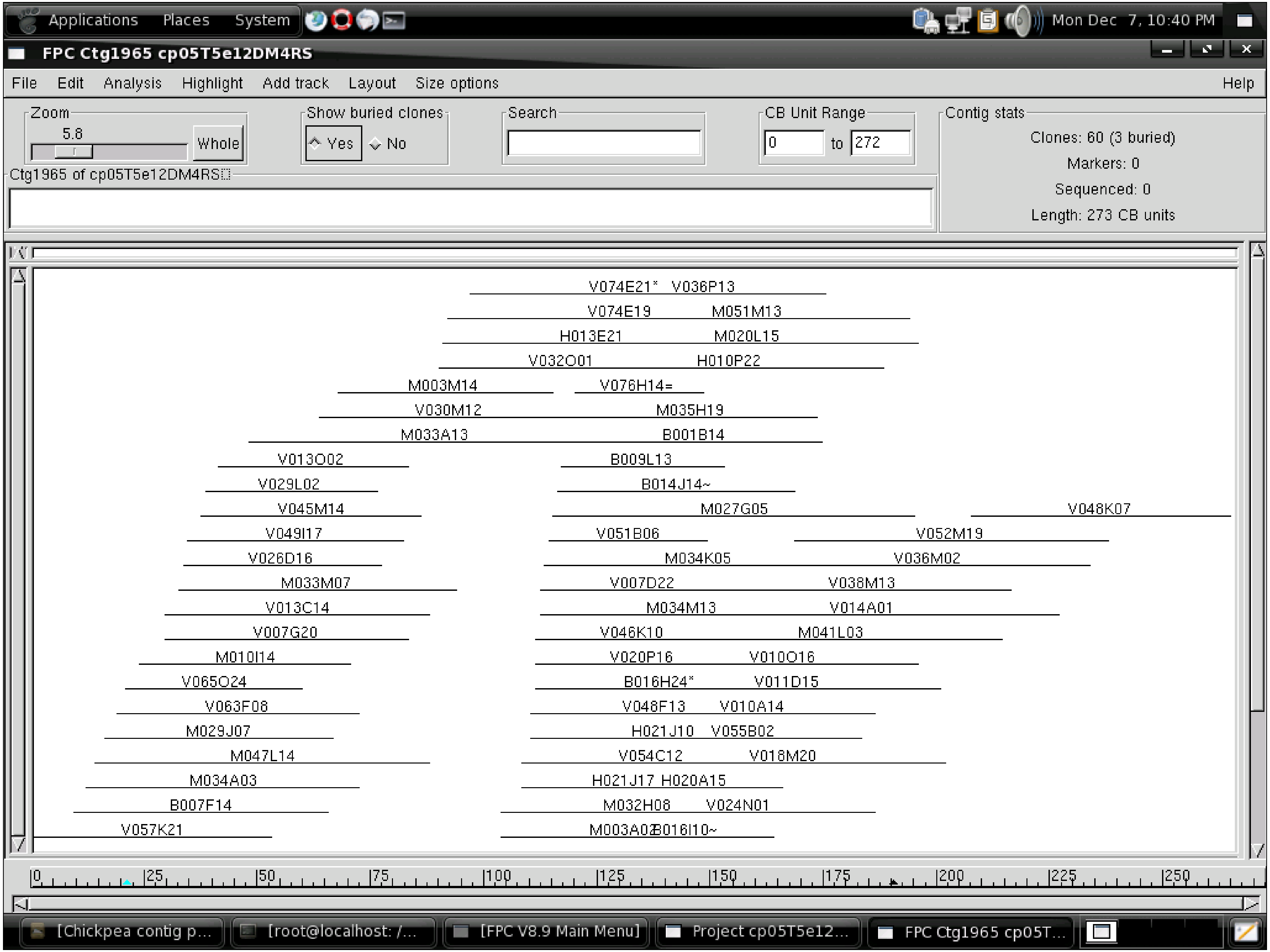
Applications Places System
Mon Dec 7, 10:40 PM
FPC Ctg1965 cp05T5e12DM4RS
File Edit Analysis Highlight Add track Layout Size options Help
Zoom
5.8
Whole
Show buried clones
Yes No
Search
CB Unit Range
0 to 272
Contig stats
Clones: 60 (3 buried)
Markers: 0
Sequenced: 0
Length: 273 CB units
Ctg1965 of cp05T5e12DM4RS
V074E21* V036P13
V074E19 M051M13
H013E21 M020L15
V032O01 H010P22
M003M14 V076H14=
V030M12 M035H19
M033A13 B001B14
V013O02 B009L13
V029L02 B014J14~
V045M14 M027G05 V048K07
V049I17 V051B06 V052M19
V026D16 M034K05 V036M02
M033M07 V007D22 V038M13
V013C14 M034M13 V014A01
V007G20 V046K10 M041L03
M010I14 V020P16 V010O16
V065O24 B016H24* V011D15
V063F08 V048F13 V010A14
M029J07 H021J10 V055B02
M047L14 V054C12 V018M20
M034A03 H021J17 H020A15
B007F14 M032H08 V024N01
V057K21 M003A02 B016I10~
0 25 50 75 100 125 150 175 200 225 250
[Chickpea contig p...
[root@localhost: /...
[FPC V8.9 Main Menu]
Project cp05T5e12...
FPC Ctg1965 cp05T...

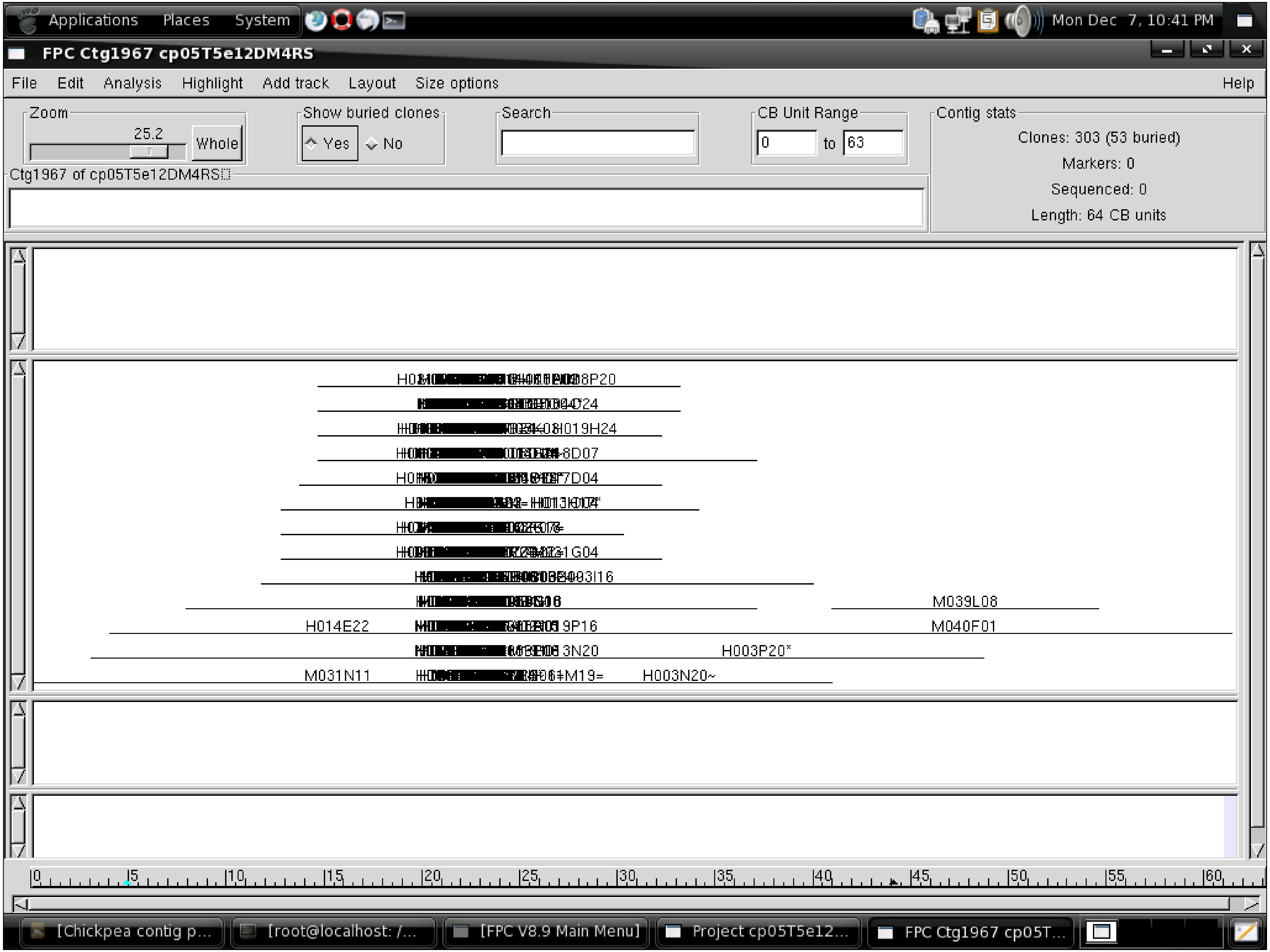
Applications Places System
Mon Dec 7, 10:41 PM
FPC Ctg1967 cp05T5e12DM4RS
File Edit Analysis Highlight Add track Layout Size options Help
Zoom
25.2
Whole
Show buried clones
Yes No
Search
CB Unit Range
0 to 63
Contig stats
Clones: 303 (53 buried)
Markers: 0
Sequenced: 0
Length: 64 CB units
Ctg1967 of cp05T5e12DM4RS
M039L08
H014E22
M040F01
H003P20*
M031N11
H003N20~
0 5 10 15 20 25 30 35 40 45 50 55 60
[Chickpea contig p...
[root@localhost: /...
[FPC V8.9 Main Menu]
Project cp05T5e12...
FPC Ctg1967 cp05T...

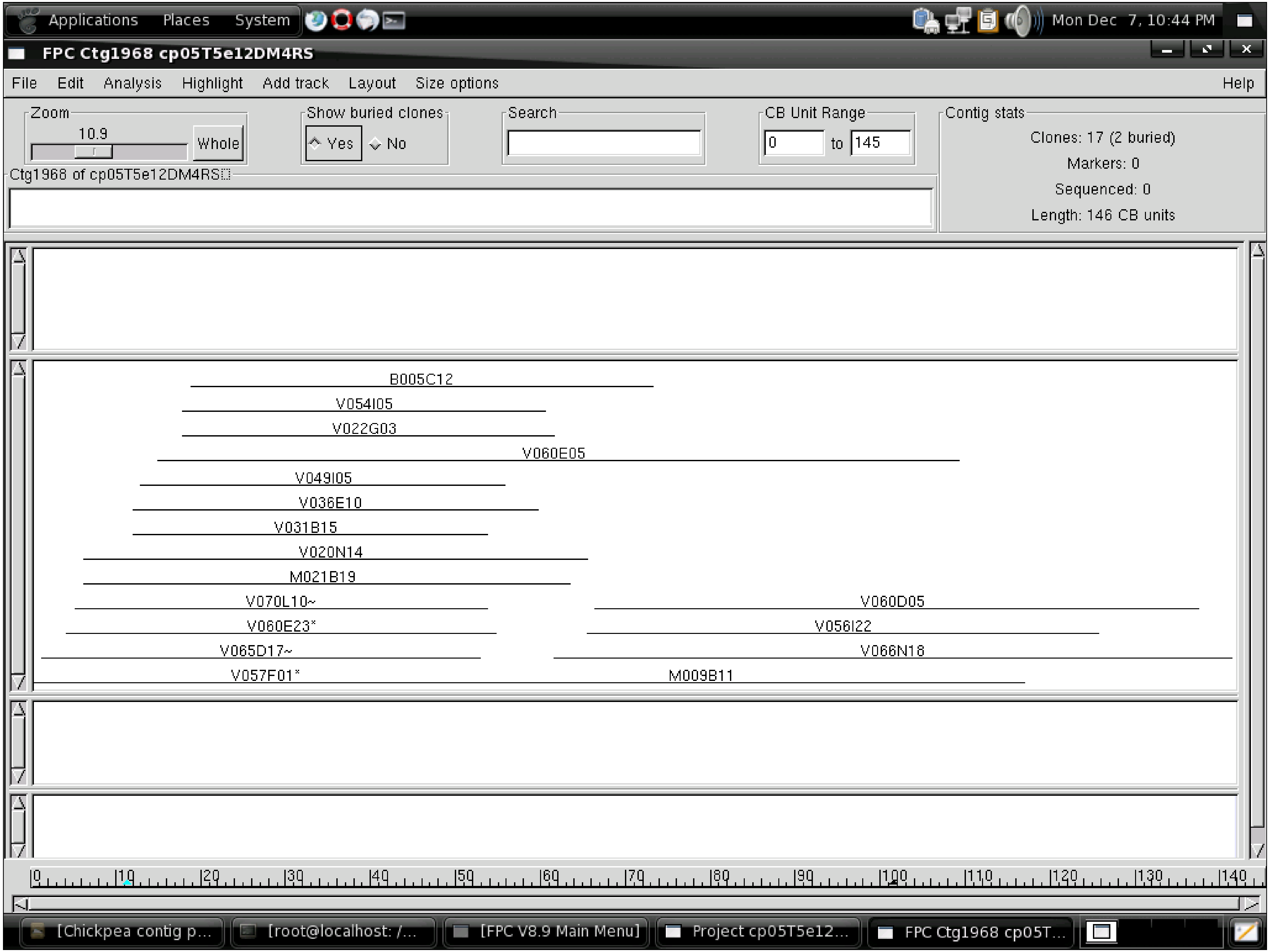
Applications Places System
Mon Dec 7, 10:44 PM
FPC Ctg1968 cp05T5e12DM4RS
File Edit Analysis Highlight Add track Layout Size options Help
Zoom
10.9
Whole
Show buried clones
Yes
No
Search
CB Unit Range
0 to 145
Contig stats
Clones: 17 (2 buried)
Markers: 0
Sequenced: 0
Length: 146 CB units
Ctg1968 of cp05T5e12DM4RS
B005C12
V054I05
V022G03
V060E05
V049I05
V036E10
V031B15
V020N14
M021B19
V070L10~
V060D05
V060E23*
V056I22
V065D17~
V066N18
V057F01*
M009B11
0 10 20 30 40 50 60 70 80 90 100 110 120 130 140
[Chickpea contig p...
[root@localhost: /...
[FPC V8.9 Main Menu]
Project cp05T5e12...
FPC Ctg1968 cp05T...

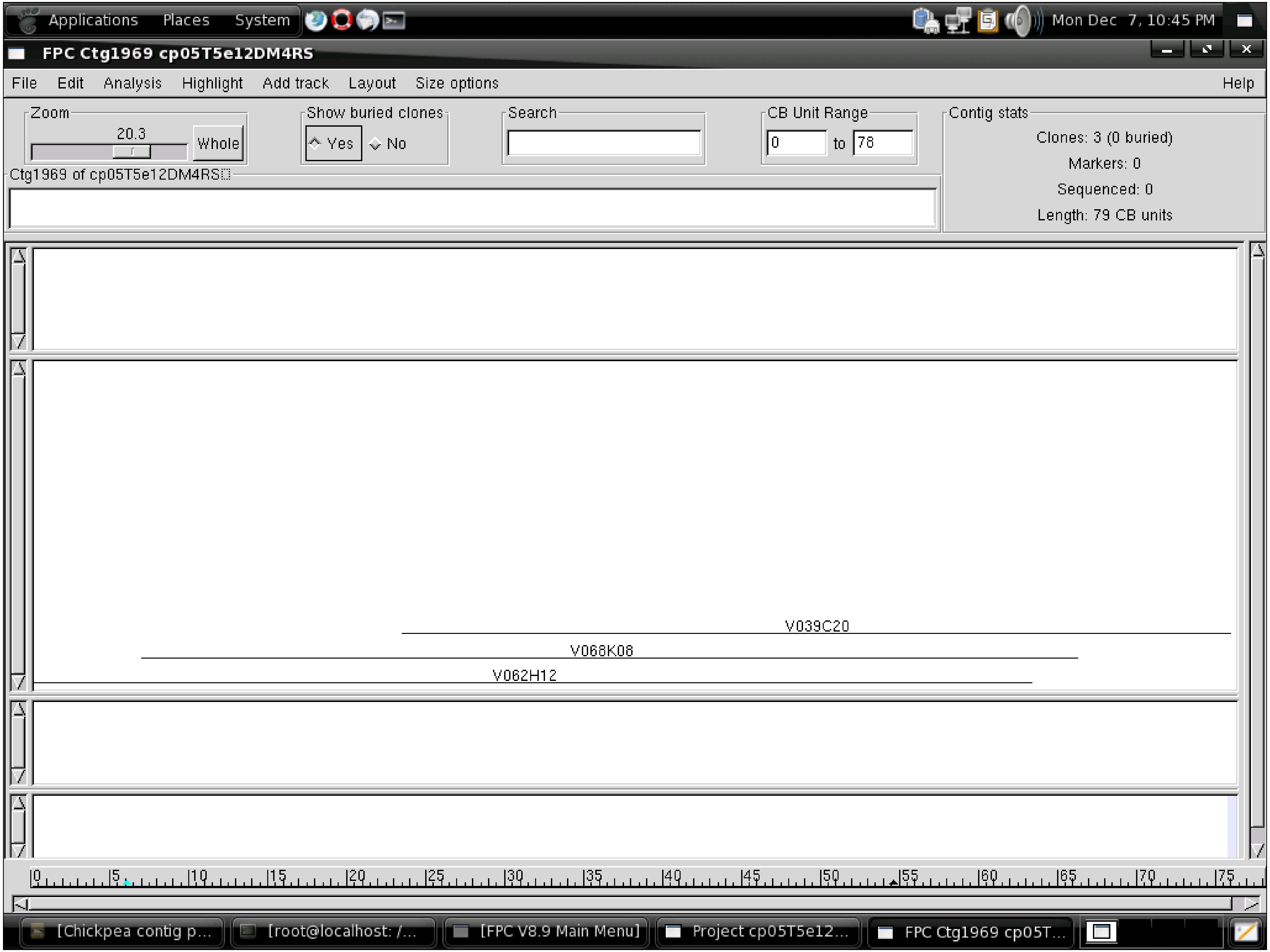
Applications Places System
Mon Dec 7, 10:45 PM
FPC Ctg1969 cp05T5e12DM4RS
File Edit Analysis Highlight Add track Layout Size options Help
Zoom
20.3
Whole
Show buried clones
Yes No
Search
CB Unit Range
0 to 78
Contig stats
Clones: 3 (0 buried)
Markers: 0
Sequenced: 0
Length: 79 CB units
Ctg1969 of cp05T5e12DM4RS
V039C20
V068K08
V062H12
0 5 10 15 20 25 30 35 40 45 50 55 60 65 70 75
[Chickpea contig p...
[root@localhost: /...
[FPC V8.9 Main Menu]
Project cp05T5e12...
FPC Ctg1969 cp05T...

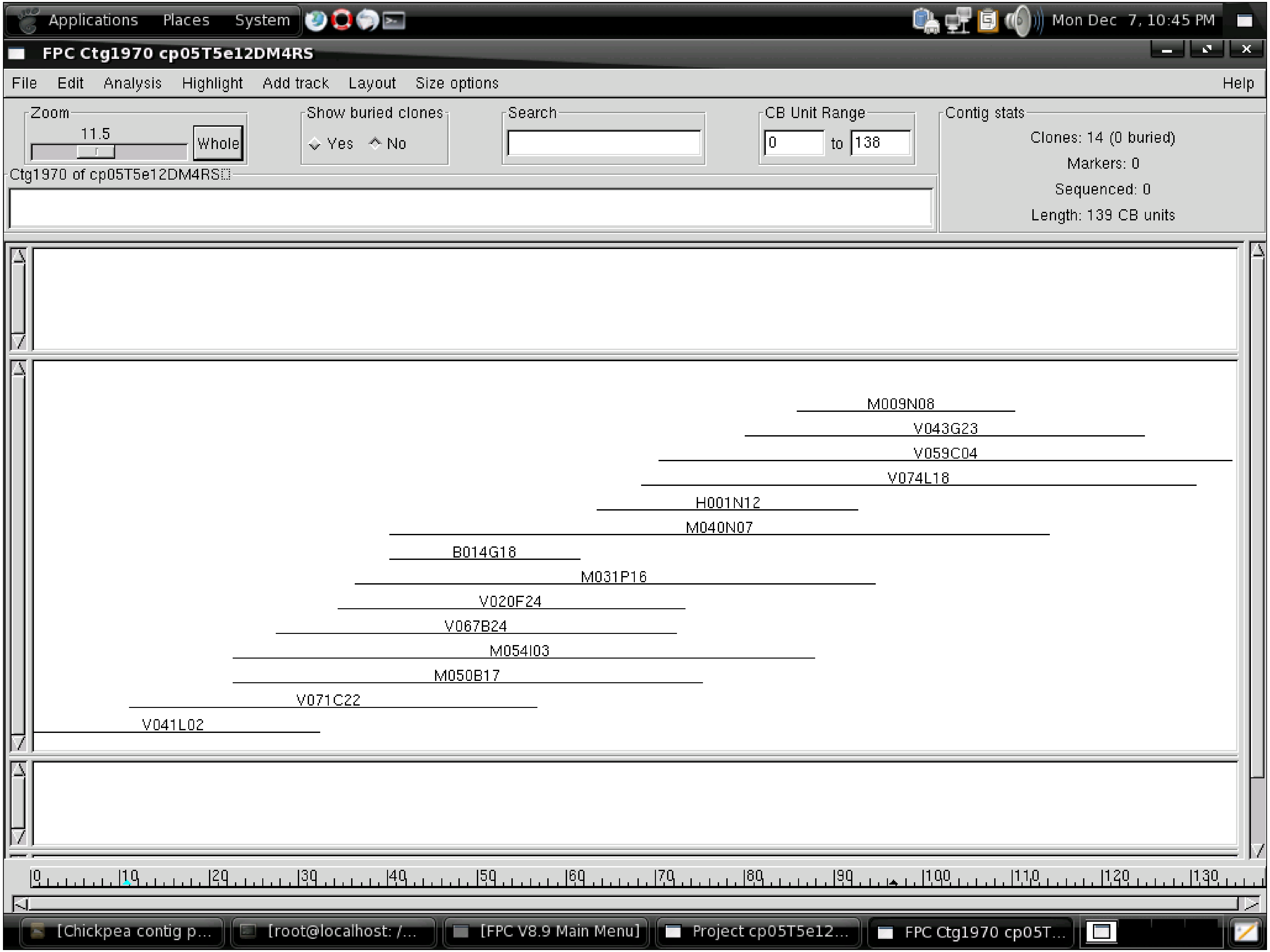
Applications
Places
System
Mon Dec 7, 10:45 PM
FPC Ctg1970 cp05T5e12DM4RS
File
Edit
Analysis
Highlight
Add track
Layout
Size options
Help
Zoom
11.5
Whole
Show buried clones
Yes
No
Search
CB Unit Range
0
to
138
Contig stats
Clones: 14 (0 buried)
Markers: 0
Sequenced: 0
Length: 139 CB units
Ctg1970 of cp05T5e12DM4RS
M009N08
V043G23
V059C04
V074L18
H001N12
M040N07
B014G18
M031P16
V020F24
V067B24
M054I03
M050B17
V071C22
V041L02
0
10
20
30
40
50
60
70
80
90
100
110
120
130
[Chickpea contig p...
[root@localhost: /...
[FPC V8.9 Main Menu]
Project cp05T5e12...
FPC Ctg1970 cp05T...

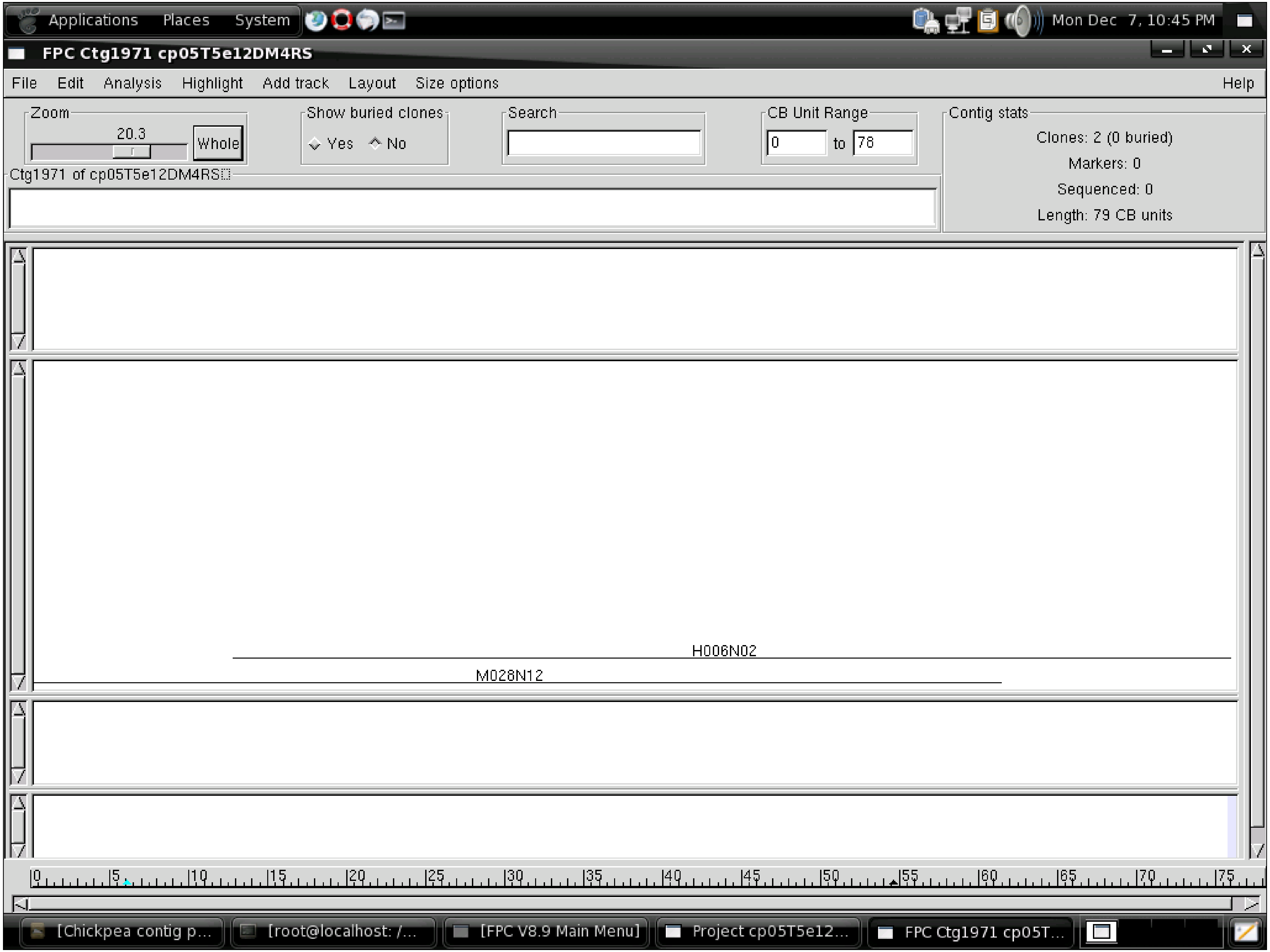
Applications
Places
System
Mon Dec 7, 10:45 PM
FPC Ctg1971 cp05T5e12DM4RS
File
Edit
Analysis
Highlight
Add track
Layout
Size options
Help
Zoom
20.3
Whole
Show buried clones
Yes
No
Search
CB Unit Range
0
to
78
Contig stats
Clones: 2 (0 buried)
Markers: 0
Sequenced: 0
Length: 79 CB units
Ctg1971 of cp05T5e12DM4RS
H006N02
M028N12
0
5
10
15
20
25
30
35
40
45
50
55
60
65
70
75
[Chickpea contig p...
[root@localhost: /...
[FPC V8.9 Main Menu]
Project cp05T5e12...
FPC Ctg1971 cp05T...

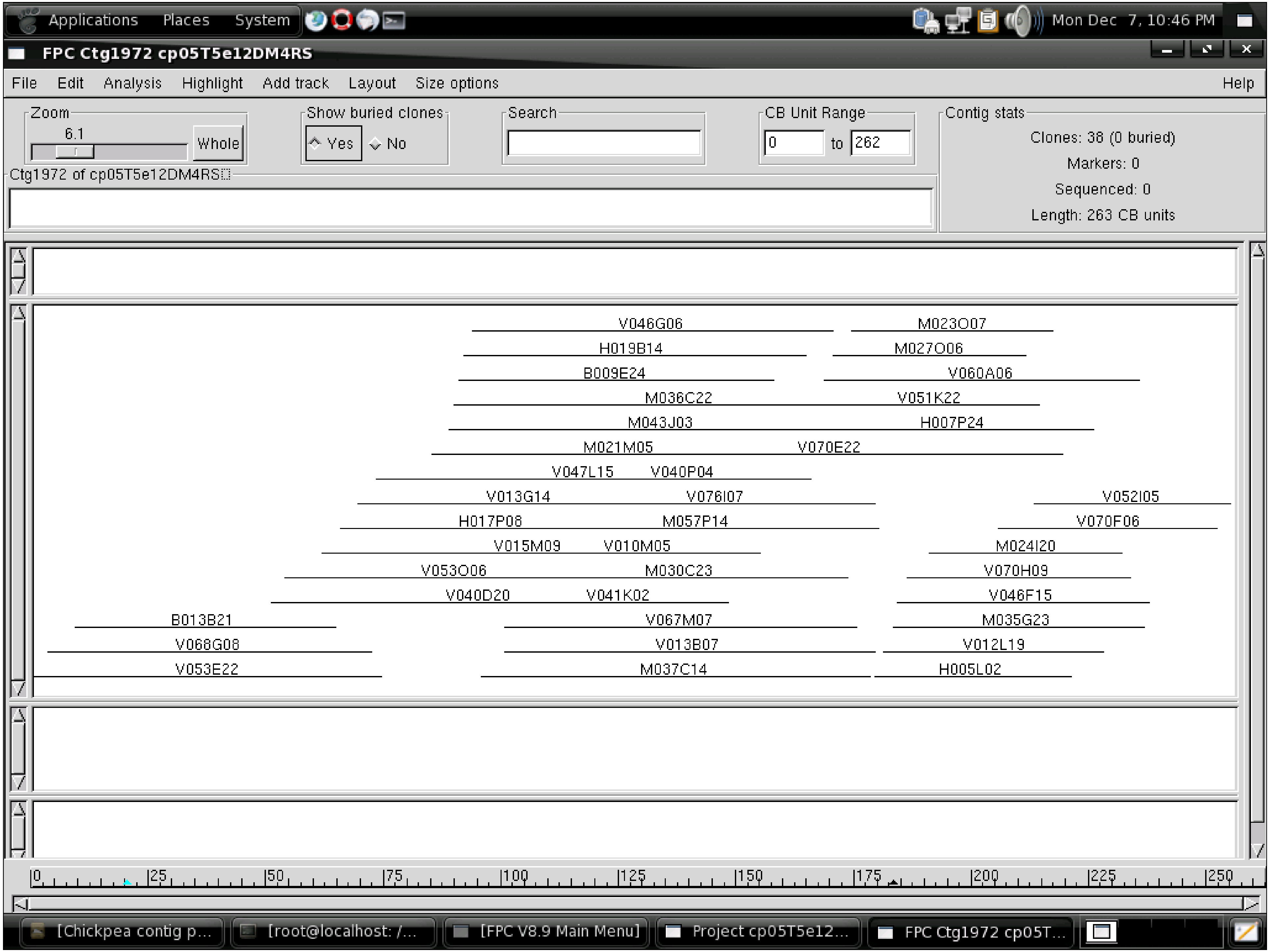
Applications Places System
Mon Dec 7, 10:46 PM
FPC Ctg1972 cp05T5e12DM4RS
File Edit Analysis Highlight Add track Layout Size options Help
Zoom
6.1
Whole
Show buried clones
Yes No
Search
CB Unit Range
0 to 262
Contig stats
Clones: 38 (0 buried)
Markers: 0
Sequenced: 0
Length: 263 CB units
Ctg1972 of cp05T5e12DM4RS
V046G06
M023O07
H019B14
M027O06
B009E24
V060A06
M036C22
V051K22
M043J03
H007P24
M021M05
V070E22
V047L15
V040P04
V013G14
V076I07
V052I05
H017P08
M057P14
V070F06
V015M09
V010M05
M024I20
V053O06
M030C23
V070H09
V040D20
V041K02
V046F15
B013B21
V067M07
M035G23
V068G08
V013B07
V012L19
V053E22
M037C14
H005L02
0 25 50 75 100 125 150 175 200 225 250
[Chickpea contig p...
[root@localhost: /...
[FPC V8.9 Main Menu]
Project cp05T5e12...
FPC Ctg1972 cp05T...

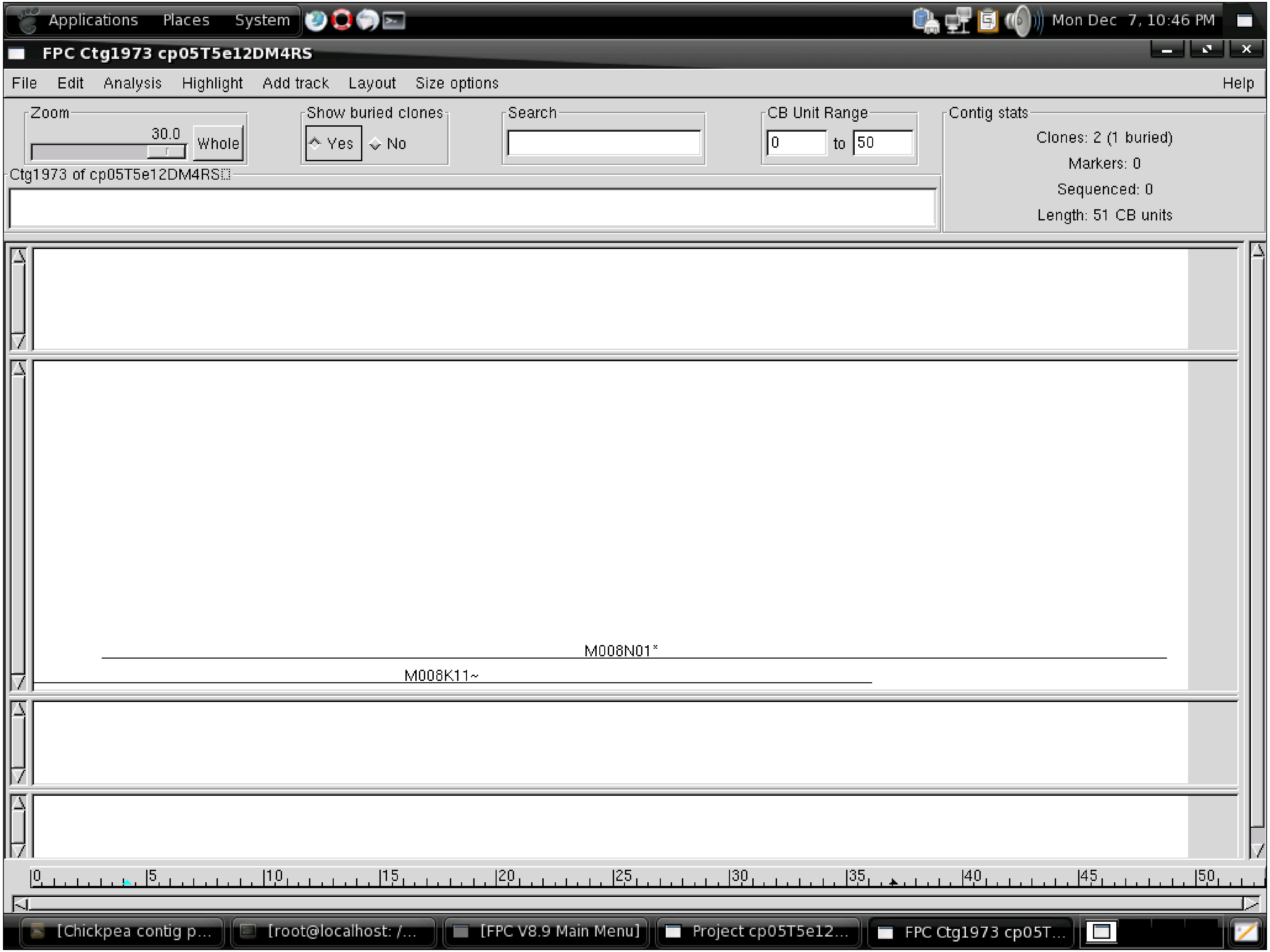
Applications Places System
Mon Dec 7, 10:46 PM
FPC Ctg1973 cp05T5e12DM4RS
File Edit Analysis Highlight Add track Layout Size options Help
Zoom
30.0
Whole
Show buried clones
Yes
No
Search
CB Unit Range
0 to 50
Contig stats
Clones: 2 (1 buried)
Markers: 0
Sequenced: 0
Length: 51 CB units
Ctg1973 of cp05T5e12DM4RS
M008N01*
M008K11~
0 5 10 15 20 25 30 35 40 45 50
[Chickpea contig p...
[root@localhost: /...
[FPC V8.9 Main Menu]
Project cp05T5e12...
FPC Ctg1973 cp05T...

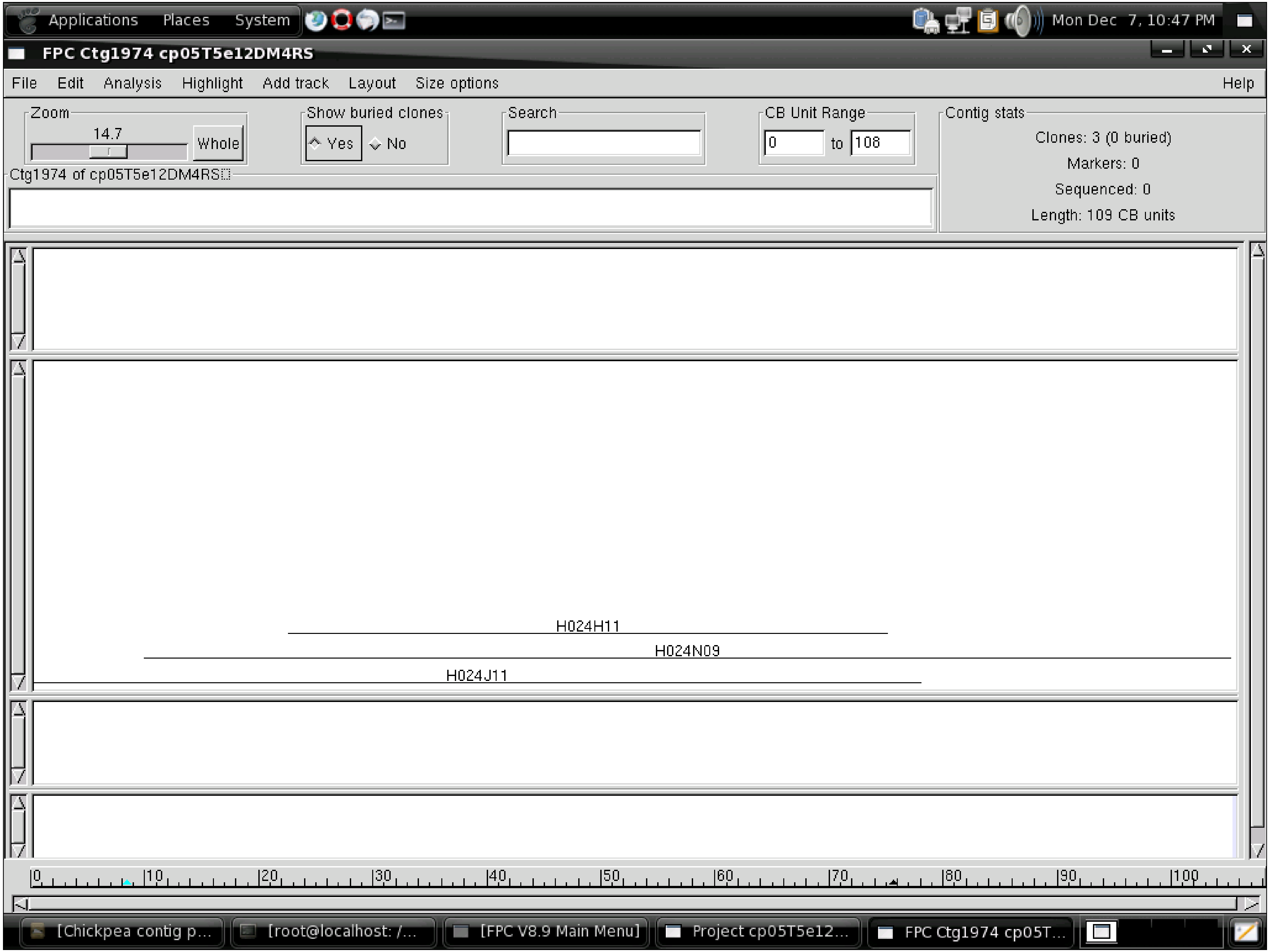
Applications Places System
Mon Dec 7, 10:47 PM
FPC Ctg1974 cp05T5e12DM4RS
File Edit Analysis Highlight Add track Layout Size options Help
Zoom
14.7
Whole
Show buried clones
Yes
No
Search
CB Unit Range
0
to
108
Contig stats
Clones: 3 (0 buried)
Markers: 0
Sequenced: 0
Length: 109 CB units
Ctg1974 of cp05T5e12DM4RS
H024H11
H024N09
H024J11
0 10 20 30 40 50 60 70 80 90 100
[Chickpea contig p...
[root@localhost: /...
[FPC V8.9 Main Menu]
Project cp05T5e12...
FPC Ctg1974 cp05T...

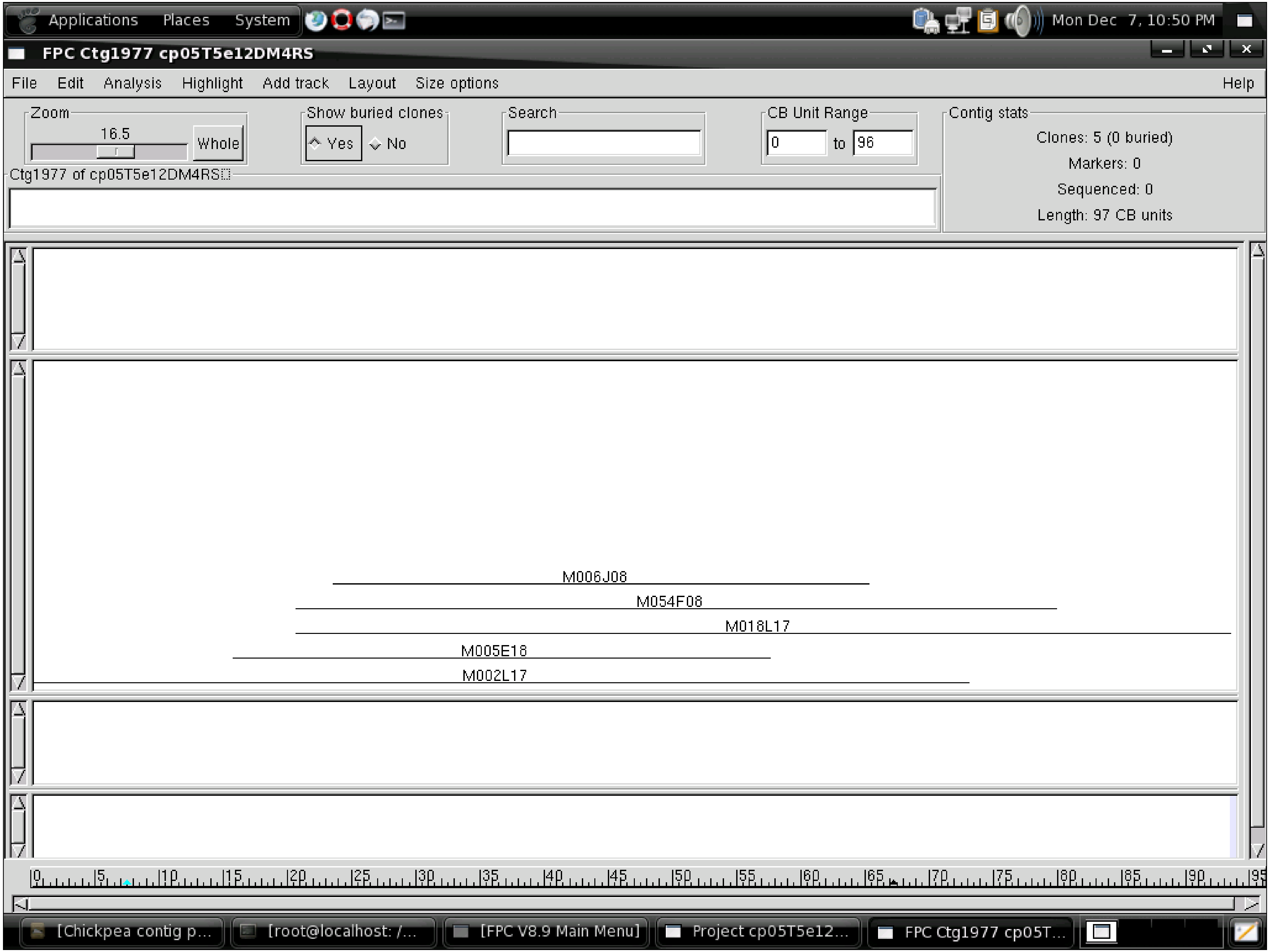
Applications Places System
Mon Dec 7, 10:50 PM
FPC Ctg1977 cp05T5e12DM4RS
File Edit Analysis Highlight Add track Layout Size options Help
Zoom
16.5
Whole
Show buried clones
Yes No
Search
CB Unit Range
0 to 96
Contig stats
Clones: 5 (0 buried)
Markers: 0
Sequenced: 0
Length: 97 CB units
Ctg1977 of cp05T5e12DM4RS
M006J08
M054F08
M018L17
M005E18
M002L17
0 5 10 15 20 25 30 35 40 45 50 55 60 65 70 75 80 85 90 95
[Chickpea contig p...
[root@localhost: /...
[FPC V8.9 Main Menu]
Project cp05T5e12...
FPC Ctg1977 cp05T...

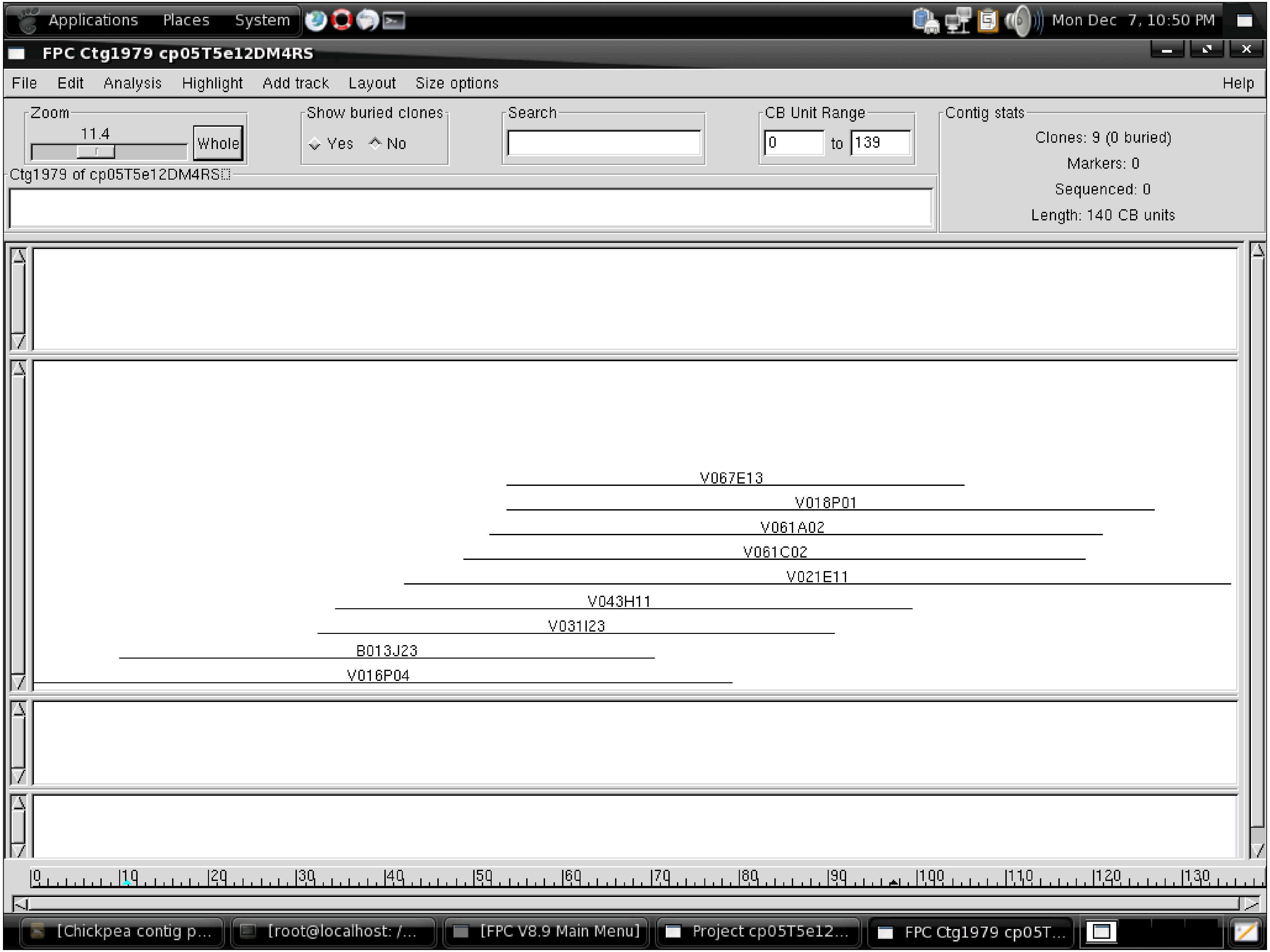
Applications Places System
Mon Dec 7, 10:50 PM
FPC Ctg1979 cp05T5e12DM4RS
File Edit Analysis Highlight Add track Layout Size options Help
Zoom
11.4
Whole
Show buried clones
Yes No
Search
CB Unit Range
0 to 139
Contig stats
Clones: 9 (0 buried)
Markers: 0
Sequenced: 0
Length: 140 CB units
Ctg1979 of cp05T5e12DM4RS
V067E13
V018P01
V061A02
V061C02
V021E11
V043H11
V031I23
B013J23
V016P04
0 10 20 30 40 50 60 70 80 90 100 110 120 130
[Chickpea contig p...
[root@localhost: /...
[FPC V8.9 Main Menu]
Project cp05T5e12...
FPC Ctg1979 cp05T...

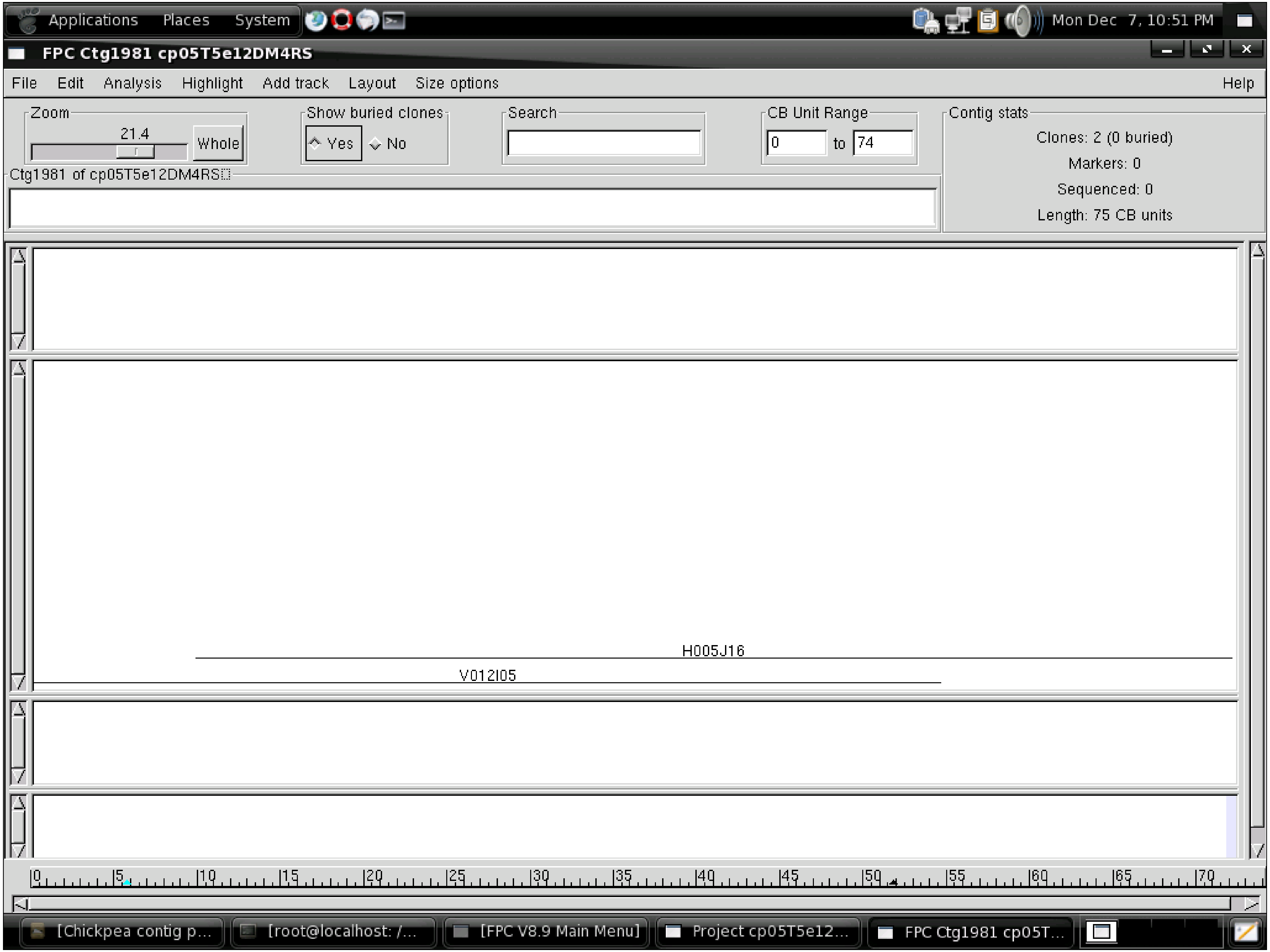
Applications
Places
System
Mon Dec 7, 10:51 PM
FPC Ctg1981 cp05T5e12DM4RS
File
Edit
Analysis
Highlight
Add track
Layout
Size options
Help
Zoom
21.4
Whole
Show buried clones
Yes
No
Search
CB Unit Range
0
to
74
Contig stats
Clones: 2 (0 buried)
Markers: 0
Sequenced: 0
Length: 75 CB units
Ctg1981 of cp05T5e12DM4RS
H005J16
V012I05
0
5
10
15
20
25
30
35
40
45
50
55
60
65
70
[Chickpea contig p...
[root@localhost: /...
[FPC V8.9 Main Menu]
Project cp05T5e12...
FPC Ctg1981 cp05T...

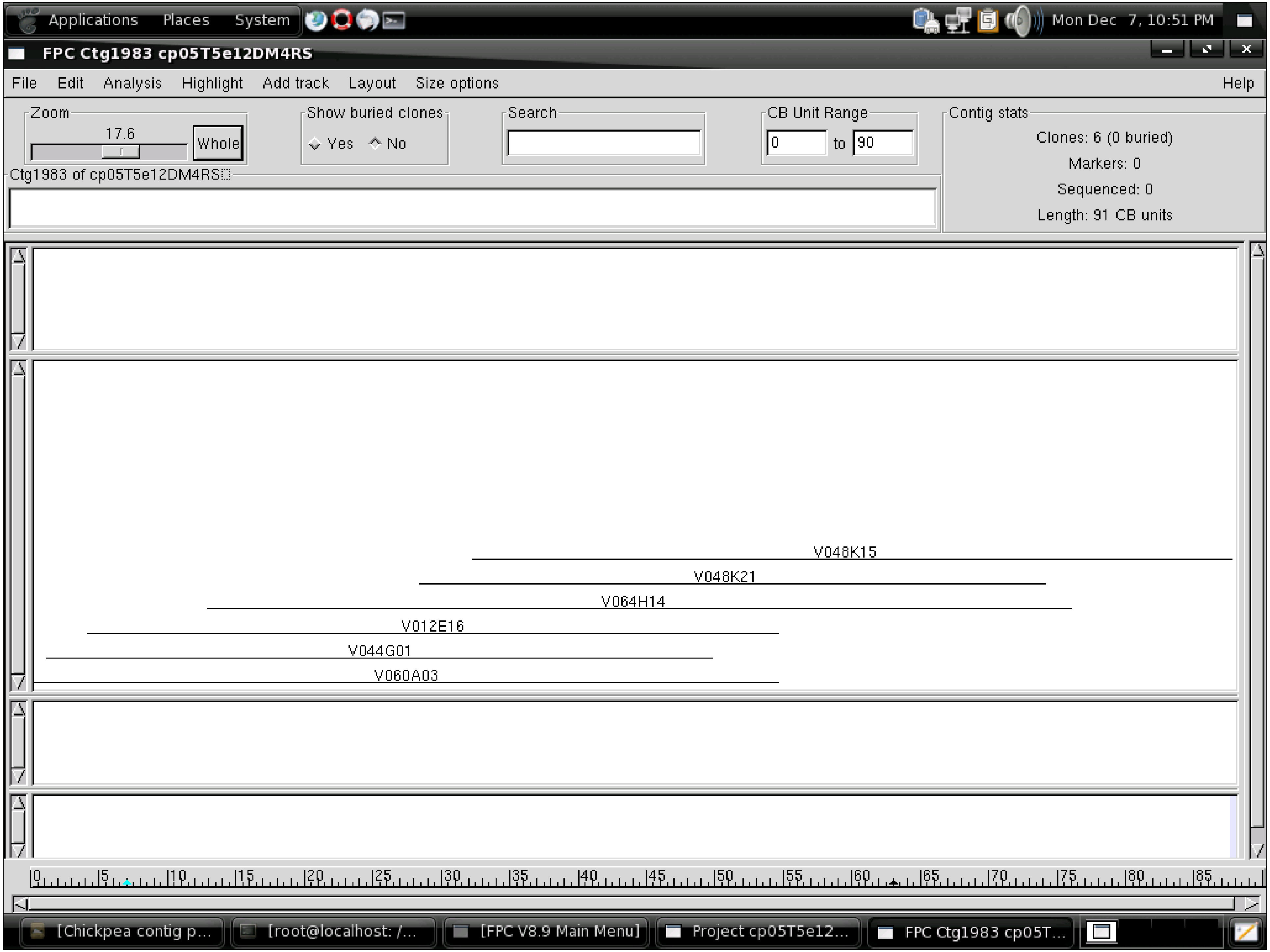
Applications Places System
Mon Dec 7, 10:51 PM
FPC Ctg1983 cp05T5e12DM4RS
File Edit Analysis Highlight Add track Layout Size options
Help
Zoom
17.6
Whole
Show buried clones
Yes No
Search
CB Unit Range
0 to 90
Contig stats
Clones: 6 (0 buried)
Markers: 0
Sequenced: 0
Length: 91 CB units
Ctg1983 of cp05T5e12DM4RS
V048K15
V048K21
V064H14
V012E16
V044G01
V060A03
0 5 10 15 20 25 30 35 40 45 50 55 60 65 70 75 80 85
[Chickpea contig p...
[root@localhost: /...
[FPC V8.9 Main Menu]
Project cp05T5e12...
FPC Ctg1983 cp05T...

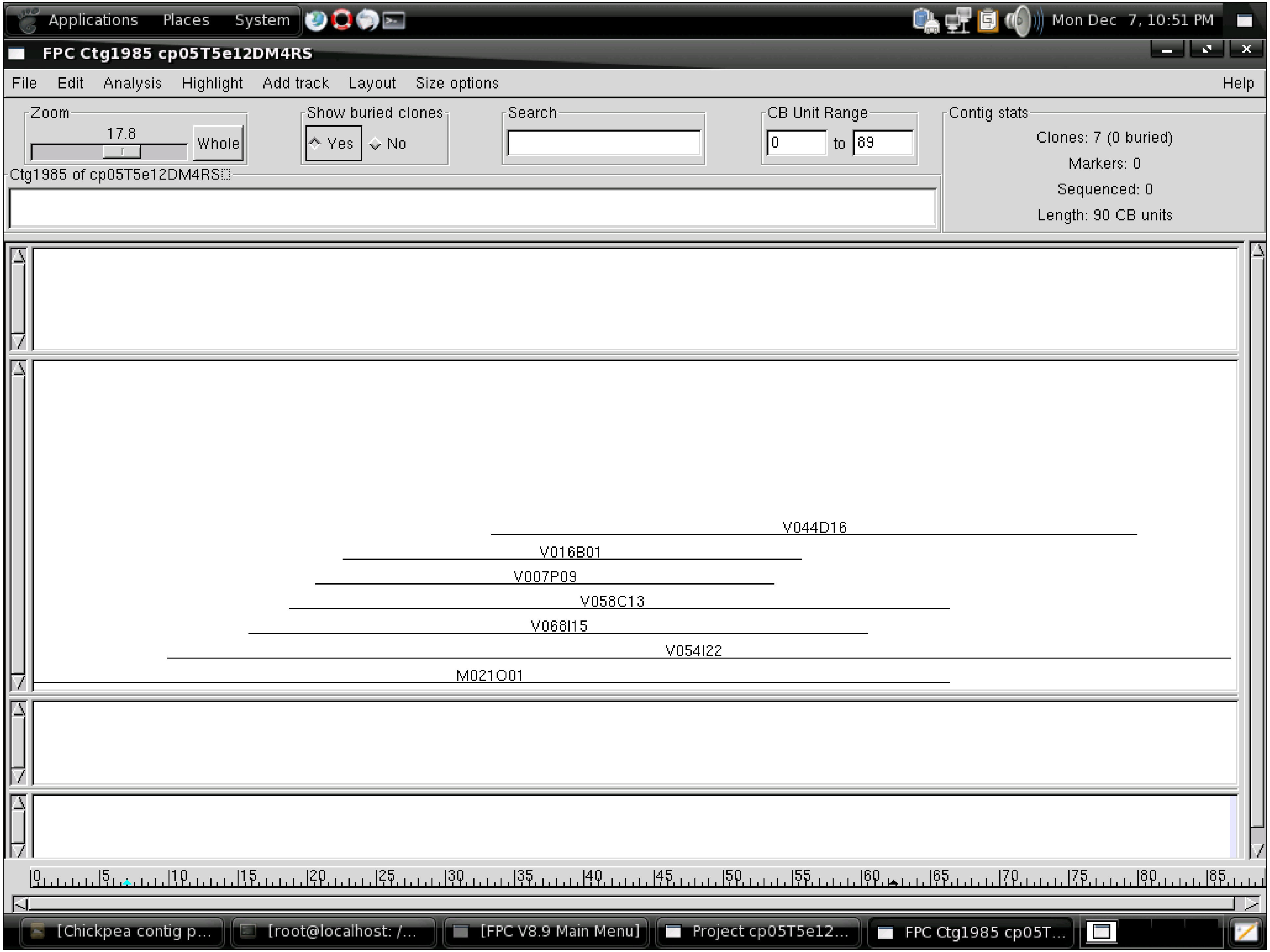
Applications Places System
Mon Dec 7, 10:51 PM
FPC Ctg1985 cp05T5e12DM4RS
File Edit Analysis Highlight Add track Layout Size options Help
Zoom
17.8
Whole
Show buried clones
Yes
No
Search
CB Unit Range
0
to
89
Contig stats
Clones: 7 (0 buried)
Markers: 0
Sequenced: 0
Length: 90 CB units
Ctg1985 of cp05T5e12DM4RS
V044D16
V016B01
V007P09
V058C13
V068I15
V054I22
M021O01
0 5 10 15 20 25 30 35 40 45 50 55 60 65 70 75 80 85
[Chickpea contig p...
[root@localhost: /...
[FPC V8.9 Main Menu]
Project cp05T5e12...
FPC Ctg1985 cp05T...

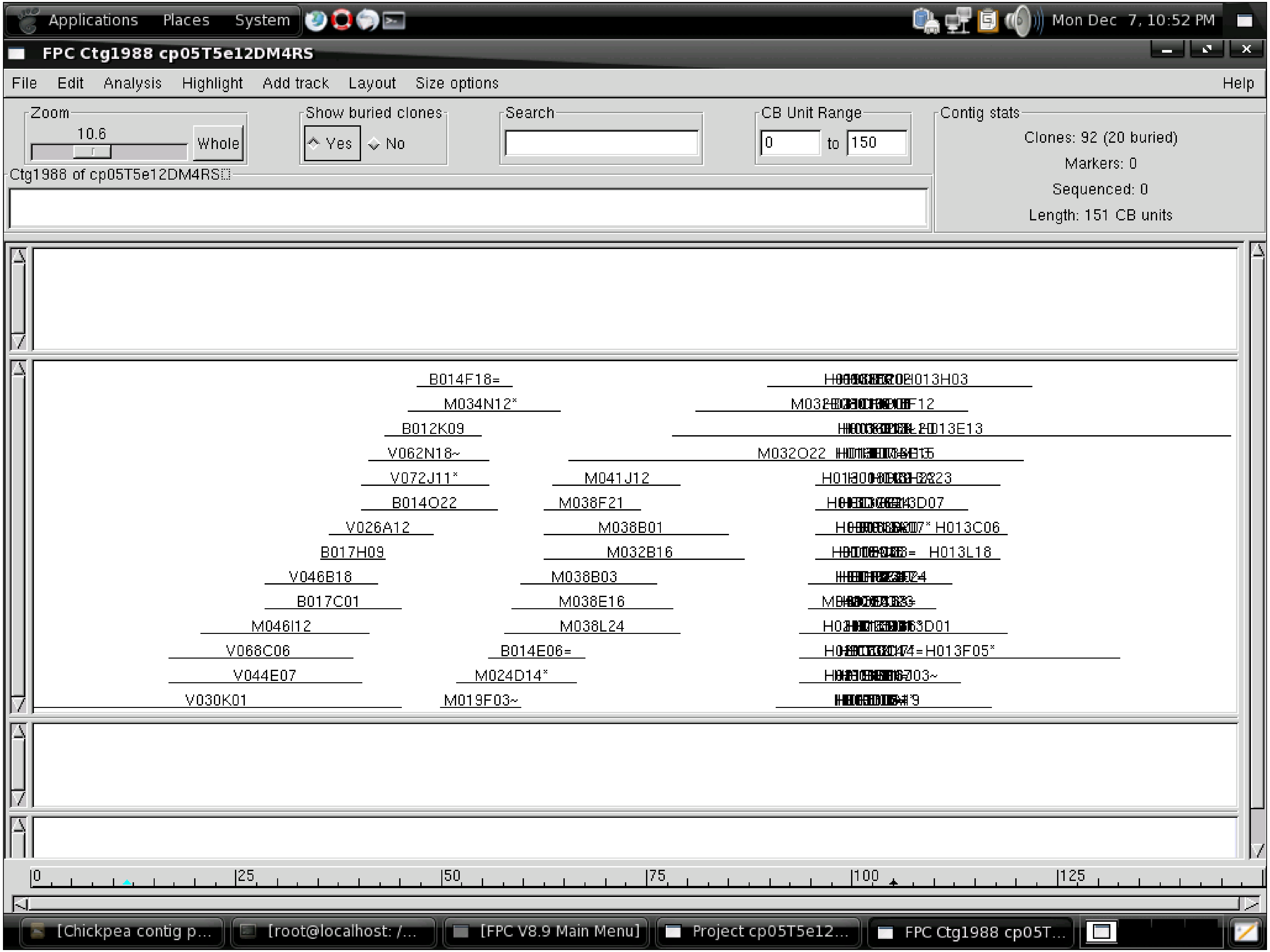
Applications
Places
System
Mon Dec 7, 10:52 PM
FPC Ctg1988 cp05T5e12DM4RS
File
Edit
Analysis
Highlight
Add track
Layout
Size options
Help
Zoom
10.6
Whole
Show buried clones
Yes
No
Search
CB Unit Range
0
to
150
Contig stats
Clones: 92 (20 buried)
Markers: 0
Sequenced: 0
Length: 151 CB units
Ctg1988 of cp05T5e12DM4RS
B014F18=
M034N12*
B012K09
V062N18~
V072J11*
B014O22
V026A12
B017H09
V046B18
B017C01
M046I12
V068C06
V044E07
V030K01
M041J12
M038F21
M038B01
M032B16
M038B03
M038E16
M038L24
B014E06=
M024D14*
M019F03~
M032O22
0
25
50
75
100
125
[Chickpea contig p...
[root@localhost: /...
[FPC V8.9 Main Menu]
Project cp05T5e12...
FPC Ctg1988 cp05T...

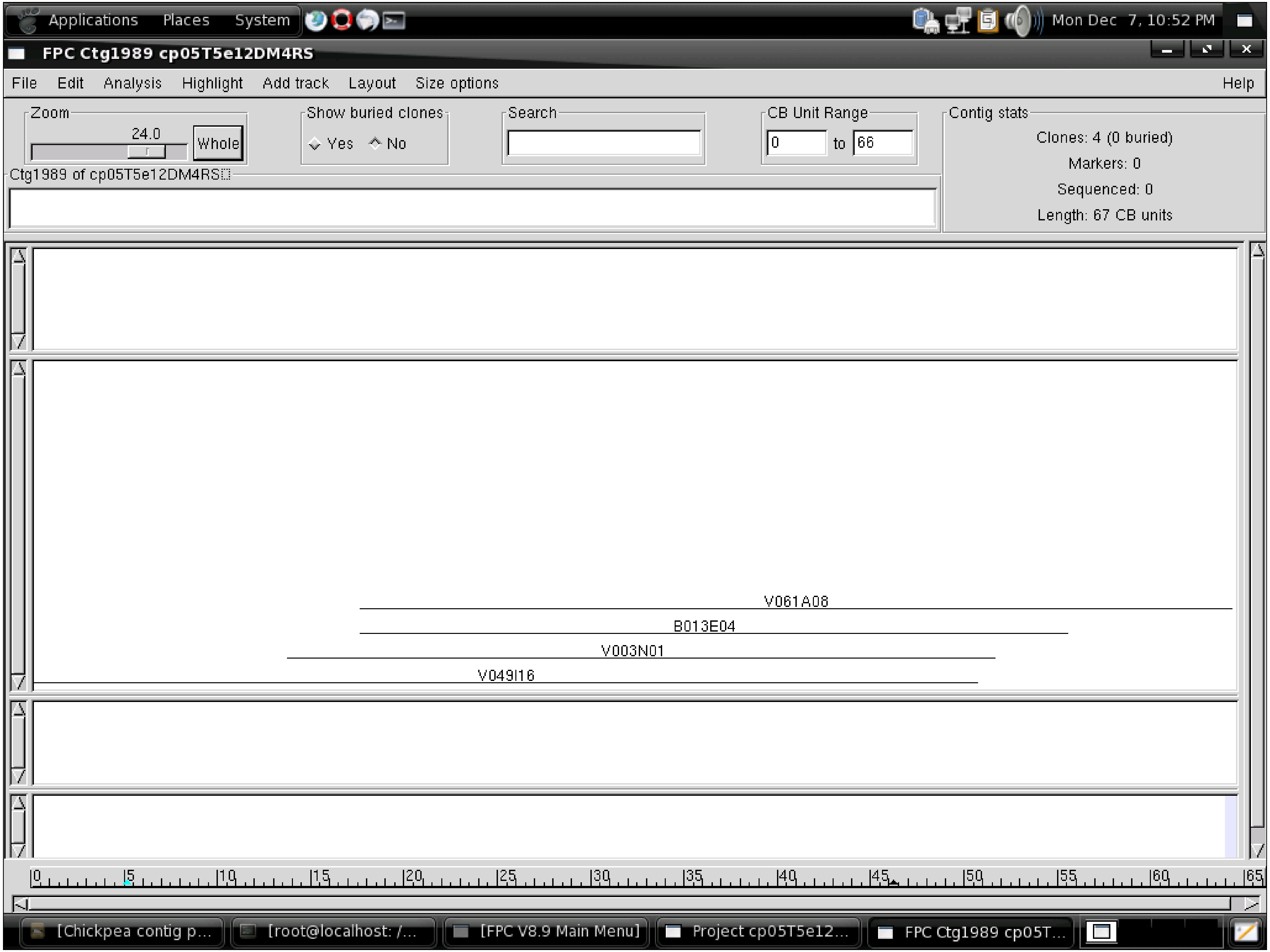
Applications Places System
Mon Dec 7, 10:52 PM
FPC Ctg1989 cp05T5e12DM4RS
File Edit Analysis Highlight Add track Layout Size options Help
Zoom
24.0
Whole
Show buried clones
Yes No
Search
CB Unit Range
0 to 66
Contig stats
Clones: 4 (0 buried)
Markers: 0
Sequenced: 0
Length: 67 CB units
Ctg1989 of cp05T5e12DM4RS
V061A08
B013E04
V003N01
V049I16
0 5 10 15 20 25 30 35 40 45 50 55 60 65
[Chickpea contig p...
[root@localhost: /...
[FPC V8.9 Main Menu]
Project cp05T5e12...
FPC Ctg1989 cp05T...

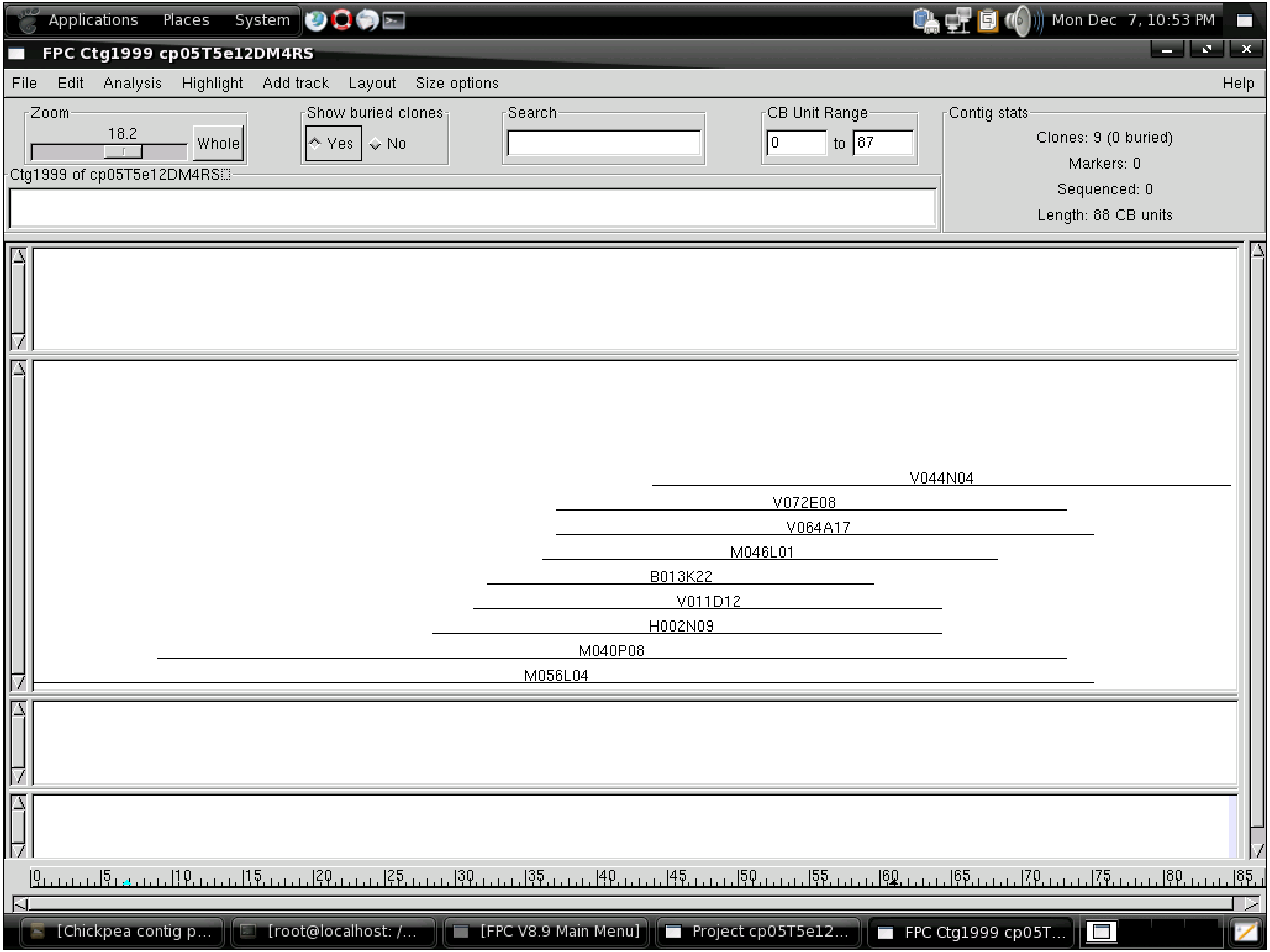
Applications
Places
System
Mon Dec 7, 10:53 PM
FPC Ctg1999 cp05T5e12DM4RS
File
Edit
Analysis
Highlight
Add track
Layout
Size options
Help
Zoom
18.2
Whole
Show buried clones
Yes
No
Search
CB Unit Range
0
to
87
Contig stats
Clones: 9 (0 buried)
Markers: 0
Sequenced: 0
Length: 88 CB units
Ctg1999 of cp05T5e12DM4RS
V044N04
V072E08
V064A17
M046L01
B013K22
V011D12
H002N09
M040P08
M056L04
0 5 10 15 20 25 30 35 40 45 50 55 60 65 70 75 80 85
[Chickpea contig p...
[root@localhost: /...
[FPC V8.9 Main Menu]
Project cp05T5e12...
FPC Ctg1999 cp05T...

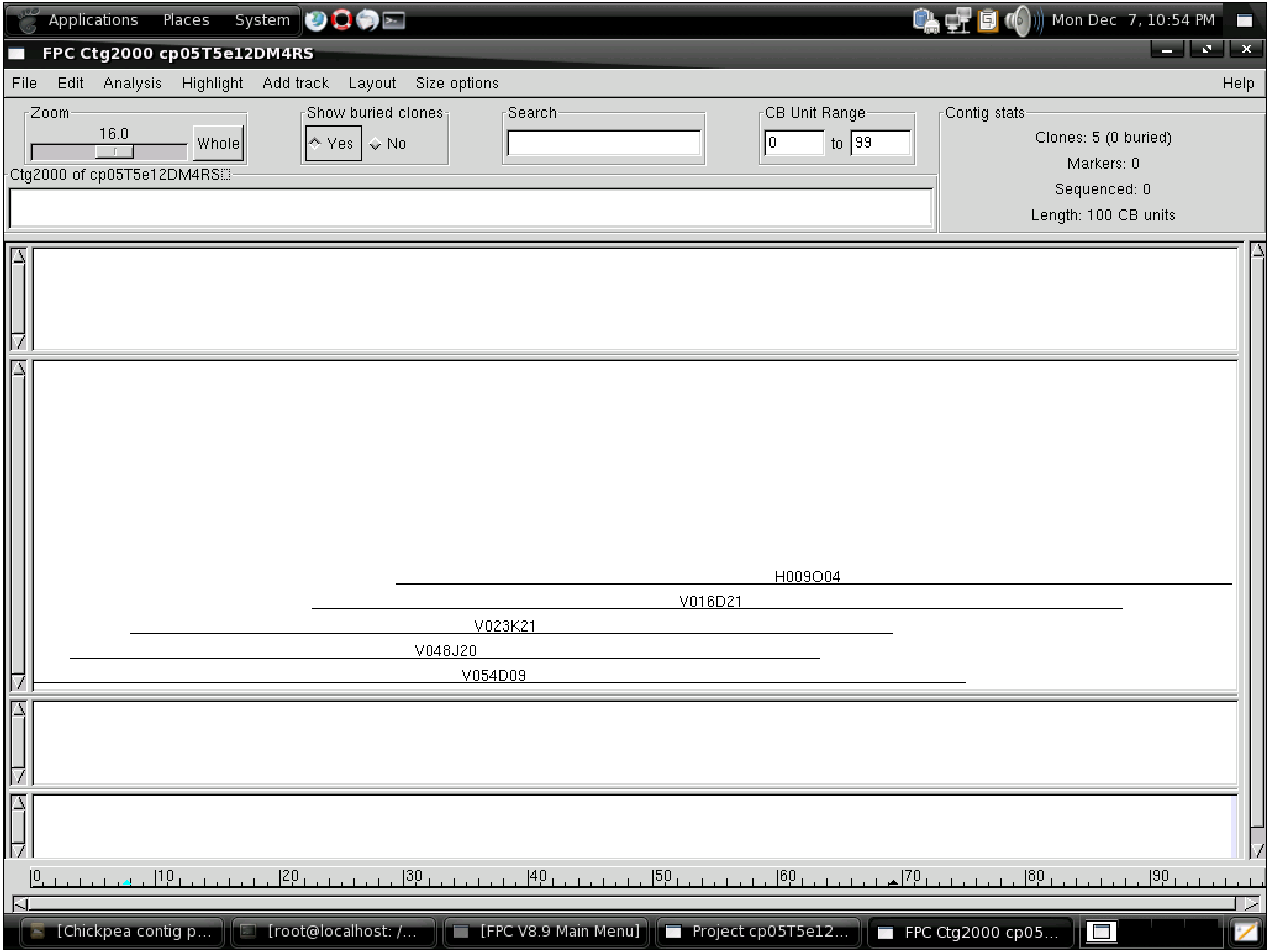
Applications Places System
Mon Dec 7, 10:54 PM
FPC Ctg2000 cp05T5e12DM4RS
File Edit Analysis Highlight Add track Layout Size options Help
Zoom
16.0
Whole
Show buried clones
Yes
No
Search
CB Unit Range
0 to 99
Contig stats
Clones: 5 (0 buried)
Markers: 0
Sequenced: 0
Length: 100 CB units
Ctg2000 of cp05T5e12DM4RS
H009O04
V016D21
V023K21
V048J20
V054D09
0 10 20 30 40 50 60 70 80 90
[Chickpea contig p...
[root@localhost: /...
[FPC V8.9 Main Menu]
Project cp05T5e12...
FPC Ctg2000 cp05...

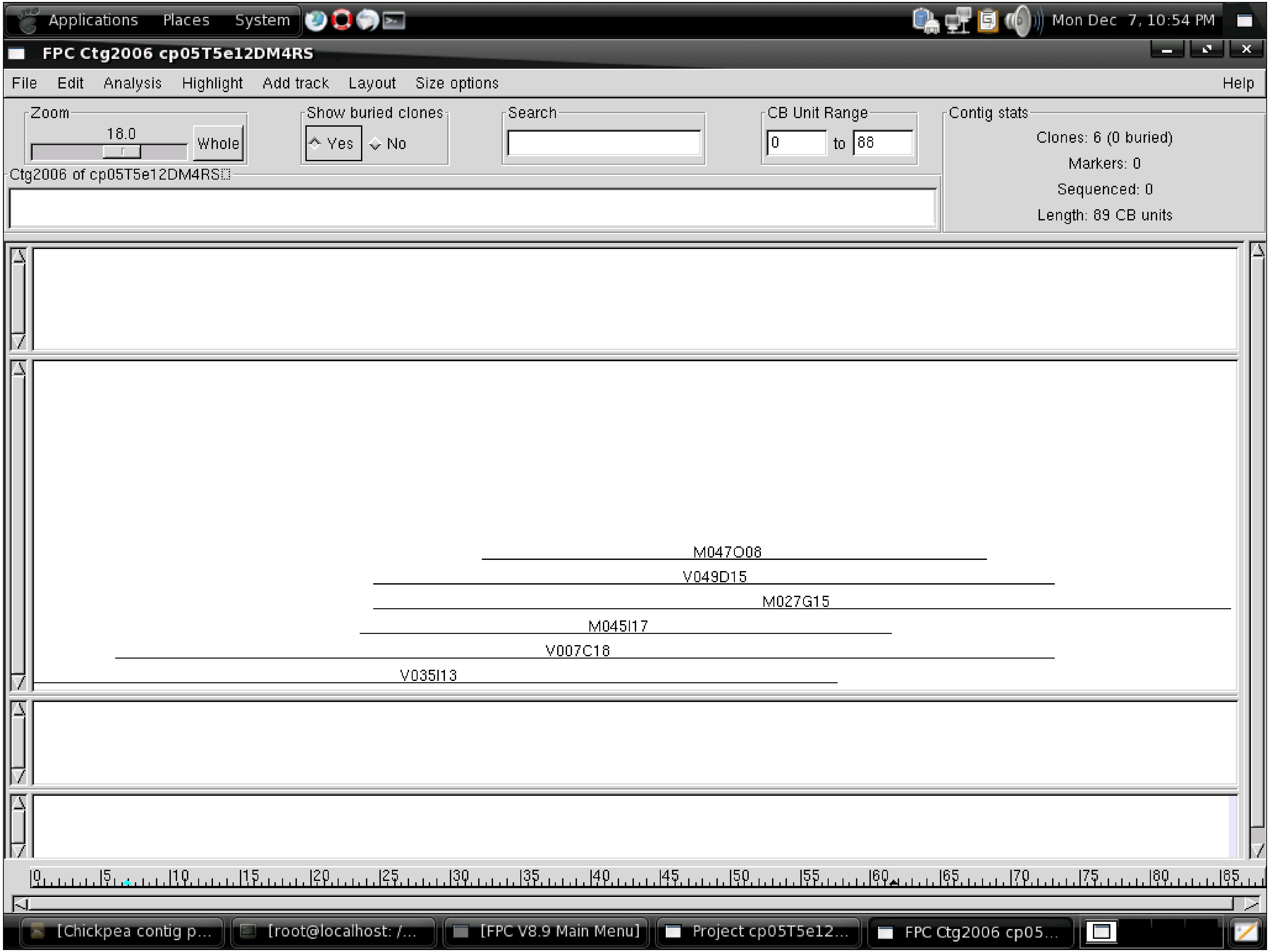
Applications
Places
System
Mon Dec 7, 10:54 PM
FPC Ctg2006 cp05T5e12DM4RS
File
Edit
Analysis
Highlight
Add track
Layout
Size options
Help
Zoom
18.0
Whole
Show buried clones
Yes
No
Search
CB Unit Range
0
to
88
Contig stats
Clones: 6 (0 buried)
Markers: 0
Sequenced: 0
Length: 89 CB units
Ctg2006 of cp05T5e12DM4RS
M047O08
V049D15
M027G15
M045I17
V007C18
V035I13
0 5 10 15 20 25 30 35 40 45 50 55 60 65 70 75 80 85
[Chickpea contig p...
[root@localhost: /...
[FPC V8.9 Main Menu]
Project cp05T5e12...
FPC Ctg2006 cp05...

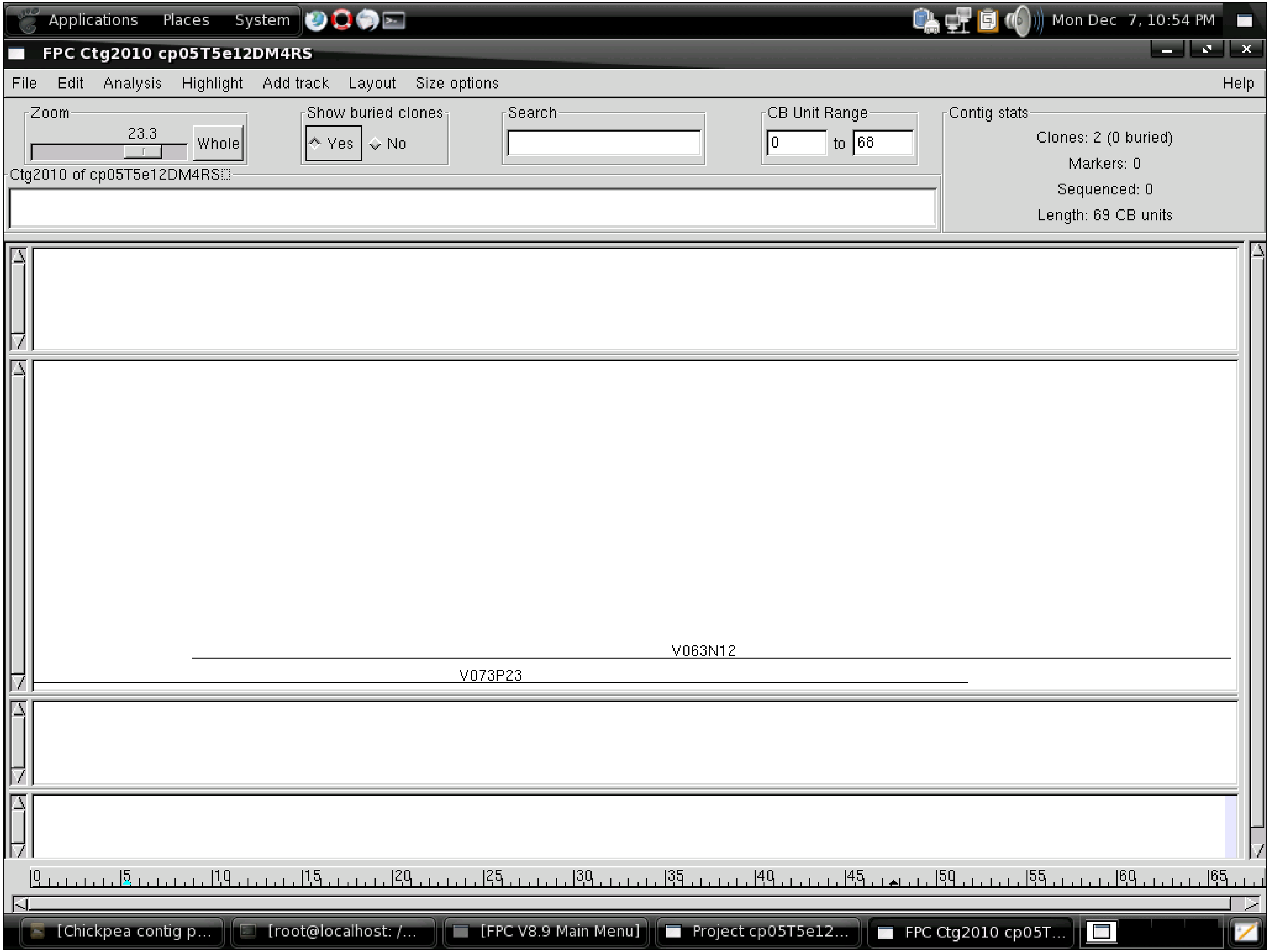
Applications
Places
System
Mon Dec 7, 10:54 PM
FPC Ctg2010 cp05T5e12DM4RS
File
Edit
Analysis
Highlight
Add track
Layout
Size options
Help
Zoom
23.3
Whole
Show buried clones
Yes
No
Search
CB Unit Range
0
to
68
Contig stats
Clones: 2 (0 buried)
Markers: 0
Sequenced: 0
Length: 69 CB units
Ctg2010 of cp05T5e12DM4RS
V063N12
V073P23
0 5 10 15 20 25 30 35 40 45 50 55 60 65
[Chickpea contig p...
[root@localhost: /...
[FPC V8.9 Main Menu]
Project cp05T5e12...
FPC Ctg2010 cp05T...

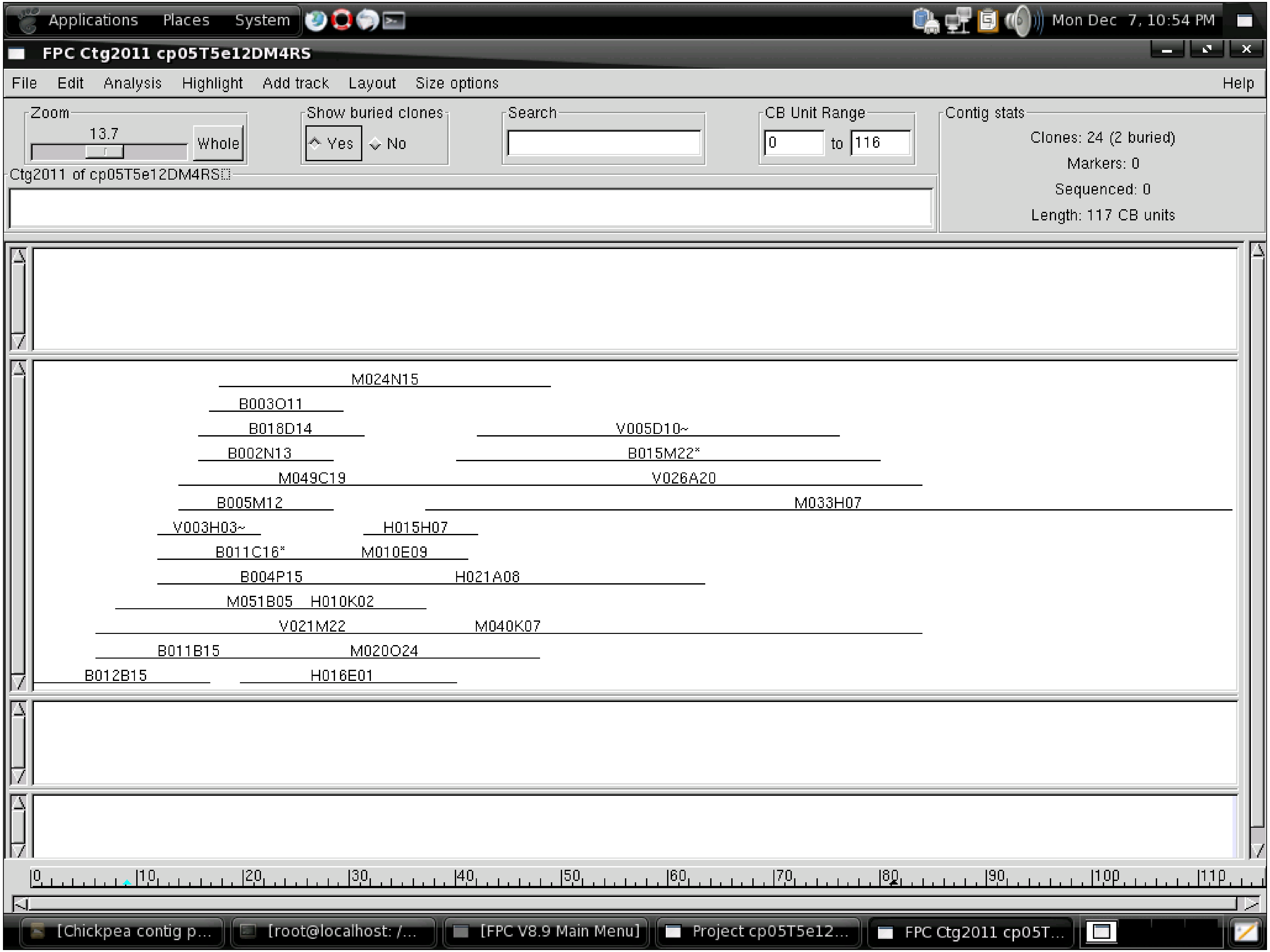
Applications Places System
Mon Dec 7, 10:54 PM
FPC Ctg2011 cp05T5e12DM4RS
File Edit Analysis Highlight Add track Layout Size options Help
Zoom
13.7
Whole
Show buried clones
Yes No
Search
CB Unit Range
0 to 116
Contig stats
Clones: 24 (2 buried)
Markers: 0
Sequenced: 0
Length: 117 CB units
Ctg2011 of cp05T5e12DM4RS
M024N15
B003O11
B018D14
V005D10~
B002N13
B015M22*
M049C19
V026A20
B005M12
M033H07
V003H03~
H015H07
B011C16*
M010E09
B004P15
H021A08
M051B05
H010K02
V021M22
M040K07
B011B15
M020O24
B012B15
H016E01
0 10 20 30 40 50 60 70 80 90 100 110
[Chickpea contig p...
[root@localhost: /...
[FPC V8.9 Main Menu]
Project cp05T5e12...
FPC Ctg2011 cp05T...

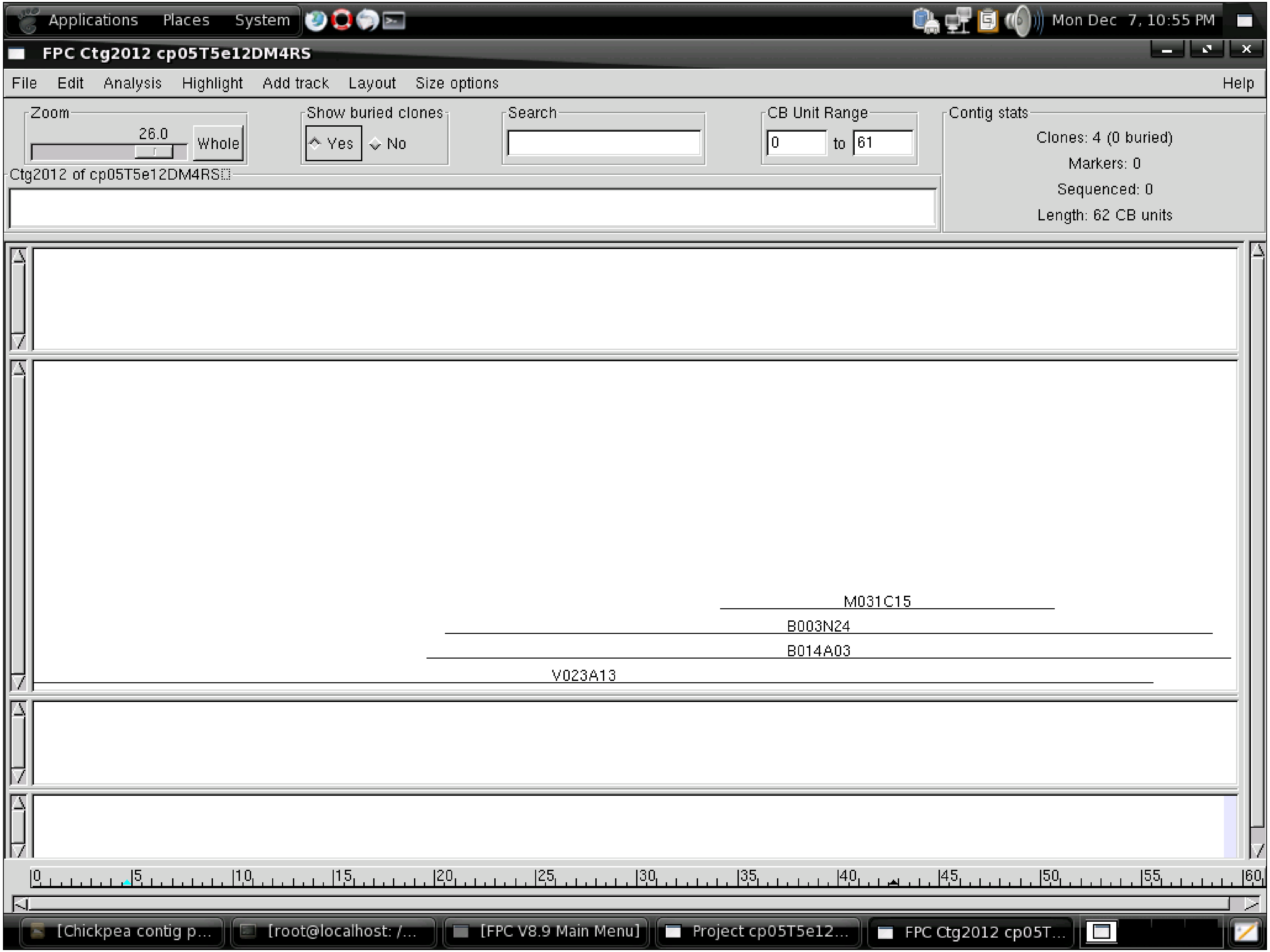
Applications Places System
Mon Dec 7, 10:55 PM
FPC Ctg2012 cp05T5e12DM4RS
File Edit Analysis Highlight Add track Layout Size options Help
Zoom
26.0
Whole
Show buried clones
Yes No
Search
CB Unit Range
0 to 61
Contig stats
Clones: 4 (0 buried)
Markers: 0
Sequenced: 0
Length: 62 CB units
Ctg2012 of cp05T5e12DM4RS
M031C15
B003N24
B014A03
V023A13
0 5 10 15 20 25 30 35 40 45 50 55 60
[Chickpea contig p...
[root@localhost: /...
[FPC V8.9 Main Menu]
Project cp05T5e12...
FPC Ctg2012 cp05T...

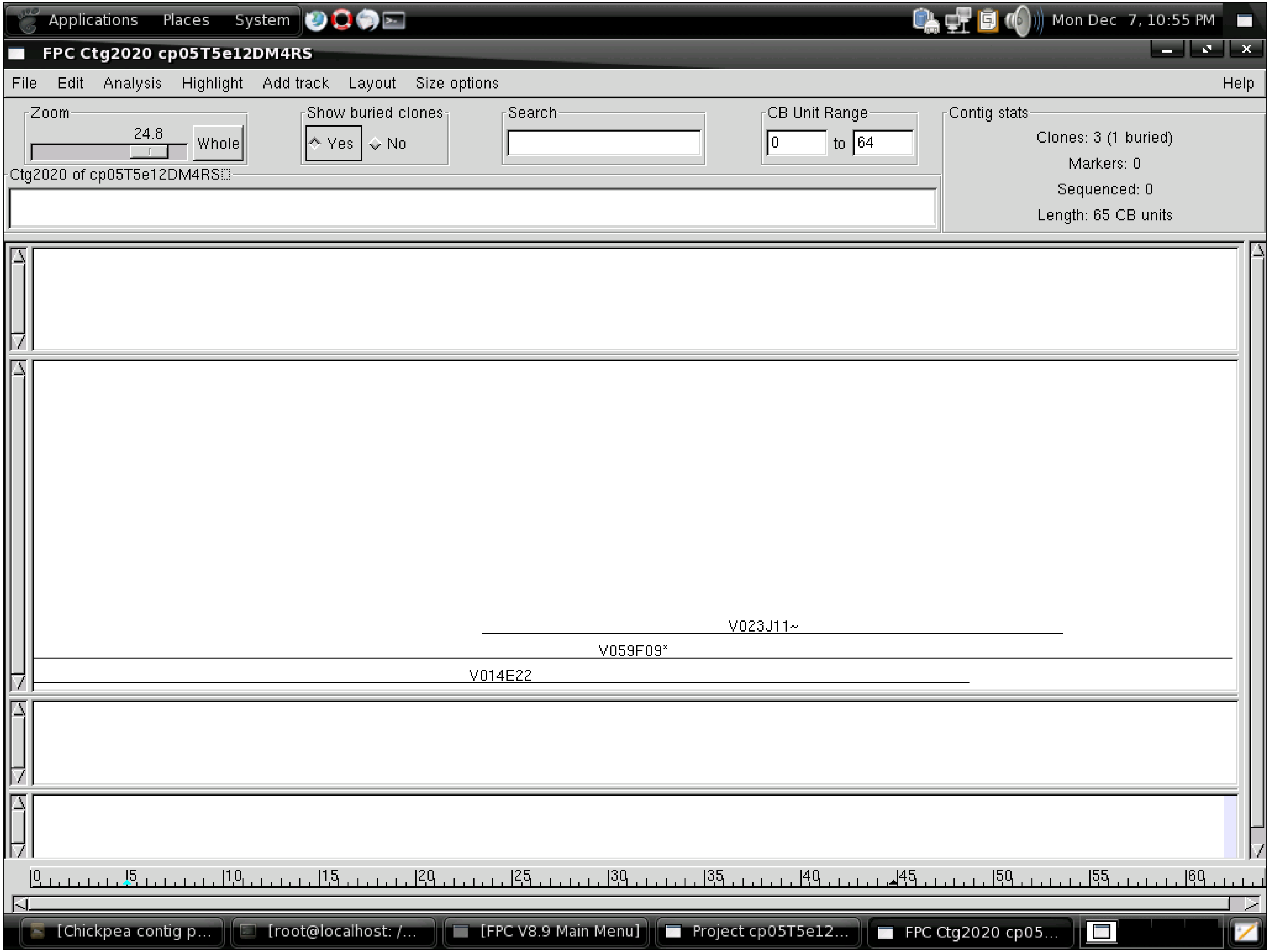
Applications
Places
System
Mon Dec 7, 10:55 PM
FPC Ctg2020 cp05T5e12DM4RS
File
Edit
Analysis
Highlight
Add track
Layout
Size options
Help
Zoom
24.8
Whole
Show buried clones
Yes
No
Search
CB Unit Range
0
to
64
Contig stats
Clones: 3 (1 buried)
Markers: 0
Sequenced: 0
Length: 65 CB units
Ctg2020 of cp05T5e12DM4RS
V023J11~
V059F09*
V014E22
0 5 10 15 20 25 30 35 40 45 50 55 60
[Chickpea contig p...
[root@localhost: /...
[FPC V8.9 Main Menu]
Project cp05T5e12...
FPC Ctg2020 cp05...

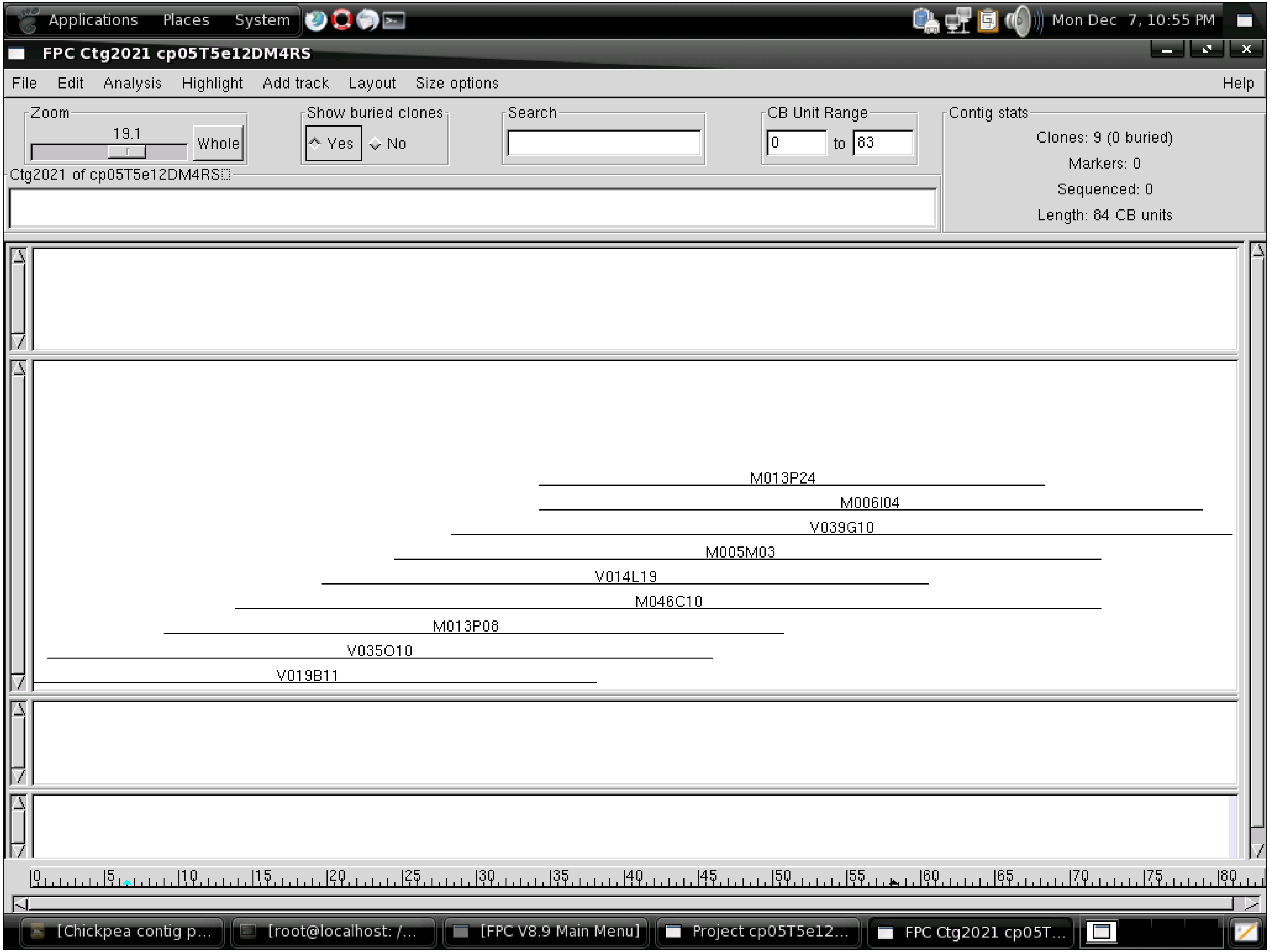

Applications
Places
System
Mon Dec 7, 10:55 PM
FPC Ctg2021 cp05T5e12DM4RS
File
Edit
Analysis
Highlight
Add track
Layout
Size options
Help
Zoom
19.1
Whole
Show buried clones
Yes
No
Search
CB Unit Range
0
to
83
Contig stats
Clones: 9 (0 buried)
Markers: 0
Sequenced: 0
Length: 84 CB units
Ctg2021 of cp05T5e12DM4RS
M013P24
M006I04
V039G10
M005M03
V014L19
M046C10
M013P08
V035O10
V019B11
0
5
10
15
20
25
30
35
40
45
50
55
60
65
70
75
80
[Chickpea contig p...
[root@localhost: /...
[FPC V8.9 Main Menu]
Project cp05T5e12...
FPC Ctg2021 cp05T...

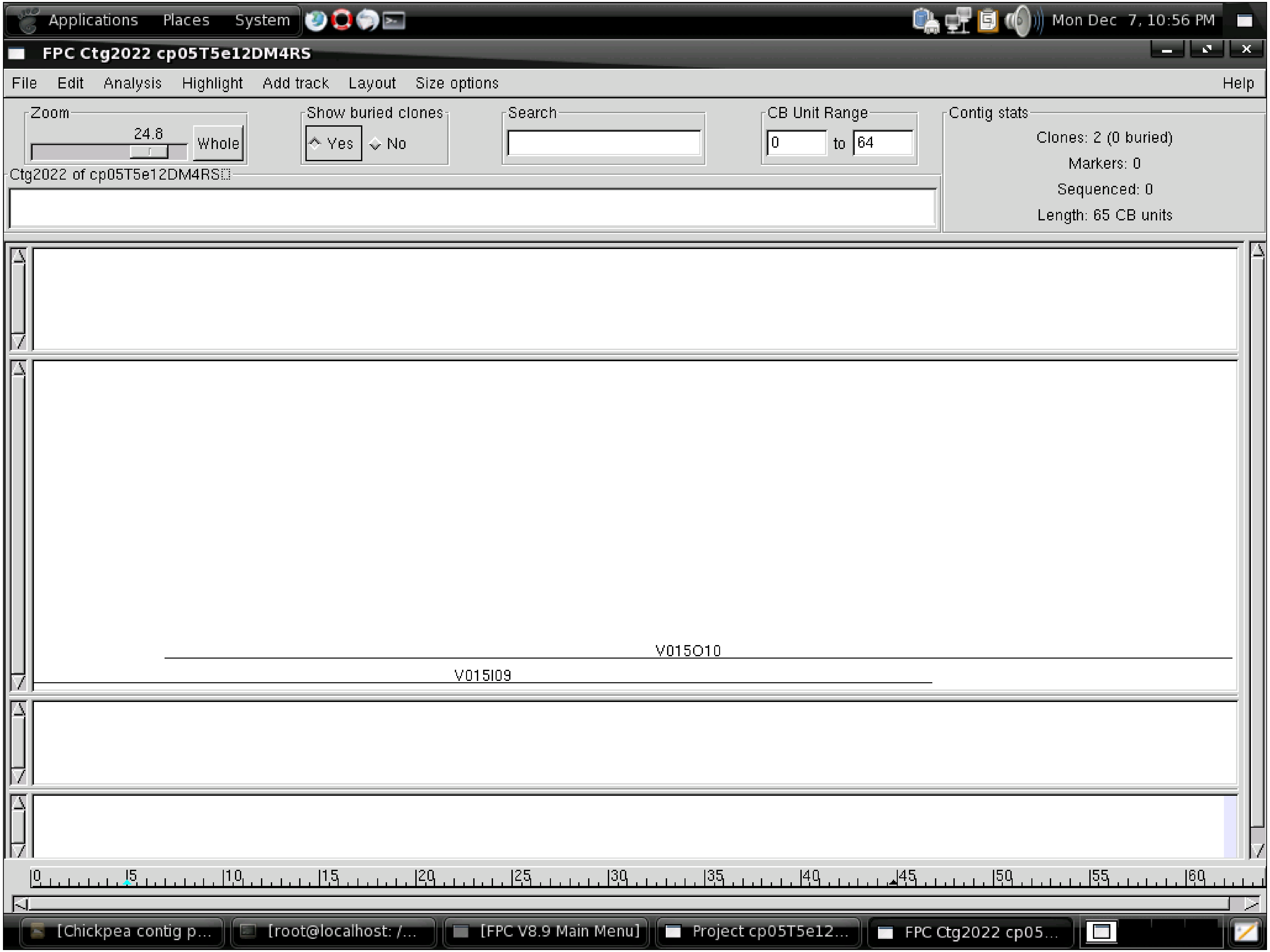
Applications Places System
Mon Dec 7, 10:56 PM
FPC Ctg2022 cp05T5e12DM4RS
File Edit Analysis Highlight Add track Layout Size options Help
Zoom
24.8
Whole
Show buried clones
Yes No
Search
CB Unit Range
0 to 64
Contig stats
Clones: 2 (0 buried)
Markers: 0
Sequenced: 0
Length: 65 CB units
Ctg2022 of cp05T5e12DM4RS
V015O10
V015I09
0 5 10 15 20 25 30 35 40 45 50 55 60
[Chickpea contig p...
[root@localhost: /...
[FPC V8.9 Main Menu]
Project cp05T5e12...
FPC Ctg2022 cp05...

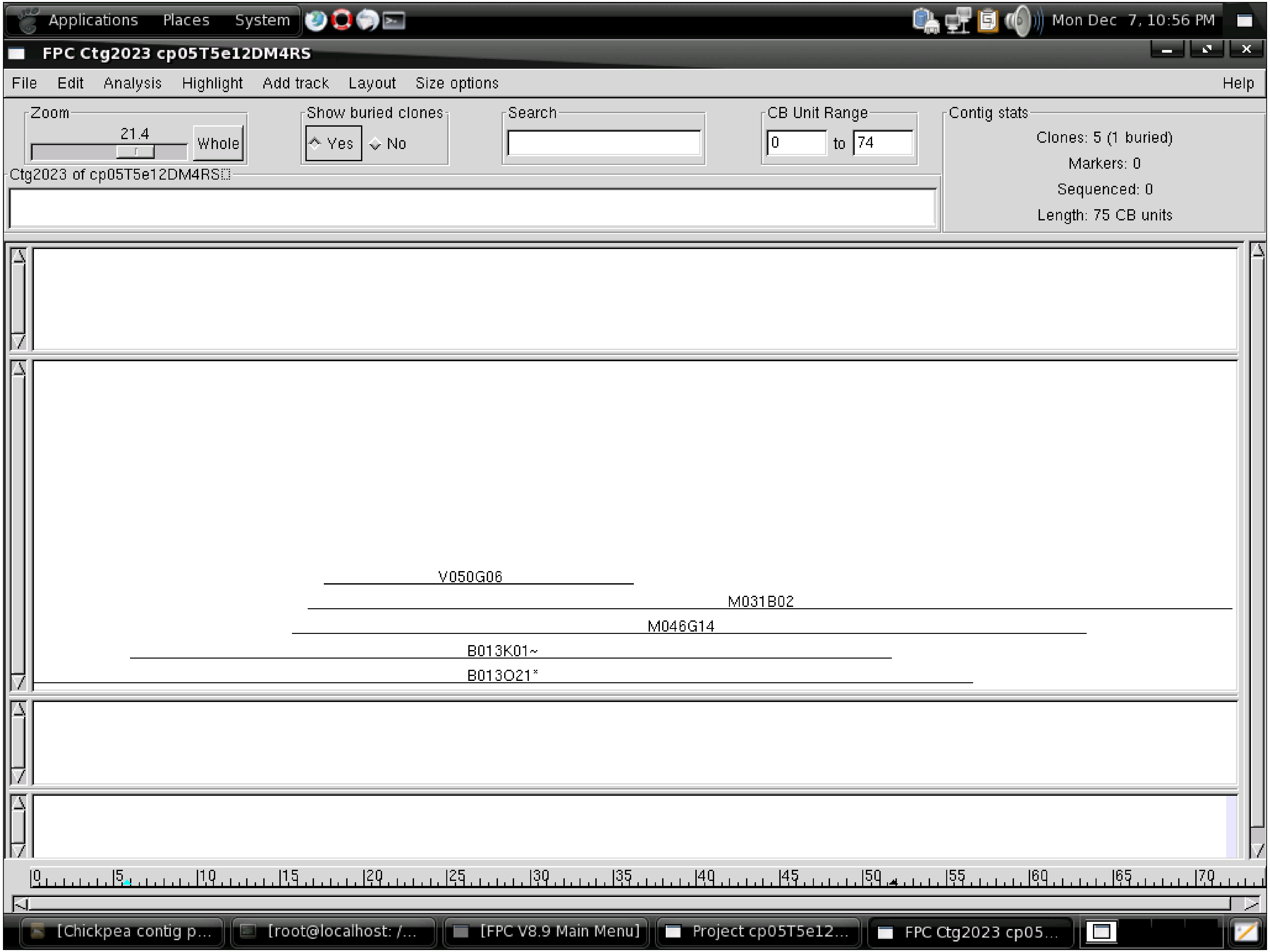
Applications Places System
Mon Dec 7, 10:56 PM
FPC Ctg2023 cp05T5e12DM4RS
File Edit Analysis Highlight Add track Layout Size options Help
Zoom
21.4
Whole
Show buried clones
Yes No
Search
CB Unit Range
0 to 74
Contig stats
Clones: 5 (1 buried)
Markers: 0
Sequenced: 0
Length: 75 CB units
Ctg2023 of cp05T5e12DM4RS
V050G06
M031B02
M046G14
B013K01~
B013O21*
0 5 10 15 20 25 30 35 40 45 50 55 60 65 70
[Chickpea contig p...
[root@localhost: /...
[FPC V8.9 Main Menu]
Project cp05T5e12...
FPC Ctg2023 cp05...

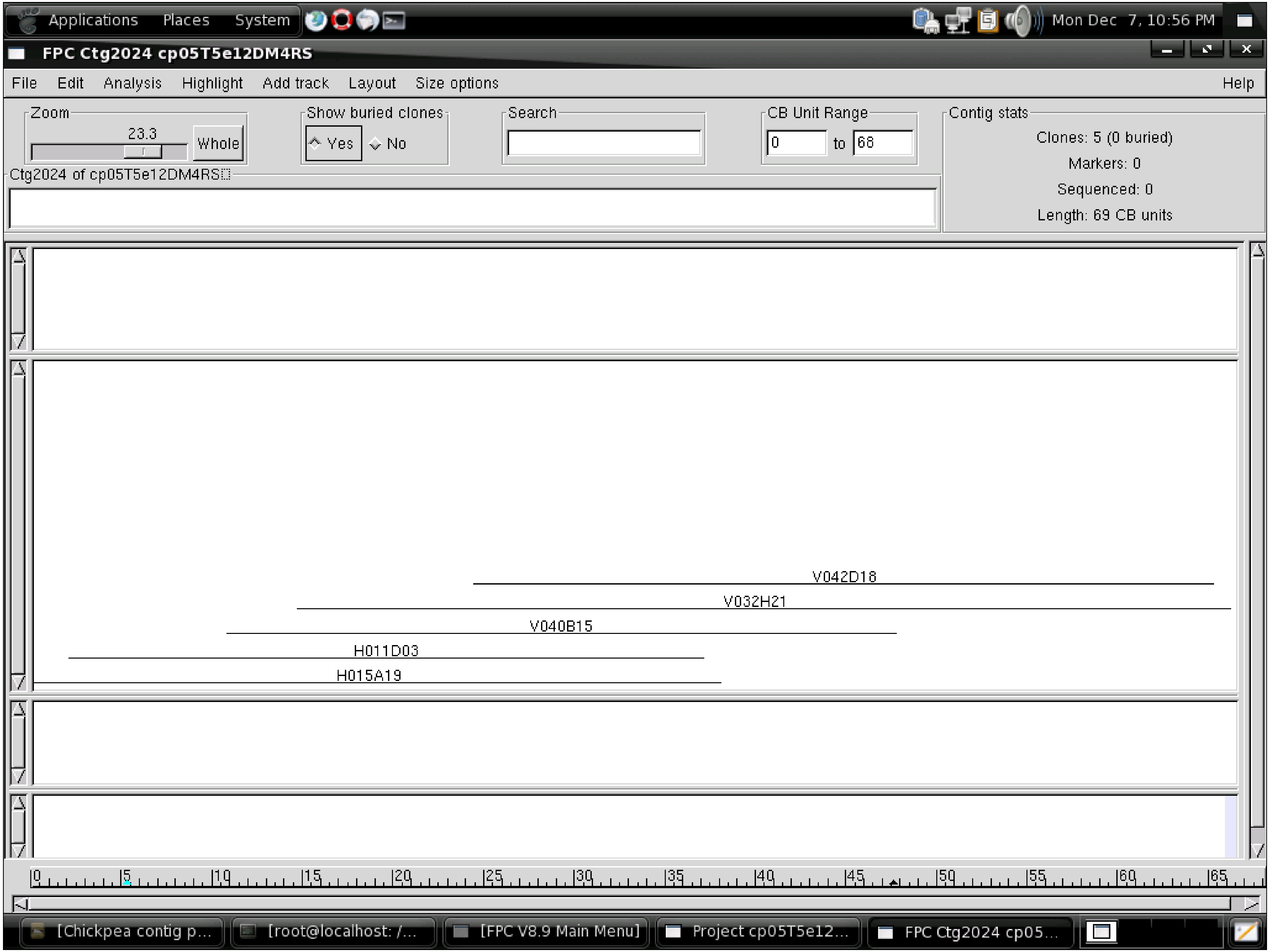
Applications
Places
System
Mon Dec 7, 10:56 PM
FPC Ctg2024 cp05T5e12DM4RS
File
Edit
Analysis
Highlight
Add track
Layout
Size options
Help
Zoom
23.3
Whole
Show buried clones
Yes
No
Search
CB Unit Range
0
to
68
Contig stats
Clones: 5 (0 buried)
Markers: 0
Sequenced: 0
Length: 69 CB units
Ctg2024 of cp05T5e12DM4RS
V042D18
V032H21
V040B15
H011D03
H015A19
0
5
10
15
20
25
30
35
40
45
50
55
60
65
[Chickpea contig p...
[root@localhost: /...
[FPC V8.9 Main Menu]
Project cp05T5e12...
FPC Ctg2024 cp05...

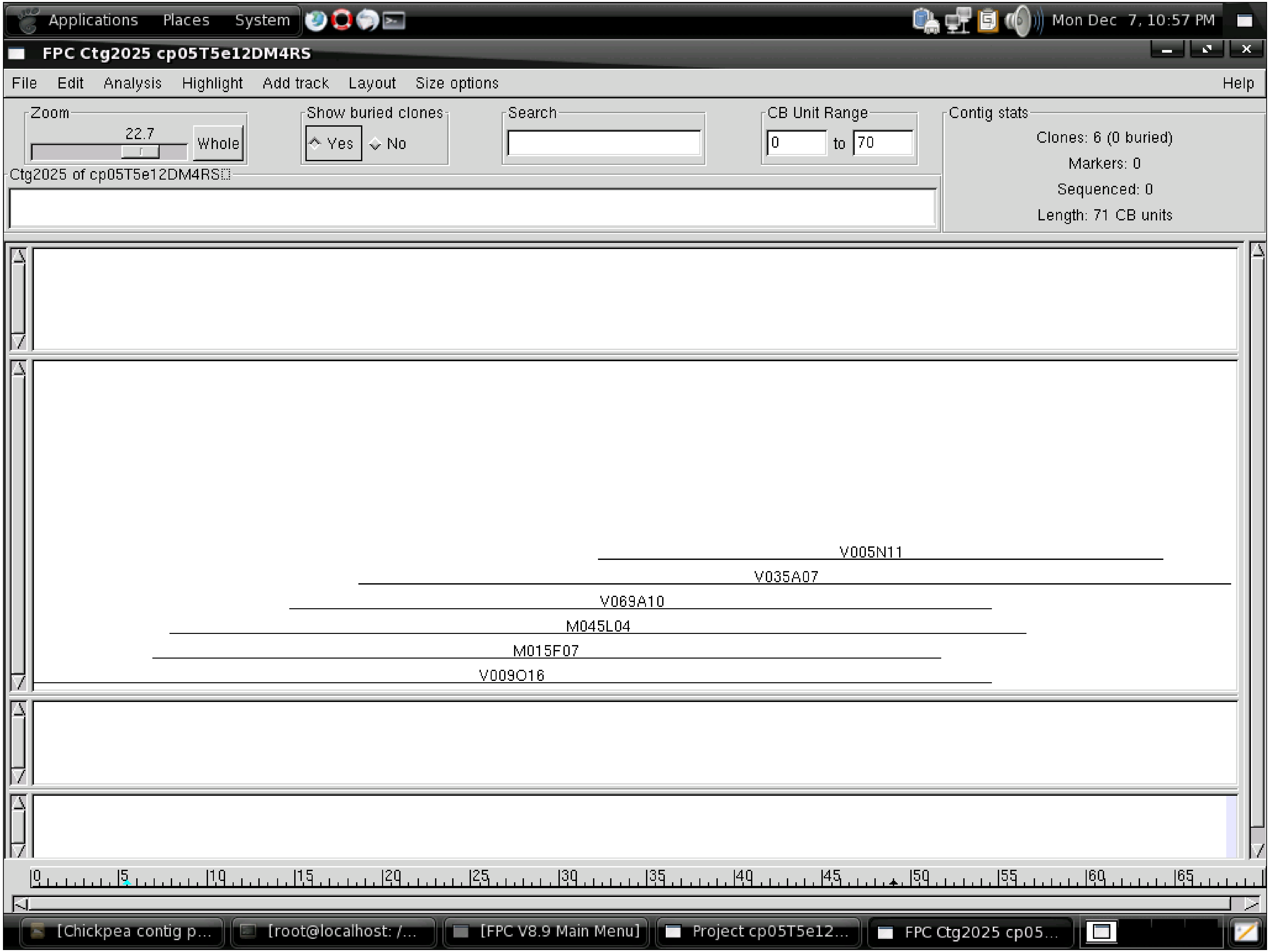
Applications Places System
Mon Dec 7, 10:57 PM
FPC Ctg2025 cp05T5e12DM4RS
File Edit Analysis Highlight Add track Layout Size options Help
Zoom
22.7
Whole
Show buried clones
Yes No
Search
CB Unit Range
0 to 70
Contig stats
Clones: 6 (0 buried)
Markers: 0
Sequenced: 0
Length: 71 CB units
Ctg2025 of cp05T5e12DM4RS
V005N11
V035A07
V069A10
M045L04
M015F07
V009O16
0 5 10 15 20 25 30 35 40 45 50 55 60 65
[Chickpea contig p...
[root@localhost: /...
[FPC V8.9 Main Menu]
Project cp05T5e12...
FPC Ctg2025 cp05...

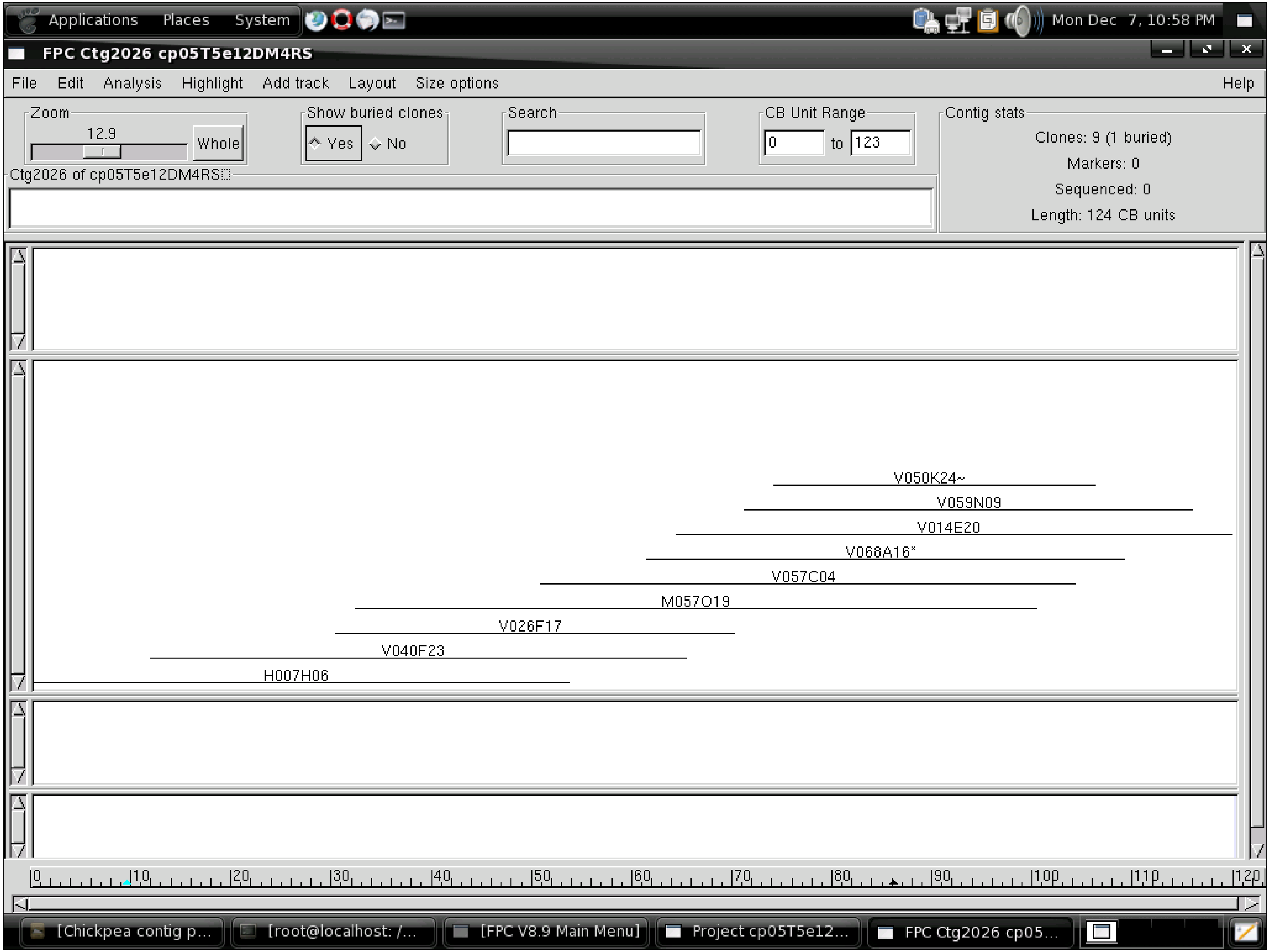
Applications Places System
Mon Dec 7, 10:58 PM
FPC Ctg2026 cp05T5e12DM4RS
File Edit Analysis Highlight Add track Layout Size options Help
Zoom
12.9
Whole
Show buried clones
Yes No
Search
CB Unit Range
0 to 123
Contig stats
Clones: 9 (1 buried)
Markers: 0
Sequenced: 0
Length: 124 CB units
Ctg2026 of cp05T5e12DM4RS
V050K24~
V059N09
V014E20
V068A16*
V057C04
M057O19
V026F17
V040F23
H007H06
0 10 20 30 40 50 60 70 80 90 100 110 120
[Chickpea contig p...
[root@localhost: /...
[FPC V8.9 Main Menu]
Project cp05T5e12...
FPC Ctg2026 cp05...

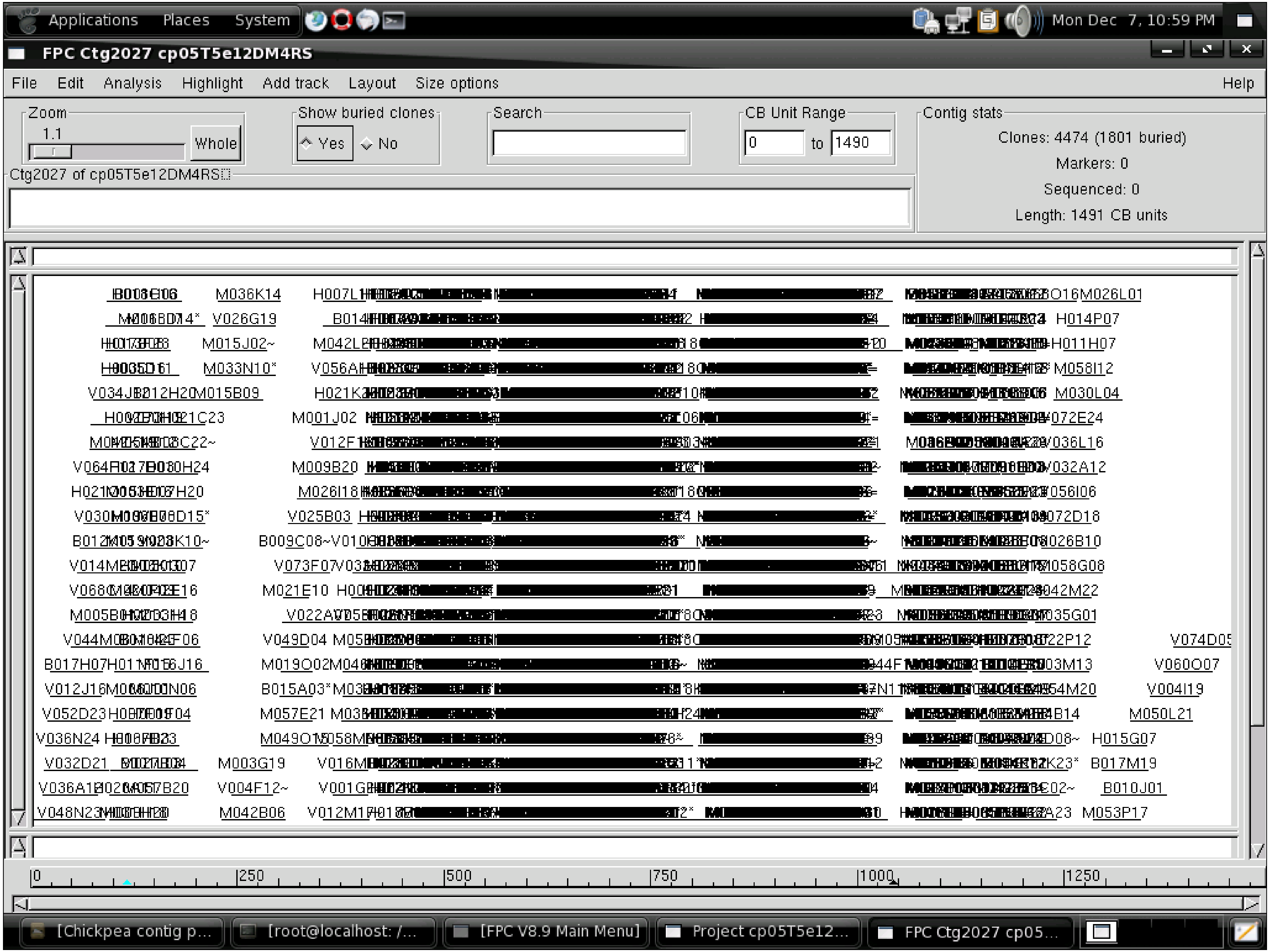

Applications Places System
Mon Dec 7, 10:59 PM
FPC Ctg2027 cp05T5e12DM4RS
File Edit Analysis Highlight Add track Layout Size options Help
Zoom
1.1
Whole
Show buried clones
Yes
No
Search
CB Unit Range
0 to 1490
Contig stats
Clones: 4474 (1801 buried)
Markers: 0
Sequenced: 0
Length: 1491 CB units
Ctg2027 of cp05T5e12DM4RS
0 250 500 750 1000 1250
[Chickpea contig p...
[root@localhost: /...
[FPC V8.9 Main Menu]
Project cp05T5e12...
FPC Ctg2027 cp05...

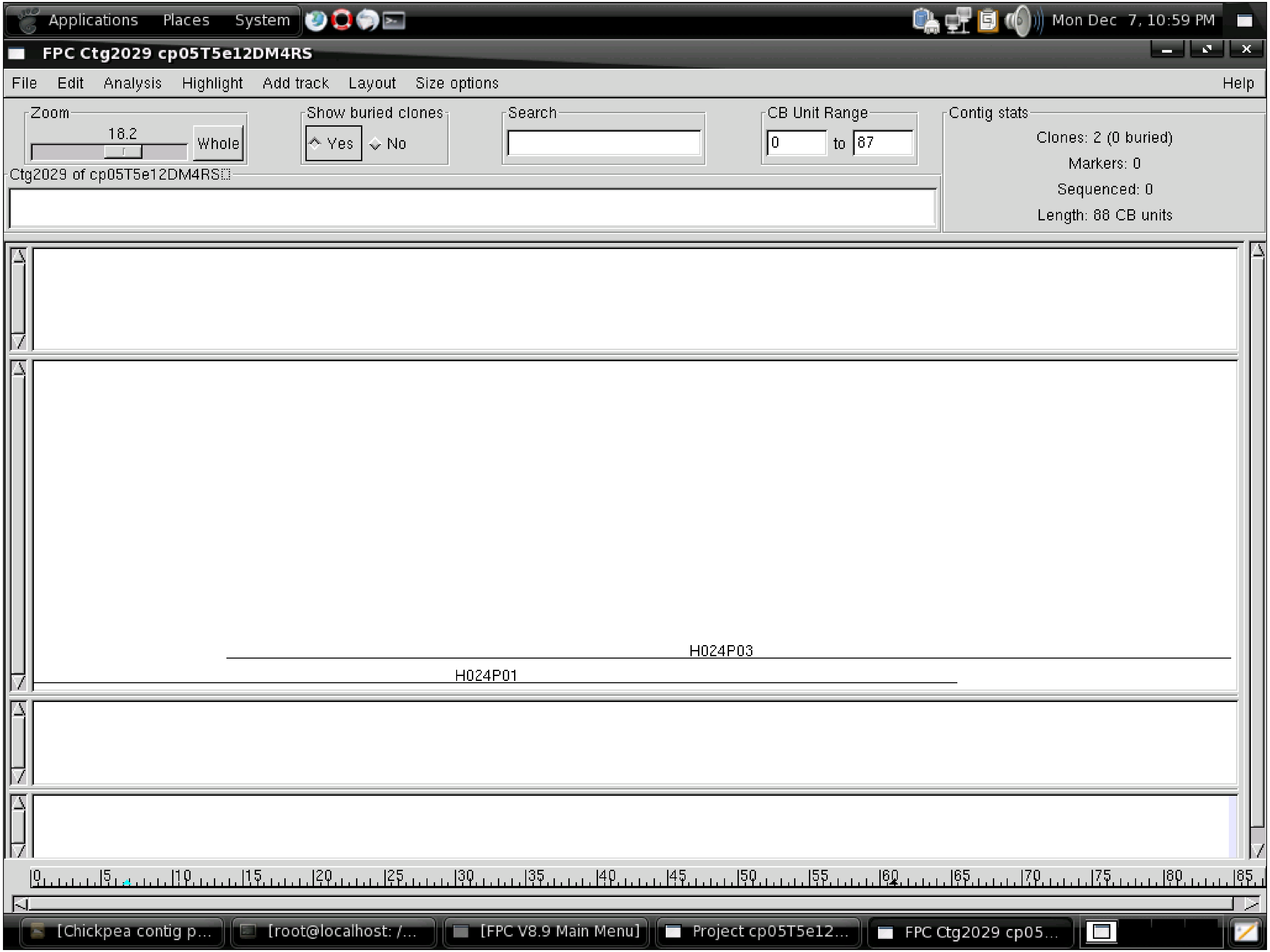
Applications Places System
Mon Dec 7, 10:59 PM
FPC Ctg2029 cp05T5e12DM4RS
File Edit Analysis Highlight Add track Layout Size options Help
Zoom
18.2
Whole
Show buried clones
Yes No
Search
CB Unit Range
0 to 87
Contig stats
Clones: 2 (0 buried)
Markers: 0
Sequenced: 0
Length: 88 CB units
Ctg2029 of cp05T5e12DM4RS
H024P03
H024P01
0 5 10 15 20 25 30 35 40 45 50 55 60 65 70 75 80 85
[Chickpea contig p...
[root@localhost: /...
[FPC V8.9 Main Menu]
Project cp05T5e12...
FPC Ctg2029 cp05...

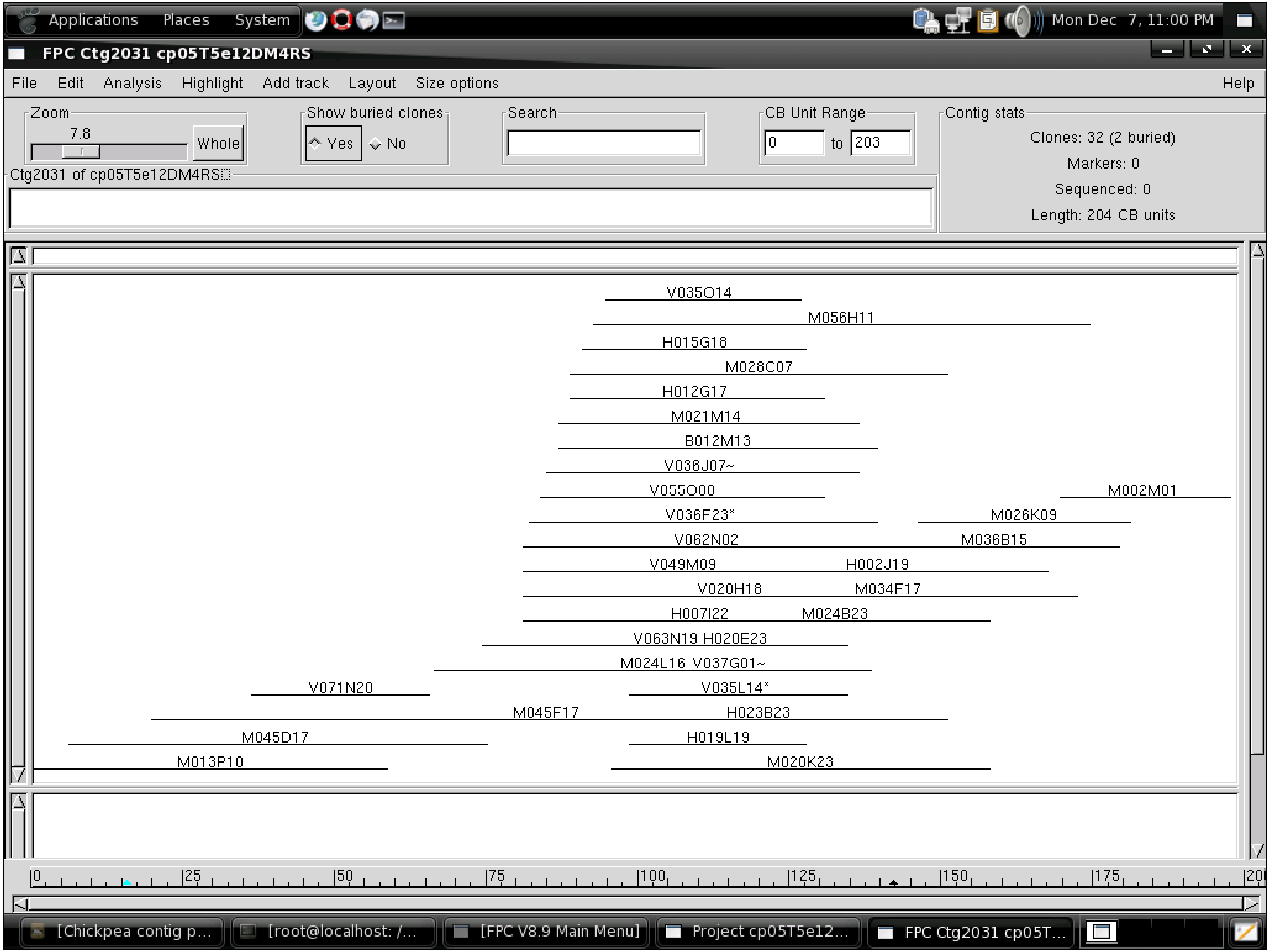
Applications Places System
Mon Dec 7, 11:00 PM
FPC Ctg2031 cp05T5e12DM4RS
File Edit Analysis Highlight Add track Layout Size options Help
Zoom
7.8
Whole
Show buried clones
Yes
No
Search
CB Unit Range
0 to 203
Contig stats
Clones: 32 (2 buried)
Markers: 0
Sequenced: 0
Length: 204 CB units
Ctg2031 of cp05T5e12DM4RS
V035O14
M056H11
H015G18
M028C07
H012G17
M021M14
B012M13
V036J07~
V055O08
M002M01
V036F23*
M026K09
V062N02
M036B15
V049M09
H002J19
V020H18
M034F17
H007I22
M024B23
V063N19 H020E23
M024L16 V037G01~
V071N20
V035L14*
M045F17
H023B23
M045D17
H019L19
M013P10
M020K23
0 25 50 75 100 125 150 175 20
[Chickpea contig p...
[root@localhost: /...
[FPC V8.9 Main Menu]
Project cp05T5e12...
FPC Ctg2031 cp05T...

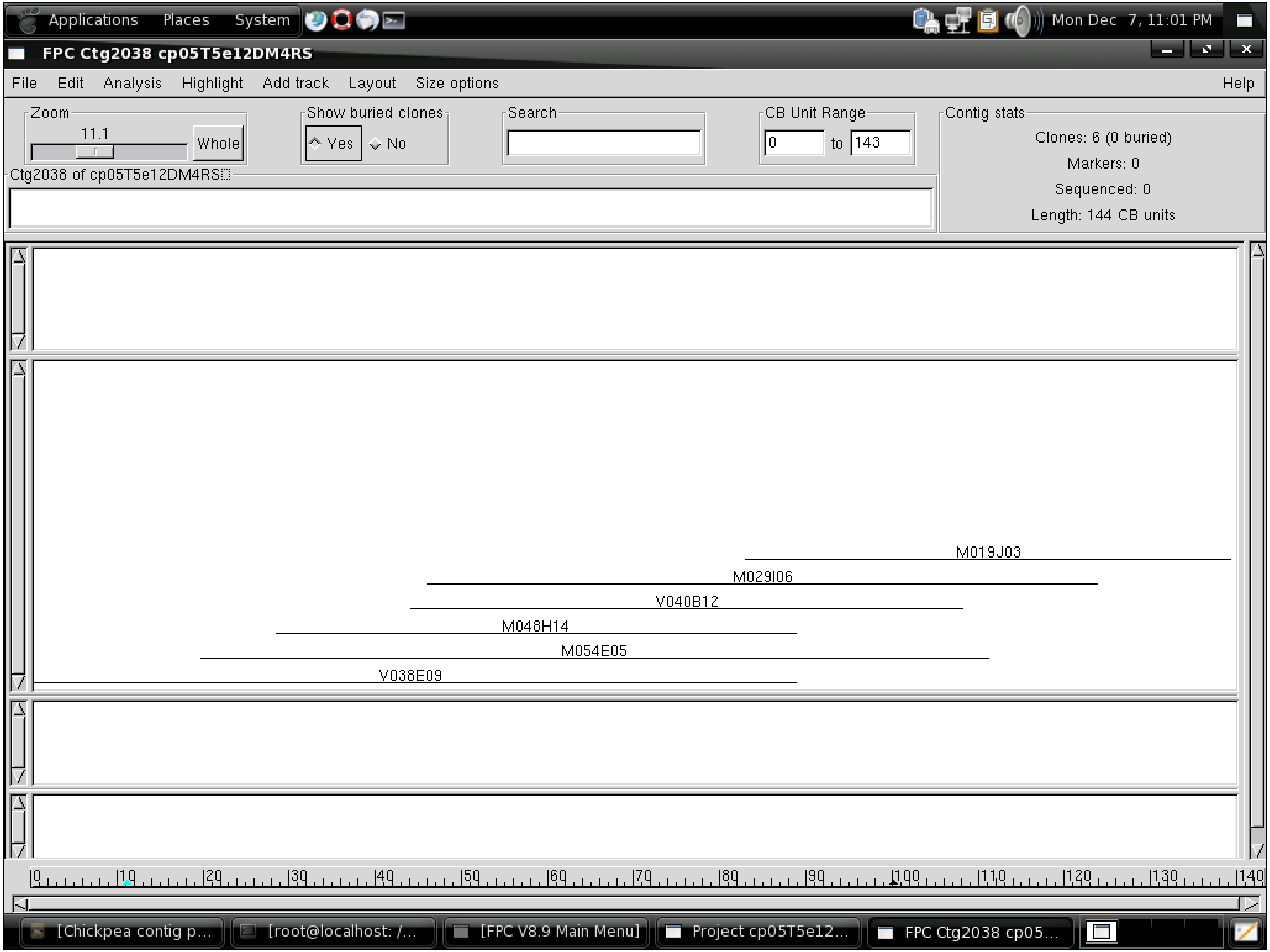
Applications
Places
System
Mon Dec 7, 11:01 PM
FPC Ctg2038 cp05T5e12DM4RS
File
Edit
Analysis
Highlight
Add track
Layout
Size options
Help
Zoom
11.1
Whole
Show buried clones
Yes
No
Search
CB Unit Range
0
to
143
Contig stats
Clones: 6 (0 buried)
Markers: 0
Sequenced: 0
Length: 144 CB units
Ctg2038 of cp05T5e12DM4RS
M019J03
M029I06
V040B12
M048H14
M054E05
V038E09
0 10 20 30 40 50 60 70 80 90 100 110 120 130 140
[Chickpea contig p...
[root@localhost: /...
[FPC V8.9 Main Menu]
Project cp05T5e12...
FPC Ctg2038 cp05...

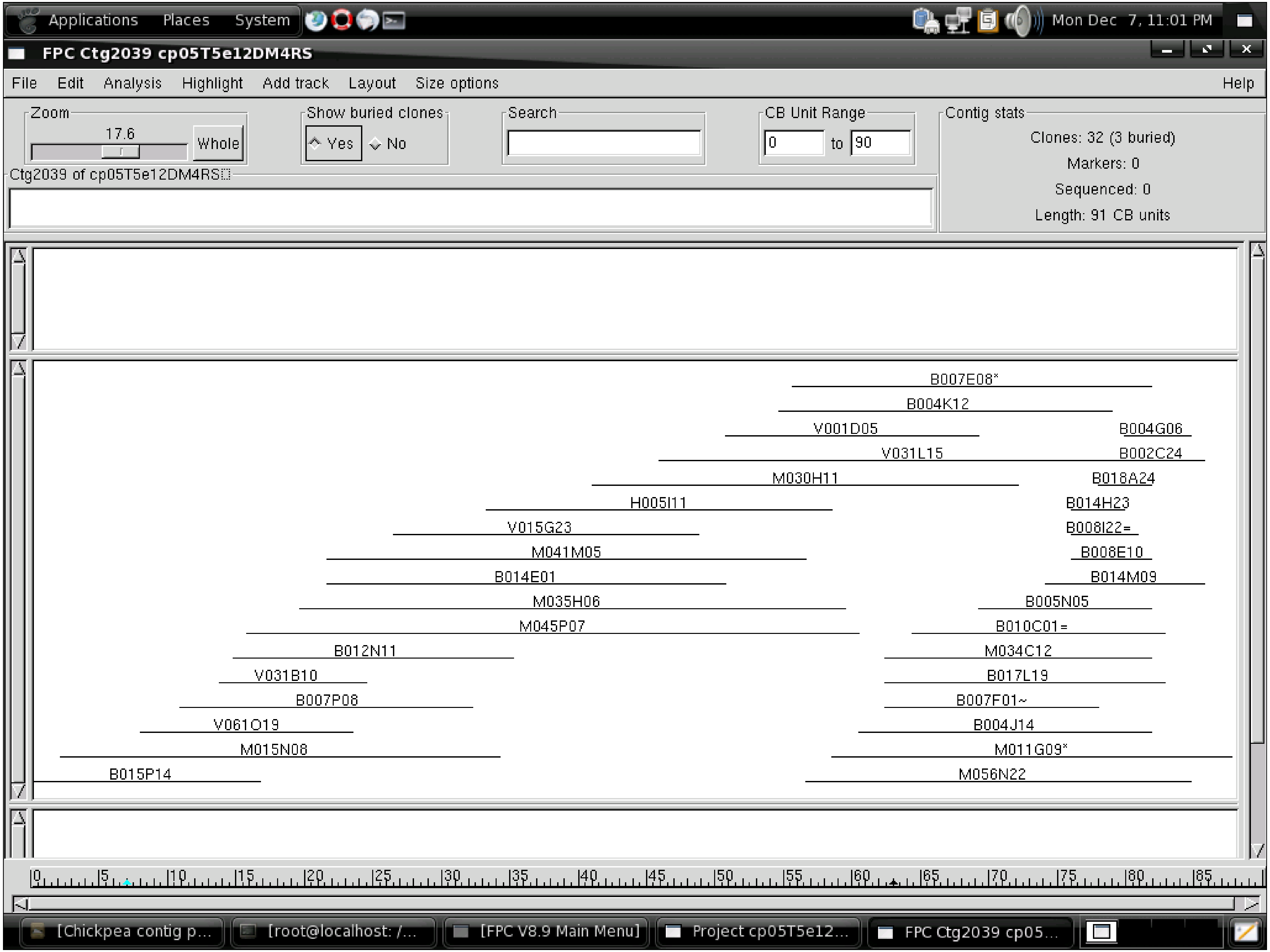

Applications Places System
Mon Dec 7, 11:01 PM
FPC Ctg2039 cp05T5e12DM4RS
File Edit Analysis Highlight Add track Layout Size options Help
Zoom
17.6
Whole
Show buried clones
Yes No
Search
CB Unit Range
0 to 90
Contig stats
Clones: 32 (3 buried)
Markers: 0
Sequenced: 0
Length: 91 CB units
Ctg2039 of cp05T5e12DM4RS
B007E08*
B004K12
V001D05
B004G06
V031L15
B002C24
M030H11
B018A24
H005I11
B014H23
V015G23
B008I22=
M041M05
B008E10
B014E01
B014M09
M035H06
B005N05
M045P07
B010C01=
B012N11
M034C12
V031B10
B017L19
B007P08
B007F01~
V061O19
B004J14
M015N08
M011G09*
B015P14
M056N22
0 5 10 15 20 25 30 35 40 45 50 55 60 65 70 75 80 85
[Chickpea contig p...
[root@localhost: /...
[FPC V8.9 Main Menu]
Project cp05T5e12...
FPC Ctg2039 cp05...

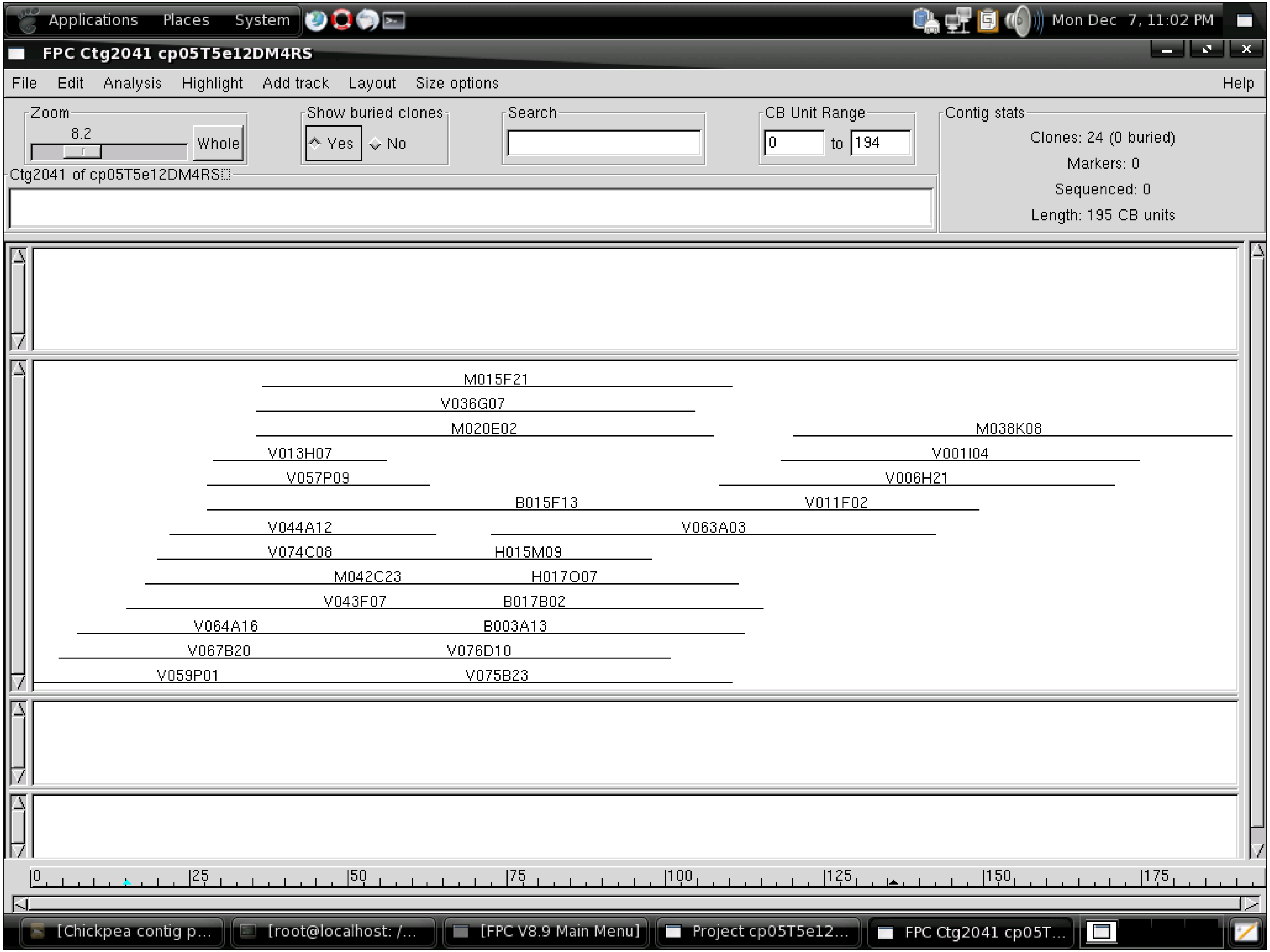
Applications Places System
Mon Dec 7, 11:02 PM
FPC Ctg2041 cp05T5e12DM4RS
File Edit Analysis Highlight Add track Layout Size options Help
Zoom
8.2
Whole
Show buried clones
Yes No
Search
CB Unit Range
0 to 194
Contig stats
Clones: 24 (0 buried)
Markers: 0
Sequenced: 0
Length: 195 CB units
Ctg2041 of cp05T5e12DM4RS
M015F21
V036G07
M020E02
M038K08
V013H07
V001I04
V057P09
V006H21
B015F13
V011F02
V044A12
V063A03
V074C08
H015M09
M042C23
H017O07
V043F07
B017B02
V064A16
B003A13
V067B20
V076D10
V059P01
V075B23
0 25 50 75 100 125 150 175
[Chickpea contig p...
[root@localhost: /...
[FPC V8.9 Main Menu]
Project cp05T5e12...
FPC Ctg2041 cp05T...

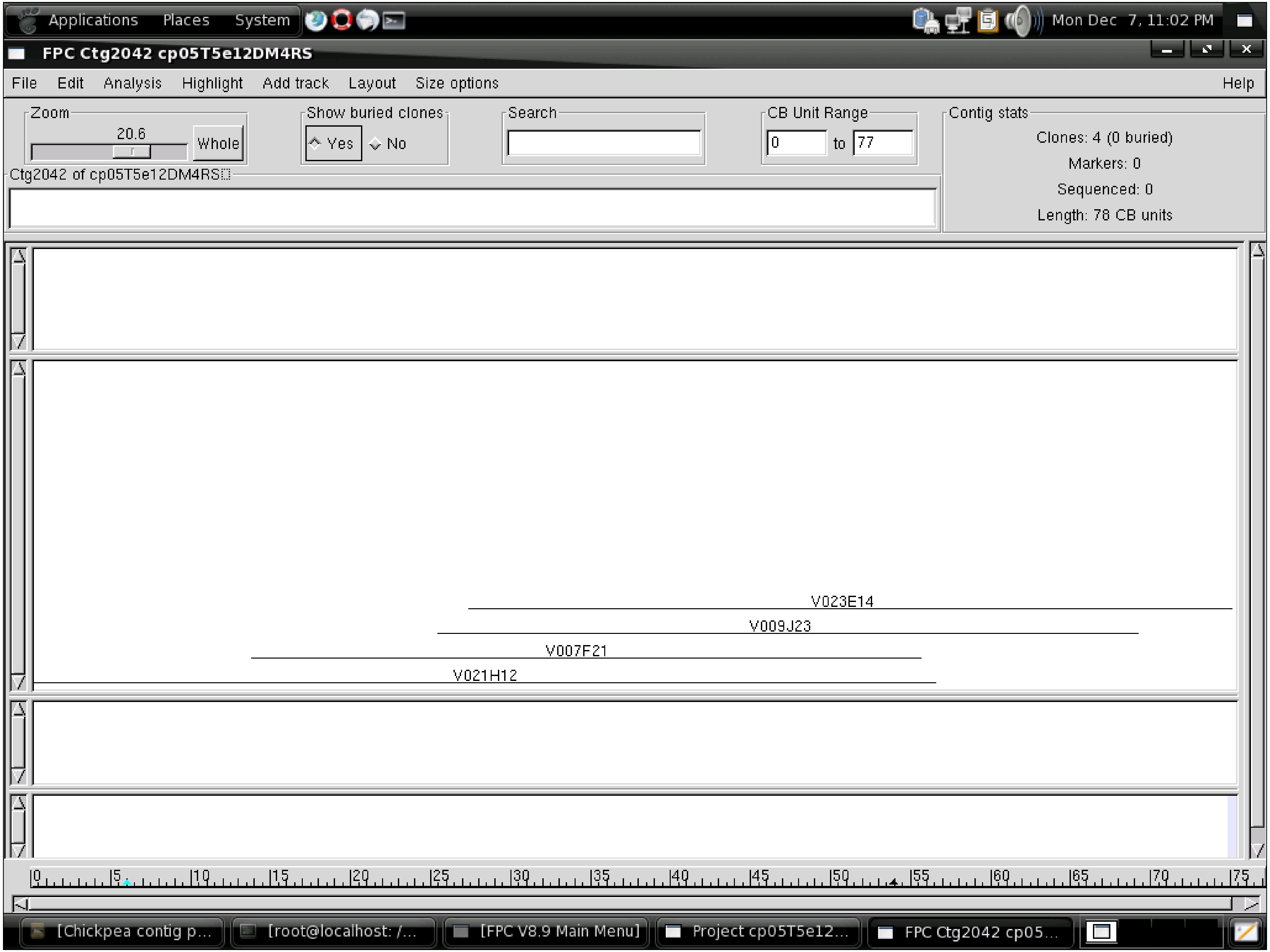
Applications Places System
Mon Dec 7, 11:02 PM
FPC Ctg2042 cp05T5e12DM4RS
File Edit Analysis Highlight Add track Layout Size options Help
Zoom
20.6
Whole
Show buried clones
Yes No
Search
CB Unit Range
0 to 77
Contig stats
Clones: 4 (0 buried)
Markers: 0
Sequenced: 0
Length: 78 CB units
Ctg2042 of cp05T5e12DM4RS
V023E14
V009J23
V007F21
V021H12
0 5 10 15 20 25 30 35 40 45 50 55 60 65 70 75
[Chickpea contig p...
[root@localhost: /...
[FPC V8.9 Main Menu]
Project cp05T5e12...
FPC Ctg2042 cp05...

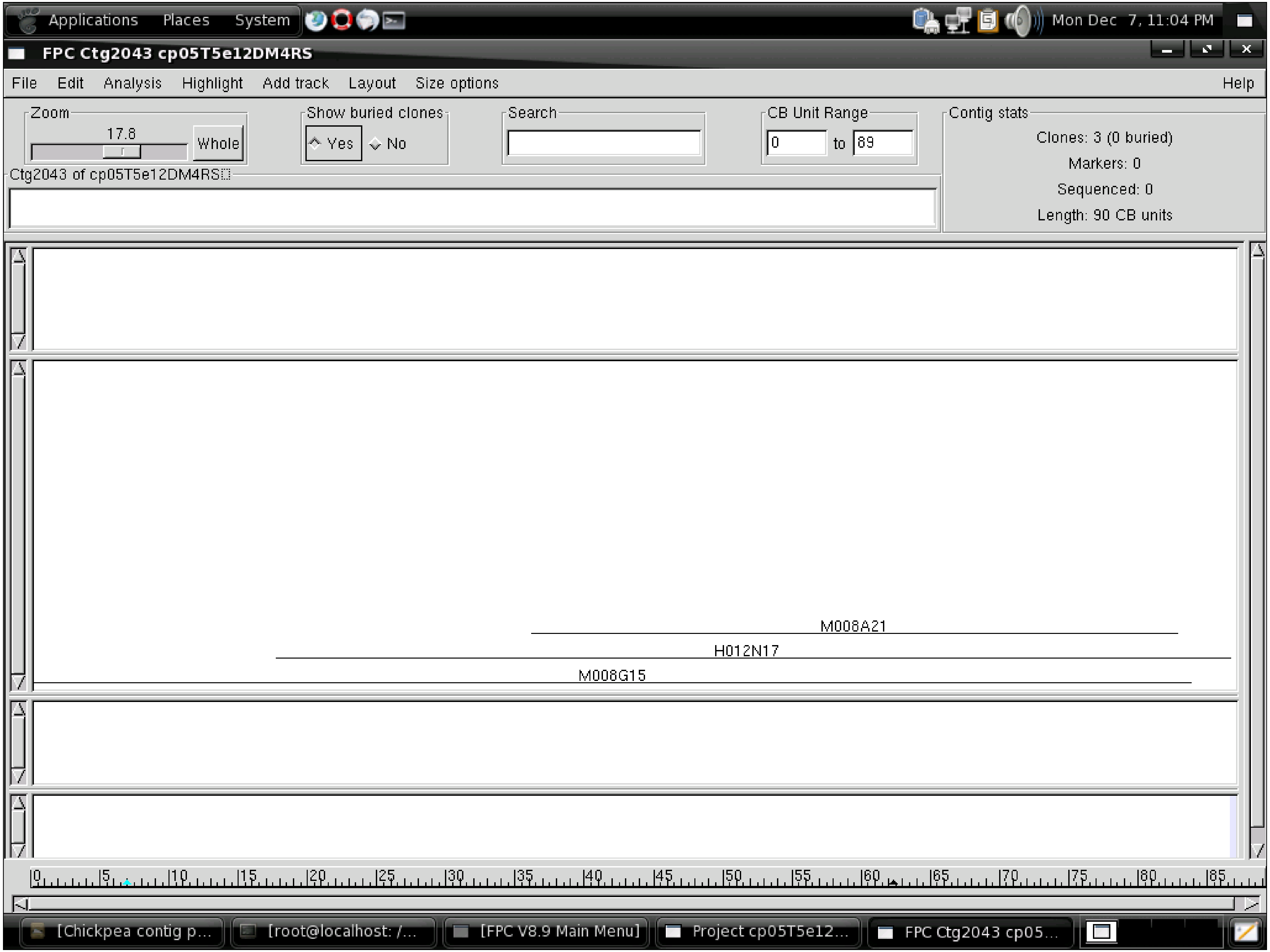
Applications Places System
Mon Dec 7, 11:04 PM
FPC Ctg2043 cp05T5e12DM4RS
File Edit Analysis Highlight Add track Layout Size options Help
Zoom
17.8
Whole
Show buried clones
Yes No
Search
CB Unit Range
0 to 89
Contig stats
Clones: 3 (0 buried)
Markers: 0
Sequenced: 0
Length: 90 CB units
Ctg2043 of cp05T5e12DM4RS
M008A21
H012N17
M008G15
0 5 10 15 20 25 30 35 40 45 50 55 60 65 70 75 80 85
[Chickpea contig p...
[root@localhost: /...
[FPC V8.9 Main Menu]
Project cp05T5e12...
FPC Ctg2043 cp05...

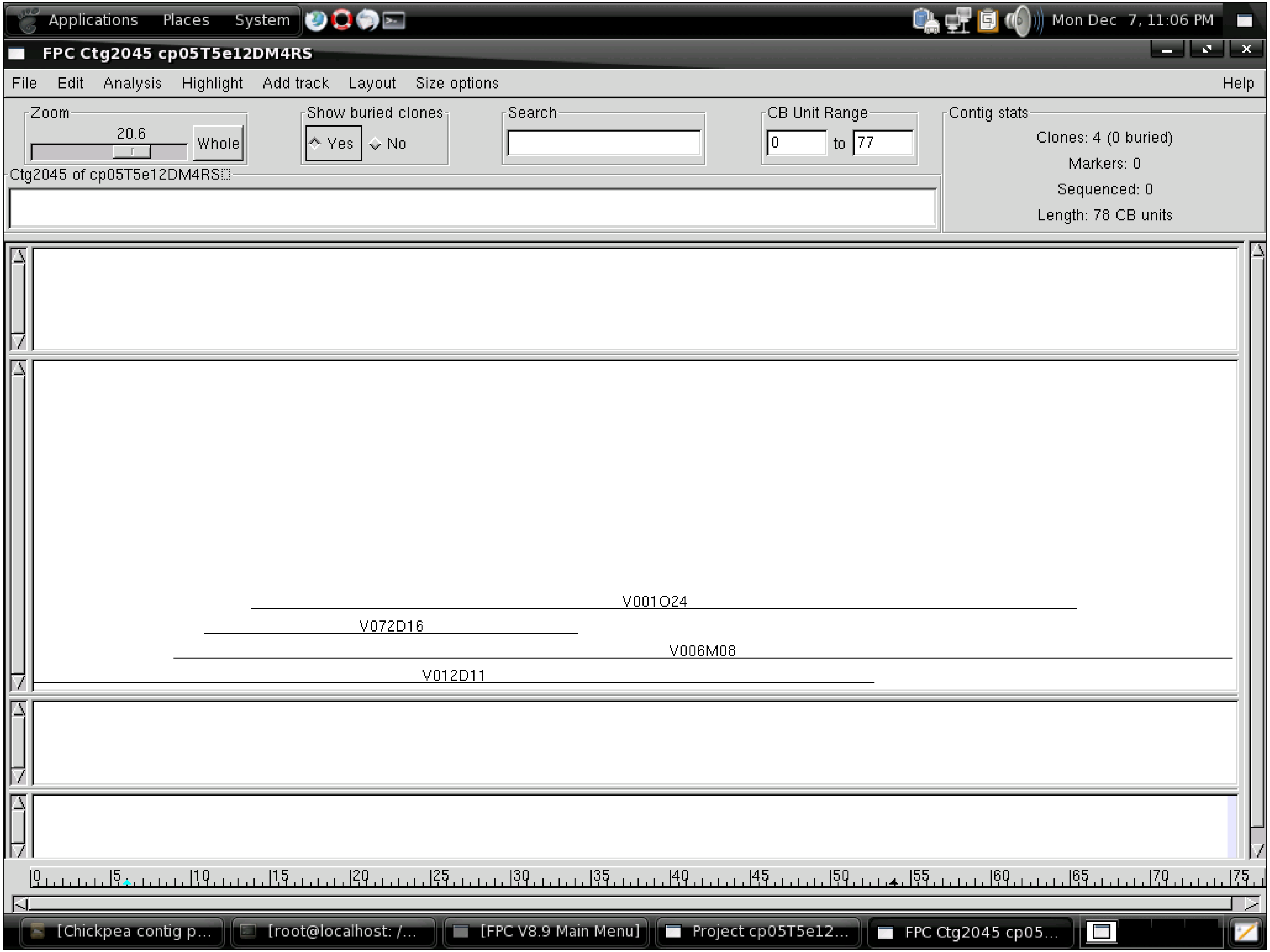

Applications
Places
System
Mon Dec 7, 11:06 PM
FPC Ctg2045 cp05T5e12DM4RS
File
Edit
Analysis
Highlight
Add track
Layout
Size options
Help
Zoom
20.6
Whole
Show buried clones
Yes
No
Search
CB Unit Range
0
to
77
Contig stats
Clones: 4 (0 buried)
Markers: 0
Sequenced: 0
Length: 78 CB units
Ctg2045 of cp05T5e12DM4RS
V001O24
V072D16
V006M08
V012D11
0 5 10 15 20 25 30 35 40 45 50 55 60 65 70 75
[Chickpea contig p...
[root@localhost: /...
[FPC V8.9 Main Menu]
Project cp05T5e12...
FPC Ctg2045 cp05...

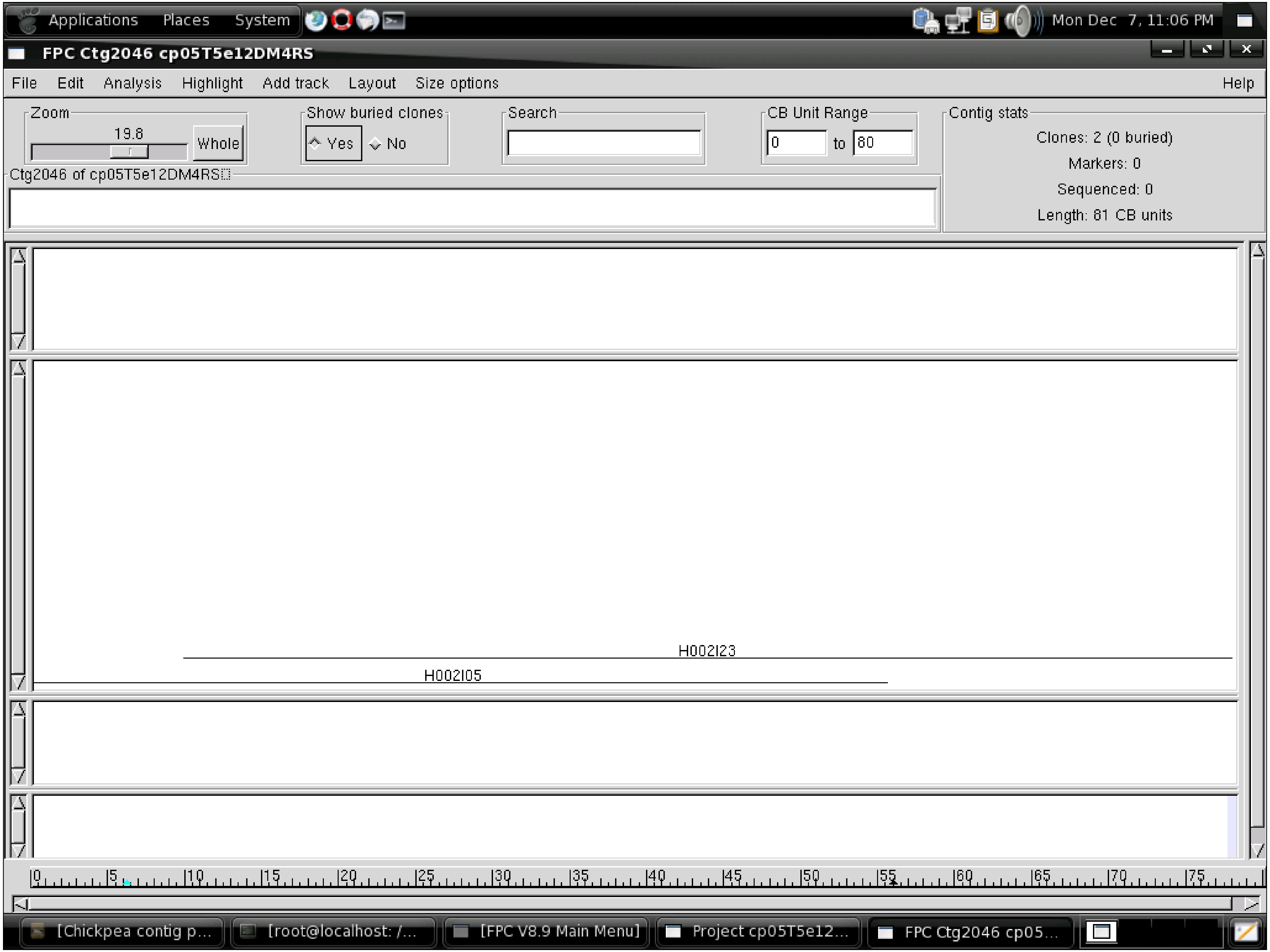
Applications Places System
Mon Dec 7, 11:06 PM
FPC Ctg2046 cp05T5e12DM4RS
File Edit Analysis Highlight Add track Layout Size options Help
Zoom
19.8
Whole
Show buried clones
Yes No
Search
CB Unit Range
0 to 80
Contig stats
Clones: 2 (0 buried)
Markers: 0
Sequenced: 0
Length: 81 CB units
Ctg2046 of cp05T5e12DM4RS
H002I23
H002I05
0 5 10 15 20 25 30 35 40 45 50 55 60 65 70 75
[Chickpea contig p...
[root@localhost: /...
[FPC V8.9 Main Menu]
Project cp05T5e12...
FPC Ctg2046 cp05...

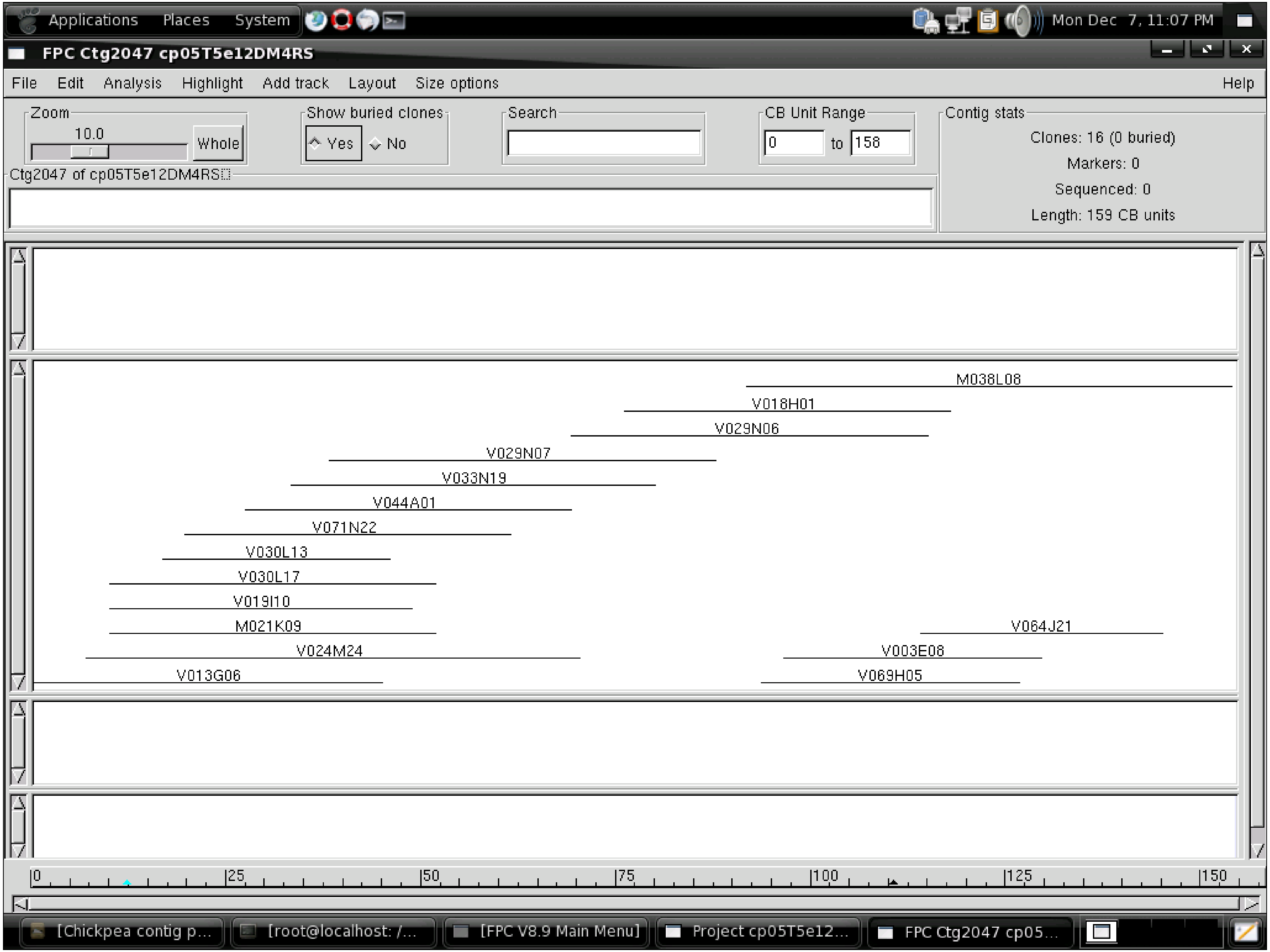
Applications Places System
Mon Dec 7, 11:07 PM
FPC Ctg2047 cp05T5e12DM4RS
File Edit Analysis Highlight Add track Layout Size options Help
Zoom
10.0
Whole
Show buried clones
Yes
No
Search
CB Unit Range
0 to 158
Contig stats
Clones: 16 (0 buried)
Markers: 0
Sequenced: 0
Length: 159 CB units
Ctg2047 of cp05T5e12DM4RS
M038L08
V018H01
V029N06
V029N07
V033N19
V044A01
V071N22
V030L13
V030L17
V019I10
M021K09
V064J21
V024M24
V003E08
V013G06
V069H05
0
25
50
75
100
125
150
[Chickpea contig p...
[root@localhost: /...
[FPC V8.9 Main Menu]
Project cp05T5e12...
FPC Ctg2047 cp05...

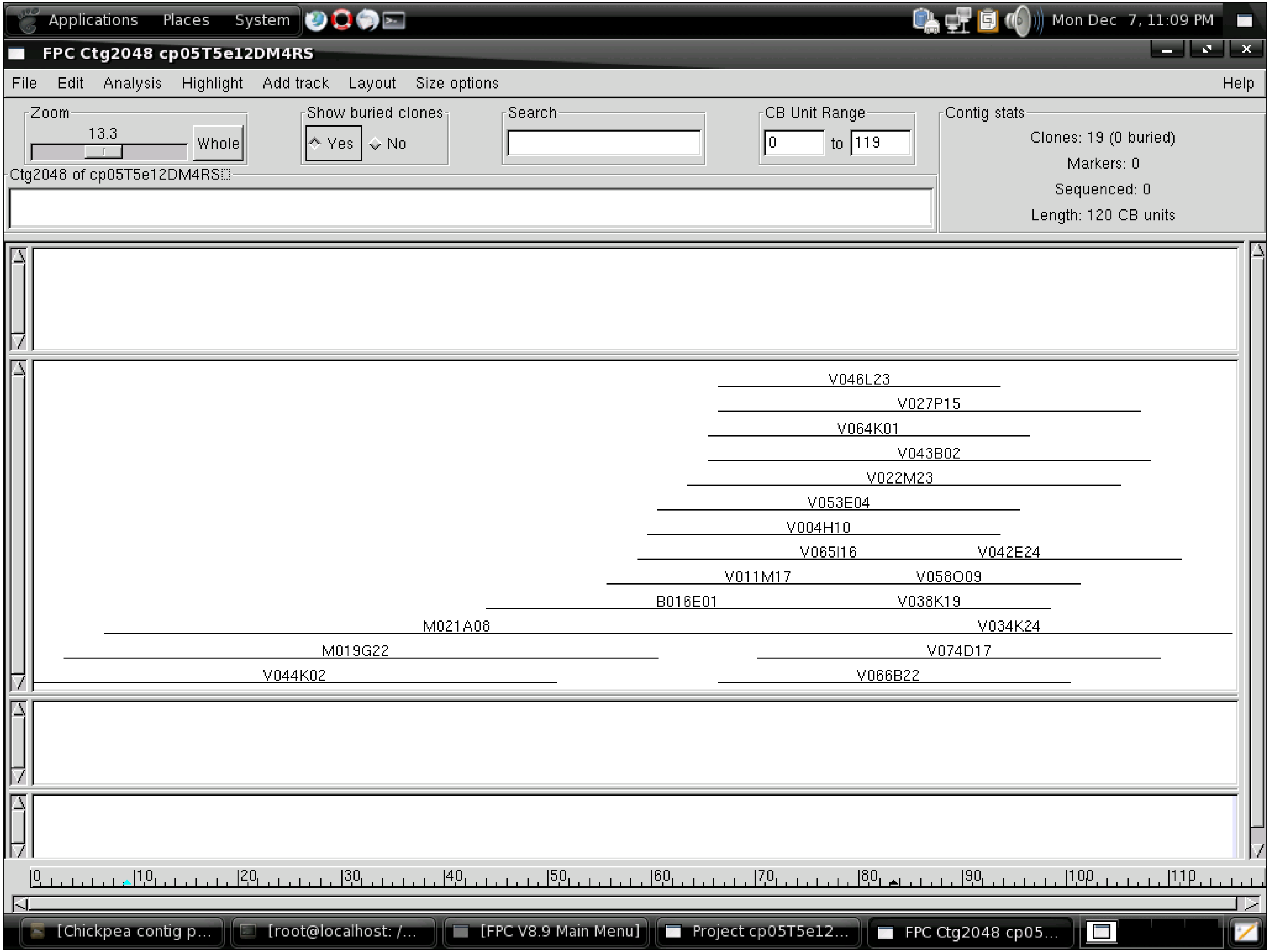
Applications Places System
Mon Dec 7, 11:09 PM
FPC Ctg2048 cp05T5e12DM4RS
File Edit Analysis Highlight Add track Layout Size options Help
Zoom
13.3
Whole
Show buried clones
Yes No
Search
CB Unit Range
0 to 119
Contig stats
Clones: 19 (0 buried)
Markers: 0
Sequenced: 0
Length: 120 CB units
Ctg2048 of cp05T5e12DM4RS
V046L23
V027P15
V064K01
V043B02
V022M23
V053E04
V004H10
V065I16
V042E24
V011M17
V058O09
B016E01
V038K19
M021A08
V034K24
M019G22
V074D17
V044K02
V066B22
0 10 20 30 40 50 60 70 80 90 100 110
[Chickpea contig p...
[root@localhost: /...
[FPC V8.9 Main Menu]
Project cp05T5e12...
FPC Ctg2048 cp05...

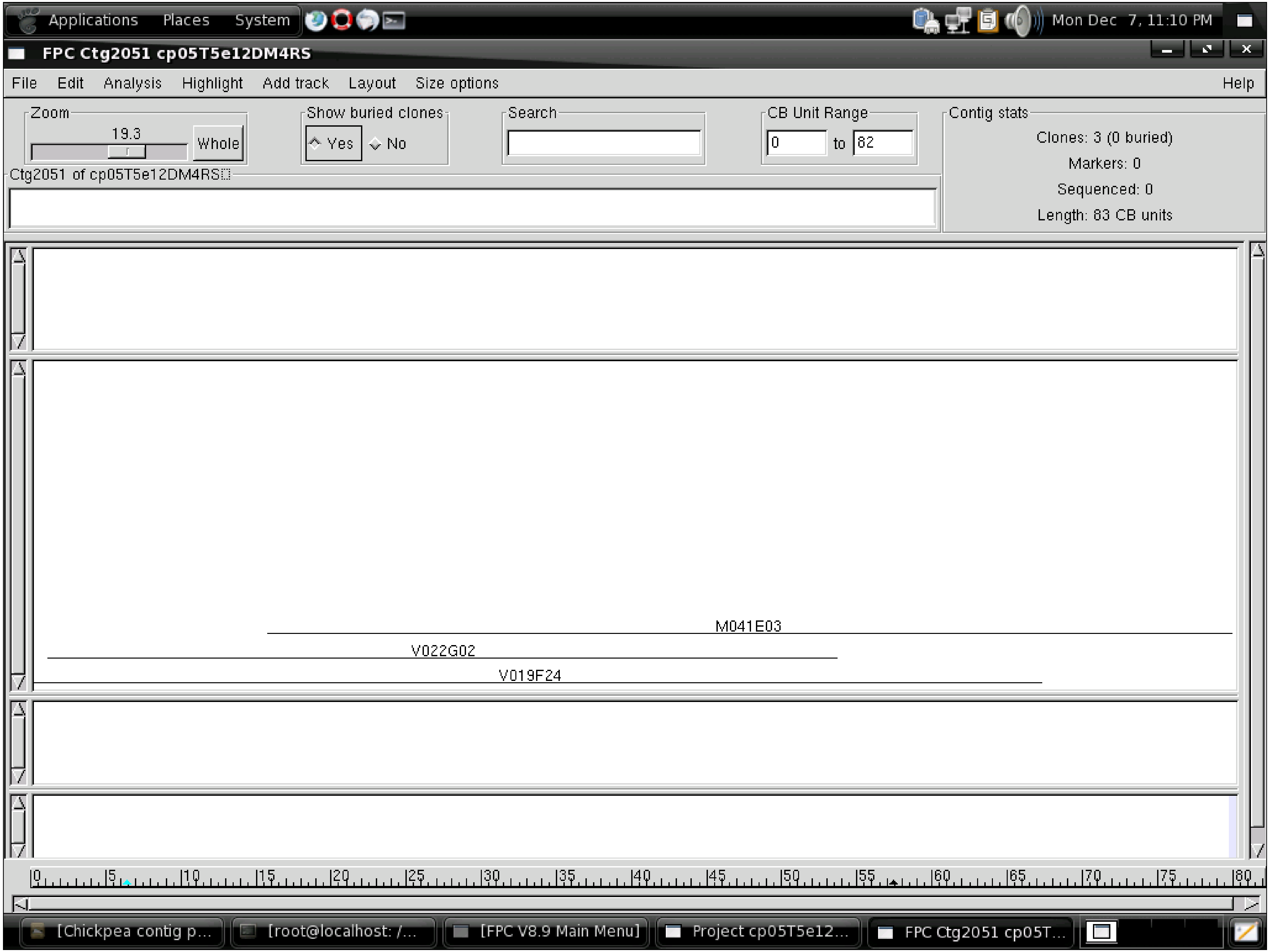
Applications Places System
Mon Dec 7, 11:10 PM
FPC Ctg2051 cp05T5e12DM4RS
File Edit Analysis Highlight Add track Layout Size options Help
Zoom
19.3
Whole
Show buried clones
Yes
No
Search
CB Unit Range
0
to
82
Contig stats
Clones: 3 (0 buried)
Markers: 0
Sequenced: 0
Length: 83 CB units
Ctg2051 of cp05T5e12DM4RS
M041E03
V022G02
V019F24
0 5 10 15 20 25 30 35 40 45 50 55 60 65 70 75 80
[Chickpea contig p...
[root@localhost: /...
[FPC V8.9 Main Menu]
Project cp05T5e12...
FPC Ctg2051 cp05T...

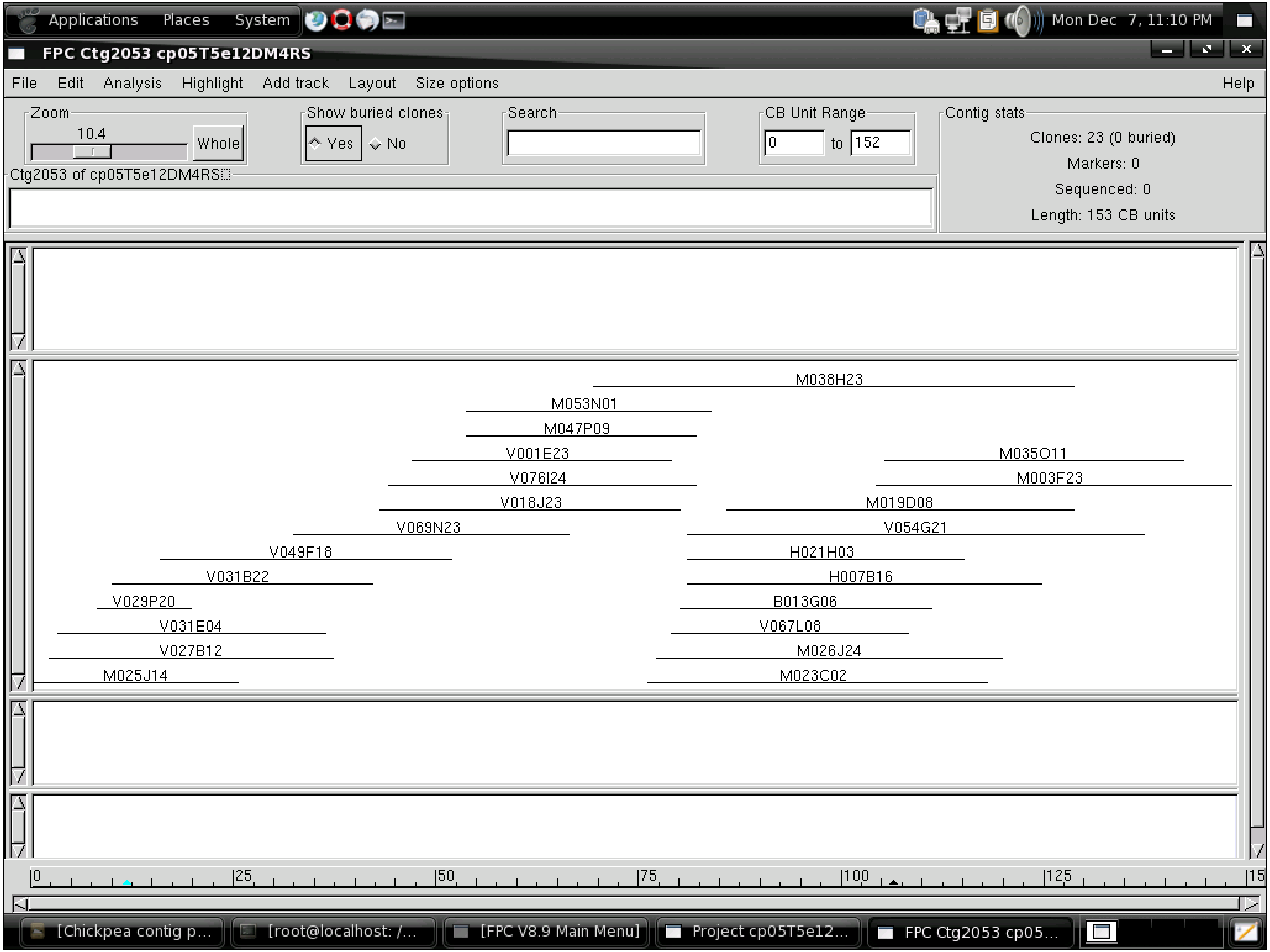
Applications Places System
Mon Dec 7, 11:10 PM
FPC Ctg2053 cp05T5e12DM4RS
File Edit Analysis Highlight Add track Layout Size options Help
Zoom
10.4
Whole
Show buried clones
Yes No
Search
CB Unit Range
0 to 152
Contig stats
Clones: 23 (0 buried)
Markers: 0
Sequenced: 0
Length: 153 CB units
Ctg2053 of cp05T5e12DM4RS
M038H23
M053N01
M047P09
V001E23
V076I24
V018J23
V069N23
V049F18
V031B22
V029P20
V031E04
V027B12
M025J14
M035O11
M003F23
M019D08
V054G21
H021H03
H007B16
B013G06
V067L08
M026J24
M023C02
0
25
50
75
100
125
[Chickpea contig p...
[root@localhost: /...
[FPC V8.9 Main Menu]
Project cp05T5e12...
FPC Ctg2053 cp05...

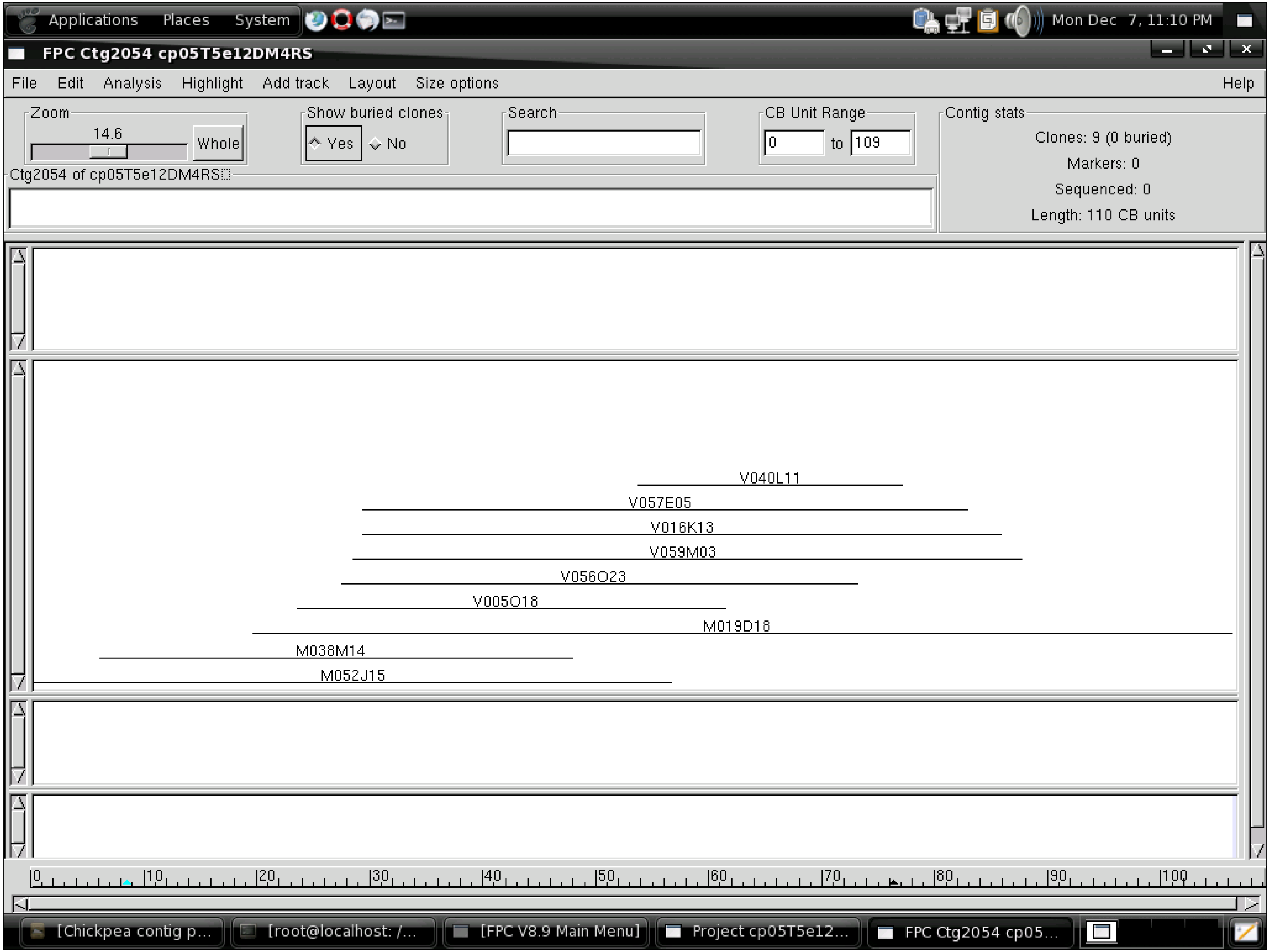
Applications
Places
System
Mon Dec 7, 11:10 PM
FPC Ctg2054 cp05T5e12DM4RS
File
Edit
Analysis
Highlight
Add track
Layout
Size options
Help
Zoom
14.6
Whole
Show buried clones
Yes
No
Search
CB Unit Range
0
to
109
Contig stats
Clones: 9 (0 buried)
Markers: 0
Sequenced: 0
Length: 110 CB units
Ctg2054 of cp05T5e12DM4RS
V040L11
V057E05
V016K13
V059M03
V056O23
V005O18
M019D18
M038M14
M052J15
0
10
20
30
40
50
60
70
80
90
100
[Chickpea contig p...
[root@localhost: /...
[FPC V8.9 Main Menu]
Project cp05T5e12...
FPC Ctg2054 cp05...

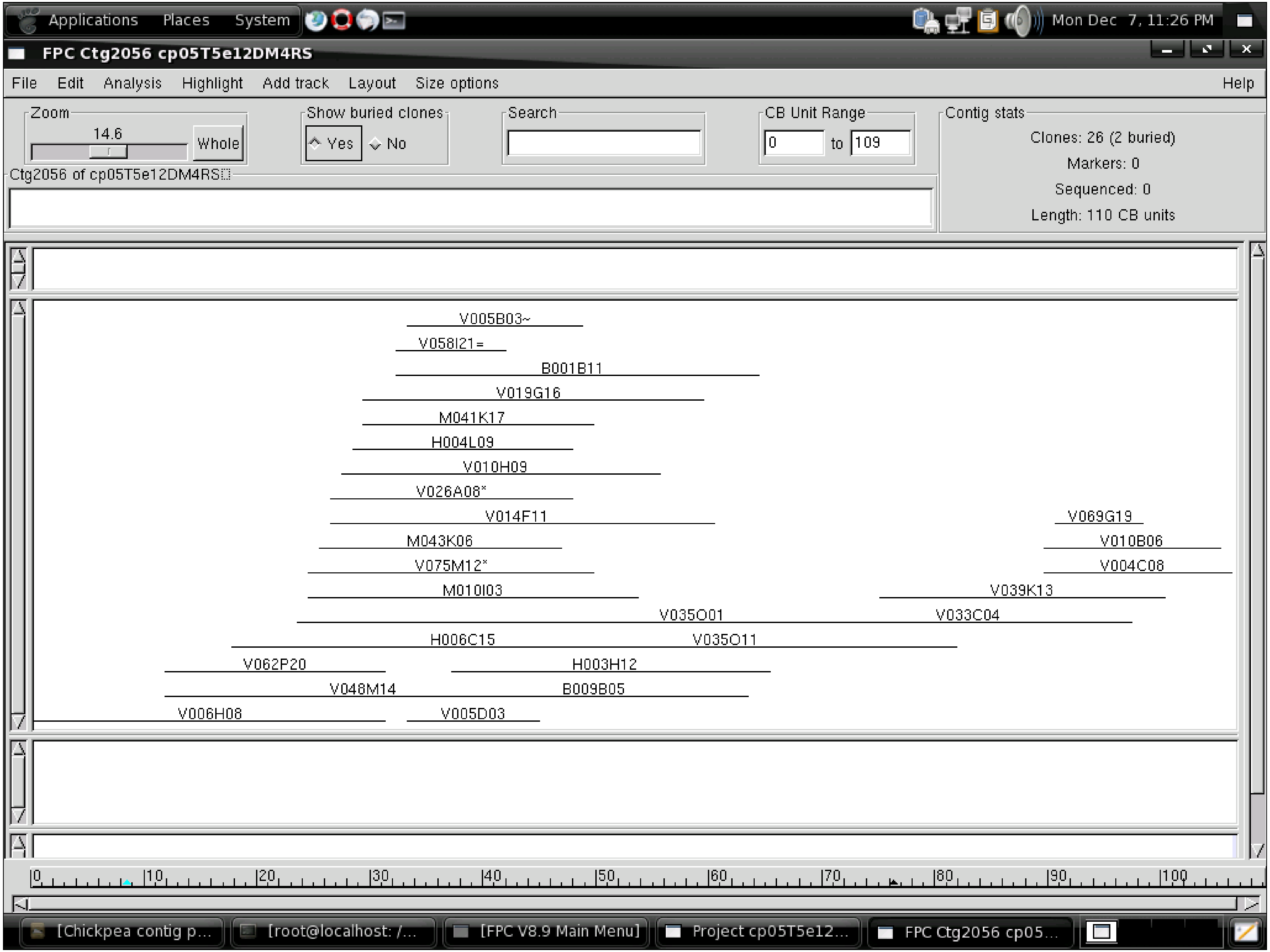
Applications Places System
Mon Dec 7, 11:26 PM
FPC Ctg2056 cp05T5e12DM4RS
File Edit Analysis Highlight Add track Layout Size options Help
Zoom
14.6
Whole
Show buried clones
Yes No
Search
CB Unit Range
0 to 109
Contig stats
Clones: 26 (2 buried)
Markers: 0
Sequenced: 0
Length: 110 CB units
Ctg2056 of cp05T5e12DM4RS
V005B03~
V058I21=
B001B11
V019G16
M041K17
H004L09
V010H09
V026A08*
V014F11
V069G19
M043K06
V010B06
V075M12*
V004C08
M010I03
V039K13
V035O01
V033C04
H006C15
V035O11
V062P20
H003H12
V048M14
B009B05
V006H08
V005D03
0 10 20 30 40 50 60 70 80 90 100
[Chickpea contig p...
[root@localhost: /...
[FPC V8.9 Main Menu]
Project cp05T5e12...
FPC Ctg2056 cp05...

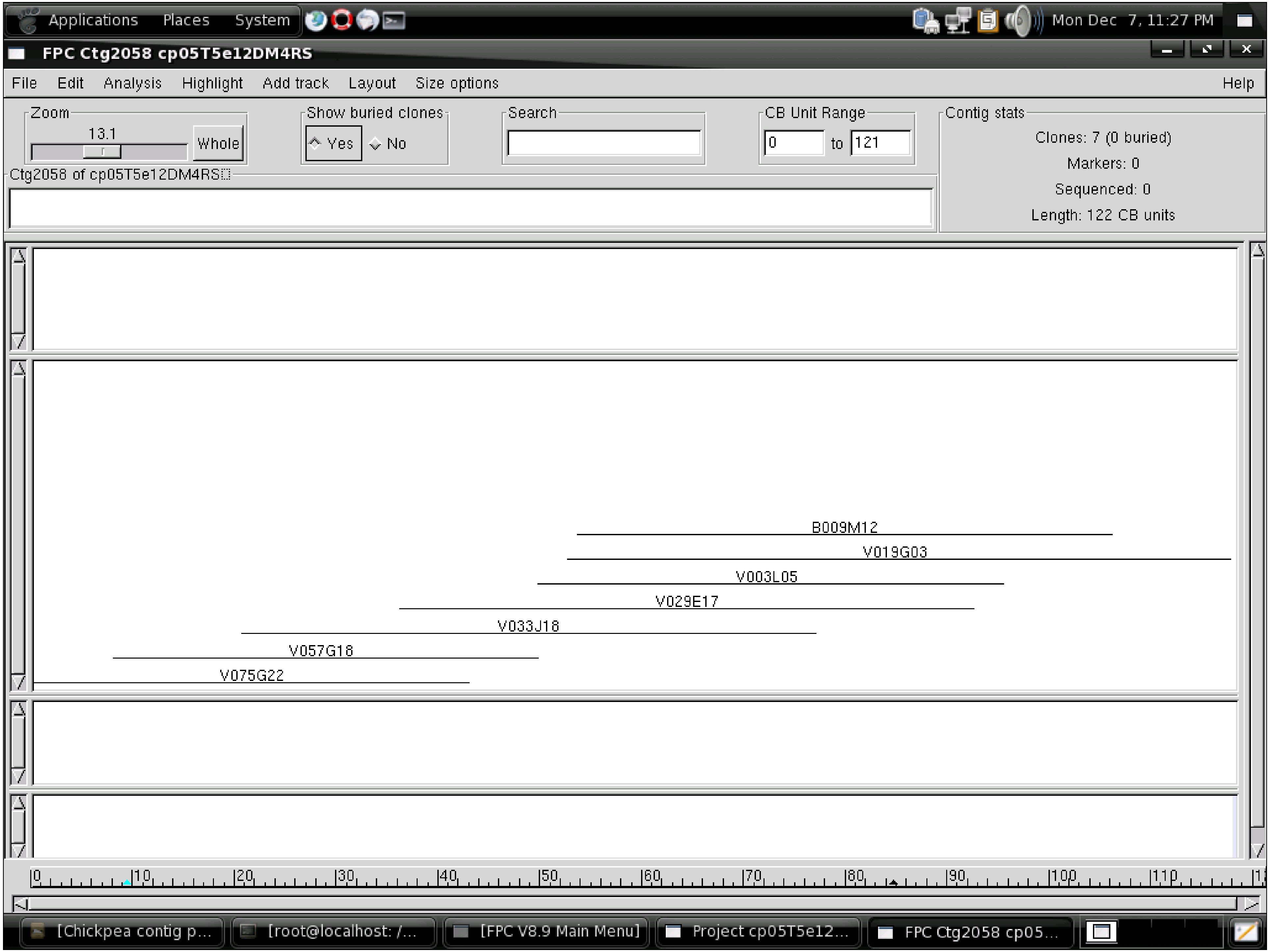

Applications Places System
Mon Dec 7, 11:27 PM
FPC Ctg2058 cp05T5e12DM4RS
File Edit Analysis Highlight Add track Layout Size options Help
Zoom
13.1
Whole
Show buried clones
Yes No
Search
CB Unit Range
0 to 121
Contig stats
Clones: 7 (0 buried)
Markers: 0
Sequenced: 0
Length: 122 CB units
Ctg2058 of cp05T5e12DM4RS
B009M12
V019G03
V003L05
V029E17
V033J18
V057G18
V075G22
0 10 20 30 40 50 60 70 80 90 100 110
[Chickpea contig p...
[root@localhost: /...
[FPC V8.9 Main Menu]
Project cp05T5e12...
FPC Ctg2058 cp05...

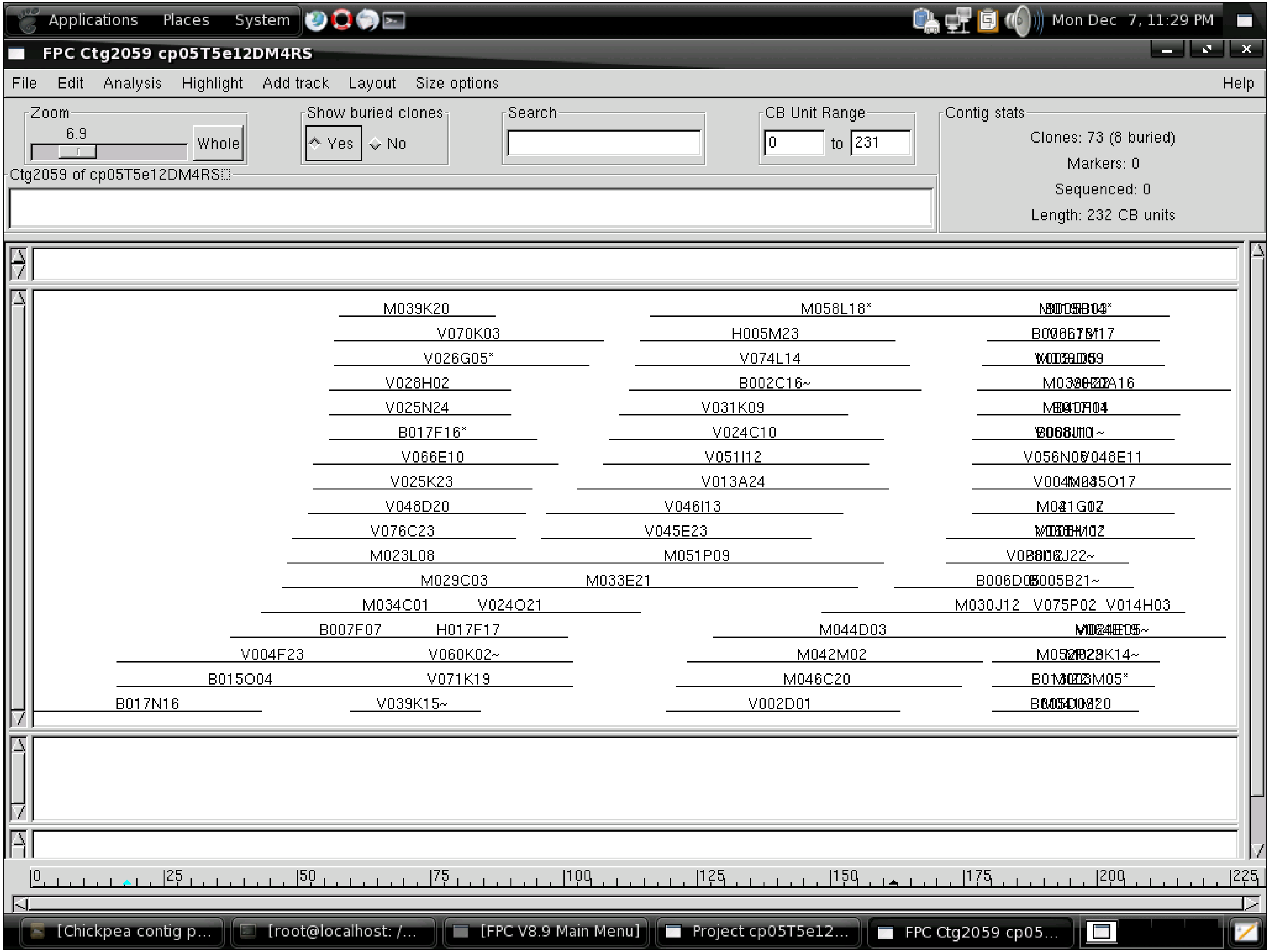
Applications Places System
Mon Dec 7, 11:29 PM
FPC Ctg2059 cp05T5e12DM4RS
File Edit Analysis Highlight Add track Layout Size options Help
Zoom 6.9 Whole
Show buried clones Yes No
Search
CB Unit Range 0 to 231
Contig stats
Clones: 73 (8 buried)
Markers: 0
Sequenced: 0
Length: 232 CB units
Ctg2059 of cp05T5e12DM4RS
M039K20
V070K03
V026G05*
V028H02
V025N24
B017F16*
V066E10
V025K23
V048D20
V076C23
M023L08
M029C03
M034C01
V024O21
B007F07
H017F17
V004F23
V060K02~
B015O04
V071K19
B017N16
V039K15~
M058L18*
H005M23
V074L14
B002C16~
V031K09
V024C10
V051I12
V013A24
V046I13
V045E23
M051P09
M033E21
M030J12
V075P02
V014H03
M044D03
M042M02
M046C20
V002D01
0 25 50 75 100 125 150 175 200 225
[Chickpea contig p...
[root@localhost: /...
[FPC V8.9 Main Menu]
Project cp05T5e12...
FPC Ctg2059 cp05...

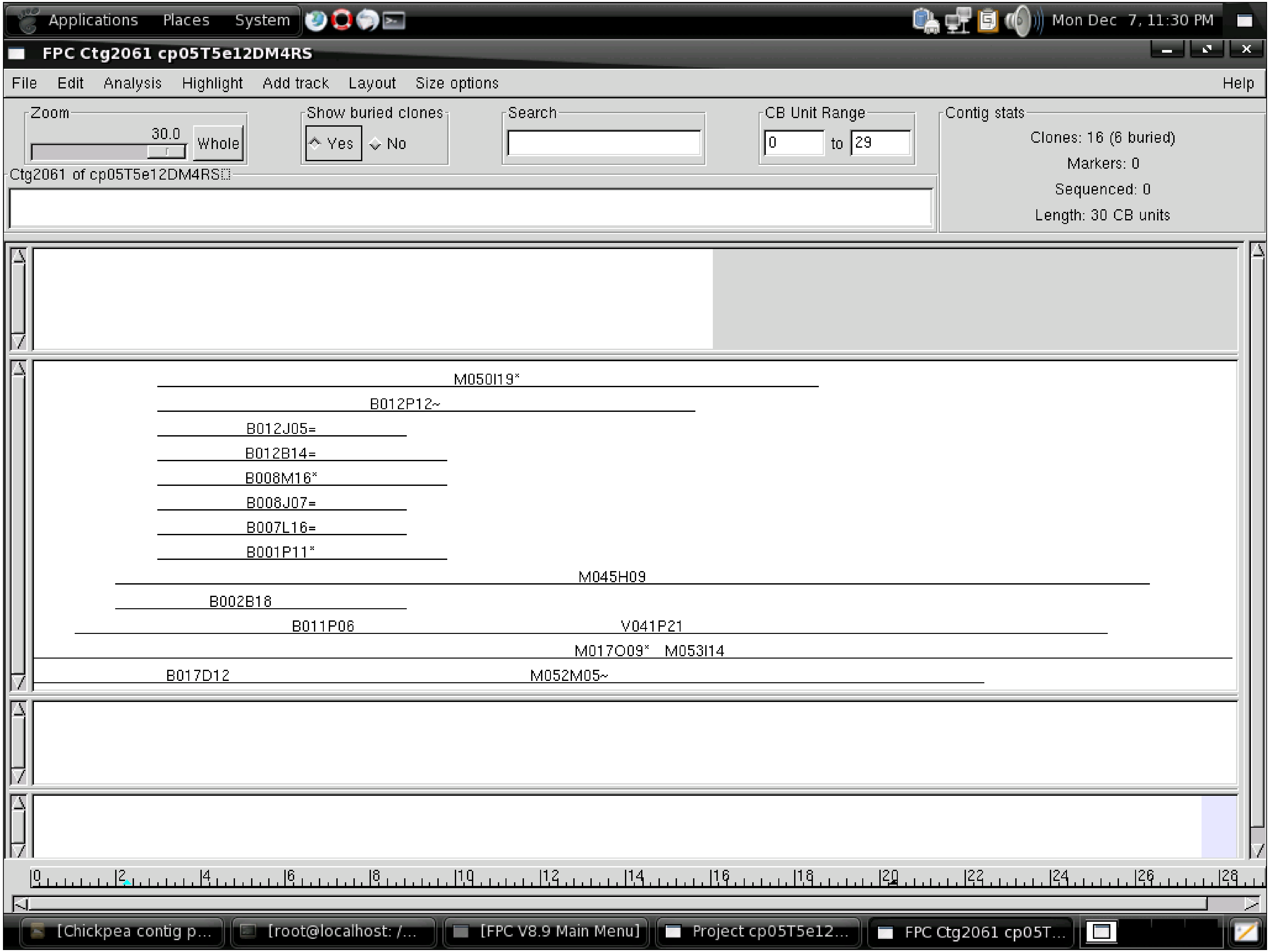
Applications Places System
Mon Dec 7, 11:30 PM
FPC Ctg2061 cp05T5e12DM4RS
File Edit Analysis Highlight Add track Layout Size options Help
Zoom
30.0
Whole
Show buried clones
Yes No
Search
CB Unit Range
0 to 29
Contig stats
Clones: 16 (6 buried)
Markers: 0
Sequenced: 0
Length: 30 CB units
Ctg2061 of cp05T5e12DM4RS
M050I19*
B012P12~
B012J05=
B012B14=
B008M16*
B008J07=
B007L16=
B001P11*
M045H09
B002B18
B011P06
V041P21
M017O09*
M053I14
B017D12
M052M05~
0 2 4 6 8 10 12 14 16 18 20 22 24 26 28
[Chickpea contig p...
[root@localhost: /...
[FPC V8.9 Main Menu]
Project cp05T5e12...
FPC Ctg2061 cp05T...

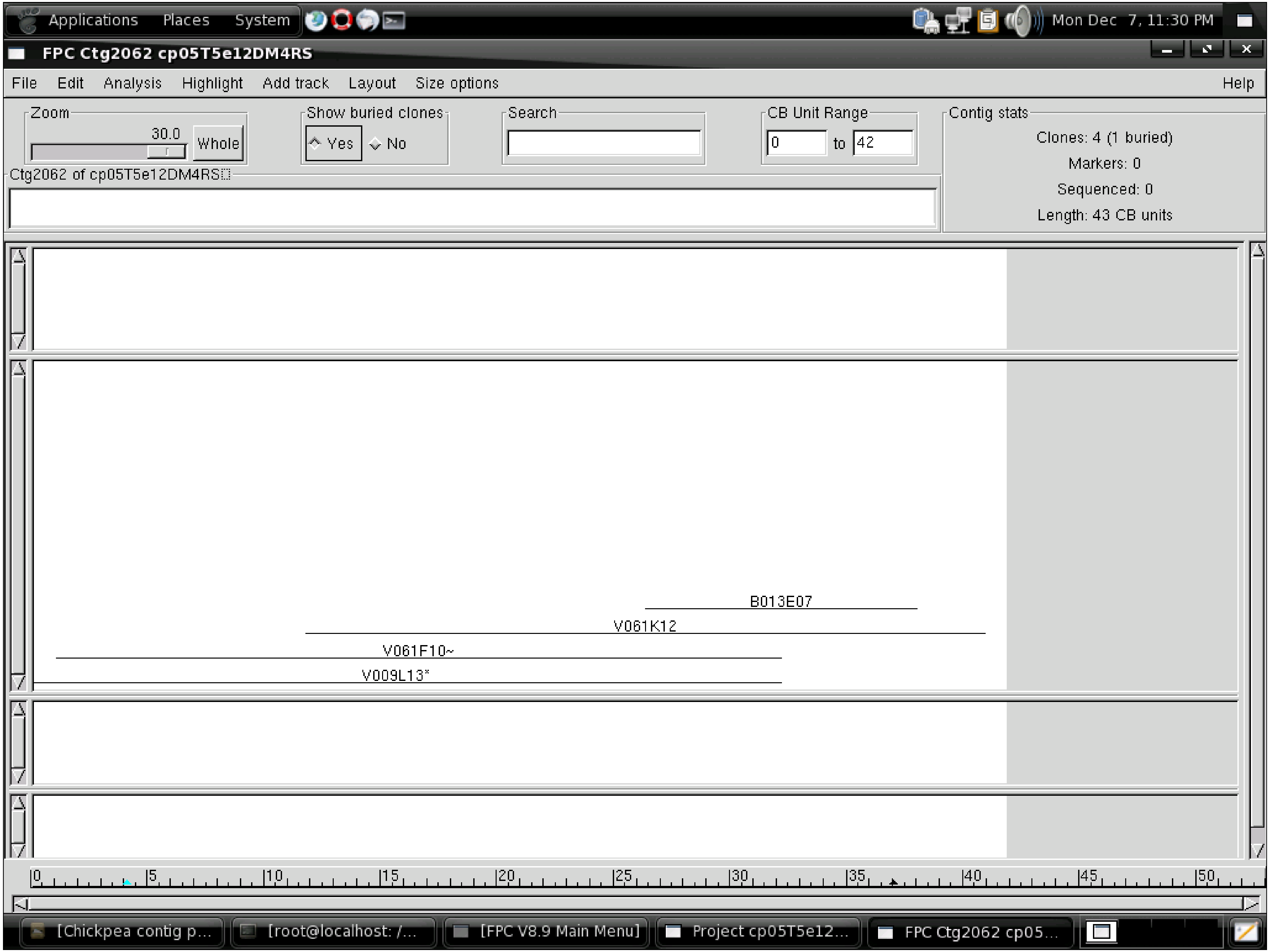
Applications Places System
Mon Dec 7, 11:30 PM
FPC Ctg2062 cp05T5e12DM4RS
File Edit Analysis Highlight Add track Layout Size options Help
Zoom
30.0
Whole
Show buried clones
Yes
No
Search
CB Unit Range
0 to 42
Contig stats
Clones: 4 (1 buried)
Markers: 0
Sequenced: 0
Length: 43 CB units
Ctg2062 of cp05T5e12DM4RS
B013E07
V061K12
V061F10~
V009L13*
0 5 10 15 20 25 30 35 40 45 50
[Chickpea contig p...
[root@localhost: /...
[FPC V8.9 Main Menu]
Project cp05T5e12...
FPC Ctg2062 cp05...

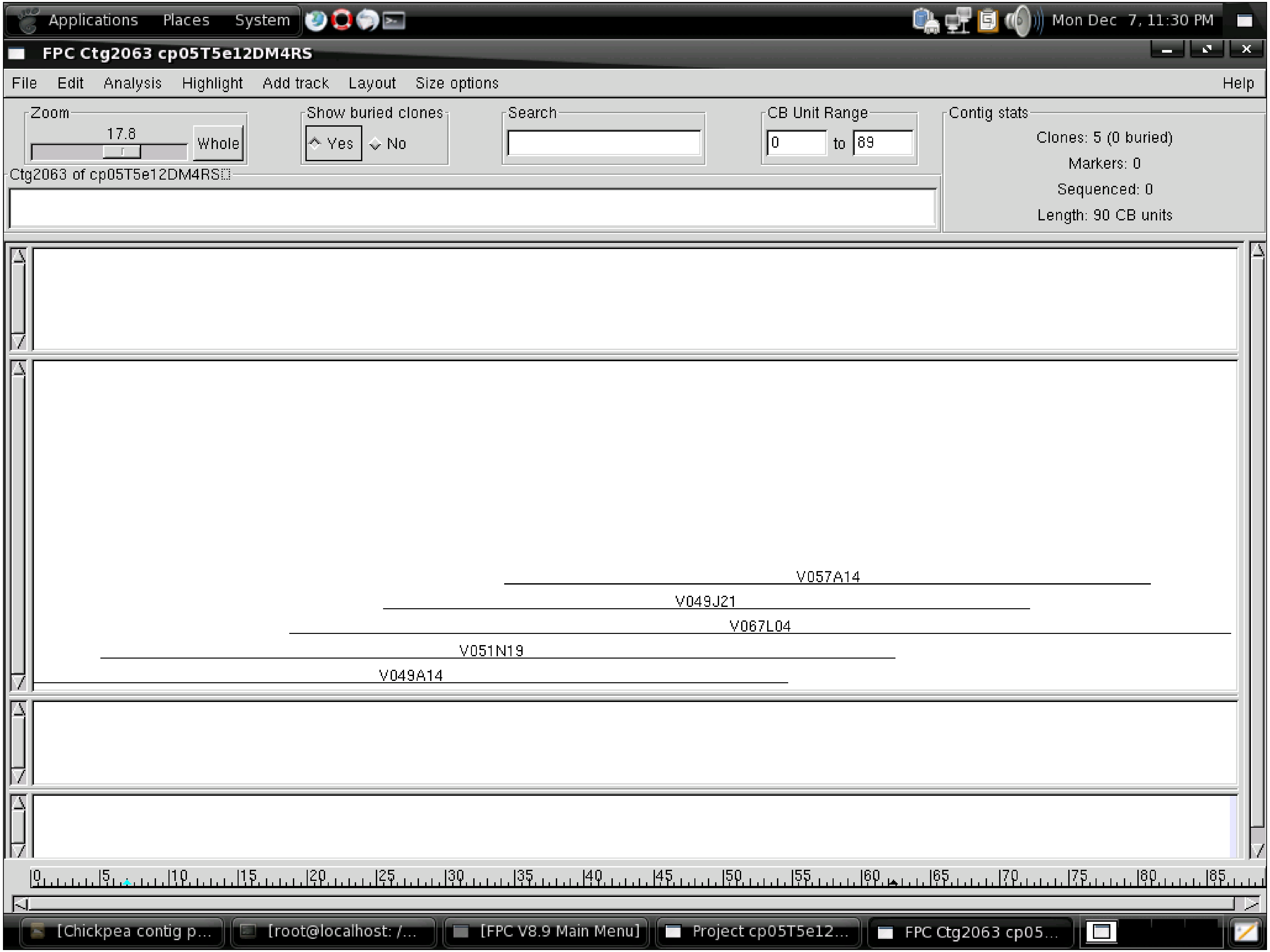
Applications Places System
Mon Dec 7, 11:30 PM
FPC Ctg2063 cp05T5e12DM4RS
File Edit Analysis Highlight Add track Layout Size options Help
Zoom
17.8
Whole
Show buried clones
Yes No
Search
CB Unit Range
0 to 89
Contig stats
Clones: 5 (0 buried)
Markers: 0
Sequenced: 0
Length: 90 CB units
Ctg2063 of cp05T5e12DM4RS
V057A14
V049J21
V067L04
V051N19
V049A14
0 5 10 15 20 25 30 35 40 45 50 55 60 65 70 75 80 85
[Chickpea contig p...
[root@localhost: /...
[FPC V8.9 Main Menu]
Project cp05T5e12...
FPC Ctg2063 cp05...

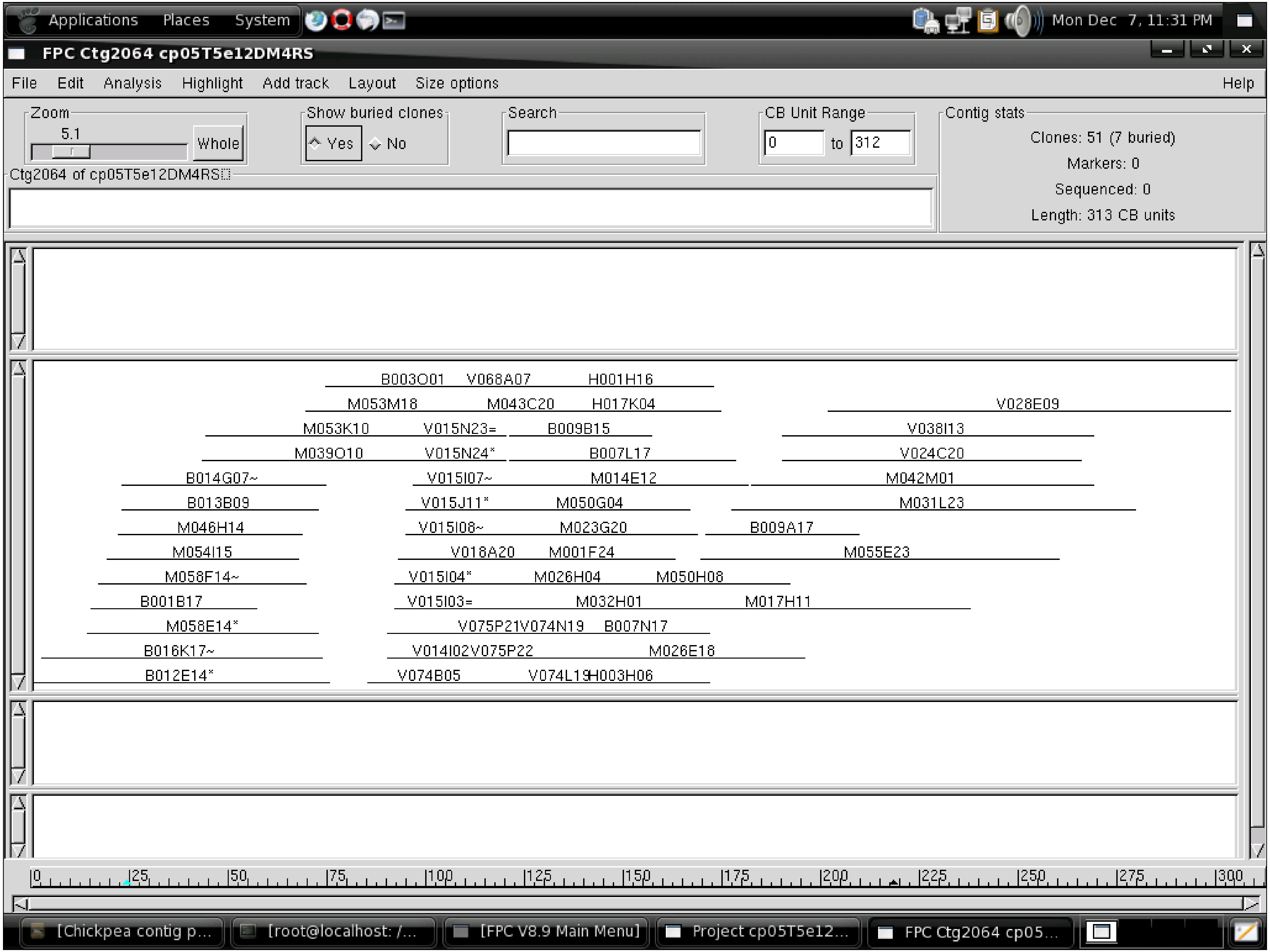
Applications Places System
Mon Dec 7, 11:31 PM
FPC Ctg2064 cp05T5e12DM4RS
File Edit Analysis Highlight Add track Layout Size options Help
Zoom
5.1
Whole
Show buried clones
Yes
No
Search
CB Unit Range
0 to 312
Contig stats
Clones: 51 (7 buried)
Markers: 0
Sequenced: 0
Length: 313 CB units
Ctg2064 of cp05T5e12DM4RS
B003O01 V068A07 H001H16
M053M18 M043C20 H017K04 V028E09
M053K10 V015N23= B009B15 V038I13
M039O10 V015N24* B007L17 V024C20
B014G07~ V015I07~ M014E12 M042M01
B013B09 V015J11* M050G04 M031L23
M046H14 V015I08~ M023G20 B009A17
M054I15 V018A20 M001F24 M055E23
M058F14~ V015I04* M026H04 M050H08
B001B17 V015I03= M032H01 M017H11
M058E14* V075P21V074N19 B007N17
B016K17~ V014I02V075P22 M026E18
B012E14* V074B05 V074L19H003H06
0 25 50 75 100 125 150 175 200 225 250 275 300
[Chickpea contig p...
[root@localhost: /...
[FPC V8.9 Main Menu]
Project cp05T5e12...
FPC Ctg2064 cp05...

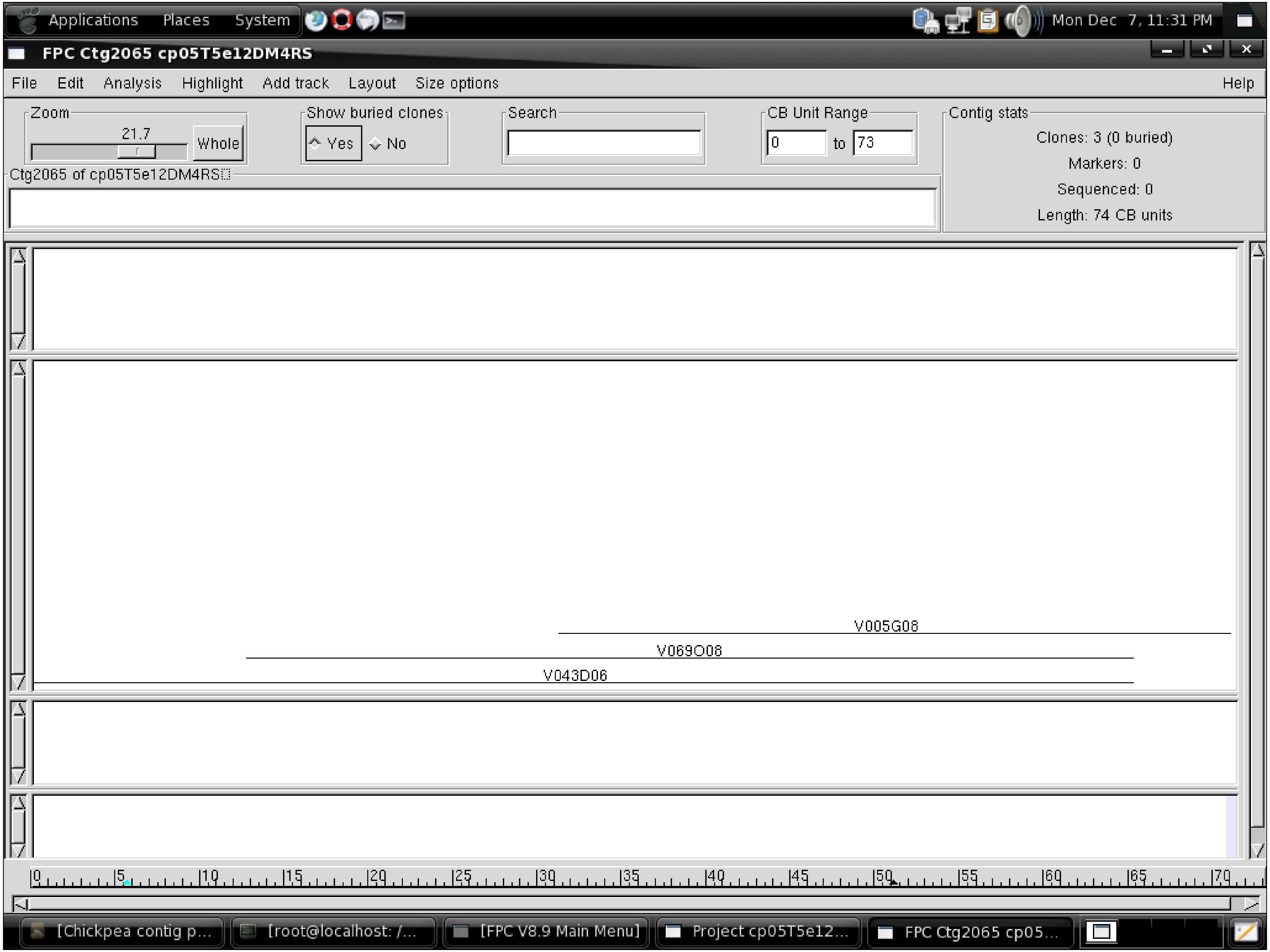
Applications Places System
Mon Dec 7, 11:31 PM
FPC Ctg2065 cp05T5e12DM4RS
File Edit Analysis Highlight Add track Layout Size options Help
Zoom
21.7
Whole
Show buried clones
Yes
No
Search
CB Unit Range
0
to
73
Contig stats
Clones: 3 (0 buried)
Markers: 0
Sequenced: 0
Length: 74 CB units
Ctg2065 of cp05T5e12DM4RS
V005G08
V069O08
V043D06
0 5 10 15 20 25 30 35 40 45 50 55 60 65 70
[Chickpea contig p...
[root@localhost: /...
[FPC V8.9 Main Menu]
Project cp05T5e12...
FPC Ctg2065 cp05...

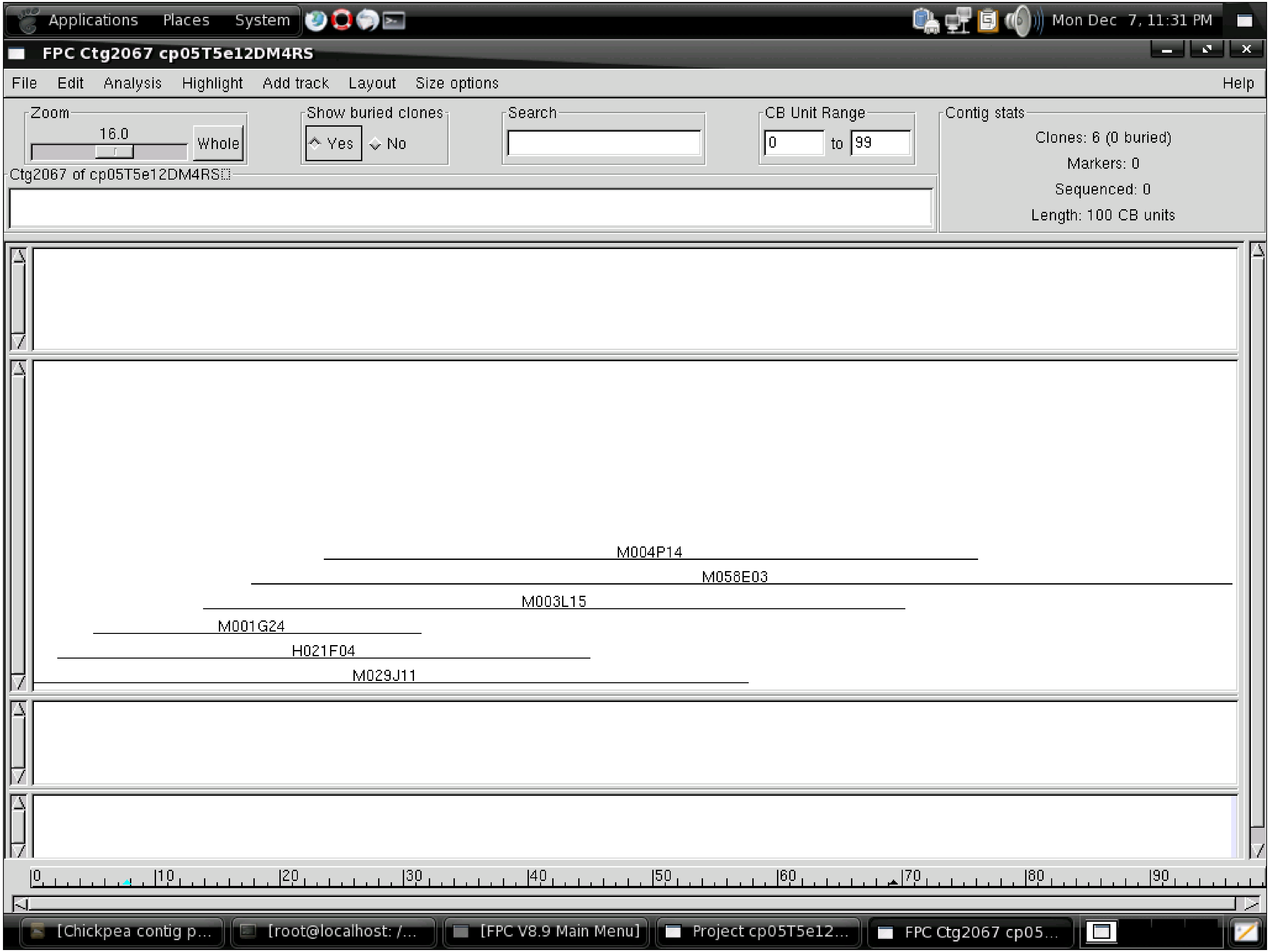
Applications Places System
Mon Dec 7, 11:31 PM
FPC Ctg2067 cp05T5e12DM4RS
File Edit Analysis Highlight Add track Layout Size options Help
Zoom
16.0
Whole
Show buried clones
Yes No
Search
CB Unit Range
0 to 99
Contig stats
Clones: 6 (0 buried)
Markers: 0
Sequenced: 0
Length: 100 CB units
Ctg2067 of cp05T5e12DM4RS
M004P14
M058E03
M003L15
M001G24
H021F04
M029J11
0 10 20 30 40 50 60 70 80 90
[Chickpea contig p...
[root@localhost: /...
[FPC V8.9 Main Menu]
Project cp05T5e12...
FPC Ctg2067 cp05...

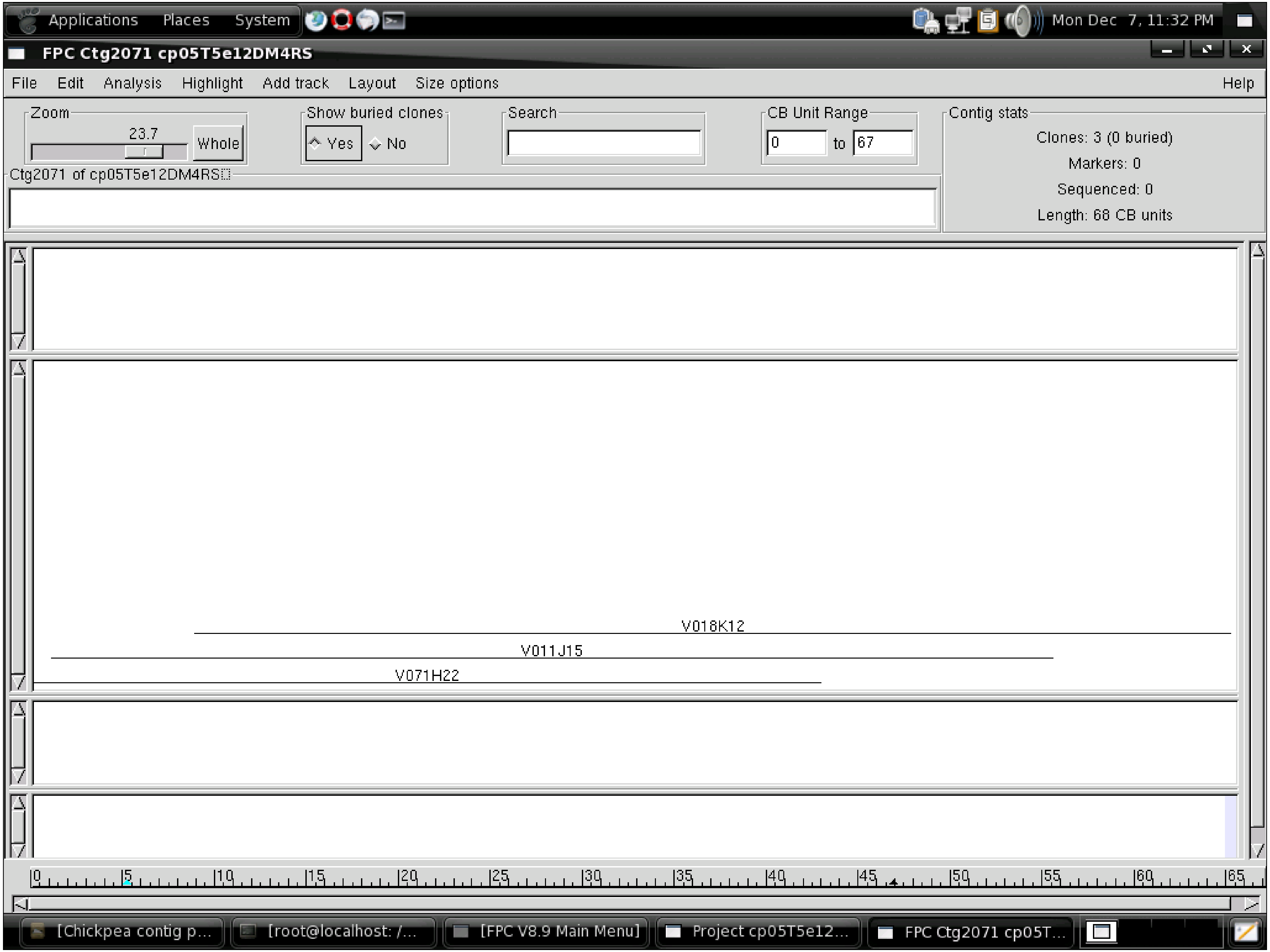
Applications Places System
Mon Dec 7, 11:32 PM
FPC Ctg2071 cp05T5e12DM4RS
File Edit Analysis Highlight Add track Layout Size options Help
Zoom
23.7
Whole
Show buried clones
Yes
No
Search
CB Unit Range
0
to
67
Contig stats
Clones: 3 (0 buried)
Markers: 0
Sequenced: 0
Length: 68 CB units
Ctg2071 of cp05T5e12DM4RS
V018K12
V011J15
V071H22
0 5 10 15 20 25 30 35 40 45 50 55 60 65
[Chickpea contig p...
[root@localhost: /...
[FPC V8.9 Main Menu]
Project cp05T5e12...
FPC Ctg2071 cp05T...

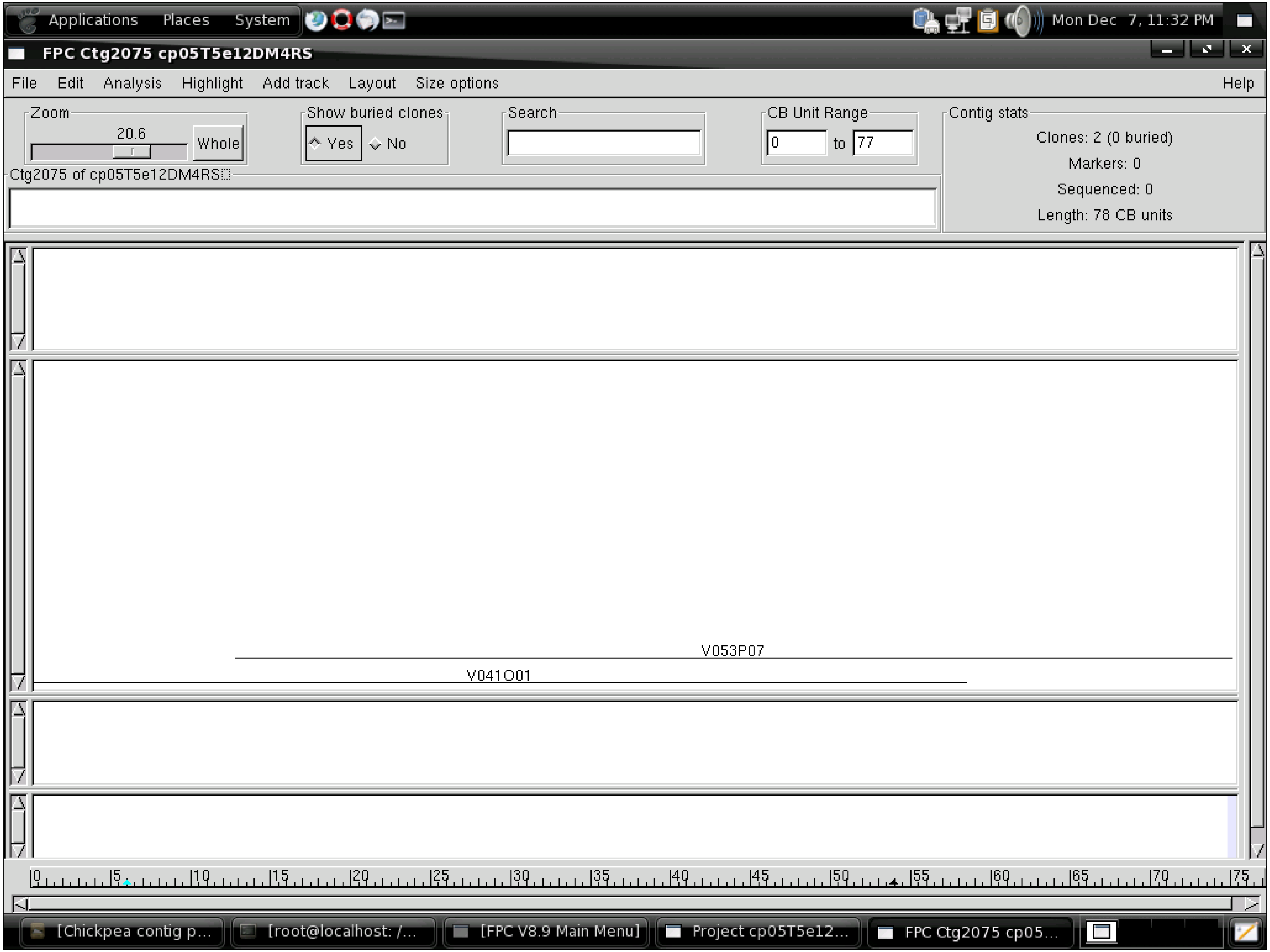
Applications Places System
Mon Dec 7, 11:32 PM
FPC Ctg2075 cp05T5e12DM4RS
File Edit Analysis Highlight Add track Layout Size options Help
Zoom
20.6
Whole
Show buried clones
Yes No
Search
CB Unit Range
0 to 77
Contig stats
Clones: 2 (0 buried)
Markers: 0
Sequenced: 0
Length: 78 CB units
Ctg2075 of cp05T5e12DM4RS
V053P07
V041O01
0 5 10 15 20 25 30 35 40 45 50 55 60 65 70 75
[Chickpea contig p...
[root@localhost: /...
[FPC V8.9 Main Menu]
Project cp05T5e12...
FPC Ctg2075 cp05...

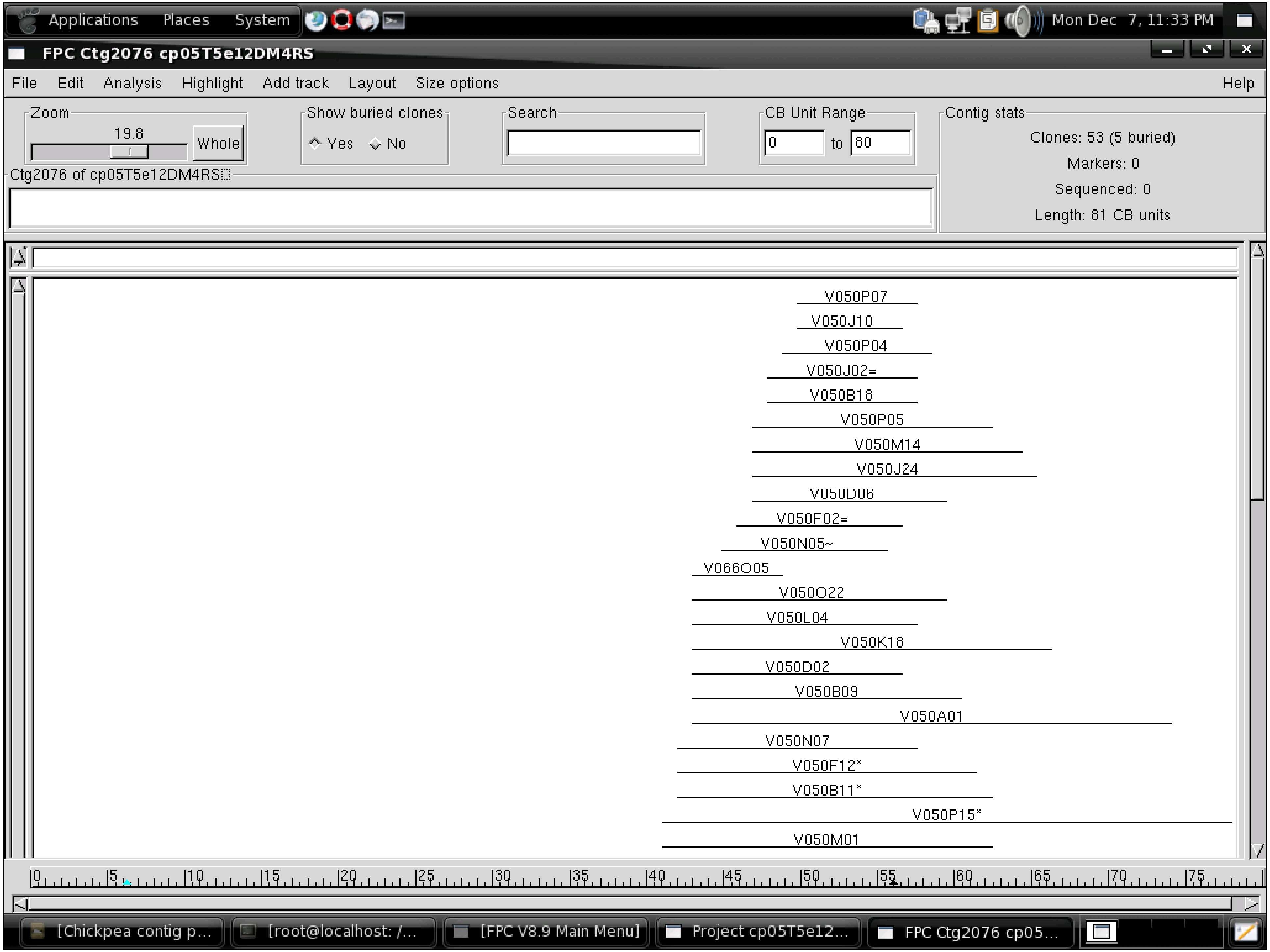
Applications Places System
Mon Dec 7, 11:33 PM
FPC Ctg2076 cp05T5e12DM4RS
File Edit Analysis Highlight Add track Layout Size options Help
Zoom
19.8
Whole
Show buried clones
Yes No
Search
CB Unit Range
0 to 80
Contig stats
Clones: 53 (5 buried)
Markers: 0
Sequenced: 0
Length: 81 CB units
Ctg2076 of cp05T5e12DM4RS
V050P07
V050J10
V050P04
V050J02=
V050B18
V050P05
V050M14
V050J24
V050D06
V050F02=
V050N05~
V066O05
V050O22
V050L04
V050K18
V050D02
V050B09
V050A01
V050N07
V050F12*
V050B11*
V050P15*
V050M01
0 5 10 15 20 25 30 35 40 45 50 55 60 65 70 75
[Chickpea contig p...
[root@localhost: /...
[FPC V8.9 Main Menu]
Project cp05T5e12...
FPC Ctg2076 cp05...

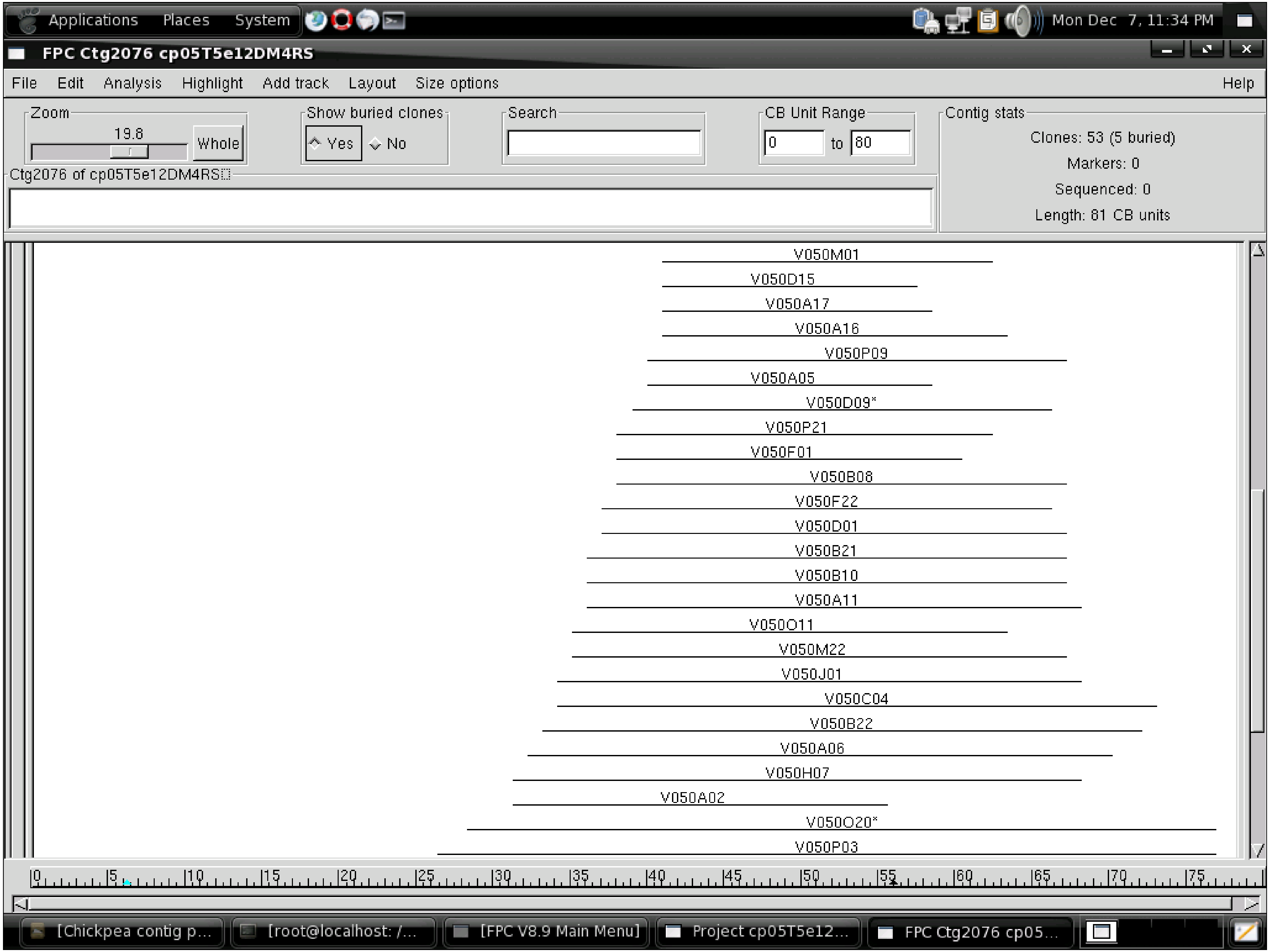
Applications Places System
Mon Dec 7, 11:34 PM
FPC Ctg2076 cp05T5e12DM4RS
File Edit Analysis Highlight Add track Layout Size options Help
Zoom
19.8
Whole
Show buried clones
Yes No
Search
CB Unit Range
0 to 80
Contig stats
Clones: 53 (5 buried)
Markers: 0
Sequenced: 0
Length: 81 CB units
Ctg2076 of cp05T5e12DM4RS
V050M01
V050D15
V050A17
V050A16
V050P09
V050A05
V050D09*
V050P21
V050F01
V050B08
V050F22
V050D01
V050B21
V050B10
V050A11
V050O11
V050M22
V050J01
V050C04
V050B22
V050A06
V050H07
V050A02
V050O20*
V050P03
0 5 10 15 20 25 30 35 40 45 50 55 60 65 70 75
[Chickpea contig p...
[root@localhost: /...
[FPC V8.9 Main Menu]
Project cp05T5e12...
FPC Ctg2076 cp05...

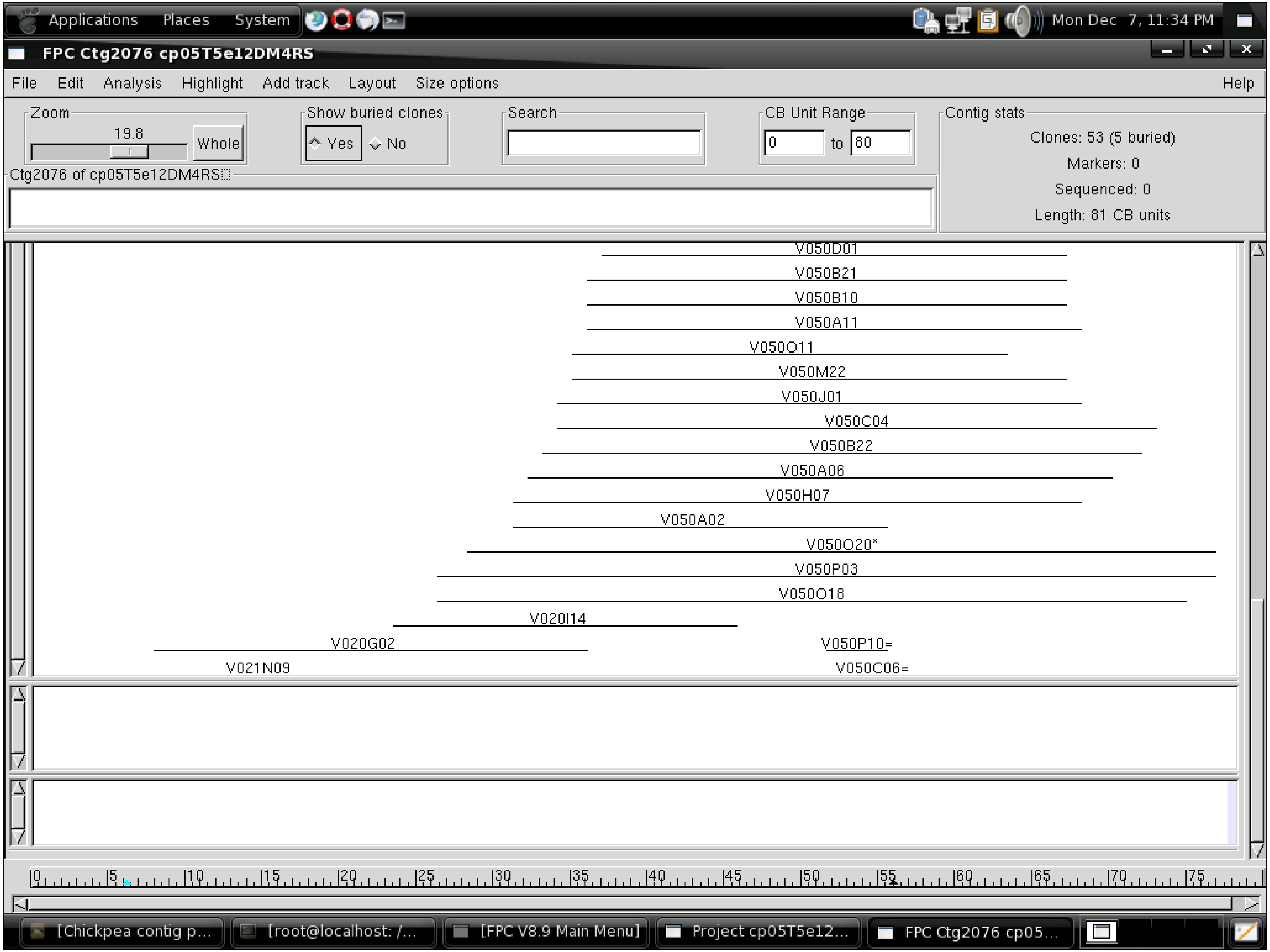
Applications
Places
System
Mon Dec 7, 11:34 PM
FPC Ctg2076 cp05T5e12DM4RS
File
Edit
Analysis
Highlight
Add track
Layout
Size options
Help
Zoom
19.8
Whole
Show buried clones
Yes
No
Search
CB Unit Range
0
to
80
Contig stats
Clones: 53 (5 buried)
Markers: 0
Sequenced: 0
Length: 81 CB units
Ctg2076 of cp05T5e12DM4RS
V050D01
V050B21
V050B10
V050A11
V050O11
V050M22
V050J01
V050C04
V050B22
V050A06
V050H07
V050A02
V050O20*
V050P03
V050O18
V020I14
V020G02
V050P10=
V021N09
V050C06=
0
5
10
15
20
25
30
35
40
45
50
55
60
65
70
75
[Chickpea contig p...
[root@localhost: /...
[FPC V8.9 Main Menu]
Project cp05T5e12...
FPC Ctg2076 cp05...

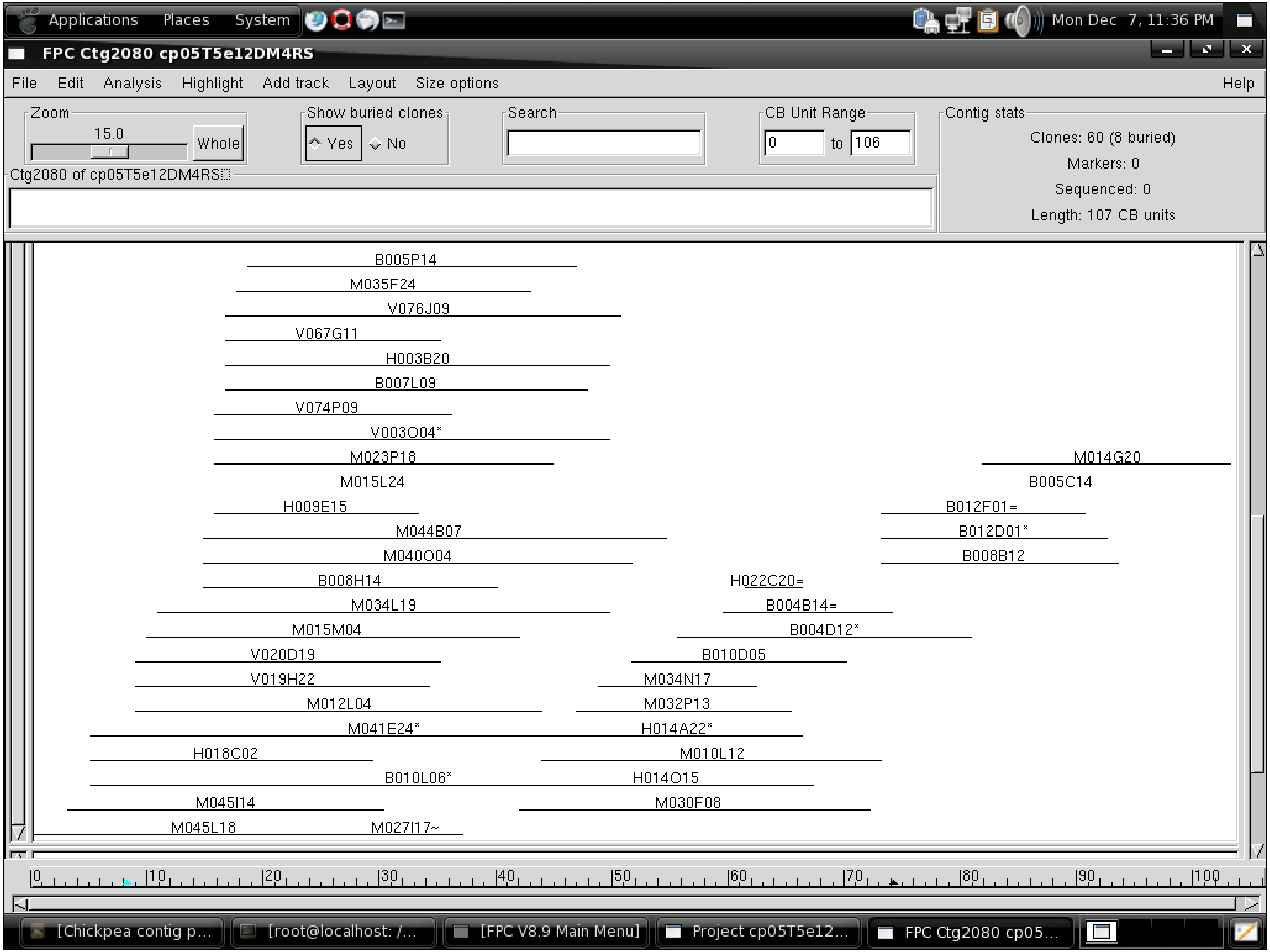
Applications Places System
Mon Dec 7, 11:36 PM
FPC Ctg2080 cp05T5e12DM4RS
File Edit Analysis Highlight Add track Layout Size options Help
Zoom
15.0
Whole
Show buried clones
Yes No
Search
CB Unit Range
0 to 106
Contig stats
Clones: 60 (8 buried)
Markers: 0
Sequenced: 0
Length: 107 CB units
Ctg2080 of cp05T5e12DM4RS
B005P14
M035F24
V076J09
V067G11
H003B20
B007L09
V074P09
V003O04*
M023P18
M015L24
H009E15
M044B07
M040O04
B008H14
M034L19
M015M04
V020D19
V019H22
M012L04
M041E24*
H018C02
B010L06*
M045I14
M045L18
M027I17~
M014G20
B005C14
B012F01=
B012D01*
B008B12
H022C20=
B004B14=
B004D12*
B010D05
M034N17
M032P13
H014A22*
M010L12
H014O15
M030F08
0 10 20 30 40 50 60 70 80 90 100
[Chickpea contig p...
[root@localhost: /...
[FPC V8.9 Main Menu]
Project cp05T5e12...
FPC Ctg2080 cp05...

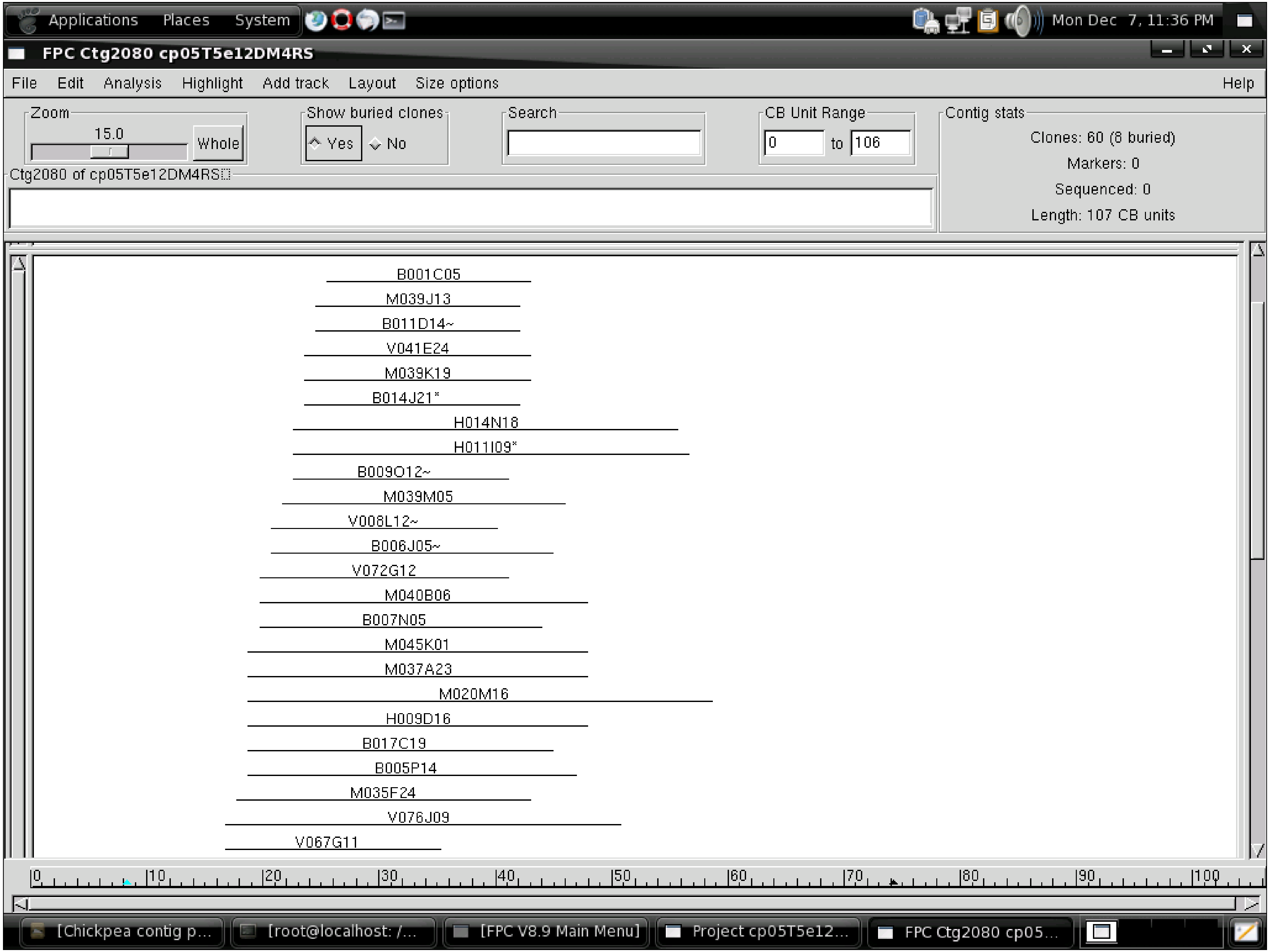
Applications Places System
Mon Dec 7, 11:36 PM
FPC Ctg2080 cp05T5e12DM4RS
File Edit Analysis Highlight Add track Layout Size options Help
Zoom
15.0
Whole
Show buried clones
Yes
No
Search
CB Unit Range
0
to
106
Contig stats
Clones: 60 (8 buried)
Markers: 0
Sequenced: 0
Length: 107 CB units
Ctg2080 of cp05T5e12DM4RS
B001C05
M039J13
B011D14~
V041E24
M039K19
B014J21*
H014N18
H011I09*
B009O12~
M039M05
V008L12~
B006J05~
V072G12
M040B06
B007N05
M045K01
M037A23
M020M16
H009D16
B017C19
B005P14
M035F24
V076J09
V067G11
0 10 20 30 40 50 60 70 80 90 100
[Chickpea contig p...
[root@localhost: /...
[FPC V8.9 Main Menu]
Project cp05T5e12...
FPC Ctg2080 cp05...

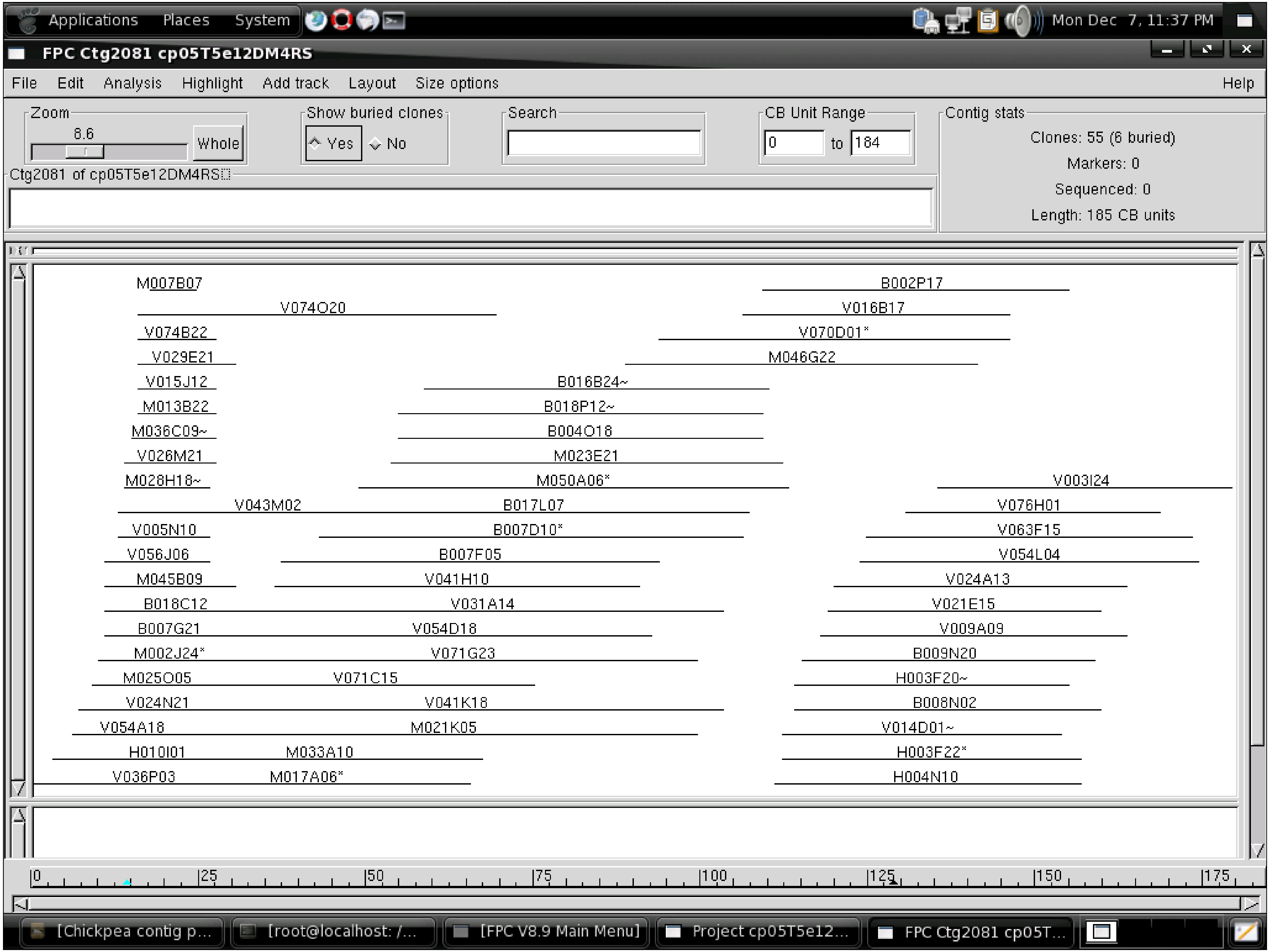
Applications Places System
Mon Dec 7, 11:37 PM
FPC Ctg2081 cp05T5e12DM4RS
File Edit Analysis Highlight Add track Layout Size options Help
Zoom
8.6
Whole
Show buried clones
Yes
No
Search
CB Unit Range
0
to
184
Contig stats
Clones: 55 (6 buried)
Markers: 0
Sequenced: 0
Length: 185 CB units
Ctg2081 of cp05T5e12DM4RS
M007B07
V074O20
V074B22
V029E21
V015J12
M013B22
M036C09~
V026M21
M028H18~
V043M02
V005N10
V056J06
M045B09
B018C12
B007G21
M002J24*
M025O05
V024N21
V054A18
H010I01
V036P03
B016B24~
B018P12~
B004O18
M023E21
M050A06*
B017L07
B007D10*
B007F05
V041H10
V031A14
V054D18
V071G23
V071C15
V041K18
M021K05
M033A10
M017A06*
B002P17
V016B17
V070D01*
M046G22
V003I24
V076H01
V063F15
V054L04
V024A13
V021E15
V009A09
B009N20
H003F20~
B008N02
V014D01~
H003F22*
H004N10
0
25
50
75
100
125
150
175
[Chickpea contig p...
[root@localhost: /...
[FPC V8.9 Main Menu]
Project cp05T5e12...
FPC Ctg2081 cp05T...

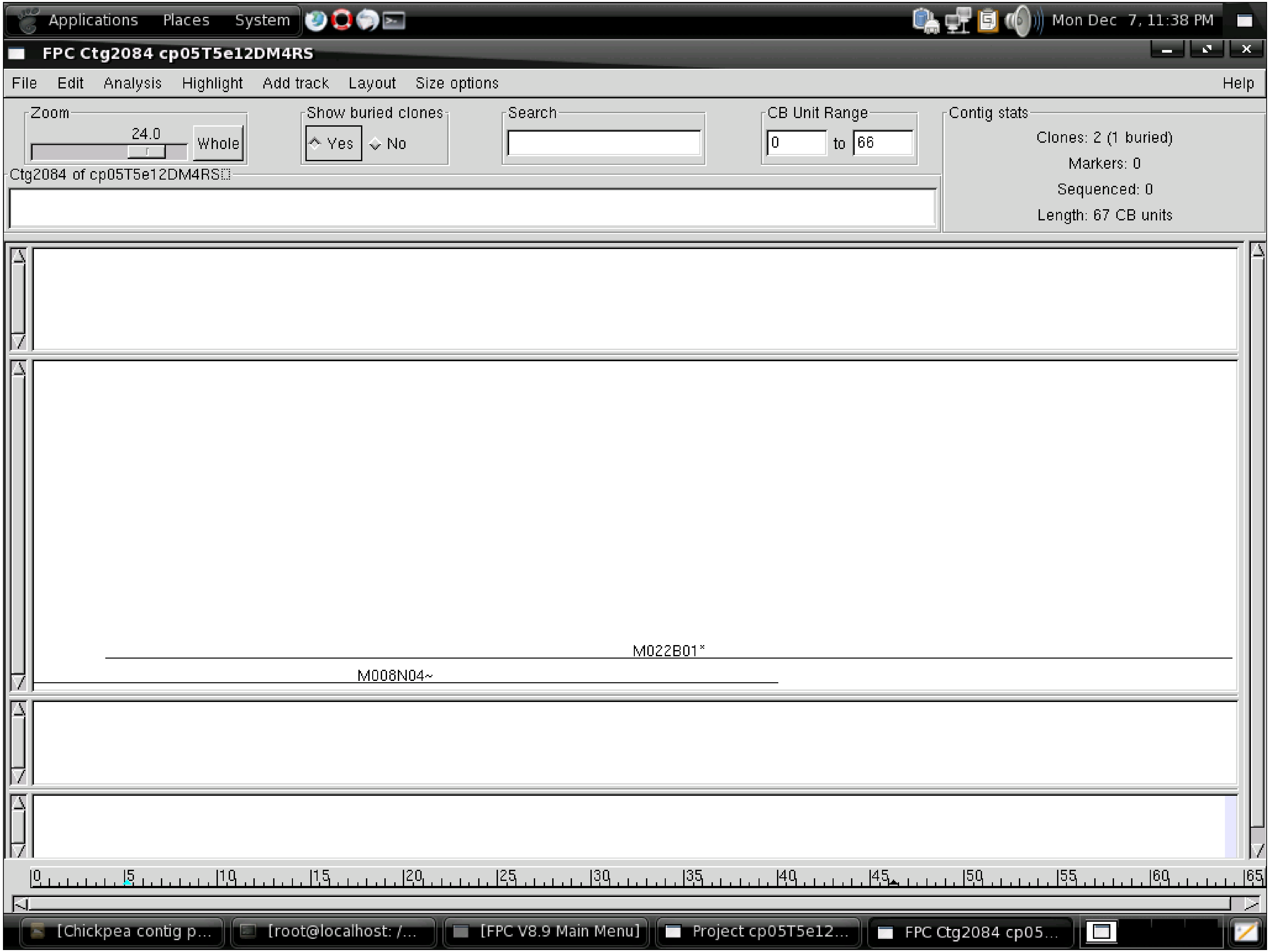
Applications Places System
Mon Dec 7, 11:38 PM
FPC Ctg2084 cp05T5e12DM4RS
File Edit Analysis Highlight Add track Layout Size options Help
Zoom
24.0
Whole
Show buried clones
Yes
No
Search
CB Unit Range
0
to
66
Contig stats
Clones: 2 (1 buried)
Markers: 0
Sequenced: 0
Length: 67 CB units
Ctg2084 of cp05T5e12DM4RS
M022B01*
M008N04~
0 5 10 15 20 25 30 35 40 45 50 55 60 65
[Chickpea contig p...
[root@localhost: /...
[FPC V8.9 Main Menu]
Project cp05T5e12...
FPC Ctg2084 cp05...

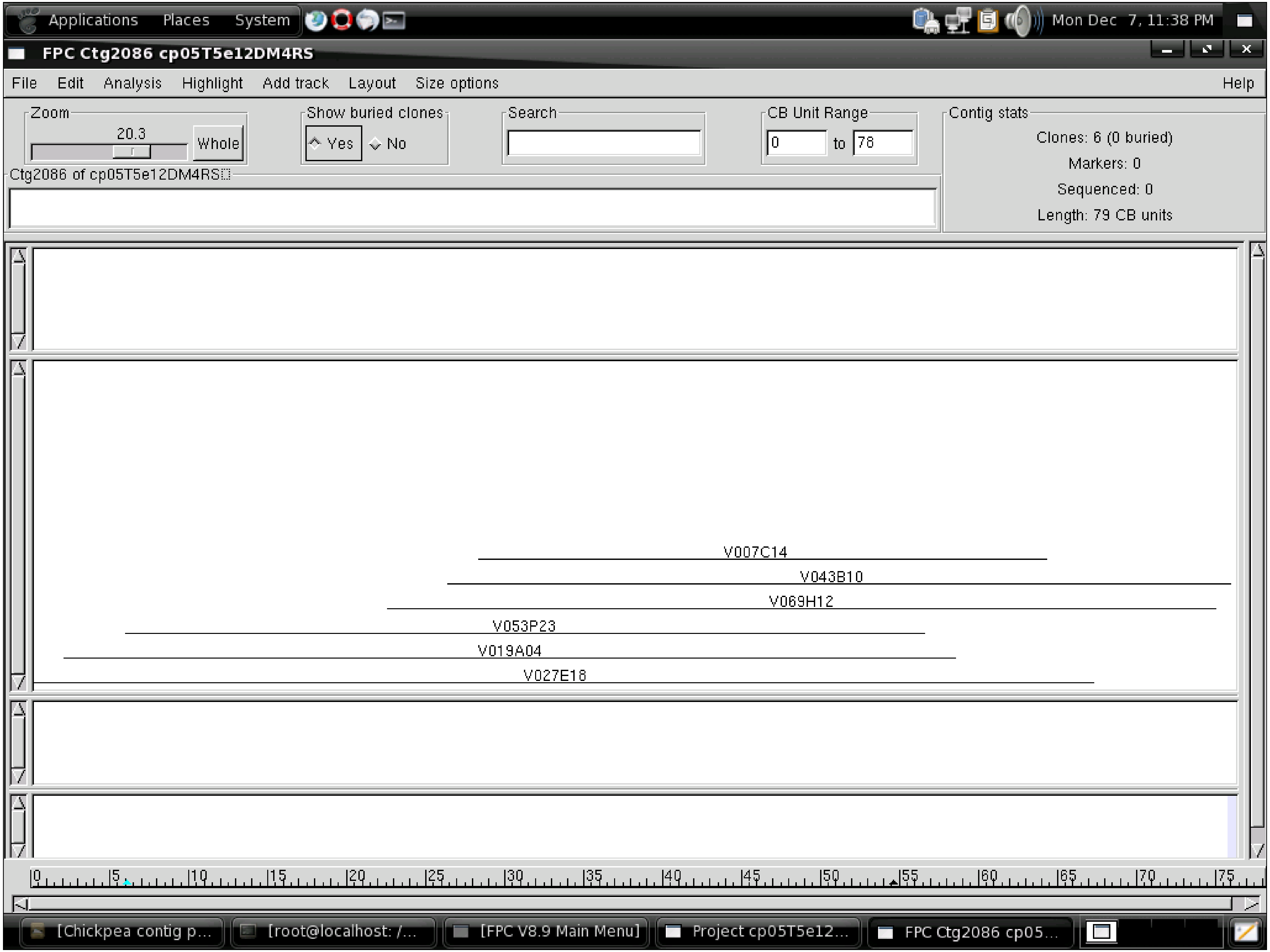
Applications Places System
Mon Dec 7, 11:38 PM
FPC Ctg2086 cp05T5e12DM4RS
File Edit Analysis Highlight Add track Layout Size options Help
Zoom
20.3
Whole
Show buried clones
Yes
No
Search
CB Unit Range
0
to
78
Contig stats
Clones: 6 (0 buried)
Markers: 0
Sequenced: 0
Length: 79 CB units
Ctg2086 of cp05T5e12DM4RS
V007C14
V043B10
V069H12
V053P23
V019A04
V027E18
0 5 10 15 20 25 30 35 40 45 50 55 60 65 70 75
[Chickpea contig p...
[root@localhost: /...
[FPC V8.9 Main Menu]
Project cp05T5e12...
FPC Ctg2086 cp05...

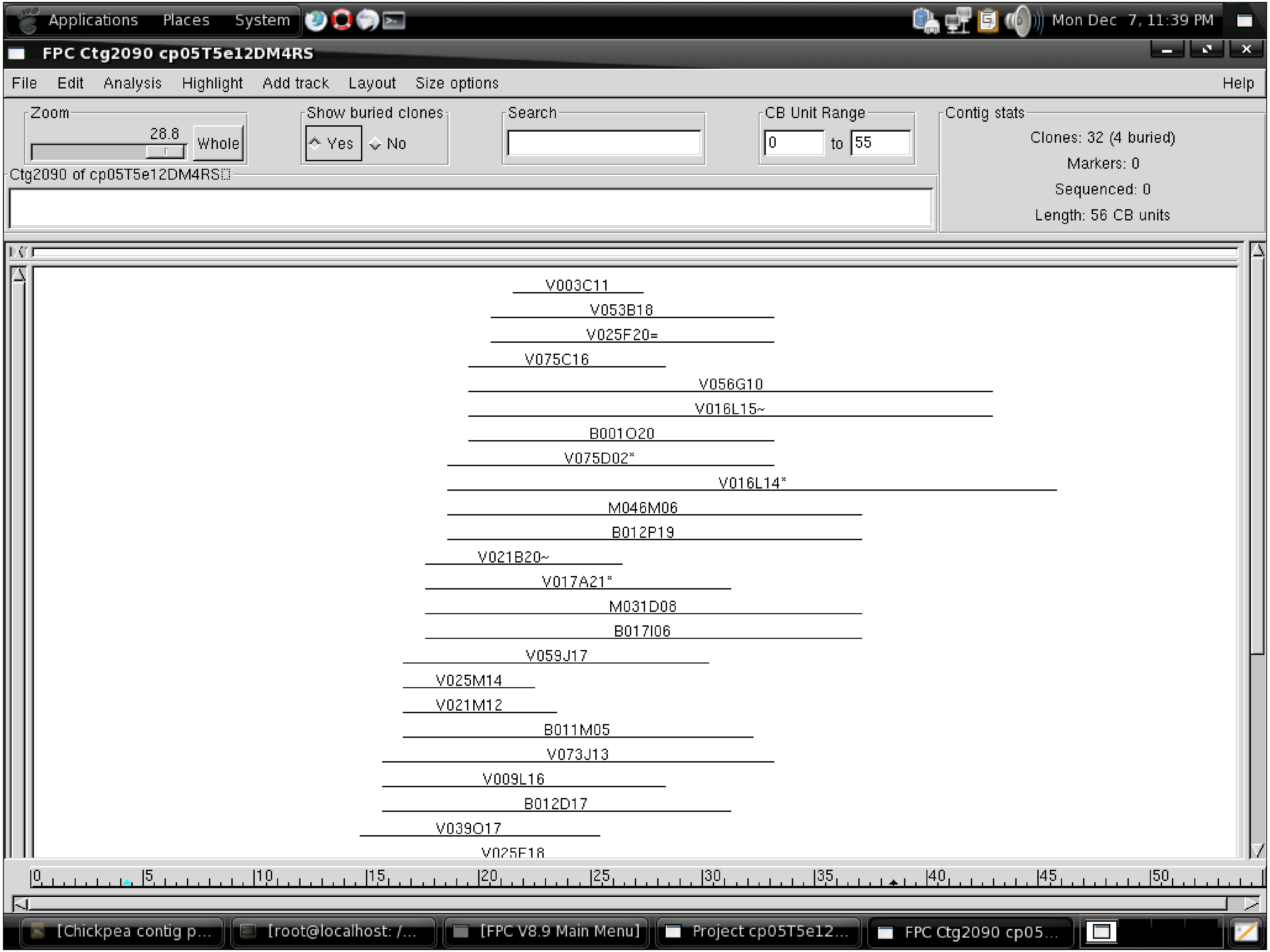
Applications Places System
Mon Dec 7, 11:39 PM
FPC Ctg2090 cp05T5e12DM4RS
File Edit Analysis Highlight Add track Layout Size options Help
Zoom
28.8
Whole
Show buried clones
Yes No
Search
CB Unit Range
0 to 55
Contig stats
Clones: 32 (4 buried)
Markers: 0
Sequenced: 0
Length: 56 CB units
Ctg2090 of cp05T5e12DM4RS
V003C11
V053B18
V025F20=
V075C16
V056G10
V016L15~
B001O20
V075D02*
V016L14*
M046M06
B012P19
V021B20~
V017A21*
M031D08
B017I06
V059J17
V025M14
V021M12
B011M05
V073J13
V009L16
B012D17
V039O17
V025F18
0 5 10 15 20 25 30 35 40 45 50
[Chickpea contig p...
[root@localhost: /...
[FPC V8.9 Main Menu]
Project cp05T5e12...
FPC Ctg2090 cp05...

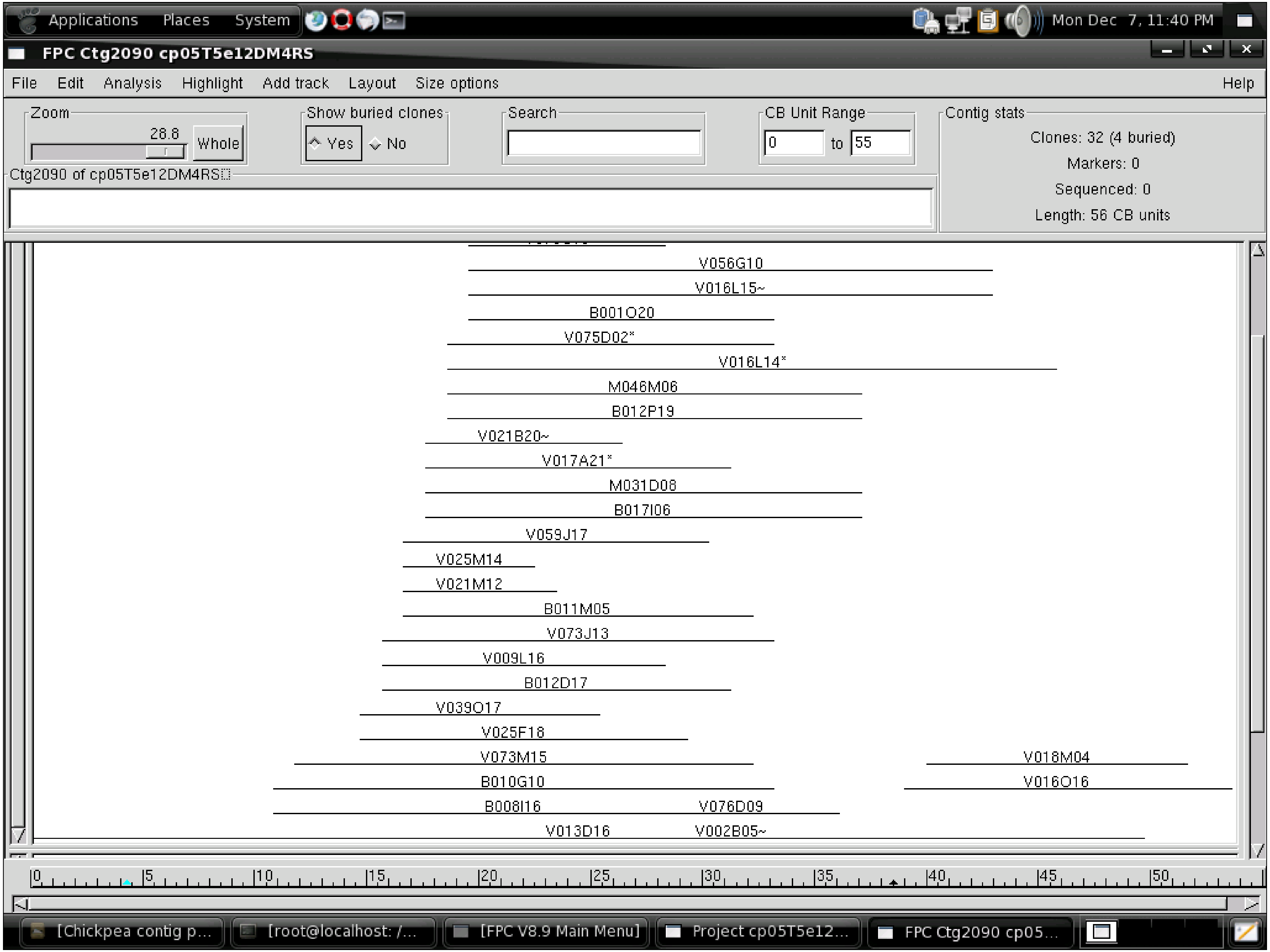
Applications Places System
Mon Dec 7, 11:40 PM
FPC Ctg2090 cp05T5e12DM4RS
File Edit Analysis Highlight Add track Layout Size options Help
Zoom
28.8
Whole
Show buried clones
Yes
No
Search
CB Unit Range
0
to
55
Contig stats
Clones: 32 (4 buried)
Markers: 0
Sequenced: 0
Length: 56 CB units
Ctg2090 of cp05T5e12DM4RS
V056G10
V016L15~
B001O20
V075D02*
V016L14*
M046M06
B012P19
V021B20~
V017A21*
M031D08
B017I06
V059J17
V025M14
V021M12
B011M05
V073J13
V009L16
B012D17
V039O17
V025F18
V073M15
V018M04
B010G10
V016O16
B008I16
V076D09
V013D16
V002B05~
0 5 10 15 20 25 30 35 40 45 50
[Chickpea contig p...
[root@localhost: /...
[FPC V8.9 Main Menu]
Project cp05T5e12...
FPC Ctg2090 cp05...

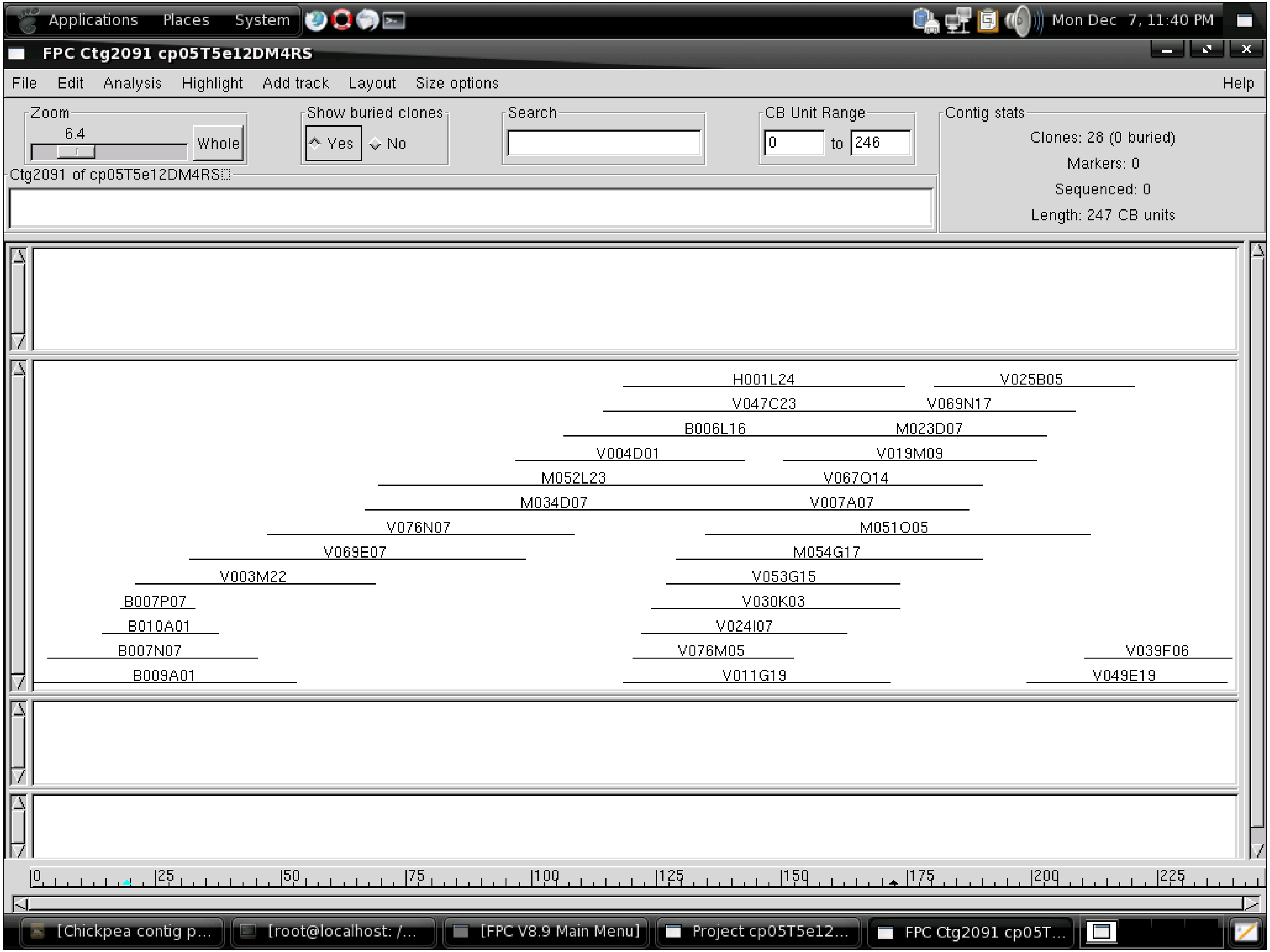
Applications Places System
Mon Dec 7, 11:40 PM
FPC Ctg2091 cp05T5e12DM4RS
File Edit Analysis Highlight Add track Layout Size options Help
Zoom
6.4
Whole
Show buried clones
Yes No
Search
CB Unit Range
0 to 246
Contig stats
Clones: 28 (0 buried)
Markers: 0
Sequenced: 0
Length: 247 CB units
Ctg2091 of cp05T5e12DM4RS
H001L24
V025B05
V047C23
V069N17
B006L16
M023D07
V004D01
V019M09
M052L23
V067O14
M034D07
V007A07
V076N07
M051O05
V069E07
M054G17
V003M22
V053G15
B007P07
V030K03
B010A01
V024I07
B007N07
V076M05
V039F06
B009A01
V011G19
V049E19
0 25 50 75 100 125 150 175 200 225
[Chickpea contig p...
[root@localhost: /...
[FPC V8.9 Main Menu]
Project cp05T5e12...
FPC Ctg2091 cp05T...

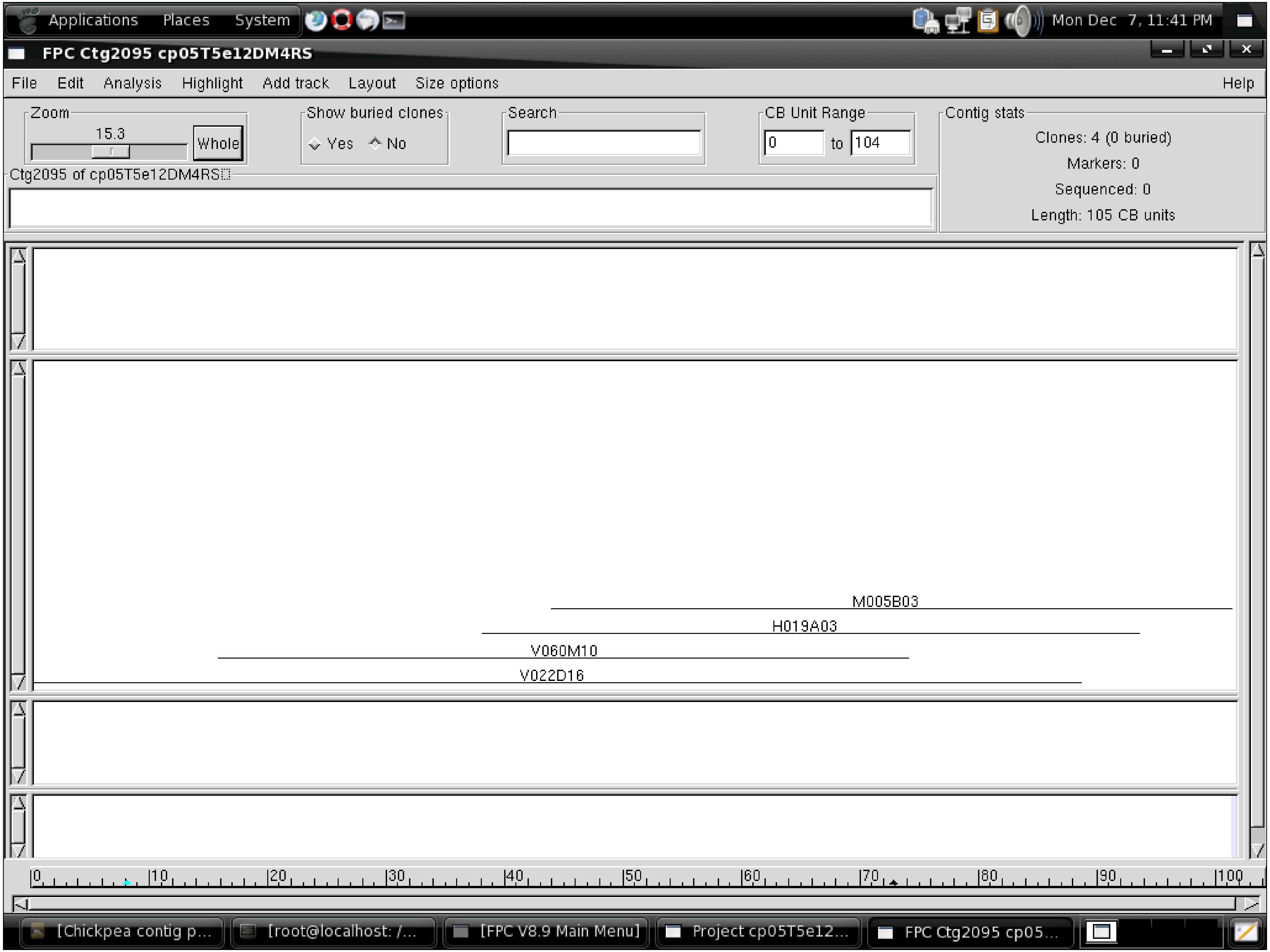
Applications Places System
Mon Dec 7, 11:41 PM
FPC Ctg2095 cp05T5e12DM4RS
File Edit Analysis Highlight Add track Layout Size options Help
Zoom
15.3
Whole
Show buried clones
Yes No
Search
CB Unit Range
0 to 104
Contig stats
Clones: 4 (0 buried)
Markers: 0
Sequenced: 0
Length: 105 CB units
Ctg2095 of cp05T5e12DM4RS
M005B03
H019A03
V060M10
V022D16
0 10 20 30 40 50 60 70 80 90 100
[Chickpea contig p...
[root@localhost: /...
[FPC V8.9 Main Menu]
Project cp05T5e12...
FPC Ctg2095 cp05...

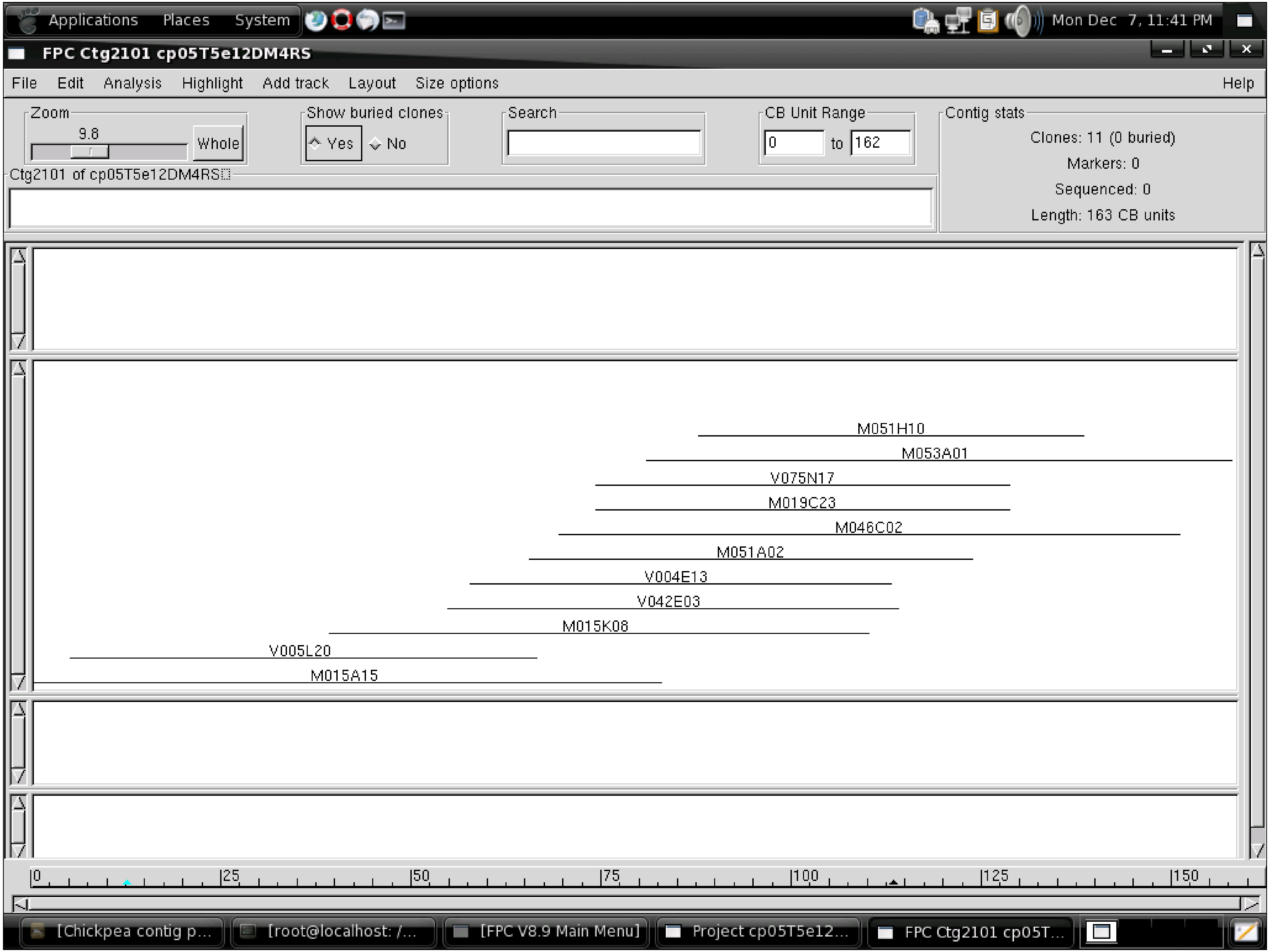
Applications
Places
System
Mon Dec 7, 11:41 PM
FPC Ctg2101 cp05T5e12DM4RS
File
Edit
Analysis
Highlight
Add track
Layout
Size options
Help
Zoom
9.8
Whole
Show buried clones
Yes
No
Search
CB Unit Range
0
to
162
Contig stats
Clones: 11 (0 buried)
Markers: 0
Sequenced: 0
Length: 163 CB units
Ctg2101 of cp05T5e12DM4RS
M051H10
M053A01
V075N17
M019C23
M046C02
M051A02
V004E13
V042E03
M015K08
V005L20
M015A15
0
25
50
75
100
125
150
[Chickpea contig p...
[root@localhost: /...
[FPC V8.9 Main Menu]
Project cp05T5e12...
FPC Ctg2101 cp05T...

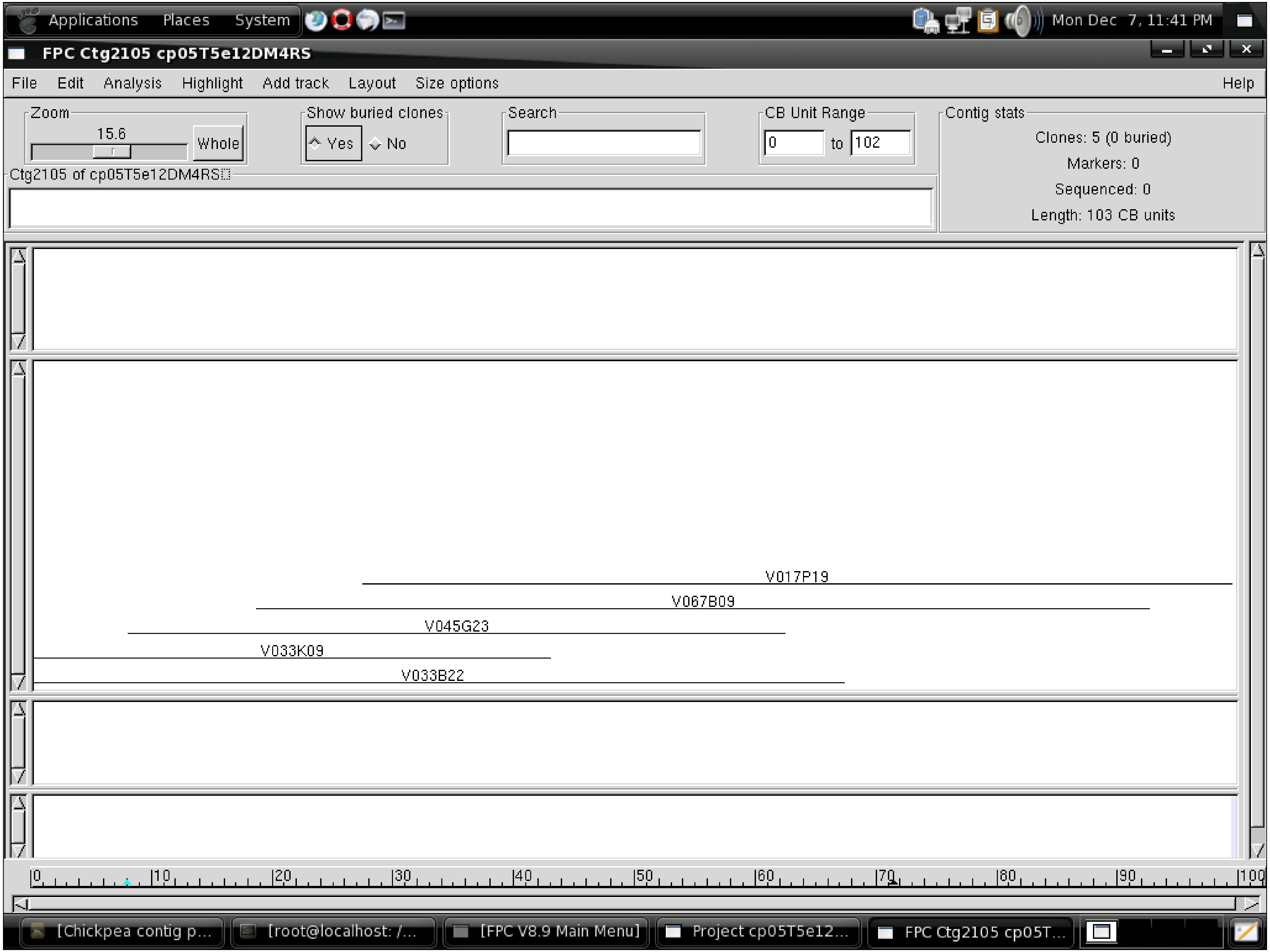
Applications
Places
System
Mon Dec 7, 11:41 PM
FPC Ctg2105 cp05T5e12DM4RS
File
Edit
Analysis
Highlight
Add track
Layout
Size options
Help
Zoom
15.6
Whole
Show buried clones
Yes
No
Search
CB Unit Range
0
to
102
Contig stats
Clones: 5 (0 buried)
Markers: 0
Sequenced: 0
Length: 103 CB units
Ctg2105 of cp05T5e12DM4RS
V017P19
V067B09
V045G23
V033K09
V033B22
0
10
20
30
40
50
60
70
80
90
100
[Chickpea contig p...
[root@localhost: /...
[FPC V8.9 Main Menu]
Project cp05T5e12...
FPC Ctg2105 cp05T...

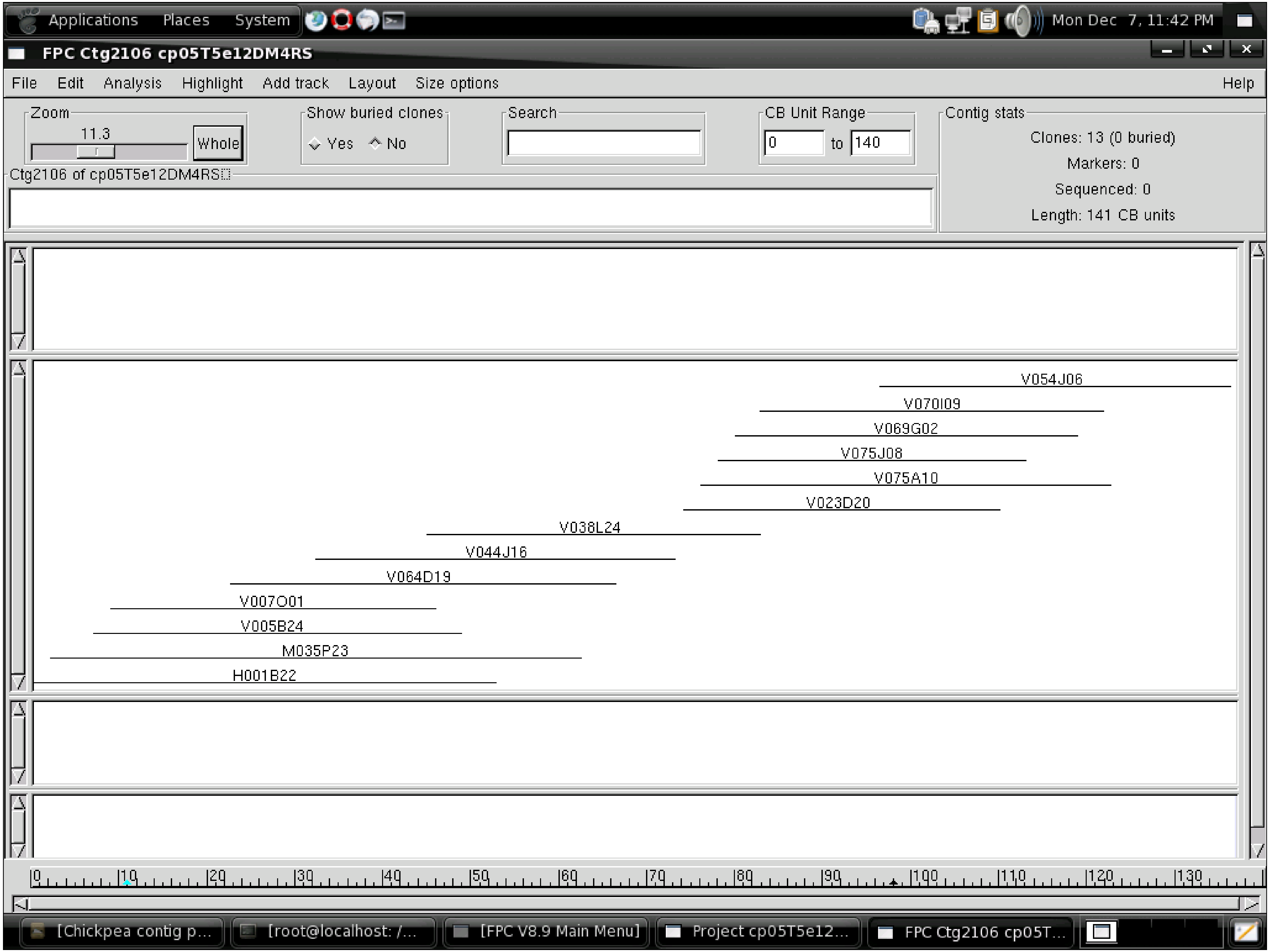
Applications
Places
System
Mon Dec 7, 11:42 PM
FPC Ctg2106 cp05T5e12DM4RS
File
Edit
Analysis
Highlight
Add track
Layout
Size options
Help
Zoom
11.3
Whole
Show buried clones
Yes
No
Search
CB Unit Range
0
to
140
Contig stats
Clones: 13 (0 buried)
Markers: 0
Sequenced: 0
Length: 141 CB units
Ctg2106 of cp05T5e12DM4RS
V054J06
V070I09
V069G02
V075J08
V075A10
V023D20
V038L24
V044J16
V064D19
V007O01
V005B24
M035P23
H001B22
0
10
20
30
40
50
60
70
80
90
100
110
120
130
[Chickpea contig p...
[root@localhost: /...
[FPC V8.9 Main Menu]
Project cp05T5e12...
FPC Ctg2106 cp05T...

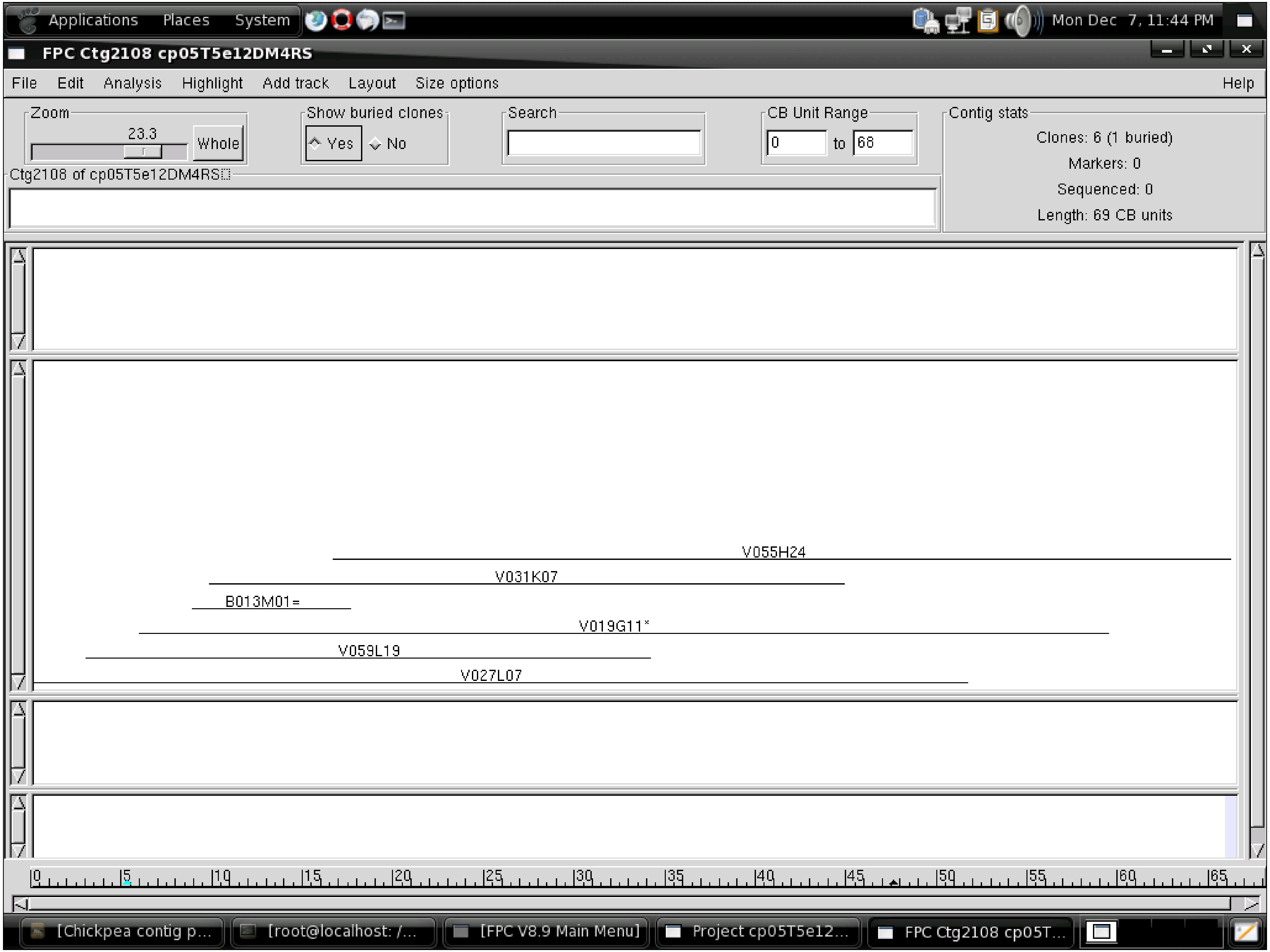
Applications
Places
System
Mon Dec 7, 11:44 PM
FPC Ctg2108 cp05T5e12DM4RS
File
Edit
Analysis
Highlight
Add track
Layout
Size options
Help
Zoom
23.3
Whole
Show buried clones
Yes
No
Search
CB Unit Range
0
to
68
Contig stats
Clones: 6 (1 buried)
Markers: 0
Sequenced: 0
Length: 69 CB units
Ctg2108 of cp05T5e12DM4RS
V055H24
V031K07
B013M01=
V019G11*
V059L19
V027L07
0
5
10
15
20
25
30
35
40
45
50
55
60
65
[Chickpea contig p...
[root@localhost: /...
[FPC V8.9 Main Menu]
Project cp05T5e12...
FPC Ctg2108 cp05T...

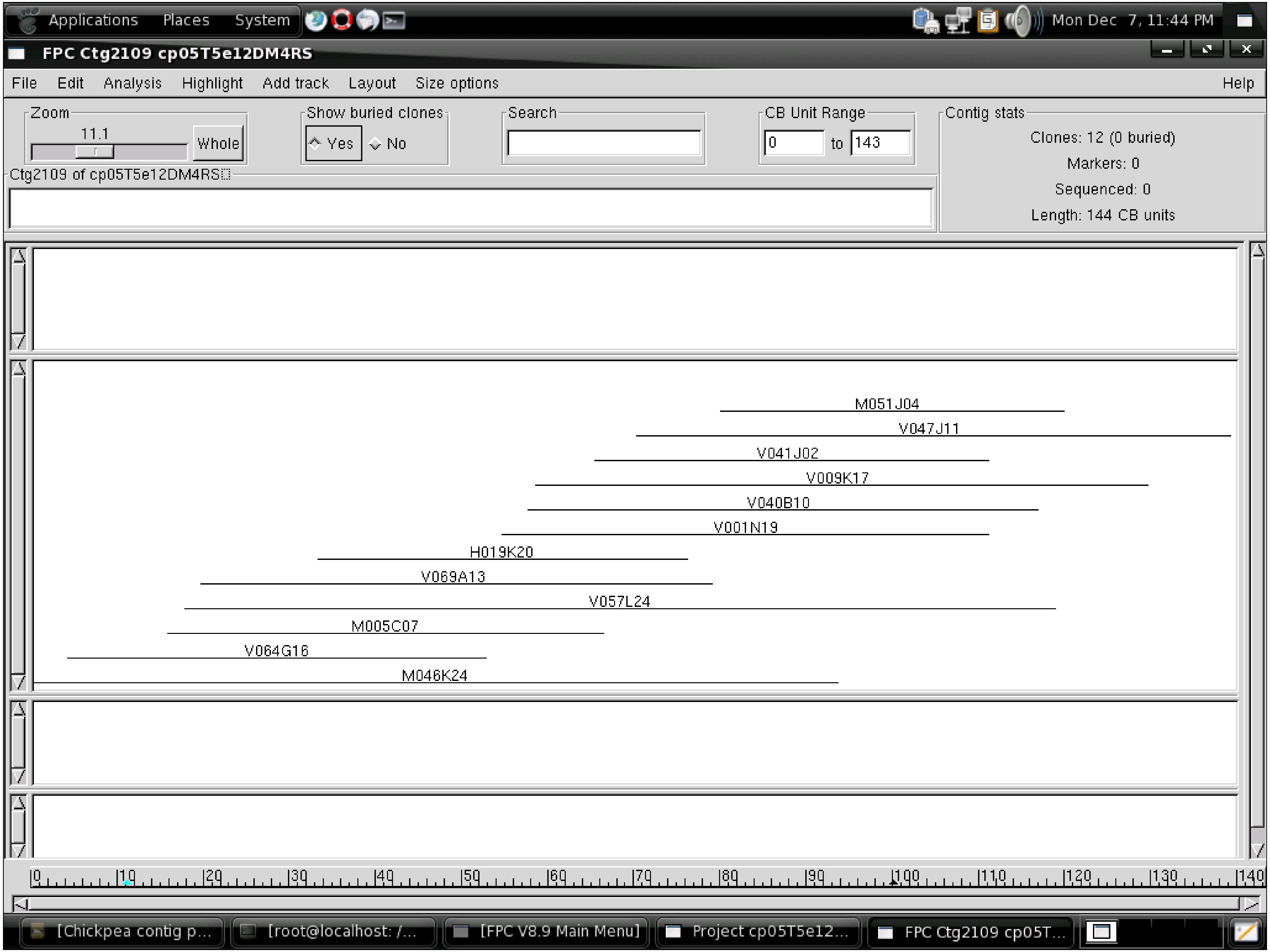
Applications
Places
System
Mon Dec 7, 11:44 PM
FPC Ctg2109 cp05T5e12DM4RS
File
Edit
Analysis
Highlight
Add track
Layout
Size options
Help
Zoom
11.1
Whole
Show buried clones
Yes
No
Search
CB Unit Range
0
to
143
Contig stats
Clones: 12 (0 buried)
Markers: 0
Sequenced: 0
Length: 144 CB units
Ctg2109 of cp05T5e12DM4RS
M051J04
V047J11
V041J02
V009K17
V040B10
V001N19
H019K20
V069A13
V057L24
M005C07
V064G16
M046K24
0
10
20
30
40
50
60
70
80
90
100
110
120
130
140
[Chickpea contig p...
[root@localhost: /...
[FPC V8.9 Main Menu]
Project cp05T5e12...
FPC Ctg2109 cp05T...

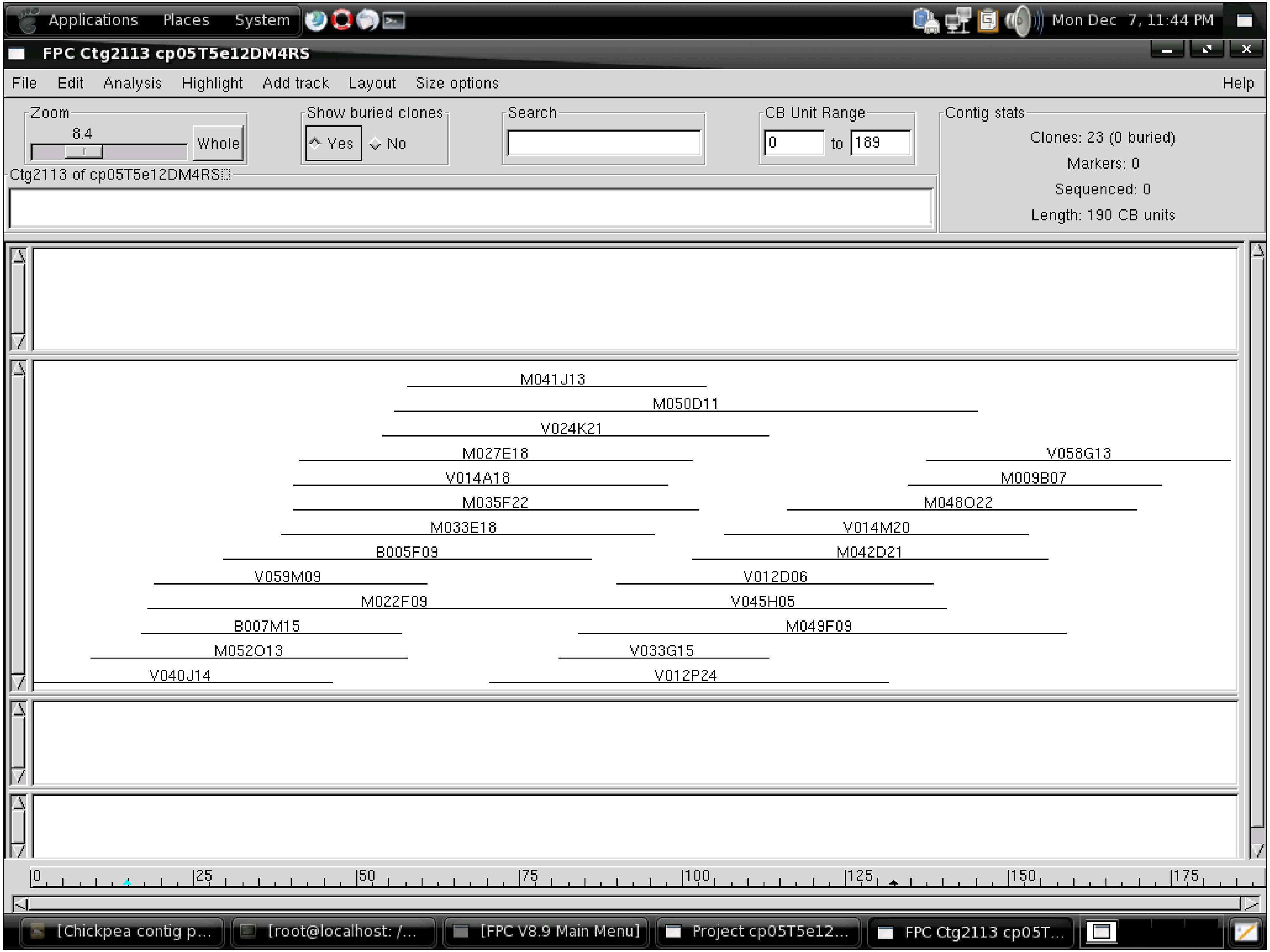
Applications
Places
System
Mon Dec 7, 11:44 PM
FPC Ctg2113 cp05T5e12DM4RS
File
Edit
Analysis
Highlight
Add track
Layout
Size options
Help
Zoom
8.4
Whole
Show buried clones
Yes
No
Search
CB Unit Range
0
to
189
Contig stats
Clones: 23 (0 buried)
Markers: 0
Sequenced: 0
Length: 190 CB units
Ctg2113 of cp05T5e12DM4RS
M041J13
M050D11
V024K21
M027E18
V058G13
V014A18
M009B07
M035F22
M048O22
M033E18
V014M20
B005F09
M042D21
V059M09
V012D06
M022F09
V045H05
B007M15
M049F09
M052O13
V033G15
V040J14
V012P24
0
25
50
75
100
125
150
175
[Chickpea contig p...
[root@localhost: /...
[FPC V8.9 Main Menu]
Project cp05T5e12...
FPC Ctg2113 cp05T...

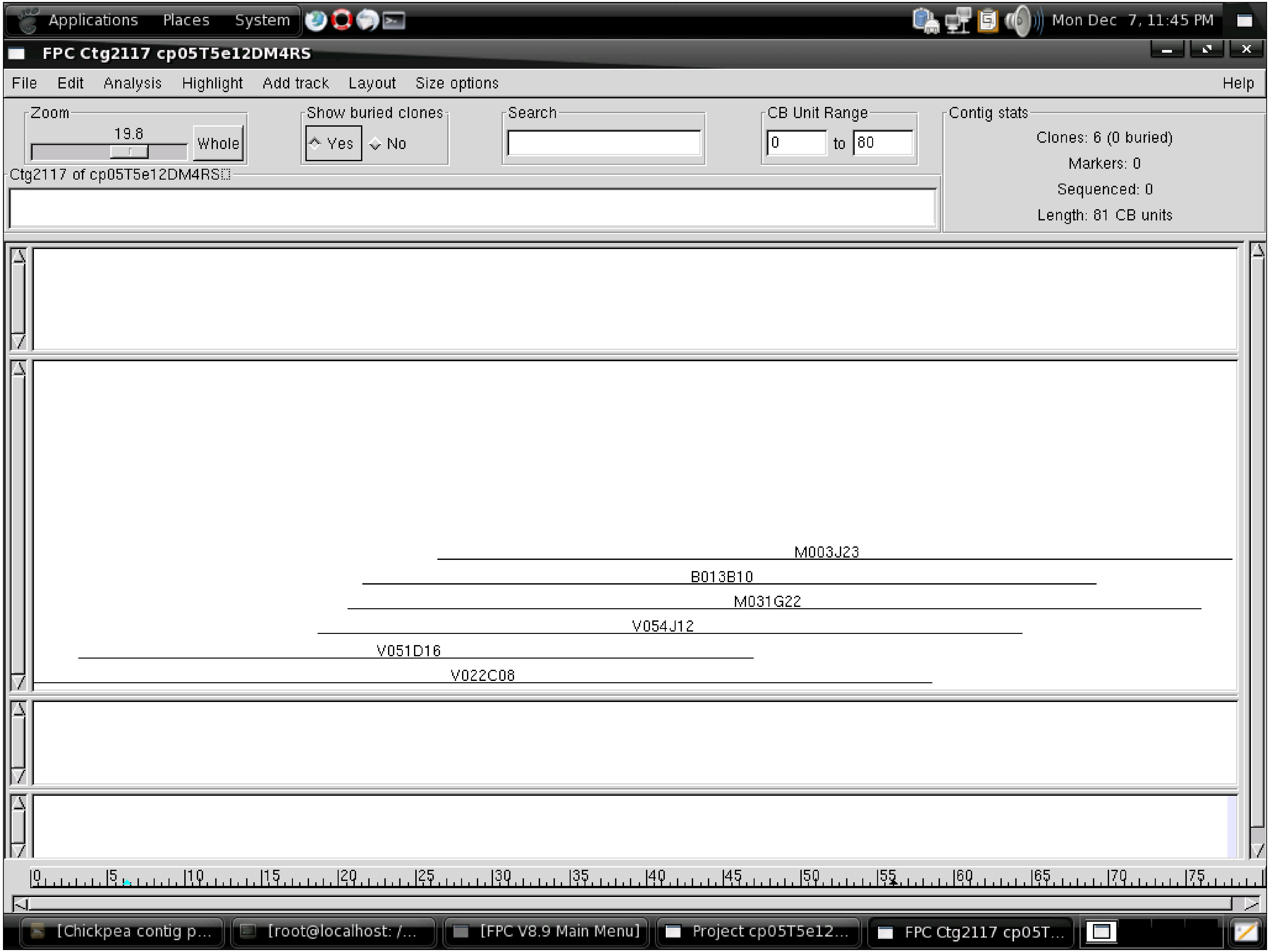
Applications Places System
Mon Dec 7, 11:45 PM
FPC Ctg2117 cp05T5e12DM4RS
File Edit Analysis Highlight Add track Layout Size options Help
Zoom
19.8
Whole
Show buried clones
Yes
No
Search
CB Unit Range
0
to
80
Contig stats
Clones: 6 (0 buried)
Markers: 0
Sequenced: 0
Length: 81 CB units
Ctg2117 of cp05T5e12DM4RS
M003J23
B013B10
M031G22
V054J12
V051D16
V022C08
0 5 10 15 20 25 30 35 40 45 50 55 60 65 70 75
[Chickpea contig p...
[root@localhost: /...
[FPC V8.9 Main Menu]
Project cp05T5e12...
FPC Ctg2117 cp05T...

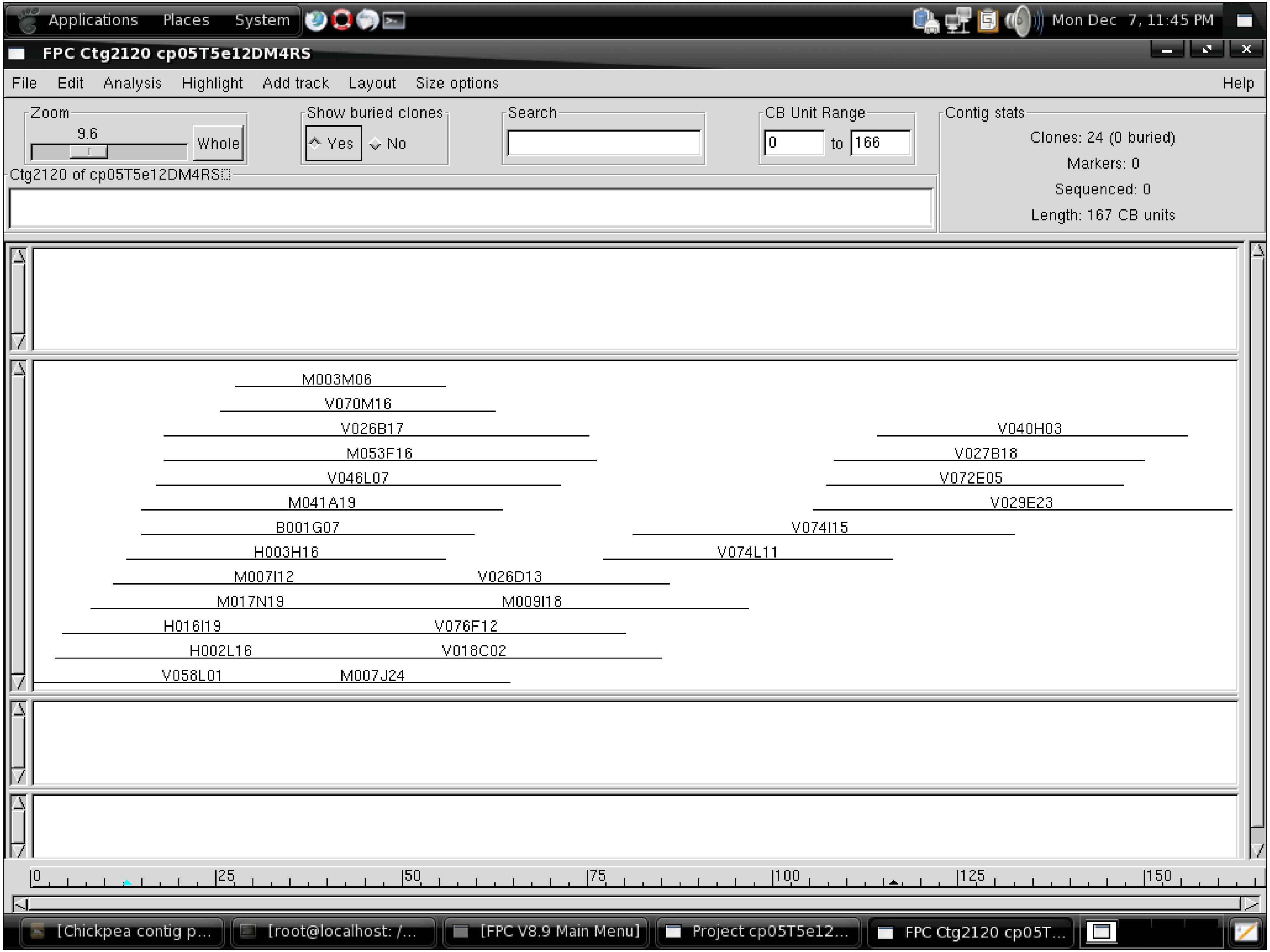
Applications Places System
Mon Dec 7, 11:45 PM
FPC Ctg2120 cp05T5e12DM4RS
File Edit Analysis Highlight Add track Layout Size options Help
Zoom
9.6
Whole
Show buried clones
Yes No
Search
CB Unit Range
0 to 166
Contig stats
Clones: 24 (0 buried)
Markers: 0
Sequenced: 0
Length: 167 CB units
Ctg2120 of cp05T5e12DM4RS
M003M06
V070M16
V026B17
V040H03
M053F16
V027B18
V046L07
V072E05
M041A19
V029E23
B001G07
V074I15
H003H16
V074L11
M007I12
V026D13
M017N19
M009I18
H016I19
V076F12
H002L16
V018C02
V058L01
M007J24
0 25 50 75 100 125 150
[Chickpea contig p...
[root@localhost: /...
[FPC V8.9 Main Menu]
Project cp05T5e12...
FPC Ctg2120 cp05T...

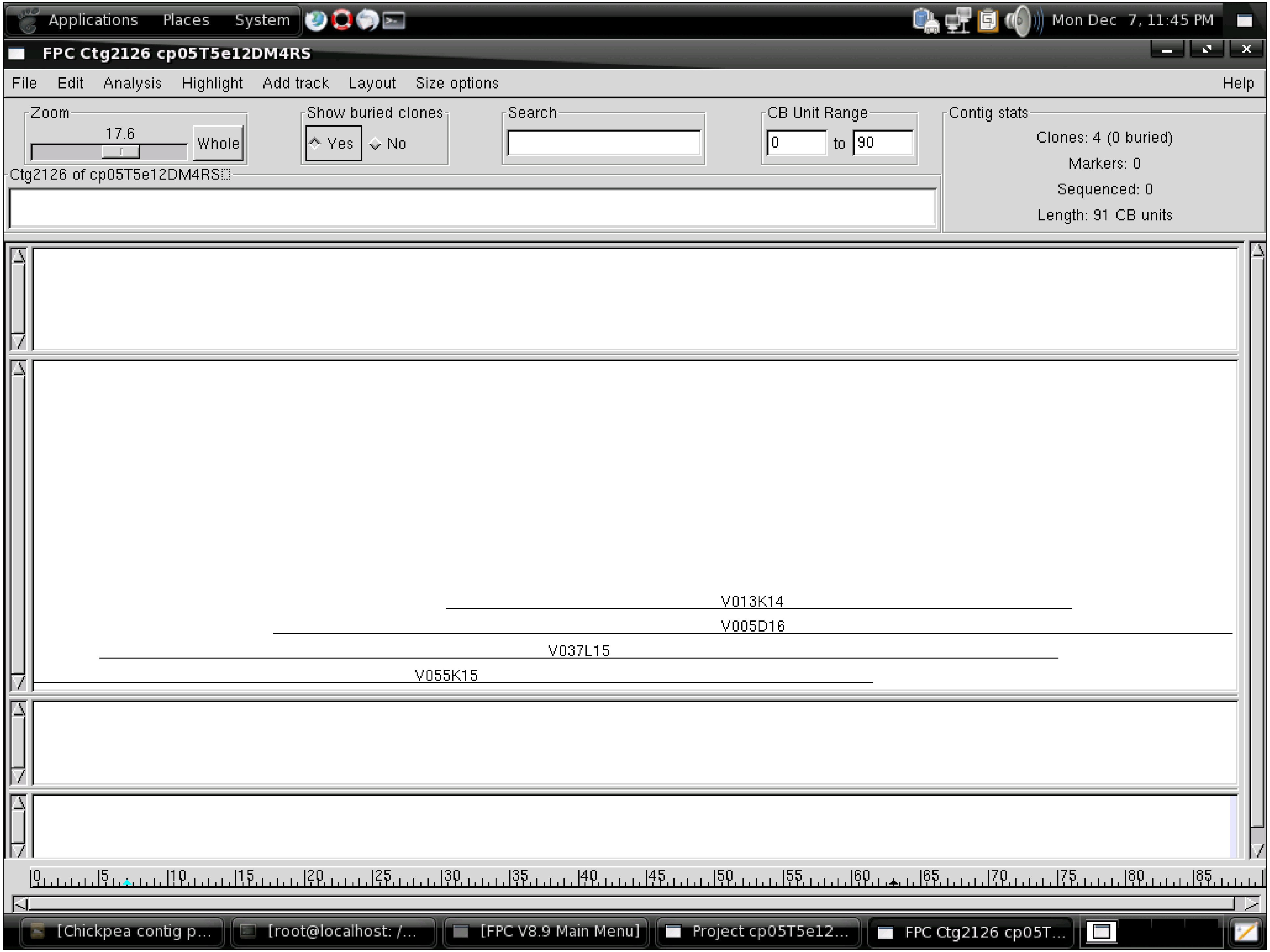
Applications Places System
Mon Dec 7, 11:45 PM
FPC Ctg2126 cp05T5e12DM4RS
File Edit Analysis Highlight Add track Layout Size options Help
Zoom
17.6
Whole
Show buried clones
Yes No
Search
CB Unit Range
0 to 90
Contig stats
Clones: 4 (0 buried)
Markers: 0
Sequenced: 0
Length: 91 CB units
Ctg2126 of cp05T5e12DM4RS
V013K14
V005D16
V037L15
V055K15
0 5 10 15 20 25 30 35 40 45 50 55 60 65 70 75 80 85
[Chickpea contig p...
[root@localhost: /...
[FPC V8.9 Main Menu]
Project cp05T5e12...
FPC Ctg2126 cp05T...

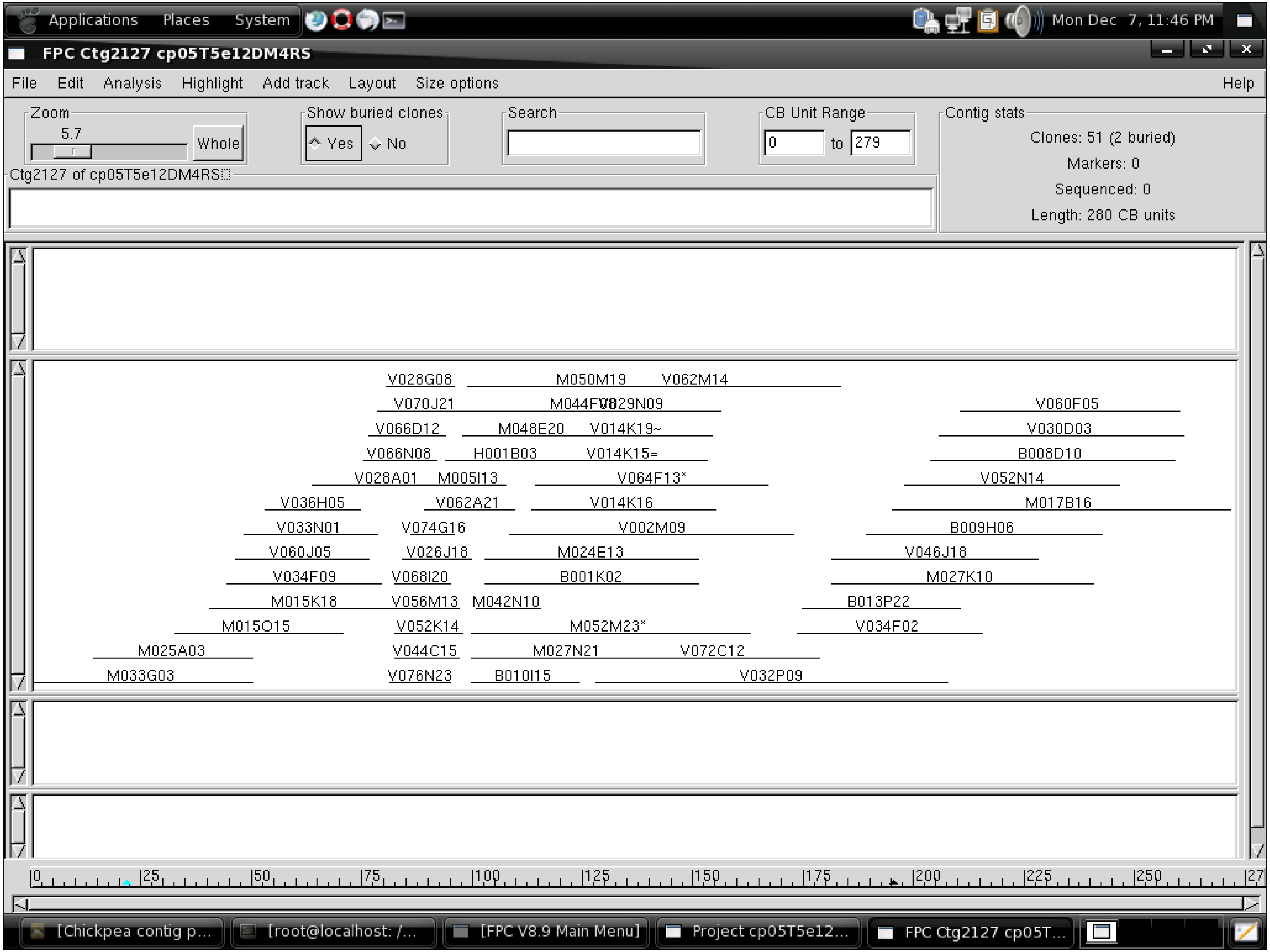
Applications Places System
Mon Dec 7, 11:46 PM
FPC Ctg2127 cp05T5e12DM4RS
File Edit Analysis Highlight Add track Layout Size options Help
Zoom
5.7
Whole
Show buried clones
Yes
No
Search
CB Unit Range
0 to 279
Contig stats
Clones: 51 (2 buried)
Markers: 0
Sequenced: 0
Length: 280 CB units
Ctg2127 of cp05T5e12DM4RS
V028G08
M050M19
V062M14
V070J21
M044F08
V029N09
V060F05
V066D12
M048E20
V014K19~
V030D03
V066N08
H001B03
V014K15=
B008D10
V028A01
M005I13
V064F13*
V052N14
V036H05
V062A21
V014K16
M017B16
V033N01
V074G16
V002M09
B009H06
V060J05
V026J18
M024E13
V046J18
V034F09
V068I20
B001K02
M027K10
M015K18
V056M13
M042N10
B013P22
M015O15
V052K14
M052M23*
V034F02
M025A03
V044C15
M027N21
V072C12
M033G03
V076N23
B010I15
V032P09
0 25 50 75 100 125 150 175 200 225 250 27
[Chickpea contig p...
[root@localhost: /...
[FPC V8.9 Main Menu]
Project cp05T5e12...
FPC Ctg2127 cp05T...

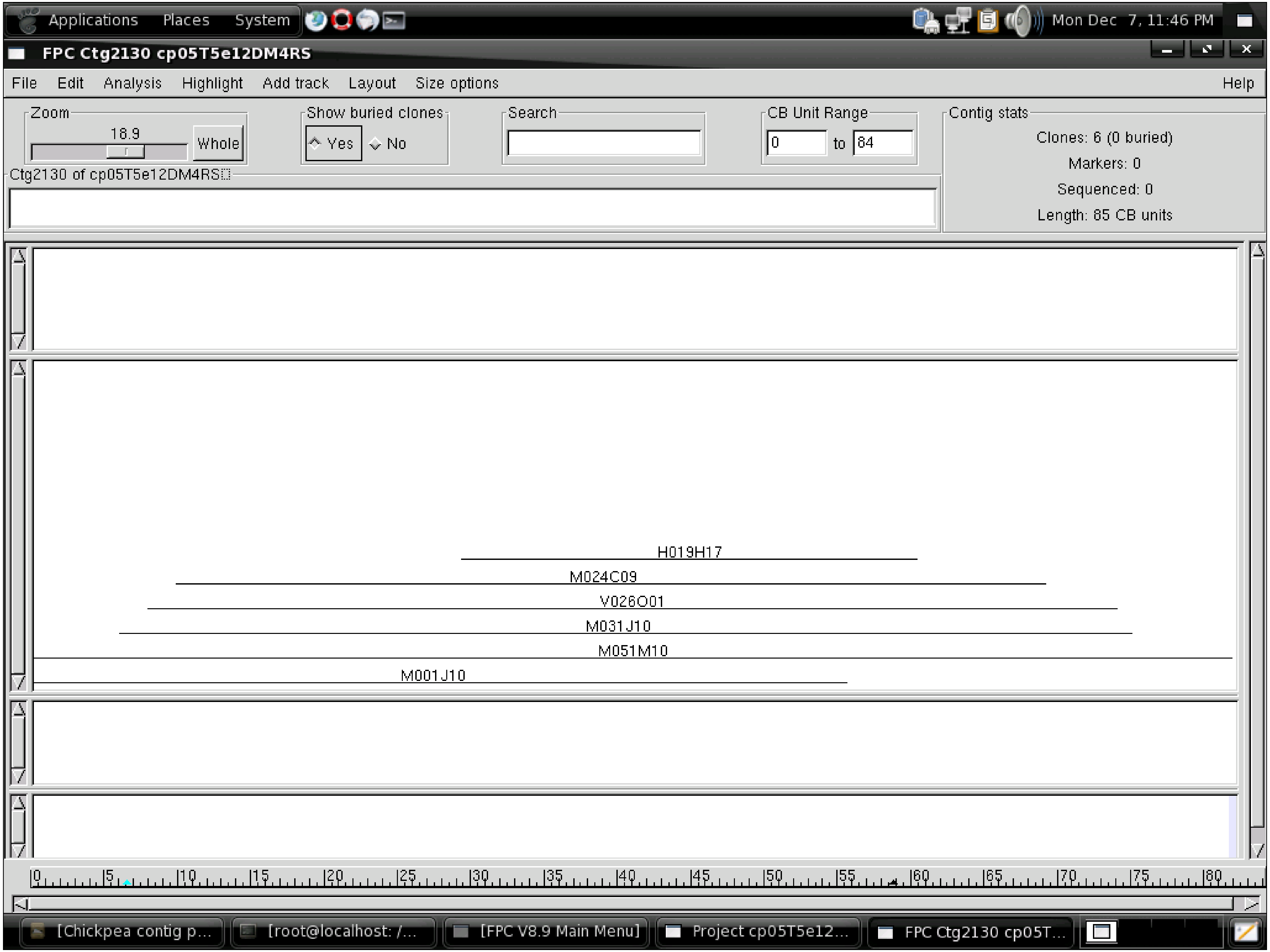

Applications Places System
Mon Dec 7, 11:46 PM
FPC Ctg2130 cp05T5e12DM4RS
File Edit Analysis Highlight Add track Layout Size options Help
Zoom
18.9
Whole
Show buried clones
Yes No
Search
CB Unit Range
0 to 84
Contig stats
Clones: 6 (0 buried)
Markers: 0
Sequenced: 0
Length: 85 CB units
Ctg2130 of cp05T5e12DM4RS
H019H17
M024C09
V026O01
M031J10
M051M10
M001J10
0 5 10 15 20 25 30 35 40 45 50 55 60 65 70 75 80
[Chickpea contig p...
[root@localhost: /...
[FPC V8.9 Main Menu]
Project cp05T5e12...
FPC Ctg2130 cp05T...

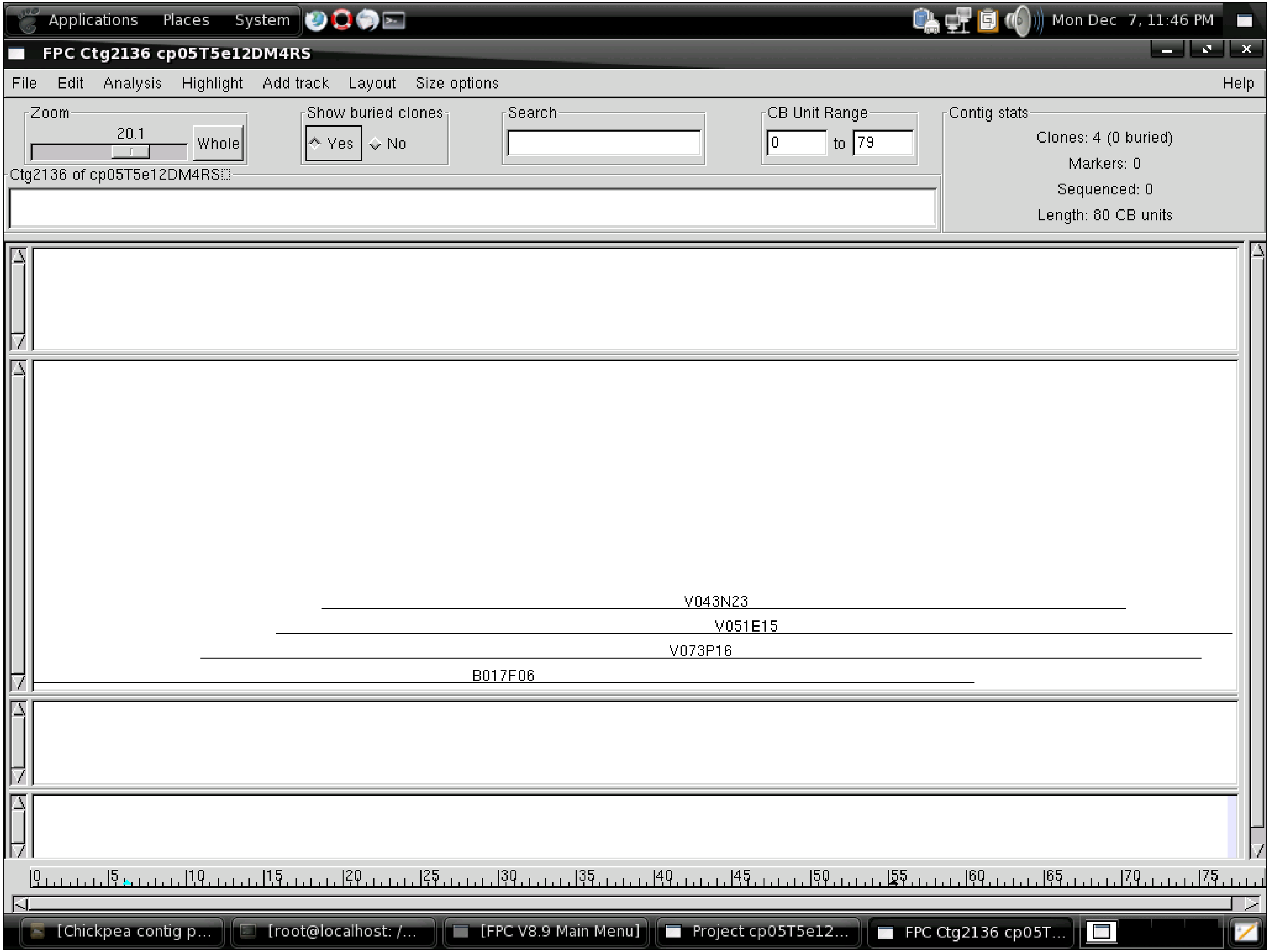
Applications Places System
Mon Dec 7, 11:46 PM
FPC Ctg2136 cp05T5e12DM4RS
File Edit Analysis Highlight Add track Layout Size options Help
Zoom
20.1
Whole
Show buried clones
Yes No
Search
CB Unit Range
0 to 79
Contig stats
Clones: 4 (0 buried)
Markers: 0
Sequenced: 0
Length: 80 CB units
Ctg2136 of cp05T5e12DM4RS
V043N23
V051E15
V073P16
B017F06
0 5 10 15 20 25 30 35 40 45 50 55 60 65 70 75
[Chickpea contig p...
[root@localhost: /...
[FPC V8.9 Main Menu]
Project cp05T5e12...
FPC Ctg2136 cp05T...

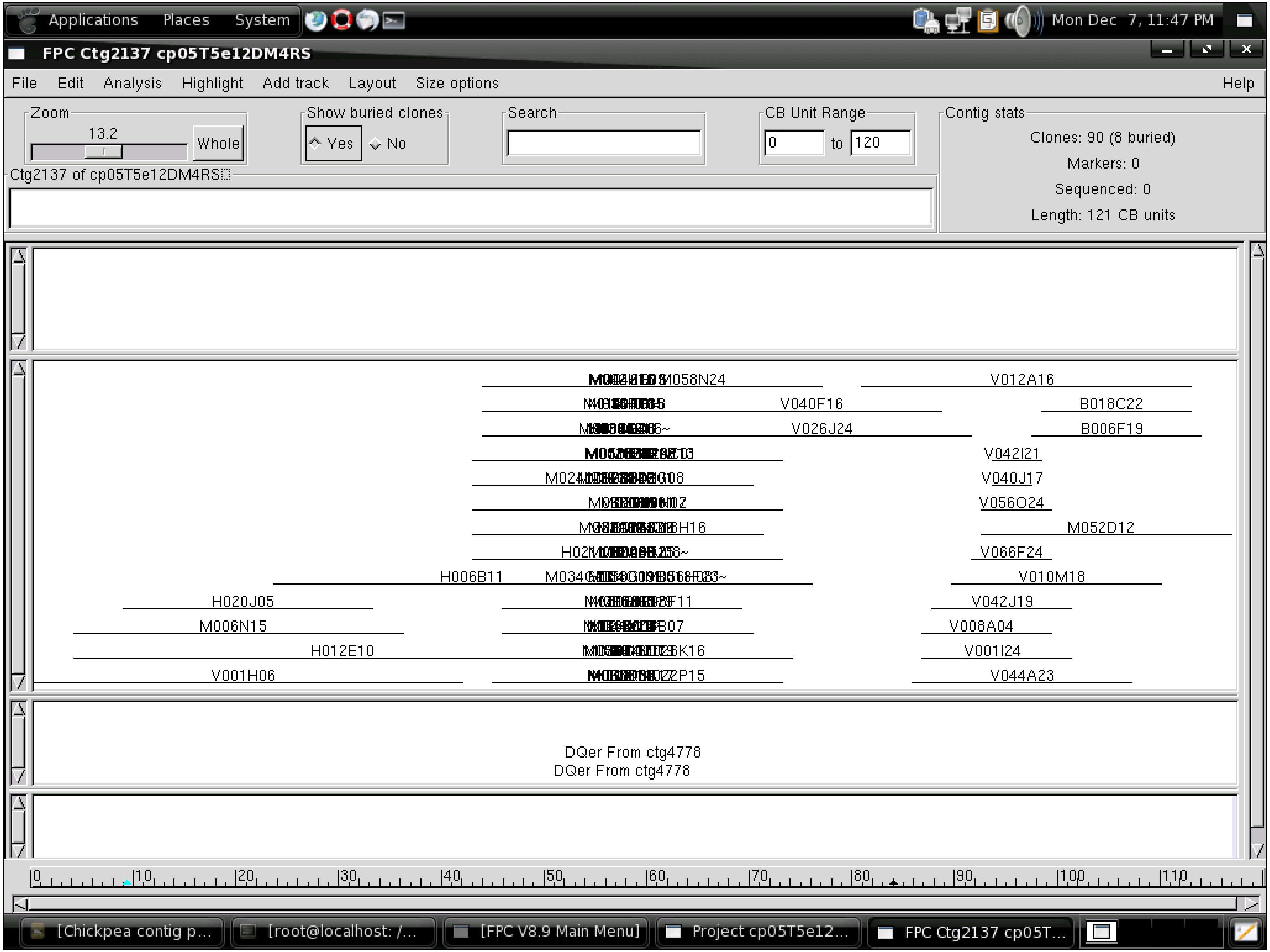
Applications Places System
Mon Dec 7, 11:47 PM
FPC Ctg2137 cp05T5e12DM4RS
File Edit Analysis Highlight Add track Layout Size options Help
Zoom
13.2
Whole
Show buried clones
Yes No
Search
CB Unit Range
0 to 120
Contig stats
Clones: 90 (8 buried)
Markers: 0
Sequenced: 0
Length: 121 CB units
Ctg2137 of cp05T5e12DM4RS
V012A16
V040F16
B018C22
V026J24
B006F19
V042I21
V040J17
V056O24
M052D12
V066F24
H006B11
V010M18
H020J05
V042J19
M006N15
V008A04
H012E10
V001I24
V001H06
V044A23
DQer From ctg4778
DQer From ctg4778
0 10 20 30 40 50 60 70 80 90 100 110
[Chickpea contig p...
[root@localhost: /...
[FPC V8.9 Main Menu]
Project cp05T5e12...
FPC Ctg2137 cp05T...

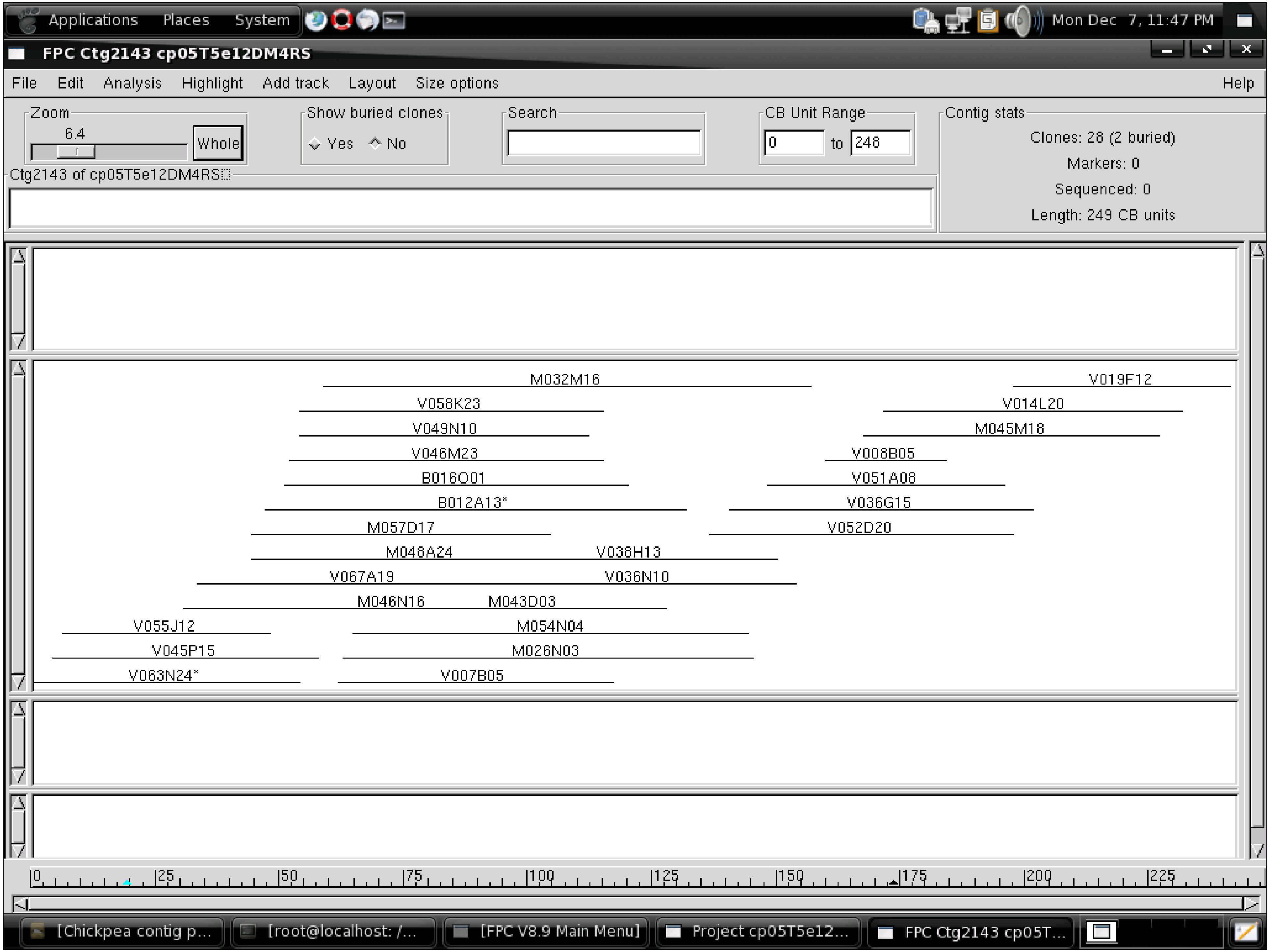
Applications Places System
Mon Dec 7, 11:47 PM
FPC Ctg2143 cp05T5e12DM4RS
File Edit Analysis Highlight Add track Layout Size options Help
Zoom 6.4 Whole
Show buried clones Yes No
Search
CB Unit Range 0 to 248
Contig stats
Clones: 28 (2 buried)
Markers: 0
Sequenced: 0
Length: 249 CB units
Ctg2143 of cp05T5e12DM4RS
M032M16
V019F12
V058K23
V014L20
V049N10
M045M18
V046M23
V008B05
B016O01
V051A08
B012A13*
V036G15
M057D17
V052D20
M048A24
V038H13
V067A19
V036N10
M046N16
M043D03
V055J12
M054N04
V045P15
M026N03
V063N24*
V007B05
0 25 50 75 100 125 150 175 200 225
[Chickpea contig p...
[root@localhost: /...
[FPC V8.9 Main Menu]
Project cp05T5e12...
FPC Ctg2143 cp05T...

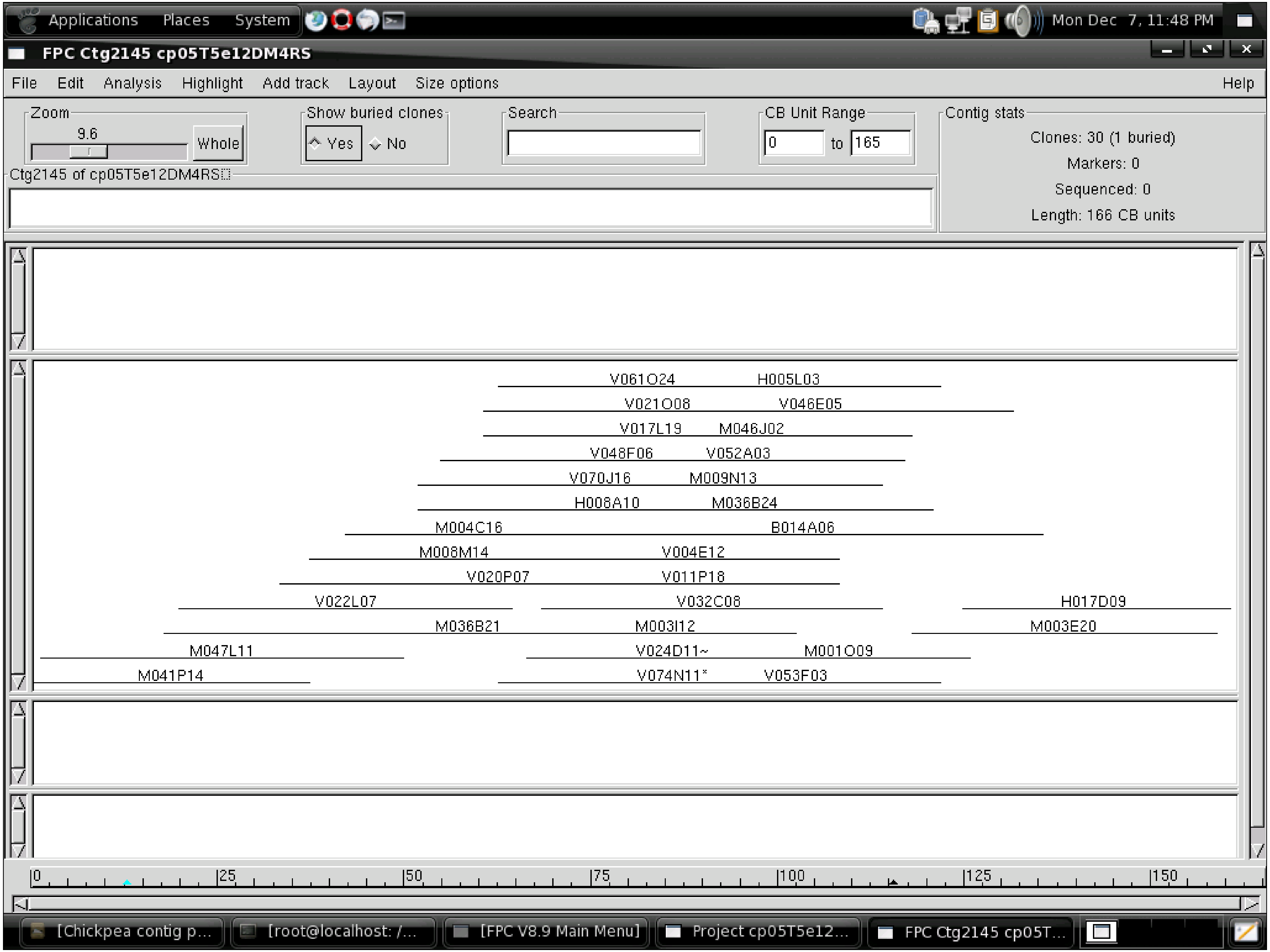
Applications Places System
Mon Dec 7, 11:48 PM
FPC Ctg2145 cp05T5e12DM4RS
File Edit Analysis Highlight Add track Layout Size options Help
Zoom
9.6
Whole
Show buried clones
Yes No
Search
CB Unit Range
0 to 165
Contig stats
Clones: 30 (1 buried)
Markers: 0
Sequenced: 0
Length: 166 CB units
Ctg2145 of cp05T5e12DM4RS
V061O24 H005L03
V021O08 V046E05
V017L19 M046J02
V048F06 V052A03
V070J16 M009N13
H008A10 M036B24
M004C16 B014A06
M008M14 V004E12
V020P07 V011P18
V022L07 V032C08 H017D09
M036B21 M003I12 M003E20
M047L11 V024D11~ M001O09
M041P14 V074N11* V053F03
0 25 50 75 100 125 150
[Chickpea contig p...
[root@localhost: /...
[FPC V8.9 Main Menu]
Project cp05T5e12...
FPC Ctg2145 cp05T...

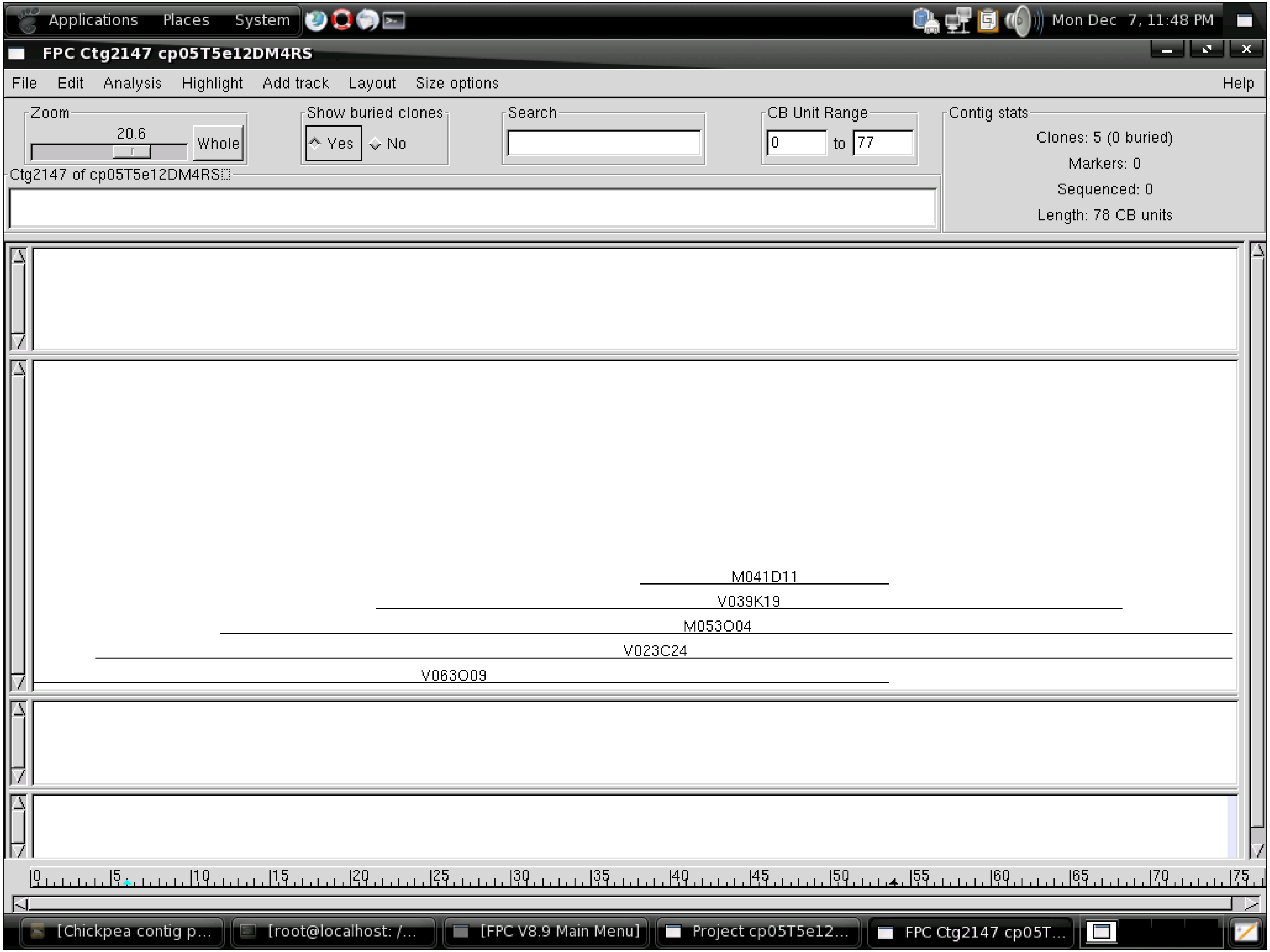
Applications Places System
Mon Dec 7, 11:48 PM
FPC Ctg2147 cp05T5e12DM4RS
File Edit Analysis Highlight Add track Layout Size options
Help
Zoom
20.6
Whole
Show buried clones
Yes
No
Search
CB Unit Range
0
to
77
Contig stats
Clones: 5 (0 buried)
Markers: 0
Sequenced: 0
Length: 78 CB units
Ctg2147 of cp05T5e12DM4RS
M041D11
V039K19
M053O04
V023C24
V063O09
0 5 10 15 20 25 30 35 40 45 50 55 60 65 70 75
[Chickpea contig p...
[root@localhost: /...
[FPC V8.9 Main Menu]
Project cp05T5e12...
FPC Ctg2147 cp05T...

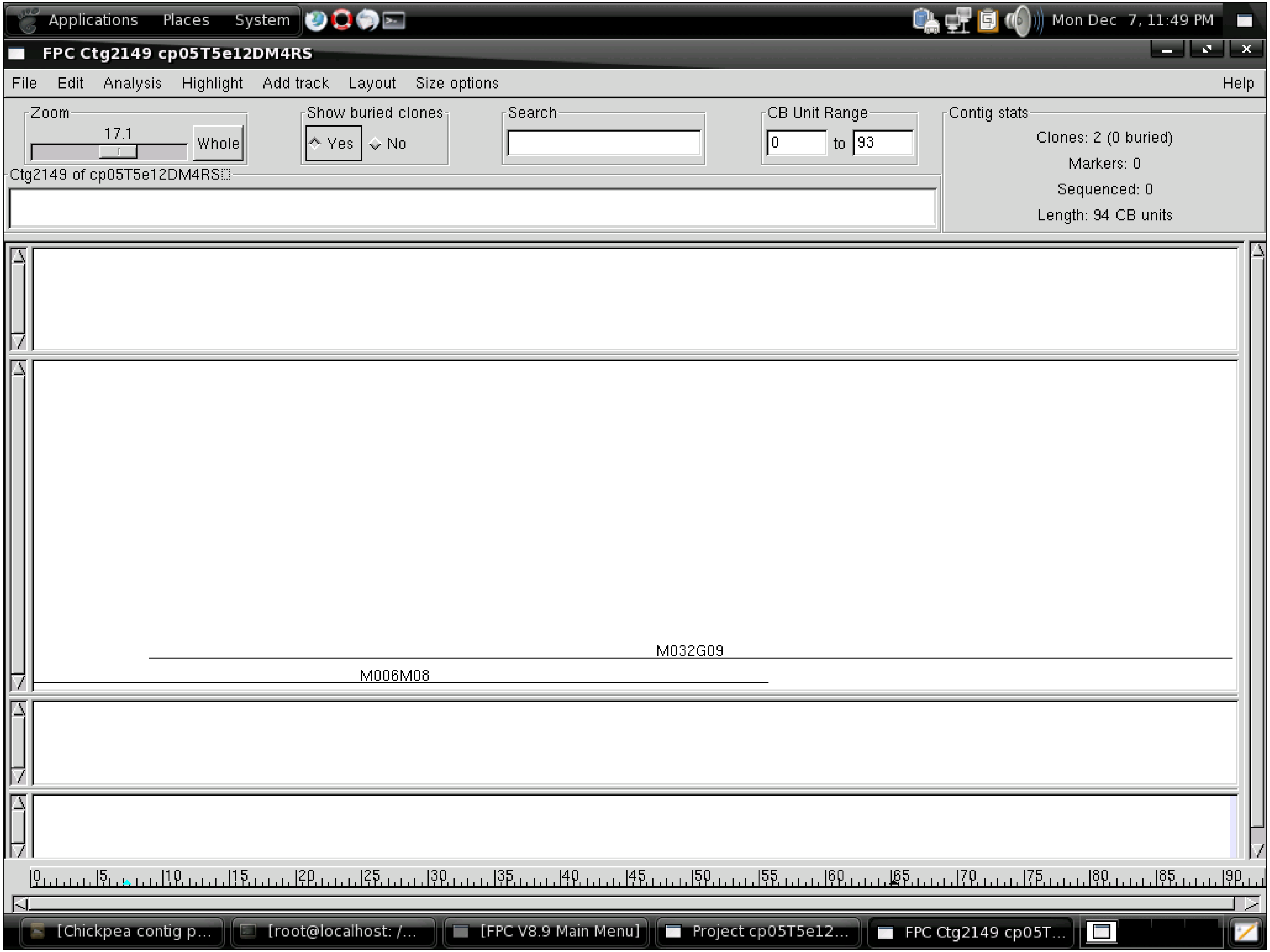
Applications Places System
Mon Dec 7, 11:49 PM
FPC Ctg2149 cp05T5e12DM4RS
File Edit Analysis Highlight Add track Layout Size options Help
Zoom
17.1
Whole
Show buried clones
Yes
No
Search
CB Unit Range
0 to 93
Contig stats
Clones: 2 (0 buried)
Markers: 0
Sequenced: 0
Length: 94 CB units
Ctg2149 of cp05T5e12DM4RS
M032G09
M006M08
0 5 10 15 20 25 30 35 40 45 50 55 60 65 70 75 80 85 90
[Chickpea contig p...
[root@localhost: /...
[FPC V8.9 Main Menu]
Project cp05T5e12...
FPC Ctg2149 cp05T...

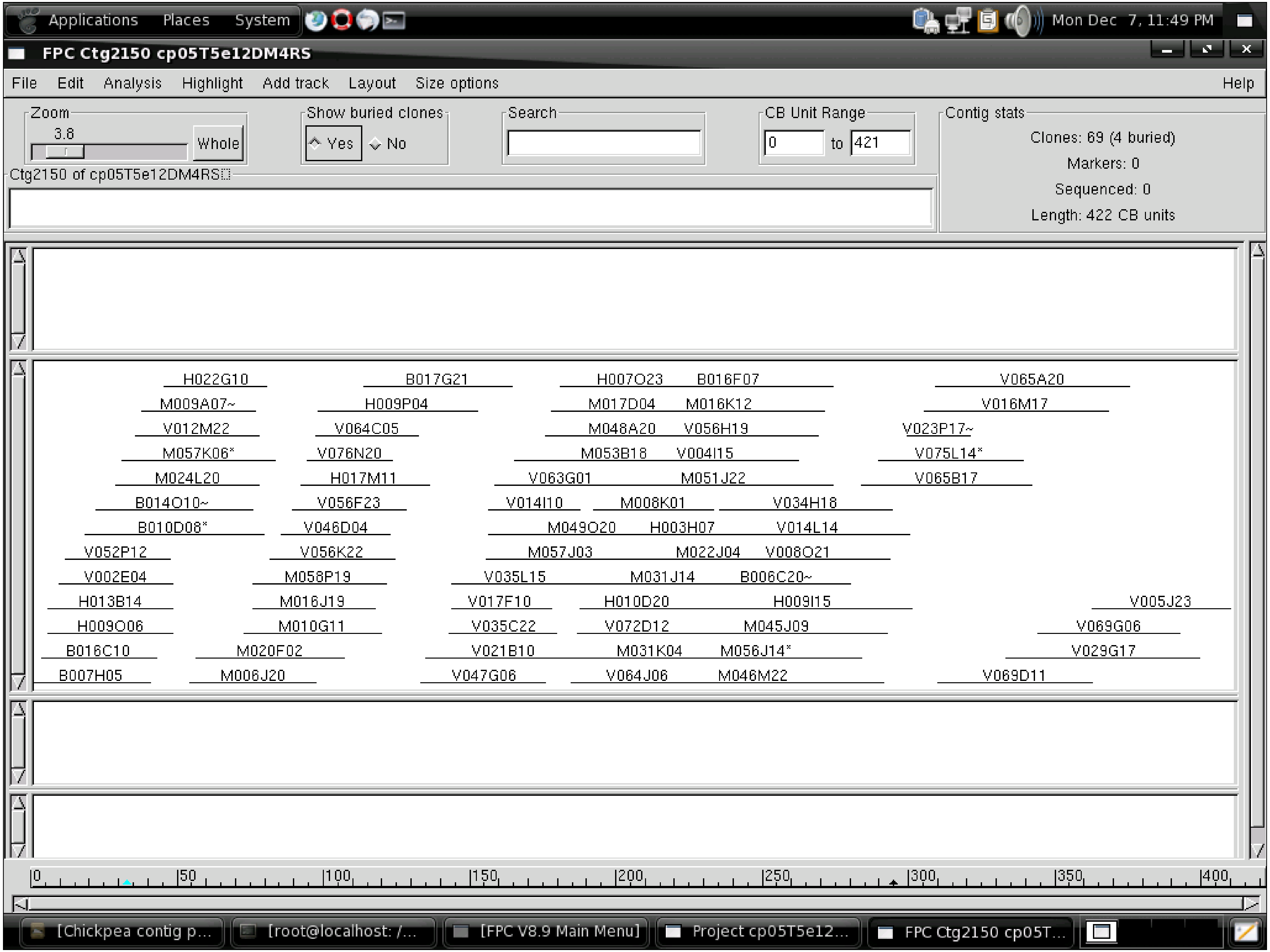
Applications Places System
Mon Dec 7, 11:49 PM
FPC Ctg2150 cp05T5e12DM4RS
File Edit Analysis Highlight Add track Layout Size options Help
Zoom
3.8
Whole
Show buried clones
Yes No
Search
CB Unit Range
0 to 421
Contig stats
Clones: 69 (4 buried)
Markers: 0
Sequenced: 0
Length: 422 CB units
Ctg2150 of cp05T5e12DM4RS
H022G10 B017G21 H007O23 B016F07 V065A20
M009A07~ H009P04 M017D04 M016K12 V016M17
V012M22 V064C05 M048A20 V056H19 V023P17~
M057K06* V076N20 M053B18 V004I15 V075L14*
M024L20 H017M11 V063G01 M051J22 V065B17
B014O10~ V056F23 V014I10 M008K01 V034H18
B010D08* V046D04 M049O20 H003H07 V014L14
V052P12 V056K22 M057J03 M022J04 V008O21
V002E04 M058P19 V035L15 M031J14 B006C20~
H013B14 M016J19 V017F10 H010D20 H009I15 V005J23
H009O06 M010G11 V035C22 V072D12 M045J09 V069G06
B016C10 M020F02 V021B10 M031K04 M056J14* V029G17
B007H05 M006J20 V047G06 V064J06 M046M22 V069D11
0 50 100 150 200 250 300 350 400
[Chickpea contig p...
[root@localhost: /...
[FPC V8.9 Main Menu]
Project cp05T5e12...
FPC Ctg2150 cp05T...

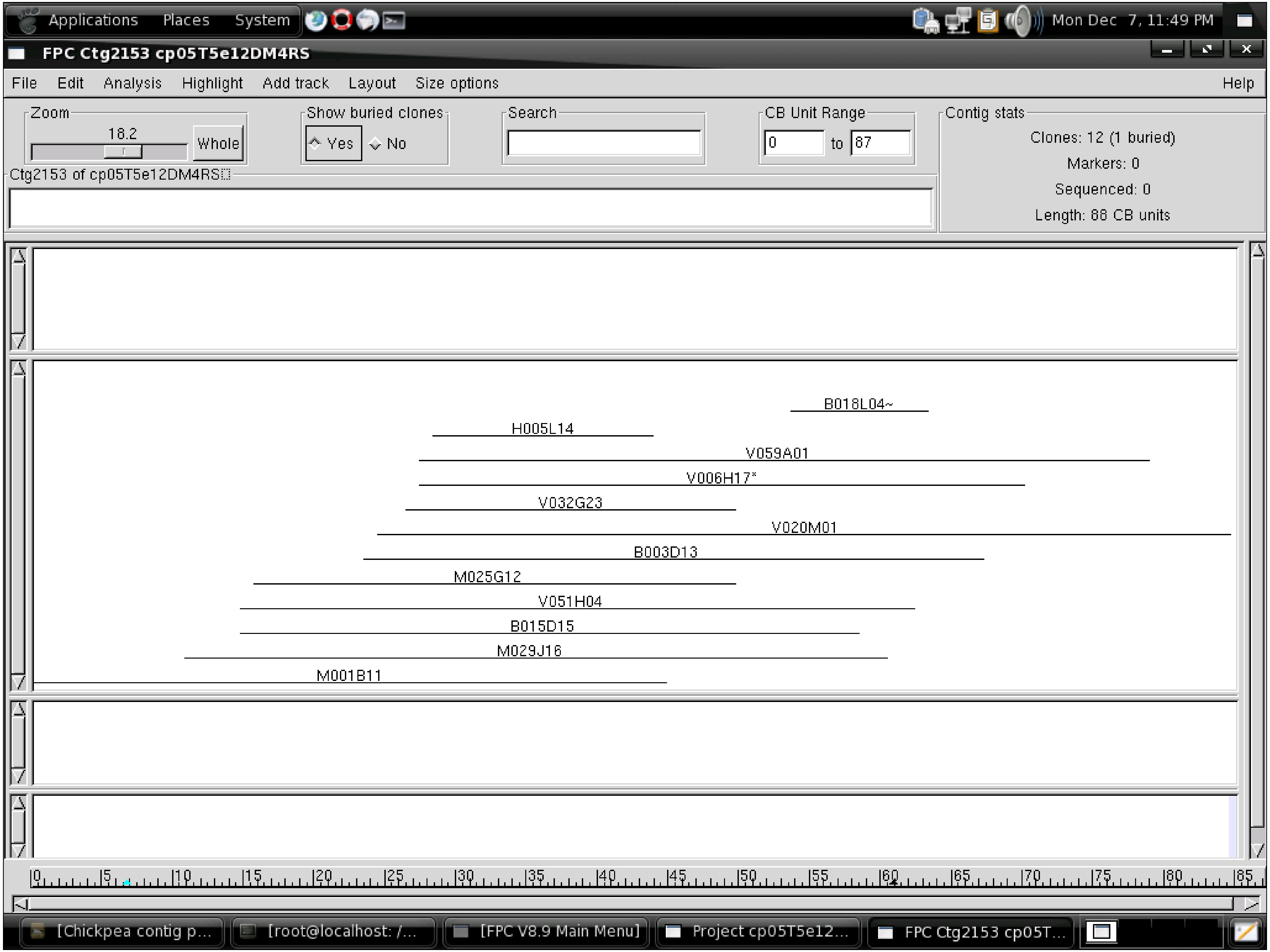
Applications Places System
Mon Dec 7, 11:49 PM
FPC Ctg2153 cp05T5e12DM4RS
File Edit Analysis Highlight Add track Layout Size options Help
Zoom
18.2
Whole
Show buried clones
Yes
No
Search
CB Unit Range
0 to 87
Contig stats
Clones: 12 (1 buried)
Markers: 0
Sequenced: 0
Length: 88 CB units
Ctg2153 of cp05T5e12DM4RS
B018L04~
H005L14
V059A01
V006H17*
V032G23
V020M01
B003D13
M025G12
V051H04
B015D15
M029J16
M001B11
0 5 10 15 20 25 30 35 40 45 50 55 60 65 70 75 80 85
[Chickpea contig p...
[root@localhost: /...
[FPC V8.9 Main Menu]
Project cp05T5e12...
FPC Ctg2153 cp05T...

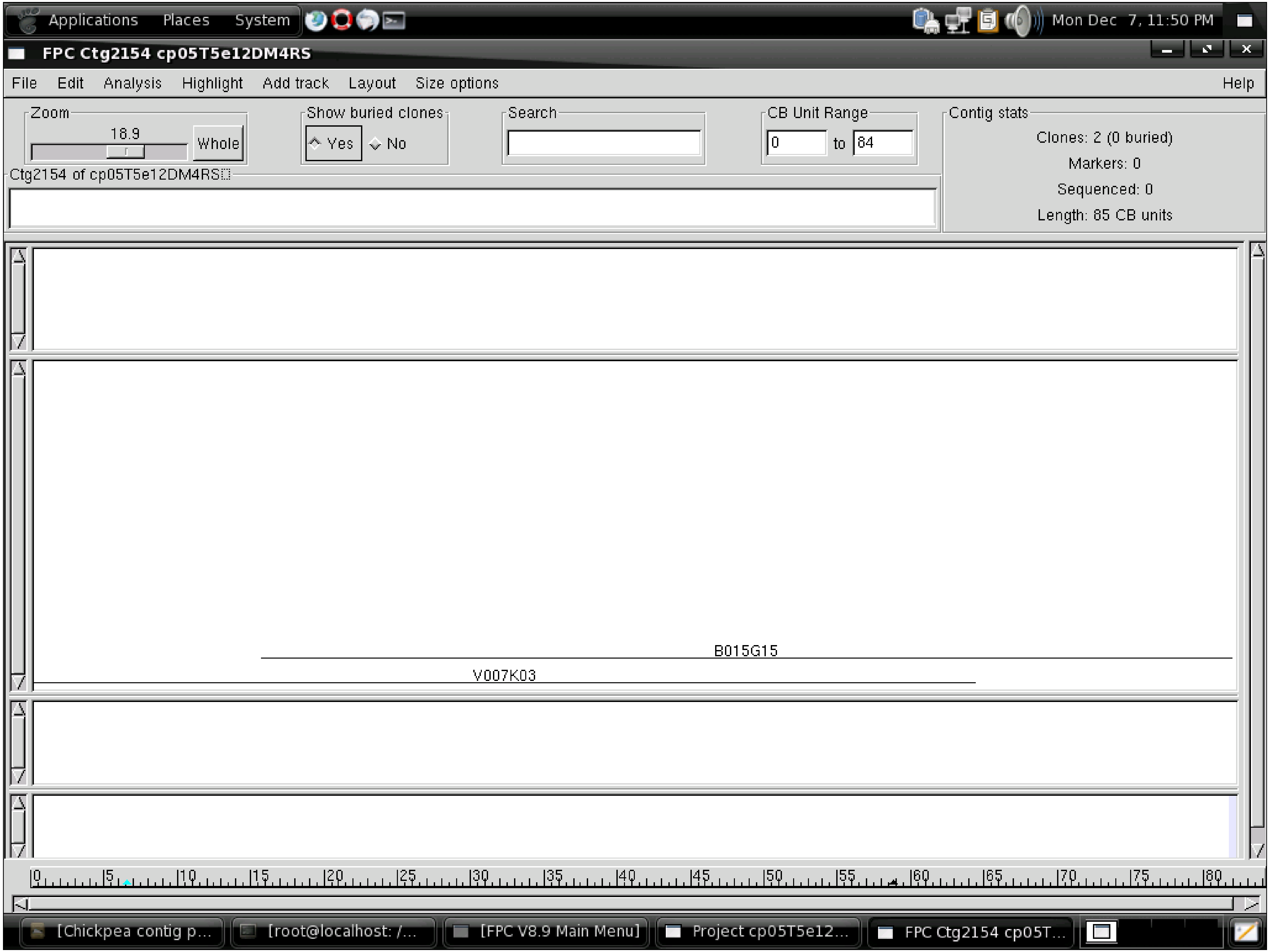
Applications Places System
Mon Dec 7, 11:50 PM
FPC Ctg2154 cp05T5e12DM4RS
File Edit Analysis Highlight Add track Layout Size options Help
Zoom
18.9
Whole
Show buried clones
Yes
No
Search
CB Unit Range
0
to
84
Contig stats
Clones: 2 (0 buried)
Markers: 0
Sequenced: 0
Length: 85 CB units
Ctg2154 of cp05T5e12DM4RS
B015G15
V007K03
0 5 10 15 20 25 30 35 40 45 50 55 60 65 70 75 80
[Chickpea contig p...
[root@localhost: /...
[FPC V8.9 Main Menu]
Project cp05T5e12...
FPC Ctg2154 cp05T...

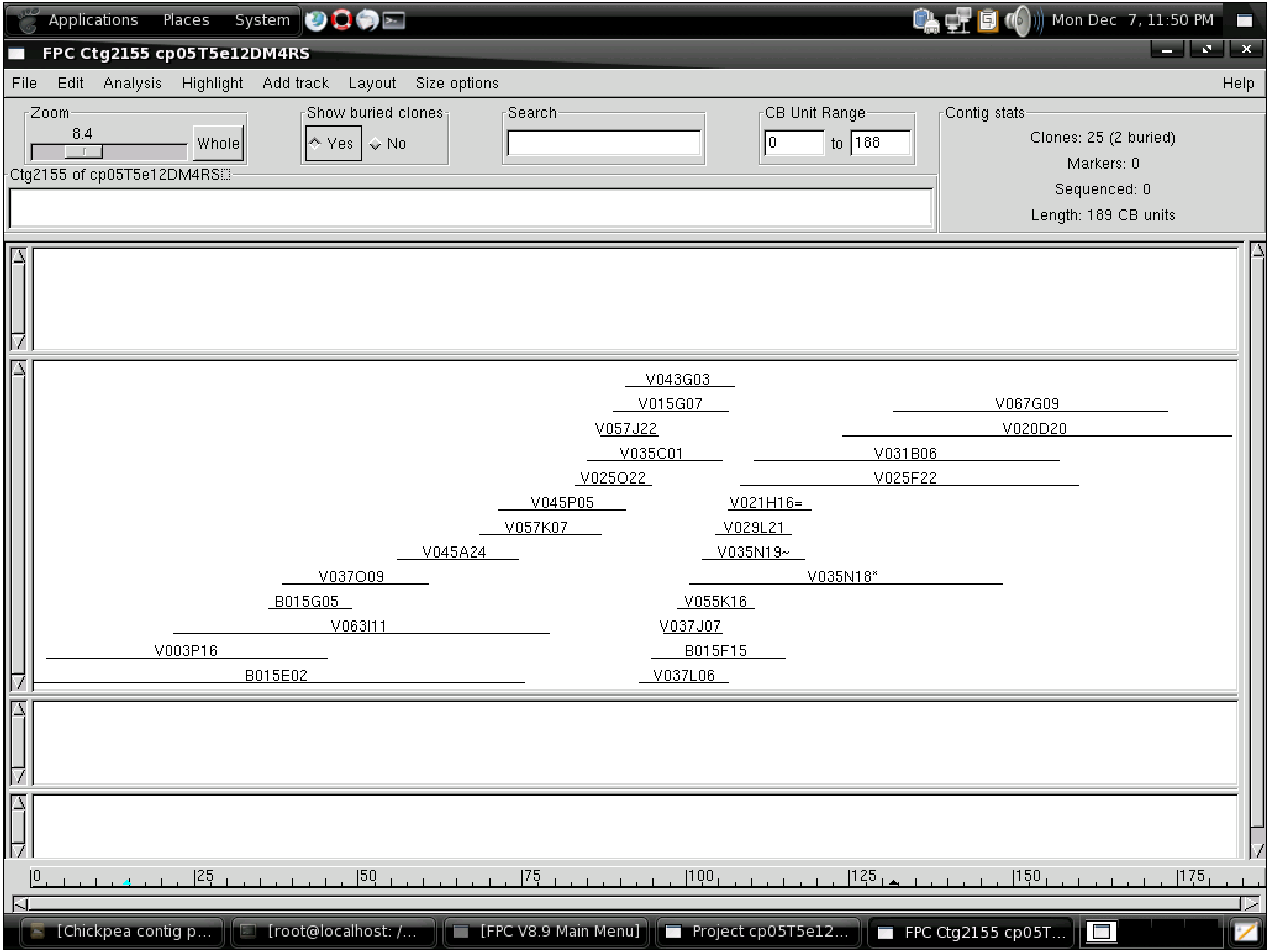
Applications Places System
Mon Dec 7, 11:50 PM
FPC Ctg2155 cp05T5e12DM4RS
File Edit Analysis Highlight Add track Layout Size options Help
Zoom
8.4
Whole
Show buried clones
Yes
No
Search
CB Unit Range
0 to 188
Contig stats
Clones: 25 (2 buried)
Markers: 0
Sequenced: 0
Length: 189 CB units
Ctg2155 of cp05T5e12DM4RS
V043G03
V015G07
V067G09
V057J22
V020D20
V035C01
V031B06
V025O22
V025F22
V045P05
V021H16=
V057K07
V029L21
V045A24
V035N19~
V037O09
V035N18*
B015G05
V055K16
V063I11
V037J07
V003P16
B015F15
B015E02
V037L06
0 25 50 75 100 125 150 175
[Chickpea contig p...
[root@localhost: /...
[FPC V8.9 Main Menu]
Project cp05T5e12...
FPC Ctg2155 cp05T...

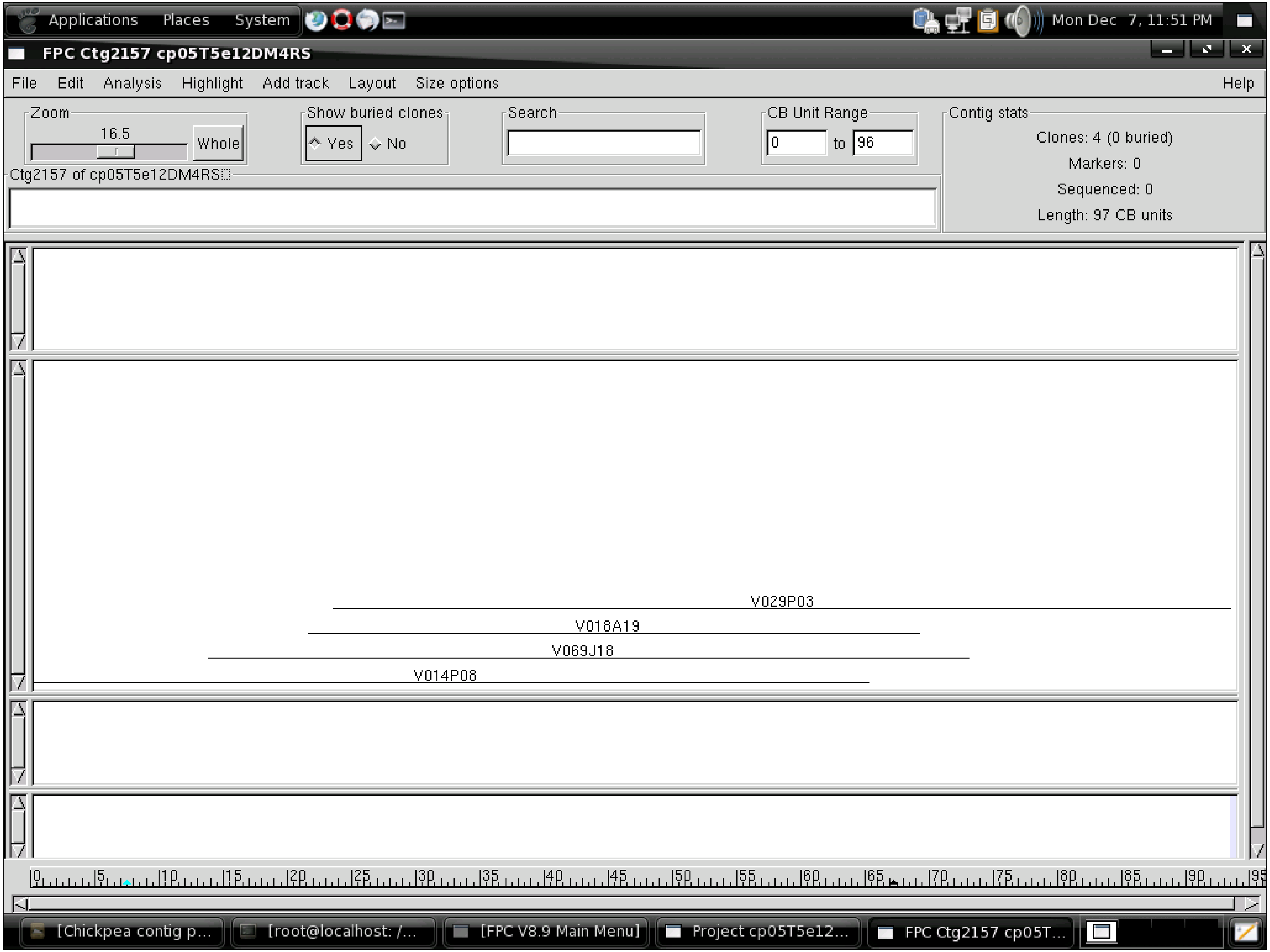
Applications Places System
Mon Dec 7, 11:51 PM
FPC Ctg2157 cp05T5e12DM4RS
File Edit Analysis Highlight Add track Layout Size options Help
Zoom
16.5
Whole
Show buried clones
Yes No
Search
CB Unit Range
0 to 96
Contig stats
Clones: 4 (0 buried)
Markers: 0
Sequenced: 0
Length: 97 CB units
Ctg2157 of cp05T5e12DM4RS
V029P03
V018A19
V069J18
V014P08
0 5 10 15 20 25 30 35 40 45 50 55 60 65 70 75 80 85 90 95
[Chickpea contig p...
[root@localhost: /...
[FPC V8.9 Main Menu]
Project cp05T5e12...
FPC Ctg2157 cp05T...

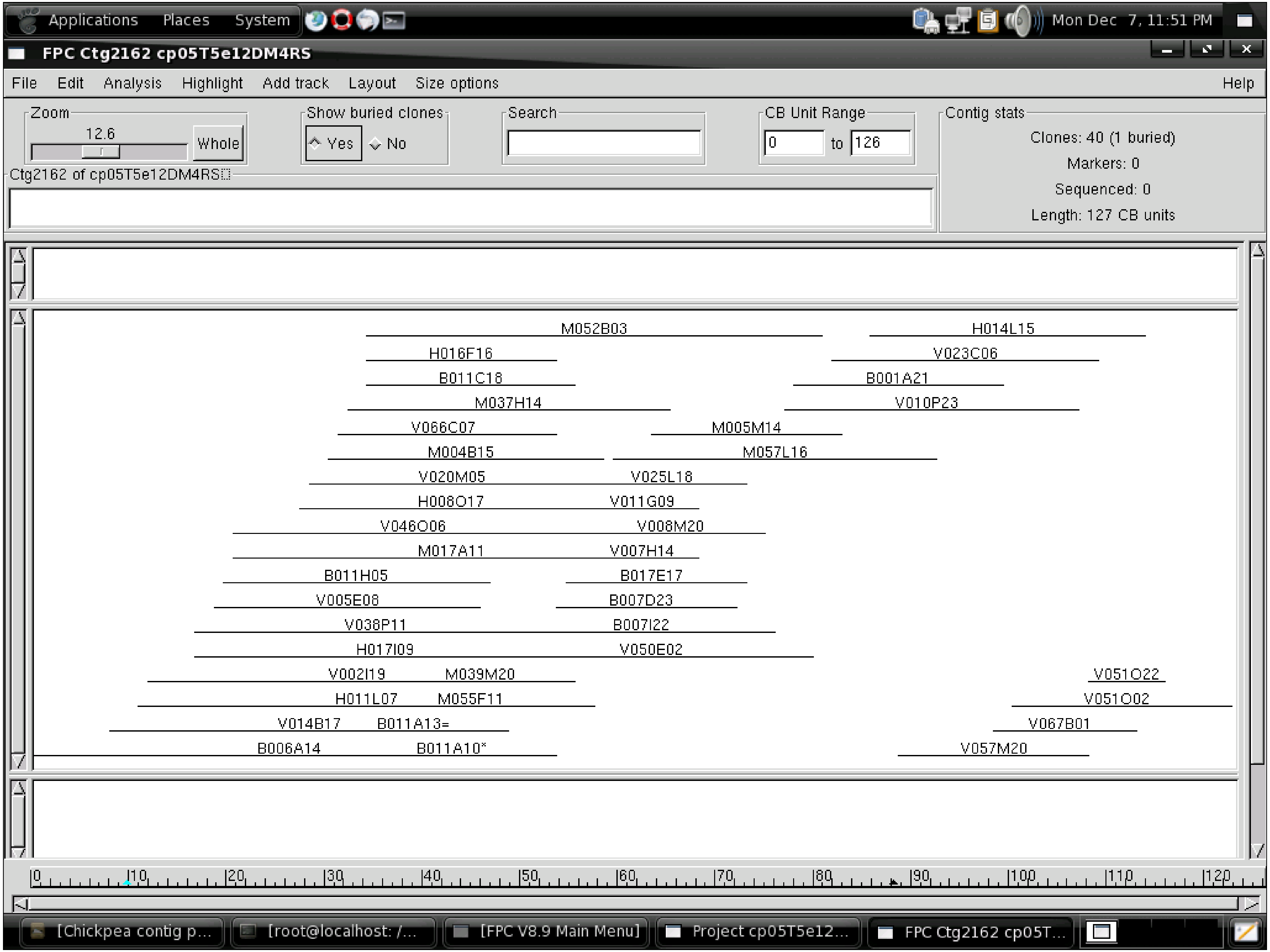
Applications Places System
Mon Dec 7, 11:51 PM
FPC Ctg2162 cp05T5e12DM4RS
File Edit Analysis Highlight Add track Layout Size options Help
Zoom
12.6
Whole
Show buried clones
Yes
No
Search
CB Unit Range
0
to
126
Contig stats
Clones: 40 (1 buried)
Markers: 0
Sequenced: 0
Length: 127 CB units
Ctg2162 of cp05T5e12DM4RS
M052B03
H014L15
H016F16
V023C06
B011C18
B001A21
M037H14
V010P23
V066C07
M005M14
M004B15
M057L16
V020M05
V025L18
H008O17
V011G09
V046O06
V008M20
M017A11
V007H14
B011H05
B017E17
V005E08
B007D23
V038P11
B007I22
H017I09
V050E02
V002I19
M039M20
V051O22
H011L07
M055F11
V051O02
V014B17
B011A13=
V067B01
B006A14
B011A10*
V057M20
0 10 20 30 40 50 60 70 80 90 100 110 120
[Chickpea contig p...
[root@localhost: /...
[FPC V8.9 Main Menu]
Project cp05T5e12...
FPC Ctg2162 cp05T...

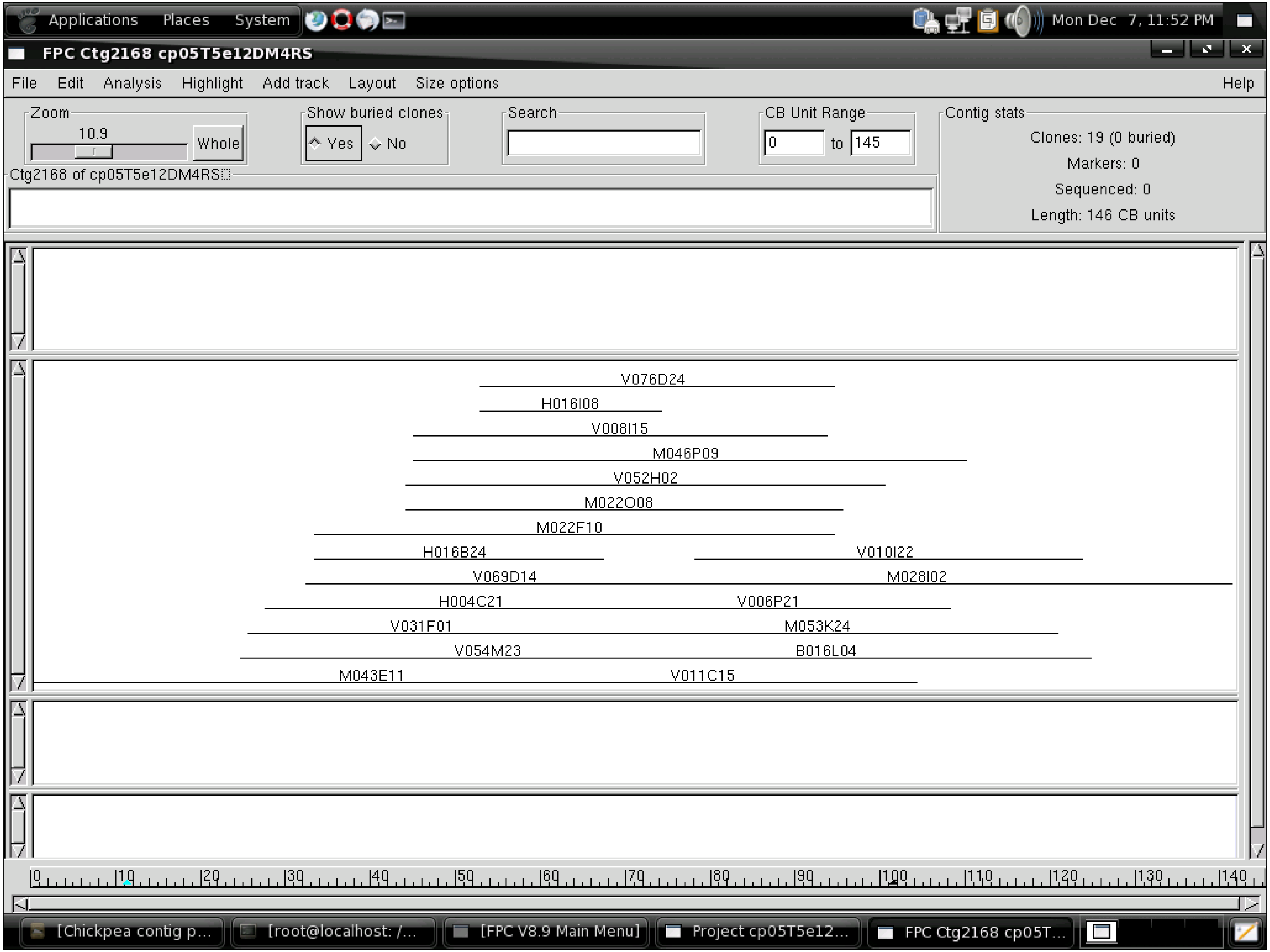
Applications Places System
Mon Dec 7, 11:52 PM
FPC Ctg2168 cp05T5e12DM4RS
File Edit Analysis Highlight Add track Layout Size options Help
Zoom
10.9
Whole
Show buried clones
Yes No
Search
CB Unit Range
0 to 145
Contig stats
Clones: 19 (0 buried)
Markers: 0
Sequenced: 0
Length: 146 CB units
Ctg2168 of cp05T5e12DM4RS
V076D24
H016I08
V008I15
M046P09
V052H02
M022O08
M022F10
H016B24
V010I22
V069D14
M028I02
H004C21
V006P21
V031F01
M053K24
V054M23
B016L04
M043E11
V011C15
0 10 20 30 40 50 60 70 80 90 100 110 120 130 140
[Chickpea contig p...
[root@localhost: /...
[FPC V8.9 Main Menu]
Project cp05T5e12...
FPC Ctg2168 cp05T...

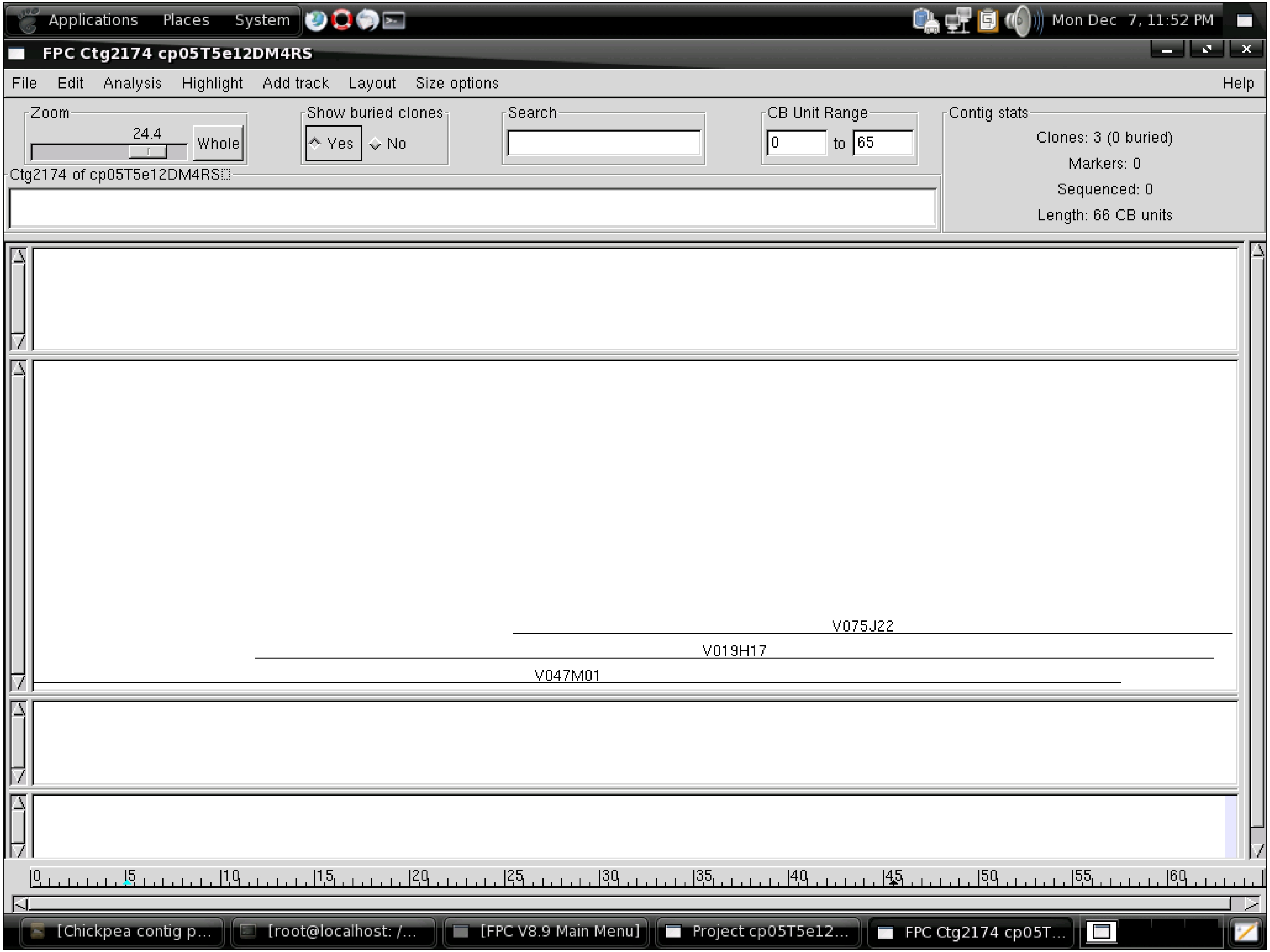
Applications Places System
Mon Dec 7, 11:52 PM
FPC Ctg2174 cp05T5e12DM4RS
File Edit Analysis Highlight Add track Layout Size options Help
Zoom
24.4
Whole
Show buried clones
Yes No
Search
CB Unit Range
0 to 65
Contig stats
Clones: 3 (0 buried)
Markers: 0
Sequenced: 0
Length: 66 CB units
Ctg2174 of cp05T5e12DM4RS
V075J22
V019H17
V047M01
0 5 10 15 20 25 30 35 40 45 50 55 60
[Chickpea contig p...
[root@localhost: /...
[FPC V8.9 Main Menu]
Project cp05T5e12...
FPC Ctg2174 cp05T...

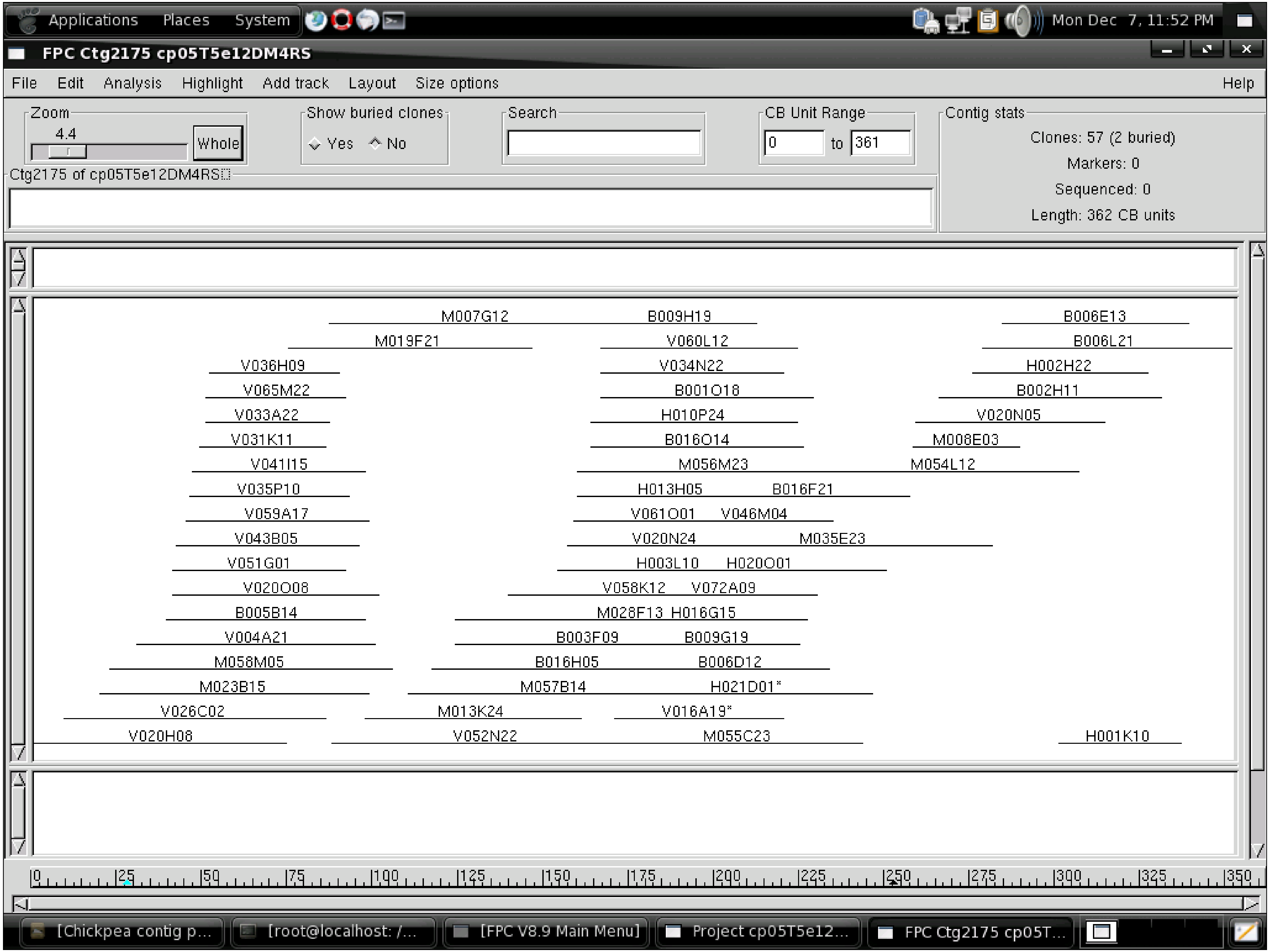
Applications Places System
Mon Dec 7, 11:52 PM
FPC Ctg2175 cp05T5e12DM4RS
File Edit Analysis Highlight Add track Layout Size options Help
Zoom
4.4
Whole
Show buried clones
Yes No
Search
CB Unit Range
0 to 361
Contig stats
Clones: 57 (2 buried)
Markers: 0
Sequenced: 0
Length: 362 CB units
Ctg2175 of cp05T5e12DM4RS
M007G12
B009H19
B006E13
M019F21
V060L12
B006L21
V036H09
V034N22
H002H22
V065M22
B001O18
B002H11
V033A22
H010P24
V020N05
V031K11
B016O14
M008E03
V041I15
M056M23
M054L12
V035P10
H013H05
B016F21
V059A17
V061O01
V046M04
V043B05
V020N24
M035E23
V051G01
H003L10
H020O01
V020O08
V058K12
V072A09
B005B14
M028F13
H016G15
V004A21
B003F09
B009G19
M058M05
B016H05
B006D12
M023B15
M057B14
H021D01*
V026C02
M013K24
V016A19*
V020H08
V052N22
M055C23
H001K10
0 25 50 75 100 125 150 175 200 225 250 275 300 325 350
[Chickpea contig p...
[root@localhost: /...
[FPC V8.9 Main Menu]
Project cp05T5e12...
FPC Ctg2175 cp05T...

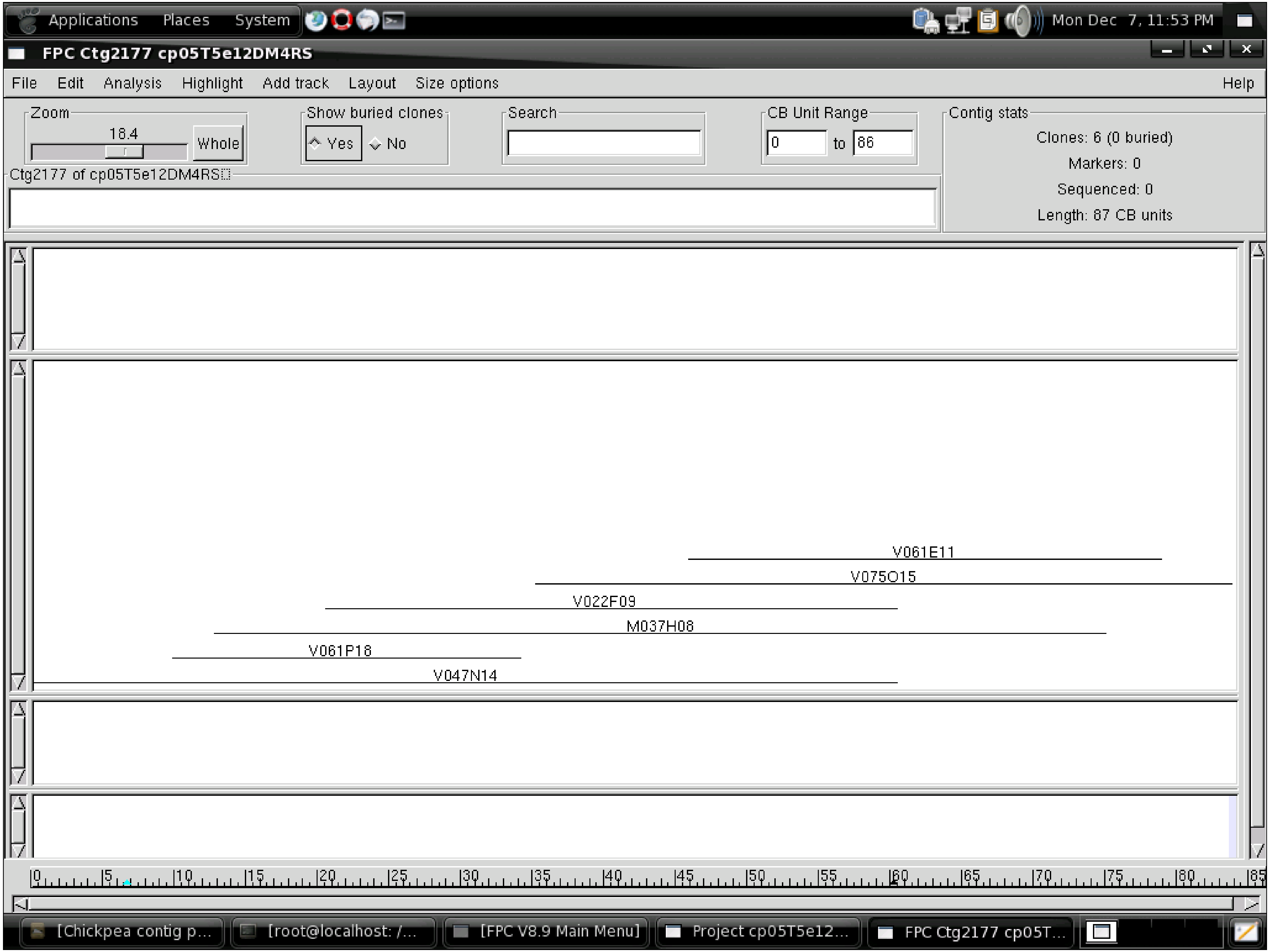
Applications Places System
Mon Dec 7, 11:53 PM
FPC Ctg2177 cp05T5e12DM4RS
File Edit Analysis Highlight Add track Layout Size options Help
Zoom
18.4
Whole
Show buried clones
Yes No
Search
CB Unit Range
0 to 86
Contig stats
Clones: 6 (0 buried)
Markers: 0
Sequenced: 0
Length: 87 CB units
Ctg2177 of cp05T5e12DM4RS
V061E11
V075O15
V022F09
M037H08
V061P18
V047N14
0 5 10 15 20 25 30 35 40 45 50 55 60 65 70 75 80 85
[Chickpea contig p...
[root@localhost: /...
[FPC V8.9 Main Menu]
Project cp05T5e12...
FPC Ctg2177 cp05T...

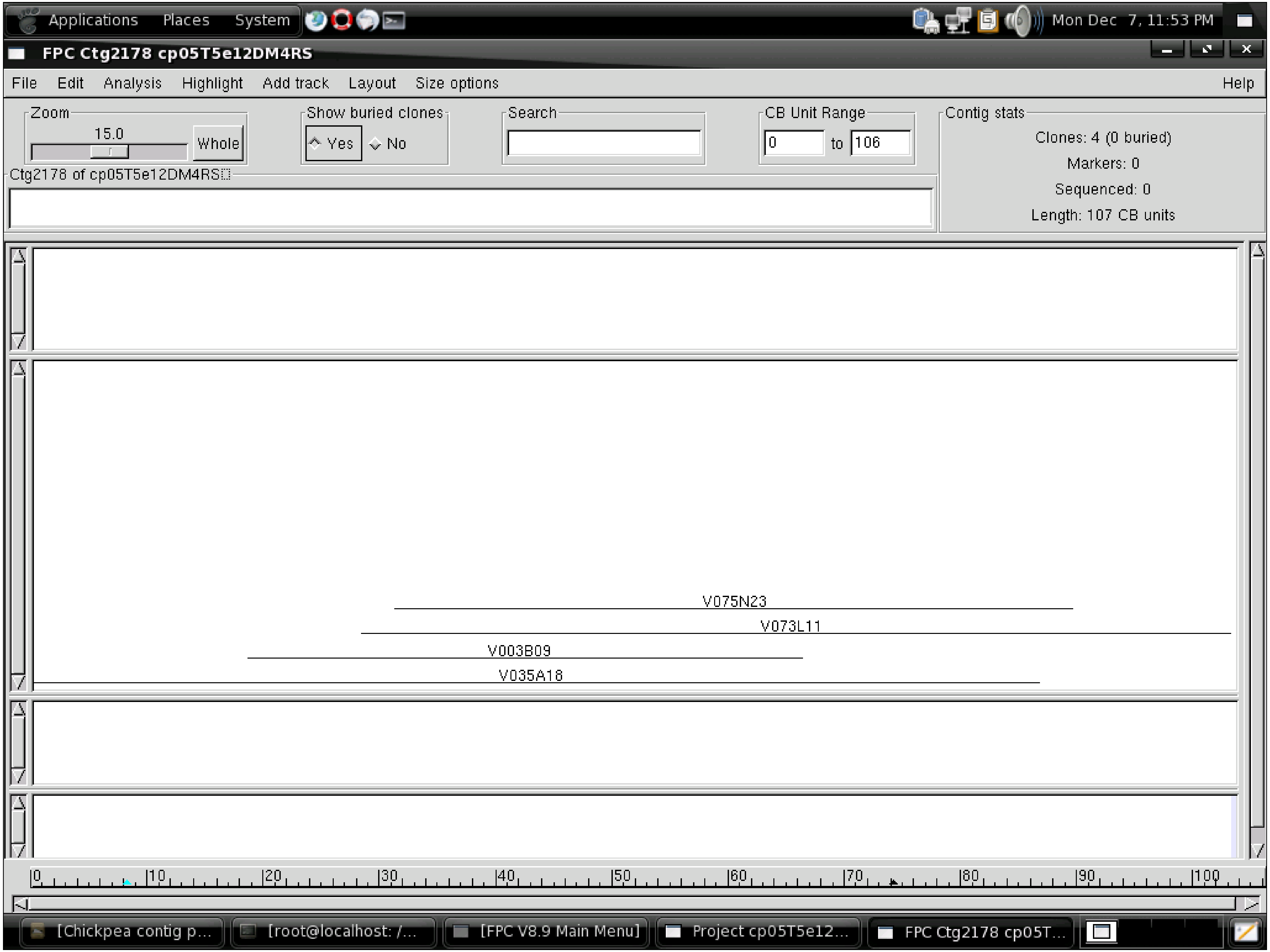
Applications Places System
Mon Dec 7, 11:53 PM
FPC Ctg2178 cp05T5e12DM4RS
File Edit Analysis Highlight Add track Layout Size options
Help
Zoom
15.0
Whole
Show buried clones
Yes
No
Search
CB Unit Range
0
to
106
Contig stats
Clones: 4 (0 buried)
Markers: 0
Sequenced: 0
Length: 107 CB units
Ctg2178 of cp05T5e12DM4RS
V075N23
V073L11
V003B09
V035A18
0 10 20 30 40 50 60 70 80 90 100
[Chickpea contig p...
[root@localhost: /...
[FPC V8.9 Main Menu]
Project cp05T5e12...
FPC Ctg2178 cp05T...

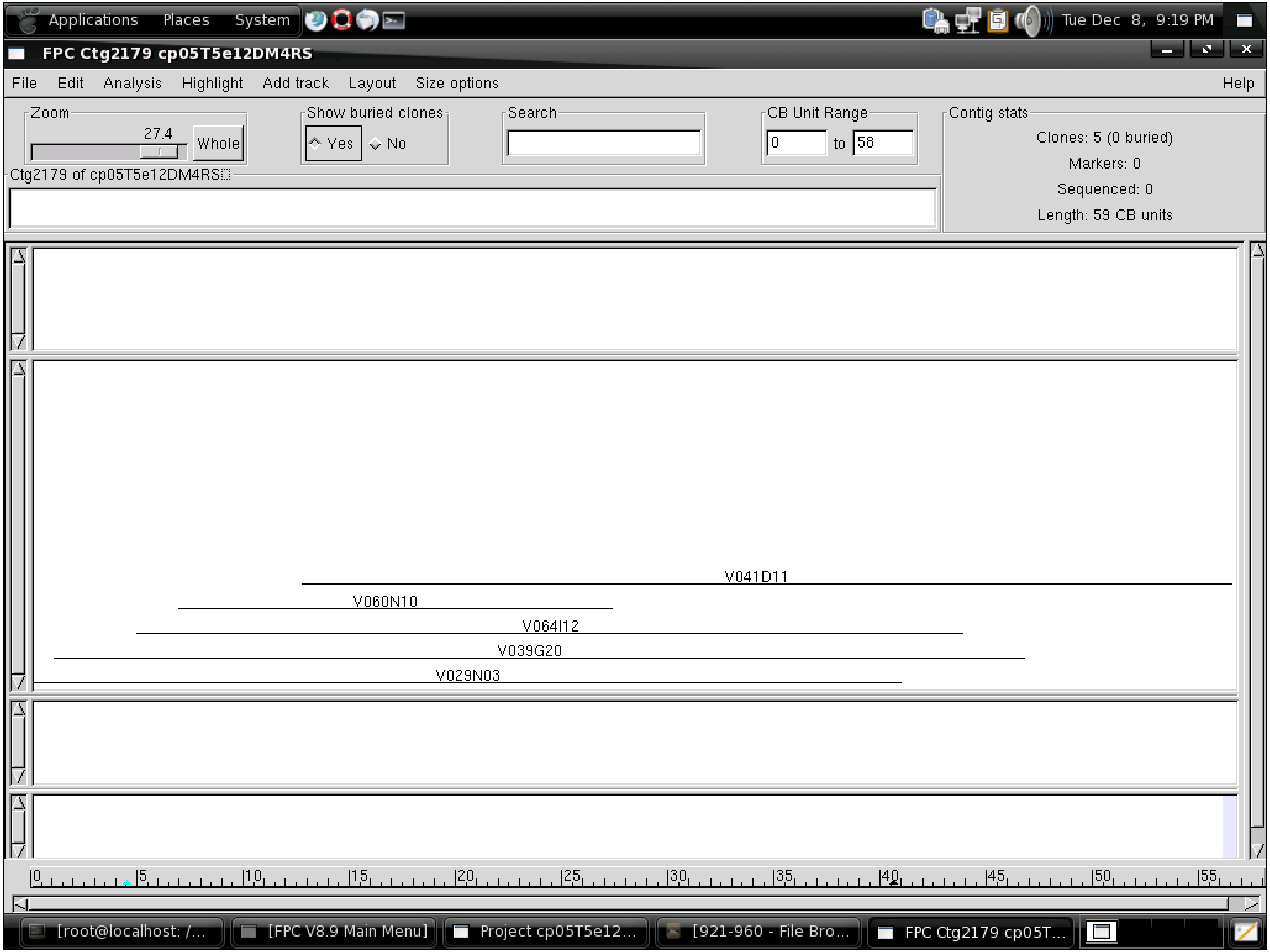
Applications Places System
Tue Dec 8, 9:19 PM
FPC Ctg2179 cp05T5e12DM4RS
File Edit Analysis Highlight Add track Layout Size options Help
Zoom
27.4
Whole
Show buried clones
Yes No
Search
CB Unit Range
0 to 58
Contig stats
Clones: 5 (0 buried)
Markers: 0
Sequenced: 0
Length: 59 CB units
Ctg2179 of cp05T5e12DM4RS
V041D11
V060N10
V064I12
V039G20
V029N03
0 5 10 15 20 25 30 35 40 45 50 55
[root@localhost: /...
[FPC V8.9 Main Menu]
Project cp05T5e12...
[921-960 - File Bro...
FPC Ctg2179 cp05T...

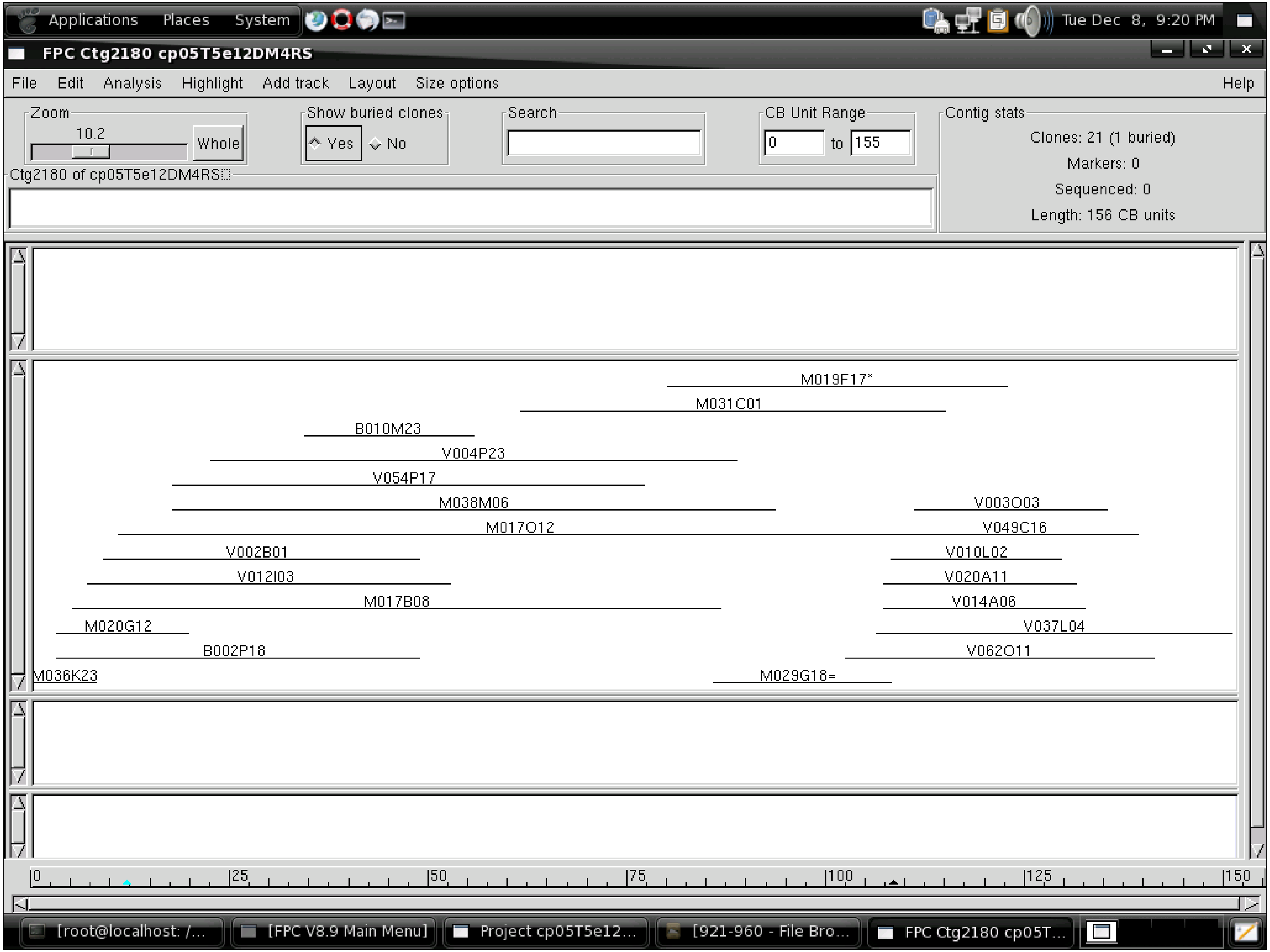
Applications Places System
Tue Dec 8, 9:20 PM
FPC Ctg2180 cp05T5e12DM4RS
File Edit Analysis Highlight Add track Layout Size options Help
Zoom
10.2
Whole
Show buried clones
Yes No
Search
CB Unit Range
0 to 155
Contig stats
Clones: 21 (1 buried)
Markers: 0
Sequenced: 0
Length: 156 CB units
Ctg2180 of cp05T5e12DM4RS
M019F17*
M031C01
B010M23
V004P23
V054P17
M038M06
V003O03
M017O12
V049C16
V002B01
V010L02
V012I03
V020A11
M017B08
V014A06
M020G12
V037L04
B002P18
V062O11
M036K23
M029G18=
0 25 50 75 100 125 150
[root@localhost: /...
[FPC V8.9 Main Menu]
Project cp05T5e12...
[921-960 - File Bro...
FPC Ctg2180 cp05T...

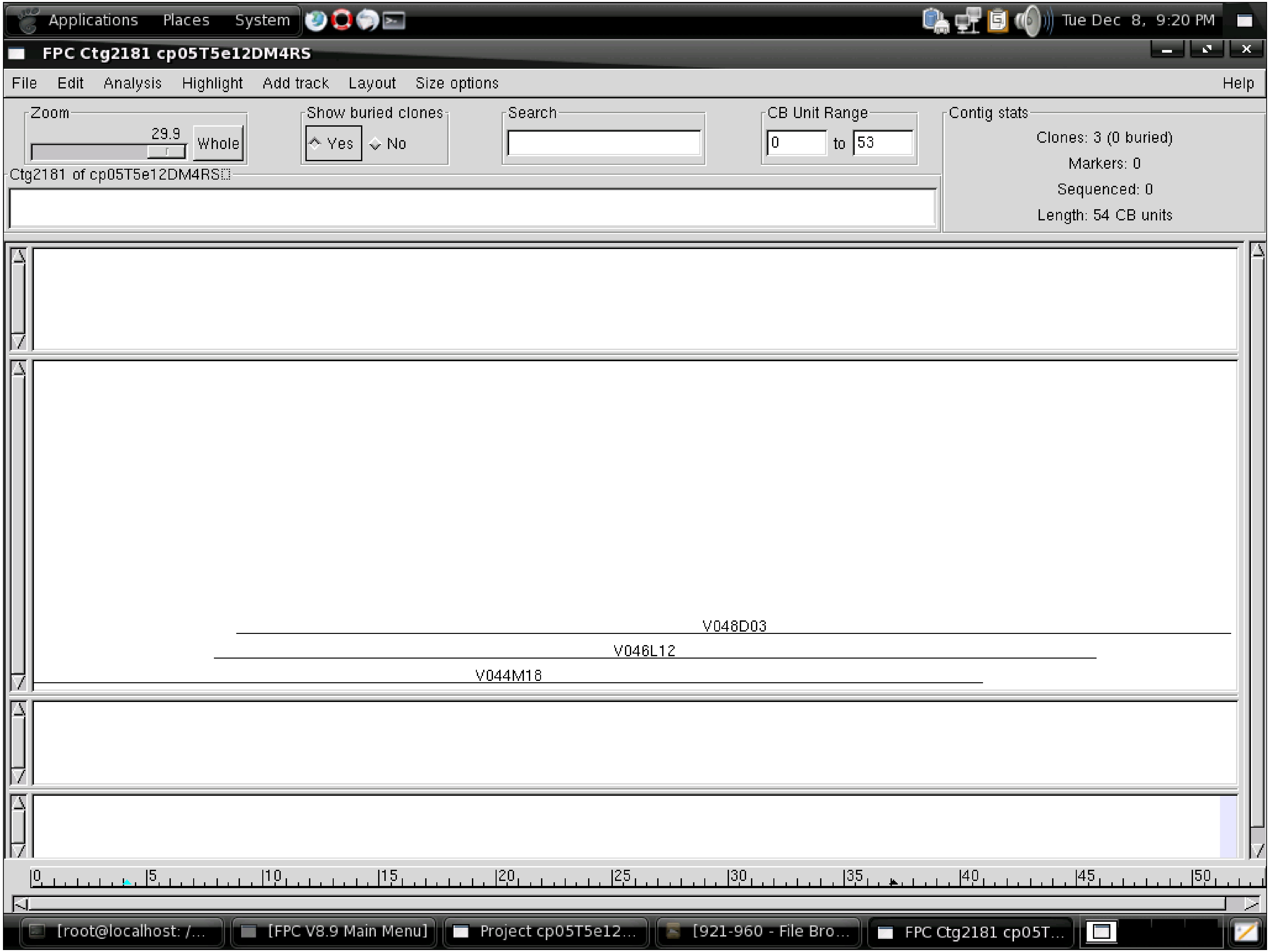

Applications
Places
System
Tue Dec 8, 9:20 PM
FPC Ctg2181 cp05T5e12DM4RS
File
Edit
Analysis
Highlight
Add track
Layout
Size options
Help
Zoom
29.9
Whole
Show buried clones
Yes
No
Search
CB Unit Range
0
to
53
Contig stats
Clones: 3 (0 buried)
Markers: 0
Sequenced: 0
Length: 54 CB units
Ctg2181 of cp05T5e12DM4RS
V048D03
V046L12
V044M18
0
5
10
15
20
25
30
35
40
45
50
[root@localhost: /...
[FPC V8.9 Main Menu]
Project cp05T5e12...
[921-960 - File Bro...
FPC Ctg2181 cp05T...

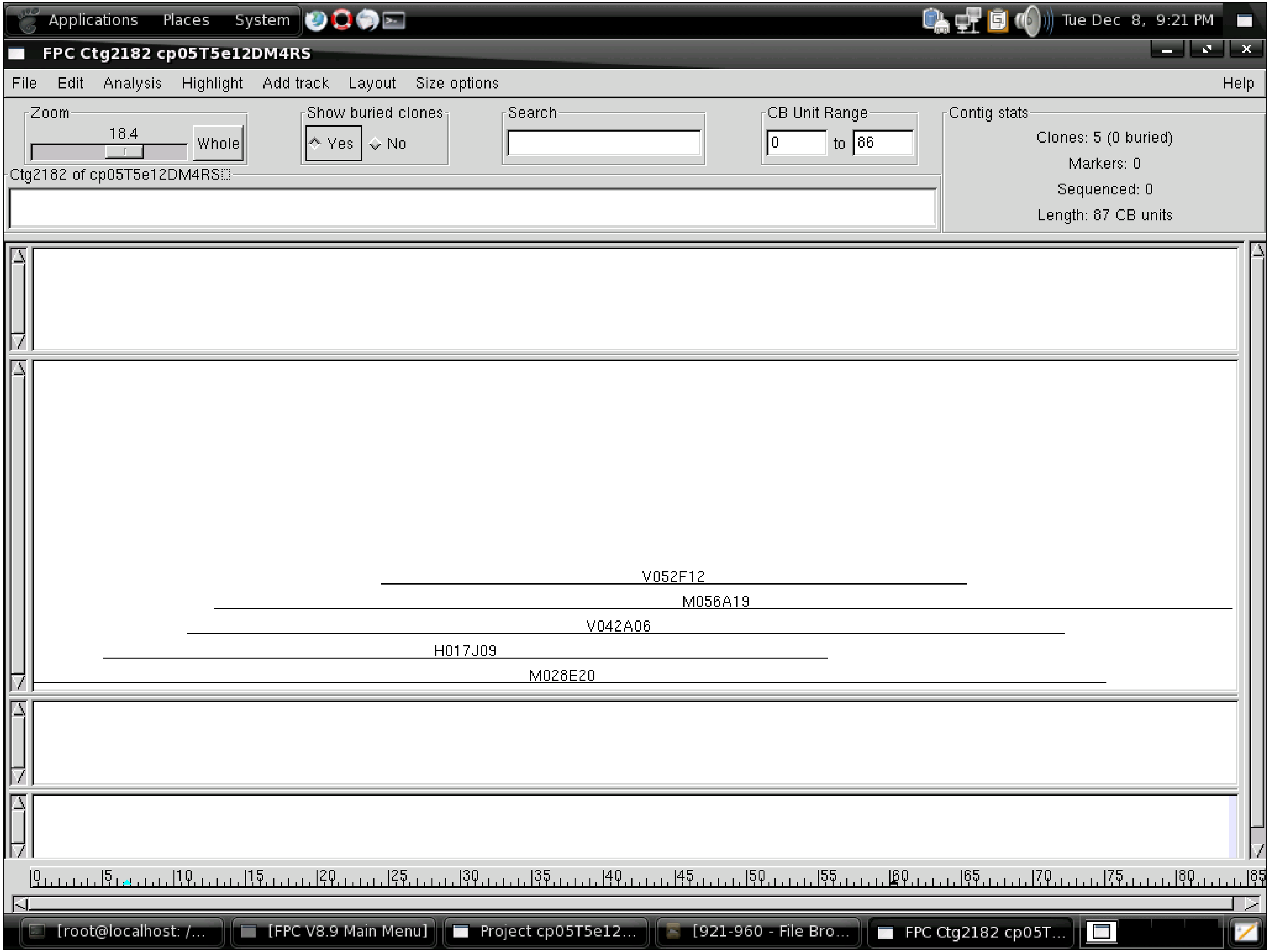
Applications Places System
Tue Dec 8, 9:21 PM
FPC Ctg2182 cp05T5e12DM4RS
File Edit Analysis Highlight Add track Layout Size options Help
Zoom
18.4
Whole
Show buried clones
Yes No
Search
CB Unit Range
0 to 86
Contig stats
Clones: 5 (0 buried)
Markers: 0
Sequenced: 0
Length: 87 CB units
Ctg2182 of cp05T5e12DM4RS
V052F12
M056A19
V042A06
H017J09
M028E20
0 5 10 15 20 25 30 35 40 45 50 55 60 65 70 75 80 85
[root@localhost: /...
[FPC V8.9 Main Menu]
Project cp05T5e12...
[921-960 - File Bro...
FPC Ctg2182 cp05T...

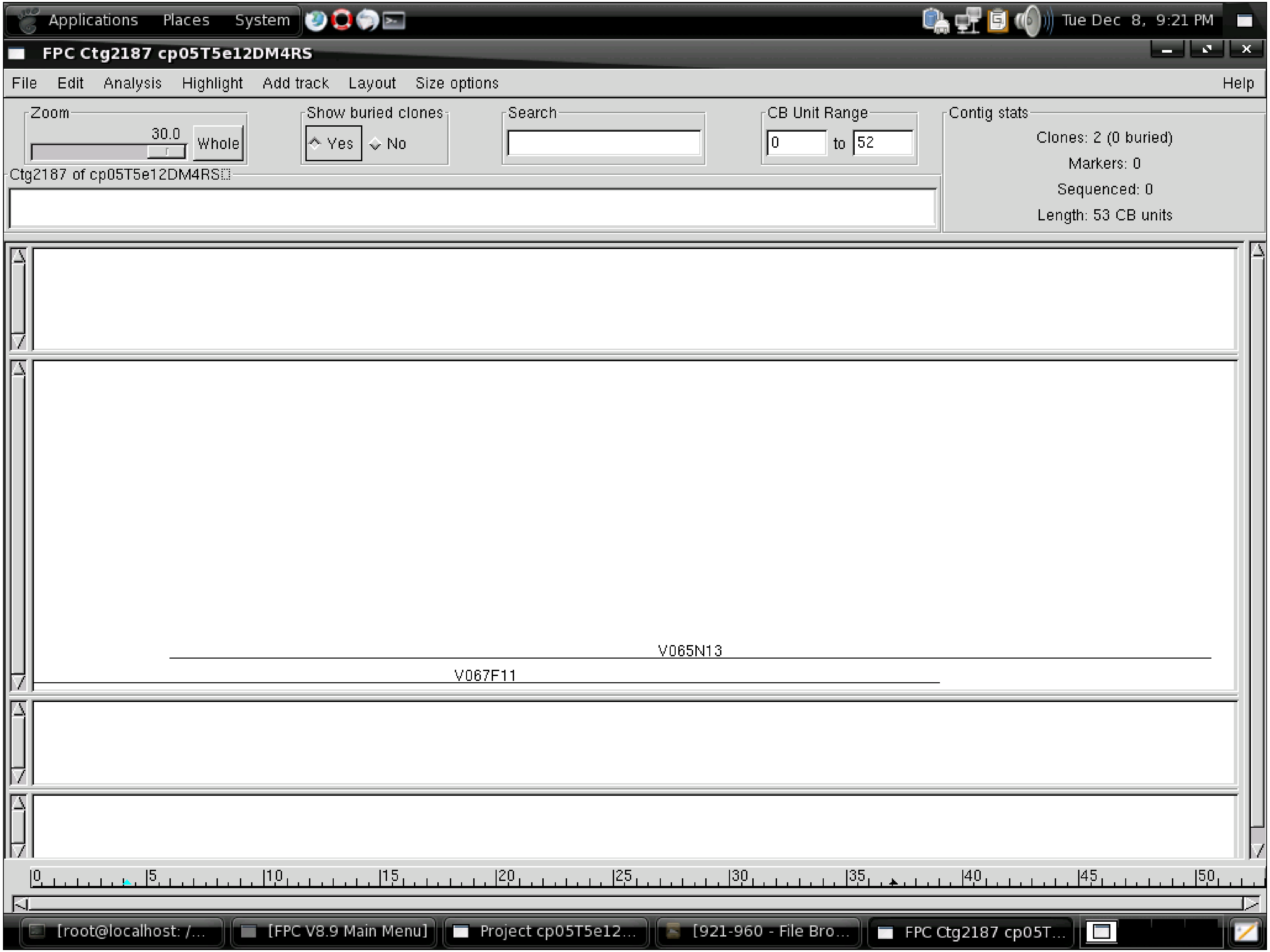
Applications
Places
System
Tue Dec 8, 9:21 PM
FPC Ctg2187 cp05T5e12DM4RS
File
Edit
Analysis
Highlight
Add track
Layout
Size options
Help
Zoom
30.0
Whole
Show buried clones
Yes
No
Search
CB Unit Range
0
to
52
Contig stats
Clones: 2 (0 buried)
Markers: 0
Sequenced: 0
Length: 53 CB units
Ctg2187 of cp05T5e12DM4RS
V065N13
V067F11
0
5
10
15
20
25
30
35
40
45
50
[root@localhost: /...
[FPC V8.9 Main Menu]
Project cp05T5e12...
[921-960 - File Bro...
FPC Ctg2187 cp05T...

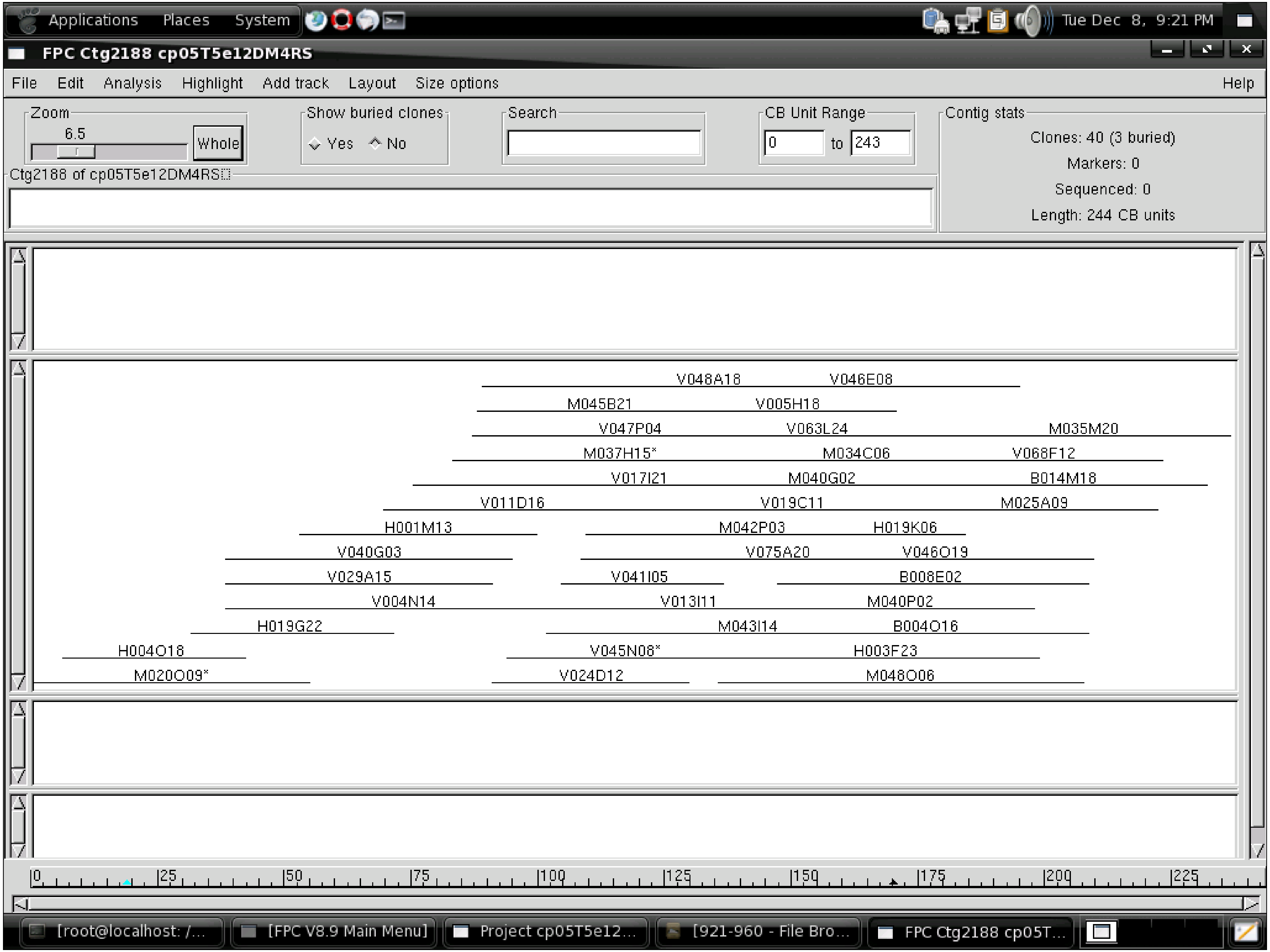
Applications Places System
Tue Dec 8, 9:21 PM
FPC Ctg2188 cp05T5e12DM4RS
File Edit Analysis Highlight Add track Layout Size options Help
Zoom
6.5
Whole
Show buried clones
Yes No
Search
CB Unit Range
0 to 243
Contig stats
Clones: 40 (3 buried)
Markers: 0
Sequenced: 0
Length: 244 CB units
Ctg2188 of cp05T5e12DM4RS
V048A18 V046E08
M045B21 V005H18
V047P04 V063L24 M035M20
M037H15* M034C06 V068F12
V017I21 M040G02 B014M18
V011D16 V019C11 M025A09
H001M13 M042P03 H019K06
V040G03 V075A20 V046O19
V029A15 V041I05 B008E02
V004N14 V013I11 M040P02
H019G22 M043I14 B004O16
H004O18 V045N08* H003F23
M020O09* V024D12 M048O06
0 25 50 75 100 125 150 175 200 225
[root@localhost: /...
[FPC V8.9 Main Menu]
Project cp05T5e12...
[921-960 - File Bro...
FPC Ctg2188 cp05T...

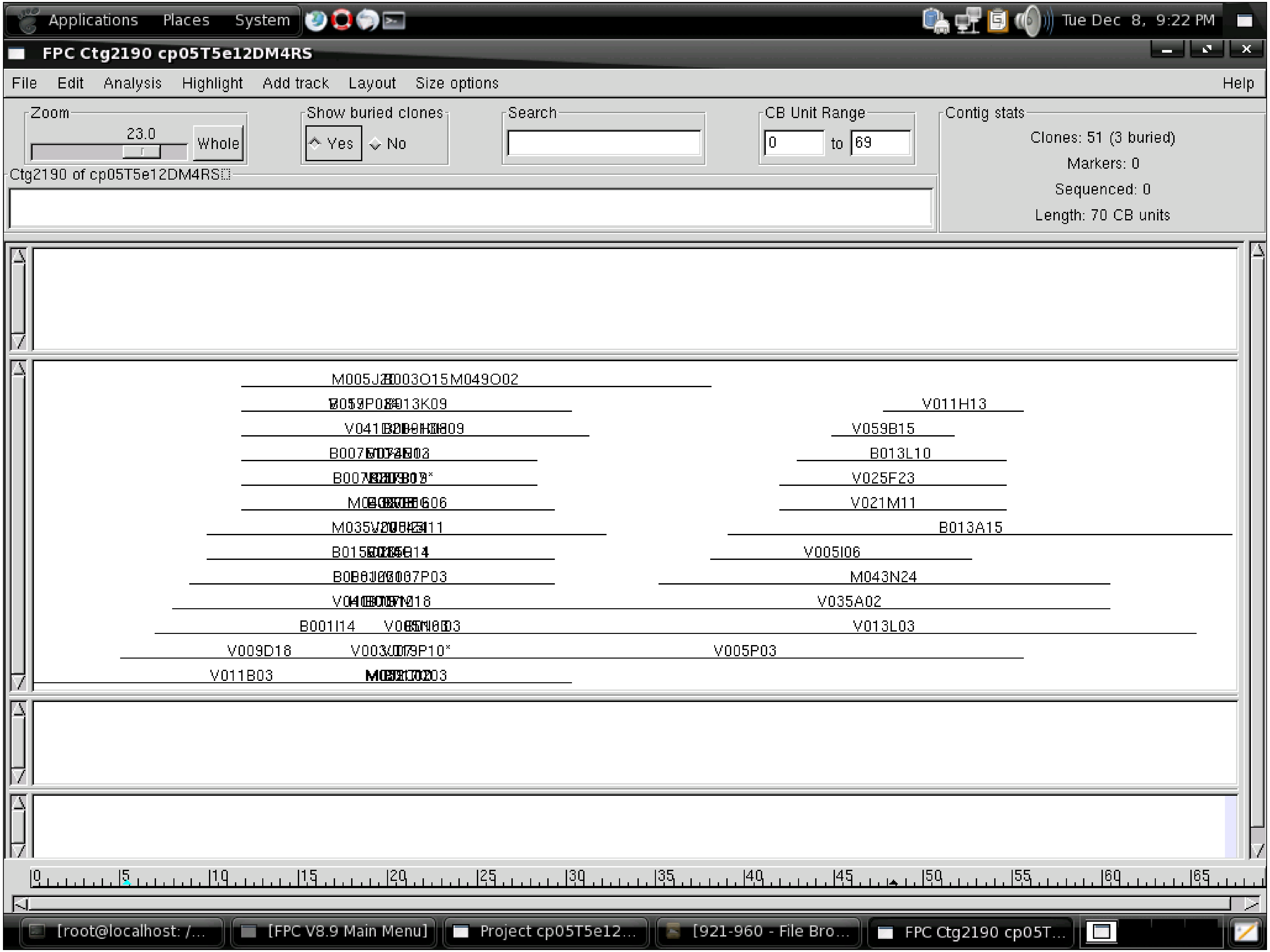
Applications Places System
Tue Dec 8, 9:22 PM
FPC Ctg2190 cp05T5e12DM4RS
File Edit Analysis Highlight Add track Layout Size options Help
Zoom
23.0
Whole
Show buried clones
Yes
No
Search
CB Unit Range
0
to
69
Contig stats
Clones: 51 (3 buried)
Markers: 0
Sequenced: 0
Length: 70 CB units
Ctg2190 of cp05T5e12DM4RS
V011H13
V059B15
B013L10
V025F23
V021M11
B013A15
V005I06
M043N24
V035A02
V013L03
B001I14
V009D18
V005P03
V011B03
0 5 10 15 20 25 30 35 40 45 50 55 60 65
[root@localhost: /...
[FPC V8.9 Main Menu]
Project cp05T5e12...
[921-960 - File Bro...
FPC Ctg2190 cp05T...

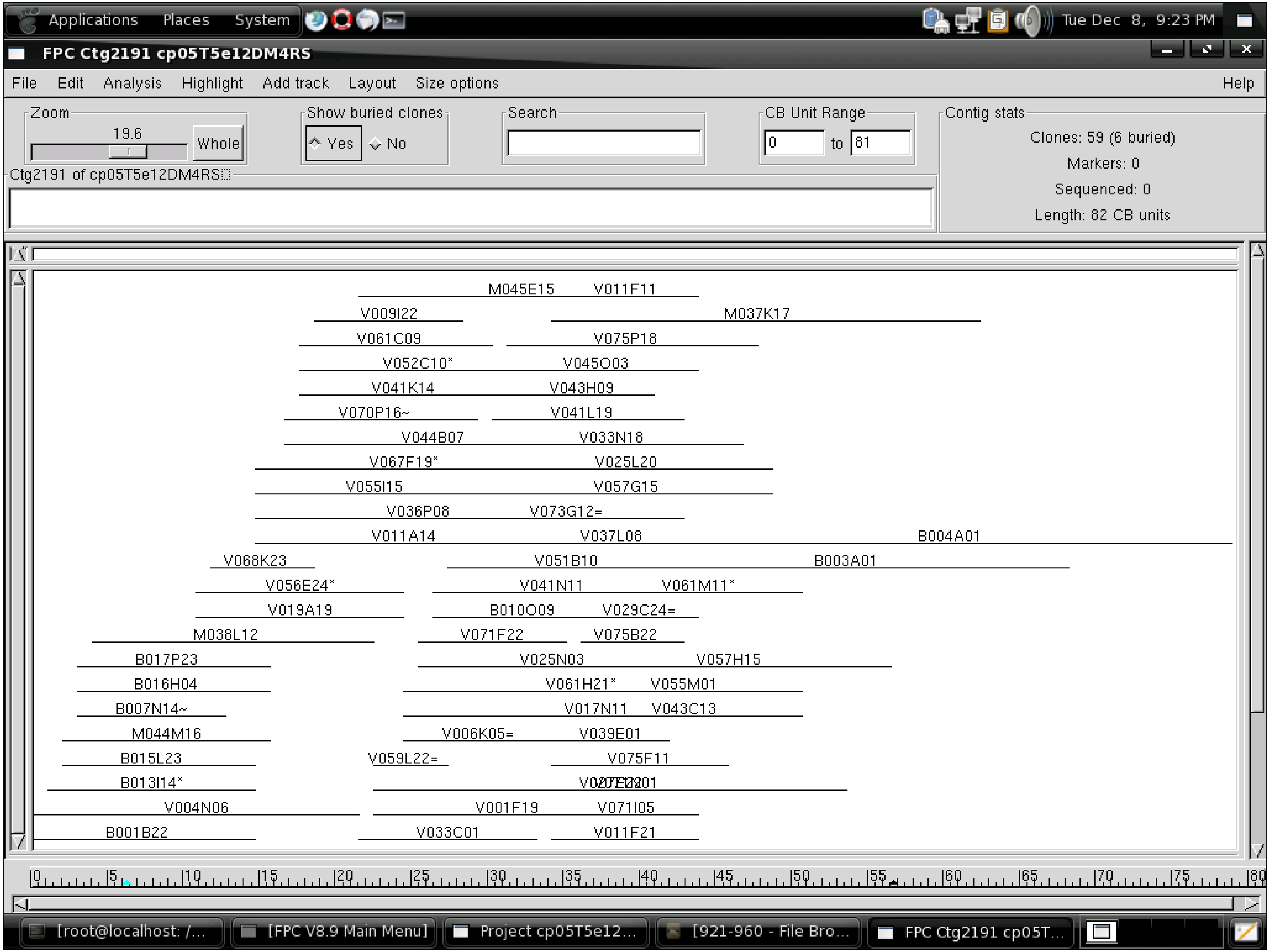

Applications Places System
Tue Dec 8, 9:23 PM
FPC Ctg2191 cp05T5e12DM4RS
File Edit Analysis Highlight Add track Layout Size options Help
Zoom
19.6
Whole
Show buried clones
Yes No
Search
CB Unit Range
0 to 81
Contig stats
Clones: 59 (6 buried)
Markers: 0
Sequenced: 0
Length: 82 CB units
Ctg2191 of cp05T5e12DM4RS
M045E15 V011F11
V009I22 M037K17
V061C09 V075P18
V052C10* V045O03
V041K14 V043H09
V070P16~ V041L19
V044B07 V033N18
V067F19* V025L20
V055I15 V057G15
V036P08 V073G12=
V011A14 V037L08 B004A01
V068K23 V051B10 B003A01
V056E24* V041N11 V061M11*
V019A19 B010O09 V029C24=
M038L12 V071F22 V075B22
B017P23 V025N03 V057H15
B016H04 V061H21* V055M01
B007N14~ V017N11 V043C13
M044M16 V006K05= V039E01
B015L23 V059L22= V075F11
B013I14*
V004N06 V001F19 V071I05
B001B22 V033C01 V011F21
0 5 10 15 20 25 30 35 40 45 50 55 60 65 70 75 80
[root@localhost: /... [FPC V8.9 Main Menu] Project cp05T5e12... [921-960 - File Bro... FPC Ctg2191 cp05T...

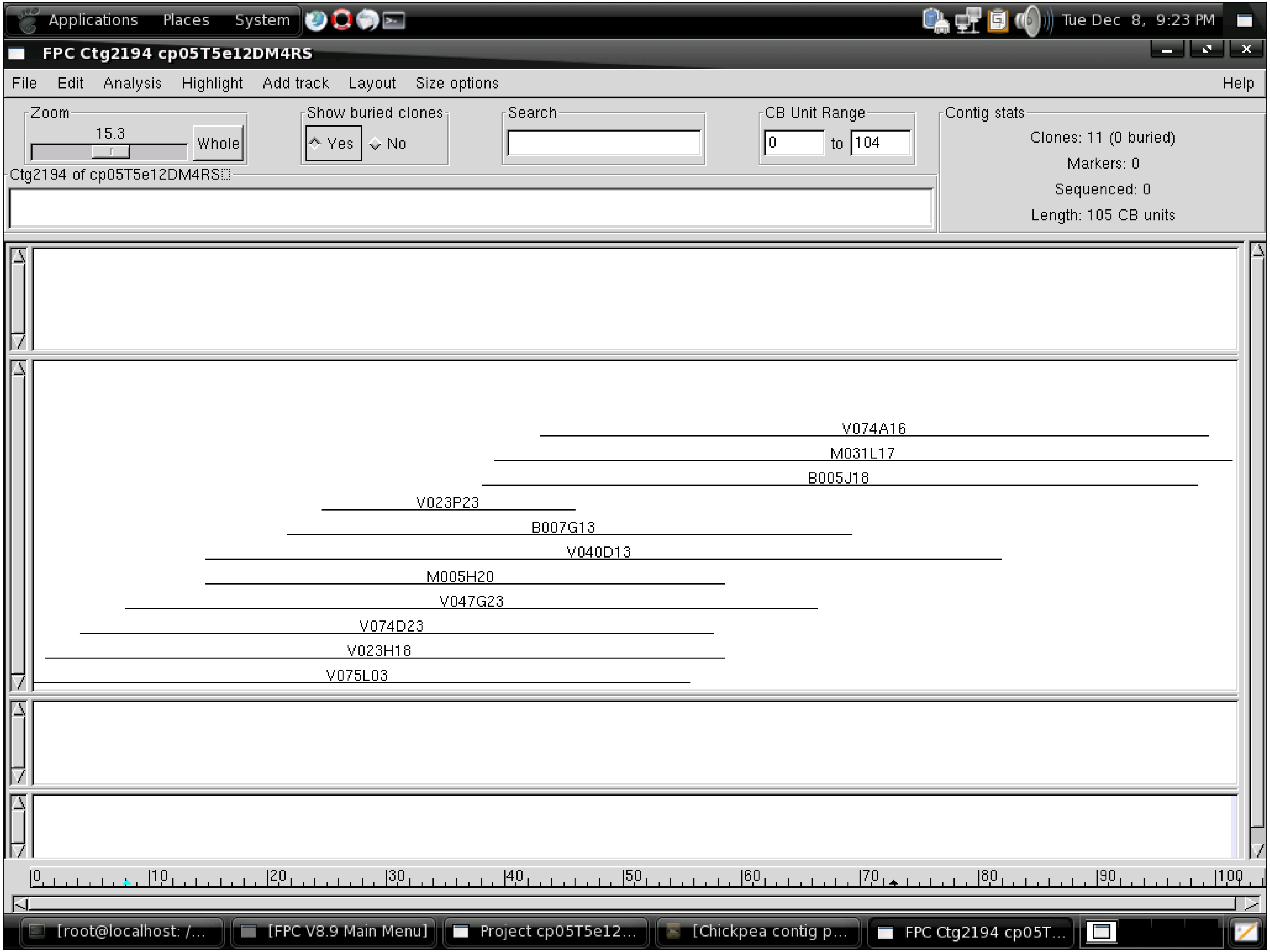
Applications Places System
Tue Dec 8, 9:23 PM
FPC Ctg2194 cp05T5e12DM4RS
File Edit Analysis Highlight Add track Layout Size options Help
Zoom
15.3
Whole
Show buried clones
Yes No
Search
CB Unit Range
0 to 104
Contig stats
Clones: 11 (0 buried)
Markers: 0
Sequenced: 0
Length: 105 CB units
Ctg2194 of cp05T5e12DM4RS
V074A16
M031L17
B005J18
V023P23
B007G13
V040D13
M005H20
V047G23
V074D23
V023H18
V075L03
0 10 20 30 40 50 60 70 80 90 100
[root@localhost: /...
[FPC V8.9 Main Menu]
Project cp05T5e12...
[Chickpea contig p...
FPC Ctg2194 cp05T...

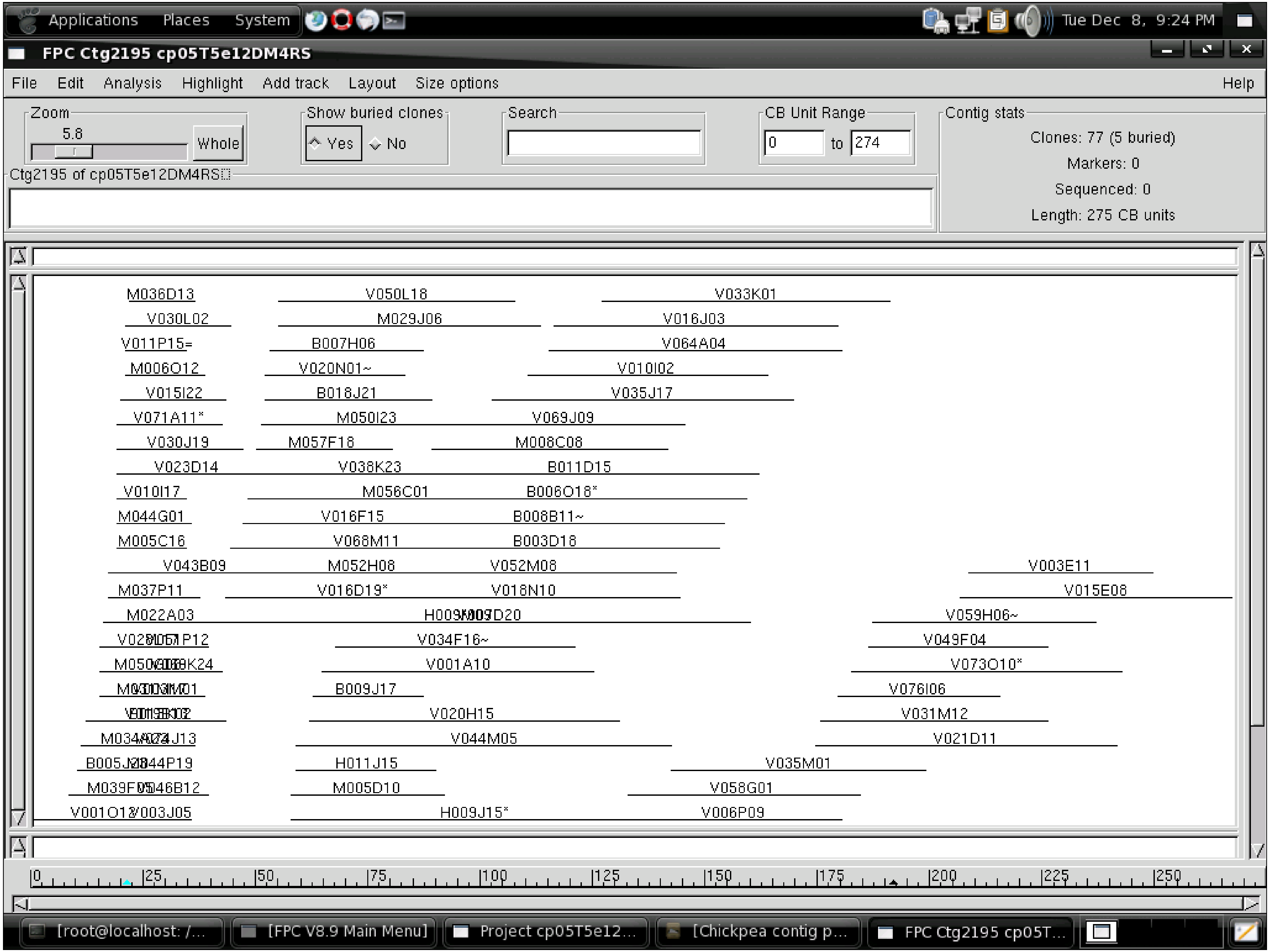
Applications
Places
System
Tue Dec 8, 9:24 PM
FPC Ctg2195 cp05T5e12DM4RS
File
Edit
Analysis
Highlight
Add track
Layout
Size options
Help
Zoom
5.8
Whole
Show buried clones
Yes
No
Search
CB Unit Range
0
to
274
Contig stats
Clones: 77 (5 buried)
Markers: 0
Sequenced: 0
Length: 275 CB units
Ctg2195 of cp05T5e12DM4RS
M036D13
V050L18
V033K01
V030L02
M029J06
V016J03
V011P15=
B007H06
V064A04
M006O12
V020N01~
V010I02
V015I22
B018J21
V035J17
V071A11*
M050I23
V069J09
V030J19
M057F18
M008C08
V023D14
V038K23
B011D15
V010I17
M056C01
B006O18*
M044G01
V016F15
B008B11~
M005C16
V068M11
B003D18
V043B09
M052H08
V052M08
V003E11
M037P11
V016D19*
V018N10
V015E08
M022A03
V059H06~
V034F16~
V049F04
V001A10
V073O10*
B009J17
V076I06
V020H15
V031M12
V044M05
V021D11
H011J15
V035M01
M005D10
V058G01
H009J15*
V006P09
0
25
50
75
100
125
150
175
200
225
250
[root@localhost: /...
[FPC V8.9 Main Menu]
Project cp05T5e12...
[Chickpea contig p...
FPC Ctg2195 cp05T...

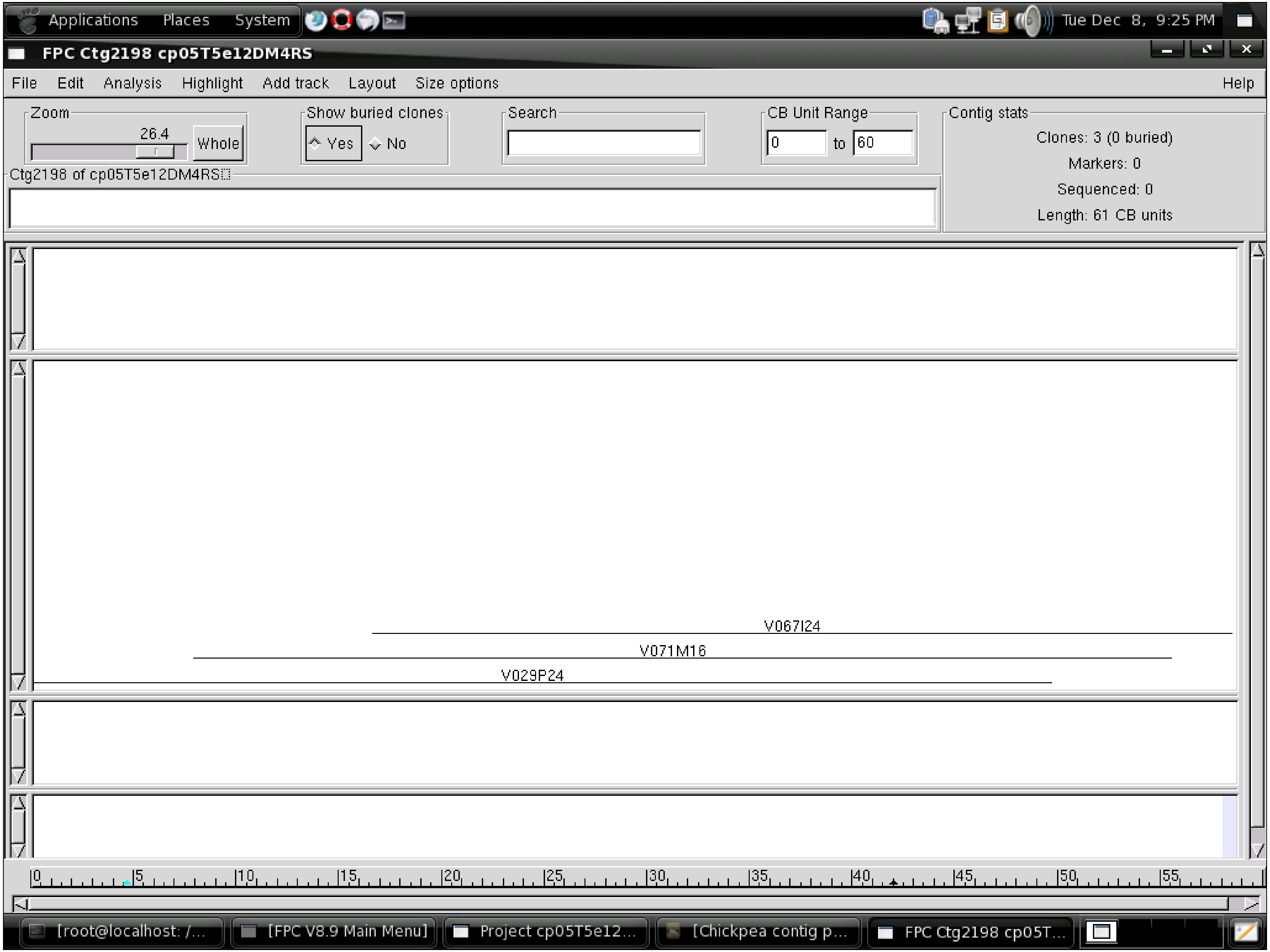
Applications Places System
Tue Dec 8, 9:25 PM
FPC Ctg2198 cp05T5e12DM4RS
File Edit Analysis Highlight Add track Layout Size options Help
Zoom
26.4
Whole
Show buried clones
Yes
No
Search
CB Unit Range
0
to
60
Contig stats
Clones: 3 (0 buried)
Markers: 0
Sequenced: 0
Length: 61 CB units
Ctg2198 of cp05T5e12DM4RS
V067I24
V071M16
V029P24
0 5 10 15 20 25 30 35 40 45 50 55
[root@localhost: /...
[FPC V8.9 Main Menu]
Project cp05T5e12...
[Chickpea contig p...
FPC Ctg2198 cp05T...

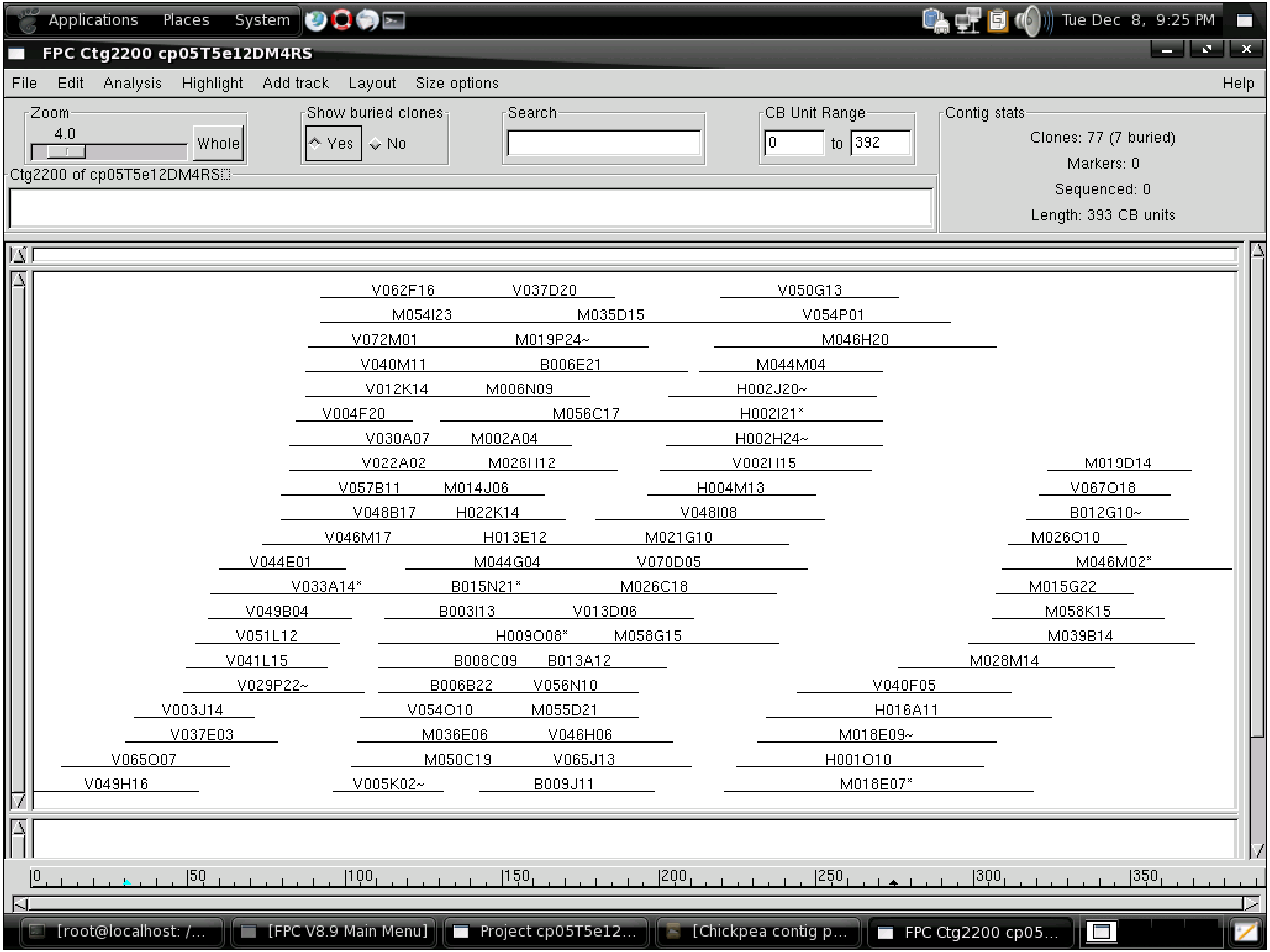
Applications Places System
Tue Dec 8, 9:25 PM
FPC Ctg2200 cp05T5e12DM4RS
File Edit Analysis Highlight Add track Layout Size options Help
Zoom
4.0
Whole
Show buried clones
Yes
No
Search
CB Unit Range
0
to
392
Contig stats
Clones: 77 (7 buried)
Markers: 0
Sequenced: 0
Length: 393 CB units
Ctg2200 of cp05T5e12DM4RS
V062F16
V037D20
V050G13
M054I23
M035D15
V054P01
V072M01
M019P24~
M046H20
V040M11
B006E21
M044M04
V012K14
M006N09
H002J20~
V004F20
M056C17
H002I21*
V030A07
M002A04
H002H24~
V022A02
M026H12
V002H15
M019D14
V057B11
M014J06
H004M13
V067O18
V048B17
H022K14
V048I08
B012G10~
V046M17
H013E12
M021G10
M026O10
V044E01
M044G04
V070D05
M046M02*
V033A14*
B015N21*
M026C18
M015G22
V049B04
B003I13
V013D06
M058K15
V051L12
H009O08*
M058G15
M039B14
V041L15
B008C09
B013A12
M028M14
V029P22~
B006B22
V056N10
V040F05
V003J14
V054O10
M055D21
H016A11
V037E03
M036E06
V046H06
M018E09~
V065O07
M050C19
V065J13
H001O10
V049H16
V005K02~
B009J11
M018E07*
0
50
100
150
200
250
300
350
[root@localhost: /...
[FPC V8.9 Main Menu]
Project cp05T5e12...
[Chickpea contig p...
FPC Ctg2200 cp05...

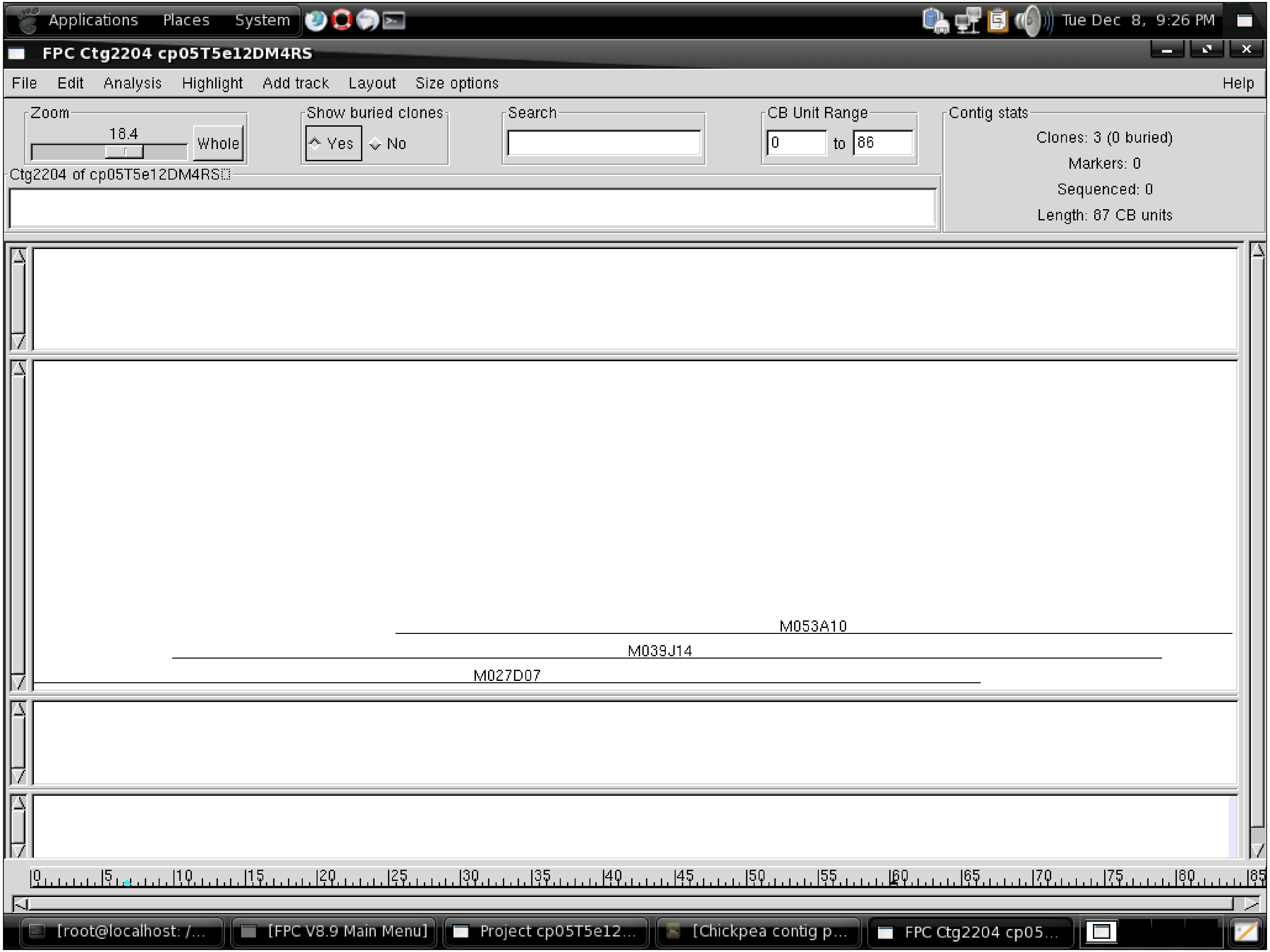
Applications Places System
Tue Dec 8, 9:26 PM
FPC Ctg2204 cp05T5e12DM4RS
File Edit Analysis Highlight Add track Layout Size options Help
Zoom
18.4
Whole
Show buried clones
Yes
No
Search
CB Unit Range
0
to
86
Contig stats
Clones: 3 (0 buried)
Markers: 0
Sequenced: 0
Length: 87 CB units
Ctg2204 of cp05T5e12DM4RS
M053A10
M039J14
M027D07
0 5 10 15 20 25 30 35 40 45 50 55 60 65 70 75 80 85
[root@localhost: /...
[FPC V8.9 Main Menu]
Project cp05T5e12...
[Chickpea contig p...
FPC Ctg2204 cp05...

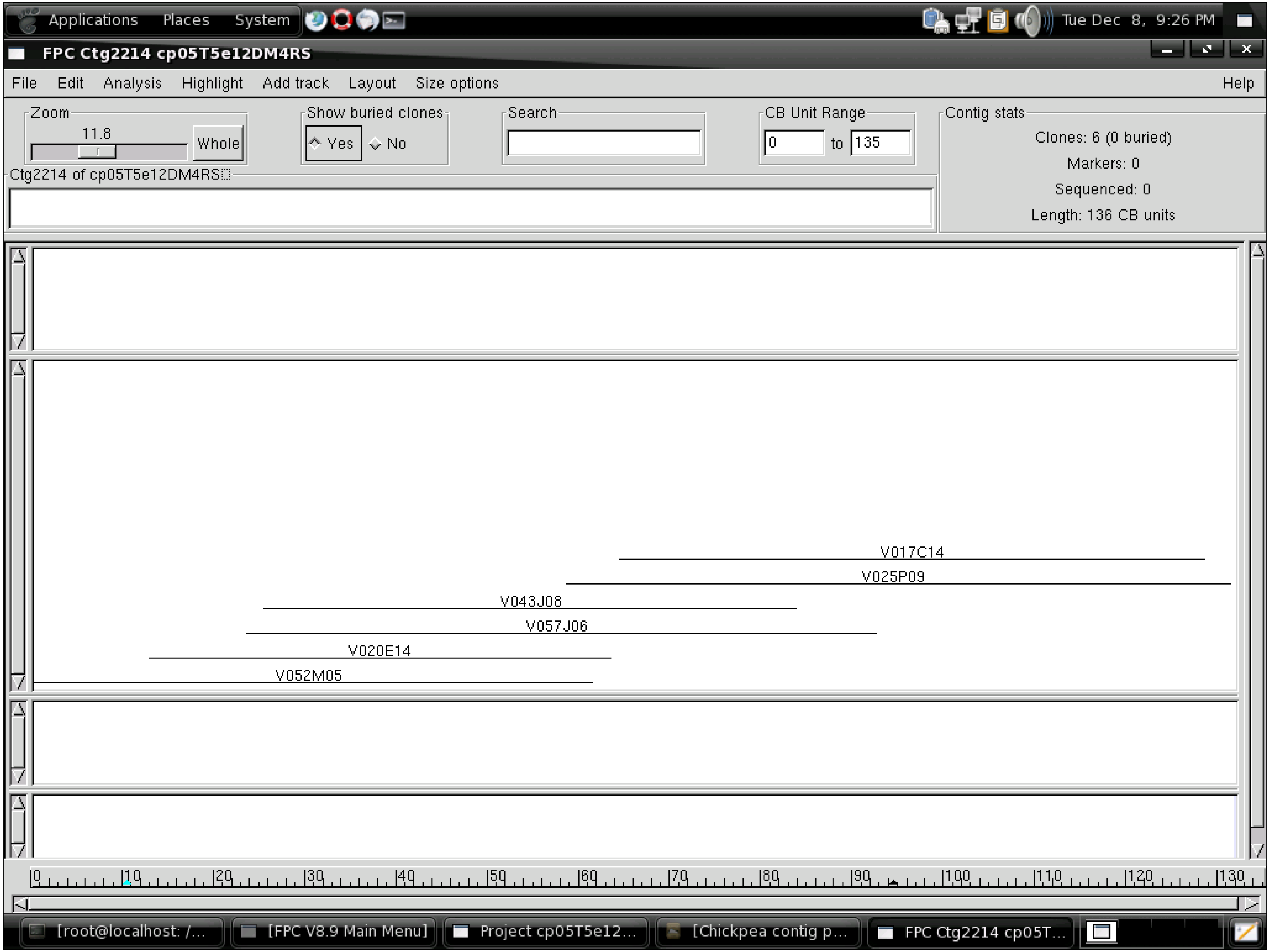

Applications
Places
System
Tue Dec 8, 9:26 PM
FPC Ctg2214 cp05T5e12DM4RS
File
Edit
Analysis
Highlight
Add track
Layout
Size options
Help
Zoom
11.8
Whole
Show buried clones
Yes
No
Search
CB Unit Range
0
to
135
Contig stats
Clones: 6 (0 buried)
Markers: 0
Sequenced: 0
Length: 136 CB units
Ctg2214 of cp05T5e12DM4RS
V017C14
V025P09
V043J08
V057J06
V020E14
V052M05
0
10
20
30
40
50
60
70
80
90
100
110
120
130
[root@localhost: /...
[FPC V8.9 Main Menu]
Project cp05T5e12...
[Chickpea contig p...
FPC Ctg2214 cp05T...

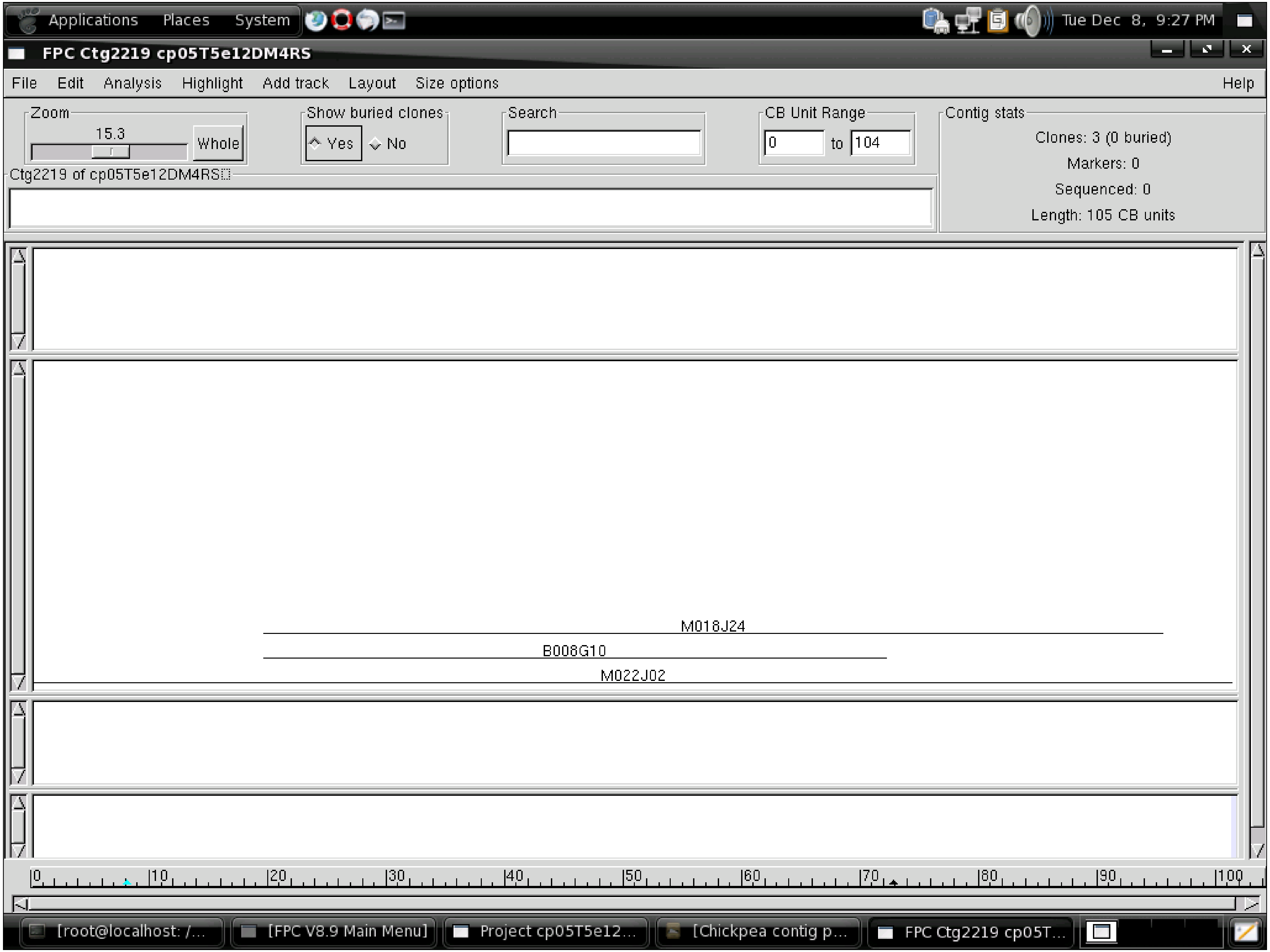
Applications Places System
Tue Dec 8, 9:27 PM
FPC Ctg2219 cp05T5e12DM4RS
File Edit Analysis Highlight Add track Layout Size options Help
Zoom
15.3
Whole
Show buried clones
Yes
No
Search
CB Unit Range
0 to 104
Contig stats
Clones: 3 (0 buried)
Markers: 0
Sequenced: 0
Length: 105 CB units
Ctg2219 of cp05T5e12DM4RS
M018J24
B008G10
M022J02
0 10 20 30 40 50 60 70 80 90 100
[root@localhost: /...
[FPC V8.9 Main Menu]
Project cp05T5e12...
[Chickpea contig p...
FPC Ctg2219 cp05T...

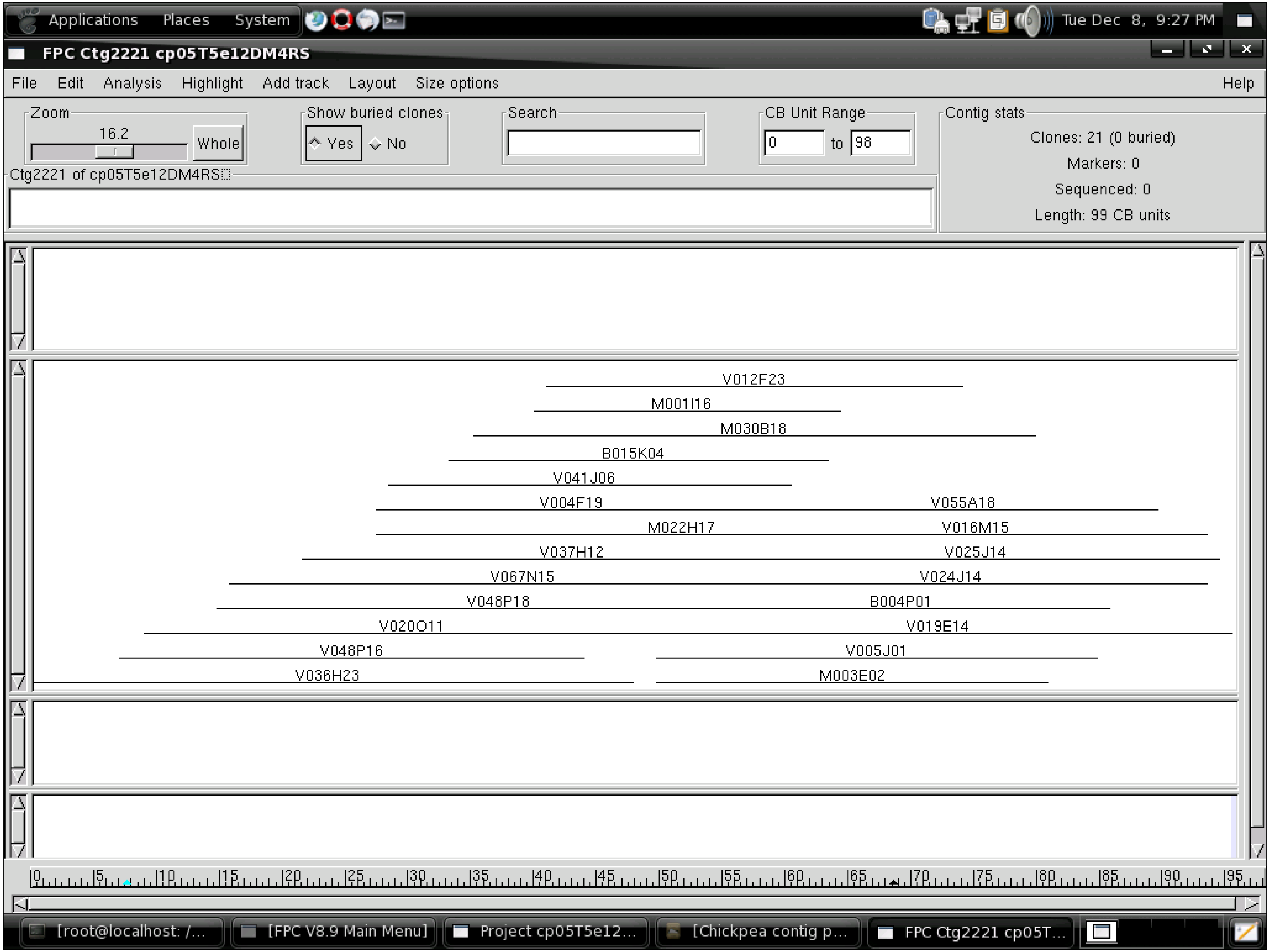
Applications
Places
System
Tue Dec 8, 9:27 PM
FPC Ctg2221 cp05T5e12DM4RS
File
Edit
Analysis
Highlight
Add track
Layout
Size options
Help
Zoom
16.2
Whole
Show buried clones
Yes
No
Search
CB Unit Range
0
to
98
Contig stats
Clones: 21 (0 buried)
Markers: 0
Sequenced: 0
Length: 99 CB units
Ctg2221 of cp05T5e12DM4RS
V012F23
M001I16
M030B18
B015K04
V041J06
V004F19
V055A18
M022H17
V016M15
V037H12
V025J14
V067N15
V024J14
V048P18
B004P01
V020O11
V019E14
V048P16
V005J01
V036H23
M003E02
0 5 10 15 20 25 30 35 40 45 50 55 60 65 70 75 80 85 90 95
[root@localhost: /...
[FPC V8.9 Main Menu]
Project cp05T5e12...
[Chickpea contig p...
FPC Ctg2221 cp05T...

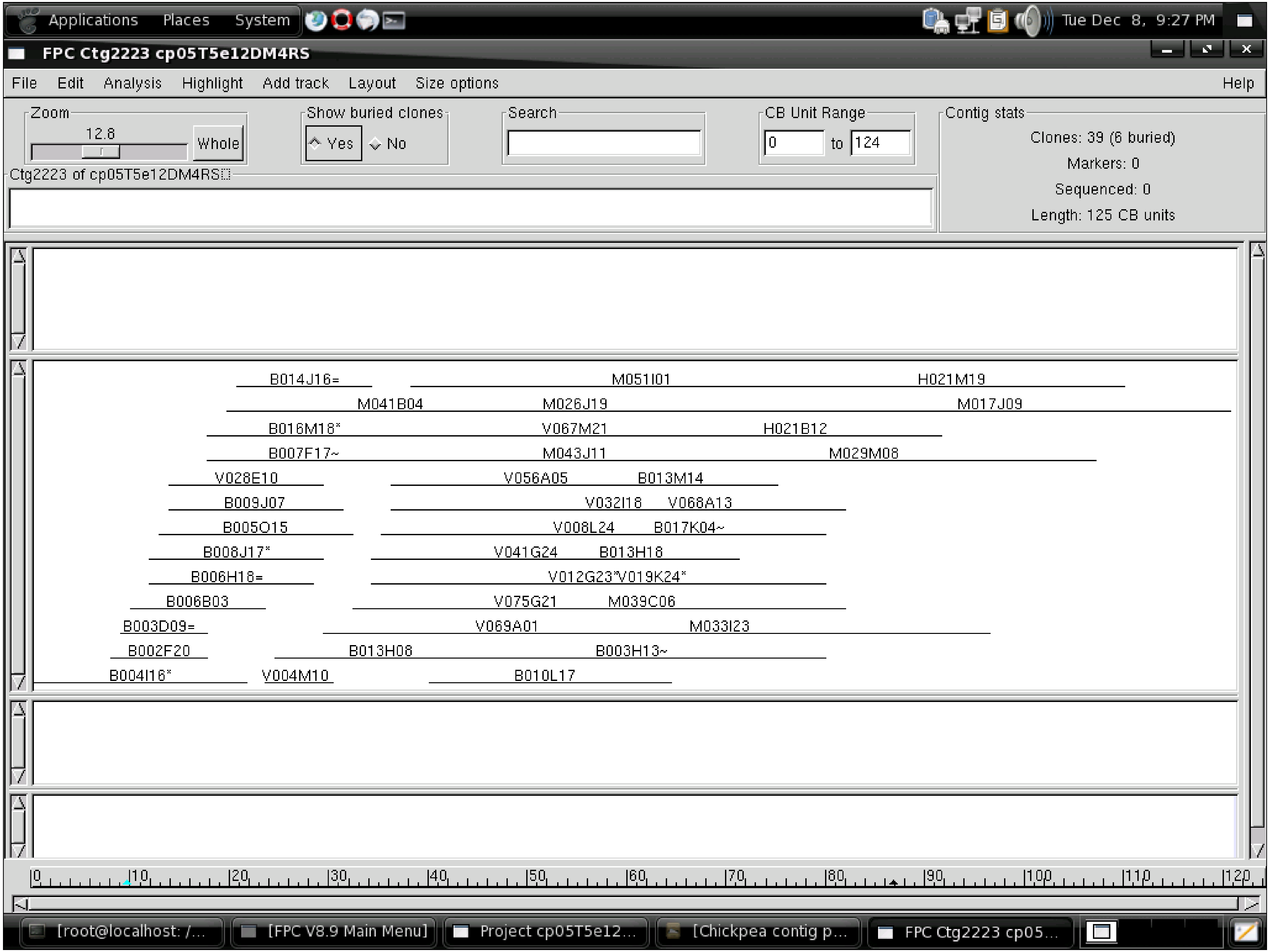

Applications Places System
Tue Dec 8, 9:27 PM
FPC Ctg2223 cp05T5e12DM4RS
File Edit Analysis Highlight Add track Layout Size options Help
Zoom 12.8 Whole
Show buried clones Yes No
Search
CB Unit Range 0 to 124
Contig stats
Clones: 39 (6 buried)
Markers: 0
Sequenced: 0
Length: 125 CB units
Ctg2223 of cp05T5e12DM4RS
B014J16= M051I01 H021M19
M041B04 M026J19 M017J09
B016M18* V067M21 H021B12
B007F17~ M043J11 M029M08
V028E10 V056A05 B013M14
B009J07 V032I18 V068A13
B005O15 V008L24 B017K04~
B008J17* V041G24 B013H18
B006H18= V012G23*V019K24*
B006B03 V075G21 M039C06
B003D09= V069A01 M033I23
B002F20 B013H08 B003H13~
B004I16* V004M10 B010L17
0 10 20 30 40 50 60 70 80 90 100 110 120
[root@localhost: /... [FPC V8.9 Main Menu] Project cp05T5e12... [Chickpea contig p... FPC Ctg2223 cp05...

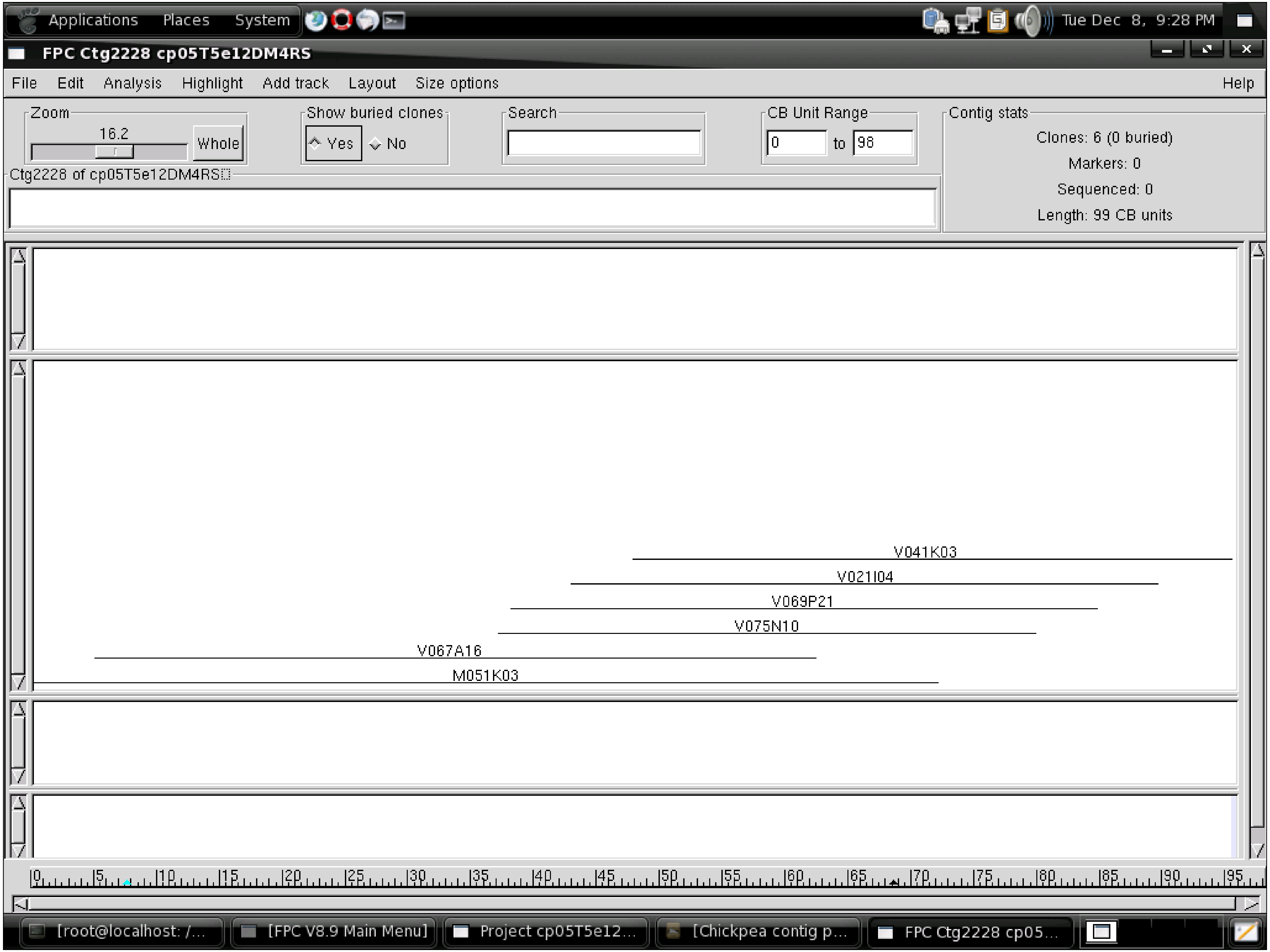
Applications Places System
Tue Dec 8, 9:28 PM
FPC Ctg2228 cp05T5e12DM4RS
File Edit Analysis Highlight Add track Layout Size options Help
Zoom
16.2
Whole
Show buried clones
Yes
No
Search
CB Unit Range
0
to
98
Contig stats
Clones: 6 (0 buried)
Markers: 0
Sequenced: 0
Length: 99 CB units
Ctg2228 of cp05T5e12DM4RS
V041K03
V021I04
V069P21
V075N10
V067A16
M051K03
0 5 10 15 20 25 30 35 40 45 50 55 60 65 70 75 80 85 90 95
[root@localhost: /...
[FPC V8.9 Main Menu]
Project cp05T5e12...
[Chickpea contig p...
FPC Ctg2228 cp05...

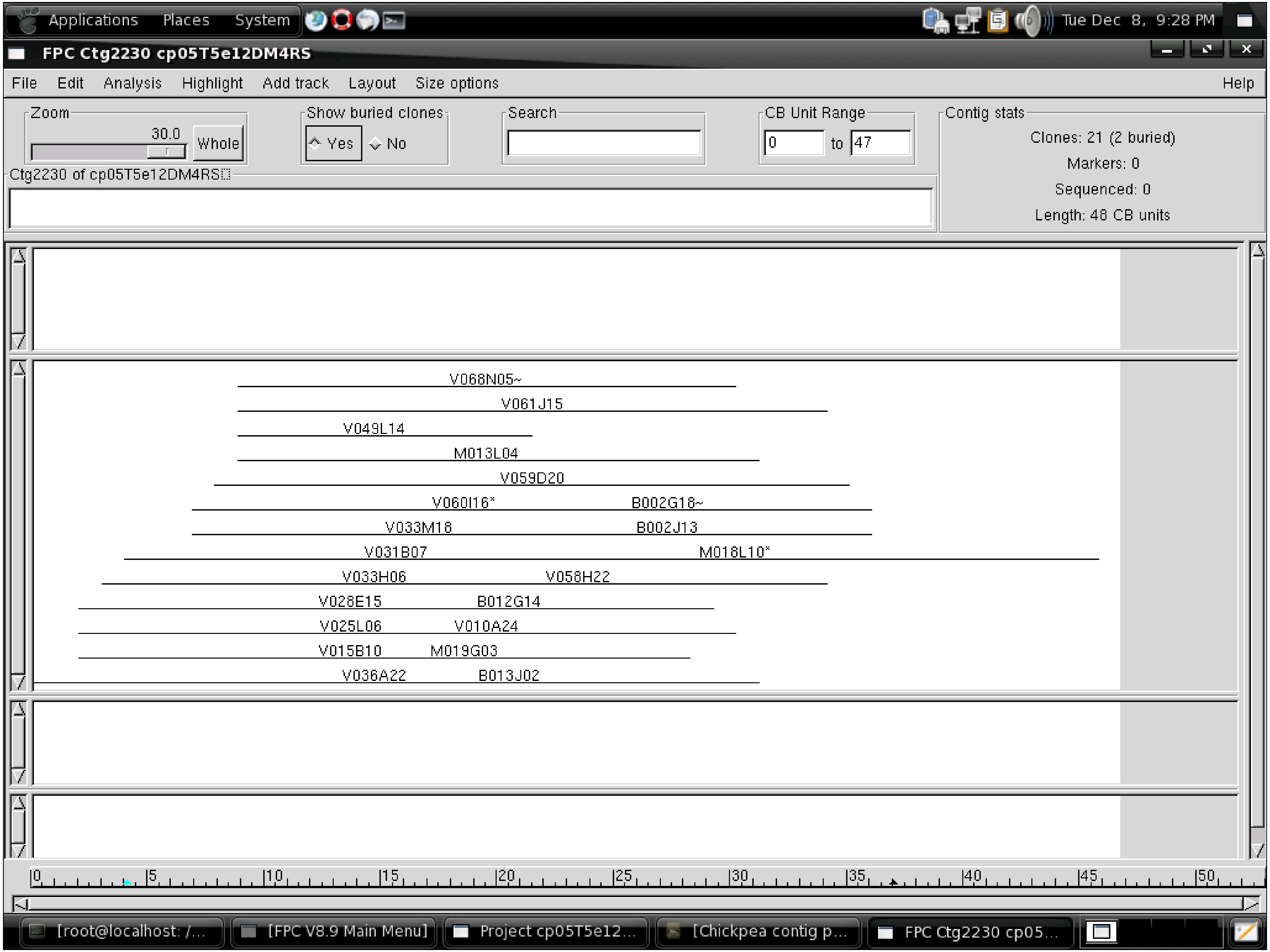
Applications Places System
Tue Dec 8, 9:28 PM
FPC Ctg2230 cp05T5e12DM4RS
File Edit Analysis Highlight Add track Layout Size options Help
Zoom
30.0
Whole
Show buried clones
Yes
No
Search
CB Unit Range
0 to 47
Contig stats
Clones: 21 (2 buried)
Markers: 0
Sequenced: 0
Length: 48 CB units
Ctg2230 of cp05T5e12DM4RS
V068N05~
V061J15
V049L14
M013L04
V059D20
V060I16* B002G18~
V033M18 B002J13
V031B07 M018L10*
V033H06 V058H22
V028E15 B012G14
V025L06 V010A24
V015B10 M019G03
V036A22 B013J02
0 5 10 15 20 25 30 35 40 45 50
[root@localhost: /...
[FPC V8.9 Main Menu]
Project cp05T5e12...
[Chickpea contig p...
FPC Ctg2230 cp05...

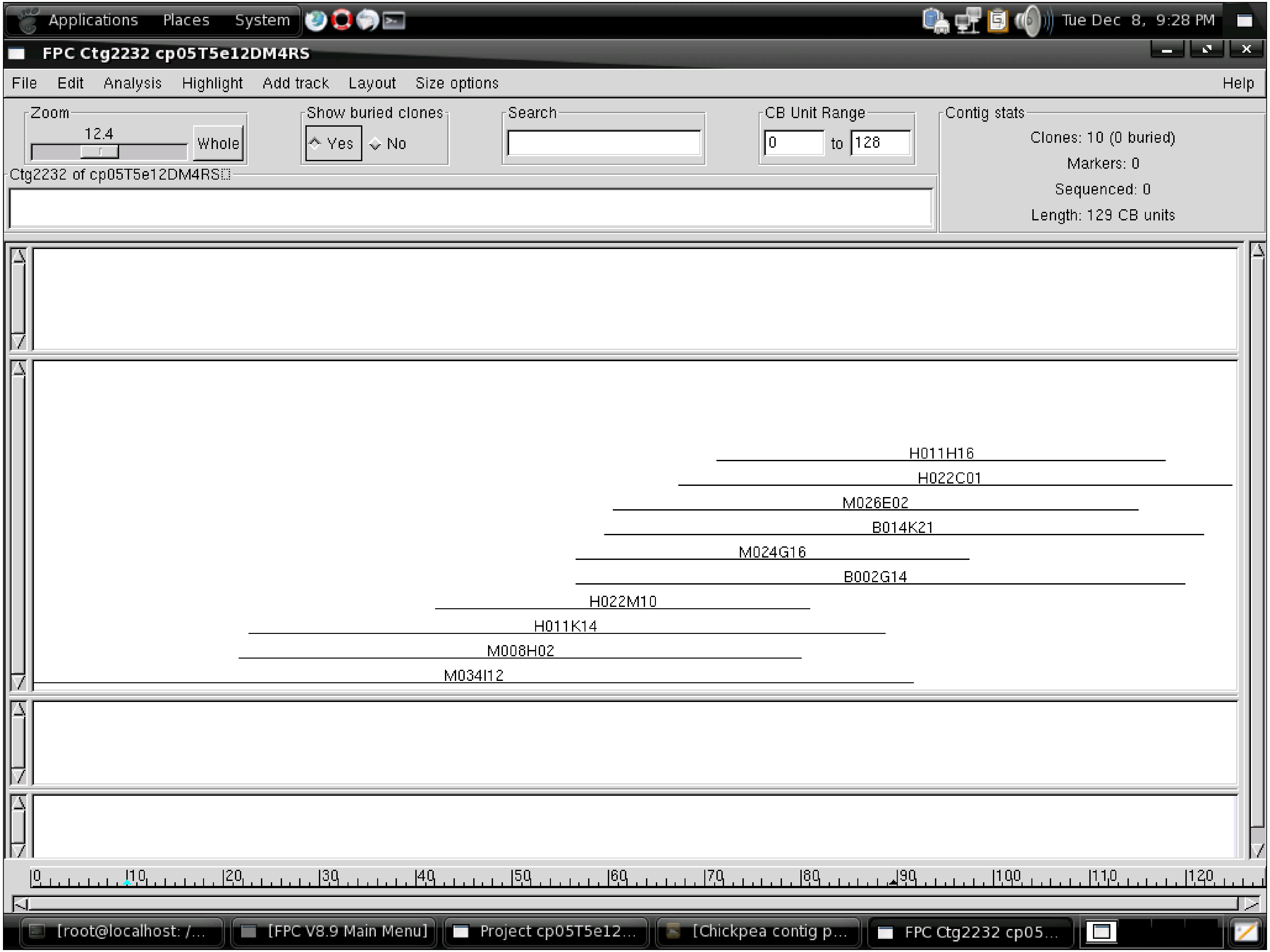
Applications Places System
Tue Dec 8, 9:28 PM
FPC Ctg2232 cp05T5e12DM4RS
File Edit Analysis Highlight Add track Layout Size options Help
Zoom
12.4
Whole
Show buried clones
Yes No
Search
CB Unit Range
0 to 128
Contig stats
Clones: 10 (0 buried)
Markers: 0
Sequenced: 0
Length: 129 CB units
Ctg2232 of cp05T5e12DM4RS
H011H16
H022C01
M026E02
B014K21
M024G16
B002G14
H022M10
H011K14
M008H02
M034I12
0 10 20 30 40 50 60 70 80 90 100 110 120
[root@localhost: /...
[FPC V8.9 Main Menu]
Project cp05T5e12...
[Chickpea contig p...
FPC Ctg2232 cp05...

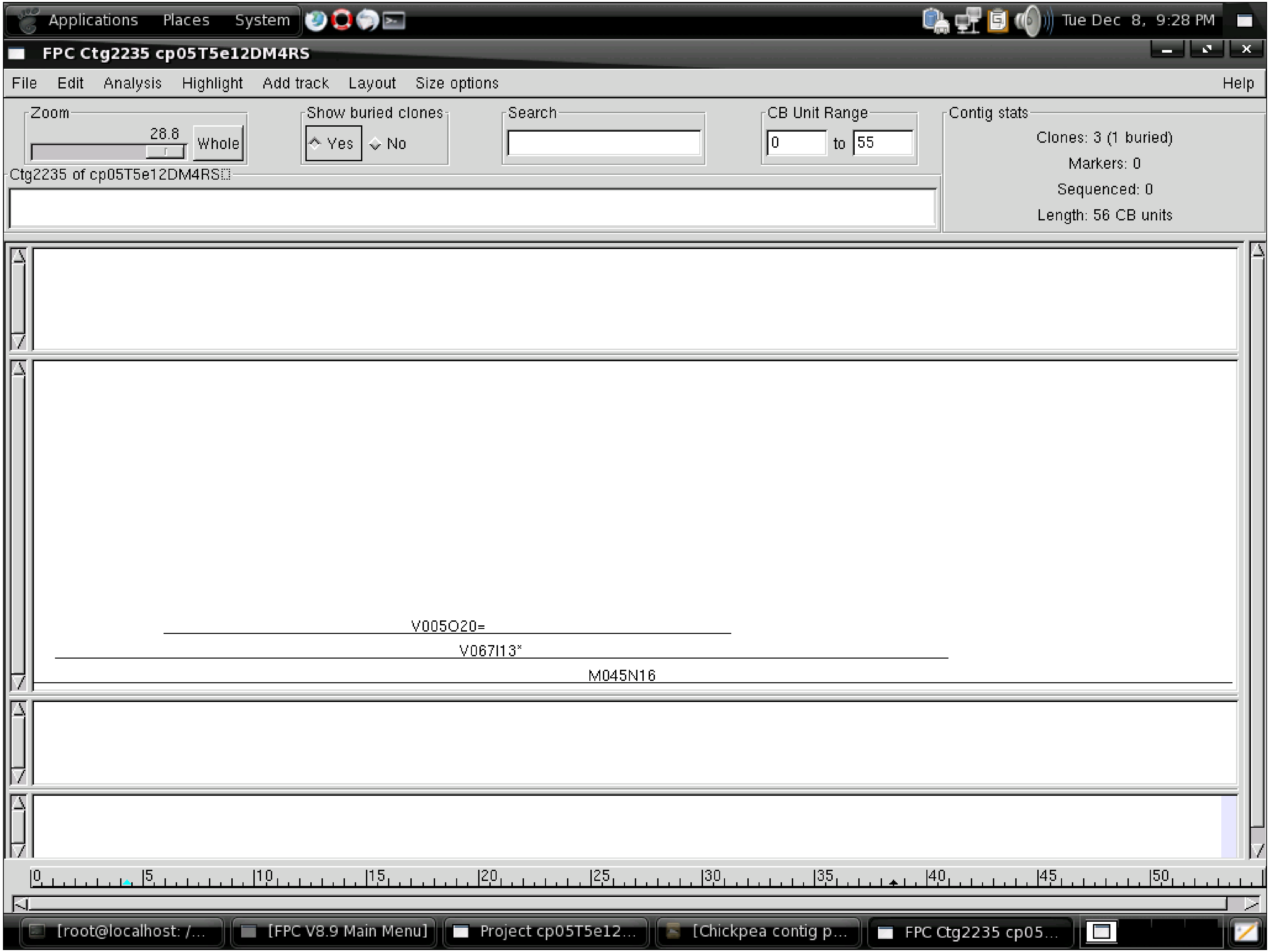
Applications Places System
Tue Dec 8, 9:28 PM
FPC Ctg2235 cp05T5e12DM4RS
File Edit Analysis Highlight Add track Layout Size options Help
Zoom
28.8
Whole
Show buried clones
Yes
No
Search
CB Unit Range
0
to
55
Contig stats
Clones: 3 (1 buried)
Markers: 0
Sequenced: 0
Length: 56 CB units
Ctg2235 of cp05T5e12DM4RS
V005O20=
V067I13*
M045N16
0 5 10 15 20 25 30 35 40 45 50
[root@localhost: /...
[FPC V8.9 Main Menu]
Project cp05T5e12...
[Chickpea contig p...
FPC Ctg2235 cp05...

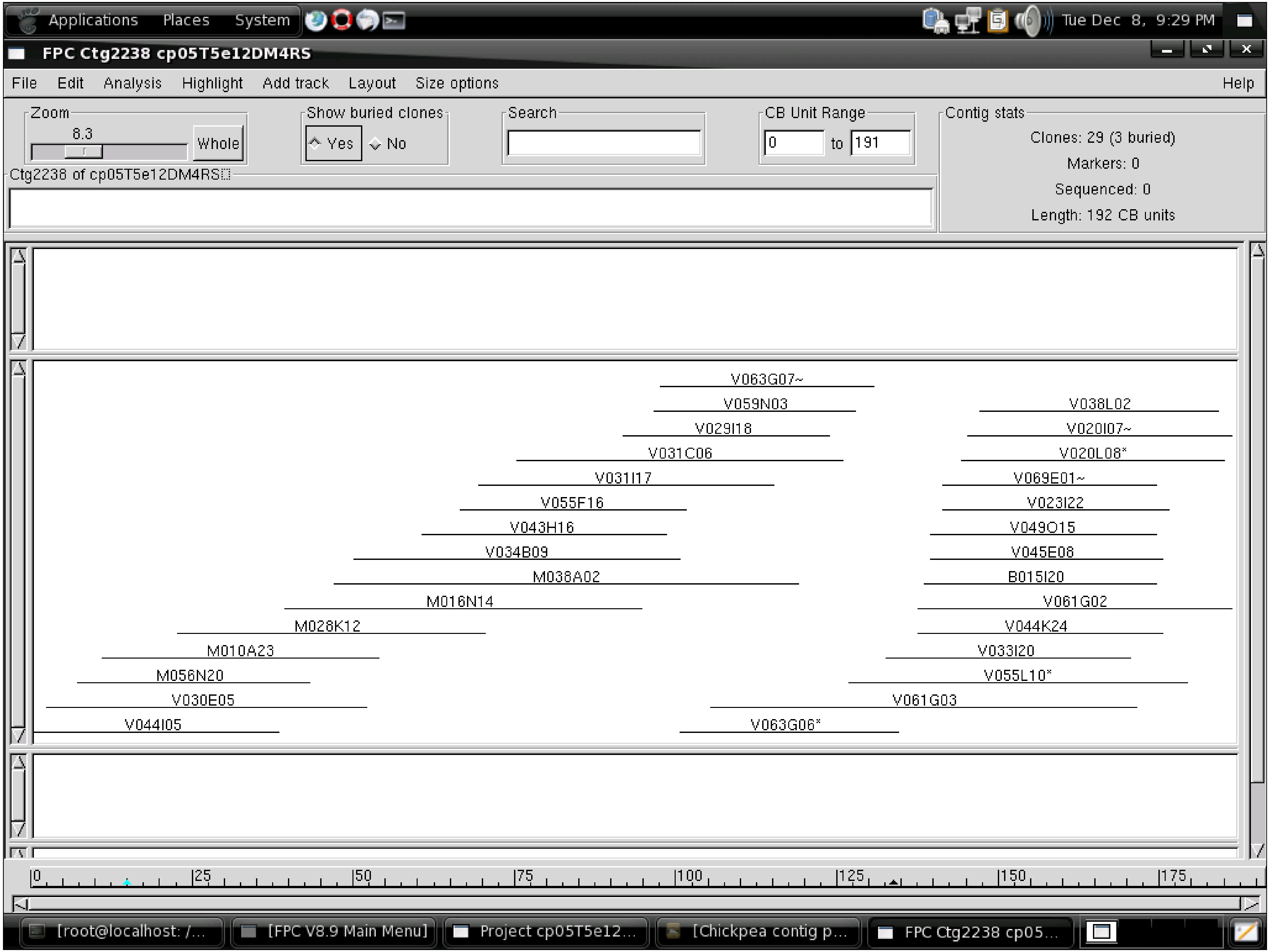
Applications Places System
Tue Dec 8, 9:29 PM
FPC Ctg2238 cp05T5e12DM4RS
File Edit Analysis Highlight Add track Layout Size options Help
Zoom
8.3
Whole
Show buried clones
Yes
No
Search
CB Unit Range
0 to 191
Contig stats
Clones: 29 (3 buried)
Markers: 0
Sequenced: 0
Length: 192 CB units
Ctg2238 of cp05T5e12DM4RS
V063G07~
V059N03
V038L02
V029I18
V020I07~
V031C06
V020L08*
V031I17
V069E01~
V055F16
V023I22
V043H16
V049O15
V034B09
V045E08
M038A02
B015I20
M016N14
V061G02
M028K12
V044K24
M010A23
V033I20
M056N20
V055L10*
V030E05
V061G03
V044I05
V063G06*
0 25 50 75 100 125 150 175
[root@localhost: /...
[FPC V8.9 Main Menu]
Project cp05T5e12...
[Chickpea contig p...
FPC Ctg2238 cp05...

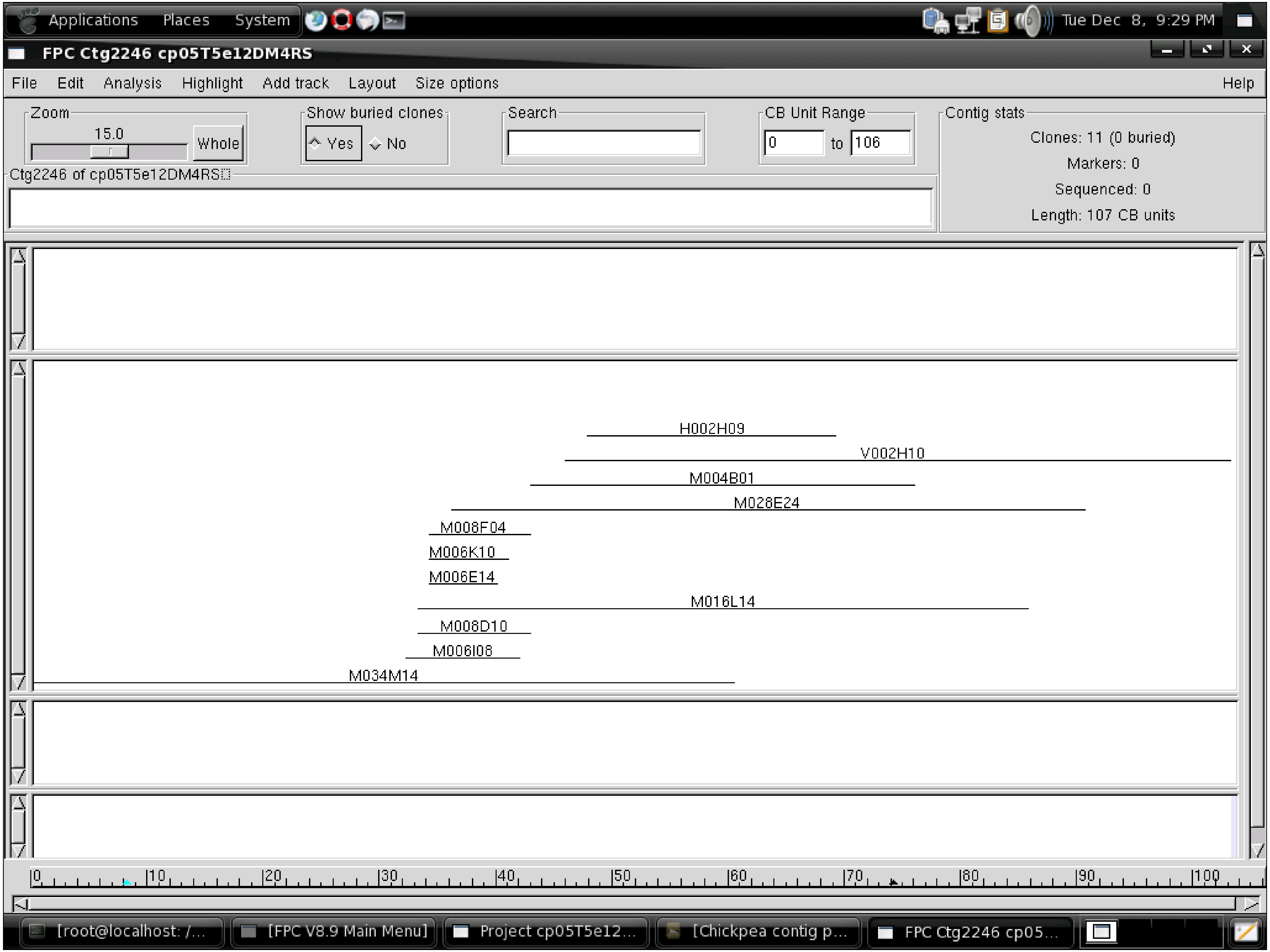
Applications Places System
Tue Dec 8, 9:29 PM
FPC Ctg2246 cp05T5e12DM4RS
File Edit Analysis Highlight Add track Layout Size options Help
Zoom
15.0
Whole
Show buried clones
Yes No
Search
CB Unit Range
0 to 106
Contig stats
Clones: 11 (0 buried)
Markers: 0
Sequenced: 0
Length: 107 CB units
Ctg2246 of cp05T5e12DM4RS
H002H09
V002H10
M004B01
M028E24
M008F04
M006K10
M006E14
M016L14
M008D10
M006I08
M034M14
0 10 20 30 40 50 60 70 80 90 100
[root@localhost: /...
[FPC V8.9 Main Menu]
Project cp05T5e12...
[Chickpea contig p...
FPC Ctg2246 cp05...

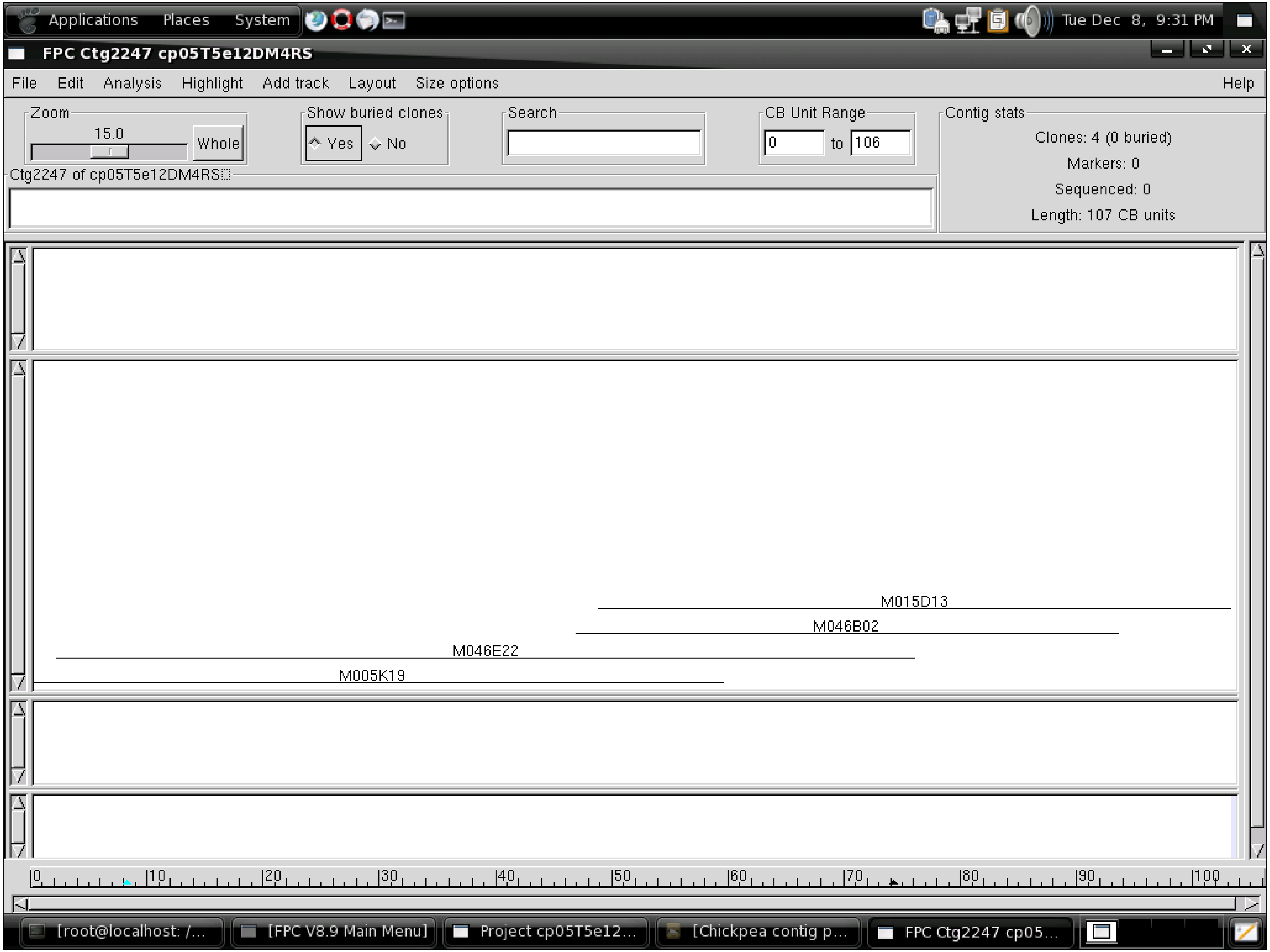
Applications Places System
Tue Dec 8, 9:31 PM
FPC Ctg2247 cp05T5e12DM4RS
File Edit Analysis Highlight Add track Layout Size options Help
Zoom
15.0
Whole
Show buried clones
Yes
No
Search
CB Unit Range
0
to
106
Contig stats
Clones: 4 (0 buried)
Markers: 0
Sequenced: 0
Length: 107 CB units
Ctg2247 of cp05T5e12DM4RS
M015D13
M046B02
M046E22
M005K19
0 10 20 30 40 50 60 70 80 90 100
[root@localhost: /...
[FPC V8.9 Main Menu]
Project cp05T5e12...
[Chickpea contig p...
FPC Ctg2247 cp05...

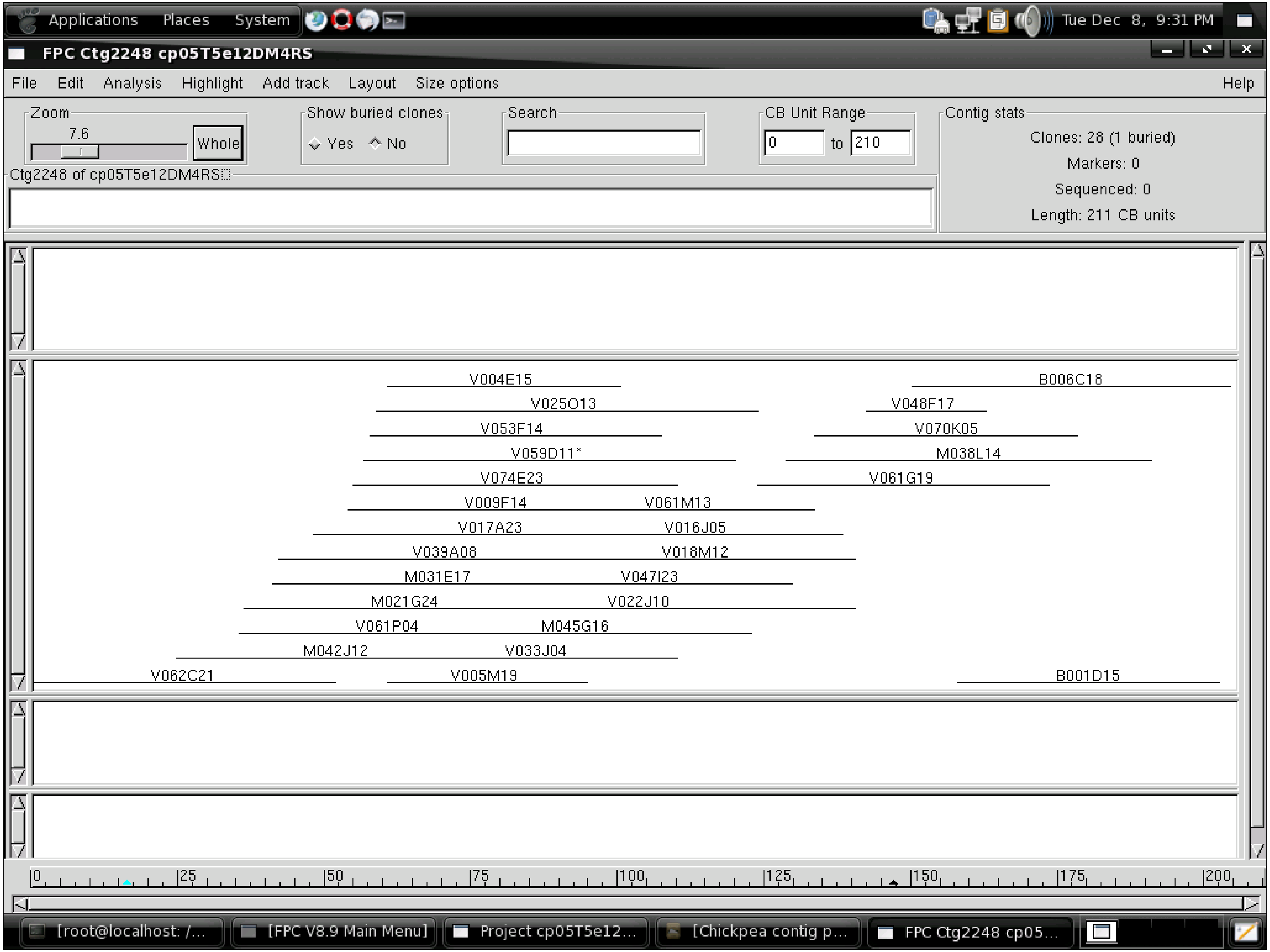
Applications Places System
Tue Dec 8, 9:31 PM
FPC Ctg2248 cp05T5e12DM4RS
File Edit Analysis Highlight Add track Layout Size options Help
Zoom
7.6
Whole
Show buried clones
Yes No
Search
CB Unit Range
0 to 210
Contig stats
Clones: 28 (1 buried)
Markers: 0
Sequenced: 0
Length: 211 CB units
Ctg2248 of cp05T5e12DM4RS
V004E15
B006C18
V025O13
V048F17
V053F14
V070K05
V059D11*
M038L14
V074E23
V061G19
V009F14
V061M13
V017A23
V016J05
V039A08
V018M12
M031E17
V047I23
M021G24
V022J10
V061P04
M045G16
M042J12
V033J04
V062C21
V005M19
B001D15
0 25 50 75 100 125 150 175 200
[root@localhost: /...
[FPC V8.9 Main Menu]
Project cp05T5e12...
[Chickpea contig p...
FPC Ctg2248 cp05...

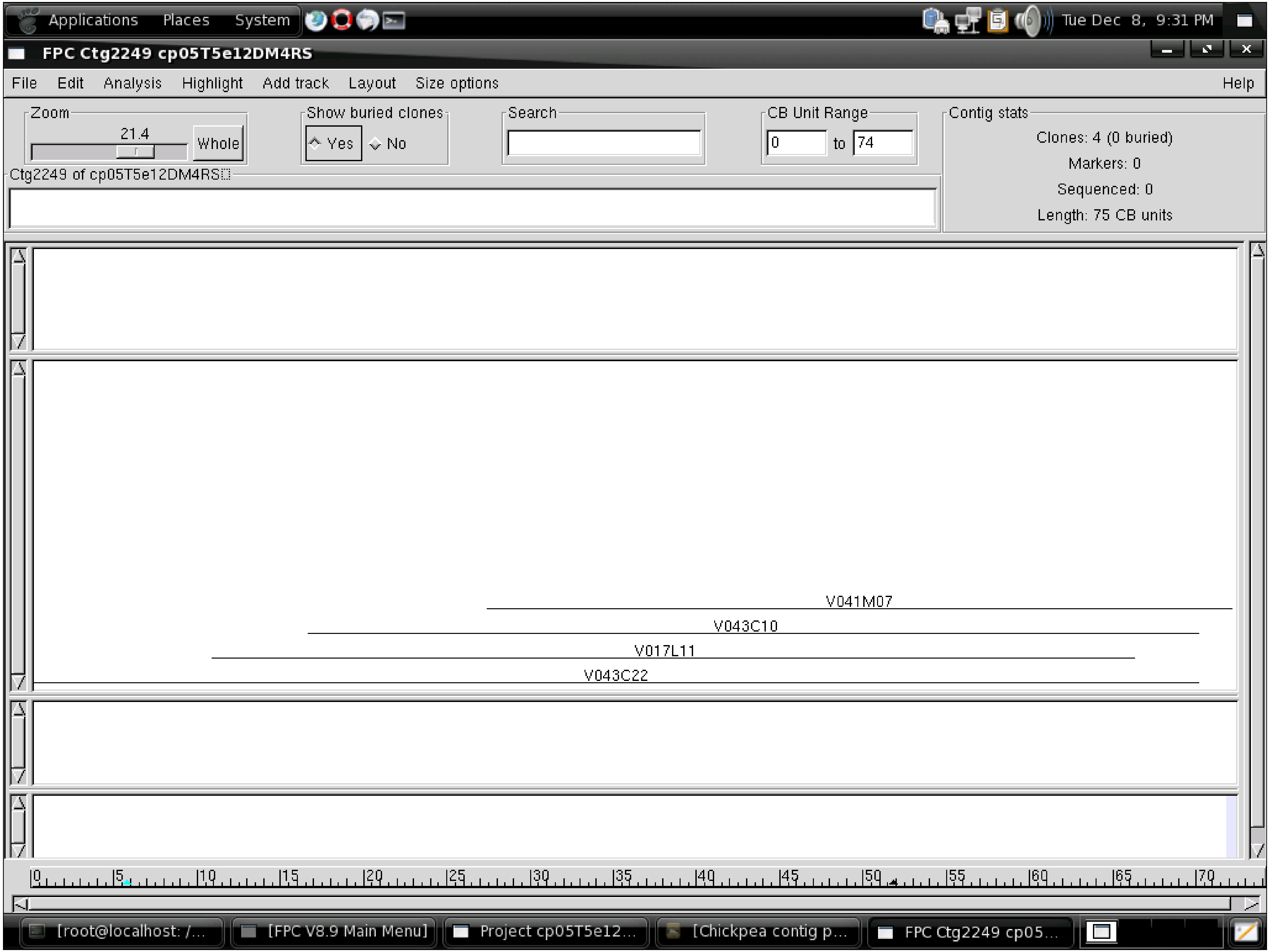
Applications
Places
System
Tue Dec 8, 9:31 PM
FPC Ctg2249 cp05T5e12DM4RS
File
Edit
Analysis
Highlight
Add track
Layout
Size options
Help
Zoom
21.4
Whole
Show buried clones
Yes
No
Search
CB Unit Range
0
to
74
Contig stats
Clones: 4 (0 buried)
Markers: 0
Sequenced: 0
Length: 75 CB units
Ctg2249 of cp05T5e12DM4RS
V041M07
V043C10
V017L11
V043C22
0
5
10
15
20
25
30
35
40
45
50
55
60
65
70
[root@localhost: /...
[FPC V8.9 Main Menu]
Project cp05T5e12...
[Chickpea contig p...
FPC Ctg2249 cp05...

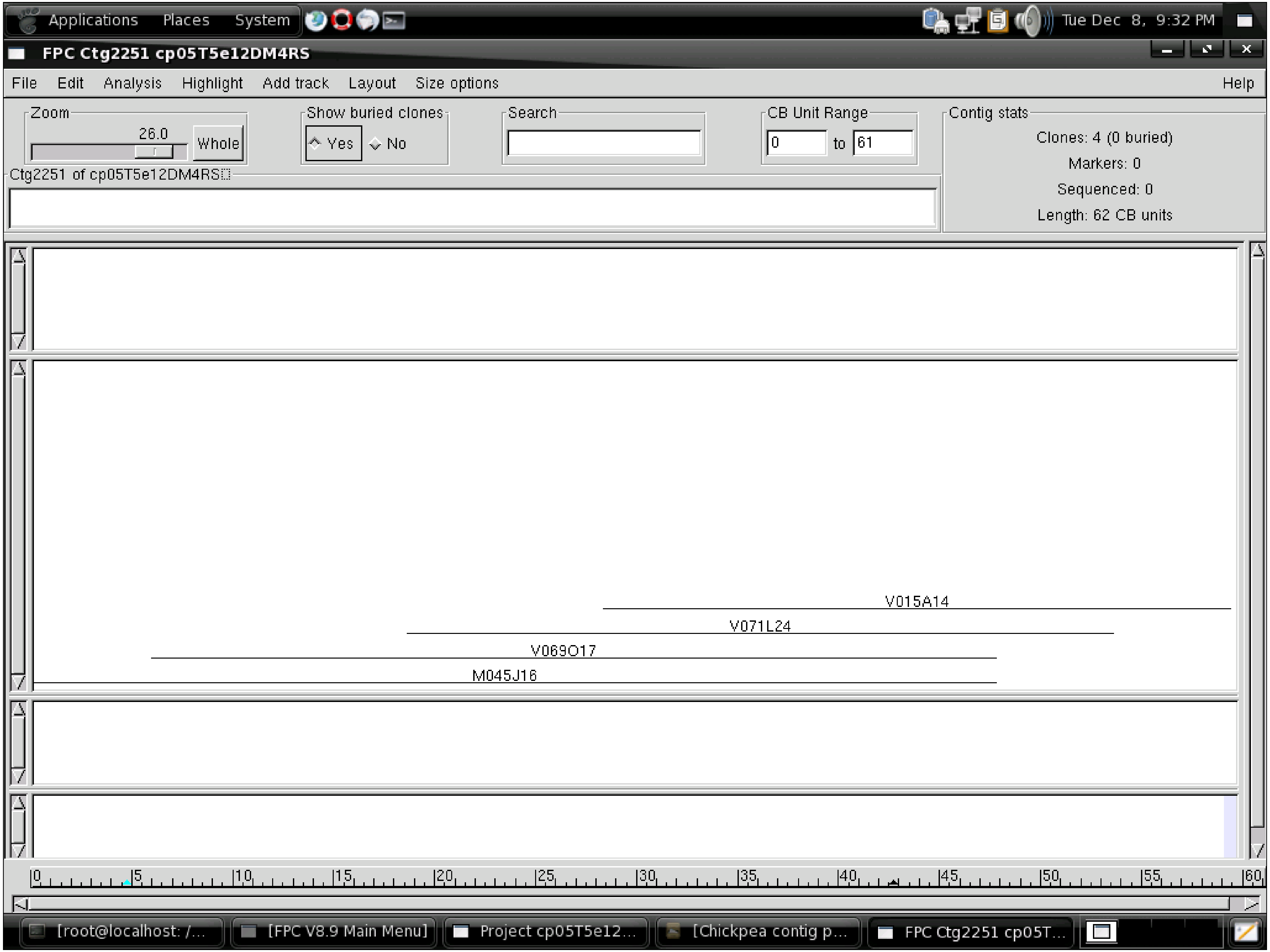
Applications Places System
Tue Dec 8, 9:32 PM
FPC Ctg2251 cp05T5e12DM4RS
File Edit Analysis Highlight Add track Layout Size options Help
Zoom
26.0
Whole
Show buried clones
Yes
No
Search
CB Unit Range
0
to
61
Contig stats
Clones: 4 (0 buried)
Markers: 0
Sequenced: 0
Length: 62 CB units
Ctg2251 of cp05T5e12DM4RS
V015A14
V071L24
V069O17
M045J16
0 5 10 15 20 25 30 35 40 45 50 55 60
[root@localhost: /...
[FPC V8.9 Main Menu]
Project cp05T5e12...
[Chickpea contig p...
FPC Ctg2251 cp05T...

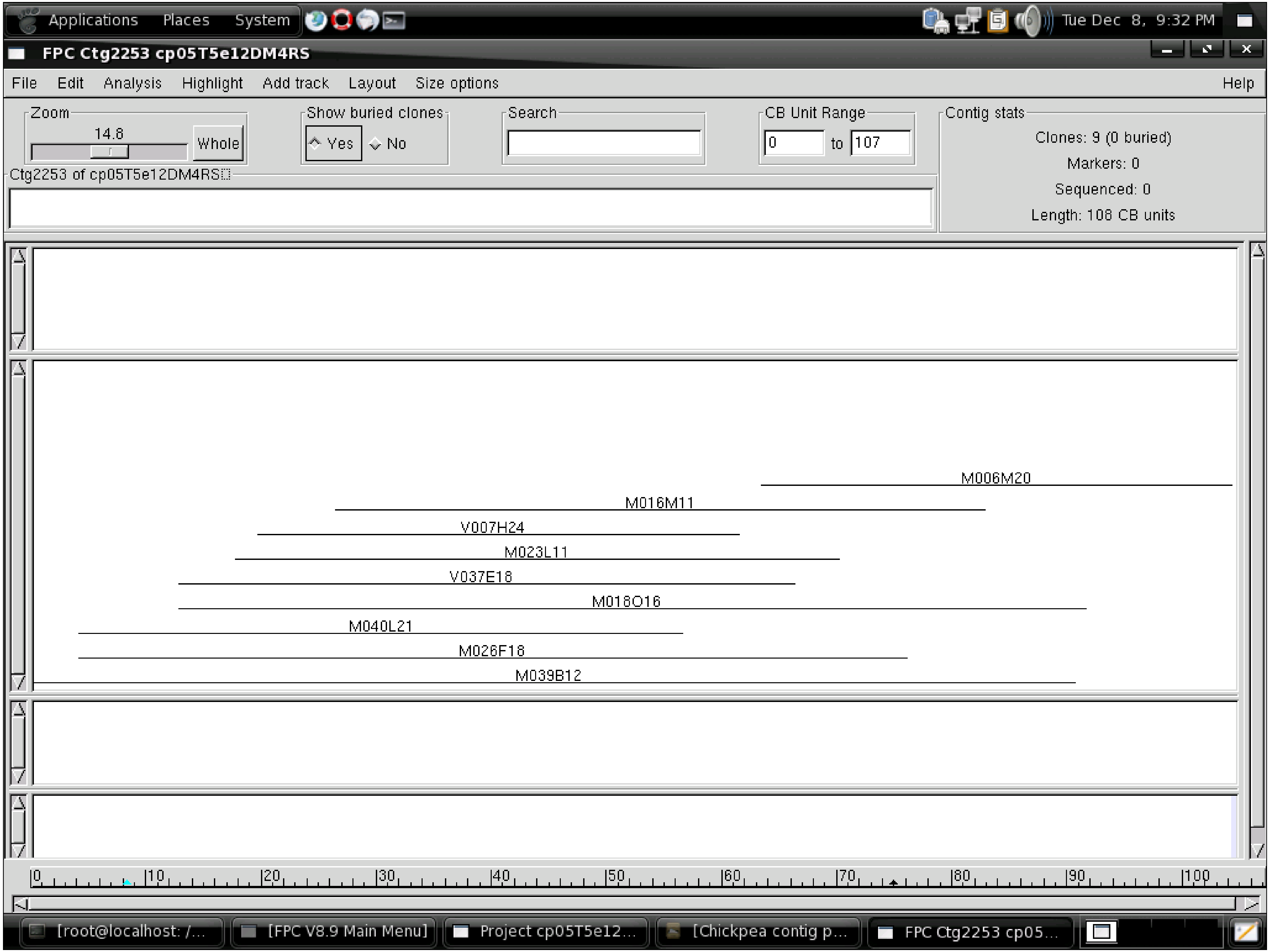
Applications Places System
Tue Dec 8, 9:32 PM
FPC Ctg2253 cp05T5e12DM4RS
File Edit Analysis Highlight Add track Layout Size options Help
Zoom
14.8
Whole
Show buried clones
Yes
No
Search
CB Unit Range
0
to
107
Contig stats
Clones: 9 (0 buried)
Markers: 0
Sequenced: 0
Length: 108 CB units
Ctg2253 of cp05T5e12DM4RS
M006M20
M016M11
V007H24
M023L11
V037E18
M018O16
M040L21
M026F18
M039B12
0 10 20 30 40 50 60 70 80 90 100
[root@localhost: /...
[FPC V8.9 Main Menu]
Project cp05T5e12...
[Chickpea contig p...
FPC Ctg2253 cp05...

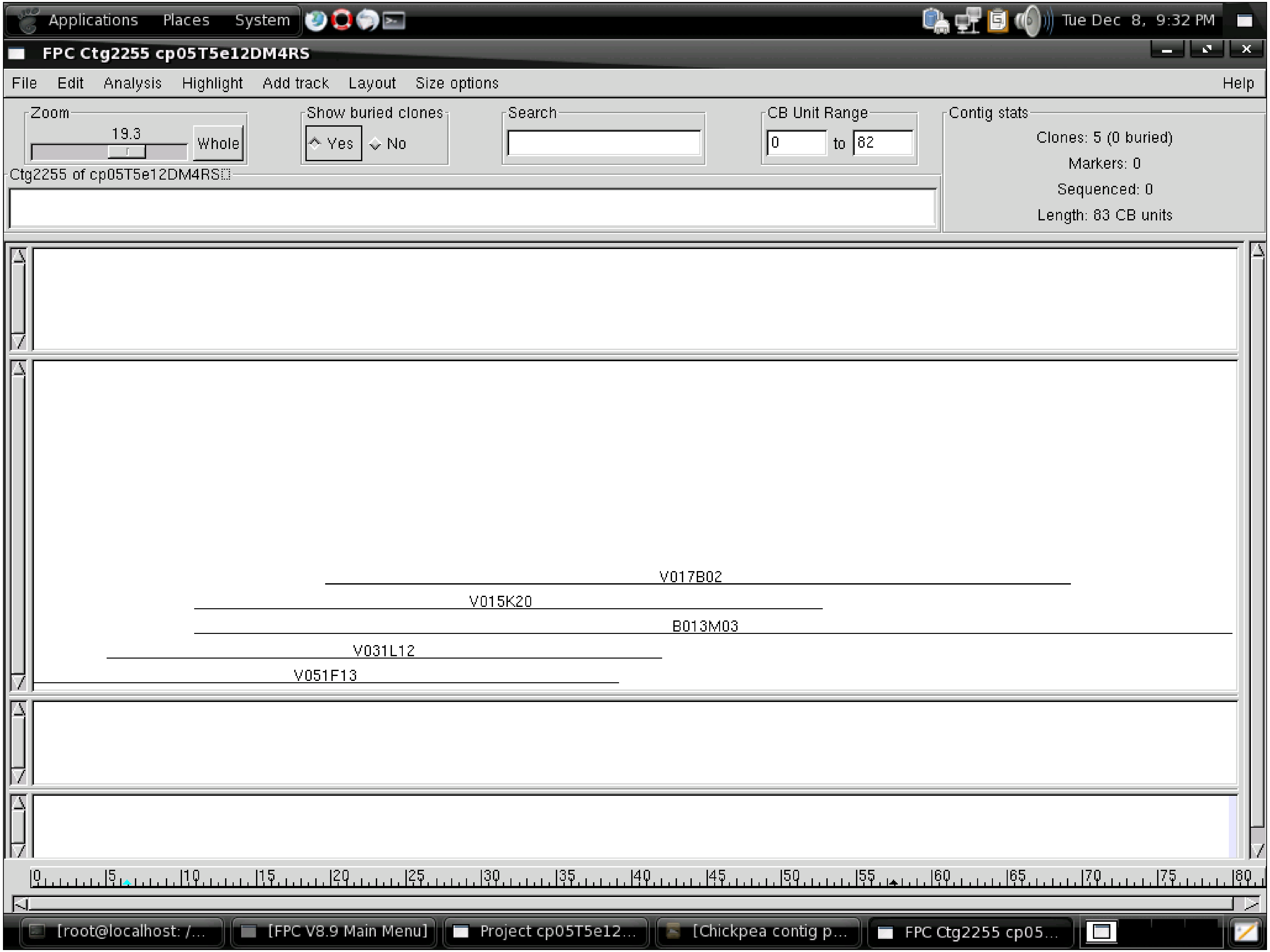
Applications
Places
System
Tue Dec 8, 9:32 PM
FPC Ctg2255 cp05T5e12DM4RS
File
Edit
Analysis
Highlight
Add track
Layout
Size options
Help
Zoom
19.3
Whole
Show buried clones
Yes
No
Search
CB Unit Range
0
to
82
Contig stats
Clones: 5 (0 buried)
Markers: 0
Sequenced: 0
Length: 83 CB units
Ctg2255 of cp05T5e12DM4RS
V017B02
V015K20
B013M03
V031L12
V051F13
0 5 10 15 20 25 30 35 40 45 50 55 60 65 70 75 80
[root@localhost: /...
[FPC V8.9 Main Menu]
Project cp05T5e12...
[Chickpea contig p...
FPC Ctg2255 cp05...

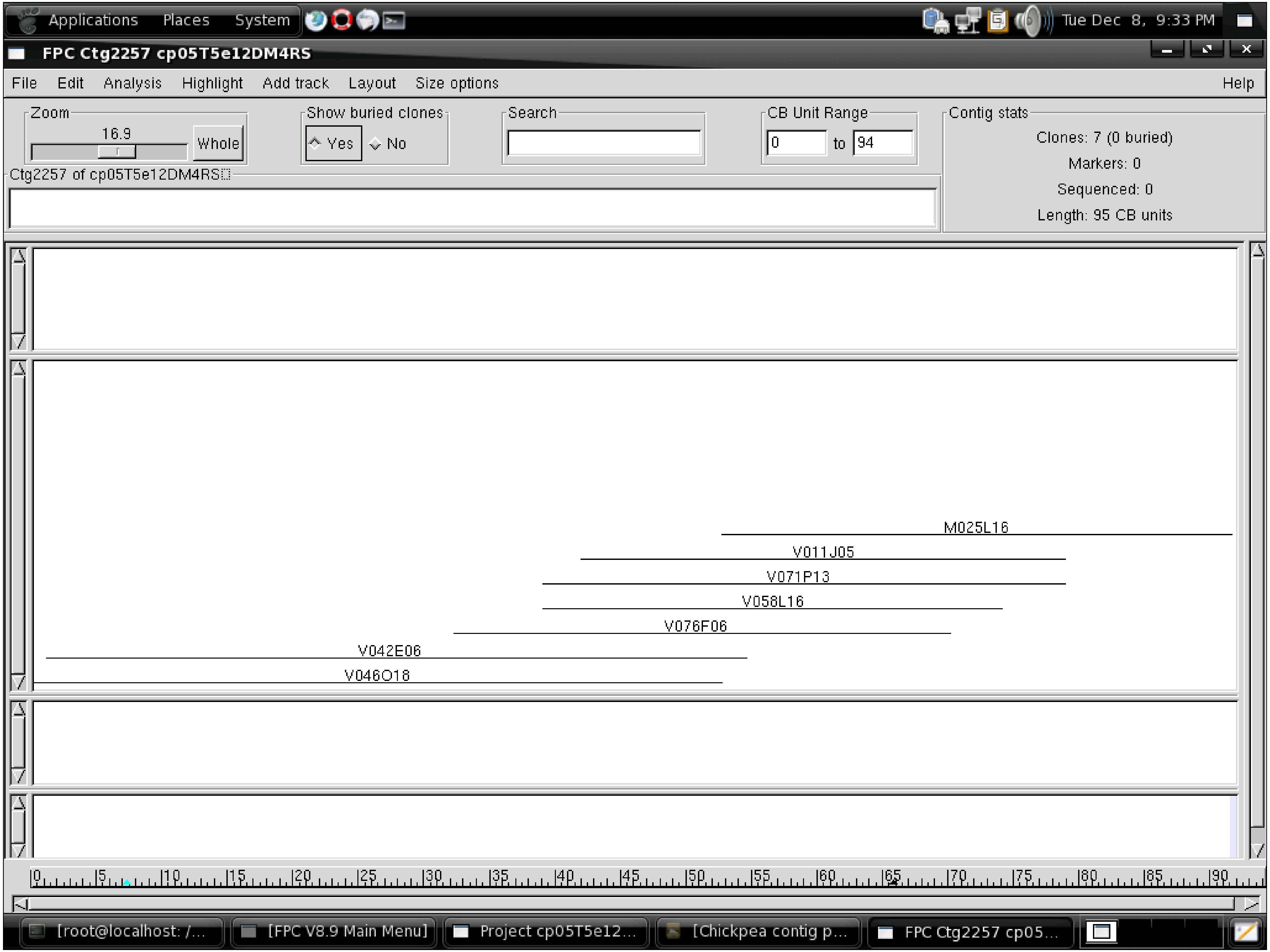
Applications Places System
Tue Dec 8, 9:33 PM
FPC Ctg2257 cp05T5e12DM4RS
File Edit Analysis Highlight Add track Layout Size options Help
Zoom
16.9
Whole
Show buried clones
Yes
No
Search
CB Unit Range
0
to
94
Contig stats
Clones: 7 (0 buried)
Markers: 0
Sequenced: 0
Length: 95 CB units
Ctg2257 of cp05T5e12DM4RS
M025L16
V011J05
V071P13
V058L16
V076F06
V042E06
V046O18
0 5 10 15 20 25 30 35 40 45 50 55 60 65 70 75 80 85 90
[root@localhost: /...
[FPC V8.9 Main Menu]
Project cp05T5e12...
[Chickpea contig p...
FPC Ctg2257 cp05...

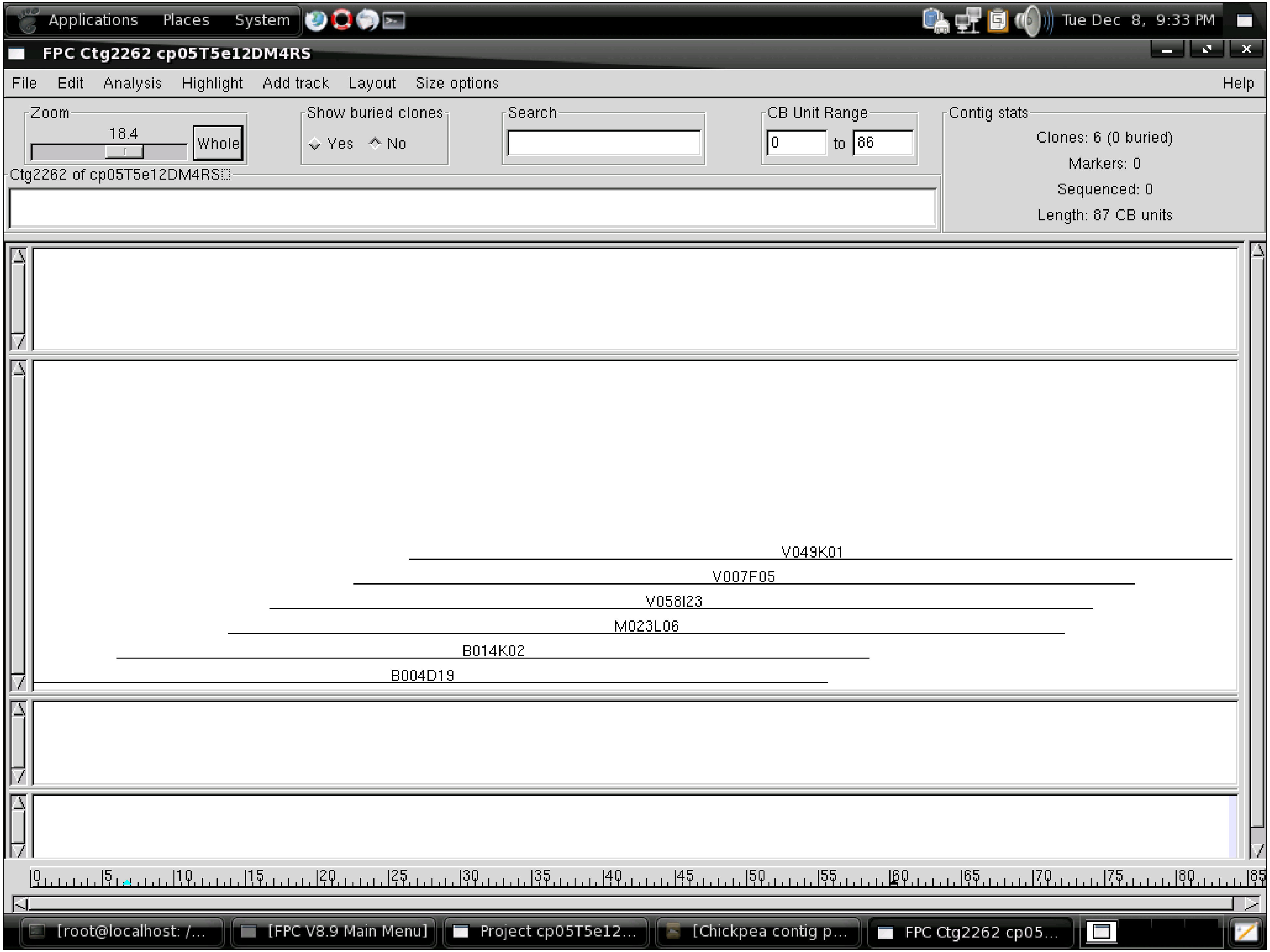
Applications Places System
Tue Dec 8, 9:33 PM
FPC Ctg2262 cp05T5e12DM4RS
File Edit Analysis Highlight Add track Layout Size options Help
Zoom
18.4
Whole
Show buried clones
Yes No
Search
CB Unit Range
0 to 86
Contig stats
Clones: 6 (0 buried)
Markers: 0
Sequenced: 0
Length: 87 CB units
Ctg2262 of cp05T5e12DM4RS
V049K01
V007F05
V058I23
M023L06
B014K02
B004D19
0 5 10 15 20 25 30 35 40 45 50 55 60 65 70 75 80 85
[root@localhost: /...
[FPC V8.9 Main Menu]
Project cp05T5e12...
[Chickpea contig p...
FPC Ctg2262 cp05...

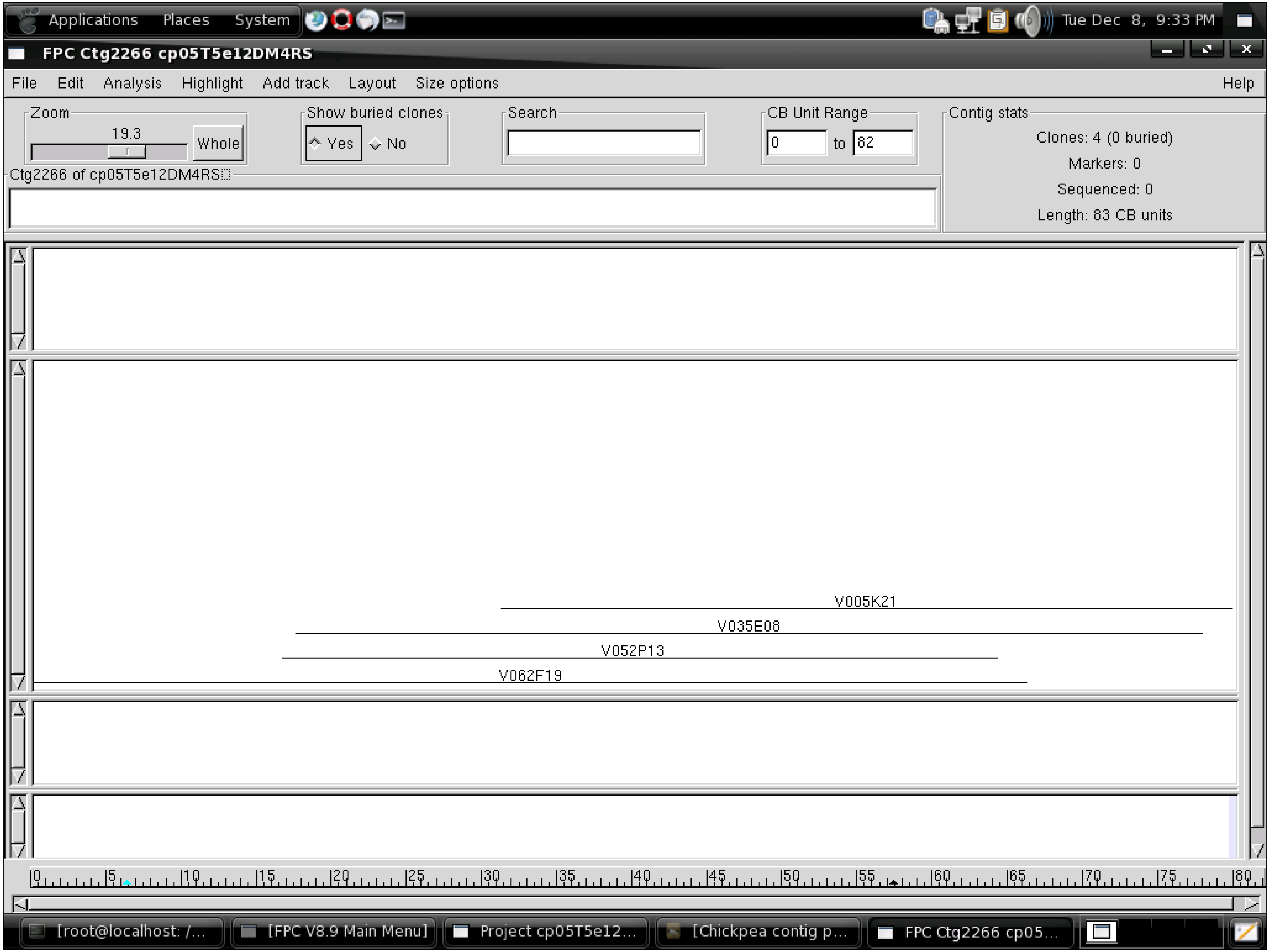
Applications Places System
Tue Dec 8, 9:33 PM
FPC Ctg2266 cp05T5e12DM4RS
File Edit Analysis Highlight Add track Layout Size options Help
Zoom
19.3
Whole
Show buried clones
Yes No
Search
CB Unit Range
0 to 82
Contig stats
Clones: 4 (0 buried)
Markers: 0
Sequenced: 0
Length: 83 CB units
Ctg2266 of cp05T5e12DM4RS
V005K21
V035E08
V052P13
V062F19
0 5 10 15 20 25 30 35 40 45 50 55 60 65 70 75 80
[root@localhost: /...
[FPC V8.9 Main Menu]
Project cp05T5e12...
[Chickpea contig p...
FPC Ctg2266 cp05...

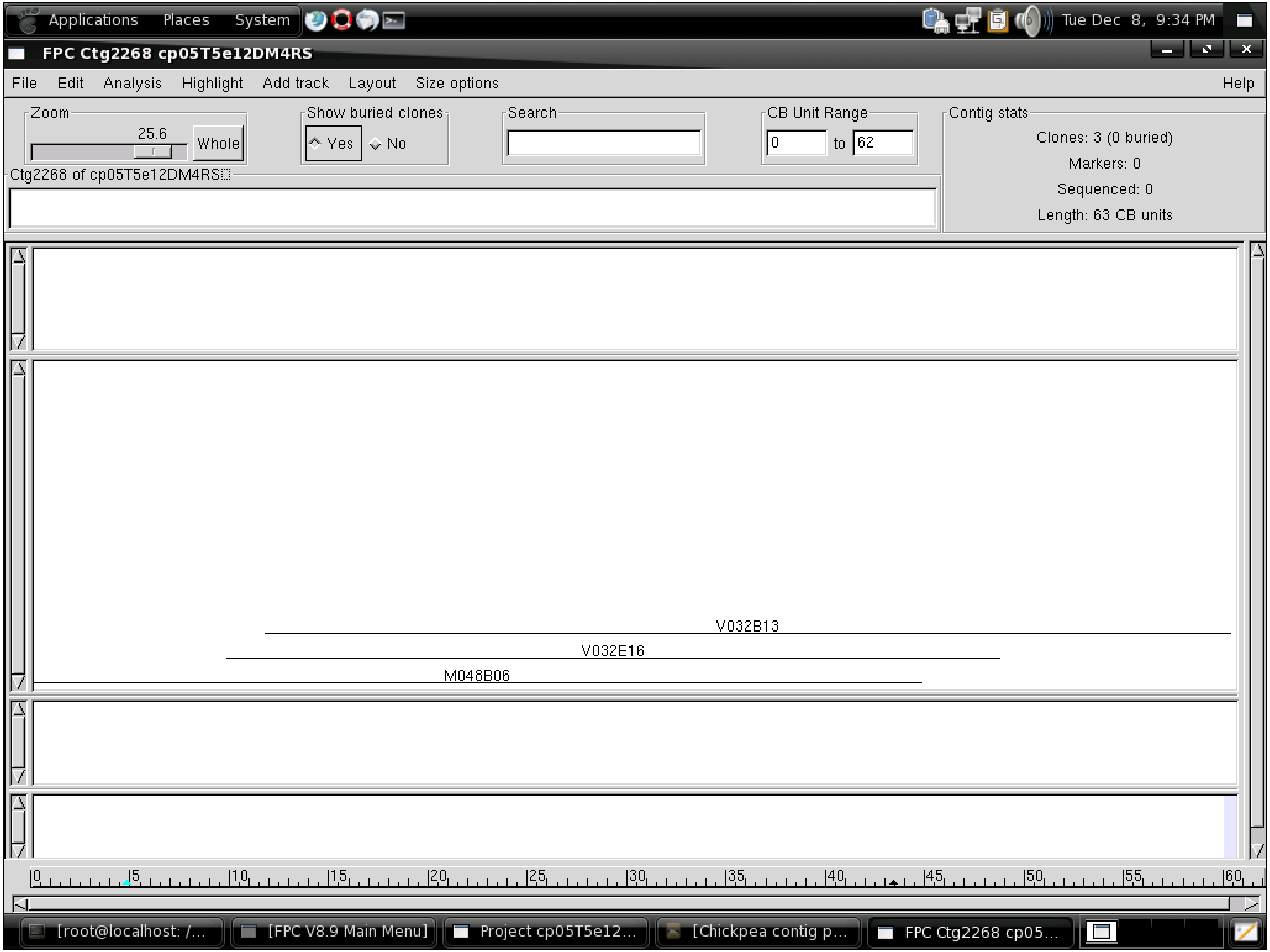
Applications
Places
System
Tue Dec 8, 9:34 PM
FPC Ctg2268 cp05T5e12DM4RS
File
Edit
Analysis
Highlight
Add track
Layout
Size options
Help
Zoom
25.6
Whole
Show buried clones
Yes
No
Search
CB Unit Range
0
to
62
Contig stats
Clones: 3 (0 buried)
Markers: 0
Sequenced: 0
Length: 63 CB units
Ctg2268 of cp05T5e12DM4RS
V032B13
V032E16
M048B06
0
5
10
15
20
25
30
35
40
45
50
55
60
[root@localhost: /...
[FPC V8.9 Main Menu]
Project cp05T5e12...
[Chickpea contig p...
FPC Ctg2268 cp05...

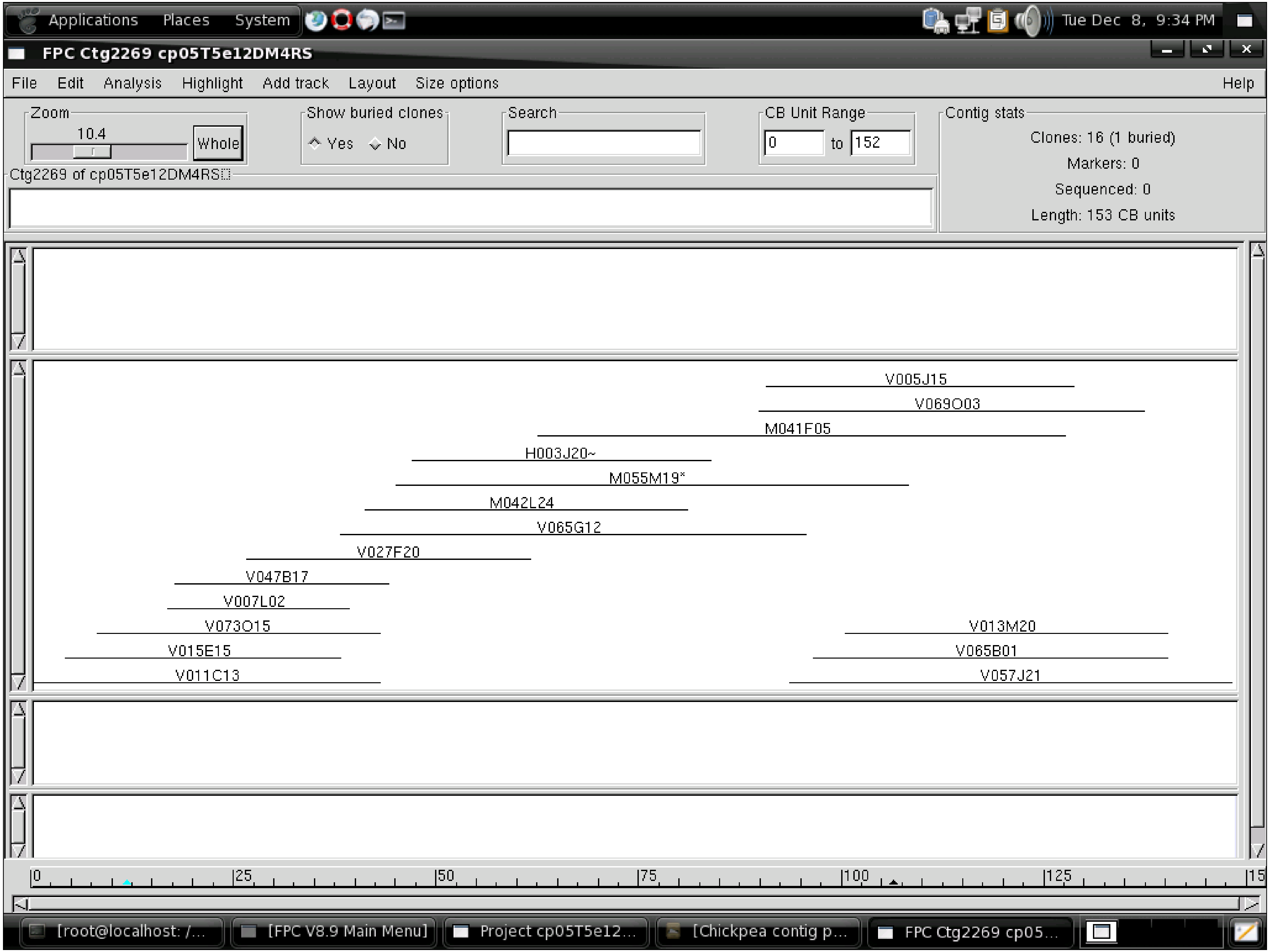
Applications Places System
Tue Dec 8, 9:34 PM
FPC Ctg2269 cp05T5e12DM4RS
File Edit Analysis Highlight Add track Layout Size options
Help
Zoom
10.4
Whole
Show buried clones
Yes No
Search
CB Unit Range
0 to 152
Contig stats
Clones: 16 (1 buried)
Markers: 0
Sequenced: 0
Length: 153 CB units
Ctg2269 of cp05T5e12DM4RS
V005J15
V069O03
M041F05
H003J20~
M055M19*
M042L24
V065G12
V027F20
V047B17
V007L02
V073O15
V013M20
V015E15
V065B01
V011C13
V057J21
0
25
50
75
100
125
[root@localhost: /...
[FPC V8.9 Main Menu]
Project cp05T5e12...
[Chickpea contig p...
FPC Ctg2269 cp05...

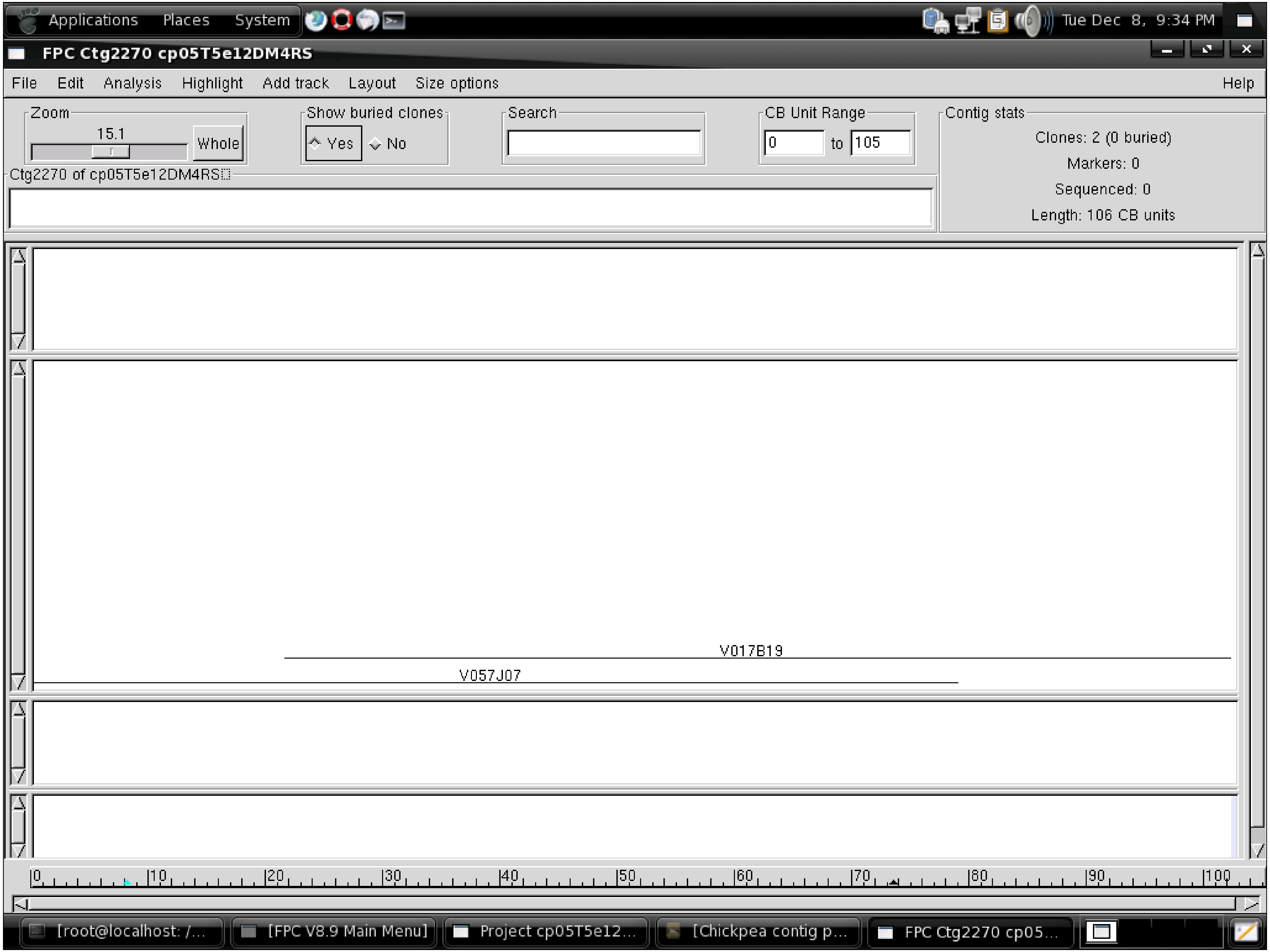
Applications Places System
Tue Dec 8, 9:34 PM
FPC Ctg2270 cp05T5e12DM4RS
File Edit Analysis Highlight Add track Layout Size options Help
Zoom
15.1
Whole
Show buried clones
Yes No
Search
CB Unit Range
0 to 105
Contig stats
Clones: 2 (0 buried)
Markers: 0
Sequenced: 0
Length: 106 CB units
Ctg2270 of cp05T5e12DM4RS
V017B19
V057J07
0 10 20 30 40 50 60 70 80 90 100
[root@localhost: /...
[FPC V8.9 Main Menu]
Project cp05T5e12...
[Chickpea contig p...
FPC Ctg2270 cp05...

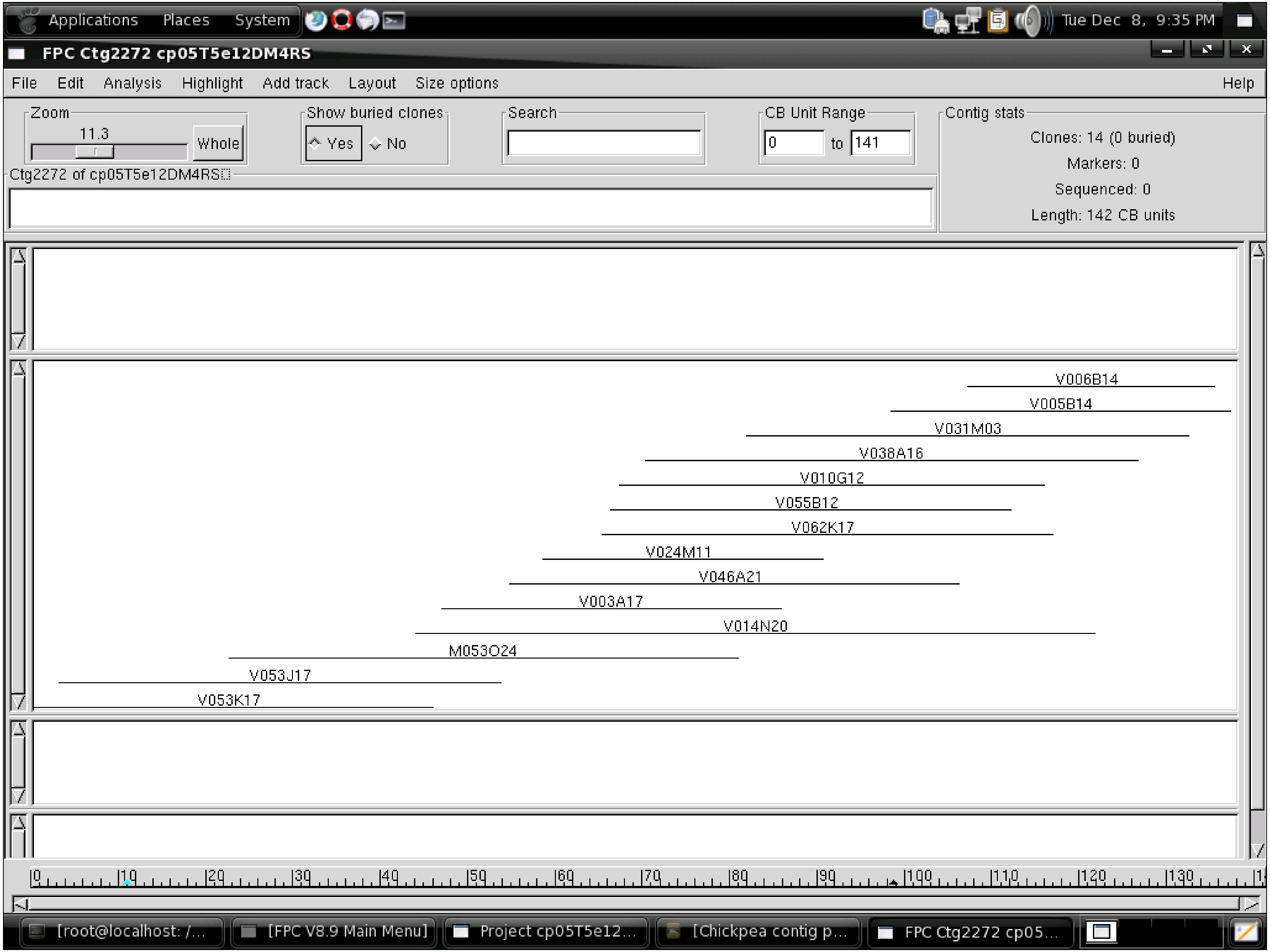
Applications Places System
Tue Dec 8, 9:35 PM
FPC Ctg2272 cp05T5e12DM4RS
File Edit Analysis Highlight Add track Layout Size options Help
Zoom
11.3
Whole
Show buried clones
Yes
No
Search
CB Unit Range
0 to 141
Contig stats
Clones: 14 (0 buried)
Markers: 0
Sequenced: 0
Length: 142 CB units
Ctg2272 of cp05T5e12DM4RS
V006B14
V005B14
V031M03
V038A16
V010G12
V055B12
V062K17
V024M11
V046A21
V003A17
V014N20
M053O24
V053J17
V053K17
0 10 20 30 40 50 60 70 80 90 100 110 120 130
[root@localhost: /...
[FPC V8.9 Main Menu]
Project cp05T5e12...
[Chickpea contig p...
FPC Ctg2272 cp05...

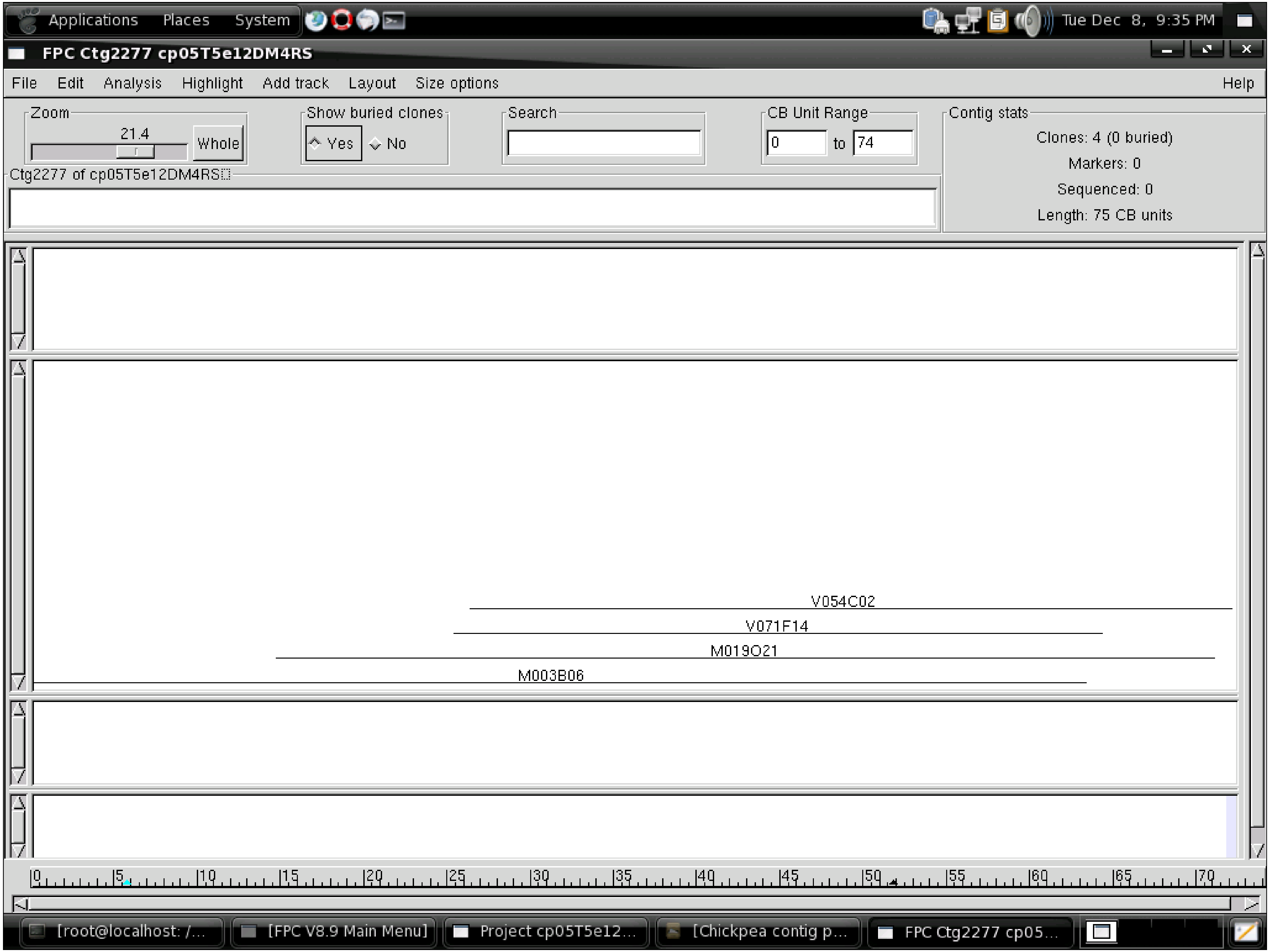
Applications Places System
Tue Dec 8, 9:35 PM
FPC Ctg2277 cp05T5e12DM4RS
File Edit Analysis Highlight Add track Layout Size options Help
Zoom
21.4
Whole
Show buried clones
Yes No
Search
CB Unit Range
0 to 74
Contig stats
Clones: 4 (0 buried)
Markers: 0
Sequenced: 0
Length: 75 CB units
Ctg2277 of cp05T5e12DM4RS
V054C02
V071F14
M019O21
M003B06
0 5 10 15 20 25 30 35 40 45 50 55 60 65 70
[root@localhost: /...
[FPC V8.9 Main Menu]
Project cp05T5e12...
[Chickpea contig p...
FPC Ctg2277 cp05...

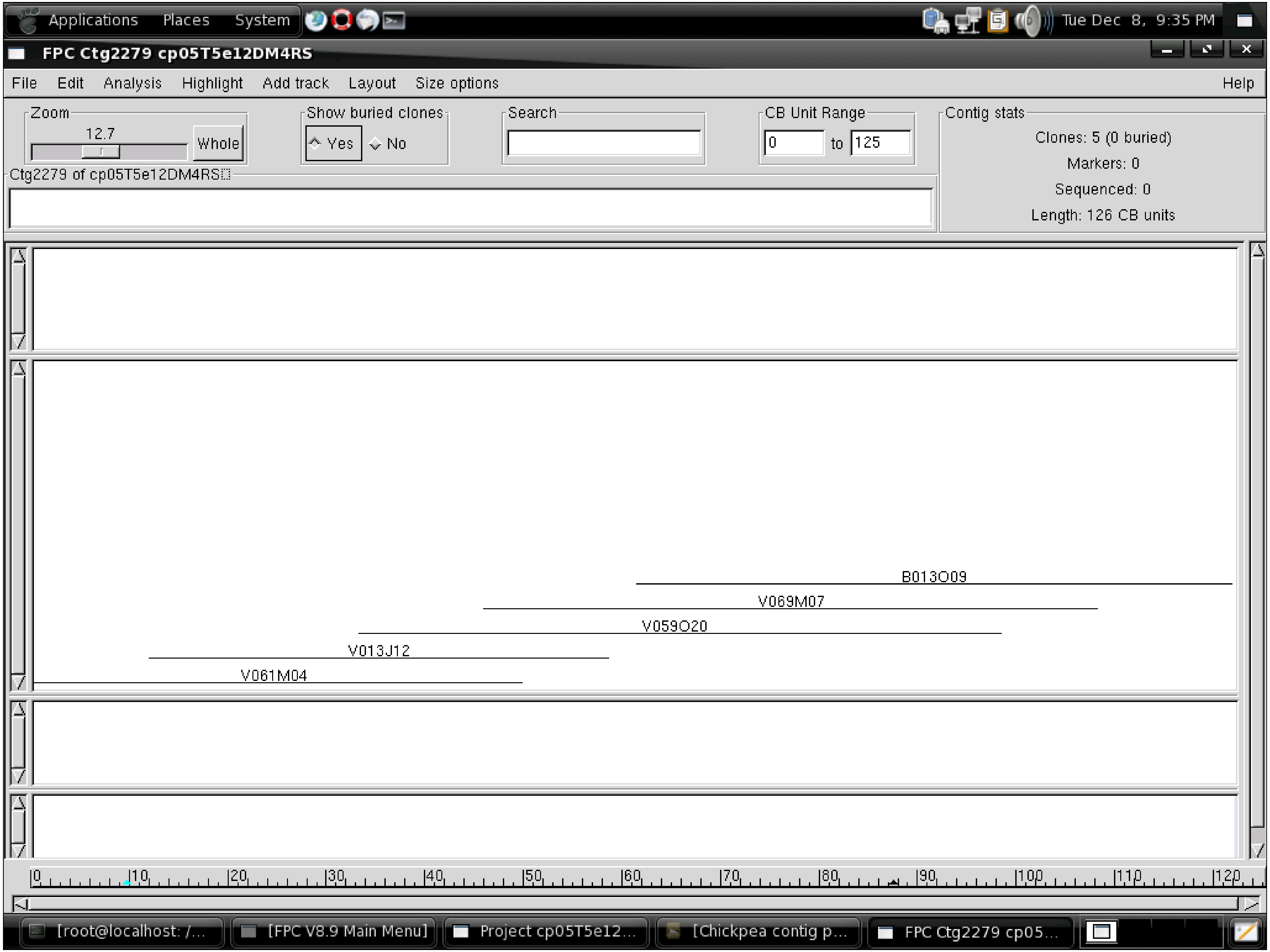
Applications
Places
System
Tue Dec 8, 9:35 PM
FPC Ctg2279 cp05T5e12DM4RS
File
Edit
Analysis
Highlight
Add track
Layout
Size options
Help
Zoom
12.7
Whole
Show buried clones
Yes
No
Search
CB Unit Range
0
to
125
Contig stats
Clones: 5 (0 buried)
Markers: 0
Sequenced: 0
Length: 126 CB units
Ctg2279 of cp05T5e12DM4RS
B013O09
V069M07
V059O20
V013J12
V061M04
0
10
20
30
40
50
60
70
80
90
100
110
120
[root@localhost: /...
[FPC V8.9 Main Menu]
Project cp05T5e12...
[Chickpea contig p...
FPC Ctg2279 cp05...

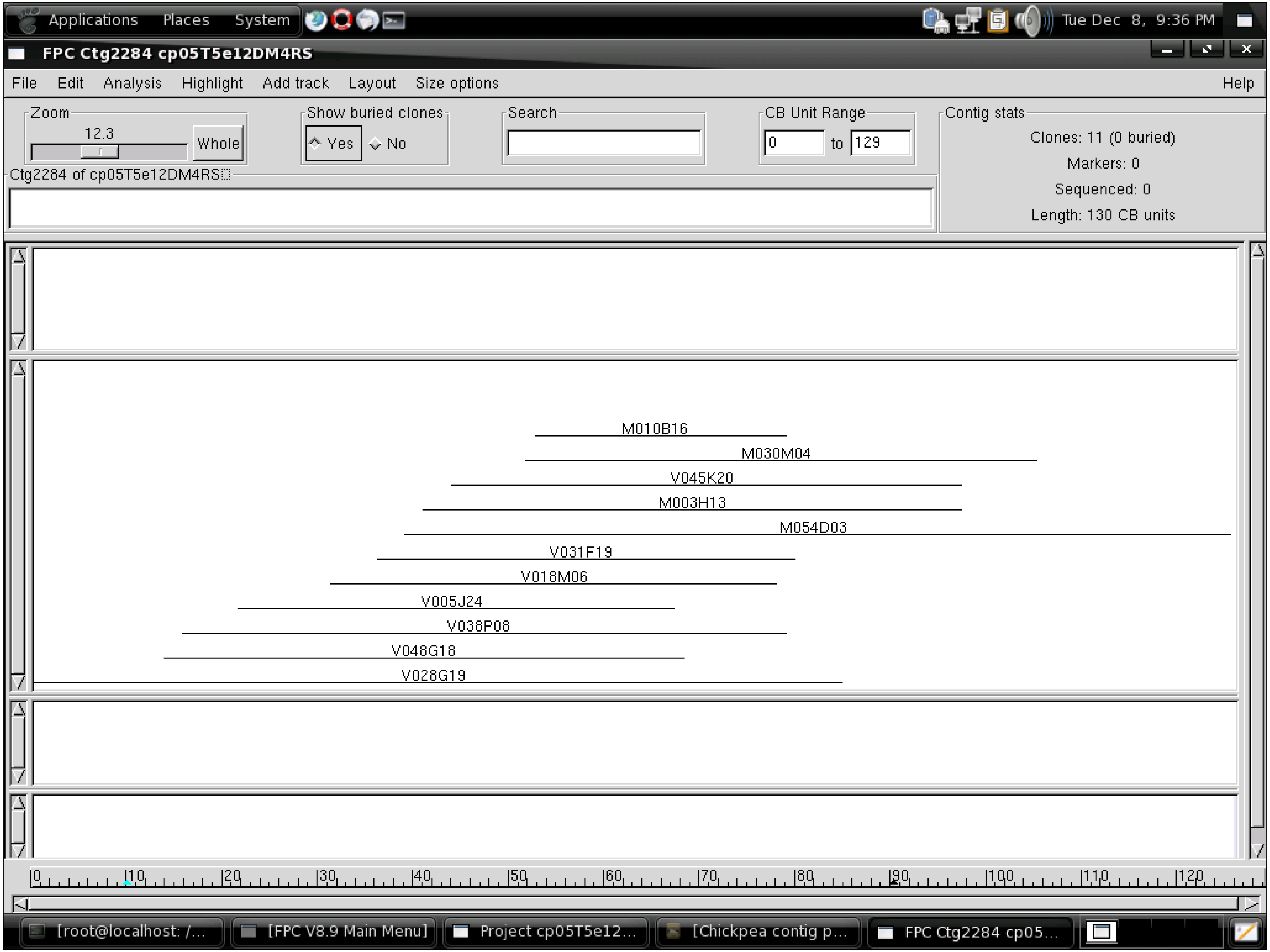
Applications
Places
System
Tue Dec 8, 9:36 PM
FPC Ctg2284 cp05T5e12DM4RS
File
Edit
Analysis
Highlight
Add track
Layout
Size options
Help
Zoom
12.3
Whole
Show buried clones
Yes
No
Search
CB Unit Range
0
to
129
Contig stats
Clones: 11 (0 buried)
Markers: 0
Sequenced: 0
Length: 130 CB units
Ctg2284 of cp05T5e12DM4RS
M010B16
M030M04
V045K20
M003H13
M054D03
V031F19
V018M06
V005J24
V038P08
V048G18
V028G19
0
10
20
30
40
50
60
70
80
90
100
110
120
[root@localhost: /...
[FPC V8.9 Main Menu]
Project cp05T5e12...
[Chickpea contig p...
FPC Ctg2284 cp05...

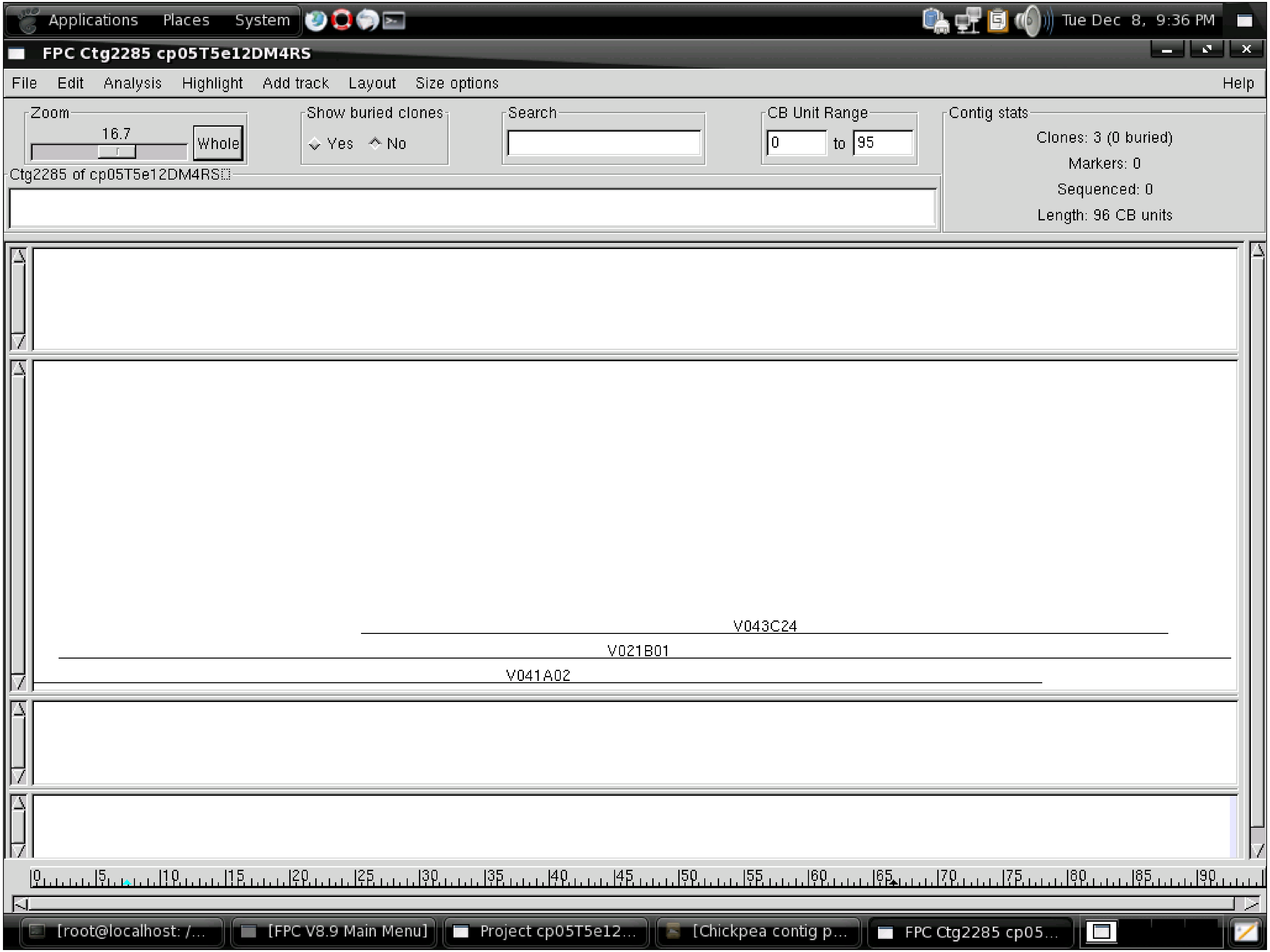

Applications Places System
Tue Dec 8, 9:36 PM
FPC Ctg2285 cp05T5e12DM4RS
File Edit Analysis Highlight Add track Layout Size options Help
Zoom
16.7
Whole
Show buried clones
Yes No
Search
CB Unit Range
0 to 95
Contig stats
Clones: 3 (0 buried)
Markers: 0
Sequenced: 0
Length: 96 CB units
Ctg2285 of cp05T5e12DM4RS
V043C24
V021B01
V041A02
0 5 10 15 20 25 30 35 40 45 50 55 60 65 70 75 80 85 90
[root@localhost: /...
[FPC V8.9 Main Menu]
Project cp05T5e12...
[Chickpea contig p...
FPC Ctg2285 cp05...

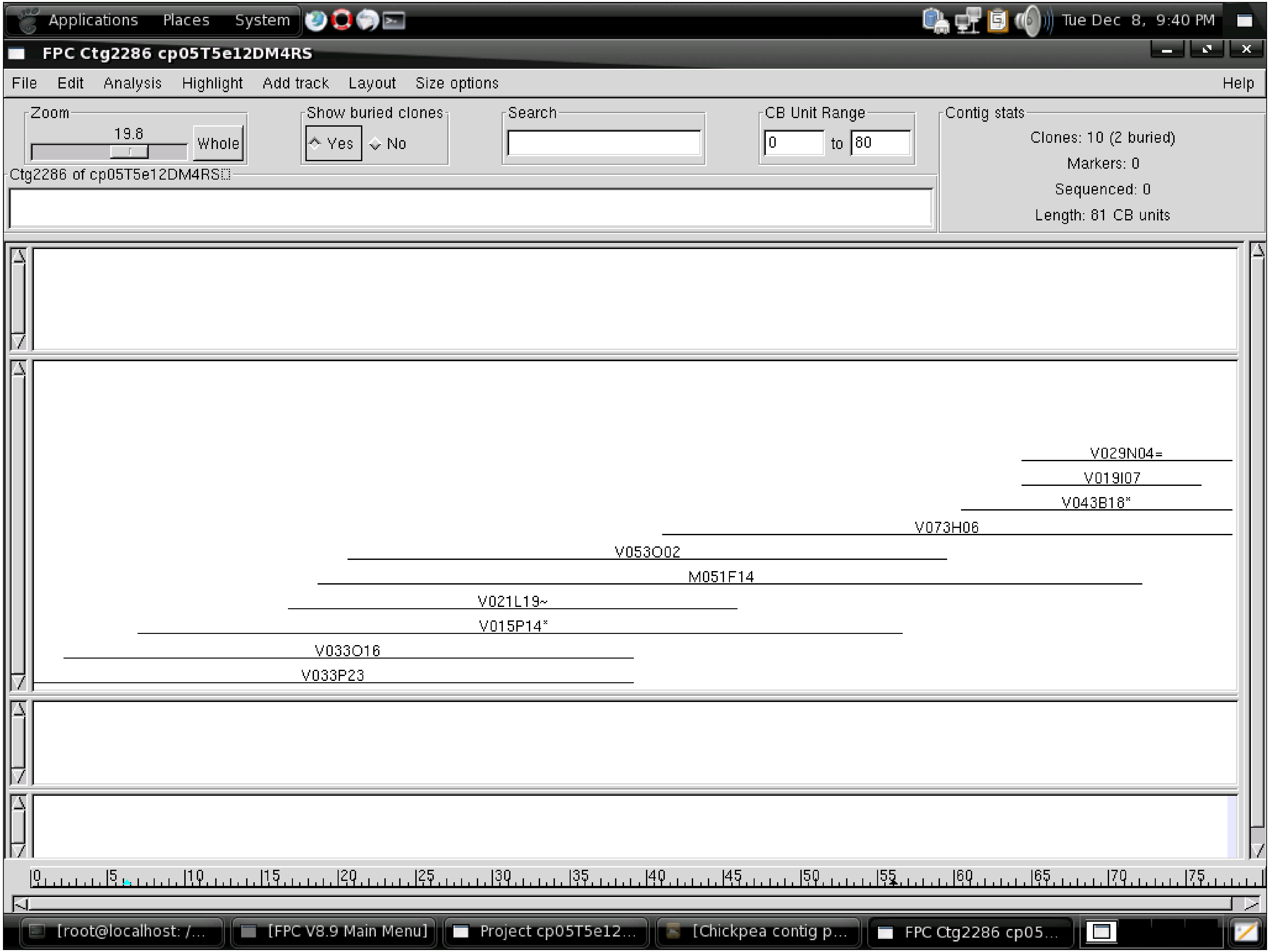
Applications Places System
Tue Dec 8, 9:40 PM
FPC Ctg2286 cp05T5e12DM4RS
File Edit Analysis Highlight Add track Layout Size options Help
Zoom
19.8
Whole
Show buried clones
Yes
No
Search
CB Unit Range
0 to 80
Contig stats
Clones: 10 (2 buried)
Markers: 0
Sequenced: 0
Length: 81 CB units
Ctg2286 of cp05T5e12DM4RS
V029N04=
V019I07
V043B18*
V073H06
V053O02
M051F14
V021L19~
V015P14*
V033O16
V033P23
0 5 10 15 20 25 30 35 40 45 50 55 60 65 70 75
[root@localhost: /...
[FPC V8.9 Main Menu]
Project cp05T5e12...
[Chickpea contig p...
FPC Ctg2286 cp05...

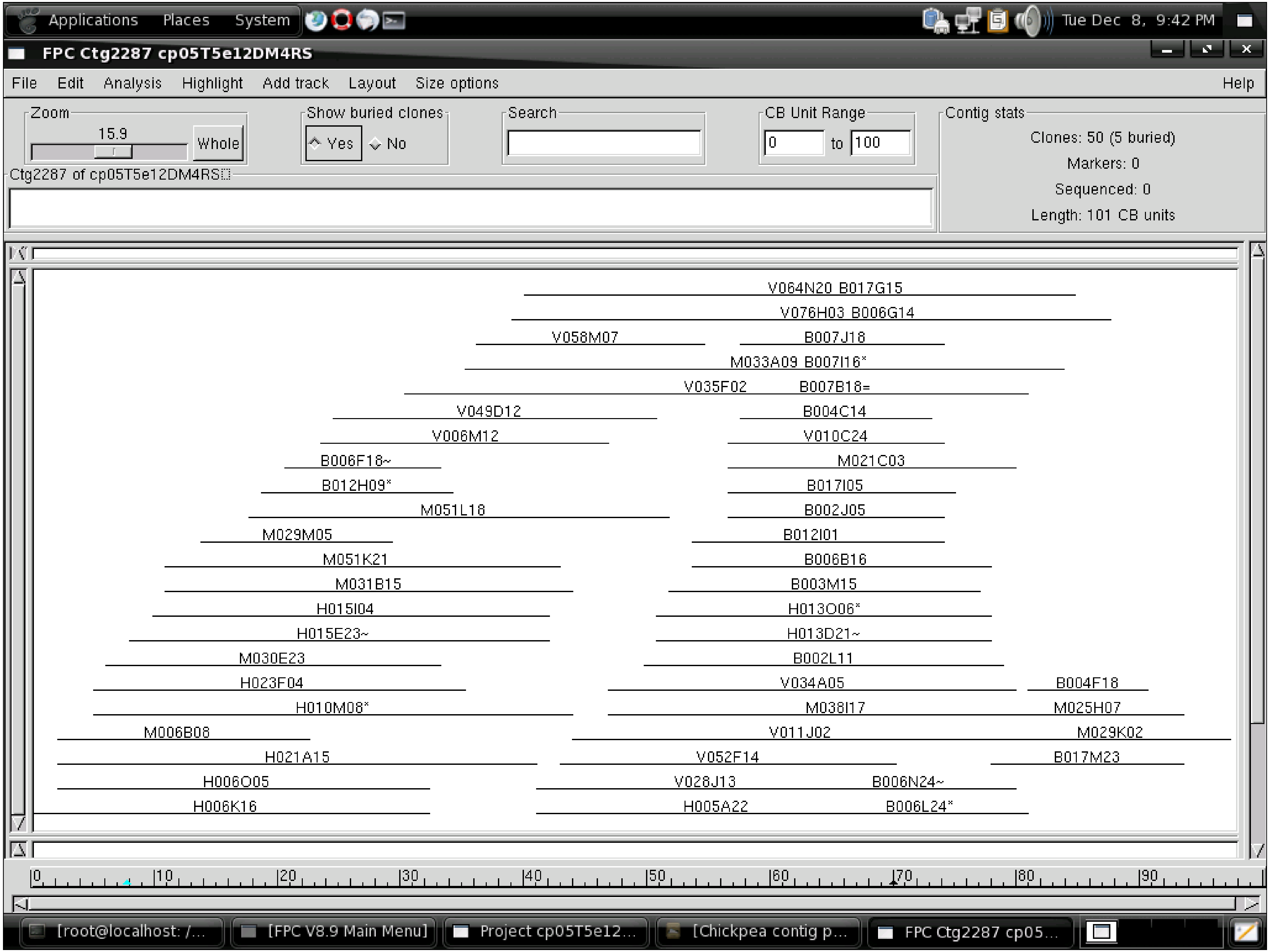
Applications Places System
Tue Dec 8, 9:42 PM
FPC Ctg2287 cp05T5e12DM4RS
File Edit Analysis Highlight Add track Layout Size options Help
Zoom
15.9
Whole
Show buried clones
Yes
No
Search
CB Unit Range
0
to
100
Contig stats
Clones: 50 (5 buried)
Markers: 0
Sequenced: 0
Length: 101 CB units
Ctg2287 of cp05T5e12DM4RS
V064N20 B017G15
V076H03 B006G14
V058M07
B007J18
M033A09 B007I16*
V035F02
B007B18=
V049D12
B004C14
V006M12
V010C24
B006F18~
M021C03
B012H09*
B017I05
M051L18
B002J05
M029M05
B012I01
M051K21
B006B16
M031B15
B003M15
H015I04
H013O06*
H015E23~
H013D21~
M030E23
B002L11
H023F04
V034A05
B004F18
H010M08*
M038I17
M025H07
M006B08
V011J02
M029K02
H021A15
V052F14
B017M23
H006O05
V028J13
B006N24~
H006K16
H005A22
B006L24*
0
10
20
30
40
50
60
70
80
90
[root@localhost: /...
[FPC V8.9 Main Menu]
Project cp05T5e12...
[Chickpea contig p...
FPC Ctg2287 cp05...

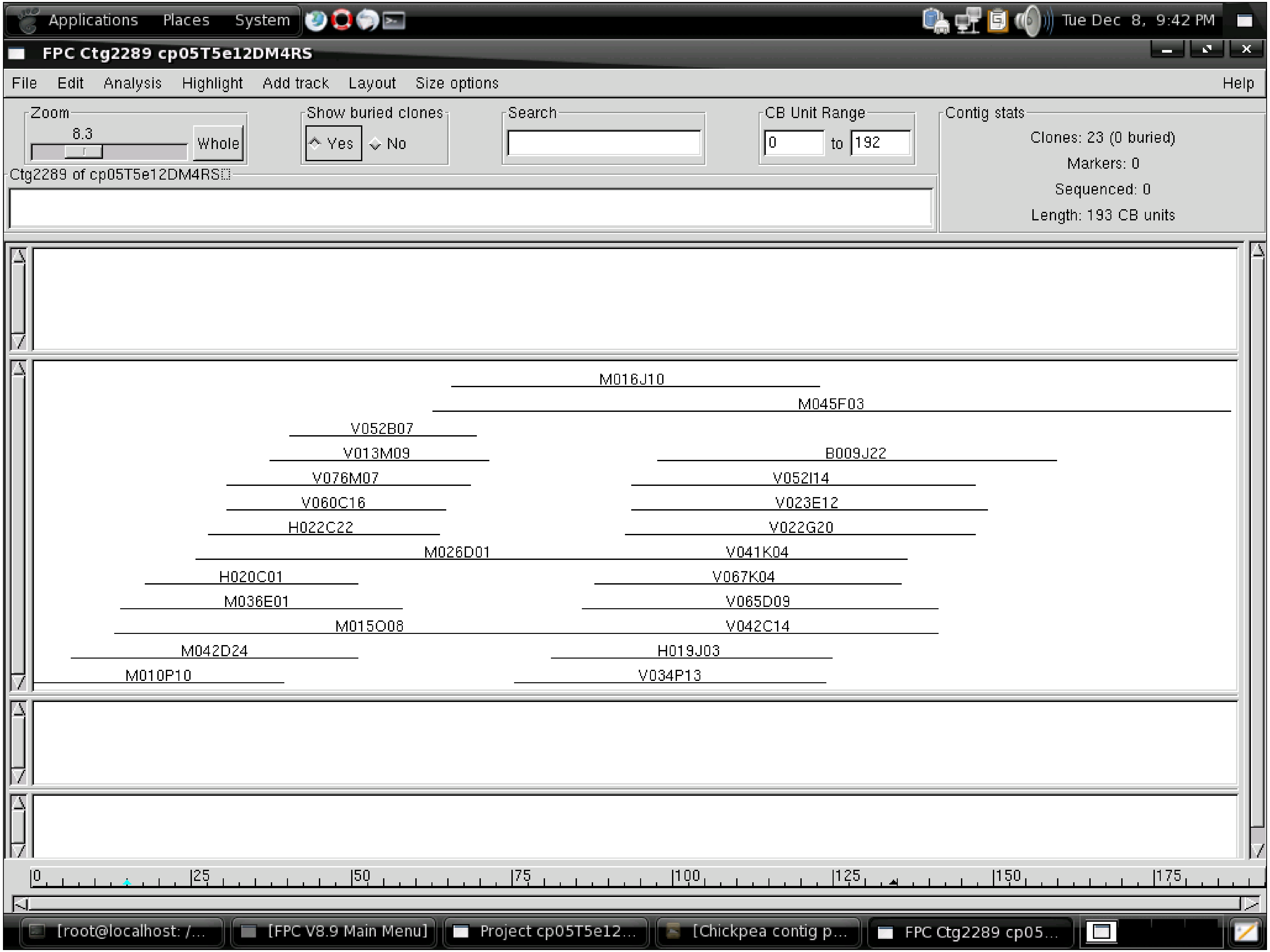
Applications Places System
Tue Dec 8, 9:42 PM
FPC Ctg2289 cp05T5e12DM4RS
File Edit Analysis Highlight Add track Layout Size options Help
Zoom
8.3
Whole
Show buried clones
Yes No
Search
CB Unit Range
0 to 192
Contig stats
Clones: 23 (0 buried)
Markers: 0
Sequenced: 0
Length: 193 CB units
Ctg2289 of cp05T5e12DM4RS
M016J10
M045F03
V052B07
V013M09
B009J22
V076M07
V052I14
V060C16
V023E12
H022C22
V022G20
M026D01
V041K04
H020C01
V067K04
M036E01
V065D09
M015O08
V042C14
M042D24
H019J03
M010P10
V034P13
0 25 50 75 100 125 150 175
[root@localhost: /...
[FPC V8.9 Main Menu]
Project cp05T5e12...
[Chickpea contig p...
FPC Ctg2289 cp05...

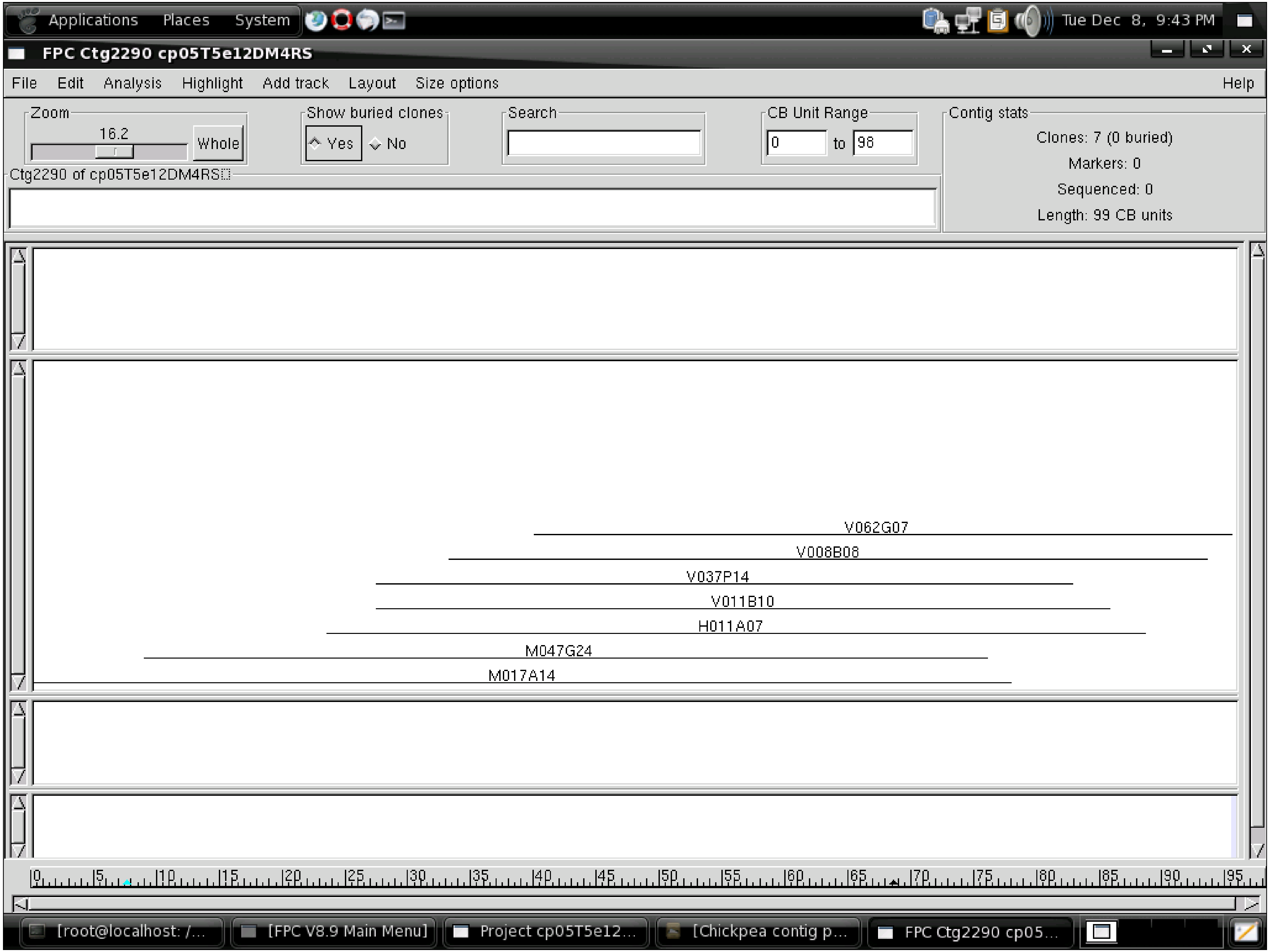
Applications Places System
Tue Dec 8, 9:43 PM
FPC Ctg2290 cp05T5e12DM4RS
File Edit Analysis Highlight Add track Layout Size options Help
Zoom
16.2
Whole
Show buried clones
Yes No
Search
CB Unit Range
0 to 98
Contig stats
Clones: 7 (0 buried)
Markers: 0
Sequenced: 0
Length: 99 CB units
Ctg2290 of cp05T5e12DM4RS
V062G07
V008B08
V037P14
V011B10
H011A07
M047G24
M017A14
0 5 10 15 20 25 30 35 40 45 50 55 60 65 70 75 80 85 90 95
[root@localhost: /...
[FPC V8.9 Main Menu]
Project cp05T5e12...
[Chickpea contig p...
FPC Ctg2290 cp05...

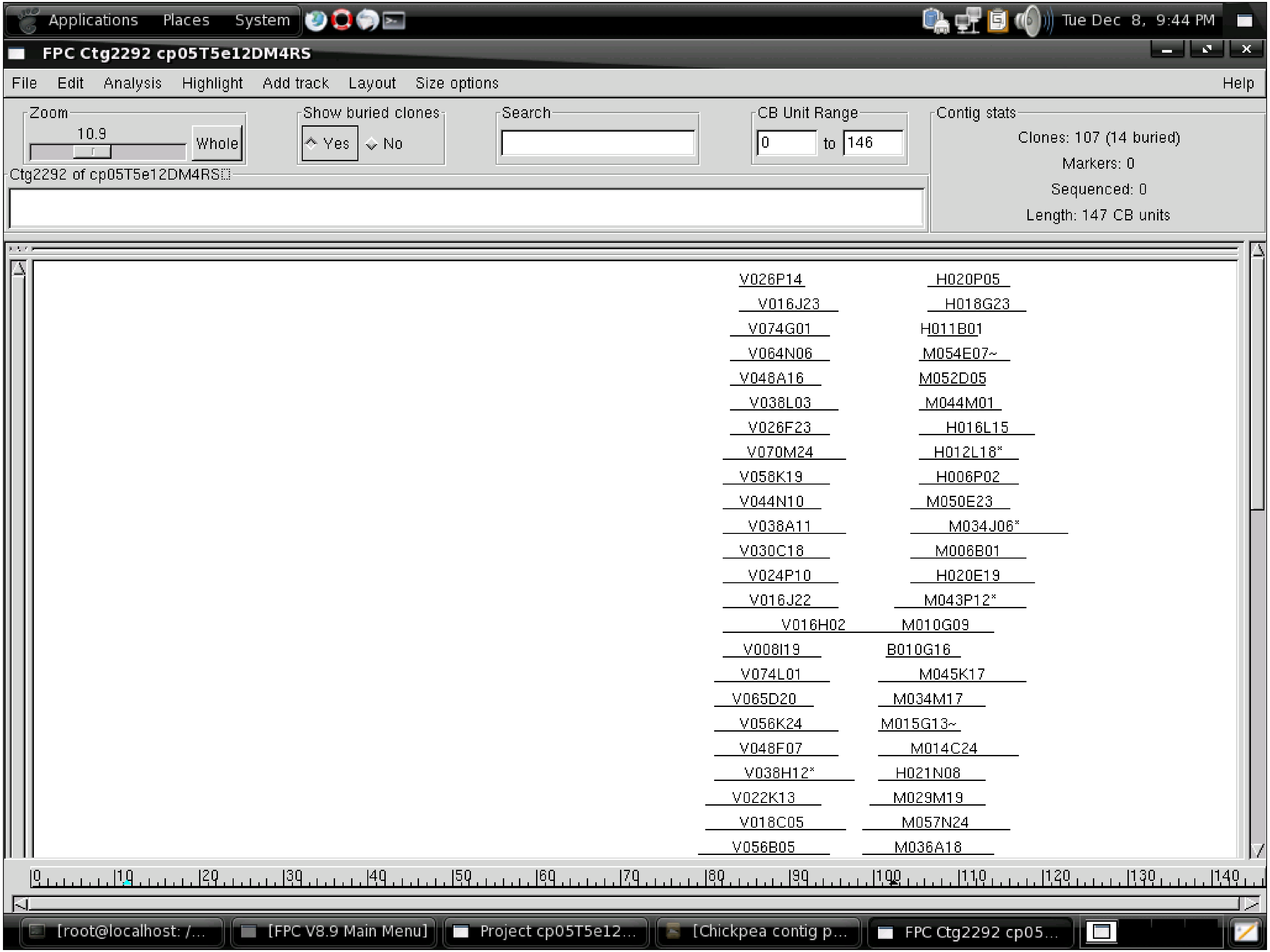

Applications Places System
Tue Dec 8, 9:44 PM
FPC Ctg2292 cp05T5e12DM4RS
File Edit Analysis Highlight Add track Layout Size options Help
Zoom
10.9
Whole
Show buried clones
Yes No
Search
CB Unit Range
0 to 146
Contig stats
Clones: 107 (14 buried)
Markers: 0
Sequenced: 0
Length: 147 CB units
Ctg2292 of cp05T5e12DM4RS
V026P14
H020P05
V016J23
H018G23
V074G01
H011B01
V064N06
M054E07~
V048A16
M052D05
V038L03
M044M01
V026F23
H016L15
V070M24
H012L18*
V058K19
H006P02
V044N10
M050E23
V038A11
M034J06*
V030C18
M006B01
V024P10
H020E19
V016J22
M043P12*
V016H02
M010G09
V008I19
B010G16
V074L01
M045K17
V065D20
M034M17
V056K24
M015G13~
V048F07
M014C24
V038H12*
H021N08
V022K13
M029M19
V018C05
M057N24
V056B05
M036A18
0 10 20 30 40 50 60 70 80 90 100 110 120 130 140
[root@localhost: /...
[FPC V8.9 Main Menu]
Project cp05T5e12...
[Chickpea contig p...
FPC Ctg2292 cp05...

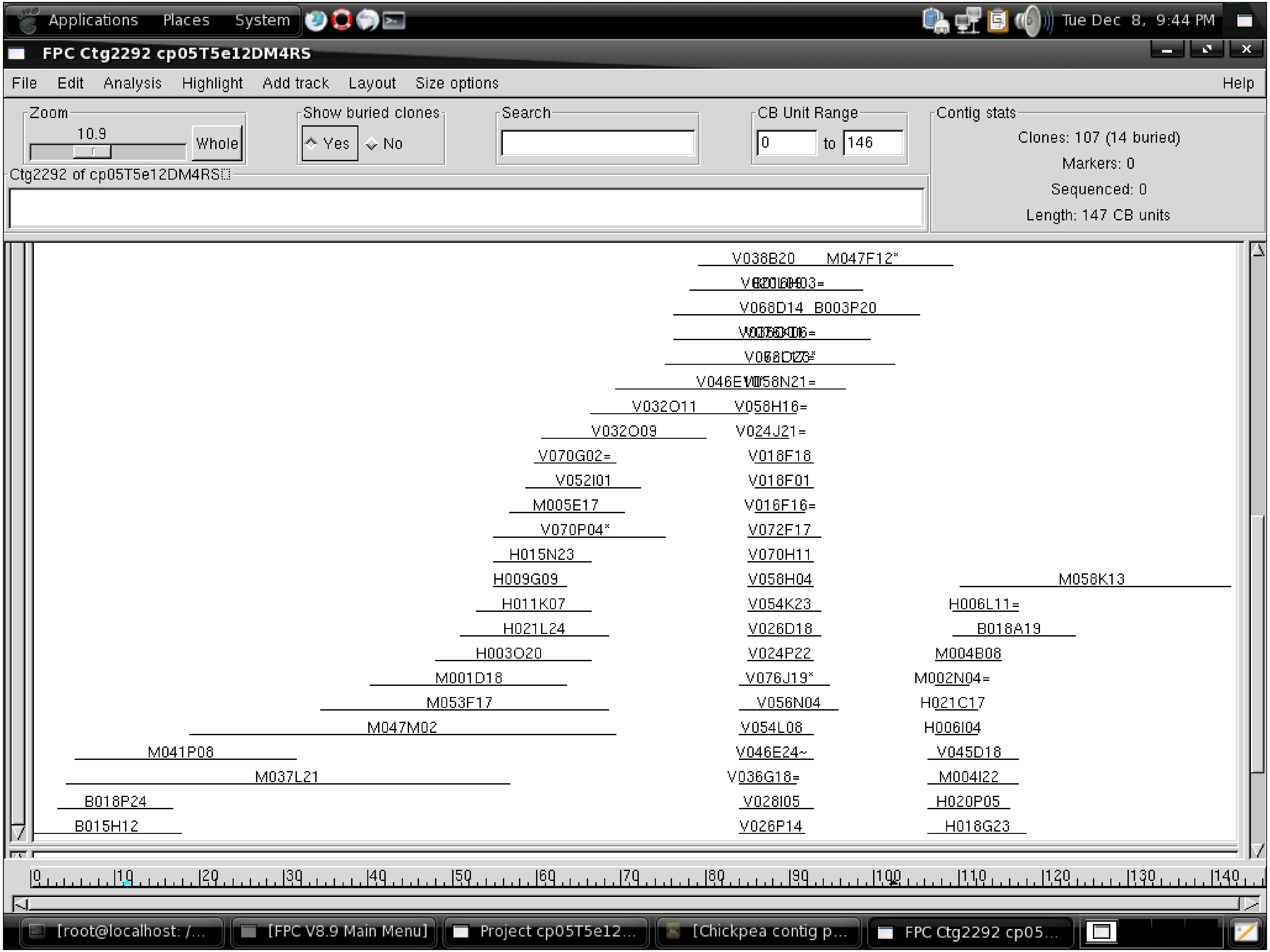
Applications Places System
Tue Dec 8, 9:44 PM
FPC Ctg2292 cp05T5e12DM4RS
File Edit Analysis Highlight Add track Layout Size options Help
Zoom
10.9
Whole
Show buried clones
Yes No
Search
CB Unit Range
0 to 146
Contig stats
Clones: 107 (14 buried)
Markers: 0
Sequenced: 0
Length: 147 CB units
Ctg2292 of cp05T5e12DM4RS
V038B20 M047F12*
V068D14 B003P20
V032O11 V058H16=
V032O09 V024J21=
V070G02= V018F18
V052I01 V018F01
M005E17 V016F16=
V070P04* V072F17
H015N23 V070H11
H009G09 V058H04 M058K13
H011K07 V054K23 H006L11=
H021L24 V026D18 B018A19
H003O20 V024P22 M004B08
M001D18 V076J19* M002N04=
M053F17 V056N04 H021C17
M047M02 V054L08 H006I04
M041P08 V046E24~ V045D18
M037L21 V036G18= M004I22
B018P24 V028I05 H020P05
B015H12 V026P14 H018G23
0 10 20 30 40 50 60 70 80 90 100 110 120 130 140
[root@localhost: /...
[FPC V8.9 Main Menu]
Project cp05T5e12...
[Chickpea contig p...
FPC Ctg2292 cp05...

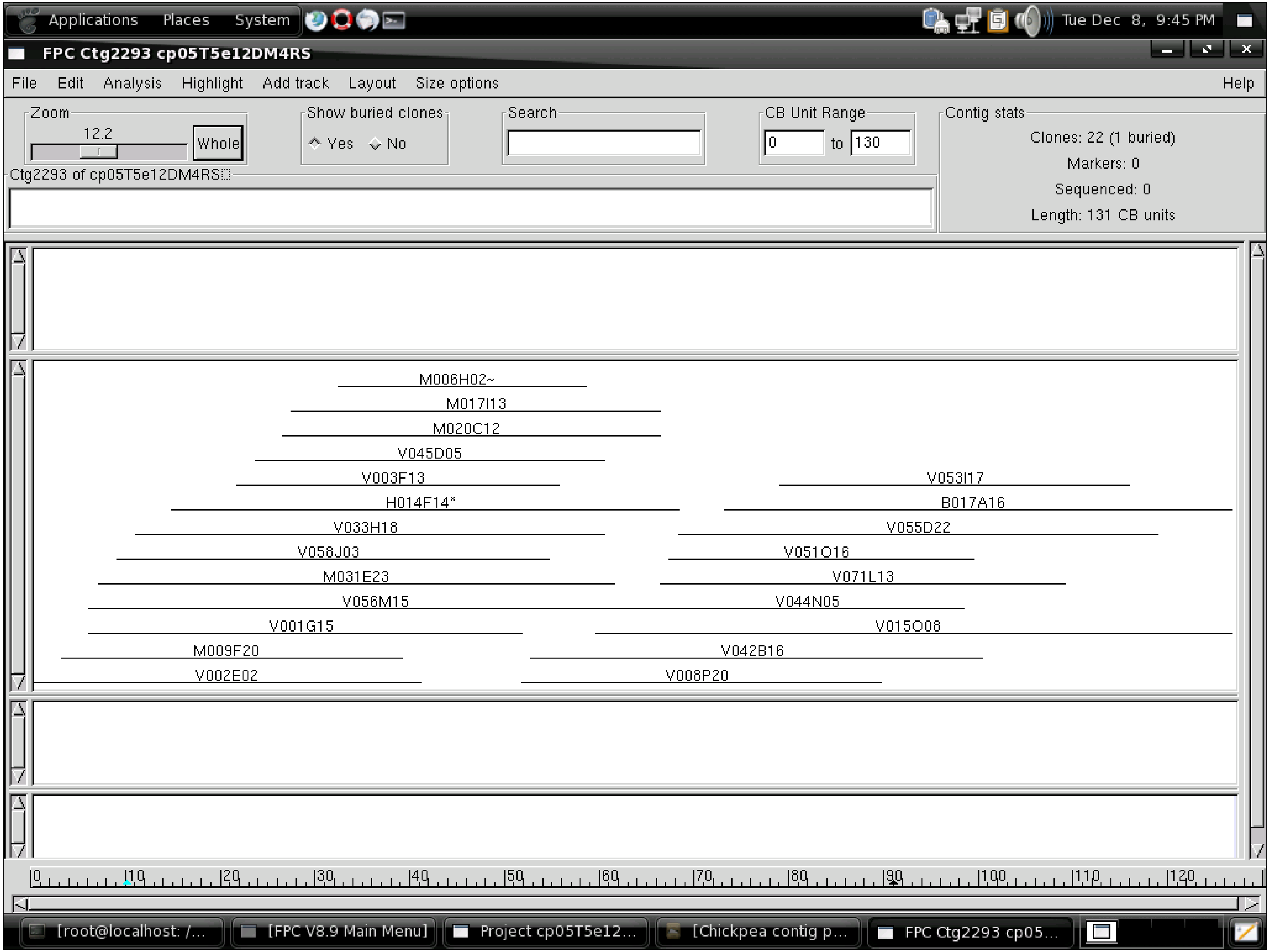
Applications Places System
Tue Dec 8, 9:45 PM
FPC Ctg2293 cp05T5e12DM4RS
File Edit Analysis Highlight Add track Layout Size options Help
Zoom
12.2
Whole
Show buried clones
Yes No
Search
CB Unit Range
0 to 130
Contig stats
Clones: 22 (1 buried)
Markers: 0
Sequenced: 0
Length: 131 CB units
Ctg2293 of cp05T5e12DM4RS
M006H02~
M017I13
M020C12
V045D05
V003F13
V053I17
H014F14*
B017A16
V033H18
V055D22
V058J03
V051O16
M031E23
V071L13
V056M15
V044N05
V001G15
V015O08
M009F20
V042B16
V002E02
V008P20
0 10 20 30 40 50 60 70 80 90 100 110 120
[root@localhost: /...
[FPC V8.9 Main Menu]
Project cp05T5e12...
[Chickpea contig p...
FPC Ctg2293 cp05...

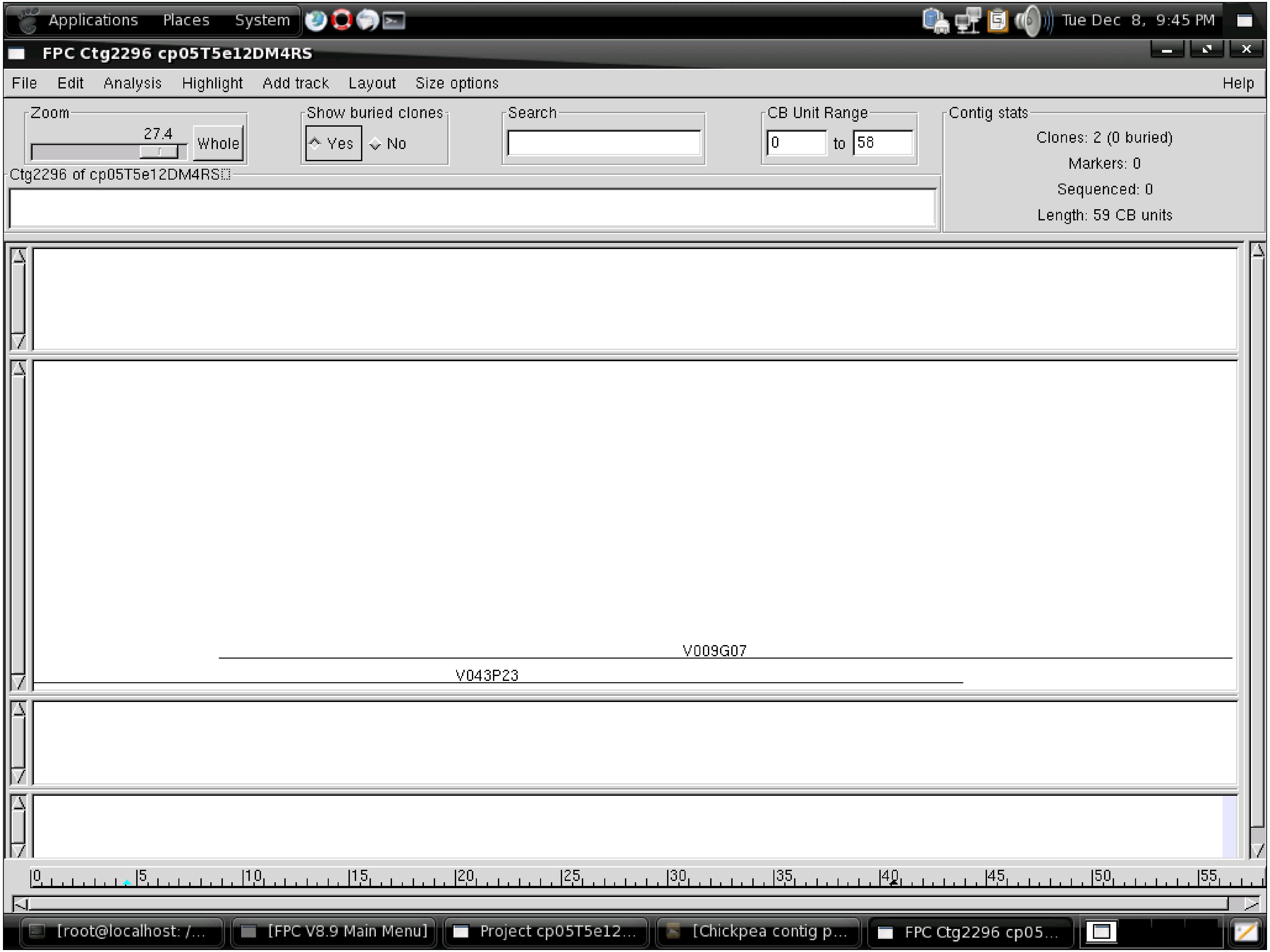
Applications Places System
Tue Dec 8, 9:45 PM
FPC Ctg2296 cp05T5e12DM4RS
File Edit Analysis Highlight Add track Layout Size options Help
Zoom
27.4
Whole
Show buried clones
Yes
No
Search
CB Unit Range
0
to
58
Contig stats
Clones: 2 (0 buried)
Markers: 0
Sequenced: 0
Length: 59 CB units
Ctg2296 of cp05T5e12DM4RS
V009G07
V043P23
0 5 10 15 20 25 30 35 40 45 50 55
[root@localhost: /...
[FPC V8.9 Main Menu]
Project cp05T5e12...
[Chickpea contig p...
FPC Ctg2296 cp05...

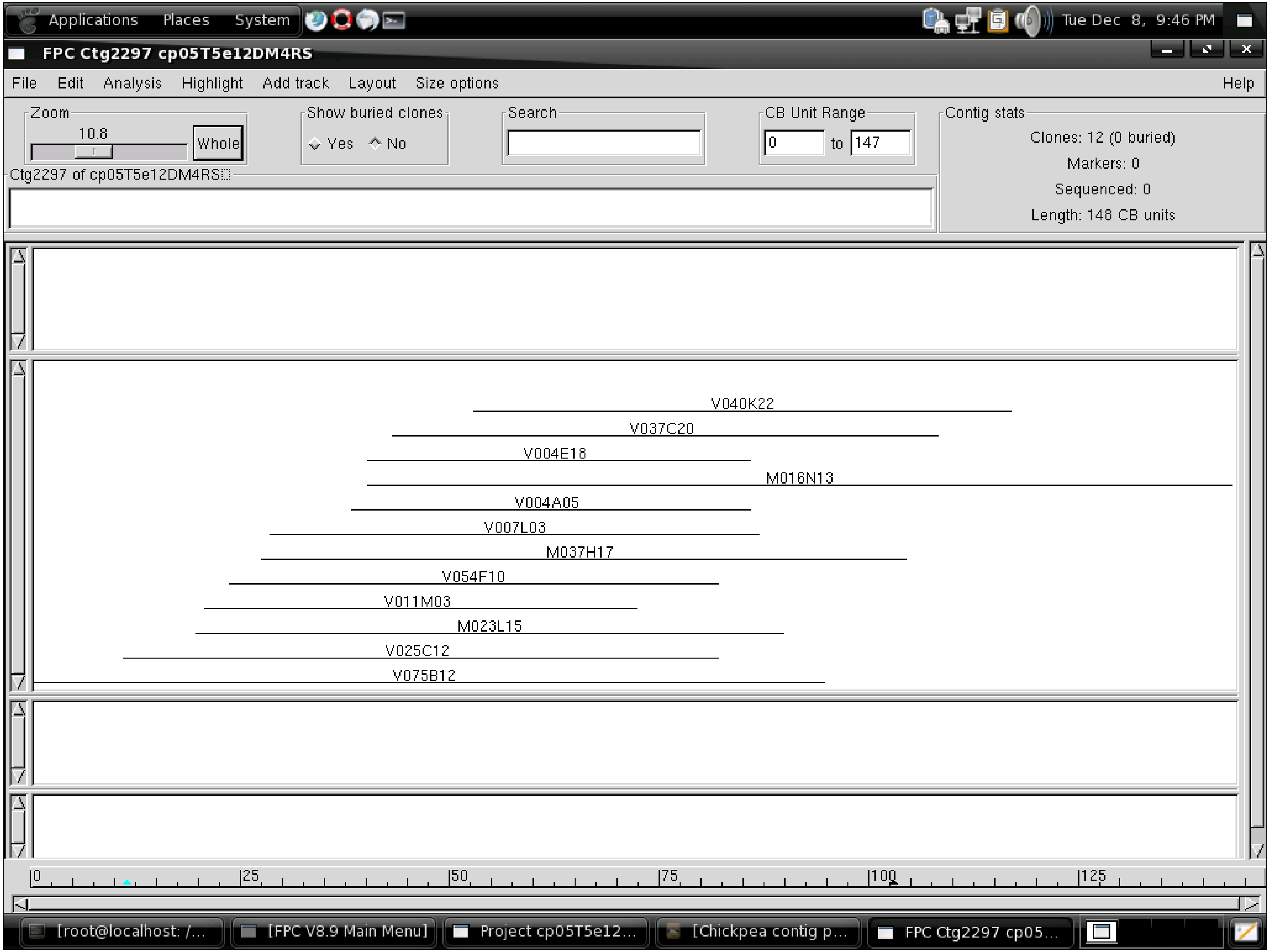
Applications Places System
Tue Dec 8, 9:46 PM
FPC Ctg2297 cp05T5e12DM4RS
File Edit Analysis Highlight Add track Layout Size options Help
Zoom
10.8
Whole
Show buried clones
Yes No
Search
CB Unit Range
0 to 147
Contig stats
Clones: 12 (0 buried)
Markers: 0
Sequenced: 0
Length: 148 CB units
Ctg2297 of cp05T5e12DM4RS
V040K22
V037C20
V004E18
M016N13
V004A05
V007L03
M037H17
V054F10
V011M03
M023L15
V025C12
V075B12
0
25
50
75
100
125
[root@localhost: /...
[FPC V8.9 Main Menu]
Project cp05T5e12...
[Chickpea contig p...
FPC Ctg2297 cp05...

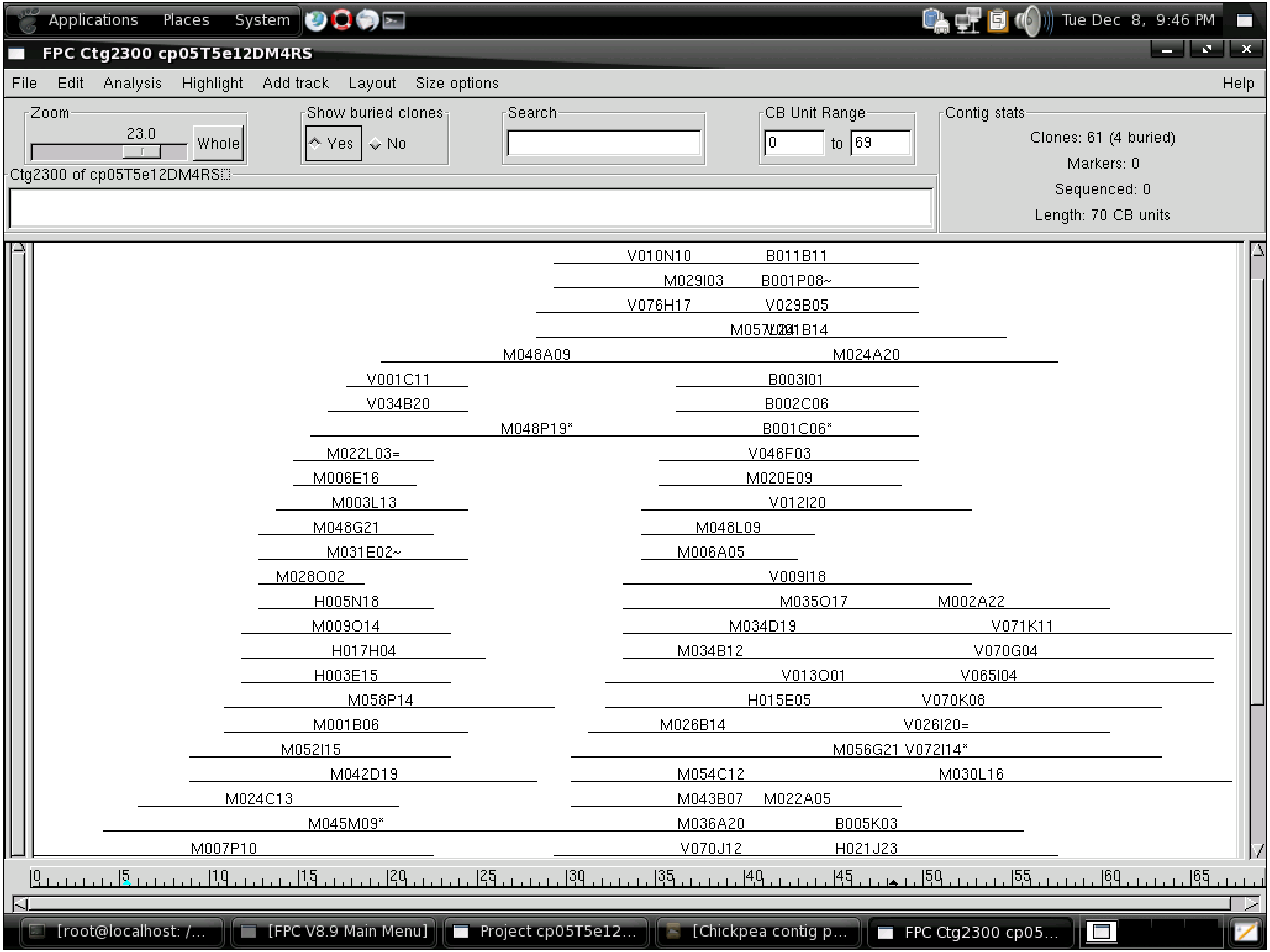
Applications Places System
Tue Dec 8, 9:46 PM
FPC Ctg2300 cp05T5e12DM4RS
File Edit Analysis Highlight Add track Layout Size options Help
Zoom
23.0
Whole
Show buried clones
Yes No
Search
CB Unit Range
0 to 69
Contig stats
Clones: 61 (4 buried)
Markers: 0
Sequenced: 0
Length: 70 CB units
Ctg2300 of cp05T5e12DM4RS
V010N10 B011B11
M029I03 B001P08~
V076H17 V029B05
M048A09 M024A20
V001C11 B003I01
V034B20 B002C06
M048P19* B001C06*
M022L03= V046F03
M006E16 M020E09
M003L13 V012I20
M048G21 M048L09
M031E02~ M006A05
M028O02 V009I18
H005N18 M035O17 M002A22
M009O14 M034D19 V071K11
H017H04 M034B12 V070G04
H003E15 V013O01 V065I04
M058P14 H015E05 V070K08
M001B06 M026B14 V026I20=
M052I15 M056G21 V072I14*
M042D19 M054C12 M030L16
M024C13 M043B07 M022A05
M045M09* M036A20 B005K03
M007P10 V070J12 H021J23
0 5 10 15 20 25 30 35 40 45 50 55 60 65
[root@localhost: /... [FPC V8.9 Main Menu] Project cp05T5e12... [Chickpea contig p... FPC Ctg2300 cp05...

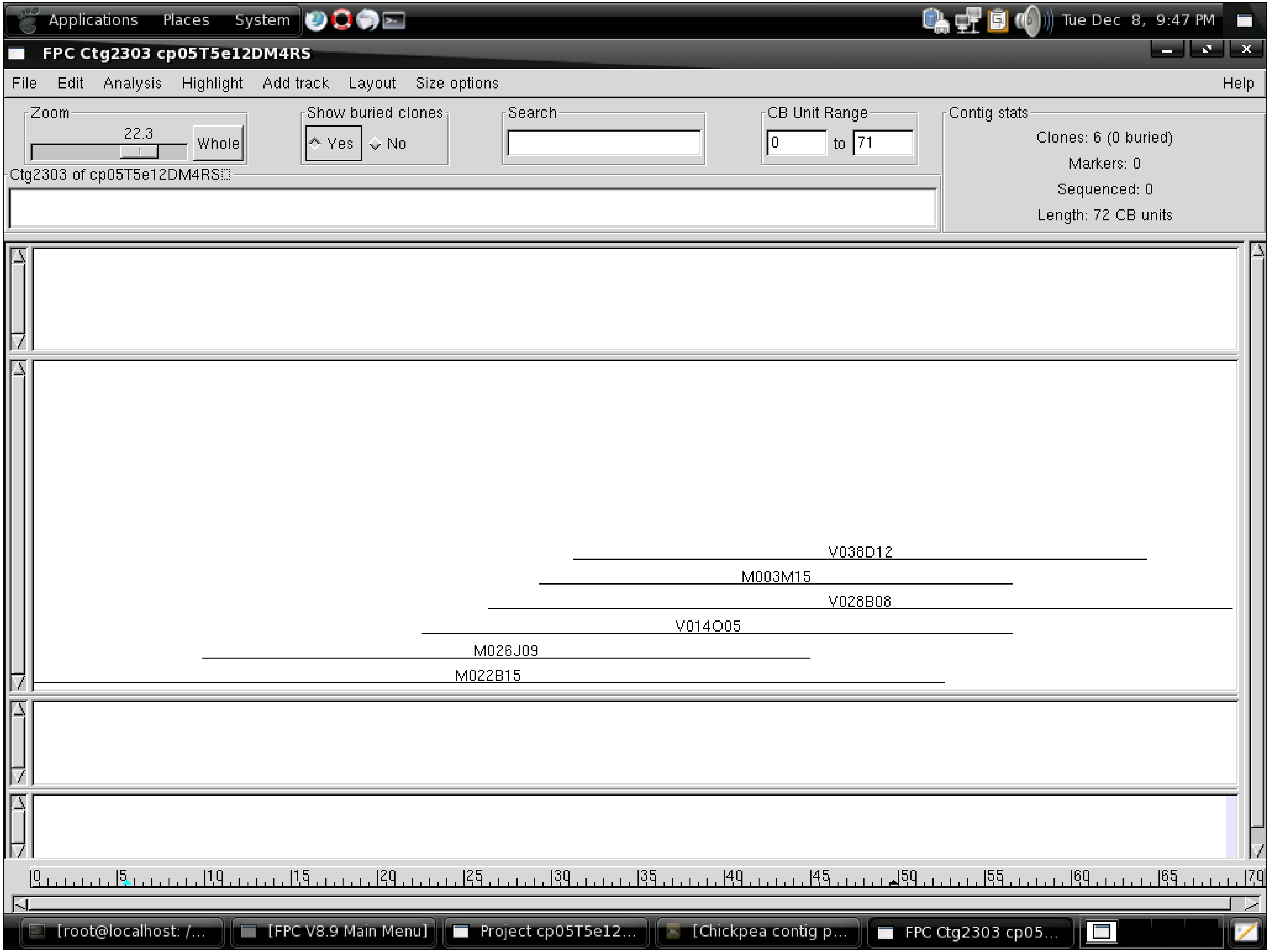
Applications Places System
Tue Dec 8, 9:47 PM
FPC Ctg2303 cp05T5e12DM4RS
File Edit Analysis Highlight Add track Layout Size options Help
Zoom
22.3
Whole
Show buried clones
Yes
No
Search
CB Unit Range
0
to
71
Contig stats
Clones: 6 (0 buried)
Markers: 0
Sequenced: 0
Length: 72 CB units
Ctg2303 of cp05T5e12DM4RS
V038D12
M003M15
V028B08
V014O05
M026J09
M022B15
0 5 10 15 20 25 30 35 40 45 50 55 60 65 70
[root@localhost: /...
[FPC V8.9 Main Menu]
Project cp05T5e12...
[Chickpea contig p...
FPC Ctg2303 cp05...

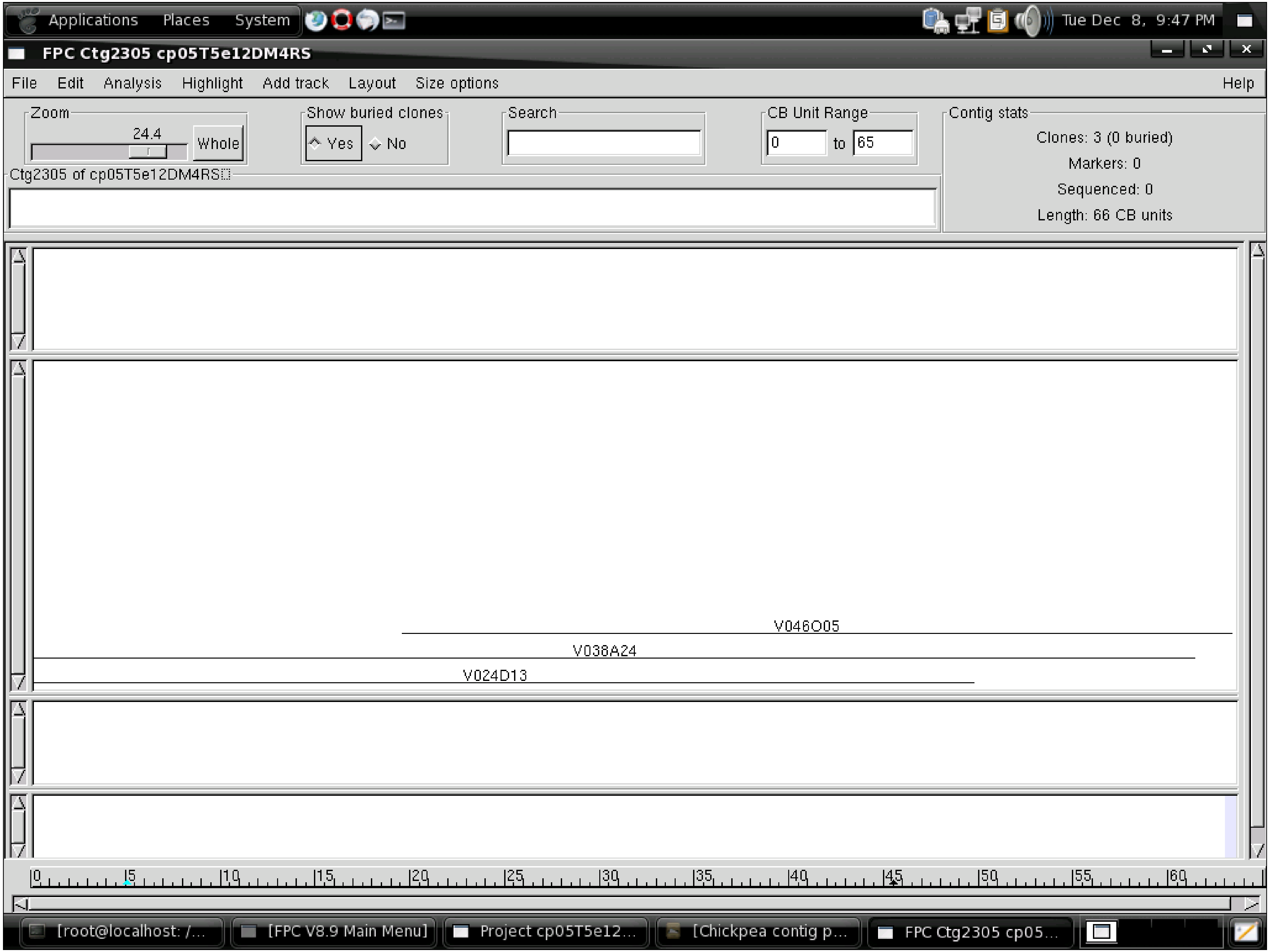
Applications Places System
Tue Dec 8, 9:47 PM
FPC Ctg2305 cp05T5e12DM4RS
File Edit Analysis Highlight Add track Layout Size options Help
Zoom
24.4
Whole
Show buried clones
Yes No
Search
CB Unit Range
0 to 65
Contig stats
Clones: 3 (0 buried)
Markers: 0
Sequenced: 0
Length: 66 CB units
Ctg2305 of cp05T5e12DM4RS
V046O05
V038A24
V024D13
0 5 10 15 20 25 30 35 40 45 50 55 60
[root@localhost: /...
[FPC V8.9 Main Menu]
Project cp05T5e12...
[Chickpea contig p...
FPC Ctg2305 cp05...

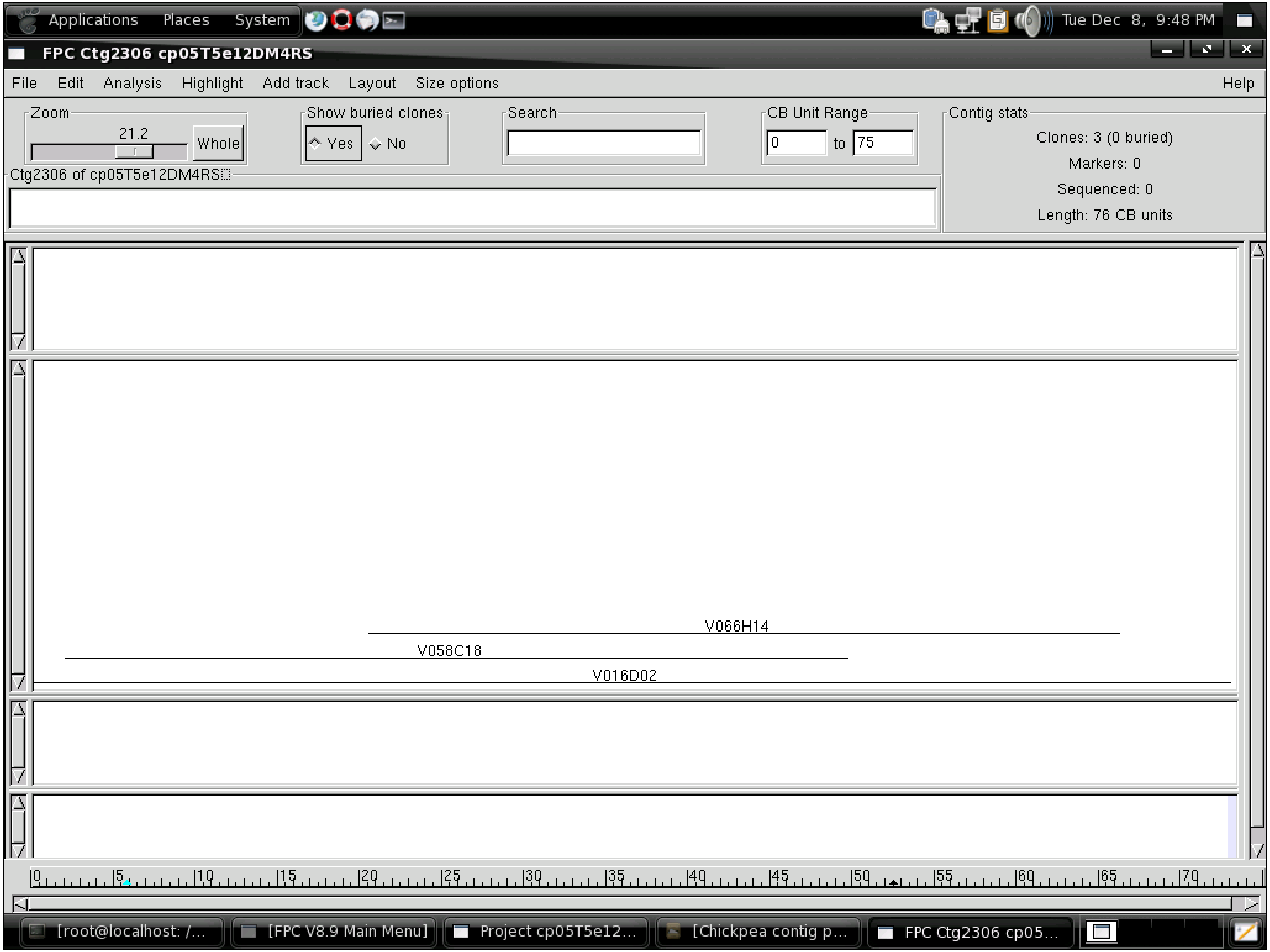
Applications Places System
Tue Dec 8, 9:48 PM
FPC Ctg2306 cp05T5e12DM4RS
File Edit Analysis Highlight Add track Layout Size options Help
Zoom
21.2
Whole
Show buried clones
Yes
No
Search
CB Unit Range
0
to
75
Contig stats
Clones: 3 (0 buried)
Markers: 0
Sequenced: 0
Length: 76 CB units
Ctg2306 of cp05T5e12DM4RS
V066H14
V058C18
V016D02
0 5 10 15 20 25 30 35 40 45 50 55 60 65 70
[root@localhost: /...
[FPC V8.9 Main Menu]
Project cp05T5e12...
[Chickpea contig p...
FPC Ctg2306 cp05...

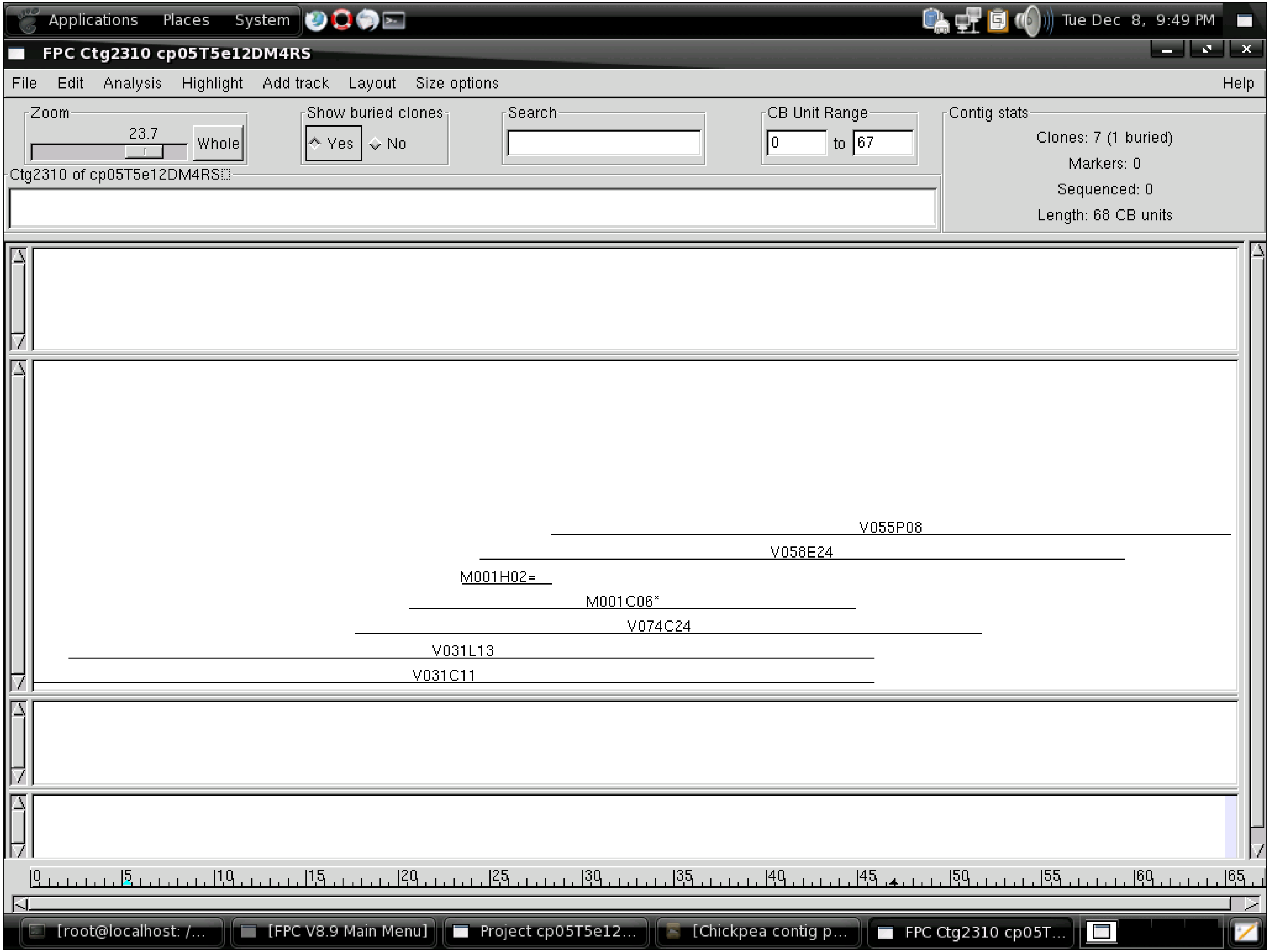

Applications
Places
System
Tue Dec 8, 9:49 PM
FPC Ctg2310 cp05T5e12DM4RS
File
Edit
Analysis
Highlight
Add track
Layout
Size options
Help
Zoom
23.7
Whole
Show buried clones
Yes
No
Search
CB Unit Range
0
to
67
Contig stats
Clones: 7 (1 buried)
Markers: 0
Sequenced: 0
Length: 68 CB units
Ctg2310 of cp05T5e12DM4RS
V055P08
V058E24
M001H02=
M001C06*
V074C24
V031L13
V031C11
0
5
10
15
20
25
30
35
40
45
50
55
60
65
[root@localhost: /...
[FPC V8.9 Main Menu]
Project cp05T5e12...
[Chickpea contig p...
FPC Ctg2310 cp05T...

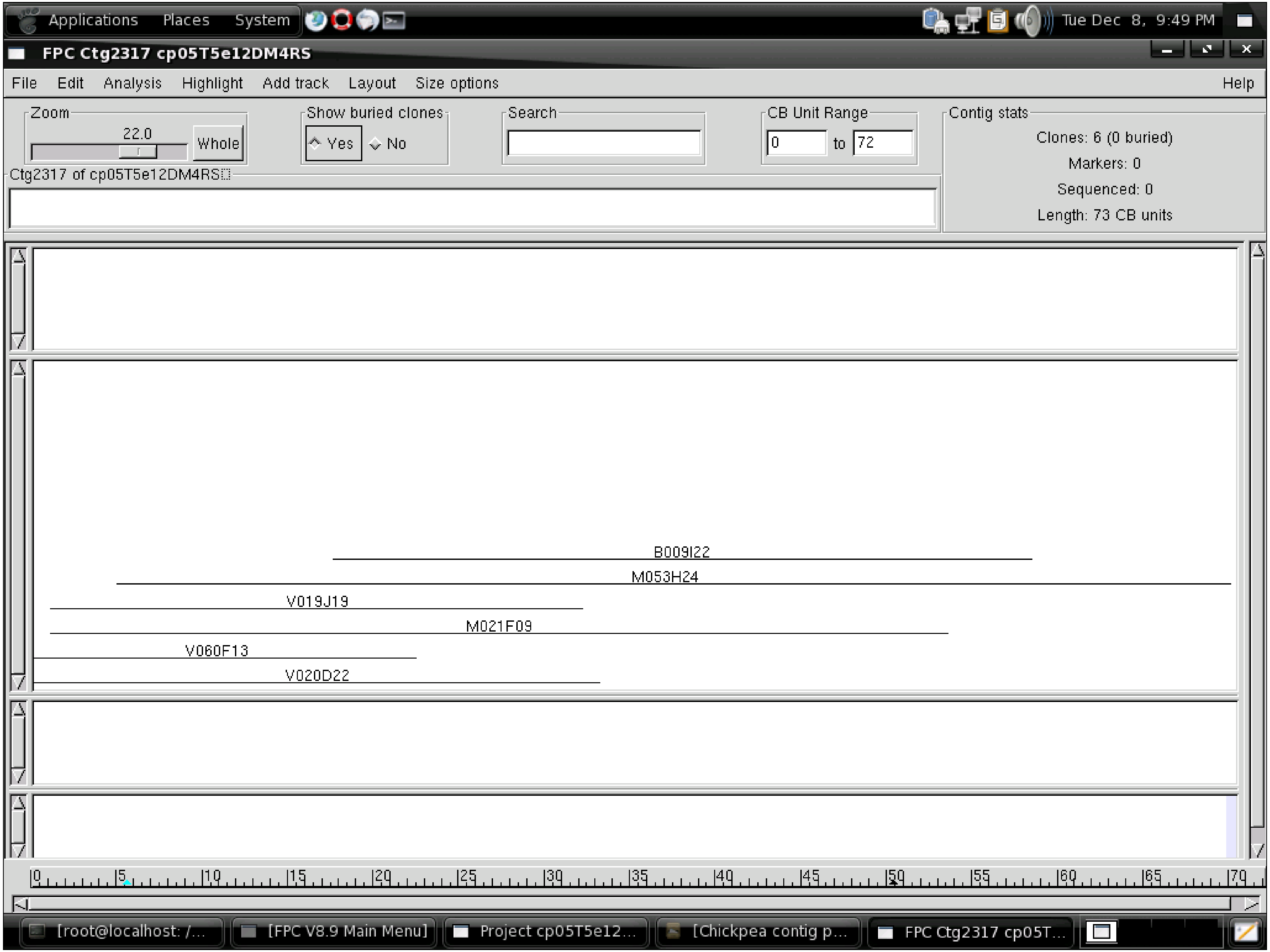
Applications Places System
Tue Dec 8, 9:49 PM
FPC Ctg2317 cp05T5e12DM4RS
File Edit Analysis Highlight Add track Layout Size options
Help
Zoom
22.0
Whole
Show buried clones
Yes
No
Search
CB Unit Range
0
to
72
Contig stats
Clones: 6 (0 buried)
Markers: 0
Sequenced: 0
Length: 73 CB units
Ctg2317 of cp05T5e12DM4RS
B009I22
M053H24
V019J19
M021F09
V060F13
V020D22
0
5
10
15
20
25
30
35
40
45
50
55
60
65
70
[root@localhost: /...
[FPC V8.9 Main Menu]
Project cp05T5e12...
[Chickpea contig p...
FPC Ctg2317 cp05T...

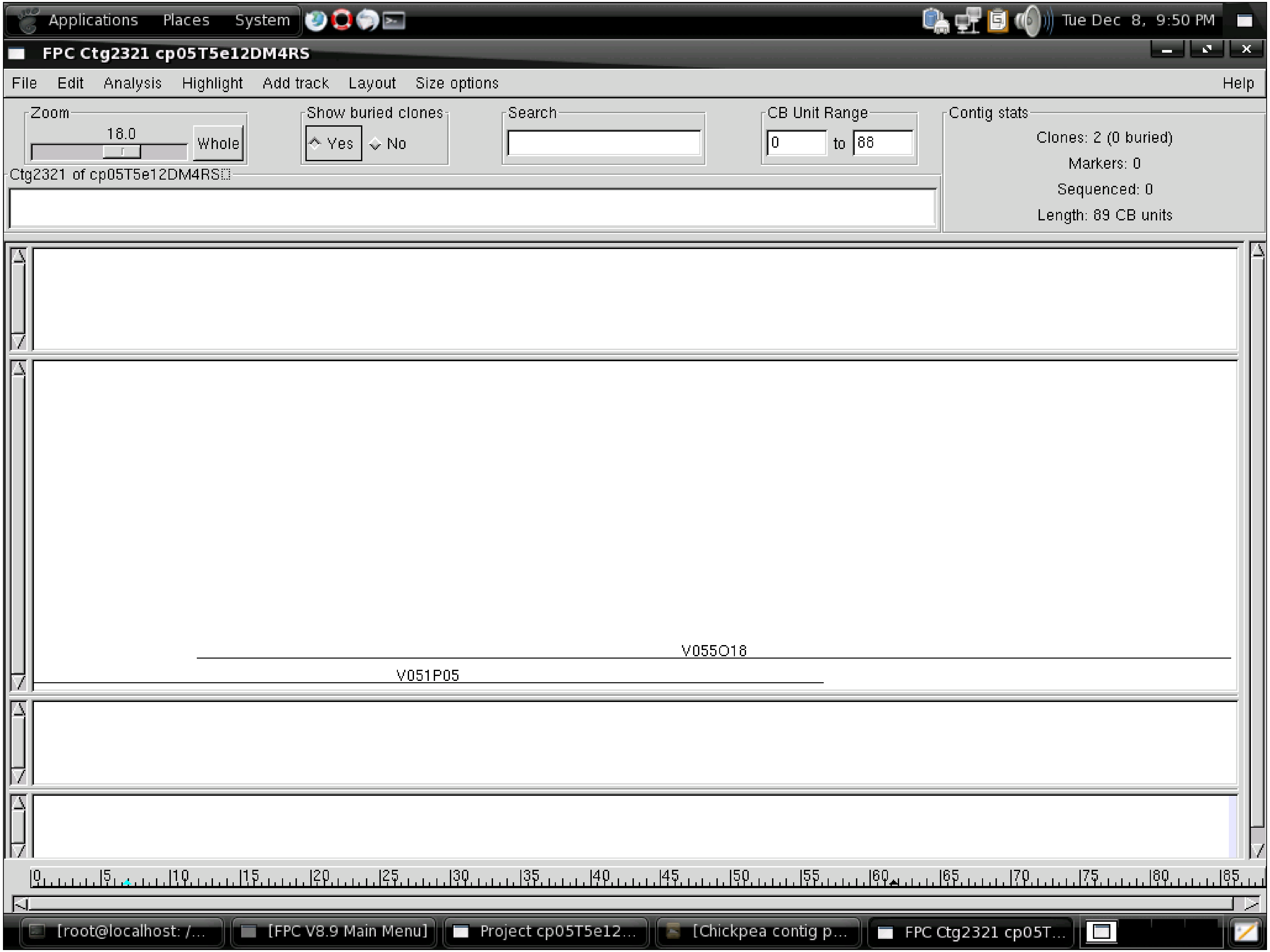
Applications Places System
Tue Dec 8, 9:50 PM
FPC Ctg2321 cp05T5e12DM4RS
File Edit Analysis Highlight Add track Layout Size options Help
Zoom
18.0
Whole
Show buried clones
Yes
No
Search
CB Unit Range
0
to
88
Contig stats
Clones: 2 (0 buried)
Markers: 0
Sequenced: 0
Length: 89 CB units
Ctg2321 of cp05T5e12DM4RS
V055O18
V051P05
0 5 10 15 20 25 30 35 40 45 50 55 60 65 70 75 80 85
[root@localhost: /...
[FPC V8.9 Main Menu]
Project cp05T5e12...
[Chickpea contig p...
FPC Ctg2321 cp05T...

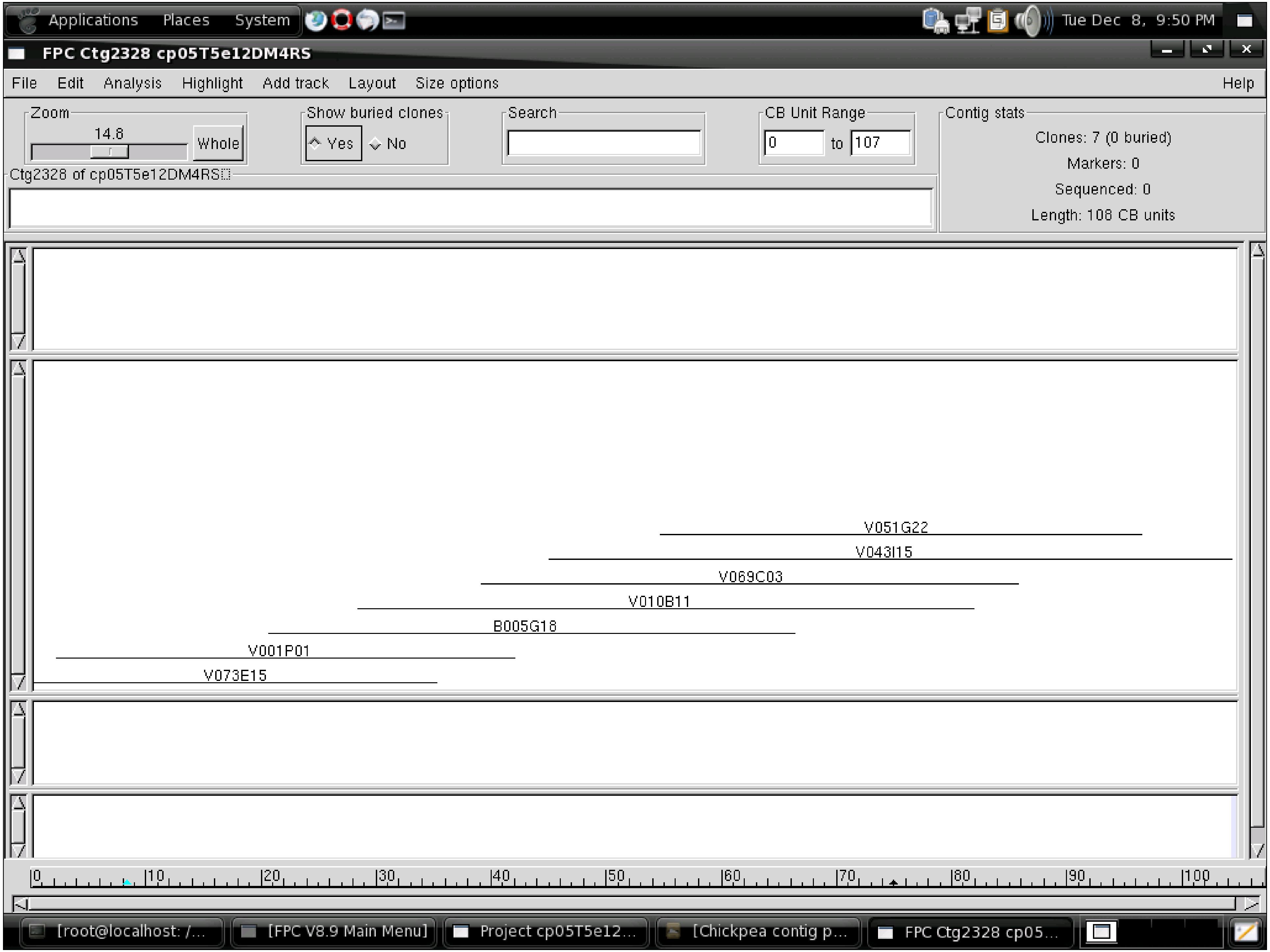
Applications Places System
Tue Dec 8, 9:50 PM
FPC Ctg2328 cp05T5e12DM4RS
File Edit Analysis Highlight Add track Layout Size options Help
Zoom
14.8
Whole
Show buried clones
Yes
No
Search
CB Unit Range
0 to 107
Contig stats
Clones: 7 (0 buried)
Markers: 0
Sequenced: 0
Length: 108 CB units
Ctg2328 of cp05T5e12DM4RS
V051G22
V043I15
V069C03
V010B11
B005G18
V001P01
V073E15
0 10 20 30 40 50 60 70 80 90 100
[root@localhost: /...
[FPC V8.9 Main Menu]
Project cp05T5e12...
[Chickpea contig p...
FPC Ctg2328 cp05...

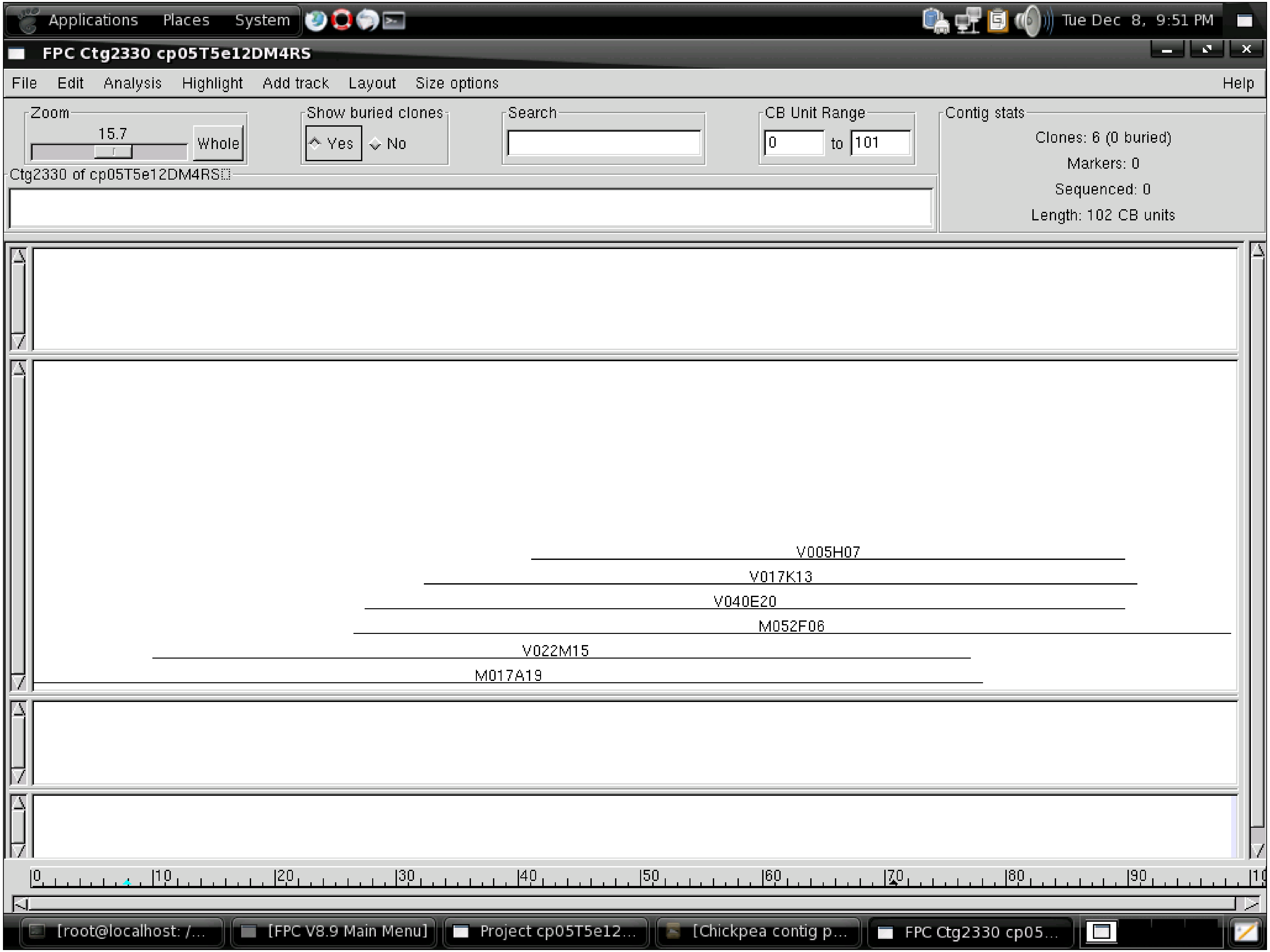

Applications
Places
System
Tue Dec 8, 9:51 PM
FPC Ctg2330 cp05T5e12DM4RS
File
Edit
Analysis
Highlight
Add track
Layout
Size options
Help
Zoom
15.7
Whole
Show buried clones
Yes
No
Search
CB Unit Range
0
to
101
Contig stats
Clones: 6 (0 buried)
Markers: 0
Sequenced: 0
Length: 102 CB units
Ctg2330 of cp05T5e12DM4RS
V005H07
V017K13
V040E20
M052F06
V022M15
M017A19
0
10
20
30
40
50
60
70
80
90
[root@localhost: /...
[FPC V8.9 Main Menu]
Project cp05T5e12...
[Chickpea contig p...
FPC Ctg2330 cp05...

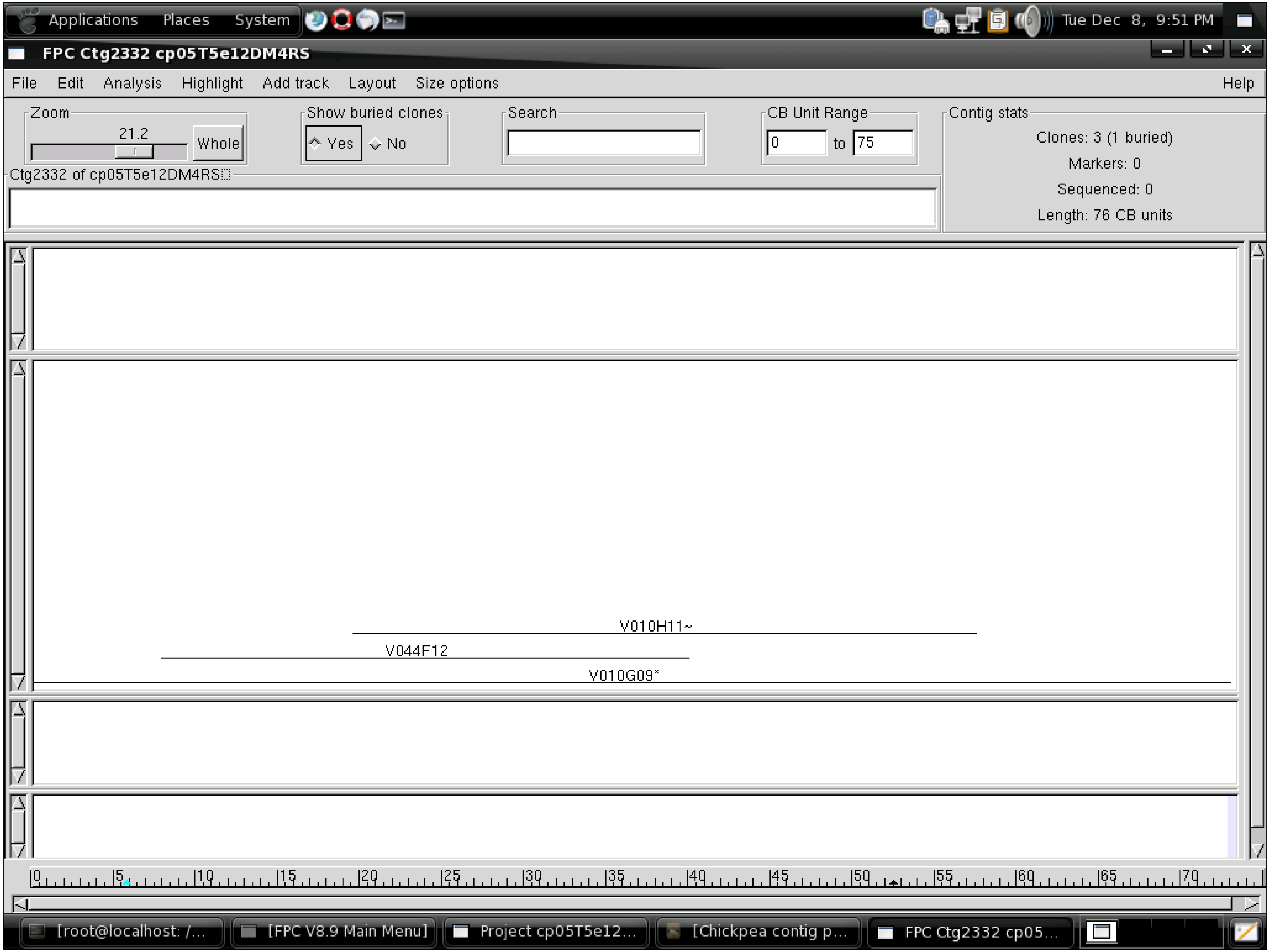
Applications Places System
Tue Dec 8, 9:51 PM
FPC Ctg2332 cp05T5e12DM4RS
File Edit Analysis Highlight Add track Layout Size options
Help
Zoom
21.2
Whole
Show buried clones
Yes
No
Search
CB Unit Range
0
to
75
Contig stats
Clones: 3 (1 buried)
Markers: 0
Sequenced: 0
Length: 76 CB units
Ctg2332 of cp05T5e12DM4RS
V010H11~
V044F12
V010G09*
0 5 10 15 20 25 30 35 40 45 50 55 60 65 70
[root@localhost: /...
[FPC V8.9 Main Menu]
Project cp05T5e12...
[Chickpea contig p...
FPC Ctg2332 cp05...

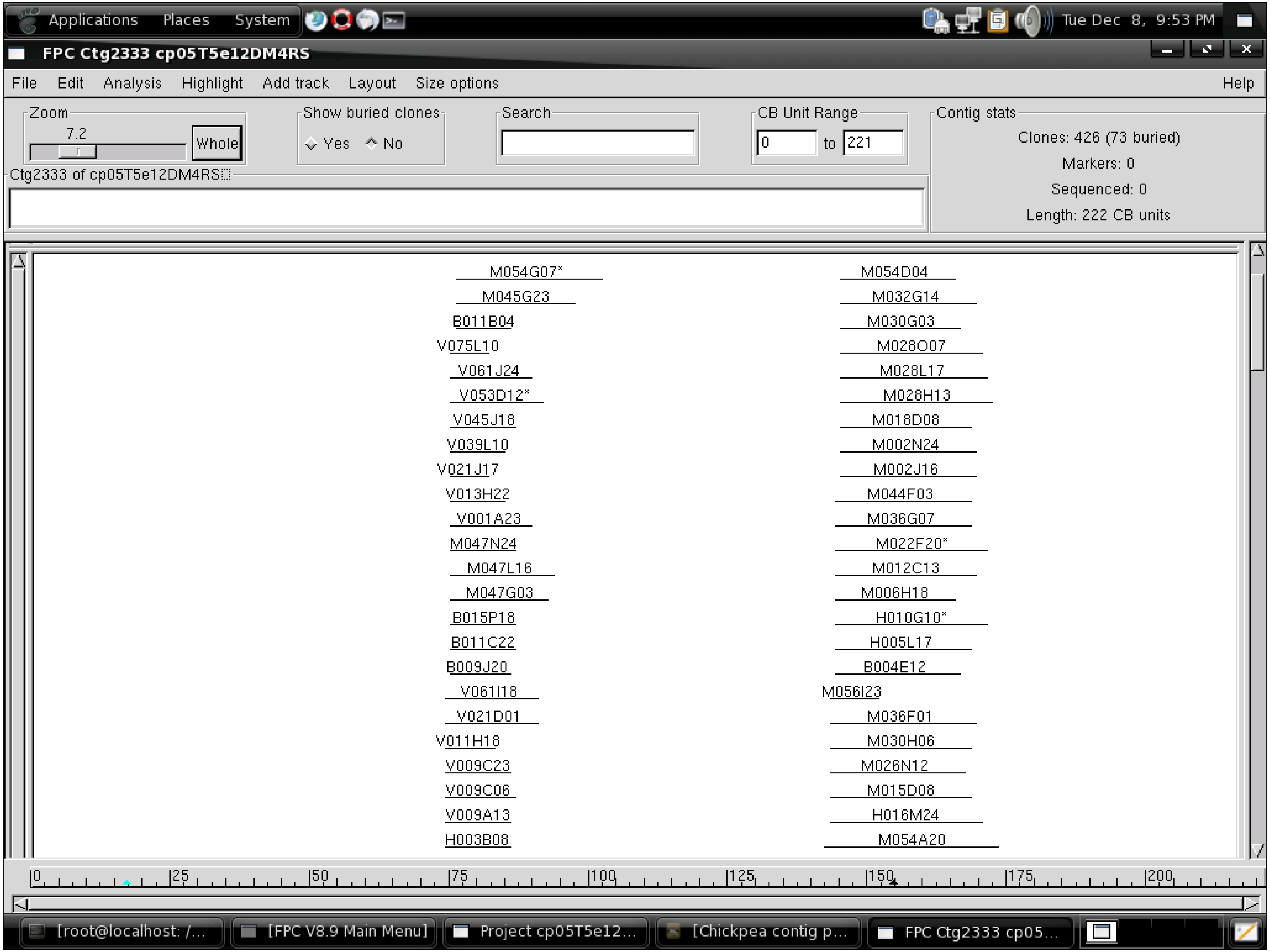

Applications Places System
Tue Dec 8, 9:53 PM
FPC Ctg2333 cp05T5e12DM4RS
File Edit Analysis Highlight Add track Layout Size options Help
Zoom
7.2
Whole
Show buried clones
Yes No
Search
CB Unit Range
0 to 221
Contig stats
Clones: 426 (73 buried)
Markers: 0
Sequenced: 0
Length: 222 CB units
Ctg2333 of cp05T5e12DM4RS
M054G07*
M045G23
B011B04
V075L10
V061J24
V053D12*
V045J18
V039L10
V021J17
V013H22
V001A23
M047N24
M047L16
M047G03
B015P18
B011C22
B009J20
V061I18
V021D01
V011H18
V009C23
V009C06
V009A13
H003B08
M054D04
M032G14
M030G03
M028O07
M028L17
M028H13
M018D08
M002N24
M002J16
M044F03
M036G07
M022F20*
M012C13
M006H18
H010G10*
H005L17
B004E12
M056I23
M036F01
M030H06
M026N12
M015D08
H016M24
M054A20
0 25 50 75 100 125 150 175 200
[root@localhost: /...
[FPC V8.9 Main Menu]
Project cp05T5e12...
[Chickpea contig p...
FPC Ctg2333 cp05...

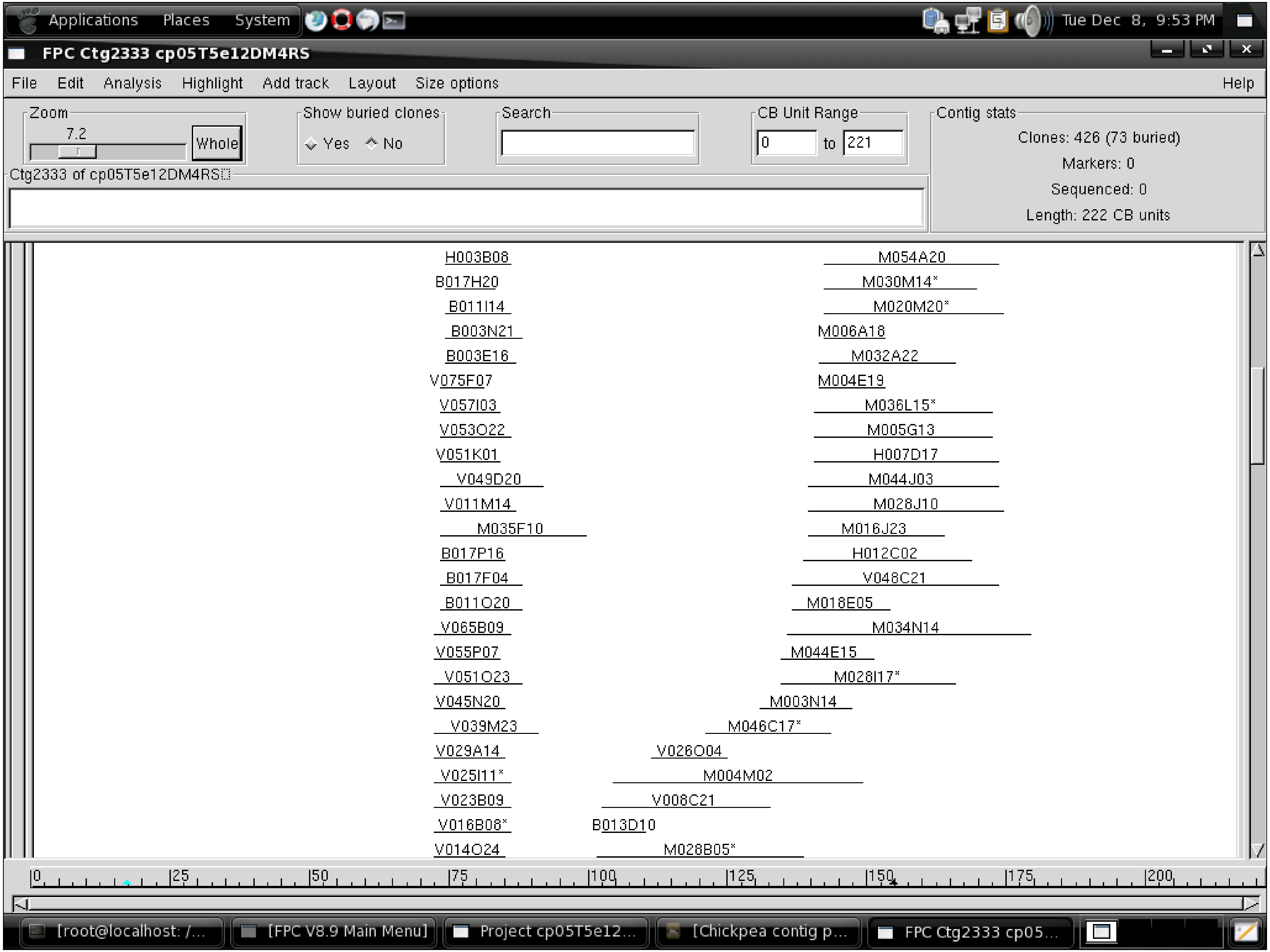
Applications Places System
Tue Dec 8, 9:53 PM
FPC Ctg2333 cp05T5e12DM4RS
File Edit Analysis Highlight Add track Layout Size options Help
Zoom
7.2
Whole
Show buried clones
Yes No
Search
CB Unit Range
0 to 221
Contig stats
Clones: 426 (73 buried)
Markers: 0
Sequenced: 0
Length: 222 CB units
Ctg2333 of cp05T5e12DM4RS
H003B08
B017H20
B011I14
B003N21
B003E16
V075F07
V057I03
V053O22
V051K01
V049D20
V011M14
M035F10
B017P16
B017F04
B011O20
V065B09
V055P07
V051O23
V045N20
V039M23
V029A14
V025I11*
V023B09
V016B08*
V014O24
M054A20
M030M14*
M020M20*
M006A18
M032A22
M004E19
M036L15*
M005G13
H007D17
M044J03
M028J10
M016J23
H012C02
V048C21
M018E05
M034N14
M044E15
M028I17*
M003N14
M046C17*
V026O04
M004M02
V008C21
B013D10
M028B05*
0 25 50 75 100 125 150 175 200
[root@localhost: /...
[FPC V8.9 Main Menu]
Project cp05T5e12...
[Chickpea contig p...
FPC Ctg2333 cp05...

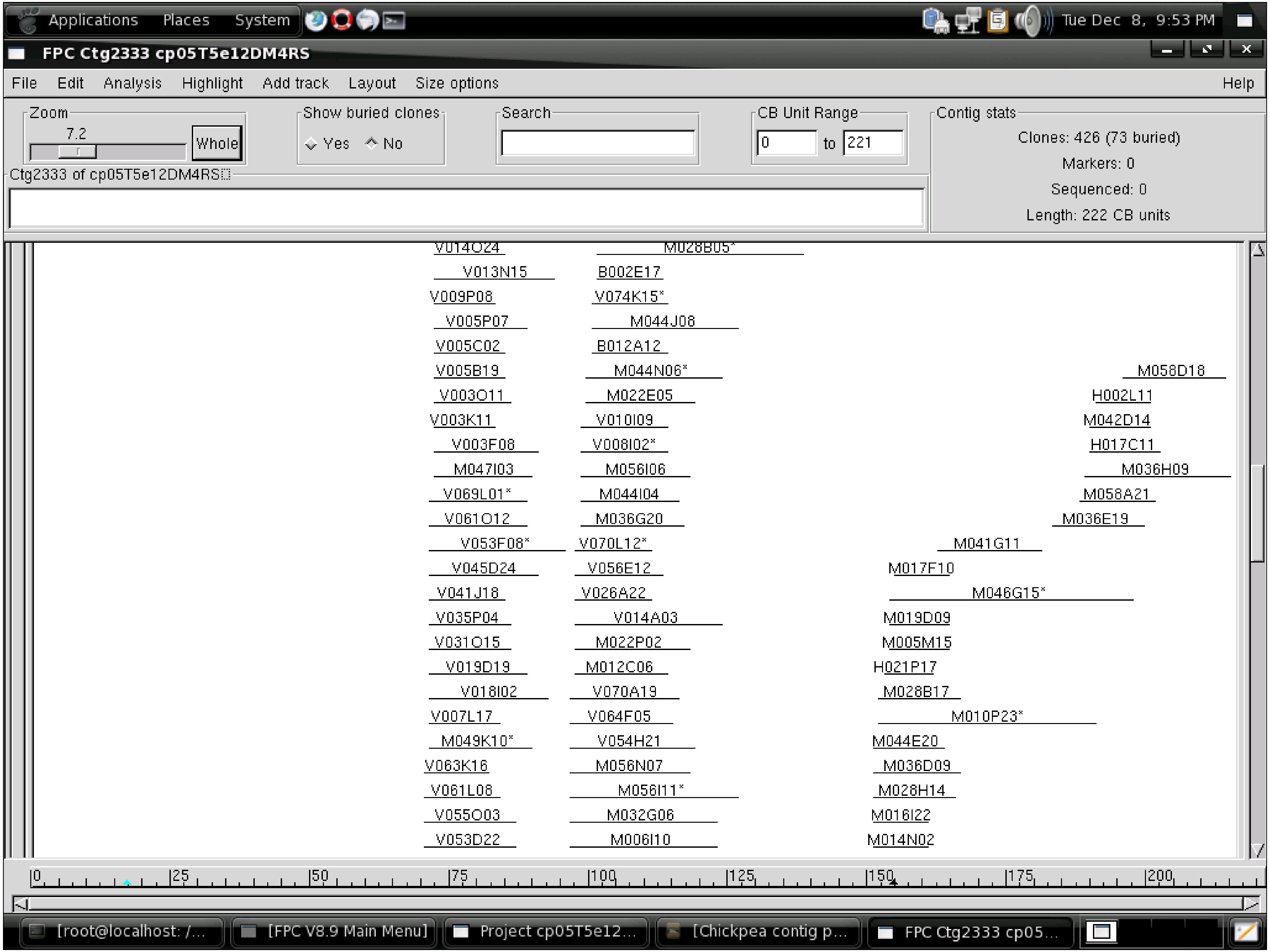
Applications Places System
Tue Dec 8, 9:53 PM
FPC Ctg2333 cp05T5e12DM4RS
File Edit Analysis Highlight Add track Layout Size options Help
Zoom
7.2
Whole
Show buried clones
Yes No
Search
CB Unit Range
0 to 221
Contig stats
Clones: 426 (73 buried)
Markers: 0
Sequenced: 0
Length: 222 CB units
Ctg2333 of cp05T5e12DM4RS
V014O24
V013N15
V009P08
V005P07
V005C02
V005B19
V003O11
V003K11
V003F08
M047I03
V069L01*
V061O12
V053F08*
V045D24
V041J18
V035P04
V031O15
V019D19
V018I02
V007L17
M049K10*
V063K16
V061L08
V055O03
V053D22
M028B05*
B002E17
V074K15*
M044J08
B012A12
M044N06*
M022E05
V010I09
V008I02*
M056I06
M044I04
M036G20
V070L12*
V056E12
V026A22
V014A03
M022P02
M012C06
V070A19
V064F05
V054H21
M056N07
M056I11*
M032G06
M006I10
M058D18
H002L11
M042D14
H017C11
M036H09
M058A21
M036E19
M041G11
M017F10
M046G15*
M019D09
M005M15
H021P17
M028B17
M010P23*
M044E20
M036D09
M028H14
M016I22
M014N02
0 25 50 75 100 125 150 175 200
[root@localhost: /...
[FPC V8.9 Main Menu]
Project cp05T5e12...
[Chickpea contig p...
FPC Ctg2333 cp05...

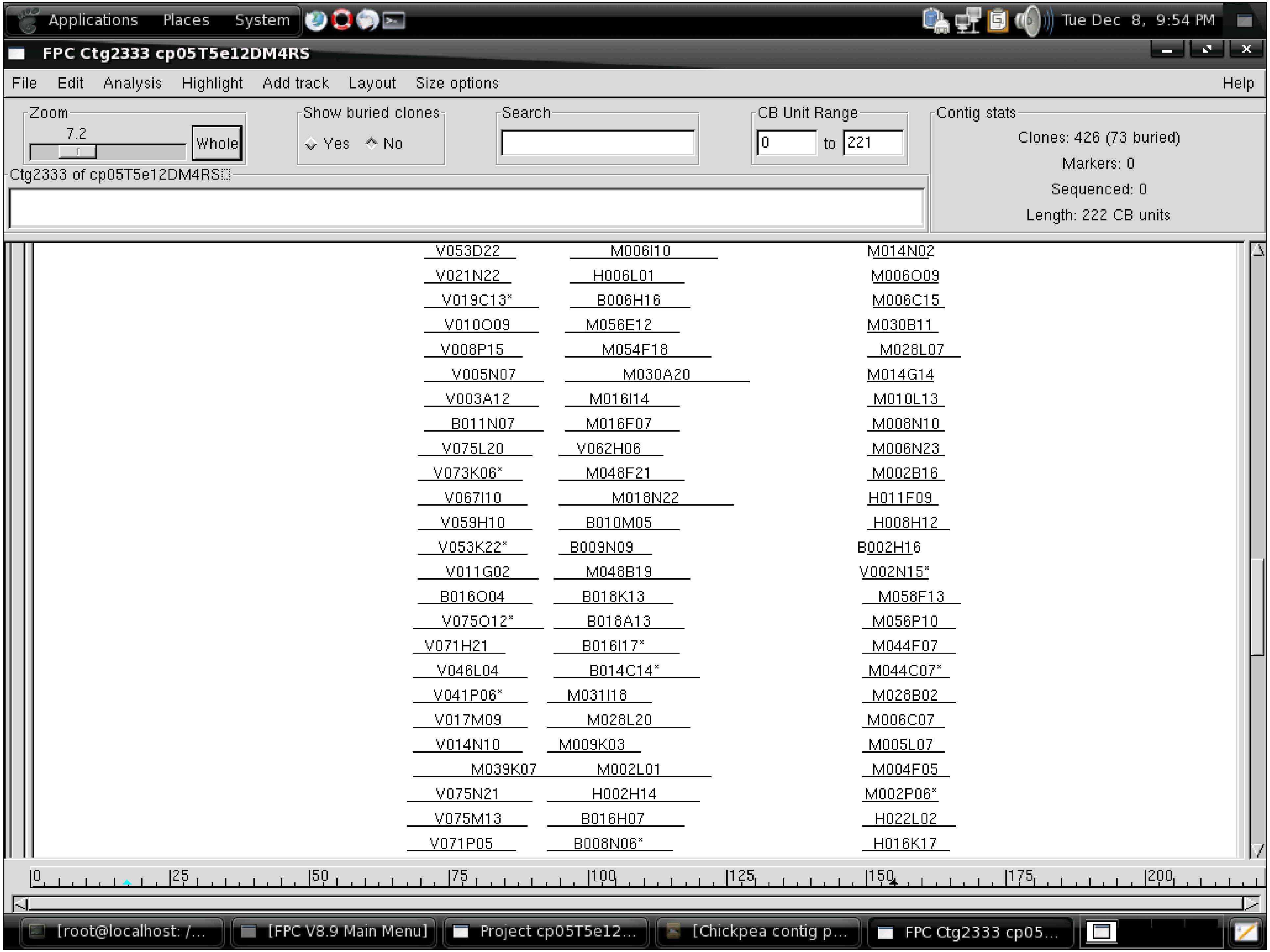
Applications Places System
Tue Dec 8, 9:54 PM
FPC Ctg2333 cp05T5e12DM4RS
File Edit Analysis Highlight Add track Layout Size options Help
Zoom
7.2
Whole
Show buried clones
Yes No
Search
CB Unit Range
0 to 221
Contig stats
Clones: 426 (73 buried)
Markers: 0
Sequenced: 0
Length: 222 CB units
Ctg2333 of cp05T5e12DM4RS
V053D22
M006I10
M014N02
V021N22
H006L01
M006O09
V019C13*
B006H16
M006C15
V010O09
M056E12
M030B11
V008P15
M054F18
M028L07
V005N07
M030A20
M014G14
V003A12
M016I14
M010L13
B011N07
M016F07
M008N10
V075L20
V062H06
M006N23
V073K06*
M048F21
M002B16
V067I10
M018N22
H011F09
V059H10
B010M05
H008H12
V053K22*
B009N09
B002H16
V011G02
M048B19
V002N15*
B016O04
B018K13
M058F13
V075O12*
B018A13
M056P10
V071H21
B016I17*
M044F07
V046L04
B014C14*
M044C07*
V041P06*
M031I18
M028B02
V017M09
M028L20
M006C07
V014N10
M009K03
M005L07
M039K07
M002L01
M004F05
V075N21
H002H14
M002P06*
V075M13
B016H07
H022L02
V071P05
B008N06*
H016K17
0 25 50 75 100 125 150 175 200
[root@localhost: /...
[FPC V8.9 Main Menu]
Project cp05T5e12...
[Chickpea contig p...
FPC Ctg2333 cp05...

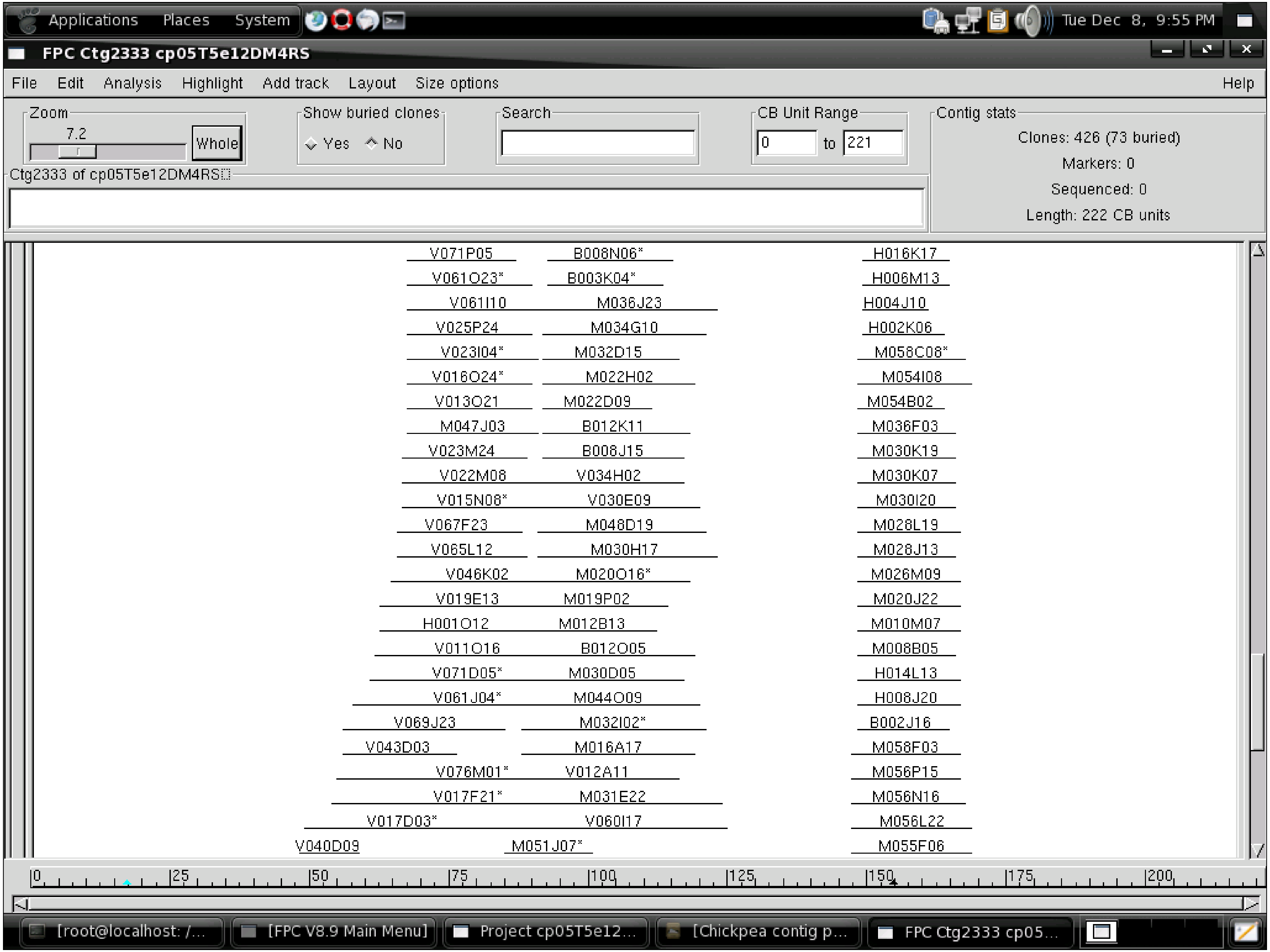
Applications Places System
Tue Dec 8, 9:55 PM
FPC Ctg2333 cp05T5e12DM4RS
File Edit Analysis Highlight Add track Layout Size options Help
Zoom 7.2 Whole
Show buried clones Yes No
Search
CB Unit Range 0 to 221
Contig stats
Clones: 426 (73 buried)
Markers: 0
Sequenced: 0
Length: 222 CB units
Ctg2333 of cp05T5e12DM4RS
V071P05 B008N06* H016K17
V061O23* B003K04* H006M13
V061I10 M036J23 H004J10
V025P24 M034G10 H002K06
V023I04* M032D15 M058C08*
V016O24* M022H02 M054I08
V013O21 M022D09 M054B02
M047J03 B012K11 M036F03
V023M24 B008J15 M030K19
V022M08 V034H02 M030K07
V015N08* V030E09 M030I20
V067F23 M048D19 M028L19
V065L12 M030H17 M028J13
V046K02 M020O16* M026M09
V019E13 M019P02 M020J22
H001O12 M012B13 M010M07
V011O16 B012O05 M008B05
V071D05* M030D05 H014L13
V061J04* M044O09 H008J20
V069J23 M032I02* B002J16
V043D03 M016A17 M058F03
V076M01* V012A11 M056P15
V017F21* M031E22 M056N16
V017D03* V060I17 M056L22
V040D09 M051J07* M055F06
0 25 50 75 100 125 150 175 200
[root@localhost: /... [FPC V8.9 Main Menu] Project cp05T5e12... [Chickpea contig p... FPC Ctg2333 cp05...

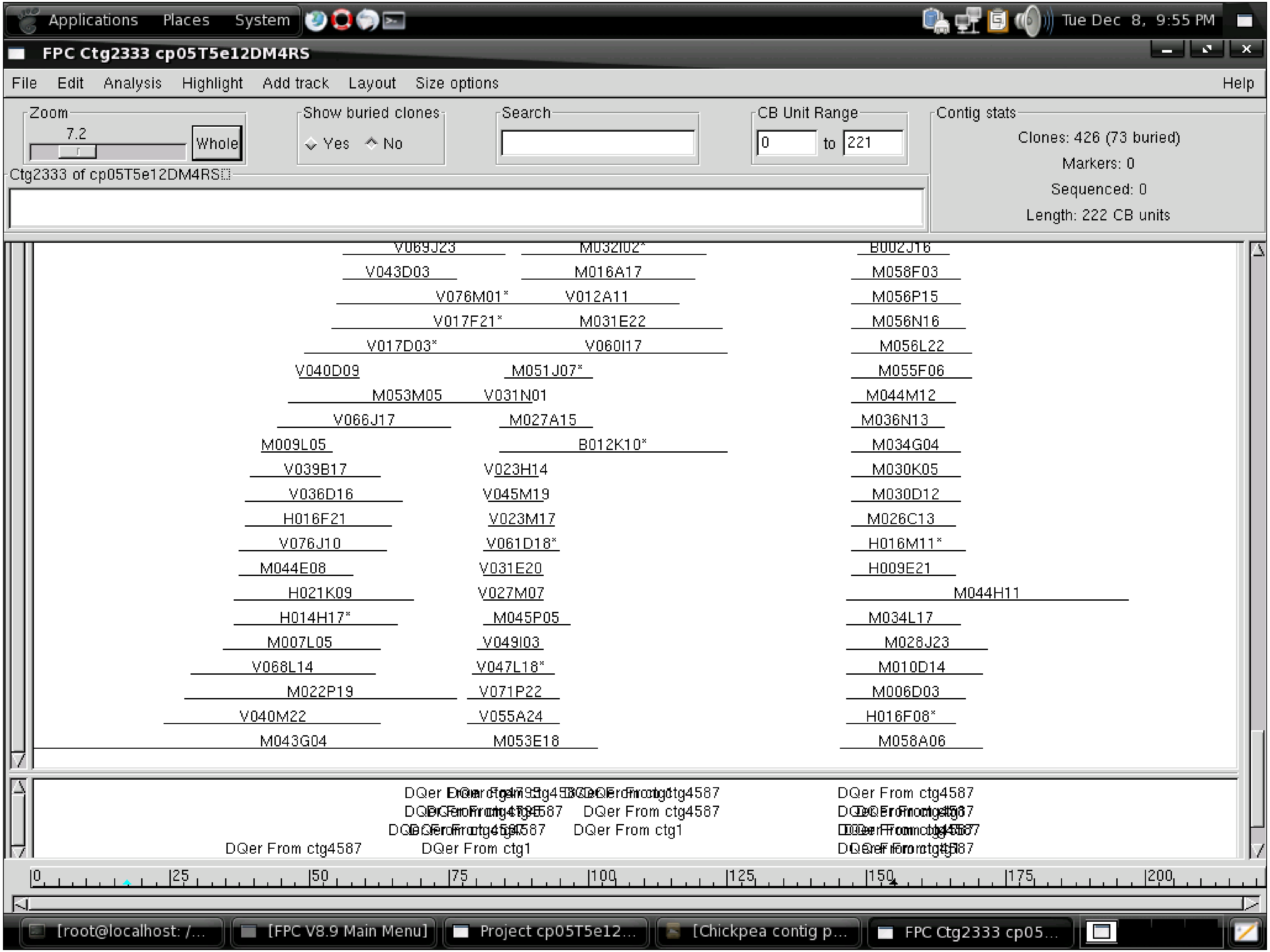

Applications Places System
Tue Dec 8, 9:55 PM
FPC Ctg2333 cp05T5e12DM4RS
File Edit Analysis Highlight Add track Layout Size options Help
Zoom
7.2
Whole
Show buried clones
Yes No
Search
CB Unit Range
0 to 221
Contig stats
Clones: 426 (73 buried)
Markers: 0
Sequenced: 0
Length: 222 CB units
Ctg2333 of cp05T5e12DM4RS
V069J23 M032I02* B002J16
V043D03 M016A17 M058F03
V076M01* V012A11 M056P15
V017F21* M031E22 M056N16
V017D03* V060I17 M056L22
V040D09 M051J07* M055F06
M053M05 V031N01 M044M12
V066J17 M027A15 M036N13
M009L05 B012K10* M034G04
V039B17 V023H14 M030K05
V036D16 V045M19 M030D12
H016F21 V023M17 M026C13
V076J10 V061D18* H016M11*
M044E08 V031E20 H009E21
H021K09 V027M07 M044H11
H014H17* M045P05 M034L17
M007L05 V049I03 M028J23
V068L14 V047L18* M010D14
M022P19 V071P22 M006D03
V040M22 V055A24 H016F08*
M043G04 M053E18 M058A06
DQer From ctg4587
DQer From ctg4587
DQer From ctg1
DQer From ctg4587
DQer From ctg1
0 25 50 75 100 125 150 175 200
[root@localhost: /...
[FPC V8.9 Main Menu]
Project cp05T5e12...
[Chickpea contig p...
FPC Ctg2333 cp05...

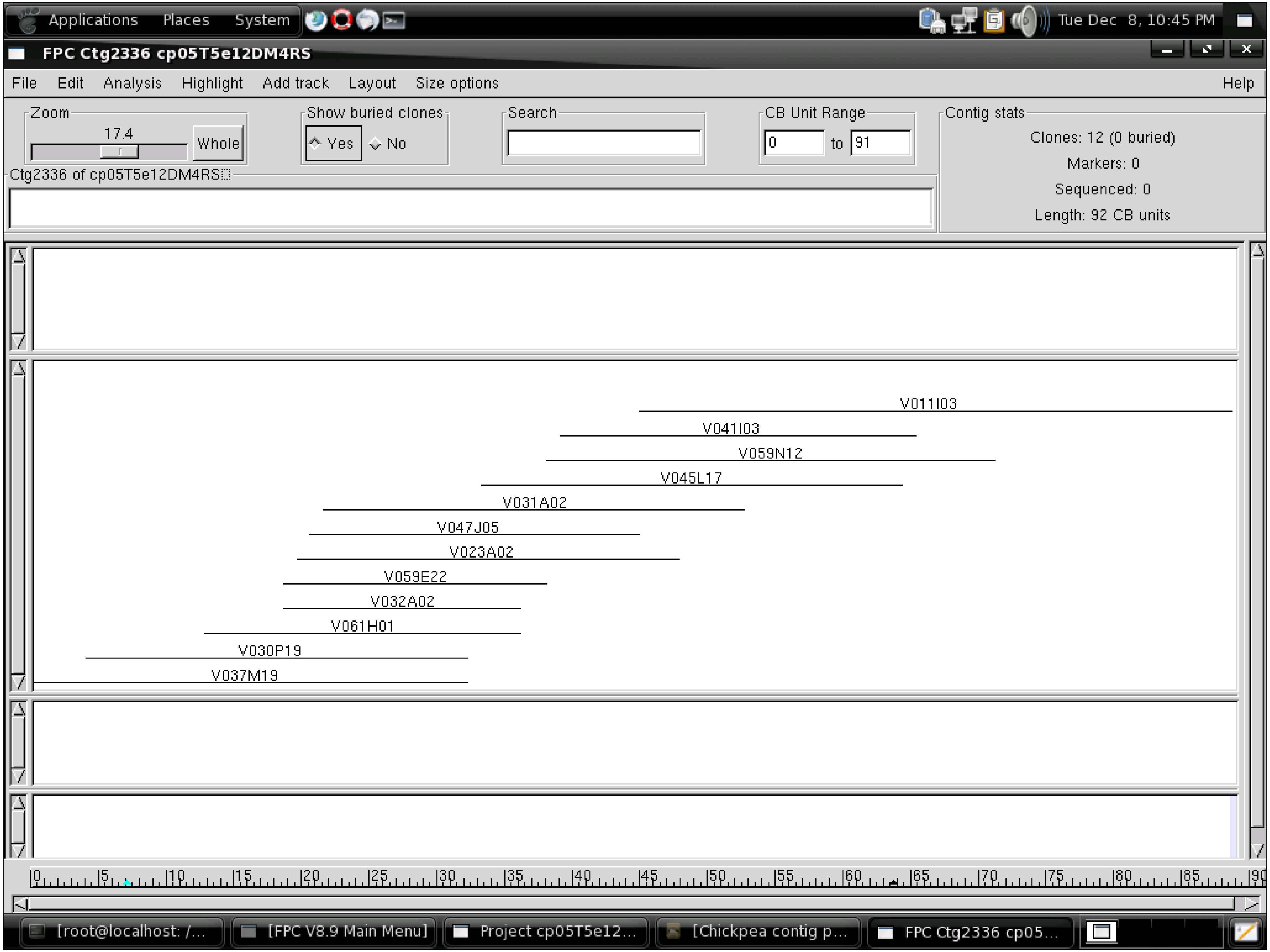

Applications Places System
Tue Dec 8, 10:45 PM
FPC Ctg2336 cp05T5e12DM4RS
File Edit Analysis Highlight Add track Layout Size options Help
Zoom
17.4
Whole
Show buried clones
Yes
No
Search
CB Unit Range
0 to 91
Contig stats
Clones: 12 (0 buried)
Markers: 0
Sequenced: 0
Length: 92 CB units
Ctg2336 of cp05T5e12DM4RS
V011I03
V041I03
V059N12
V045L17
V031A02
V047J05
V023A02
V059E22
V032A02
V061H01
V030P19
V037M19
[root@localhost: /...
[FPC V8.9 Main Menu]
Project cp05T5e12...
[Chickpea contig p...
FPC Ctg2336 cp05...

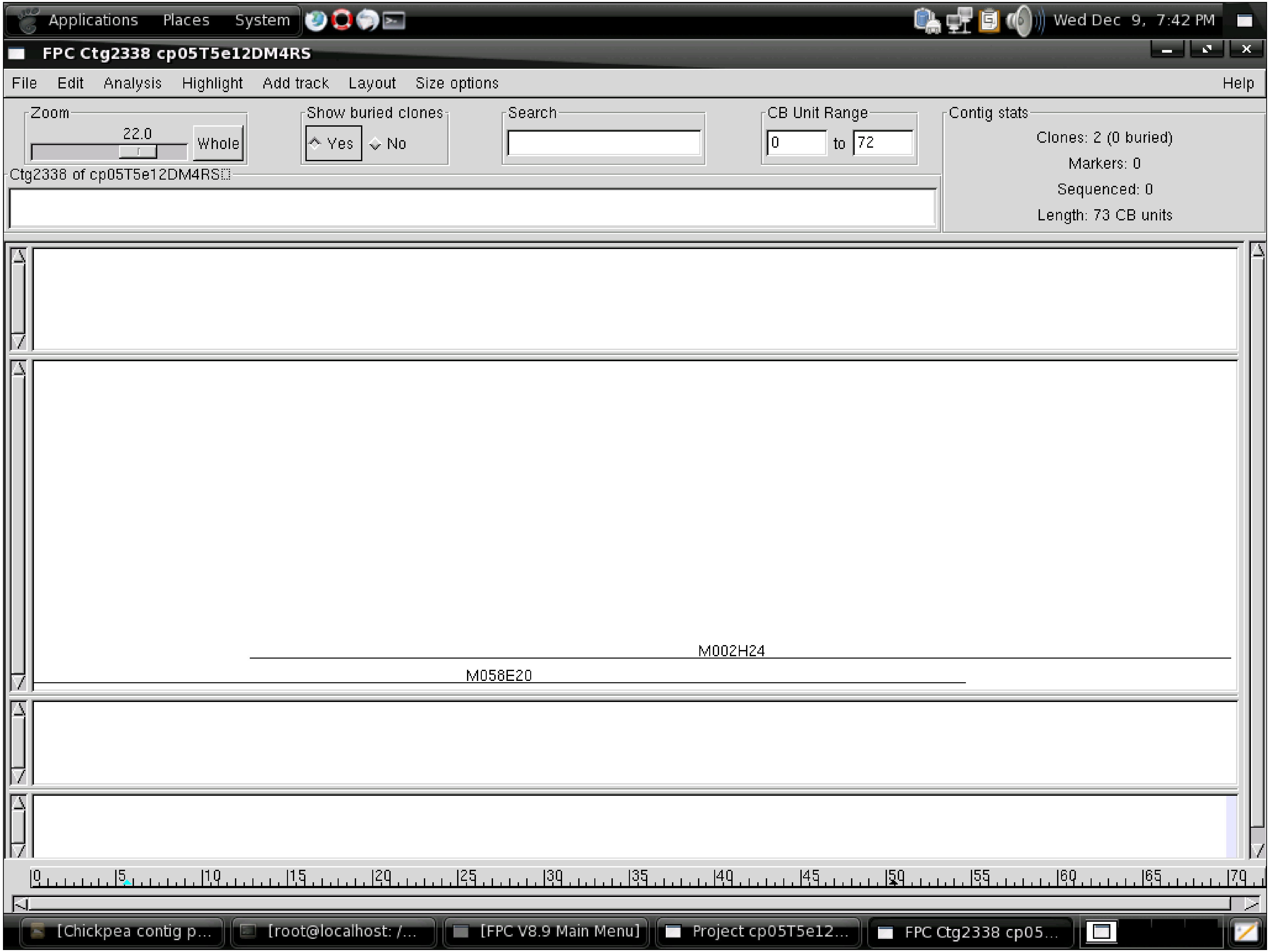
Applications Places System
Wed Dec 9, 7:42 PM
FPC Ctg2338 cp05T5e12DM4RS
File Edit Analysis Highlight Add track Layout Size options Help
Zoom
22.0
Whole
Show buried clones
Yes No
Search
CB Unit Range
0 to 72
Contig stats
Clones: 2 (0 buried)
Markers: 0
Sequenced: 0
Length: 73 CB units
Ctg2338 of cp05T5e12DM4RS
M002H24
M058E20
0 5 10 15 20 25 30 35 40 45 50 55 60 65 70
[Chickpea contig p...
[root@localhost: /...
[FPC V8.9 Main Menu]
Project cp05T5e12...
FPC Ctg2338 cp05...

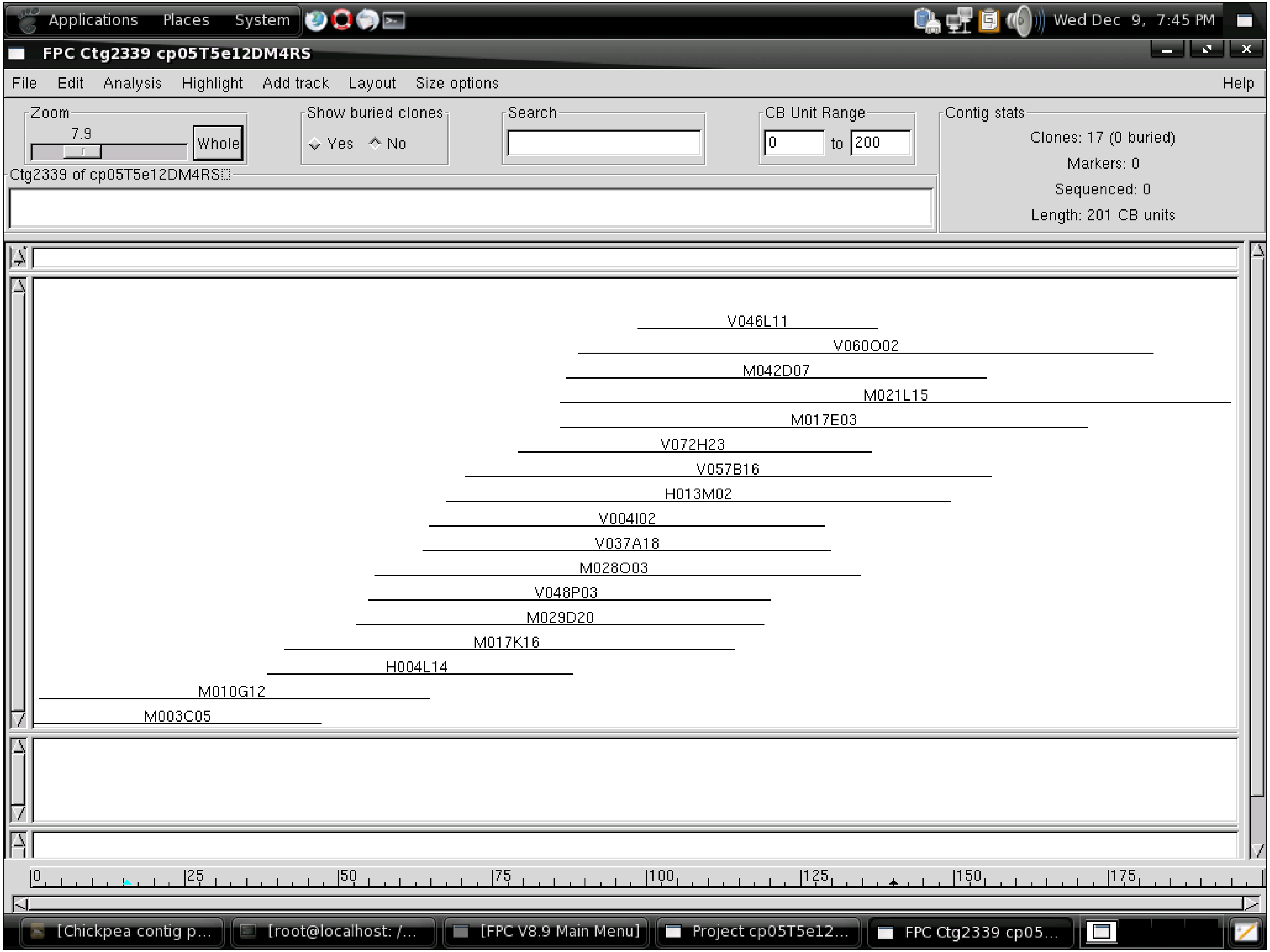
Applications Places System
Wed Dec 9, 7:45 PM
FPC Ctg2339 cp05T5e12DM4RS
File Edit Analysis Highlight Add track Layout Size options Help
Zoom
7.9
Whole
Show buried clones
Yes No
Search
CB Unit Range
0 to 200
Contig stats
Clones: 17 (0 buried)
Markers: 0
Sequenced: 0
Length: 201 CB units
Ctg2339 of cp05T5e12DM4RS
V046L11
V060O02
M042D07
M021L15
M017E03
V072H23
V057B16
H013M02
V004I02
V037A18
M028O03
V048P03
M029D20
M017K16
H004L14
M010G12
M003C05
0
25
50
75
100
125
150
175
[Chickpea contig p...
[root@localhost: /...
[FPC V8.9 Main Menu]
Project cp05T5e12...
FPC Ctg2339 cp05...

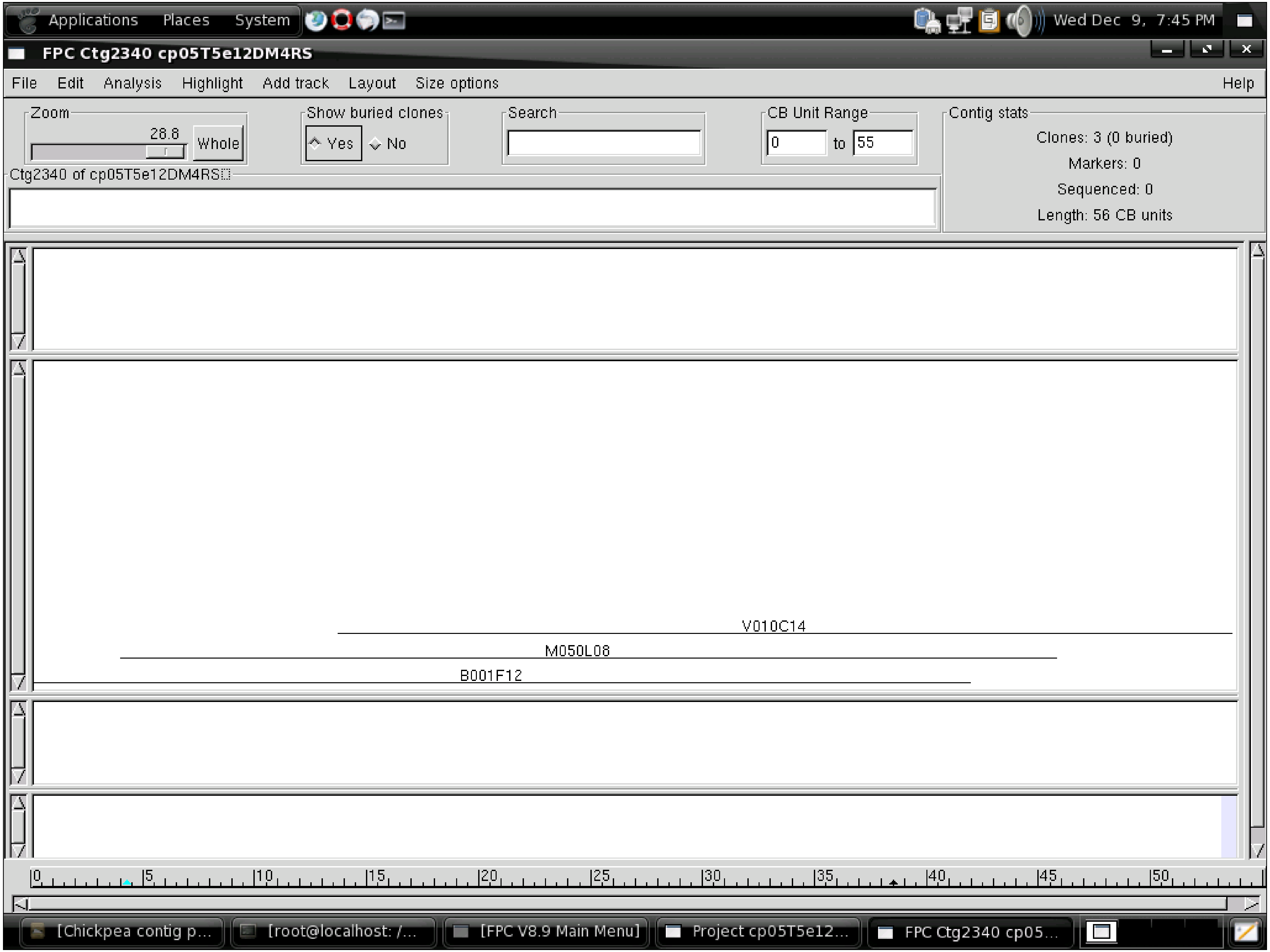
Applications Places System
Wed Dec 9, 7:45 PM
FPC Ctg2340 cp05T5e12DM4RS
File Edit Analysis Highlight Add track Layout Size options Help
Zoom
28.8
Whole
Show buried clones
Yes
No
Search
CB Unit Range
0
to
55
Contig stats
Clones: 3 (0 buried)
Markers: 0
Sequenced: 0
Length: 56 CB units
Ctg2340 of cp05T5e12DM4RS
V010C14
M050L08
B001F12
0 5 10 15 20 25 30 35 40 45 50
[Chickpea contig p...
[root@localhost: /...
[FPC V8.9 Main Menu]
Project cp05T5e12...
FPC Ctg2340 cp05...

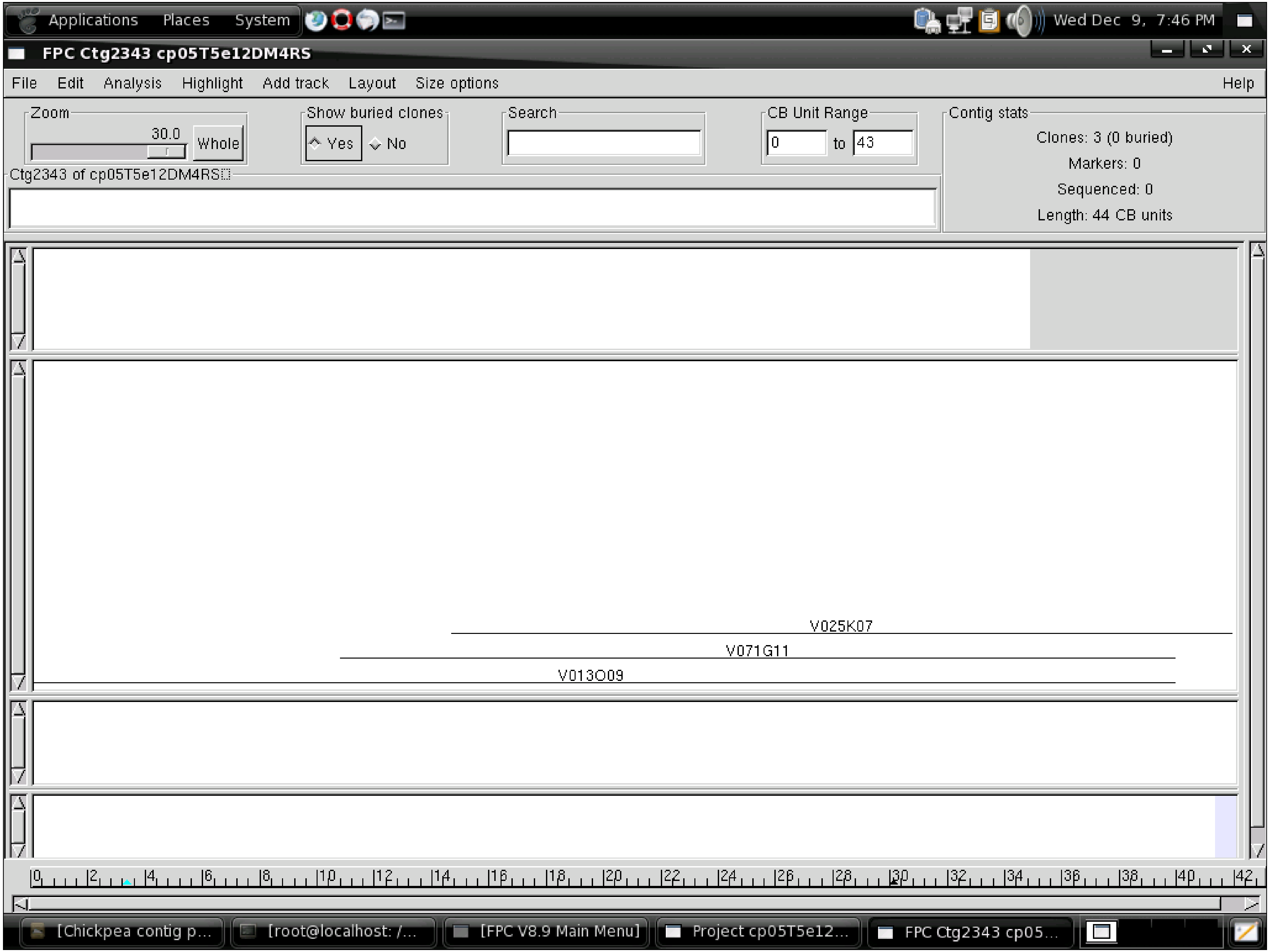
Applications Places System
Wed Dec 9, 7:46 PM
FPC Ctg2343 cp05T5e12DM4RS
File Edit Analysis Highlight Add track Layout Size options Help
Zoom
30.0
Whole
Show buried clones
Yes
No
Search
CB Unit Range
0
to
43
Contig stats
Clones: 3 (0 buried)
Markers: 0
Sequenced: 0
Length: 44 CB units
Ctg2343 of cp05T5e12DM4RS
V025K07
V071G11
V013O09
0 2 4 6 8 10 12 14 16 18 20 22 24 26 28 30 32 34 36 38 40 42
[Chickpea contig p...
[root@localhost: /...
[FPC V8.9 Main Menu]
Project cp05T5e12...
FPC Ctg2343 cp05...

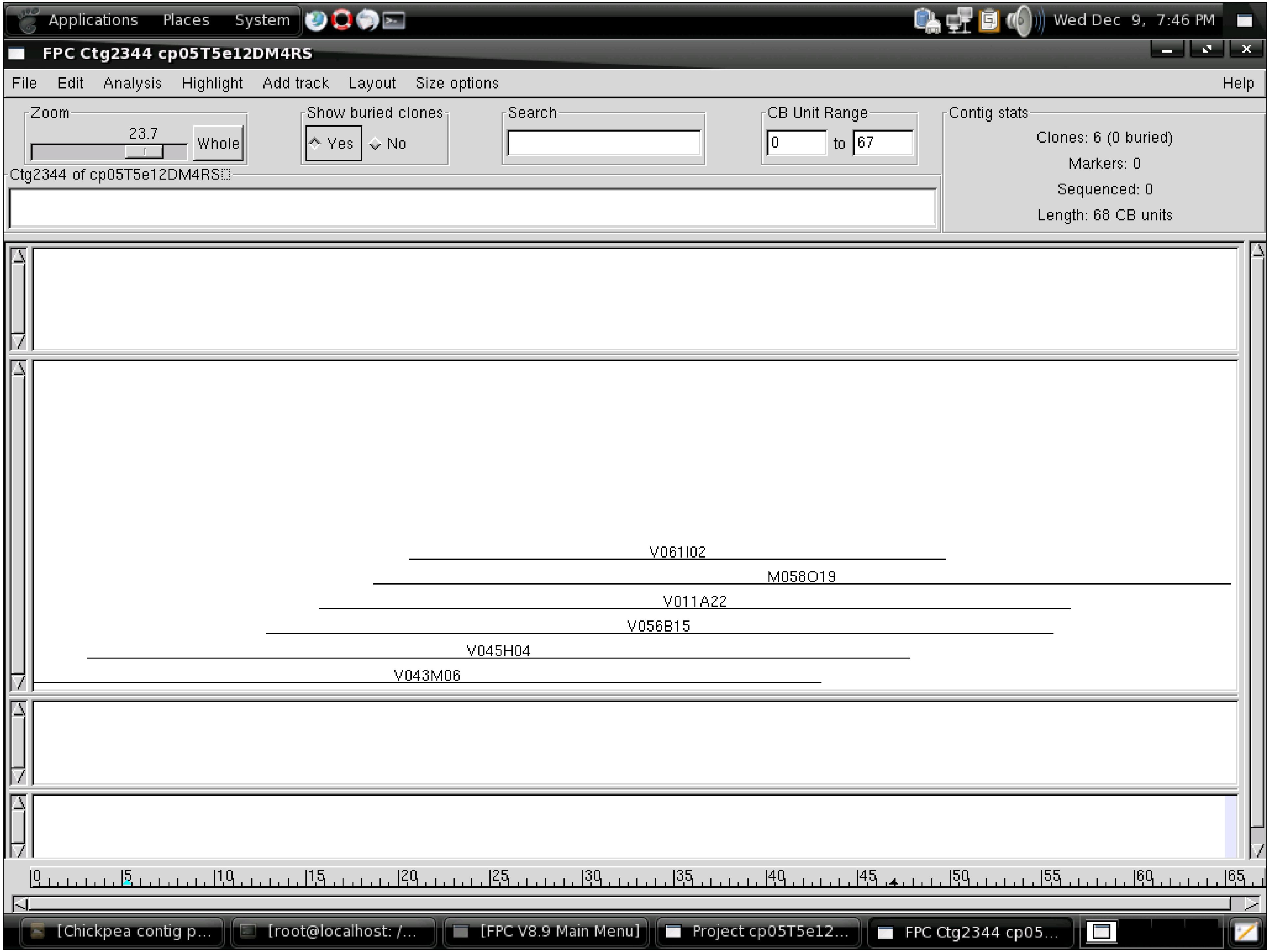
Applications Places System
Wed Dec 9, 7:46 PM
FPC Ctg2344 cp05T5e12DM4RS
File Edit Analysis Highlight Add track Layout Size options Help
Zoom
23.7
Whole
Show buried clones
Yes
No
Search
CB Unit Range
0
to
67
Contig stats
Clones: 6 (0 buried)
Markers: 0
Sequenced: 0
Length: 68 CB units
Ctg2344 of cp05T5e12DM4RS
V061I02
M058O19
V011A22
V056B15
V045H04
V043M06
0 5 10 15 20 25 30 35 40 45 50 55 60 65
[Chickpea contig p...
[root@localhost: /...
[FPC V8.9 Main Menu]
Project cp05T5e12...
FPC Ctg2344 cp05...

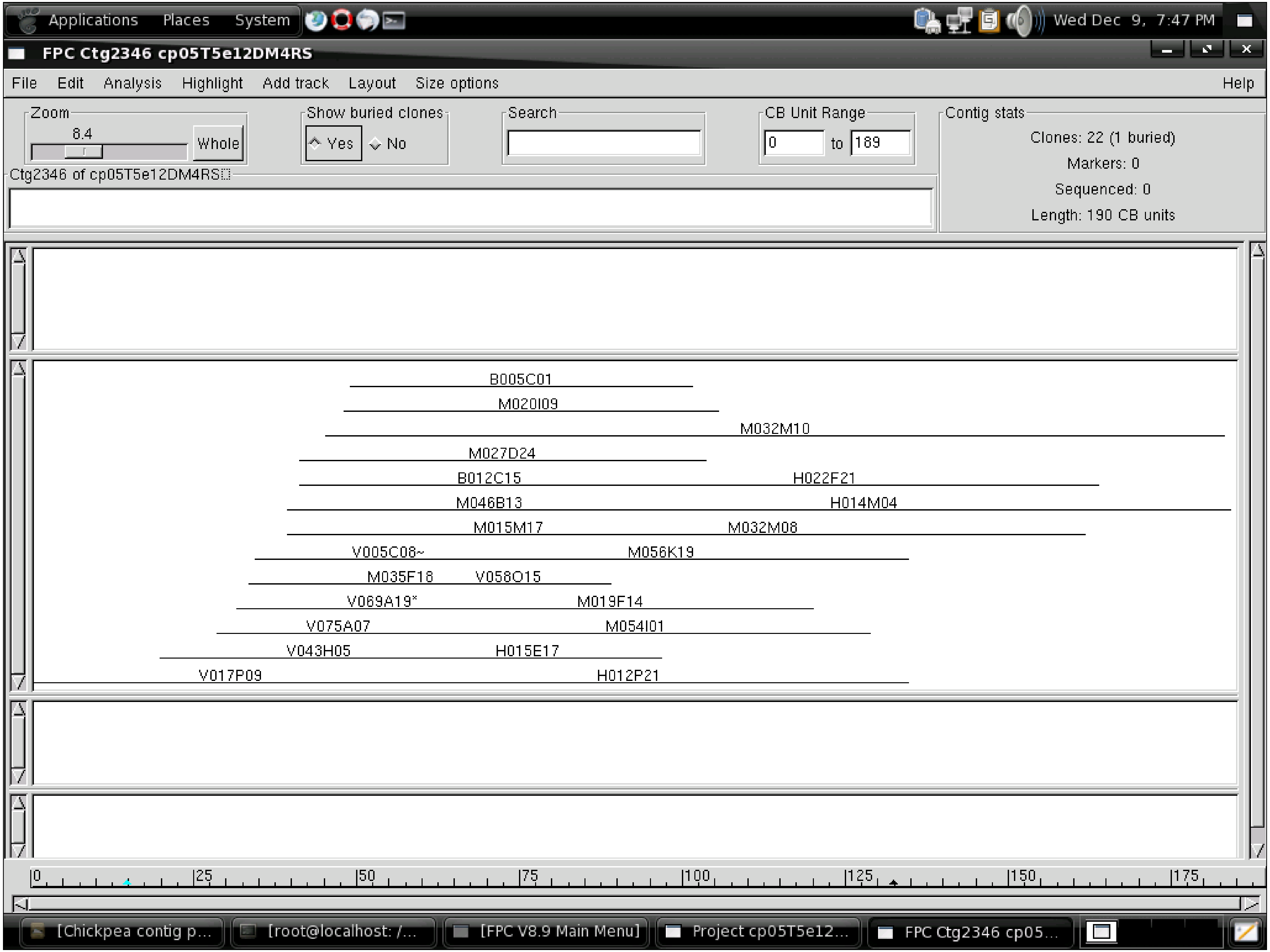
Applications Places System
Wed Dec 9, 7:47 PM
FPC Ctg2346 cp05T5e12DM4RS
File Edit Analysis Highlight Add track Layout Size options Help
Zoom
8.4
Whole
Show buried clones
Yes
No
Search
CB Unit Range
0
to
189
Contig stats
Clones: 22 (1 buried)
Markers: 0
Sequenced: 0
Length: 190 CB units
Ctg2346 of cp05T5e12DM4RS
B005C01
M020I09
M032M10
M027D24
B012C15
H022F21
M046B13
H014M04
M015M17
M032M08
V005C08~
M056K19
M035F18
V058O15
V069A19*
M019F14
V075A07
M054I01
V043H05
H015E17
V017P09
H012P21
0
25
50
75
100
125
150
175
[Chickpea contig p...
[root@localhost: /...
[FPC V8.9 Main Menu]
Project cp05T5e12...
FPC Ctg2346 cp05...

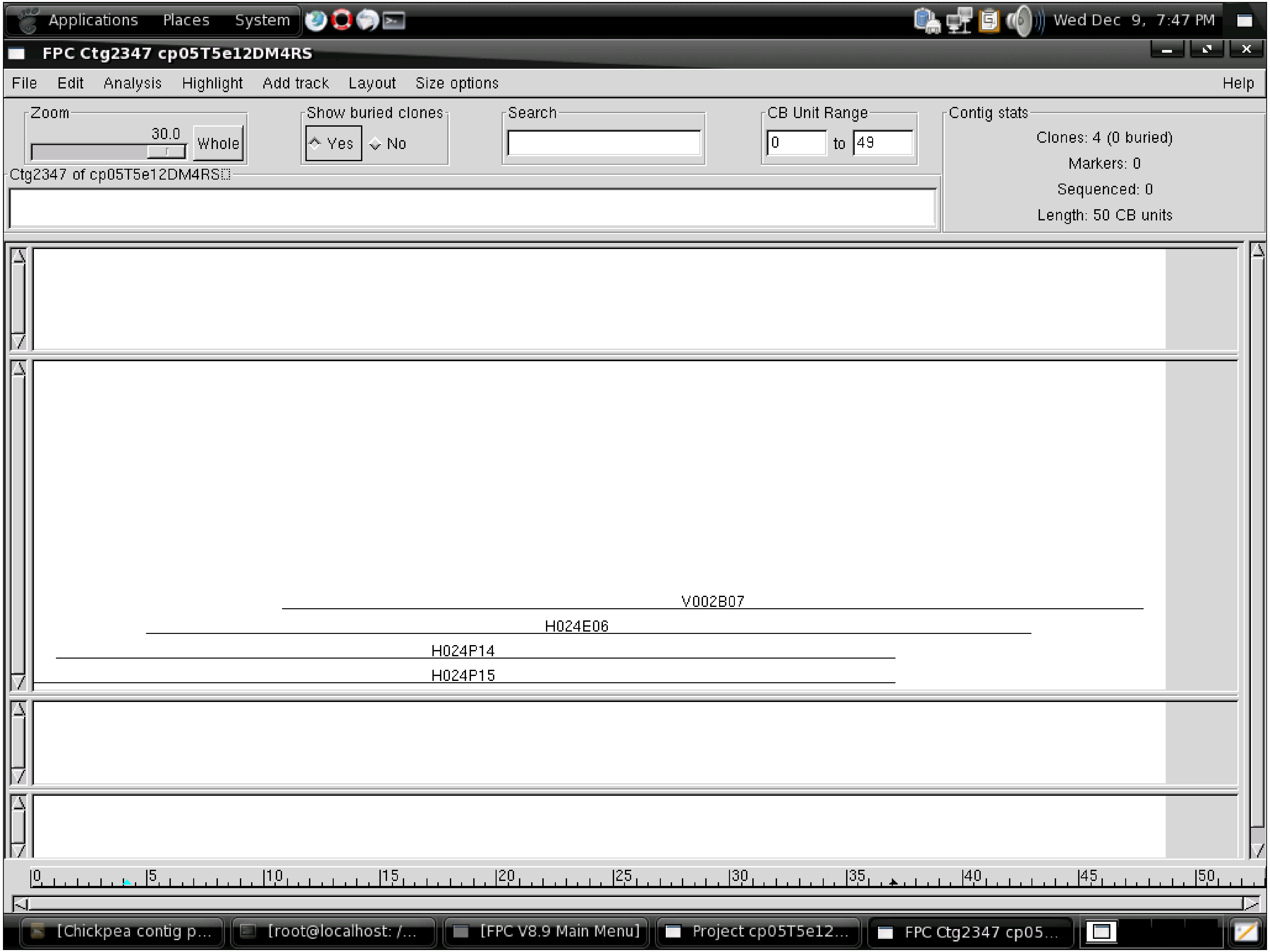
Applications Places System
Wed Dec 9, 7:47 PM
FPC Ctg2347 cp05T5e12DM4RS
File Edit Analysis Highlight Add track Layout Size options Help
Zoom
30.0
Whole
Show buried clones
Yes
No
Search
CB Unit Range
0
to
49
Contig stats
Clones: 4 (0 buried)
Markers: 0
Sequenced: 0
Length: 50 CB units
Ctg2347 of cp05T5e12DM4RS
V002B07
H024E06
H024P14
H024P15
0 5 10 15 20 25 30 35 40 45 50
[Chickpea contig p...
[root@localhost: /...
[FPC V8.9 Main Menu]
Project cp05T5e12...
FPC Ctg2347 cp05...

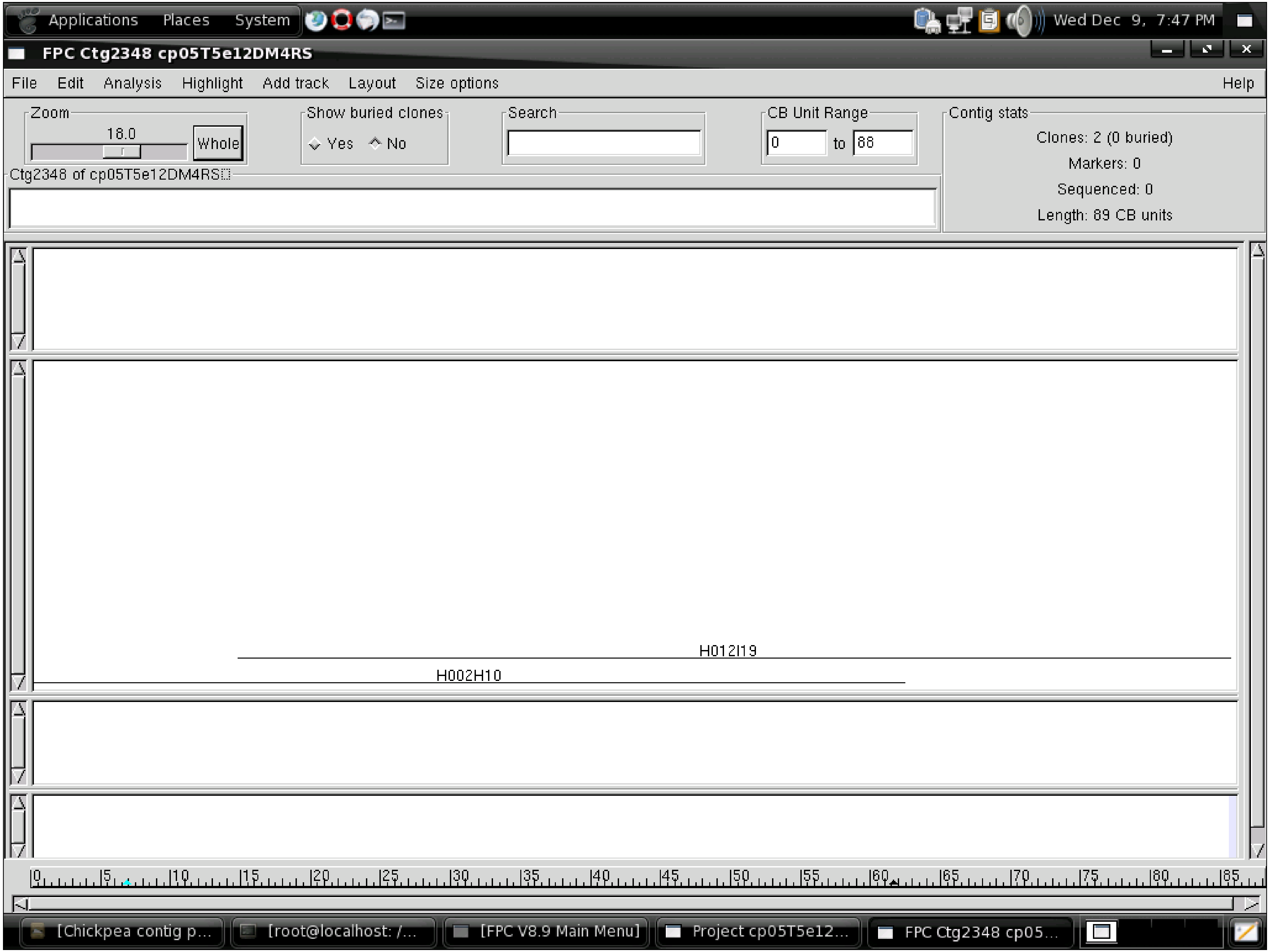
Applications Places System
Wed Dec 9, 7:47 PM
FPC Ctg2348 cp05T5e12DM4RS
File Edit Analysis Highlight Add track Layout Size options Help
Zoom
18.0
Whole
Show buried clones
Yes No
Search
CB Unit Range
0 to 88
Contig stats
Clones: 2 (0 buried)
Markers: 0
Sequenced: 0
Length: 89 CB units
Ctg2348 of cp05T5e12DM4RS
H012I19
H002H10
0 5 10 15 20 25 30 35 40 45 50 55 60 65 70 75 80 85
[Chickpea contig p...
[root@localhost: /...
[FPC V8.9 Main Menu]
Project cp05T5e12...
FPC Ctg2348 cp05...

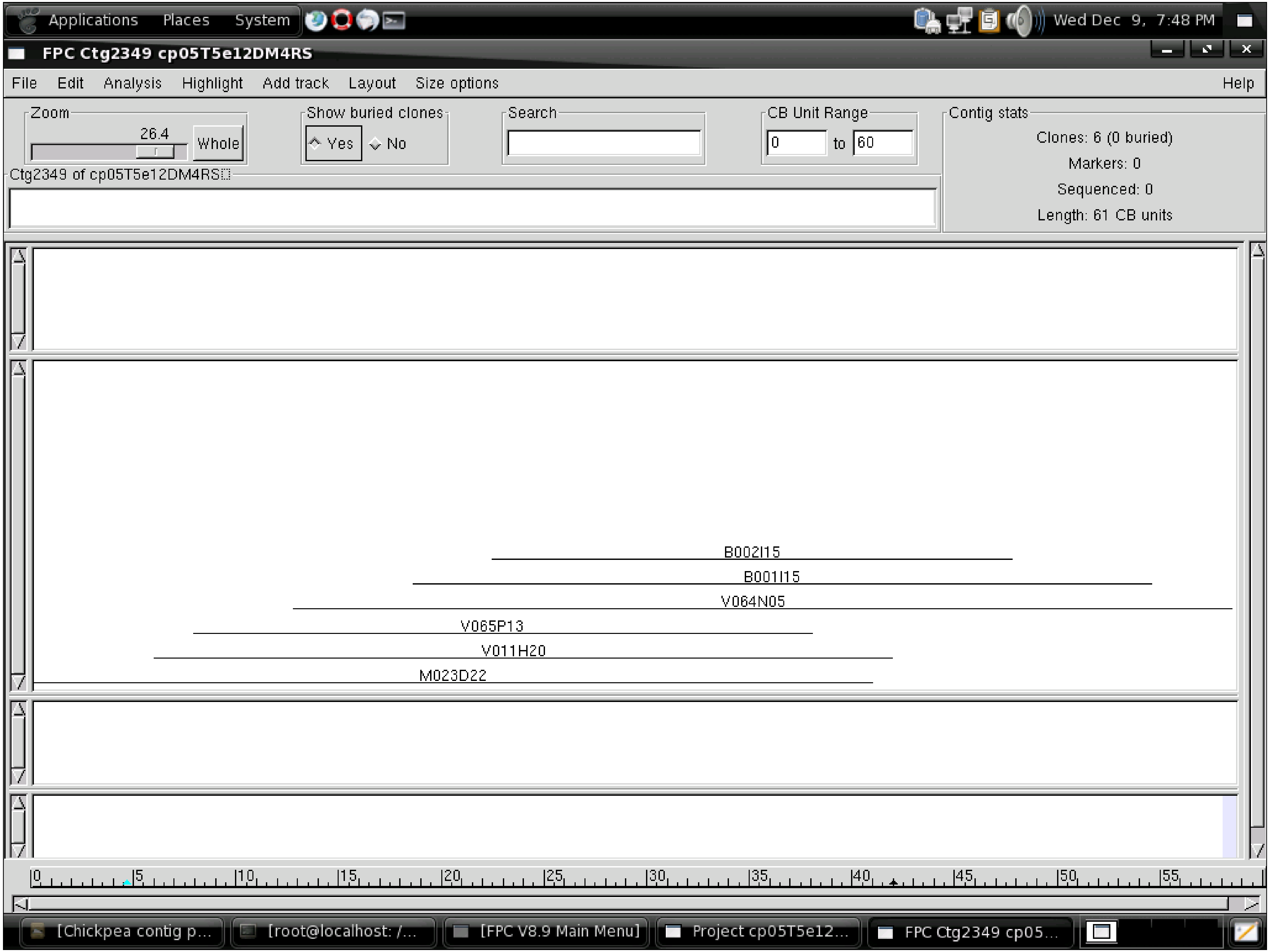
Applications Places System
Wed Dec 9, 7:48 PM
FPC Ctg2349 cp05T5e12DM4RS
File Edit Analysis Highlight Add track Layout Size options Help
Zoom
26.4
Whole
Show buried clones
Yes
No
Search
CB Unit Range
0 to 60
Contig stats
Clones: 6 (0 buried)
Markers: 0
Sequenced: 0
Length: 61 CB units
Ctg2349 of cp05T5e12DM4RS
B002I15
B001I15
V064N05
V065P13
V011H20
M023D22
0 5 10 15 20 25 30 35 40 45 50 55
[Chickpea contig p...
[root@localhost: /...
[FPC V8.9 Main Menu]
Project cp05T5e12...
FPC Ctg2349 cp05...

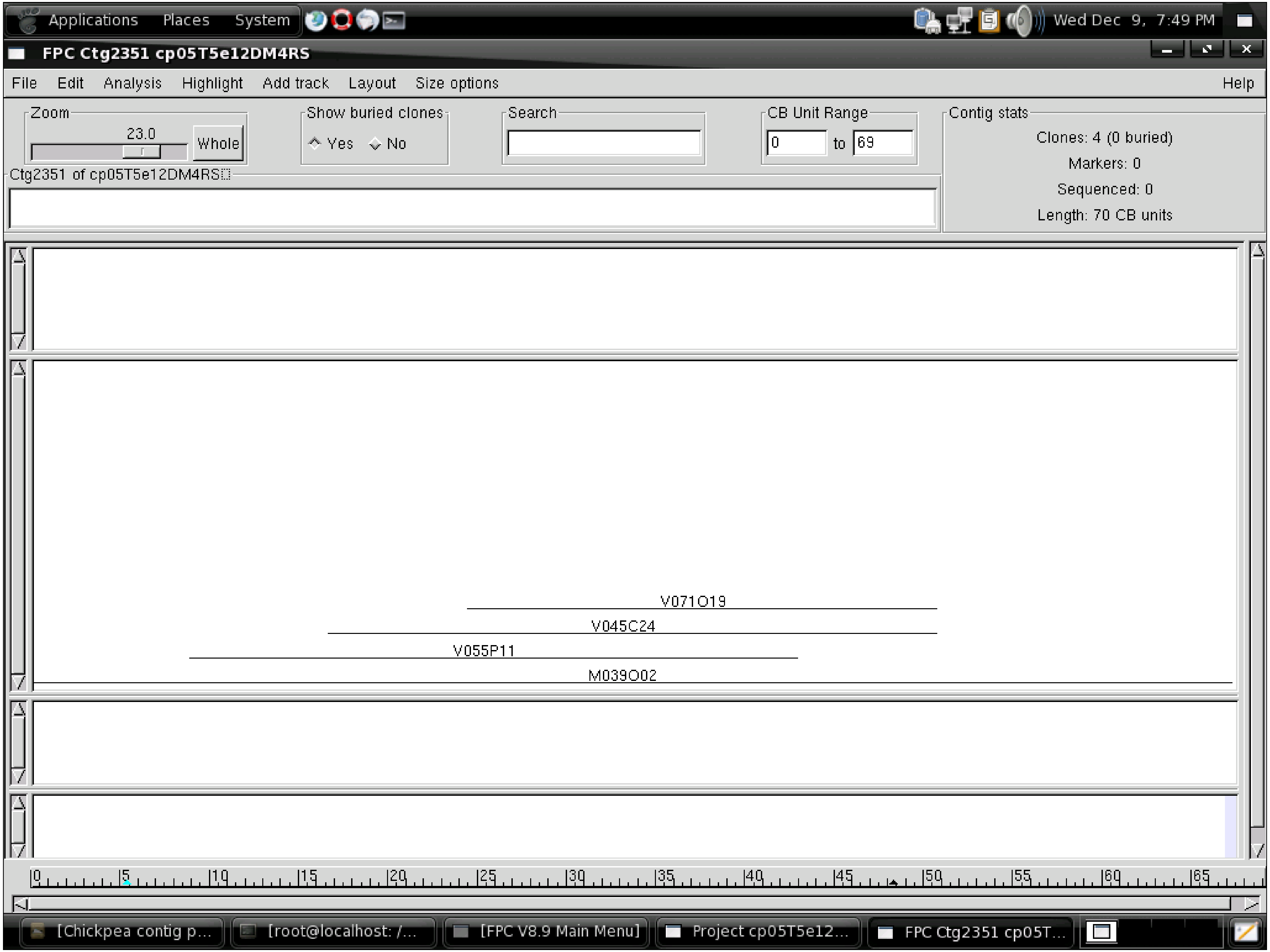
Applications Places System
Wed Dec 9, 7:49 PM
FPC Ctg2351 cp05T5e12DM4RS
File Edit Analysis Highlight Add track Layout Size options Help
Zoom
23.0
Whole
Show buried clones
Yes No
Search
CB Unit Range
0 to 69
Contig stats
Clones: 4 (0 buried)
Markers: 0
Sequenced: 0
Length: 70 CB units
Ctg2351 of cp05T5e12DM4RS
V071O19
V045C24
V055P11
M039O02
0 5 10 15 20 25 30 35 40 45 50 55 60 65
[Chickpea contig p...
[root@localhost: /...
[FPC V8.9 Main Menu]
Project cp05T5e12...
FPC Ctg2351 cp05T...

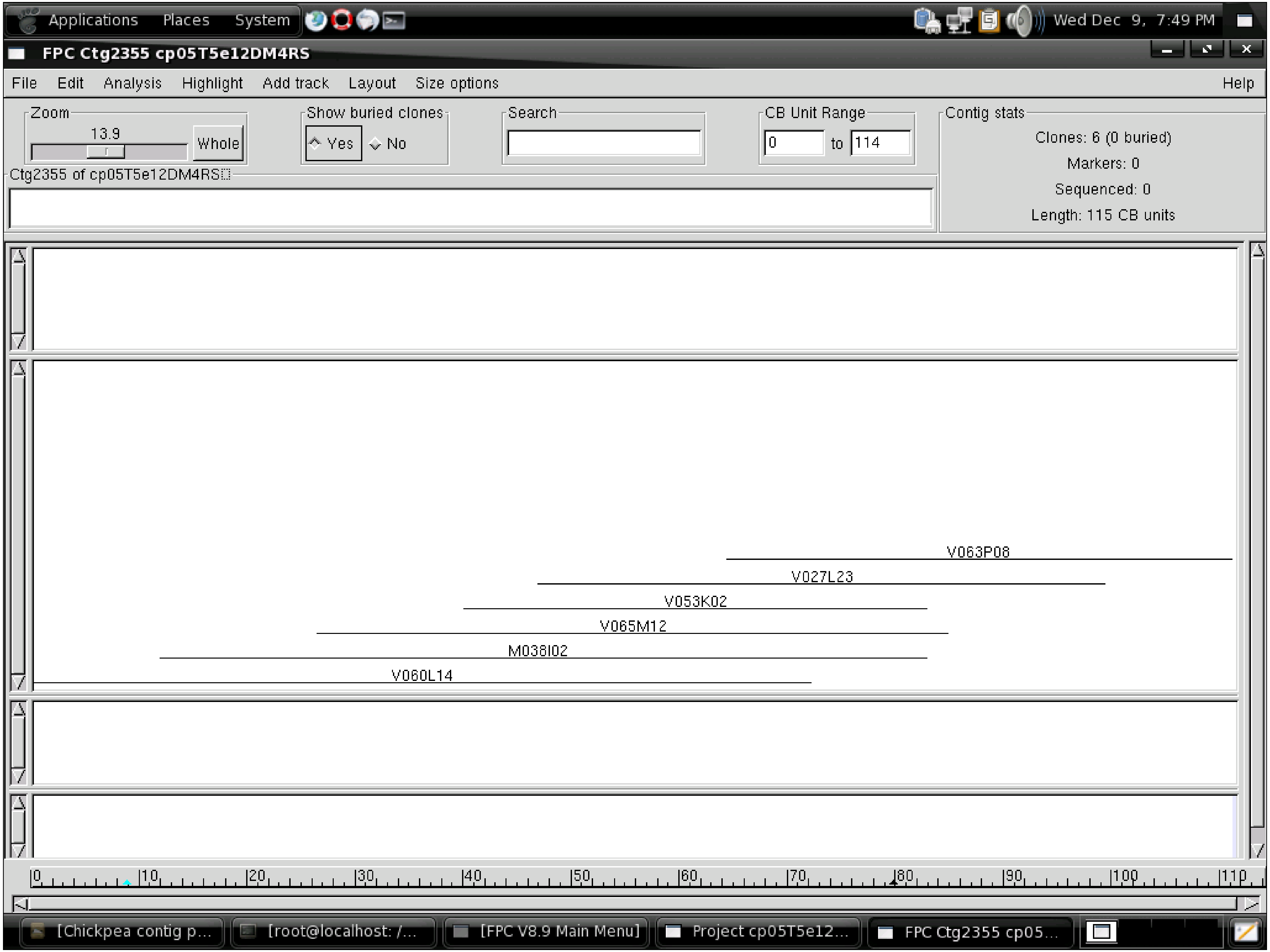
Applications Places System
Wed Dec 9, 7:49 PM
FPC Ctg2355 cp05T5e12DM4RS
File Edit Analysis Highlight Add track Layout Size options Help
Zoom
13.9
Whole
Show buried clones
Yes
No
Search
CB Unit Range
0 to 114
Contig stats
Clones: 6 (0 buried)
Markers: 0
Sequenced: 0
Length: 115 CB units
Ctg2355 of cp05T5e12DM4RS
V063P08
V027L23
V053K02
V065M12
M038I02
V060L14
0 10 20 30 40 50 60 70 80 90 100 110
[Chickpea contig p...
[root@localhost: /...
[FPC V8.9 Main Menu]
Project cp05T5e12...
FPC Ctg2355 cp05...

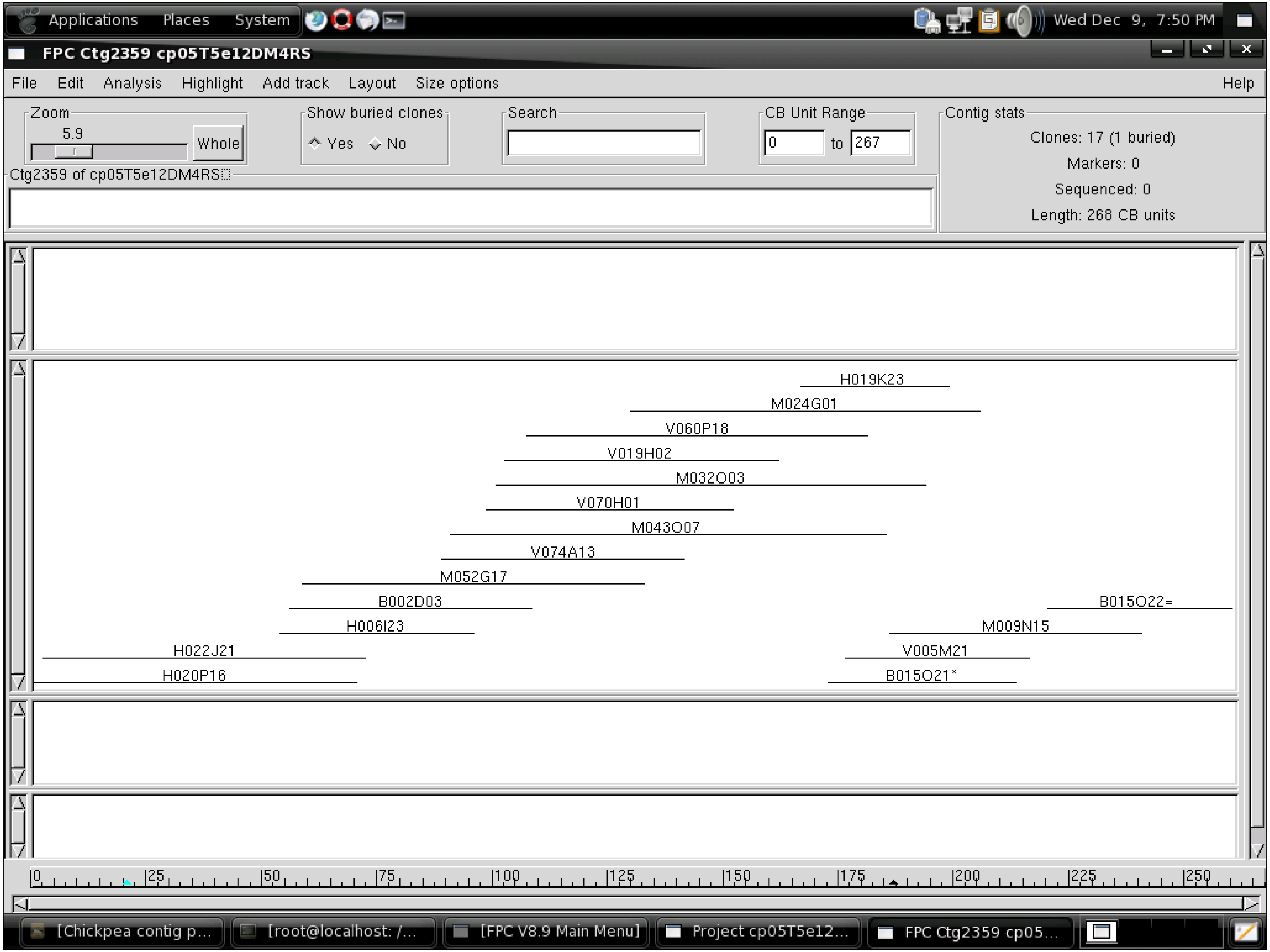
Applications Places System
Wed Dec 9, 7:50 PM
FPC Ctg2359 cp05T5e12DM4RS
File Edit Analysis Highlight Add track Layout Size options
Help
Zoom
5.9
Whole
Show buried clones
Yes No
Search
CB Unit Range
0 to 267
Contig stats
Clones: 17 (1 buried)
Markers: 0
Sequenced: 0
Length: 268 CB units
Ctg2359 of cp05T5e12DM4RS
H019K23
M024G01
V060P18
V019H02
M032O03
V070H01
M043O07
V074A13
M052G17
B002D03
B015O22=
H006I23
M009N15
H022J21
V005M21
H020P16
B015O21*
0 25 50 75 100 125 150 175 200 225 250
[Chickpea contig p...
[root@localhost: /...
[FPC V8.9 Main Menu]
Project cp05T5e12...
FPC Ctg2359 cp05...

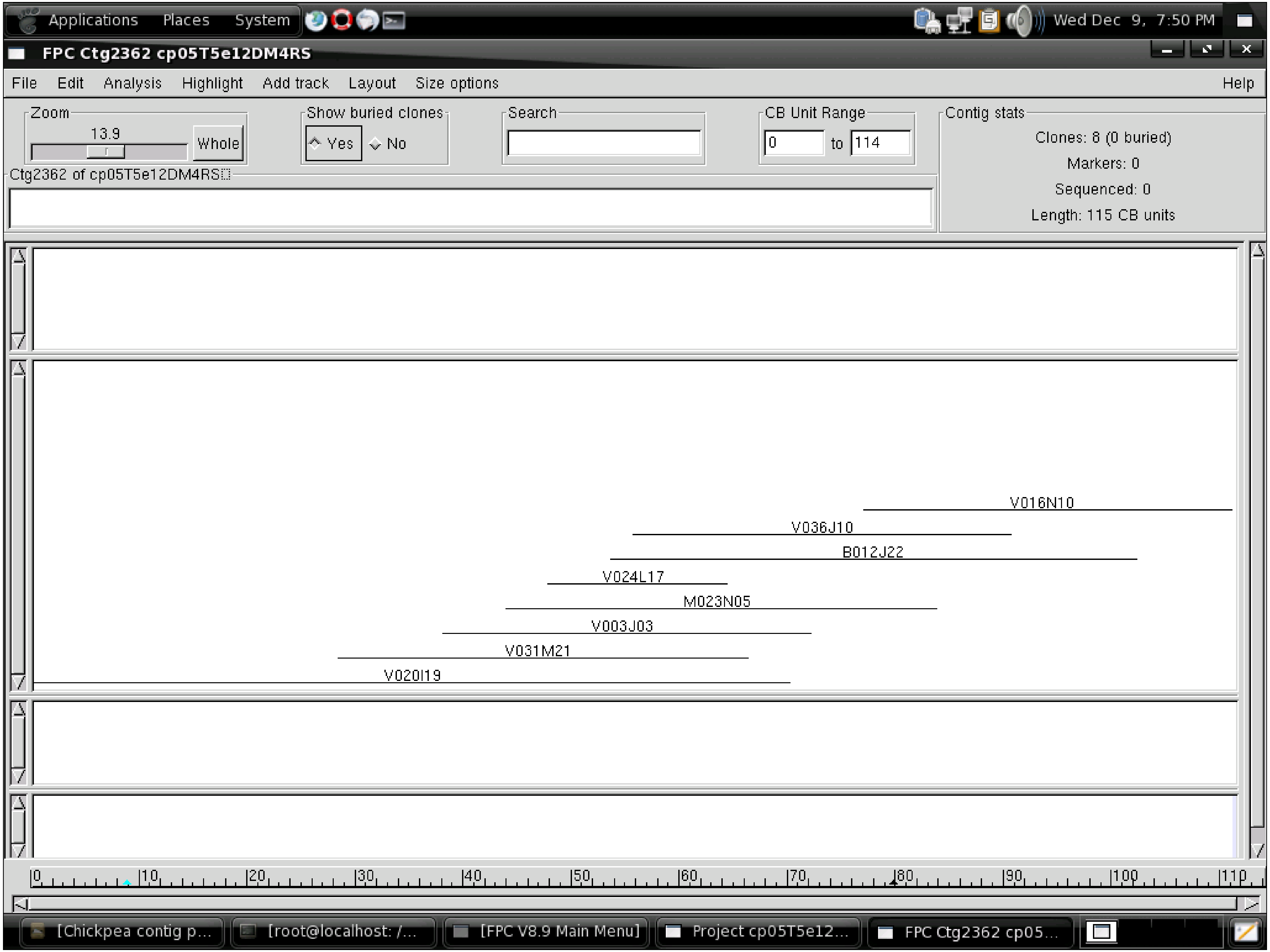
Applications
Places
System
Wed Dec 9, 7:50 PM
FPC Ctg2362 cp05T5e12DM4RS
File
Edit
Analysis
Highlight
Add track
Layout
Size options
Help
Zoom
13.9
Whole
Show buried clones
Yes
No
Search
CB Unit Range
0
to
114
Contig stats
Clones: 8 (0 buried)
Markers: 0
Sequenced: 0
Length: 115 CB units
Ctg2362 of cp05T5e12DM4RS
V016N10
V036J10
B012J22
V024L17
M023N05
V003J03
V031M21
V020I19
0
10
20
30
40
50
60
70
80
90
100
110
[Chickpea contig p...
[root@localhost: /...
[FPC V8.9 Main Menu]
Project cp05T5e12...
FPC Ctg2362 cp05...

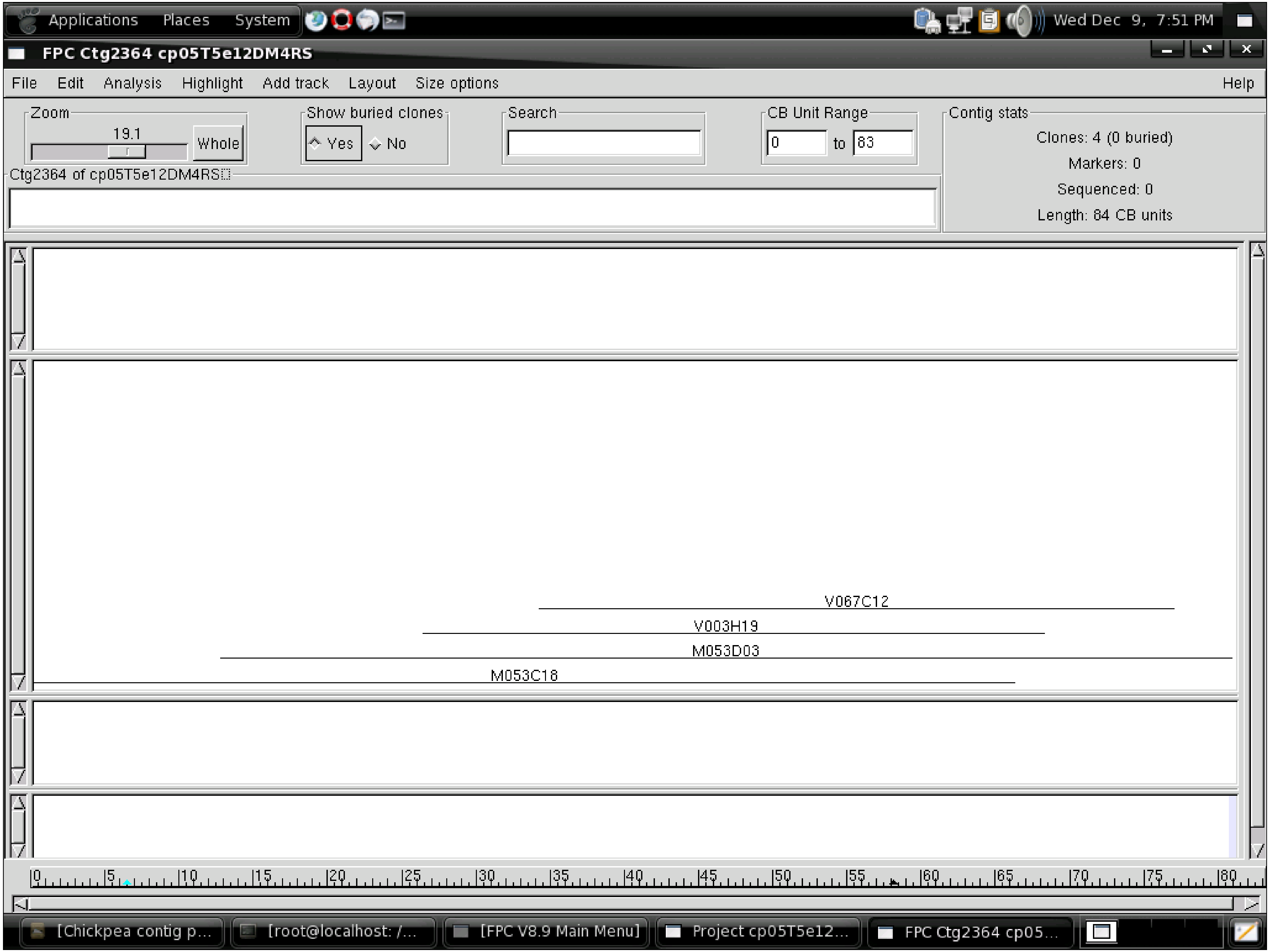
Applications Places System
Wed Dec 9, 7:51 PM
FPC Ctg2364 cp05T5e12DM4RS
File Edit Analysis Highlight Add track Layout Size options Help
Zoom
19.1
Whole
Show buried clones
Yes
No
Search
CB Unit Range
0
to
83
Contig stats
Clones: 4 (0 buried)
Markers: 0
Sequenced: 0
Length: 84 CB units
Ctg2364 of cp05T5e12DM4RS
V067C12
V003H19
M053D03
M053C18
0 5 10 15 20 25 30 35 40 45 50 55 60 65 70 75 80
[Chickpea contig p...
[root@localhost: /...
[FPC V8.9 Main Menu]
Project cp05T5e12...
FPC Ctg2364 cp05...

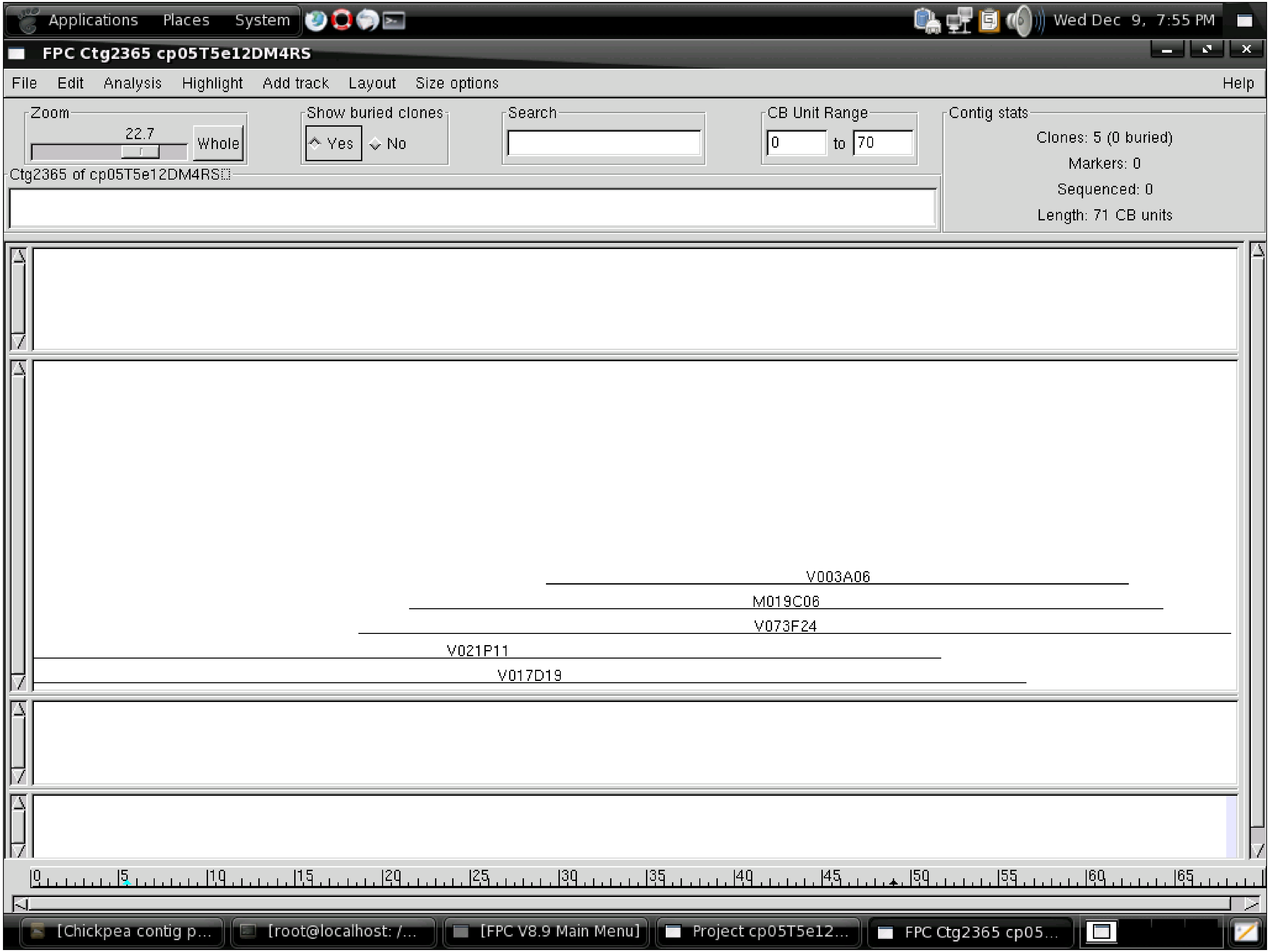
Applications Places System
Wed Dec 9, 7:55 PM
FPC Ctg2365 cp05T5e12DM4RS
File Edit Analysis Highlight Add track Layout Size options Help
Zoom
22.7
Whole
Show buried clones
Yes No
Search
CB Unit Range
0 to 70
Contig stats
Clones: 5 (0 buried)
Markers: 0
Sequenced: 0
Length: 71 CB units
Ctg2365 of cp05T5e12DM4RS
V003A06
M019C06
V073F24
V021P11
V017D19
0 5 10 15 20 25 30 35 40 45 50 55 60 65
[Chickpea contig p...
[root@localhost: /...
[FPC V8.9 Main Menu]
Project cp05T5e12...
FPC Ctg2365 cp05...

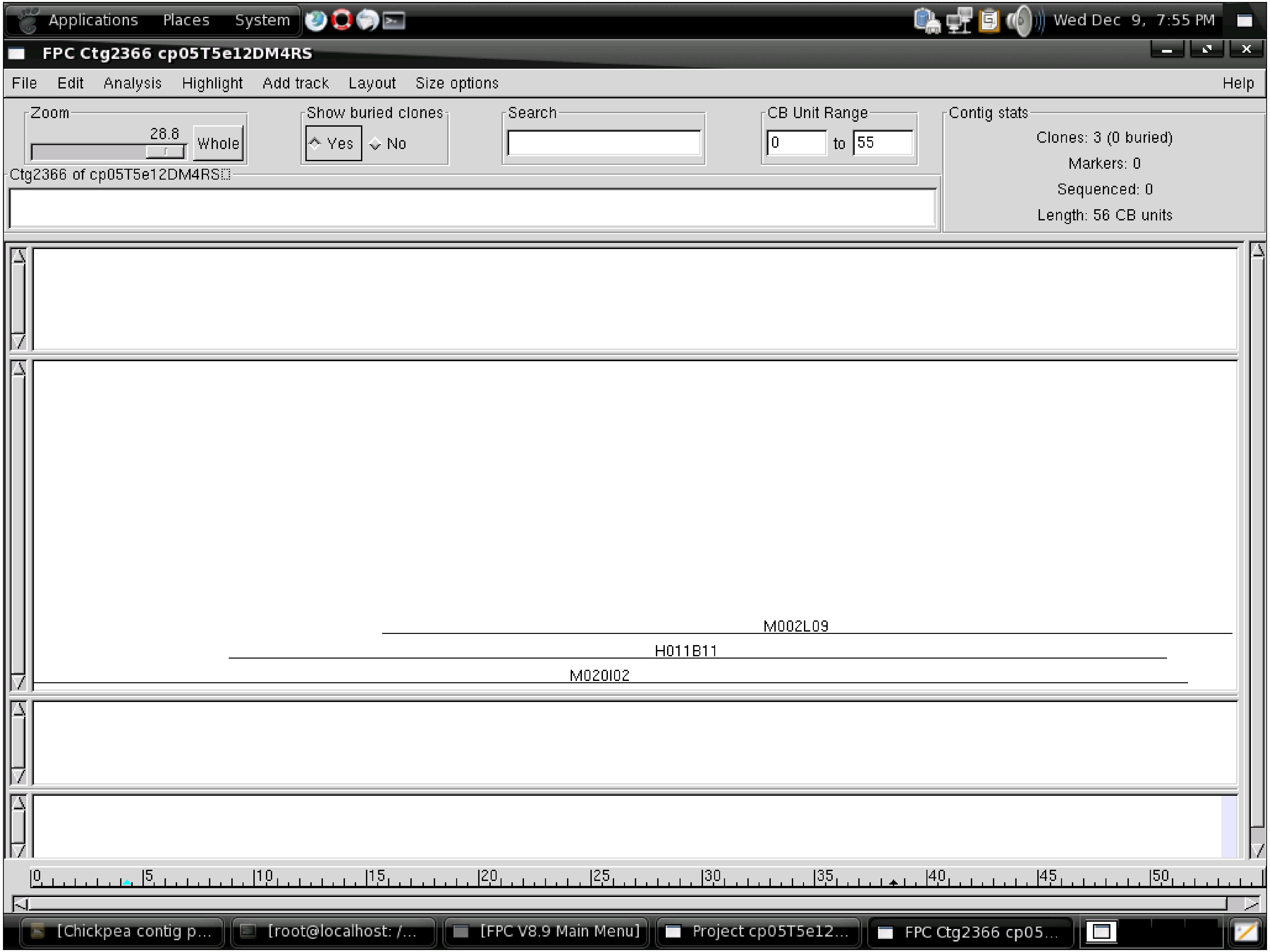
Applications Places System
Wed Dec 9, 7:55 PM
FPC Ctg2366 cp05T5e12DM4RS
File Edit Analysis Highlight Add track Layout Size options Help
Zoom
28.8
Whole
Show buried clones
Yes
No
Search
CB Unit Range
0
to
55
Contig stats
Clones: 3 (0 buried)
Markers: 0
Sequenced: 0
Length: 56 CB units
Ctg2366 of cp05T5e12DM4RS
M002L09
H011B11
M020I02
0 5 10 15 20 25 30 35 40 45 50
[Chickpea contig p...
[root@localhost: /...
[FPC V8.9 Main Menu]
Project cp05T5e12...
FPC Ctg2366 cp05...

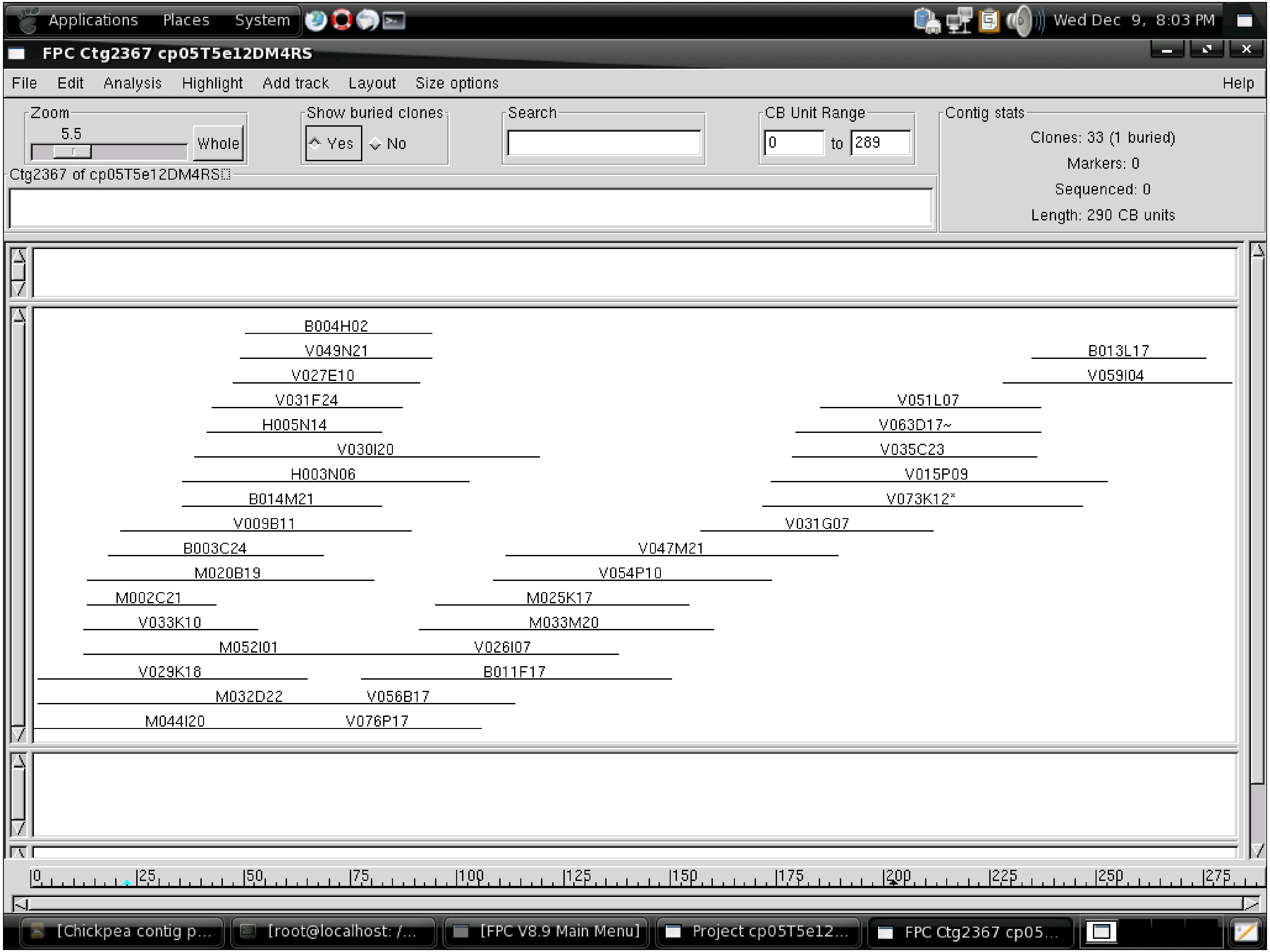
Applications Places System
Wed Dec 9, 8:03 PM
FPC Ctg2367 cp05T5e12DM4RS
File Edit Analysis Highlight Add track Layout Size options Help
Zoom
5.5
Whole
Show buried clones
Yes No
Search
CB Unit Range
0 to 289
Contig stats
Clones: 33 (1 buried)
Markers: 0
Sequenced: 0
Length: 290 CB units
Ctg2367 of cp05T5e12DM4RS
B004H02
V049N21
V027E10
V031F24
H005N14
V030I20
H003N06
B014M21
V009B11
B003C24
M020B19
M002C21
V033K10
M052I01
V029K18
M032D22
M044I20
B013L17
V059I04
V051L07
V063D17~
V035C23
V015P09
V073K12*
V031G07
V047M21
V054P10
M025K17
M033M20
V026I07
B011F17
V056B17
V076P17
0 25 50 75 100 125 150 175 200 225 250 275
[Chickpea contig p...
[root@localhost: /...
[FPC V8.9 Main Menu]
Project cp05T5e12...
FPC Ctg2367 cp05...

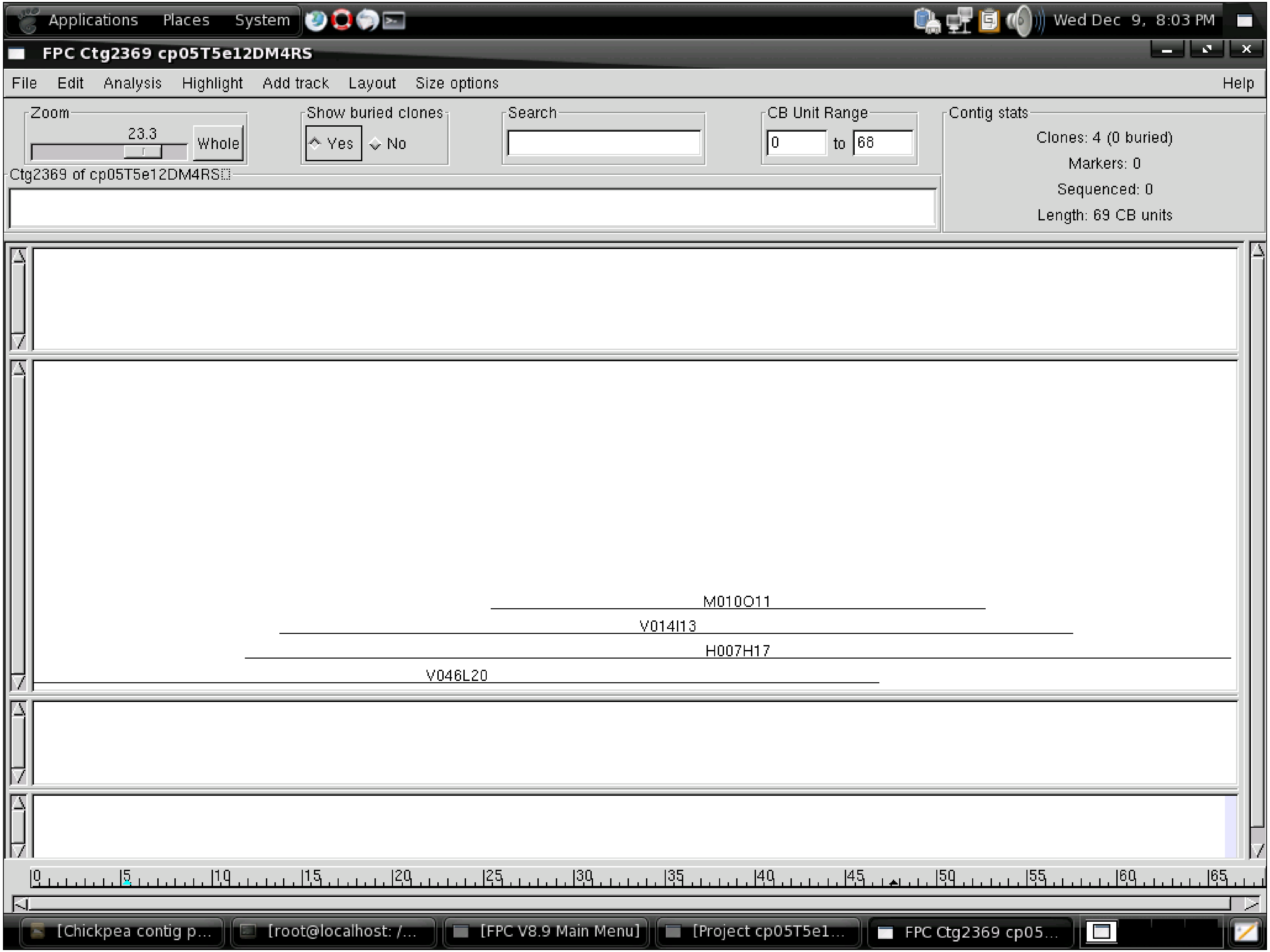
Applications Places System
Wed Dec 9, 8:03 PM
FPC Ctg2369 cp05T5e12DM4RS
File Edit Analysis Highlight Add track Layout Size options Help
Zoom
23.3
Whole
Show buried clones
Yes No
Search
CB Unit Range
0 to 68
Contig stats
Clones: 4 (0 buried)
Markers: 0
Sequenced: 0
Length: 69 CB units
Ctg2369 of cp05T5e12DM4RS
M010O11
V014I13
H007H17
V046L20
0 5 10 15 20 25 30 35 40 45 50 55 60 65
[Chickpea contig p...
[root@localhost: /...
[FPC V8.9 Main Menu]
[Project cp05T5e1...
FPC Ctg2369 cp05...

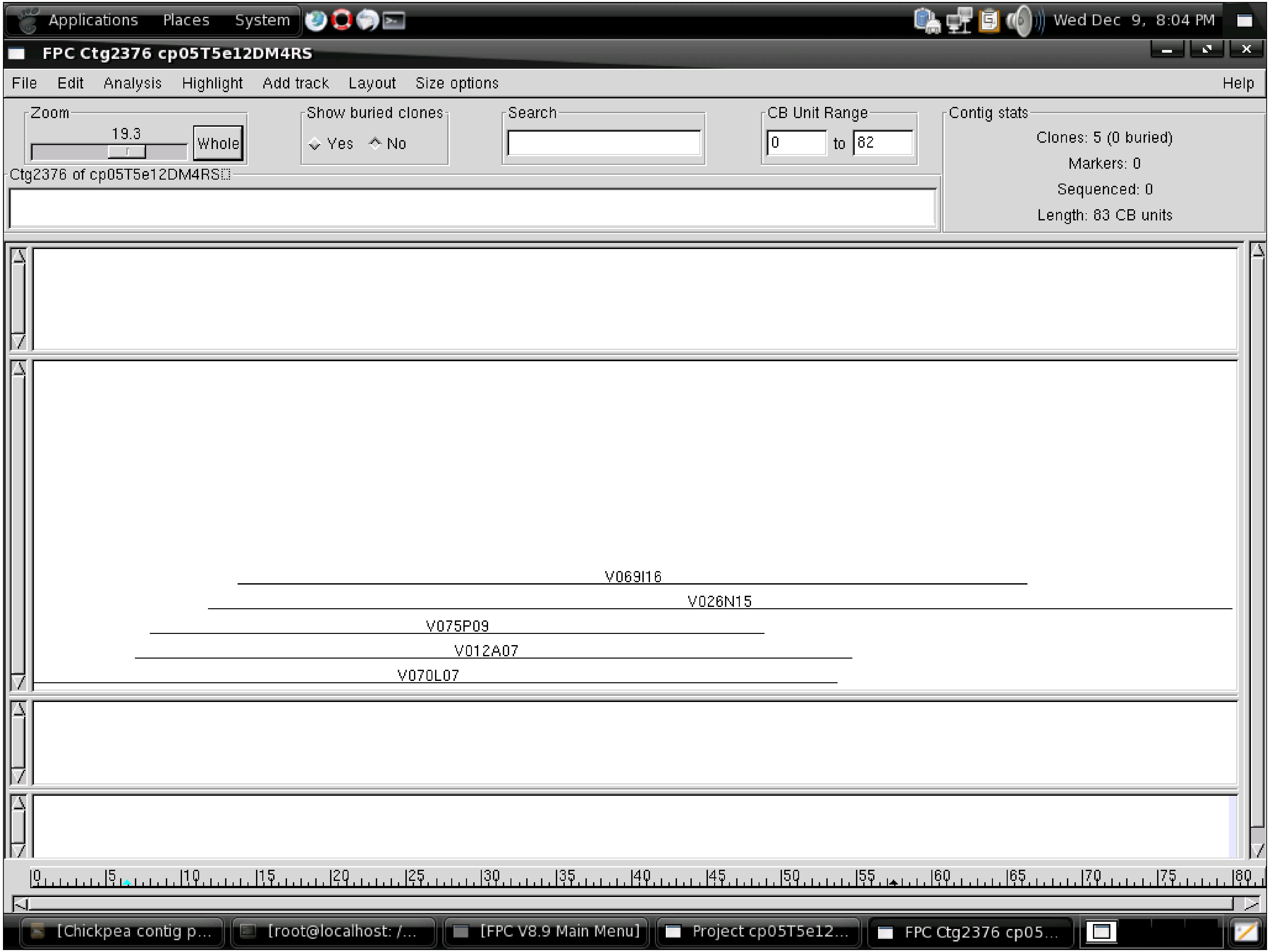
Applications Places System
Wed Dec 9, 8:04 PM
FPC Ctg2376 cp05T5e12DM4RS
File Edit Analysis Highlight Add track Layout Size options Help
Zoom
19.3
Whole
Show buried clones
Yes No
Search
CB Unit Range
0 to 82
Contig stats
Clones: 5 (0 buried)
Markers: 0
Sequenced: 0
Length: 83 CB units
Ctg2376 of cp05T5e12DM4RS
V069I16
V026N15
V075P09
V012A07
V070L07
0 5 10 15 20 25 30 35 40 45 50 55 60 65 70 75 80
[Chickpea contig p...
[root@localhost: /...
[FPC V8.9 Main Menu]
Project cp05T5e12...
FPC Ctg2376 cp05...

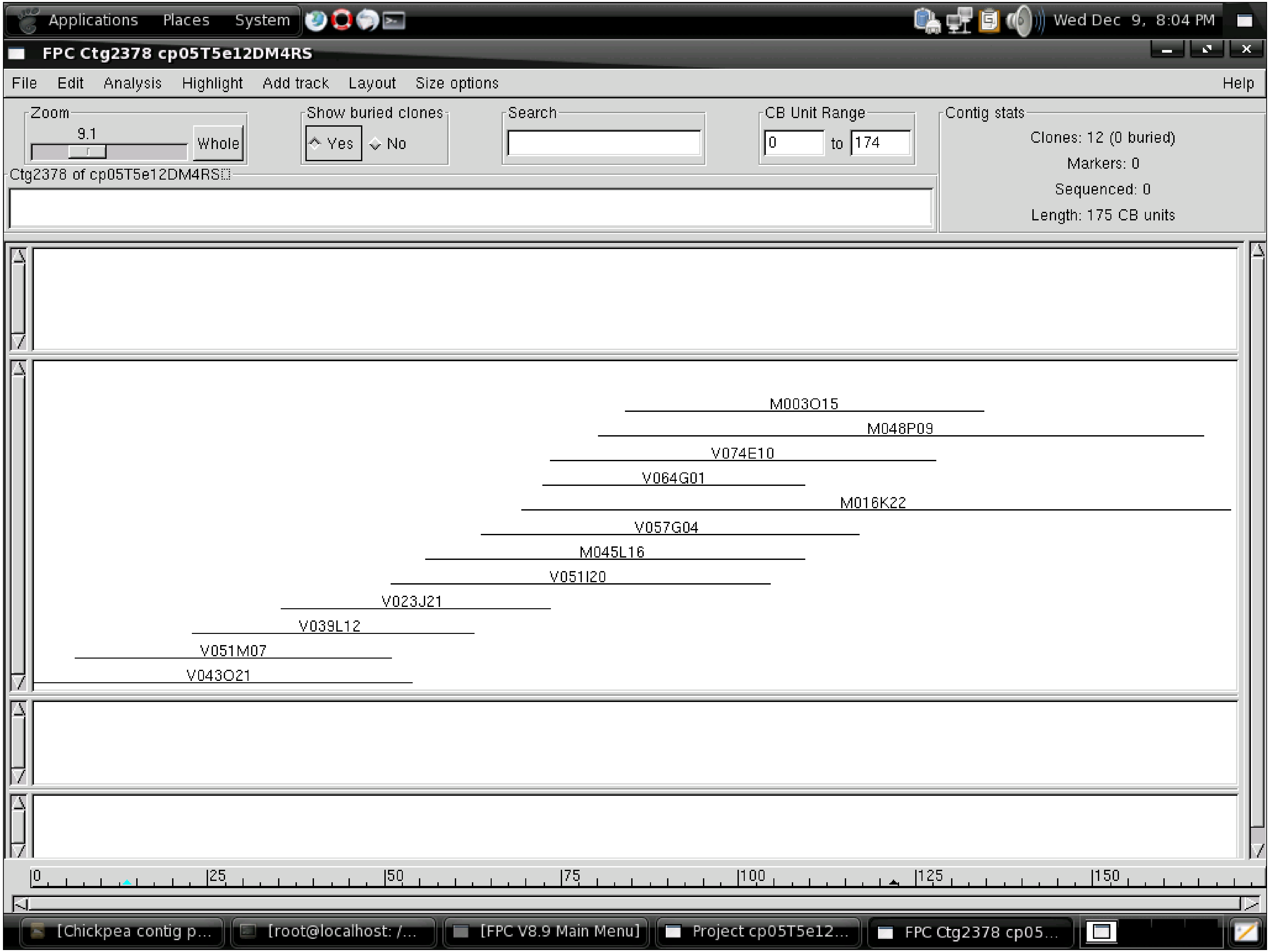
Applications Places System
Wed Dec 9, 8:04 PM
FPC Ctg2378 cp05T5e12DM4RS
File Edit Analysis Highlight Add track Layout Size options Help
Zoom
9.1
Whole
Show buried clones
Yes No
Search
CB Unit Range
0 to 174
Contig stats
Clones: 12 (0 buried)
Markers: 0
Sequenced: 0
Length: 175 CB units
Ctg2378 of cp05T5e12DM4RS
M003O15
M048P09
V074E10
V064G01
M016K22
V057G04
M045L16
V051I20
V023J21
V039L12
V051M07
V043O21
0 25 50 75 100 125 150
[Chickpea contig p...
[root@localhost: /...
[FPC V8.9 Main Menu]
Project cp05T5e12...
FPC Ctg2378 cp05...

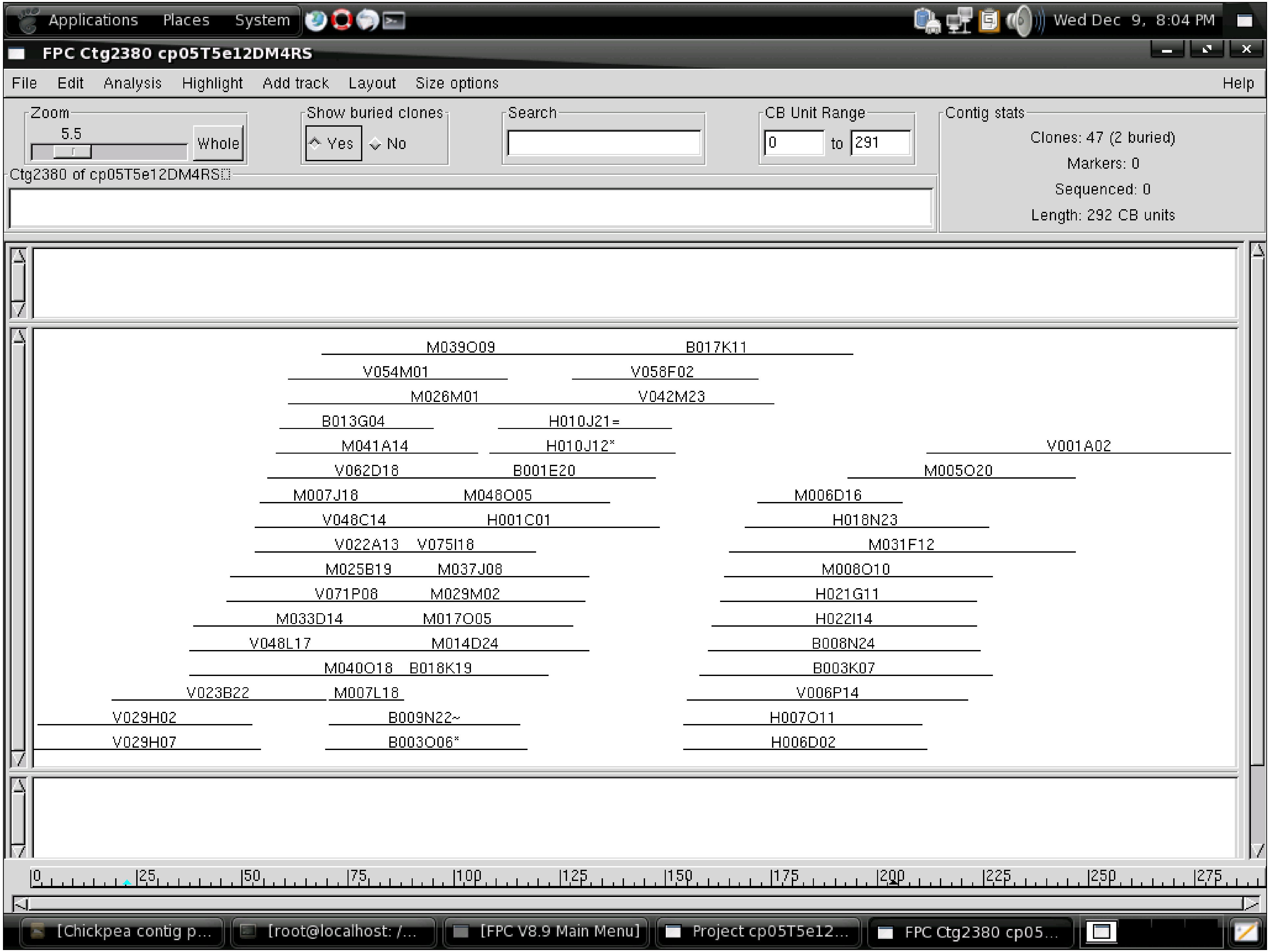

Applications Places System
Wed Dec 9, 8:04 PM
FPC Ctg2380 cp05T5e12DM4RS
File Edit Analysis Highlight Add track Layout Size options Help
Zoom
5.5
Whole
Show buried clones
Yes No
Search
CB Unit Range
0 to 291
Contig stats
Clones: 47 (2 buried)
Markers: 0
Sequenced: 0
Length: 292 CB units
Ctg2380 of cp05T5e12DM4RS
M039O09
B017K11
V054M01
V058F02
M026M01
V042M23
B013G04
H010J21=
M041A14
H010J12*
V001A02
V062D18
B001E20
M005O20
M007J18
M048O05
M006D16
V048C14
H001C01
H018N23
V022A13
V075I18
M031F12
M025B19
M037J08
M008O10
V071P08
M029M02
H021G11
M033D14
M017O05
H022I14
V048L17
M014D24
B008N24
M040O18
B018K19
B003K07
V023B22
M007L18
V006P14
V029H02
B009N22~
H007O11
V029H07
B003O06*
H006D02
0 25 50 75 100 125 150 175 200 225 250 275
[Chickpea contig p...
[root@localhost: /...
[FPC V8.9 Main Menu]
Project cp05T5e12...
FPC Ctg2380 cp05...

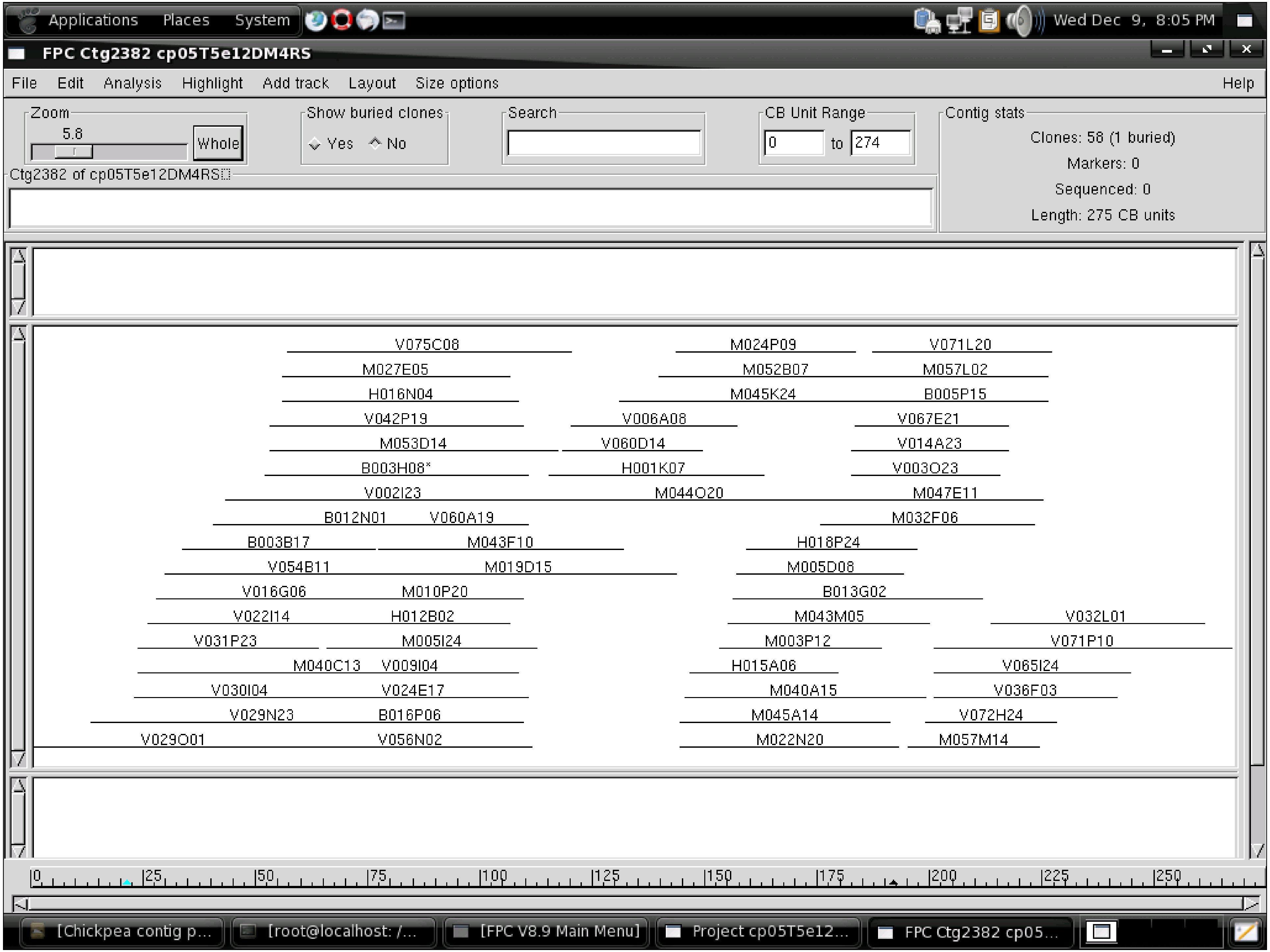
Applications Places System
Wed Dec 9, 8:05 PM
FPC Ctg2382 cp05T5e12DM4RS
File Edit Analysis Highlight Add track Layout Size options Help
Zoom 5.8 Whole
Show buried clones Yes No
Search
CB Unit Range 0 to 274
Contig stats
Clones: 58 (1 buried)
Markers: 0
Sequenced: 0
Length: 275 CB units
Ctg2382 of cp05T5e12DM4RS
V075C08 M024P09 V071L20
M027E05 M052B07 M057L02
H016N04 M045K24 B005P15
V042P19 V006A08 V067E21
M053D14 V060D14 V014A23
B003H08* H001K07 V003O23
V002I23 M044O20 M047E11
B012N01 V060A19 M032F06
B003B17 M043F10 H018P24
V054B11 M019D15 M005D08
V016G06 M010P20 B013G02
V022I14 H012B02 M043M05 V032L01
V031P23 M005I24 M003P12 V071P10
M040C13 V009I04 H015A06 V065I24
V030I04 V024E17 M040A15 V036F03
V029N23 B016P06 M045A14 V072H24
V029O01 V056N02 M022N20 M057M14
0 25 50 75 100 125 150 175 200 225 250
[Chickpea contig p... [root@localhost: /... [FPC V8.9 Main Menu] Project cp05T5e12... FPC Ctg2382 cp05...

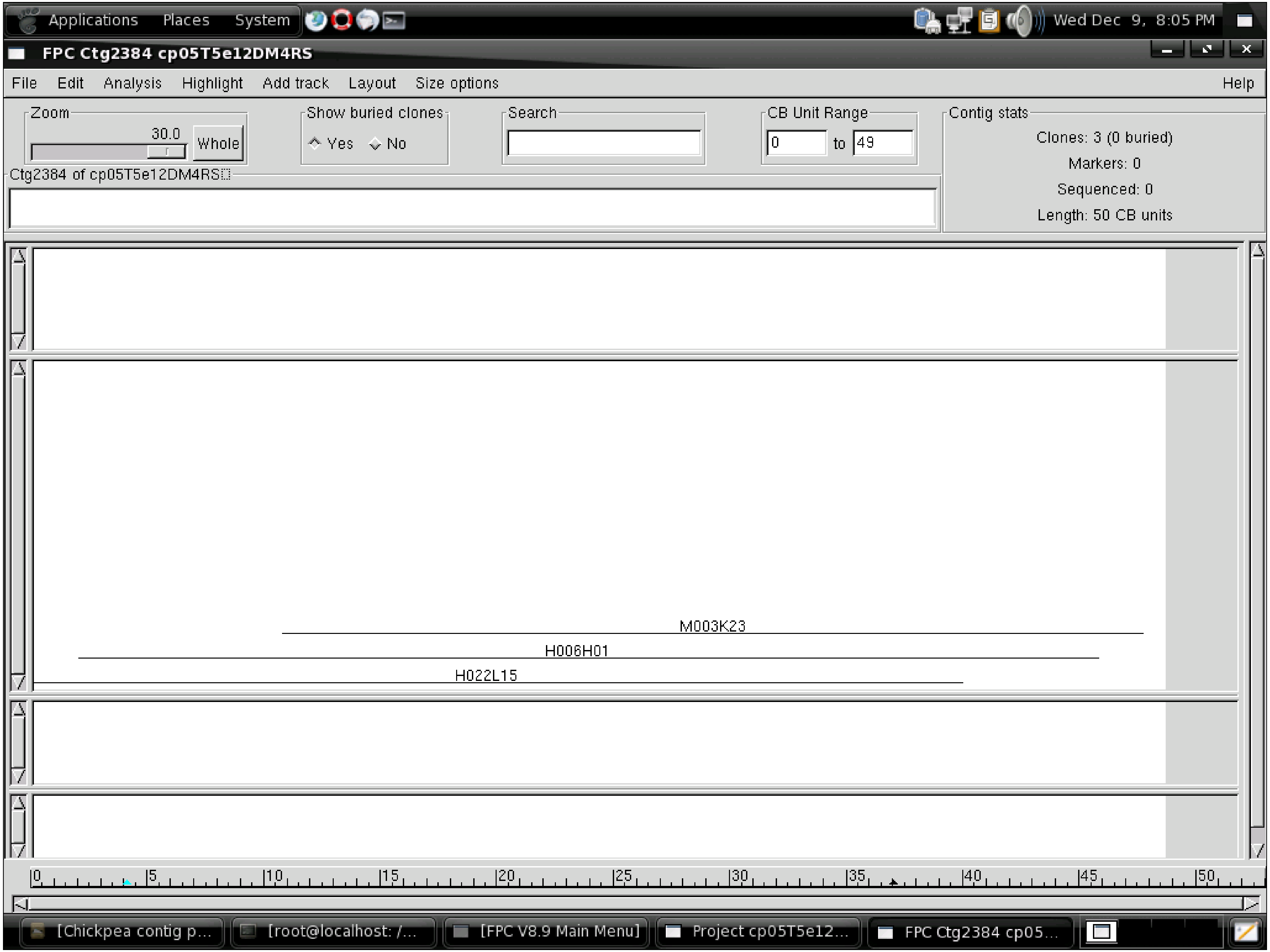
Applications Places System
Wed Dec 9, 8:05 PM
FPC Ctg2384 cp05T5e12DM4RS
File Edit Analysis Highlight Add track Layout Size options Help
Zoom
30.0
Whole
Show buried clones
Yes No
Search
CB Unit Range
0 to 49
Contig stats
Clones: 3 (0 buried)
Markers: 0
Sequenced: 0
Length: 50 CB units
Ctg2384 of cp05T5e12DM4RS
M003K23
H006H01
H022L15
0 5 10 15 20 25 30 35 40 45 50
[Chickpea contig p...
[root@localhost: /...
[FPC V8.9 Main Menu]
Project cp05T5e12...
FPC Ctg2384 cp05...

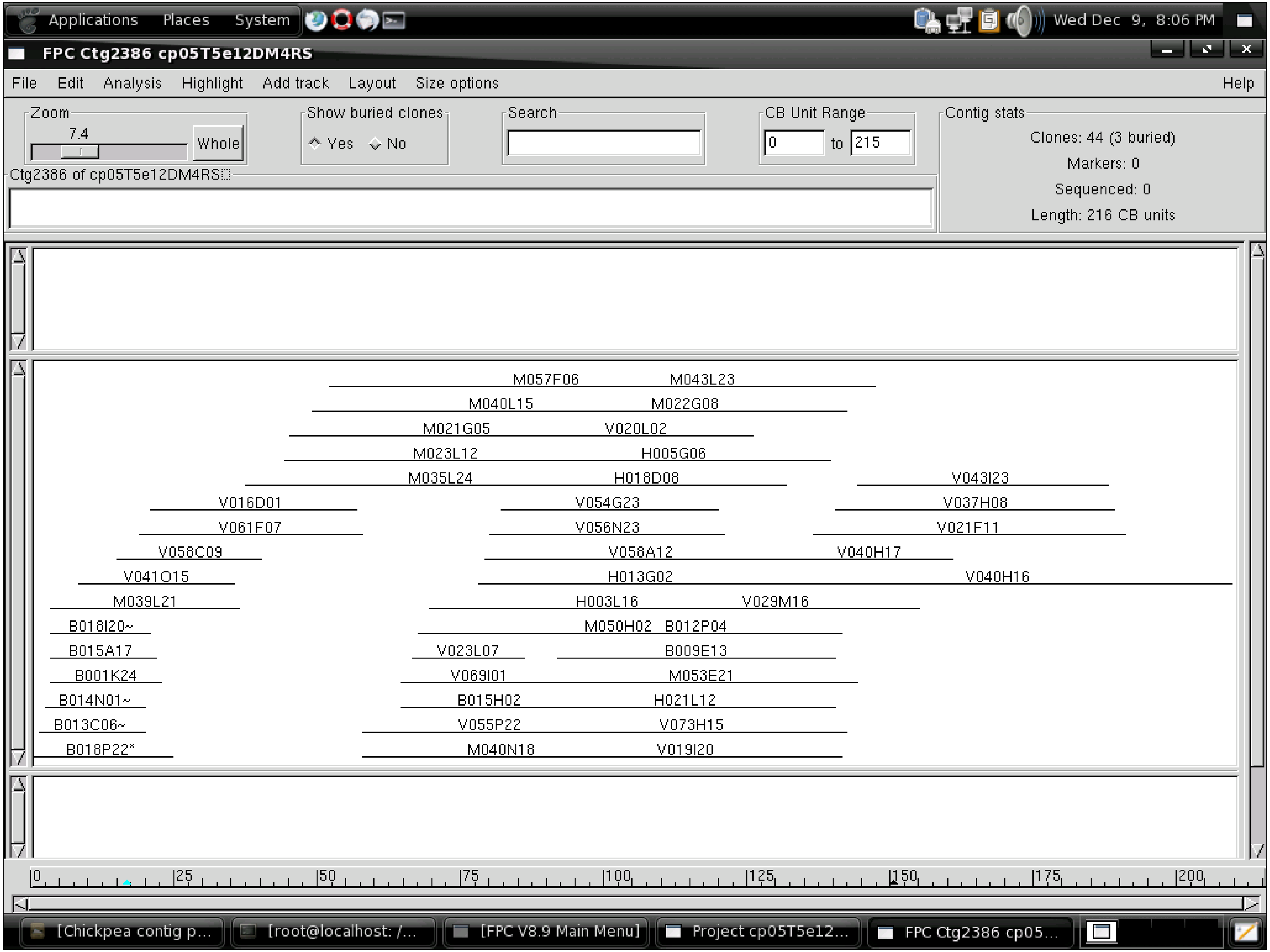
Applications Places System
Wed Dec 9, 8:06 PM
FPC Ctg2386 cp05T5e12DM4RS
File Edit Analysis Highlight Add track Layout Size options Help
Zoom
7.4
Whole
Show buried clones
Yes No
Search
CB Unit Range
0 to 215
Contig stats
Clones: 44 (3 buried)
Markers: 0
Sequenced: 0
Length: 216 CB units
Ctg2386 of cp05T5e12DM4RS
M057F06 M043L23
M040L15 M022G08
M021G05 V020L02
M023L12 H005G06
M035L24 H018D08 V043I23
V016D01 V054G23 V037H08
V061F07 V056N23 V021F11
V058C09 V058A12 V040H17
V041O15 H013G02 V040H16
M039L21 H003L16 V029M16
B018I20~ M050H02 B012P04
B015A17 V023L07 B009E13
B001K24 V069I01 M053E21
B014N01~ B015H02 H021L12
B013C06~ V055P22 V073H15
B018P22* M040N18 V019I20
0 25 50 75 100 125 150 175 200
[Chickpea contig p...
[root@localhost: /...
[FPC V8.9 Main Menu]
Project cp05T5e12...
FPC Ctg2386 cp05...

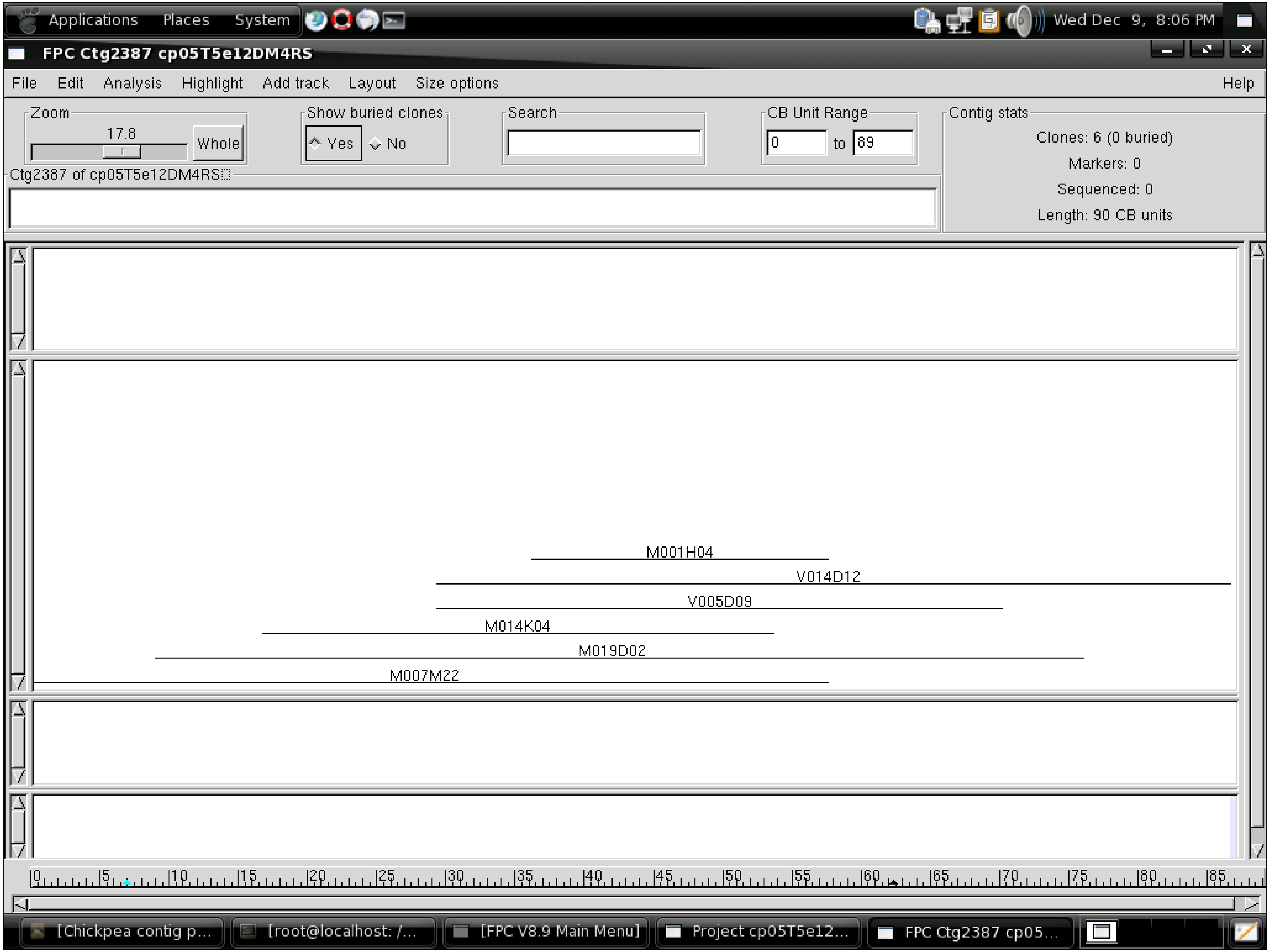
Applications Places System
Wed Dec 9, 8:06 PM
FPC Ctg2387 cp05T5e12DM4RS
File Edit Analysis Highlight Add track Layout Size options Help
Zoom
17.8
Whole
Show buried clones
Yes
No
Search
CB Unit Range
0 to 89
Contig stats
Clones: 6 (0 buried)
Markers: 0
Sequenced: 0
Length: 90 CB units
Ctg2387 of cp05T5e12DM4RS
M001H04
V014D12
V005D09
M014K04
M019D02
M007M22
[Chickpea contig p...
[root@localhost: /...
[FPC V8.9 Main Menu]
Project cp05T5e12...
FPC Ctg2387 cp05...

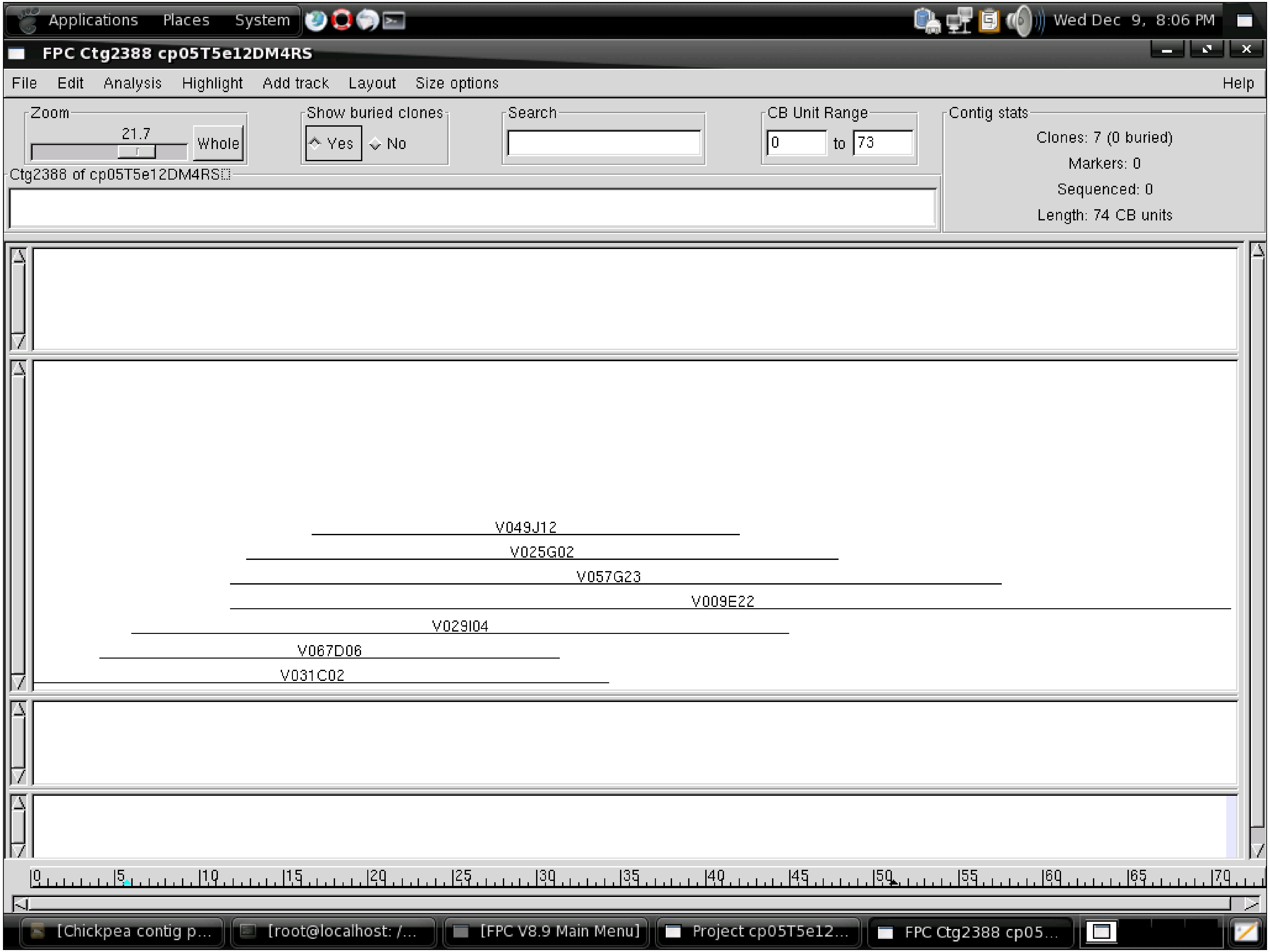
Applications Places System
Wed Dec 9, 8:06 PM
FPC Ctg2388 cp05T5e12DM4RS
File Edit Analysis Highlight Add track Layout Size options Help
Zoom
21.7
Whole
Show buried clones
Yes
No
Search
CB Unit Range
0 to 73
Contig stats
Clones: 7 (0 buried)
Markers: 0
Sequenced: 0
Length: 74 CB units
Ctg2388 of cp05T5e12DM4RS
V049J12
V025G02
V057G23
V009E22
V029I04
V067D06
V031C02
0 5 10 15 20 25 30 35 40 45 50 55 60 65 70
[Chickpea contig p...
[root@localhost: /...
[FPC V8.9 Main Menu]
Project cp05T5e12...
FPC Ctg2388 cp05...

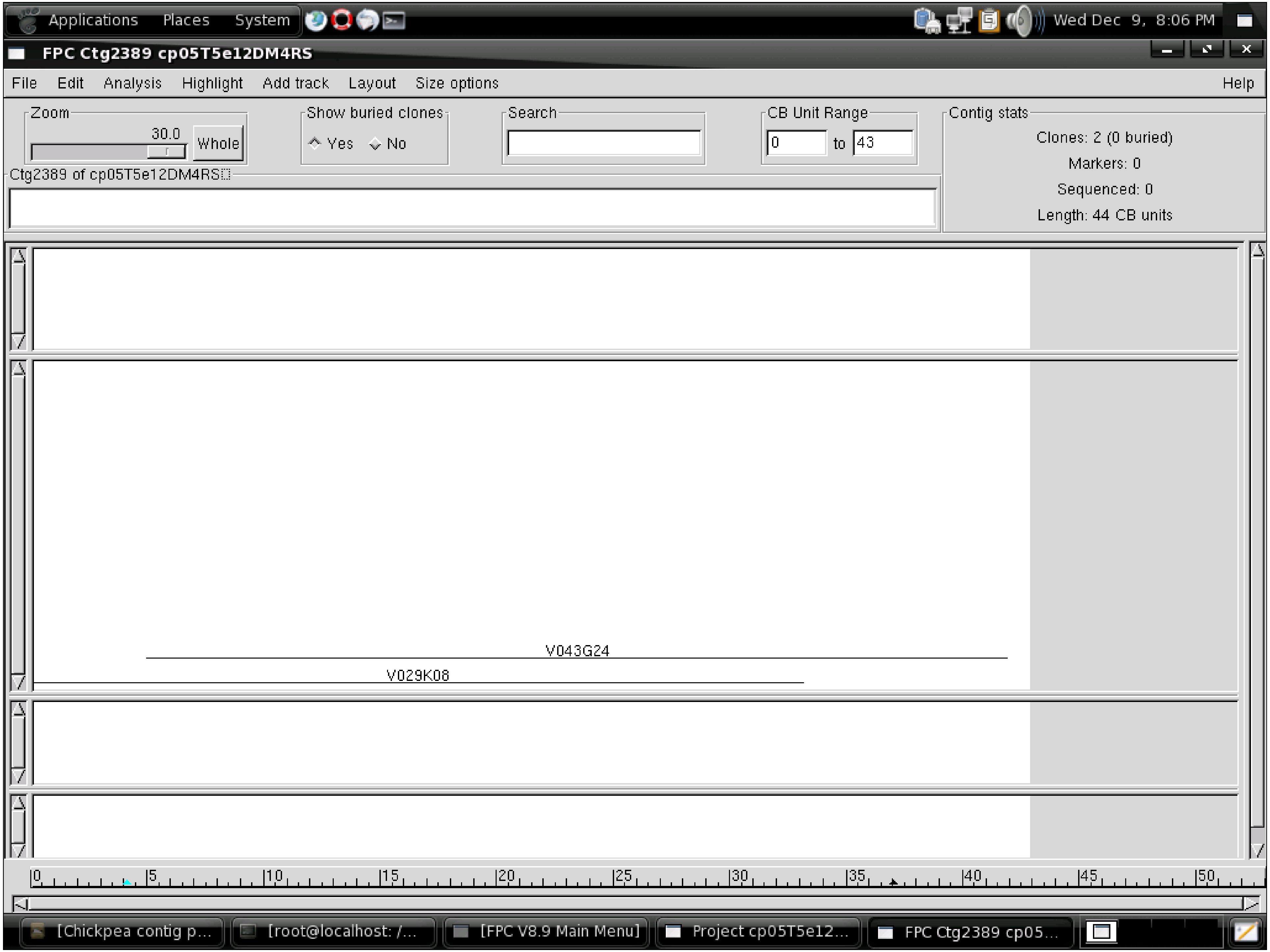
Applications Places System
Wed Dec 9, 8:06 PM
FPC Ctg2389 cp05T5e12DM4RS
File Edit Analysis Highlight Add track Layout Size options Help
Zoom
30.0
Whole
Show buried clones
Yes No
Search
CB Unit Range
0 to 43
Contig stats
Clones: 2 (0 buried)
Markers: 0
Sequenced: 0
Length: 44 CB units
Ctg2389 of cp05T5e12DM4RS
V043G24
V029K08
0 5 10 15 20 25 30 35 40 45 50
[Chickpea contig p...
[root@localhost: /...
[FPC V8.9 Main Menu]
Project cp05T5e12...
FPC Ctg2389 cp05...

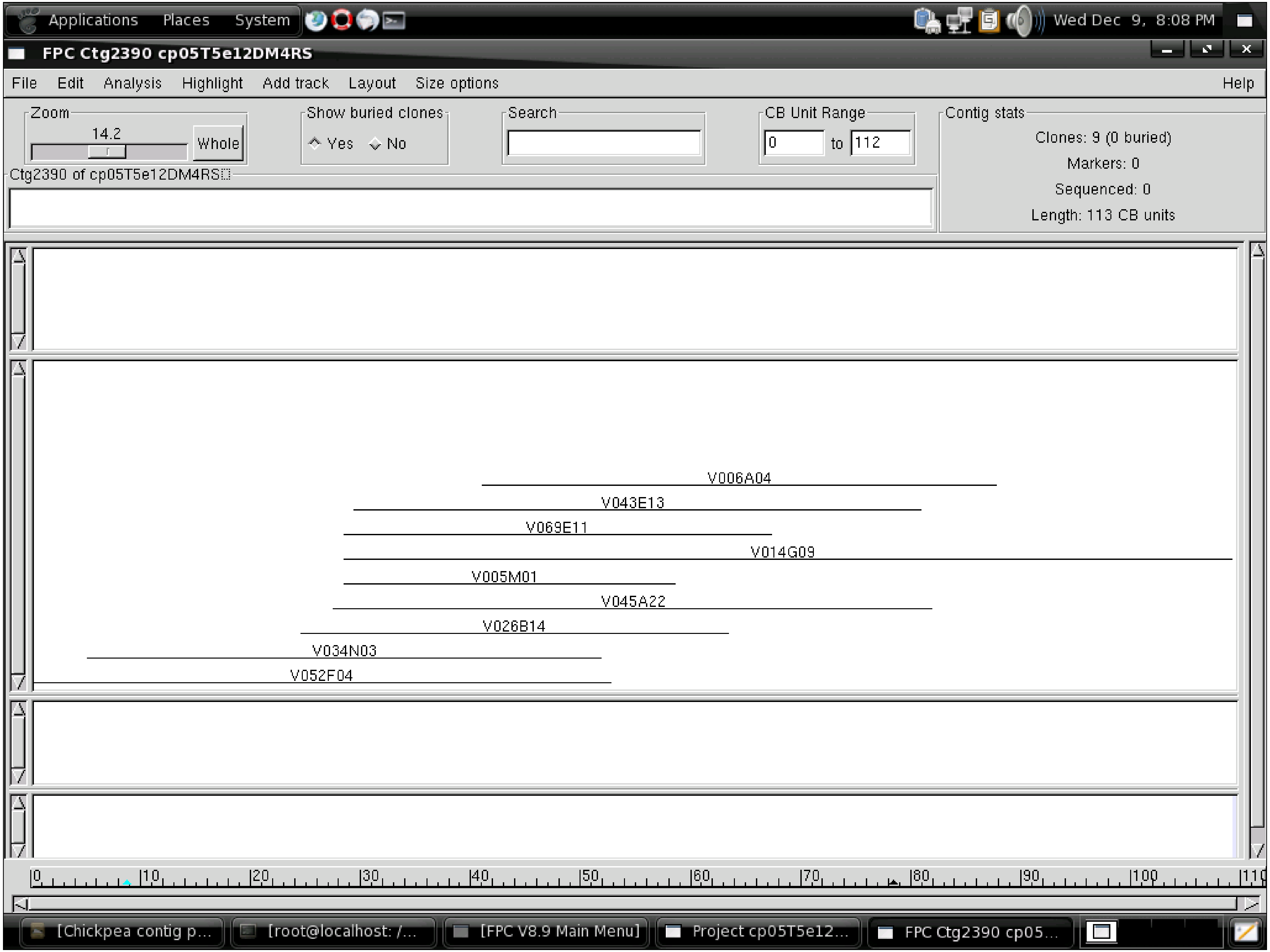
Applications Places System
Wed Dec 9, 8:08 PM
FPC Ctg2390 cp05T5e12DM4RS
File Edit Analysis Highlight Add track Layout Size options Help
Zoom
14.2
Whole
Show buried clones
Yes No
Search
CB Unit Range
0 to 112
Contig stats
Clones: 9 (0 buried)
Markers: 0
Sequenced: 0
Length: 113 CB units
Ctg2390 of cp05T5e12DM4RS
V006A04
V043E13
V069E11
V014G09
V005M01
V045A22
V026B14
V034N03
V052F04
0 10 20 30 40 50 60 70 80 90 100 110
[Chickpea contig p...
[root@localhost: /...
[FPC V8.9 Main Menu]
Project cp05T5e12...
FPC Ctg2390 cp05...

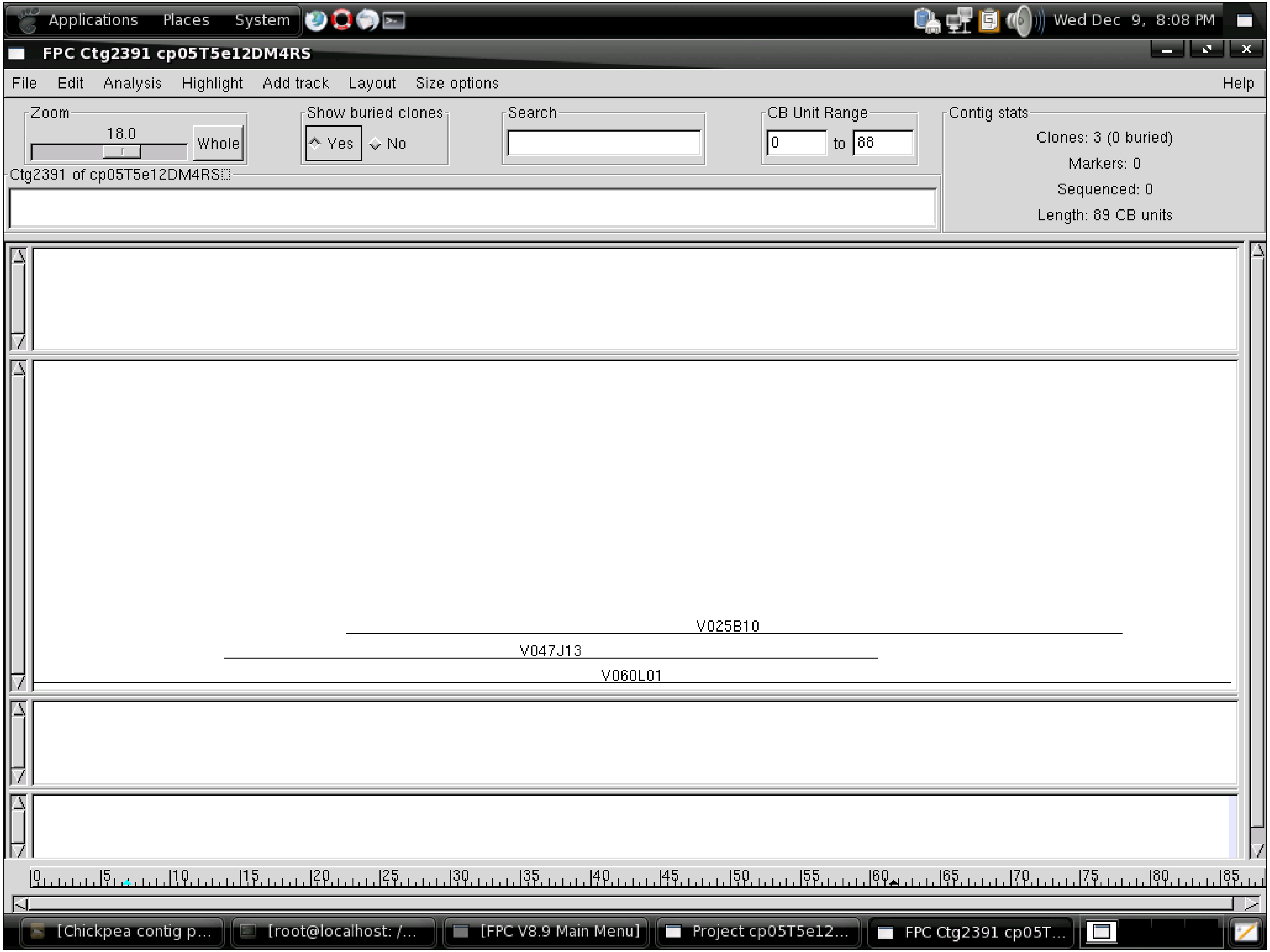
Applications Places System
Wed Dec 9, 8:08 PM
FPC Ctg2391 cp05T5e12DM4RS
File Edit Analysis Highlight Add track Layout Size options Help
Zoom
18.0
Whole
Show buried clones
Yes
No
Search
CB Unit Range
0 to 88
Contig stats
Clones: 3 (0 buried)
Markers: 0
Sequenced: 0
Length: 89 CB units
Ctg2391 of cp05T5e12DM4RS
V025B10
V047J13
V060L01
0 5 10 15 20 25 30 35 40 45 50 55 60 65 70 75 80 85
[Chickpea contig p...
[root@localhost: /...
[FPC V8.9 Main Menu]
Project cp05T5e12...
FPC Ctg2391 cp05T...

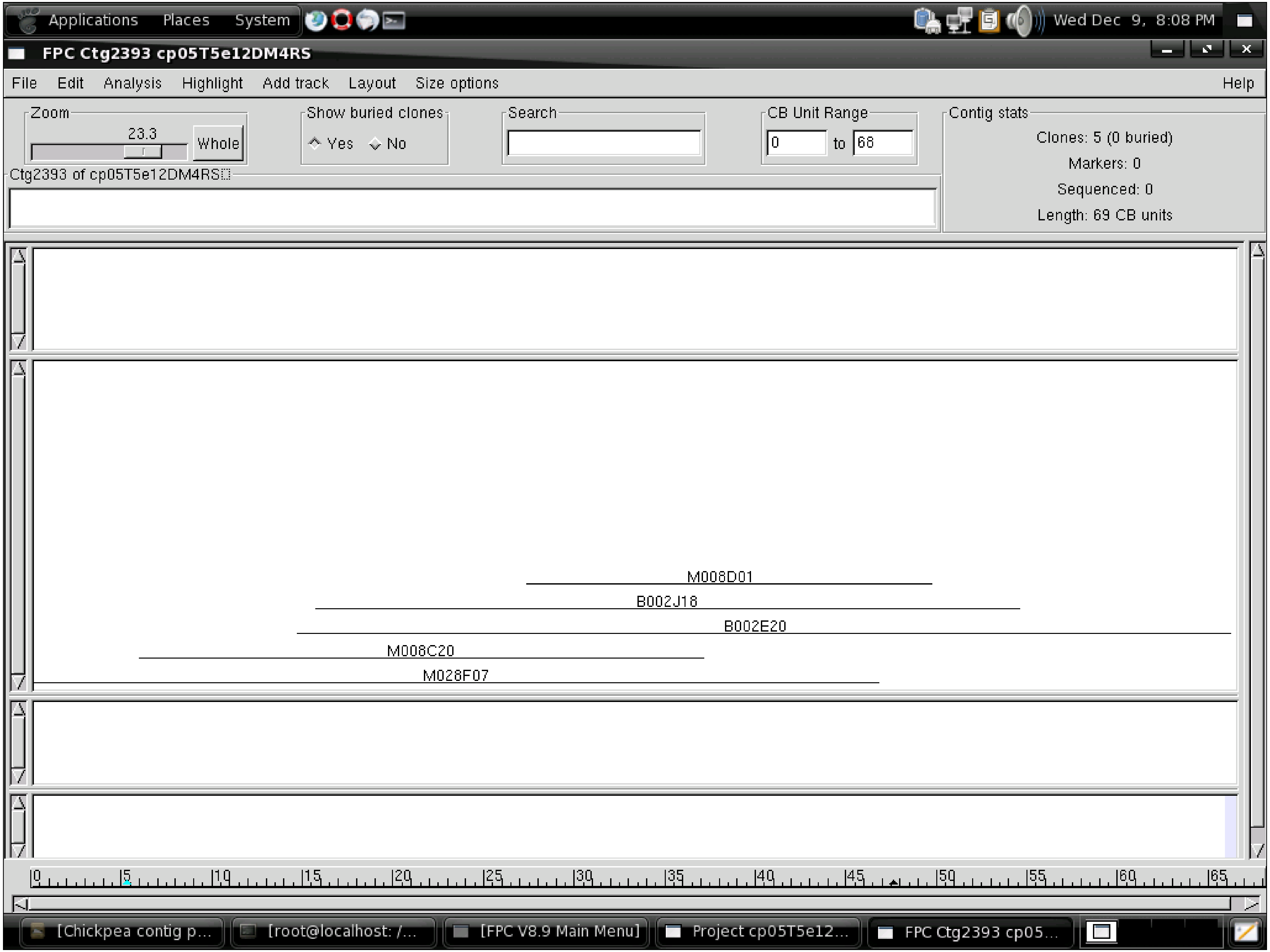
Applications Places System
Wed Dec 9, 8:08 PM
FPC Ctg2393 cp05T5e12DM4RS
File Edit Analysis Highlight Add track Layout Size options Help
Zoom
23.3
Whole
Show buried clones
Yes No
Search
CB Unit Range
0 to 68
Contig stats
Clones: 5 (0 buried)
Markers: 0
Sequenced: 0
Length: 69 CB units
Ctg2393 of cp05T5e12DM4RS
M008D01
B002J18
B002E20
M008C20
M028F07
0 5 10 15 20 25 30 35 40 45 50 55 60 65
[Chickpea contig p...
[root@localhost: /...
[FPC V8.9 Main Menu]
Project cp05T5e12...
FPC Ctg2393 cp05...

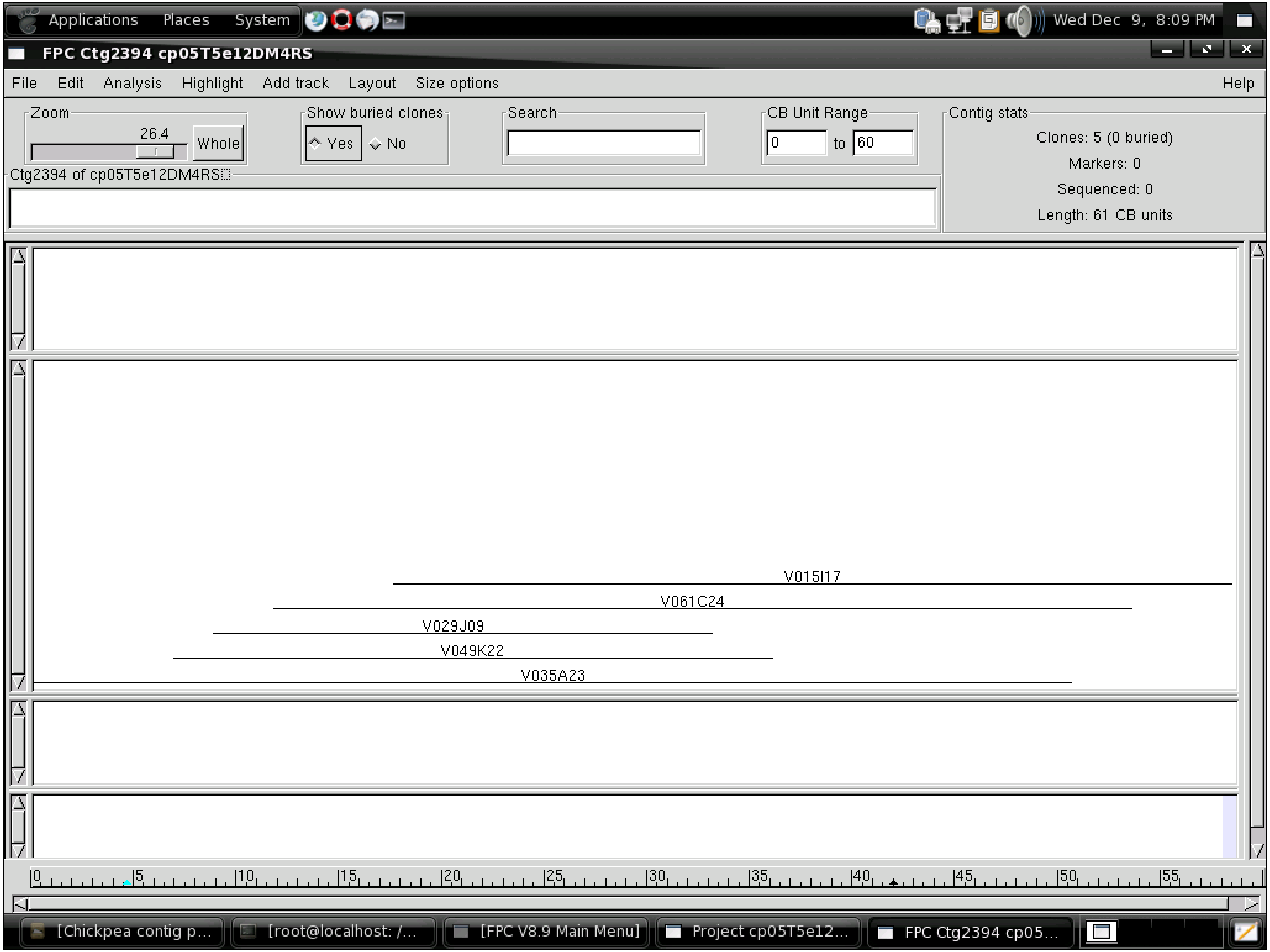
Applications Places System
Wed Dec 9, 8:09 PM
FPC Ctg2394 cp05T5e12DM4RS
File Edit Analysis Highlight Add track Layout Size options
Help
Zoom
26.4
Whole
Show buried clones
Yes
No
Search
CB Unit Range
0
to
60
Contig stats
Clones: 5 (0 buried)
Markers: 0
Sequenced: 0
Length: 61 CB units
Ctg2394 of cp05T5e12DM4RS
V015I17
V061C24
V029J09
V049K22
V035A23
0 5 10 15 20 25 30 35 40 45 50 55
[Chickpea contig p...
[root@localhost: /...
[FPC V8.9 Main Menu]
Project cp05T5e12...
FPC Ctg2394 cp05...

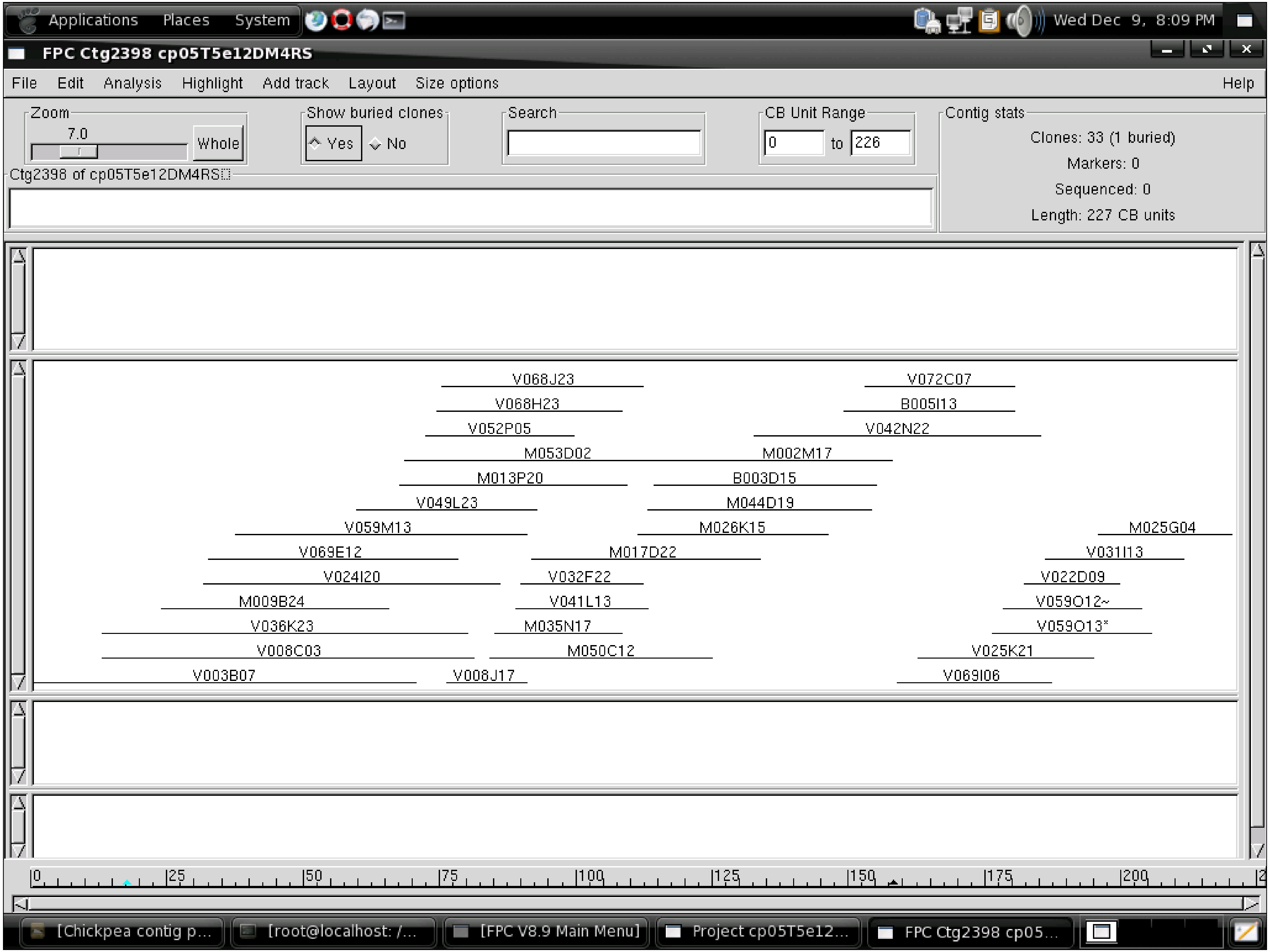

Applications Places System
Wed Dec 9, 8:09 PM
FPC Ctg2398 cp05T5e12DM4RS
File Edit Analysis Highlight Add track Layout Size options Help
Zoom
7.0
Whole
Show buried clones
Yes No
Search
CB Unit Range
0 to 226
Contig stats
Clones: 33 (1 buried)
Markers: 0
Sequenced: 0
Length: 227 CB units
Ctg2398 of cp05T5e12DM4RS
V068J23
V072C07
V068H23
B005I13
V052P05
V042N22
M053D02
M002M17
M013P20
B003D15
V049L23
M044D19
V059M13
M026K15
M025G04
V069E12
M017D22
V031I13
V024I20
V032F22
V022D09
M009B24
V041L13
V059O12~
V036K23
M035N17
V059O13*
V008C03
M050C12
V025K21
V003B07
V008J17
V069I06
0 25 50 75 100 125 150 175 200
[Chickpea contig p...
[root@localhost: /...
[FPC V8.9 Main Menu]
Project cp05T5e12...
FPC Ctg2398 cp05...

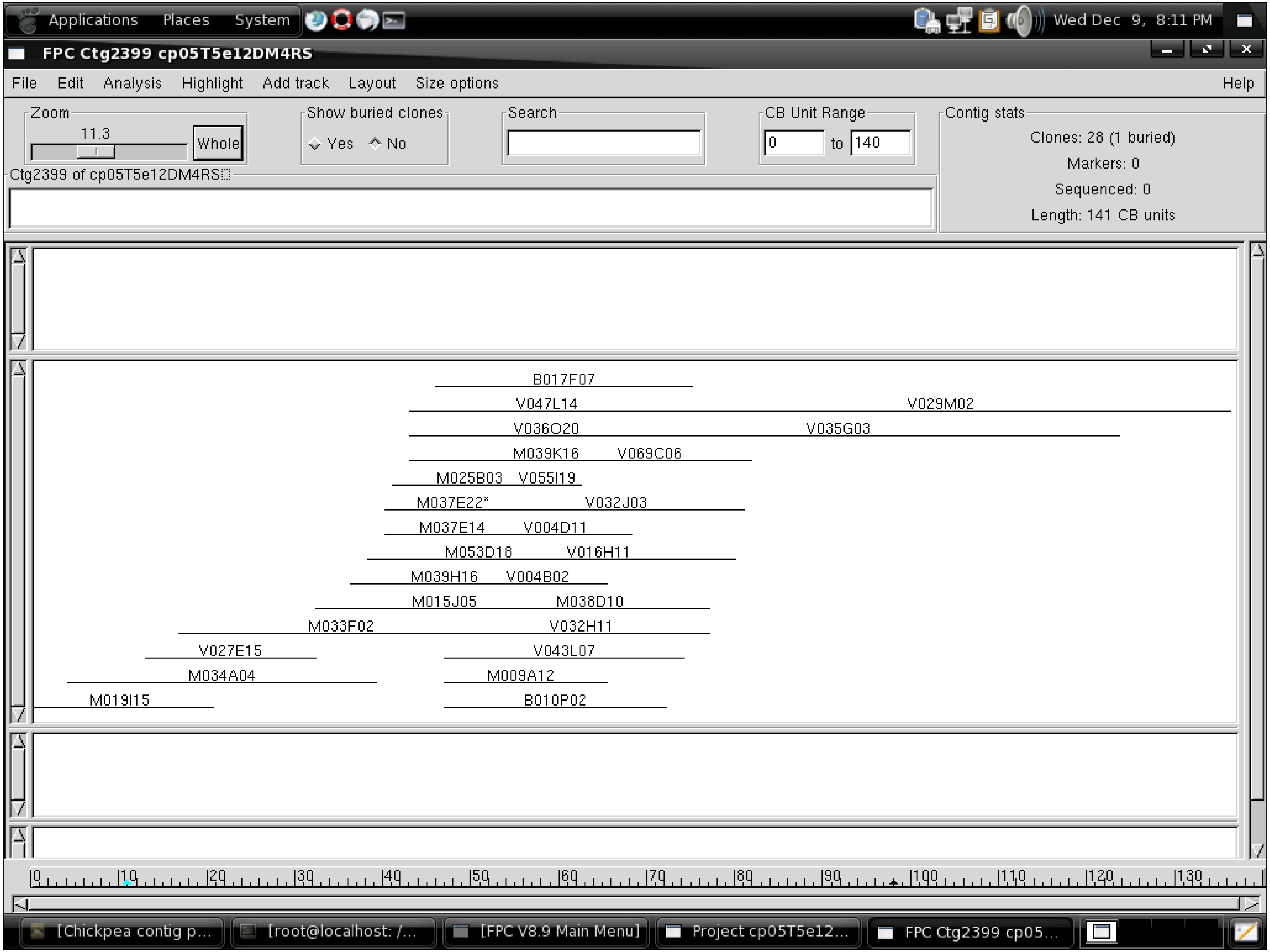

Applications
Places
System
Wed Dec 9, 8:11 PM
FPC Ctg2399 cp05T5e12DM4RS
File
Edit
Analysis
Highlight
Add track
Layout
Size options
Help
Zoom
11.3
Whole
Show buried clones
Yes
No
Search
CB Unit Range
0
to
140
Contig stats
Clones: 28 (1 buried)
Markers: 0
Sequenced: 0
Length: 141 CB units
Ctg2399 of cp05T5e12DM4RS
B017F07
V047L14
V029M02
V036O20
V035G03
M039K16
V069C06
M025B03
V055I19
M037E22*
V032J03
M037E14
V004D11
M053D18
V016H11
M039H16
V004B02
M015J05
M038D10
M033F02
V032H11
V027E15
V043L07
M034A04
M009A12
M019I15
B010P02
0
10
20
30
40
50
60
70
80
90
100
110
120
130
[Chickpea contig p...
[root@localhost: /...
[FPC V8.9 Main Menu]
Project cp05T5e12...
FPC Ctg2399 cp05...

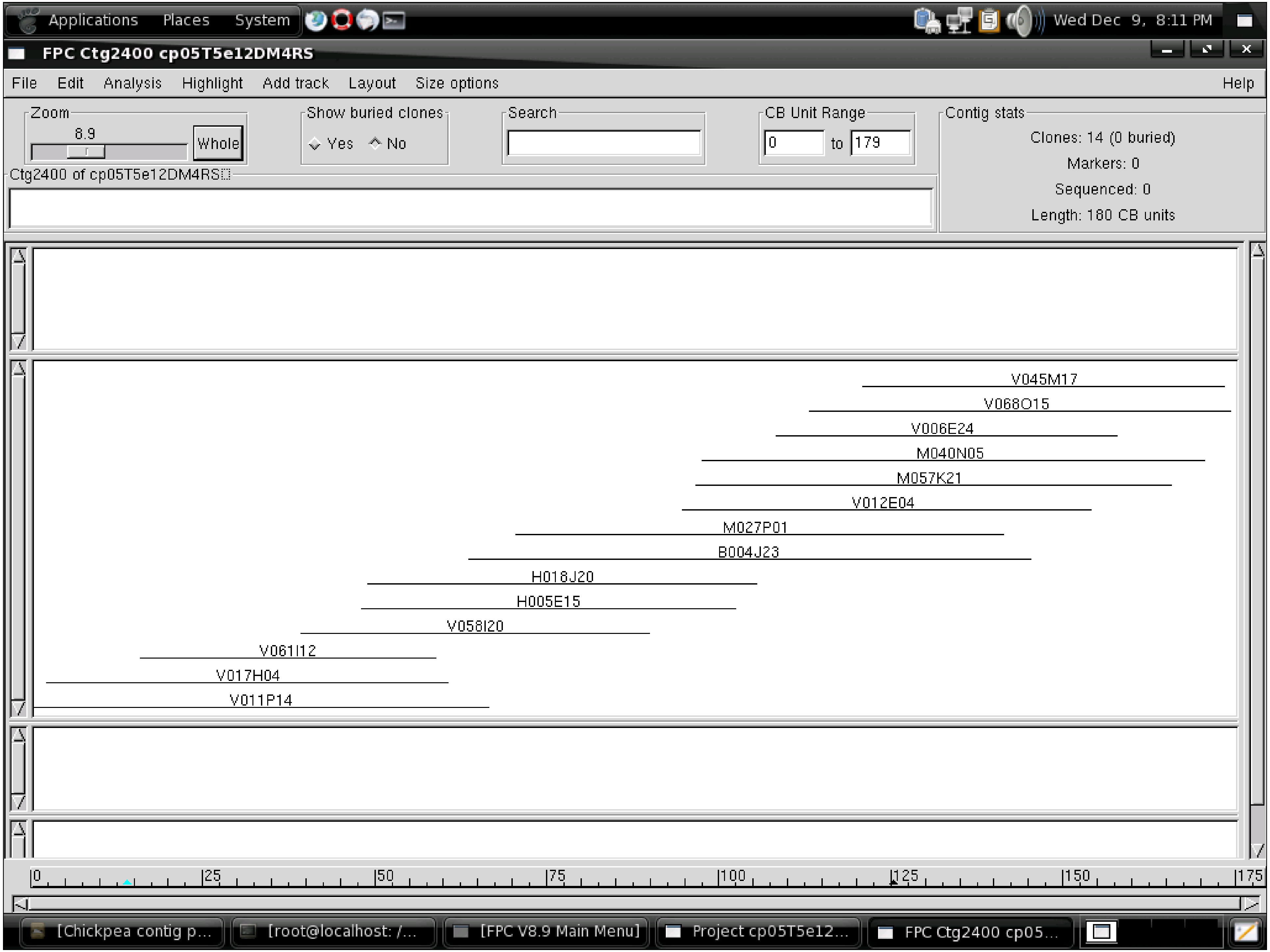

Applications Places System
Wed Dec 9, 8:11 PM
FPC Ctg2400 cp05T5e12DM4RS
File Edit Analysis Highlight Add track Layout Size options Help
Zoom
8.9
Whole
Show buried clones
Yes No
Search
CB Unit Range
0 to 179
Contig stats
Clones: 14 (0 buried)
Markers: 0
Sequenced: 0
Length: 180 CB units
Ctg2400 of cp05T5e12DM4RS
V045M17
V068O15
V006E24
M040N05
M057K21
V012E04
M027P01
B004J23
H018J20
H005E15
V058I20
V061I12
V017H04
V011P14
0 25 50 75 100 125 150 175
[Chickpea contig p...
[root@localhost: /...
[FPC V8.9 Main Menu]
Project cp05T5e12...
FPC Ctg2400 cp05...

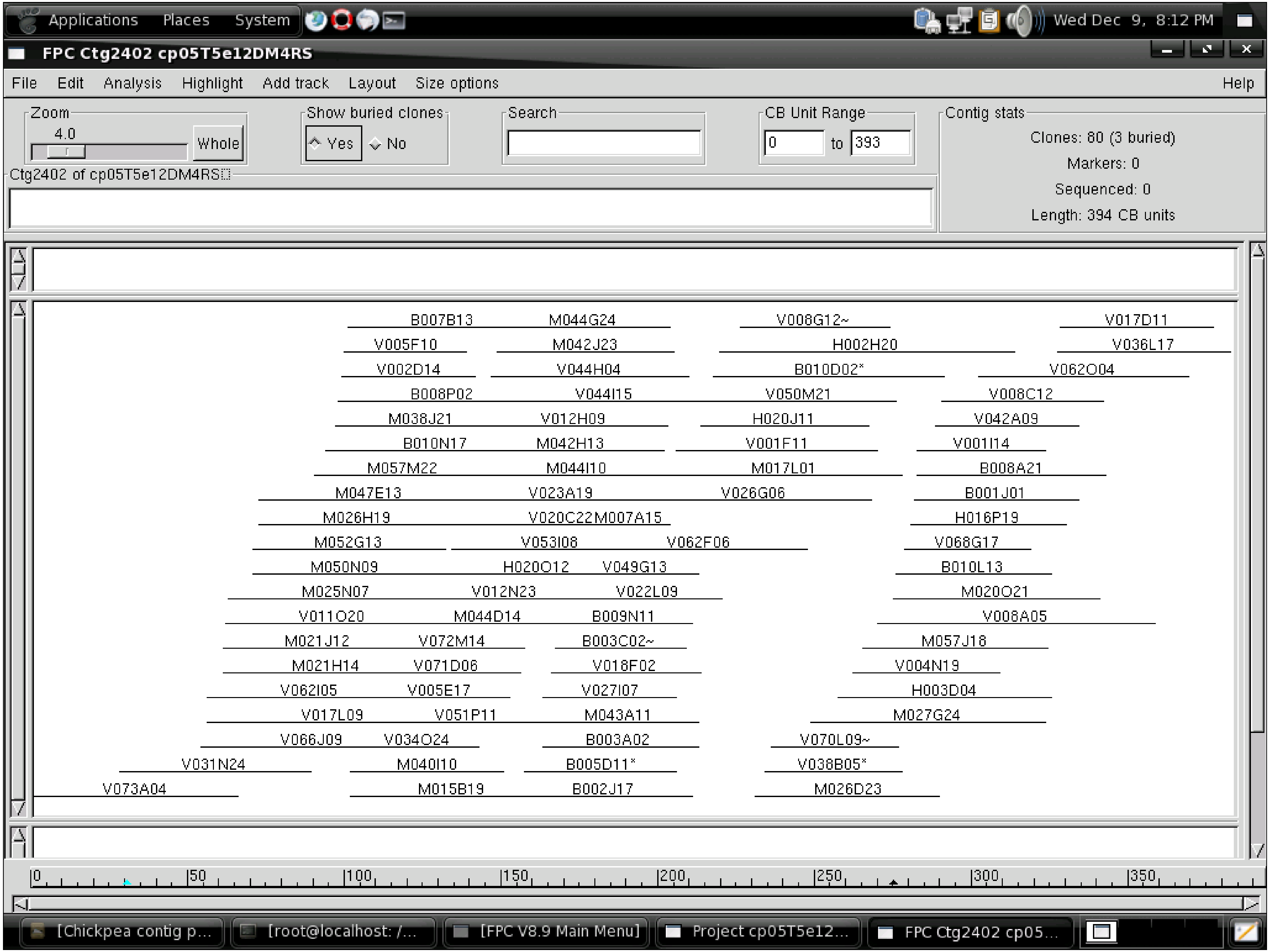
Applications Places System
Wed Dec 9, 8:12 PM
FPC Ctg2402 cp05T5e12DM4RS
File Edit Analysis Highlight Add track Layout Size options Help
Zoom 4.0 Whole
Show buried clones Yes No
Search
CB Unit Range 0 to 393
Contig stats
Clones: 80 (3 buried)
Markers: 0
Sequenced: 0
Length: 394 CB units
Ctg2402 of cp05T5e12DM4RS
B007B13 M044G24 V008G12~ V017D11
V005F10 M042J23 H002H20 V036L17
V002D14 V044H04 B010D02* V062O04
B008P02 V044I15 V050M21 V008C12
M038J21 V012H09 H020J11 V042A09
B010N17 M042H13 V001F11 V001I14
M057M22 M044I10 M017L01 B008A21
M047E13 V023A19 V026G06 B001J01
M026H19 V020C22M007A15 H016P19
M052G13 V053I08 V062F06 V068G17
M050N09 H020O12 V049G13 B010L13
M025N07 V012N23 V022L09 M020O21
V011O20 M044D14 B009N11 V008A05
M021J12 V072M14 B003C02~ M057J18
M021H14 V071D06 V018F02 V004N19
V062I05 V005E17 V027I07 H003D04
V017L09 V051P11 M043A11 M027G24
V066J09 V034O24 B003A02 V070L09~
V031N24 M040I10 B005D11* V038B05*
V073A04 M015B19 B002J17 M026D23
0 50 100 150 200 250 300 350
[Chickpea contig p... [root@localhost: /... [FPC V8.9 Main Menu] Project cp05T5e12... FPC Ctg2402 cp05...

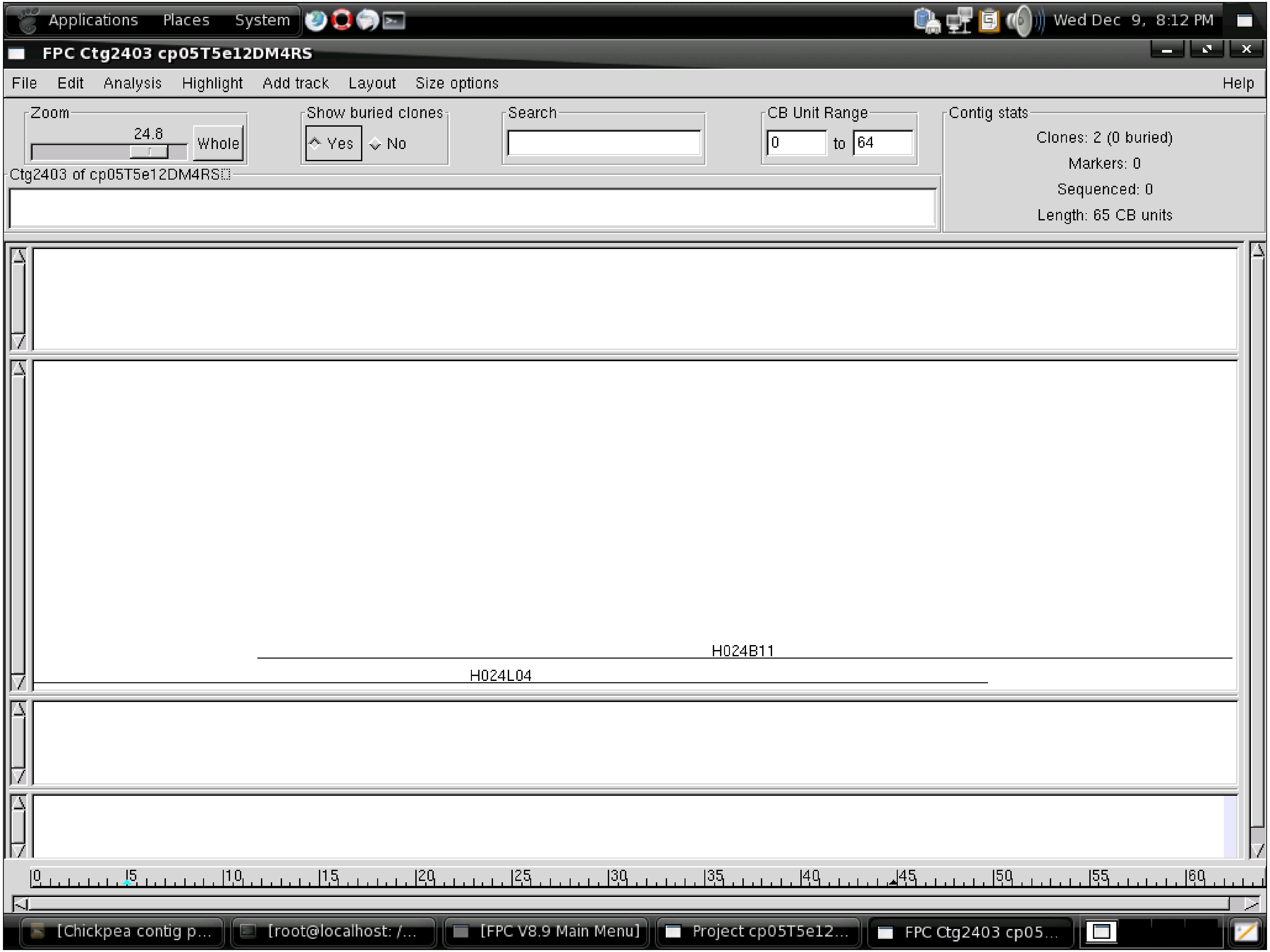
Applications Places System
Wed Dec 9, 8:12 PM
FPC Ctg2403 cp05T5e12DM4RS
File Edit Analysis Highlight Add track Layout Size options Help
Zoom
24.8
Whole
Show buried clones
Yes
No
Search
CB Unit Range
0
to
64
Contig stats
Clones: 2 (0 buried)
Markers: 0
Sequenced: 0
Length: 65 CB units
Ctg2403 of cp05T5e12DM4RS
H024B11
H024L04
0 5 10 15 20 25 30 35 40 45 50 55 60
[Chickpea contig p...
[root@localhost: /...
[FPC V8.9 Main Menu]
Project cp05T5e12...
FPC Ctg2403 cp05...

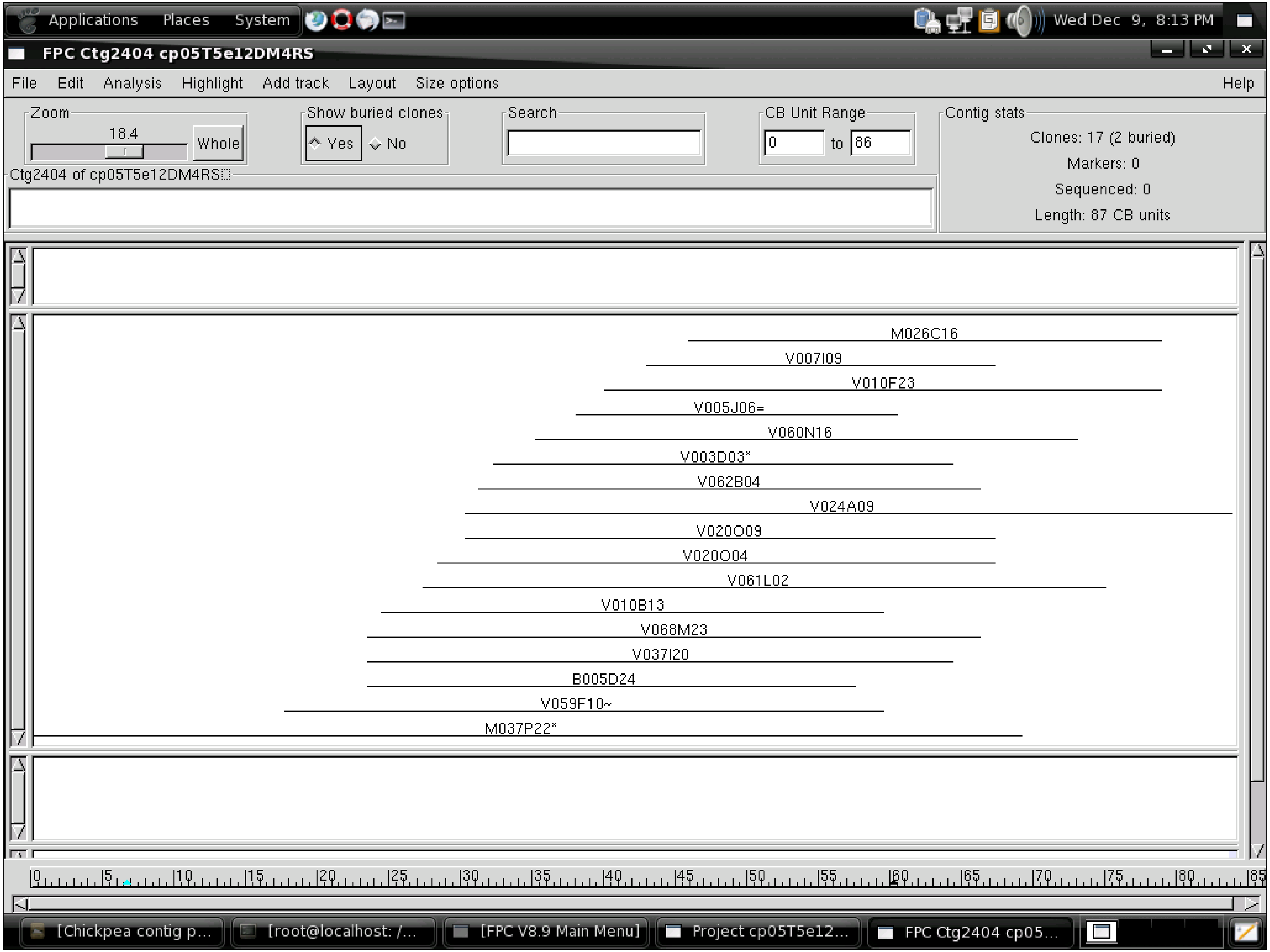

Applications
Places
System
Wed Dec 9, 8:13 PM
FPC Ctg2404 cp05T5e12DM4RS
File
Edit
Analysis
Highlight
Add track
Layout
Size options
Help
Zoom
18.4
Whole
Show buried clones
Yes
No
Search
CB Unit Range
0
to
86
Contig stats
Clones: 17 (2 buried)
Markers: 0
Sequenced: 0
Length: 87 CB units
Ctg2404 of cp05T5e12DM4RS
M026C16
V007I09
V010F23
V005J06=
V060N16
V003D03*
V062B04
V024A09
V020O09
V020O04
V061L02
V010B13
V068M23
V037I20
B005D24
V059F10~
M037P22*
0
5
10
15
20
25
30
35
40
45
50
55
60
65
70
75
80
85
[Chickpea contig p...
[root@localhost: /...
[FPC V8.9 Main Menu]
Project cp05T5e12...
FPC Ctg2404 cp05...

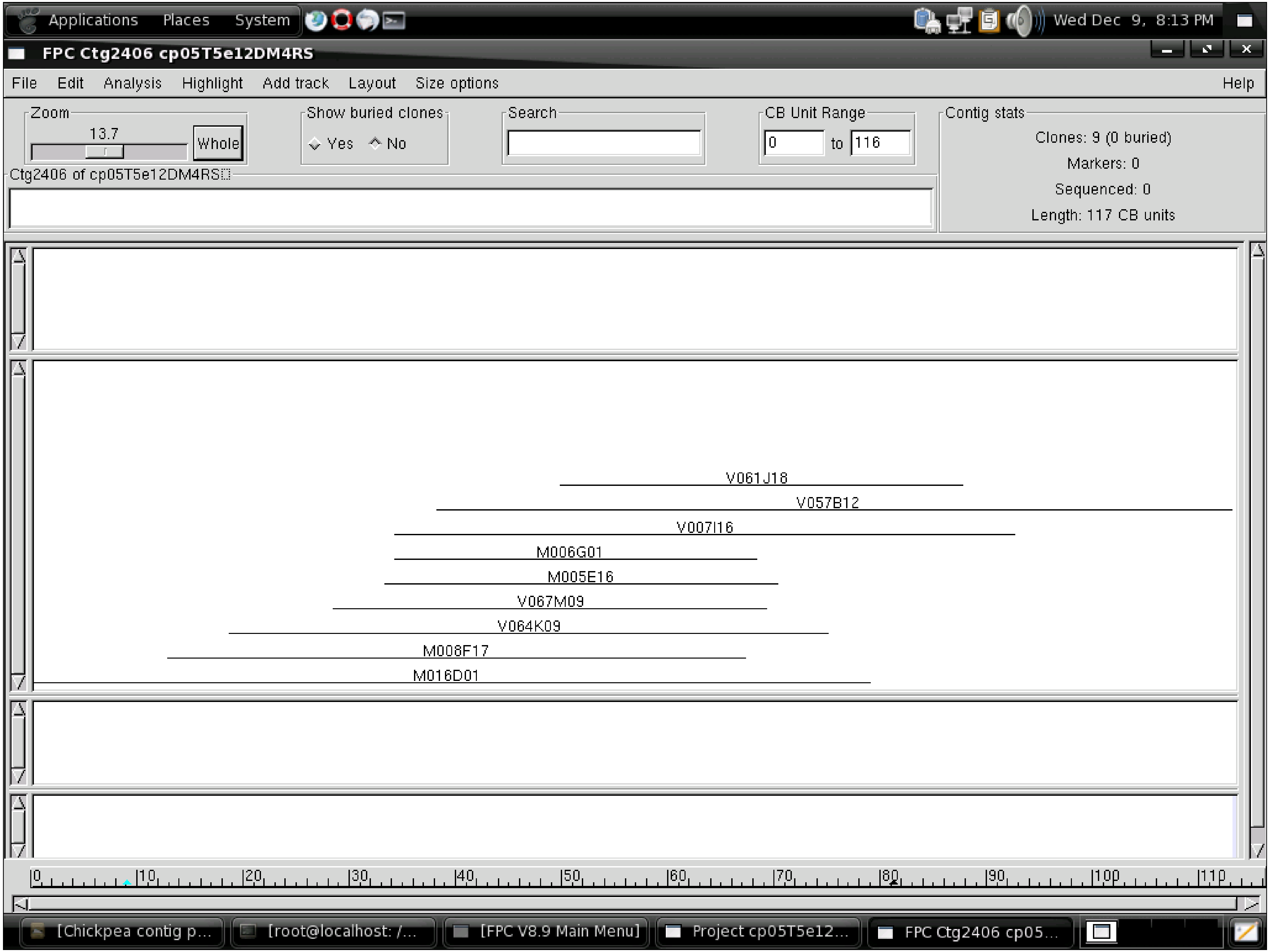
Applications Places System
Wed Dec 9, 8:13 PM
FPC Ctg2406 cp05T5e12DM4RS
File Edit Analysis Highlight Add track Layout Size options Help
Zoom
13.7
Whole
Show buried clones
Yes No
Search
CB Unit Range
0 to 116
Contig stats
Clones: 9 (0 buried)
Markers: 0
Sequenced: 0
Length: 117 CB units
Ctg2406 of cp05T5e12DM4RS
V061J18
V057B12
V007I16
M006G01
M005E16
V067M09
V064K09
M008F17
M016D01
0 10 20 30 40 50 60 70 80 90 100 110
[Chickpea contig p...
[root@localhost: /...
[FPC V8.9 Main Menu]
Project cp05T5e12...
FPC Ctg2406 cp05...

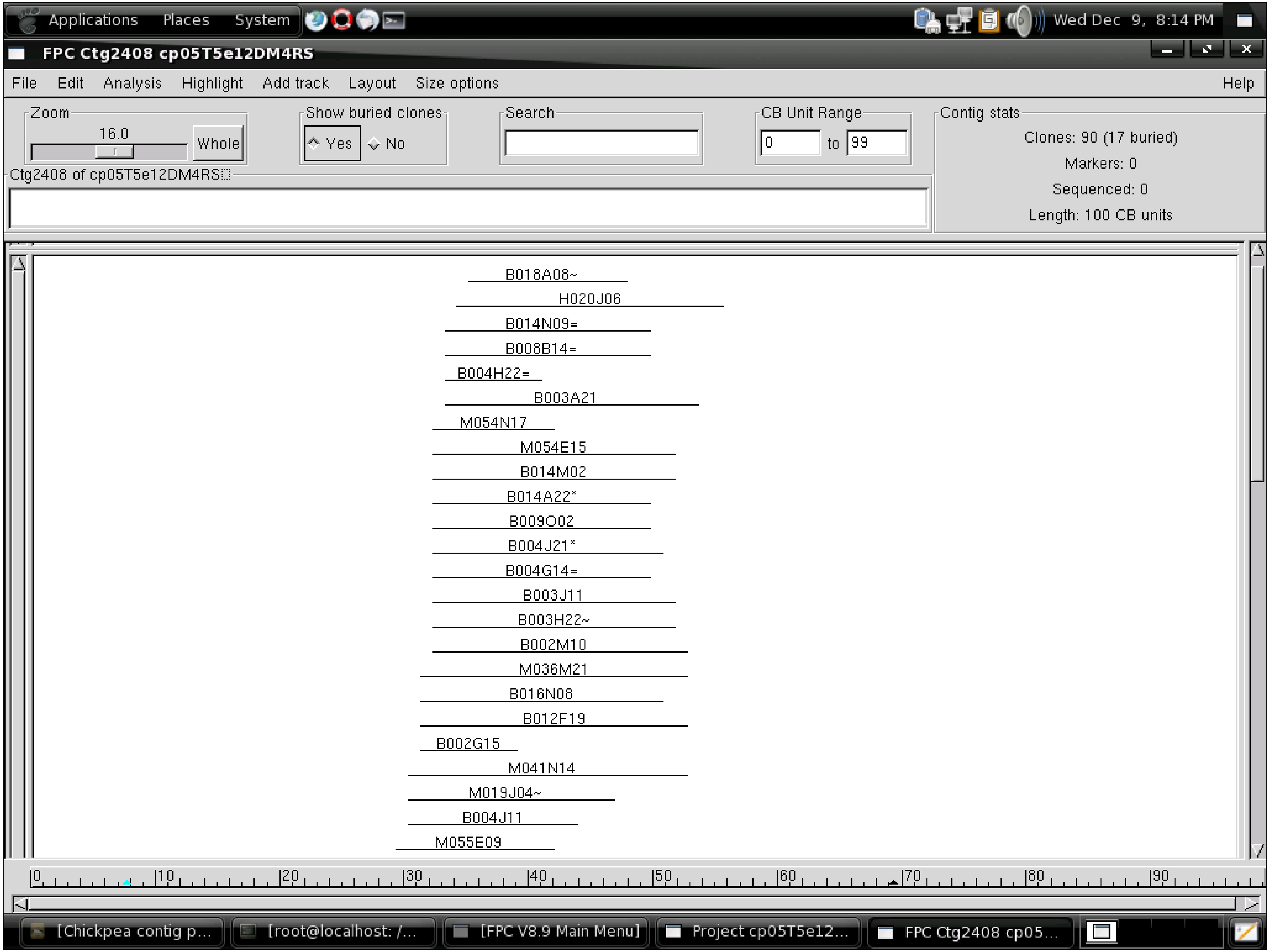

Applications Places System
Wed Dec 9, 8:14 PM
FPC Ctg2408 cp05T5e12DM4RS
File Edit Analysis Highlight Add track Layout Size options Help
Zoom
16.0
Whole
Show buried clones
Yes No
Search
CB Unit Range
0 to 99
Contig stats
Clones: 90 (17 buried)
Markers: 0
Sequenced: 0
Length: 100 CB units
Ctg2408 of cp05T5e12DM4RS
B018A08~
H020J06
B014N09=
B008B14=
B004H22=
B003A21
M054N17
M054E15
B014M02
B014A22*
B009O02
B004J21*
B004G14=
B003J11
B003H22~
B002M10
M036M21
B016N08
B012F19
B002G15
M041N14
M019J04~
B004J11
M055E09
0 10 20 30 40 50 60 70 80 90
[Chickpea contig p...
[root@localhost: /...
[FPC V8.9 Main Menu]
Project cp05T5e12...
FPC Ctg2408 cp05...

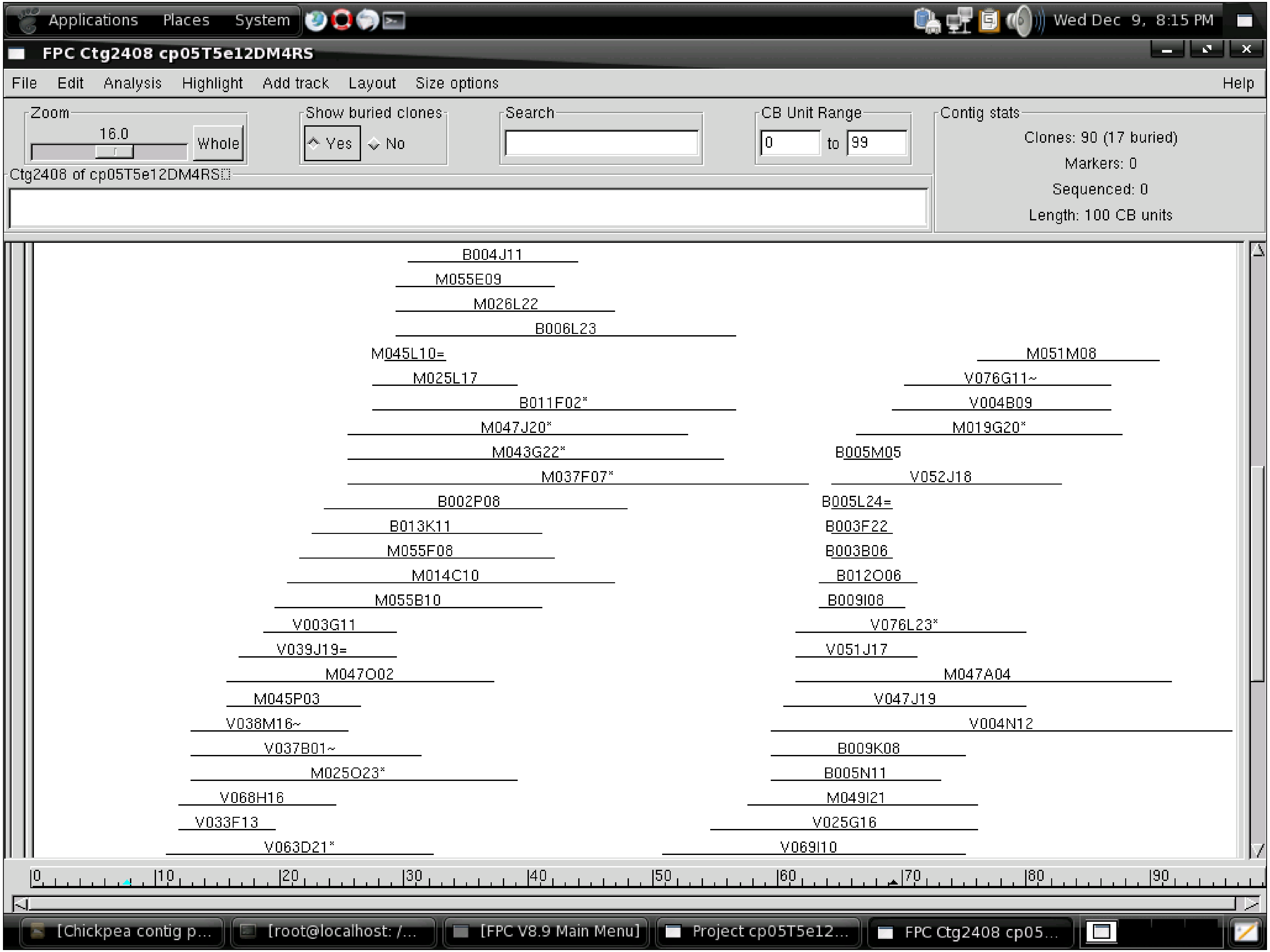
Applications Places System
Wed Dec 9, 8:15 PM
FPC Ctg2408 cp05T5e12DM4RS
File Edit Analysis Highlight Add track Layout Size options Help
Zoom
16.0
Whole
Show buried clones
Yes No
Search
CB Unit Range
0 to 99
Contig stats
Clones: 90 (17 buried)
Markers: 0
Sequenced: 0
Length: 100 CB units
Ctg2408 of cp05T5e12DM4RS
B004J11
M055E09
M026L22
B006L23
M045L10=
M051M08
M025L17
V076G11~
B011F02*
V004B09
M047J20*
M019G20*
M043G22*
B005M05
M037F07*
V052J18
B002P08
B005L24=
B013K11
B003F22
M055F08
B003B06
M014C10
B012O06
M055B10
B009I08
V003G11
V076L23*
V039J19=
V051J17
M047O02
M047A04
M045P03
V047J19
V038M16~
V004N12
V037B01~
B009K08
M025O23*
B005N11
V068H16
M049I21
V033F13
V025G16
V063D21*
V069I10
0 10 20 30 40 50 60 70 80 90
[Chickpea contig p...
[root@localhost: /...
[FPC V8.9 Main Menu]
Project cp05T5e12...
FPC Ctg2408 cp05...

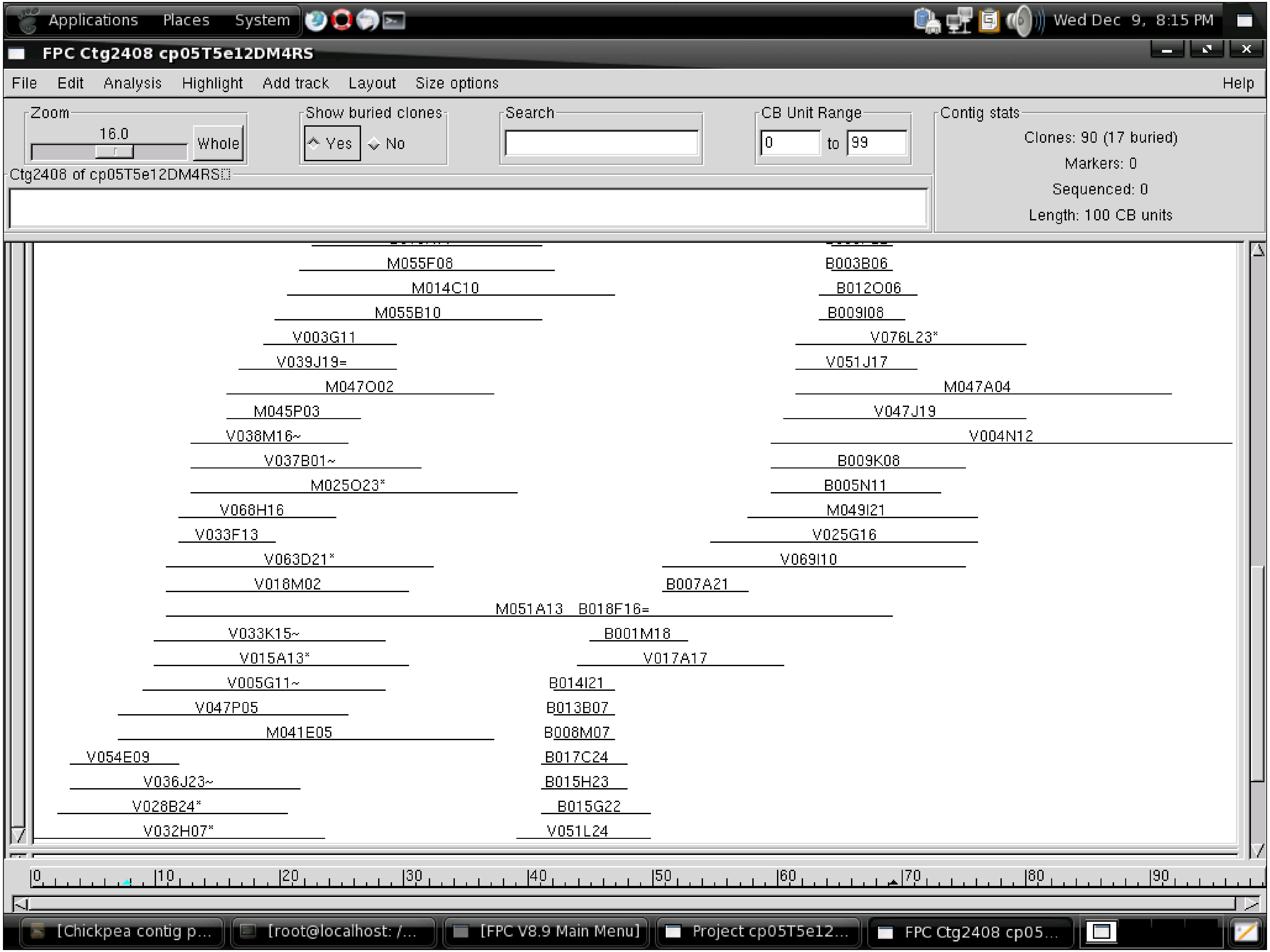
Applications Places System
Wed Dec 9, 8:15 PM
FPC Ctg2408 cp05T5e12DM4RS
File Edit Analysis Highlight Add track Layout Size options Help
Zoom
16.0
Whole
Show buried clones
Yes No
Search
CB Unit Range
0 to 99
Contig stats
Clones: 90 (17 buried)
Markers: 0
Sequenced: 0
Length: 100 CB units
Ctg2408 of cp05T5e12DM4RS
M055F08
B003B06
M014C10
B012O06
M055B10
B009I08
V003G11
V076L23*
V039J19=
V051J17
M047O02
M047A04
M045P03
V047J19
V038M16~
V004N12
V037B01~
B009K08
M025O23*
B005N11
V068H16
M049I21
V033F13
V025G16
V063D21*
V069I10
V018M02
B007A21
M051A13 B018F16=
V033K15~
B001M18
V015A13*
V017A17
V005G11~
B014I21
V047P05
B013B07
M041E05
B008M07
V054E09
B017C24
V036J23~
B015H23
V028B24*
B015G22
V032H07*
V051L24
0 10 20 30 40 50 60 70 80 90
[Chickpea contig p...
[root@localhost: /...
[FPC V8.9 Main Menu]
Project cp05T5e12...
FPC Ctg2408 cp05...

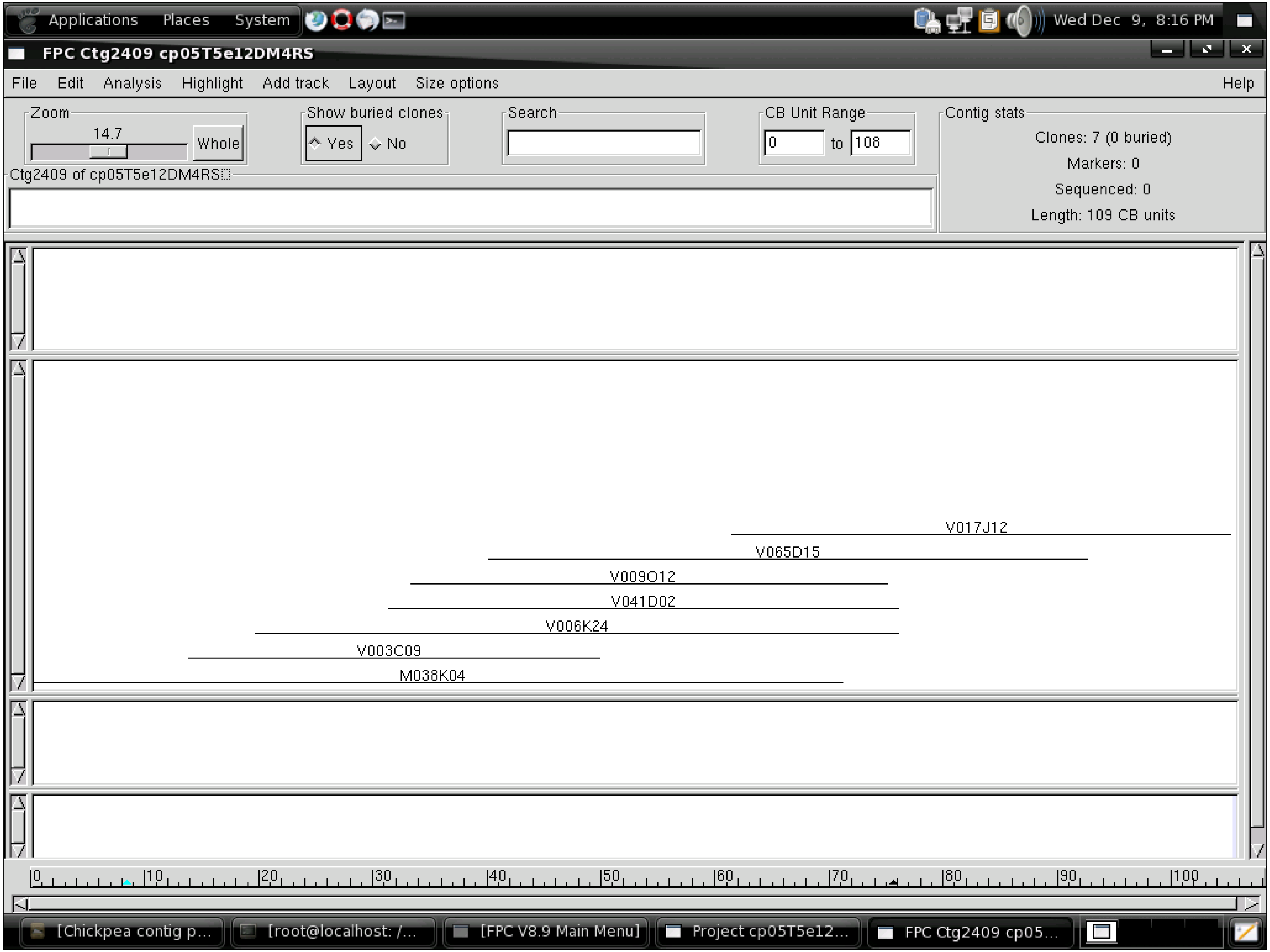
Applications
Places
System
Wed Dec 9, 8:16 PM
FPC Ctg2409 cp05T5e12DM4RS
File
Edit
Analysis
Highlight
Add track
Layout
Size options
Help
Zoom
14.7
Whole
Show buried clones
Yes
No
Search
CB Unit Range
0
to
108
Contig stats
Clones: 7 (0 buried)
Markers: 0
Sequenced: 0
Length: 109 CB units
Ctg2409 of cp05T5e12DM4RS
V017J12
V065D15
V009O12
V041D02
V006K24
V003C09
M038K04
0
10
20
30
40
50
60
70
80
90
100
[Chickpea contig p...
[root@localhost: /...
[FPC V8.9 Main Menu]
Project cp05T5e12...
FPC Ctg2409 cp05...

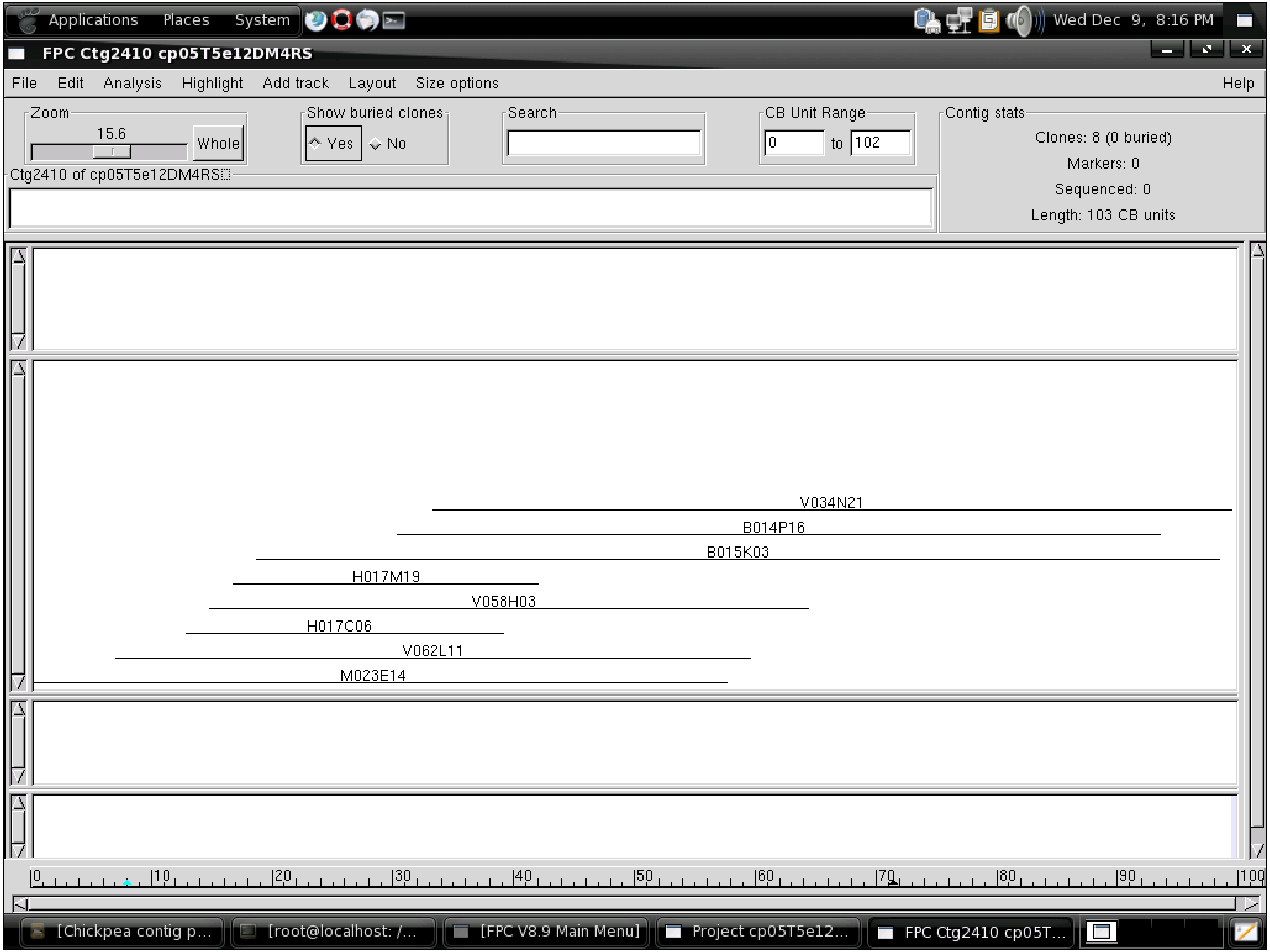
Applications Places System
Wed Dec 9, 8:16 PM
FPC Ctg2410 cp05T5e12DM4RS
File Edit Analysis Highlight Add track Layout Size options Help
Zoom
15.6
Whole
Show buried clones
Yes
No
Search
CB Unit Range
0 to 102
Contig stats
Clones: 8 (0 buried)
Markers: 0
Sequenced: 0
Length: 103 CB units
Ctg2410 of cp05T5e12DM4RS
V034N21
B014P16
B015K03
H017M19
V058H03
H017C06
V062L11
M023E14
0 10 20 30 40 50 60 70 80 90 100
[Chickpea contig p...
[root@localhost: /...
[FPC V8.9 Main Menu]
Project cp05T5e12...
FPC Ctg2410 cp05T...

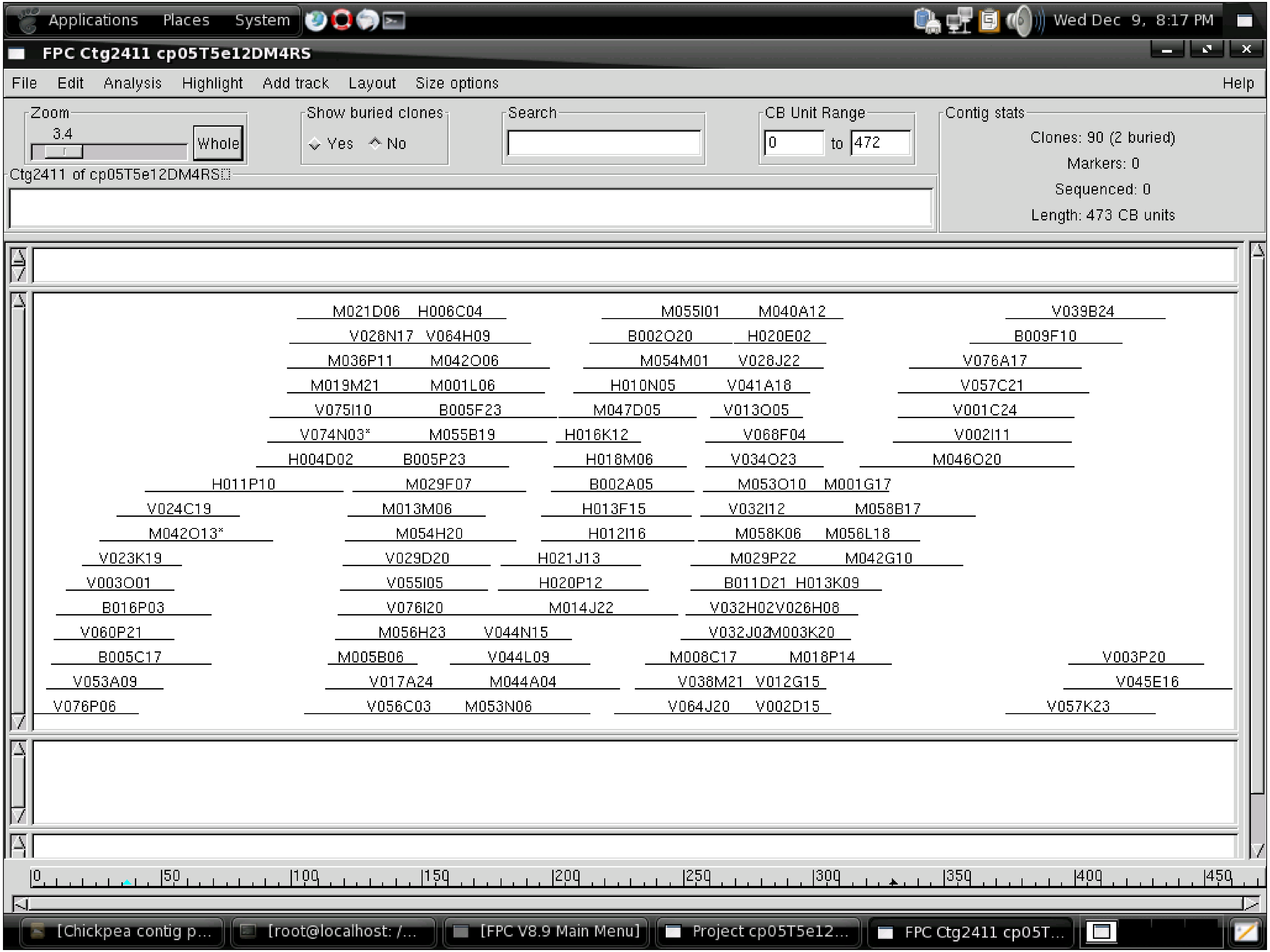
Applications Places System
Wed Dec 9, 8:17 PM
FPC Ctg2411 cp05T5e12DM4RS
File Edit Analysis Highlight Add track Layout Size options Help
Zoom 3.4 Whole
Show buried clones Yes No
Search
CB Unit Range 0 to 472
Contig stats
Clones: 90 (2 buried)
Markers: 0
Sequenced: 0
Length: 473 CB units
Ctg2411 of cp05T5e12DM4RS
M021D06 H006C04 M055I01 M040A12 V039B24
V028N17 V064H09 B002O20 H020E02 B009F10
M036P11 M042O06 M054M01 V028J22 V076A17
M019M21 M001L06 H010N05 V041A18 V057C21
V075I10 B005F23 M047D05 V013O05 V001C24
V074N03* M055B19 H016K12 V068F04 V002I11
H004D02 B005P23 H018M06 V034O23 M046O20
H011P10 M029F07 B002A05 M053O10 M001G17
V024C19 M013M06 H013F15 V032I12 M058B17
M042O13* M054H20 H012I16 M058K06 M056L18
V023K19 V029D20 H021J13 M029P22 M042G10
V003O01 V055I05 H020P12 B011D21 H013K09
B016P03 V076I20 M014J22 V032H02 V026H08
V060P21 M056H23 V044N15 V032J02 M003K20
B005C17 M005B06 V044L09 M008C17 M018P14 V003P20
V053A09 V017A24 M044A04 V038M21 V012G15 V045E16
V076P06 V056C03 M053N06 V064J20 V002D15 V057K23
0 50 100 150 200 250 300 350 400 450
[Chickpea contig p... [root@localhost: /... [FPC V8.9 Main Menu] Project cp05T5e12... FPC Ctg2411 cp05T...

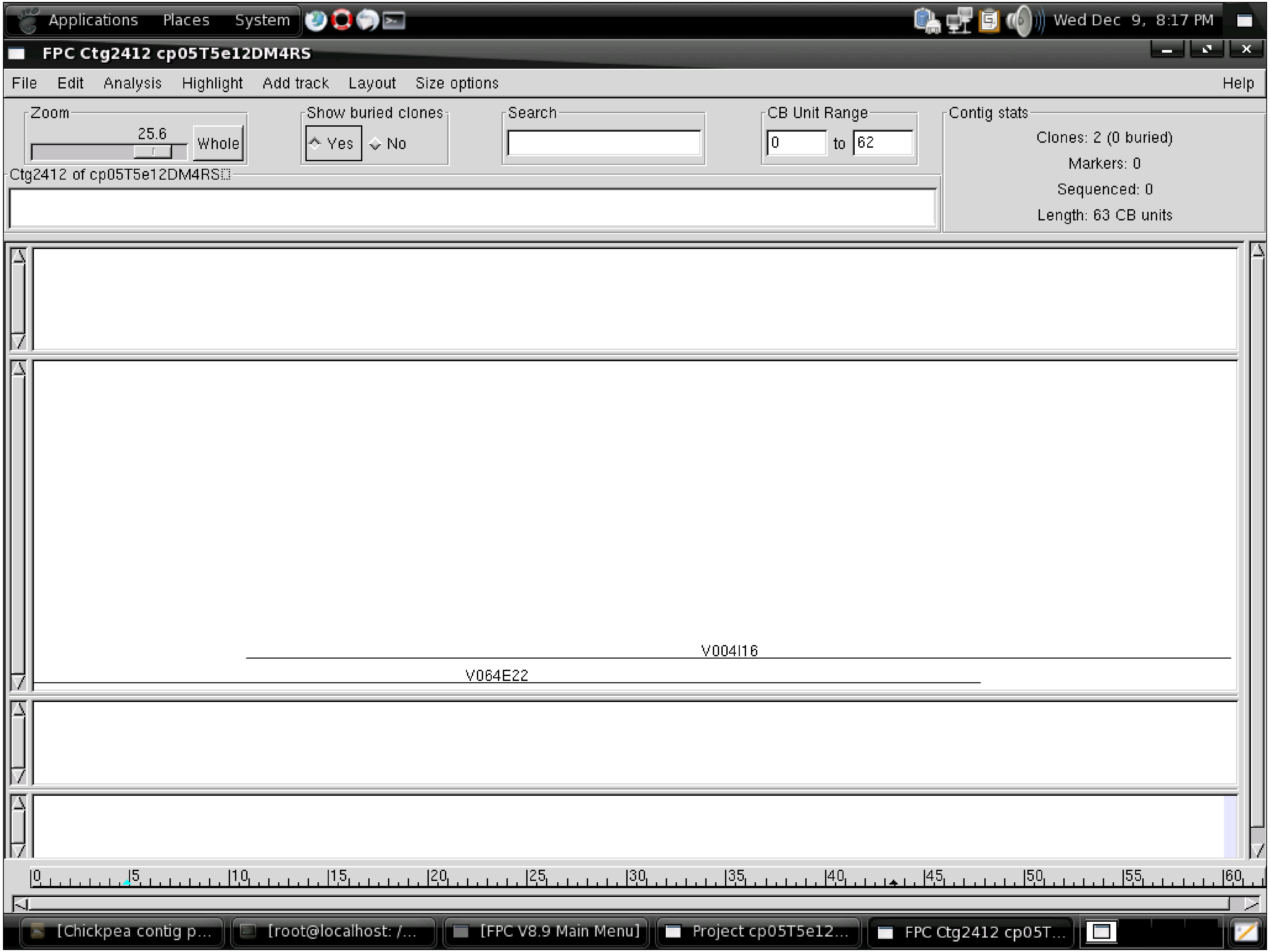
Applications Places System
Wed Dec 9, 8:17 PM
FPC Ctg2412 cp05T5e12DM4RS
File Edit Analysis Highlight Add track Layout Size options Help
Zoom
25.6
Whole
Show buried clones
Yes
No
Search
CB Unit Range
0 to 62
Contig stats
Clones: 2 (0 buried)
Markers: 0
Sequenced: 0
Length: 63 CB units
Ctg2412 of cp05T5e12DM4RS
V004I16
V064E22
0 5 10 15 20 25 30 35 40 45 50 55 60
[Chickpea contig p...
[root@localhost: /...
[FPC V8.9 Main Menu]
Project cp05T5e12...
FPC Ctg2412 cp05T...

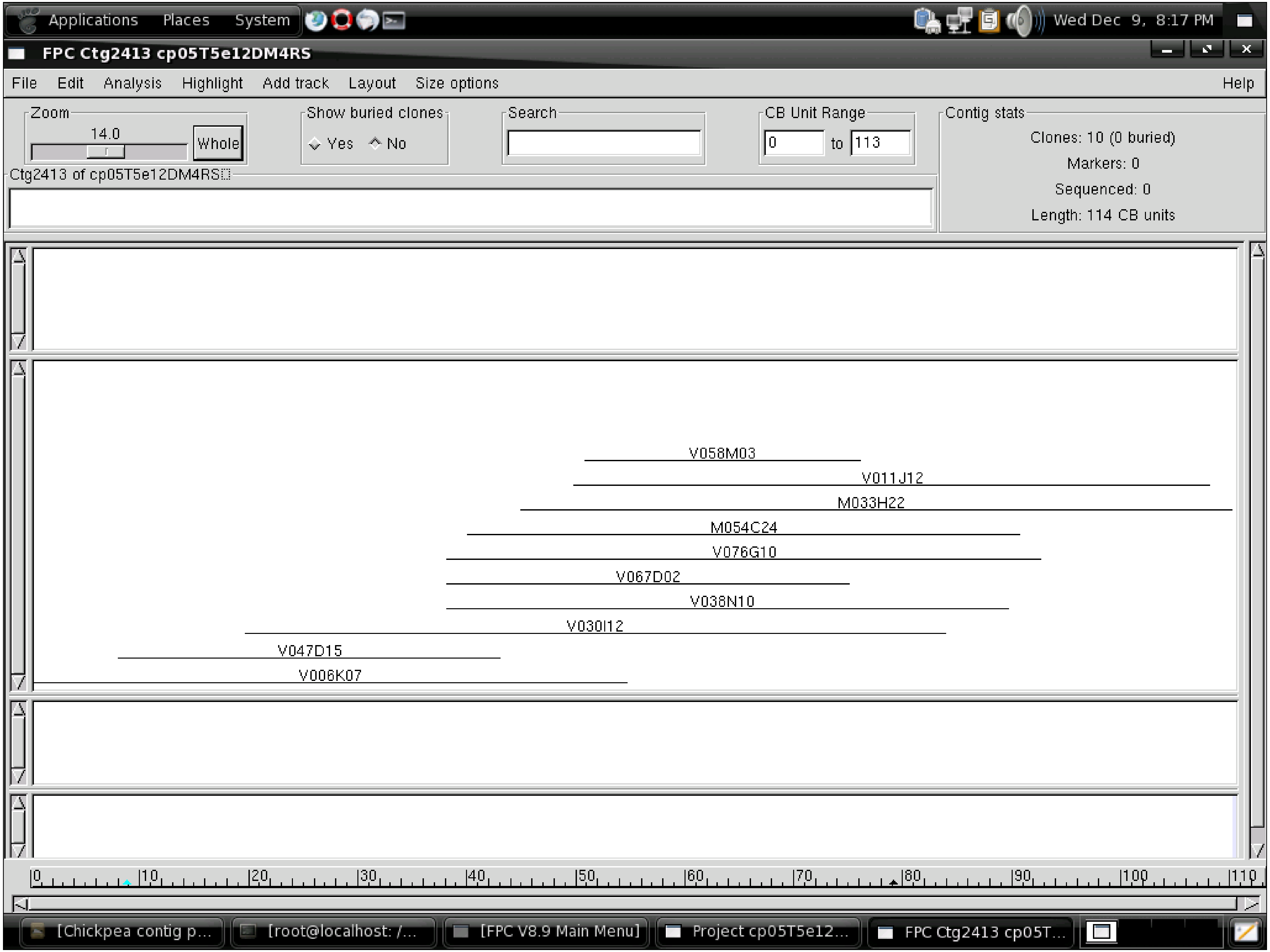
Applications Places System
Wed Dec 9, 8:17 PM
FPC Ctg2413 cp05T5e12DM4RS
File Edit Analysis Highlight Add track Layout Size options Help
Zoom
14.0
Whole
Show buried clones
Yes No
Search
CB Unit Range
0 to 113
Contig stats
Clones: 10 (0 buried)
Markers: 0
Sequenced: 0
Length: 114 CB units
Ctg2413 of cp05T5e12DM4RS
V058M03
V011J12
M033H22
M054C24
V076G10
V067D02
V038N10
V030I12
V047D15
V006K07
0 10 20 30 40 50 60 70 80 90 100 110
[Chickpea contig p...
[root@localhost: /...
[FPC V8.9 Main Menu]
Project cp05T5e12...
FPC Ctg2413 cp05T...

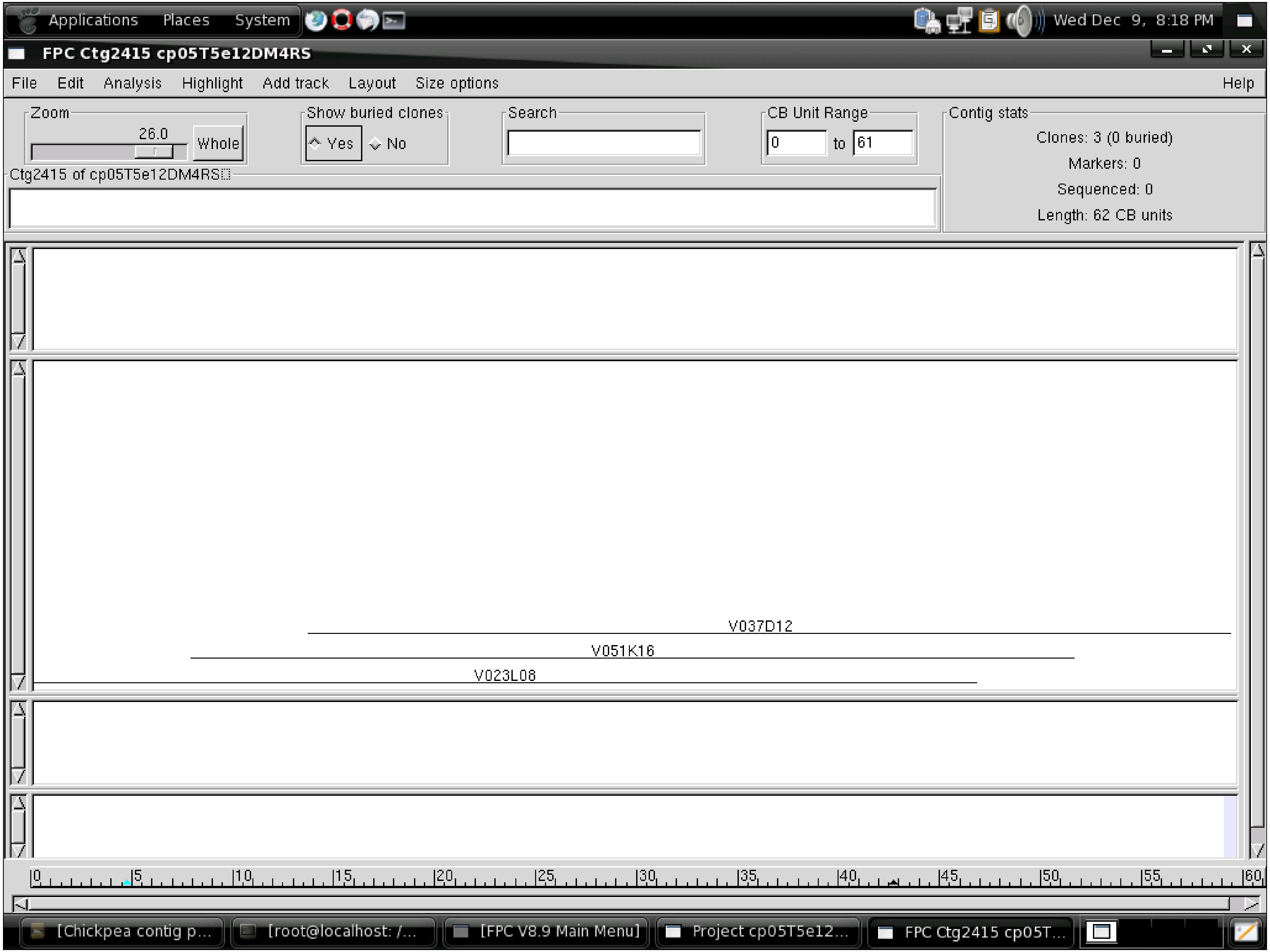
Applications
Places
System
Wed Dec 9, 8:18 PM
FPC Ctg2415 cp05T5e12DM4RS
File
Edit
Analysis
Highlight
Add track
Layout
Size options
Help
Zoom
26.0
Whole
Show buried clones
Yes
No
Search
CB Unit Range
0
to
61
Contig stats
Clones: 3 (0 buried)
Markers: 0
Sequenced: 0
Length: 62 CB units
Ctg2415 of cp05T5e12DM4RS
V037D12
V051K16
V023L08
0 5 10 15 20 25 30 35 40 45 50 55 60
[Chickpea contig p...
[root@localhost: /...
[FPC V8.9 Main Menu]
Project cp05T5e12...
FPC Ctg2415 cp05T...

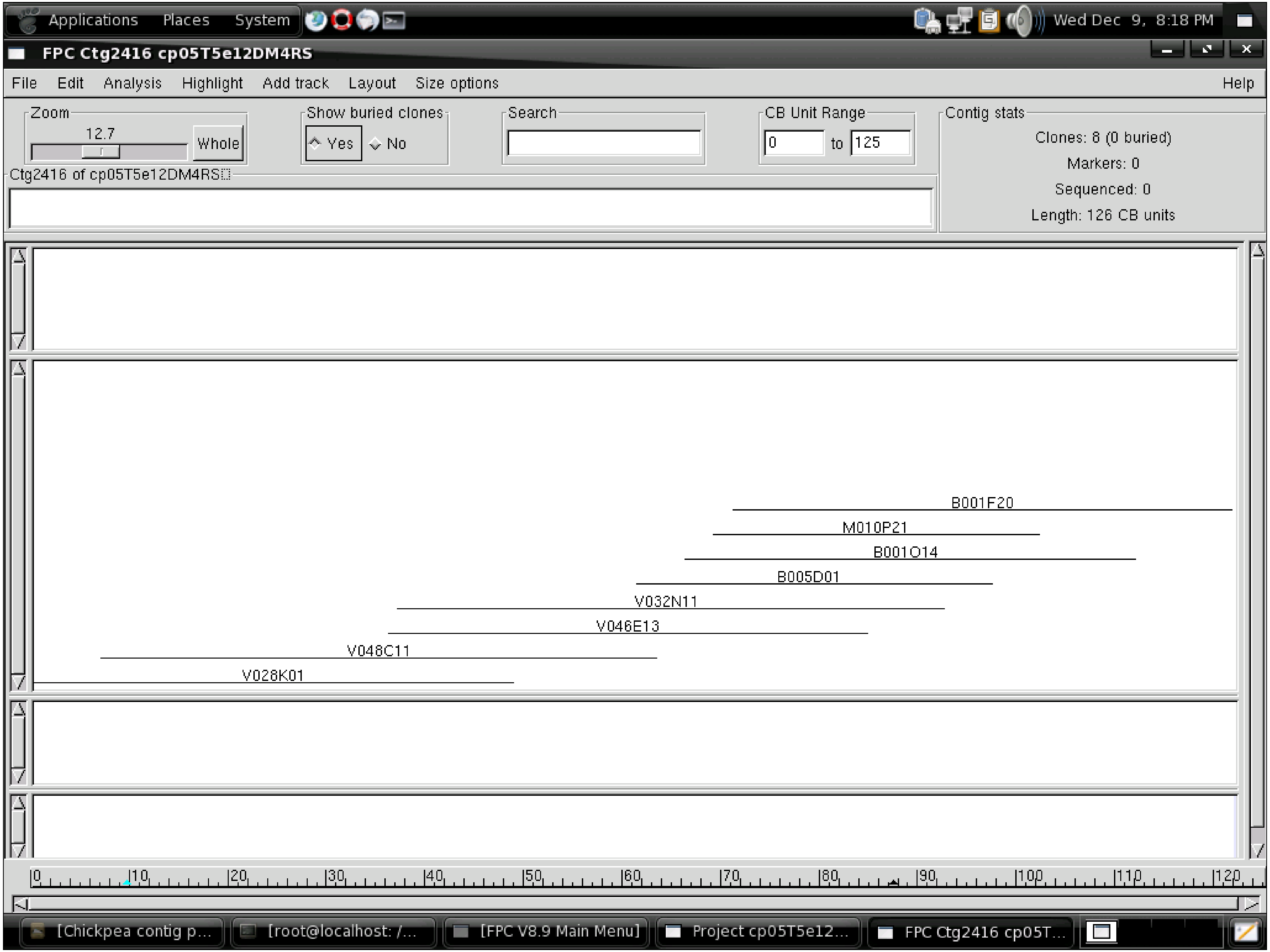
Applications Places System
Wed Dec 9, 8:18 PM
FPC Ctg2416 cp05T5e12DM4RS
File Edit Analysis Highlight Add track Layout Size options Help
Zoom
12.7
Whole
Show buried clones
Yes No
Search
CB Unit Range
0 to 125
Contig stats
Clones: 8 (0 buried)
Markers: 0
Sequenced: 0
Length: 126 CB units
Ctg2416 of cp05T5e12DM4RS
B001F20
M010P21
B001O14
B005D01
V032N11
V046E13
V048C11
V028K01
0 10 20 30 40 50 60 70 80 90 100 110 120
[Chickpea contig p...
[root@localhost: /...
[FPC V8.9 Main Menu]
Project cp05T5e12...
FPC Ctg2416 cp05T...

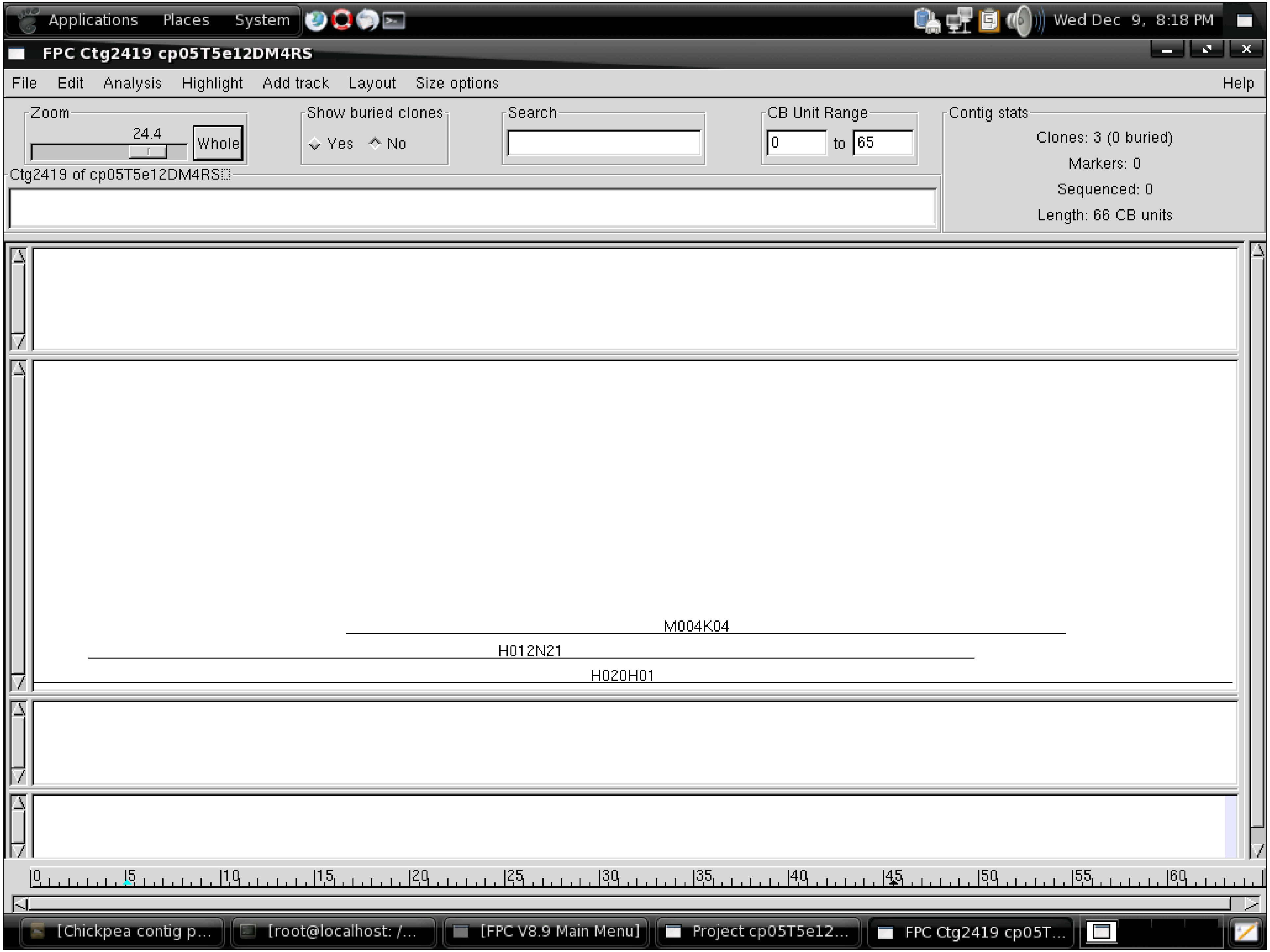
Applications Places System
Wed Dec 9, 8:18 PM
FPC Ctg2419 cp05T5e12DM4RS
File Edit Analysis Highlight Add track Layout Size options Help
Zoom
24.4
Whole
Show buried clones
Yes No
Search
CB Unit Range
0 to 65
Contig stats
Clones: 3 (0 buried)
Markers: 0
Sequenced: 0
Length: 66 CB units
Ctg2419 of cp05T5e12DM4RS
M004K04
H012N21
H020H01
0 5 10 15 20 25 30 35 40 45 50 55 60
[Chickpea contig p...
[root@localhost: /...
[FPC V8.9 Main Menu]
Project cp05T5e12...
FPC Ctg2419 cp05T...

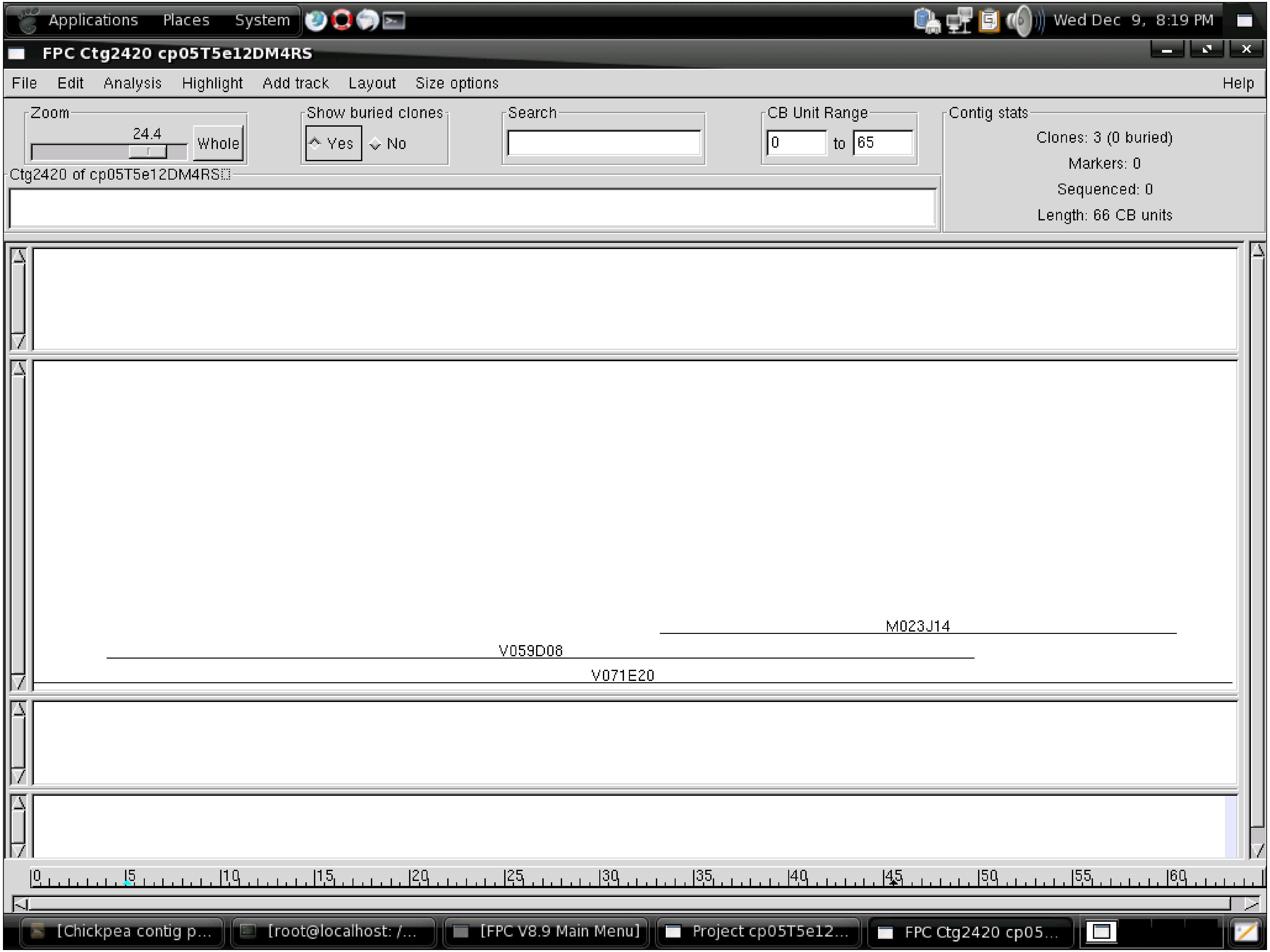
Applications Places System
Wed Dec 9, 8:19 PM
FPC Ctg2420 cp05T5e12DM4RS
File Edit Analysis Highlight Add track Layout Size options
Help
Zoom
24.4
Whole
Show buried clones
Yes
No
Search
CB Unit Range
0
to
65
Contig stats
Clones: 3 (0 buried)
Markers: 0
Sequenced: 0
Length: 66 CB units
Ctg2420 of cp05T5e12DM4RS
M023J14
V059D08
V071E20
0 5 10 15 20 25 30 35 40 45 50 55 60
[Chickpea contig p...
[root@localhost: /...
[FPC V8.9 Main Menu]
Project cp05T5e12...
FPC Ctg2420 cp05...

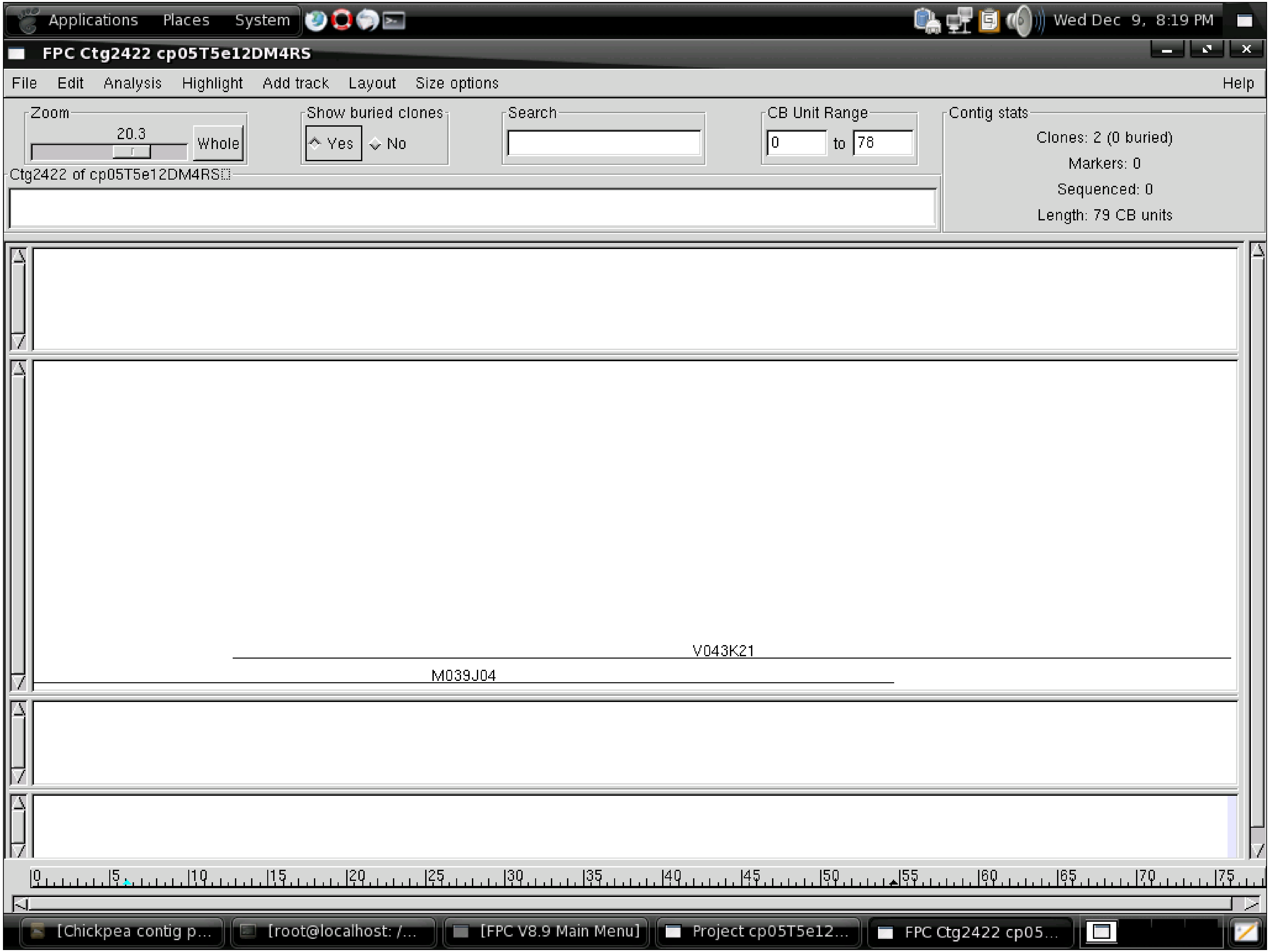
Applications Places System
Wed Dec 9, 8:19 PM
FPC Ctg2422 cp05T5e12DM4RS
File Edit Analysis Highlight Add track Layout Size options Help
Zoom
20.3
Whole
Show buried clones
Yes No
Search
CB Unit Range
0 to 78
Contig stats
Clones: 2 (0 buried)
Markers: 0
Sequenced: 0
Length: 79 CB units
Ctg2422 of cp05T5e12DM4RS
V043K21
M039J04
0 5 10 15 20 25 30 35 40 45 50 55 60 65 70 75
[Chickpea contig p...
[root@localhost: /...
[FPC V8.9 Main Menu]
Project cp05T5e12...
FPC Ctg2422 cp05...

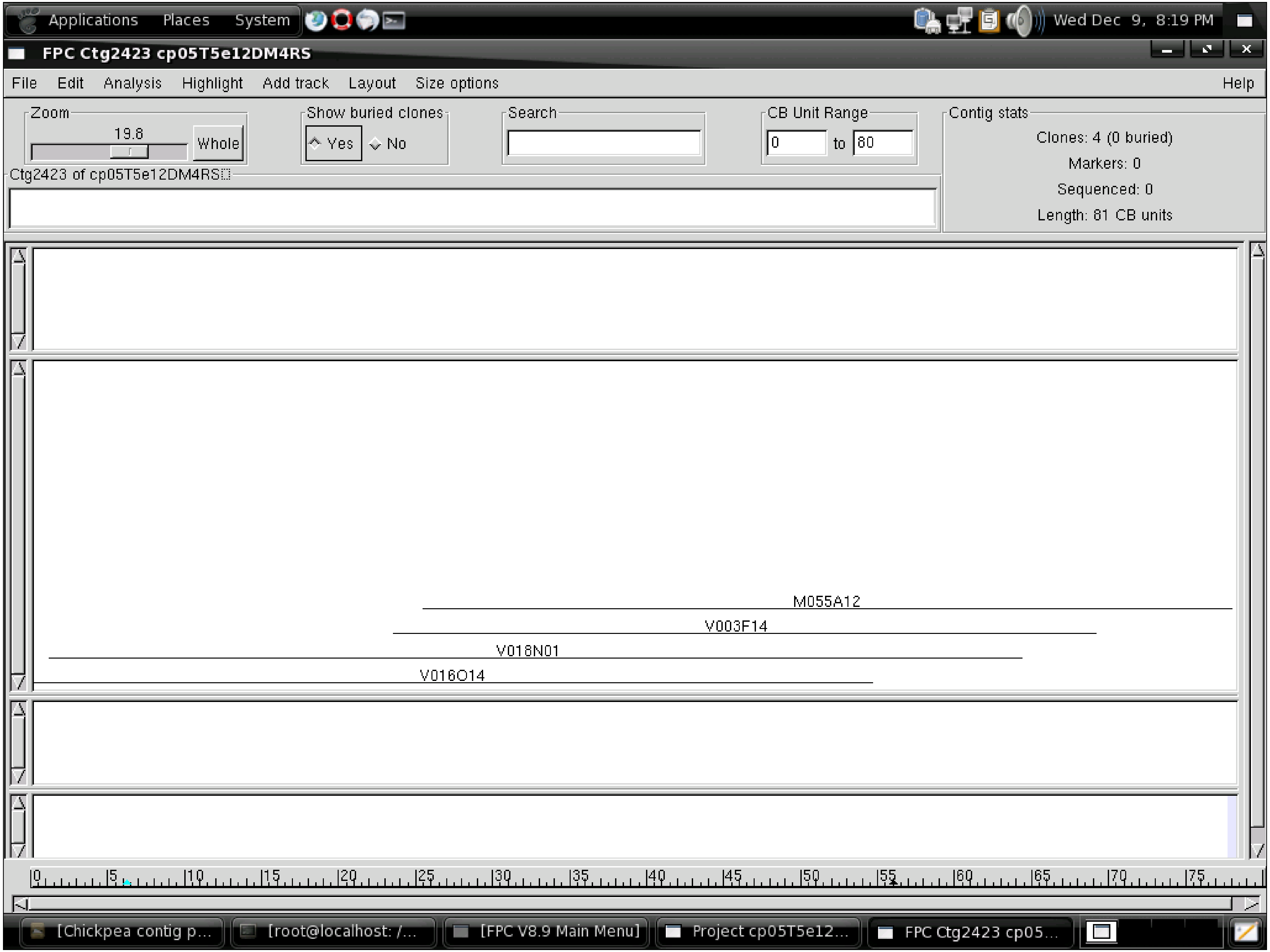
Applications Places System
Wed Dec 9, 8:19 PM
FPC Ctg2423 cp05T5e12DM4RS
File Edit Analysis Highlight Add track Layout Size options Help
Zoom
19.8
Whole
Show buried clones
Yes
No
Search
CB Unit Range
0
to
80
Contig stats
Clones: 4 (0 buried)
Markers: 0
Sequenced: 0
Length: 81 CB units
Ctg2423 of cp05T5e12DM4RS
M055A12
V003F14
V018N01
V016O14
0 5 10 15 20 25 30 35 40 45 50 55 60 65 70 75
[Chickpea contig p...
[root@localhost: /...
[FPC V8.9 Main Menu]
Project cp05T5e12...
FPC Ctg2423 cp05...

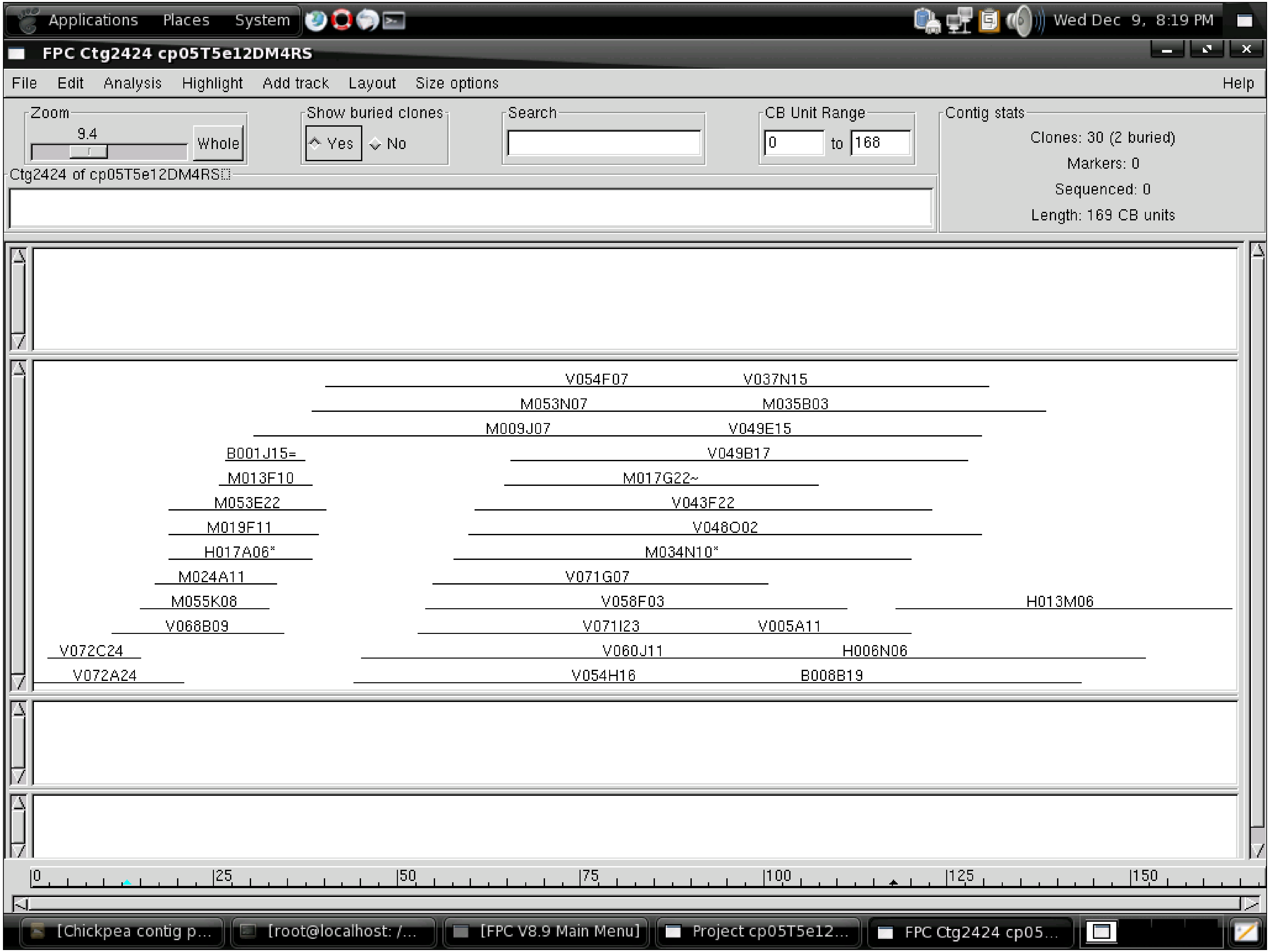
Applications Places System
Wed Dec 9, 8:19 PM
FPC Ctg2424 cp05T5e12DM4RS
File Edit Analysis Highlight Add track Layout Size options Help
Zoom
9.4
Whole
Show buried clones
Yes No
Search
CB Unit Range
0 to 168
Contig stats
Clones: 30 (2 buried)
Markers: 0
Sequenced: 0
Length: 169 CB units
Ctg2424 of cp05T5e12DM4RS
V054F07
V037N15
M053N07
M035B03
M009J07
V049E15
B001J15=
V049B17
M013F10
M017G22~
M053E22
V043F22
M019F11
V048O02
H017A06*
M034N10*
M024A11
V071G07
M055K08
V058F03
H013M06
V068B09
V071I23
V005A11
V072C24
V060J11
H006N06
V072A24
V054H16
B008B19
0 25 50 75 100 125 150
[Chickpea contig p...
[root@localhost: /...
[FPC V8.9 Main Menu]
Project cp05T5e12...
FPC Ctg2424 cp05...

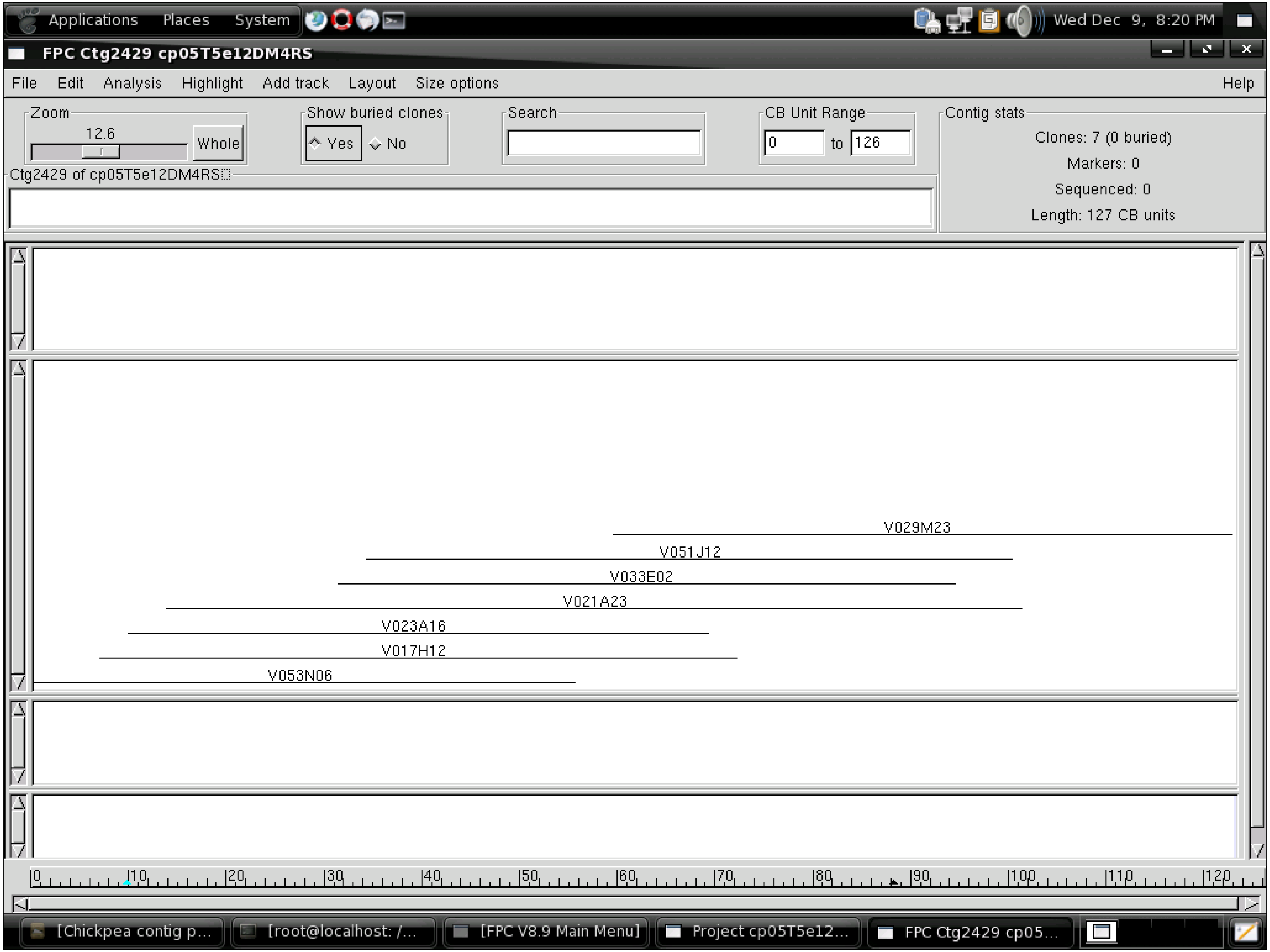
Applications Places System
Wed Dec 9, 8:20 PM
FPC Ctg2429 cp05T5e12DM4RS
File Edit Analysis Highlight Add track Layout Size options Help
Zoom
12.6
Whole
Show buried clones
Yes No
Search
CB Unit Range
0 to 126
Contig stats
Clones: 7 (0 buried)
Markers: 0
Sequenced: 0
Length: 127 CB units
Ctg2429 of cp05T5e12DM4RS
V029M23
V051J12
V033E02
V021A23
V023A16
V017H12
V053N06
0 10 20 30 40 50 60 70 80 90 100 110 120
[Chickpea contig p...
[root@localhost: /...
[FPC V8.9 Main Menu]
Project cp05T5e12...
FPC Ctg2429 cp05...

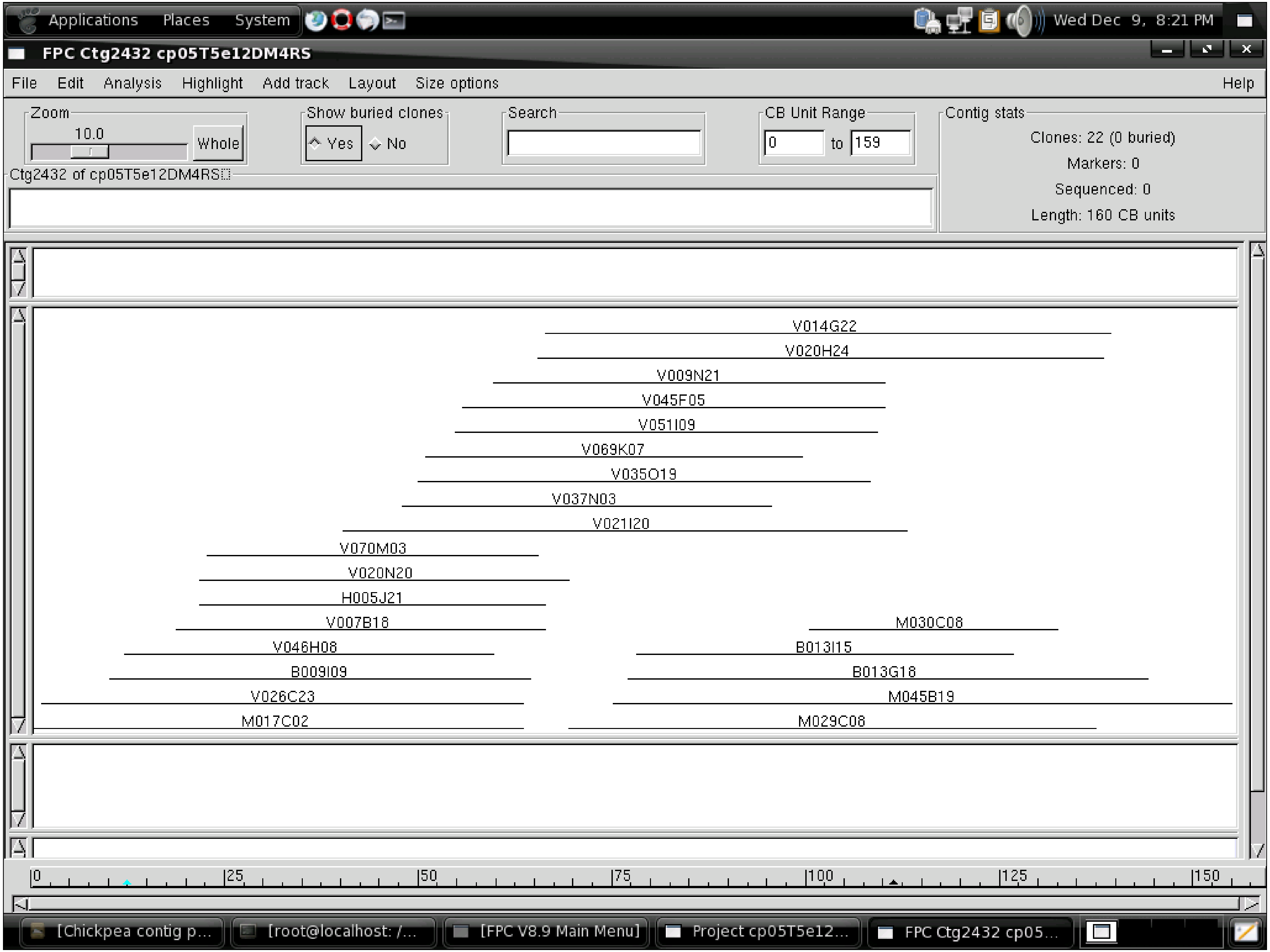
Applications Places System
Wed Dec 9, 8:21 PM
FPC Ctg2432 cp05T5e12DM4RS
File Edit Analysis Highlight Add track Layout Size options Help
Zoom
10.0
Whole
Show buried clones
Yes No
Search
CB Unit Range
0 to 159
Contig stats
Clones: 22 (0 buried)
Markers: 0
Sequenced: 0
Length: 160 CB units
Ctg2432 of cp05T5e12DM4RS
V014G22
V020H24
V009N21
V045F05
V051I09
V069K07
V035O19
V037N03
V021I20
V070M03
V020N20
H005J21
V007B18
M030C08
V046H08
B013I15
B009I09
B013G18
V026C23
M045B19
M017C02
M029C08
0
25
50
75
100
125
150
[Chickpea contig p...
[root@localhost: /...
[FPC V8.9 Main Menu]
Project cp05T5e12...
FPC Ctg2432 cp05...

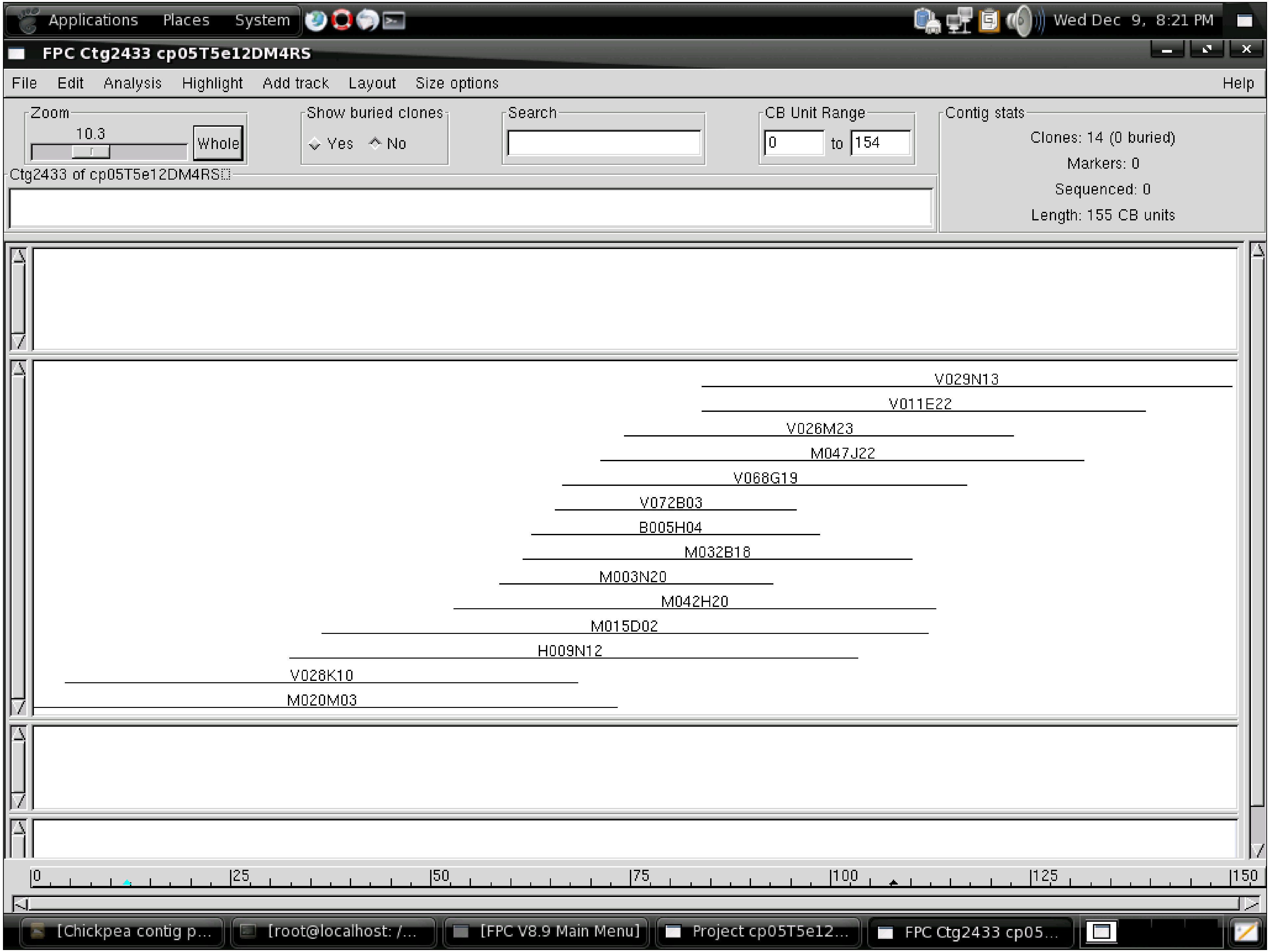
Applications
Places
System
Wed Dec 9, 8:21 PM
FPC Ctg2433 cp05T5e12DM4RS
File
Edit
Analysis
Highlight
Add track
Layout
Size options
Help
Zoom
10.3
Whole
Show buried clones
Yes
No
Search
CB Unit Range
0
to
154
Contig stats
Clones: 14 (0 buried)
Markers: 0
Sequenced: 0
Length: 155 CB units
Ctg2433 of cp05T5e12DM4RS
V029N13
V011E22
V026M23
M047J22
V068G19
V072B03
B005H04
M032B18
M003N20
M042H20
M015D02
H009N12
V028K10
M020M03
0
25
50
75
100
125
150
[Chickpea contig p...
[root@localhost: /...
[FPC V8.9 Main Menu]
Project cp05T5e12...
FPC Ctg2433 cp05...

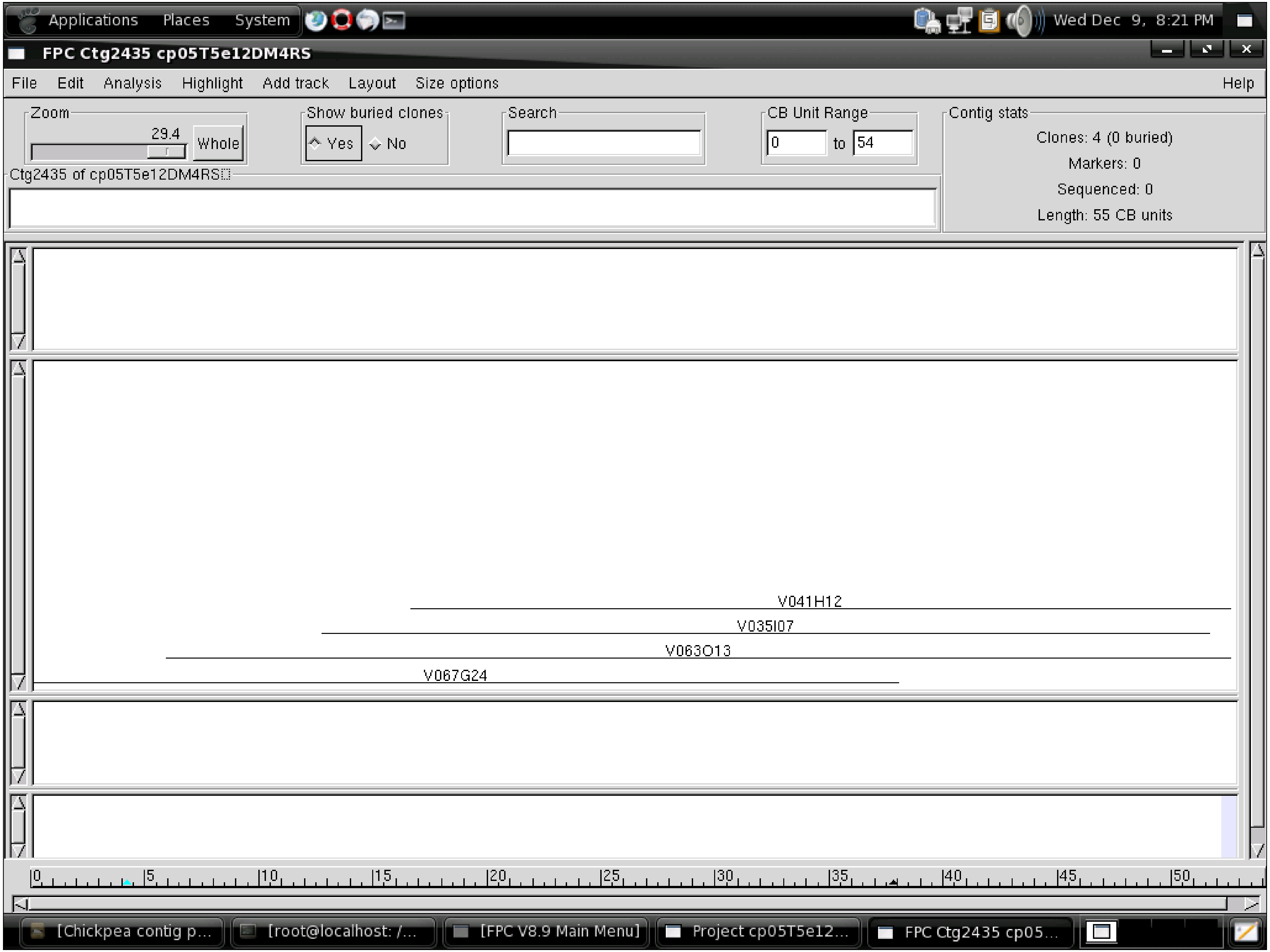
Applications Places System
Wed Dec 9, 8:21 PM
FPC Ctg2435 cp05T5e12DM4RS
File Edit Analysis Highlight Add track Layout Size options Help
Zoom
29.4
Whole
Show buried clones
Yes
No
Search
CB Unit Range
0
to
54
Contig stats
Clones: 4 (0 buried)
Markers: 0
Sequenced: 0
Length: 55 CB units
Ctg2435 of cp05T5e12DM4RS
V041H12
V035I07
V063O13
V067G24
0 5 10 15 20 25 30 35 40 45 50
[Chickpea contig p...
[root@localhost: /...
[FPC V8.9 Main Menu]
Project cp05T5e12...
FPC Ctg2435 cp05...

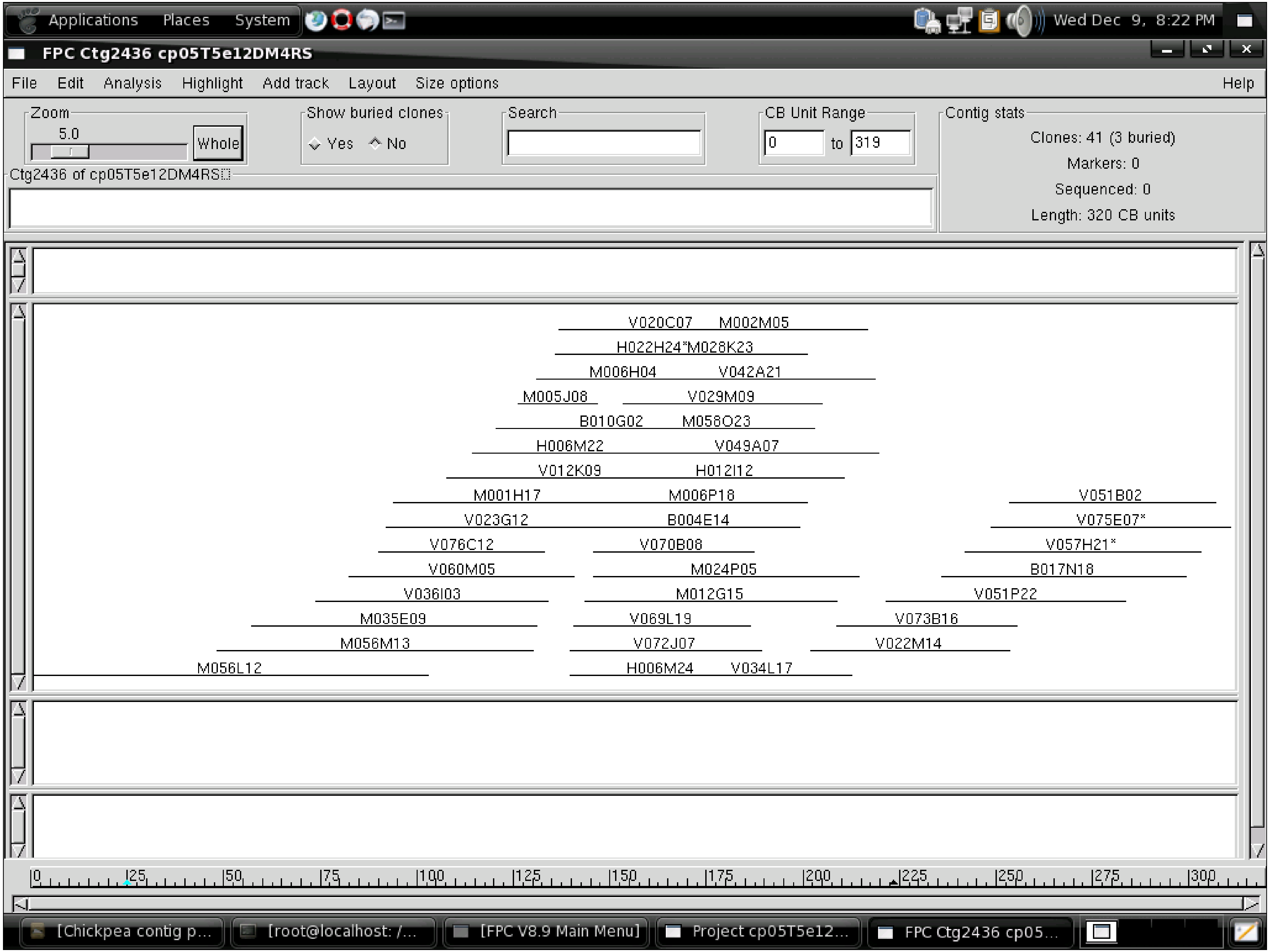
Applications Places System
Wed Dec 9, 8:22 PM
FPC Ctg2436 cp05T5e12DM4RS
File Edit Analysis Highlight Add track Layout Size options Help
Zoom
5.0
Whole
Show buried clones
Yes No
Search
CB Unit Range
0 to 319
Contig stats
Clones: 41 (3 buried)
Markers: 0
Sequenced: 0
Length: 320 CB units
Ctg2436 of cp05T5e12DM4RS
V020C07 M002M05
H022H24*M028K23
M006H04 V042A21
M005J08 V029M09
B010G02 M058O23
H006M22 V049A07
V012K09 H012I12
M001H17 M006P18 V051B02
V023G12 B004E14 V075E07*
V076C12 V070B08 V057H21*
V060M05 M024P05 B017N18
V036I03 M012G15 V051P22
M035E09 V069L19 V073B16
M056M13 V072J07 V022M14
M056L12 H006M24 V034L17
0 25 50 75 100 125 150 175 200 225 250 275 300
[Chickpea contig p...
[root@localhost: /...
[FPC V8.9 Main Menu]
Project cp05T5e12...
FPC Ctg2436 cp05...

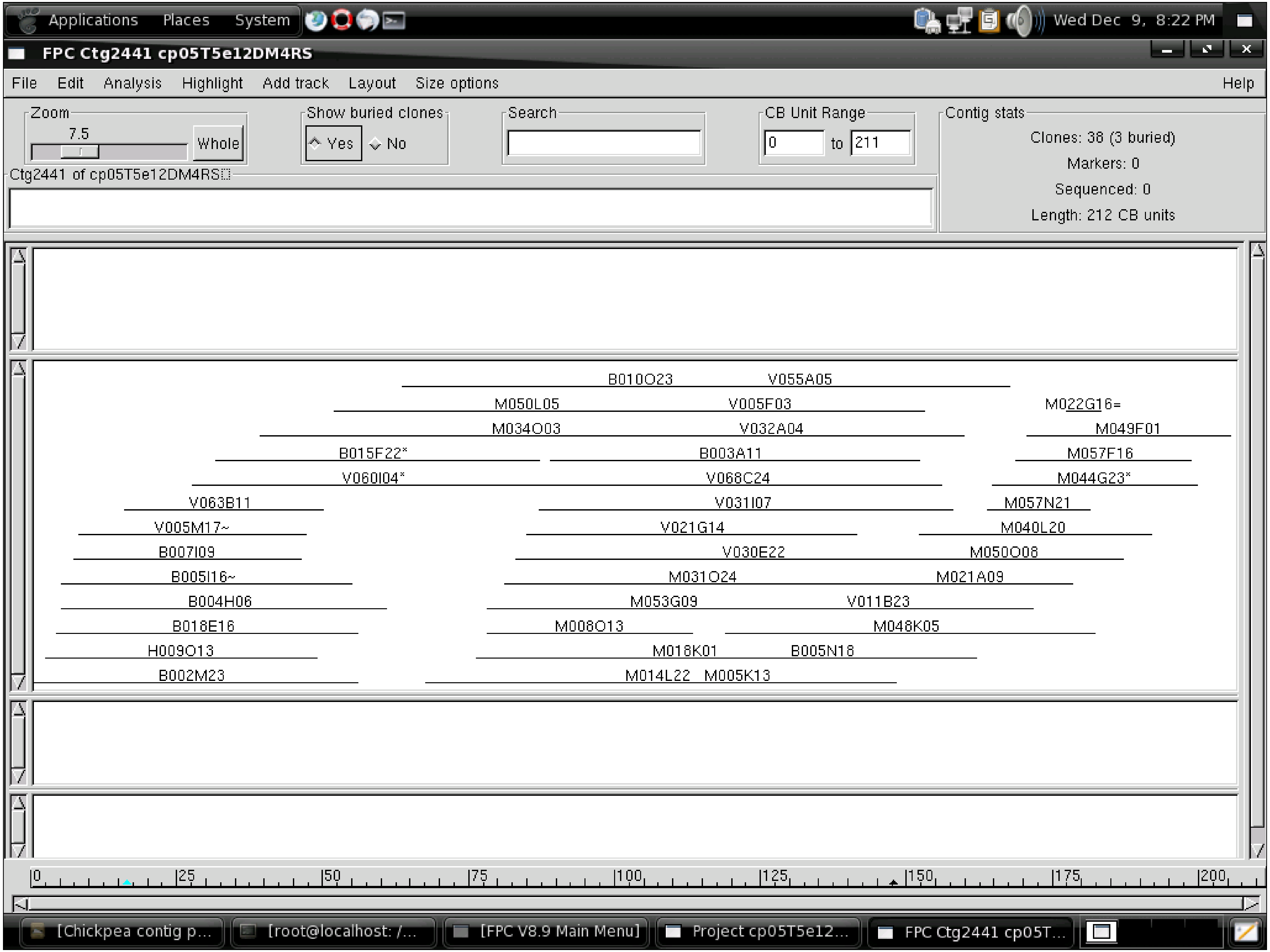
Applications Places System
Wed Dec 9, 8:22 PM
FPC Ctg2441 cp05T5e12DM4RS
File Edit Analysis Highlight Add track Layout Size options Help
Zoom
7.5
Whole
Show buried clones
Yes
No
Search
CB Unit Range
0 to 211
Contig stats
Clones: 38 (3 buried)
Markers: 0
Sequenced: 0
Length: 212 CB units
Ctg2441 of cp05T5e12DM4RS
B010O23 V055A05
M050L05 V005F03 M022G16=
M034O03 V032A04 M049F01
B015F22* B003A11 M057F16
V060I04* V068C24 M044G23*
V063B11 V031I07 M057N21
V005M17~ V021G14 M040L20
B007I09 V030E22 M050O08
B005I16~ M031O24 M021A09
B004H06 M053G09 V011B23
B018E16 M008O13 M048K05
H009O13 M018K01 B005N18
B002M23 M014L22 M005K13
0 25 50 75 100 125 150 175 200
[Chickpea contig p...
[root@localhost: /...
[FPC V8.9 Main Menu]
Project cp05T5e12...
FPC Ctg2441 cp05T...

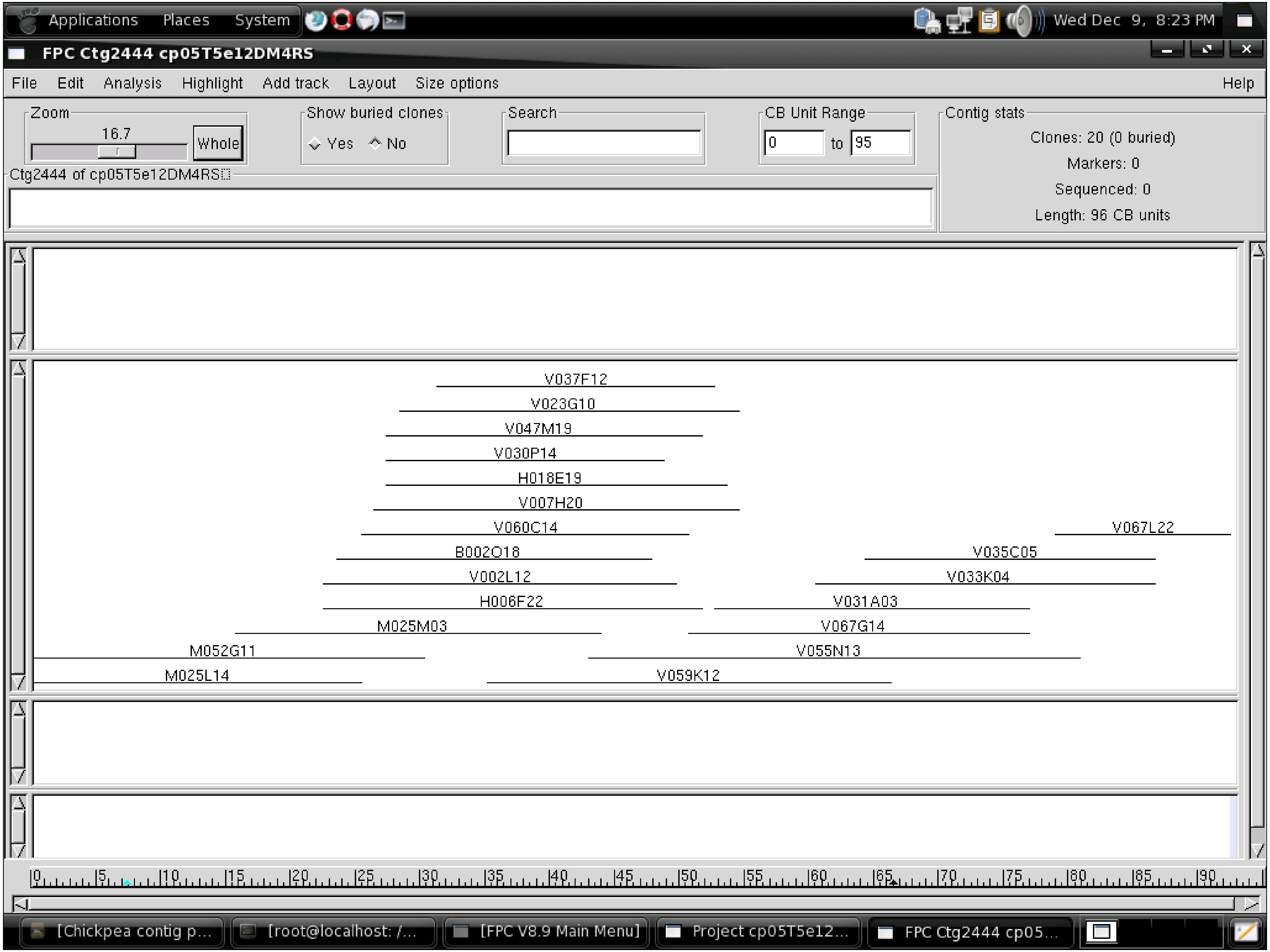
Applications Places System
Wed Dec 9, 8:23 PM
FPC Ctg2444 cp05T5e12DM4RS
File Edit Analysis Highlight Add track Layout Size options Help
Zoom
16.7
Whole
Show buried clones
Yes No
Search
CB Unit Range
0 to 95
Contig stats
Clones: 20 (0 buried)
Markers: 0
Sequenced: 0
Length: 96 CB units
Ctg2444 of cp05T5e12DM4RS
V037F12
V023G10
V047M19
V030P14
H018E19
V007H20
V060C14
V067L22
B002O18
V035C05
V002L12
V033K04
H006F22
V031A03
M025M03
V067G14
M052G11
V055N13
M025L14
V059K12
0 5 10 15 20 25 30 35 40 45 50 55 60 65 70 75 80 85 90
[Chickpea contig p...
[root@localhost: /...
[FPC V8.9 Main Menu]
Project cp05T5e12...
FPC Ctg2444 cp05...

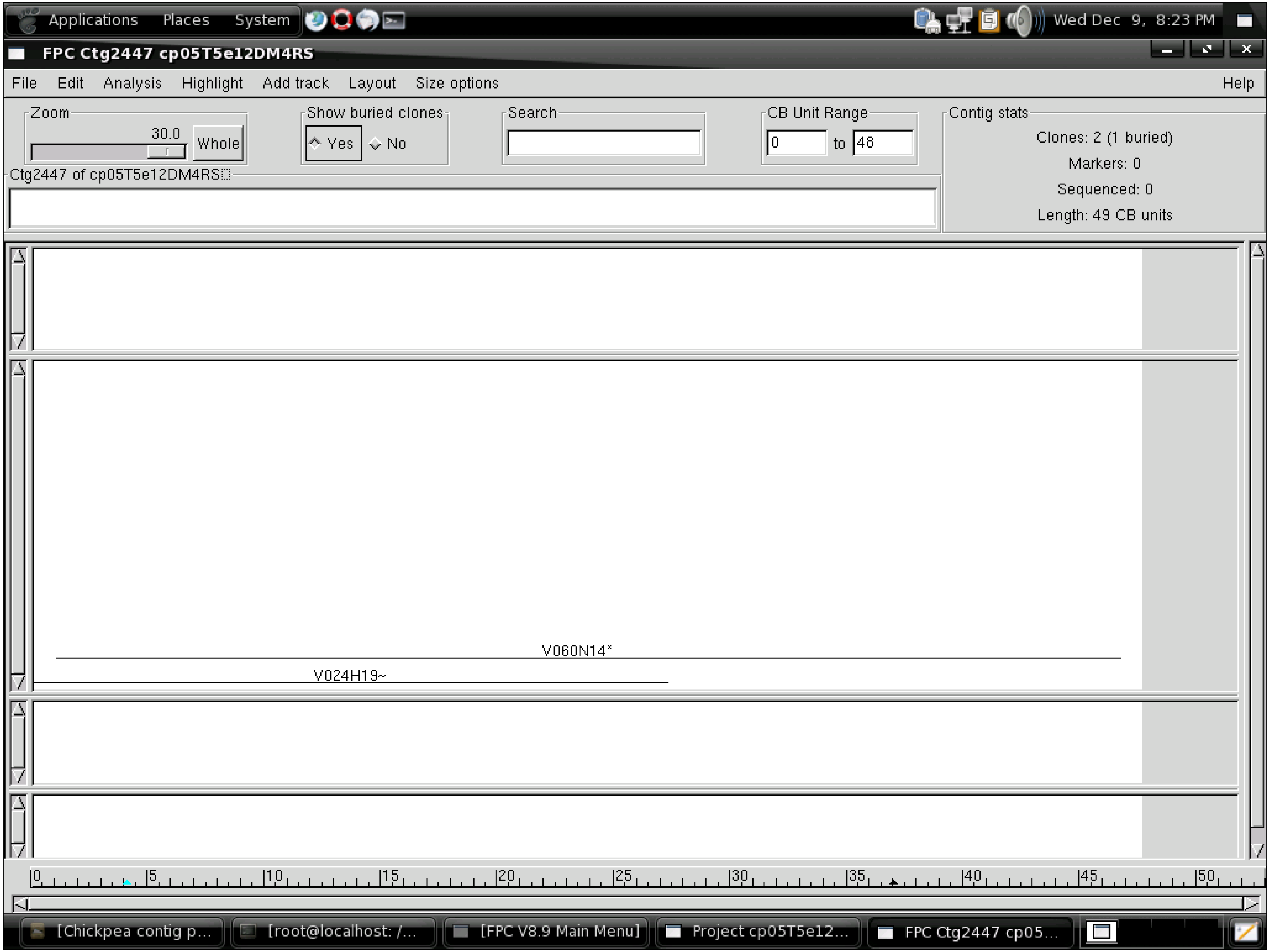
Applications Places System
Wed Dec 9, 8:23 PM
FPC Ctg2447 cp05T5e12DM4RS
File Edit Analysis Highlight Add track Layout Size options Help
Zoom
30.0
Whole
Show buried clones
Yes
No
Search
CB Unit Range
0 to 48
Contig stats
Clones: 2 (1 buried)
Markers: 0
Sequenced: 0
Length: 49 CB units
Ctg2447 of cp05T5e12DM4RS
V060N14*
V024H19~
0 5 10 15 20 25 30 35 40 45 50
[Chickpea contig p...
[root@localhost: /...
[FPC V8.9 Main Menu]
Project cp05T5e12...
FPC Ctg2447 cp05...

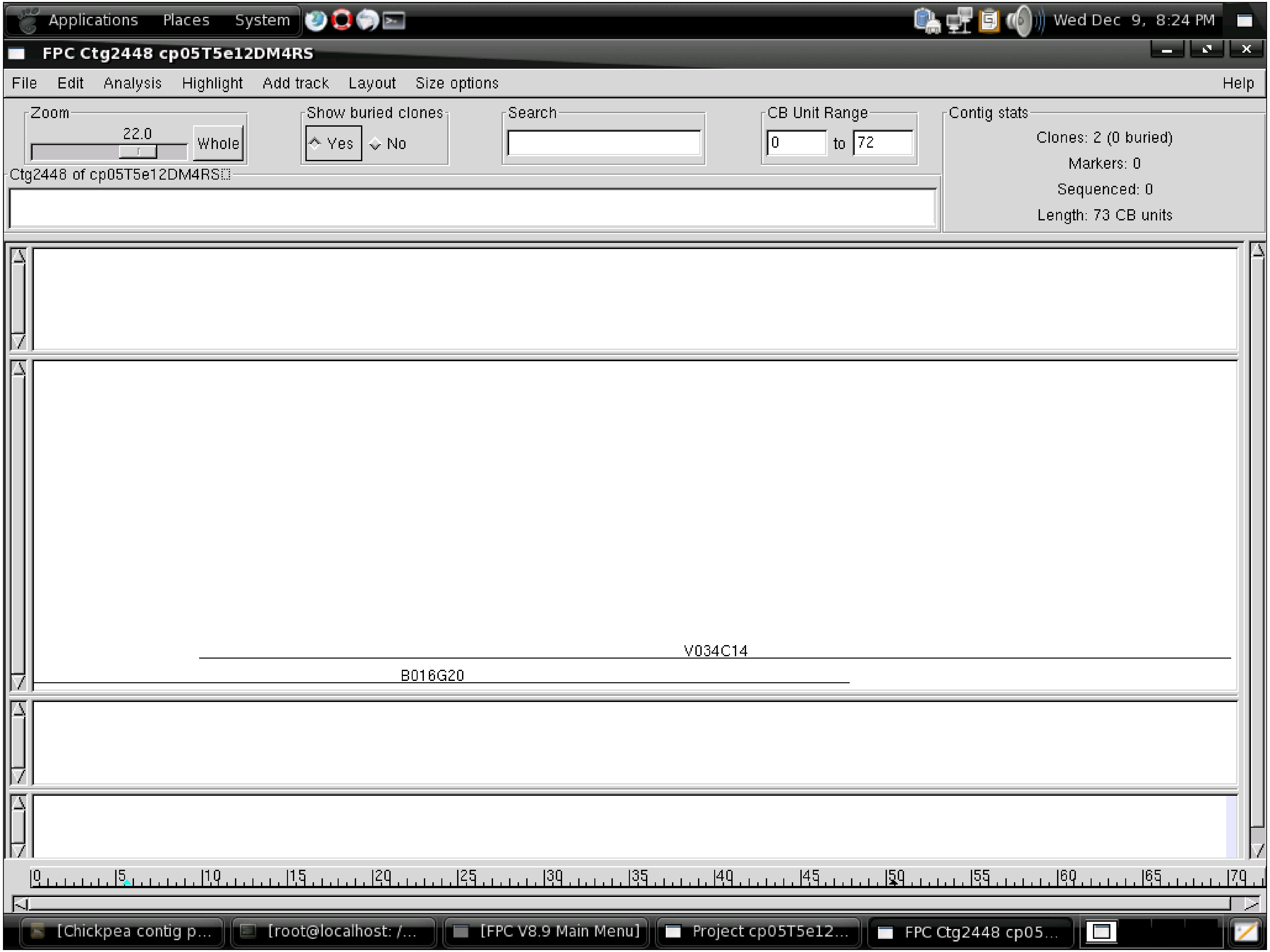

Applications Places System
Wed Dec 9, 8:24 PM
FPC Ctg2448 cp05T5e12DM4RS
File Edit Analysis Highlight Add track Layout Size options Help
Zoom
22.0
Whole
Show buried clones
Yes
No
Search
CB Unit Range
0
to
72
Contig stats
Clones: 2 (0 buried)
Markers: 0
Sequenced: 0
Length: 73 CB units
Ctg2448 of cp05T5e12DM4RS
V034C14
B016G20
0 5 10 15 20 25 30 35 40 45 50 55 60 65 70
[Chickpea contig p...
[root@localhost: /...
[FPC V8.9 Main Menu]
Project cp05T5e12...
FPC Ctg2448 cp05...

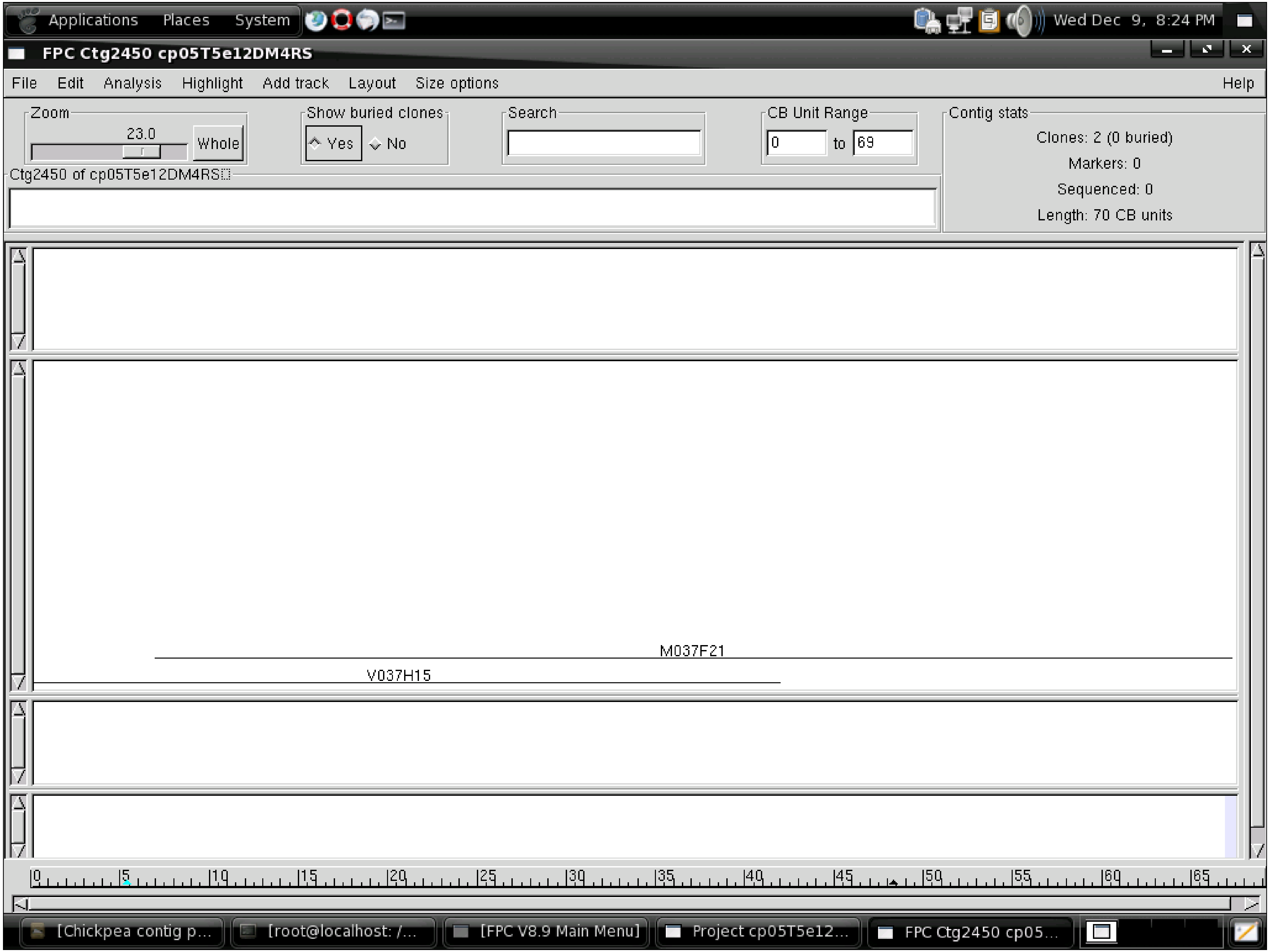
Applications Places System
Wed Dec 9, 8:24 PM
FPC Ctg2450 cp05T5e12DM4RS
File Edit Analysis Highlight Add track Layout Size options Help
Zoom
23.0
Whole
Show buried clones
Yes
No
Search
CB Unit Range
0 to 69
Contig stats
Clones: 2 (0 buried)
Markers: 0
Sequenced: 0
Length: 70 CB units
Ctg2450 of cp05T5e12DM4RS
M037F21
V037H15
0 5 10 15 20 25 30 35 40 45 50 55 60 65
[Chickpea contig p...
[root@localhost: /...
[FPC V8.9 Main Menu]
Project cp05T5e12...
FPC Ctg2450 cp05...

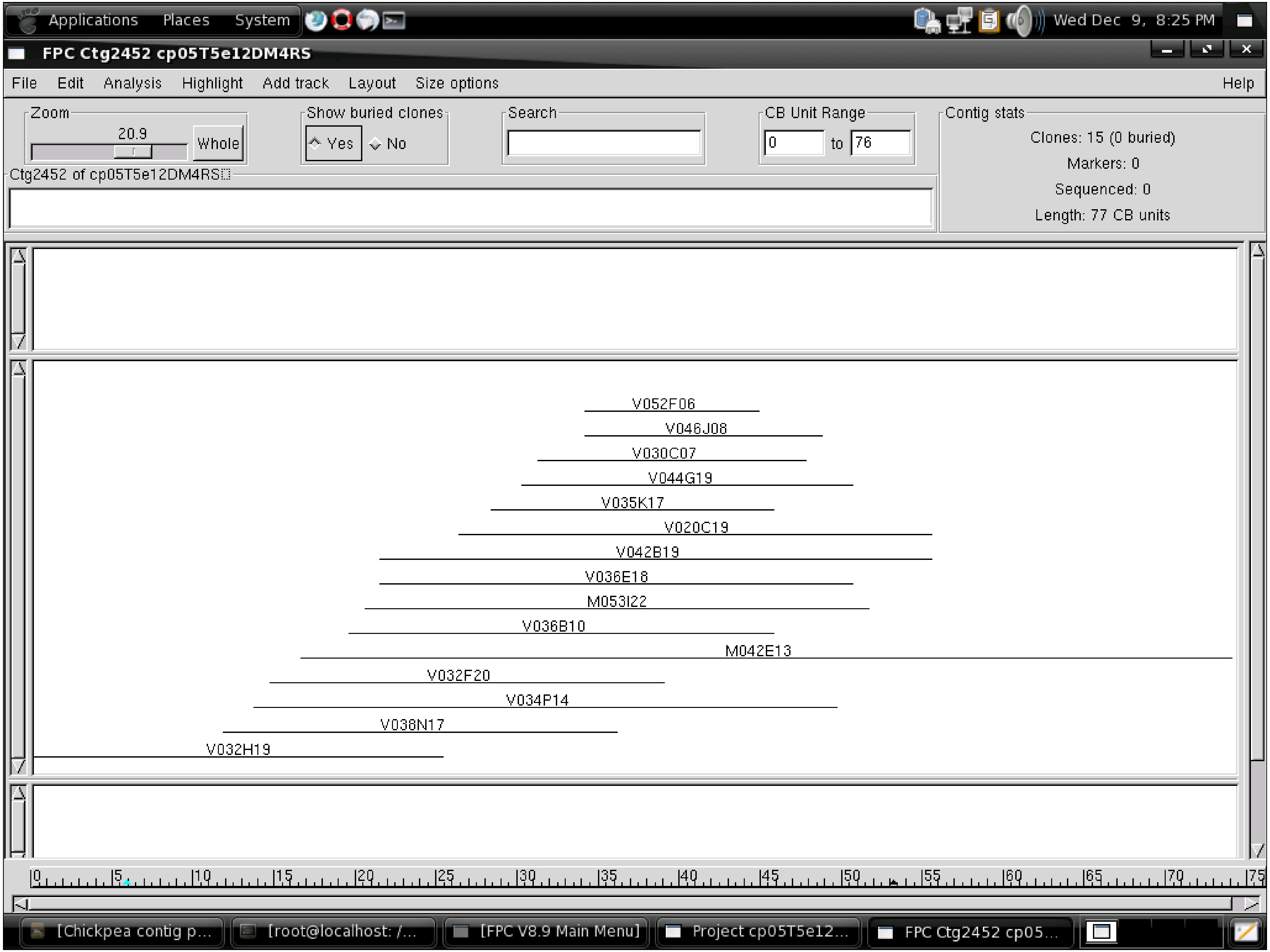
Applications Places System
Wed Dec 9, 8:25 PM
FPC Ctg2452 cp05T5e12DM4RS
File Edit Analysis Highlight Add track Layout Size options Help
Zoom
20.9
Whole
Show buried clones
Yes
No
Search
CB Unit Range
0 to 76
Contig stats
Clones: 15 (0 buried)
Markers: 0
Sequenced: 0
Length: 77 CB units
Ctg2452 of cp05T5e12DM4RS
V052F06
V046J08
V030C07
V044G19
V035K17
V020C19
V042B19
V036E18
M053I22
V036B10
M042E13
V032F20
V034P14
V038N17
V032H19
0 5 10 15 20 25 30 35 40 45 50 55 60 65 70 75
[Chickpea contig p...
[root@localhost: /...
[FPC V8.9 Main Menu]
Project cp05T5e12...
FPC Ctg2452 cp05...

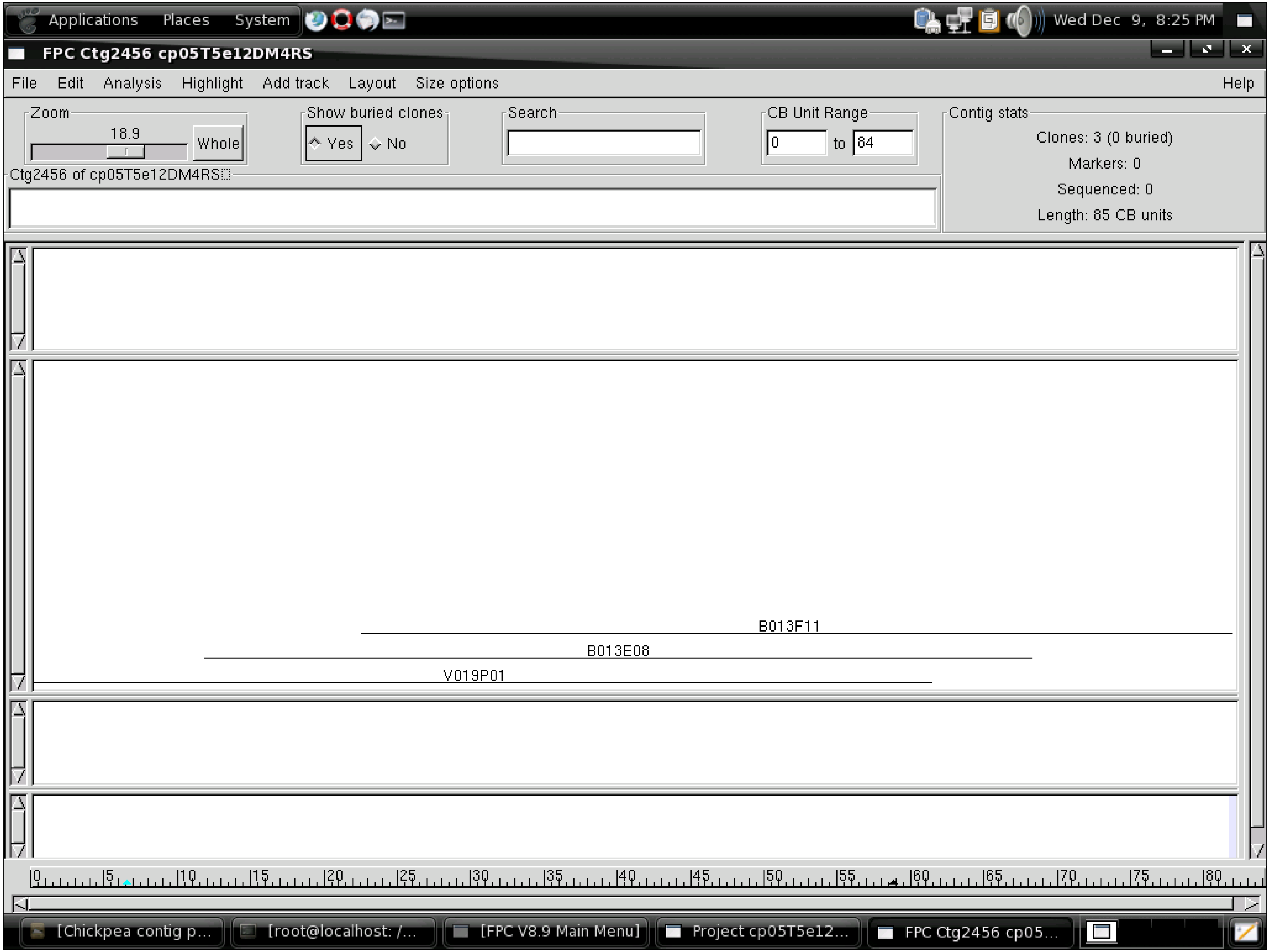
Applications Places System
Wed Dec 9, 8:25 PM
FPC Ctg2456 cp05T5e12DM4RS
File Edit Analysis Highlight Add track Layout Size options Help
Zoom
18.9
Whole
Show buried clones
Yes No
Search
CB Unit Range
0 to 84
Contig stats
Clones: 3 (0 buried)
Markers: 0
Sequenced: 0
Length: 85 CB units
Ctg2456 of cp05T5e12DM4RS
B013F11
B013E08
V019P01
0 5 10 15 20 25 30 35 40 45 50 55 60 65 70 75 80
[Chickpea contig p...
[root@localhost: /...
[FPC V8.9 Main Menu]
Project cp05T5e12...
FPC Ctg2456 cp05...

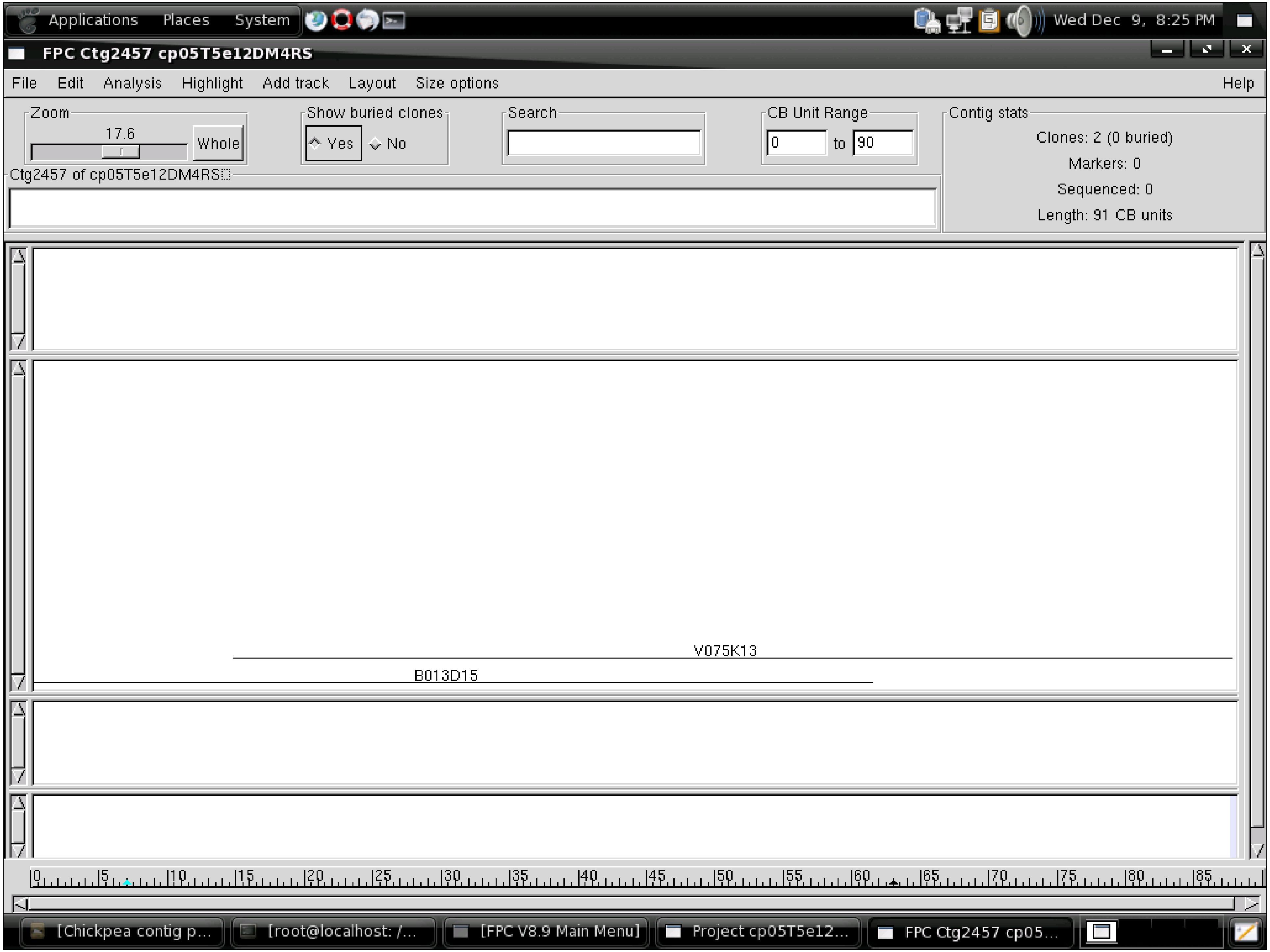
Applications Places System
Wed Dec 9, 8:25 PM
FPC Ctg2457 cp05T5e12DM4RS
File Edit Analysis Highlight Add track Layout Size options Help
Zoom
17.6
Whole
Show buried clones
Yes
No
Search
CB Unit Range
0
to
90
Contig stats
Clones: 2 (0 buried)
Markers: 0
Sequenced: 0
Length: 91 CB units
Ctg2457 of cp05T5e12DM4RS
V075K13
B013D15
0 5 10 15 20 25 30 35 40 45 50 55 60 65 70 75 80 85
[Chickpea contig p...
[root@localhost: /...
[FPC V8.9 Main Menu]
Project cp05T5e12...
FPC Ctg2457 cp05...

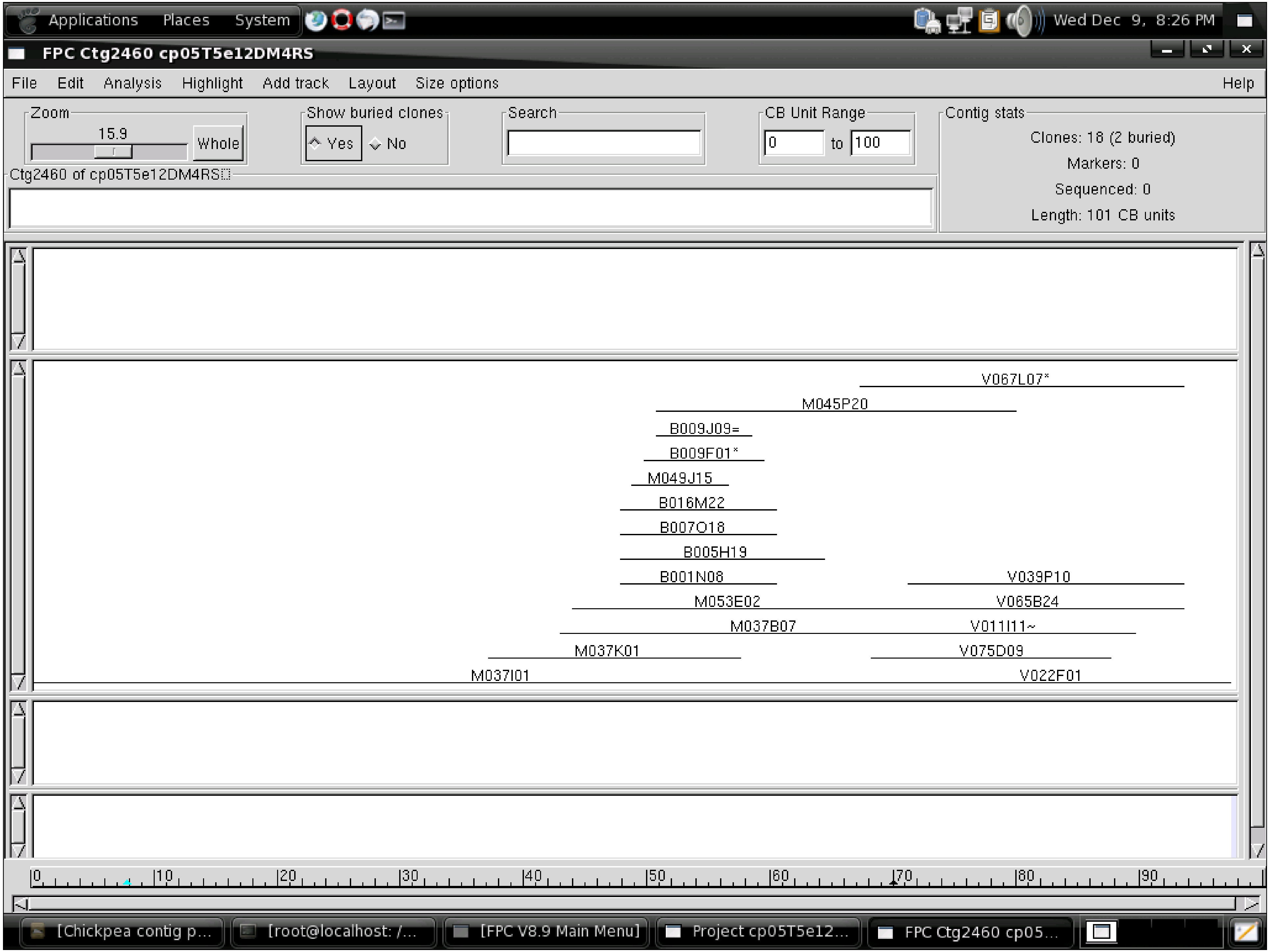
Applications Places System
Wed Dec 9, 8:26 PM
FPC Ctg2460 cp05T5e12DM4RS
File Edit Analysis Highlight Add track Layout Size options Help
Zoom
15.9
Whole
Show buried clones
Yes
No
Search
CB Unit Range
0 to 100
Contig stats
Clones: 18 (2 buried)
Markers: 0
Sequenced: 0
Length: 101 CB units
Ctg2460 of cp05T5e12DM4RS
V067L07*
M045P20
B009J09=
B009F01*
M049J15
B016M22
B007O18
B005H19
B001N08
V039P10
M053E02
V065B24
M037B07
V011I11~
M037K01
V075D09
M037I01
V022F01
0 10 20 30 40 50 60 70 80 90
[Chickpea contig p...
[root@localhost: /...
[FPC V8.9 Main Menu]
Project cp05T5e12...
FPC Ctg2460 cp05...

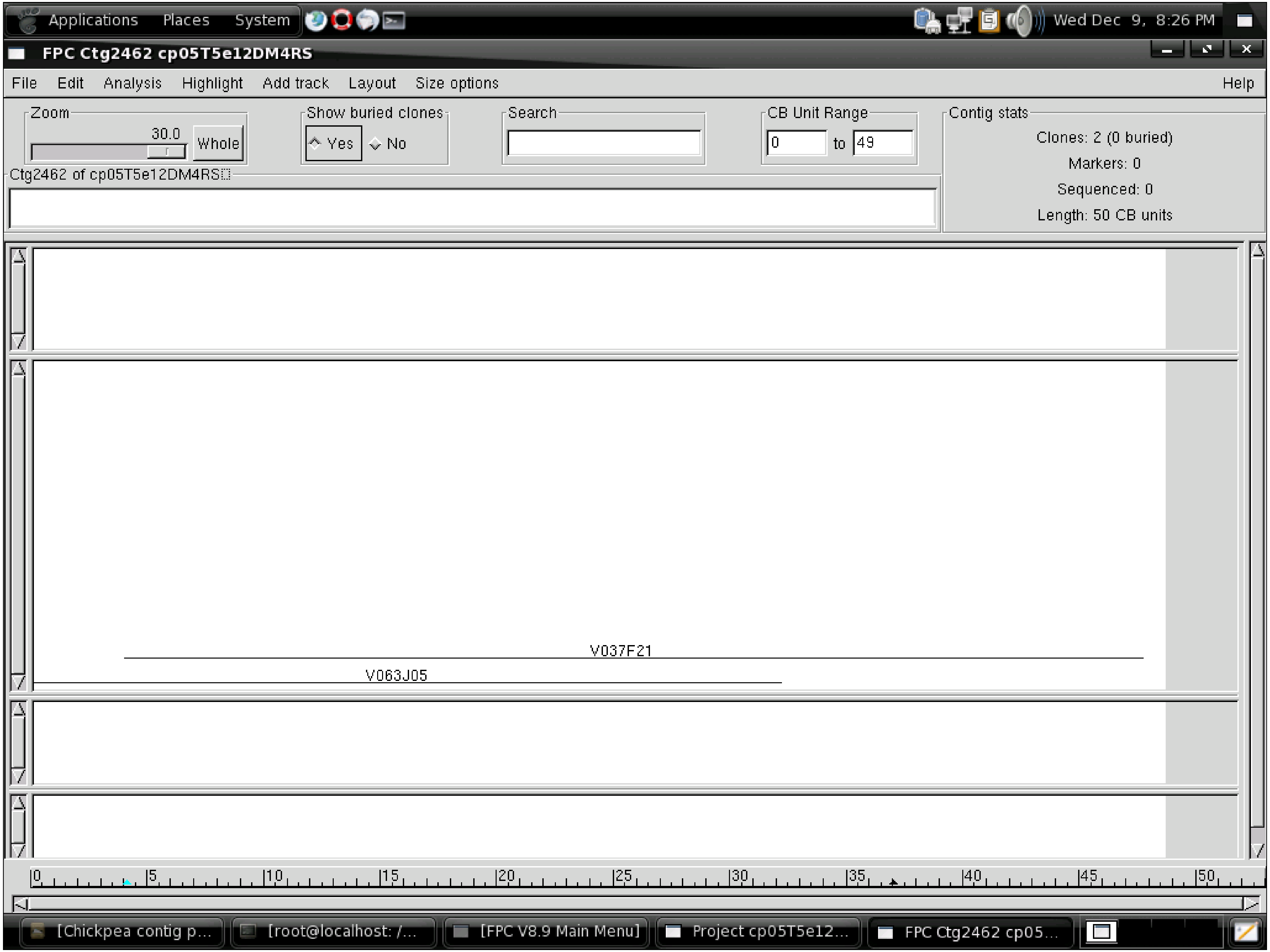
Applications Places System
Wed Dec 9, 8:26 PM
FPC Ctg2462 cp05T5e12DM4RS
File Edit Analysis Highlight Add track Layout Size options Help
Zoom
30.0
Whole
Show buried clones
Yes
No
Search
CB Unit Range
0 to 49
Contig stats
Clones: 2 (0 buried)
Markers: 0
Sequenced: 0
Length: 50 CB units
Ctg2462 of cp05T5e12DM4RS
V037F21
V063J05
0 5 10 15 20 25 30 35 40 45 50
[Chickpea contig p...
[root@localhost: /...
[FPC V8.9 Main Menu]
Project cp05T5e12...
FPC Ctg2462 cp05...

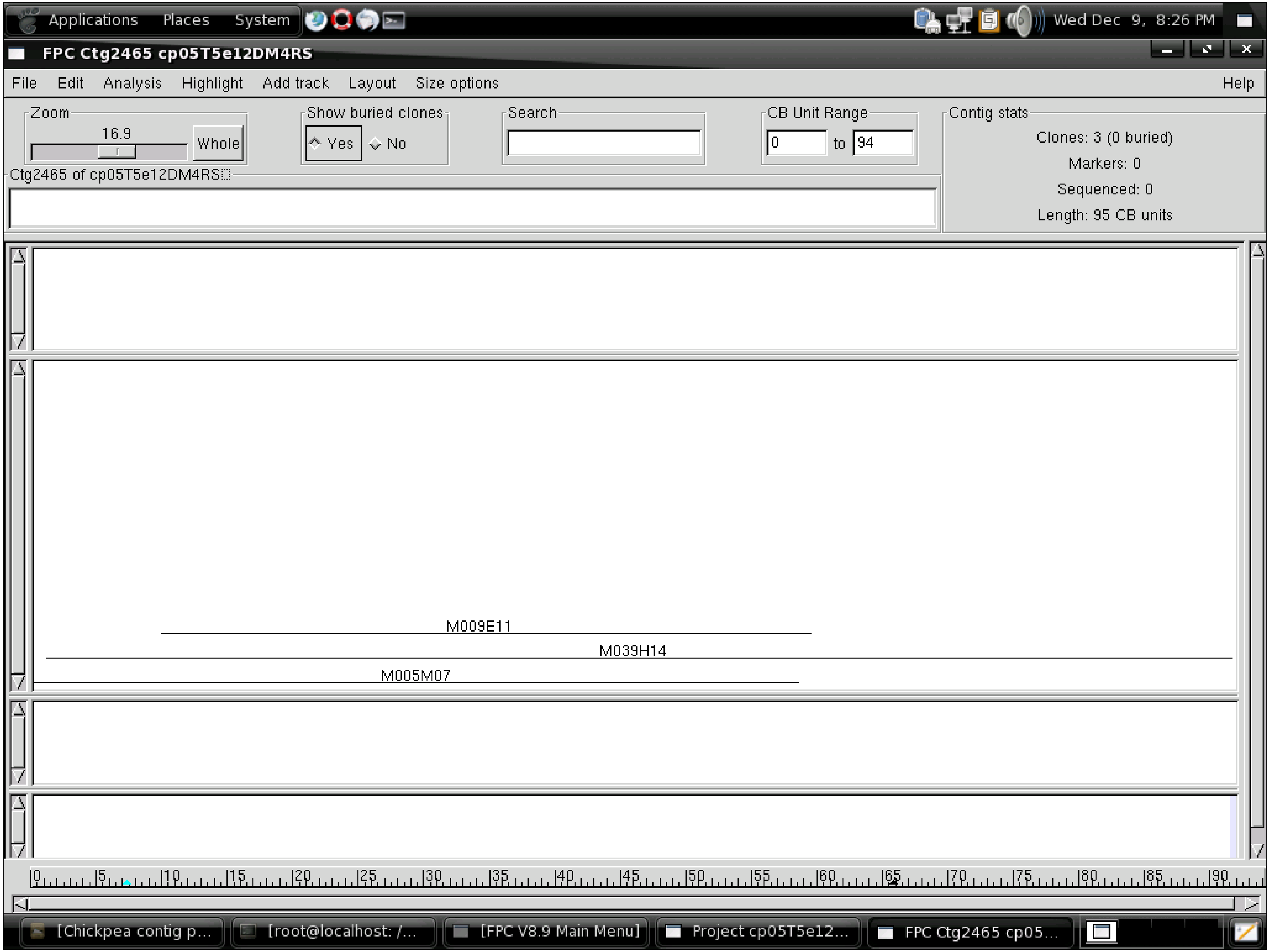
Applications Places System
Wed Dec 9, 8:26 PM
FPC Ctg2465 cp05T5e12DM4RS
File Edit Analysis Highlight Add track Layout Size options Help
Zoom
16.9
Whole
Show buried clones
Yes
No
Search
CB Unit Range
0
to
94
Contig stats
Clones: 3 (0 buried)
Markers: 0
Sequenced: 0
Length: 95 CB units
Ctg2465 of cp05T5e12DM4RS
M009E11
M039H14
M005M07
0 5 10 15 20 25 30 35 40 45 50 55 60 65 70 75 80 85 90
[Chickpea contig p...
[root@localhost: /...
[FPC V8.9 Main Menu]
Project cp05T5e12...
FPC Ctg2465 cp05...

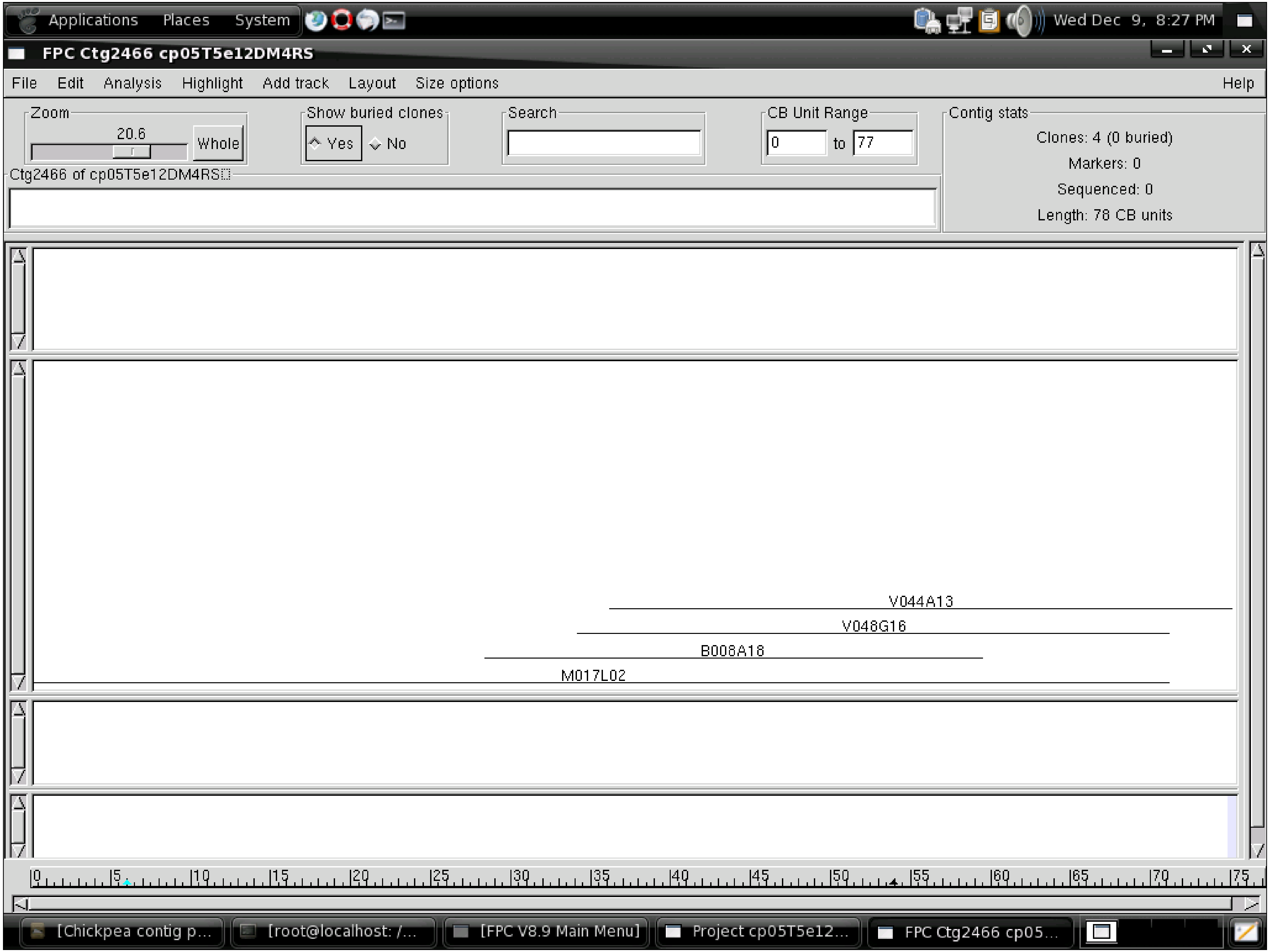
Applications Places System
Wed Dec 9, 8:27 PM
FPC Ctg2466 cp05T5e12DM4RS
File Edit Analysis Highlight Add track Layout Size options Help
Zoom
20.6
Whole
Show buried clones
Yes
No
Search
CB Unit Range
0
to
77
Contig stats
Clones: 4 (0 buried)
Markers: 0
Sequenced: 0
Length: 78 CB units
Ctg2466 of cp05T5e12DM4RS
V044A13
V048G16
B008A18
M017L02
0 5 10 15 20 25 30 35 40 45 50 55 60 65 70 75
[Chickpea contig p...
[root@localhost: /...
[FPC V8.9 Main Menu]
Project cp05T5e12...
FPC Ctg2466 cp05...

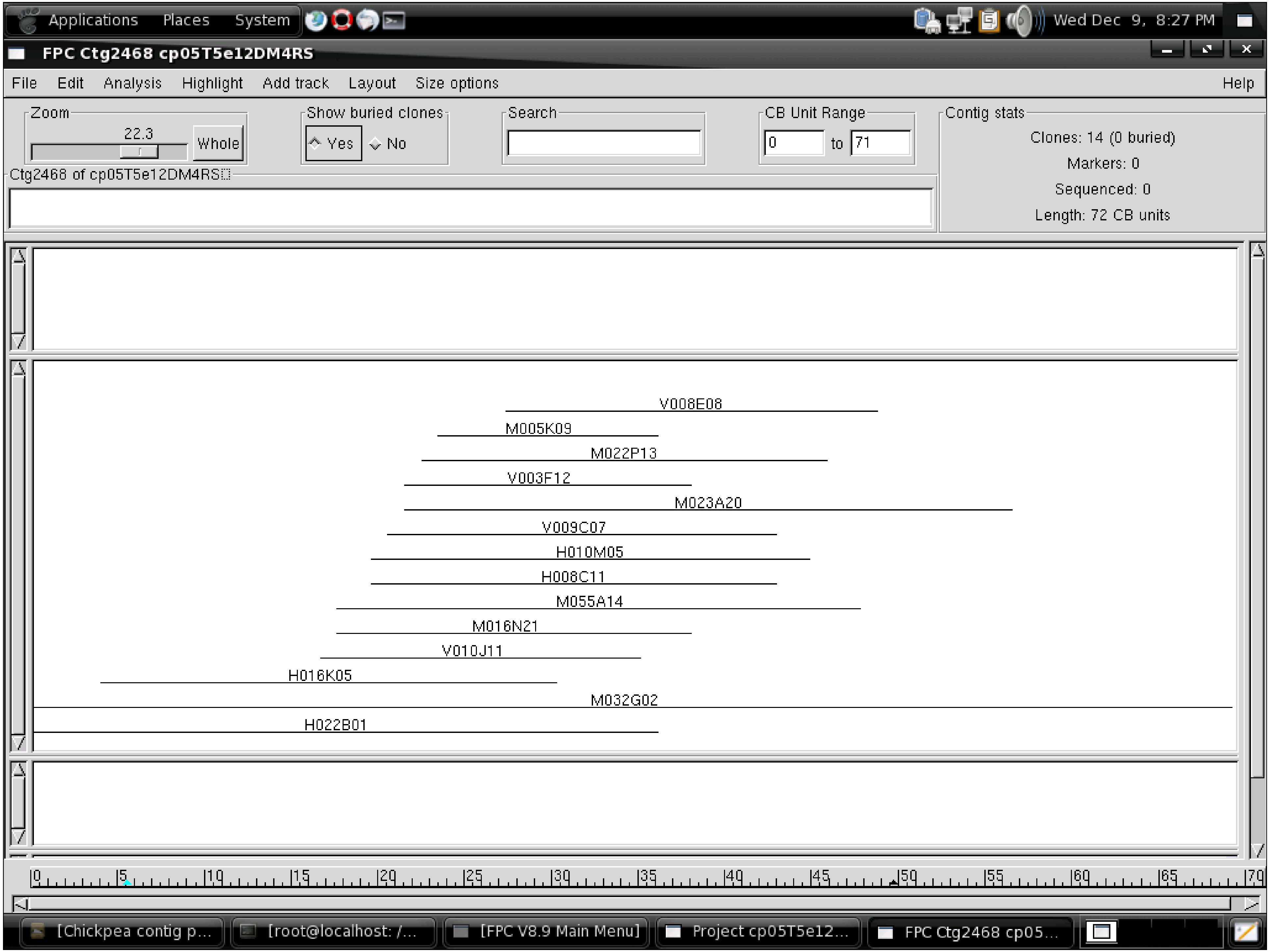
Applications Places System
Wed Dec 9, 8:27 PM
FPC Ctg2468 cp05T5e12DM4RS
File Edit Analysis Highlight Add track Layout Size options Help
Zoom
22.3
Whole
Show buried clones
Yes No
Search
CB Unit Range
0 to 71
Contig stats
Clones: 14 (0 buried)
Markers: 0
Sequenced: 0
Length: 72 CB units
Ctg2468 of cp05T5e12DM4RS
V008E08
M005K09
M022P13
V003F12
M023A20
V009C07
H010M05
H008C11
M055A14
M016N21
V010J11
H016K05
M032G02
H022B01
0 5 10 15 20 25 30 35 40 45 50 55 60 65 70
[Chickpea contig p...
[root@localhost: /...
[FPC V8.9 Main Menu]
Project cp05T5e12...
FPC Ctg2468 cp05...

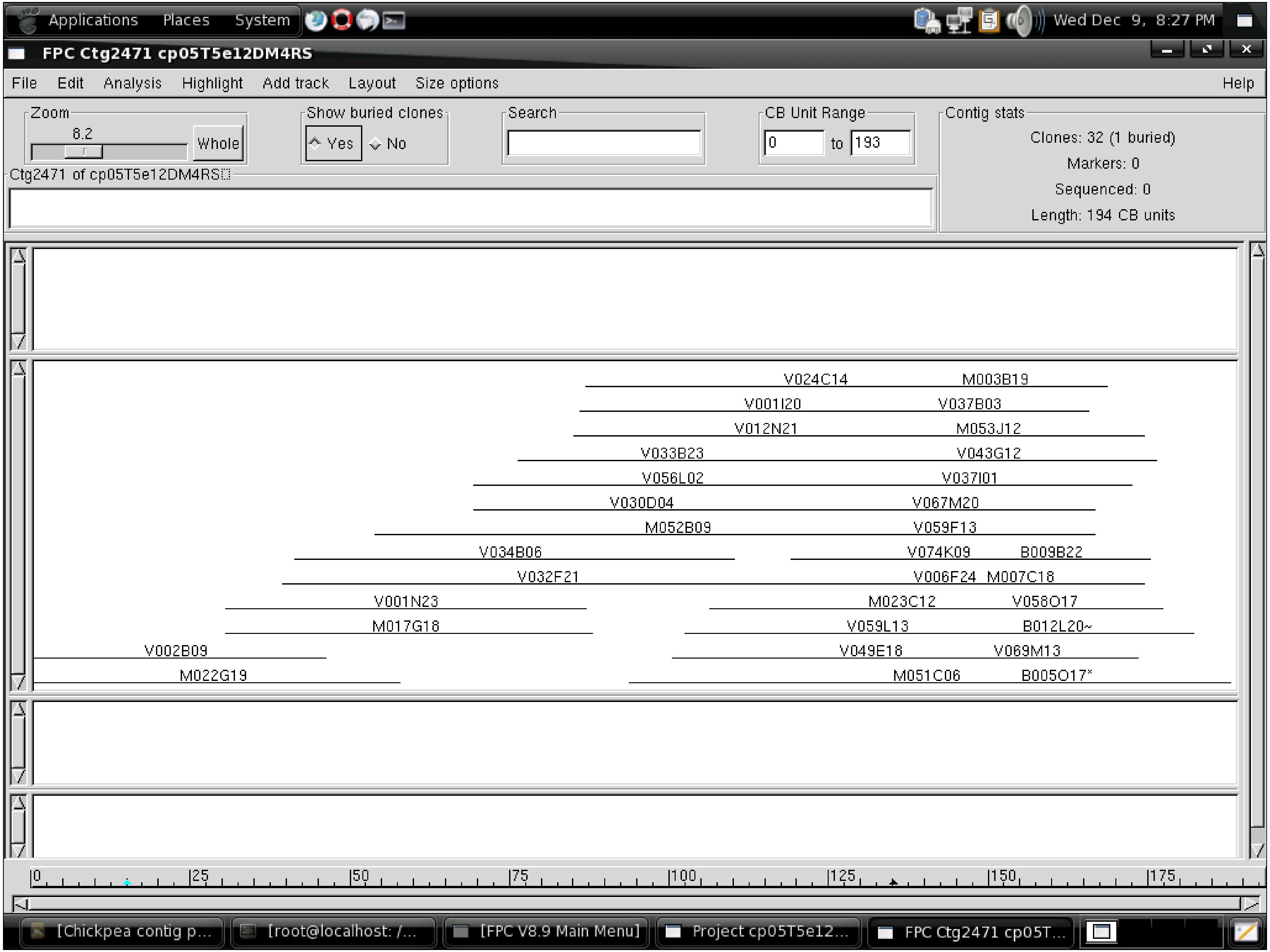
Applications Places System
Wed Dec 9, 8:27 PM
FPC Ctg2471 cp05T5e12DM4RS
File Edit Analysis Highlight Add track Layout Size options Help
Zoom
8.2
Whole
Show buried clones
Yes
No
Search
CB Unit Range
0 to 193
Contig stats
Clones: 32 (1 buried)
Markers: 0
Sequenced: 0
Length: 194 CB units
Ctg2471 of cp05T5e12DM4RS
V024C14
M003B19
V001I20
V037B03
V012N21
M053J12
V033B23
V043G12
V056L02
V037I01
V030D04
V067M20
M052B09
V059F13
V034B06
V074K09
B009B22
V032F21
V006F24
M007C18
V001N23
M023C12
V058O17
M017G18
V059L13
B012L20~
V002B09
V049E18
V069M13
M022G19
M051C06
B005O17*
0 25 50 75 100 125 150 175
[Chickpea contig p...
[root@localhost: /...
[FPC V8.9 Main Menu]
Project cp05T5e12...
FPC Ctg2471 cp05T...

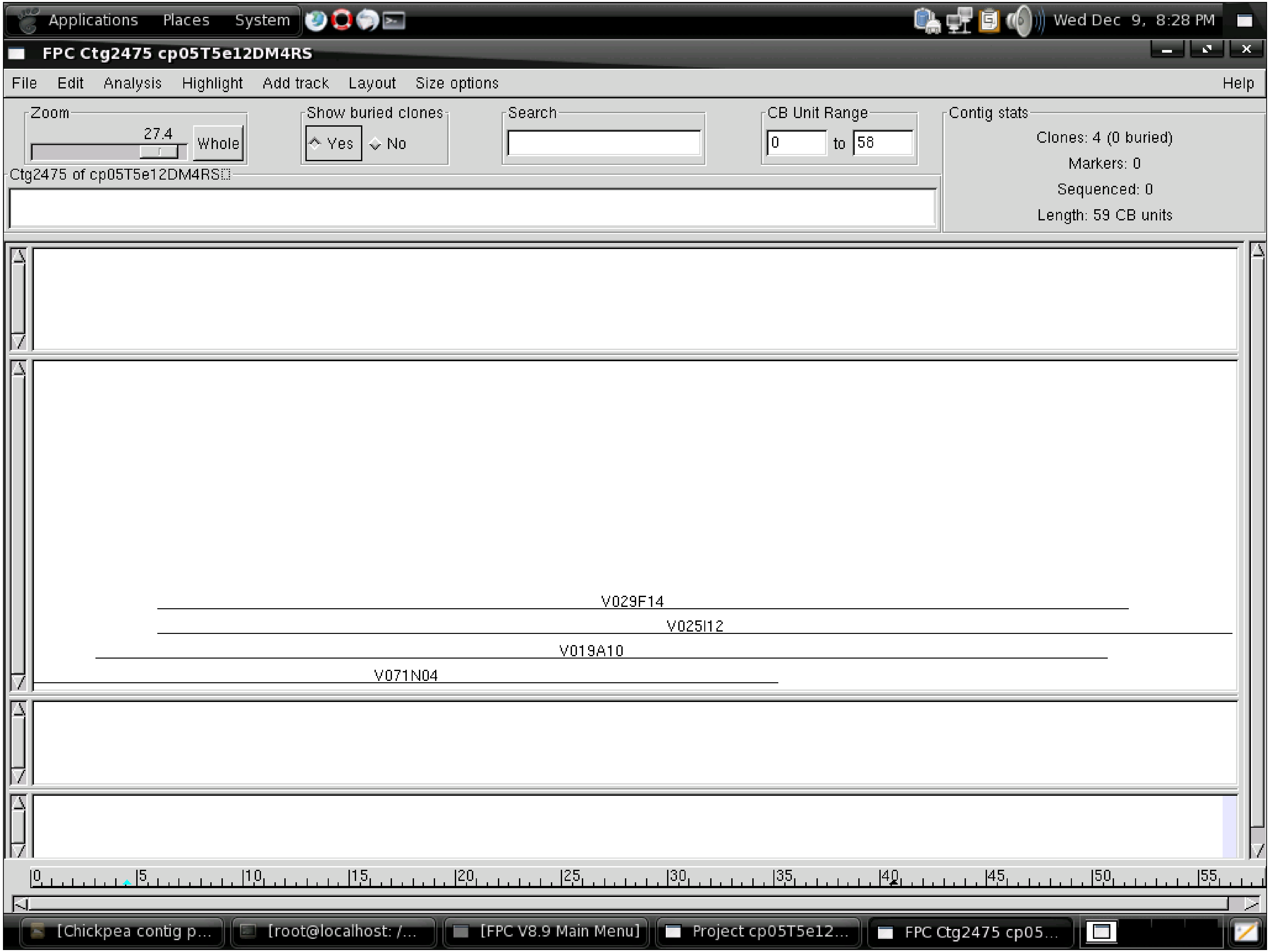
Applications Places System
Wed Dec 9, 8:28 PM
FPC Ctg2475 cp05T5e12DM4RS
File Edit Analysis Highlight Add track Layout Size options Help
Zoom
27.4
Whole
Show buried clones
Yes No
Search
CB Unit Range
0 to 58
Contig stats
Clones: 4 (0 buried)
Markers: 0
Sequenced: 0
Length: 59 CB units
Ctg2475 of cp05T5e12DM4RS
V029F14
V025I12
V019A10
V071N04
0 5 10 15 20 25 30 35 40 45 50 55
[Chickpea contig p...
[root@localhost: /...
[FPC V8.9 Main Menu]
Project cp05T5e12...
FPC Ctg2475 cp05...

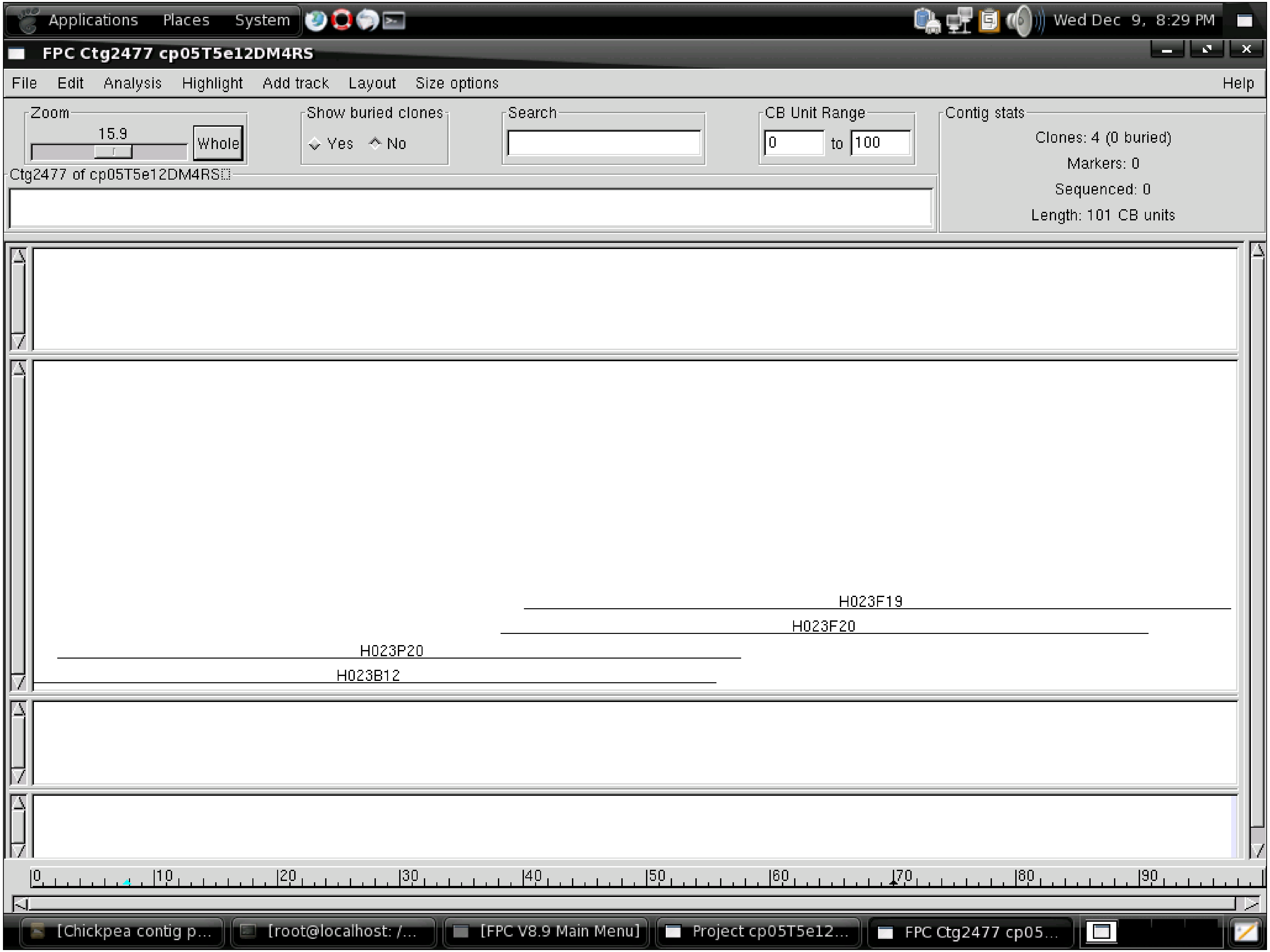

Applications Places System
Wed Dec 9, 8:29 PM
FPC Ctg2477 cp05T5e12DM4RS
File Edit Analysis Highlight Add track Layout Size options Help
Zoom
15.9
Whole
Show buried clones
Yes No
Search
CB Unit Range
0 to 100
Contig stats
Clones: 4 (0 buried)
Markers: 0
Sequenced: 0
Length: 101 CB units
Ctg2477 of cp05T5e12DM4RS
H023F19
H023F20
H023P20
H023B12
0 10 20 30 40 50 60 70 80 90
[Chickpea contig p...
[root@localhost: /...
[FPC V8.9 Main Menu]
Project cp05T5e12...
FPC Ctg2477 cp05...

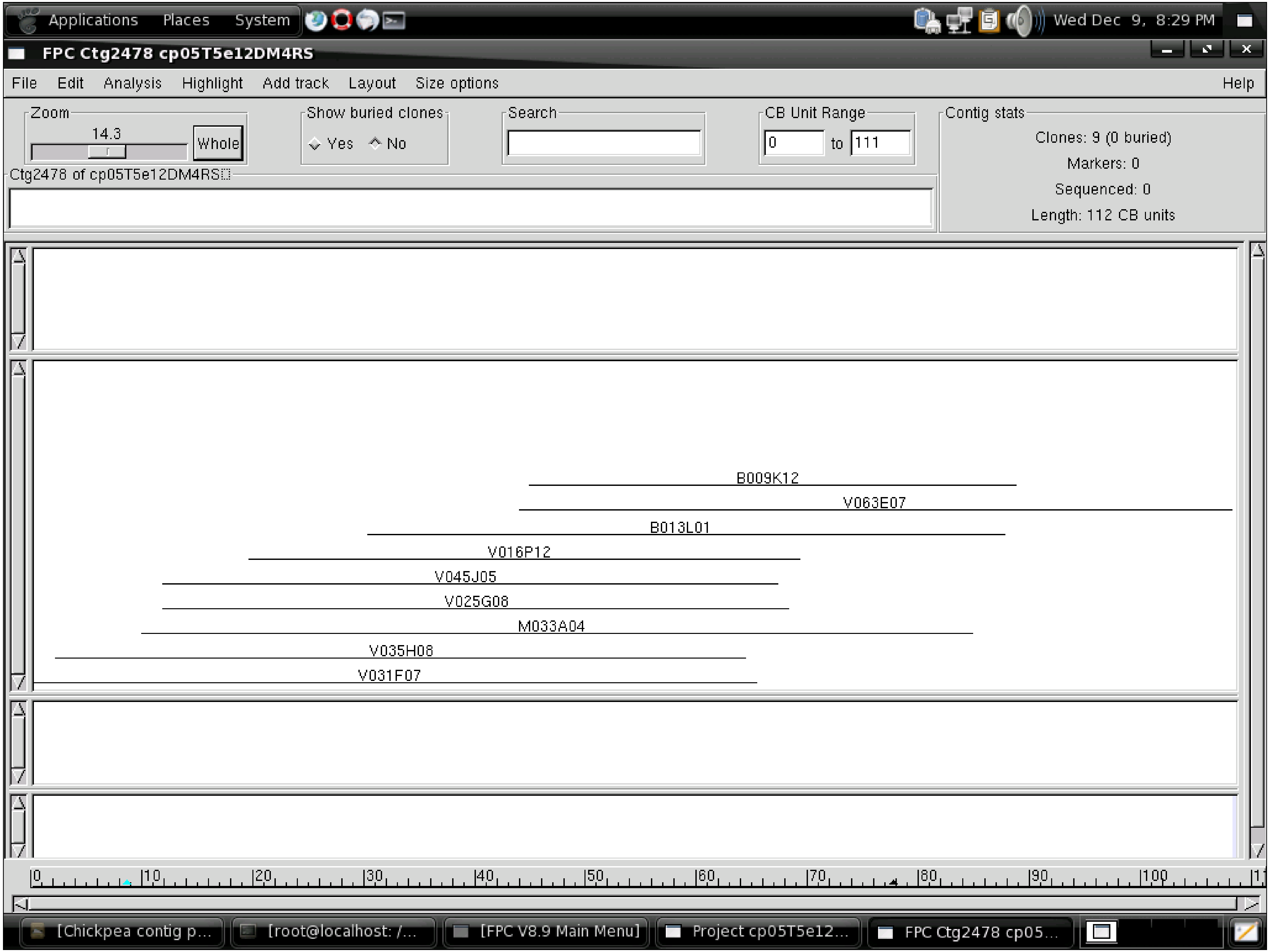
Applications Places System
Wed Dec 9, 8:29 PM
FPC Ctg2478 cp05T5e12DM4RS
File Edit Analysis Highlight Add track Layout Size options Help
Zoom
14.3
Whole
Show buried clones
Yes No
Search
CB Unit Range
0 to 111
Contig stats
Clones: 9 (0 buried)
Markers: 0
Sequenced: 0
Length: 112 CB units
Ctg2478 of cp05T5e12DM4RS
B009K12
V063E07
B013L01
V016P12
V045J05
V025G08
M033A04
V035H08
V031F07
0 10 20 30 40 50 60 70 80 90 100
[Chickpea contig p...
[root@localhost: /...
[FPC V8.9 Main Menu]
Project cp05T5e12...
FPC Ctg2478 cp05...

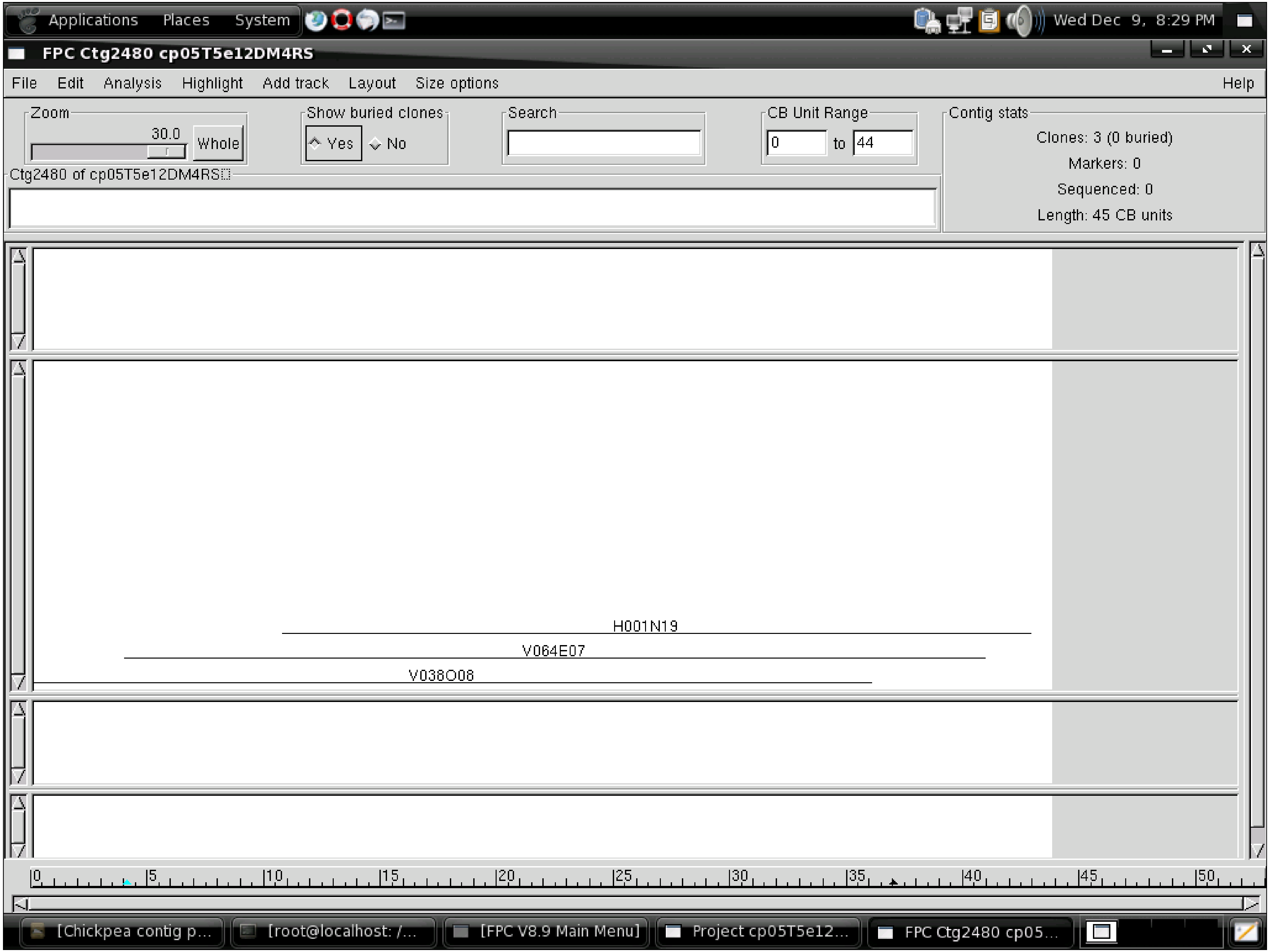
Applications Places System
Wed Dec 9, 8:29 PM
FPC Ctg2480 cp05T5e12DM4RS
File Edit Analysis Highlight Add track Layout Size options
Help
Zoom
30.0
Whole
Show buried clones
Yes
No
Search
CB Unit Range
0
to
44
Contig stats
Clones: 3 (0 buried)
Markers: 0
Sequenced: 0
Length: 45 CB units
Ctg2480 of cp05T5e12DM4RS
H001N19
V064E07
V038O08
0 5 10 15 20 25 30 35 40 45 50
[Chickpea contig p...
[root@localhost: /...
[FPC V8.9 Main Menu]
Project cp05T5e12...
FPC Ctg2480 cp05...

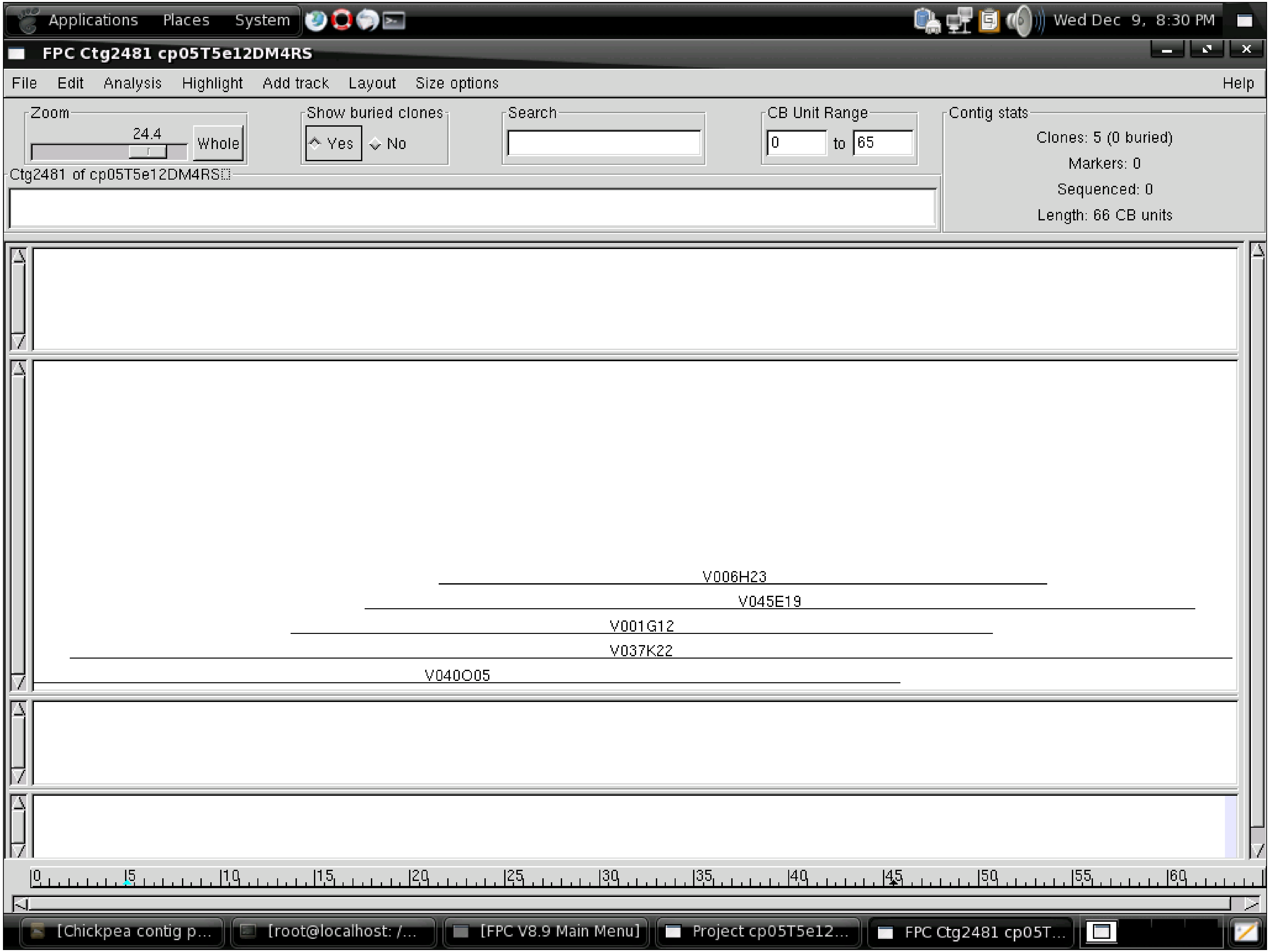
Applications Places System
Wed Dec 9, 8:30 PM
FPC Ctg2481 cp05T5e12DM4RS
File Edit Analysis Highlight Add track Layout Size options Help
Zoom
24.4
Whole
Show buried clones
Yes No
Search
CB Unit Range
0 to 65
Contig stats
Clones: 5 (0 buried)
Markers: 0
Sequenced: 0
Length: 66 CB units
Ctg2481 of cp05T5e12DM4RS
V006H23
V045E19
V001G12
V037K22
V040O05
0 5 10 15 20 25 30 35 40 45 50 55 60
[Chickpea contig p...
[root@localhost: /...
[FPC V8.9 Main Menu]
Project cp05T5e12...
FPC Ctg2481 cp05T...

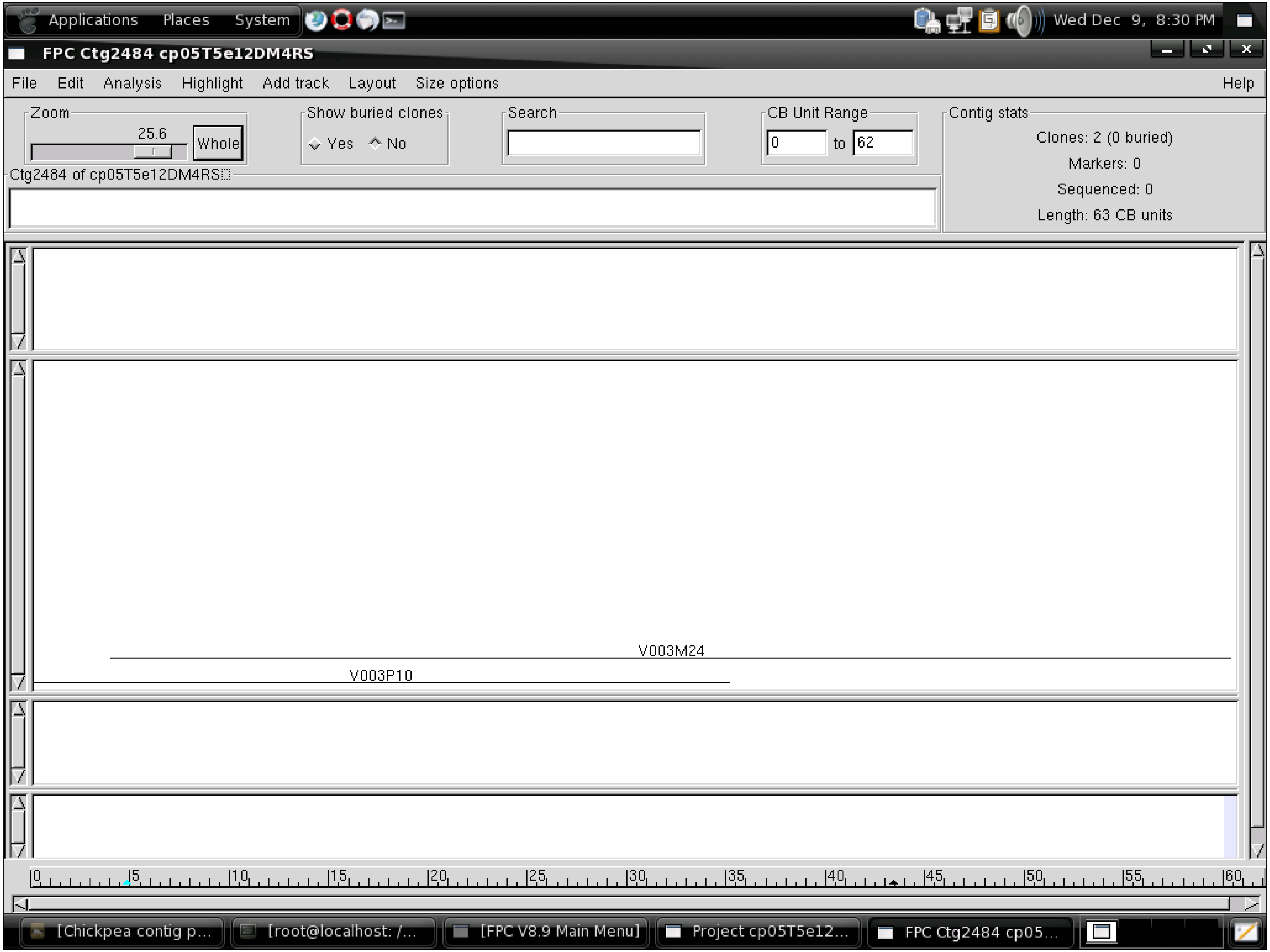
Applications
Places
System
Wed Dec 9, 8:30 PM
FPC Ctg2484 cp05T5e12DM4RS
File
Edit
Analysis
Highlight
Add track
Layout
Size options
Help
Zoom
25.6
Whole
Show buried clones
Yes
No
Search
CB Unit Range
0
to
62
Contig stats
Clones: 2 (0 buried)
Markers: 0
Sequenced: 0
Length: 63 CB units
Ctg2484 of cp05T5e12DM4RS
V003M24
V003P10
0 5 10 15 20 25 30 35 40 45 50 55 60
[Chickpea contig p...
[root@localhost: /...
[FPC V8.9 Main Menu]
Project cp05T5e12...
FPC Ctg2484 cp05...

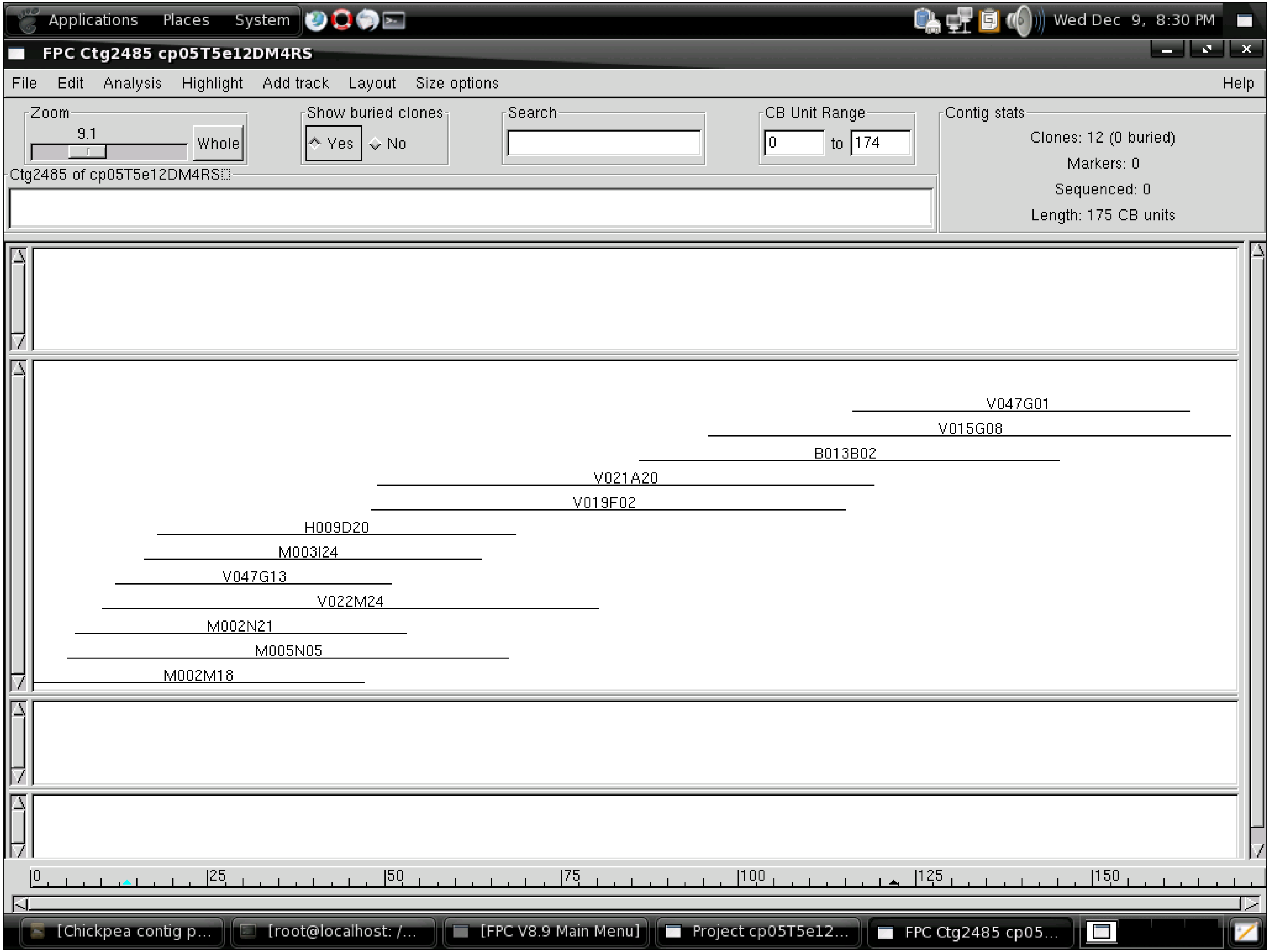

Applications Places System
Wed Dec 9, 8:30 PM
FPC Ctg2485 cp05T5e12DM4RS
File Edit Analysis Highlight Add track Layout Size options Help
Zoom
9.1
Whole
Show buried clones
Yes
No
Search
CB Unit Range
0 to 174
Contig stats
Clones: 12 (0 buried)
Markers: 0
Sequenced: 0
Length: 175 CB units
Ctg2485 of cp05T5e12DM4RS
V047G01
V015G08
B013B02
V021A20
V019F02
H009D20
M003I24
V047G13
V022M24
M002N21
M005N05
M002M18
0 25 50 75 100 125 150
[Chickpea contig p...
[root@localhost: /...
[FPC V8.9 Main Menu]
Project cp05T5e12...
FPC Ctg2485 cp05...

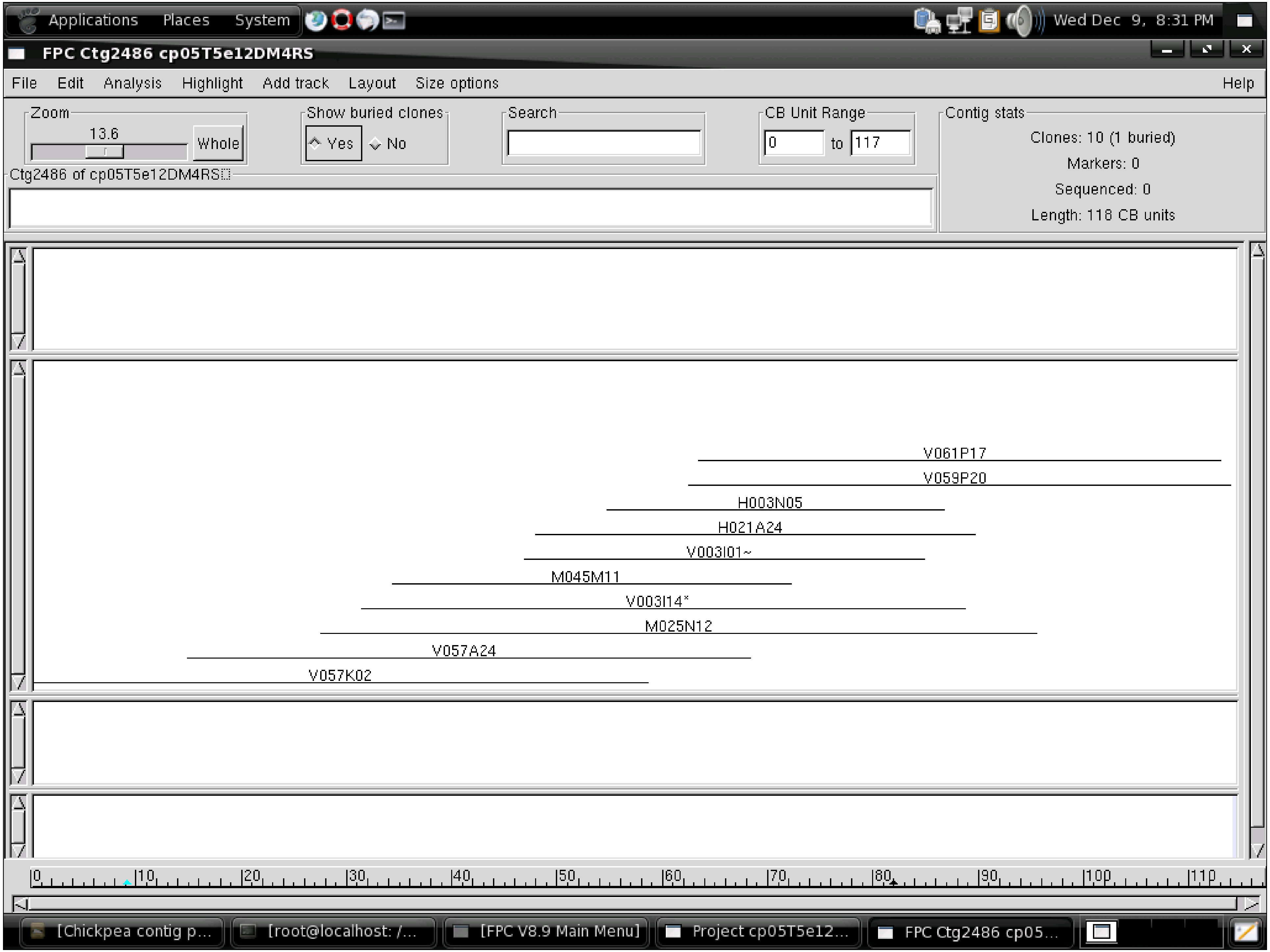
Applications Places System
Wed Dec 9, 8:31 PM
FPC Ctg2486 cp05T5e12DM4RS
File Edit Analysis Highlight Add track Layout Size options Help
Zoom
13.6
Whole
Show buried clones
Yes
No
Search
CB Unit Range
0 to 117
Contig stats
Clones: 10 (1 buried)
Markers: 0
Sequenced: 0
Length: 118 CB units
Ctg2486 of cp05T5e12DM4RS
V061P17
V059P20
H003N05
H021A24
V003I01~
M045M11
V003I14*
M025N12
V057A24
V057K02
0 10 20 30 40 50 60 70 80 90 100 110
[Chickpea contig p...
[root@localhost: /...
[FPC V8.9 Main Menu]
Project cp05T5e12...
FPC Ctg2486 cp05...

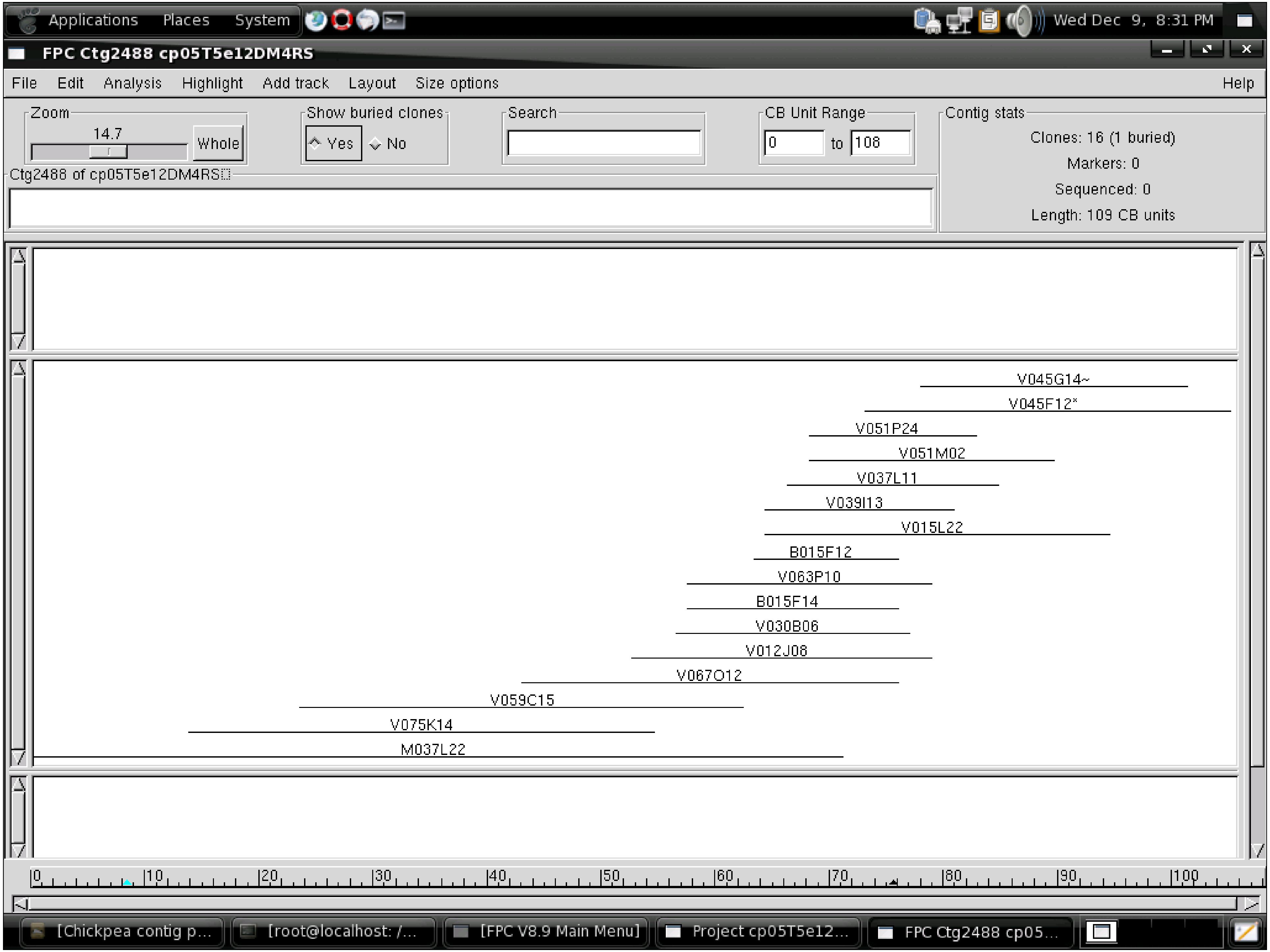
Applications Places System
Wed Dec 9, 8:31 PM
FPC Ctg2488 cp05T5e12DM4RS
File Edit Analysis Highlight Add track Layout Size options Help
Zoom
14.7
Whole
Show buried clones
Yes No
Search
CB Unit Range
0 to 108
Contig stats
Clones: 16 (1 buried)
Markers: 0
Sequenced: 0
Length: 109 CB units
Ctg2488 of cp05T5e12DM4RS
V045G14~
V045F12*
V051P24
V051M02
V037L11
V039I13
V015L22
B015F12
V063P10
B015F14
V030B06
V012J08
V067O12
V059C15
V075K14
M037L22
0 10 20 30 40 50 60 70 80 90 100
[Chickpea contig p...
[root@localhost: /...
[FPC V8.9 Main Menu]
Project cp05T5e12...
FPC Ctg2488 cp05...

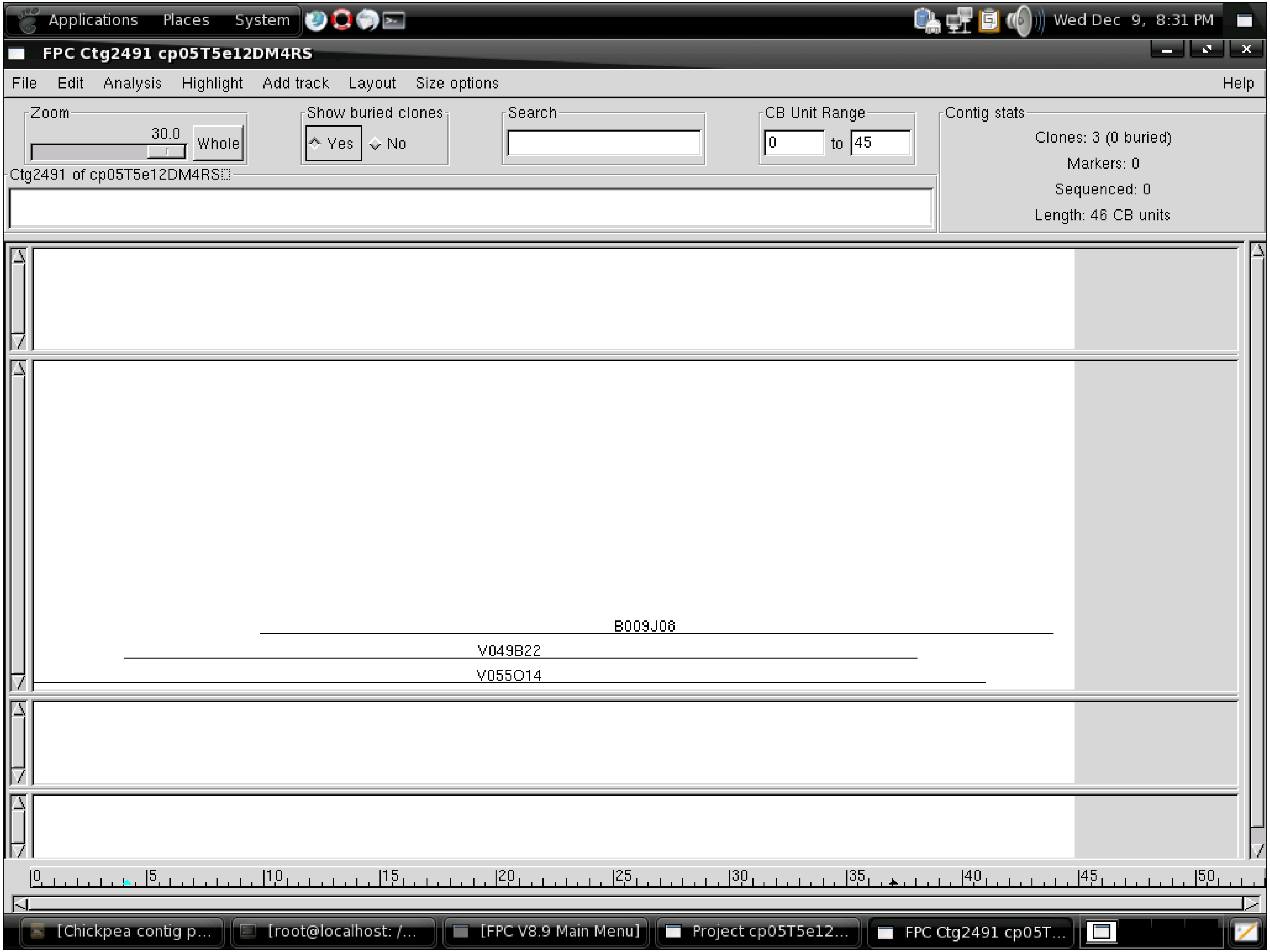
Applications Places System
Wed Dec 9, 8:31 PM
FPC Ctg2491 cp05T5e12DM4RS
File Edit Analysis Highlight Add track Layout Size options Help
Zoom
30.0
Whole
Show buried clones
Yes No
Search
CB Unit Range
0 to 45
Contig stats
Clones: 3 (0 buried)
Markers: 0
Sequenced: 0
Length: 46 CB units
Ctg2491 of cp05T5e12DM4RS
B009J08
V049B22
V055O14
0 5 10 15 20 25 30 35 40 45 50
[Chickpea contig p...
[root@localhost: /...
[FPC V8.9 Main Menu]
Project cp05T5e12...
FPC Ctg2491 cp05T...

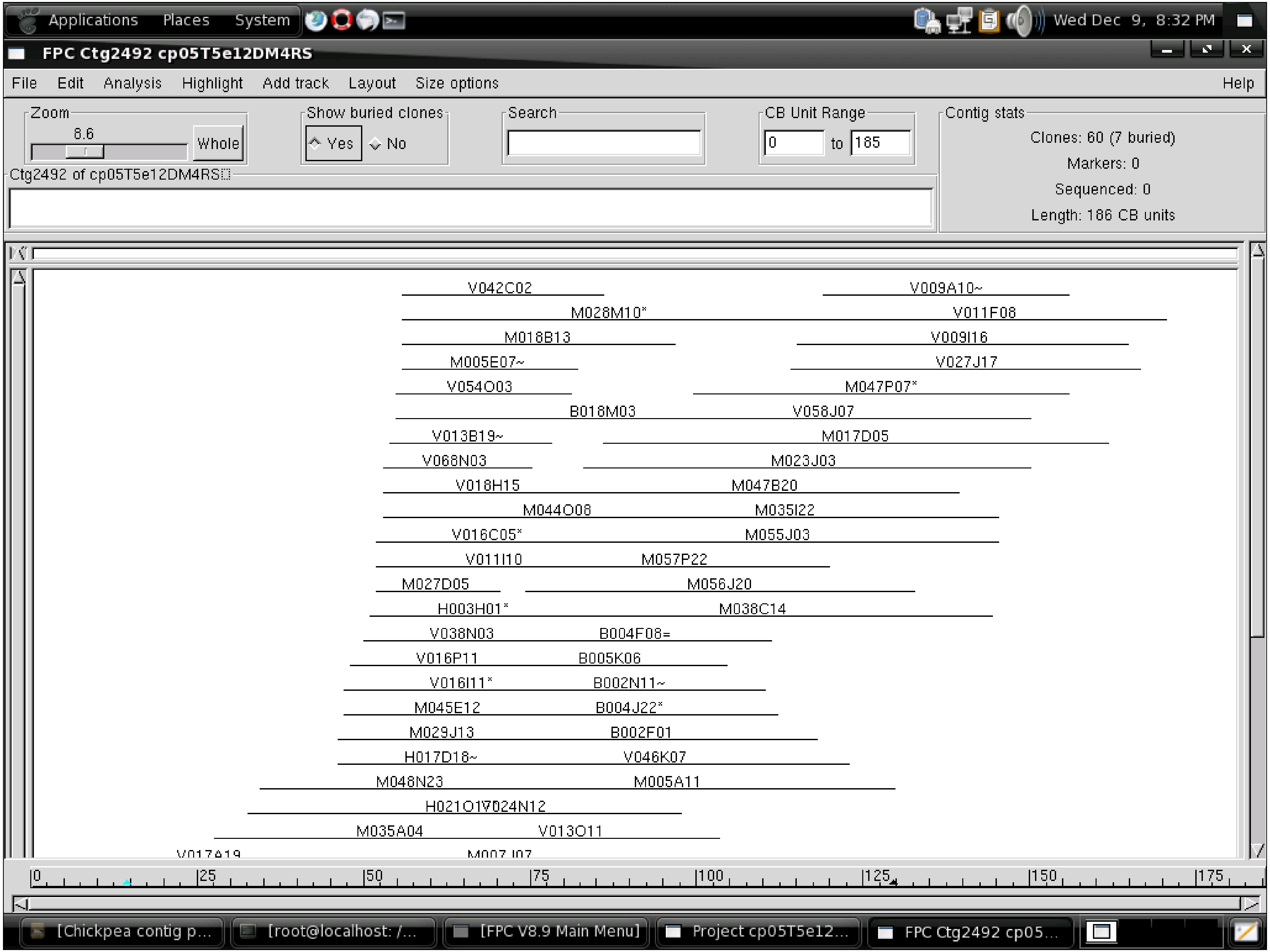
Applications Places System
Wed Dec 9, 8:32 PM
FPC Ctg2492 cp05T5e12DM4RS
File Edit Analysis Highlight Add track Layout Size options Help
Zoom 8.6 Whole
Show buried clones Yes No
Search
CB Unit Range 0 to 185
Contig stats
Clones: 60 (7 buried)
Markers: 0
Sequenced: 0
Length: 186 CB units
Ctg2492 of cp05T5e12DM4RS
V042C02
V009A10~
M028M10*
V011F08
M018B13
V009I16
M005E07~
V027J17
V054O03
M047P07*
B018M03
V058J07
V013B19~
M017D05
V068N03
M023J03
V018H15
M047B20
M044O08
M035I22
V016C05*
M055J03
V011I10
M057P22
M027D05
M056J20
H003H01*
M038C14
V038N03
B004F08=
V016P11
B005K06
V016I11*
B002N11~
M045E12
B004J22*
M029J13
B002F01
H017D18~
V046K07
M048N23
M005A11
H021O17V024N12
M035A04
V013O11
0 25 50 75 100 125 150 175
[Chickpea contig p...
[root@localhost: /...
[FPC V8.9 Main Menu]
Project cp05T5e12...
FPC Ctg2492 cp05...

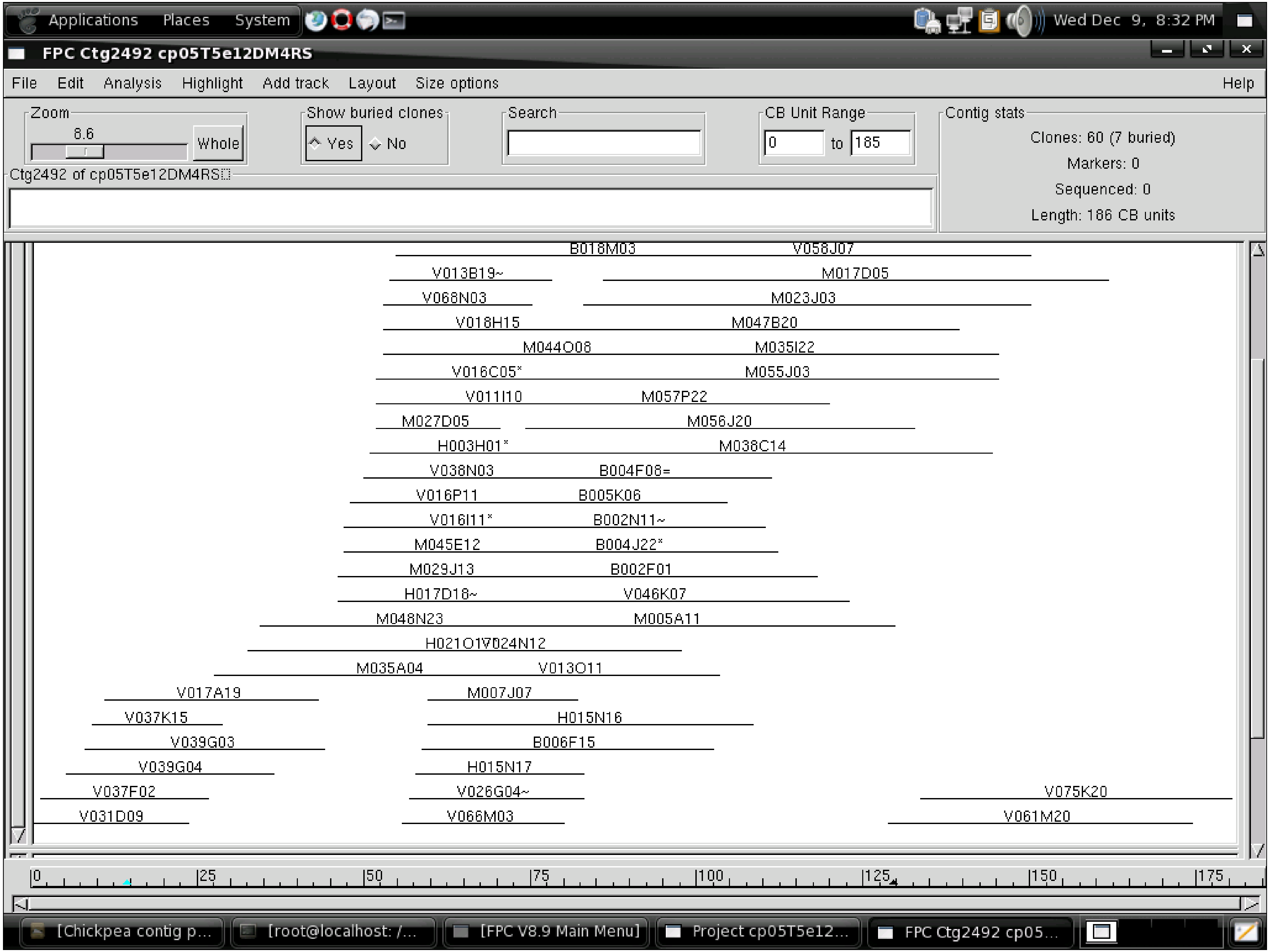
Applications Places System
Wed Dec 9, 8:32 PM
FPC Ctg2492 cp05T5e12DM4RS
File Edit Analysis Highlight Add track Layout Size options Help
Zoom
8.6
Whole
Show buried clones
Yes No
Search
CB Unit Range
0 to 185
Contig stats
Clones: 60 (7 buried)
Markers: 0
Sequenced: 0
Length: 186 CB units
Ctg2492 of cp05T5e12DM4RS
B018M03
V058J07
V013B19~
M017D05
V068N03
M023J03
V018H15
M047B20
M044O08
M035I22
V016C05*
M055J03
V011I10
M057P22
M027D05
M056J20
H003H01*
M038C14
V038N03
B004F08=
V016P11
B005K06
V016I11*
B002N11~
M045E12
B004J22*
M029J13
B002F01
H017D18~
V046K07
M048N23
M005A11
H021O1V024N12
M035A04
V013O11
V017A19
M007J07
V037K15
H015N16
V039G03
B006F15
V039G04
H015N17
V037F02
V026G04~
V075K20
V031D09
V066M03
V061M20
0 25 50 75 100 125 150 175
[Chickpea contig p...
[root@localhost: /...
[FPC V8.9 Main Menu]
Project cp05T5e12...
FPC Ctg2492 cp05...

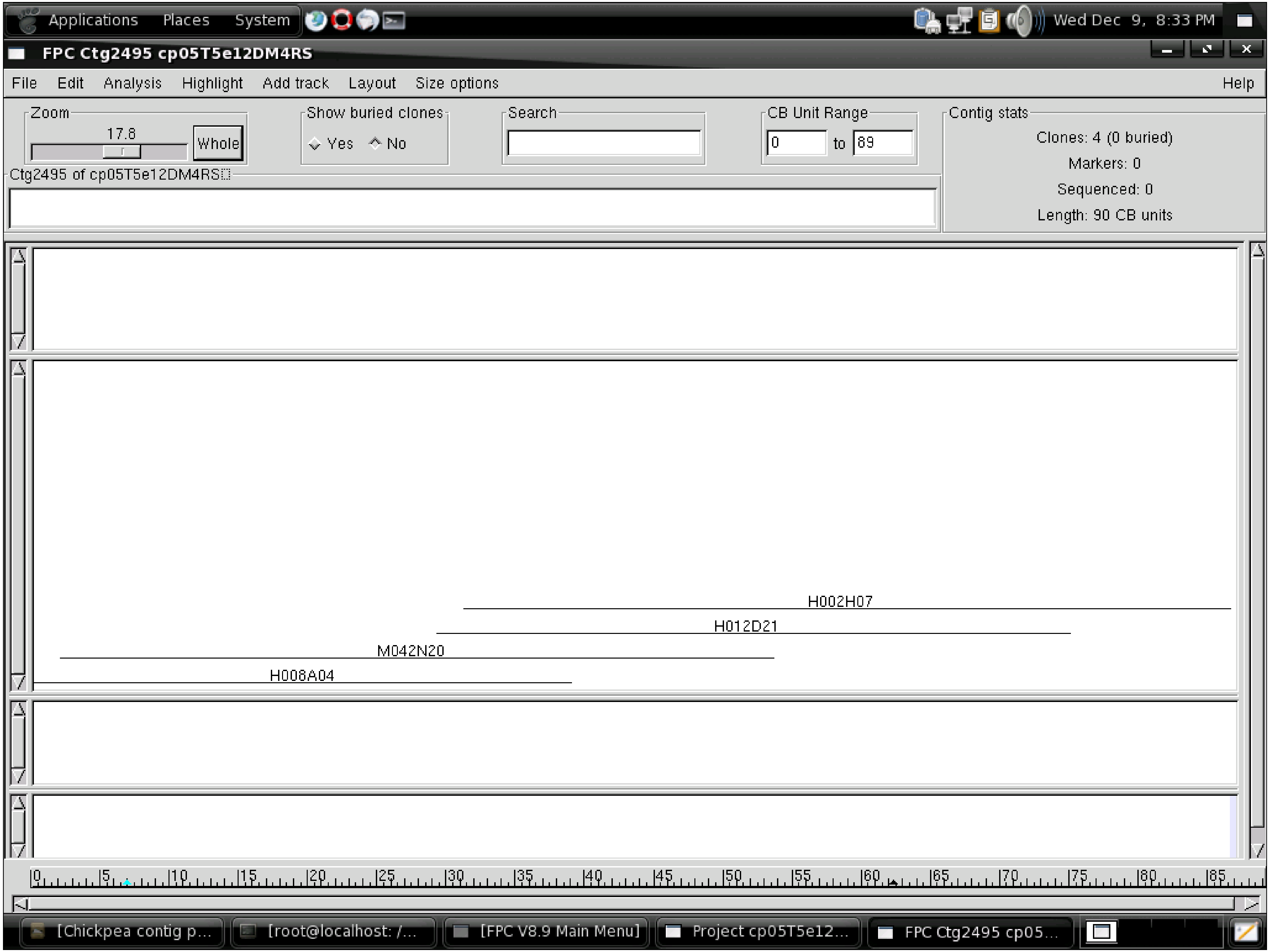
Applications Places System
Wed Dec 9, 8:33 PM
FPC Ctg2495 cp05T5e12DM4RS
File Edit Analysis Highlight Add track Layout Size options Help
Zoom
17.8
Whole
Show buried clones
Yes No
Search
CB Unit Range
0 to 89
Contig stats
Clones: 4 (0 buried)
Markers: 0
Sequenced: 0
Length: 90 CB units
Ctg2495 of cp05T5e12DM4RS
H002H07
H012D21
M042N20
H008A04
0 5 10 15 20 25 30 35 40 45 50 55 60 65 70 75 80 85
[Chickpea contig p...
[root@localhost: /...
[FPC V8.9 Main Menu]
Project cp05T5e12...
FPC Ctg2495 cp05...

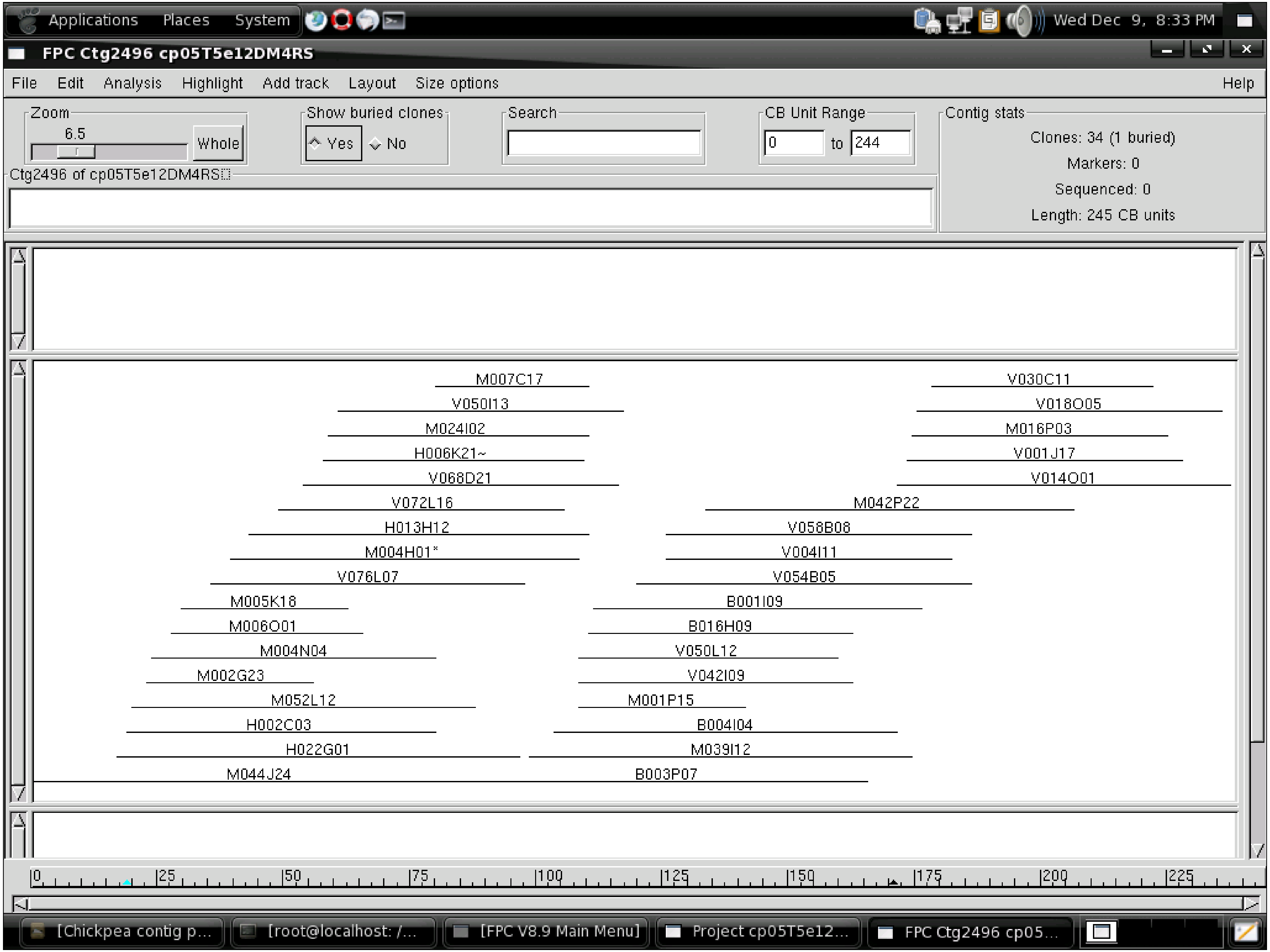
Applications Places System
Wed Dec 9, 8:33 PM
FPC Ctg2496 cp05T5e12DM4RS
File Edit Analysis Highlight Add track Layout Size options Help
Zoom
6.5
Whole
Show buried clones
Yes No
Search
CB Unit Range
0 to 244
Contig stats
Clones: 34 (1 buried)
Markers: 0
Sequenced: 0
Length: 245 CB units
Ctg2496 of cp05T5e12DM4RS
M007C17
V050I13
M024I02
H006K21~
V068D21
V072L16
H013H12
M004H01*
V076L07
M005K18
M006O01
M004N04
M002G23
M052L12
H002C03
H022G01
M044J24
V030C11
V018O05
M016P03
V001J17
V014O01
M042P22
V058B08
V004I11
V054B05
B001I09
B016H09
V050L12
V042I09
M001P15
B004I04
M039I12
B003P07
0 25 50 75 100 125 150 175 200 225
[Chickpea contig p...
[root@localhost: /...
[FPC V8.9 Main Menu]
Project cp05T5e12...
FPC Ctg2496 cp05...

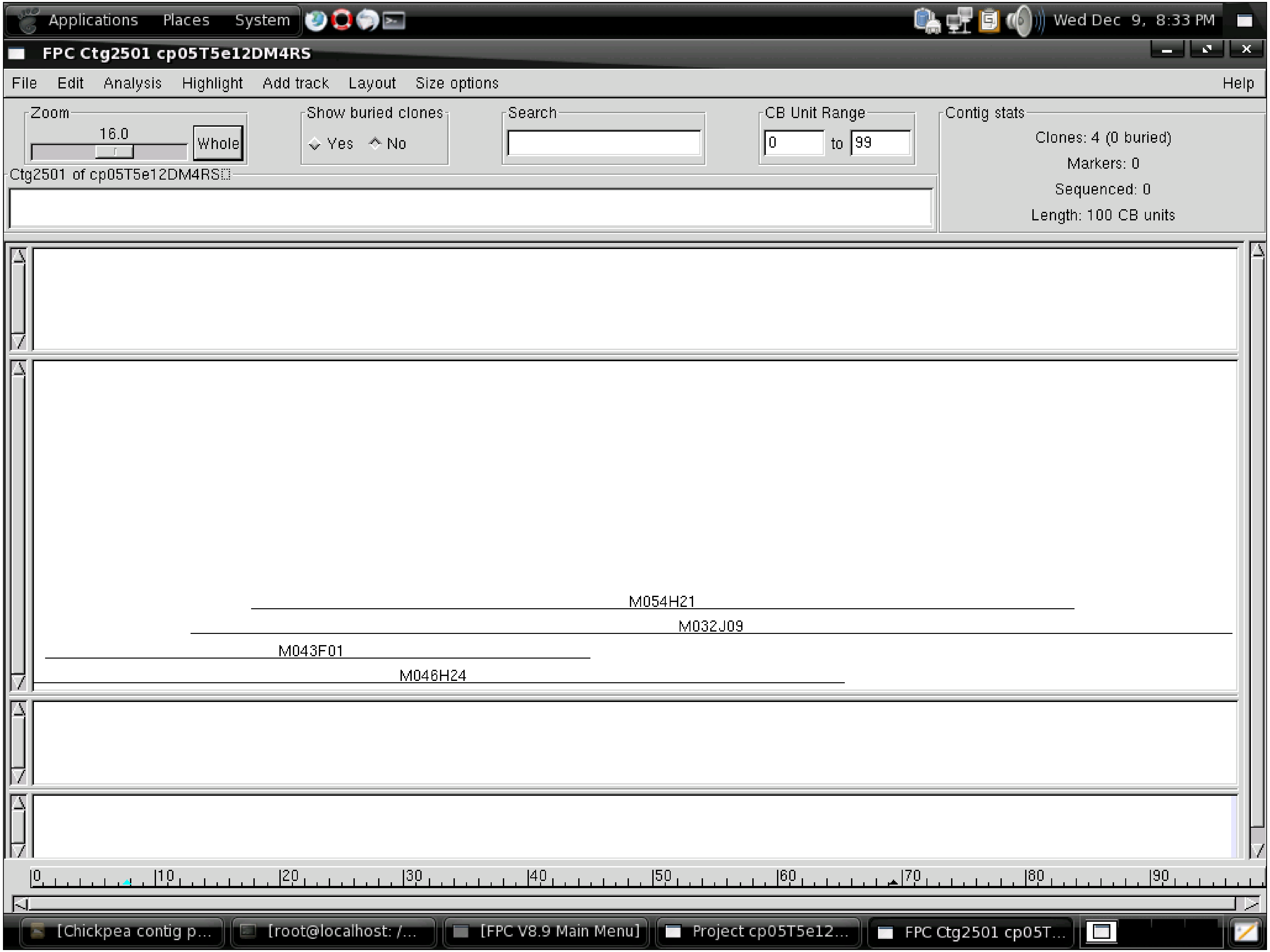
Applications Places System
Wed Dec 9, 8:33 PM
FPC Ctg2501 cp05T5e12DM4RS
File Edit Analysis Highlight Add track Layout Size options Help
Zoom
16.0
Whole
Show buried clones
Yes No
Search
CB Unit Range
0 to 99
Contig stats
Clones: 4 (0 buried)
Markers: 0
Sequenced: 0
Length: 100 CB units
Ctg2501 of cp05T5e12DM4RS
M054H21
M032J09
M043F01
M046H24
0 10 20 30 40 50 60 70 80 90
[Chickpea contig p...
[root@localhost: /...
[FPC V8.9 Main Menu]
Project cp05T5e12...
FPC Ctg2501 cp05T...

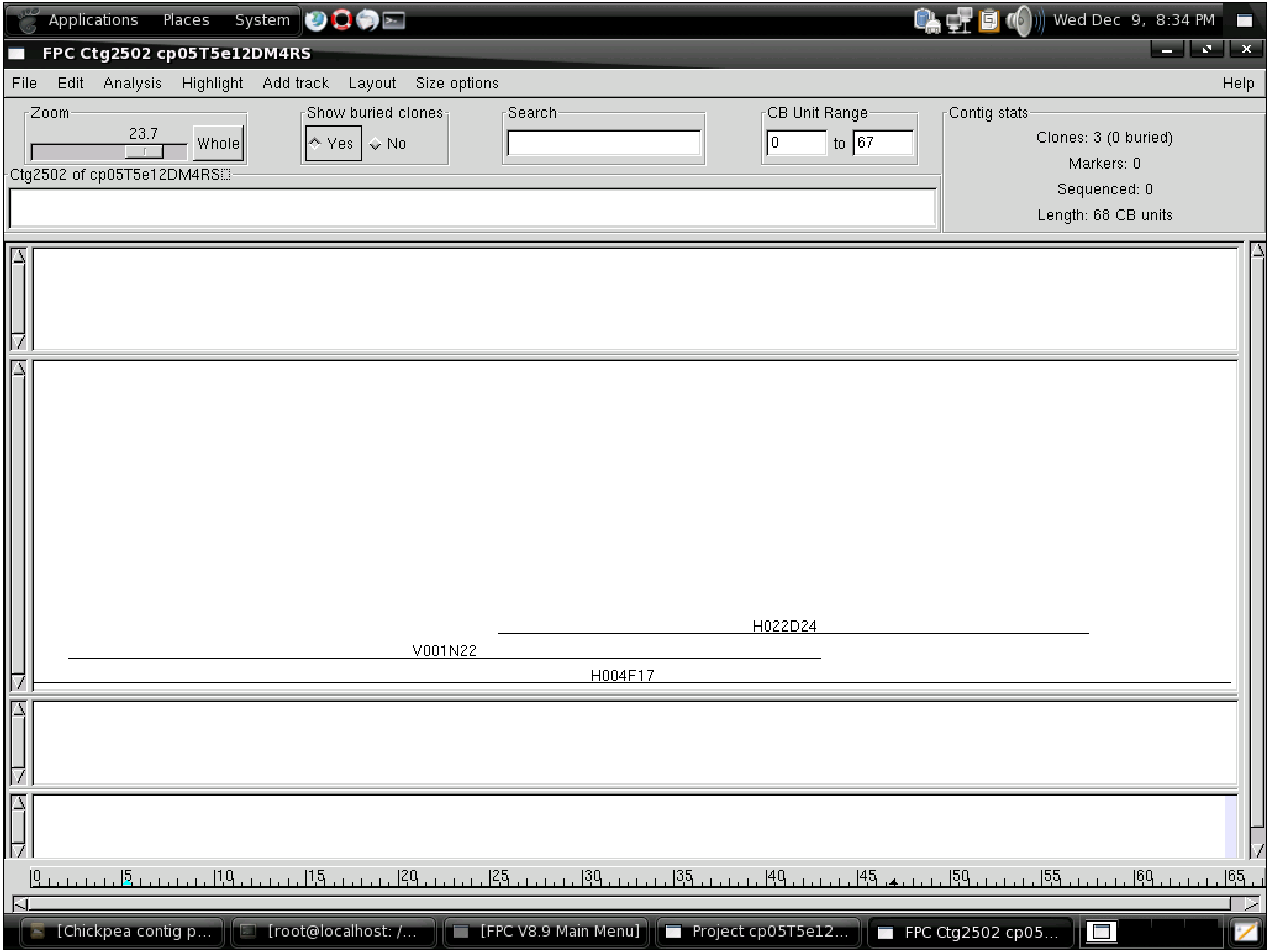
Applications Places System
Wed Dec 9, 8:34 PM
FPC Ctg2502 cp05T5e12DM4RS
File Edit Analysis Highlight Add track Layout Size options Help
Zoom
23.7
Whole
Show buried clones
Yes No
Search
CB Unit Range
0 to 67
Contig stats
Clones: 3 (0 buried)
Markers: 0
Sequenced: 0
Length: 68 CB units
Ctg2502 of cp05T5e12DM4RS
H022D24
V001N22
H004F17
0 5 10 15 20 25 30 35 40 45 50 55 60 65
[Chickpea contig p...
[root@localhost: /...
[FPC V8.9 Main Menu]
Project cp05T5e12...
FPC Ctg2502 cp05...

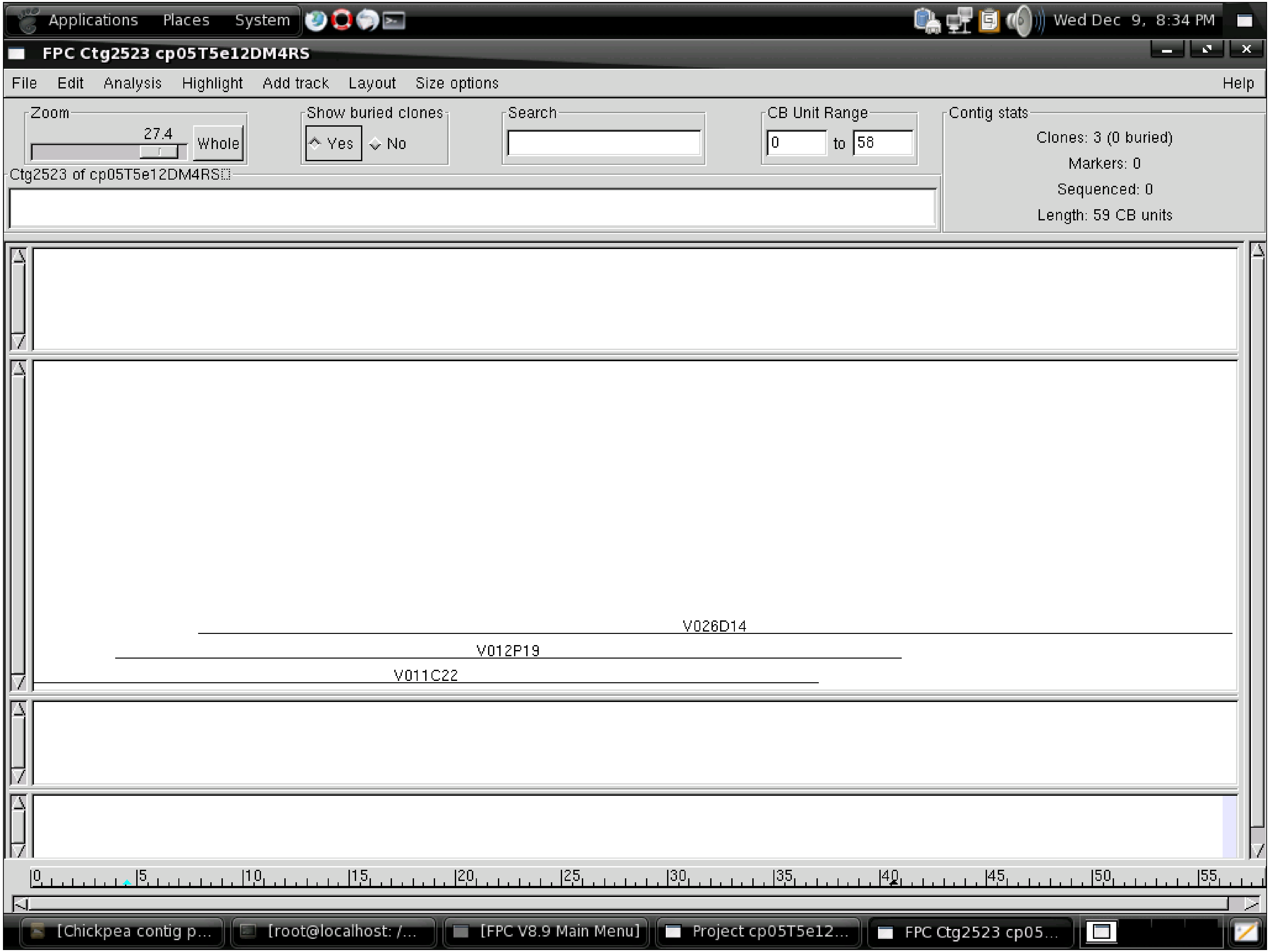

Applications Places System
Wed Dec 9, 8:34 PM
FPC Ctg2523 cp05T5e12DM4RS
File Edit Analysis Highlight Add track Layout Size options Help
Zoom
27.4
Whole
Show buried clones
Yes No
Search
CB Unit Range
0 to 58
Contig stats
Clones: 3 (0 buried)
Markers: 0
Sequenced: 0
Length: 59 CB units
Ctg2523 of cp05T5e12DM4RS
V026D14
V012P19
V011C22
0 5 10 15 20 25 30 35 40 45 50 55
[Chickpea contig p...
[root@localhost: /...
[FPC V8.9 Main Menu]
Project cp05T5e12...
FPC Ctg2523 cp05...

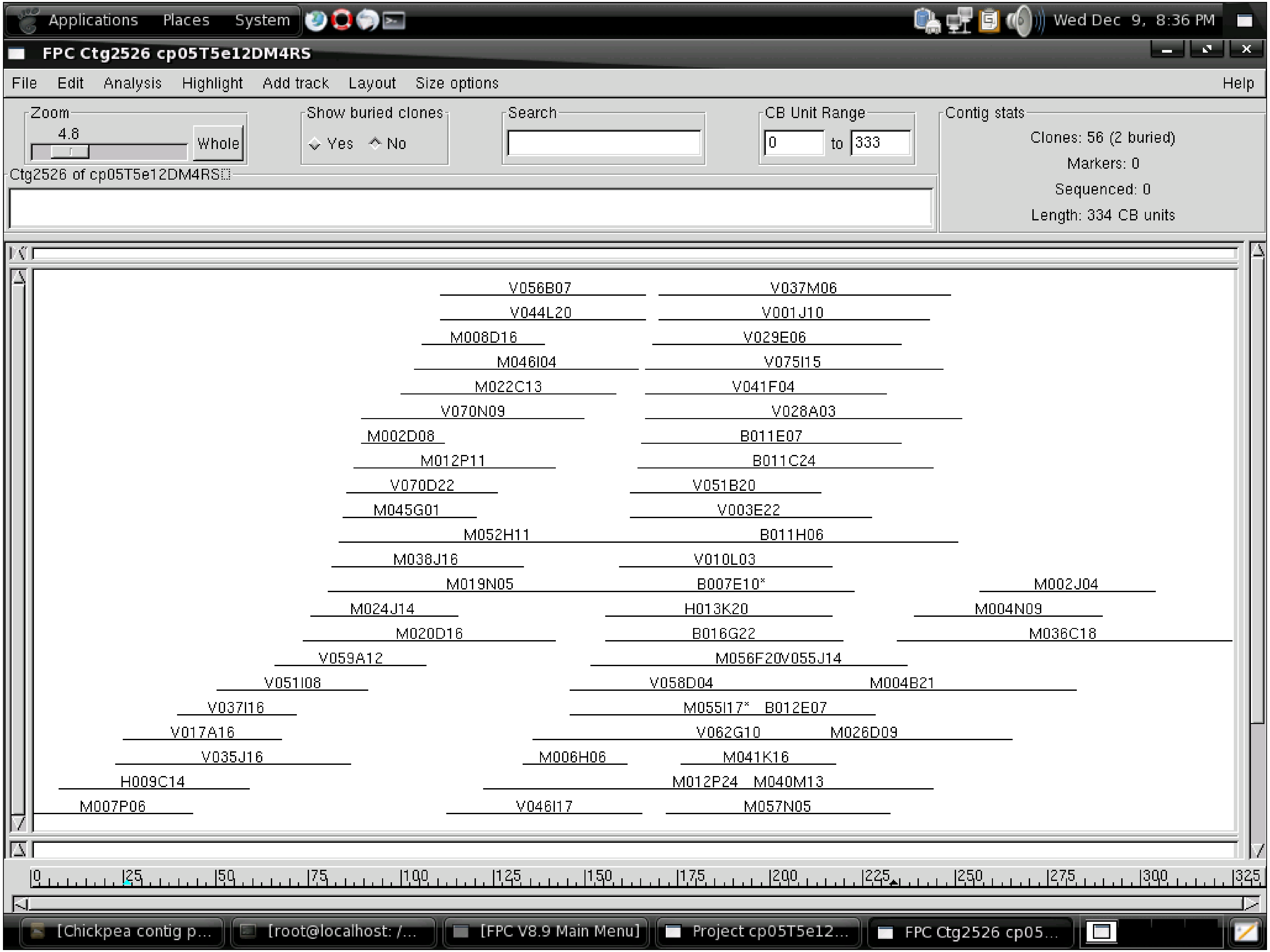
Applications Places System
Wed Dec 9, 8:36 PM
FPC Ctg2526 cp05T5e12DM4RS
File Edit Analysis Highlight Add track Layout Size options Help
Zoom 4.8 Whole
Show buried clones Yes No
Search
CB Unit Range 0 to 333
Contig stats
Clones: 56 (2 buried)
Markers: 0
Sequenced: 0
Length: 334 CB units
Ctg2526 of cp05T5e12DM4RS
V056B07
V037M06
V044L20
V001J10
M008D16
V029E06
M046I04
V075I15
M022C13
V041F04
V070N09
V028A03
M002D08
B011E07
M012P11
B011C24
V070D22
V051B20
M045G01
V003E22
M052H11
B011H06
M038J16
V010L03
M019N05
B007E10*
M002J04
M024J14
H013K20
M004N09
M020D16
B016G22
M036C18
V059A12
M056F20V055J14
V051I08
V058D04
M004B21
V037I16
M055I17* B012E07
V017A16
V062G10
M026D09
V035J16
M006H06
M041K16
H009C14
M012P24 M040M13
M007P06
V046I17
M057N05
0 25 50 75 100 125 150 175 200 225 250 275 300 325
[Chickpea contig p...
[root@localhost: /...
[FPC V8.9 Main Menu]
Project cp05T5e12...
FPC Ctg2526 cp05...

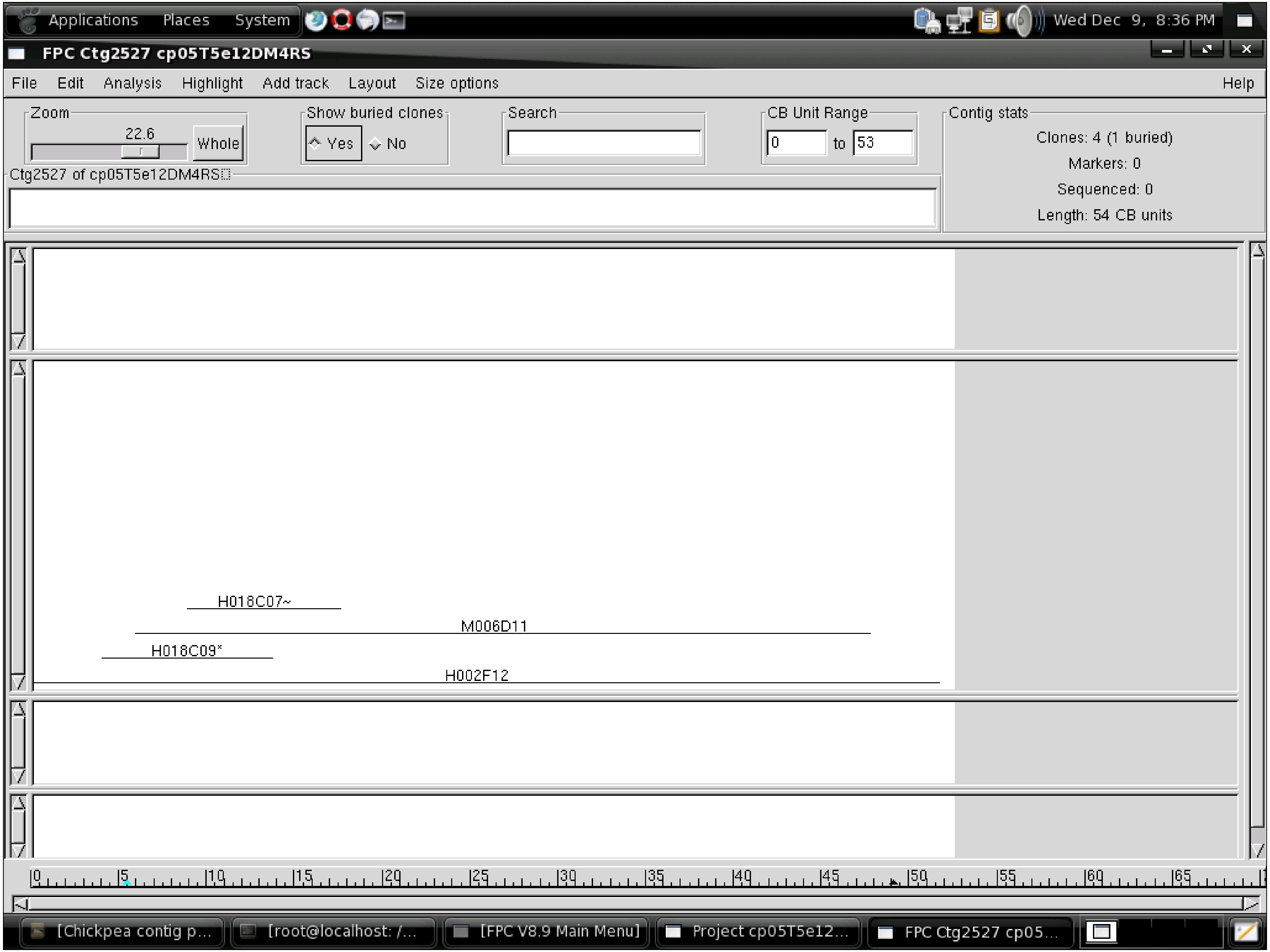
Applications Places System
Wed Dec 9, 8:36 PM
FPC Ctg2527 cp05T5e12DM4RS
File Edit Analysis Highlight Add track Layout Size options Help
Zoom
22.6
Whole
Show buried clones
Yes No
Search
CB Unit Range
0 to 53
Contig stats
Clones: 4 (1 buried)
Markers: 0
Sequenced: 0
Length: 54 CB units
Ctg2527 of cp05T5e12DM4RS
H018C07~
M006D11
H018C09*
H002F12
0 5 10 15 20 25 30 35 40 45 50 55 60 65
[Chickpea contig p...
[root@localhost: /...
[FPC V8.9 Main Menu]
Project cp05T5e12...
FPC Ctg2527 cp05...

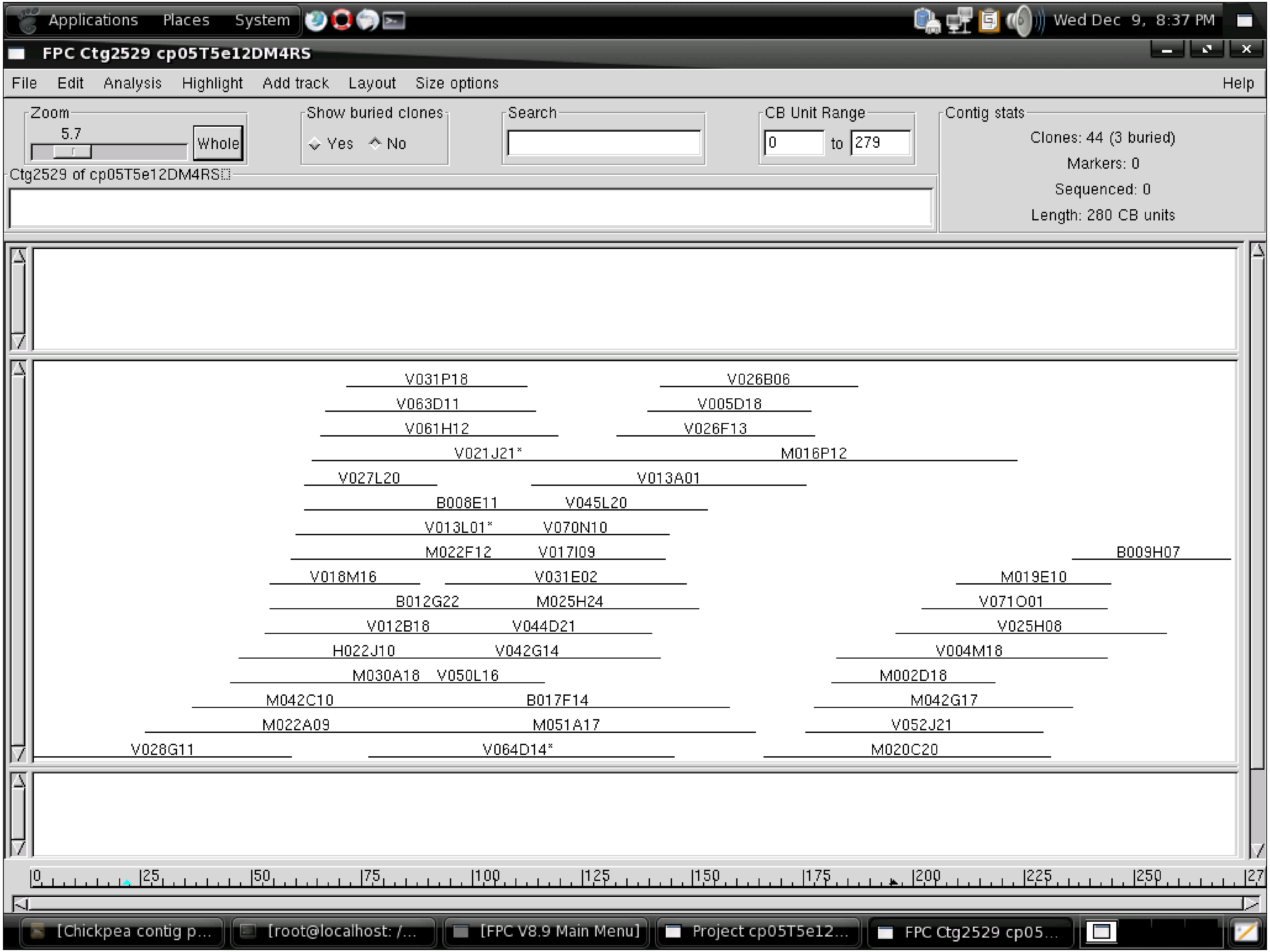
Applications Places System
Wed Dec 9, 8:37 PM
FPC Ctg2529 cp05T5e12DM4RS
File Edit Analysis Highlight Add track Layout Size options Help
Zoom
5.7
Whole
Show buried clones
Yes No
Search
CB Unit Range
0 to 279
Contig stats
Clones: 44 (3 buried)
Markers: 0
Sequenced: 0
Length: 280 CB units
Ctg2529 of cp05T5e12DM4RS
V031P18
V026B06
V063D11
V005D18
V061H12
V026F13
V021J21*
M016P12
V027L20
V013A01
B008E11
V045L20
V013L01*
V070N10
M022F12
V017I09
B009H07
V018M16
V031E02
M019E10
B012G22
M025H24
V071O01
V012B18
V044D21
V025H08
H022J10
V042G14
V004M18
M030A18
V050L16
M002D18
M042C10
B017F14
M042G17
M022A09
M051A17
V052J21
V028G11
V064D14*
M020C20
0 25 50 75 100 125 150 175 200 225 250 27
[Chickpea contig p...
[root@localhost: /...
[FPC V8.9 Main Menu]
Project cp05T5e12...
FPC Ctg2529 cp05...

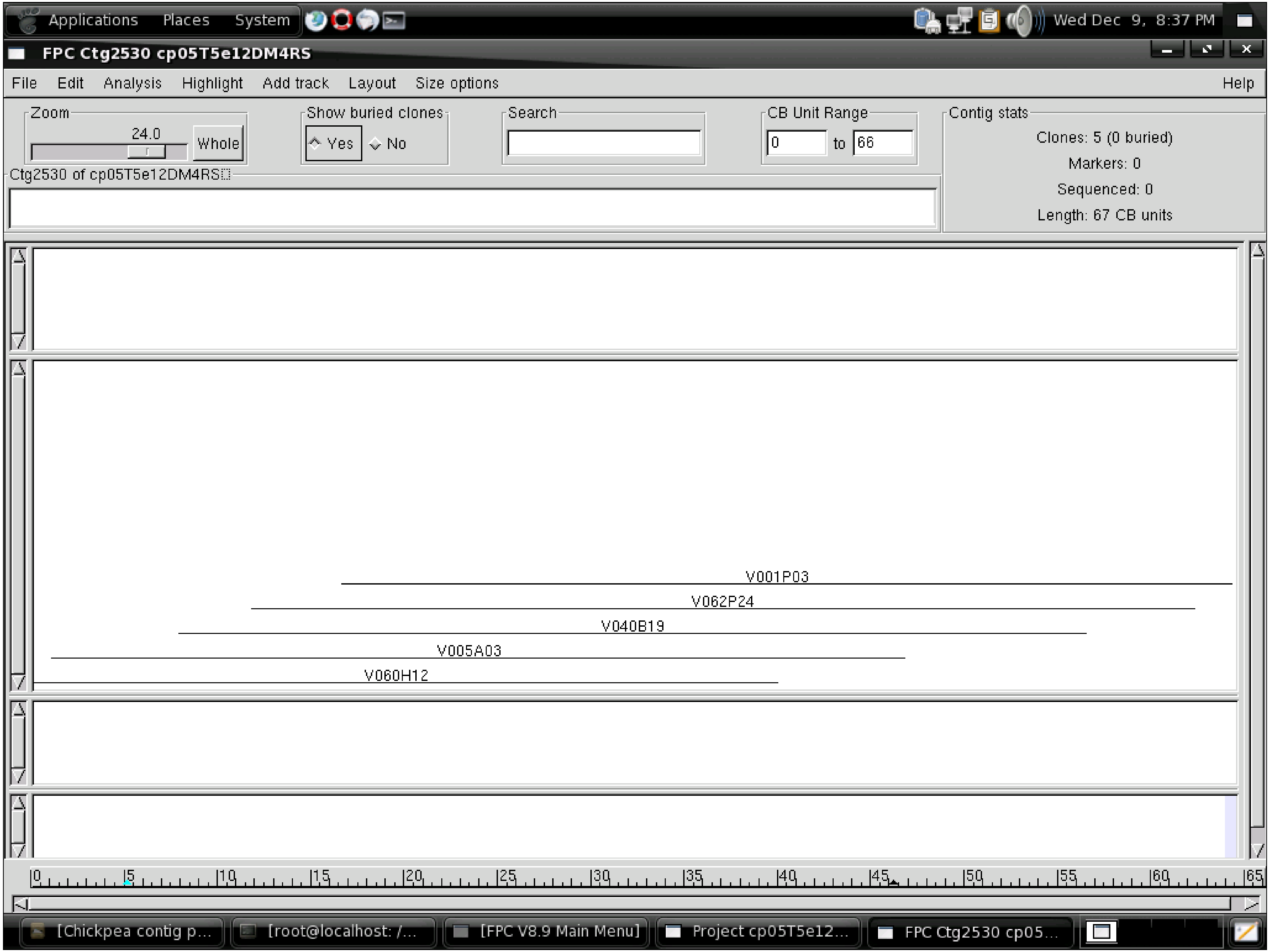
Applications Places System
Wed Dec 9, 8:37 PM
FPC Ctg2530 cp05T5e12DM4RS
File Edit Analysis Highlight Add track Layout Size options Help
Zoom
24.0
Whole
Show buried clones
Yes
No
Search
CB Unit Range
0
to
66
Contig stats
Clones: 5 (0 buried)
Markers: 0
Sequenced: 0
Length: 67 CB units
Ctg2530 of cp05T5e12DM4RS
V001P03
V062P24
V040B19
V005A03
V060H12
0 5 10 15 20 25 30 35 40 45 50 55 60 65
[Chickpea contig p...
[root@localhost: /...
[FPC V8.9 Main Menu]
Project cp05T5e12...
FPC Ctg2530 cp05...

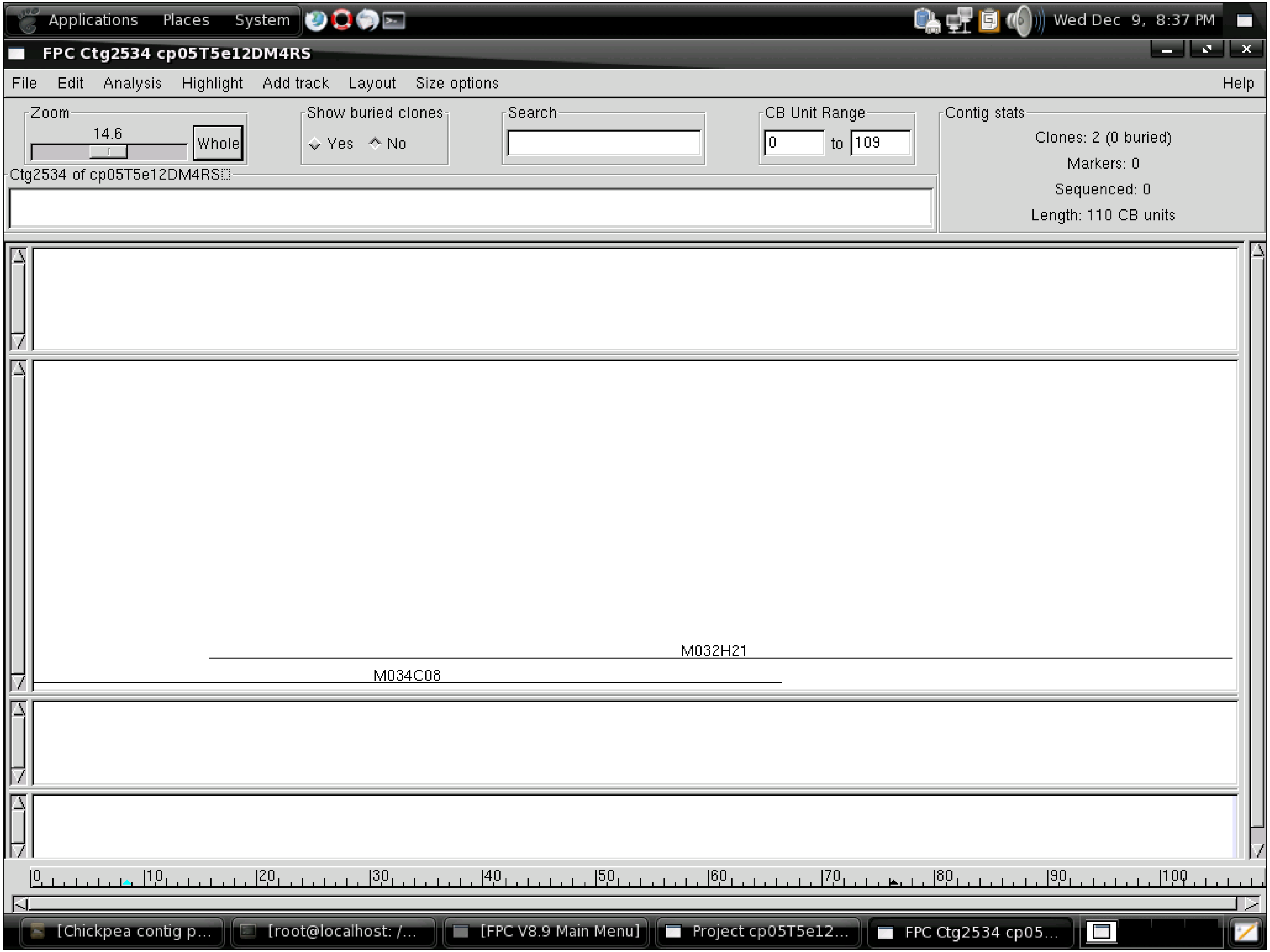
Applications Places System
Wed Dec 9, 8:37 PM
FPC Ctg2534 cp05T5e12DM4RS
File Edit Analysis Highlight Add track Layout Size options Help
Zoom
14.6
Whole
Show buried clones
Yes No
Search
CB Unit Range
0 to 109
Contig stats
Clones: 2 (0 buried)
Markers: 0
Sequenced: 0
Length: 110 CB units
Ctg2534 of cp05T5e12DM4RS
M032H21
M034C08
0 10 20 30 40 50 60 70 80 90 100
[Chickpea contig p...
[root@localhost: /...
[FPC V8.9 Main Menu]
Project cp05T5e12...
FPC Ctg2534 cp05...

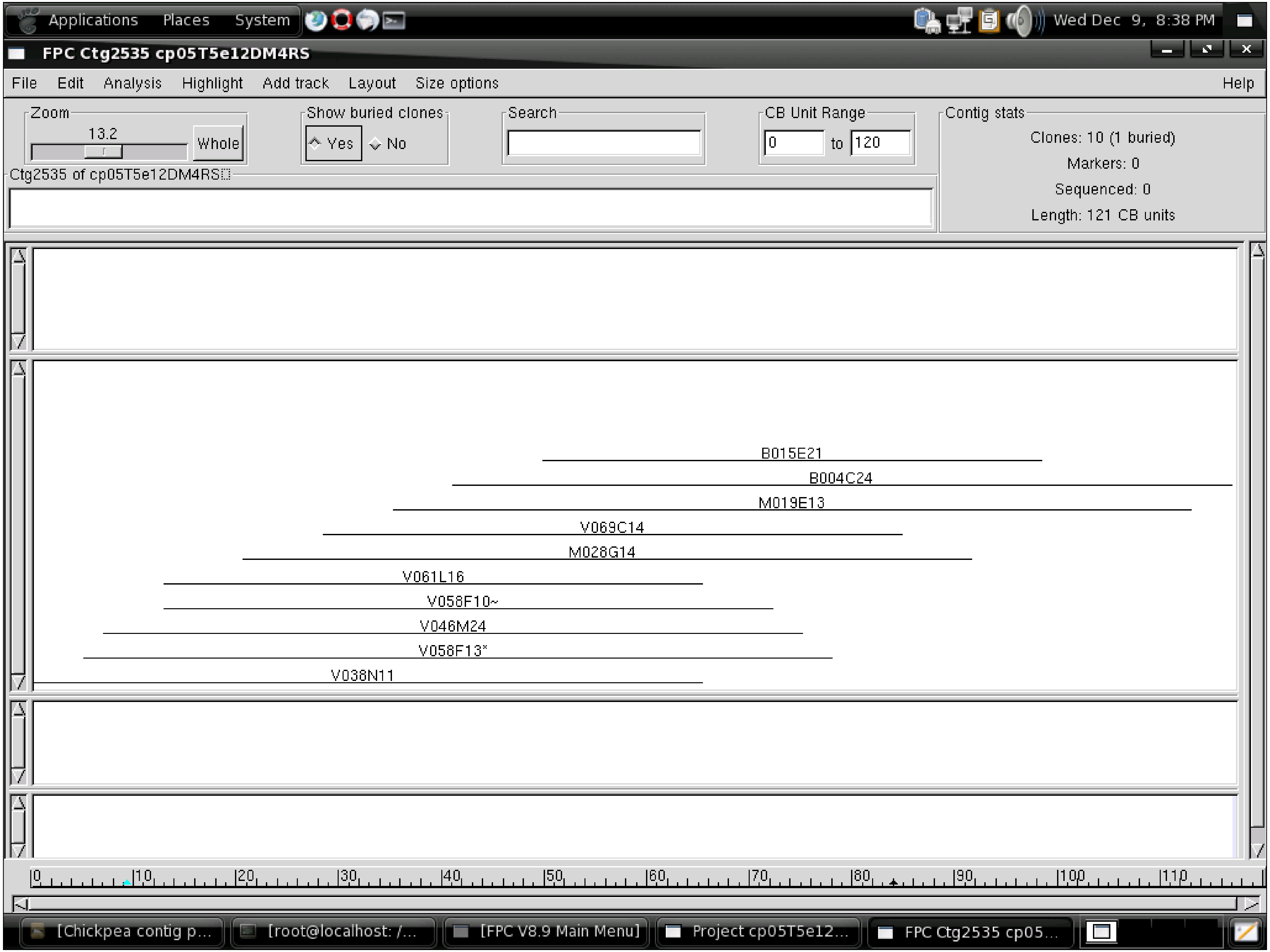
Applications Places System
Wed Dec 9, 8:38 PM
FPC Ctg2535 cp05T5e12DM4RS
File Edit Analysis Highlight Add track Layout Size options Help
Zoom
13.2
Whole
Show buried clones
Yes
No
Search
CB Unit Range
0 to 120
Contig stats
Clones: 10 (1 buried)
Markers: 0
Sequenced: 0
Length: 121 CB units
Ctg2535 of cp05T5e12DM4RS
B015E21
B004C24
M019E13
V069C14
M028G14
V061L16
V058F10~
V046M24
V058F13*
V038N11
0 10 20 30 40 50 60 70 80 90 100 110
[Chickpea contig p...
[root@localhost: /...
[FPC V8.9 Main Menu]
Project cp05T5e12...
FPC Ctg2535 cp05...

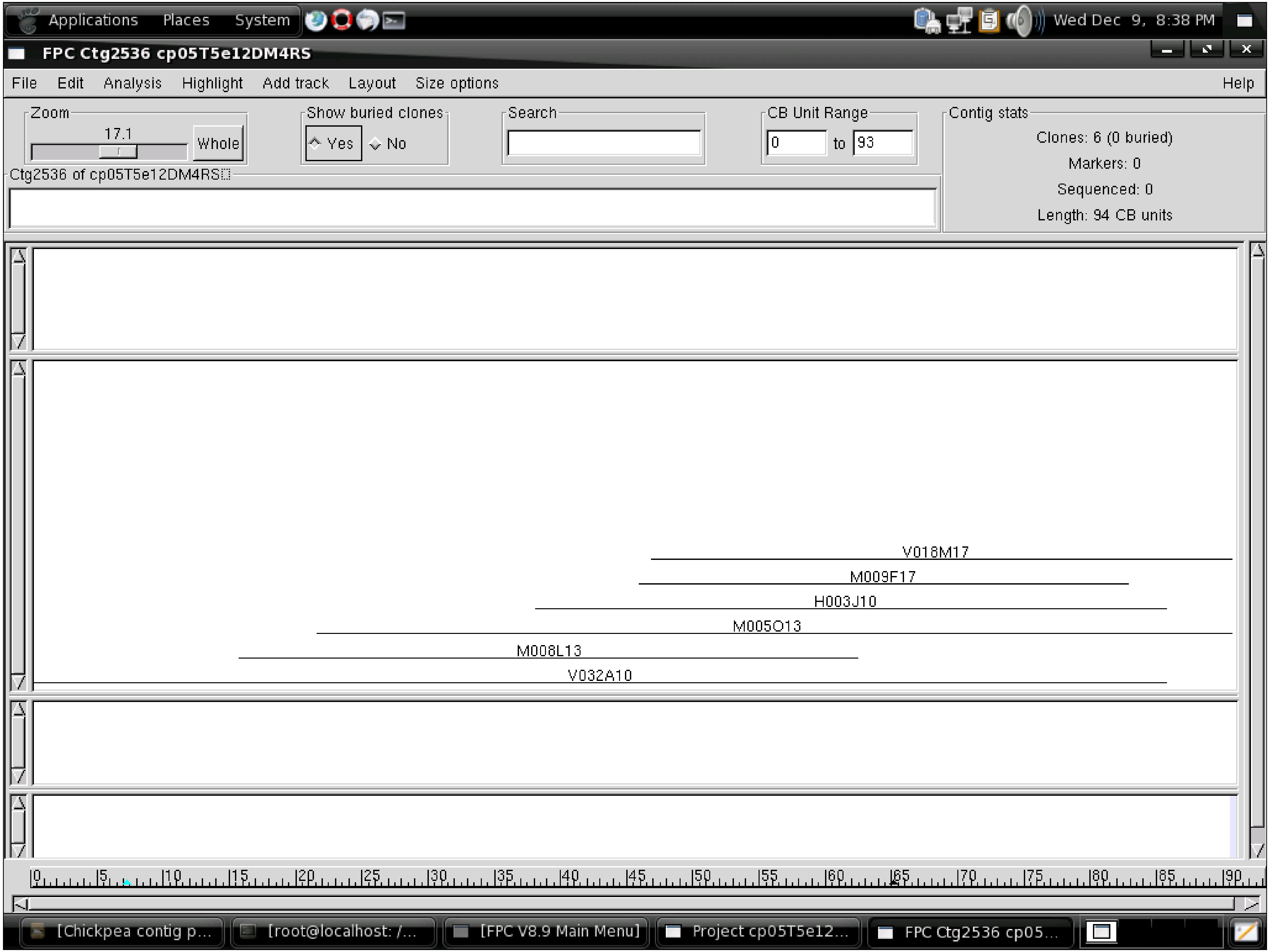

Applications Places System
Wed Dec 9, 8:38 PM
FPC Ctg2536 cp05T5e12DM4RS
File Edit Analysis Highlight Add track Layout Size options Help
Zoom
17.1
Whole
Show buried clones
Yes No
Search
CB Unit Range
0 to 93
Contig stats
Clones: 6 (0 buried)
Markers: 0
Sequenced: 0
Length: 94 CB units
Ctg2536 of cp05T5e12DM4RS
V018M17
M009F17
H003J10
M005O13
M008L13
V032A10
0 5 10 15 20 25 30 35 40 45 50 55 60 65 70 75 80 85 90
[Chickpea contig p...
[root@localhost: /...
[FPC V8.9 Main Menu]
Project cp05T5e12...
FPC Ctg2536 cp05...

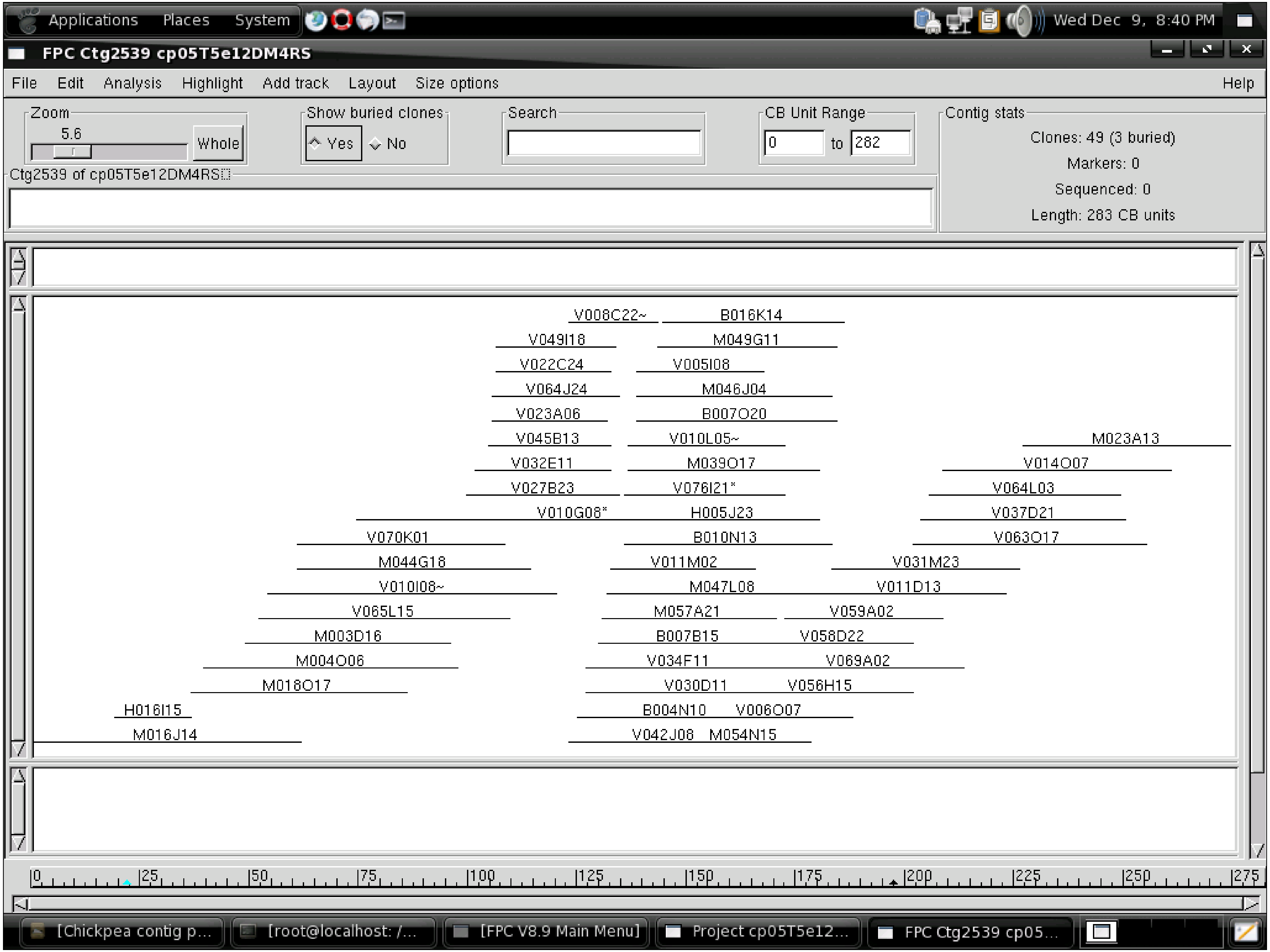
Applications Places System
Wed Dec 9, 8:40 PM
FPC Ctg2539 cp05T5e12DM4RS
File Edit Analysis Highlight Add track Layout Size options Help
Zoom
5.6
Whole
Show buried clones
Yes No
Search
CB Unit Range
0 to 282
Contig stats
Clones: 49 (3 buried)
Markers: 0
Sequenced: 0
Length: 283 CB units
Ctg2539 of cp05T5e12DM4RS
V008C22~ B016K14
V049I18 M049G11
V022C24 V005I08
V064J24 M046J04
V023A06 B007O20
V045B13 V010L05~ M023A13
V032E11 M039O17 V014O07
V027B23 V076I21* V064L03
V010G08* H005J23 V037D21
V070K01 B010N13 V063O17
M044G18 V011M02 V031M23
V010I08~ M047L08 V011D13
V065L15 M057A21 V059A02
M003D16 B007B15 V058D22
M004O06 V034F11 V069A02
M018O17 V030D11 V056H15
H016I15 B004N10 V006O07
M016J14 V042J08 M054N15
0 25 50 75 100 125 150 175 200 225 250 275
[Chickpea contig p... [root@localhost: /... [FPC V8.9 Main Menu] Project cp05T5e12... FPC Ctg2539 cp05...

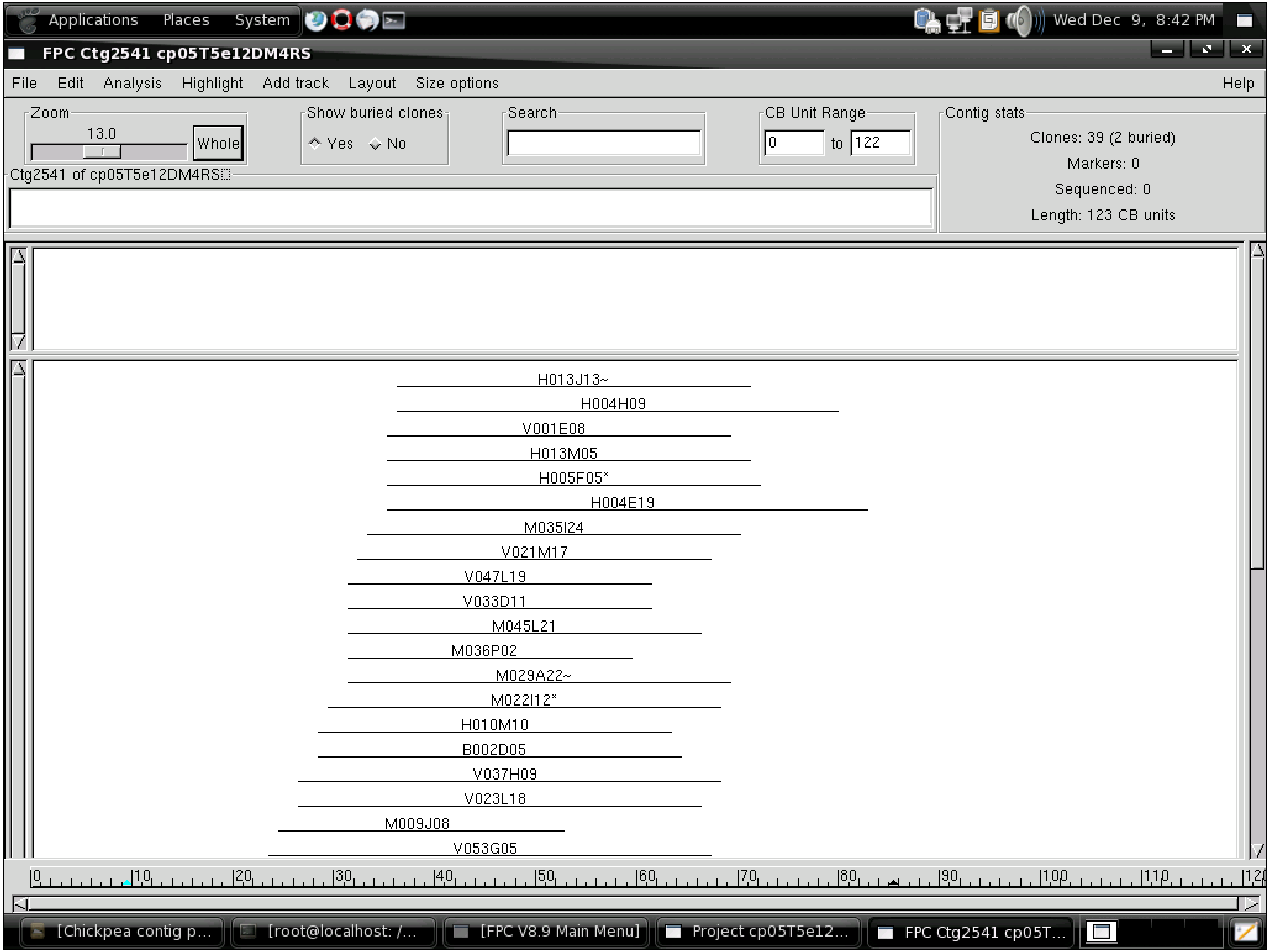
Applications Places System
Wed Dec 9, 8:42 PM
FPC Ctg2541 cp05T5e12DM4RS
File Edit Analysis Highlight Add track Layout Size options Help
Zoom
13.0
Whole
Show buried clones
Yes No
Search
CB Unit Range
0 to 122
Contig stats
Clones: 39 (2 buried)
Markers: 0
Sequenced: 0
Length: 123 CB units
Ctg2541 of cp05T5e12DM4RS
H013J13~
H004H09
V001E08
H013M05
H005F05*
H004E19
M035I24
V021M17
V047L19
V033D11
M045L21
M036P02
M029A22~
M022I12*
H010M10
B002D05
V037H09
V023L18
M009J08
V053G05
0 10 20 30 40 50 60 70 80 90 100 110
[Chickpea contig p...
[root@localhost: /...
[FPC V8.9 Main Menu]
Project cp05T5e12...
FPC Ctg2541 cp05T...

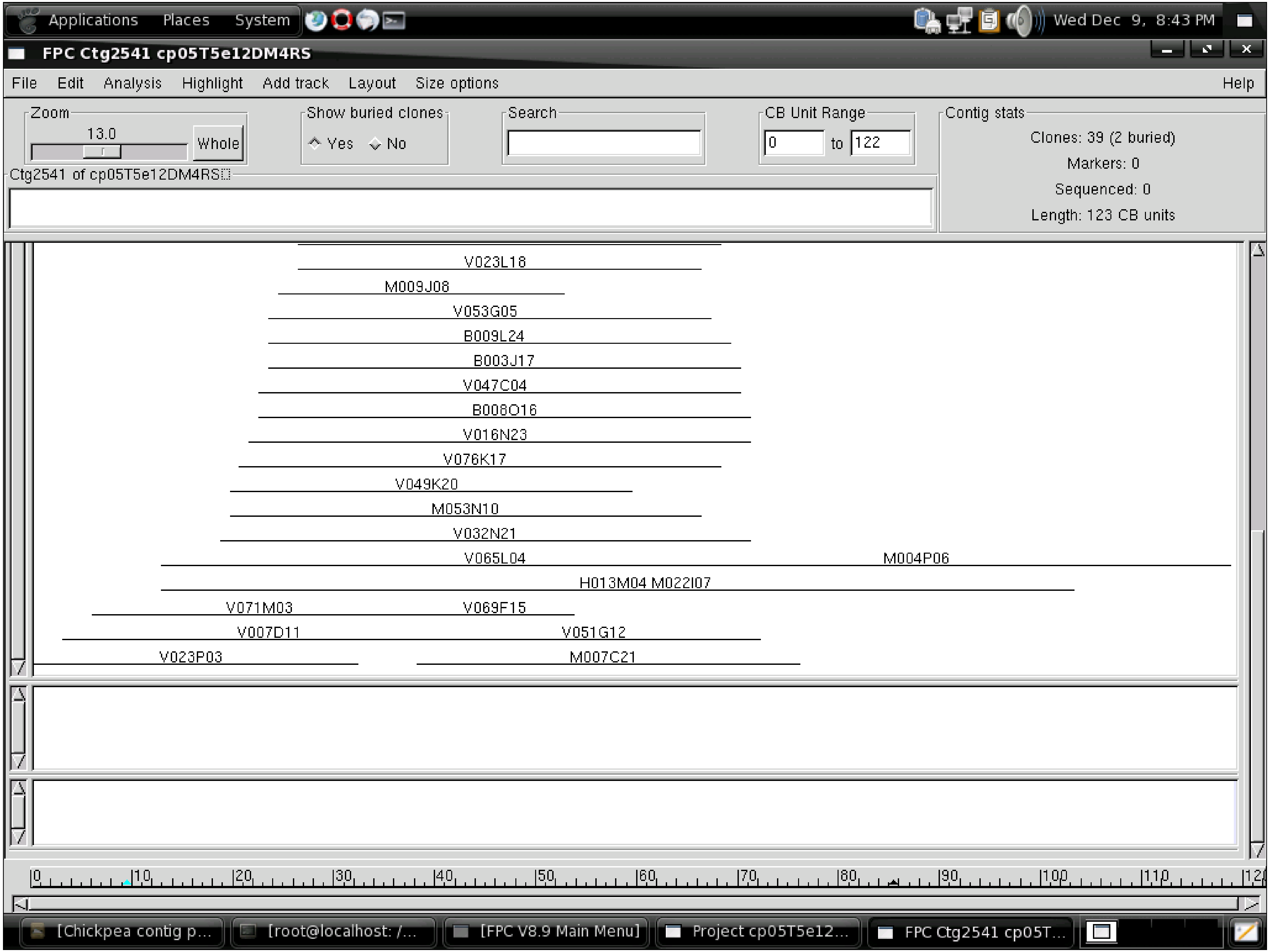
Applications Places System
Wed Dec 9, 8:43 PM
FPC Ctg2541 cp05T5e12DM4RS
File Edit Analysis Highlight Add track Layout Size options Help
Zoom
13.0
Whole
Show buried clones
Yes No
Search
CB Unit Range
0 to 122
Contig stats
Clones: 39 (2 buried)
Markers: 0
Sequenced: 0
Length: 123 CB units
Ctg2541 of cp05T5e12DM4RS
V023L18
M009J08
V053G05
B009L24
B003J17
V047C04
B008O16
V016N23
V076K17
V049K20
M053N10
V032N21
V065L04
M004P06
H013M04 M022I07
V071M03
V069F15
V007D11
V051G12
V023P03
M007C21
0 10 20 30 40 50 60 70 80 90 100 110 12
[Chickpea contig p...
[root@localhost: /...
[FPC V8.9 Main Menu]
Project cp05T5e12...
FPC Ctg2541 cp05T...

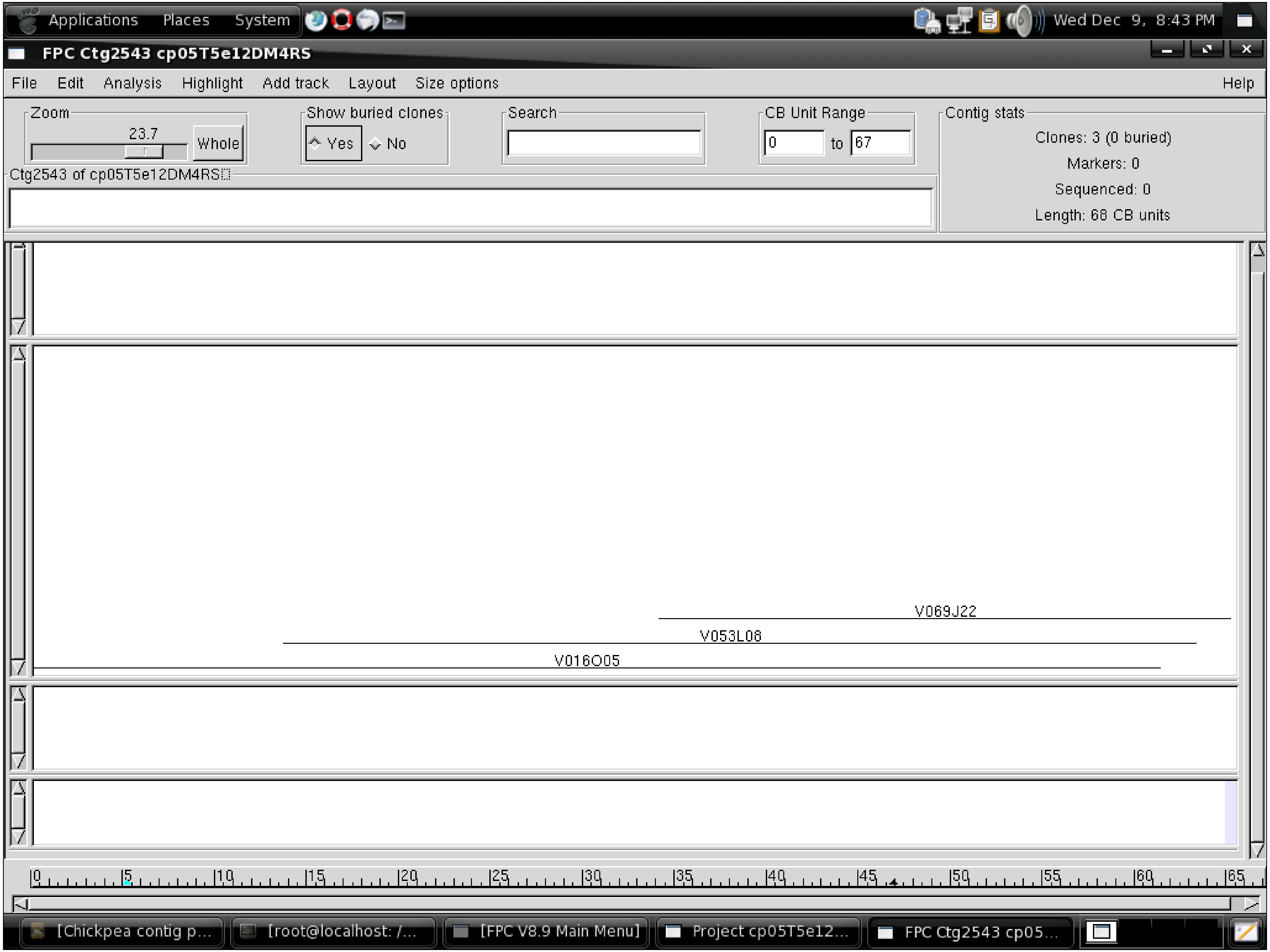
Applications Places System
Wed Dec 9, 8:43 PM
FPC Ctg2543 cp05T5e12DM4RS
File Edit Analysis Highlight Add track Layout Size options Help
Zoom
23.7
Whole
Show buried clones
Yes No
Search
CB Unit Range
0 to 67
Contig stats
Clones: 3 (0 buried)
Markers: 0
Sequenced: 0
Length: 68 CB units
Ctg2543 of cp05T5e12DM4RS
V069J22
V053L08
V016O05
0 5 10 15 20 25 30 35 40 45 50 55 60 65
[Chickpea contig p...
[root@localhost: /...
[FPC V8.9 Main Menu]
Project cp05T5e12...
FPC Ctg2543 cp05...

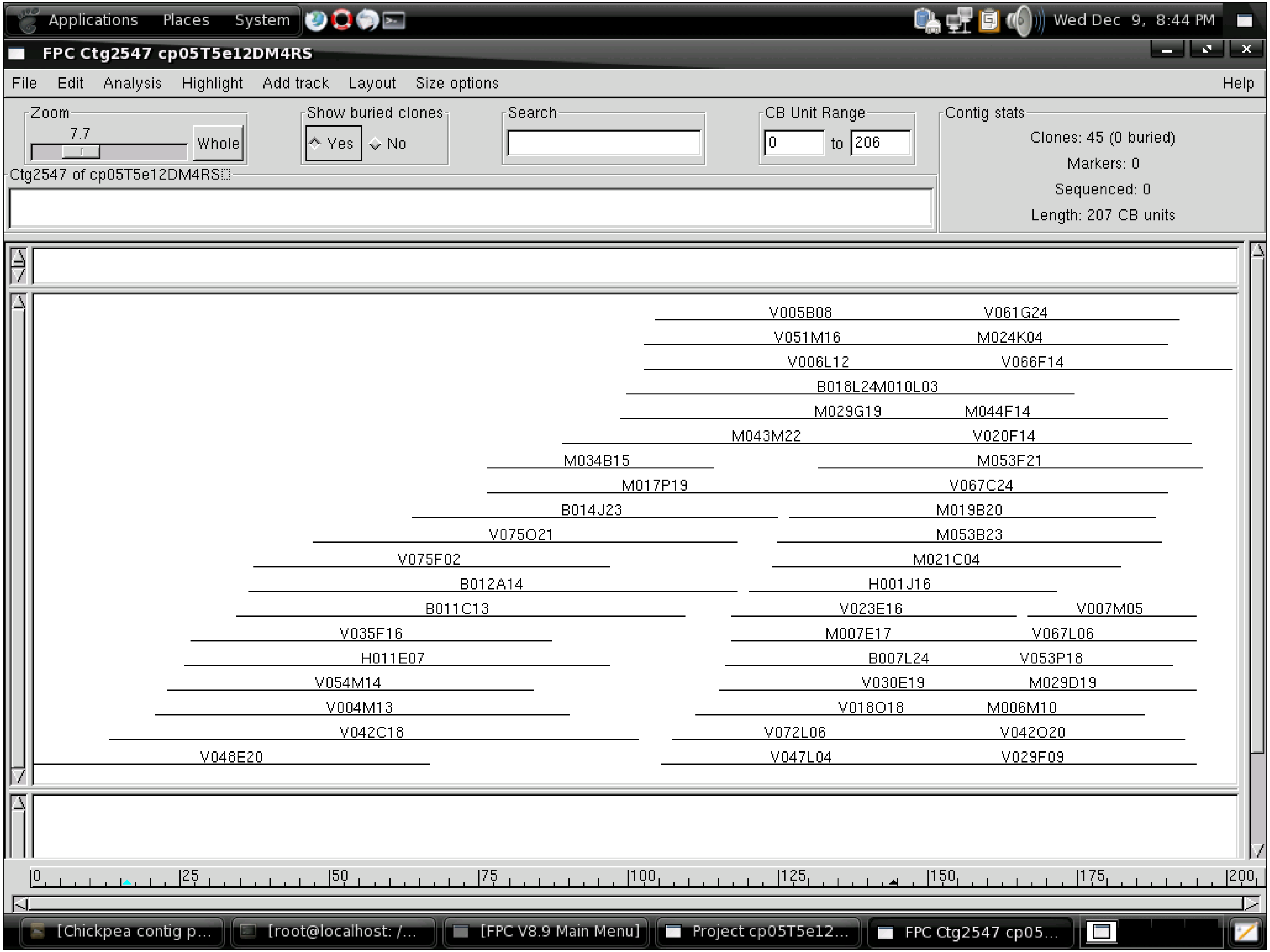

Applications Places System
Wed Dec 9, 8:44 PM
FPC Ctg2547 cp05T5e12DM4RS
File Edit Analysis Highlight Add track Layout Size options Help
Zoom 7.7 Whole
Show buried clones Yes No
Search
CB Unit Range 0 to 206
Contig stats
Clones: 45 (0 buried)
Markers: 0
Sequenced: 0
Length: 207 CB units
Ctg2547 of cp05T5e12DM4RS
V005B08
V061G24
V051M16
M024K04
V006L12
V066F14
B018L24M010L03
M029G19
M044F14
M043M22
V020F14
M034B15
M053F21
M017P19
V067C24
B014J23
M019B20
V075O21
M053B23
V075F02
M021C04
B012A14
H001J16
B011C13
V023E16
V007M05
V035F16
M007E17
V067L06
H011E07
B007L24
V053P18
V054M14
V030E19
M029D19
V004M13
V018O18
M006M10
V042C18
V072L06
V042O20
V048E20
V047L04
V029F09
0 25 50 75 100 125 150 175 200
[Chickpea contig p...
[root@localhost: /...
[FPC V8.9 Main Menu]
Project cp05T5e12...
FPC Ctg2547 cp05...

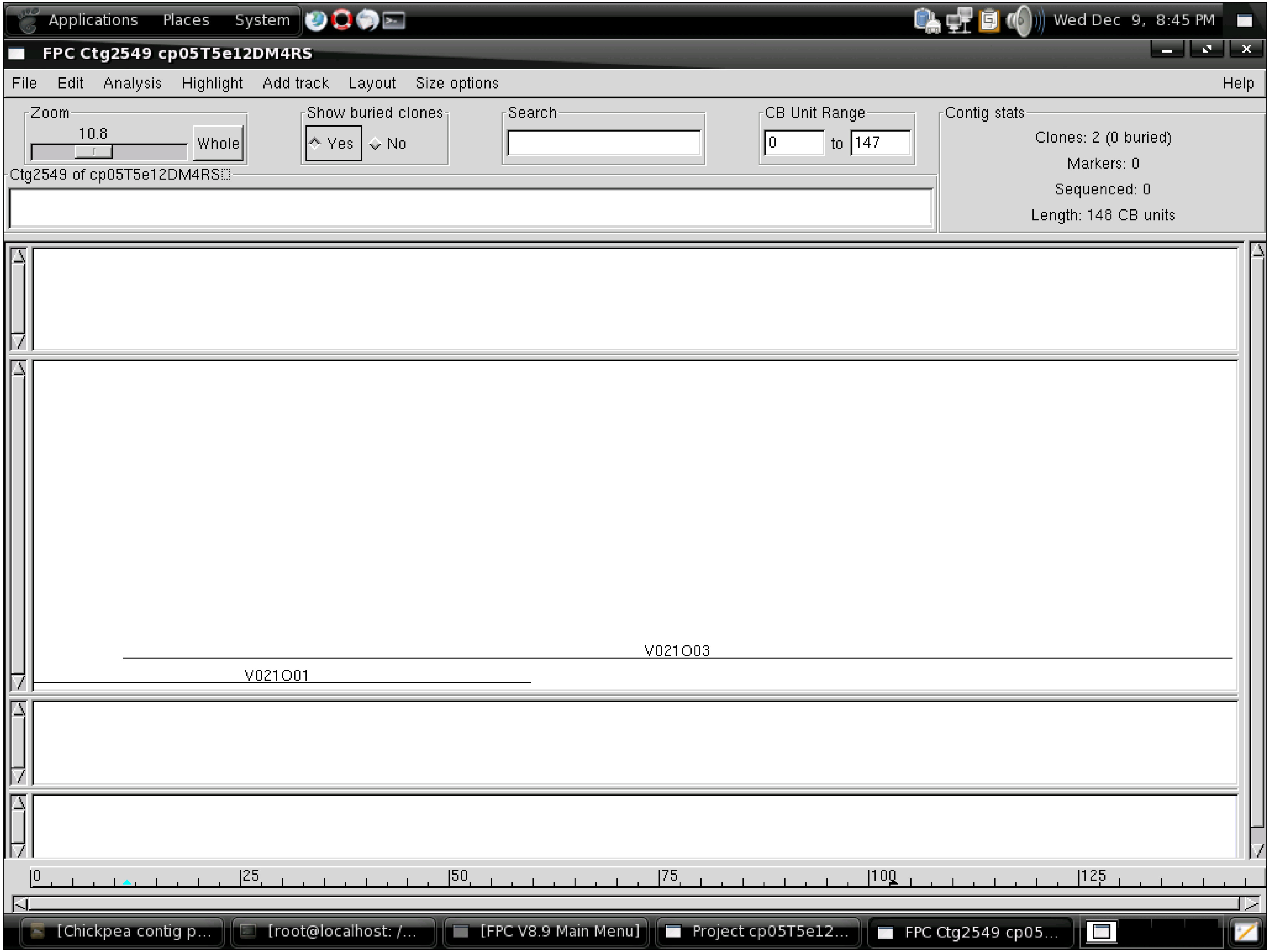
Applications Places System
Wed Dec 9, 8:45 PM
FPC Ctg2549 cp05T5e12DM4RS
File Edit Analysis Highlight Add track Layout Size options Help
Zoom
10.8
Whole
Show buried clones
Yes
No
Search
CB Unit Range
0
to
147
Contig stats
Clones: 2 (0 buried)
Markers: 0
Sequenced: 0
Length: 148 CB units
Ctg2549 of cp05T5e12DM4RS
V021O03
V021O01
0 25 50 75 100 125
[Chickpea contig p...
[root@localhost: /...
[FPC V8.9 Main Menu]
Project cp05T5e12...
FPC Ctg2549 cp05...

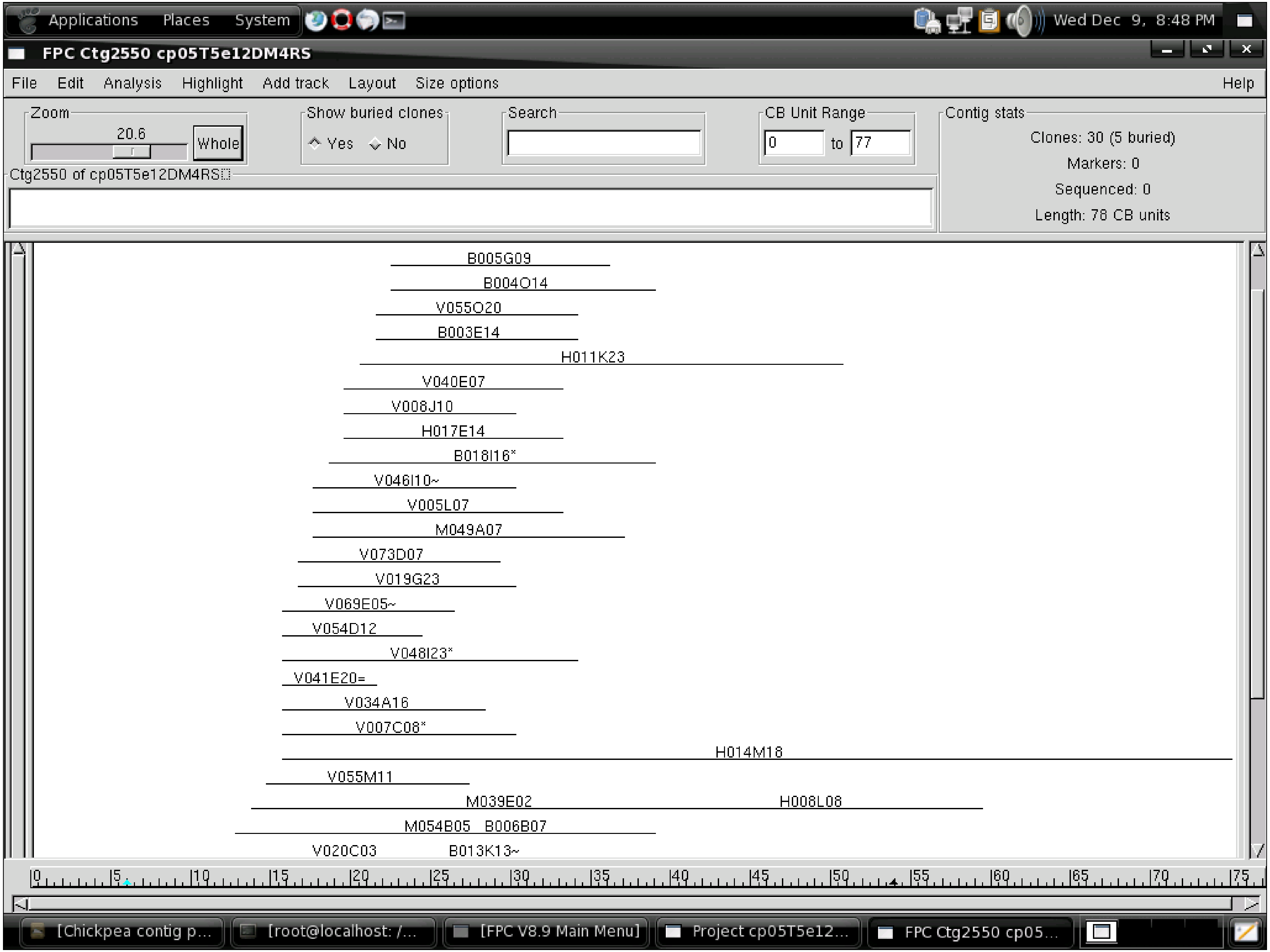
Applications Places System
Wed Dec 9, 8:48 PM
FPC Ctg2550 cp05T5e12DM4RS
File Edit Analysis Highlight Add track Layout Size options Help
Zoom
20.6
Whole
Show buried clones
Yes No
Search
CB Unit Range
0 to 77
Contig stats
Clones: 30 (5 buried)
Markers: 0
Sequenced: 0
Length: 78 CB units
Ctg2550 of cp05T5e12DM4RS
B005G09
B004O14
V055O20
B003E14
H011K23
V040E07
V008J10
H017E14
B018I16*
V046I10~
V005L07
M049A07
V073D07
V019G23
V069E05~
V054D12
V048I23*
V041E20=
V034A16
V007C08*
H014M18
V055M11
M039E02
H008L08
M054B05
B006B07
V020C03
B013K13~
0 5 10 15 20 25 30 35 40 45 50 55 60 65 70 75
[Chickpea contig p...
[root@localhost: /...
[FPC V8.9 Main Menu]
Project cp05T5e12...
FPC Ctg2550 cp05...

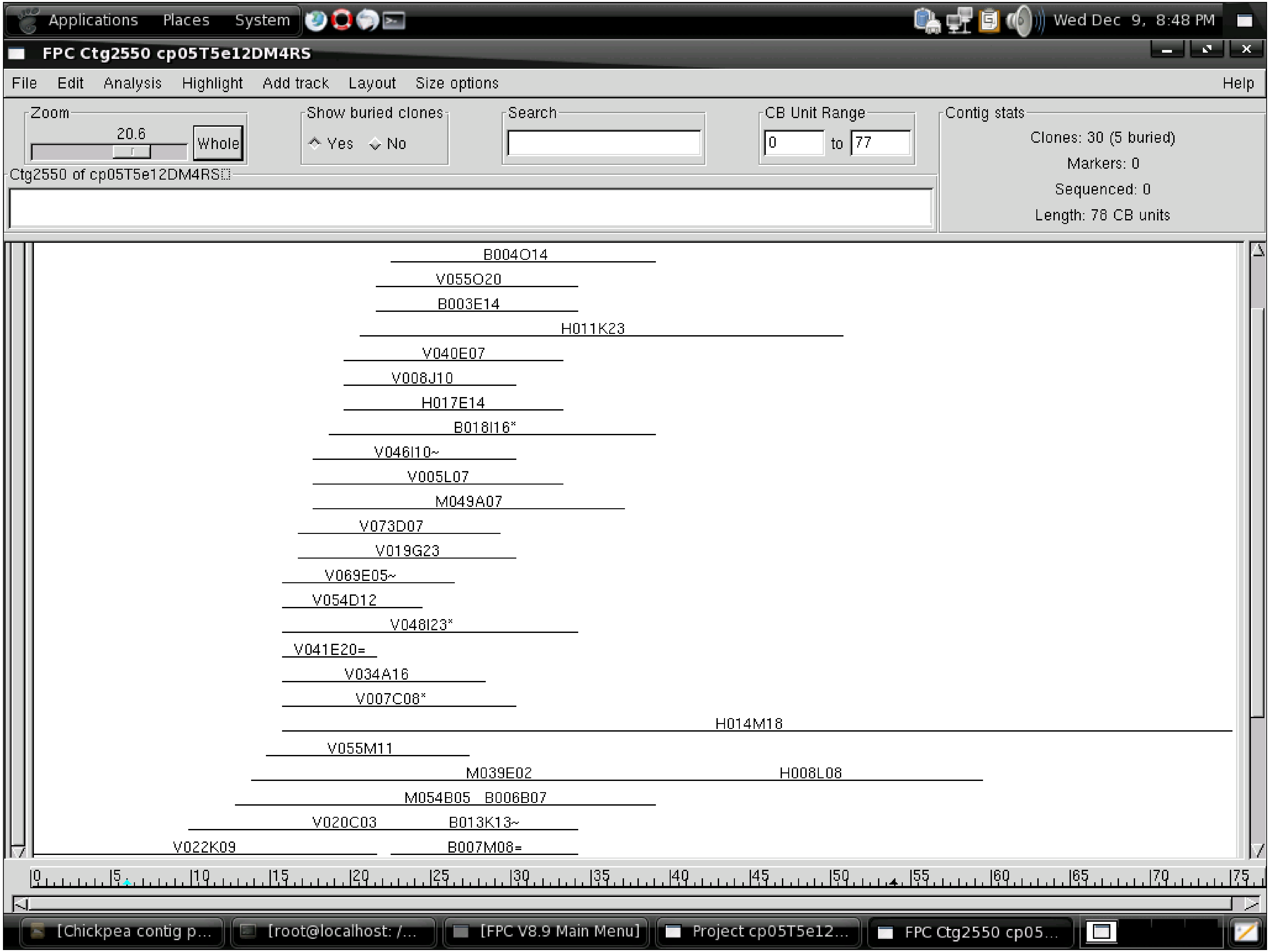
Applications Places System
Wed Dec 9, 8:48 PM
FPC Ctg2550 cp05T5e12DM4RS
File Edit Analysis Highlight Add track Layout Size options Help
Zoom
20.6
Whole
Show buried clones
Yes No
Search
CB Unit Range
0 to 77
Contig stats
Clones: 30 (5 buried)
Markers: 0
Sequenced: 0
Length: 78 CB units
Ctg2550 of cp05T5e12DM4RS
B004O14
V055O20
B003E14
H011K23
V040E07
V008J10
H017E14
B018I16*
V046I10~
V005L07
M049A07
V073D07
V019G23
V069E05~
V054D12
V048I23*
V041E20=
V034A16
V007C08*
H014M18
V055M11
M039E02
H008L08
M054B05
B006B07
V020C03
B013K13~
V022K09
B007M08=
0 5 10 15 20 25 30 35 40 45 50 55 60 65 70 75
[Chickpea contig p...
[root@localhost: /...
[FPC V8.9 Main Menu]
Project cp05T5e12...
FPC Ctg2550 cp05...

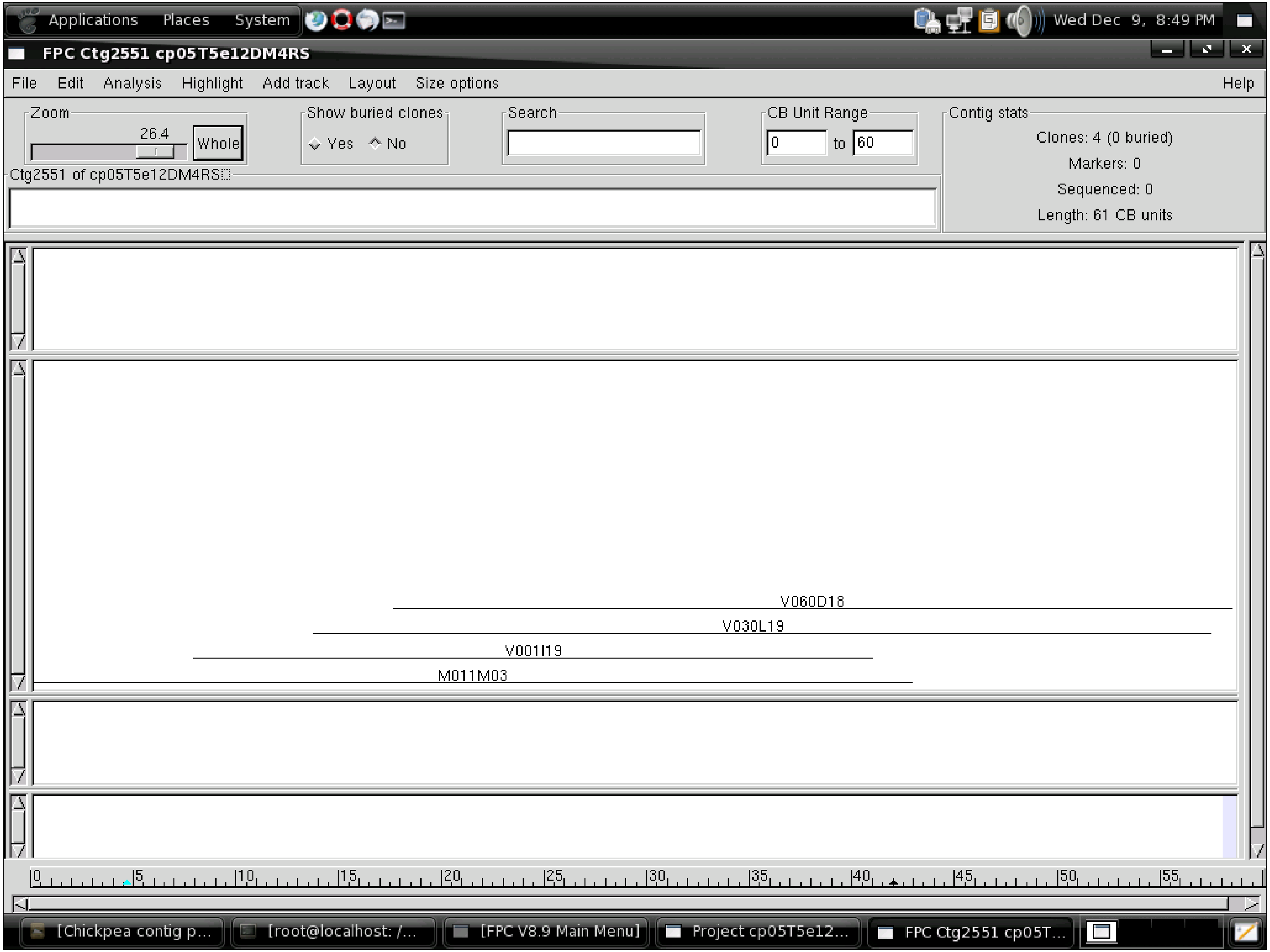
Applications Places System
Wed Dec 9, 8:49 PM
FPC Ctg2551 cp05T5e12DM4RS
File Edit Analysis Highlight Add track Layout Size options Help
Zoom
26.4
Whole
Show buried clones
Yes No
Search
CB Unit Range
0 to 60
Contig stats
Clones: 4 (0 buried)
Markers: 0
Sequenced: 0
Length: 61 CB units
Ctg2551 of cp05T5e12DM4RS
V060D18
V030L19
V001I19
M011M03
0 5 10 15 20 25 30 35 40 45 50 55
[Chickpea contig p...
[root@localhost: /...
[FPC V8.9 Main Menu]
Project cp05T5e12...
FPC Ctg2551 cp05T...

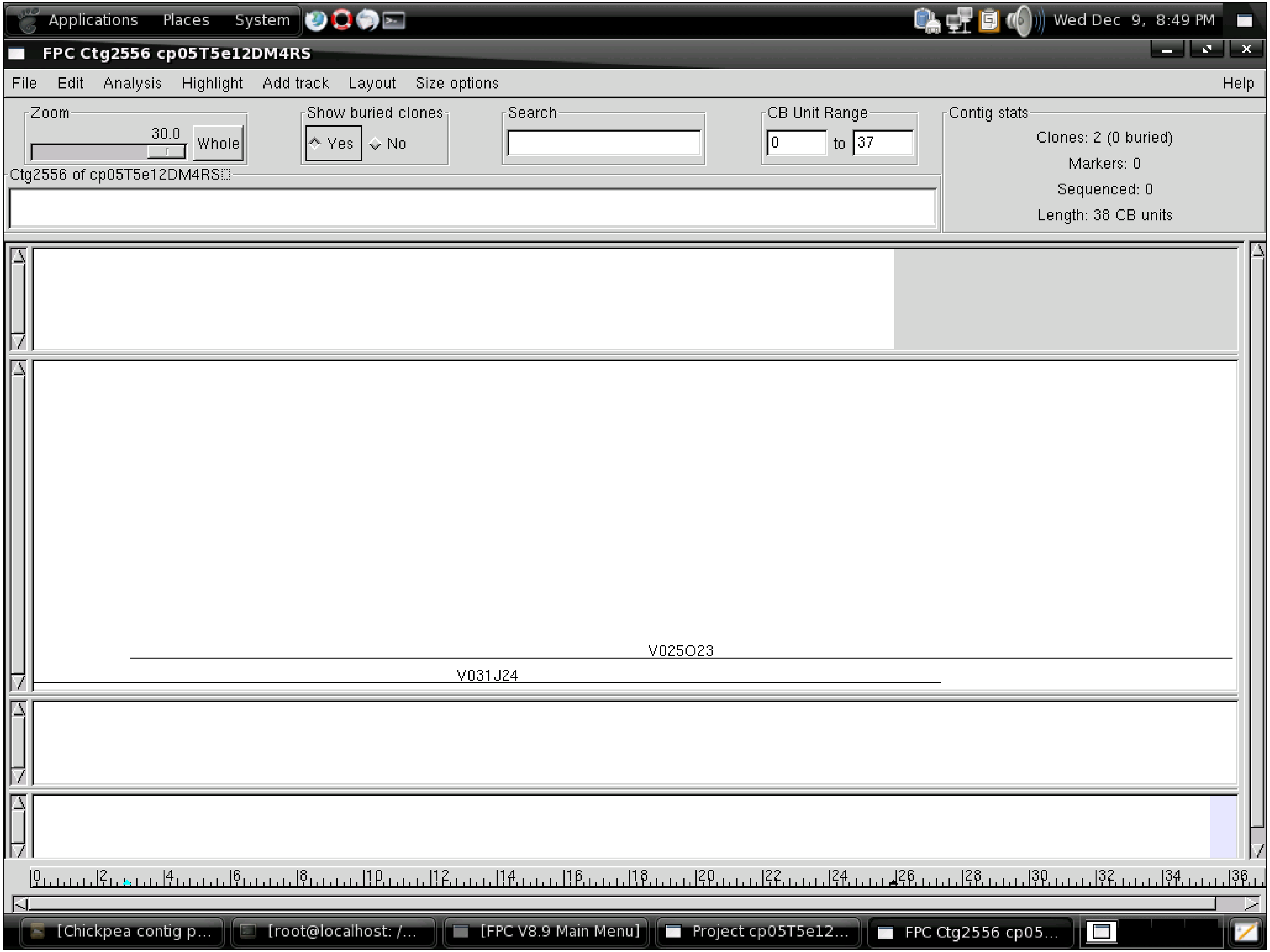
Applications Places System
Wed Dec 9, 8:49 PM
FPC Ctg2556 cp05T5e12DM4RS
File Edit Analysis Highlight Add track Layout Size options
Help
Zoom
30.0
Whole
Show buried clones
Yes
No
Search
CB Unit Range
0
to
37
Contig stats
Clones: 2 (0 buried)
Markers: 0
Sequenced: 0
Length: 38 CB units
Ctg2556 of cp05T5e12DM4RS
V025O23
V031J24
0 2 4 6 8 10 12 14 16 18 20 22 24 26 28 30 32 34 36
[Chickpea contig p...
[root@localhost: /...
[FPC V8.9 Main Menu]
Project cp05T5e12...
FPC Ctg2556 cp05...

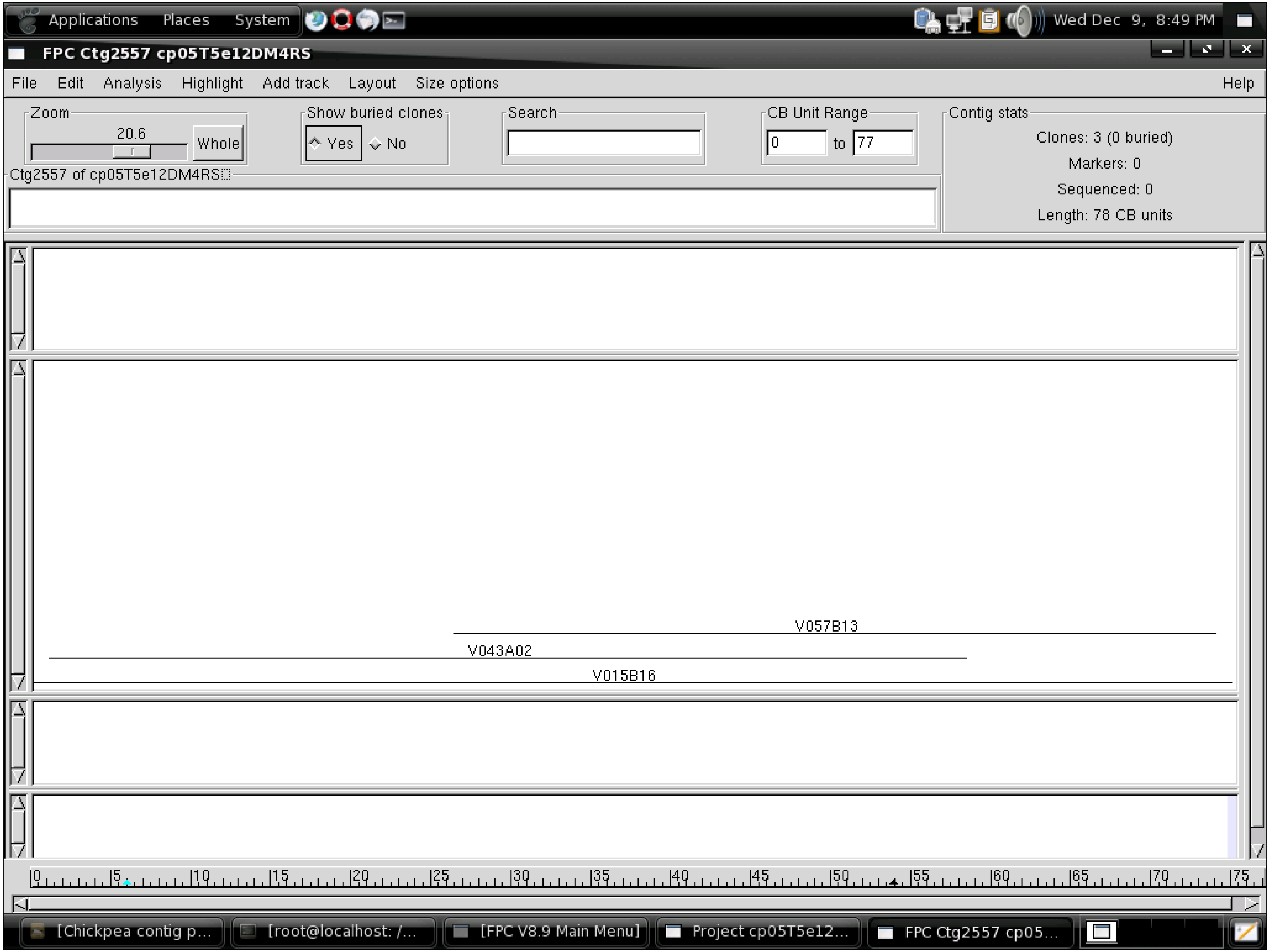
Applications Places System
Wed Dec 9, 8:49 PM
FPC Ctg2557 cp05T5e12DM4RS
File Edit Analysis Highlight Add track Layout Size options Help
Zoom
20.6
Whole
Show buried clones
Yes
No
Search
CB Unit Range
0
to
77
Contig stats
Clones: 3 (0 buried)
Markers: 0
Sequenced: 0
Length: 78 CB units
Ctg2557 of cp05T5e12DM4RS
V057B13
V043A02
V015B16
0 5 10 15 20 25 30 35 40 45 50 55 60 65 70 75
[Chickpea contig p...
[root@localhost: /...
[FPC V8.9 Main Menu]
Project cp05T5e12...
FPC Ctg2557 cp05...

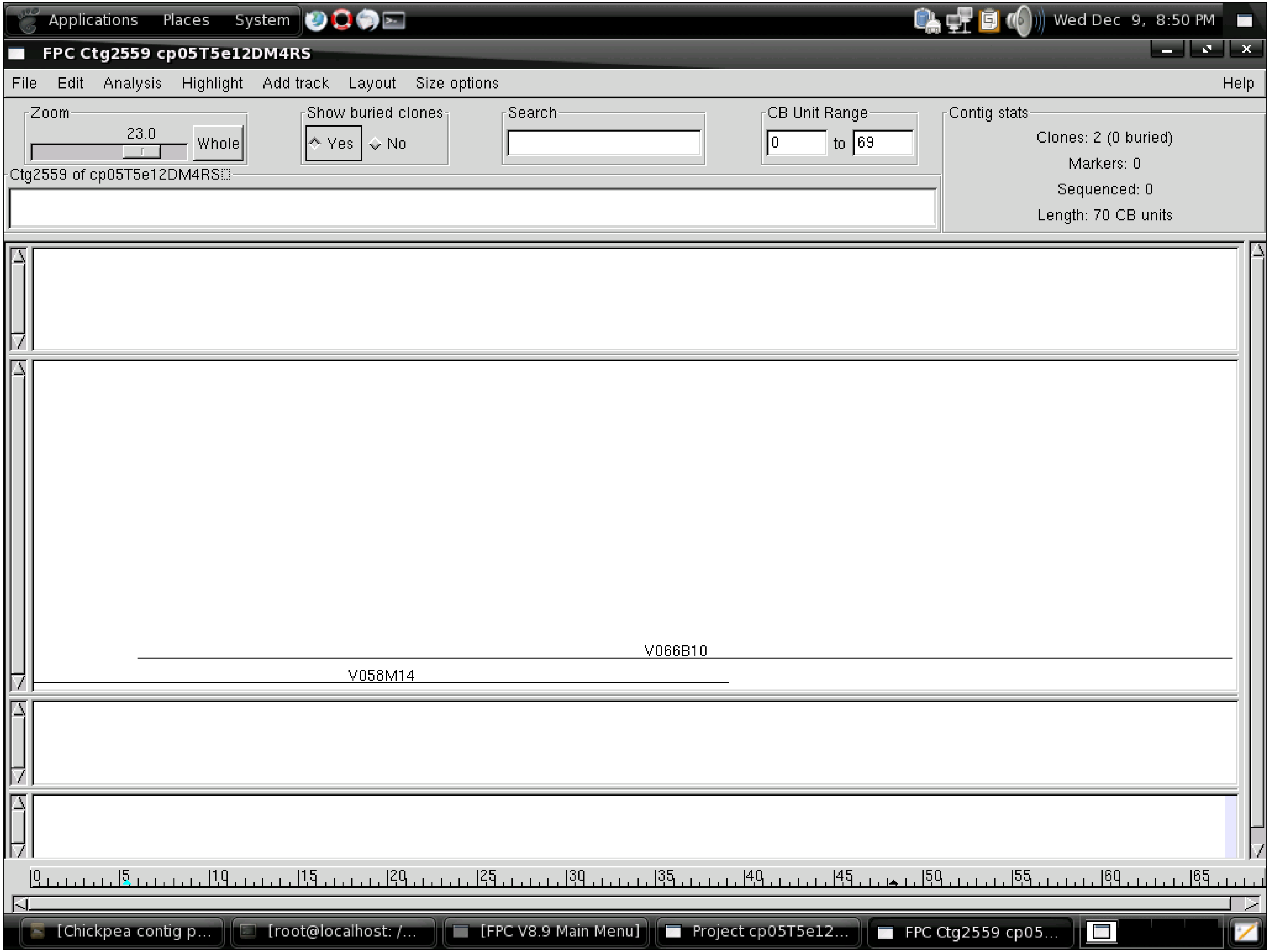
Applications Places System
Wed Dec 9, 8:50 PM
FPC Ctg2559 cp05T5e12DM4RS
File Edit Analysis Highlight Add track Layout Size options Help
Zoom
23.0
Whole
Show buried clones
Yes
No
Search
CB Unit Range
0 to 69
Contig stats
Clones: 2 (0 buried)
Markers: 0
Sequenced: 0
Length: 70 CB units
Ctg2559 of cp05T5e12DM4RS
V066B10
V058M14
0 5 10 15 20 25 30 35 40 45 50 55 60 65
[Chickpea contig p...
[root@localhost: /...
[FPC V8.9 Main Menu]
Project cp05T5e12...
FPC Ctg2559 cp05...

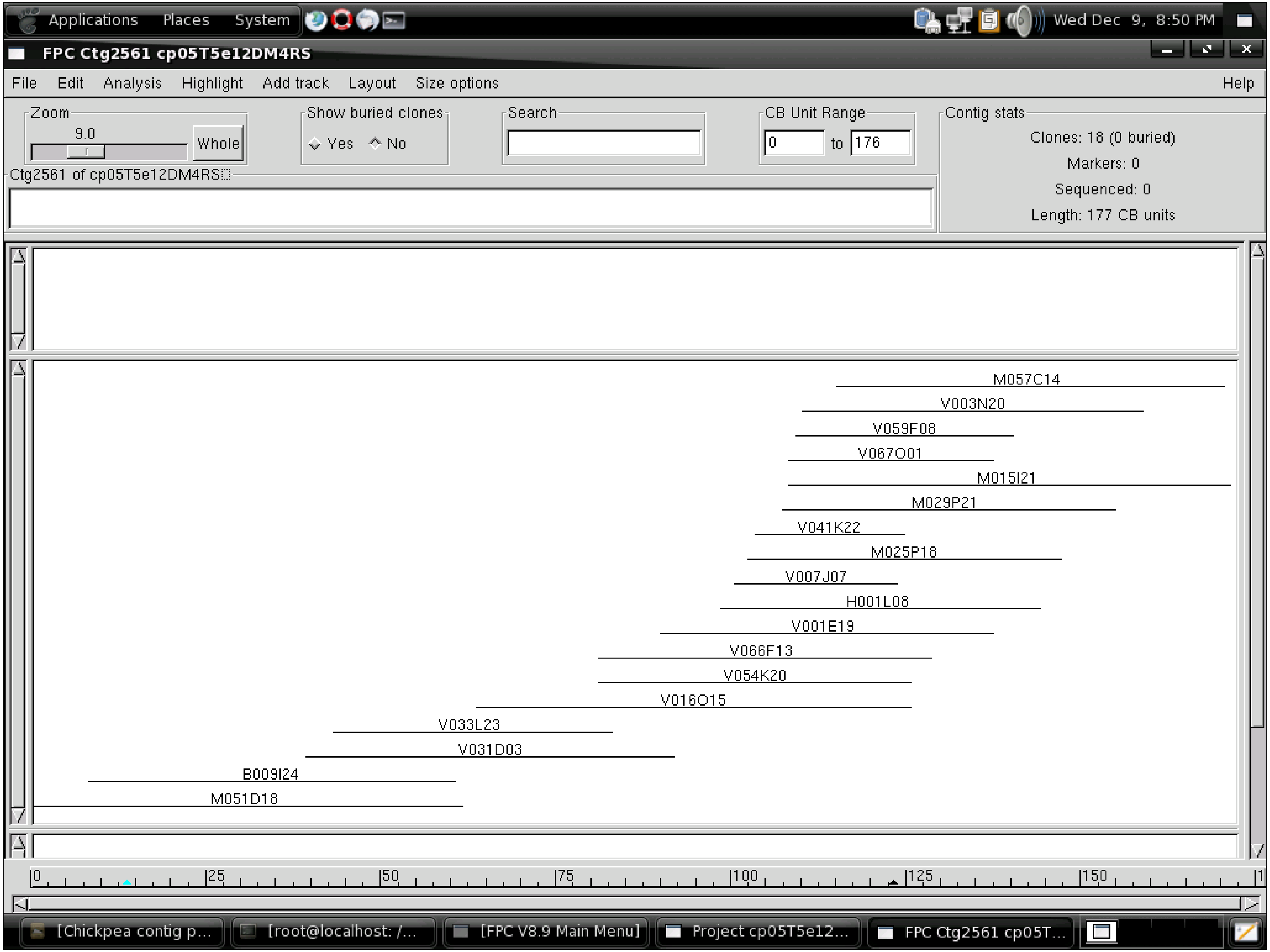
Applications Places System
Wed Dec 9, 8:50 PM
FPC Ctg2561 cp05T5e12DM4RS
File Edit Analysis Highlight Add track Layout Size options Help
Zoom
9.0
Whole
Show buried clones
Yes No
Search
CB Unit Range
0 to 176
Contig stats
Clones: 18 (0 buried)
Markers: 0
Sequenced: 0
Length: 177 CB units
Ctg2561 of cp05T5e12DM4RS
M057C14
V003N20
V059F08
V067O01
M015I21
M029P21
V041K22
M025P18
V007J07
H001L08
V001E19
V066F13
V054K20
V016O15
V033L23
V031D03
B009I24
M051D18
0 25 50 75 100 125 150
[Chickpea contig p...
[root@localhost: /...
[FPC V8.9 Main Menu]
Project cp05T5e12...
FPC Ctg2561 cp05T...

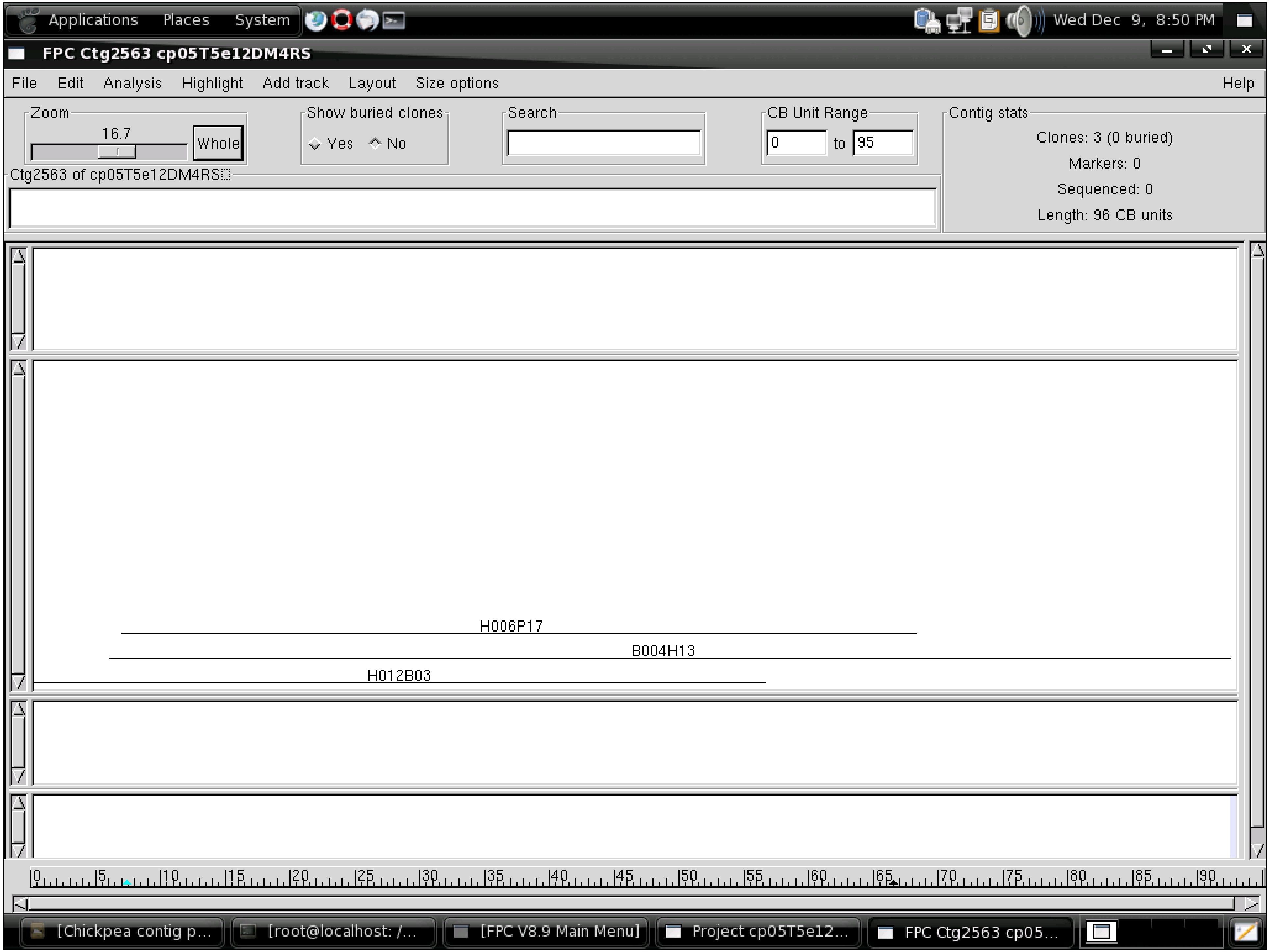

Applications Places System
Wed Dec 9, 8:50 PM
FPC Ctg2563 cp05T5e12DM4RS
File Edit Analysis Highlight Add track Layout Size options Help
Zoom
16.7
Whole
Show buried clones
Yes No
Search
CB Unit Range
0 to 95
Contig stats
Clones: 3 (0 buried)
Markers: 0
Sequenced: 0
Length: 96 CB units
Ctg2563 of cp05T5e12DM4RS
H006P17
B004H13
H012B03
0 5 10 15 20 25 30 35 40 45 50 55 60 65 70 75 80 85 90
[Chickpea contig p...
[root@localhost: /...
[FPC V8.9 Main Menu]
Project cp05T5e12...
FPC Ctg2563 cp05...

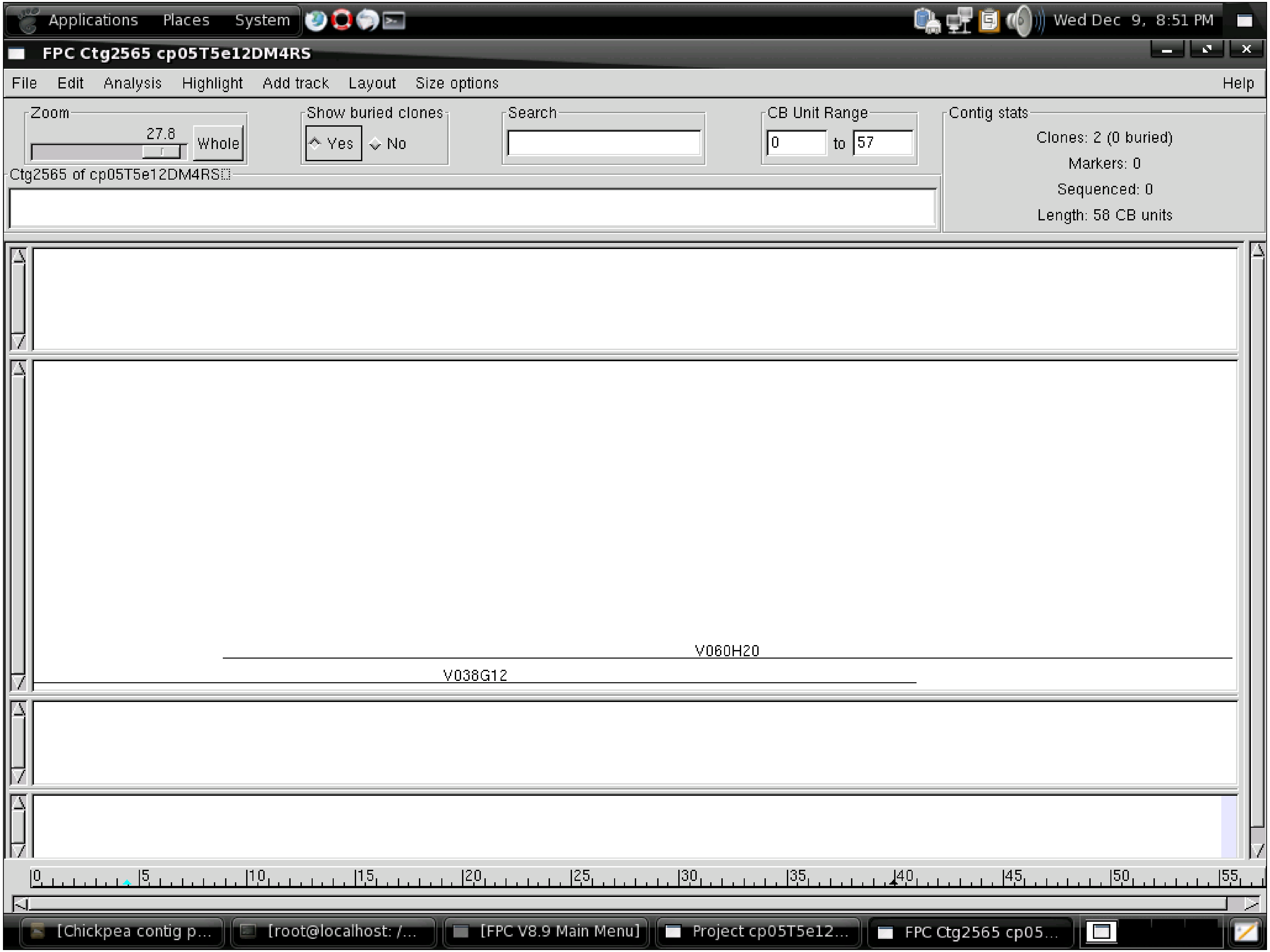
Applications Places System
Wed Dec 9, 8:51 PM
FPC Ctg2565 cp05T5e12DM4RS
File Edit Analysis Highlight Add track Layout Size options Help
Zoom
27.8
Whole
Show buried clones
Yes No
Search
CB Unit Range
0 to 57
Contig stats
Clones: 2 (0 buried)
Markers: 0
Sequenced: 0
Length: 58 CB units
Ctg2565 of cp05T5e12DM4RS
V060H20
V038G12
0 5 10 15 20 25 30 35 40 45 50 55
[Chickpea contig p...
[root@localhost: /...
[FPC V8.9 Main Menu]
Project cp05T5e12...
FPC Ctg2565 cp05...

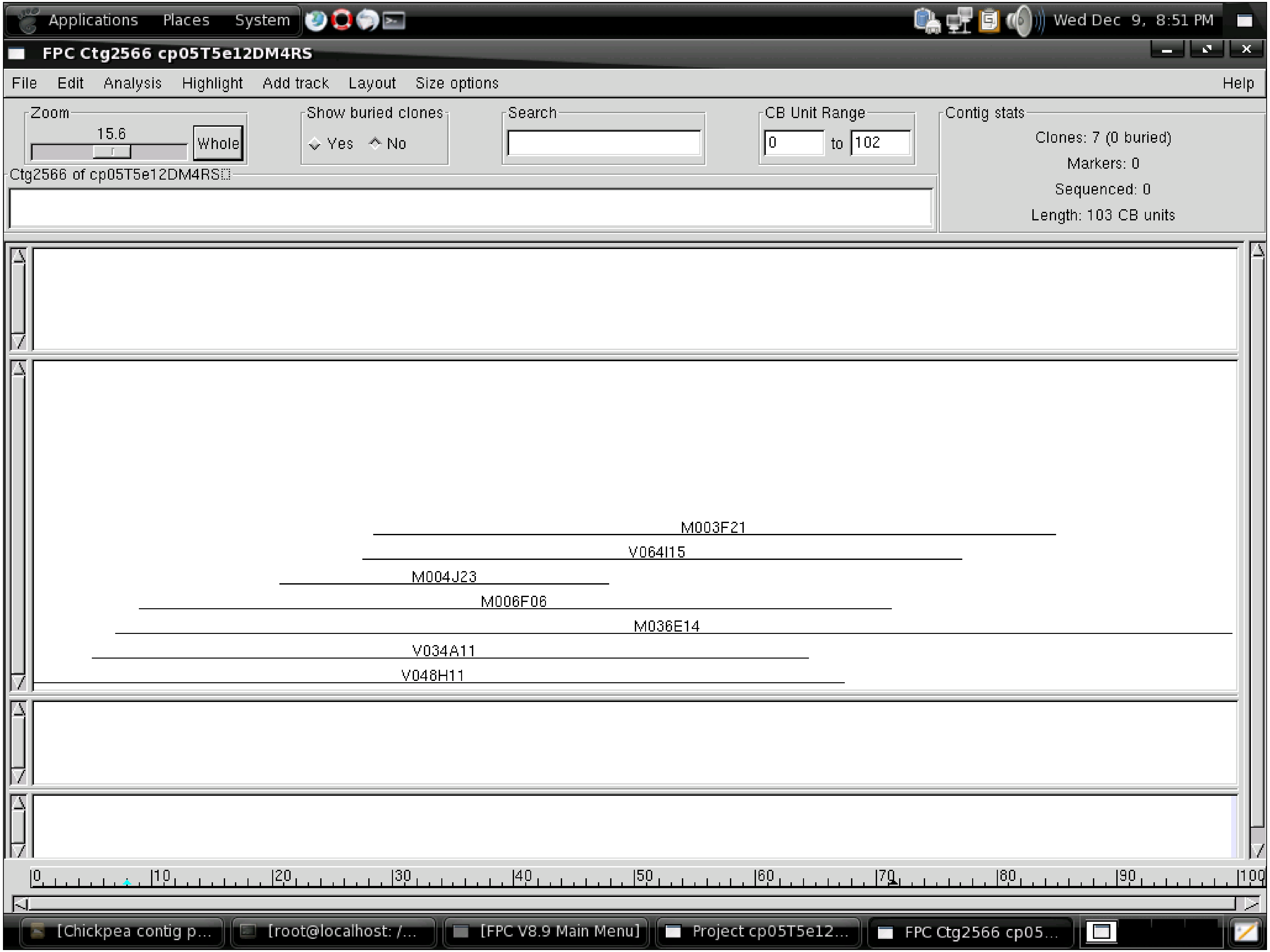
Applications Places System
Wed Dec 9, 8:51 PM
FPC Ctg2566 cp05T5e12DM4RS
File Edit Analysis Highlight Add track Layout Size options Help
Zoom
15.6
Whole
Show buried clones
Yes No
Search
CB Unit Range
0 to 102
Contig stats
Clones: 7 (0 buried)
Markers: 0
Sequenced: 0
Length: 103 CB units
Ctg2566 of cp05T5e12DM4RS
M003F21
V064I15
M004J23
M006F06
M036E14
V034A11
V048H11
0 10 20 30 40 50 60 70 80 90 100
[Chickpea contig p...
[root@localhost: /...
[FPC V8.9 Main Menu]
Project cp05T5e12...
FPC Ctg2566 cp05...

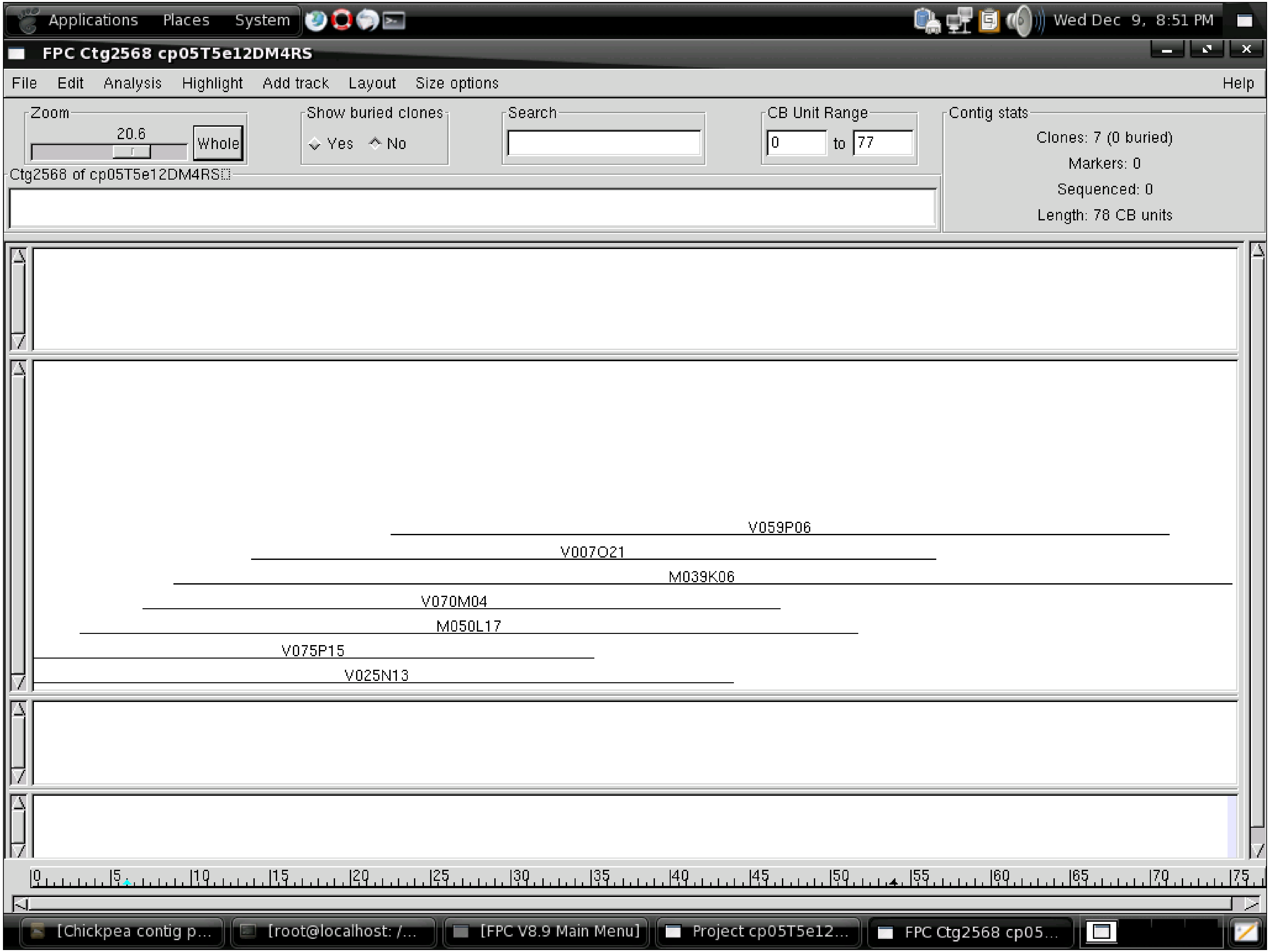
Applications Places System
Wed Dec 9, 8:51 PM
FPC Ctg2568 cp05T5e12DM4RS
File Edit Analysis Highlight Add track Layout Size options Help
Zoom
20.6
Whole
Show buried clones
Yes No
Search
CB Unit Range
0 to 77
Contig stats
Clones: 7 (0 buried)
Markers: 0
Sequenced: 0
Length: 78 CB units
Ctg2568 of cp05T5e12DM4RS
V059P06
V007O21
M039K06
V070M04
M050L17
V075P15
V025N13
0 5 10 15 20 25 30 35 40 45 50 55 60 65 70 75
[Chickpea contig p...
[root@localhost: /...
[FPC V8.9 Main Menu]
Project cp05T5e12...
FPC Ctg2568 cp05...

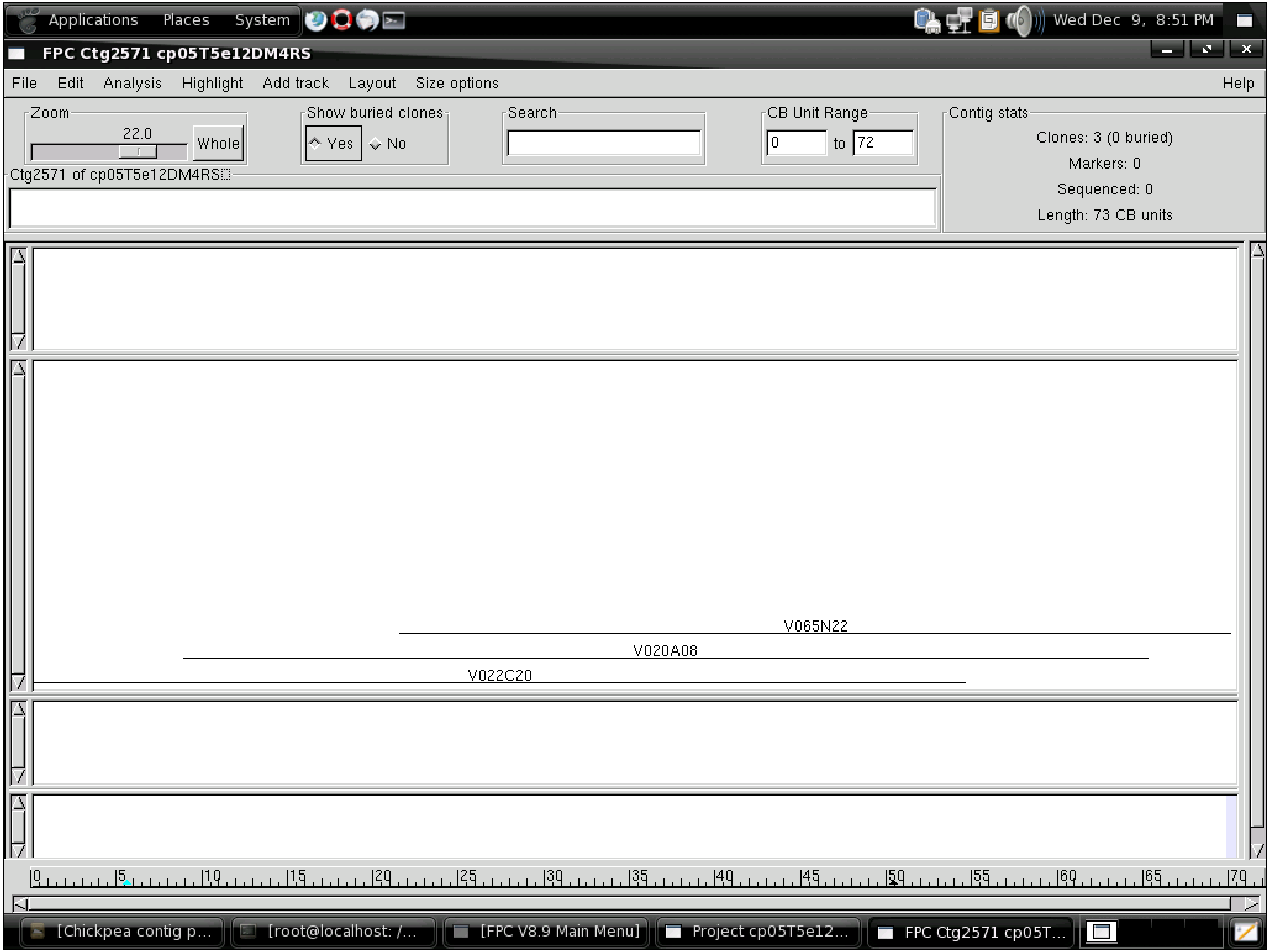
Applications Places System
Wed Dec 9, 8:51 PM
FPC Ctg2571 cp05T5e12DM4RS
File Edit Analysis Highlight Add track Layout Size options Help
Zoom
22.0
Whole
Show buried clones
Yes
No
Search
CB Unit Range
0 to 72
Contig stats
Clones: 3 (0 buried)
Markers: 0
Sequenced: 0
Length: 73 CB units
Ctg2571 of cp05T5e12DM4RS
V065N22
V020A08
V022C20
0 5 10 15 20 25 30 35 40 45 50 55 60 65 70
[Chickpea contig p...
[root@localhost: /...
[FPC V8.9 Main Menu]
Project cp05T5e12...
FPC Ctg2571 cp05T...

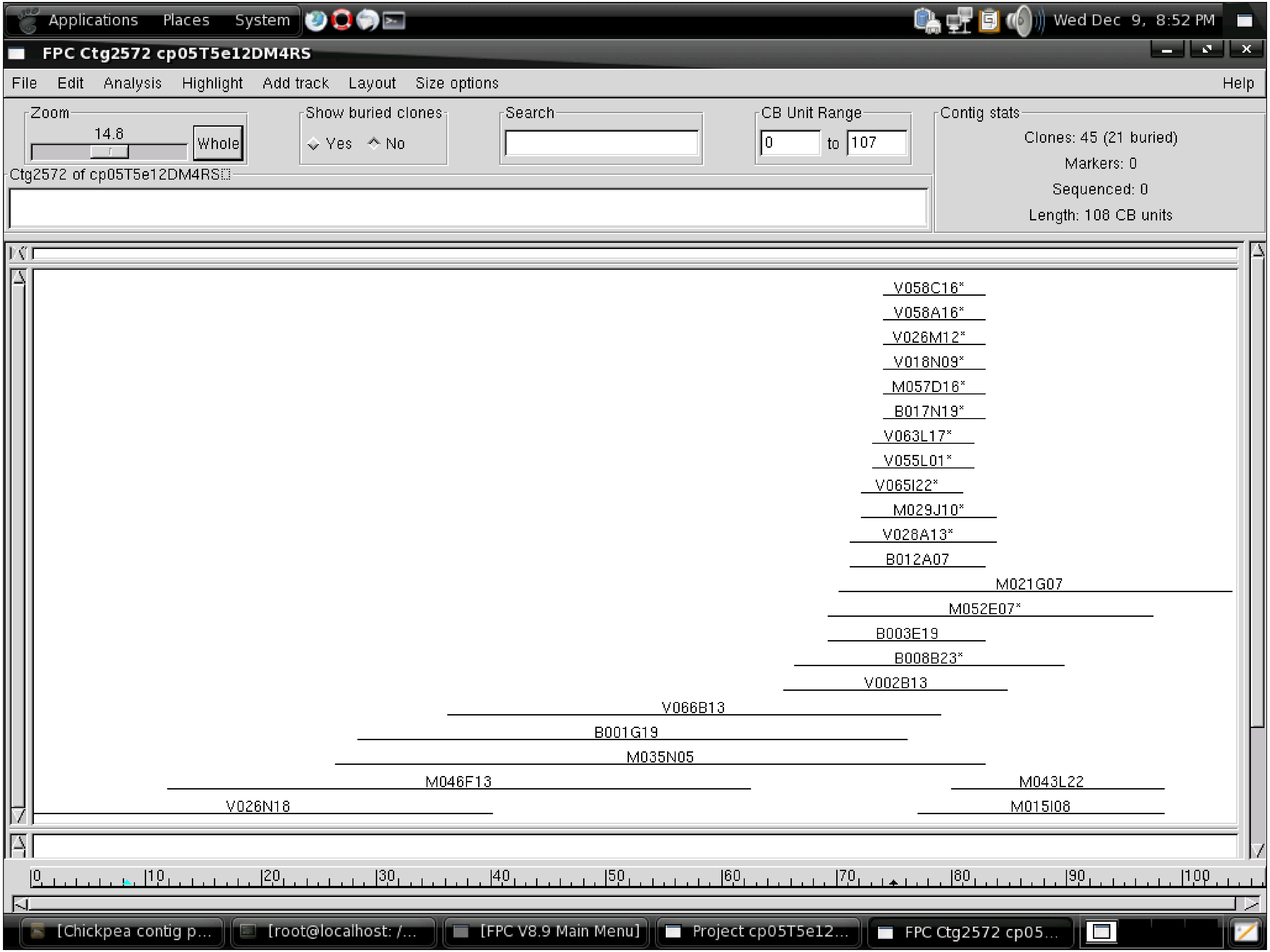
Applications Places System
Wed Dec 9, 8:52 PM
FPC Ctg2572 cp05T5e12DM4RS
File Edit Analysis Highlight Add track Layout Size options Help
Zoom
14.8
Whole
Show buried clones
Yes No
Search
CB Unit Range
0 to 107
Contig stats
Clones: 45 (21 buried)
Markers: 0
Sequenced: 0
Length: 108 CB units
Ctg2572 of cp05T5e12DM4RS
V058C16*
V058A16*
V026M12*
V018N09*
M057D16*
B017N19*
V063L17*
V055L01*
V065I22*
M029J10*
V028A13*
B012A07
M021G07
M052E07*
B003E19
B008B23*
V002B13
V066B13
B001G19
M035N05
M046F13
M043L22
V026N18
M015I08
0 10 20 30 40 50 60 70 80 90 100
[Chickpea contig p...
[root@localhost: /...
[FPC V8.9 Main Menu]
Project cp05T5e12...
FPC Ctg2572 cp05...

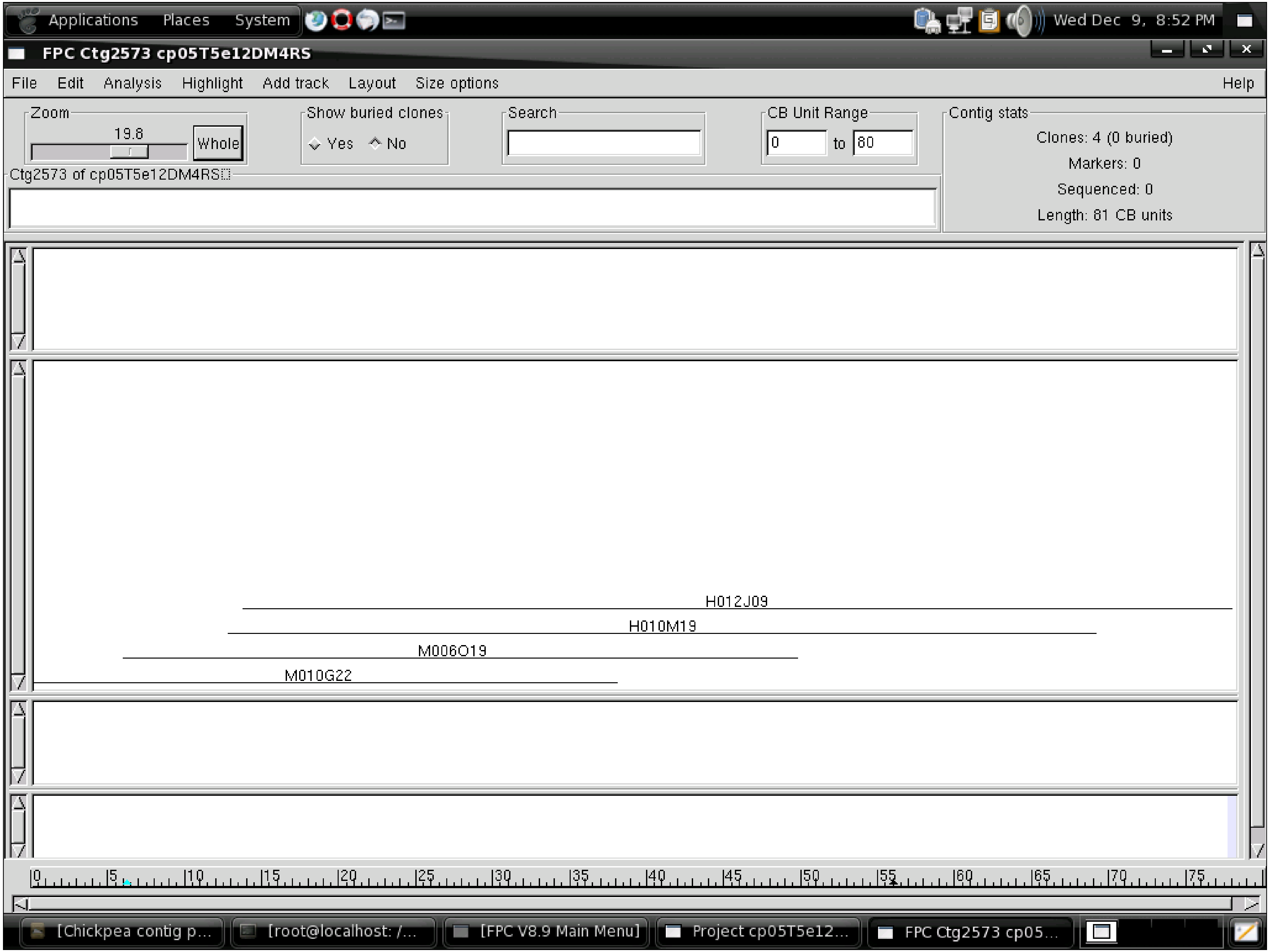
Applications Places System
Wed Dec 9, 8:52 PM
FPC Ctg2573 cp05T5e12DM4RS
File Edit Analysis Highlight Add track Layout Size options Help
Zoom
19.8
Whole
Show buried clones
Yes No
Search
CB Unit Range
0 to 80
Contig stats
Clones: 4 (0 buried)
Markers: 0
Sequenced: 0
Length: 81 CB units
Ctg2573 of cp05T5e12DM4RS
H012J09
H010M19
M006O19
M010G22
0 5 10 15 20 25 30 35 40 45 50 55 60 65 70 75
[Chickpea contig p...
[root@localhost: /...
[FPC V8.9 Main Menu]
Project cp05T5e12...
FPC Ctg2573 cp05...

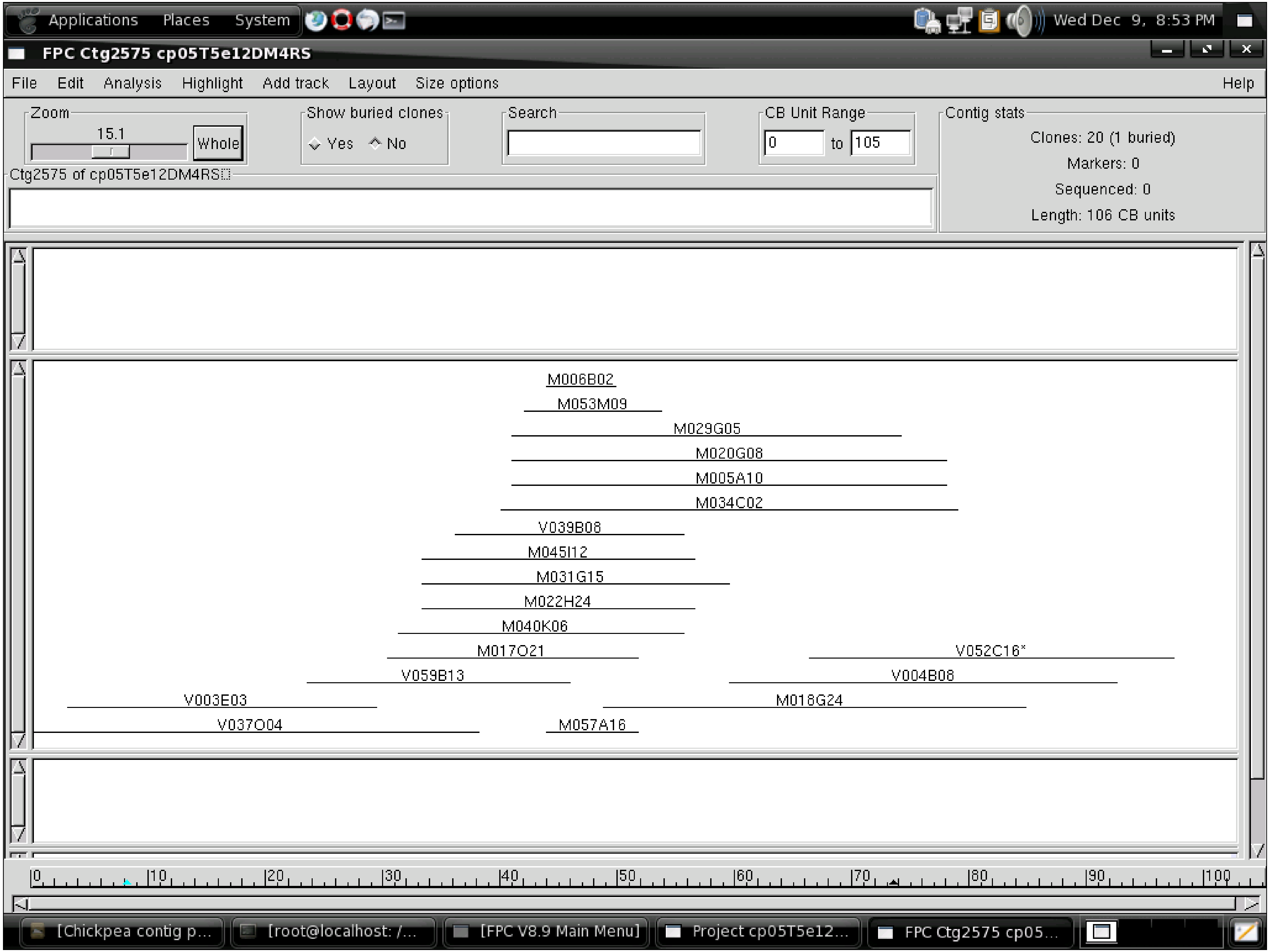

Applications Places System
Wed Dec 9, 8:53 PM
FPC Ctg2575 cp05T5e12DM4RS
File Edit Analysis Highlight Add track Layout Size options Help
Zoom
15.1
Whole
Show buried clones
Yes No
Search
CB Unit Range
0 to 105
Contig stats
Clones: 20 (1 buried)
Markers: 0
Sequenced: 0
Length: 106 CB units
Ctg2575 of cp05T5e12DM4RS
M006B02
M053M09
M029G05
M020G08
M005A10
M034C02
V039B08
M045I12
M031G15
M022H24
M040K06
M017O21
V052C16*
V059B13
V004B08
V003E03
M018G24
V037O04
M057A16
0 10 20 30 40 50 60 70 80 90 100
[Chickpea contig p...
[root@localhost: /...
[FPC V8.9 Main Menu]
Project cp05T5e12...
FPC Ctg2575 cp05...

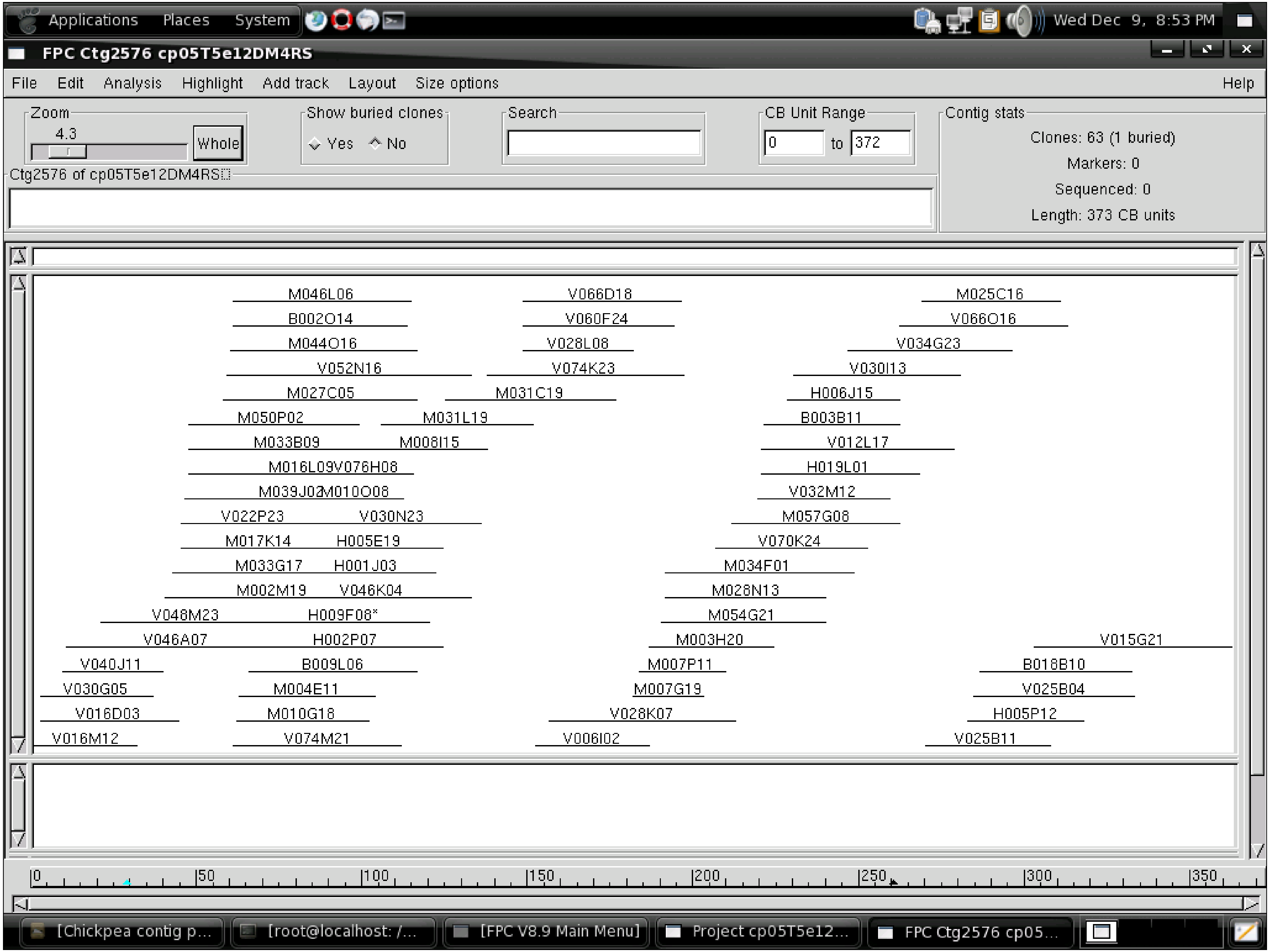
Applications Places System
Wed Dec 9, 8:53 PM
FPC Ctg2576 cp05T5e12DM4RS
File Edit Analysis Highlight Add track Layout Size options Help
Zoom
4.3
Whole
Show buried clones
Yes No
Search
CB Unit Range
0 to 372
Contig stats
Clones: 63 (1 buried)
Markers: 0
Sequenced: 0
Length: 373 CB units
Ctg2576 of cp05T5e12DM4RS
M046L06
B002O14
M044O16
V052N16
M027C05
M050P02
M033B09
M016L09V076H08
M039J02M010O08
V022P23
M017K14
M033G17
M002M19
V048M23
V046A07
V040J11
V030G05
V016D03
V016M12
V030N23
H005E19
H001J03
V046K04
H009F08*
H002P07
B009L06
M004E11
M010G18
V074M21
V066D18
V060F24
V028L08
V074K23
M031C19
M031L19
M008I15
V028K07
V006I02
M025C16
V066O16
V034G23
V030I13
H006J15
B003B11
V012L17
H019L01
V032M12
M057G08
V070K24
M034F01
M028N13
M054G21
M003H20
M007P11
M007G19
V015G21
B018B10
V025B04
H005P12
V025B11
0
50
100
150
200
250
300
350
[Chickpea contig p...
[root@localhost: /...
[FPC V8.9 Main Menu]
Project cp05T5e12...
FPC Ctg2576 cp05...

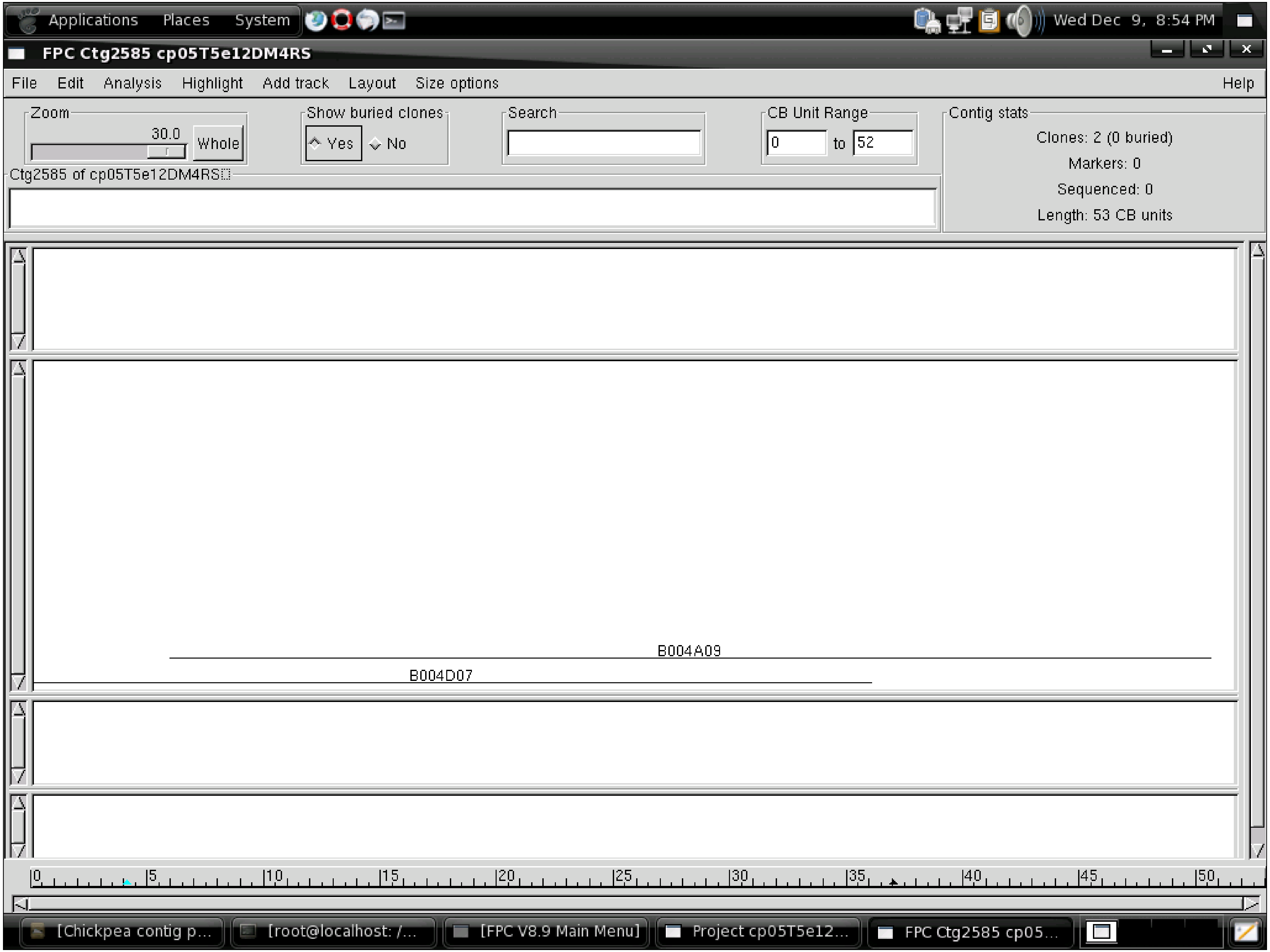
Applications Places System
Wed Dec 9, 8:54 PM
FPC Ctg2585 cp05T5e12DM4RS
File Edit Analysis Highlight Add track Layout Size options Help
Zoom
30.0
Whole
Show buried clones
Yes No
Search
CB Unit Range
0 to 52
Contig stats
Clones: 2 (0 buried)
Markers: 0
Sequenced: 0
Length: 53 CB units
Ctg2585 of cp05T5e12DM4RS
B004A09
B004D07
0 5 10 15 20 25 30 35 40 45 50
[Chickpea contig p...
[root@localhost: /...
[FPC V8.9 Main Menu]
Project cp05T5e12...
FPC Ctg2585 cp05...

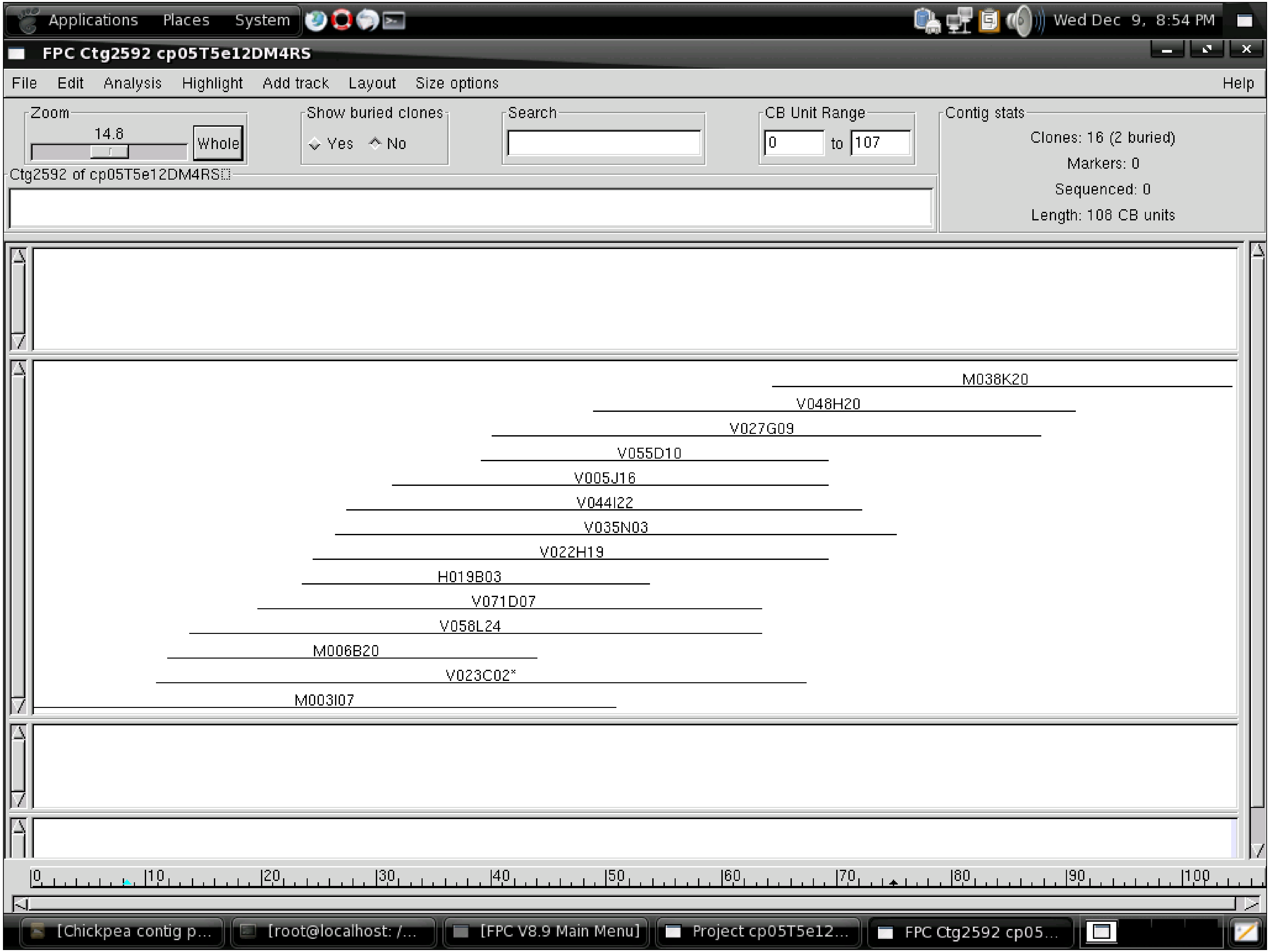
Applications Places System
Wed Dec 9, 8:54 PM
FPC Ctg2592 cp05T5e12DM4RS
File Edit Analysis Highlight Add track Layout Size options Help
Zoom
14.8
Whole
Show buried clones
Yes No
Search
CB Unit Range
0 to 107
Contig stats
Clones: 16 (2 buried)
Markers: 0
Sequenced: 0
Length: 108 CB units
Ctg2592 of cp05T5e12DM4RS
M038K20
V048H20
V027G09
V055D10
V005J16
V044I22
V035N03
V022H19
H019B03
V071D07
V058L24
M006B20
V023C02*
M003I07
0 10 20 30 40 50 60 70 80 90 100
[Chickpea contig p...
[root@localhost: /...
[FPC V8.9 Main Menu]
Project cp05T5e12...
FPC Ctg2592 cp05...

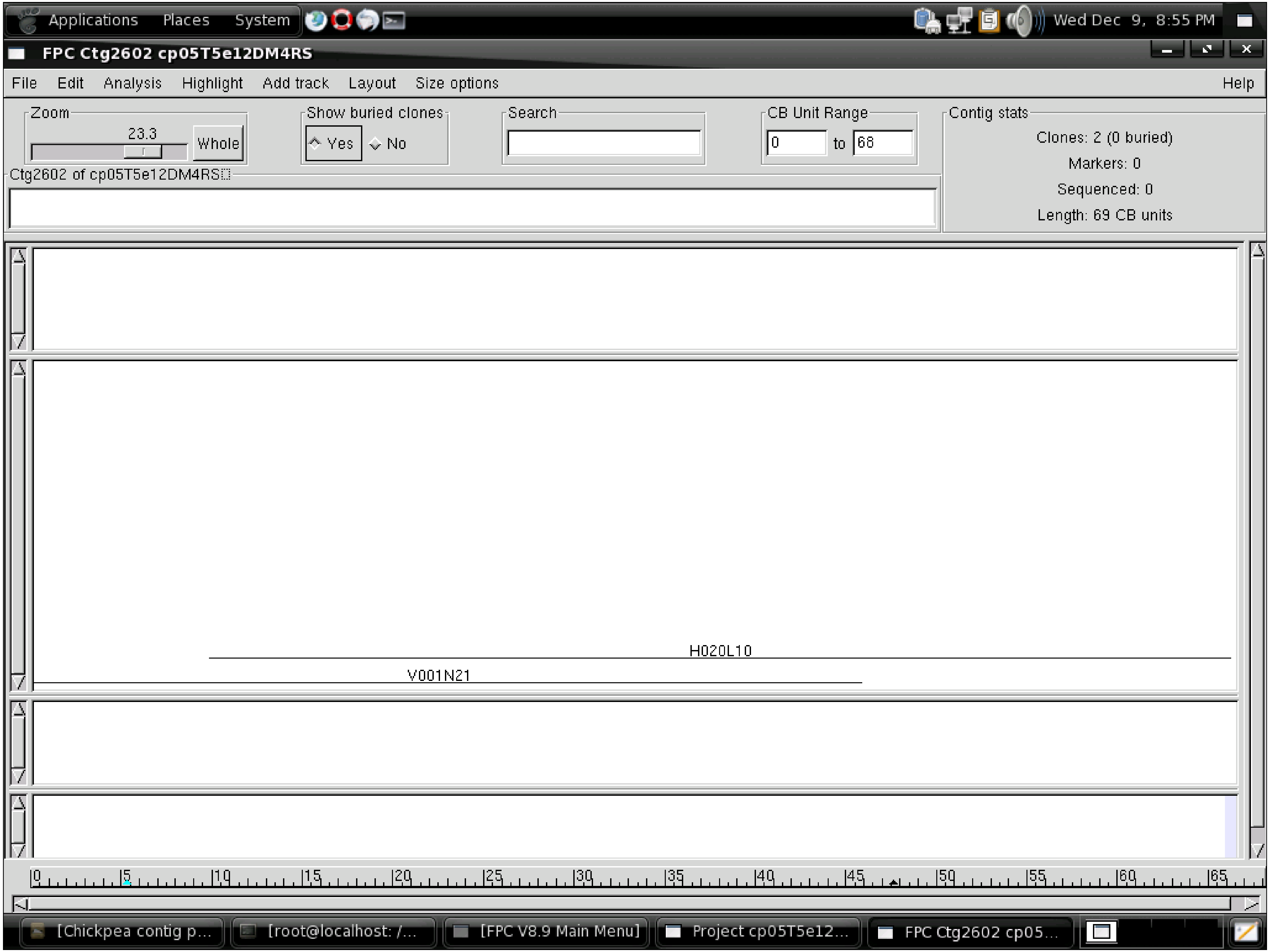
Applications Places System
Wed Dec 9, 8:55 PM
FPC Ctg2602 cp05T5e12DM4RS
File Edit Analysis Highlight Add track Layout Size options Help
Zoom
23.3
Whole
Show buried clones
Yes
No
Search
CB Unit Range
0
to
68
Contig stats
Clones: 2 (0 buried)
Markers: 0
Sequenced: 0
Length: 69 CB units
Ctg2602 of cp05T5e12DM4RS
H020L10
V001N21
0 5 10 15 20 25 30 35 40 45 50 55 60 65
[Chickpea contig p...
[root@localhost: /...
[FPC V8.9 Main Menu]
Project cp05T5e12...
FPC Ctg2602 cp05...

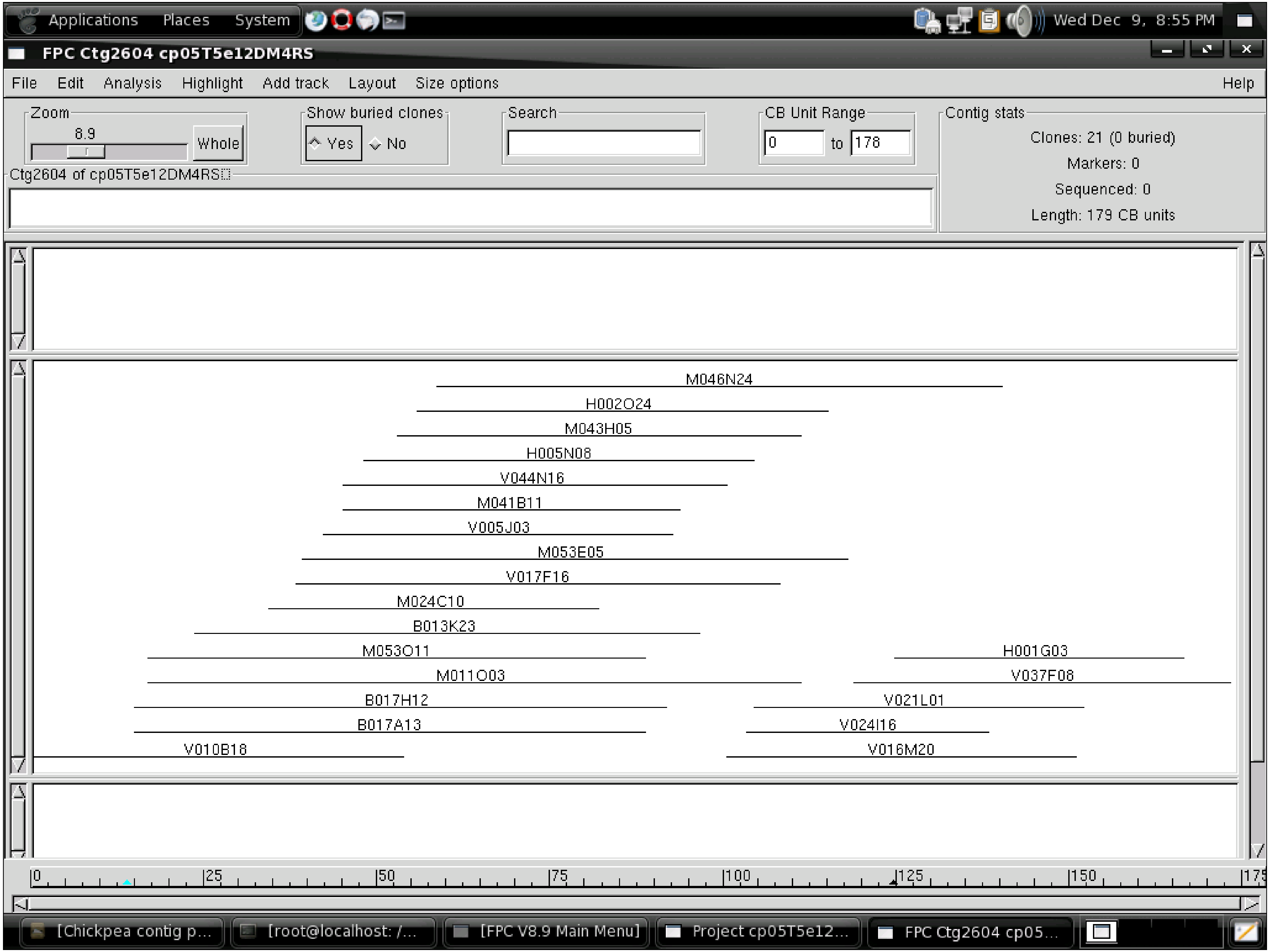
Applications Places System
Wed Dec 9, 8:55 PM
FPC Ctg2604 cp05T5e12DM4RS
File Edit Analysis Highlight Add track Layout Size options Help
Zoom
8.9
Whole
Show buried clones
Yes No
Search
CB Unit Range
0 to 178
Contig stats
Clones: 21 (0 buried)
Markers: 0
Sequenced: 0
Length: 179 CB units
Ctg2604 of cp05T5e12DM4RS
M046N24
H002O24
M043H05
H005N08
V044N16
M041B11
V005J03
M053E05
V017F16
M024C10
B013K23
M053O11
H001G03
M011O03
V037F08
B017H12
V021L01
B017A13
V024I16
V010B18
V016M20
0 25 50 75 100 125 150 175
[Chickpea contig p...
[root@localhost: /...
[FPC V8.9 Main Menu]
Project cp05T5e12...
FPC Ctg2604 cp05...

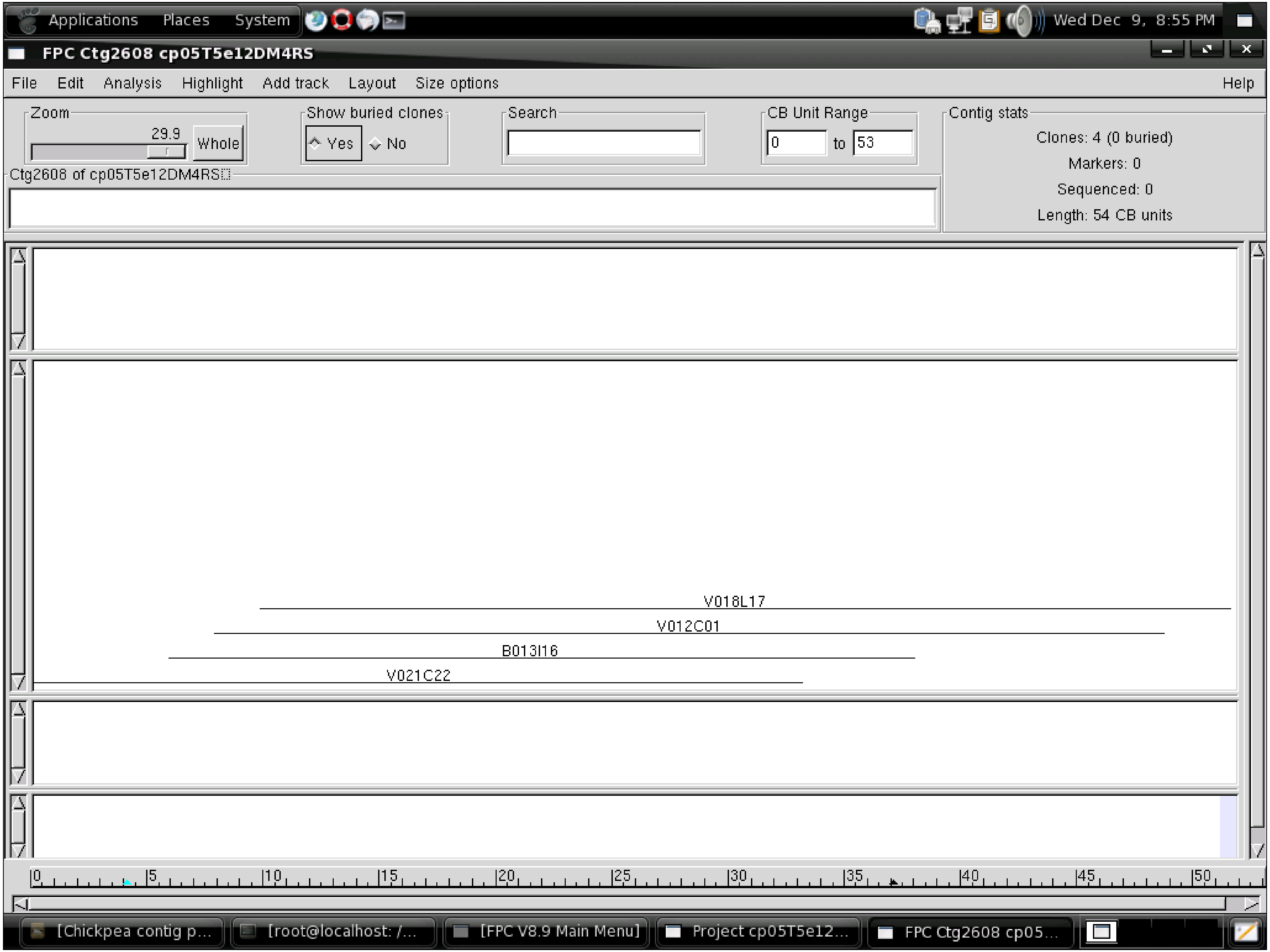
Applications
Places
System
Wed Dec 9, 8:55 PM
FPC Ctg2608 cp05T5e12DM4RS
File
Edit
Analysis
Highlight
Add track
Layout
Size options
Help
Zoom
29.9
Whole
Show buried clones
Yes
No
Search
CB Unit Range
0
to
53
Contig stats
Clones: 4 (0 buried)
Markers: 0
Sequenced: 0
Length: 54 CB units
Ctg2608 of cp05T5e12DM4RS
V018L17
V012C01
B013I16
V021C22
0
5
10
15
20
25
30
35
40
45
50
[Chickpea contig p...
[root@localhost: /...
[FPC V8.9 Main Menu]
Project cp05T5e12...
FPC Ctg2608 cp05...

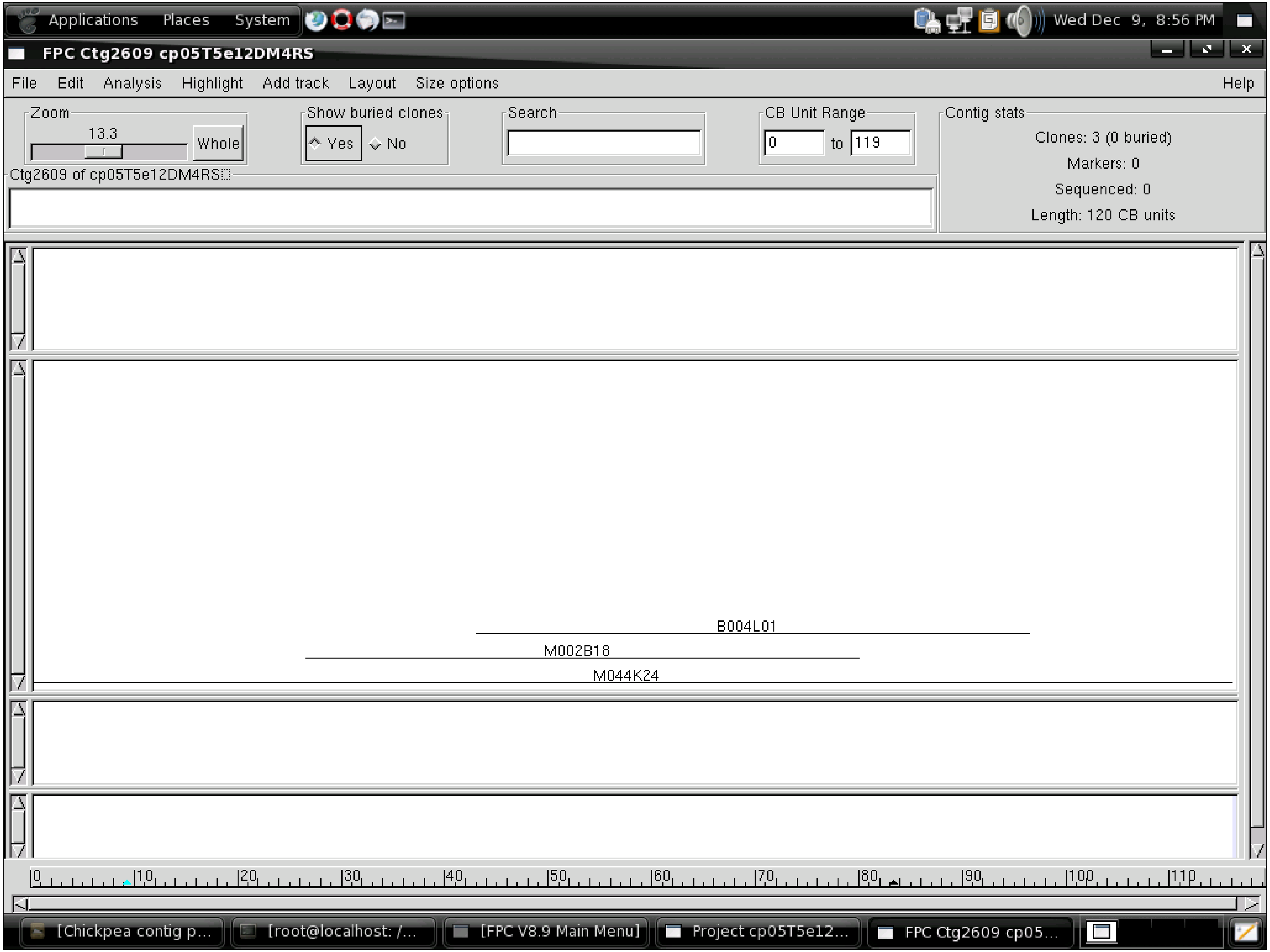
Applications
Places
System
Wed Dec 9, 8:56 PM
FPC Ctg2609 cp05T5e12DM4RS
File
Edit
Analysis
Highlight
Add track
Layout
Size options
Help
Zoom
13.3
Whole
Show buried clones
Yes
No
Search
CB Unit Range
0
to
119
Contig stats
Clones: 3 (0 buried)
Markers: 0
Sequenced: 0
Length: 120 CB units
Ctg2609 of cp05T5e12DM4RS
B004L01
M002B18
M044K24
0
10
20
30
40
50
60
70
80
90
100
110
[Chickpea contig p...
[root@localhost: /...
[FPC V8.9 Main Menu]
Project cp05T5e12...
FPC Ctg2609 cp05...

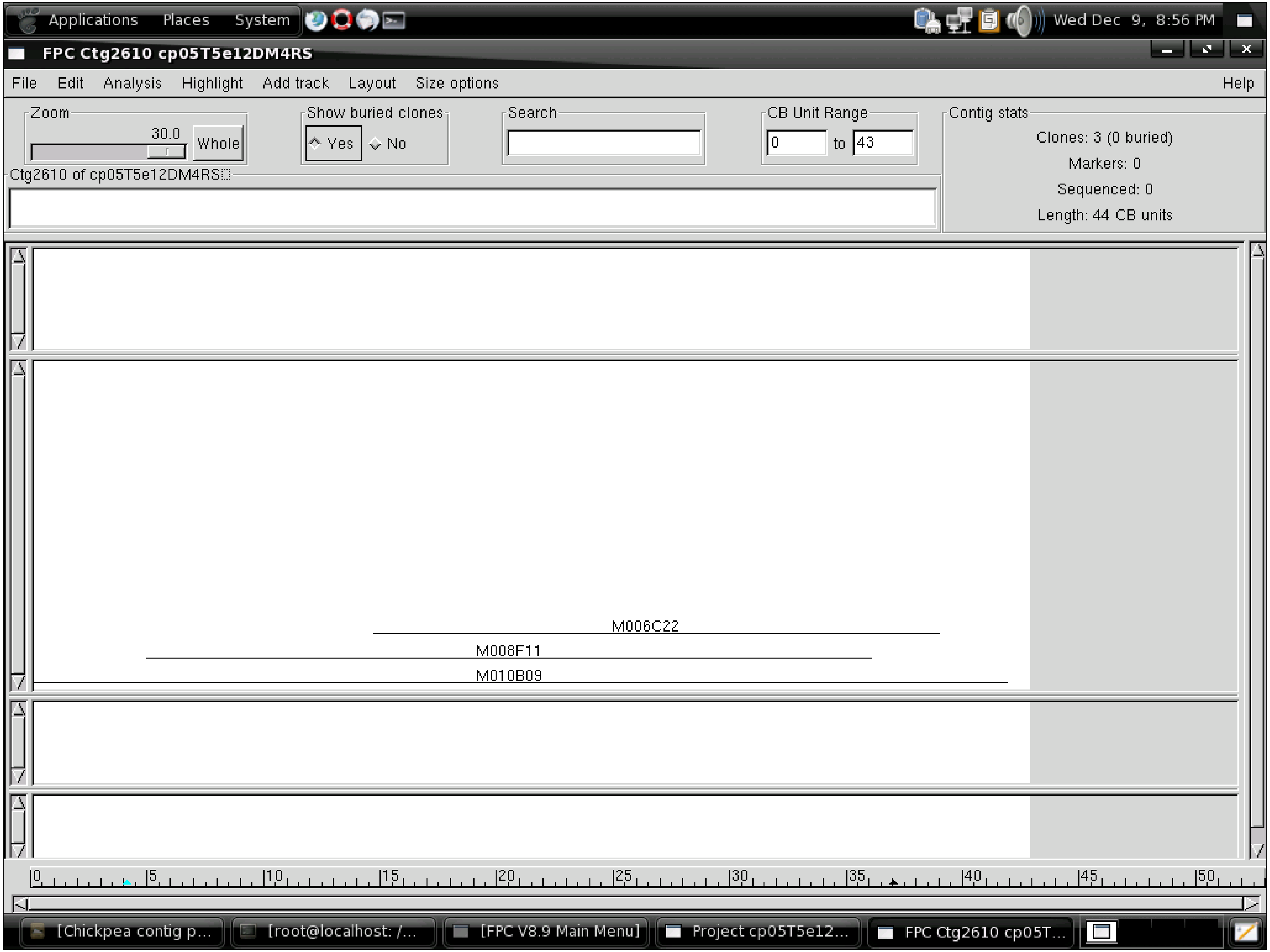
Applications Places System
Wed Dec 9, 8:56 PM
FPC Ctg2610 cp05T5e12DM4RS
File Edit Analysis Highlight Add track Layout Size options Help
Zoom
30.0
Whole
Show buried clones
Yes No
Search
CB Unit Range
0 to 43
Contig stats
Clones: 3 (0 buried)
Markers: 0
Sequenced: 0
Length: 44 CB units
Ctg2610 of cp05T5e12DM4RS
M006C22
M008F11
M010B09
0 5 10 15 20 25 30 35 40 45 50
[Chickpea contig p...
[root@localhost: /...
[FPC V8.9 Main Menu]
Project cp05T5e12...
FPC Ctg2610 cp05T...

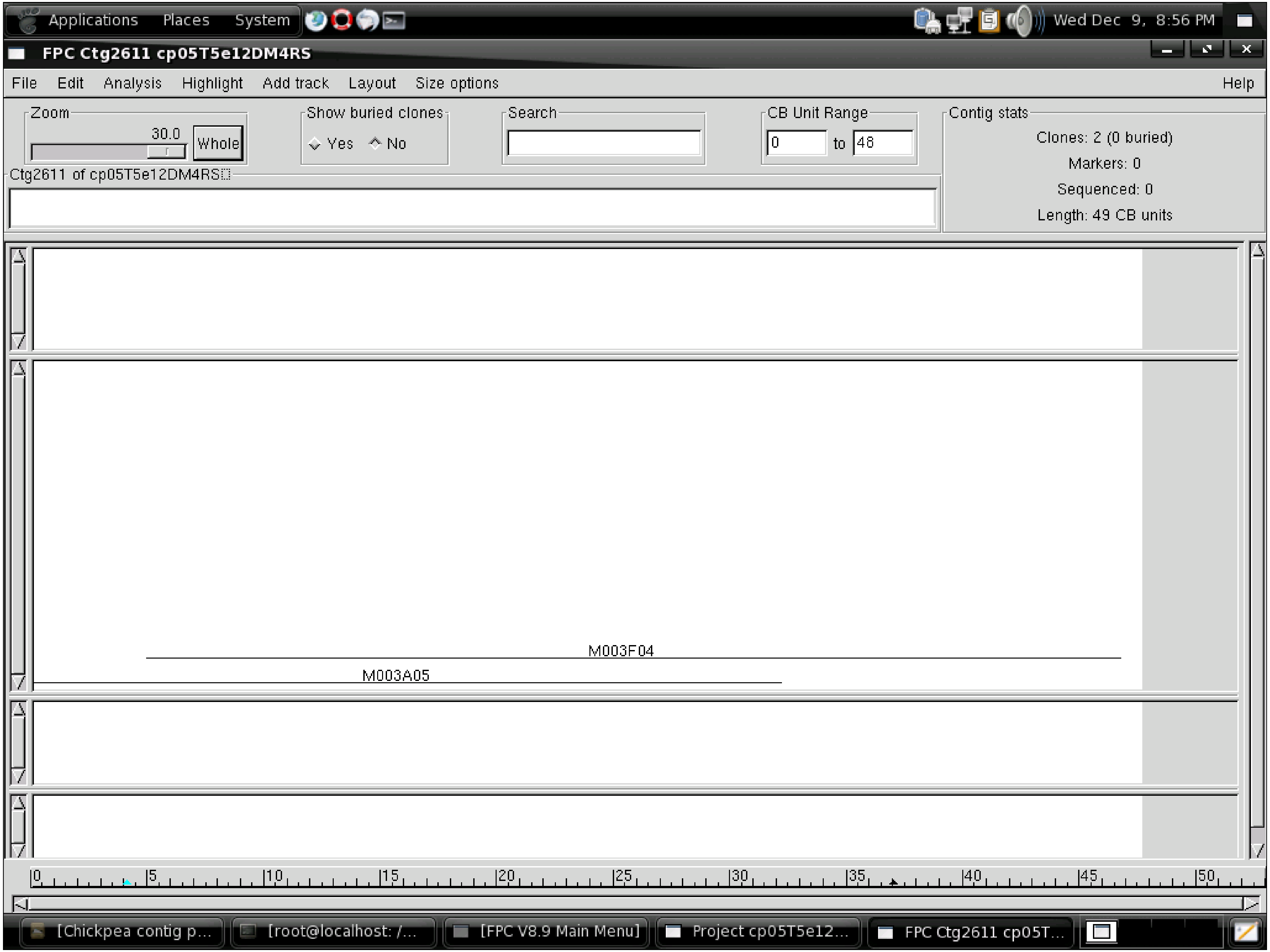
Applications Places System
Wed Dec 9, 8:56 PM
FPC Ctg2611 cp05T5e12DM4RS
File Edit Analysis Highlight Add track Layout Size options Help
Zoom
30.0
Whole
Show buried clones
Yes No
Search
CB Unit Range
0 to 48
Contig stats
Clones: 2 (0 buried)
Markers: 0
Sequenced: 0
Length: 49 CB units
Ctg2611 of cp05T5e12DM4RS
M003F04
M003A05
0 5 10 15 20 25 30 35 40 45 50
[Chickpea contig p...
[root@localhost: /...
[FPC V8.9 Main Menu]
Project cp05T5e12...
FPC Ctg2611 cp05T...

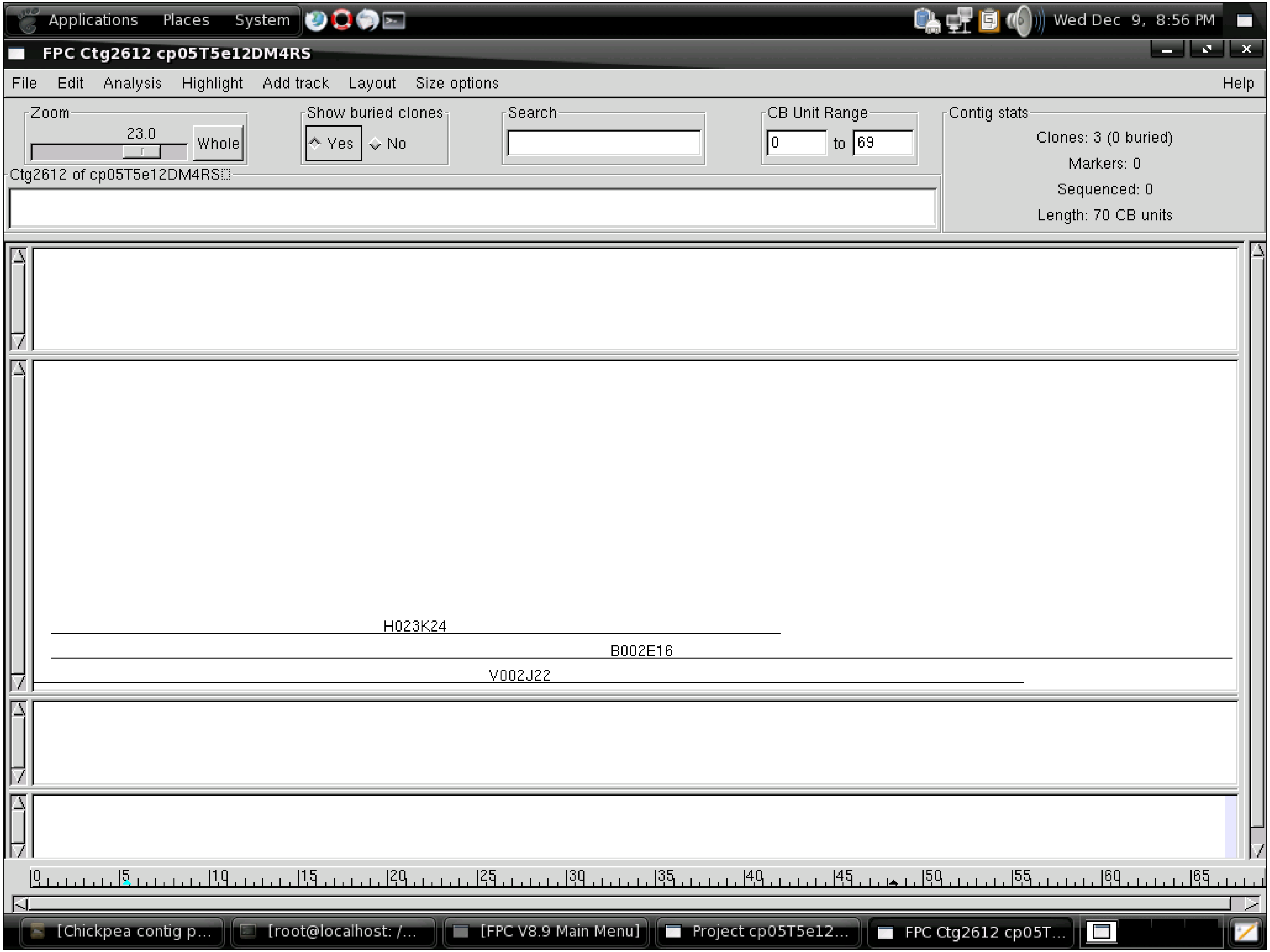
Applications Places System
Wed Dec 9, 8:56 PM
FPC Ctg2612 cp05T5e12DM4RS
File Edit Analysis Highlight Add track Layout Size options Help
Zoom
23.0
Whole
Show buried clones
Yes No
Search
CB Unit Range
0 to 69
Contig stats
Clones: 3 (0 buried)
Markers: 0
Sequenced: 0
Length: 70 CB units
Ctg2612 of cp05T5e12DM4RS
H023K24
B002E16
V002J22
0 5 10 15 20 25 30 35 40 45 50 55 60 65
[Chickpea contig p...
[root@localhost: /...
[FPC V8.9 Main Menu]
Project cp05T5e12...
FPC Ctg2612 cp05T...

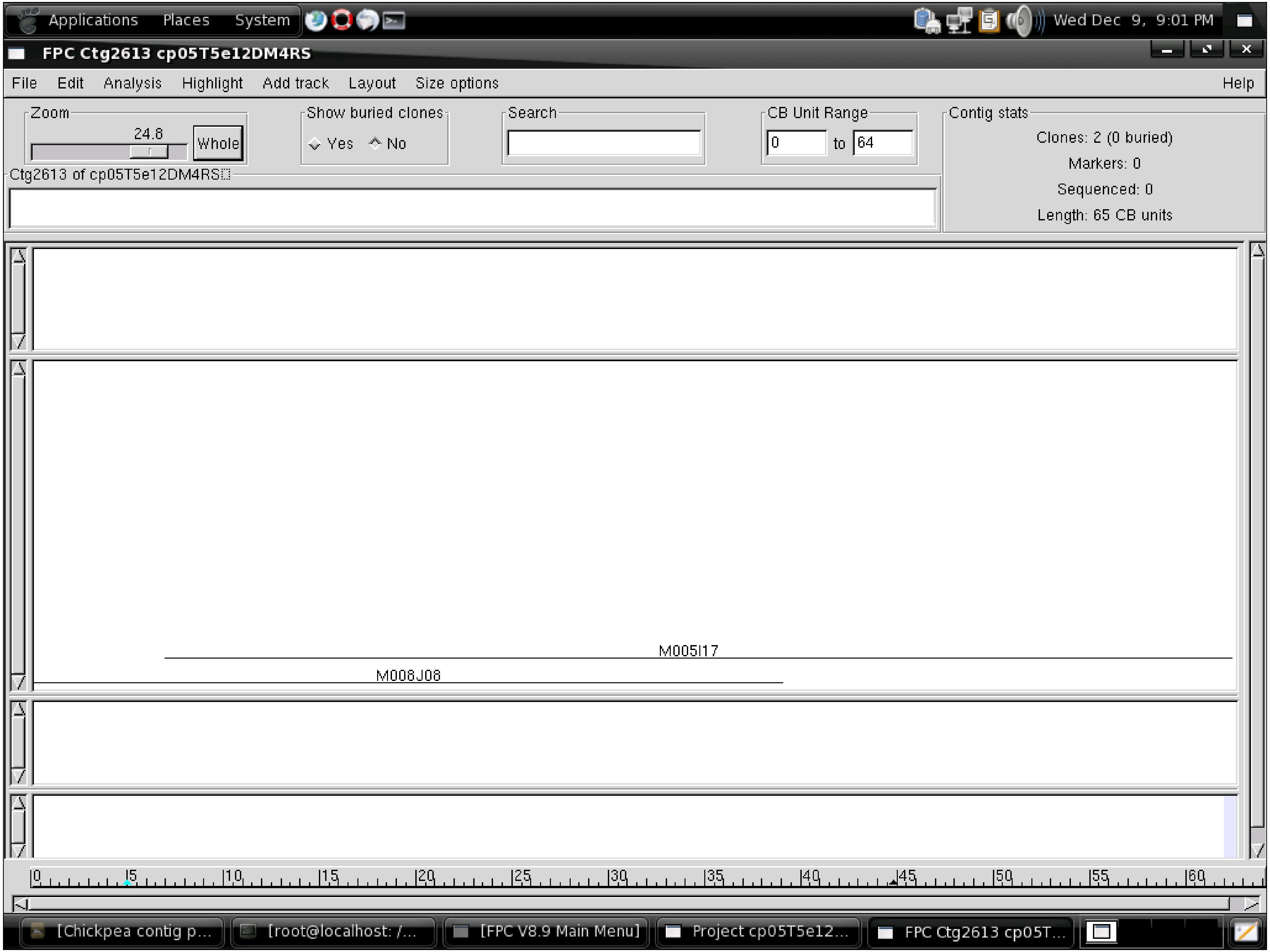
Applications Places System
Wed Dec 9, 9:01 PM
FPC Ctg2613 cp05T5e12DM4RS
File Edit Analysis Highlight Add track Layout Size options Help
Zoom
24.8
Whole
Show buried clones
Yes No
Search
CB Unit Range
0 to 64
Contig stats
Clones: 2 (0 buried)
Markers: 0
Sequenced: 0
Length: 65 CB units
Ctg2613 of cp05T5e12DM4RS
M005I17
M008J08
0 5 10 15 20 25 30 35 40 45 50 55 60
[Chickpea contig p...
[root@localhost: /...
[FPC V8.9 Main Menu]
Project cp05T5e12...
FPC Ctg2613 cp05T...

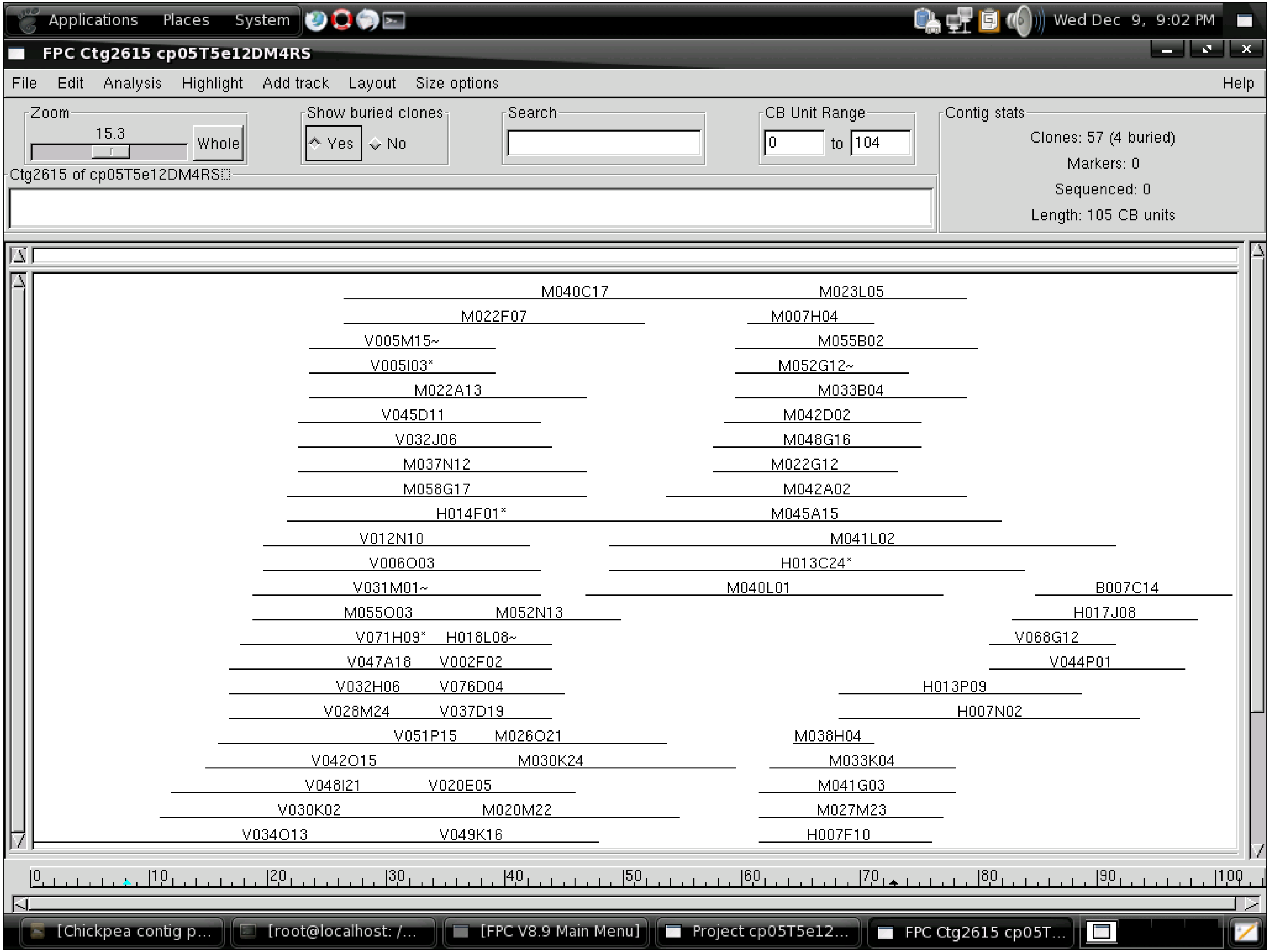
Applications Places System
Wed Dec 9, 9:02 PM
FPC Ctg2615 cp05T5e12DM4RS
File Edit Analysis Highlight Add track Layout Size options Help
Zoom 15.3 Whole
Show buried clones Yes No
Search
CB Unit Range 0 to 104
Contig stats
Clones: 57 (4 buried)
Markers: 0
Sequenced: 0
Length: 105 CB units
Ctg2615 of cp05T5e12DM4RS
M040C17
M023L05
M022F07
M007H04
V005M15~
M055B02
V005I03*
M052G12~
M022A13
M033B04
V045D11
M042D02
V032J06
M048G16
M037N12
M022G12
M058G17
M042A02
H014F01*
M045A15
V012N10
M041L02
V006O03
H013C24*
V031M01~
M040L01
B007C14
M055O03
M052N13
H017J08
V071H09*
H018L08~
V068G12
V047A18
V002F02
V044P01
V032H06
V076D04
H013P09
V028M24
V037D19
H007N02
V051P15
M026O21
M038H04
V042O15
M030K24
M033K04
V048I21
V020E05
M041G03
V030K02
M020M22
M027M23
V034O13
V049K16
H007F10
0 10 20 30 40 50 60 70 80 90 100
[Chickpea contig p...
[root@localhost: /...
[FPC V8.9 Main Menu]
Project cp05T5e12...
FPC Ctg2615 cp05T...

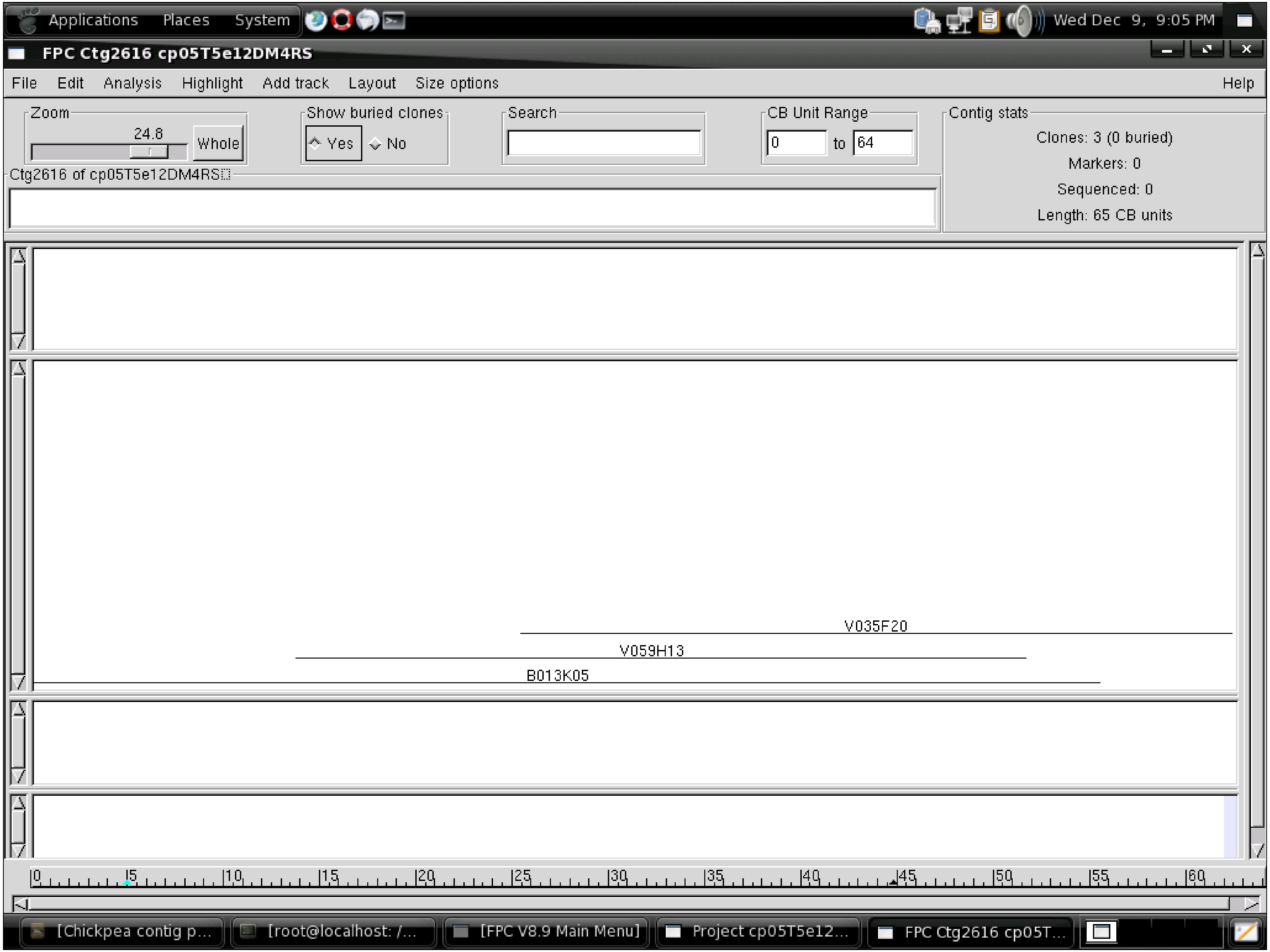
Applications Places System
Wed Dec 9, 9:05 PM
FPC Ctg2616 cp05T5e12DM4RS
File Edit Analysis Highlight Add track Layout Size options Help
Zoom
24.8
Whole
Show buried clones
Yes No
Search
CB Unit Range
0 to 64
Contig stats
Clones: 3 (0 buried)
Markers: 0
Sequenced: 0
Length: 65 CB units
Ctg2616 of cp05T5e12DM4RS
V035F20
V059H13
B013K05
0 5 10 15 20 25 30 35 40 45 50 55 60
[Chickpea contig p...
[root@localhost: /...
[FPC V8.9 Main Menu]
Project cp05T5e12...
FPC Ctg2616 cp05T...

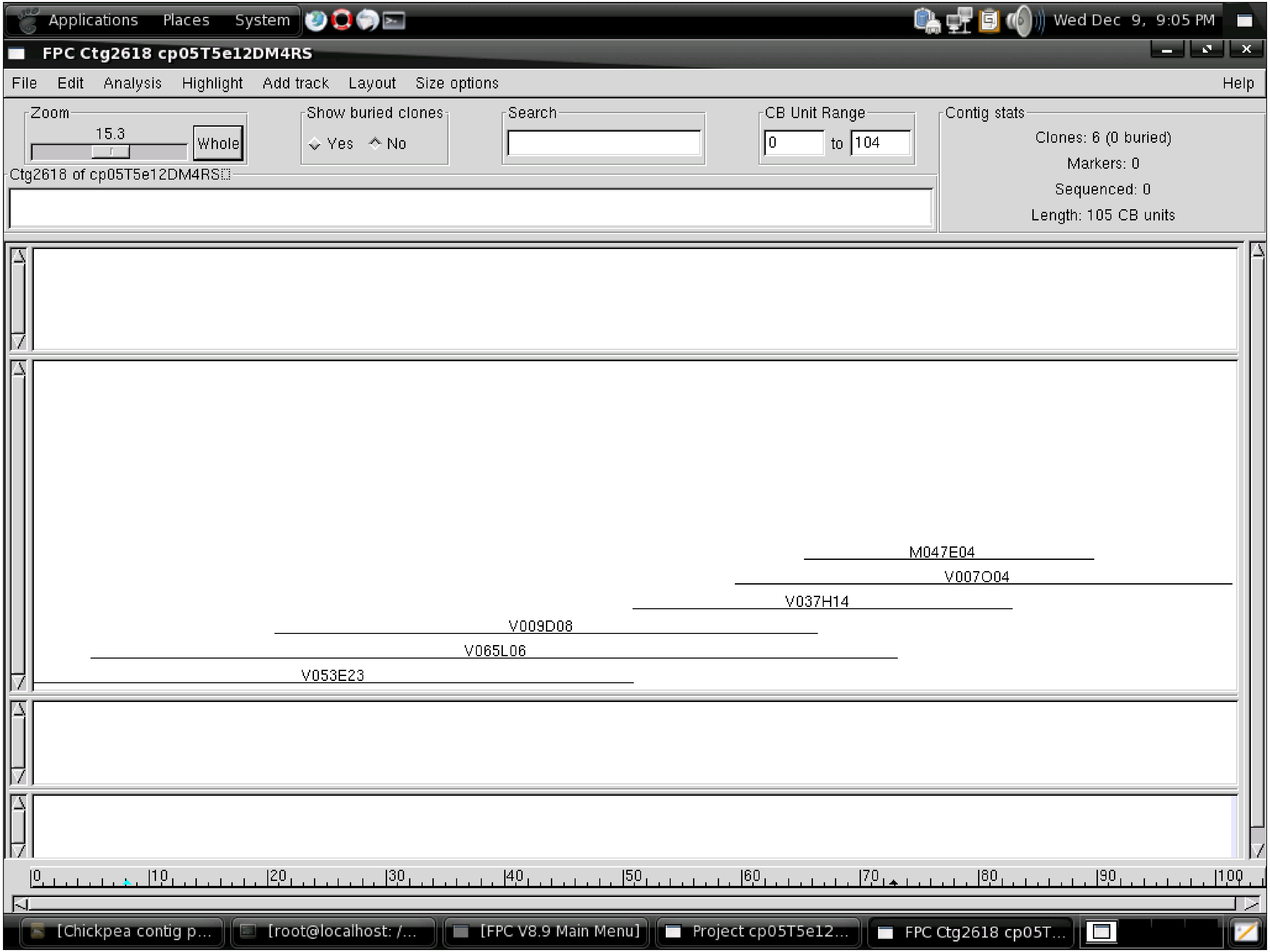

Applications Places System
Wed Dec 9, 9:05 PM
FPC Ctg2618 cp05T5e12DM4RS
File Edit Analysis Highlight Add track Layout Size options Help
Zoom
15.3
Whole
Show buried clones
Yes No
Search
CB Unit Range
0 to 104
Contig stats
Clones: 6 (0 buried)
Markers: 0
Sequenced: 0
Length: 105 CB units
Ctg2618 of cp05T5e12DM4RS
M047E04
V007O04
V037H14
V009D08
V065L06
V053E23
0 10 20 30 40 50 60 70 80 90 100
[Chickpea contig p...
[root@localhost: /...
[FPC V8.9 Main Menu]
Project cp05T5e12...
FPC Ctg2618 cp05T...

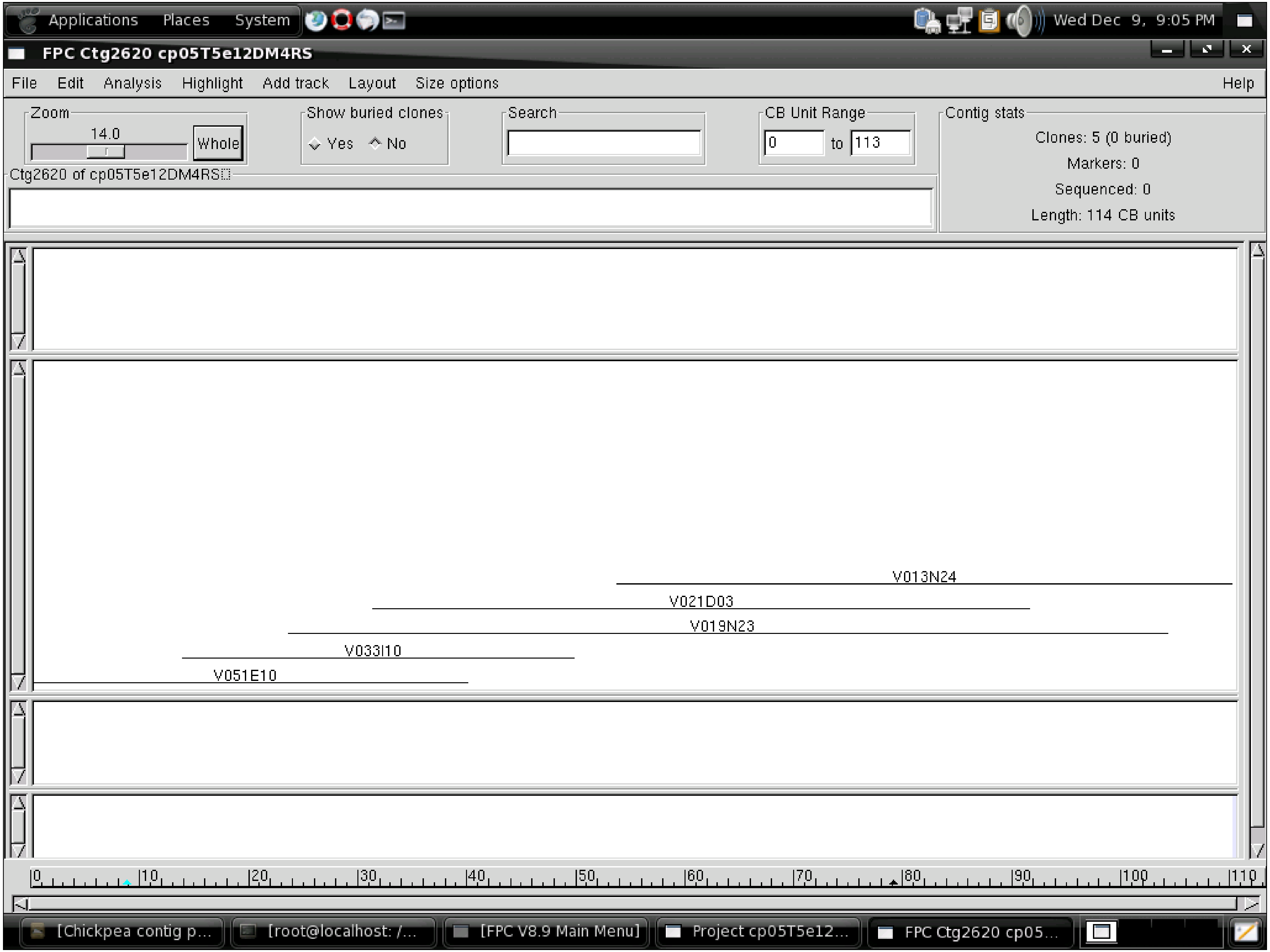
Applications Places System
Wed Dec 9, 9:05 PM
FPC Ctg2620 cp05T5e12DM4RS
File Edit Analysis Highlight Add track Layout Size options Help
Zoom
14.0
Whole
Show buried clones
Yes No
Search
CB Unit Range
0 to 113
Contig stats
Clones: 5 (0 buried)
Markers: 0
Sequenced: 0
Length: 114 CB units
Ctg2620 of cp05T5e12DM4RS
V013N24
V021D03
V019N23
V033I10
V051E10
0 10 20 30 40 50 60 70 80 90 100 110
[Chickpea contig p...
[root@localhost: /...
[FPC V8.9 Main Menu]
Project cp05T5e12...
FPC Ctg2620 cp05...

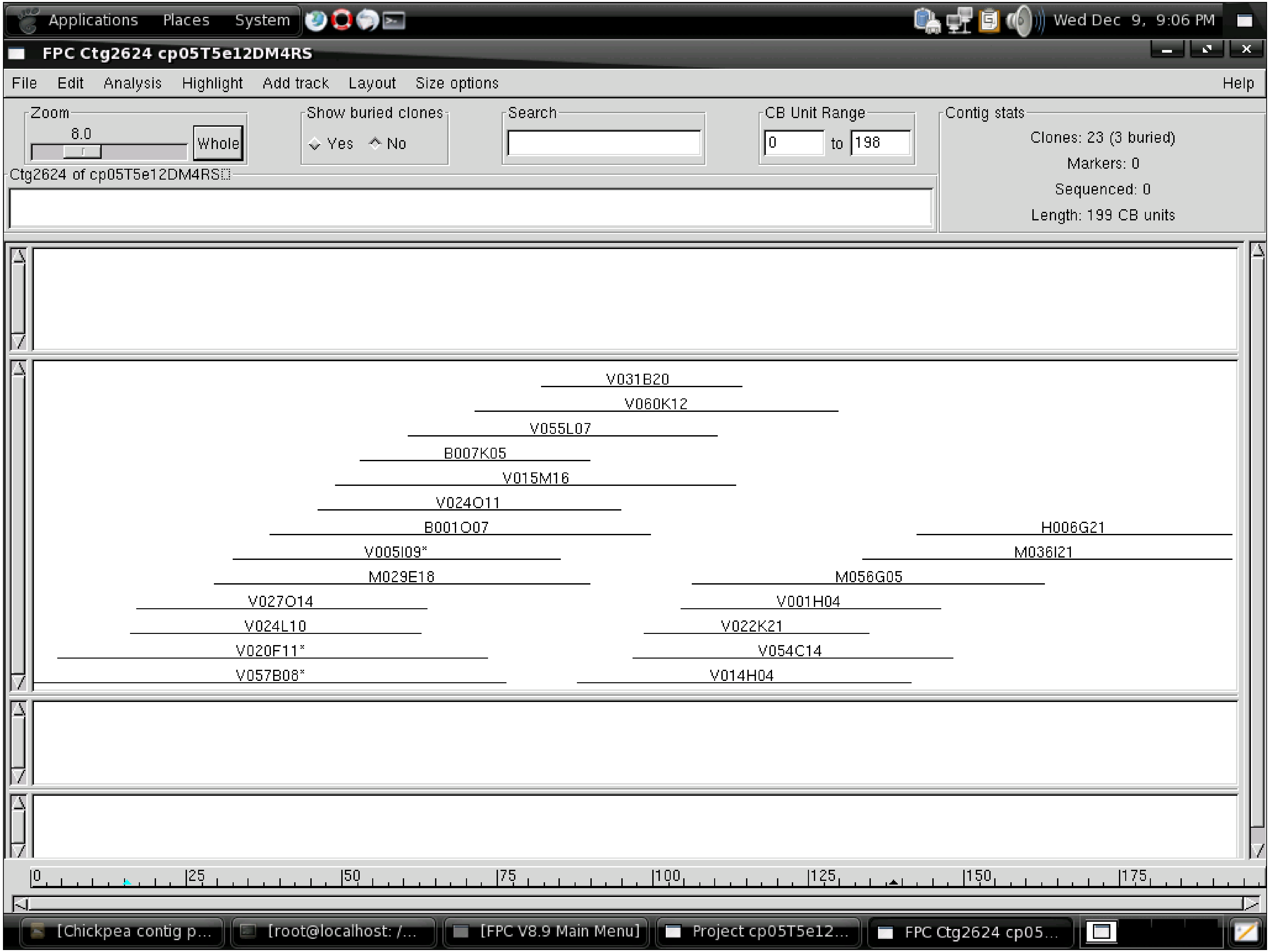
Applications Places System
Wed Dec 9, 9:06 PM
FPC Ctg2624 cp05T5e12DM4RS
File Edit Analysis Highlight Add track Layout Size options Help
Zoom
8.0
Whole
Show buried clones
Yes No
Search
CB Unit Range
0 to 198
Contig stats
Clones: 23 (3 buried)
Markers: 0
Sequenced: 0
Length: 199 CB units
Ctg2624 of cp05T5e12DM4RS
V031B20
V060K12
V055L07
B007K05
V015M16
V024O11
B001O07
H006G21
V005I09*
M036I21
M029E18
M056G05
V027O14
V001H04
V024L10
V022K21
V020F11*
V054C14
V057B08*
V014H04
0 25 50 75 100 125 150 175
[Chickpea contig p...
[root@localhost: /...
[FPC V8.9 Main Menu]
Project cp05T5e12...
FPC Ctg2624 cp05...

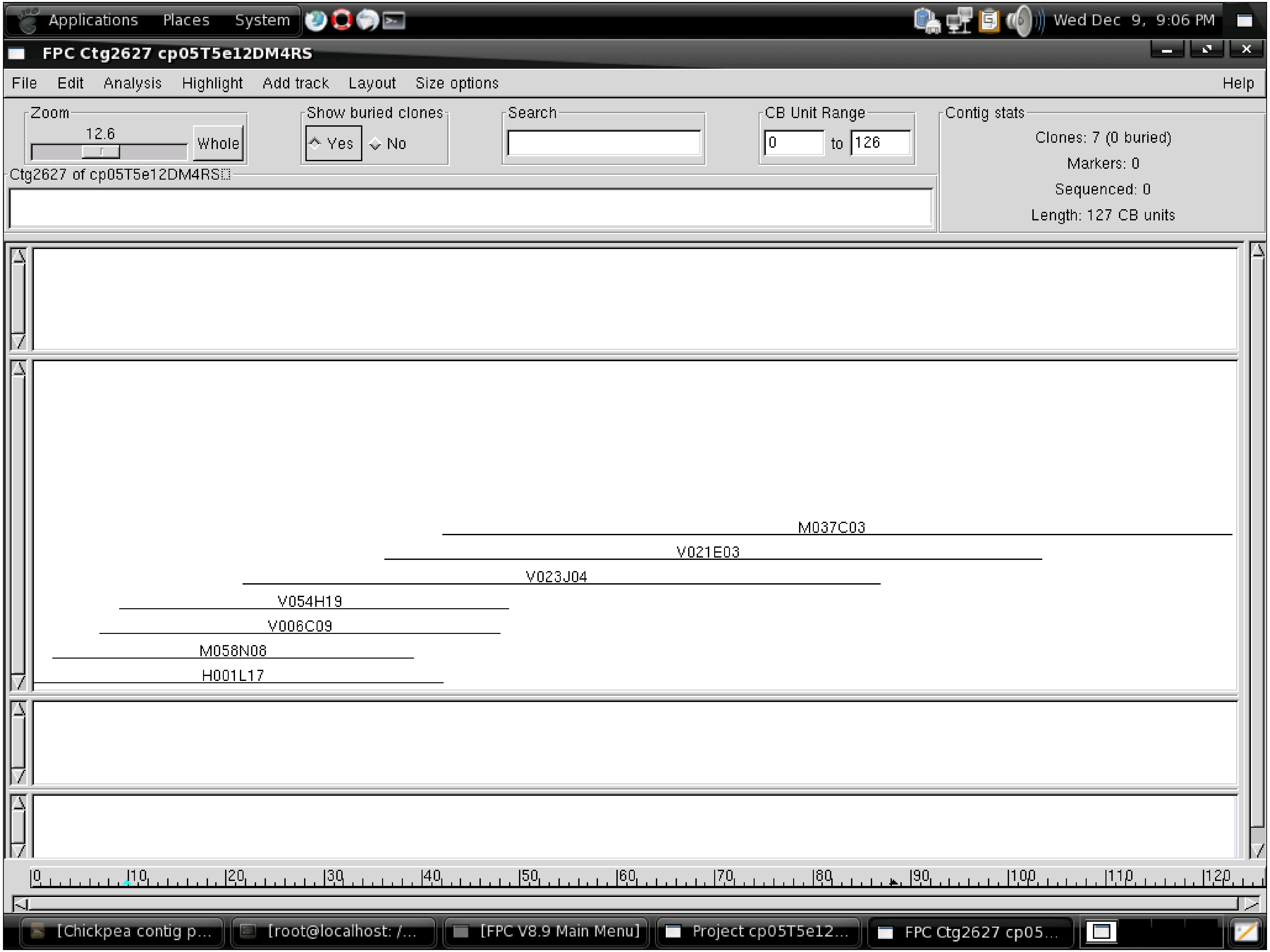
Applications Places System
Wed Dec 9, 9:06 PM
FPC Ctg2627 cp05T5e12DM4RS
File Edit Analysis Highlight Add track Layout Size options Help
Zoom
12.6
Whole
Show buried clones
Yes
No
Search
CB Unit Range
0
to
126
Contig stats
Clones: 7 (0 buried)
Markers: 0
Sequenced: 0
Length: 127 CB units
Ctg2627 of cp05T5e12DM4RS
M037C03
V021E03
V023J04
V054H19
V006C09
M058N08
H001L17
0 10 20 30 40 50 60 70 80 90 100 110 120
[Chickpea contig p...
[root@localhost: /...
[FPC V8.9 Main Menu]
Project cp05T5e12...
FPC Ctg2627 cp05...

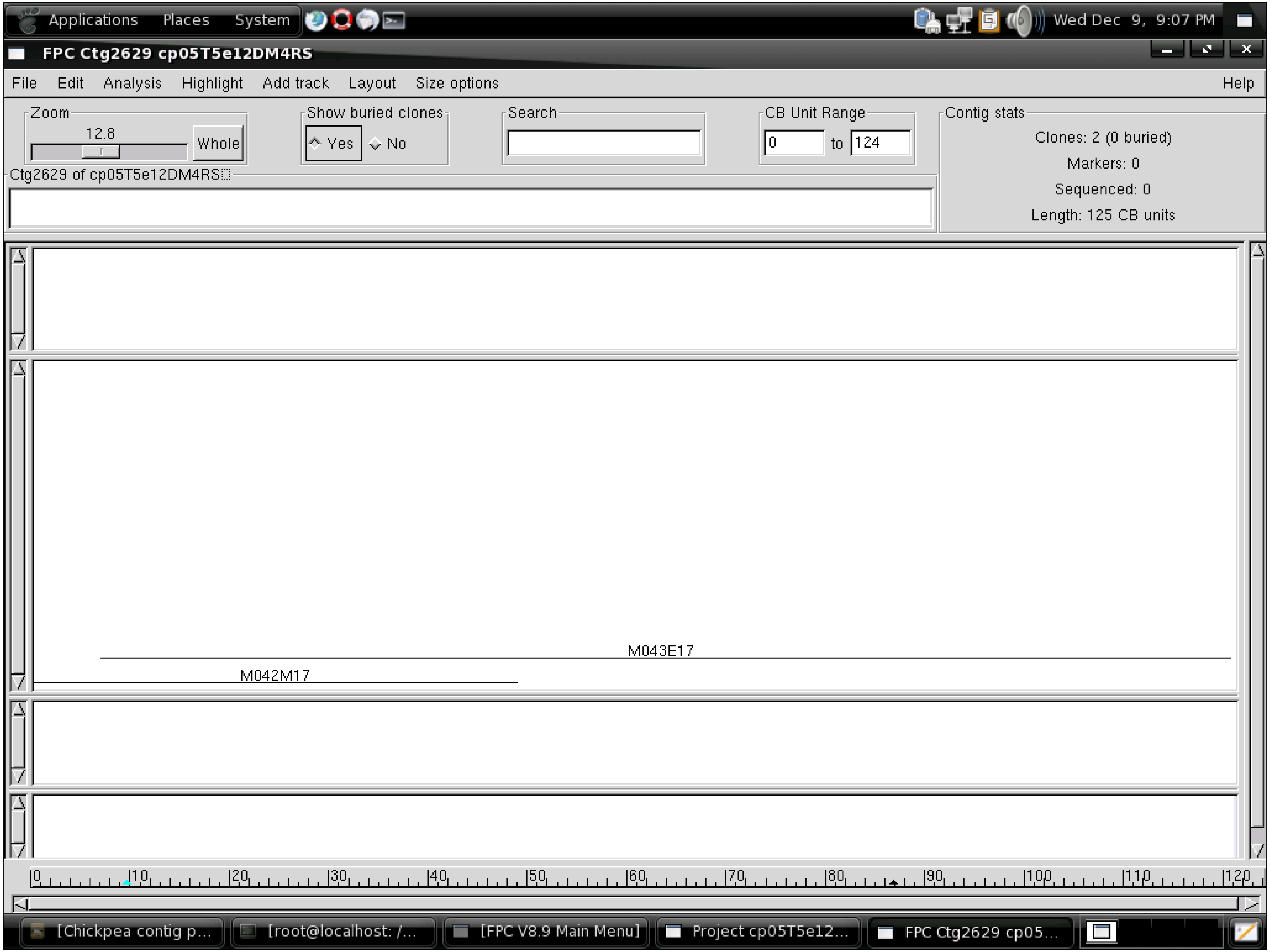
Applications Places System
Wed Dec 9, 9:07 PM
FPC Ctg2629 cp05T5e12DM4RS
File Edit Analysis Highlight Add track Layout Size options Help
Zoom
12.8
Whole
Show buried clones
Yes No
Search
CB Unit Range
0 to 124
Contig stats
Clones: 2 (0 buried)
Markers: 0
Sequenced: 0
Length: 125 CB units
Ctg2629 of cp05T5e12DM4RS
M043E17
M042M17
0 10 20 30 40 50 60 70 80 90 100 110 120
[Chickpea contig p...
[root@localhost: /...
[FPC V8.9 Main Menu]
Project cp05T5e12...
FPC Ctg2629 cp05...

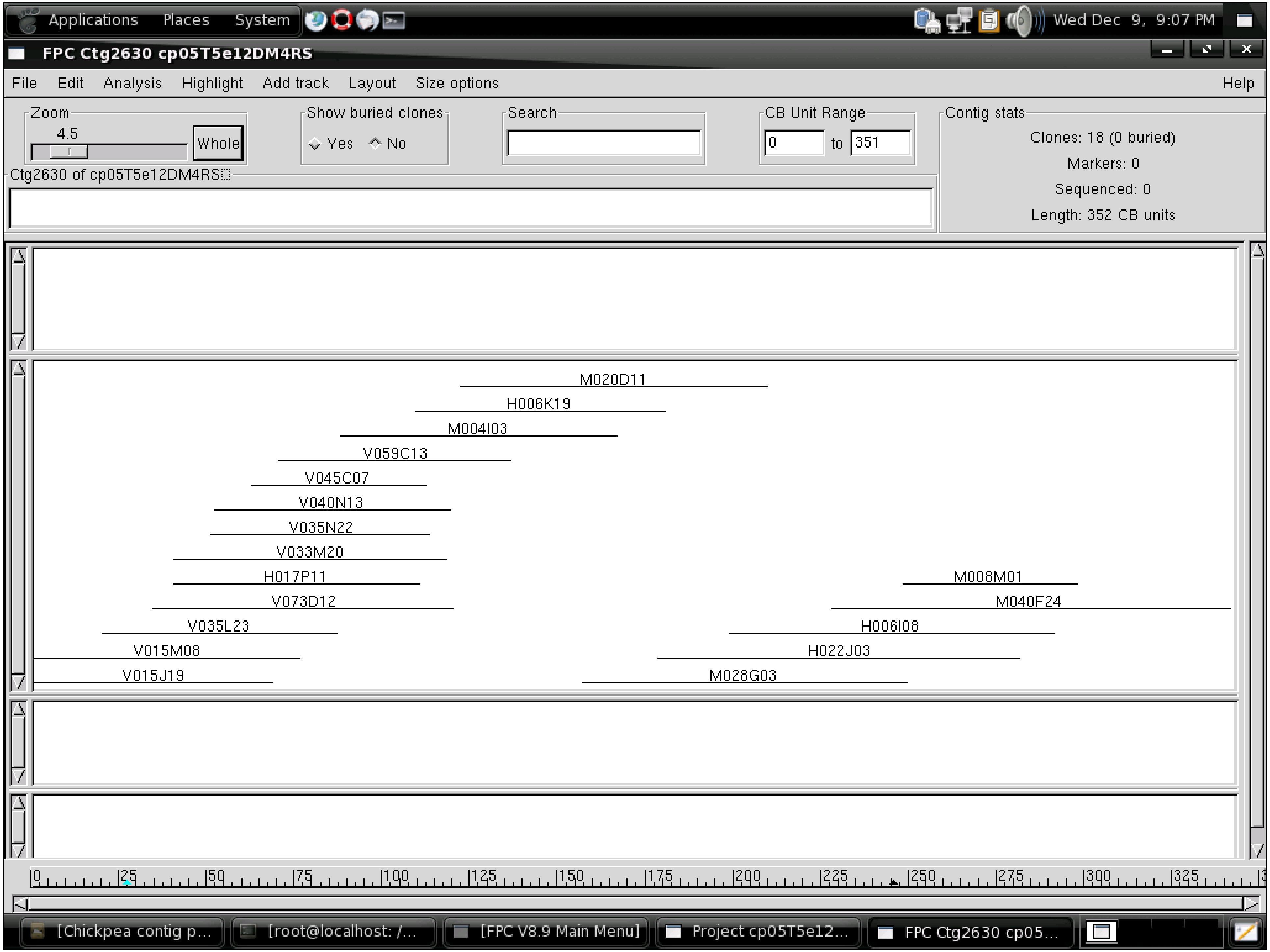
Applications
Places
System
Wed Dec 9, 9:07 PM
FPC Ctg2630 cp05T5e12DM4RS
File
Edit
Analysis
Highlight
Add track
Layout
Size options
Help
Zoom
4.5
Whole
Show buried clones
Yes
No
Search
CB Unit Range
0
to
351
Contig stats
Clones: 18 (0 buried)
Markers: 0
Sequenced: 0
Length: 352 CB units
Ctg2630 of cp05T5e12DM4RS
M020D11
H006K19
M004I03
V059C13
V045C07
V040N13
V035N22
V033M20
H017P11
M008M01
V073D12
M040F24
V035L23
H006I08
V015M08
H022J03
V015J19
M028G03
0
25
50
75
100
125
150
175
200
225
250
275
300
325
[Chickpea contig p...
[root@localhost: /...
[FPC V8.9 Main Menu]
Project cp05T5e12...
FPC Ctg2630 cp05...

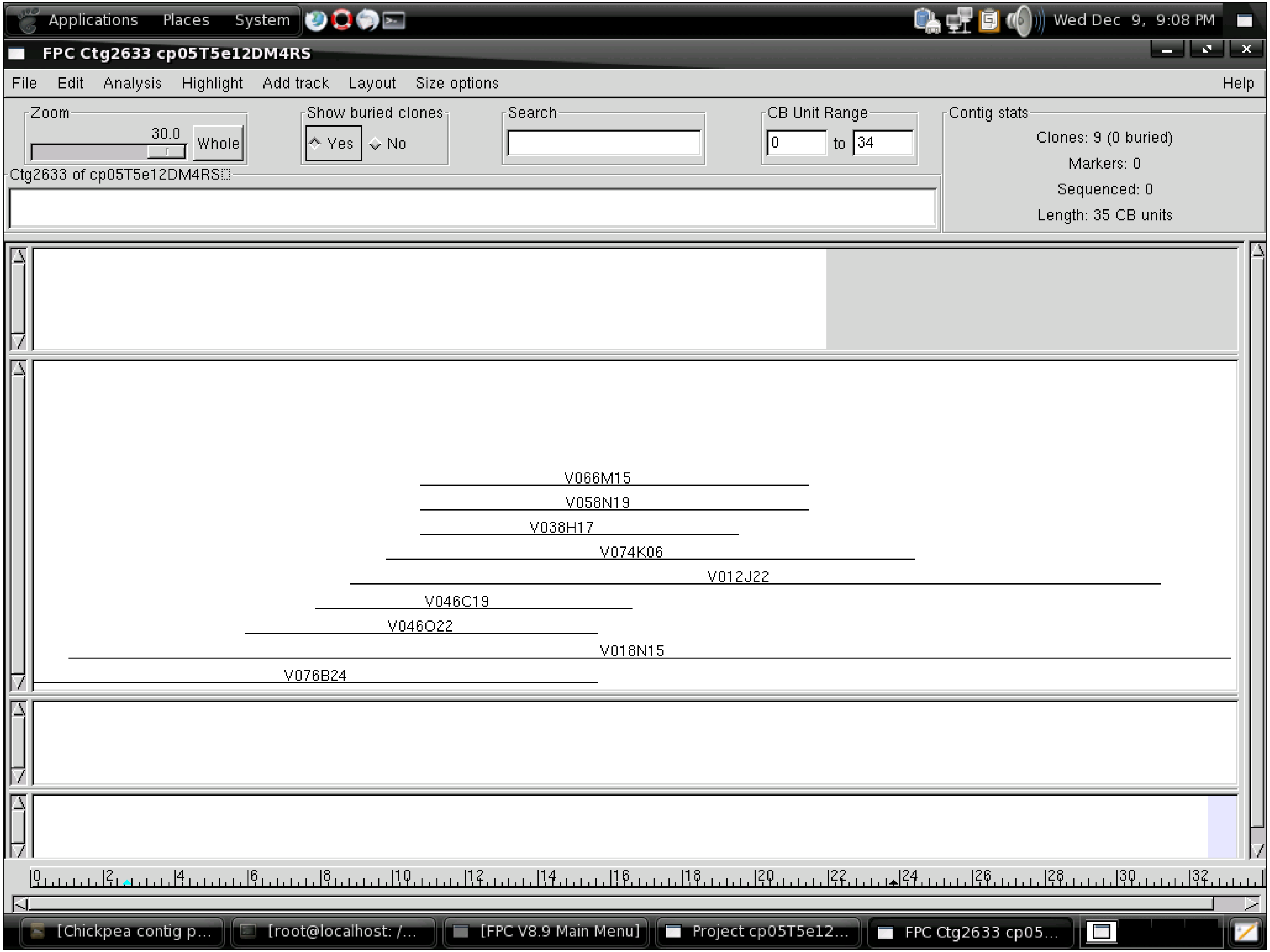
Applications Places System
Wed Dec 9, 9:08 PM
FPC Ctg2633 cp05T5e12DM4RS
File Edit Analysis Highlight Add track Layout Size options Help
Zoom
30.0
Whole
Show buried clones
Yes
No
Search
CB Unit Range
0
to
34
Contig stats
Clones: 9 (0 buried)
Markers: 0
Sequenced: 0
Length: 35 CB units
Ctg2633 of cp05T5e12DM4RS
V066M15
V058N19
V038H17
V074K06
V012J22
V046C19
V046O22
V018N15
V076B24
0 2 4 6 8 10 12 14 16 18 20 22 24 26 28 30 32
[Chickpea contig p...
[root@localhost: /...
[FPC V8.9 Main Menu]
Project cp05T5e12...
FPC Ctg2633 cp05...

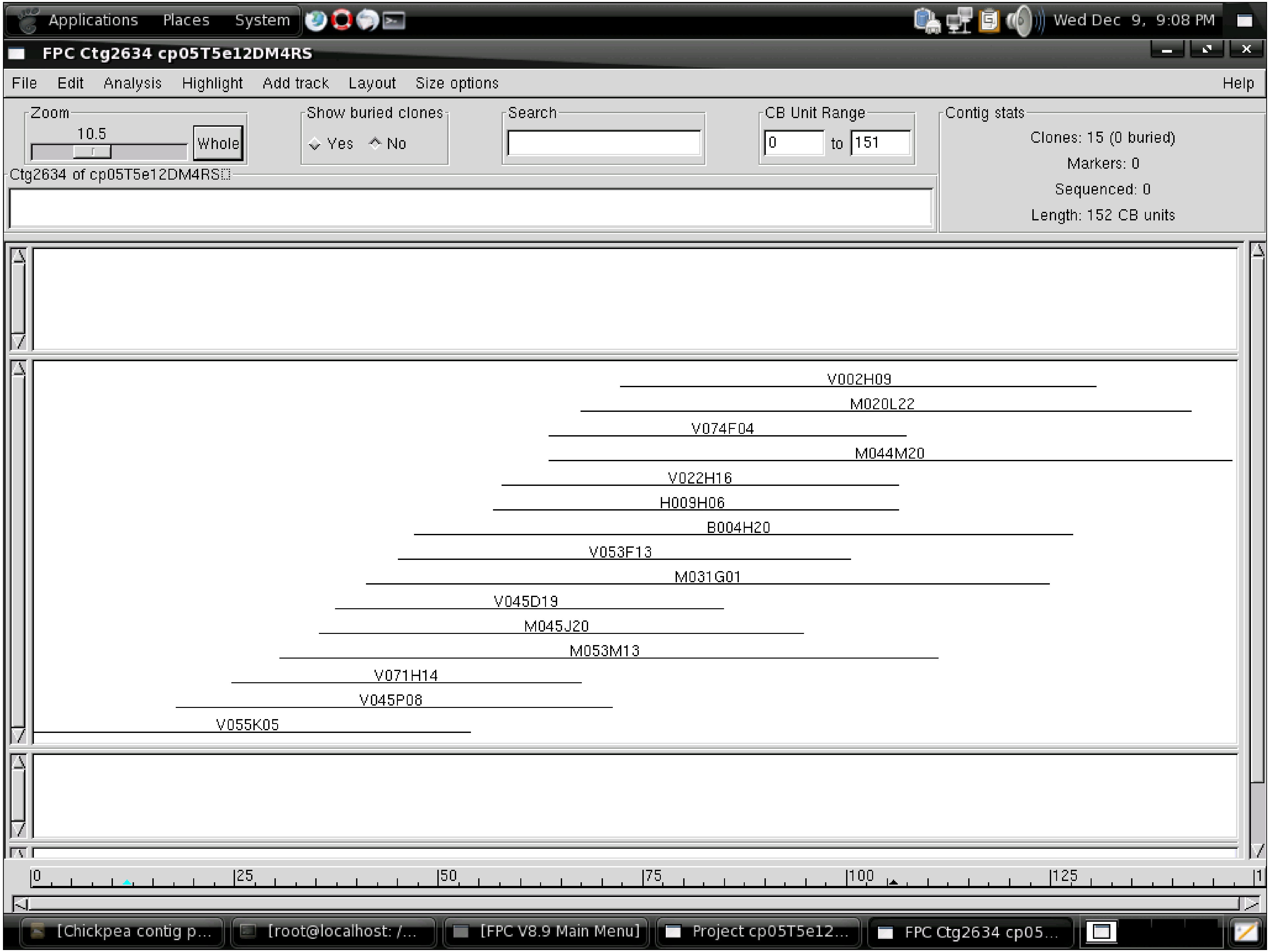
Applications Places System
Wed Dec 9, 9:08 PM
FPC Ctg2634 cp05T5e12DM4RS
File Edit Analysis Highlight Add track Layout Size options Help
Zoom
10.5
Whole
Show buried clones
Yes No
Search
CB Unit Range
0 to 151
Contig stats
Clones: 15 (0 buried)
Markers: 0
Sequenced: 0
Length: 152 CB units
Ctg2634 of cp05T5e12DM4RS
V002H09
M020L22
V074F04
M044M20
V022H16
H009H06
B004H20
V053F13
M031G01
V045D19
M045J20
M053M13
V071H14
V045P08
V055K05
0 25 50 75 100 125
[Chickpea contig p...
[root@localhost: /...
[FPC V8.9 Main Menu]
Project cp05T5e12...
FPC Ctg2634 cp05...

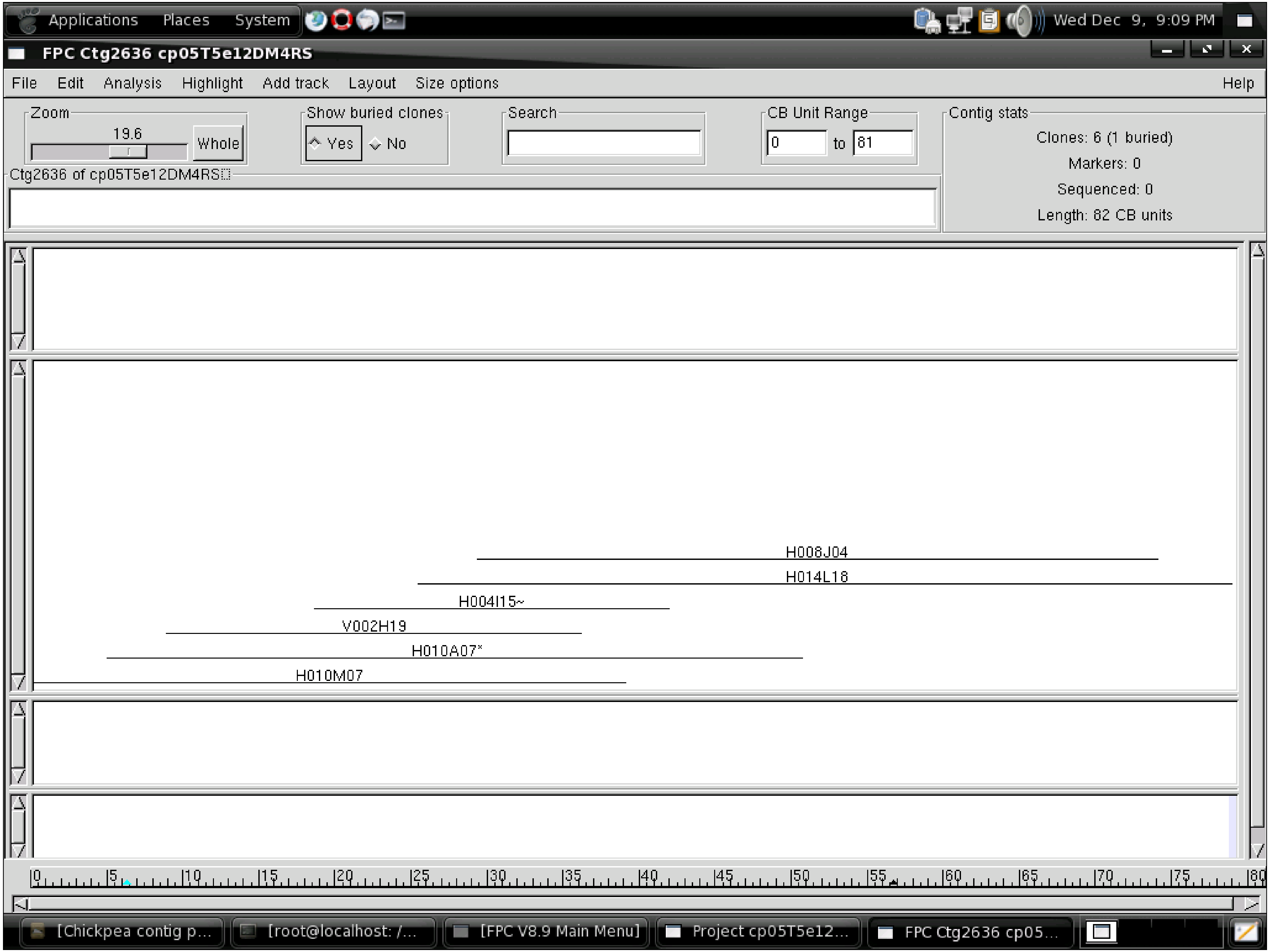
Applications Places System
Wed Dec 9, 9:09 PM
FPC Ctg2636 cp05T5e12DM4RS
File Edit Analysis Highlight Add track Layout Size options Help
Zoom
19.6
Whole
Show buried clones
Yes No
Search
CB Unit Range
0 to 81
Contig stats
Clones: 6 (1 buried)
Markers: 0
Sequenced: 0
Length: 82 CB units
Ctg2636 of cp05T5e12DM4RS
H008J04
H014L18
H004I15~
V002H19
H010A07*
H010M07
0 5 10 15 20 25 30 35 40 45 50 55 60 65 70 75 80
[Chickpea contig p...
[root@localhost: /...
[FPC V8.9 Main Menu]
Project cp05T5e12...
FPC Ctg2636 cp05...

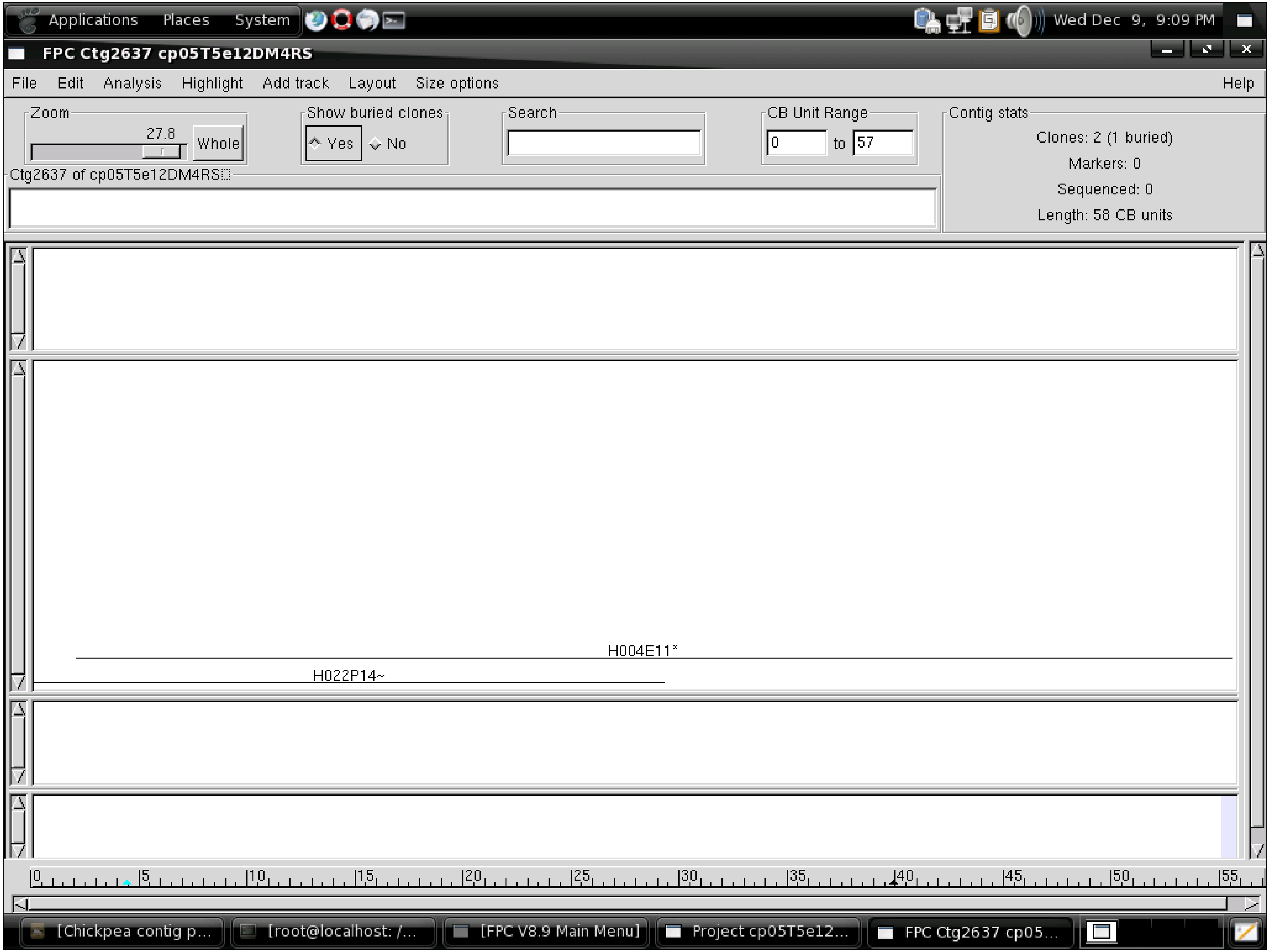
Applications Places System
Wed Dec 9, 9:09 PM
FPC Ctg2637 cp05T5e12DM4RS
File Edit Analysis Highlight Add track Layout Size options Help
Zoom
27.8
Whole
Show buried clones
Yes
No
Search
CB Unit Range
0
to
57
Contig stats
Clones: 2 (1 buried)
Markers: 0
Sequenced: 0
Length: 58 CB units
Ctg2637 of cp05T5e12DM4RS
H004E11*
H022P14~
0 5 10 15 20 25 30 35 40 45 50 55
[Chickpea contig p...
[root@localhost: /...
[FPC V8.9 Main Menu]
Project cp05T5e12...
FPC Ctg2637 cp05...

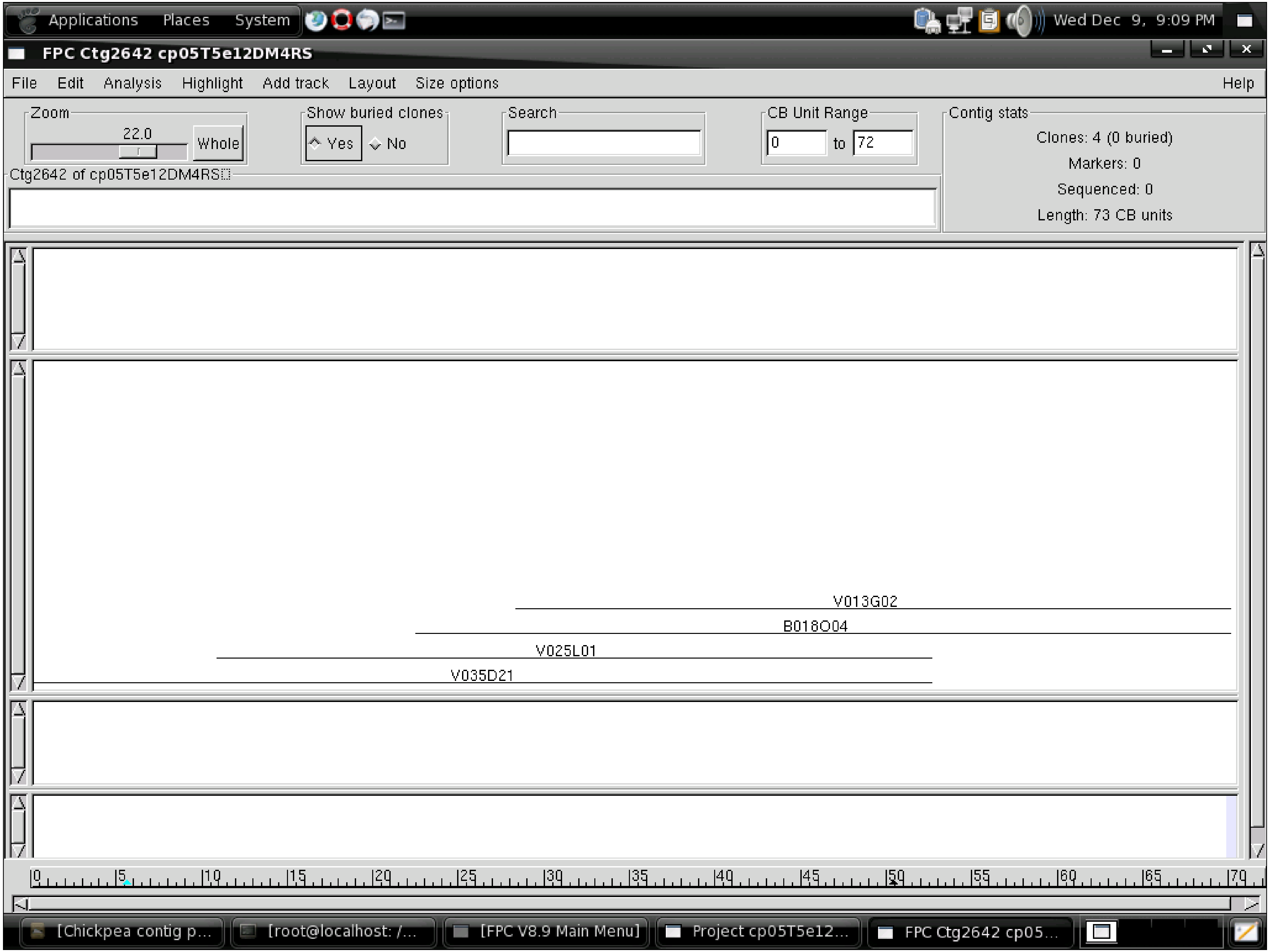
Applications Places System
Wed Dec 9, 9:09 PM
FPC Ctg2642 cp05T5e12DM4RS
File Edit Analysis Highlight Add track Layout Size options Help
Zoom
22.0
Whole
Show buried clones
Yes No
Search
CB Unit Range
0 to 72
Contig stats
Clones: 4 (0 buried)
Markers: 0
Sequenced: 0
Length: 73 CB units
Ctg2642 of cp05T5e12DM4RS
V013G02
B018O04
V025L01
V035D21
0 5 10 15 20 25 30 35 40 45 50 55 60 65 70
[Chickpea contig p...
[root@localhost: /...
[FPC V8.9 Main Menu]
Project cp05T5e12...
FPC Ctg2642 cp05...

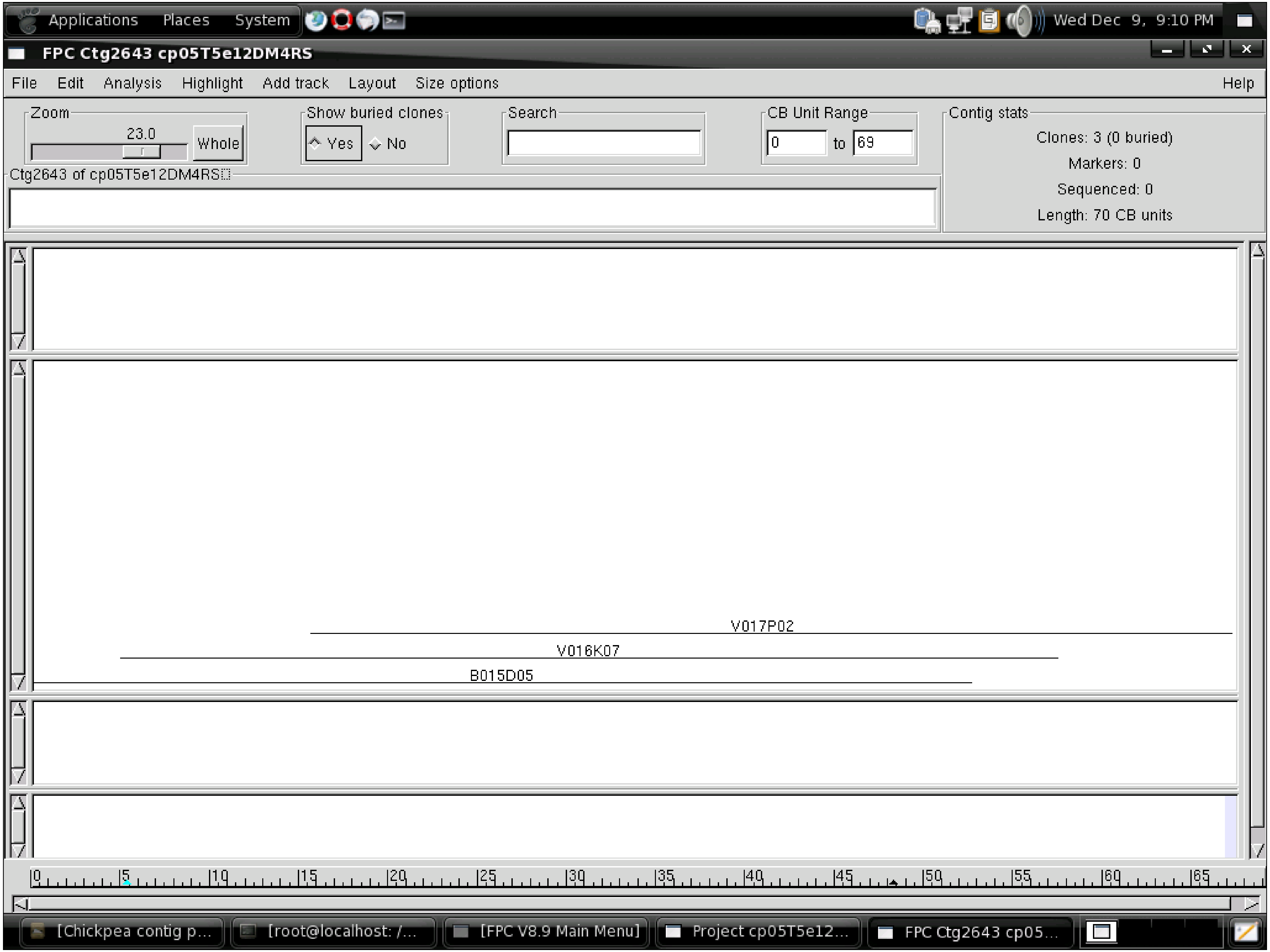
Applications Places System
Wed Dec 9, 9:10 PM
FPC Ctg2643 cp05T5e12DM4RS
File Edit Analysis Highlight Add track Layout Size options
Help
Zoom
23.0
Whole
Show buried clones
Yes
No
Search
CB Unit Range
0
to
69
Contig stats
Clones: 3 (0 buried)
Markers: 0
Sequenced: 0
Length: 70 CB units
Ctg2643 of cp05T5e12DM4RS
V017P02
V016K07
B015D05
0 5 10 15 20 25 30 35 40 45 50 55 60 65
[Chickpea contig p...
[root@localhost: /...
[FPC V8.9 Main Menu]
Project cp05T5e12...
FPC Ctg2643 cp05...

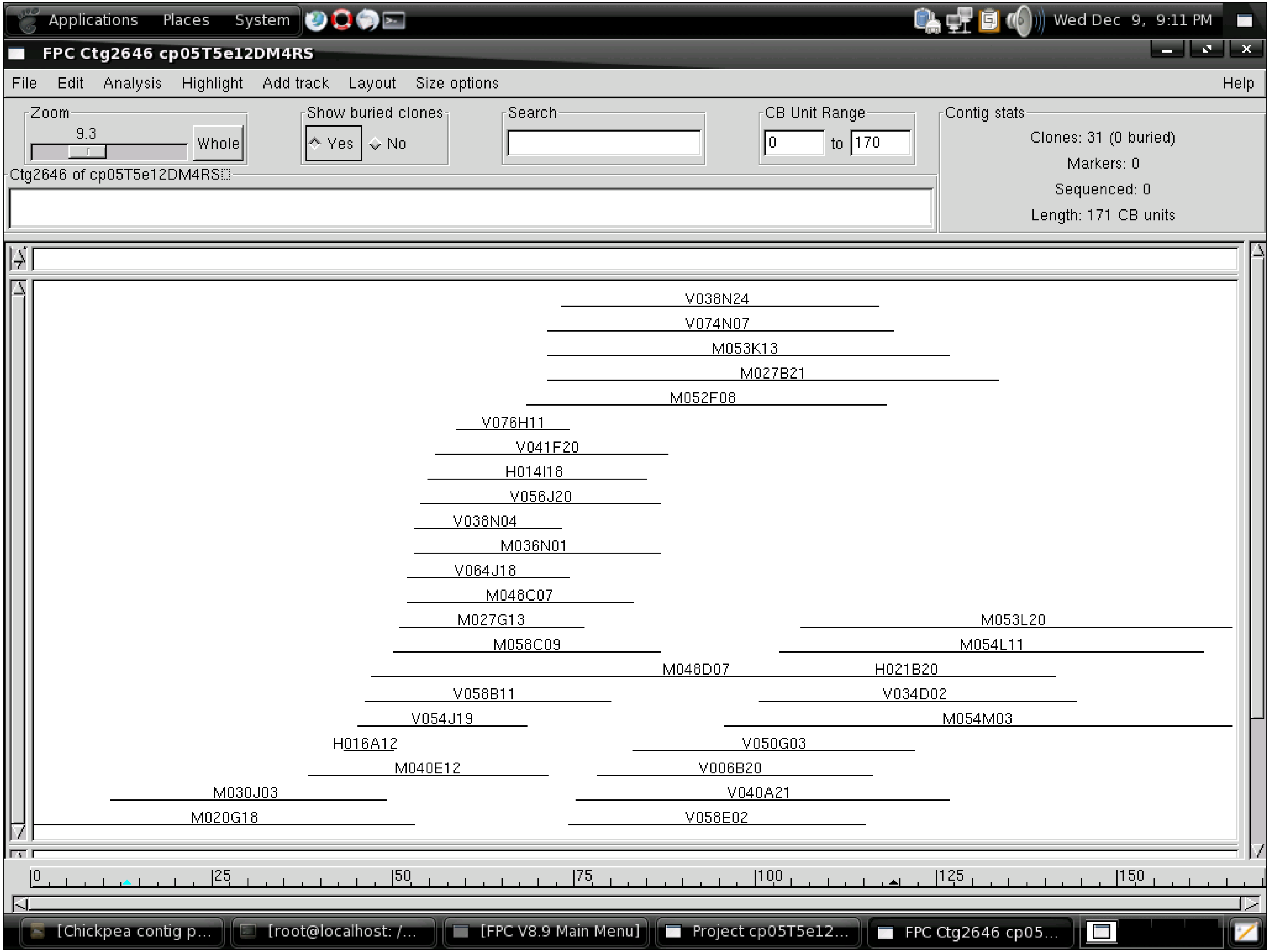
Applications Places System
Wed Dec 9, 9:11 PM
FPC Ctg2646 cp05T5e12DM4RS
File Edit Analysis Highlight Add track Layout Size options Help
Zoom
9.3
Whole
Show buried clones
Yes No
Search
CB Unit Range
0 to 170
Contig stats
Clones: 31 (0 buried)
Markers: 0
Sequenced: 0
Length: 171 CB units
Ctg2646 of cp05T5e12DM4RS
V038N24
V074N07
M053K13
M027B21
M052F08
V076H11
V041F20
H014I18
V056J20
V038N04
M036N01
V064J18
M048C07
M027G13
M053L20
M058C09
M054L11
M048D07
H021B20
V058B11
V034D02
V054J19
M054M03
H016A12
V050G03
M040E12
V006B20
M030J03
V040A21
M020G18
V058E02
0
25
50
75
100
125
150
[Chickpea contig p...
[root@localhost: /...
[FPC V8.9 Main Menu]
Project cp05T5e12...
FPC Ctg2646 cp05...

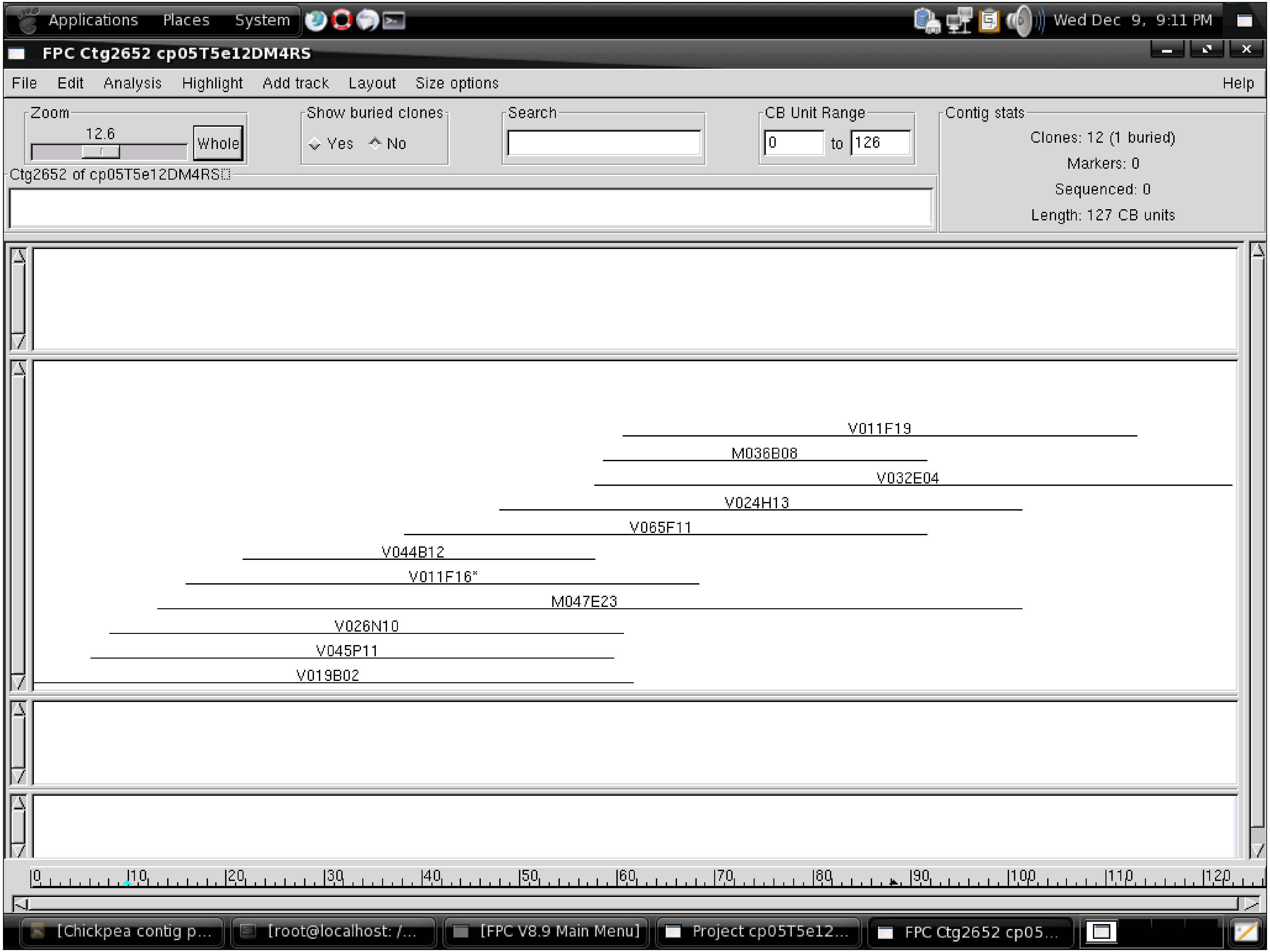
Applications Places System
Wed Dec 9, 9:11 PM
FPC Ctg2652 cp05T5e12DM4RS
File Edit Analysis Highlight Add track Layout Size options Help
Zoom
12.6
Whole
Show buried clones
Yes No
Search
CB Unit Range
0 to 126
Contig stats
Clones: 12 (1 buried)
Markers: 0
Sequenced: 0
Length: 127 CB units
Ctg2652 of cp05T5e12DM4RS
V011F19
M036B08
V032E04
V024H13
V065F11
V044B12
V011F16*
M047E23
V026N10
V045P11
V019B02
0 10 20 30 40 50 60 70 80 90 100 110 120
[Chickpea contig p...
[root@localhost: /...
[FPC V8.9 Main Menu]
Project cp05T5e12...
FPC Ctg2652 cp05...

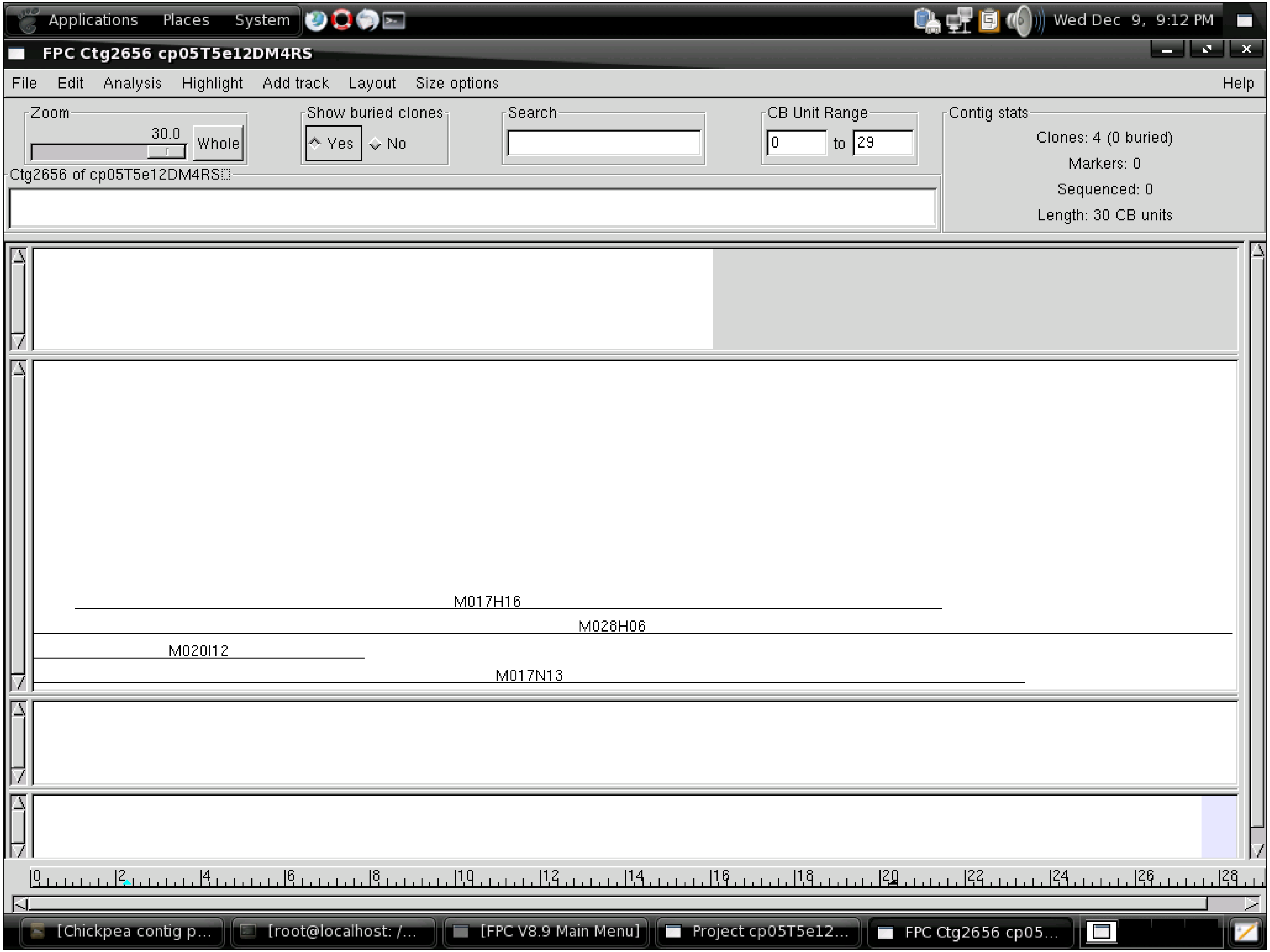

Applications Places System
Wed Dec 9, 9:12 PM
FPC Ctg2656 cp05T5e12DM4RS
File Edit Analysis Highlight Add track Layout Size options Help
Zoom
30.0
Whole
Show buried clones
Yes
No
Search
CB Unit Range
0 to 29
Contig stats
Clones: 4 (0 buried)
Markers: 0
Sequenced: 0
Length: 30 CB units
Ctg2656 of cp05T5e12DM4RS
M017H16
M028H06
M020I12
M017N13
0 2 4 6 8 10 12 14 16 18 20 22 24 26 28
[Chickpea contig p...
[root@localhost: /...
[FPC V8.9 Main Menu]
Project cp05T5e12...
FPC Ctg2656 cp05...

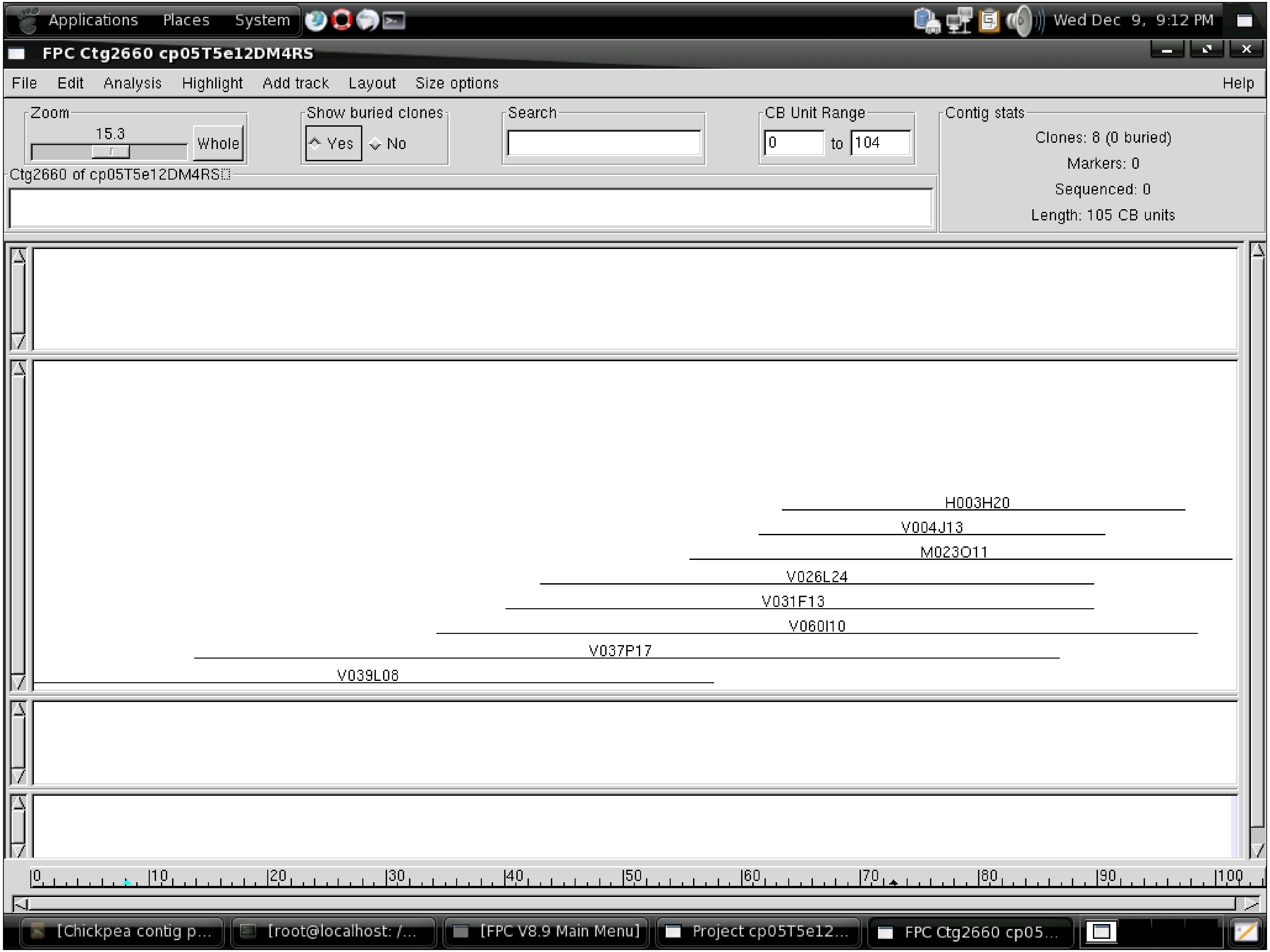

Applications Places System
Wed Dec 9, 9:12 PM
FPC Ctg2660 cp05T5e12DM4RS
File Edit Analysis Highlight Add track Layout Size options Help
Zoom
15.3
Whole
Show buried clones
Yes
No
Search
CB Unit Range
0 to 104
Contig stats
Clones: 8 (0 buried)
Markers: 0
Sequenced: 0
Length: 105 CB units
Ctg2660 of cp05T5e12DM4RS
H003H20
V004J13
M023O11
V026L24
V031F13
V060I10
V037P17
V039L08
0 10 20 30 40 50 60 70 80 90 100
[Chickpea contig p...
[root@localhost: /...
[FPC V8.9 Main Menu]
Project cp05T5e12...
FPC Ctg2660 cp05...

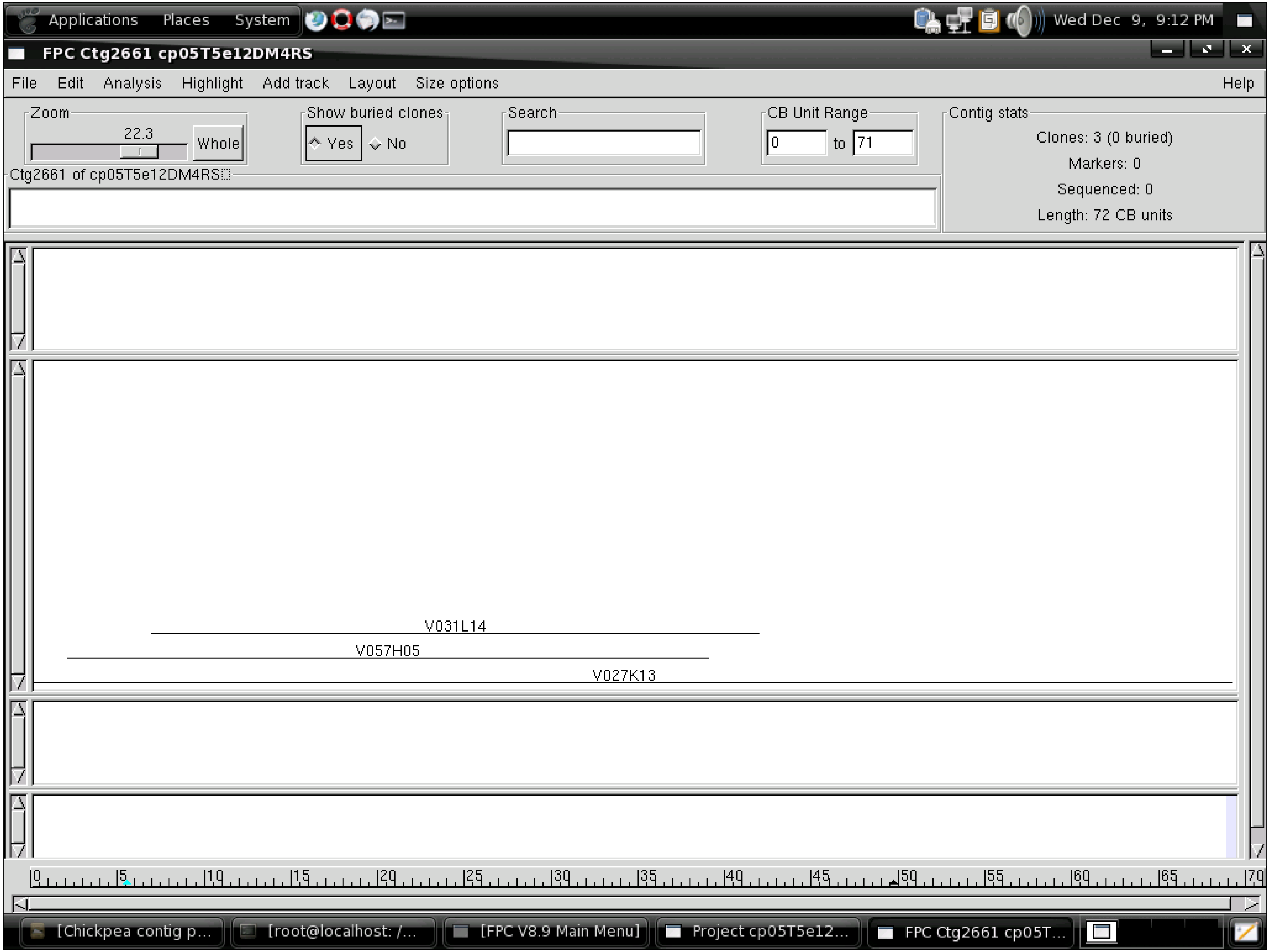
Applications Places System
Wed Dec 9, 9:12 PM
FPC Ctg2661 cp05T5e12DM4RS
File Edit Analysis Highlight Add track Layout Size options Help
Zoom
22.3
Whole
Show buried clones
Yes No
Search
CB Unit Range
0 to 71
Contig stats
Clones: 3 (0 buried)
Markers: 0
Sequenced: 0
Length: 72 CB units
Ctg2661 of cp05T5e12DM4RS
V031L14
V057H05
V027K13
0 5 10 15 20 25 30 35 40 45 50 55 60 65 70
[Chickpea contig p...
[root@localhost: /...
[FPC V8.9 Main Menu]
Project cp05T5e12...
FPC Ctg2661 cp05T...

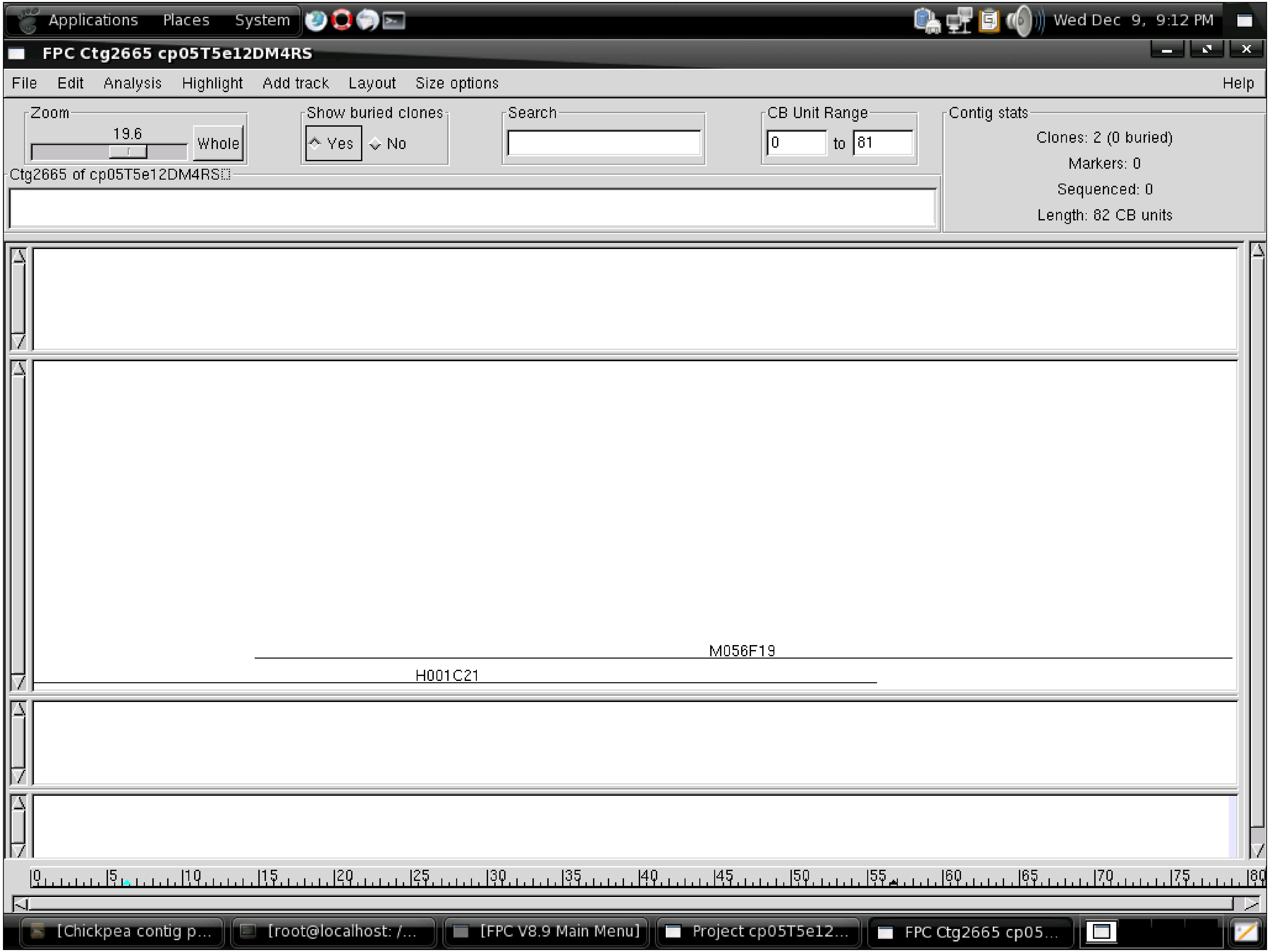

Applications Places System
Wed Dec 9, 9:12 PM
FPC Ctg2665 cp05T5e12DM4RS
File Edit Analysis Highlight Add track Layout Size options Help
Zoom
19.6
Whole
Show buried clones
Yes
No
Search
CB Unit Range
0 to 81
Contig stats
Clones: 2 (0 buried)
Markers: 0
Sequenced: 0
Length: 82 CB units
Ctg2665 of cp05T5e12DM4RS
M056F19
H001C21
0 5 10 15 20 25 30 35 40 45 50 55 60 65 70 75 80
[Chickpea contig p...
[root@localhost: /...
[FPC V8.9 Main Menu]
Project cp05T5e12...
FPC Ctg2665 cp05...

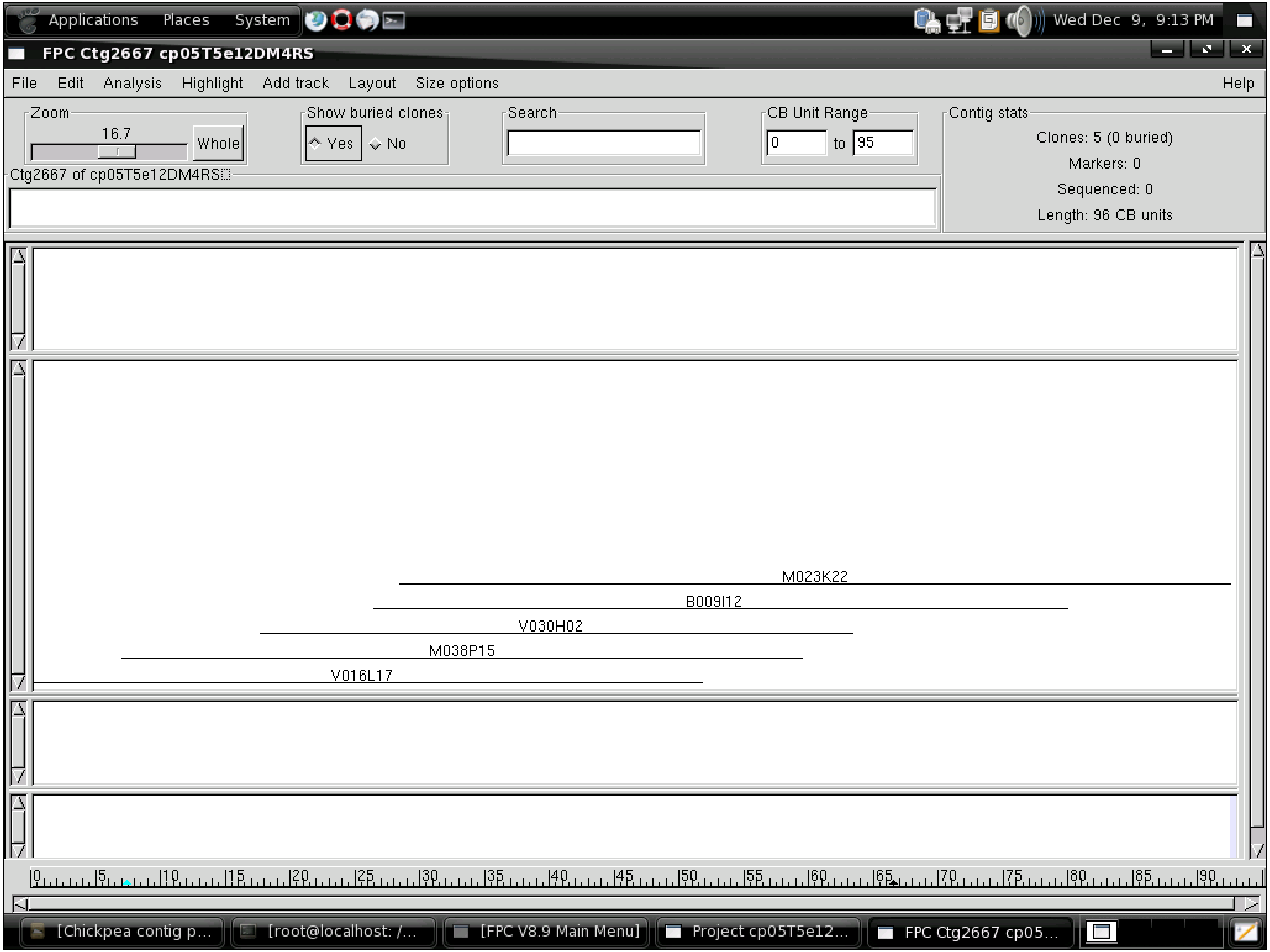
Applications Places System
Wed Dec 9, 9:13 PM
FPC Ctg2667 cp05T5e12DM4RS
File Edit Analysis Highlight Add track Layout Size options Help
Zoom
16.7
Whole
Show buried clones
Yes
No
Search
CB Unit Range
0
to
95
Contig stats
Clones: 5 (0 buried)
Markers: 0
Sequenced: 0
Length: 96 CB units
Ctg2667 of cp05T5e12DM4RS
M023K22
B009I12
V030H02
M038P15
V016L17
0 5 10 15 20 25 30 35 40 45 50 55 60 65 70 75 80 85 90
[Chickpea contig p...
[root@localhost: /...
[FPC V8.9 Main Menu]
Project cp05T5e12...
FPC Ctg2667 cp05...

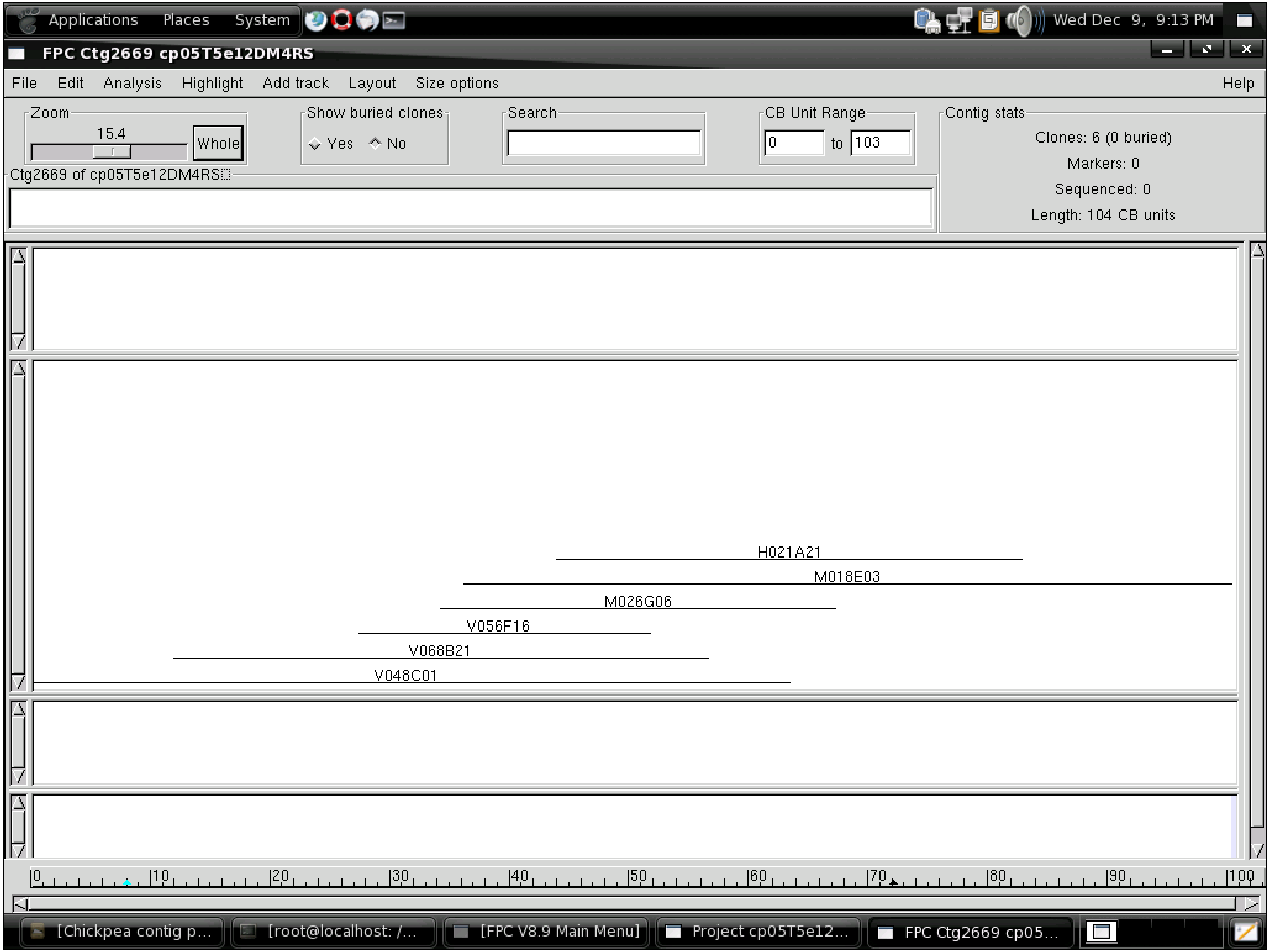
Applications Places System
Wed Dec 9, 9:13 PM
FPC Ctg2669 cp05T5e12DM4RS
File Edit Analysis Highlight Add track Layout Size options Help
Zoom
15.4
Whole
Show buried clones
Yes No
Search
CB Unit Range
0 to 103
Contig stats
Clones: 6 (0 buried)
Markers: 0
Sequenced: 0
Length: 104 CB units
Ctg2669 of cp05T5e12DM4RS
H021A21
M018E03
M026G06
V056F16
V068B21
V048C01
0 10 20 30 40 50 60 70 80 90 100
[Chickpea contig p...
[root@localhost: /...
[FPC V8.9 Main Menu]
Project cp05T5e12...
FPC Ctg2669 cp05...

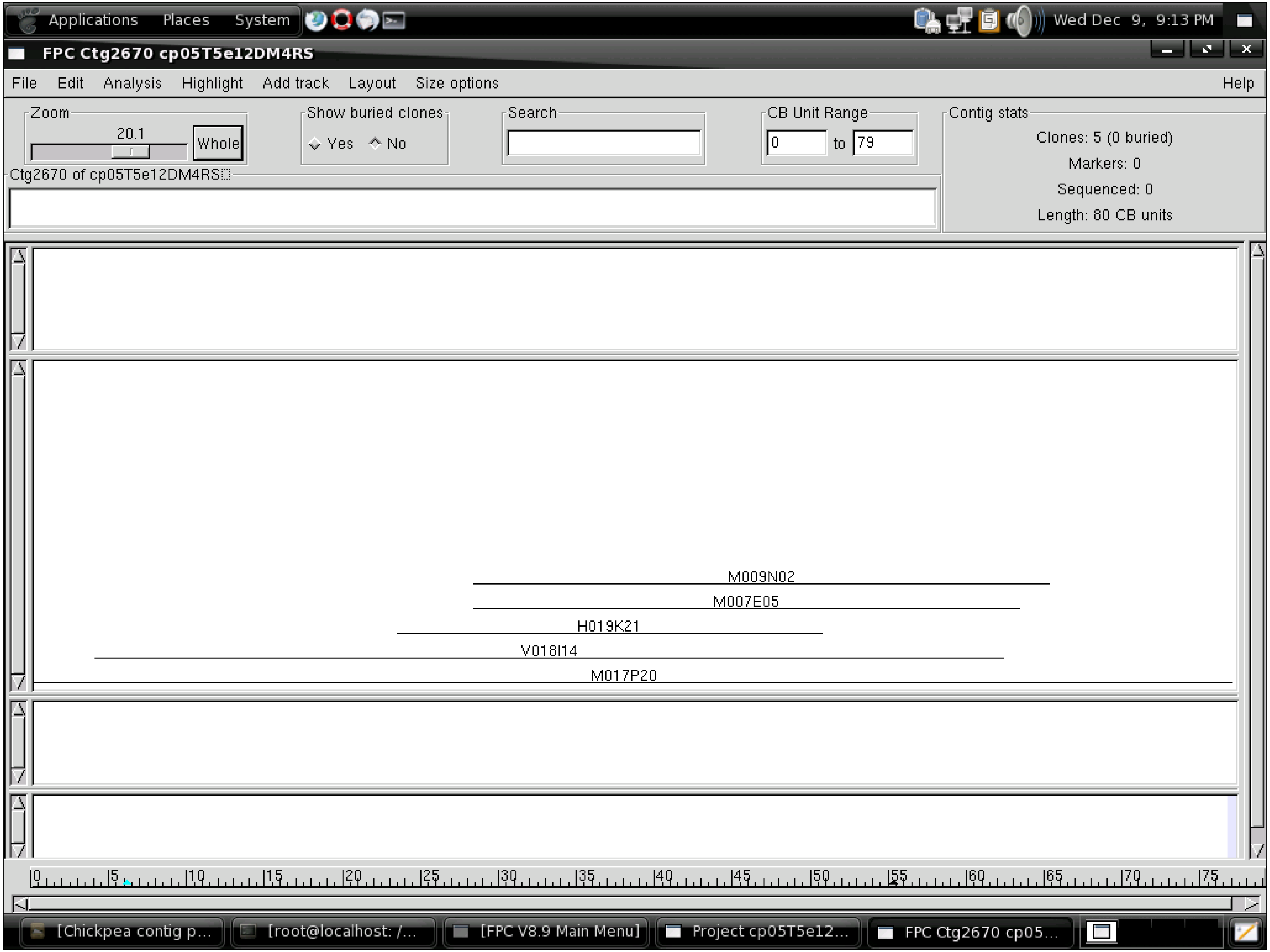
Applications Places System
Wed Dec 9, 9:13 PM
FPC Ctg2670 cp05T5e12DM4RS
File Edit Analysis Highlight Add track Layout Size options Help
Zoom
20.1
Whole
Show buried clones
Yes No
Search
CB Unit Range
0 to 79
Contig stats
Clones: 5 (0 buried)
Markers: 0
Sequenced: 0
Length: 80 CB units
Ctg2670 of cp05T5e12DM4RS
M009N02
M007E05
H019K21
V018I14
M017P20
0 5 10 15 20 25 30 35 40 45 50 55 60 65 70 75
[Chickpea contig p...
[root@localhost: /...
[FPC V8.9 Main Menu]
Project cp05T5e12...
FPC Ctg2670 cp05...

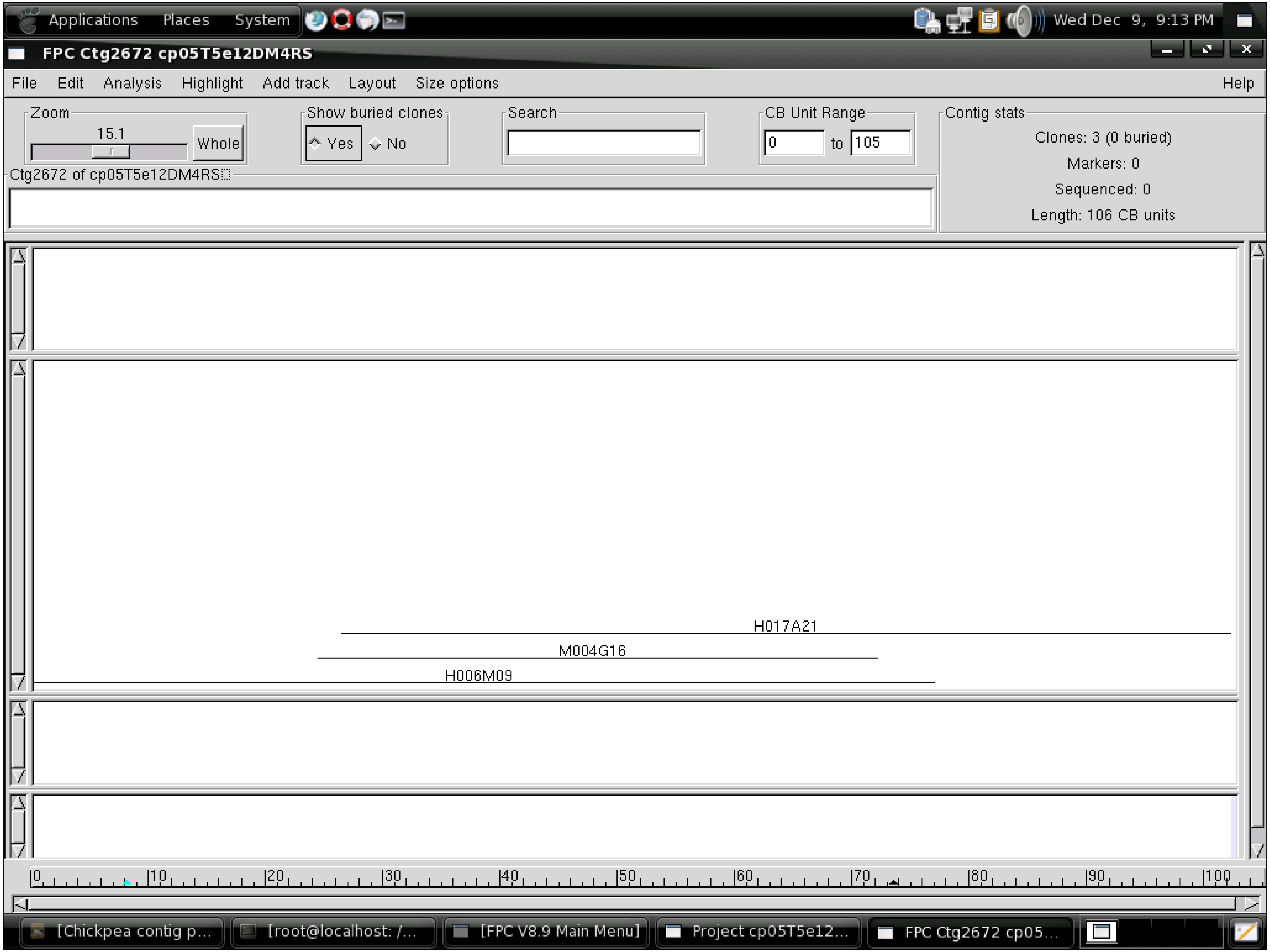
Applications Places System
Wed Dec 9, 9:13 PM
FPC Ctg2672 cp05T5e12DM4RS
File Edit Analysis Highlight Add track Layout Size options Help
Zoom
15.1
Whole
Show buried clones
Yes
No
Search
CB Unit Range
0 to 105
Contig stats
Clones: 3 (0 buried)
Markers: 0
Sequenced: 0
Length: 106 CB units
Ctg2672 of cp05T5e12DM4RS
H017A21
M004G16
H006M09
0 10 20 30 40 50 60 70 80 90 100
[Chickpea contig p...
[root@localhost: /...
[FPC V8.9 Main Menu]
Project cp05T5e12...
FPC Ctg2672 cp05...

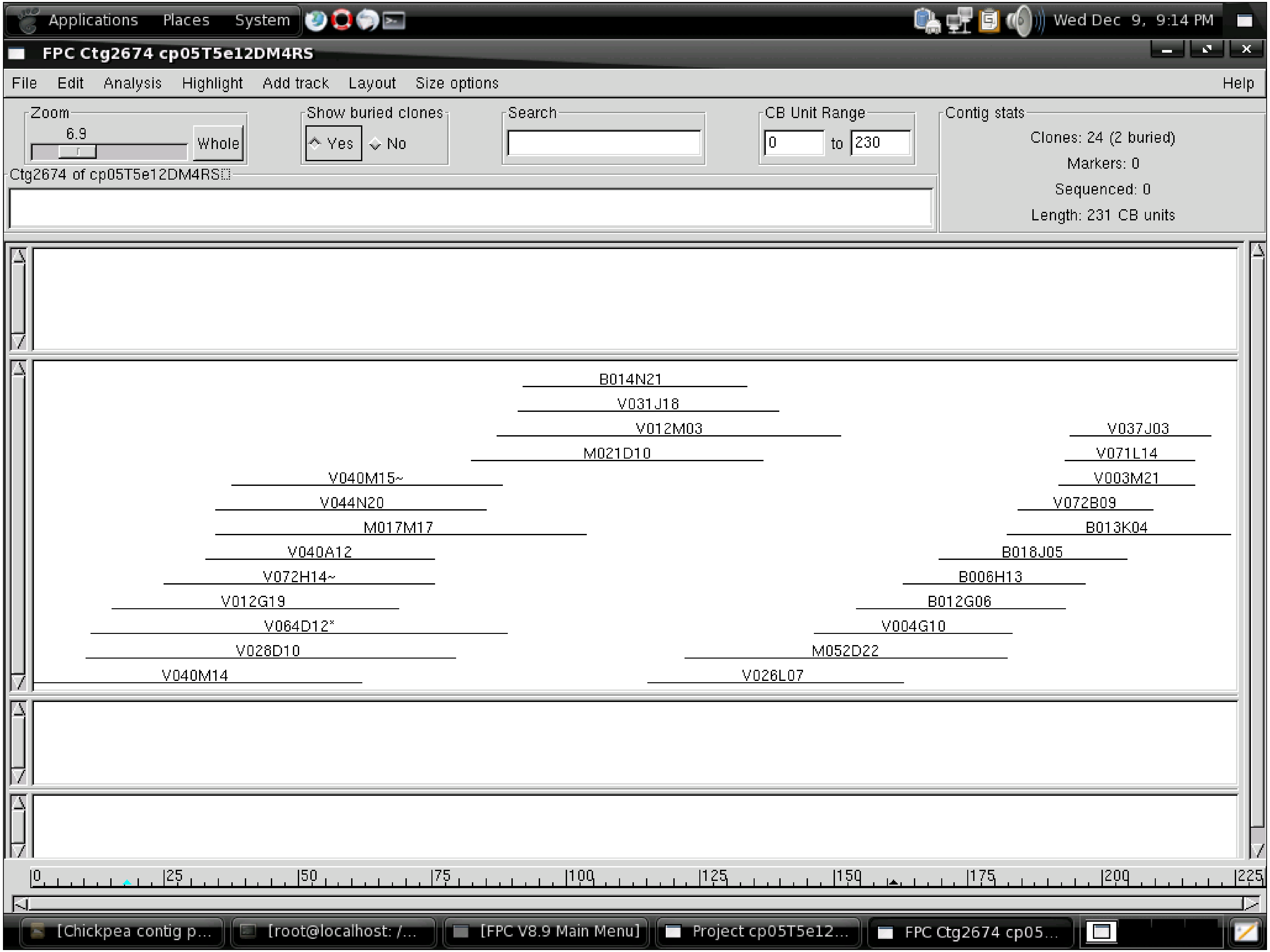
Applications Places System
Wed Dec 9, 9:14 PM
FPC Ctg2674 cp05T5e12DM4RS
File Edit Analysis Highlight Add track Layout Size options Help
Zoom
6.9
Whole
Show buried clones
Yes No
Search
CB Unit Range
0 to 230
Contig stats
Clones: 24 (2 buried)
Markers: 0
Sequenced: 0
Length: 231 CB units
Ctg2674 of cp05T5e12DM4RS
B014N21
V031J18
V012M03
V037J03
M021D10
V071L14
V040M15~
V003M21
V044N20
V072B09
M017M17
B013K04
V040A12
B018J05
V072H14~
B006H13
V012G19
B012G06
V064D12*
V004G10
V028D10
M052D22
V040M14
V026L07
0 25 50 75 100 125 150 175 200 225
[Chickpea contig p...
[root@localhost: /...
[FPC V8.9 Main Menu]
Project cp05T5e12...
FPC Ctg2674 cp05...

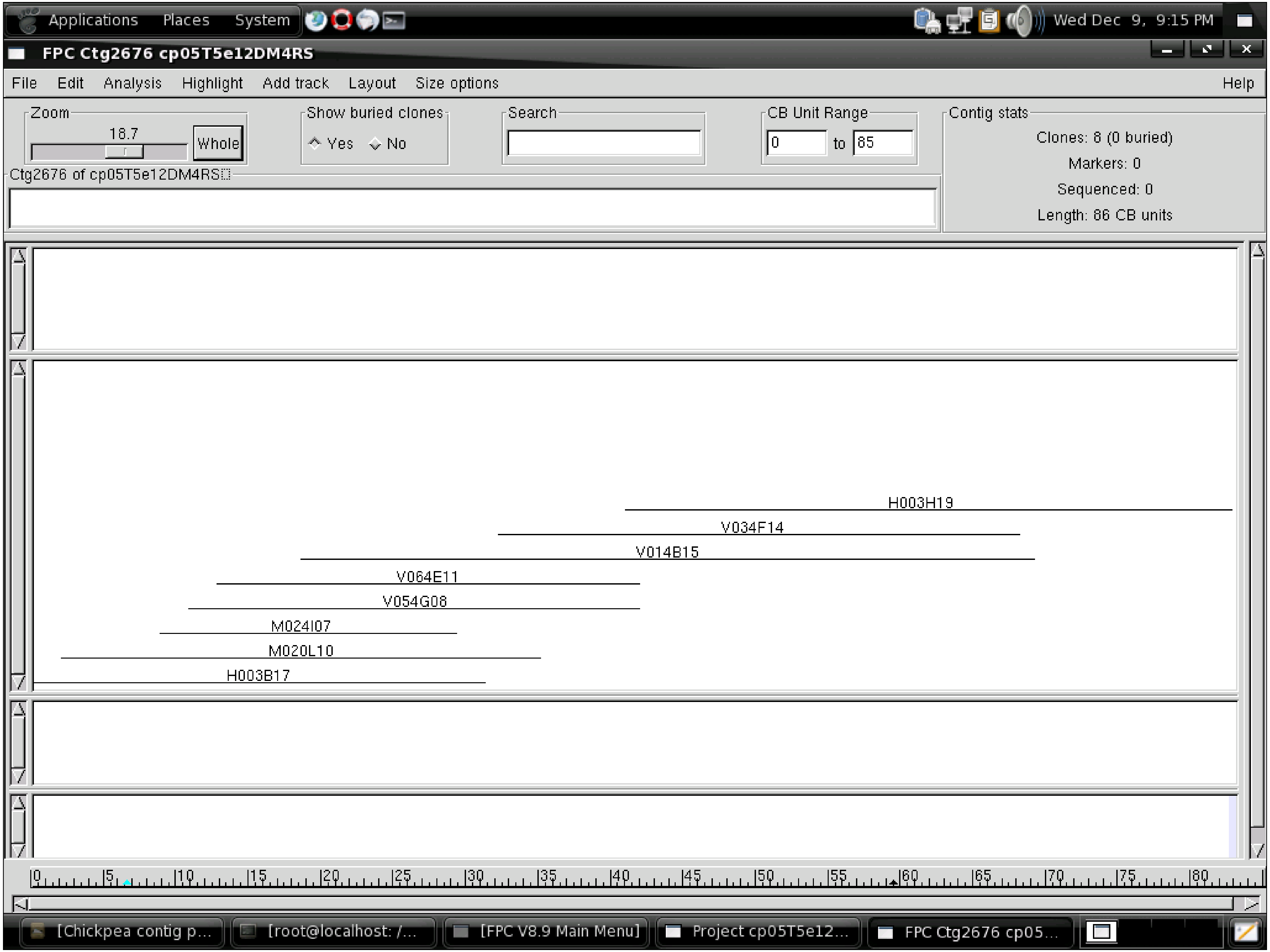
Applications Places System
Wed Dec 9, 9:15 PM
FPC Ctg2676 cp05T5e12DM4RS
File Edit Analysis Highlight Add track Layout Size options Help
Zoom
18.7
Whole
Show buried clones
Yes No
Search
CB Unit Range
0 to 85
Contig stats
Clones: 8 (0 buried)
Markers: 0
Sequenced: 0
Length: 86 CB units
Ctg2676 of cp05T5e12DM4RS
H003H19
V034F14
V014B15
V064E11
V054G08
M024I07
M020L10
H003B17
0 5 10 15 20 25 30 35 40 45 50 55 60 65 70 75 80
[Chickpea contig p...
[root@localhost: /...
[FPC V8.9 Main Menu]
Project cp05T5e12...
FPC Ctg2676 cp05...

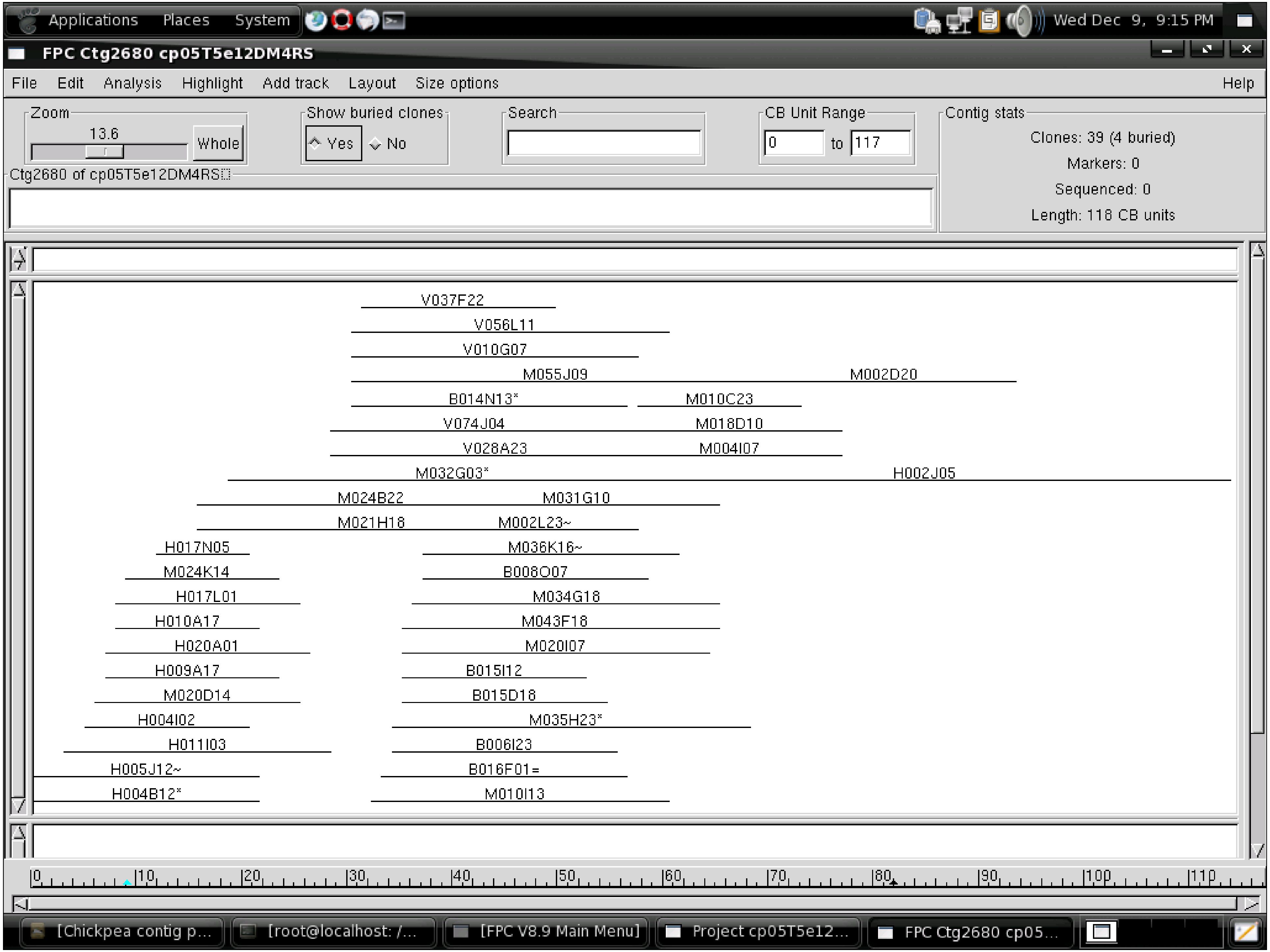
Applications Places System
Wed Dec 9, 9:15 PM
FPC Ctg2680 cp05T5e12DM4RS
File Edit Analysis Highlight Add track Layout Size options Help
Zoom
13.6
Whole
Show buried clones
Yes No
Search
CB Unit Range
0 to 117
Contig stats
Clones: 39 (4 buried)
Markers: 0
Sequenced: 0
Length: 118 CB units
Ctg2680 of cp05T5e12DM4RS
V037F22
V056L11
V010G07
M055J09
M002D20
B014N13*
M010C23
V074J04
M018D10
V028A23
M004I07
M032G03*
H002J05
M024B22
M031G10
M021H18
M002L23~
H017N05
M036K16~
M024K14
B008O07
H017L01
M034G18
H010A17
M043F18
H020A01
M020I07
H009A17
B015I12
M020D14
B015D18
H004I02
M035H23*
H011I03
B006I23
H005J12~
B016F01=
H004B12*
M010I13
0 10 20 30 40 50 60 70 80 90 100 110
[Chickpea contig p...
[root@localhost: /...
[FPC V8.9 Main Menu]
Project cp05T5e12...
FPC Ctg2680 cp05...

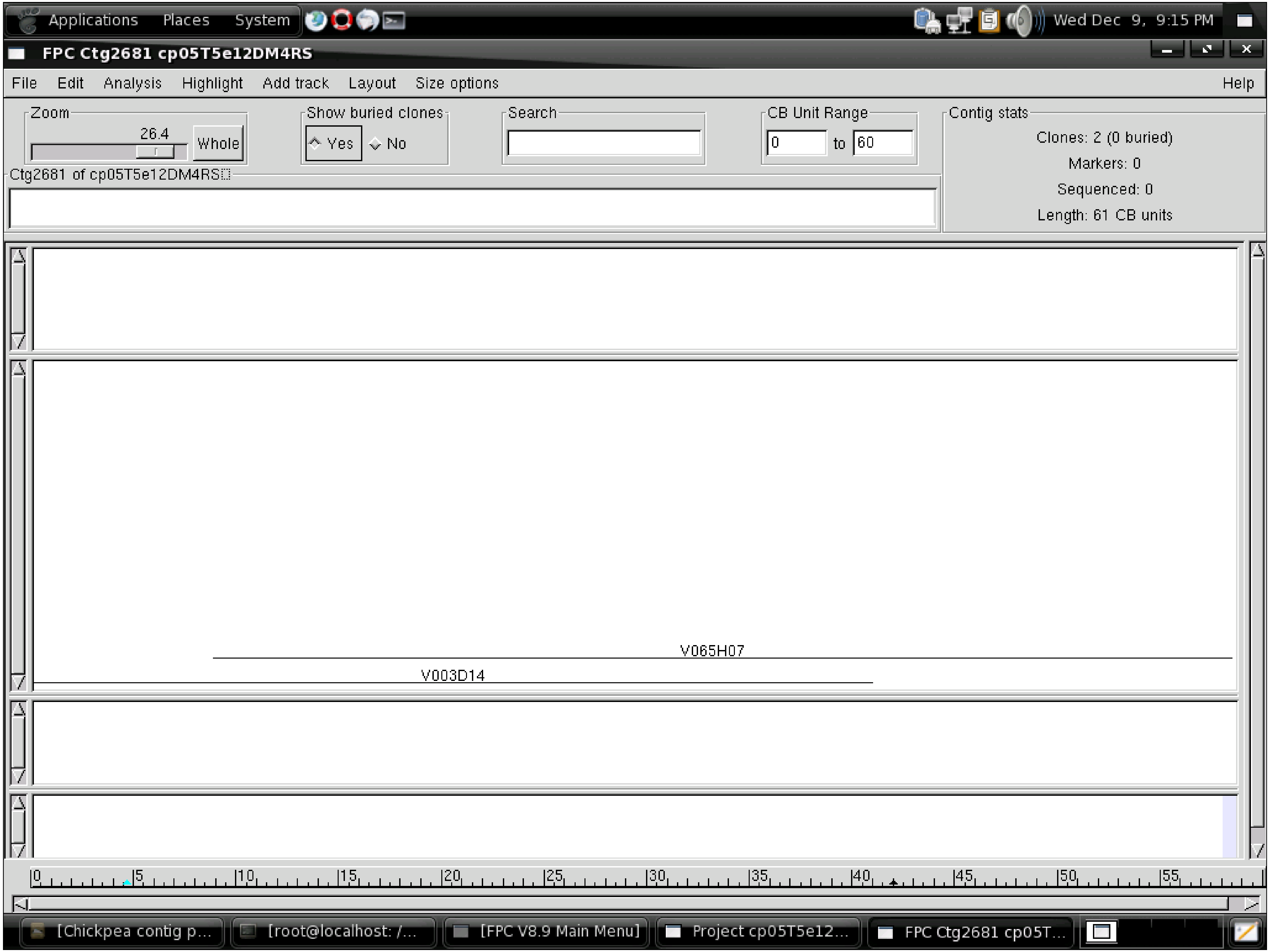
Applications Places System
Wed Dec 9, 9:15 PM
FPC Ctg2681 cp05T5e12DM4RS
File Edit Analysis Highlight Add track Layout Size options Help
Zoom
26.4
Whole
Show buried clones
Yes
No
Search
CB Unit Range
0 to 60
Contig stats
Clones: 2 (0 buried)
Markers: 0
Sequenced: 0
Length: 61 CB units
Ctg2681 of cp05T5e12DM4RS
V065H07
V003D14
0 5 10 15 20 25 30 35 40 45 50 55
[Chickpea contig p...
[root@localhost: /...
[FPC V8.9 Main Menu]
Project cp05T5e12...
FPC Ctg2681 cp05T...

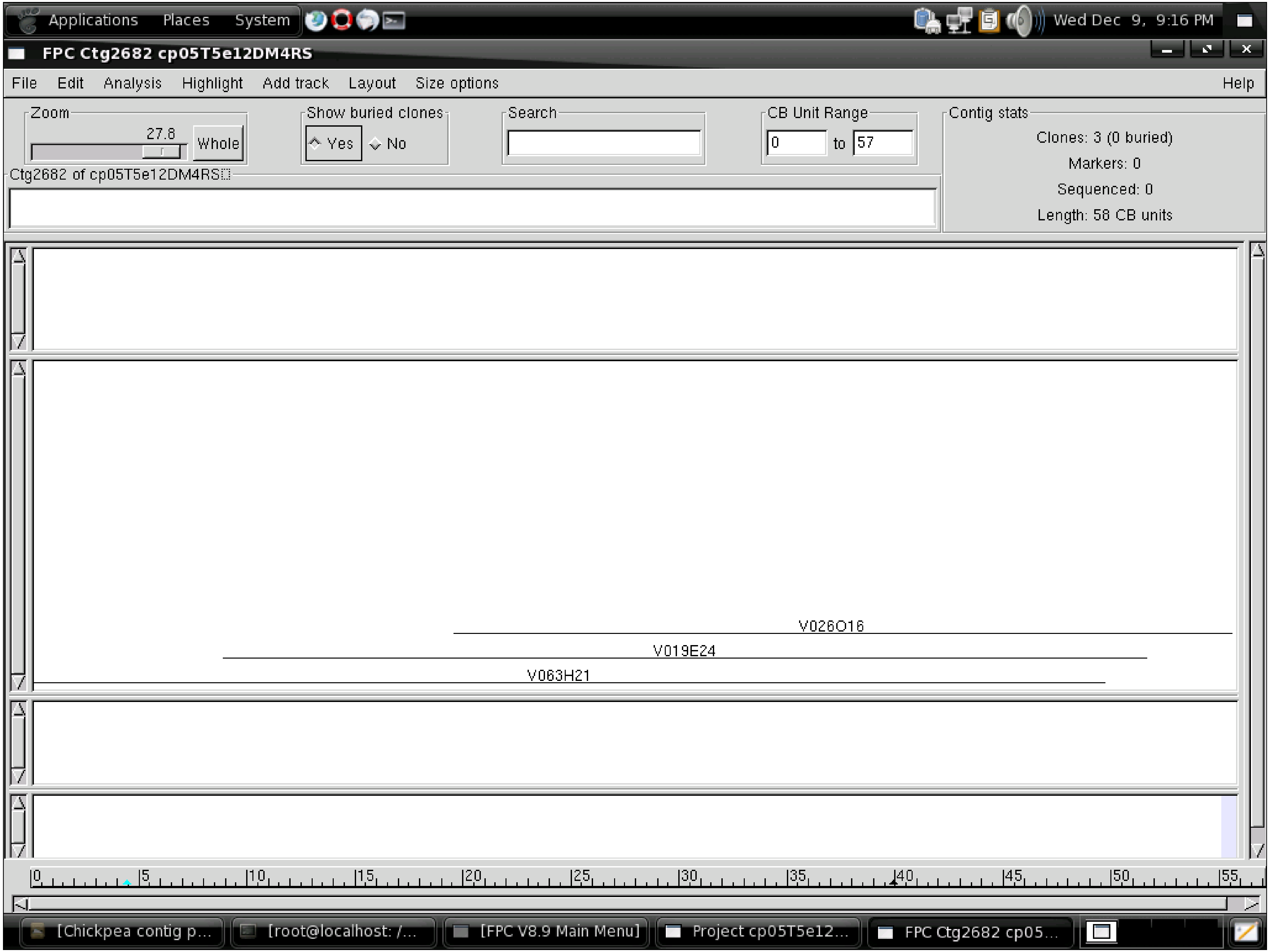
Applications Places System
Wed Dec 9, 9:16 PM
FPC Ctg2682 cp05T5e12DM4RS
File Edit Analysis Highlight Add track Layout Size options Help
Zoom
27.8
Whole
Show buried clones
Yes
No
Search
CB Unit Range
0
to
57
Contig stats
Clones: 3 (0 buried)
Markers: 0
Sequenced: 0
Length: 58 CB units
Ctg2682 of cp05T5e12DM4RS
V026O16
V019E24
V063H21
0 5 10 15 20 25 30 35 40 45 50 55
[Chickpea contig p...
[root@localhost: /...
[FPC V8.9 Main Menu]
Project cp05T5e12...
FPC Ctg2682 cp05...

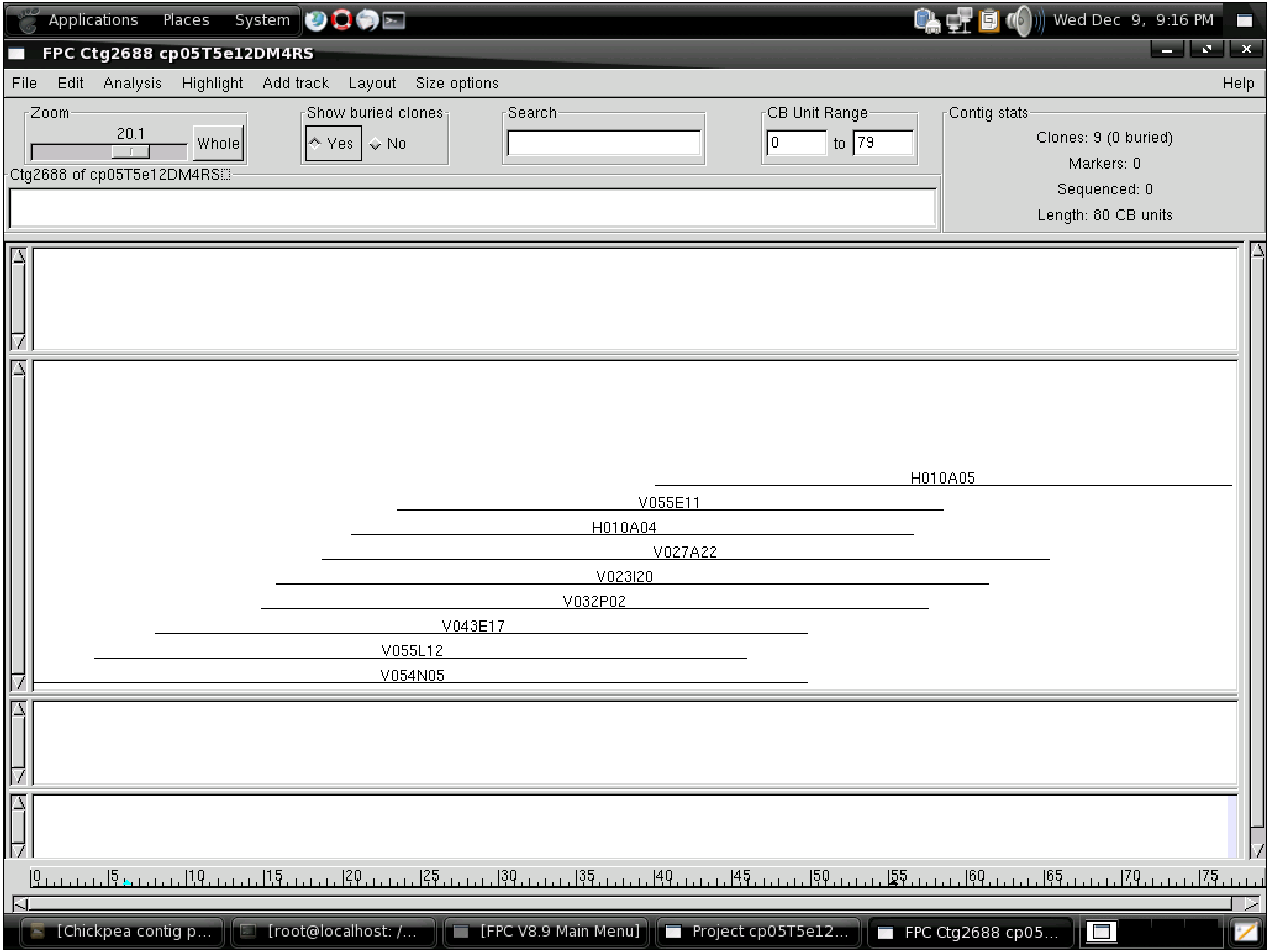
Applications Places System
Wed Dec 9, 9:16 PM
FPC Ctg2688 cp05T5e12DM4RS
File Edit Analysis Highlight Add track Layout Size options Help
Zoom
20.1
Whole
Show buried clones
Yes No
Search
CB Unit Range
0 to 79
Contig stats
Clones: 9 (0 buried)
Markers: 0
Sequenced: 0
Length: 80 CB units
Ctg2688 of cp05T5e12DM4RS
H010A05
V055E11
H010A04
V027A22
V023I20
V032P02
V043E17
V055L12
V054N05
0 5 10 15 20 25 30 35 40 45 50 55 60 65 70 75
[Chickpea contig p...
[root@localhost: /...
[FPC V8.9 Main Menu]
Project cp05T5e12...
FPC Ctg2688 cp05...

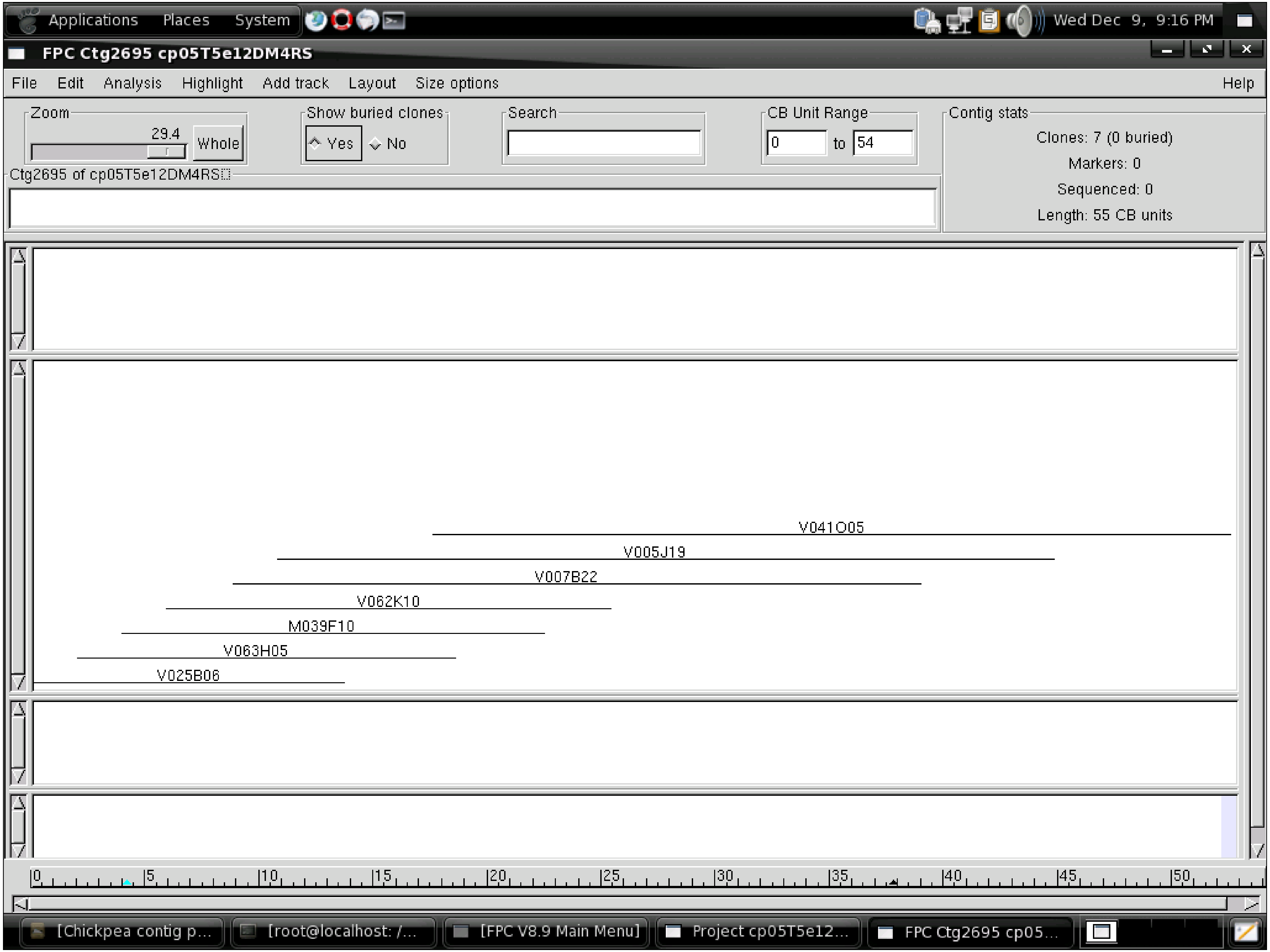

Applications
Places
System
Wed Dec 9, 9:16 PM
FPC Ctg2695 cp05T5e12DM4RS
File
Edit
Analysis
Highlight
Add track
Layout
Size options
Help
Zoom
29.4
Whole
Show buried clones
Yes
No
Search
CB Unit Range
0
to
54
Contig stats
Clones: 7 (0 buried)
Markers: 0
Sequenced: 0
Length: 55 CB units
Ctg2695 of cp05T5e12DM4RS
V041O05
V005J19
V007B22
V062K10
M039F10
V063H05
V025B06
0
5
10
15
20
25
30
35
40
45
50
[Chickpea contig p...
[root@localhost: /...
[FPC V8.9 Main Menu]
Project cp05T5e12...
FPC Ctg2695 cp05...

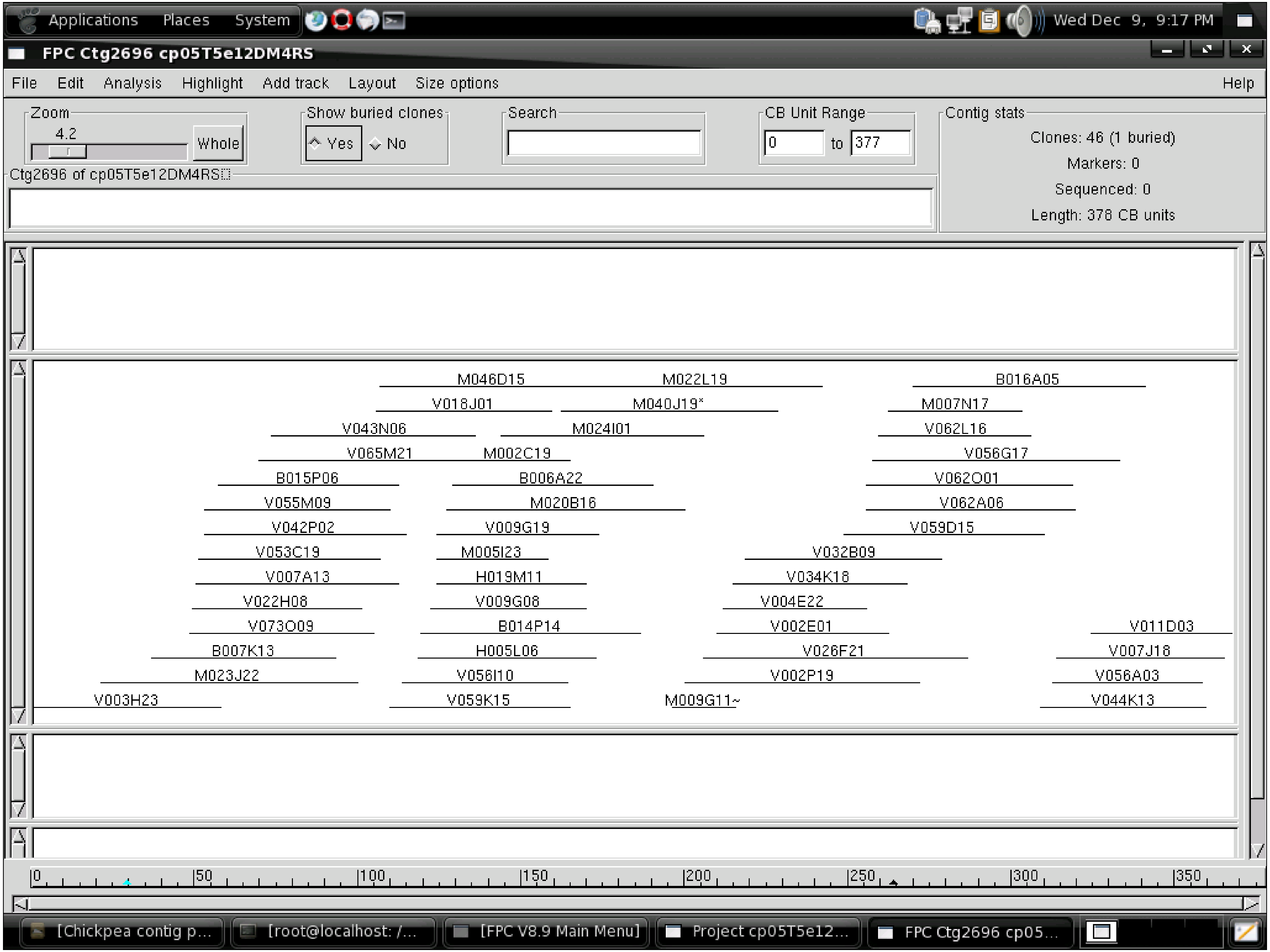

Applications
Places
System
Wed Dec 9, 9:17 PM
FPC Ctg2696 cp05T5e12DM4RS
File
Edit
Analysis
Highlight
Add track
Layout
Size options
Help
Zoom
4.2
Whole
Show buried clones
Yes
No
Search
CB Unit Range
0
to
377
Contig stats
Clones: 46 (1 buried)
Markers: 0
Sequenced: 0
Length: 378 CB units
Ctg2696 of cp05T5e12DM4RS
M046D15
M022L19
B016A05
V018J01
M040J19*
M007N17
V043N06
M024I01
V062L16
V065M21
M002C19
V056G17
B015P06
B006A22
V062O01
V055M09
M020B16
V062A06
V042P02
V009G19
V059D15
V053C19
M005I23
V032B09
V007A13
H019M11
V034K18
V022H08
V009G08
V004E22
V073O09
B014P14
V002E01
V011D03
B007K13
H005L06
V026F21
V007J18
M023J22
V056I10
V002P19
V056A03
V003H23
V059K15
M009G11~
V044K13
0
50
100
150
200
250
300
350
[Chickpea contig p...
[root@localhost: /...
[FPC V8.9 Main Menu]
Project cp05T5e12...
FPC Ctg2696 cp05...

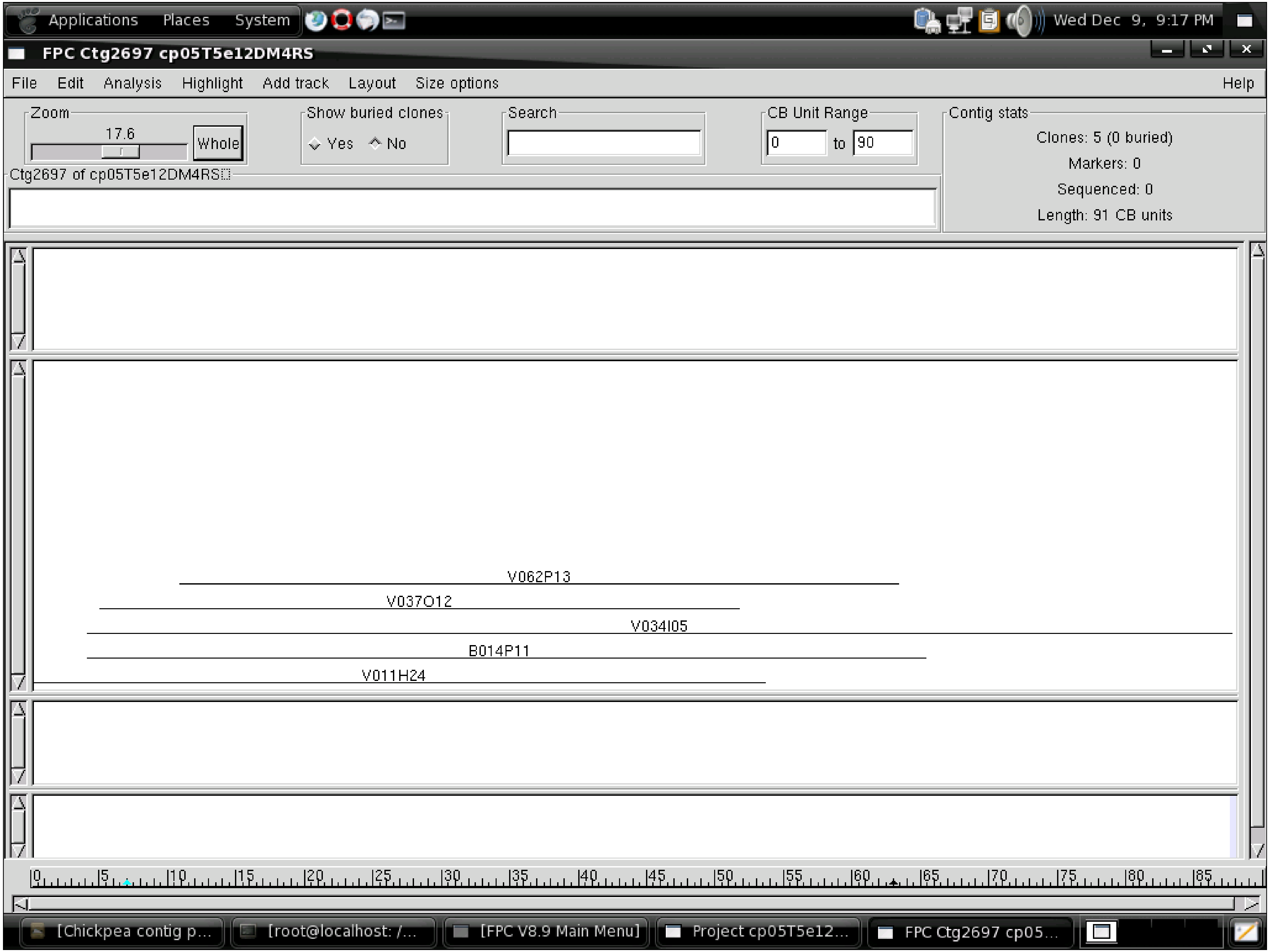
Applications Places System
Wed Dec 9, 9:17 PM
FPC Ctg2697 cp05T5e12DM4RS
File Edit Analysis Highlight Add track Layout Size options Help
Zoom
17.6
Whole
Show buried clones
Yes No
Search
CB Unit Range
0 to 90
Contig stats
Clones: 5 (0 buried)
Markers: 0
Sequenced: 0
Length: 91 CB units
Ctg2697 of cp05T5e12DM4RS
V062P13
V037O12
V034I05
B014P11
V011H24
0 5 10 15 20 25 30 35 40 45 50 55 60 65 70 75 80 85
[Chickpea contig p...
[root@localhost: /...
[FPC V8.9 Main Menu]
Project cp05T5e12...
FPC Ctg2697 cp05...

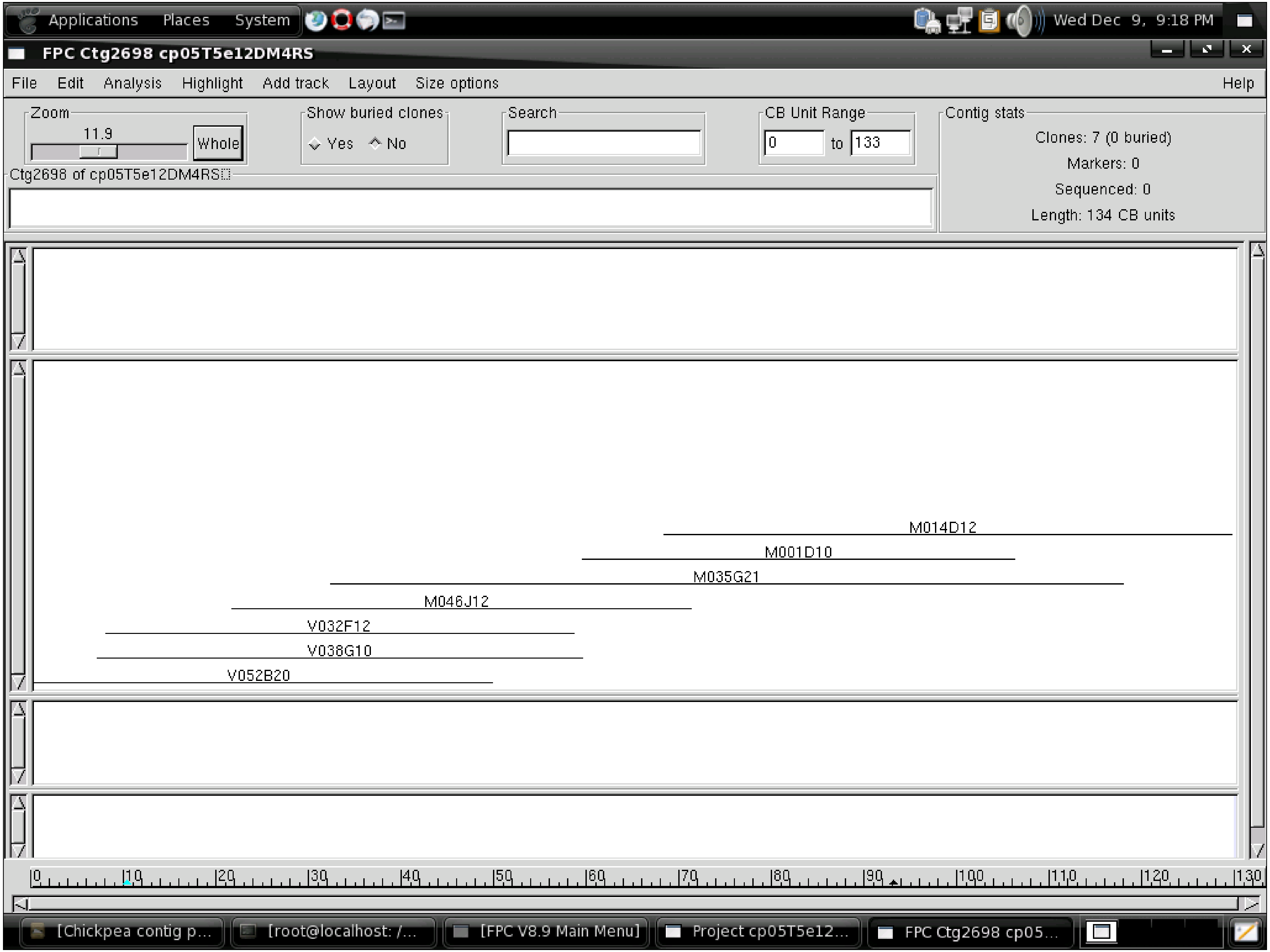
Applications Places System
Wed Dec 9, 9:18 PM
FPC Ctg2698 cp05T5e12DM4RS
File Edit Analysis Highlight Add track Layout Size options Help
Zoom
11.9
Whole
Show buried clones
Yes No
Search
CB Unit Range
0 to 133
Contig stats
Clones: 7 (0 buried)
Markers: 0
Sequenced: 0
Length: 134 CB units
Ctg2698 of cp05T5e12DM4RS
M014D12
M001D10
M035G21
M046J12
V032F12
V038G10
V052B20
0 10 20 30 40 50 60 70 80 90 100 110 120 130
[Chickpea contig p...
[root@localhost: /...
[FPC V8.9 Main Menu]
Project cp05T5e12...
FPC Ctg2698 cp05...

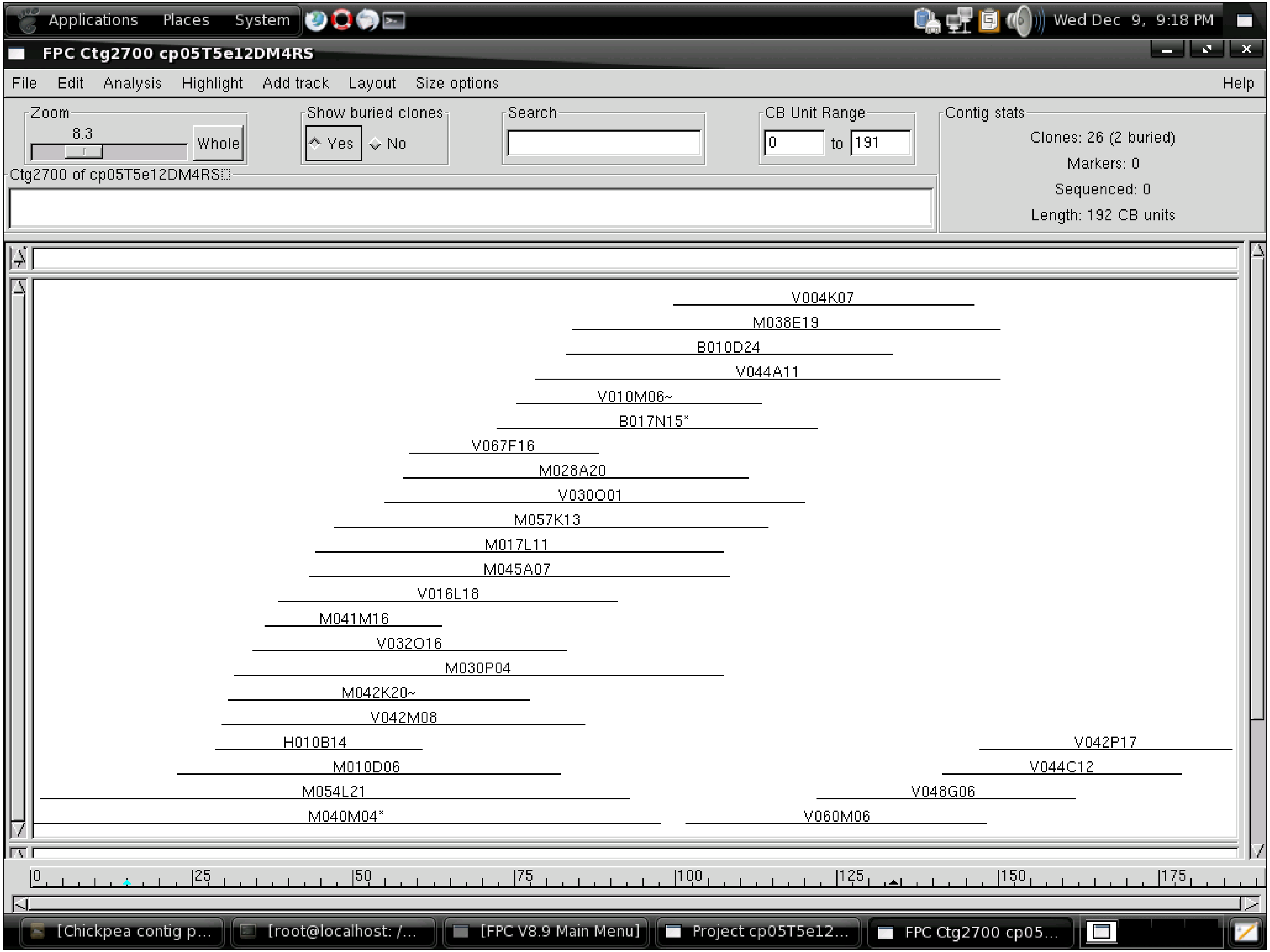
Applications Places System
Wed Dec 9, 9:18 PM
FPC Ctg2700 cp05T5e12DM4RS
File Edit Analysis Highlight Add track Layout Size options Help
Zoom
8.3
Whole
Show buried clones
Yes No
Search
CB Unit Range
0 to 191
Contig stats
Clones: 26 (2 buried)
Markers: 0
Sequenced: 0
Length: 192 CB units
Ctg2700 of cp05T5e12DM4RS
V004K07
M038E19
B010D24
V044A11
V010M06~
B017N15*
V067F16
M028A20
V030O01
M057K13
M017L11
M045A07
V016L18
M041M16
V032O16
M030P04
M042K20~
V042M08
H010B14
V042P17
M010D06
V044C12
M054L21
V048G06
M040M04*
V060M06
0 25 50 75 100 125 150 175
[Chickpea contig p...
[root@localhost: /...
[FPC V8.9 Main Menu]
Project cp05T5e12...
FPC Ctg2700 cp05...

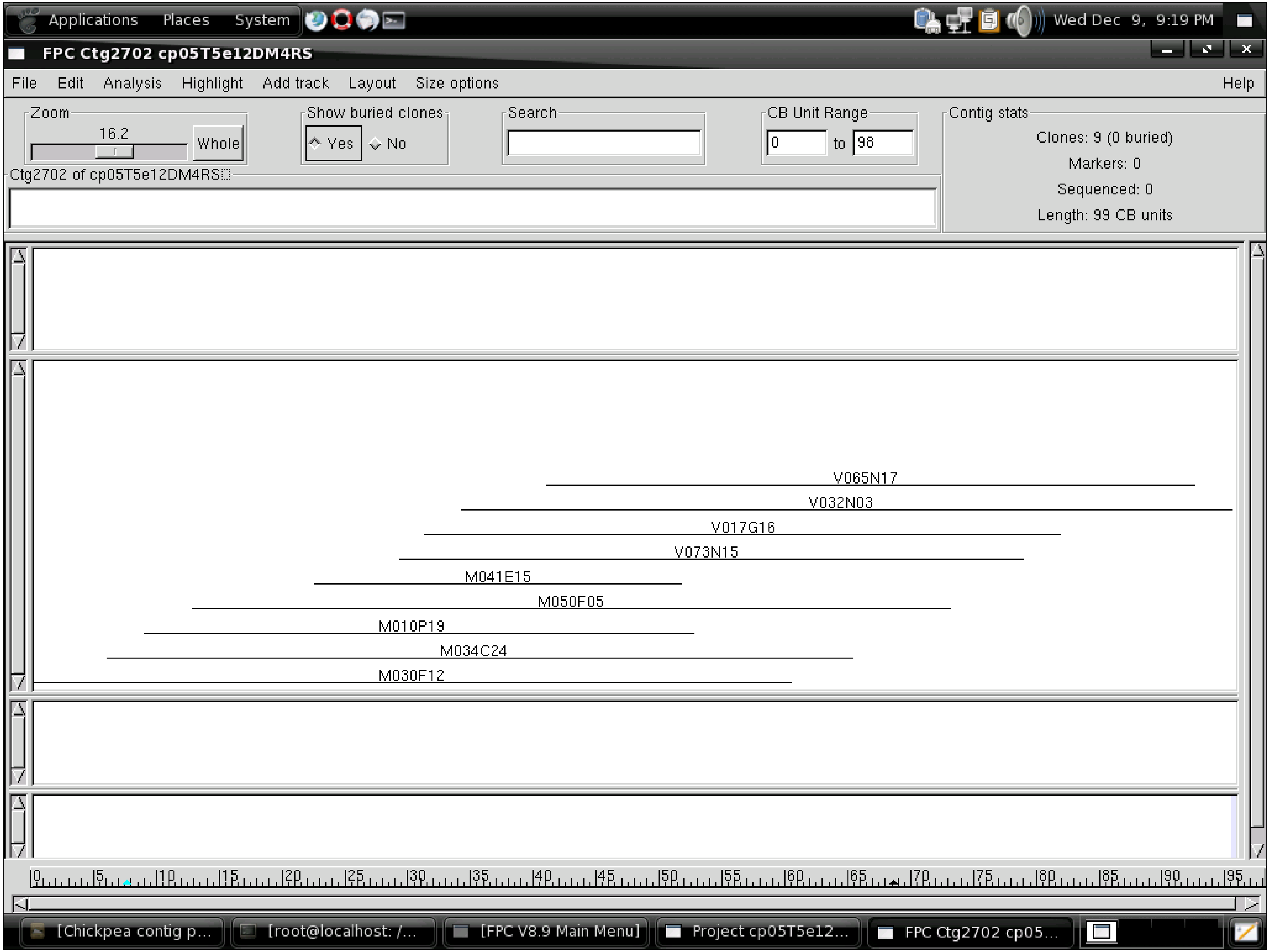
Applications Places System
Wed Dec 9, 9:19 PM
FPC Ctg2702 cp05T5e12DM4RS
File Edit Analysis Highlight Add track Layout Size options Help
Zoom
16.2
Whole
Show buried clones
Yes
No
Search
CB Unit Range
0
to
98
Contig stats
Clones: 9 (0 buried)
Markers: 0
Sequenced: 0
Length: 99 CB units
Ctg2702 of cp05T5e12DM4RS
V065N17
V032N03
V017G16
V073N15
M041E15
M050F05
M010P19
M034C24
M030F12
0 5 10 15 20 25 30 35 40 45 50 55 60 65 70 75 80 85 90 95
[Chickpea contig p...
[root@localhost: /...
[FPC V8.9 Main Menu]
Project cp05T5e12...
FPC Ctg2702 cp05...

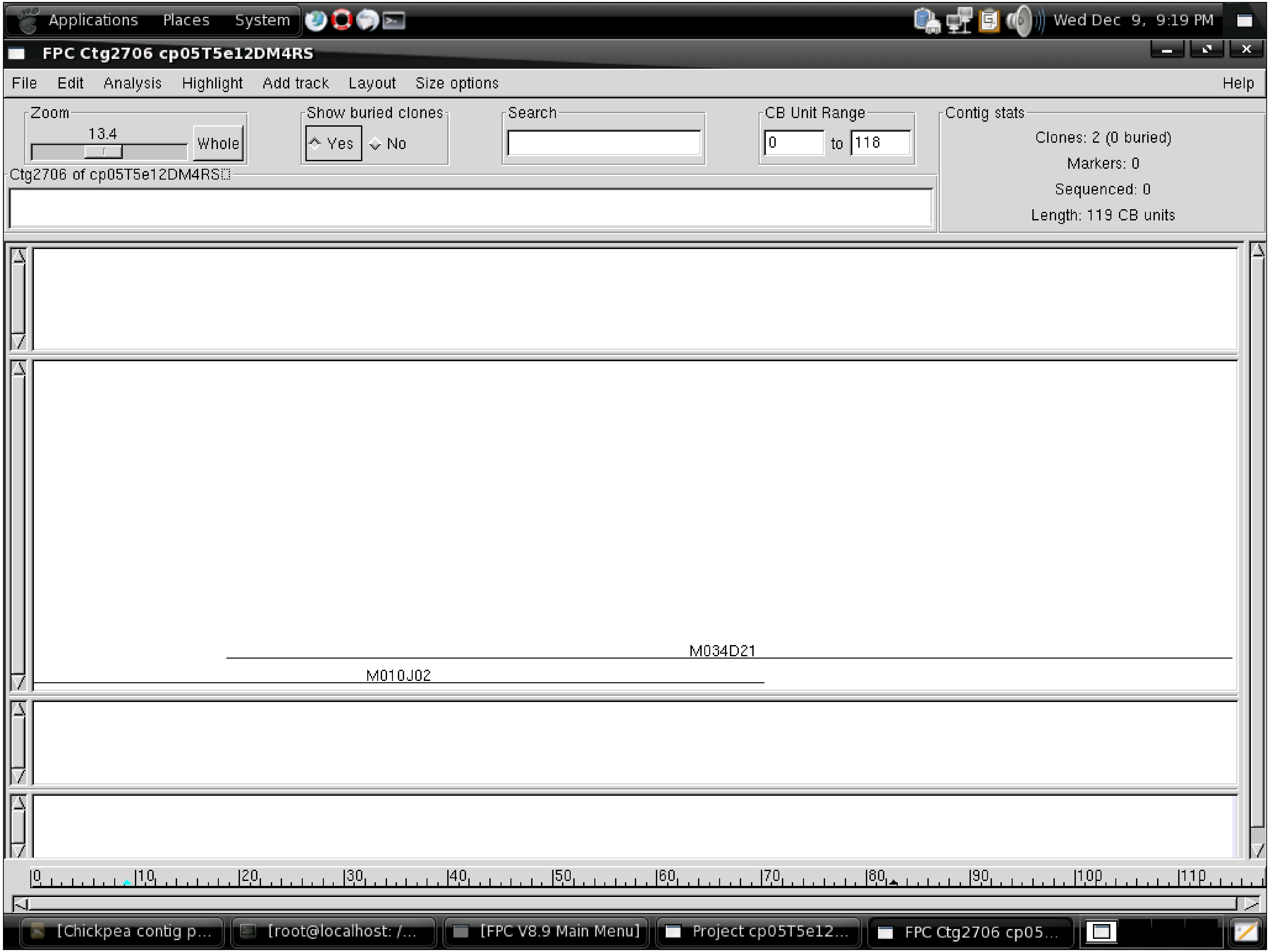
Applications Places System
Wed Dec 9, 9:19 PM
FPC Ctg2706 cp05T5e12DM4RS
File Edit Analysis Highlight Add track Layout Size options Help
Zoom
13.4
Whole
Show buried clones
Yes No
Search
CB Unit Range
0 to 118
Contig stats
Clones: 2 (0 buried)
Markers: 0
Sequenced: 0
Length: 119 CB units
Ctg2706 of cp05T5e12DM4RS
M034D21
M010J02
0 10 20 30 40 50 60 70 80 90 100 110
[Chickpea contig p...
[root@localhost: /...
[FPC V8.9 Main Menu]
Project cp05T5e12...
FPC Ctg2706 cp05...

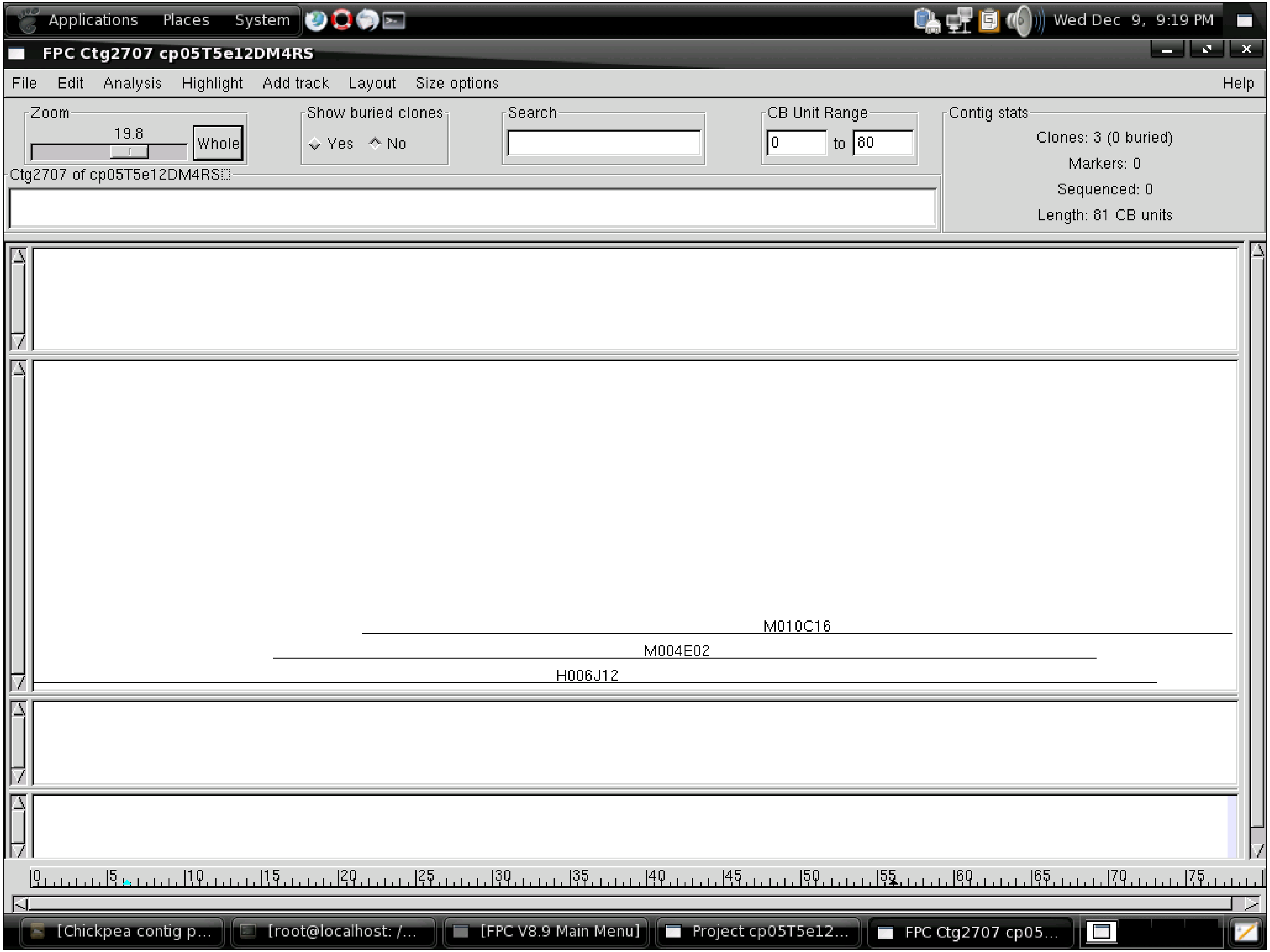
Applications
Places
System
Wed Dec 9, 9:19 PM
FPC Ctg2707 cp05T5e12DM4RS
File
Edit
Analysis
Highlight
Add track
Layout
Size options
Help
Zoom
19.8
Whole
Show buried clones
Yes
No
Search
CB Unit Range
0
to
80
Contig stats
Clones: 3 (0 buried)
Markers: 0
Sequenced: 0
Length: 81 CB units
Ctg2707 of cp05T5e12DM4RS
M010C16
M004E02
H006J12
0
5
10
15
20
25
30
35
40
45
50
55
60
65
70
75
[Chickpea contig p...
[root@localhost: /...
[FPC V8.9 Main Menu]
Project cp05T5e12...
FPC Ctg2707 cp05...

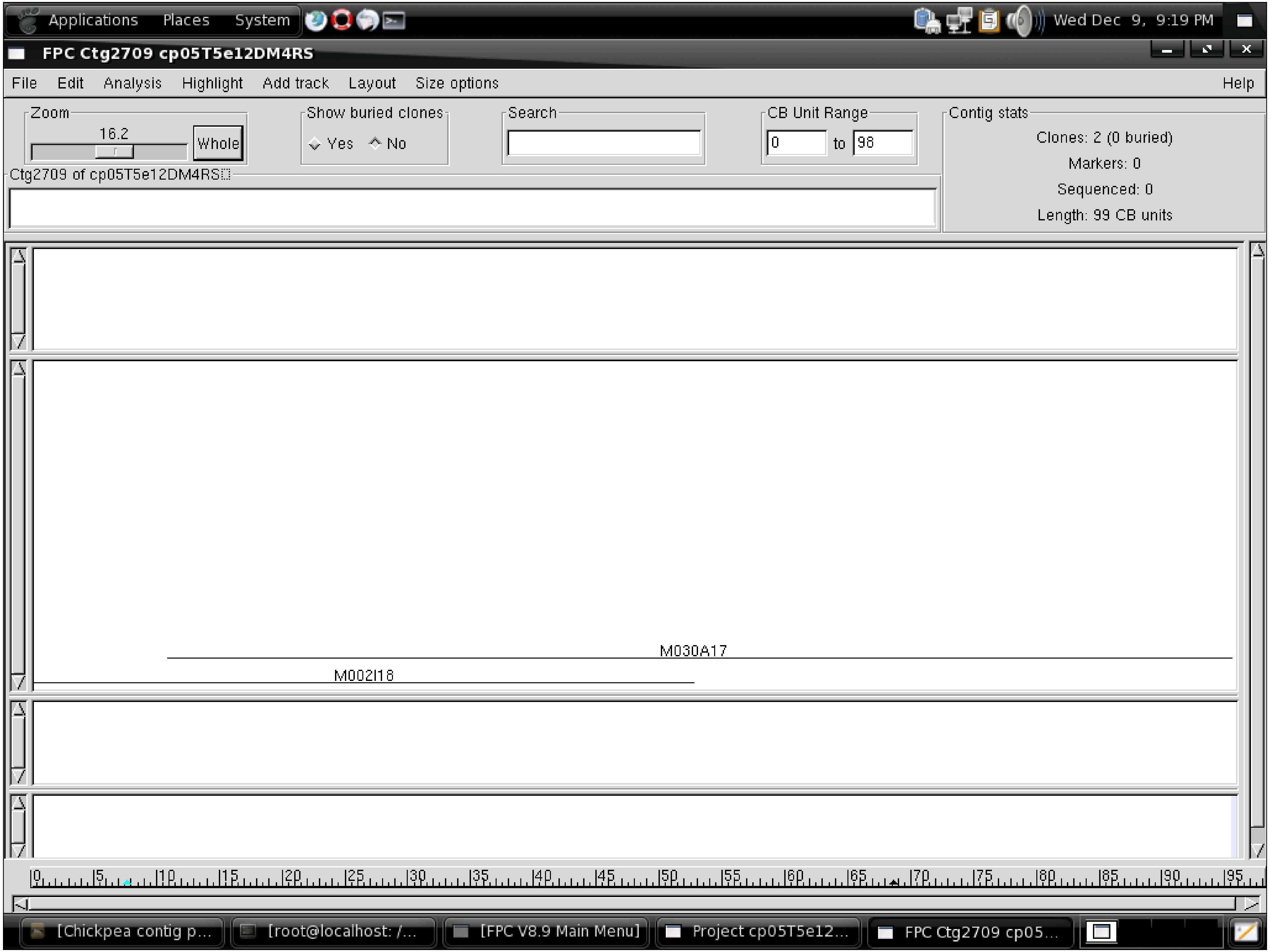
Applications Places System
Wed Dec 9, 9:19 PM
FPC Ctg2709 cp05T5e12DM4RS
File Edit Analysis Highlight Add track Layout Size options Help
Zoom
16.2
Whole
Show buried clones
Yes No
Search
CB Unit Range
0 to 98
Contig stats
Clones: 2 (0 buried)
Markers: 0
Sequenced: 0
Length: 99 CB units
Ctg2709 of cp05T5e12DM4RS
M030A17
M002I18
0 5 10 15 20 25 30 35 40 45 50 55 60 65 70 75 80 85 90 95
[Chickpea contig p...
[root@localhost: /...
[FPC V8.9 Main Menu]
Project cp05T5e12...
FPC Ctg2709 cp05...

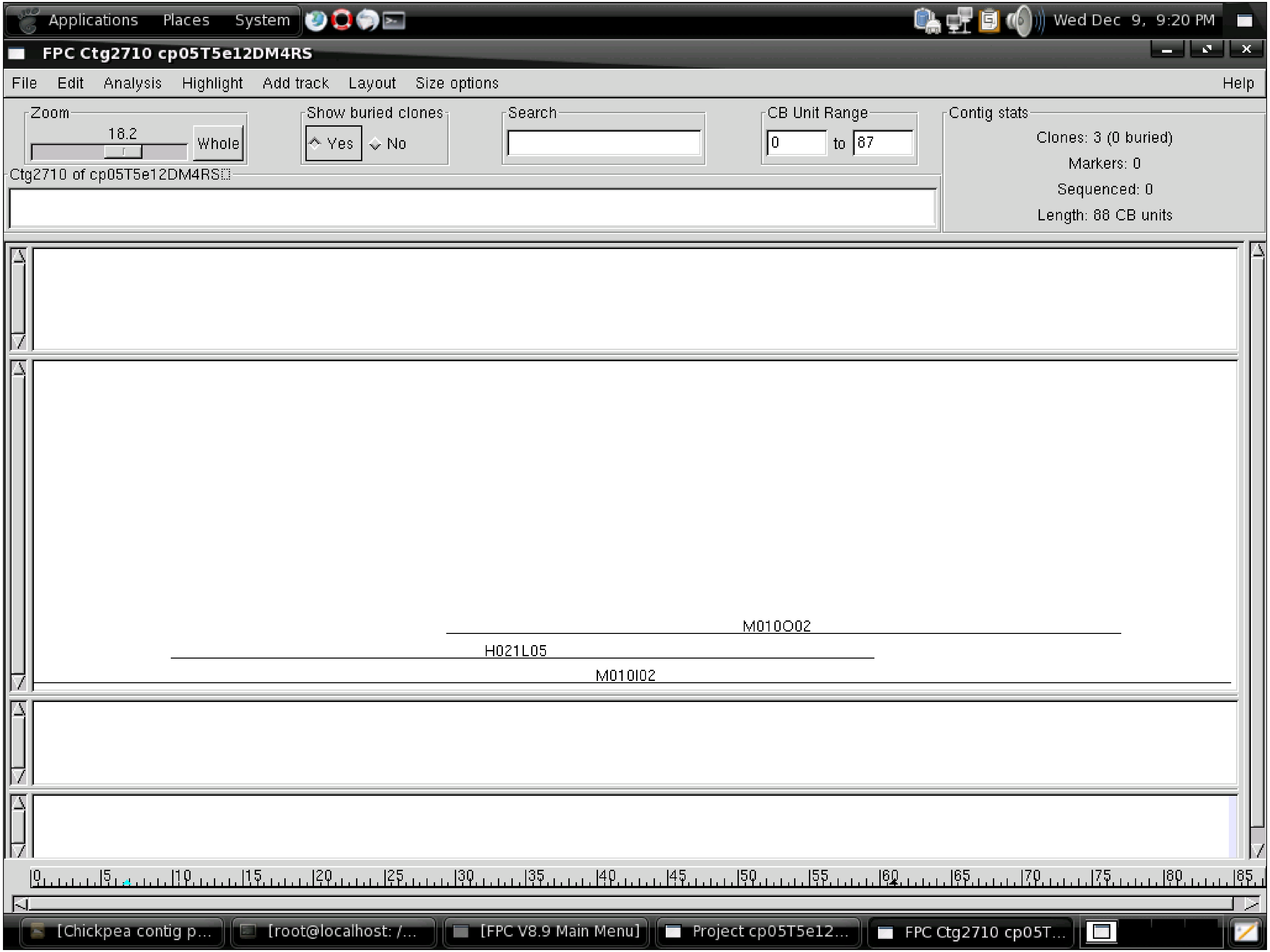
Applications Places System
Wed Dec 9, 9:20 PM
FPC Ctg2710 cp05T5e12DM4RS
File Edit Analysis Highlight Add track Layout Size options Help
Zoom
18.2
Whole
Show buried clones
Yes No
Search
CB Unit Range
0 to 87
Contig stats
Clones: 3 (0 buried)
Markers: 0
Sequenced: 0
Length: 88 CB units
Ctg2710 of cp05T5e12DM4RS
M010O02
H021L05
M010I02
0 5 10 15 20 25 30 35 40 45 50 55 60 65 70 75 80 85
[Chickpea contig p...
[root@localhost: /...
[FPC V8.9 Main Menu]
Project cp05T5e12...
FPC Ctg2710 cp05T...

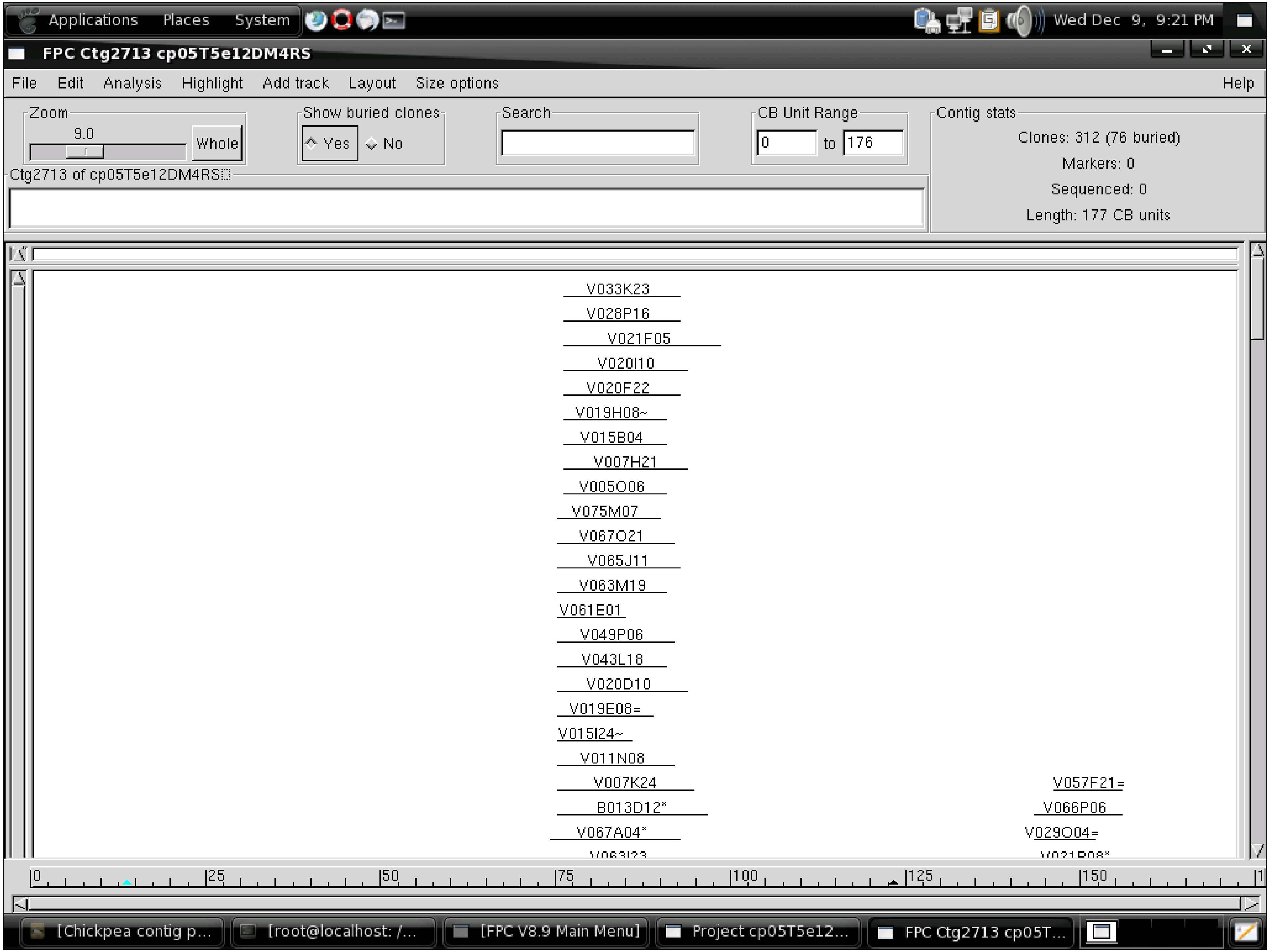
Applications Places System
Wed Dec 9, 9:21 PM
FPC Ctg2713 cp05T5e12DM4RS
File Edit Analysis Highlight Add track Layout Size options Help
Zoom
9.0
Whole
Show buried clones
Yes
No
Search
CB Unit Range
0
to
176
Contig stats
Clones: 312 (76 buried)
Markers: 0
Sequenced: 0
Length: 177 CB units
Ctg2713 of cp05T5e12DM4RS
V033K23
V028P16
V021F05
V020I10
V020F22
V019H08~
V015B04
V007H21
V005O06
V075M07
V067O21
V065J11
V063M19
V061E01
V049P06
V043L18
V020D10
V019E08=
V015I24~
V011N08
V007K24
B013D12*
V067A04*
V057F21=
V066P06
V029O04=
0
25
50
75
100
125
150
[Chickpea contig p...
[root@localhost: /...
[FPC V8.9 Main Menu]
Project cp05T5e12...
FPC Ctg2713 cp05T...

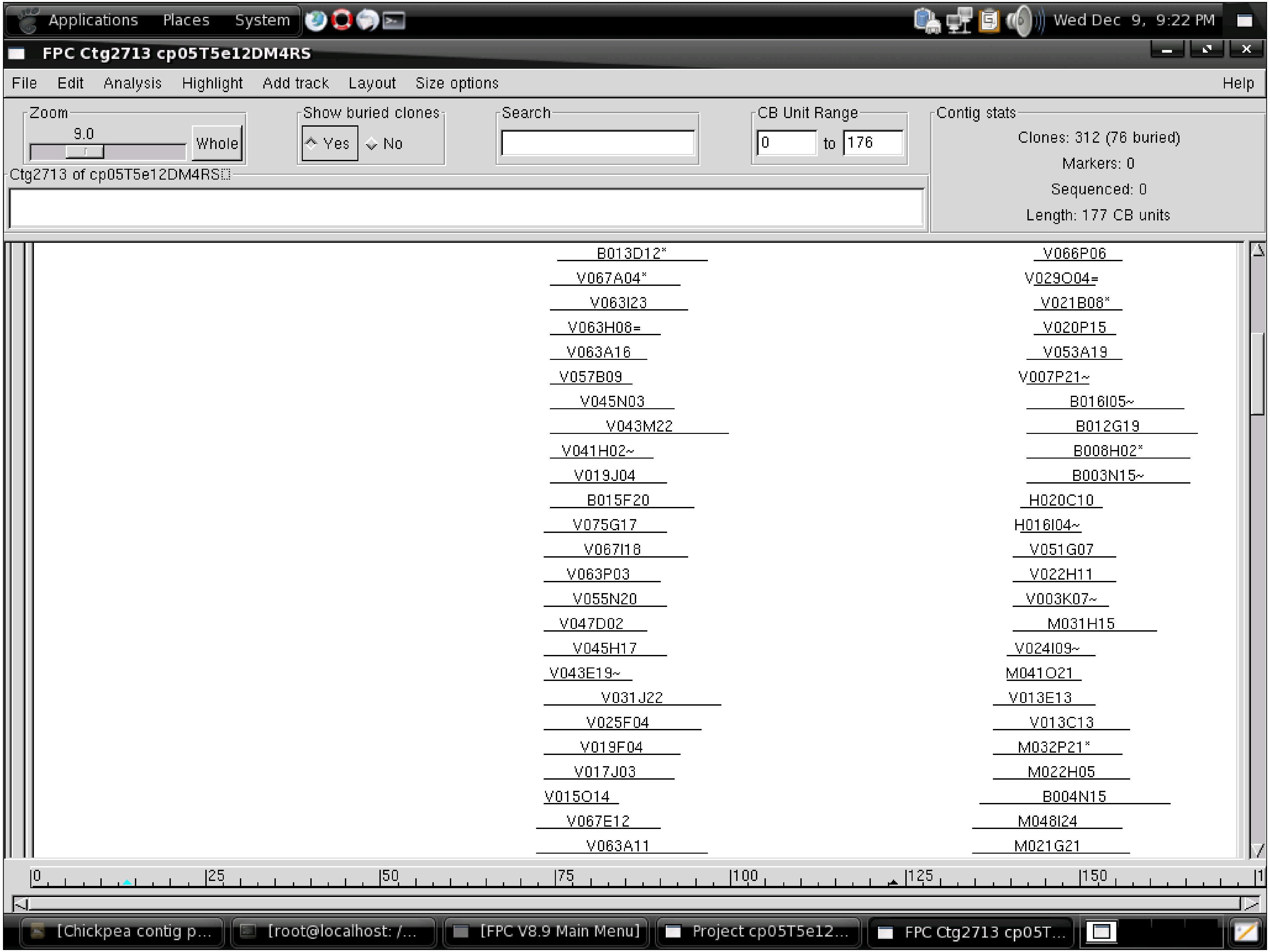
Applications Places System
Wed Dec 9, 9:22 PM
FPC Ctg2713 cp05T5e12DM4RS
File Edit Analysis Highlight Add track Layout Size options Help
Zoom
9.0
Whole
Show buried clones
Yes No
Search
CB Unit Range
0 to 176
Contig stats
Clones: 312 (76 buried)
Markers: 0
Sequenced: 0
Length: 177 CB units
Ctg2713 of cp05T5e12DM4RS
B013D12*
V067A04*
V063I23
V063H08=
V063A16
V057B09
V045N03
V043M22
V041H02~
V019J04
B015F20
V075G17
V067I18
V063P03
V055N20
V047D02
V045H17
V043E19~
V031J22
V025F04
V019F04
V017J03
V015O14
V067E12
V063A11
V066P06
V029O04=
V021B08*
V020P15
V053A19
V007P21~
B016I05~
B012G19
B008H02*
B003N15~
H020C10
H016I04~
V051G07
V022H11
V003K07~
M031H15
V024I09~
M041O21
V013E13
V013C13
M032P21*
M022H05
B004N15
M048I24
M021G21
0 25 50 75 100 125 150
[Chickpea contig p...
[root@localhost: /...
[FPC V8.9 Main Menu]
Project cp05T5e12...
FPC Ctg2713 cp05T...

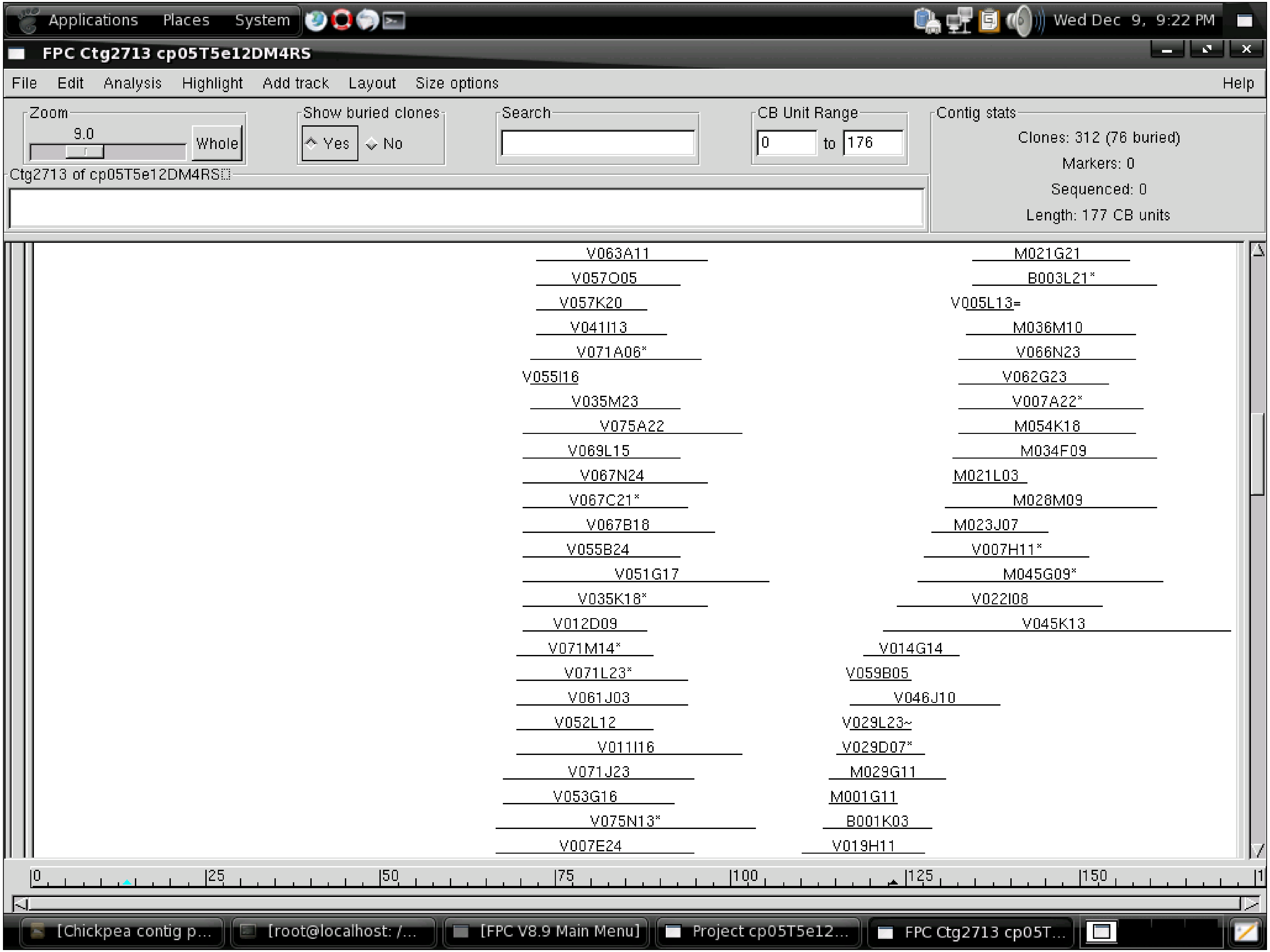
Applications Places System
Wed Dec 9, 9:22 PM
FPC Ctg2713 cp05T5e12DM4RS
File Edit Analysis Highlight Add track Layout Size options Help
Zoom
9.0
Whole
Show buried clones
Yes No
Search
CB Unit Range
0 to 176
Contig stats
Clones: 312 (76 buried)
Markers: 0
Sequenced: 0
Length: 177 CB units
Ctg2713 of cp05T5e12DM4RS
V063A11
V057O05
V057K20
V041I13
V071A06*
V055I16
V035M23
V075A22
V069L15
V067N24
V067C21*
V067B18
V055B24
V051G17
V035K18*
V012D09
V071M14*
V071L23*
V061J03
V052L12
V011I16
V071J23
V053G16
V075N13*
V007E24
M021G21
B003L21*
V005L13=
M036M10
V066N23
V062G23
V007A22*
M054K18
M034F09
M021L03
M028M09
M023J07
V007H11*
M045G09*
V022I08
V045K13
V014G14
V059B05
V046J10
V029L23~
V029D07*
M029G11
M001G11
B001K03
V019H11
0 25 50 75 100 125 150
[Chickpea contig p...
[root@localhost: /...
[FPC V8.9 Main Menu]
Project cp05T5e12...
FPC Ctg2713 cp05T...

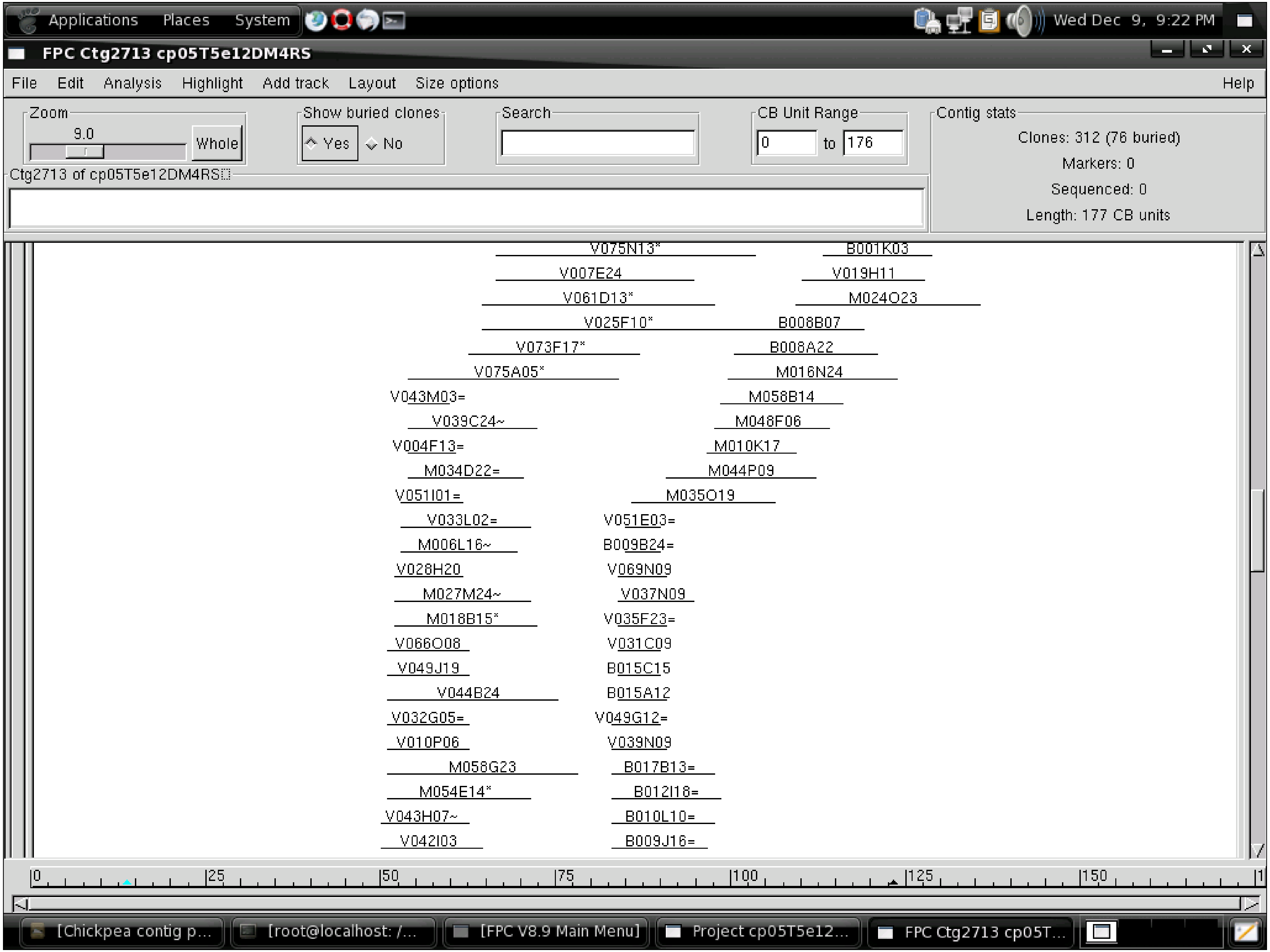
Applications Places System
Wed Dec 9, 9:22 PM
FPC Ctg2713 cp05T5e12DM4RS
File Edit Analysis Highlight Add track Layout Size options Help
Zoom
9.0
Whole
Show buried clones
Yes No
Search
CB Unit Range
0 to 176
Contig stats
Clones: 312 (76 buried)
Markers: 0
Sequenced: 0
Length: 177 CB units
Ctg2713 of cp05T5e12DM4RS
V075N13* B001K03
V007E24 V019H11
V061D13* M024O23
V025F10* B008B07
V073F17* B008A22
V075A05* M016N24
V043M03= M058B14
V039C24~ M048F06
V004F13= M010K17
M034D22= M044P09
V051I01= M035O19
V033L02= V051E03=
M006L16~ B009B24=
V028H20 V069N09
M027M24~ V037N09
M018B15* V035F23=
V066O08 V031C09
V049J19 B015C15
V044B24 B015A12
V032G05= V049G12=
V010P06 V039N09
M058G23 B017B13=
M054E14* B012I18=
V043H07~ B010L10=
V042I03 B009J16=
0 25 50 75 100 125 150
[Chickpea contig p...
[root@localhost: /...
[FPC V8.9 Main Menu]
Project cp05T5e12...
FPC Ctg2713 cp05T...

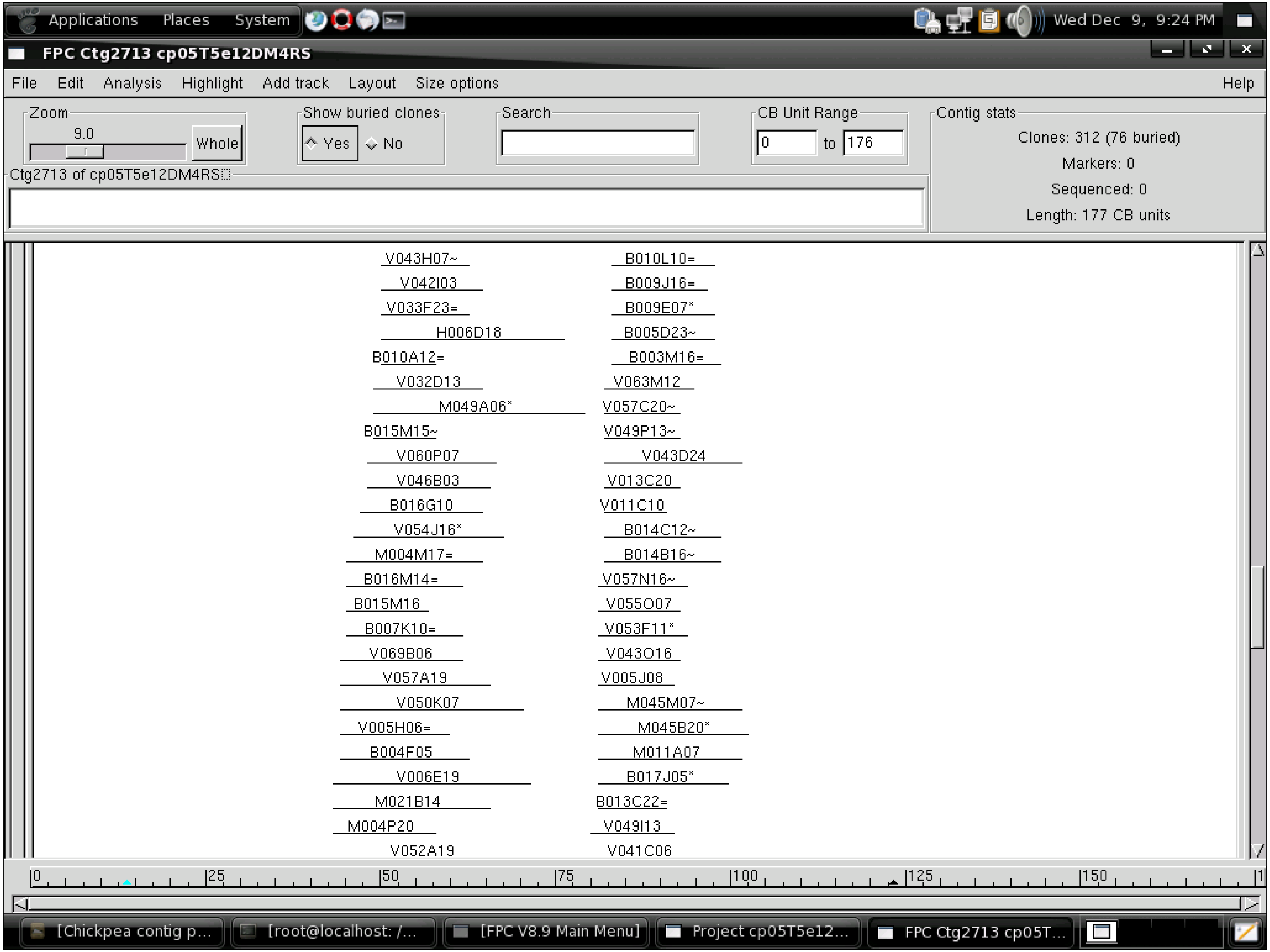
Applications Places System
Wed Dec 9, 9:24 PM
FPC Ctg2713 cp05T5e12DM4RS
File Edit Analysis Highlight Add track Layout Size options Help
Zoom
9.0
Whole
Show buried clones
Yes No
Search
CB Unit Range
0 to 176
Contig stats
Clones: 312 (76 buried)
Markers: 0
Sequenced: 0
Length: 177 CB units
Ctg2713 of cp05T5e12DM4RS
V043H07~
B010L10=
V042I03
B009J16=
V033F23=
B009E07*
H006D18
B005D23~
B010A12=
B003M16=
V032D13
V063M12
M049A06*
V057C20~
B015M15~
V049P13~
V060P07
V043D24
V046B03
V013C20
B016G10
V011C10
V054J16*
B014C12~
M004M17=
B014B16~
B016M14=
V057N16~
B015M16
V055O07
B007K10=
V053F11*
V069B06
V043O16
V057A19
V005J08
V050K07
M045M07~
V005H06=
M045B20*
B004F05
M011A07
V006E19
B017J05*
M021B14
B013C22=
M004P20
V049I13
V052A19
V041C06
0 25 50 75 100 125 150
[Chickpea contig p...
[root@localhost: /...
[FPC V8.9 Main Menu]
Project cp05T5e12...
FPC Ctg2713 cp05T...

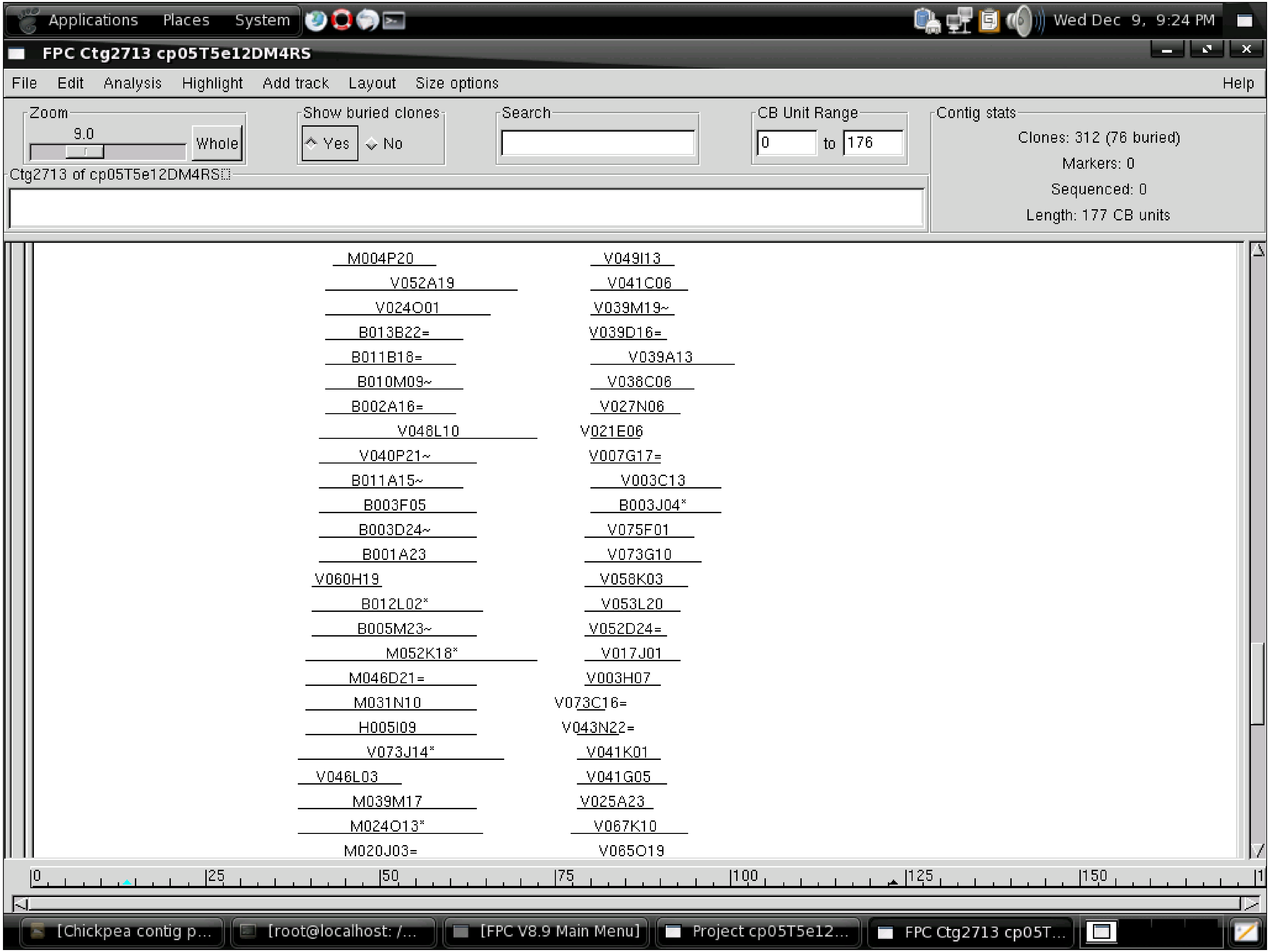
Applications Places System
Wed Dec 9, 9:24 PM
FPC Ctg2713 cp05T5e12DM4RS
File Edit Analysis Highlight Add track Layout Size options Help
Zoom
9.0
Whole
Show buried clones
Yes No
Search
CB Unit Range
0 to 176
Contig stats
Clones: 312 (76 buried)
Markers: 0
Sequenced: 0
Length: 177 CB units
Ctg2713 of cp05T5e12DM4RS
M004P20
V052A19
V024O01
B013B22=
B011B18=
B010M09~
B002A16=
V048L10
V040P21~
B011A15~
B003F05
B003D24~
B001A23
V060H19
B012L02*
B005M23~
M052K18*
M046D21=
M031N10
H005I09
V073J14*
V046L03
M039M17
M024O13*
M020J03=
V049I13
V041C06
V039M19~
V039D16=
V039A13
V038C06
V027N06
V021E06
V007G17=
V003C13
B003J04*
V075F01
V073G10
V058K03
V053L20
V052D24=
V017J01
V003H07
V073C16=
V043N22=
V041K01
V041G05
V025A23
V067K10
V065O19
0 25 50 75 100 125 150
[Chickpea contig p...
[root@localhost: /...
[FPC V8.9 Main Menu]
Project cp05T5e12...
FPC Ctg2713 cp05T...

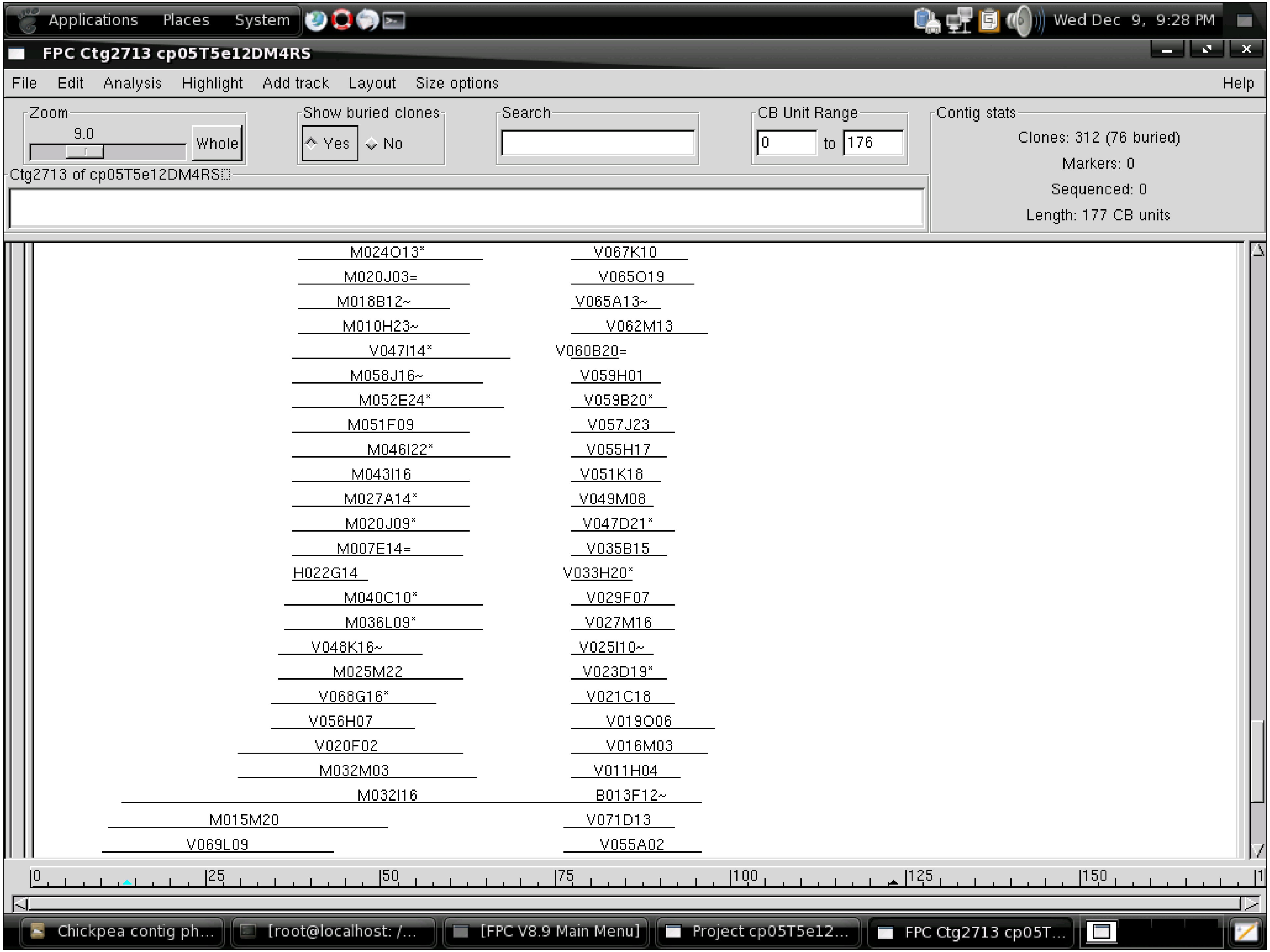
Applications Places System
Wed Dec 9, 9:28 PM
FPC Ctg2713 cp05T5e12DM4RS
File Edit Analysis Highlight Add track Layout Size options Help
Zoom
9.0
Whole
Show buried clones
Yes No
Search
CB Unit Range
0 to 176
Contig stats
Clones: 312 (76 buried)
Markers: 0
Sequenced: 0
Length: 177 CB units
Ctg2713 of cp05T5e12DM4RS
M024O13*
V067K10
M020J03=
V065O19
M018B12~
V065A13~
M010H23~
V062M13
V047I14*
V060B20=
M058J16~
V059H01
M052E24*
V059B20*
M051F09
V057J23
M046I22*
V055H17
M043I16
V051K18
M027A14*
V049M08
M020J09*
V047D21*
M007E14=
V035B15
H022G14
V033H20*
M040C10*
V029F07
M036L09*
V027M16
V048K16~
V025I10~
M025M22
V023D19*
V068G16*
V021C18
V056H07
V019O06
V020F02
V016M03
M032M03
V011H04
M032I16
B013F12~
M015M20
V071D13
V069L09
V055A02
0 25 50 75 100 125 150
Chickpea contig ph...
[root@localhost: /...
[FPC V8.9 Main Menu]
Project cp05T5e12...
FPC Ctg2713 cp05T...

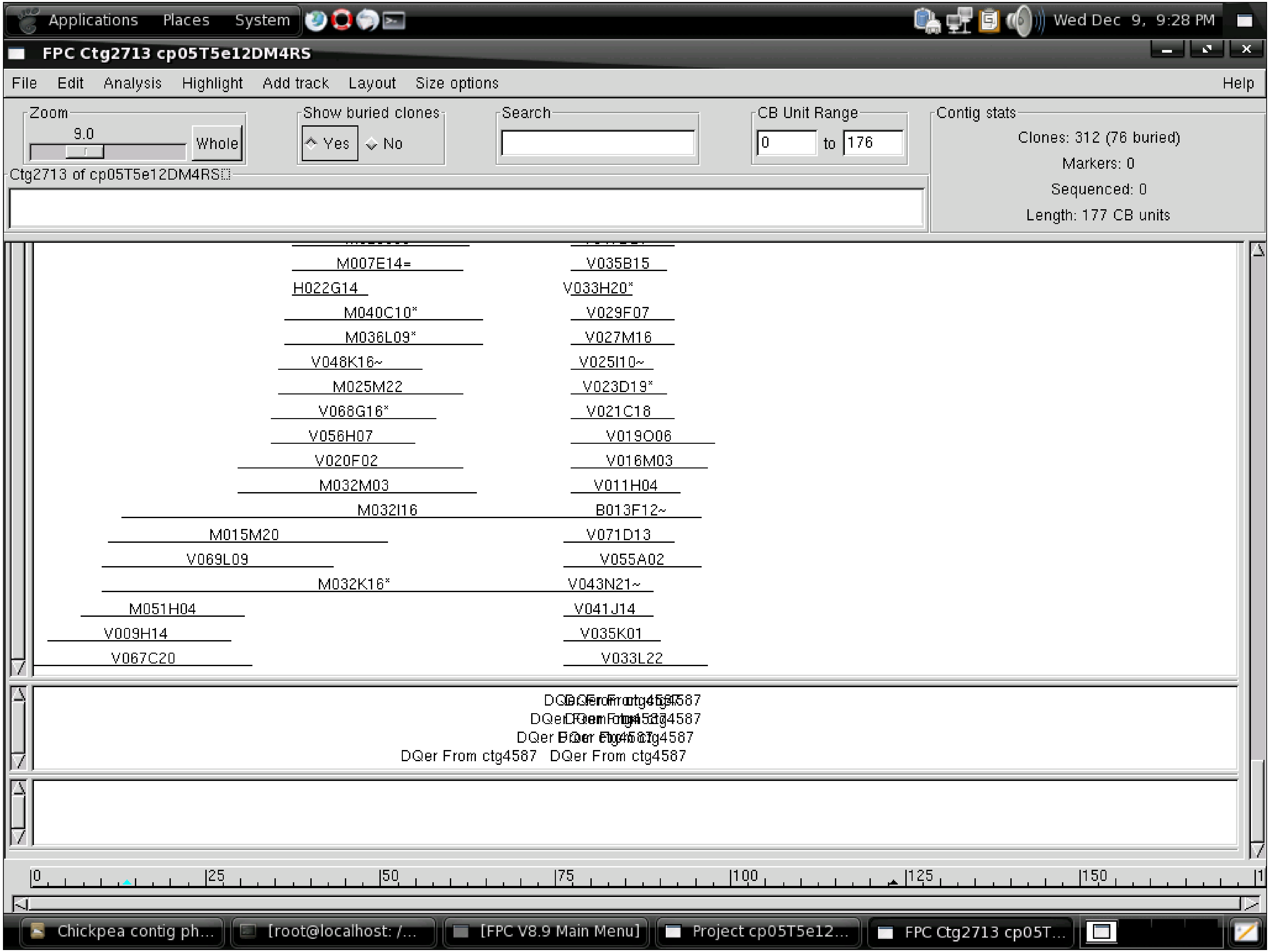
Applications Places System
Wed Dec 9, 9:28 PM
FPC Ctg2713 cp05T5e12DM4RS
File Edit Analysis Highlight Add track Layout Size options Help
Zoom
9.0
Whole
Show buried clones
Yes No
Search
CB Unit Range
0 to 176
Contig stats
Clones: 312 (76 buried)
Markers: 0
Sequenced: 0
Length: 177 CB units
Ctg2713 of cp05T5e12DM4RS
M007E14=
V035B15
H022G14
V033H20*
M040C10*
V029F07
M036L09*
V027M16
V048K16~
V025I10~
M025M22
V023D19*
V068G16*
V021C18
V056H07
V019O06
V020F02
V016M03
M032M03
V011H04
M032I16
B013F12~
M015M20
V071D13
V069L09
V055A02
M032K16*
V043N21~
M051H04
V041J14
V009H14
V035K01
V067C20
V033L22
DQer From ctg4587
DQer From ctg4587
0 25 50 75 100 125 150
Chickpea contig ph...
[root@localhost: /...
[FPC V8.9 Main Menu]
Project cp05T5e12...
FPC Ctg2713 cp05T...

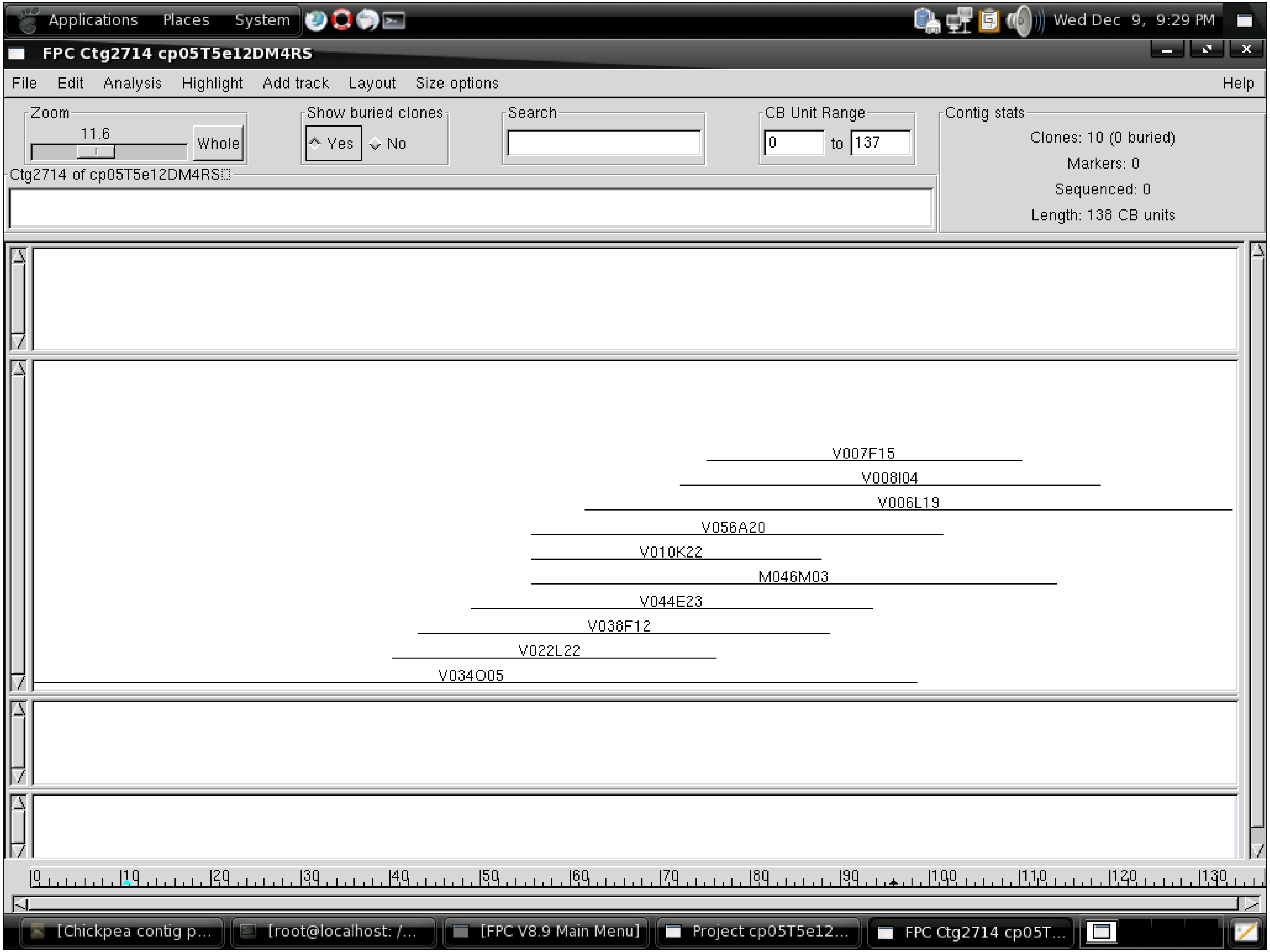
Applications Places System
Wed Dec 9, 9:29 PM
FPC Ctg2714 cp05T5e12DM4RS
File Edit Analysis Highlight Add track Layout Size options Help
Zoom
11.6
Whole
Show buried clones
Yes
No
Search
CB Unit Range
0 to 137
Contig stats
Clones: 10 (0 buried)
Markers: 0
Sequenced: 0
Length: 138 CB units
Ctg2714 of cp05T5e12DM4RS
V007F15
V008I04
V006L19
V056A20
V010K22
M046M03
V044E23
V038F12
V022L22
V034O05
0 10 20 30 40 50 60 70 80 90 100 110 120 130
[Chickpea contig p...
[root@localhost: /...
[FPC V8.9 Main Menu]
Project cp05T5e12...
FPC Ctg2714 cp05T...

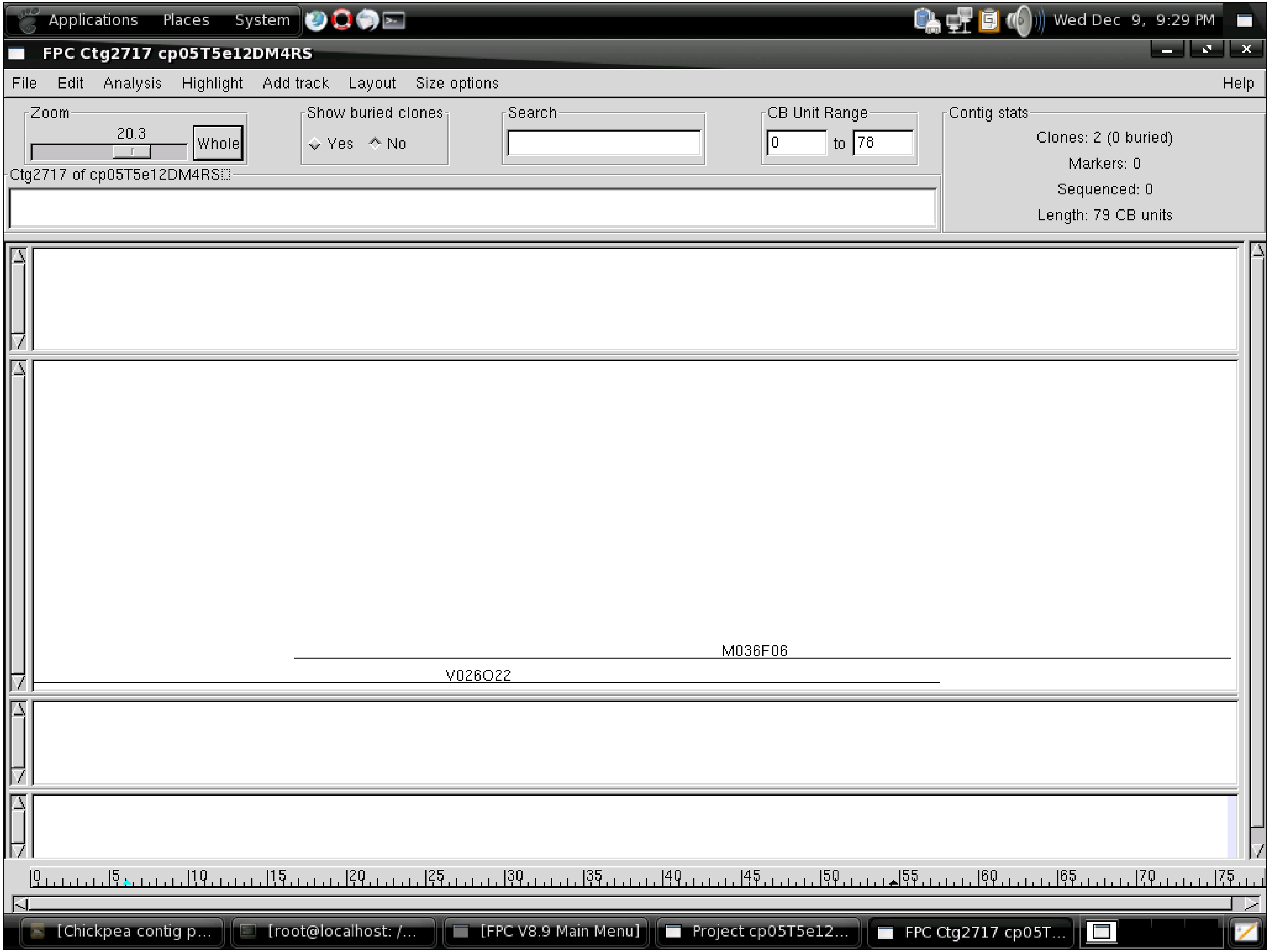
Applications Places System
Wed Dec 9, 9:29 PM
FPC Ctg2717 cp05T5e12DM4RS
File Edit Analysis Highlight Add track Layout Size options
Help
Zoom
20.3
Whole
Show buried clones
Yes
No
Search
CB Unit Range
0
to
78
Contig stats
Clones: 2 (0 buried)
Markers: 0
Sequenced: 0
Length: 79 CB units
Ctg2717 of cp05T5e12DM4RS
M036F06
V026O22
0 5 10 15 20 25 30 35 40 45 50 55 60 65 70 75
[Chickpea contig p...
[root@localhost: /...
[FPC V8.9 Main Menu]
Project cp05T5e12...
FPC Ctg2717 cp05T...

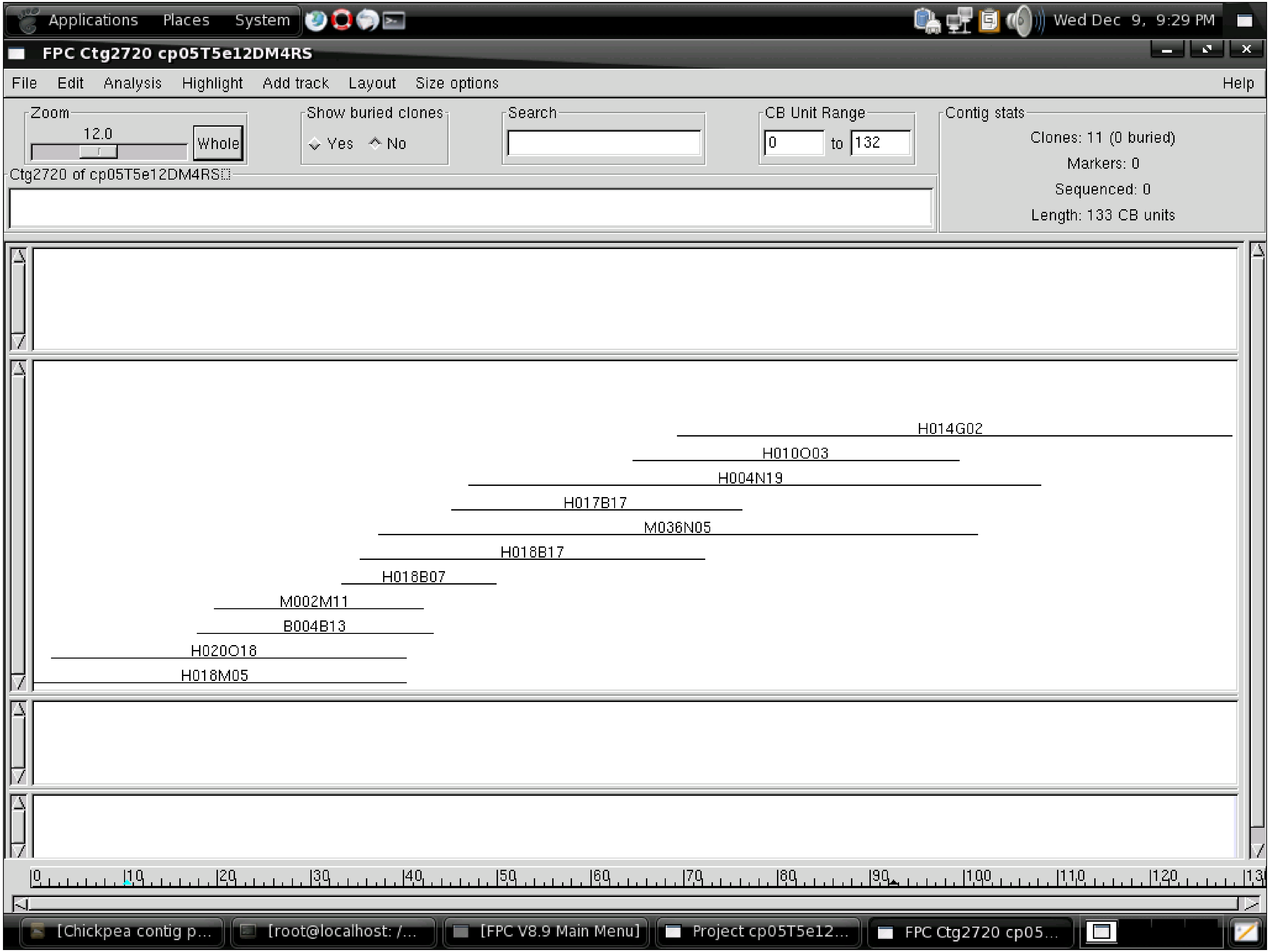
Applications Places System
Wed Dec 9, 9:29 PM
FPC Ctg2720 cp05T5e12DM4RS
File Edit Analysis Highlight Add track Layout Size options Help
Zoom
12.0
Whole
Show buried clones
Yes No
Search
CB Unit Range
0 to 132
Contig stats
Clones: 11 (0 buried)
Markers: 0
Sequenced: 0
Length: 133 CB units
Ctg2720 of cp05T5e12DM4RS
H014G02
H010O03
H004N19
H017B17
M036N05
H018B17
H018B07
M002M11
B004B13
H020O18
H018M05
0 10 20 30 40 50 60 70 80 90 100 110 120
[Chickpea contig p...
[root@localhost: /...
[FPC V8.9 Main Menu]
Project cp05T5e12...
FPC Ctg2720 cp05...

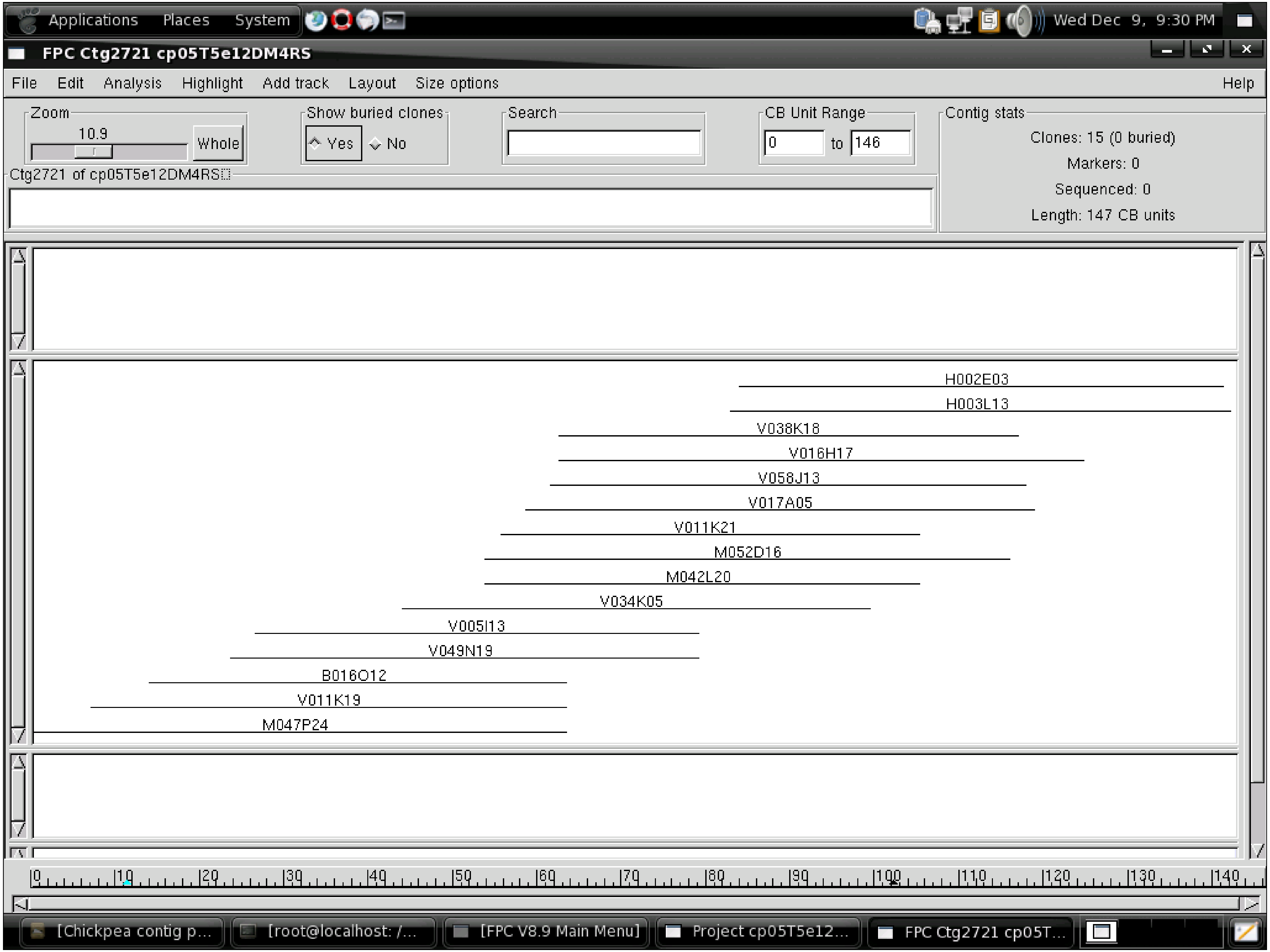
Applications Places System
Wed Dec 9, 9:30 PM
FPC Ctg2721 cp05T5e12DM4RS
File Edit Analysis Highlight Add track Layout Size options Help
Zoom
10.9
Whole
Show buried clones
Yes
No
Search
CB Unit Range
0
to
146
Contig stats
Clones: 15 (0 buried)
Markers: 0
Sequenced: 0
Length: 147 CB units
Ctg2721 of cp05T5e12DM4RS
H002E03
H003L13
V038K18
V016H17
V058J13
V017A05
V011K21
M052D16
M042L20
V034K05
V005I13
V049N19
B016O12
V011K19
M047P24
0
10
20
30
40
50
60
70
80
90
100
110
120
130
140
[Chickpea contig p...
[root@localhost: /...
[FPC V8.9 Main Menu]
Project cp05T5e12...
FPC Ctg2721 cp05T...

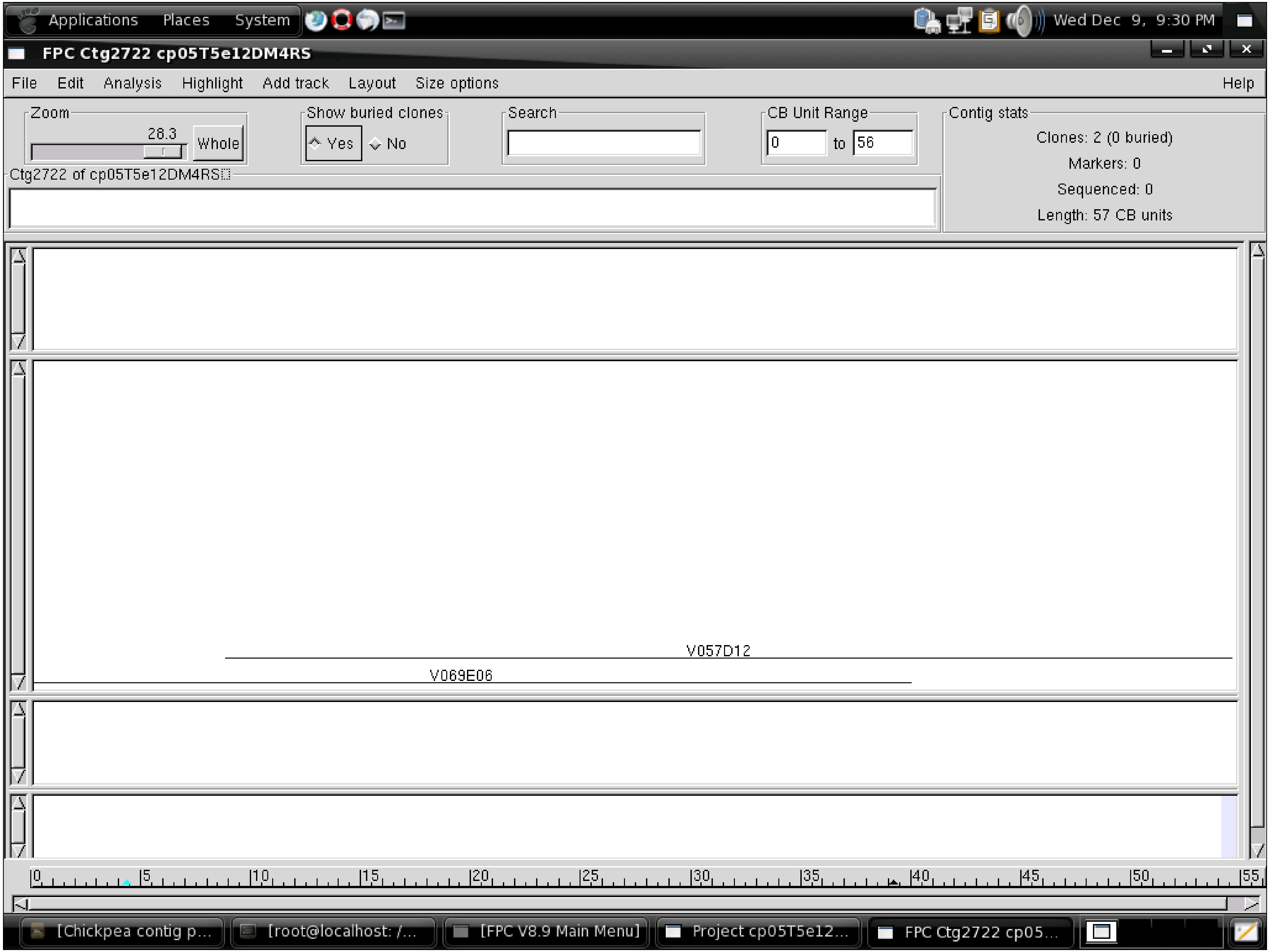
Applications Places System
Wed Dec 9, 9:30 PM
FPC Ctg2722 cp05T5e12DM4RS
File Edit Analysis Highlight Add track Layout Size options Help
Zoom
28.3
Whole
Show buried clones
Yes
No
Search
CB Unit Range
0 to 56
Contig stats
Clones: 2 (0 buried)
Markers: 0
Sequenced: 0
Length: 57 CB units
Ctg2722 of cp05T5e12DM4RS
V057D12
V069E06
0 5 10 15 20 25 30 35 40 45 50 55
[Chickpea contig p...
[root@localhost: /...
[FPC V8.9 Main Menu]
Project cp05T5e12...
FPC Ctg2722 cp05...

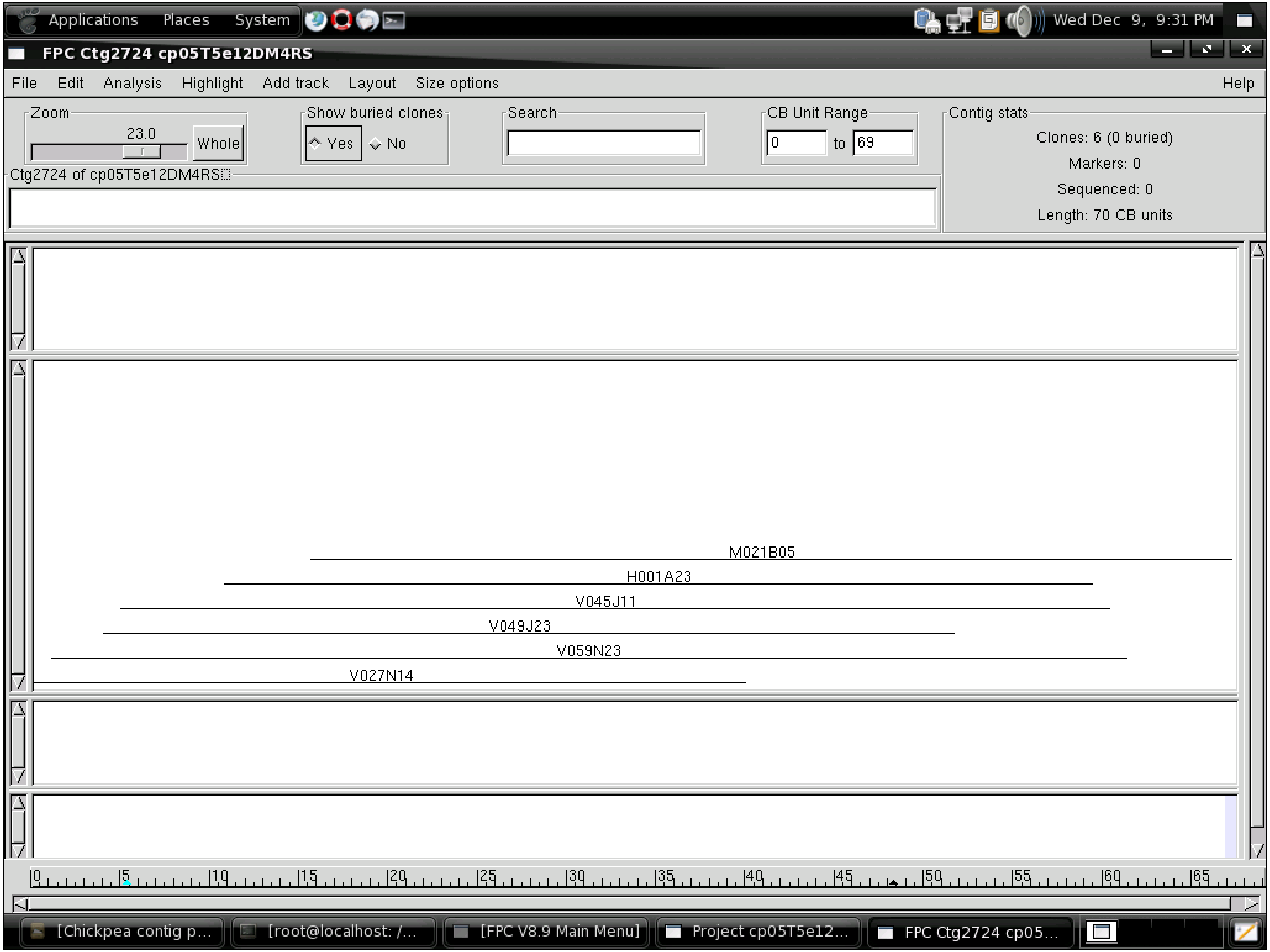
Applications Places System
Wed Dec 9, 9:31 PM
FPC Ctg2724 cp05T5e12DM4RS
File Edit Analysis Highlight Add track Layout Size options Help
Zoom
23.0
Whole
Show buried clones
Yes No
Search
CB Unit Range
0 to 69
Contig stats
Clones: 6 (0 buried)
Markers: 0
Sequenced: 0
Length: 70 CB units
Ctg2724 of cp05T5e12DM4RS
M021B05
H001A23
V045J11
V049J23
V059N23
V027N14
0 5 10 15 20 25 30 35 40 45 50 55 60 65
[Chickpea contig p...
[root@localhost: /...
[FPC V8.9 Main Menu]
Project cp05T5e12...
FPC Ctg2724 cp05...

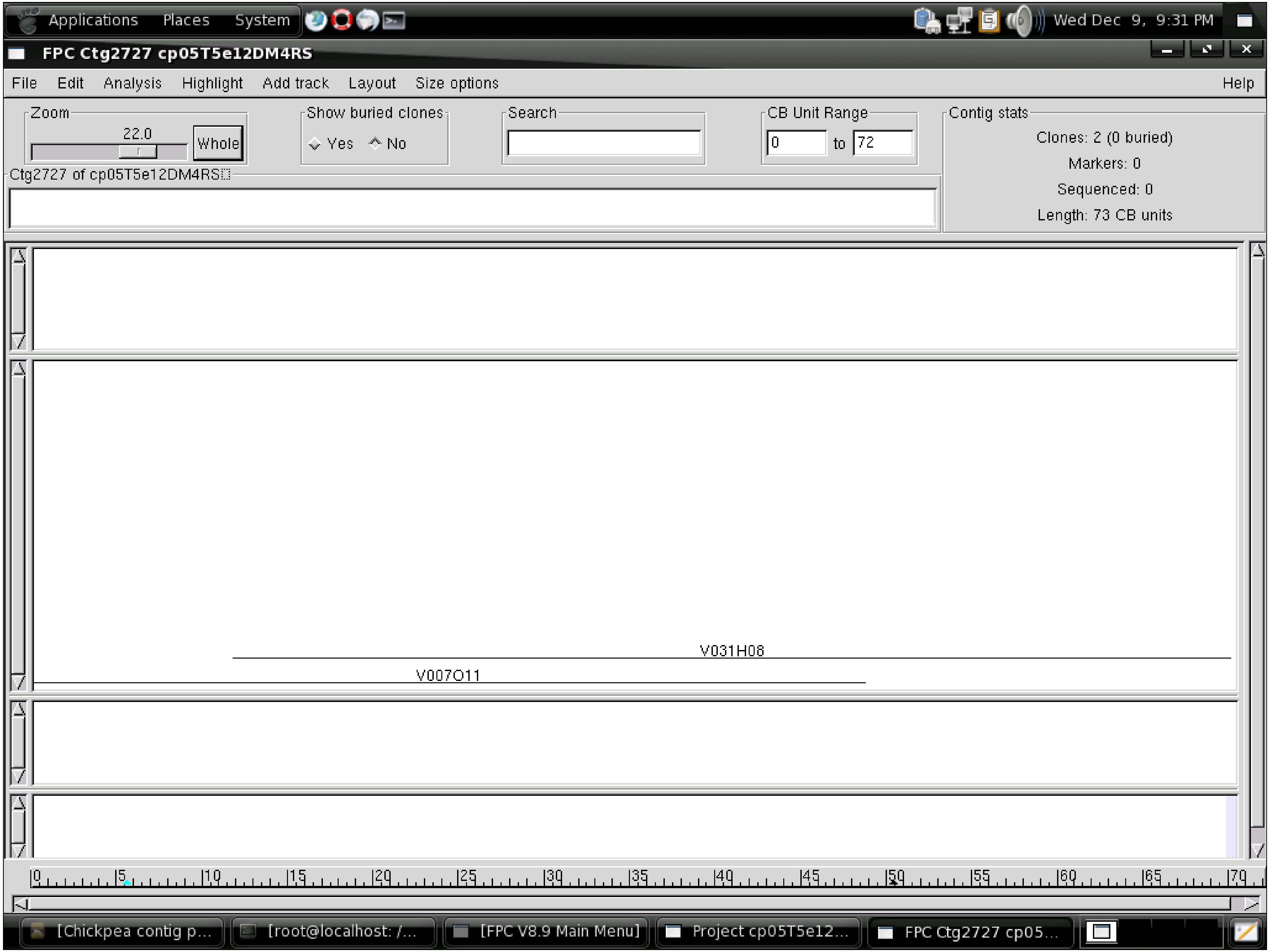
Applications Places System
Wed Dec 9, 9:31 PM
FPC Ctg2727 cp05T5e12DM4RS
File Edit Analysis Highlight Add track Layout Size options Help
Zoom
22.0
Whole
Show buried clones
Yes No
Search
CB Unit Range
0 to 72
Contig stats
Clones: 2 (0 buried)
Markers: 0
Sequenced: 0
Length: 73 CB units
Ctg2727 of cp05T5e12DM4RS
V031H08
V007O11
0 5 10 15 20 25 30 35 40 45 50 55 60 65 70
[Chickpea contig p...
[root@localhost: /...
[FPC V8.9 Main Menu]
Project cp05T5e12...
FPC Ctg2727 cp05...

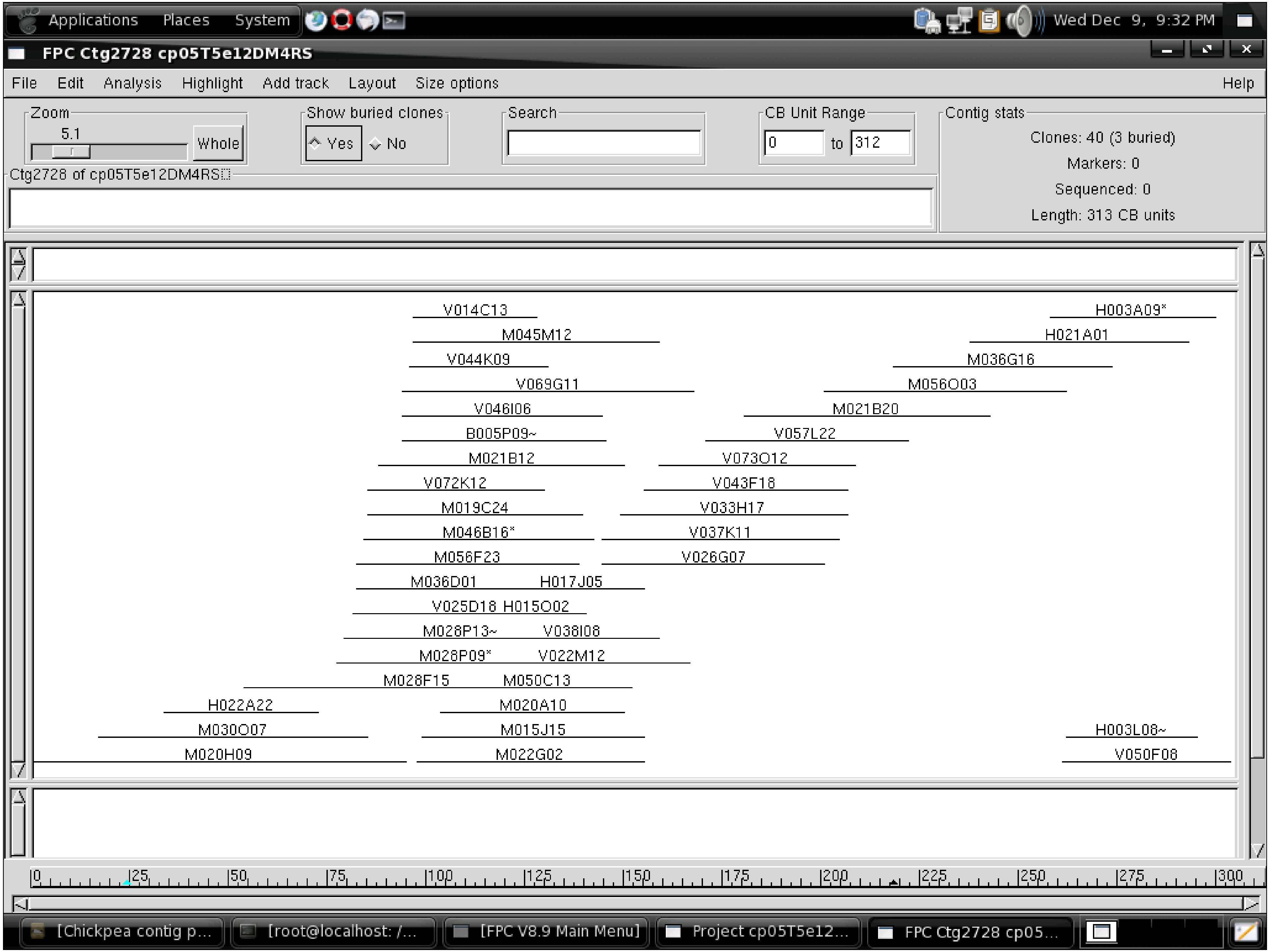
Applications Places System
Wed Dec 9, 9:32 PM
FPC Ctg2728 cp05T5e12DM4RS
File Edit Analysis Highlight Add track Layout Size options Help
Zoom
5.1
Whole
Show buried clones
Yes
No
Search
CB Unit Range
0 to 312
Contig stats
Clones: 40 (3 buried)
Markers: 0
Sequenced: 0
Length: 313 CB units
Ctg2728 of cp05T5e12DM4RS
V014C13
H003A09*
M045M12
H021A01
V044K09
M036G16
V069G11
M056O03
V046I06
M021B20
B005P09~
V057L22
M021B12
V073O12
V072K12
V043F18
M019C24
V033H17
M046B16*
V037K11
M056F23
V026G07
M036D01
H017J05
V025D18
H015O02
M028P13~
V038I08
M028P09*
V022M12
M028F15
M050C13
H022A22
M020A10
M030O07
M015J15
H003L08~
M020H09
M022G02
V050F08
0 25 50 75 100 125 150 175 200 225 250 275 300
[Chickpea contig p...
[root@localhost: /...
[FPC V8.9 Main Menu]
Project cp05T5e12...
FPC Ctg2728 cp05...

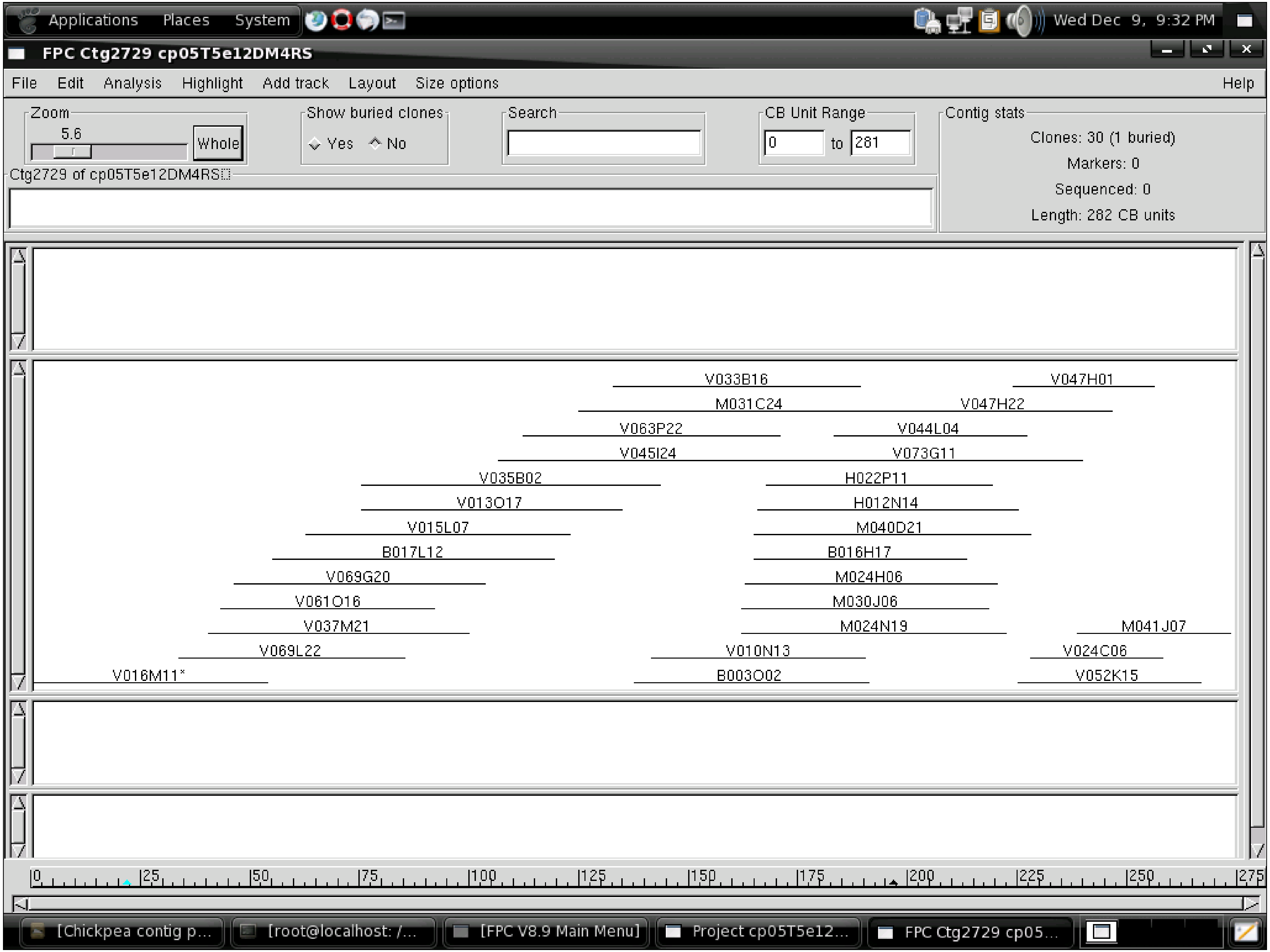
Applications Places System
Wed Dec 9, 9:32 PM
FPC Ctg2729 cp05T5e12DM4RS
File Edit Analysis Highlight Add track Layout Size options Help
Zoom
5.6
Whole
Show buried clones
Yes No
Search
CB Unit Range
0 to 281
Contig stats
Clones: 30 (1 buried)
Markers: 0
Sequenced: 0
Length: 282 CB units
Ctg2729 of cp05T5e12DM4RS
V033B16
V047H01
M031C24
V047H22
V063P22
V044L04
V045I24
V073G11
V035B02
H022P11
V013O17
H012N14
V015L07
M040D21
B017L12
B016H17
V069G20
M024H06
V061O16
M030J06
V037M21
M024N19
M041J07
V069L22
V010N13
V024C06
V016M11*
B003O02
V052K15
0 25 50 75 100 125 150 175 200 225 250 275
[Chickpea contig p...
[root@localhost: /...
[FPC V8.9 Main Menu]
Project cp05T5e12...
FPC Ctg2729 cp05...

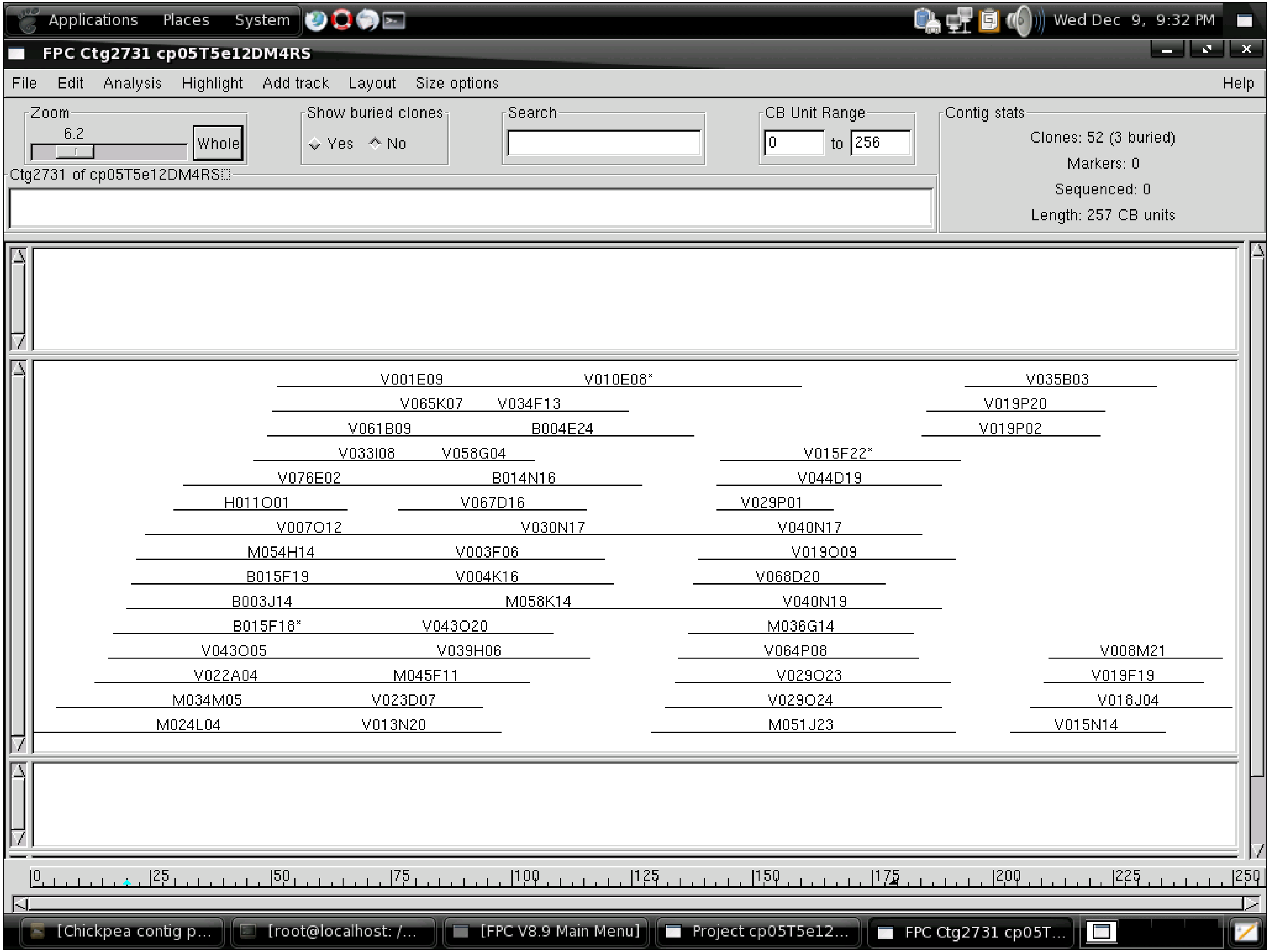
Applications Places System
Wed Dec 9, 9:32 PM
FPC Ctg2731 cp05T5e12DM4RS
File Edit Analysis Highlight Add track Layout Size options Help
Zoom 6.2 Whole
Show buried clones Yes No
Search
CB Unit Range 0 to 256
Contig stats
Clones: 52 (3 buried)
Markers: 0
Sequenced: 0
Length: 257 CB units
Ctg2731 of cp05T5e12DM4RS
V001E09 V010E08* V035B03
V065K07 V034F13 V019P20
V061B09 B004E24 V019P02
V033I08 V058G04 V015F22*
V076E02 B014N16 V044D19
H011O01 V067D16 V029P01
V007O12 V030N17 V040N17
M054H14 V003F06 V019O09
B015F19 V004K16 V068D20
B003J14 M058K14 V040N19
B015F18* V043O20 M036G14
V043O05 V039H06 V064P08 V008M21
V022A04 M045F11 V029O23 V019F19
M034M05 V023D07 V029O24 V018J04
M024L04 V013N20 M051J23 V015N14
0 25 50 75 100 125 150 175 200 225 250
[Chickpea contig p... [root@localhost: /... [FPC V8.9 Main Menu] Project cp05T5e12... FPC Ctg2731 cp05T...

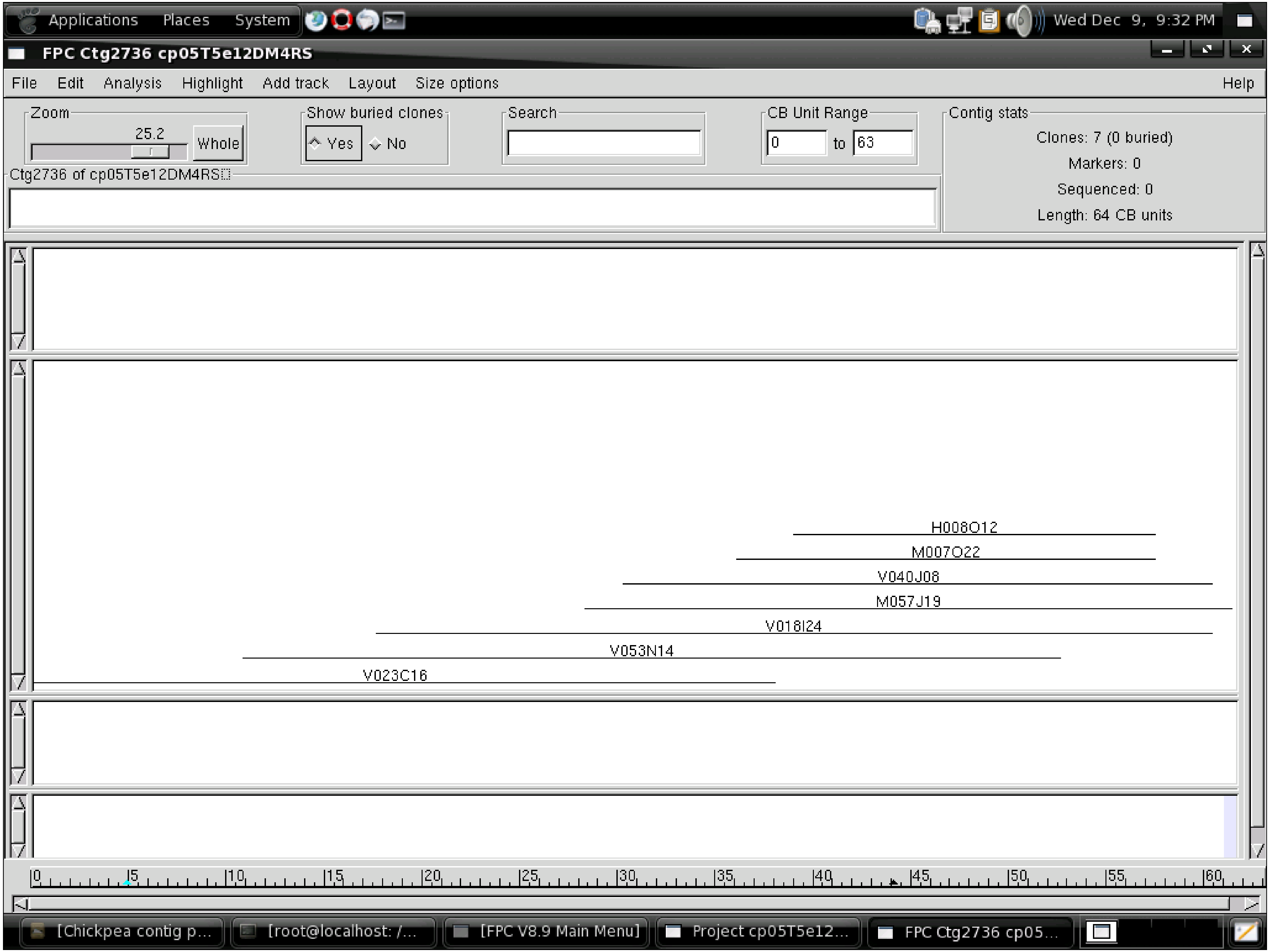
Applications Places System
Wed Dec 9, 9:32 PM
FPC Ctg2736 cp05T5e12DM4RS
File Edit Analysis Highlight Add track Layout Size options Help
Zoom
25.2
Whole
Show buried clones
Yes
No
Search
CB Unit Range
0
to
63
Contig stats
Clones: 7 (0 buried)
Markers: 0
Sequenced: 0
Length: 64 CB units
Ctg2736 of cp05T5e12DM4RS
H008O12
M007O22
V040J08
M057J19
V018I24
V053N14
V023C16
0 5 10 15 20 25 30 35 40 45 50 55 60
[Chickpea contig p...
[root@localhost: /...
[FPC V8.9 Main Menu]
Project cp05T5e12...
FPC Ctg2736 cp05...

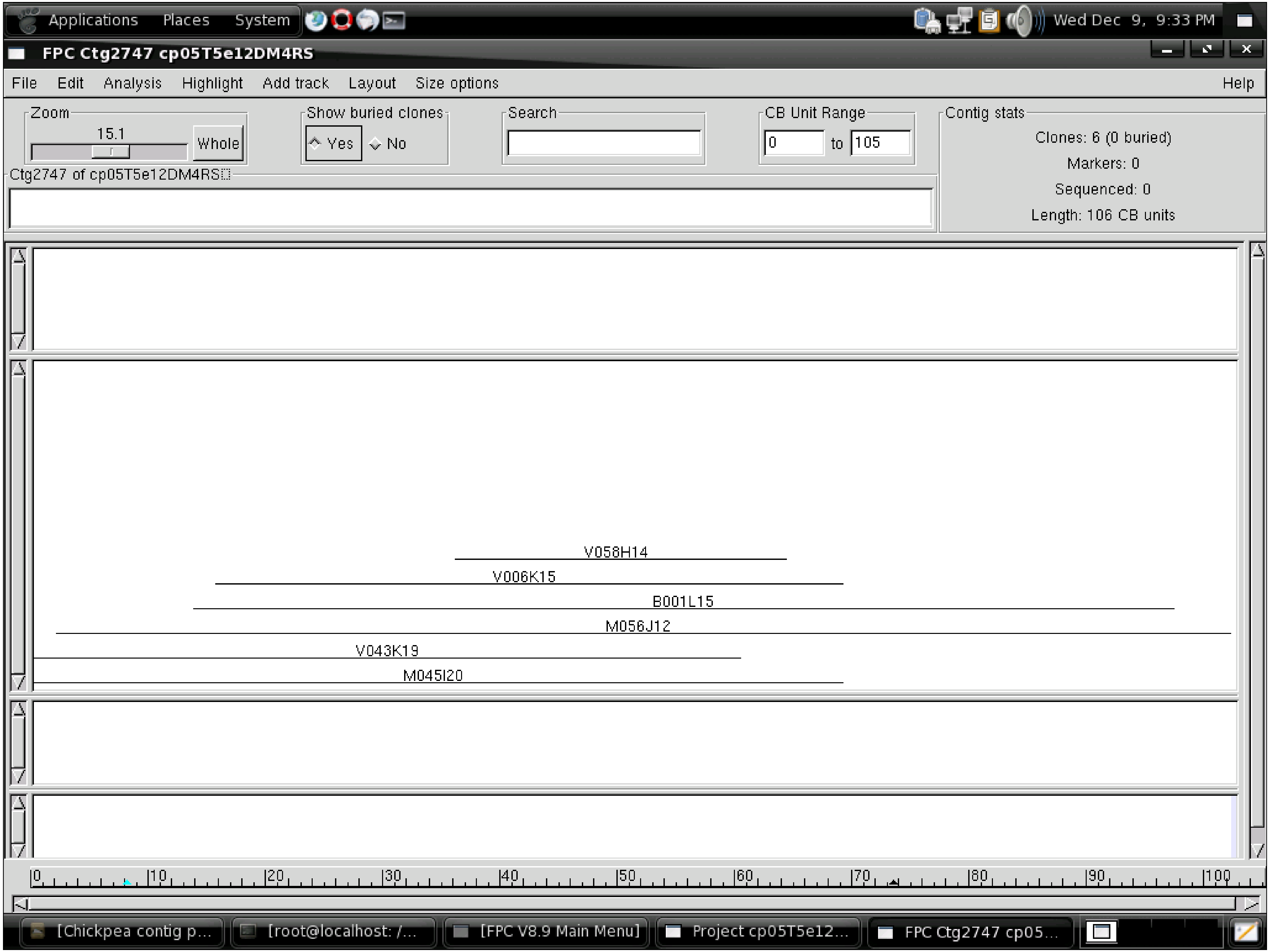
Applications Places System
Wed Dec 9, 9:33 PM
FPC Ctg2747 cp05T5e12DM4RS
File Edit Analysis Highlight Add track Layout Size options Help
Zoom
15.1
Whole
Show buried clones
Yes No
Search
CB Unit Range
0 to 105
Contig stats
Clones: 6 (0 buried)
Markers: 0
Sequenced: 0
Length: 106 CB units
Ctg2747 of cp05T5e12DM4RS
V058H14
V006K15
B001L15
M056J12
V043K19
M045I20
0 10 20 30 40 50 60 70 80 90 100
[Chickpea contig p...
[root@localhost: /...
[FPC V8.9 Main Menu]
Project cp05T5e12...
FPC Ctg2747 cp05...

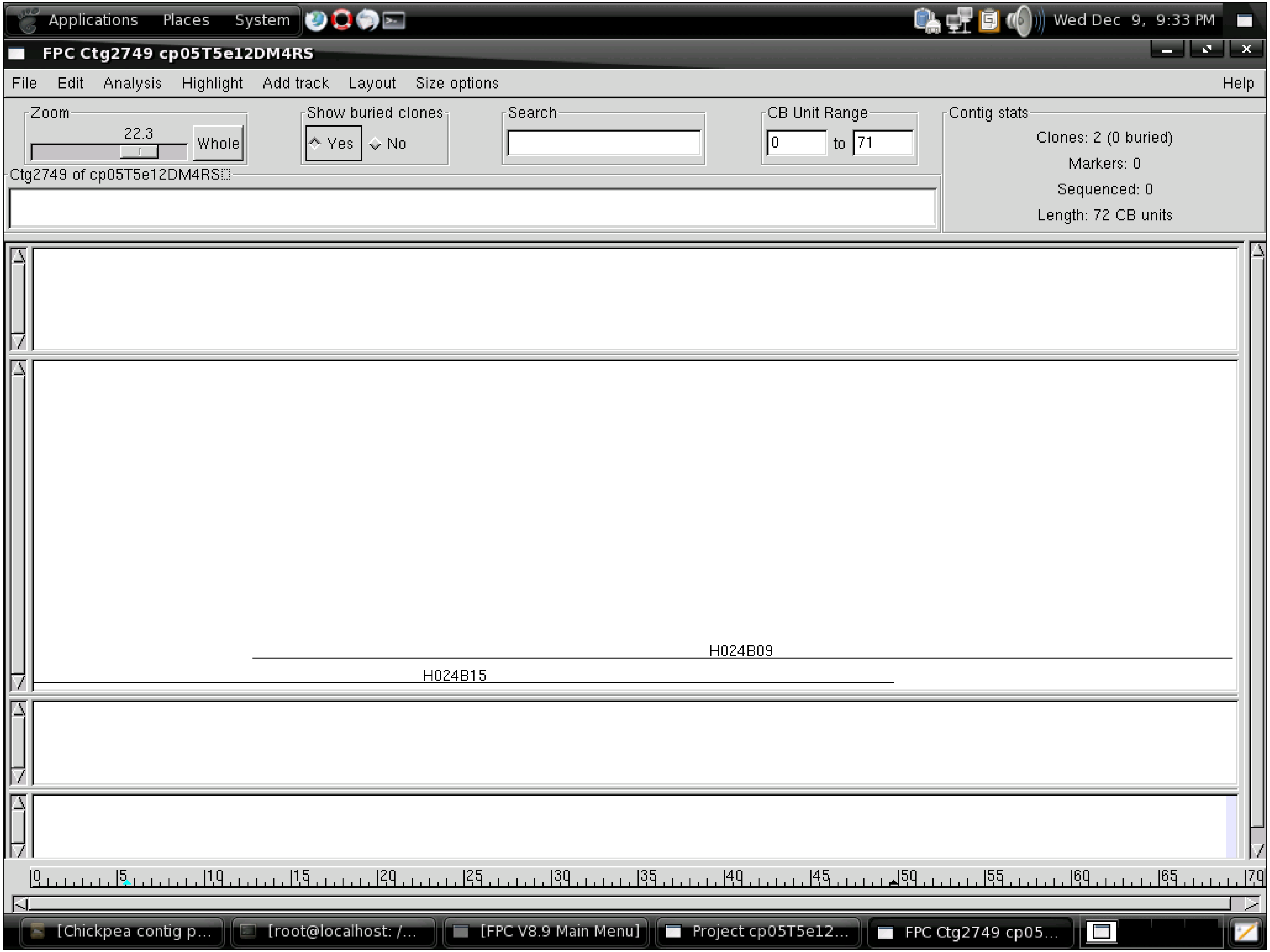
Applications Places System
Wed Dec 9, 9:33 PM
FPC Ctg2749 cp05T5e12DM4RS
File Edit Analysis Highlight Add track Layout Size options Help
Zoom
22.3
Whole
Show buried clones
Yes No
Search
CB Unit Range
0 to 71
Contig stats
Clones: 2 (0 buried)
Markers: 0
Sequenced: 0
Length: 72 CB units
Ctg2749 of cp05T5e12DM4RS
H024B09
H024B15
0 5 10 15 20 25 30 35 40 45 50 55 60 65 70
[Chickpea contig p...
[root@localhost: /...
[FPC V8.9 Main Menu]
Project cp05T5e12...
FPC Ctg2749 cp05...

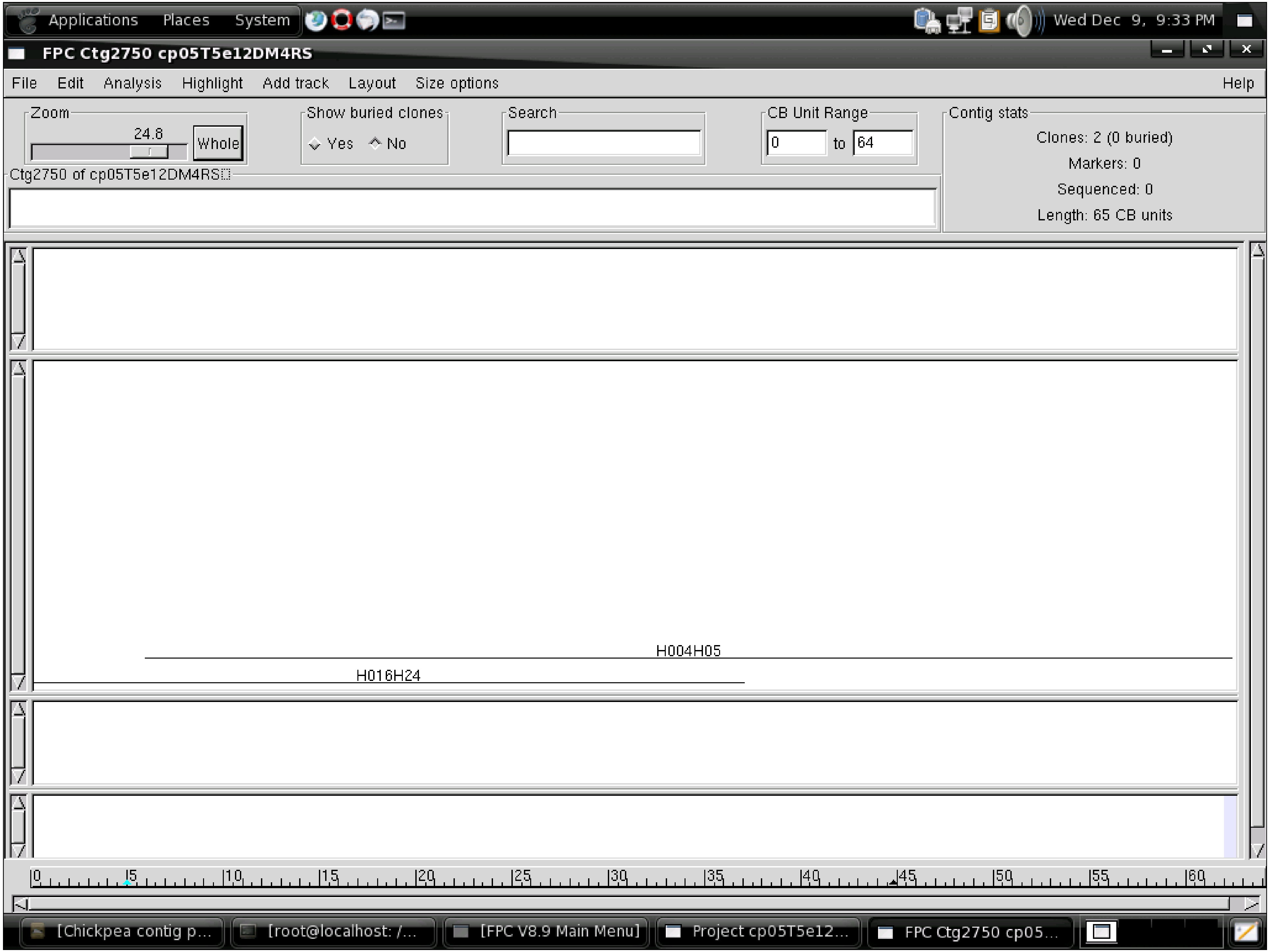
Applications Places System
Wed Dec 9, 9:33 PM
FPC Ctg2750 cp05T5e12DM4RS
File Edit Analysis Highlight Add track Layout Size options Help
Zoom
24.8
Whole
Show buried clones
Yes No
Search
CB Unit Range
0 to 64
Contig stats
Clones: 2 (0 buried)
Markers: 0
Sequenced: 0
Length: 65 CB units
Ctg2750 of cp05T5e12DM4RS
H004H05
H016H24
0 5 10 15 20 25 30 35 40 45 50 55 60
[Chickpea contig p...
[root@localhost: /...
[FPC V8.9 Main Menu]
Project cp05T5e12...
FPC Ctg2750 cp05...

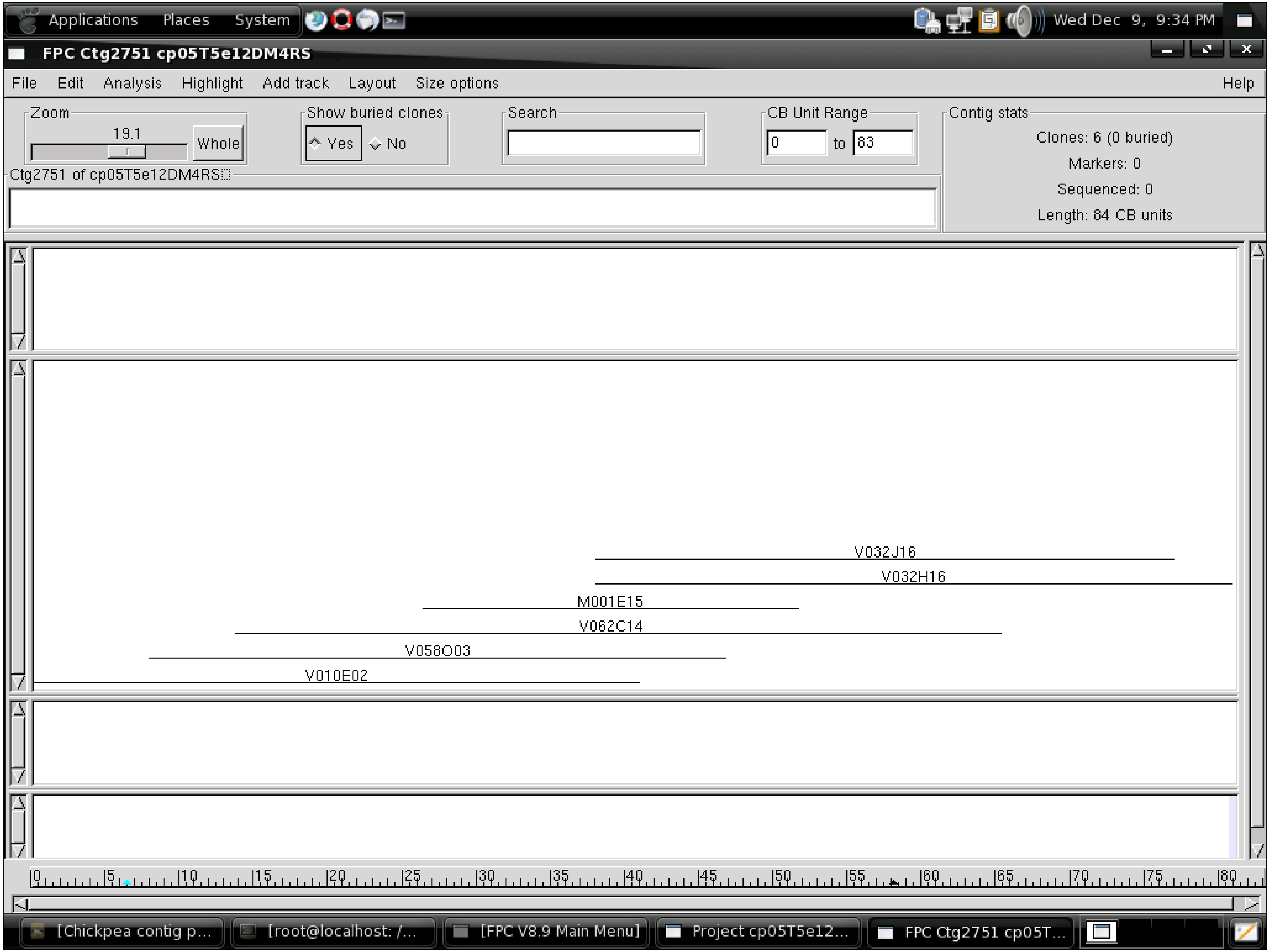
Applications Places System
Wed Dec 9, 9:34 PM
FPC Ctg2751 cp05T5e12DM4RS
File Edit Analysis Highlight Add track Layout Size options Help
Zoom
19.1
Whole
Show buried clones
Yes
No
Search
CB Unit Range
0
to
83
Contig stats
Clones: 6 (0 buried)
Markers: 0
Sequenced: 0
Length: 84 CB units
Ctg2751 of cp05T5e12DM4RS
V032J16
V032H16
M001E15
V062C14
V058O03
V010E02
0 5 10 15 20 25 30 35 40 45 50 55 60 65 70 75 80
[Chickpea contig p...
[root@localhost: /...
[FPC V8.9 Main Menu]
Project cp05T5e12...
FPC Ctg2751 cp05T...

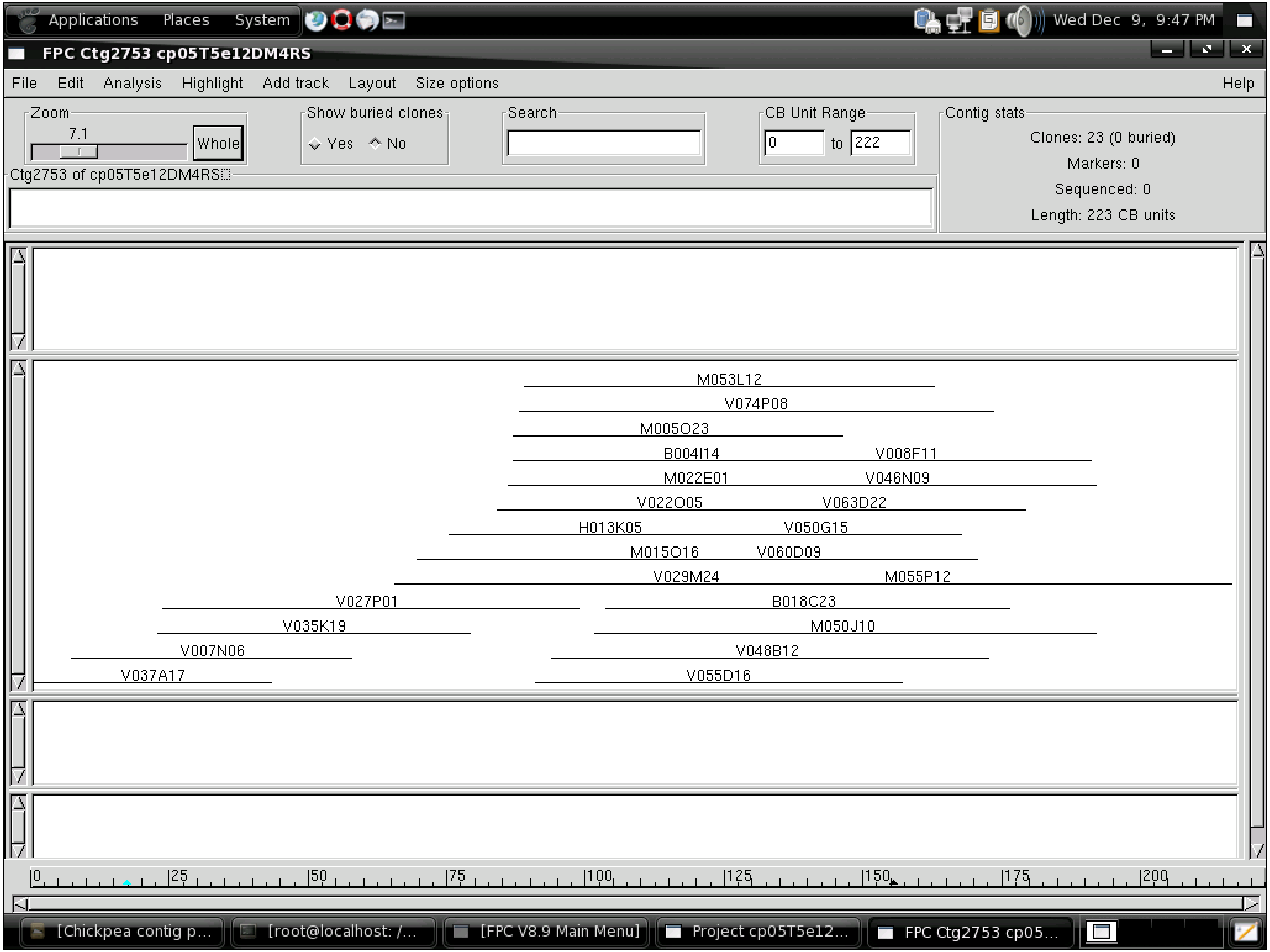
Applications Places System
Wed Dec 9, 9:47 PM
FPC Ctg2753 cp05T5e12DM4RS
File Edit Analysis Highlight Add track Layout Size options Help
Zoom
7.1
Whole
Show buried clones
Yes No
Search
CB Unit Range
0 to 222
Contig stats
Clones: 23 (0 buried)
Markers: 0
Sequenced: 0
Length: 223 CB units
Ctg2753 of cp05T5e12DM4RS
M053L12
V074P08
M005O23
B004I14
V008F11
M022E01
V046N09
V022O05
V063D22
H013K05
V050G15
M015O16
V060D09
V029M24
M055P12
V027P01
B018C23
V035K19
M050J10
V007N06
V048B12
V037A17
V055D16
0 25 50 75 100 125 150 175 200
[Chickpea contig p...
[root@localhost: /...
[FPC V8.9 Main Menu]
Project cp05T5e12...
FPC Ctg2753 cp05...

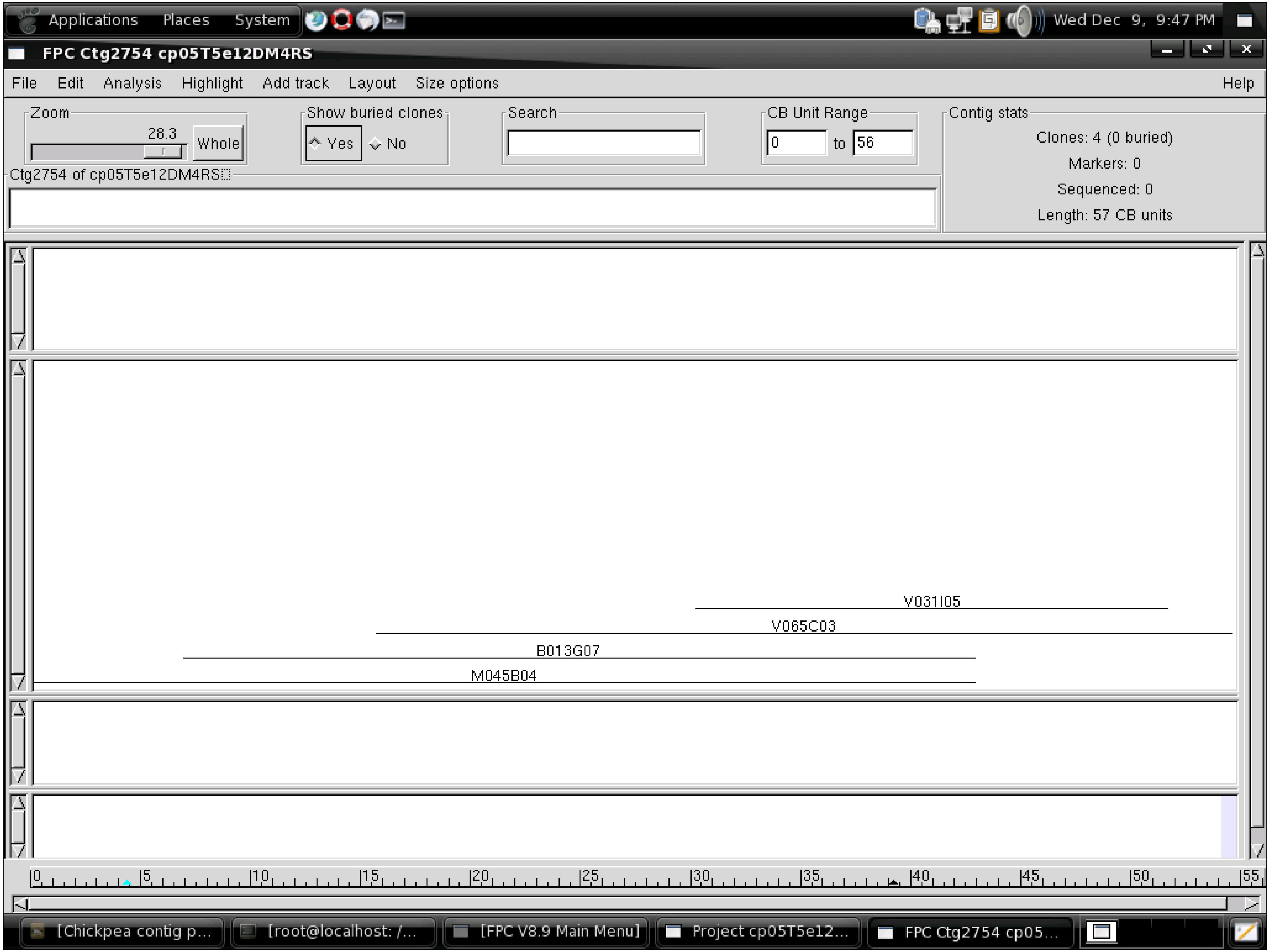
Applications Places System
Wed Dec 9, 9:47 PM
FPC Ctg2754 cp05T5e12DM4RS
File Edit Analysis Highlight Add track Layout Size options
Help
Zoom
28.3
Whole
Show buried clones
Yes
No
Search
CB Unit Range
0
to
56
Contig stats
Clones: 4 (0 buried)
Markers: 0
Sequenced: 0
Length: 57 CB units
Ctg2754 of cp05T5e12DM4RS
V031I05
V065C03
B013G07
M045B04
0 5 10 15 20 25 30 35 40 45 50 55
[Chickpea contig p...
[root@localhost: /...
[FPC V8.9 Main Menu]
Project cp05T5e12...
FPC Ctg2754 cp05...

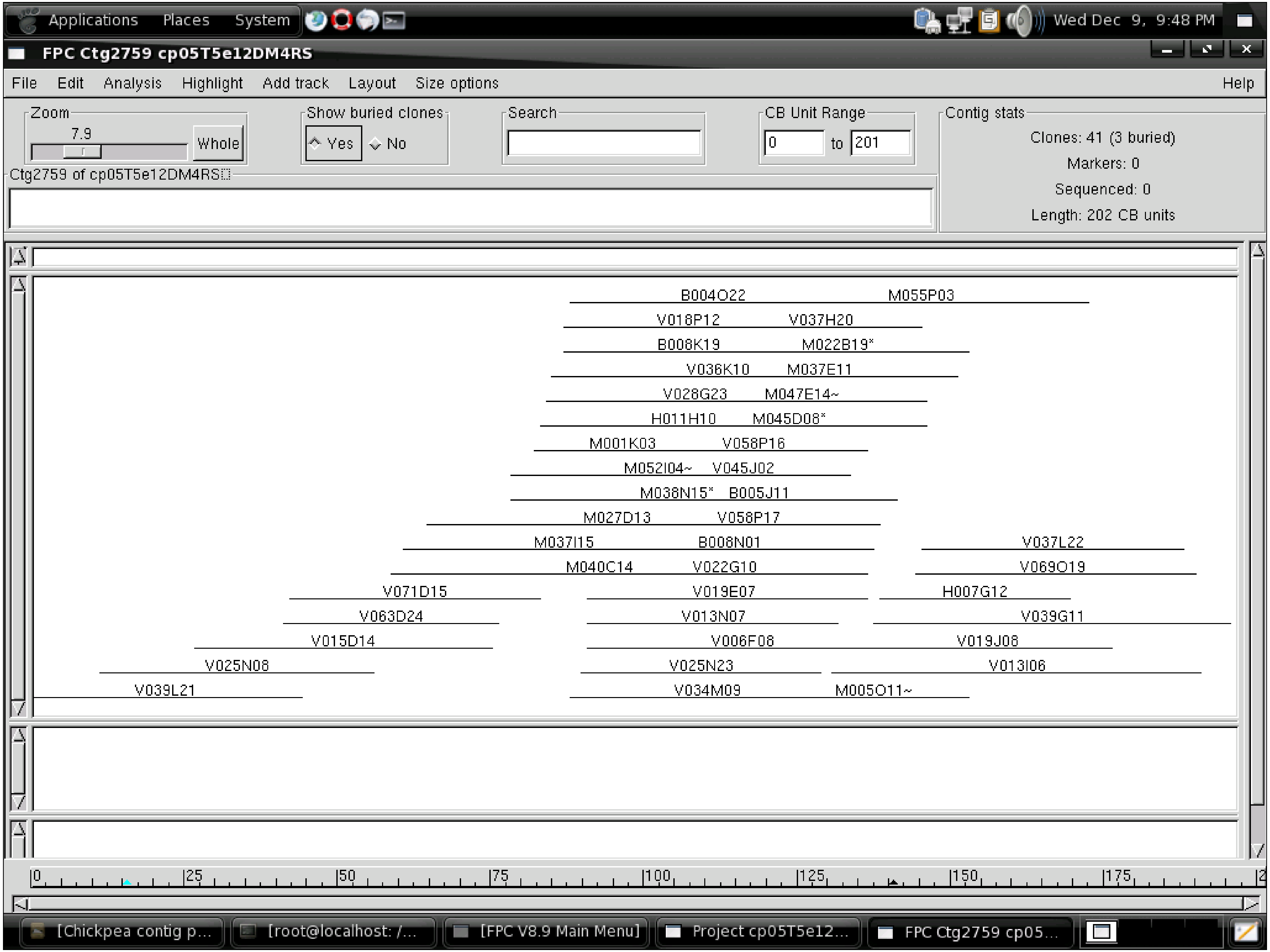
Applications Places System
Wed Dec 9, 9:48 PM
FPC Ctg2759 cp05T5e12DM4RS
File Edit Analysis Highlight Add track Layout Size options Help
Zoom
7.9
Whole
Show buried clones
Yes No
Search
CB Unit Range
0 to 201
Contig stats
Clones: 41 (3 buried)
Markers: 0
Sequenced: 0
Length: 202 CB units
Ctg2759 of cp05T5e12DM4RS
B004O22 M055P03
V018P12 V037H20
B008K19 M022B19*
V036K10 M037E11
V028G23 M047E14~
H011H10 M045D08*
M001K03 V058P16
M052I04~ V045J02
M038N15* B005J11
M027D13 V058P17
M037I15 B008N01 V037L22
M040C14 V022G10 V069O19
V071D15 V019E07 H007G12
V063D24 V013N07 V039G11
V015D14 V006F08 V019J08
V025N08 V025N23 V013I06
V039L21 V034M09 M005O11~
0 25 50 75 100 125 150 175
[Chickpea contig p... [root@localhost: /... [FPC V8.9 Main Menu] Project cp05T5e12... FPC Ctg2759 cp05...

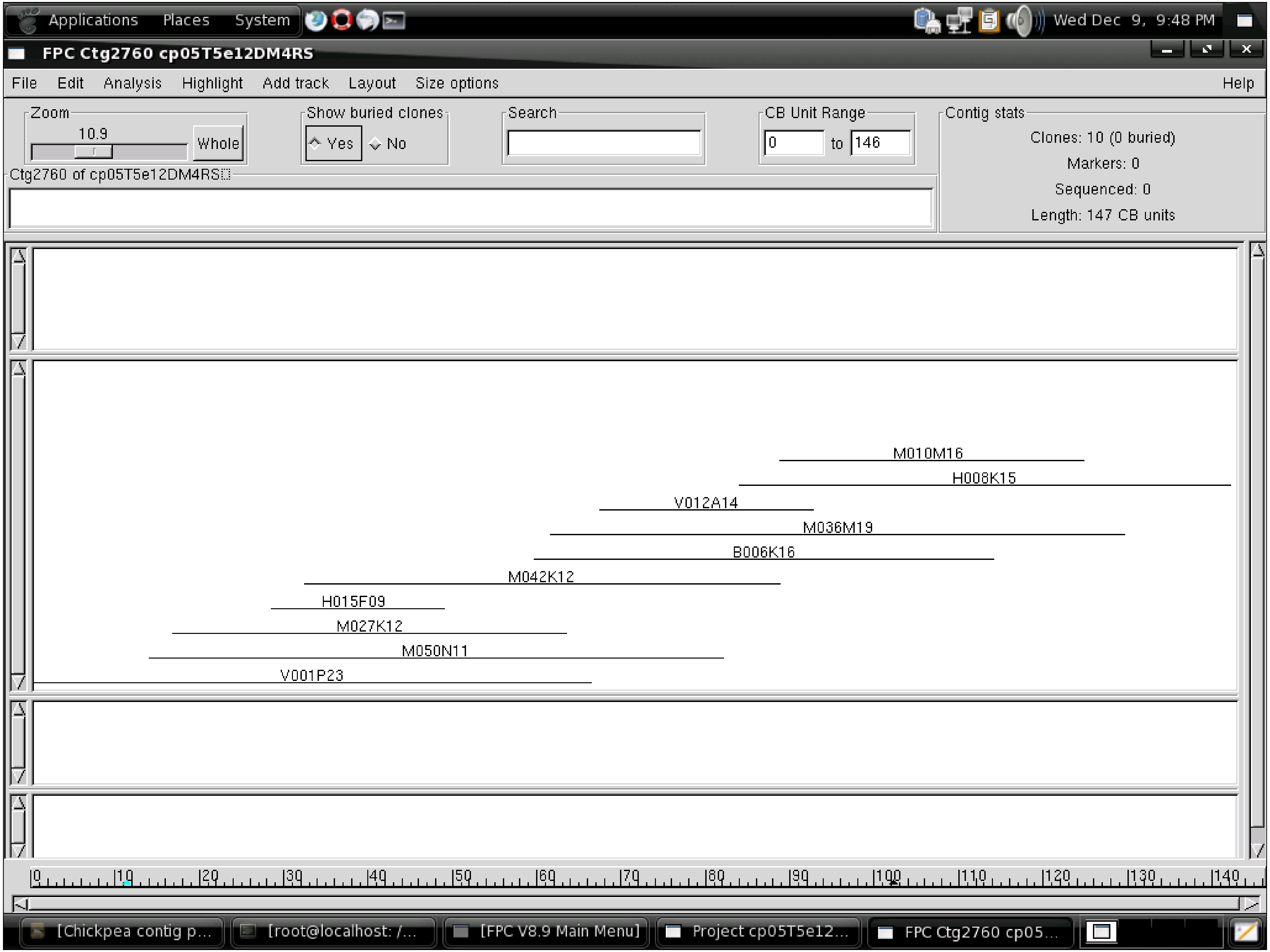
Applications Places System
Wed Dec 9, 9:48 PM
FPC Ctg2760 cp05T5e12DM4RS
File Edit Analysis Highlight Add track Layout Size options Help
Zoom
10.9
Whole
Show buried clones
Yes No
Search
CB Unit Range
0 to 146
Contig stats
Clones: 10 (0 buried)
Markers: 0
Sequenced: 0
Length: 147 CB units
Ctg2760 of cp05T5e12DM4RS
M010M16
H008K15
V012A14
M036M19
B006K16
M042K12
H015F09
M027K12
M050N11
V001P23
0 10 20 30 40 50 60 70 80 90 100 110 120 130 140
[Chickpea contig p...
[root@localhost: /...
[FPC V8.9 Main Menu]
Project cp05T5e12...
FPC Ctg2760 cp05...

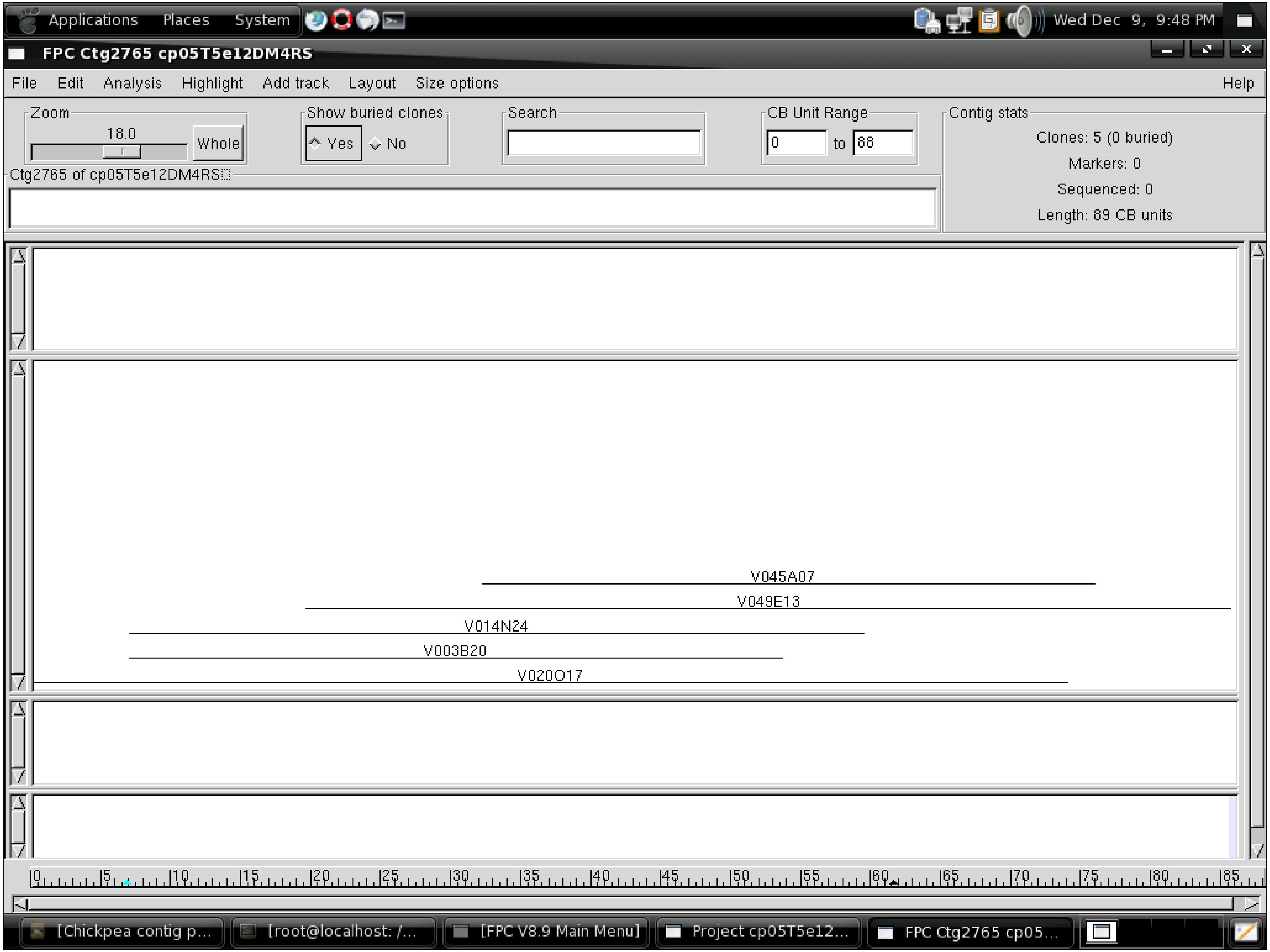

Applications
Places
System
Wed Dec 9, 9:48 PM
FPC Ctg2765 cp05T5e12DM4RS
File
Edit
Analysis
Highlight
Add track
Layout
Size options
Help
Zoom
18.0
Whole
Show buried clones
Yes
No
Search
CB Unit Range
0
to
88
Contig stats
Clones: 5 (0 buried)
Markers: 0
Sequenced: 0
Length: 89 CB units
Ctg2765 of cp05T5e12DM4RS
V045A07
V049E13
V014N24
V003B20
V020O17
0 5 10 15 20 25 30 35 40 45 50 55 60 65 70 75 80 85
[Chickpea contig p...
[root@localhost: /...
[FPC V8.9 Main Menu]
Project cp05T5e12...
FPC Ctg2765 cp05...

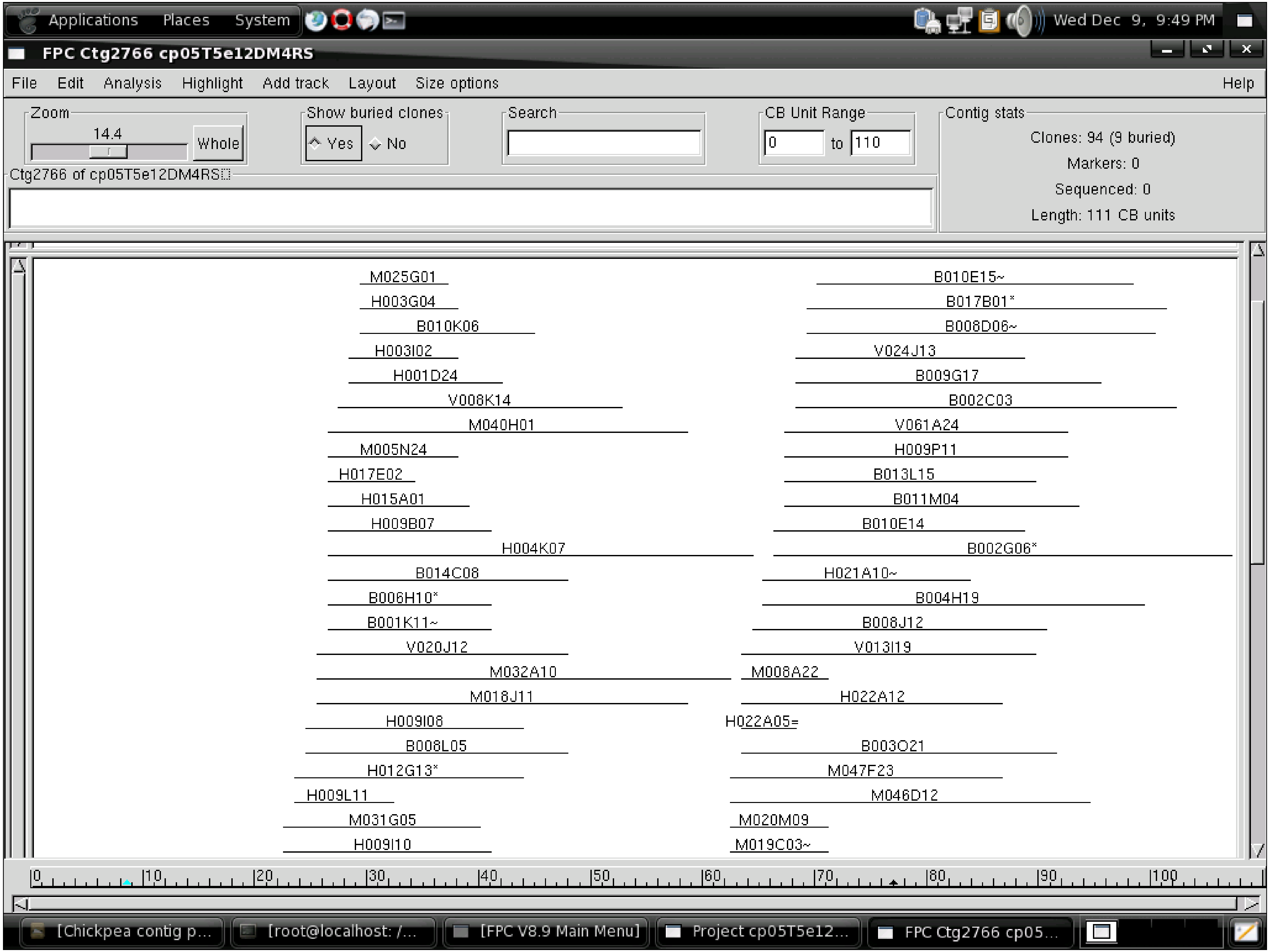
Applications Places System
Wed Dec 9, 9:49 PM
FPC Ctg2766 cp05T5e12DM4RS
File Edit Analysis Highlight Add track Layout Size options Help
Zoom
14.4
Whole
Show buried clones
Yes No
Search
CB Unit Range
0 to 110
Contig stats
Clones: 94 (9 buried)
Markers: 0
Sequenced: 0
Length: 111 CB units
Ctg2766 of cp05T5e12DM4RS
M025G01
H003G04
B010K06
H003I02
H001D24
V008K14
M040H01
M005N24
H017E02
H015A01
H009B07
H004K07
B014C08
B006H10*
B001K11~
V020J12
M032A10
M018J11
H009I08
B008L05
H012G13*
H009L11
M031G05
H009I10
B010E15~
B017B01*
B008D06~
V024J13
B009G17
B002C03
V061A24
H009P11
B013L15
B011M04
B010E14
B002G06*
H021A10~
B004H19
B008J12
V013I19
M008A22
H022A12
H022A05=
B003O21
M047F23
M046D12
M020M09
M019C03~
0 10 20 30 40 50 60 70 80 90 100
[Chickpea contig p...
[root@localhost: /...
[FPC V8.9 Main Menu]
Project cp05T5e12...
FPC Ctg2766 cp05...

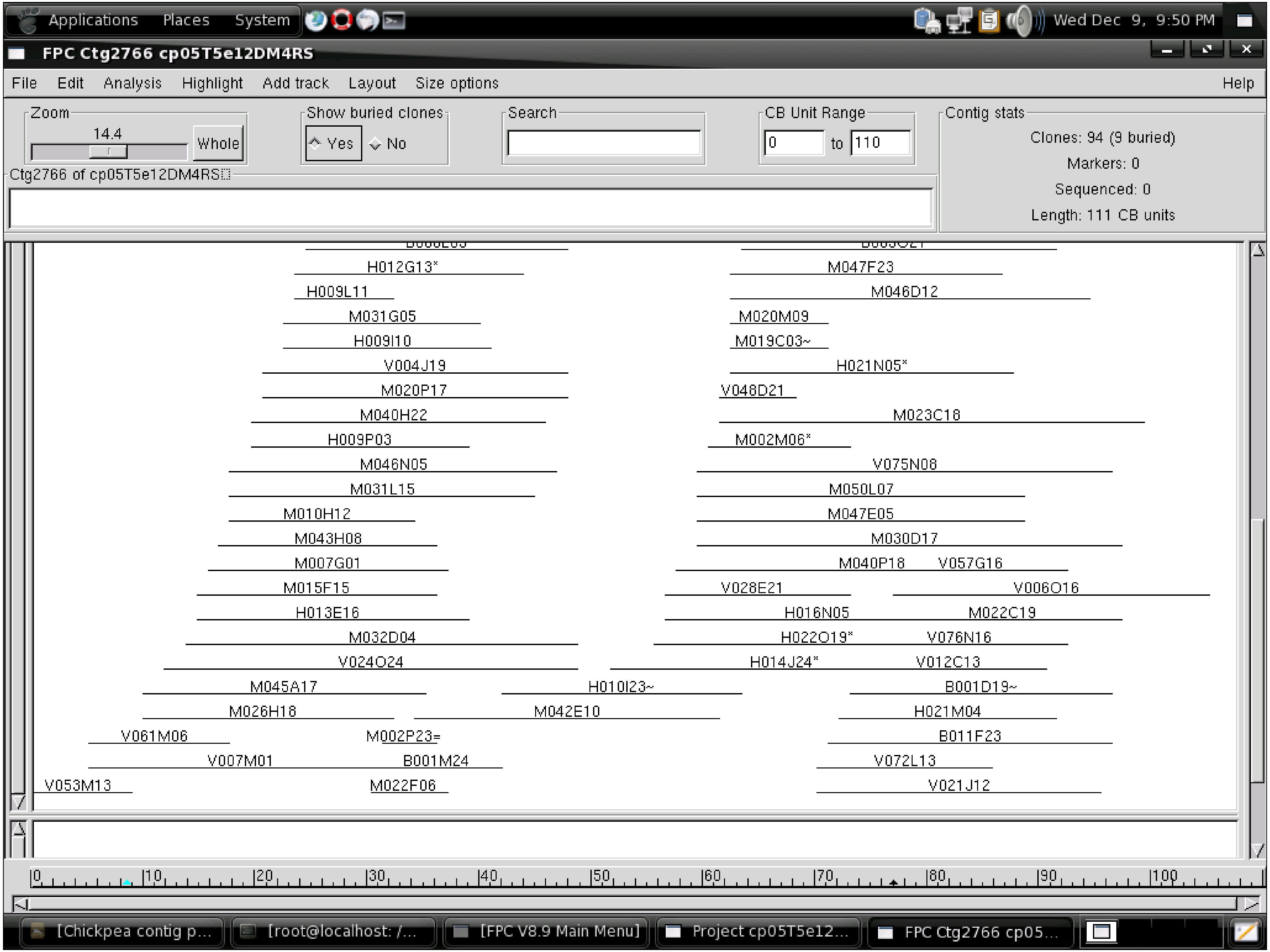
Applications Places System
Wed Dec 9, 9:50 PM
FPC Ctg2766 cp05T5e12DM4RS
File Edit Analysis Highlight Add track Layout Size options Help
Zoom
14.4
Whole
Show buried clones
Yes
No
Search
CB Unit Range
0
to
110
Contig stats
Clones: 94 (9 buried)
Markers: 0
Sequenced: 0
Length: 111 CB units
Ctg2766 of cp05T5e12DM4RS
H012G13*
H009L11
M031G05
H009I10
V004J19
M020P17
M040H22
H009P03
M046N05
M031L15
M010H12
M043H08
M007G01
M015F15
H013E16
M032D04
V024O24
M045A17
M026H18
V061M06
V007M01
V053M13
M047F23
M046D12
M020M09
M019C03~
H021N05*
V048D21
M023C18
M002M06*
V075N08
M050L07
M047E05
M030D17
M040P18
V057G16
V028E21
V006O16
H016N05
M022C19
H022O19*
V076N16
H014J24*
V012C13
H010I23~
B001D19~
M042E10
H021M04
M002P23=
B011F23
B001M24
V072L13
M022F06
V021J12
0 10 20 30 40 50 60 70 80 90 100
[Chickpea contig p...
[root@localhost: /...
[FPC V8.9 Main Menu]
Project cp05T5e12...
FPC Ctg2766 cp05...

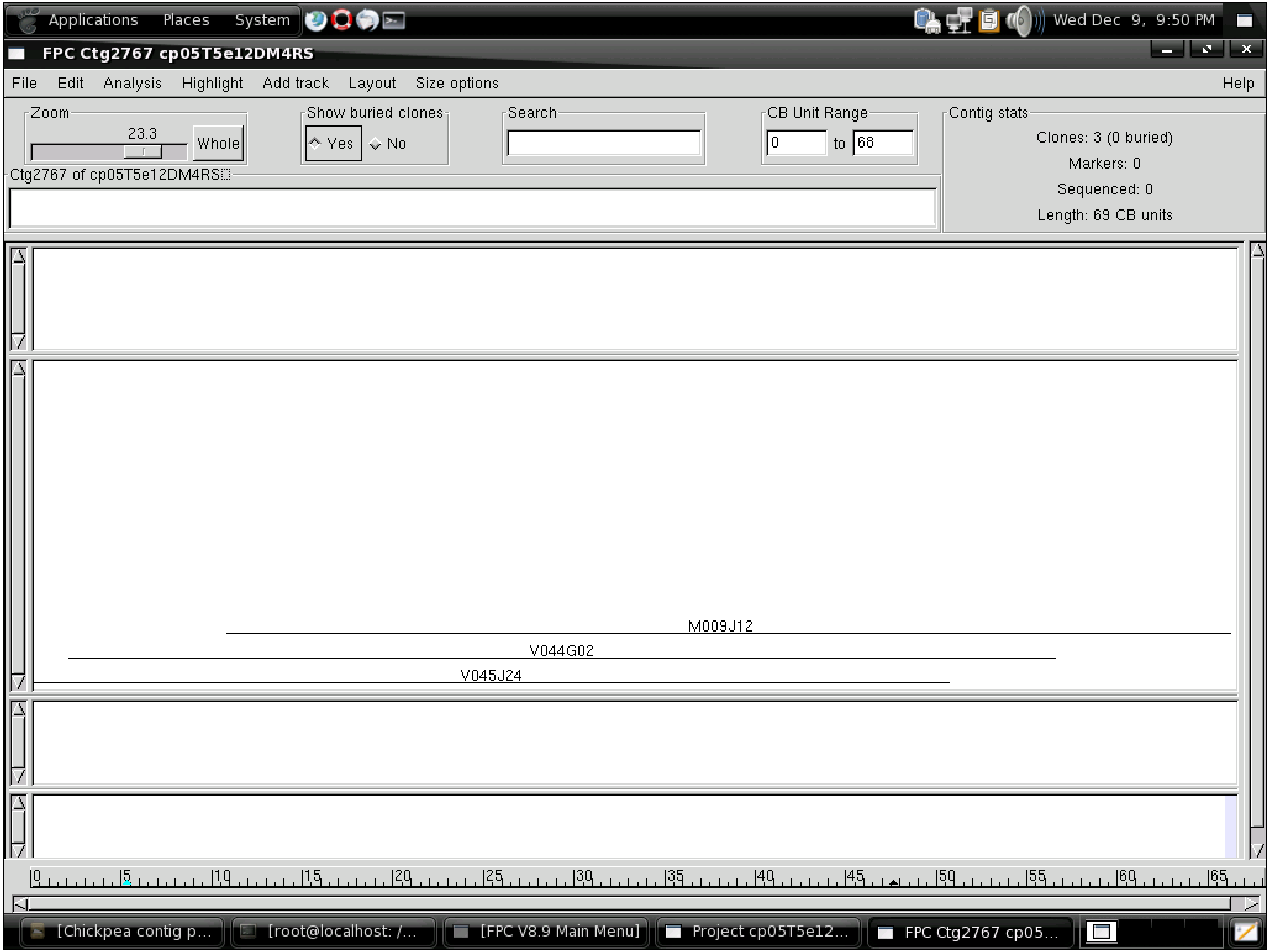

Applications Places System
Wed Dec 9, 9:50 PM
FPC Ctg2767 cp05T5e12DM4RS
File Edit Analysis Highlight Add track Layout Size options
Help
Zoom
23.3
Whole
Show buried clones
Yes
No
Search
CB Unit Range
0
to
68
Contig stats
Clones: 3 (0 buried)
Markers: 0
Sequenced: 0
Length: 69 CB units
Ctg2767 of cp05T5e12DM4RS
M009J12
V044G02
V045J24
0 5 10 15 20 25 30 35 40 45 50 55 60 65
[Chickpea contig p...
[root@localhost: /...
[FPC V8.9 Main Menu]
Project cp05T5e12...
FPC Ctg2767 cp05...

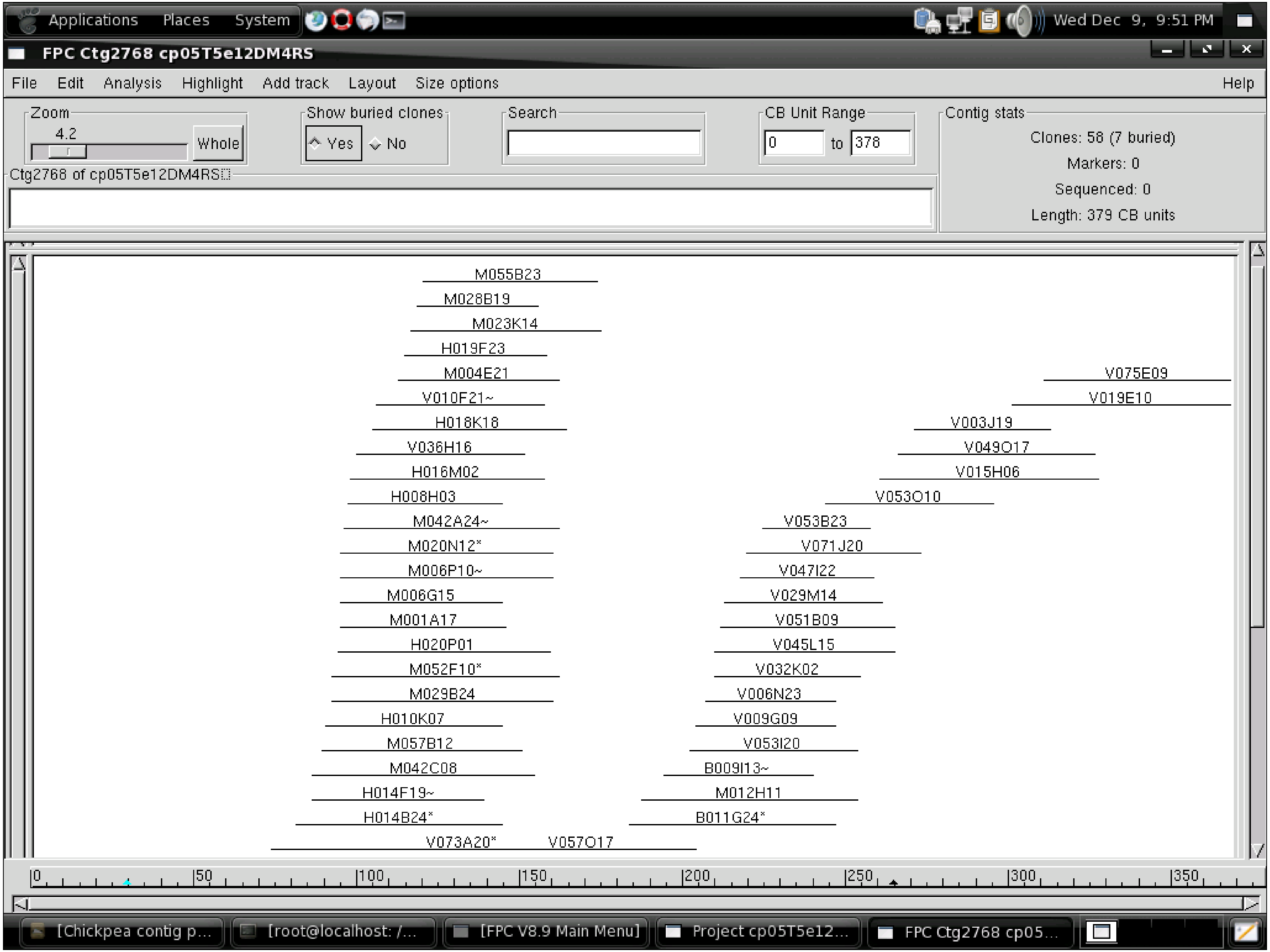
Applications Places System
Wed Dec 9, 9:51 PM
FPC Ctg2768 cp05T5e12DM4RS
File Edit Analysis Highlight Add track Layout Size options Help
Zoom
4.2
Whole
Show buried clones
Yes No
Search
CB Unit Range
0 to 378
Contig stats
Clones: 58 (7 buried)
Markers: 0
Sequenced: 0
Length: 379 CB units
Ctg2768 of cp05T5e12DM4RS
M055B23
M028B19
M023K14
H019F23
M004E21
V010F21~
H018K18
V036H16
H016M02
H008H03
M042A24~
M020N12*
M006P10~
M006G15
M001A17
H020P01
M052F10*
M029B24
H010K07
M057B12
M042C08
H014F19~
H014B24*
V073A20*
V057O17
V075E09
V019E10
V003J19
V049O17
V015H06
V053O10
V053B23
V071J20
V047I22
V029M14
V051B09
V045L15
V032K02
V006N23
V009G09
V053I20
B009I13~
M012H11
B011G24*
0 50 100 150 200 250 300 350
[Chickpea contig p...
[root@localhost: /...
[FPC V8.9 Main Menu]
Project cp05T5e12...
FPC Ctg2768 cp05...

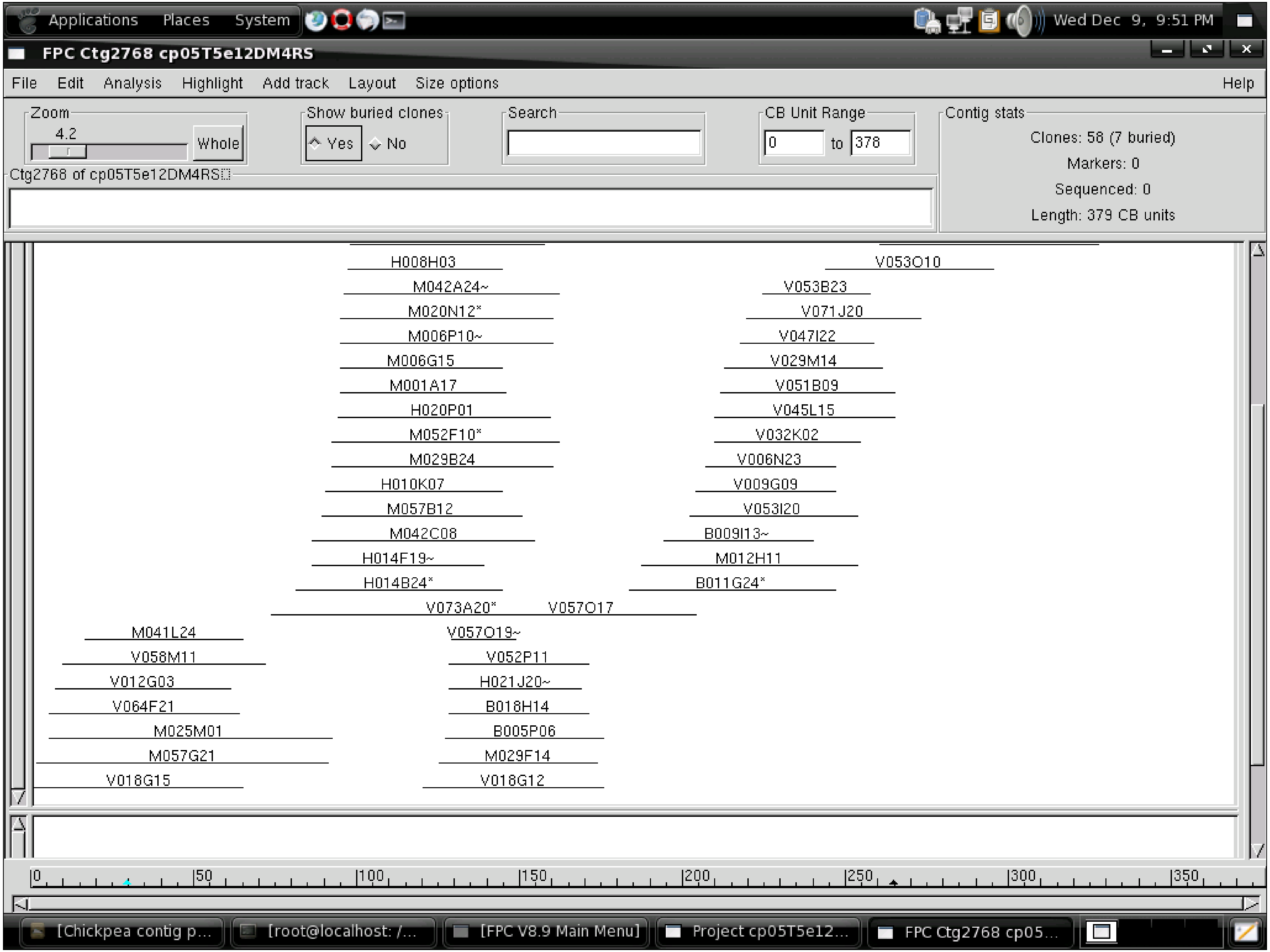
Applications Places System
Wed Dec 9, 9:51 PM
FPC Ctg2768 cp05T5e12DM4RS
File Edit Analysis Highlight Add track Layout Size options Help
Zoom
4.2
Whole
Show buried clones
Yes
No
Search
CB Unit Range
0
to
378
Contig stats
Clones: 58 (7 buried)
Markers: 0
Sequenced: 0
Length: 379 CB units
Ctg2768 of cp05T5e12DM4RS
H008H03
V053O10
M042A24~
V053B23
M020N12*
V071J20
M006P10~
V047I22
M006G15
V029M14
M001A17
V051B09
H020P01
V045L15
M052F10*
V032K02
M029B24
V006N23
H010K07
V009G09
M057B12
V053I20
M042C08
B009I13~
H014F19~
M012H11
H014B24*
B011G24*
V073A20*
V057O17
M041L24
V057O19~
V058M11
V052P11
V012G03
H021J20~
V064F21
B018H14
M025M01
B005P06
M057G21
M029F14
V018G15
V018G12
0
50
100
150
200
250
300
350
[Chickpea contig p...
[root@localhost: /...
[FPC V8.9 Main Menu]
Project cp05T5e12...
FPC Ctg2768 cp05...

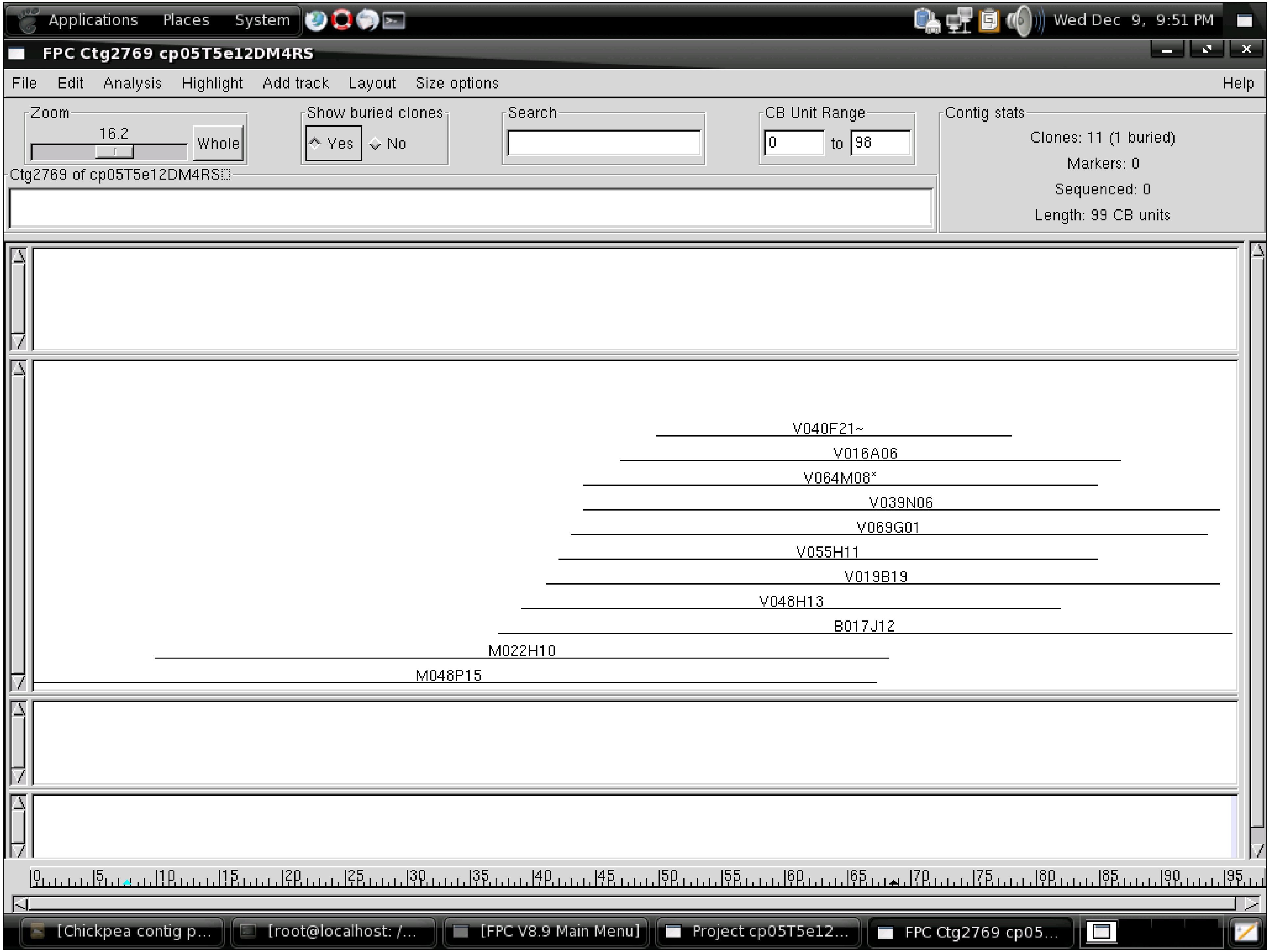
Applications Places System
Wed Dec 9, 9:51 PM
FPC Ctg2769 cp05T5e12DM4RS
File Edit Analysis Highlight Add track Layout Size options Help
Zoom
16.2
Whole
Show buried clones
Yes
No
Search
CB Unit Range
0
to
98
Contig stats
Clones: 11 (1 buried)
Markers: 0
Sequenced: 0
Length: 99 CB units
Ctg2769 of cp05T5e12DM4RS
V040F21~
V016A06
V064M08*
V039N06
V069G01
V055H11
V019B19
V048H13
B017J12
M022H10
M048P15
0 5 10 15 20 25 30 35 40 45 50 55 60 65 70 75 80 85 90 95
[Chickpea contig p...
[root@localhost: /...
[FPC V8.9 Main Menu]
Project cp05T5e12...
FPC Ctg2769 cp05...

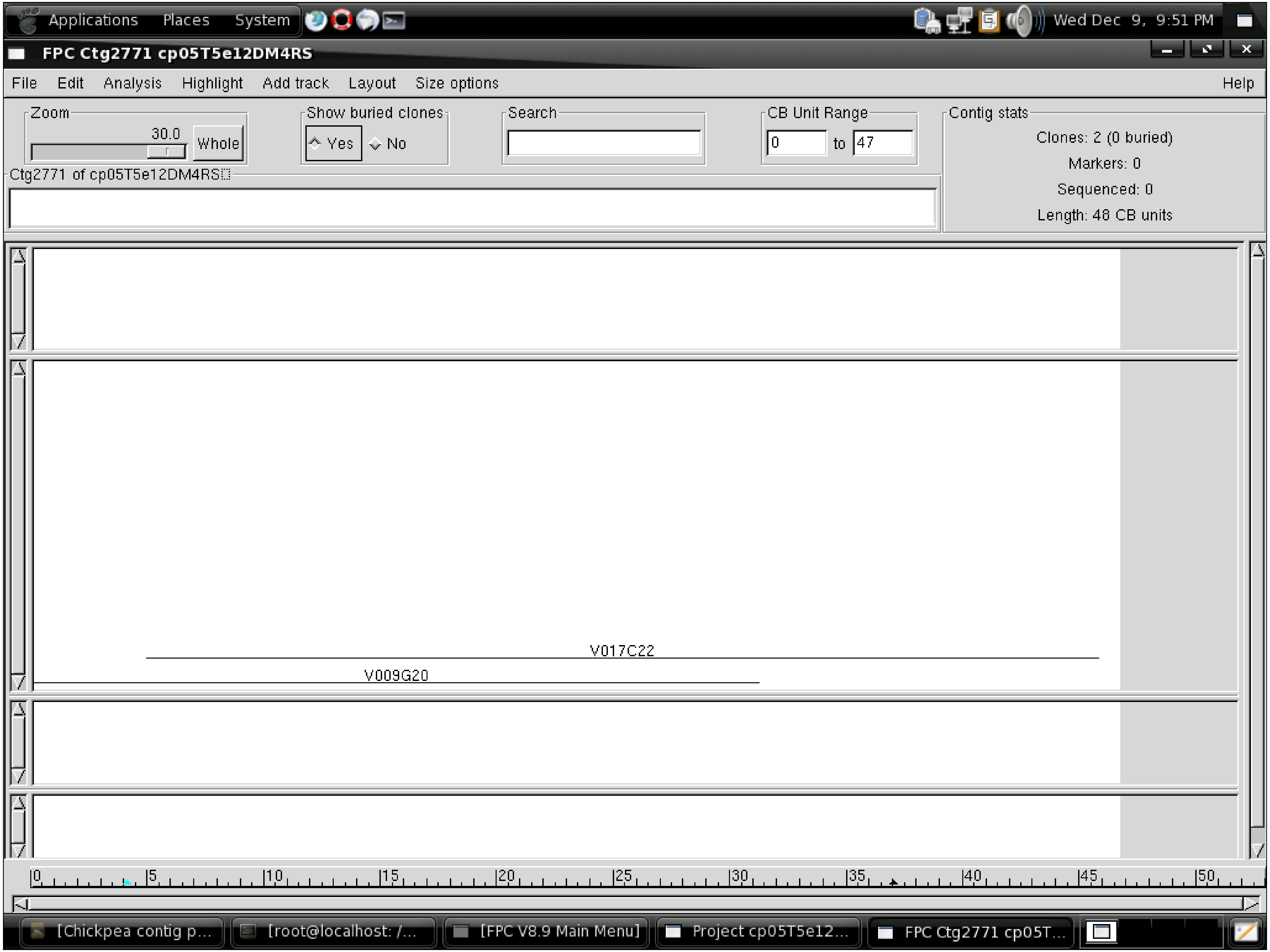
Applications
Places
System
Wed Dec 9, 9:51 PM
FPC Ctg2771 cp05T5e12DM4RS
File
Edit
Analysis
Highlight
Add track
Layout
Size options
Help
Zoom
30.0
Whole
Show buried clones
Yes
No
Search
CB Unit Range
0
to
47
Contig stats
Clones: 2 (0 buried)
Markers: 0
Sequenced: 0
Length: 48 CB units
Ctg2771 of cp05T5e12DM4RS
V017C22
V009G20
0
5
10
15
20
25
30
35
40
45
50
[Chickpea contig p...
[root@localhost: /...
[FPC V8.9 Main Menu]
Project cp05T5e12...
FPC Ctg2771 cp05T...

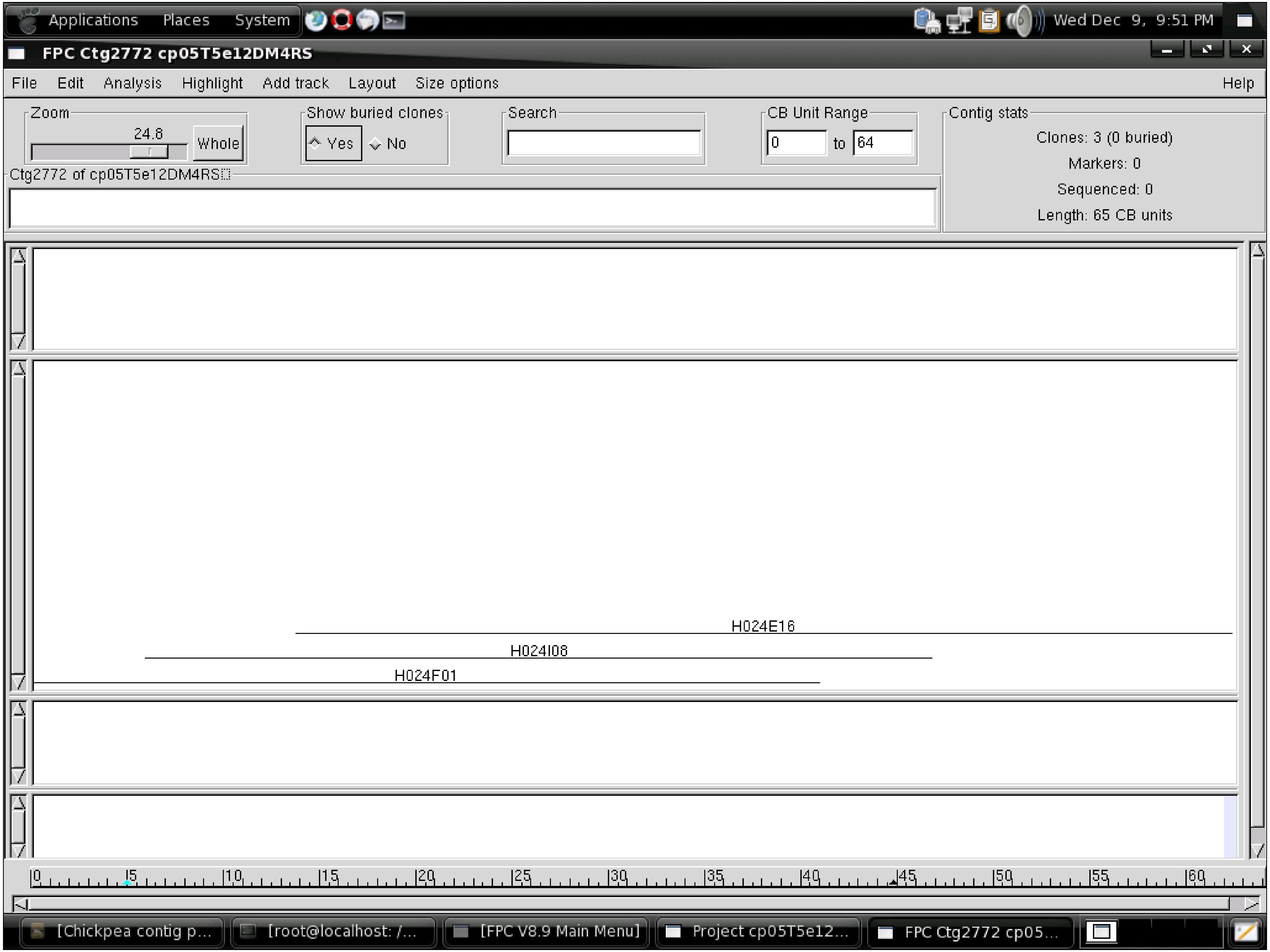
Applications Places System
Wed Dec 9, 9:51 PM
FPC Ctg2772 cp05T5e12DM4RS
File Edit Analysis Highlight Add track Layout Size options Help
Zoom
24.8
Whole
Show buried clones
Yes No
Search
CB Unit Range
0 to 64
Contig stats
Clones: 3 (0 buried)
Markers: 0
Sequenced: 0
Length: 65 CB units
Ctg2772 of cp05T5e12DM4RS
H024E16
H024I08
H024F01
0 5 10 15 20 25 30 35 40 45 50 55 60
[Chickpea contig p...
[root@localhost: /...
[FPC V8.9 Main Menu]
Project cp05T5e12...
FPC Ctg2772 cp05...

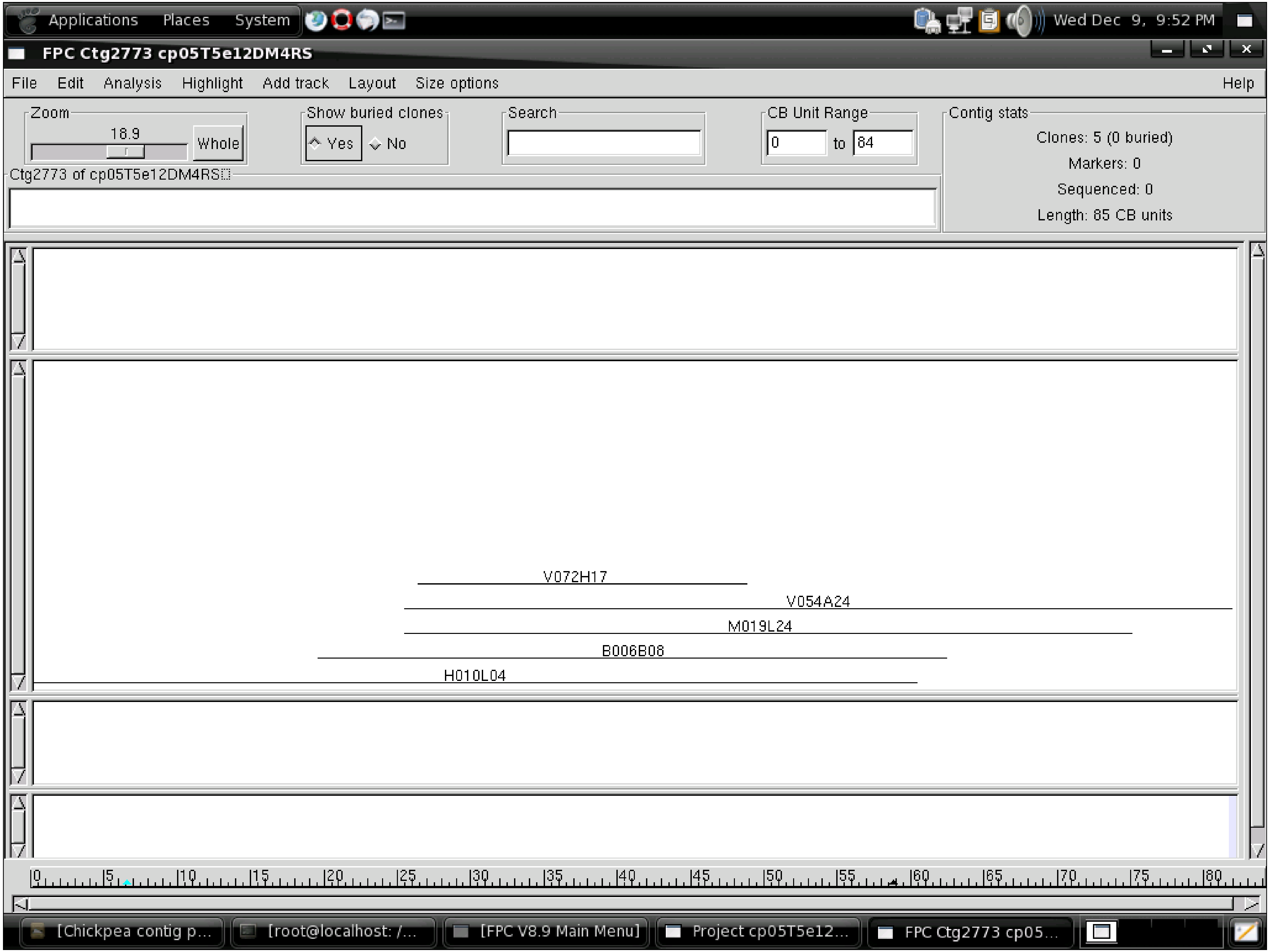
Applications Places System
Wed Dec 9, 9:52 PM
FPC Ctg2773 cp05T5e12DM4RS
File Edit Analysis Highlight Add track Layout Size options Help
Zoom
18.9
Whole
Show buried clones
Yes No
Search
CB Unit Range
0 to 84
Contig stats
Clones: 5 (0 buried)
Markers: 0
Sequenced: 0
Length: 85 CB units
Ctg2773 of cp05T5e12DM4RS
V072H17
V054A24
M019L24
B006B08
H010L04
0 5 10 15 20 25 30 35 40 45 50 55 60 65 70 75 80
[Chickpea contig p...
[root@localhost: /...
[FPC V8.9 Main Menu]
Project cp05T5e12...
FPC Ctg2773 cp05...

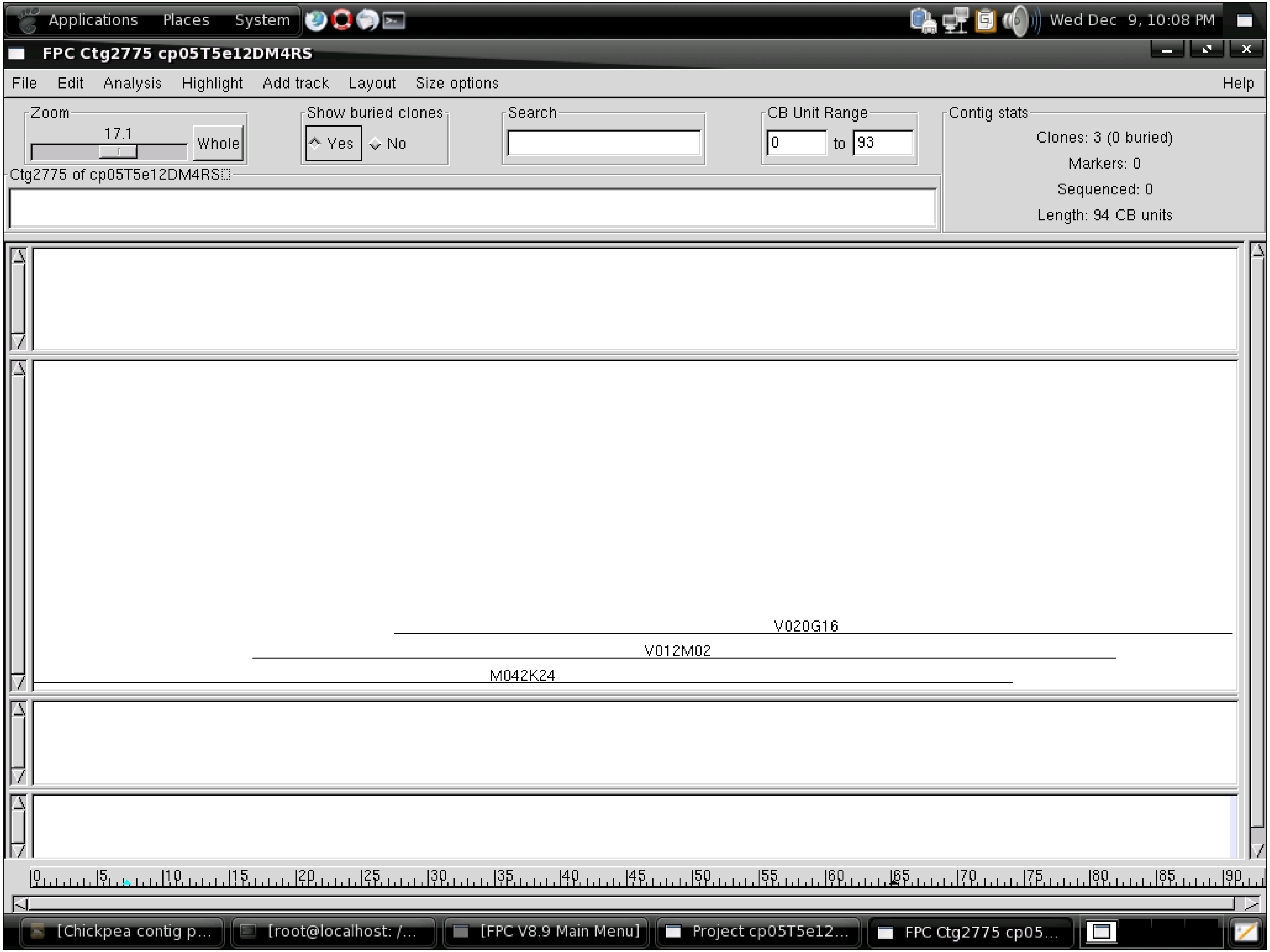
Applications Places System
Wed Dec 9, 10:08 PM
FPC Ctg2775 cp05T5e12DM4RS
File Edit Analysis Highlight Add track Layout Size options Help
Zoom
17.1
Whole
Show buried clones
Yes
No
Search
CB Unit Range
0 to 93
Contig stats
Clones: 3 (0 buried)
Markers: 0
Sequenced: 0
Length: 94 CB units
Ctg2775 of cp05T5e12DM4RS
V020G16
V012M02
M042K24
0 5 10 15 20 25 30 35 40 45 50 55 60 65 70 75 80 85 90
[Chickpea contig p...
[root@localhost: /...
[FPC V8.9 Main Menu]
Project cp05T5e12...
FPC Ctg2775 cp05...

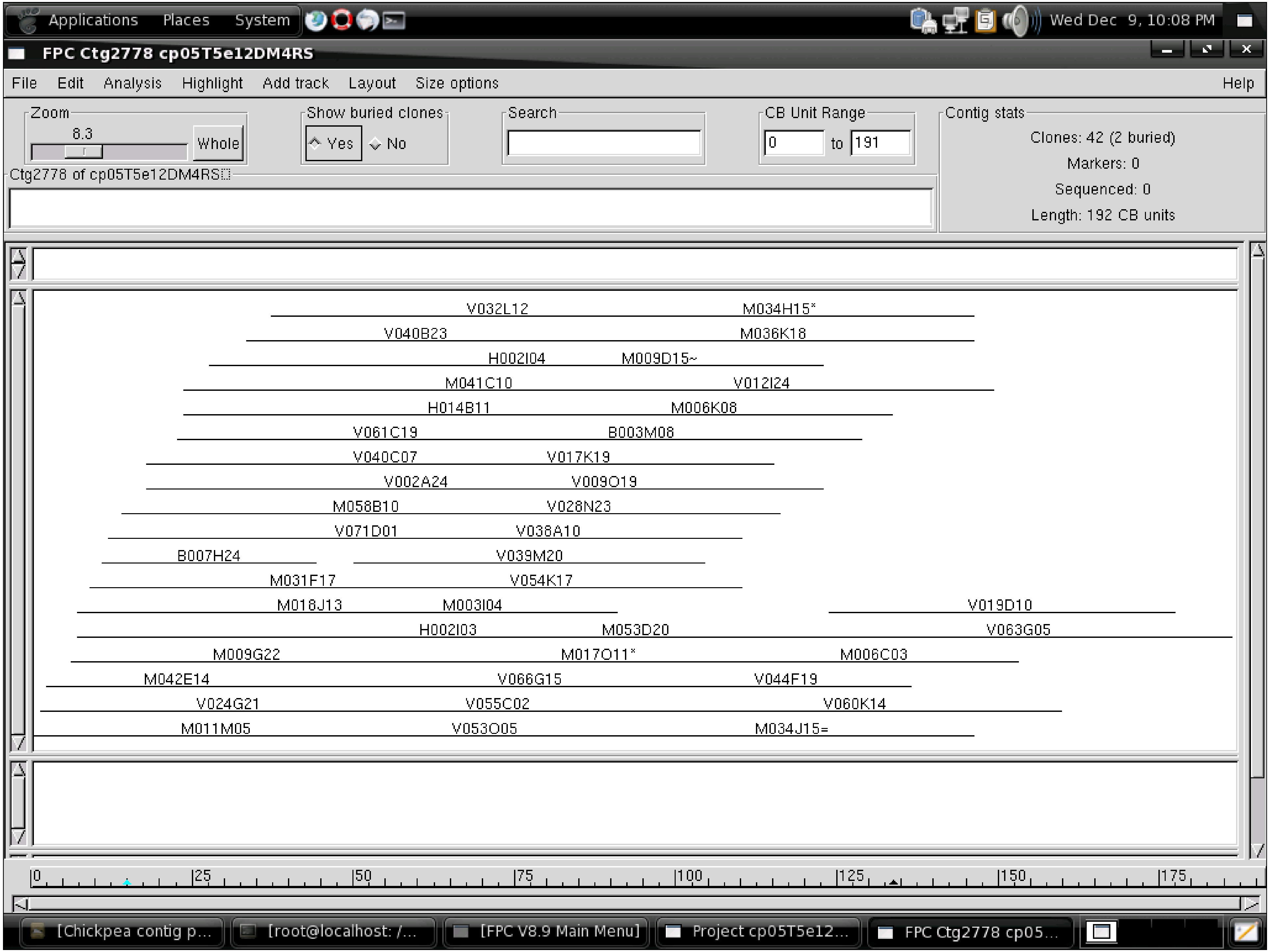
Applications Places System
Wed Dec 9, 10:08 PM
FPC Ctg2778 cp05T5e12DM4RS
File Edit Analysis Highlight Add track Layout Size options Help
Zoom
8.3
Whole
Show buried clones
Yes No
Search
CB Unit Range
0 to 191
Contig stats
Clones: 42 (2 buried)
Markers: 0
Sequenced: 0
Length: 192 CB units
Ctg2778 of cp05T5e12DM4RS
V032L12 M034H15*
V040B23 M036K18
H002I04 M009D15~
M041C10 V012I24
H014B11 M006K08
V061C19 B003M08
V040C07 V017K19
V002A24 V009O19
M058B10 V028N23
V071D01 V038A10
B007H24 V039M20
M031F17 V054K17
M018J13 M003I04 V019D10
H002I03 M053D20 V063G05
M009G22 M017O11* M006C03
M042E14 V066G15 V044F19
V024G21 V055C02 V060K14
M011M05 V053O05 M034J15=
0 25 50 75 100 125 150 175
[Chickpea contig p...
[root@localhost: /...
[FPC V8.9 Main Menu]
Project cp05T5e12...
FPC Ctg2778 cp05...

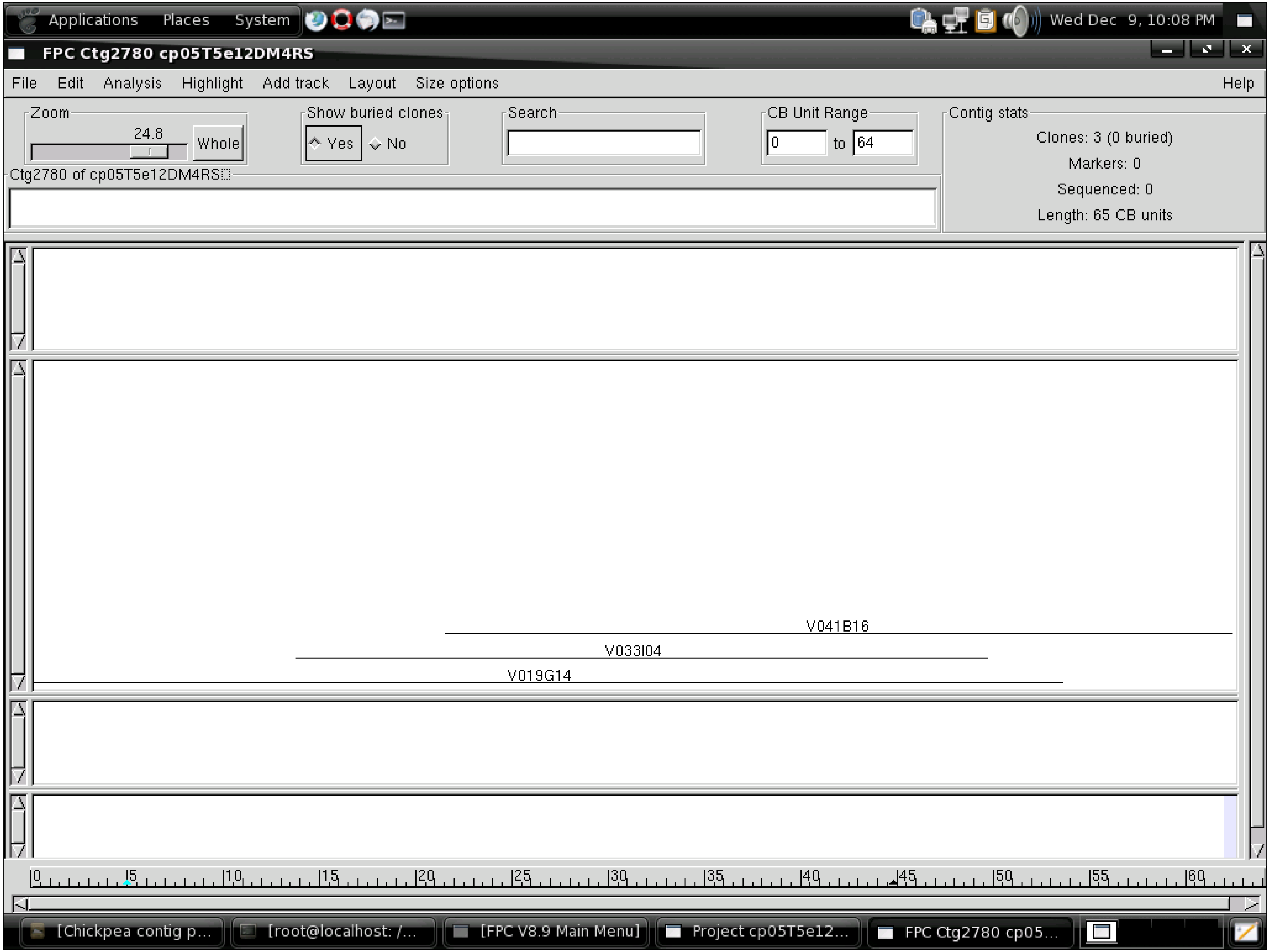
Applications Places System
Wed Dec 9, 10:08 PM
FPC Ctg2780 cp05T5e12DM4RS
File Edit Analysis Highlight Add track Layout Size options Help
Zoom
24.8
Whole
Show buried clones
Yes
No
Search
CB Unit Range
0
to
64
Contig stats
Clones: 3 (0 buried)
Markers: 0
Sequenced: 0
Length: 65 CB units
Ctg2780 of cp05T5e12DM4RS
V041B16
V033I04
V019G14
0 5 10 15 20 25 30 35 40 45 50 55 60
[Chickpea contig p...
[root@localhost: /...
[FPC V8.9 Main Menu]
Project cp05T5e12...
FPC Ctg2780 cp05...

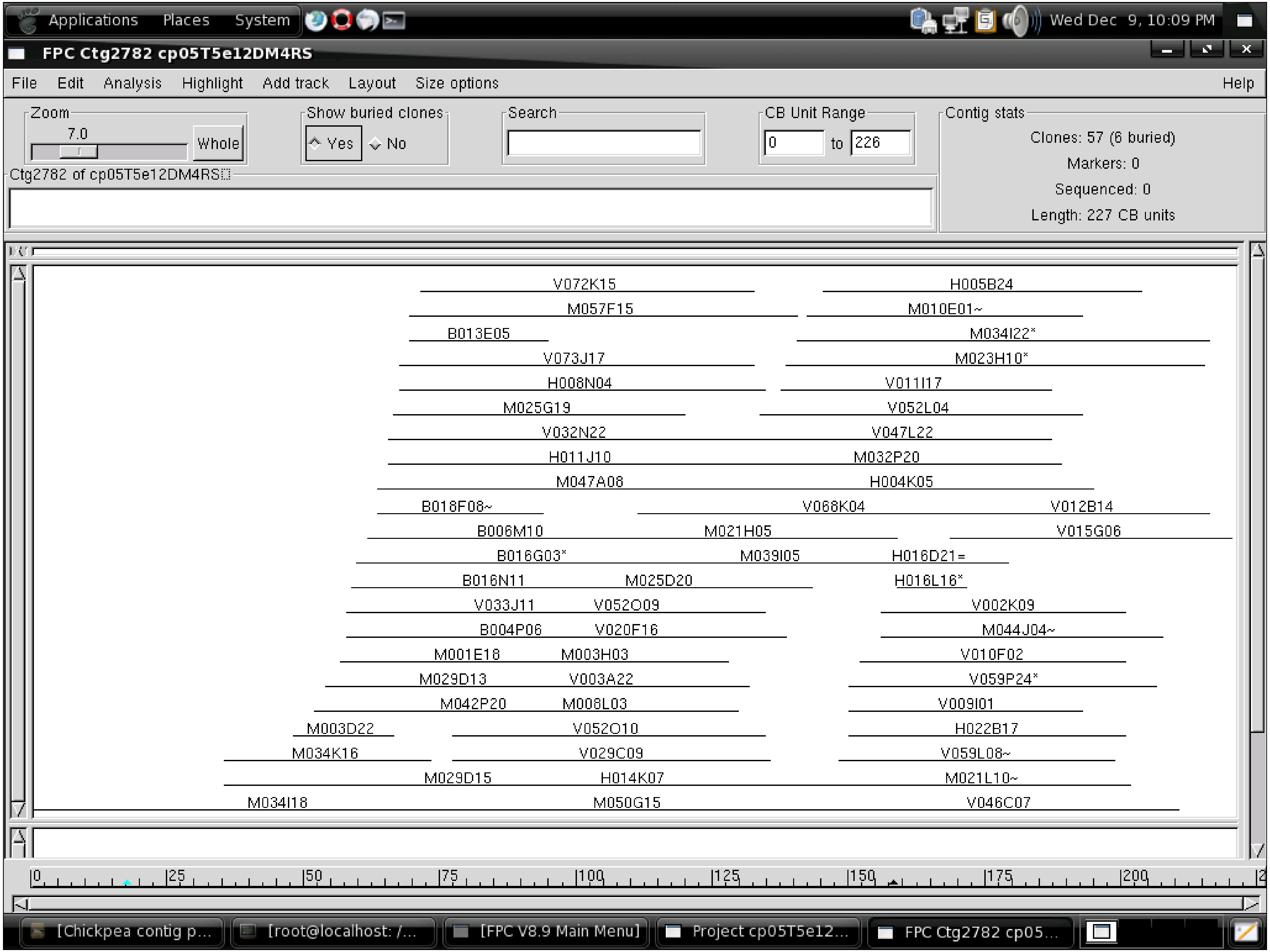
Applications Places System
Wed Dec 9, 10:09 PM
FPC Ctg2782 cp05T5e12DM4RS
File Edit Analysis Highlight Add track Layout Size options Help
Zoom
7.0
Whole
Show buried clones
Yes
No
Search
CB Unit Range
0
to
226
Contig stats
Clones: 57 (6 buried)
Markers: 0
Sequenced: 0
Length: 227 CB units
Ctg2782 of cp05T5e12DM4RS
V072K15
H005B24
M057F15
M010E01~
B013E05
M034I22*
V073J17
M023H10*
H008N04
V011I17
M025G19
V052L04
V032N22
V047L22
H011J10
M032P20
M047A08
H004K05
B018F08~
V068K04
V012B14
B006M10
M021H05
V015G06
B016G03*
M039I05
H016D21=
B016N11
M025D20
H016L16*
V033J11
V052O09
V002K09
B004P06
V020F16
M044J04~
M001E18
M003H03
V010F02
M029D13
V003A22
V059P24*
M042P20
M008L03
V009I01
M003D22
V052O10
H022B17
M034K16
V029C09
V059L08~
M029D15
H014K07
M021L10~
M034I18
M050G15
V046C07
0
25
50
75
100
125
150
175
200
[Chickpea contig p...
[root@localhost: /...
[FPC V8.9 Main Menu]
Project cp05T5e12...
FPC Ctg2782 cp05...

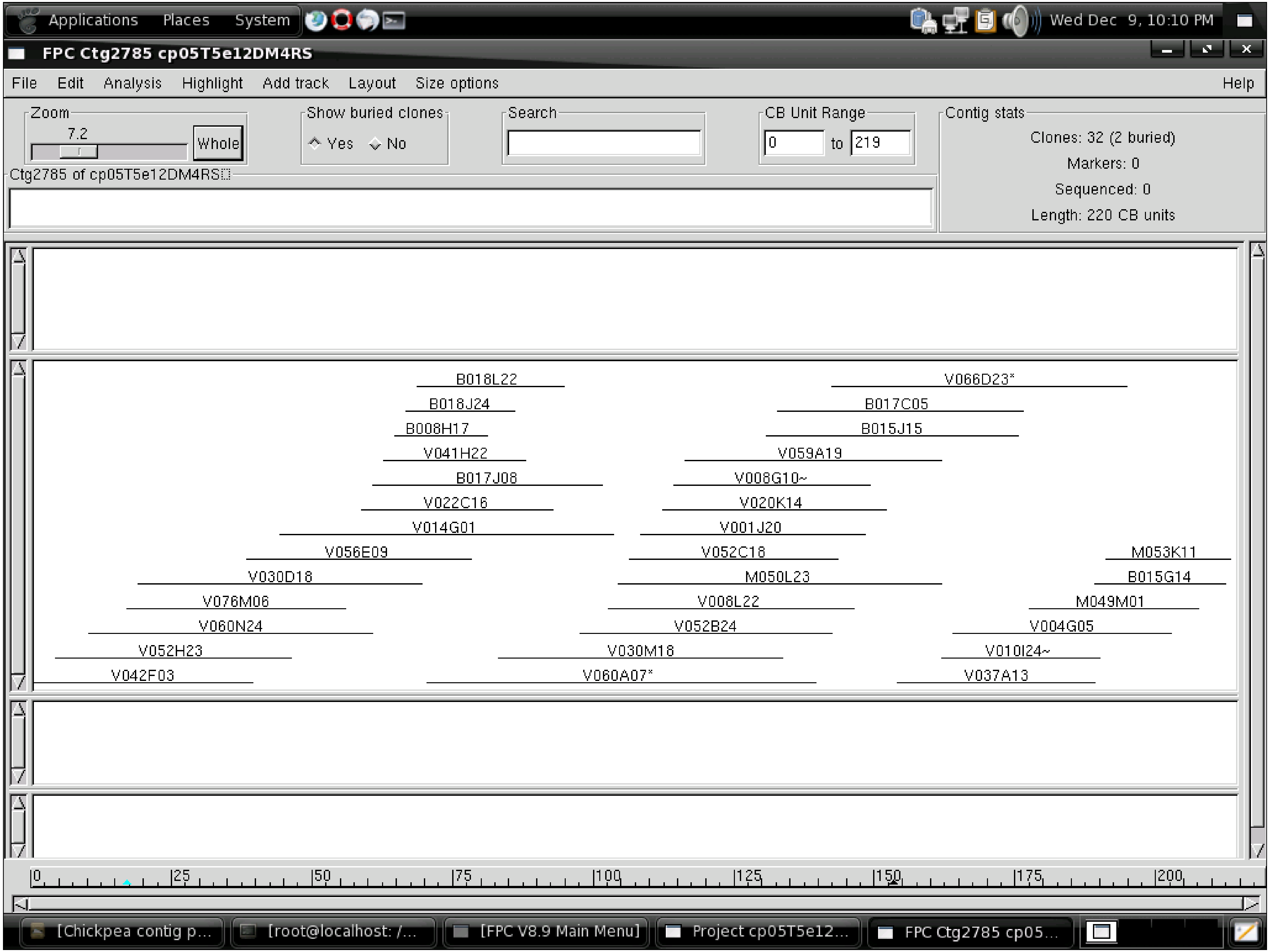
Applications Places System
Wed Dec 9, 10:10 PM
FPC Ctg2785 cp05T5e12DM4RS
File Edit Analysis Highlight Add track Layout Size options Help
Zoom
7.2
Whole
Show buried clones
Yes No
Search
CB Unit Range
0 to 219
Contig stats
Clones: 32 (2 buried)
Markers: 0
Sequenced: 0
Length: 220 CB units
Ctg2785 of cp05T5e12DM4RS
B018L22
V066D23*
B018J24
B017C05
B008H17
B015J15
V041H22
V059A19
B017J08
V008G10~
V022C16
V020K14
V014G01
V001J20
V056E09
V052C18
M053K11
V030D18
M050L23
B015G14
V076M06
V008L22
M049M01
V060N24
V052B24
V004G05
V052H23
V030M18
V010I24~
V042F03
V060A07*
V037A13
0 25 50 75 100 125 150 175 200
[Chickpea contig p...
[root@localhost: /...
[FPC V8.9 Main Menu]
Project cp05T5e12...
FPC Ctg2785 cp05...

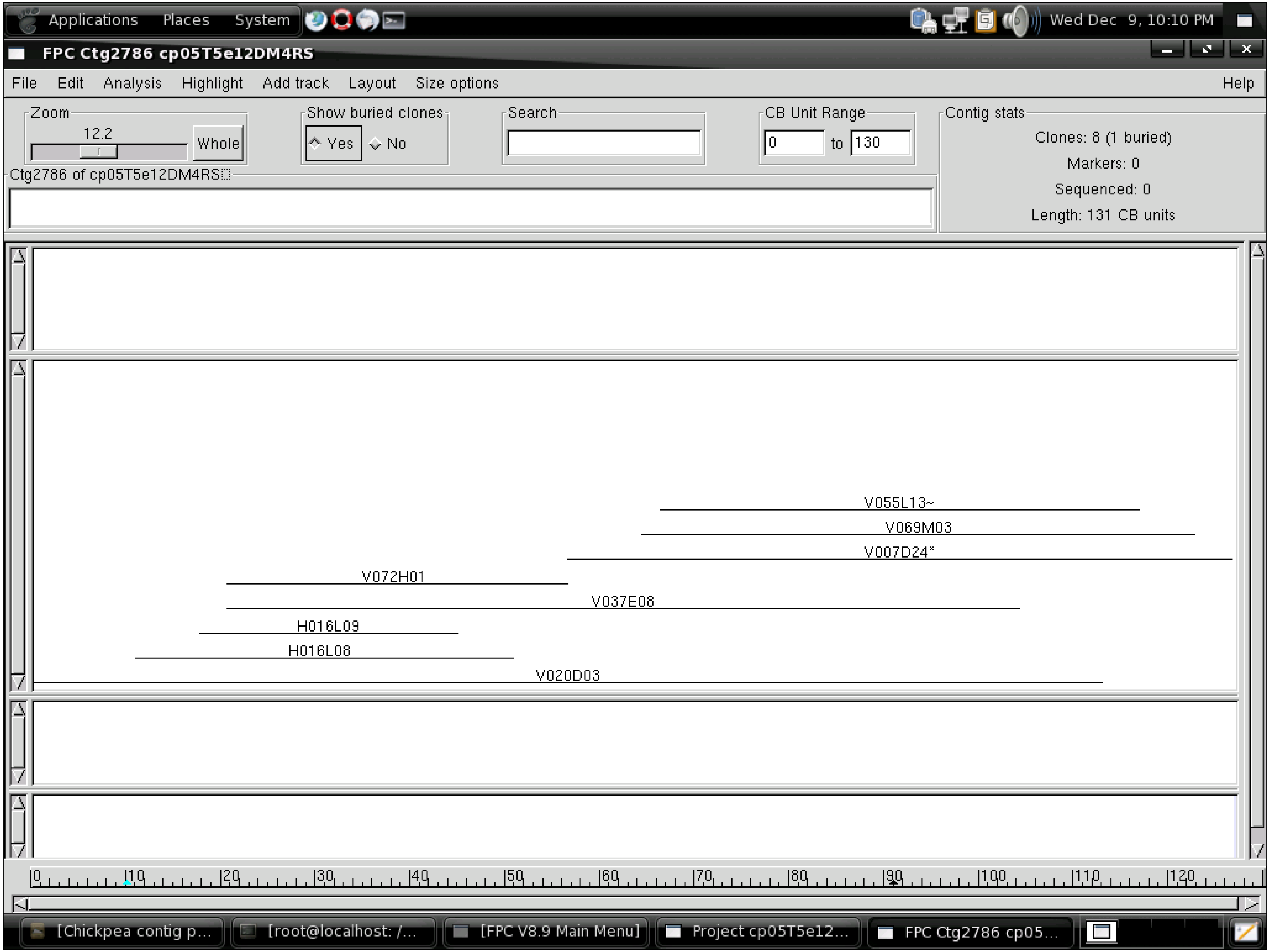
Applications Places System
Wed Dec 9, 10:10 PM
FPC Ctg2786 cp05T5e12DM4RS
File Edit Analysis Highlight Add track Layout Size options Help
Zoom
12.2
Whole
Show buried clones
Yes No
Search
CB Unit Range
0 to 130
Contig stats
Clones: 8 (1 buried)
Markers: 0
Sequenced: 0
Length: 131 CB units
Ctg2786 of cp05T5e12DM4RS
V055L13~
V069M03
V007D24*
V072H01
V037E08
H016L09
H016L08
V020D03
0 10 20 30 40 50 60 70 80 90 100 110 120
[Chickpea contig p...
[root@localhost: /...
[FPC V8.9 Main Menu]
Project cp05T5e12...
FPC Ctg2786 cp05...

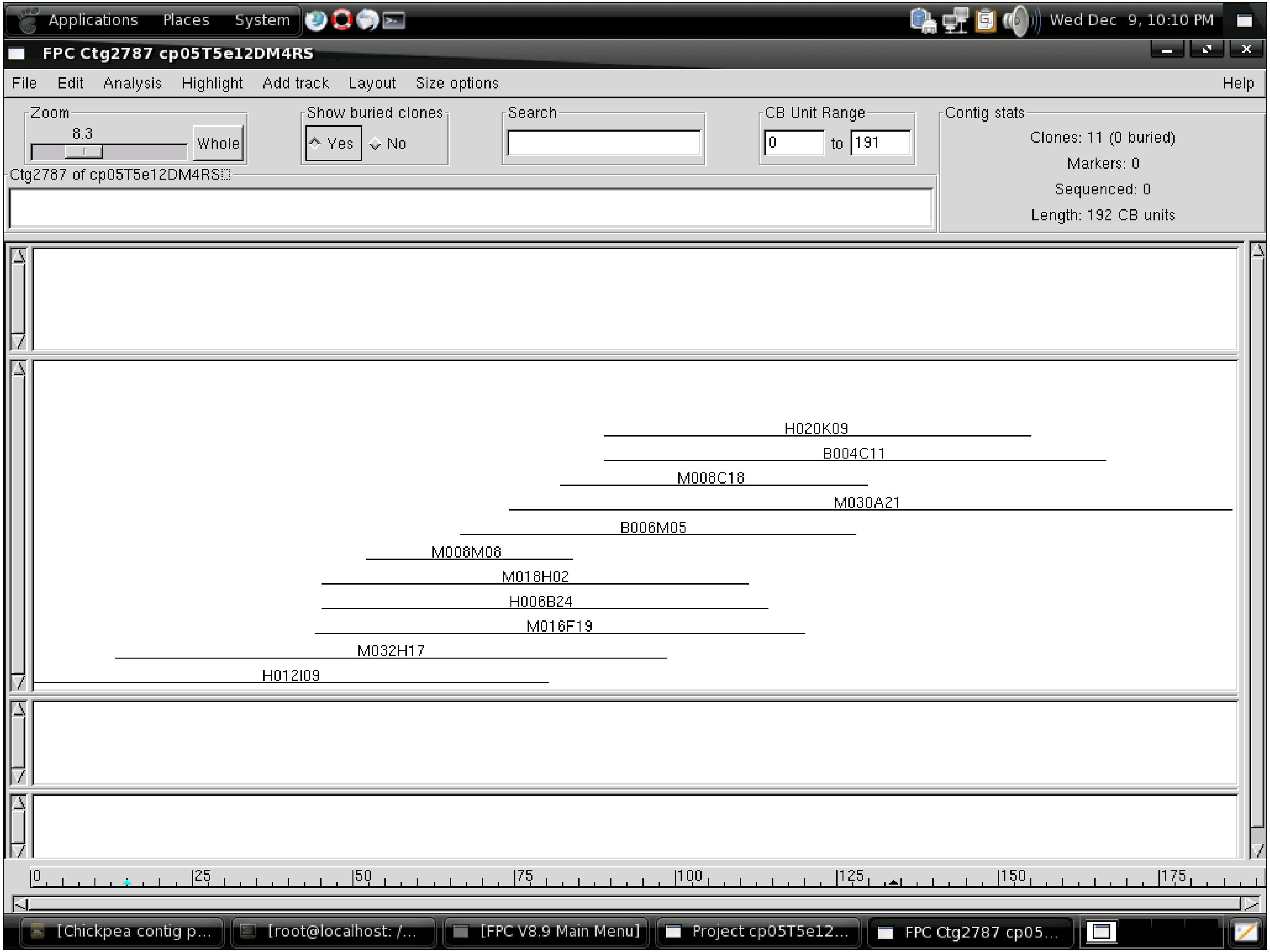
Applications Places System
Wed Dec 9, 10:10 PM
FPC Ctg2787 cp05T5e12DM4RS
File Edit Analysis Highlight Add track Layout Size options Help
Zoom
8.3
Whole
Show buried clones
Yes
No
Search
CB Unit Range
0
to
191
Contig stats
Clones: 11 (0 buried)
Markers: 0
Sequenced: 0
Length: 192 CB units
Ctg2787 of cp05T5e12DM4RS
H020K09
B004C11
M008C18
M030A21
B006M05
M008M08
M018H02
H006B24
M016F19
M032H17
H012I09
0
25
50
75
100
125
150
175
[Chickpea contig p...
[root@localhost: /...
[FPC V8.9 Main Menu]
Project cp05T5e12...
FPC Ctg2787 cp05...

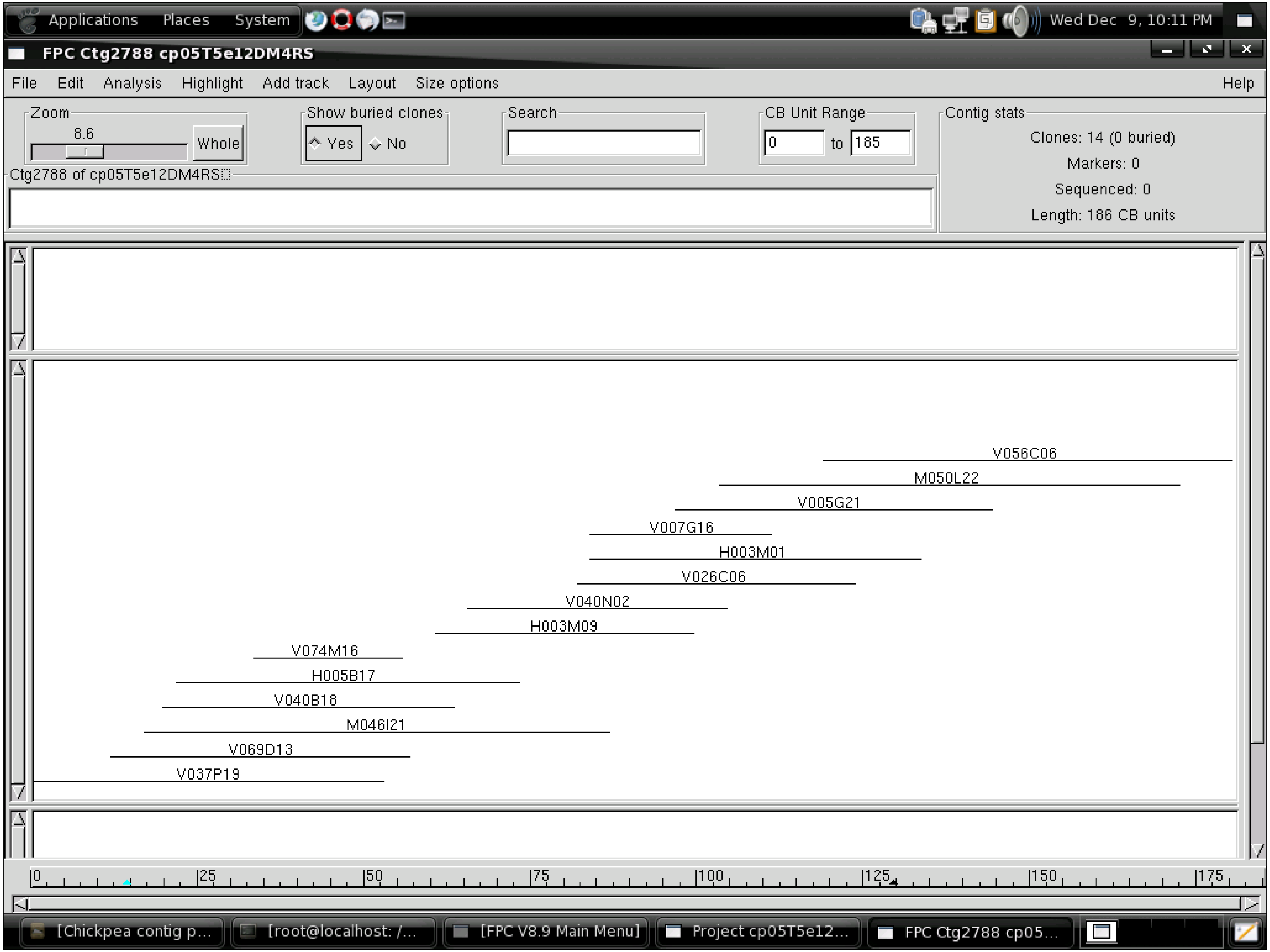
Applications Places System
Wed Dec 9, 10:11 PM
FPC Ctg2788 cp05T5e12DM4RS
File Edit Analysis Highlight Add track Layout Size options Help
Zoom
8.6
Whole
Show buried clones
Yes No
Search
CB Unit Range
0 to 185
Contig stats
Clones: 14 (0 buried)
Markers: 0
Sequenced: 0
Length: 186 CB units
Ctg2788 of cp05T5e12DM4RS
V056C06
M050L22
V005G21
V007G16
H003M01
V026C06
V040N02
H003M09
V074M16
H005B17
V040B18
M046I21
V069D13
V037P19
0 25 50 75 100 125 150 175
[Chickpea contig p...
[root@localhost: /...
[FPC V8.9 Main Menu]
Project cp05T5e12...
FPC Ctg2788 cp05...

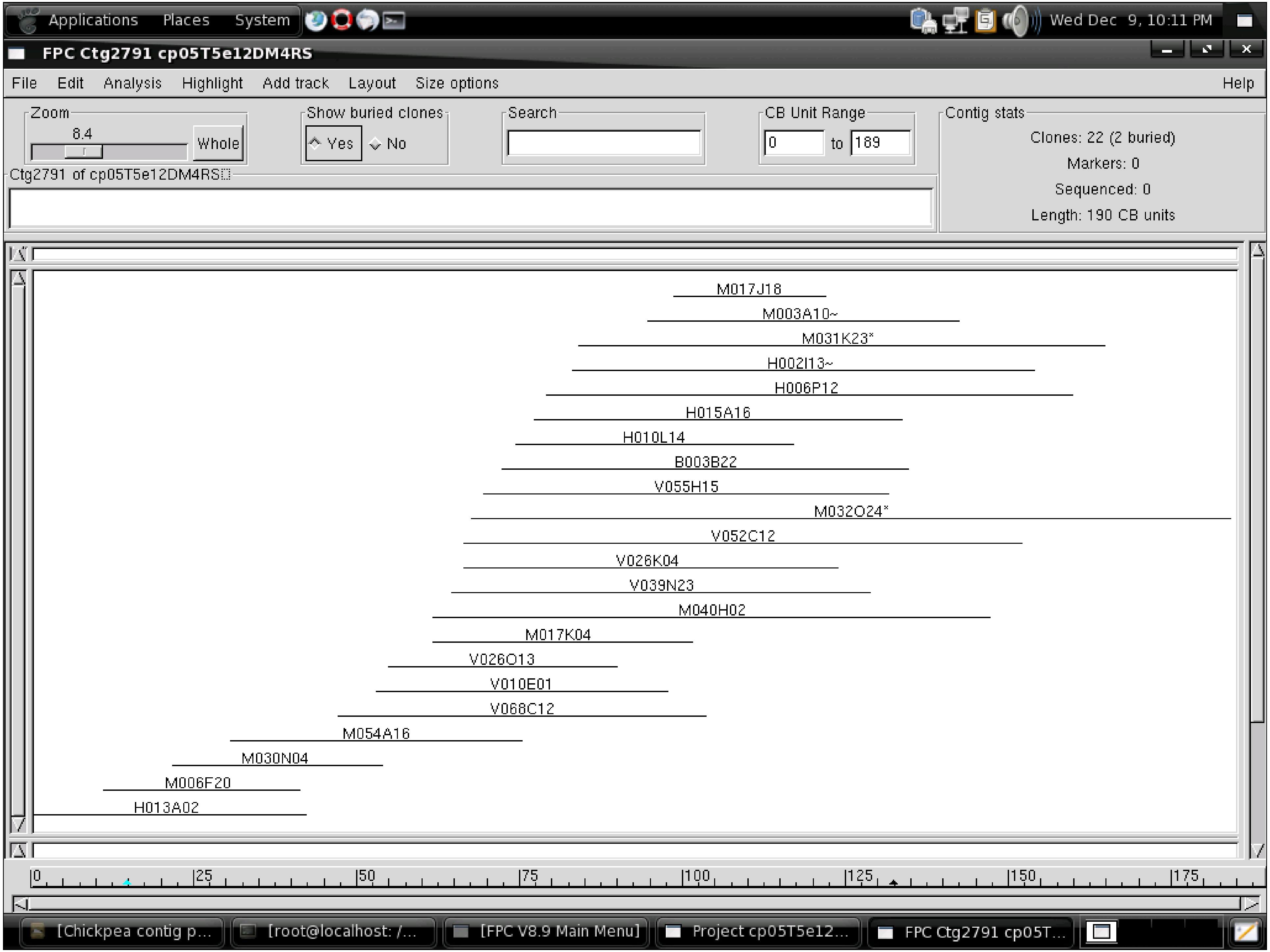
Applications Places System
Wed Dec 9, 10:11 PM
FPC Ctg2791 cp05T5e12DM4RS
File Edit Analysis Highlight Add track Layout Size options Help
Zoom
8.4
Whole
Show buried clones
Yes No
Search
CB Unit Range
0 to 189
Contig stats
Clones: 22 (2 buried)
Markers: 0
Sequenced: 0
Length: 190 CB units
Ctg2791 of cp05T5e12DM4RS
M017J18
M003A10~
M031K23*
H002I13~
H006P12
H015A16
H010L14
B003B22
V055H15
M032O24*
V052C12
V026K04
V039N23
M040H02
M017K04
V026O13
V010E01
V068C12
M054A16
M030N04
M006F20
H013A02
0
25
50
75
100
125
150
175
[Chickpea contig p...
[root@localhost: /...
[FPC V8.9 Main Menu]
Project cp05T5e12...
FPC Ctg2791 cp05T...

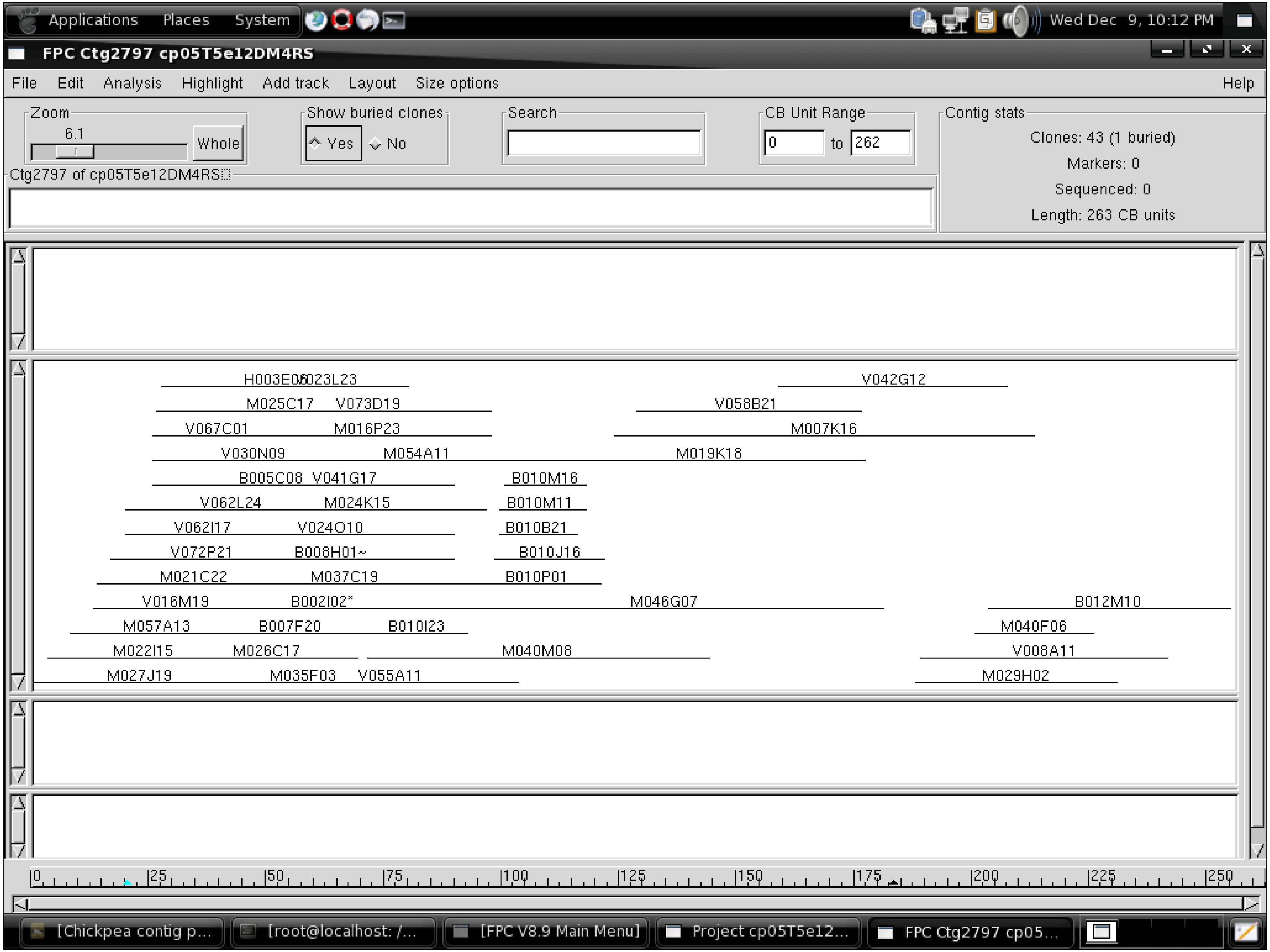
Applications Places System
Wed Dec 9, 10:12 PM
FPC Ctg2797 cp05T5e12DM4RS
File Edit Analysis Highlight Add track Layout Size options Help
Zoom
6.1
Whole
Show buried clones
Yes No
Search
CB Unit Range
0 to 262
Contig stats
Clones: 43 (1 buried)
Markers: 0
Sequenced: 0
Length: 263 CB units
Ctg2797 of cp05T5e12DM4RS
V042G12
M025C17 V073D19
V058B21
V067C01 M016P23
M007K16
V030N09 M054A11
M019K18
B005C08 V041G17
B010M16
V062L24 M024K15
B010M11
V062I17 V024O10
B010B21
V072P21 B008H01~
B010J16
M021C22 M037C19
B010P01
V016M19 B002I02*
M046G07
B012M10
M057A13 B007F20 B010I23
M040F06
M022I15 M026C17
M040M08
V008A11
M027J19 M035F03 V055A11
M029H02
0 25 50 75 100 125 150 175 200 225 250
[Chickpea contig p...
[root@localhost: /...
[FPC V8.9 Main Menu]
Project cp05T5e12...
FPC Ctg2797 cp05...

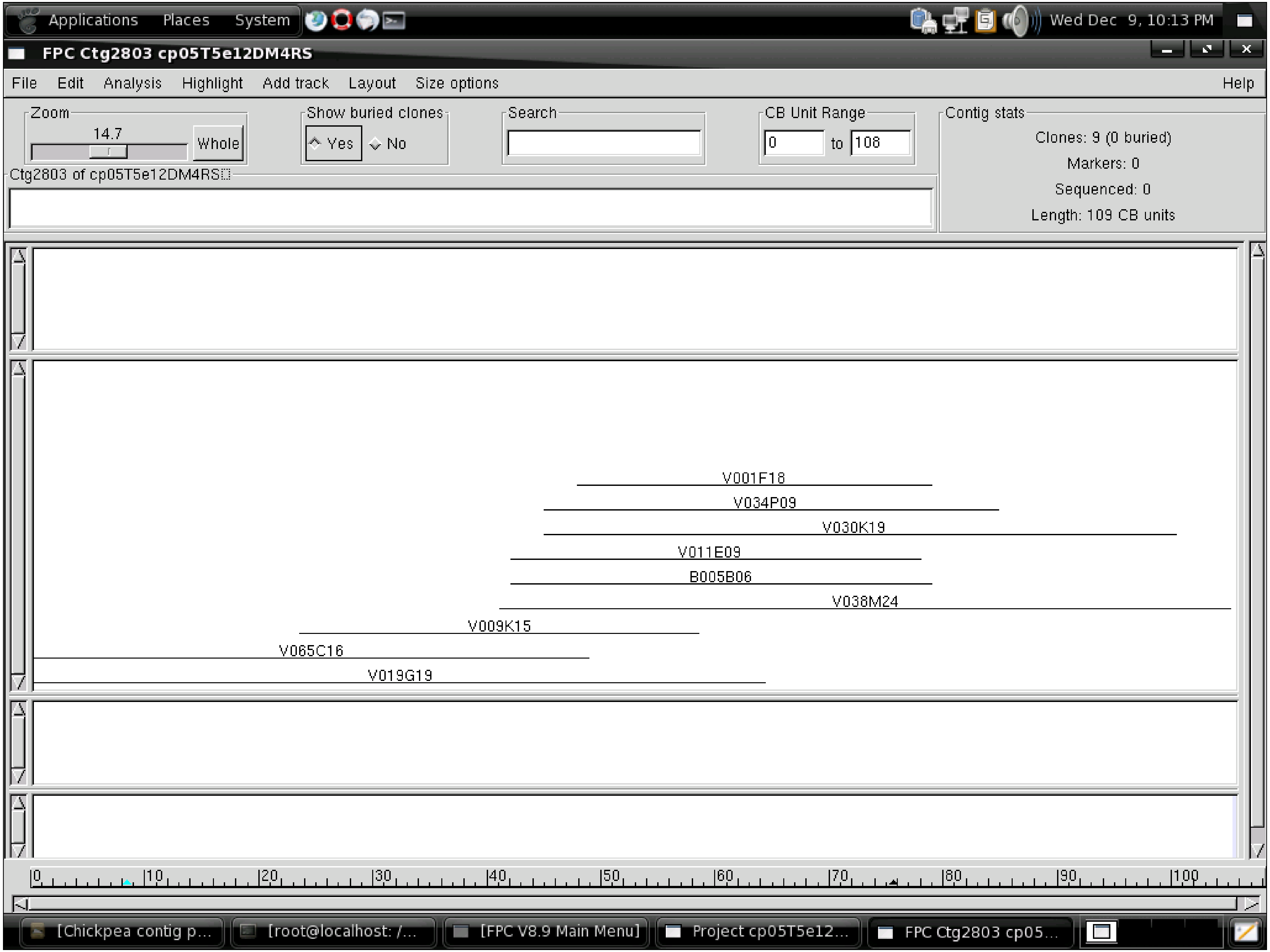
Applications Places System
Wed Dec 9, 10:13 PM
FPC Ctg2803 cp05T5e12DM4RS
File Edit Analysis Highlight Add track Layout Size options Help
Zoom
14.7
Whole
Show buried clones
Yes No
Search
CB Unit Range
0 to 108
Contig stats
Clones: 9 (0 buried)
Markers: 0
Sequenced: 0
Length: 109 CB units
Ctg2803 of cp05T5e12DM4RS
V001F18
V034P09
V030K19
V011E09
B005B06
V038M24
V009K15
V065C16
V019G19
0 10 20 30 40 50 60 70 80 90 100
[Chickpea contig p...
[root@localhost: /...
[FPC V8.9 Main Menu]
Project cp05T5e12...
FPC Ctg2803 cp05...

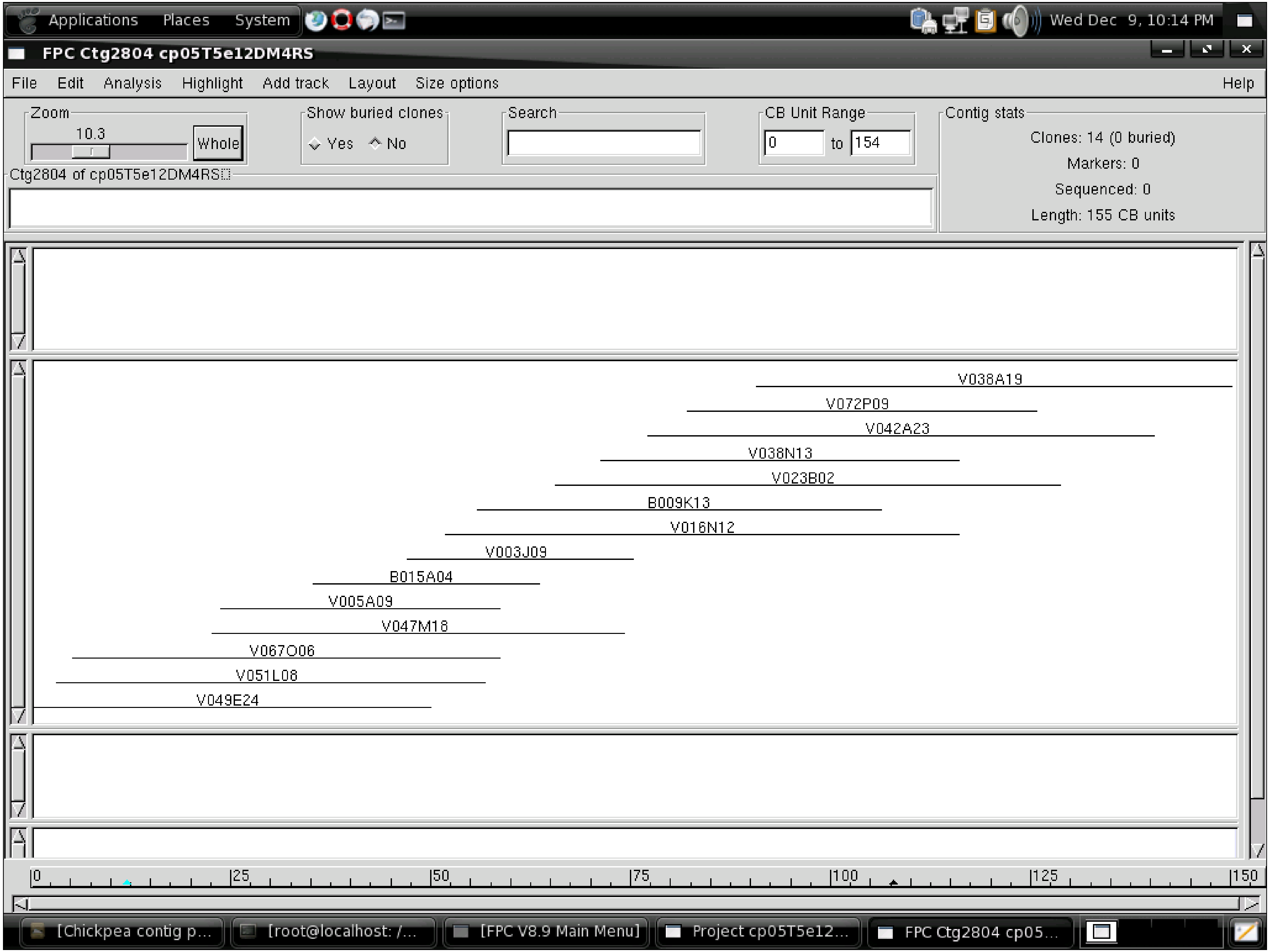
Applications Places System
Wed Dec 9, 10:14 PM
FPC Ctg2804 cp05T5e12DM4RS
File Edit Analysis Highlight Add track Layout Size options Help
Zoom
10.3
Whole
Show buried clones
Yes No
Search
CB Unit Range
0 to 154
Contig stats
Clones: 14 (0 buried)
Markers: 0
Sequenced: 0
Length: 155 CB units
Ctg2804 of cp05T5e12DM4RS
V038A19
V072P09
V042A23
V038N13
V023B02
B009K13
V016N12
V003J09
B015A04
V005A09
V047M18
V067O06
V051L08
V049E24
0 25 50 75 100 125 150
[Chickpea contig p...
[root@localhost: /...
[FPC V8.9 Main Menu]
Project cp05T5e12...
FPC Ctg2804 cp05...

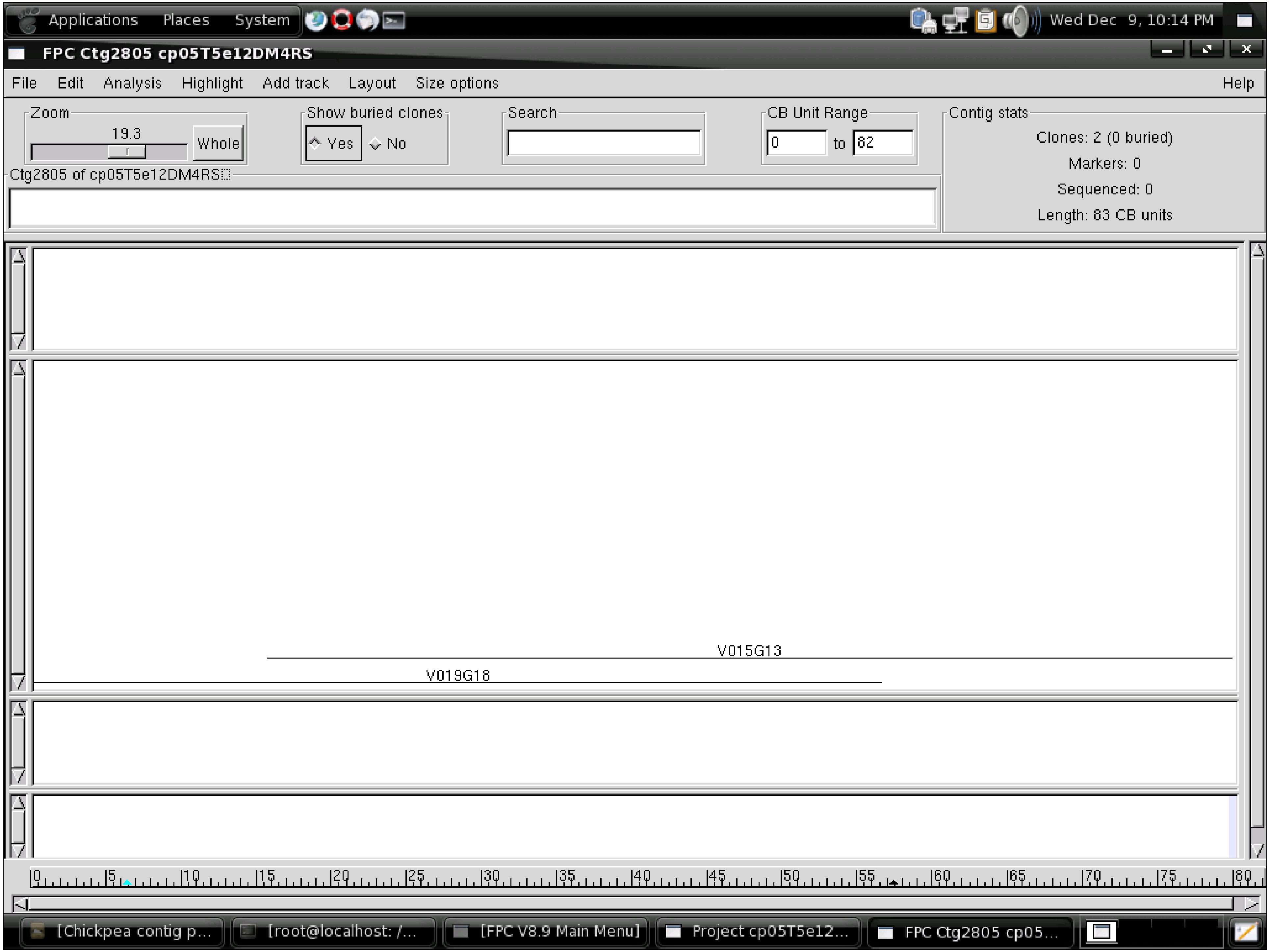
Applications Places System
Wed Dec 9, 10:14 PM
FPC Ctg2805 cp05T5e12DM4RS
File Edit Analysis Highlight Add track Layout Size options Help
Zoom
19.3
Whole
Show buried clones
Yes No
Search
CB Unit Range
0 to 82
Contig stats
Clones: 2 (0 buried)
Markers: 0
Sequenced: 0
Length: 83 CB units
Ctg2805 of cp05T5e12DM4RS
V015G13
V019G18
0 5 10 15 20 25 30 35 40 45 50 55 60 65 70 75 80
[Chickpea contig p...
[root@localhost: /...
[FPC V8.9 Main Menu]
Project cp05T5e12...
FPC Ctg2805 cp05...

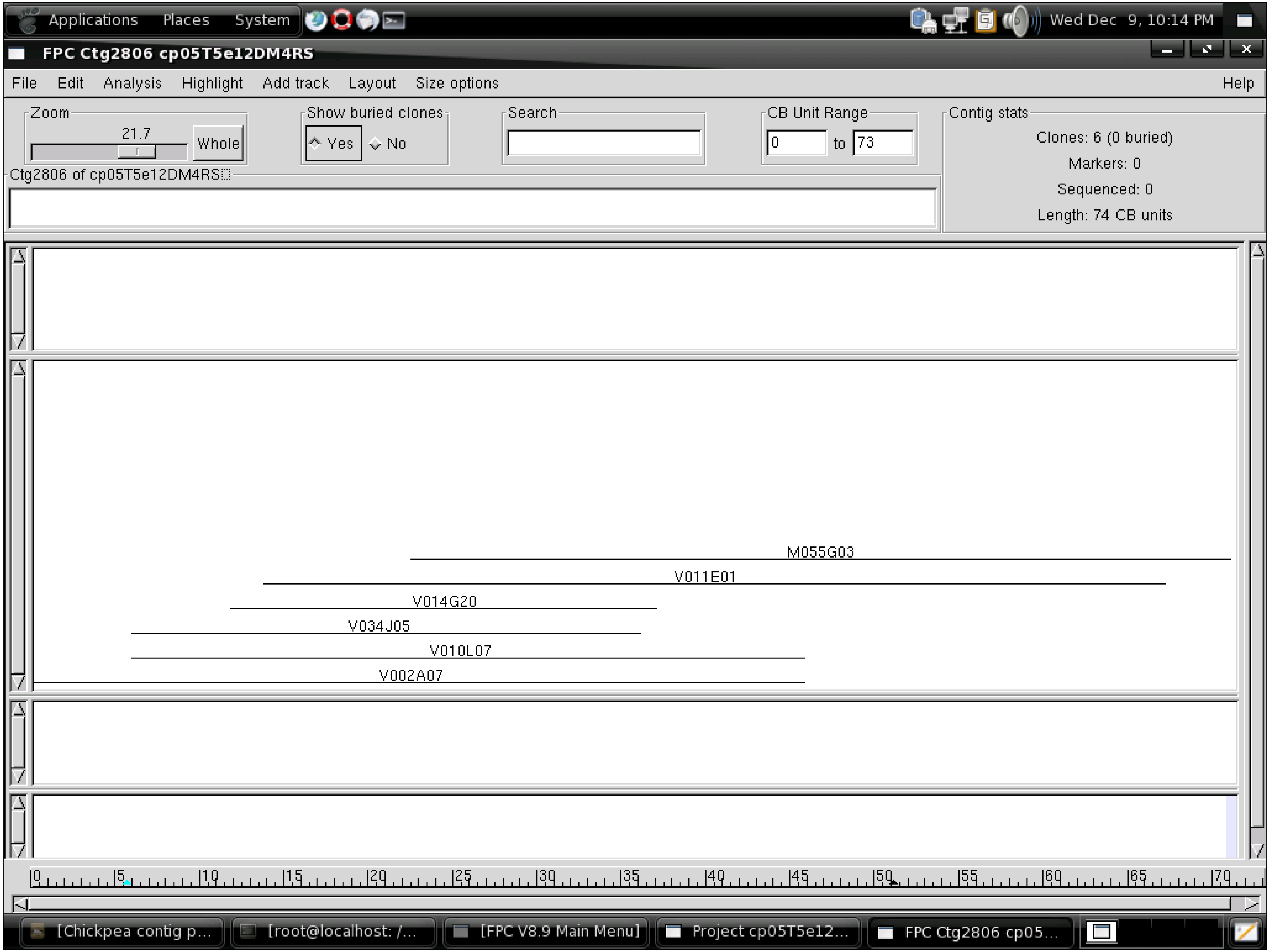
Applications Places System
Wed Dec 9, 10:14 PM
FPC Ctg2806 cp05T5e12DM4RS
File Edit Analysis Highlight Add track Layout Size options
Help
Zoom
21.7
Whole
Show buried clones
Yes
No
Search
CB Unit Range
0
to
73
Contig stats
Clones: 6 (0 buried)
Markers: 0
Sequenced: 0
Length: 74 CB units
Ctg2806 of cp05T5e12DM4RS
M055G03
V011E01
V014G20
V034J05
V010L07
V002A07
0 5 10 15 20 25 30 35 40 45 50 55 60 65 70
[Chickpea contig p...
[root@localhost: /...
[FPC V8.9 Main Menu]
Project cp05T5e12...
FPC Ctg2806 cp05...

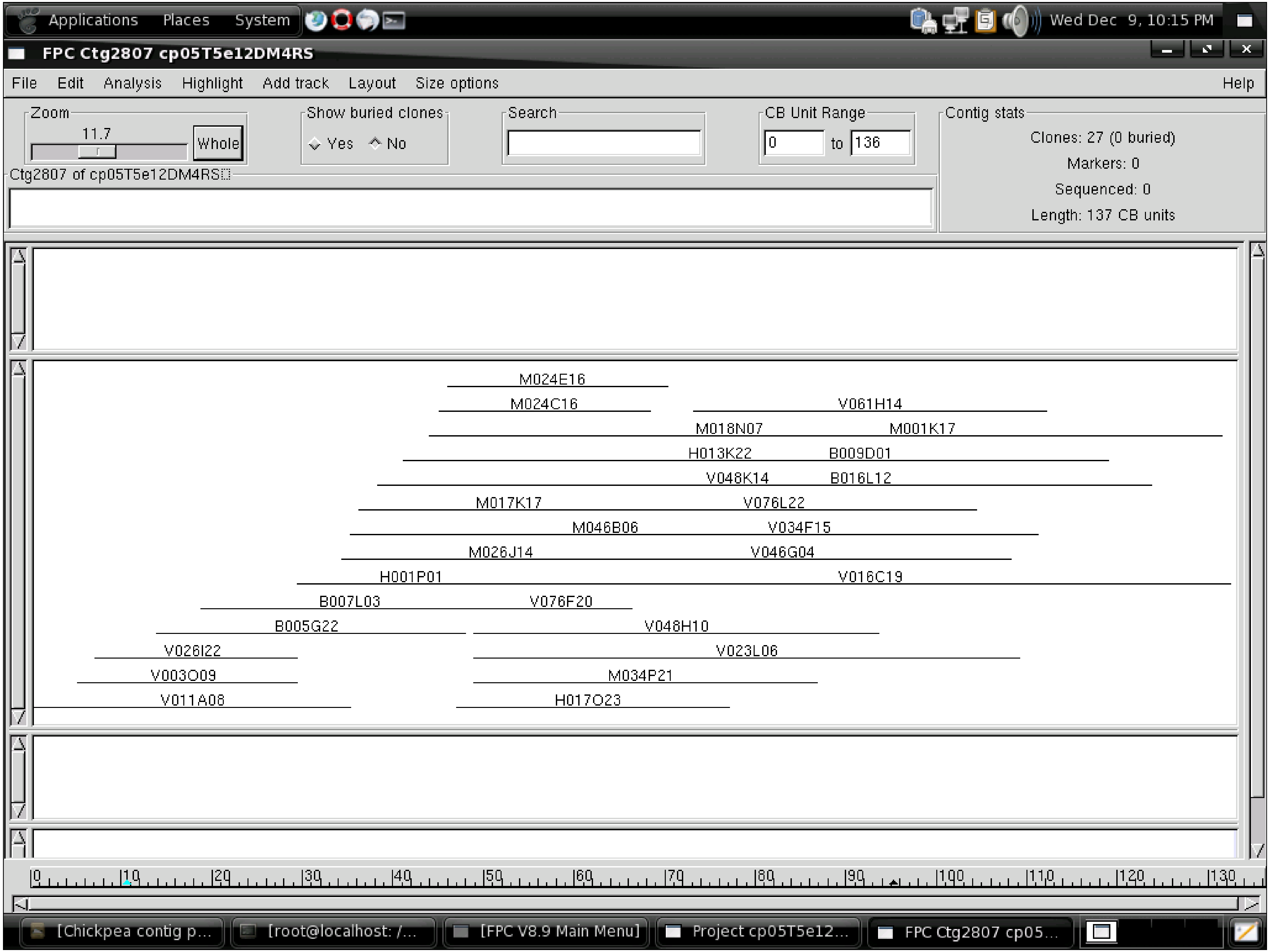
Applications Places System
Wed Dec 9, 10:15 PM
FPC Ctg2807 cp05T5e12DM4RS
File Edit Analysis Highlight Add track Layout Size options Help
Zoom
11.7
Whole
Show buried clones
Yes No
Search
CB Unit Range
0 to 136
Contig stats
Clones: 27 (0 buried)
Markers: 0
Sequenced: 0
Length: 137 CB units
Ctg2807 of cp05T5e12DM4RS
M024E16
M024C16
V061H14
M018N07
M001K17
H013K22
B009D01
V048K14
B016L12
M017K17
V076L22
M046B06
V034F15
M026J14
V046G04
H001P01
V016C19
B007L03
V076F20
B005G22
V048H10
V026I22
V023L06
V003O09
M034P21
V011A08
H017O23
0 10 20 30 40 50 60 70 80 90 100 110 120 130
[Chickpea contig p...
[root@localhost: /...
[FPC V8.9 Main Menu]
Project cp05T5e12...
FPC Ctg2807 cp05...

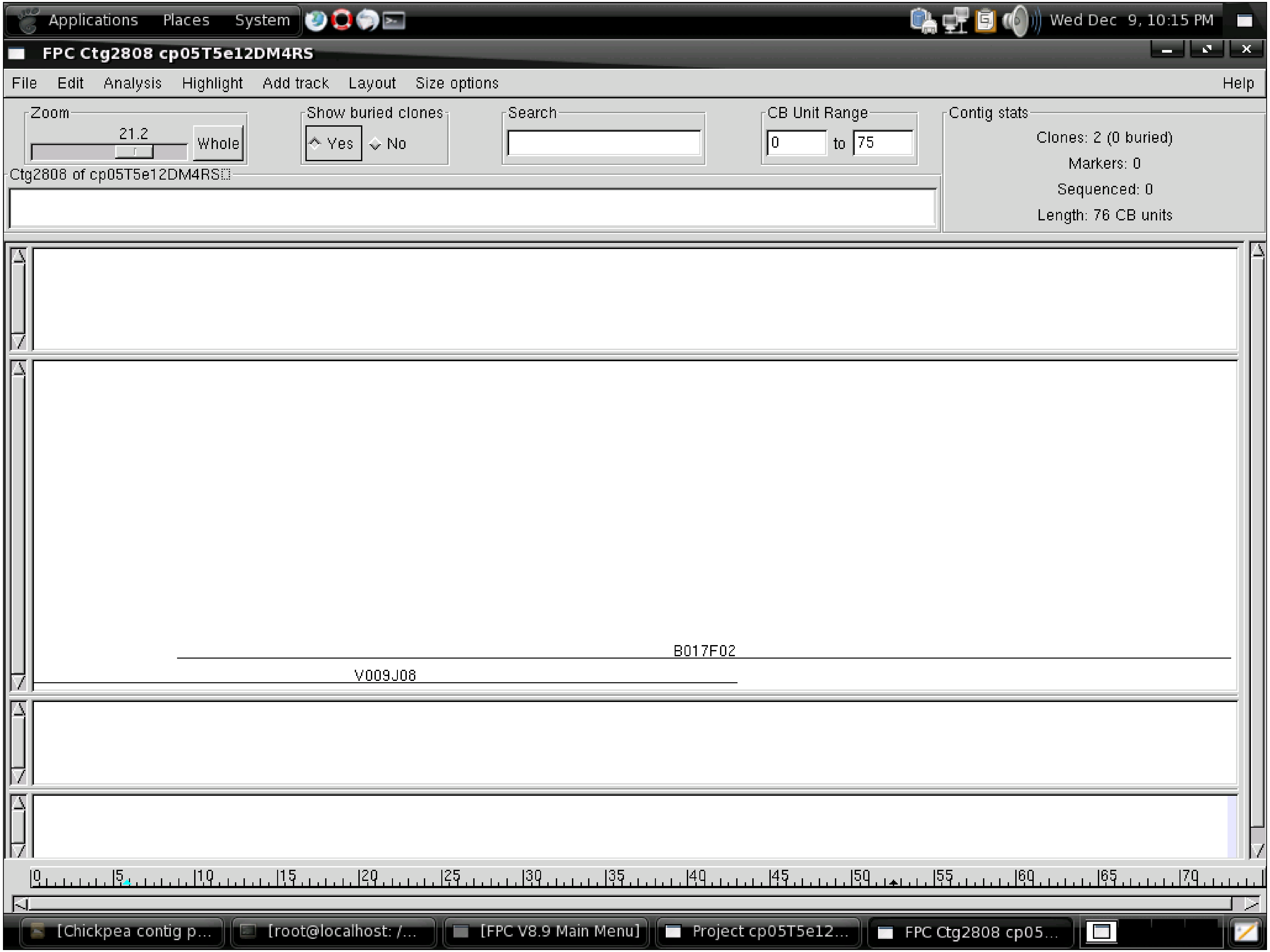
Applications Places System
Wed Dec 9, 10:15 PM
FPC Ctg2808 cp05T5e12DM4RS
File Edit Analysis Highlight Add track Layout Size options Help
Zoom
21.2
Whole
Show buried clones
Yes No
Search
CB Unit Range
0 to 75
Contig stats
Clones: 2 (0 buried)
Markers: 0
Sequenced: 0
Length: 76 CB units
Ctg2808 of cp05T5e12DM4RS
B017F02
V009J08
0 5 10 15 20 25 30 35 40 45 50 55 60 65 70
[Chickpea contig p...
[root@localhost: /...
[FPC V8.9 Main Menu]
Project cp05T5e12...
FPC Ctg2808 cp05...

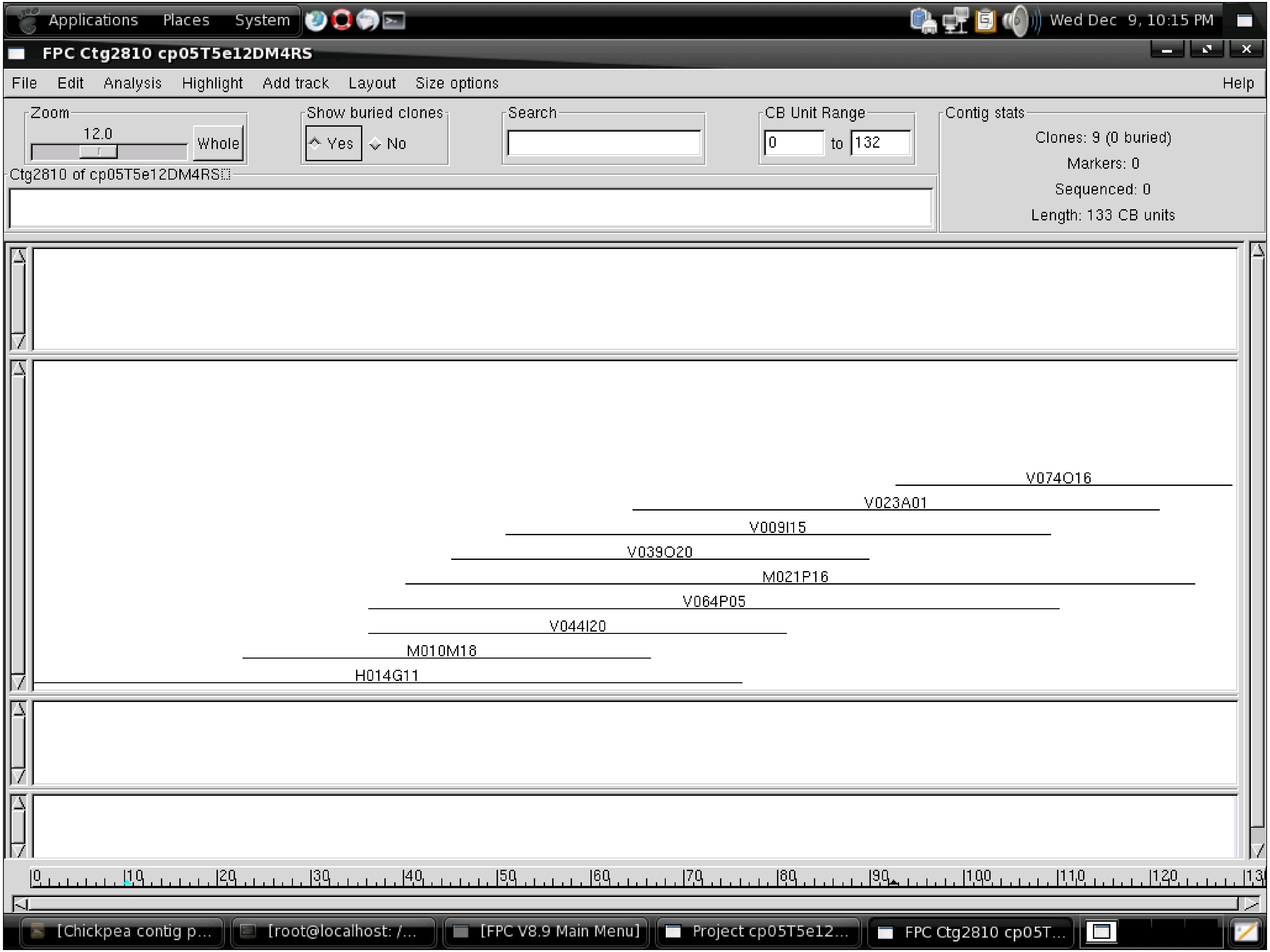
Applications Places System
Wed Dec 9, 10:15 PM
FPC Ctg2810 cp05T5e12DM4RS
File Edit Analysis Highlight Add track Layout Size options Help
Zoom
12.0
Whole
Show buried clones
Yes No
Search
CB Unit Range
0 to 132
Contig stats
Clones: 9 (0 buried)
Markers: 0
Sequenced: 0
Length: 133 CB units
Ctg2810 of cp05T5e12DM4RS
V074O16
V023A01
V009I15
V039O20
M021P16
V064P05
V044I20
M010M18
H014G11
0 10 20 30 40 50 60 70 80 90 100 110 120
[Chickpea contig p...
[root@localhost: /...
[FPC V8.9 Main Menu]
Project cp05T5e12...
FPC Ctg2810 cp05T...

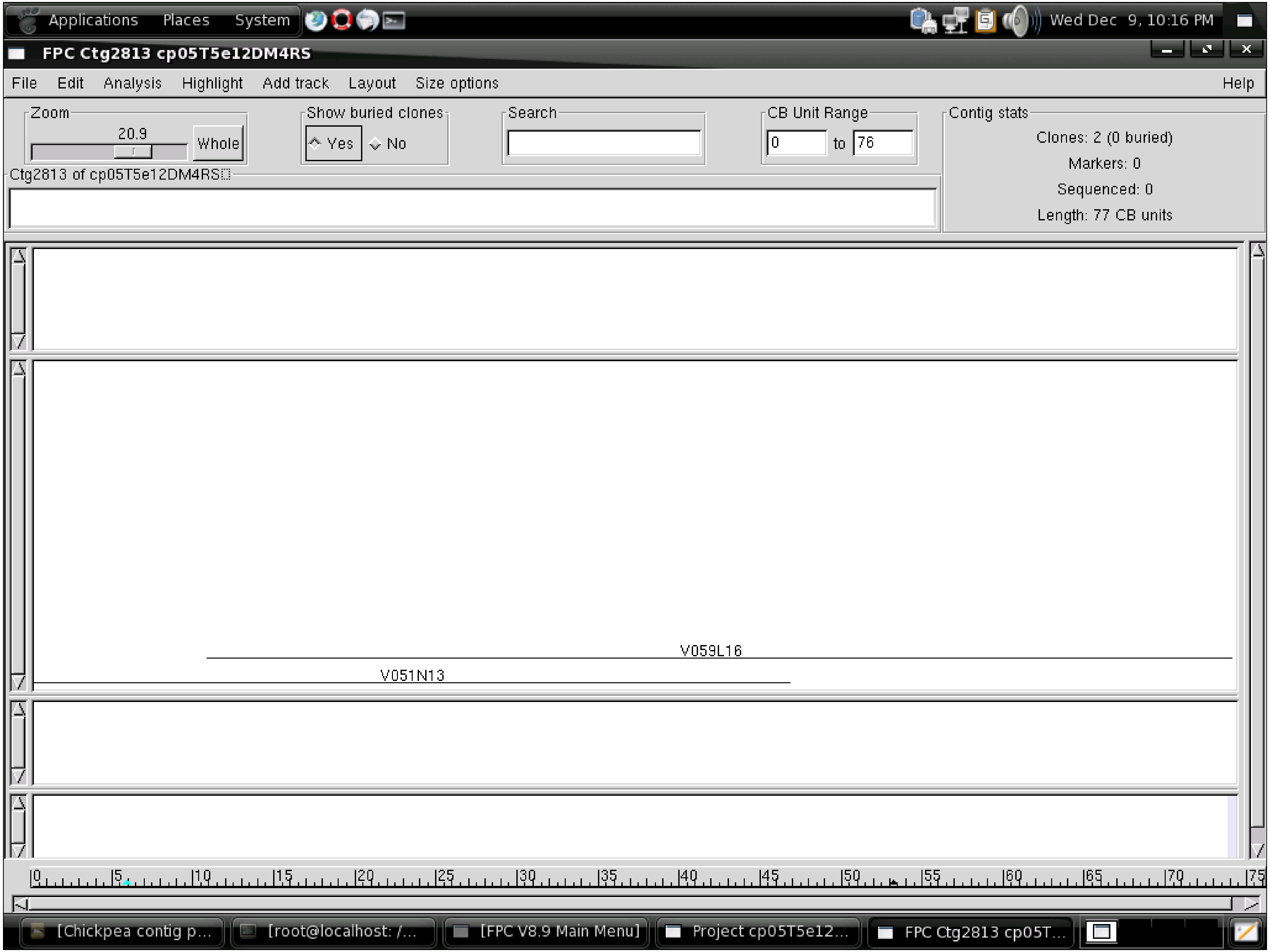
Applications Places System
Wed Dec 9, 10:16 PM
FPC Ctg2813 cp05T5e12DM4RS
File Edit Analysis Highlight Add track Layout Size options Help
Zoom
20.9
Whole
Show buried clones
Yes
No
Search
CB Unit Range
0
to
76
Contig stats
Clones: 2 (0 buried)
Markers: 0
Sequenced: 0
Length: 77 CB units
Ctg2813 of cp05T5e12DM4RS
V059L16
V051N13
0 5 10 15 20 25 30 35 40 45 50 55 60 65 70 75
[Chickpea contig p...
[root@localhost: /...
[FPC V8.9 Main Menu]
Project cp05T5e12...
FPC Ctg2813 cp05T...

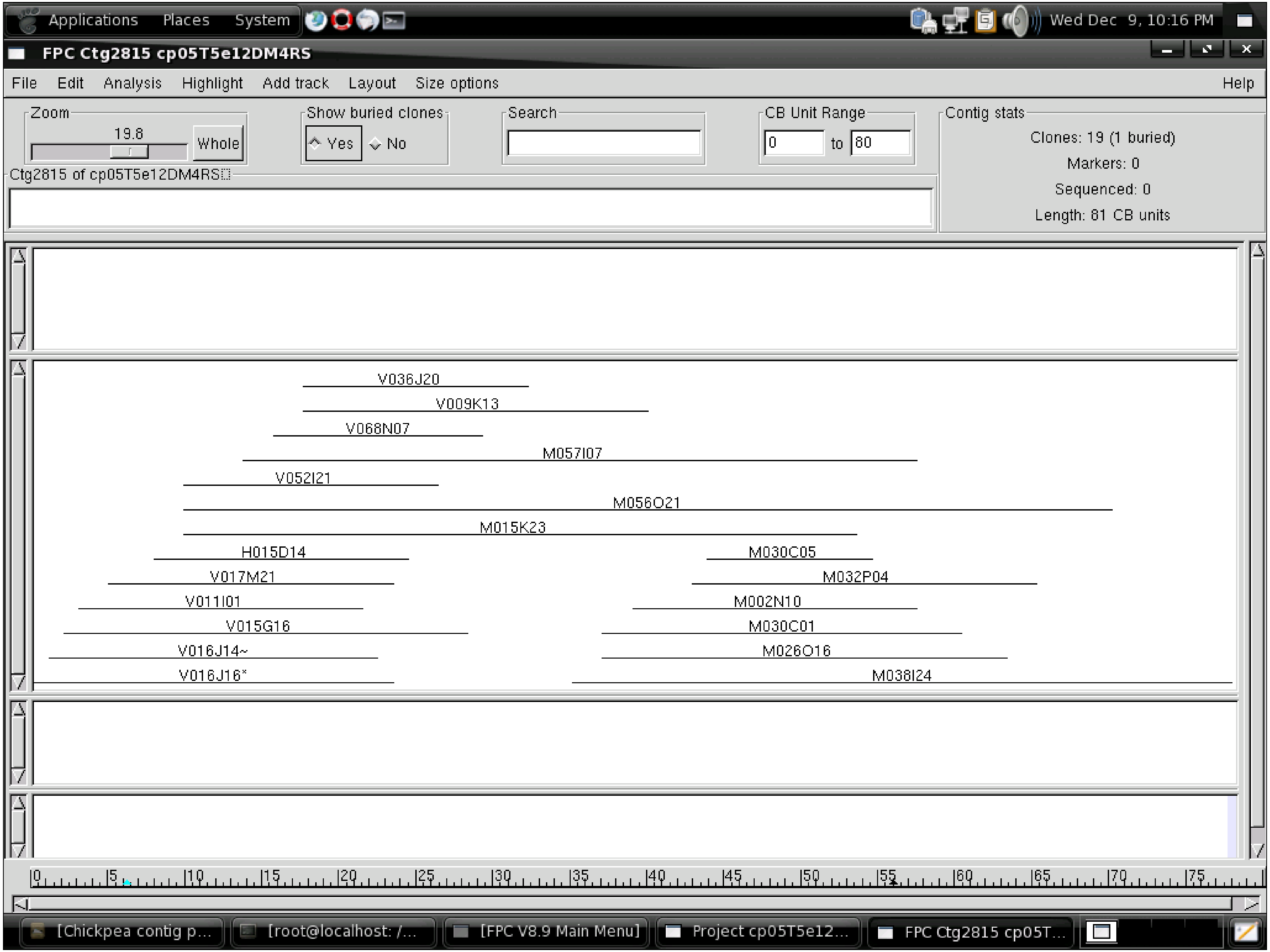
Applications Places System
Wed Dec 9, 10:16 PM
FPC Ctg2815 cp05T5e12DM4RS
File Edit Analysis Highlight Add track Layout Size options Help
Zoom
19.8
Whole
Show buried clones
Yes
No
Search
CB Unit Range
0 to 80
Contig stats
Clones: 19 (1 buried)
Markers: 0
Sequenced: 0
Length: 81 CB units
Ctg2815 of cp05T5e12DM4RS
V036J20
V009K13
V068N07
M057I07
V052I21
M056O21
M015K23
H015D14
M030C05
V017M21
M032P04
V011I01
M002N10
V015G16
M030C01
V016J14~
M026O16
V016J16*
M038I24
0 5 10 15 20 25 30 35 40 45 50 55 60 65 70 75
[Chickpea contig p...
[root@localhost: /...
[FPC V8.9 Main Menu]
Project cp05T5e12...
FPC Ctg2815 cp05T...

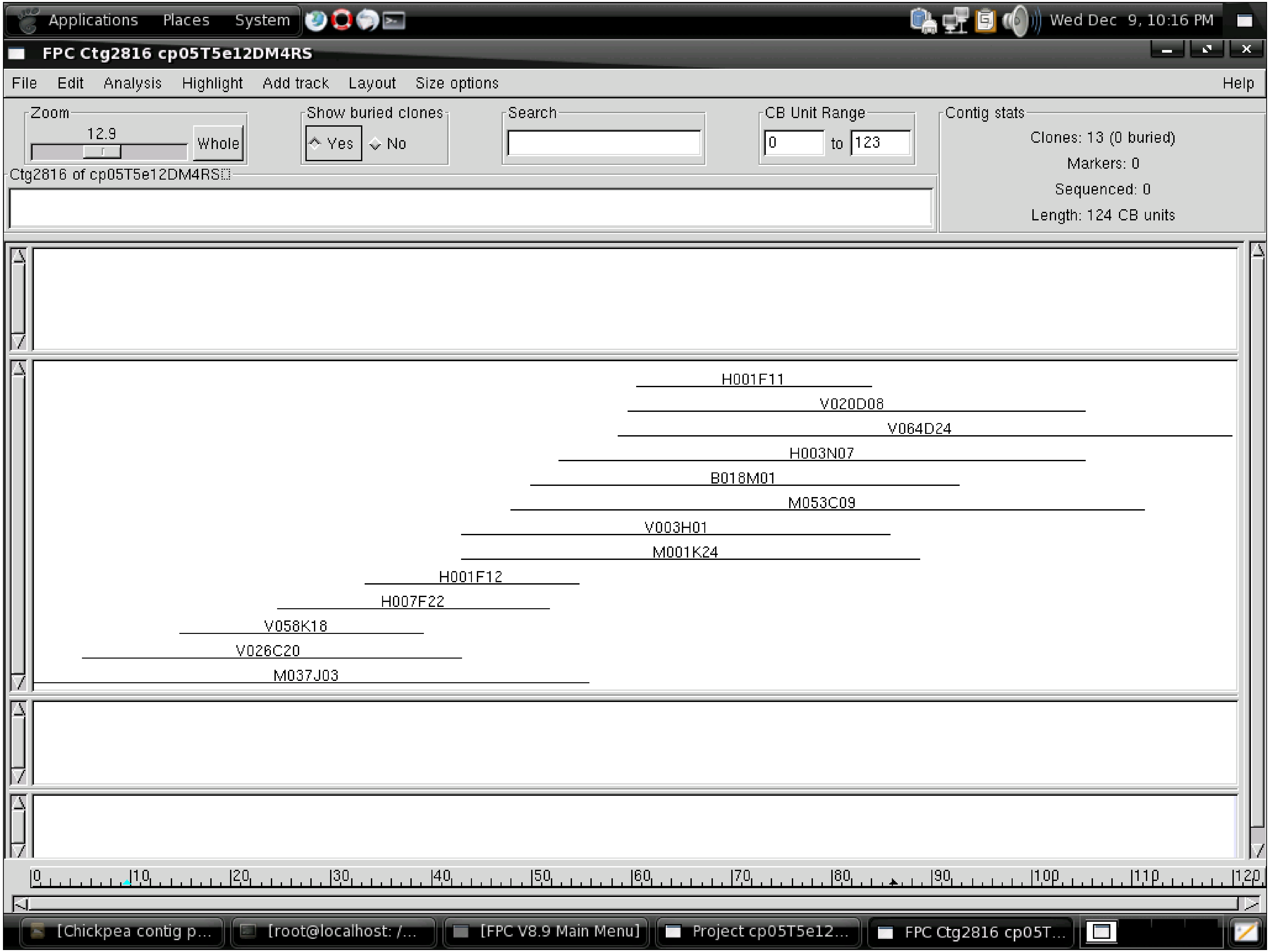
Applications Places System
Wed Dec 9, 10:16 PM
FPC Ctg2816 cp05T5e12DM4RS
File Edit Analysis Highlight Add track Layout Size options Help
Zoom
12.9
Whole
Show buried clones
Yes
No
Search
CB Unit Range
0
to
123
Contig stats
Clones: 13 (0 buried)
Markers: 0
Sequenced: 0
Length: 124 CB units
Ctg2816 of cp05T5e12DM4RS
H001F11
V020D08
V064D24
H003N07
B018M01
M053C09
V003H01
M001K24
H001F12
H007F22
V058K18
V026C20
M037J03
0 10 20 30 40 50 60 70 80 90 100 110 120
[Chickpea contig p...
[root@localhost: /...
[FPC V8.9 Main Menu]
Project cp05T5e12...
FPC Ctg2816 cp05T...

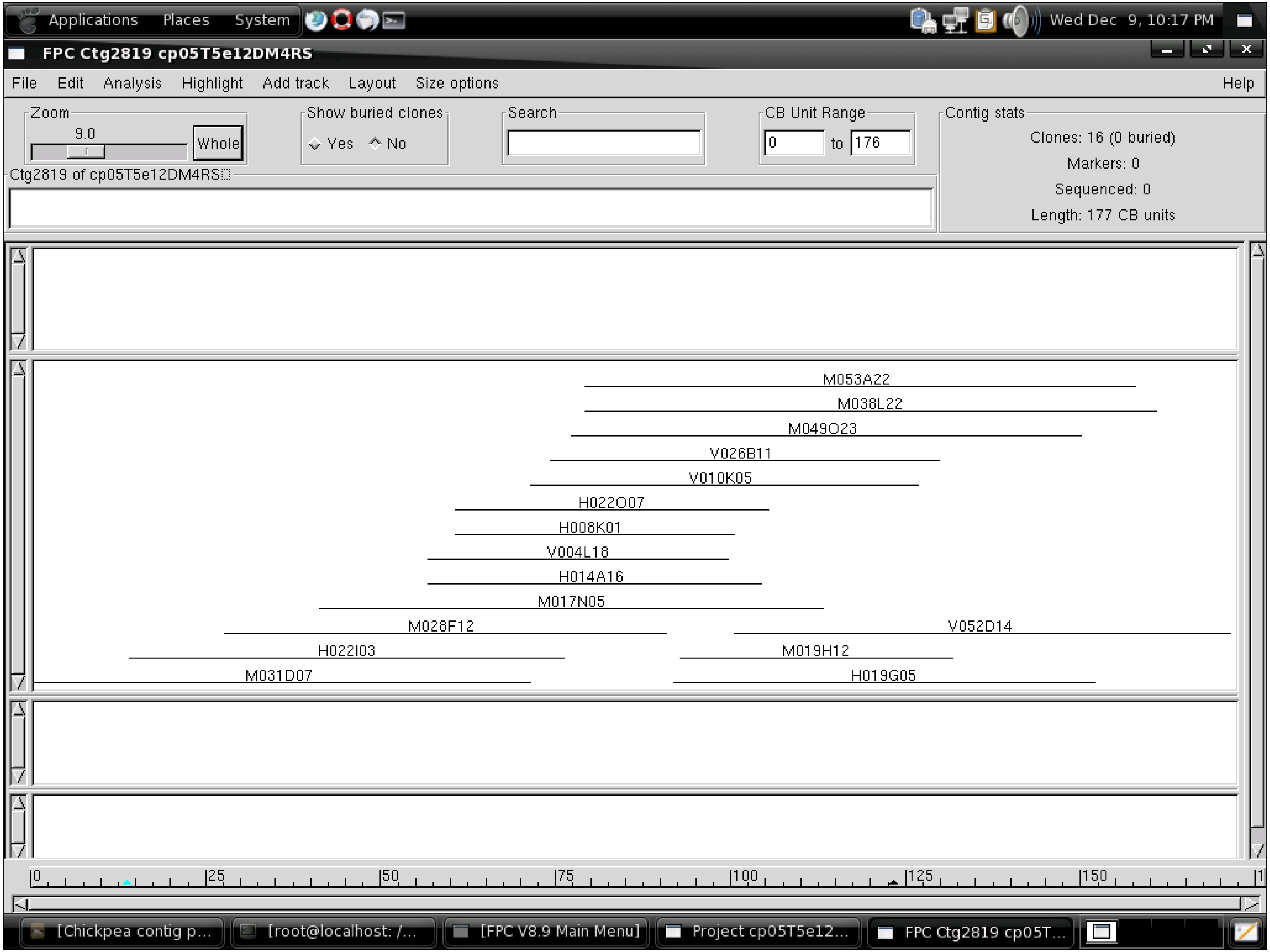
Applications Places System
Wed Dec 9, 10:17 PM
FPC Ctg2819 cp05T5e12DM4RS
File Edit Analysis Highlight Add track Layout Size options Help
Zoom
9.0
Whole
Show buried clones
Yes No
Search
CB Unit Range
0 to 176
Contig stats
Clones: 16 (0 buried)
Markers: 0
Sequenced: 0
Length: 177 CB units
Ctg2819 of cp05T5e12DM4RS
M053A22
M038L22
M049O23
V026B11
V010K05
H022O07
H008K01
V004L18
H014A16
M017N05
M028F12
V052D14
H022I03
M019H12
M031D07
H019G05
0 25 50 75 100 125 150
[Chickpea contig p...
[root@localhost: /...
[FPC V8.9 Main Menu]
Project cp05T5e12...
FPC Ctg2819 cp05T...

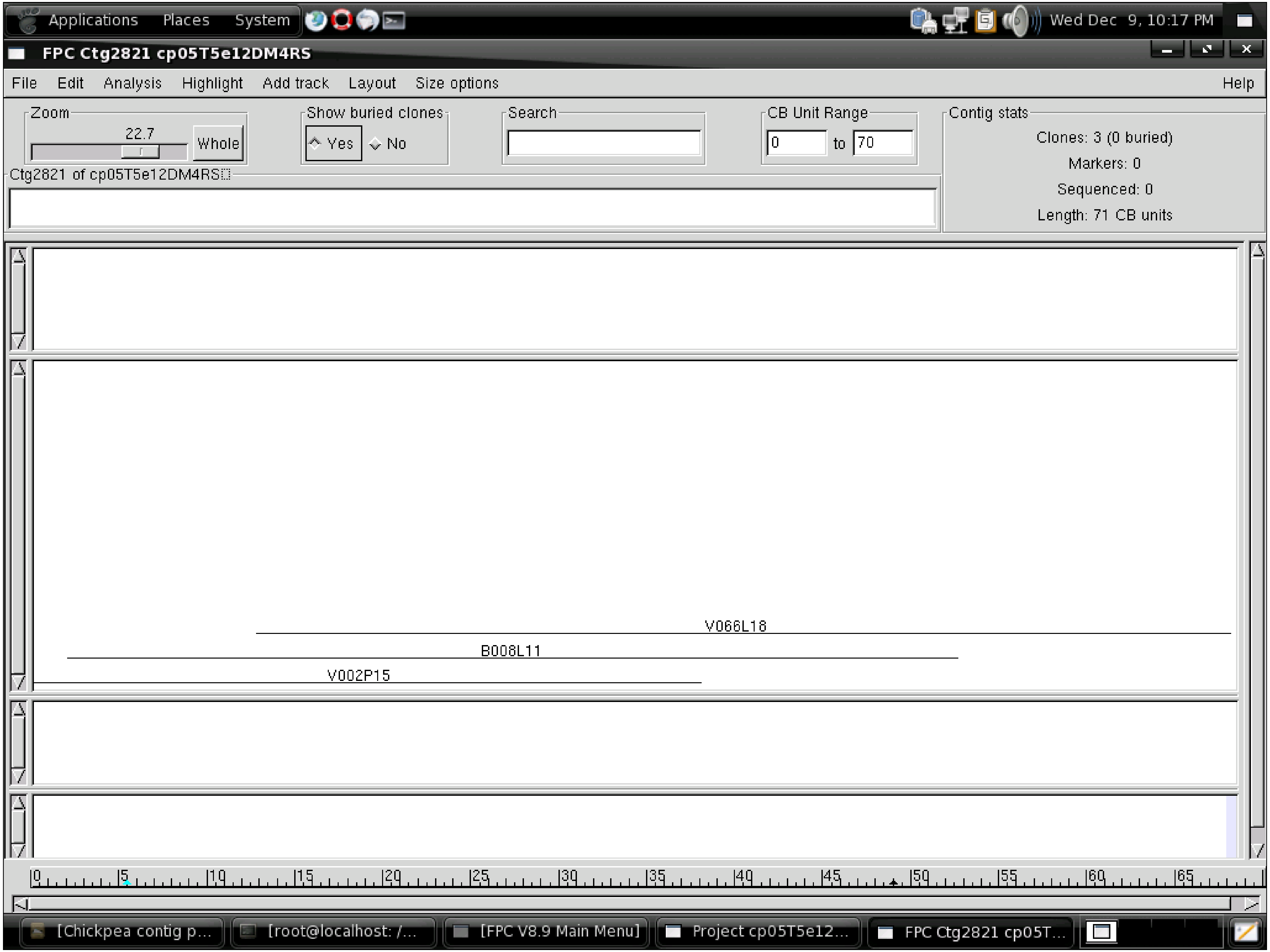
Applications Places System
Wed Dec 9, 10:17 PM
FPC Ctg2821 cp05T5e12DM4RS
File Edit Analysis Highlight Add track Layout Size options Help
Zoom
22.7
Whole
Show buried clones
Yes
No
Search
CB Unit Range
0
to
70
Contig stats
Clones: 3 (0 buried)
Markers: 0
Sequenced: 0
Length: 71 CB units
Ctg2821 of cp05T5e12DM4RS
V066L18
B008L11
V002P15
0 5 10 15 20 25 30 35 40 45 50 55 60 65
[Chickpea contig p...
[root@localhost: /...
[FPC V8.9 Main Menu]
Project cp05T5e12...
FPC Ctg2821 cp05T...

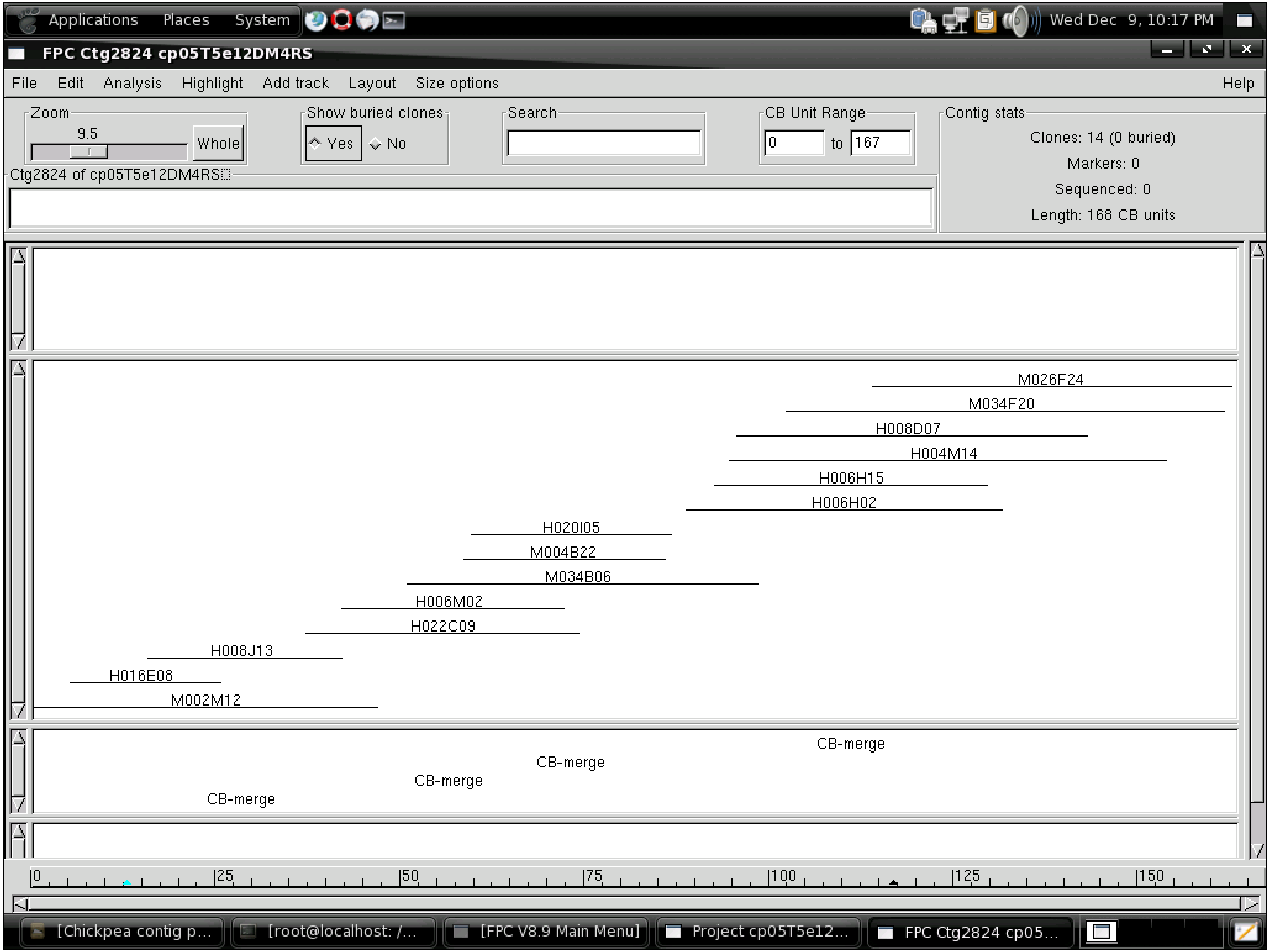

Applications Places System
Wed Dec 9, 10:17 PM
FPC Ctg2824 cp05T5e12DM4RS
File Edit Analysis Highlight Add track Layout Size options Help
Zoom 9.5 Whole
Show buried clones Yes No
Search
CB Unit Range 0 to 167
Contig stats
Clones: 14 (0 buried)
Markers: 0
Sequenced: 0
Length: 168 CB units
Ctg2824 of cp05T5e12DM4RS
M026F24
M034F20
H008D07
H004M14
H006H15
H006H02
H020I05
M004B22
M034B06
H006M02
H022C09
H008J13
H016E08
M002M12
CB-merge
CB-merge
CB-merge
CB-merge
0 25 50 75 100 125 150
[Chickpea contig p...
[root@localhost: /...
[FPC V8.9 Main Menu]
Project cp05T5e12...
FPC Ctg2824 cp05...

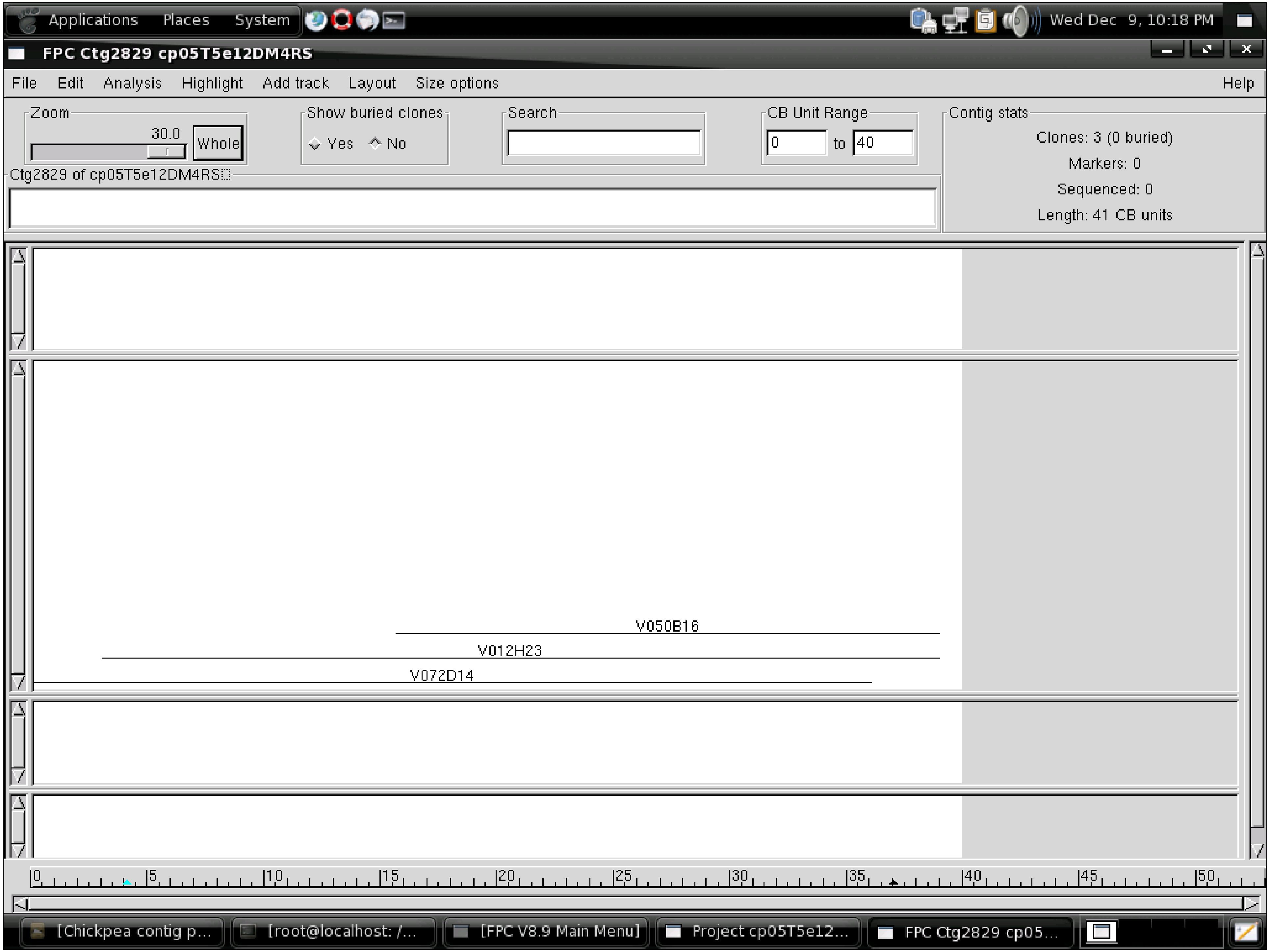
Applications Places System
Wed Dec 9, 10:18 PM
FPC Ctg2829 cp05T5e12DM4RS
File Edit Analysis Highlight Add track Layout Size options Help
Zoom
30.0
Whole
Show buried clones
Yes No
Search
CB Unit Range
0 to 40
Contig stats
Clones: 3 (0 buried)
Markers: 0
Sequenced: 0
Length: 41 CB units
Ctg2829 of cp05T5e12DM4RS
V050B16
V012H23
V072D14
0 5 10 15 20 25 30 35 40 45 50
[Chickpea contig p...
[root@localhost: /...
[FPC V8.9 Main Menu]
Project cp05T5e12...
FPC Ctg2829 cp05...

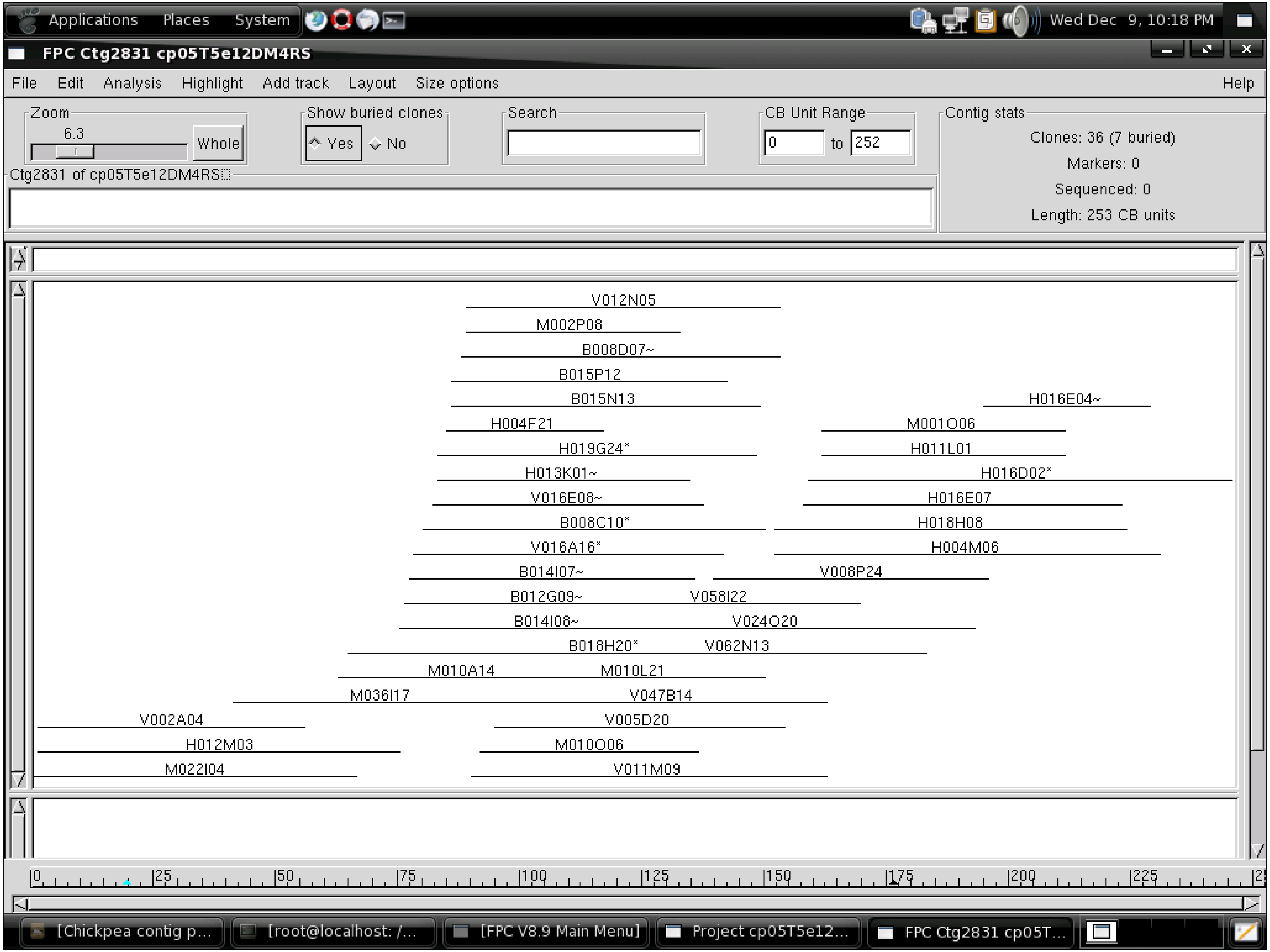
Applications Places System
Wed Dec 9, 10:18 PM
FPC Ctg2831 cp05T5e12DM4RS
File Edit Analysis Highlight Add track Layout Size options Help
Zoom
6.3
Whole
Show buried clones
Yes No
Search
CB Unit Range
0 to 252
Contig stats
Clones: 36 (7 buried)
Markers: 0
Sequenced: 0
Length: 253 CB units
Ctg2831 of cp05T5e12DM4RS
V012N05
M002P08
B008D07~
B015P12
B015N13
H016E04~
H004F21
M001O06
H019G24*
H011L01
H013K01~
H016D02*
V016E08~
H016E07
B008C10*
H018H08
V016A16*
H004M06
B014I07~
V008P24
B012G09~
V058I22
B014I08~
V024O20
B018H20*
V062N13
M010A14
M010L21
M036I17
V047B14
V002A04
V005D20
H012M03
M010O06
M022I04
V011M09
0 25 50 75 100 125 150 175 200 225
[Chickpea contig p...
[root@localhost: /...
[FPC V8.9 Main Menu]
Project cp05T5e12...
FPC Ctg2831 cp05T...

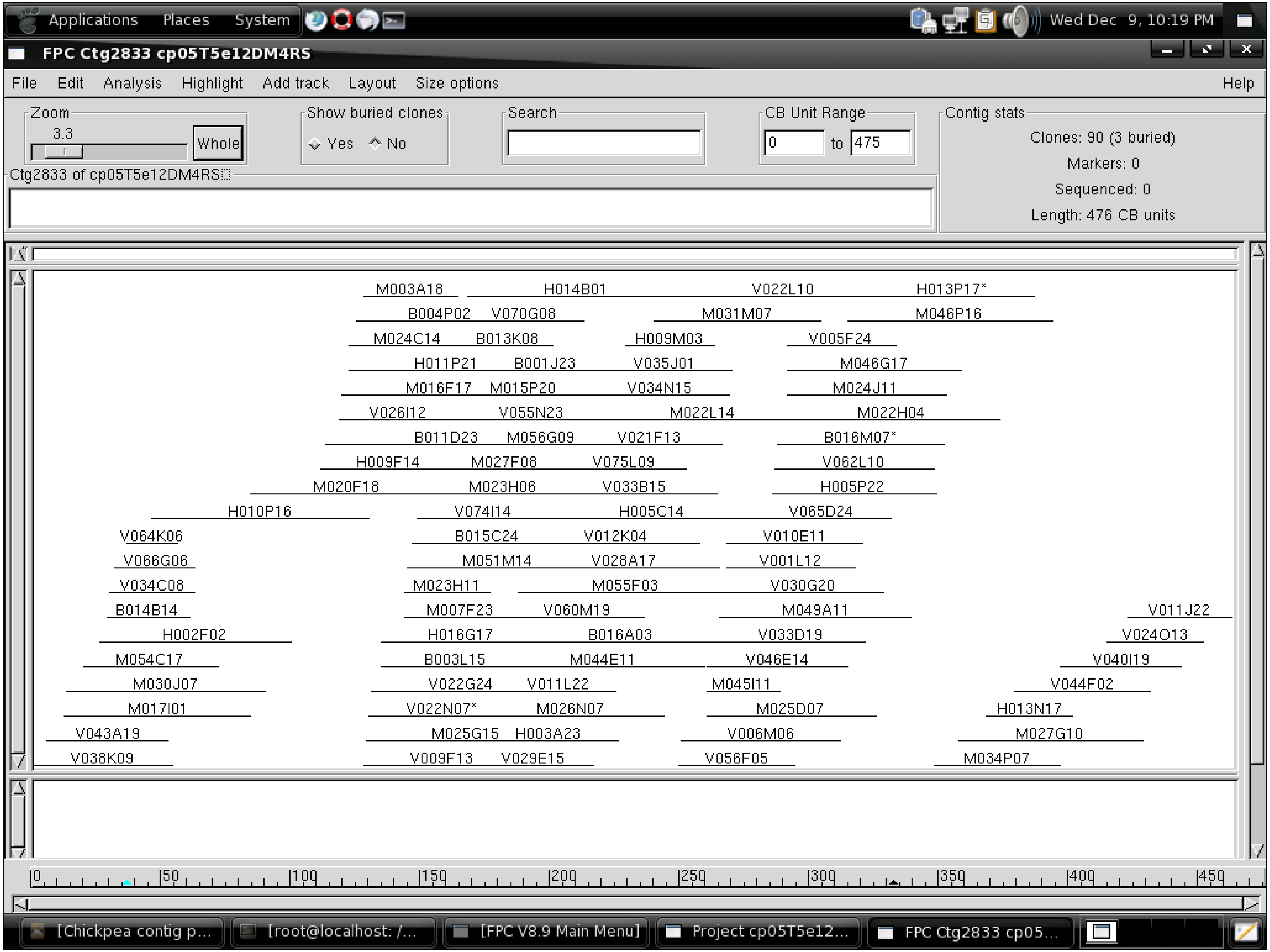
Applications Places System
Wed Dec 9, 10:19 PM
FPC Ctg2833 cp05T5e12DM4RS
File Edit Analysis Highlight Add track Layout Size options Help
Zoom
3.3
Whole
Show buried clones
Yes No
Search
CB Unit Range
0 to 475
Contig stats
Clones: 90 (3 buried)
Markers: 0
Sequenced: 0
Length: 476 CB units
Ctg2833 of cp05T5e12DM4RS
M003A18 H014B01 V022L10 H013P17*
B004P02 V070G08 M031M07 M046P16
M024C14 B013K08 H009M03 V005F24
H011P21 B001J23 V035J01 M046G17
M016F17 M015P20 V034N15 M024J11
V026I12 V055N23 M022L14 M022H04
B011D23 M056G09 V021F13 B016M07*
H009F14 M027F08 V075L09 V062L10
M020F18 M023H06 V033B15 H005P22
H010P16 V074I14 H005C14 V065D24
V064K06 B015C24 V012K04 V010E11
V066G06 M051M14 V028A17 V001L12
V034C08 M023H11 M055F03 V030G20
B014B14 M007F23 V060M19 M049A11 V011J22
H002F02 H016G17 B016A03 V033D19 V024O13
M054C17 B003L15 M044E11 V046E14 V040I19
M030J07 V022G24 V011L22 M045I11 V044F02
M017I01 V022N07* M026N07 M025D07 H013N17
V043A19 M025G15 H003A23 V006M06 M027G10
V038K09 V009F13 V029E15 V056F05 M034P07
0 50 100 150 200 250 300 350 400 450
[Chickpea contig p...
[root@localhost: /...
[FPC V8.9 Main Menu]
Project cp05T5e12...
FPC Ctg2833 cp05...

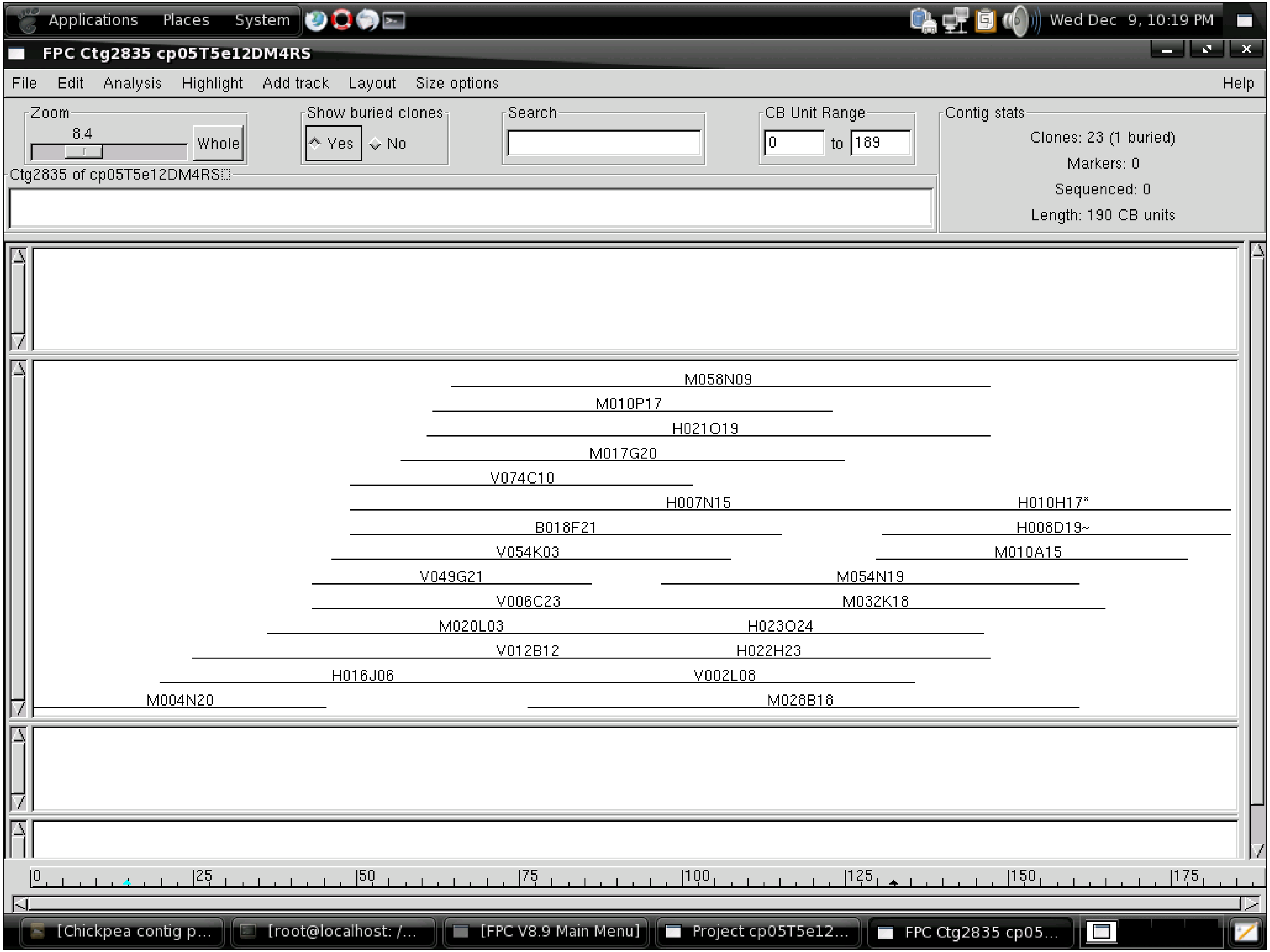
Applications Places System
Wed Dec 9, 10:19 PM
FPC Ctg2835 cp05T5e12DM4RS
File Edit Analysis Highlight Add track Layout Size options Help
Zoom
8.4
Whole
Show buried clones
Yes No
Search
CB Unit Range
0 to 189
Contig stats
Clones: 23 (1 buried)
Markers: 0
Sequenced: 0
Length: 190 CB units
Ctg2835 of cp05T5e12DM4RS
M058N09
M010P17
H021O19
M017G20
V074C10
H007N15
H010H17*
B018F21
H008D19~
V054K03
M010A15
V049G21
M054N19
V006C23
M032K18
M020L03
H023O24
V012B12
H022H23
H016J06
V002L08
M004N20
M028B18
0
25
50
75
100
125
150
175
[Chickpea contig p...
[root@localhost: /...
[FPC V8.9 Main Menu]
Project cp05T5e12...
FPC Ctg2835 cp05...

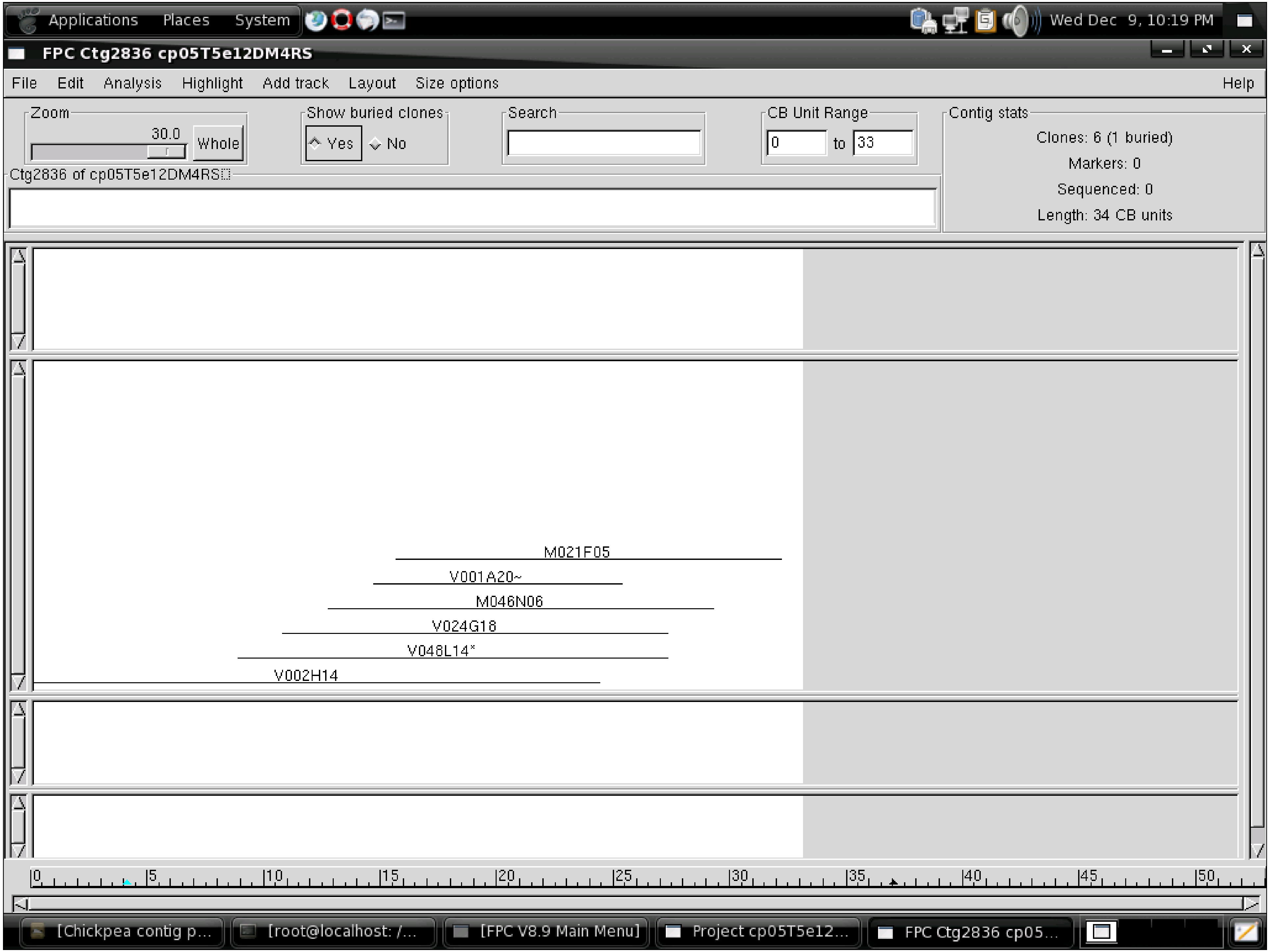
Applications Places System
Wed Dec 9, 10:19 PM
FPC Ctg2836 cp05T5e12DM4RS
File Edit Analysis Highlight Add track Layout Size options Help
Zoom
30.0
Whole
Show buried clones
Yes
No
Search
CB Unit Range
0
to
33
Contig stats
Clones: 6 (1 buried)
Markers: 0
Sequenced: 0
Length: 34 CB units
Ctg2836 of cp05T5e12DM4RS
M021F05
V001A20~
M046N06
V024G18
V048L14*
V002H14
0 5 10 15 20 25 30 35 40 45 50
[Chickpea contig p...
[root@localhost: /...
[FPC V8.9 Main Menu]
Project cp05T5e12...
FPC Ctg2836 cp05...

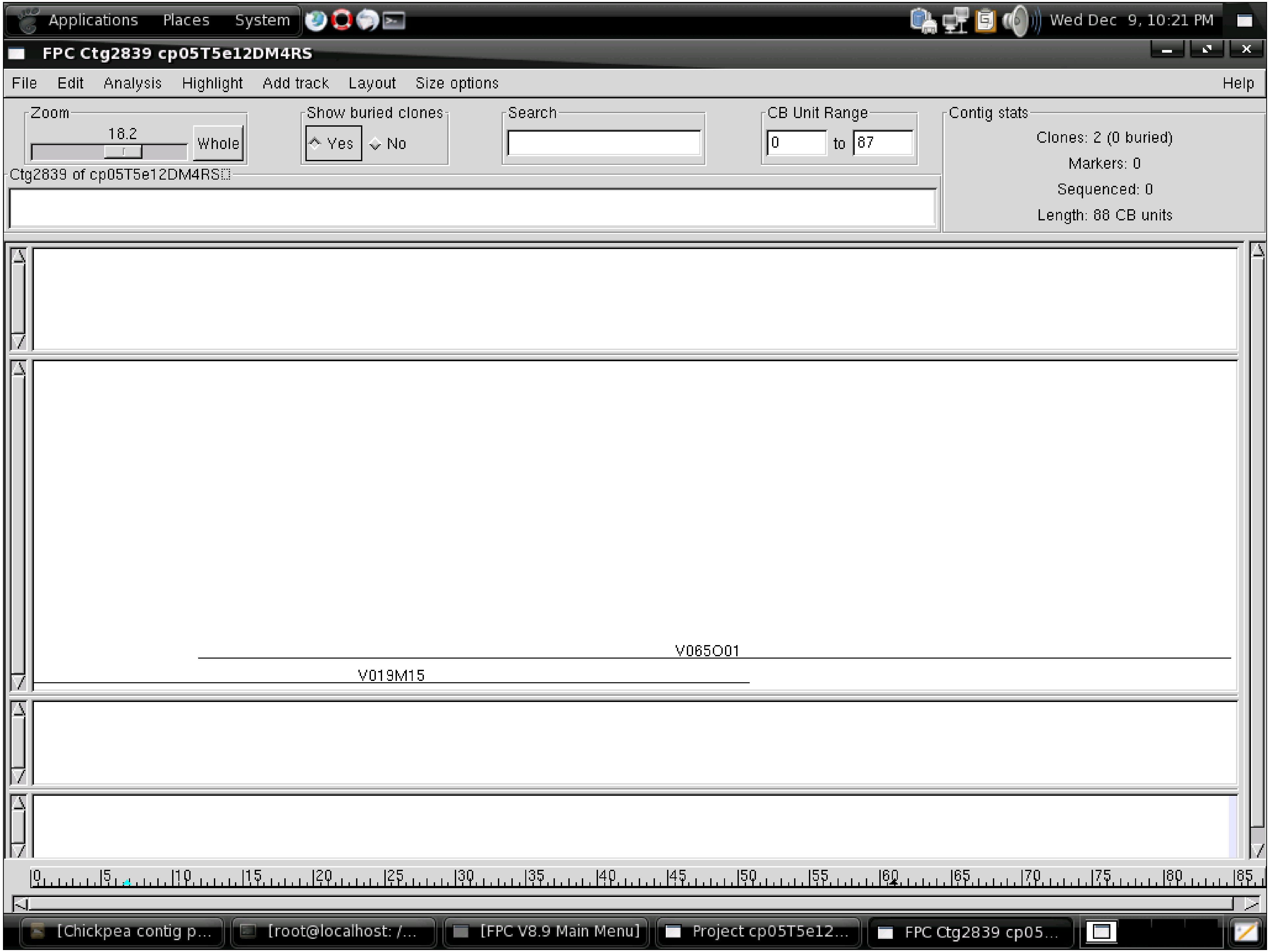
Applications Places System
Wed Dec 9, 10:21 PM
FPC Ctg2839 cp05T5e12DM4RS
File Edit Analysis Highlight Add track Layout Size options Help
Zoom
18.2
Whole
Show buried clones
Yes No
Search
CB Unit Range
0 to 87
Contig stats
Clones: 2 (0 buried)
Markers: 0
Sequenced: 0
Length: 88 CB units
Ctg2839 of cp05T5e12DM4RS
V065O01
V019M15
0 5 10 15 20 25 30 35 40 45 50 55 60 65 70 75 80 85
[Chickpea contig p...
[root@localhost: /...
[FPC V8.9 Main Menu]
Project cp05T5e12...
FPC Ctg2839 cp05...

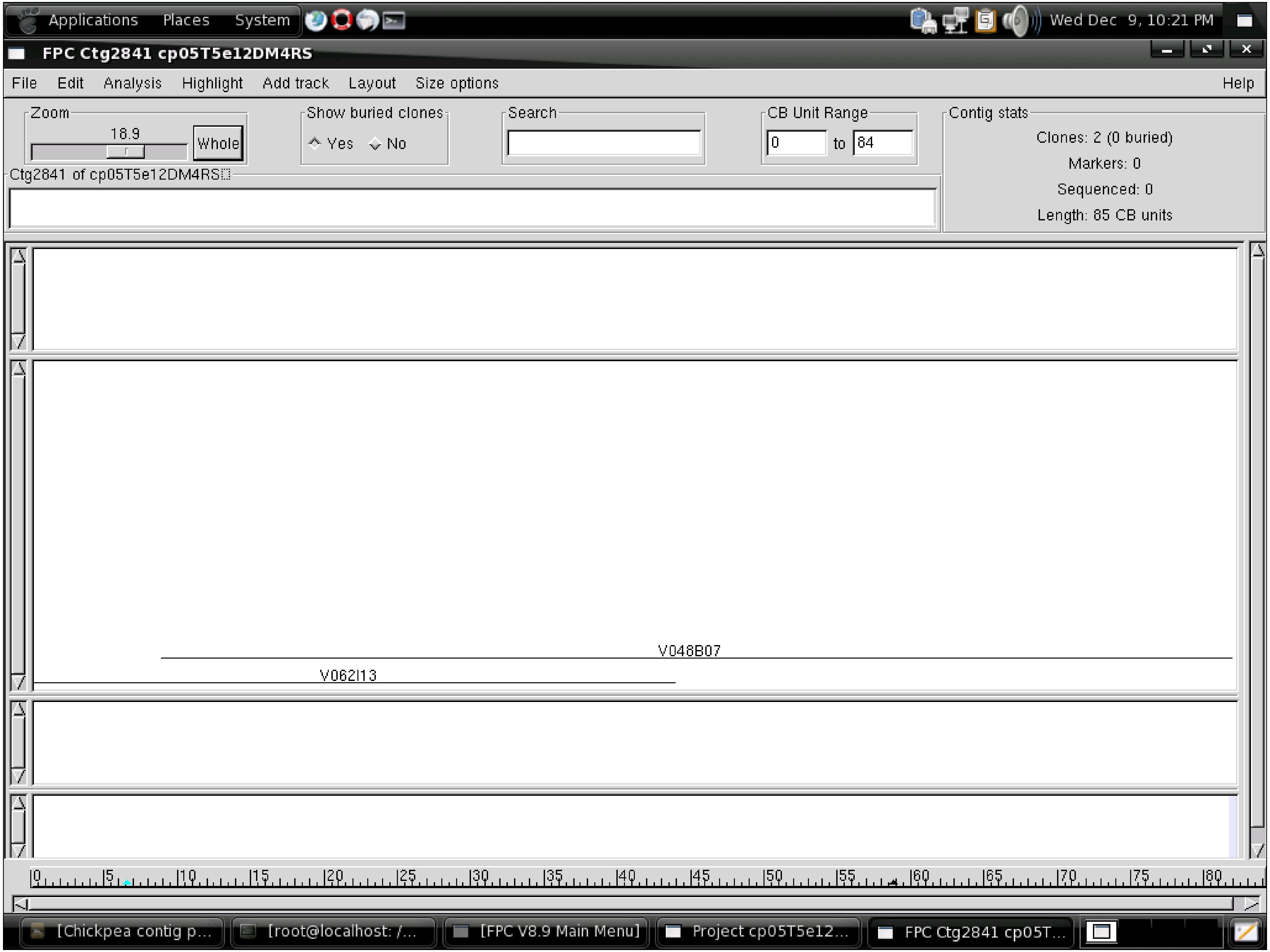
Applications
Places
System
Wed Dec 9, 10:21 PM
FPC Ctg2841 cp05T5e12DM4RS
File
Edit
Analysis
Highlight
Add track
Layout
Size options
Help
Zoom
18.9
Whole
Show buried clones
Yes
No
Search
CB Unit Range
0
to
84
Contig stats
Clones: 2 (0 buried)
Markers: 0
Sequenced: 0
Length: 85 CB units
Ctg2841 of cp05T5e12DM4RS
V048B07
V062I13
0 5 10 15 20 25 30 35 40 45 50 55 60 65 70 75 80
[Chickpea contig p...
[root@localhost: /...
[FPC V8.9 Main Menu]
Project cp05T5e12...
FPC Ctg2841 cp05T...

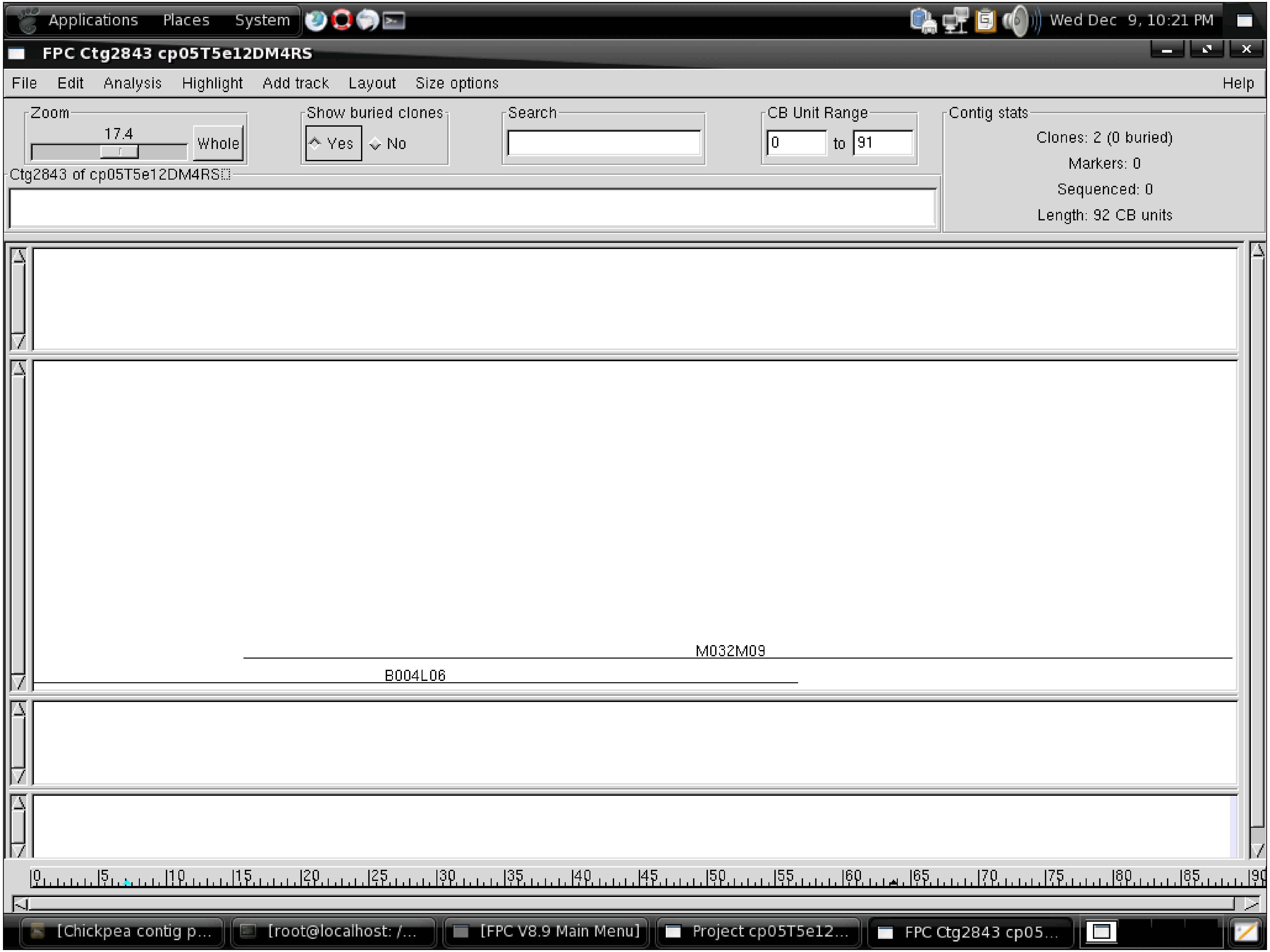
Applications
Places
System
Wed Dec 9, 10:21 PM
FPC Ctg2843 cp05T5e12DM4RS
File
Edit
Analysis
Highlight
Add track
Layout
Size options
Help
Zoom
17.4
Whole
Show buried clones
Yes
No
Search
CB Unit Range
0
to
91
Contig stats
Clones: 2 (0 buried)
Markers: 0
Sequenced: 0
Length: 92 CB units
Ctg2843 of cp05T5e12DM4RS
M032M09
B004L06
0 5 10 15 20 25 30 35 40 45 50 55 60 65 70 75 80 85 90
[Chickpea contig p...
[root@localhost: /...
[FPC V8.9 Main Menu]
Project cp05T5e12...
FPC Ctg2843 cp05...

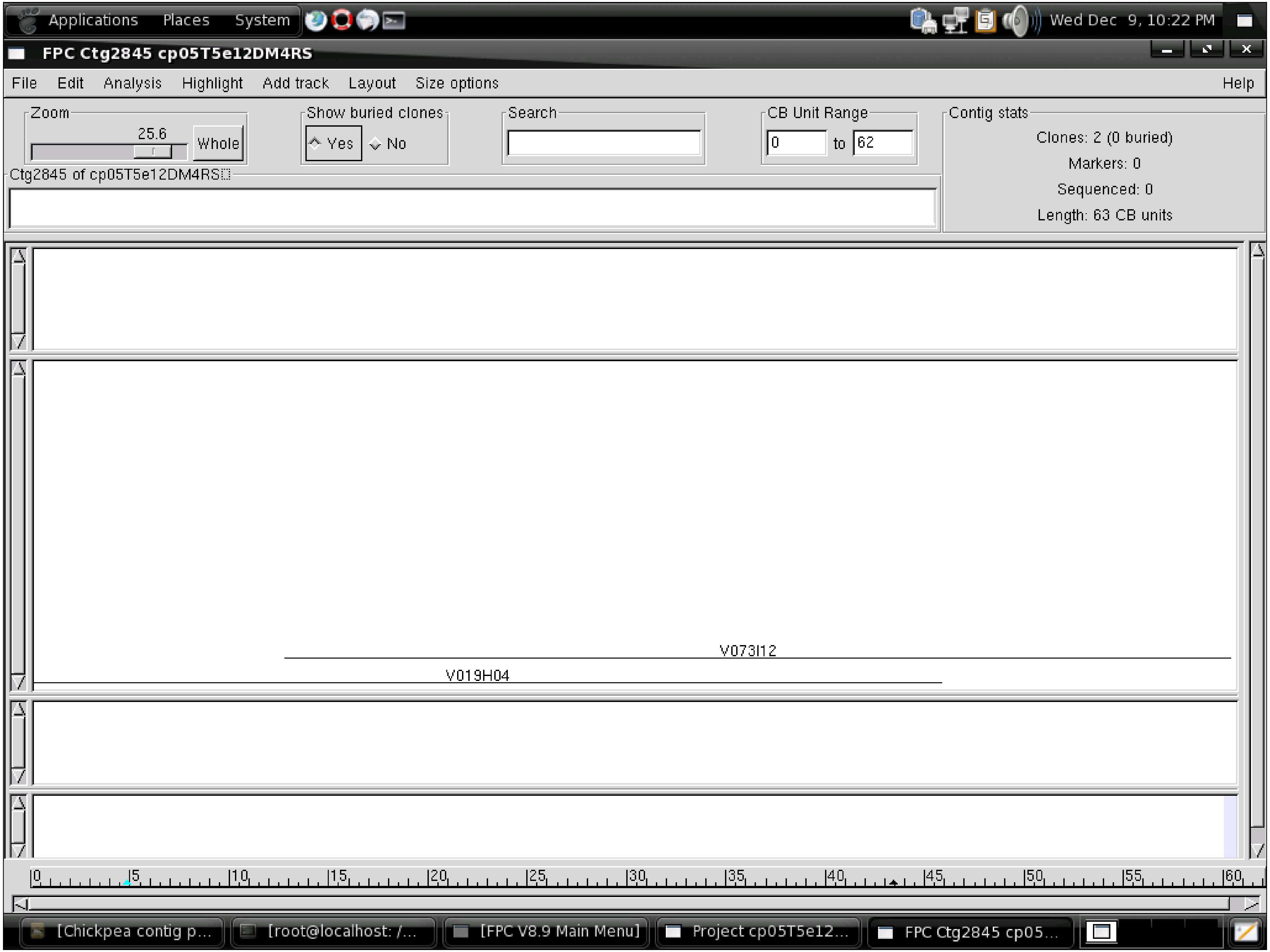
Applications Places System
Wed Dec 9, 10:22 PM
FPC Ctg2845 cp05T5e12DM4RS
File Edit Analysis Highlight Add track Layout Size options Help
Zoom
25.6
Whole
Show buried clones
Yes No
Search
CB Unit Range
0 to 62
Contig stats
Clones: 2 (0 buried)
Markers: 0
Sequenced: 0
Length: 63 CB units
Ctg2845 of cp05T5e12DM4RS
V073I12
V019H04
0 5 10 15 20 25 30 35 40 45 50 55 60
[Chickpea contig p...
[root@localhost: /...
[FPC V8.9 Main Menu]
Project cp05T5e12...
FPC Ctg2845 cp05...
