# Supplementary material for: A BAC/BIBAC-based physical map of chickpea, Cicer arietinum L
Source: BMC Genomics. 2010 Sep 17;11:501. doi: 10.1186/1471-2164-11-501 (PMC2996997; doi:10.1186/1471-2164-11-501)
Supplement: Additional file 4 — Shows the contigs constituting the physical map of the chickpea genome 4 (ctg2849-3727). [file 1471-2164-11-501-S4.PDF]

A BAC/BIBAC-based physical map of chickpea, *Cicer arietinum* L.

Xiaojun Zhang[1,6], Chantel F Scheuring[1], Meiping Zhang[1,2], Jennifer J Dong[1], Yang Zhang[1], James J Huang[1], Mi-Kyung Lee[1], Shahal Abbo[3], Amir Sherman[4], Dani Shtienberg[4], Weidong Chen[5], Fred Muehlbauer[5], Hong-Bin Zhang[1,*]

[1] Department of Soil and Crop Sciences, Texas A&M University, College Station, Texas 77843-2474, USA
[2] College of Life Science, Jilin Agricultural University, Changchun, Jilin 130118, China
[3] Institute of Plant Science and Genetics in Agriculture, The Hebrew University of Jerusalem, Rehovot, 76100, Israel
[4] The Volcani Center, P.O. Box 6, Bet-Dagan, 50250, Israel
[5] USDA-ARS and Department of Crop and Soil Sciences, Washington State University, Pullman, WA 99164-6434, USA
[6] The Key Laboratory of Experimental Marine Biology, Institute of Oceanology, Chinese Academy of Sciences, Qingdao 266071, China

***Corresponding author:** H.-B. Zhang, Tel.: +1-979-862-2244; Fax: +1-979-845-0456; E-mail: hbz7049@tamu.edu

Additional file 4. The contigs constituting the physical map of the chickpea genome 4(ctg2849-3727)

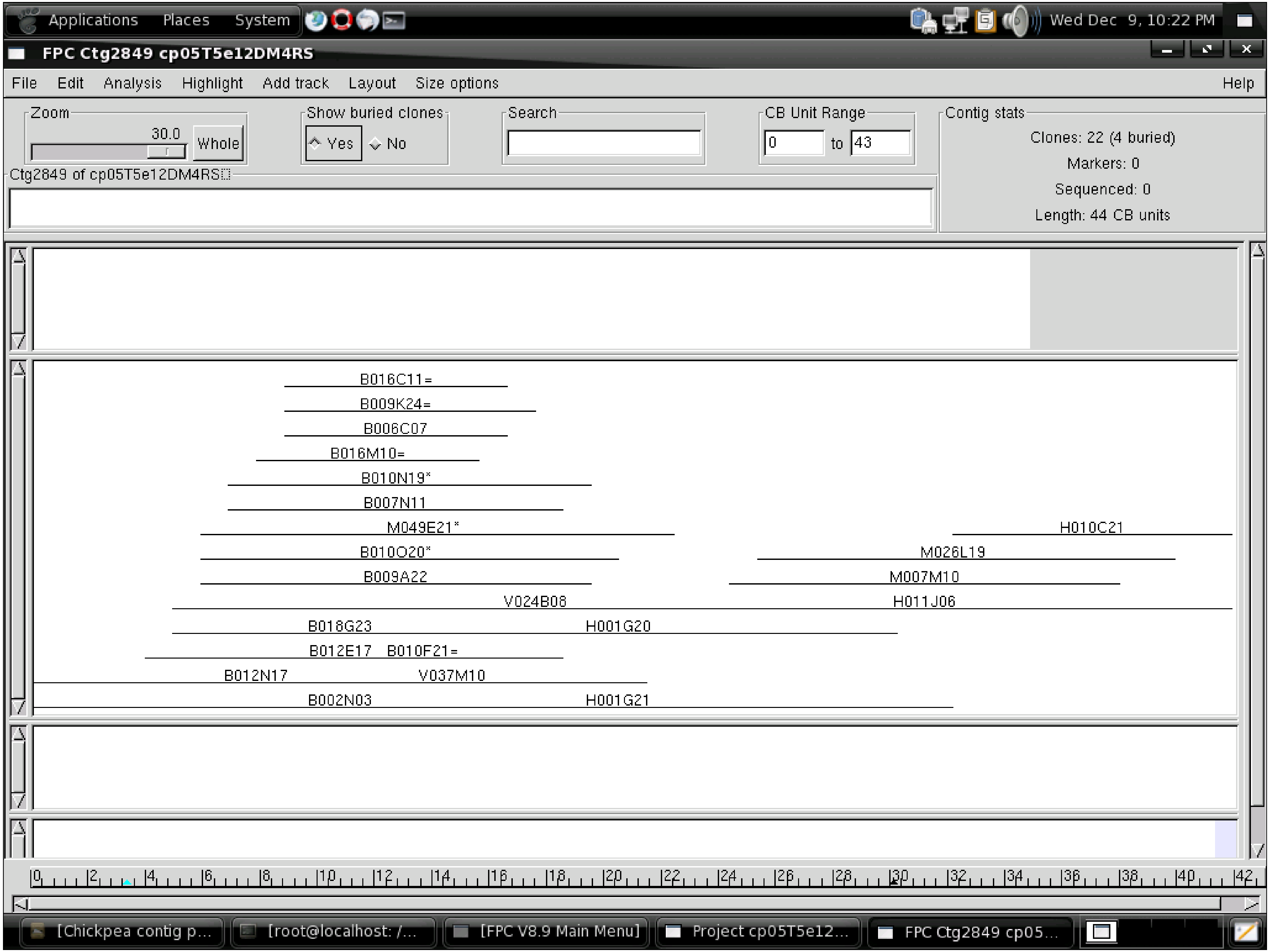
Applications Places System
Wed Dec 9, 10:22 PM
FPC Ctg2849 cp05T5e12DM4RS
File Edit Analysis Highlight Add track Layout Size options Help
Zoom
30.0
Whole
Show buried clones
Yes No
Search
CB Unit Range
0 to 43
Contig stats
Clones: 22 (4 buried)
Markers: 0
Sequenced: 0
Length: 44 CB units
Ctg2849 of cp05T5e12DM4RS
B016C11=
B009K24=
B006C07
B016M10=
B010N19*
B007N11
M049E21*
H010C21
B010O20*
M026L19
B009A22
M007M10
V024B08
H011J06
B018G23
H001G20
B012E17 B010F21=
B012N17
V037M10
B002N03
H001G21
0 2 4 6 8 10 12 14 16 18 20 22 24 26 28 30 32 34 36 38 40 42
[Chickpea contig p...
[root@localhost: /...
[FPC V8.9 Main Menu]
Project cp05T5e12...
FPC Ctg2849 cp05...

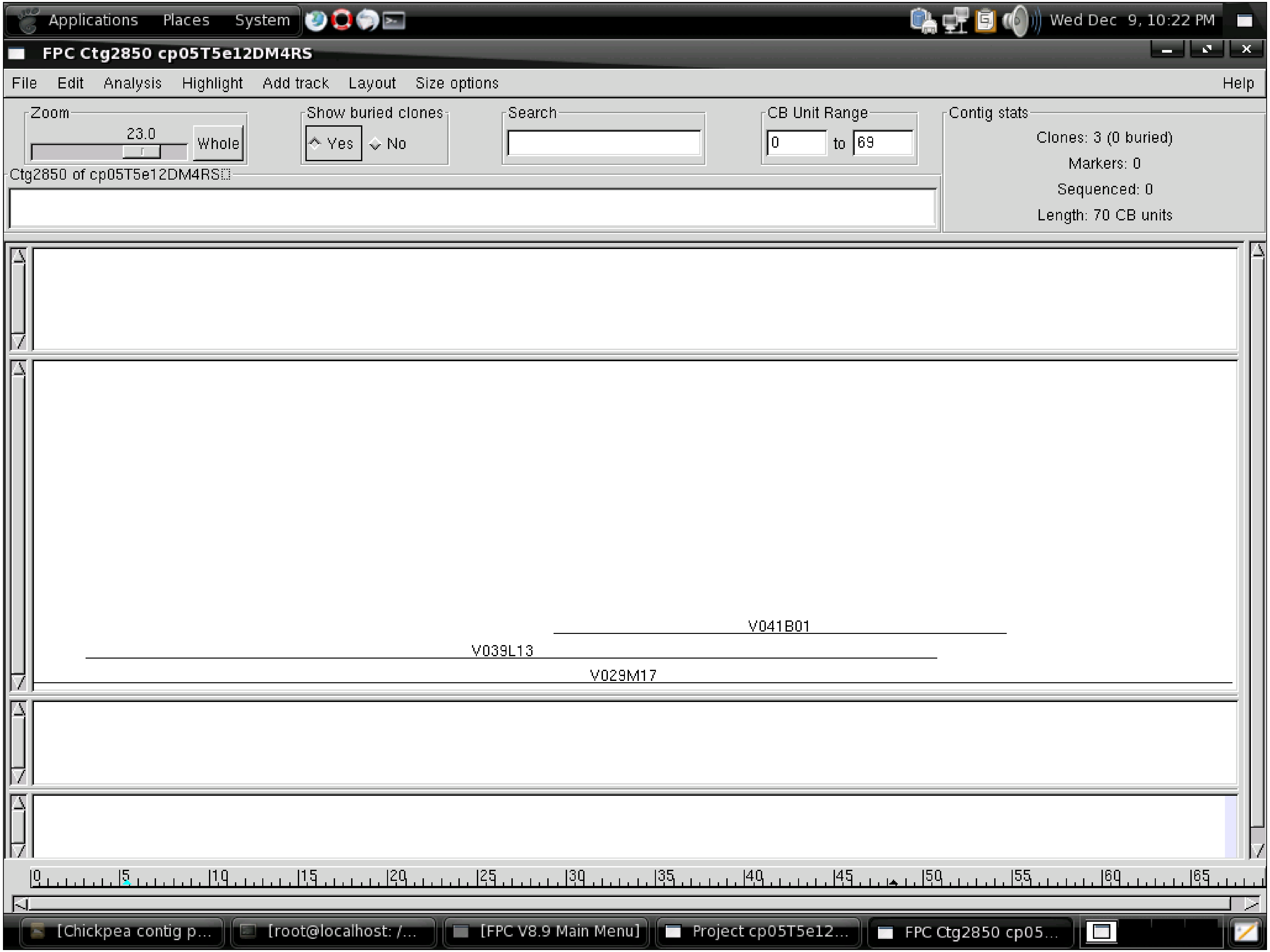
Applications Places System
Wed Dec 9, 10:22 PM
FPC Ctg2850 cp05T5e12DM4RS
File Edit Analysis Highlight Add track Layout Size options Help
Zoom
23.0
Whole
Show buried clones
Yes No
Search
CB Unit Range
0 to 69
Contig stats
Clones: 3 (0 buried)
Markers: 0
Sequenced: 0
Length: 70 CB units
Ctg2850 of cp05T5e12DM4RS
V041B01
V039L13
V029M17
0 5 10 15 20 25 30 35 40 45 50 55 60 65
[Chickpea contig p...
[root@localhost: /...
[FPC V8.9 Main Menu]
Project cp05T5e12...
FPC Ctg2850 cp05...

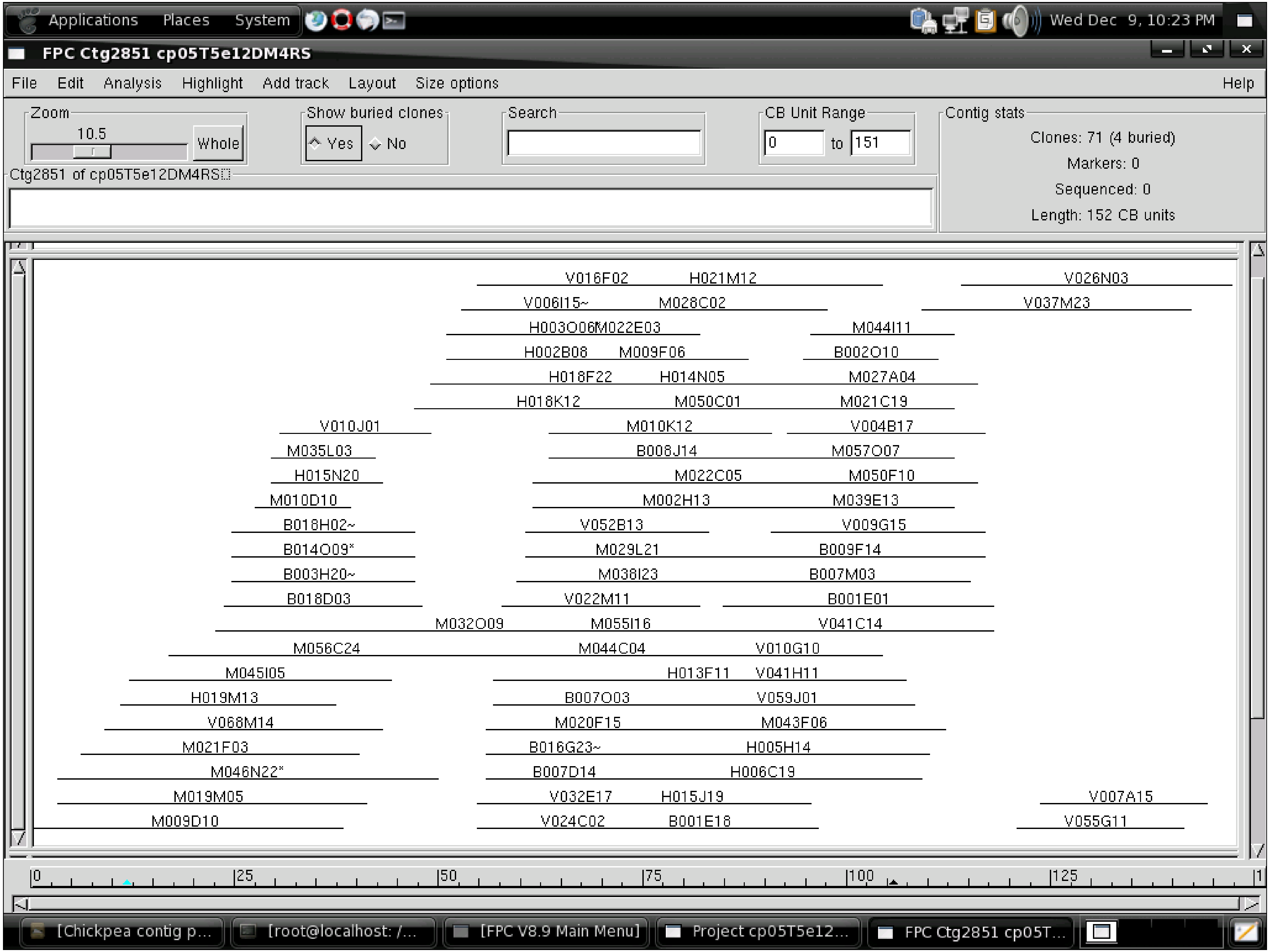
Applications Places System
Wed Dec 9, 10:23 PM
FPC Ctg2851 cp05T5e12DM4RS
File Edit Analysis Highlight Add track Layout Size options Help
Zoom 10.5 Whole
Show buried clones Yes No
Search
CB Unit Range 0 to 151
Contig stats
Clones: 71 (4 buried)
Markers: 0
Sequenced: 0
Length: 152 CB units
Ctg2851 of cp05T5e12DM4RS
V016F02 H021M12 V026N03
V006I15~ M028C02 V037M23
H003O06 M022E03 M044I11
H002B08 M009F06 B002O10
H018F22 H014N05 M027A04
H018K12 M050C01 M021C19
V010J01 M010K12 V004B17
M035L03 B008J14 M057O07
H015N20 M022C05 M050F10
M010D10 M002H13 M039E13
B018H02~ V052B13 V009G15
B014O09* M029L21 B009F14
B003H20~ M038I23 B007M03
B018D03 V022M11 B001E01
M032O09 M055I16 V041C14
M056C24 M044C04 V010G10
M045I05 H013F11 V041H11
H019M13 B007O03 V059J01
V068M14 M020F15 M043F06
M021F03 B016G23~ H005H14
M046N22* B007D14 H006C19
M019M05 V032E17 H015J19 V007A15
M009D10 V024C02 B001E18 V055G11
0 25 50 75 100 125
[Chickpea contig p... [root@localhost: /... [FPC V8.9 Main Menu] Project cp05T5e12... FPC Ctg2851 cp05T...

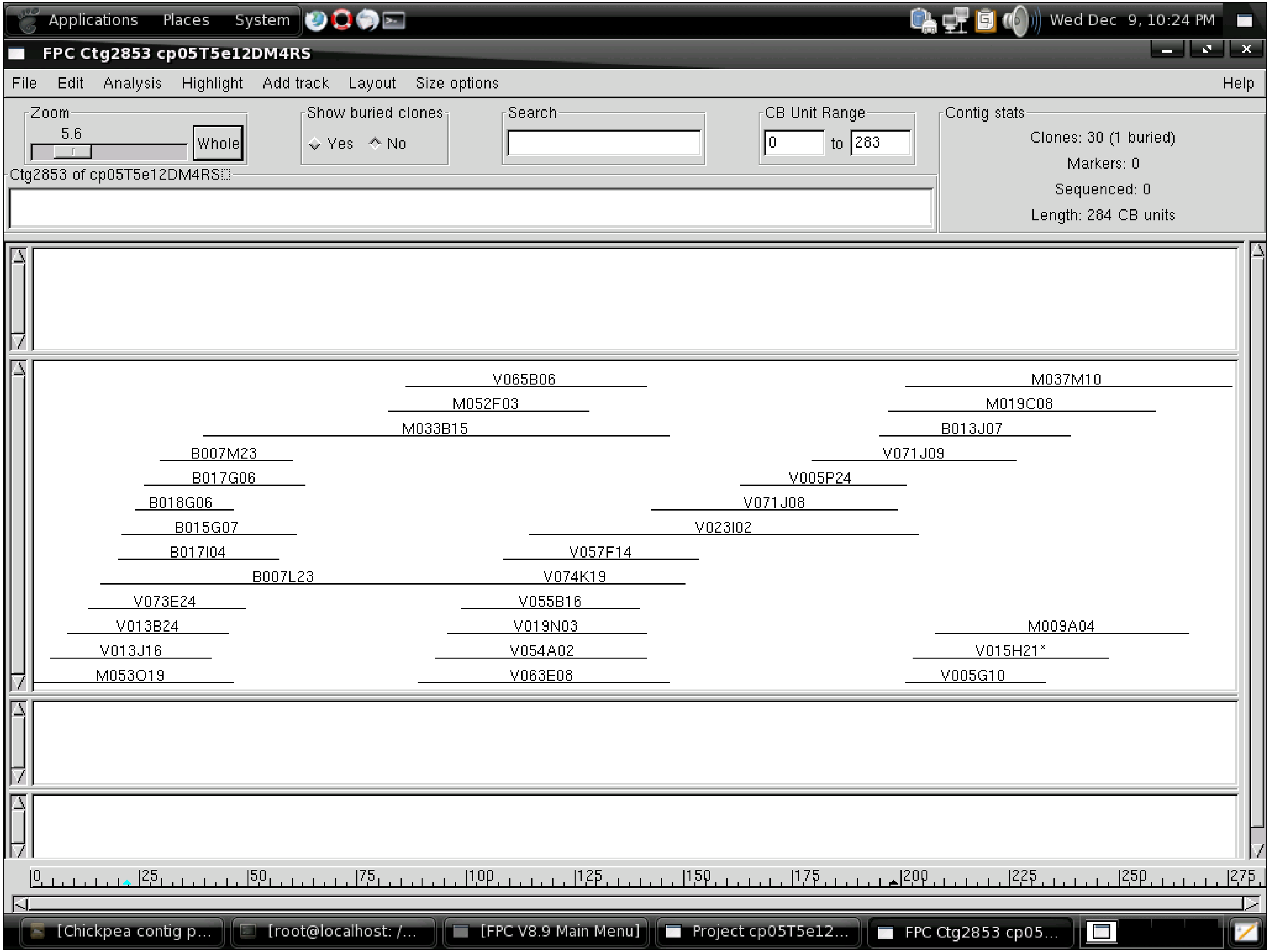
Applications Places System
Wed Dec 9, 10:24 PM
FPC Ctg2853 cp05T5e12DM4RS
File Edit Analysis Highlight Add track Layout Size options Help
Zoom 5.6 Whole
Show buried clones Yes No
Search
CB Unit Range 0 to 283
Contig stats
Clones: 30 (1 buried)
Markers: 0
Sequenced: 0
Length: 284 CB units
Ctg2853 of cp05T5e12DM4RS
V065B06
M037M10
M052F03
M019C08
M033B15
B013J07
B007M23
V071J09
B017G06
V005P24
B018G06
V071J08
B015G07
V023I02
B017I04
V057F14
B007L23
V074K19
V073E24
V055B16
V013B24
V019N03
M009A04
V013J16
V054A02
V015H21*
M053O19
V063E08
V005G10
0 25 50 75 100 125 150 175 200 225 250 275
[Chickpea contig p...
[root@localhost: /...
[FPC V8.9 Main Menu]
Project cp05T5e12...
FPC Ctg2853 cp05...

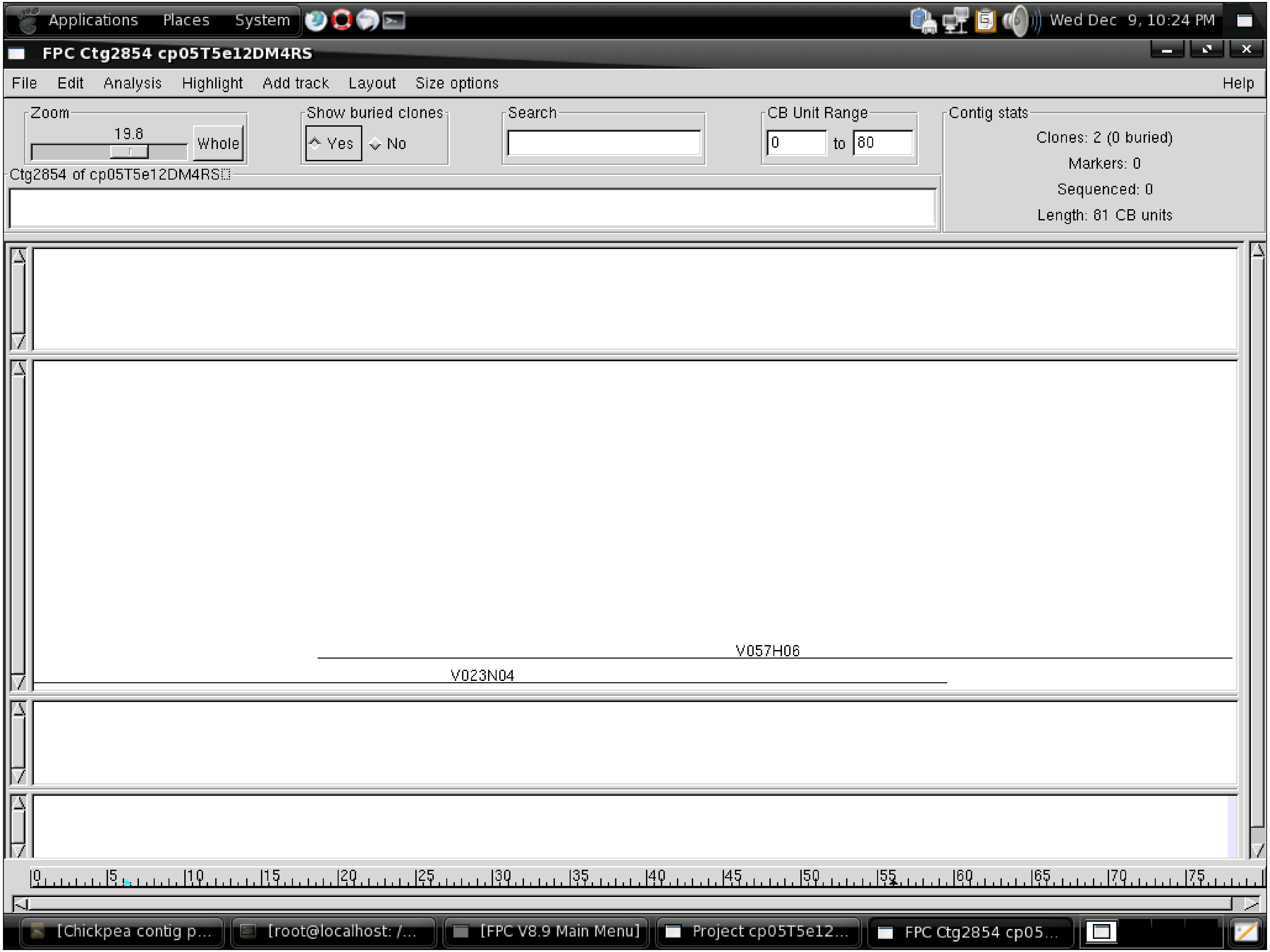

Applications Places System
Wed Dec 9, 10:24 PM
FPC Ctg2854 cp05T5e12DM4RS
File Edit Analysis Highlight Add track Layout Size options Help
Zoom
19.8
Whole
Show buried clones
Yes No
Search
CB Unit Range
0 to 80
Contig stats
Clones: 2 (0 buried)
Markers: 0
Sequenced: 0
Length: 81 CB units
Ctg2854 of cp05T5e12DM4RS
V057H06
V023N04
0 5 10 15 20 25 30 35 40 45 50 55 60 65 70 75
[Chickpea contig p...
[root@localhost: /...
[FPC V8.9 Main Menu]
Project cp05T5e12...
FPC Ctg2854 cp05...

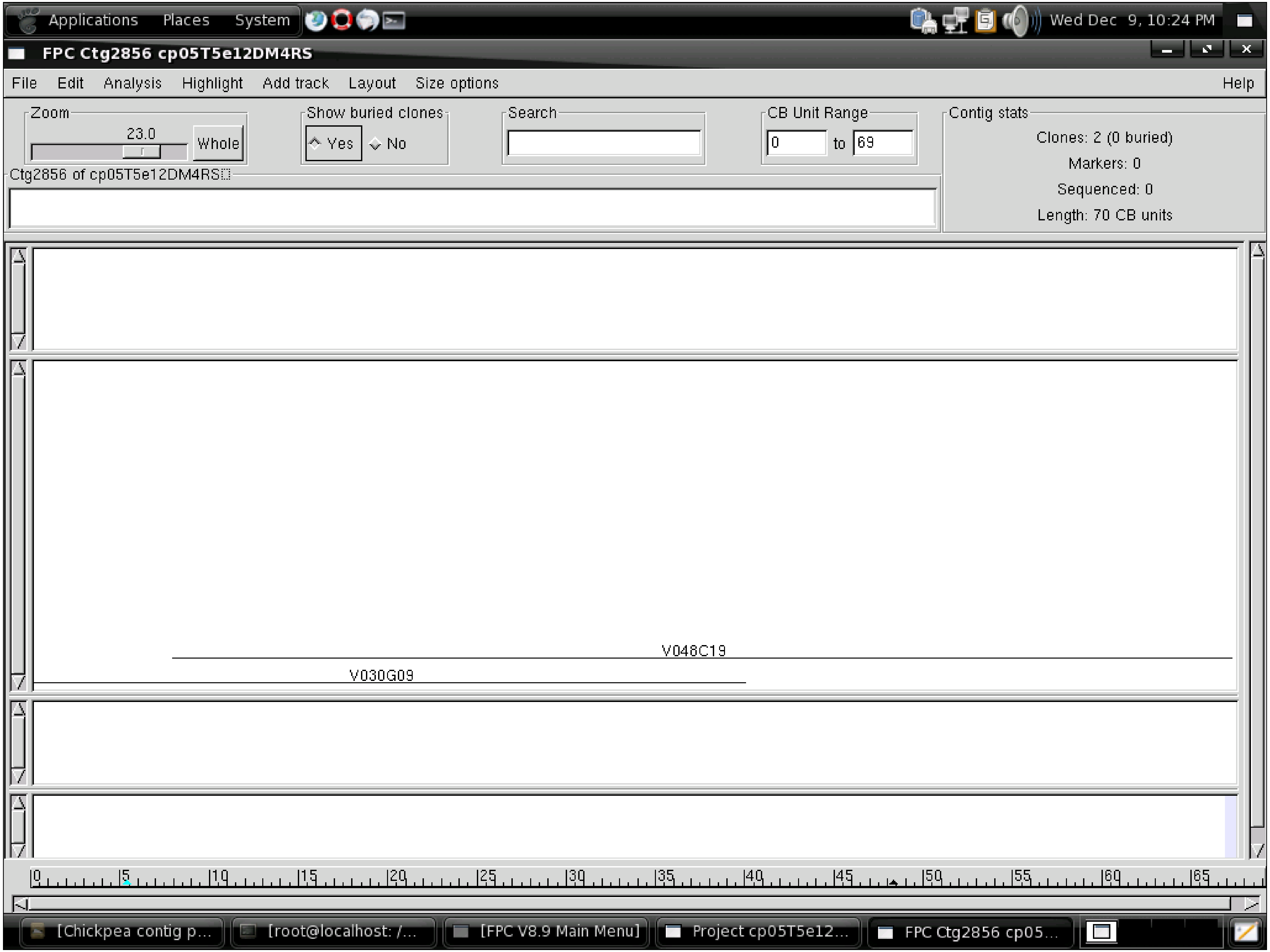
Applications
Places
System
Wed Dec 9, 10:24 PM
FPC Ctg2856 cp05T5e12DM4RS
File
Edit
Analysis
Highlight
Add track
Layout
Size options
Help
Zoom
23.0
Whole
Show buried clones
Yes
No
Search
CB Unit Range
0
to
69
Contig stats
Clones: 2 (0 buried)
Markers: 0
Sequenced: 0
Length: 70 CB units
Ctg2856 of cp05T5e12DM4RS
V048C19
V030G09
0
5
10
15
20
25
30
35
40
45
50
55
60
65
[Chickpea contig p...
[root@localhost: /...
[FPC V8.9 Main Menu]
Project cp05T5e12...
FPC Ctg2856 cp05...

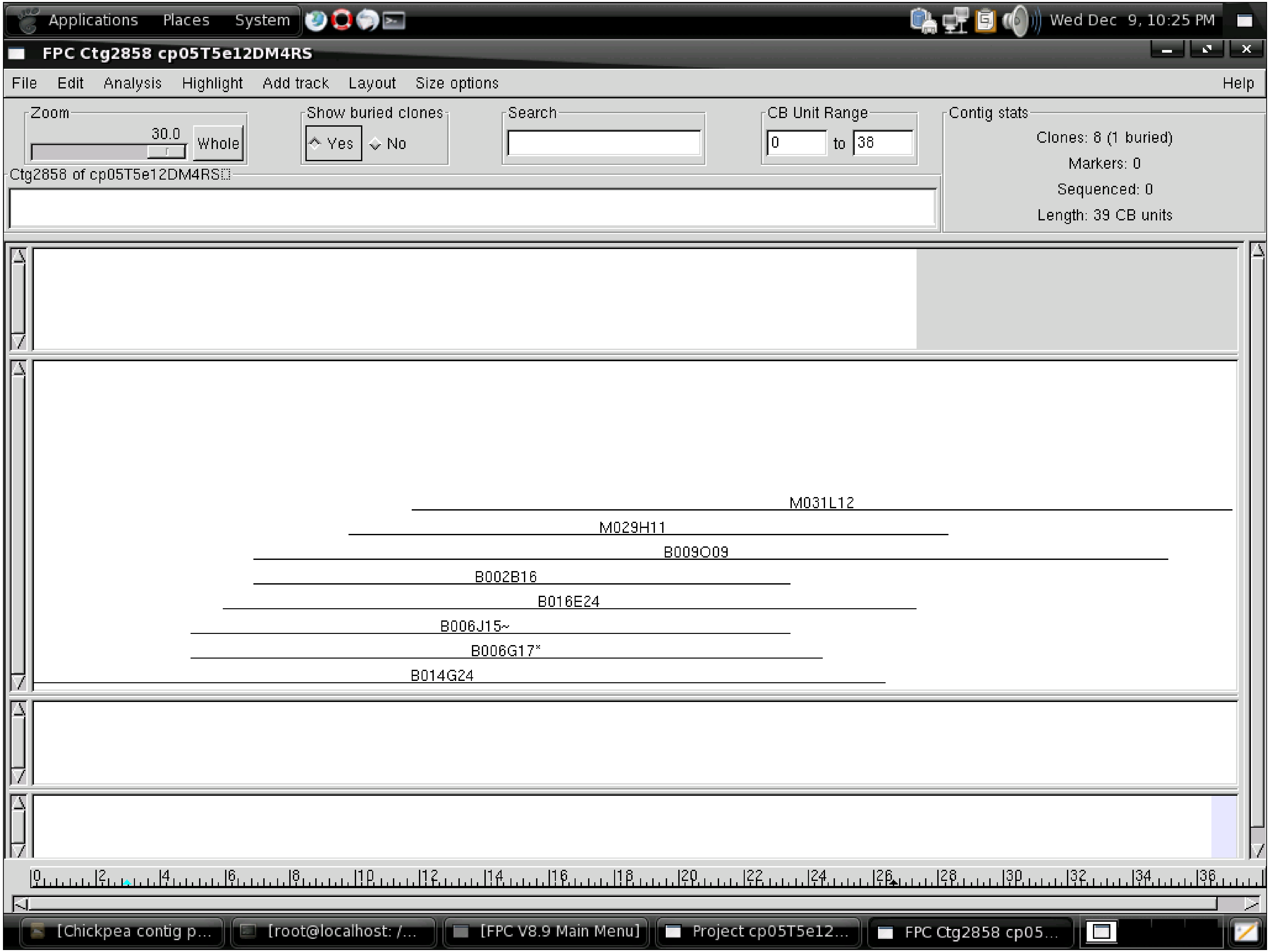
Applications Places System
Wed Dec 9, 10:25 PM
FPC Ctg2858 cp05T5e12DM4RS
File Edit Analysis Highlight Add track Layout Size options Help
Zoom
30.0
Whole
Show buried clones
Yes
No
Search
CB Unit Range
0
to
38
Contig stats
Clones: 8 (1 buried)
Markers: 0
Sequenced: 0
Length: 39 CB units
Ctg2858 of cp05T5e12DM4RS
M031L12
M029H11
B009O09
B002B16
B016E24
B006J15~
B006G17*
B014G24
0 2 4 6 8 10 12 14 16 18 20 22 24 26 28 30 32 34 36
[Chickpea contig p...
[root@localhost: /...
[FPC V8.9 Main Menu]
Project cp05T5e12...
FPC Ctg2858 cp05...

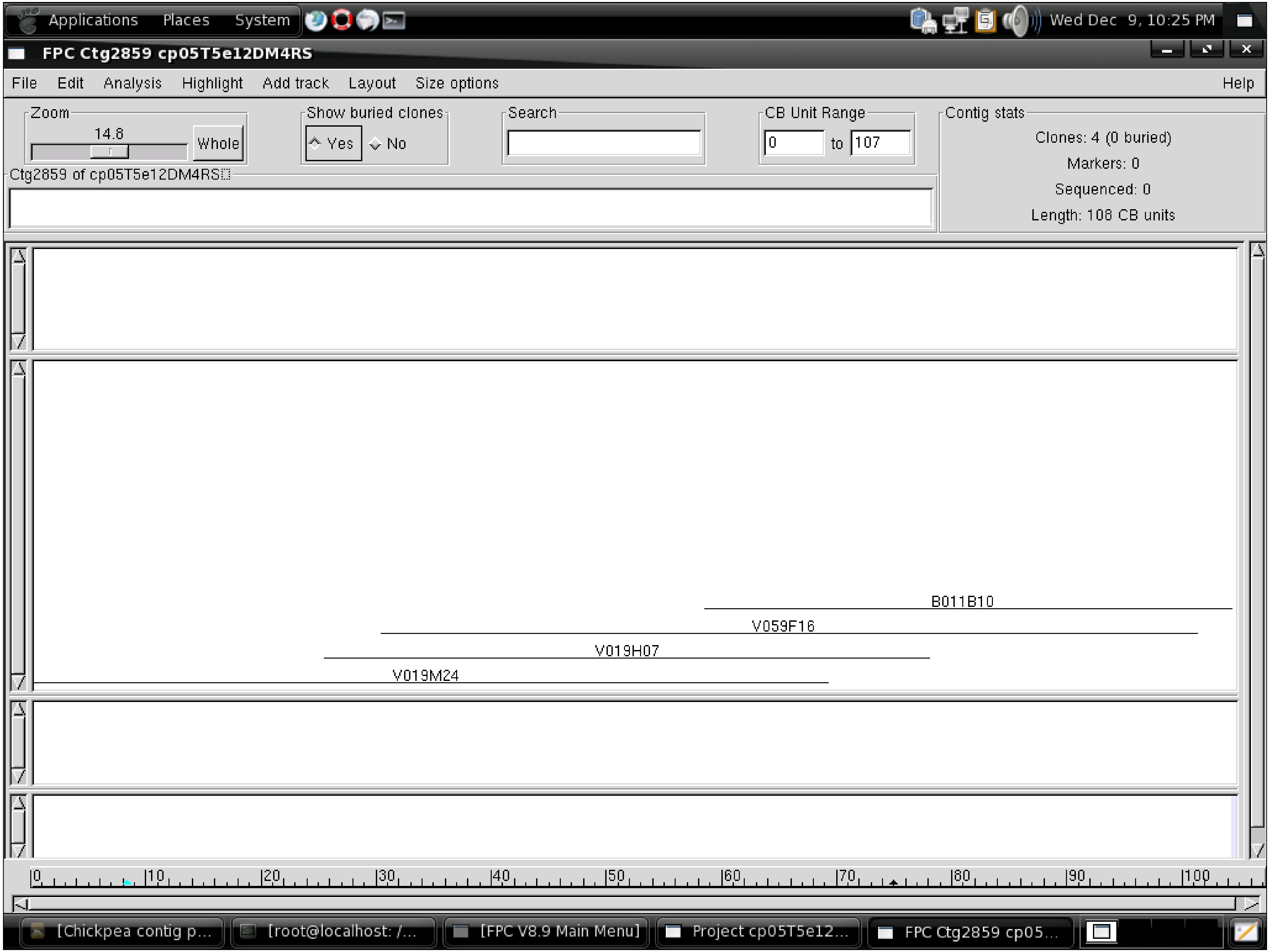
Applications Places System
Wed Dec 9, 10:25 PM
FPC Ctg2859 cp05T5e12DM4RS
File Edit Analysis Highlight Add track Layout Size options Help
Zoom
14.8
Whole
Show buried clones
Yes No
Search
CB Unit Range
0 to 107
Contig stats
Clones: 4 (0 buried)
Markers: 0
Sequenced: 0
Length: 108 CB units
Ctg2859 of cp05T5e12DM4RS
B011B10
V059F16
V019H07
V019M24
0 10 20 30 40 50 60 70 80 90 100
[Chickpea contig p...
[root@localhost: /...
[FPC V8.9 Main Menu]
Project cp05T5e12...
FPC Ctg2859 cp05...

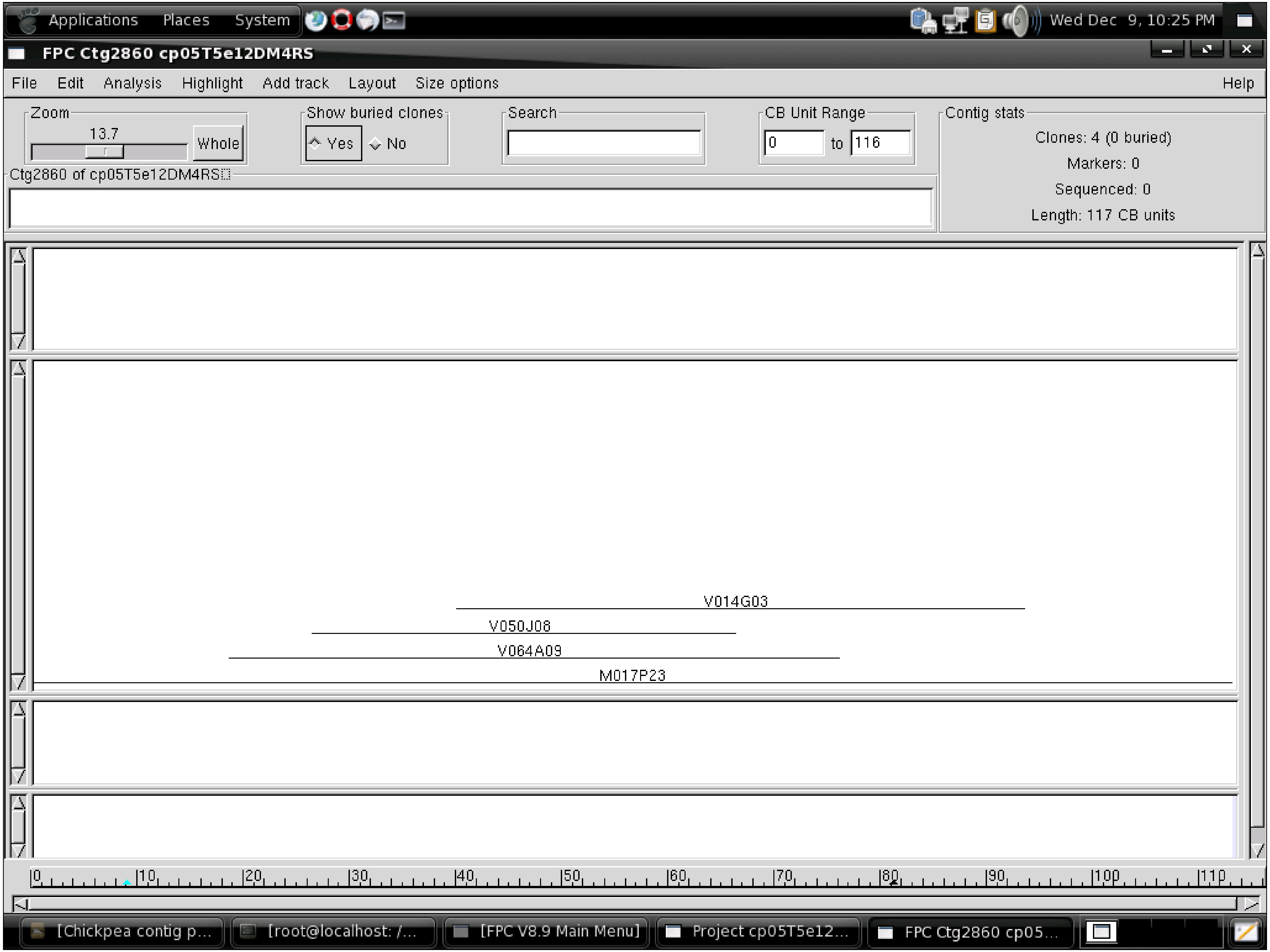
Applications
Places
System
Wed Dec 9, 10:25 PM
FPC Ctg2860 cp05T5e12DM4RS
File
Edit
Analysis
Highlight
Add track
Layout
Size options
Help
Zoom
13.7
Whole
Show buried clones
Yes
No
Search
CB Unit Range
0
to
116
Contig stats
Clones: 4 (0 buried)
Markers: 0
Sequenced: 0
Length: 117 CB units
Ctg2860 of cp05T5e12DM4RS
V014G03
V050J08
V064A09
M017P23
0
10
20
30
40
50
60
70
80
90
100
110
[Chickpea contig p...
[root@localhost: /...
[FPC V8.9 Main Menu]
Project cp05T5e12...
FPC Ctg2860 cp05...

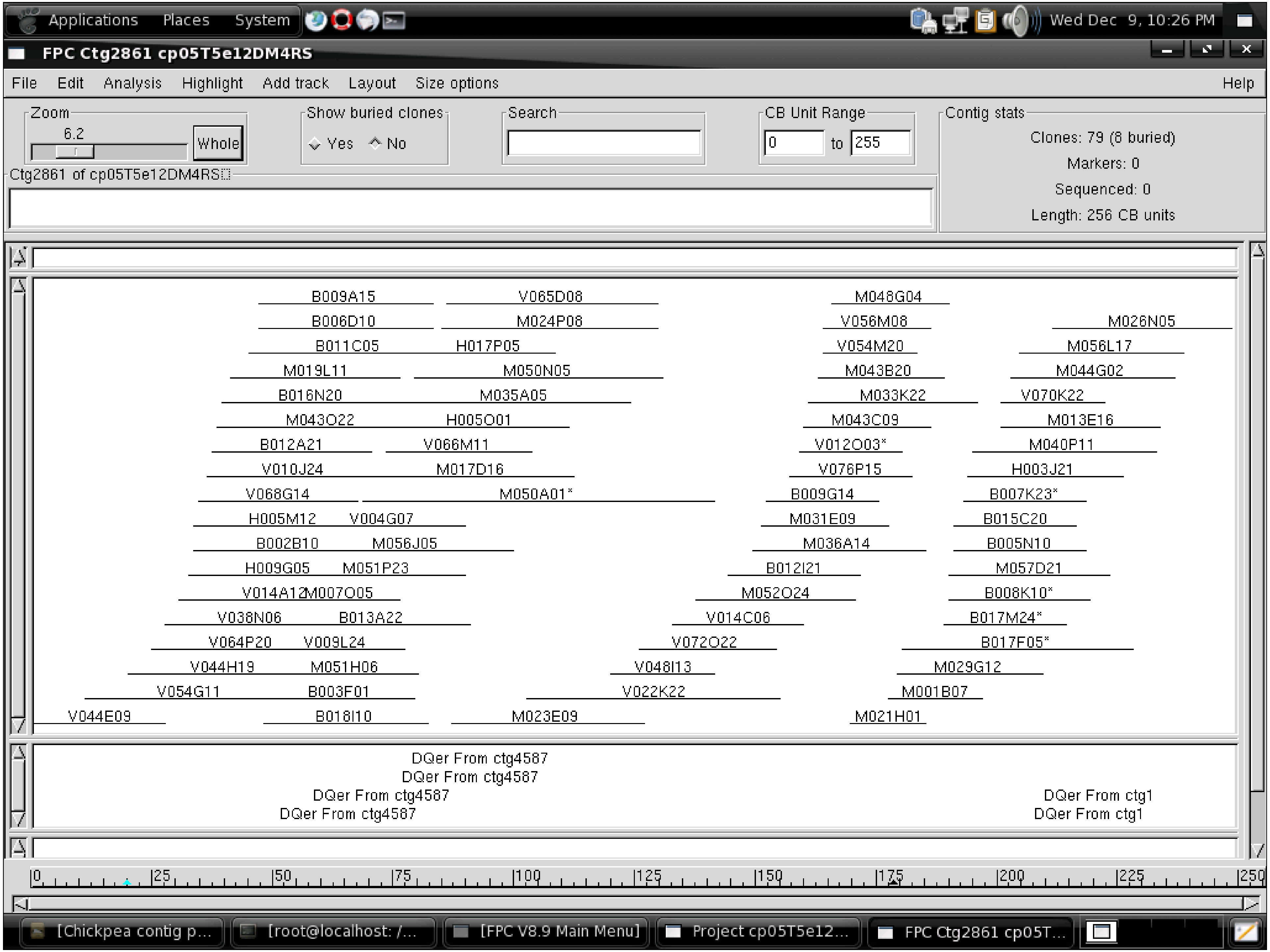
Applications Places System
Wed Dec 9, 10:26 PM
FPC Ctg2861 cp05T5e12DM4RS
File Edit Analysis Highlight Add track Layout Size options Help
Zoom
6.2
Whole
Show buried clones
Yes No
Search
CB Unit Range
0 to 255
Contig stats
Clones: 79 (8 buried)
Markers: 0
Sequenced: 0
Length: 256 CB units
Ctg2861 of cp05T5e12DM4RS
B009A15 V065D08 M048G04
B006D10 M024P08 V056M08 M026N05
B011C05 H017P05 V054M20 M056L17
M019L11 M050N05 M043B20 M044G02
B016N20 M035A05 M033K22 V070K22
M043O22 H005O01 M043C09 M013E16
B012A21 V066M11 V012O03* M040P11
V010J24 M017D16 V076P15 H003J21
V068G14 M050A01* B009G14 B007K23*
H005M12 V004G07 M031E09 B015C20
B002B10 M056J05 M036A14 B005N10
H009G05 M051P23 B012I21 M057D21
V014A12 M007O05 M052O24 B008K10*
V038N06 B013A22 V014C06 B017M24*
V064P20 V009L24 V072O22 B017F05*
V044H19 M051H06 V048I13 M029G12
V054G11 B003F01 V022K22 M001B07
V044E09 B018I10 M023E09 M021H01
DQer From ctg4587
DQer From ctg4587
DQer From ctg4587
DQer From ctg4587
DQer From ctg1
DQer From ctg1
0 25 50 75 100 125 150 175 200 225 250
[Chickpea contig p...
[root@localhost: /...
[FPC V8.9 Main Menu]
Project cp05T5e12...
FPC Ctg2861 cp05T...

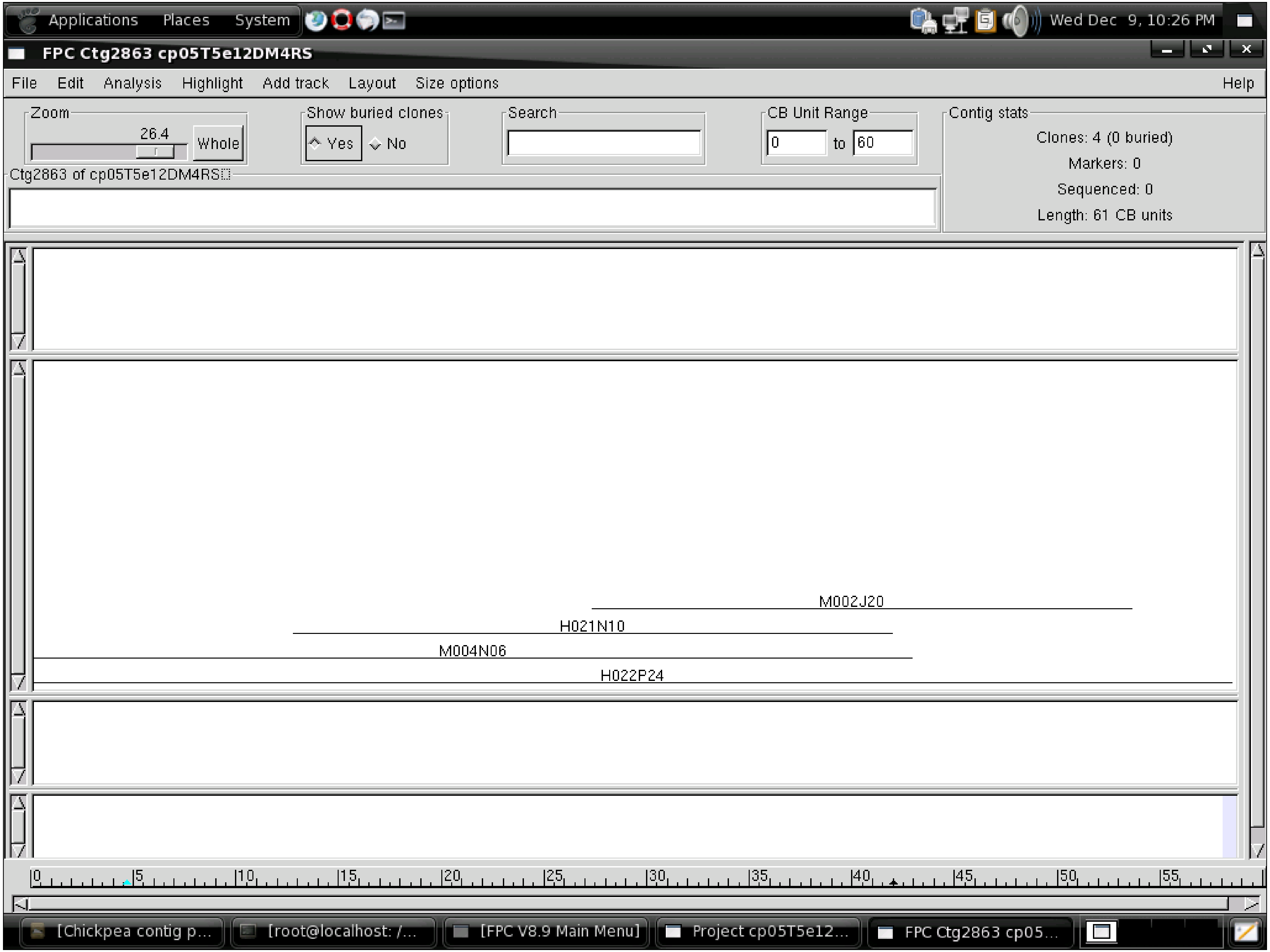
Applications
Places
System
Wed Dec 9, 10:26 PM
FPC Ctg2863 cp05T5e12DM4RS
File
Edit
Analysis
Highlight
Add track
Layout
Size options
Help
Zoom
26.4
Whole
Show buried clones
Yes
No
Search
CB Unit Range
0
to
60
Contig stats
Clones: 4 (0 buried)
Markers: 0
Sequenced: 0
Length: 61 CB units
Ctg2863 of cp05T5e12DM4RS
M002J20
H021N10
M004N06
H022P24
0
5
10
15
20
25
30
35
40
45
50
55
[Chickpea contig p...
[root@localhost: /...
[FPC V8.9 Main Menu]
Project cp05T5e12...
FPC Ctg2863 cp05...

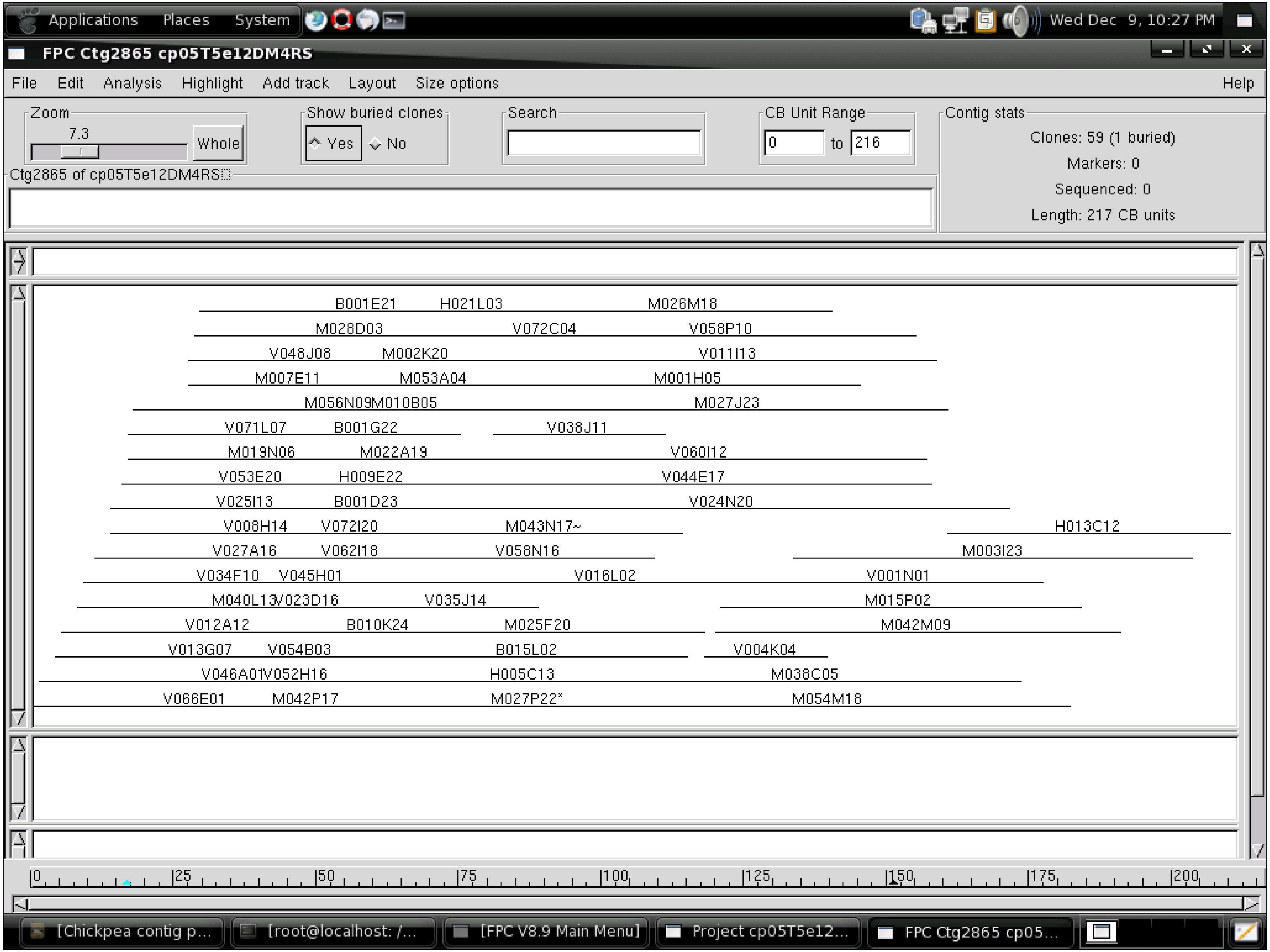
Applications Places System
Wed Dec 9, 10:27 PM
FPC Ctg2865 cp05T5e12DM4RS
File Edit Analysis Highlight Add track Layout Size options Help
Zoom
7.3
Whole
Show buried clones
Yes No
Search
CB Unit Range
0 to 216
Contig stats
Clones: 59 (1 buried)
Markers: 0
Sequenced: 0
Length: 217 CB units
Ctg2865 of cp05T5e12DM4RS
B001E21 H021L03 M026M18
M028D03 V072C04 V058P10
V048J08 M002K20 V011I13
M007E11 M053A04 M001H05
M056N09M010B05 M027J23
V071L07 B001G22 V038J11
M019N06 M022A19 V060I12
V053E20 H009E22 V044E17
V025I13 B001D23 V024N20
V008H14 V072I20 M043N17~ H013C12
V027A16 V062I18 V058N16 M003I23
V034F10 V045H01 V016L02 V001N01
M040L13V023D16 V035J14 M015P02
V012A12 B010K24 M025F20 M042M09
V013G07 V054B03 B015L02 V004K04
V046A01V052H16 H005C13 M038C05
V066E01 M042P17 M027P22* M054M18
0 25 50 75 100 125 150 175 200
[Chickpea contig p... [root@localhost: /... [FPC V8.9 Main Menu] Project cp05T5e12... FPC Ctg2865 cp05...

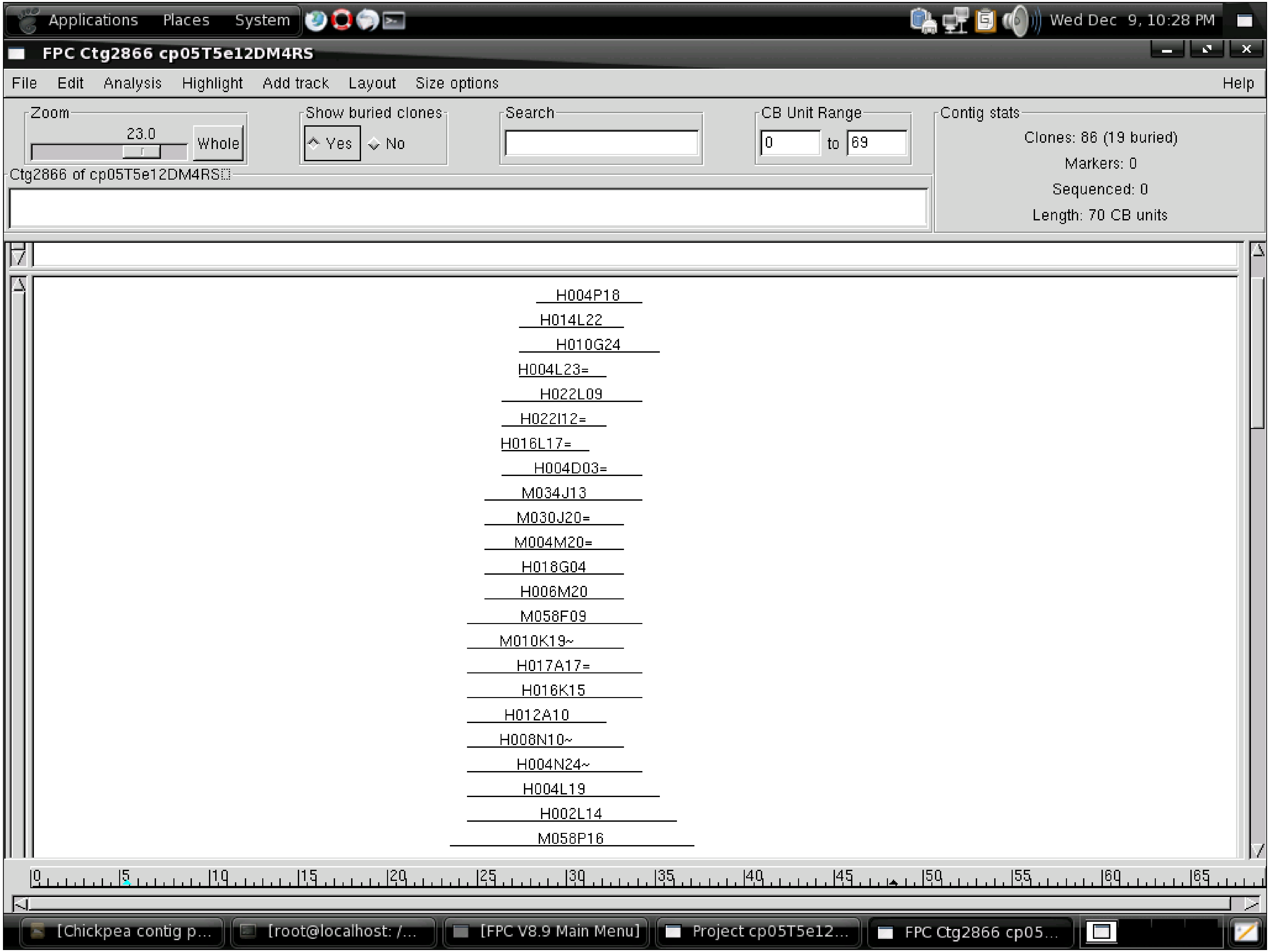

Applications Places System
Wed Dec 9, 10:28 PM
FPC Ctg2866 cp05T5e12DM4RS
File Edit Analysis Highlight Add track Layout Size options Help
Zoom
23.0
Whole
Show buried clones
Yes No
Search
CB Unit Range
0 to 69
Contig stats
Clones: 86 (19 buried)
Markers: 0
Sequenced: 0
Length: 70 CB units
Ctg2866 of cp05T5e12DM4RS
H004P18
H014L22
H010G24
H004L23=
H022L09
H022I12=
H016L17=
H004D03=
M034J13
M030J20=
M004M20=
H018G04
H006M20
M058F09
M010K19~
H017A17=
H016K15
H012A10
H008N10~
H004N24~
H004L19
H002L14
M058P16
0 5 10 15 20 25 30 35 40 45 50 55 60 65
[Chickpea contig p...
[root@localhost: /...
[FPC V8.9 Main Menu]
Project cp05T5e12...
FPC Ctg2866 cp05...

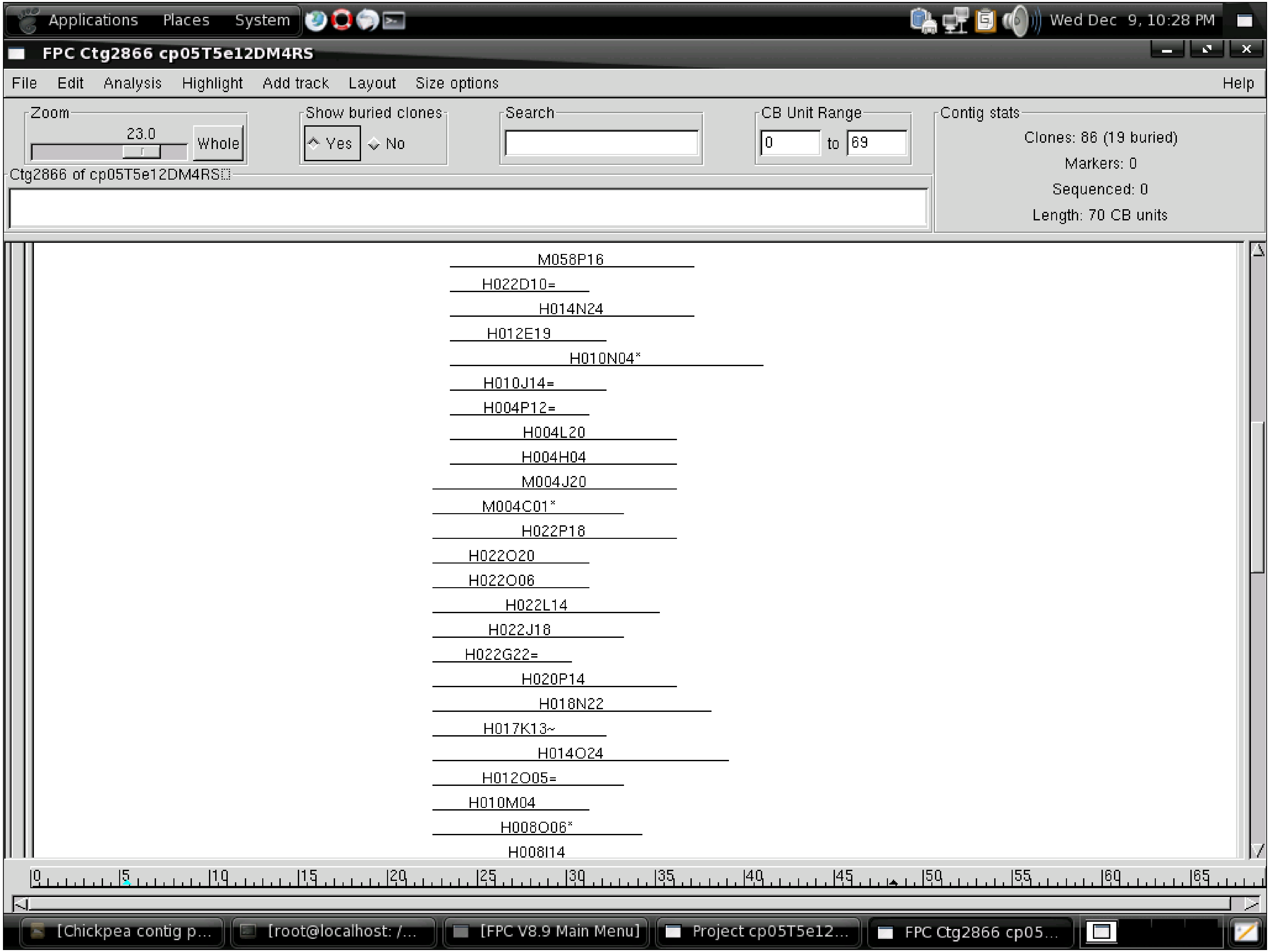
Applications Places System
Wed Dec 9, 10:28 PM
FPC Ctg2866 cp05T5e12DM4RS
File Edit Analysis Highlight Add track Layout Size options Help
Zoom
23.0
Whole
Show buried clones
Yes No
Search
CB Unit Range
0 to 69
Contig stats
Clones: 86 (19 buried)
Markers: 0
Sequenced: 0
Length: 70 CB units
Ctg2866 of cp05T5e12DM4RS
M058P16
H022D10=
H014N24
H012E19
H010N04*
H010J14=
H004P12=
H004L20
H004H04
M004J20
M004C01*
H022P18
H022O20
H022O06
H022L14
H022J18
H022G22=
H020P14
H018N22
H017K13~
H014O24
H012O05=
H010M04
H008O06*
H008I14
0 5 10 15 20 25 30 35 40 45 50 55 60 65
[Chickpea contig p...
[root@localhost: /...
[FPC V8.9 Main Menu]
Project cp05T5e12...
FPC Ctg2866 cp05...

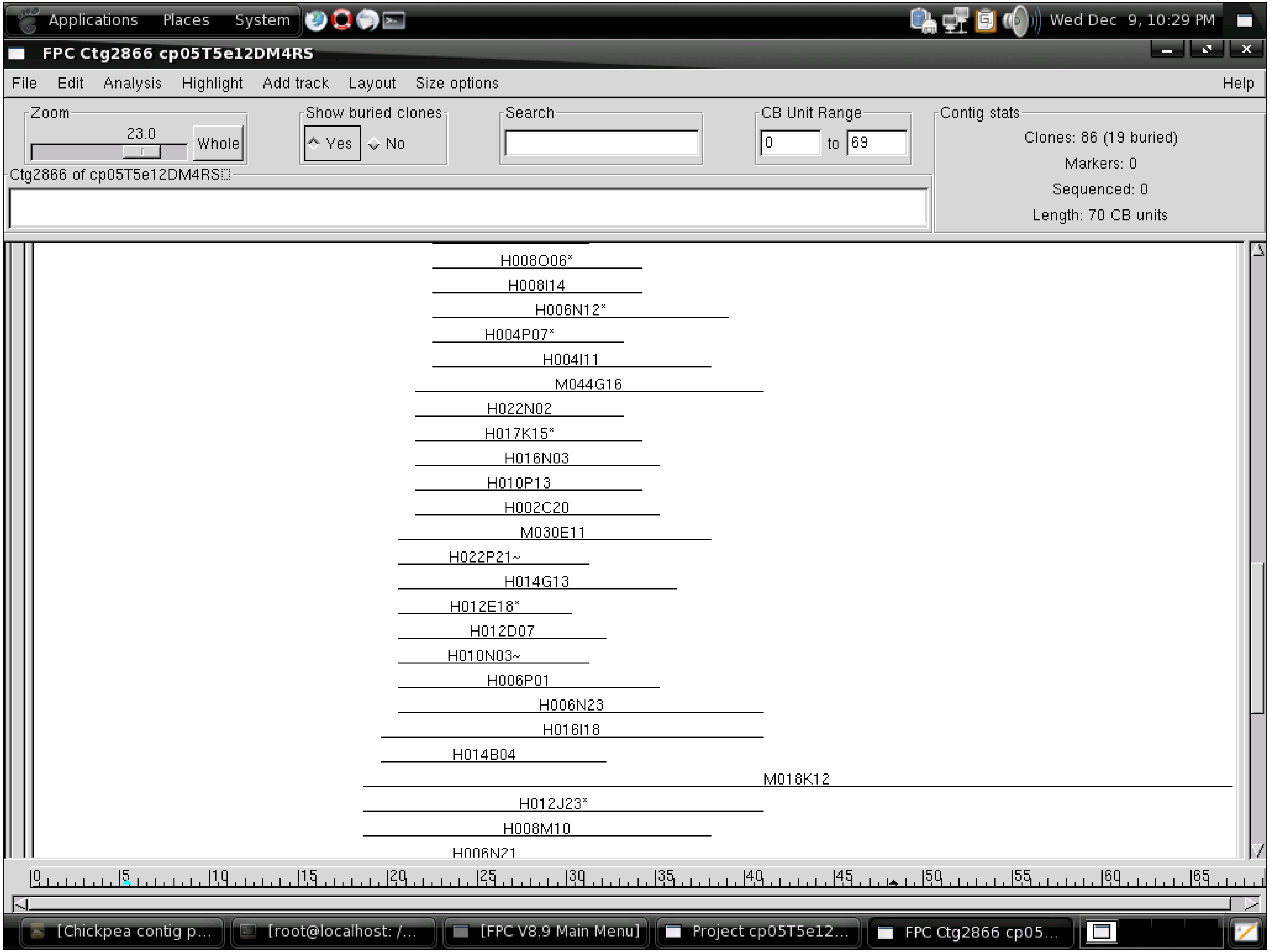
Applications Places System
Wed Dec 9, 10:29 PM
FPC Ctg2866 cp05T5e12DM4RS
File Edit Analysis Highlight Add track Layout Size options Help
Zoom
23.0
Whole
Show buried clones
Yes No
Search
CB Unit Range
0 to 69
Contig stats
Clones: 86 (19 buried)
Markers: 0
Sequenced: 0
Length: 70 CB units
Ctg2866 of cp05T5e12DM4RS
H008O06*
H008I14
H006N12*
H004P07*
H004I11
M044G16
H022N02
H017K15*
H016N03
H010P13
H002C20
M030E11
H022P21~
H014G13
H012E18*
H012D07
H010N03~
H006P01
H006N23
H016I18
H014B04
M018K12
H012J23*
H008M10
H006N21
0 5 10 15 20 25 30 35 40 45 50 55 60 65
[Chickpea contig p...
[root@localhost: /...
[FPC V8.9 Main Menu]
Project cp05T5e12...
FPC Ctg2866 cp05...

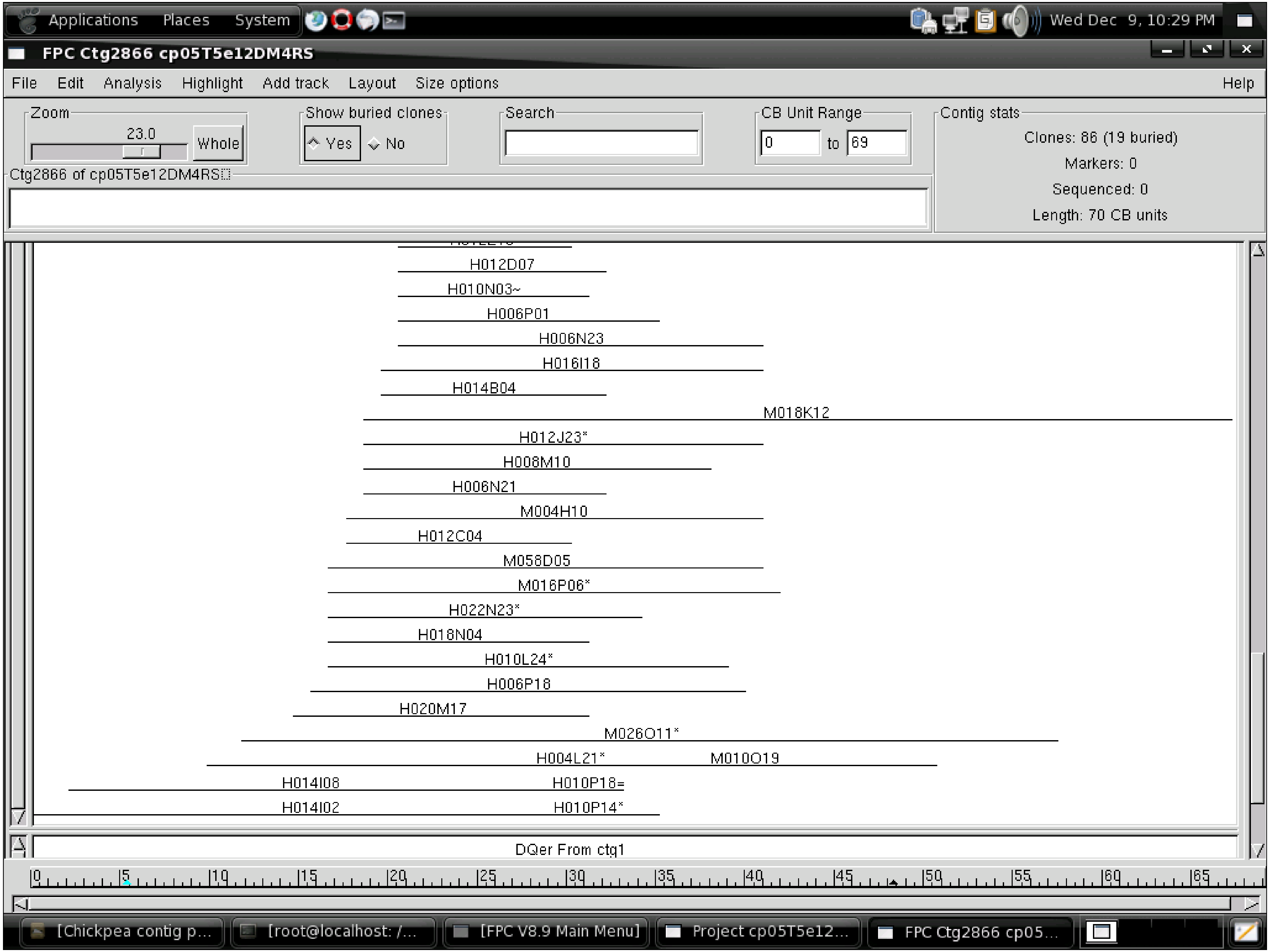
Applications Places System
Wed Dec 9, 10:29 PM
FPC Ctg2866 cp05T5e12DM4RS
File Edit Analysis Highlight Add track Layout Size options Help
Zoom
23.0
Whole
Show buried clones
Yes No
Search
CB Unit Range
0 to 69
Contig stats
Clones: 86 (19 buried)
Markers: 0
Sequenced: 0
Length: 70 CB units
Ctg2866 of cp05T5e12DM4RS
H012D07
H010N03~
H006P01
H006N23
H016I18
H014B04
M018K12
H012J23*
H008M10
H006N21
M004H10
H012C04
M058D05
M016P06*
H022N23*
H018N04
H010L24*
H006P18
H020M17
M026O11*
H004L21*
M010O19
H014I08
H010P18=
H014I02
H010P14*
DQer From ctg1
0 5 10 15 20 25 30 35 40 45 50 55 60 65
[Chickpea contig p...
[root@localhost: /...
[FPC V8.9 Main Menu]
Project cp05T5e12...
FPC Ctg2866 cp05...

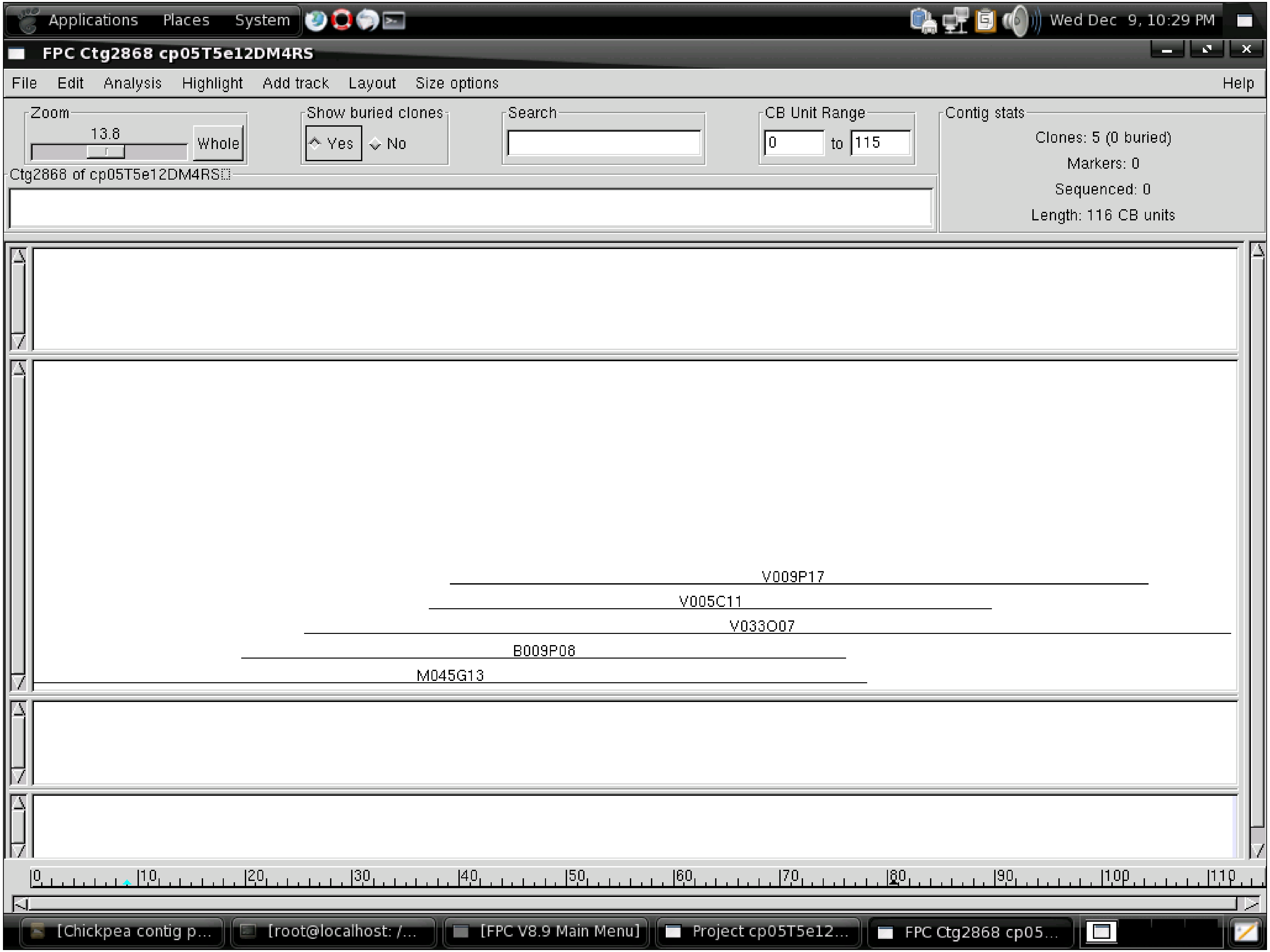
Applications Places System
Wed Dec 9, 10:29 PM
FPC Ctg2868 cp05T5e12DM4RS
File Edit Analysis Highlight Add track Layout Size options Help
Zoom
13.8
Whole
Show buried clones
Yes
No
Search
CB Unit Range
0 to 115
Contig stats
Clones: 5 (0 buried)
Markers: 0
Sequenced: 0
Length: 116 CB units
Ctg2868 of cp05T5e12DM4RS
V009P17
V005C11
V033O07
B009P08
M045G13
0 10 20 30 40 50 60 70 80 90 100 110
[Chickpea contig p...
[root@localhost: /...
[FPC V8.9 Main Menu]
Project cp05T5e12...
FPC Ctg2868 cp05...

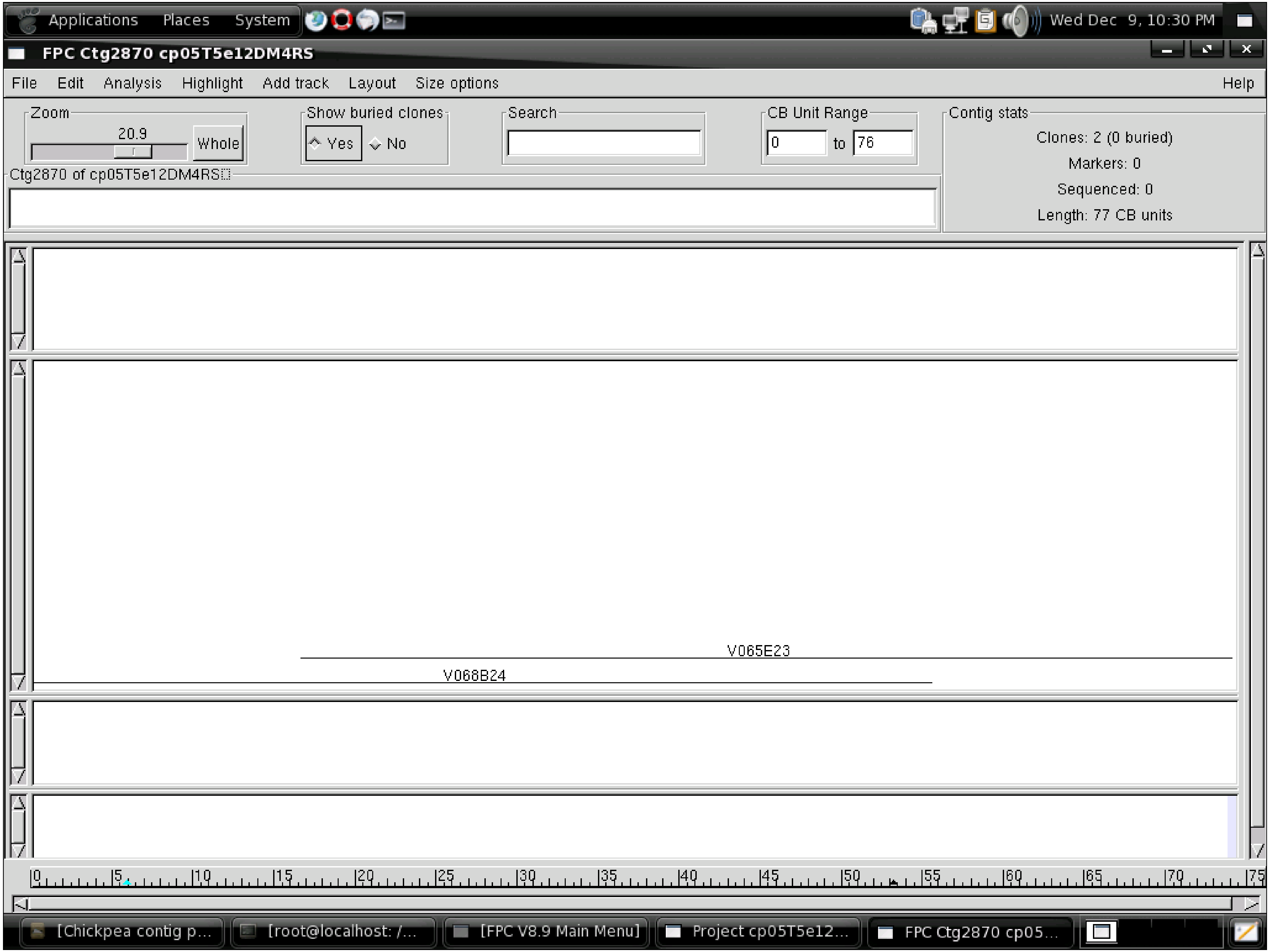
Applications
Places
System
Wed Dec 9, 10:30 PM
FPC Ctg2870 cp05T5e12DM4RS
File
Edit
Analysis
Highlight
Add track
Layout
Size options
Help
Zoom
20.9
Whole
Show buried clones
Yes
No
Search
CB Unit Range
0
to
76
Contig stats
Clones: 2 (0 buried)
Markers: 0
Sequenced: 0
Length: 77 CB units
Ctg2870 of cp05T5e12DM4RS
V065E23
V068B24
0 5 10 15 20 25 30 35 40 45 50 55 60 65 70 75
[Chickpea contig p...
[root@localhost: /...
[FPC V8.9 Main Menu]
Project cp05T5e12...
FPC Ctg2870 cp05...

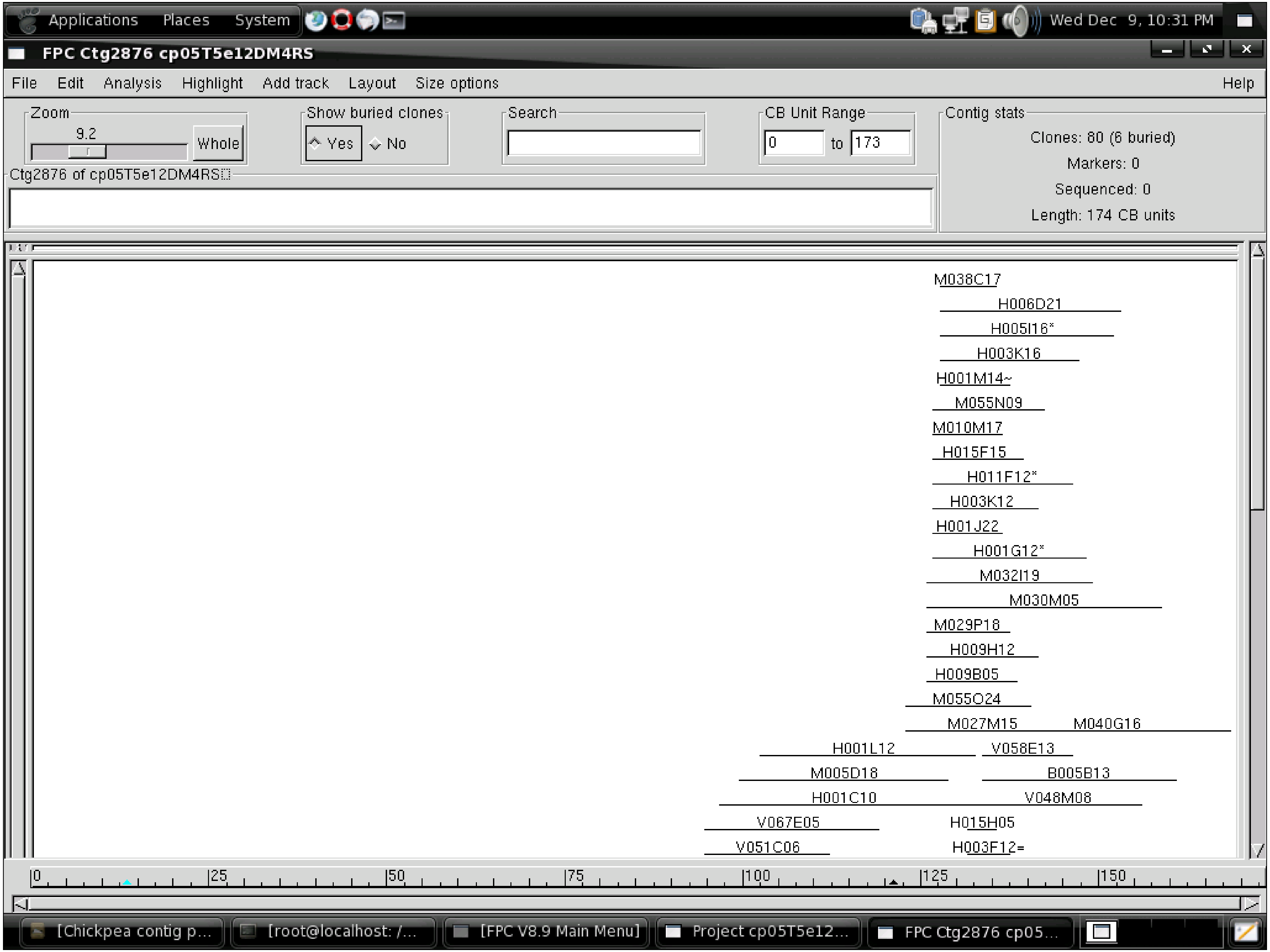
Applications Places System
Wed Dec 9, 10:31 PM
FPC Ctg2876 cp05T5e12DM4RS
File Edit Analysis Highlight Add track Layout Size options Help
Zoom
9.2
Whole
Show buried clones
Yes
No
Search
CB Unit Range
0
to
173
Contig stats
Clones: 80 (6 buried)
Markers: 0
Sequenced: 0
Length: 174 CB units
Ctg2876 of cp05T5e12DM4RS
M038C17
H006D21
H005I16*
H003K16
H001M14~
M055N09
M010M17
H015F15
H011F12*
H003K12
H001J22
H001G12*
M032I19
M030M05
M029P18
H009H12
H009B05
M055O24
M027M15
M040G16
H001L12
V058E13
M005D18
B005B13
H001C10
V048M08
V067E05
H015H05
V051C06
H003F12=
0
25
50
75
100
125
150
[Chickpea contig p...
[root@localhost: /...
[FPC V8.9 Main Menu]
Project cp05T5e12...
FPC Ctg2876 cp05...

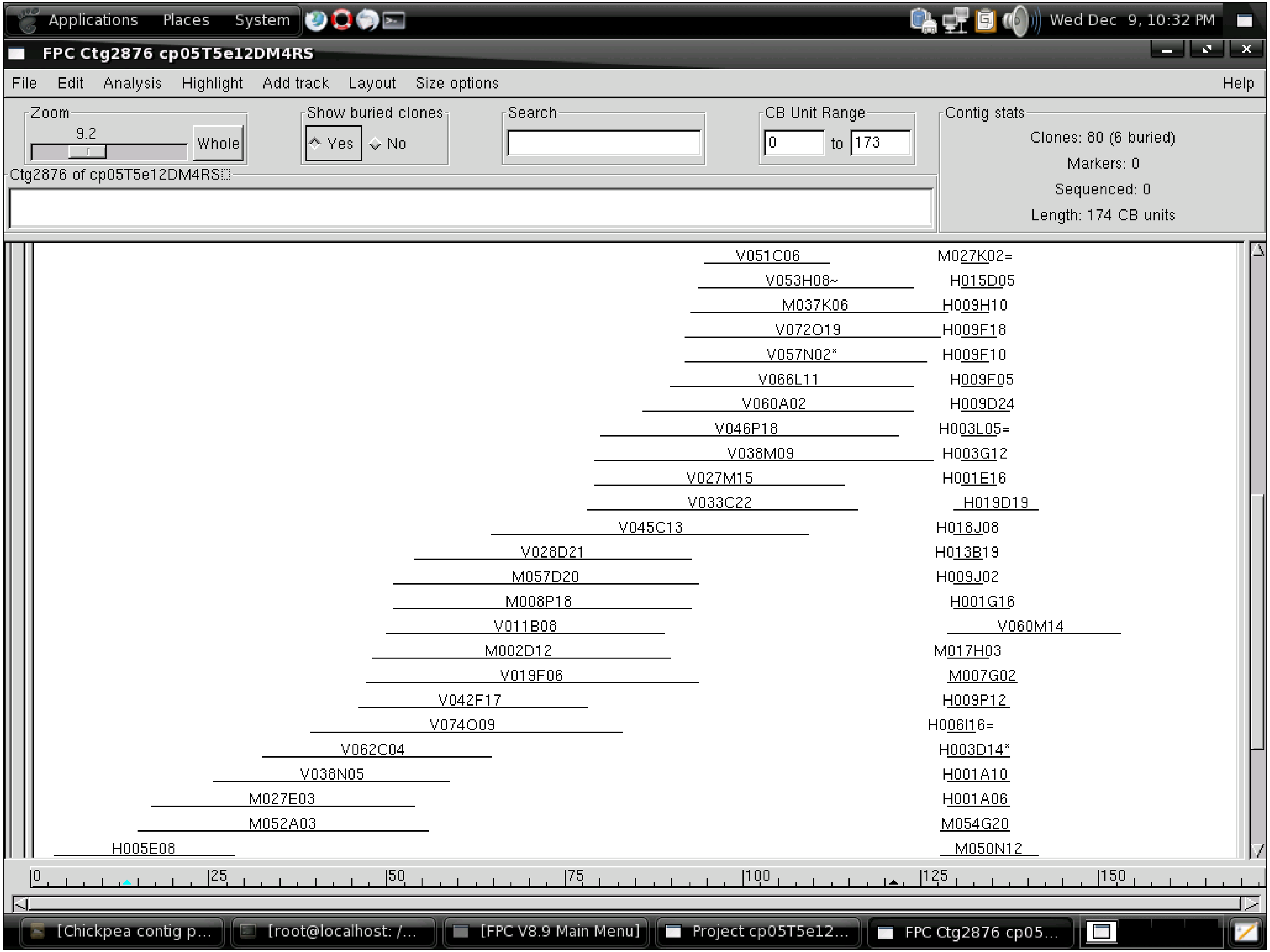
Applications Places System
Wed Dec 9, 10:32 PM
FPC Ctg2876 cp05T5e12DM4RS
File Edit Analysis Highlight Add track Layout Size options Help
Zoom
9.2
Whole
Show buried clones
Yes
No
Search
CB Unit Range
0 to 173
Contig stats
Clones: 80 (6 buried)
Markers: 0
Sequenced: 0
Length: 174 CB units
Ctg2876 of cp05T5e12DM4RS
V051C06
V053H08~
M037K06
V072O19
V057N02*
V066L11
V060A02
V046P18
V038M09
V027M15
V033C22
V045C13
V028D21
M057D20
M008P18
V011B08
M002D12
V019F06
V042F17
V074O09
V062C04
V038N05
M027E03
M052A03
H005E08
M027K02=
H015D05
H009H10
H009F18
H009F10
H009F05
H009D24
H003L05=
H003G12
H001E16
H019D19
H018J08
H013B19
H009J02
H001G16
V060M14
M017H03
M007G02
H009P12
H006I16=
H003D14*
H001A10
H001A06
M054G20
M050N12
0 25 50 75 100 125 150
[Chickpea contig p...
[root@localhost: /...
[FPC V8.9 Main Menu]
Project cp05T5e12...
FPC Ctg2876 cp05...

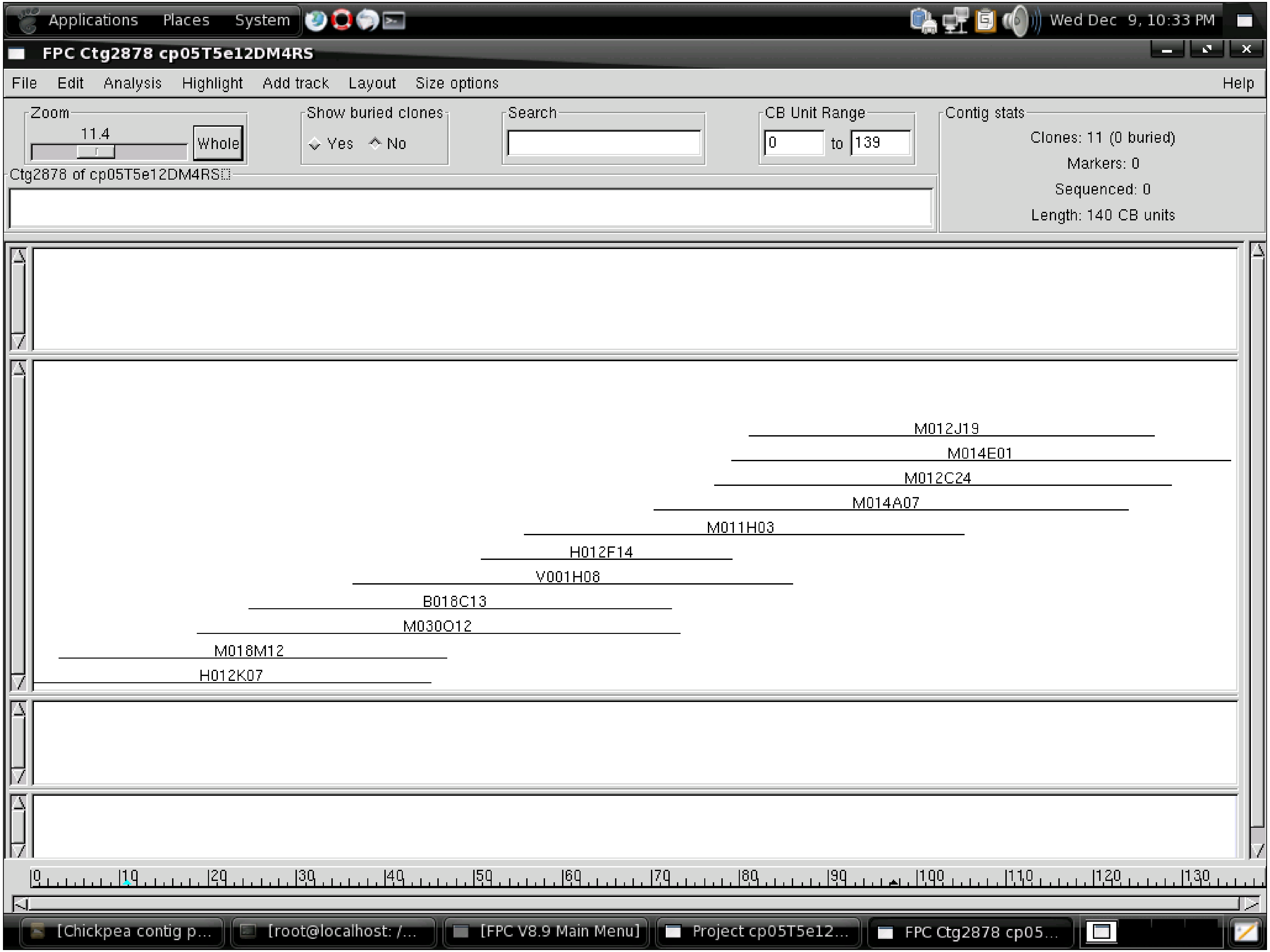

Applications
Places
System
Wed Dec 9, 10:33 PM
FPC Ctg2878 cp05T5e12DM4RS
File
Edit
Analysis
Highlight
Add track
Layout
Size options
Help
Zoom
11.4
Whole
Show buried clones
Yes
No
Search
CB Unit Range
0
to
139
Contig stats
Clones: 11 (0 buried)
Markers: 0
Sequenced: 0
Length: 140 CB units
Ctg2878 of cp05T5e12DM4RS
M012J19
M014E01
M012C24
M014A07
M011H03
H012F14
V001H08
B018C13
M030O12
M018M12
H012K07
0 10 20 30 40 50 60 70 80 90 100 110 120 130
[Chickpea contig p...
[root@localhost: /...
[FPC V8.9 Main Menu]
Project cp05T5e12...
FPC Ctg2878 cp05...

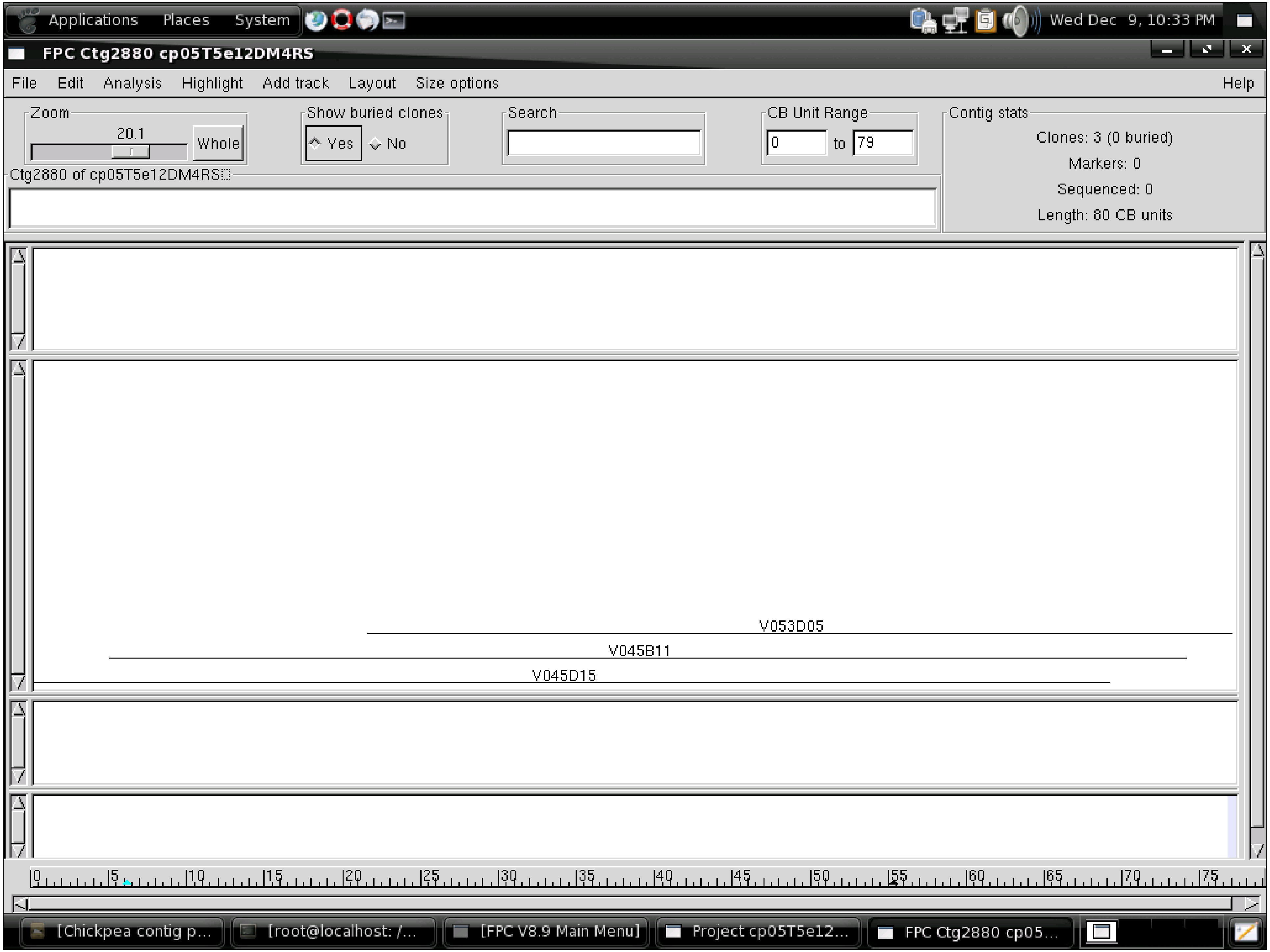

Applications
Places
System
Wed Dec 9, 10:33 PM
FPC Ctg2880 cp05T5e12DM4RS
File
Edit
Analysis
Highlight
Add track
Layout
Size options
Help
Zoom
20.1
Whole
Show buried clones
Yes
No
Search
CB Unit Range
0
to
79
Contig stats
Clones: 3 (0 buried)
Markers: 0
Sequenced: 0
Length: 80 CB units
Ctg2880 of cp05T5e12DM4RS
V053D05
V045B11
V045D15
0 5 10 15 20 25 30 35 40 45 50 55 60 65 70 75
[Chickpea contig p...
[root@localhost: /...
[FPC V8.9 Main Menu]
Project cp05T5e12...
FPC Ctg2880 cp05...

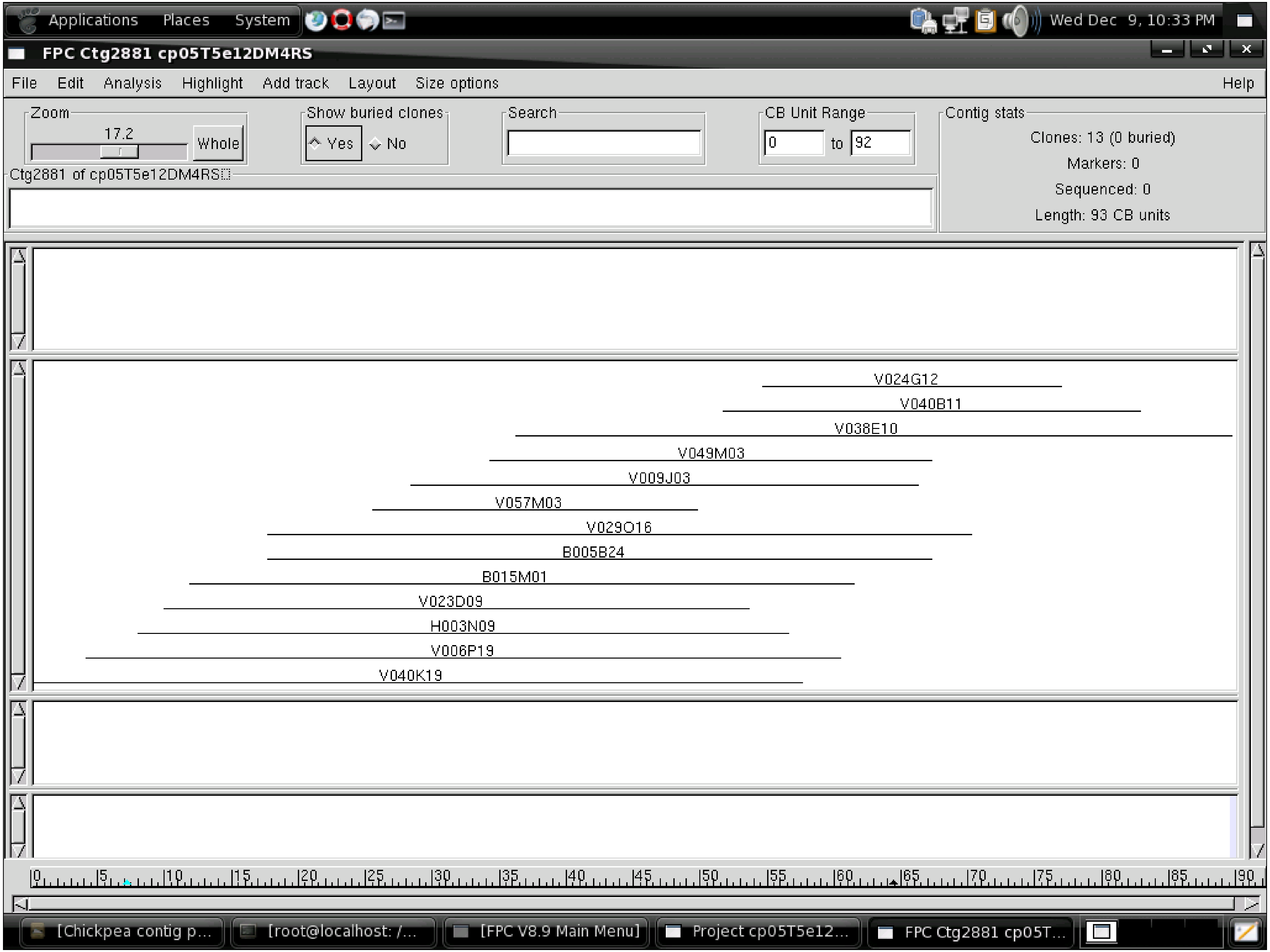

Applications Places System
Wed Dec 9, 10:33 PM
FPC Ctg2881 cp05T5e12DM4RS
File Edit Analysis Highlight Add track Layout Size options Help
Zoom
17.2
Whole
Show buried clones
Yes No
Search
CB Unit Range
0 to 92
Contig stats
Clones: 13 (0 buried)
Markers: 0
Sequenced: 0
Length: 93 CB units
Ctg2881 of cp05T5e12DM4RS
V024G12
V040B11
V038E10
V049M03
V009J03
V057M03
V029O16
B005B24
B015M01
V023D09
H003N09
V006P19
V040K19
0 5 10 15 20 25 30 35 40 45 50 55 60 65 70 75 80 85 90
[Chickpea contig p...
[root@localhost: /...
[FPC V8.9 Main Menu]
Project cp05T5e12...
FPC Ctg2881 cp05T...

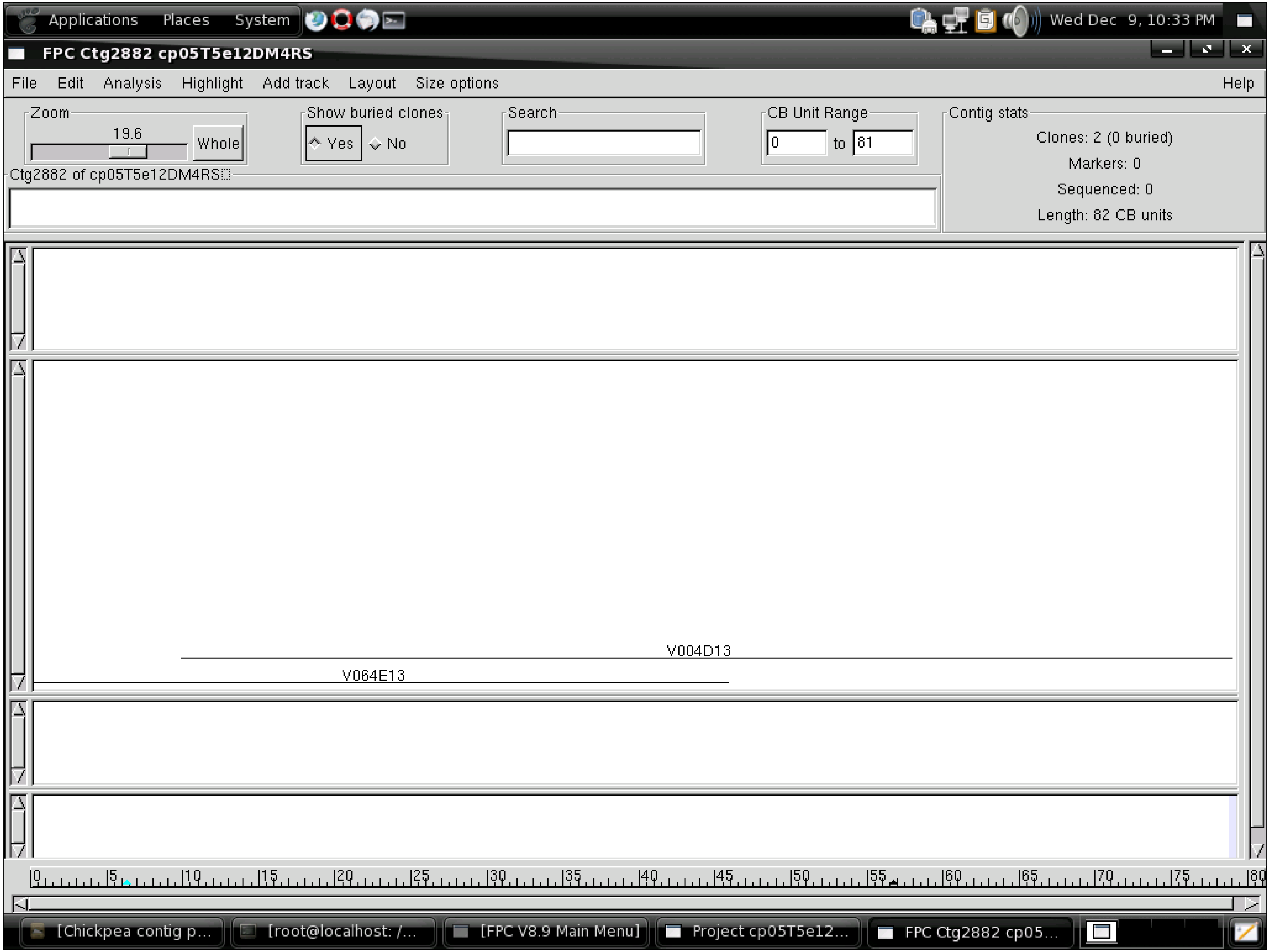
Applications Places System
Wed Dec 9, 10:33 PM
FPC Ctg2882 cp05T5e12DM4RS
File Edit Analysis Highlight Add track Layout Size options Help
Zoom
19.6
Whole
Show buried clones
Yes No
Search
CB Unit Range
0 to 81
Contig stats
Clones: 2 (0 buried)
Markers: 0
Sequenced: 0
Length: 82 CB units
Ctg2882 of cp05T5e12DM4RS
V004D13
V064E13
0 5 10 15 20 25 30 35 40 45 50 55 60 65 70 75 80
[Chickpea contig p...
[root@localhost: /...
[FPC V8.9 Main Menu]
Project cp05T5e12...
FPC Ctg2882 cp05...

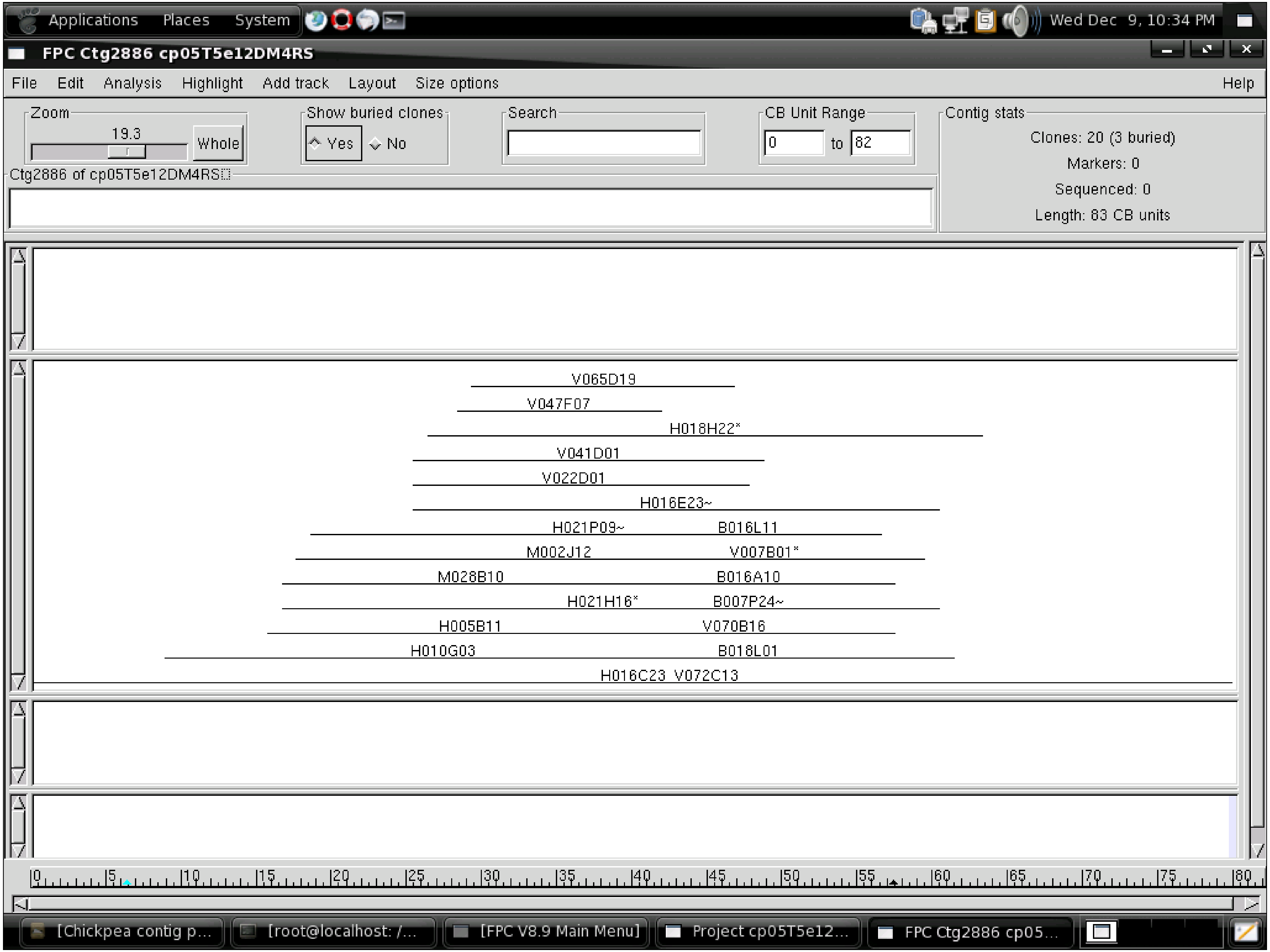
Applications Places System
Wed Dec 9, 10:34 PM
FPC Ctg2886 cp05T5e12DM4RS
File Edit Analysis Highlight Add track Layout Size options Help
Zoom
19.3
Whole
Show buried clones
Yes No
Search
CB Unit Range
0 to 82
Contig stats
Clones: 20 (3 buried)
Markers: 0
Sequenced: 0
Length: 83 CB units
Ctg2886 of cp05T5e12DM4RS
V065D19
V047F07
H018H22*
V041D01
V022D01
H016E23~
H021P09~ B016L11
M002J12 V007B01*
M028B10 B016A10
H021H16* B007P24~
H005B11 V070B16
H010G03 B018L01
H016C23 V072C13
0 5 10 15 20 25 30 35 40 45 50 55 60 65 70 75 80
[Chickpea contig p...
[root@localhost: /...
[FPC V8.9 Main Menu]
Project cp05T5e12...
FPC Ctg2886 cp05...

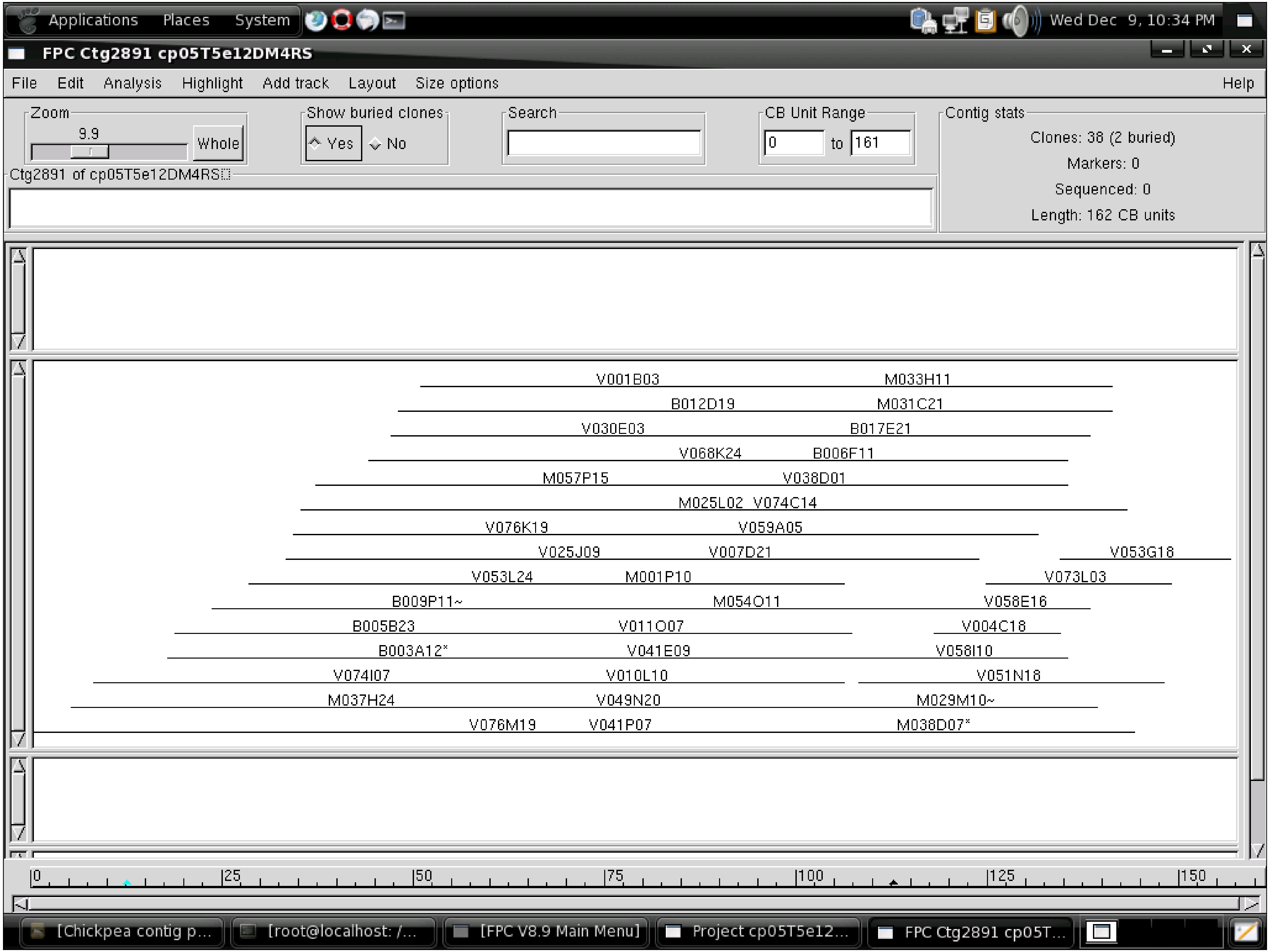
Applications Places System
Wed Dec 9, 10:34 PM
FPC Ctg2891 cp05T5e12DM4RS
File Edit Analysis Highlight Add track Layout Size options Help
Zoom
9.9
Whole
Show buried clones
Yes No
Search
CB Unit Range
0 to 161
Contig stats
Clones: 38 (2 buried)
Markers: 0
Sequenced: 0
Length: 162 CB units
Ctg2891 of cp05T5e12DM4RS
V001B03 M033H11
B012D19 M031C21
V030E03 B017E21
V068K24 B006F11
M057P15 V038D01
M025L02 V074C14
V076K19 V059A05
V025J09 V007D21 V053G18
V053L24 M001P10 V073L03
B009P11~ M054O11 V058E16
B005B23 V011O07 V004C18
B003A12* V041E09 V058I10
V074I07 V010L10 V051N18
M037H24 V049N20 M029M10~
V076M19 V041P07 M038D07*
0 25 50 75 100 125 150
[Chickpea contig p...
[root@localhost: /...
[FPC V8.9 Main Menu]
Project cp05T5e12...
FPC Ctg2891 cp05T...

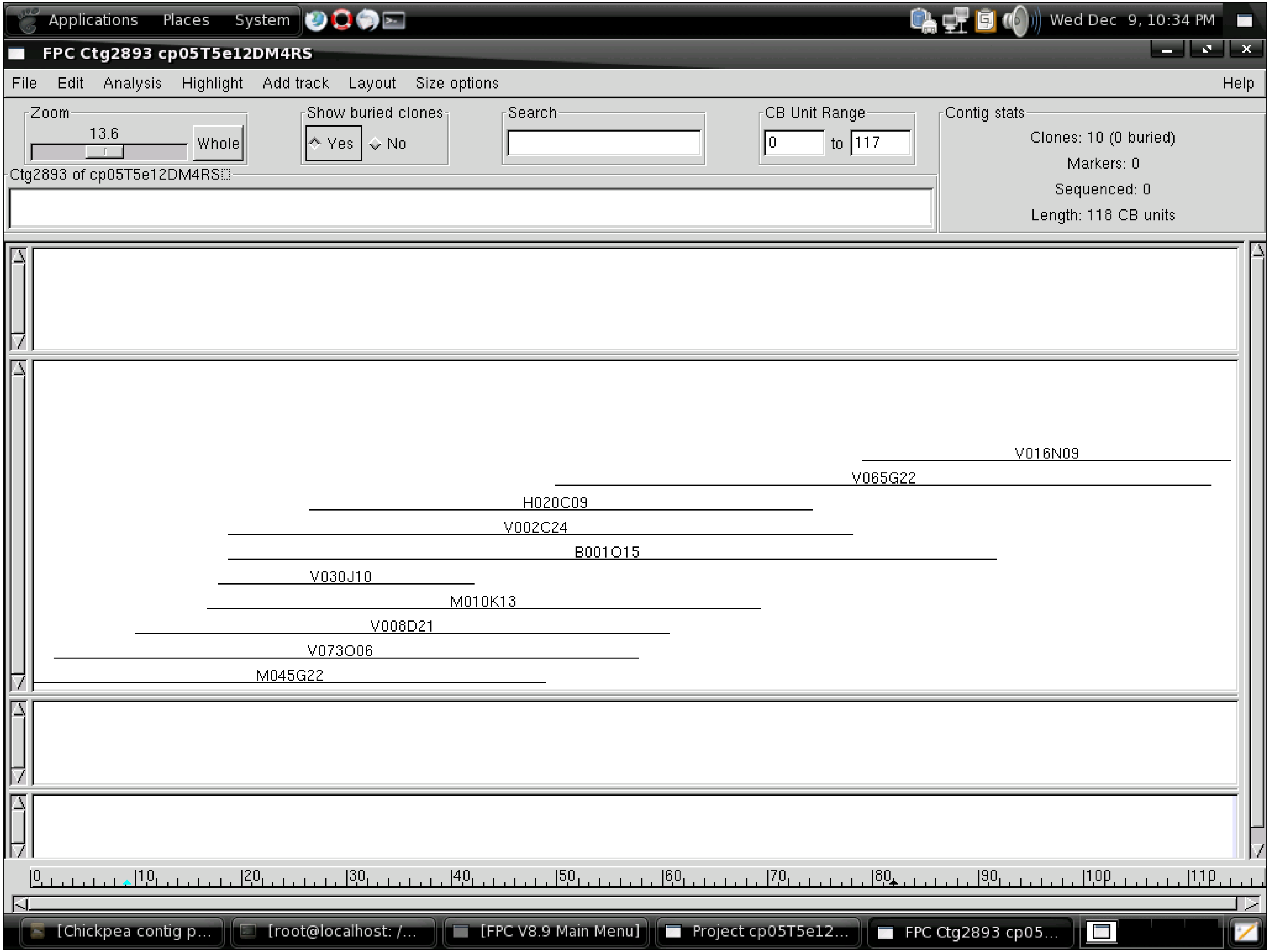
Applications
Places
System
Wed Dec 9, 10:34 PM
FPC Ctg2893 cp05T5e12DM4RS
File
Edit
Analysis
Highlight
Add track
Layout
Size options
Help
Zoom
13.6
Whole
Show buried clones
Yes
No
Search
CB Unit Range
0
to
117
Contig stats
Clones: 10 (0 buried)
Markers: 0
Sequenced: 0
Length: 118 CB units
Ctg2893 of cp05T5e12DM4RS
V016N09
V065G22
H020C09
V002C24
B001O15
V030J10
M010K13
V008D21
V073O06
M045G22
0
10
20
30
40
50
60
70
80
90
100
110
[Chickpea contig p...
[root@localhost: /...
[FPC V8.9 Main Menu]
Project cp05T5e12...
FPC Ctg2893 cp05...

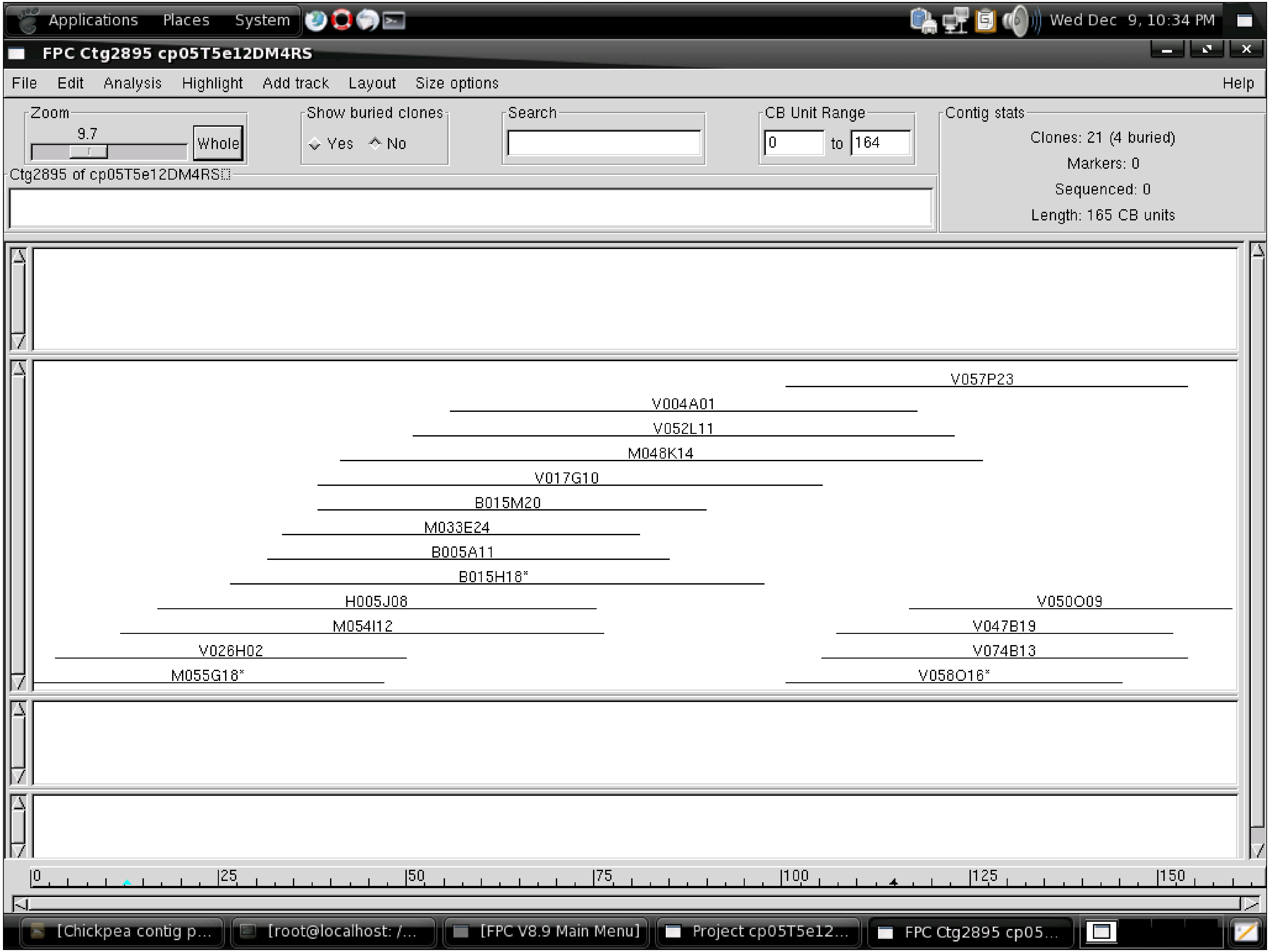
Applications Places System
Wed Dec 9, 10:34 PM
FPC Ctg2895 cp05T5e12DM4RS
File Edit Analysis Highlight Add track Layout Size options Help
Zoom
9.7
Whole
Show buried clones
Yes No
Search
CB Unit Range
0 to 164
Contig stats
Clones: 21 (4 buried)
Markers: 0
Sequenced: 0
Length: 165 CB units
Ctg2895 of cp05T5e12DM4RS
V057P23
V004A01
V052L11
M048K14
V017G10
B015M20
M033E24
B005A11
B015H18*
H005J08
V050O09
M054I12
V047B19
V026H02
V074B13
M055G18*
V058O16*
0
25
50
75
100
125
150
[Chickpea contig p...
[root@localhost: /...
[FPC V8.9 Main Menu]
Project cp05T5e12...
FPC Ctg2895 cp05...

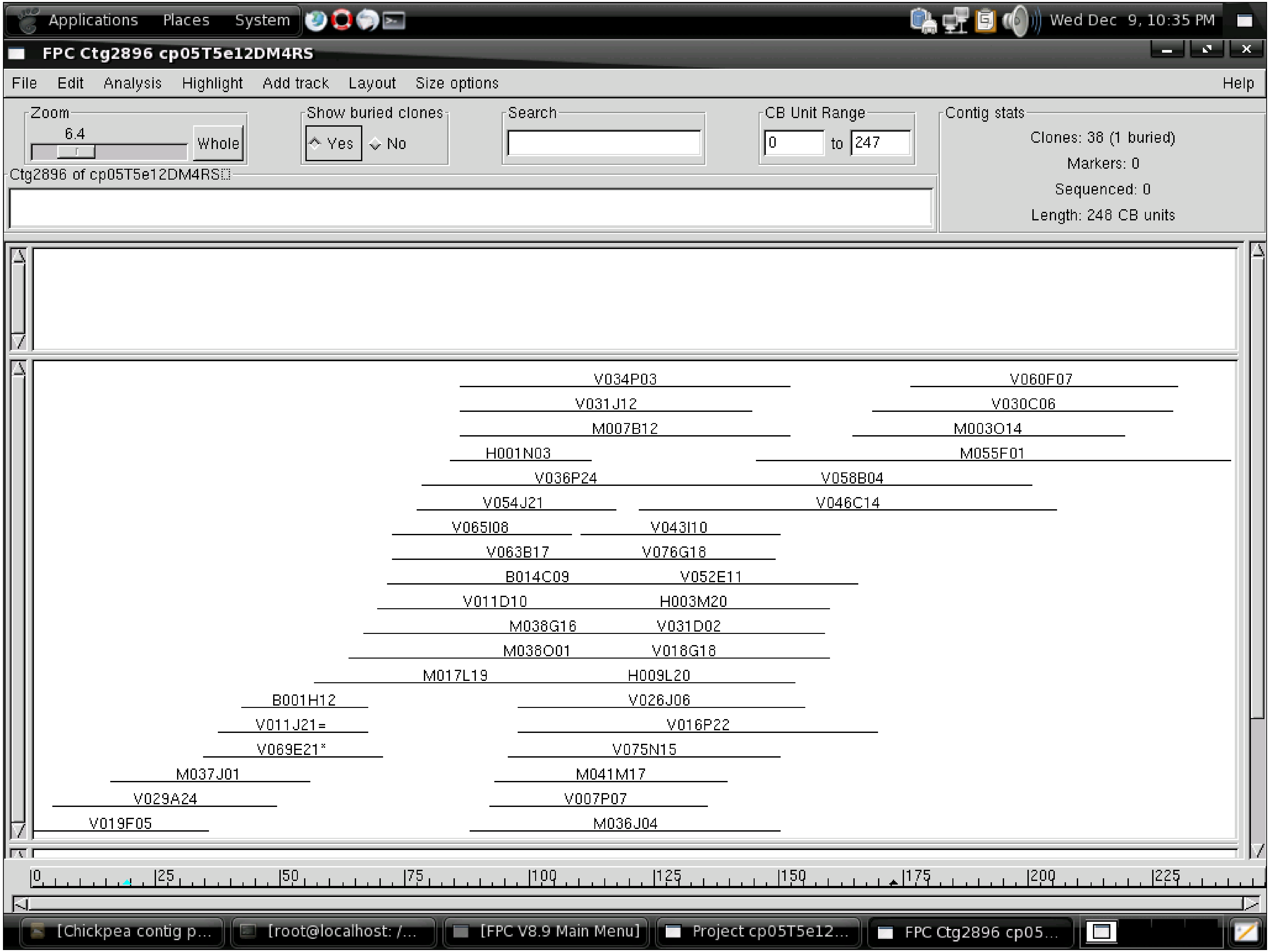
Applications
Places
System
Wed Dec 9, 10:35 PM
FPC Ctg2896 cp05T5e12DM4RS
File
Edit
Analysis
Highlight
Add track
Layout
Size options
Help
Zoom
6.4
Whole
Show buried clones
Yes
No
Search
CB Unit Range
0
to
247
Contig stats
Clones: 38 (1 buried)
Markers: 0
Sequenced: 0
Length: 248 CB units
Ctg2896 of cp05T5e12DM4RS
V034P03
V060F07
V031J12
V030C06
M007B12
M003O14
H001N03
M055F01
V036P24
V058B04
V054J21
V046C14
V065I08
V043I10
V063B17
V076G18
B014C09
V052E11
V011D10
H003M20
M038G16
V031D02
M038O01
V018G18
M017L19
H009L20
B001H12
V026J06
V011J21=
V016P22
V069E21*
V075N15
M037J01
M041M17
V029A24
V007P07
V019F05
M036J04
0
25
50
75
100
125
150
175
200
225
[Chickpea contig p...
[root@localhost: /...
[FPC V8.9 Main Menu]
Project cp05T5e12...
FPC Ctg2896 cp05...

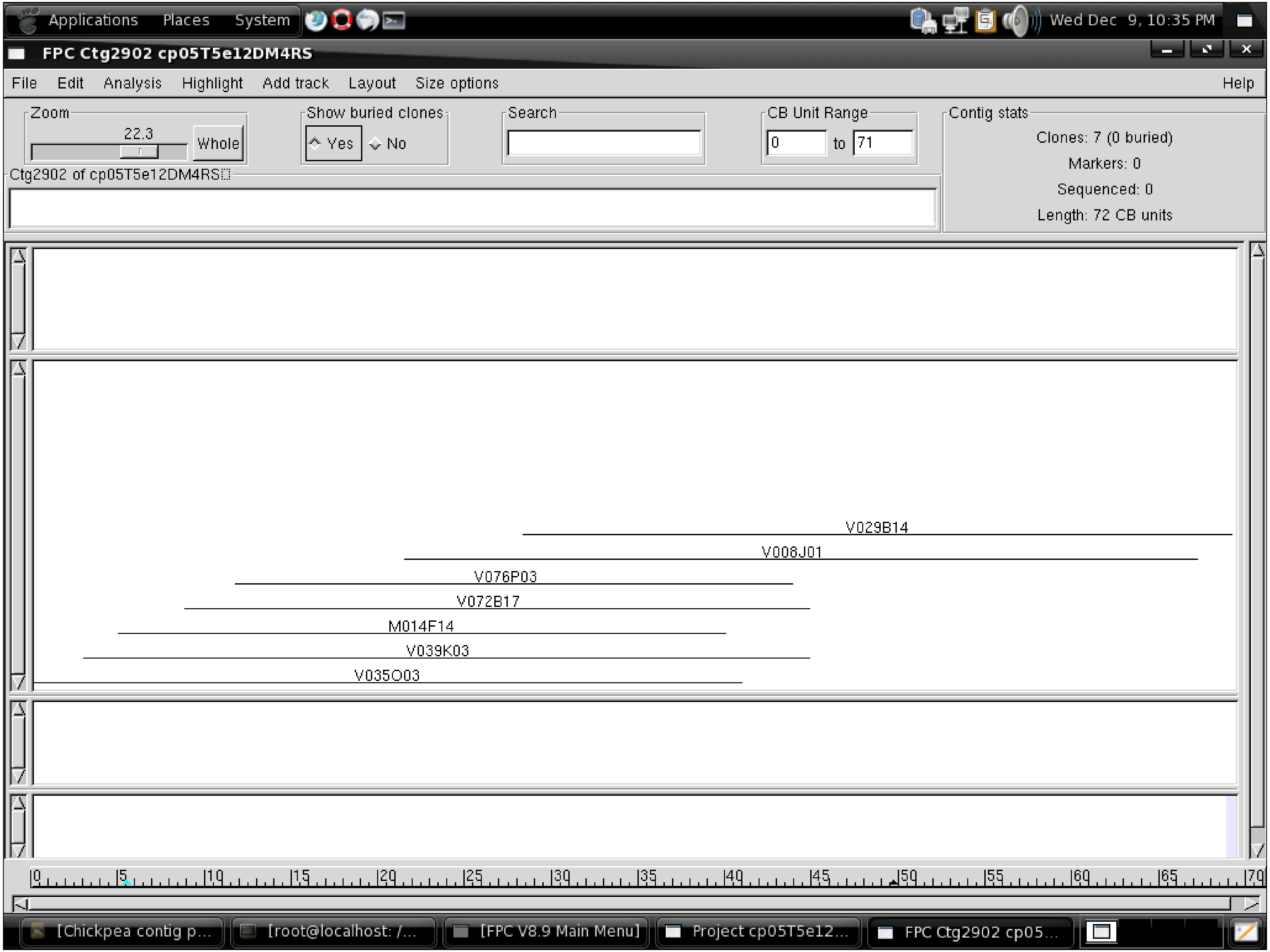
Applications
Places
System
Wed Dec 9, 10:35 PM
FPC Ctg2902 cp05T5e12DM4RS
File
Edit
Analysis
Highlight
Add track
Layout
Size options
Help
Zoom
22.3
Whole
Show buried clones
Yes
No
Search
CB Unit Range
0
to
71
Contig stats
Clones: 7 (0 buried)
Markers: 0
Sequenced: 0
Length: 72 CB units
Ctg2902 of cp05T5e12DM4RS
V029B14
V008J01
V076P03
V072B17
M014F14
V039K03
V035O03
0 5 10 15 20 25 30 35 40 45 50 55 60 65 70
[Chickpea contig p...
[root@localhost: /...
[FPC V8.9 Main Menu]
Project cp05T5e12...
FPC Ctg2902 cp05...

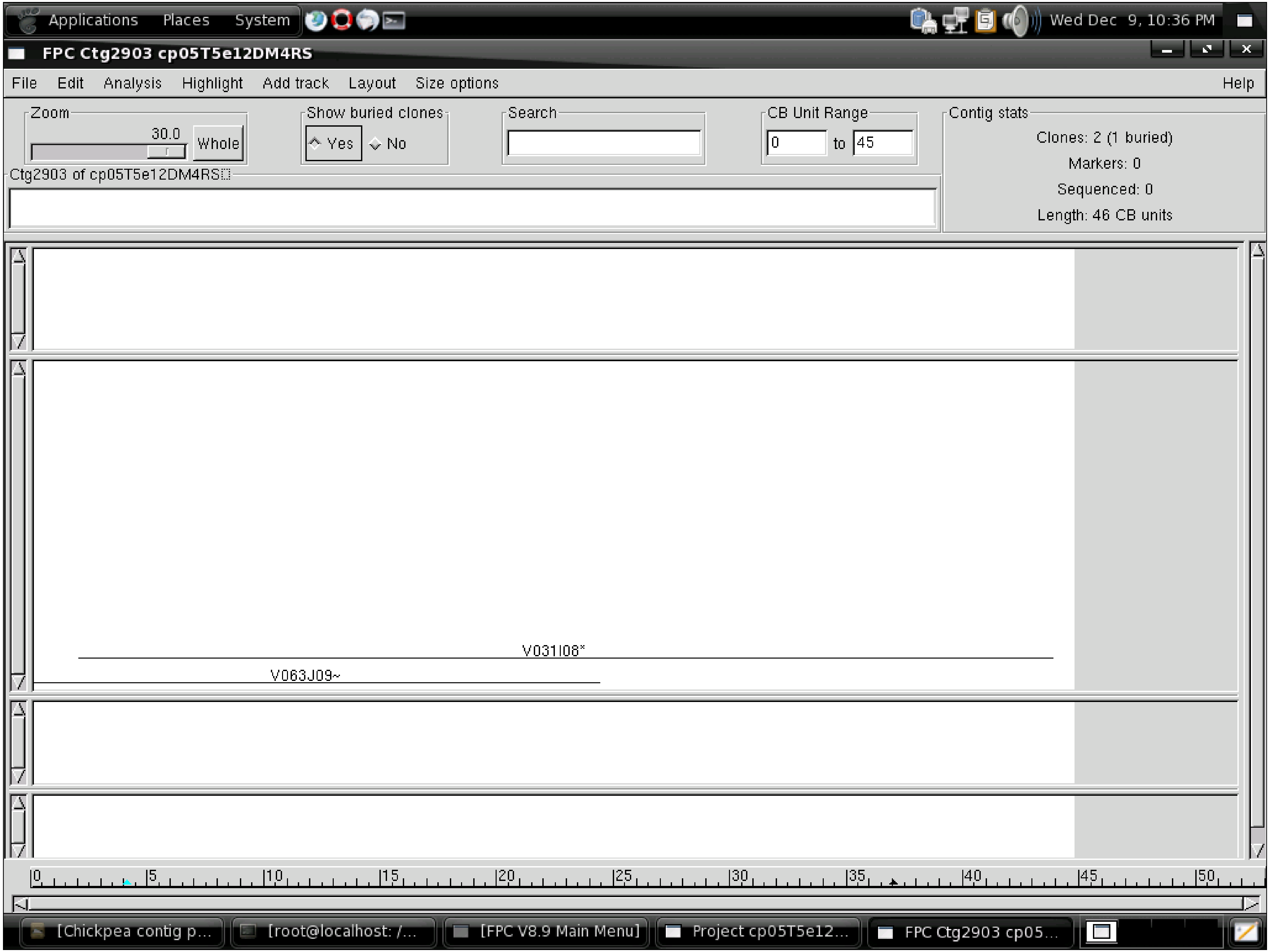
Applications Places System
Wed Dec 9, 10:36 PM
FPC Ctg2903 cp05T5e12DM4RS
File Edit Analysis Highlight Add track Layout Size options Help
Zoom
30.0
Whole
Show buried clones
Yes
No
Search
CB Unit Range
0
to
45
Contig stats
Clones: 2 (1 buried)
Markers: 0
Sequenced: 0
Length: 46 CB units
Ctg2903 of cp05T5e12DM4RS
V031I08*
V063J09~
0 5 10 15 20 25 30 35 40 45 50
[Chickpea contig p...
[root@localhost: /...
[FPC V8.9 Main Menu]
Project cp05T5e12...
FPC Ctg2903 cp05...

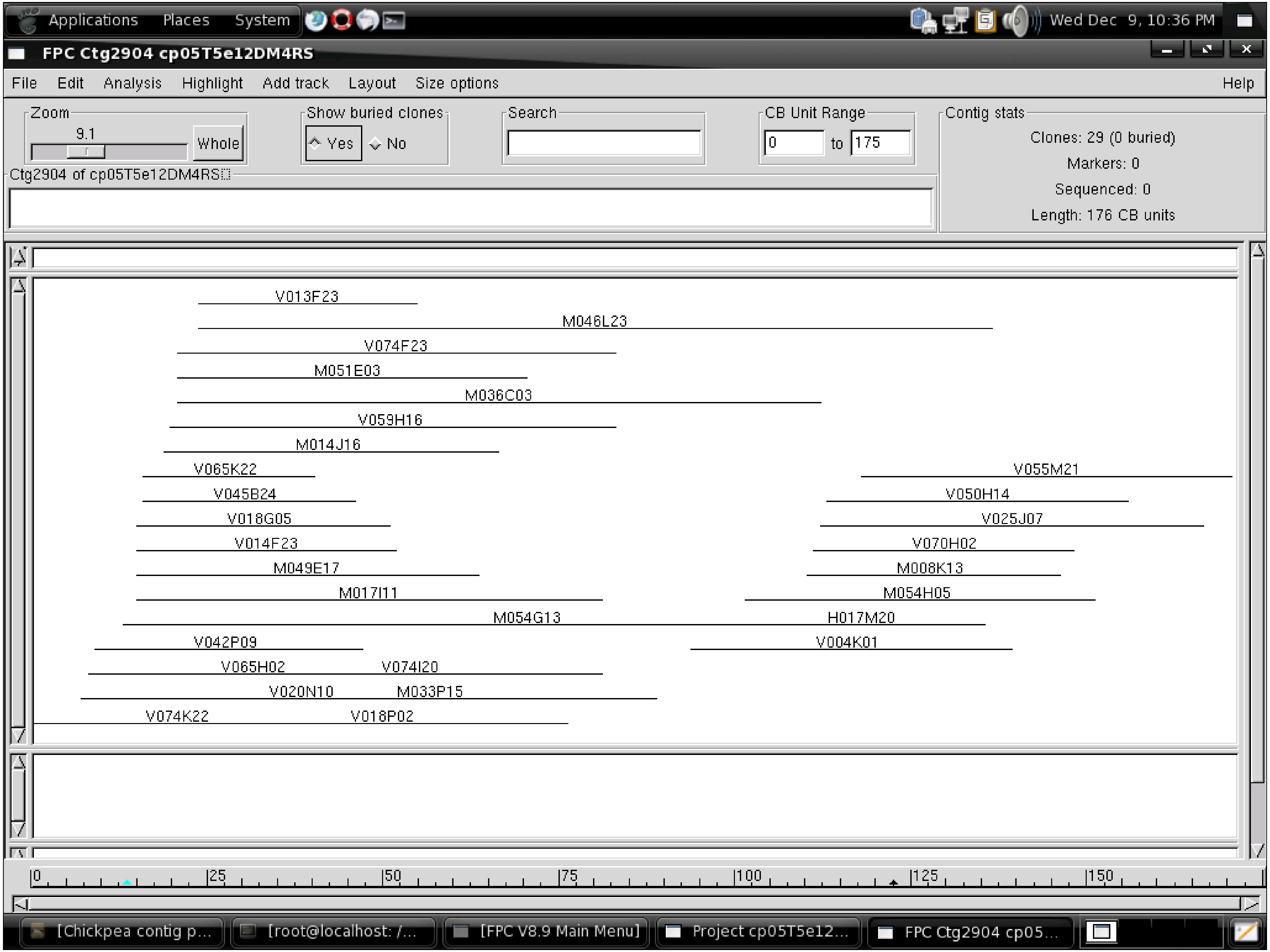
Applications Places System
Wed Dec 9, 10:36 PM
FPC Ctg2904 cp05T5e12DM4RS
File Edit Analysis Highlight Add track Layout Size options Help
Zoom
9.1
Whole
Show buried clones
Yes No
Search
CB Unit Range
0 to 175
Contig stats
Clones: 29 (0 buried)
Markers: 0
Sequenced: 0
Length: 176 CB units
Ctg2904 of cp05T5e12DM4RS
V013F23
M046L23
V074F23
M051E03
M036C03
V059H16
M014J16
V065K22
V055M21
V045B24
V050H14
V018G05
V025J07
V014F23
V070H02
M049E17
M008K13
M017I11
M054H05
M054G13
H017M20
V042P09
V004K01
V065H02
V074I20
V020N10
M033P15
V074K22
V018P02
0 25 50 75 100 125 150
[Chickpea contig p...
[root@localhost: /...
[FPC V8.9 Main Menu]
Project cp05T5e12...
FPC Ctg2904 cp05...

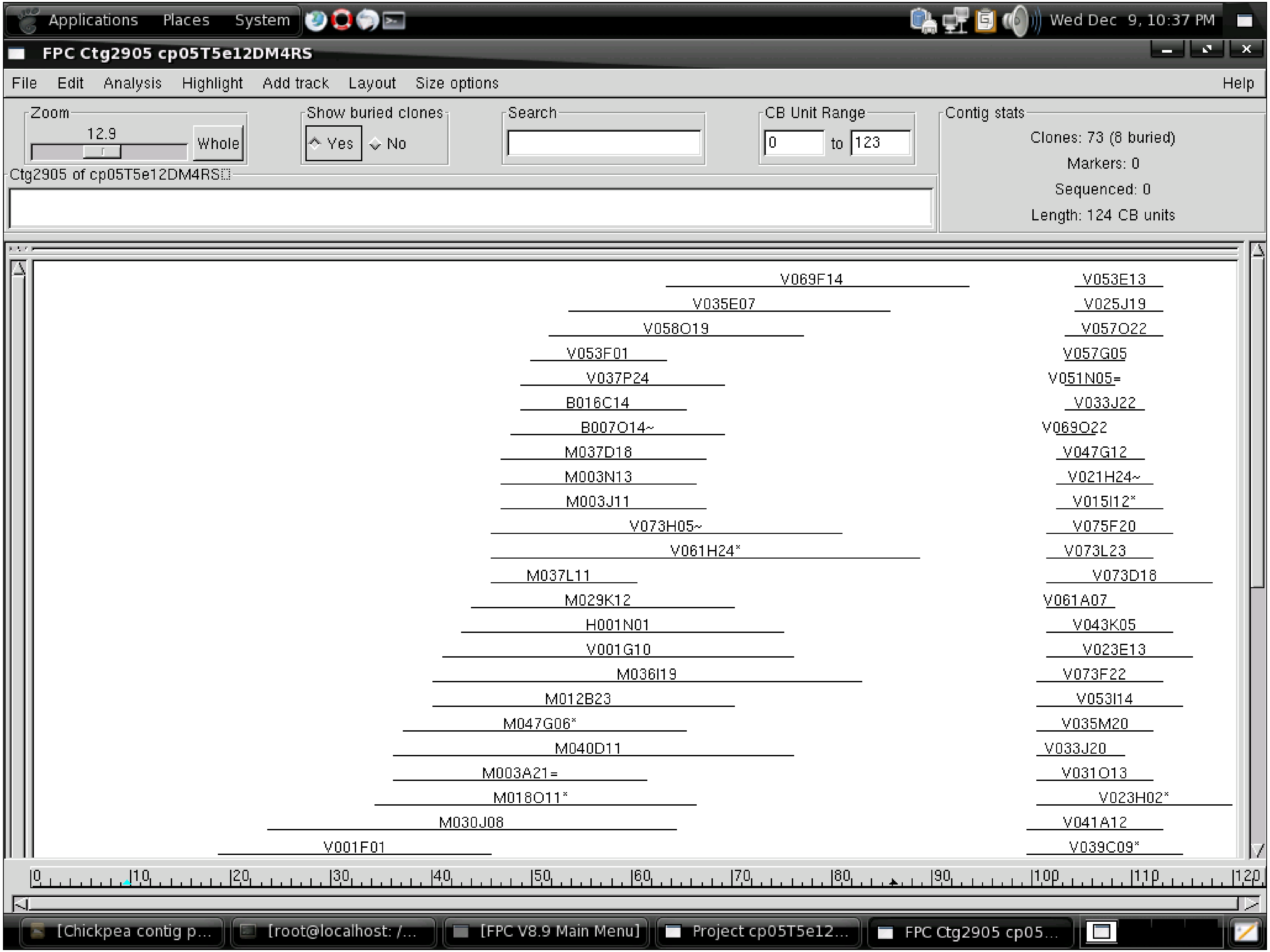
Applications Places System
Wed Dec 9, 10:37 PM
FPC Ctg2905 cp05T5e12DM4RS
File Edit Analysis Highlight Add track Layout Size options Help
Zoom
12.9
Whole
Show buried clones
Yes
No
Search
CB Unit Range
0
to
123
Contig stats
Clones: 73 (8 buried)
Markers: 0
Sequenced: 0
Length: 124 CB units
Ctg2905 of cp05T5e12DM4RS
V069F14
V035E07
V058O19
V053F01
V037P24
B016C14
B007O14~
M037D18
M003N13
M003J11
V073H05~
V061H24*
M037L11
M029K12
H001N01
V001G10
M036I19
M012B23
M047G06*
M040D11
M003A21=
M018O11*
M030J08
V001F01
V053E13
V025J19
V057O22
V057G05
V051N05=
V033J22
V069O22
V047G12
V021H24~
V015I12*
V075F20
V073L23
V073D18
V061A07
V043K05
V023E13
V073F22
V053I14
V035M20
V033J20
V031O13
V023H02*
V041A12
V039C09*
0 10 20 30 40 50 60 70 80 90 100 110 120
[Chickpea contig p...
[root@localhost: /...
[FPC V8.9 Main Menu]
Project cp05T5e12...
FPC Ctg2905 cp05...

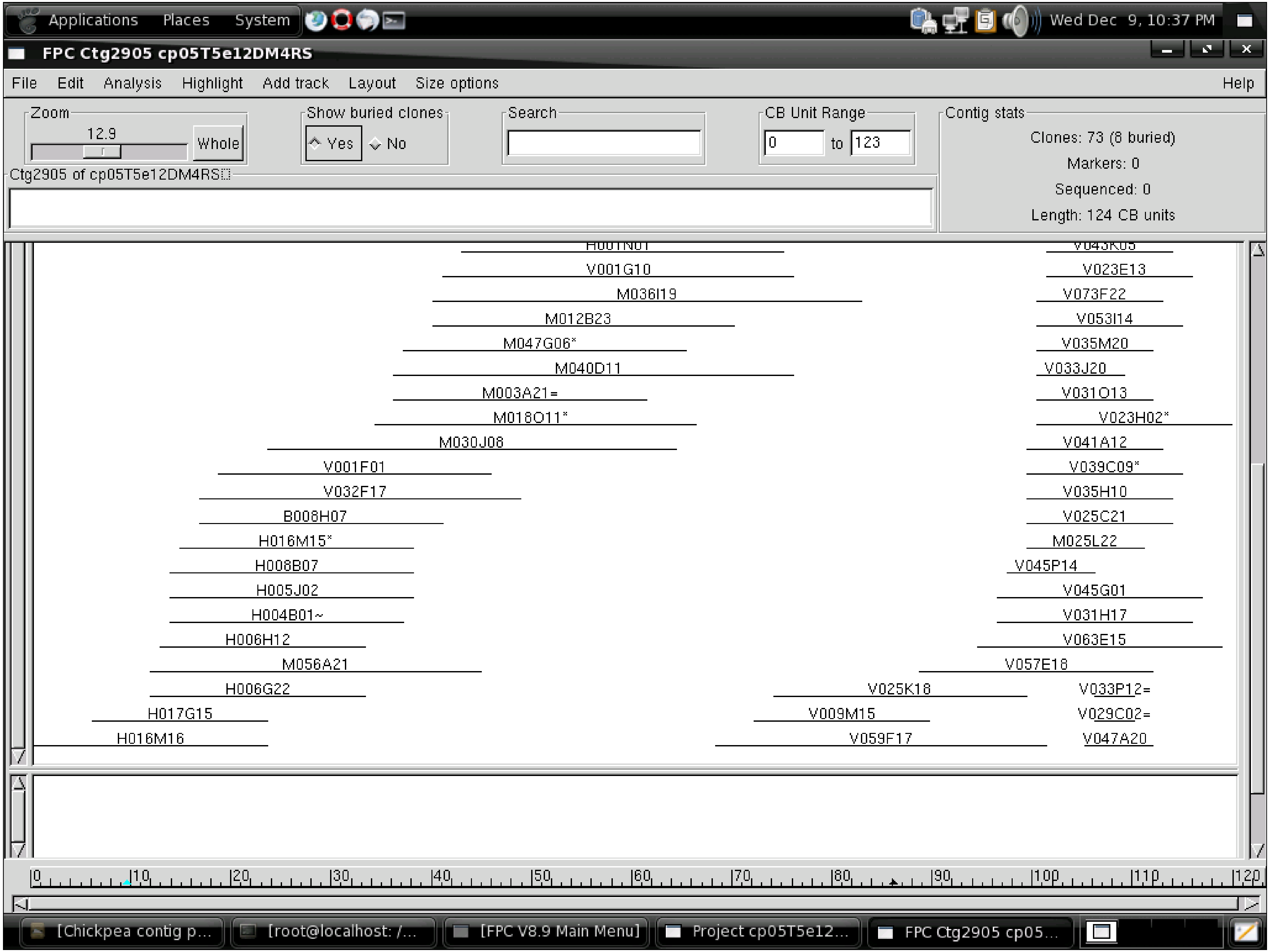

Applications Places System
Wed Dec 9, 10:37 PM
FPC Ctg2905 cp05T5e12DM4RS
File Edit Analysis Highlight Add track Layout Size options Help
Zoom
12.9
Whole
Show buried clones
Yes
No
Search
CB Unit Range
0
to
123
Contig stats
Clones: 73 (8 buried)
Markers: 0
Sequenced: 0
Length: 124 CB units
Ctg2905 of cp05T5e12DM4RS
V001G10
M036I19
M012B23
M047G06*
M040D11
M003A21=
M018O11*
M030J08
V001F01
V032F17
B008H07
H016M15*
H008B07
H005J02
H004B01~
H006H12
M056A21
H006G22
H017G15
H016M16
V023E13
V073F22
V053I14
V035M20
V033J20
V031O13
V023H02*
V041A12
V039C09*
V035H10
V025C21
M025L22
V045P14
V045G01
V031H17
V063E15
V057E18
V025K18
V033P12=
V009M15
V029C02=
V059F17
V047A20
0 10 20 30 40 50 60 70 80 90 100 110 120
[Chickpea contig p...
[root@localhost: /...
[FPC V8.9 Main Menu]
Project cp05T5e12...
FPC Ctg2905 cp05...

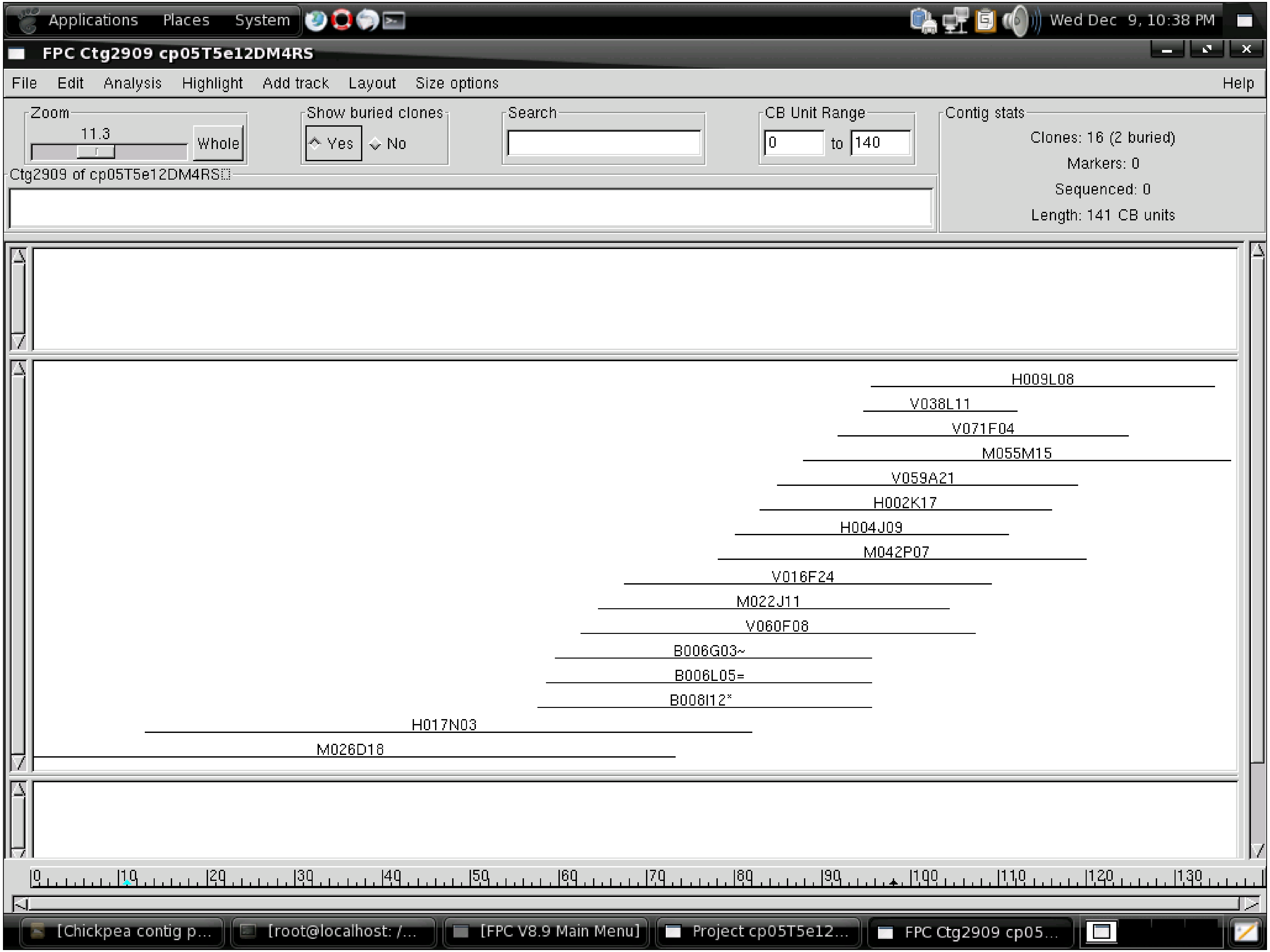
Applications Places System
Wed Dec 9, 10:38 PM
FPC Ctg2909 cp05T5e12DM4RS
File Edit Analysis Highlight Add track Layout Size options Help
Zoom
11.3
Whole
Show buried clones
Yes
No
Search
CB Unit Range
0 to 140
Contig stats
Clones: 16 (2 buried)
Markers: 0
Sequenced: 0
Length: 141 CB units
Ctg2909 of cp05T5e12DM4RS
H009L08
V038L11
V071F04
M055M15
V059A21
H002K17
H004J09
M042P07
V016F24
M022J11
V060F08
B006G03~
B006L05=
B008I12*
H017N03
M026D18
0 10 20 30 40 50 60 70 80 90 100 110 120 130
[Chickpea contig p...
[root@localhost: /...
[FPC V8.9 Main Menu]
Project cp05T5e12...
FPC Ctg2909 cp05...

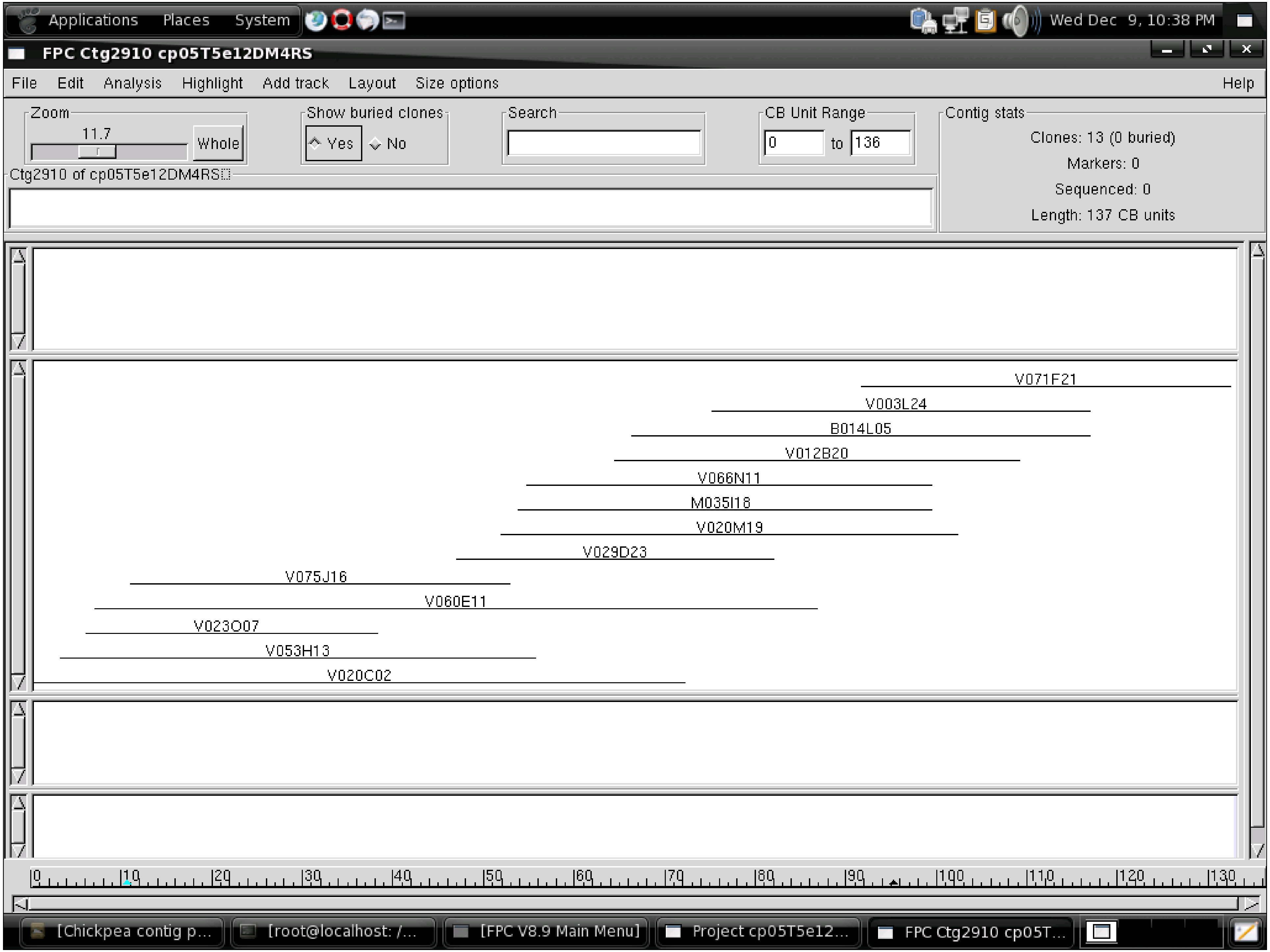
Applications Places System
Wed Dec 9, 10:38 PM
FPC Ctg2910 cp05T5e12DM4RS
File Edit Analysis Highlight Add track Layout Size options Help
Zoom
11.7
Whole
Show buried clones
Yes No
Search
CB Unit Range
0 to 136
Contig stats
Clones: 13 (0 buried)
Markers: 0
Sequenced: 0
Length: 137 CB units
Ctg2910 of cp05T5e12DM4RS
V071F21
V003L24
B014L05
V012B20
V066N11
M035I18
V020M19
V029D23
V075J16
V060E11
V023O07
V053H13
V020C02
0 10 20 30 40 50 60 70 80 90 100 110 120 130
[Chickpea contig p...
[root@localhost: /...
[FPC V8.9 Main Menu]
Project cp05T5e12...
FPC Ctg2910 cp05T...

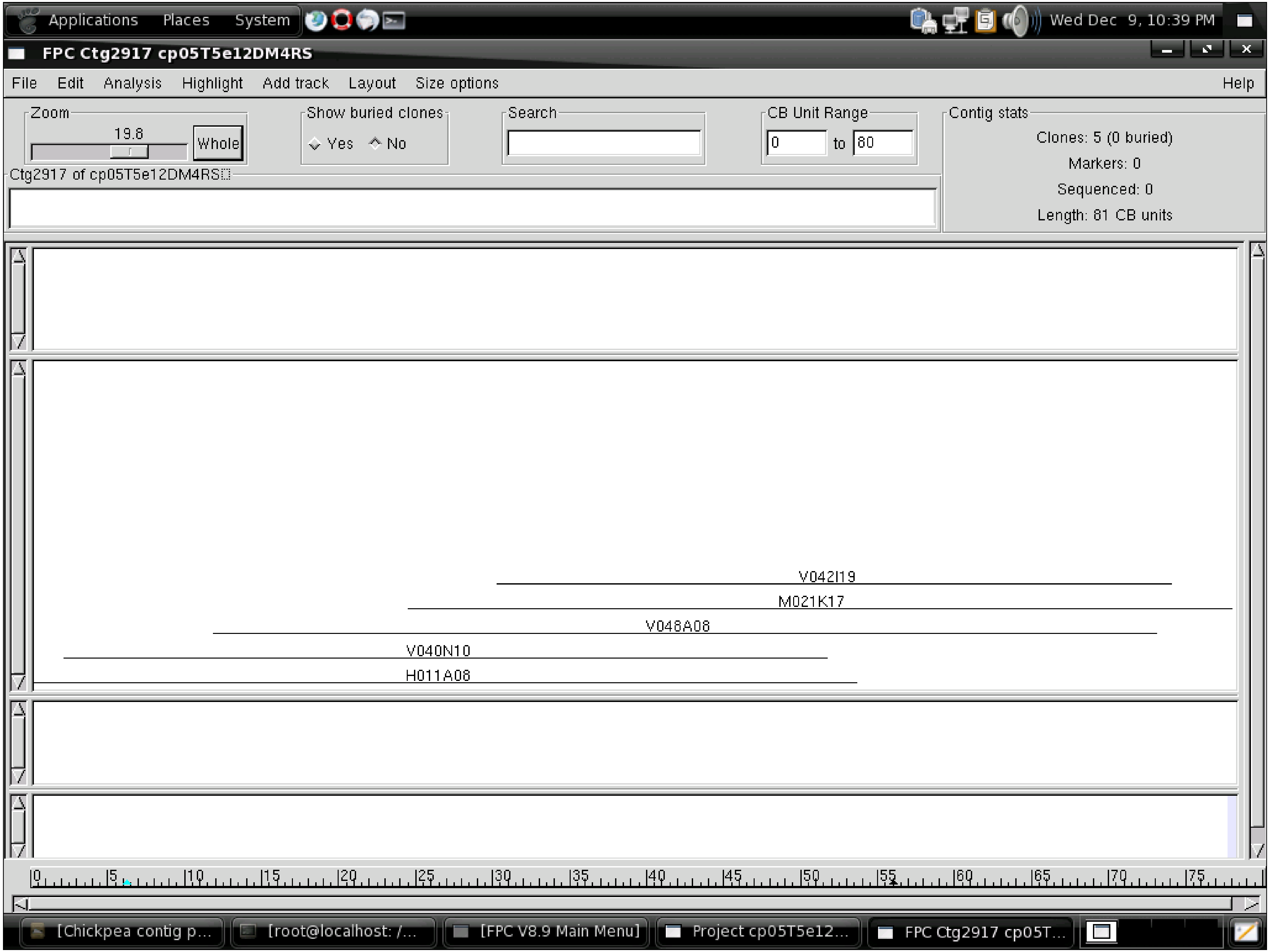
Applications Places System
Wed Dec 9, 10:39 PM
FPC Ctg2917 cp05T5e12DM4RS
File Edit Analysis Highlight Add track Layout Size options Help
Zoom
19.8
Whole
Show buried clones
Yes No
Search
CB Unit Range
0 to 80
Contig stats
Clones: 5 (0 buried)
Markers: 0
Sequenced: 0
Length: 81 CB units
Ctg2917 of cp05T5e12DM4RS
V042I19
M021K17
V048A08
V040N10
H011A08
0 5 10 15 20 25 30 35 40 45 50 55 60 65 70 75
[Chickpea contig p...
[root@localhost: /...
[FPC V8.9 Main Menu]
Project cp05T5e12...
FPC Ctg2917 cp05T...

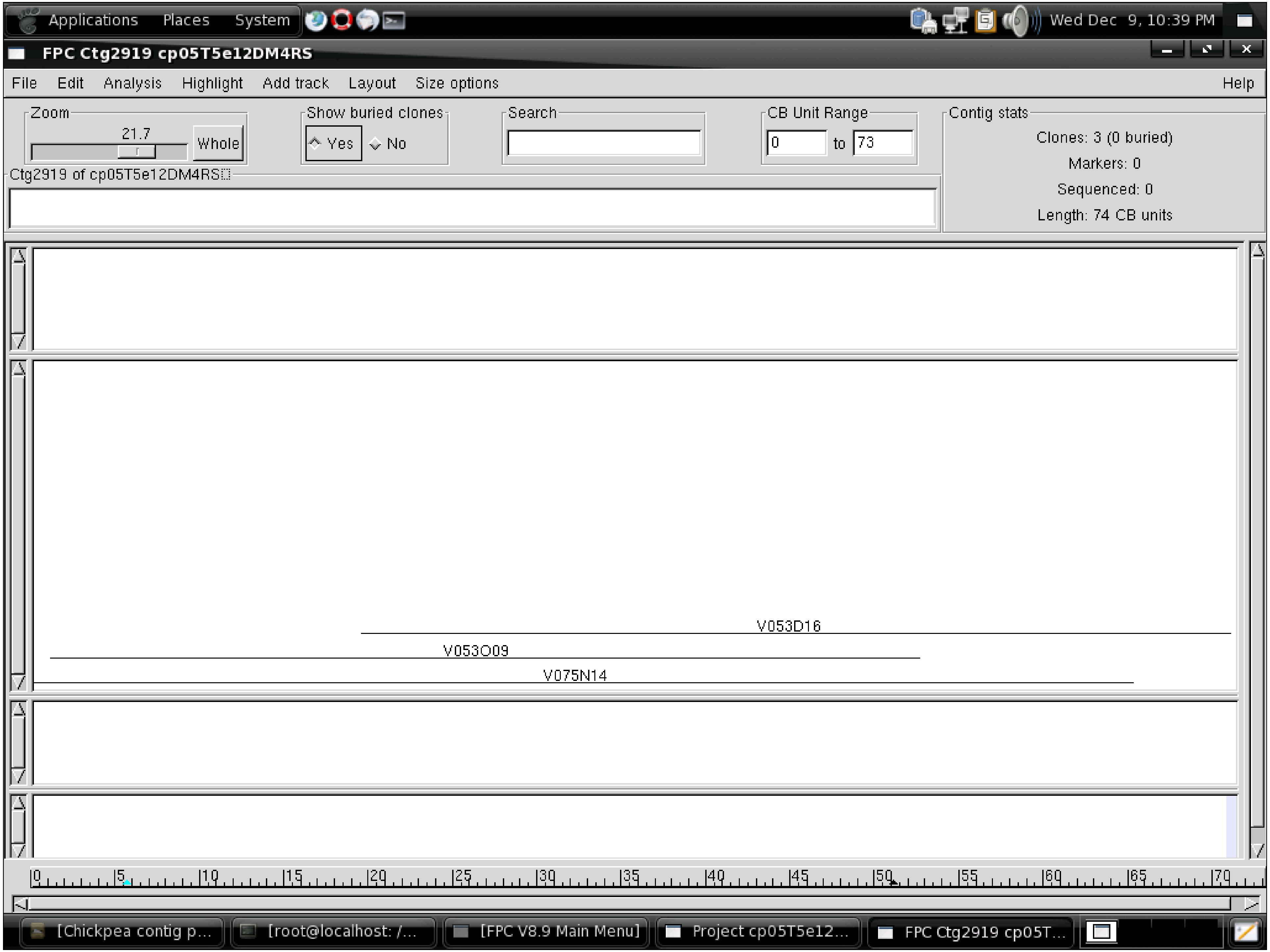
Applications Places System
Wed Dec 9, 10:39 PM
FPC Ctg2919 cp05T5e12DM4RS
File Edit Analysis Highlight Add track Layout Size options Help
Zoom
21.7
Whole
Show buried clones
Yes No
Search
CB Unit Range
0 to 73
Contig stats
Clones: 3 (0 buried)
Markers: 0
Sequenced: 0
Length: 74 CB units
Ctg2919 of cp05T5e12DM4RS
V053D16
V053O09
V075N14
0 5 10 15 20 25 30 35 40 45 50 55 60 65 70
[Chickpea contig p...
[root@localhost: /...
[FPC V8.9 Main Menu]
Project cp05T5e12...
FPC Ctg2919 cp05T...

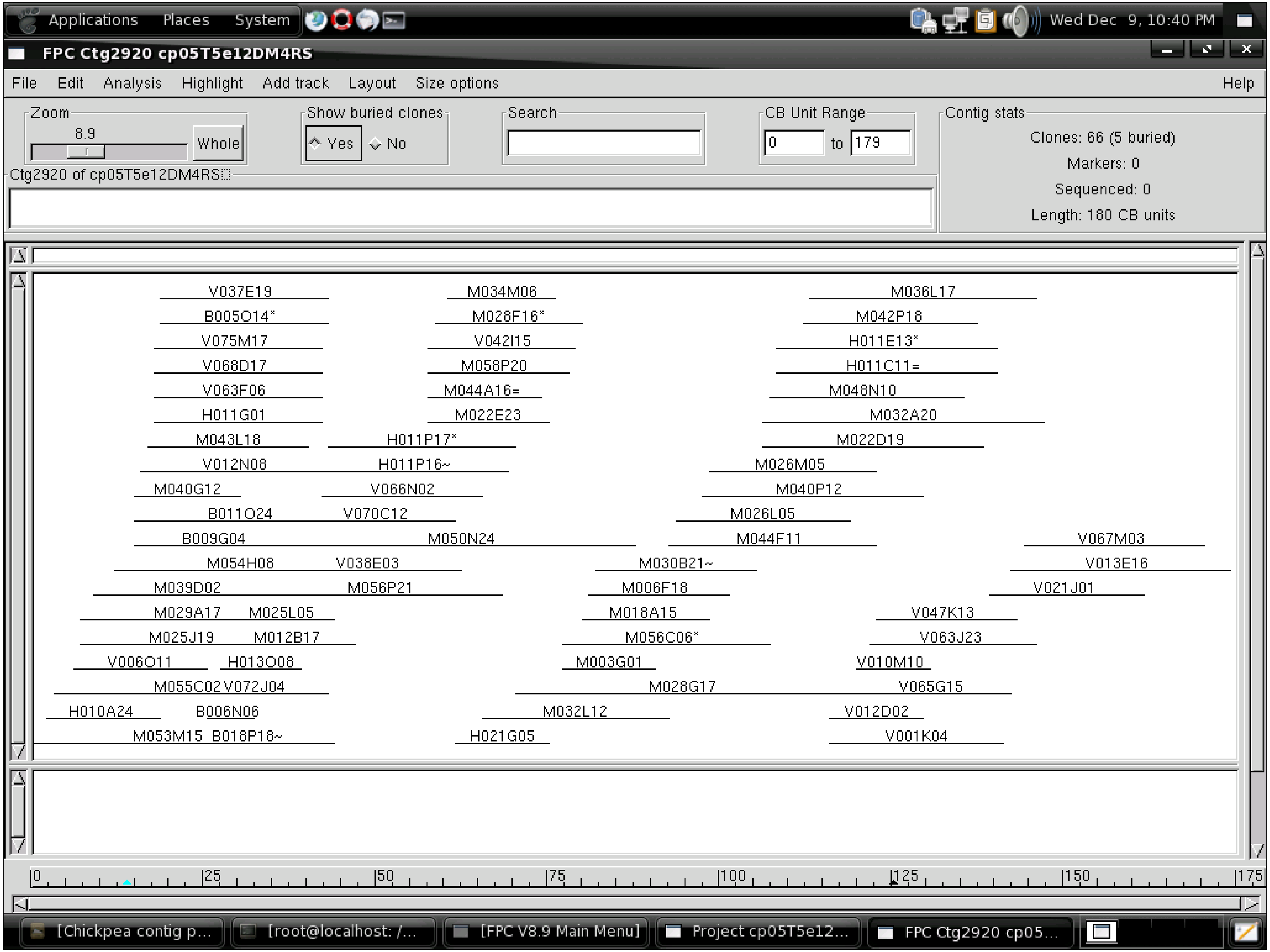
Applications
Places
System
Wed Dec 9, 10:40 PM
FPC Ctg2920 cp05T5e12DM4RS
File
Edit
Analysis
Highlight
Add track
Layout
Size options
Help
Zoom
8.9
Whole
Show buried clones
Yes
No
Search
CB Unit Range
0
to
179
Contig stats
Clones: 66 (5 buried)
Markers: 0
Sequenced: 0
Length: 180 CB units
Ctg2920 of cp05T5e12DM4RS
V037E19
M034M06
M036L17
B005O14*
M028F16*
M042P18
V075M17
V042I15
H011E13*
V068D17
M058P20
H011C11=
V063F06
M044A16=
M048N10
H011G01
M022E23
M032A20
M043L18
H011P17*
M022D19
V012N08
H011P16~
M026M05
M040G12
V066N02
M040P12
B011O24
V070C12
M026L05
B009G04
M050N24
M044F11
V067M03
M054H08
V038E03
M030B21~
V013E16
M039D02
M056P21
M006F18
V021J01
M029A17
M025L05
M018A15
V047K13
M025J19
M012B17
M056C06*
V063J23
V006O11
H013O08
M003G01
V010M10
M055C02
V072J04
M028G17
V065G15
H010A24
B006N06
M032L12
V012D02
M053M15
B018P18~
H021G05
V001K04
0
25
50
75
100
125
150
175
[Chickpea contig p...
[root@localhost: /...
[FPC V8.9 Main Menu]
Project cp05T5e12...
FPC Ctg2920 cp05...

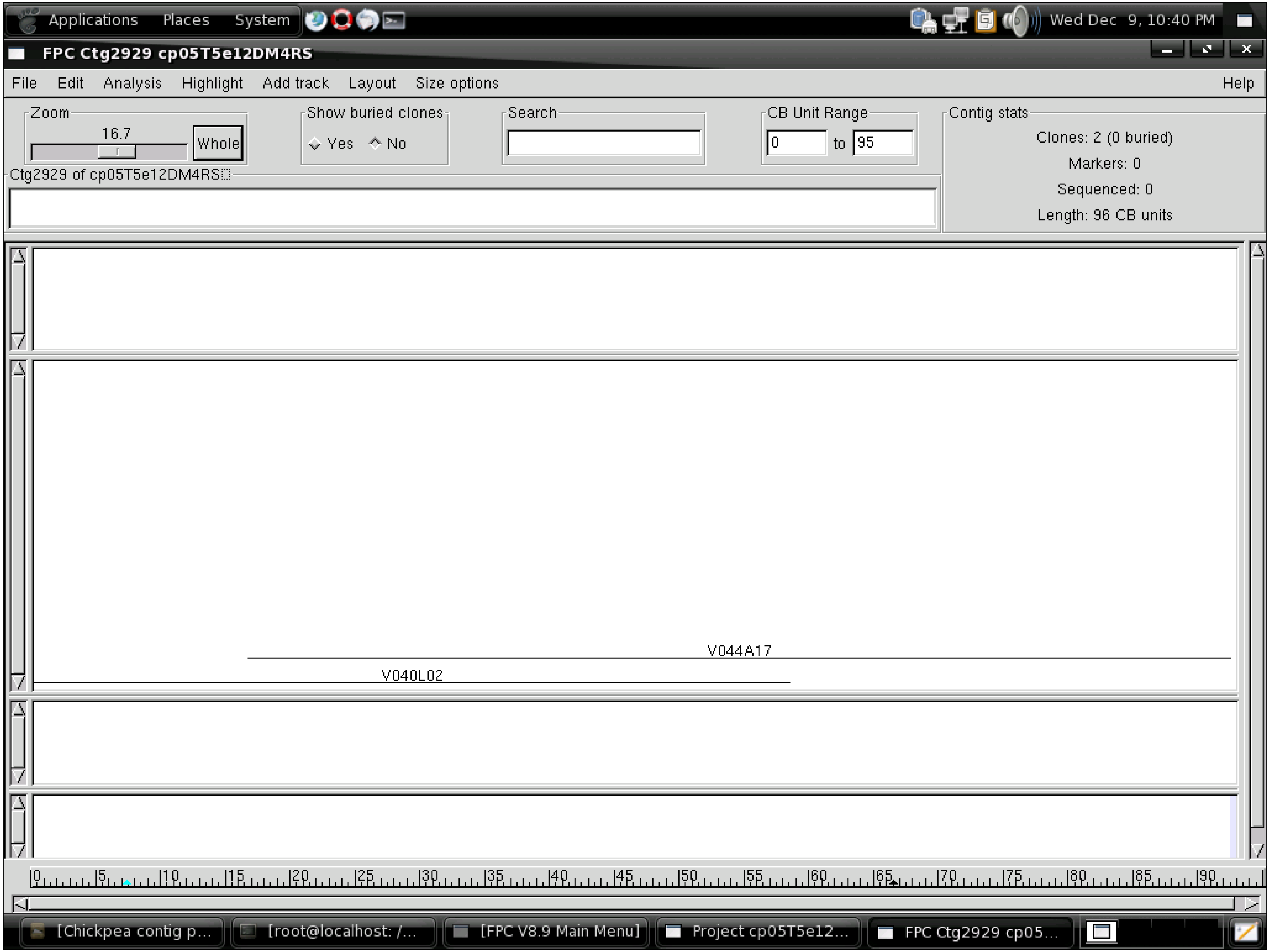
Applications
Places
System
Wed Dec 9, 10:40 PM
FPC Ctg2929 cp05T5e12DM4RS
File
Edit
Analysis
Highlight
Add track
Layout
Size options
Help
Zoom
16.7
Whole
Show buried clones
Yes
No
Search
CB Unit Range
0
to
95
Contig stats
Clones: 2 (0 buried)
Markers: 0
Sequenced: 0
Length: 96 CB units
Ctg2929 of cp05T5e12DM4RS
V044A17
V040L02
0 5 10 15 20 25 30 35 40 45 50 55 60 65 70 75 80 85 90
[Chickpea contig p...
[root@localhost: /...
[FPC V8.9 Main Menu]
Project cp05T5e12...
FPC Ctg2929 cp05...

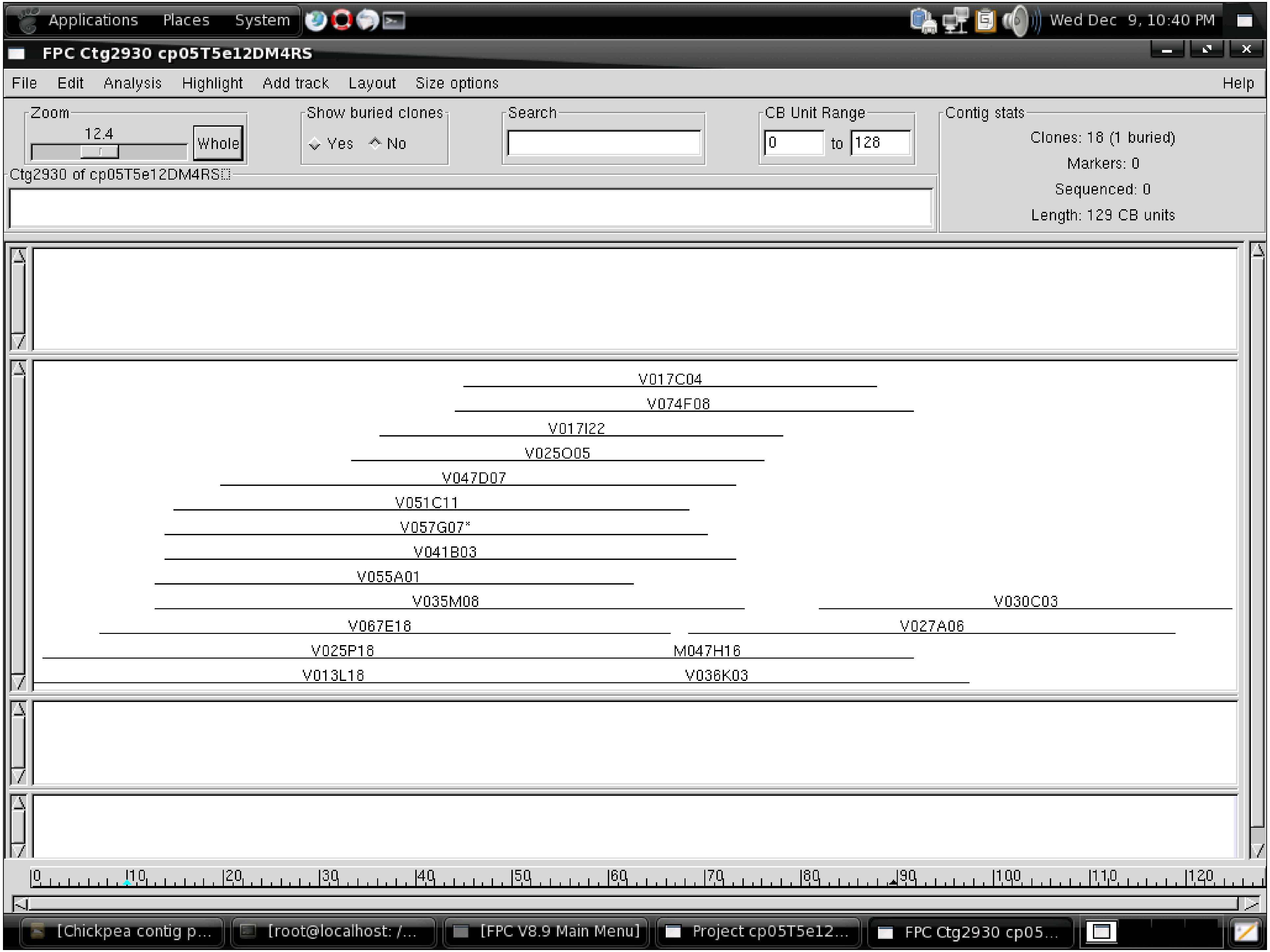
Applications Places System
Wed Dec 9, 10:40 PM
FPC Ctg2930 cp05T5e12DM4RS
File Edit Analysis Highlight Add track Layout Size options Help
Zoom
12.4
Whole
Show buried clones
Yes No
Search
CB Unit Range
0 to 128
Contig stats
Clones: 18 (1 buried)
Markers: 0
Sequenced: 0
Length: 129 CB units
Ctg2930 of cp05T5e12DM4RS
V017C04
V074F08
V017I22
V025O05
V047D07
V051C11
V057G07*
V041B03
V055A01
V035M08
V030C03
V067E18
V027A06
V025P18
M047H16
V013L18
V036K03
0 10 20 30 40 50 60 70 80 90 100 110 120
[Chickpea contig p...
[root@localhost: /...
[FPC V8.9 Main Menu]
Project cp05T5e12...
FPC Ctg2930 cp05...

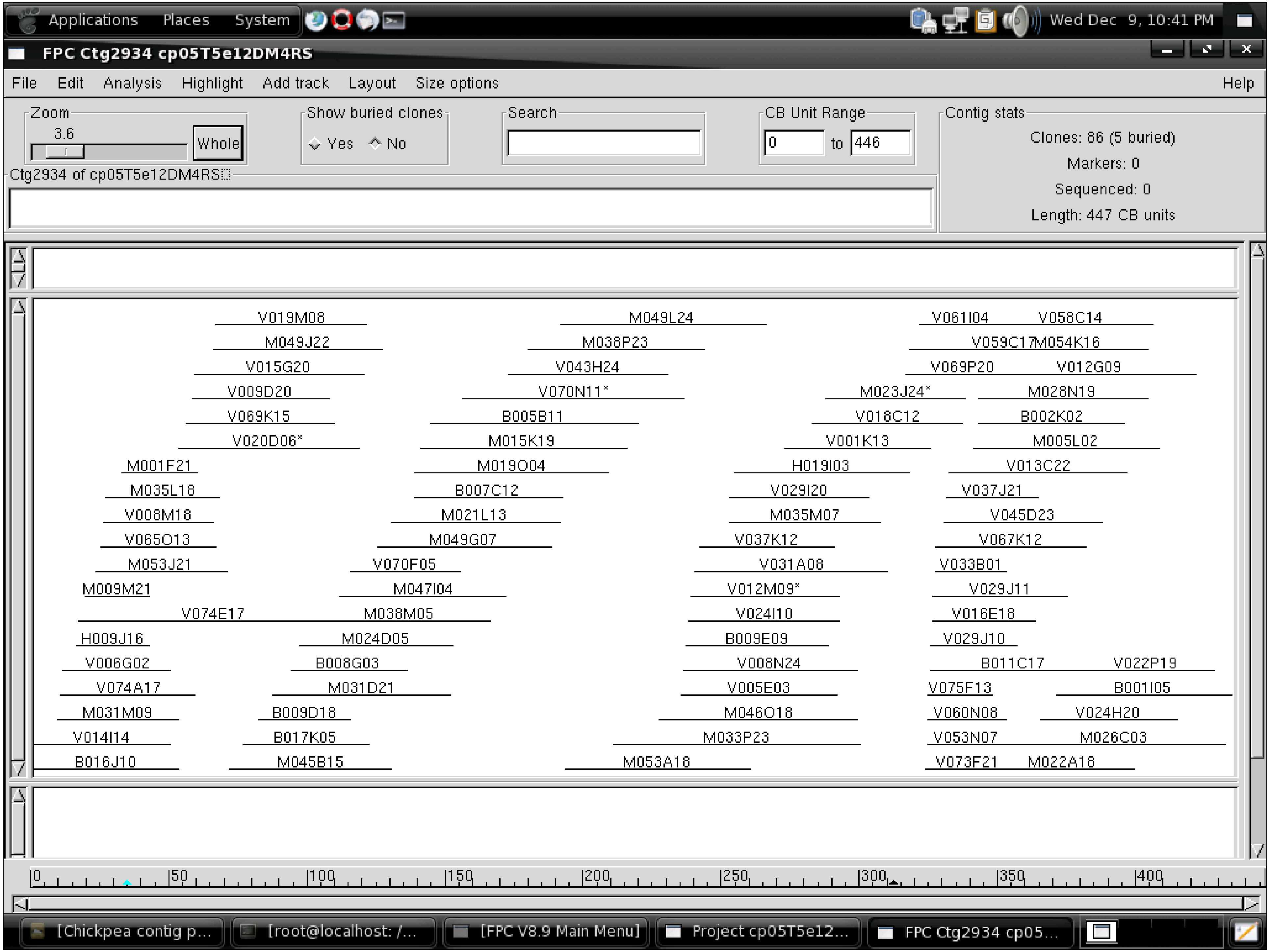

Applications Places System
Wed Dec 9, 10:41 PM
FPC Ctg2934 cp05T5e12DM4RS
File Edit Analysis Highlight Add track Layout Size options Help
Zoom
3.6
Whole
Show buried clones
Yes No
Search
CB Unit Range
0 to 446
Contig stats
Clones: 86 (5 buried)
Markers: 0
Sequenced: 0
Length: 447 CB units
Ctg2934 of cp05T5e12DM4RS
V019M08 M049L24 V061I04 V058C14
M049J22 M038P23 V059C17 M054K16
V015G20 V043H24 V069P20 V012G09
V009D20 V070N11* M023J24* M028N19
V069K15 B005B11 V018C12 B002K02
V020D06* M015K19 V001K13 M005L02
M001F21 M019O04 H019I03 V013C22
M035L18 B007C12 V029I20 V037J21
V008M18 M021L13 M035M07 V045D23
V065O13 M049G07 V037K12 V067K12
M053J21 V070F05 V031A08 V033B01
M009M21 M047I04 V012M09* V029J11
V074E17 M038M05 V024I10 V016E18
H009J16 M024D05 B009E09 V029J10
V006G02 B008G03 V008N24 B011C17 V022P19
V074A17 M031D21 V005E03 V075F13 B001I05
M031M09 B009D18 M046O18 V060N08 V024H20
V014I14 B017K05 M033P23 V053N07 M026C03
B016J10 M045B15 M053A18 V073F21 M022A18
0 50 100 150 200 250 300 350 400
[Chickpea contig p... [root@localhost: /... [FPC V8.9 Main Menu] Project cp05T5e12... FPC Ctg2934 cp05...

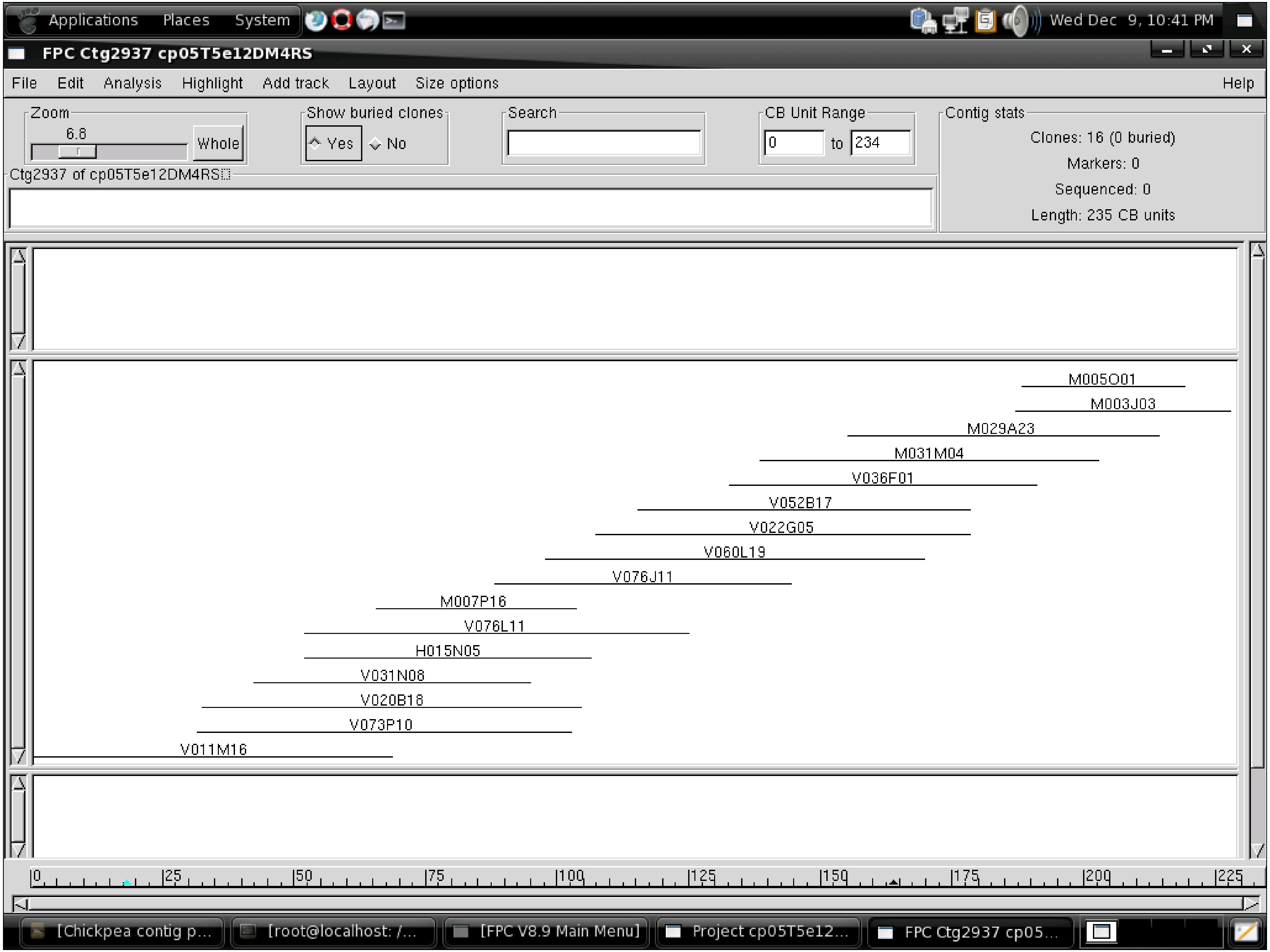
Applications
Places
System
Wed Dec 9, 10:41 PM
FPC Ctg2937 cp05T5e12DM4RS
File
Edit
Analysis
Highlight
Add track
Layout
Size options
Help
Zoom
6.8
Whole
Show buried clones
Yes
No
Search
CB Unit Range
0
to
234
Contig stats
Clones: 16 (0 buried)
Markers: 0
Sequenced: 0
Length: 235 CB units
Ctg2937 of cp05T5e12DM4RS
M005O01
M003J03
M029A23
M031M04
V036F01
V052B17
V022G05
V060L19
V076J11
M007P16
V076L11
H015N05
V031N08
V020B18
V073P10
V011M16
0
25
50
75
100
125
150
175
200
225
[Chickpea contig p...
[root@localhost: /...
[FPC V8.9 Main Menu]
Project cp05T5e12...
FPC Ctg2937 cp05...

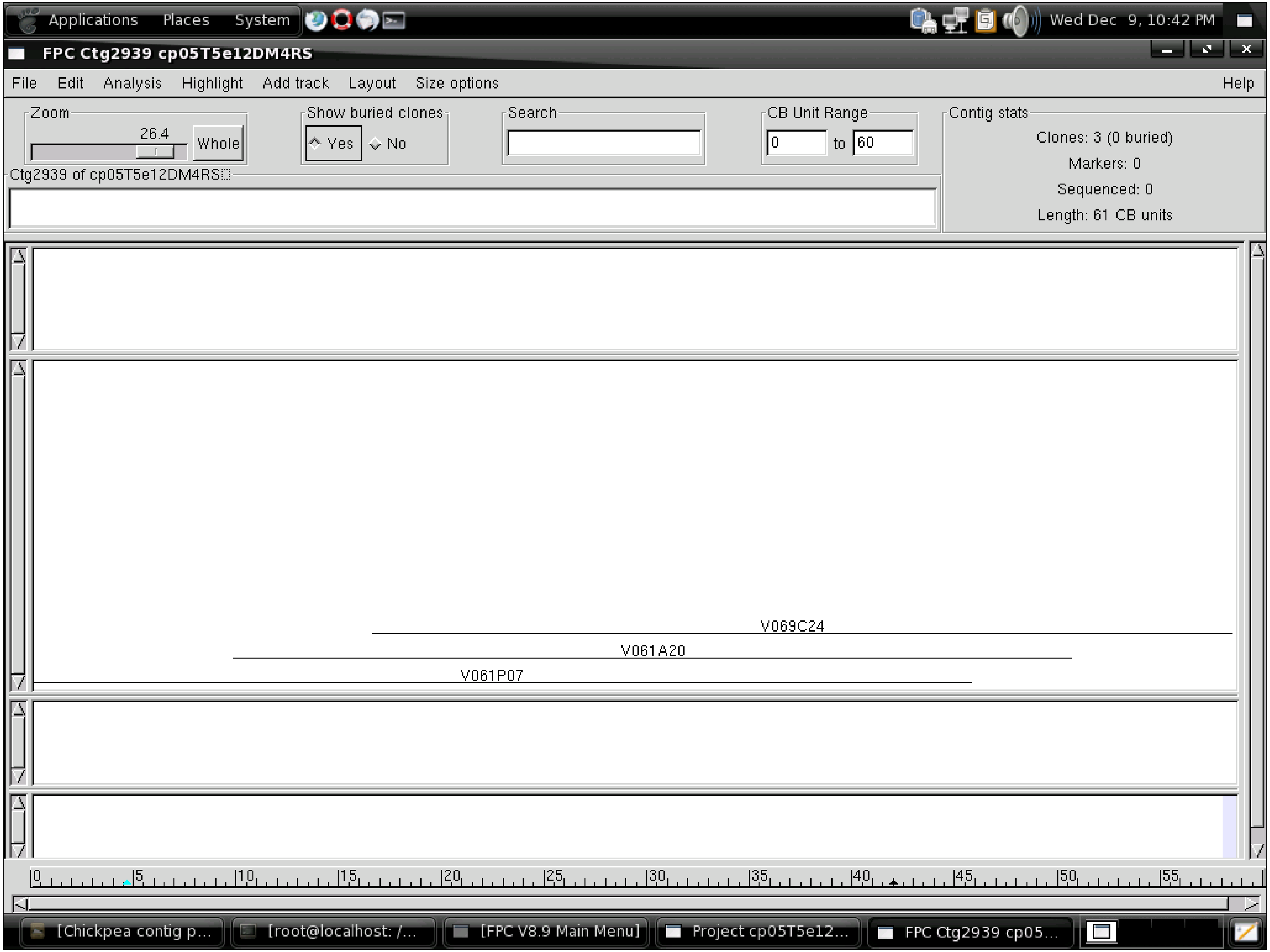
Applications Places System
Wed Dec 9, 10:42 PM
FPC Ctg2939 cp05T5e12DM4RS
File Edit Analysis Highlight Add track Layout Size options
Help
Zoom
26.4
Whole
Show buried clones
Yes
No
Search
CB Unit Range
0
to
60
Contig stats
Clones: 3 (0 buried)
Markers: 0
Sequenced: 0
Length: 61 CB units
Ctg2939 of cp05T5e12DM4RS
V069C24
V061A20
V061P07
0 5 10 15 20 25 30 35 40 45 50 55
[Chickpea contig p...
[root@localhost: /...
[FPC V8.9 Main Menu]
Project cp05T5e12...
FPC Ctg2939 cp05...

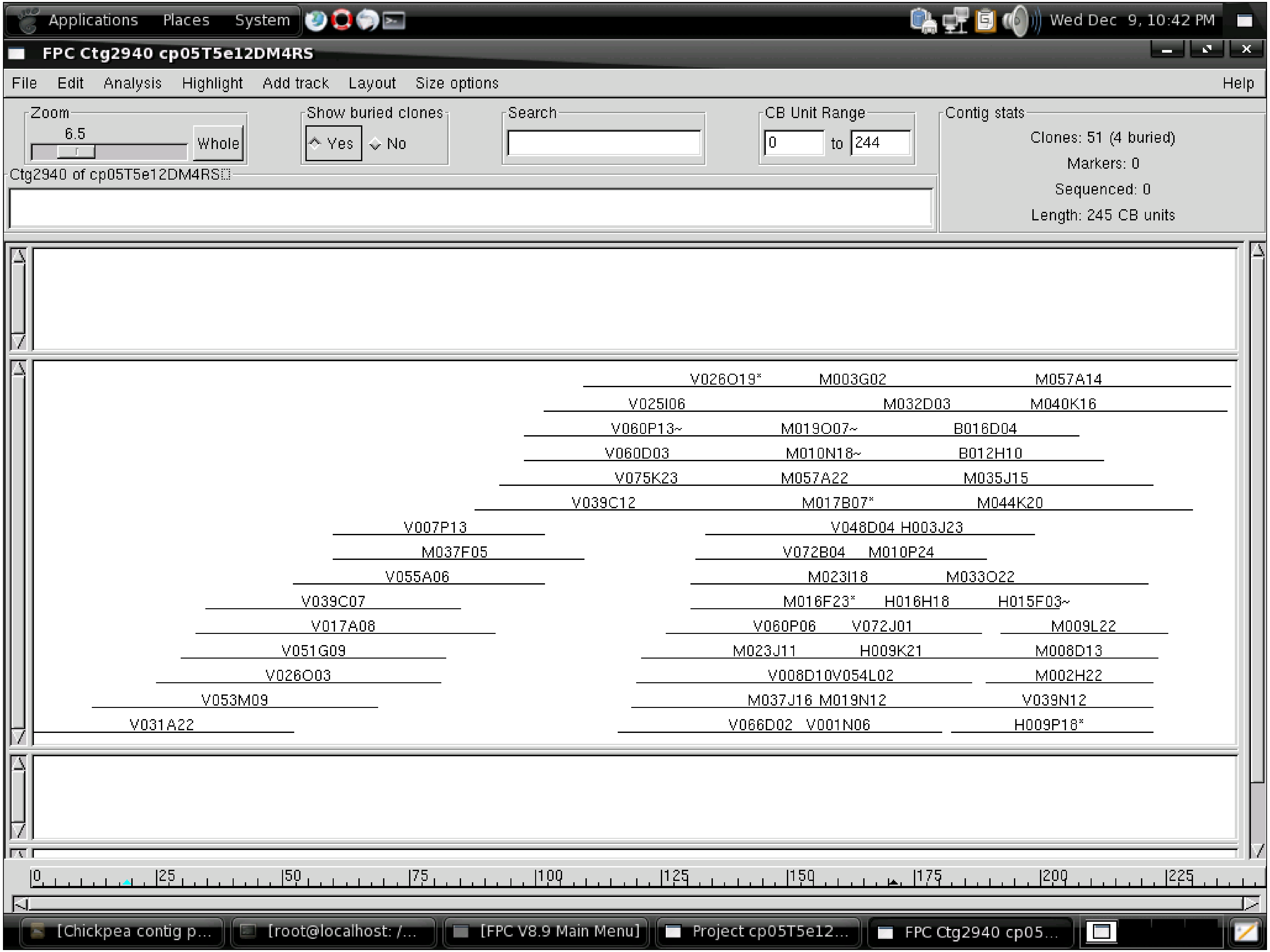
Applications Places System
Wed Dec 9, 10:42 PM
FPC Ctg2940 cp05T5e12DM4RS
File Edit Analysis Highlight Add track Layout Size options Help
Zoom
6.5
Whole
Show buried clones
Yes No
Search
CB Unit Range
0 to 244
Contig stats
Clones: 51 (4 buried)
Markers: 0
Sequenced: 0
Length: 245 CB units
Ctg2940 of cp05T5e12DM4RS
V026O19* M003G02 M057A14
V025I06 M032D03 M040K16
V060P13~ M019O07~ B016D04
V060D03 M010N18~ B012H10
V075K23 M057A22 M035J15
V039C12 M017B07* M044K20
V007P13 V048D04 H003J23
M037F05 V072B04 M010P24
V055A06 M023I18 M033O22
V039C07 M016F23* H016H18 H015F03~
V017A08 V060P06 V072J01 M009L22
V051G09 M023J11 H009K21 M008D13
V026O03 V008D10V054L02 M002H22
V053M09 M037J16 M019N12 V039N12
V031A22 V066D02 V001N06 H009P18*
0 25 50 75 100 125 150 175 200 225
[Chickpea contig p...
[root@localhost: /...
[FPC V8.9 Main Menu]
Project cp05T5e12...
FPC Ctg2940 cp05...

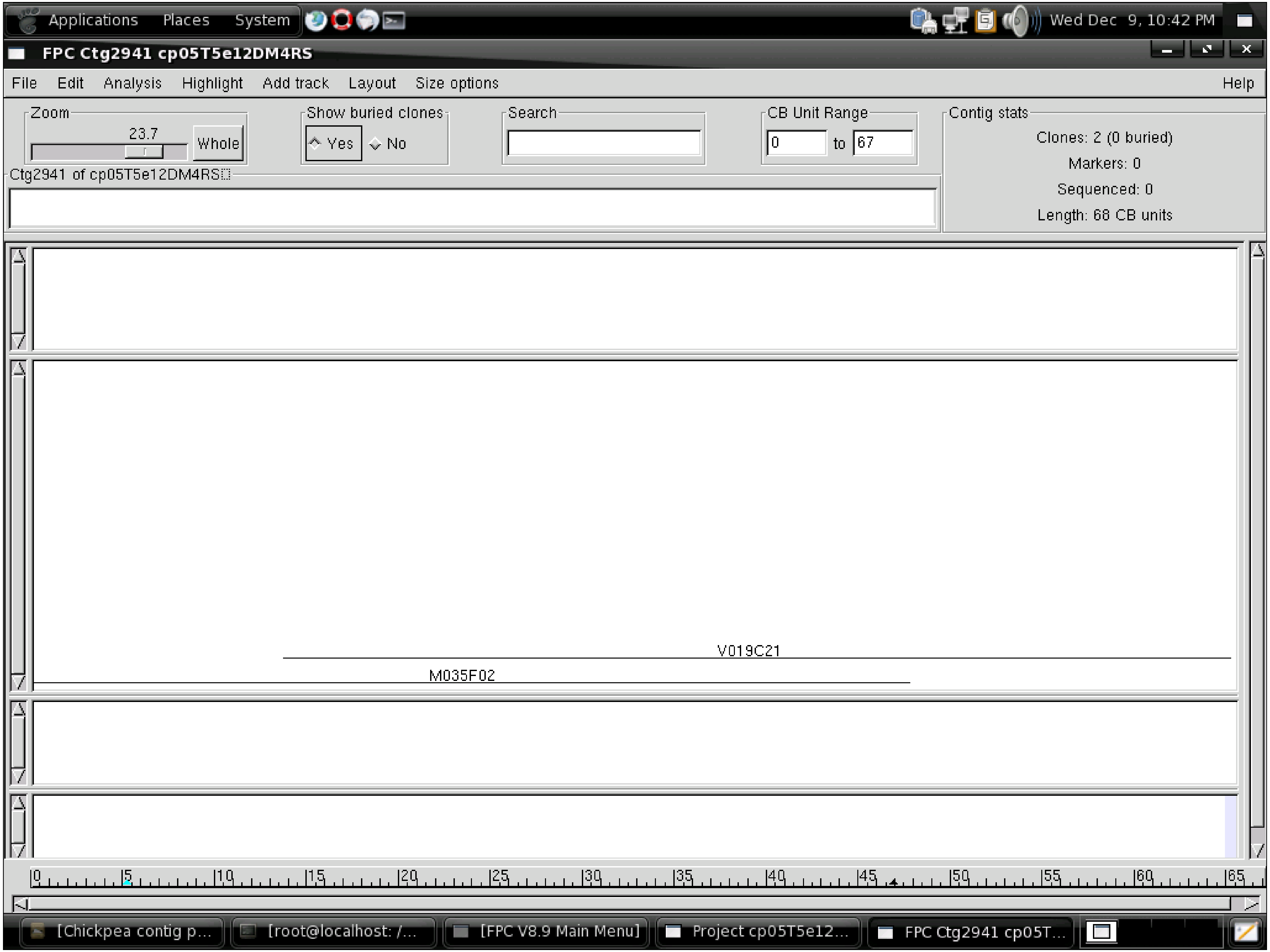
Applications Places System
Wed Dec 9, 10:42 PM
FPC Ctg2941 cp05T5e12DM4RS
File Edit Analysis Highlight Add track Layout Size options Help
Zoom
23.7
Whole
Show buried clones
Yes
No
Search
CB Unit Range
0
to
67
Contig stats
Clones: 2 (0 buried)
Markers: 0
Sequenced: 0
Length: 68 CB units
Ctg2941 of cp05T5e12DM4RS
V019C21
M035F02
0 5 10 15 20 25 30 35 40 45 50 55 60 65
[Chickpea contig p...
[root@localhost: /...
[FPC V8.9 Main Menu]
Project cp05T5e12...
FPC Ctg2941 cp05T...

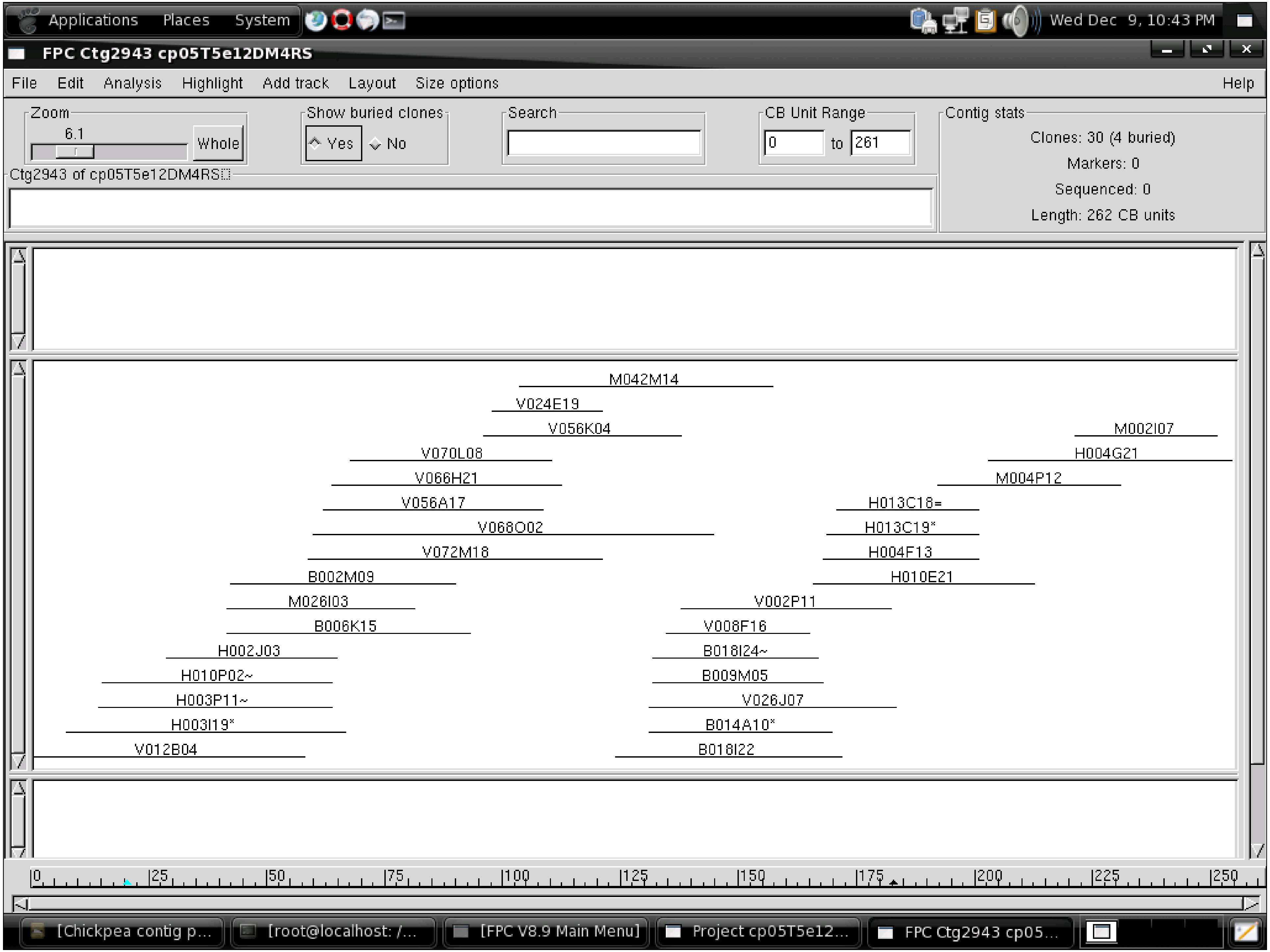
Applications Places System
Wed Dec 9, 10:43 PM
FPC Ctg2943 cp05T5e12DM4RS
File Edit Analysis Highlight Add track Layout Size options Help
Zoom
6.1
Whole
Show buried clones
Yes
No
Search
CB Unit Range
0
to
261
Contig stats
Clones: 30 (4 buried)
Markers: 0
Sequenced: 0
Length: 262 CB units
Ctg2943 of cp05T5e12DM4RS
M042M14
V024E19
V056K04
M002I07
V070L08
H004G21
V066H21
M004P12
V056A17
H013C18=
V068O02
H013C19*
V072M18
H004F13
B002M09
H010E21
M026I03
V002P11
B006K15
V008F16
H002J03
B018I24~
H010P02~
B009M05
H003P11~
V026J07
H003I19*
B014A10*
V012B04
B018I22
0
25
50
75
100
125
150
175
200
225
250
[Chickpea contig p...
[root@localhost: /...
[FPC V8.9 Main Menu]
Project cp05T5e12...
FPC Ctg2943 cp05...

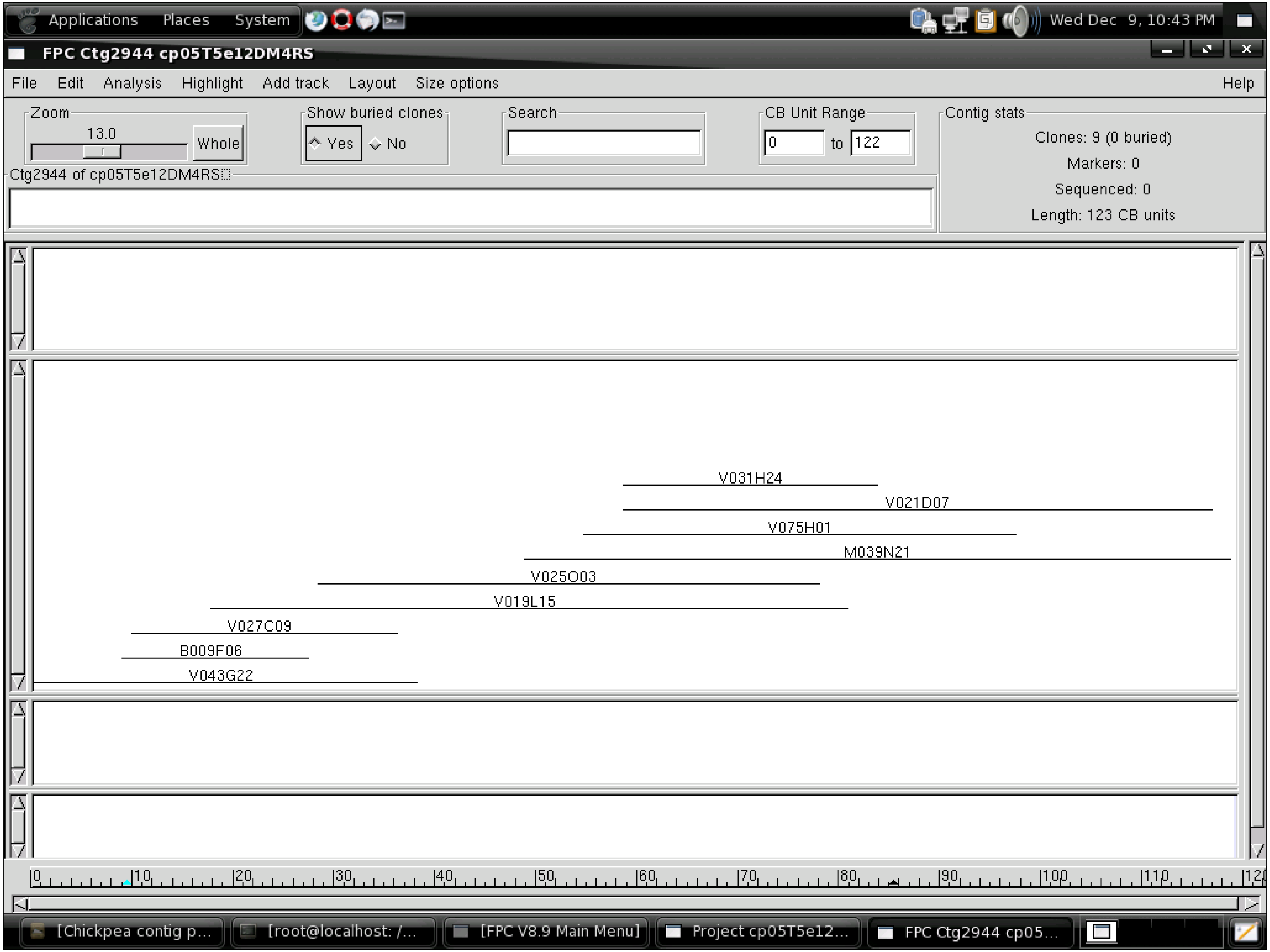
Applications Places System
Wed Dec 9, 10:43 PM
FPC Ctg2944 cp05T5e12DM4RS
File Edit Analysis Highlight Add track Layout Size options Help
Zoom
13.0
Whole
Show buried clones
Yes No
Search
CB Unit Range
0 to 122
Contig stats
Clones: 9 (0 buried)
Markers: 0
Sequenced: 0
Length: 123 CB units
Ctg2944 of cp05T5e12DM4RS
V031H24
V021D07
V075H01
M039N21
V025O03
V019L15
V027C09
B009F06
V043G22
0 10 20 30 40 50 60 70 80 90 100 110 12
[Chickpea contig p...
[root@localhost: /...
[FPC V8.9 Main Menu]
Project cp05T5e12...
FPC Ctg2944 cp05...

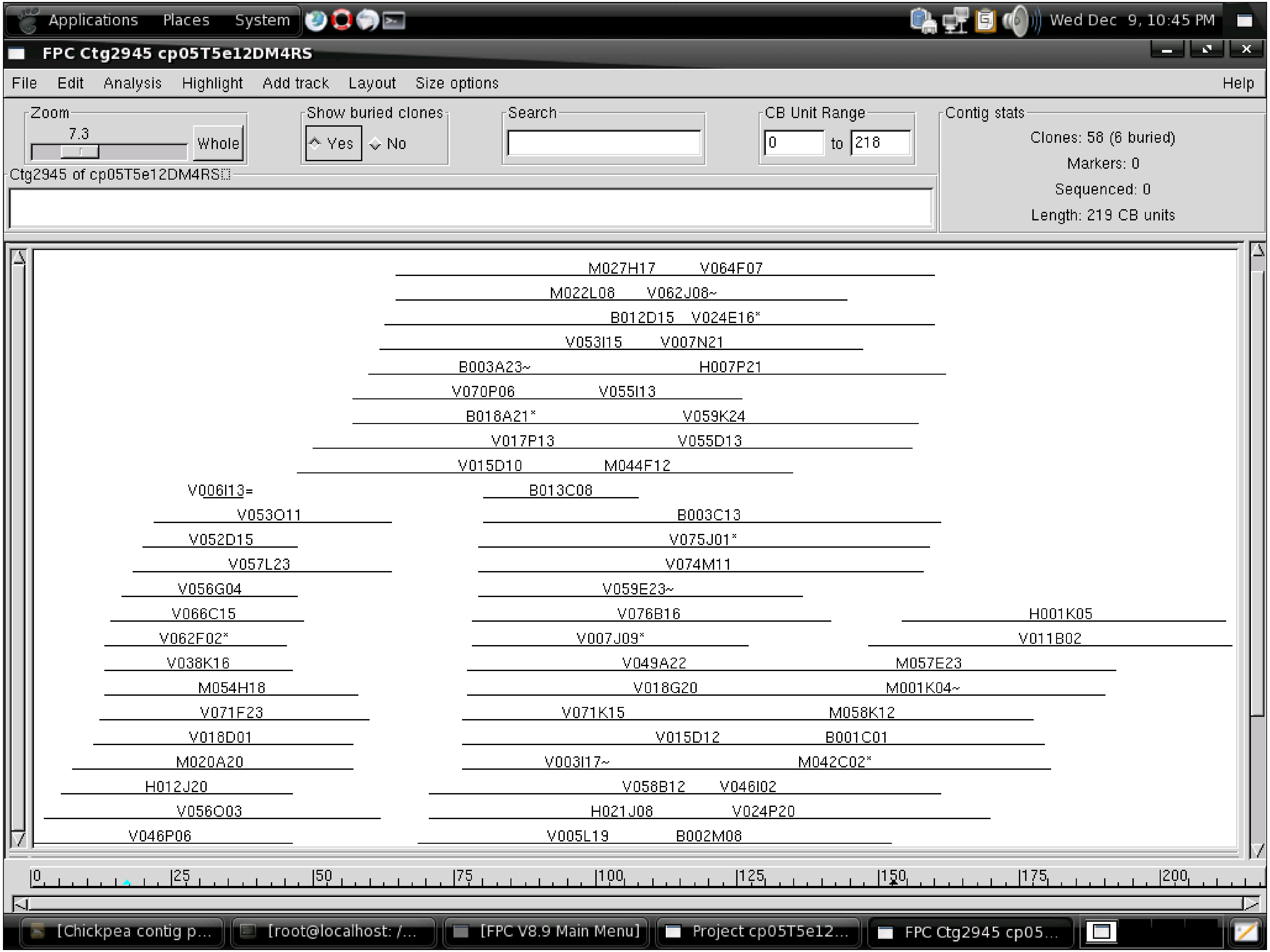
Applications Places System
Wed Dec 9, 10:45 PM
FPC Ctg2945 cp05T5e12DM4RS
File Edit Analysis Highlight Add track Layout Size options Help
Zoom
7.3
Whole
Show buried clones
Yes No
Search
CB Unit Range
0 to 218
Contig stats
Clones: 58 (6 buried)
Markers: 0
Sequenced: 0
Length: 219 CB units
Ctg2945 of cp05T5e12DM4RS
M027H17 V064F07
M022L08 V062J08~
B012D15 V024E16*
V053I15 V007N21
B003A23~ H007P21
V070P06 V055I13
B018A21* V059K24
V017P13 V055D13
V015D10 M044F12
V006I13= B013C08
V053O11 B003C13
V052D15 V075J01*
V057L23 V074M11
V056G04 V059E23~
V066C15 V076B16 H001K05
V062F02* V007J09* V011B02
V038K16 V049A22 M057E23
M054H18 V018G20 M001K04~
V071F23 V071K15 M058K12
V018D01 V015D12 B001C01
M020A20 V003I17~ M042C02*
H012J20 V058B12 V046I02
V056O03 H021J08 V024P20
V046P06 V005L19 B002M08
0 25 50 75 100 125 150 175 200
[Chickpea contig p...
[root@localhost: /...
[FPC V8.9 Main Menu]
Project cp05T5e12...
FPC Ctg2945 cp05...

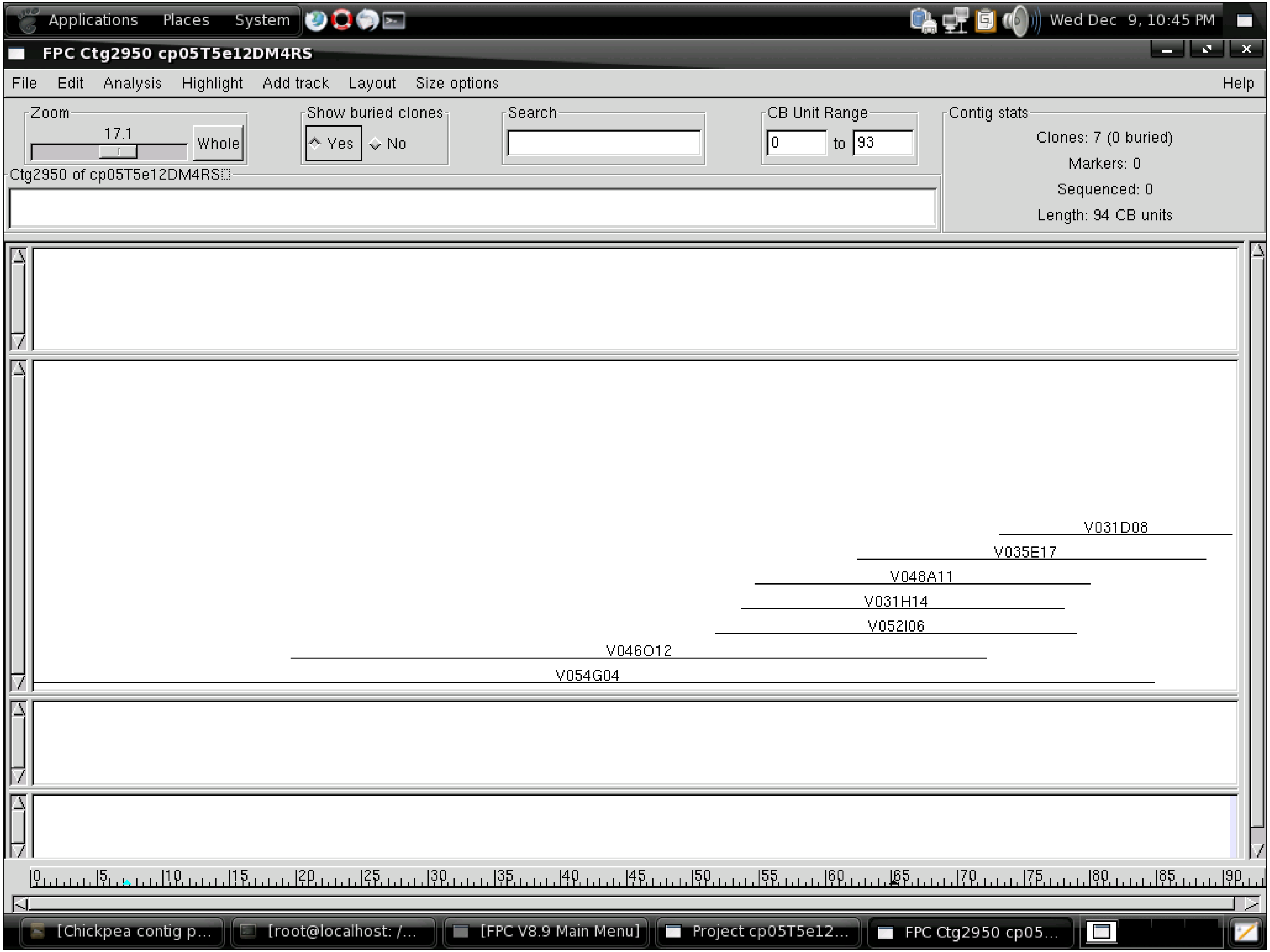
Applications Places System
Wed Dec 9, 10:45 PM
FPC Ctg2950 cp05T5e12DM4RS
File Edit Analysis Highlight Add track Layout Size options Help
Zoom
17.1
Whole
Show buried clones
Yes No
Search
CB Unit Range
0 to 93
Contig stats
Clones: 7 (0 buried)
Markers: 0
Sequenced: 0
Length: 94 CB units
Ctg2950 of cp05T5e12DM4RS
V031D08
V035E17
V048A11
V031H14
V052I06
V046O12
V054G04
0 5 10 15 20 25 30 35 40 45 50 55 60 65 70 75 80 85 90
[Chickpea contig p...
[root@localhost: /...
[FPC V8.9 Main Menu]
Project cp05T5e12...
FPC Ctg2950 cp05...

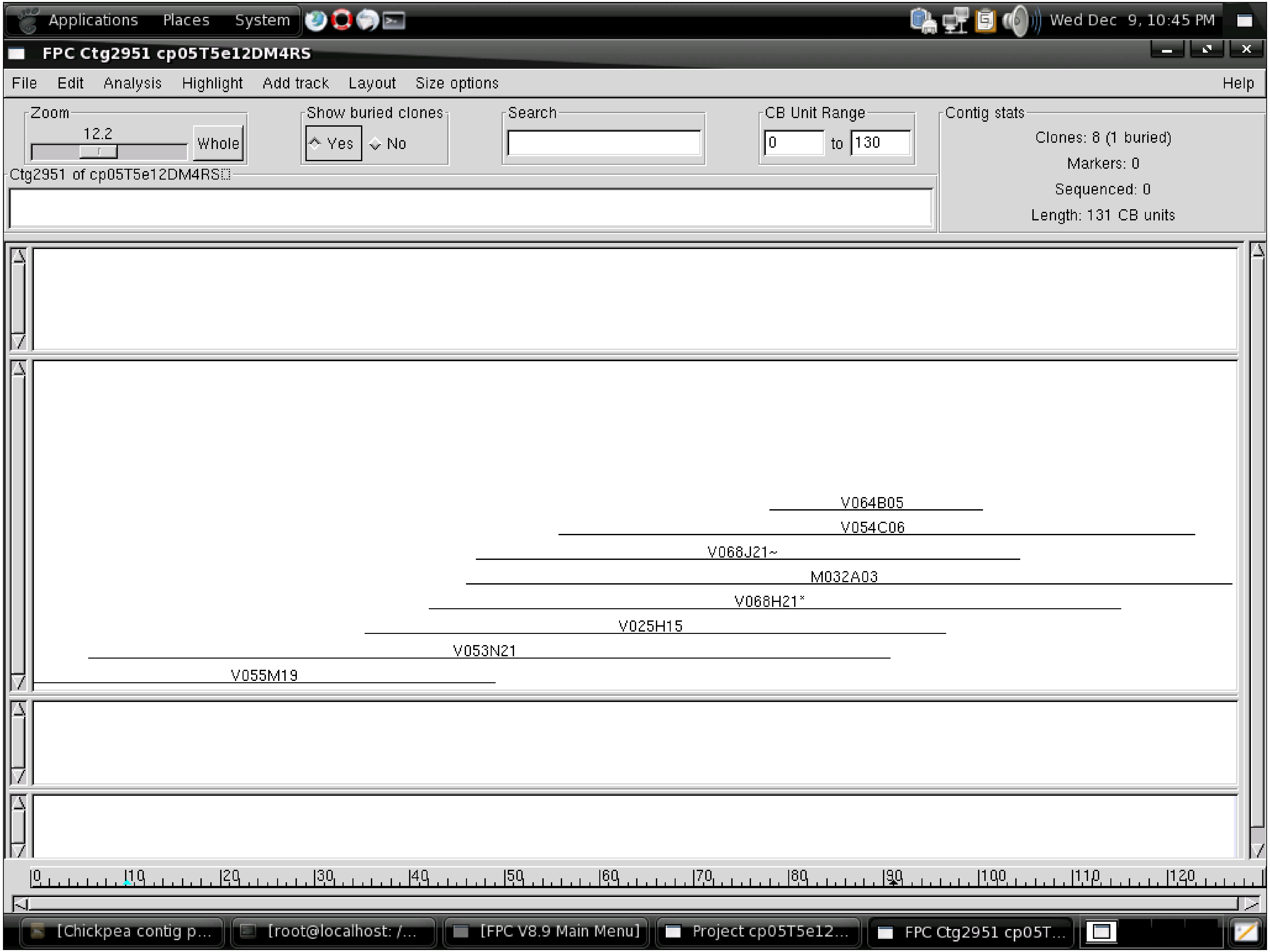
Applications Places System
Wed Dec 9, 10:45 PM
FPC Ctg2951 cp05T5e12DM4RS
File Edit Analysis Highlight Add track Layout Size options Help
Zoom
12.2
Whole
Show buried clones
Yes
No
Search
CB Unit Range
0 to 130
Contig stats
Clones: 8 (1 buried)
Markers: 0
Sequenced: 0
Length: 131 CB units
Ctg2951 of cp05T5e12DM4RS
V064B05
V054C06
V068J21~
M032A03
V068H21*
V025H15
V053N21
V055M19
0 10 20 30 40 50 60 70 80 90 100 110 120
[Chickpea contig p...
[root@localhost: /...
[FPC V8.9 Main Menu]
Project cp05T5e12...
FPC Ctg2951 cp05T...

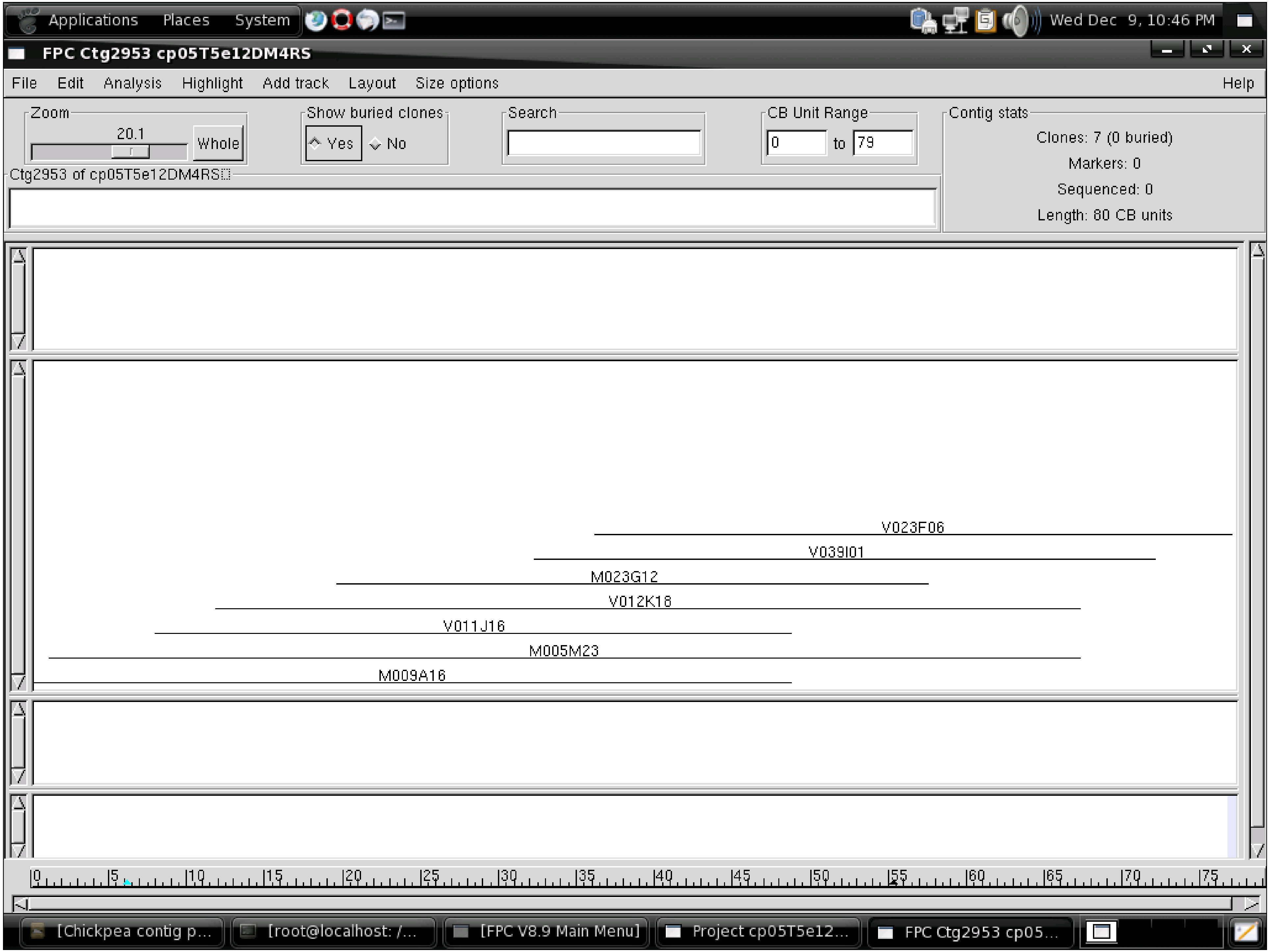
Applications Places System
Wed Dec 9, 10:46 PM
FPC Ctg2953 cp05T5e12DM4RS
File Edit Analysis Highlight Add track Layout Size options Help
Zoom
20.1
Whole
Show buried clones
Yes
No
Search
CB Unit Range
0
to
79
Contig stats
Clones: 7 (0 buried)
Markers: 0
Sequenced: 0
Length: 80 CB units
Ctg2953 of cp05T5e12DM4RS
V023F06
V039I01
M023G12
V012K18
V011J16
M005M23
M009A16
0 5 10 15 20 25 30 35 40 45 50 55 60 65 70 75
[Chickpea contig p...
[root@localhost: /...
[FPC V8.9 Main Menu]
Project cp05T5e12...
FPC Ctg2953 cp05...

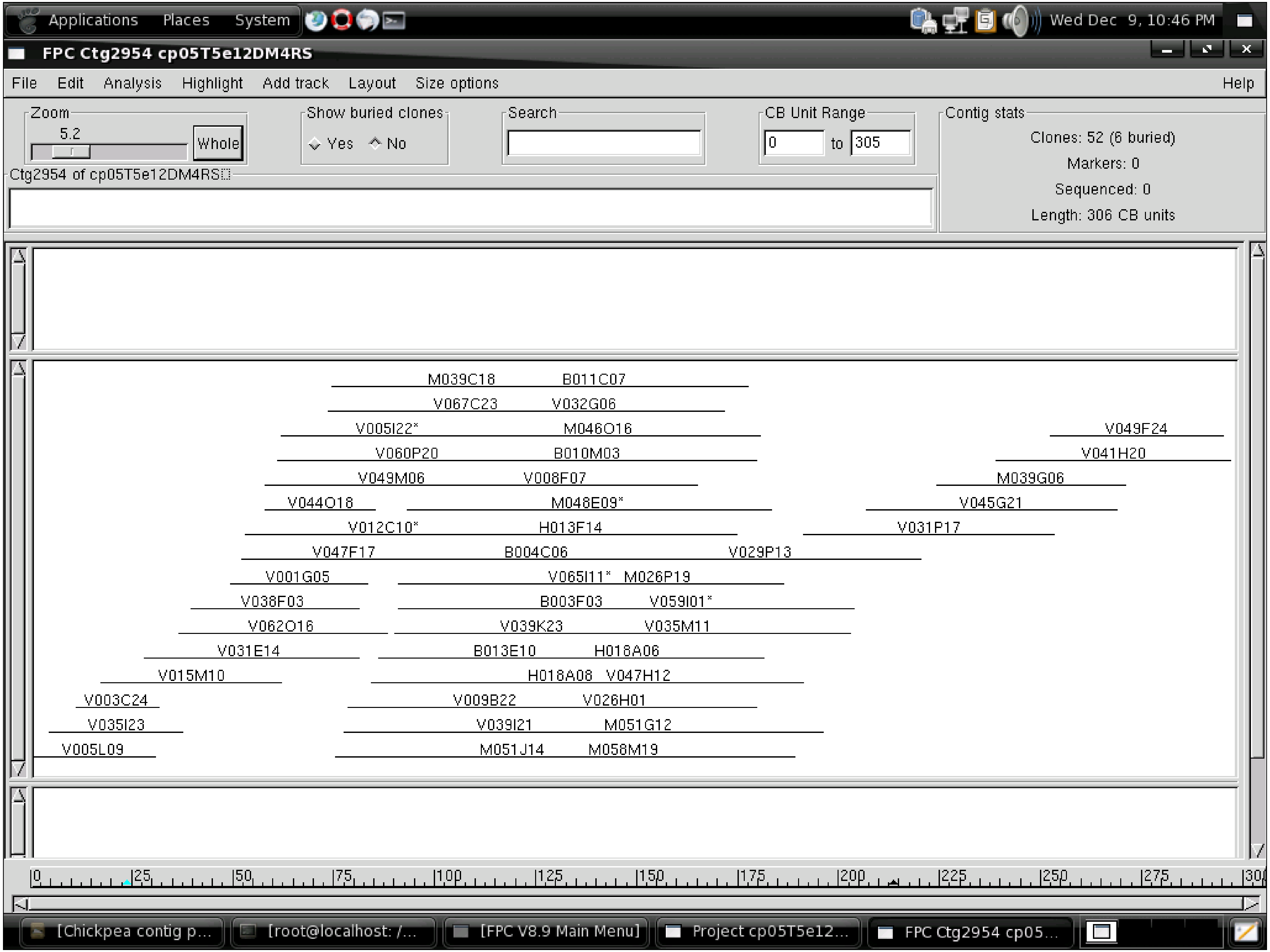
Applications Places System
Wed Dec 9, 10:46 PM
FPC Ctg2954 cp05T5e12DM4RS
File Edit Analysis Highlight Add track Layout Size options Help
Zoom 5.2 Whole
Show buried clones Yes No
Search
CB Unit Range 0 to 305
Contig stats
Clones: 52 (6 buried)
Markers: 0
Sequenced: 0
Length: 306 CB units
Ctg2954 of cp05T5e12DM4RS
M039C18 B011C07
V067C23 V032G06
V005I22* M046O16 V049F24
V060P20 B010M03 V041H20
V049M06 V008F07 M039G06
V044O18 M048E09* V045G21
V012C10* H013F14 V031P17
V047F17 B004C06 V029P13
V001G05 V065I11* M026P19
V038F03 B003F03 V059I01*
V062O16 V039K23 V035M11
V031E14 B013E10 H018A06
V015M10 H018A08 V047H12
V003C24 V009B22 V026H01
V035I23 V039I21 M051G12
V005L09 M051J14 M058M19
0 25 50 75 100 125 150 175 200 225 250 275 30
[Chickpea contig p... [root@localhost: /... [FPC V8.9 Main Menu] Project cp05T5e12... FPC Ctg2954 cp05...

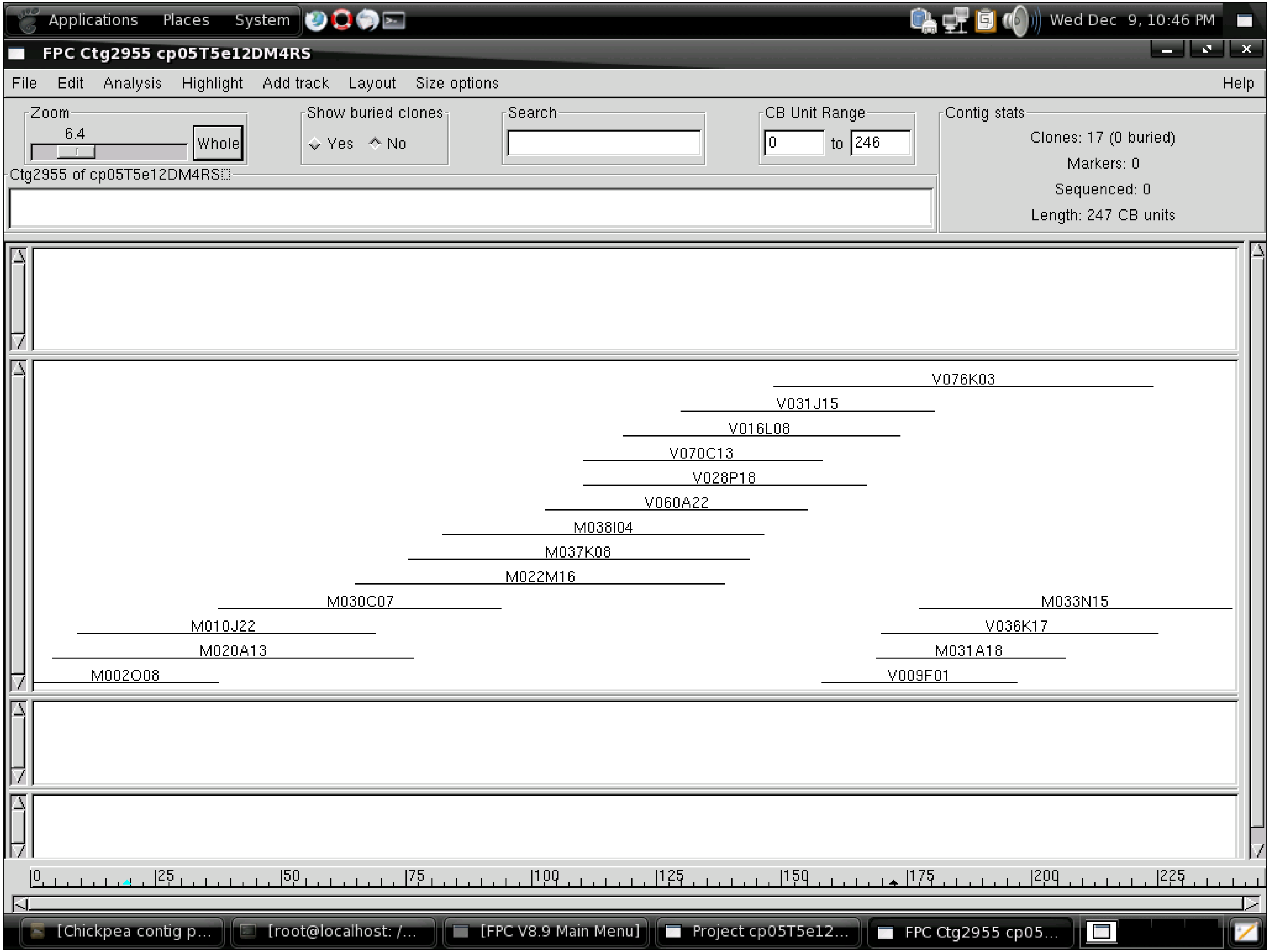
Applications
Places
System
Wed Dec 9, 10:46 PM
FPC Ctg2955 cp05T5e12DM4RS
File
Edit
Analysis
Highlight
Add track
Layout
Size options
Help
Zoom
6.4
Whole
Show buried clones
Yes
No
Search
CB Unit Range
0
to
246
Contig stats
Clones: 17 (0 buried)
Markers: 0
Sequenced: 0
Length: 247 CB units
Ctg2955 of cp05T5e12DM4RS
V076K03
V031J15
V016L08
V070C13
V028P18
V060A22
M038I04
M037K08
M022M16
M030C07
M033N15
M010J22
V036K17
M020A13
M031A18
M002O08
V009F01
0
25
50
75
100
125
150
175
200
225
[Chickpea contig p...
[root@localhost: /...
[FPC V8.9 Main Menu]
Project cp05T5e12...
FPC Ctg2955 cp05...

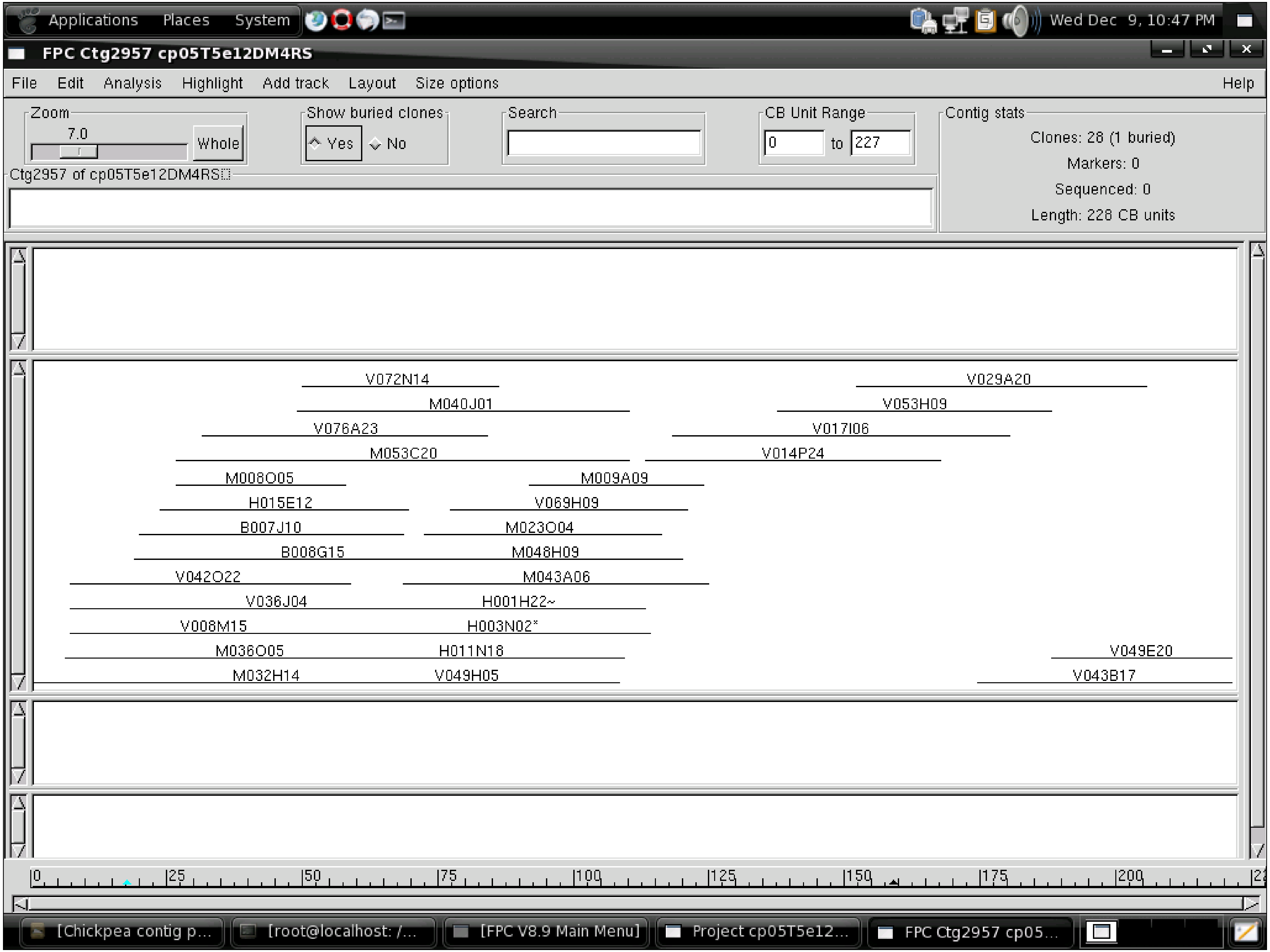
Applications Places System
Wed Dec 9, 10:47 PM
FPC Ctg2957 cp05T5e12DM4RS
File Edit Analysis Highlight Add track Layout Size options Help
Zoom
7.0
Whole
Show buried clones
Yes No
Search
CB Unit Range
0 to 227
Contig stats
Clones: 28 (1 buried)
Markers: 0
Sequenced: 0
Length: 228 CB units
Ctg2957 of cp05T5e12DM4RS
V072N14
V029A20
M040J01
V053H09
V076A23
V017I06
M053C20
V014P24
M008O05
M009A09
H015E12
V069H09
B007J10
M023O04
B008G15
M048H09
V042O22
M043A06
V036J04
H001H22~
V008M15
H003N02*
M036O05
H011N18
V049E20
M032H14
V049H05
V043B17
0 25 50 75 100 125 150 175 200
[Chickpea contig p...
[root@localhost: /...
[FPC V8.9 Main Menu]
Project cp05T5e12...
FPC Ctg2957 cp05...

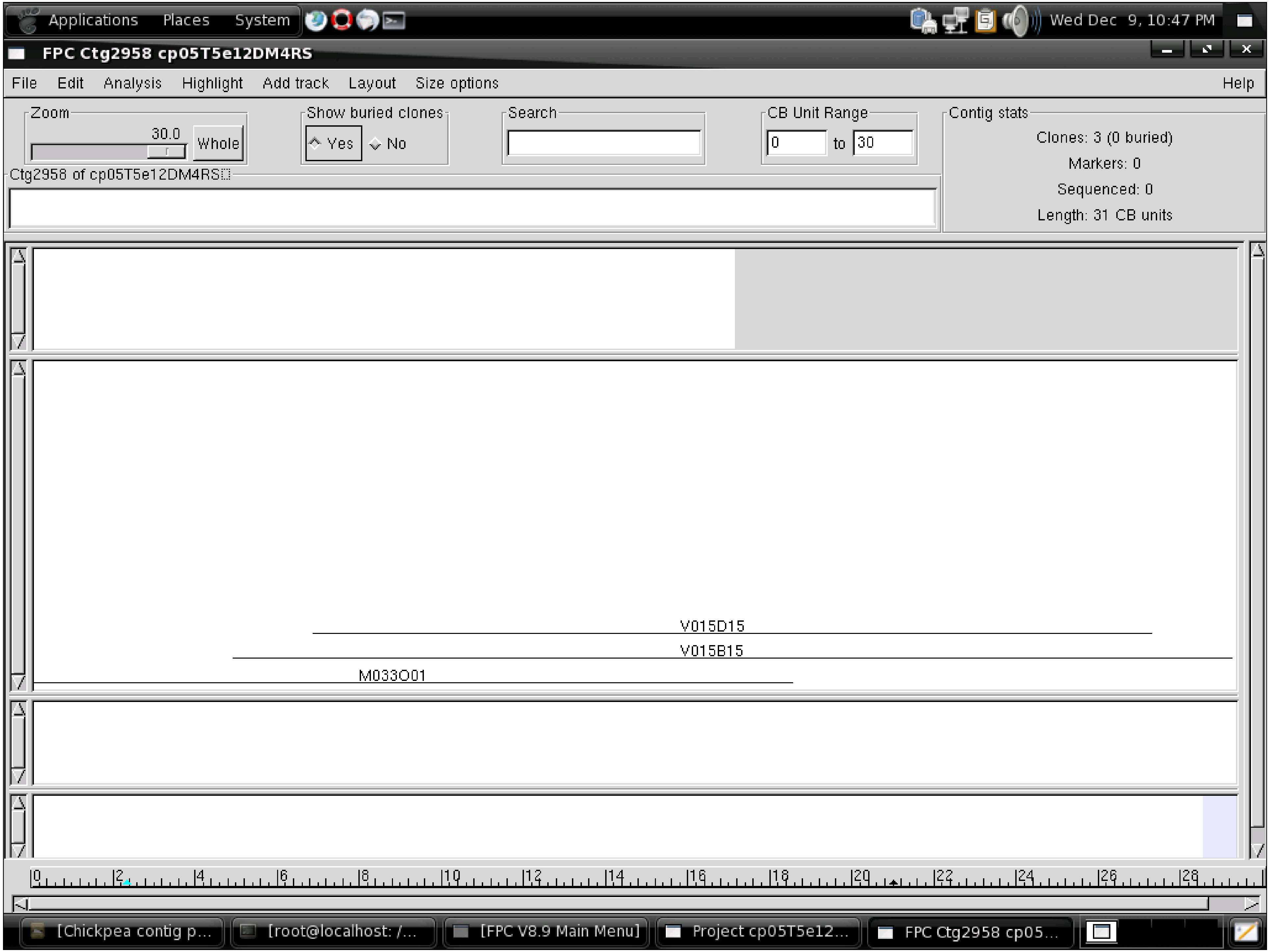
Applications Places System
Wed Dec 9, 10:47 PM
FPC Ctg2958 cp05T5e12DM4RS
File Edit Analysis Highlight Add track Layout Size options Help
Zoom
30.0
Whole
Show buried clones
Yes No
Search
CB Unit Range
0 to 30
Contig stats
Clones: 3 (0 buried)
Markers: 0
Sequenced: 0
Length: 31 CB units
Ctg2958 of cp05T5e12DM4RS
V015D15
V015B15
M033O01
0 2 4 6 8 10 12 14 16 18 20 22 24 26 28
[Chickpea contig p...
[root@localhost: /...
[FPC V8.9 Main Menu]
Project cp05T5e12...
FPC Ctg2958 cp05...

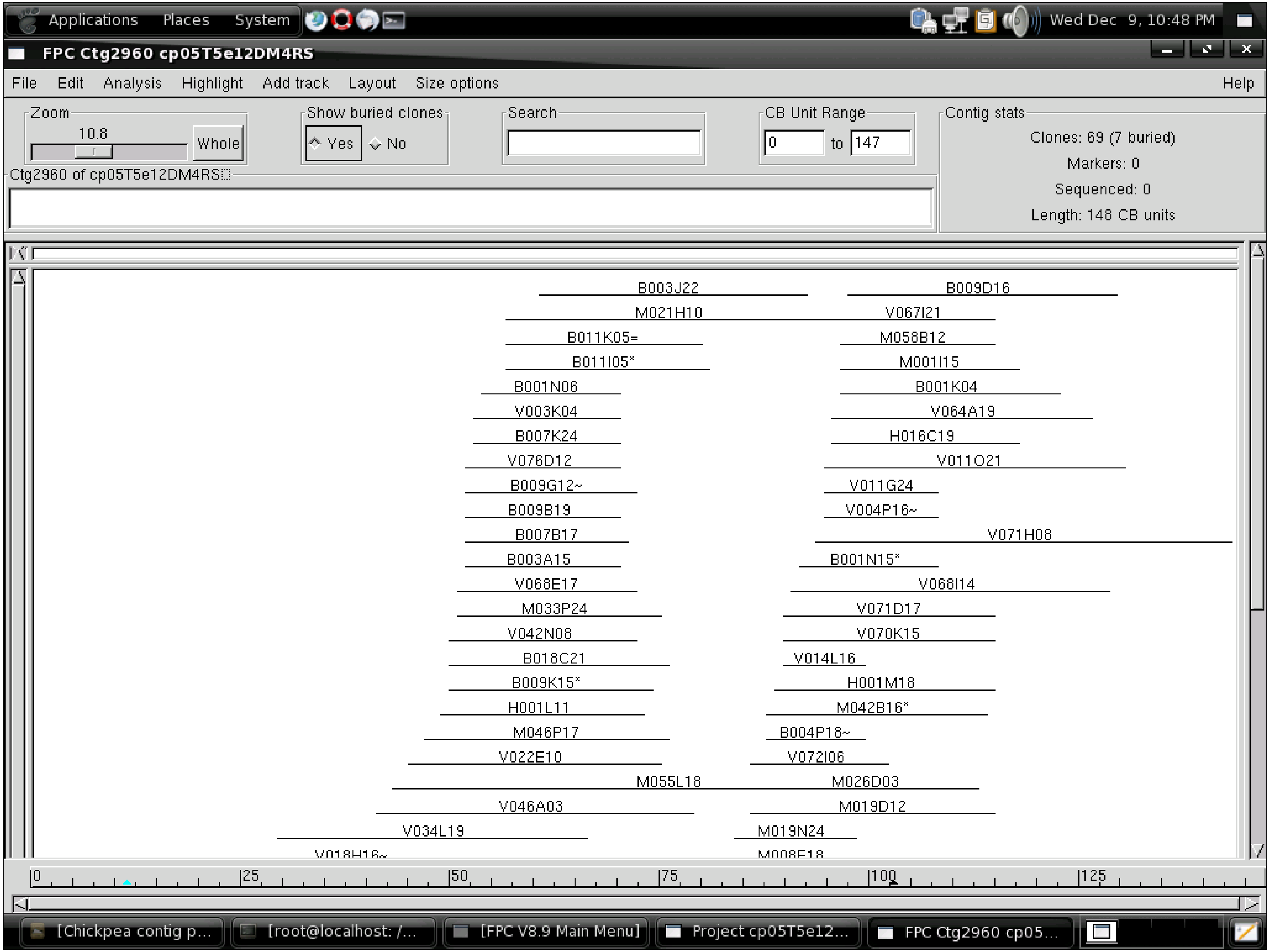
Applications Places System
Wed Dec 9, 10:48 PM
FPC Ctg2960 cp05T5e12DM4RS
File Edit Analysis Highlight Add track Layout Size options Help
Zoom
10.8
Whole
Show buried clones
Yes
No
Search
CB Unit Range
0 to 147
Contig stats
Clones: 69 (7 buried)
Markers: 0
Sequenced: 0
Length: 148 CB units
Ctg2960 of cp05T5e12DM4RS
B003J22
B009D16
M021H10
V067I21
B011K05=
M058B12
B011I05*
M001I15
B001N06
B001K04
V003K04
V064A19
B007K24
H016C19
V076D12
V011O21
B009G12~
V011G24
B009B19
V004P16~
B007B17
V071H08
B003A15
B001N15*
V068E17
V068I14
M033P24
V071D17
V042N08
V070K15
B018C21
V014L16
B009K15*
H001M18
H001L11
M042B16*
M046P17
B004P18~
V022E10
V072I06
M055L18
M026D03
V046A03
M019D12
V034L19
M019N24
0
25
50
75
100
125
[Chickpea contig p...
[root@localhost: /...
[FPC V8.9 Main Menu]
Project cp05T5e12...
FPC Ctg2960 cp05...

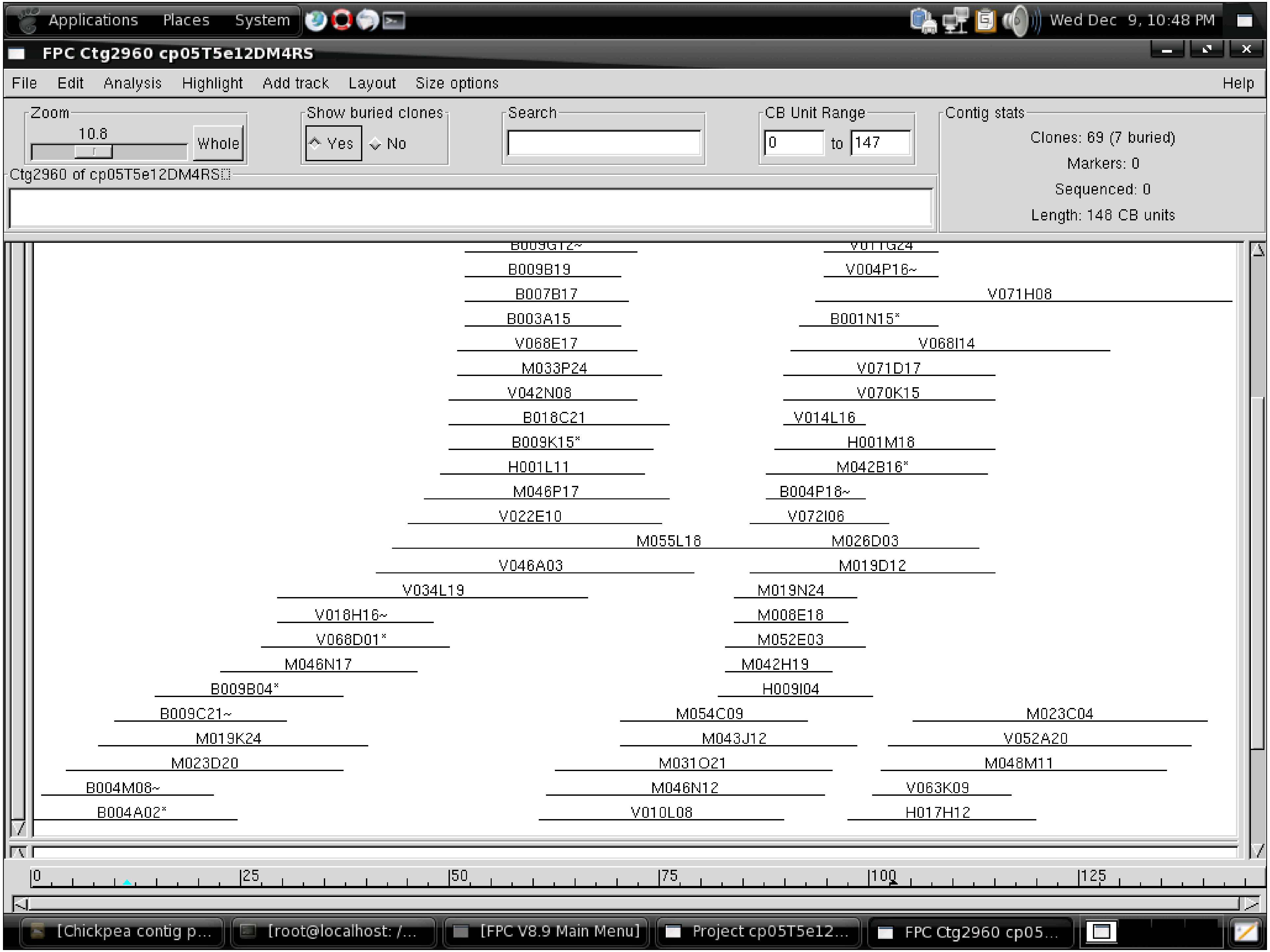
Applications Places System
Wed Dec 9, 10:48 PM
FPC Ctg2960 cp05T5e12DM4RS
File Edit Analysis Highlight Add track Layout Size options Help
Zoom
10.8
Whole
Show buried clones
Yes
No
Search
CB Unit Range
0 to 147
Contig stats
Clones: 69 (7 buried)
Markers: 0
Sequenced: 0
Length: 148 CB units
Ctg2960 of cp05T5e12DM4RS
B009B19
B007B17
B003A15
V068E17
M033P24
V042N08
B018C21
B009K15*
H001L11
M046P17
V022E10
M055L18
V046A03
V034L19
V018H16~
V068D01*
M046N17
B009B04*
B009C21~
M019K24
M023D20
B004M08~
B004A02*
V004P16~
V071H08
B001N15*
V068I14
V071D17
V070K15
V014L16
H001M18
M042B16*
B004P18~
V072I06
M026D03
M019D12
M019N24
M008E18
M052E03
M042H19
H009I04
M054C09
M043J12
M031O21
M046N12
V010L08
M023C04
V052A20
M048M11
V063K09
H017H12
0
25
50
75
100
125
[Chickpea contig p...
[root@localhost: /...
[FPC V8.9 Main Menu]
Project cp05T5e12...
FPC Ctg2960 cp05...

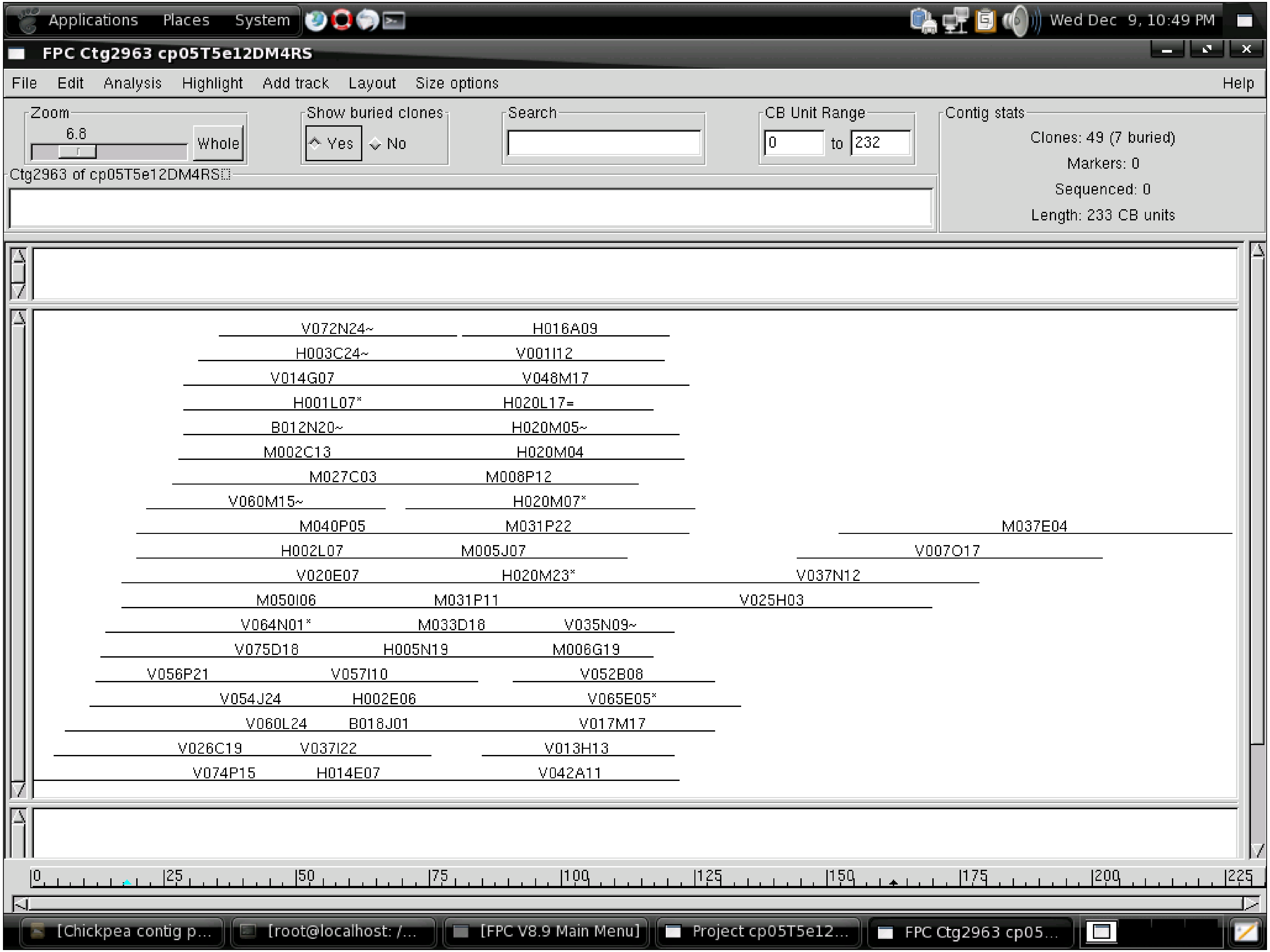

Applications Places System
Wed Dec 9, 10:49 PM
FPC Ctg2963 cp05T5e12DM4RS
File Edit Analysis Highlight Add track Layout Size options Help
Zoom
6.8
Whole
Show buried clones
Yes
No
Search
CB Unit Range
0 to 232
Contig stats
Clones: 49 (7 buried)
Markers: 0
Sequenced: 0
Length: 233 CB units
Ctg2963 of cp05T5e12DM4RS
V072N24~
H016A09
H003C24~
V001I12
V014G07
V048M17
H001L07*
H020L17=
B012N20~
H020M05~
M002C13
H020M04
M027C03
M008P12
V060M15~
H020M07*
M040P05
M031P22
M037E04
H002L07
M005J07
V007O17
V020E07
H020M23*
V037N12
M050I06
M031P11
V025H03
V064N01*
M033D18
V035N09~
V075D18
H005N19
M006G19
V056P21
V057I10
V052B08
V054J24
H002E06
V065E05*
V060L24
B018J01
V017M17
V026C19
V037I22
V013H13
V074P15
H014E07
V042A11
0 25 50 75 100 125 150 175 200 225
[Chickpea contig p...
[root@localhost: /...
[FPC V8.9 Main Menu]
Project cp05T5e12...
FPC Ctg2963 cp05...

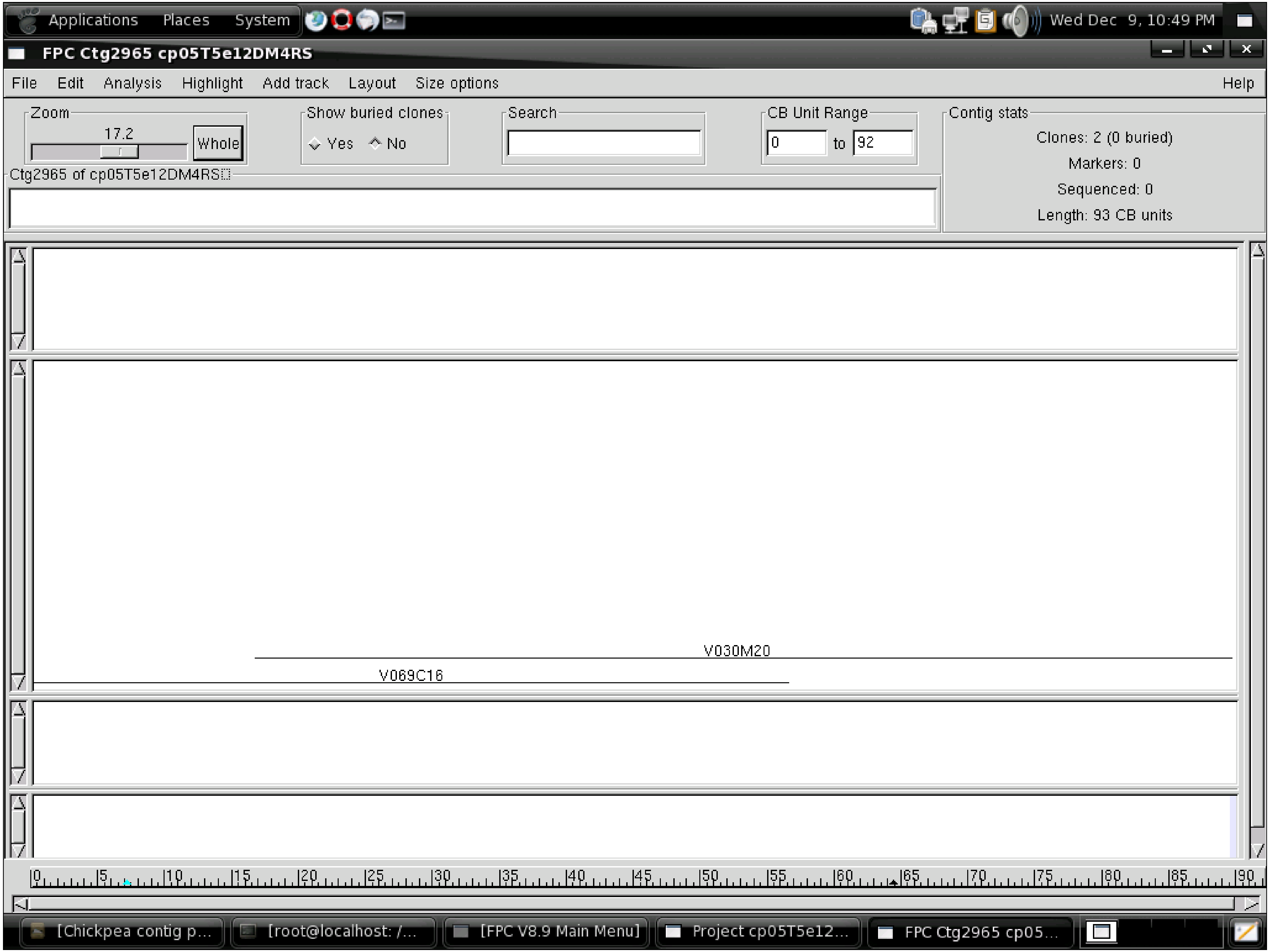
Applications Places System
Wed Dec 9, 10:49 PM
FPC Ctg2965 cp05T5e12DM4RS
File Edit Analysis Highlight Add track Layout Size options Help
Zoom
17.2
Whole
Show buried clones
Yes No
Search
CB Unit Range
0 to 92
Contig stats
Clones: 2 (0 buried)
Markers: 0
Sequenced: 0
Length: 93 CB units
Ctg2965 of cp05T5e12DM4RS
V030M20
V069C16
0 5 10 15 20 25 30 35 40 45 50 55 60 65 70 75 80 85 90
[Chickpea contig p...
[root@localhost: /...
[FPC V8.9 Main Menu]
Project cp05T5e12...
FPC Ctg2965 cp05...

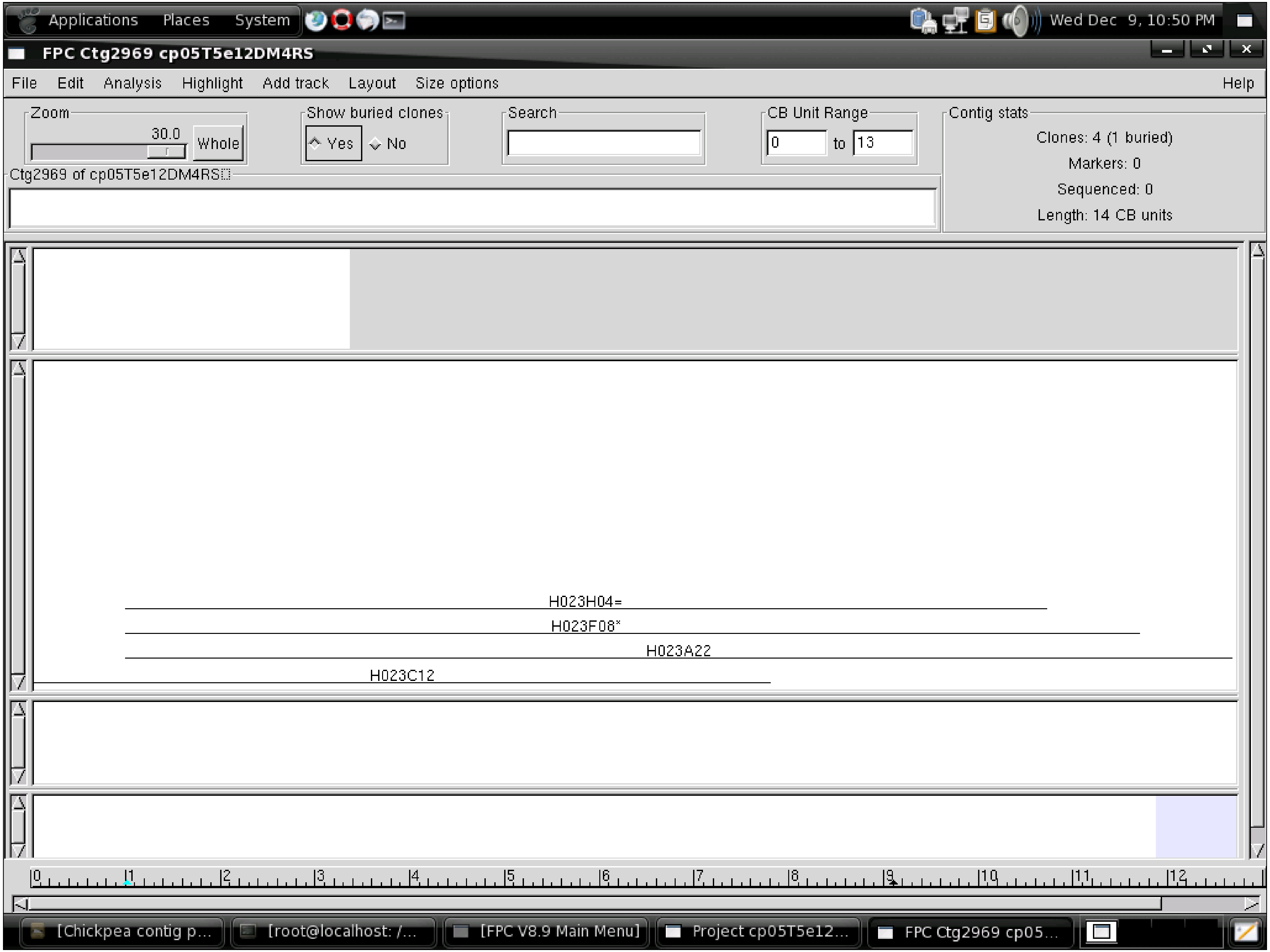
Applications Places System
Wed Dec 9, 10:50 PM
FPC Ctg2969 cp05T5e12DM4RS
File Edit Analysis Highlight Add track Layout Size options Help
Zoom
30.0
Whole
Show buried clones
Yes No
Search
CB Unit Range
0 to 13
Contig stats
Clones: 4 (1 buried)
Markers: 0
Sequenced: 0
Length: 14 CB units
Ctg2969 of cp05T5e12DM4RS
H023H04=
H023F08*
H023A22
H023C12
0 1 2 3 4 5 6 7 8 9 10 11 12
[Chickpea contig p...
[root@localhost: /...
[FPC V8.9 Main Menu]
Project cp05T5e12...
FPC Ctg2969 cp05...

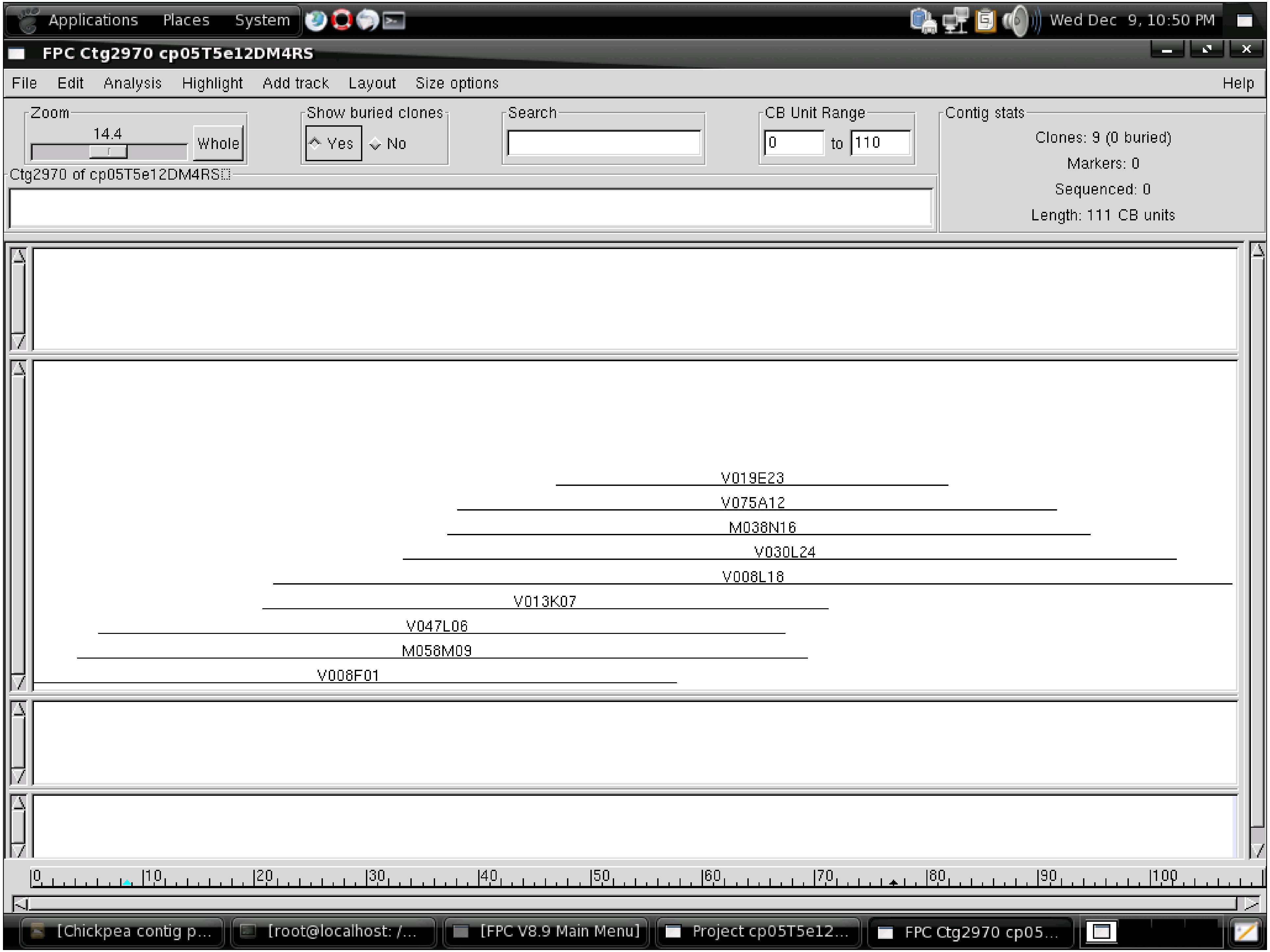
Applications Places System
Wed Dec 9, 10:50 PM
FPC Ctg2970 cp05T5e12DM4RS
File Edit Analysis Highlight Add track Layout Size options Help
Zoom
14.4
Whole
Show buried clones
Yes No
Search
CB Unit Range
0 to 110
Contig stats
Clones: 9 (0 buried)
Markers: 0
Sequenced: 0
Length: 111 CB units
Ctg2970 of cp05T5e12DM4RS
V019E23
V075A12
M038N16
V030L24
V008L18
V013K07
V047L06
M058M09
V008F01
0 10 20 30 40 50 60 70 80 90 100
[Chickpea contig p...
[root@localhost: /...
[FPC V8.9 Main Menu]
Project cp05T5e12...
FPC Ctg2970 cp05...

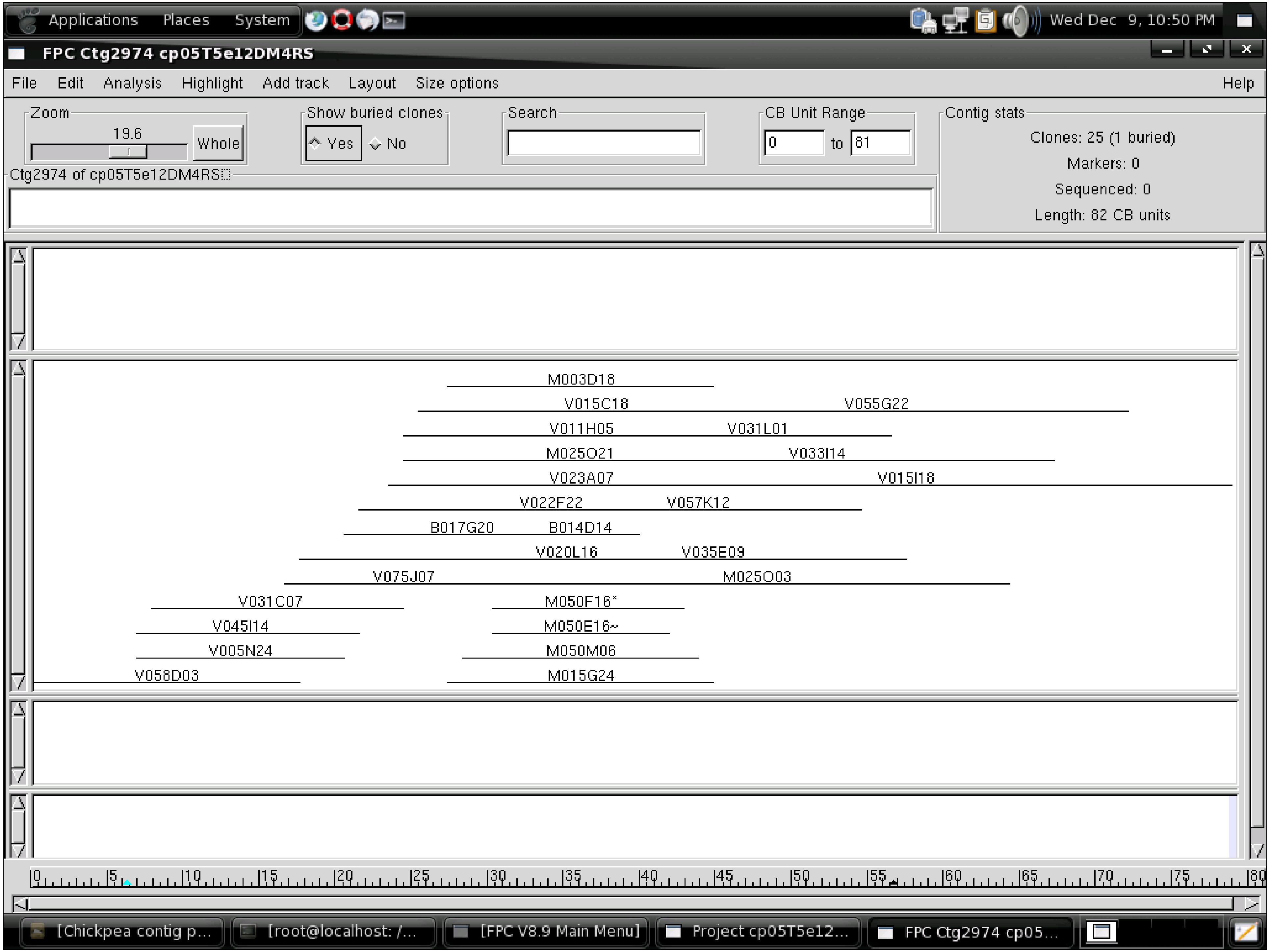
Applications Places System
Wed Dec 9, 10:50 PM
FPC Ctg2974 cp05T5e12DM4RS
File Edit Analysis Highlight Add track Layout Size options Help
Zoom
19.6
Whole
Show buried clones
Yes
No
Search
CB Unit Range
0
to
81
Contig stats
Clones: 25 (1 buried)
Markers: 0
Sequenced: 0
Length: 82 CB units
Ctg2974 of cp05T5e12DM4RS
M003D18
V015C18
V055G22
V011H05
V031L01
M025O21
V033I14
V023A07
V015I18
V022F22
V057K12
B017G20
B014D14
V020L16
V035E09
V075J07
M025O03
V031C07
M050F16*
V045I14
M050E16~
V005N24
M050M06
V058D03
M015G24
0 5 10 15 20 25 30 35 40 45 50 55 60 65 70 75 80
[Chickpea contig p...
[root@localhost: /...
[FPC V8.9 Main Menu]
Project cp05T5e12...
FPC Ctg2974 cp05...

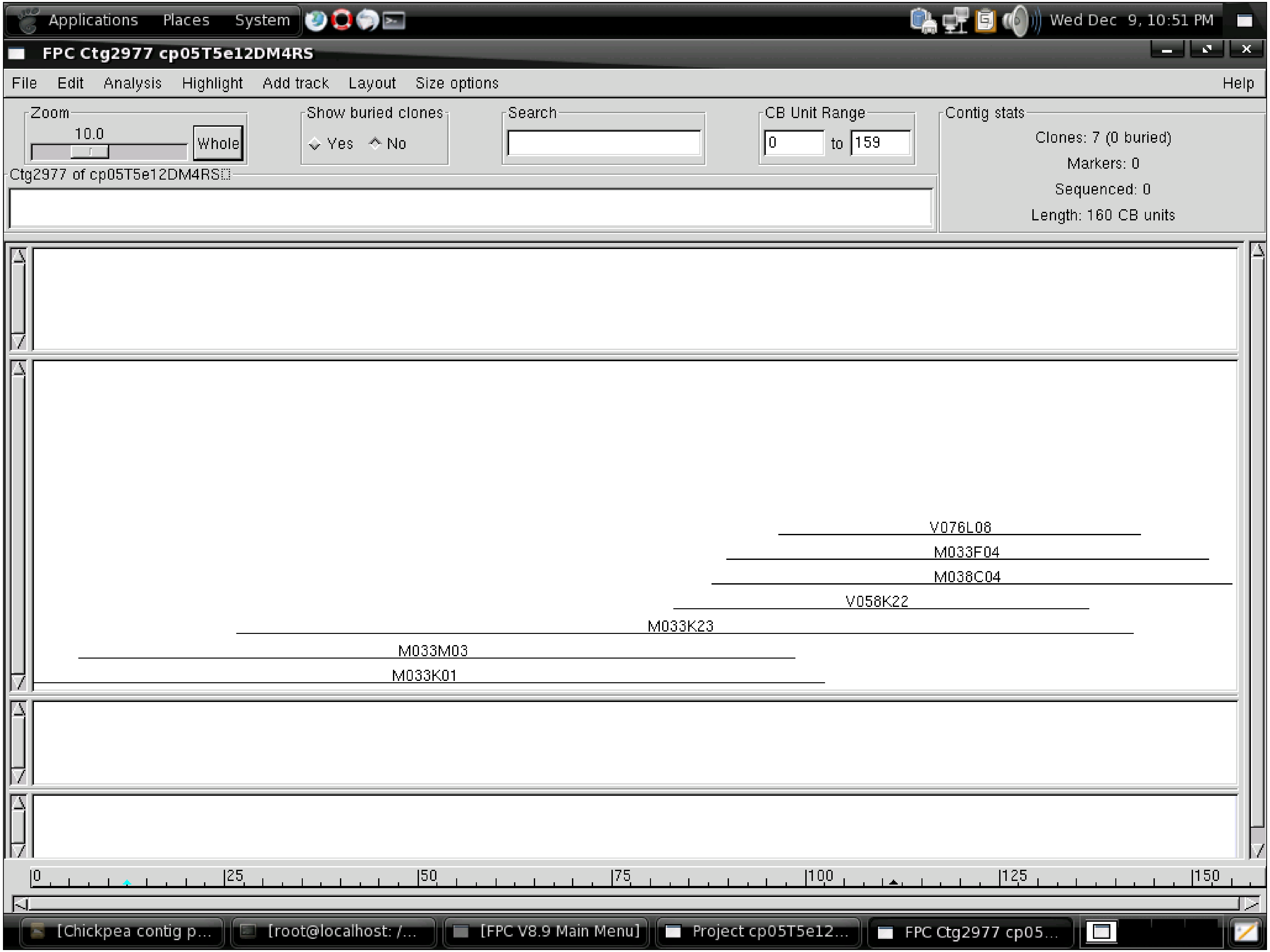
Applications
Places
System
Wed Dec 9, 10:51 PM
FPC Ctg2977 cp05T5e12DM4RS
File
Edit
Analysis
Highlight
Add track
Layout
Size options
Help
Zoom
10.0
Whole
Show buried clones
Yes
No
Search
CB Unit Range
0
to
159
Contig stats
Clones: 7 (0 buried)
Markers: 0
Sequenced: 0
Length: 160 CB units
Ctg2977 of cp05T5e12DM4RS
V076L08
M033F04
M038C04
V058K22
M033K23
M033M03
M033K01
0
25
50
75
100
125
150
[Chickpea contig p...
[root@localhost: /...
[FPC V8.9 Main Menu]
Project cp05T5e12...
FPC Ctg2977 cp05...

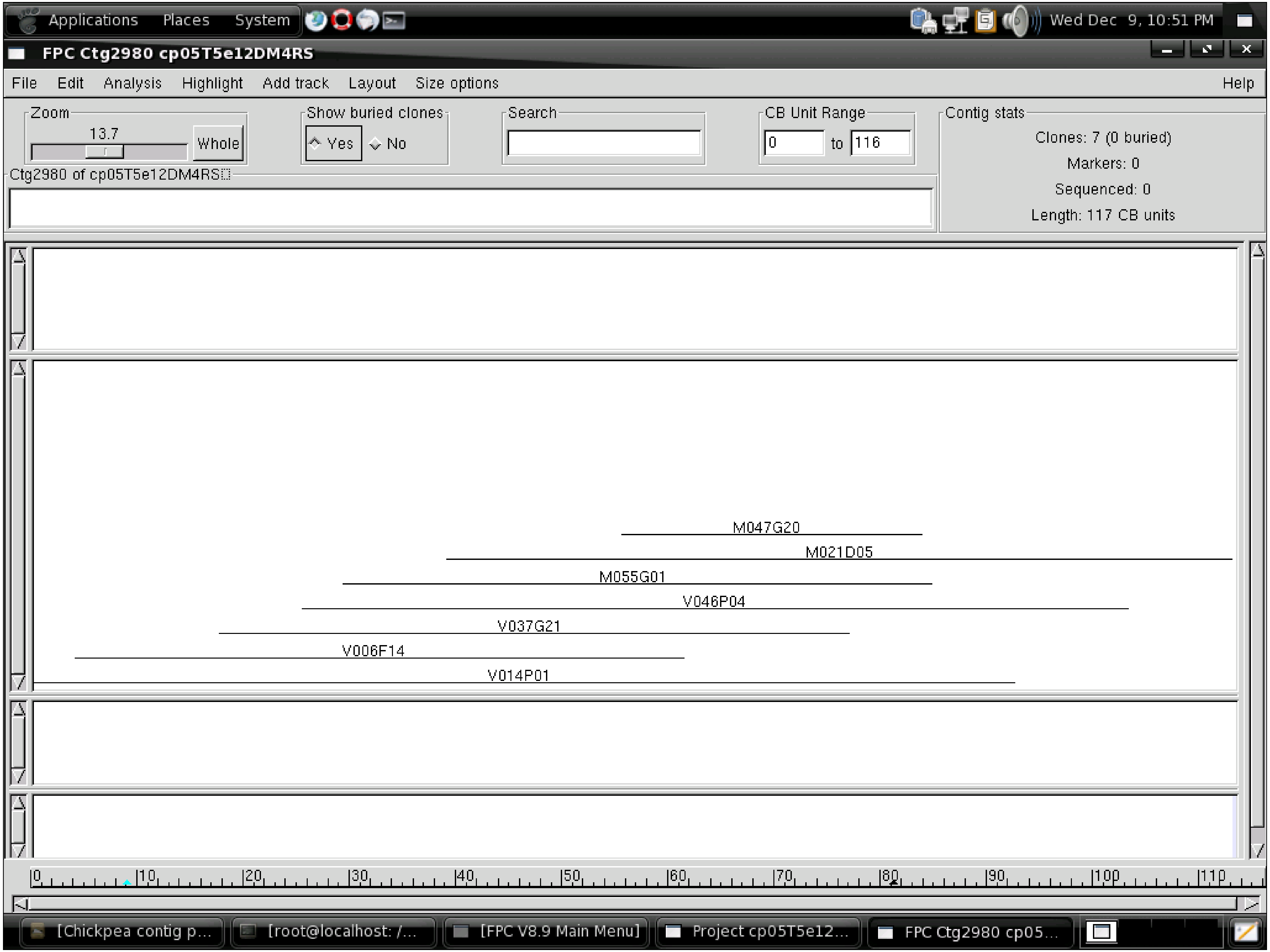
Applications Places System
Wed Dec 9, 10:51 PM
FPC Ctg2980 cp05T5e12DM4RS
File Edit Analysis Highlight Add track Layout Size options Help
Zoom
13.7
Whole
Show buried clones
Yes No
Search
CB Unit Range
0 to 116
Contig stats
Clones: 7 (0 buried)
Markers: 0
Sequenced: 0
Length: 117 CB units
Ctg2980 of cp05T5e12DM4RS
M047G20
M021D05
M055G01
V046P04
V037G21
V006F14
V014P01
0 10 20 30 40 50 60 70 80 90 100 110
[Chickpea contig p...
[root@localhost: /...
[FPC V8.9 Main Menu]
Project cp05T5e12...
FPC Ctg2980 cp05...

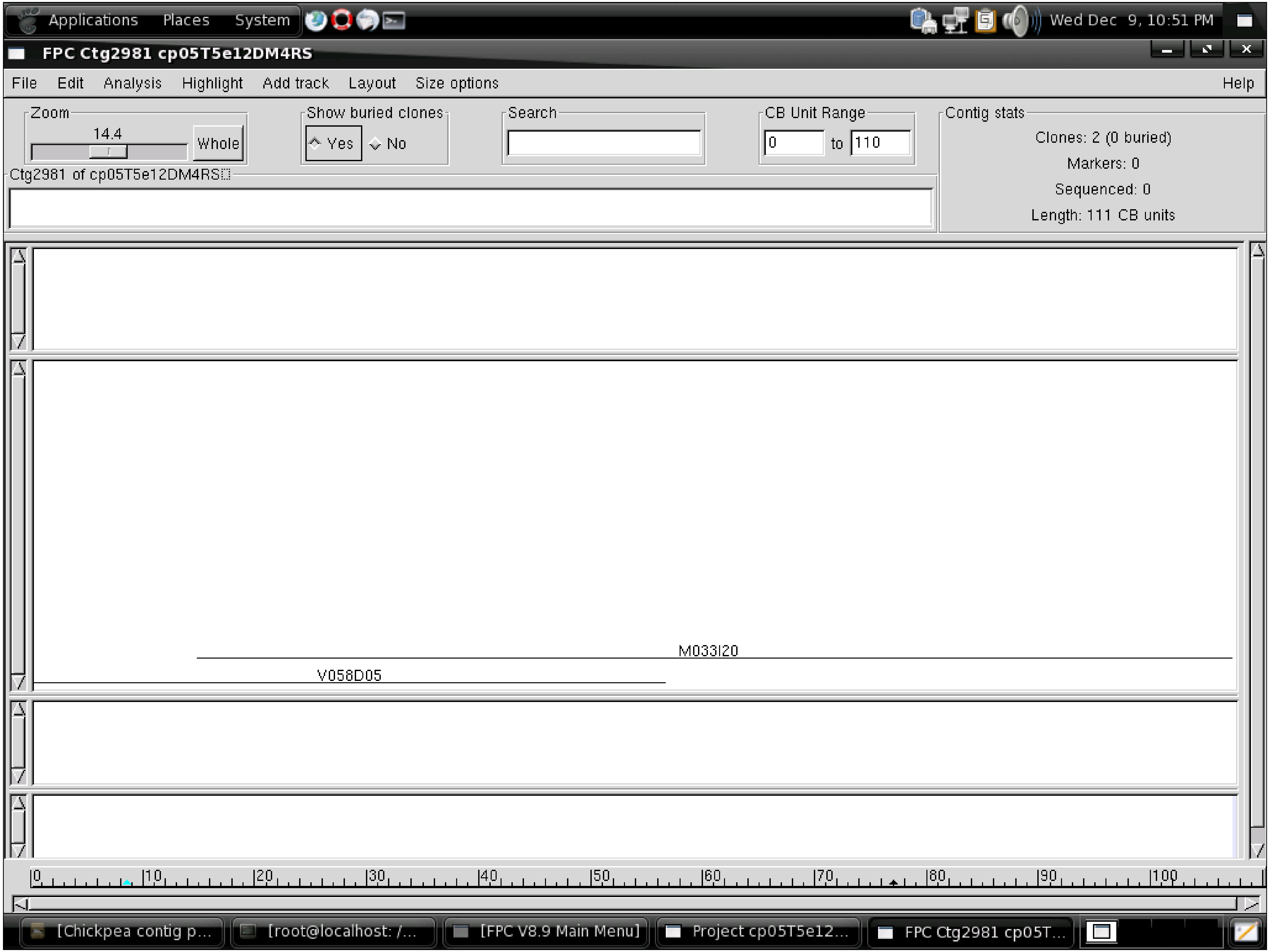
Applications Places System
Wed Dec 9, 10:51 PM
FPC Ctg2981 cp05T5e12DM4RS
File Edit Analysis Highlight Add track Layout Size options
Help
Zoom
14.4
Whole
Show buried clones
Yes No
Search
CB Unit Range
0 to 110
Contig stats
Clones: 2 (0 buried)
Markers: 0
Sequenced: 0
Length: 111 CB units
Ctg2981 of cp05T5e12DM4RS
M033I20
V058D05
0 10 20 30 40 50 60 70 80 90 100
[Chickpea contig p...
[root@localhost: /...
[FPC V8.9 Main Menu]
Project cp05T5e12...
FPC Ctg2981 cp05T...

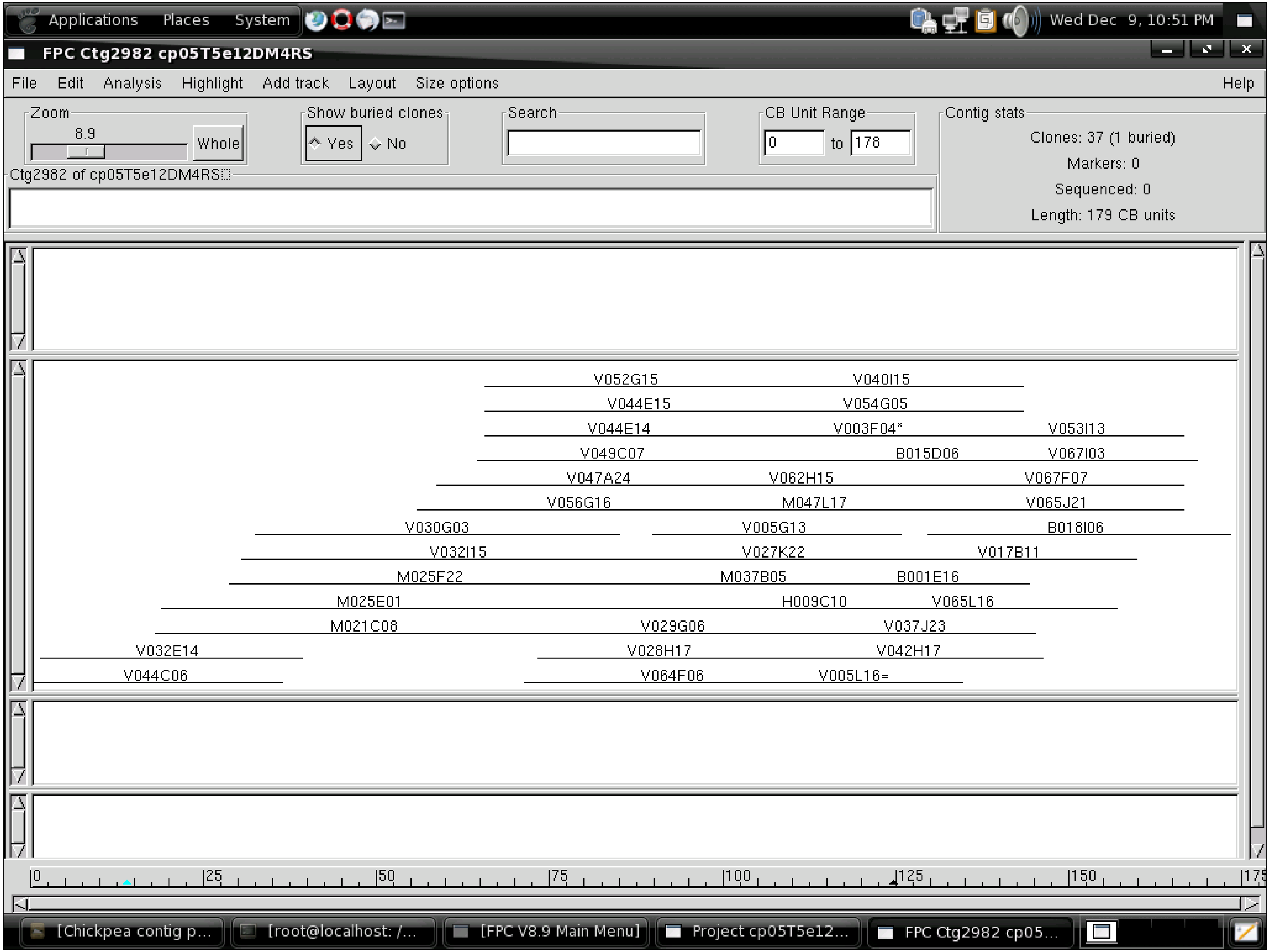

Applications
Places
System
Wed Dec 9, 10:51 PM
FPC Ctg2982 cp05T5e12DM4RS
File
Edit
Analysis
Highlight
Add track
Layout
Size options
Help
Zoom
8.9
Whole
Show buried clones
Yes
No
Search
CB Unit Range
0
to
178
Contig stats
Clones: 37 (1 buried)
Markers: 0
Sequenced: 0
Length: 179 CB units
Ctg2982 of cp05T5e12DM4RS
V052G15
V040I15
V044E15
V054G05
V044E14
V003F04*
V053I13
V049C07
B015D06
V067I03
V047A24
V062H15
V067F07
V056G16
M047L17
V065J21
V030G03
V005G13
B018I06
V032I15
V027K22
V017B11
M025F22
M037B05
B001E16
M025E01
H009C10
V065L16
M021C08
V029G06
V037J23
V032E14
V028H17
V042H17
V044C06
V064F06
V005L16=
0
25
50
75
100
125
150
175
[Chickpea contig p...
[root@localhost: /...
[FPC V8.9 Main Menu]
Project cp05T5e12...
FPC Ctg2982 cp05...

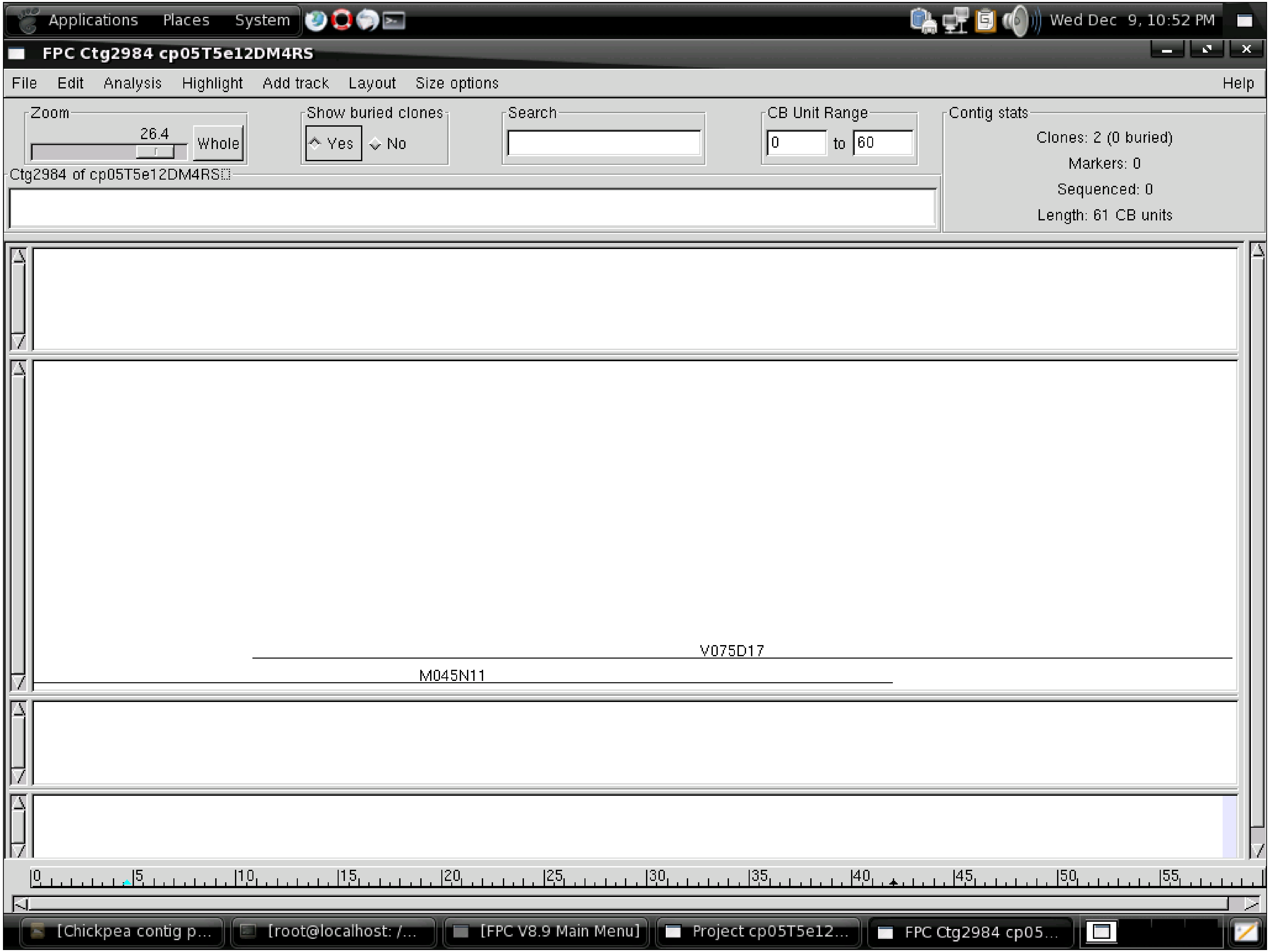
Applications Places System
Wed Dec 9, 10:52 PM
FPC Ctg2984 cp05T5e12DM4RS
File Edit Analysis Highlight Add track Layout Size options Help
Zoom
26.4
Whole
Show buried clones
Yes
No
Search
CB Unit Range
0 to 60
Contig stats
Clones: 2 (0 buried)
Markers: 0
Sequenced: 0
Length: 61 CB units
Ctg2984 of cp05T5e12DM4RS
V075D17
M045N11
0 5 10 15 20 25 30 35 40 45 50 55
[Chickpea contig p...
[root@localhost: /...
[FPC V8.9 Main Menu]
Project cp05T5e12...
FPC Ctg2984 cp05...

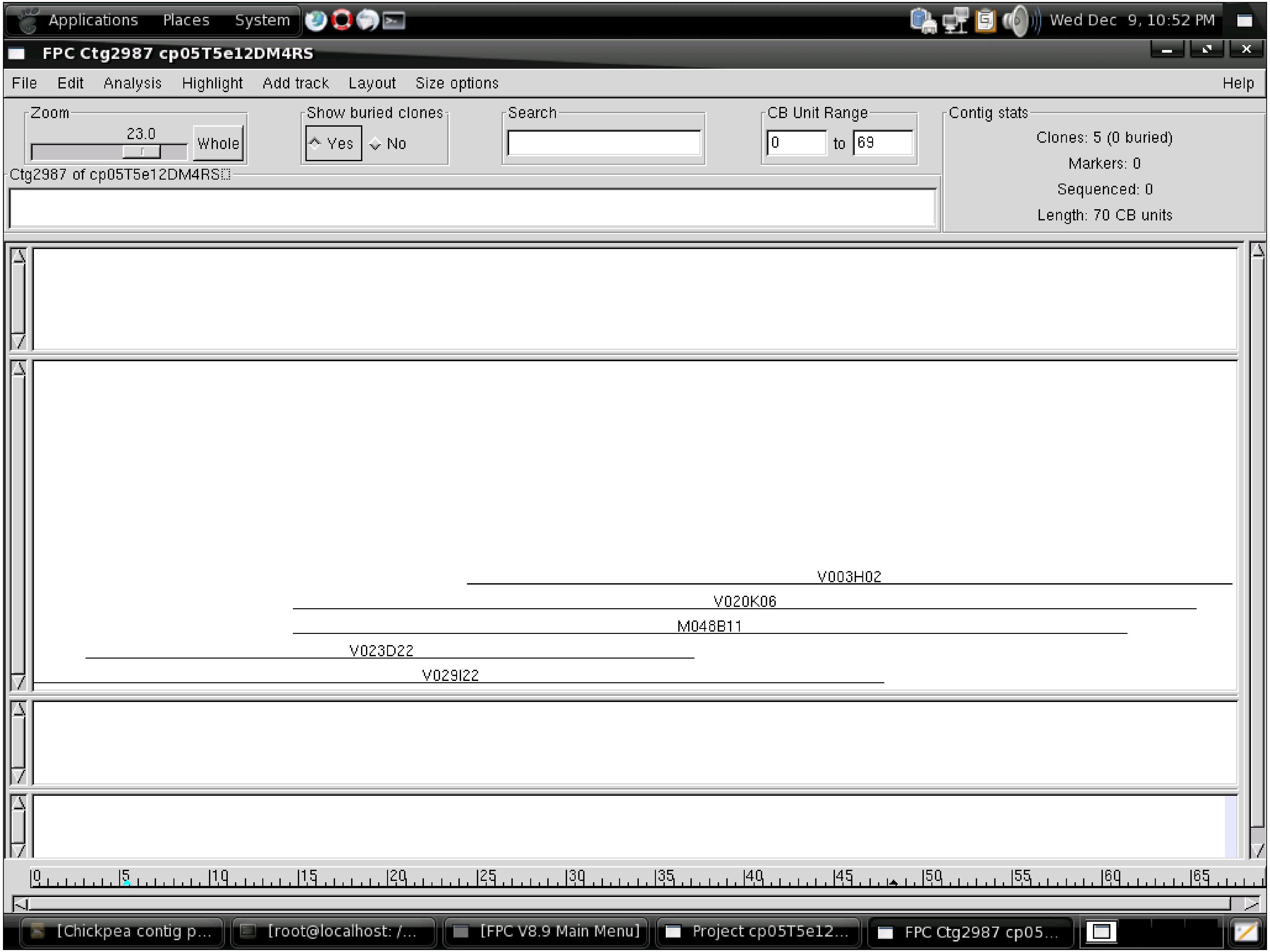
Applications
Places
System
Wed Dec 9, 10:52 PM
FPC Ctg2987 cp05T5e12DM4RS
File
Edit
Analysis
Highlight
Add track
Layout
Size options
Help
Zoom
23.0
Whole
Show buried clones
Yes
No
Search
CB Unit Range
0
to
69
Contig stats
Clones: 5 (0 buried)
Markers: 0
Sequenced: 0
Length: 70 CB units
Ctg2987 of cp05T5e12DM4RS
V003H02
V020K06
M048B11
V023D22
V029I22
0
5
10
15
20
25
30
35
40
45
50
55
60
65
[Chickpea contig p...
[root@localhost: /...
[FPC V8.9 Main Menu]
Project cp05T5e12...
FPC Ctg2987 cp05...

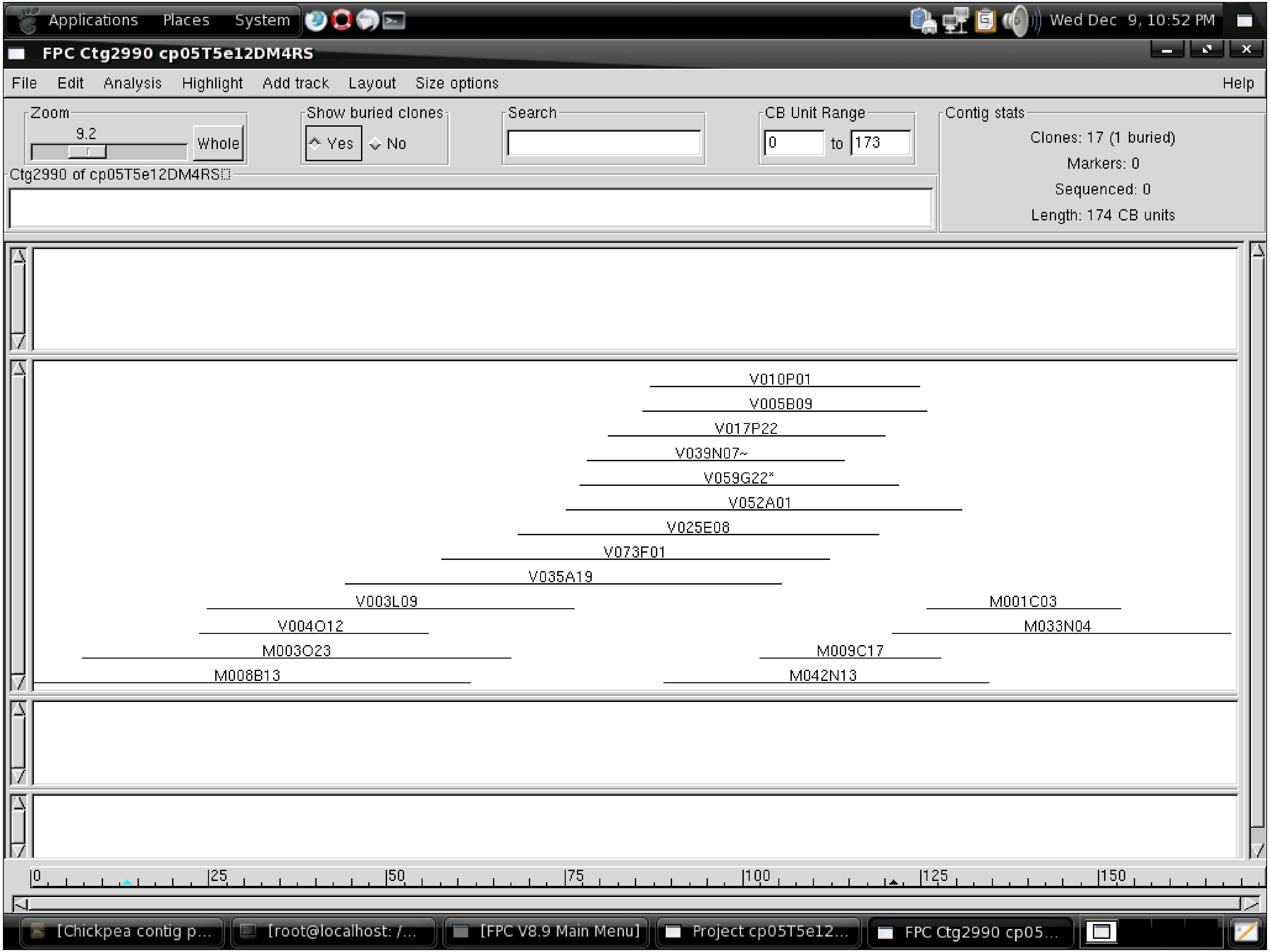
Applications Places System
Wed Dec 9, 10:52 PM
FPC Ctg2990 cp05T5e12DM4RS
File Edit Analysis Highlight Add track Layout Size options Help
Zoom
9.2
Whole
Show buried clones
Yes
No
Search
CB Unit Range
0
to
173
Contig stats
Clones: 17 (1 buried)
Markers: 0
Sequenced: 0
Length: 174 CB units
Ctg2990 of cp05T5e12DM4RS
V010P01
V005B09
V017P22
V039N07~
V059G22*
V052A01
V025E08
V073F01
V035A19
V003L09
M001C03
V004O12
M033N04
M003O23
M009C17
M008B13
M042N13
0
25
50
75
100
125
150
[Chickpea contig p...
[root@localhost: /...
[FPC V8.9 Main Menu]
Project cp05T5e12...
FPC Ctg2990 cp05...

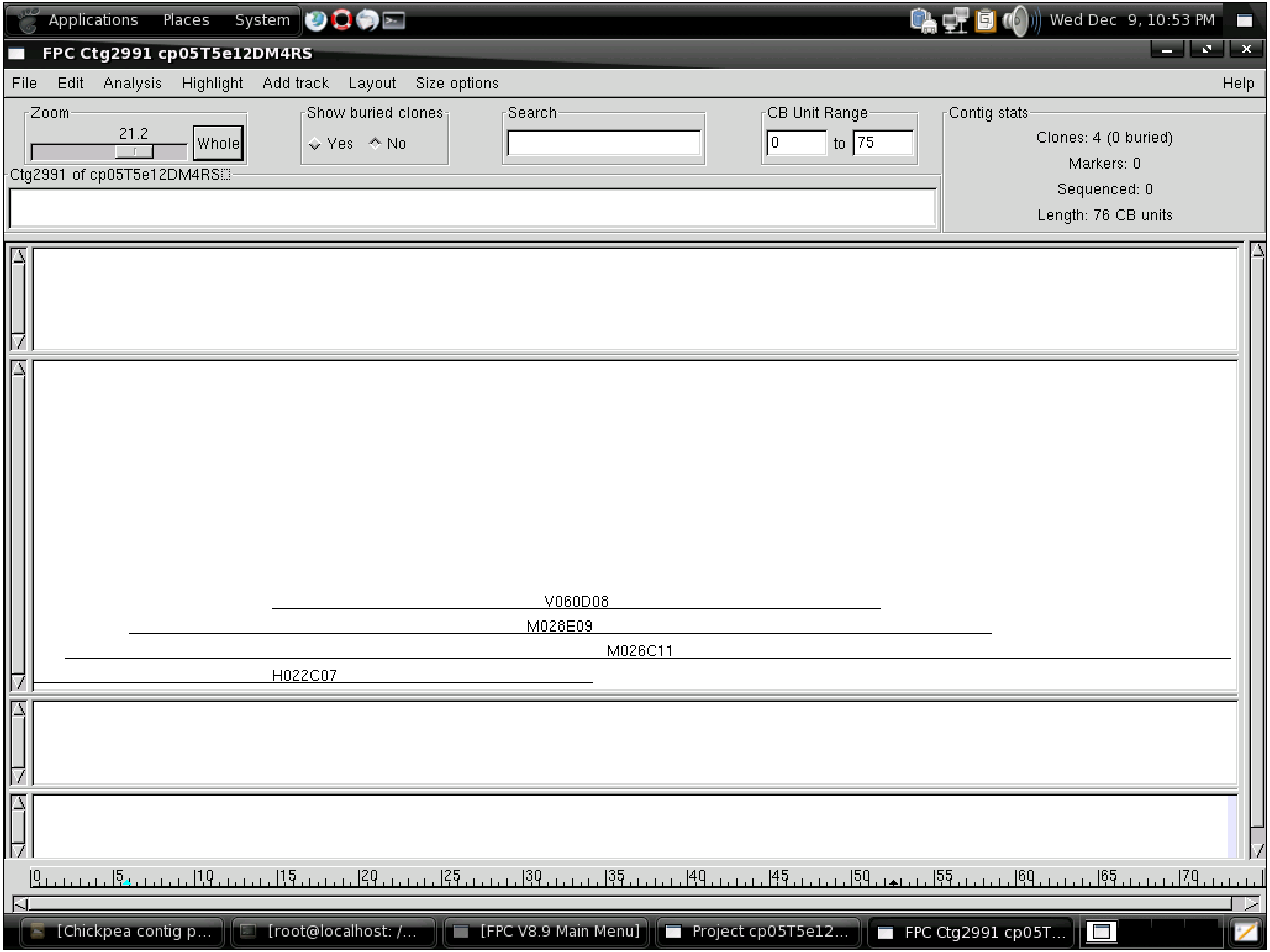
Applications
Places
System
Wed Dec 9, 10:53 PM
FPC Ctg2991 cp05T5e12DM4RS
File
Edit
Analysis
Highlight
Add track
Layout
Size options
Help
Zoom
21.2
Whole
Show buried clones
Yes
No
Search
CB Unit Range
0
to
75
Contig stats
Clones: 4 (0 buried)
Markers: 0
Sequenced: 0
Length: 76 CB units
Ctg2991 of cp05T5e12DM4RS
V060D08
M028E09
M026C11
H022C07
0 5 10 15 20 25 30 35 40 45 50 55 60 65 70
[Chickpea contig p...
[root@localhost: /...
[FPC V8.9 Main Menu]
Project cp05T5e12...
FPC Ctg2991 cp05T...

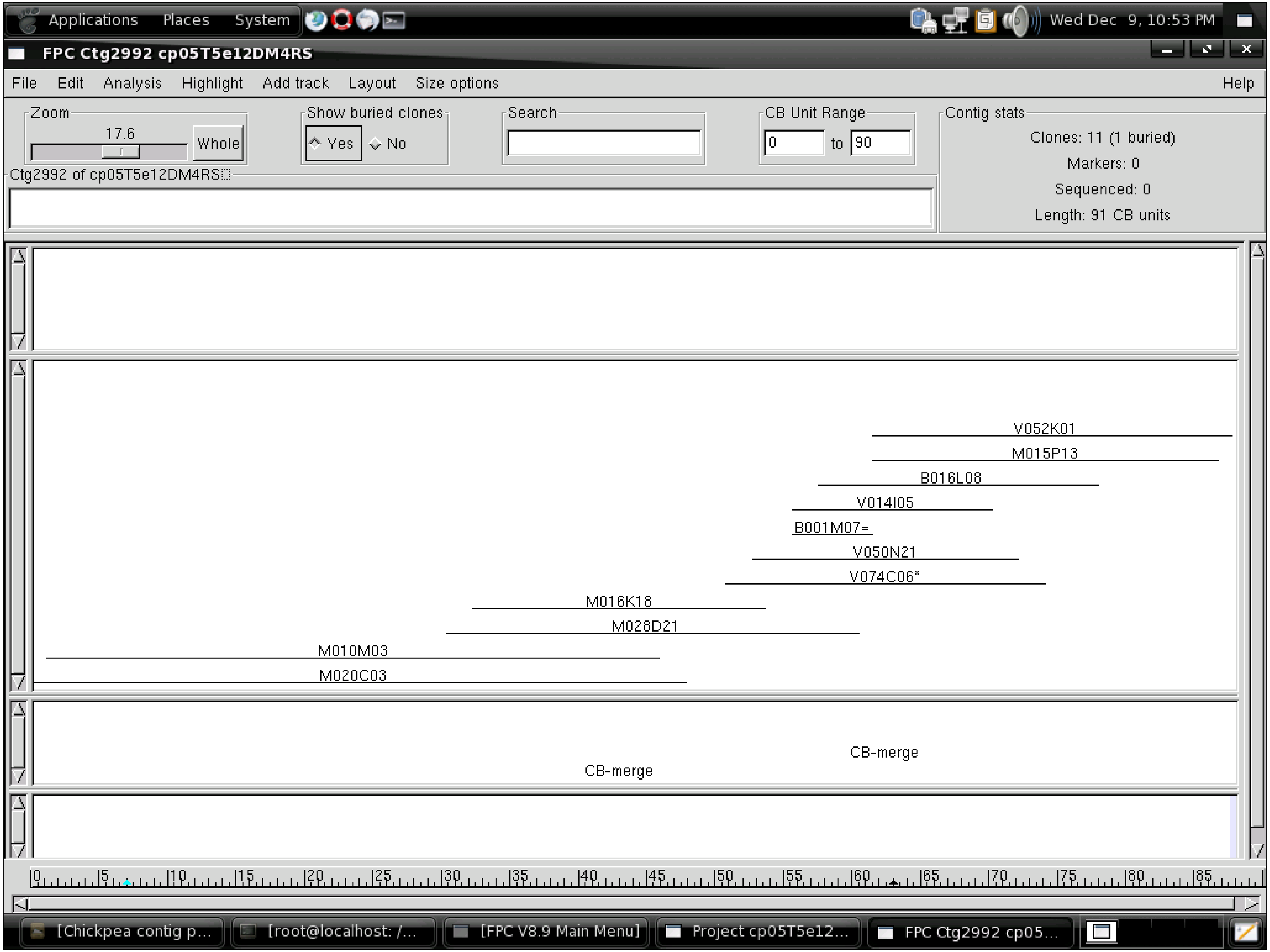
Applications Places System
Wed Dec 9, 10:53 PM
FPC Ctg2992 cp05T5e12DM4RS
File Edit Analysis Highlight Add track Layout Size options Help
Zoom
17.6
Whole
Show buried clones
Yes
No
Search
CB Unit Range
0 to 90
Contig stats
Clones: 11 (1 buried)
Markers: 0
Sequenced: 0
Length: 91 CB units
Ctg2992 of cp05T5e12DM4RS
V052K01
M015P13
B016L08
V014I05
B001M07=
V050N21
V074C06*
M016K18
M028D21
M010M03
M020C03
CB-merge
CB-merge
0 5 10 15 20 25 30 35 40 45 50 55 60 65 70 75 80 85
[Chickpea contig p...
[root@localhost: /...
[FPC V8.9 Main Menu]
Project cp05T5e12...
FPC Ctg2992 cp05...

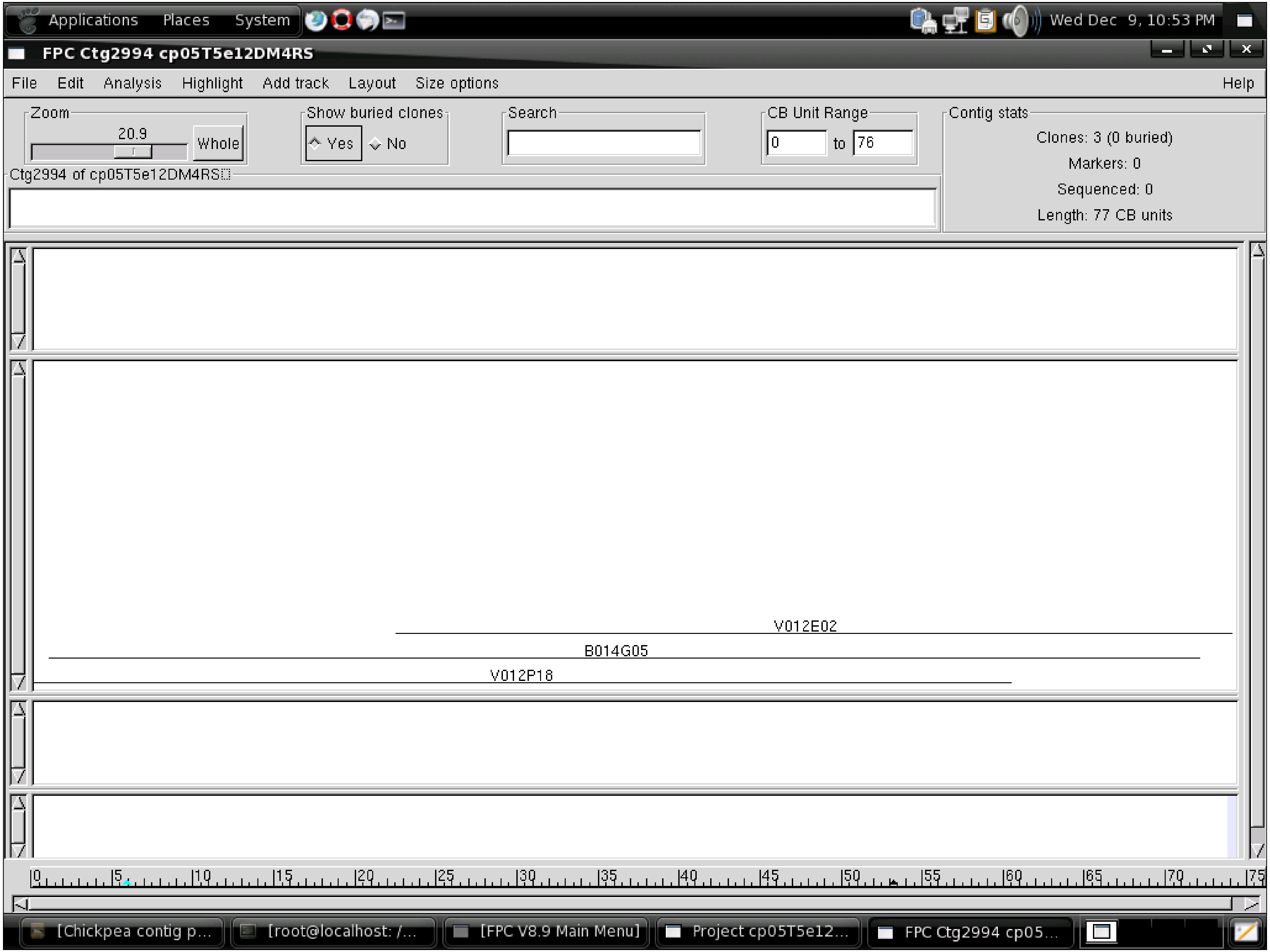
Applications Places System
Wed Dec 9, 10:53 PM
FPC Ctg2994 cp05T5e12DM4RS
File Edit Analysis Highlight Add track Layout Size options Help
Zoom
20.9
Whole
Show buried clones
Yes No
Search
CB Unit Range
0 to 76
Contig stats
Clones: 3 (0 buried)
Markers: 0
Sequenced: 0
Length: 77 CB units
Ctg2994 of cp05T5e12DM4RS
V012E02
B014G05
V012P18
0 5 10 15 20 25 30 35 40 45 50 55 60 65 70 75
[Chickpea contig p...
[root@localhost: /...
[FPC V8.9 Main Menu]
Project cp05T5e12...
FPC Ctg2994 cp05...

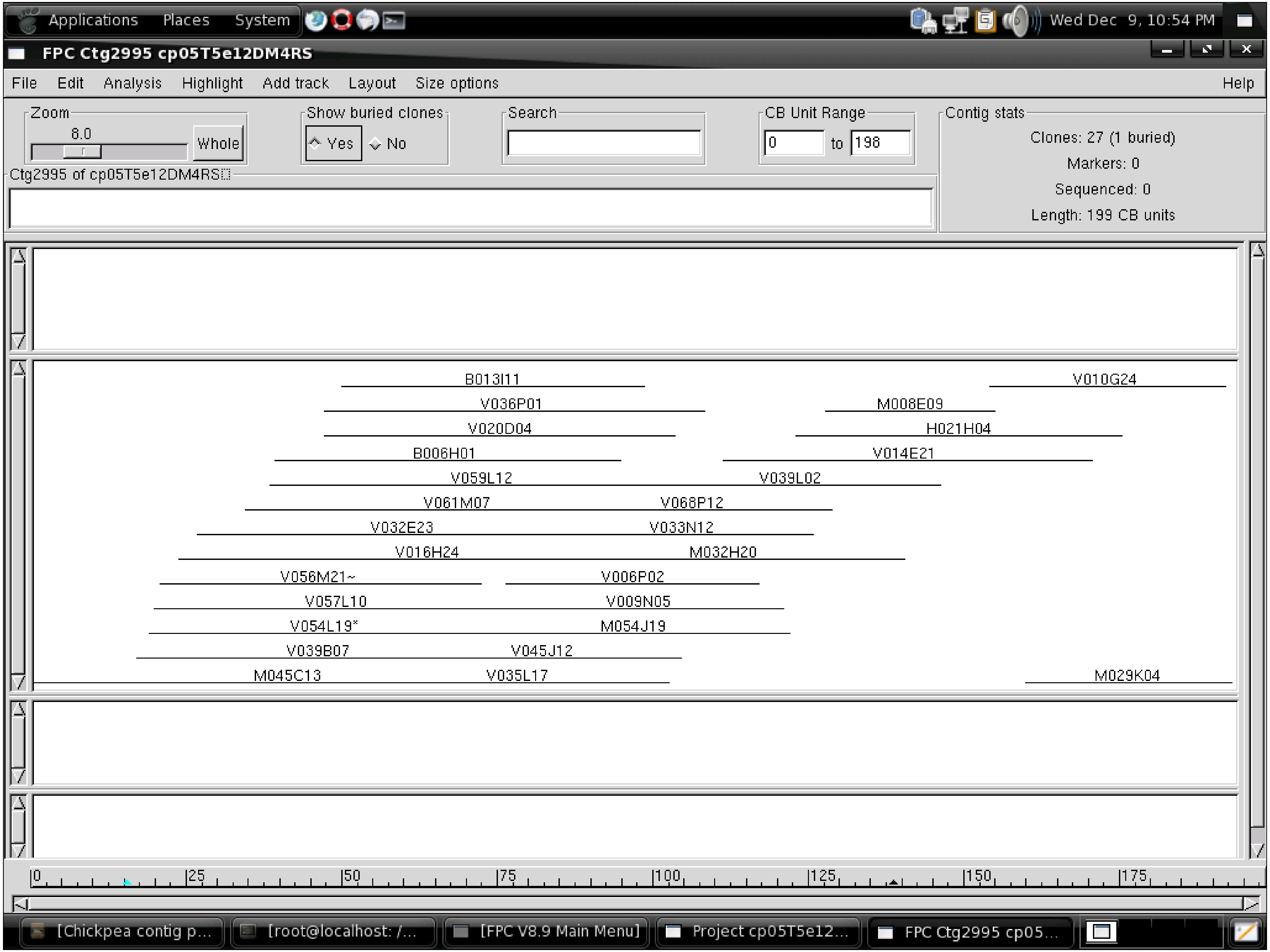
Applications Places System
Wed Dec 9, 10:54 PM
FPC Ctg2995 cp05T5e12DM4RS
File Edit Analysis Highlight Add track Layout Size options Help
Zoom
8.0
Whole
Show buried clones
Yes
No
Search
CB Unit Range
0 to 198
Contig stats
Clones: 27 (1 buried)
Markers: 0
Sequenced: 0
Length: 199 CB units
Ctg2995 of cp05T5e12DM4RS
B013I11
V010G24
V036P01
M008E09
V020D04
H021H04
B006H01
V014E21
V059L12
V039L02
V061M07
V068P12
V032E23
V033N12
V016H24
M032H20
V056M21~
V006P02
V057L10
V009N05
V054L19*
M054J19
V039B07
V045J12
M045C13
V035L17
M029K04
0 25 50 75 100 125 150 175
[Chickpea contig p...
[root@localhost: /...
[FPC V8.9 Main Menu]
Project cp05T5e12...
FPC Ctg2995 cp05...

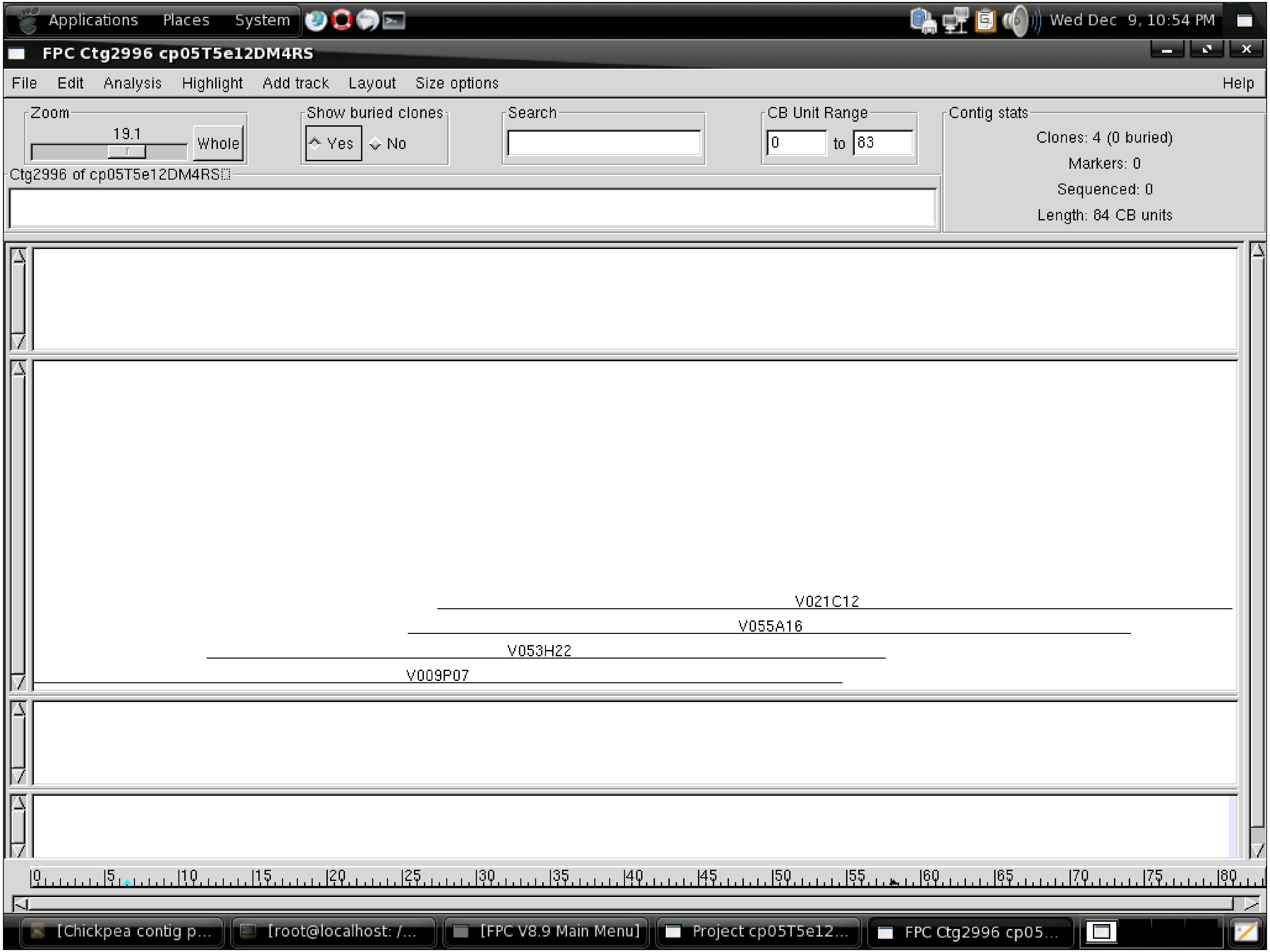
Applications Places System
Wed Dec 9, 10:54 PM
FPC Ctg2996 cp05T5e12DM4RS
File Edit Analysis Highlight Add track Layout Size options Help
Zoom
19.1
Whole
Show buried clones
Yes
No
Search
CB Unit Range
0 to 83
Contig stats
Clones: 4 (0 buried)
Markers: 0
Sequenced: 0
Length: 84 CB units
Ctg2996 of cp05T5e12DM4RS
V021C12
V055A16
V053H22
V009P07
0 5 10 15 20 25 30 35 40 45 50 55 60 65 70 75 80
[Chickpea contig p...
[root@localhost: /...
[FPC V8.9 Main Menu]
Project cp05T5e12...
FPC Ctg2996 cp05...

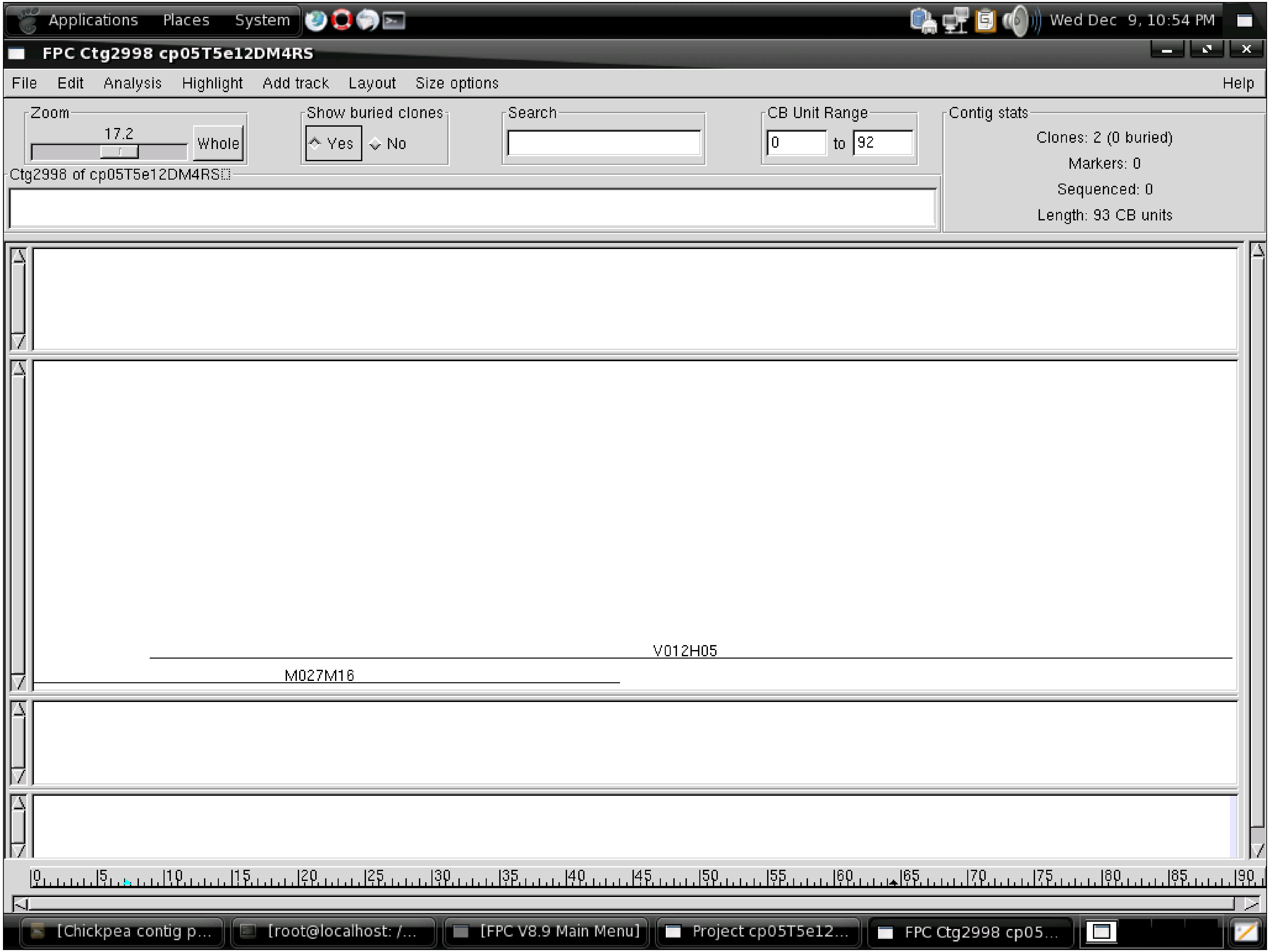
Applications Places System
Wed Dec 9, 10:54 PM
FPC Ctg2998 cp05T5e12DM4RS
File Edit Analysis Highlight Add track Layout Size options Help
Zoom
17.2
Whole
Show buried clones
Yes No
Search
CB Unit Range
0 to 92
Contig stats
Clones: 2 (0 buried)
Markers: 0
Sequenced: 0
Length: 93 CB units
Ctg2998 of cp05T5e12DM4RS
V012H05
M027M16
0 5 10 15 20 25 30 35 40 45 50 55 60 65 70 75 80 85 90
[Chickpea contig p...
[root@localhost: /...
[FPC V8.9 Main Menu]
Project cp05T5e12...
FPC Ctg2998 cp05...

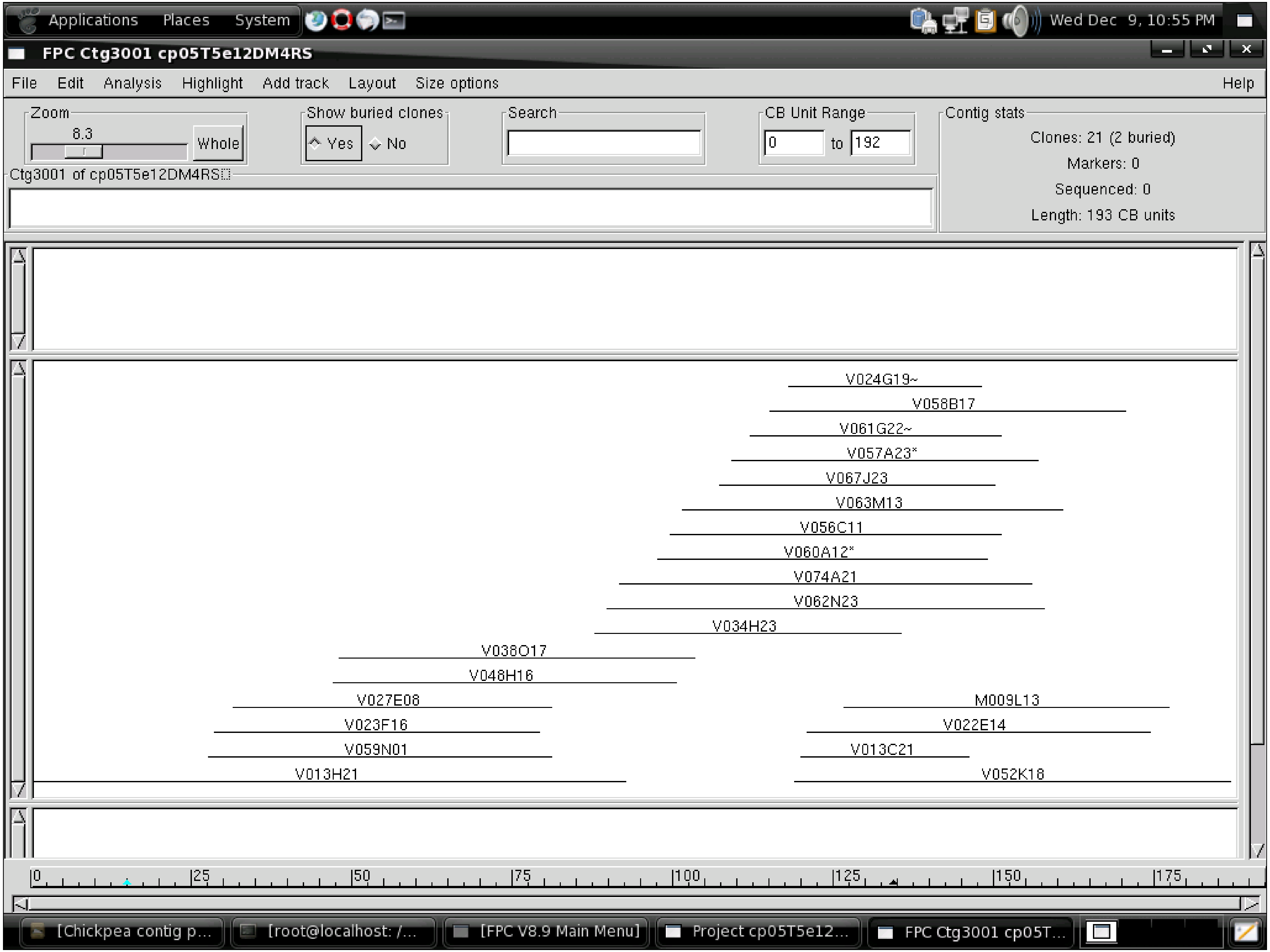
Applications Places System
Wed Dec 9, 10:55 PM
FPC Ctg3001 cp05T5e12DM4RS
File Edit Analysis Highlight Add track Layout Size options Help
Zoom
8.3
Whole
Show buried clones
Yes
No
Search
CB Unit Range
0 to 192
Contig stats
Clones: 21 (2 buried)
Markers: 0
Sequenced: 0
Length: 193 CB units
Ctg3001 of cp05T5e12DM4RS
V024G19~
V058B17
V061G22~
V057A23*
V067J23
V063M13
V056C11
V060A12*
V074A21
V062N23
V034H23
V038O17
V048H16
V027E08
M009L13
V023F16
V022E14
V059N01
V013C21
V013H21
V052K18
0
25
50
75
100
125
150
175
[Chickpea contig p...
[root@localhost: /...
[FPC V8.9 Main Menu]
Project cp05T5e12...
FPC Ctg3001 cp05T...

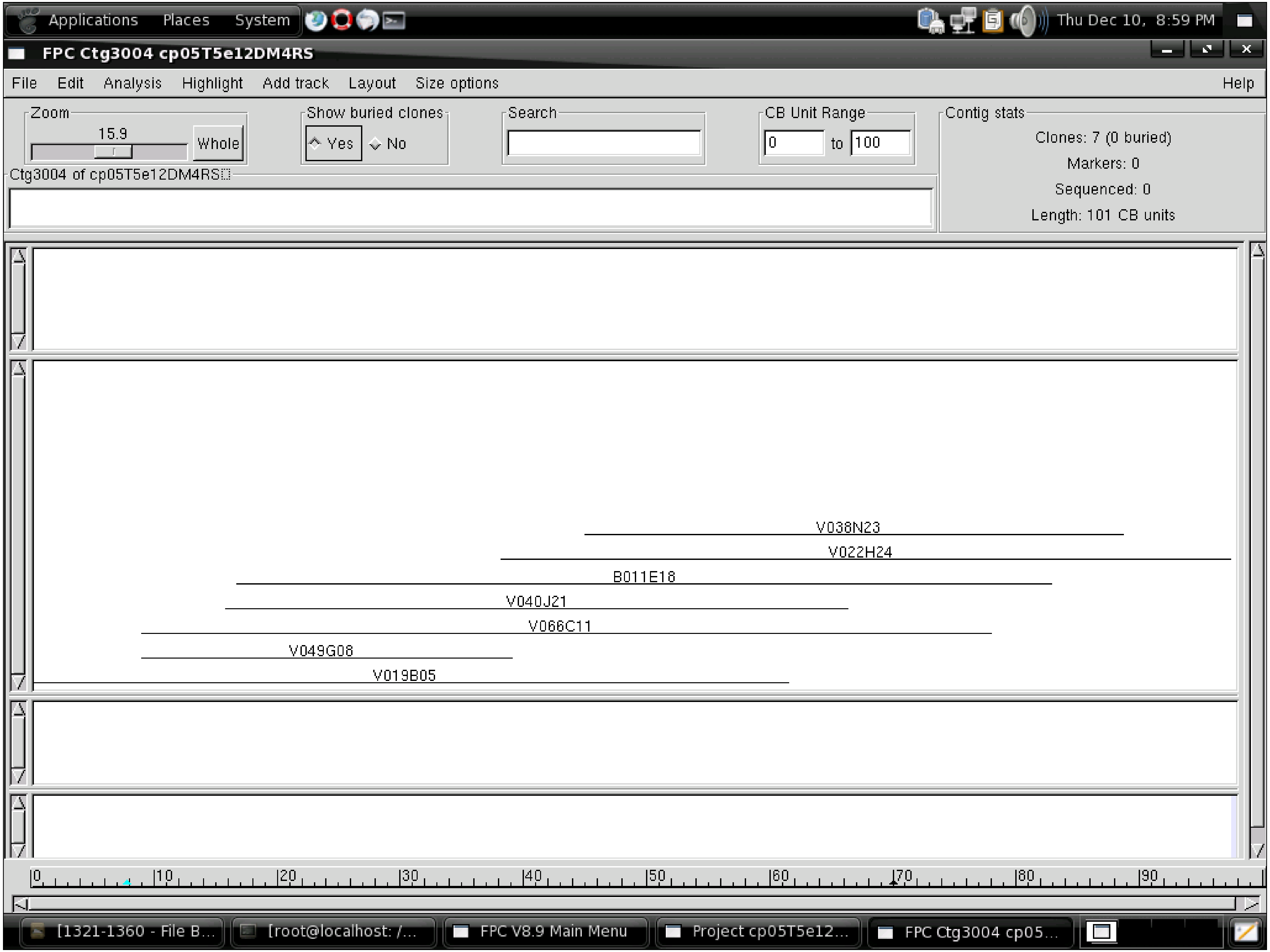
Applications Places System
Thu Dec 10, 8:59 PM
FPC Ctg3004 cp05T5e12DM4RS
File Edit Analysis Highlight Add track Layout Size options Help
Zoom
15.9
Whole
Show buried clones
Yes
No
Search
CB Unit Range
0 to 100
Contig stats
Clones: 7 (0 buried)
Markers: 0
Sequenced: 0
Length: 101 CB units
Ctg3004 of cp05T5e12DM4RS
V038N23
V022H24
B011E18
V040J21
V066C11
V049G08
V019B05
0 10 20 30 40 50 60 70 80 90
[1321-1360 - File B...
[root@localhost: /...
FPC V8.9 Main Menu
Project cp05T5e12...
FPC Ctg3004 cp05...

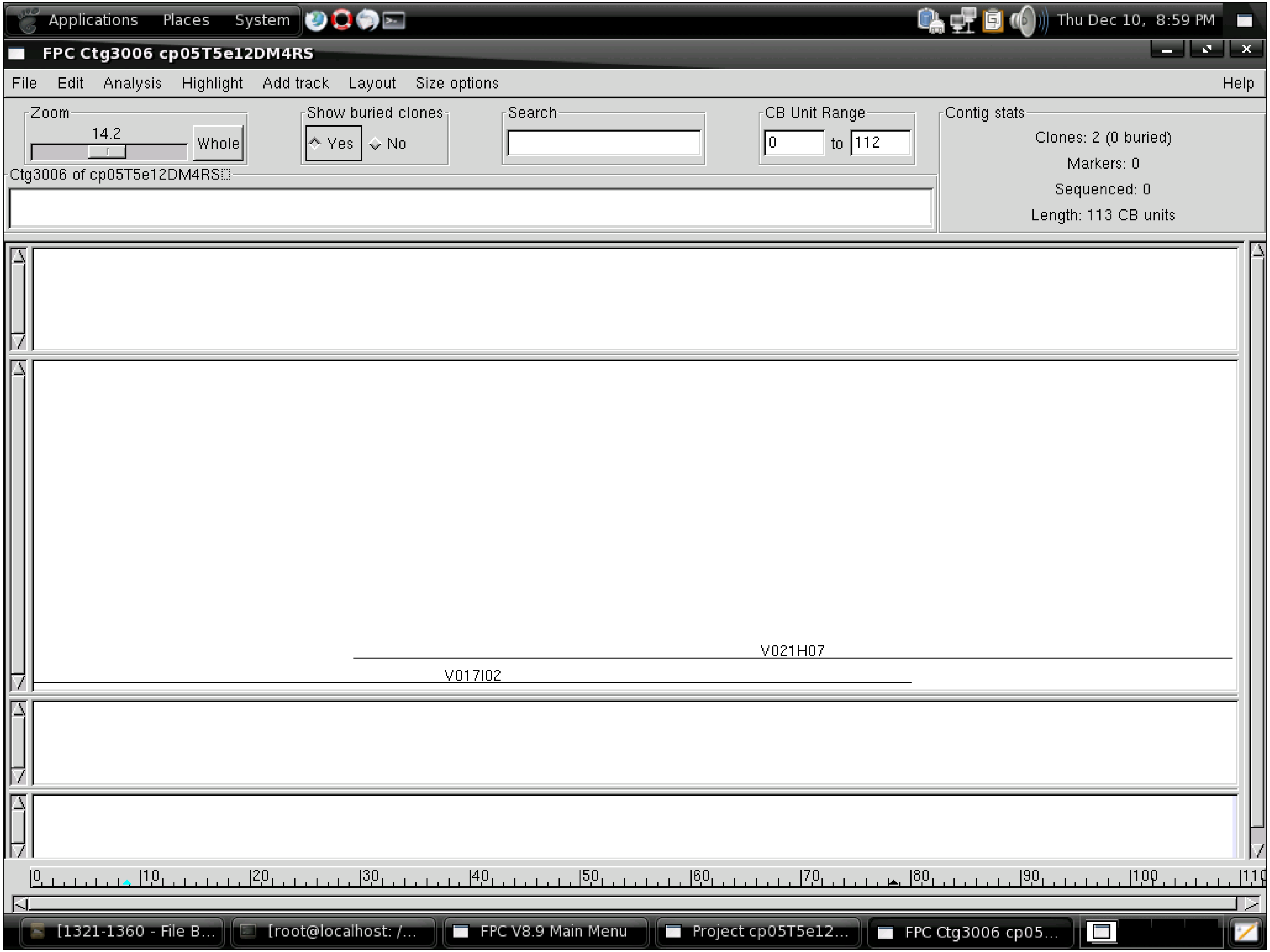
Applications
Places
System
Thu Dec 10, 8:59 PM
FPC Ctg3006 cp05T5e12DM4RS
File
Edit
Analysis
Highlight
Add track
Layout
Size options
Help
Zoom
14.2
Whole
Show buried clones
Yes
No
Search
CB Unit Range
0
to
112
Contig stats
Clones: 2 (0 buried)
Markers: 0
Sequenced: 0
Length: 113 CB units
Ctg3006 of cp05T5e12DM4RS
V021H07
V017I02
0
10
20
30
40
50
60
70
80
90
100
[1321-1360 - File B...
[root@localhost: /...
FPC V8.9 Main Menu
Project cp05T5e12...
FPC Ctg3006 cp05...

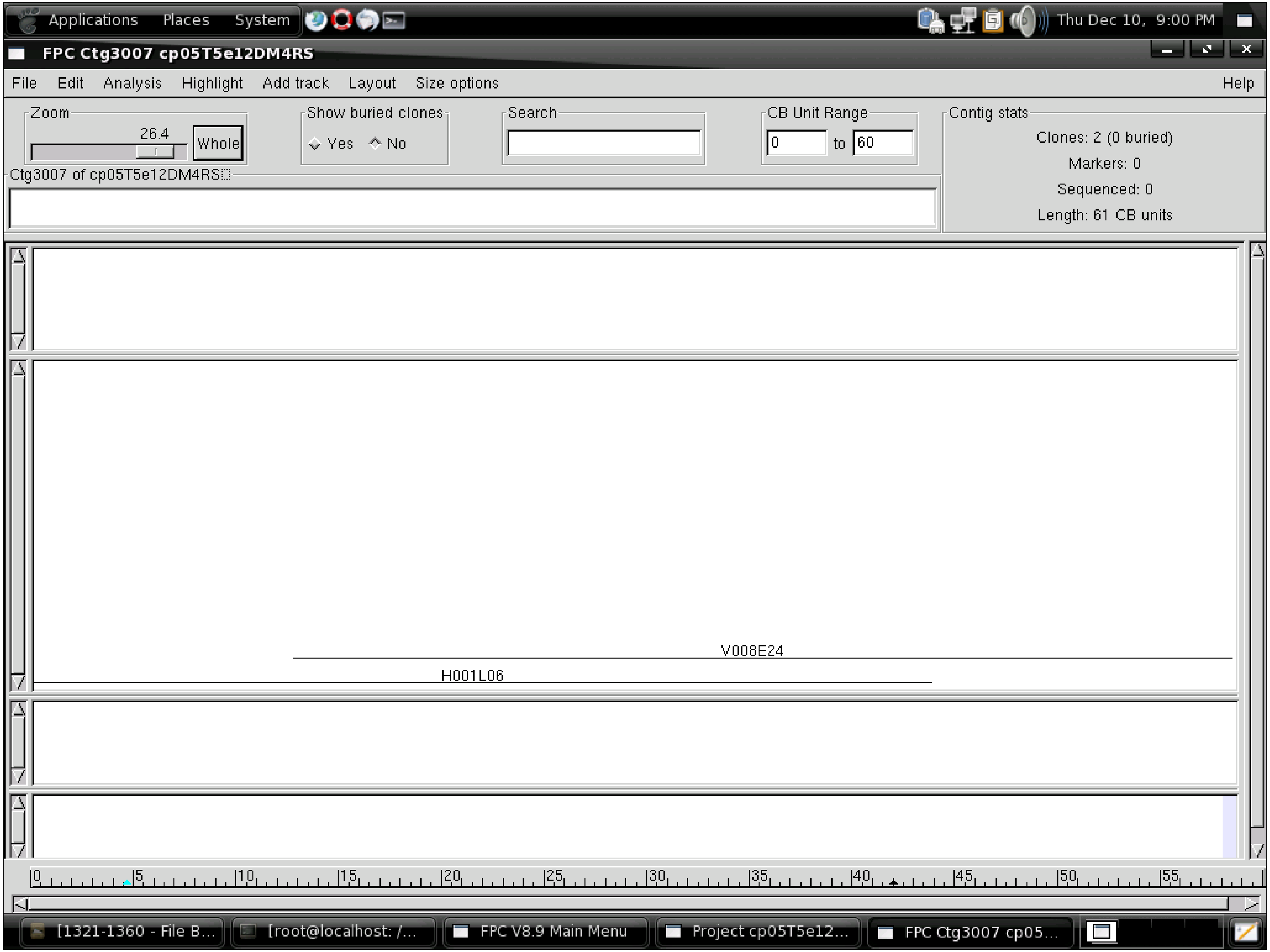
Applications Places System
Thu Dec 10, 9:00 PM
FPC Ctg3007 cp05T5e12DM4RS
File Edit Analysis Highlight Add track Layout Size options Help
Zoom
26.4
Whole
Show buried clones
Yes No
Search
CB Unit Range
0 to 60
Contig stats
Clones: 2 (0 buried)
Markers: 0
Sequenced: 0
Length: 61 CB units
Ctg3007 of cp05T5e12DM4RS
V008E24
H001L06
0 5 10 15 20 25 30 35 40 45 50 55
[1321-1360 - File B...
[root@localhost: /...
FPC V8.9 Main Menu
Project cp05T5e12...
FPC Ctg3007 cp05...

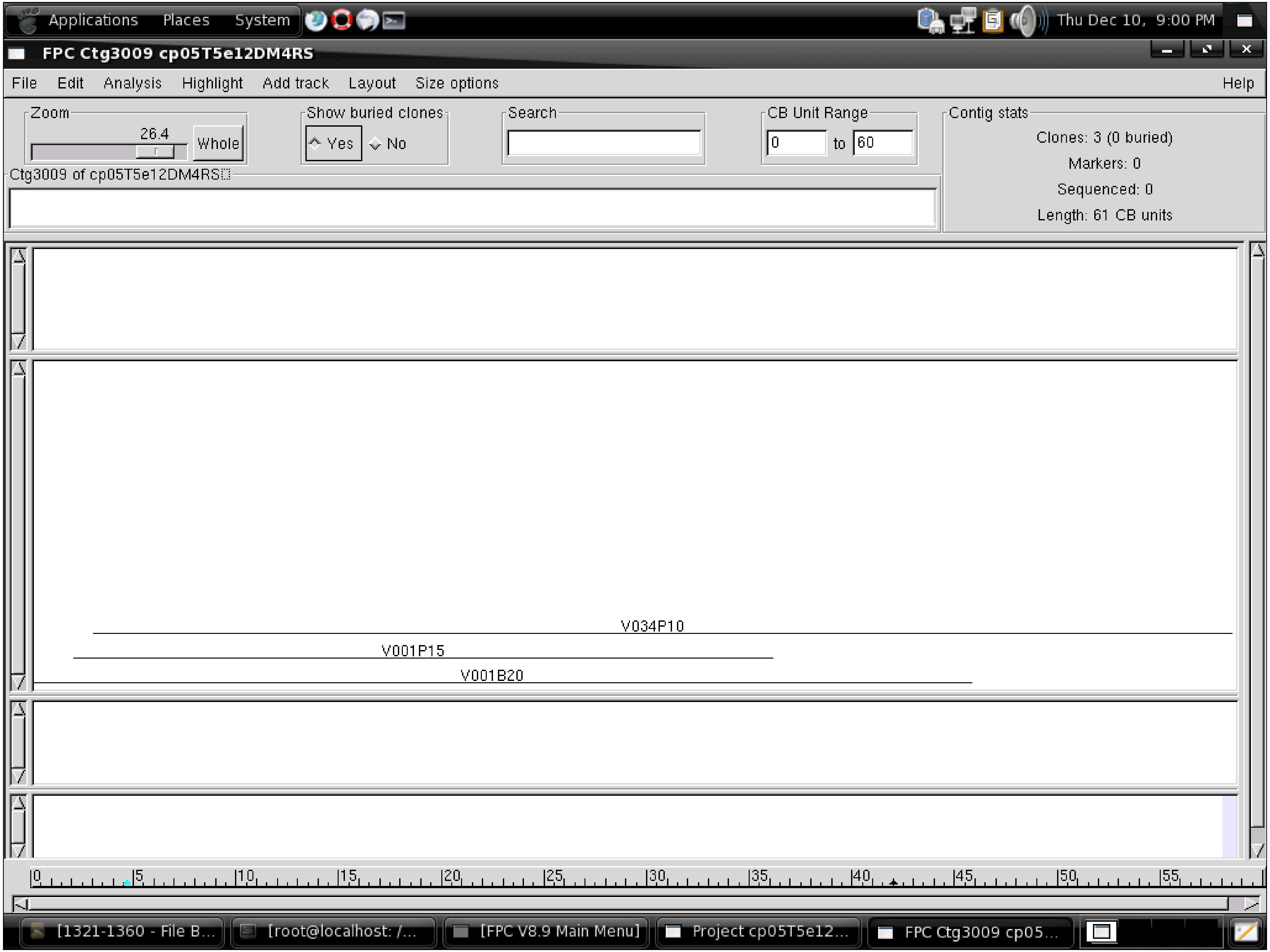
Applications Places System
Thu Dec 10, 9:00 PM
FPC Ctg3009 cp05T5e12DM4RS
File Edit Analysis Highlight Add track Layout Size options Help
Zoom
26.4
Whole
Show buried clones
Yes
No
Search
CB Unit Range
0 to 60
Contig stats
Clones: 3 (0 buried)
Markers: 0
Sequenced: 0
Length: 61 CB units
Ctg3009 of cp05T5e12DM4RS
V034P10
V001P15
V001B20
0 5 10 15 20 25 30 35 40 45 50 55
[1321-1360 - File B...
[root@localhost: /...
[FPC V8.9 Main Menu]
Project cp05T5e12...
FPC Ctg3009 cp05...

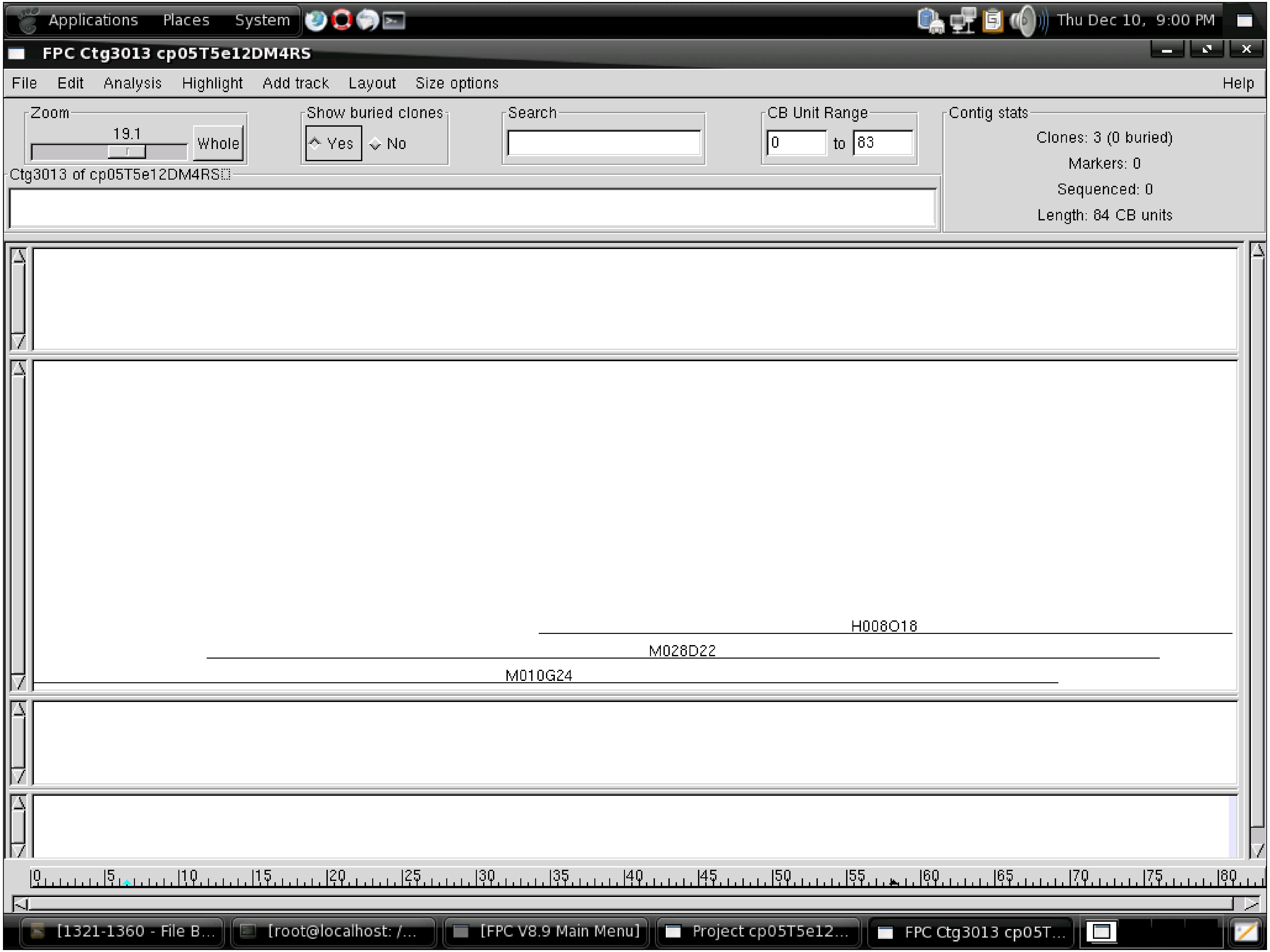
Applications
Places
System
Thu Dec 10, 9:00 PM
FPC Ctg3013 cp05T5e12DM4RS
File
Edit
Analysis
Highlight
Add track
Layout
Size options
Help
Zoom
19.1
Whole
Show buried clones
Yes
No
Search
CB Unit Range
0
to
83
Contig stats
Clones: 3 (0 buried)
Markers: 0
Sequenced: 0
Length: 84 CB units
Ctg3013 of cp05T5e12DM4RS
H008O18
M028D22
M010G24
0 5 10 15 20 25 30 35 40 45 50 55 60 65 70 75 80
[1321-1360 - File B...
[root@localhost: /...
[FPC V8.9 Main Menu]
Project cp05T5e12...
FPC Ctg3013 cp05T...

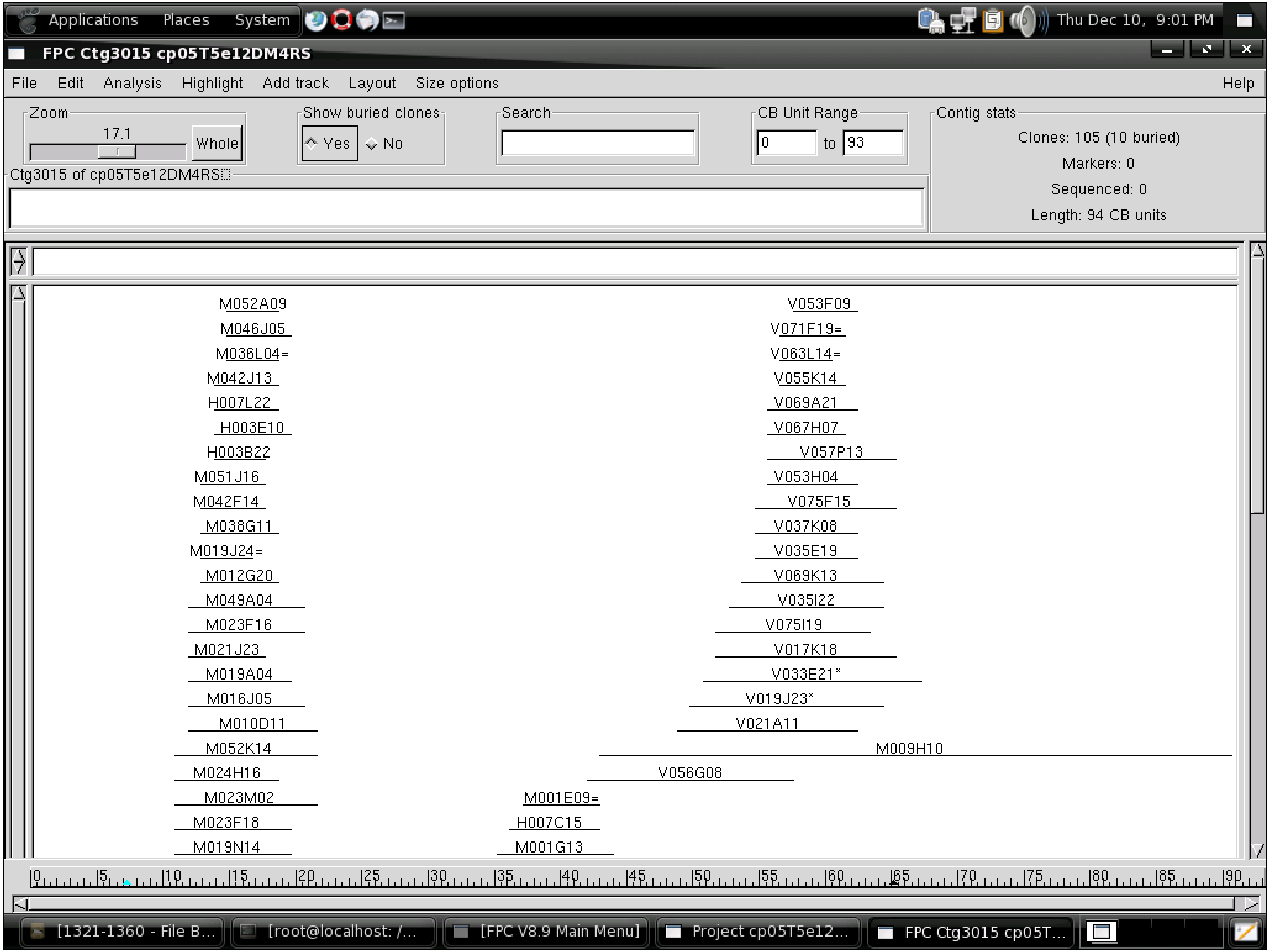

Applications Places System
Thu Dec 10, 9:01 PM
FPC Ctg3015 cp05T5e12DM4RS
File Edit Analysis Highlight Add track Layout Size options Help
Zoom
17.1
Whole
Show buried clones
Yes
No
Search
CB Unit Range
0 to 93
Contig stats
Clones: 105 (10 buried)
Markers: 0
Sequenced: 0
Length: 94 CB units
Ctg3015 of cp05T5e12DM4RS
M052A09
M046J05
M036L04=
M042J13
H007L22
H003E10
H003B22
M051J16
M042F14
M038G11
M019J24=
M012G20
M049A04
M023F16
M021J23
M019A04
M016J05
M010D11
M052K14
M024H16
M023M02
M023F18
M019N14
V053F09
V071F19=
V063L14=
V055K14
V069A21
V067H07
V057P13
V053H04
V075F15
V037K08
V035E19
V069K13
V035I22
V075I19
V017K18
V033E21*
V019J23*
V021A11
M009H10
V056G08
M001E09=
H007C15
M001G13
0 5 10 15 20 25 30 35 40 45 50 55 60 65 70 75 80 85 90
[1321-1360 - File B...
[root@localhost: /...
[FPC V8.9 Main Menu]
Project cp05T5e12...
FPC Ctg3015 cp05T...

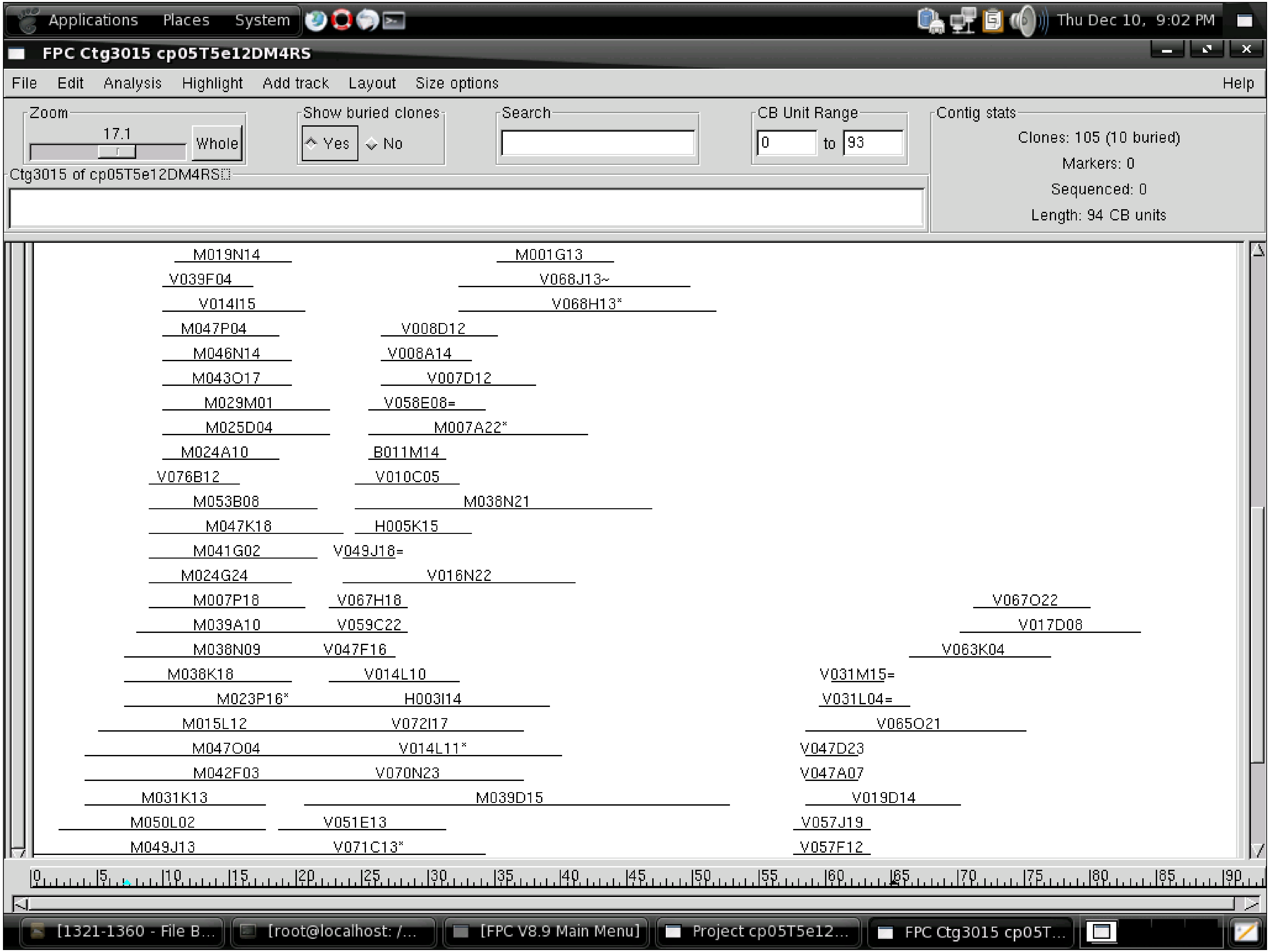
Applications Places System
Thu Dec 10, 9:02 PM
FPC Ctg3015 cp05T5e12DM4RS
File Edit Analysis Highlight Add track Layout Size options Help
Zoom
17.1
Whole
Show buried clones
Yes No
Search
CB Unit Range
0 to 93
Contig stats
Clones: 105 (10 buried)
Markers: 0
Sequenced: 0
Length: 94 CB units
Ctg3015 of cp05T5e12DM4RS
M019N14
M001G13
V039F04
V068J13~
V014I15
V068H13*
M047P04
V008D12
M046N14
V008A14
M043O17
V007D12
M029M01
V058E08=
M025D04
M007A22*
M024A10
B011M14
V076B12
V010C05
M053B08
M038N21
M047K18
H005K15
M041G02
V049J18=
M024G24
V016N22
M007P18
V067H18
V067O22
M039A10
V059C22
V017D08
M038N09
V047F16
V063K04
M038K18
V014L10
V031M15=
M023P16*
H003I14
V031L04=
M015L12
V072I17
V065O21
M047O04
V014L11*
V047D23
M042F03
V070N23
V047A07
M031K13
M039D15
V019D14
M050L02
V051E13
V057J19
M049J13
V071C13*
V057F12
0 5 10 15 20 25 30 35 40 45 50 55 60 65 70 75 80 85 90
[1321-1360 - File B...
[root@localhost: /...
[FPC V8.9 Main Menu]
Project cp05T5e12...
FPC Ctg3015 cp05T...

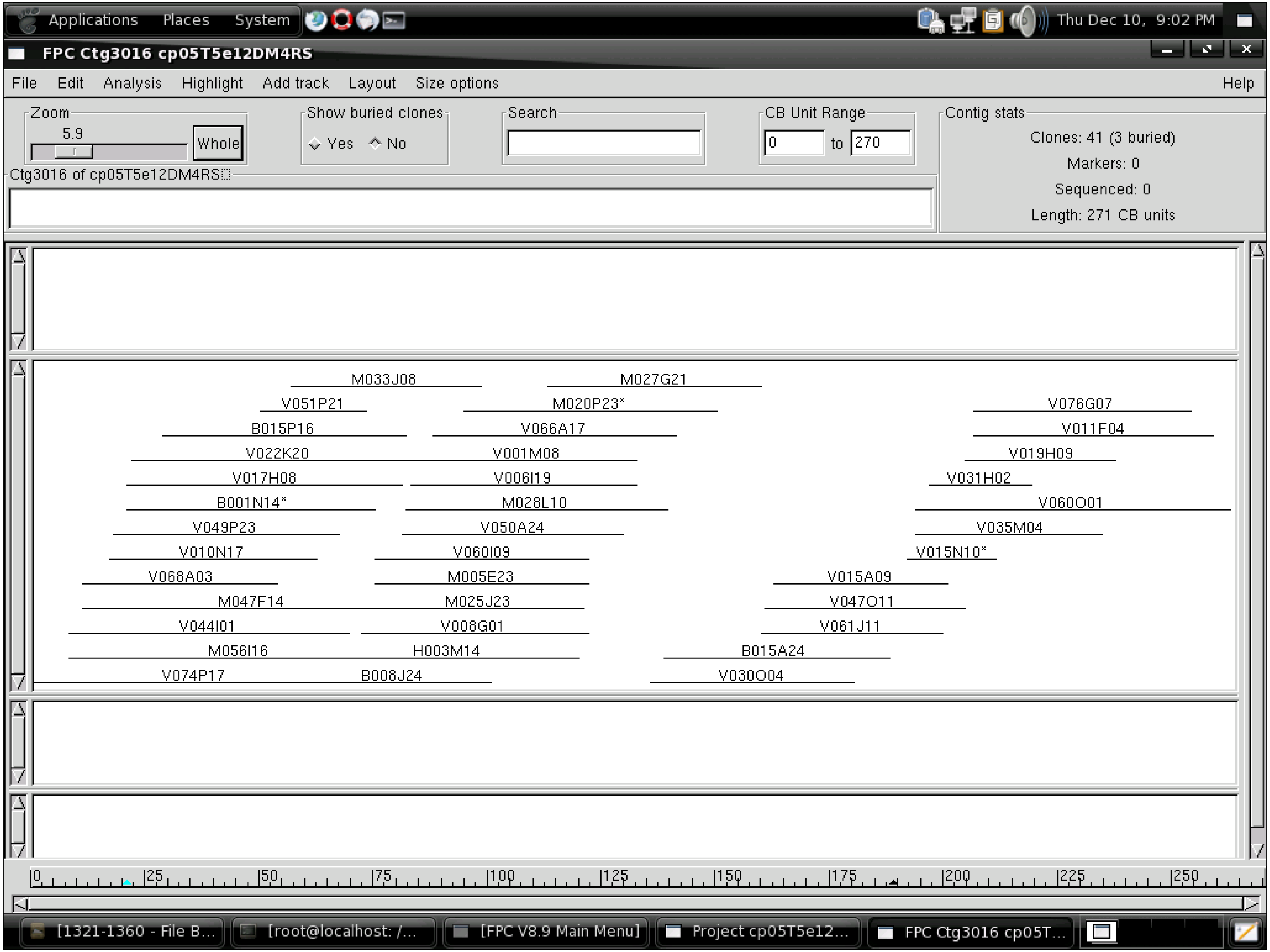
Applications Places System
Thu Dec 10, 9:02 PM
FPC Ctg3016 cp05T5e12DM4RS
File Edit Analysis Highlight Add track Layout Size options Help
Zoom
5.9
Whole
Show buried clones
Yes No
Search
CB Unit Range
0 to 270
Contig stats
Clones: 41 (3 buried)
Markers: 0
Sequenced: 0
Length: 271 CB units
Ctg3016 of cp05T5e12DM4RS
M033J08
M027G21
V051P21
M020P23*
V076G07
B015P16
V066A17
V011F04
V022K20
V001M08
V019H09
V017H08
V006I19
V031H02
B001N14*
M028L10
V060O01
V049P23
V050A24
V035M04
V010N17
V060I09
V015N10*
V068A03
M005E23
V015A09
M047F14
M025J23
V047O11
V044I01
V008G01
V061J11
M056I16
H003M14
B015A24
V074P17
B008J24
V030O04
0 25 50 75 100 125 150 175 200 225 250
[1321-1360 - File B...
[root@localhost: /...
[FPC V8.9 Main Menu]
Project cp05T5e12...
FPC Ctg3016 cp05T...

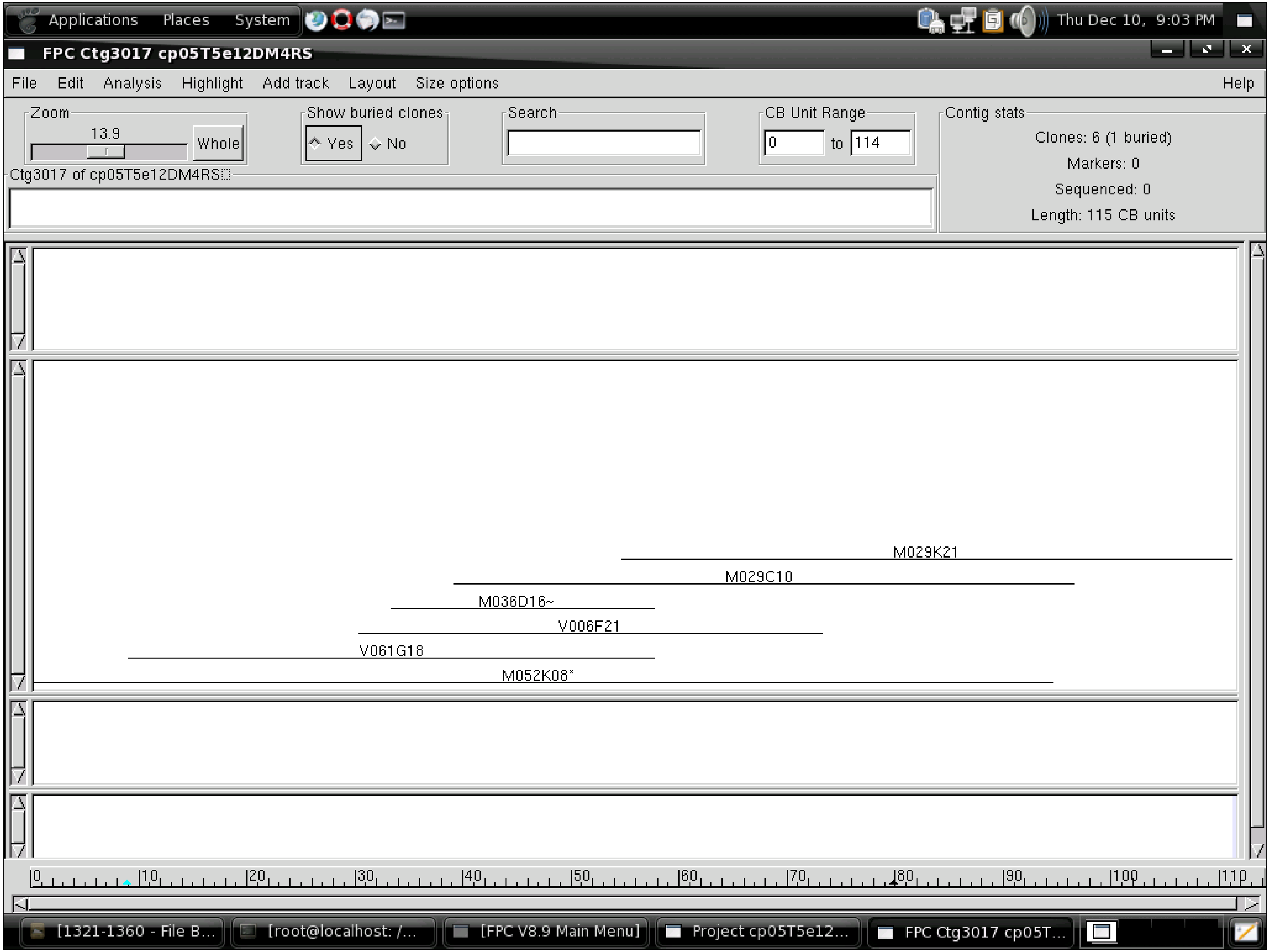
Applications Places System
Thu Dec 10, 9:03 PM
FPC Ctg3017 cp05T5e12DM4RS
File Edit Analysis Highlight Add track Layout Size options Help
Zoom
13.9
Whole
Show buried clones
Yes No
Search
CB Unit Range
0 to 114
Contig stats
Clones: 6 (1 buried)
Markers: 0
Sequenced: 0
Length: 115 CB units
Ctg3017 of cp05T5e12DM4RS
M029K21
M029C10
M036D16~
V006F21
V061G18
M052K08*
0 10 20 30 40 50 60 70 80 90 100 110
[1321-1360 - File B...
[root@localhost: /...
[FPC V8.9 Main Menu]
Project cp05T5e12...
FPC Ctg3017 cp05T...

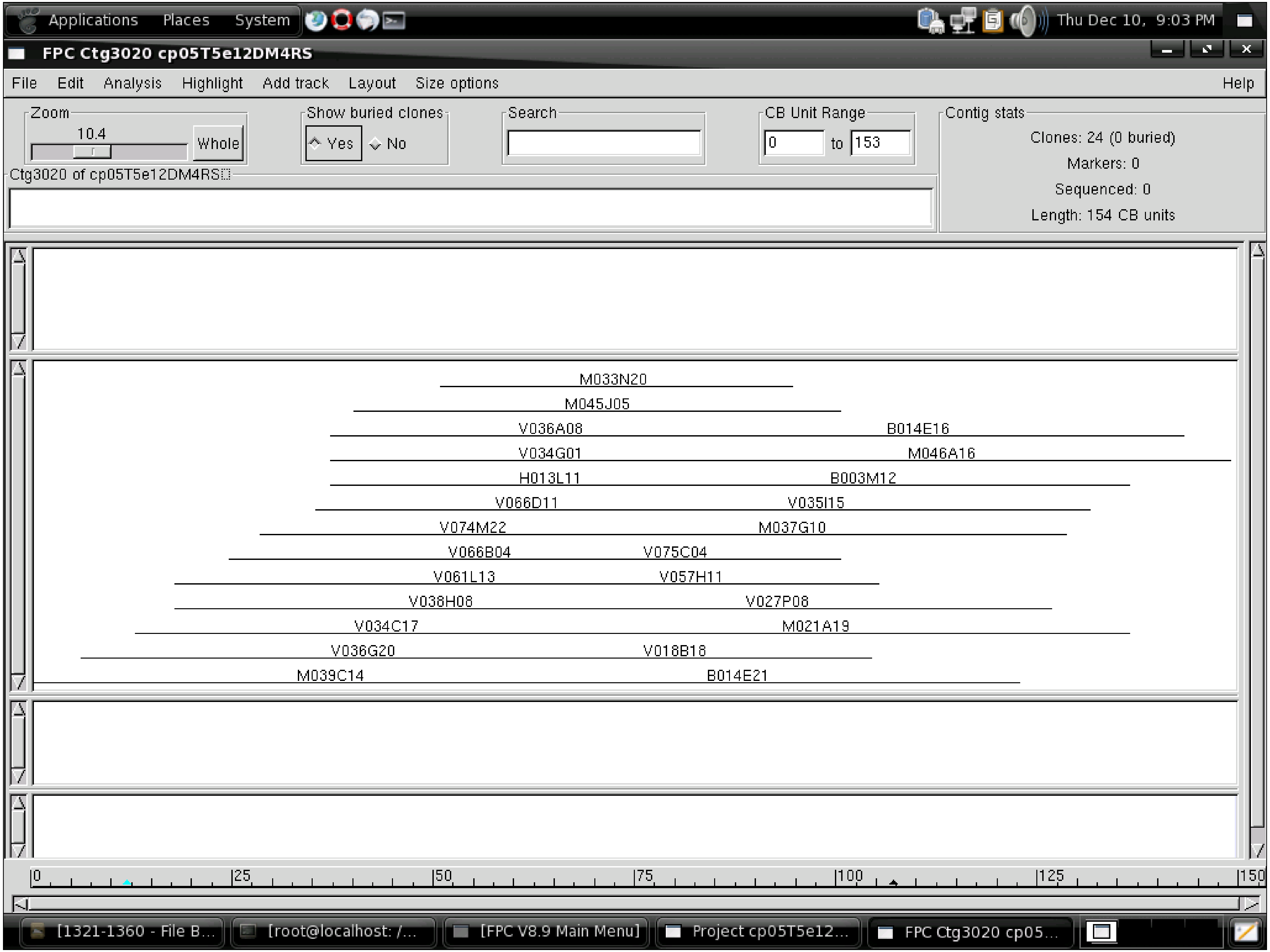
Applications Places System
Thu Dec 10, 9:03 PM
FPC Ctg3020 cp05T5e12DM4RS
File Edit Analysis Highlight Add track Layout Size options Help
Zoom
10.4
Whole
Show buried clones
Yes No
Search
CB Unit Range
0 to 153
Contig stats
Clones: 24 (0 buried)
Markers: 0
Sequenced: 0
Length: 154 CB units
Ctg3020 of cp05T5e12DM4RS
M033N20
M045J05
V036A08 B014E16
V034G01 M046A16
H013L11 B003M12
V066D11 V035I15
V074M22 M037G10
V066B04 V075C04
V061L13 V057H11
V038H08 V027P08
V034C17 M021A19
V036G20 V018B18
M039C14 B014E21
0 25 50 75 100 125 150
[1321-1360 - File B...
[root@localhost: /...
[FPC V8.9 Main Menu]
Project cp05T5e12...
FPC Ctg3020 cp05...

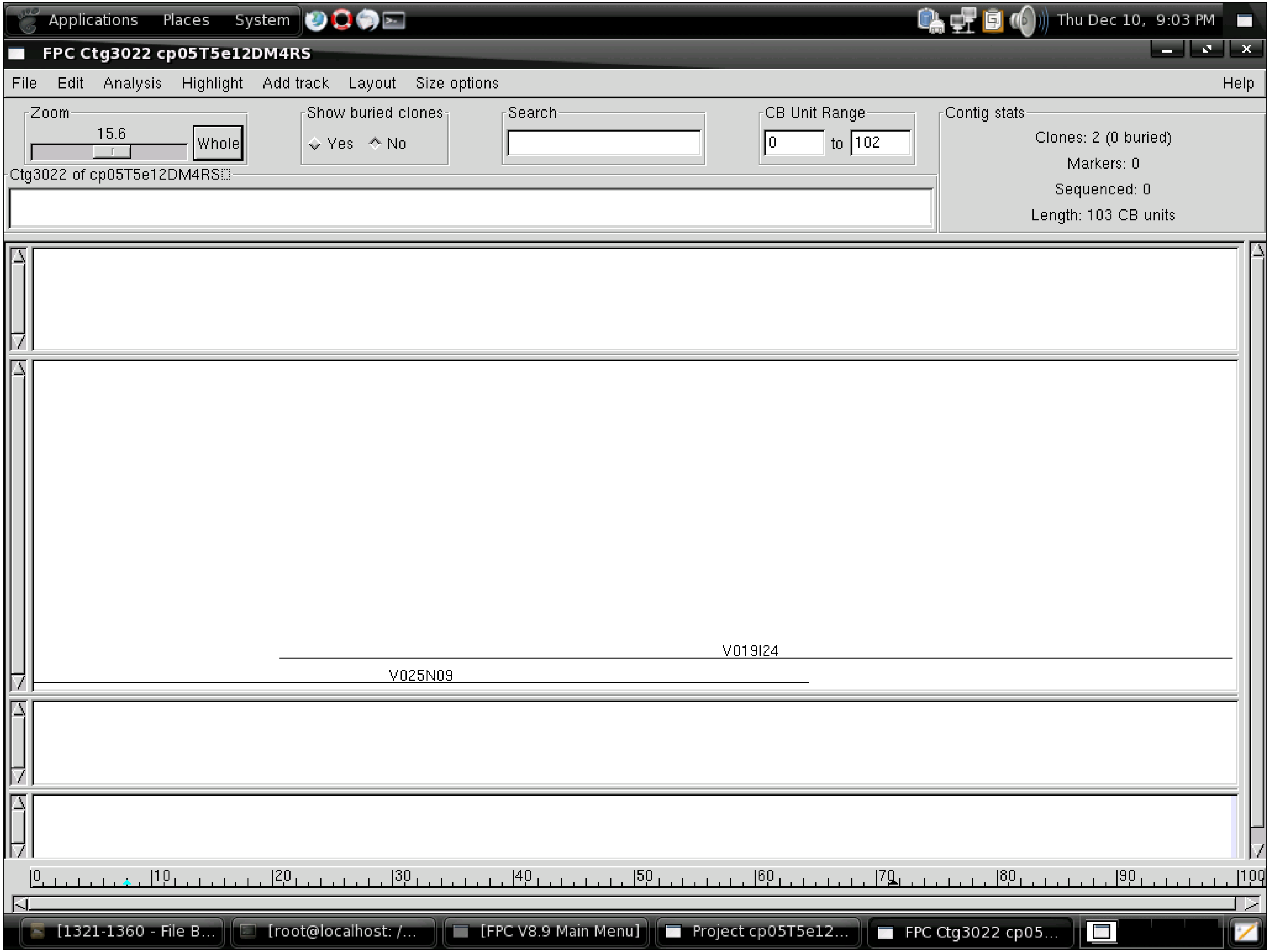
Applications
Places
System
Thu Dec 10, 9:03 PM
FPC Ctg3022 cp05T5e12DM4RS
File
Edit
Analysis
Highlight
Add track
Layout
Size options
Help
Zoom
15.6
Whole
Show buried clones
Yes
No
Search
CB Unit Range
0
to
102
Contig stats
Clones: 2 (0 buried)
Markers: 0
Sequenced: 0
Length: 103 CB units
Ctg3022 of cp05T5e12DM4RS
V019I24
V025N09
0
10
20
30
40
50
60
70
80
90
100
[1321-1360 - File B...
[root@localhost: /...
[FPC V8.9 Main Menu]
Project cp05T5e12...
FPC Ctg3022 cp05...

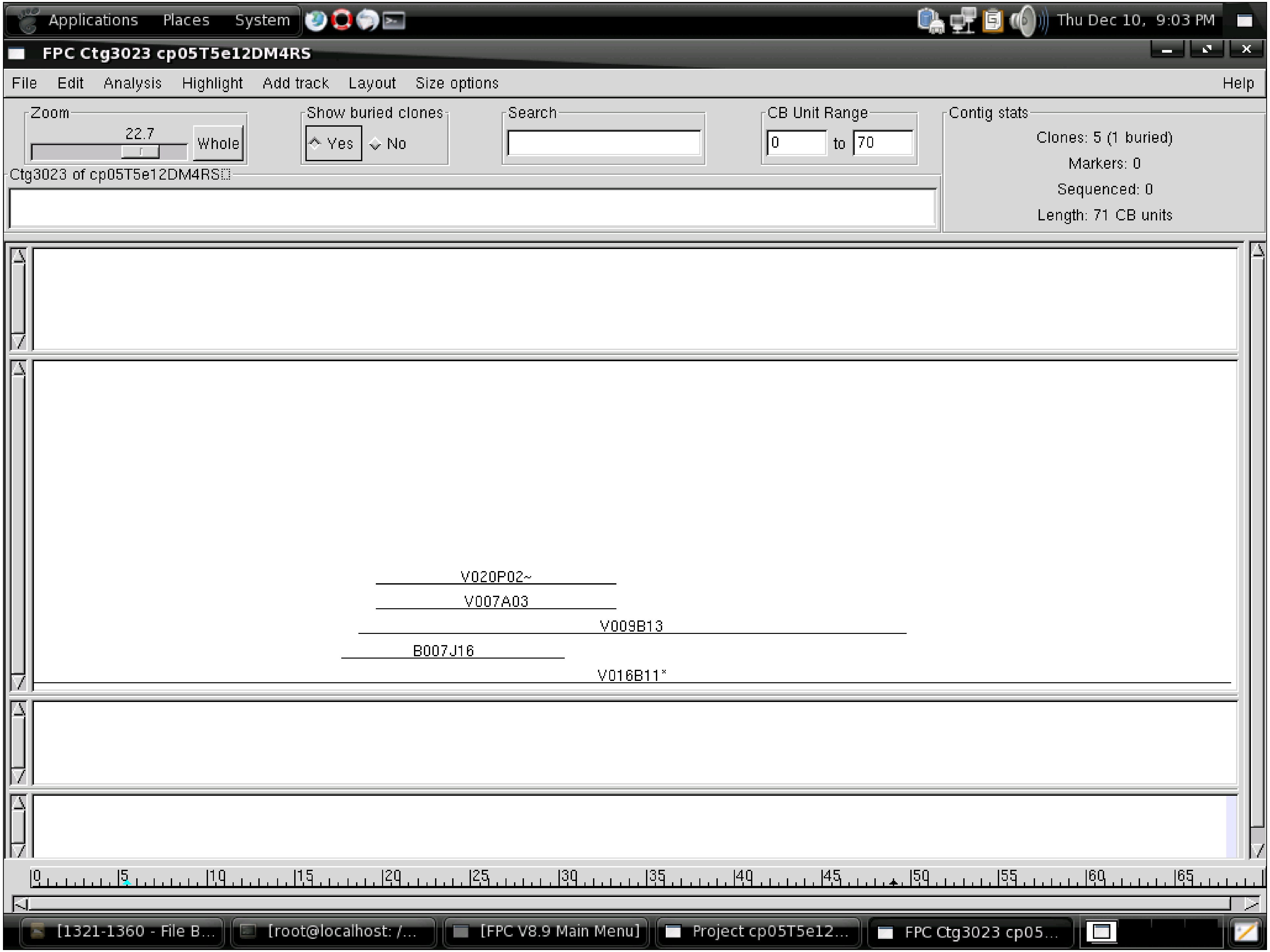
Applications Places System
Thu Dec 10, 9:03 PM
FPC Ctg3023 cp05T5e12DM4RS
File Edit Analysis Highlight Add track Layout Size options Help
Zoom
22.7
Whole
Show buried clones
Yes
No
Search
CB Unit Range
0
to
70
Contig stats
Clones: 5 (1 buried)
Markers: 0
Sequenced: 0
Length: 71 CB units
Ctg3023 of cp05T5e12DM4RS
V020P02~
V007A03
V009B13
B007J16
V016B11*
0 5 10 15 20 25 30 35 40 45 50 55 60 65
[1321-1360 - File B...
[root@localhost: /...
[FPC V8.9 Main Menu]
Project cp05T5e12...
FPC Ctg3023 cp05...

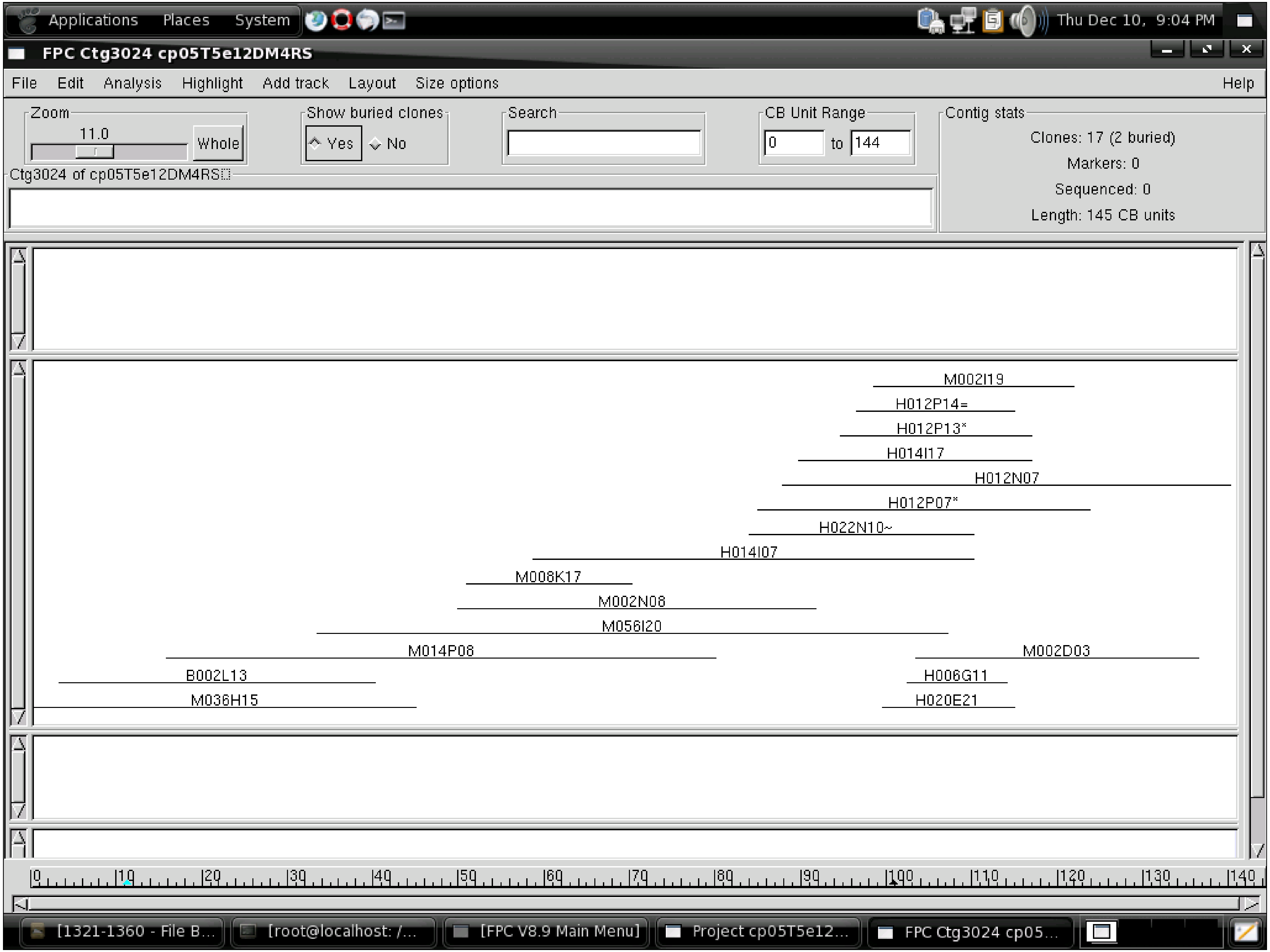
Applications Places System
Thu Dec 10, 9:04 PM
FPC Ctg3024 cp05T5e12DM4RS
File Edit Analysis Highlight Add track Layout Size options Help
Zoom
11.0
Whole
Show buried clones
Yes No
Search
CB Unit Range
0 to 144
Contig stats
Clones: 17 (2 buried)
Markers: 0
Sequenced: 0
Length: 145 CB units
Ctg3024 of cp05T5e12DM4RS
M002I19
H012P14=
H012P13*
H014I17
H012N07
H012P07*
H022N10~
H014I07
M008K17
M002N08
M056I20
M014P08
M002D03
B002L13
H006G11
M036H15
H020E21
0 10 20 30 40 50 60 70 80 90 100 110 120 130 140
[1321-1360 - File B...
[root@localhost: /...
[FPC V8.9 Main Menu]
Project cp05T5e12...
FPC Ctg3024 cp05...

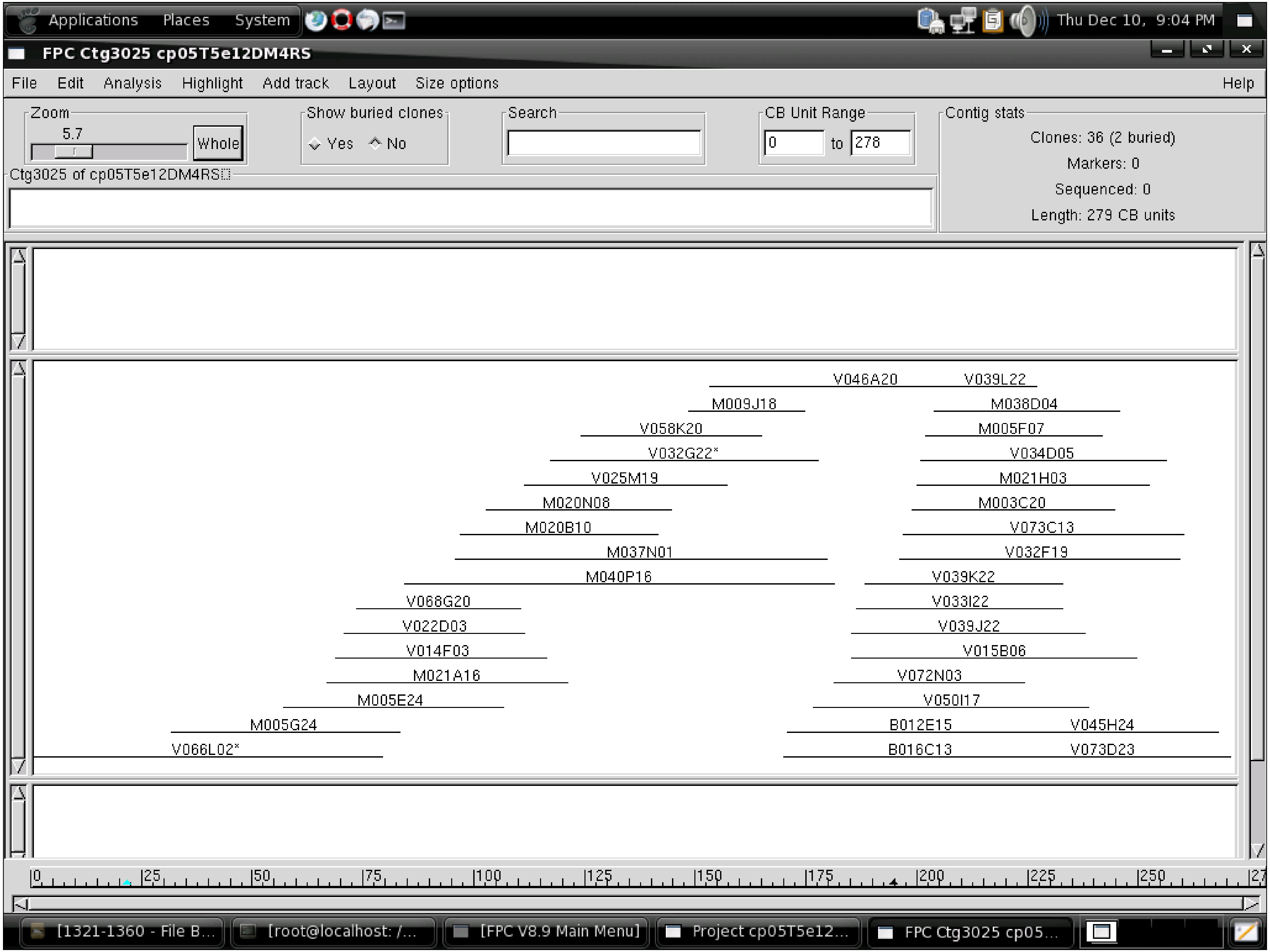
Applications Places System
Thu Dec 10, 9:04 PM
FPC Ctg3025 cp05T5e12DM4RS
File Edit Analysis Highlight Add track Layout Size options Help
Zoom
5.7
Whole
Show buried clones
Yes No
Search
CB Unit Range
0 to 278
Contig stats
Clones: 36 (2 buried)
Markers: 0
Sequenced: 0
Length: 279 CB units
Ctg3025 of cp05T5e12DM4RS
V046A20
V039L22
M009J18
M038D04
V058K20
M005F07
V032G22*
V034D05
V025M19
M021H03
M020N08
M003C20
M020B10
V073C13
M037N01
V032F19
M040P16
V039K22
V068G20
V033I22
V022D03
V039J22
V014F03
V015B06
M021A16
V072N03
M005E24
V050I17
M005G24
B012E15
V045H24
V066L02*
B016C13
V073D23
0 25 50 75 100 125 150 175 200 225 250
[1321-1360 - File B...
[root@localhost: /...
[FPC V8.9 Main Menu]
Project cp05T5e12...
FPC Ctg3025 cp05...

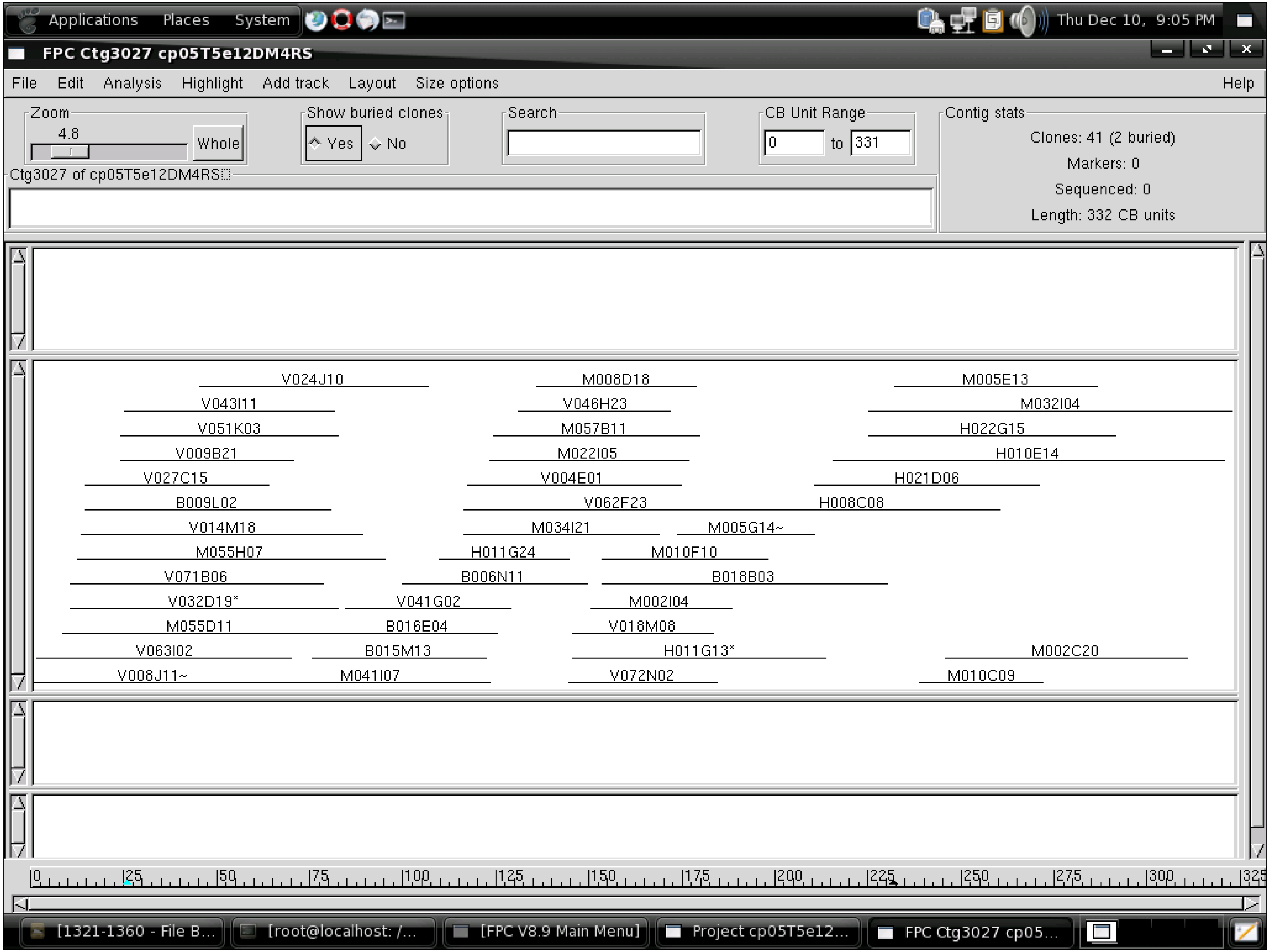
Applications Places System
Thu Dec 10, 9:05 PM
FPC Ctg3027 cp05T5e12DM4RS
File Edit Analysis Highlight Add track Layout Size options Help
Zoom
4.8
Whole
Show buried clones
Yes No
Search
CB Unit Range
0 to 331
Contig stats
Clones: 41 (2 buried)
Markers: 0
Sequenced: 0
Length: 332 CB units
Ctg3027 of cp05T5e12DM4RS
V024J10
M008D18
M005E13
V043I11
V046H23
M032I04
V051K03
M057B11
H022G15
V009B21
M022I05
H010E14
V027C15
V004E01
H021D06
B009L02
V062F23
H008C08
V014M18
M034I21
M005G14~
M055H07
H011G24
M010F10
V071B06
B006N11
B018B03
V032D19*
V041G02
M002I04
M055D11
B016E04
V018M08
V063I02
B015M13
H011G13*
M002C20
V008J11~
M041I07
V072N02
M010C09
0 25 50 75 100 125 150 175 200 225 250 275 300 325
[1321-1360 - File B...
[root@localhost: /...
[FPC V8.9 Main Menu]
Project cp05T5e12...
FPC Ctg3027 cp05...

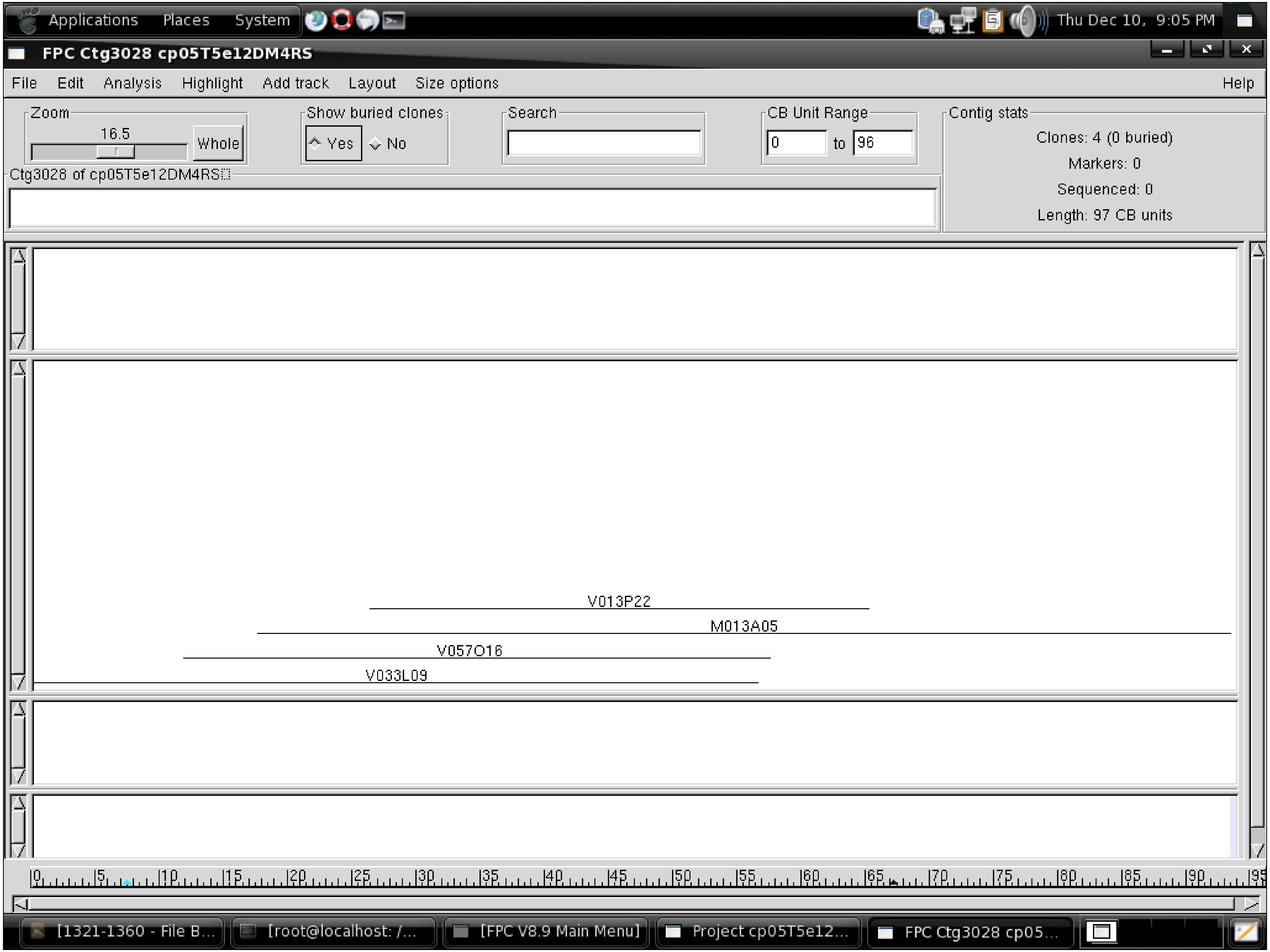
Applications Places System
Thu Dec 10, 9:05 PM
FPC Ctg3028 cp05T5e12DM4RS
File Edit Analysis Highlight Add track Layout Size options Help
Zoom
16.5
Whole
Show buried clones
Yes No
Search
CB Unit Range
0 to 96
Contig stats
Clones: 4 (0 buried)
Markers: 0
Sequenced: 0
Length: 97 CB units
Ctg3028 of cp05T5e12DM4RS
V013P22
M013A05
V057O16
V033L09
0 5 10 15 20 25 30 35 40 45 50 55 60 65 70 75 80 85 90 95
[1321-1360 - File B...
[root@localhost: /...
[FPC V8.9 Main Menu]
Project cp05T5e12...
FPC Ctg3028 cp05...

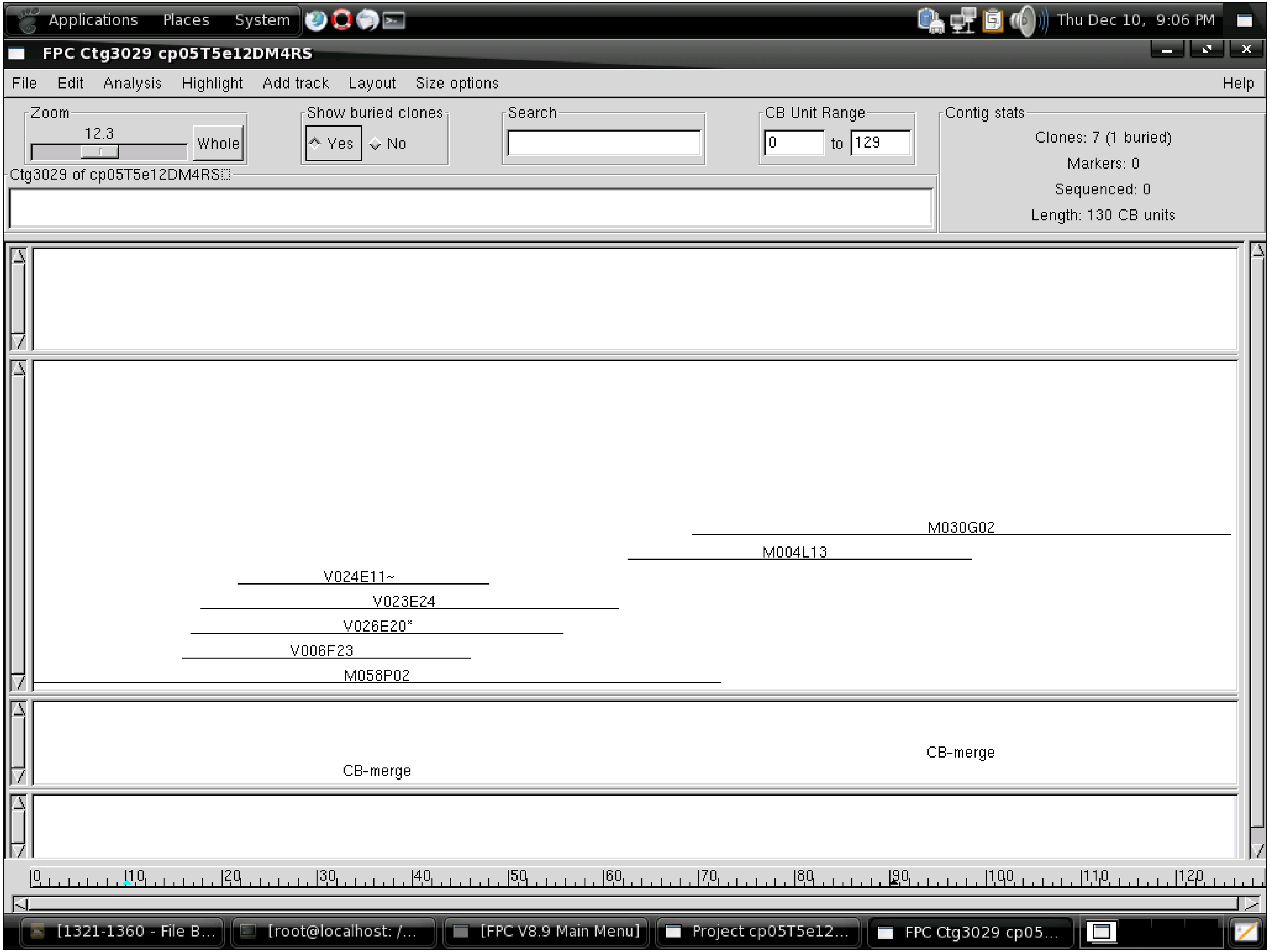
Applications Places System
Thu Dec 10, 9:06 PM
FPC Ctg3029 cp05T5e12DM4RS
File Edit Analysis Highlight Add track Layout Size options Help
Zoom
12.3
Whole
Show buried clones
Yes No
Search
CB Unit Range
0 to 129
Contig stats
Clones: 7 (1 buried)
Markers: 0
Sequenced: 0
Length: 130 CB units
Ctg3029 of cp05T5e12DM4RS
M030G02
M004L13
V024E11~
V023E24
V026E20*
V006F23
M058P02
CB-merge
CB-merge
0 10 20 30 40 50 60 70 80 90 100 110 120
[1321-1360 - File B...
[root@localhost: /...
[FPC V8.9 Main Menu]
Project cp05T5e12...
FPC Ctg3029 cp05...

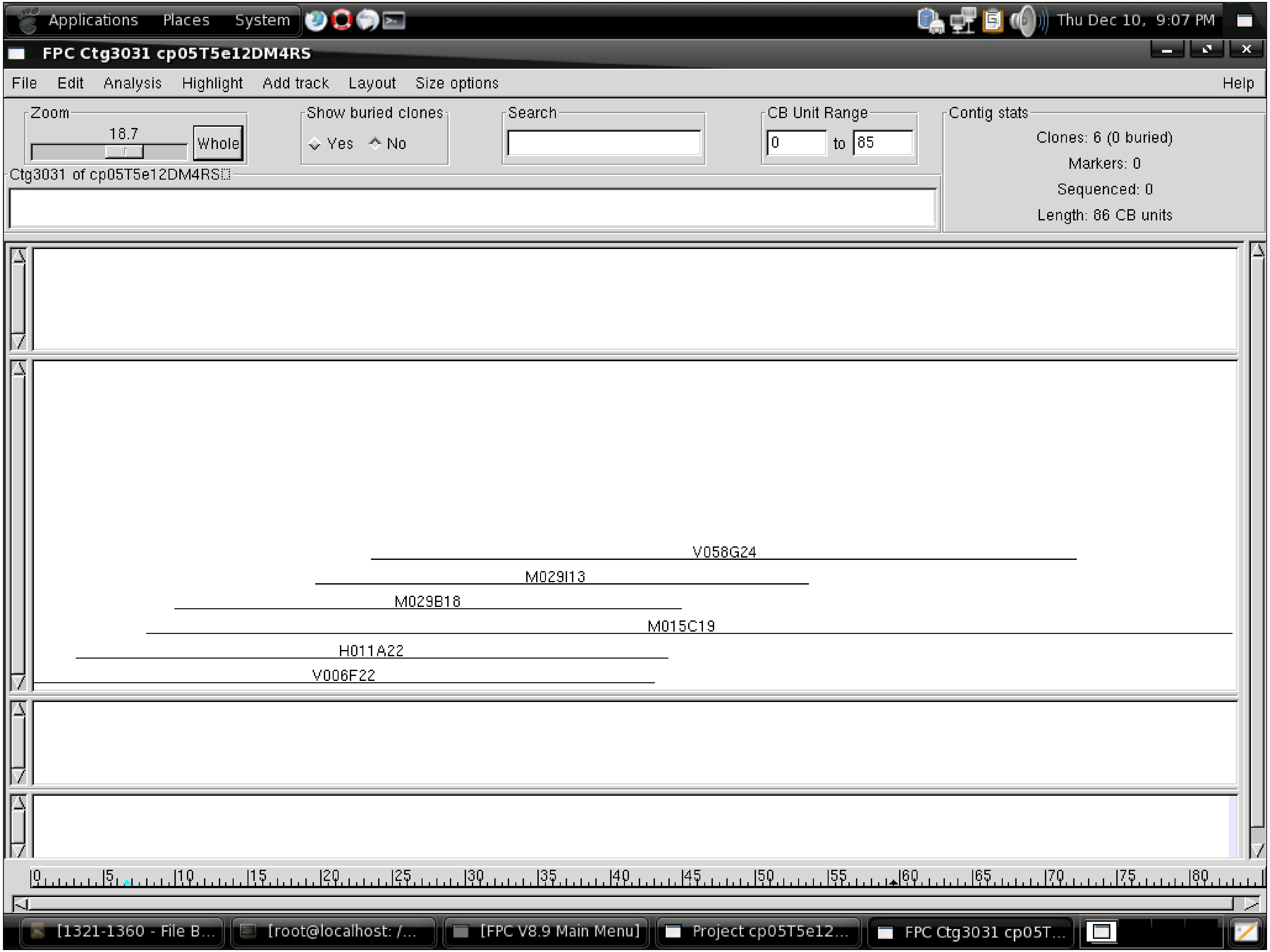
Applications Places System
Thu Dec 10, 9:07 PM
FPC Ctg3031 cp05T5e12DM4RS
File Edit Analysis Highlight Add track Layout Size options Help
Zoom
18.7
Whole
Show buried clones
Yes No
Search
CB Unit Range
0 to 85
Contig stats
Clones: 6 (0 buried)
Markers: 0
Sequenced: 0
Length: 86 CB units
Ctg3031 of cp05T5e12DM4RS
V058G24
M029I13
M029B18
M015C19
H011A22
V006F22
0 5 10 15 20 25 30 35 40 45 50 55 60 65 70 75 80
[1321-1360 - File B...
[root@localhost: /...
[FPC V8.9 Main Menu]
Project cp05T5e12...
FPC Ctg3031 cp05T...

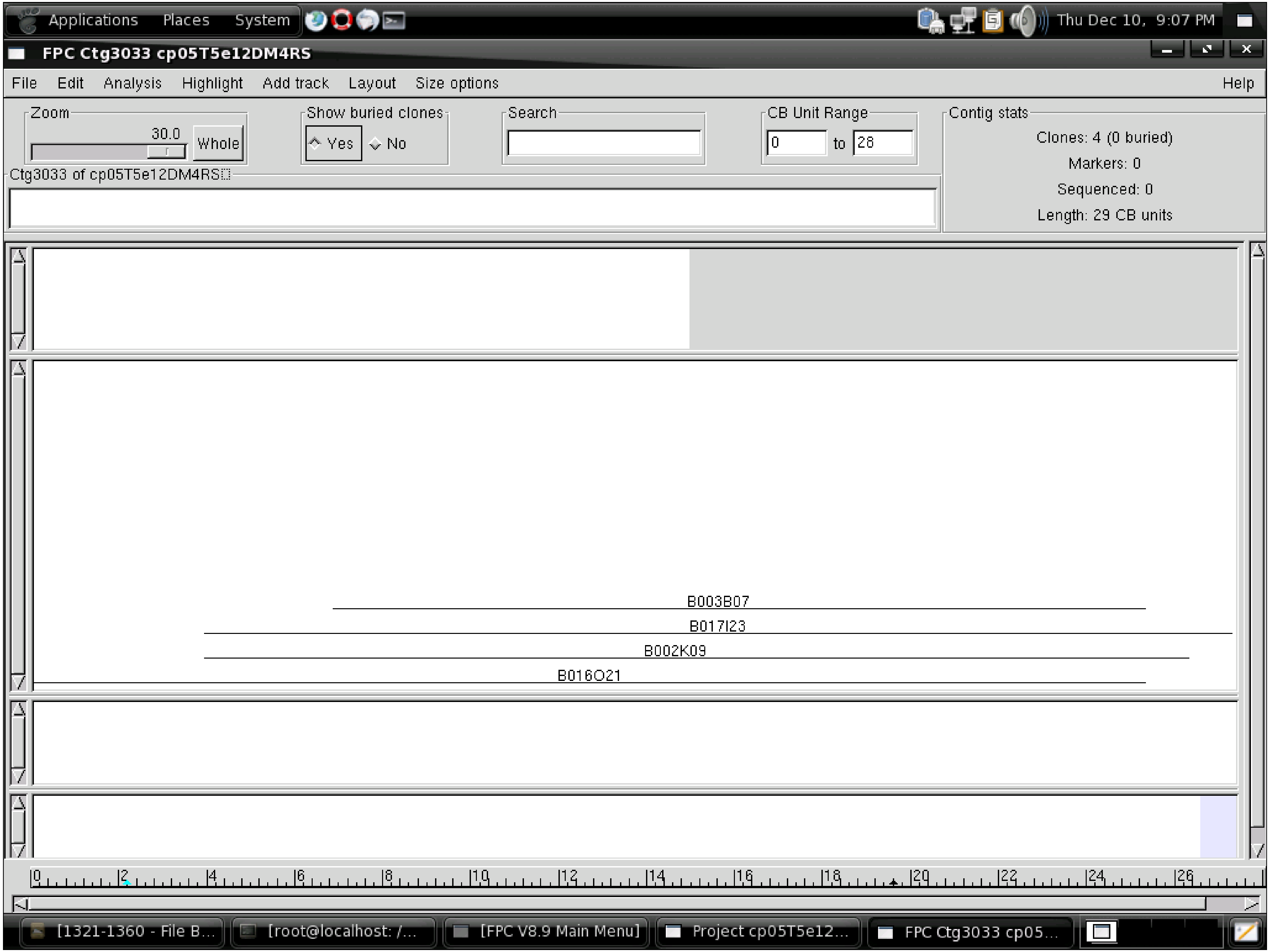
Applications
Places
System
Thu Dec 10, 9:07 PM
FPC Ctg3033 cp05T5e12DM4RS
File
Edit
Analysis
Highlight
Add track
Layout
Size options
Help
Zoom
30.0
Whole
Show buried clones
Yes
No
Search
CB Unit Range
0
to
28
Contig stats
Clones: 4 (0 buried)
Markers: 0
Sequenced: 0
Length: 29 CB units
Ctg3033 of cp05T5e12DM4RS
B003B07
B017I23
B002K09
B016O21
0 2 4 6 8 10 12 14 16 18 20 22 24 26
[1321-1360 - File B...
[root@localhost: /...
[FPC V8.9 Main Menu]
Project cp05T5e12...
FPC Ctg3033 cp05...

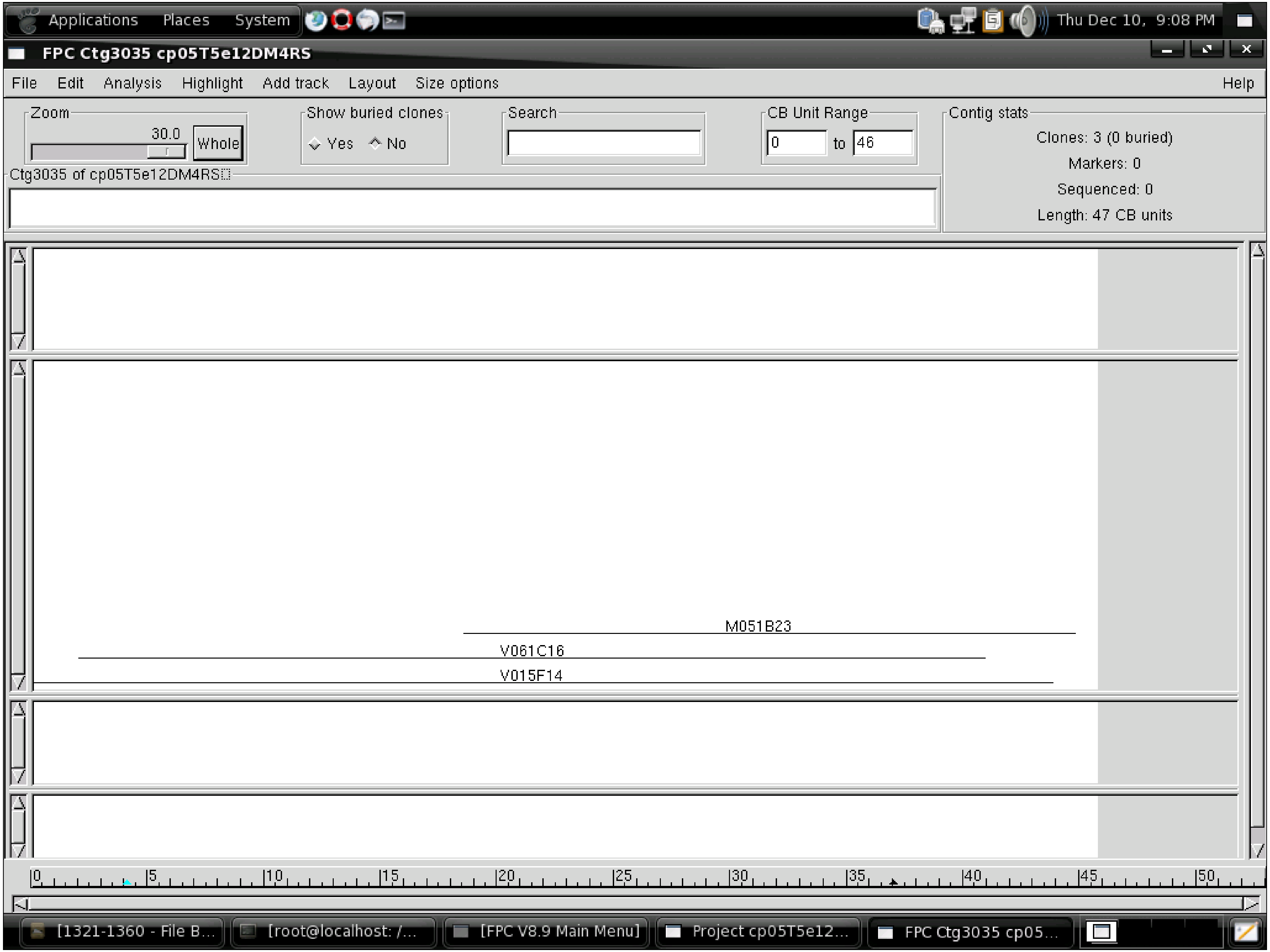
Applications
Places
System
Thu Dec 10, 9:08 PM
FPC Ctg3035 cp05T5e12DM4RS
File
Edit
Analysis
Highlight
Add track
Layout
Size options
Help
Zoom
30.0
Whole
Show buried clones
Yes
No
Search
CB Unit Range
0
to
46
Contig stats
Clones: 3 (0 buried)
Markers: 0
Sequenced: 0
Length: 47 CB units
Ctg3035 of cp05T5e12DM4RS
M051B23
V061C16
V015F14
0
5
10
15
20
25
30
35
40
45
50
[1321-1360 - File B...
[root@localhost: /...
[FPC V8.9 Main Menu]
Project cp05T5e12...
FPC Ctg3035 cp05...

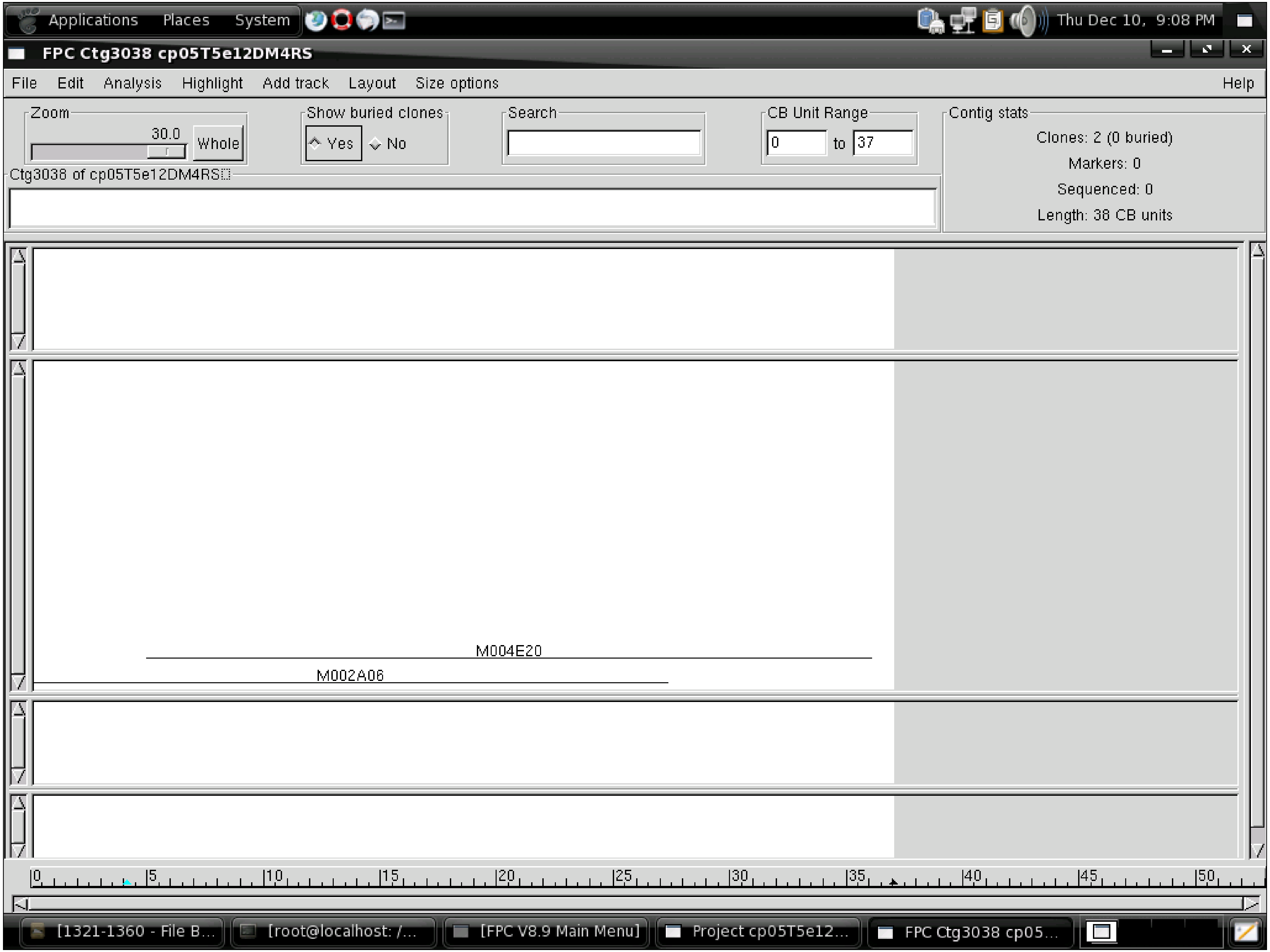
Applications Places System
Thu Dec 10, 9:08 PM
FPC Ctg3038 cp05T5e12DM4RS
File Edit Analysis Highlight Add track Layout Size options Help
Zoom
30.0
Whole
Show buried clones
Yes No
Search
CB Unit Range
0 to 37
Contig stats
Clones: 2 (0 buried)
Markers: 0
Sequenced: 0
Length: 38 CB units
Ctg3038 of cp05T5e12DM4RS
M004E20
M002A06
0 5 10 15 20 25 30 35 40 45 50
[1321-1360 - File B...
[root@localhost: /...
[FPC V8.9 Main Menu]
Project cp05T5e12...
FPC Ctg3038 cp05...

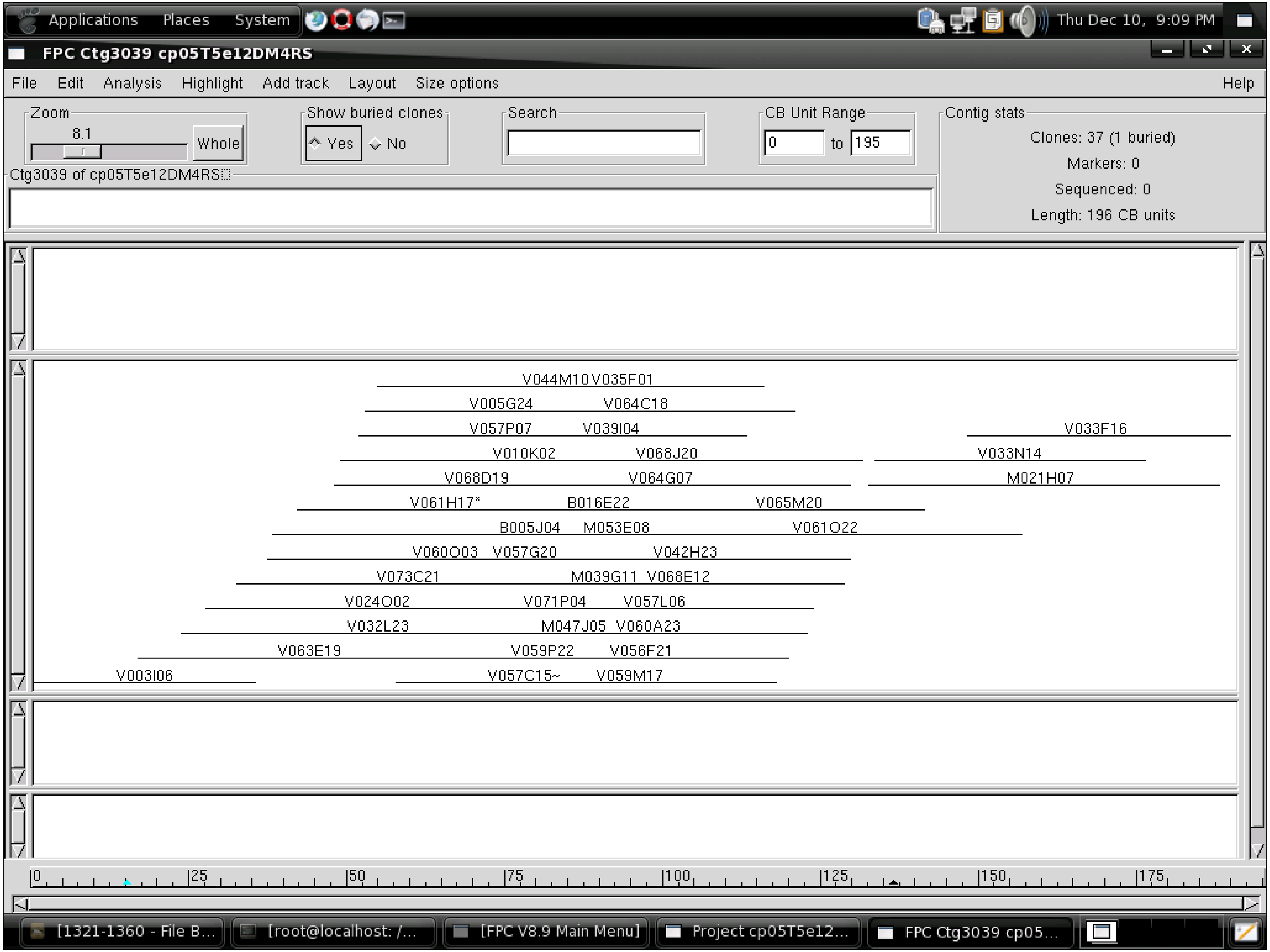

Applications Places System
Thu Dec 10, 9:09 PM
FPC Ctg3039 cp05T5e12DM4RS
File Edit Analysis Highlight Add track Layout Size options Help
Zoom
8.1
Whole
Show buried clones
Yes No
Search
CB Unit Range
0 to 195
Contig stats
Clones: 37 (1 buried)
Markers: 0
Sequenced: 0
Length: 196 CB units
Ctg3039 of cp05T5e12DM4RS
V044M10 V035F01
V005G24 V064C18
V057P07 V039I04 V033F16
V010K02 V068J20 V033N14
V068D19 V064G07 M021H07
V061H17* B016E22 V065M20
B005J04 M053E08 V061O22
V060O03 V057G20 V042H23
V073C21 M039G11 V068E12
V024O02 V071P04 V057L06
V032L23 M047J05 V060A23
V063E19 V059P22 V056F21
V003I06 V057C15~ V059M17
0 25 50 75 100 125 150 175
[1321-1360 - File B...
[root@localhost: /...
[FPC V8.9 Main Menu]
Project cp05T5e12...
FPC Ctg3039 cp05...

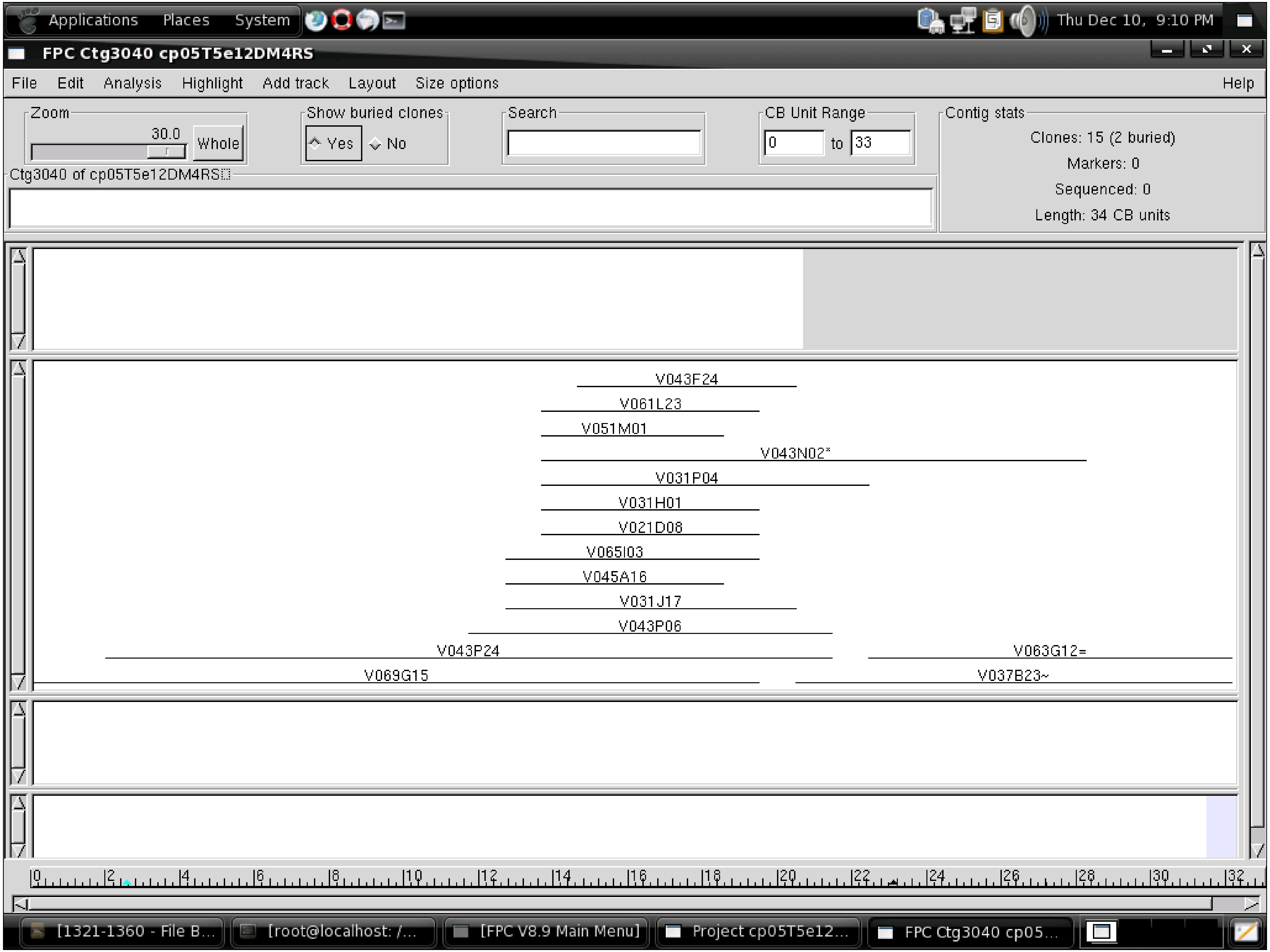
Applications Places System
Thu Dec 10, 9:10 PM
FPC Ctg3040 cp05T5e12DM4RS
File Edit Analysis Highlight Add track Layout Size options Help
Zoom
30.0
Whole
Show buried clones
Yes
No
Search
CB Unit Range
0
to
33
Contig stats
Clones: 15 (2 buried)
Markers: 0
Sequenced: 0
Length: 34 CB units
Ctg3040 of cp05T5e12DM4RS
V043F24
V061L23
V051M01
V043N02*
V031P04
V031H01
V021D08
V065I03
V045A16
V031J17
V043P06
V043P24
V063G12=
V069G15
V037B23~
0 2 4 6 8 10 12 14 16 18 20 22 24 26 28 30 32
[1321-1360 - File B...
[root@localhost: /...
[FPC V8.9 Main Menu]
Project cp05T5e12...
FPC Ctg3040 cp05...

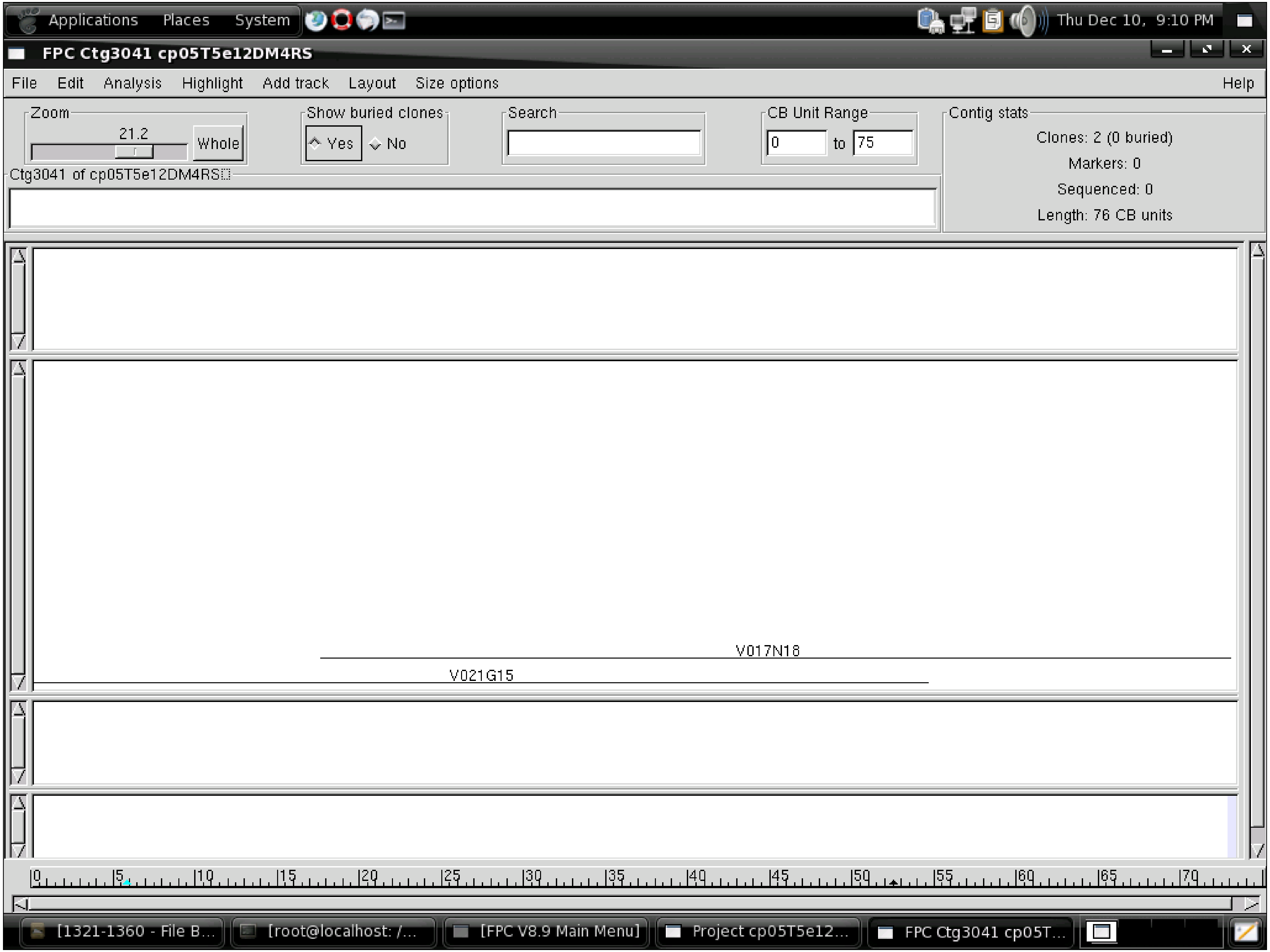
Applications Places System
Thu Dec 10, 9:10 PM
FPC Ctg3041 cp05T5e12DM4RS
File Edit Analysis Highlight Add track Layout Size options Help
Zoom
21.2
Whole
Show buried clones
Yes No
Search
CB Unit Range
0 to 75
Contig stats
Clones: 2 (0 buried)
Markers: 0
Sequenced: 0
Length: 76 CB units
Ctg3041 of cp05T5e12DM4RS
V017N18
V021G15
0 5 10 15 20 25 30 35 40 45 50 55 60 65 70
[1321-1360 - File B...
[root@localhost: /...
[FPC V8.9 Main Menu]
Project cp05T5e12...
FPC Ctg3041 cp05T...

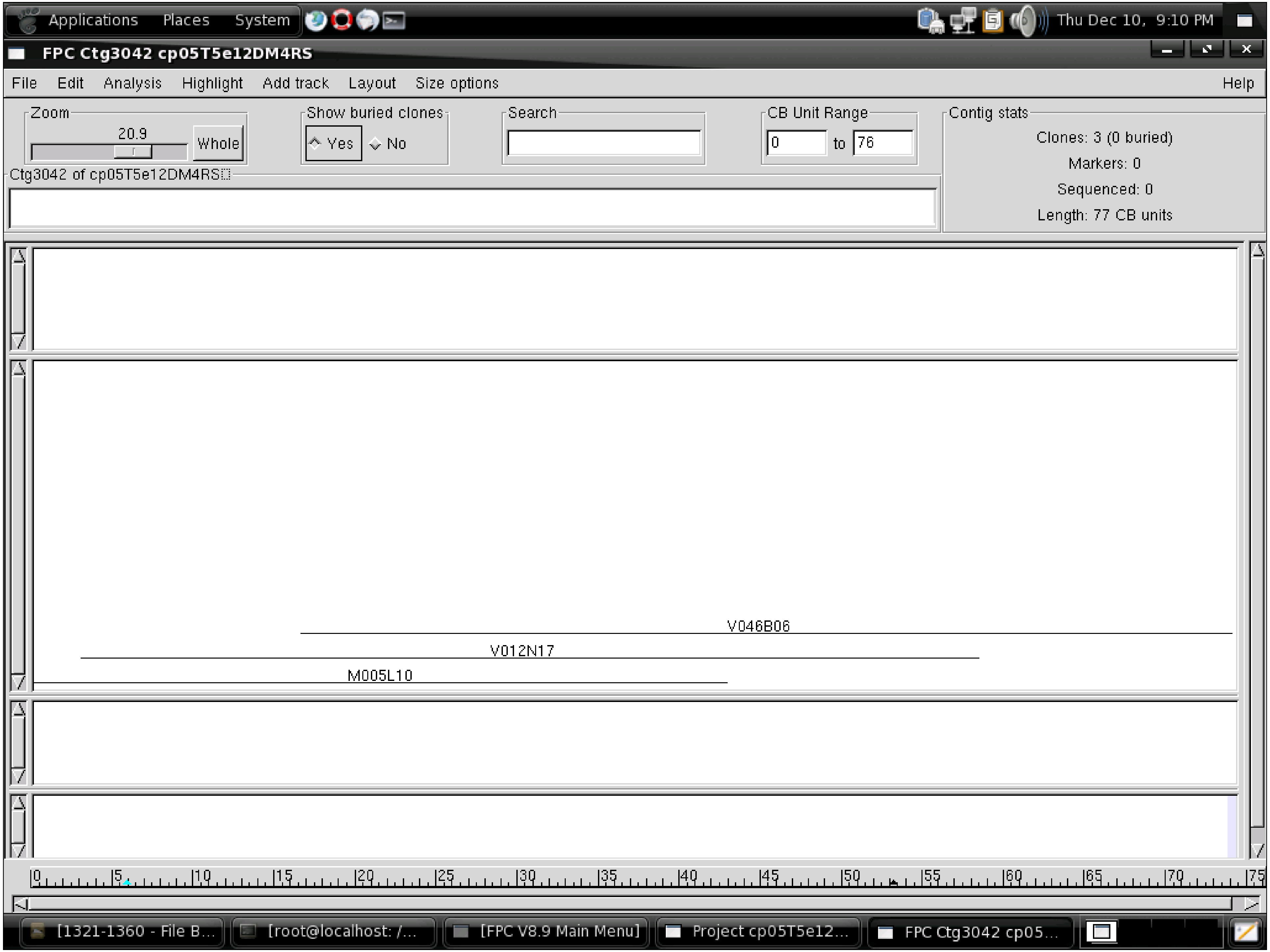
Applications
Places
System
Thu Dec 10, 9:10 PM
FPC Ctg3042 cp05T5e12DM4RS
File
Edit
Analysis
Highlight
Add track
Layout
Size options
Help
Zoom
20.9
Whole
Show buried clones
Yes
No
Search
CB Unit Range
0
to
76
Contig stats
Clones: 3 (0 buried)
Markers: 0
Sequenced: 0
Length: 77 CB units
Ctg3042 of cp05T5e12DM4RS
V046B06
V012N17
M005L10
0 5 10 15 20 25 30 35 40 45 50 55 60 65 70 75
[1321-1360 - File B...
[root@localhost: /...
[FPC V8.9 Main Menu]
Project cp05T5e12...
FPC Ctg3042 cp05...

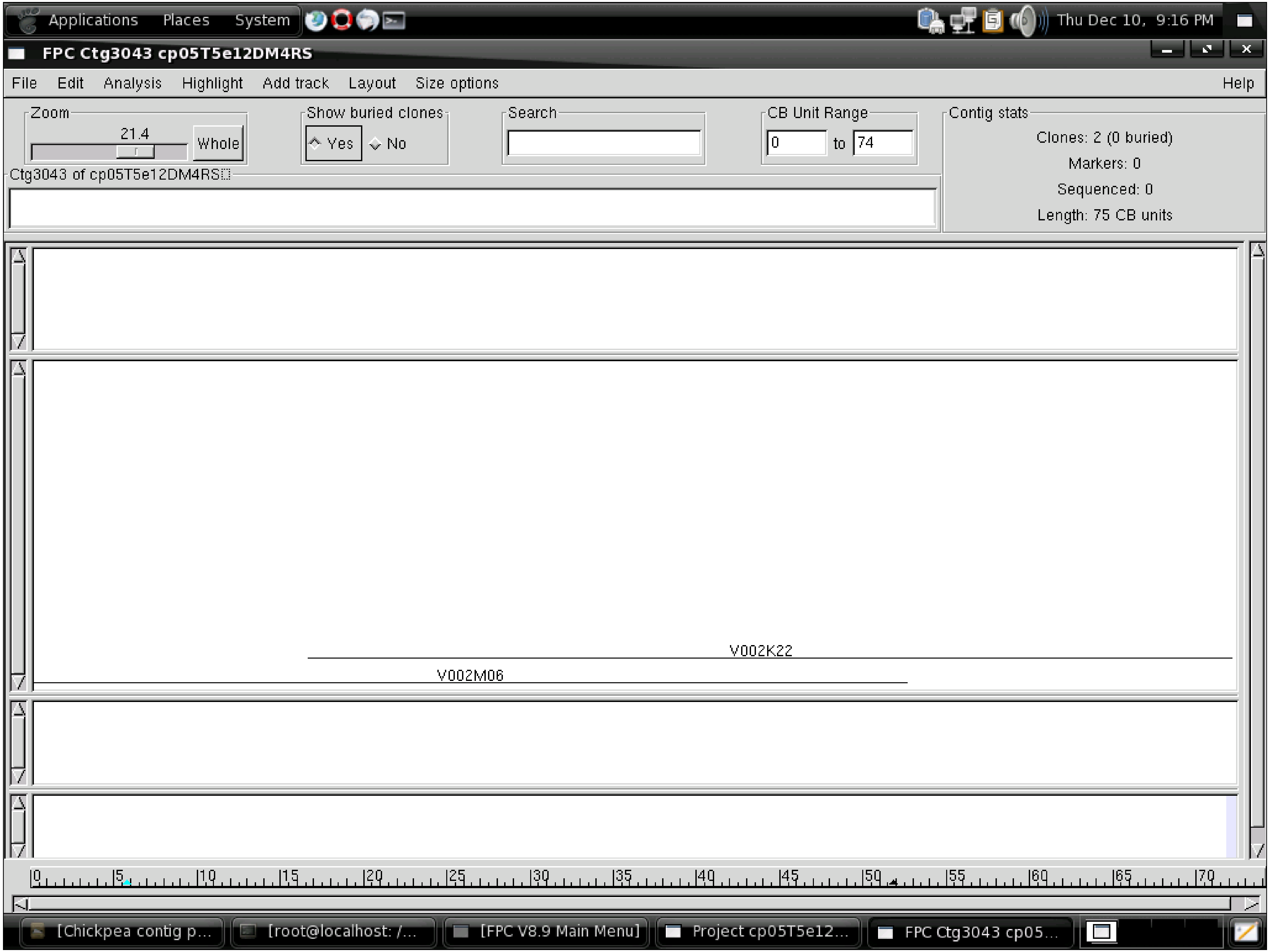
Applications Places System
Thu Dec 10, 9:16 PM
FPC Ctg3043 cp05T5e12DM4RS
File Edit Analysis Highlight Add track Layout Size options Help
Zoom
21.4
Whole
Show buried clones
Yes No
Search
CB Unit Range
0 to 74
Contig stats
Clones: 2 (0 buried)
Markers: 0
Sequenced: 0
Length: 75 CB units
Ctg3043 of cp05T5e12DM4RS
V002K22
V002M06
0 5 10 15 20 25 30 35 40 45 50 55 60 65 70
[Chickpea contig p...
[root@localhost: /...
[FPC V8.9 Main Menu]
Project cp05T5e12...
FPC Ctg3043 cp05...

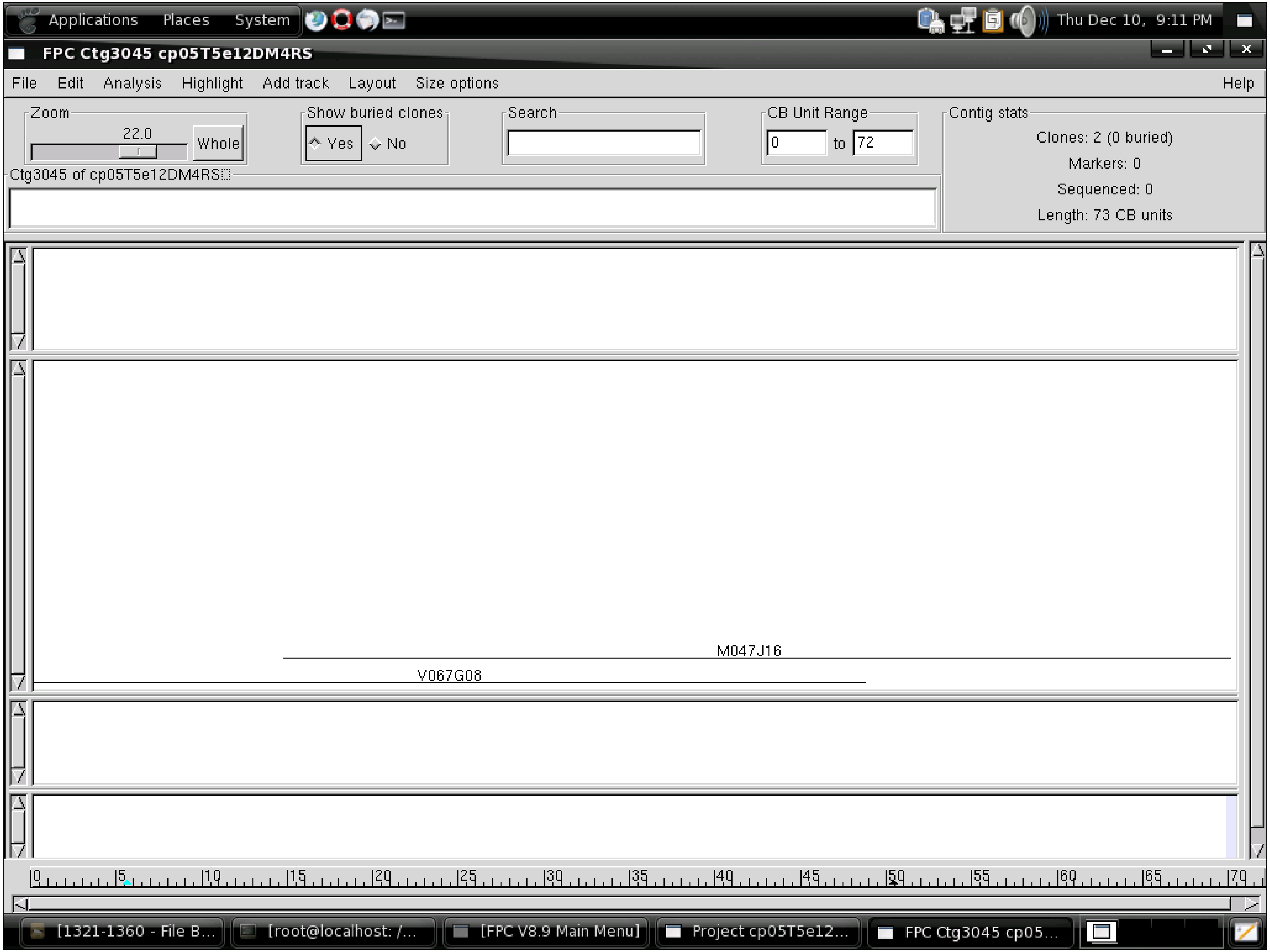
Applications
Places
System
Thu Dec 10, 9:11 PM
FPC Ctg3045 cp05T5e12DM4RS
File
Edit
Analysis
Highlight
Add track
Layout
Size options
Help
Zoom
22.0
Whole
Show buried clones
Yes
No
Search
CB Unit Range
0
to
72
Contig stats
Clones: 2 (0 buried)
Markers: 0
Sequenced: 0
Length: 73 CB units
Ctg3045 of cp05T5e12DM4RS
M047J16
V067G08
0 5 10 15 20 25 30 35 40 45 50 55 60 65 70
[1321-1360 - File B...
[root@localhost: /...
[FPC V8.9 Main Menu]
Project cp05T5e12...
FPC Ctg3045 cp05...

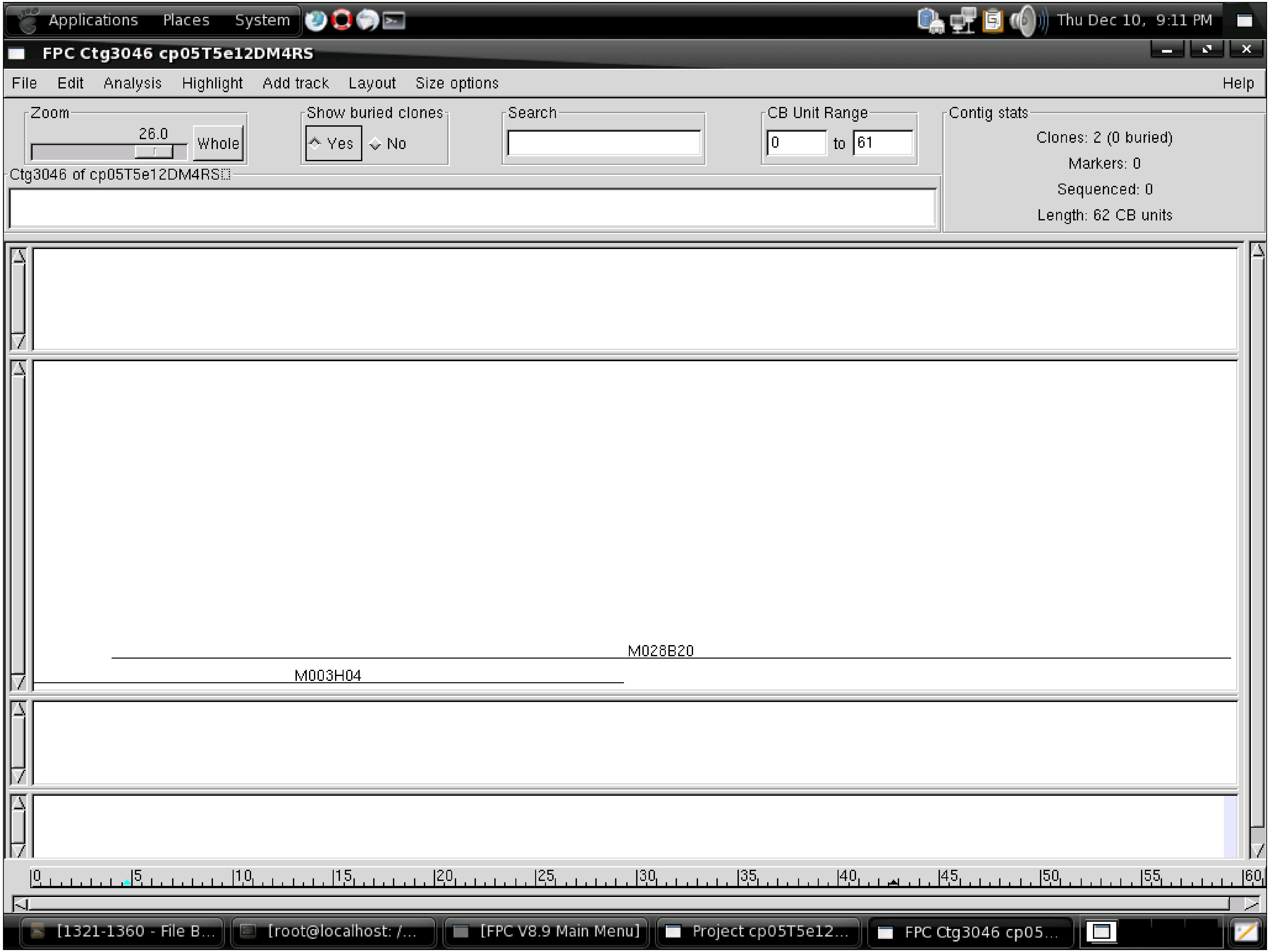
Applications
Places
System
Thu Dec 10, 9:11 PM
FPC Ctg3046 cp05T5e12DM4RS
File
Edit
Analysis
Highlight
Add track
Layout
Size options
Help
Zoom
26.0
Whole
Show buried clones
Yes
No
Search
CB Unit Range
0
to
61
Contig stats
Clones: 2 (0 buried)
Markers: 0
Sequenced: 0
Length: 62 CB units
Ctg3046 of cp05T5e12DM4RS
M028B20
M003H04
0 5 10 15 20 25 30 35 40 45 50 55 60
[1321-1360 - File B...
[root@localhost: /...
[FPC V8.9 Main Menu]
Project cp05T5e12...
FPC Ctg3046 cp05...

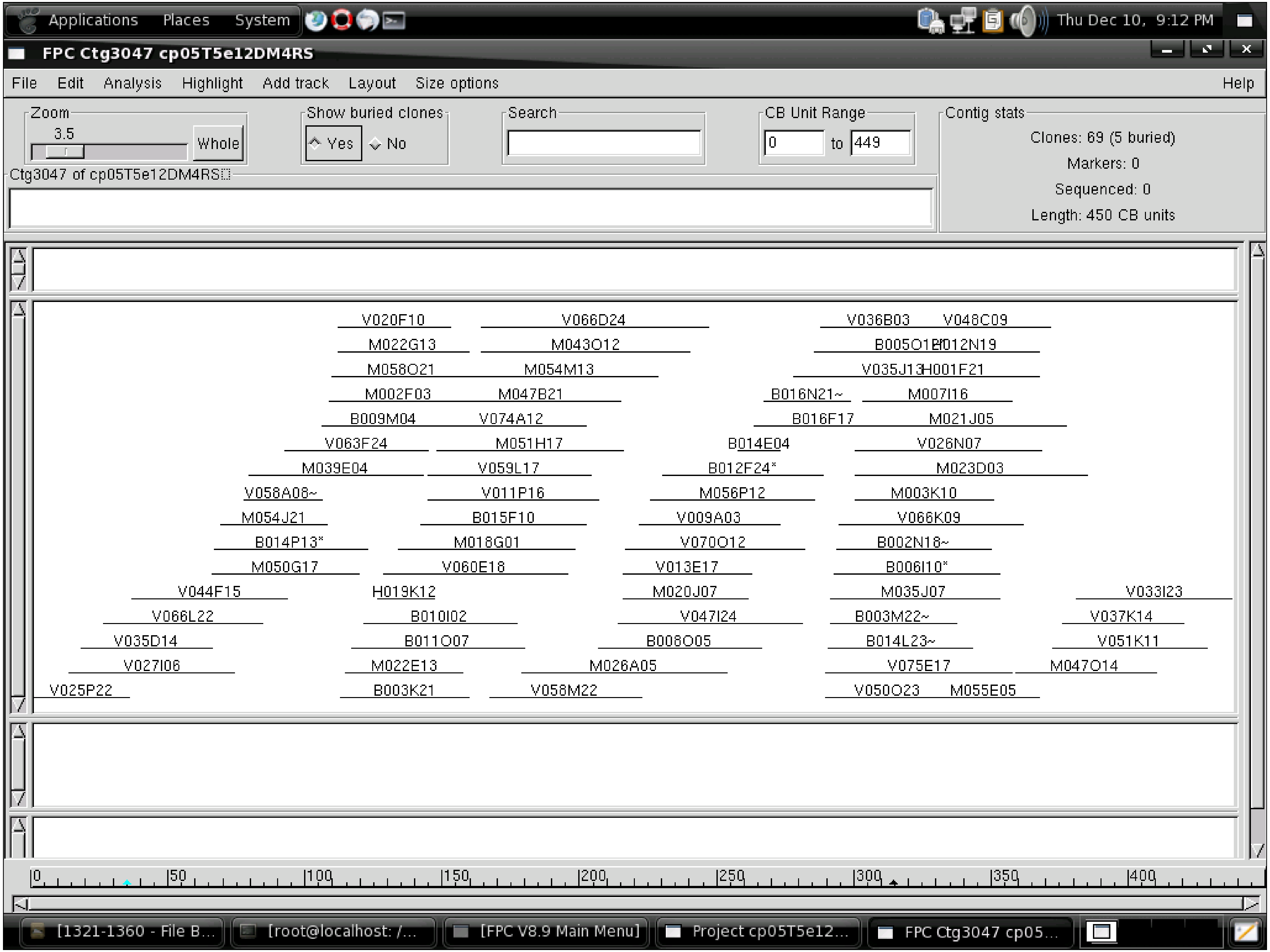

Applications Places System
Thu Dec 10, 9:12 PM
FPC Ctg3047 cp05T5e12DM4RS
File Edit Analysis Highlight Add track Layout Size options Help
Zoom
3.5
Whole
Show buried clones
Yes
No
Search
CB Unit Range
0 to 449
Contig stats
Clones: 69 (5 buried)
Markers: 0
Sequenced: 0
Length: 450 CB units
Ctg3047 of cp05T5e12DM4RS
V020F10
V066D24
V036B03
V048C09
M022G13
M043O12
B005O12
H012N19
M058O21
M054M13
V035J13
H001F21
M002F03
M047B21
B016N21~
M007I16
B009M04
V074A12
B016F17
M021J05
V063F24
M051H17
B014E04
V026N07
M039E04
V059L17
B012F24*
M023D03
V058A08~
V011P16
M056P12
M003K10
M054J21
B015F10
V009A03
V066K09
B014P13*
M018G01
V070O12
B002N18~
M050G17
V060E18
V013E17
B006I10*
V044F15
H019K12
M020J07
M035J07
V033I23
V066L22
B010I02
V047I24
B003M22~
V037K14
V035D14
B011O07
B008O05
B014L23~
V051K11
V027I06
M022E13
M026A05
V075E17
M047O14
V025P22
B003K21
V058M22
V050O23
M055E05
0 50 100 150 200 250 300 350 400
[1321-1360 - File B...
[root@localhost: /...
[FPC V8.9 Main Menu]
Project cp05T5e12...
FPC Ctg3047 cp05...

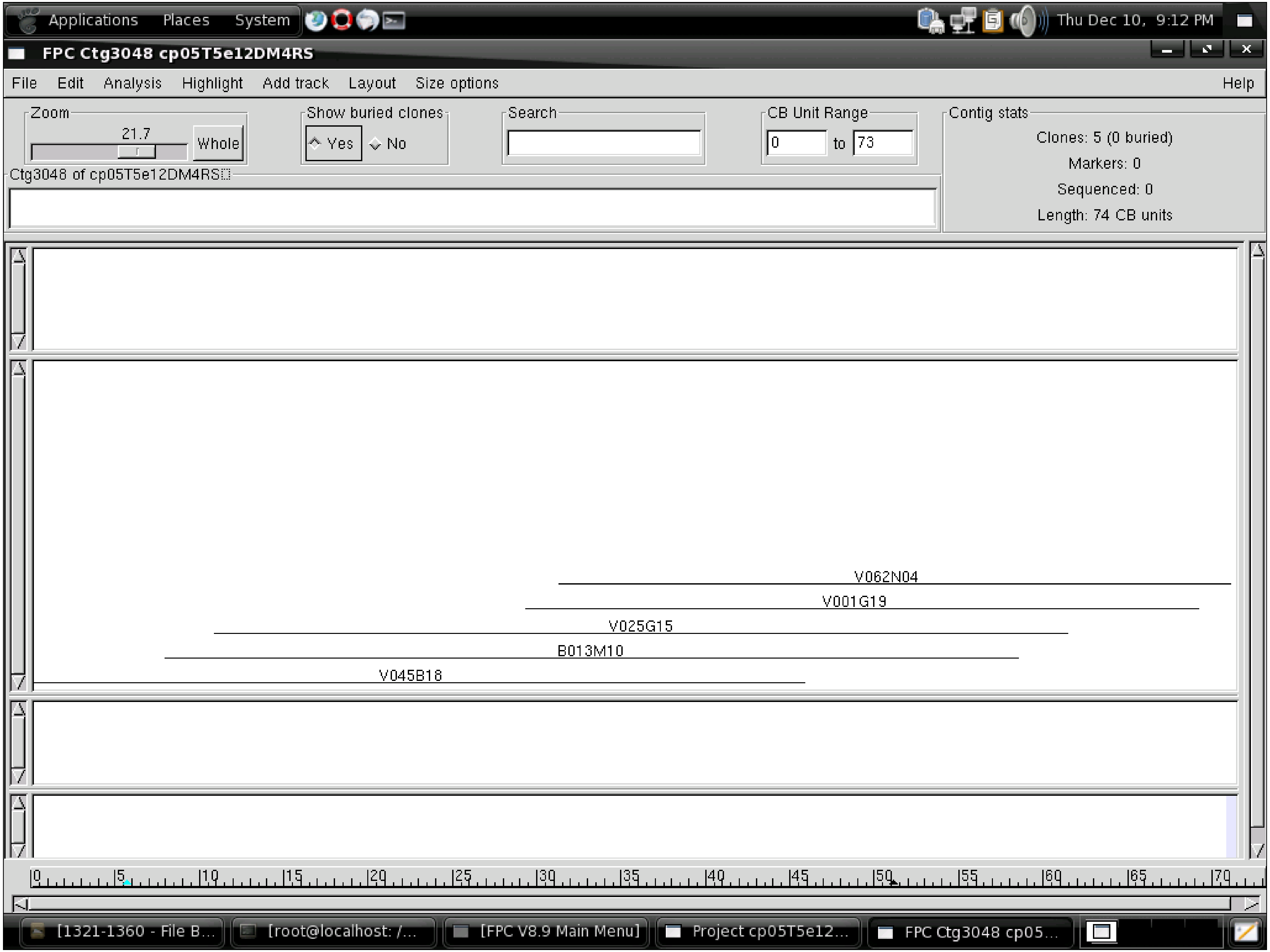
Applications Places System
Thu Dec 10, 9:12 PM
FPC Ctg3048 cp05T5e12DM4RS
File Edit Analysis Highlight Add track Layout Size options Help
Zoom
21.7
Whole
Show buried clones
Yes No
Search
CB Unit Range
0 to 73
Contig stats
Clones: 5 (0 buried)
Markers: 0
Sequenced: 0
Length: 74 CB units
Ctg3048 of cp05T5e12DM4RS
V062N04
V001G19
V025G15
B013M10
V045B18
0 5 10 15 20 25 30 35 40 45 50 55 60 65 70
[1321-1360 - File B...
[root@localhost: /...
[FPC V8.9 Main Menu]
Project cp05T5e12...
FPC Ctg3048 cp05...

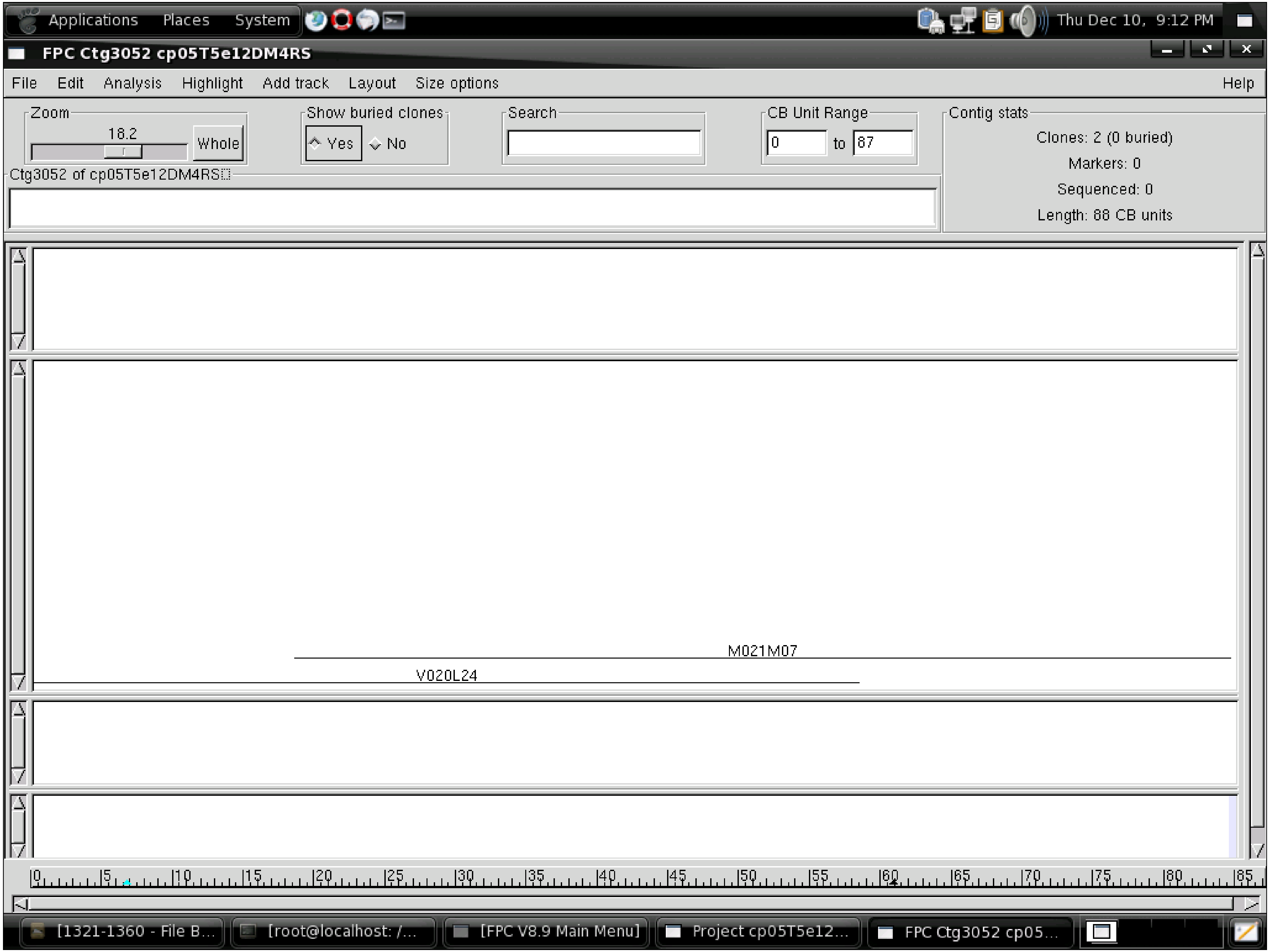
Applications Places System
Thu Dec 10, 9:12 PM
FPC Ctg3052 cp05T5e12DM4RS
File Edit Analysis Highlight Add track Layout Size options Help
Zoom
18.2
Whole
Show buried clones
Yes
No
Search
CB Unit Range
0 to 87
Contig stats
Clones: 2 (0 buried)
Markers: 0
Sequenced: 0
Length: 88 CB units
Ctg3052 of cp05T5e12DM4RS
M021M07
V020L24
0 5 10 15 20 25 30 35 40 45 50 55 60 65 70 75 80 85
[1321-1360 - File B...
[root@localhost: /...
[FPC V8.9 Main Menu]
Project cp05T5e12...
FPC Ctg3052 cp05...

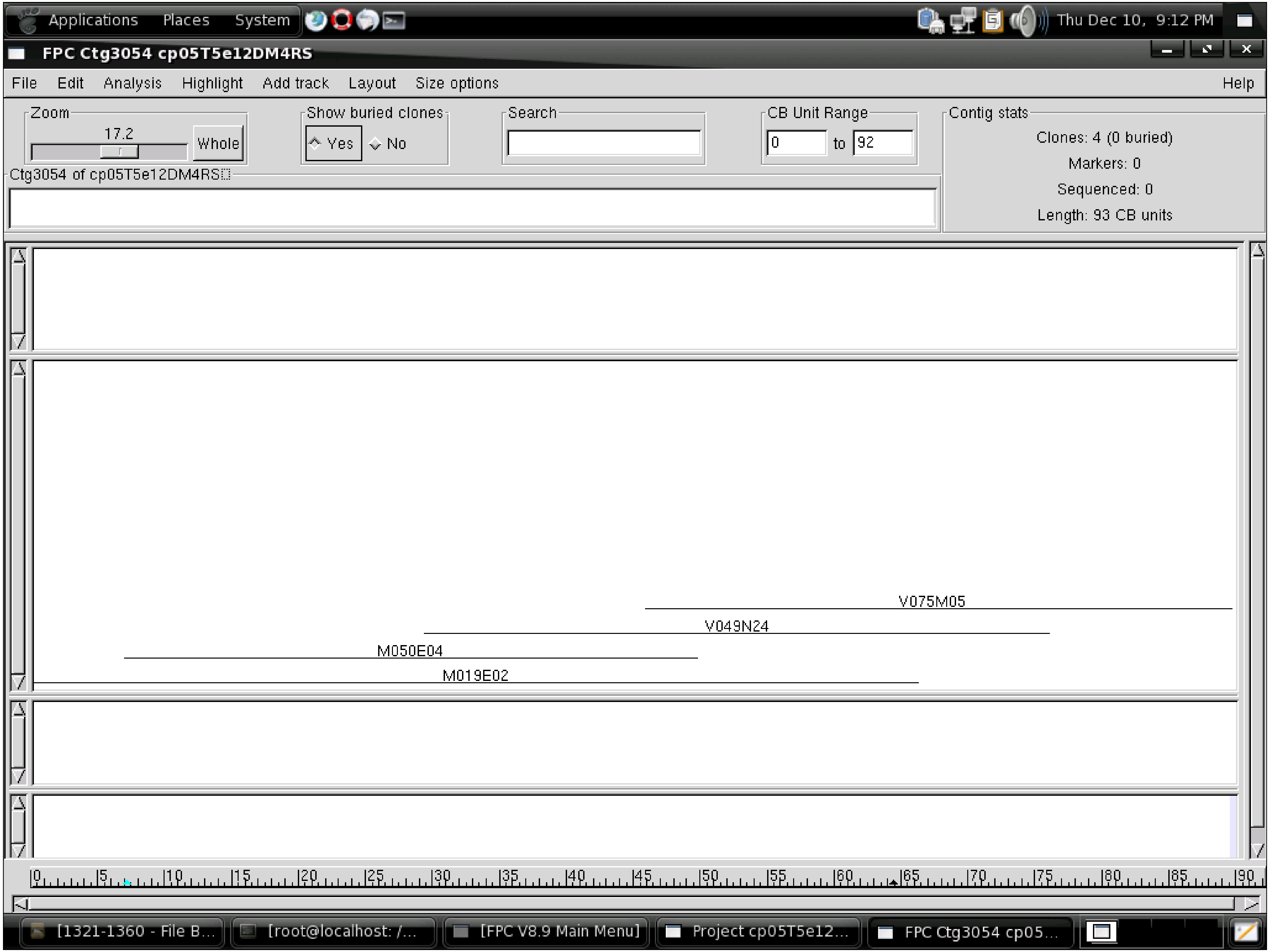

Applications Places System
Thu Dec 10, 9:12 PM
FPC Ctg3054 cp05T5e12DM4RS
File Edit Analysis Highlight Add track Layout Size options Help
Zoom
17.2
Whole
Show buried clones
Yes No
Search
CB Unit Range
0 to 92
Contig stats
Clones: 4 (0 buried)
Markers: 0
Sequenced: 0
Length: 93 CB units
Ctg3054 of cp05T5e12DM4RS
V075M05
V049N24
M050E04
M019E02
0 5 10 15 20 25 30 35 40 45 50 55 60 65 70 75 80 85 90
[1321-1360 - File B...
[root@localhost: /...
[FPC V8.9 Main Menu]
Project cp05T5e12...
FPC Ctg3054 cp05...

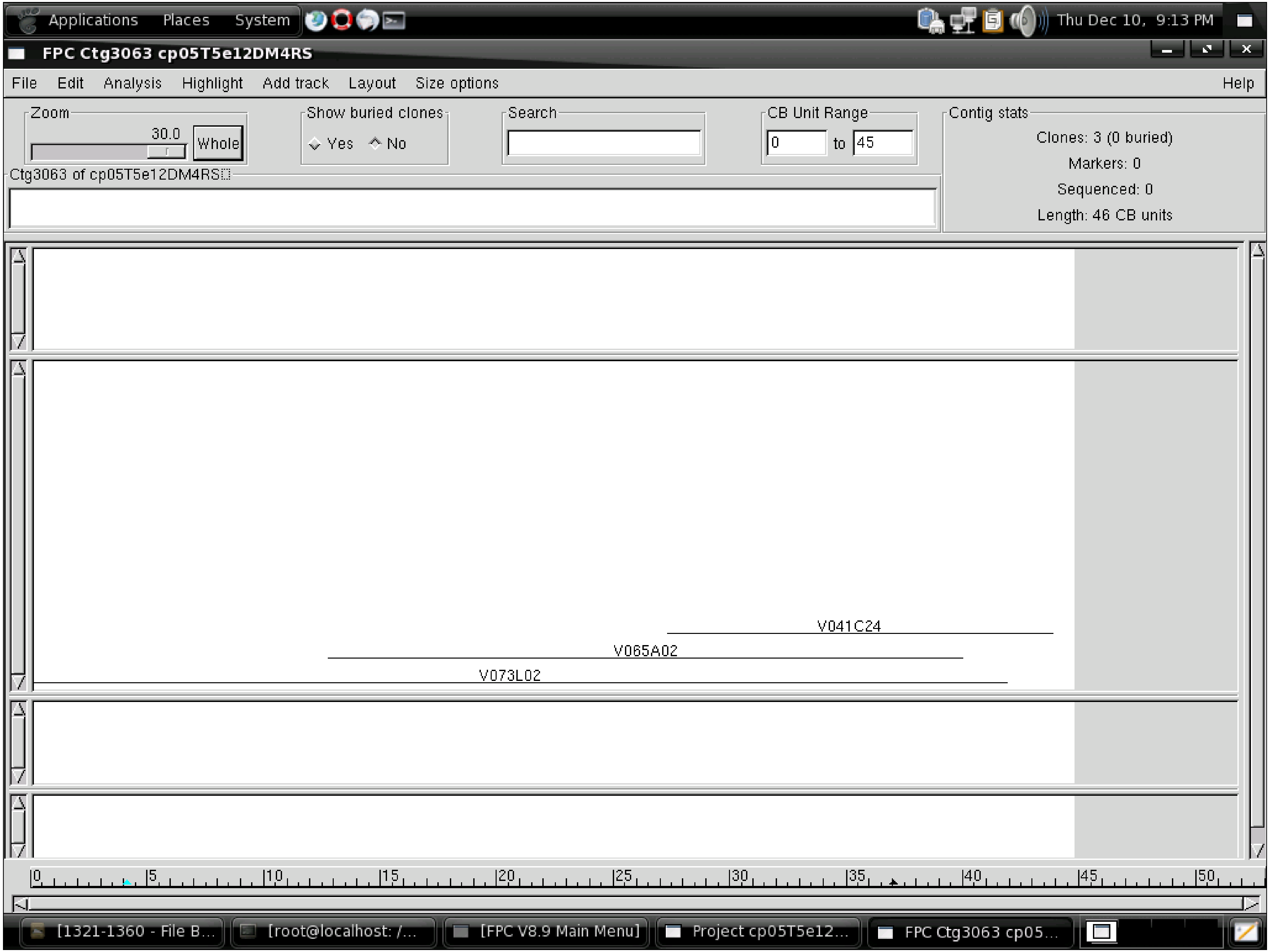
Applications Places System
Thu Dec 10, 9:13 PM
FPC Ctg3063 cp05T5e12DM4RS
File Edit Analysis Highlight Add track Layout Size options Help
Zoom
30.0
Whole
Show buried clones
Yes No
Search
CB Unit Range
0 to 45
Contig stats
Clones: 3 (0 buried)
Markers: 0
Sequenced: 0
Length: 46 CB units
Ctg3063 of cp05T5e12DM4RS
V041C24
V065A02
V073L02
0 5 10 15 20 25 30 35 40 45 50
[1321-1360 - File B...
[root@localhost: /...
[FPC V8.9 Main Menu]
Project cp05T5e12...
FPC Ctg3063 cp05...

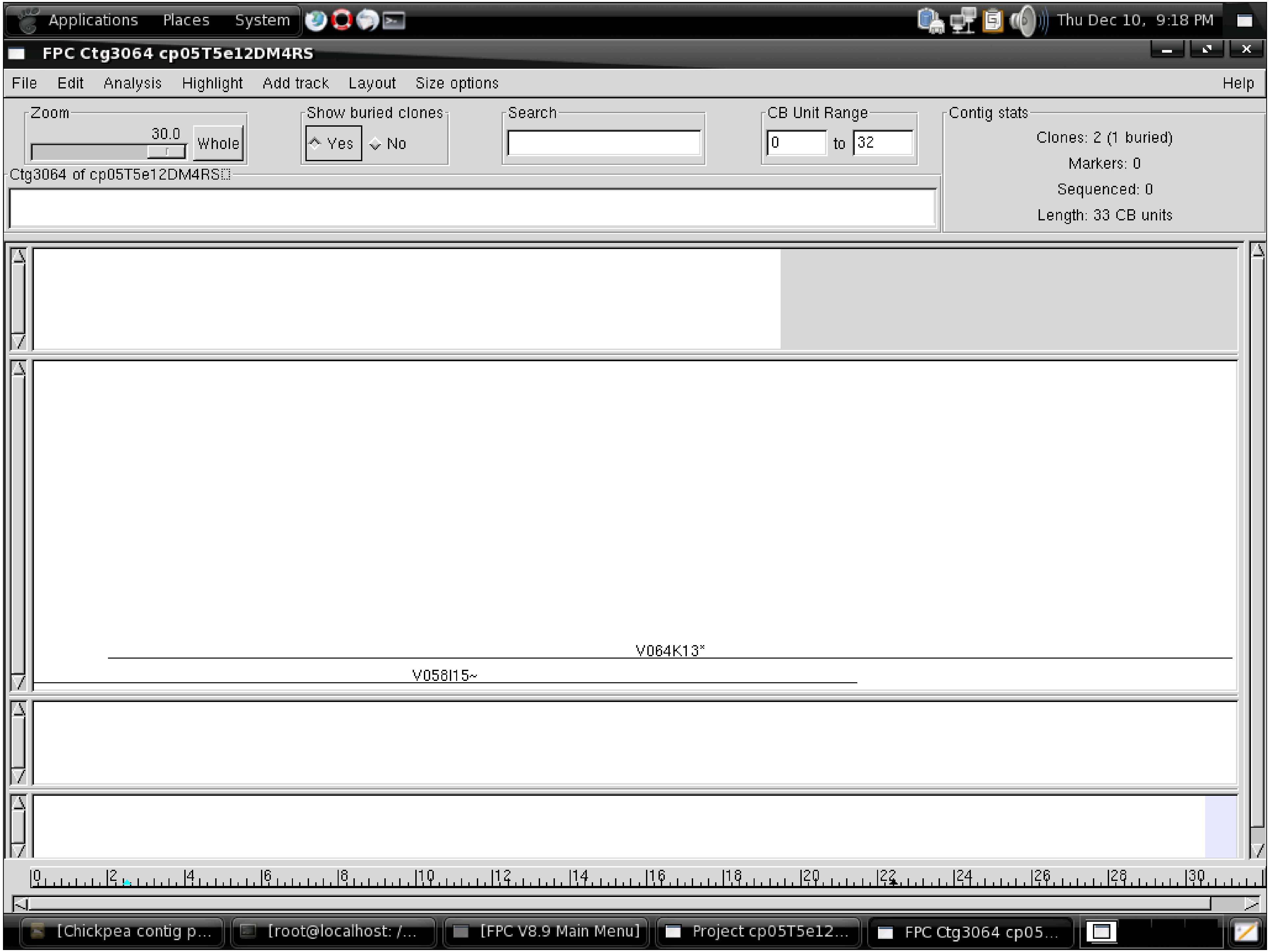
Applications Places System
Thu Dec 10, 9:18 PM
FPC Ctg3064 cp05T5e12DM4RS
File Edit Analysis Highlight Add track Layout Size options Help
Zoom
30.0
Whole
Show buried clones
Yes No
Search
CB Unit Range
0 to 32
Contig stats
Clones: 2 (1 buried)
Markers: 0
Sequenced: 0
Length: 33 CB units
Ctg3064 of cp05T5e12DM4RS
V064K13*
V058I15~
[Chickpea contig p...
[root@localhost: /...
[FPC V8.9 Main Menu]
Project cp05T5e12...
FPC Ctg3064 cp05...

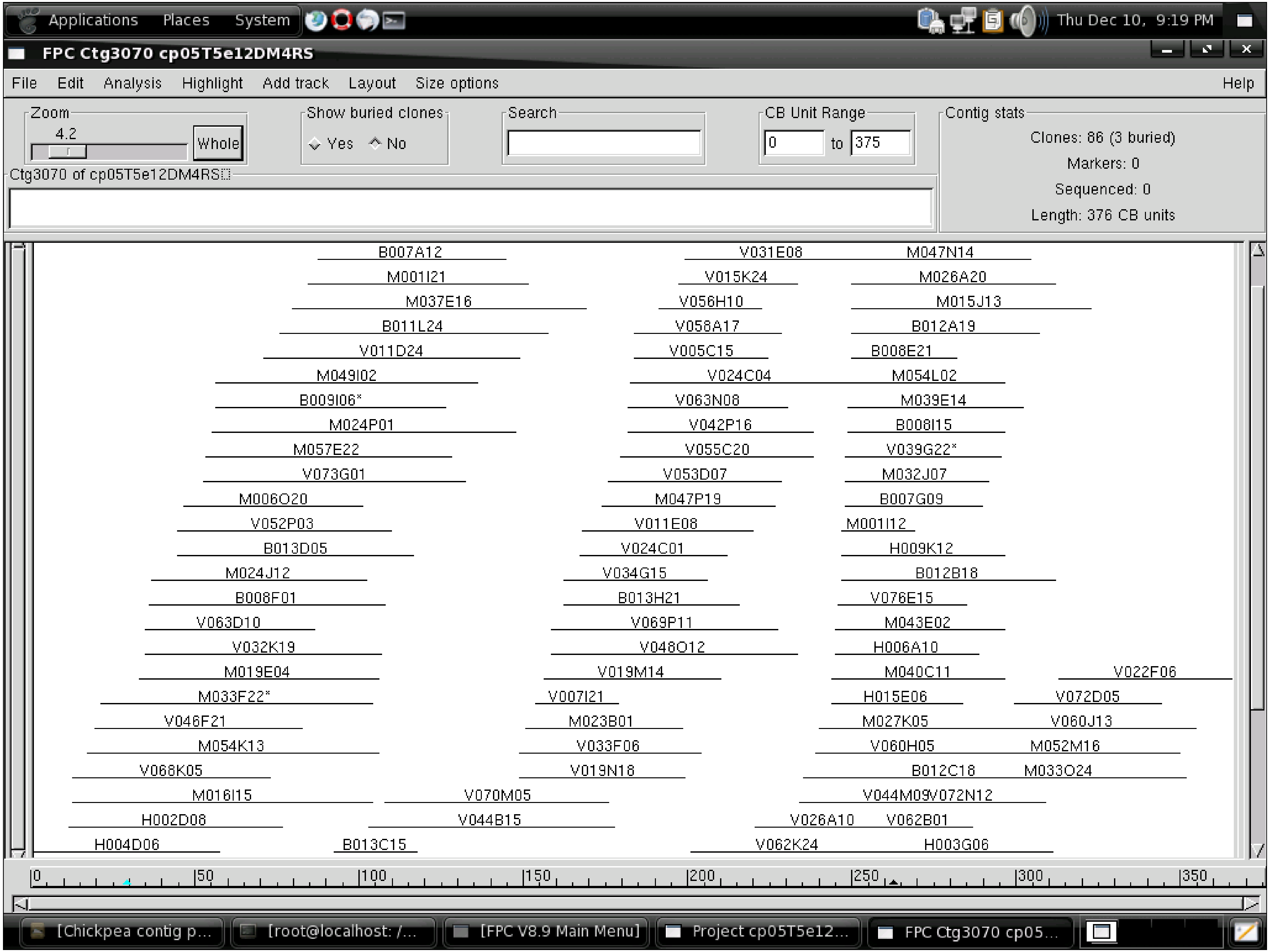
Applications Places System
Thu Dec 10, 9:19 PM
FPC Ctg3070 cp05T5e12DM4RS
File Edit Analysis Highlight Add track Layout Size options Help
Zoom
4.2
Whole
Show buried clones
Yes No
Search
CB Unit Range
0 to 375
Contig stats
Clones: 86 (3 buried)
Markers: 0
Sequenced: 0
Length: 376 CB units
Ctg3070 of cp05T5e12DM4RS
B007A12 V031E08 M047N14
M001I21 V015K24 M026A20
M037E16 V056H10 M015J13
B011L24 V058A17 B012A19
V011D24 V005C15 B008E21
M049I02 V024C04 M054L02
B009I06* V063N08 M039E14
M024P01 V042P16 B008I15
M057E22 V055C20 V039G22*
V073G01 V053D07 M032J07
M006O20 M047P19 B007G09
V052P03 V011E08 M001I12
B013D05 V024C01 H009K12
M024J12 V034G15 B012B18
B008F01 B013H21 V076E15
V063D10 V069P11 M043E02
V032K19 V048O12 H006A10
M019E04 V019M14 M040C11 V022F06
M033F22* V007I21 H015E06 V072D05
V046F21 M023B01 M027K05 V060J13
M054K13 V033F06 V060H05 M052M16
V068K05 V019N18 B012C18 M033O24
M016I15 V070M05 V044M09V072N12
H002D08 V044B15 V026A10 V062B01
H004D06 B013C15 V062K24 H003G06
0 50 100 150 200 250 300 350
[Chickpea contig p...
[root@localhost: /...
[FPC V8.9 Main Menu]
Project cp05T5e12...
FPC Ctg3070 cp05...

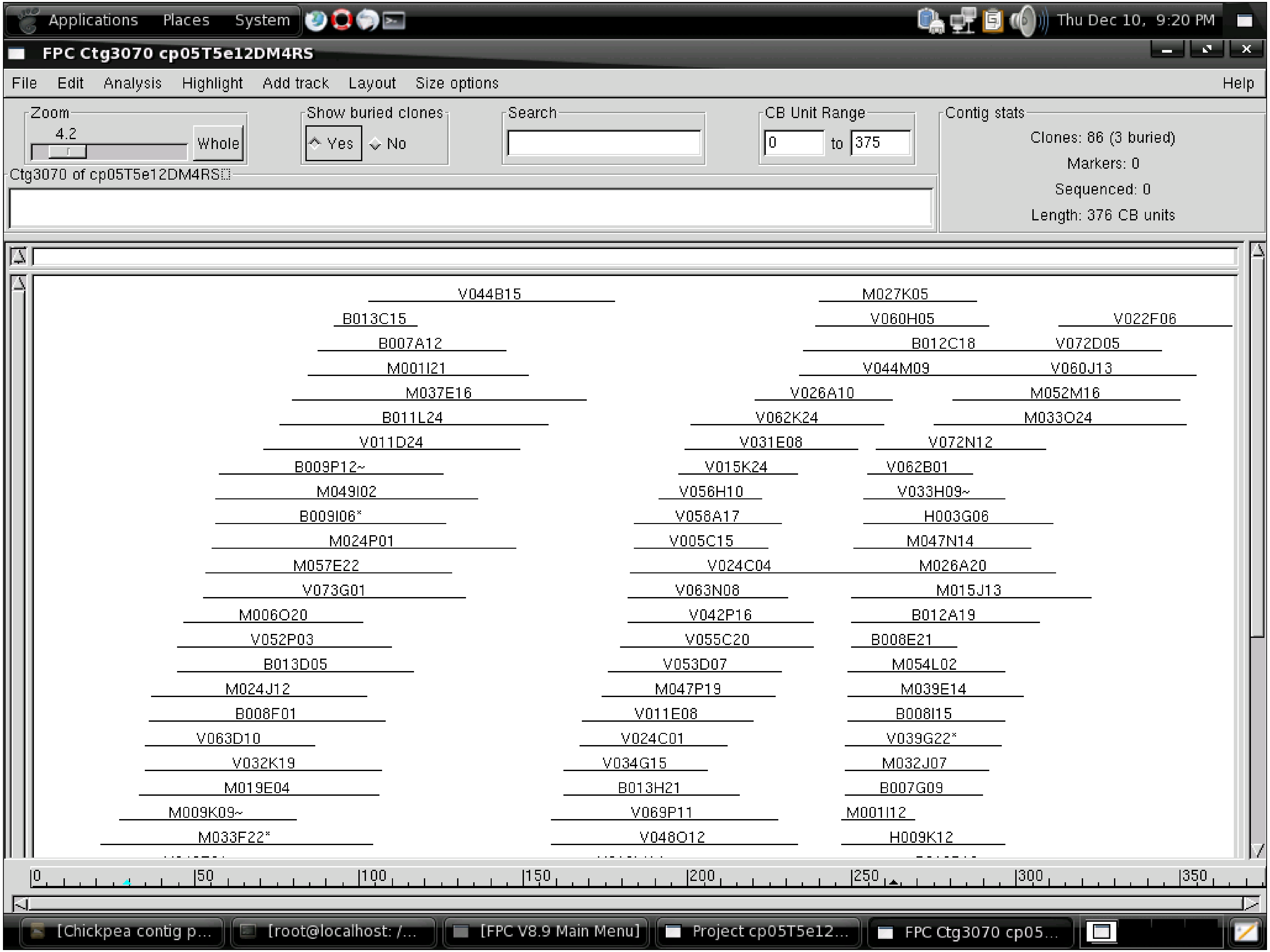
Applications Places System
Thu Dec 10, 9:20 PM
FPC Ctg3070 cp05T5e12DM4RS
File Edit Analysis Highlight Add track Layout Size options Help
Zoom
4.2
Whole
Show buried clones
Yes No
Search
CB Unit Range
0 to 375
Contig stats
Clones: 86 (3 buried)
Markers: 0
Sequenced: 0
Length: 376 CB units
Ctg3070 of cp05T5e12DM4RS
V044B15
M027K05
B013C15
V060H05
V022F06
B007A12
B012C18
V072D05
M001I21
V044M09
V060J13
M037E16
V026A10
M052M16
B011L24
V062K24
M033O24
V011D24
V031E08
V072N12
B009P12~
V015K24
V062B01
M049I02
V056H10
V033H09~
B009I06*
V058A17
H003G06
M024P01
V005C15
M047N14
M057E22
V024C04
M026A20
V073G01
V063N08
M015J13
M006O20
V042P16
B012A19
V052P03
V055C20
B008E21
B013D05
V053D07
M054L02
M024J12
M047P19
M039E14
B008F01
V011E08
B008I15
V063D10
V024C01
V039G22*
V032K19
V034G15
M032J07
M019E04
B013H21
B007G09
M009K09~
V069P11
M001I12
M033F22*
V048O12
H009K12
0 50 100 150 200 250 300 350
[Chickpea contig p...
[root@localhost: /...
[FPC V8.9 Main Menu]
Project cp05T5e12...
FPC Ctg3070 cp05...

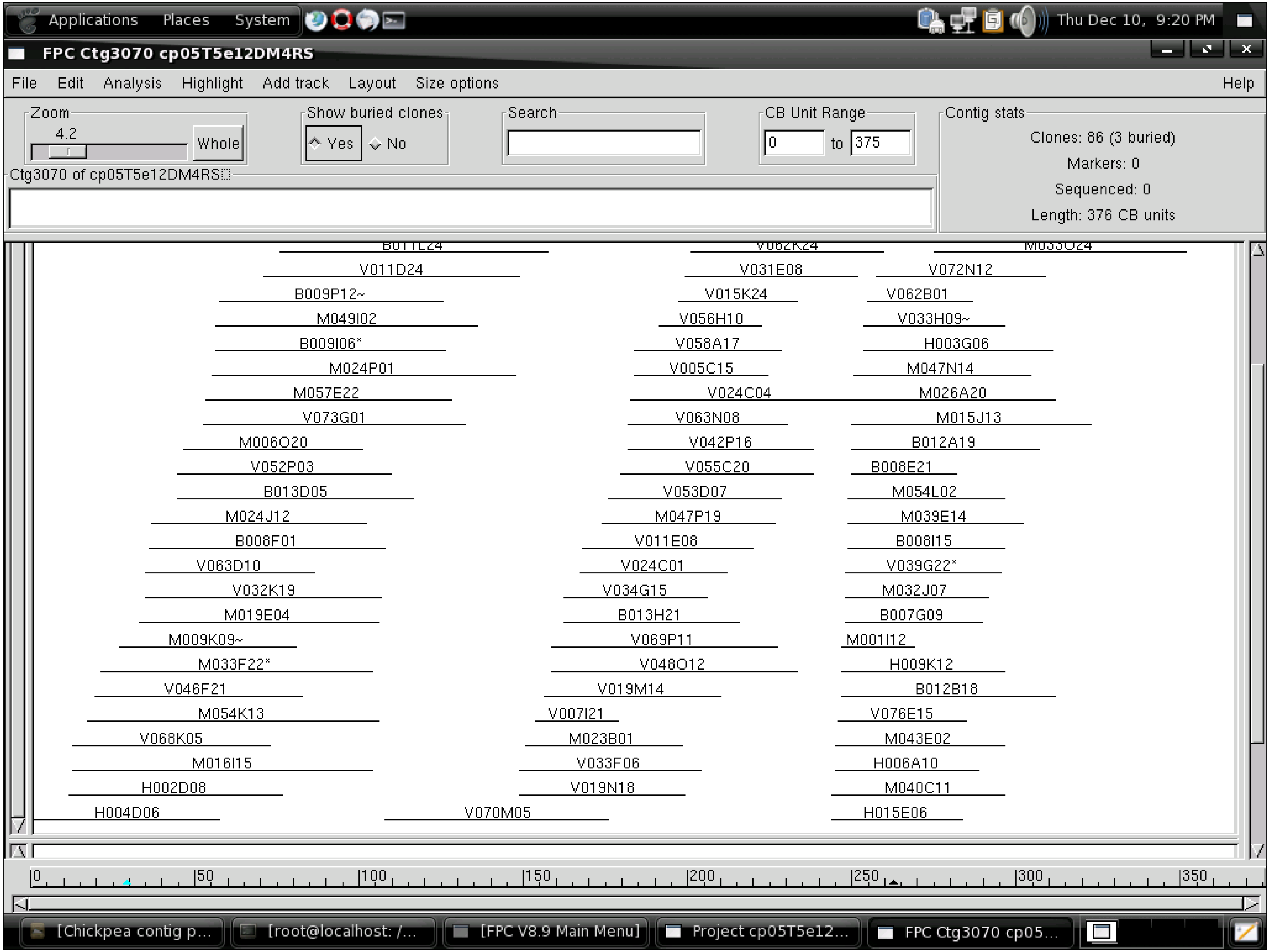

Applications Places System
Thu Dec 10, 9:20 PM
FPC Ctg3070 cp05T5e12DM4RS
File Edit Analysis Highlight Add track Layout Size options Help
Zoom
4.2
Whole
Show buried clones
Yes
No
Search
CB Unit Range
0 to 375
Contig stats
Clones: 86 (3 buried)
Markers: 0
Sequenced: 0
Length: 376 CB units
Ctg3070 of cp05T5e12DM4RS
V011D24
V031E08
V072N12
B009P12~
V015K24
V062B01
M049I02
V056H10
V033H09~
B009I06*
V058A17
H003G06
M024P01
V005C15
M047N14
M057E22
V024C04
M026A20
V073G01
V063N08
M015J13
M006O20
V042P16
B012A19
V052P03
V055C20
B008E21
B013D05
V053D07
M054L02
M024J12
M047P19
M039E14
B008F01
V011E08
B008I15
V063D10
V024C01
V039G22*
V032K19
V034G15
M032J07
M019E04
B013H21
B007G09
M009K09~
V069P11
M001I12
M033F22*
V048O12
H009K12
V046F21
V019M14
B012B18
M054K13
V007I21
V076E15
V068K05
M023B01
M043E02
M016I15
V033F06
H006A10
H002D08
V019N18
M040C11
H004D06
V070M05
H015E06
0 50 100 150 200 250 300 350
[Chickpea contig p...
[root@localhost: /...
[FPC V8.9 Main Menu]
Project cp05T5e12...
FPC Ctg3070 cp05...

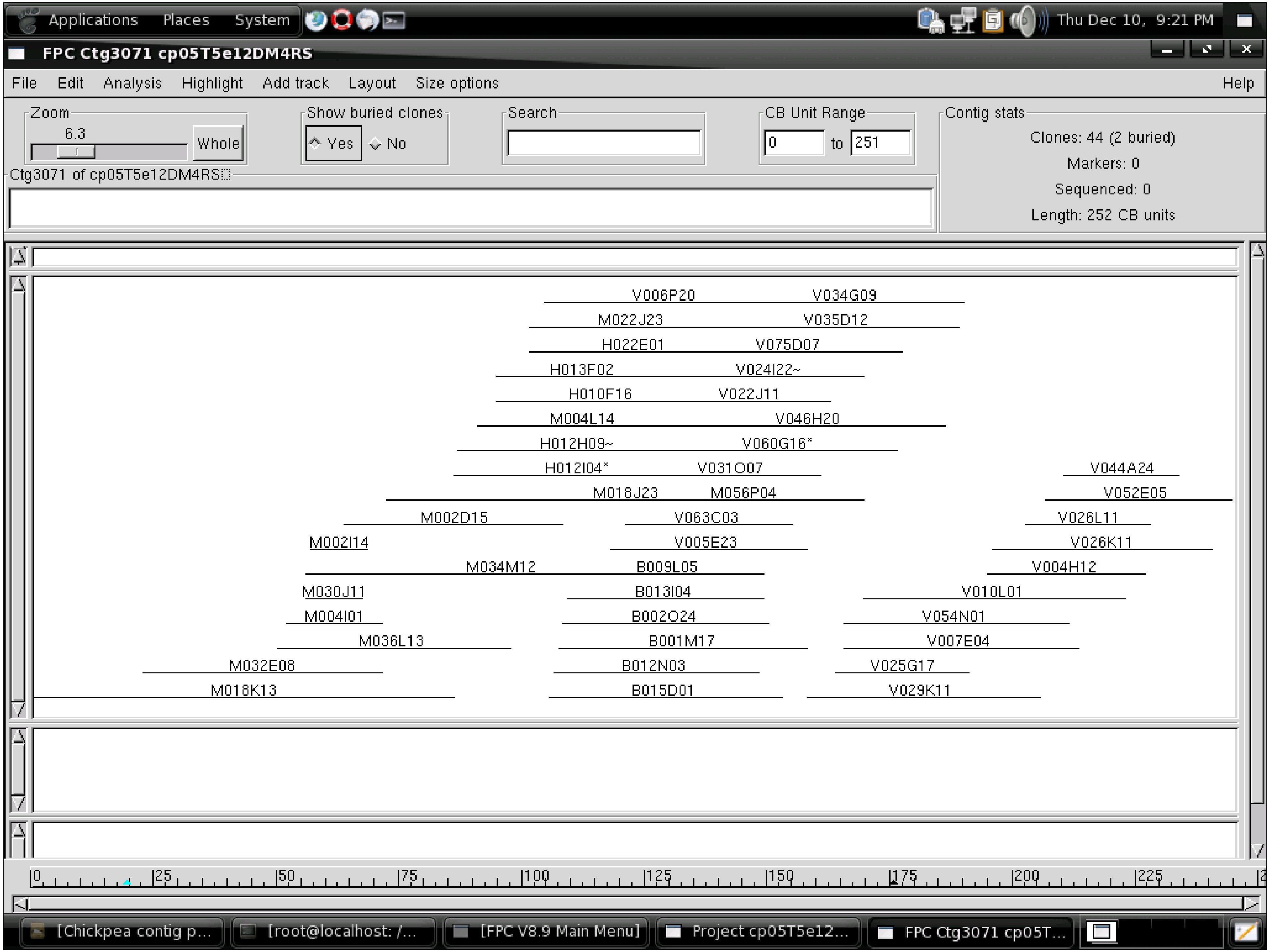
Applications Places System
Thu Dec 10, 9:21 PM
FPC Ctg3071 cp05T5e12DM4RS
File Edit Analysis Highlight Add track Layout Size options Help
Zoom
6.3
Whole
Show buried clones
Yes
No
Search
CB Unit Range
0
to
251
Contig stats
Clones: 44 (2 buried)
Markers: 0
Sequenced: 0
Length: 252 CB units
Ctg3071 of cp05T5e12DM4RS
V006P20
V034G09
M022J23
V035D12
H022E01
V075D07
H013F02
V024I22~
H010F16
V022J11
M004L14
V046H20
H012H09~
V060G16*
H012I04*
V031O07
V044A24
M018J23
M056P04
V052E05
M002D15
V063C03
V026L11
M002I14
V005E23
V026K11
M034M12
B009L05
V004H12
M030J11
B013I04
V010L01
M004I01
B002O24
V054N01
M036L13
B001M17
V007E04
M032E08
B012N03
V025G17
M018K13
B015D01
V029K11
0 25 50 75 100 125 150 175 200 225
[Chickpea contig p...
[root@localhost: /...
[FPC V8.9 Main Menu]
Project cp05T5e12...
FPC Ctg3071 cp05T...

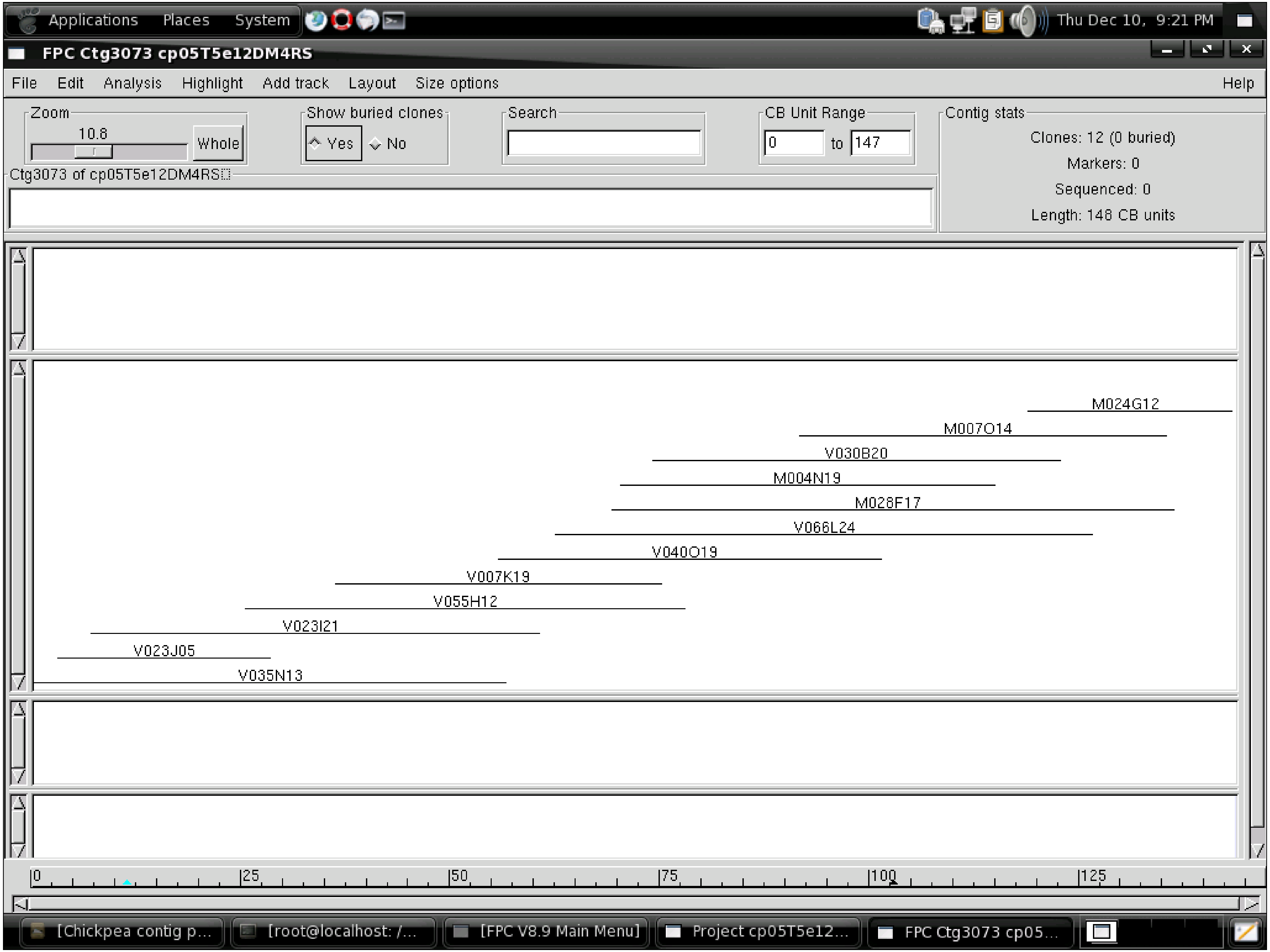
Applications
Places
System
Thu Dec 10, 9:21 PM
FPC Ctg3073 cp05T5e12DM4RS
File
Edit
Analysis
Highlight
Add track
Layout
Size options
Help
Zoom
10.8
Whole
Show buried clones
Yes
No
Search
CB Unit Range
0
to
147
Contig stats
Clones: 12 (0 buried)
Markers: 0
Sequenced: 0
Length: 148 CB units
Ctg3073 of cp05T5e12DM4RS
M024G12
M007O14
V030B20
M004N19
M028F17
V066L24
V040O19
V007K19
V055H12
V023I21
V023J05
V035N13
0
25
50
75
100
125
[Chickpea contig p...
[root@localhost: /...
[FPC V8.9 Main Menu]
Project cp05T5e12...
FPC Ctg3073 cp05...

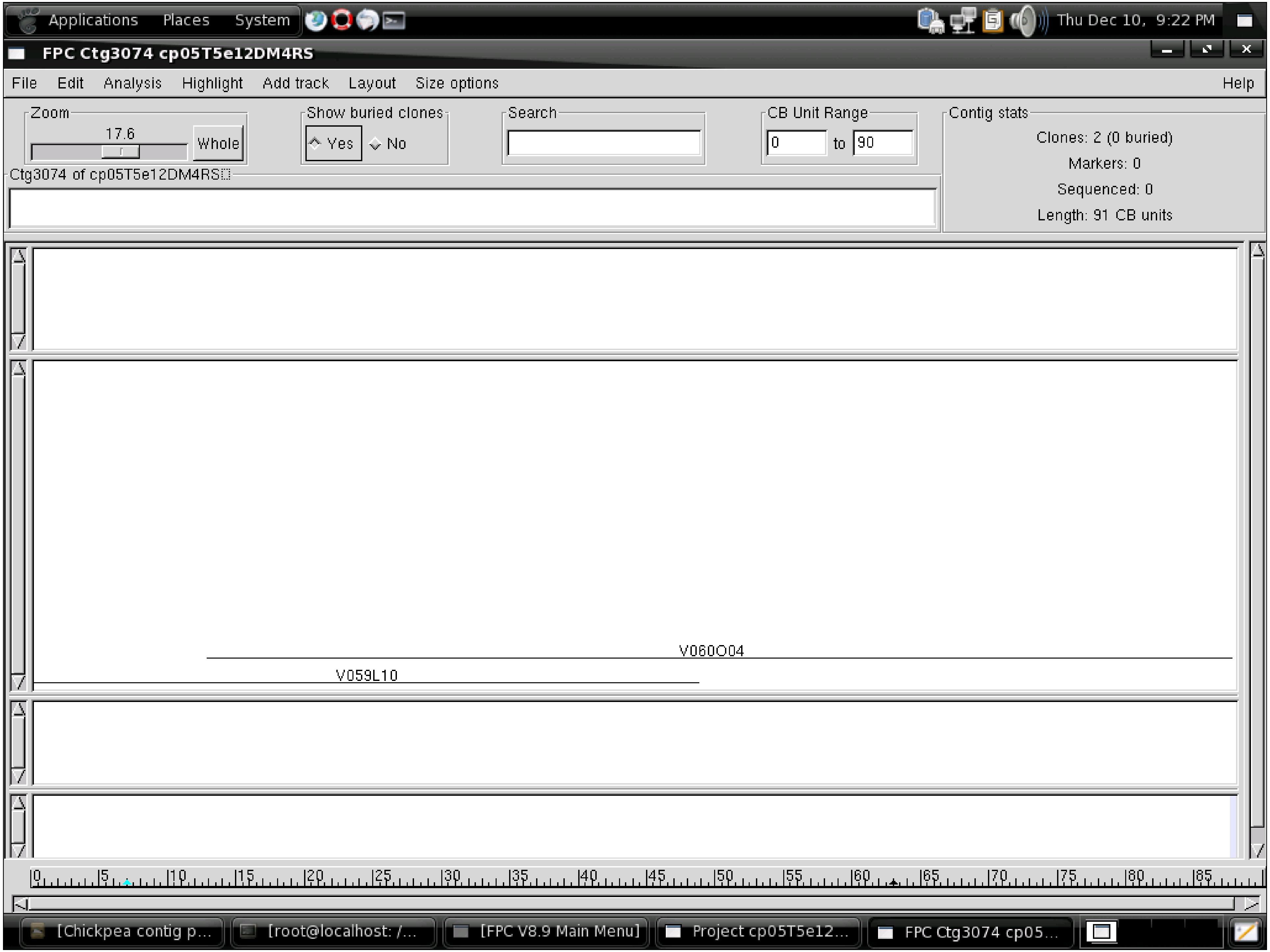
Applications
Places
System
Thu Dec 10, 9:22 PM
FPC Ctg3074 cp05T5e12DM4RS
File
Edit
Analysis
Highlight
Add track
Layout
Size options
Help
Zoom
17.6
Whole
Show buried clones
Yes
No
Search
CB Unit Range
0
to
90
Contig stats
Clones: 2 (0 buried)
Markers: 0
Sequenced: 0
Length: 91 CB units
Ctg3074 of cp05T5e12DM4RS
V060O04
V059L10
0 5 10 15 20 25 30 35 40 45 50 55 60 65 70 75 80 85
[Chickpea contig p...
[root@localhost: /...
[FPC V8.9 Main Menu]
Project cp05T5e12...
FPC Ctg3074 cp05...

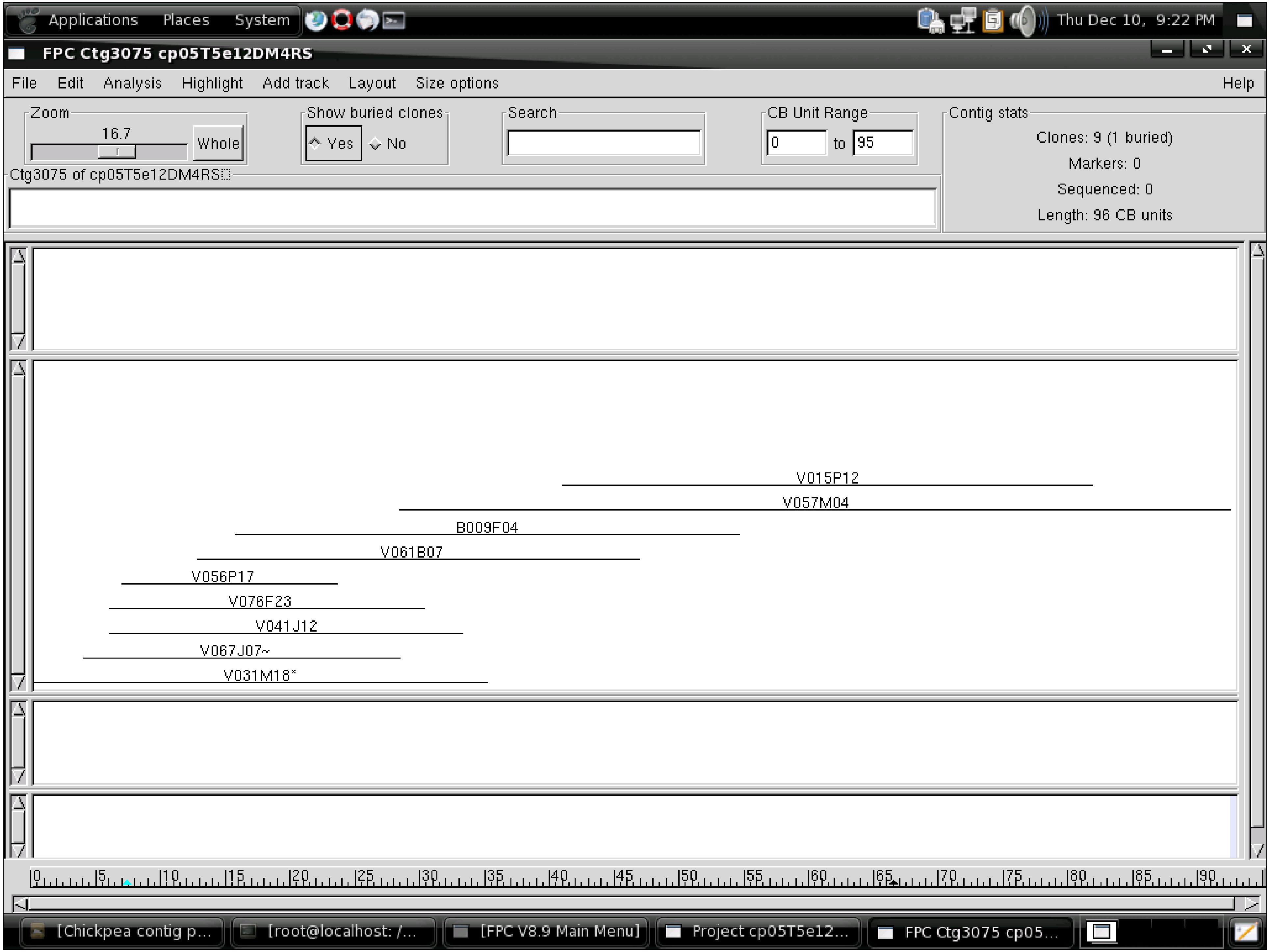
Applications Places System
Thu Dec 10, 9:22 PM
FPC Ctg3075 cp05T5e12DM4RS
File Edit Analysis Highlight Add track Layout Size options Help
Zoom
16.7
Whole
Show buried clones
Yes No
Search
CB Unit Range
0 to 95
Contig stats
Clones: 9 (1 buried)
Markers: 0
Sequenced: 0
Length: 96 CB units
Ctg3075 of cp05T5e12DM4RS
V015P12
V057M04
B009F04
V061B07
V056P17
V076F23
V041J12
V067J07~
V031M18*
0 5 10 15 20 25 30 35 40 45 50 55 60 65 70 75 80 85 90
[Chickpea contig p...
[root@localhost: /...
[FPC V8.9 Main Menu]
Project cp05T5e12...
FPC Ctg3075 cp05...

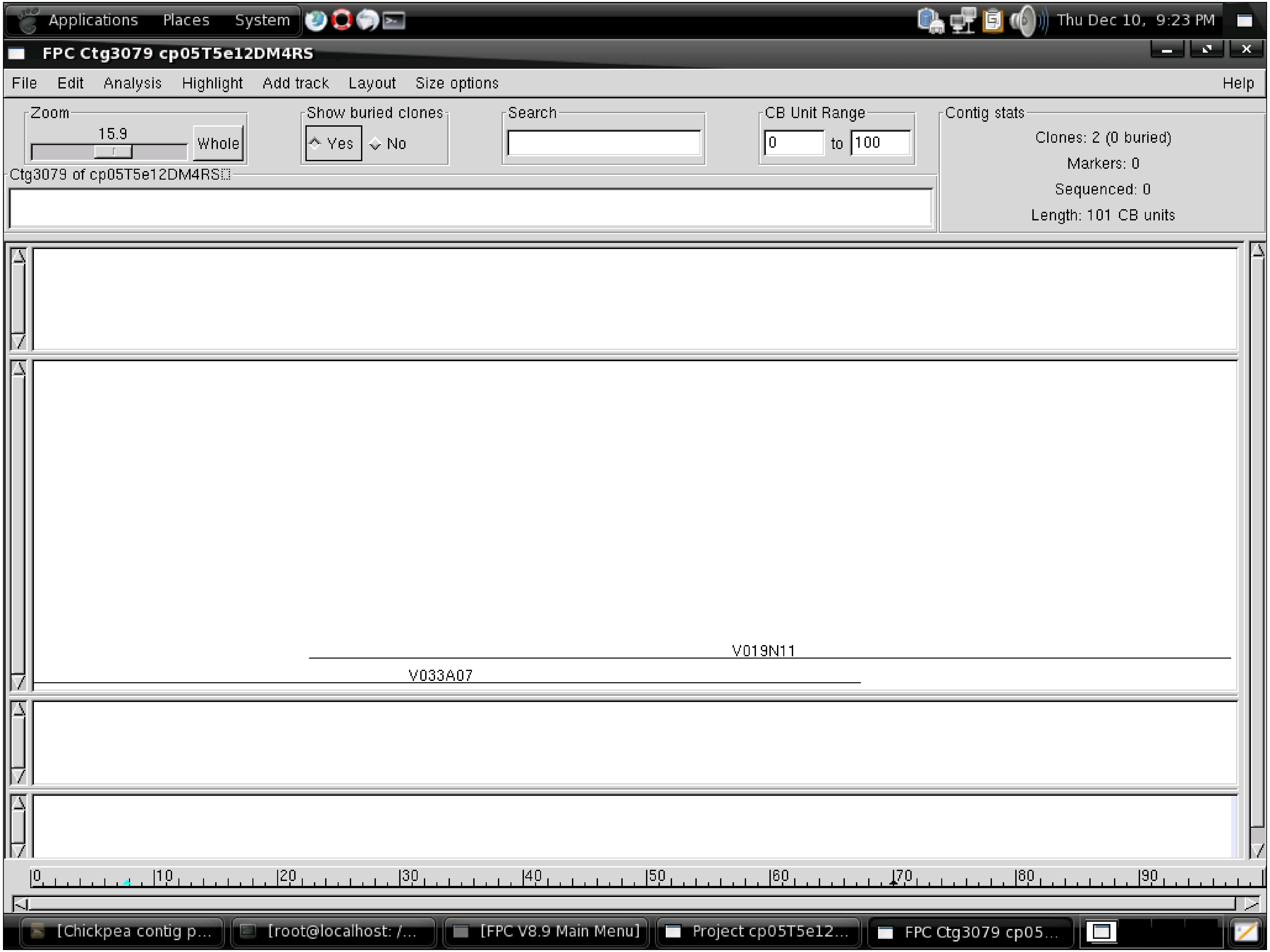
Applications Places System
Thu Dec 10, 9:23 PM
FPC Ctg3079 cp05T5e12DM4RS
File Edit Analysis Highlight Add track Layout Size options
Help
Zoom
15.9
Whole
Show buried clones
Yes
No
Search
CB Unit Range
0
to
100
Contig stats
Clones: 2 (0 buried)
Markers: 0
Sequenced: 0
Length: 101 CB units
Ctg3079 of cp05T5e12DM4RS
V019N11
V033A07
0 10 20 30 40 50 60 70 80 90
[Chickpea contig p...
[root@localhost: /...
[FPC V8.9 Main Menu]
Project cp05T5e12...
FPC Ctg3079 cp05...

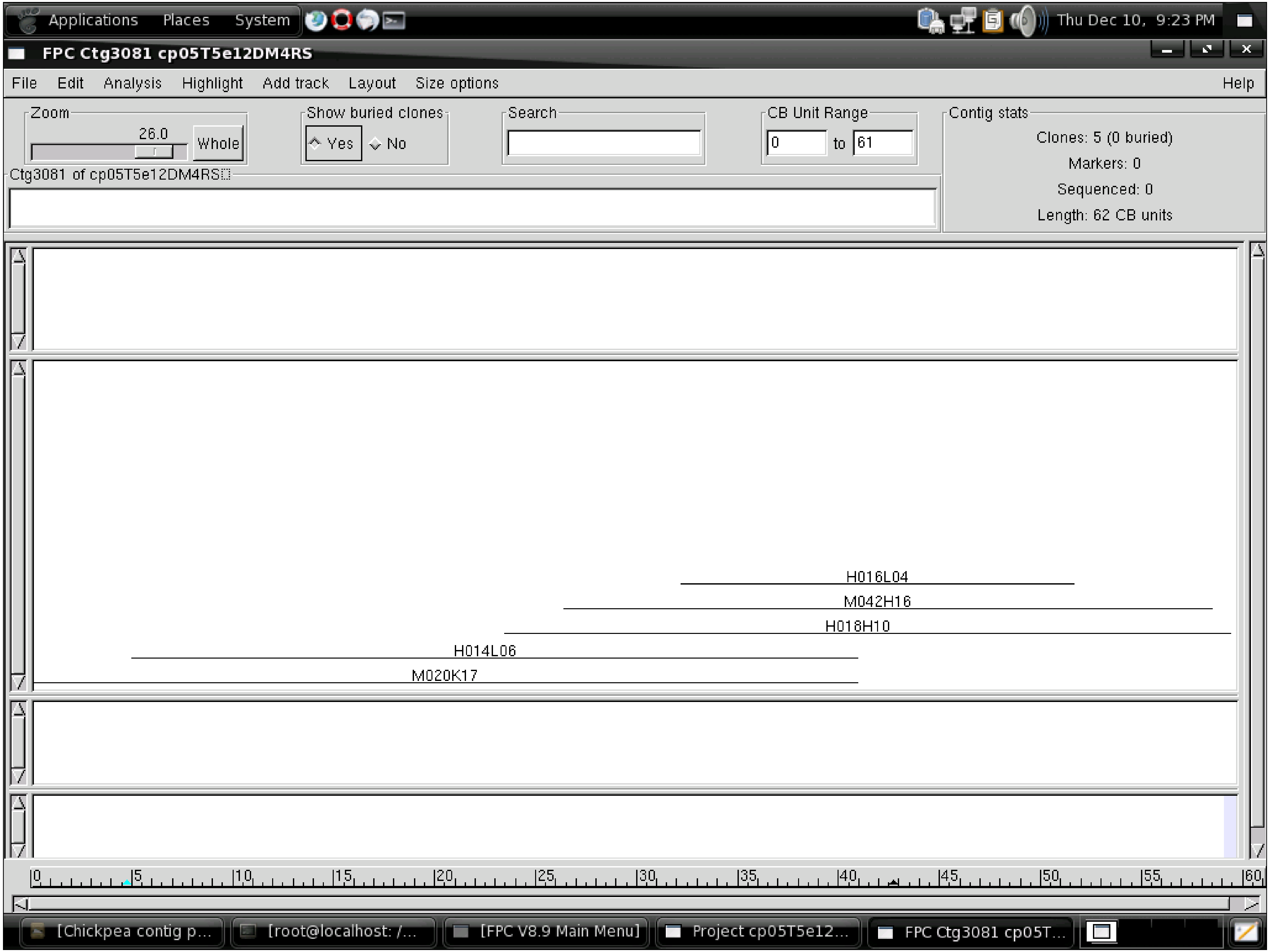
Applications
Places
System
Thu Dec 10, 9:23 PM
FPC Ctg3081 cp05T5e12DM4RS
File
Edit
Analysis
Highlight
Add track
Layout
Size options
Help
Zoom
26.0
Whole
Show buried clones
Yes
No
Search
CB Unit Range
0
to
61
Contig stats
Clones: 5 (0 buried)
Markers: 0
Sequenced: 0
Length: 62 CB units
Ctg3081 of cp05T5e12DM4RS
H016L04
M042H16
H018H10
H014L06
M020K17
0 5 10 15 20 25 30 35 40 45 50 55 60
[Chickpea contig p...
[root@localhost: /...
[FPC V8.9 Main Menu]
Project cp05T5e12...
FPC Ctg3081 cp05T...

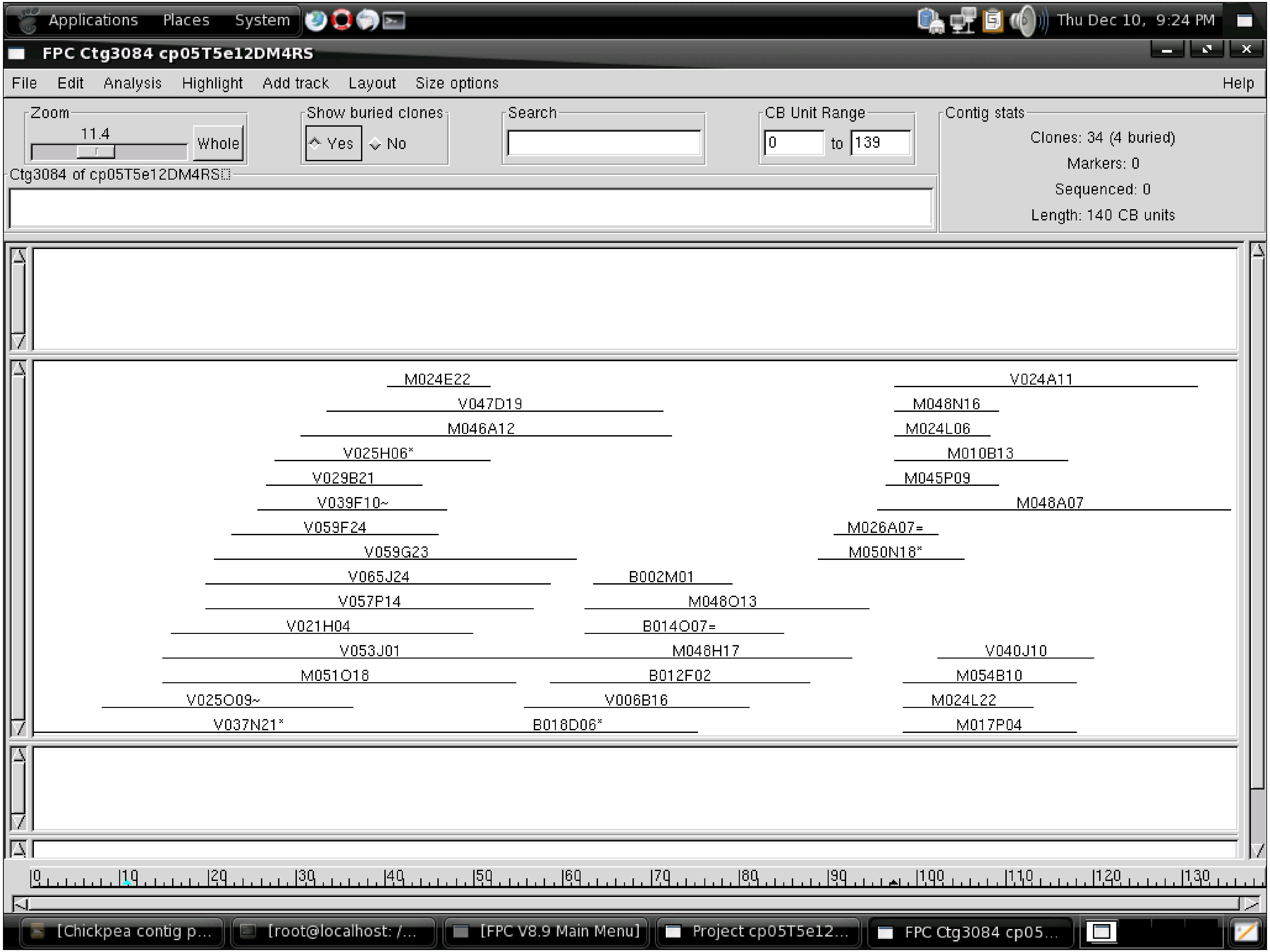
Applications
Places
System
Thu Dec 10, 9:24 PM
FPC Ctg3084 cp05T5e12DM4RS
File
Edit
Analysis
Highlight
Add track
Layout
Size options
Help
Zoom
11.4
Whole
Show buried clones
Yes
No
Search
CB Unit Range
0
to
139
Contig stats
Clones: 34 (4 buried)
Markers: 0
Sequenced: 0
Length: 140 CB units
Ctg3084 of cp05T5e12DM4RS
M024E22
V047D19
M046A12
V025H06*
V029B21
V039F10~
V059F24
V059G23
V065J24
V057P14
V021H04
V053J01
M051O18
V025O09~
V037N21*
B002M01
M048O13
B014O07=
M048H17
B012F02
V006B16
B018D06*
V024A11
M048N16
M024L06
M010B13
M045P09
M048A07
M026A07=
M050N18*
V040J10
M054B10
M024L22
M017P04
0
10
20
30
40
50
60
70
80
90
100
110
120
130
[Chickpea contig p...
[root@localhost: /...
[FPC V8.9 Main Menu]
Project cp05T5e12...
FPC Ctg3084 cp05...

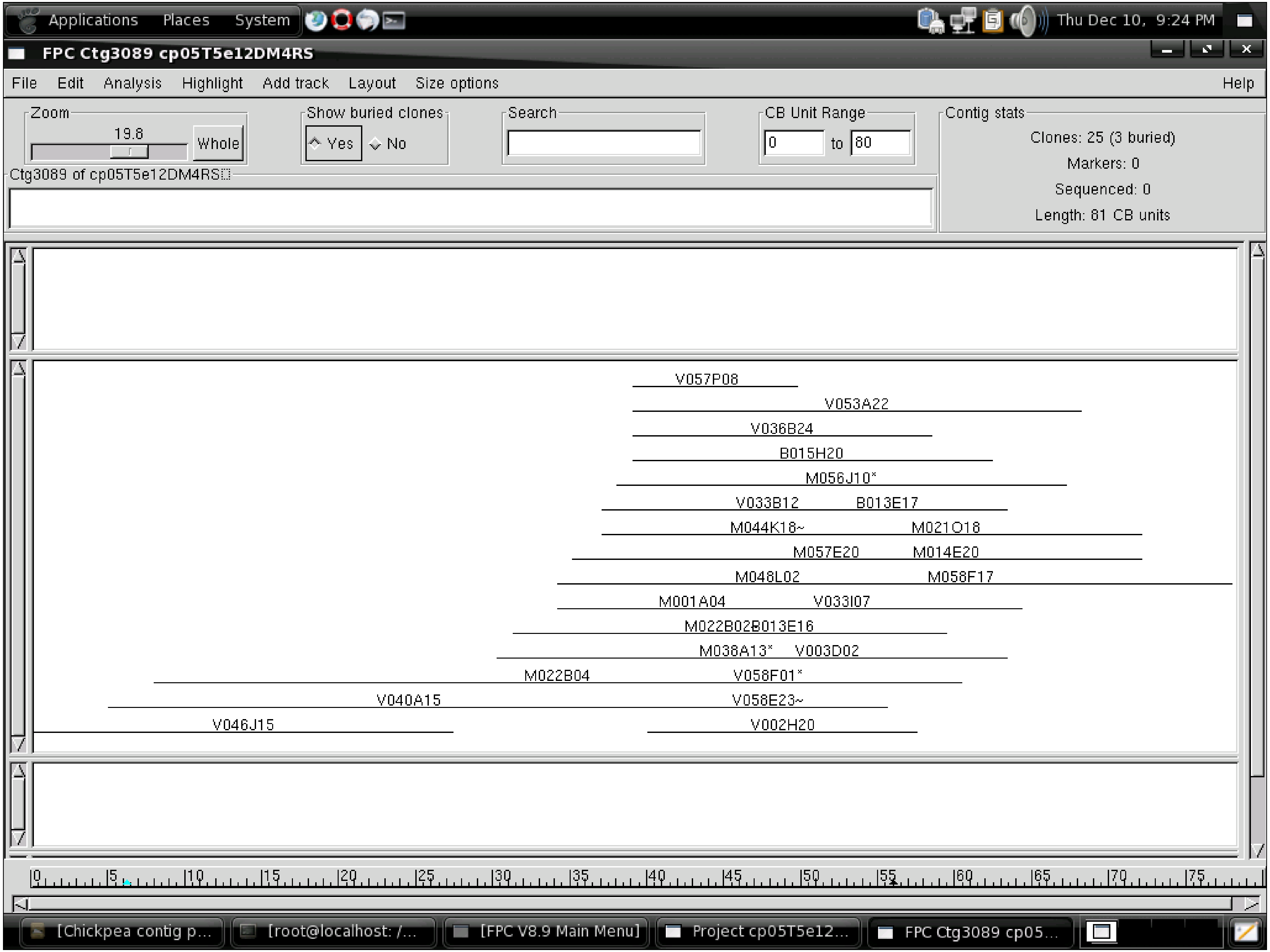
Applications Places System
Thu Dec 10, 9:24 PM
FPC Ctg3089 cp05T5e12DM4RS
File Edit Analysis Highlight Add track Layout Size options Help
Zoom
19.8
Whole
Show buried clones
Yes No
Search
CB Unit Range
0 to 80
Contig stats
Clones: 25 (3 buried)
Markers: 0
Sequenced: 0
Length: 81 CB units
Ctg3089 of cp05T5e12DM4RS
V057P08
V053A22
V036B24
B015H20
M056J10*
V033B12 B013E17
M044K18~ M021O18
M057E20 M014E20
M048L02 M058F17
M001A04 V033I07
M022B02B013E16
M038A13* V003D02
M022B04 V058F01*
V040A15 V058E23~
V046J15 V002H20
0 5 10 15 20 25 30 35 40 45 50 55 60 65 70 75
[Chickpea contig p...
[root@localhost: /...
[FPC V8.9 Main Menu]
Project cp05T5e12...
FPC Ctg3089 cp05...

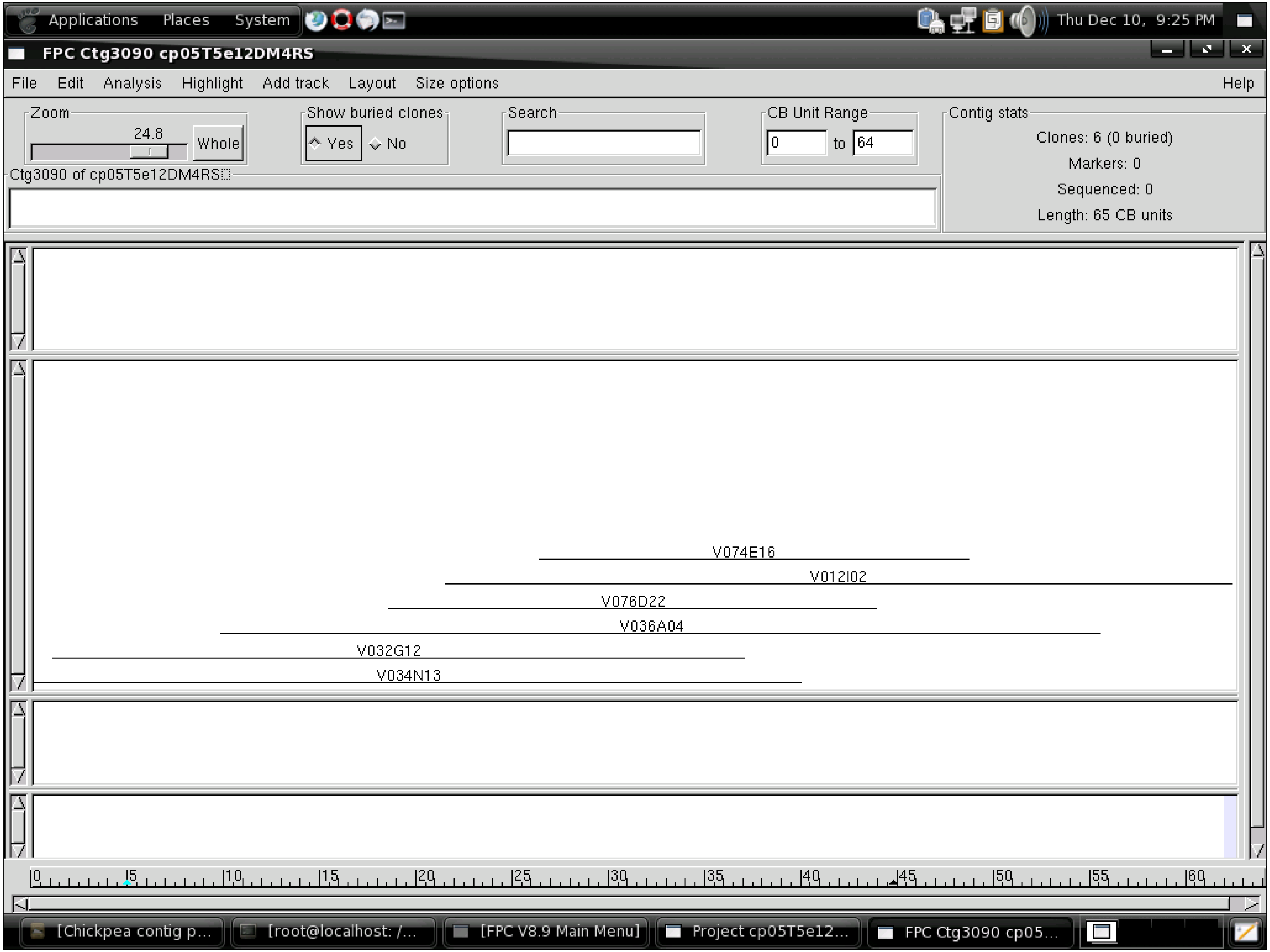
Applications Places System
Thu Dec 10, 9:25 PM
FPC Ctg3090 cp05T5e12DM4RS
File Edit Analysis Highlight Add track Layout Size options Help
Zoom
24.8
Whole
Show buried clones
Yes
No
Search
CB Unit Range
0
to
64
Contig stats
Clones: 6 (0 buried)
Markers: 0
Sequenced: 0
Length: 65 CB units
Ctg3090 of cp05T5e12DM4RS
V074E16
V012I02
V076D22
V036A04
V032G12
V034N13
0 5 10 15 20 25 30 35 40 45 50 55 60
[Chickpea contig p...
[root@localhost: /...
[FPC V8.9 Main Menu]
Project cp05T5e12...
FPC Ctg3090 cp05...

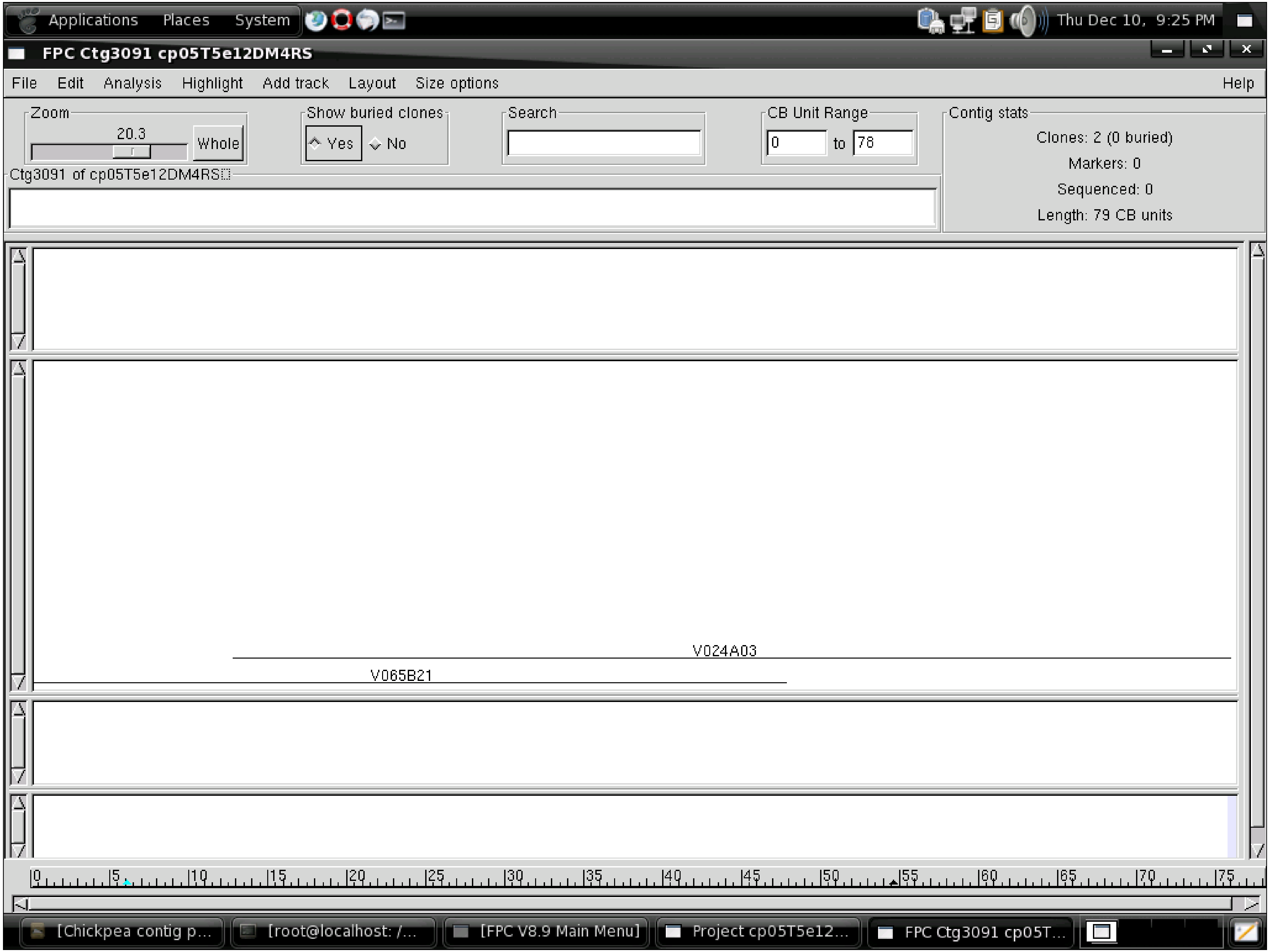
Applications
Places
System
Thu Dec 10, 9:25 PM
FPC Ctg3091 cp05T5e12DM4RS
File
Edit
Analysis
Highlight
Add track
Layout
Size options
Help
Zoom
20.3
Whole
Show buried clones
Yes
No
Search
CB Unit Range
0
to
78
Contig stats
Clones: 2 (0 buried)
Markers: 0
Sequenced: 0
Length: 79 CB units
Ctg3091 of cp05T5e12DM4RS
V024A03
V065B21
0 5 10 15 20 25 30 35 40 45 50 55 60 65 70 75
[Chickpea contig p...
[root@localhost: /...
[FPC V8.9 Main Menu]
Project cp05T5e12...
FPC Ctg3091 cp05T...

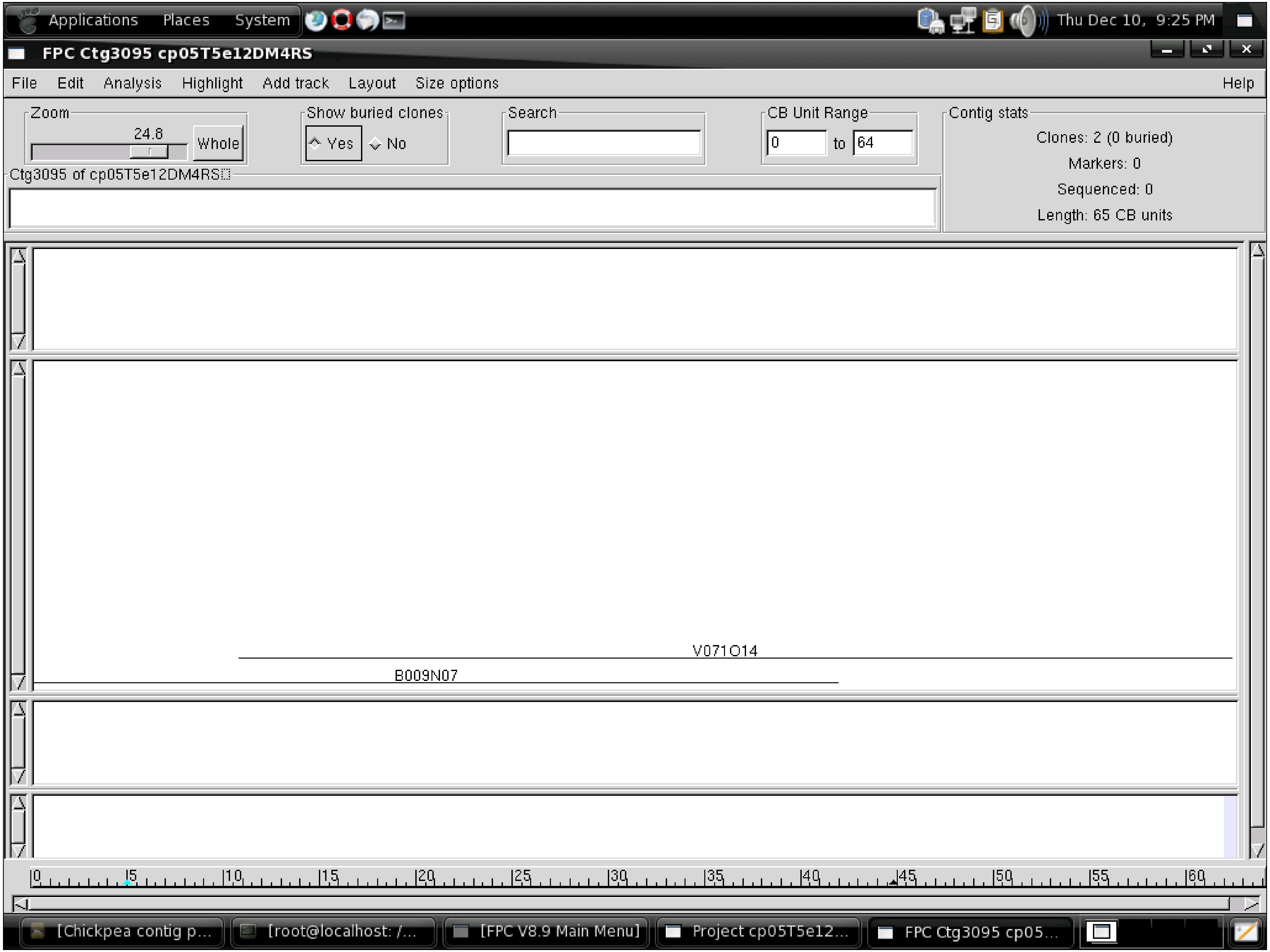
Applications
Places
System
Thu Dec 10, 9:25 PM
FPC Ctg3095 cp05T5e12DM4RS
File
Edit
Analysis
Highlight
Add track
Layout
Size options
Help
Zoom
24.8
Whole
Show buried clones
Yes
No
Search
CB Unit Range
0
to
64
Contig stats
Clones: 2 (0 buried)
Markers: 0
Sequenced: 0
Length: 65 CB units
Ctg3095 of cp05T5e12DM4RS
V071O14
B009N07
0
5
10
15
20
25
30
35
40
45
50
55
60
[Chickpea contig p...
[root@localhost: /...
[FPC V8.9 Main Menu]
Project cp05T5e12...
FPC Ctg3095 cp05...

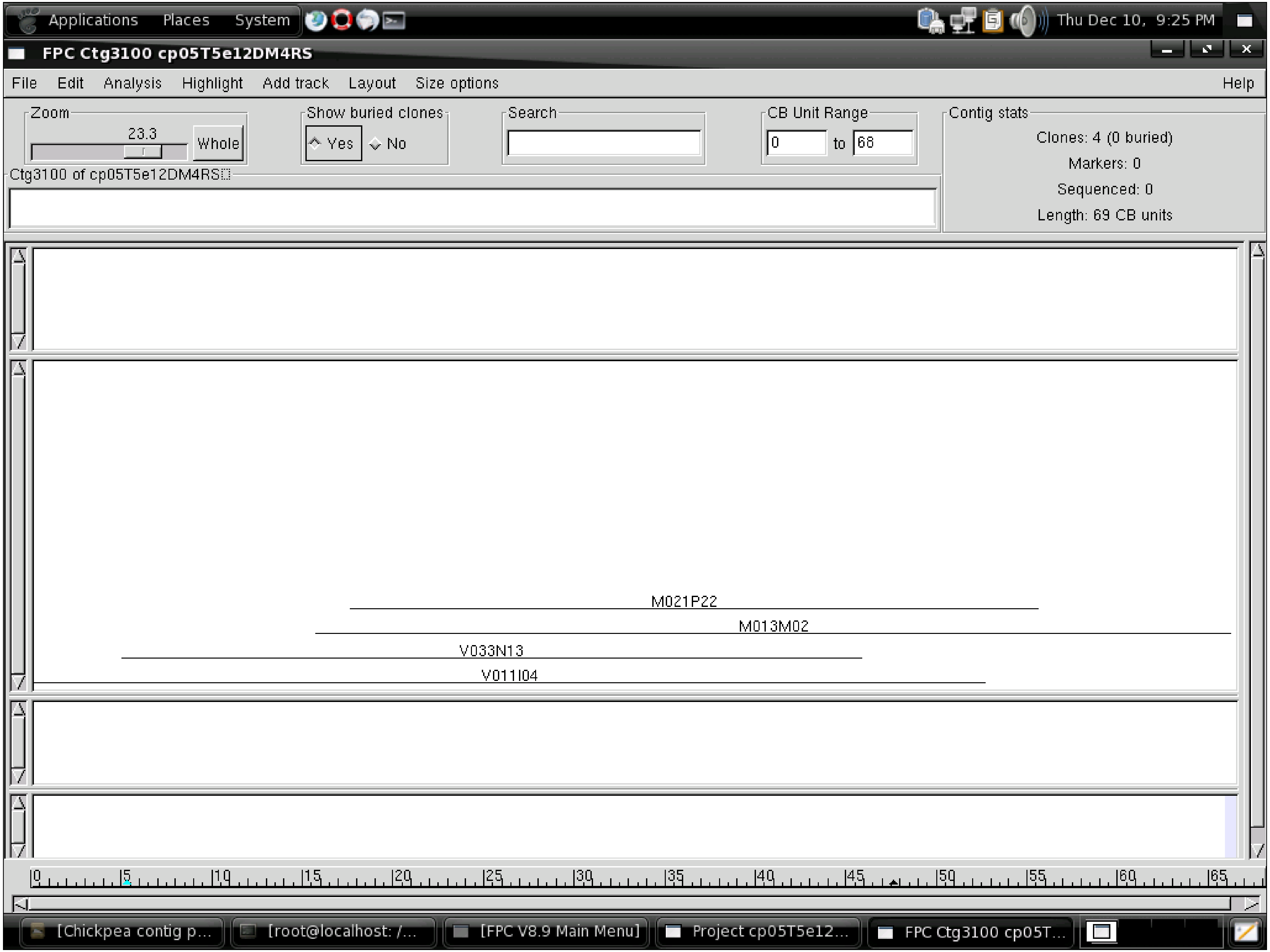
Applications Places System
Thu Dec 10, 9:25 PM
FPC Ctg3100 cp05T5e12DM4RS
File Edit Analysis Highlight Add track Layout Size options Help
Zoom
23.3
Whole
Show buried clones
Yes
No
Search
CB Unit Range
0 to 68
Contig stats
Clones: 4 (0 buried)
Markers: 0
Sequenced: 0
Length: 69 CB units
Ctg3100 of cp05T5e12DM4RS
M021P22
M013M02
V033N13
V011I04
0 5 10 15 20 25 30 35 40 45 50 55 60 65
[Chickpea contig p...
[root@localhost: /...
[FPC V8.9 Main Menu]
Project cp05T5e12...
FPC Ctg3100 cp05T...

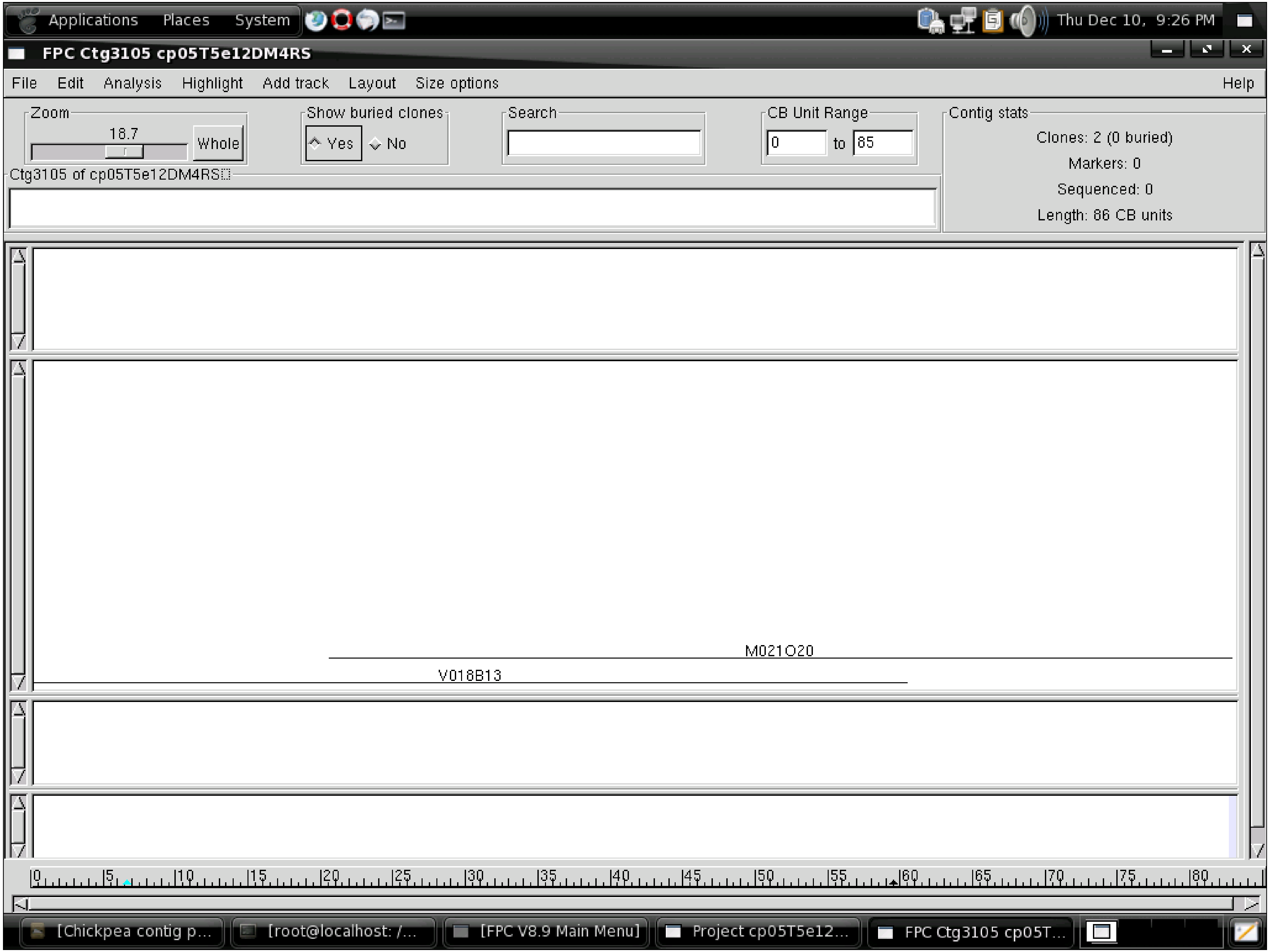
Applications Places System
Thu Dec 10, 9:26 PM
FPC Ctg3105 cp05T5e12DM4RS
File Edit Analysis Highlight Add track Layout Size options Help
Zoom
18.7
Whole
Show buried clones
Yes
No
Search
CB Unit Range
0 to 85
Contig stats
Clones: 2 (0 buried)
Markers: 0
Sequenced: 0
Length: 86 CB units
Ctg3105 of cp05T5e12DM4RS
M021O20
V018B13
0 5 10 15 20 25 30 35 40 45 50 55 60 65 70 75 80
[Chickpea contig p...
[root@localhost: /...
[FPC V8.9 Main Menu]
Project cp05T5e12...
FPC Ctg3105 cp05T...

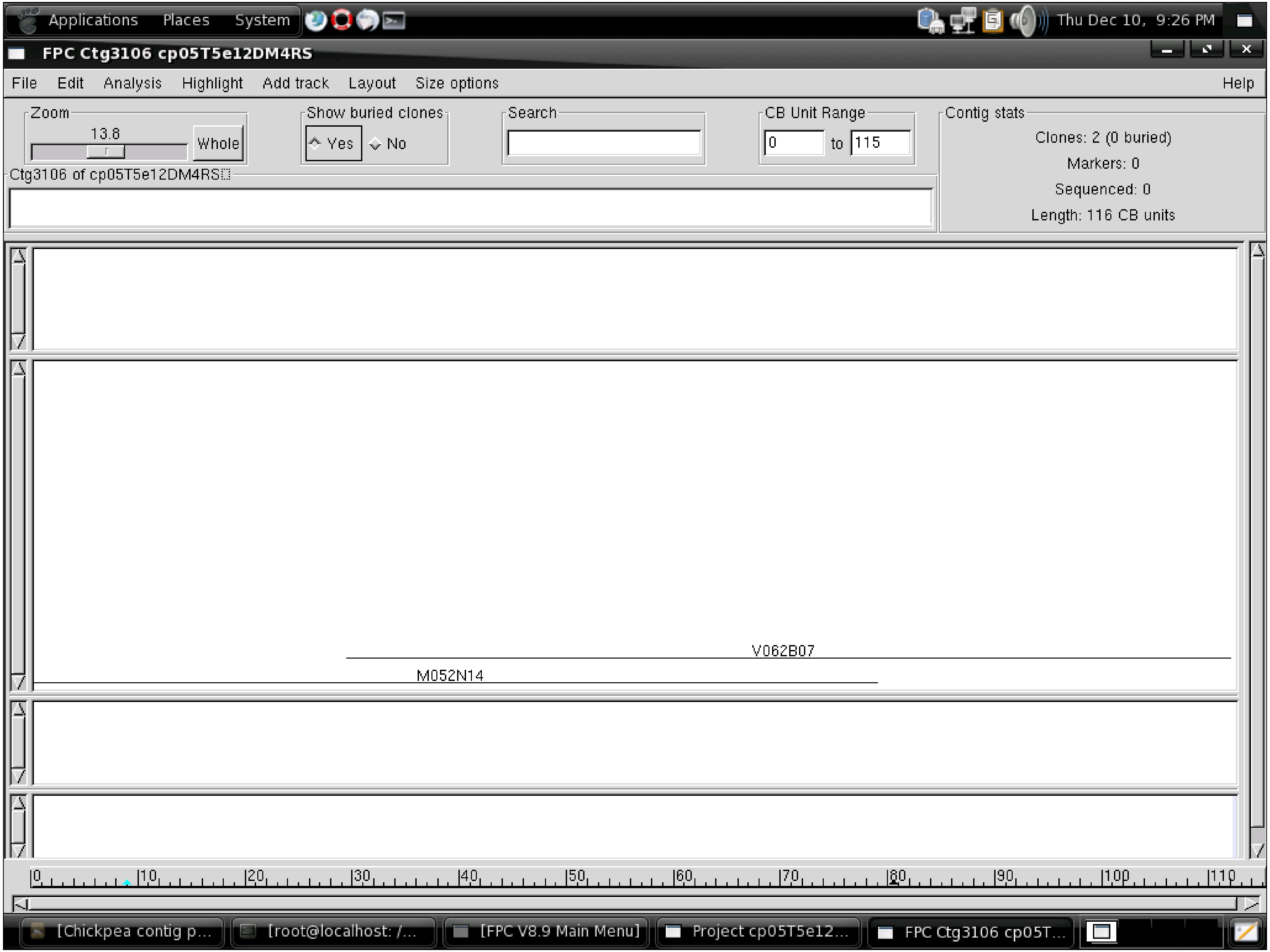

Applications Places System
Thu Dec 10, 9:26 PM
FPC Ctg3106 cp05T5e12DM4RS
File Edit Analysis Highlight Add track Layout Size options
Help
Zoom
13.8
Whole
Show buried clones
Yes
No
Search
CB Unit Range
0
to
115
Contig stats
Clones: 2 (0 buried)
Markers: 0
Sequenced: 0
Length: 116 CB units
Ctg3106 of cp05T5e12DM4RS
V062B07
M052N14
0 10 20 30 40 50 60 70 80 90 100 110
[Chickpea contig p...
[root@localhost: /...
[FPC V8.9 Main Menu]
Project cp05T5e12...
FPC Ctg3106 cp05T...

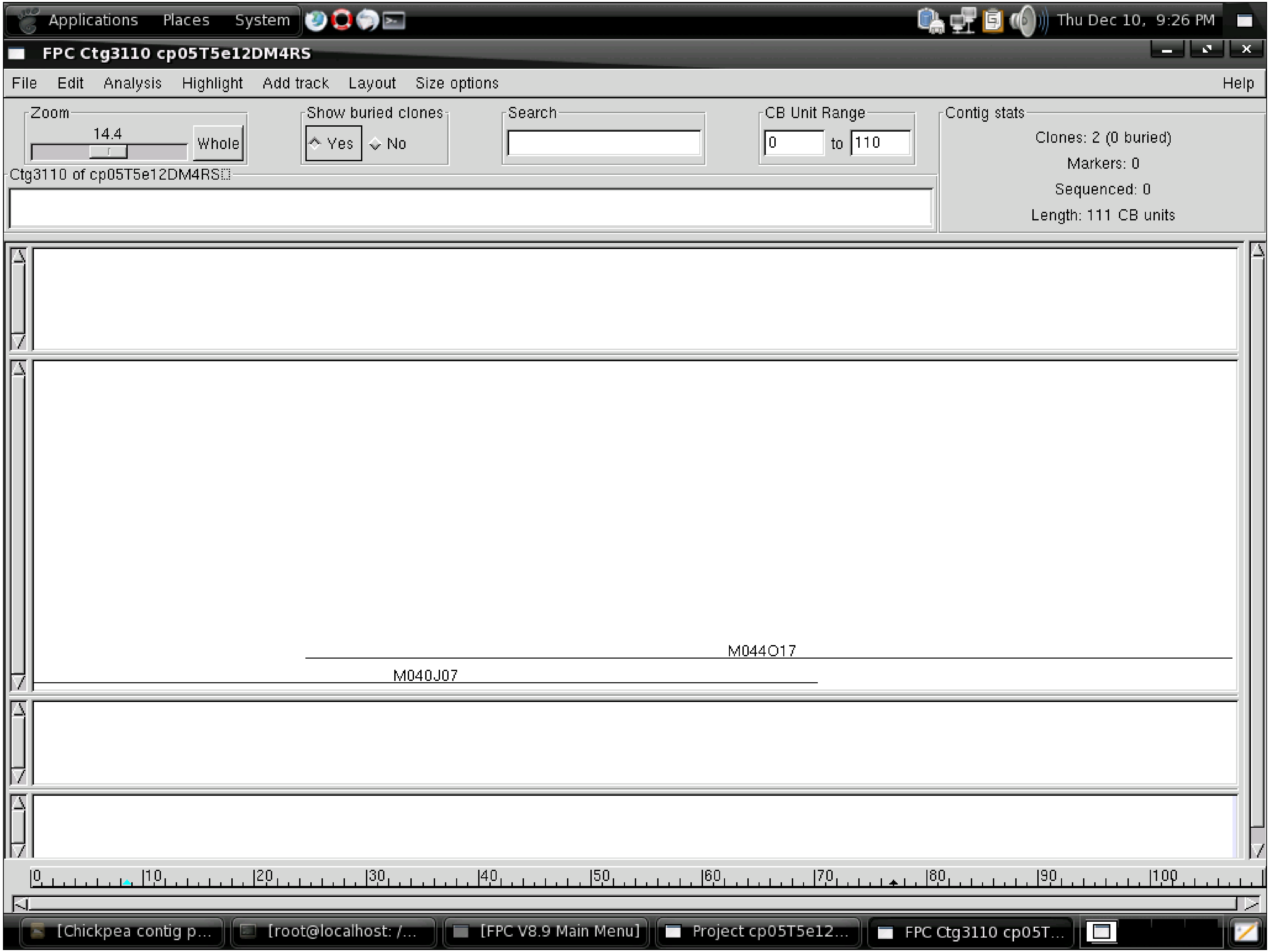
Applications Places System
Thu Dec 10, 9:26 PM
FPC Ctg3110 cp05T5e12DM4RS
File Edit Analysis Highlight Add track Layout Size options
Help
Zoom
14.4
Whole
Show buried clones
Yes
No
Search
CB Unit Range
0
to
110
Contig stats
Clones: 2 (0 buried)
Markers: 0
Sequenced: 0
Length: 111 CB units
Ctg3110 of cp05T5e12DM4RS
M044O17
M040J07
0 10 20 30 40 50 60 70 80 90 100
[Chickpea contig p...
[root@localhost: /...
[FPC V8.9 Main Menu]
Project cp05T5e12...
FPC Ctg3110 cp05T...

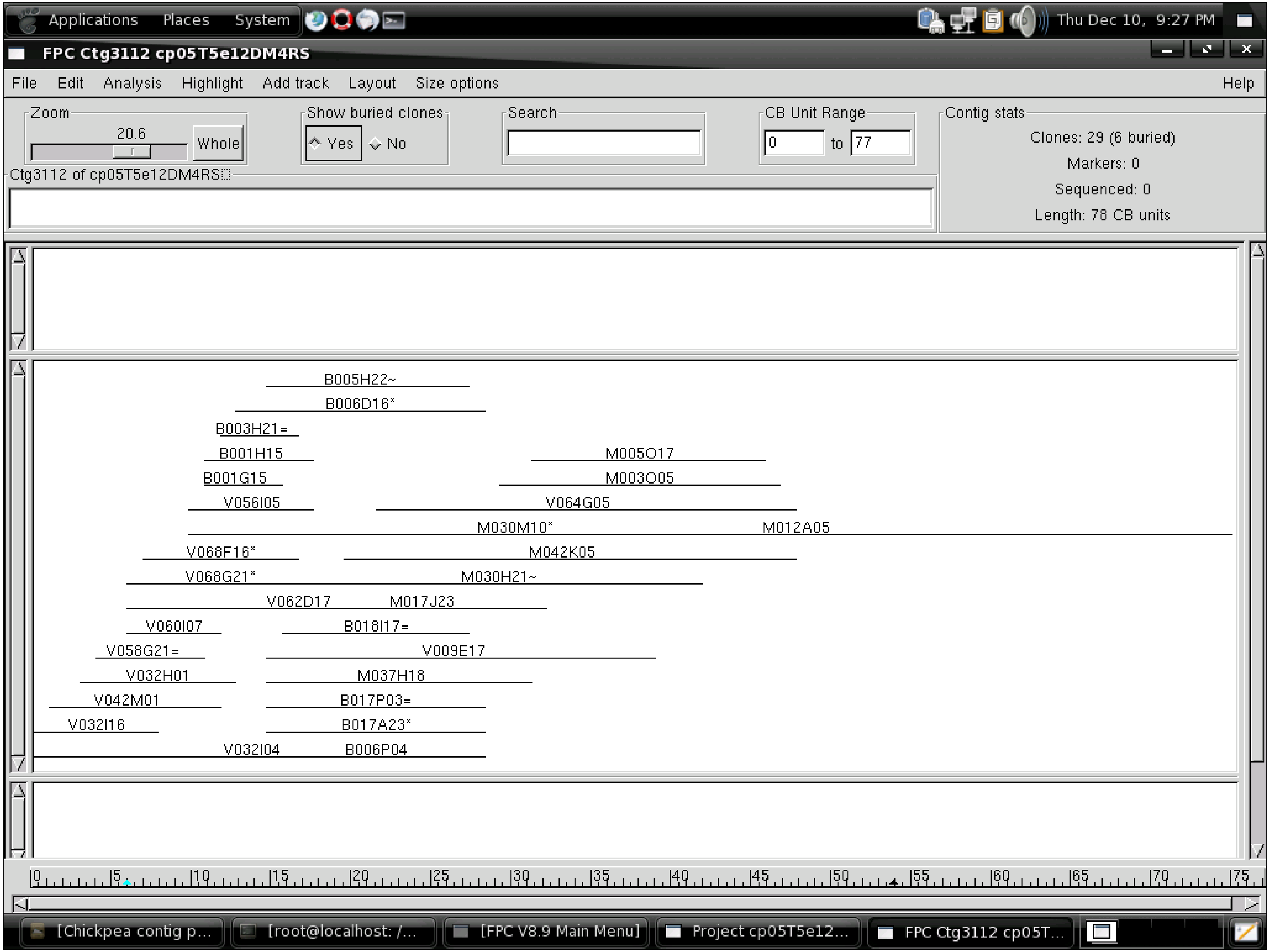
Applications Places System
Thu Dec 10, 9:27 PM
FPC Ctg3112 cp05T5e12DM4RS
File Edit Analysis Highlight Add track Layout Size options Help
Zoom
20.6
Whole
Show buried clones
Yes
No
Search
CB Unit Range
0 to 77
Contig stats
Clones: 29 (6 buried)
Markers: 0
Sequenced: 0
Length: 78 CB units
Ctg3112 of cp05T5e12DM4RS
B005H22~
B006D16*
B003H21=
B001H15
M005O17
B001G15
M003O05
V056I05
V064G05
M030M10*
M012A05
V068F16*
M042K05
V068G21*
M030H21~
V062D17
M017J23
V060I07
B018I17=
V058G21=
V009E17
V032H01
M037H18
V042M01
B017P03=
V032I16
B017A23*
V032I04
B006P04
0 5 10 15 20 25 30 35 40 45 50 55 60 65 70 75
[Chickpea contig p...
[root@localhost: /...
[FPC V8.9 Main Menu]
Project cp05T5e12...
FPC Ctg3112 cp05T...

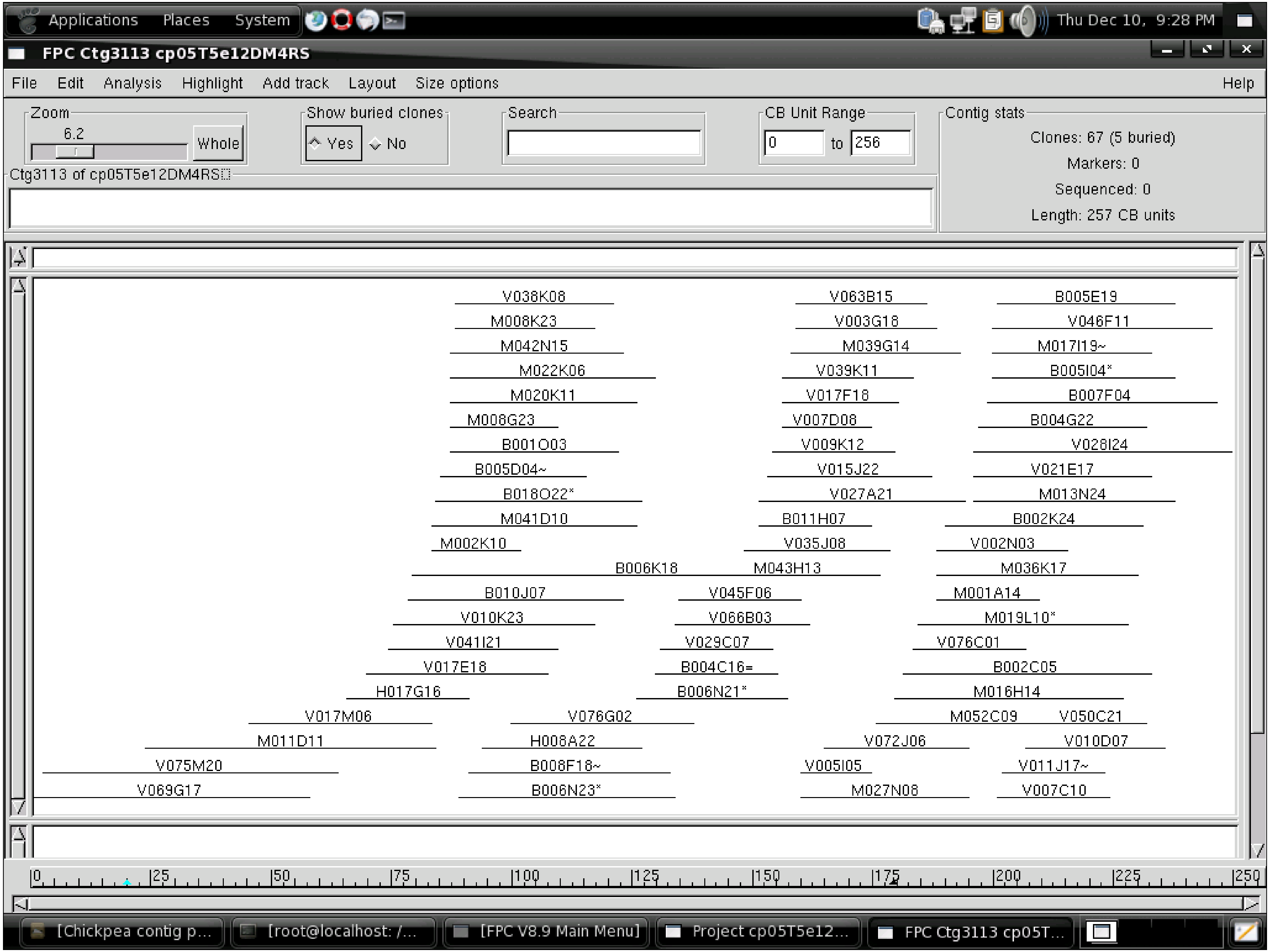
Applications Places System
Thu Dec 10, 9:28 PM
FPC Ctg3113 cp05T5e12DM4RS
File Edit Analysis Highlight Add track Layout Size options Help
Zoom
6.2
Whole
Show buried clones
Yes No
Search
CB Unit Range
0 to 256
Contig stats
Clones: 67 (5 buried)
Markers: 0
Sequenced: 0
Length: 257 CB units
Ctg3113 of cp05T5e12DM4RS
V038K08 V063B15 B005E19
M008K23 V003G18 V046F11
M042N15 M039G14 M017I19~
M022K06 V039K11 B005I04*
M020K11 V017F18 B007F04
M008G23 V007D08 B004G22
B001O03 V009K12 V028I24
B005D04~ V015J22 V021E17
B018O22* V027A21 M013N24
M041D10 B011H07 B002K24
M002K10 V035J08 V002N03
B006K18 M043H13 M036K17
B010J07 V045F06 M001A14
V010K23 V066B03 M019L10*
V041I21 V029C07 V076C01
V017E18 B004C16= B002C05
H017G16 B006N21* M016H14
V017M06 V076G02 M052C09 V050C21
M011D11 H008A22 V072J06 V010D07
V075M20 B008F18~ V005I05 V011J17~
V069G17 B006N23* M027N08 V007C10
0 25 50 75 100 125 150 175 200 225 250
[Chickpea contig p...
[root@localhost: /...
[FPC V8.9 Main Menu]
Project cp05T5e12...
FPC Ctg3113 cp05T...

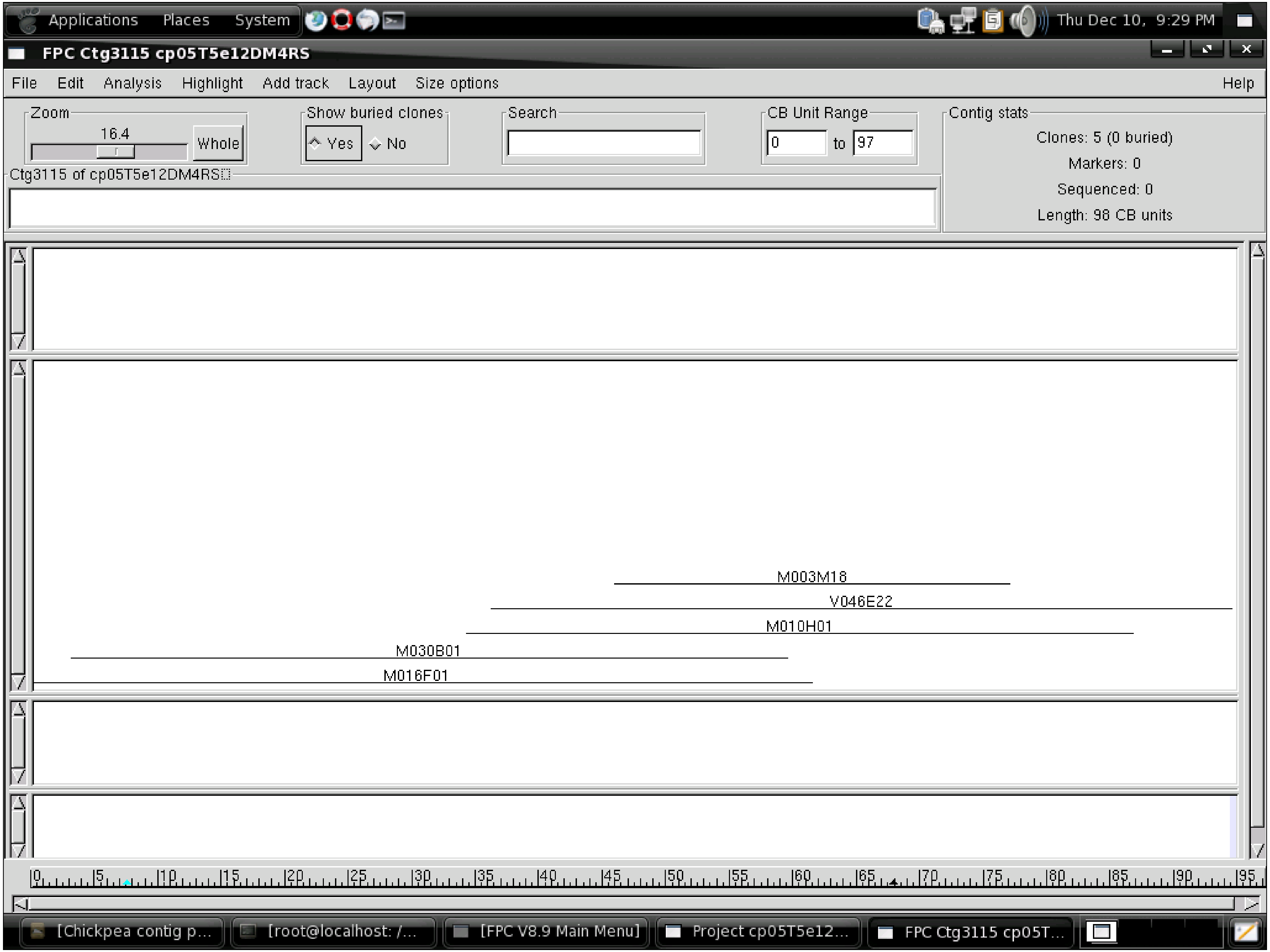
Applications Places System
Thu Dec 10, 9:29 PM
FPC Ctg3115 cp05T5e12DM4RS
File Edit Analysis Highlight Add track Layout Size options Help
Zoom
16.4
Whole
Show buried clones
Yes No
Search
CB Unit Range
0 to 97
Contig stats
Clones: 5 (0 buried)
Markers: 0
Sequenced: 0
Length: 98 CB units
Ctg3115 of cp05T5e12DM4RS
M003M18
V046E22
M010H01
M030B01
M016F01
0 5 10 15 20 25 30 35 40 45 50 55 60 65 70 75 80 85 90 95
[Chickpea contig p...
[root@localhost: /...
[FPC V8.9 Main Menu]
Project cp05T5e12...
FPC Ctg3115 cp05T...

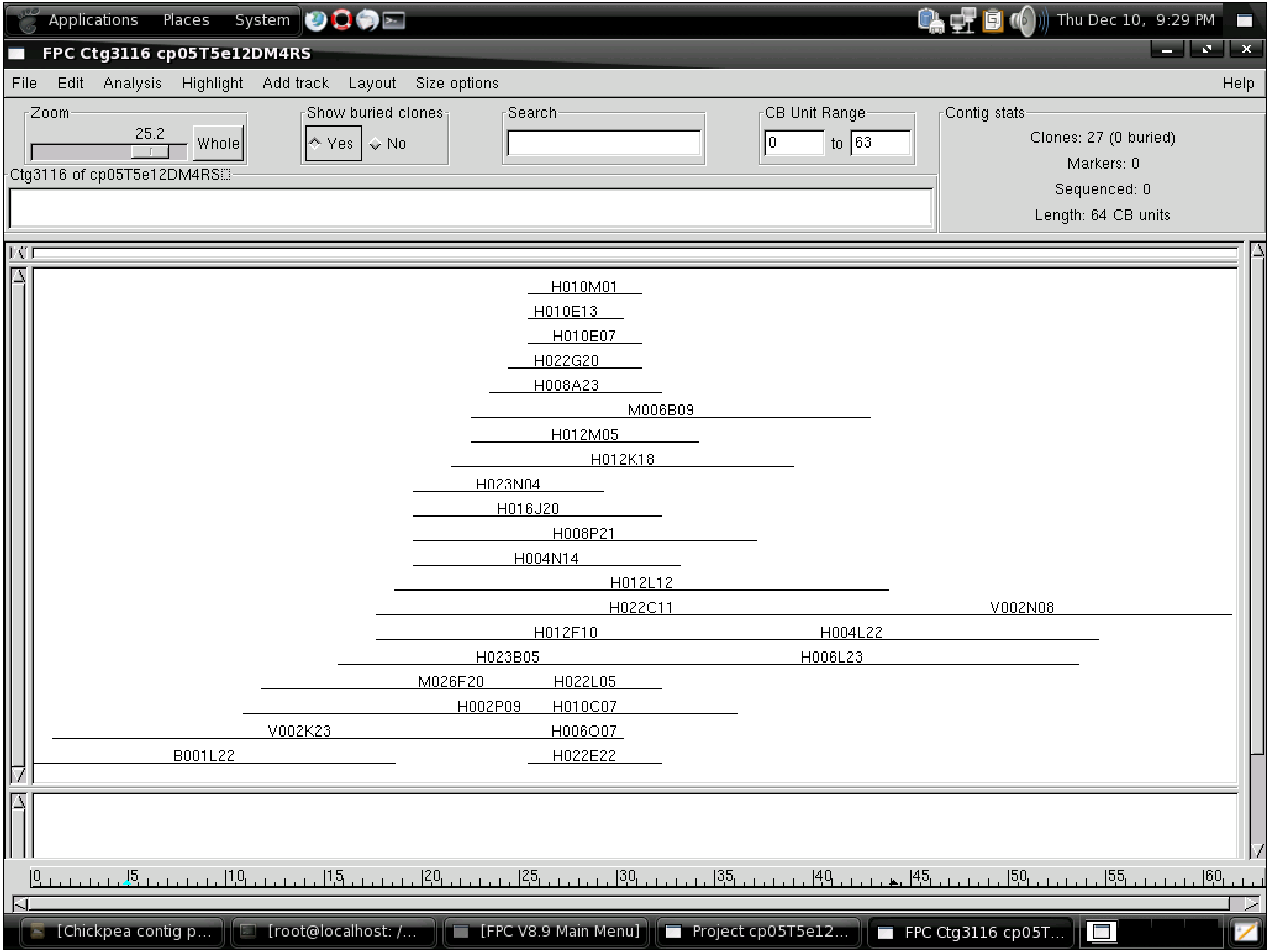
Applications Places System
Thu Dec 10, 9:29 PM
FPC Ctg3116 cp05T5e12DM4RS
File Edit Analysis Highlight Add track Layout Size options Help
Zoom
25.2
Whole
Show buried clones
Yes No
Search
CB Unit Range
0 to 63
Contig stats
Clones: 27 (0 buried)
Markers: 0
Sequenced: 0
Length: 64 CB units
Ctg3116 of cp05T5e12DM4RS
H010M01
H010E13
H010E07
H022G20
H008A23
M006B09
H012M05
H012K18
H023N04
H016J20
H008P21
H004N14
H012L12
H022C11
V002N08
H012F10
H004L22
H023B05
H006L23
M026F20
H022L05
H002P09
H010C07
V002K23
H006O07
B001L22
H022E22
0 5 10 15 20 25 30 35 40 45 50 55 60
[Chickpea contig p...
[root@localhost: /...
[FPC V8.9 Main Menu]
Project cp05T5e12...
FPC Ctg3116 cp05T...

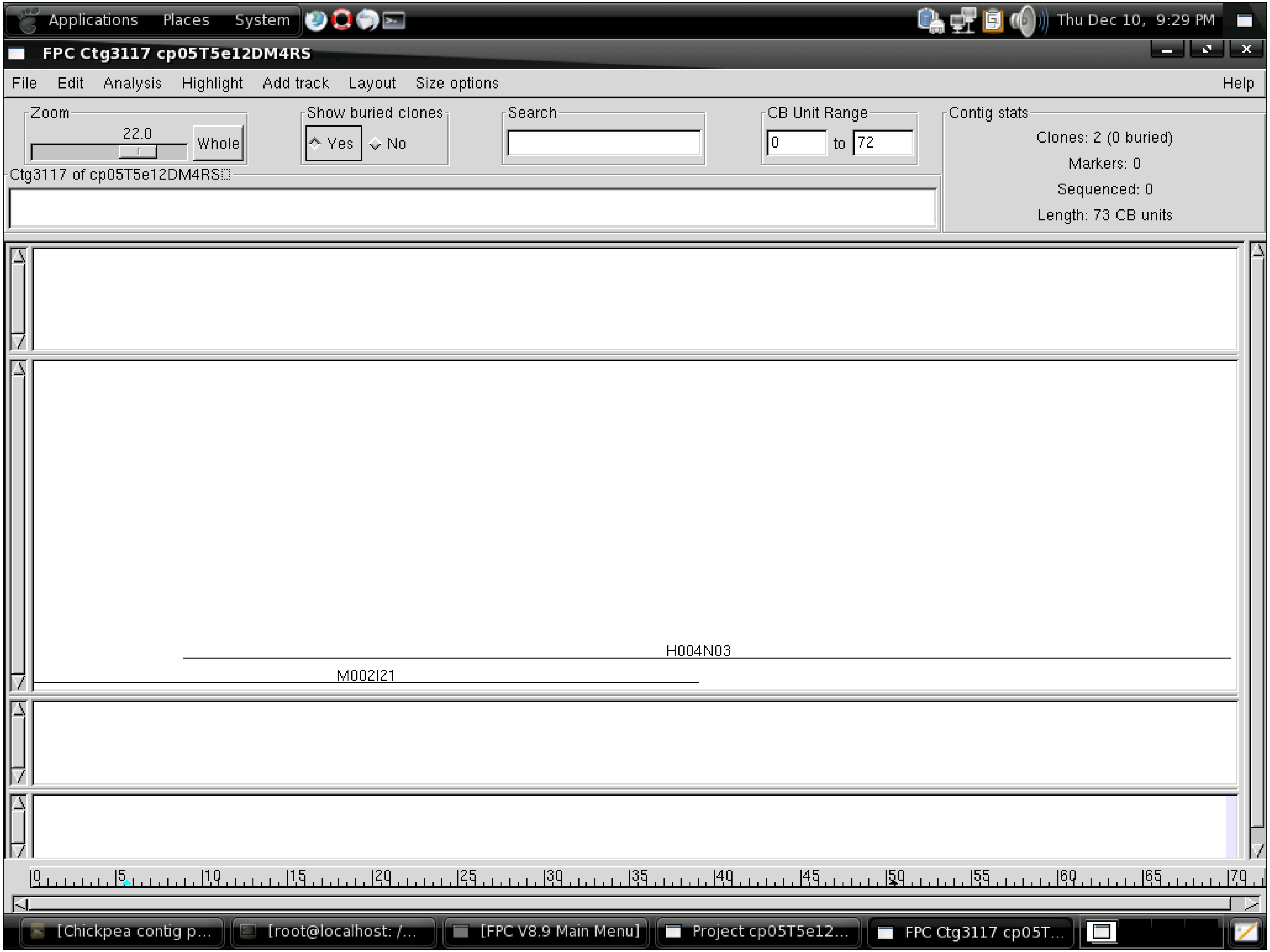
Applications
Places
System
Thu Dec 10, 9:29 PM
FPC Ctg3117 cp05T5e12DM4RS
File
Edit
Analysis
Highlight
Add track
Layout
Size options
Help
Zoom
22.0
Whole
Show buried clones
Yes
No
Search
CB Unit Range
0
to
72
Contig stats
Clones: 2 (0 buried)
Markers: 0
Sequenced: 0
Length: 73 CB units
Ctg3117 of cp05T5e12DM4RS
H004N03
M002I21
0
5
10
15
20
25
30
35
40
45
50
55
60
65
70
[Chickpea contig p...
[root@localhost: /...
[FPC V8.9 Main Menu]
Project cp05T5e12...
FPC Ctg3117 cp05T...

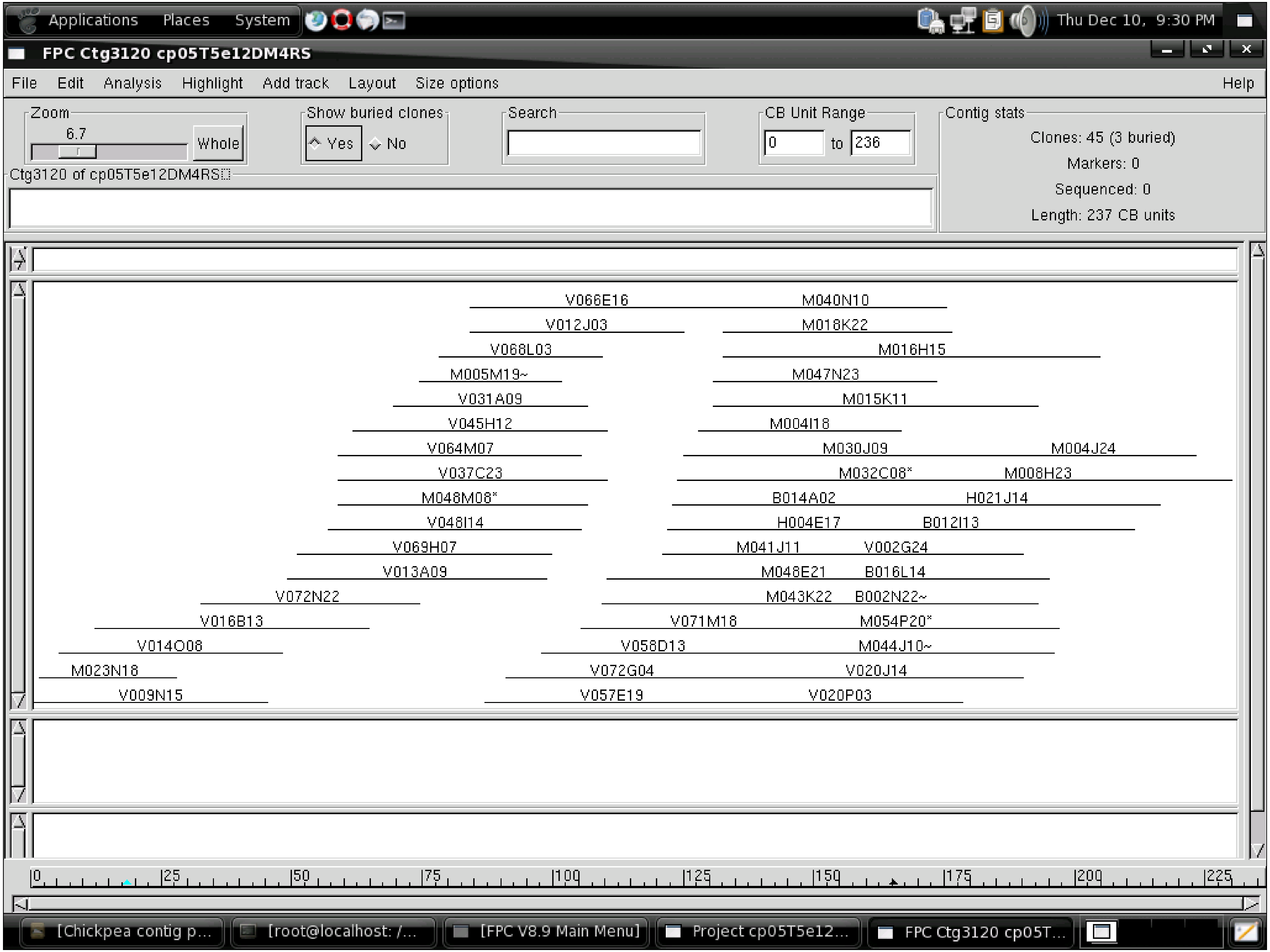
Applications Places System
Thu Dec 10, 9:30 PM
FPC Ctg3120 cp05T5e12DM4RS
File Edit Analysis Highlight Add track Layout Size options Help
Zoom
6.7
Whole
Show buried clones
Yes
No
Search
CB Unit Range
0
to
236
Contig stats
Clones: 45 (3 buried)
Markers: 0
Sequenced: 0
Length: 237 CB units
Ctg3120 of cp05T5e12DM4RS
V066E16
M040N10
V012J03
M018K22
V068L03
M016H15
M005M19~
M047N23
V031A09
M015K11
V045H12
M004I18
V064M07
M030J09
M004J24
V037C23
M032C08*
M008H23
M048M08*
B014A02
H021J14
V048I14
H004E17
B012I13
V069H07
M041J11
V002G24
V013A09
M048E21
B016L14
V072N22
M043K22
B002N22~
V016B13
V071M18
M054P20*
V014O08
V058D13
M044J10~
M023N18
V072G04
V020J14
V009N15
V057E19
V020P03
0
25
50
75
100
125
150
175
200
225
[Chickpea contig p...
[root@localhost: /...
[FPC V8.9 Main Menu]
Project cp05T5e12...
FPC Ctg3120 cp05T...

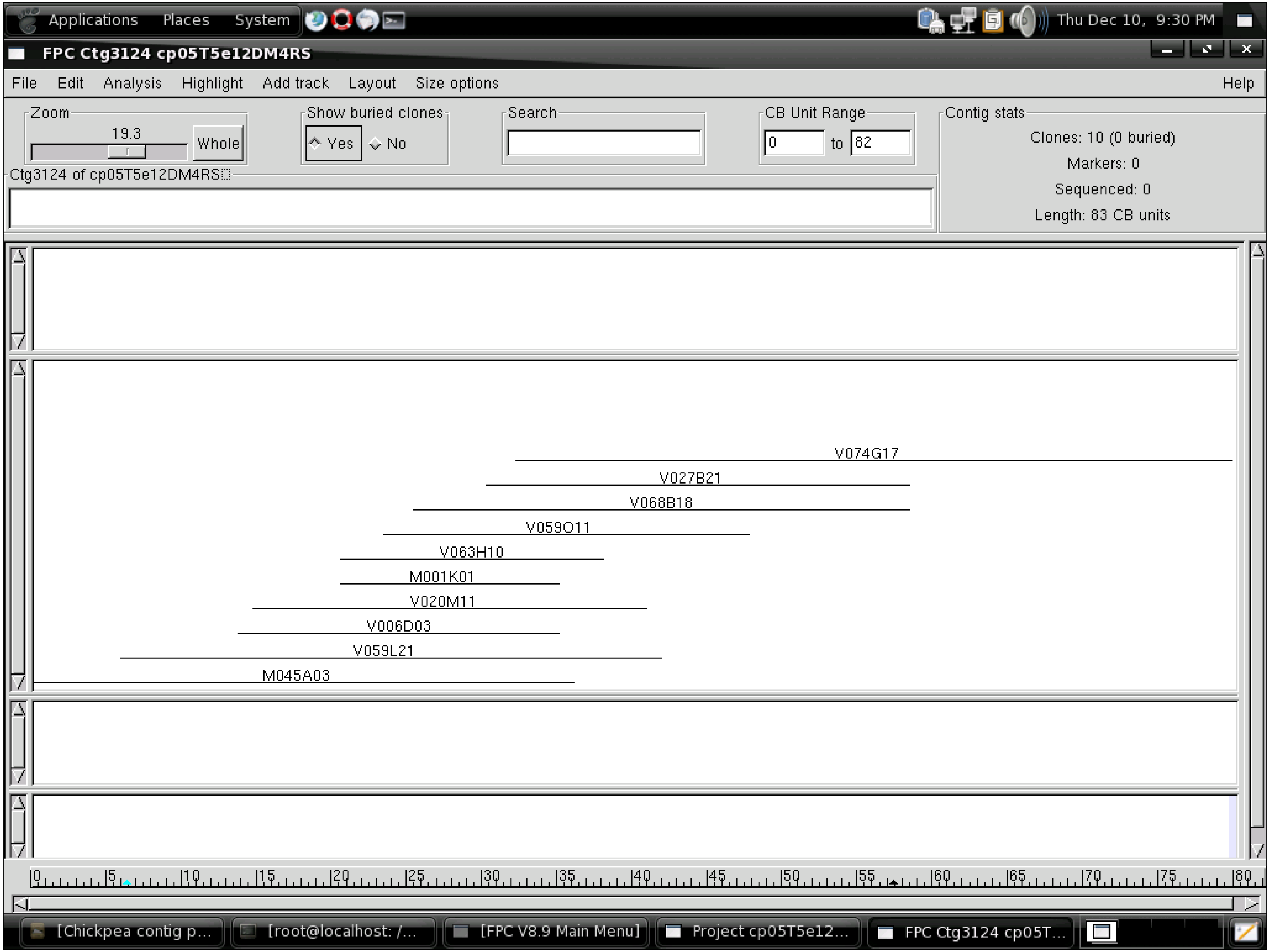
Applications Places System
Thu Dec 10, 9:30 PM
FPC Ctg3124 cp05T5e12DM4RS
File Edit Analysis Highlight Add track Layout Size options Help
Zoom
19.3
Whole
Show buried clones
Yes No
Search
CB Unit Range
0 to 82
Contig stats
Clones: 10 (0 buried)
Markers: 0
Sequenced: 0
Length: 83 CB units
Ctg3124 of cp05T5e12DM4RS
V074G17
V027B21
V068B18
V059O11
V063H10
M001K01
V020M11
V006D03
V059L21
M045A03
0 5 10 15 20 25 30 35 40 45 50 55 60 65 70 75 80
[Chickpea contig p...
[root@localhost: /...
[FPC V8.9 Main Menu]
Project cp05T5e12...
FPC Ctg3124 cp05T...

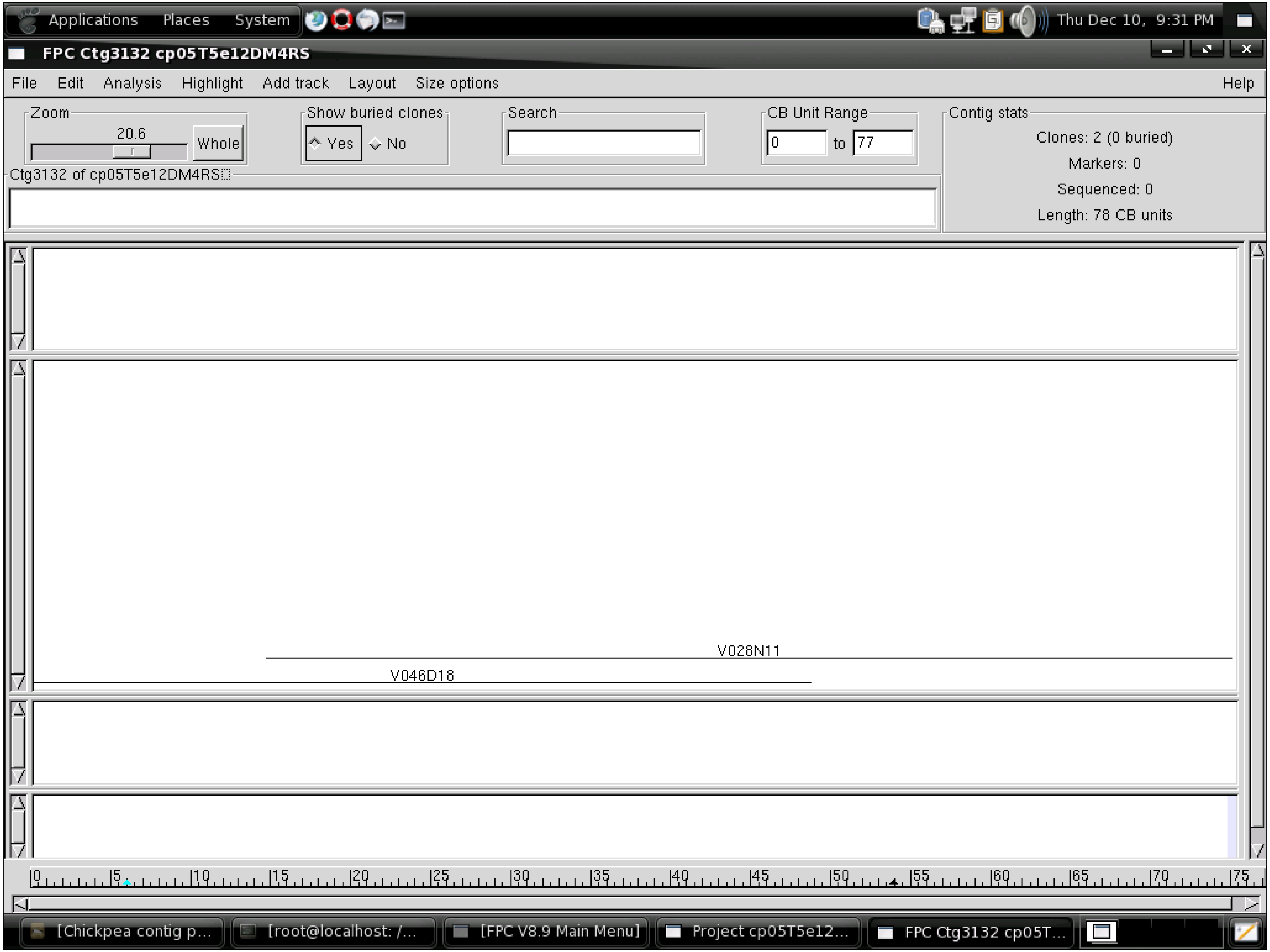
Applications Places System
Thu Dec 10, 9:31 PM
FPC Ctg3132 cp05T5e12DM4RS
File Edit Analysis Highlight Add track Layout Size options
Help
Zoom
20.6
Whole
Show buried clones
Yes
No
Search
CB Unit Range
0
to
77
Contig stats
Clones: 2 (0 buried)
Markers: 0
Sequenced: 0
Length: 78 CB units
Ctg3132 of cp05T5e12DM4RS
V028N11
V046D18
0 5 10 15 20 25 30 35 40 45 50 55 60 65 70 75
[Chickpea contig p...
[root@localhost: /...
[FPC V8.9 Main Menu]
Project cp05T5e12...
FPC Ctg3132 cp05T...

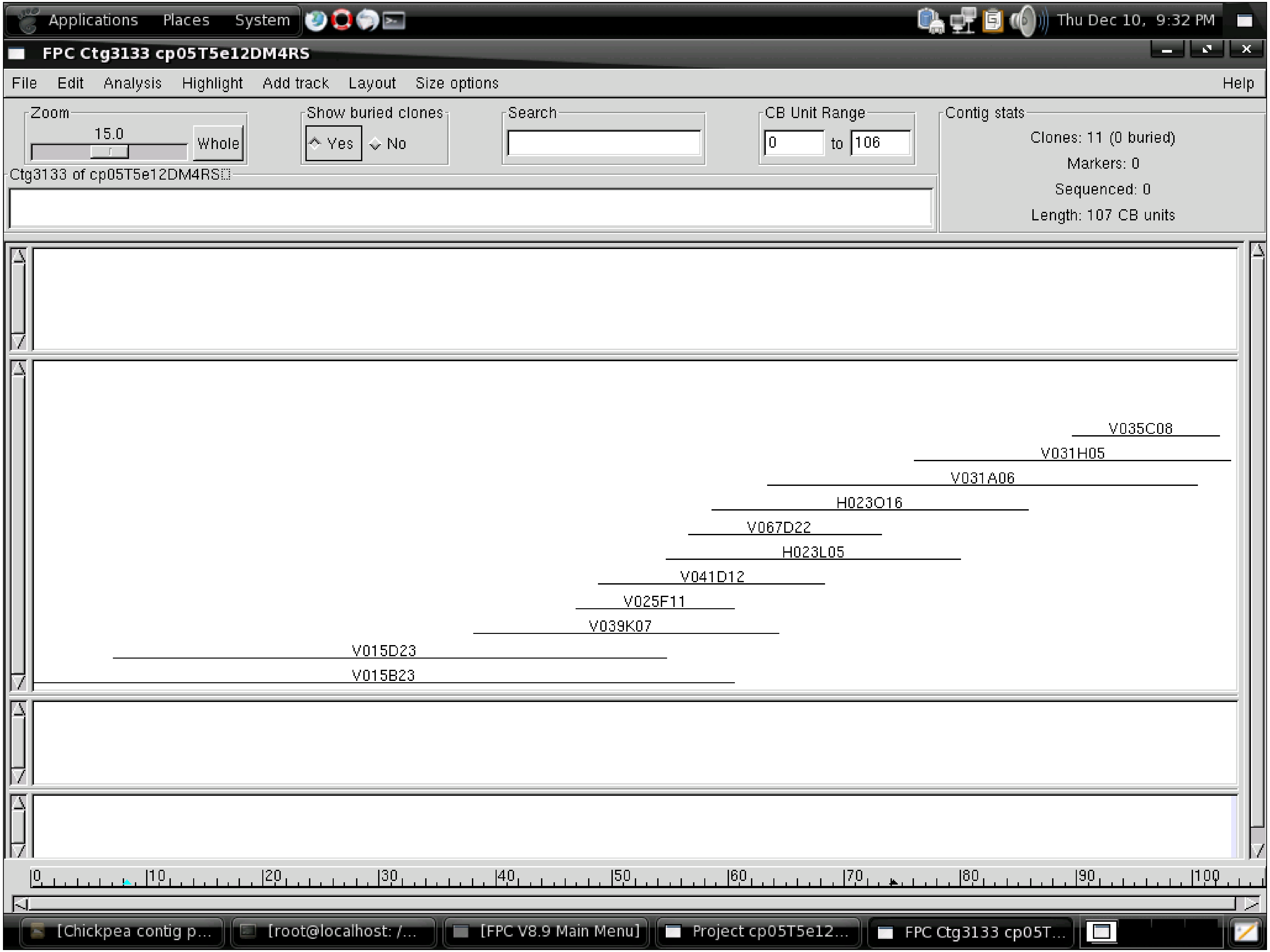
Applications Places System
Thu Dec 10, 9:32 PM
FPC Ctg3133 cp05T5e12DM4RS
File Edit Analysis Highlight Add track Layout Size options Help
Zoom
15.0
Whole
Show buried clones
Yes No
Search
CB Unit Range
0 to 106
Contig stats
Clones: 11 (0 buried)
Markers: 0
Sequenced: 0
Length: 107 CB units
Ctg3133 of cp05T5e12DM4RS
V035C08
V031H05
V031A06
H023O16
V067D22
H023L05
V041D12
V025F11
V039K07
V015D23
V015B23
0 10 20 30 40 50 60 70 80 90 100
[Chickpea contig p...
[root@localhost: /...
[FPC V8.9 Main Menu]
Project cp05T5e12...
FPC Ctg3133 cp05T...

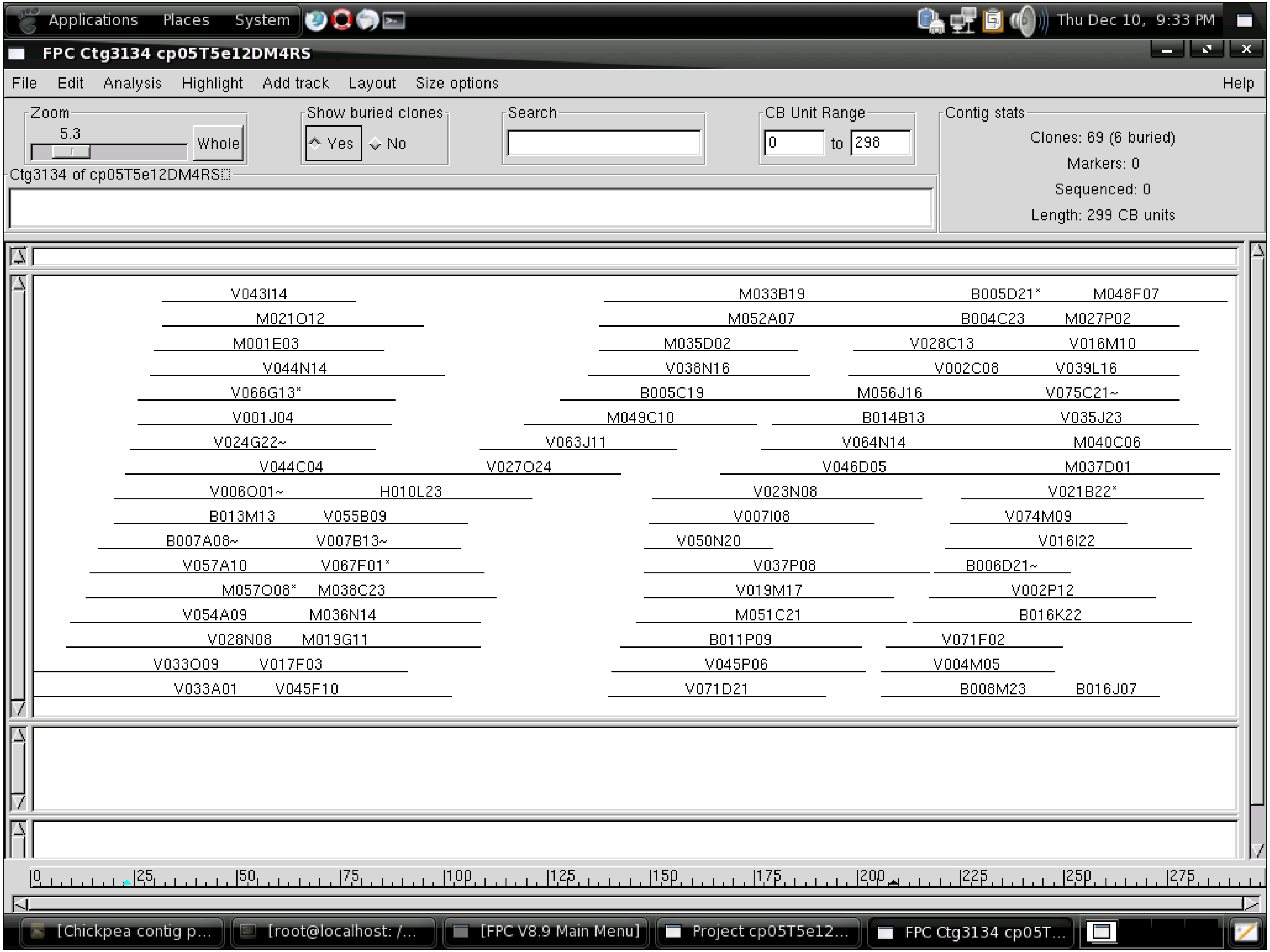
Applications Places System
Thu Dec 10, 9:33 PM
FPC Ctg3134 cp05T5e12DM4RS
File Edit Analysis Highlight Add track Layout Size options Help
Zoom
5.3
Whole
Show buried clones
Yes No
Search
CB Unit Range
0 to 298
Contig stats
Clones: 69 (6 buried)
Markers: 0
Sequenced: 0
Length: 299 CB units
Ctg3134 of cp05T5e12DM4RS
V043I14
M021O12
M001E03
V044N14
V066G13*
V001J04
V024G22~
V044C04
V006O01~
H010L23
B013M13
V055B09
B007A08~
V007B13~
V057A10
V067F01*
M057O08*
M038C23
V054A09
M036N14
V028N08
M019G11
V033O09
V017F03
V033A01
V045F10
V063J11
V027O24
M033B19
M052A07
M035D02
V038N16
B005C19
M049C10
B005D21*
M048F07
B004C23
M027P02
V028C13
V016M10
V002C08
V039L16
M056J16
V075C21~
B014B13
V035J23
V064N14
M040C06
V046D05
M037D01
V023N08
V021B22*
V007I08
V074M09
V050N20
V016I22
V037P08
B006D21~
V019M17
V002P12
M051C21
B016K22
B011P09
V071F02
V045P06
V004M05
V071D21
B008M23
B016J07
0 25 50 75 100 125 150 175 200 225 250 275
[Chickpea contig p...
[root@localhost: /...
[FPC V8.9 Main Menu]
Project cp05T5e12...
FPC Ctg3134 cp05T...

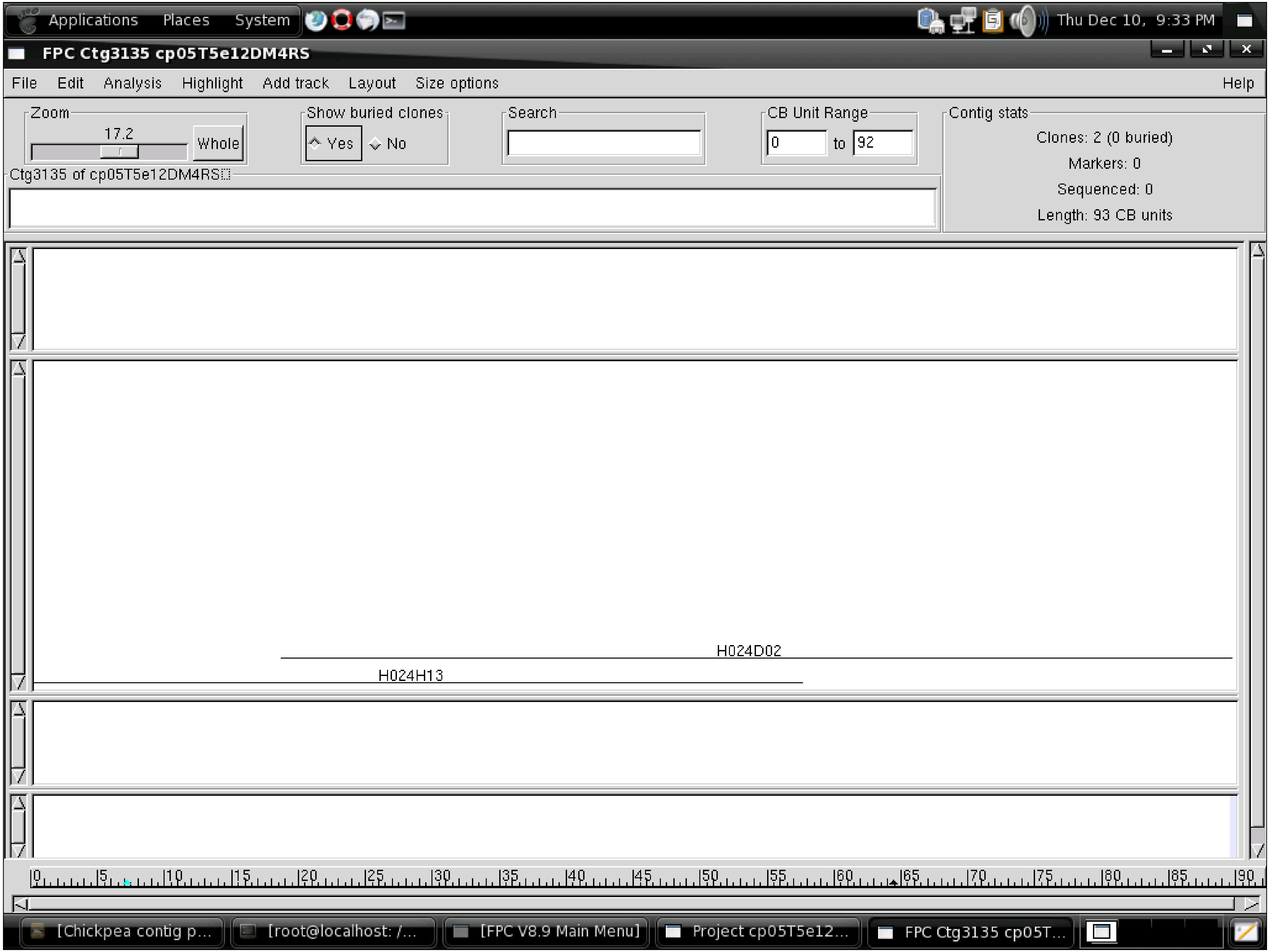
Applications Places System
Thu Dec 10, 9:33 PM
FPC Ctg3135 cp05T5e12DM4RS
File Edit Analysis Highlight Add track Layout Size options Help
Zoom
17.2
Whole
Show buried clones
Yes No
Search
CB Unit Range
0 to 92
Contig stats
Clones: 2 (0 buried)
Markers: 0
Sequenced: 0
Length: 93 CB units
Ctg3135 of cp05T5e12DM4RS
H024D02
H024H13
0 5 10 15 20 25 30 35 40 45 50 55 60 65 70 75 80 85 90
[Chickpea contig p...
[root@localhost: /...
[FPC V8.9 Main Menu]
Project cp05T5e12...
FPC Ctg3135 cp05T...

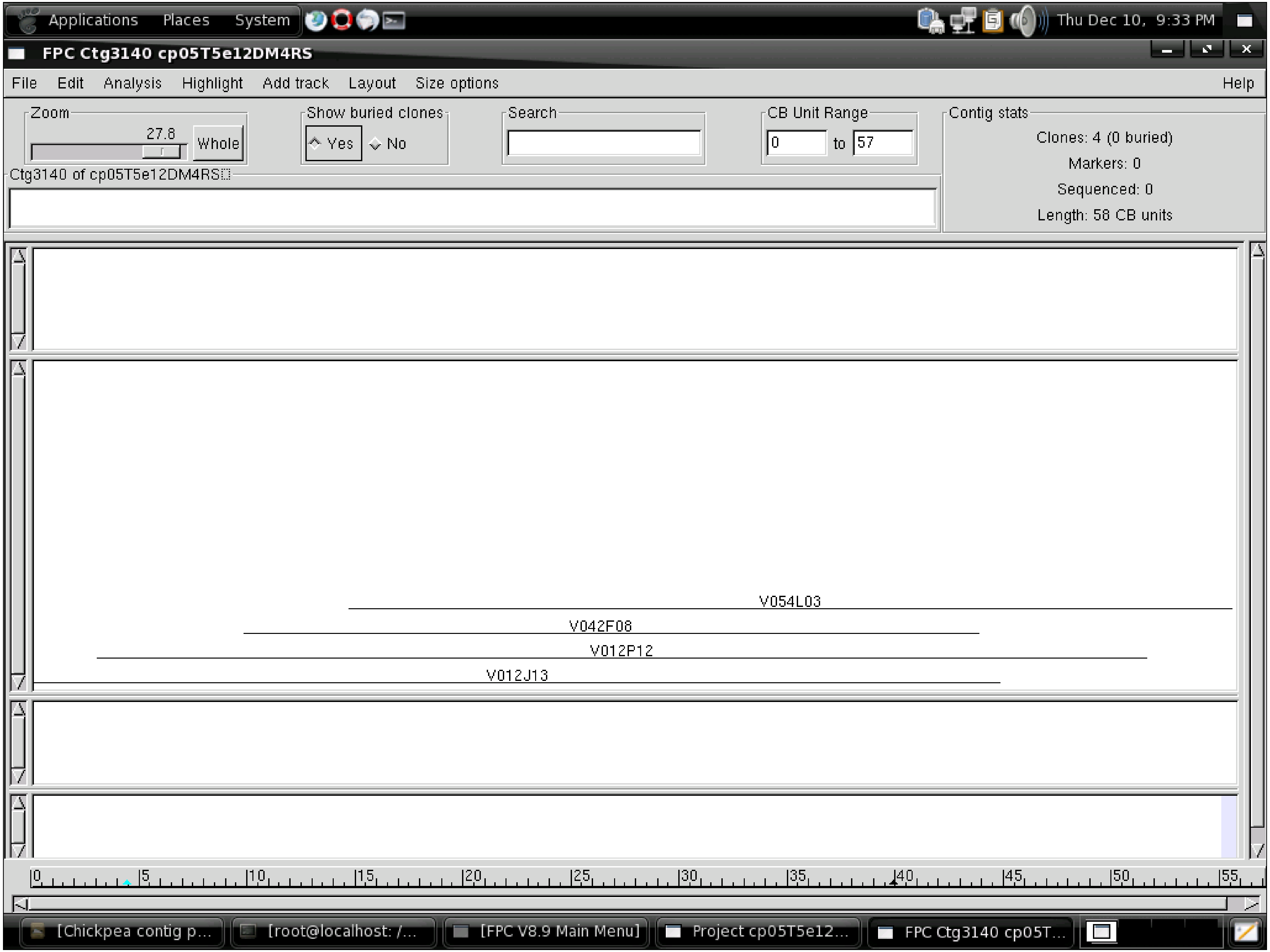
Applications
Places
System
Thu Dec 10, 9:33 PM
FPC Ctg3140 cp05T5e12DM4RS
File
Edit
Analysis
Highlight
Add track
Layout
Size options
Help
Zoom
27.8
Whole
Show buried clones
Yes
No
Search
CB Unit Range
0
to
57
Contig stats
Clones: 4 (0 buried)
Markers: 0
Sequenced: 0
Length: 58 CB units
Ctg3140 of cp05T5e12DM4RS
V054L03
V042F08
V012P12
V012J13
0
5
10
15
20
25
30
35
40
45
50
55
[Chickpea contig p...
[root@localhost: /...
[FPC V8.9 Main Menu]
Project cp05T5e12...
FPC Ctg3140 cp05T...

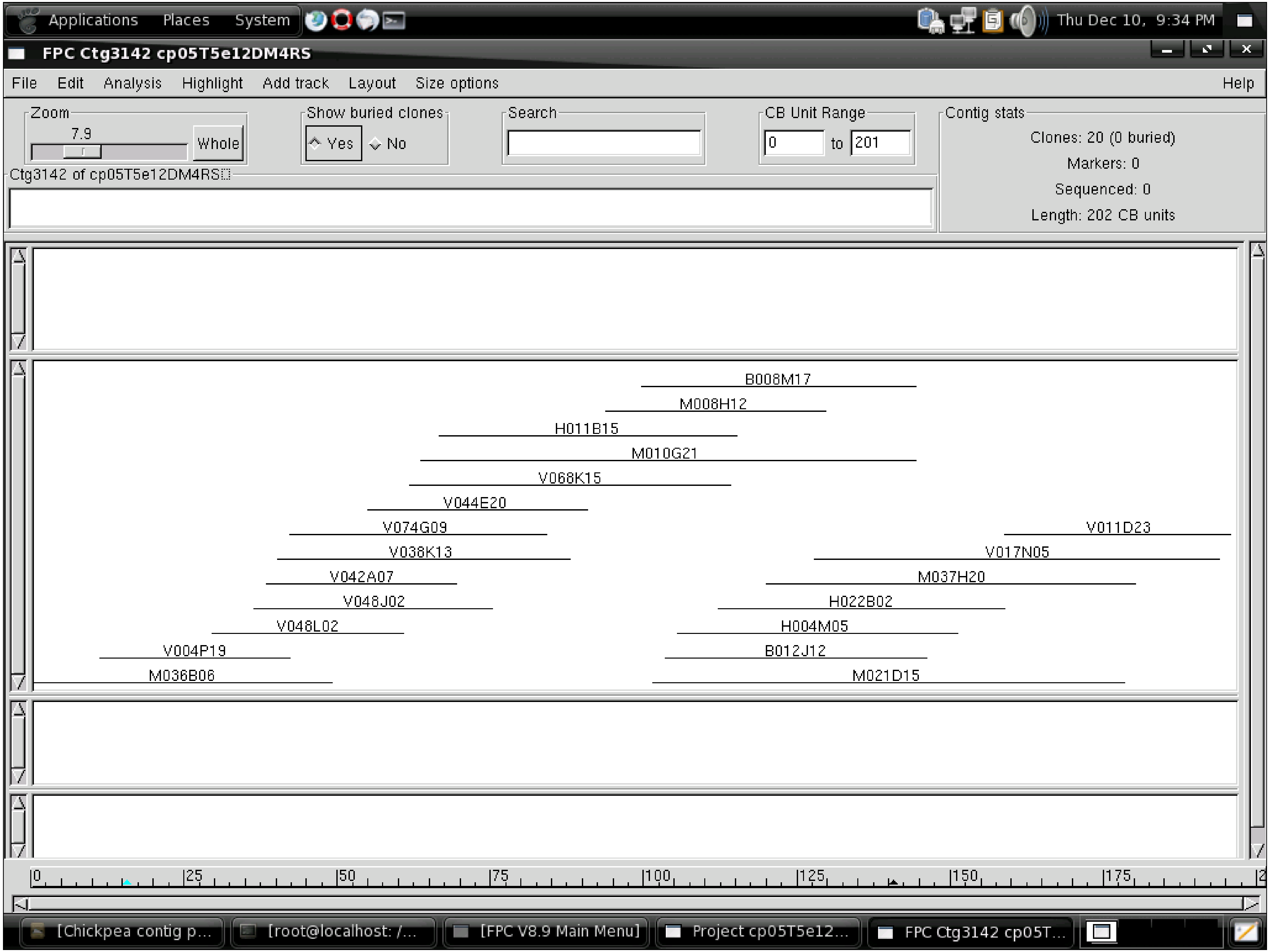
Applications Places System
Thu Dec 10, 9:34 PM
FPC Ctg3142 cp05T5e12DM4RS
File Edit Analysis Highlight Add track Layout Size options Help
Zoom
7.9
Whole
Show buried clones
Yes
No
Search
CB Unit Range
0
to
201
Contig stats
Clones: 20 (0 buried)
Markers: 0
Sequenced: 0
Length: 202 CB units
Ctg3142 of cp05T5e12DM4RS
B008M17
M008H12
H011B15
M010G21
V068K15
V044E20
V074G09
V011D23
V038K13
V017N05
V042A07
M037H20
V048J02
H022B02
V048L02
H004M05
V004P19
B012J12
M036B06
M021D15
0
25
50
75
100
125
150
175
[Chickpea contig p...
[root@localhost: /...
[FPC V8.9 Main Menu]
Project cp05T5e12...
FPC Ctg3142 cp05T...

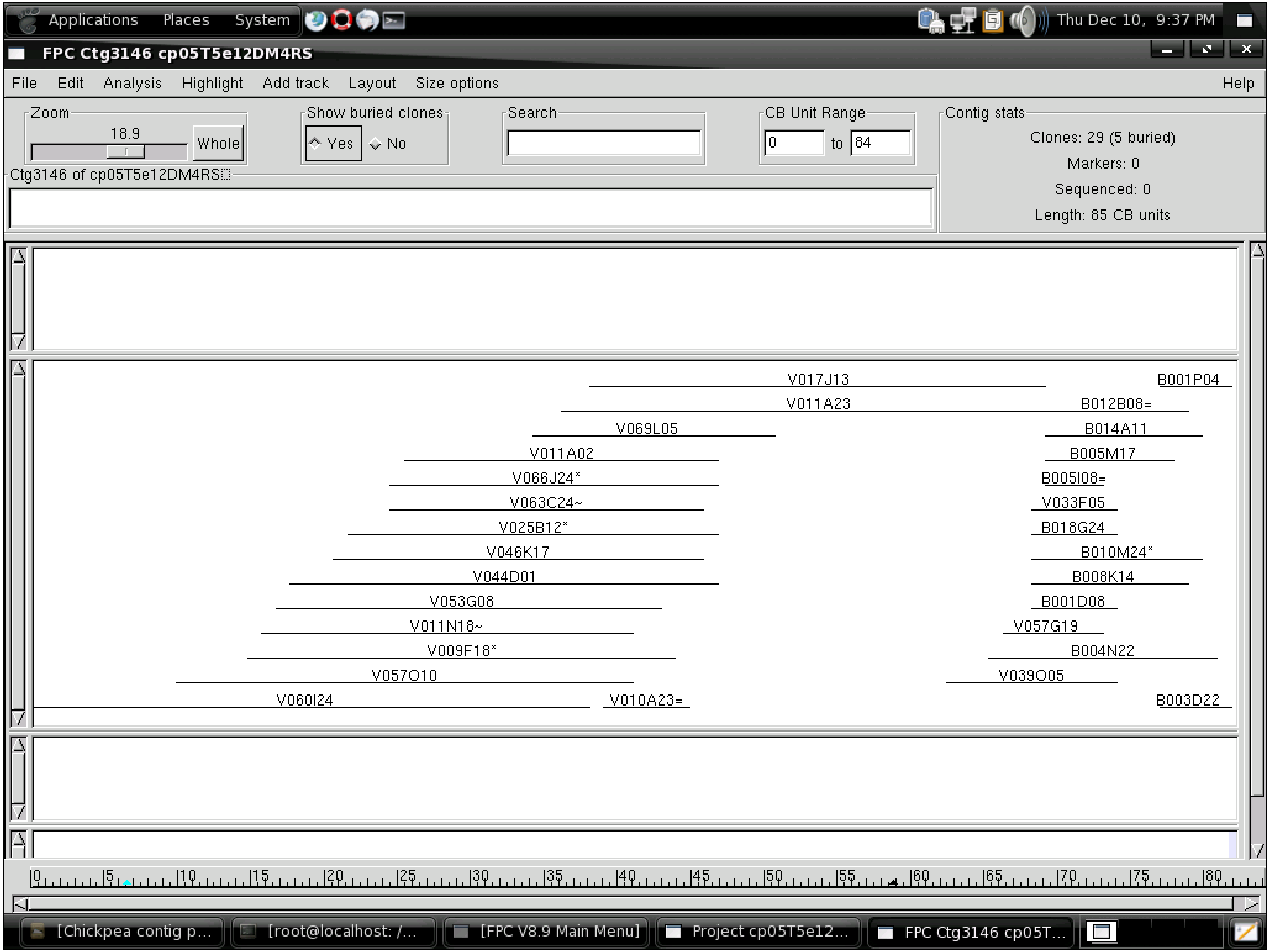
Applications Places System
Thu Dec 10, 9:37 PM
FPC Ctg3146 cp05T5e12DM4RS
File Edit Analysis Highlight Add track Layout Size options Help
Zoom
18.9
Whole
Show buried clones
Yes
No
Search
CB Unit Range
0 to 84
Contig stats
Clones: 29 (5 buried)
Markers: 0
Sequenced: 0
Length: 85 CB units
Ctg3146 of cp05T5e12DM4RS
V017J13
B001P04
V011A23
B012B08=
V069L05
B014A11
V011A02
B005M17
V066J24*
B005I08=
V063C24~
V033F05
V025B12*
B018G24
V046K17
B010M24*
V044D01
B008K14
V053G08
B001D08
V011N18~
V057G19
V009F18*
B004N22
V057O10
V039O05
V060I24
V010A23=
B003D22
0 5 10 15 20 25 30 35 40 45 50 55 60 65 70 75 80
[Chickpea contig p...
[root@localhost: /...
[FPC V8.9 Main Menu]
Project cp05T5e12...
FPC Ctg3146 cp05T...

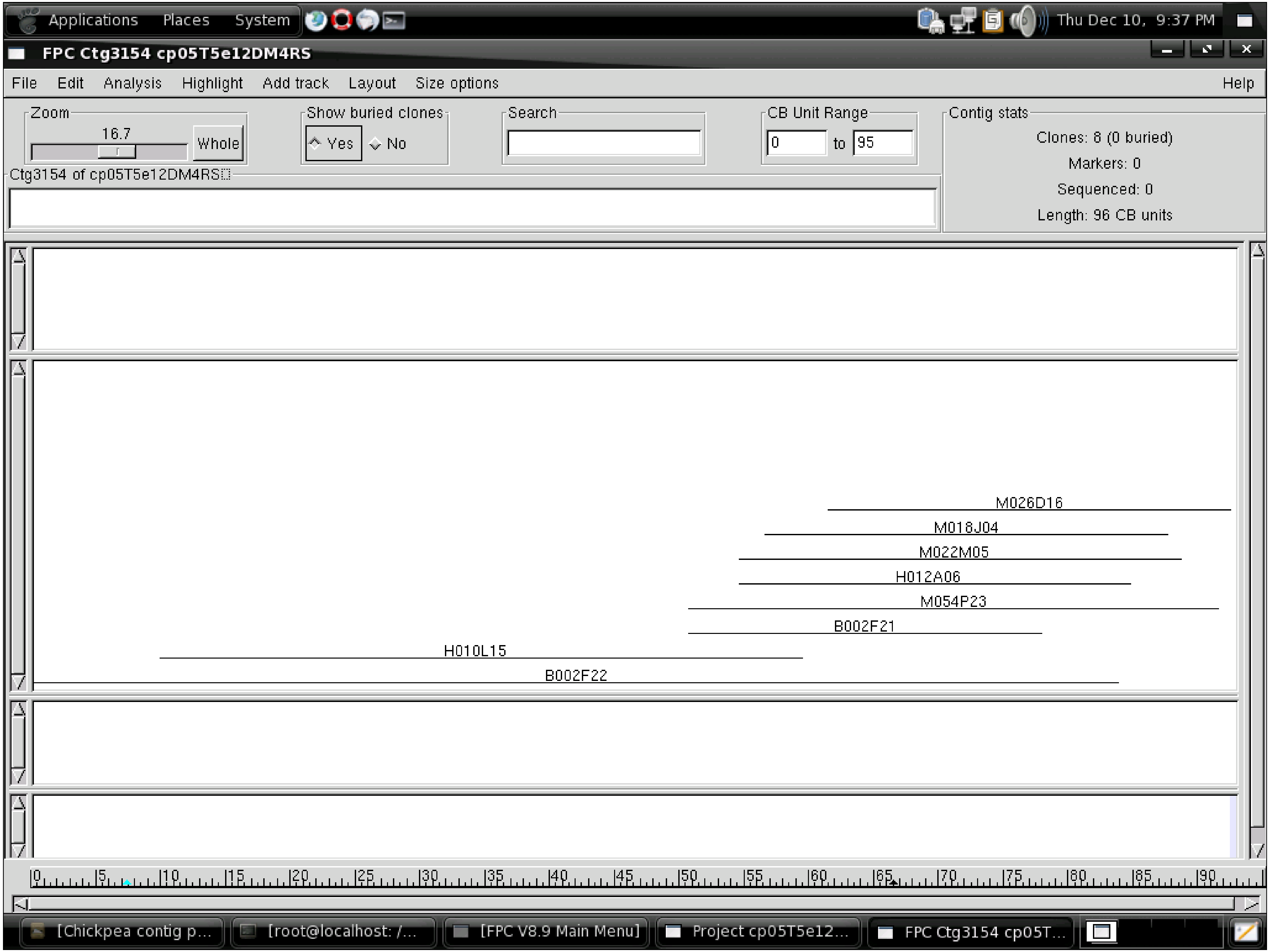
Applications Places System
Thu Dec 10, 9:37 PM
FPC Ctg3154 cp05T5e12DM4RS
File Edit Analysis Highlight Add track Layout Size options Help
Zoom
16.7
Whole
Show buried clones
Yes No
Search
CB Unit Range
0 to 95
Contig stats
Clones: 8 (0 buried)
Markers: 0
Sequenced: 0
Length: 96 CB units
Ctg3154 of cp05T5e12DM4RS
M026D16
M018J04
M022M05
H012A06
M054P23
B002F21
H010L15
B002F22
0 5 10 15 20 25 30 35 40 45 50 55 60 65 70 75 80 85 90
[Chickpea contig p...
[root@localhost: /...
[FPC V8.9 Main Menu]
Project cp05T5e12...
FPC Ctg3154 cp05T...

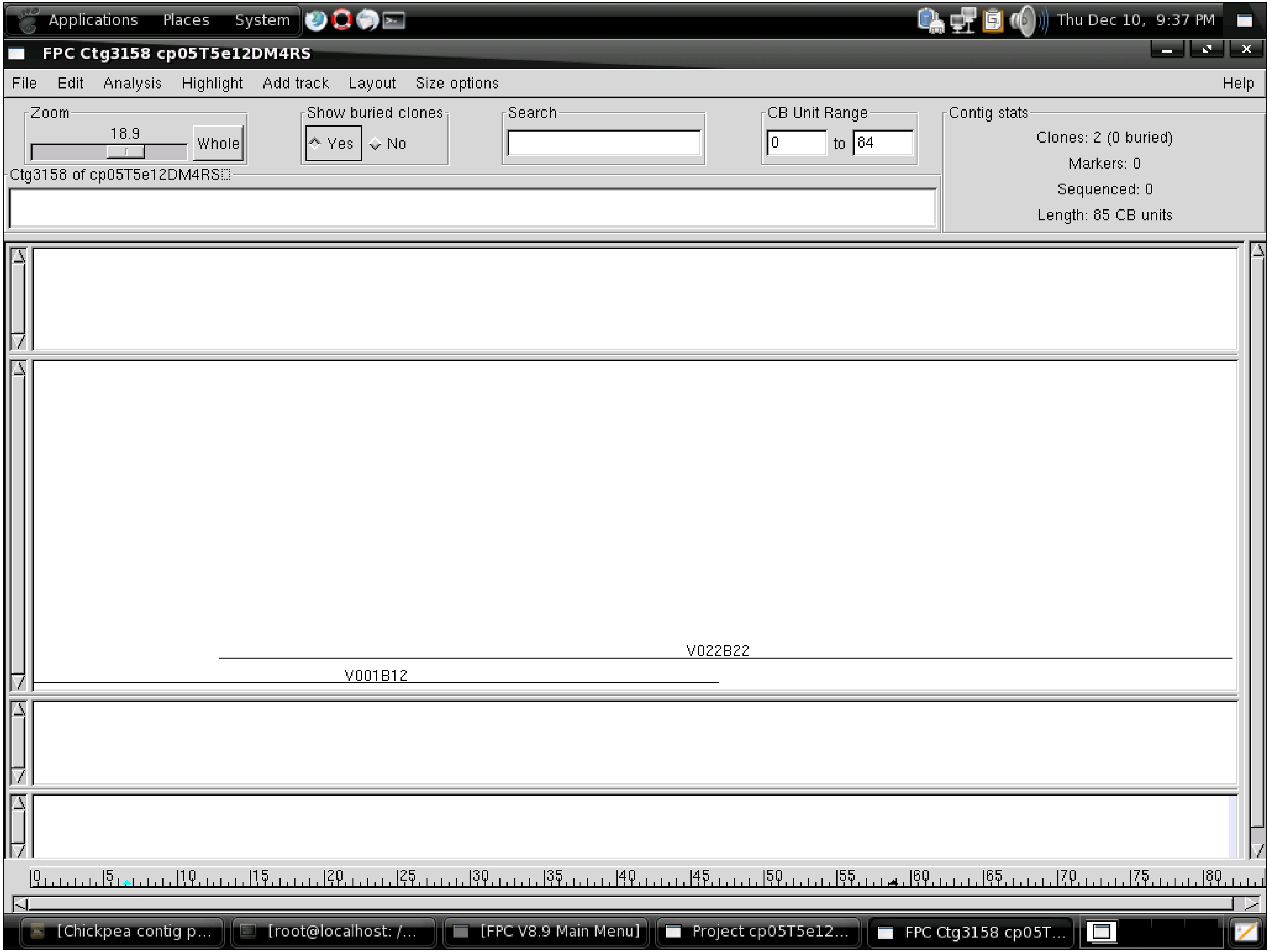
Applications
Places
System
Thu Dec 10, 9:37 PM
FPC Ctg3158 cp05T5e12DM4RS
File
Edit
Analysis
Highlight
Add track
Layout
Size options
Help
Zoom
18.9
Whole
Show buried clones
Yes
No
Search
CB Unit Range
0
to
84
Contig stats
Clones: 2 (0 buried)
Markers: 0
Sequenced: 0
Length: 85 CB units
Ctg3158 of cp05T5e12DM4RS
V022B22
V001B12
0 5 10 15 20 25 30 35 40 45 50 55 60 65 70 75 80
[Chickpea contig p...
[root@localhost: /...
[FPC V8.9 Main Menu]
Project cp05T5e12...
FPC Ctg3158 cp05T...

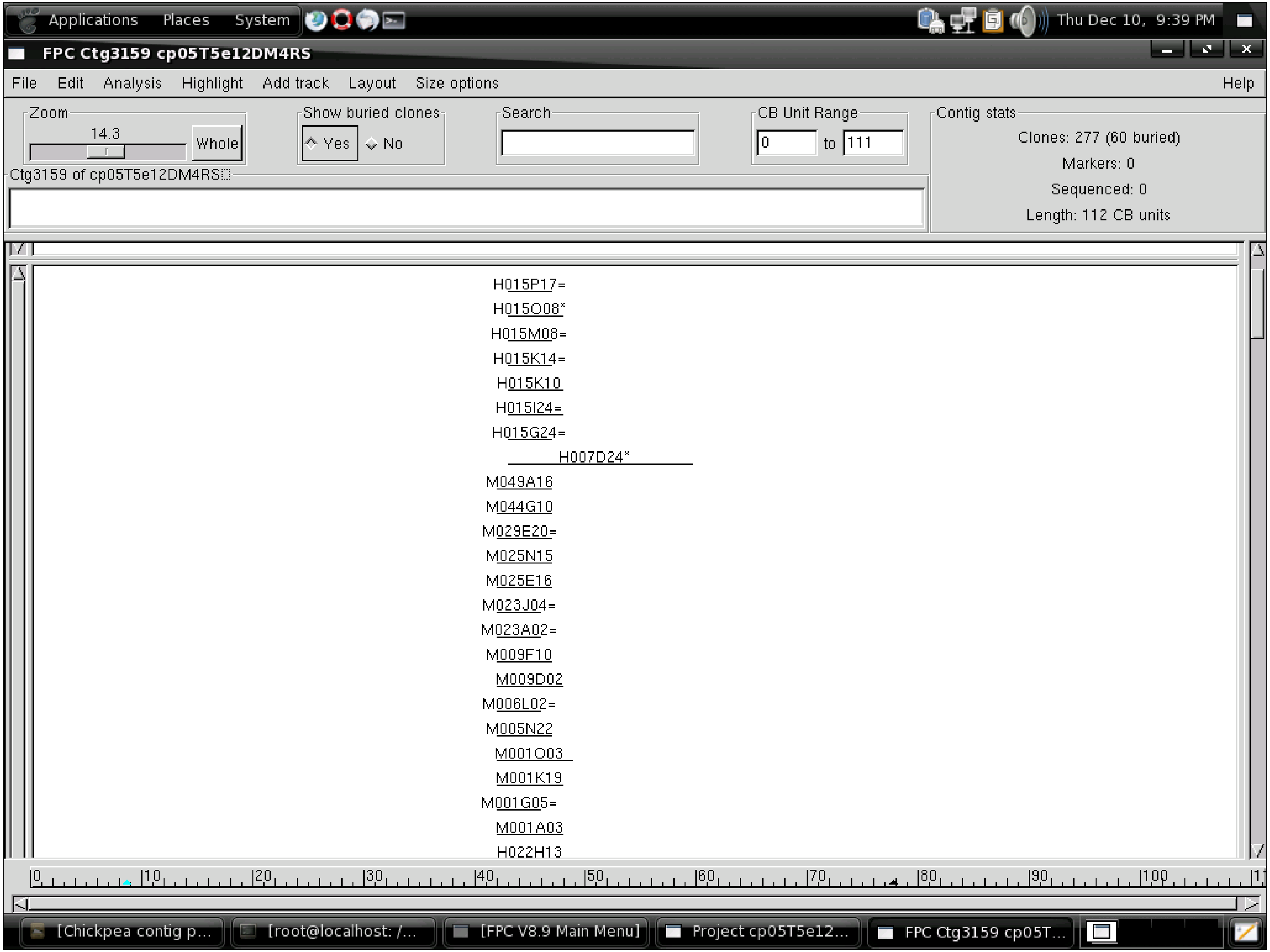
Applications Places System
Thu Dec 10, 9:39 PM
FPC Ctg3159 cp05T5e12DM4RS
File Edit Analysis Highlight Add track Layout Size options Help
Zoom
14.3
Whole
Show buried clones
Yes
No
Search
CB Unit Range
0 to 111
Contig stats
Clones: 277 (60 buried)
Markers: 0
Sequenced: 0
Length: 112 CB units
Ctg3159 of cp05T5e12DM4RS
H015P17=
H015O08*
H015M08=
H015K14=
H015K10
H015I24=
H015G24=
H007D24*
M049A16
M044G10
M029E20=
M025N15
M025E16
M023J04=
M023A02=
M009F10
M009D02
M006L02=
M005N22
M001O03
M001K19
M001G05=
M001A03
H022H13
0 10 20 30 40 50 60 70 80 90 100
[Chickpea contig p...
[root@localhost: /...
[FPC V8.9 Main Menu]
Project cp05T5e12...
FPC Ctg3159 cp05T...

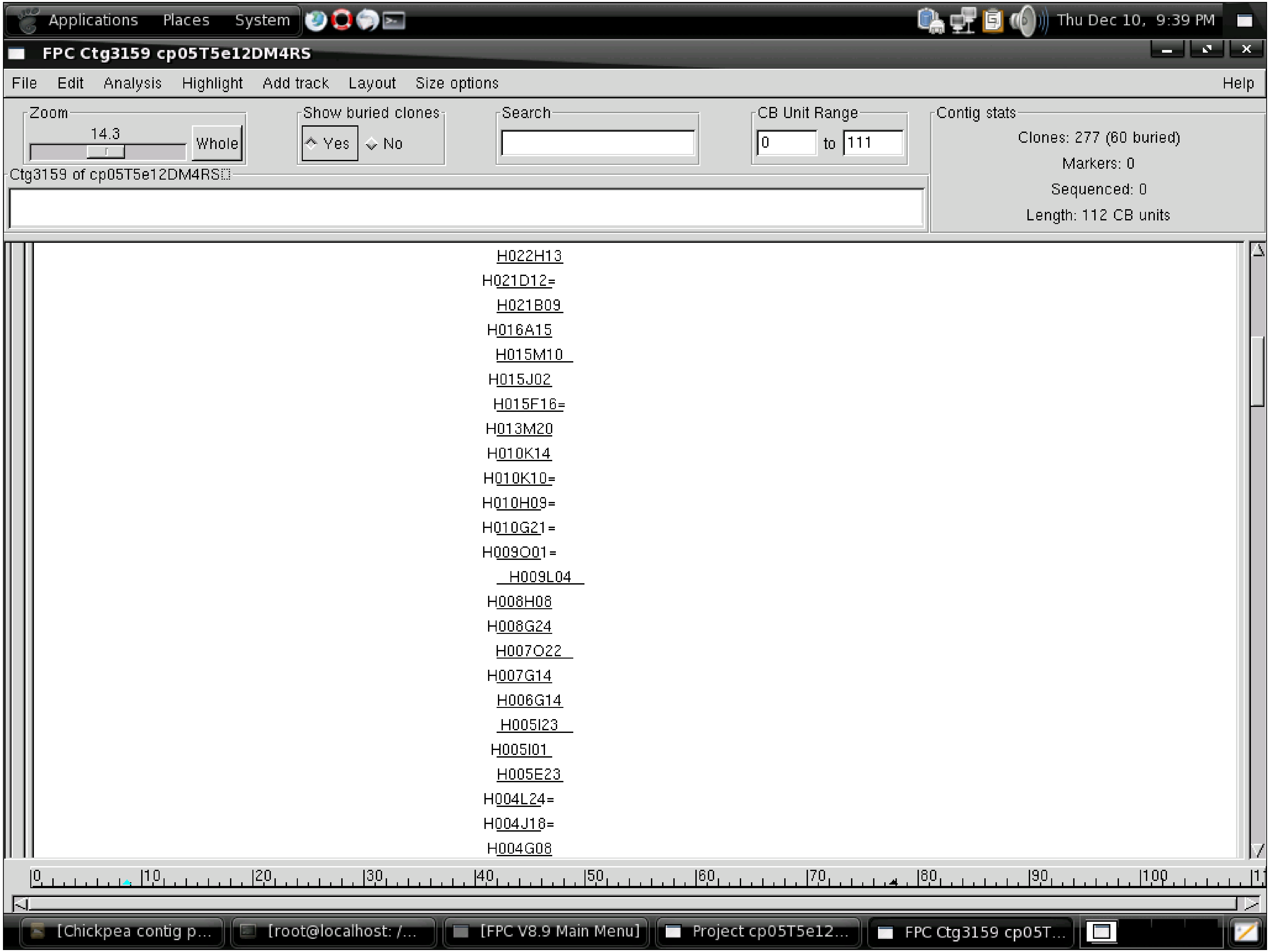
Applications Places System
Thu Dec 10, 9:39 PM
FPC Ctg3159 cp05T5e12DM4RS
File Edit Analysis Highlight Add track Layout Size options Help
Zoom
14.3
Whole
Show buried clones
Yes No
Search
CB Unit Range
0 to 111
Contig stats
Clones: 277 (60 buried)
Markers: 0
Sequenced: 0
Length: 112 CB units
Ctg3159 of cp05T5e12DM4RS
H022H13
H021D12=
H021B09
H016A15
H015M10
H015J02
H015F16=
H013M20
H010K14
H010K10=
H010H09=
H010G21=
H009O01=
H009L04
H008H08
H008G24
H007O22
H007G14
H006G14
H005I23
H005I01
H005E23
H004L24=
H004J18=
H004G08
0 10 20 30 40 50 60 70 80 90 100
[Chickpea contig p...
[root@localhost: /...
[FPC V8.9 Main Menu]
Project cp05T5e12...
FPC Ctg3159 cp05T...

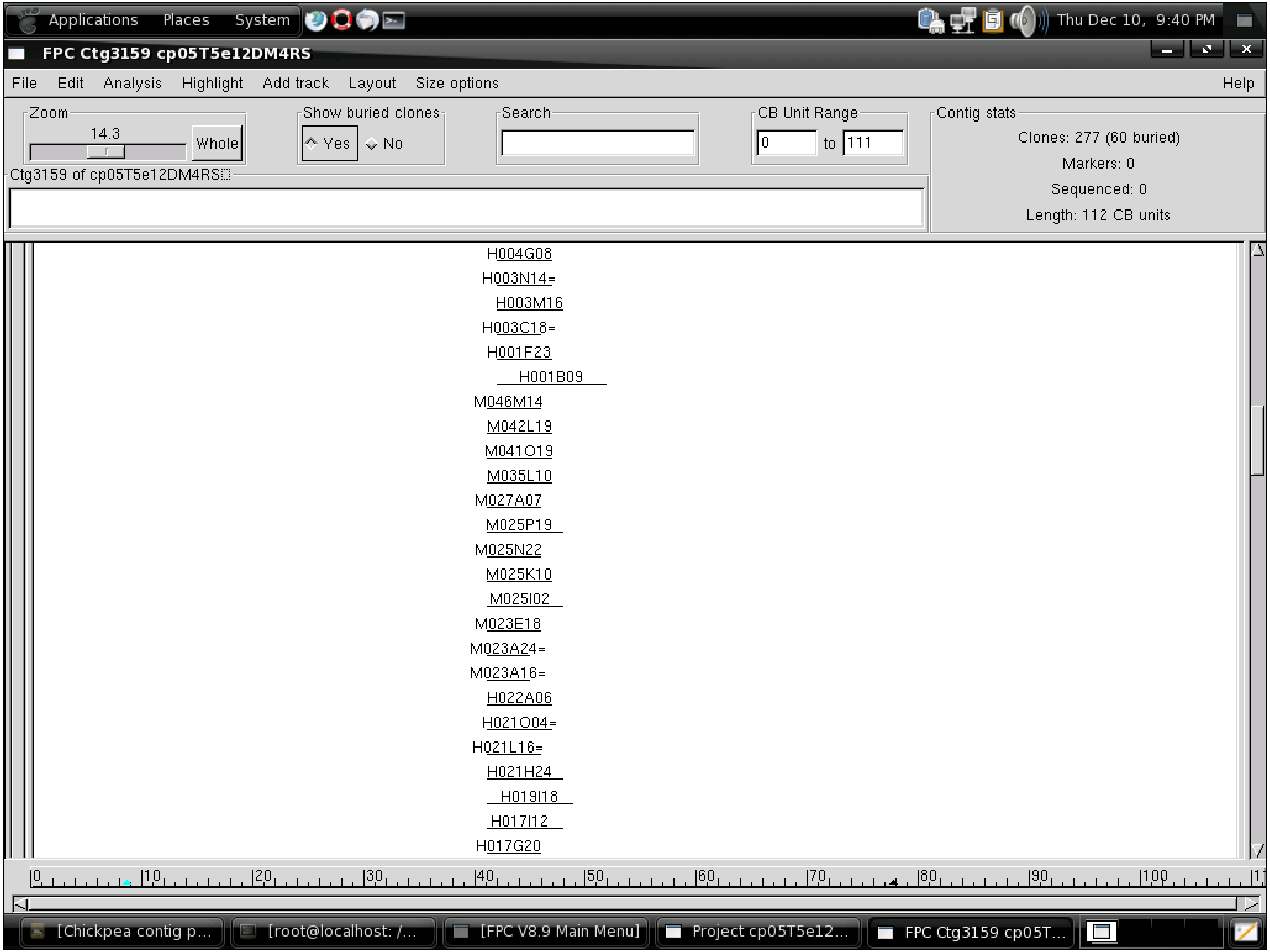
Applications Places System
Thu Dec 10, 9:40 PM
FPC Ctg3159 cp05T5e12DM4RS
File Edit Analysis Highlight Add track Layout Size options Help
Zoom
14.3
Whole
Show buried clones
Yes No
Search
CB Unit Range
0 to 111
Contig stats
Clones: 277 (60 buried)
Markers: 0
Sequenced: 0
Length: 112 CB units
Ctg3159 of cp05T5e12DM4RS
H004G08
H003N14=
H003M16
H003C18=
H001F23
H001B09
M046M14
M042L19
M041O19
M035L10
M027A07
M025P19
M025N22
M025K10
M025I02
M023E18
M023A24=
M023A16=
H022A06
H021O04=
H021L16=
H021H24
H019I18
H017I12
H017G20
0 10 20 30 40 50 60 70 80 90 100
[Chickpea contig p...
[root@localhost: /...
[FPC V8.9 Main Menu]
Project cp05T5e12...
FPC Ctg3159 cp05T...

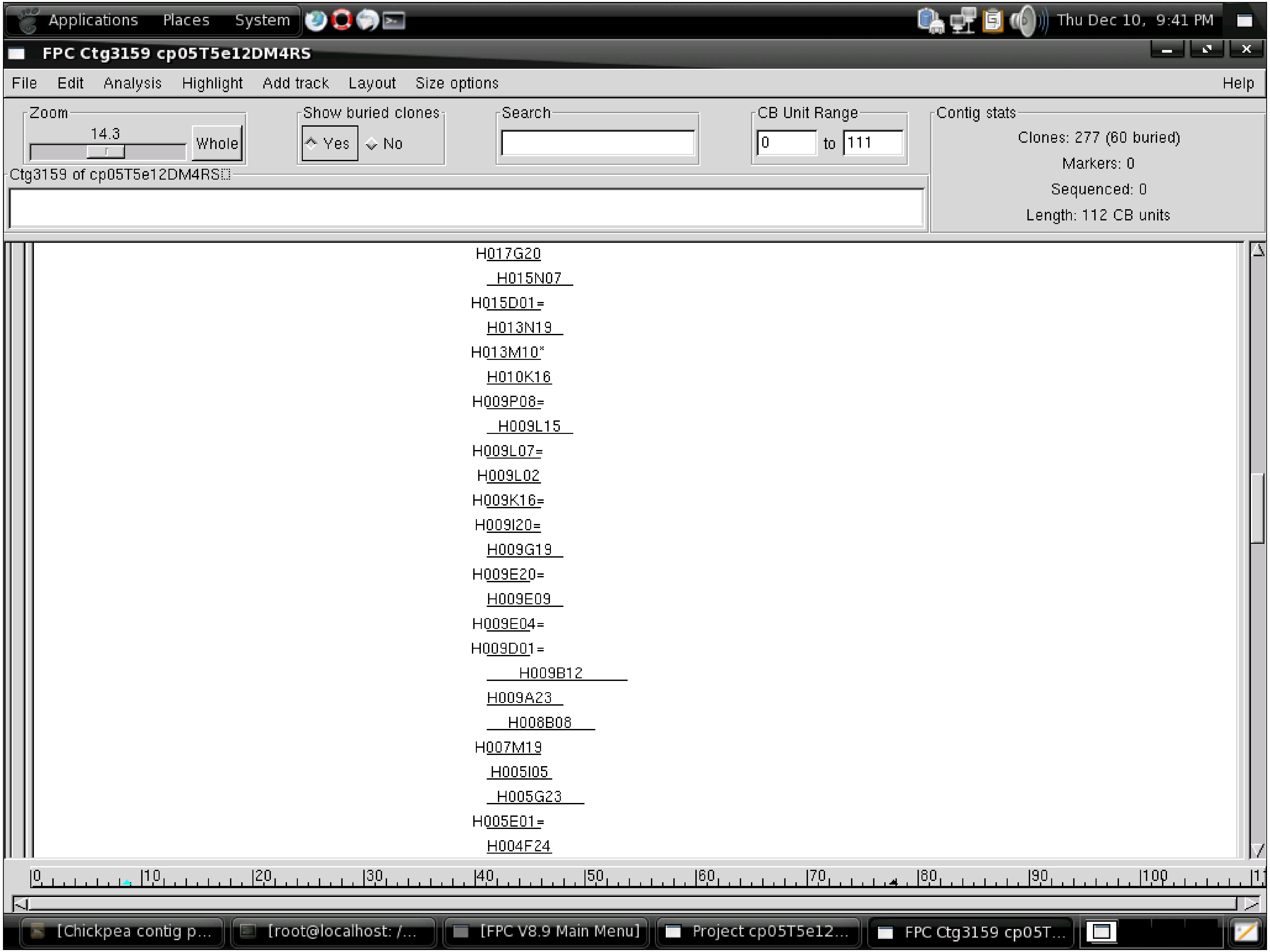
Applications Places System
Thu Dec 10, 9:41 PM
FPC Ctg3159 cp05T5e12DM4RS
File Edit Analysis Highlight Add track Layout Size options Help
Zoom
14.3
Whole
Show buried clones
Yes No
Search
CB Unit Range
0 to 111
Contig stats
Clones: 277 (60 buried)
Markers: 0
Sequenced: 0
Length: 112 CB units
Ctg3159 of cp05T5e12DM4RS
H017G20
H015N07
H015D01=
H013N19
H013M10*
H010K16
H009P08=
H009L15
H009L07=
H009L02
H009K16=
H009I20=
H009G19
H009E20=
H009E09
H009E04=
H009D01=
H009B12
H009A23
H008B08
H007M19
H005I05
H005G23
H005E01=
H004F24
0 10 20 30 40 50 60 70 80 90 100 11
[Chickpea contig p...
[root@localhost: /...
[FPC V8.9 Main Menu]
Project cp05T5e12...
FPC Ctg3159 cp05T...

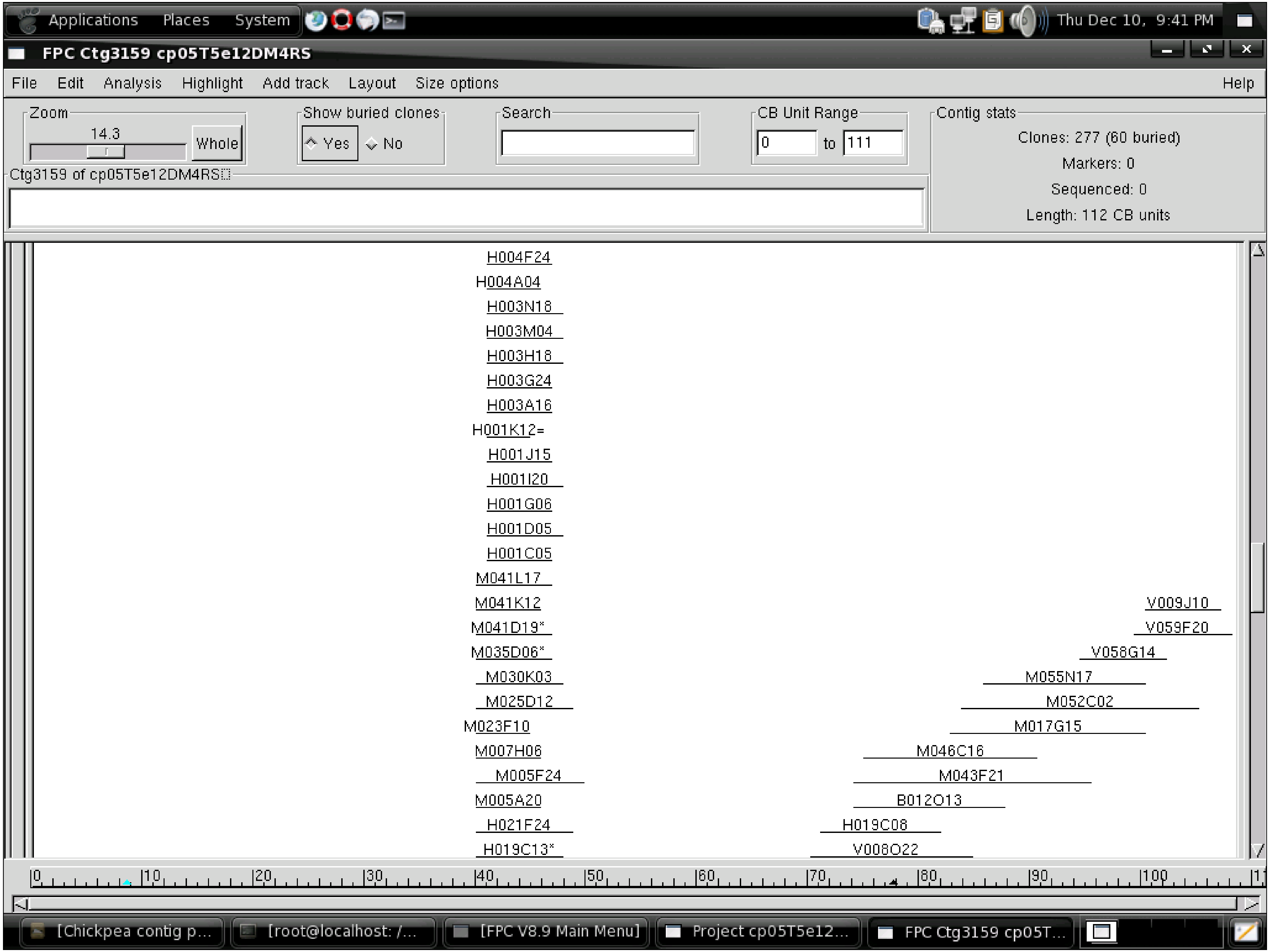
Applications Places System
Thu Dec 10, 9:41 PM
FPC Ctg3159 cp05T5e12DM4RS
File Edit Analysis Highlight Add track Layout Size options Help
Zoom
14.3
Whole
Show buried clones
Yes No
Search
CB Unit Range
0 to 111
Contig stats
Clones: 277 (60 buried)
Markers: 0
Sequenced: 0
Length: 112 CB units
Ctg3159 of cp05T5e12DM4RS
H004F24
H004A04
H003N18
H003M04
H003H18
H003G24
H003A16
H001K12=
H001J15
H001I20
H001G06
H001D05
H001C05
M041L17
M041K12
M041D19*
M035D06*
M030K03
M025D12
M023F10
M007H06
M005F24
M005A20
H021F24
H019C13*
V009J10
V059F20
V058G14
M055N17
M052C02
M017G15
M046C16
M043F21
B012O13
H019C08
V008O22
0 10 20 30 40 50 60 70 80 90 100
[Chickpea contig p...
[root@localhost: /...
[FPC V8.9 Main Menu]
Project cp05T5e12...
FPC Ctg3159 cp05T...

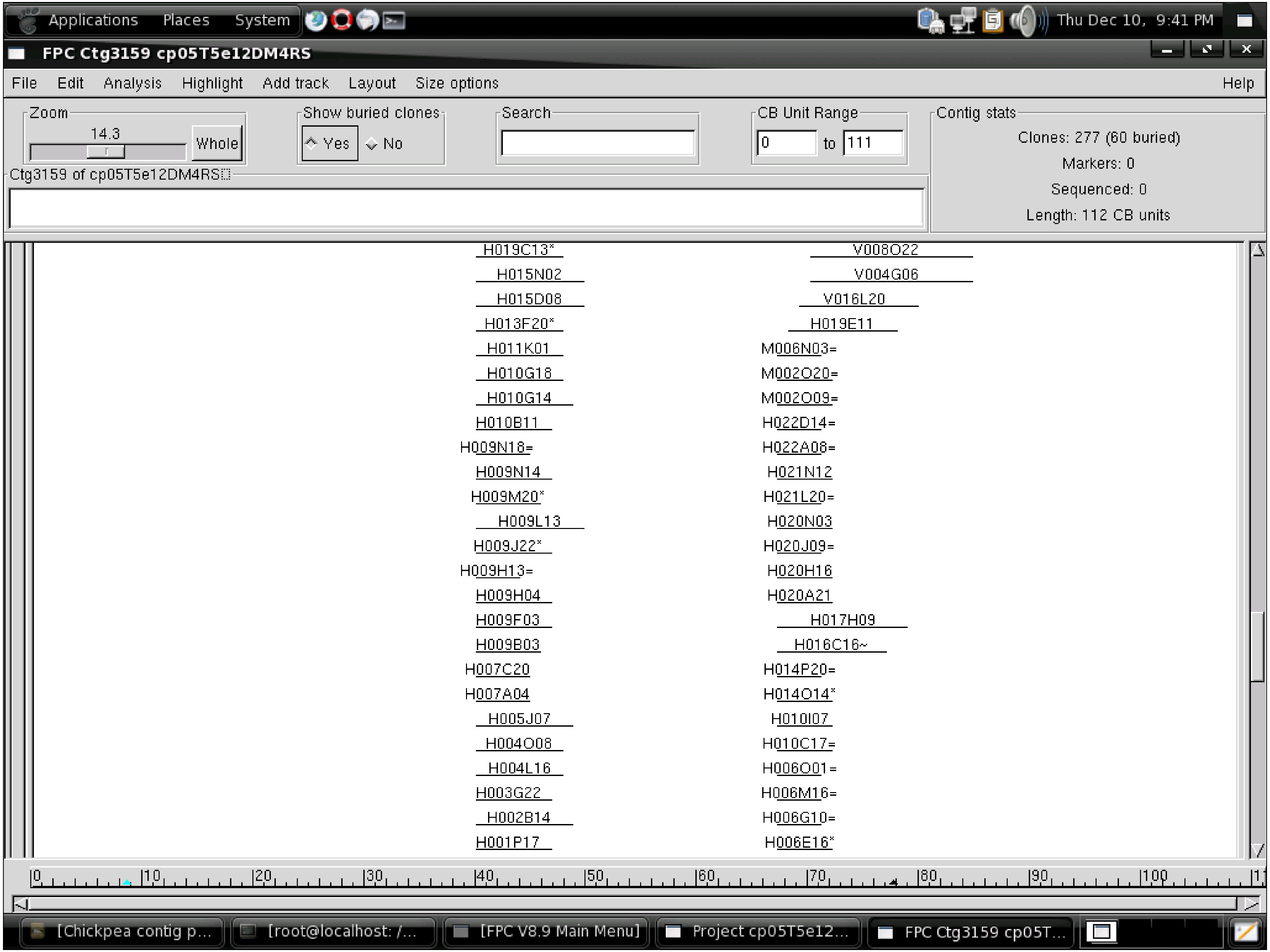
Applications Places System
Thu Dec 10, 9:41 PM
FPC Ctg3159 cp05T5e12DM4RS
File Edit Analysis Highlight Add track Layout Size options Help
Zoom
14.3
Whole
Show buried clones
Yes No
Search
CB Unit Range
0 to 111
Contig stats
Clones: 277 (60 buried)
Markers: 0
Sequenced: 0
Length: 112 CB units
Ctg3159 of cp05T5e12DM4RS
H019C13*
H015N02
H015D08
H013F20*
H011K01
H010G18
H010G14
H010B11
H009N18=
H009N14
H009M20*
H009L13
H009J22*
H009H13=
H009H04
H009F03
H009B03
H007C20
H007A04
H005J07
H004O08
H004L16
H003G22
H002B14
H001P17
V008O22
V004G06
V016L20
H019E11
M006N03=
M002O20=
M002O09=
H022D14=
H022A08=
H021N12
H021L20=
H020N03
H020J09=
H020H16
H020A21
H017H09
H016C16~
H014P20=
H014O14*
H010I07
H010C17=
H006O01=
H006M16=
H006G10=
H006E16*
0 10 20 30 40 50 60 70 80 90 100
[Chickpea contig p...
[root@localhost: /...
[FPC V8.9 Main Menu]
Project cp05T5e12...
FPC Ctg3159 cp05T...

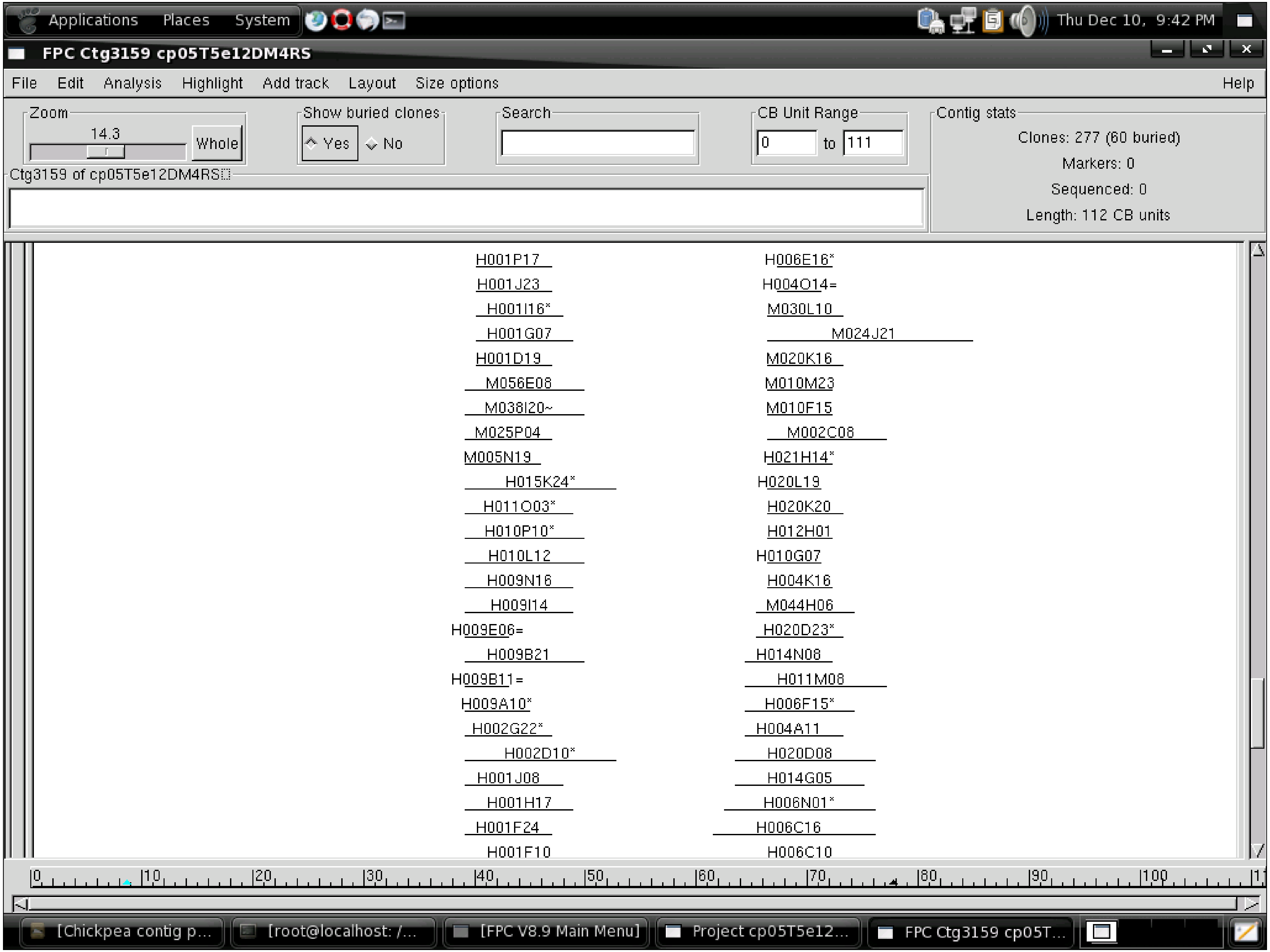
Applications Places System
Thu Dec 10, 9:42 PM
FPC Ctg3159 cp05T5e12DM4RS
File Edit Analysis Highlight Add track Layout Size options Help
Zoom
14.3
Whole
Show buried clones
Yes No
Search
CB Unit Range
0 to 111
Contig stats
Clones: 277 (60 buried)
Markers: 0
Sequenced: 0
Length: 112 CB units
Ctg3159 of cp05T5e12DM4RS
H001P17
H001J23
H001I16*
H001G07
H001D19
M056E08
M038I20~
M025P04
M005N19
H015K24*
H011O03*
H010P10*
H010L12
H009N16
H009I14
H009E06=
H009B21
H009B11=
H009A10*
H002G22*
H002D10*
H001J08
H001H17
H001F24
H001F10
H006E16*
H004O14=
M030L10
M024J21
M020K16
M010M23
M010F15
M002C08
H021H14*
H020L19
H020K20
H012H01
H010G07
H004K16
M044H06
H020D23*
H014N08
H011M08
H006F15*
H004A11
H020D08
H014G05
H006N01*
H006C16
H006C10
0 10 20 30 40 50 60 70 80 90 100
[Chickpea contig p...
[root@localhost: /...
[FPC V8.9 Main Menu]
Project cp05T5e12...
FPC Ctg3159 cp05T...

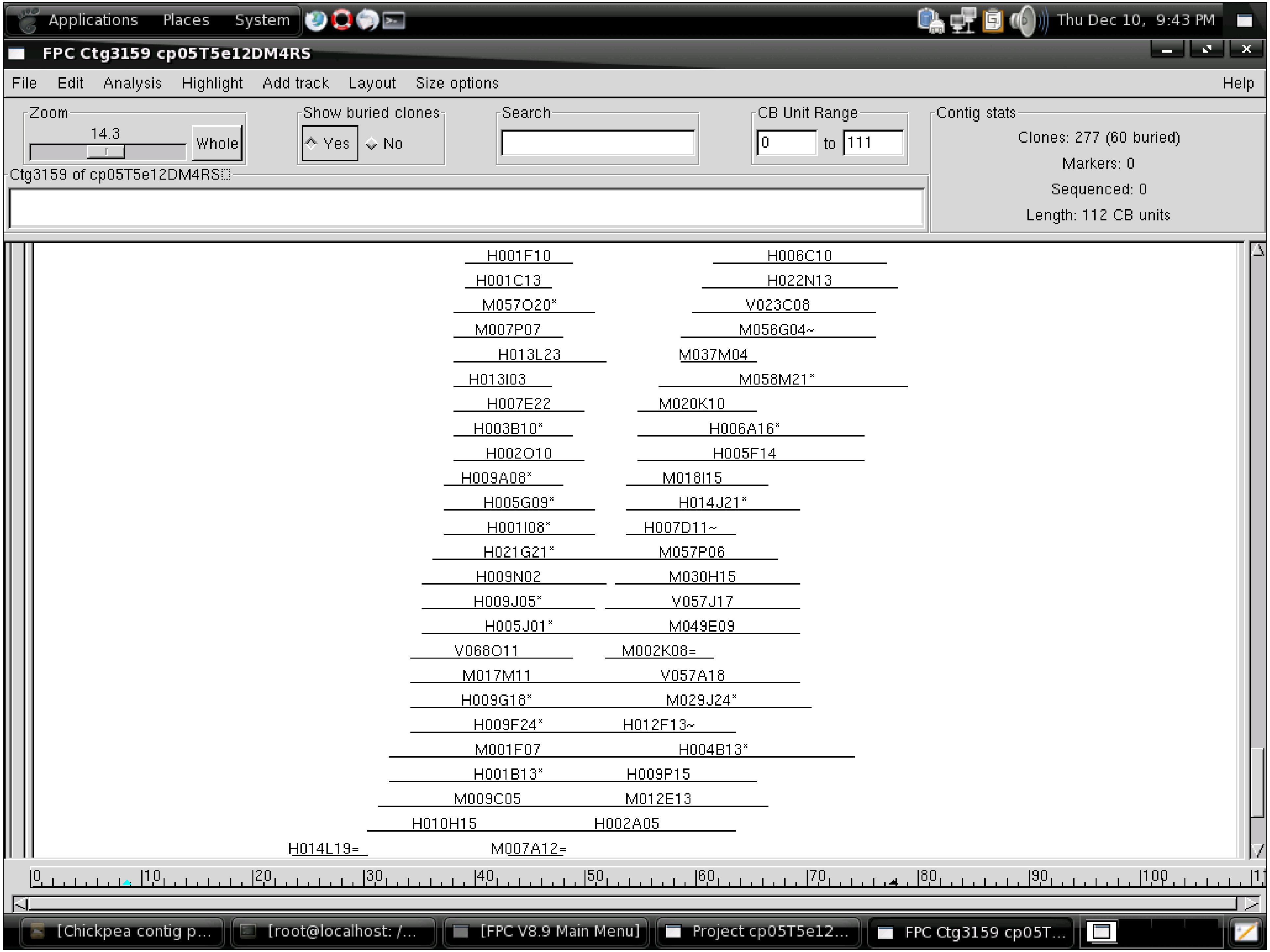
Applications Places System
Thu Dec 10, 9:43 PM
FPC Ctg3159 cp05T5e12DM4RS
File Edit Analysis Highlight Add track Layout Size options Help
Zoom
14.3
Whole
Show buried clones
Yes No
Search
CB Unit Range
0 to 111
Contig stats
Clones: 277 (60 buried)
Markers: 0
Sequenced: 0
Length: 112 CB units
Ctg3159 of cp05T5e12DM4RS
H001F10
H006C10
H001C13
H022N13
M057O20*
V023C08
M007P07
M056G04~
H013L23
M037M04
H013I03
M058M21*
H007E22
M020K10
H003B10*
H006A16*
H002O10
H005F14
H009A08*
M018I15
H005G09*
H014J21*
H001I08*
H007D11~
H021G21*
M057P06
H009N02
M030H15
H009J05*
V057J17
H005J01*
M049E09
V068O11
M002K08=
M017M11
V057A18
H009G18*
M029J24*
H009F24*
H012F13~
M001F07
H004B13*
H001B13*
H009P15
M009C05
M012E13
H010H15
H002A05
H014L19=
M007A12=
0 10 20 30 40 50 60 70 80 90 100
[Chickpea contig p...
[root@localhost: /...
[FPC V8.9 Main Menu]
Project cp05T5e12...
FPC Ctg3159 cp05T...

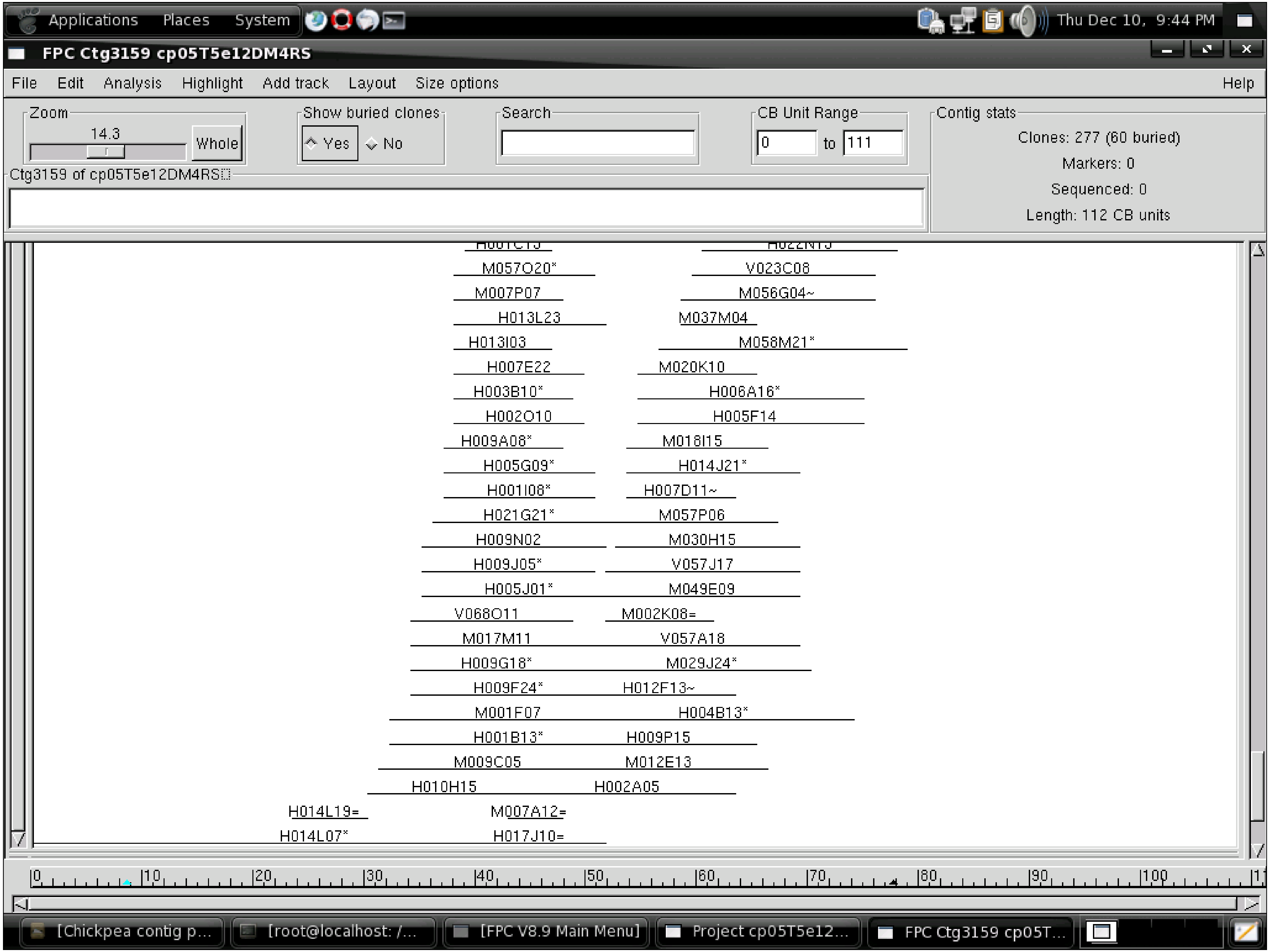
Applications Places System
Thu Dec 10, 9:44 PM
FPC Ctg3159 cp05T5e12DM4RS
File Edit Analysis Highlight Add track Layout Size options Help
Zoom
14.3
Whole
Show buried clones
Yes No
Search
CB Unit Range
0 to 111
Contig stats
Clones: 277 (60 buried)
Markers: 0
Sequenced: 0
Length: 112 CB units
Ctg3159 of cp05T5e12DM4RS
M057O20*
V023C08
M007P07
M056G04~
H013L23
M037M04
H013I03
M058M21*
H007E22
M020K10
H003B10*
H006A16*
H002O10
H005F14
H009A08*
M018I15
H005G09*
H014J21*
H001I08*
H007D11~
H021G21*
M057P06
H009N02
M030H15
H009J05*
V057J17
H005J01*
M049E09
V068O11
M002K08=
M017M11
V057A18
H009G18*
M029J24*
H009F24*
H012F13~
M001F07
H004B13*
H001B13*
H009P15
M009C05
M012E13
H010H15
H002A05
H014L19=
M007A12=
H014L07*
H017J10=
0 10 20 30 40 50 60 70 80 90 100
[Chickpea contig p...
[root@localhost: /...
[FPC V8.9 Main Menu]
Project cp05T5e12...
FPC Ctg3159 cp05T...

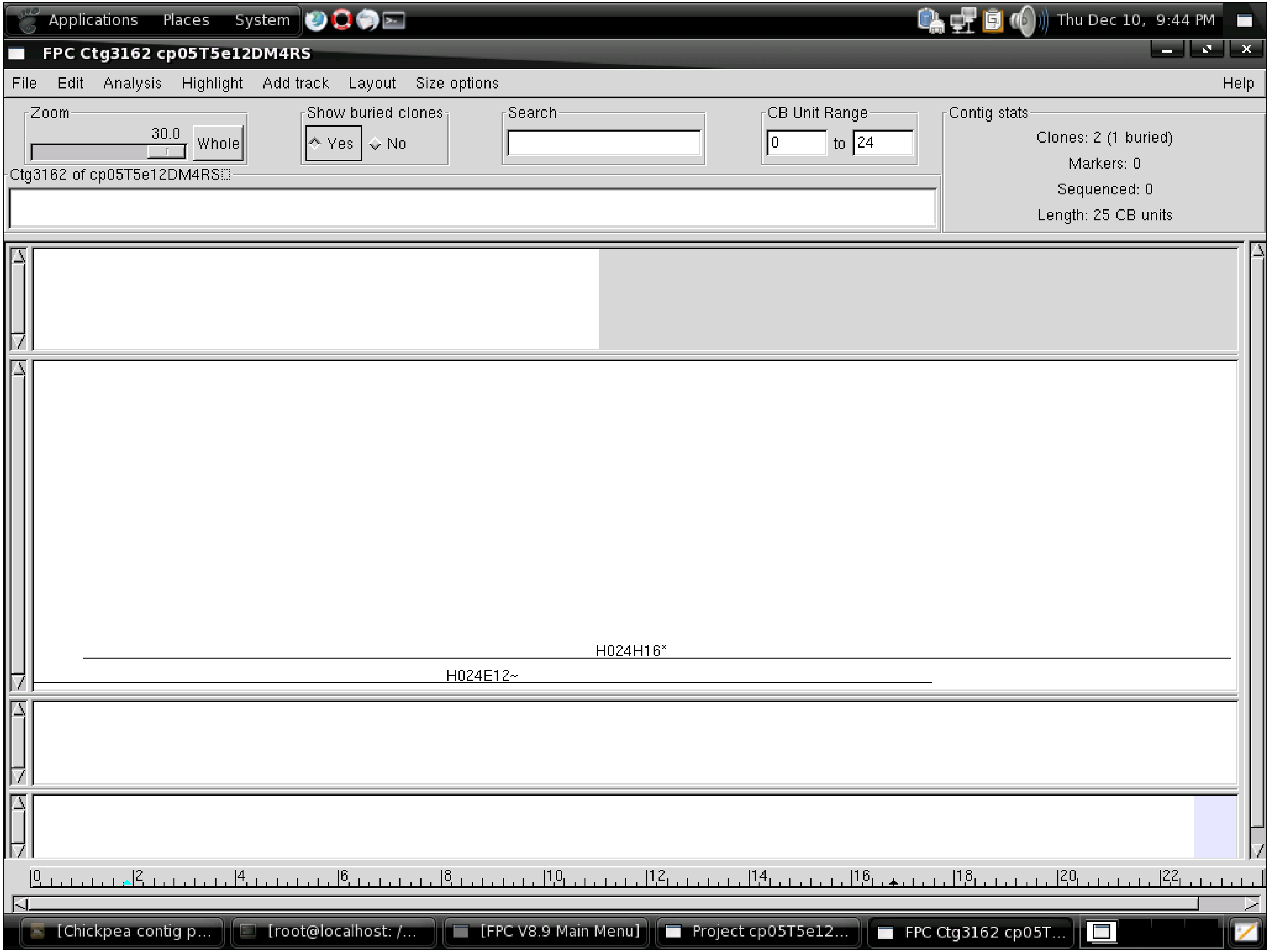

Applications
Places
System
Thu Dec 10, 9:44 PM
FPC Ctg3162 cp05T5e12DM4RS
File
Edit
Analysis
Highlight
Add track
Layout
Size options
Help
Zoom
30.0
Whole
Show buried clones
Yes
No
Search
CB Unit Range
0
to
24
Contig stats
Clones: 2 (1 buried)
Markers: 0
Sequenced: 0
Length: 25 CB units
Ctg3162 of cp05T5e12DM4RS
H024H16*
H024E12~
0
2
4
6
8
10
12
14
16
18
20
22
[Chickpea contig p...
[root@localhost: /...
[FPC V8.9 Main Menu]
Project cp05T5e12...
FPC Ctg3162 cp05T...

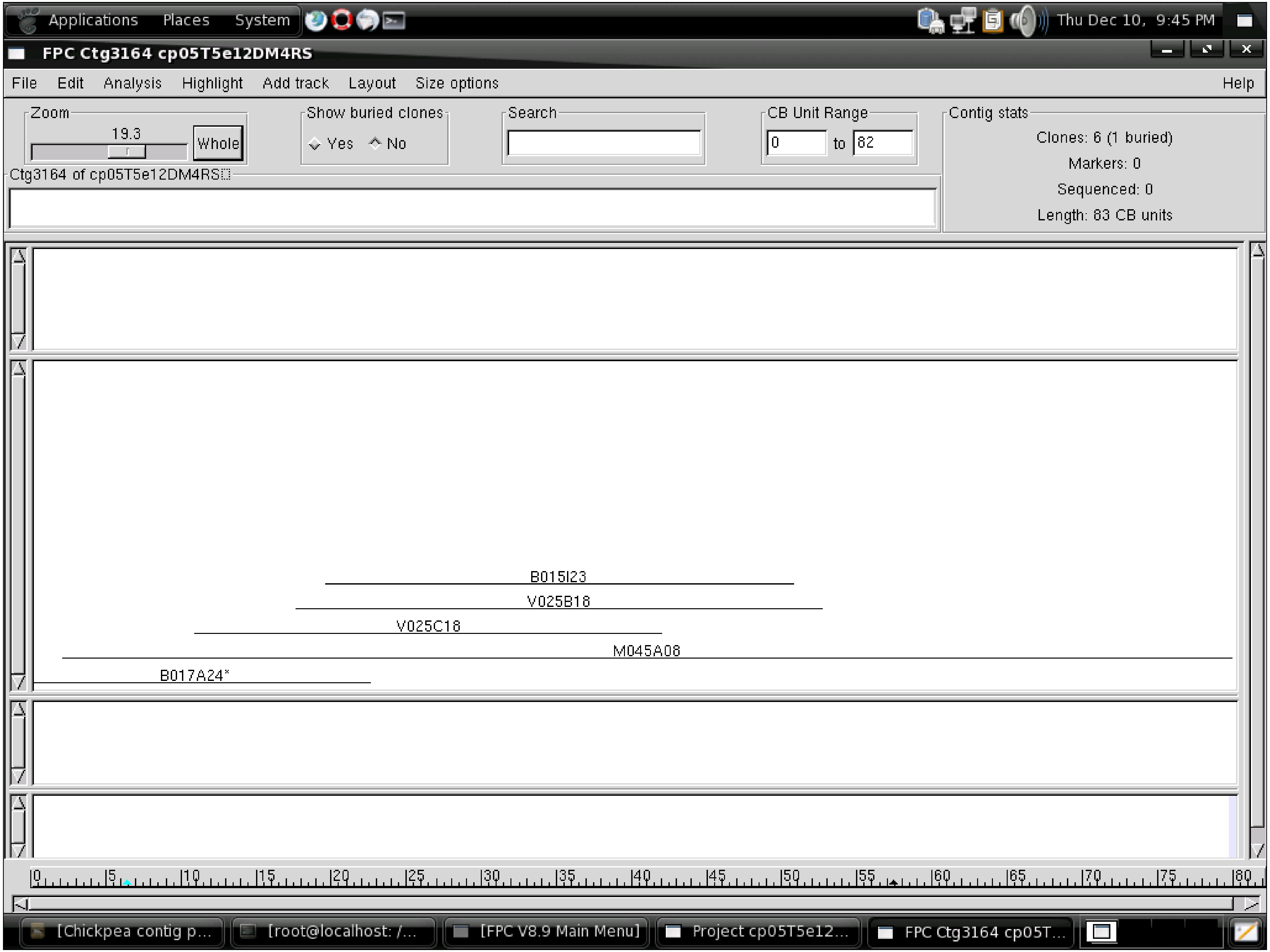
Applications Places System
Thu Dec 10, 9:45 PM
FPC Ctg3164 cp05T5e12DM4RS
File Edit Analysis Highlight Add track Layout Size options Help
Zoom
19.3
Whole
Show buried clones
Yes No
Search
CB Unit Range
0 to 82
Contig stats
Clones: 6 (1 buried)
Markers: 0
Sequenced: 0
Length: 83 CB units
Ctg3164 of cp05T5e12DM4RS
B015I23
V025B18
V025C18
M045A08
B017A24*
0 5 10 15 20 25 30 35 40 45 50 55 60 65 70 75 80
[Chickpea contig p...
[root@localhost: /...
[FPC V8.9 Main Menu]
Project cp05T5e12...
FPC Ctg3164 cp05T...

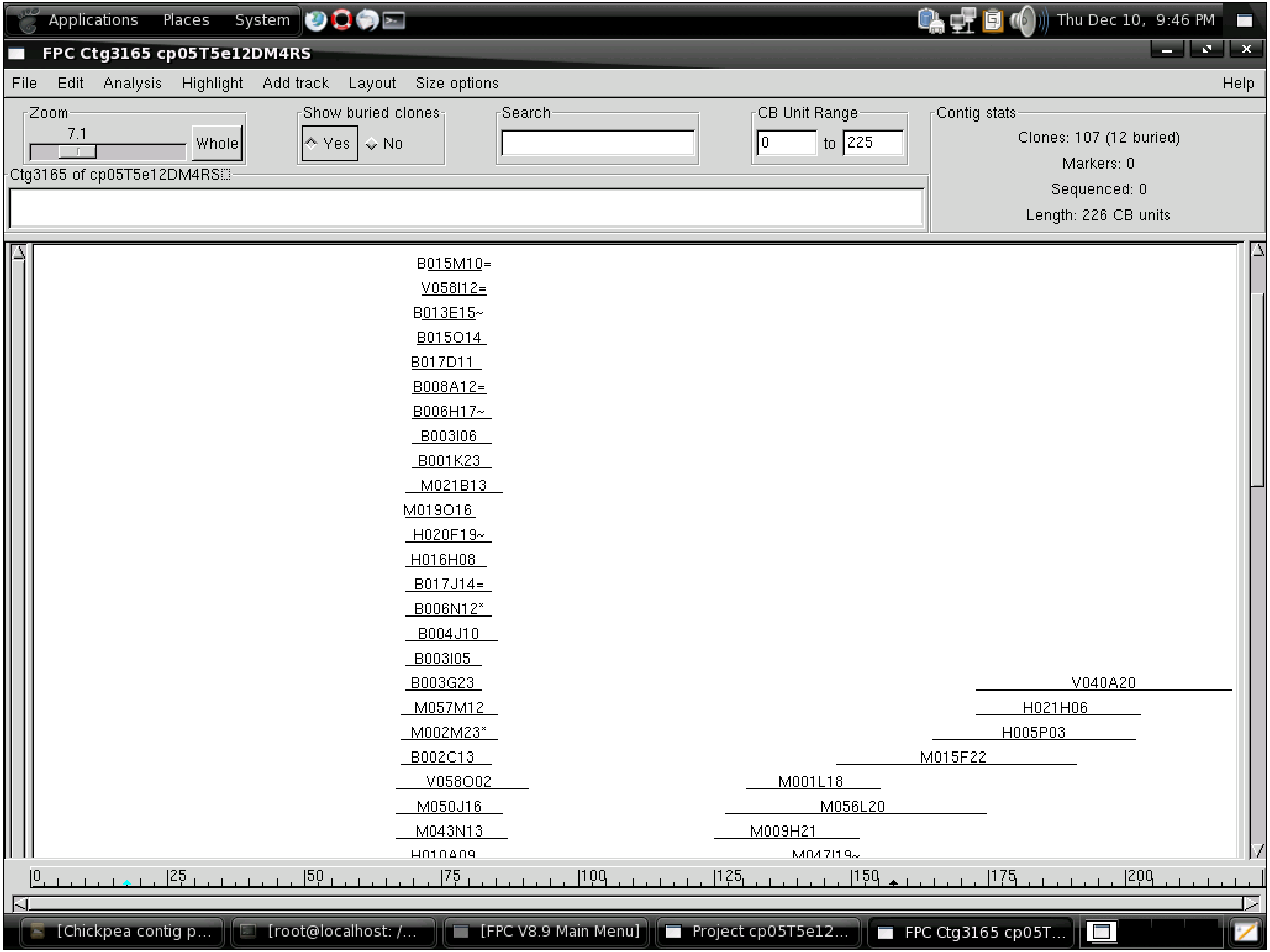

Applications
Places
System
Thu Dec 10, 9:46 PM
FPC Ctg3165 cp05T5e12DM4RS
File
Edit
Analysis
Highlight
Add track
Layout
Size options
Help
Zoom
7.1
Whole
Show buried clones
Yes
No
Search
CB Unit Range
0
to
225
Contig stats
Clones: 107 (12 buried)
Markers: 0
Sequenced: 0
Length: 226 CB units
Ctg3165 of cp05T5e12DM4RS
B015M10=
V058I12=
B013E15~
B015O14
B017D11
B008A12=
B006H17~
B003I06
B001K23
M021B13
M019O16
H020F19~
H016H08
B017J14=
B006N12*
B004J10
B003I05
B003G23
V040A20
M057M12
H021H06
M002M23*
H005P03
B002C13
M015F22
V058O02
M001L18
M050J16
M056L20
M043N13
M009H21
0
25
50
75
100
125
150
175
200
[Chickpea contig p...
[root@localhost: /...
[FPC V8.9 Main Menu]
Project cp05T5e12...
FPC Ctg3165 cp05T...

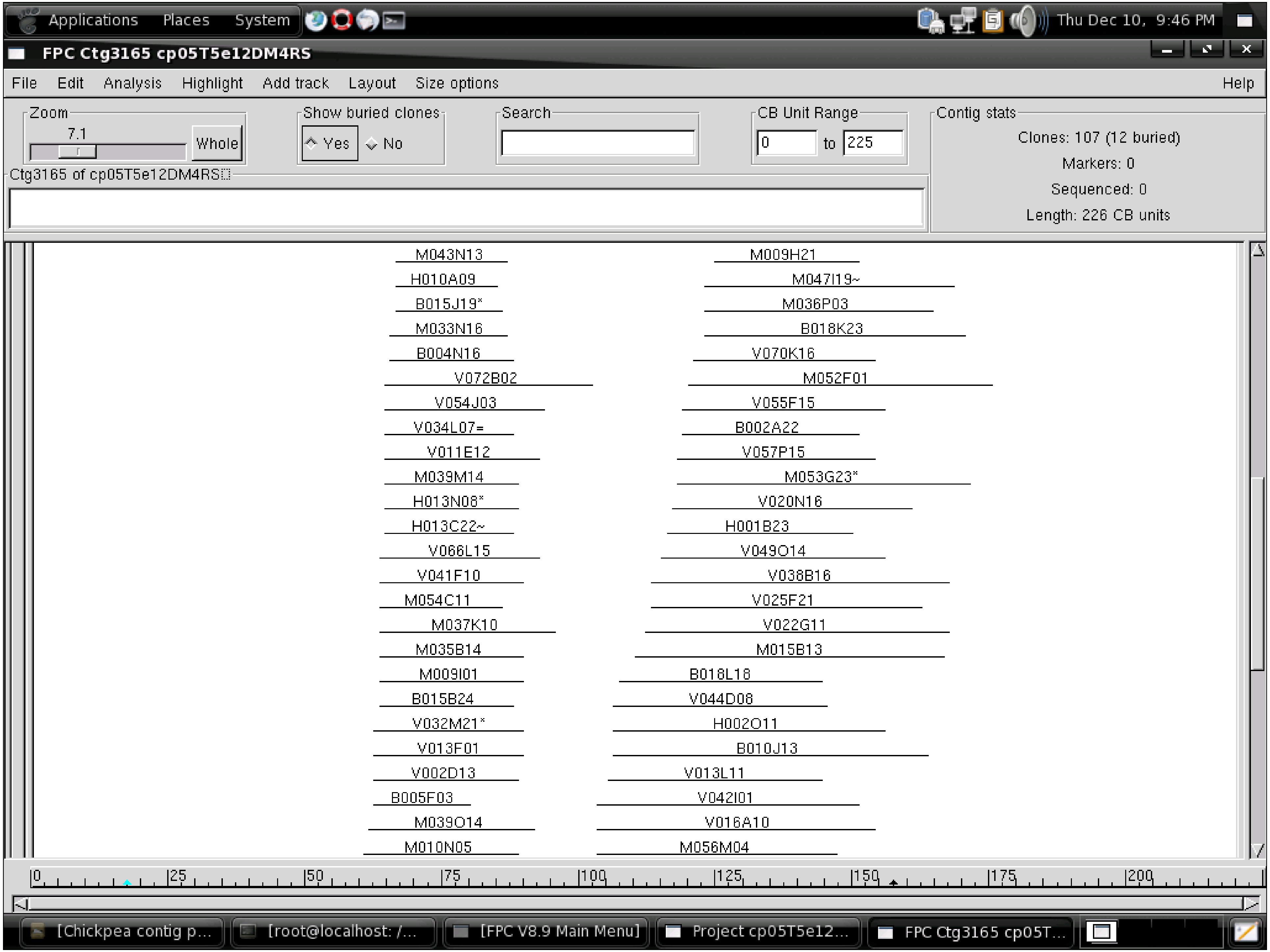
Applications Places System
Thu Dec 10, 9:46 PM
FPC Ctg3165 cp05T5e12DM4RS
File Edit Analysis Highlight Add track Layout Size options Help
Zoom
7.1
Whole
Show buried clones
Yes No
Search
CB Unit Range
0 to 225
Contig stats
Clones: 107 (12 buried)
Markers: 0
Sequenced: 0
Length: 226 CB units
Ctg3165 of cp05T5e12DM4RS
M043N13
M009H21
H010A09
M047I19~
B015J19*
M036P03
M033N16
B018K23
B004N16
V070K16
V072B02
M052F01
V054J03
V055F15
V034L07=
B002A22
V011E12
V057P15
M039M14
M053G23*
H013N08*
V020N16
H013C22~
H001B23
V066L15
V049O14
V041F10
V038B16
M054C11
V025F21
M037K10
V022G11
M035B14
M015B13
M009I01
B018L18
B015B24
V044D08
V032M21*
H002O11
V013F01
B010J13
V002D13
V013L11
B005F03
V042I01
M039O14
V016A10
M010N05
M056M04
0 25 50 75 100 125 150 175 200
[Chickpea contig p...
[root@localhost: /...
[FPC V8.9 Main Menu]
Project cp05T5e12...
FPC Ctg3165 cp05T...

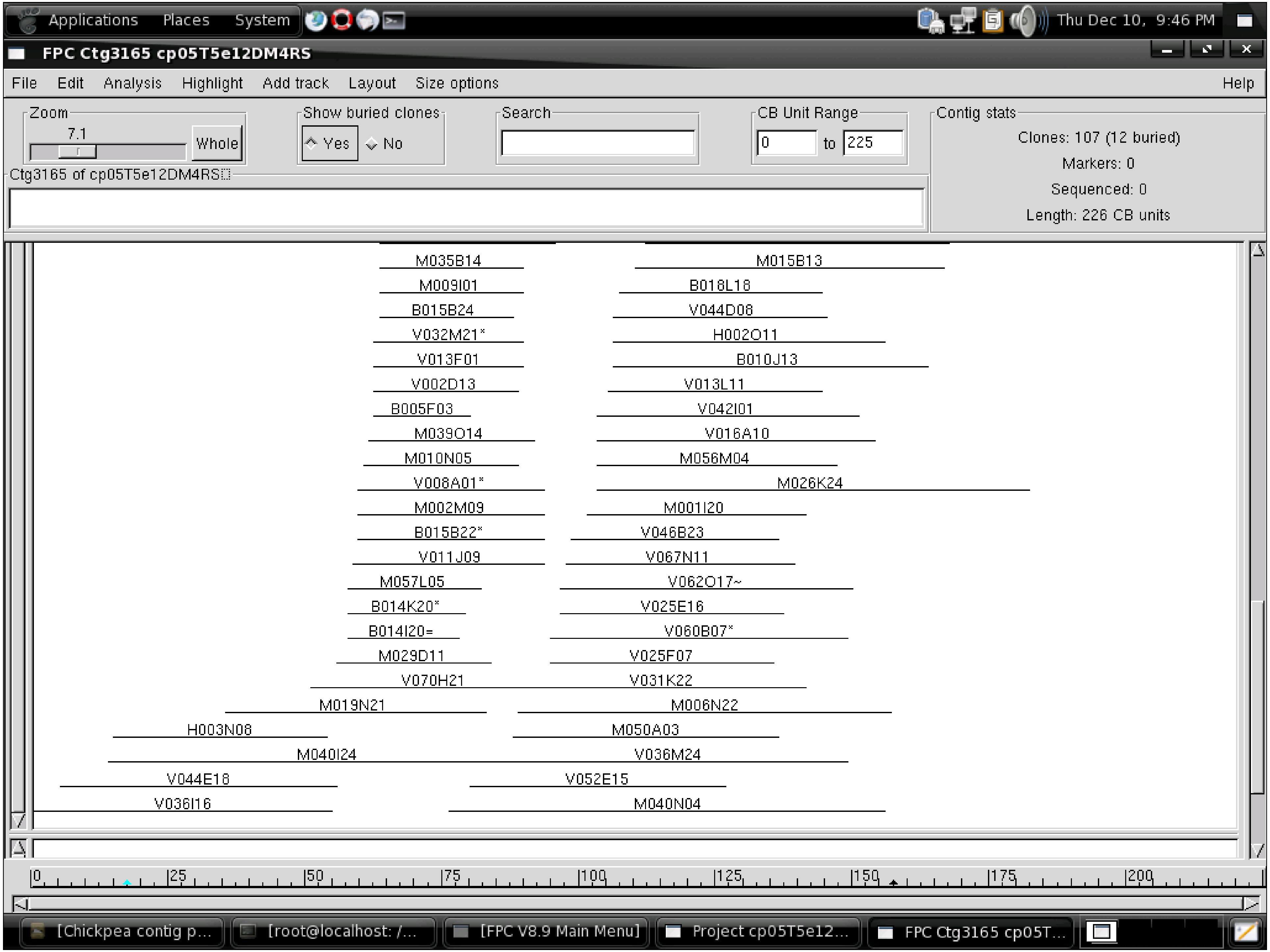
Applications Places System
Thu Dec 10, 9:46 PM
FPC Ctg3165 cp05T5e12DM4RS
File Edit Analysis Highlight Add track Layout Size options Help
Zoom
7.1
Whole
Show buried clones
Yes No
Search
CB Unit Range
0 to 225
Contig stats
Clones: 107 (12 buried)
Markers: 0
Sequenced: 0
Length: 226 CB units
Ctg3165 of cp05T5e12DM4RS
M035B14
M015B13
M009I01
B018L18
B015B24
V044D08
V032M21*
H002O11
V013F01
B010J13
V002D13
V013L11
B005F03
V042I01
M039O14
V016A10
M010N05
M056M04
V008A01*
M026K24
M002M09
M001I20
B015B22*
V046B23
V011J09
V067N11
M057L05
V062O17~
B014K20*
V025E16
B014I20=
V060B07*
M029D11
V025F07
V070H21
V031K22
M019N21
M006N22
H003N08
M050A03
M040I24
V036M24
V044E18
V052E15
V036I16
M040N04
0 25 50 75 100 125 150 175 200
[Chickpea contig p...
[root@localhost: /...
[FPC V8.9 Main Menu]
Project cp05T5e12...
FPC Ctg3165 cp05T...

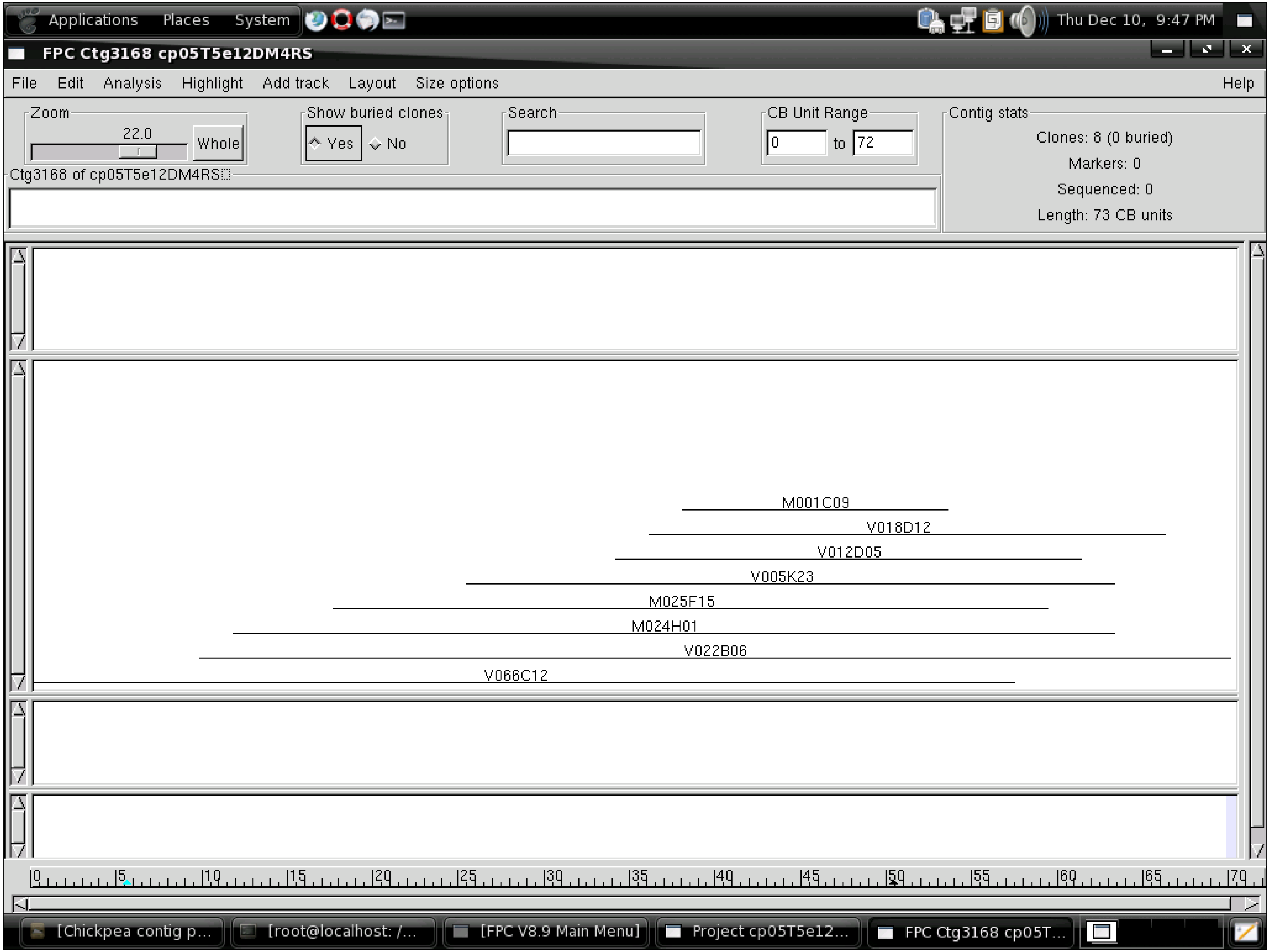
Applications
Places
System
Thu Dec 10, 9:47 PM
FPC Ctg3168 cp05T5e12DM4RS
File
Edit
Analysis
Highlight
Add track
Layout
Size options
Help
Zoom
22.0
Whole
Show buried clones
Yes
No
Search
CB Unit Range
0
to
72
Contig stats
Clones: 8 (0 buried)
Markers: 0
Sequenced: 0
Length: 73 CB units
Ctg3168 of cp05T5e12DM4RS
M001C09
V018D12
V012D05
V005K23
M025F15
M024H01
V022B06
V066C12
0
5
10
15
20
25
30
35
40
45
50
55
60
65
70
[Chickpea contig p...
[root@localhost: /...
[FPC V8.9 Main Menu]
Project cp05T5e12...
FPC Ctg3168 cp05T...

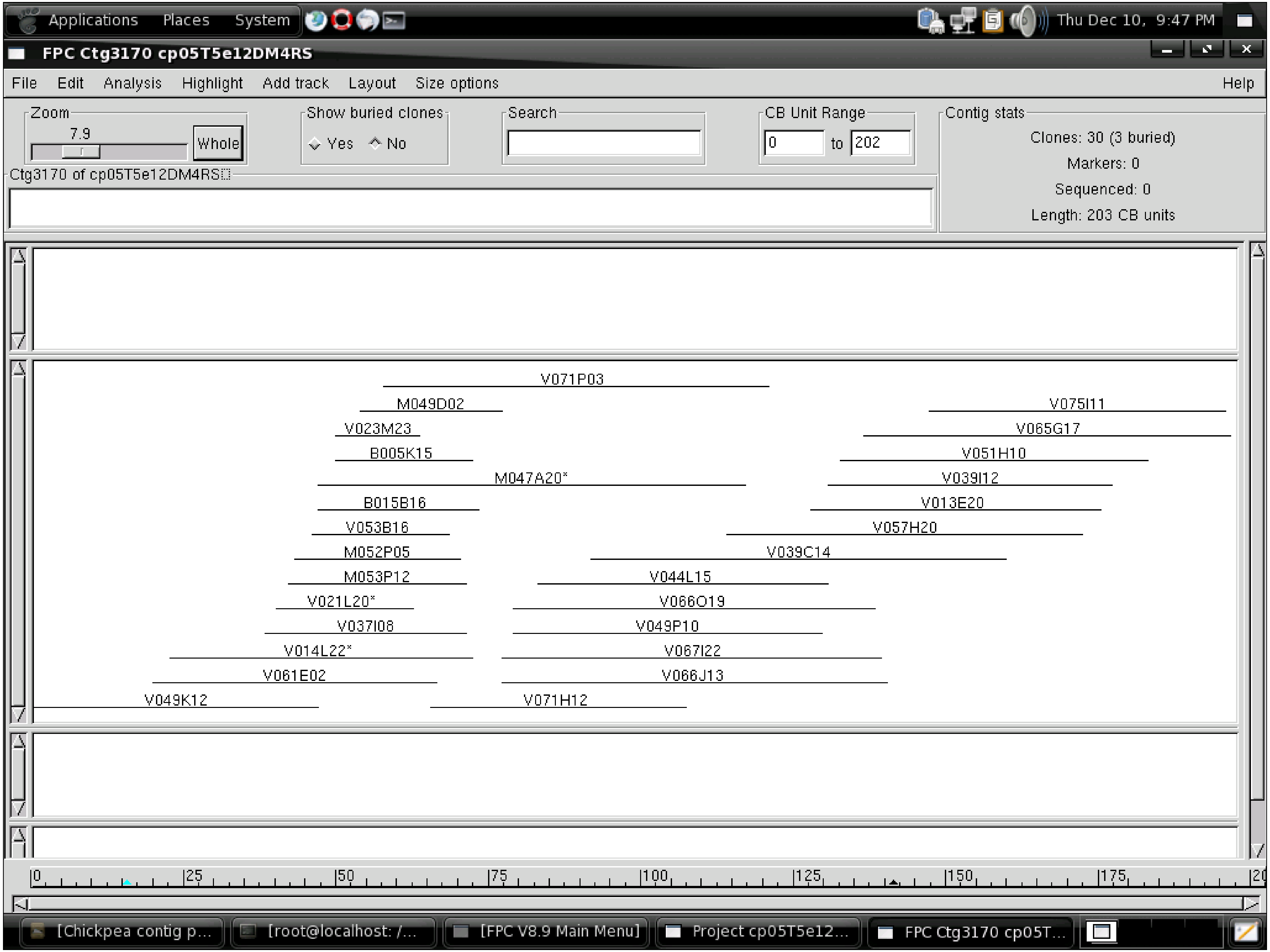
Applications Places System
Thu Dec 10, 9:47 PM
FPC Ctg3170 cp05T5e12DM4RS
File Edit Analysis Highlight Add track Layout Size options Help
Zoom
7.9
Whole
Show buried clones
Yes No
Search
CB Unit Range
0 to 202
Contig stats
Clones: 30 (3 buried)
Markers: 0
Sequenced: 0
Length: 203 CB units
Ctg3170 of cp05T5e12DM4RS
V071P03
M049D02
V075I11
V023M23
V065G17
B005K15
V051H10
M047A20*
V039I12
B015B16
V013E20
V053B16
V057H20
M052P05
V039C14
M053P12
V044L15
V021L20*
V066O19
V037I08
V049P10
V014L22*
V067I22
V061E02
V066J13
V049K12
V071H12
0 25 50 75 100 125 150 175
[Chickpea contig p...
[root@localhost: /...
[FPC V8.9 Main Menu]
Project cp05T5e12...
FPC Ctg3170 cp05T...

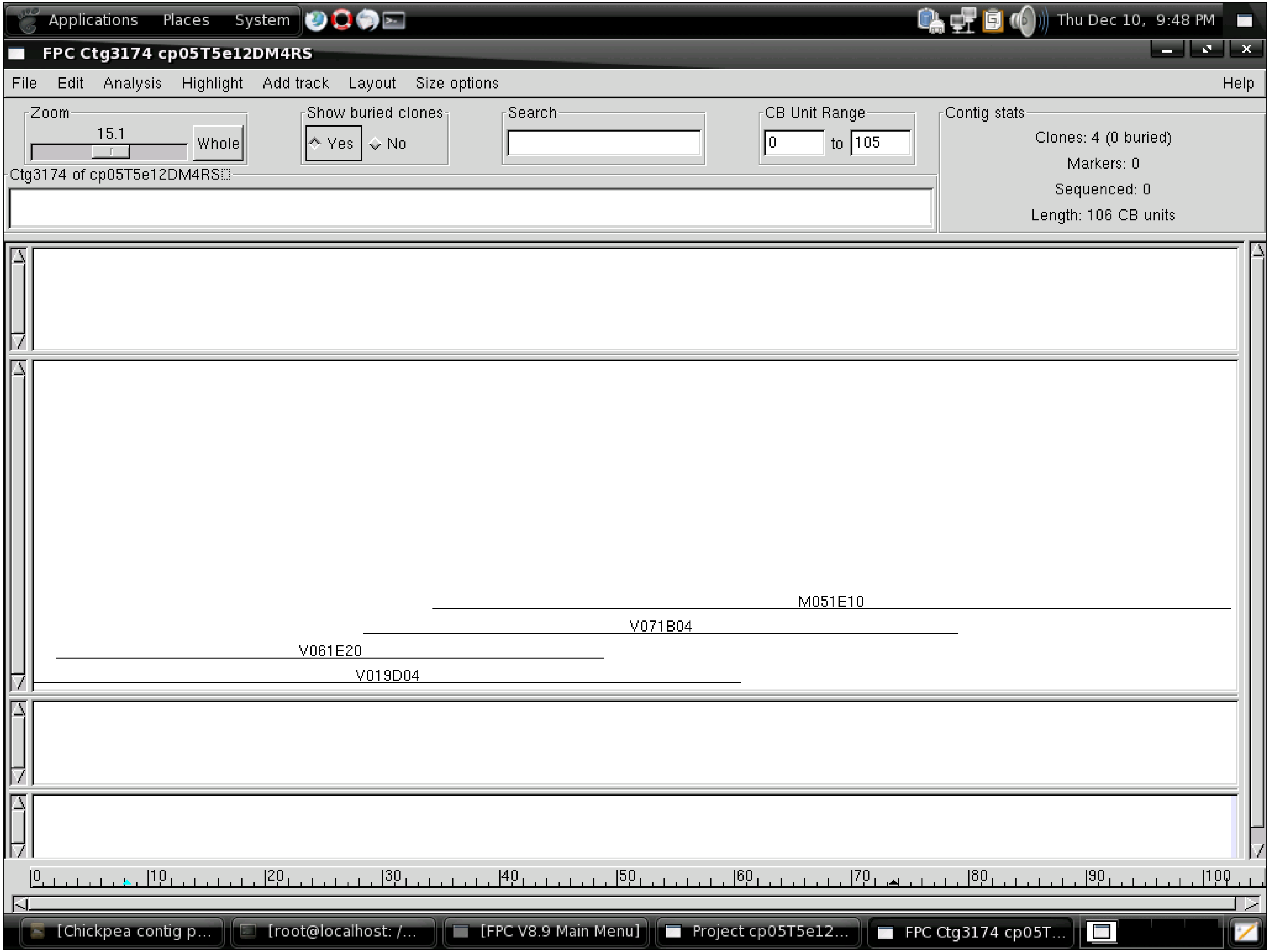
Applications Places System
Thu Dec 10, 9:48 PM
FPC Ctg3174 cp05T5e12DM4RS
File Edit Analysis Highlight Add track Layout Size options Help
Zoom
15.1
Whole
Show buried clones
Yes
No
Search
CB Unit Range
0 to 105
Contig stats
Clones: 4 (0 buried)
Markers: 0
Sequenced: 0
Length: 106 CB units
Ctg3174 of cp05T5e12DM4RS
M051E10
V071B04
V061E20
V019D04
0 10 20 30 40 50 60 70 80 90 100
[Chickpea contig p...
[root@localhost: /...
[FPC V8.9 Main Menu]
Project cp05T5e12...
FPC Ctg3174 cp05T...

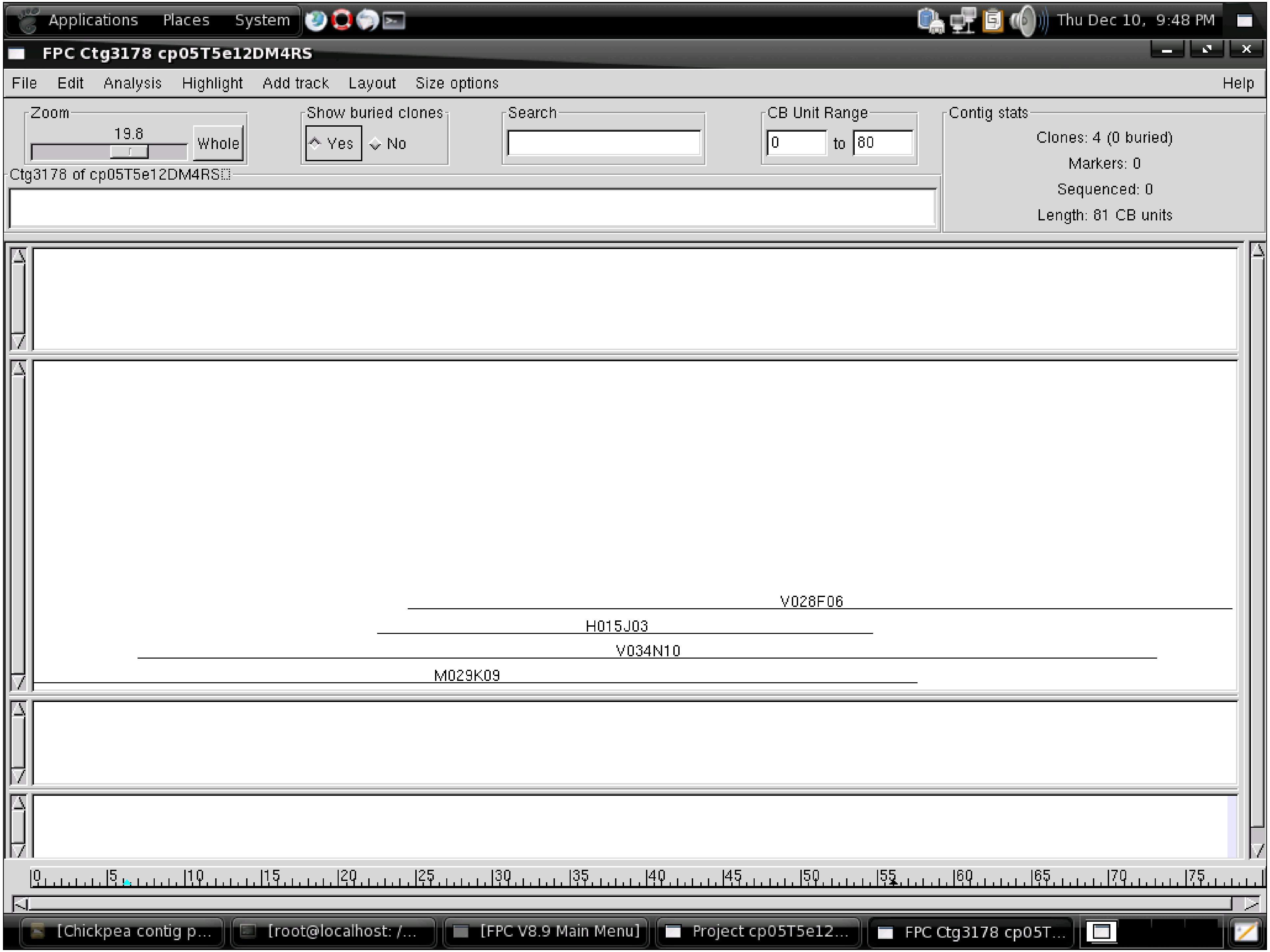
Applications Places System
Thu Dec 10, 9:48 PM
FPC Ctg3178 cp05T5e12DM4RS
File Edit Analysis Highlight Add track Layout Size options Help
Zoom
19.8
Whole
Show buried clones
Yes
No
Search
CB Unit Range
0
to
80
Contig stats
Clones: 4 (0 buried)
Markers: 0
Sequenced: 0
Length: 81 CB units
Ctg3178 of cp05T5e12DM4RS
V028F06
H015J03
V034N10
M029K09
0 5 10 15 20 25 30 35 40 45 50 55 60 65 70 75
[Chickpea contig p...
[root@localhost: /...
[FPC V8.9 Main Menu]
Project cp05T5e12...
FPC Ctg3178 cp05T...

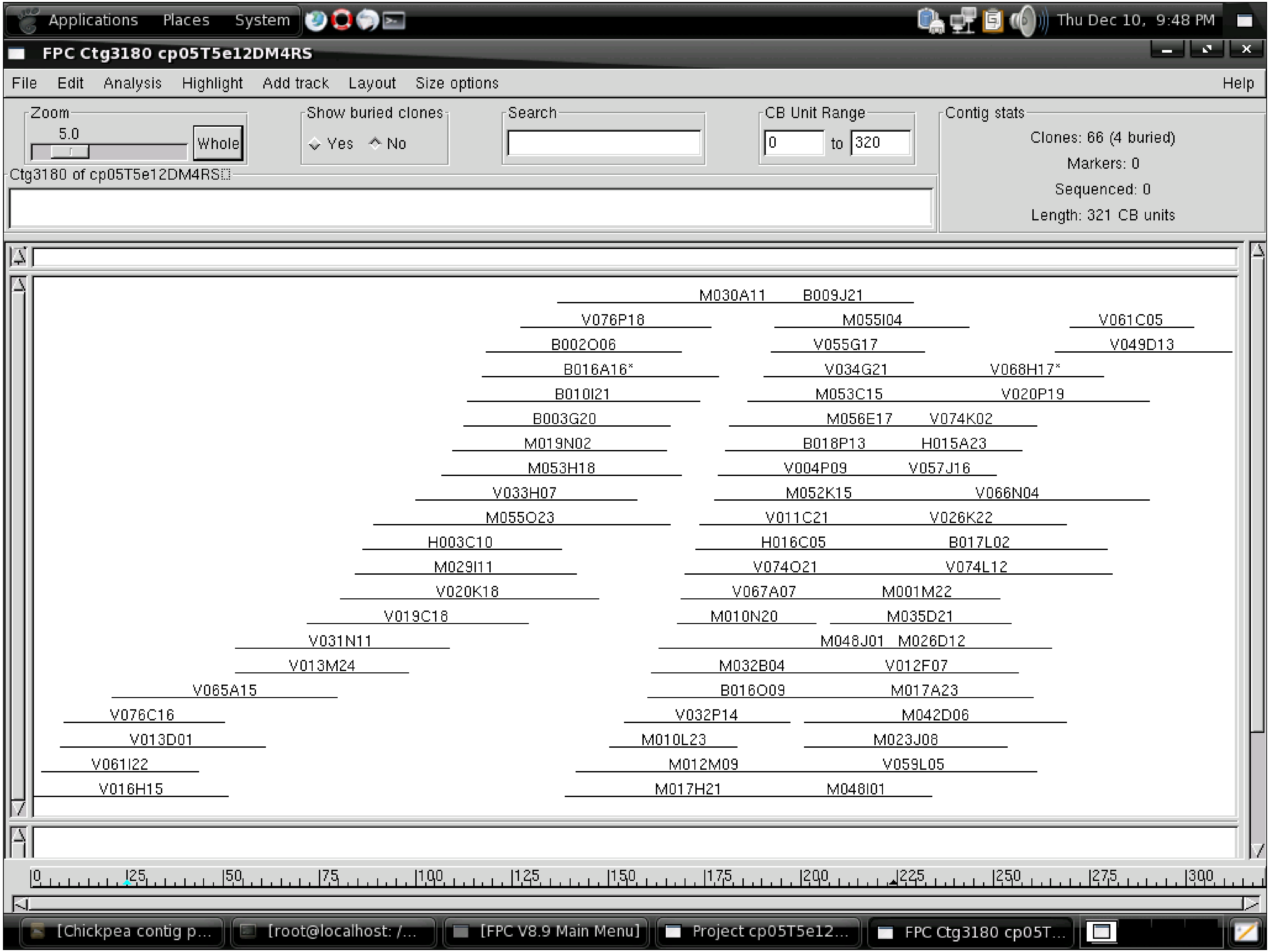
Applications Places System
Thu Dec 10, 9:48 PM
FPC Ctg3180 cp05T5e12DM4RS
File Edit Analysis Highlight Add track Layout Size options Help
Zoom
5.0
Whole
Show buried clones
Yes No
Search
CB Unit Range
0 to 320
Contig stats
Clones: 66 (4 buried)
Markers: 0
Sequenced: 0
Length: 321 CB units
Ctg3180 of cp05T5e12DM4RS
M030A11 B009J21
V076P18 M055I04 V061C05
B002O06 V055G17 V049D13
B016A16* V034G21 V068H17*
B010I21 M053C15 V020P19
B003G20 M056E17 V074K02
M019N02 B018P13 H015A23
M053H18 V004P09 V057J16
V033H07 M052K15 V066N04
M055O23 V011C21 V026K22
H003C10 H016C05 B017L02
M029I11 V074O21 V074L12
V020K18 V067A07 M001M22
V019C18 M010N20 M035D21
V031N11 M048J01 M026D12
V013M24 M032B04 V012F07
V065A15 B016O09 M017A23
V076C16 V032P14 M042D06
V013D01 M010L23 M023J08
V061I22 M012M09 V059L05
V016H15 M017H21 M048I01
0 25 50 75 100 125 150 175 200 225 250 275 300
[Chickpea contig p...
[root@localhost: /...
[FPC V8.9 Main Menu]
Project cp05T5e12...
FPC Ctg3180 cp05T...

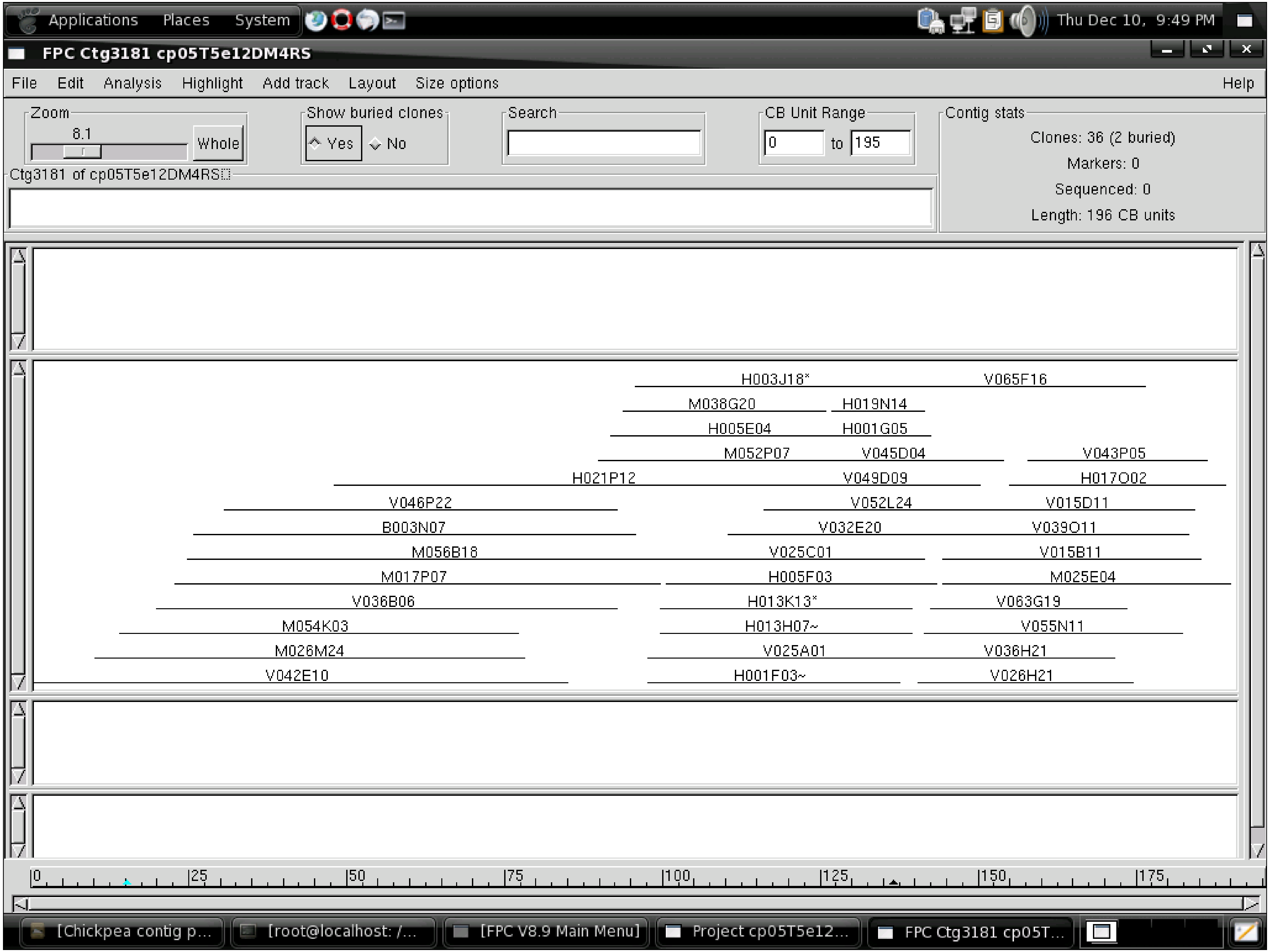

Applications Places System
Thu Dec 10, 9:49 PM
FPC Ctg3181 cp05T5e12DM4RS
File Edit Analysis Highlight Add track Layout Size options Help
Zoom
8.1
Whole
Show buried clones
Yes
No
Search
CB Unit Range
0 to 195
Contig stats
Clones: 36 (2 buried)
Markers: 0
Sequenced: 0
Length: 196 CB units
Ctg3181 of cp05T5e12DM4RS
H003J18*
V065F16
M038G20
H019N14
H005E04
H001G05
M052P07
V045D04
V043P05
H021P12
V049D09
H017O02
V046P22
V052L24
V015D11
B003N07
V032E20
V039O11
M056B18
V025C01
V015B11
M017P07
H005F03
M025E04
V036B06
H013K13*
V063G19
M054K03
H013H07~
V055N11
M026M24
V025A01
V036H21
V042E10
H001F03~
V026H21
0
25
50
75
100
125
150
175
[Chickpea contig p...
[root@localhost: /...
[FPC V8.9 Main Menu]
Project cp05T5e12...
FPC Ctg3181 cp05T...

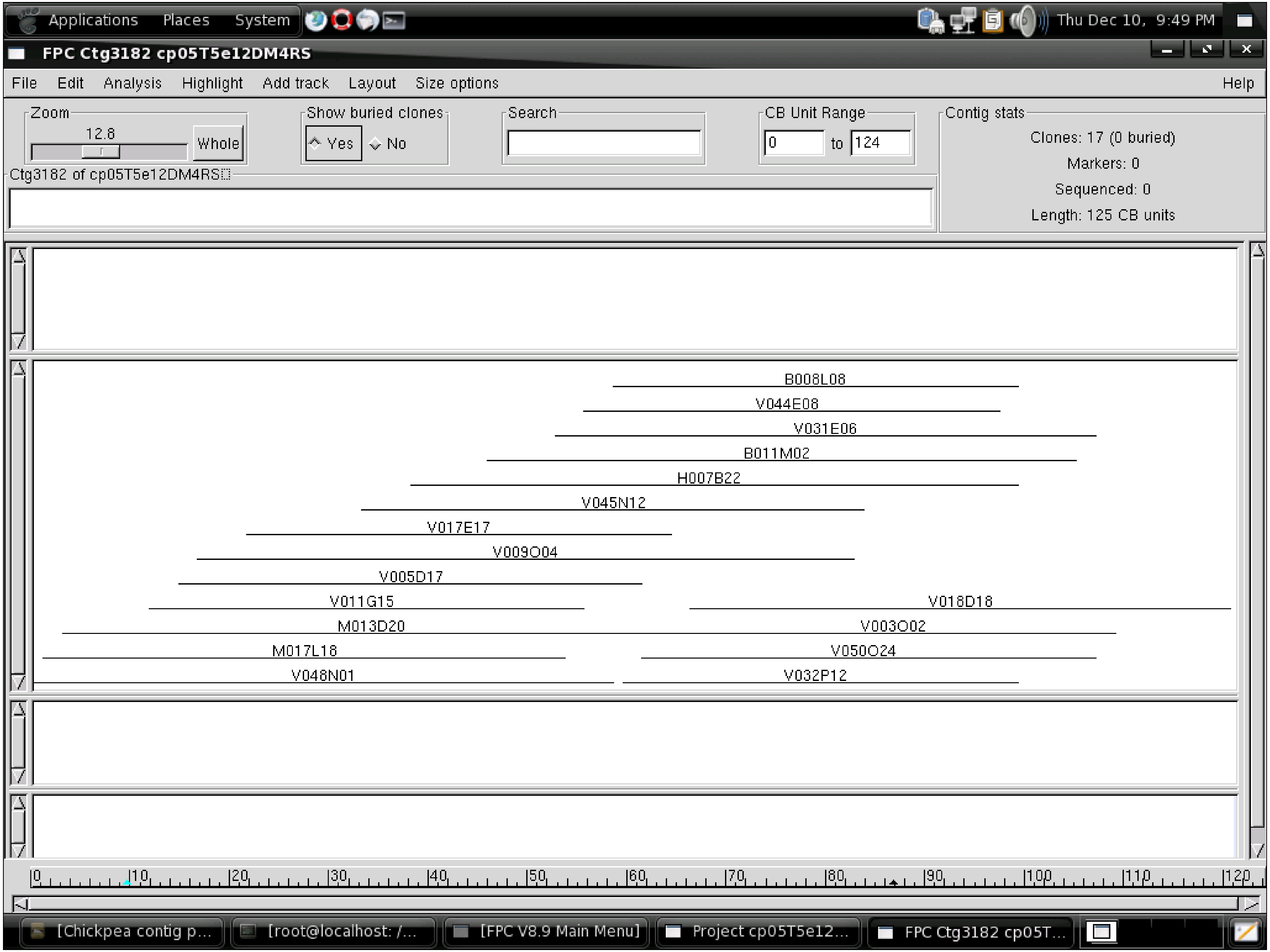
Applications Places System
Thu Dec 10, 9:49 PM
FPC Ctg3182 cp05T5e12DM4RS
File Edit Analysis Highlight Add track Layout Size options Help
Zoom
12.8
Whole
Show buried clones
Yes
No
Search
CB Unit Range
0
to
124
Contig stats
Clones: 17 (0 buried)
Markers: 0
Sequenced: 0
Length: 125 CB units
Ctg3182 of cp05T5e12DM4RS
B008L08
V044E08
V031E06
B011M02
H007B22
V045N12
V017E17
V009O04
V005D17
V011G15
V018D18
M013D20
V003O02
M017L18
V050O24
V048N01
V032P12
0
10
20
30
40
50
60
70
80
90
100
110
120
[Chickpea contig p...
[root@localhost: /...
[FPC V8.9 Main Menu]
Project cp05T5e12...
FPC Ctg3182 cp05T...

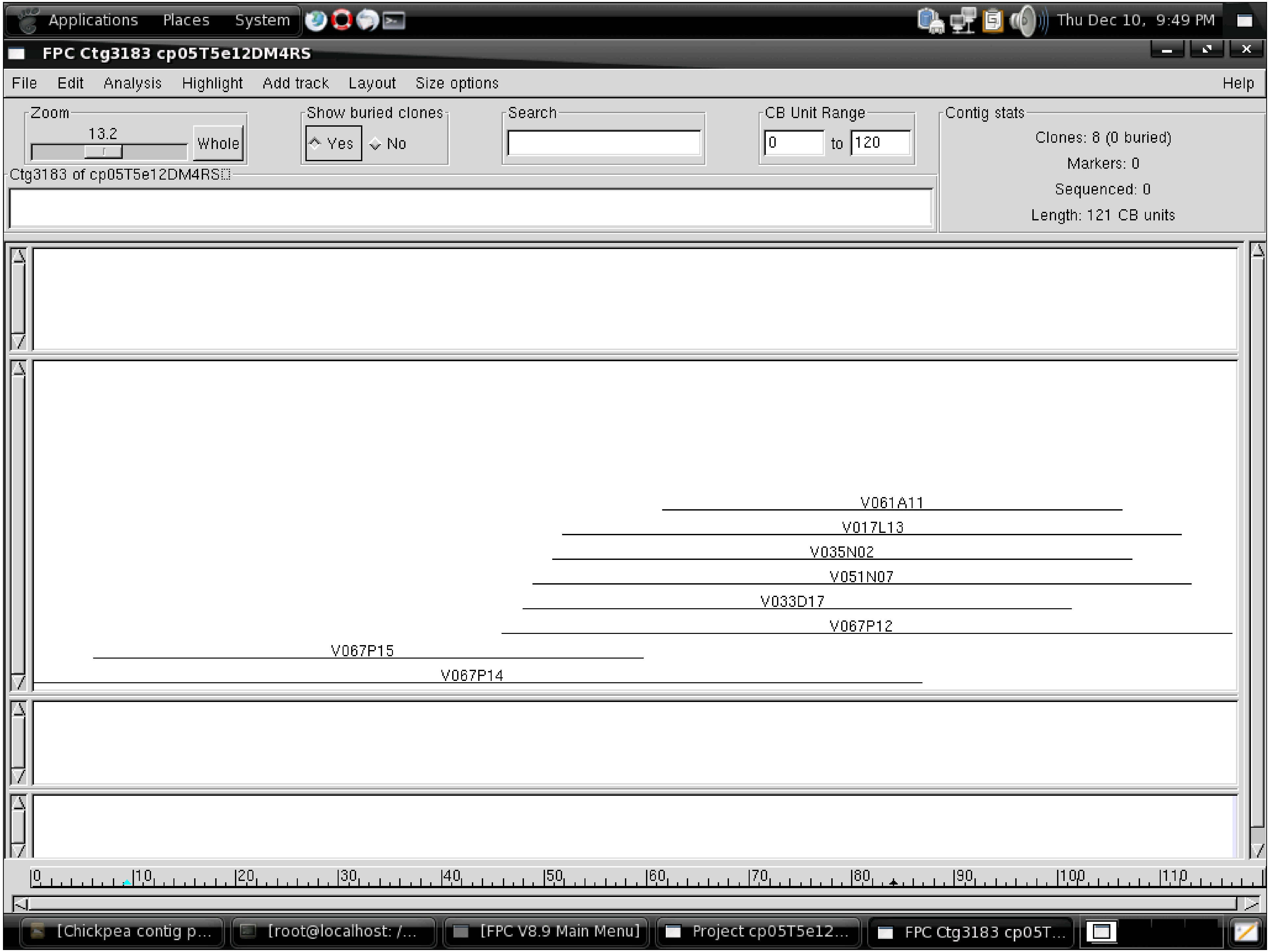
Applications Places System
Thu Dec 10, 9:49 PM
FPC Ctg3183 cp05T5e12DM4RS
File Edit Analysis Highlight Add track Layout Size options Help
Zoom
13.2
Whole
Show buried clones
Yes
No
Search
CB Unit Range
0 to 120
Contig stats
Clones: 8 (0 buried)
Markers: 0
Sequenced: 0
Length: 121 CB units
Ctg3183 of cp05T5e12DM4RS
V061A11
V017L13
V035N02
V051N07
V033D17
V067P12
V067P15
V067P14
0 10 20 30 40 50 60 70 80 90 100 110
[Chickpea contig p...
[root@localhost: /...
[FPC V8.9 Main Menu]
Project cp05T5e12...
FPC Ctg3183 cp05T...

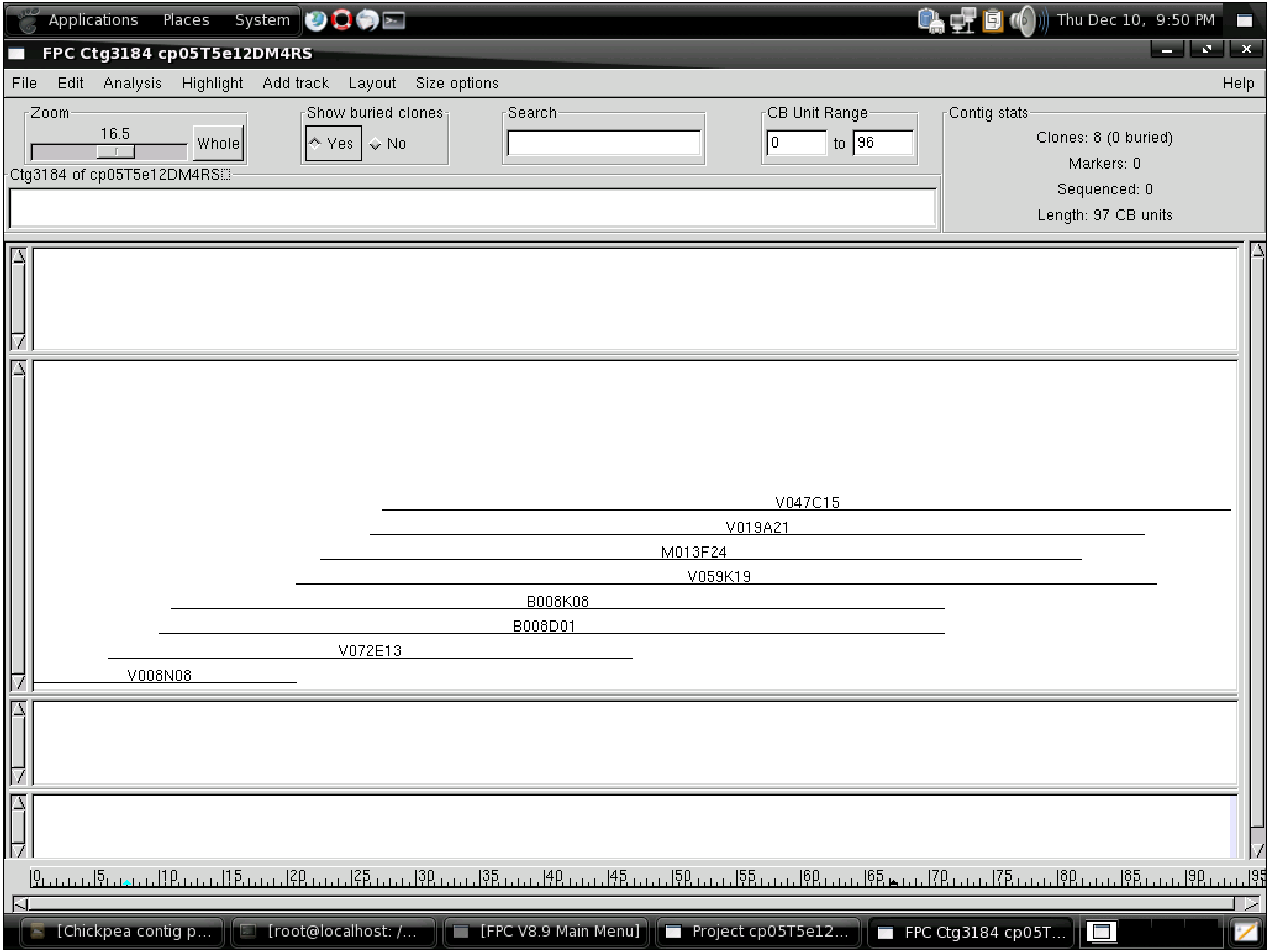
Applications Places System
Thu Dec 10, 9:50 PM
FPC Ctg3184 cp05T5e12DM4RS
File Edit Analysis Highlight Add track Layout Size options Help
Zoom
16.5
Whole
Show buried clones
Yes
No
Search
CB Unit Range
0
to
96
Contig stats
Clones: 8 (0 buried)
Markers: 0
Sequenced: 0
Length: 97 CB units
Ctg3184 of cp05T5e12DM4RS
V047C15
V019A21
M013F24
V059K19
B008K08
B008D01
V072E13
V008N08
0 5 10 15 20 25 30 35 40 45 50 55 60 65 70 75 80 85 90 95
[Chickpea contig p...
[root@localhost: /...
[FPC V8.9 Main Menu]
Project cp05T5e12...
FPC Ctg3184 cp05T...

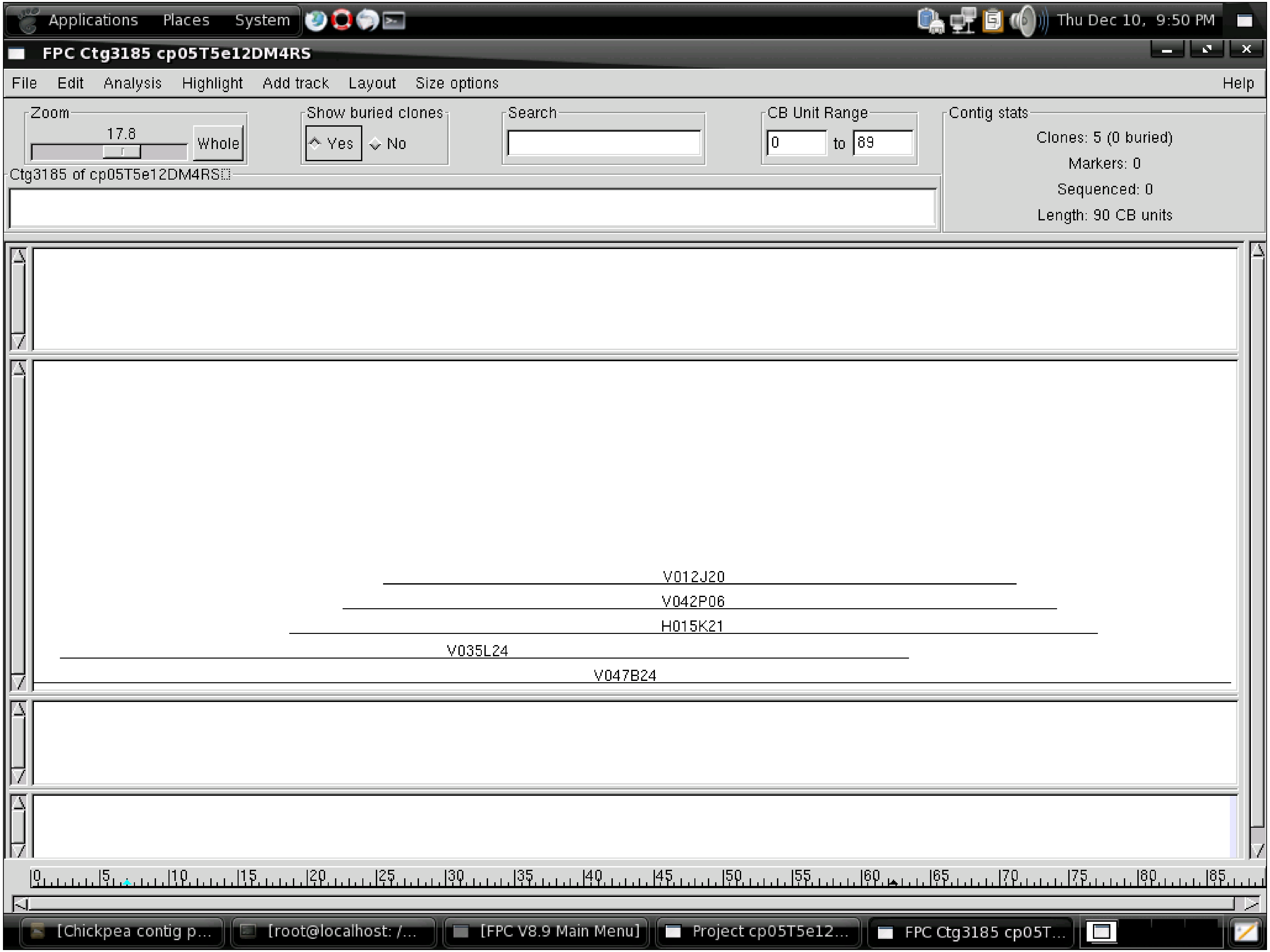
Applications Places System
Thu Dec 10, 9:50 PM
FPC Ctg3185 cp05T5e12DM4RS
File Edit Analysis Highlight Add track Layout Size options Help
Zoom
17.8
Whole
Show buried clones
Yes
No
Search
CB Unit Range
0
to
89
Contig stats
Clones: 5 (0 buried)
Markers: 0
Sequenced: 0
Length: 90 CB units
Ctg3185 of cp05T5e12DM4RS
V012J20
V042P06
H015K21
V035L24
V047B24
0 5 10 15 20 25 30 35 40 45 50 55 60 65 70 75 80 85
[Chickpea contig p...
[root@localhost: /...
[FPC V8.9 Main Menu]
Project cp05T5e12...
FPC Ctg3185 cp05T...

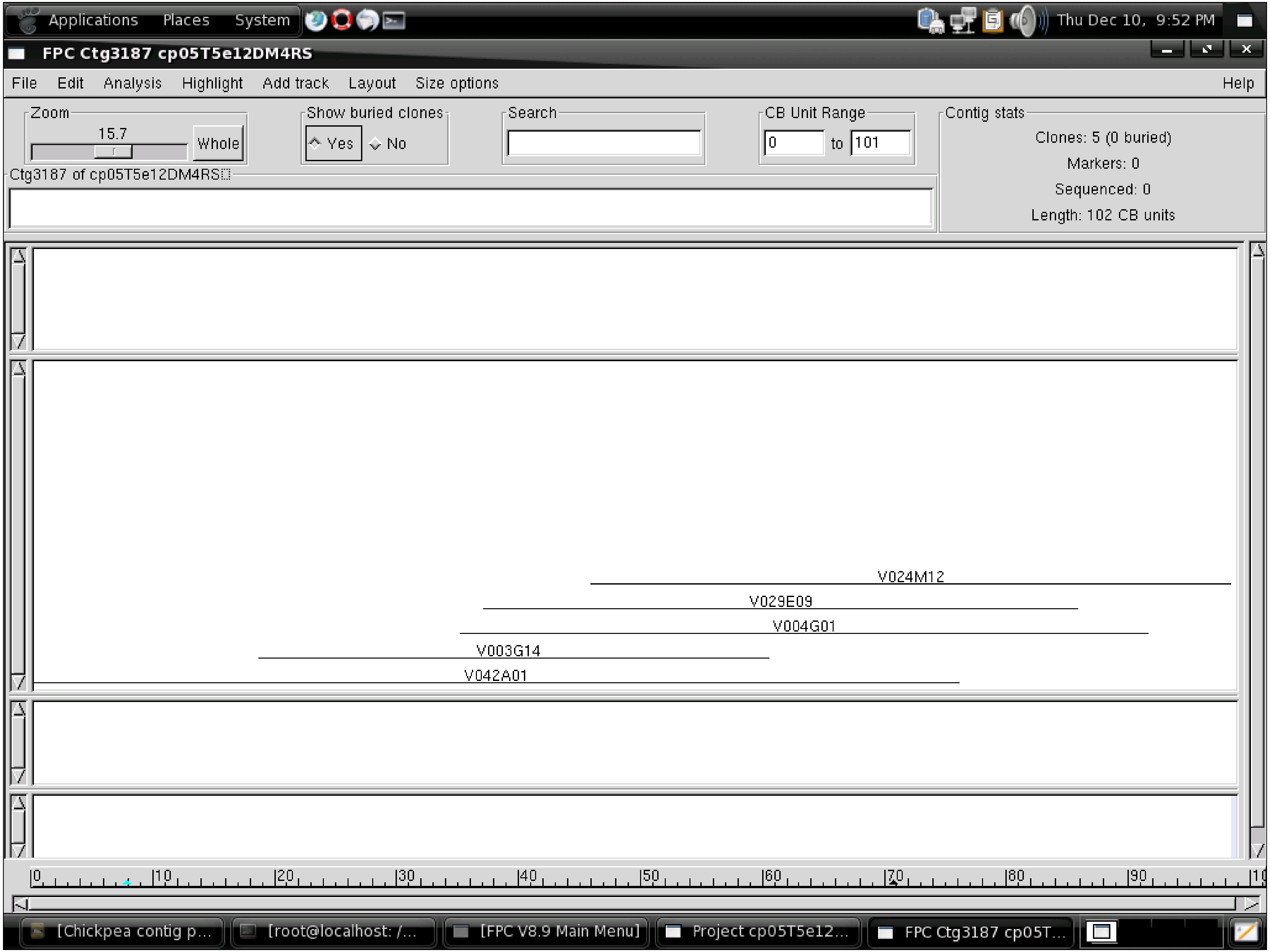
Applications Places System
Thu Dec 10, 9:52 PM
FPC Ctg3187 cp05T5e12DM4RS
File Edit Analysis Highlight Add track Layout Size options Help
Zoom
15.7
Whole
Show buried clones
Yes
No
Search
CB Unit Range
0 to 101
Contig stats
Clones: 5 (0 buried)
Markers: 0
Sequenced: 0
Length: 102 CB units
Ctg3187 of cp05T5e12DM4RS
V024M12
V029E09
V004G01
V003G14
V042A01
0 10 20 30 40 50 60 70 80 90
[Chickpea contig p...
[root@localhost: /...
[FPC V8.9 Main Menu]
Project cp05T5e12...
FPC Ctg3187 cp05T...

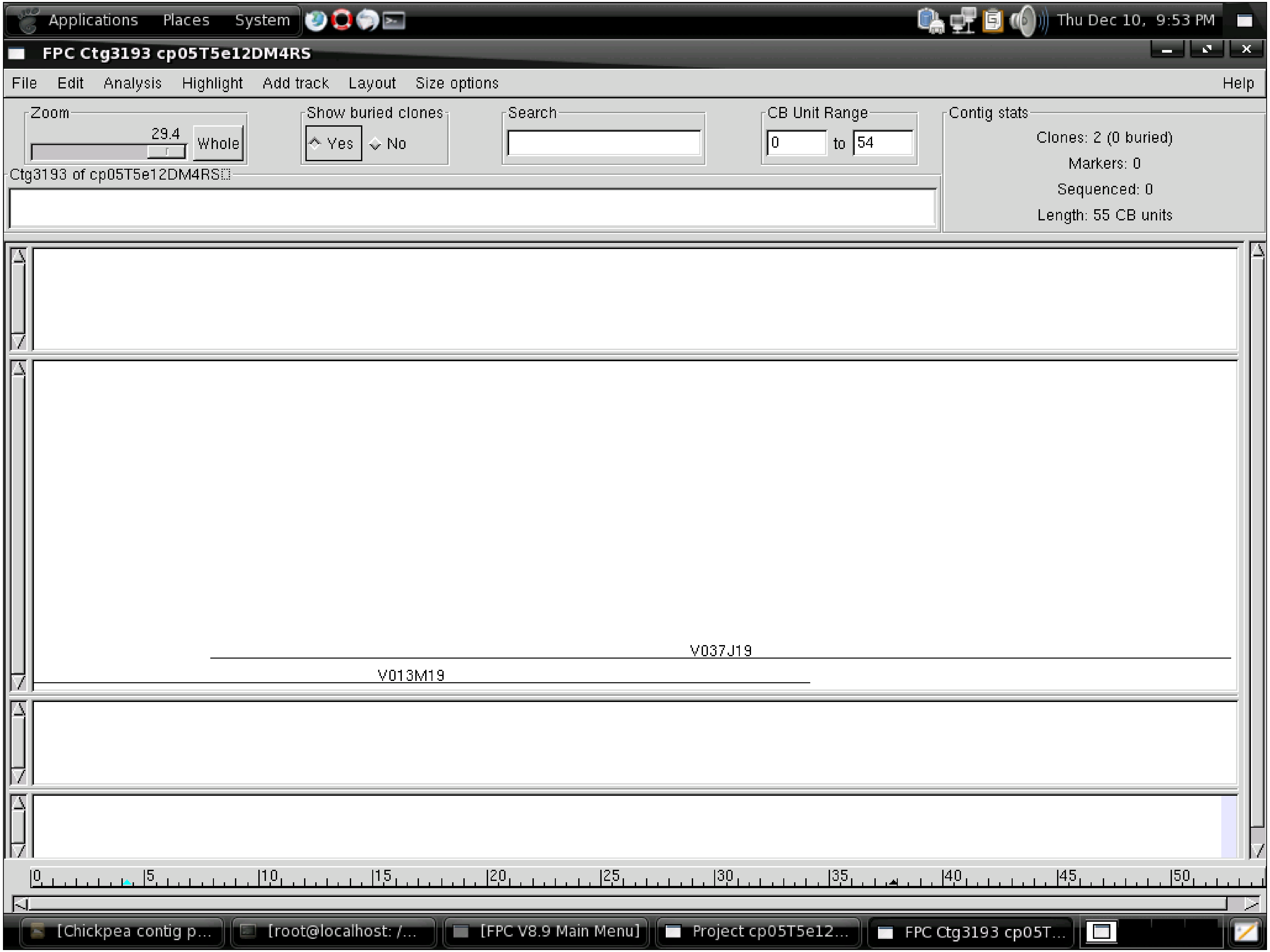
Applications Places System
Thu Dec 10, 9:53 PM
FPC Ctg3193 cp05T5e12DM4RS
File Edit Analysis Highlight Add track Layout Size options Help
Zoom
29.4
Whole
Show buried clones
Yes No
Search
CB Unit Range
0 to 54
Contig stats
Clones: 2 (0 buried)
Markers: 0
Sequenced: 0
Length: 55 CB units
Ctg3193 of cp05T5e12DM4RS
V037J19
V013M19
0 5 10 15 20 25 30 35 40 45 50
[Chickpea contig p...
[root@localhost: /...
[FPC V8.9 Main Menu]
Project cp05T5e12...
FPC Ctg3193 cp05T...

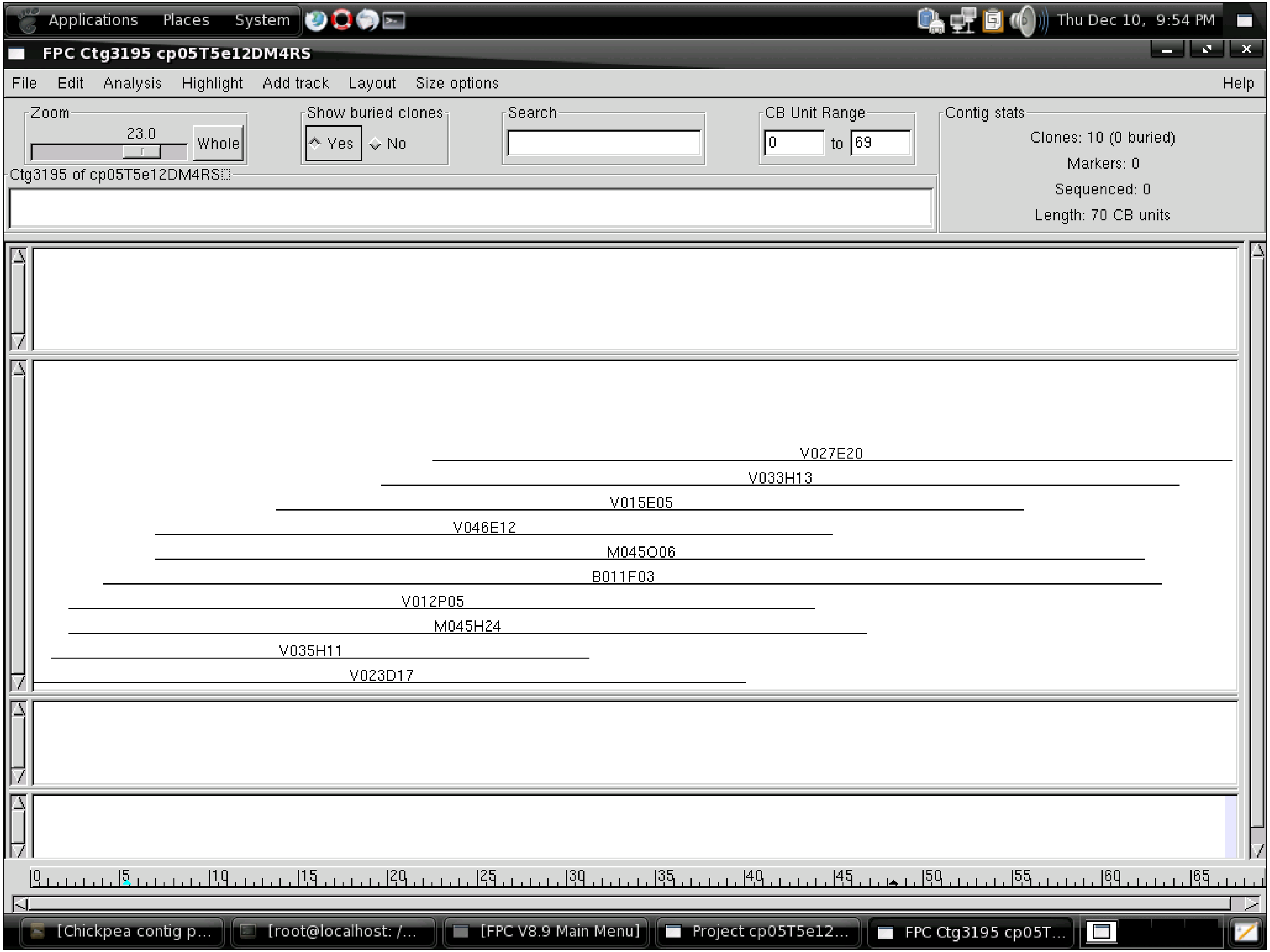
Applications Places System
Thu Dec 10, 9:54 PM
FPC Ctg3195 cp05T5e12DM4RS
File Edit Analysis Highlight Add track Layout Size options Help
Zoom
23.0
Whole
Show buried clones
Yes No
Search
CB Unit Range
0 to 69
Contig stats
Clones: 10 (0 buried)
Markers: 0
Sequenced: 0
Length: 70 CB units
Ctg3195 of cp05T5e12DM4RS
V027E20
V033H13
V015E05
V046E12
M045O06
B011F03
V012P05
M045H24
V035H11
V023D17
0 5 10 15 20 25 30 35 40 45 50 55 60 65
[Chickpea contig p...
[root@localhost: /...
[FPC V8.9 Main Menu]
Project cp05T5e12...
FPC Ctg3195 cp05T...

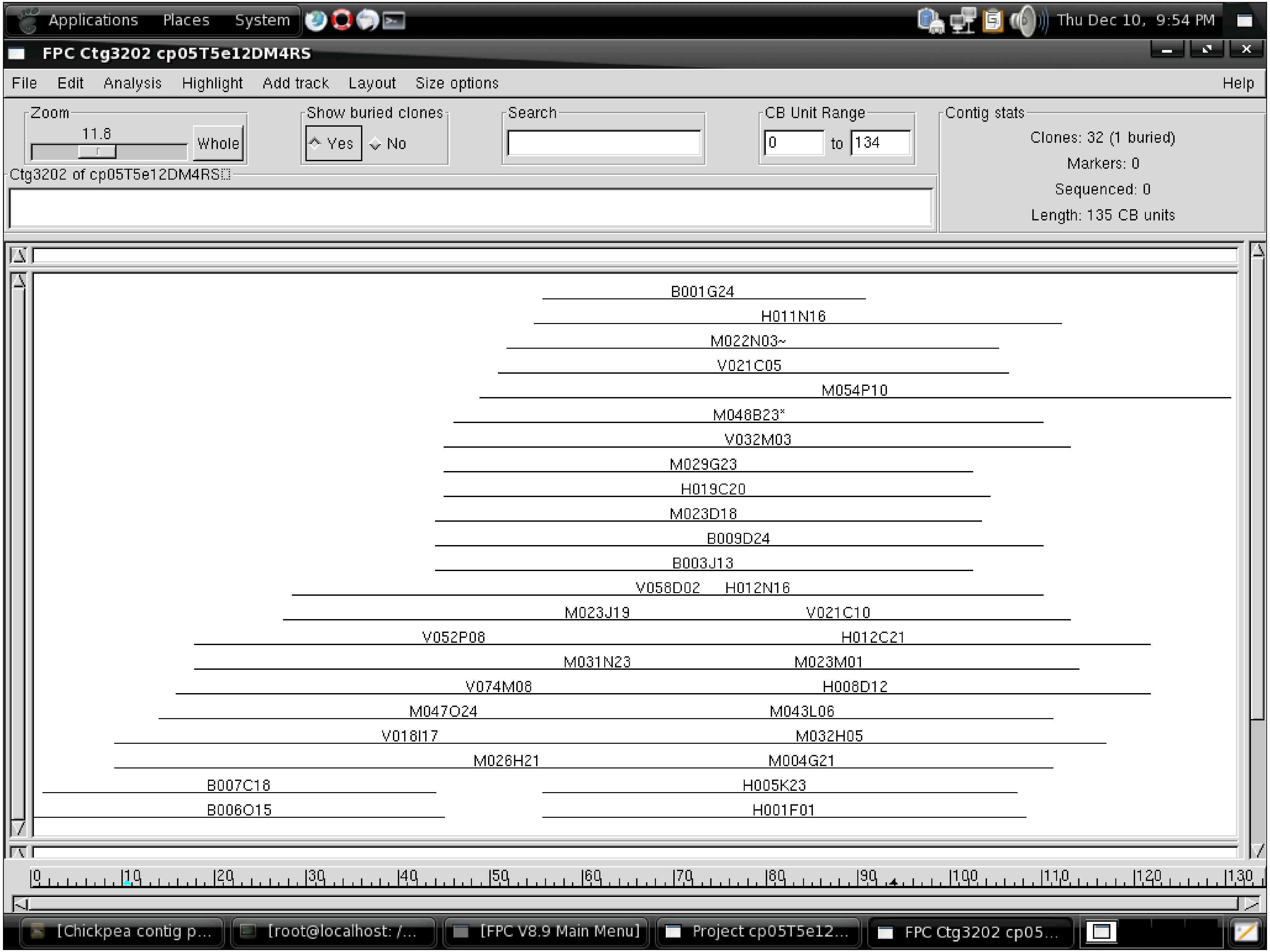
Applications Places System
Thu Dec 10, 9:54 PM
FPC Ctg3202 cp05T5e12DM4RS
File Edit Analysis Highlight Add track Layout Size options Help
Zoom
11.8
Whole
Show buried clones
Yes No
Search
CB Unit Range
0 to 134
Contig stats
Clones: 32 (1 buried)
Markers: 0
Sequenced: 0
Length: 135 CB units
Ctg3202 of cp05T5e12DM4RS
B001G24
H011N16
M022N03~
V021C05
M054P10
M048B23*
V032M03
M029G23
H019C20
M023D18
B009D24
B003J13
V058D02 H012N16
M023J19 V021C10
V052P08 H012C21
M031N23 M023M01
V074M08 H008D12
M047O24 M043L06
V018I17 M032H05
M026H21 M004G21
B007C18 H005K23
B006O15 H001F01
0 10 20 30 40 50 60 70 80 90 100 110 120 130
[Chickpea contig p...
[root@localhost: /...
[FPC V8.9 Main Menu]
Project cp05T5e12...
FPC Ctg3202 cp05...

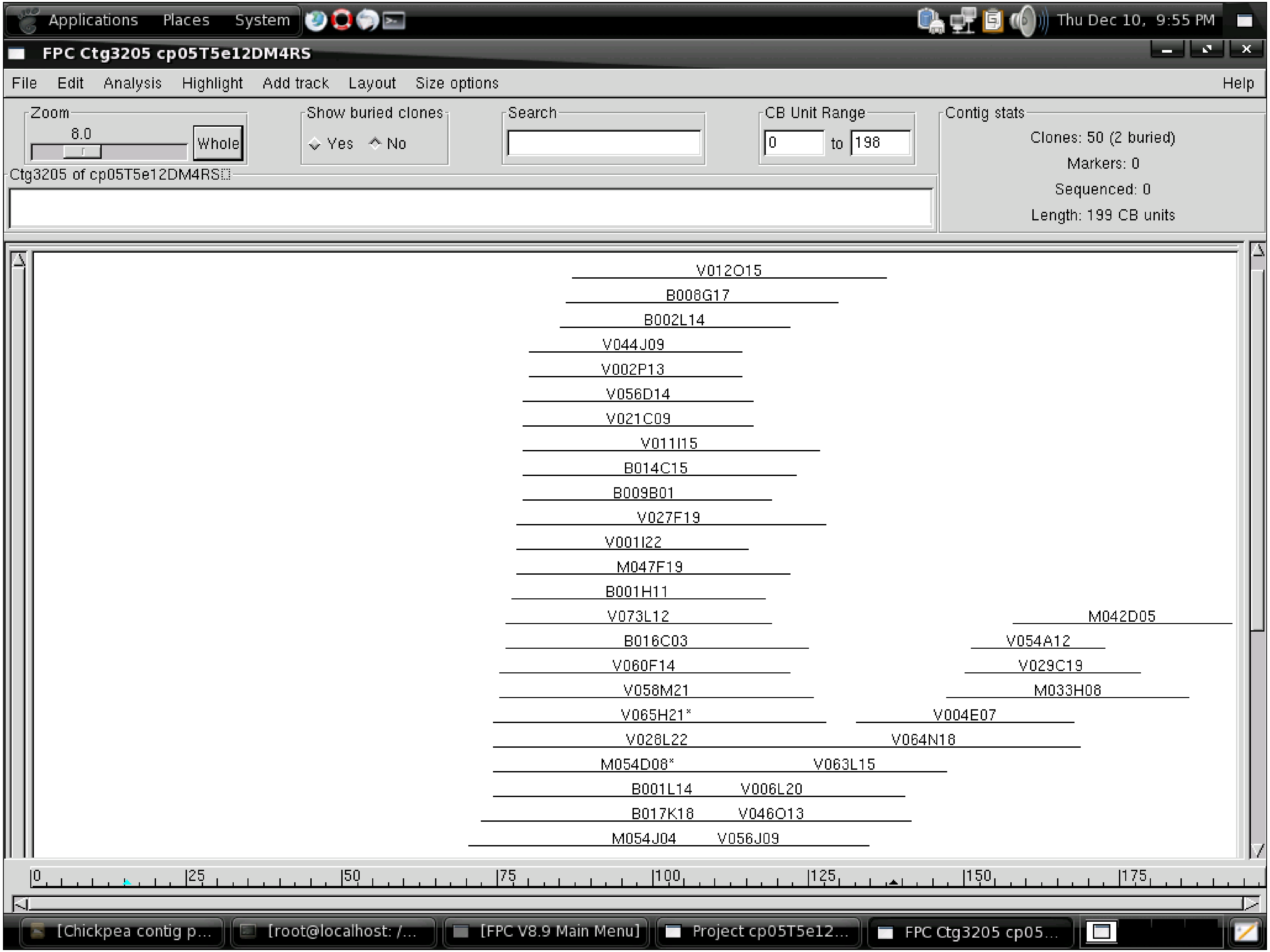
Applications Places System
Thu Dec 10, 9:55 PM
FPC Ctg3205 cp05T5e12DM4RS
File Edit Analysis Highlight Add track Layout Size options Help
Zoom
8.0
Whole
Show buried clones
Yes No
Search
CB Unit Range
0 to 198
Contig stats
Clones: 50 (2 buried)
Markers: 0
Sequenced: 0
Length: 199 CB units
Ctg3205 of cp05T5e12DM4RS
V012O15
B008G17
B002L14
V044J09
V002P13
V056D14
V021C09
V011I15
B014C15
B009B01
V027F19
V001I22
M047F19
B001H11
V073L12
M042D05
B016C03
V054A12
V060F14
V029C19
V058M21
M033H08
V065H21*
V004E07
V028L22
V064N18
M054D08*
V063L15
B001L14
V006L20
B017K18
V046O13
M054J04
V056J09
0
25
50
75
100
125
150
175
[Chickpea contig p...
[root@localhost: /...
[FPC V8.9 Main Menu]
Project cp05T5e12...
FPC Ctg3205 cp05...

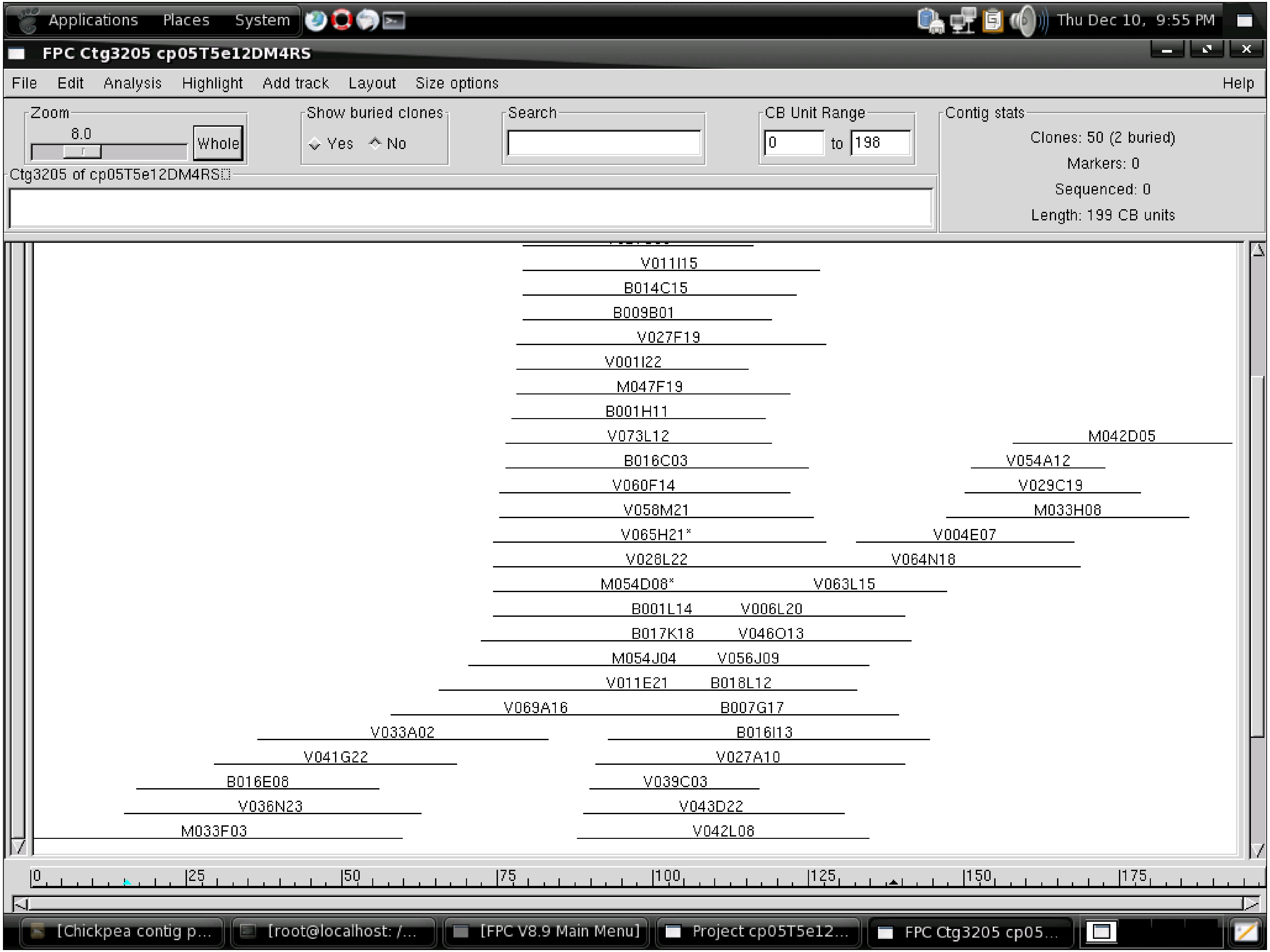
Applications Places System
Thu Dec 10, 9:55 PM
FPC Ctg3205 cp05T5e12DM4RS
File Edit Analysis Highlight Add track Layout Size options Help
Zoom
8.0
Whole
Show buried clones
Yes No
Search
CB Unit Range
0 to 198
Contig stats
Clones: 50 (2 buried)
Markers: 0
Sequenced: 0
Length: 199 CB units
Ctg3205 of cp05T5e12DM4RS
V011I15
B014C15
B009B01
V027F19
V001I22
M047F19
B001H11
V073L12
M042D05
B016C03
V054A12
V060F14
V029C19
V058M21
M033H08
V065H21*
V004E07
V028L22
V064N18
M054D08*
V063L15
B001L14
V006L20
B017K18
V046O13
M054J04
V056J09
V011E21
B018L12
V069A16
B007G17
V033A02
B016I13
V041G22
V027A10
B016E08
V039C03
V036N23
V043D22
M033F03
V042L08
0 25 50 75 100 125 150 175
[Chickpea contig p...
[root@localhost: /...
[FPC V8.9 Main Menu]
Project cp05T5e12...
FPC Ctg3205 cp05...

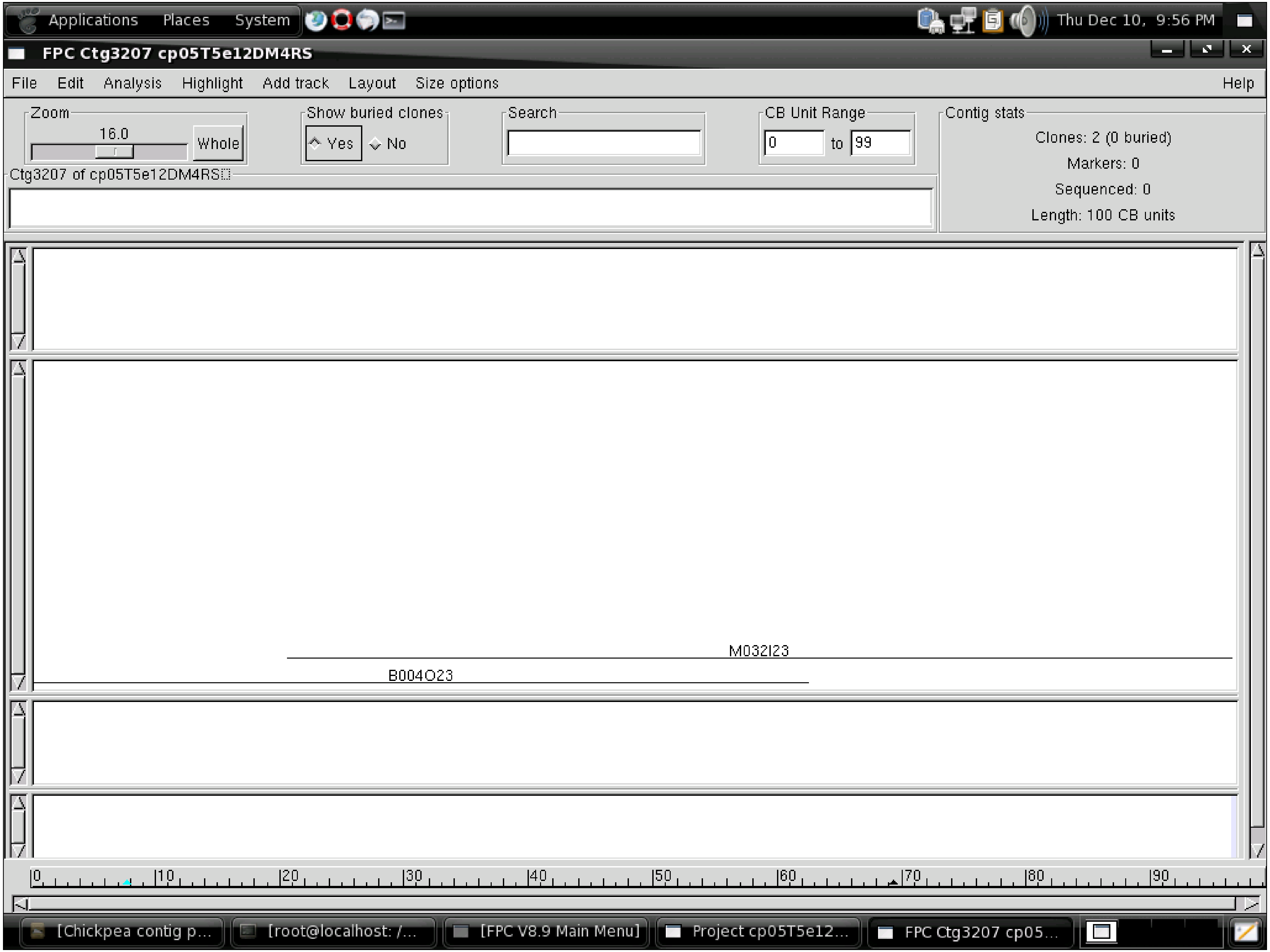

Applications
Places
System
Thu Dec 10, 9:56 PM
FPC Ctg3207 cp05T5e12DM4RS
File
Edit
Analysis
Highlight
Add track
Layout
Size options
Help
Zoom
16.0
Whole
Show buried clones
Yes
No
Search
CB Unit Range
0
to
99
Contig stats
Clones: 2 (0 buried)
Markers: 0
Sequenced: 0
Length: 100 CB units
Ctg3207 of cp05T5e12DM4RS
M032I23
B004O23
0
10
20
30
40
50
60
70
80
90
[Chickpea contig p...
[root@localhost: /...
[FPC V8.9 Main Menu]
Project cp05T5e12...
FPC Ctg3207 cp05...

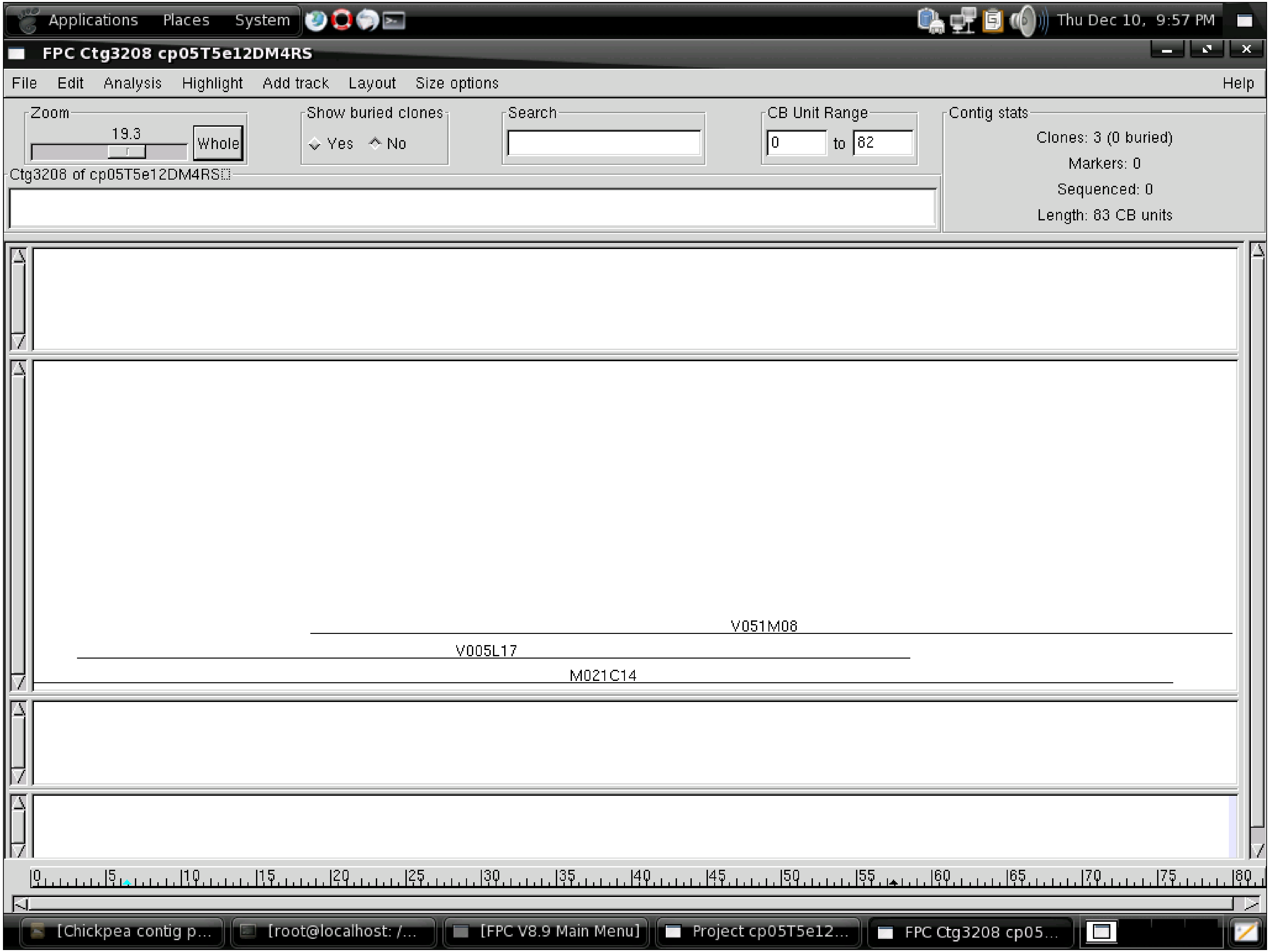
Applications
Places
System
Thu Dec 10, 9:57 PM
FPC Ctg3208 cp05T5e12DM4RS
File
Edit
Analysis
Highlight
Add track
Layout
Size options
Help
Zoom
19.3
Whole
Show buried clones
Yes
No
Search
CB Unit Range
0
to
82
Contig stats
Clones: 3 (0 buried)
Markers: 0
Sequenced: 0
Length: 83 CB units
Ctg3208 of cp05T5e12DM4RS
V051M08
V005L17
M021C14
0 5 10 15 20 25 30 35 40 45 50 55 60 65 70 75 80
[Chickpea contig p...
[root@localhost: /...
[FPC V8.9 Main Menu]
Project cp05T5e12...
FPC Ctg3208 cp05...

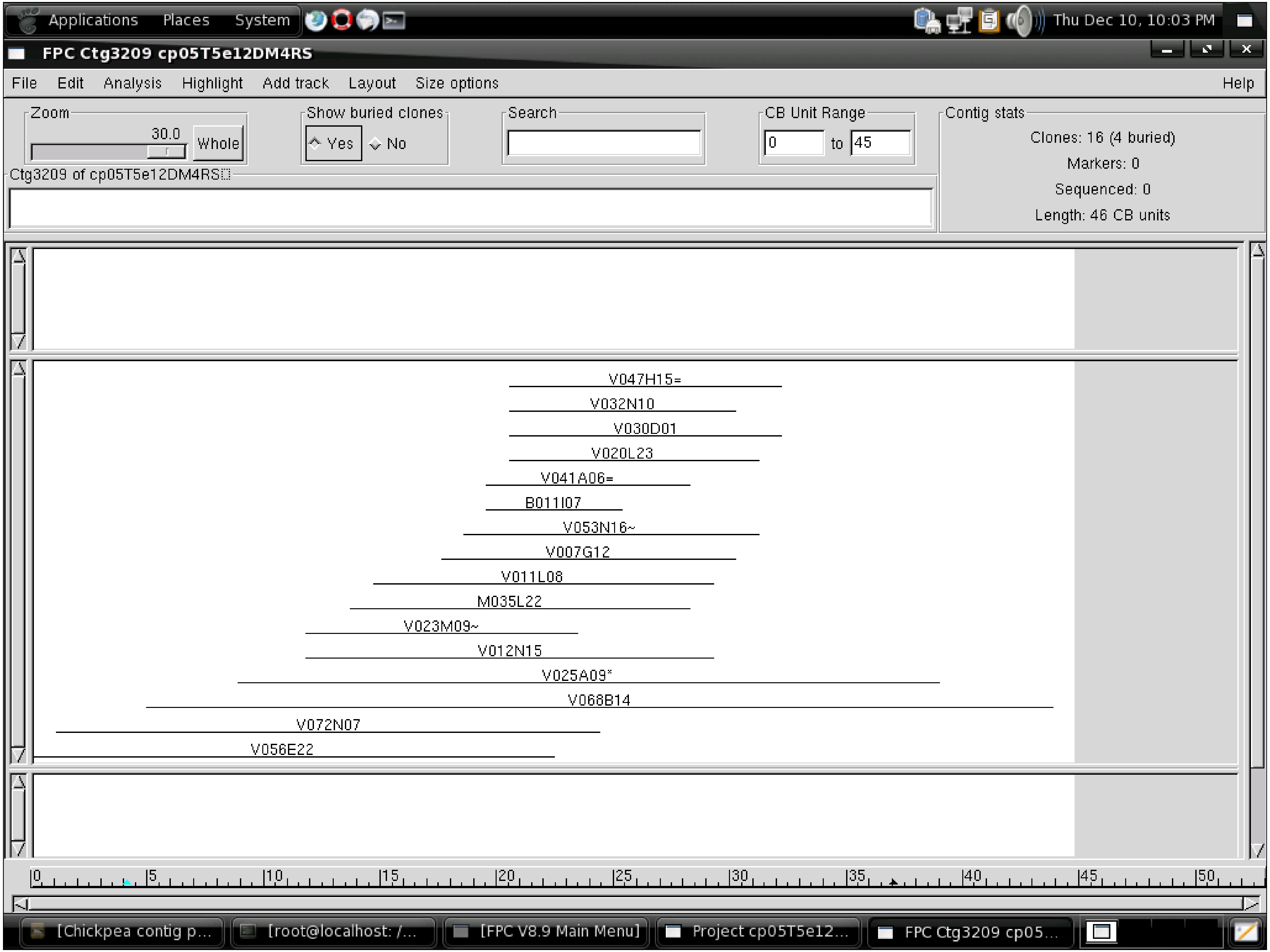
Applications
Places
System
Thu Dec 10, 10:03 PM
FPC Ctg3209 cp05T5e12DM4RS
File
Edit
Analysis
Highlight
Add track
Layout
Size options
Help
Zoom
30.0
Whole
Show buried clones
Yes
No
Search
CB Unit Range
0
to
45
Contig stats
Clones: 16 (4 buried)
Markers: 0
Sequenced: 0
Length: 46 CB units
Ctg3209 of cp05T5e12DM4RS
V047H15=
V032N10
V030D01
V020L23
V041A06=
B011I07
V053N16~
V007G12
V011L08
M035L22
V023M09~
V012N15
V025A09*
V068B14
V072N07
V056E22
0
5
10
15
20
25
30
35
40
45
50
[Chickpea contig p...
[root@localhost: /...
[FPC V8.9 Main Menu]
Project cp05T5e12...
FPC Ctg3209 cp05...

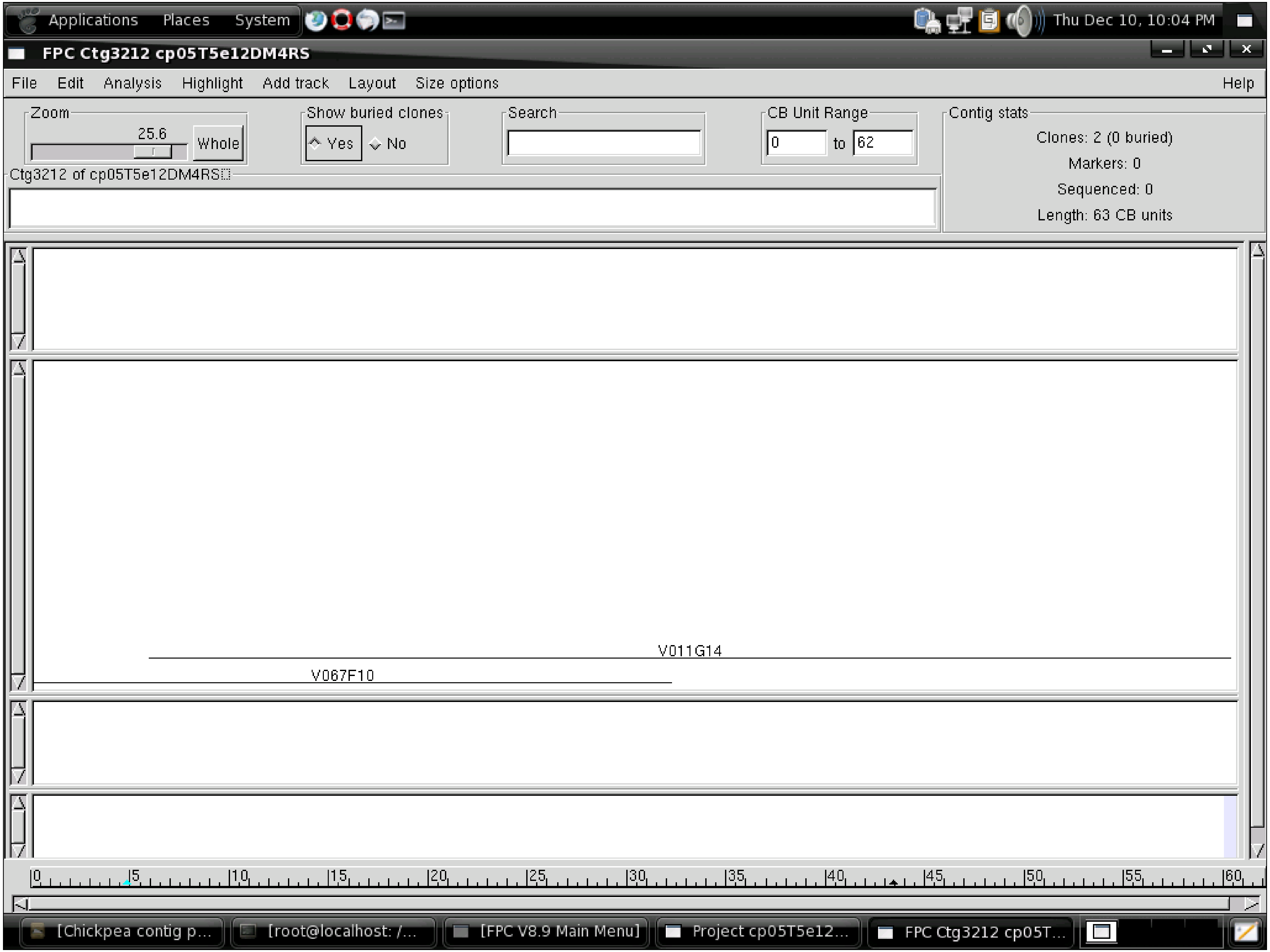

Applications
Places
System
Thu Dec 10, 10:04 PM
FPC Ctg3212 cp05T5e12DM4RS
File
Edit
Analysis
Highlight
Add track
Layout
Size options
Help
Zoom
25.6
Whole
Show buried clones
Yes
No
Search
CB Unit Range
0
to
62
Contig stats
Clones: 2 (0 buried)
Markers: 0
Sequenced: 0
Length: 63 CB units
Ctg3212 of cp05T5e12DM4RS
V011G14
V067F10
0 5 10 15 20 25 30 35 40 45 50 55 60
[Chickpea contig p...
[root@localhost: /...
[FPC V8.9 Main Menu]
Project cp05T5e12...
FPC Ctg3212 cp05T...

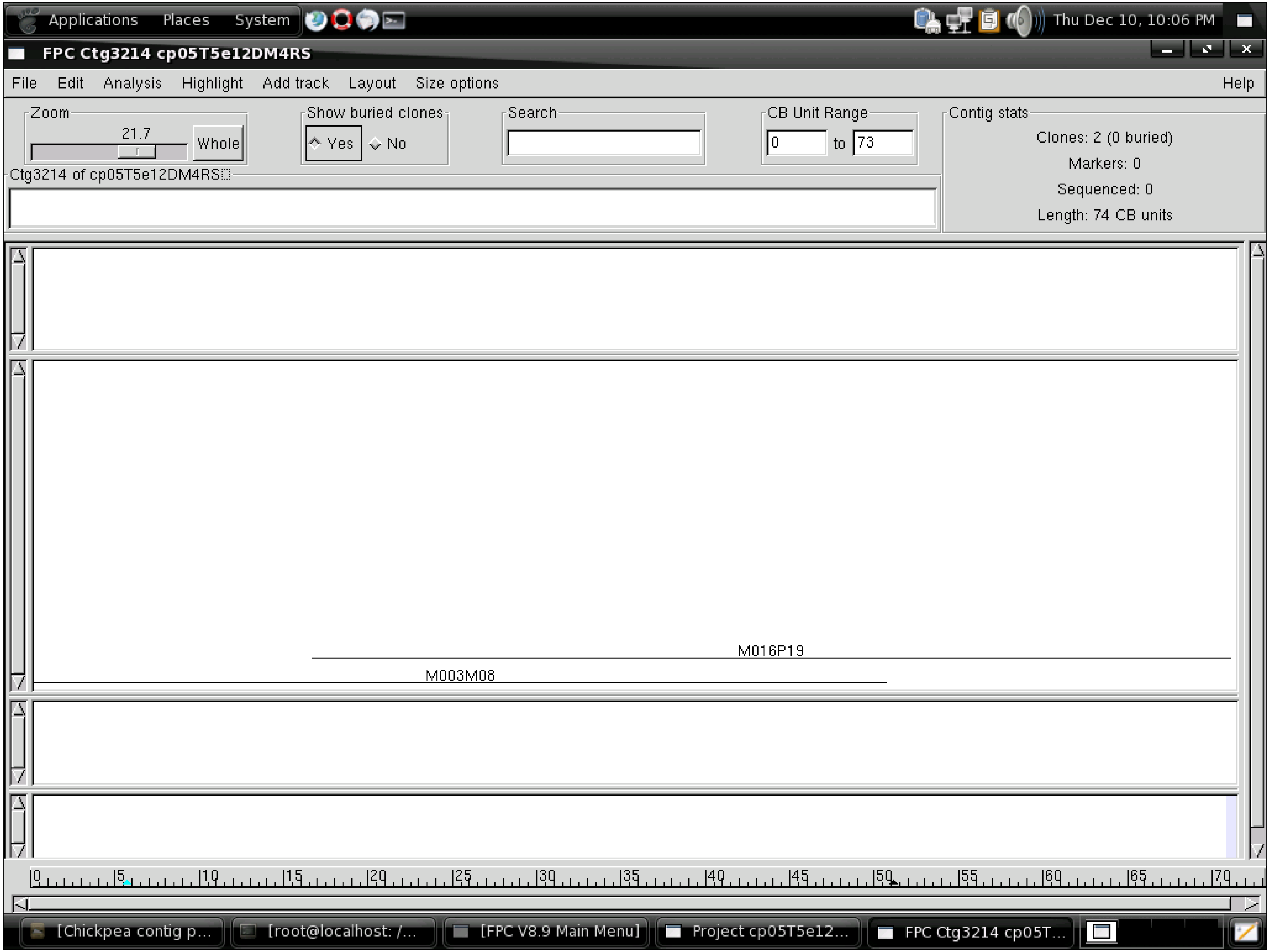
Applications Places System
Thu Dec 10, 10:06 PM
FPC Ctg3214 cp05T5e12DM4RS
File Edit Analysis Highlight Add track Layout Size options Help
Zoom
21.7
Whole
Show buried clones
Yes
No
Search
CB Unit Range
0 to 73
Contig stats
Clones: 2 (0 buried)
Markers: 0
Sequenced: 0
Length: 74 CB units
Ctg3214 of cp05T5e12DM4RS
M016P19
M003M08
0 5 10 15 20 25 30 35 40 45 50 55 60 65 70
[Chickpea contig p...
[root@localhost: /...
[FPC V8.9 Main Menu]
Project cp05T5e12...
FPC Ctg3214 cp05T...

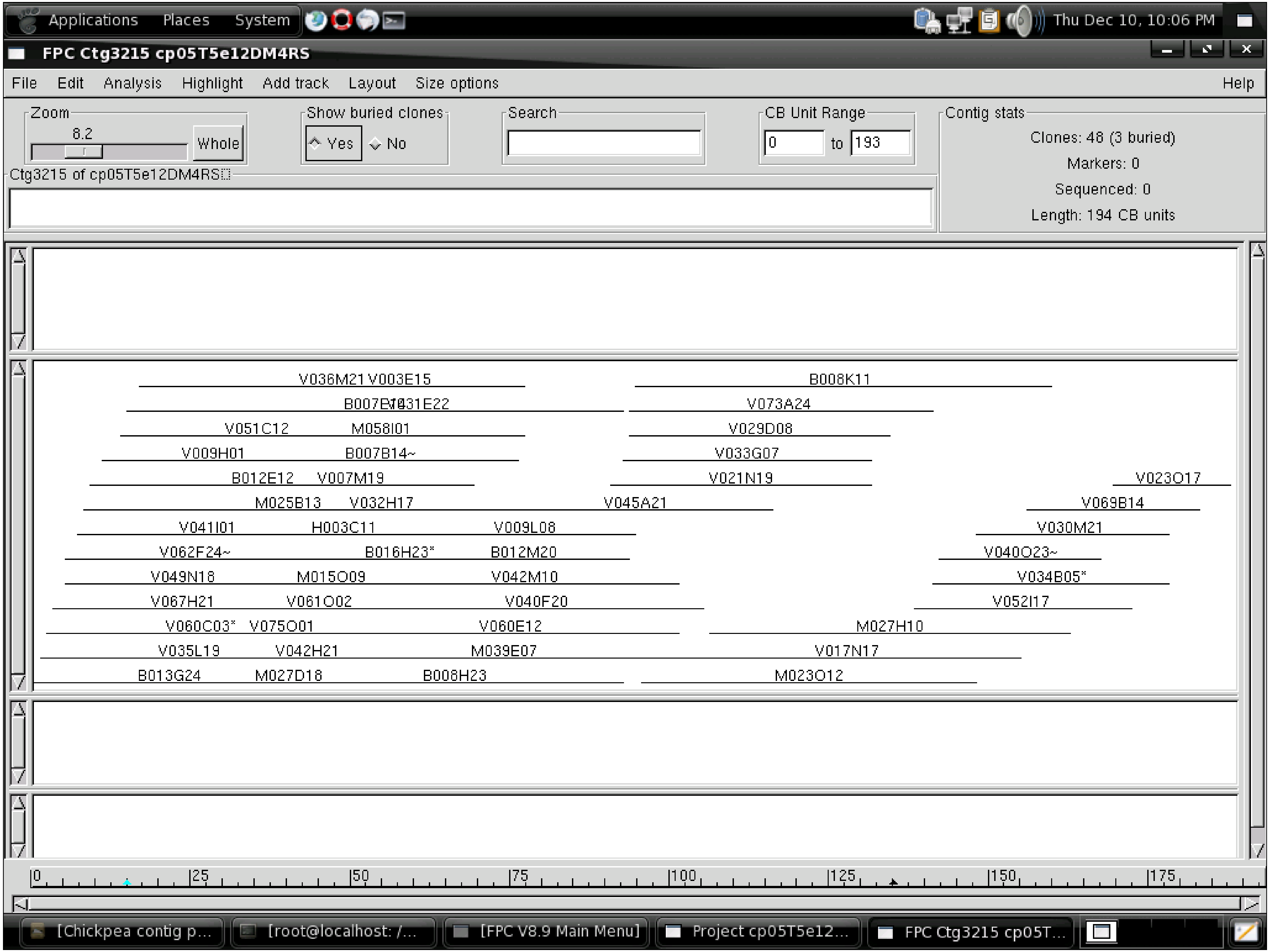

Applications Places System
Thu Dec 10, 10:06 PM
FPC Ctg3215 cp05T5e12DM4RS
File Edit Analysis Highlight Add track Layout Size options Help
Zoom
8.2
Whole
Show buried clones
Yes No
Search
CB Unit Range
0 to 193
Contig stats
Clones: 48 (3 buried)
Markers: 0
Sequenced: 0
Length: 194 CB units
Ctg3215 of cp05T5e12DM4RS
V036M21 V003E15
B008K11
V073A24
V051C12
M058I01
V029D08
V009H01
B007B14~
V033G07
B012E12
V007M19
V021N19
V023O17
M025B13
V032H17
V045A21
V069B14
V041I01
H003C11
V009L08
V030M21
V062F24~
B016H23*
B012M20
V040O23~
V049N18
M015O09
V042M10
V034B05*
V067H21
V061O02
V040F20
V052I17
V060C03*
V075O01
V060E12
M027H10
V035L19
V042H21
M039E07
V017N17
B013G24
M027D18
B008H23
M023O12
0 25 50 75 100 125 150 175
[Chickpea contig p...
[root@localhost: /...
[FPC V8.9 Main Menu]
Project cp05T5e12...
FPC Ctg3215 cp05T...

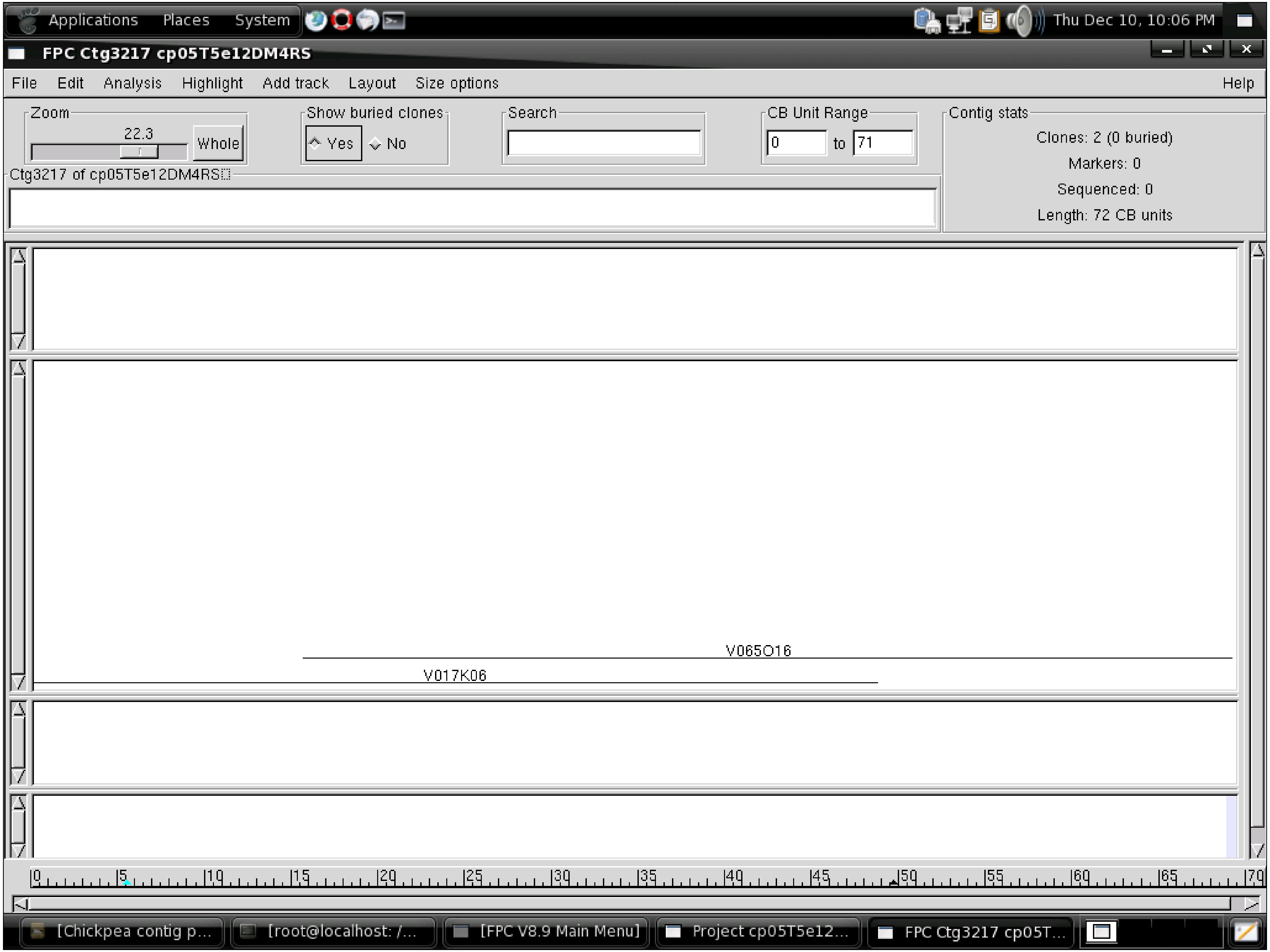
Applications Places System
Thu Dec 10, 10:06 PM
FPC Ctg3217 cp05T5e12DM4RS
File Edit Analysis Highlight Add track Layout Size options
Help
Zoom
22.3
Whole
Show buried clones
Yes
No
Search
CB Unit Range
0
to
71
Contig stats
Clones: 2 (0 buried)
Markers: 0
Sequenced: 0
Length: 72 CB units
Ctg3217 of cp05T5e12DM4RS
V065O16
V017K06
0 5 10 15 20 25 30 35 40 45 50 55 60 65 70
[Chickpea contig p...
[root@localhost: /...
[FPC V8.9 Main Menu]
Project cp05T5e12...
FPC Ctg3217 cp05T...

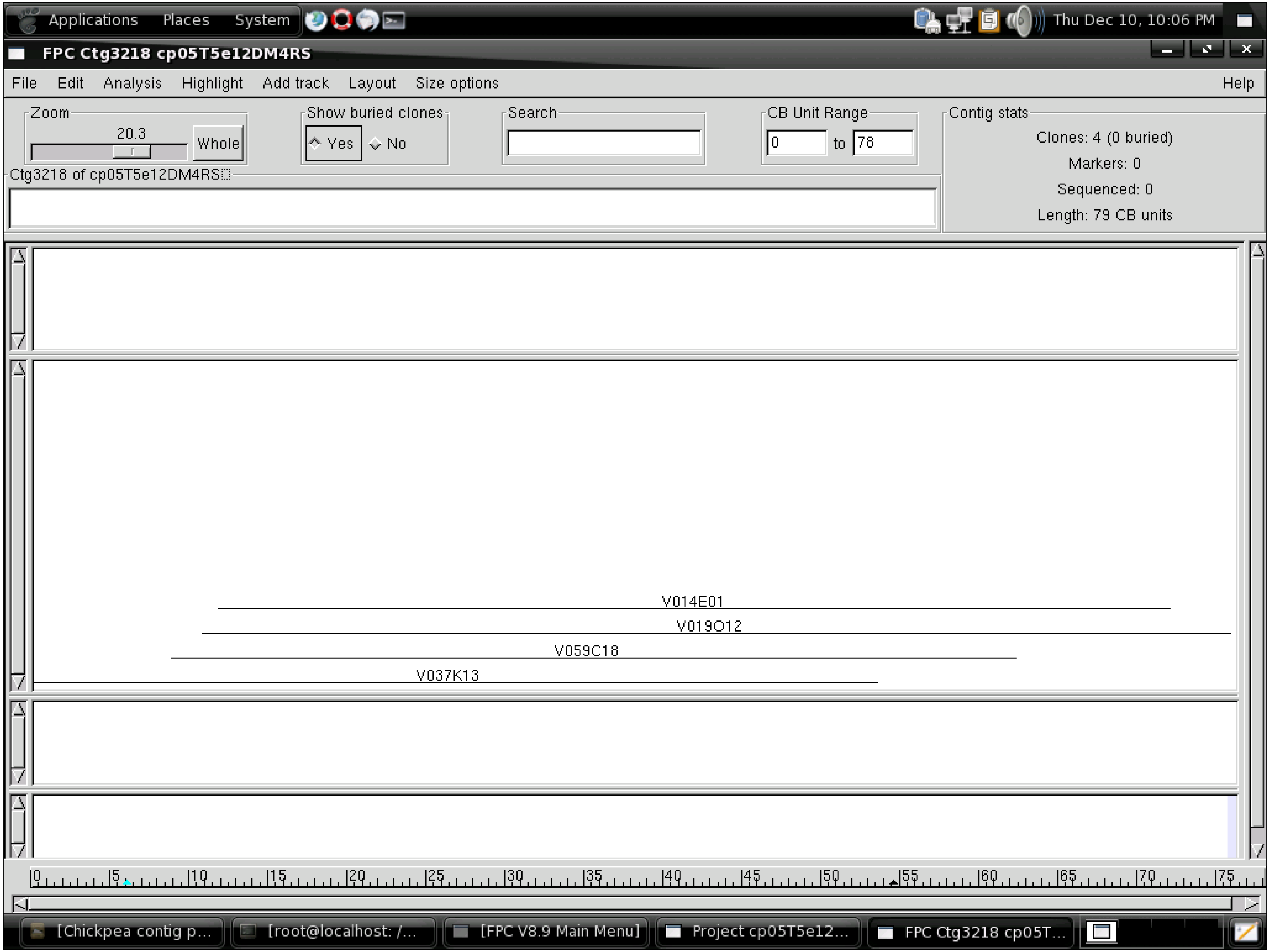
Applications Places System
Thu Dec 10, 10:06 PM
FPC Ctg3218 cp05T5e12DM4RS
File Edit Analysis Highlight Add track Layout Size options Help
Zoom
20.3
Whole
Show buried clones
Yes
No
Search
CB Unit Range
0 to 78
Contig stats
Clones: 4 (0 buried)
Markers: 0
Sequenced: 0
Length: 79 CB units
Ctg3218 of cp05T5e12DM4RS
V014E01
V019O12
V059C18
V037K13
0 5 10 15 20 25 30 35 40 45 50 55 60 65 70 75
[Chickpea contig p...
[root@localhost: /...
[FPC V8.9 Main Menu]
Project cp05T5e12...
FPC Ctg3218 cp05T...

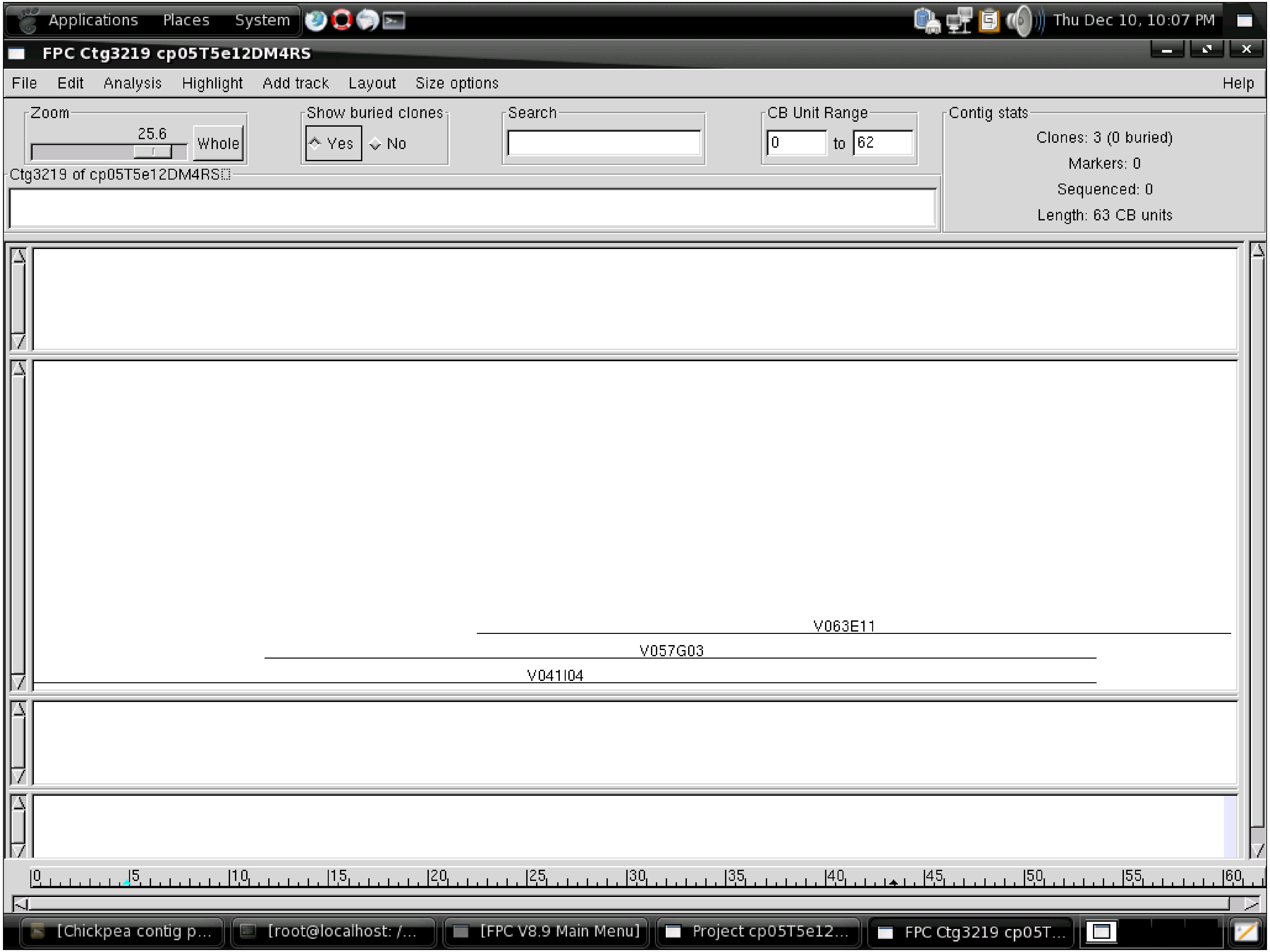
Applications Places System
Thu Dec 10, 10:07 PM
FPC Ctg3219 cp05T5e12DM4RS
File Edit Analysis Highlight Add track Layout Size options
Help
Zoom
25.6
Whole
Show buried clones
Yes
No
Search
CB Unit Range
0
to
62
Contig stats
Clones: 3 (0 buried)
Markers: 0
Sequenced: 0
Length: 63 CB units
Ctg3219 of cp05T5e12DM4RS
V063E11
V057G03
V041I04
0 5 10 15 20 25 30 35 40 45 50 55 60
[Chickpea contig p...
[root@localhost: /...
[FPC V8.9 Main Menu]
Project cp05T5e12...
FPC Ctg3219 cp05T...

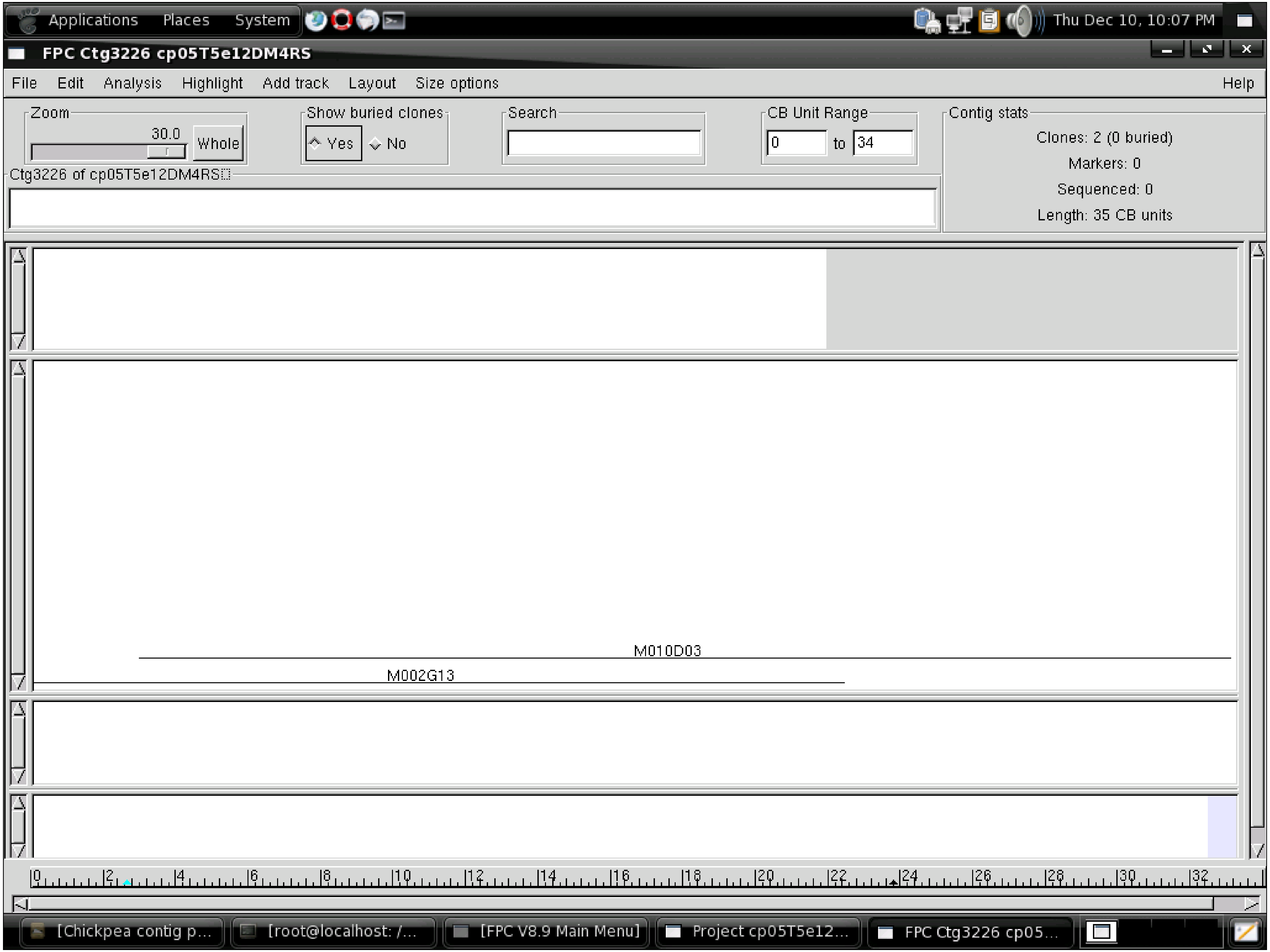

Applications
Places
System
Thu Dec 10, 10:07 PM
FPC Ctg3226 cp05T5e12DM4RS
File
Edit
Analysis
Highlight
Add track
Layout
Size options
Help
Zoom
30.0
Whole
Show buried clones
Yes
No
Search
CB Unit Range
0
to
34
Contig stats
Clones: 2 (0 buried)
Markers: 0
Sequenced: 0
Length: 35 CB units
Ctg3226 of cp05T5e12DM4RS
M010D03
M002G13
0 2 4 6 8 10 12 14 16 18 20 22 24 26 28 30 32
[Chickpea contig p...
[root@localhost: /...
[FPC V8.9 Main Menu]
Project cp05T5e12...
FPC Ctg3226 cp05...

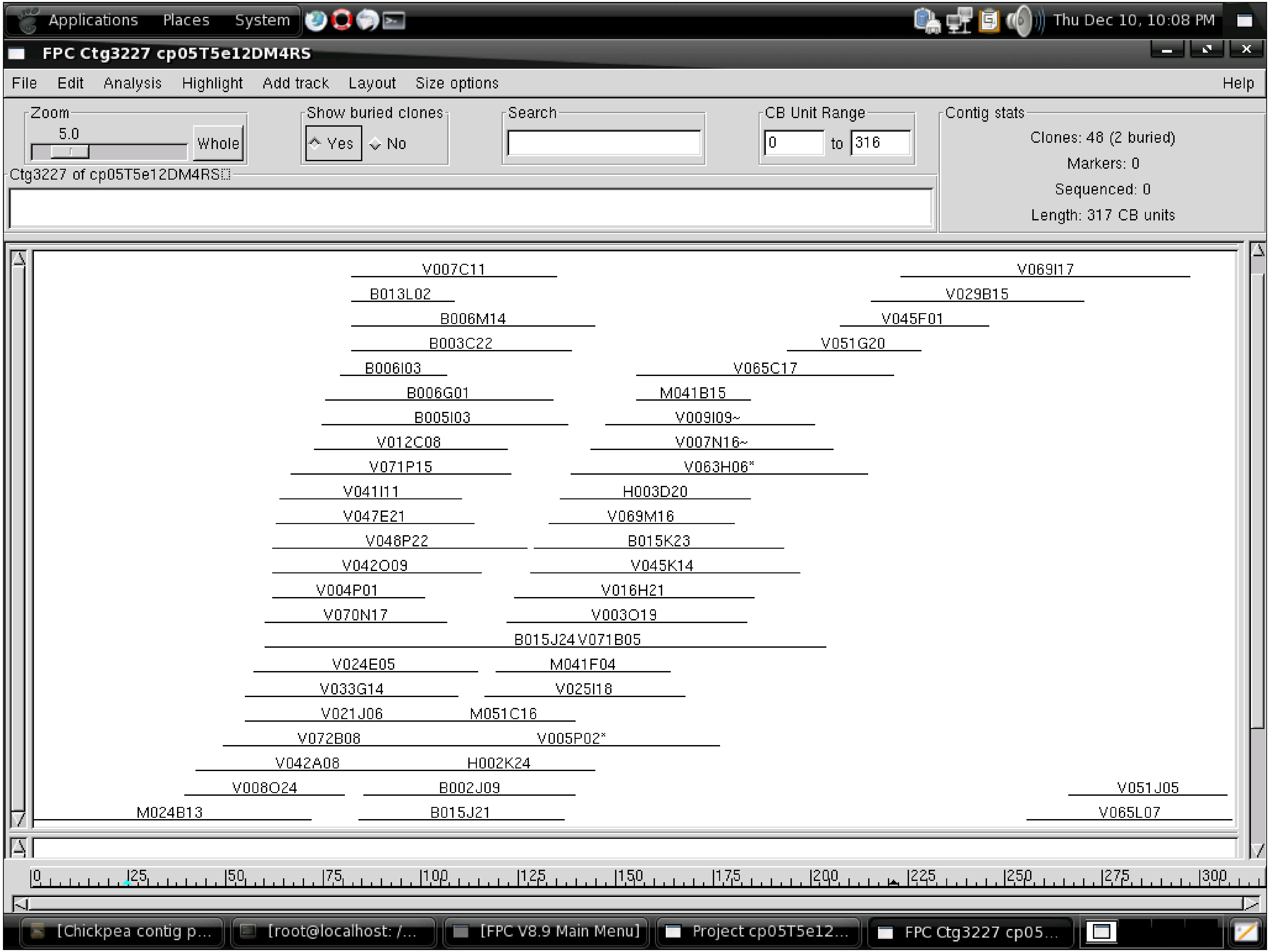
Applications
Places
System
Thu Dec 10, 10:08 PM
FPC Ctg3227 cp05T5e12DM4RS
File
Edit
Analysis
Highlight
Add track
Layout
Size options
Help
Zoom
5.0
Whole
Show buried clones
Yes
No
Search
CB Unit Range
0
to
316
Contig stats
Clones: 48 (2 buried)
Markers: 0
Sequenced: 0
Length: 317 CB units
Ctg3227 of cp05T5e12DM4RS
V007C11
V069I17
B013L02
V029B15
B006M14
V045F01
B003C22
V051G20
B006I03
V065C17
B006G01
M041B15
B005I03
V009I09~
V012C08
V007N16~
V071P15
V063H06*
V041I11
H003D20
V047E21
V069M16
V048P22
B015K23
V042O09
V045K14
V004P01
V016H21
V070N17
V003O19
B015J24 V071B05
V024E05
M041F04
V033G14
V025I18
V021J06
M051C16
V072B08
V005P02*
V042A08
H002K24
V008O24
B002J09
V051J05
M024B13
B015J21
V065L07
0
25
50
75
100
125
150
175
200
225
250
275
300
[Chickpea contig p...
[root@localhost: /...
[FPC V8.9 Main Menu]
Project cp05T5e12...
FPC Ctg3227 cp05...

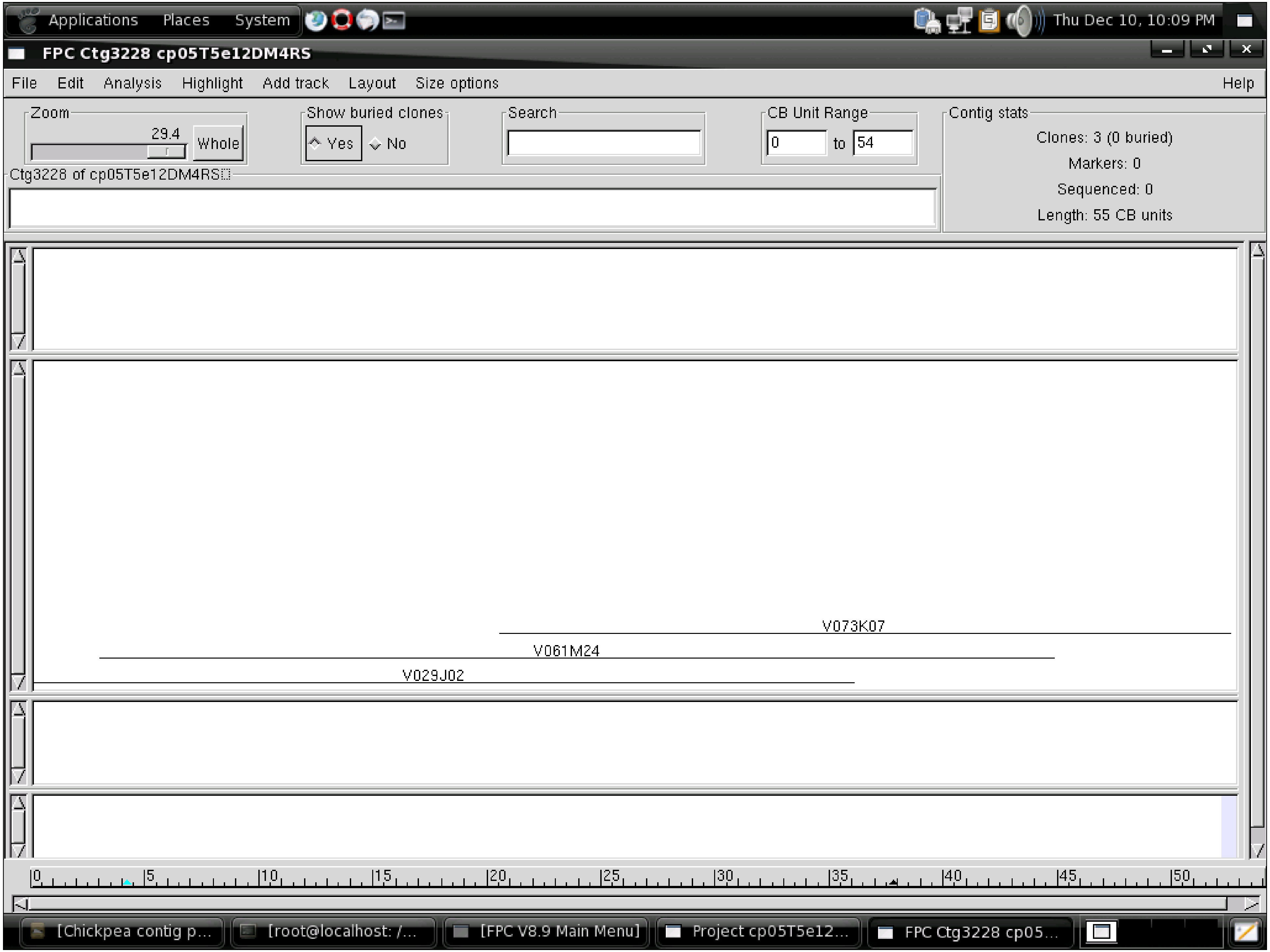
Applications
Places
System
Thu Dec 10, 10:09 PM
FPC Ctg3228 cp05T5e12DM4RS
File
Edit
Analysis
Highlight
Add track
Layout
Size options
Help
Zoom
29.4
Whole
Show buried clones
Yes
No
Search
CB Unit Range
0
to
54
Contig stats
Clones: 3 (0 buried)
Markers: 0
Sequenced: 0
Length: 55 CB units
Ctg3228 of cp05T5e12DM4RS
V073K07
V061M24
V029J02
0
5
10
15
20
25
30
35
40
45
50
[Chickpea contig p...
[root@localhost: /...
[FPC V8.9 Main Menu]
Project cp05T5e12...
FPC Ctg3228 cp05...

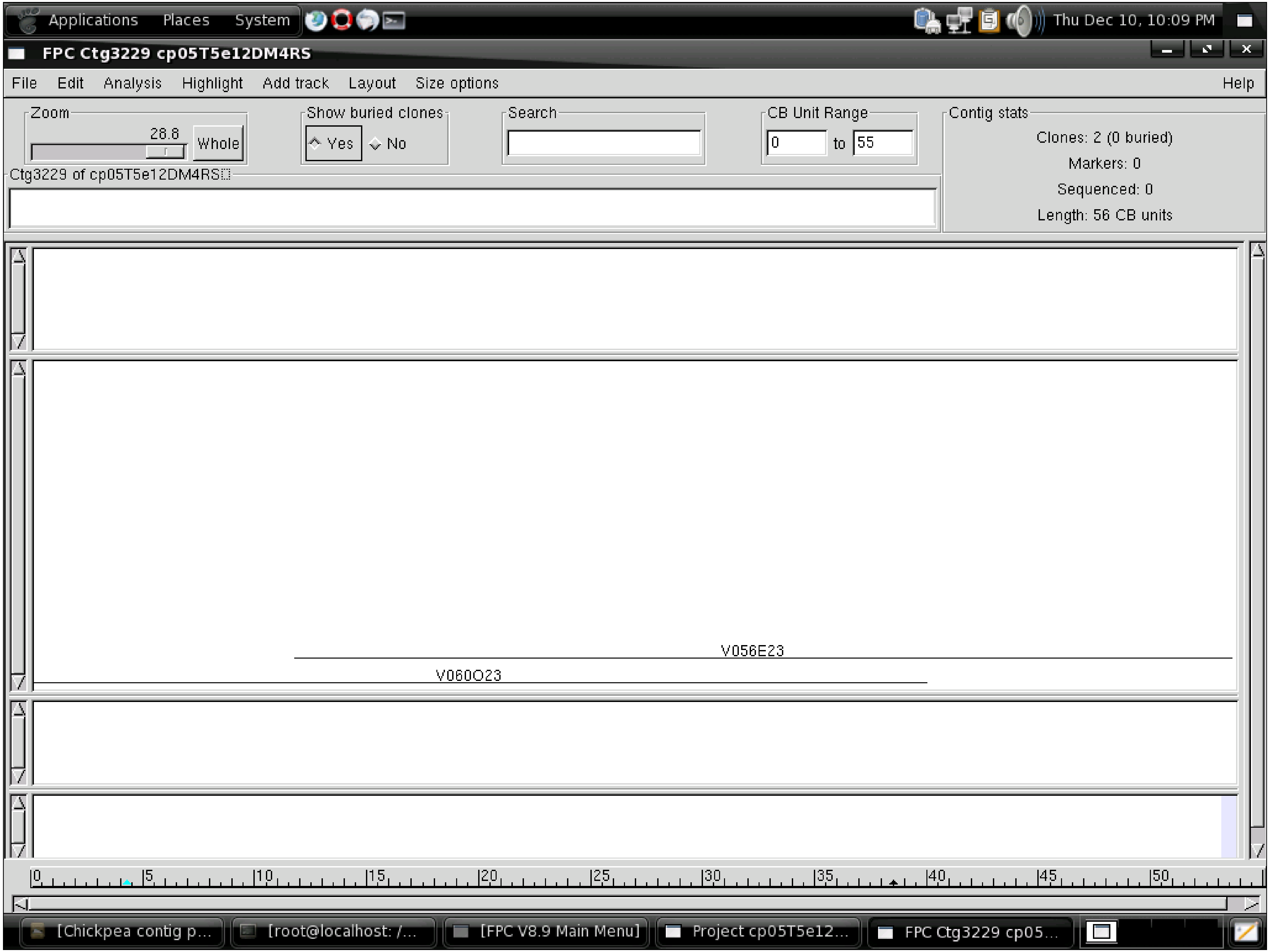
Applications Places System
Thu Dec 10, 10:09 PM
FPC Ctg3229 cp05T5e12DM4RS
File Edit Analysis Highlight Add track Layout Size options Help
Zoom
28.8
Whole
Show buried clones
Yes No
Search
CB Unit Range
0 to 55
Contig stats
Clones: 2 (0 buried)
Markers: 0
Sequenced: 0
Length: 56 CB units
Ctg3229 of cp05T5e12DM4RS
V056E23
V060O23
0 5 10 15 20 25 30 35 40 45 50
[Chickpea contig p...
[root@localhost: /...
[FPC V8.9 Main Menu]
Project cp05T5e12...
FPC Ctg3229 cp05...

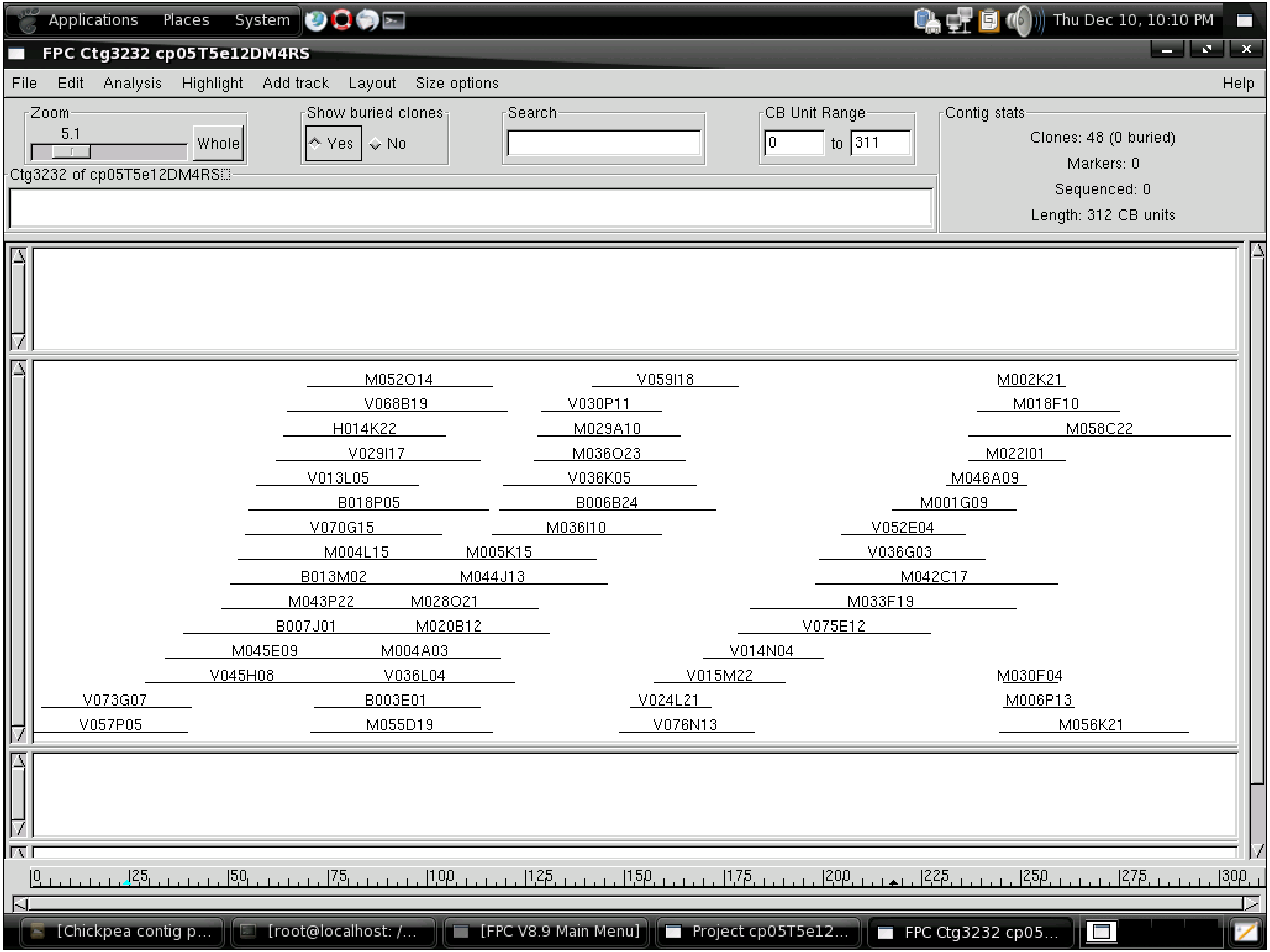
Applications Places System
Thu Dec 10, 10:10 PM
FPC Ctg3232 cp05T5e12DM4RS
File Edit Analysis Highlight Add track Layout Size options Help
Zoom
5.1
Whole
Show buried clones
Yes No
Search
CB Unit Range
0 to 311
Contig stats
Clones: 48 (0 buried)
Markers: 0
Sequenced: 0
Length: 312 CB units
Ctg3232 of cp05T5e12DM4RS
M052O14
V059I18
M002K21
V068B19
V030P11
M018F10
H014K22
M029A10
M058C22
V029I17
M036O23
M022I01
V013L05
V036K05
M046A09
B018P05
B006B24
M001G09
V070G15
M036I10
V052E04
M004L15
M005K15
V036G03
B013M02
M044J13
M042C17
M043P22
M028O21
M033F19
B007J01
M020B12
V075E12
M045E09
M004A03
V014N04
V045H08
V036L04
V015M22
M030F04
V073G07
B003E01
V024L21
M006P13
V057P05
M055D19
V076N13
M056K21
0 25 50 75 100 125 150 175 200 225 250 275 300
[Chickpea contig p...
[root@localhost: /...
[FPC V8.9 Main Menu]
Project cp05T5e12...
FPC Ctg3232 cp05...

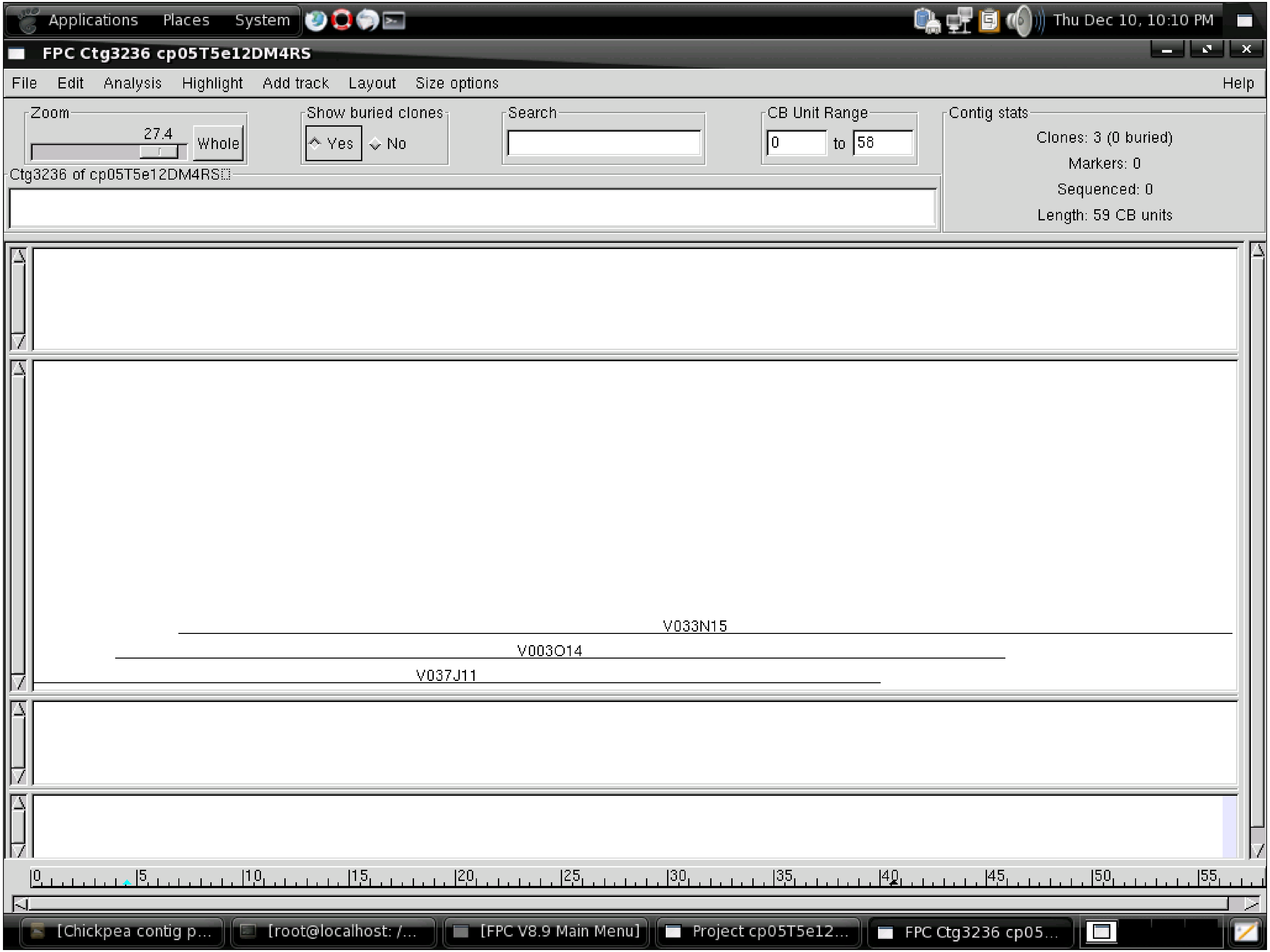
Applications Places System
Thu Dec 10, 10:10 PM
FPC Ctg3236 cp05T5e12DM4RS
File Edit Analysis Highlight Add track Layout Size options Help
Zoom
27.4
Whole
Show buried clones
Yes No
Search
CB Unit Range
0 to 58
Contig stats
Clones: 3 (0 buried)
Markers: 0
Sequenced: 0
Length: 59 CB units
Ctg3236 of cp05T5e12DM4RS
V033N15
V003O14
V037J11
0 5 10 15 20 25 30 35 40 45 50 55
[Chickpea contig p...
[root@localhost: /...
[FPC V8.9 Main Menu]
Project cp05T5e12...
FPC Ctg3236 cp05...

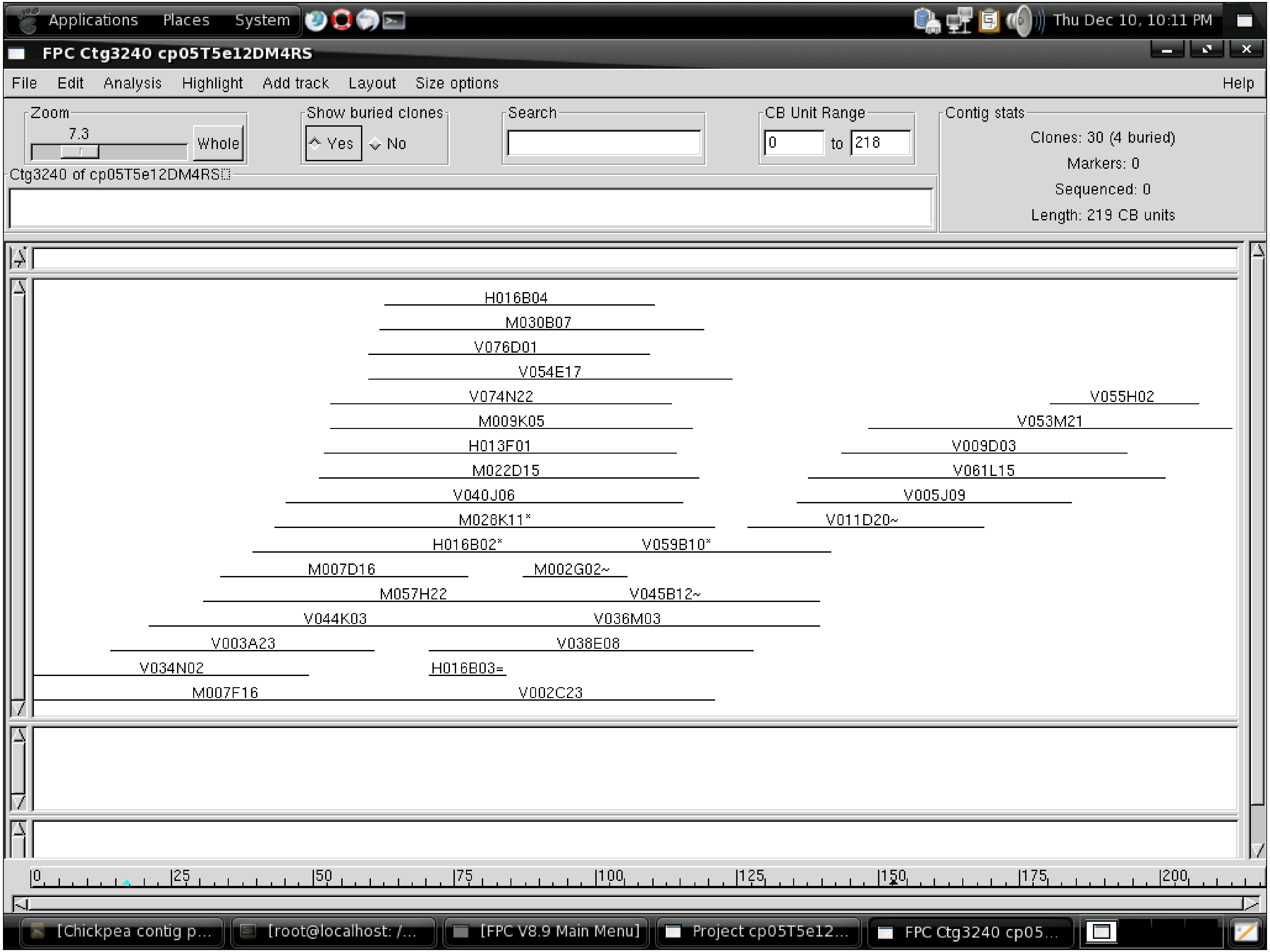
Applications
Places
System
Thu Dec 10, 10:11 PM
FPC Ctg3240 cp05T5e12DM4RS
File
Edit
Analysis
Highlight
Add track
Layout
Size options
Help
Zoom
7.3
Whole
Show buried clones
Yes
No
Search
CB Unit Range
0
to
218
Contig stats
Clones: 30 (4 buried)
Markers: 0
Sequenced: 0
Length: 219 CB units
Ctg3240 of cp05T5e12DM4RS
H016B04
M030B07
V076D01
V054E17
V074N22
V055H02
M009K05
V053M21
H013F01
V009D03
M022D15
V061L15
V040J06
V005J09
M028K11*
V011D20~
H016B02*
V059B10*
M007D16
M002G02~
M057H22
V045B12~
V044K03
V036M03
V003A23
V038E08
V034N02
H016B03=
M007F16
V002C23
0
25
50
75
100
125
150
175
200
[Chickpea contig p...
[root@localhost: /...
[FPC V8.9 Main Menu]
Project cp05T5e12...
FPC Ctg3240 cp05...

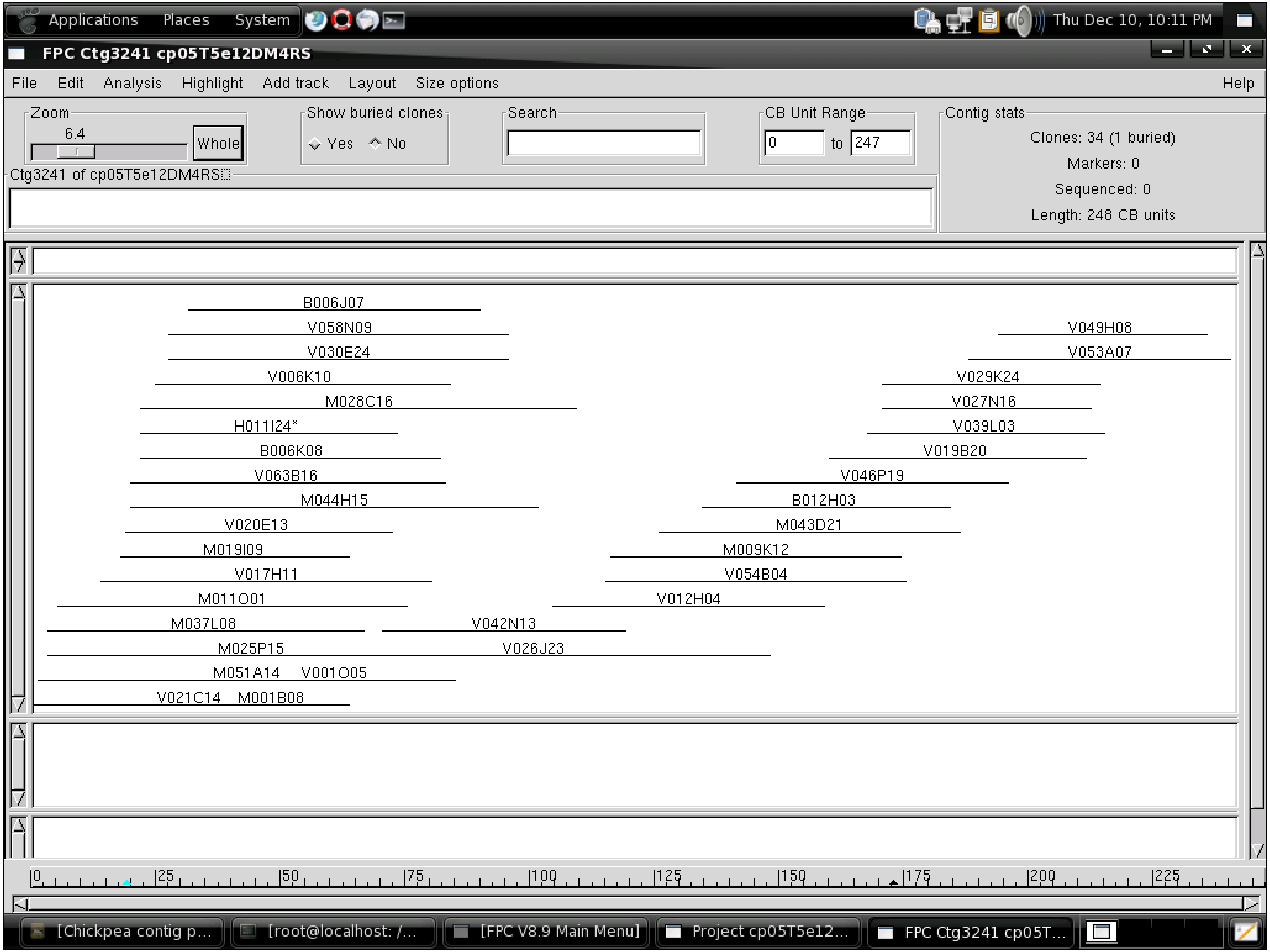
Applications Places System
Thu Dec 10, 10:11 PM
FPC Ctg3241 cp05T5e12DM4RS
File Edit Analysis Highlight Add track Layout Size options Help
Zoom
6.4
Whole
Show buried clones
Yes No
Search
CB Unit Range
0 to 247
Contig stats
Clones: 34 (1 buried)
Markers: 0
Sequenced: 0
Length: 248 CB units
Ctg3241 of cp05T5e12DM4RS
B006J07
V058N09
V030E24
V006K10
M028C16
H011I24*
B006K08
V063B16
M044H15
V020E13
M019I09
V017H11
M011O01
M037L08
M025P15
M051A14
V001O05
V021C14
M001B08
V049H08
V053A07
V029K24
V027N16
V039L03
V019B20
V046P19
B012H03
M043D21
M009K12
V054B04
V012H04
V042N13
V026J23
0 25 50 75 100 125 150 175 200 225
[Chickpea contig p...
[root@localhost: /...
[FPC V8.9 Main Menu]
Project cp05T5e12...
FPC Ctg3241 cp05T...

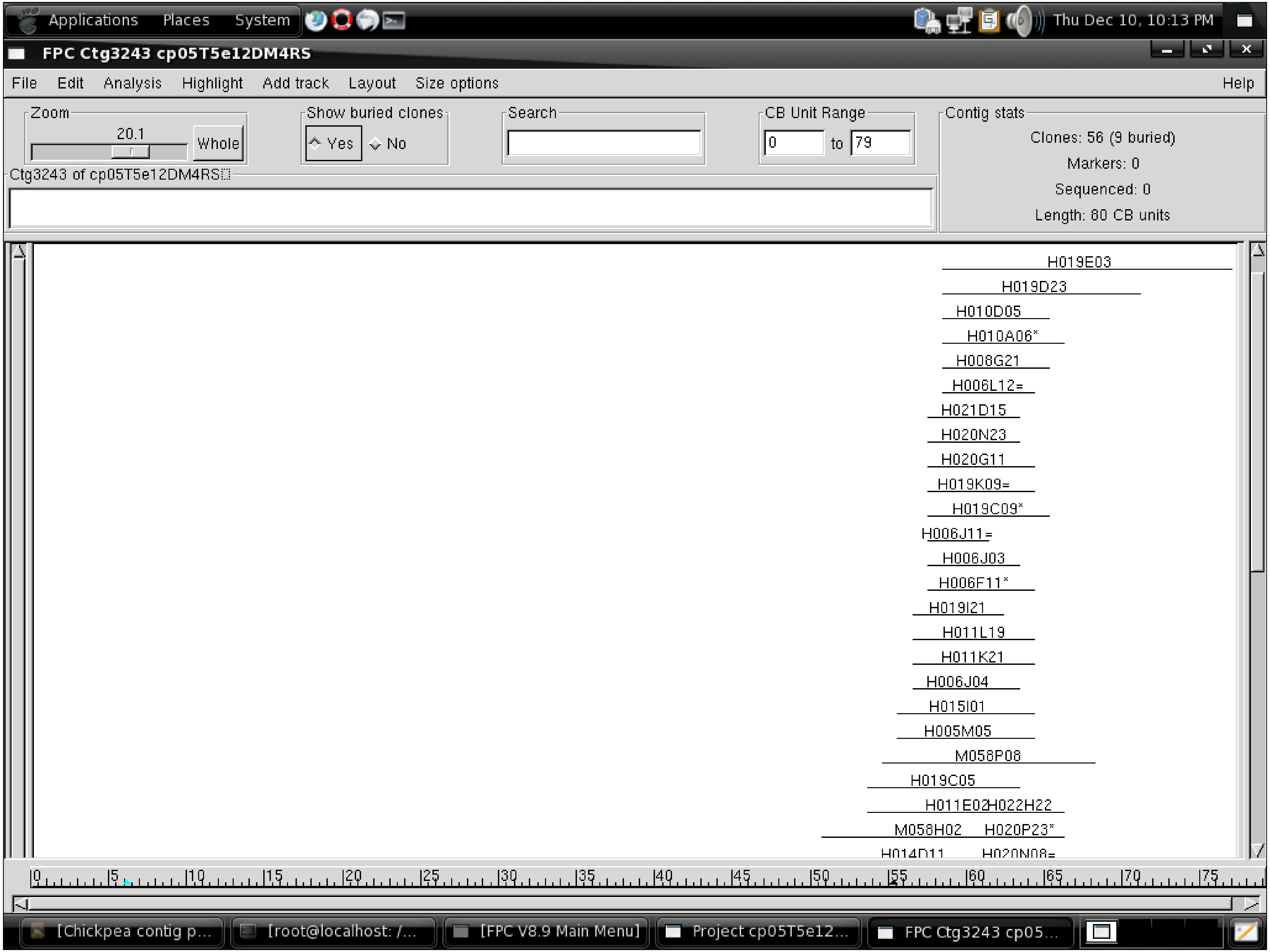
Applications Places System
Thu Dec 10, 10:13 PM
FPC Ctg3243 cp05T5e12DM4RS
File Edit Analysis Highlight Add track Layout Size options
Help
Zoom
20.1
Whole
Show buried clones
Yes
No
Search
CB Unit Range
0
to
79
Contig stats
Clones: 56 (9 buried)
Markers: 0
Sequenced: 0
Length: 80 CB units
Ctg3243 of cp05T5e12DM4RS
H019E03
H019D23
H010D05
H010A06*
H008G21
H006L12=
H021D15
H020N23
H020G11
H019K09=
H019C09*
H006J11=
H006J03
H006F11*
H019I21
H011L19
H011K21
H006J04
H015I01
H005M05
M058P08
H019C05
H011E02
H022H22
M058H02
H020P23*
H014D11
H020N08=
0 5 10 15 20 25 30 35 40 45 50 55 60 65 70 75
[Chickpea contig p...
[root@localhost: /...
[FPC V8.9 Main Menu]
Project cp05T5e12...
FPC Ctg3243 cp05...

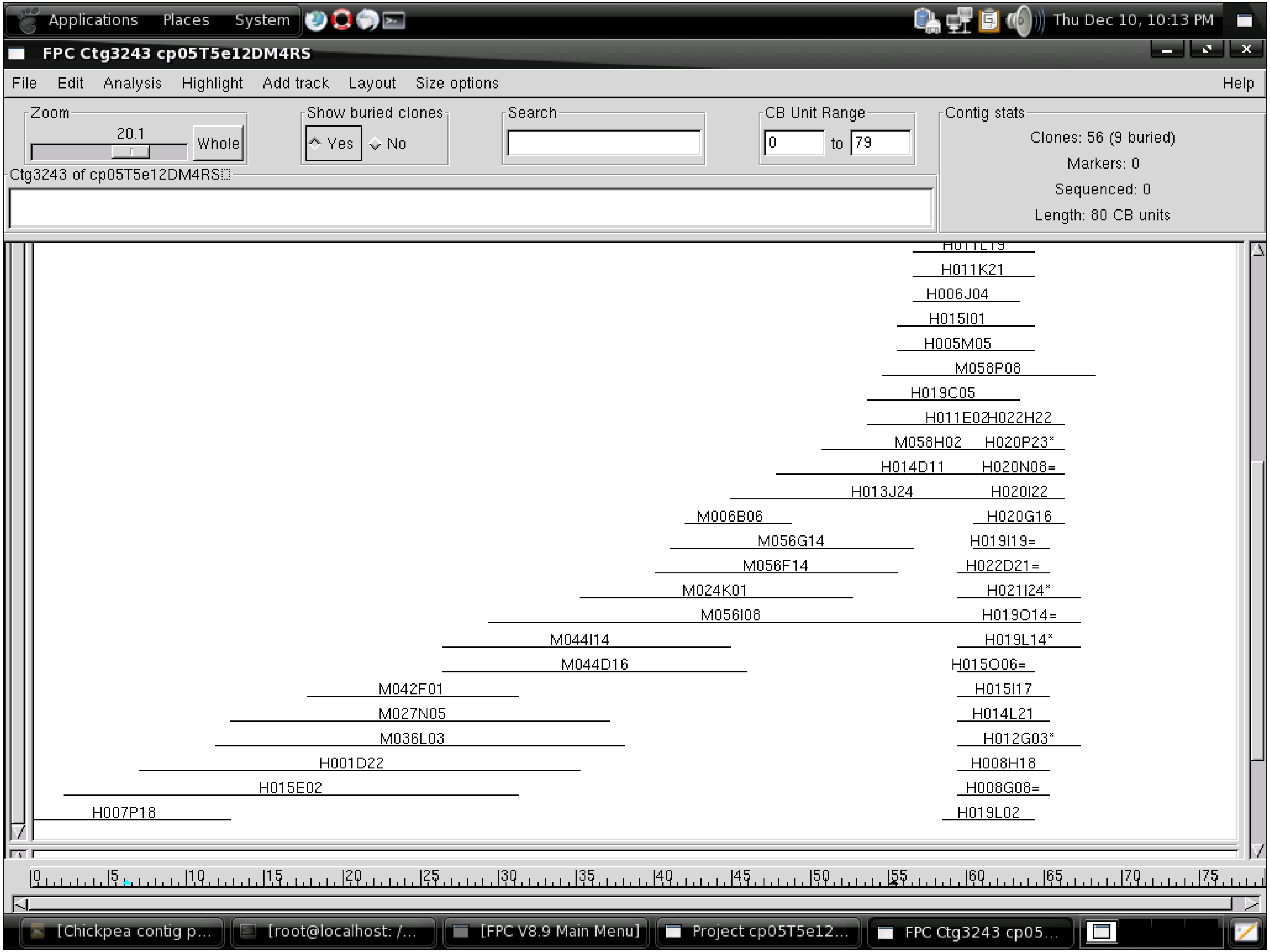
Applications Places System
Thu Dec 10, 10:13 PM
FPC Ctg3243 cp05T5e12DM4RS
File Edit Analysis Highlight Add track Layout Size options Help
Zoom
20.1
Whole
Show buried clones
Yes No
Search
CB Unit Range
0 to 79
Contig stats
Clones: 56 (9 buried)
Markers: 0
Sequenced: 0
Length: 80 CB units
Ctg3243 of cp05T5e12DM4RS
H011K21
H006J04
H015I01
H005M05
M058P08
H019C05
H011E02
H022H22
M058H02
H020P23*
H014D11
H020N08=
H013J24
H020I22
M006B06
H020G16
M056G14
H019I19=
M056F14
H022D21=
M024K01
H021I24*
M056I08
H019O14=
M044I14
H019L14*
M044D16
H015O06=
M042F01
H015I17
M027N05
H014L21
M036L03
H012G03*
H001D22
H008H18
H015E02
H008G08=
H007P18
H019L02
0 5 10 15 20 25 30 35 40 45 50 55 60 65 70 75
[Chickpea contig p...
[root@localhost: /...
[FPC V8.9 Main Menu]
Project cp05T5e12...
FPC Ctg3243 cp05...

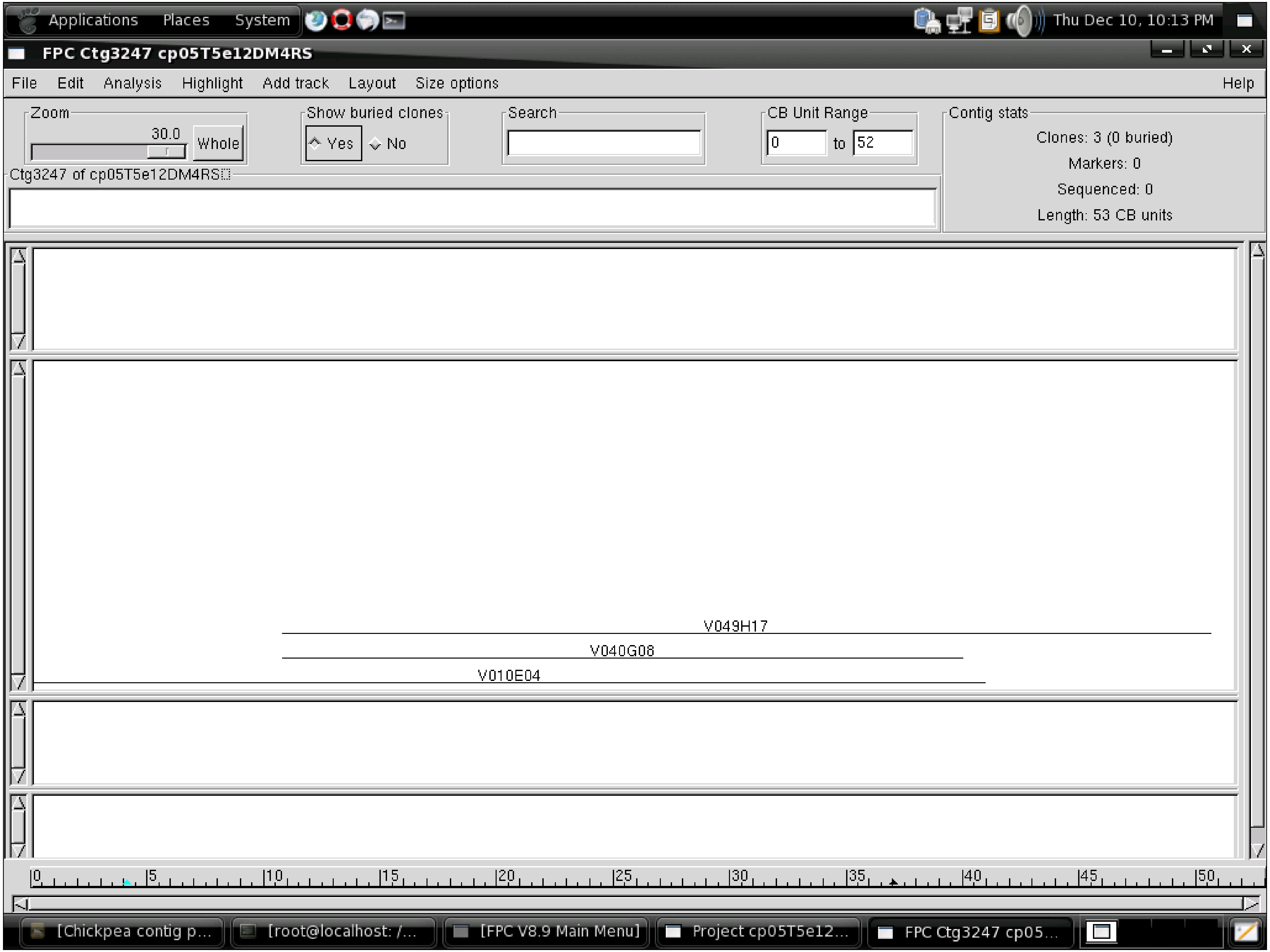
Applications
Places
System
Thu Dec 10, 10:13 PM
FPC Ctg3247 cp05T5e12DM4RS
File
Edit
Analysis
Highlight
Add track
Layout
Size options
Help
Zoom
30.0
Whole
Show buried clones
Yes
No
Search
CB Unit Range
0
to
52
Contig stats
Clones: 3 (0 buried)
Markers: 0
Sequenced: 0
Length: 53 CB units
Ctg3247 of cp05T5e12DM4RS
V049H17
V040G08
V010E04
0 5 10 15 20 25 30 35 40 45 50
[Chickpea contig p...
[root@localhost: /...
[FPC V8.9 Main Menu]
Project cp05T5e12...
FPC Ctg3247 cp05...

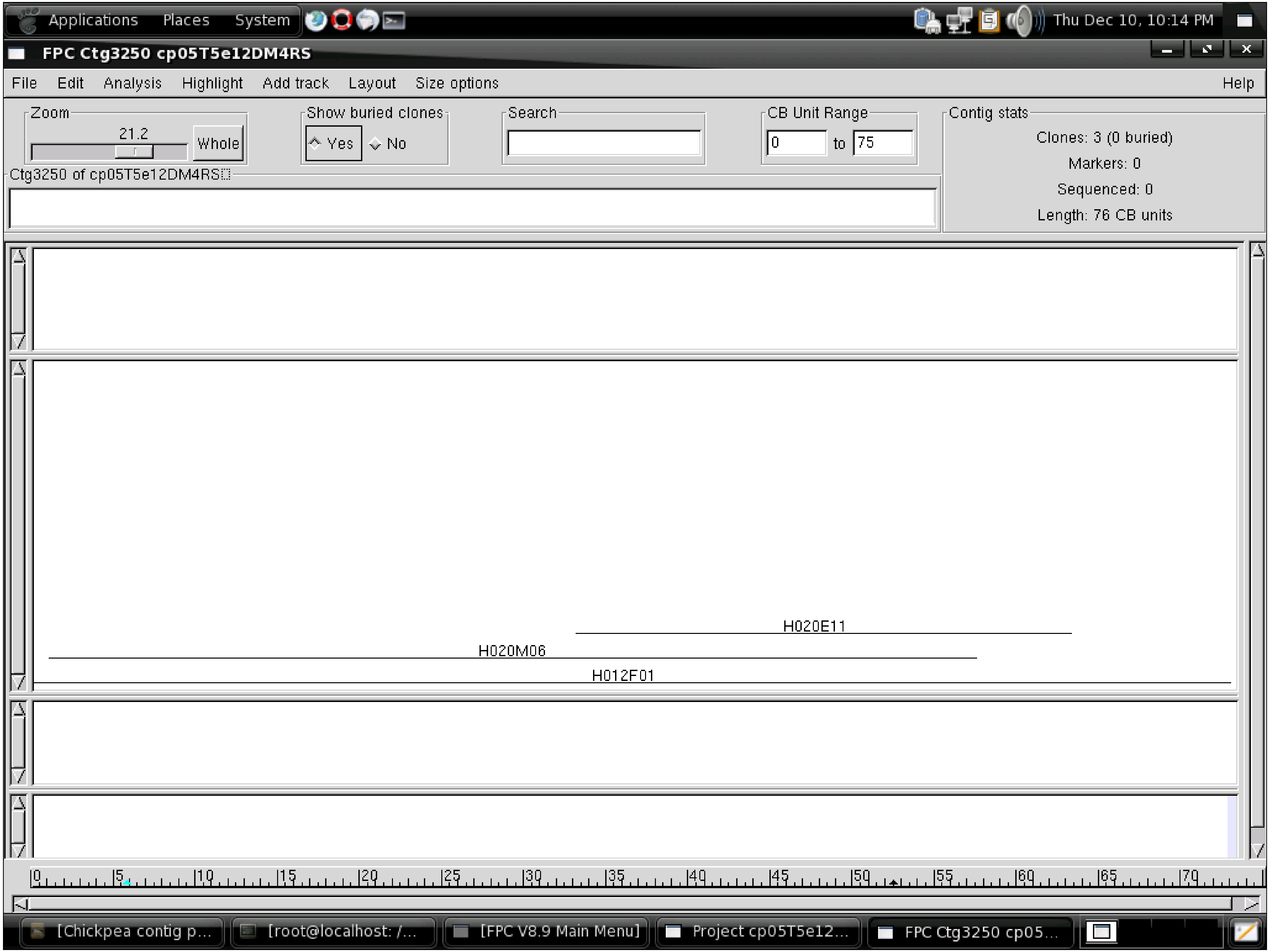
Applications Places System
Thu Dec 10, 10:14 PM
FPC Ctg3250 cp05T5e12DM4RS
File Edit Analysis Highlight Add track Layout Size options Help
Zoom
21.2
Whole
Show buried clones
Yes No
Search
CB Unit Range
0 to 75
Contig stats
Clones: 3 (0 buried)
Markers: 0
Sequenced: 0
Length: 76 CB units
Ctg3250 of cp05T5e12DM4RS
H020E11
H020M06
H012F01
0 5 10 15 20 25 30 35 40 45 50 55 60 65 70
[Chickpea contig p...
[root@localhost: /...
[FPC V8.9 Main Menu]
Project cp05T5e12...
FPC Ctg3250 cp05...

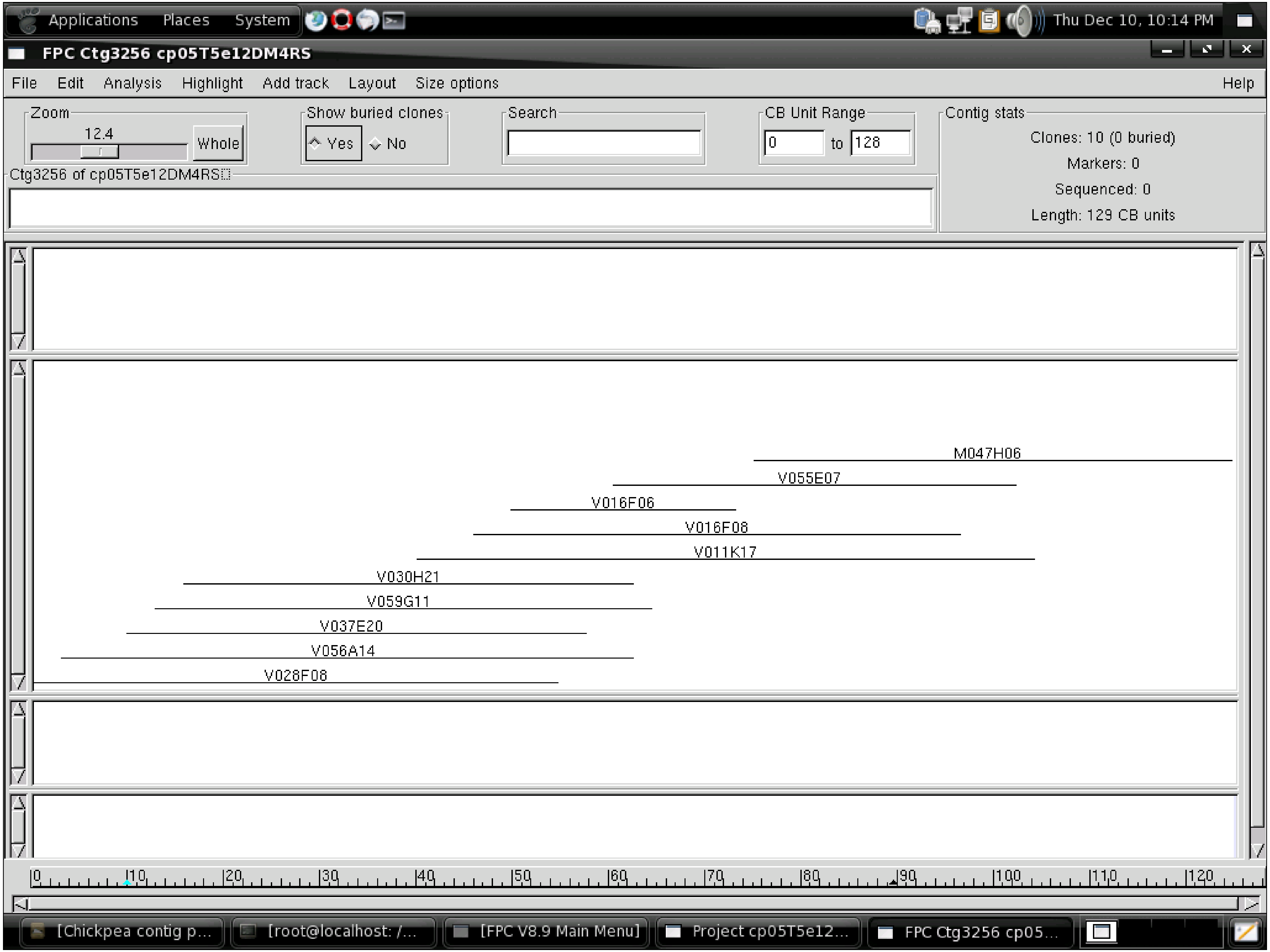
Applications
Places
System
Thu Dec 10, 10:14 PM
FPC Ctg3256 cp05T5e12DM4RS
File
Edit
Analysis
Highlight
Add track
Layout
Size options
Help
Zoom
12.4
Whole
Show buried clones
Yes
No
Search
CB Unit Range
0
to
128
Contig stats
Clones: 10 (0 buried)
Markers: 0
Sequenced: 0
Length: 129 CB units
Ctg3256 of cp05T5e12DM4RS
M047H06
V055E07
V016F06
V016F08
V011K17
V030H21
V059G11
V037E20
V056A14
V028F08
0
10
20
30
40
50
60
70
80
90
100
110
120
[Chickpea contig p...
[root@localhost: /...
[FPC V8.9 Main Menu]
Project cp05T5e12...
FPC Ctg3256 cp05...

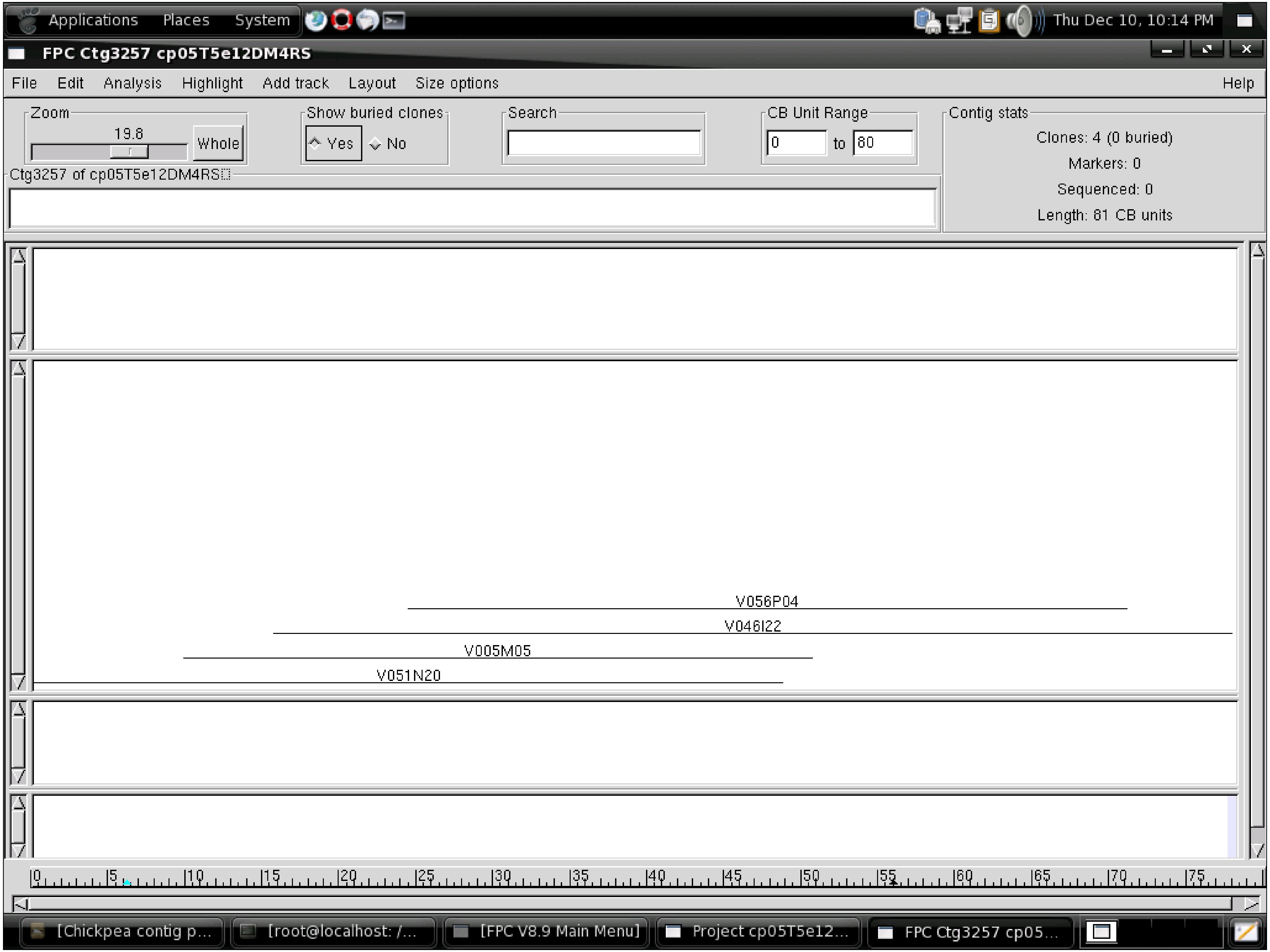
Applications Places System
Thu Dec 10, 10:14 PM
FPC Ctg3257 cp05T5e12DM4RS
File Edit Analysis Highlight Add track Layout Size options Help
Zoom
19.8
Whole
Show buried clones
Yes
No
Search
CB Unit Range
0 to 80
Contig stats
Clones: 4 (0 buried)
Markers: 0
Sequenced: 0
Length: 81 CB units
Ctg3257 of cp05T5e12DM4RS
V056P04
V046I22
V005M05
V051N20
0 5 10 15 20 25 30 35 40 45 50 55 60 65 70 75
[Chickpea contig p...
[root@localhost: /...
[FPC V8.9 Main Menu]
Project cp05T5e12...
FPC Ctg3257 cp05...

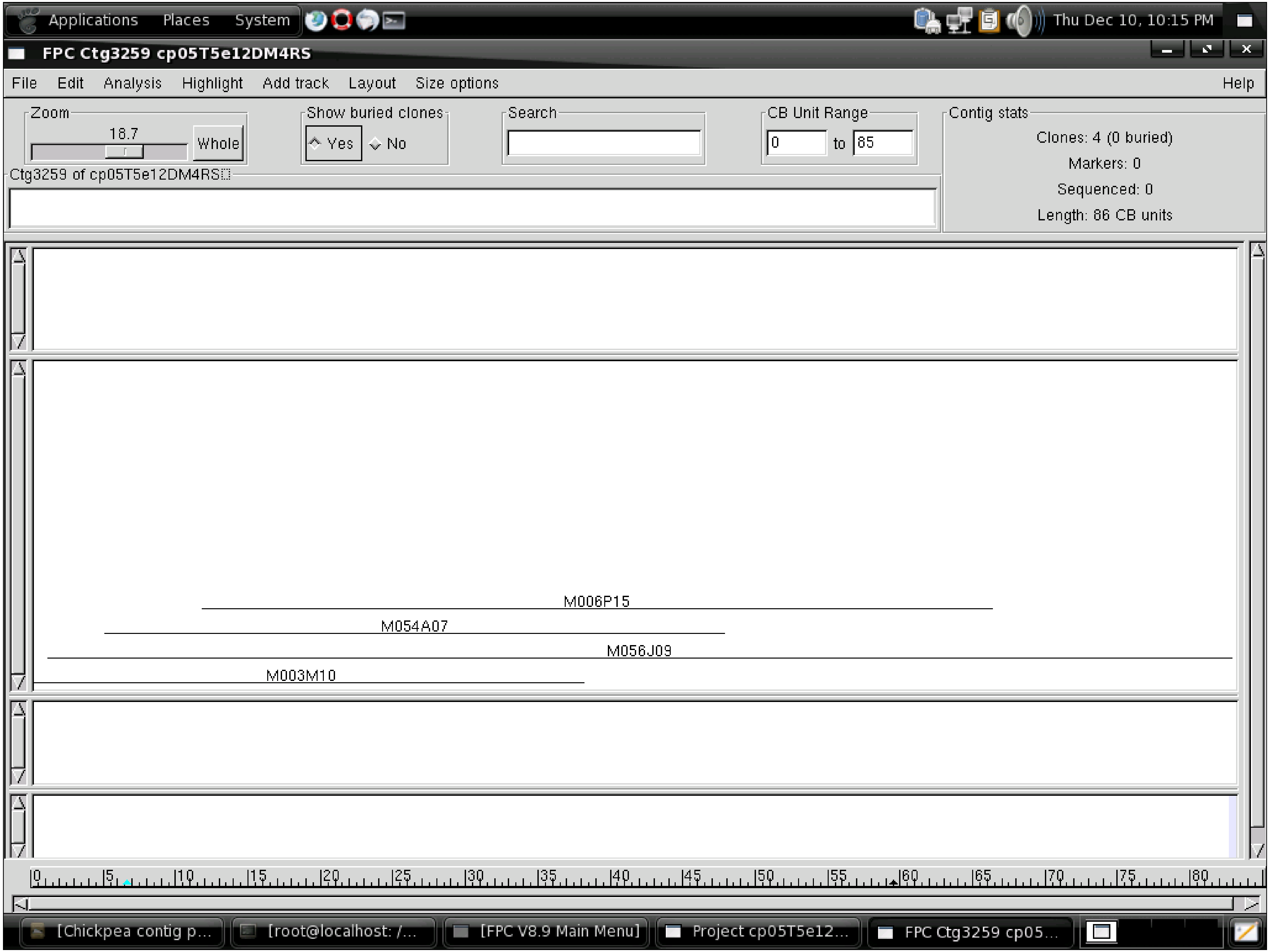

Applications Places System
Thu Dec 10, 10:15 PM
FPC Ctg3259 cp05T5e12DM4RS
File Edit Analysis Highlight Add track Layout Size options
Help
Zoom
18.7
Whole
Show buried clones
Yes
No
Search
CB Unit Range
0
to
85
Contig stats
Clones: 4 (0 buried)
Markers: 0
Sequenced: 0
Length: 86 CB units
Ctg3259 of cp05T5e12DM4RS
M006P15
M054A07
M056J09
M003M10
0 5 10 15 20 25 30 35 40 45 50 55 60 65 70 75 80
[Chickpea contig p...
[root@localhost: /...
[FPC V8.9 Main Menu]
Project cp05T5e12...
FPC Ctg3259 cp05...

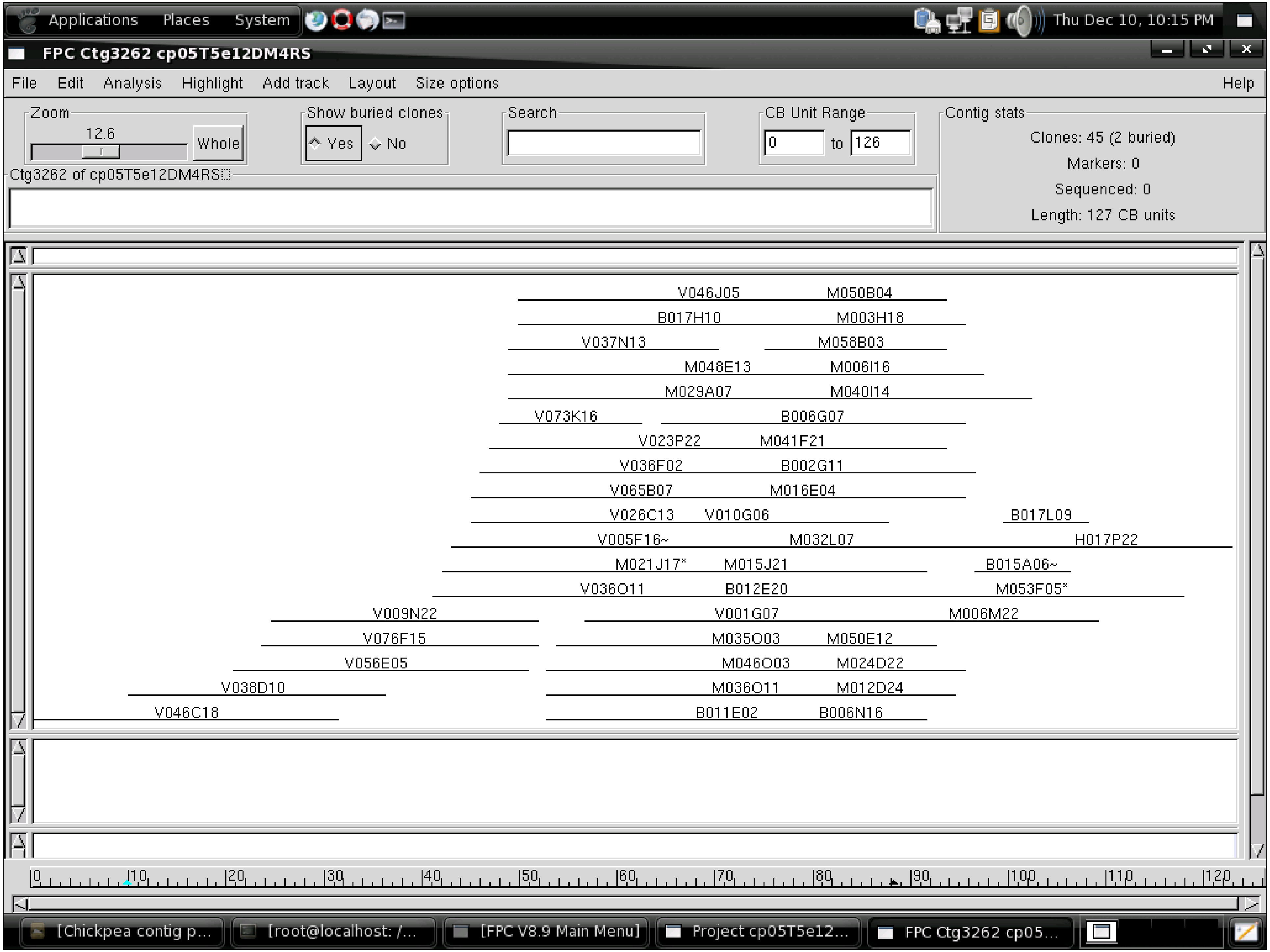
Applications Places System
Thu Dec 10, 10:15 PM
FPC Ctg3262 cp05T5e12DM4RS
File Edit Analysis Highlight Add track Layout Size options Help
Zoom
12.6
Whole
Show buried clones
Yes
No
Search
CB Unit Range
0
to
126
Contig stats
Clones: 45 (2 buried)
Markers: 0
Sequenced: 0
Length: 127 CB units
Ctg3262 of cp05T5e12DM4RS
V046J05 M050B04
B017H10 M003H18
V037N13 M058B03
M048E13 M006I16
M029A07 M040I14
V073K16 B006G07
V023P22 M041F21
V036F02 B002G11
V065B07 M016E04
V026C13 V010G06 B017L09
V005F16~ M032L07 H017P22
M021J17* M015J21 B015A06~
V036O11 B012E20 M053F05*
V009N22 V001G07 M006M22
V076F15 M035O03 M050E12
V056E05 M046O03 M024D22
V038D10 M036O11 M012D24
V046C18 B011E02 B006N16
0 10 20 30 40 50 60 70 80 90 100 110 120
[Chickpea contig p...
[root@localhost: /...
[FPC V8.9 Main Menu]
Project cp05T5e12...
FPC Ctg3262 cp05...

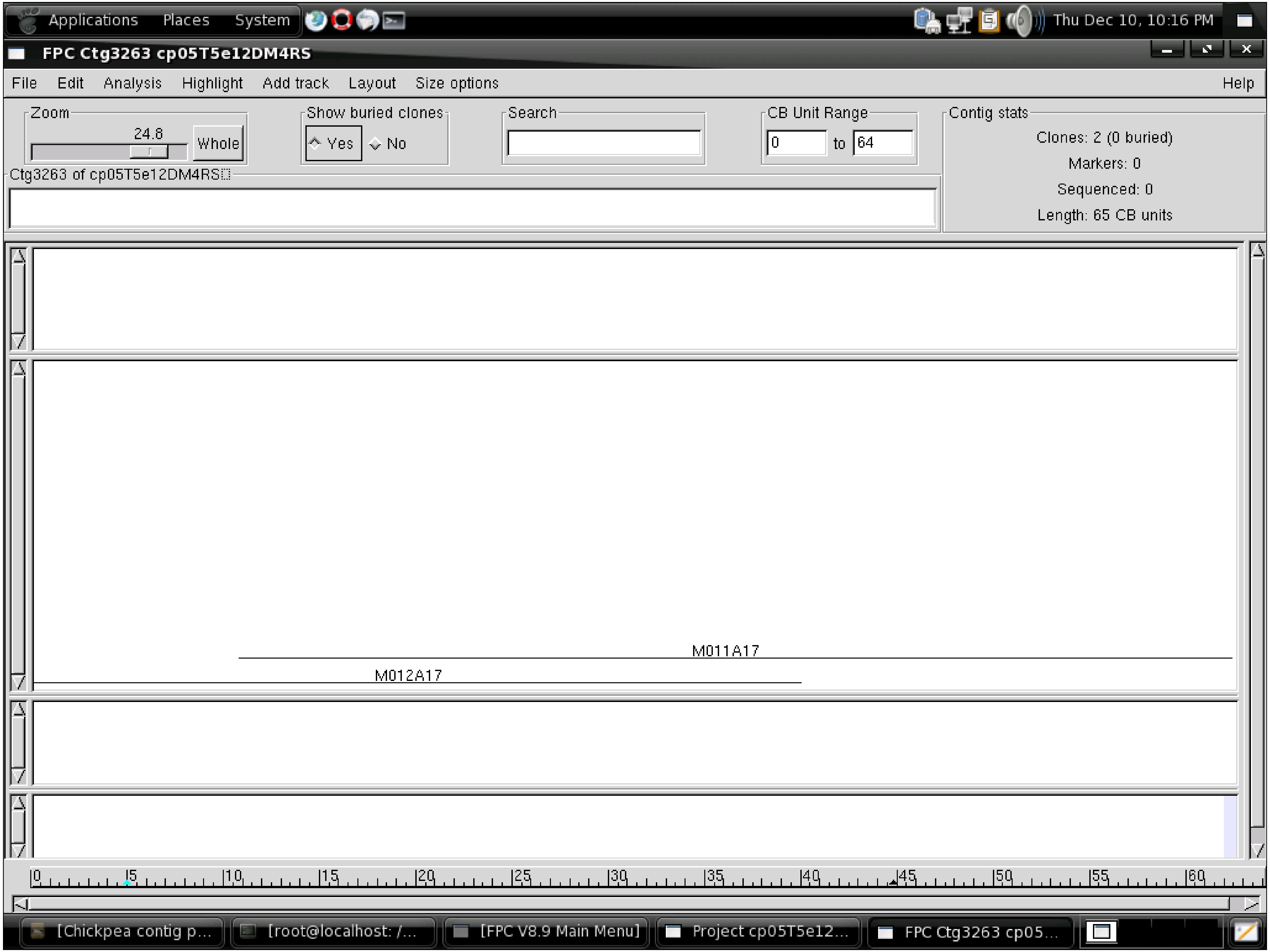
Applications Places System
Thu Dec 10, 10:16 PM
FPC Ctg3263 cp05T5e12DM4RS
File Edit Analysis Highlight Add track Layout Size options Help
Zoom
24.8
Whole
Show buried clones
Yes No
Search
CB Unit Range
0 to 64
Contig stats
Clones: 2 (0 buried)
Markers: 0
Sequenced: 0
Length: 65 CB units
Ctg3263 of cp05T5e12DM4RS
M011A17
M012A17
0 5 10 15 20 25 30 35 40 45 50 55 60
[Chickpea contig p...
[root@localhost: /...
[FPC V8.9 Main Menu]
Project cp05T5e12...
FPC Ctg3263 cp05...

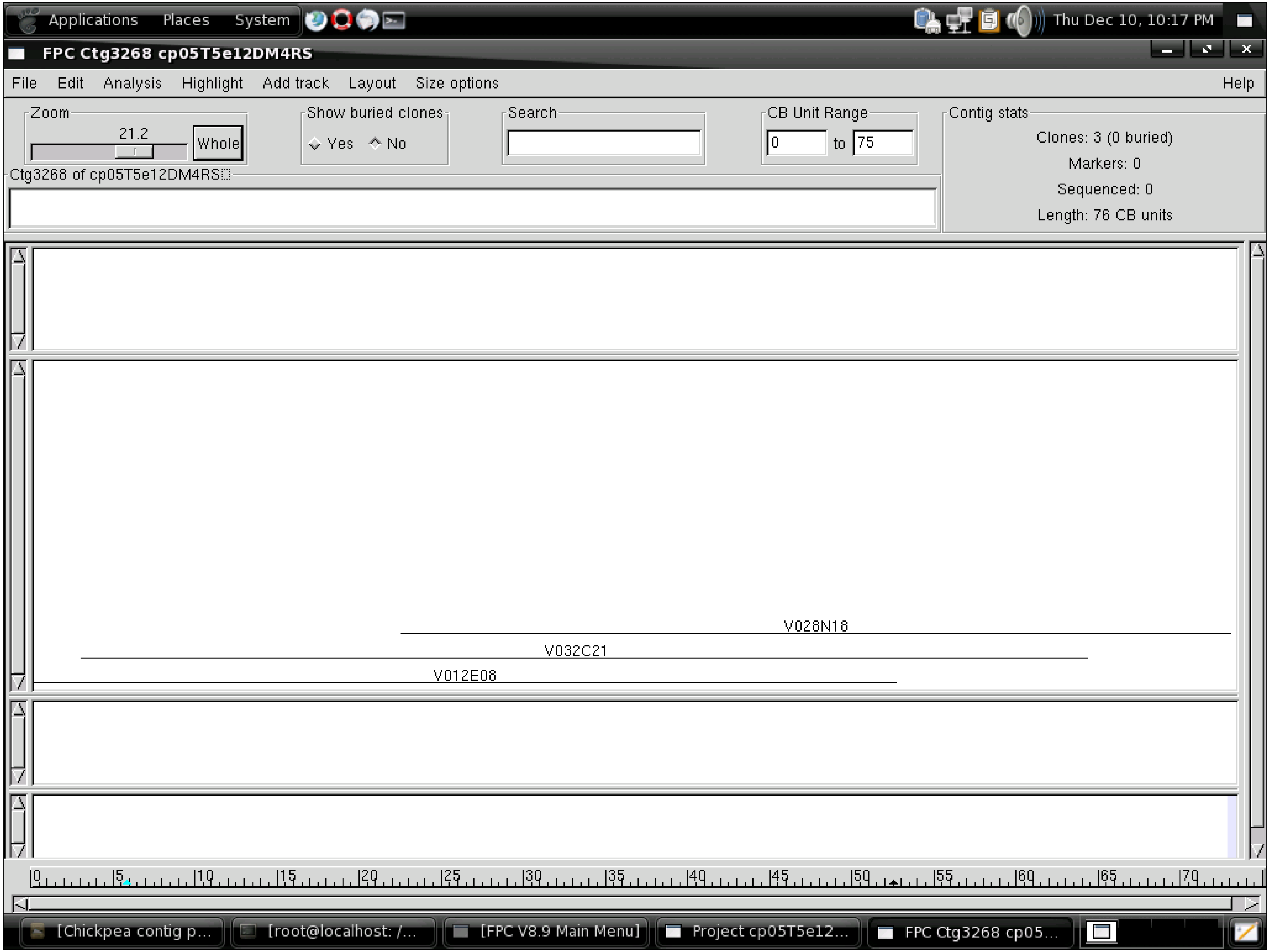
Applications Places System
Thu Dec 10, 10:17 PM
FPC Ctg3268 cp05T5e12DM4RS
File Edit Analysis Highlight Add track Layout Size options Help
Zoom
21.2
Whole
Show buried clones
Yes No
Search
CB Unit Range
0 to 75
Contig stats
Clones: 3 (0 buried)
Markers: 0
Sequenced: 0
Length: 76 CB units
Ctg3268 of cp05T5e12DM4RS
V028N18
V032C21
V012E08
0 5 10 15 20 25 30 35 40 45 50 55 60 65 70
[Chickpea contig p...
[root@localhost: /...
[FPC V8.9 Main Menu]
Project cp05T5e12...
FPC Ctg3268 cp05...

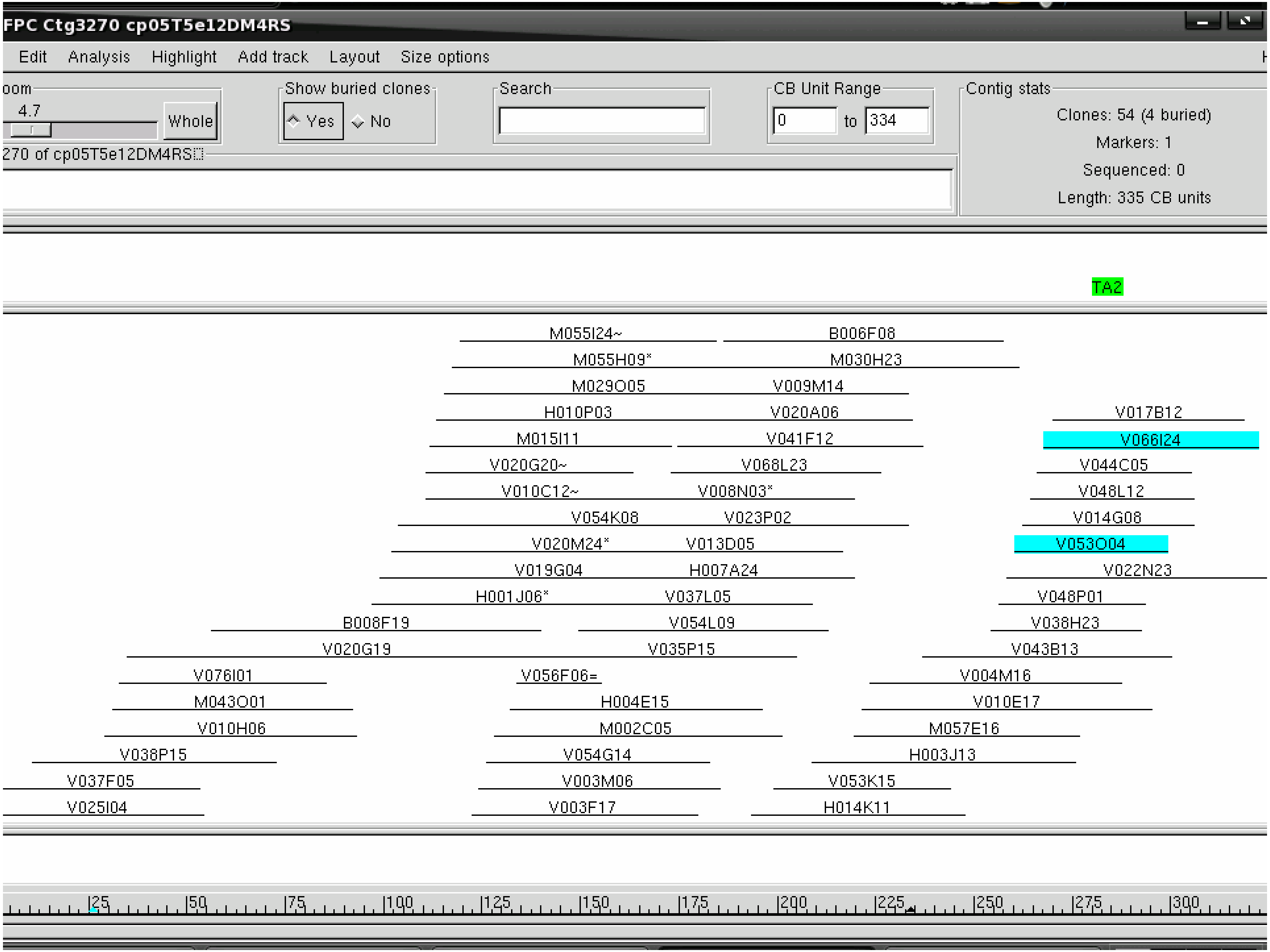
FPC Ctg3270 cp05T5e12DM4RS
Edit
Analysis
Highlight
Add track
Layout
Size options
oom
4.7
Whole
Show buried clones
Yes
No
Search
CB Unit Range
0
to
334
Contig stats
Clones: 54 (4 buried)
Markers: 1
Sequenced: 0
Length: 335 CB units
270 of cp05T5e12DM4RS
TA2
M055I24~
B006F08
M055H09*
M030H23
M029O05
V009M14
H010P03
V020A06
V017B12
M015I11
V041F12
V066I24
V020G20~
V068L23
V044C05
V010C12~
V008N03*
V048L12
V054K08
V023P02
V014G08
V020M24*
V013D05
V053O04
V019G04
H007A24
V022N23
H001J06*
V037L05
V048P01
B008F19
V054L09
V038H23
V020G19
V035P15
V043B13
V076I01
V056F06=
V004M16
M043O01
H004E15
V010E17
V010H06
M002C05
M057E16
V038P15
V054G14
H003J13
V037F05
V003M06
V053K15
V025I04
V003F17
H014K11
25
50
75
100
125
150
175
200
225
250
275
300

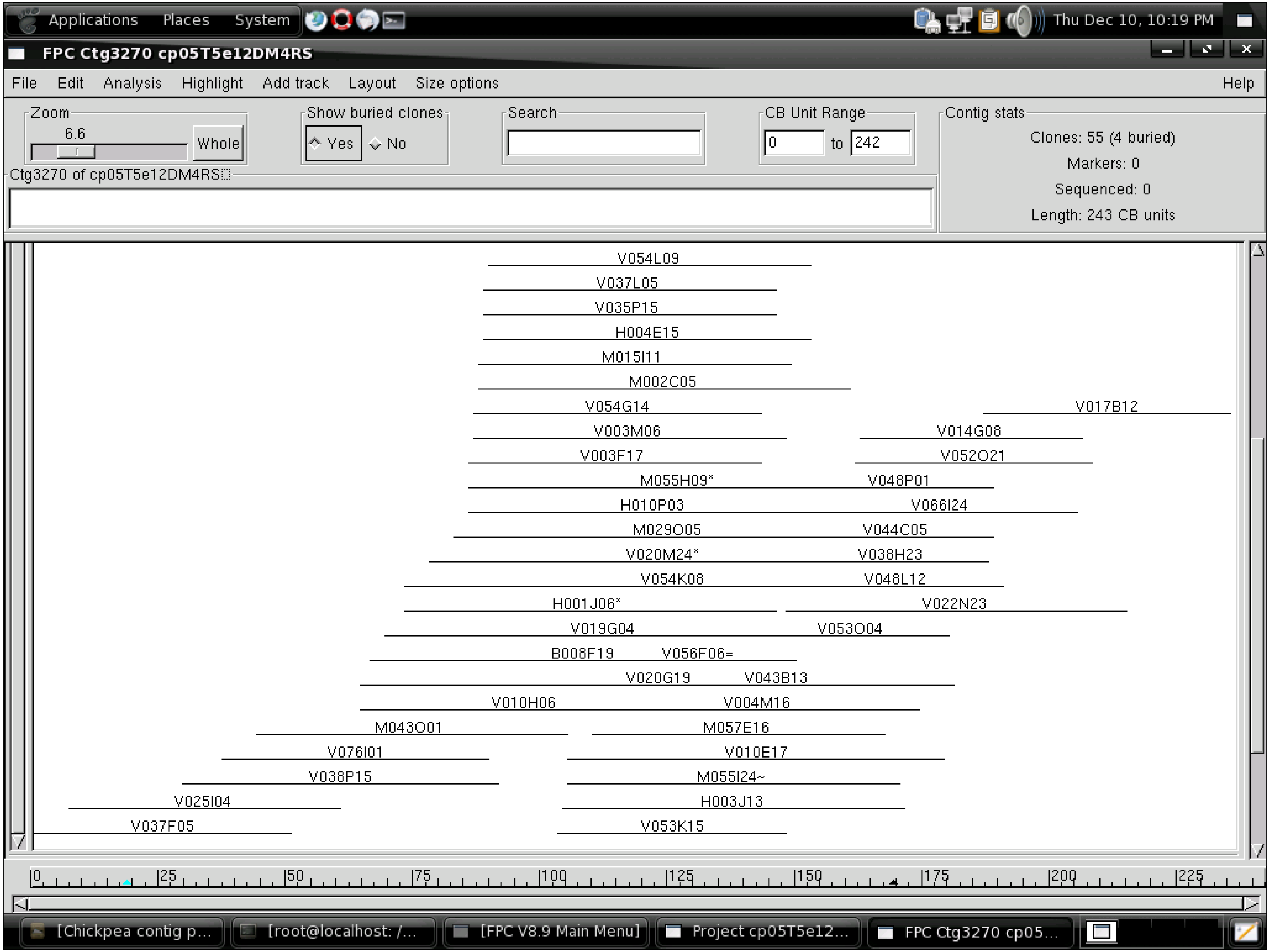
Applications Places System
Thu Dec 10, 10:19 PM
FPC Ctg3270 cp05T5e12DM4RS
File Edit Analysis Highlight Add track Layout Size options Help
Zoom
6.6
Whole
Show buried clones
Yes
No
Search
CB Unit Range
0
to
242
Contig stats
Clones: 55 (4 buried)
Markers: 0
Sequenced: 0
Length: 243 CB units
Ctg3270 of cp05T5e12DM4RS
V054L09
V037L05
V035P15
H004E15
M015I11
M002C05
V054G14
V017B12
V003M06
V014G08
V003F17
V052O21
M055H09*
V048P01
H010P03
V066I24
M029O05
V044C05
V020M24*
V038H23
V054K08
V048L12
H001J06*
V022N23
V019G04
V053O04
B008F19
V056F06=
V020G19
V043B13
V010H06
V004M16
M043O01
M057E16
V076I01
V010E17
V038P15
M055I24~
V025I04
H003J13
V037F05
V053K15
0 25 50 75 100 125 150 175 200 225
[Chickpea contig p...
[root@localhost: /...
[FPC V8.9 Main Menu]
Project cp05T5e12...
FPC Ctg3270 cp05...

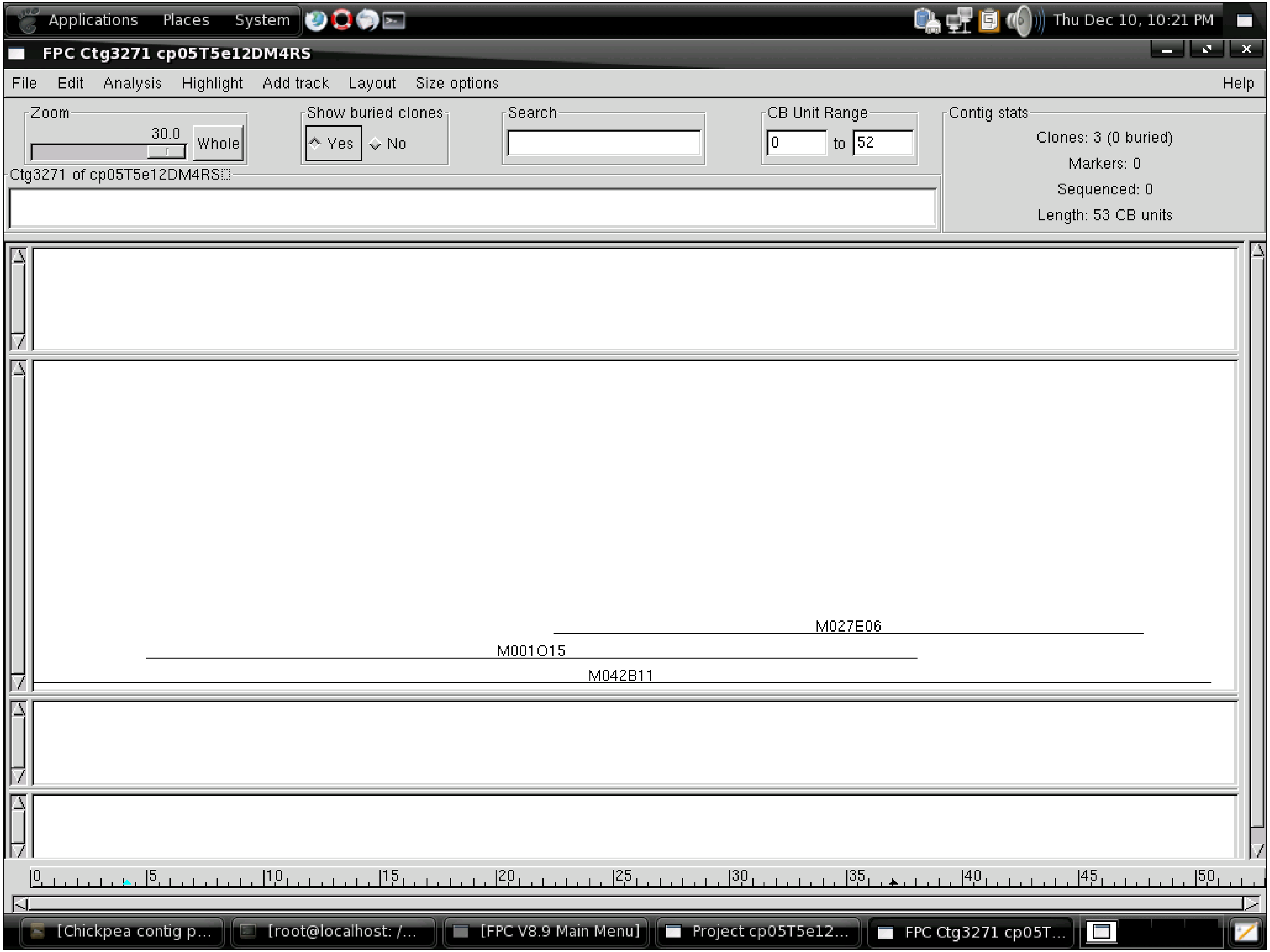
Applications Places System
Thu Dec 10, 10:21 PM
FPC Ctg3271 cp05T5e12DM4RS
File Edit Analysis Highlight Add track Layout Size options Help
Zoom
30.0
Whole
Show buried clones
Yes
No
Search
CB Unit Range
0 to 52
Contig stats
Clones: 3 (0 buried)
Markers: 0
Sequenced: 0
Length: 53 CB units
Ctg3271 of cp05T5e12DM4RS
M027E06
M001O15
M042B11
0 5 10 15 20 25 30 35 40 45 50
[Chickpea contig p...
[root@localhost: /...
[FPC V8.9 Main Menu]
Project cp05T5e12...
FPC Ctg3271 cp05T...

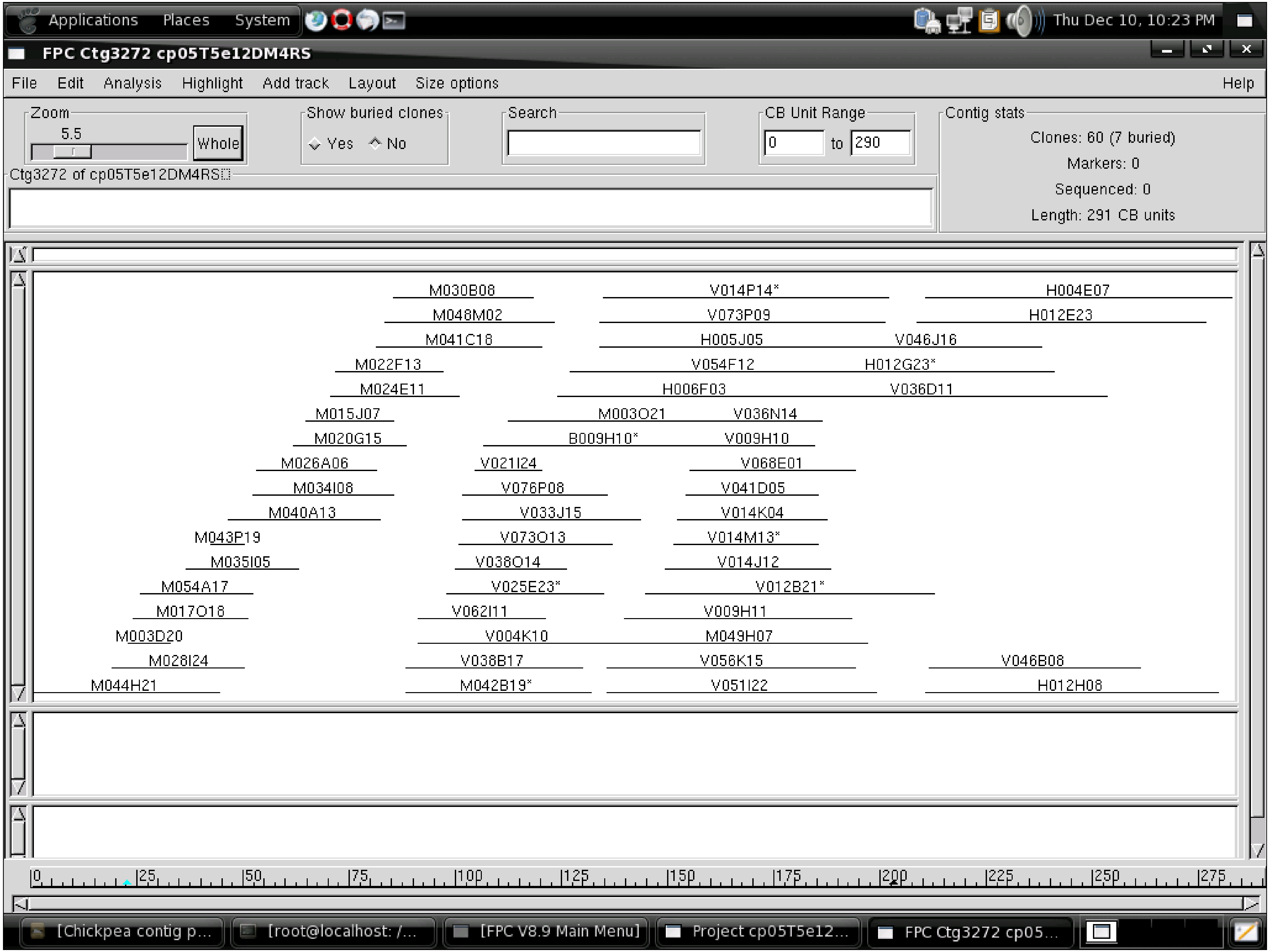
Applications Places System
Thu Dec 10, 10:23 PM
FPC Ctg3272 cp05T5e12DM4RS
File Edit Analysis Highlight Add track Layout Size options Help
Zoom 5.5 Whole
Show buried clones Yes No
Search
CB Unit Range 0 to 290
Contig stats
Clones: 60 (7 buried)
Markers: 0
Sequenced: 0
Length: 291 CB units
Ctg3272 of cp05T5e12DM4RS
M030B08 V014P14* H004E07
M048M02 V073P09 H012E23
M041C18 H005J05 V046J16
M022F13 V054F12 H012G23*
M024E11 H006F03 V036D11
M015J07 M003O21 V036N14
M020G15 B009H10* V009H10
M026A06 V021I24 V068E01
M034I08 V076P08 V041D05
M040A13 V033J15 V014K04
M043P19 V073O13 V014M13*
M035I05 V038O14 V014J12
M054A17 V025E23* V012B21*
M017O18 V062I11 V009H11
M003D20 V004K10 M049H07
M028I24 V038B17 V056K15 V046B08
M044H21 V042B19* V051I22 H012H08
0 25 50 75 100 125 150 175 200 225 250 275
[Chickpea contig p... [root@localhost: /... [FPC V8.9 Main Menu] Project cp05T5e12... FPC Ctg3272 cp05...

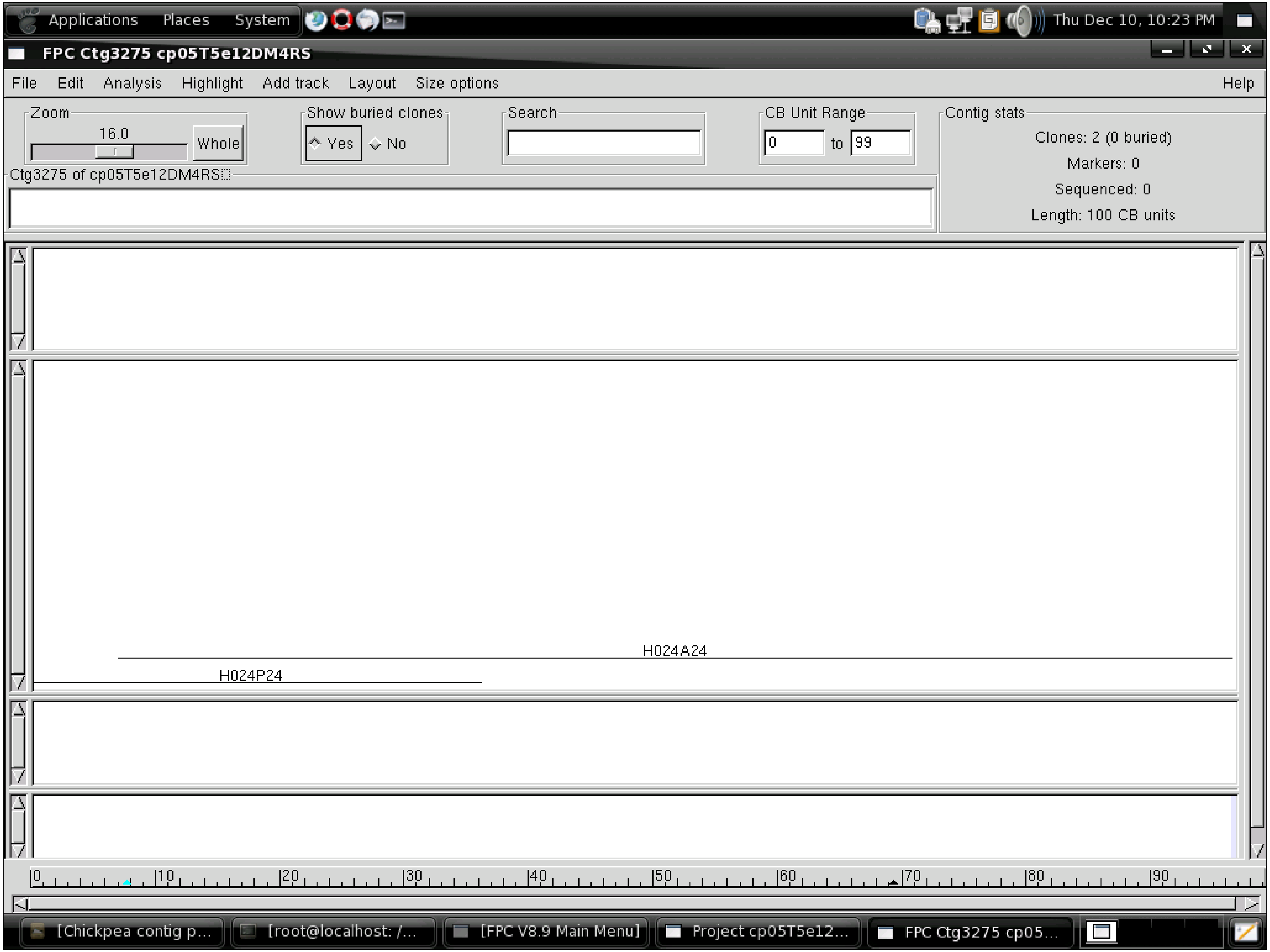

Applications
Places
System
Thu Dec 10, 10:23 PM
FPC Ctg3275 cp05T5e12DM4RS
File
Edit
Analysis
Highlight
Add track
Layout
Size options
Help
Zoom
16.0
Whole
Show buried clones
Yes
No
Search
CB Unit Range
0
to
99
Contig stats
Clones: 2 (0 buried)
Markers: 0
Sequenced: 0
Length: 100 CB units
Ctg3275 of cp05T5e12DM4RS
H024A24
H024P24
0
10
20
30
40
50
60
70
80
90
[Chickpea contig p...
[root@localhost: /...
[FPC V8.9 Main Menu]
Project cp05T5e12...
FPC Ctg3275 cp05...

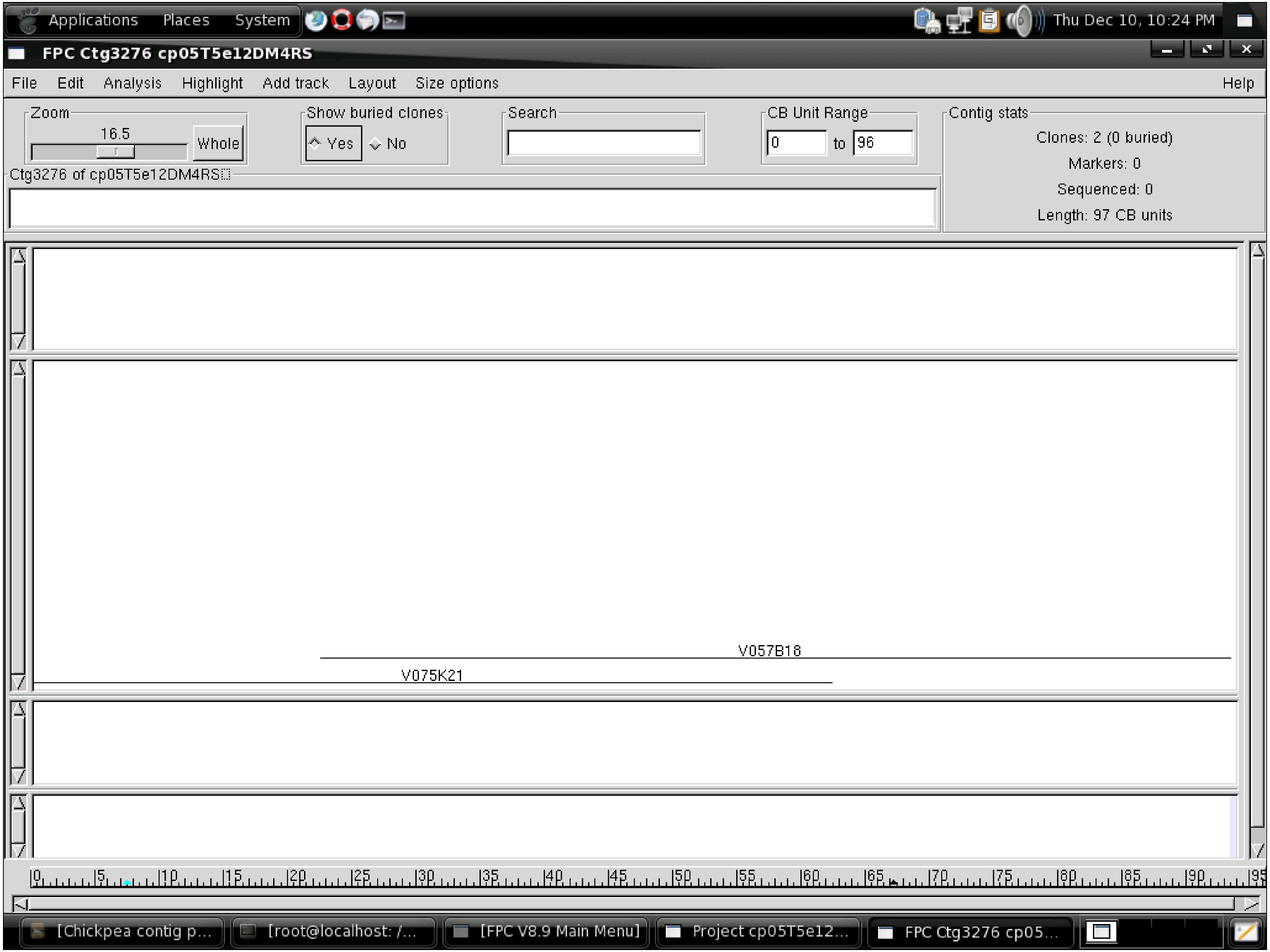
Applications Places System
Thu Dec 10, 10:24 PM
FPC Ctg3276 cp05T5e12DM4RS
File Edit Analysis Highlight Add track Layout Size options Help
Zoom
16.5
Whole
Show buried clones
Yes
No
Search
CB Unit Range
0
to
96
Contig stats
Clones: 2 (0 buried)
Markers: 0
Sequenced: 0
Length: 97 CB units
Ctg3276 of cp05T5e12DM4RS
V057B18
V075K21
0 5 10 15 20 25 30 35 40 45 50 55 60 65 70 75 80 85 90 95
[Chickpea contig p...
[root@localhost: /...
[FPC V8.9 Main Menu]
Project cp05T5e12...
FPC Ctg3276 cp05...

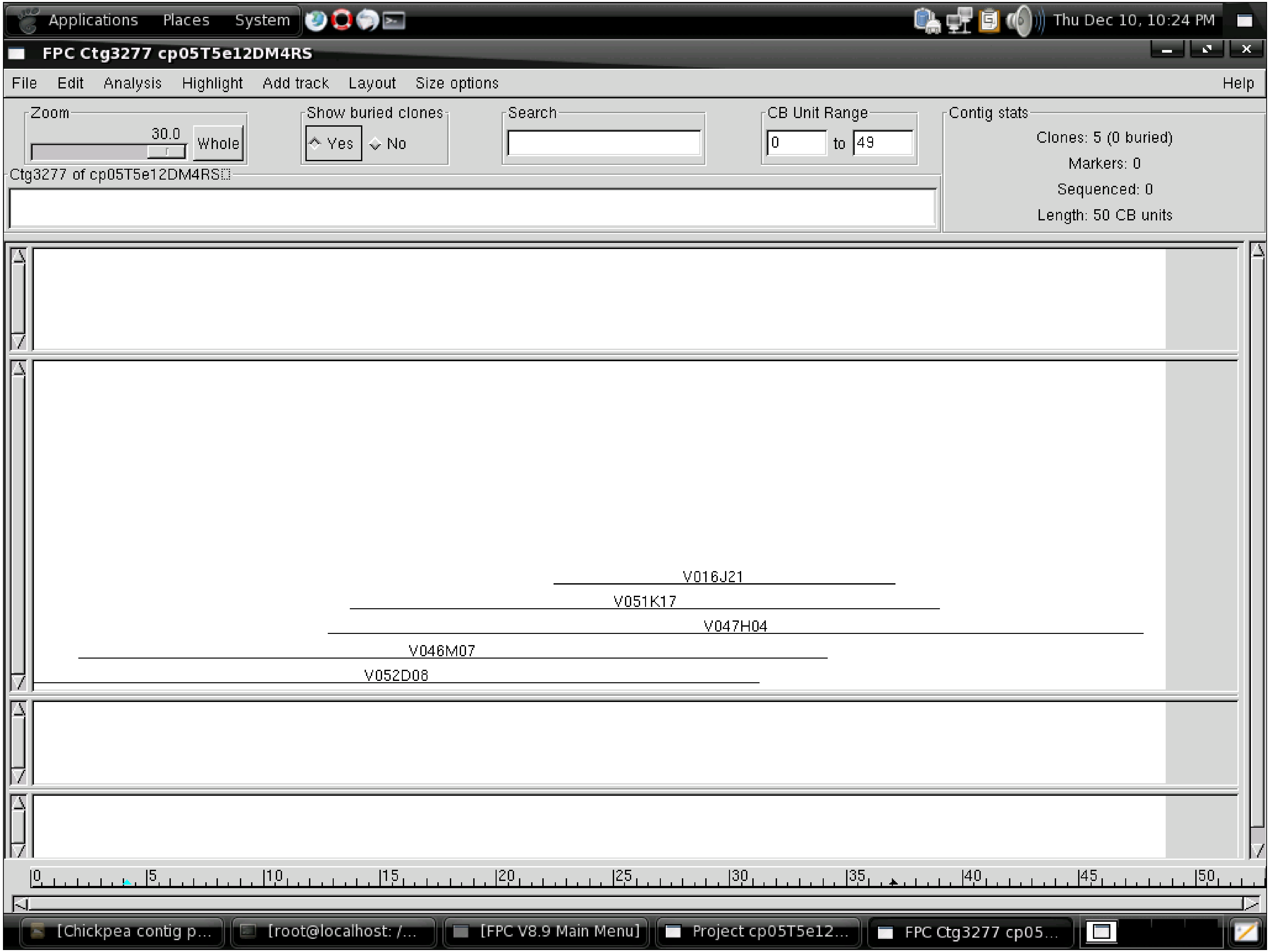
Applications Places System
Thu Dec 10, 10:24 PM
FPC Ctg3277 cp05T5e12DM4RS
File Edit Analysis Highlight Add track Layout Size options Help
Zoom
30.0
Whole
Show buried clones
Yes No
Search
CB Unit Range
0 to 49
Contig stats
Clones: 5 (0 buried)
Markers: 0
Sequenced: 0
Length: 50 CB units
Ctg3277 of cp05T5e12DM4RS
V016J21
V051K17
V047H04
V046M07
V052D08
0 5 10 15 20 25 30 35 40 45 50
[Chickpea contig p...
[root@localhost: /...
[FPC V8.9 Main Menu]
Project cp05T5e12...
FPC Ctg3277 cp05...

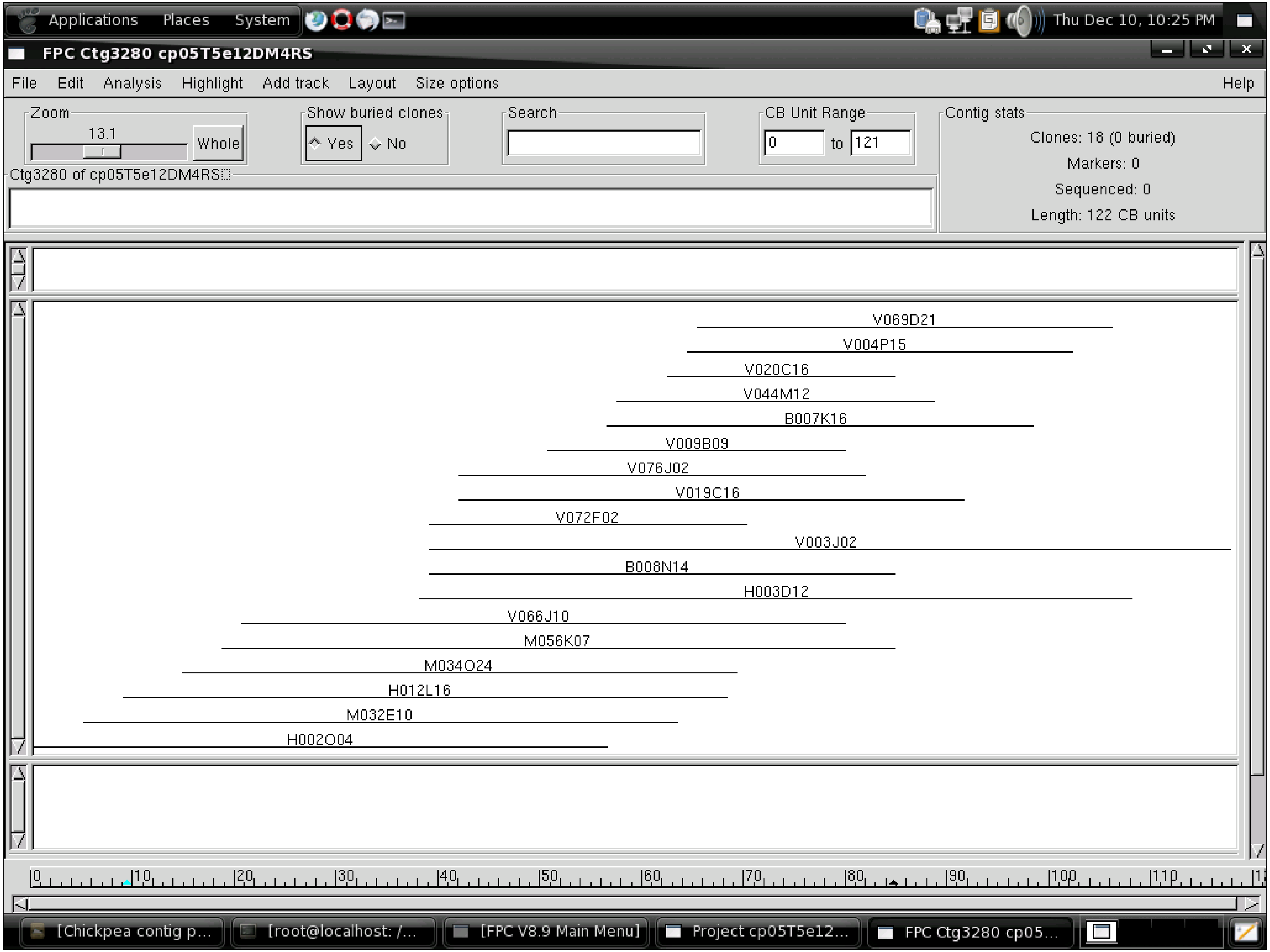
Applications Places System
Thu Dec 10, 10:25 PM
FPC Ctg3280 cp05T5e12DM4RS
File Edit Analysis Highlight Add track Layout Size options Help
Zoom
13.1
Whole
Show buried clones
Yes
No
Search
CB Unit Range
0 to 121
Contig stats
Clones: 18 (0 buried)
Markers: 0
Sequenced: 0
Length: 122 CB units
Ctg3280 of cp05T5e12DM4RS
V069D21
V004P15
V020C16
V044M12
B007K16
V009B09
V076J02
V019C16
V072F02
V003J02
B008N14
H003D12
V066J10
M056K07
M034O24
H012L16
M032E10
H002O04
0 10 20 30 40 50 60 70 80 90 100 110
[Chickpea contig p...
[root@localhost: /...
[FPC V8.9 Main Menu]
Project cp05T5e12...
FPC Ctg3280 cp05...

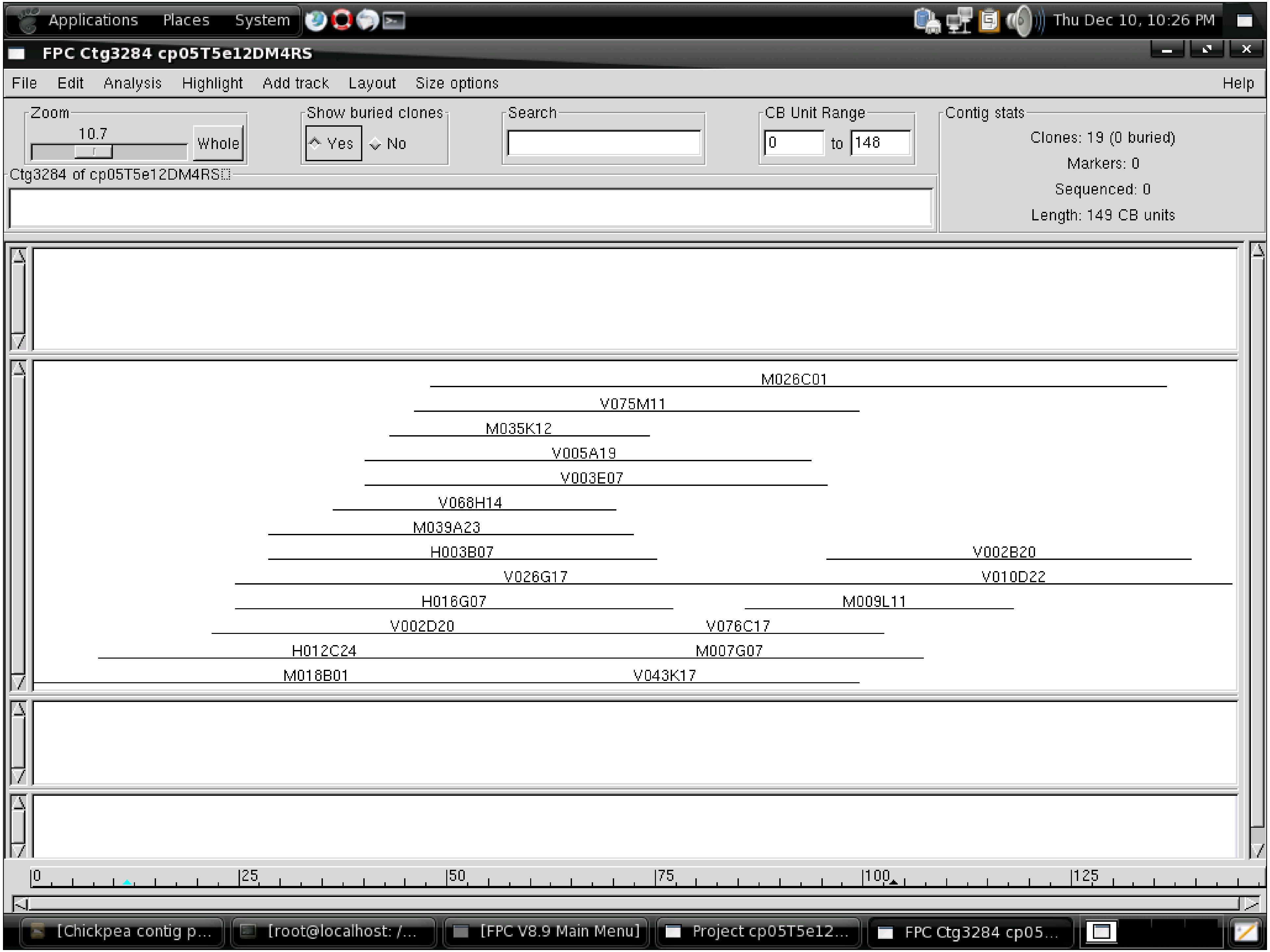
Applications Places System
Thu Dec 10, 10:26 PM
FPC Ctg3284 cp05T5e12DM4RS
File Edit Analysis Highlight Add track Layout Size options Help
Zoom
10.7
Whole
Show buried clones
Yes
No
Search
CB Unit Range
0
to
148
Contig stats
Clones: 19 (0 buried)
Markers: 0
Sequenced: 0
Length: 149 CB units
Ctg3284 of cp05T5e12DM4RS
M026C01
V075M11
M035K12
V005A19
V003E07
V068H14
M039A23
H003B07
V002B20
V026G17
V010D22
H016G07
M009L11
V002D20
V076C17
H012C24
M007G07
M018B01
V043K17
0
25
50
75
100
125
[Chickpea contig p...
[root@localhost: /...
[FPC V8.9 Main Menu]
Project cp05T5e12...
FPC Ctg3284 cp05...

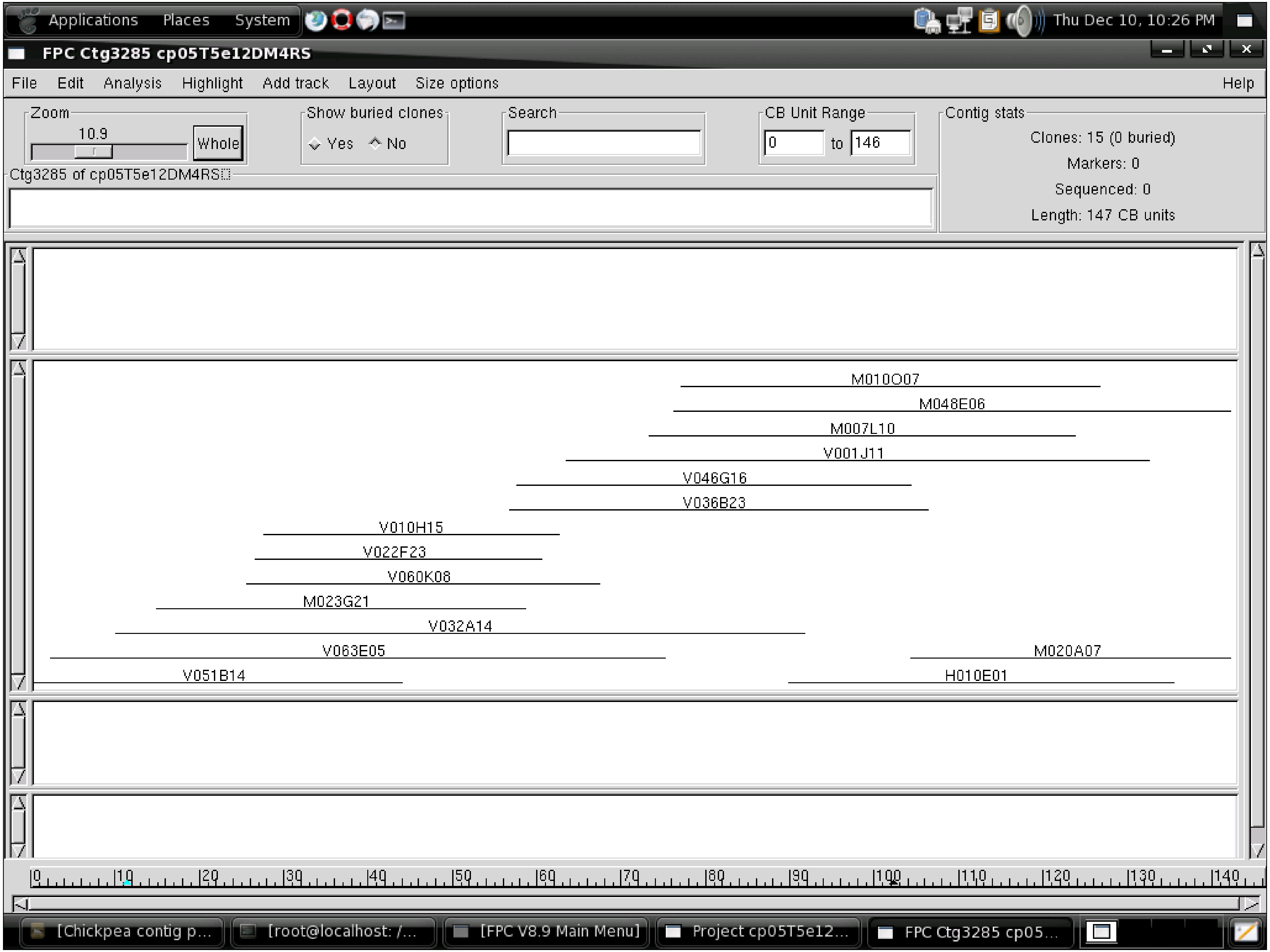
Applications
Places
System
Thu Dec 10, 10:26 PM
FPC Ctg3285 cp05T5e12DM4RS
File
Edit
Analysis
Highlight
Add track
Layout
Size options
Help
Zoom
10.9
Whole
Show buried clones
Yes
No
Search
CB Unit Range
0
to
146
Contig stats
Clones: 15 (0 buried)
Markers: 0
Sequenced: 0
Length: 147 CB units
Ctg3285 of cp05T5e12DM4RS
M010O07
M048E06
M007L10
V001J11
V046G16
V036B23
V010H15
V022F23
V060K08
M023G21
V032A14
V063E05
M020A07
V051B14
H010E01
0
10
20
30
40
50
60
70
80
90
100
110
120
130
140
[Chickpea contig p...
[root@localhost: /...
[FPC V8.9 Main Menu]
Project cp05T5e12...
FPC Ctg3285 cp05...

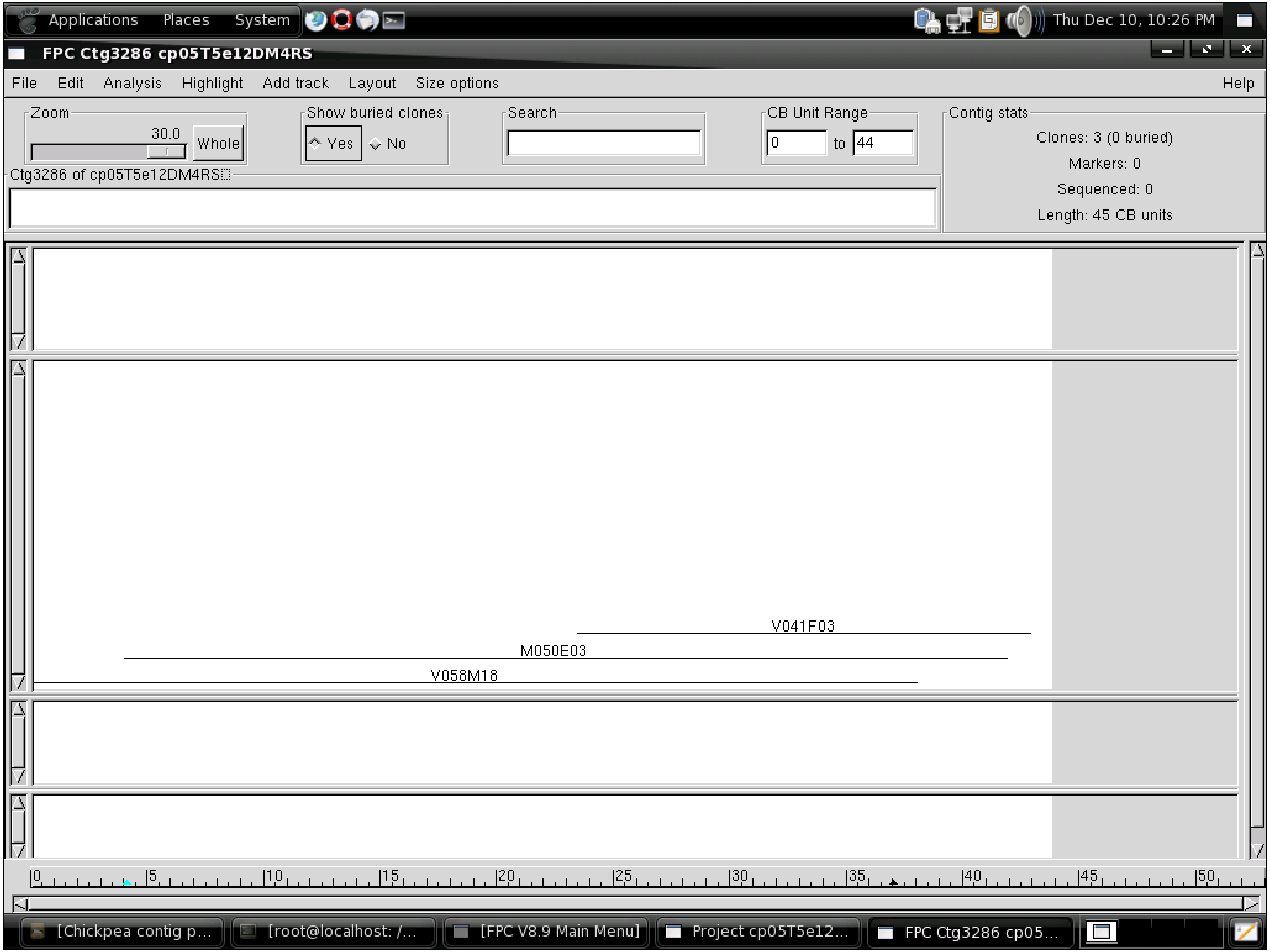
Applications Places System
Thu Dec 10, 10:26 PM
FPC Ctg3286 cp05T5e12DM4RS
File Edit Analysis Highlight Add track Layout Size options Help
Zoom
30.0
Whole
Show buried clones
Yes
No
Search
CB Unit Range
0
to
44
Contig stats
Clones: 3 (0 buried)
Markers: 0
Sequenced: 0
Length: 45 CB units
Ctg3286 of cp05T5e12DM4RS
V041F03
M050E03
V058M18
0 5 10 15 20 25 30 35 40 45 50
[Chickpea contig p...
[root@localhost: /...
[FPC V8.9 Main Menu]
Project cp05T5e12...
FPC Ctg3286 cp05...

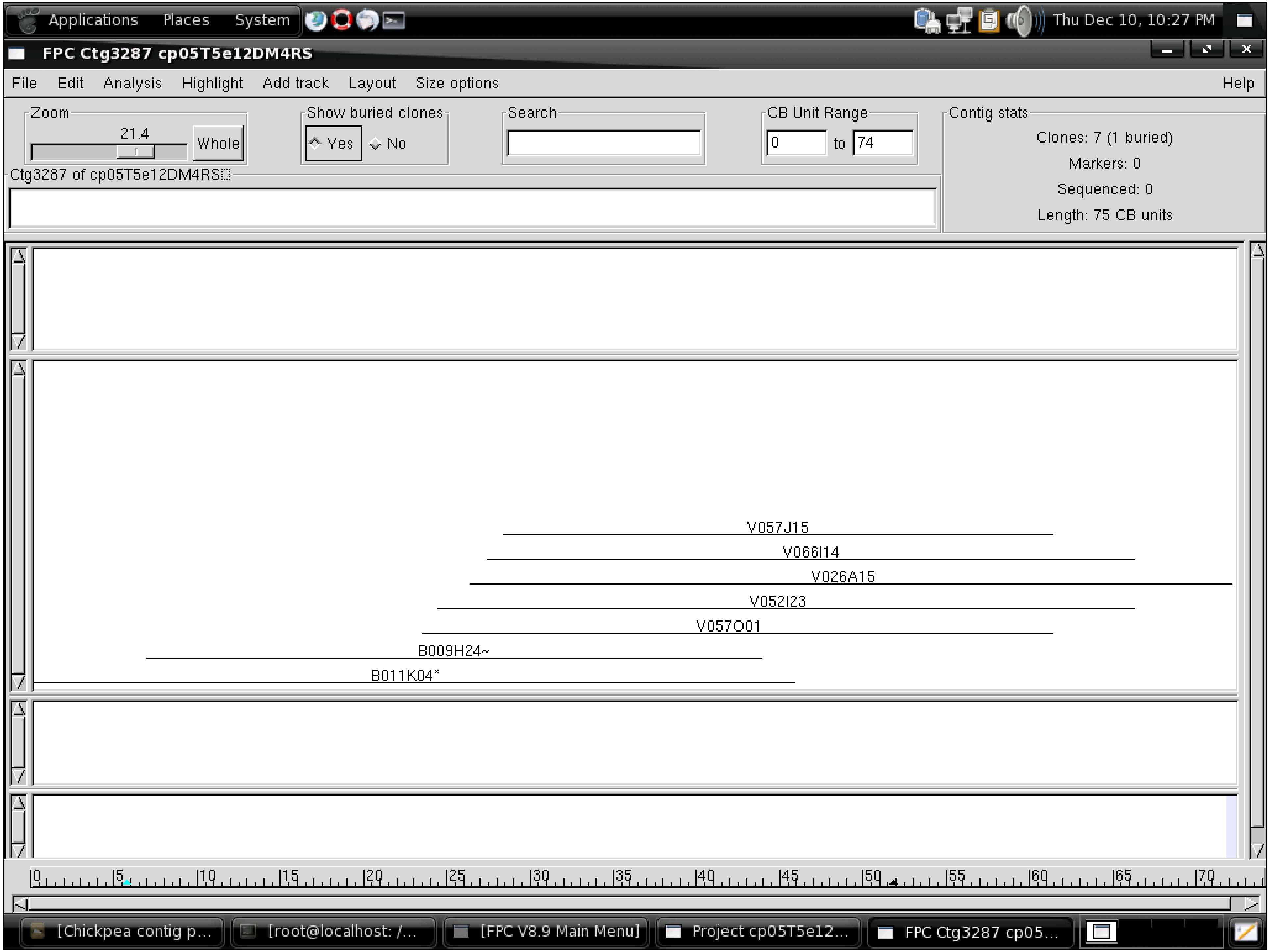
Applications Places System
Thu Dec 10, 10:27 PM
FPC Ctg3287 cp05T5e12DM4RS
File Edit Analysis Highlight Add track Layout Size options Help
Zoom
21.4
Whole
Show buried clones
Yes
No
Search
CB Unit Range
0 to 74
Contig stats
Clones: 7 (1 buried)
Markers: 0
Sequenced: 0
Length: 75 CB units
Ctg3287 of cp05T5e12DM4RS
V057J15
V066I14
V026A15
V052I23
V057O01
B009H24~
B011K04*
0 5 10 15 20 25 30 35 40 45 50 55 60 65 70
[Chickpea contig p...
[root@localhost: /...
[FPC V8.9 Main Menu]
Project cp05T5e12...
FPC Ctg3287 cp05...

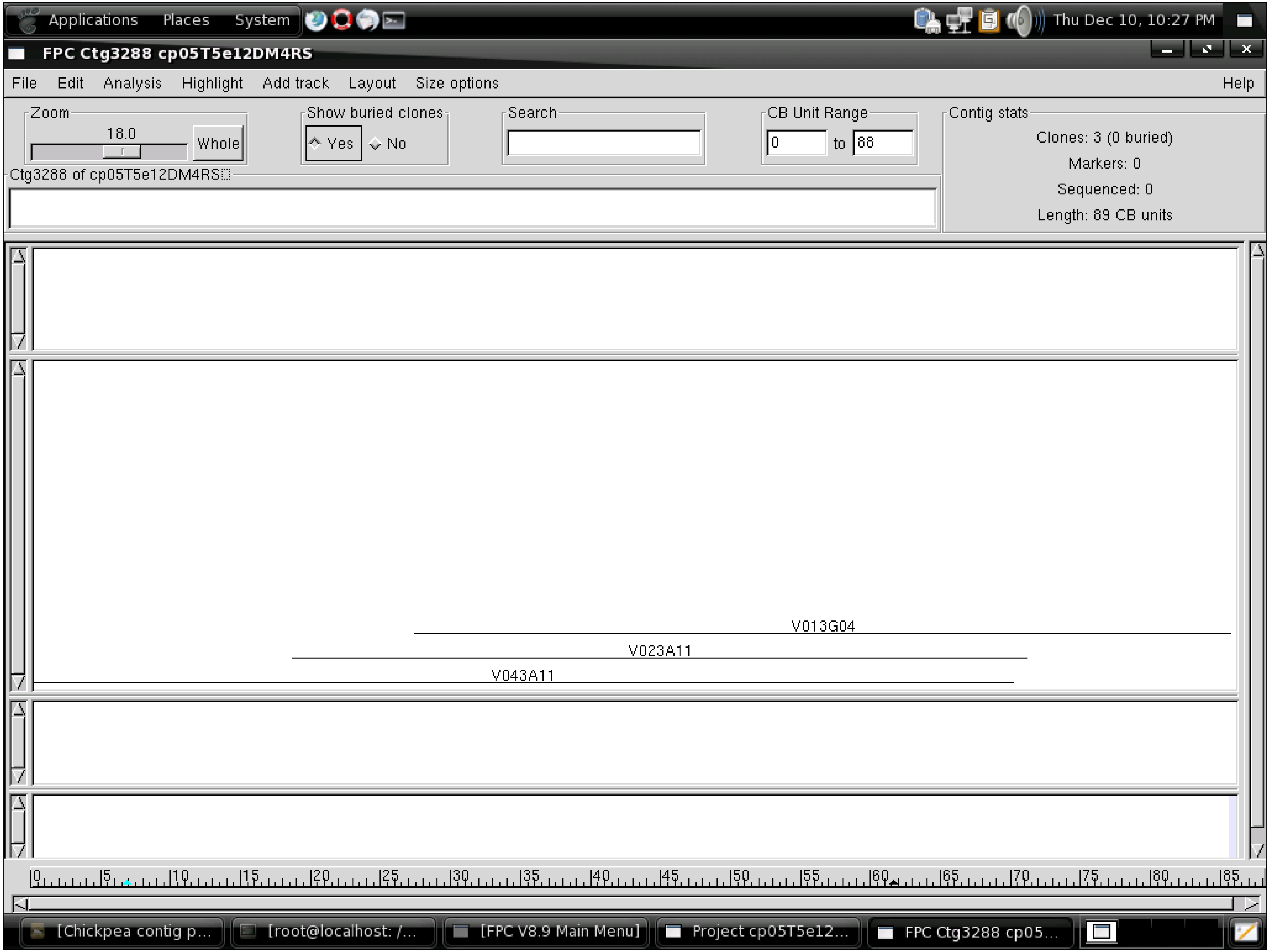
Applications
Places
System
Thu Dec 10, 10:27 PM
FPC Ctg3288 cp05T5e12DM4RS
File
Edit
Analysis
Highlight
Add track
Layout
Size options
Help
Zoom
18.0
Whole
Show buried clones
Yes
No
Search
CB Unit Range
0
to
88
Contig stats
Clones: 3 (0 buried)
Markers: 0
Sequenced: 0
Length: 89 CB units
Ctg3288 of cp05T5e12DM4RS
V013G04
V023A11
V043A11
0 5 10 15 20 25 30 35 40 45 50 55 60 65 70 75 80 85
[Chickpea contig p...
[root@localhost: /...
[FPC V8.9 Main Menu]
Project cp05T5e12...
FPC Ctg3288 cp05...

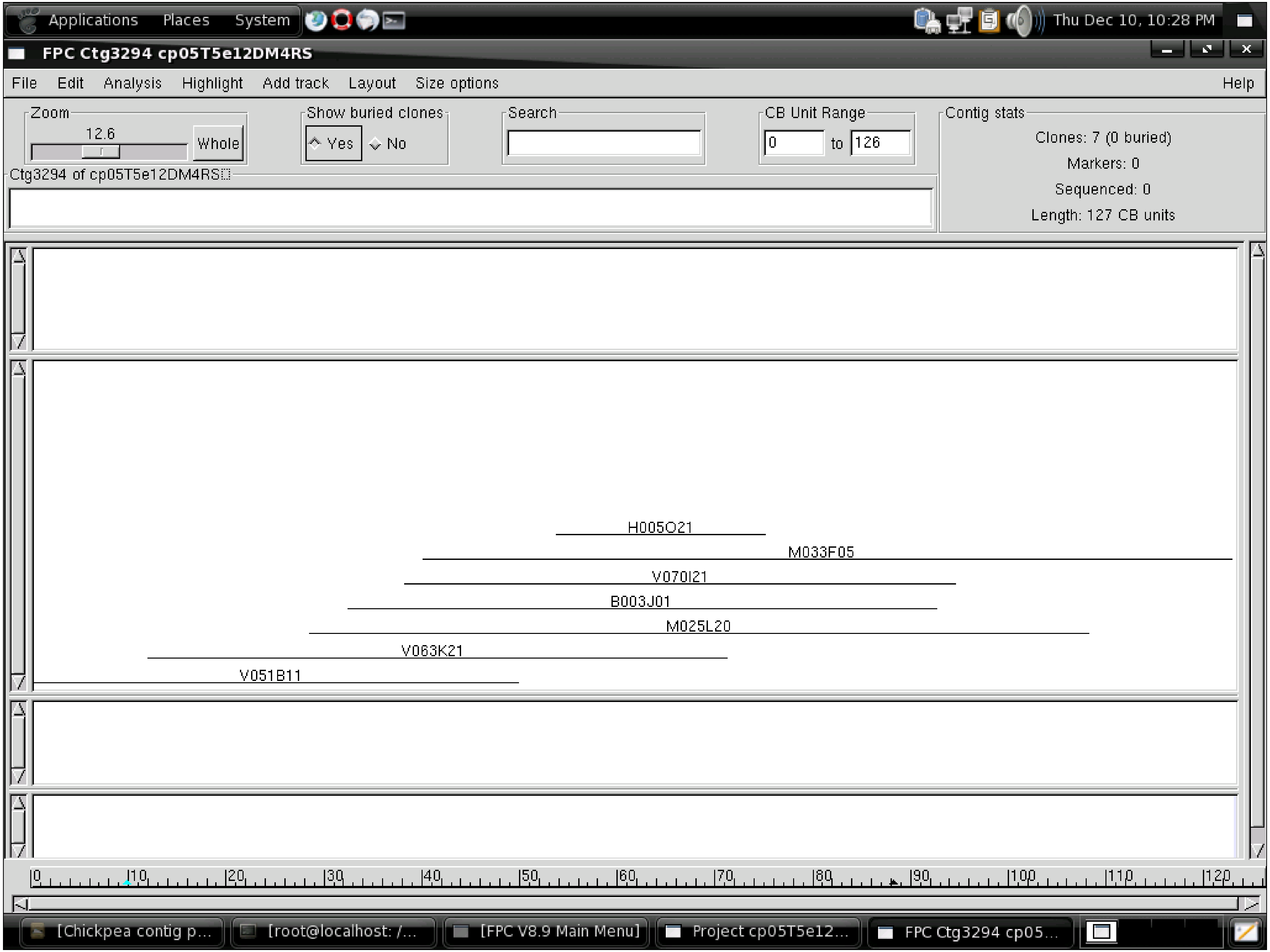
Applications Places System
Thu Dec 10, 10:28 PM
FPC Ctg3294 cp05T5e12DM4RS
File Edit Analysis Highlight Add track Layout Size options Help
Zoom
12.6
Whole
Show buried clones
Yes No
Search
CB Unit Range
0 to 126
Contig stats
Clones: 7 (0 buried)
Markers: 0
Sequenced: 0
Length: 127 CB units
Ctg3294 of cp05T5e12DM4RS
H005O21
M033F05
V070I21
B003J01
M025L20
V063K21
V051B11
0 10 20 30 40 50 60 70 80 90 100 110 120
[Chickpea contig p...
[root@localhost: /...
[FPC V8.9 Main Menu]
Project cp05T5e12...
FPC Ctg3294 cp05...

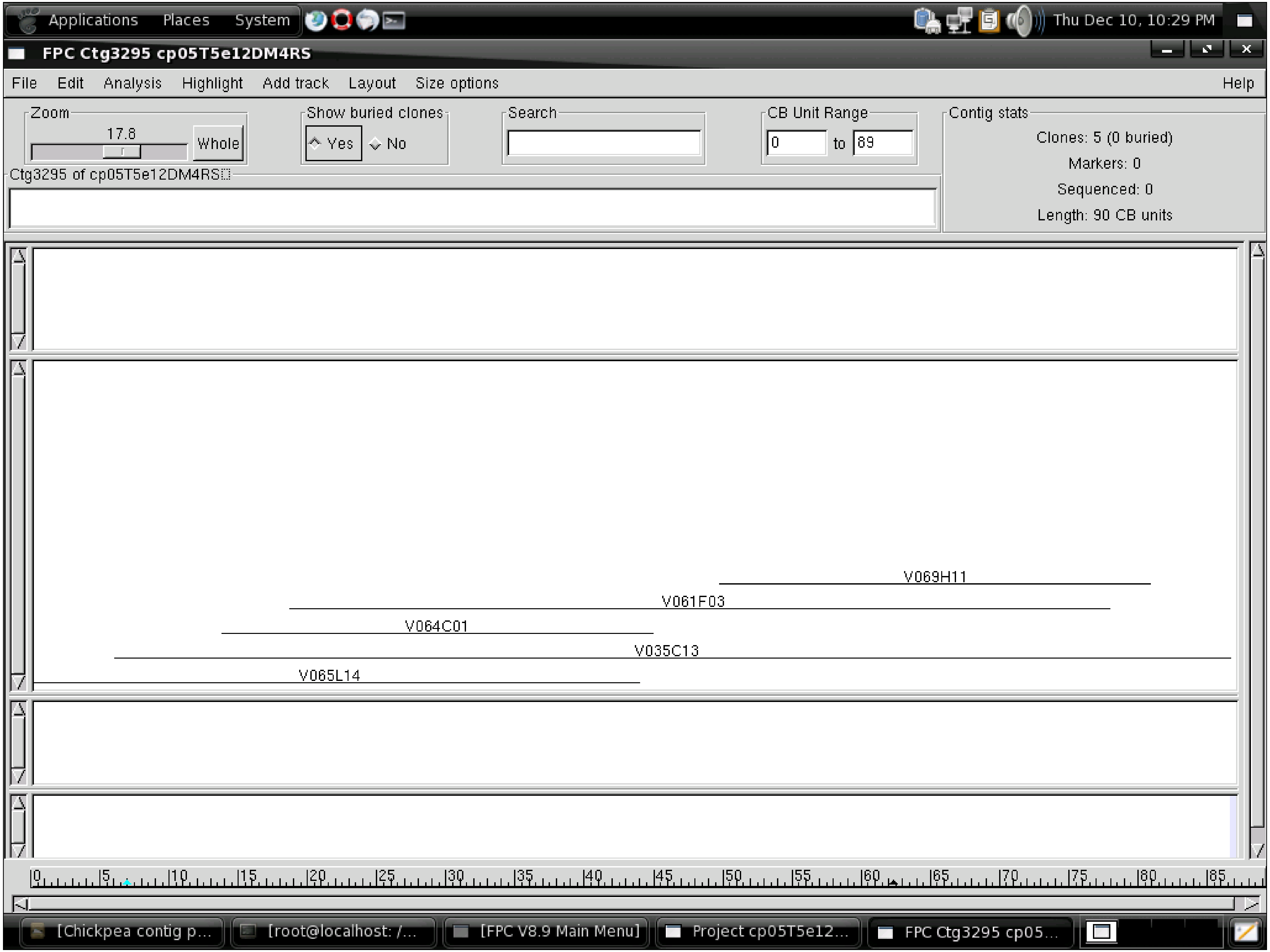
Applications
Places
System
Thu Dec 10, 10:29 PM
FPC Ctg3295 cp05T5e12DM4RS
File
Edit
Analysis
Highlight
Add track
Layout
Size options
Help
Zoom
17.8
Whole
Show buried clones
Yes
No
Search
CB Unit Range
0
to
89
Contig stats
Clones: 5 (0 buried)
Markers: 0
Sequenced: 0
Length: 90 CB units
Ctg3295 of cp05T5e12DM4RS
V069H11
V061F03
V064C01
V035C13
V065L14
0
5
10
15
20
25
30
35
40
45
50
55
60
65
70
75
80
85
[Chickpea contig p...
[root@localhost: /...
[FPC V8.9 Main Menu]
Project cp05T5e12...
FPC Ctg3295 cp05...

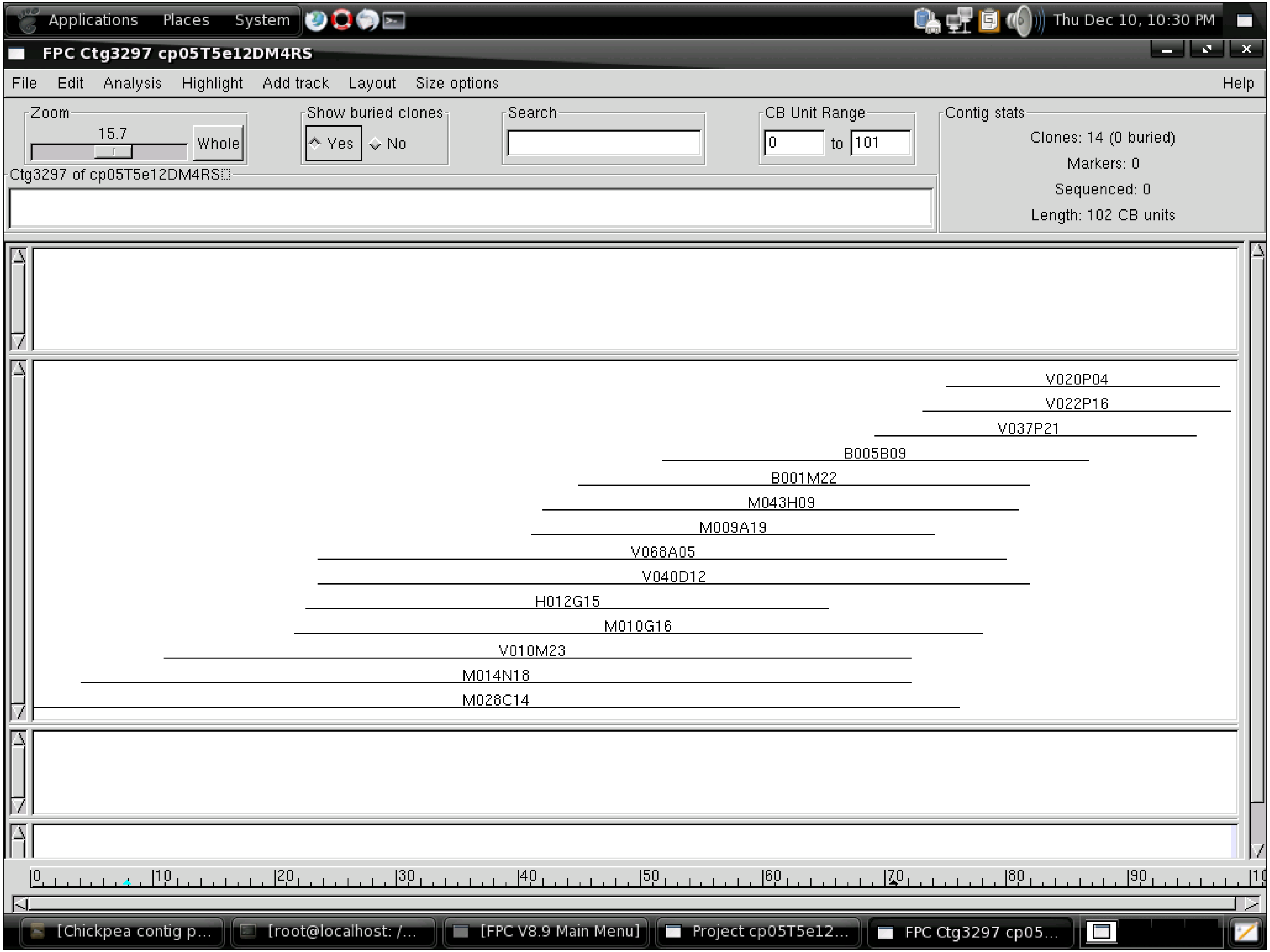
Applications Places System
Thu Dec 10, 10:30 PM
FPC Ctg3297 cp05T5e12DM4RS
File Edit Analysis Highlight Add track Layout Size options Help
Zoom
15.7
Whole
Show buried clones
Yes No
Search
CB Unit Range
0 to 101
Contig stats
Clones: 14 (0 buried)
Markers: 0
Sequenced: 0
Length: 102 CB units
Ctg3297 of cp05T5e12DM4RS
V020P04
V022P16
V037P21
B005B09
B001M22
M043H09
M009A19
V068A05
V040D12
H012G15
M010G16
V010M23
M014N18
M028C14
0 10 20 30 40 50 60 70 80 90
[Chickpea contig p...
[root@localhost: /...
[FPC V8.9 Main Menu]
Project cp05T5e12...
FPC Ctg3297 cp05...

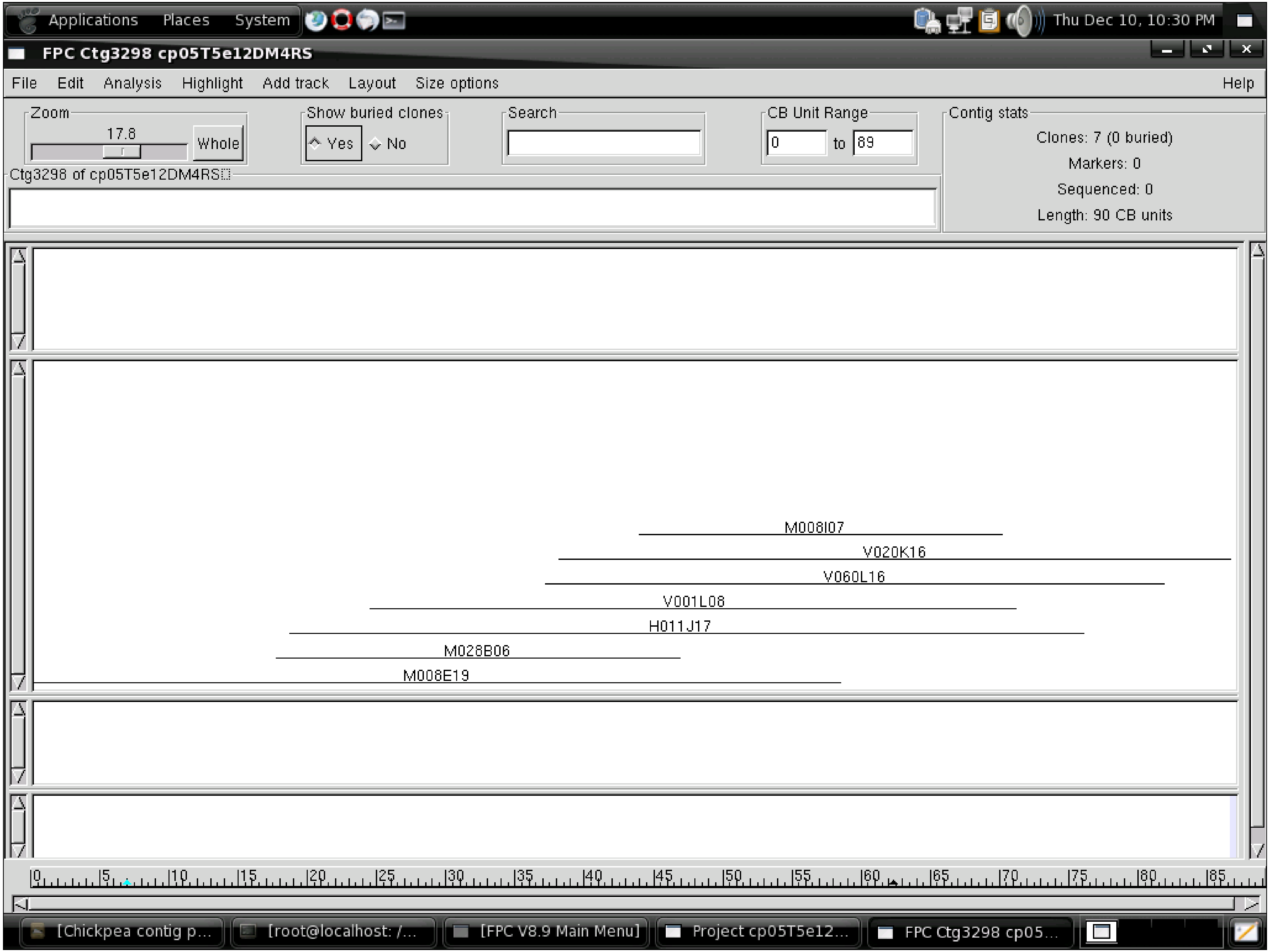
Applications Places System
Thu Dec 10, 10:30 PM
FPC Ctg3298 cp05T5e12DM4RS
File Edit Analysis Highlight Add track Layout Size options Help
Zoom
17.8
Whole
Show buried clones
Yes No
Search
CB Unit Range
0 to 89
Contig stats
Clones: 7 (0 buried)
Markers: 0
Sequenced: 0
Length: 90 CB units
Ctg3298 of cp05T5e12DM4RS
M008I07
V020K16
V060L16
V001L08
H011J17
M028B06
M008E19
0 5 10 15 20 25 30 35 40 45 50 55 60 65 70 75 80 85
[Chickpea contig p...
[root@localhost: /...
[FPC V8.9 Main Menu]
Project cp05T5e12...
FPC Ctg3298 cp05...

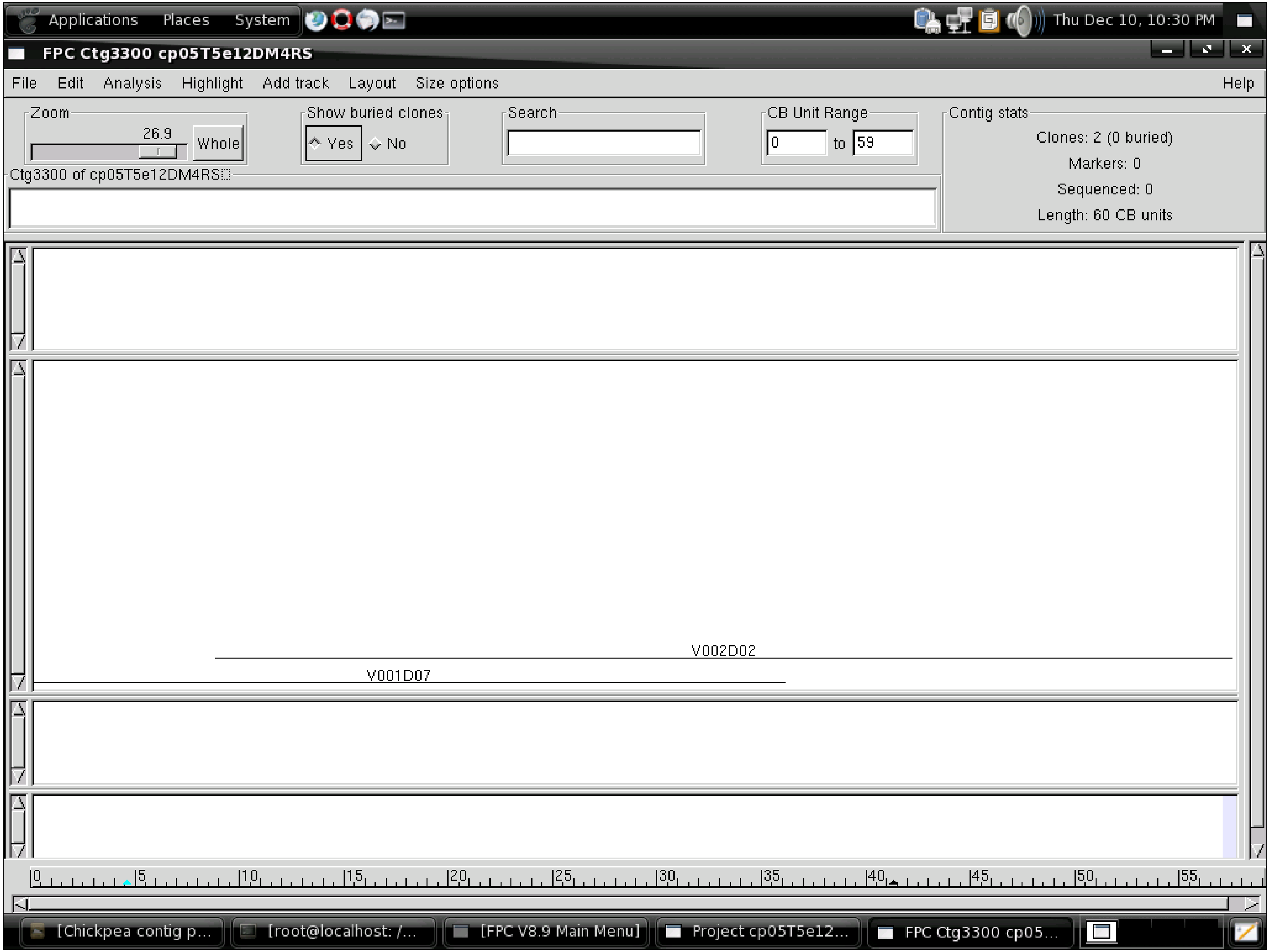
Applications Places System
Thu Dec 10, 10:30 PM
FPC Ctg3300 cp05T5e12DM4RS
File Edit Analysis Highlight Add track Layout Size options Help
Zoom
26.9
Whole
Show buried clones
Yes
No
Search
CB Unit Range
0
to
59
Contig stats
Clones: 2 (0 buried)
Markers: 0
Sequenced: 0
Length: 60 CB units
Ctg3300 of cp05T5e12DM4RS
V002D02
V001D07
0 5 10 15 20 25 30 35 40 45 50 55
[Chickpea contig p...
[root@localhost: /...
[FPC V8.9 Main Menu]
Project cp05T5e12...
FPC Ctg3300 cp05...

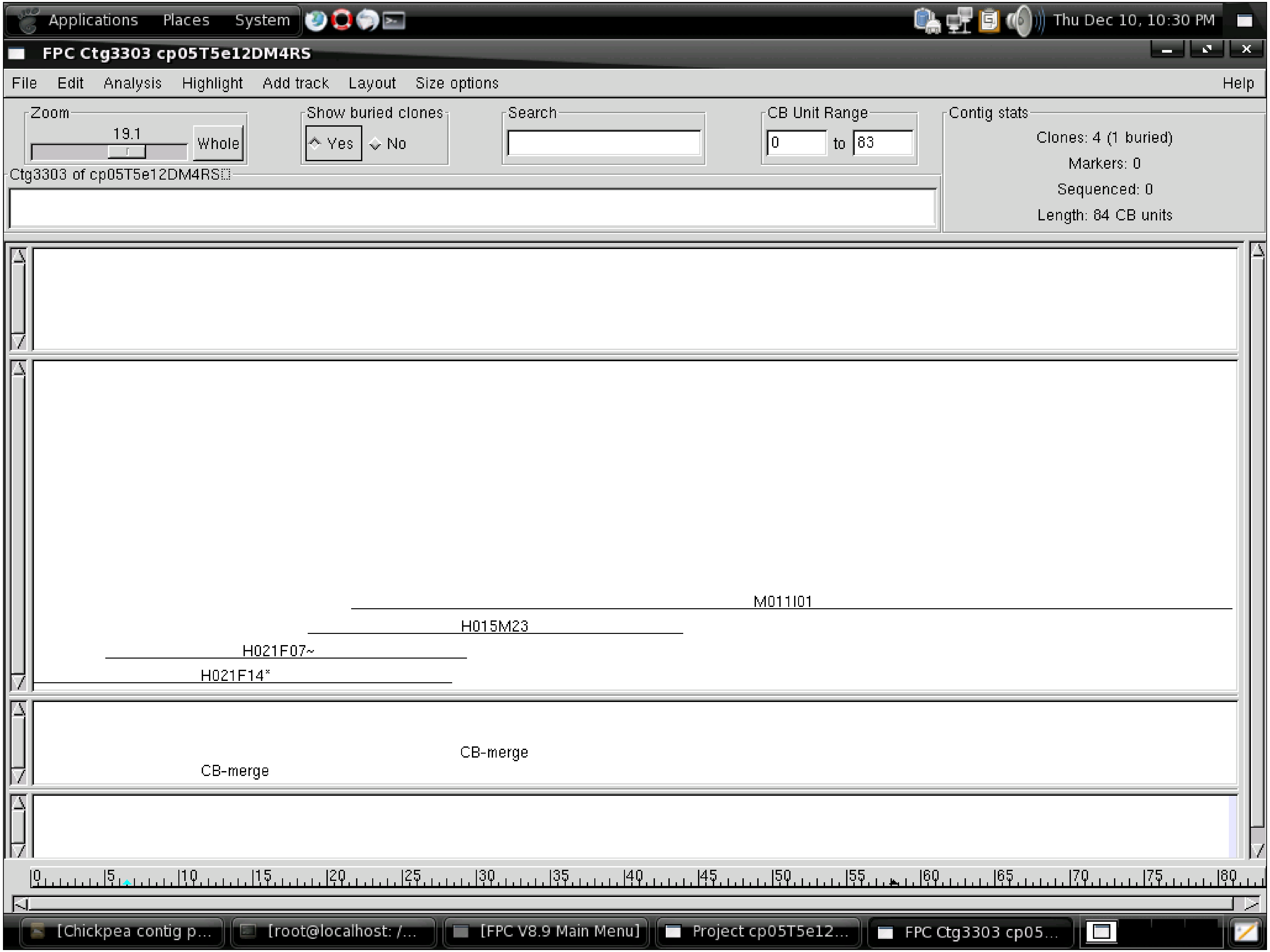

Applications Places System
Thu Dec 10, 10:30 PM
FPC Ctg3303 cp05T5e12DM4RS
File Edit Analysis Highlight Add track Layout Size options Help
Zoom
19.1
Whole
Show buried clones
Yes No
Search
CB Unit Range
0 to 83
Contig stats
Clones: 4 (1 buried)
Markers: 0
Sequenced: 0
Length: 84 CB units
Ctg3303 of cp05T5e12DM4RS
M011I01
H015M23
H021F07~
H021F14*
CB-merge
CB-merge
0 5 10 15 20 25 30 35 40 45 50 55 60 65 70 75 80
[Chickpea contig p...
[root@localhost: /...
[FPC V8.9 Main Menu]
Project cp05T5e12...
FPC Ctg3303 cp05...

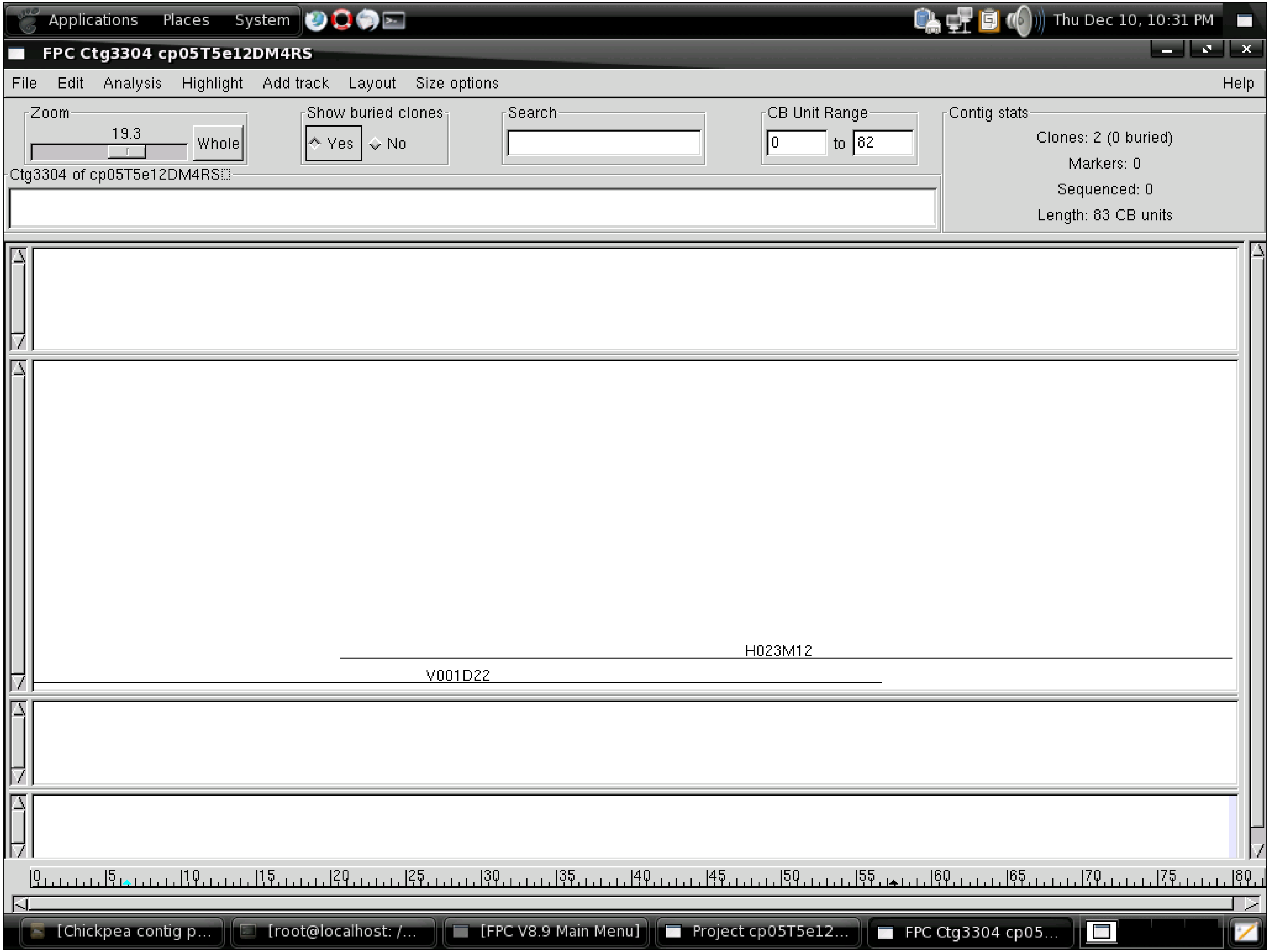
Applications Places System
Thu Dec 10, 10:31 PM
FPC Ctg3304 cp05T5e12DM4RS
File Edit Analysis Highlight Add track Layout Size options Help
Zoom
19.3
Whole
Show buried clones
Yes No
Search
CB Unit Range
0 to 82
Contig stats
Clones: 2 (0 buried)
Markers: 0
Sequenced: 0
Length: 83 CB units
Ctg3304 of cp05T5e12DM4RS
H023M12
V001D22
0 5 10 15 20 25 30 35 40 45 50 55 60 65 70 75 80
[Chickpea contig p...
[root@localhost: /...
[FPC V8.9 Main Menu]
Project cp05T5e12...
FPC Ctg3304 cp05...

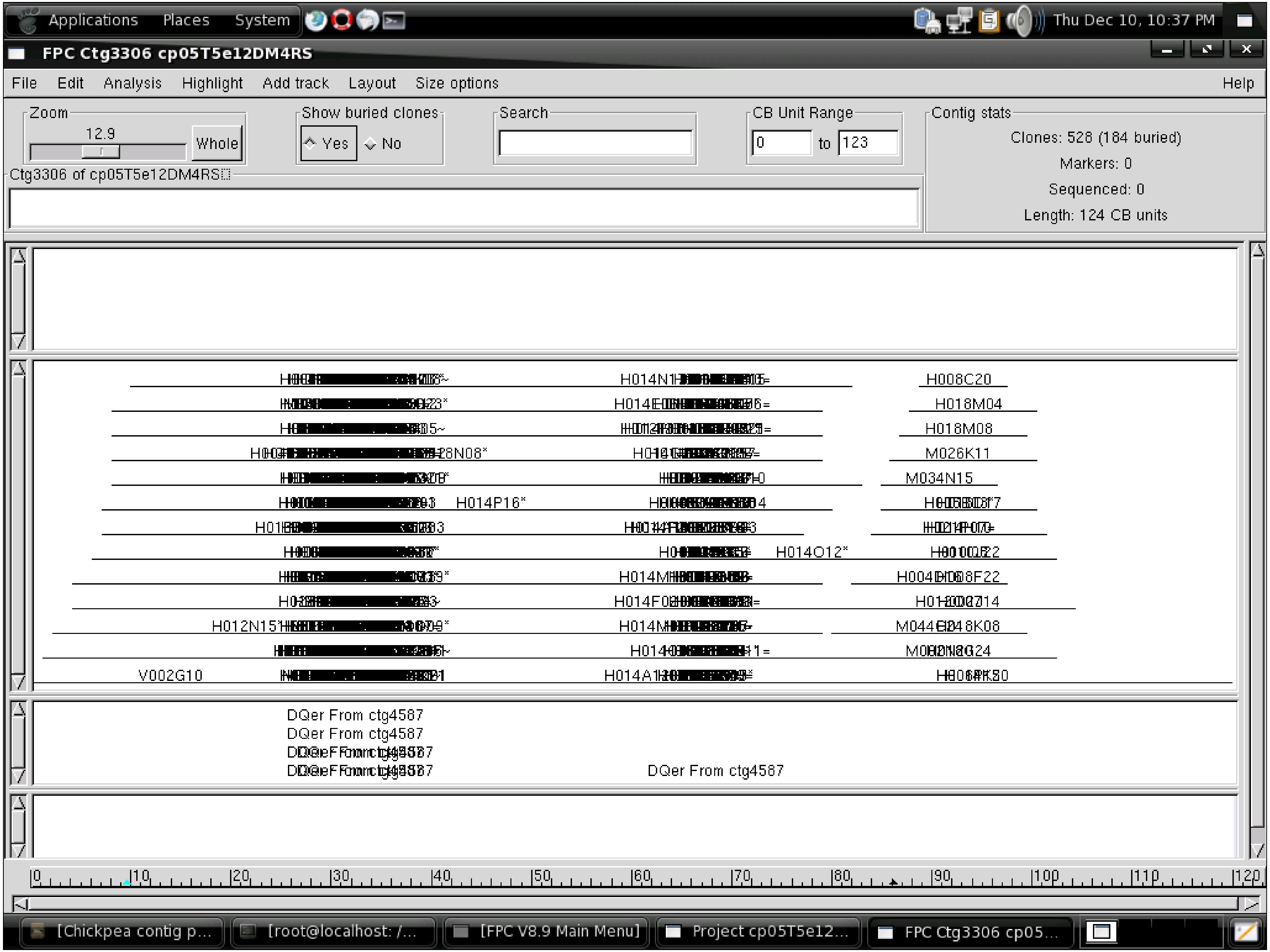
Applications Places System
Thu Dec 10, 10:37 PM
FPC Ctg3306 cp05T5e12DM4RS
File Edit Analysis Highlight Add track Layout Size options Help
Zoom
12.9
Whole
Show buried clones
Yes
No
Search
CB Unit Range
0 to 123
Contig stats
Clones: 528 (184 buried)
Markers: 0
Sequenced: 0
Length: 124 CB units
Ctg3306 of cp05T5e12DM4RS
H014P16*
H008C20
H018M04
H018M08
M026K11
M034N15
H014O12*
V002G10
DQer From ctg4587
DQer From ctg4587
DQer From ctg4587
0 10 20 30 40 50 60 70 80 90 100 110 120
[Chickpea contig p...
[root@localhost: /...
[FPC V8.9 Main Menu]
Project cp05T5e12...
FPC Ctg3306 cp05...

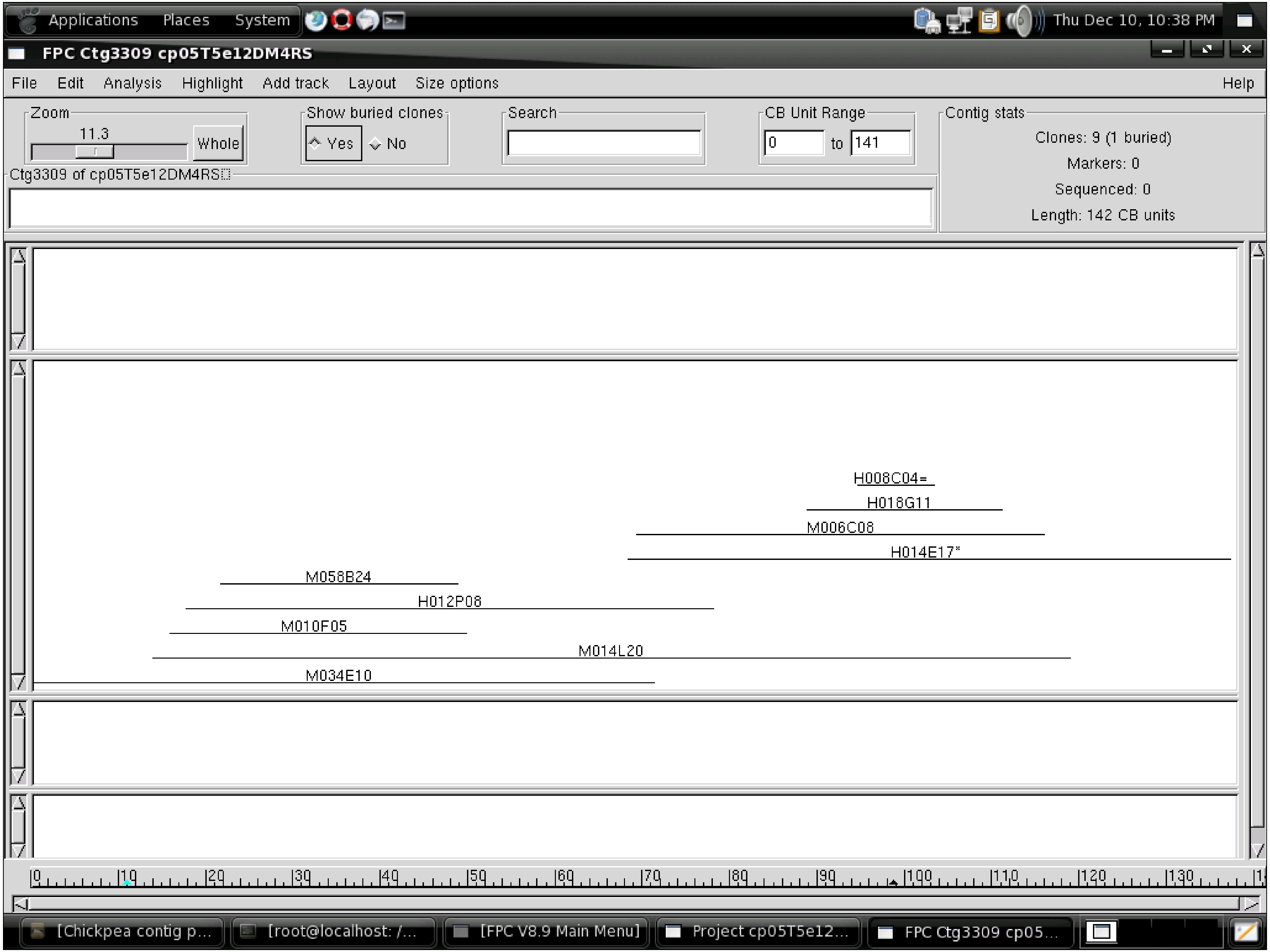
Applications Places System
Thu Dec 10, 10:38 PM
FPC Ctg3309 cp05T5e12DM4RS
File Edit Analysis Highlight Add track Layout Size options Help
Zoom
11.3
Whole
Show buried clones
Yes
No
Search
CB Unit Range
0
to
141
Contig stats
Clones: 9 (1 buried)
Markers: 0
Sequenced: 0
Length: 142 CB units
Ctg3309 of cp05T5e12DM4RS
H008C04=
H018G11
M006C08
H014E17*
M058B24
H012P08
M010F05
M014L20
M034E10
0 10 20 30 40 50 60 70 80 90 100 110 120 130
[Chickpea contig p...
[root@localhost: /...
[FPC V8.9 Main Menu]
Project cp05T5e12...
FPC Ctg3309 cp05...

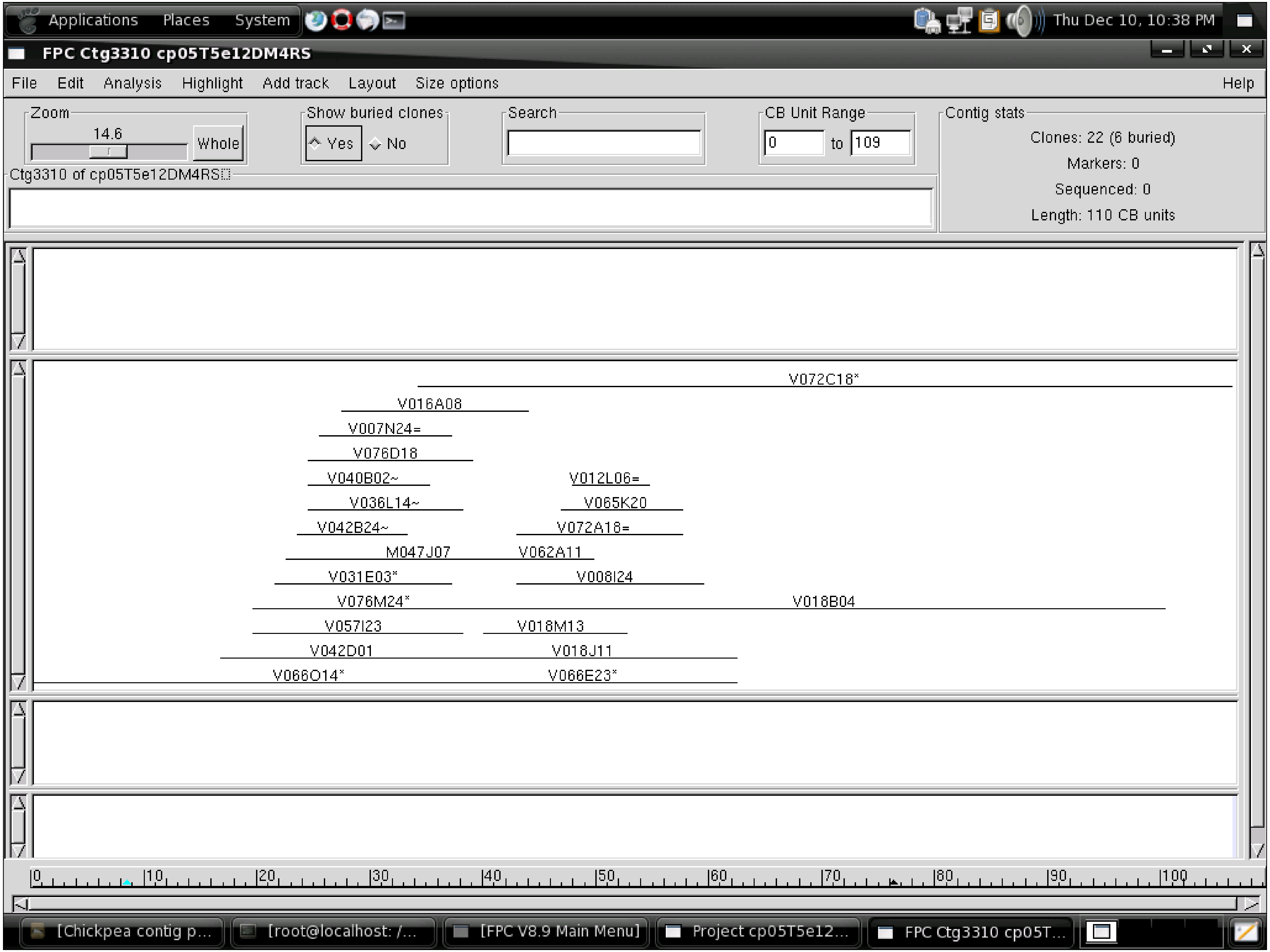
Applications Places System
Thu Dec 10, 10:38 PM
FPC Ctg3310 cp05T5e12DM4RS
File Edit Analysis Highlight Add track Layout Size options Help
Zoom
14.6
Whole
Show buried clones
Yes No
Search
CB Unit Range
0 to 109
Contig stats
Clones: 22 (6 buried)
Markers: 0
Sequenced: 0
Length: 110 CB units
Ctg3310 of cp05T5e12DM4RS
V072C18*
V016A08
V007N24=
V076D18
V040B02~
V012L06=
V036L14~
V065K20
V042B24~
V072A18=
M047J07
V062A11
V031E03*
V008I24
V076M24*
V018B04
V057I23
V018M13
V042D01
V018J11
V066O14*
V066E23*
0 10 20 30 40 50 60 70 80 90 100
[Chickpea contig p...
[root@localhost: /...
[FPC V8.9 Main Menu]
Project cp05T5e12...
FPC Ctg3310 cp05T...

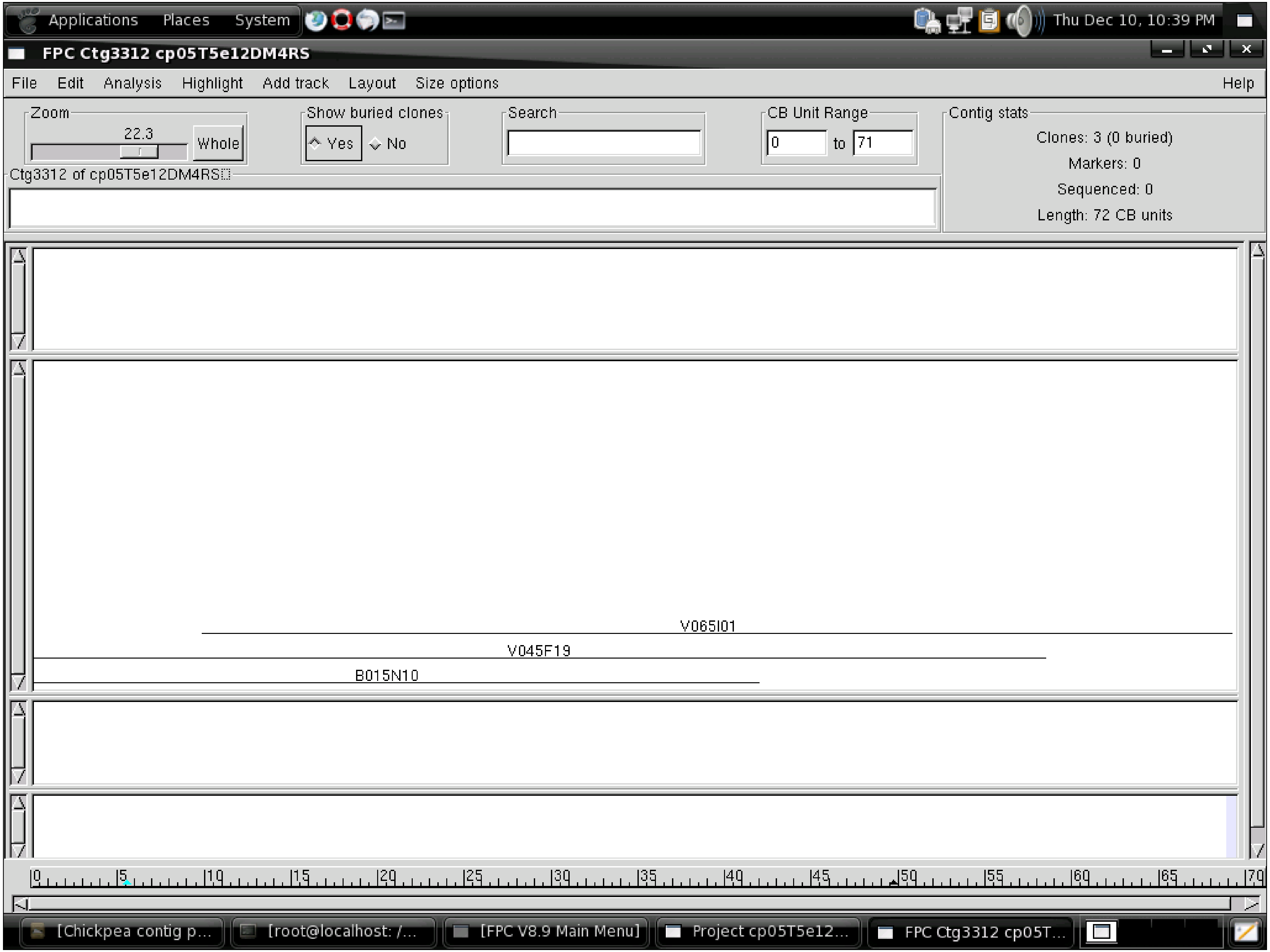
Applications Places System
Thu Dec 10, 10:39 PM
FPC Ctg3312 cp05T5e12DM4RS
File Edit Analysis Highlight Add track Layout Size options Help
Zoom
22.3
Whole
Show buried clones
Yes
No
Search
CB Unit Range
0 to 71
Contig stats
Clones: 3 (0 buried)
Markers: 0
Sequenced: 0
Length: 72 CB units
Ctg3312 of cp05T5e12DM4RS
V065I01
V045F19
B015N10
0 5 10 15 20 25 30 35 40 45 50 55 60 65 70
[Chickpea contig p...
[root@localhost: /...
[FPC V8.9 Main Menu]
Project cp05T5e12...
FPC Ctg3312 cp05T...

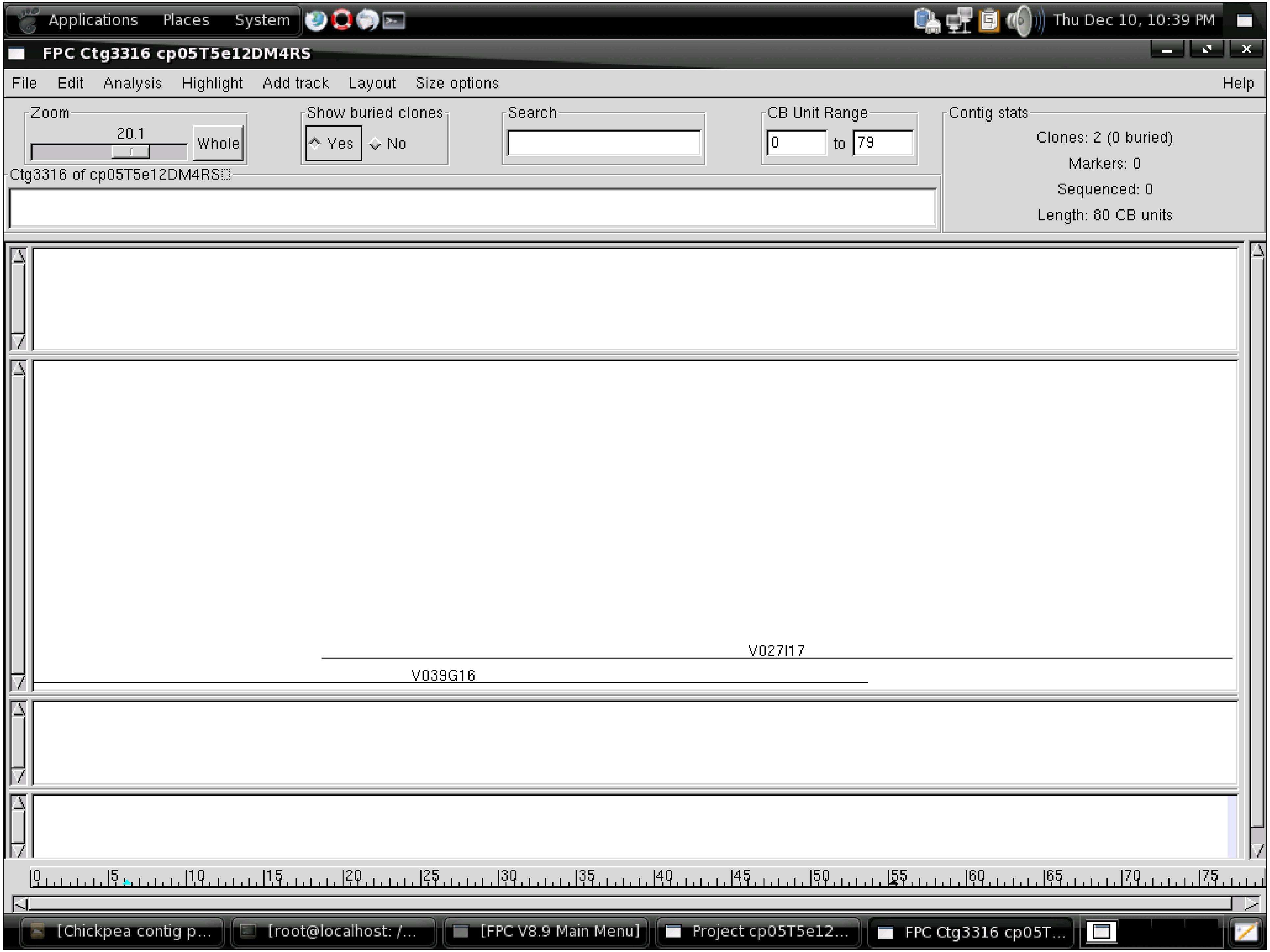
Applications Places System
Thu Dec 10, 10:39 PM
FPC Ctg3316 cp05T5e12DM4RS
File Edit Analysis Highlight Add track Layout Size options
Help
Zoom
20.1
Whole
Show buried clones
Yes
No
Search
CB Unit Range
0
to
79
Contig stats
Clones: 2 (0 buried)
Markers: 0
Sequenced: 0
Length: 80 CB units
Ctg3316 of cp05T5e12DM4RS
V027I17
V039G16
0 5 10 15 20 25 30 35 40 45 50 55 60 65 70 75
[Chickpea contig p...
[root@localhost: /...
[FPC V8.9 Main Menu]
Project cp05T5e12...
FPC Ctg3316 cp05T...

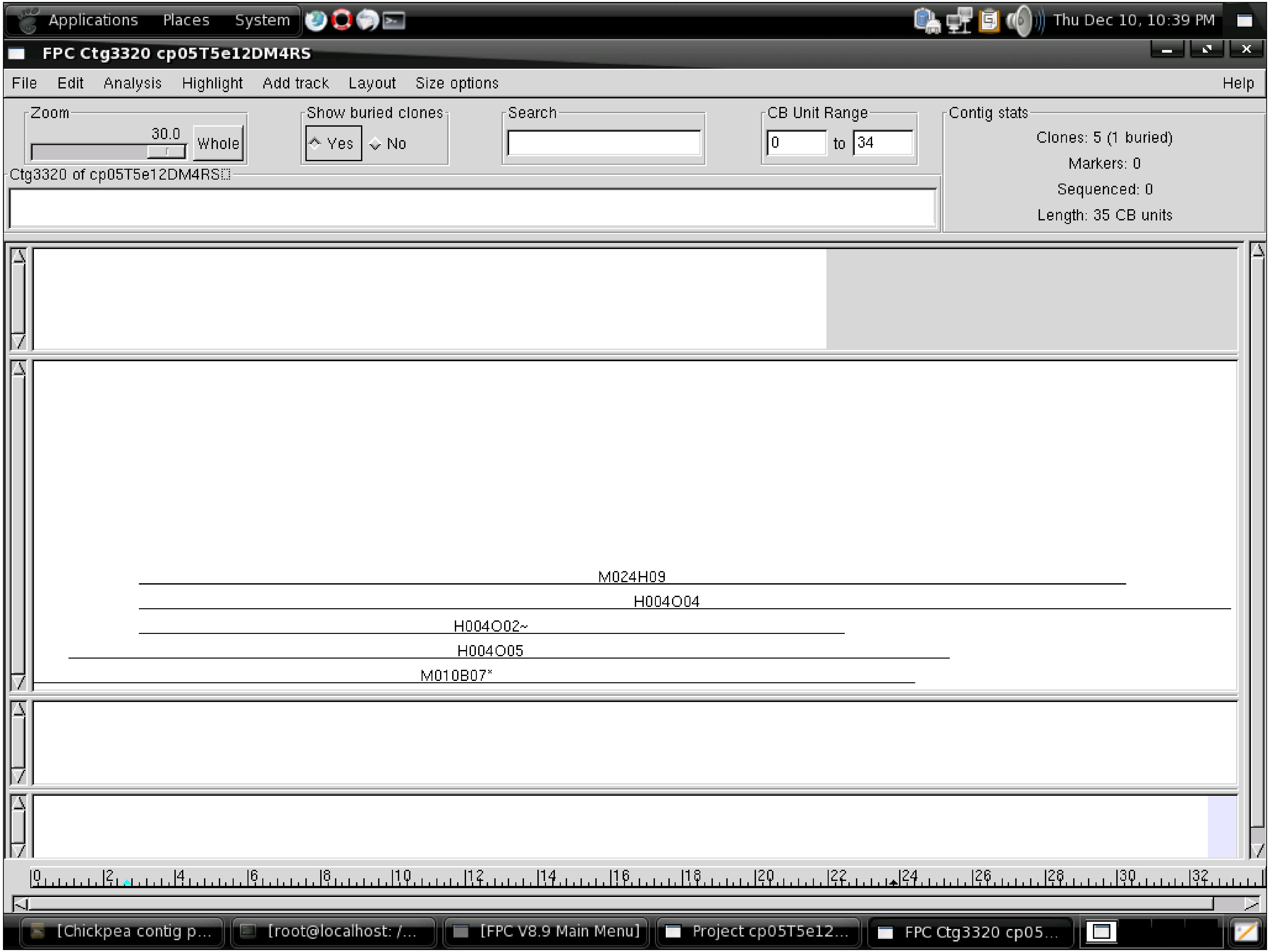
Applications Places System
Thu Dec 10, 10:39 PM
FPC Ctg3320 cp05T5e12DM4RS
File Edit Analysis Highlight Add track Layout Size options Help
Zoom
30.0
Whole
Show buried clones
Yes No
Search
CB Unit Range
0 to 34
Contig stats
Clones: 5 (1 buried)
Markers: 0
Sequenced: 0
Length: 35 CB units
Ctg3320 of cp05T5e12DM4RS
M024H09
H004O04
H004O02~
H004O05
M010B07*
0 2 4 6 8 10 12 14 16 18 20 22 24 26 28 30 32
[Chickpea contig p...
[root@localhost: /...
[FPC V8.9 Main Menu]
Project cp05T5e12...
FPC Ctg3320 cp05...

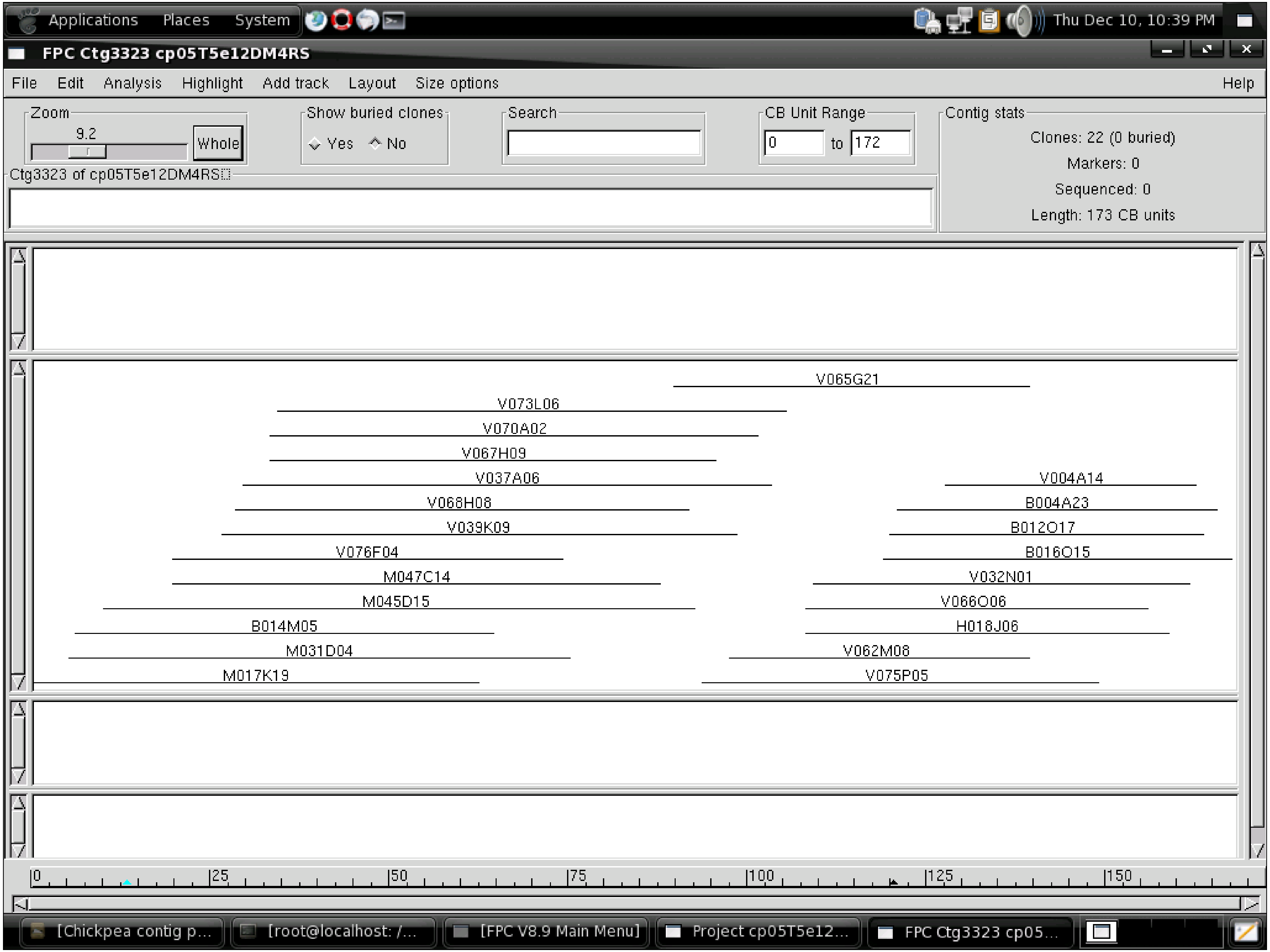
Applications Places System
Thu Dec 10, 10:39 PM
FPC Ctg3323 cp05T5e12DM4RS
File Edit Analysis Highlight Add track Layout Size options Help
Zoom
9.2
Whole
Show buried clones
Yes No
Search
CB Unit Range
0 to 172
Contig stats
Clones: 22 (0 buried)
Markers: 0
Sequenced: 0
Length: 173 CB units
Ctg3323 of cp05T5e12DM4RS
V065G21
V073L06
V070A02
V067H09
V037A06
V004A14
V068H08
B004A23
V039K09
B012O17
V076F04
B016O15
M047C14
V032N01
M045D15
V066O06
B014M05
H018J06
M031D04
V062M08
M017K19
V075P05
0 25 50 75 100 125 150
[Chickpea contig p...
[root@localhost: /...
[FPC V8.9 Main Menu]
Project cp05T5e12...
FPC Ctg3323 cp05...

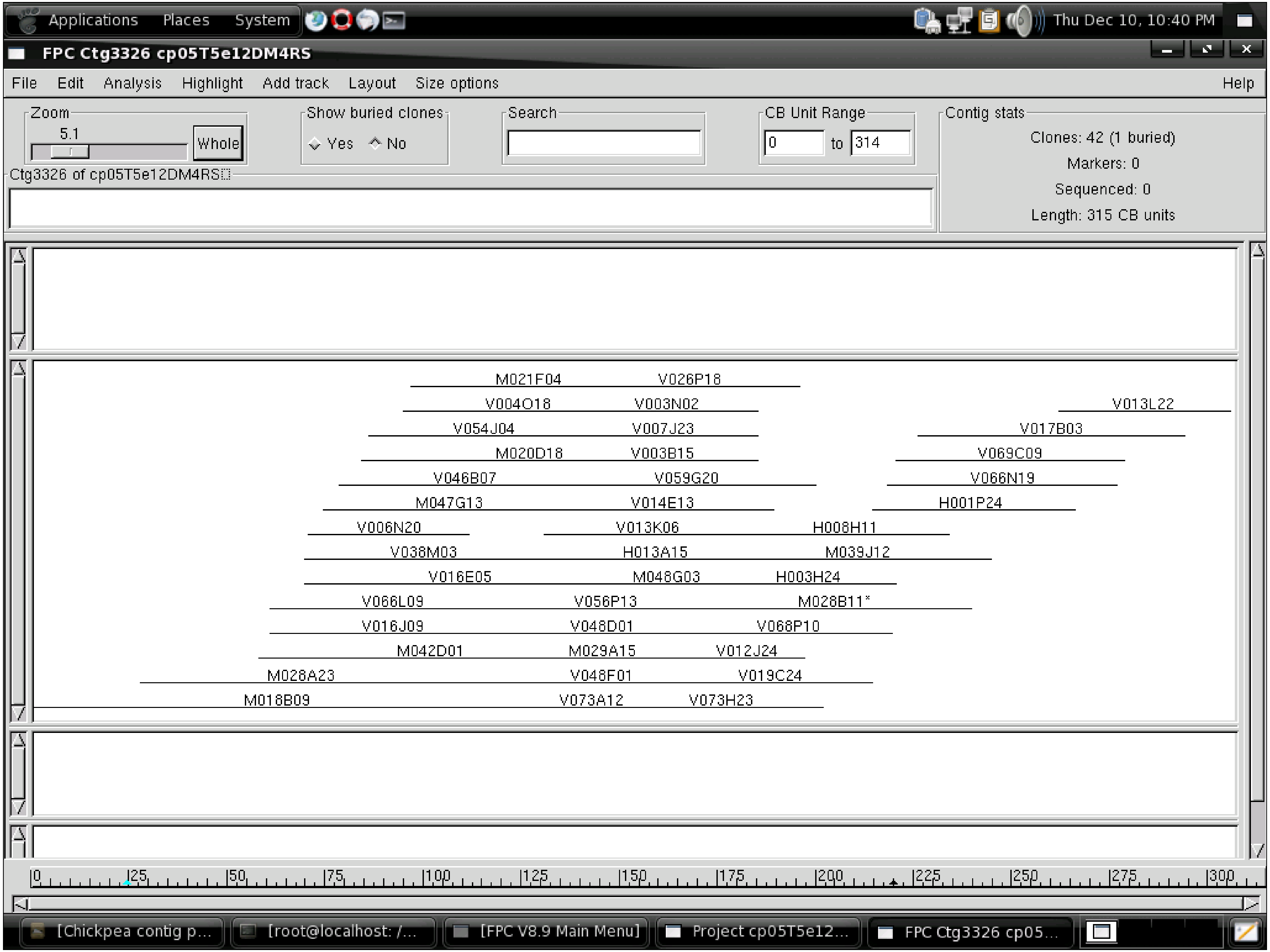
Applications Places System
Thu Dec 10, 10:40 PM
FPC Ctg3326 cp05T5e12DM4RS
File Edit Analysis Highlight Add track Layout Size options Help
Zoom
5.1
Whole
Show buried clones
Yes No
Search
CB Unit Range
0 to 314
Contig stats
Clones: 42 (1 buried)
Markers: 0
Sequenced: 0
Length: 315 CB units
Ctg3326 of cp05T5e12DM4RS
M021F04 V026P18
V004O18 V003N02 V013L22
V054J04 V007J23 V017B03
M020D18 V003B15 V069C09
V046B07 V059G20 V066N19
M047G13 V014E13 H001P24
V006N20 V013K06 H008H11
V038M03 H013A15 M039J12
V016E05 M048G03 H003H24
V066L09 V056P13 M028B11*
V016J09 V048D01 V068P10
M042D01 M029A15 V012J24
M028A23 V048F01 V019C24
M018B09 V073A12 V073H23
0 25 50 75 100 125 150 175 200 225 250 275 300
[Chickpea contig p... [root@localhost: /... [FPC V8.9 Main Menu] Project cp05T5e12... FPC Ctg3326 cp05...

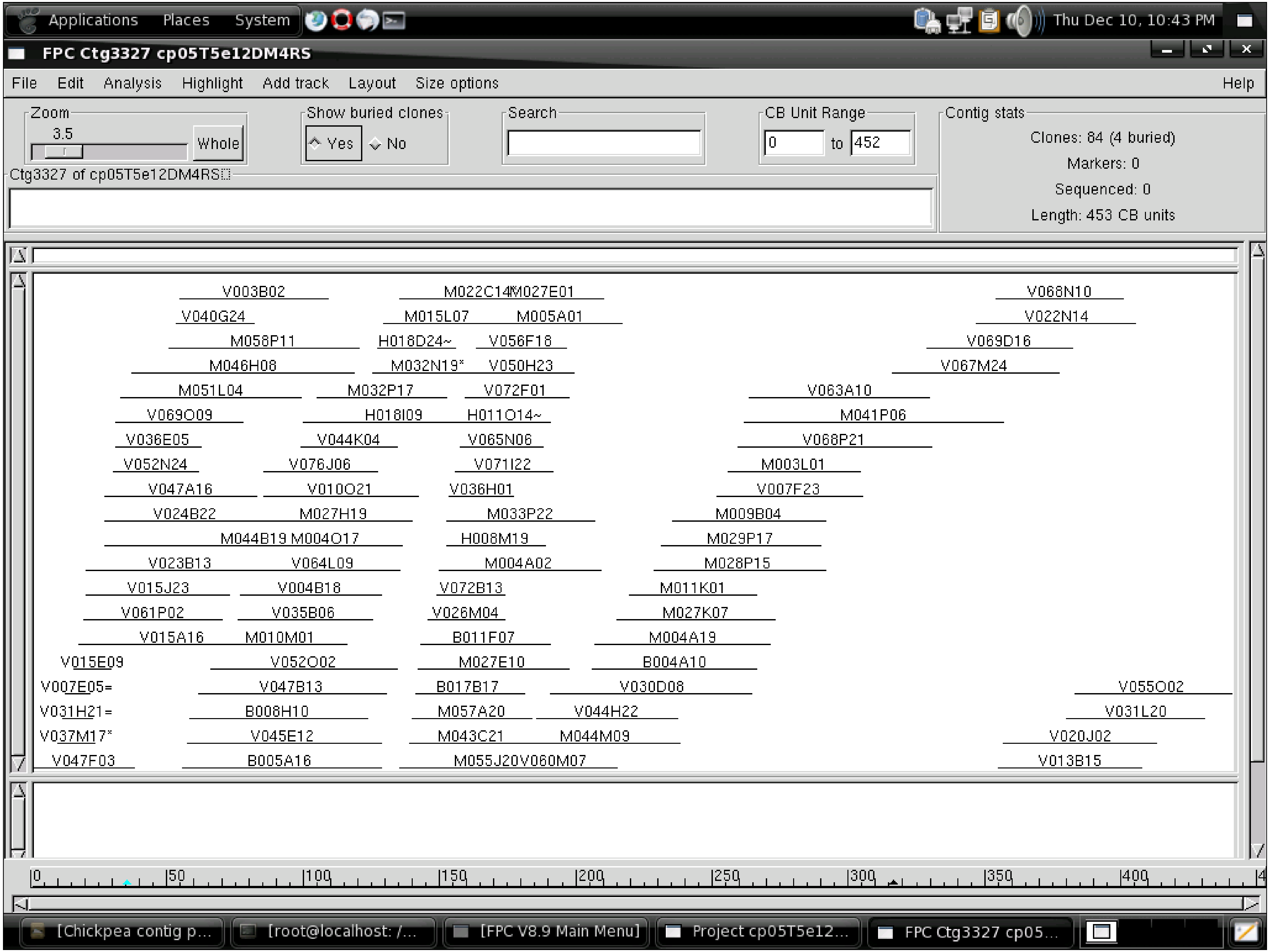
Applications Places System
Thu Dec 10, 10:43 PM
FPC Ctg3327 cp05T5e12DM4RS
File Edit Analysis Highlight Add track Layout Size options Help
Zoom
3.5
Whole
Show buried clones
Yes No
Search
CB Unit Range
0 to 452
Contig stats
Clones: 84 (4 buried)
Markers: 0
Sequenced: 0
Length: 453 CB units
Ctg3327 of cp05T5e12DM4RS
V003B02
M022C14M027E01
V068N10
V040G24
M015L07
M005A01
V022N14
M058P11
H018D24~
V056F18
V069D16
M046H08
M032N19*
V050H23
V067M24
M051L04
M032P17
V072F01
V063A10
V069O09
H018I09
H011O14~
M041P06
V036E05
V044K04
V065N06
V068P21
V052N24
V076J06
V071I22
M003L01
V047A16
V010O21
V036H01
V007F23
V024B22
M027H19
M033P22
M009B04
M044B19 M004O17
H008M19
M029P17
V023B13
V064L09
M004A02
M028P15
V015J23
V004B18
V072B13
M011K01
V061P02
V035B06
V026M04
M027K07
V015A16
M010M01
B011F07
M004A19
V015E09
V052O02
M027E10
B004A10
V007E05=
V047B13
B017B17
V030D08
V055O02
V031H21=
B008H10
M057A20
V044H22
V031L20
V037M17*
V045E12
M043C21
M044M09
V020J02
V047F03
B005A16
M055J20V060M07
V013B15
0 50 100 150 200 250 300 350 400
[Chickpea contig p...
[root@localhost: /...
[FPC V8.9 Main Menu]
Project cp05T5e12...
FPC Ctg3327 cp05...

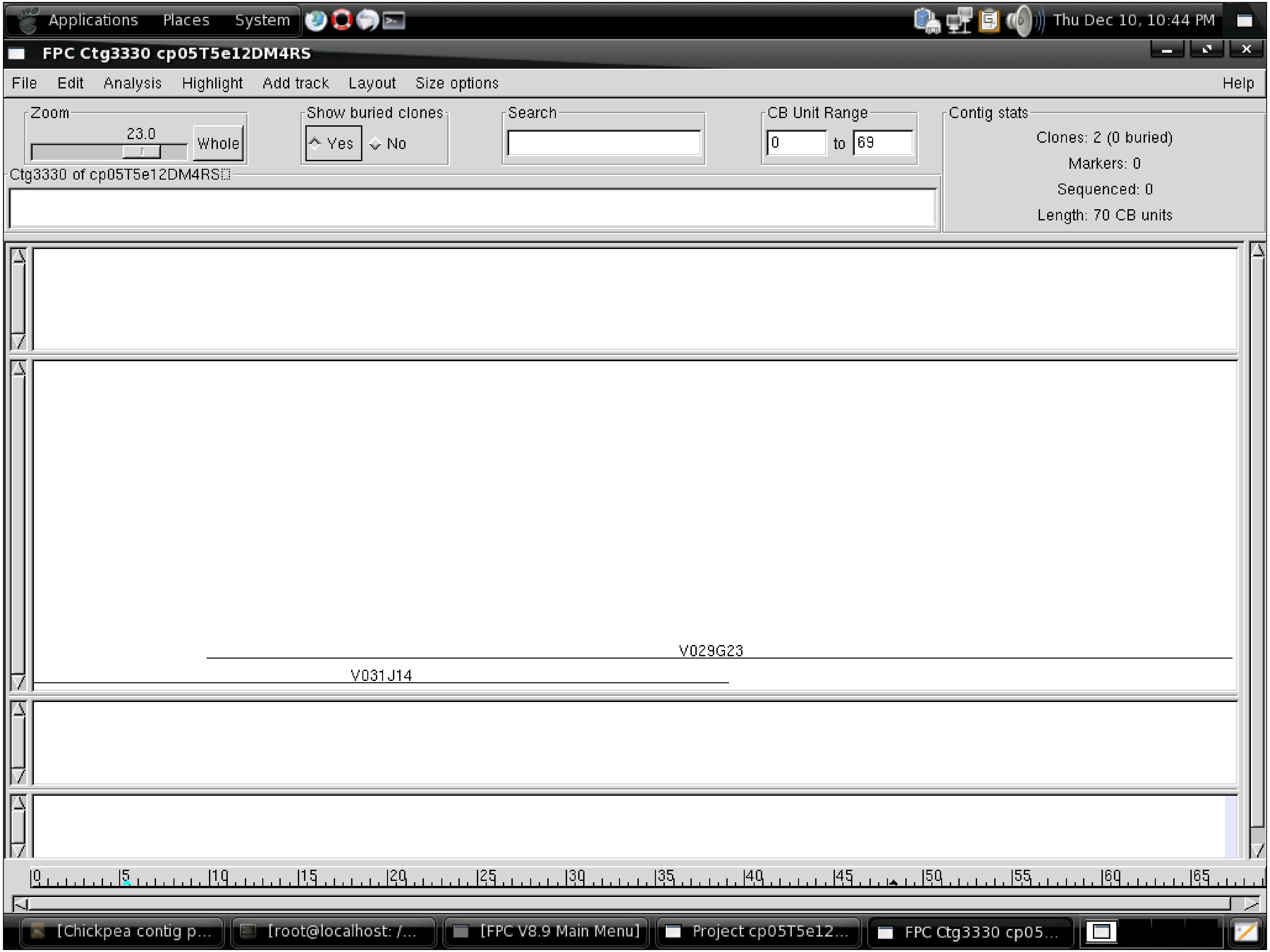
Applications Places System
Thu Dec 10, 10:44 PM
FPC Ctg3330 cp05T5e12DM4RS
File Edit Analysis Highlight Add track Layout Size options Help
Zoom
23.0
Whole
Show buried clones
Yes
No
Search
CB Unit Range
0 to 69
Contig stats
Clones: 2 (0 buried)
Markers: 0
Sequenced: 0
Length: 70 CB units
Ctg3330 of cp05T5e12DM4RS
V029G23
V031J14
0 5 10 15 20 25 30 35 40 45 50 55 60 65
[Chickpea contig p...
[root@localhost: /...
[FPC V8.9 Main Menu]
Project cp05T5e12...
FPC Ctg3330 cp05...

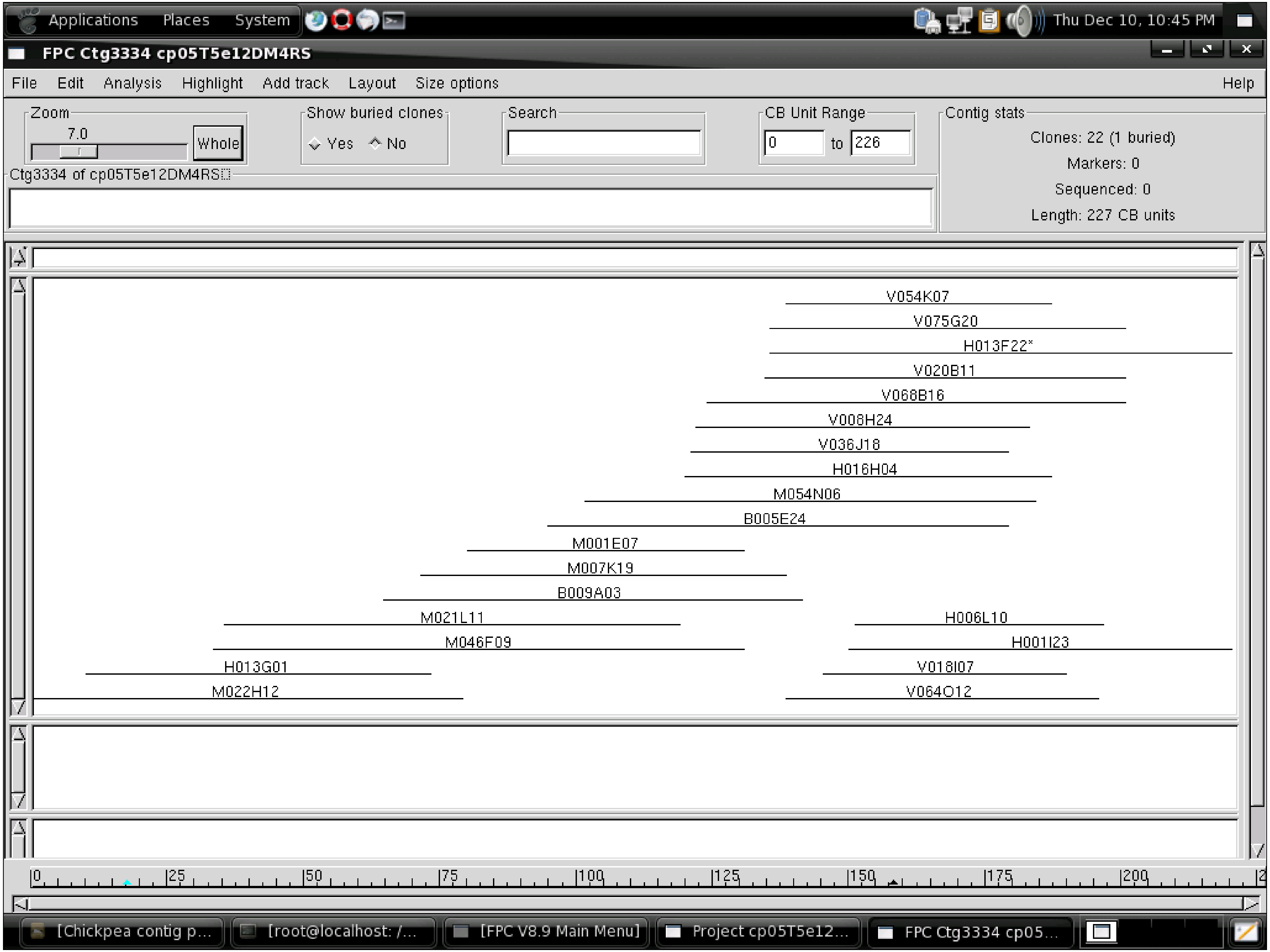

Applications
Places
System
Thu Dec 10, 10:45 PM
FPC Ctg3334 cp05T5e12DM4RS
File
Edit
Analysis
Highlight
Add track
Layout
Size options
Help
Zoom
7.0
Whole
Show buried clones
Yes
No
Search
CB Unit Range
0
to
226
Contig stats
Clones: 22 (1 buried)
Markers: 0
Sequenced: 0
Length: 227 CB units
Ctg3334 of cp05T5e12DM4RS
V054K07
V075G20
H013F22*
V020B11
V068B16
V008H24
V036J18
H016H04
M054N06
B005E24
M001E07
M007K19
B009A03
M021L11
H006L10
M046F09
H001I23
H013G01
V018I07
M022H12
V064O12
0
25
50
75
100
125
150
175
200
[Chickpea contig p...
[root@localhost: /...
[FPC V8.9 Main Menu]
Project cp05T5e12...
FPC Ctg3334 cp05...

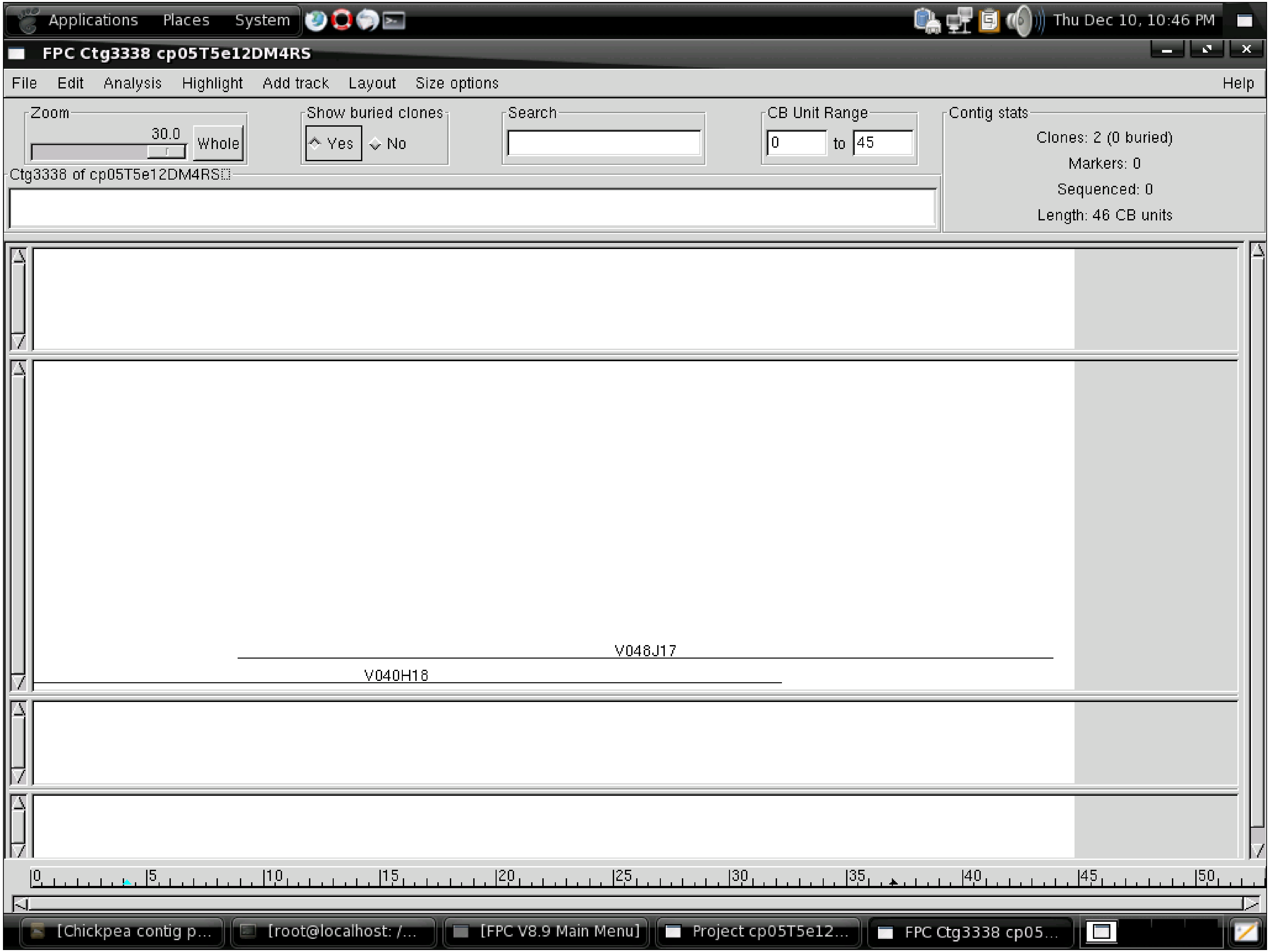

Applications
Places
System
Thu Dec 10, 10:46 PM
FPC Ctg3338 cp05T5e12DM4RS
File
Edit
Analysis
Highlight
Add track
Layout
Size options
Help
Zoom
30.0
Whole
Show buried clones
Yes
No
Search
CB Unit Range
0
to
45
Contig stats
Clones: 2 (0 buried)
Markers: 0
Sequenced: 0
Length: 46 CB units
Ctg3338 of cp05T5e12DM4RS
V048J17
V040H18
0 5 10 15 20 25 30 35 40 45 50
[Chickpea contig p...
[root@localhost: /...
[FPC V8.9 Main Menu]
Project cp05T5e12...
FPC Ctg3338 cp05...

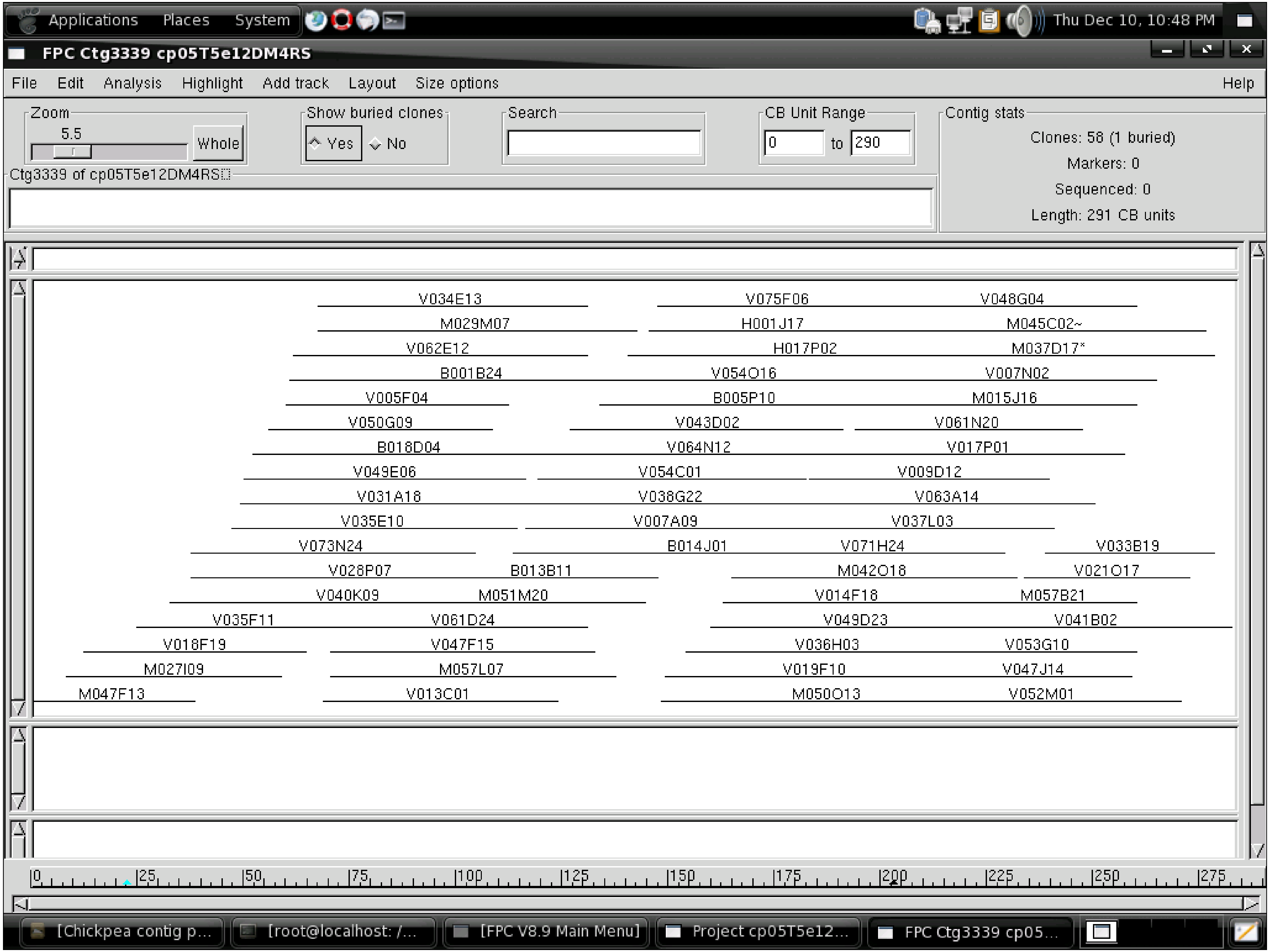
Applications Places System
Thu Dec 10, 10:48 PM
FPC Ctg3339 cp05T5e12DM4RS
File Edit Analysis Highlight Add track Layout Size options Help
Zoom 5.5 Whole
Show buried clones Yes No
Search
CB Unit Range 0 to 290
Contig stats
Clones: 58 (1 buried)
Markers: 0
Sequenced: 0
Length: 291 CB units
Ctg3339 of cp05T5e12DM4RS
V034E13 V075F06 V048G04
M029M07 H001J17 M045C02~
V062E12 H017P02 M037D17*
B001B24 V054O16 V007N02
V005F04 B005P10 M015J16
V050G09 V043D02 V061N20
B018D04 V064N12 V017P01
V049E06 V054C01 V009D12
V031A18 V038G22 V063A14
V035E10 V007A09 V037L03
V073N24 B014J01 V071H24 V033B19
V028P07 B013B11 M042O18 V021O17
V040K09 M051M20 V014F18 M057B21
V035F11 V061D24 V049D23 V041B02
V018F19 V047F15 V036H03 V053G10
M027I09 M057L07 V019F10 V047J14
M047F13 V013C01 M050O13 V052M01
0 25 50 75 100 125 150 175 200 225 250 275
[Chickpea contig p... [root@localhost: /... [FPC V8.9 Main Menu] Project cp05T5e12... FPC Ctg3339 cp05...

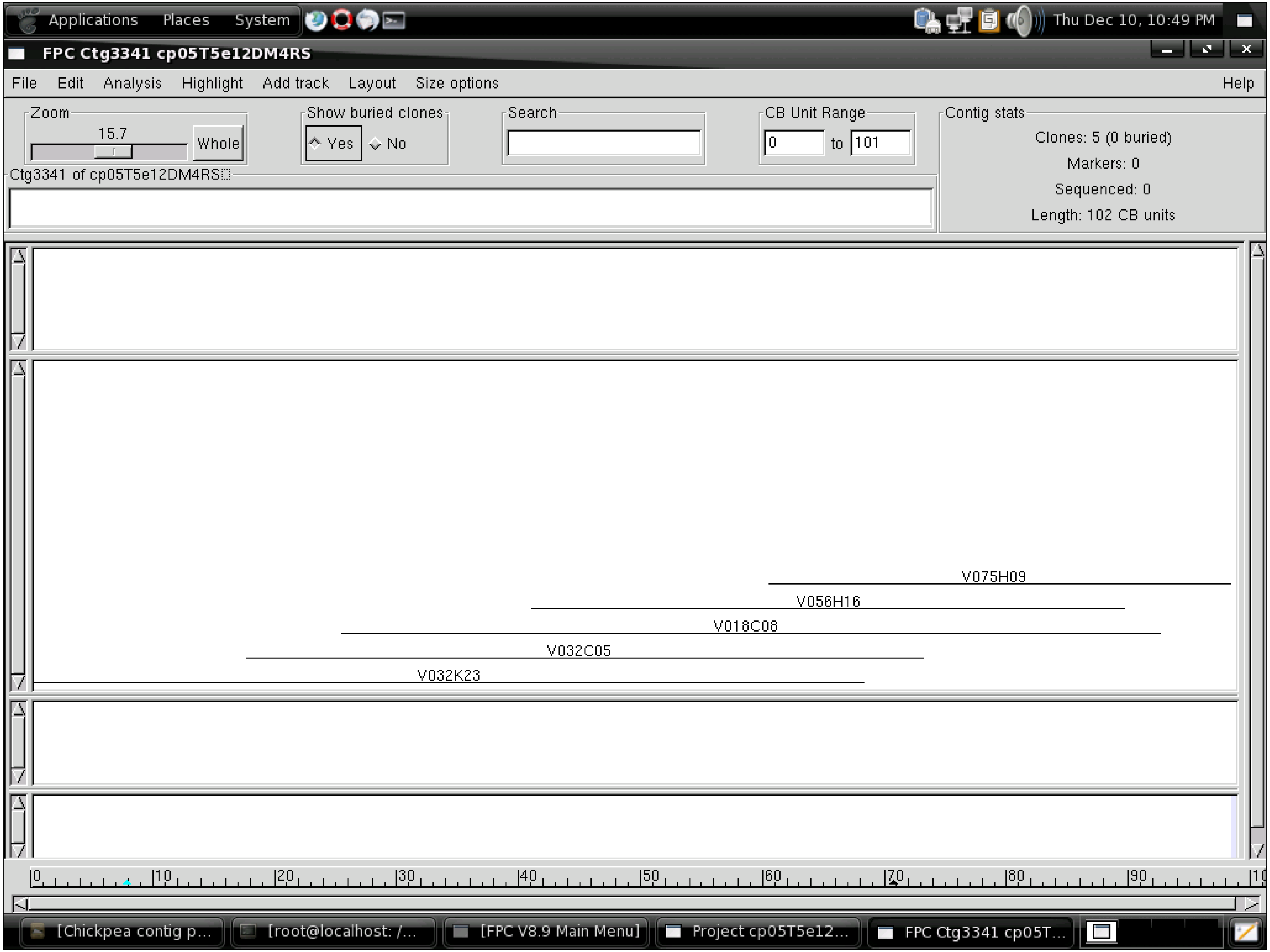
Applications Places System
Thu Dec 10, 10:49 PM
FPC Ctg3341 cp05T5e12DM4RS
File Edit Analysis Highlight Add track Layout Size options Help
Zoom
15.7
Whole
Show buried clones
Yes No
Search
CB Unit Range
0 to 101
Contig stats
Clones: 5 (0 buried)
Markers: 0
Sequenced: 0
Length: 102 CB units
Ctg3341 of cp05T5e12DM4RS
V075H09
V056H16
V018C08
V032C05
V032K23
0 10 20 30 40 50 60 70 80 90
[Chickpea contig p...
[root@localhost: /...
[FPC V8.9 Main Menu]
Project cp05T5e12...
FPC Ctg3341 cp05T...

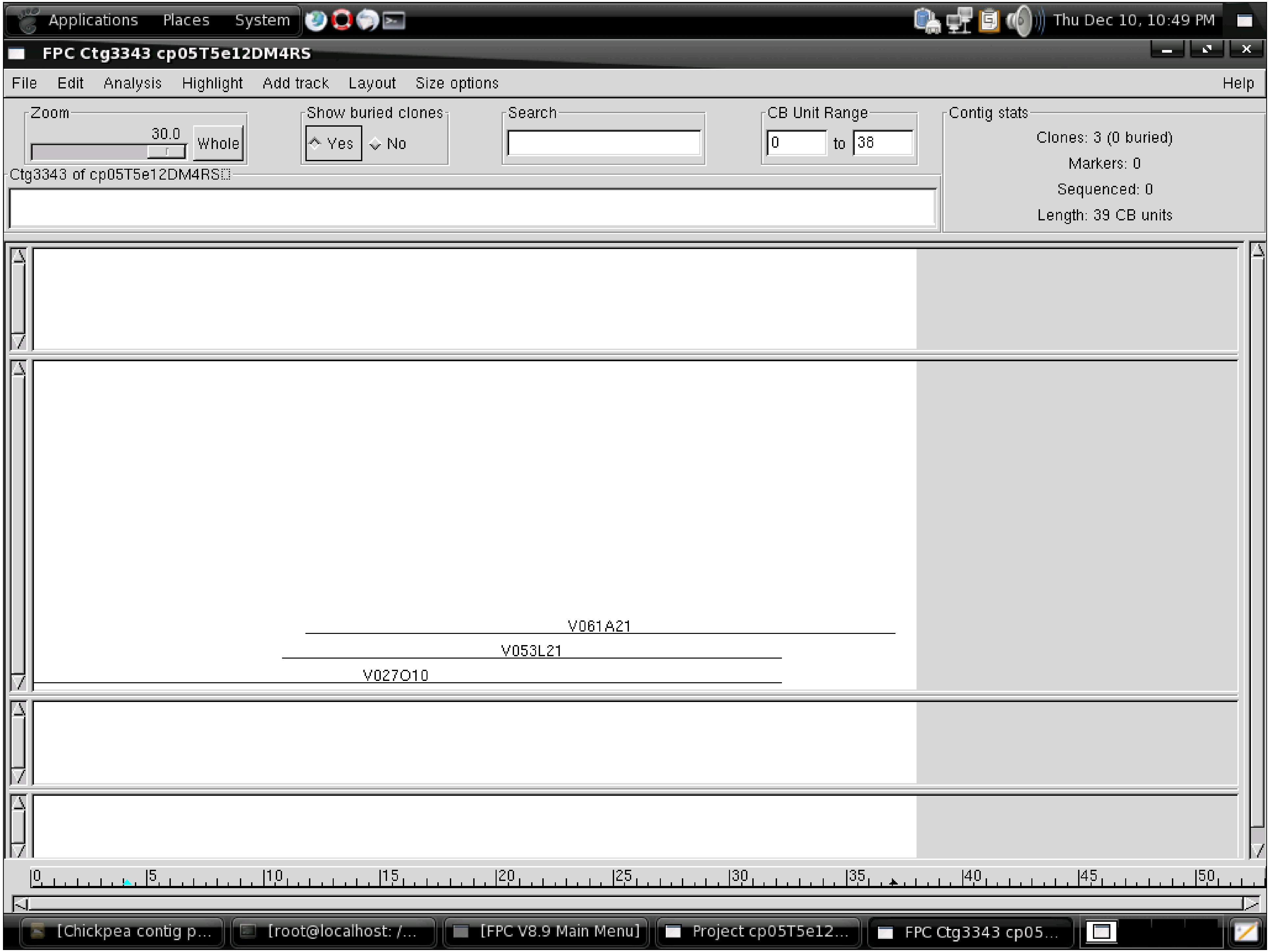
Applications Places System
Thu Dec 10, 10:49 PM
FPC Ctg3343 cp05T5e12DM4RS
File Edit Analysis Highlight Add track Layout Size options
Help
Zoom
30.0
Whole
Show buried clones
Yes
No
Search
CB Unit Range
0
to
38
Contig stats
Clones: 3 (0 buried)
Markers: 0
Sequenced: 0
Length: 39 CB units
Ctg3343 of cp05T5e12DM4RS
V061A21
V053L21
V027O10
0 5 10 15 20 25 30 35 40 45 50
[Chickpea contig p...
[root@localhost: /...
[FPC V8.9 Main Menu]
Project cp05T5e12...
FPC Ctg3343 cp05...

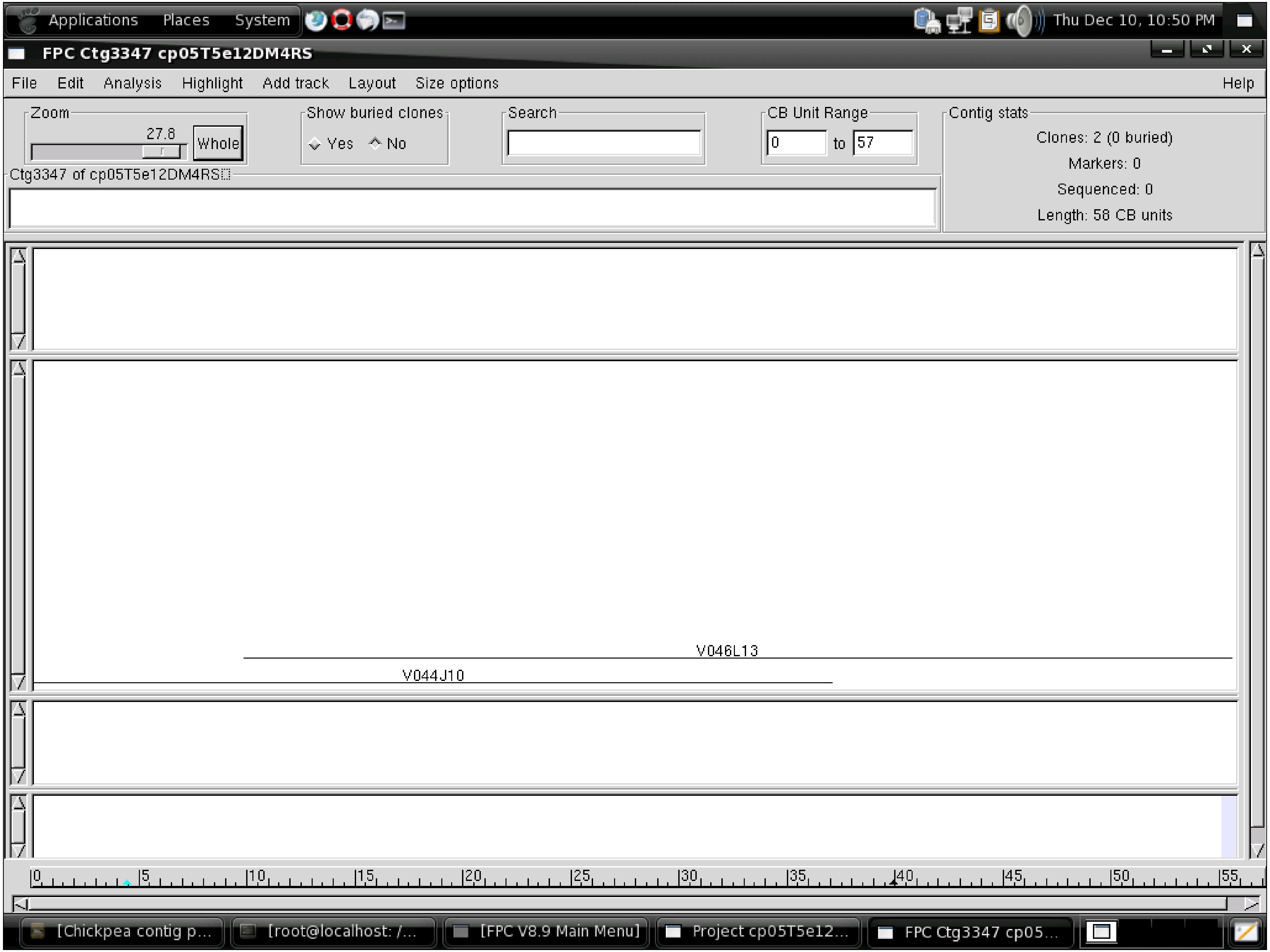
Applications Places System
Thu Dec 10, 10:50 PM
FPC Ctg3347 cp05T5e12DM4RS
File Edit Analysis Highlight Add track Layout Size options Help
Zoom
27.8
Whole
Show buried clones
Yes No
Search
CB Unit Range
0 to 57
Contig stats
Clones: 2 (0 buried)
Markers: 0
Sequenced: 0
Length: 58 CB units
Ctg3347 of cp05T5e12DM4RS
V046L13
V044J10
0 5 10 15 20 25 30 35 40 45 50 55
[Chickpea contig p...
[root@localhost: /...
[FPC V8.9 Main Menu]
Project cp05T5e12...
FPC Ctg3347 cp05...

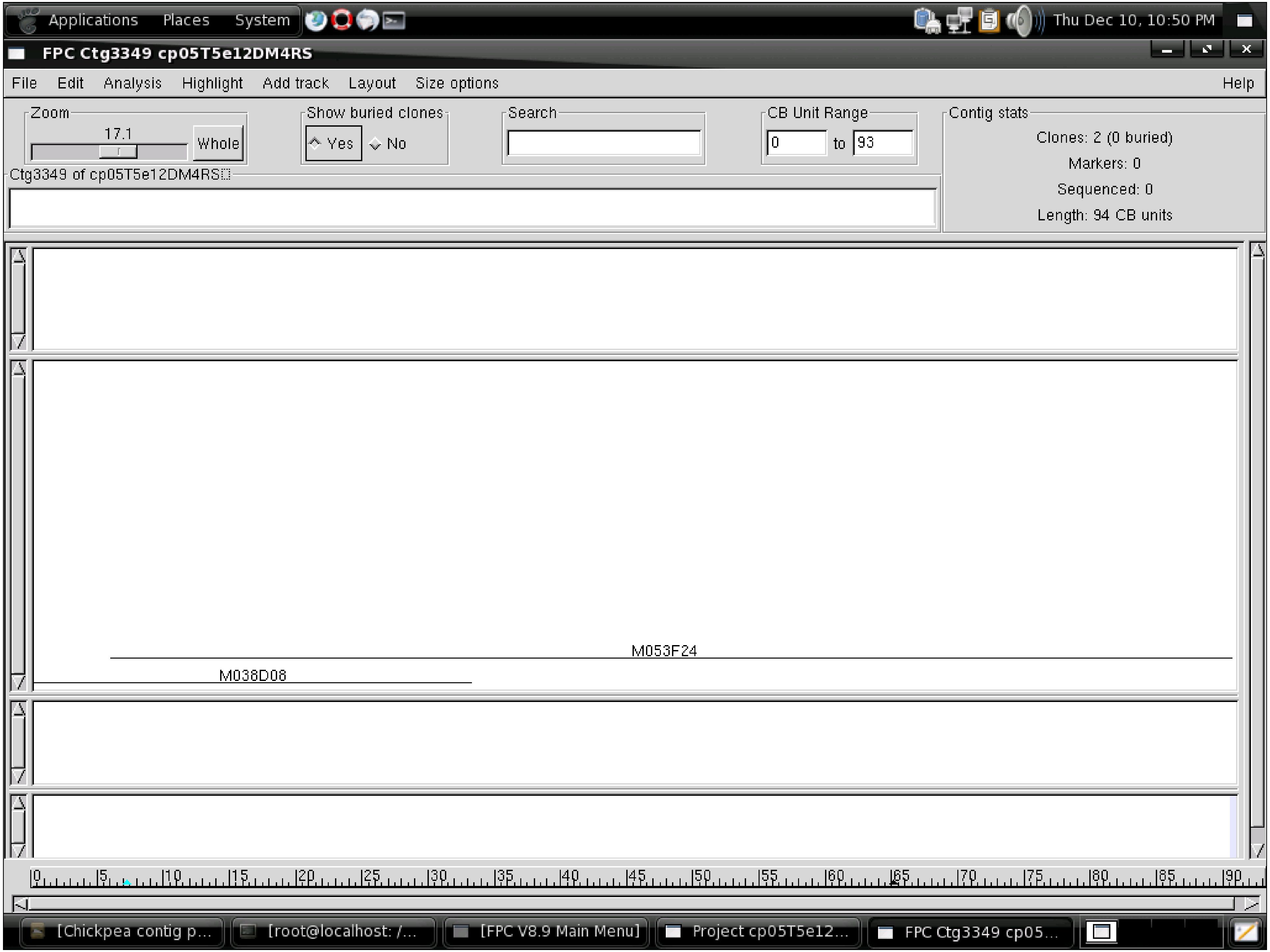
Applications
Places
System
Thu Dec 10, 10:50 PM
FPC Ctg3349 cp05T5e12DM4RS
File
Edit
Analysis
Highlight
Add track
Layout
Size options
Help
Zoom
17.1
Whole
Show buried clones
Yes
No
Search
CB Unit Range
0
to
93
Contig stats
Clones: 2 (0 buried)
Markers: 0
Sequenced: 0
Length: 94 CB units
Ctg3349 of cp05T5e12DM4RS
M053F24
M038D08
0 5 10 15 20 25 30 35 40 45 50 55 60 65 70 75 80 85 90
[Chickpea contig p...
[root@localhost: /...
[FPC V8.9 Main Menu]
Project cp05T5e12...
FPC Ctg3349 cp05...

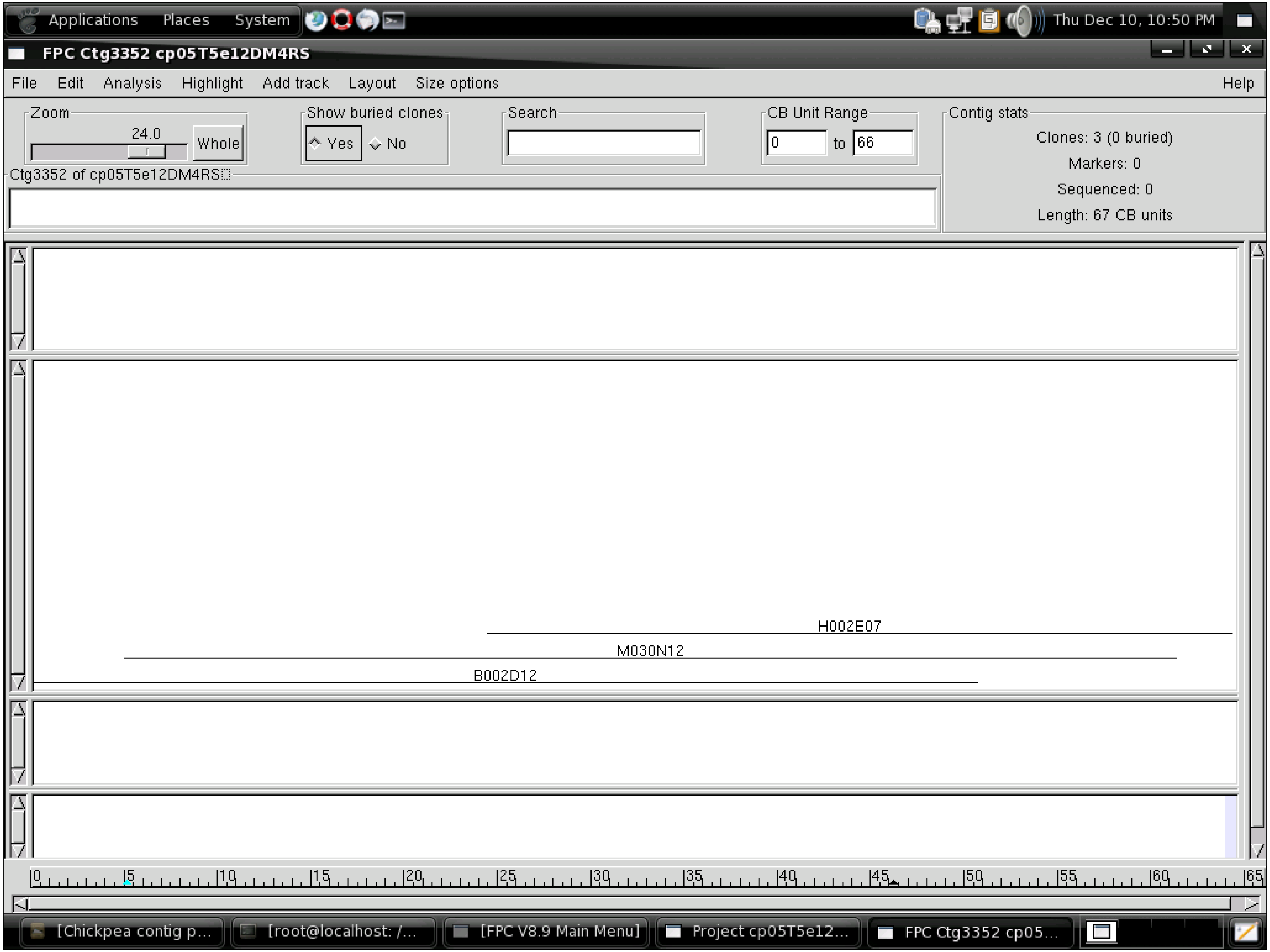
Applications Places System
Thu Dec 10, 10:50 PM
FPC Ctg3352 cp05T5e12DM4RS
File Edit Analysis Highlight Add track Layout Size options Help
Zoom
24.0
Whole
Show buried clones
Yes No
Search
CB Unit Range
0 to 66
Contig stats
Clones: 3 (0 buried)
Markers: 0
Sequenced: 0
Length: 67 CB units
Ctg3352 of cp05T5e12DM4RS
H002E07
M030N12
B002D12
0 5 10 15 20 25 30 35 40 45 50 55 60 65
[Chickpea contig p...
[root@localhost: /...
[FPC V8.9 Main Menu]
Project cp05T5e12...
FPC Ctg3352 cp05...

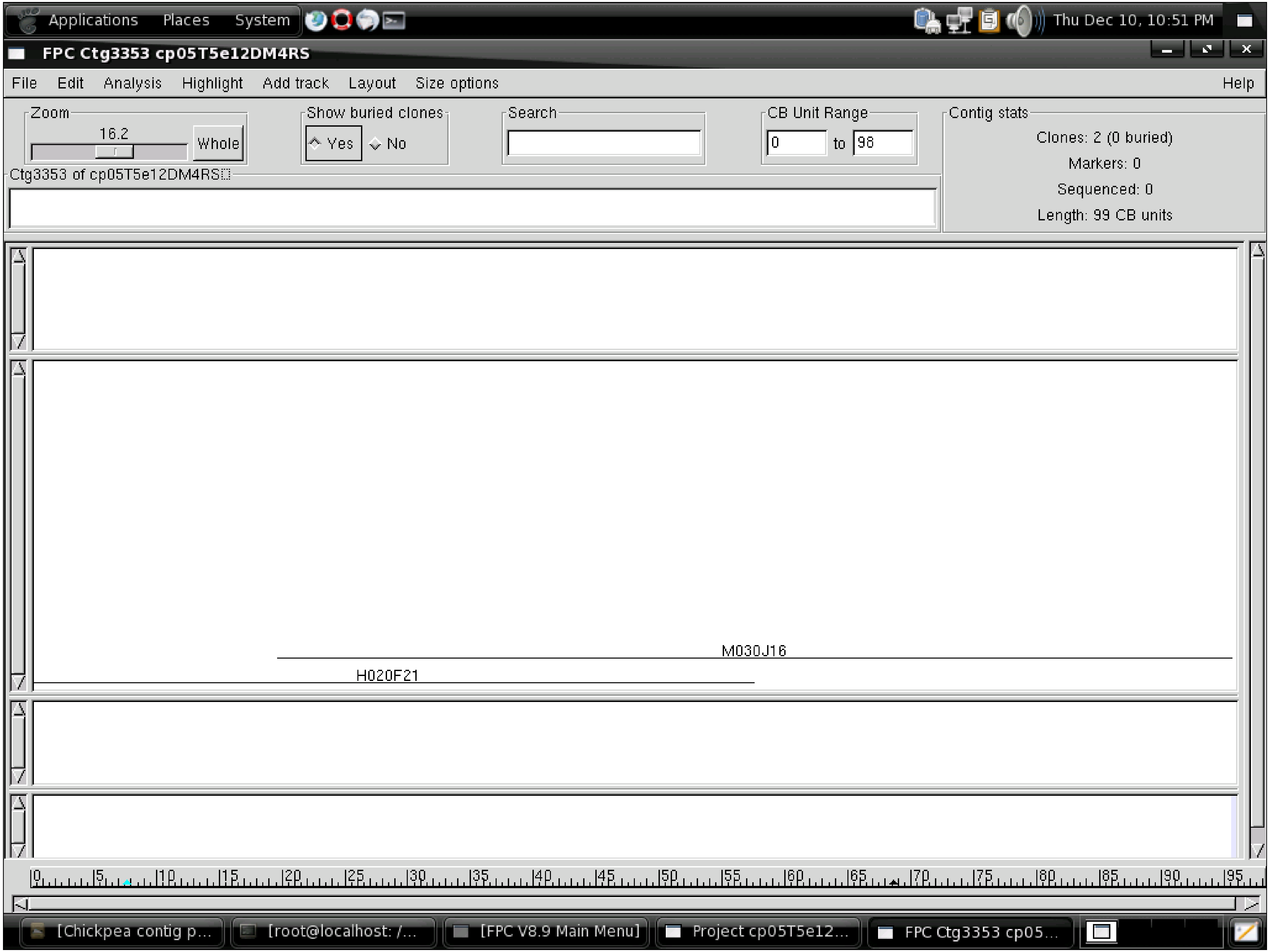
Applications Places System
Thu Dec 10, 10:51 PM
FPC Ctg3353 cp05T5e12DM4RS
File Edit Analysis Highlight Add track Layout Size options
Help
Zoom
16.2
Whole
Show buried clones
Yes
No
Search
CB Unit Range
0
to
98
Contig stats
Clones: 2 (0 buried)
Markers: 0
Sequenced: 0
Length: 99 CB units
Ctg3353 of cp05T5e12DM4RS
M030J16
H020F21
0 5 10 15 20 25 30 35 40 45 50 55 60 65 70 75 80 85 90 95
[Chickpea contig p...
[root@localhost: /...
[FPC V8.9 Main Menu]
Project cp05T5e12...
FPC Ctg3353 cp05...

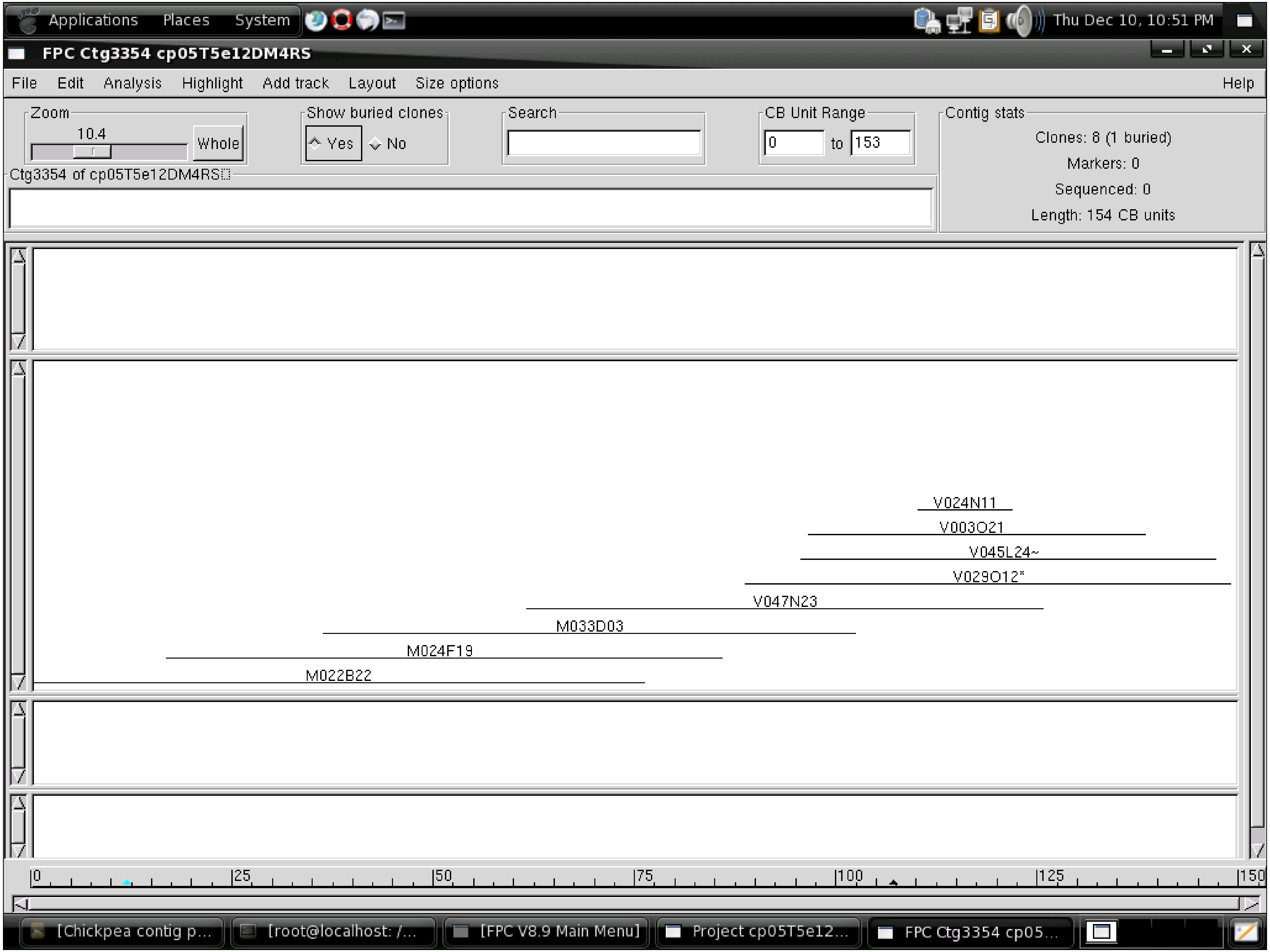
Applications Places System
Thu Dec 10, 10:51 PM
FPC Ctg3354 cp05T5e12DM4RS
File Edit Analysis Highlight Add track Layout Size options Help
Zoom
10.4
Whole
Show buried clones
Yes
No
Search
CB Unit Range
0 to 153
Contig stats
Clones: 8 (1 buried)
Markers: 0
Sequenced: 0
Length: 154 CB units
Ctg3354 of cp05T5e12DM4RS
V024N11
V003O21
V045L24~
V029O12*
V047N23
M033D03
M024F19
M022B22
0 25 50 75 100 125 150
[Chickpea contig p...
[root@localhost: /...
[FPC V8.9 Main Menu]
Project cp05T5e12...
FPC Ctg3354 cp05...

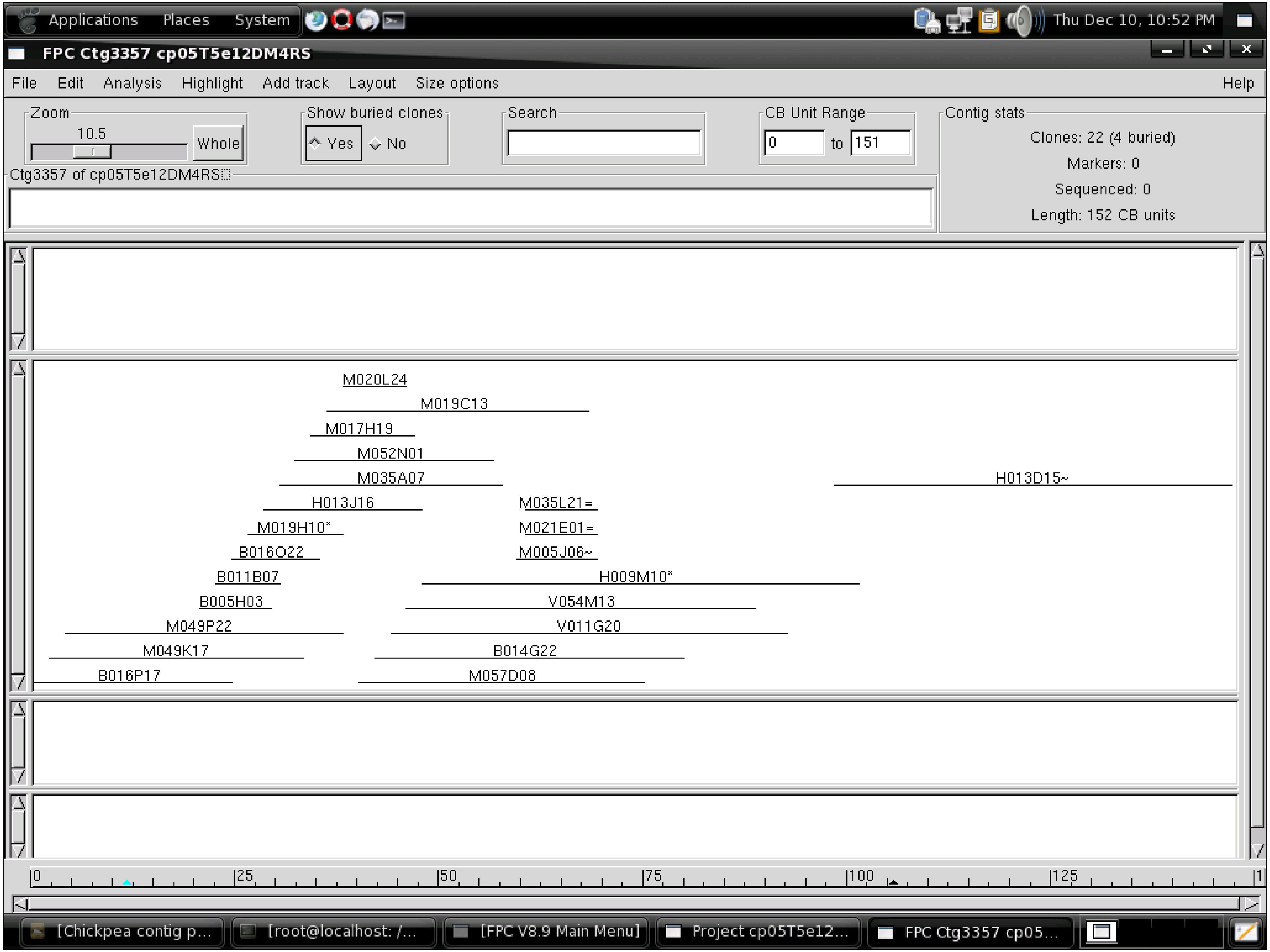
Applications Places System
Thu Dec 10, 10:52 PM
FPC Ctg3357 cp05T5e12DM4RS
File Edit Analysis Highlight Add track Layout Size options Help
Zoom
10.5
Whole
Show buried clones
Yes
No
Search
CB Unit Range
0
to
151
Contig stats
Clones: 22 (4 buried)
Markers: 0
Sequenced: 0
Length: 152 CB units
Ctg3357 of cp05T5e12DM4RS
M020L24
M019C13
M017H19
M052N01
M035A07
H013D15~
H013J16
M035L21=
M019H10*
M021E01=
B016O22
M005J06~
B011B07
H009M10*
B005H03
V054M13
M049P22
V011G20
M049K17
B014G22
B016P17
M057D08
0
25
50
75
100
125
[Chickpea contig p...
[root@localhost: /...
[FPC V8.9 Main Menu]
Project cp05T5e12...
FPC Ctg3357 cp05...

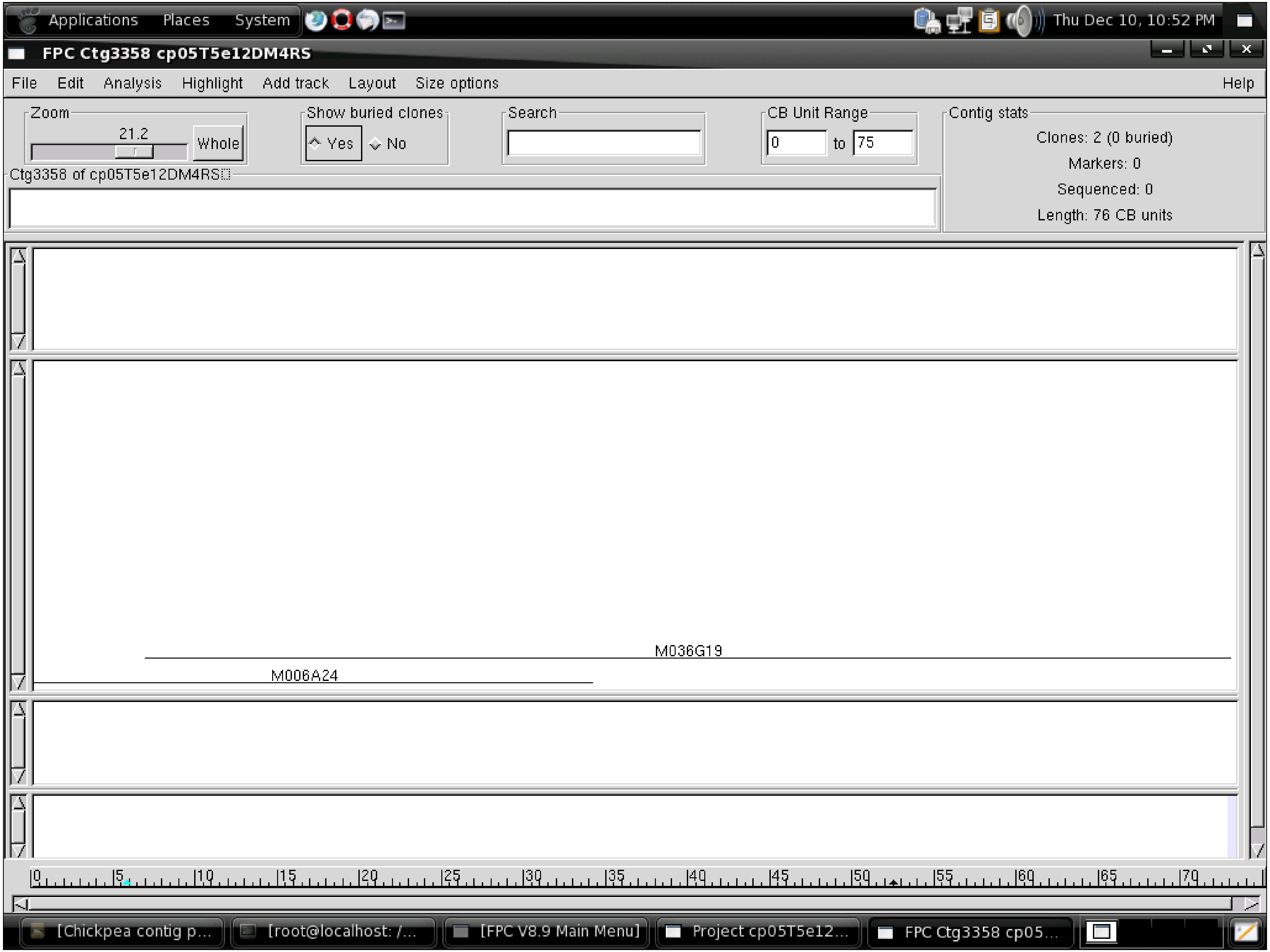
Applications
Places
System
Thu Dec 10, 10:52 PM
FPC Ctg3358 cp05T5e12DM4RS
File
Edit
Analysis
Highlight
Add track
Layout
Size options
Help
Zoom
21.2
Whole
Show buried clones
Yes
No
Search
CB Unit Range
0
to
75
Contig stats
Clones: 2 (0 buried)
Markers: 0
Sequenced: 0
Length: 76 CB units
Ctg3358 of cp05T5e12DM4RS
M036G19
M006A24
0
5
10
15
20
25
30
35
40
45
50
55
60
65
70
[Chickpea contig p...
[root@localhost: /...
[FPC V8.9 Main Menu]
Project cp05T5e12...
FPC Ctg3358 cp05...

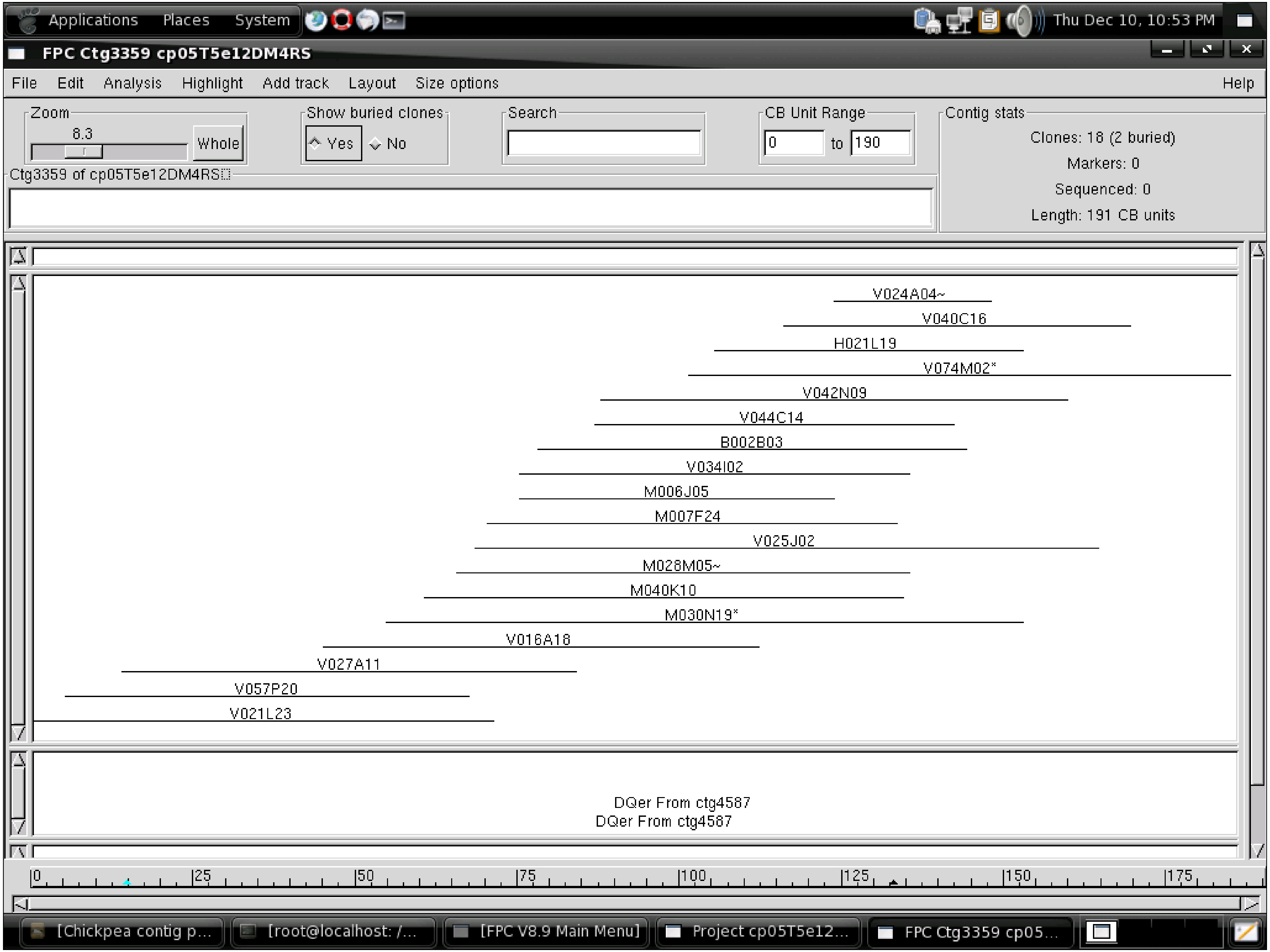
Applications Places System
Thu Dec 10, 10:53 PM
FPC Ctg3359 cp05T5e12DM4RS
File Edit Analysis Highlight Add track Layout Size options Help
Zoom
8.3
Whole
Show buried clones
Yes No
Search
CB Unit Range
0 to 190
Contig stats
Clones: 18 (2 buried)
Markers: 0
Sequenced: 0
Length: 191 CB units
Ctg3359 of cp05T5e12DM4RS
V024A04~
V040C16
H021L19
V074M02*
V042N09
V044C14
B002B03
V034I02
M006J05
M007F24
V025J02
M028M05~
M040K10
M030N19*
V016A18
V027A11
V057P20
V021L23
DQer From ctg4587
DQer From ctg4587
0 25 50 75 100 125 150 175
[Chickpea contig p...
[root@localhost: /...
[FPC V8.9 Main Menu]
Project cp05T5e12...
FPC Ctg3359 cp05...

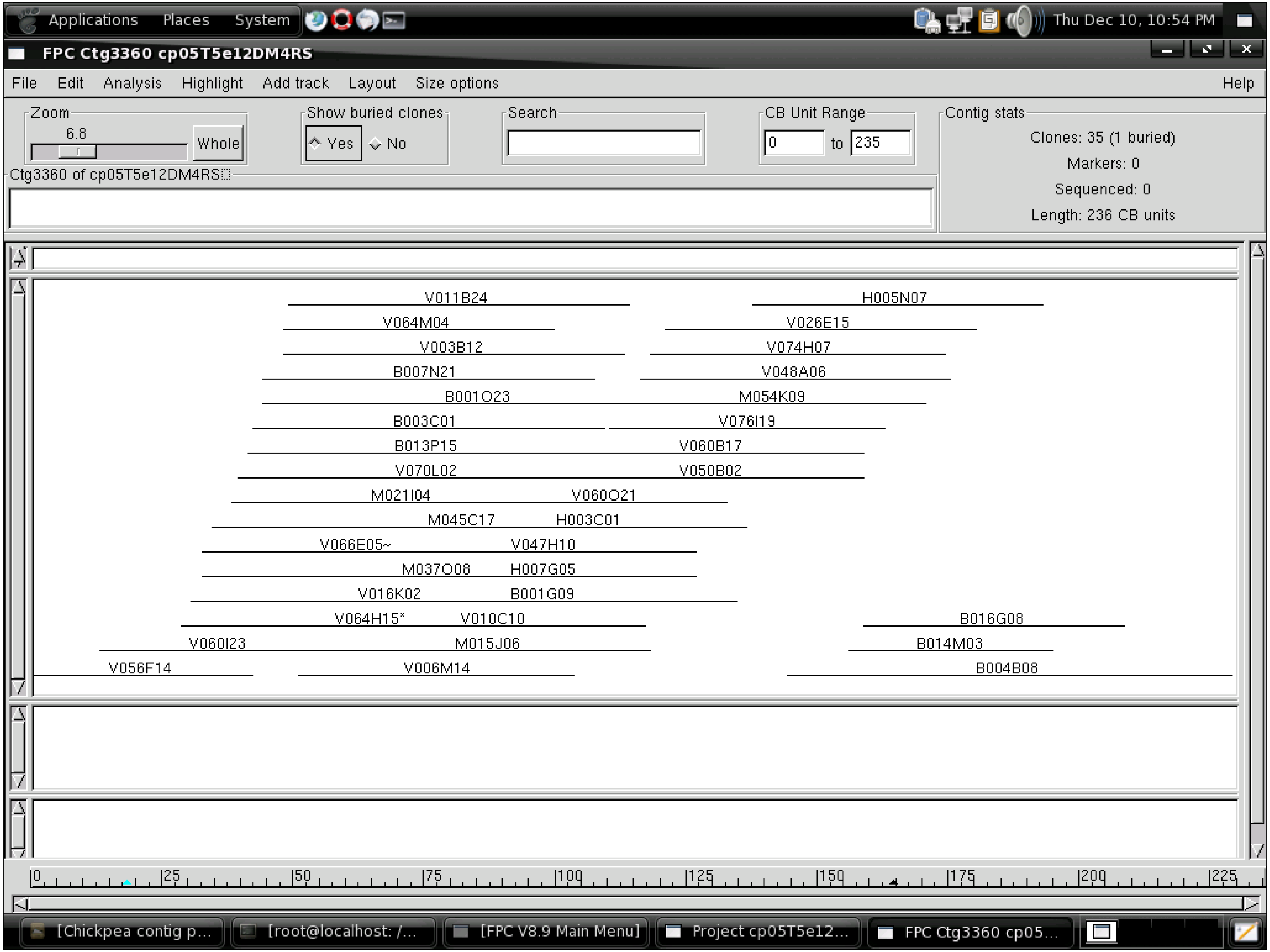
Applications Places System
Thu Dec 10, 10:54 PM
FPC Ctg3360 cp05T5e12DM4RS
File Edit Analysis Highlight Add track Layout Size options Help
Zoom
6.8
Whole
Show buried clones
Yes
No
Search
CB Unit Range
0 to 235
Contig stats
Clones: 35 (1 buried)
Markers: 0
Sequenced: 0
Length: 236 CB units
Ctg3360 of cp05T5e12DM4RS
V011B24
H005N07
V064M04
V026E15
V003B12
V074H07
B007N21
V048A06
B001O23
M054K09
B003C01
V076I19
B013P15
V060B17
V070L02
V050B02
M021I04
V060O21
M045C17
H003C01
V066E05~
V047H10
M037O08
H007G05
V016K02
B001G09
V064H15*
V010C10
B016G08
V060I23
M015J06
B014M03
V056F14
V006M14
B004B08
0 25 50 75 100 125 150 175 200 225
[Chickpea contig p...
[root@localhost: /...
[FPC V8.9 Main Menu]
Project cp05T5e12...
FPC Ctg3360 cp05...

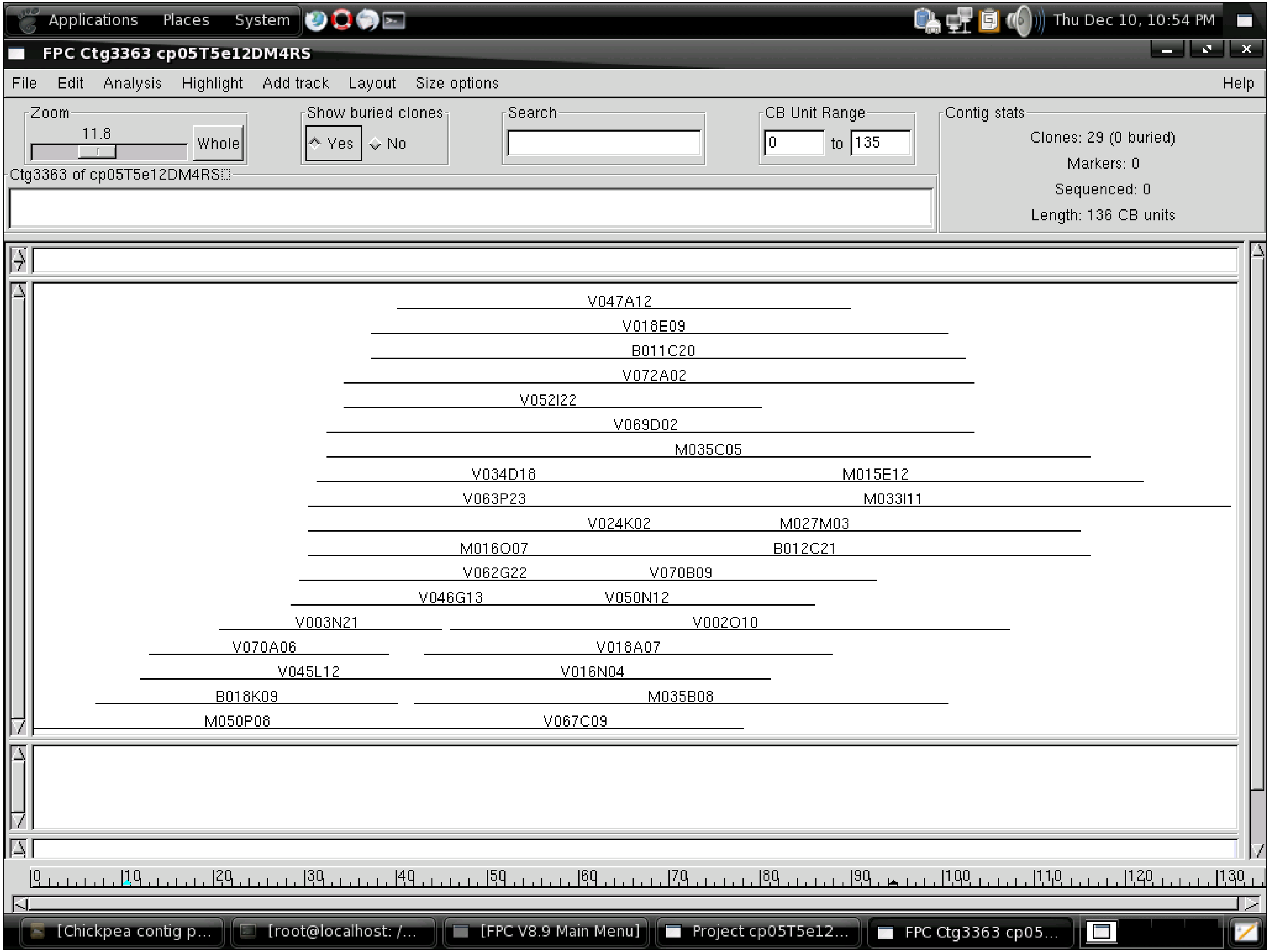
Applications
Places
System
Thu Dec 10, 10:54 PM
FPC Ctg3363 cp05T5e12DM4RS
File
Edit
Analysis
Highlight
Add track
Layout
Size options
Help
Zoom
11.8
Whole
Show buried clones
Yes
No
Search
CB Unit Range
0
to
135
Contig stats
Clones: 29 (0 buried)
Markers: 0
Sequenced: 0
Length: 136 CB units
Ctg3363 of cp05T5e12DM4RS
V047A12
V018E09
B011C20
V072A02
V052I22
V069D02
M035C05
V034D18
M015E12
V063P23
M033I11
V024K02
M027M03
M016O07
B012C21
V062G22
V070B09
V046G13
V050N12
V003N21
V002O10
V070A06
V018A07
V045L12
V016N04
B018K09
M035B08
M050P08
V067C09
0
10
20
30
40
50
60
70
80
90
100
110
120
130
[Chickpea contig p...
[root@localhost: /...
[FPC V8.9 Main Menu]
Project cp05T5e12...
FPC Ctg3363 cp05...

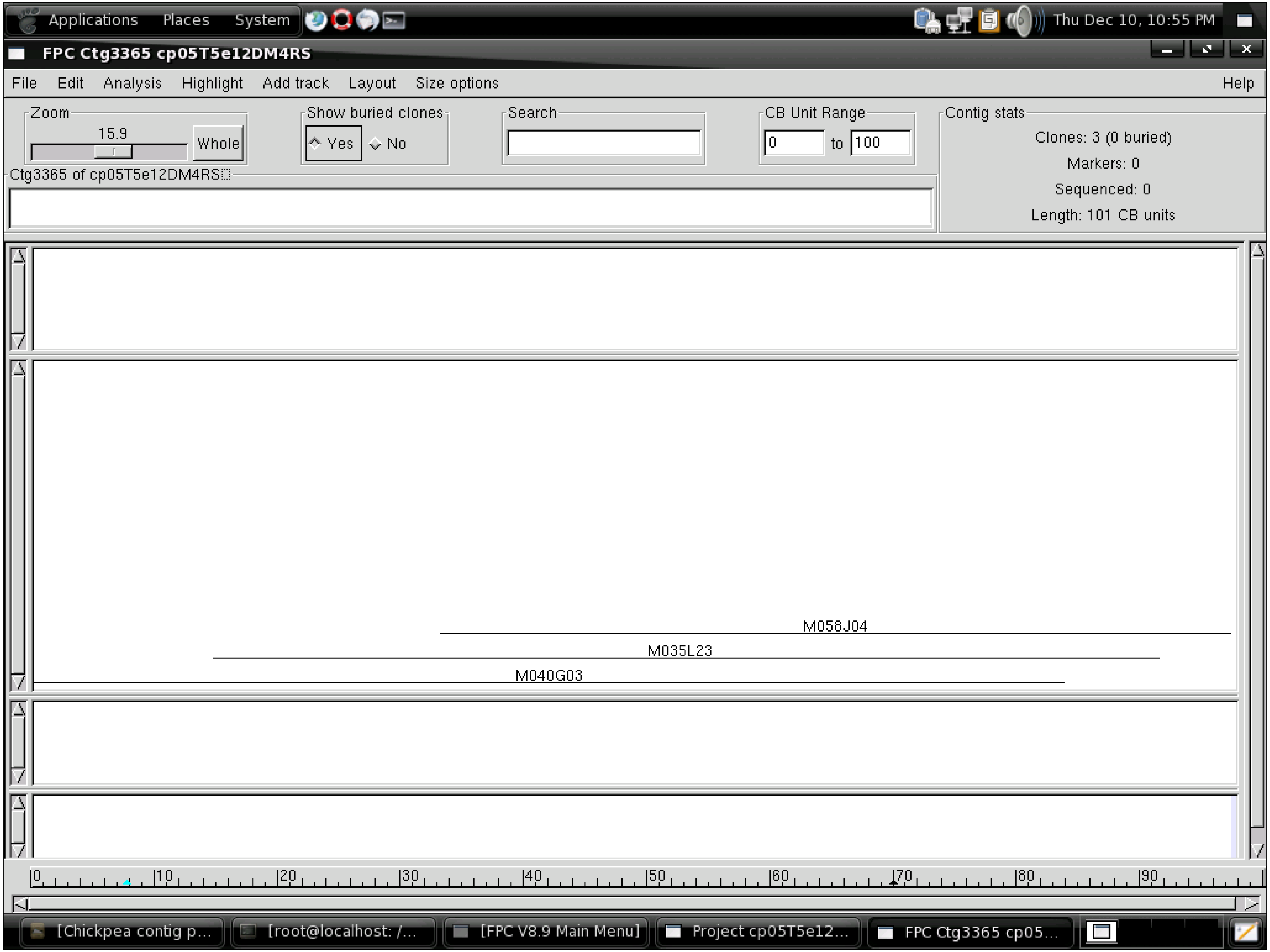
Applications
Places
System
Thu Dec 10, 10:55 PM
FPC Ctg3365 cp05T5e12DM4RS
File
Edit
Analysis
Highlight
Add track
Layout
Size options
Help
Zoom
15.9
Whole
Show buried clones
Yes
No
Search
CB Unit Range
0
to
100
Contig stats
Clones: 3 (0 buried)
Markers: 0
Sequenced: 0
Length: 101 CB units
Ctg3365 of cp05T5e12DM4RS
M058J04
M035L23
M040G03
0
10
20
30
40
50
60
70
80
90
[Chickpea contig p...
[root@localhost: /...
[FPC V8.9 Main Menu]
Project cp05T5e12...
FPC Ctg3365 cp05...

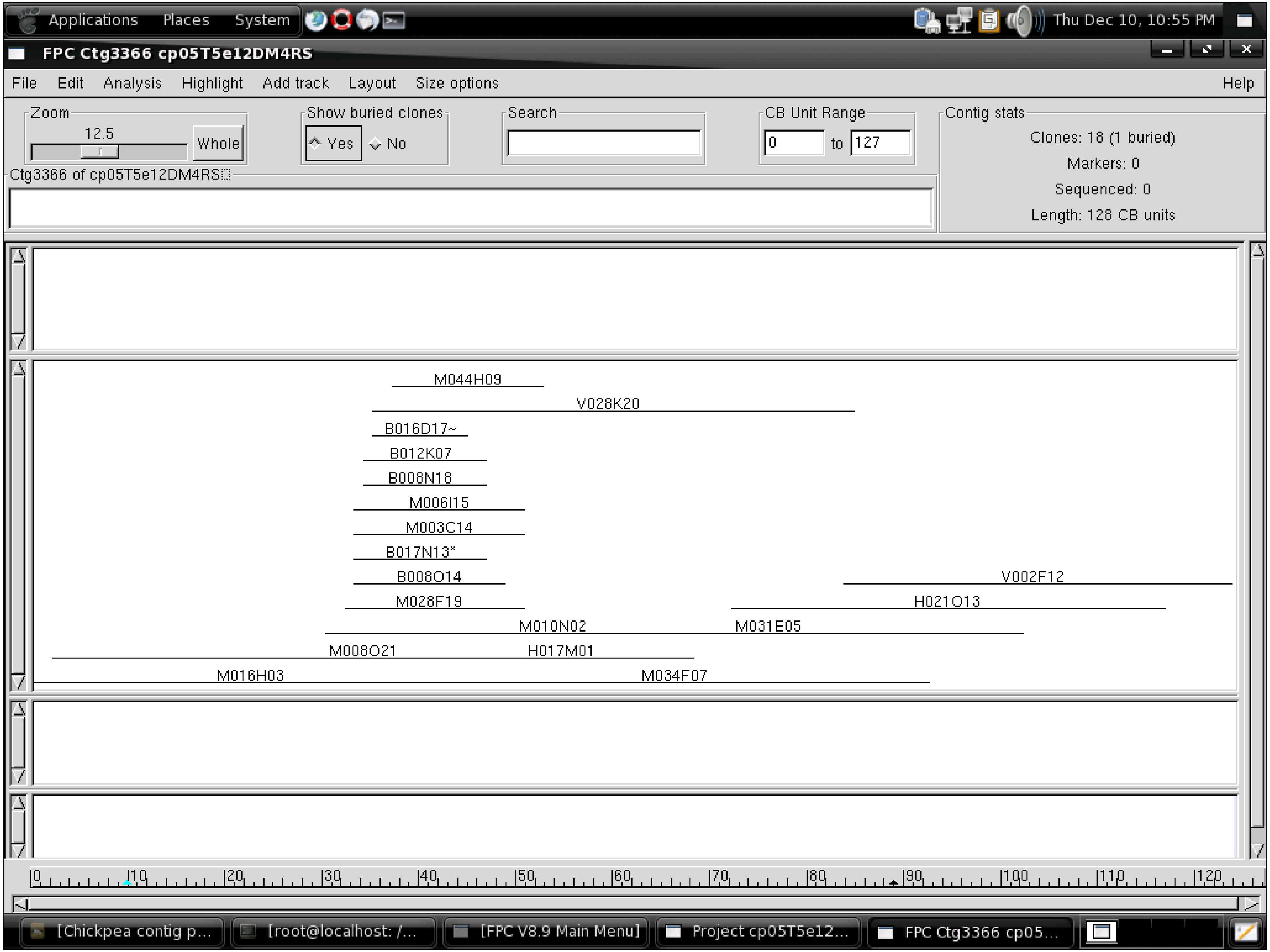
Applications Places System
Thu Dec 10, 10:55 PM
FPC Ctg3366 cp05T5e12DM4RS
File Edit Analysis Highlight Add track Layout Size options Help
Zoom
12.5
Whole
Show buried clones
Yes No
Search
CB Unit Range
0 to 127
Contig stats
Clones: 18 (1 buried)
Markers: 0
Sequenced: 0
Length: 128 CB units
Ctg3366 of cp05T5e12DM4RS
M044H09
V028K20
B016D17~
B012K07
B008N18
M006I15
M003C14
B017N13*
B008O14
V002F12
M028F19
H021O13
M010N02
M031E05
M008O21
H017M01
M016H03
M034F07
0 10 20 30 40 50 60 70 80 90 100 110 120
[Chickpea contig p...
[root@localhost: /...
[FPC V8.9 Main Menu]
Project cp05T5e12...
FPC Ctg3366 cp05...

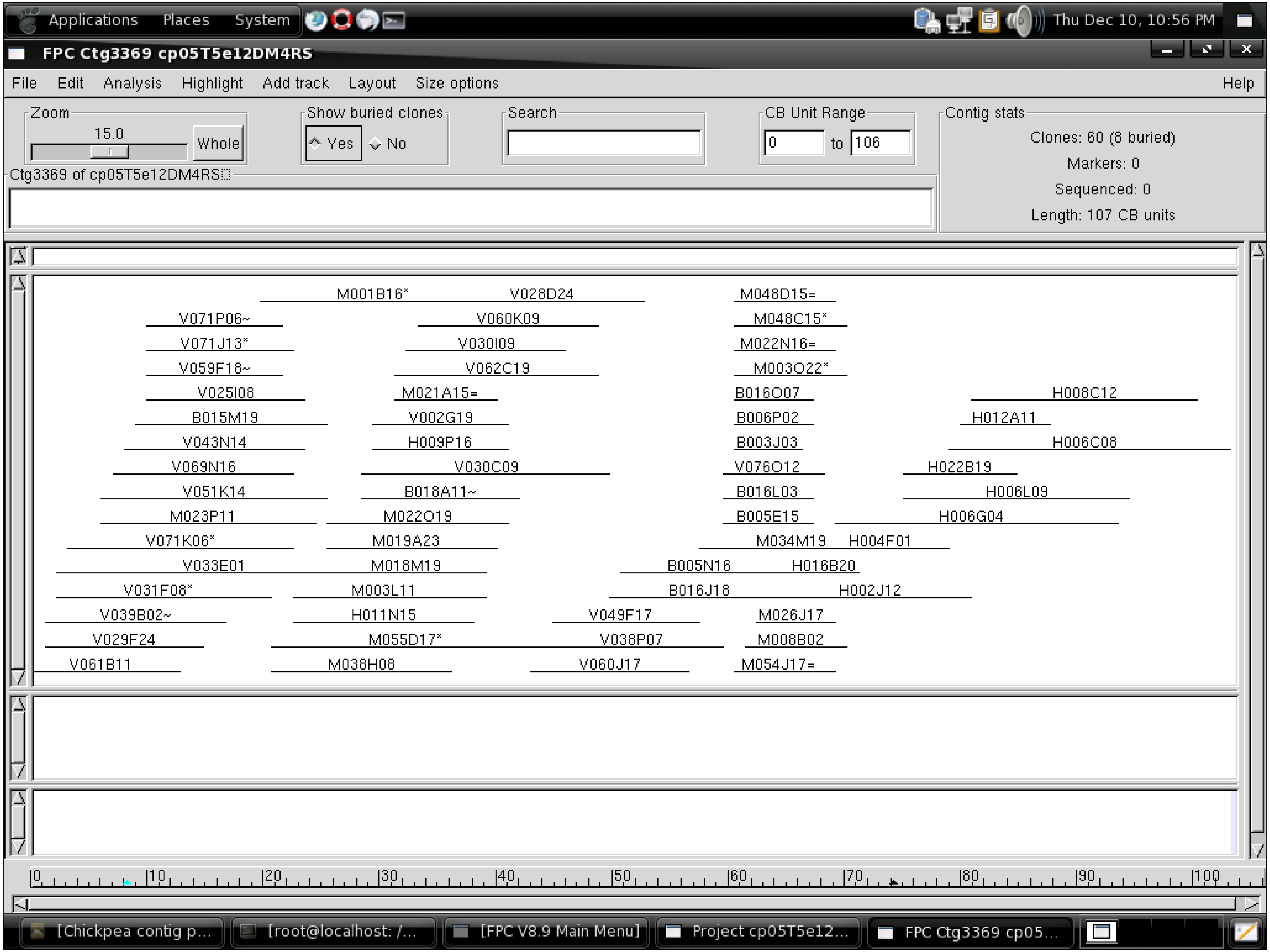
Applications
Places
System
Thu Dec 10, 10:56 PM
FPC Ctg3369 cp05T5e12DM4RS
File
Edit
Analysis
Highlight
Add track
Layout
Size options
Help
Zoom
15.0
Whole
Show buried clones
Yes
No
Search
CB Unit Range
0
to
106
Contig stats
Clones: 60 (8 buried)
Markers: 0
Sequenced: 0
Length: 107 CB units
Ctg3369 of cp05T5e12DM4RS
M001B16*
V028D24
M048D15=
V071P06~
V060K09
M048C15*
V071J13*
V030I09
M022N16=
V059F18~
V062C19
M003O22*
V025I08
M021A15=
B016O07
H008C12
B015M19
V002G19
B006P02
H012A11
V043N14
H009P16
B003J03
H006C08
V069N16
V030C09
V076O12
H022B19
V051K14
B018A11~
B016L03
H006L09
M023P11
M022O19
B005E15
H006G04
V071K06*
M019A23
M034M19
H004F01
V033E01
M018M19
B005N16
H016B20
V031F08*
M003L11
B016J18
H002J12
V039B02~
H011N15
V049F17
M026J17
V029F24
M055D17*
V038P07
M008B02
V061B11
M038H08
V060J17
M054J17=
0
10
20
30
40
50
60
70
80
90
100
[Chickpea contig p...
[root@localhost: /...
[FPC V8.9 Main Menu]
Project cp05T5e12...
FPC Ctg3369 cp05...

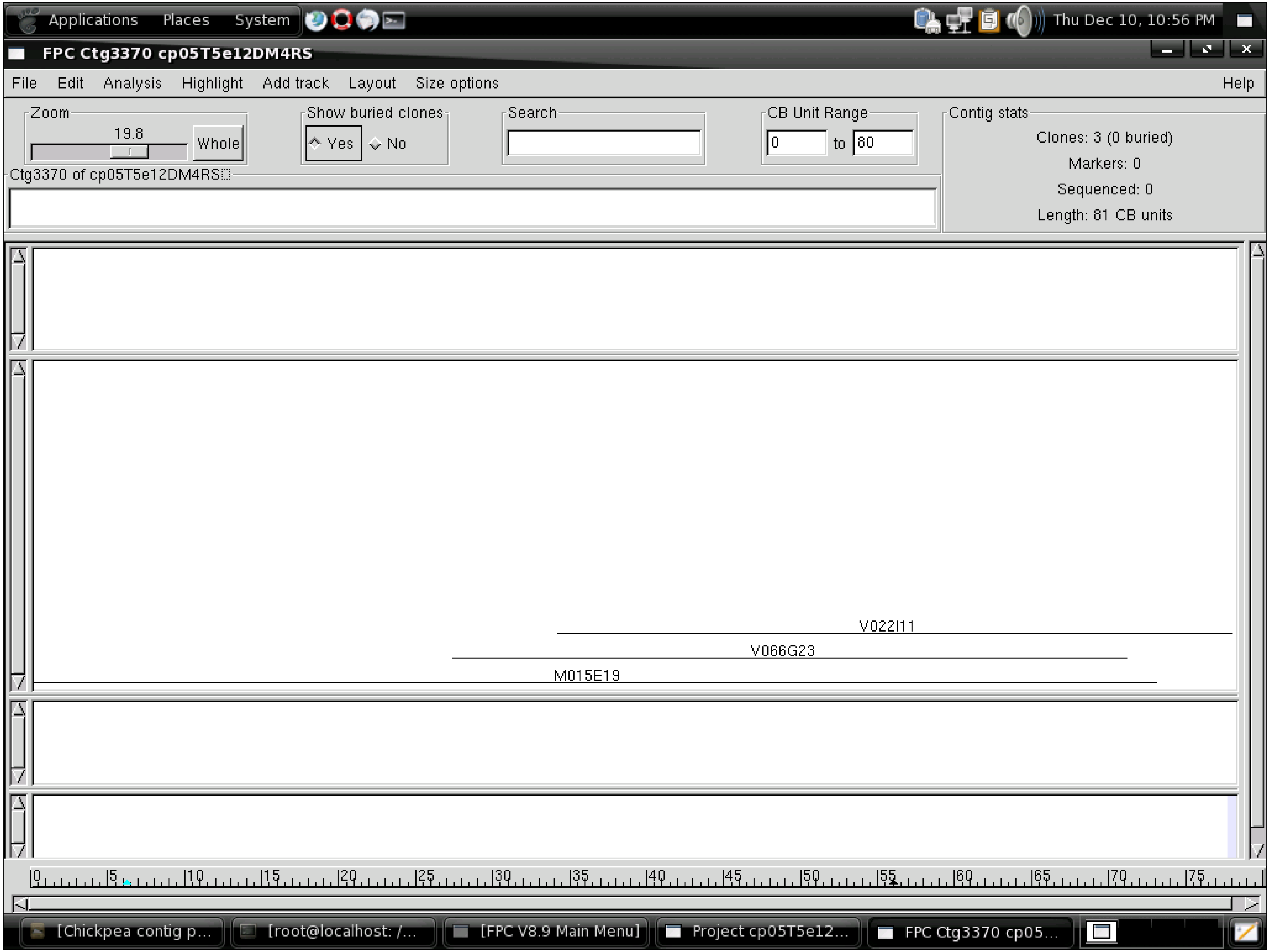
Applications
Places
System
Thu Dec 10, 10:56 PM
FPC Ctg3370 cp05T5e12DM4RS
File
Edit
Analysis
Highlight
Add track
Layout
Size options
Help
Zoom
19.8
Whole
Show buried clones
Yes
No
Search
CB Unit Range
0
to
80
Contig stats
Clones: 3 (0 buried)
Markers: 0
Sequenced: 0
Length: 81 CB units
Ctg3370 of cp05T5e12DM4RS
V022I11
V066G23
M015E19
0
5
10
15
20
25
30
35
40
45
50
55
60
65
70
75
[Chickpea contig p...
[root@localhost: /...
[FPC V8.9 Main Menu]
Project cp05T5e12...
FPC Ctg3370 cp05...

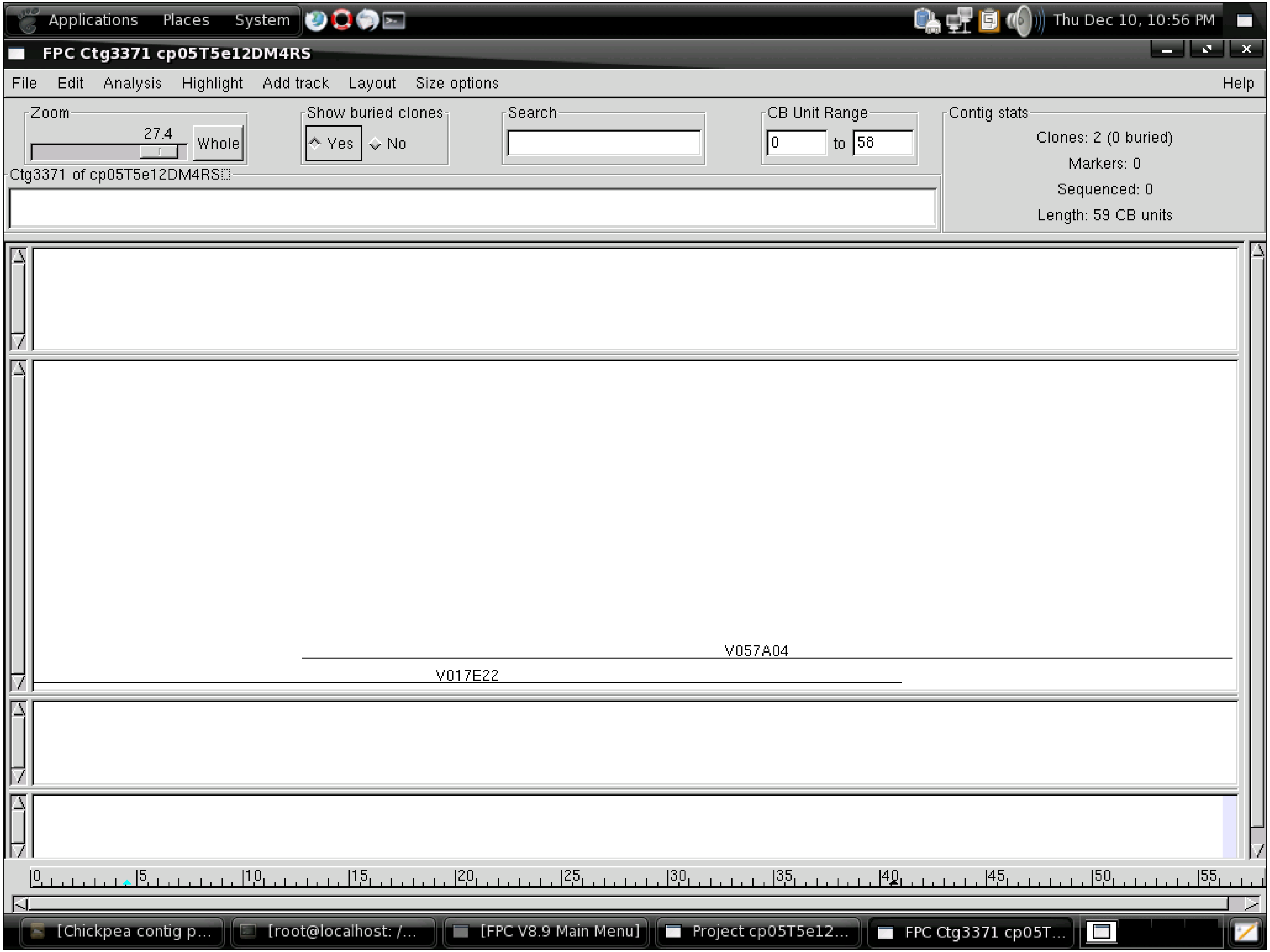
Applications Places System
Thu Dec 10, 10:56 PM
FPC Ctg3371 cp05T5e12DM4RS
File Edit Analysis Highlight Add track Layout Size options Help
Zoom
27.4
Whole
Show buried clones
Yes No
Search
CB Unit Range
0 to 58
Contig stats
Clones: 2 (0 buried)
Markers: 0
Sequenced: 0
Length: 59 CB units
Ctg3371 of cp05T5e12DM4RS
V057A04
V017E22
0 5 10 15 20 25 30 35 40 45 50 55
[Chickpea contig p...
[root@localhost: /...
[FPC V8.9 Main Menu]
Project cp05T5e12...
FPC Ctg3371 cp05T...

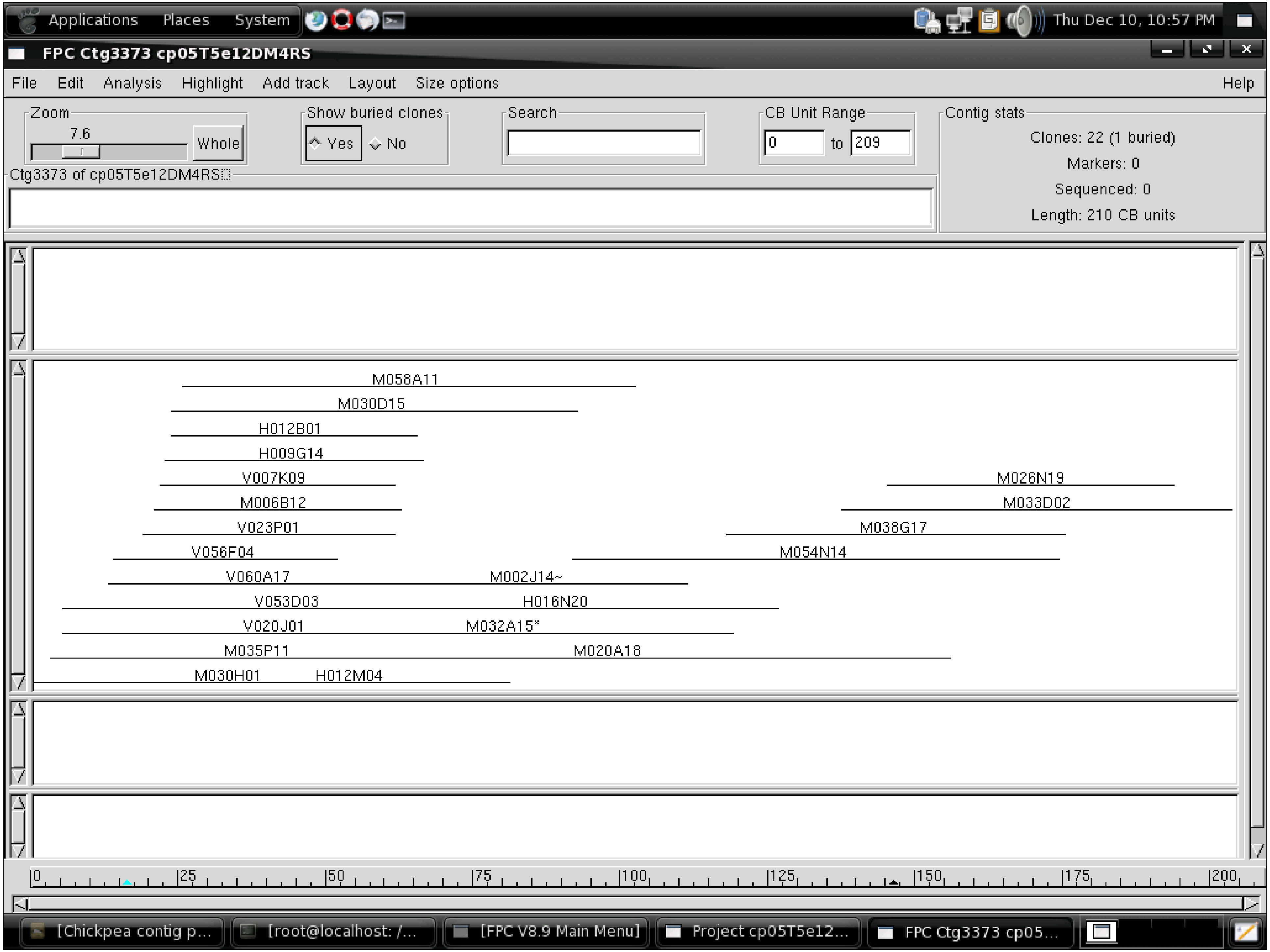
Applications Places System
Thu Dec 10, 10:57 PM
FPC Ctg3373 cp05T5e12DM4RS
File Edit Analysis Highlight Add track Layout Size options Help
Zoom
7.6
Whole
Show buried clones
Yes No
Search
CB Unit Range
0 to 209
Contig stats
Clones: 22 (1 buried)
Markers: 0
Sequenced: 0
Length: 210 CB units
Ctg3373 of cp05T5e12DM4RS
M058A11
M030D15
H012B01
H009G14
V007K09
M006B12
V023P01
V056F04
V060A17
V053D03
V020J01
M035P11
M030H01
H012M04
M026N19
M033D02
M038G17
M054N14
M002J14~
H016N20
M032A15*
M020A18
0 25 50 75 100 125 150 175 200
[Chickpea contig p...
[root@localhost: /...
[FPC V8.9 Main Menu]
Project cp05T5e12...
FPC Ctg3373 cp05...

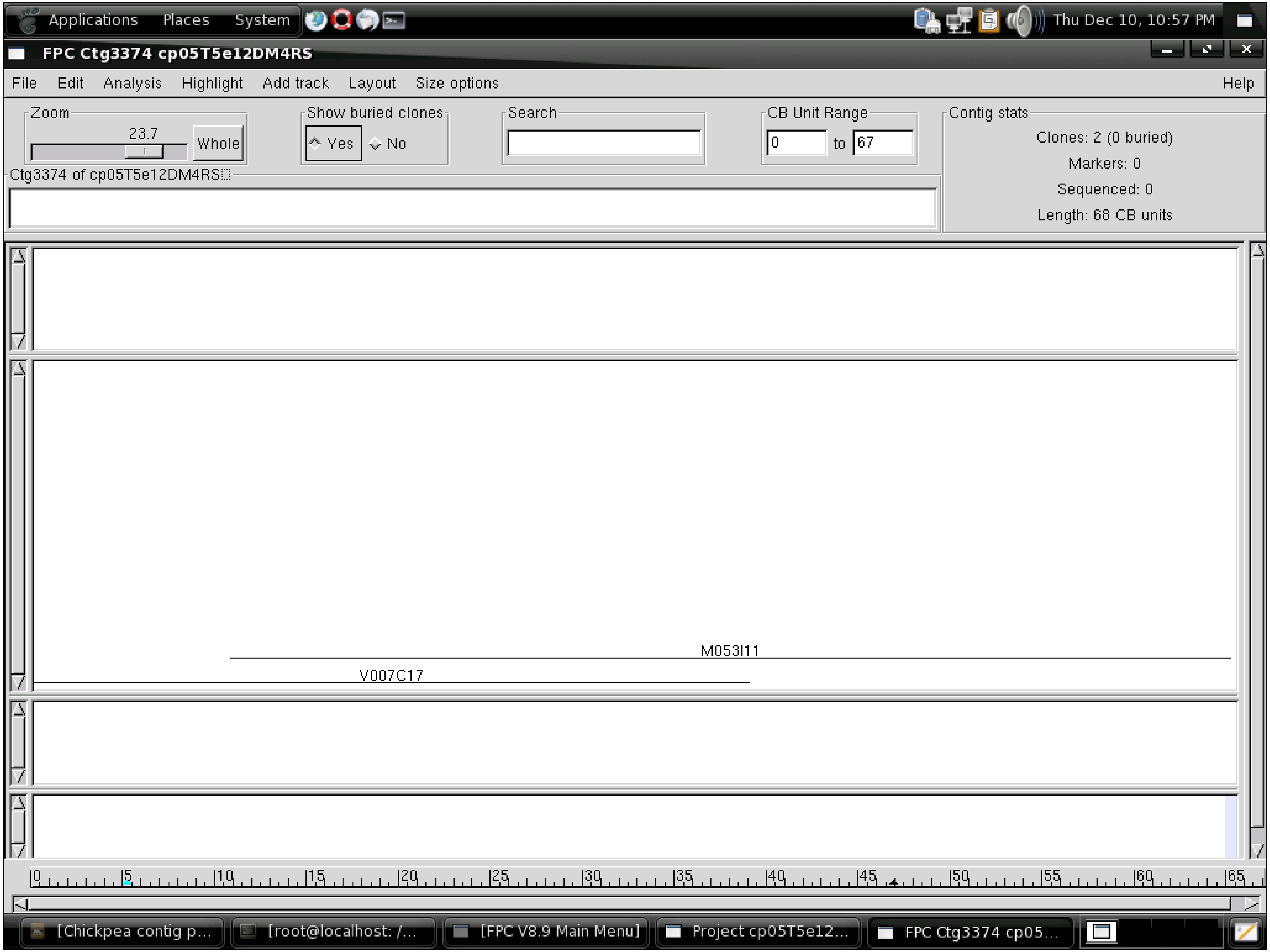
Applications
Places
System
Thu Dec 10, 10:57 PM
FPC Ctg3374 cp05T5e12DM4RS
File
Edit
Analysis
Highlight
Add track
Layout
Size options
Help
Zoom
23.7
Whole
Show buried clones
Yes
No
Search
CB Unit Range
0
to
67
Contig stats
Clones: 2 (0 buried)
Markers: 0
Sequenced: 0
Length: 68 CB units
Ctg3374 of cp05T5e12DM4RS
M053I11
V007C17
0
5
10
15
20
25
30
35
40
45
50
55
60
65
[Chickpea contig p...
[root@localhost: /...
[FPC V8.9 Main Menu]
Project cp05T5e12...
FPC Ctg3374 cp05...

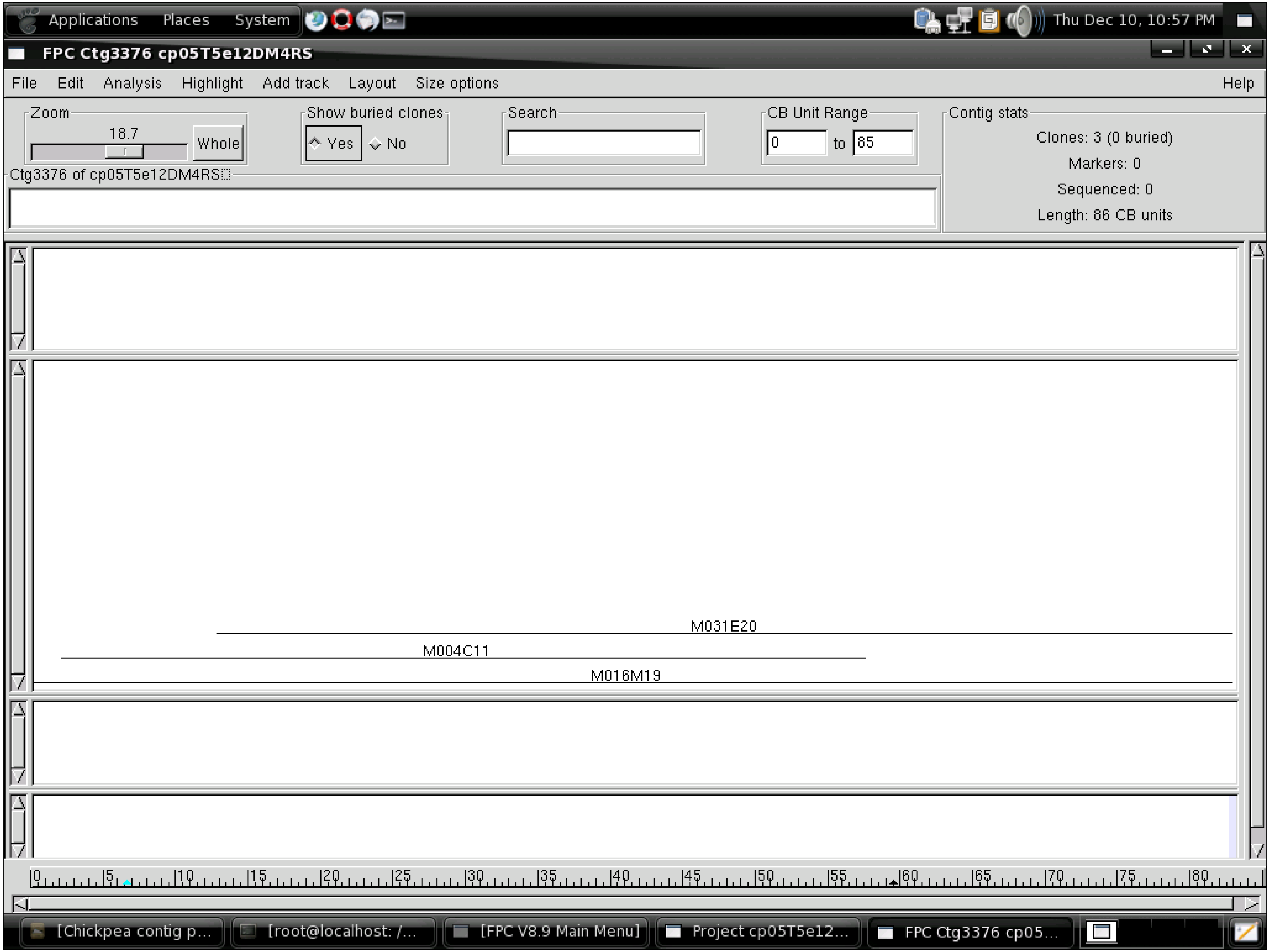
Applications Places System
Thu Dec 10, 10:57 PM
FPC Ctg3376 cp05T5e12DM4RS
File Edit Analysis Highlight Add track Layout Size options Help
Zoom
18.7
Whole
Show buried clones
Yes No
Search
CB Unit Range
0 to 85
Contig stats
Clones: 3 (0 buried)
Markers: 0
Sequenced: 0
Length: 86 CB units
Ctg3376 of cp05T5e12DM4RS
M031E20
M004C11
M016M19
0 5 10 15 20 25 30 35 40 45 50 55 60 65 70 75 80
[Chickpea contig p...
[root@localhost: /...
[FPC V8.9 Main Menu]
Project cp05T5e12...
FPC Ctg3376 cp05...

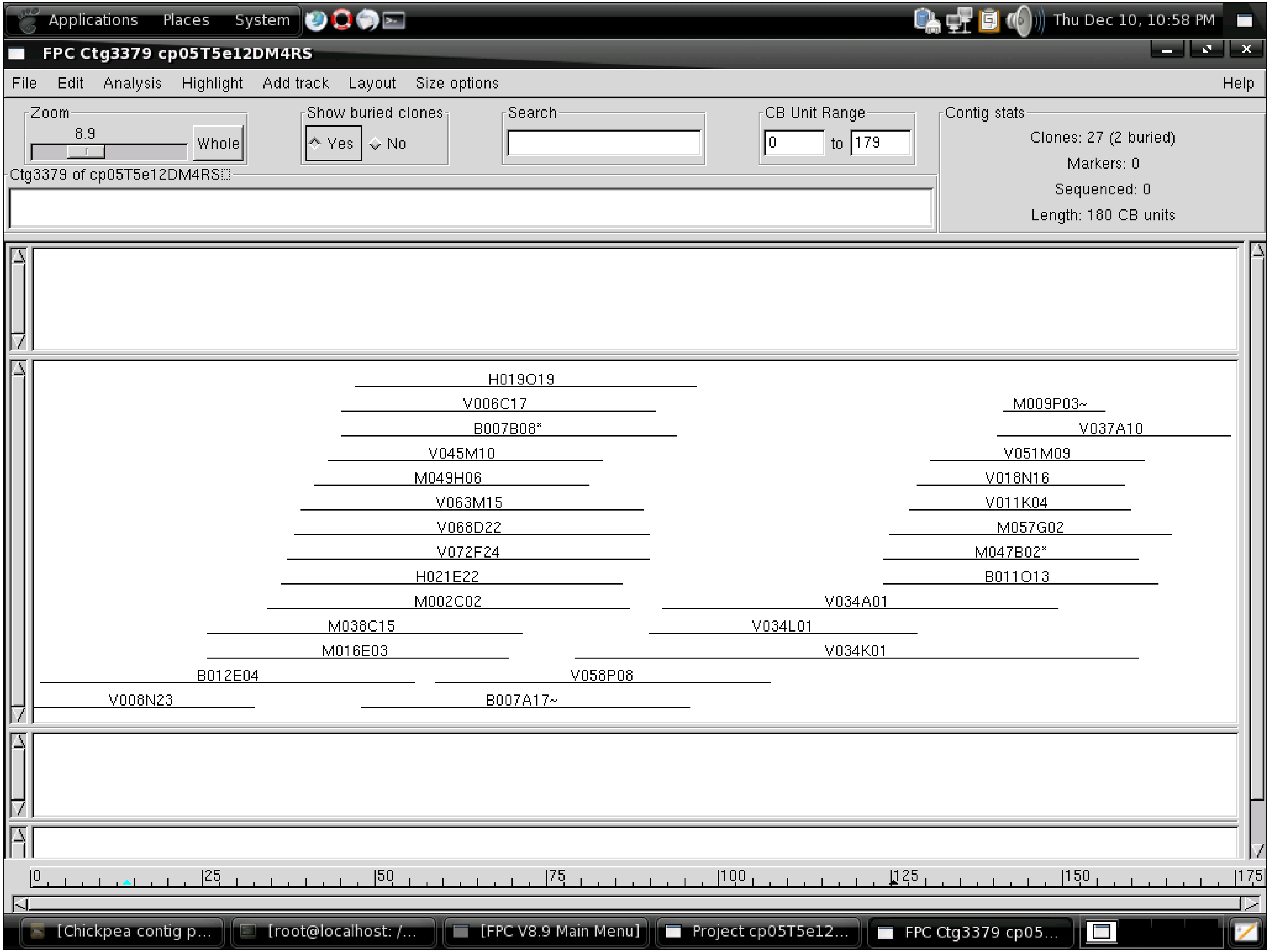

Applications Places System
Thu Dec 10, 10:58 PM
FPC Ctg3379 cp05T5e12DM4RS
File Edit Analysis Highlight Add track Layout Size options Help
Zoom
8.9
Whole
Show buried clones
Yes
No
Search
CB Unit Range
0 to 179
Contig stats
Clones: 27 (2 buried)
Markers: 0
Sequenced: 0
Length: 180 CB units
Ctg3379 of cp05T5e12DM4RS
H019O19
V006C17
M009P03~
B007B08*
V037A10
V045M10
V051M09
M049H06
V018N16
V063M15
V011K04
V068D22
M057G02
V072F24
M047B02*
H021E22
B011O13
M002C02
V034A01
M038C15
V034L01
M016E03
V034K01
B012E04
V058P08
V008N23
B007A17~
0 25 50 75 100 125 150 175
[Chickpea contig p...
[root@localhost: /...
[FPC V8.9 Main Menu]
Project cp05T5e12...
FPC Ctg3379 cp05...

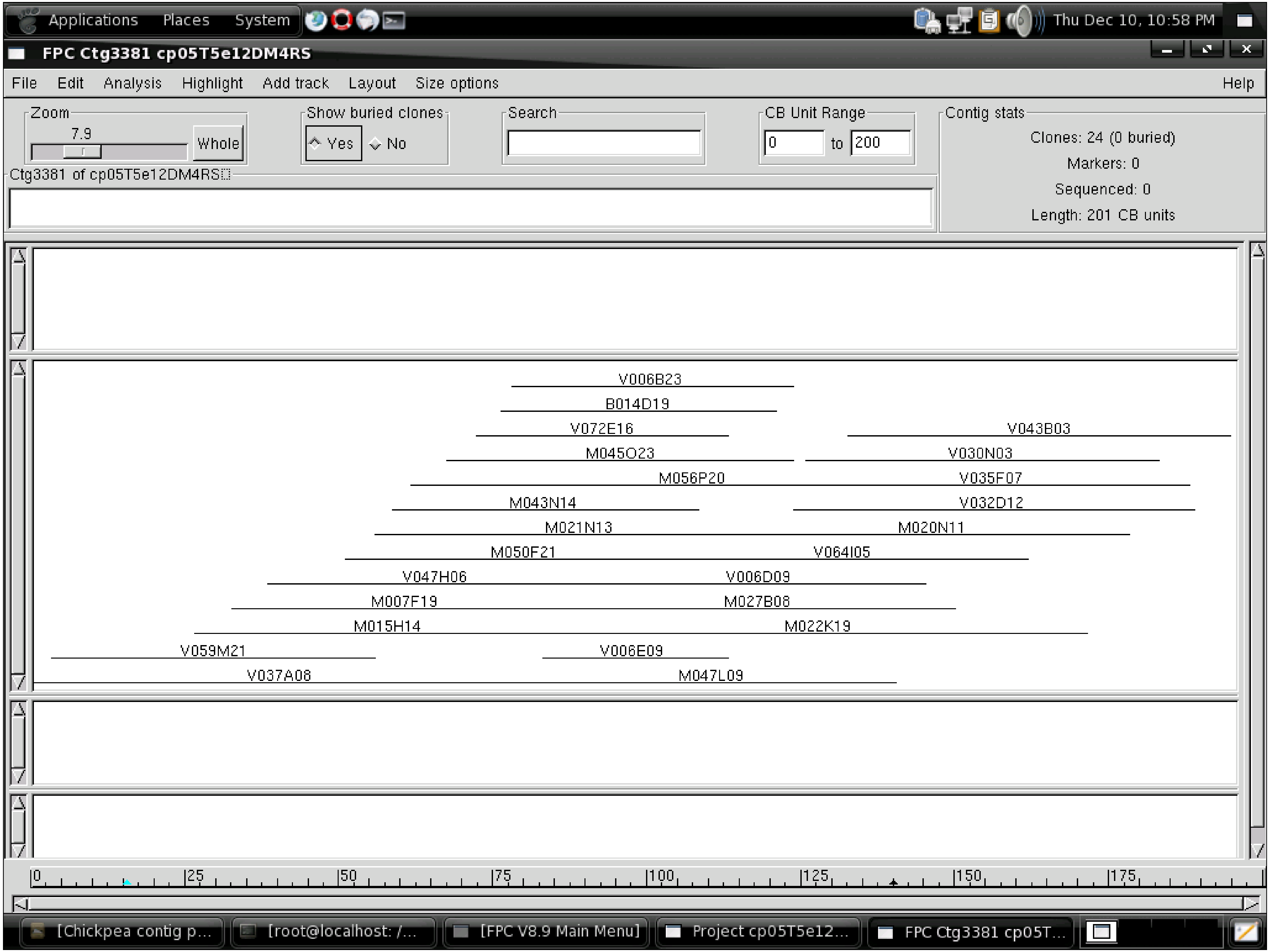
Applications Places System
Thu Dec 10, 10:58 PM
FPC Ctg3381 cp05T5e12DM4RS
File Edit Analysis Highlight Add track Layout Size options Help
Zoom
7.9
Whole
Show buried clones
Yes No
Search
CB Unit Range
0 to 200
Contig stats
Clones: 24 (0 buried)
Markers: 0
Sequenced: 0
Length: 201 CB units
Ctg3381 of cp05T5e12DM4RS
V006B23
B014D19
V072E16
V043B03
M045O23
V030N03
M056P20
V035F07
M043N14
V032D12
M021N13
M020N11
M050F21
V064I05
V047H06
V006D09
M007F19
M027B08
M015H14
M022K19
V059M21
V006E09
V037A08
M047L09
0 25 50 75 100 125 150 175
[Chickpea contig p...
[root@localhost: /...
[FPC V8.9 Main Menu]
Project cp05T5e12...
FPC Ctg3381 cp05T...

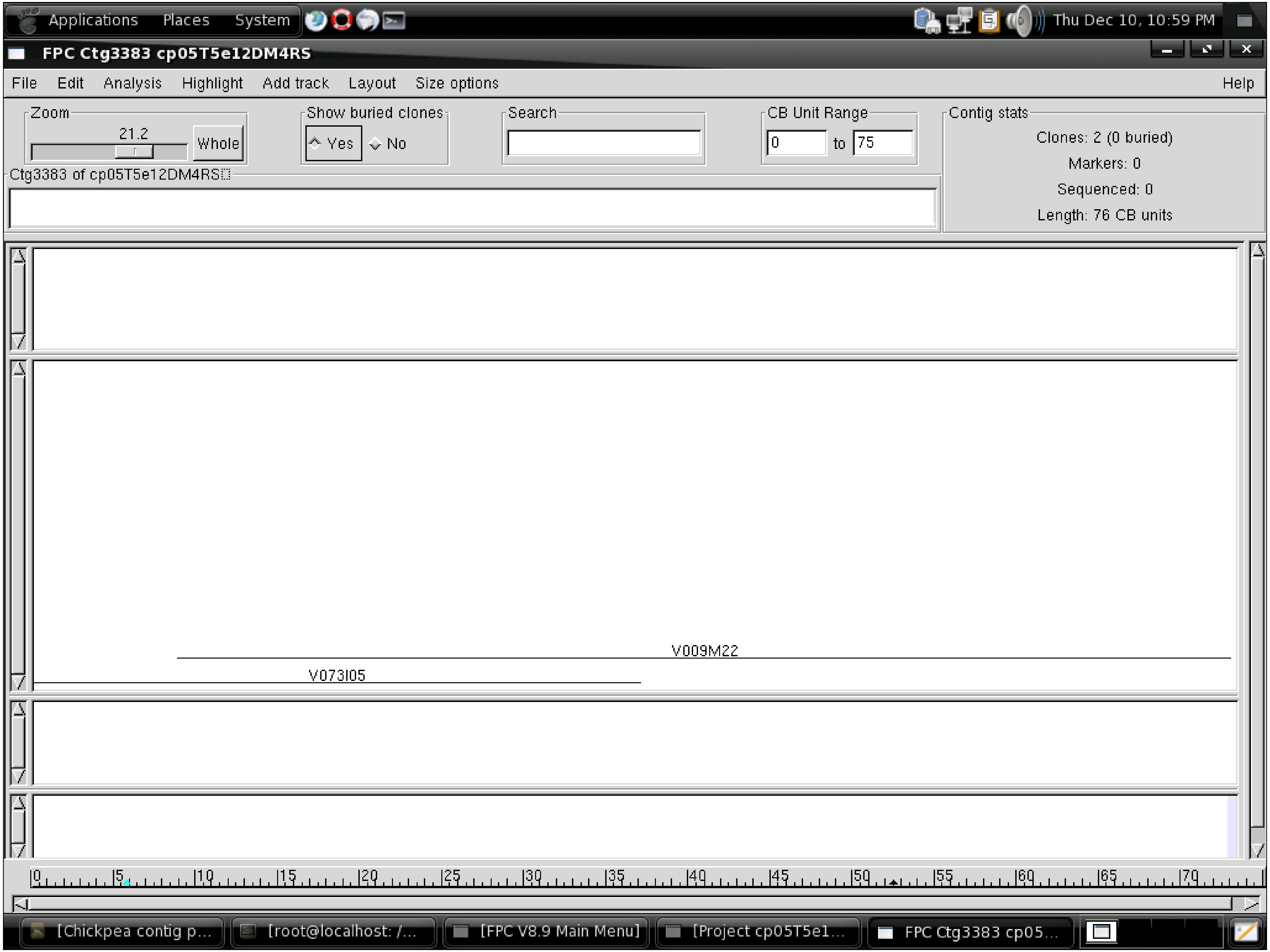
Applications Places System
Thu Dec 10, 10:59 PM
FPC Ctg3383 cp05T5e12DM4RS
File Edit Analysis Highlight Add track Layout Size options Help
Zoom
21.2
Whole
Show buried clones
Yes No
Search
CB Unit Range
0 to 75
Contig stats
Clones: 2 (0 buried)
Markers: 0
Sequenced: 0
Length: 76 CB units
Ctg3383 of cp05T5e12DM4RS
V009M22
V073I05
0 5 10 15 20 25 30 35 40 45 50 55 60 65 70
[Chickpea contig p...
[root@localhost: /...
[FPC V8.9 Main Menu]
[Project cp05T5e1...
FPC Ctg3383 cp05...

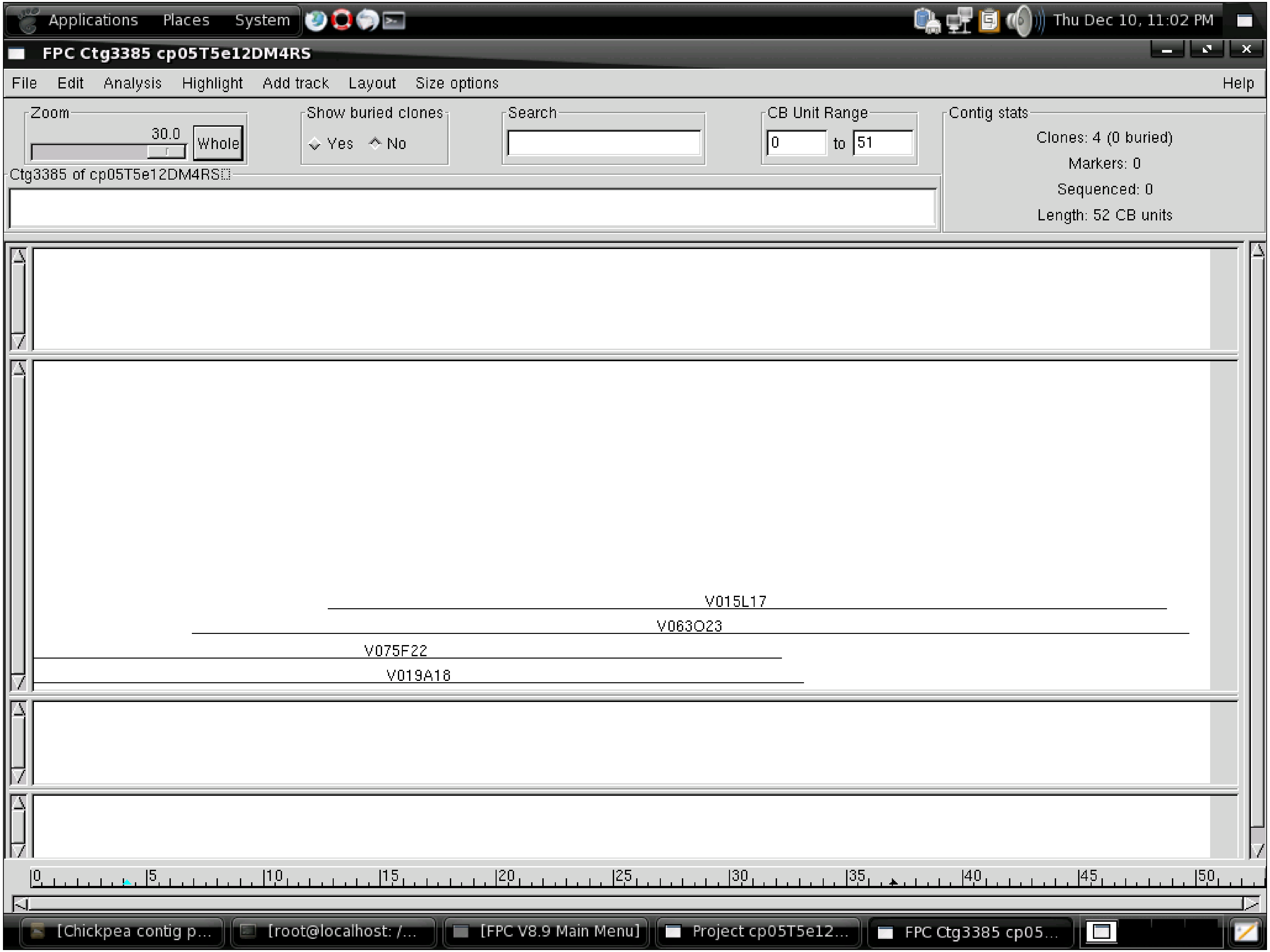
Applications
Places
System
Thu Dec 10, 11:02 PM
FPC Ctg3385 cp05T5e12DM4RS
File
Edit
Analysis
Highlight
Add track
Layout
Size options
Help
Zoom
30.0
Whole
Show buried clones
Yes
No
Search
CB Unit Range
0
to
51
Contig stats
Clones: 4 (0 buried)
Markers: 0
Sequenced: 0
Length: 52 CB units
Ctg3385 of cp05T5e12DM4RS
V015L17
V063O23
V075F22
V019A18
0
5
10
15
20
25
30
35
40
45
50
[Chickpea contig p...
[root@localhost: /...
[FPC V8.9 Main Menu]
Project cp05T5e12...
FPC Ctg3385 cp05...

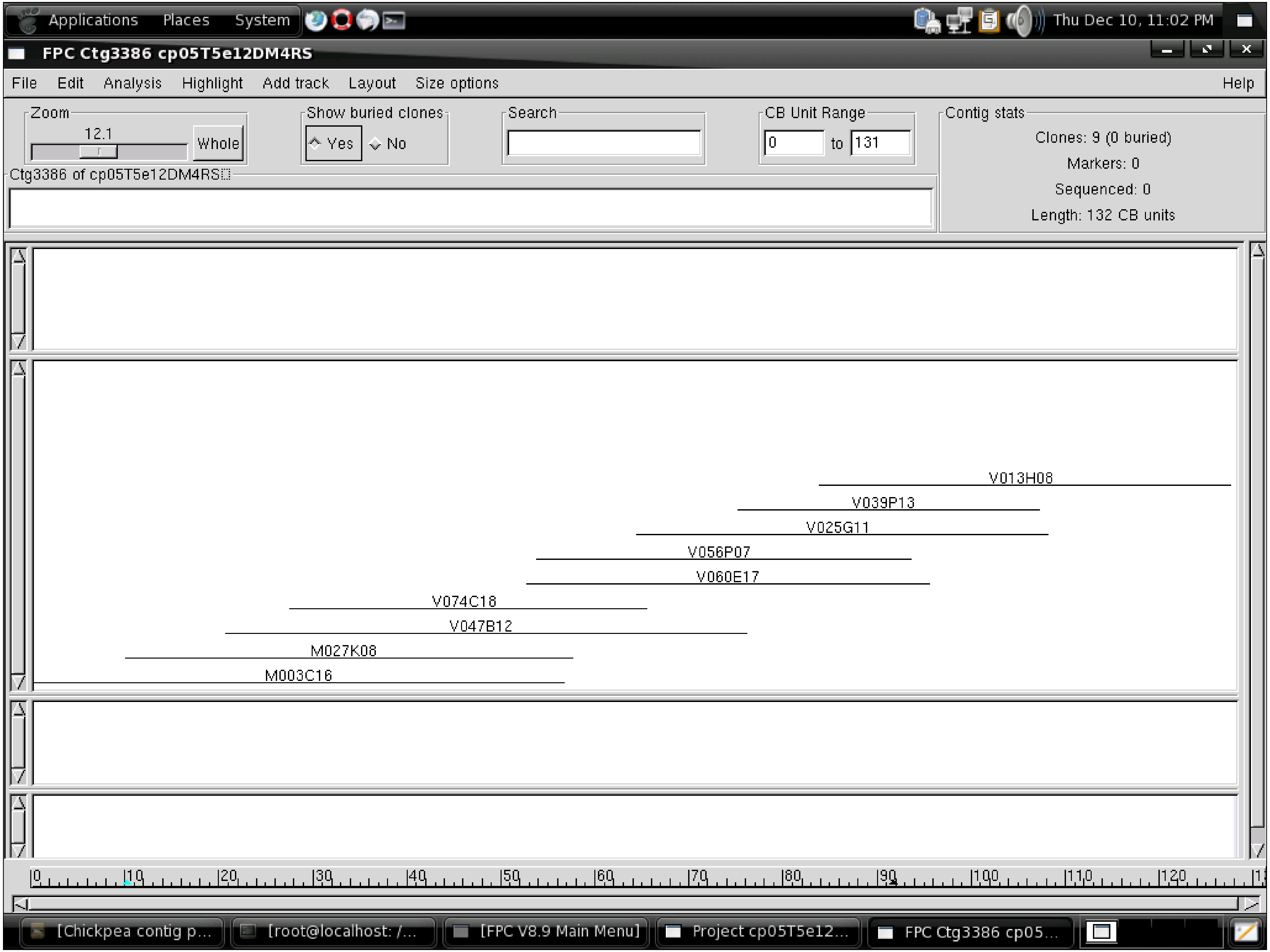
Applications Places System
Thu Dec 10, 11:02 PM
FPC Ctg3386 cp05T5e12DM4RS
File Edit Analysis Highlight Add track Layout Size options Help
Zoom
12.1
Whole
Show buried clones
Yes No
Search
CB Unit Range
0 to 131
Contig stats
Clones: 9 (0 buried)
Markers: 0
Sequenced: 0
Length: 132 CB units
Ctg3386 of cp05T5e12DM4RS
V013H08
V039P13
V025G11
V056P07
V060E17
V074C18
V047B12
M027K08
M003C16
0 10 20 30 40 50 60 70 80 90 100 110 120
[Chickpea contig p...
[root@localhost: /...
[FPC V8.9 Main Menu]
Project cp05T5e12...
FPC Ctg3386 cp05...

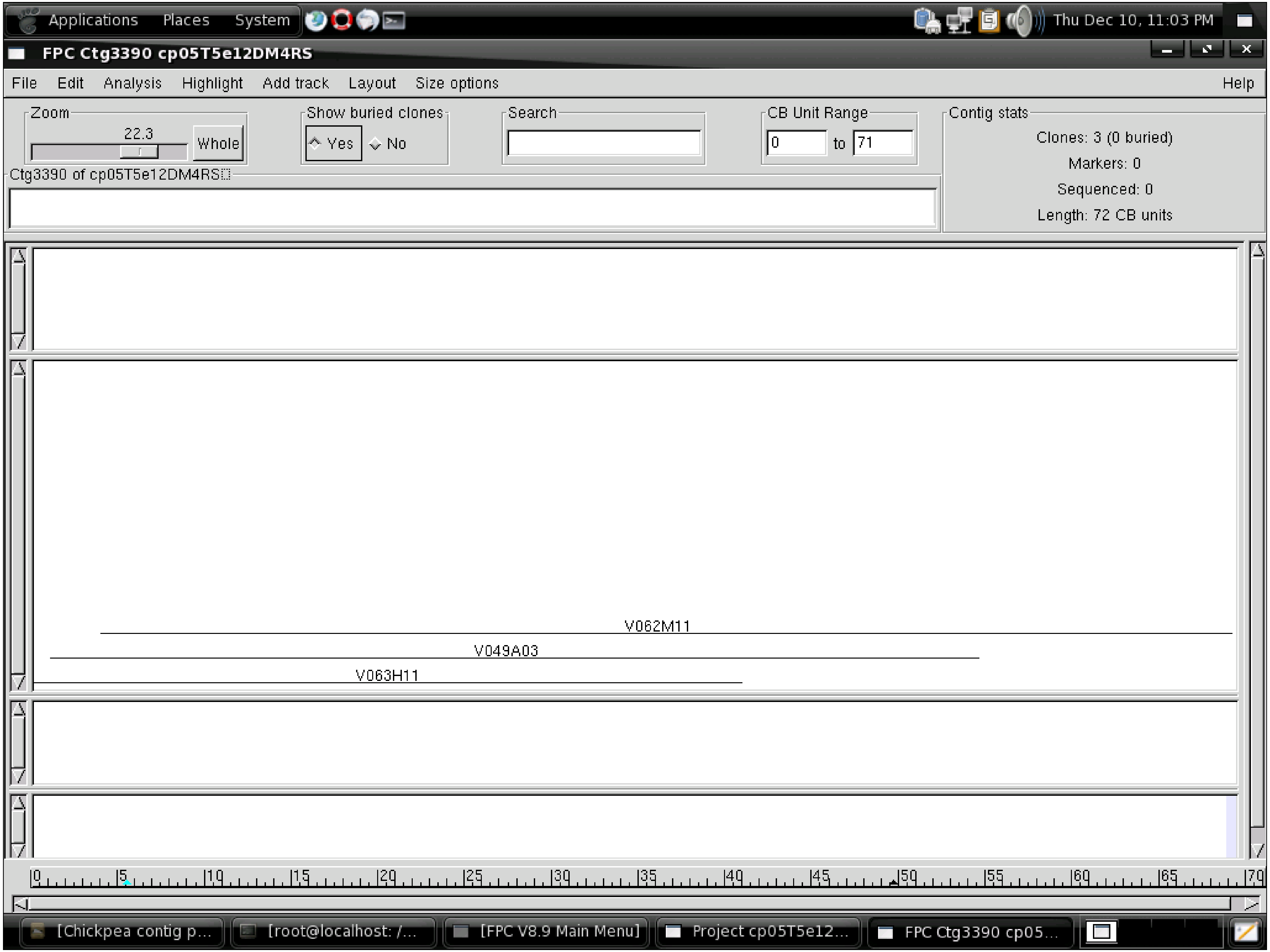
Applications Places System
Thu Dec 10, 11:03 PM
FPC Ctg3390 cp05T5e12DM4RS
File Edit Analysis Highlight Add track Layout Size options Help
Zoom
22.3
Whole
Show buried clones
Yes
No
Search
CB Unit Range
0
to
71
Contig stats
Clones: 3 (0 buried)
Markers: 0
Sequenced: 0
Length: 72 CB units
Ctg3390 of cp05T5e12DM4RS
V062M11
V049A03
V063H11
0 5 10 15 20 25 30 35 40 45 50 55 60 65 70
[Chickpea contig p...
[root@localhost: /...
[FPC V8.9 Main Menu]
Project cp05T5e12...
FPC Ctg3390 cp05...

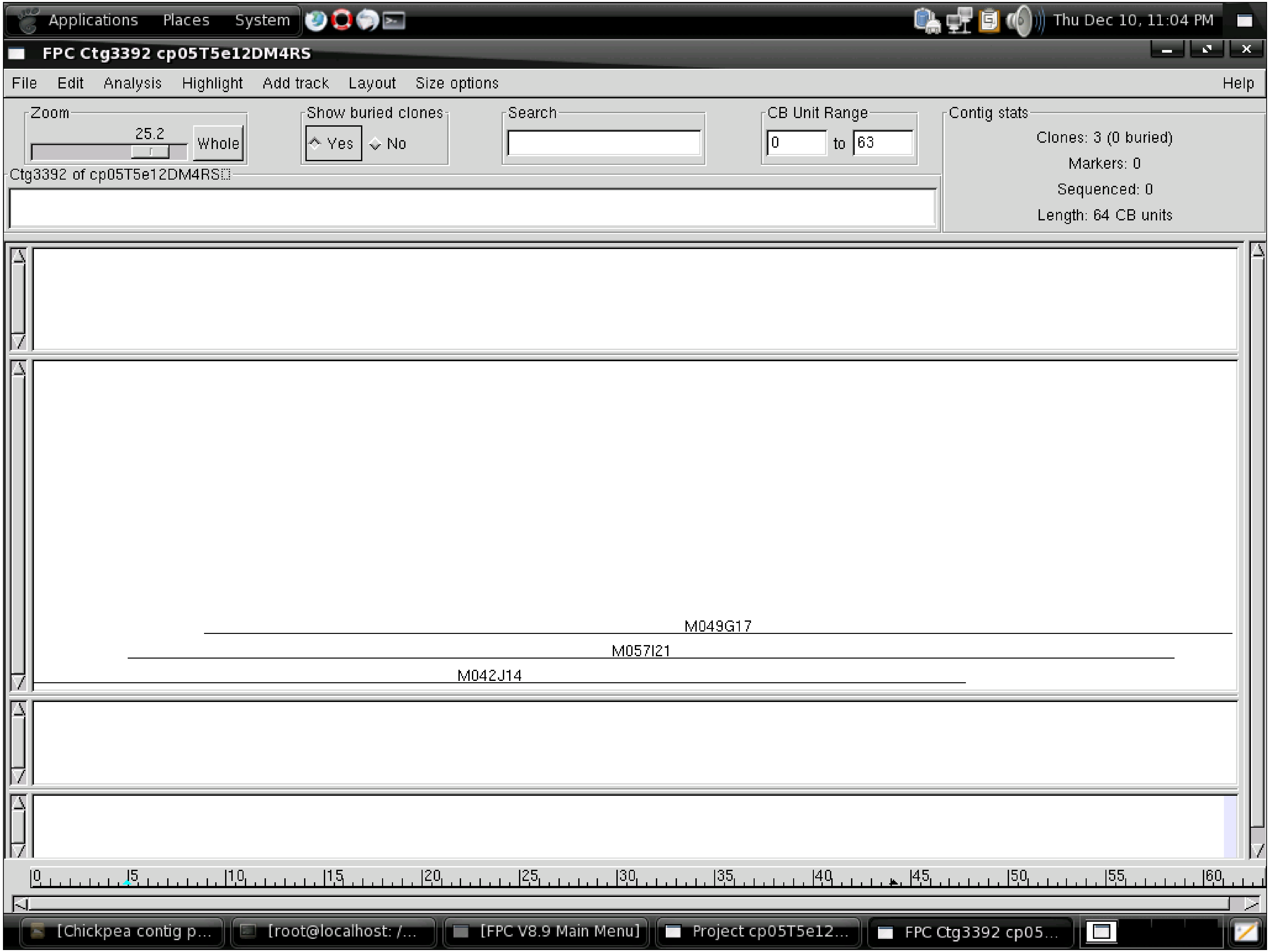
Applications
Places
System
Thu Dec 10, 11:04 PM
FPC Ctg3392 cp05T5e12DM4RS
File
Edit
Analysis
Highlight
Add track
Layout
Size options
Help
Zoom
25.2
Whole
Show buried clones
Yes
No
Search
CB Unit Range
0
to
63
Contig stats
Clones: 3 (0 buried)
Markers: 0
Sequenced: 0
Length: 64 CB units
Ctg3392 of cp05T5e12DM4RS
M049G17
M057I21
M042J14
0 5 10 15 20 25 30 35 40 45 50 55 60
[Chickpea contig p...
[root@localhost: /...
[FPC V8.9 Main Menu]
Project cp05T5e12...
FPC Ctg3392 cp05...

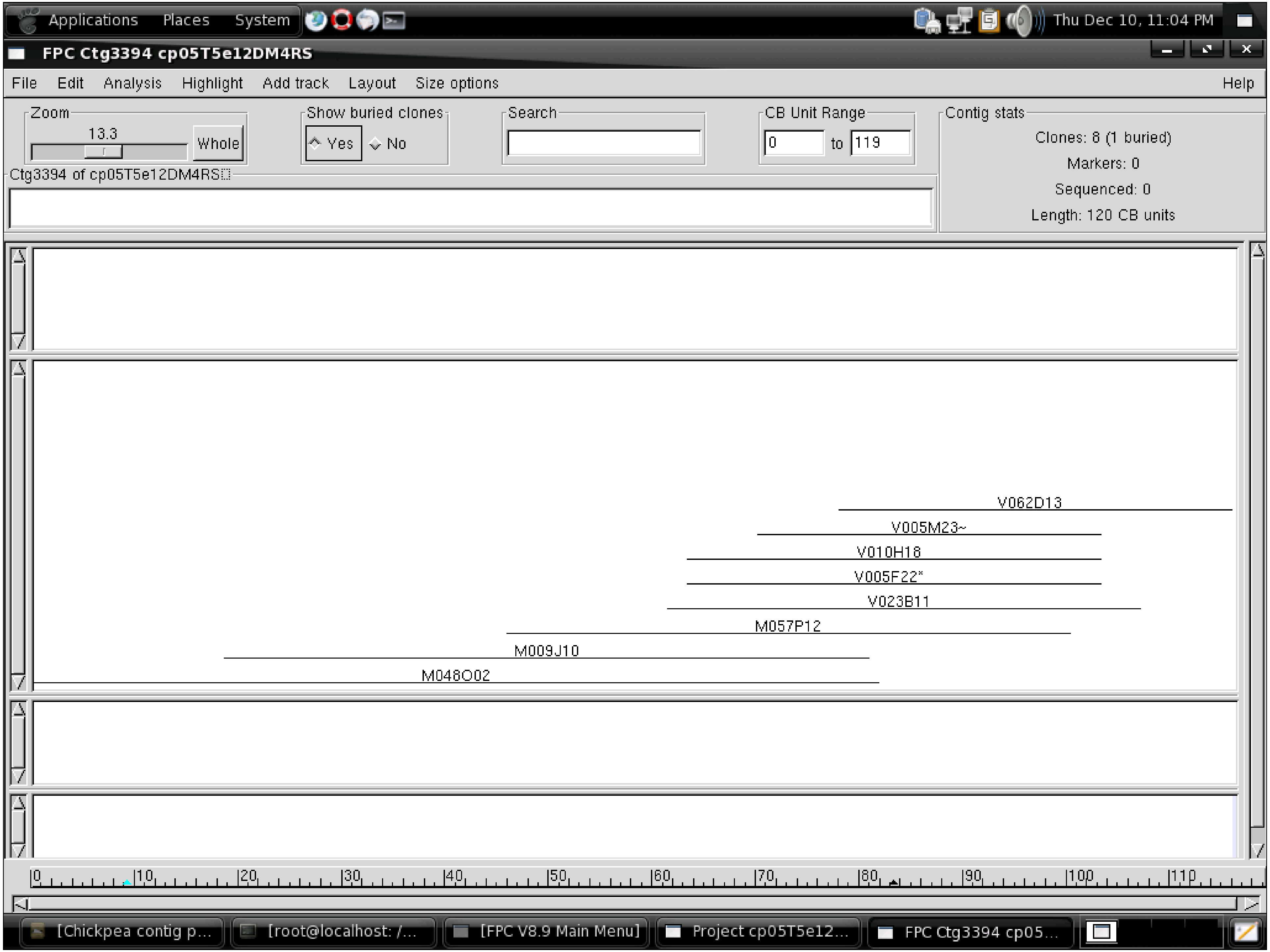

Applications
Places
System
Thu Dec 10, 11:04 PM
FPC Ctg3394 cp05T5e12DM4RS
File
Edit
Analysis
Highlight
Add track
Layout
Size options
Help
Zoom
13.3
Whole
Show buried clones
Yes
No
Search
CB Unit Range
0
to
119
Contig stats
Clones: 8 (1 buried)
Markers: 0
Sequenced: 0
Length: 120 CB units
Ctg3394 of cp05T5e12DM4RS
V062D13
V005M23~
V010H18
V005F22*
V023B11
M057P12
M009J10
M048O02
0 10 20 30 40 50 60 70 80 90 100 110
[Chickpea contig p...
[root@localhost: /...
[FPC V8.9 Main Menu]
Project cp05T5e12...
FPC Ctg3394 cp05...

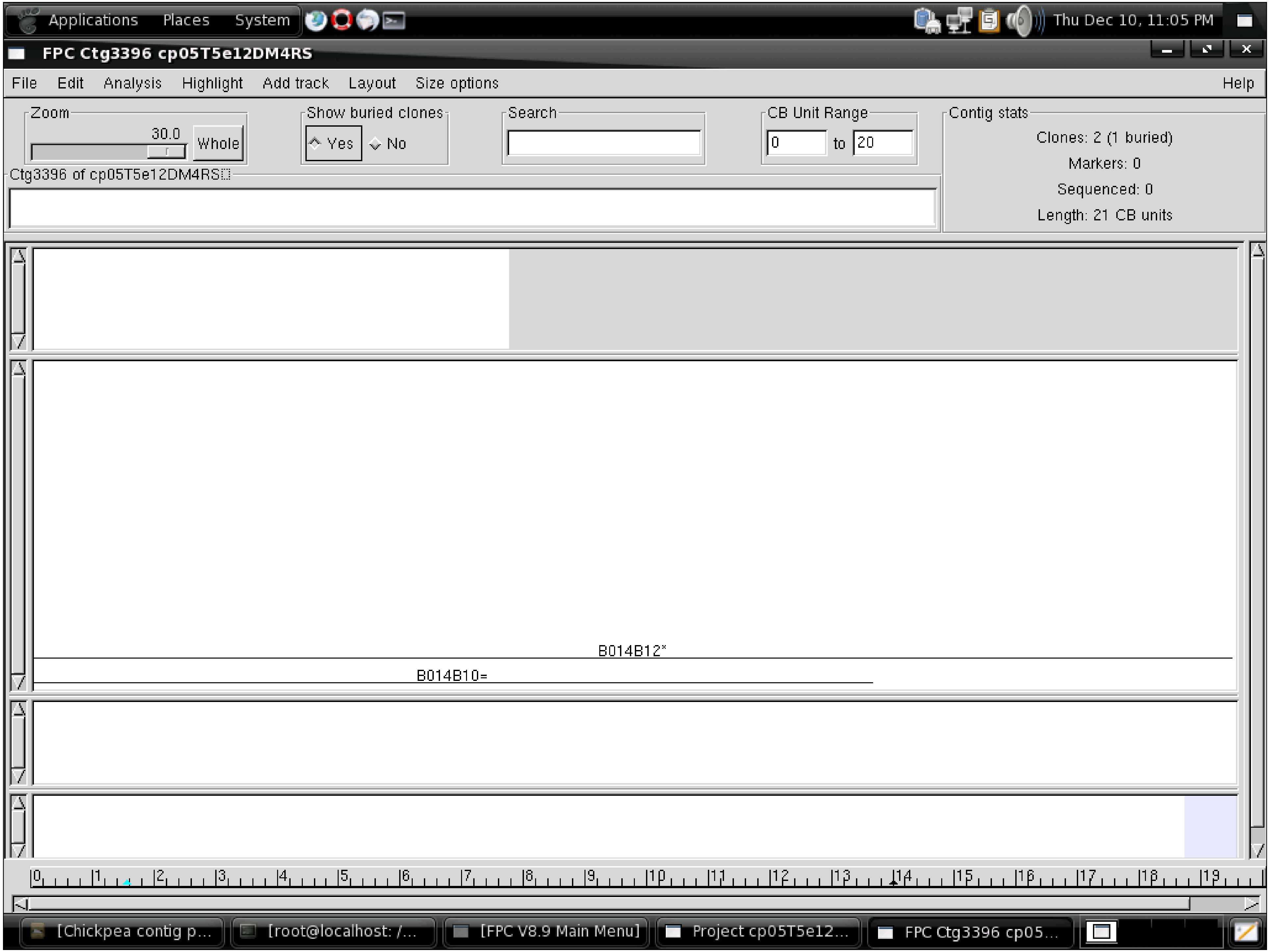
Applications Places System
Thu Dec 10, 11:05 PM
FPC Ctg3396 cp05T5e12DM4RS
File Edit Analysis Highlight Add track Layout Size options Help
Zoom
30.0
Whole
Show buried clones
Yes No
Search
CB Unit Range
0 to 20
Contig stats
Clones: 2 (1 buried)
Markers: 0
Sequenced: 0
Length: 21 CB units
Ctg3396 of cp05T5e12DM4RS
B014B12*
B014B10=
0 1 2 3 4 5 6 7 8 9 10 11 12 13 14 15 16 17 18 19
[Chickpea contig p...
[root@localhost: /...
[FPC V8.9 Main Menu]
Project cp05T5e12...
FPC Ctg3396 cp05...

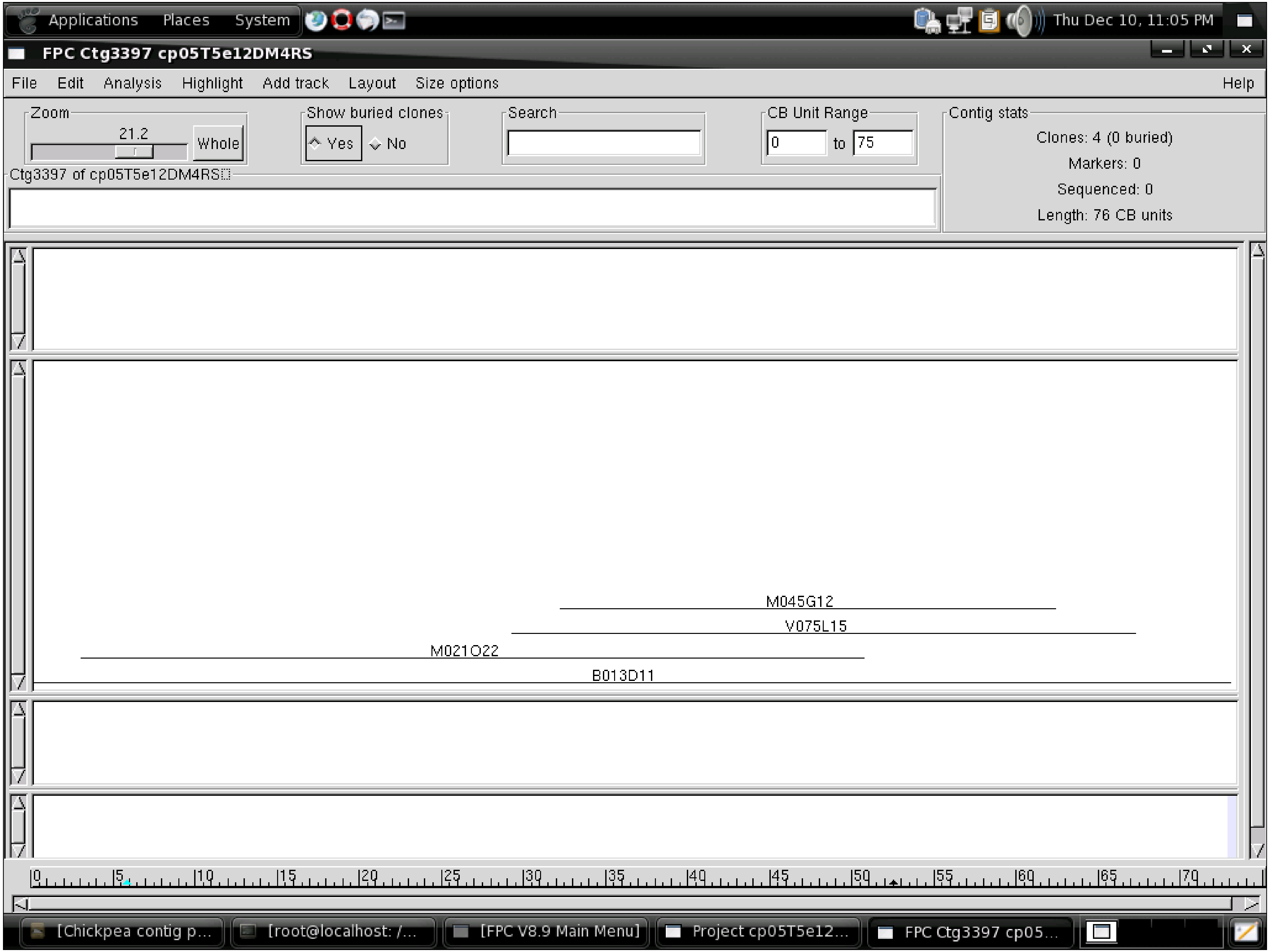
Applications Places System
Thu Dec 10, 11:05 PM
FPC Ctg3397 cp05T5e12DM4RS
File Edit Analysis Highlight Add track Layout Size options Help
Zoom
21.2
Whole
Show buried clones
Yes No
Search
CB Unit Range
0 to 75
Contig stats
Clones: 4 (0 buried)
Markers: 0
Sequenced: 0
Length: 76 CB units
Ctg3397 of cp05T5e12DM4RS
M045G12
V075L15
M021O22
B013D11
0 5 10 15 20 25 30 35 40 45 50 55 60 65 70
[Chickpea contig p...
[root@localhost: /...
[FPC V8.9 Main Menu]
Project cp05T5e12...
FPC Ctg3397 cp05...

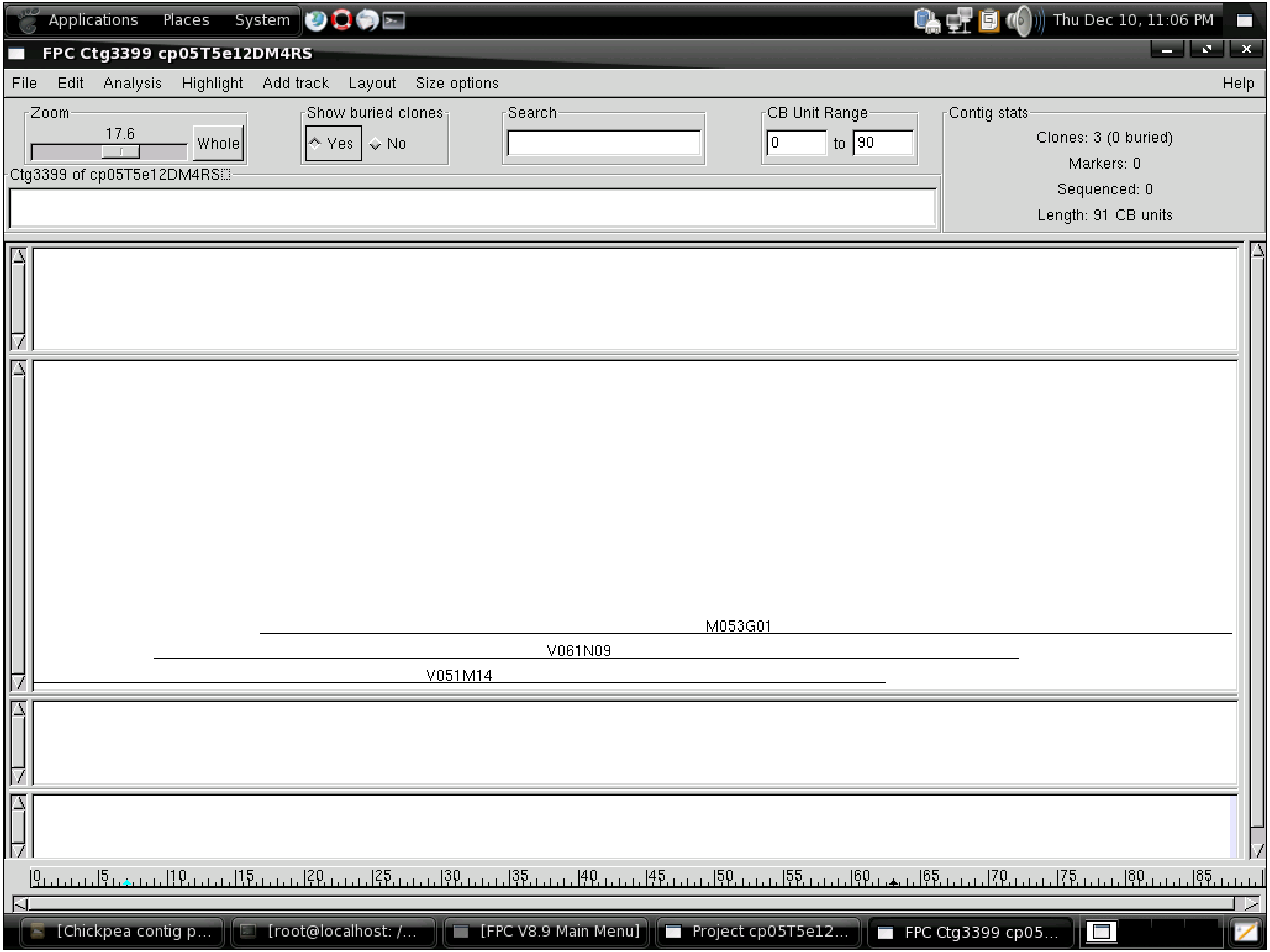
Applications Places System
Thu Dec 10, 11:06 PM
FPC Ctg3399 cp05T5e12DM4RS
File Edit Analysis Highlight Add track Layout Size options Help
Zoom
17.6
Whole
Show buried clones
Yes No
Search
CB Unit Range
0 to 90
Contig stats
Clones: 3 (0 buried)
Markers: 0
Sequenced: 0
Length: 91 CB units
Ctg3399 of cp05T5e12DM4RS
M053G01
V061N09
V051M14
0 5 10 15 20 25 30 35 40 45 50 55 60 65 70 75 80 85
[Chickpea contig p...
[root@localhost: /...
[FPC V8.9 Main Menu]
Project cp05T5e12...
FPC Ctg3399 cp05...

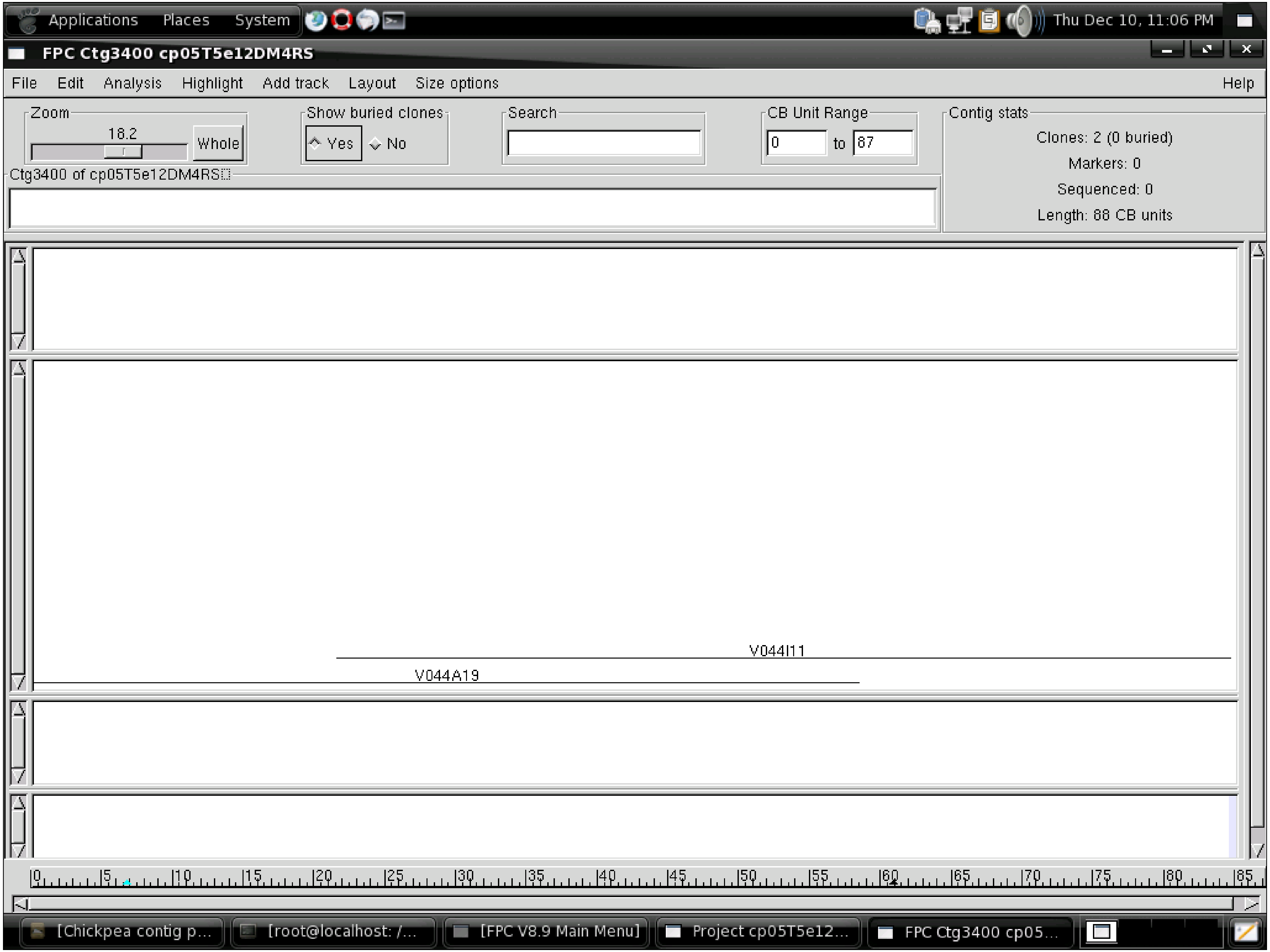
Applications Places System
Thu Dec 10, 11:06 PM
FPC Ctg3400 cp05T5e12DM4RS
File Edit Analysis Highlight Add track Layout Size options Help
Zoom
18.2
Whole
Show buried clones
Yes No
Search
CB Unit Range
0 to 87
Contig stats
Clones: 2 (0 buried)
Markers: 0
Sequenced: 0
Length: 88 CB units
Ctg3400 of cp05T5e12DM4RS
V044I11
V044A19
0 5 10 15 20 25 30 35 40 45 50 55 60 65 70 75 80 85
[Chickpea contig p...
[root@localhost: /...
[FPC V8.9 Main Menu]
Project cp05T5e12...
FPC Ctg3400 cp05...

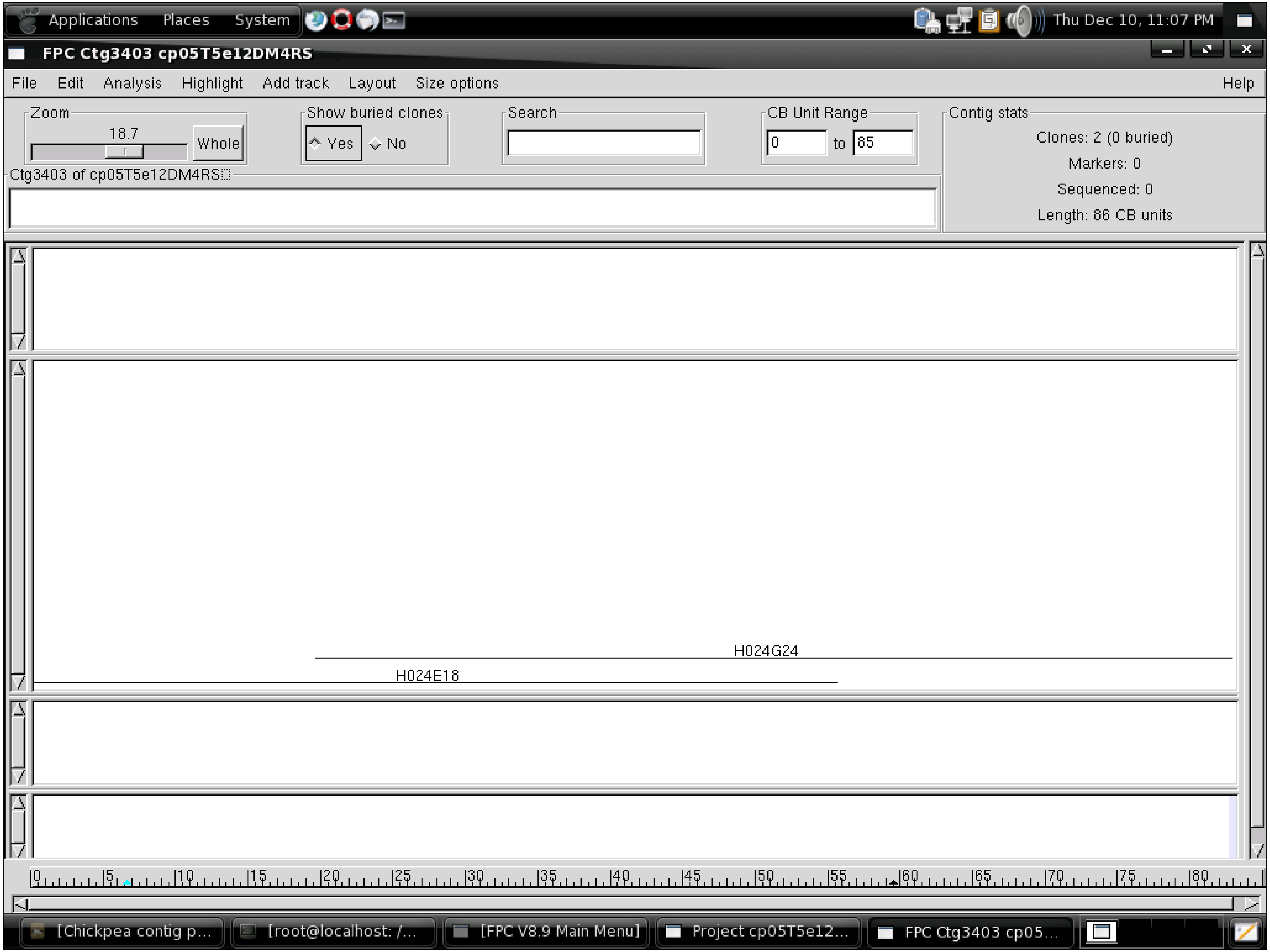
Applications
Places
System
Thu Dec 10, 11:07 PM
FPC Ctg3403 cp05T5e12DM4RS
File
Edit
Analysis
Highlight
Add track
Layout
Size options
Help
Zoom
18.7
Whole
Show buried clones
Yes
No
Search
CB Unit Range
0
to
85
Contig stats
Clones: 2 (0 buried)
Markers: 0
Sequenced: 0
Length: 86 CB units
Ctg3403 of cp05T5e12DM4RS
H024G24
H024E18
0 5 10 15 20 25 30 35 40 45 50 55 60 65 70 75 80
[Chickpea contig p...
[root@localhost: /...
[FPC V8.9 Main Menu]
Project cp05T5e12...
FPC Ctg3403 cp05...

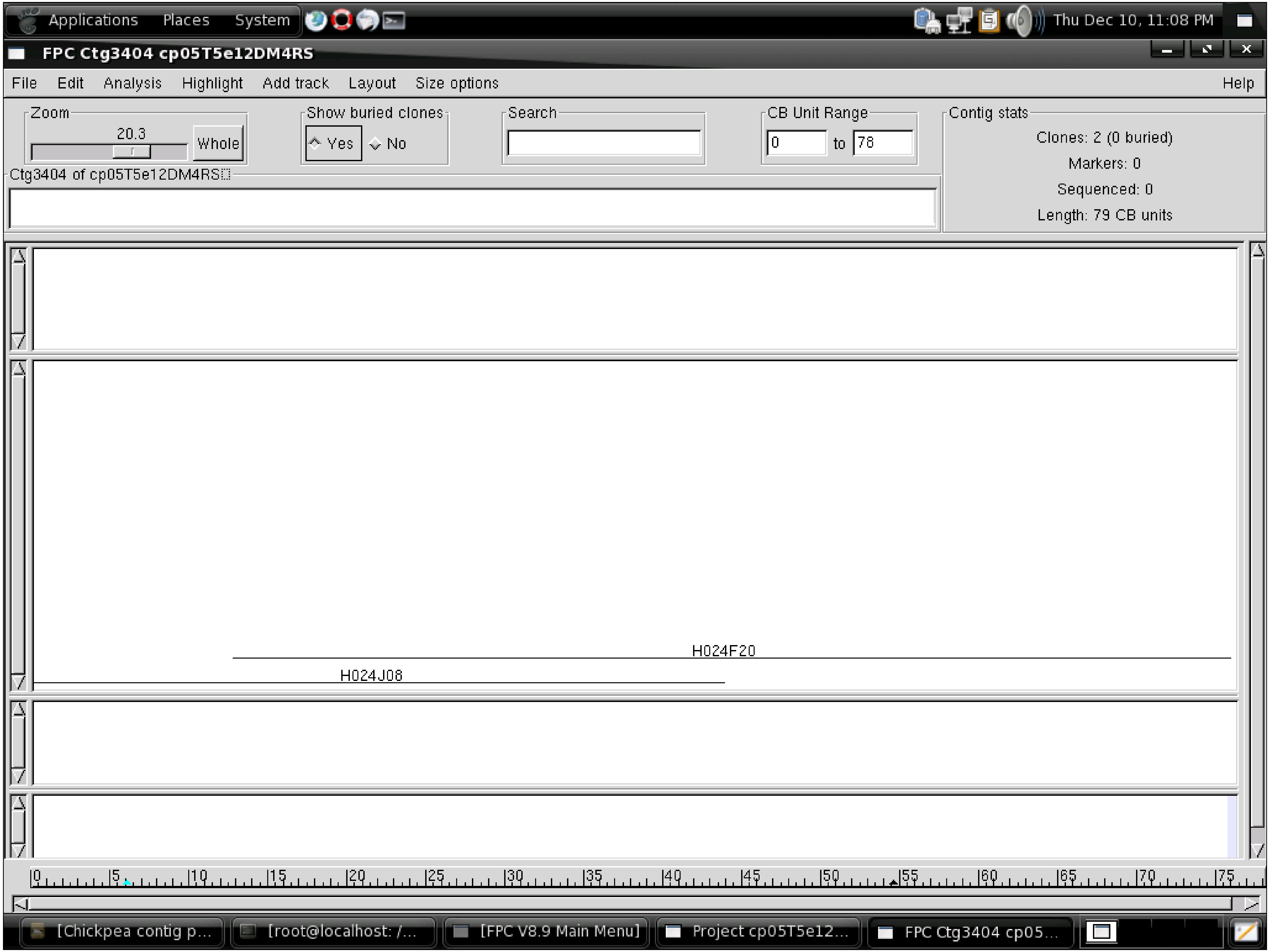
Applications Places System
Thu Dec 10, 11:08 PM
FPC Ctg3404 cp05T5e12DM4RS
File Edit Analysis Highlight Add track Layout Size options Help
Zoom
20.3
Whole
Show buried clones
Yes No
Search
CB Unit Range
0 to 78
Contig stats
Clones: 2 (0 buried)
Markers: 0
Sequenced: 0
Length: 79 CB units
Ctg3404 of cp05T5e12DM4RS
H024F20
H024J08
0 5 10 15 20 25 30 35 40 45 50 55 60 65 70 75
[Chickpea contig p...
[root@localhost: /...
[FPC V8.9 Main Menu]
Project cp05T5e12...
FPC Ctg3404 cp05...

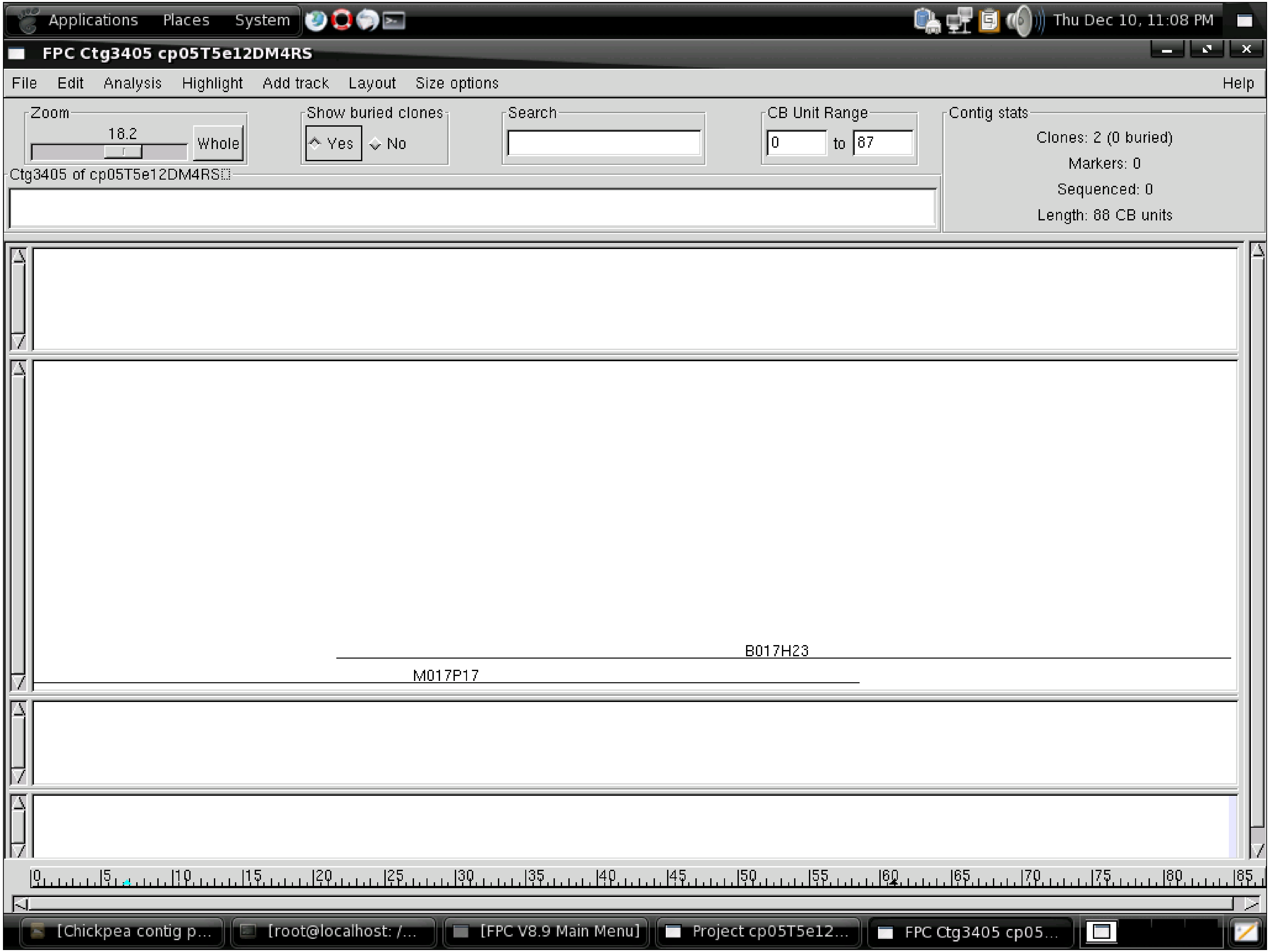
Applications Places System
Thu Dec 10, 11:08 PM
FPC Ctg3405 cp05T5e12DM4RS
File Edit Analysis Highlight Add track Layout Size options Help
Zoom
18.2
Whole
Show buried clones
Yes No
Search
CB Unit Range
0 to 87
Contig stats
Clones: 2 (0 buried)
Markers: 0
Sequenced: 0
Length: 88 CB units
Ctg3405 of cp05T5e12DM4RS
B017H23
M017P17
0 5 10 15 20 25 30 35 40 45 50 55 60 65 70 75 80 85
[Chickpea contig p...
[root@localhost: /...
[FPC V8.9 Main Menu]
Project cp05T5e12...
FPC Ctg3405 cp05...

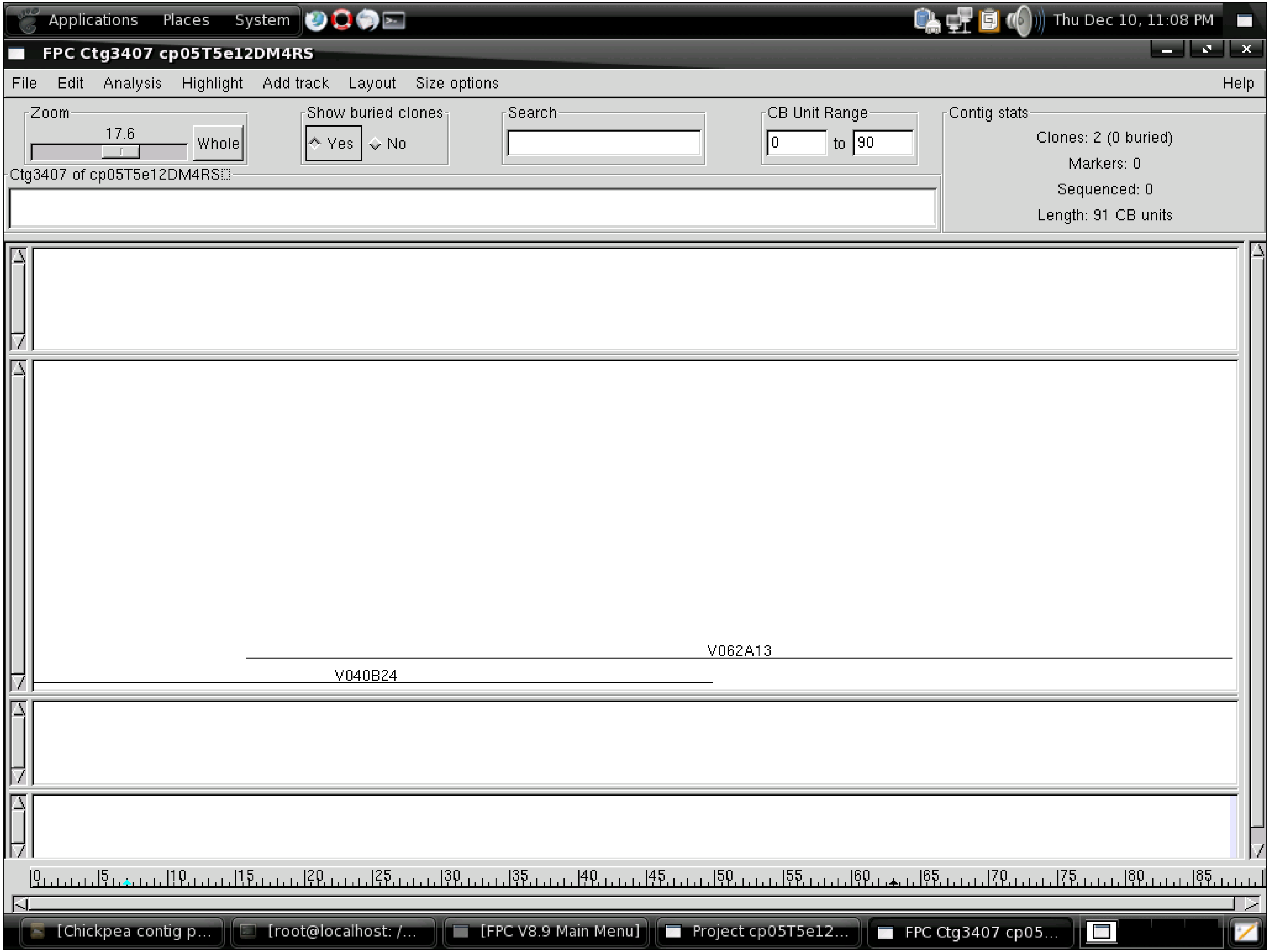

Applications
Places
System
Thu Dec 10, 11:08 PM
FPC Ctg3407 cp05T5e12DM4RS
File
Edit
Analysis
Highlight
Add track
Layout
Size options
Help
Zoom
17.6
Whole
Show buried clones
Yes
No
Search
CB Unit Range
0
to
90
Contig stats
Clones: 2 (0 buried)
Markers: 0
Sequenced: 0
Length: 91 CB units
Ctg3407 of cp05T5e12DM4RS
V062A13
V040B24
0 5 10 15 20 25 30 35 40 45 50 55 60 65 70 75 80 85
[Chickpea contig p...
[root@localhost: /...
[FPC V8.9 Main Menu]
Project cp05T5e12...
FPC Ctg3407 cp05...

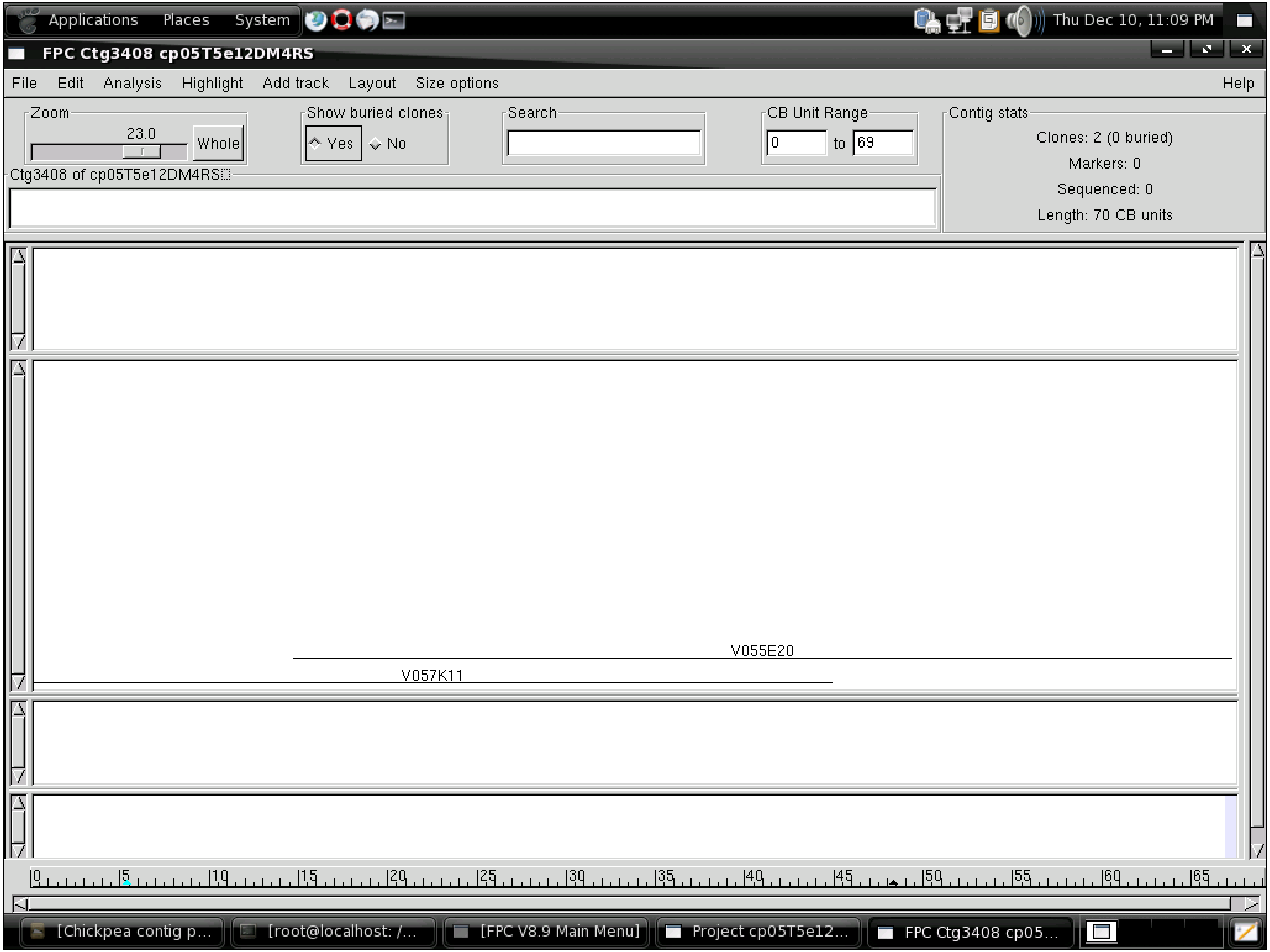
Applications Places System
Thu Dec 10, 11:09 PM
FPC Ctg3408 cp05T5e12DM4RS
File Edit Analysis Highlight Add track Layout Size options Help
Zoom
23.0
Whole
Show buried clones
Yes No
Search
CB Unit Range
0 to 69
Contig stats
Clones: 2 (0 buried)
Markers: 0
Sequenced: 0
Length: 70 CB units
Ctg3408 of cp05T5e12DM4RS
V055E20
V057K11
0 5 10 15 20 25 30 35 40 45 50 55 60 65
[Chickpea contig p...
[root@localhost: /...
[FPC V8.9 Main Menu]
Project cp05T5e12...
FPC Ctg3408 cp05...

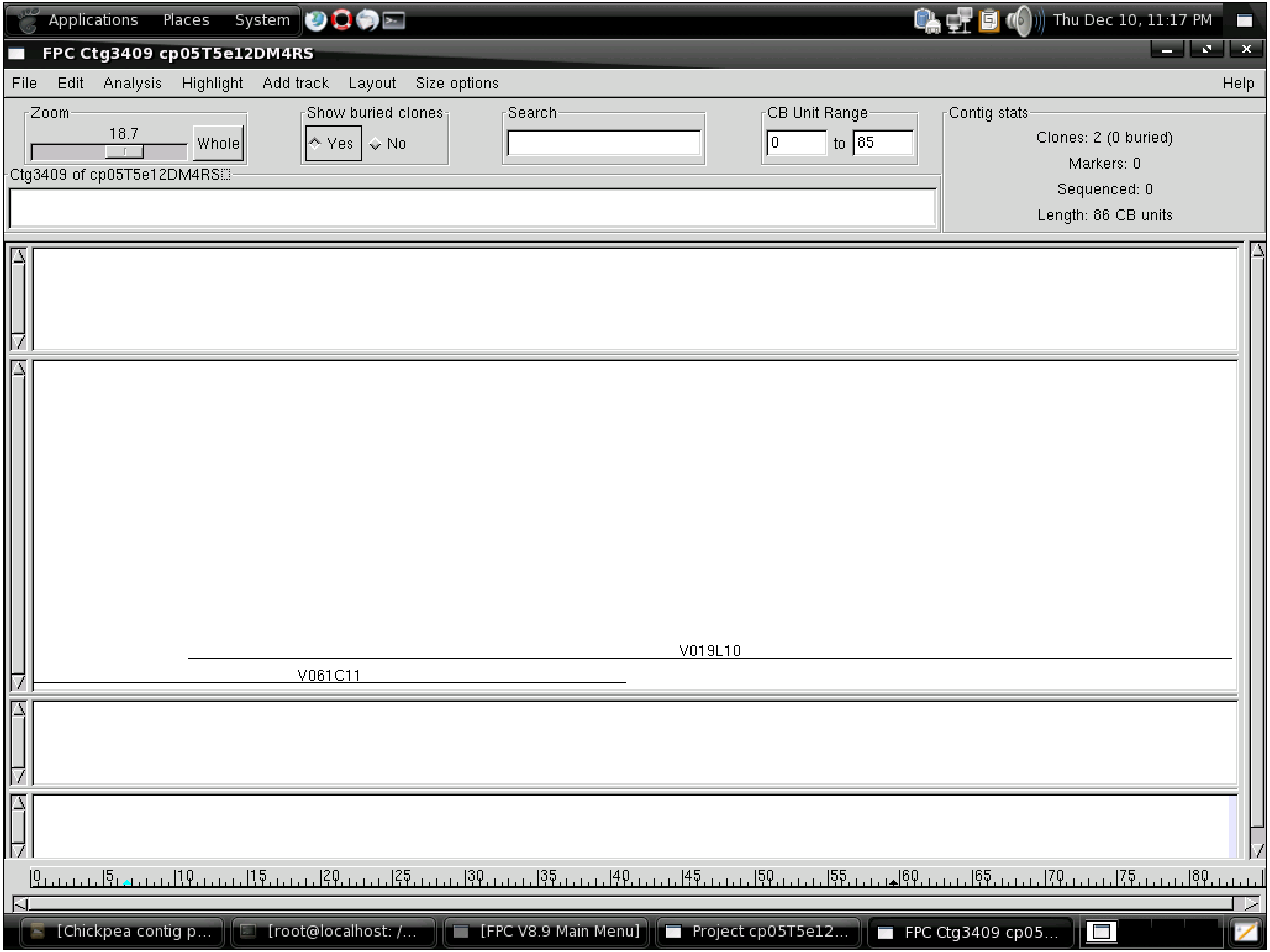
Applications
Places
System
Thu Dec 10, 11:17 PM
FPC Ctg3409 cp05T5e12DM4RS
File
Edit
Analysis
Highlight
Add track
Layout
Size options
Help
Zoom
18.7
Whole
Show buried clones
Yes
No
Search
CB Unit Range
0
to
85
Contig stats
Clones: 2 (0 buried)
Markers: 0
Sequenced: 0
Length: 86 CB units
Ctg3409 of cp05T5e12DM4RS
V019L10
V061C11
0
5
10
15
20
25
30
35
40
45
50
55
60
65
70
75
80
[Chickpea contig p...
[root@localhost: /...
[FPC V8.9 Main Menu]
Project cp05T5e12...
FPC Ctg3409 cp05...

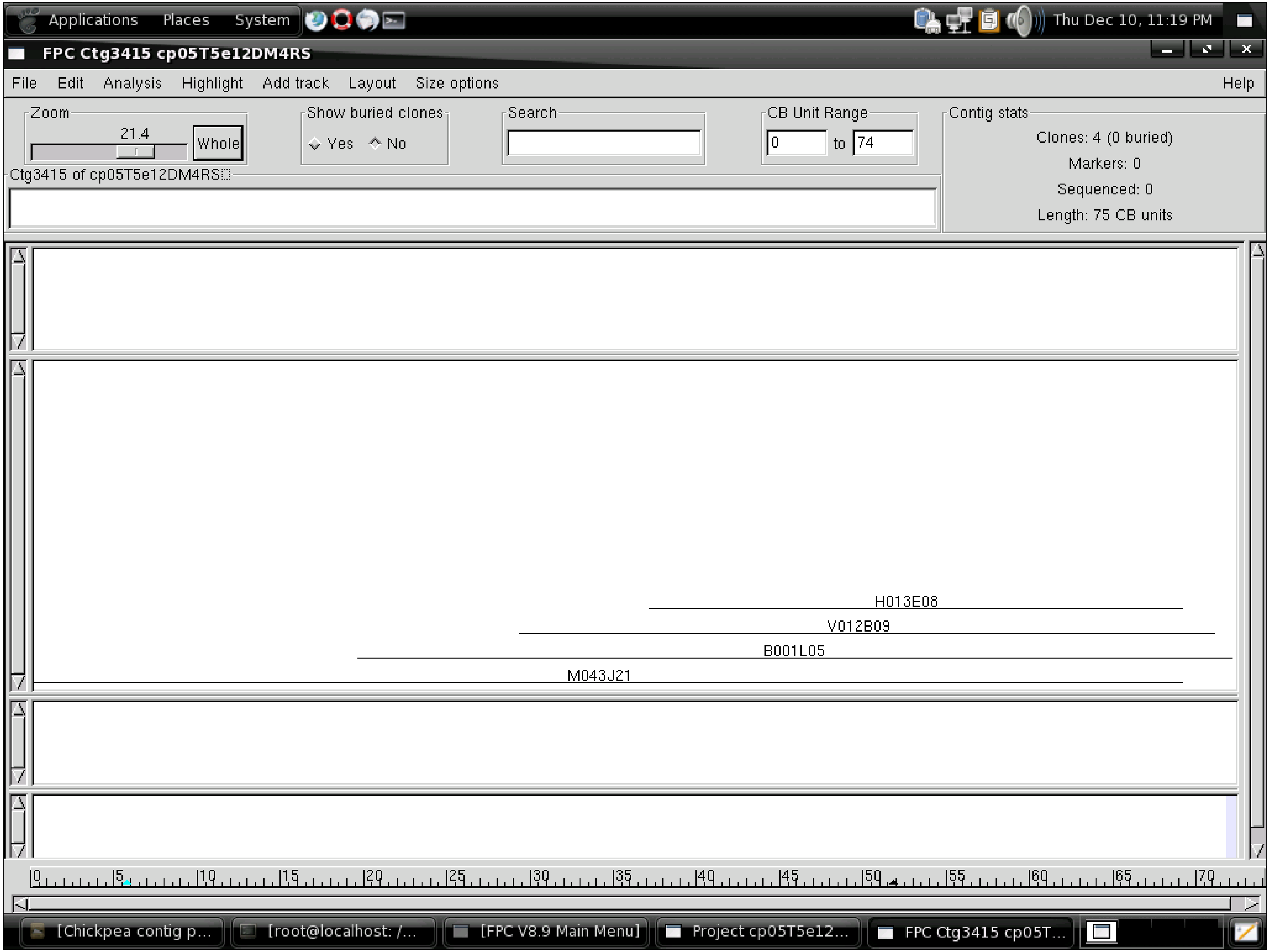

Applications
Places
System
Thu Dec 10, 11:19 PM
FPC Ctg3415 cp05T5e12DM4RS
File
Edit
Analysis
Highlight
Add track
Layout
Size options
Help
Zoom
21.4
Whole
Show buried clones
Yes
No
Search
CB Unit Range
0
to
74
Contig stats
Clones: 4 (0 buried)
Markers: 0
Sequenced: 0
Length: 75 CB units
Ctg3415 of cp05T5e12DM4RS
H013E08
V012B09
B001L05
M043J21
0
5
10
15
20
25
30
35
40
45
50
55
60
65
70
[Chickpea contig p...
[root@localhost: /...
[FPC V8.9 Main Menu]
Project cp05T5e12...
FPC Ctg3415 cp05T...

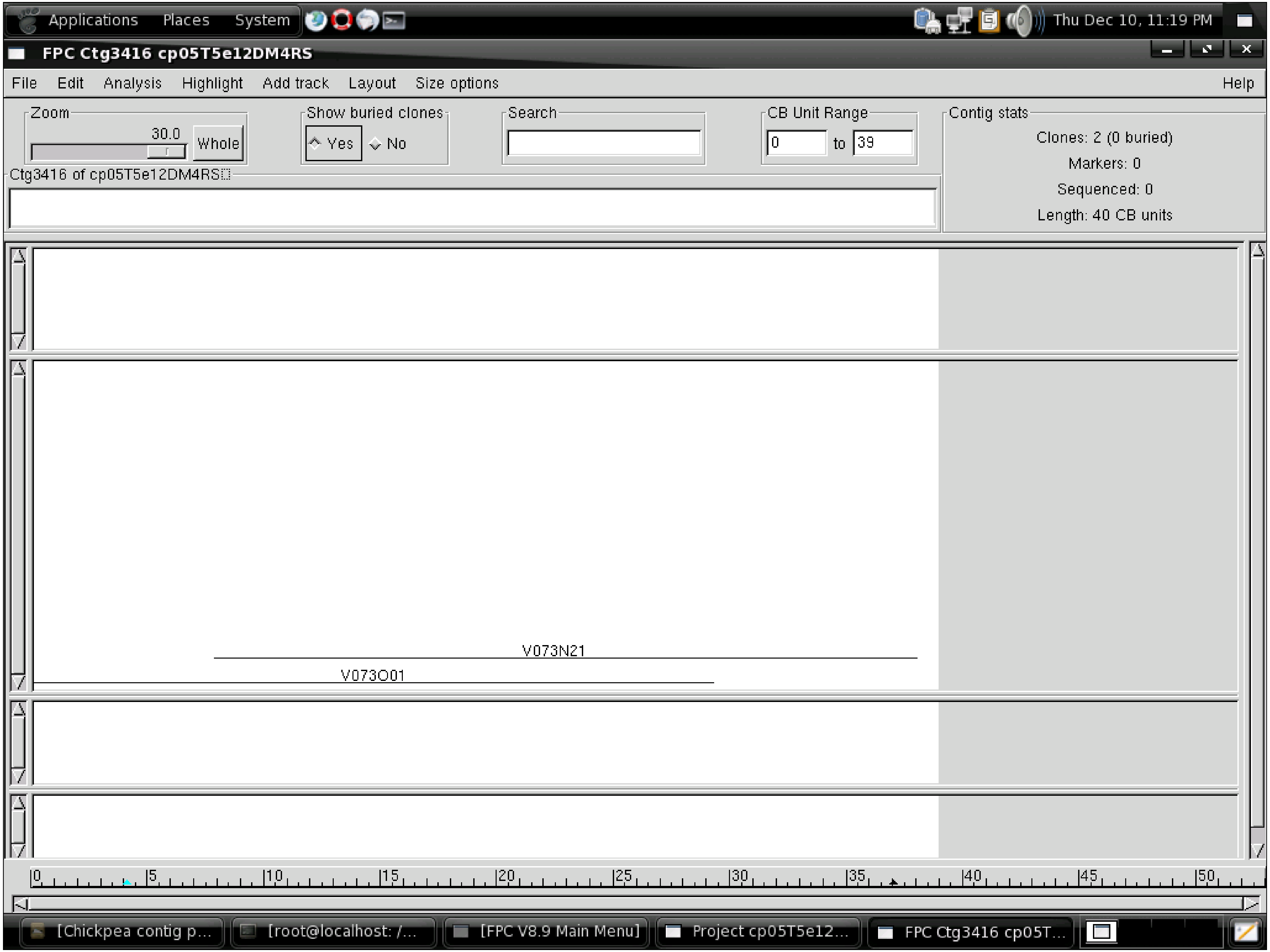

Applications
Places
System
Thu Dec 10, 11:19 PM
FPC Ctg3416 cp05T5e12DM4RS
File
Edit
Analysis
Highlight
Add track
Layout
Size options
Help
Zoom
30.0
Whole
Show buried clones
Yes
No
Search
CB Unit Range
0
to
39
Contig stats
Clones: 2 (0 buried)
Markers: 0
Sequenced: 0
Length: 40 CB units
Ctg3416 of cp05T5e12DM4RS
V073N21
V073O01
0
5
10
15
20
25
30
35
40
45
50
[Chickpea contig p...
[root@localhost: /...
[FPC V8.9 Main Menu]
Project cp05T5e12...
FPC Ctg3416 cp05T...

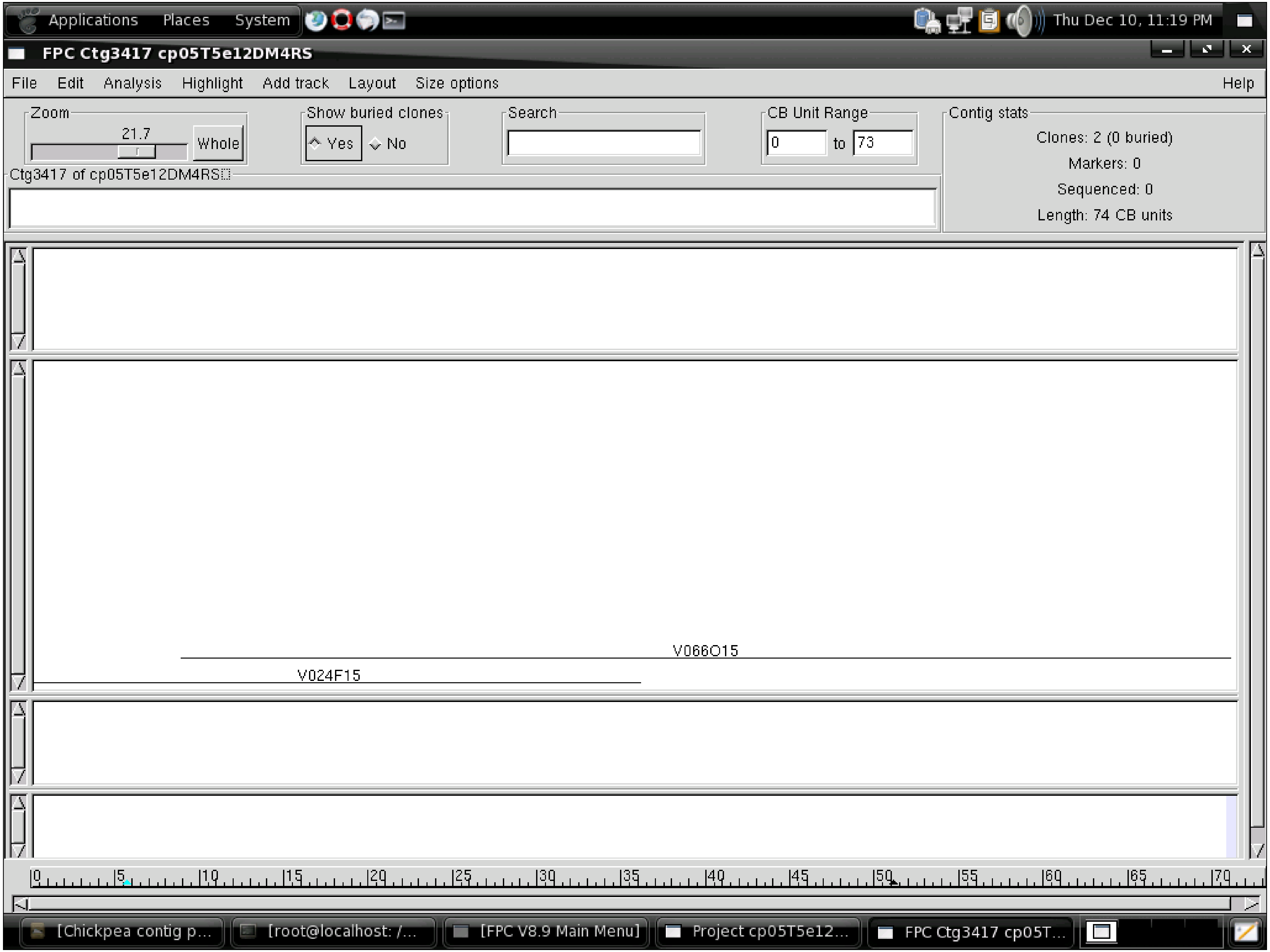
Applications
Places
System
Thu Dec 10, 11:19 PM
FPC Ctg3417 cp05T5e12DM4RS
File
Edit
Analysis
Highlight
Add track
Layout
Size options
Help
Zoom
21.7
Whole
Show buried clones
Yes
No
Search
CB Unit Range
0
to
73
Contig stats
Clones: 2 (0 buried)
Markers: 0
Sequenced: 0
Length: 74 CB units
Ctg3417 of cp05T5e12DM4RS
V066O15
V024F15
0 5 10 15 20 25 30 35 40 45 50 55 60 65 70
[Chickpea contig p...
[root@localhost: /...
[FPC V8.9 Main Menu]
Project cp05T5e12...
FPC Ctg3417 cp05T...

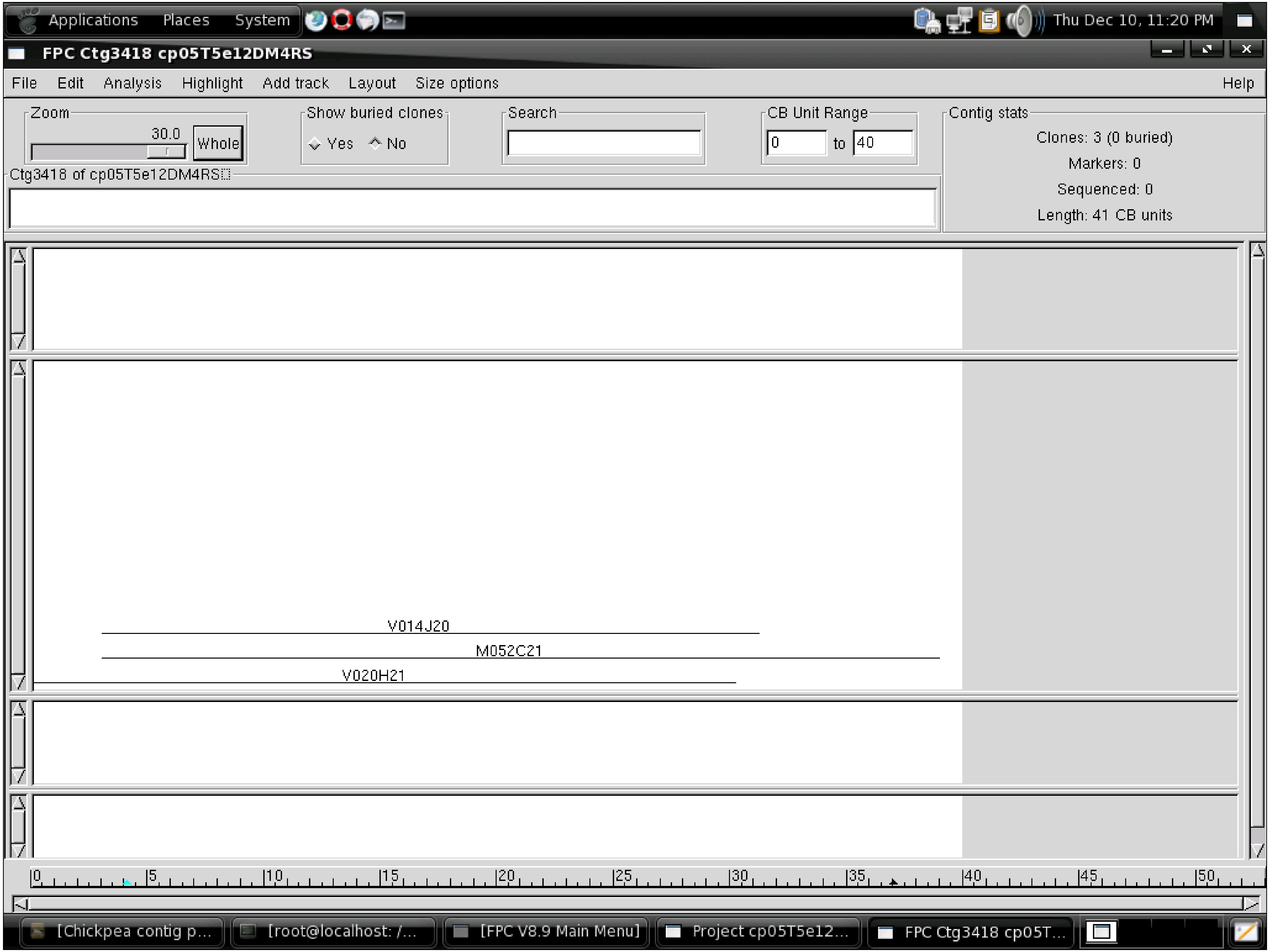
Applications
Places
System
Thu Dec 10, 11:20 PM
FPC Ctg3418 cp05T5e12DM4RS
File
Edit
Analysis
Highlight
Add track
Layout
Size options
Help
Zoom
30.0
Whole
Show buried clones
Yes
No
Search
CB Unit Range
0
to
40
Contig stats
Clones: 3 (0 buried)
Markers: 0
Sequenced: 0
Length: 41 CB units
Ctg3418 of cp05T5e12DM4RS
V014J20
M052C21
V020H21
0
5
10
15
20
25
30
35
40
45
50
[Chickpea contig p...
[root@localhost: /...
[FPC V8.9 Main Menu]
Project cp05T5e12...
FPC Ctg3418 cp05T...

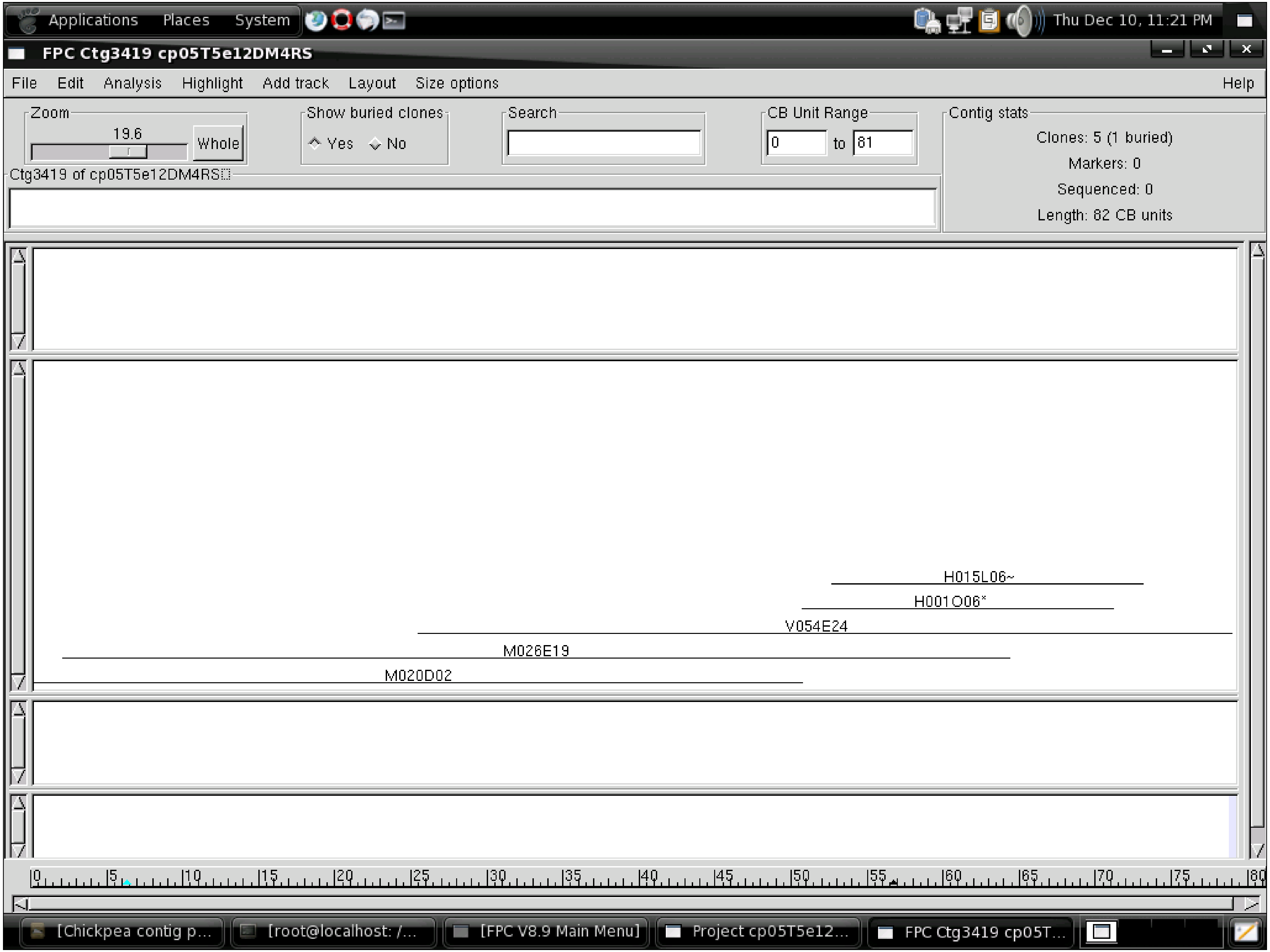
Applications Places System
Thu Dec 10, 11:21 PM
FPC Ctg3419 cp05T5e12DM4RS
File Edit Analysis Highlight Add track Layout Size options Help
Zoom
19.6
Whole
Show buried clones
Yes No
Search
CB Unit Range
0 to 81
Contig stats
Clones: 5 (1 buried)
Markers: 0
Sequenced: 0
Length: 82 CB units
Ctg3419 of cp05T5e12DM4RS
H015L06~
H001O06*
V054E24
M026E19
M020D02
0 5 10 15 20 25 30 35 40 45 50 55 60 65 70 75 80
[Chickpea contig p...
[root@localhost: /...
[FPC V8.9 Main Menu]
Project cp05T5e12...
FPC Ctg3419 cp05T...

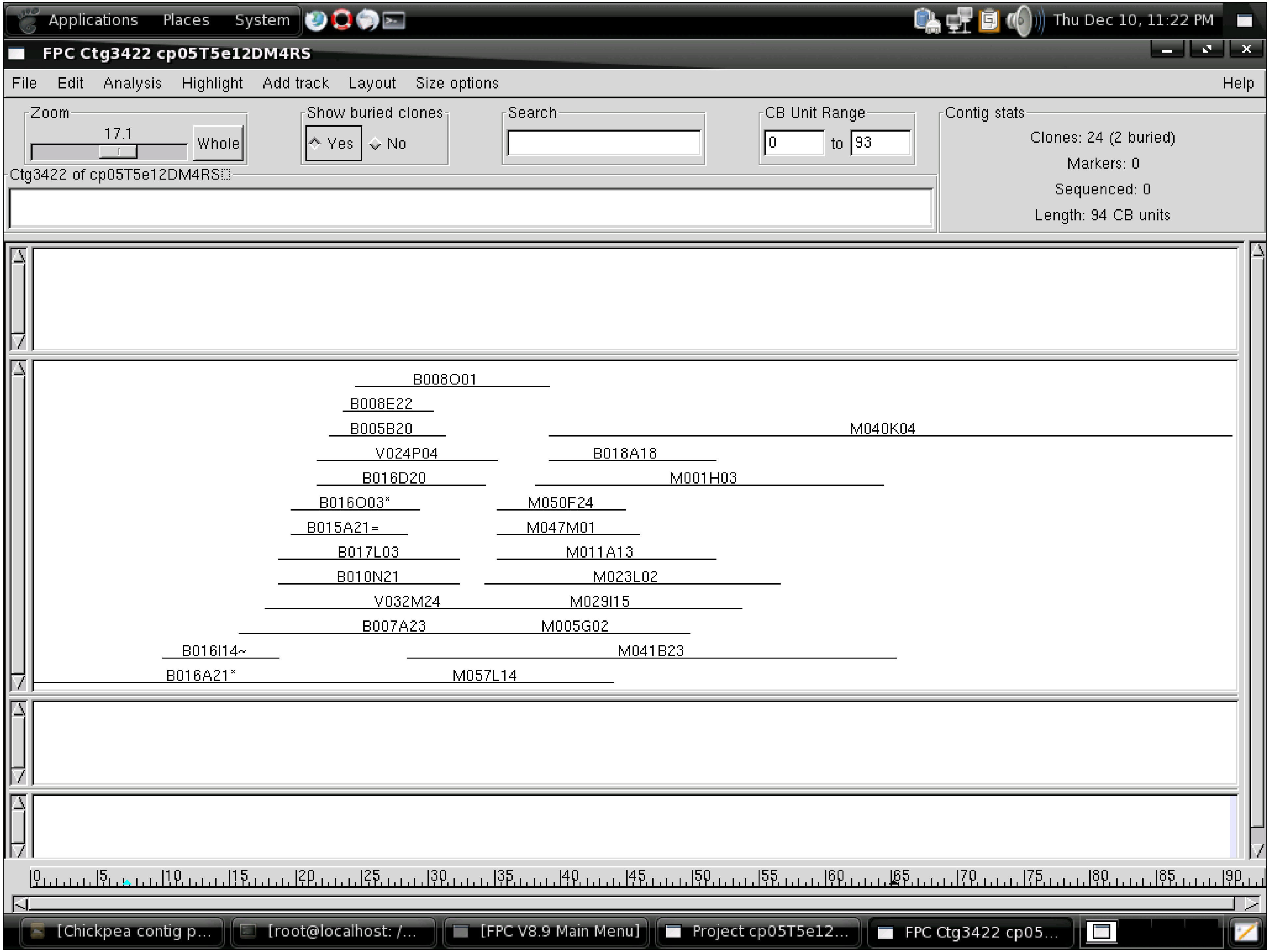
Applications Places System
Thu Dec 10, 11:22 PM
FPC Ctg3422 cp05T5e12DM4RS
File Edit Analysis Highlight Add track Layout Size options Help
Zoom
17.1
Whole
Show buried clones
Yes No
Search
CB Unit Range
0 to 93
Contig stats
Clones: 24 (2 buried)
Markers: 0
Sequenced: 0
Length: 94 CB units
Ctg3422 of cp05T5e12DM4RS
B008O01
B008E22
B005B20
M040K04
V024P04
B018A18
B016D20
M001H03
B016O03*
M050F24
B015A21=
M047M01
B017L03
M011A13
B010N21
M023L02
V032M24
M029I15
B007A23
M005G02
B016I14~
M041B23
B016A21*
M057L14
0 5 10 15 20 25 30 35 40 45 50 55 60 65 70 75 80 85 90
[Chickpea contig p...
[root@localhost: /...
[FPC V8.9 Main Menu]
Project cp05T5e12...
FPC Ctg3422 cp05...

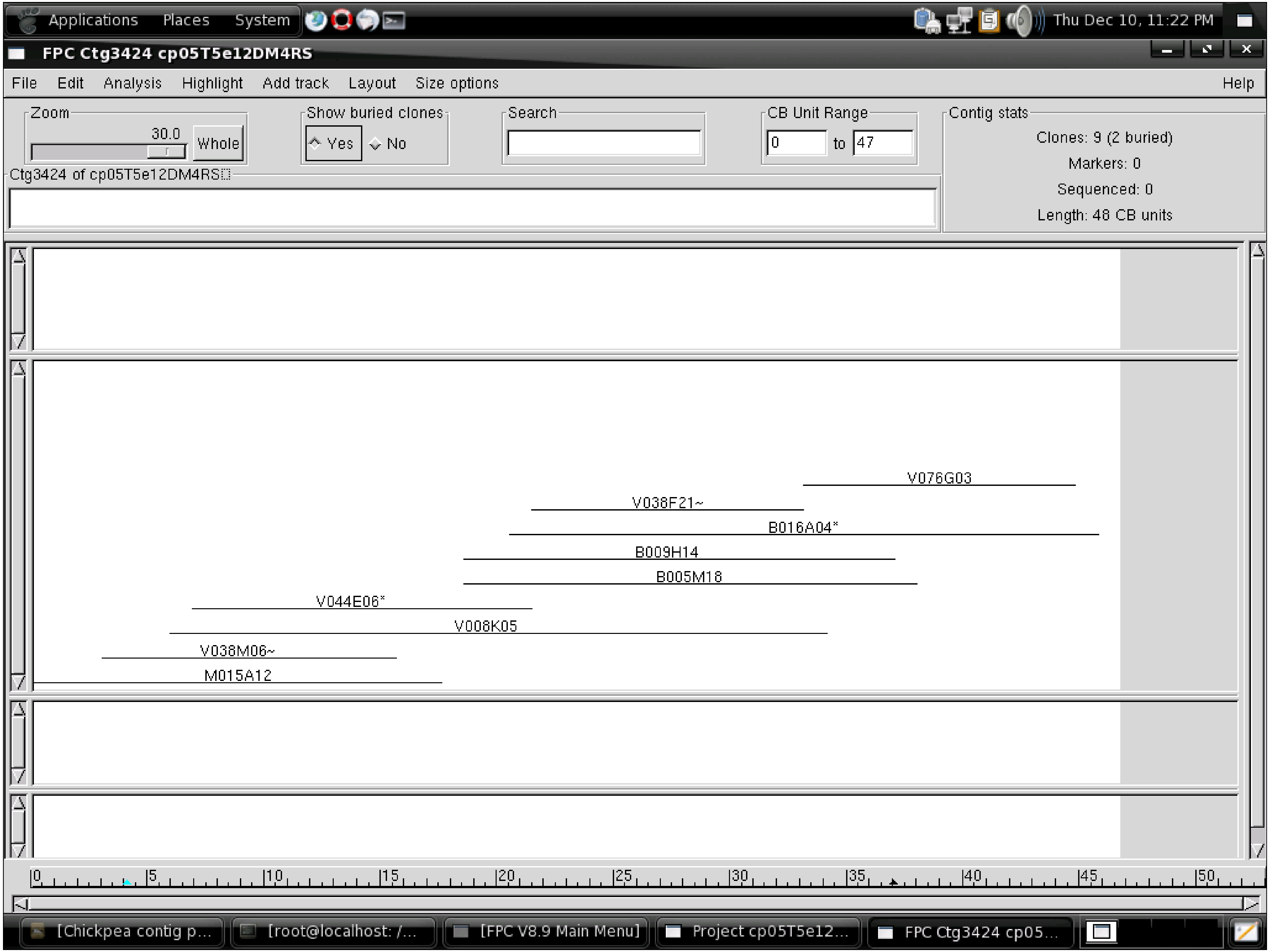
Applications Places System
Thu Dec 10, 11:22 PM
FPC Ctg3424 cp05T5e12DM4RS
File Edit Analysis Highlight Add track Layout Size options Help
Zoom
30.0
Whole
Show buried clones
Yes
No
Search
CB Unit Range
0
to
47
Contig stats
Clones: 9 (2 buried)
Markers: 0
Sequenced: 0
Length: 48 CB units
Ctg3424 of cp05T5e12DM4RS
V076G03
V038F21~
B016A04*
B009H14
B005M18
V044E06*
V008K05
V038M06~
M015A12
0 5 10 15 20 25 30 35 40 45 50
[Chickpea contig p...
[root@localhost: /...
[FPC V8.9 Main Menu]
Project cp05T5e12...
FPC Ctg3424 cp05...

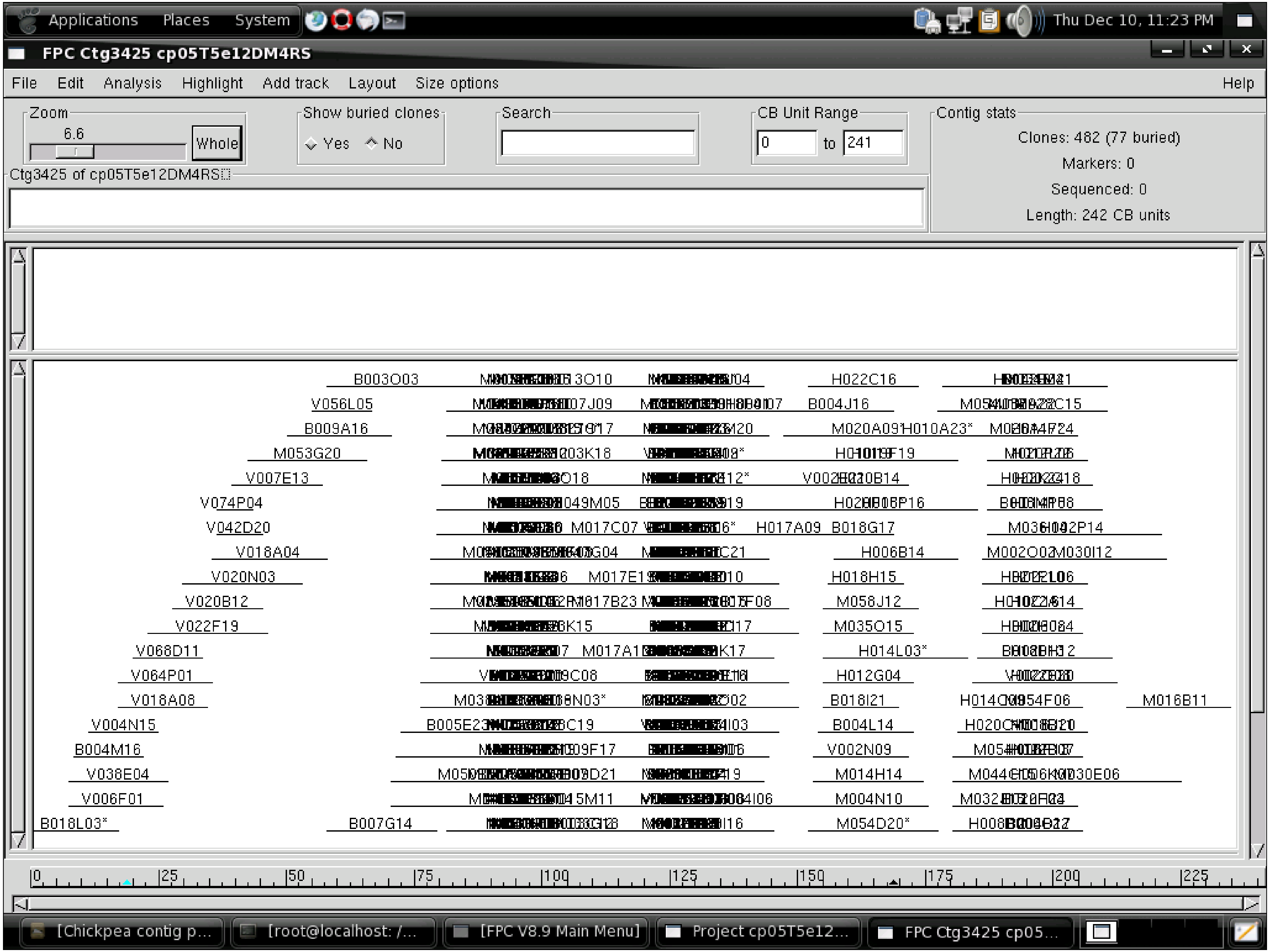
Applications Places System
Thu Dec 10, 11:23 PM
FPC Ctg3425 cp05T5e12DM4RS
File Edit Analysis Highlight Add track Layout Size options Help
Zoom
6.6
Whole
Show buried clones
Yes No
Search
CB Unit Range
0 to 241
Contig stats
Clones: 482 (77 buried)
Markers: 0
Sequenced: 0
Length: 242 CB units
Ctg3425 of cp05T5e12DM4RS
B003O03
V056L05
B009A16
M053G20
V007E13
V074P04
V042D20
V018A04
V020N03
V020B12
V022F19
V068D11
V064P01
V018A08
V004N15
B004M16
V038E04
V006F01
B018L03*
B007G14
H022C16
B004J16
H018H15
M058J12
M035O15
H014L03*
H012G04
B018I21
B004L14
V002N09
M014H14
M004N10
M054D20*
M016B11
M002O02
M030I12
H017A09
B018G17
H006B14
0 25 50 75 100 125 150 175 200 225
[Chickpea contig p...
[root@localhost: /...
[FPC V8.9 Main Menu]
Project cp05T5e12...
FPC Ctg3425 cp05...

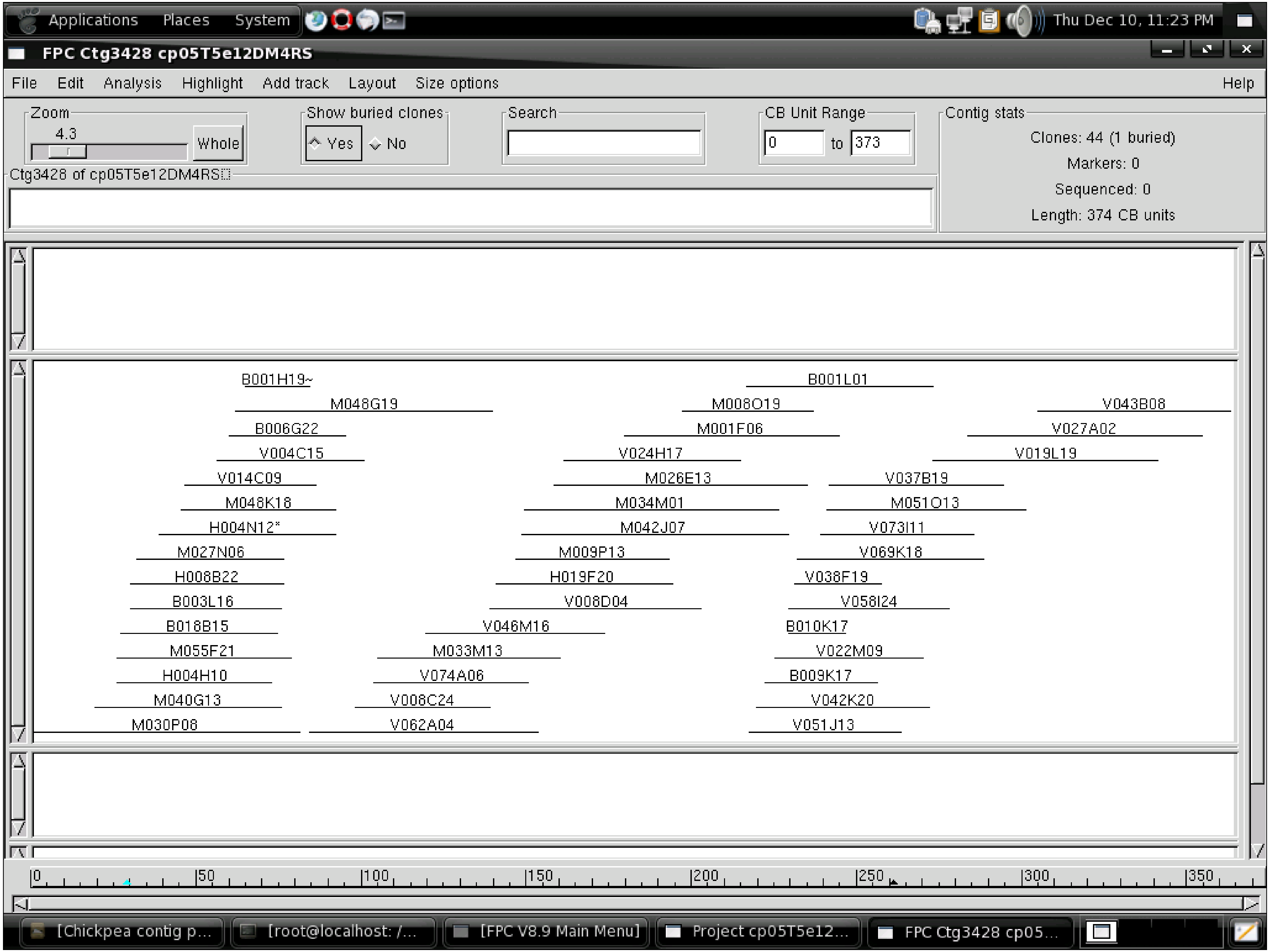
Applications
Places
System
Thu Dec 10, 11:23 PM
FPC Ctg3428 cp05T5e12DM4RS
File
Edit
Analysis
Highlight
Add track
Layout
Size options
Help
Zoom
4.3
Whole
Show buried clones
Yes
No
Search
CB Unit Range
0
to
373
Contig stats
Clones: 44 (1 buried)
Markers: 0
Sequenced: 0
Length: 374 CB units
Ctg3428 of cp05T5e12DM4RS
B001H19~
B001L01
M048G19
M008O19
V043B08
B006G22
M001F06
V027A02
V004C15
V024H17
V019L19
V014C09
M026E13
V037B19
M048K18
M034M01
M051O13
H004N12*
M042J07
V073I11
M027N06
M009P13
V069K18
H008B22
H019F20
V038F19
B003L16
V008D04
V058I24
B018B15
V046M16
B010K17
M055F21
M033M13
V022M09
H004H10
V074A06
B009K17
M040G13
V008C24
V042K20
M030P08
V062A04
V051J13
0
50
100
150
200
250
300
350
[Chickpea contig p...
[root@localhost: /...
[FPC V8.9 Main Menu]
Project cp05T5e12...
FPC Ctg3428 cp05...

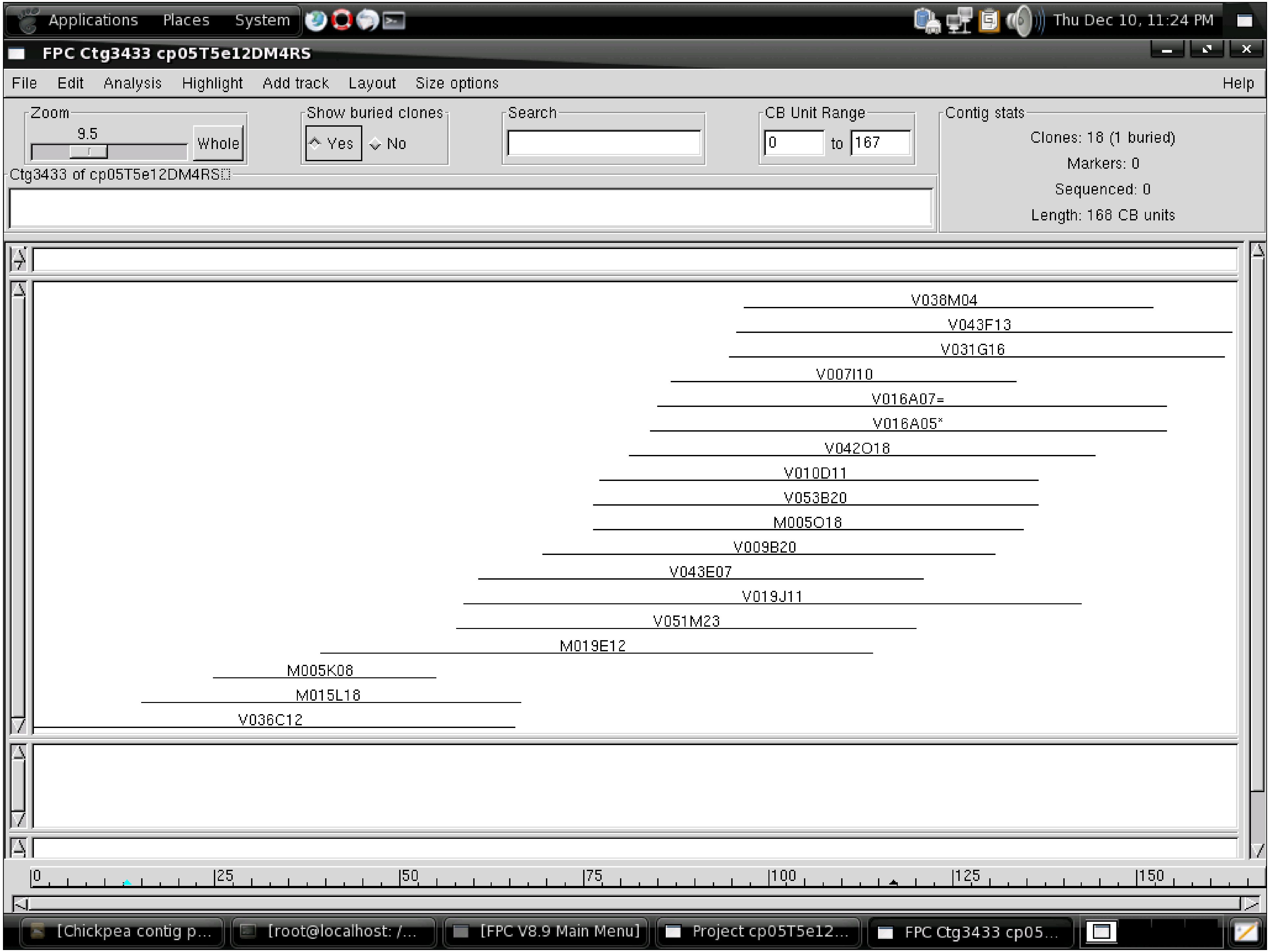
Applications
Places
System
Thu Dec 10, 11:24 PM
FPC Ctg3433 cp05T5e12DM4RS
File
Edit
Analysis
Highlight
Add track
Layout
Size options
Help
Zoom
9.5
Whole
Show buried clones
Yes
No
Search
CB Unit Range
0
to
167
Contig stats
Clones: 18 (1 buried)
Markers: 0
Sequenced: 0
Length: 168 CB units
Ctg3433 of cp05T5e12DM4RS
V038M04
V043F13
V031G16
V007I10
V016A07=
V016A05*
V042O18
V010D11
V053B20
M005O18
V009B20
V043E07
V019J11
V051M23
M019E12
M005K08
M015L18
V036C12
0
25
50
75
100
125
150
[Chickpea contig p...
[root@localhost: /...
[FPC V8.9 Main Menu]
Project cp05T5e12...
FPC Ctg3433 cp05...

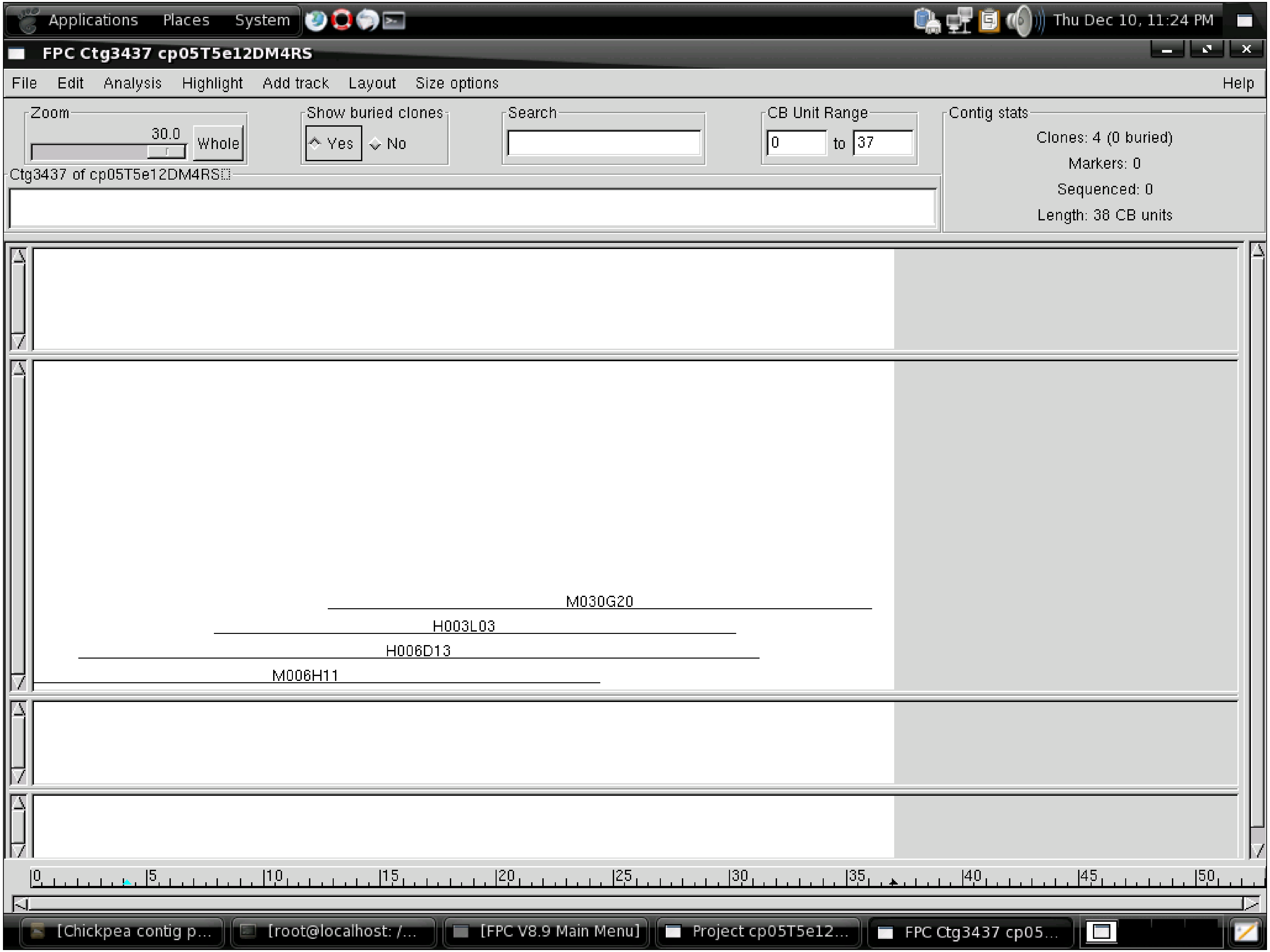
Applications Places System
Thu Dec 10, 11:24 PM
FPC Ctg3437 cp05T5e12DM4RS
File Edit Analysis Highlight Add track Layout Size options Help
Zoom
30.0
Whole
Show buried clones
Yes
No
Search
CB Unit Range
0
to
37
Contig stats
Clones: 4 (0 buried)
Markers: 0
Sequenced: 0
Length: 38 CB units
Ctg3437 of cp05T5e12DM4RS
M030G20
H003L03
H006D13
M006H11
0 5 10 15 20 25 30 35 40 45 50
[Chickpea contig p...
[root@localhost: /...
[FPC V8.9 Main Menu]
Project cp05T5e12...
FPC Ctg3437 cp05...

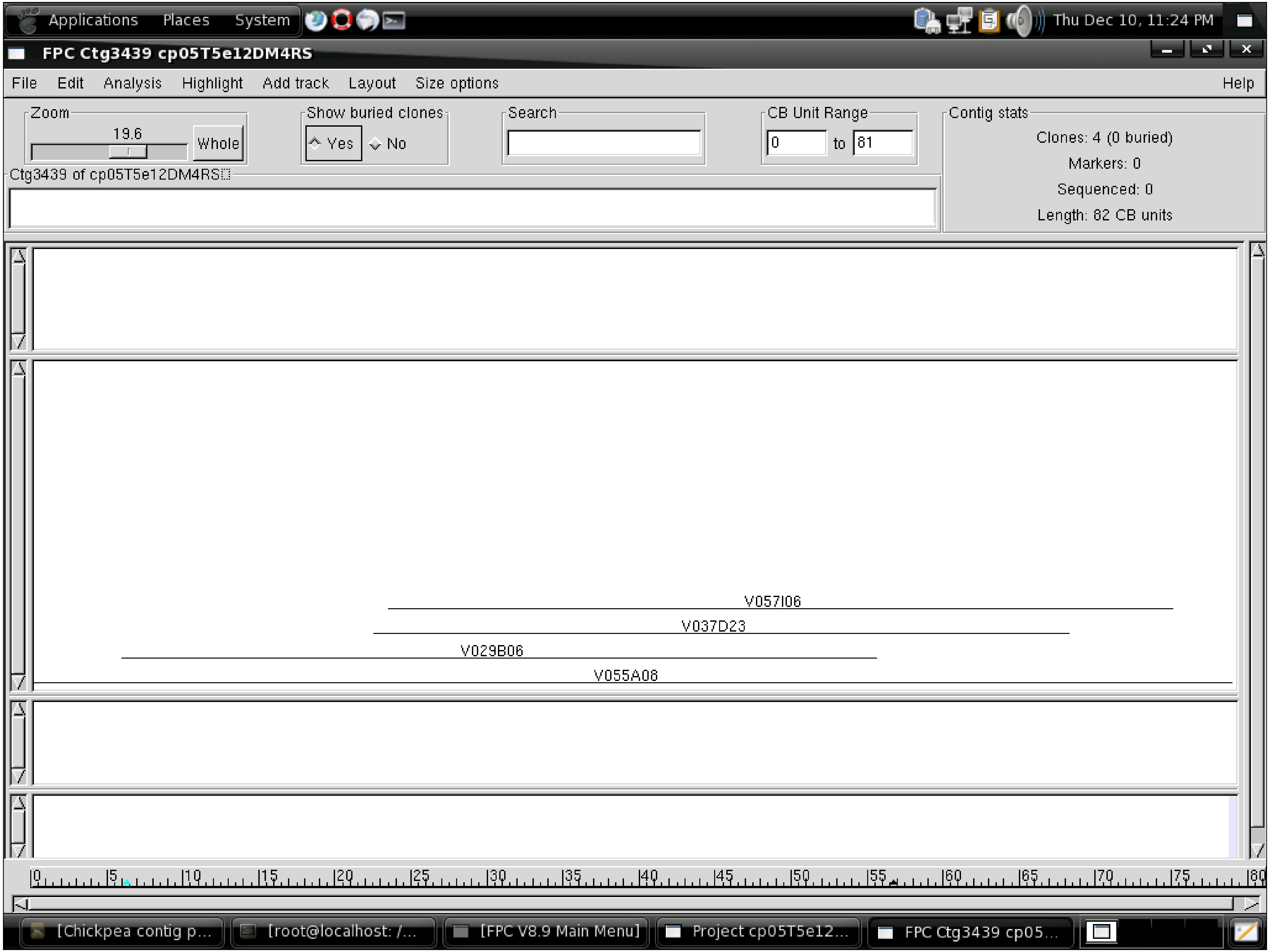
Applications
Places
System
Thu Dec 10, 11:24 PM
FPC Ctg3439 cp05T5e12DM4RS
File
Edit
Analysis
Highlight
Add track
Layout
Size options
Help
Zoom
19.6
Whole
Show buried clones
Yes
No
Search
CB Unit Range
0
to
81
Contig stats
Clones: 4 (0 buried)
Markers: 0
Sequenced: 0
Length: 82 CB units
Ctg3439 of cp05T5e12DM4RS
V057I06
V037D23
V029B06
V055A08
0 5 10 15 20 25 30 35 40 45 50 55 60 65 70 75 80
[Chickpea contig p...
[root@localhost: /...
[FPC V8.9 Main Menu]
Project cp05T5e12...
FPC Ctg3439 cp05...

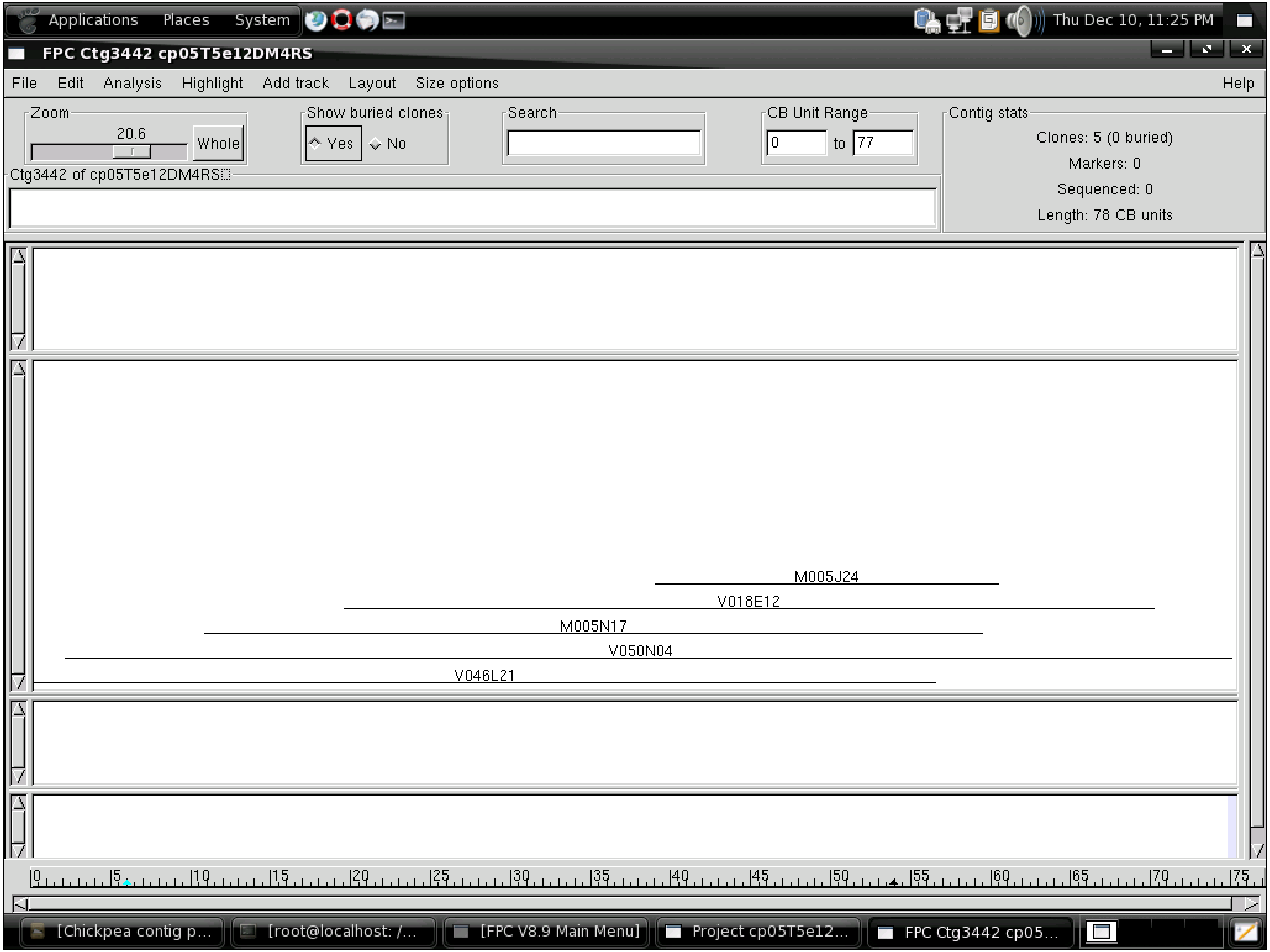
Applications Places System
Thu Dec 10, 11:25 PM
FPC Ctg3442 cp05T5e12DM4RS
File Edit Analysis Highlight Add track Layout Size options Help
Zoom
20.6
Whole
Show buried clones
Yes
No
Search
CB Unit Range
0 to 77
Contig stats
Clones: 5 (0 buried)
Markers: 0
Sequenced: 0
Length: 78 CB units
Ctg3442 of cp05T5e12DM4RS
M005J24
V018E12
M005N17
V050N04
V046L21
0 5 10 15 20 25 30 35 40 45 50 55 60 65 70 75
[Chickpea contig p...
[root@localhost: /...
[FPC V8.9 Main Menu]
Project cp05T5e12...
FPC Ctg3442 cp05...

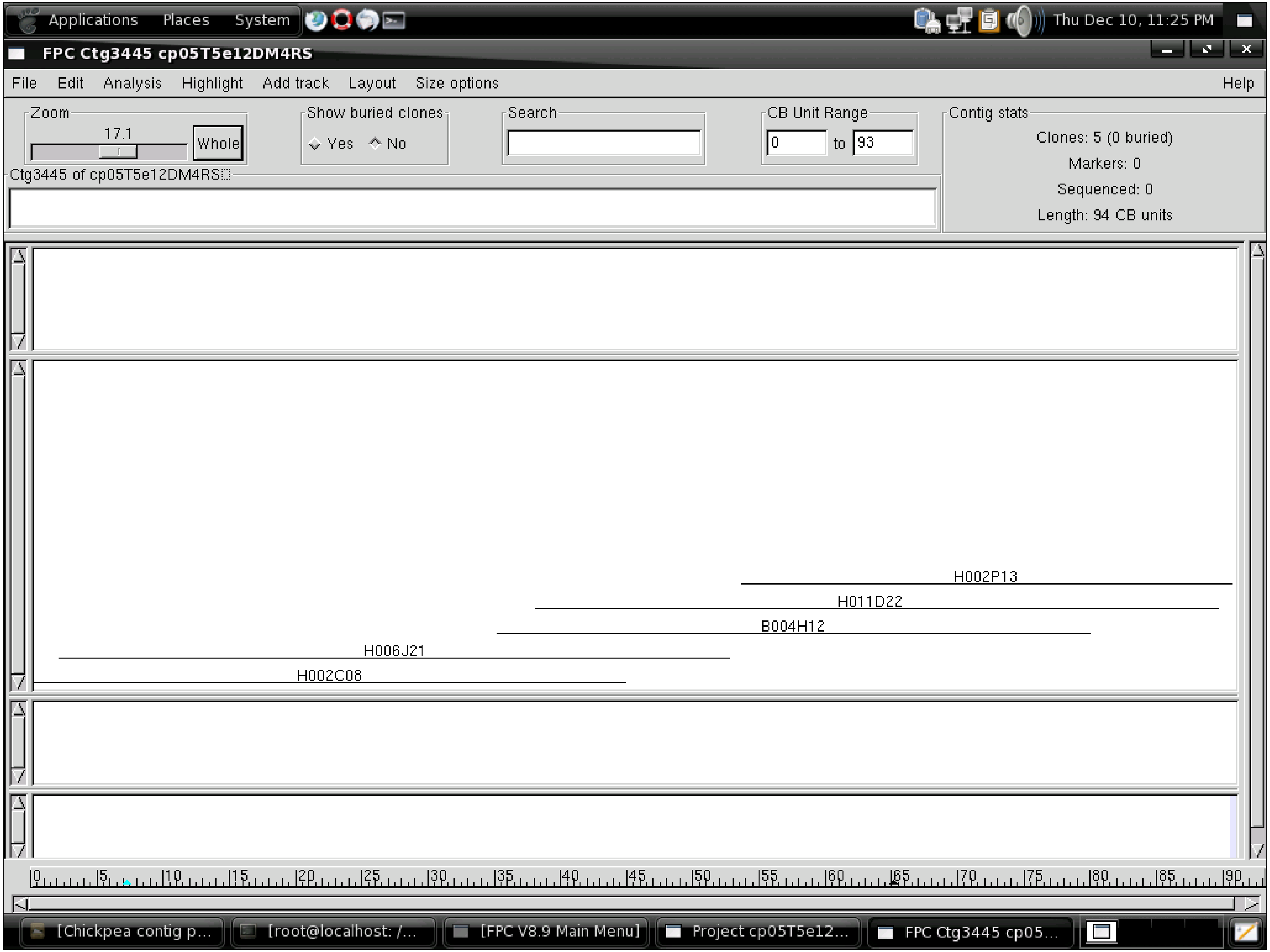
Applications Places System
Thu Dec 10, 11:25 PM
FPC Ctg3445 cp05T5e12DM4RS
File Edit Analysis Highlight Add track Layout Size options Help
Zoom
17.1
Whole
Show buried clones
Yes No
Search
CB Unit Range
0 to 93
Contig stats
Clones: 5 (0 buried)
Markers: 0
Sequenced: 0
Length: 94 CB units
Ctg3445 of cp05T5e12DM4RS
H002P13
H011D22
B004H12
H006J21
H002C08
0 5 10 15 20 25 30 35 40 45 50 55 60 65 70 75 80 85 90
[Chickpea contig p...
[root@localhost: /...
[FPC V8.9 Main Menu]
Project cp05T5e12...
FPC Ctg3445 cp05...

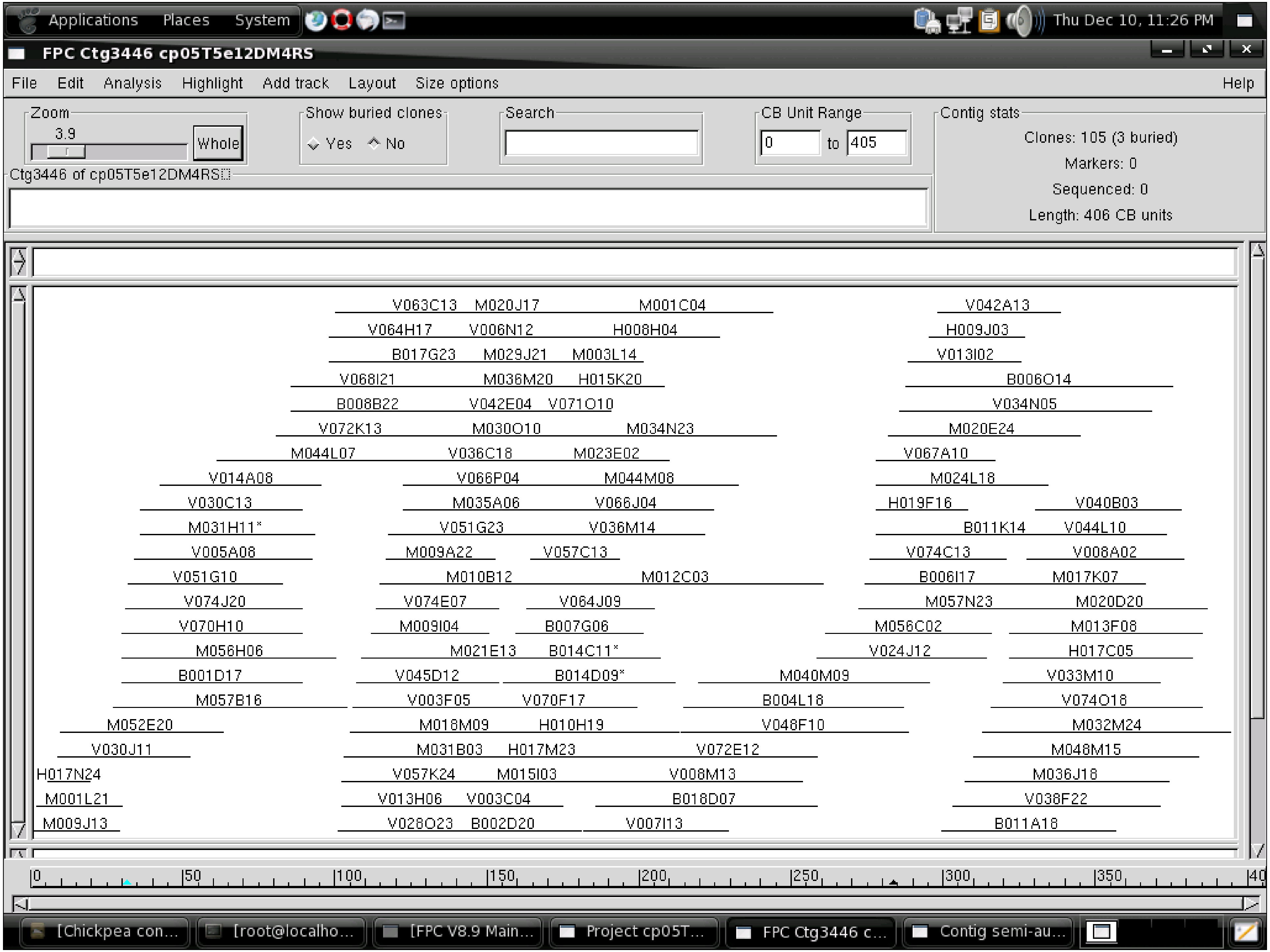

Applications Places System
Thu Dec 10, 11:26 PM
FPC Ctg3446 cp05T5e12DM4RS
File Edit Analysis Highlight Add track Layout Size options Help
Zoom
3.9
Whole
Show buried clones
Yes No
Search
CB Unit Range
0 to 405
Contig stats
Clones: 105 (3 buried)
Markers: 0
Sequenced: 0
Length: 406 CB units
Ctg3446 of cp05T5e12DM4RS
V063C13 M020J17 M001C04 V042A13
V064H17 V006N12 H008H04 H009J03
B017G23 M029J21 M003L14 V013I02
V068I21 M036M20 H015K20 B006O14
B008B22 V042E04 V071O10 V034N05
V072K13 M030O10 M034N23 M020E24
M044L07 V036C18 M023E02 V067A10
V014A08 V066P04 M044M08 M024L18
V030C13 M035A06 V066J04 H019F16 V040B03
M031H11* V051G23 V036M14 B011K14 V044L10
V005A08 M009A22 V057C13 V074C13 V008A02
V051G10 M010B12 M012C03 B006I17 M017K07
V074J20 V074E07 V064J09 M057N23 M020D20
V070H10 M009I04 B007G06 M056C02 M013F08
M056H06 M021E13 B014C11* V024J12 H017C05
B001D17 V045D12 B014D09* M040M09 V033M10
M057B16 V003F05 V070F17 B004L18 V074O18
M052E20 M018M09 H010H19 V048F10 M032M24
V030J11 M031B03 H017M23 V072E12 M048M15
H017N24 V057K24 M015I03 V008M13 M036J18
M001L21 V013H06 V003C04 B018D07 V038F22
M009J13 V028O23 B002D20 V007I13 B011A18
0 50 100 150 200 250 300 350 40
[Chickpea con... [root@localho... [FPC V8.9 Main... Project cp05T... FPC Ctg3446 c... Contig semi-au...

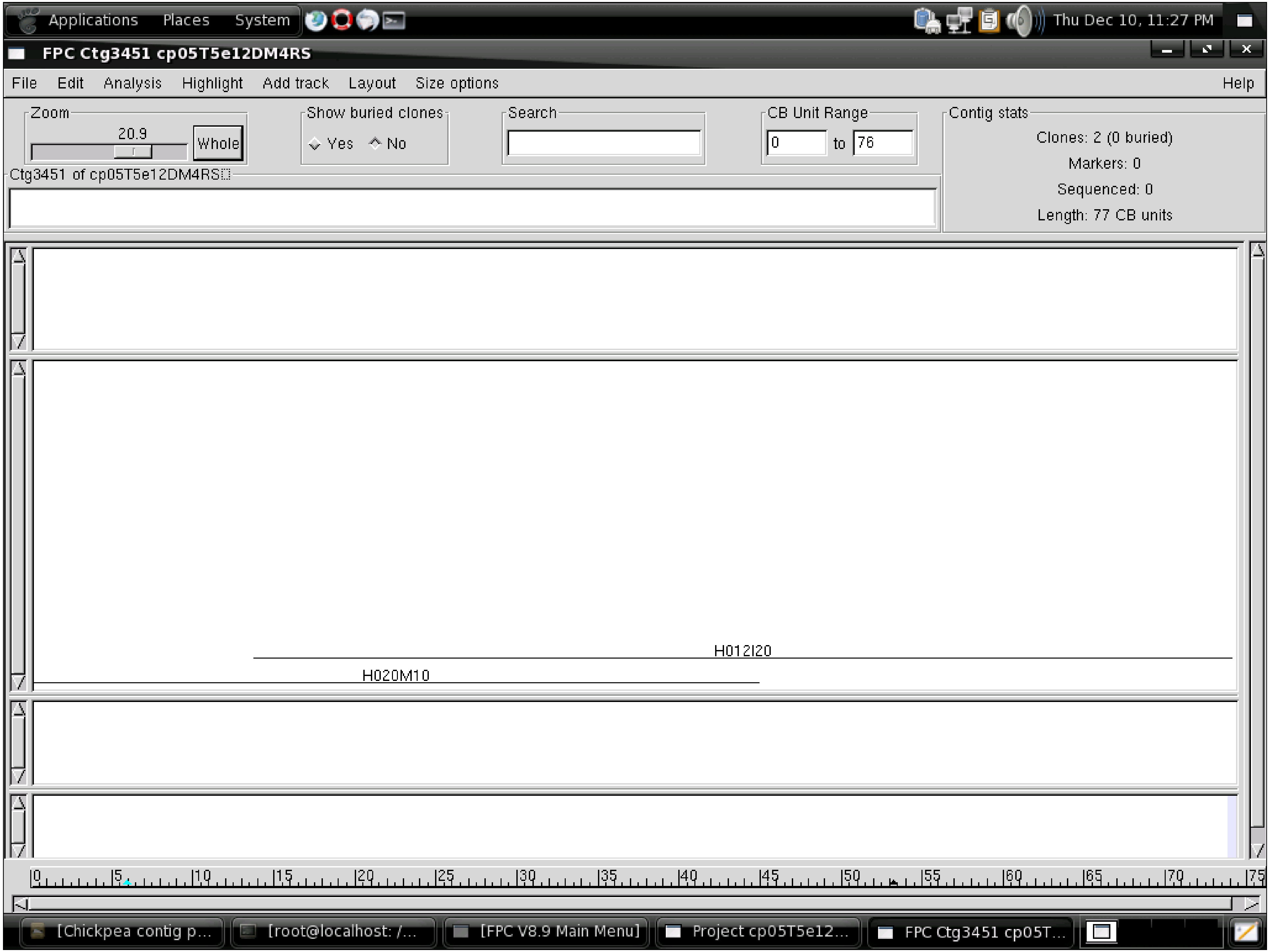
Applications Places System
Thu Dec 10, 11:27 PM
FPC Ctg3451 cp05T5e12DM4RS
File Edit Analysis Highlight Add track Layout Size options
Help
Zoom
20.9
Whole
Show buried clones
Yes No
Search
CB Unit Range
0 to 76
Contig stats
Clones: 2 (0 buried)
Markers: 0
Sequenced: 0
Length: 77 CB units
Ctg3451 of cp05T5e12DM4RS
H012I20
H020M10
0 5 10 15 20 25 30 35 40 45 50 55 60 65 70 75
[Chickpea contig p...
[root@localhost: /...
[FPC V8.9 Main Menu]
Project cp05T5e12...
FPC Ctg3451 cp05T...

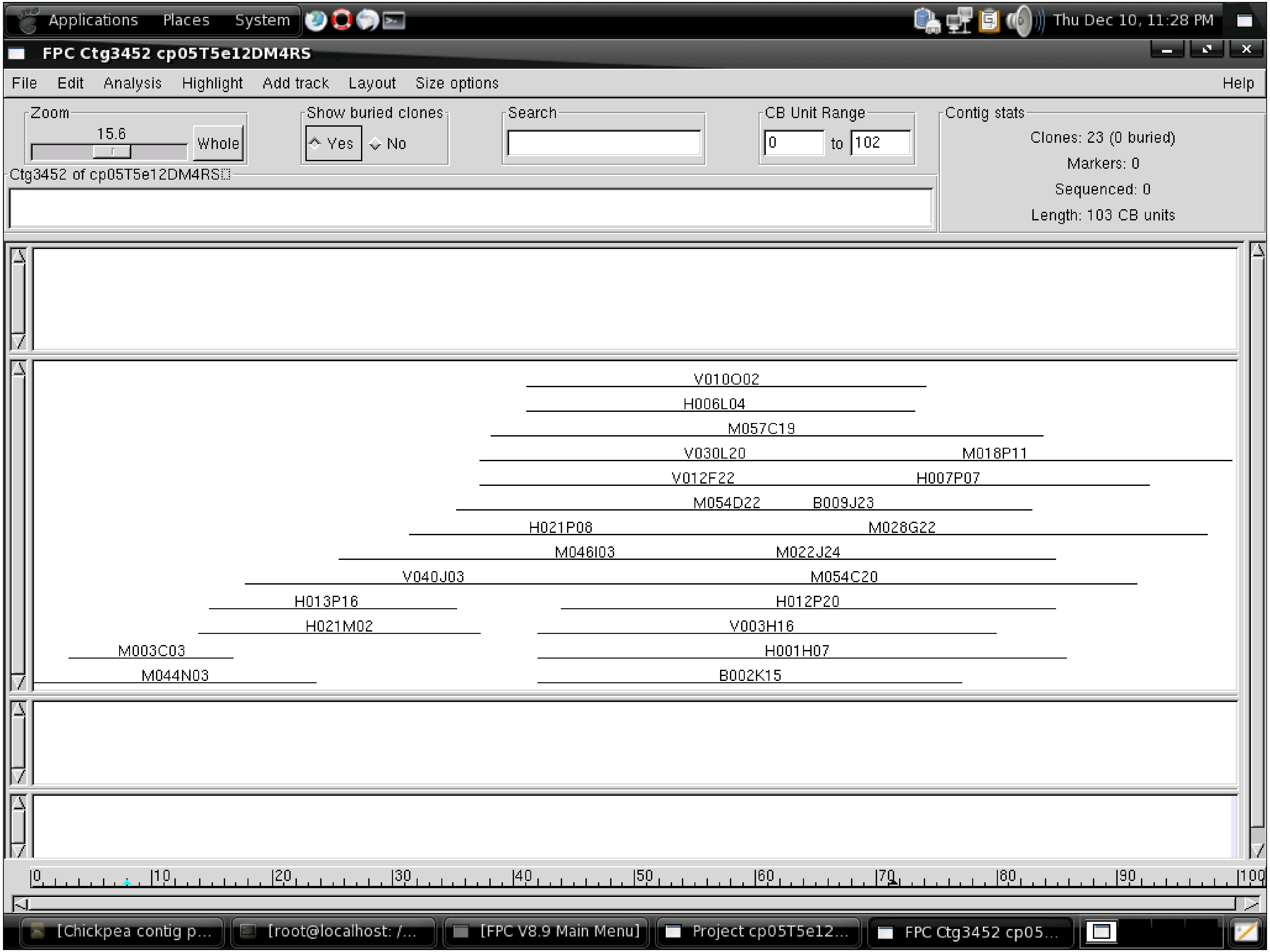

Applications Places System
Thu Dec 10, 11:28 PM
FPC Ctg3452 cp05T5e12DM4RS
File Edit Analysis Highlight Add track Layout Size options Help
Zoom
15.6
Whole
Show buried clones
Yes No
Search
CB Unit Range
0 to 102
Contig stats
Clones: 23 (0 buried)
Markers: 0
Sequenced: 0
Length: 103 CB units
Ctg3452 of cp05T5e12DM4RS
V010O02
H006L04
M057C19
V030L20
M018P11
V012F22
H007P07
M054D22
B009J23
H021P08
M028G22
M046I03
M022J24
V040J03
M054C20
H013P16
H012P20
H021M02
V003H16
M003C03
H001H07
M044N03
B002K15
0 10 20 30 40 50 60 70 80 90 100
[Chickpea contig p...
[root@localhost: /...
[FPC V8.9 Main Menu]
Project cp05T5e12...
FPC Ctg3452 cp05...

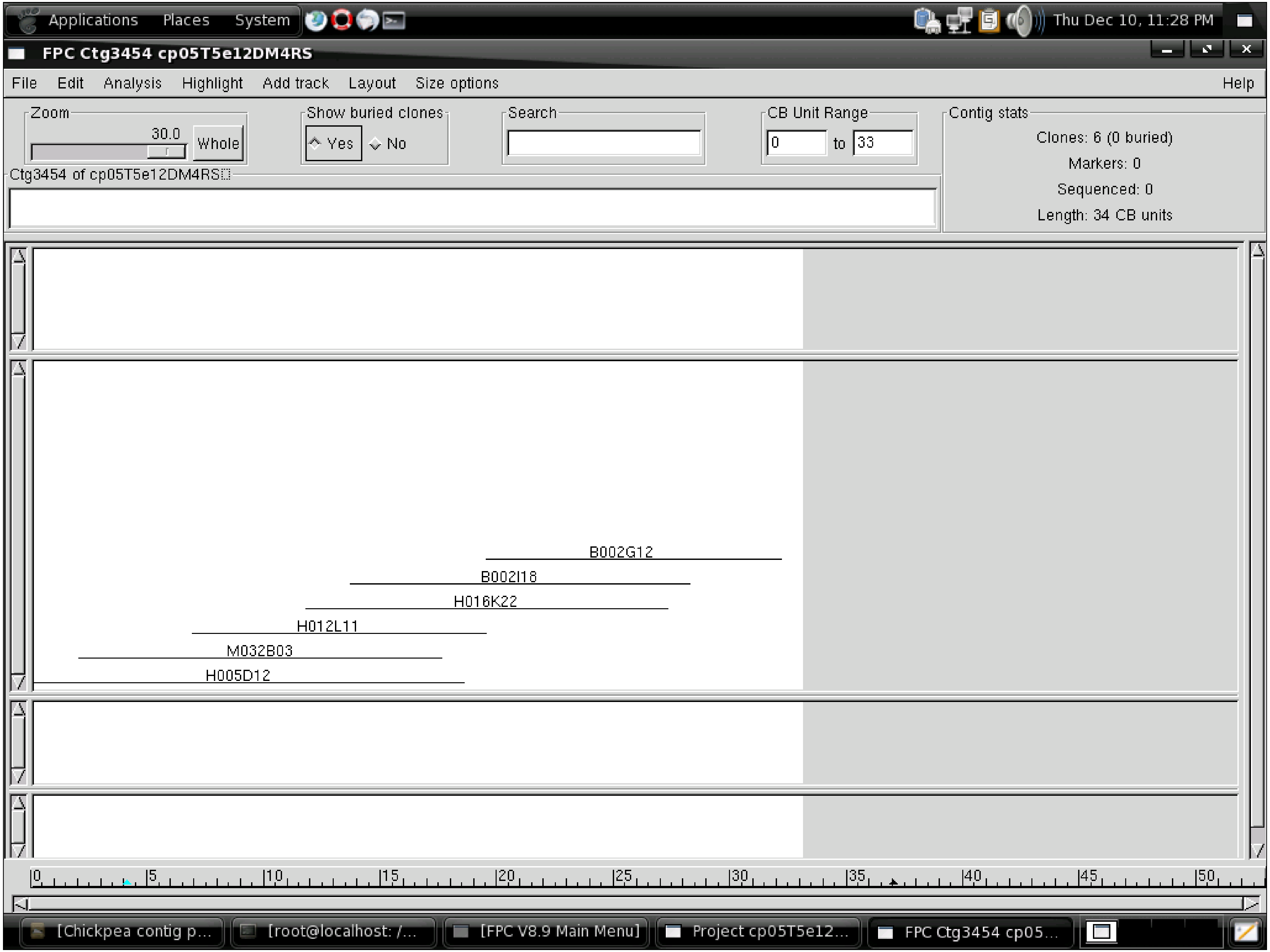
Applications
Places
System
Thu Dec 10, 11:28 PM
FPC Ctg3454 cp05T5e12DM4RS
File
Edit
Analysis
Highlight
Add track
Layout
Size options
Help
Zoom
30.0
Whole
Show buried clones
Yes
No
Search
CB Unit Range
0
to
33
Contig stats
Clones: 6 (0 buried)
Markers: 0
Sequenced: 0
Length: 34 CB units
Ctg3454 of cp05T5e12DM4RS
B002G12
B002I18
H016K22
H012L11
M032B03
H005D12
0
5
10
15
20
25
30
35
40
45
50
[Chickpea contig p...
[root@localhost: /...
[FPC V8.9 Main Menu]
Project cp05T5e12...
FPC Ctg3454 cp05...

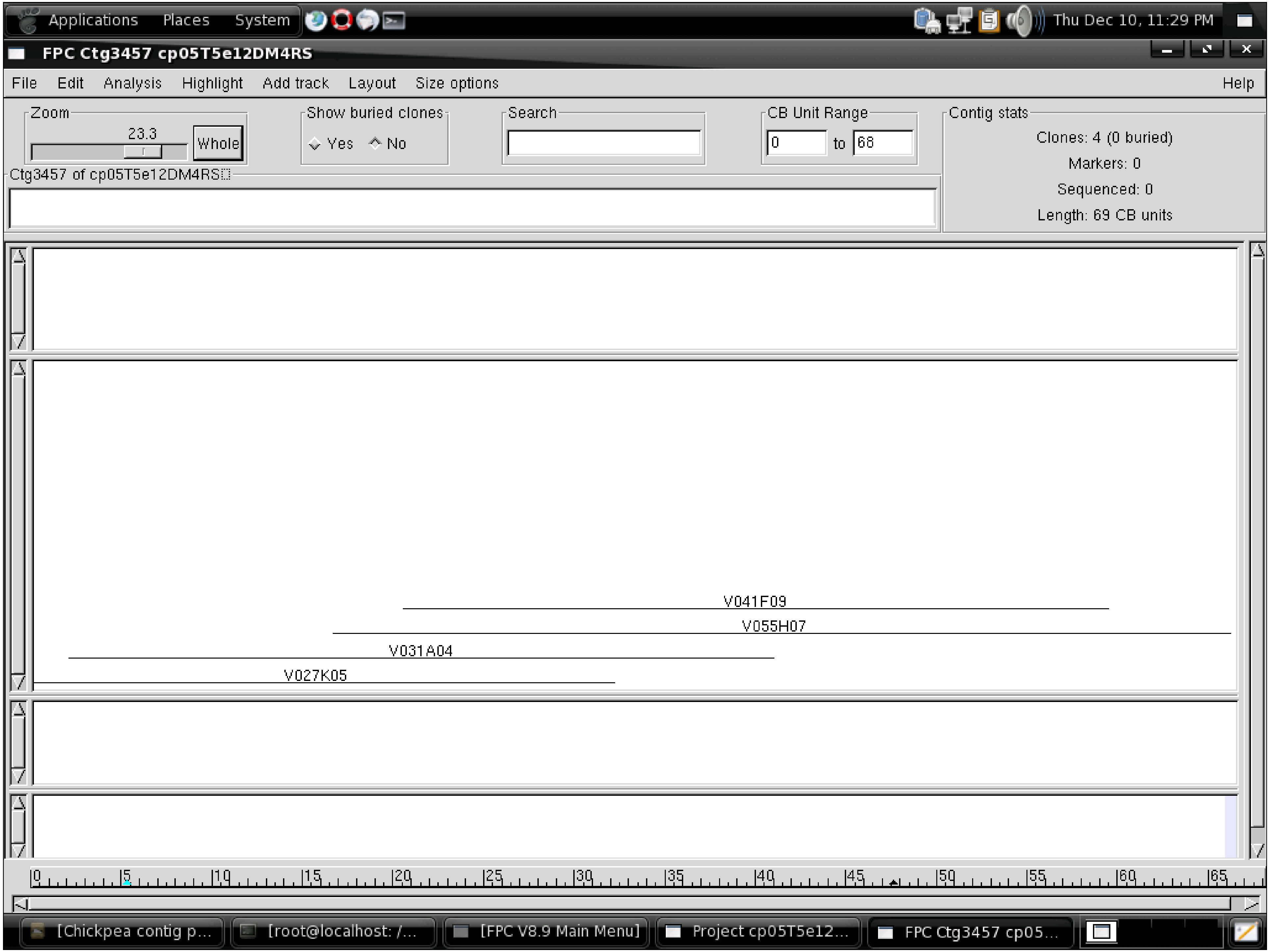
Applications
Places
System
Thu Dec 10, 11:29 PM
FPC Ctg3457 cp05T5e12DM4RS
File
Edit
Analysis
Highlight
Add track
Layout
Size options
Help
Zoom
23.3
Whole
Show buried clones
Yes
No
Search
CB Unit Range
0
to
68
Contig stats
Clones: 4 (0 buried)
Markers: 0
Sequenced: 0
Length: 69 CB units
Ctg3457 of cp05T5e12DM4RS
V041F09
V055H07
V031A04
V027K05
0
5
10
15
20
25
30
35
40
45
50
55
60
65
[Chickpea contig p...
[root@localhost: /...
[FPC V8.9 Main Menu]
Project cp05T5e12...
FPC Ctg3457 cp05...

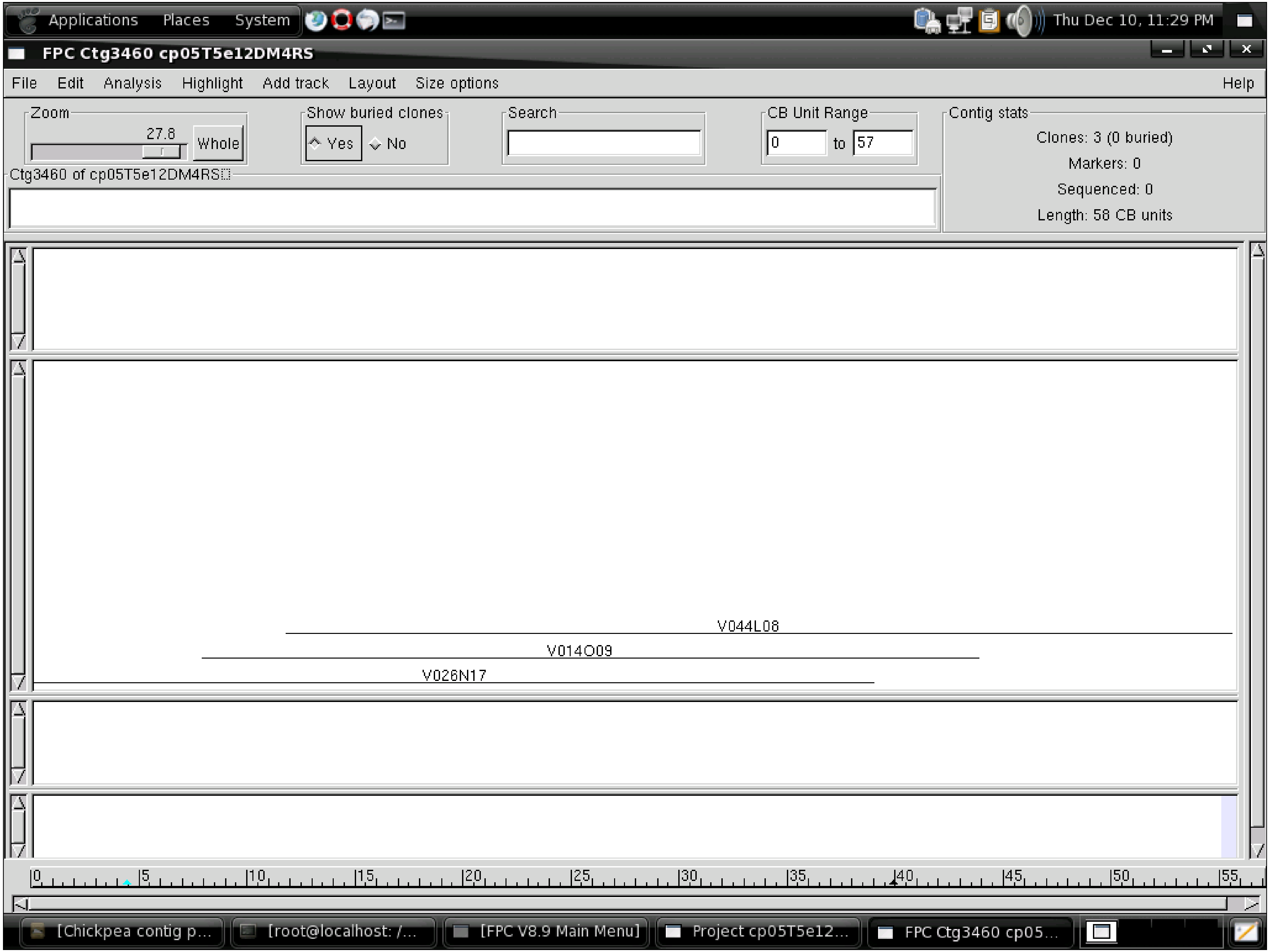
Applications Places System
Thu Dec 10, 11:29 PM
FPC Ctg3460 cp05T5e12DM4RS
File Edit Analysis Highlight Add track Layout Size options Help
Zoom
27.8
Whole
Show buried clones
Yes No
Search
CB Unit Range
0 to 57
Contig stats
Clones: 3 (0 buried)
Markers: 0
Sequenced: 0
Length: 58 CB units
Ctg3460 of cp05T5e12DM4RS
V044L08
V014O09
V026N17
0 5 10 15 20 25 30 35 40 45 50 55
[Chickpea contig p...
[root@localhost: /...
[FPC V8.9 Main Menu]
Project cp05T5e12...
FPC Ctg3460 cp05...

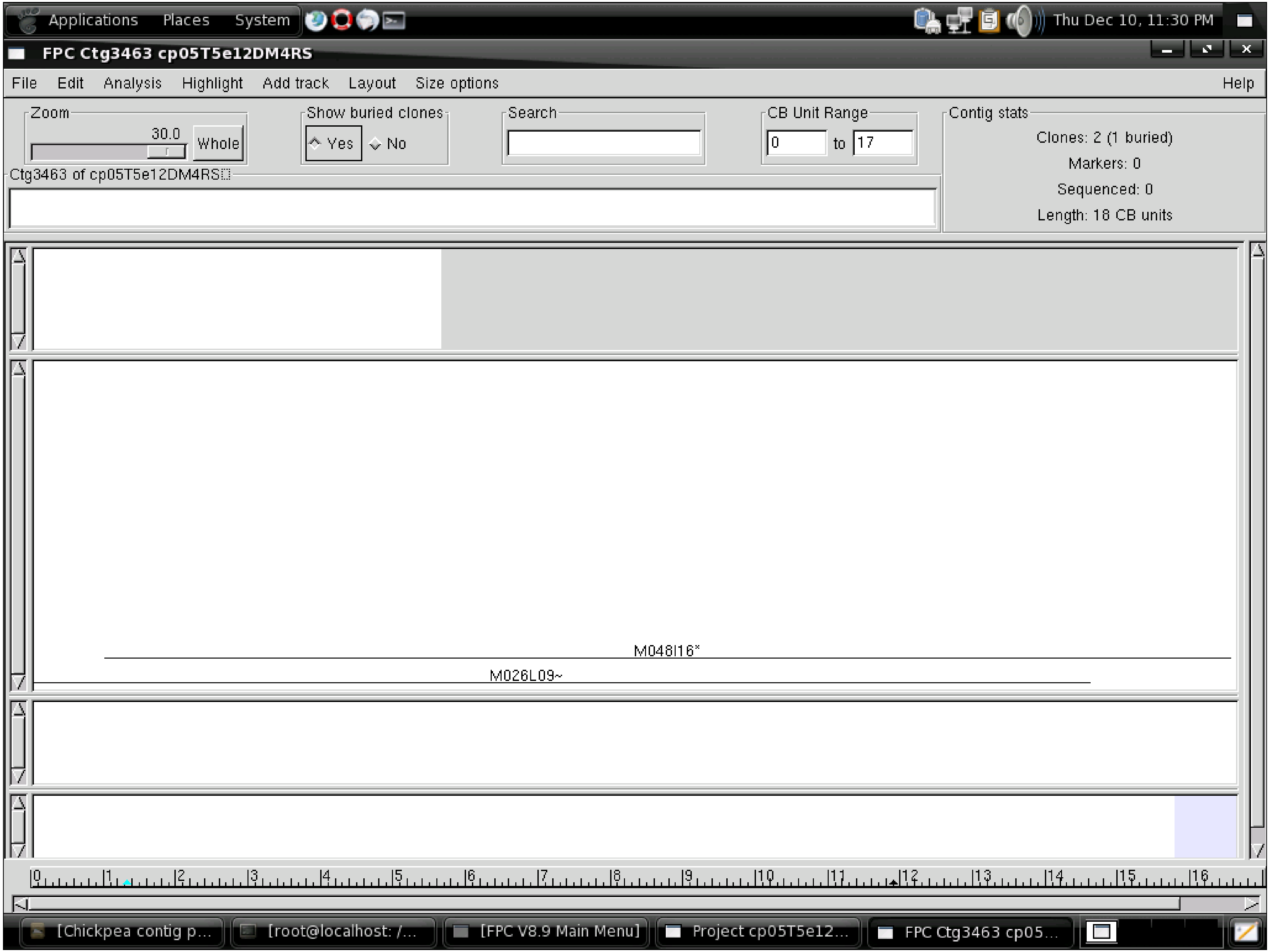
Applications Places System
Thu Dec 10, 11:30 PM
FPC Ctg3463 cp05T5e12DM4RS
File Edit Analysis Highlight Add track Layout Size options Help
Zoom
30.0
Whole
Show buried clones
Yes No
Search
CB Unit Range
0 to 17
Contig stats
Clones: 2 (1 buried)
Markers: 0
Sequenced: 0
Length: 18 CB units
Ctg3463 of cp05T5e12DM4RS
M048I16*
M026L09~
0 1 2 3 4 5 6 7 8 9 10 11 12 13 14 15 16
[Chickpea contig p...
[root@localhost: /...
[FPC V8.9 Main Menu]
Project cp05T5e12...
FPC Ctg3463 cp05...

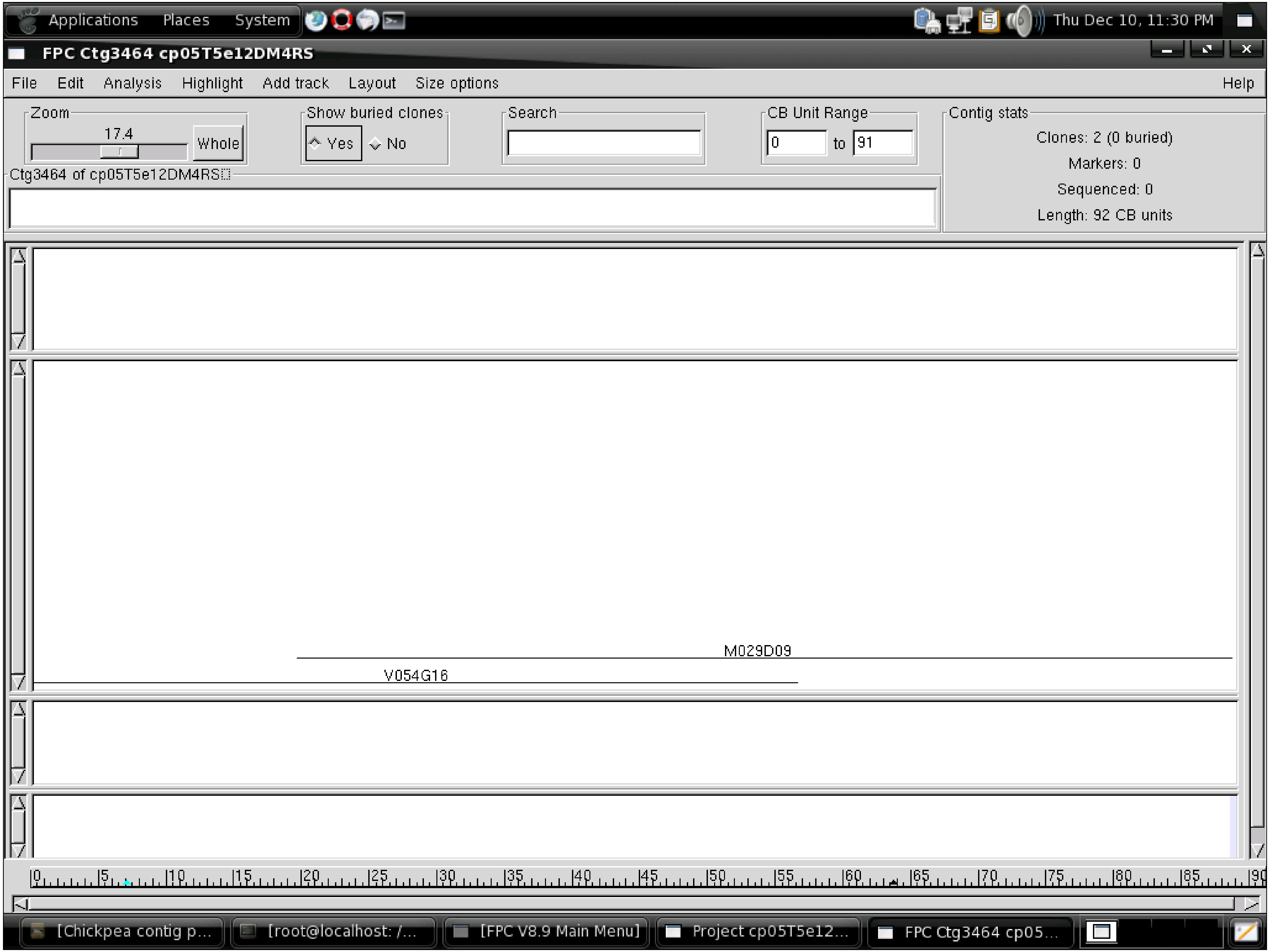
Applications Places System
Thu Dec 10, 11:30 PM
FPC Ctg3464 cp05T5e12DM4RS
File Edit Analysis Highlight Add track Layout Size options Help
Zoom
17.4
Whole
Show buried clones
Yes No
Search
CB Unit Range
0 to 91
Contig stats
Clones: 2 (0 buried)
Markers: 0
Sequenced: 0
Length: 92 CB units
Ctg3464 of cp05T5e12DM4RS
M029D09
V054G16
[Chickpea contig p...
[root@localhost: /...
[FPC V8.9 Main Menu]
Project cp05T5e12...
FPC Ctg3464 cp05...

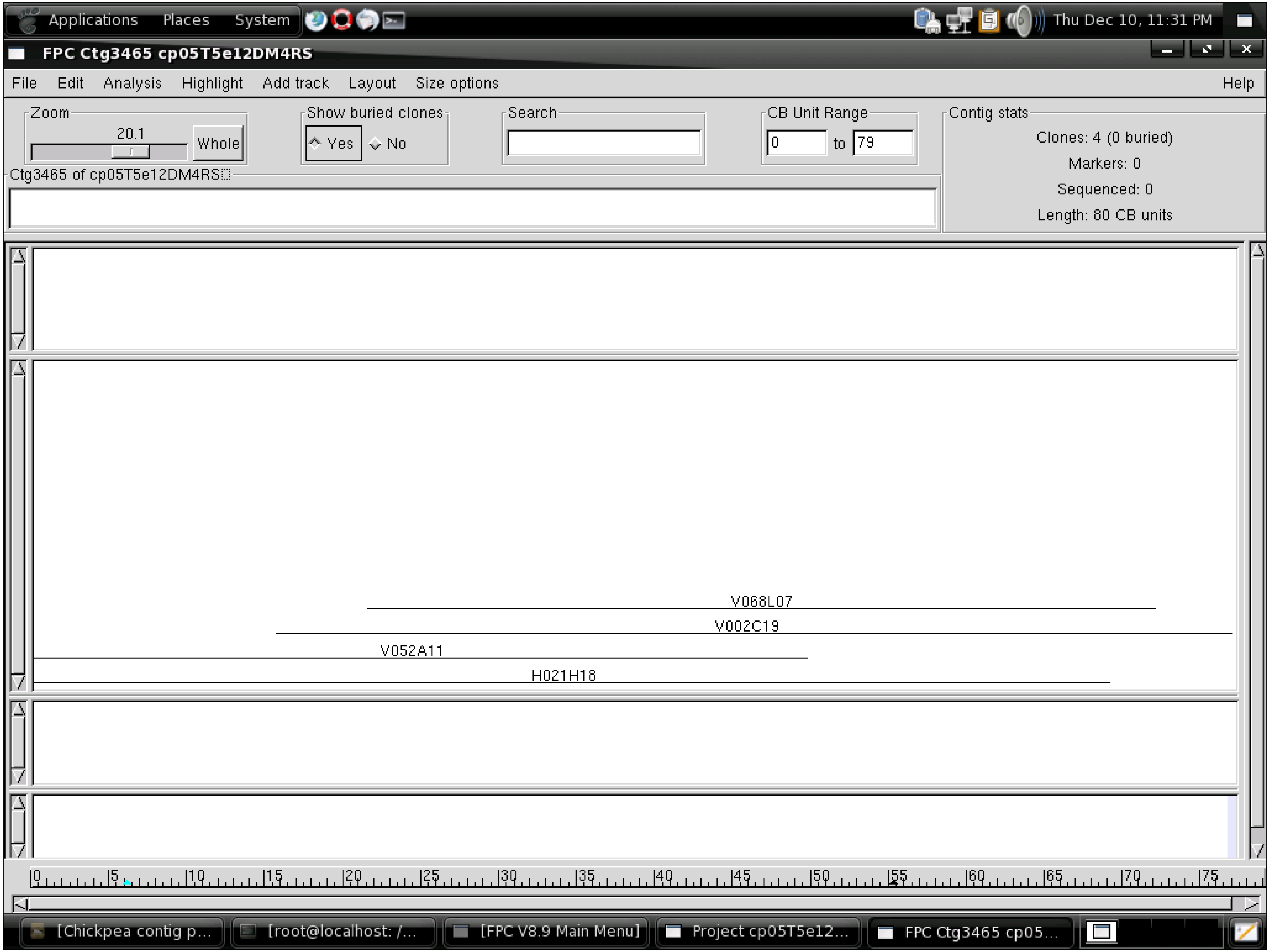
Applications
Places
System
Thu Dec 10, 11:31 PM
FPC Ctg3465 cp05T5e12DM4RS
File
Edit
Analysis
Highlight
Add track
Layout
Size options
Help
Zoom
20.1
Whole
Show buried clones
Yes
No
Search
CB Unit Range
0
to
79
Contig stats
Clones: 4 (0 buried)
Markers: 0
Sequenced: 0
Length: 80 CB units
Ctg3465 of cp05T5e12DM4RS
V068L07
V002C19
V052A11
H021H18
0
5
10
15
20
25
30
35
40
45
50
55
60
65
70
75
[Chickpea contig p...
[root@localhost: /...
[FPC V8.9 Main Menu]
Project cp05T5e12...
FPC Ctg3465 cp05...

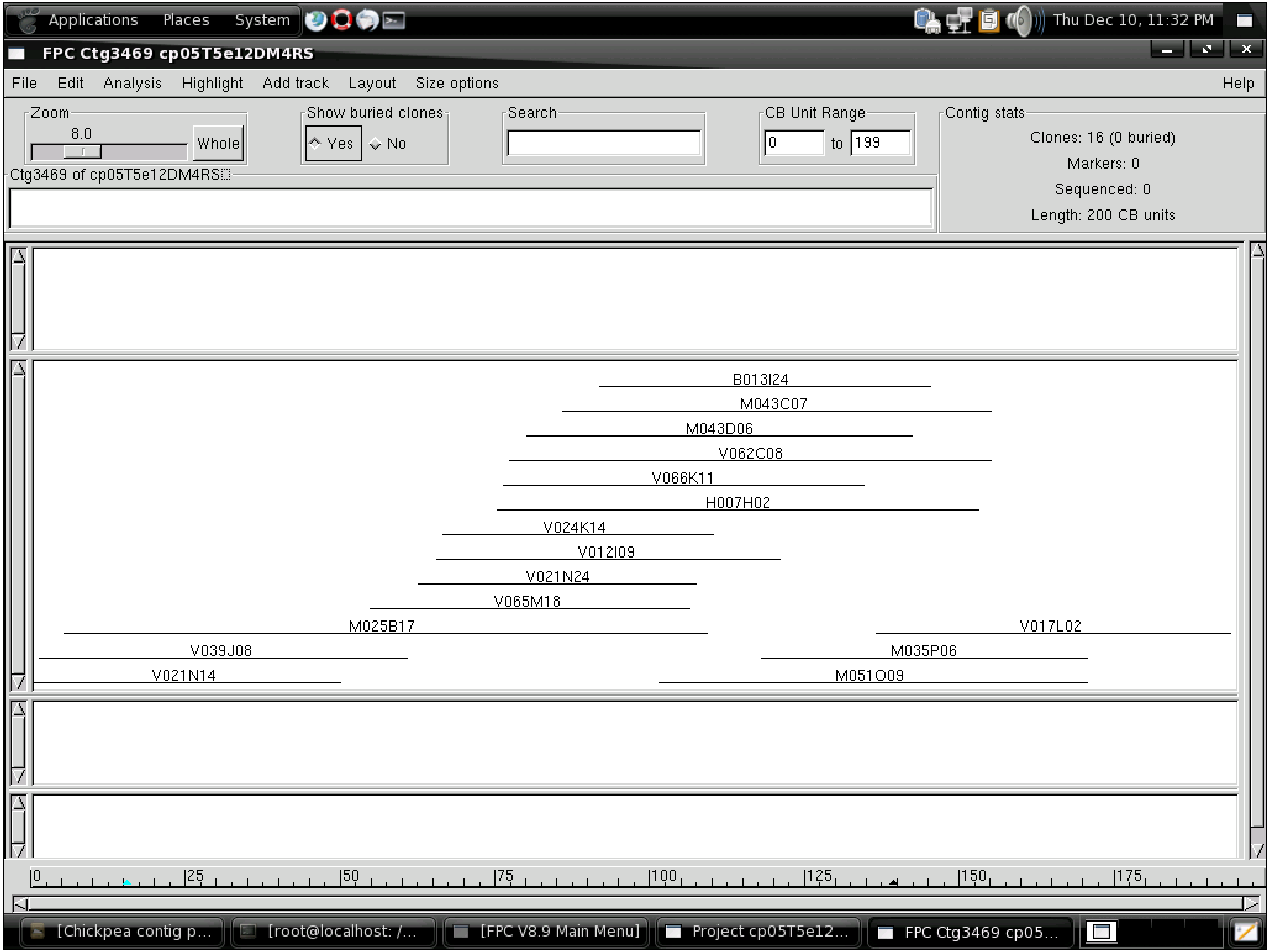
Applications Places System
Thu Dec 10, 11:32 PM
FPC Ctg3469 cp05T5e12DM4RS
File Edit Analysis Highlight Add track Layout Size options Help
Zoom
8.0
Whole
Show buried clones
Yes
No
Search
CB Unit Range
0 to 199
Contig stats
Clones: 16 (0 buried)
Markers: 0
Sequenced: 0
Length: 200 CB units
Ctg3469 of cp05T5e12DM4RS
B013I24
M043C07
M043D06
V062C08
V066K11
H007H02
V024K14
V012I09
V021N24
V065M18
M025B17
V017L02
V039J08
M035P06
V021N14
M051O09
0 25 50 75 100 125 150 175
[Chickpea contig p...
[root@localhost: /...
[FPC V8.9 Main Menu]
Project cp05T5e12...
FPC Ctg3469 cp05...

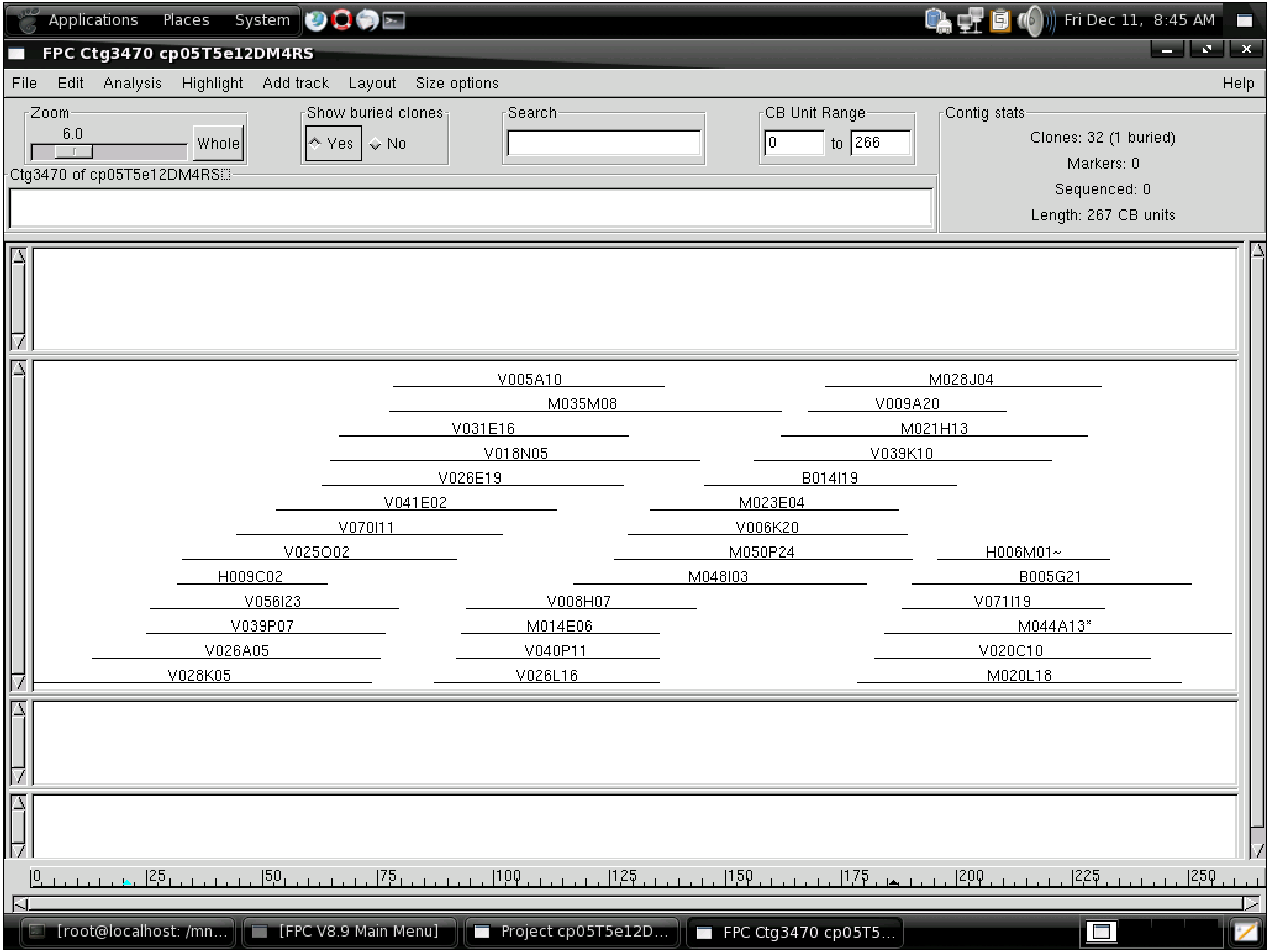
Applications Places System
Fri Dec 11, 8:45 AM
FPC Ctg3470 cp05T5e12DM4RS
File Edit Analysis Highlight Add track Layout Size options Help
Zoom
6.0
Whole
Show buried clones
Yes
No
Search
CB Unit Range
0
to
266
Contig stats
Clones: 32 (1 buried)
Markers: 0
Sequenced: 0
Length: 267 CB units
Ctg3470 of cp05T5e12DM4RS
V005A10
M028J04
M035M08
V009A20
V031E16
M021H13
V018N05
V039K10
V026E19
B014I19
V041E02
M023E04
V070I11
V006K20
V025O02
M050P24
H006M01~
H009C02
M048I03
B005G21
V056I23
V008H07
V071I19
V039P07
M014E06
M044A13*
V026A05
V040P11
V020C10
V028K05
V026L16
M020L18
0 25 50 75 100 125 150 175 200 225 250
[root@localhost: /mn...
[FPC V8.9 Main Menu]
Project cp05T5e12D...
FPC Ctg3470 cp05T5...

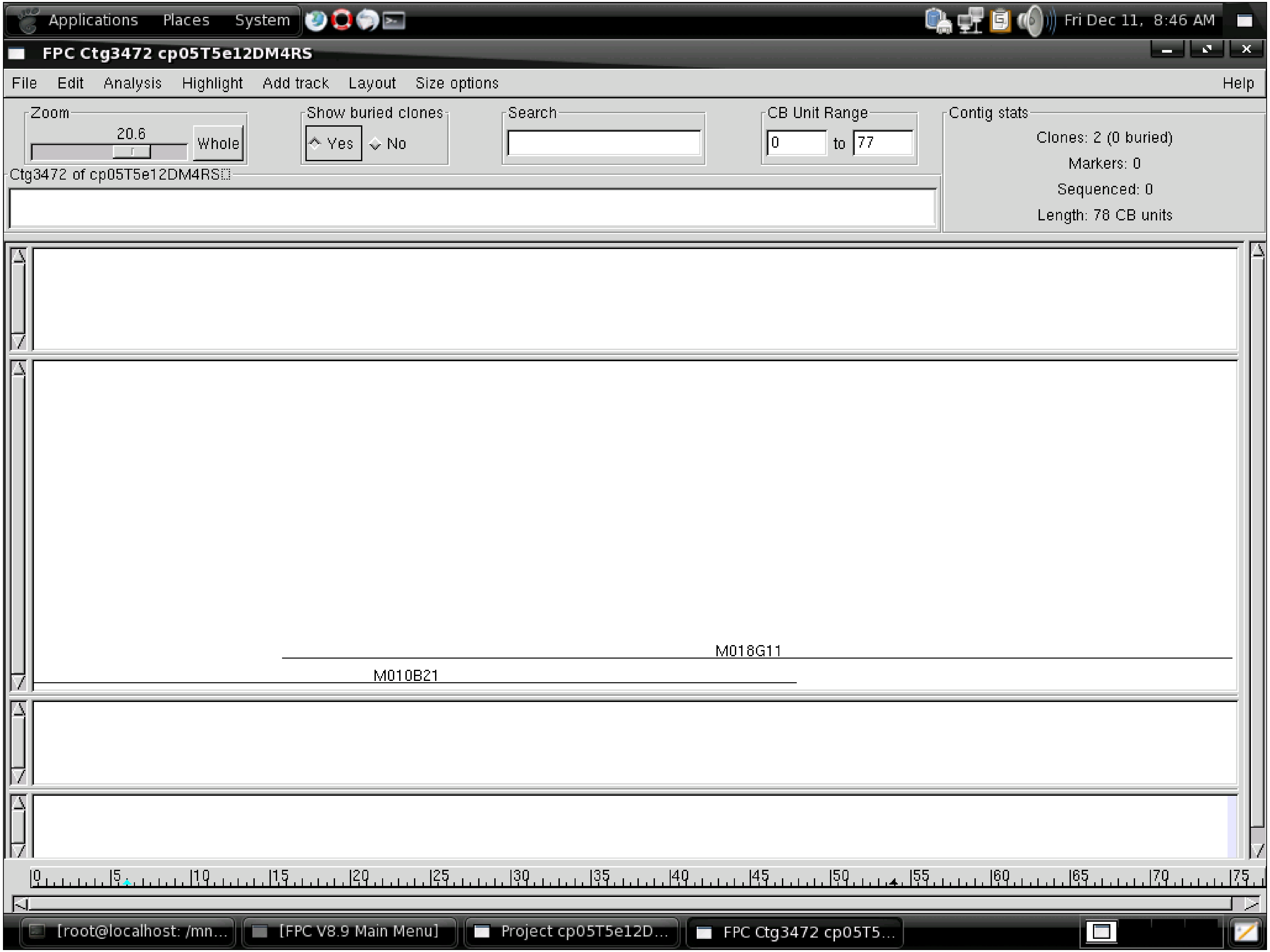
Applications
Places
System
Fri Dec 11, 8:46 AM
FPC Ctg3472 cp05T5e12DM4RS
File
Edit
Analysis
Highlight
Add track
Layout
Size options
Help
Zoom
20.6
Whole
Show buried clones
Yes
No
Search
CB Unit Range
0
to
77
Contig stats
Clones: 2 (0 buried)
Markers: 0
Sequenced: 0
Length: 78 CB units
Ctg3472 of cp05T5e12DM4RS
M018G11
M010B21
0 5 10 15 20 25 30 35 40 45 50 55 60 65 70 75
[root@localhost: /mn...
[FPC V8.9 Main Menu]
Project cp05T5e12D...
FPC Ctg3472 cp05T5...

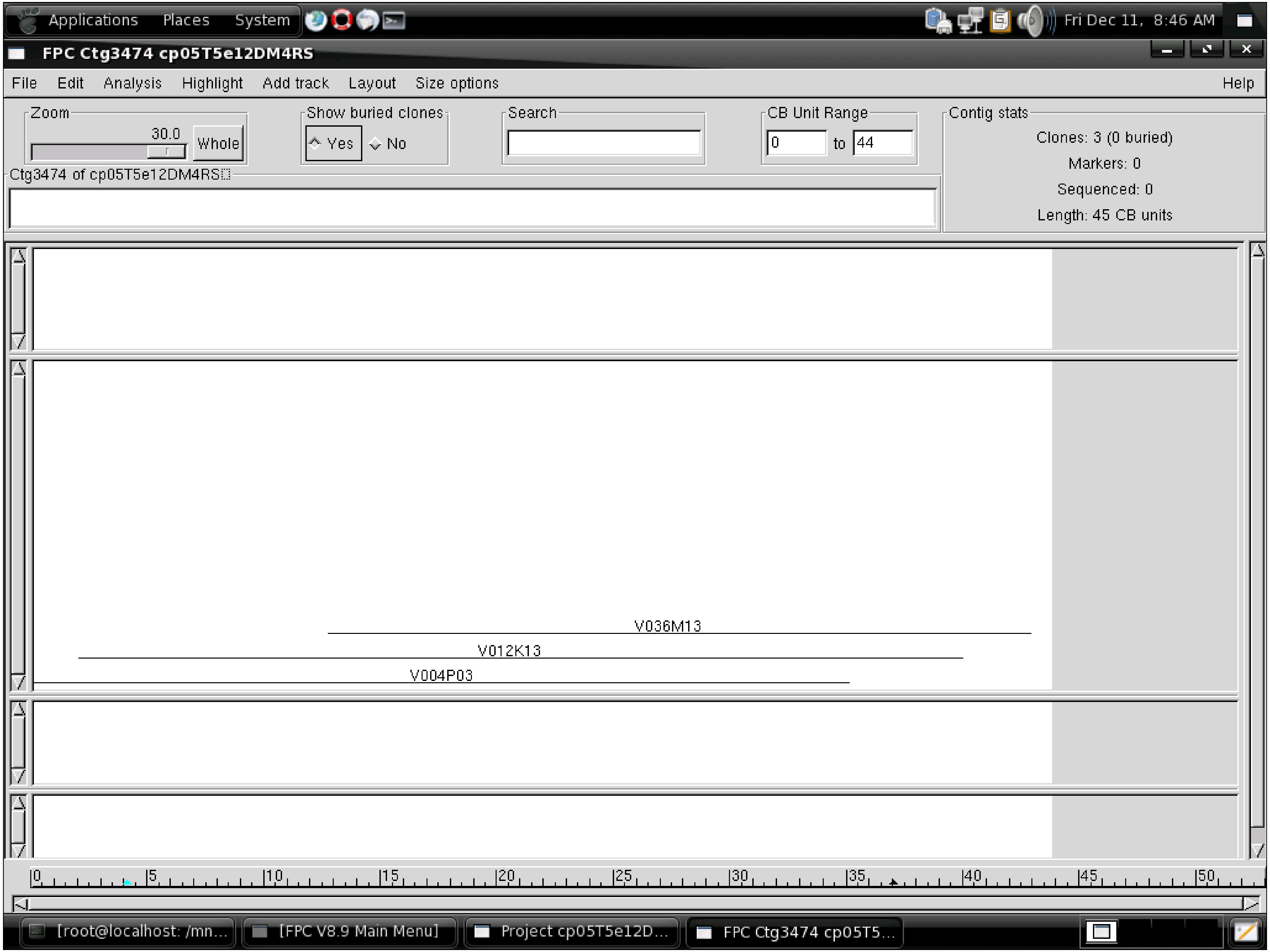
Applications
Places
System
Fri Dec 11, 8:46 AM
FPC Ctg3474 cp05T5e12DM4RS
File
Edit
Analysis
Highlight
Add track
Layout
Size options
Help
Zoom
30.0
Whole
Show buried clones
Yes
No
Search
CB Unit Range
0
to
44
Contig stats
Clones: 3 (0 buried)
Markers: 0
Sequenced: 0
Length: 45 CB units
Ctg3474 of cp05T5e12DM4RS
V036M13
V012K13
V004P03
0
5
10
15
20
25
30
35
40
45
50
[root@localhost: /mn...
[FPC V8.9 Main Menu]
Project cp05T5e12D...
FPC Ctg3474 cp05T5...

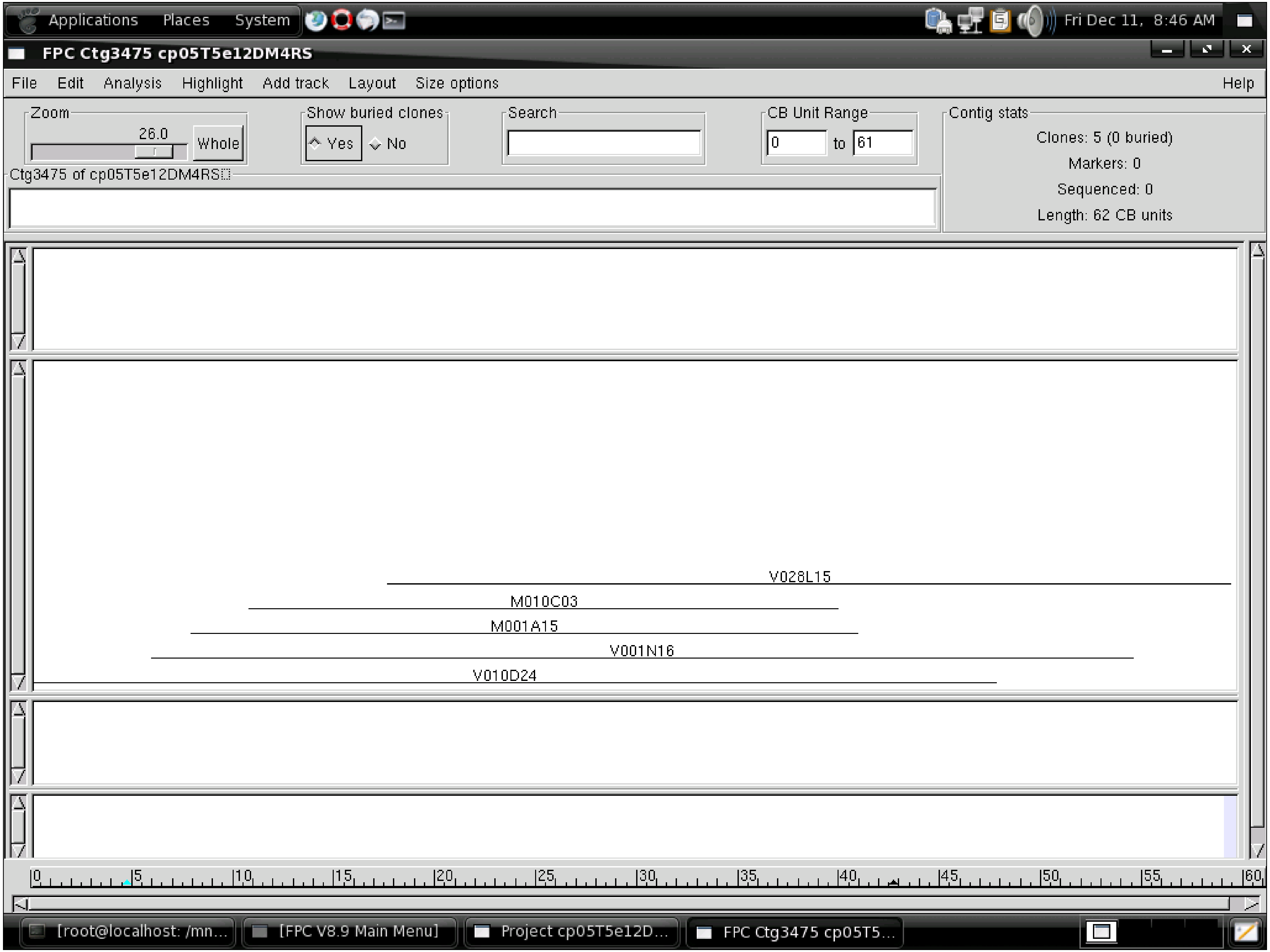
Applications Places System
Fri Dec 11, 8:46 AM
FPC Ctg3475 cp05T5e12DM4RS
File Edit Analysis Highlight Add track Layout Size options Help
Zoom
26.0
Whole
Show buried clones
Yes No
Search
CB Unit Range
0 to 61
Contig stats
Clones: 5 (0 buried)
Markers: 0
Sequenced: 0
Length: 62 CB units
Ctg3475 of cp05T5e12DM4RS
V028L15
M010C03
M001A15
V001N16
V010D24
0 5 10 15 20 25 30 35 40 45 50 55 60
[root@localhost: /mn...
[FPC V8.9 Main Menu]
Project cp05T5e12D...
FPC Ctg3475 cp05T5...

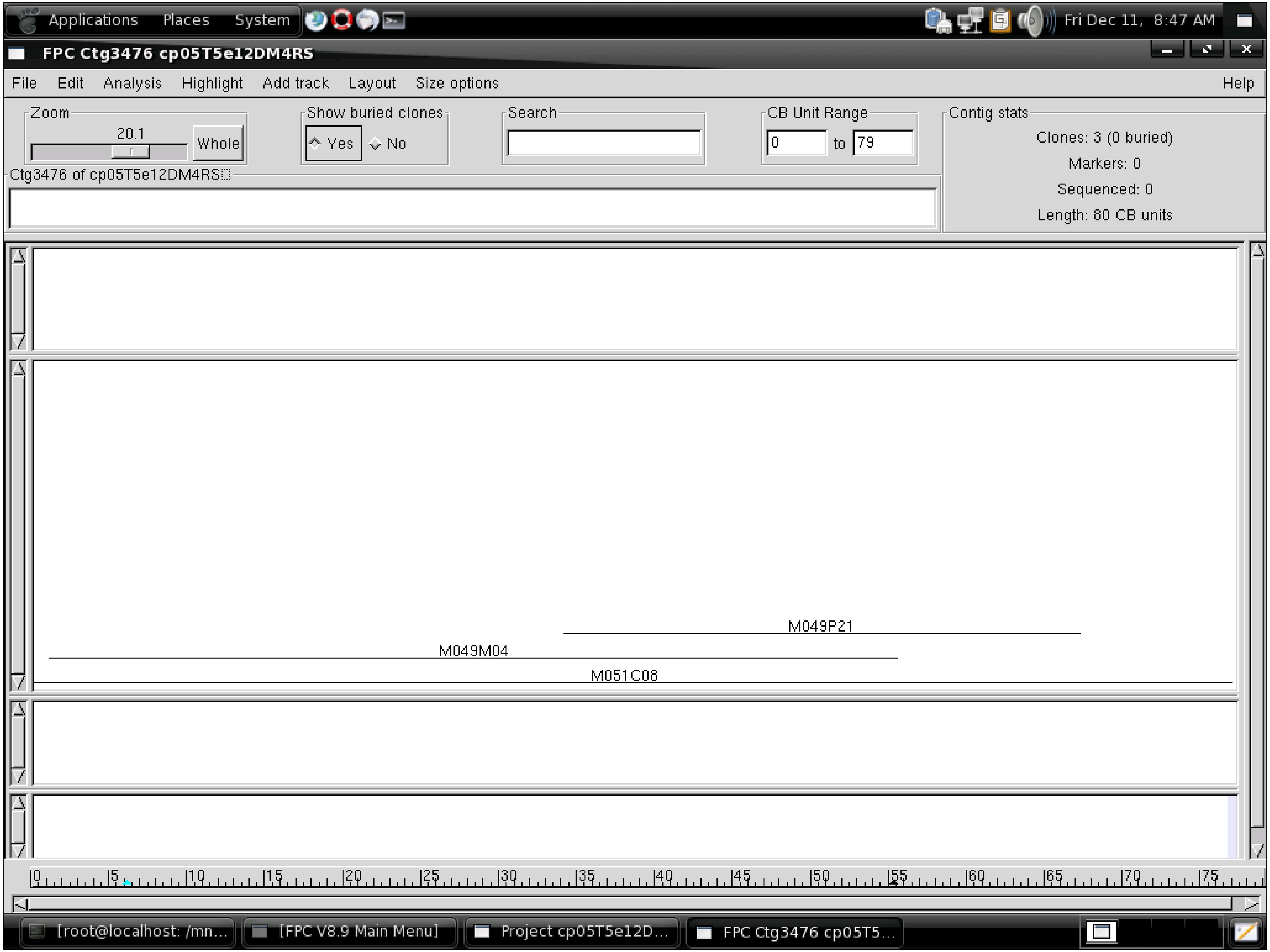
Applications Places System
Fri Dec 11, 8:47 AM
FPC Ctg3476 cp05T5e12DM4RS
File Edit Analysis Highlight Add track Layout Size options Help
Zoom
20.1
Whole
Show buried clones
Yes No
Search
CB Unit Range
0 to 79
Contig stats
Clones: 3 (0 buried)
Markers: 0
Sequenced: 0
Length: 80 CB units
Ctg3476 of cp05T5e12DM4RS
M049P21
M049M04
M051C08
0 5 10 15 20 25 30 35 40 45 50 55 60 65 70 75
[root@localhost: /mn...
[FPC V8.9 Main Menu]
Project cp05T5e12D...
FPC Ctg3476 cp05T5...

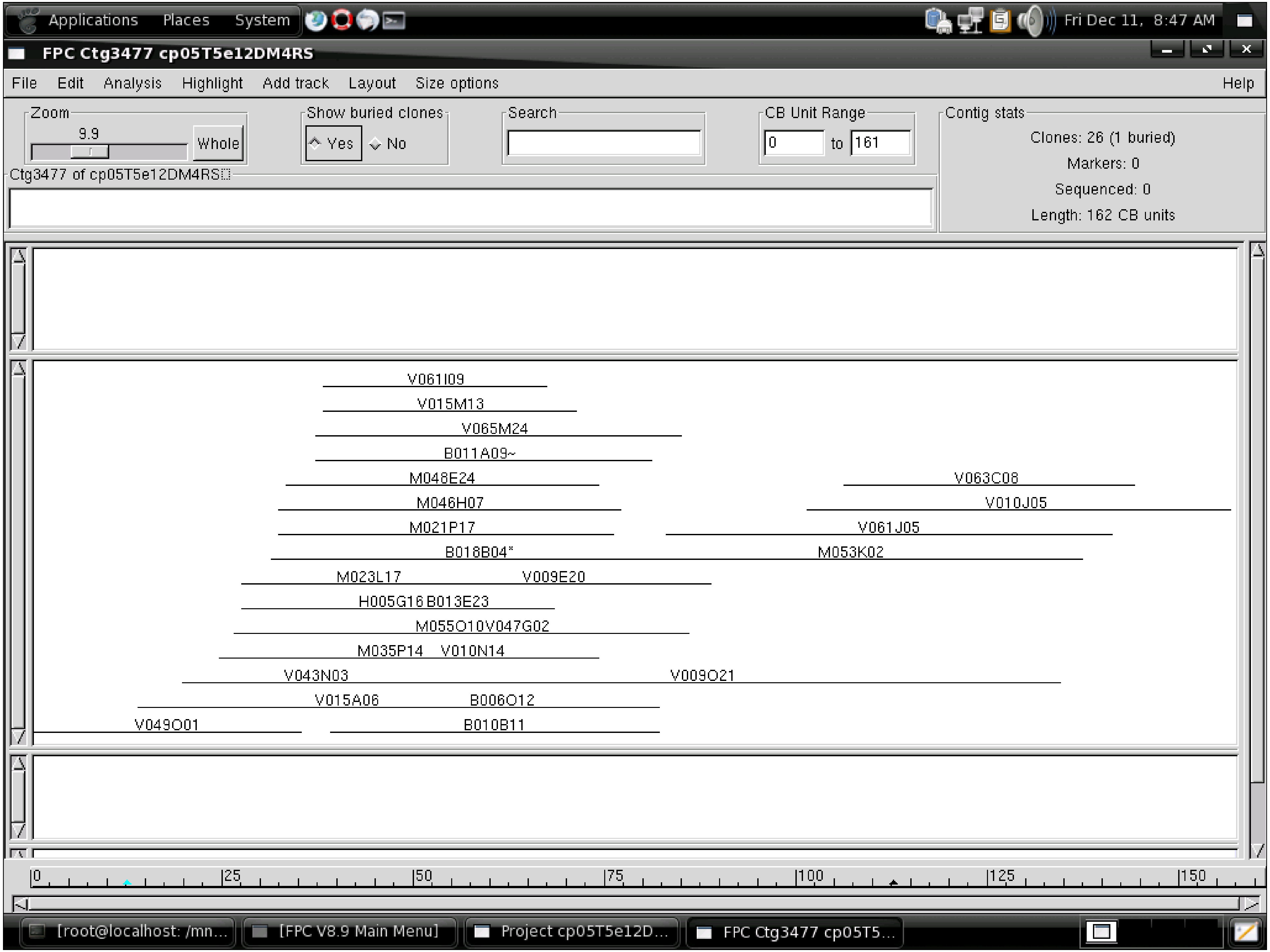
Applications Places System
Fri Dec 11, 8:47 AM
FPC Ctg3477 cp05T5e12DM4RS
File Edit Analysis Highlight Add track Layout Size options Help
Zoom
9.9
Whole
Show buried clones
Yes
No
Search
CB Unit Range
0 to 161
Contig stats
Clones: 26 (1 buried)
Markers: 0
Sequenced: 0
Length: 162 CB units
Ctg3477 of cp05T5e12DM4RS
V061I09
V015M13
V065M24
B011A09~
M048E24
V063C08
M046H07
V010J05
M021P17
V061J05
B018B04*
M053K02
M023L17
V009E20
H005G16 B013E23
M055O10V047G02
M035P14 V010N14
V043N03
V009O21
V015A06
B006O12
V049O01
B010B11
0 25 50 75 100 125 150
[root@localhost: /mn...
[FPC V8.9 Main Menu]
Project cp05T5e12D...
FPC Ctg3477 cp05T5...

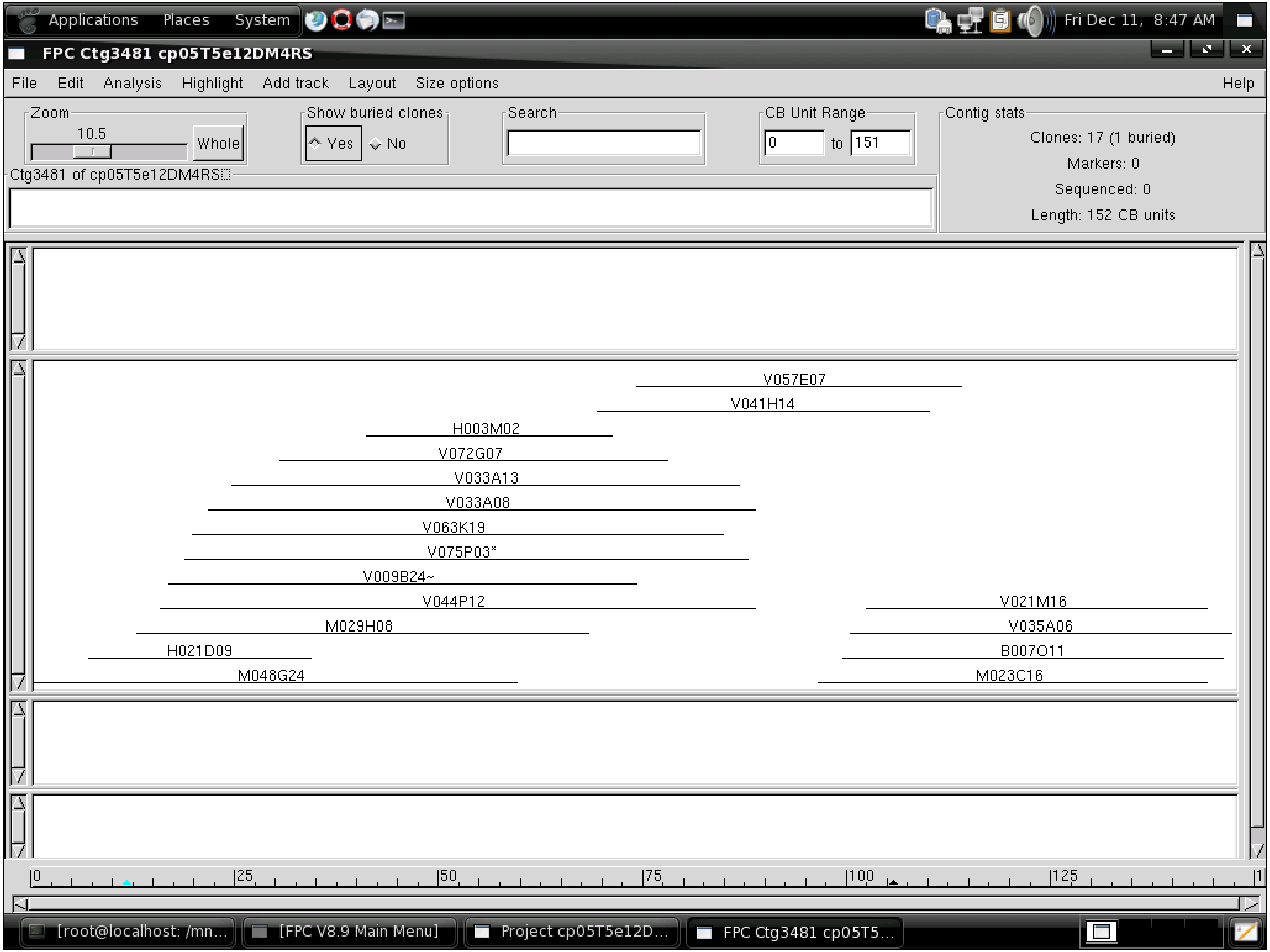
Applications Places System
Fri Dec 11, 8:47 AM
FPC Ctg3481 cp05T5e12DM4RS
File Edit Analysis Highlight Add track Layout Size options Help
Zoom
10.5
Whole
Show buried clones
Yes No
Search
CB Unit Range
0 to 151
Contig stats
Clones: 17 (1 buried)
Markers: 0
Sequenced: 0
Length: 152 CB units
Ctg3481 of cp05T5e12DM4RS
V057E07
V041H14
H003M02
V072G07
V033A13
V033A08
V063K19
V075P03*
V009B24~
V044P12
V021M16
M029H08
V035A06
H021D09
B007O11
M048G24
M023C16
0 25 50 75 100 125
[root@localhost: /mn...
[FPC V8.9 Main Menu]
Project cp05T5e12D...
FPC Ctg3481 cp05T5...

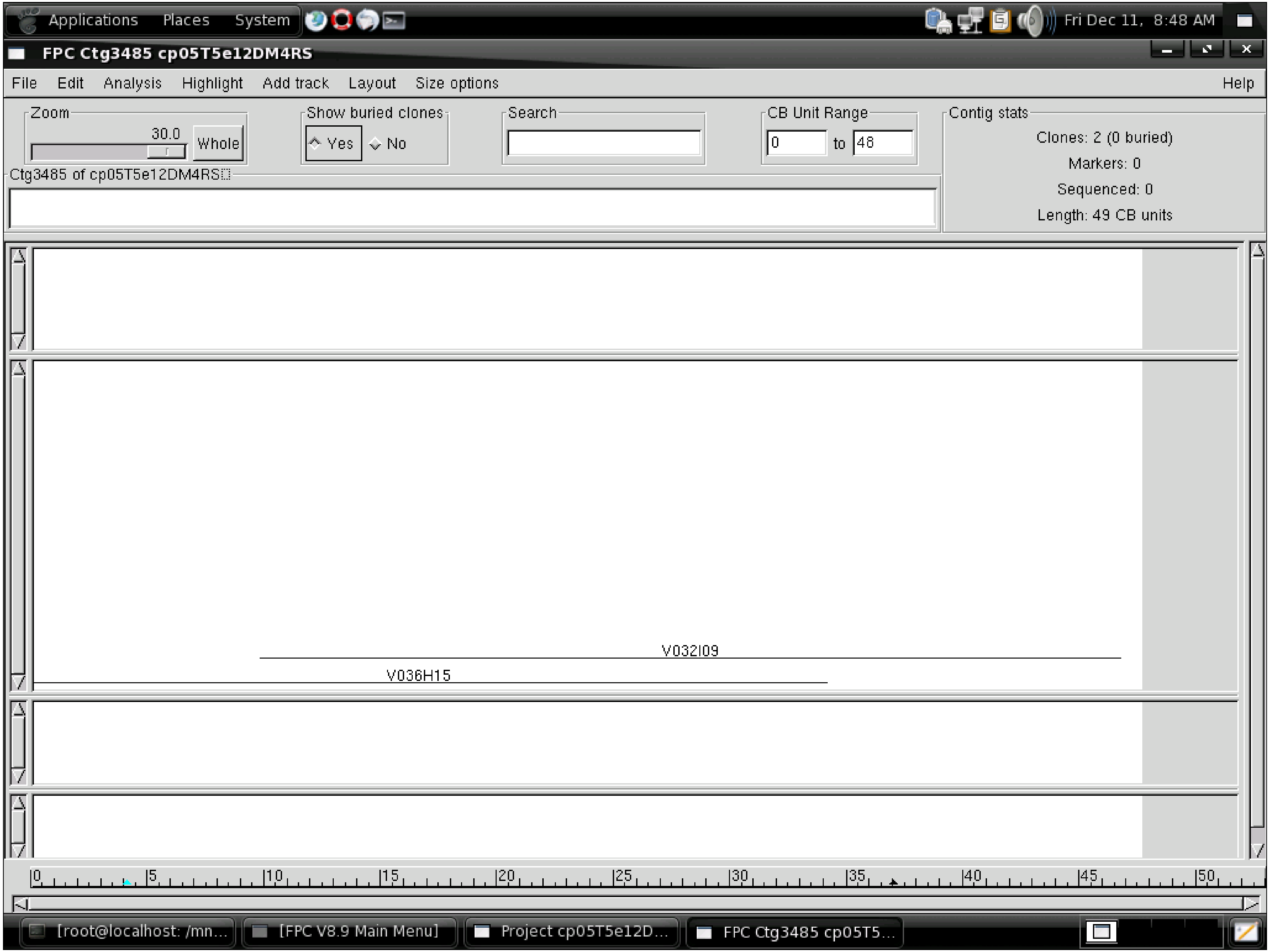
Applications Places System
Fri Dec 11, 8:48 AM
FPC Ctg3485 cp05T5e12DM4RS
File Edit Analysis Highlight Add track Layout Size options
Help
Zoom
30.0
Whole
Show buried clones
Yes
No
Search
CB Unit Range
0
to
48
Contig stats
Clones: 2 (0 buried)
Markers: 0
Sequenced: 0
Length: 49 CB units
Ctg3485 of cp05T5e12DM4RS
V032I09
V036H15
0 5 10 15 20 25 30 35 40 45 50
[root@localhost: /mn...
[FPC V8.9 Main Menu]
Project cp05T5e12D...
FPC Ctg3485 cp05T5...

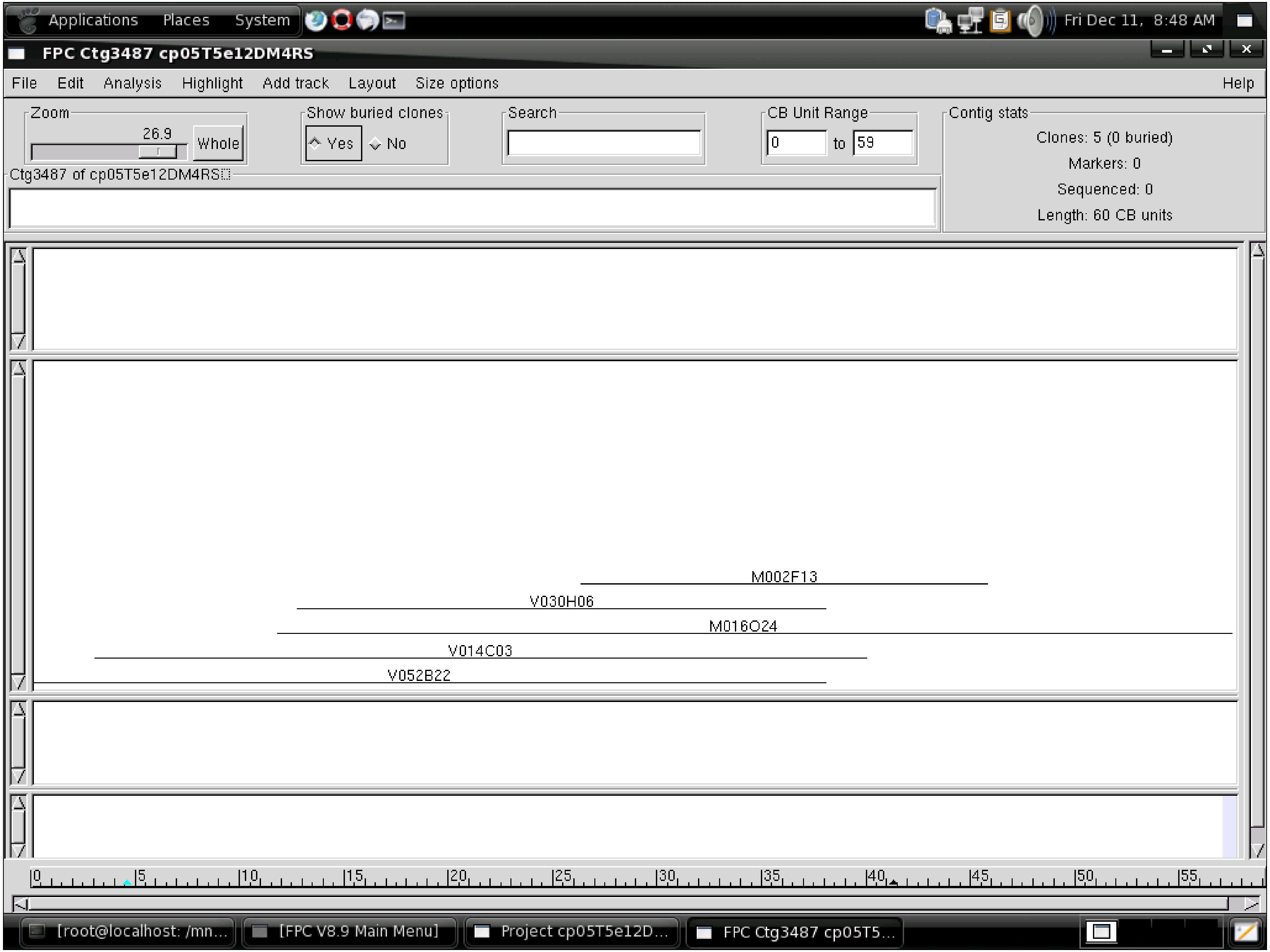
Applications
Places
System
Fri Dec 11, 8:48 AM
FPC Ctg3487 cp05T5e12DM4RS
File
Edit
Analysis
Highlight
Add track
Layout
Size options
Help
Zoom
26.9
Whole
Show buried clones
Yes
No
Search
CB Unit Range
0
to
59
Contig stats
Clones: 5 (0 buried)
Markers: 0
Sequenced: 0
Length: 60 CB units
Ctg3487 of cp05T5e12DM4RS
M002F13
V030H06
M016O24
V014C03
V052B22
0
5
10
15
20
25
30
35
40
45
50
55
[root@localhost: /mn...
[FPC V8.9 Main Menu]
Project cp05T5e12D...
FPC Ctg3487 cp05T5...

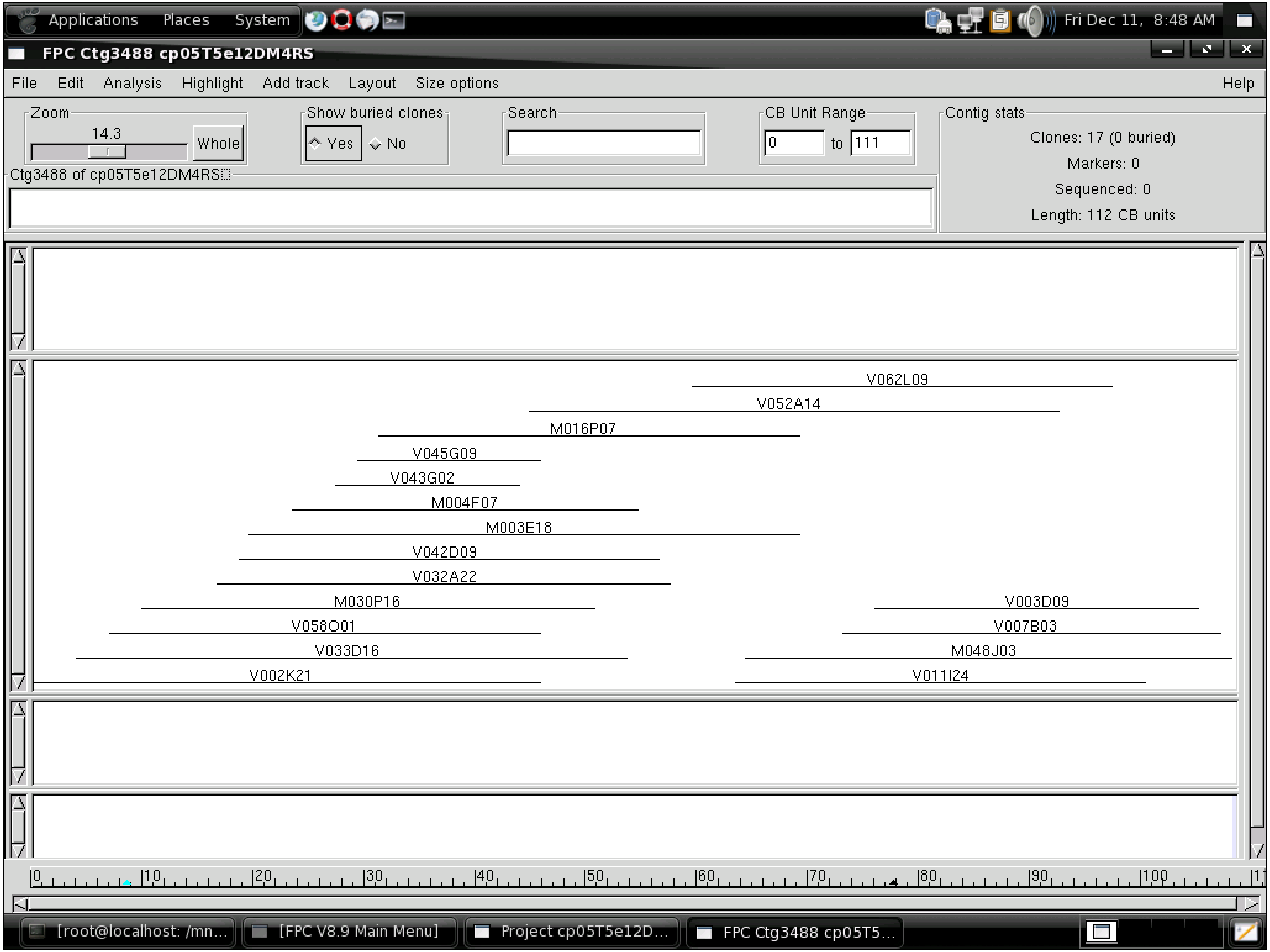
Applications Places System
Fri Dec 11, 8:48 AM
FPC Ctg3488 cp05T5e12DM4RS
File Edit Analysis Highlight Add track Layout Size options Help
Zoom
14.3
Whole
Show buried clones
Yes No
Search
CB Unit Range
0 to 111
Contig stats
Clones: 17 (0 buried)
Markers: 0
Sequenced: 0
Length: 112 CB units
Ctg3488 of cp05T5e12DM4RS
V062L09
V052A14
M016P07
V045G09
V043G02
M004F07
M003E18
V042D09
V032A22
M030P16
V003D09
V058O01
V007B03
V033D16
M048J03
V002K21
V011I24
0 10 20 30 40 50 60 70 80 90 100
[root@localhost: /mn...
[FPC V8.9 Main Menu]
Project cp05T5e12D...
FPC Ctg3488 cp05T5...

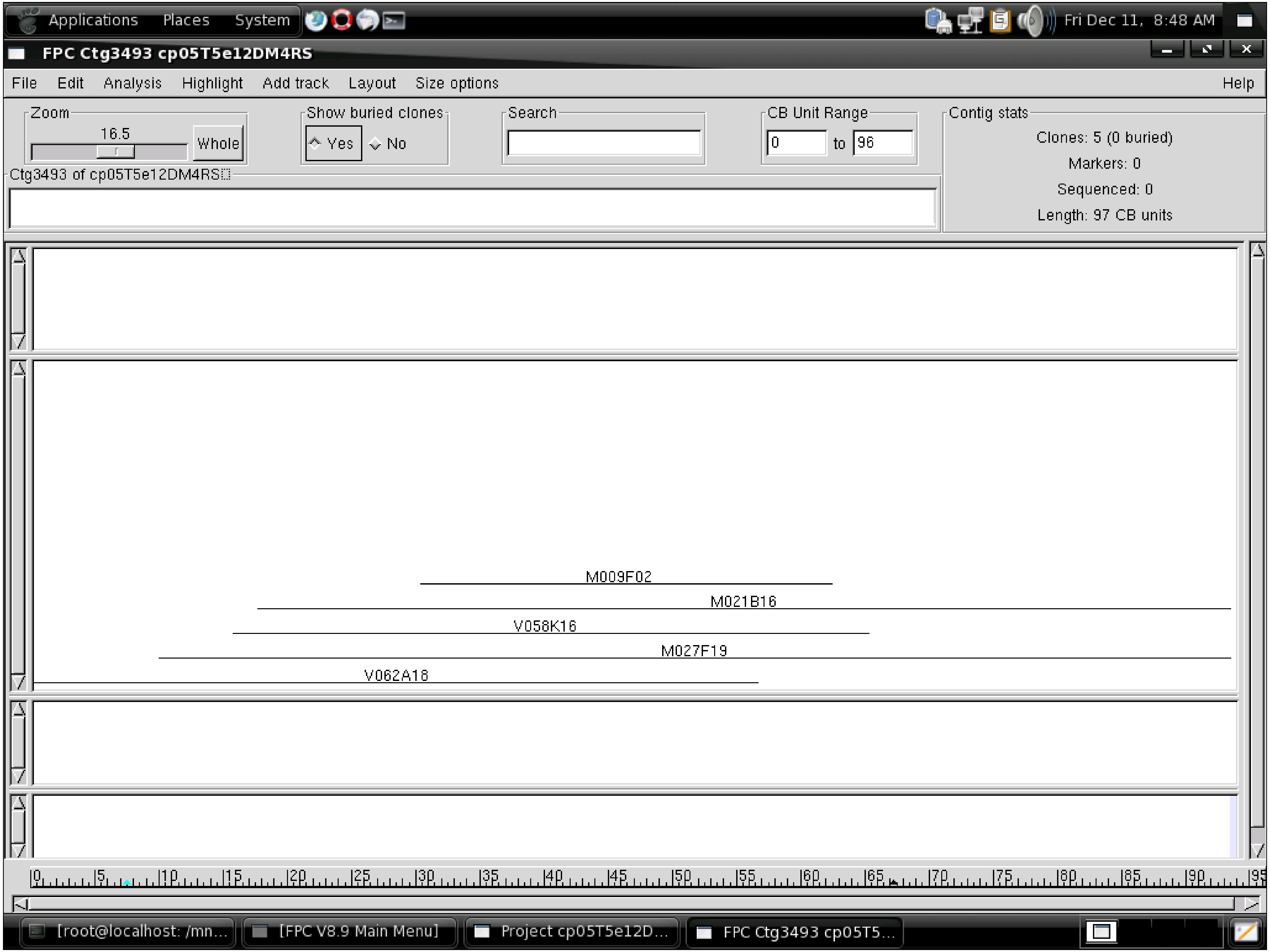
Applications Places System
Fri Dec 11, 8:48 AM
FPC Ctg3493 cp05T5e12DM4RS
File Edit Analysis Highlight Add track Layout Size options Help
Zoom
16.5
Whole
Show buried clones
Yes No
Search
CB Unit Range
0 to 96
Contig stats
Clones: 5 (0 buried)
Markers: 0
Sequenced: 0
Length: 97 CB units
Ctg3493 of cp05T5e12DM4RS
M009F02
M021B16
V058K16
M027F19
V062A18
0 5 10 15 20 25 30 35 40 45 50 55 60 65 70 75 80 85 90 95
[root@localhost: /mn...
[FPC V8.9 Main Menu]
Project cp05T5e12D...
FPC Ctg3493 cp05T5...

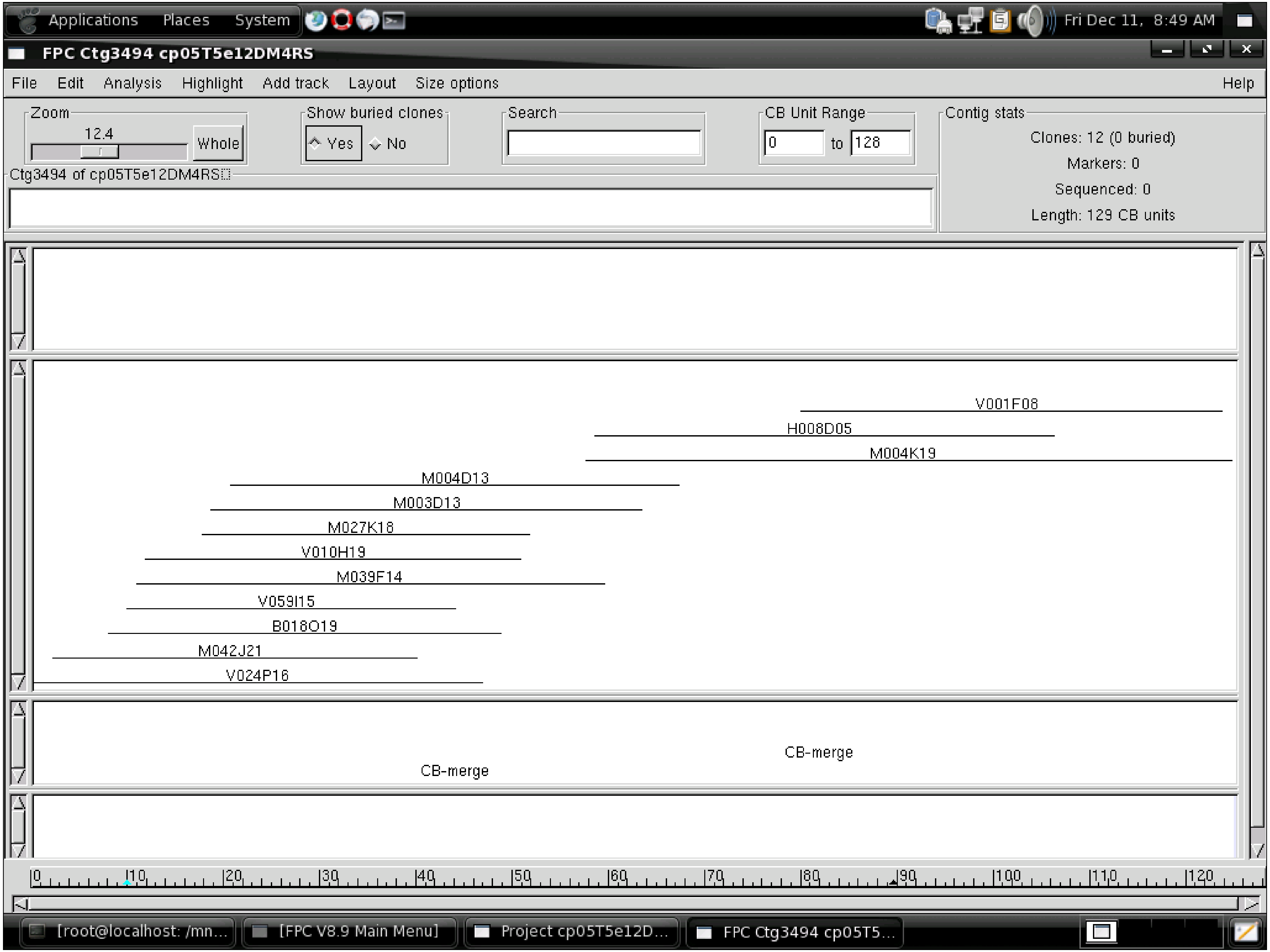
Applications Places System
Fri Dec 11, 8:49 AM
FPC Ctg3494 cp05T5e12DM4RS
File Edit Analysis Highlight Add track Layout Size options
Help
Zoom
12.4
Whole
Show buried clones
Yes
No
Search
CB Unit Range
0
to
128
Contig stats
Clones: 12 (0 buried)
Markers: 0
Sequenced: 0
Length: 129 CB units
Ctg3494 of cp05T5e12DM4RS
V001F08
H008D05
M004K19
M004D13
M003D13
M027K18
V010H19
M039F14
V059I15
B018O19
M042J21
V024P16
CB-merge
CB-merge
0 10 20 30 40 50 60 70 80 90 100 110 120
[root@localhost: /mn...
[FPC V8.9 Main Menu]
Project cp05T5e12D...
FPC Ctg3494 cp05T5...

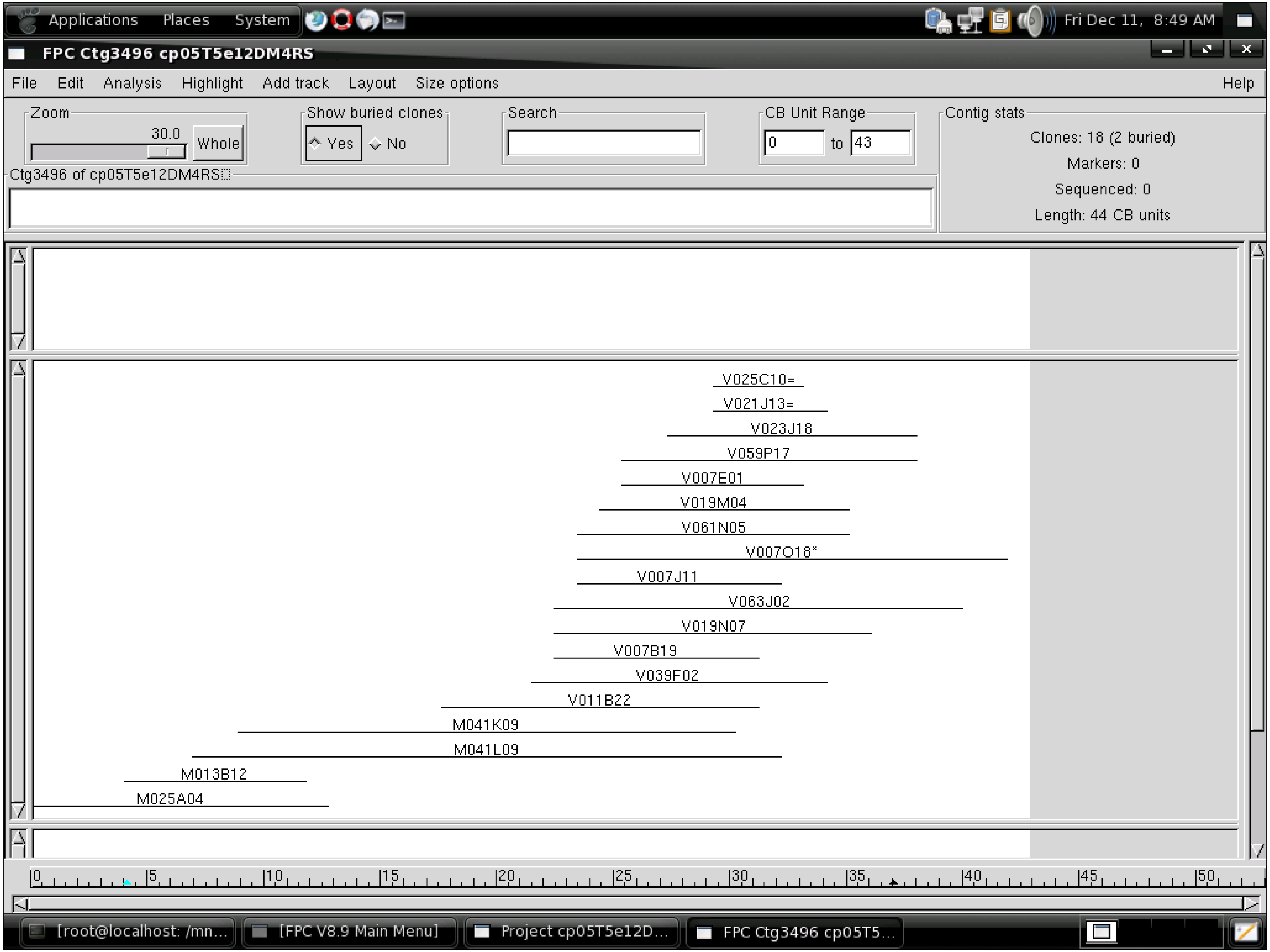
Applications Places System
Fri Dec 11, 8:49 AM
FPC Ctg3496 cp05T5e12DM4RS
File Edit Analysis Highlight Add track Layout Size options Help
Zoom
30.0
Whole
Show buried clones
Yes
No
Search
CB Unit Range
0 to 43
Contig stats
Clones: 18 (2 buried)
Markers: 0
Sequenced: 0
Length: 44 CB units
Ctg3496 of cp05T5e12DM4RS
V025C10=
V021J13=
V023J18
V059P17
V007E01
V019M04
V061N05
V007O18*
V007J11
V063J02
V019N07
V007B19
V039F02
V011B22
M041K09
M041L09
M013B12
M025A04
0 5 10 15 20 25 30 35 40 45 50
[root@localhost: /mn...
[FPC V8.9 Main Menu]
Project cp05T5e12D...
FPC Ctg3496 cp05T5...

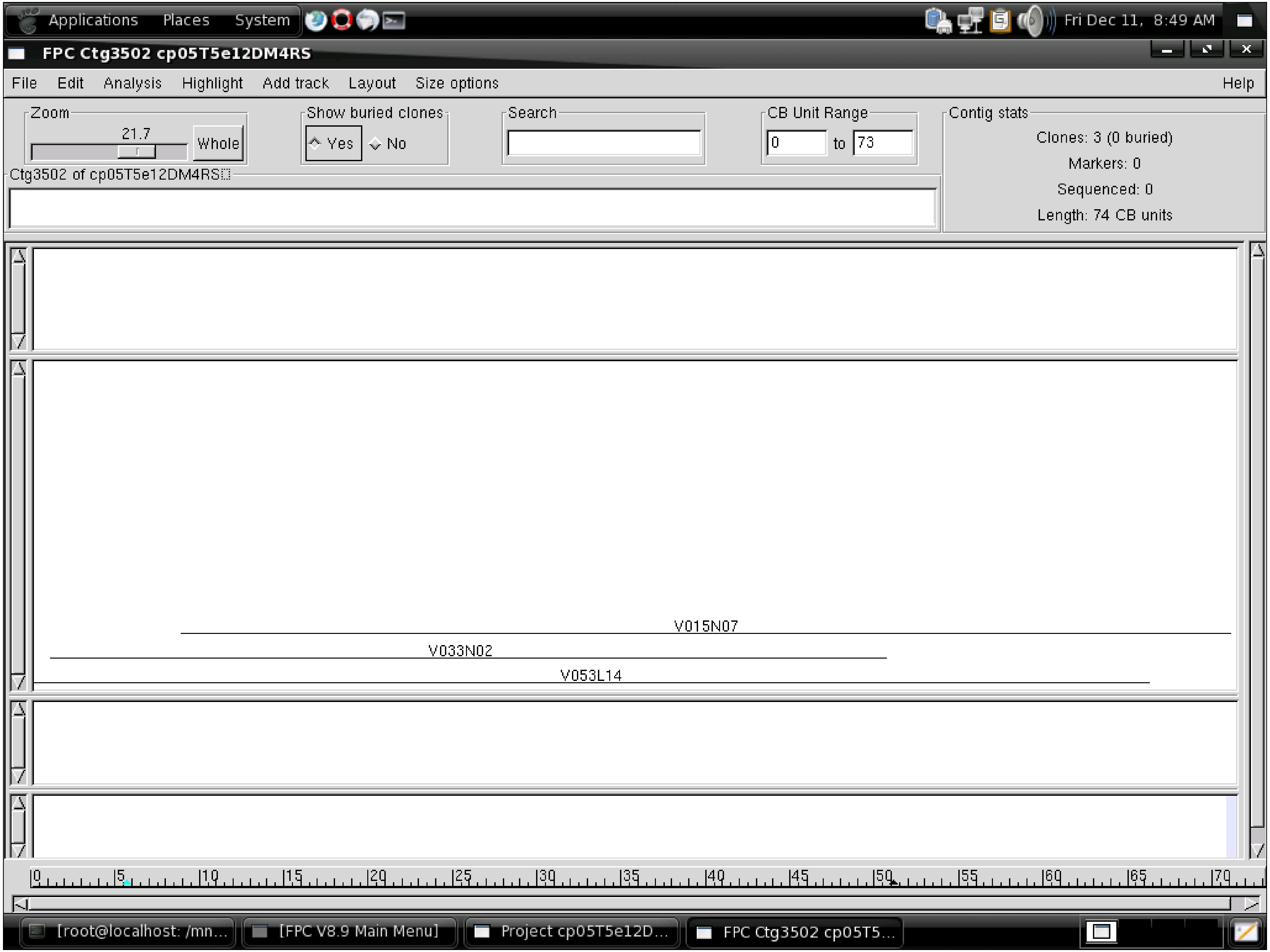
Applications Places System
Fri Dec 11, 8:49 AM
FPC Ctg3502 cp05T5e12DM4RS
File Edit Analysis Highlight Add track Layout Size options Help
Zoom
21.7
Whole
Show buried clones
Yes No
Search
CB Unit Range
0 to 73
Contig stats
Clones: 3 (0 buried)
Markers: 0
Sequenced: 0
Length: 74 CB units
Ctg3502 of cp05T5e12DM4RS
V015N07
V033N02
V053L14
0 5 10 15 20 25 30 35 40 45 50 55 60 65 70
[root@localhost: /mn...
[FPC V8.9 Main Menu]
Project cp05T5e12D...
FPC Ctg3502 cp05T5...

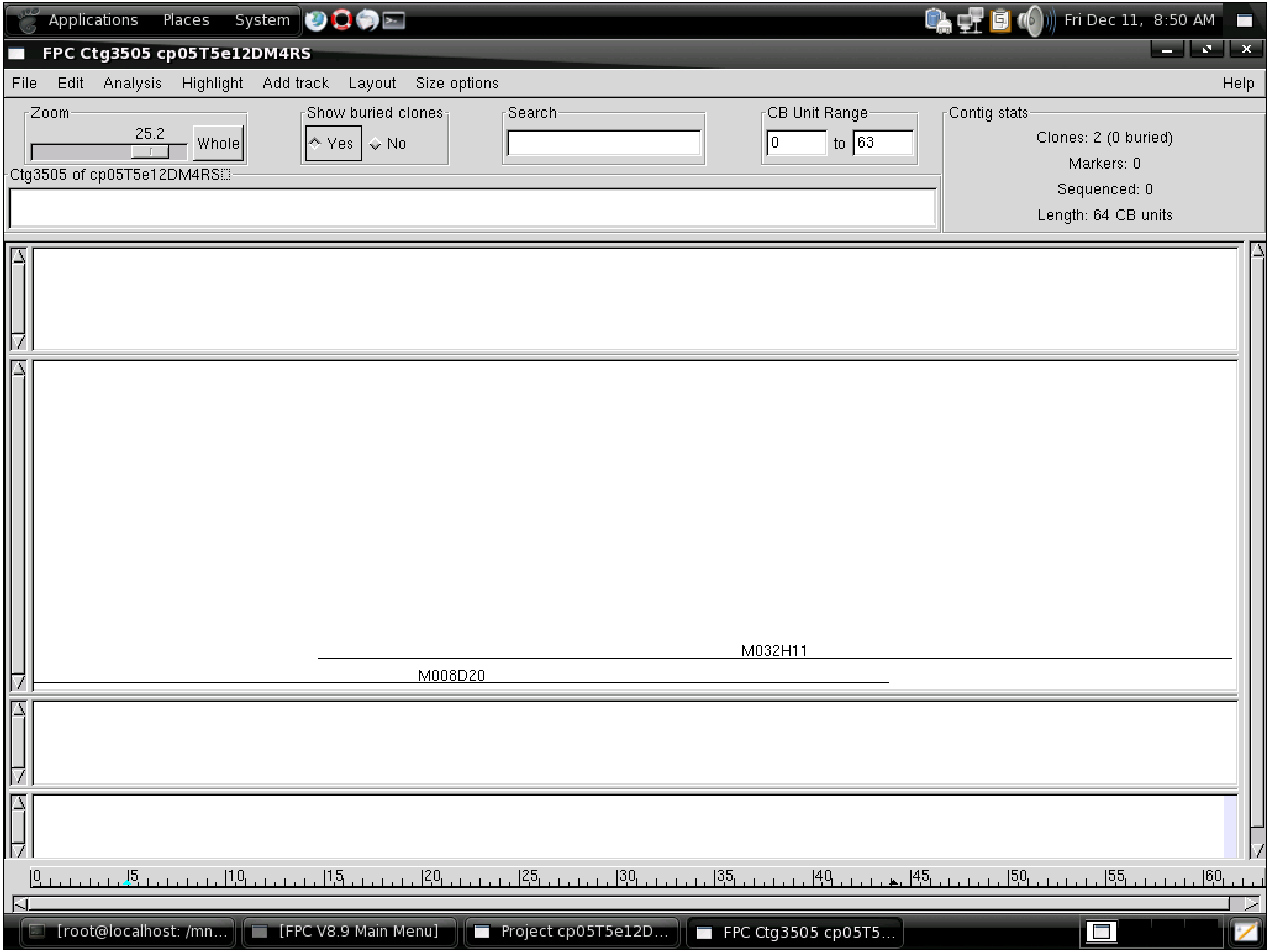
Applications Places System
Fri Dec 11, 8:50 AM
FPC Ctg3505 cp05T5e12DM4RS
File Edit Analysis Highlight Add track Layout Size options Help
Zoom
25.2
Whole
Show buried clones
Yes No
Search
CB Unit Range
0 to 63
Contig stats
Clones: 2 (0 buried)
Markers: 0
Sequenced: 0
Length: 64 CB units
Ctg3505 of cp05T5e12DM4RS
M032H11
M008D20
0 5 10 15 20 25 30 35 40 45 50 55 60
[root@localhost: /mn...
[FPC V8.9 Main Menu]
Project cp05T5e12D...
FPC Ctg3505 cp05T5...

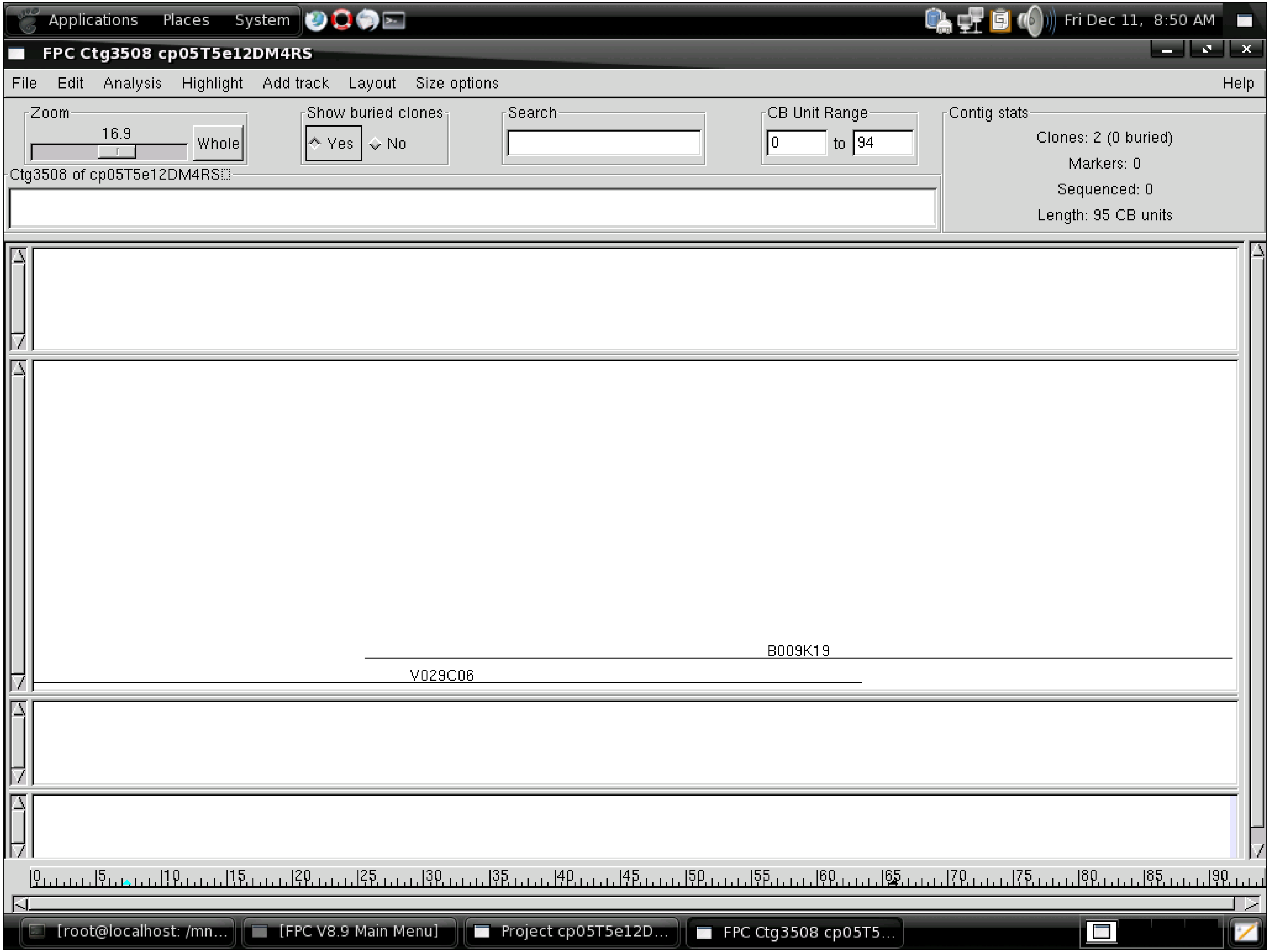
Applications Places System
Fri Dec 11, 8:50 AM
FPC Ctg3508 cp05T5e12DM4RS
File Edit Analysis Highlight Add track Layout Size options Help
Zoom
16.9
Whole
Show buried clones
Yes
No
Search
CB Unit Range
0 to 94
Contig stats
Clones: 2 (0 buried)
Markers: 0
Sequenced: 0
Length: 95 CB units
Ctg3508 of cp05T5e12DM4RS
B009K19
V029C06
0 5 10 15 20 25 30 35 40 45 50 55 60 65 70 75 80 85 90
[root@localhost: /mn...
[FPC V8.9 Main Menu]
Project cp05T5e12D...
FPC Ctg3508 cp05T5...

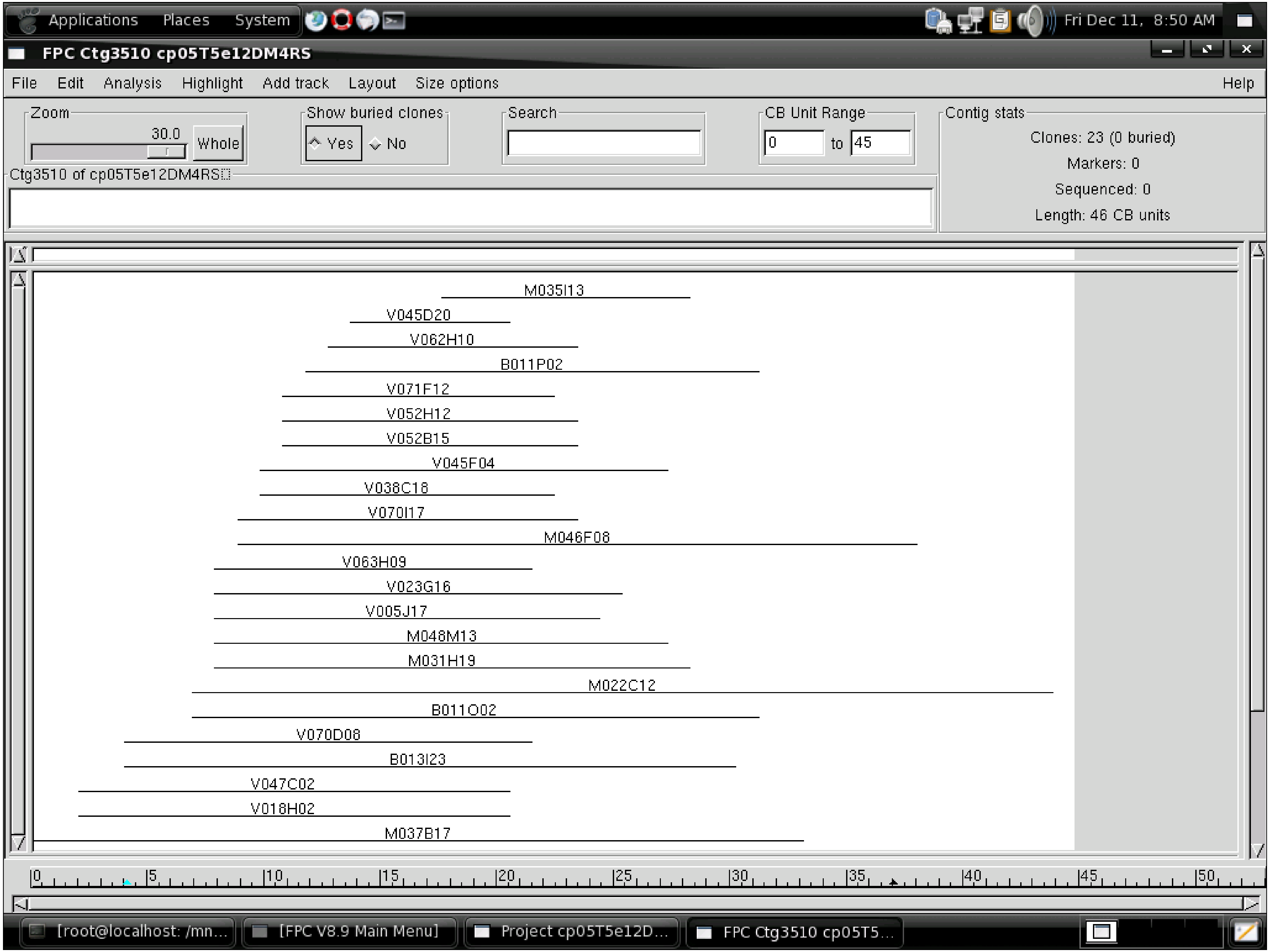
Applications Places System
Fri Dec 11, 8:50 AM
FPC Ctg3510 cp05T5e12DM4RS
File Edit Analysis Highlight Add track Layout Size options Help
Zoom
30.0
Whole
Show buried clones
Yes
No
Search
CB Unit Range
0 to 45
Contig stats
Clones: 23 (0 buried)
Markers: 0
Sequenced: 0
Length: 46 CB units
Ctg3510 of cp05T5e12DM4RS
M035I13
V045D20
V062H10
B011P02
V071F12
V052H12
V052B15
V045F04
V038C18
V070I17
M046F08
V063H09
V023G16
V005J17
M048M13
M031H19
M022C12
B011O02
V070D08
B013I23
V047C02
V018H02
M037B17
0 5 10 15 20 25 30 35 40 45 50
[root@localhost: /mn...
[FPC V8.9 Main Menu]
Project cp05T5e12D...
FPC Ctg3510 cp05T5...

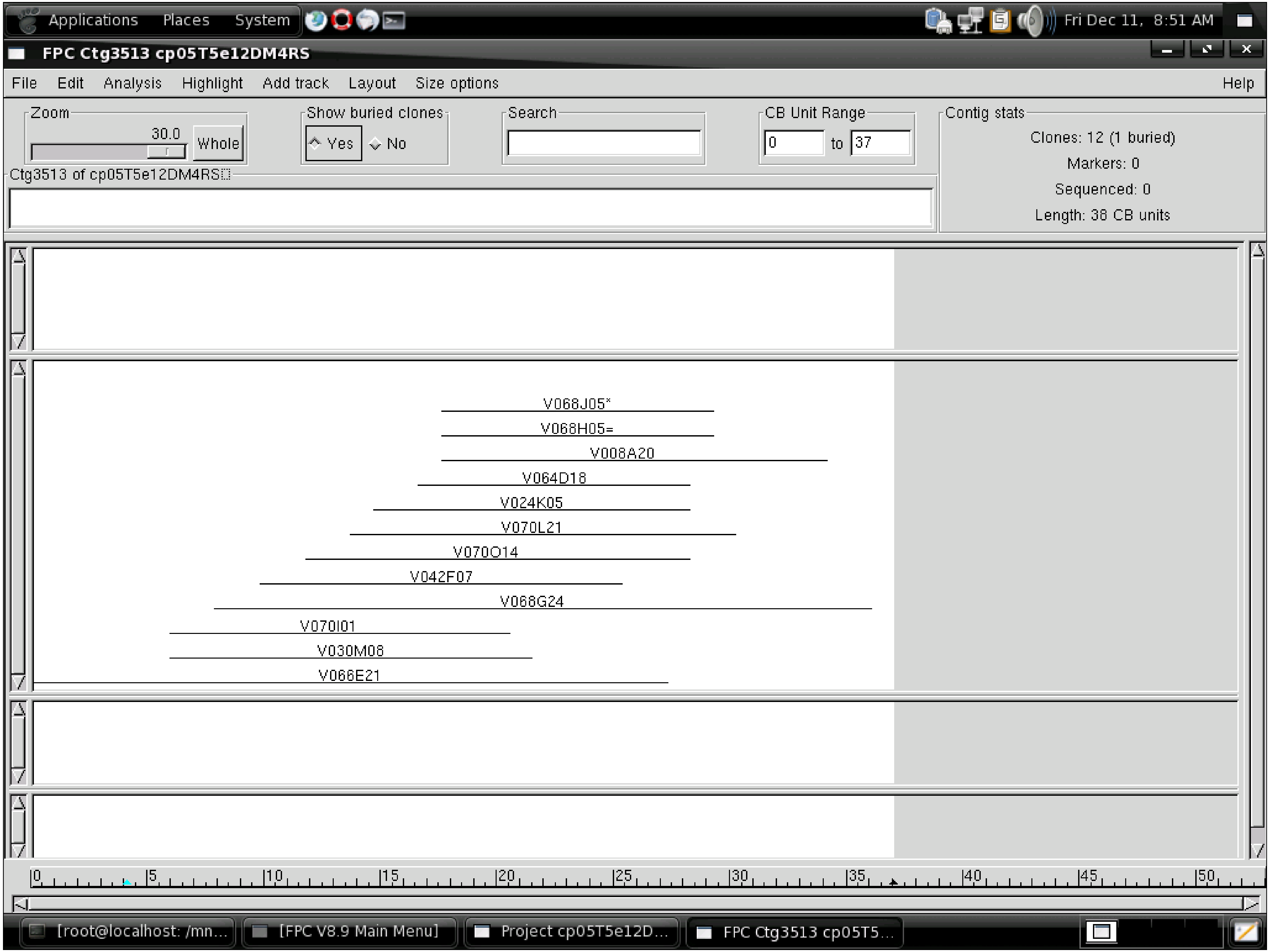
Applications Places System
Fri Dec 11, 8:51 AM
FPC Ctg3513 cp05T5e12DM4RS
File Edit Analysis Highlight Add track Layout Size options Help
Zoom
30.0
Whole
Show buried clones
Yes
No
Search
CB Unit Range
0
to
37
Contig stats
Clones: 12 (1 buried)
Markers: 0
Sequenced: 0
Length: 38 CB units
Ctg3513 of cp05T5e12DM4RS
V068J05*
V068H05=
V008A20
V064D18
V024K05
V070L21
V070O14
V042F07
V068G24
V070I01
V030M08
V066E21
0 5 10 15 20 25 30 35 40 45 50
[root@localhost: /mn...
[FPC V8.9 Main Menu]
Project cp05T5e12D...
FPC Ctg3513 cp05T5...

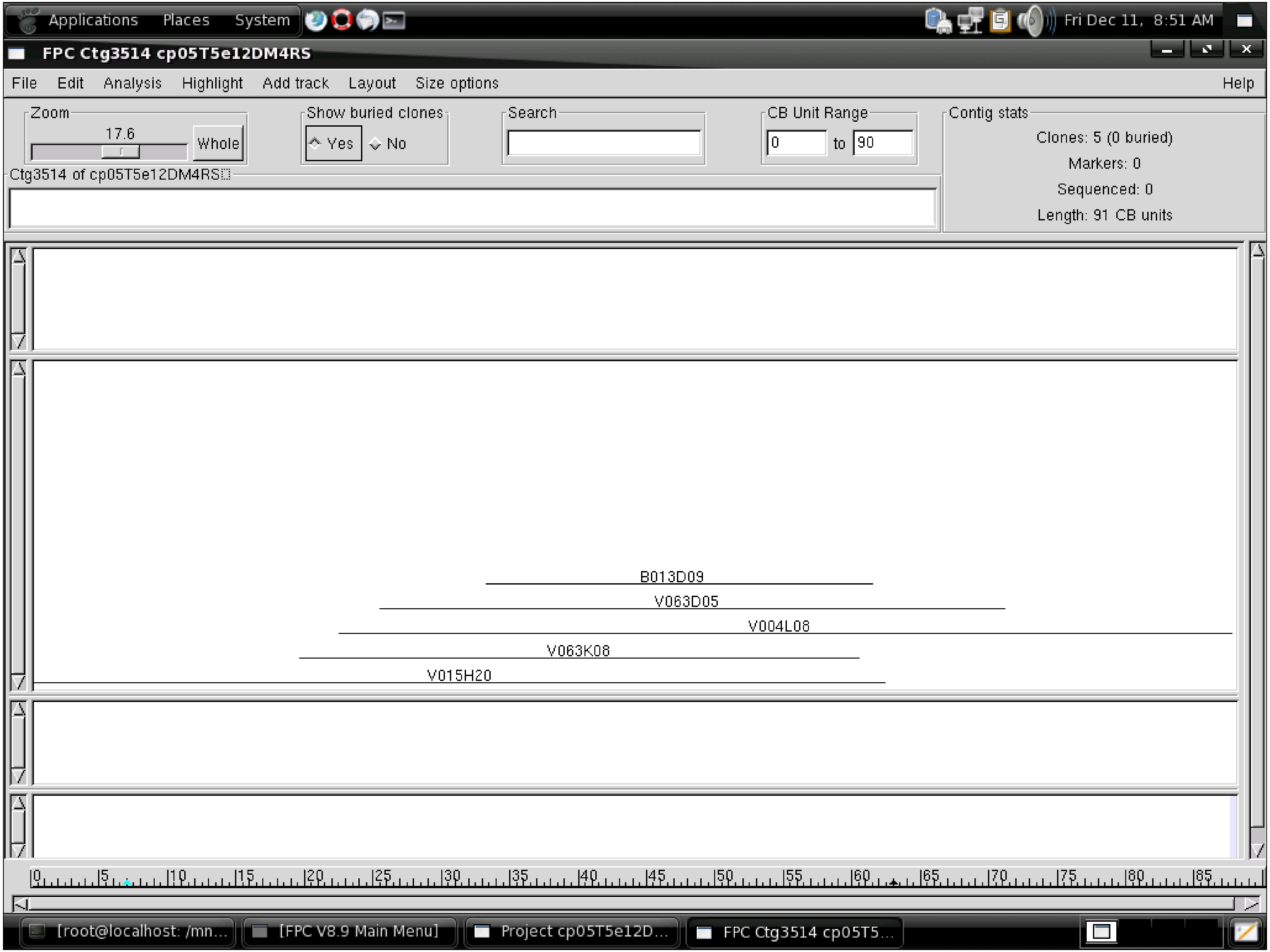
Applications Places System
Fri Dec 11, 8:51 AM
FPC Ctg3514 cp05T5e12DM4RS
File Edit Analysis Highlight Add track Layout Size options Help
Zoom
17.6
Whole
Show buried clones
Yes No
Search
CB Unit Range
0 to 90
Contig stats
Clones: 5 (0 buried)
Markers: 0
Sequenced: 0
Length: 91 CB units
Ctg3514 of cp05T5e12DM4RS
B013D09
V063D05
V004L08
V063K08
V015H20
0 5 10 15 20 25 30 35 40 45 50 55 60 65 70 75 80 85
[root@localhost: /mn...
[FPC V8.9 Main Menu]
Project cp05T5e12D...
FPC Ctg3514 cp05T5...

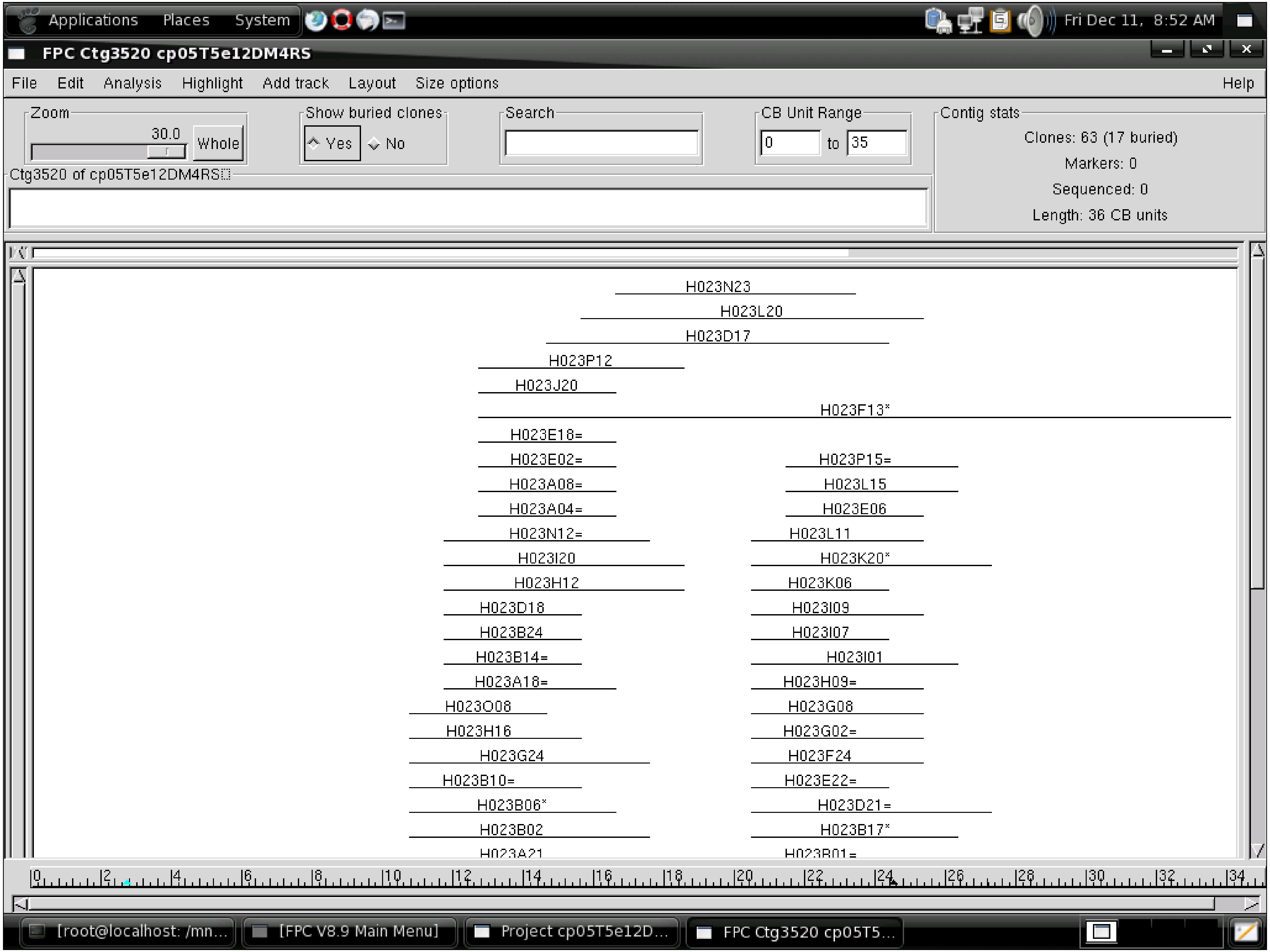
Applications Places System
Fri Dec 11, 8:52 AM
FPC Ctg3520 cp05T5e12DM4RS
File Edit Analysis Highlight Add track Layout Size options Help
Zoom
30.0
Whole
Show buried clones
Yes No
Search
CB Unit Range
0 to 35
Contig stats
Clones: 63 (17 buried)
Markers: 0
Sequenced: 0
Length: 36 CB units
Ctg3520 of cp05T5e12DM4RS
H023N23
H023L20
H023D17
H023P12
H023J20
H023F13*
H023E18=
H023E02=
H023P15=
H023A08=
H023L15
H023A04=
H023E06
H023N12=
H023L11
H023I20
H023K20*
H023H12
H023K06
H023D18
H023I09
H023B24
H023I07
H023B14=
H023I01
H023A18=
H023H09=
H023O08
H023G08
H023H16
H023G02=
H023G24
H023F24
H023B10=
H023E22=
H023B06*
H023D21=
H023B02
H023B17*
H023A21
H023B01=
0 2 4 6 8 10 12 14 16 18 20 22 24 26 28 30 32 34
[root@localhost: /mn...
[FPC V8.9 Main Menu]
Project cp05T5e12D...
FPC Ctg3520 cp05T5...

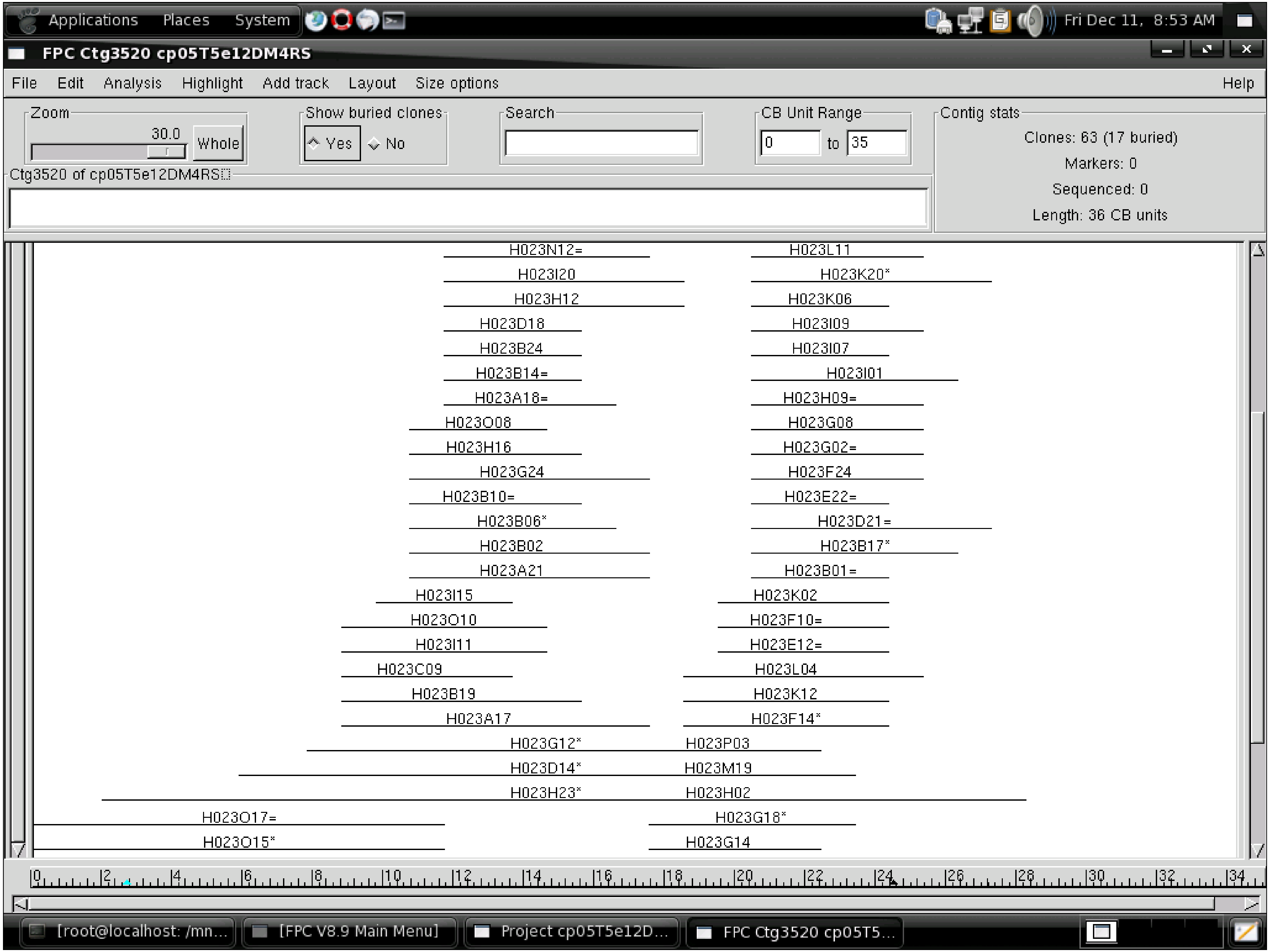
Applications Places System
Fri Dec 11, 8:53 AM
FPC Ctg3520 cp05T5e12DM4RS
File Edit Analysis Highlight Add track Layout Size options Help
Zoom
30.0
Whole
Show buried clones
Yes No
Search
CB Unit Range
0 to 35
Contig stats
Clones: 63 (17 buried)
Markers: 0
Sequenced: 0
Length: 36 CB units
Ctg3520 of cp05T5e12DM4RS
H023N12=
H023L11
H023I20
H023K20*
H023H12
H023K06
H023D18
H023I09
H023B24
H023I07
H023B14=
H023I01
H023A18=
H023H09=
H023O08
H023G08
H023H16
H023G02=
H023G24
H023F24
H023B10=
H023E22=
H023B06*
H023D21=
H023B02
H023B17*
H023A21
H023B01=
H023I15
H023K02
H023O10
H023F10=
H023I11
H023E12=
H023C09
H023L04
H023B19
H023K12
H023A17
H023F14*
H023G12*
H023P03
H023D14*
H023M19
H023H23*
H023H02
H023O17=
H023G18*
H023O15*
H023G14
0 2 4 6 8 10 12 14 16 18 20 22 24 26 28 30 32 34
[root@localhost: /mn...
[FPC V8.9 Main Menu]
Project cp05T5e12D...
FPC Ctg3520 cp05T5...

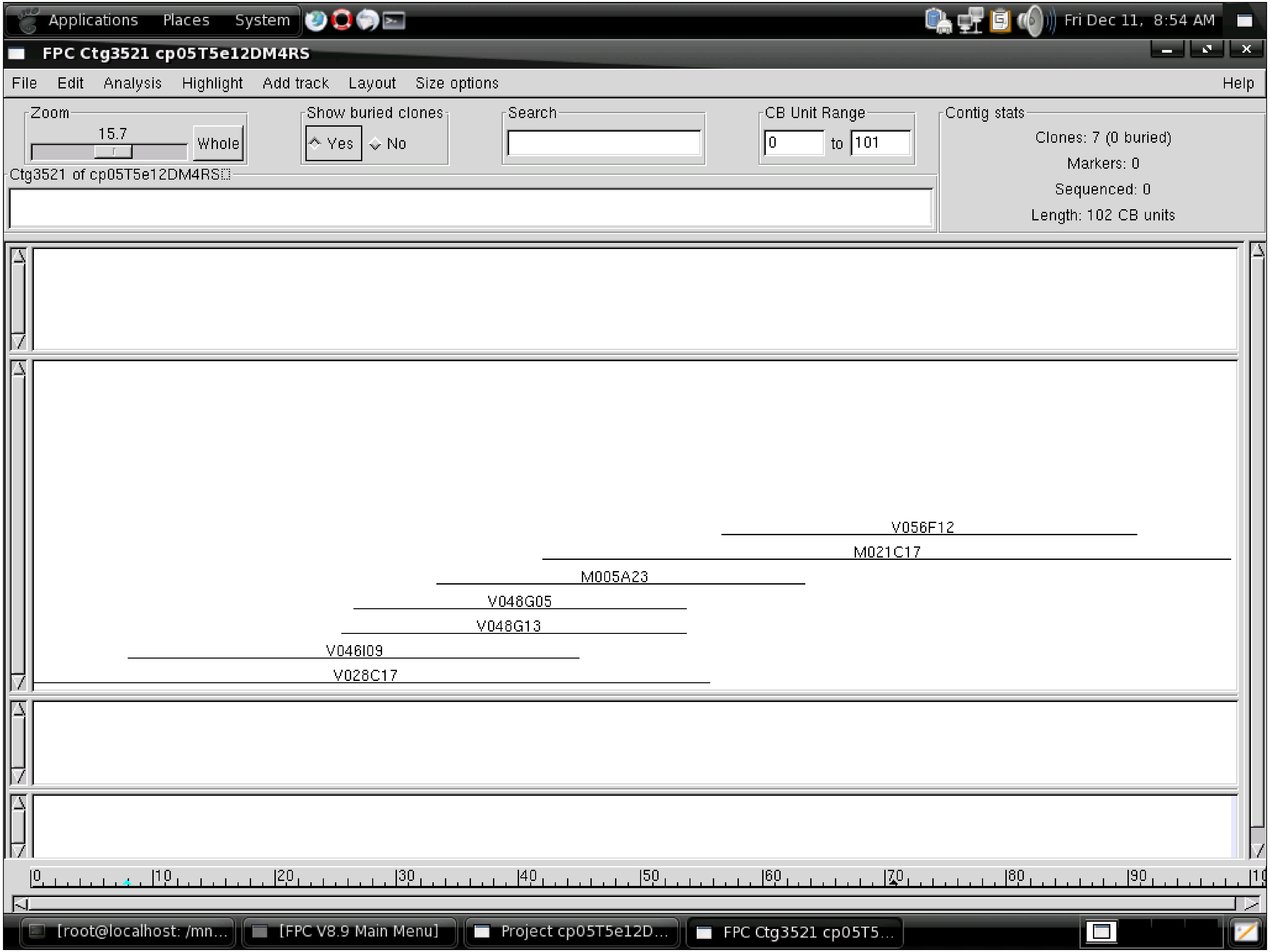
Applications Places System
Fri Dec 11, 8:54 AM
FPC Ctg3521 cp05T5e12DM4RS
File Edit Analysis Highlight Add track Layout Size options Help
Zoom
15.7
Whole
Show buried clones
Yes No
Search
CB Unit Range
0 to 101
Contig stats
Clones: 7 (0 buried)
Markers: 0
Sequenced: 0
Length: 102 CB units
Ctg3521 of cp05T5e12DM4RS
V056F12
M021C17
M005A23
V048G05
V048G13
V046I09
V028C17
0 10 20 30 40 50 60 70 80 90
[root@localhost: /mn...
[FPC V8.9 Main Menu]
Project cp05T5e12D...
FPC Ctg3521 cp05T5...

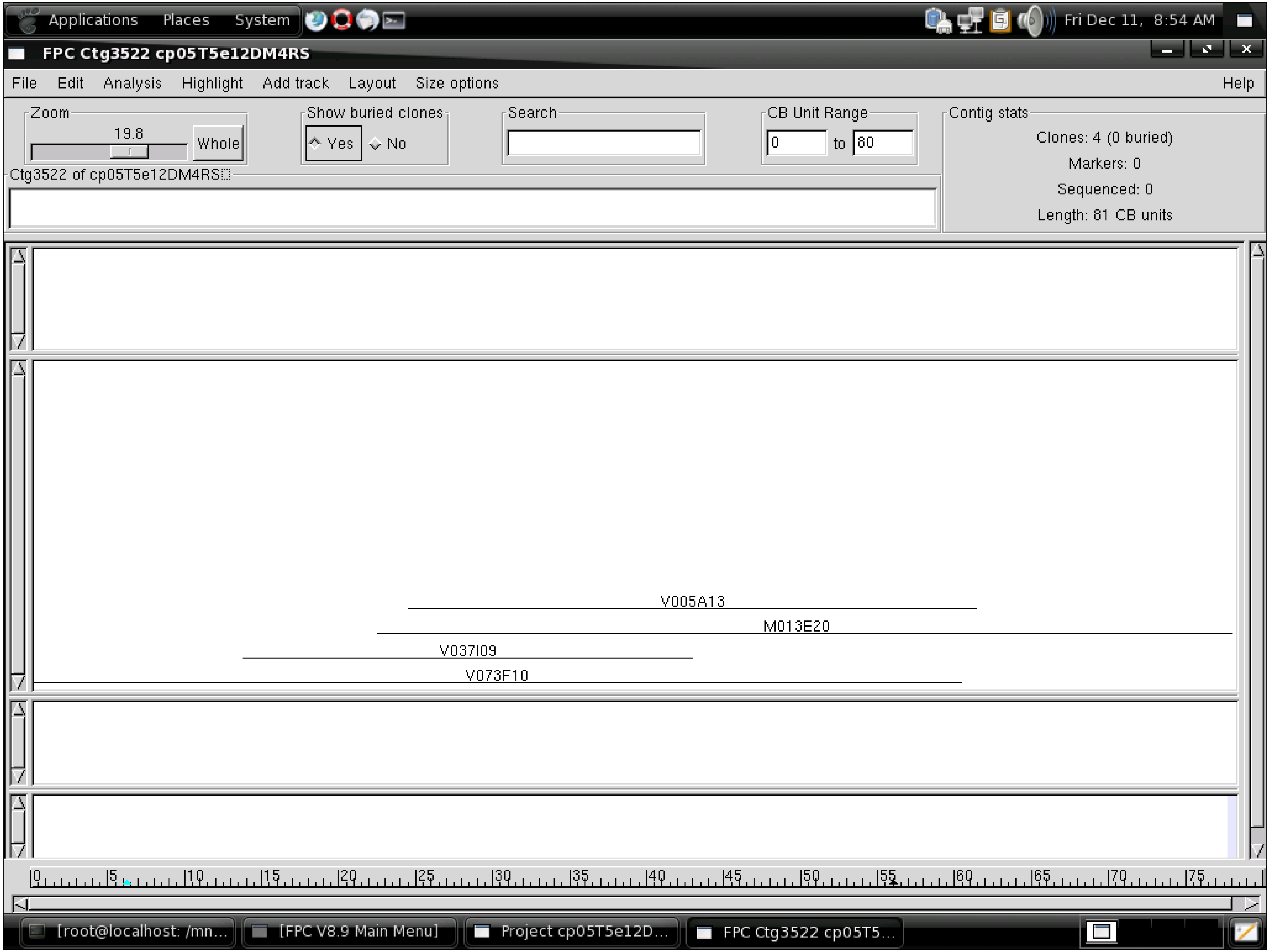
Applications
Places
System
Fri Dec 11, 8:54 AM
FPC Ctg3522 cp05T5e12DM4RS
File
Edit
Analysis
Highlight
Add track
Layout
Size options
Help
Zoom
19.8
Whole
Show buried clones
Yes
No
Search
CB Unit Range
0
to
80
Contig stats
Clones: 4 (0 buried)
Markers: 0
Sequenced: 0
Length: 81 CB units
Ctg3522 of cp05T5e12DM4RS
V005A13
M013E20
V037I09
V073F10
0 5 10 15 20 25 30 35 40 45 50 55 60 65 70 75
[root@localhost: /mn...
[FPC V8.9 Main Menu]
Project cp05T5e12D...
FPC Ctg3522 cp05T5...

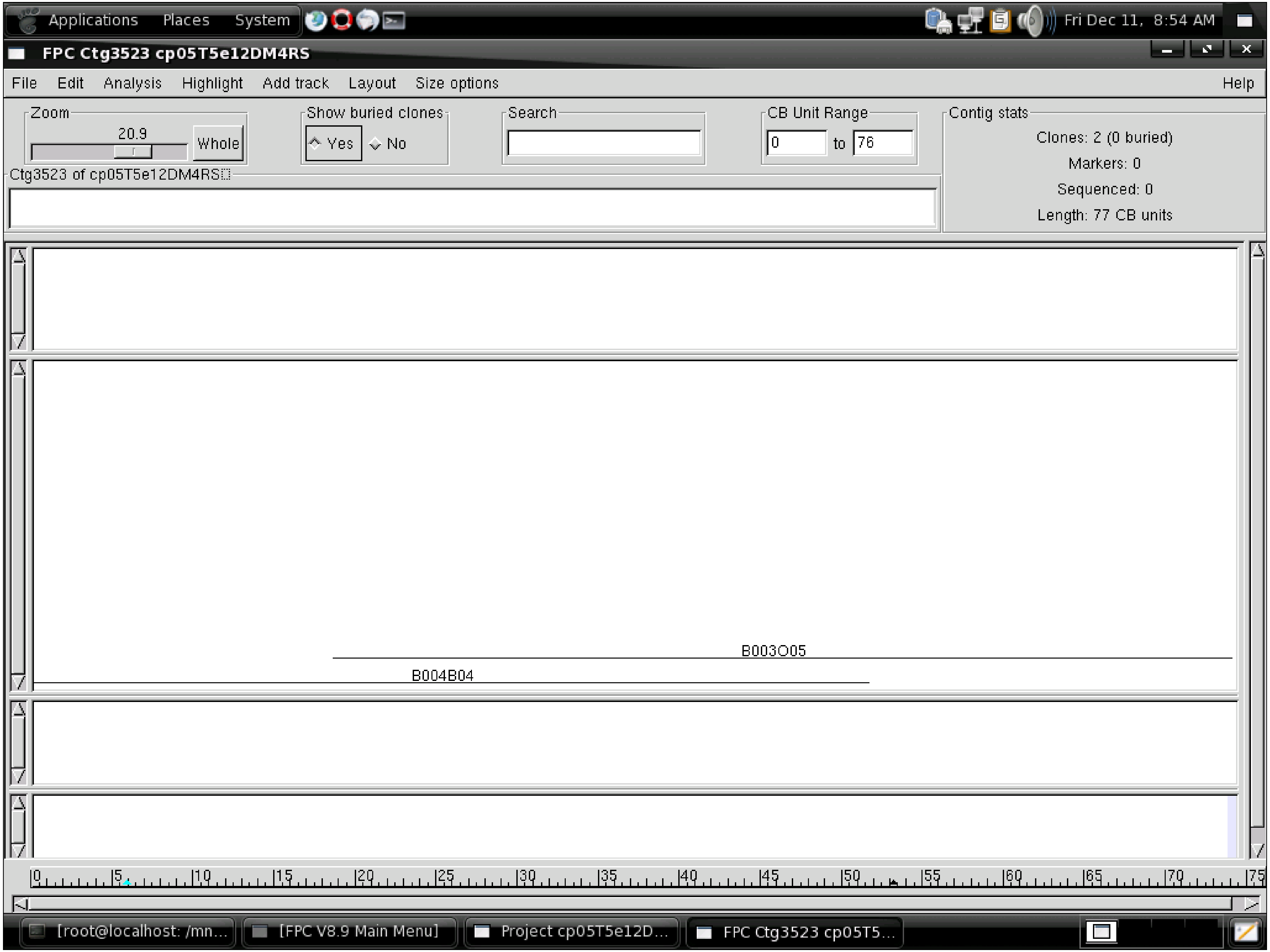
Applications Places System
Fri Dec 11, 8:54 AM
FPC Ctg3523 cp05T5e12DM4RS
File Edit Analysis Highlight Add track Layout Size options Help
Zoom
20.9
Whole
Show buried clones
Yes
No
Search
CB Unit Range
0
to
76
Contig stats
Clones: 2 (0 buried)
Markers: 0
Sequenced: 0
Length: 77 CB units
Ctg3523 of cp05T5e12DM4RS
B003O05
B004B04
0 5 10 15 20 25 30 35 40 45 50 55 60 65 70 75
[root@localhost: /mn...
[FPC V8.9 Main Menu]
Project cp05T5e12D...
FPC Ctg3523 cp05T5...

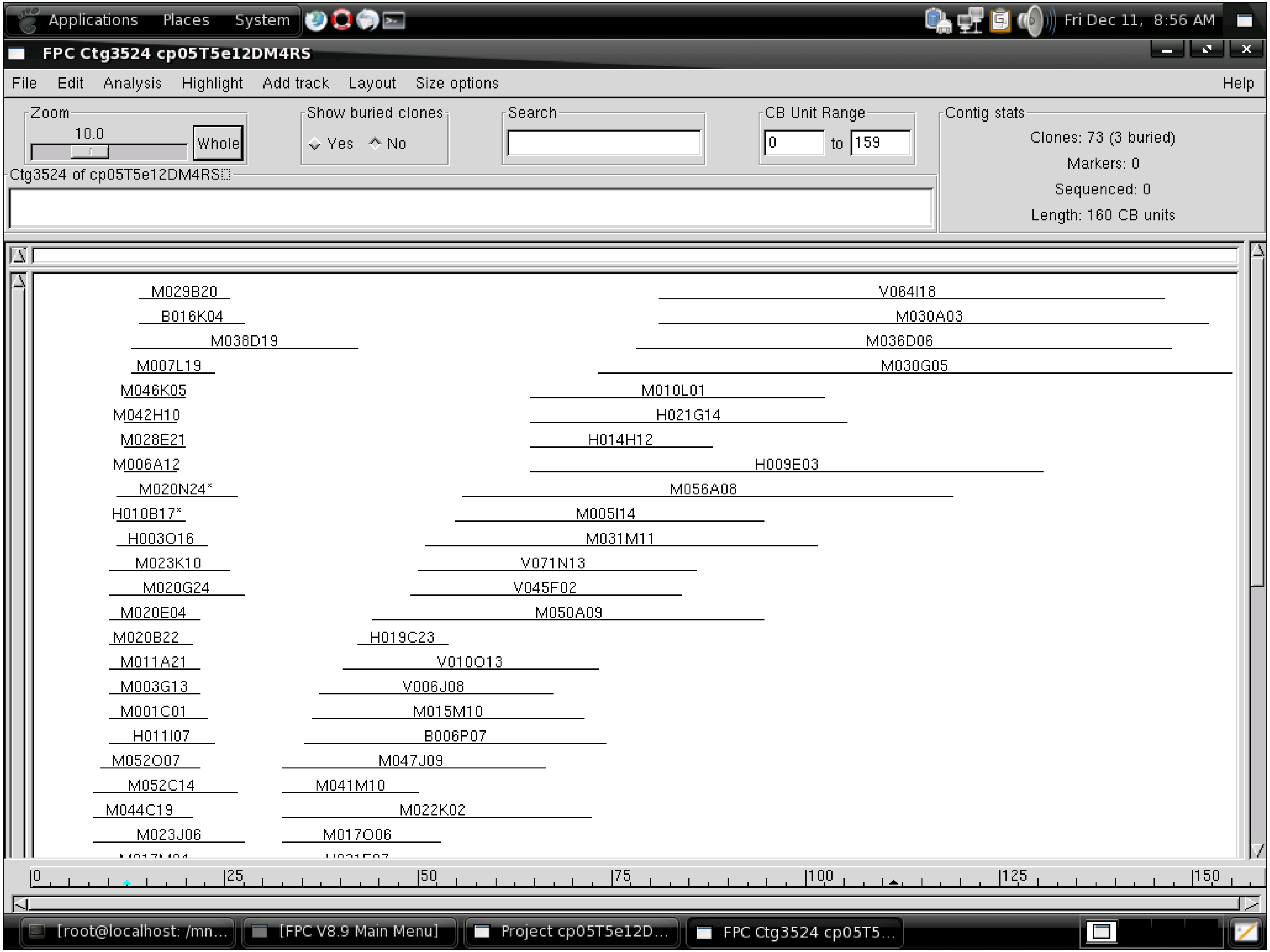
Applications Places System
Fri Dec 11, 8:56 AM
FPC Ctg3524 cp05T5e12DM4RS
File Edit Analysis Highlight Add track Layout Size options
Help
Zoom
10.0
Whole
Show buried clones
Yes No
Search
CB Unit Range
0 to 159
Contig stats
Clones: 73 (3 buried)
Markers: 0
Sequenced: 0
Length: 160 CB units
Ctg3524 of cp05T5e12DM4RS
M029B20
B016K04
M038D19
M007L19
M046K05
M042H10
M028E21
M006A12
M020N24*
H010B17*
H003O16
M023K10
M020G24
M020E04
M020B22
M011A21
M003G13
M001C01
H011I07
M052O07
M052C14
M044C19
M023J06
V064I18
M030A03
M036D06
M030G05
M010L01
H021G14
H014H12
H009E03
M056A08
M005I14
M031M11
V071N13
V045F02
M050A09
H019C23
V010O13
V006J08
M015M10
B006P07
M047J09
M041M10
M022K02
M017O06
0
25
50
75
100
125
150
[root@localhost: /mn...
[FPC V8.9 Main Menu]
Project cp05T5e12D...
FPC Ctg3524 cp05T5...

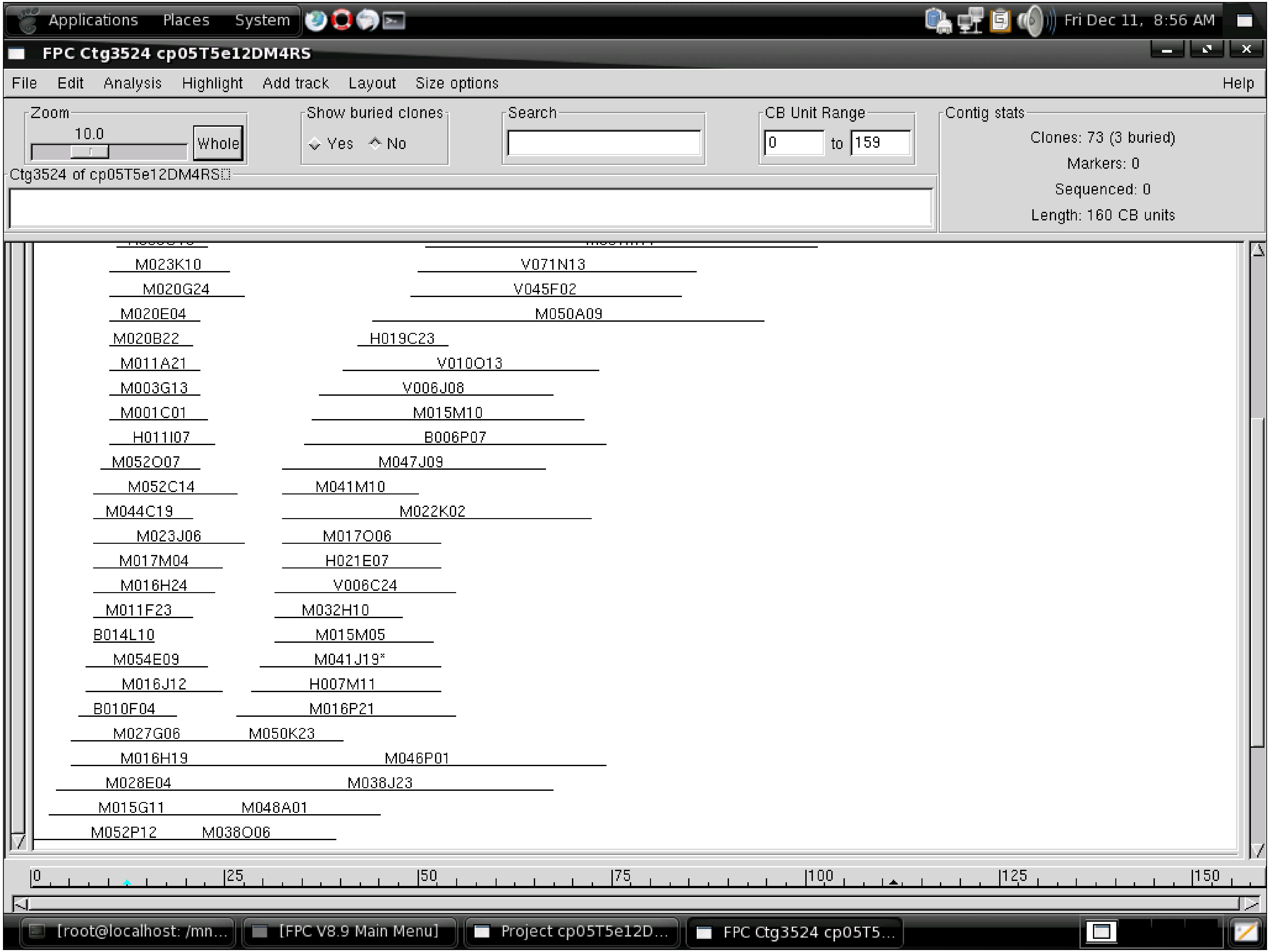
Applications Places System
Fri Dec 11, 8:56 AM
FPC Ctg3524 cp05T5e12DM4RS
File Edit Analysis Highlight Add track Layout Size options Help
Zoom
10.0
Whole
Show buried clones
Yes No
Search
CB Unit Range
0 to 159
Contig stats
Clones: 73 (3 buried)
Markers: 0
Sequenced: 0
Length: 160 CB units
Ctg3524 of cp05T5e12DM4RS
M023K10
V071N13
M020G24
V045F02
M020E04
M050A09
M020B22
H019C23
M011A21
V010O13
M003G13
V006J08
M001C01
M015M10
H011I07
B006P07
M052O07
M047J09
M052C14
M041M10
M044C19
M022K02
M023J06
M017O06
M017M04
H021E07
M016H24
V006C24
M011F23
M032H10
B014L10
M015M05
M054E09
M041J19*
M016J12
H007M11
B010F04
M016P21
M027G06
M050K23
M016H19
M046P01
M028E04
M038J23
M015G11
M048A01
M052P12
M038O06
0
25
50
75
100
125
150
[root@localhost: /mn...
[FPC V8.9 Main Menu]
Project cp05T5e12D...
FPC Ctg3524 cp05T5...

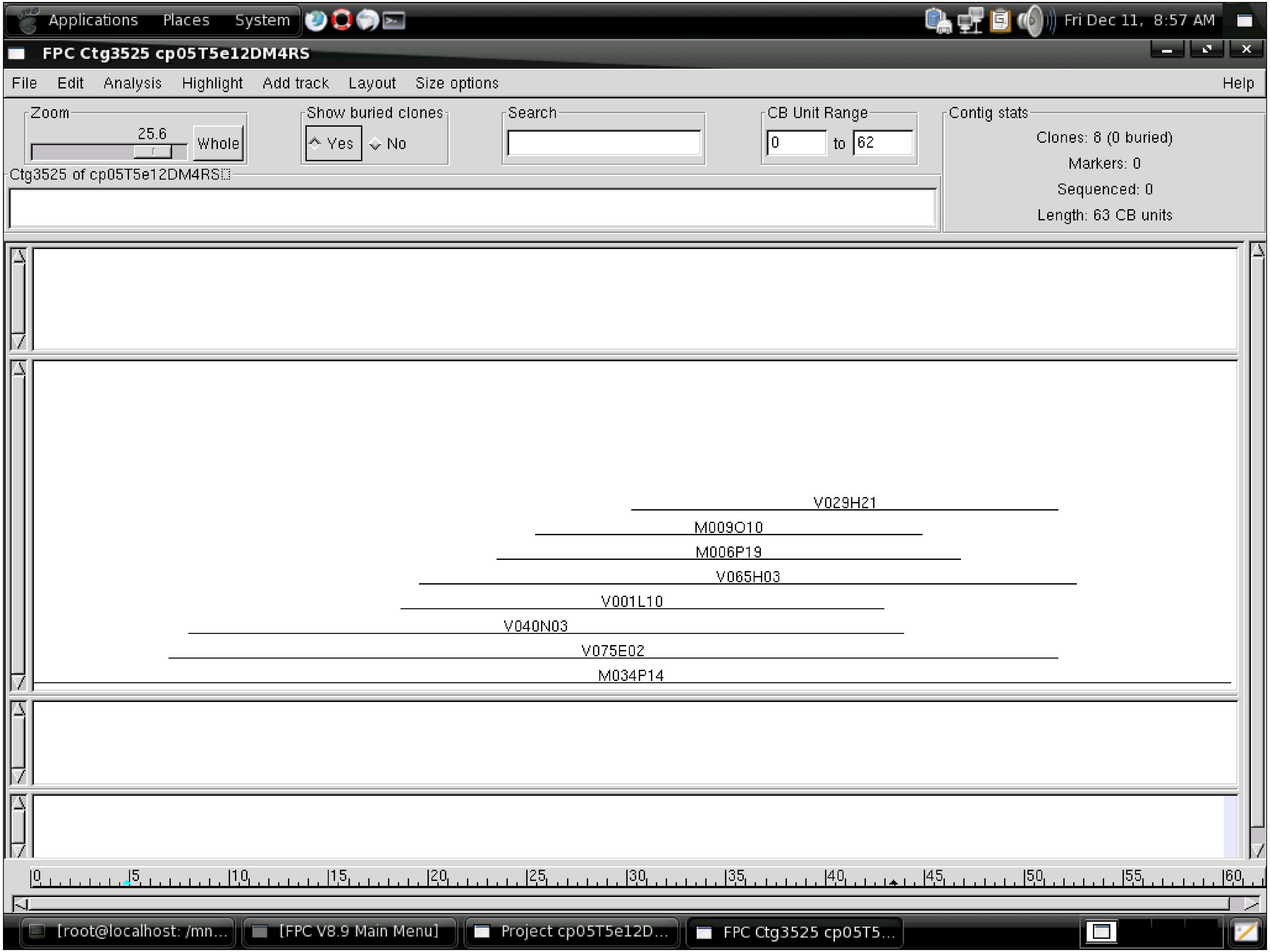
Applications Places System
Fri Dec 11, 8:57 AM
FPC Ctg3525 cp05T5e12DM4RS
File Edit Analysis Highlight Add track Layout Size options Help
Zoom
25.6
Whole
Show buried clones
Yes
No
Search
CB Unit Range
0
to
62
Contig stats
Clones: 8 (0 buried)
Markers: 0
Sequenced: 0
Length: 63 CB units
Ctg3525 of cp05T5e12DM4RS
V029H21
M009O10
M006P19
V065H03
V001L10
V040N03
V075E02
M034P14
0 5 10 15 20 25 30 35 40 45 50 55 60
[root@localhost: /mn...
[FPC V8.9 Main Menu]
Project cp05T5e12D...
FPC Ctg3525 cp05T5...

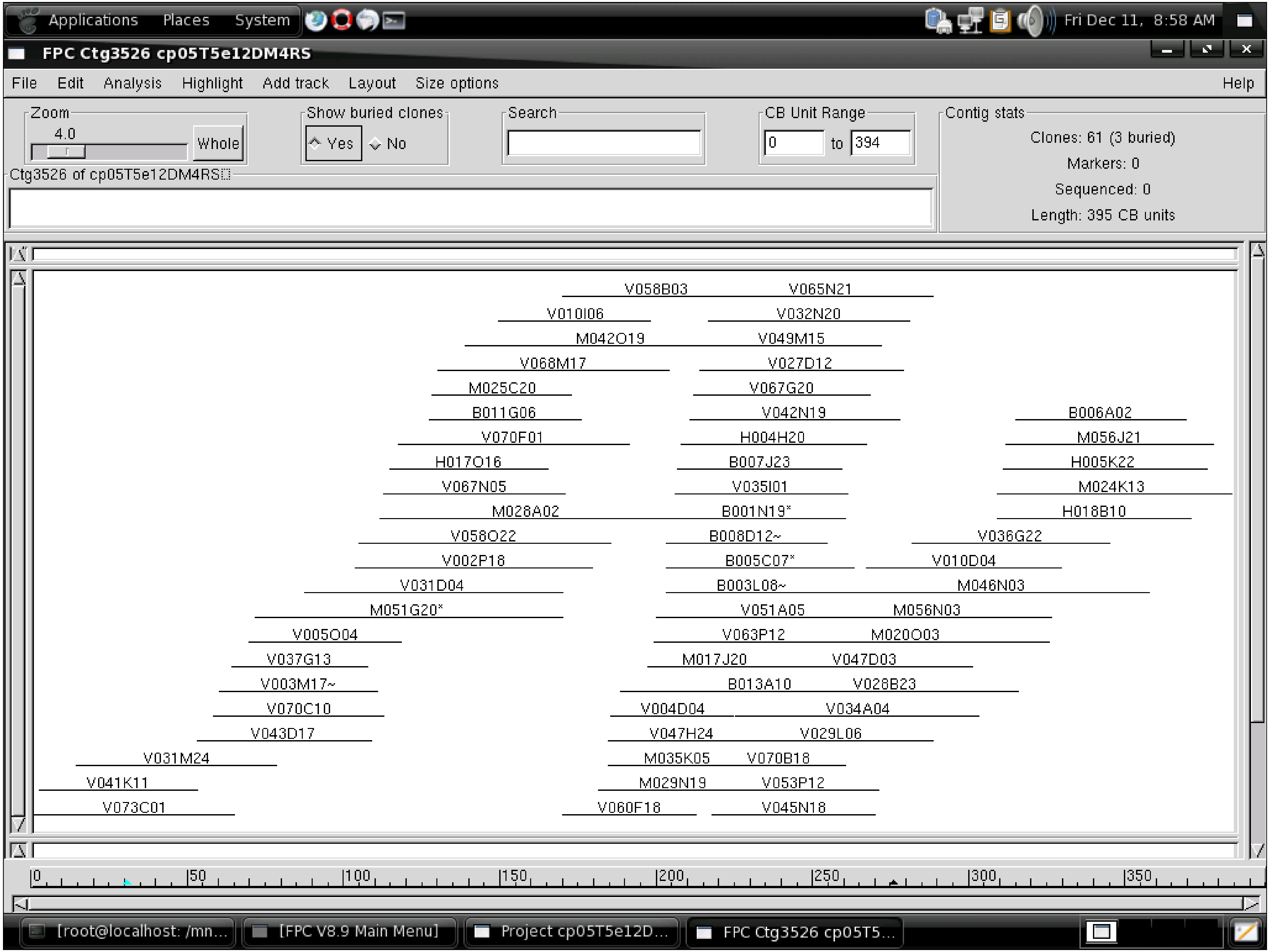
Applications
Places
System
Fri Dec 11, 8:58 AM
FPC Ctg3526 cp05T5e12DM4RS
File
Edit
Analysis
Highlight
Add track
Layout
Size options
Help
Zoom
4.0
Whole
Show buried clones
Yes
No
Search
CB Unit Range
0
to
394
Contig stats
Clones: 61 (3 buried)
Markers: 0
Sequenced: 0
Length: 395 CB units
Ctg3526 of cp05T5e12DM4RS
V058B03
V065N21
V010I06
V032N20
M042O19
V049M15
V068M17
V027D12
M025C20
V067G20
B011G06
V042N19
B006A02
V070F01
H004H20
M056J21
H017O16
B007J23
H005K22
V067N05
V035I01
M024K13
M028A02
B001N19*
H018B10
V058O22
B008D12~
V036G22
V002P18
B005C07*
V010D04
V031D04
B003L08~
M046N03
M051G20*
V051A05
M056N03
V005O04
V063P12
M020O03
V037G13
M017J20
V047D03
V003M17~
B013A10
V028B23
V070C10
V004D04
V034A04
V043D17
V047H24
V029L06
V031M24
M035K05
V070B18
V041K11
M029N19
V053P12
V073C01
V060F18
V045N18
0
50
100
150
200
250
300
350
[root@localhost: /mn...
[FPC V8.9 Main Menu]
Project cp05T5e12D...
FPC Ctg3526 cp05T5...

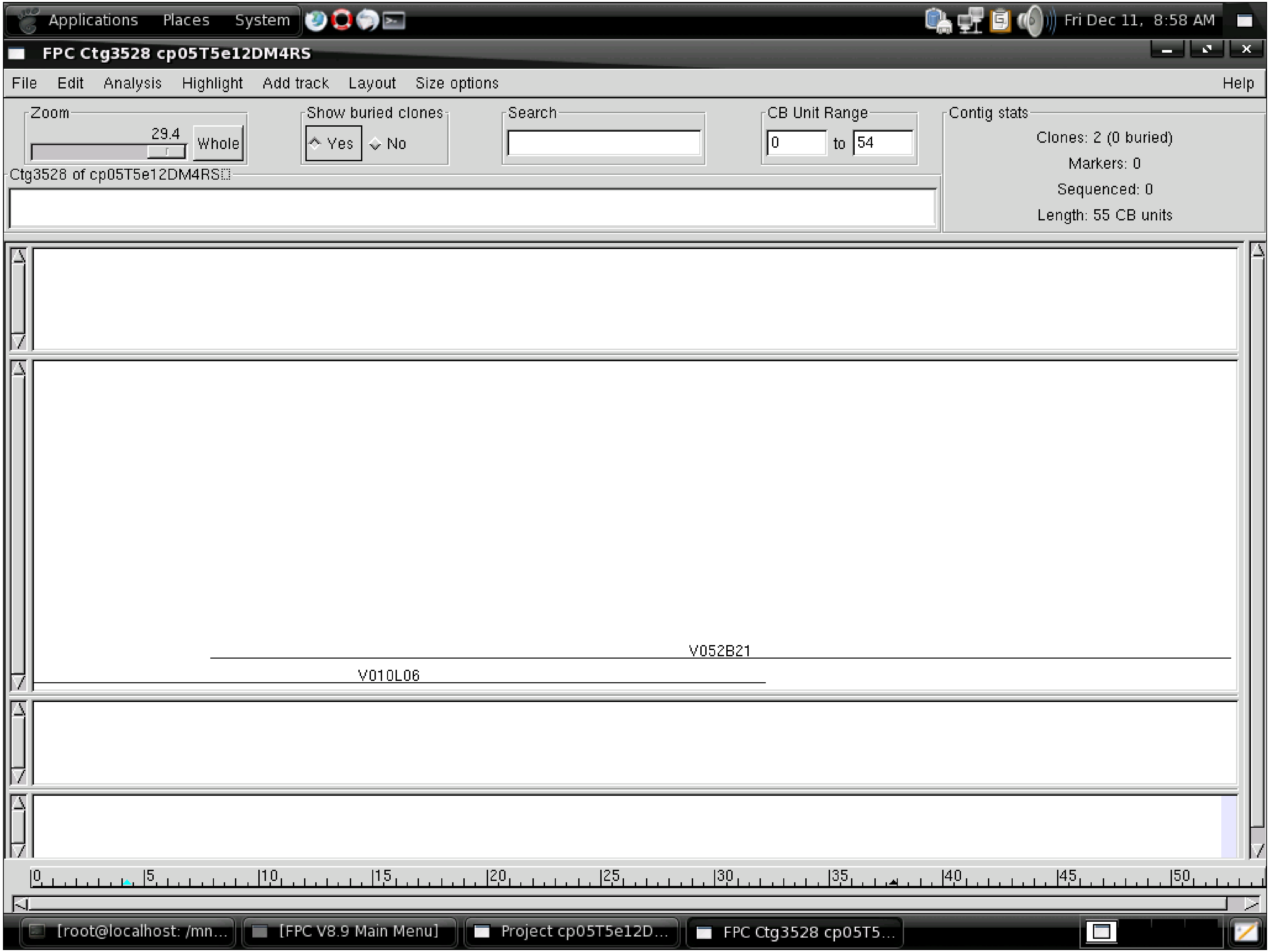
Applications Places System
Fri Dec 11, 8:58 AM
FPC Ctg3528 cp05T5e12DM4RS
File Edit Analysis Highlight Add track Layout Size options Help
Zoom
29.4
Whole
Show buried clones
Yes No
Search
CB Unit Range
0 to 54
Contig stats
Clones: 2 (0 buried)
Markers: 0
Sequenced: 0
Length: 55 CB units
Ctg3528 of cp05T5e12DM4RS
V052B21
V010L06
0 5 10 15 20 25 30 35 40 45 50
[root@localhost: /mn...
[FPC V8.9 Main Menu]
Project cp05T5e12D...
FPC Ctg3528 cp05T5...

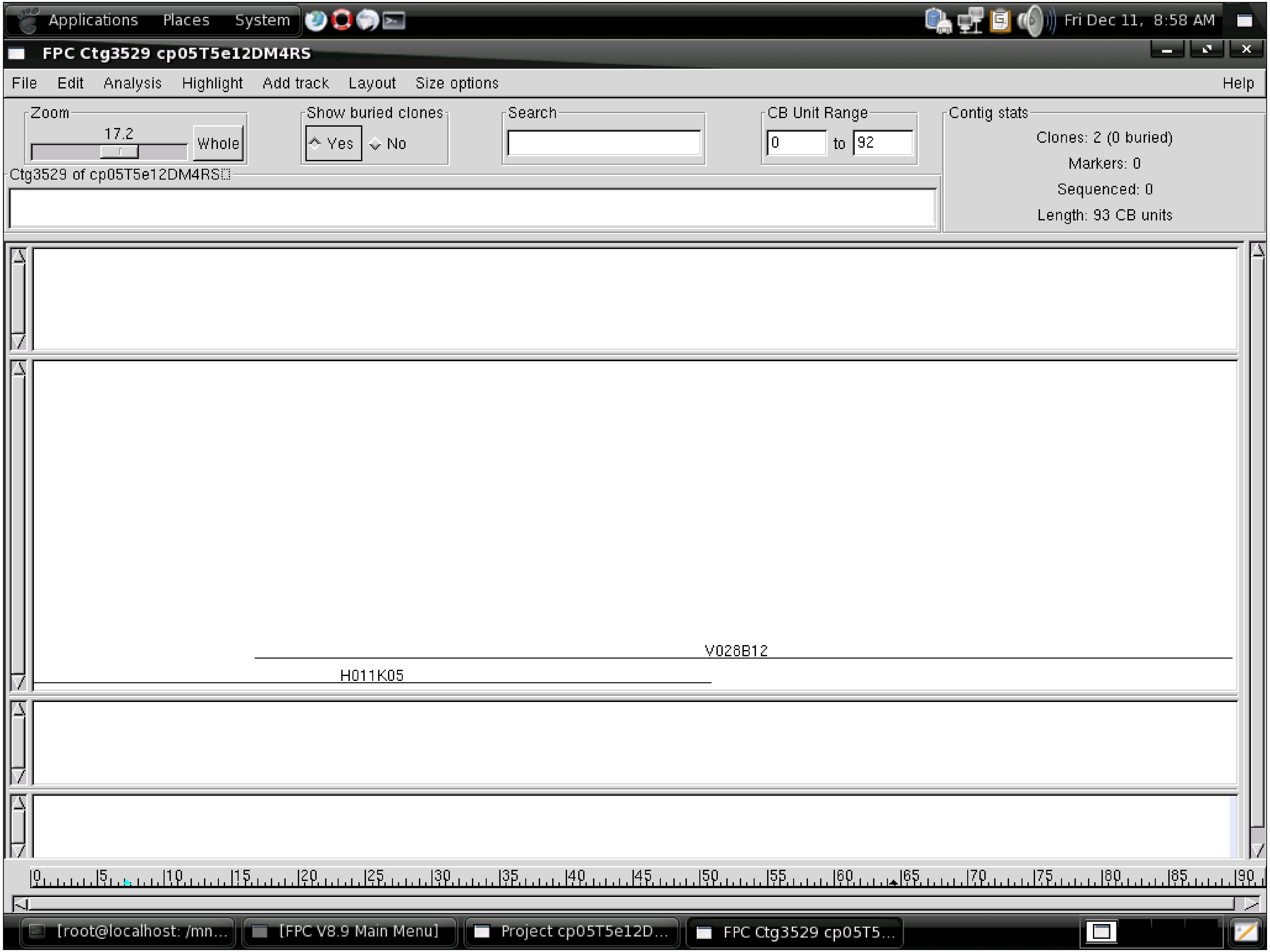
Applications Places System
Fri Dec 11, 8:58 AM
FPC Ctg3529 cp05T5e12DM4RS
File Edit Analysis Highlight Add track Layout Size options Help
Zoom
17.2
Whole
Show buried clones
Yes No
Search
CB Unit Range
0 to 92
Contig stats
Clones: 2 (0 buried)
Markers: 0
Sequenced: 0
Length: 93 CB units
Ctg3529 of cp05T5e12DM4RS
V028B12
H011K05
0 5 10 15 20 25 30 35 40 45 50 55 60 65 70 75 80 85 90
[root@localhost: /mn...
[FPC V8.9 Main Menu]
Project cp05T5e12D...
FPC Ctg3529 cp05T5...

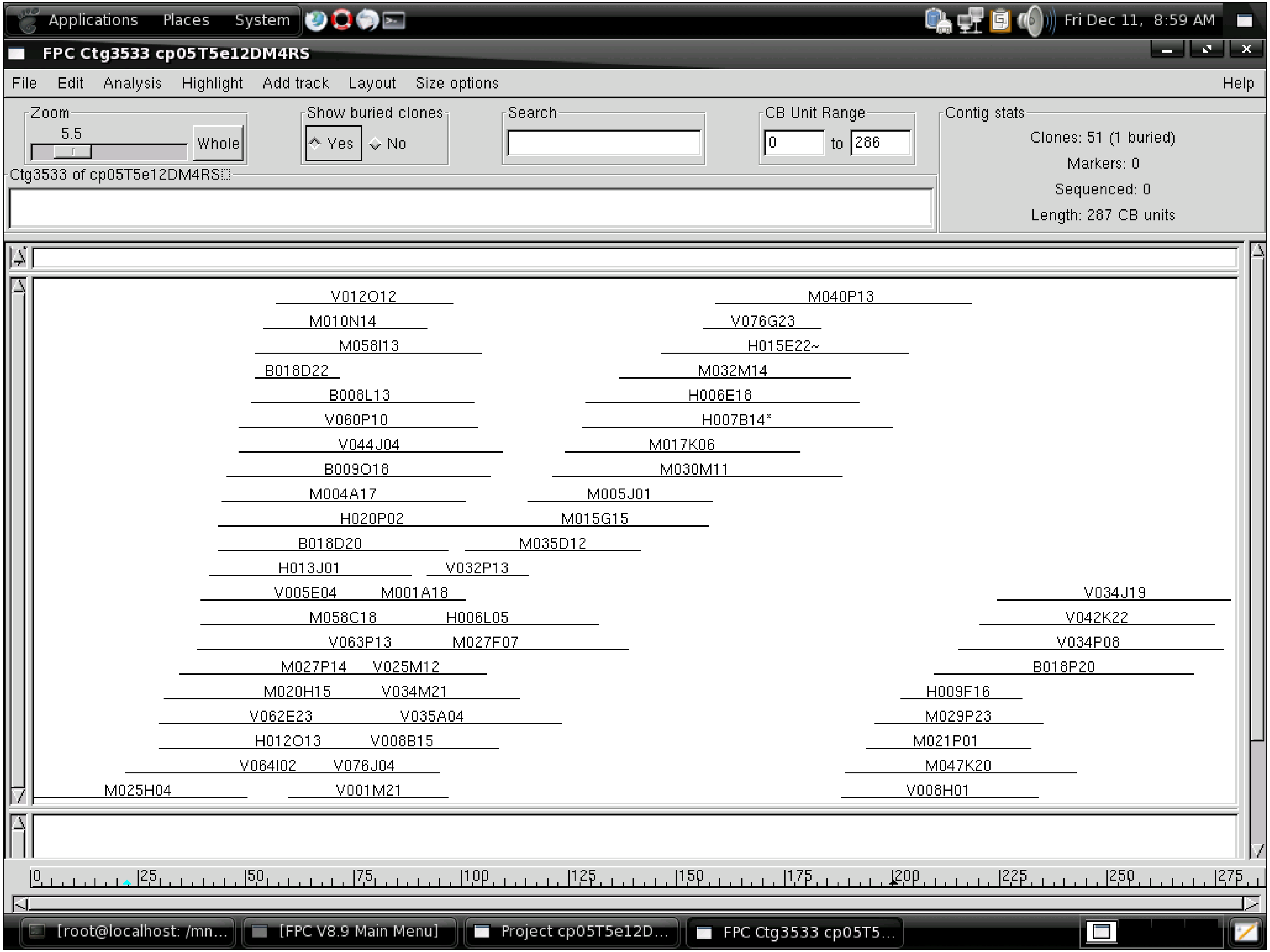
Applications Places System
Fri Dec 11, 8:59 AM
FPC Ctg3533 cp05T5e12DM4RS
File Edit Analysis Highlight Add track Layout Size options Help
Zoom
5.5
Whole
Show buried clones
Yes
No
Search
CB Unit Range
0 to 286
Contig stats
Clones: 51 (1 buried)
Markers: 0
Sequenced: 0
Length: 287 CB units
Ctg3533 of cp05T5e12DM4RS
V012O12
M040P13
M010N14
V076G23
M058I13
H015E22~
B018D22
M032M14
B008L13
H006E18
V060P10
H007B14*
V044J04
M017K06
B009O18
M030M11
M004A17
M005J01
H020P02
M015G15
B018D20
M035D12
H013J01
V032P13
V005E04
M001A18
V034J19
M058C18
H006L05
V042K22
V063P13
M027F07
V034P08
M027P14
V025M12
B018P20
M020H15
V034M21
H009F16
V062E23
V035A04
M029P23
H012O13
V008B15
M021P01
V064I02
V076J04
M047K20
M025H04
V001M21
V008H01
0 25 50 75 100 125 150 175 200 225 250 275
[root@localhost: /mn...
[FPC V8.9 Main Menu]
Project cp05T5e12D...
FPC Ctg3533 cp05T5...

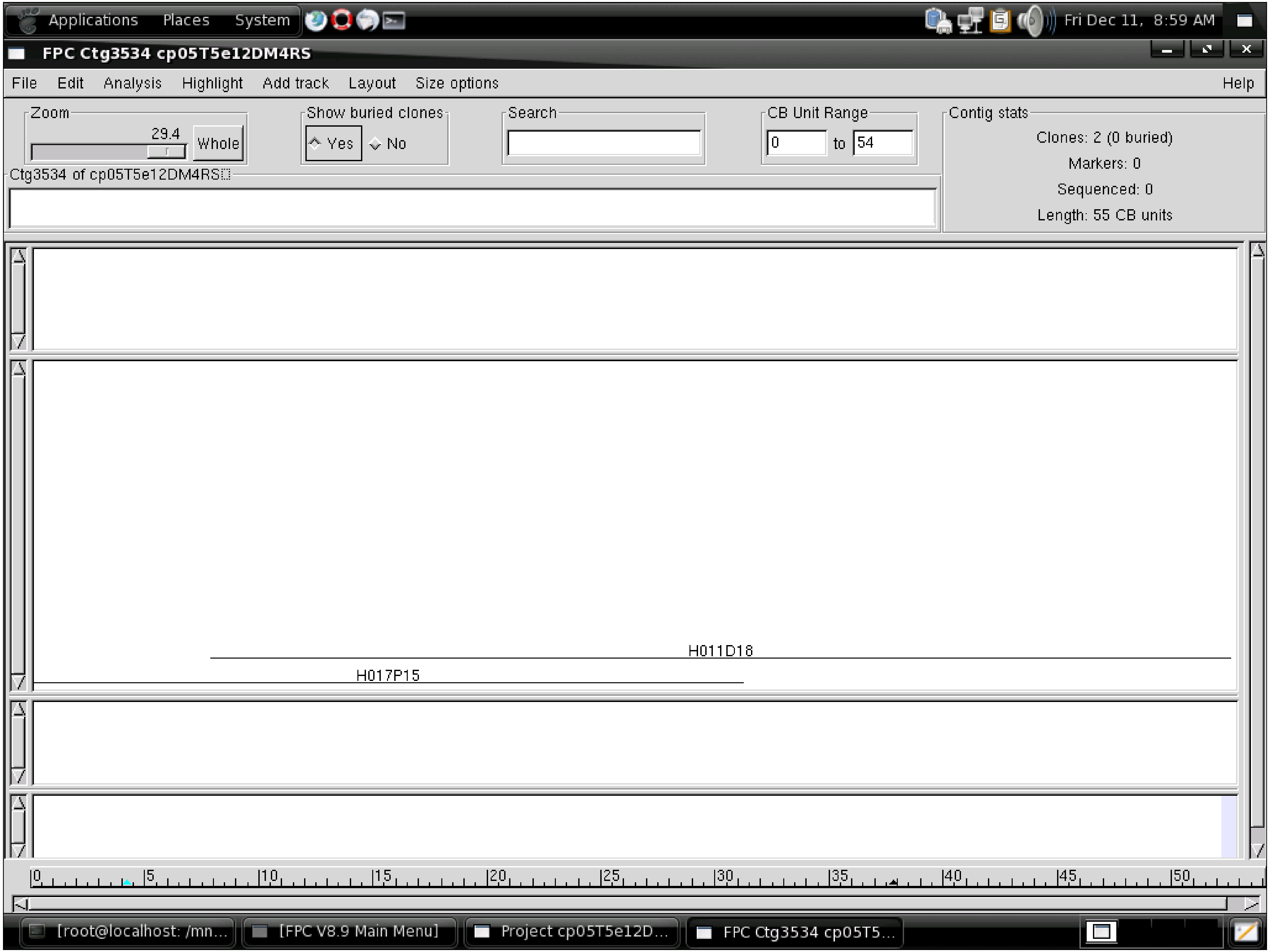

Applications Places System
Fri Dec 11, 8:59 AM
FPC Ctg3534 cp05T5e12DM4RS
File Edit Analysis Highlight Add track Layout Size options Help
Zoom
29.4
Whole
Show buried clones
Yes No
Search
CB Unit Range
0 to 54
Contig stats
Clones: 2 (0 buried)
Markers: 0
Sequenced: 0
Length: 55 CB units
Ctg3534 of cp05T5e12DM4RS
H011D18
H017P15
0 5 10 15 20 25 30 35 40 45 50
[root@localhost: /mn...
[FPC V8.9 Main Menu]
Project cp05T5e12D...
FPC Ctg3534 cp05T5...

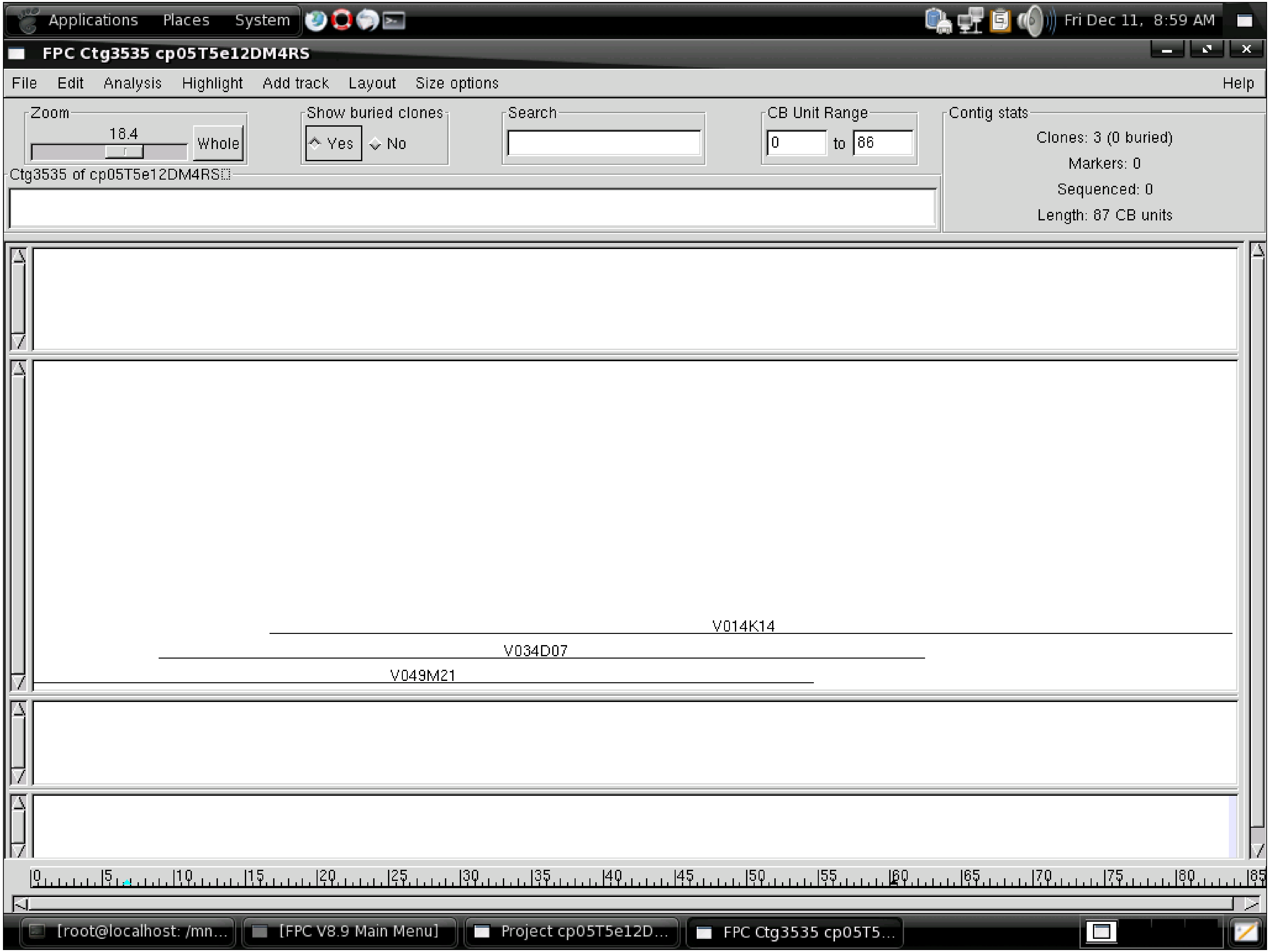
Applications
Places
System
Fri Dec 11, 8:59 AM
FPC Ctg3535 cp05T5e12DM4RS
File
Edit
Analysis
Highlight
Add track
Layout
Size options
Help
Zoom
18.4
Whole
Show buried clones
Yes
No
Search
CB Unit Range
0
to
86
Contig stats
Clones: 3 (0 buried)
Markers: 0
Sequenced: 0
Length: 87 CB units
Ctg3535 of cp05T5e12DM4RS
V014K14
V034D07
V049M21
0 5 10 15 20 25 30 35 40 45 50 55 60 65 70 75 80 85
[root@localhost: /mn...
[FPC V8.9 Main Menu]
Project cp05T5e12D...
FPC Ctg3535 cp05T5...

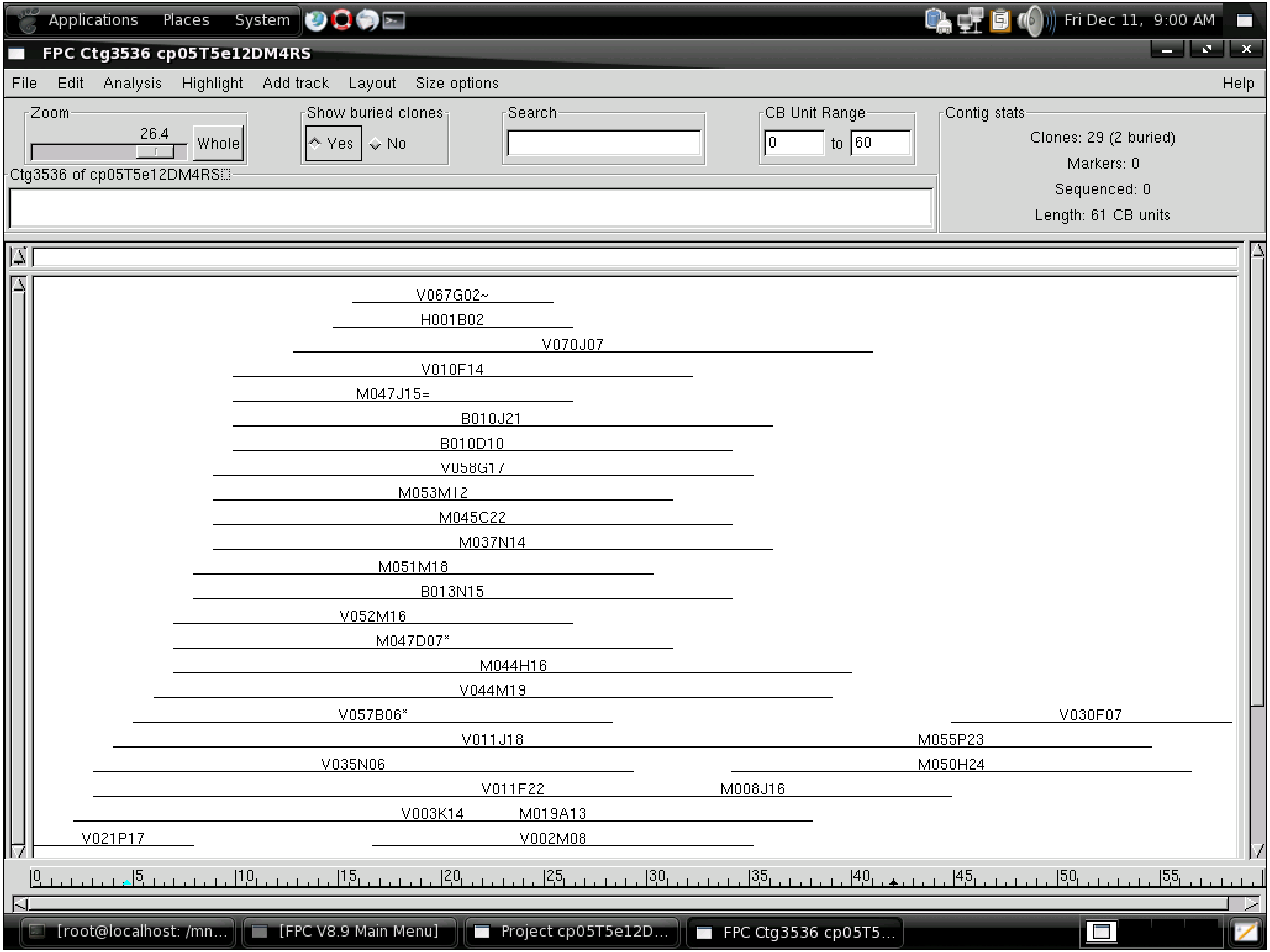
Applications Places System
Fri Dec 11, 9:00 AM
FPC Ctg3536 cp05T5e12DM4RS
File Edit Analysis Highlight Add track Layout Size options Help
Zoom
26.4
Whole
Show buried clones
Yes No
Search
CB Unit Range
0 to 60
Contig stats
Clones: 29 (2 buried)
Markers: 0
Sequenced: 0
Length: 61 CB units
Ctg3536 of cp05T5e12DM4RS
V067G02~
H001B02
V070J07
V010F14
M047J15=
B010J21
B010D10
V058G17
M053M12
M045C22
M037N14
M051M18
B013N15
V052M16
M047D07*
M044H16
V044M19
V057B06*
V030F07
V011J18
M055P23
V035N06
M050H24
V011F22
M008J16
V003K14
M019A13
V021P17
V002M08
0 5 10 15 20 25 30 35 40 45 50 55
[root@localhost: /mn...
[FPC V8.9 Main Menu]
Project cp05T5e12D...
FPC Ctg3536 cp05T5...

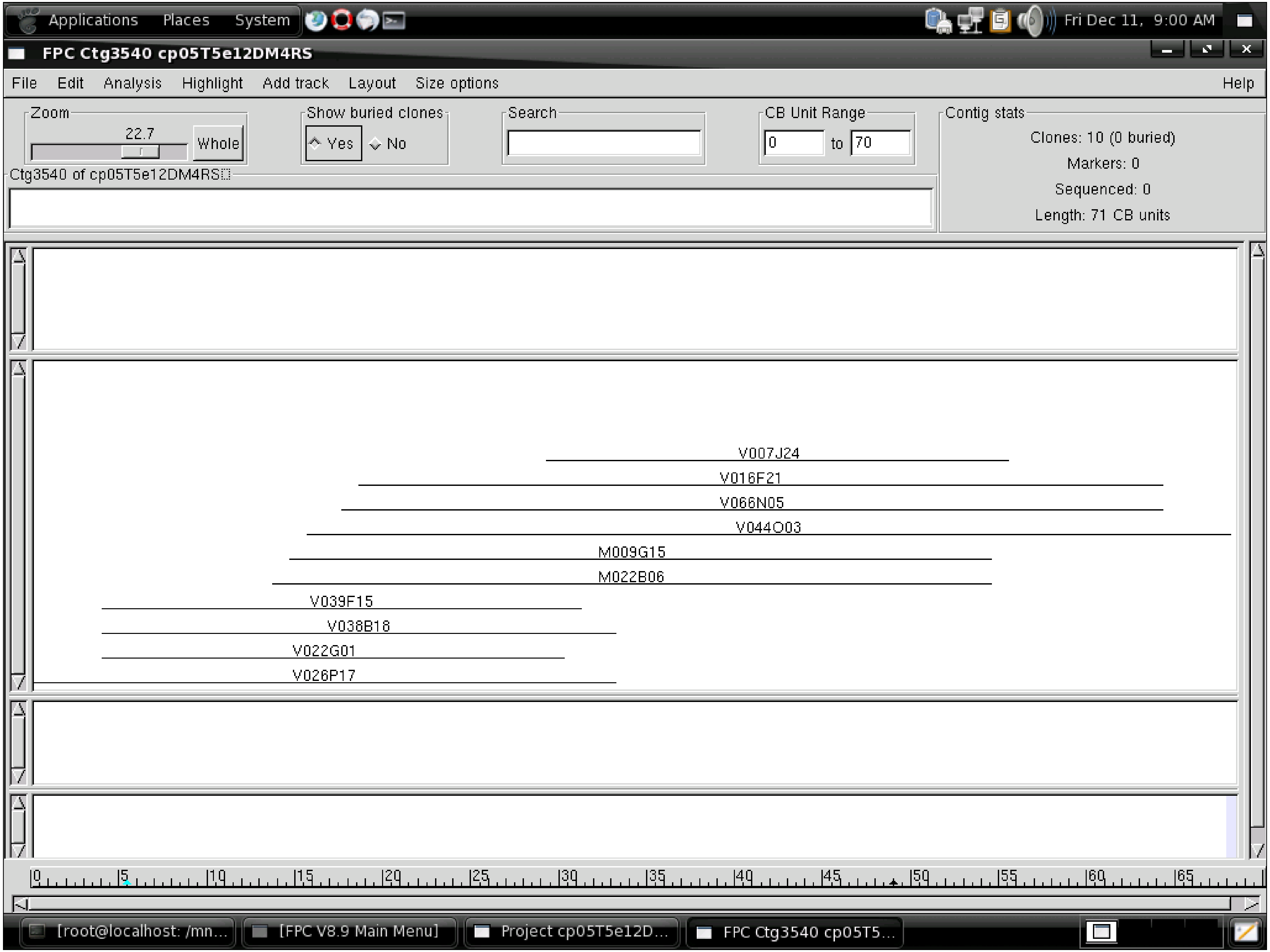
Applications
Places
System
Fri Dec 11, 9:00 AM
FPC Ctg3540 cp05T5e12DM4RS
File
Edit
Analysis
Highlight
Add track
Layout
Size options
Help
Zoom
22.7
Whole
Show buried clones
Yes
No
Search
CB Unit Range
0
to
70
Contig stats
Clones: 10 (0 buried)
Markers: 0
Sequenced: 0
Length: 71 CB units
Ctg3540 of cp05T5e12DM4RS
V007J24
V016F21
V066N05
V044O03
M009G15
M022B06
V039F15
V038B18
V022G01
V026P17
0
5
10
15
20
25
30
35
40
45
50
55
60
65
[root@localhost: /mn...
[FPC V8.9 Main Menu]
Project cp05T5e12D...
FPC Ctg3540 cp05T5...

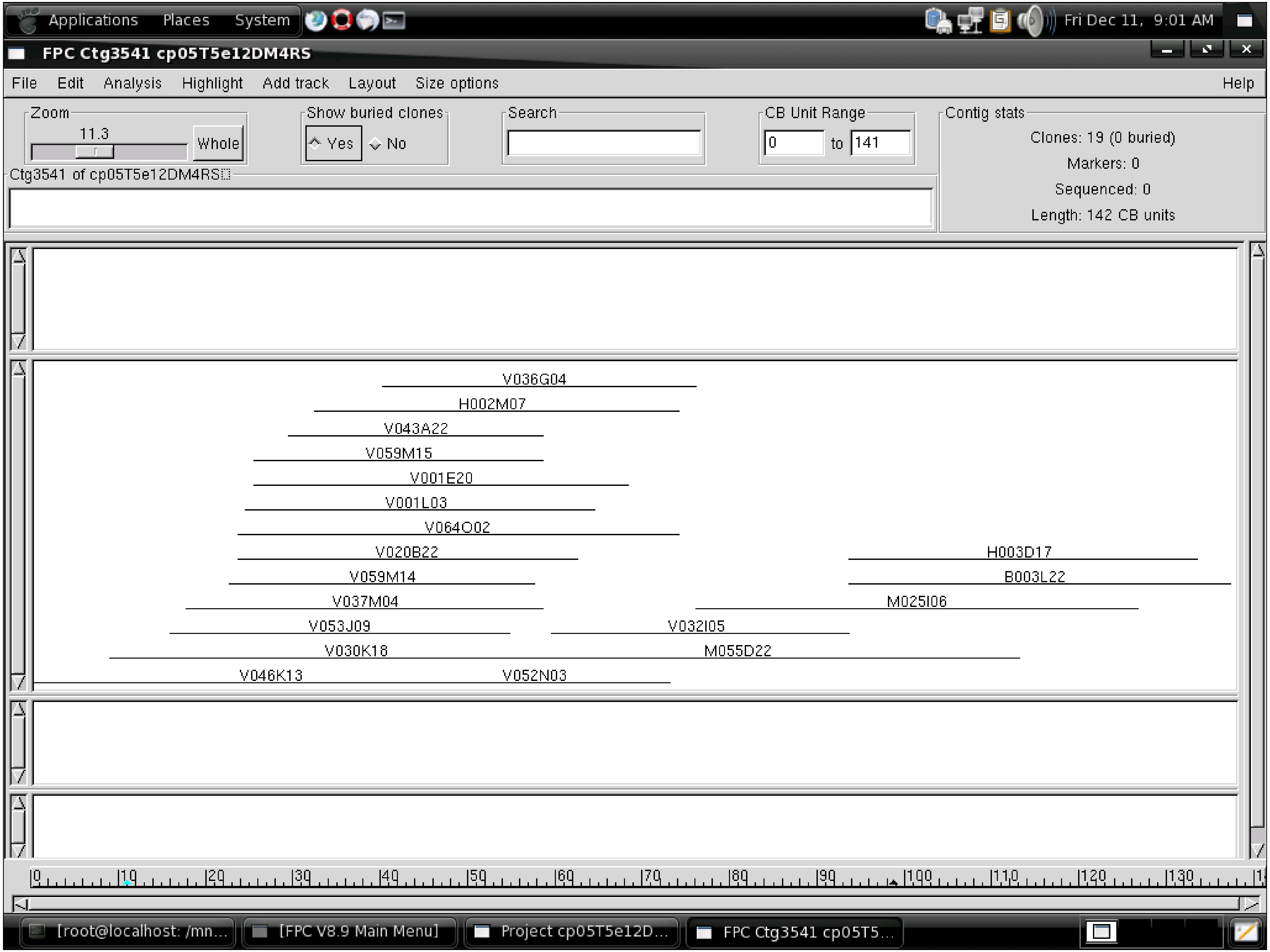
Applications Places System
Fri Dec 11, 9:01 AM
FPC Ctg3541 cp05T5e12DM4RS
File Edit Analysis Highlight Add track Layout Size options Help
Zoom
11.3
Whole
Show buried clones
Yes
No
Search
CB Unit Range
0 to 141
Contig stats
Clones: 19 (0 buried)
Markers: 0
Sequenced: 0
Length: 142 CB units
Ctg3541 of cp05T5e12DM4RS
V036G04
H002M07
V043A22
V059M15
V001E20
V001L03
V064O02
V020B22
H003D17
V059M14
B003L22
V037M04
M025I06
V053J09
V032I05
V030K18
M055D22
V046K13
V052N03
0 10 20 30 40 50 60 70 80 90 100 110 120 130
[root@localhost: /mn...
[FPC V8.9 Main Menu]
Project cp05T5e12D...
FPC Ctg3541 cp05T5...

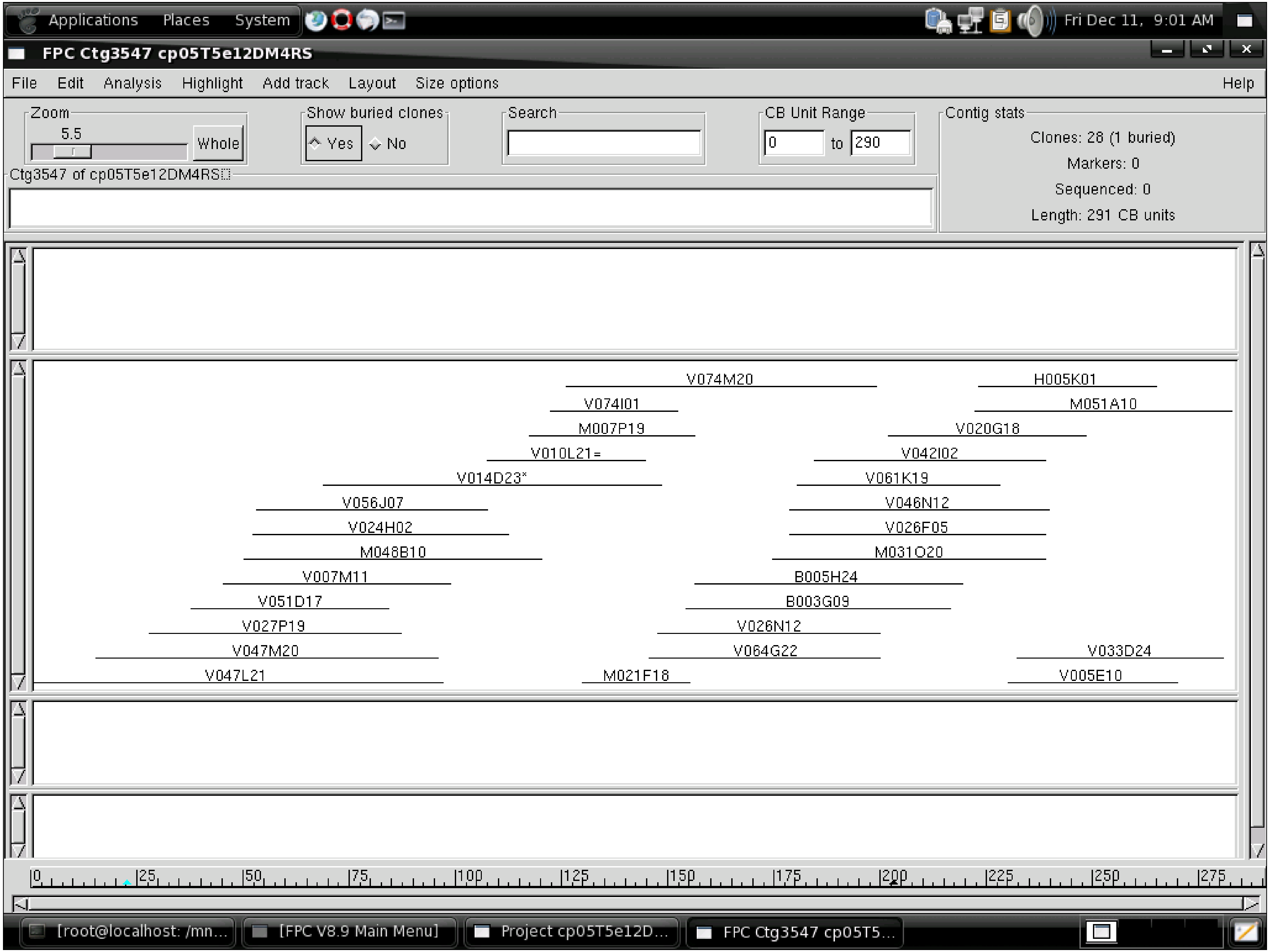
Applications Places System
Fri Dec 11, 9:01 AM
FPC Ctg3547 cp05T5e12DM4RS
File Edit Analysis Highlight Add track Layout Size options Help
Zoom
5.5
Whole
Show buried clones
Yes No
Search
CB Unit Range
0 to 290
Contig stats
Clones: 28 (1 buried)
Markers: 0
Sequenced: 0
Length: 291 CB units
Ctg3547 of cp05T5e12DM4RS
V074M20
H005K01
V074I01
M051A10
M007P19
V020G18
V010L21=
V042I02
V014D23*
V061K19
V056J07
V046N12
V024H02
V026F05
M048B10
M031O20
V007M11
B005H24
V051D17
B003G09
V027P19
V026N12
V047M20
V064G22
V033D24
V047L21
M021F18
V005E10
0 25 50 75 100 125 150 175 200 225 250 275
[root@localhost: /mn...
[FPC V8.9 Main Menu]
Project cp05T5e12D...
FPC Ctg3547 cp05T5...

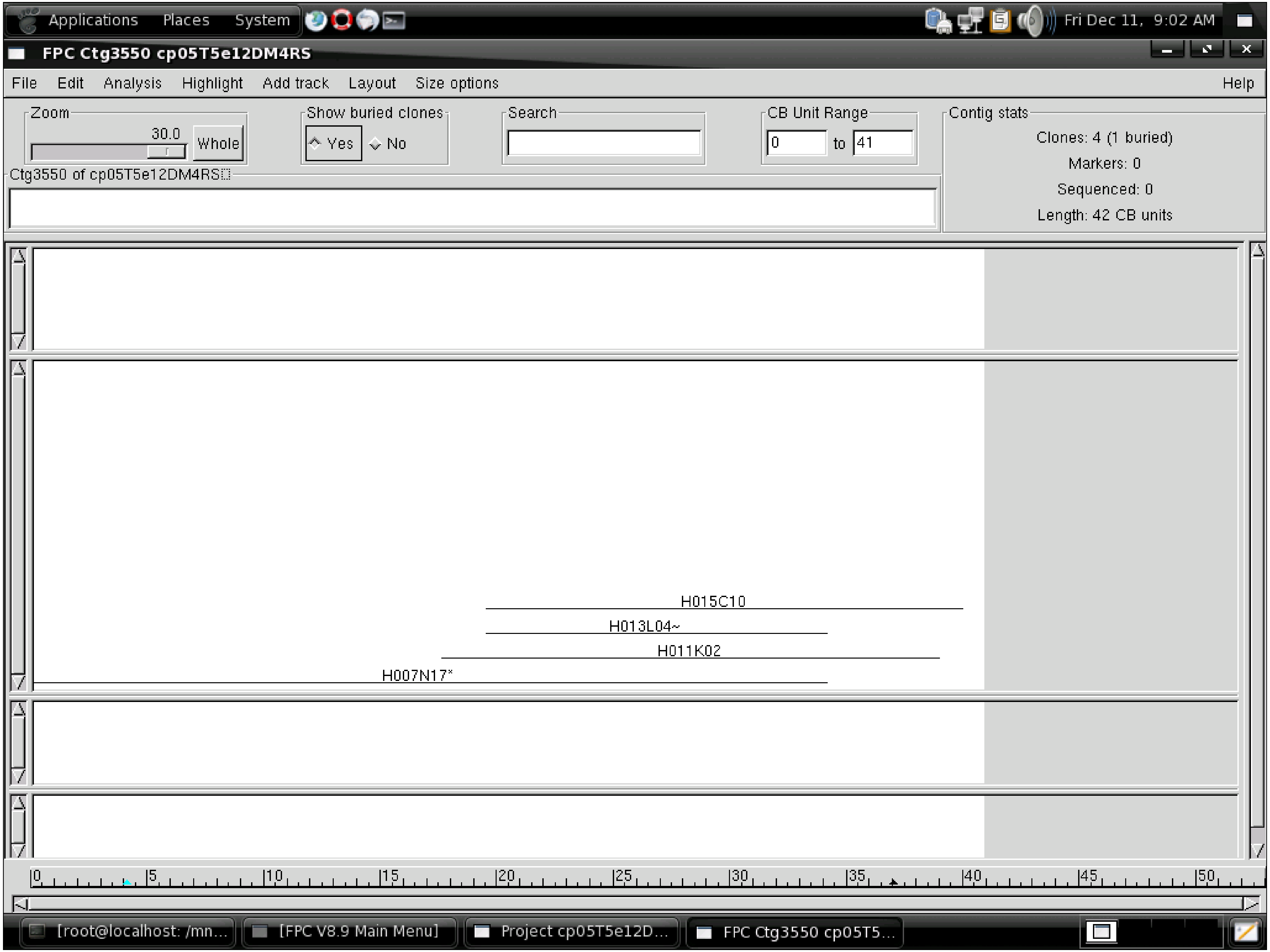
Applications Places System
Fri Dec 11, 9:02 AM
FPC Ctg3550 cp05T5e12DM4RS
File Edit Analysis Highlight Add track Layout Size options Help
Zoom
30.0
Whole
Show buried clones
Yes
No
Search
CB Unit Range
0
to
41
Contig stats
Clones: 4 (1 buried)
Markers: 0
Sequenced: 0
Length: 42 CB units
Ctg3550 of cp05T5e12DM4RS
H015C10
H013L04~
H011K02
H007N17*
0 5 10 15 20 25 30 35 40 45 50
[root@localhost: /mn...
[FPC V8.9 Main Menu]
Project cp05T5e12D...
FPC Ctg3550 cp05T5...

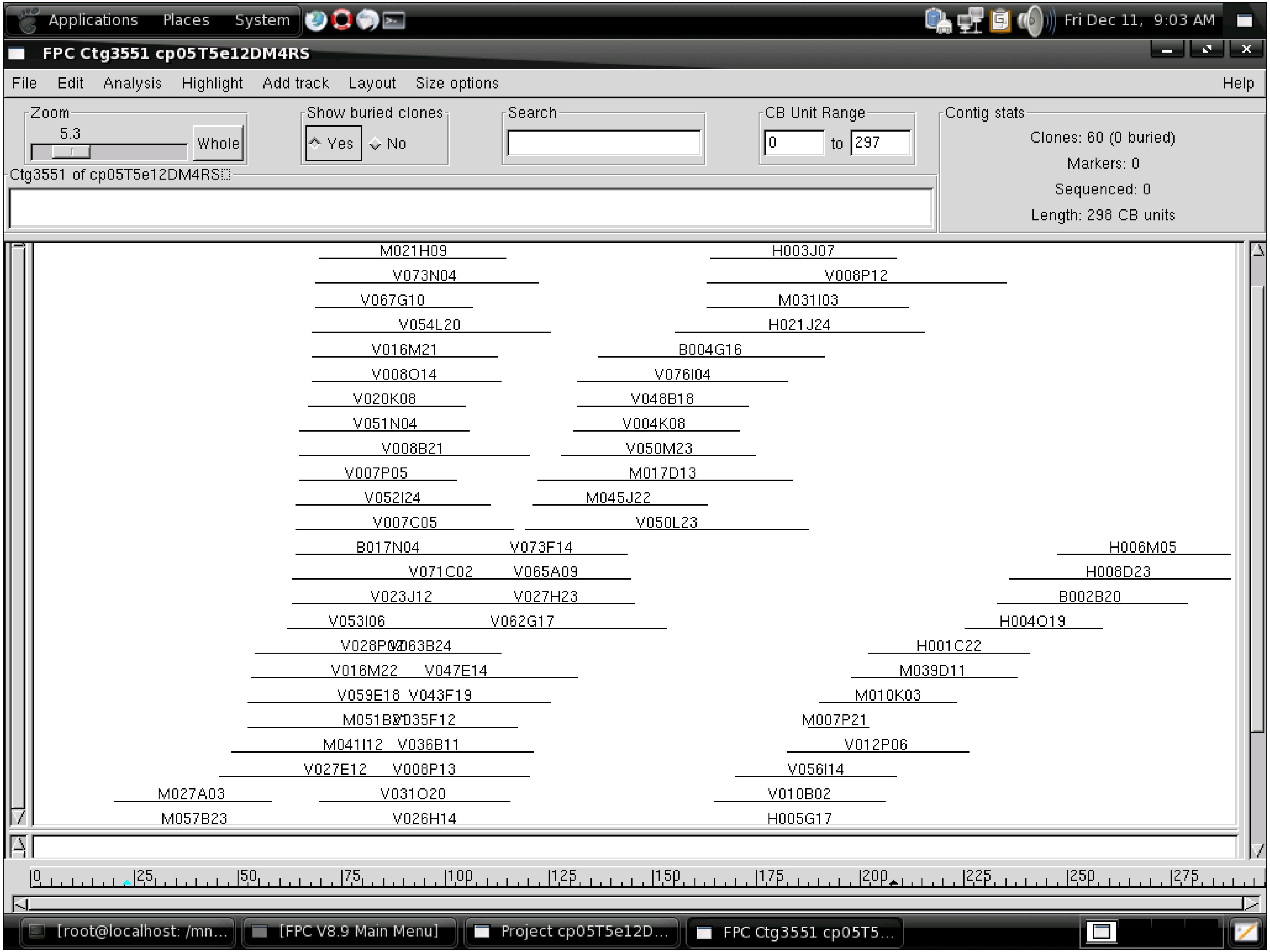
Applications Places System
Fri Dec 11, 9:03 AM
FPC Ctg3551 cp05T5e12DM4RS
File Edit Analysis Highlight Add track Layout Size options Help
Zoom
5.3
Whole
Show buried clones
Yes
No
Search
CB Unit Range
0
to
297
Contig stats
Clones: 60 (0 buried)
Markers: 0
Sequenced: 0
Length: 298 CB units
Ctg3551 of cp05T5e12DM4RS
M021H09
H003J07
V073N04
V008P12
V067G10
M031I03
V054L20
H021J24
V016M21
B004G16
V008O14
V076I04
V020K08
V048B18
V051N04
V004K08
V008B21
V050M23
V007P05
M017D13
V052I24
M045J22
V007C05
V050L23
B017N04
V073F14
H006M05
V071C02
V065A09
H008D23
V023J12
V027H23
B002B20
V053I06
V062G17
H004O19
H001C22
V016M22
V047E14
M039D11
V059E18
V043F19
M010K03
M007P21
M041I12
V036B11
V012P06
V027E12
V008P13
V056I14
M027A03
V031O20
V010B02
M057B23
V026H14
H005G17
0 25 50 75 100 125 150 175 200 225 250 275
[root@localhost: /mn...
[FPC V8.9 Main Menu]
Project cp05T5e12D...
FPC Ctg3551 cp05T5...

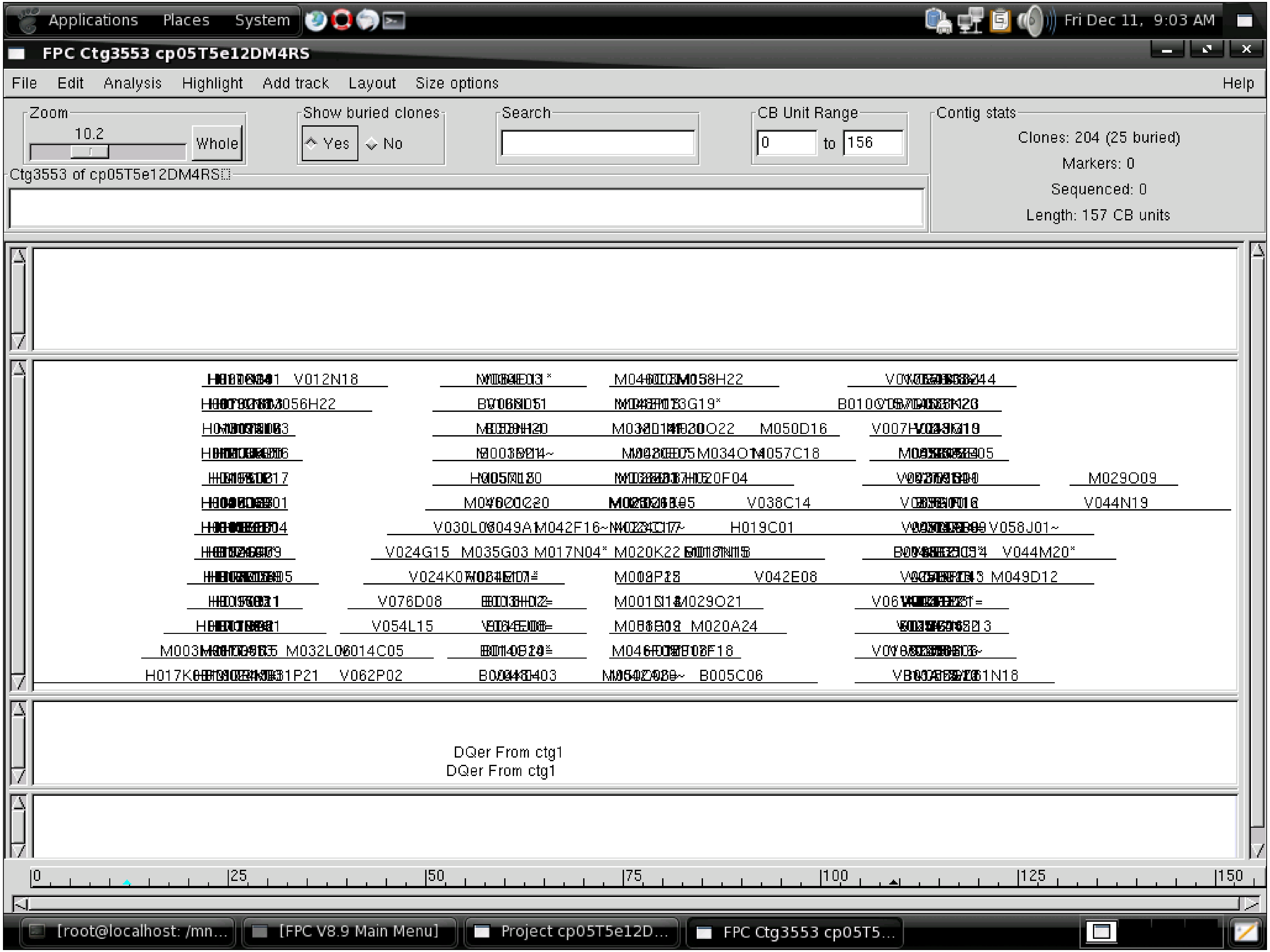
Applications Places System
Fri Dec 11, 9:03 AM
FPC Ctg3553 cp05T5e12DM4RS
File Edit Analysis Highlight Add track Layout Size options Help
Zoom
10.2
Whole
Show buried clones
Yes
No
Search
CB Unit Range
0 to 156
Contig stats
Clones: 204 (25 buried)
Markers: 0
Sequenced: 0
Length: 157 CB units
Ctg3553 of cp05T5e12DM4RS
V012N18
M029O09
V044N19
V038C14
H019C01
V058J01~
V024G15 M035G03 M017N04* M020K22
V044M20*
V042E08
M049D12
V076D08
V054L15
M020A24
V062P02
B005C06
M050D16
DQer From ctg1
DQer From ctg1
0 25 50 75 100 125 150
[root@localhost: /mn...
[FPC V8.9 Main Menu]
Project cp05T5e12D...
FPC Ctg3553 cp05T5...

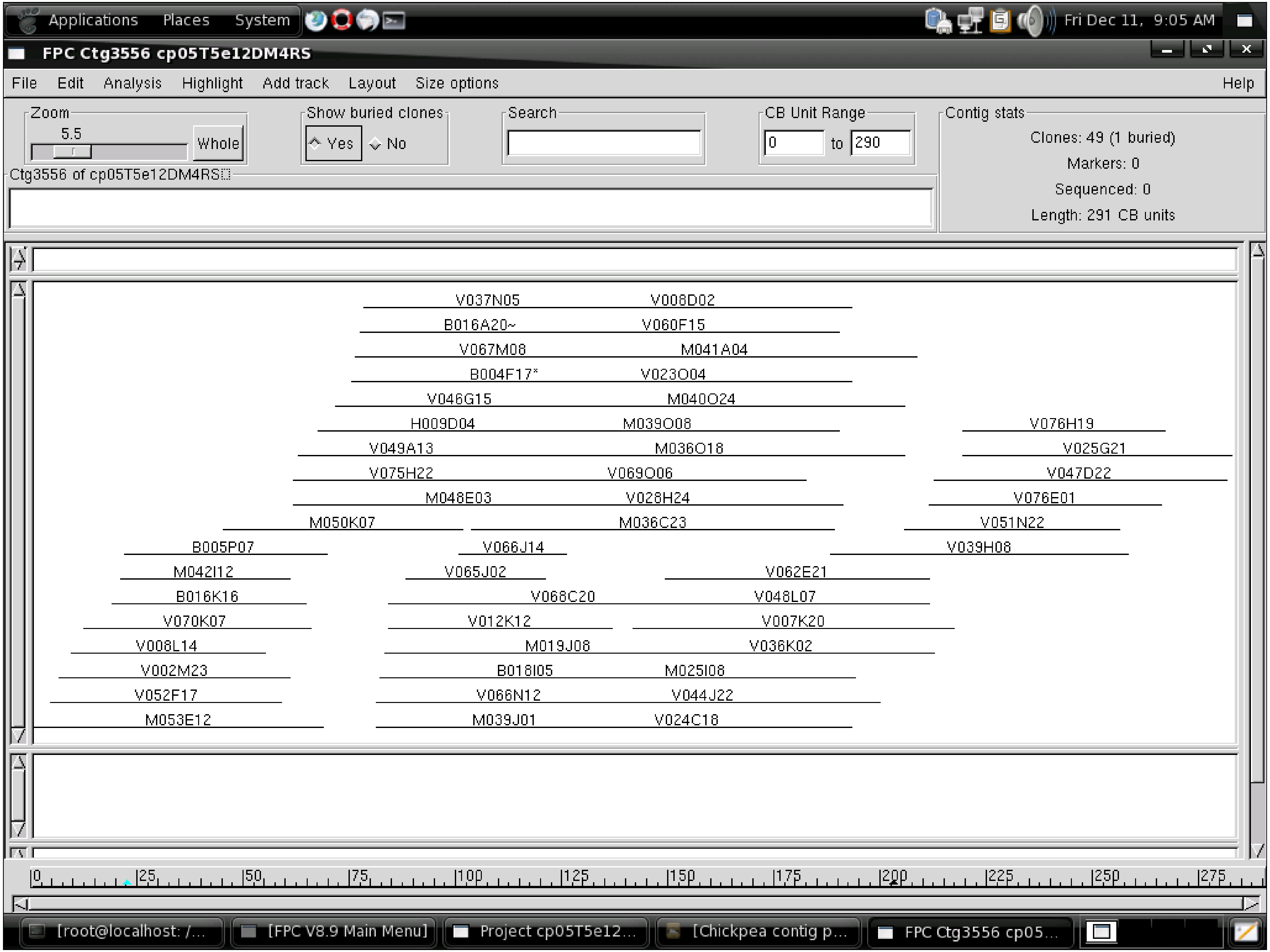
Applications Places System
Fri Dec 11, 9:05 AM
FPC Ctg3556 cp05T5e12DM4RS
File Edit Analysis Highlight Add track Layout Size options Help
Zoom 5.5 Whole
Show buried clones Yes No
Search
CB Unit Range 0 to 290
Contig stats
Clones: 49 (1 buried)
Markers: 0
Sequenced: 0
Length: 291 CB units
Ctg3556 of cp05T5e12DM4RS
V037N05
V008D02
B016A20~
V060F15
V067M08
M041A04
B004F17*
V023O04
V046G15
M040O24
H009D04
M039O08
V076H19
V049A13
M036O18
V025G21
V075H22
V069O06
V047D22
M048E03
V028H24
V076E01
M050K07
M036C23
V051N22
B005P07
V066J14
V039H08
M042I12
V065J02
V062E21
B016K16
V068C20
V048L07
V070K07
V012K12
V007K20
V008L14
M019J08
V036K02
V002M23
B018I05
M025I08
V052F17
V066N12
V044J22
M053E12
M039J01
V024C18
0 25 50 75 100 125 150 175 200 225 250 275
[root@localhost: /...
[FPC V8.9 Main Menu]
Project cp05T5e12...
[Chickpea contig p...
FPC Ctg3556 cp05...

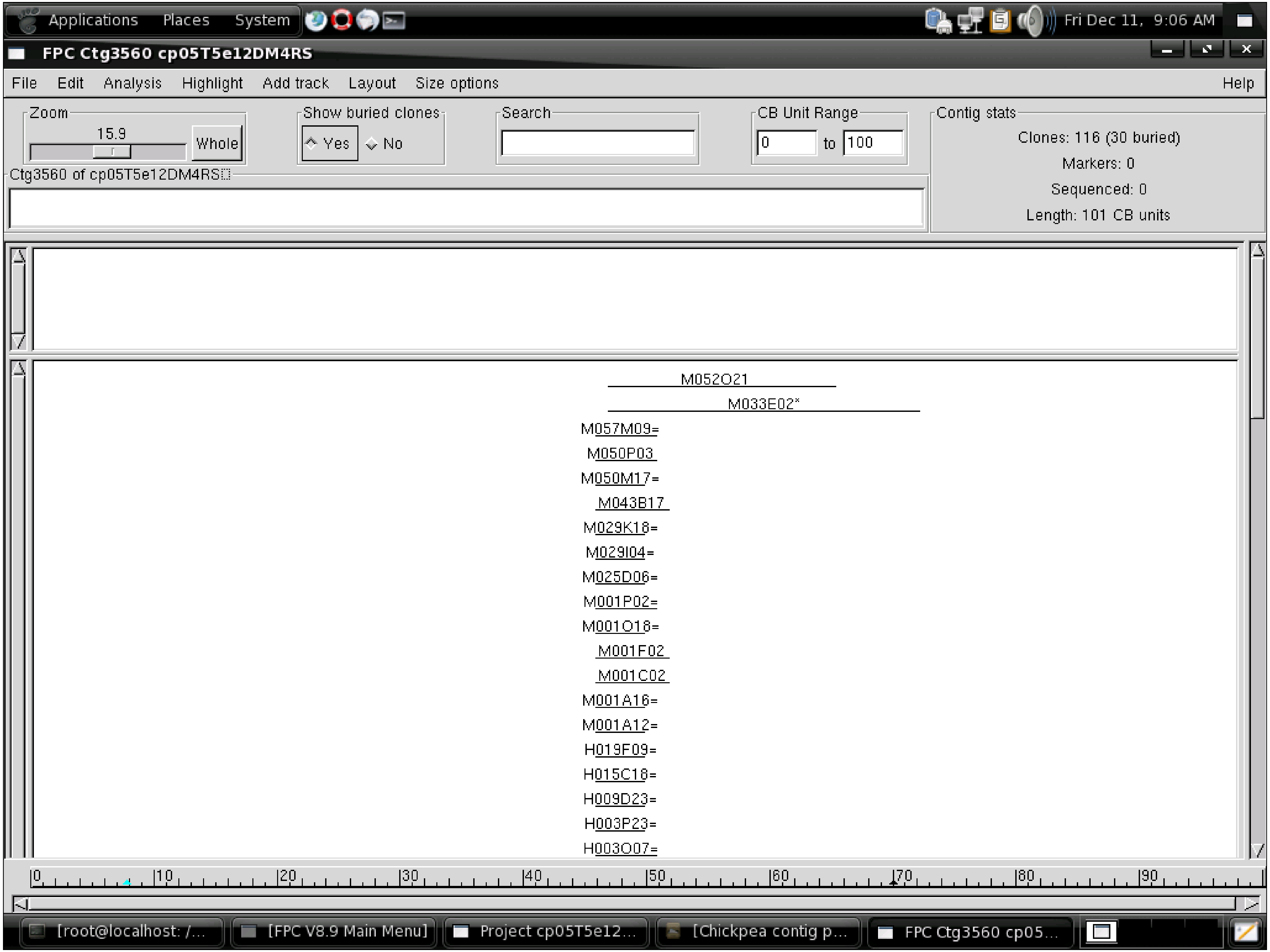
Applications Places System
Fri Dec 11, 9:06 AM
FPC Ctg3560 cp05T5e12DM4RS
File Edit Analysis Highlight Add track Layout Size options Help
Zoom
15.9
Whole
Show buried clones
Yes
No
Search
CB Unit Range
0 to 100
Contig stats
Clones: 116 (30 buried)
Markers: 0
Sequenced: 0
Length: 101 CB units
Ctg3560 of cp05T5e12DM4RS
M052O21
M033E02*
M057M09=
M050P03
M050M17=
M043B17
M029K18=
M029I04=
M025D06=
M001P02=
M001O18=
M001F02
M001C02
M001A16=
M001A12=
H019F09=
H015C18=
H009D23=
H003P23=
H003O07=
0 10 20 30 40 50 60 70 80 90
[root@localhost: /...
[FPC V8.9 Main Menu]
Project cp05T5e12...
[Chickpea contig p...
FPC Ctg3560 cp05...

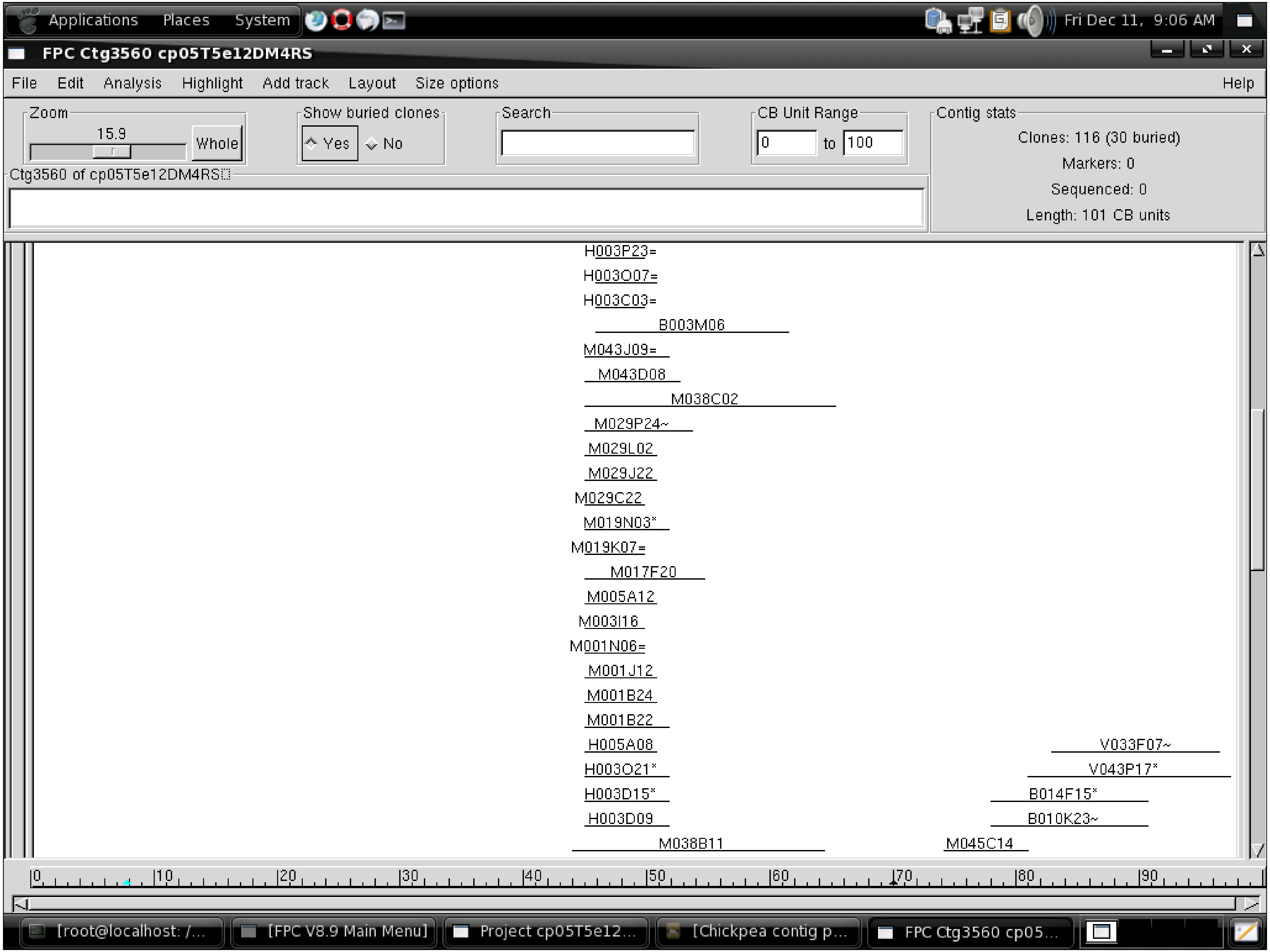
Applications Places System
Fri Dec 11, 9:06 AM
FPC Ctg3560 cp05T5e12DM4RS
File Edit Analysis Highlight Add track Layout Size options Help
Zoom
15.9
Whole
Show buried clones
Yes No
Search
CB Unit Range
0 to 100
Contig stats
Clones: 116 (30 buried)
Markers: 0
Sequenced: 0
Length: 101 CB units
Ctg3560 of cp05T5e12DM4RS
H003P23=
H003O07=
H003C03=
B003M06
M043J09=
M043D08
M038C02
M029P24~
M029L02
M029J22
M029C22
M019N03*
M019K07=
M017F20
M005A12
M003I16
M001N06=
M001J12
M001B24
M001B22
H005A08
V033F07~
H003O21*
V043P17*
H003D15*
B014F15*
H003D09
B010K23~
M038B11
M045C14
0 10 20 30 40 50 60 70 80 90
[root@localhost: /...
[FPC V8.9 Main Menu]
Project cp05T5e12...
[Chickpea contig p...
FPC Ctg3560 cp05...

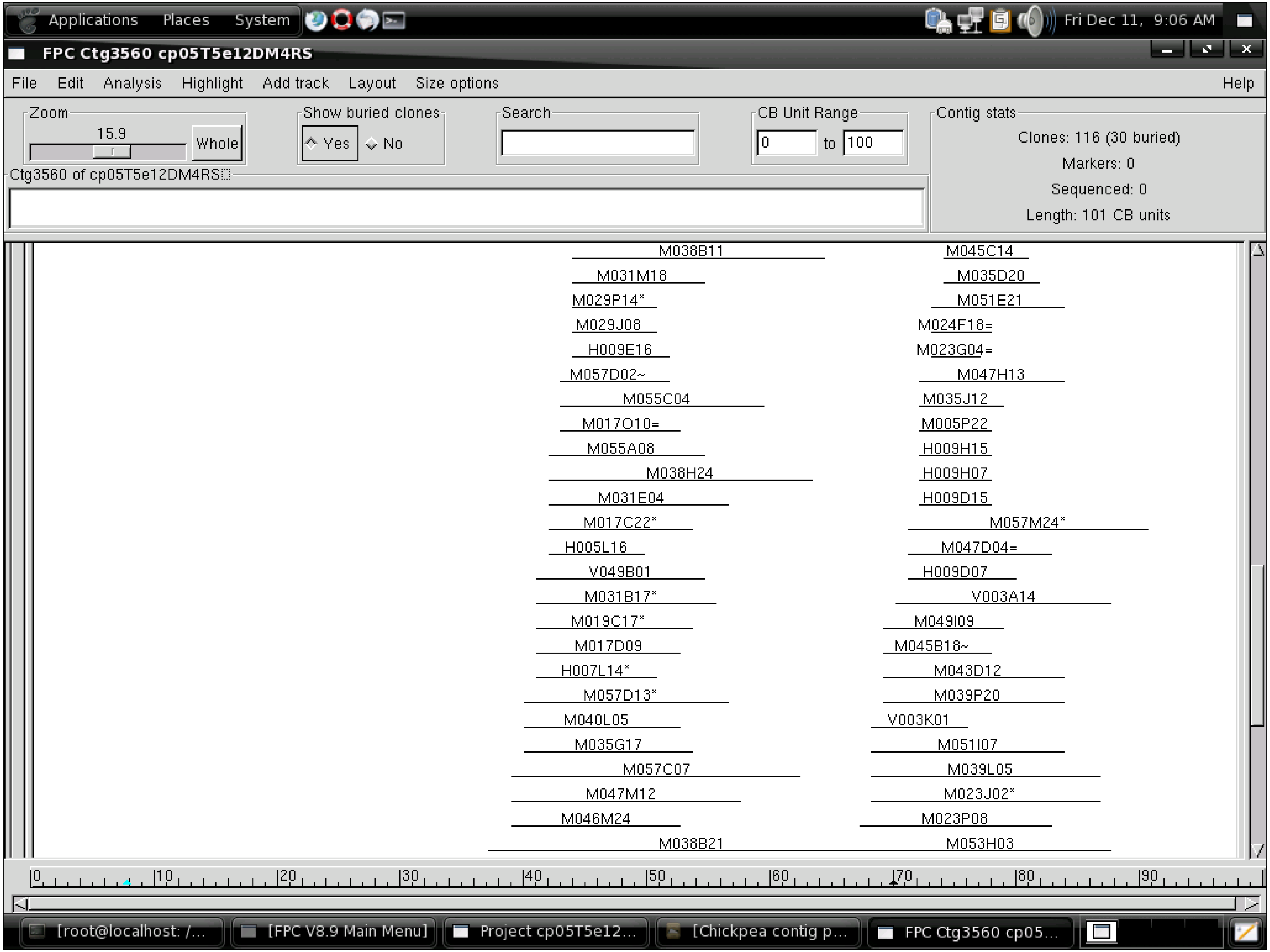
Applications Places System
Fri Dec 11, 9:06 AM
FPC Ctg3560 cp05T5e12DM4RS
File Edit Analysis Highlight Add track Layout Size options Help
Zoom
15.9
Whole
Show buried clones
Yes No
Search
CB Unit Range
0 to 100
Contig stats
Clones: 116 (30 buried)
Markers: 0
Sequenced: 0
Length: 101 CB units
Ctg3560 of cp05T5e12DM4RS
M038B11
M031M18
M029P14*
M029J08
H009E16
M057D02~
M055C04
M017O10=
M055A08
M038H24
M031E04
M017C22*
H005L16
V049B01
M031B17*
M019C17*
M017D09
H007L14*
M057D13*
M040L05
M035G17
M057C07
M047M12
M046M24
M038B21
M045C14
M035D20
M051E21
M024F18=
M023G04=
M047H13
M035J12
M005P22
H009H15
H009H07
H009D15
M057M24*
M047D04=
H009D07
V003A14
M049I09
M045B18~
M043D12
M039P20
V003K01
M051I07
M039L05
M023J02*
M023P08
M053H03
0 10 20 30 40 50 60 70 80 90
[root@localhost: /...
[FPC V8.9 Main Menu]
Project cp05T5e12...
[Chickpea contig p...
FPC Ctg3560 cp05...

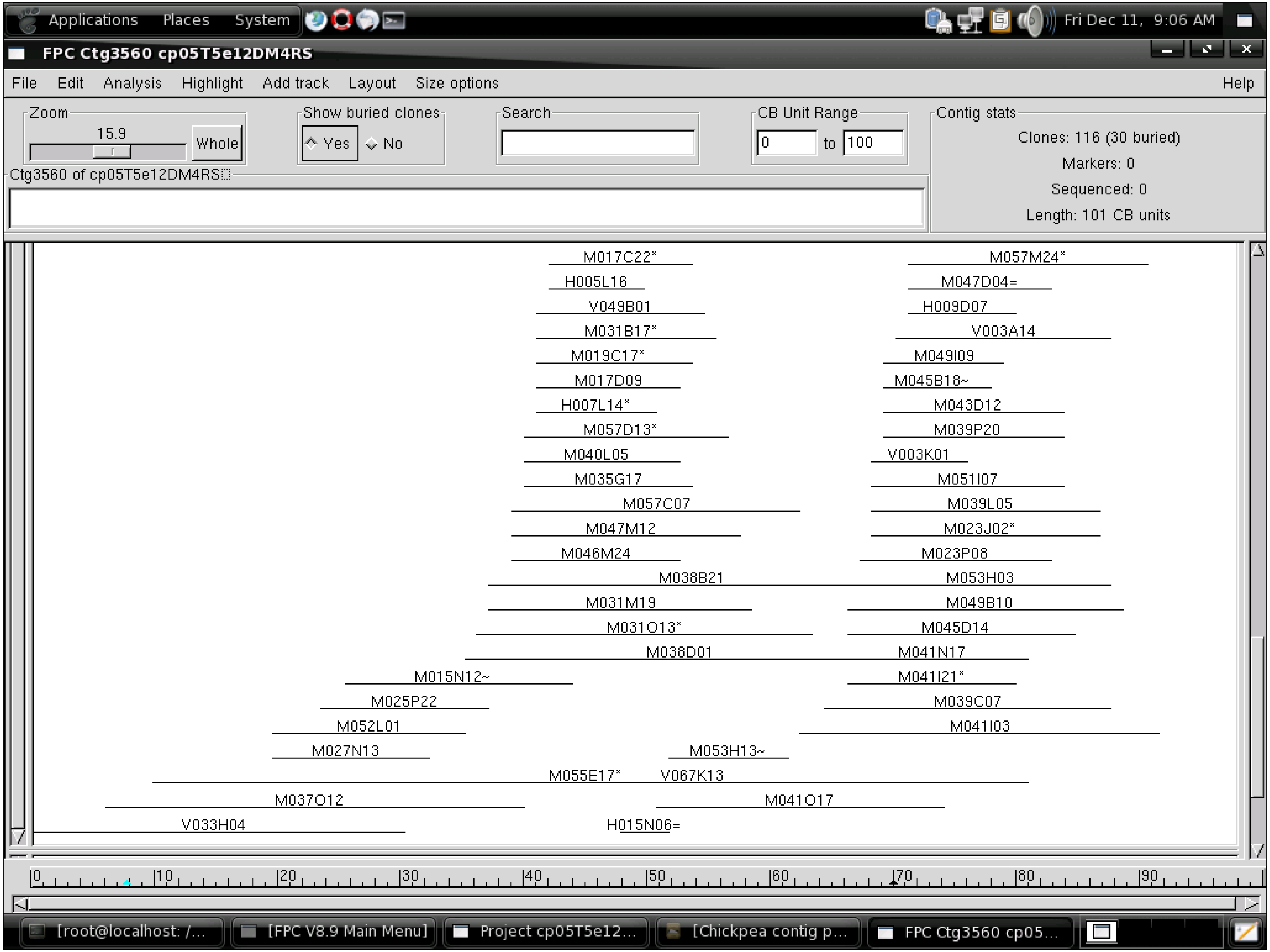
Applications Places System
Fri Dec 11, 9:06 AM
FPC Ctg3560 cp05T5e12DM4RS
File Edit Analysis Highlight Add track Layout Size options Help
Zoom
15.9
Whole
Show buried clones
Yes No
Search
CB Unit Range
0 to 100
Contig stats
Clones: 116 (30 buried)
Markers: 0
Sequenced: 0
Length: 101 CB units
Ctg3560 of cp05T5e12DM4RS
M017C22*
H005L16
V049B01
M031B17*
M019C17*
M017D09
H007L14*
M057D13*
M040L05
M035G17
M057C07
M047M12
M046M24
M038B21
M031M19
M031O13*
M038D01
M015N12~
M025P22
M052L01
M027N13
M055E17*
M037O12
V033H04
M057M24*
M047D04=
H009D07
V003A14
M049I09
M045B18~
M043D12
M039P20
V003K01
M051I07
M039L05
M023J02*
M023P08
M053H03
M049B10
M045D14
M041N17
M041I21*
M039C07
M041I03
M053H13~
V067K13
M041O17
H015N06=
0 10 20 30 40 50 60 70 80 90
[root@localhost: /...
[FPC V8.9 Main Menu]
Project cp05T5e12...
[Chickpea contig p...
FPC Ctg3560 cp05...

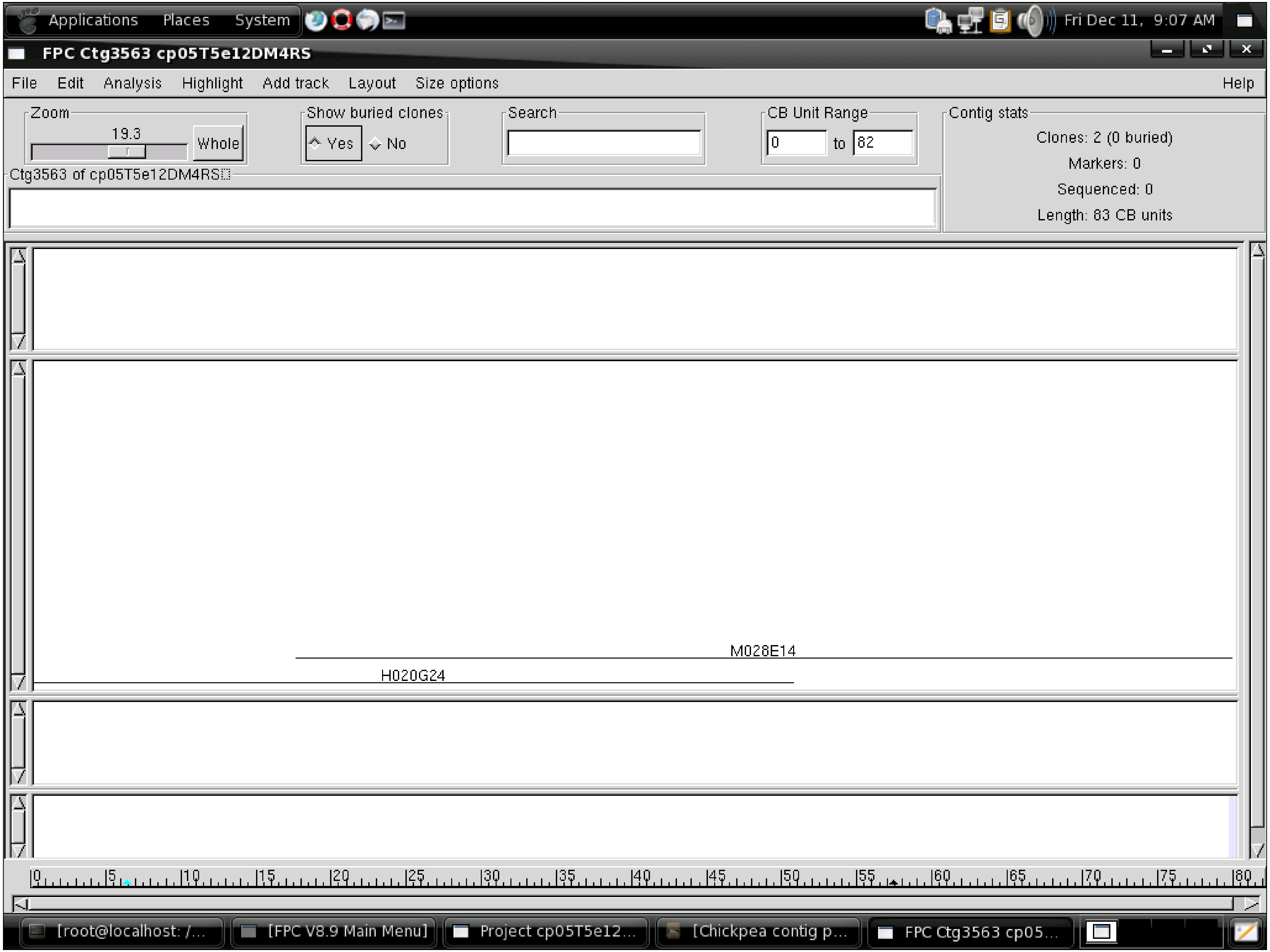
Applications Places System
Fri Dec 11, 9:07 AM
FPC Ctg3563 cp05T5e12DM4RS
File Edit Analysis Highlight Add track Layout Size options
Help
Zoom
19.3
Whole
Show buried clones
Yes
No
Search
CB Unit Range
0
to
82
Contig stats
Clones: 2 (0 buried)
Markers: 0
Sequenced: 0
Length: 83 CB units
Ctg3563 of cp05T5e12DM4RS
M028E14
H020G24
0 5 10 15 20 25 30 35 40 45 50 55 60 65 70 75 80
[root@localhost: /...
[FPC V8.9 Main Menu]
Project cp05T5e12...
[Chickpea contig p...
FPC Ctg3563 cp05...

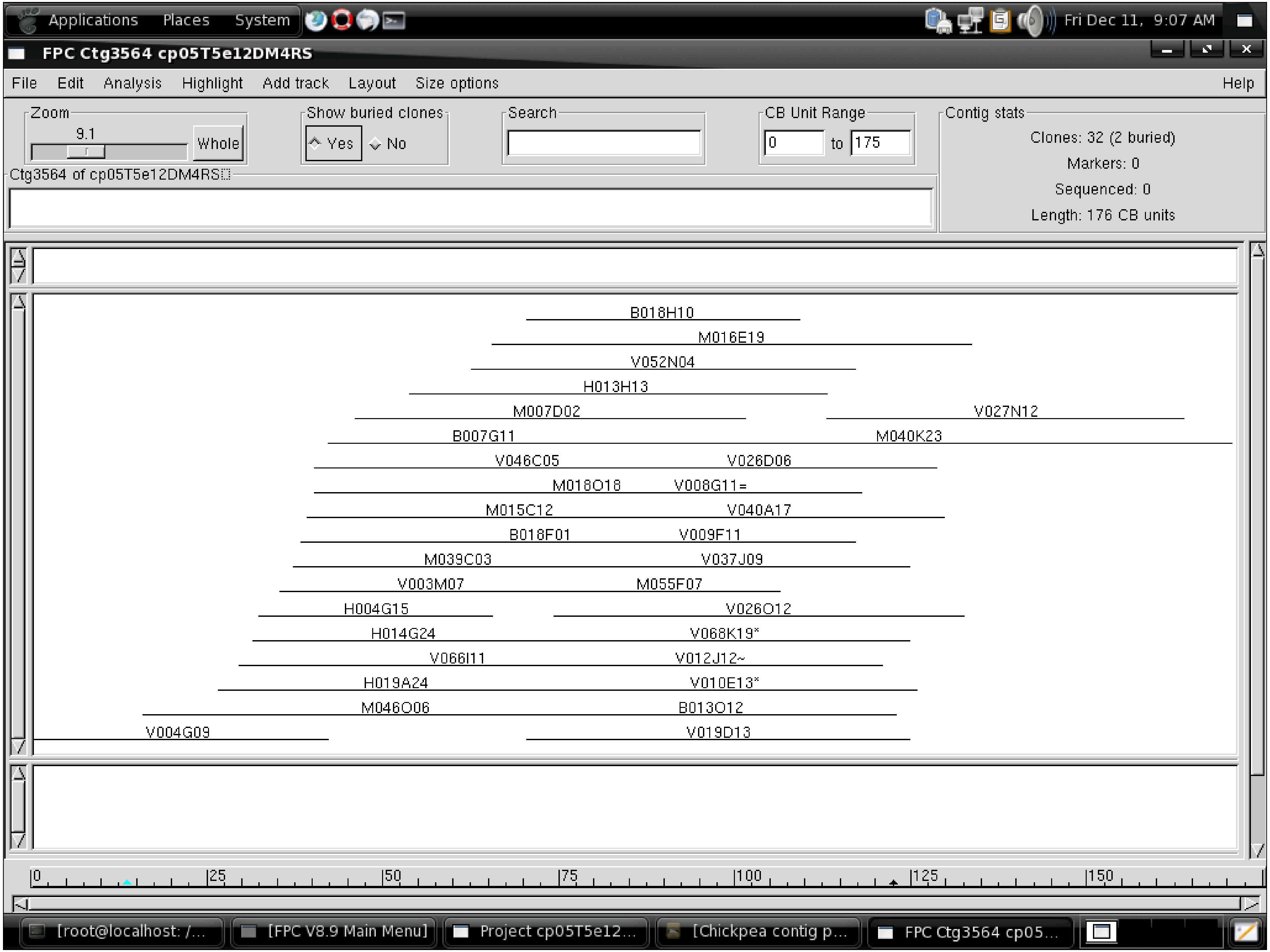

Applications
Places
System
Fri Dec 11, 9:07 AM
FPC Ctg3564 cp05T5e12DM4RS
File
Edit
Analysis
Highlight
Add track
Layout
Size options
Help
Zoom
9.1
Whole
Show buried clones
Yes
No
Search
CB Unit Range
0
to
175
Contig stats
Clones: 32 (2 buried)
Markers: 0
Sequenced: 0
Length: 176 CB units
Ctg3564 of cp05T5e12DM4RS
B018H10
M016E19
V052N04
H013H13
M007D02
V027N12
B007G11
M040K23
V046C05
V026D06
M018O18
V008G11=
M015C12
V040A17
B018F01
V009F11
M039C03
V037J09
V003M07
M055F07
H004G15
V026O12
H014G24
V068K19*
V066I11
V012J12~
H019A24
V010E13*
M046O06
B013O12
V004G09
V019D13
0
25
50
75
100
125
150
[root@localhost: /...
[FPC V8.9 Main Menu]
Project cp05T5e12...
[Chickpea contig p...
FPC Ctg3564 cp05...

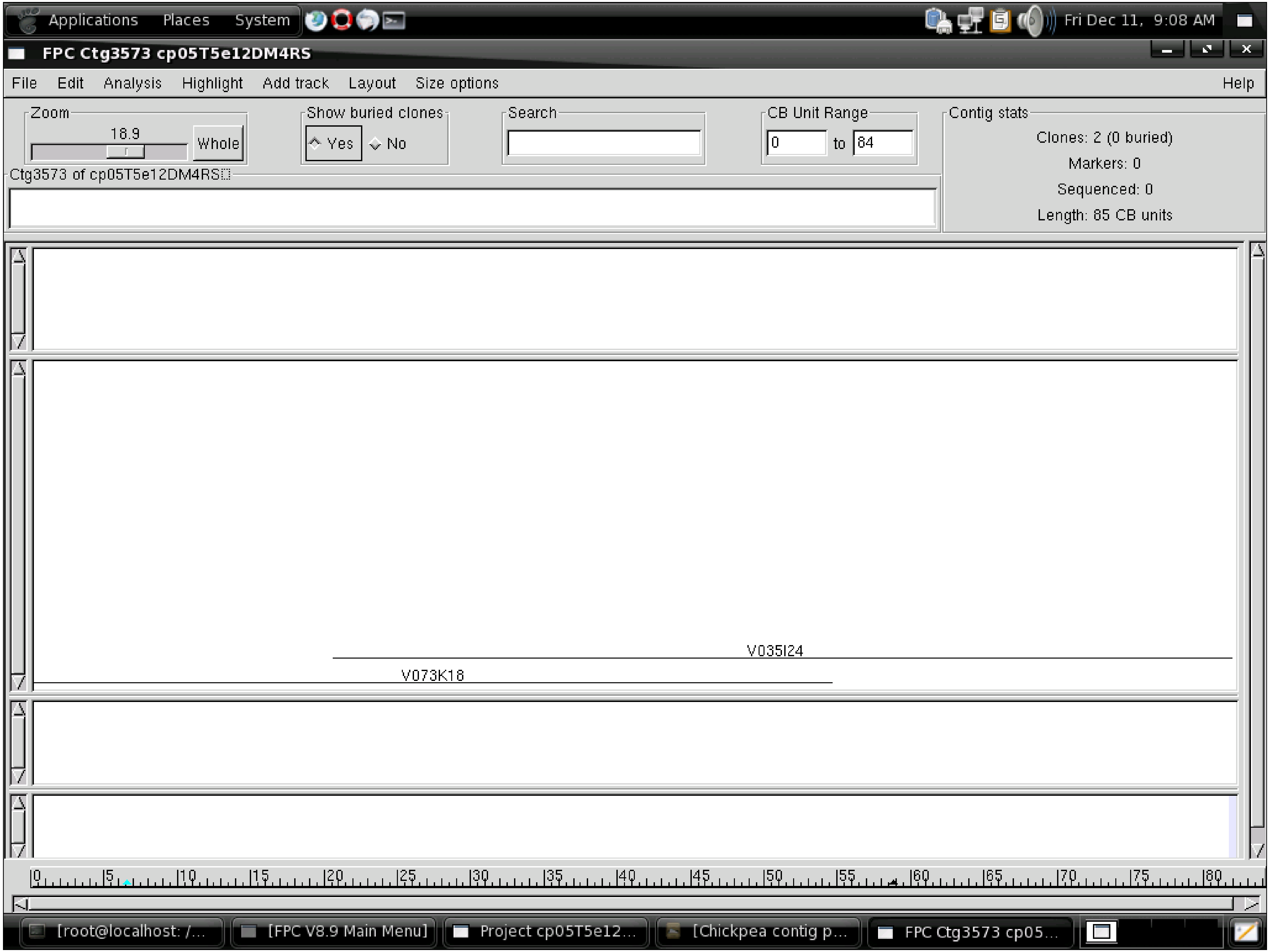
Applications Places System
Fri Dec 11, 9:08 AM
FPC Ctg3573 cp05T5e12DM4RS
File Edit Analysis Highlight Add track Layout Size options Help
Zoom
18.9
Whole
Show buried clones
Yes No
Search
CB Unit Range
0 to 84
Contig stats
Clones: 2 (0 buried)
Markers: 0
Sequenced: 0
Length: 85 CB units
Ctg3573 of cp05T5e12DM4RS
V035I24
V073K18
0 5 10 15 20 25 30 35 40 45 50 55 60 65 70 75 80
[root@localhost: /...
[FPC V8.9 Main Menu]
Project cp05T5e12...
[Chickpea contig p...
FPC Ctg3573 cp05...

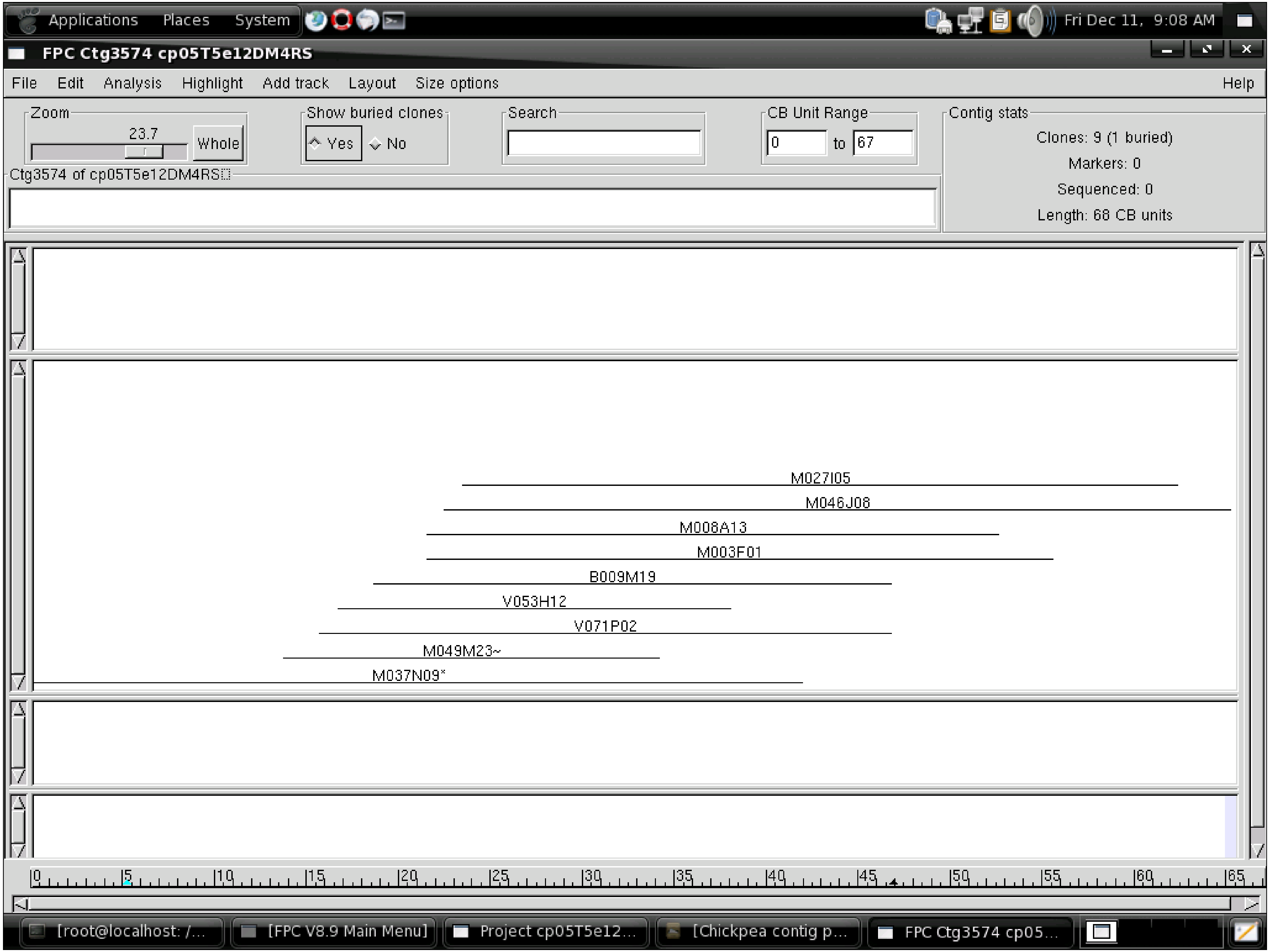
Applications
Places
System
Fri Dec 11, 9:08 AM
FPC Ctg3574 cp05T5e12DM4RS
File
Edit
Analysis
Highlight
Add track
Layout
Size options
Help
Zoom
23.7
Whole
Show buried clones
Yes
No
Search
CB Unit Range
0
to
67
Contig stats
Clones: 9 (1 buried)
Markers: 0
Sequenced: 0
Length: 68 CB units
Ctg3574 of cp05T5e12DM4RS
M027I05
M046J08
M008A13
M003F01
B009M19
V053H12
V071P02
M049M23~
M037N09*
0
5
10
15
20
25
30
35
40
45
50
55
60
65
[root@localhost: /...
[FPC V8.9 Main Menu]
Project cp05T5e12...
[Chickpea contig p...
FPC Ctg3574 cp05...

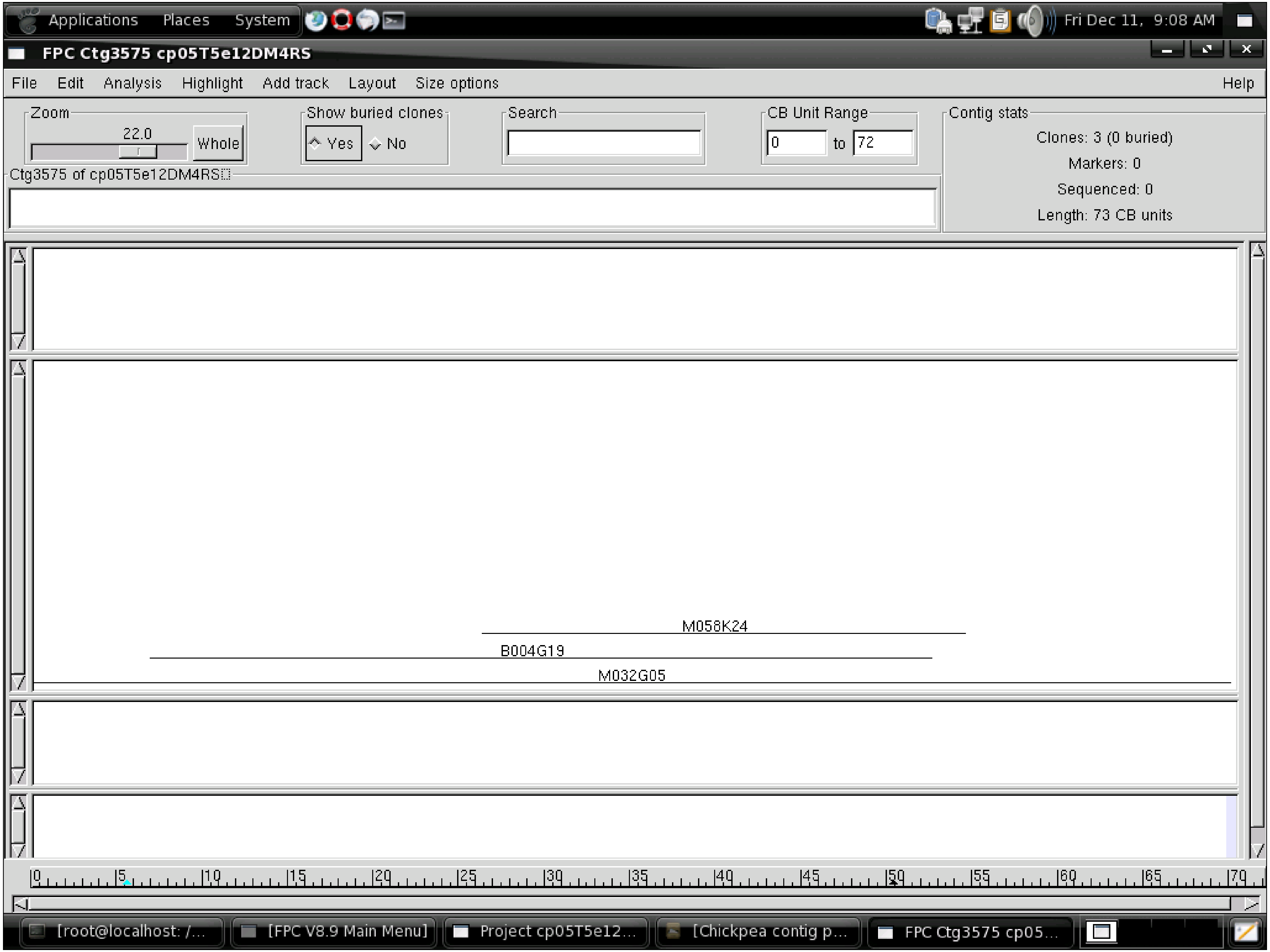
Applications Places System
Fri Dec 11, 9:08 AM
FPC Ctg3575 cp05T5e12DM4RS
File Edit Analysis Highlight Add track Layout Size options Help
Zoom
22.0
Whole
Show buried clones
Yes No
Search
CB Unit Range
0 to 72
Contig stats
Clones: 3 (0 buried)
Markers: 0
Sequenced: 0
Length: 73 CB units
Ctg3575 of cp05T5e12DM4RS
M058K24
B004G19
M032G05
0 5 10 15 20 25 30 35 40 45 50 55 60 65 70
[root@localhost: /...
[FPC V8.9 Main Menu]
Project cp05T5e12...
[Chickpea contig p...
FPC Ctg3575 cp05...

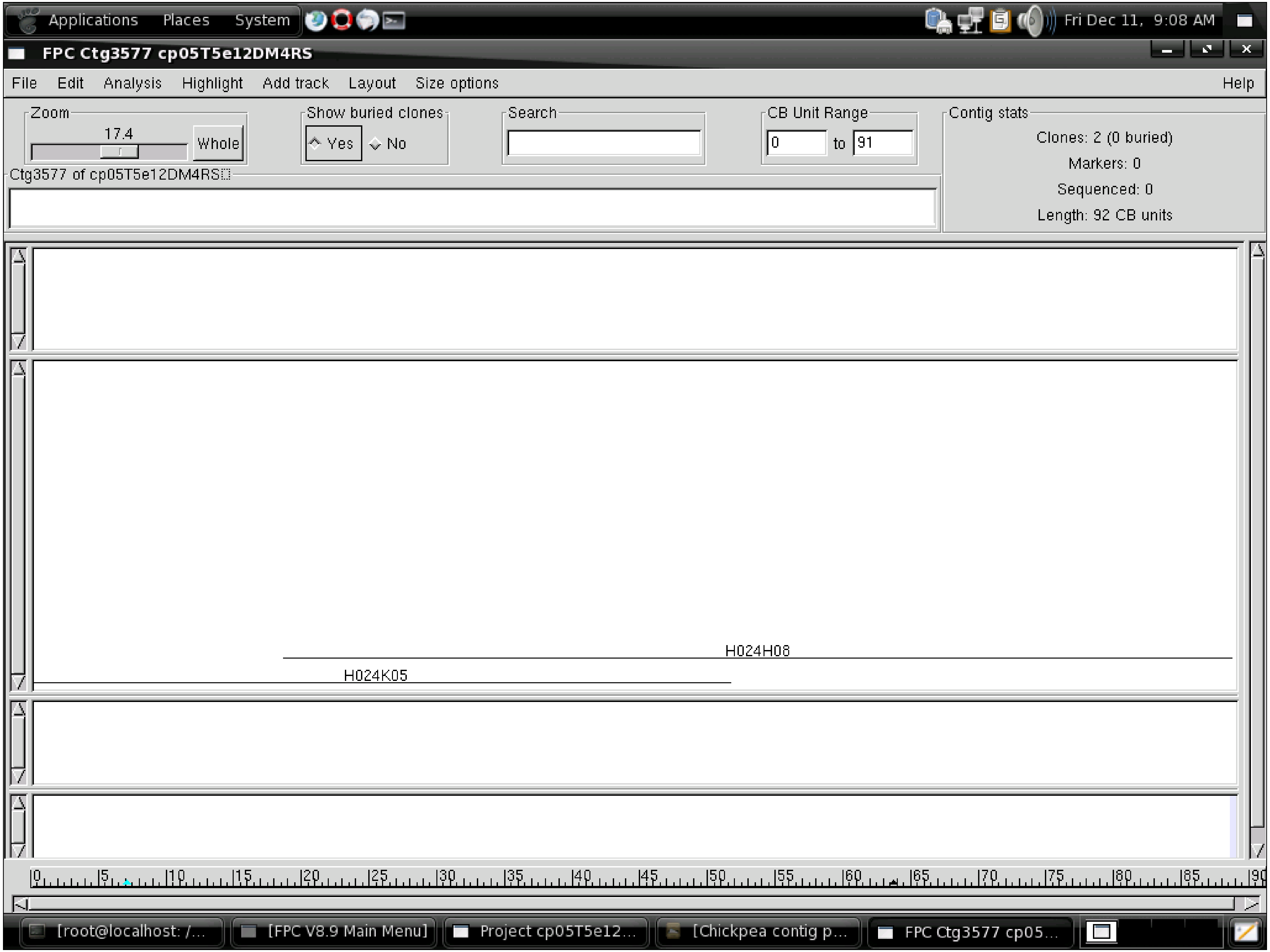
Applications
Places
System
Fri Dec 11, 9:08 AM
FPC Ctg3577 cp05T5e12DM4RS
File
Edit
Analysis
Highlight
Add track
Layout
Size options
Help
Zoom
17.4
Whole
Show buried clones
Yes
No
Search
CB Unit Range
0
to
91
Contig stats
Clones: 2 (0 buried)
Markers: 0
Sequenced: 0
Length: 92 CB units
Ctg3577 of cp05T5e12DM4RS
H024H08
H024K05
0 5 10 15 20 25 30 35 40 45 50 55 60 65 70 75 80 85 90
[root@localhost: /...
[FPC V8.9 Main Menu]
Project cp05T5e12...
[Chickpea contig p...
FPC Ctg3577 cp05...

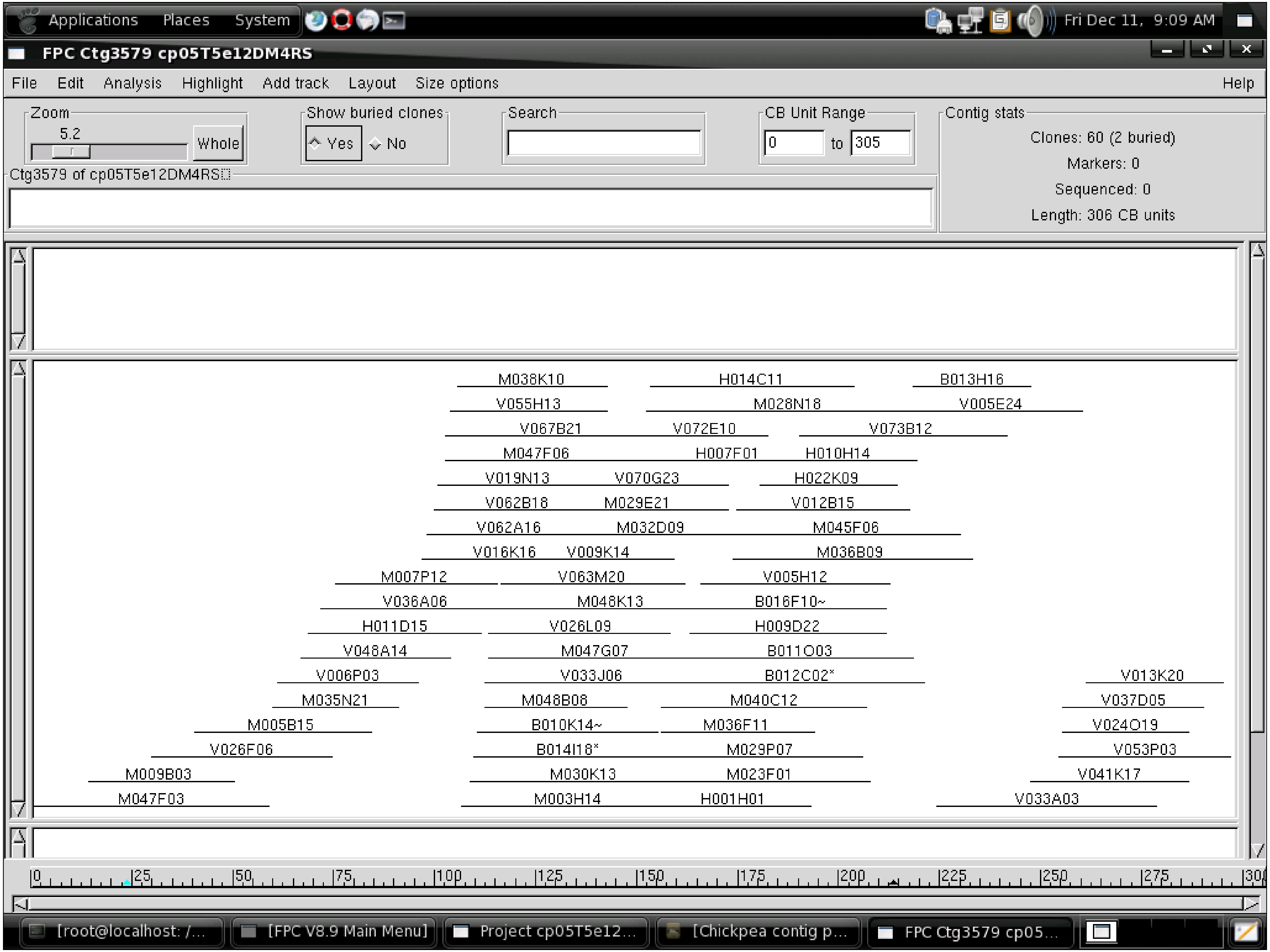
Applications Places System
Fri Dec 11, 9:09 AM
FPC Ctg3579 cp05T5e12DM4RS
File Edit Analysis Highlight Add track Layout Size options Help
Zoom
5.2
Whole
Show buried clones
Yes
No
Search
CB Unit Range
0 to 305
Contig stats
Clones: 60 (2 buried)
Markers: 0
Sequenced: 0
Length: 306 CB units
Ctg3579 of cp05T5e12DM4RS
M038K10
H014C11
B013H16
V055H13
M028N18
V005E24
V067B21
V072E10
V073B12
M047F06
H007F01
H010H14
V019N13
V070G23
H022K09
V062B18
M029E21
V012B15
V062A16
M032D09
M045F06
V016K16
V009K14
M036B09
M007P12
V063M20
V005H12
V036A06
M048K13
B016F10~
H011D15
V026L09
H009D22
V048A14
M047G07
B011O03
V006P03
V033J06
B012C02*
V013K20
M035N21
M048B08
M040C12
V037D05
M005B15
B010K14~
M036F11
V024O19
V026F06
B014I18*
M029P07
V053P03
M009B03
M030K13
M023F01
V041K17
M047F03
M003H14
H001H01
V033A03
0 25 50 75 100 125 150 175 200 225 250 275 30
[root@localhost: /...
[FPC V8.9 Main Menu]
Project cp05T5e12...
[Chickpea contig p...
FPC Ctg3579 cp05...

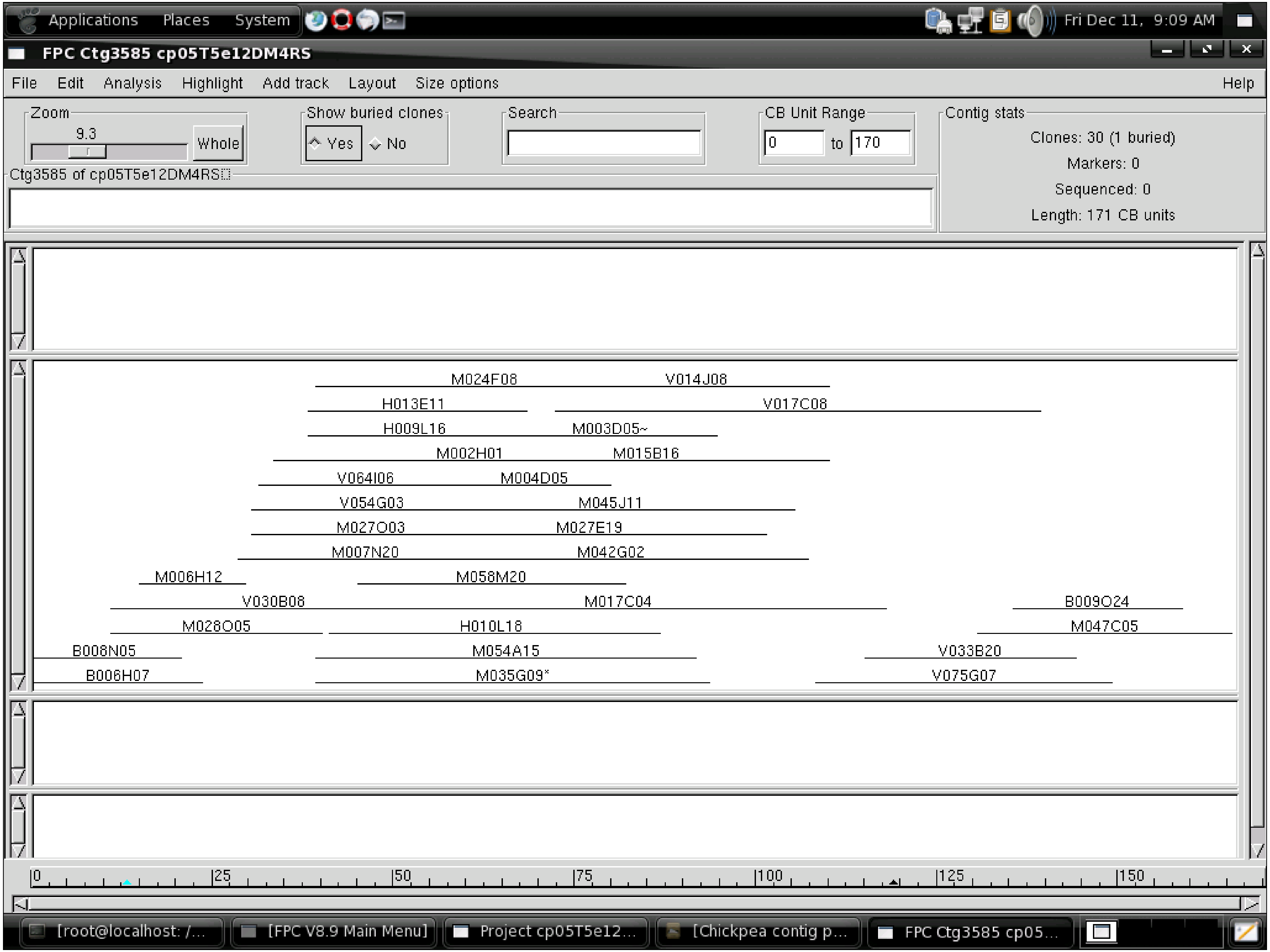
Applications Places System
Fri Dec 11, 9:09 AM
FPC Ctg3585 cp05T5e12DM4RS
File Edit Analysis Highlight Add track Layout Size options Help
Zoom
9.3
Whole
Show buried clones
Yes No
Search
CB Unit Range
0 to 170
Contig stats
Clones: 30 (1 buried)
Markers: 0
Sequenced: 0
Length: 171 CB units
Ctg3585 of cp05T5e12DM4RS
M024F08
V014J08
H013E11
V017C08
H009L16
M003D05~
M002H01
M015B16
V064I06
M004D05
V054G03
M045J11
M027O03
M027E19
M007N20
M042G02
M006H12
M058M20
V030B08
M017C04
B009O24
M028O05
H010L18
M047C05
B008N05
M054A15
V033B20
B006H07
M035G09*
V075G07
0 25 50 75 100 125 150
[root@localhost: /...
[FPC V8.9 Main Menu]
Project cp05T5e12...
[Chickpea contig p...
FPC Ctg3585 cp05...

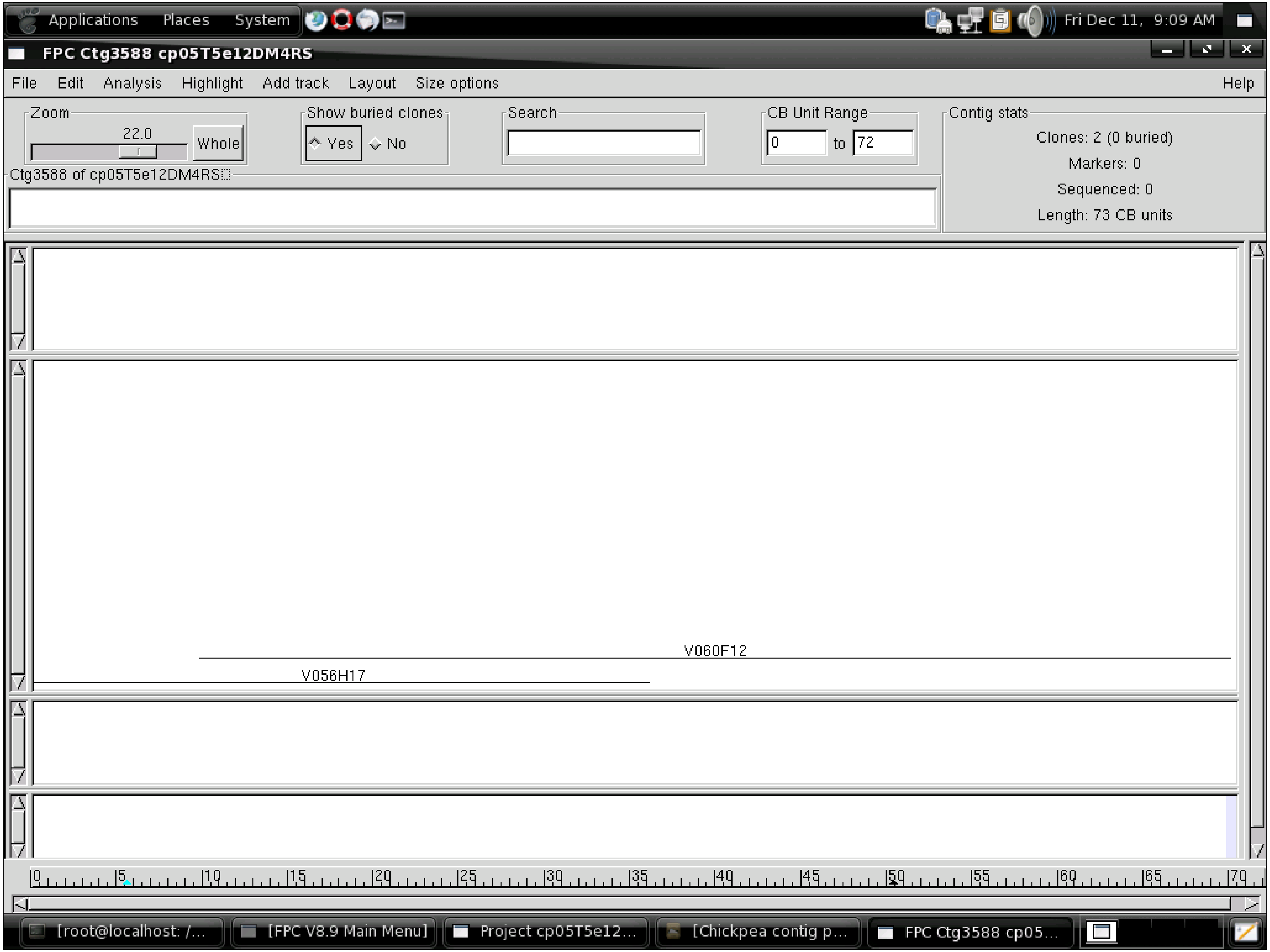
Applications Places System
Fri Dec 11, 9:09 AM
FPC Ctg3588 cp05T5e12DM4RS
File Edit Analysis Highlight Add track Layout Size options Help
Zoom
22.0
Whole
Show buried clones
Yes
No
Search
CB Unit Range
0
to
72
Contig stats
Clones: 2 (0 buried)
Markers: 0
Sequenced: 0
Length: 73 CB units
Ctg3588 of cp05T5e12DM4RS
V060F12
V056H17
0 5 10 15 20 25 30 35 40 45 50 55 60 65 70
[root@localhost: /...
[FPC V8.9 Main Menu]
Project cp05T5e12...
[Chickpea contig p...
FPC Ctg3588 cp05...

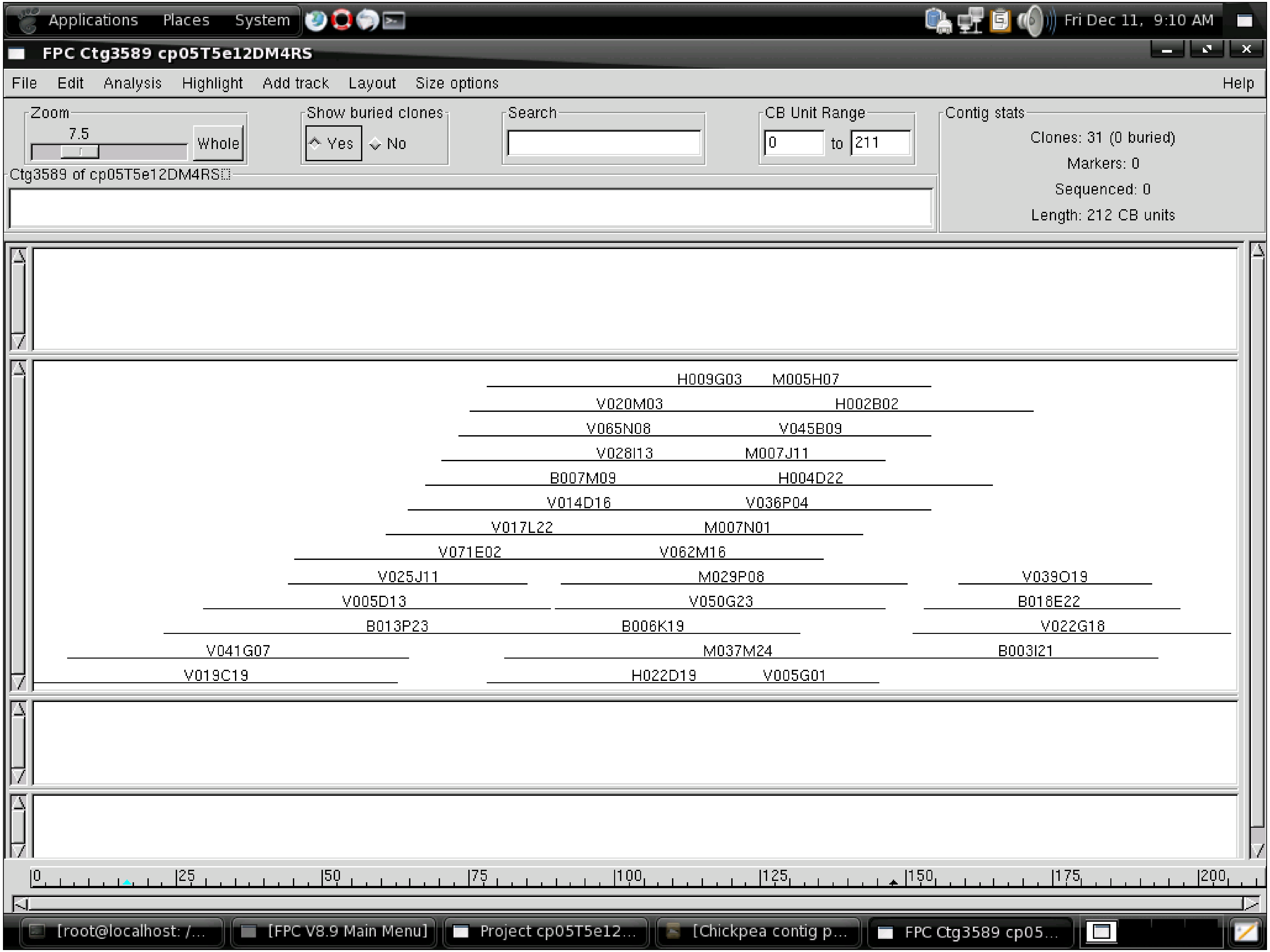
Applications
Places
System
Fri Dec 11, 9:10 AM
FPC Ctg3589 cp05T5e12DM4RS
File
Edit
Analysis
Highlight
Add track
Layout
Size options
Help
Zoom
7.5
Whole
Show buried clones
Yes
No
Search
CB Unit Range
0
to
211
Contig stats
Clones: 31 (0 buried)
Markers: 0
Sequenced: 0
Length: 212 CB units
Ctg3589 of cp05T5e12DM4RS
H009G03
M005H07
V020M03
H002B02
V065N08
V045B09
V028I13
M007J11
B007M09
H004D22
V014D16
V036P04
V017L22
M007N01
V071E02
V062M16
V025J11
M029P08
V039O19
V005D13
V050G23
B018E22
B013P23
B006K19
V022G18
V041G07
M037M24
B003I21
V019C19
H022D19
V005G01
0
25
50
75
100
125
150
175
200
[root@localhost: /...
[FPC V8.9 Main Menu]
Project cp05T5e12...
[Chickpea contig p...
FPC Ctg3589 cp05...

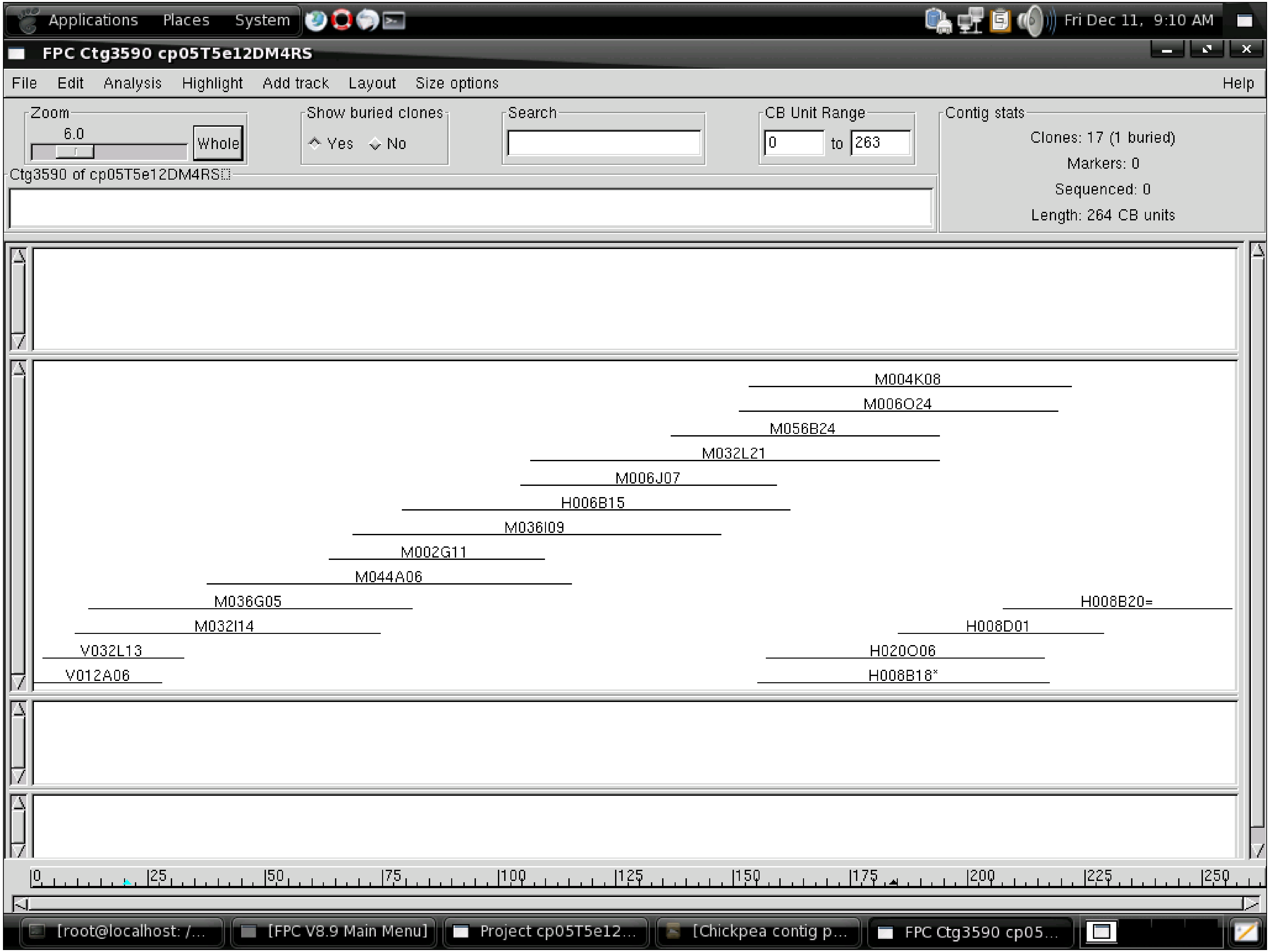
Applications Places System
Fri Dec 11, 9:10 AM
FPC Ctg3590 cp05T5e12DM4RS
File Edit Analysis Highlight Add track Layout Size options Help
Zoom
6.0
Whole
Show buried clones
Yes No
Search
CB Unit Range
0 to 263
Contig stats
Clones: 17 (1 buried)
Markers: 0
Sequenced: 0
Length: 264 CB units
Ctg3590 of cp05T5e12DM4RS
M004K08
M006O24
M056B24
M032L21
M006J07
H006B15
M036I09
M002G11
M044A06
M036G05
H008B20=
M032I14
H008D01
V032L13
H020O06
V012A06
H008B18*
0 25 50 75 100 125 150 175 200 225 250
[root@localhost: /...
[FPC V8.9 Main Menu]
Project cp05T5e12...
[Chickpea contig p...
FPC Ctg3590 cp05...

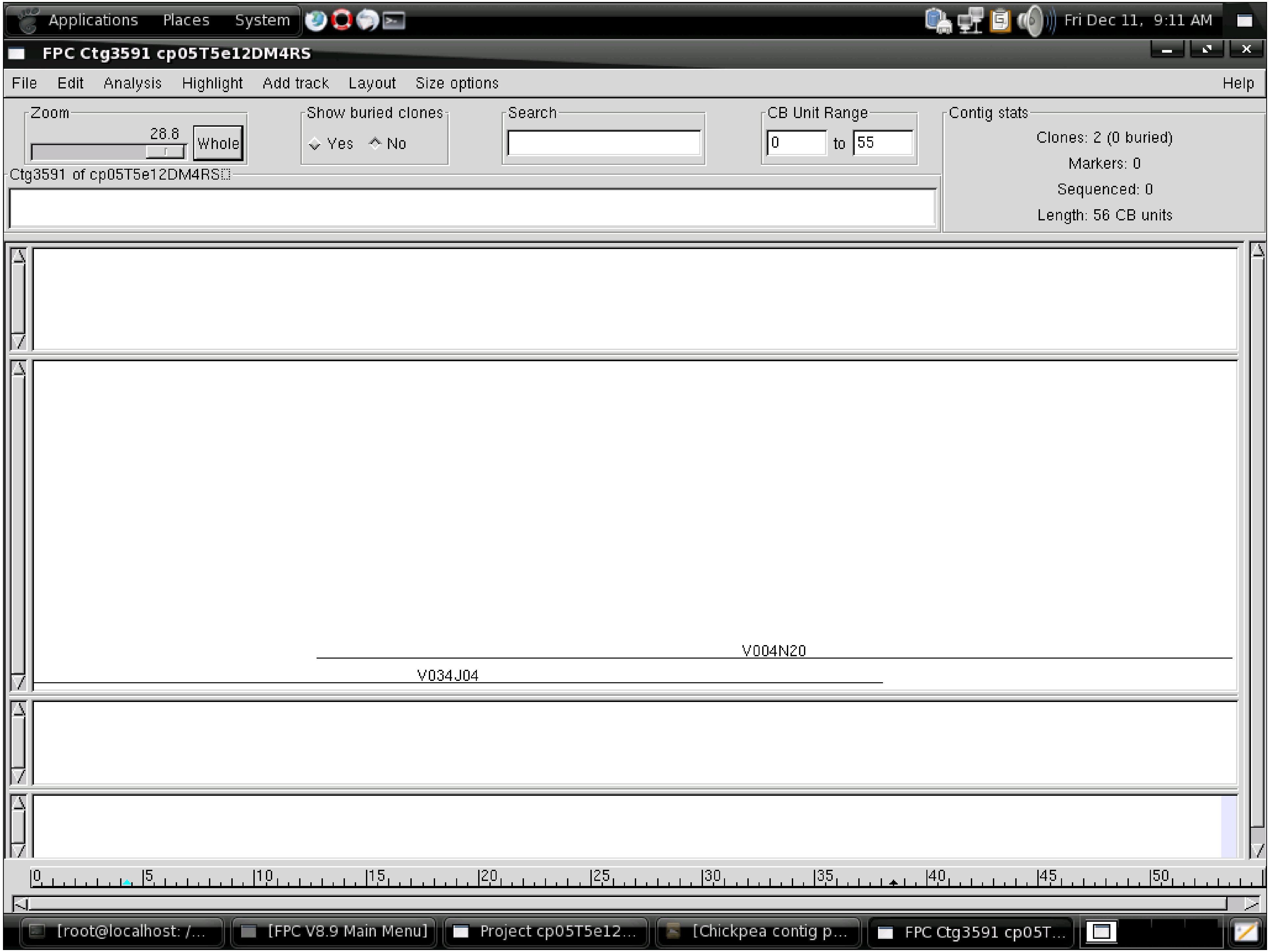
Applications
Places
System
Fri Dec 11, 9:11 AM
FPC Ctg3591 cp05T5e12DM4RS
File
Edit
Analysis
Highlight
Add track
Layout
Size options
Help
Zoom
28.8
Whole
Show buried clones
Yes
No
Search
CB Unit Range
0
to
55
Contig stats
Clones: 2 (0 buried)
Markers: 0
Sequenced: 0
Length: 56 CB units
Ctg3591 of cp05T5e12DM4RS
V004N20
V034J04
0
5
10
15
20
25
30
35
40
45
50
[root@localhost: /...
[FPC V8.9 Main Menu]
Project cp05T5e12...
[Chickpea contig p...
FPC Ctg3591 cp05T...

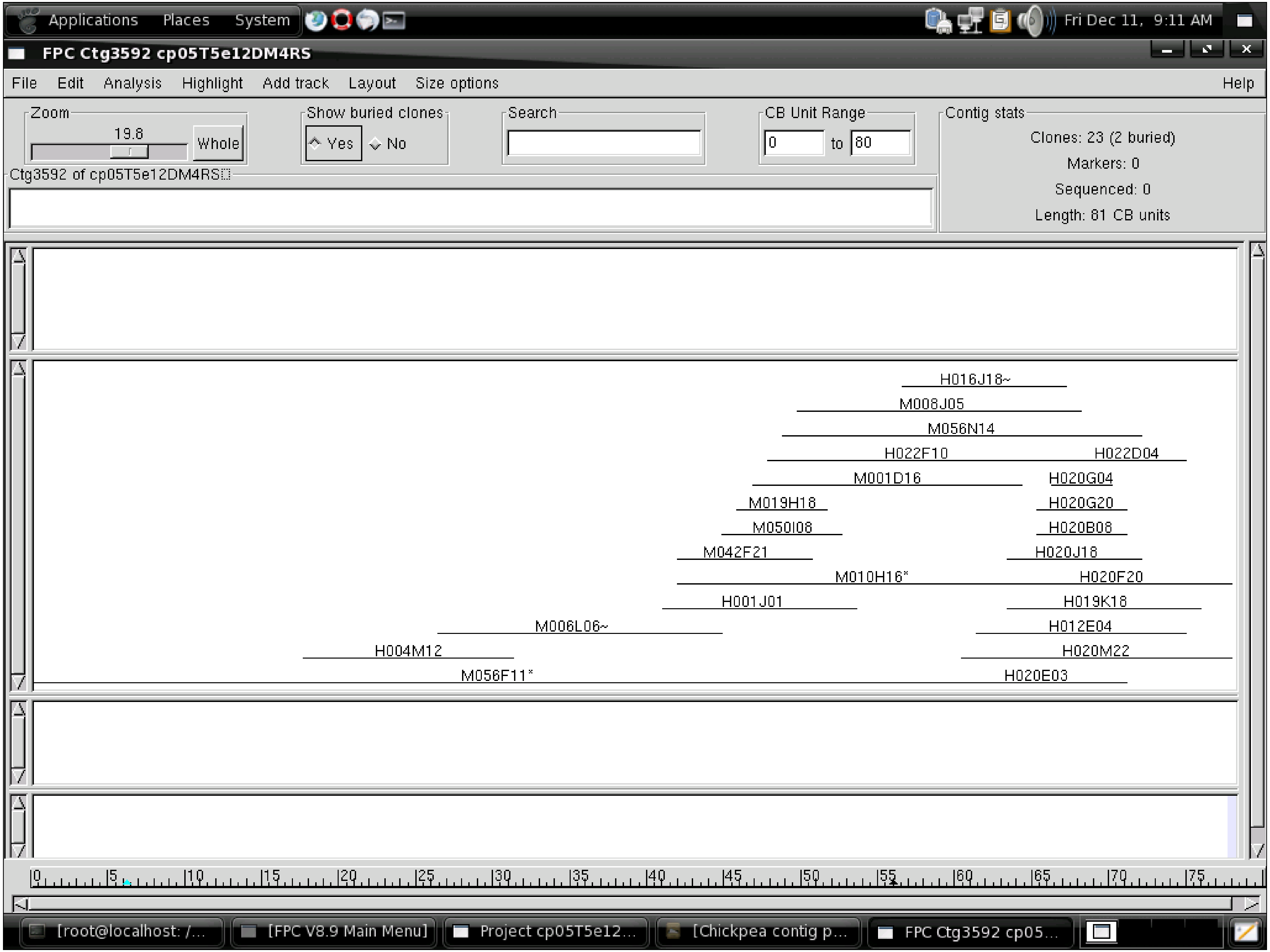
Applications Places System
Fri Dec 11, 9:11 AM
FPC Ctg3592 cp05T5e12DM4RS
File Edit Analysis Highlight Add track Layout Size options Help
Zoom
19.8
Whole
Show buried clones
Yes
No
Search
CB Unit Range
0
to
80
Contig stats
Clones: 23 (2 buried)
Markers: 0
Sequenced: 0
Length: 81 CB units
Ctg3592 of cp05T5e12DM4RS
H016J18~
M008J05
M056N14
H022F10
H022D04
M001D16
H020G04
M019H18
H020G20
M050I08
H020B08
M042F21
H020J18
M010H16*
H020F20
H001J01
H019K18
M006L06~
H012E04
H004M12
H020M22
M056F11*
H020E03
0 5 10 15 20 25 30 35 40 45 50 55 60 65 70 75
[root@localhost: /...
[FPC V8.9 Main Menu]
Project cp05T5e12...
[Chickpea contig p...
FPC Ctg3592 cp05...

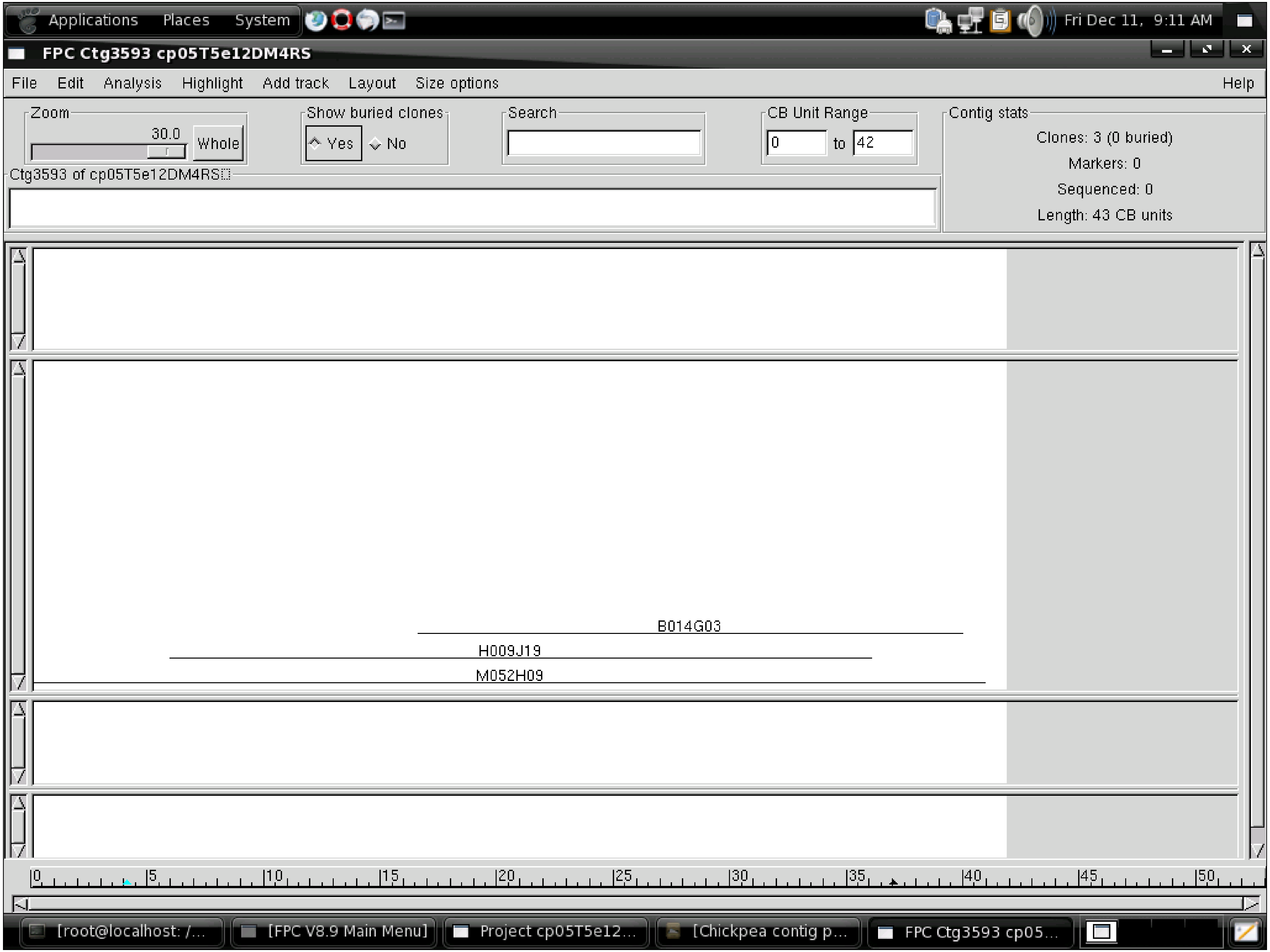
Applications
Places
System
Fri Dec 11, 9:11 AM
FPC Ctg3593 cp05T5e12DM4RS
File
Edit
Analysis
Highlight
Add track
Layout
Size options
Help
Zoom
30.0
Whole
Show buried clones
Yes
No
Search
CB Unit Range
0
to
42
Contig stats
Clones: 3 (0 buried)
Markers: 0
Sequenced: 0
Length: 43 CB units
Ctg3593 of cp05T5e12DM4RS
B014G03
H009J19
M052H09
0
5
10
15
20
25
30
35
40
45
50
[root@localhost: /...
[FPC V8.9 Main Menu]
Project cp05T5e12...
[Chickpea contig p...
FPC Ctg3593 cp05...

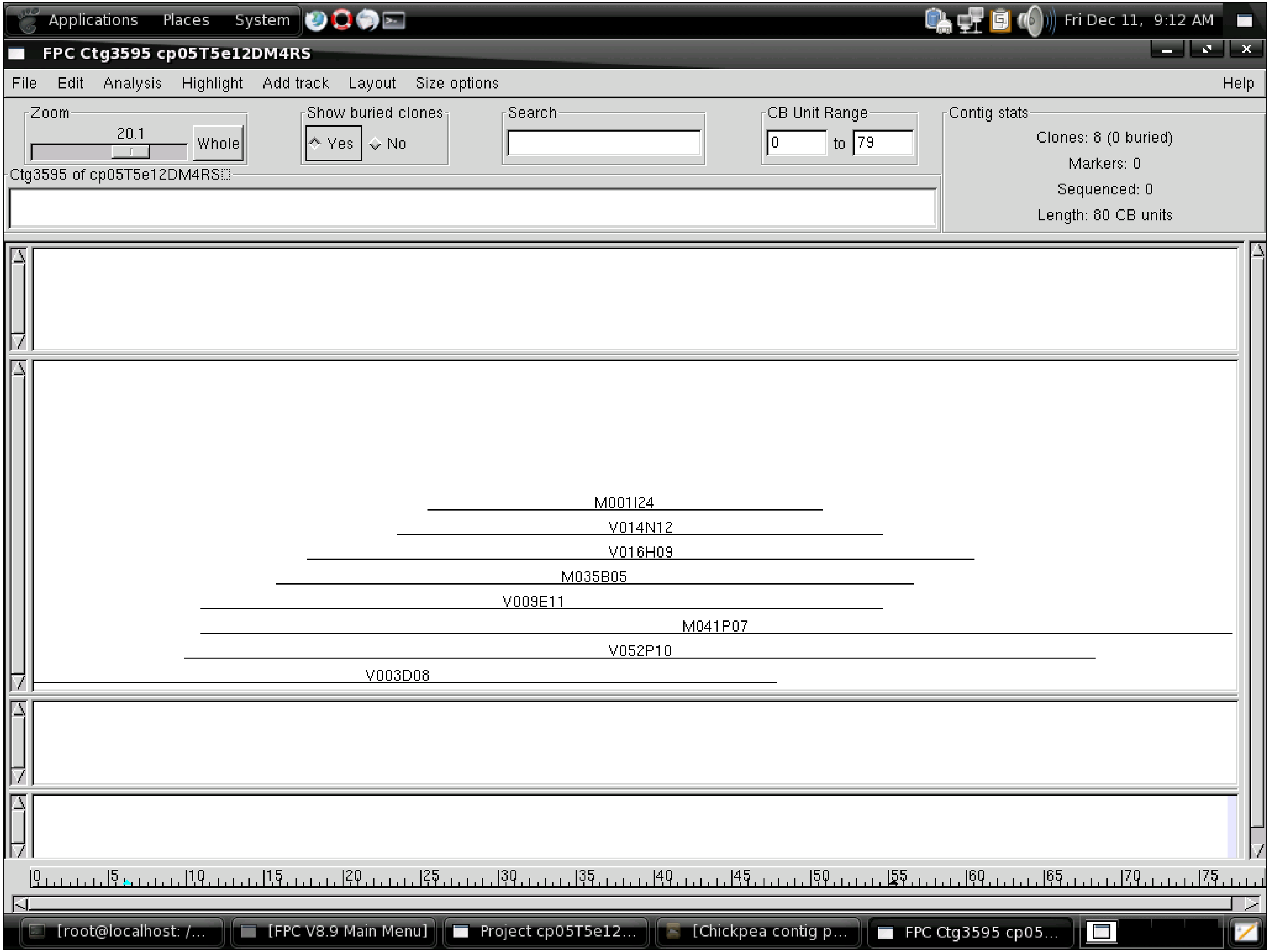
Applications
Places
System
Fri Dec 11, 9:12 AM
FPC Ctg3595 cp05T5e12DM4RS
File
Edit
Analysis
Highlight
Add track
Layout
Size options
Help
Zoom
20.1
Whole
Show buried clones
Yes
No
Search
CB Unit Range
0
to
79
Contig stats
Clones: 8 (0 buried)
Markers: 0
Sequenced: 0
Length: 80 CB units
Ctg3595 of cp05T5e12DM4RS
M001I24
V014N12
V016H09
M035B05
V009E11
M041P07
V052P10
V003D08
0
5
10
15
20
25
30
35
40
45
50
55
60
65
70
75
[root@localhost: /...
[FPC V8.9 Main Menu]
Project cp05T5e12...
[Chickpea contig p...
FPC Ctg3595 cp05...

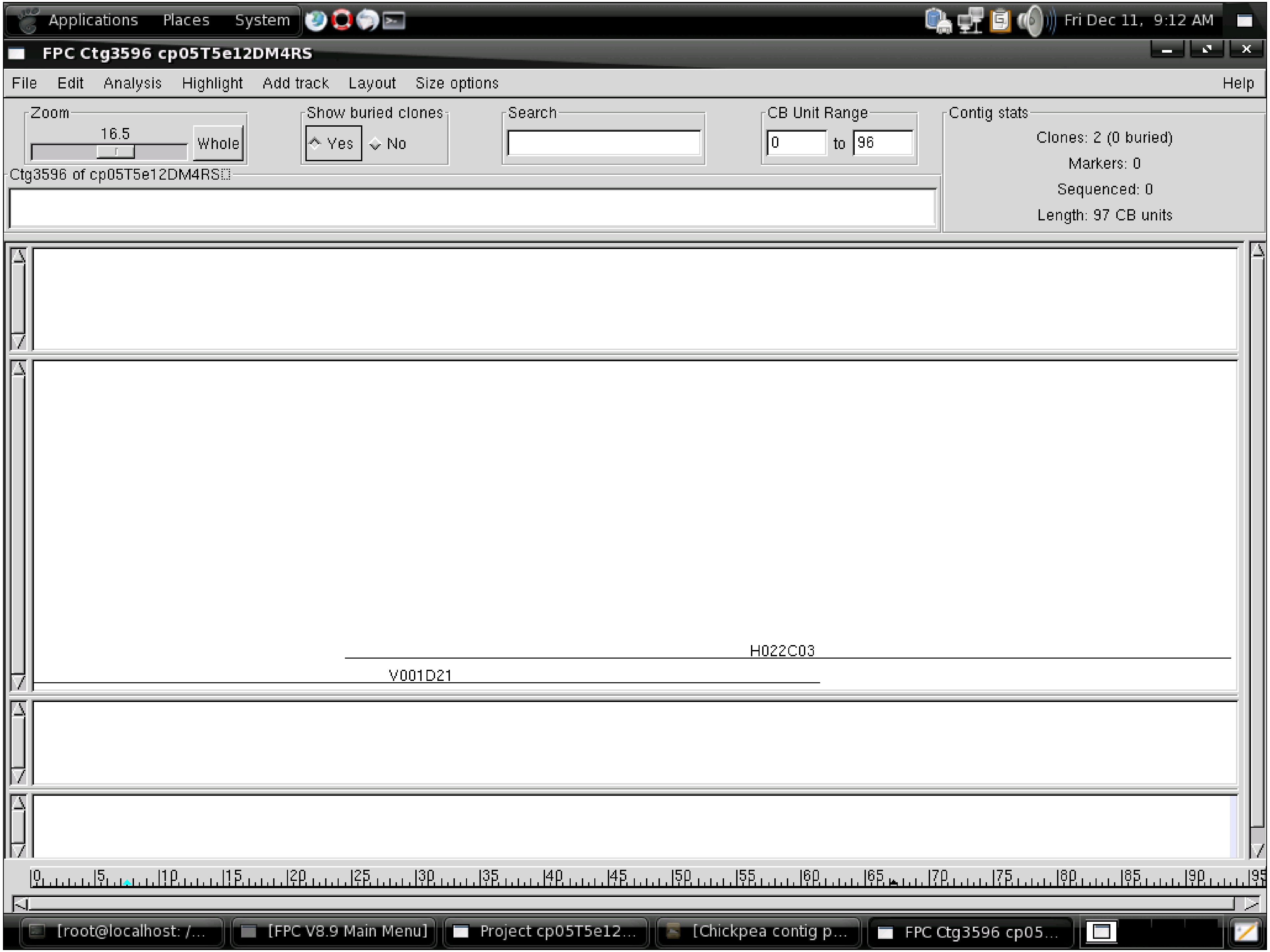
Applications Places System
Fri Dec 11, 9:12 AM
FPC Ctg3596 cp05T5e12DM4RS
File Edit Analysis Highlight Add track Layout Size options Help
Zoom
16.5
Whole
Show buried clones
Yes
No
Search
CB Unit Range
0 to 96
Contig stats
Clones: 2 (0 buried)
Markers: 0
Sequenced: 0
Length: 97 CB units
Ctg3596 of cp05T5e12DM4RS
H022C03
V001D21
0 5 10 15 20 25 30 35 40 45 50 55 60 65 70 75 80 85 90 95
[root@localhost: /...
[FPC V8.9 Main Menu]
Project cp05T5e12...
[Chickpea contig p...
FPC Ctg3596 cp05...

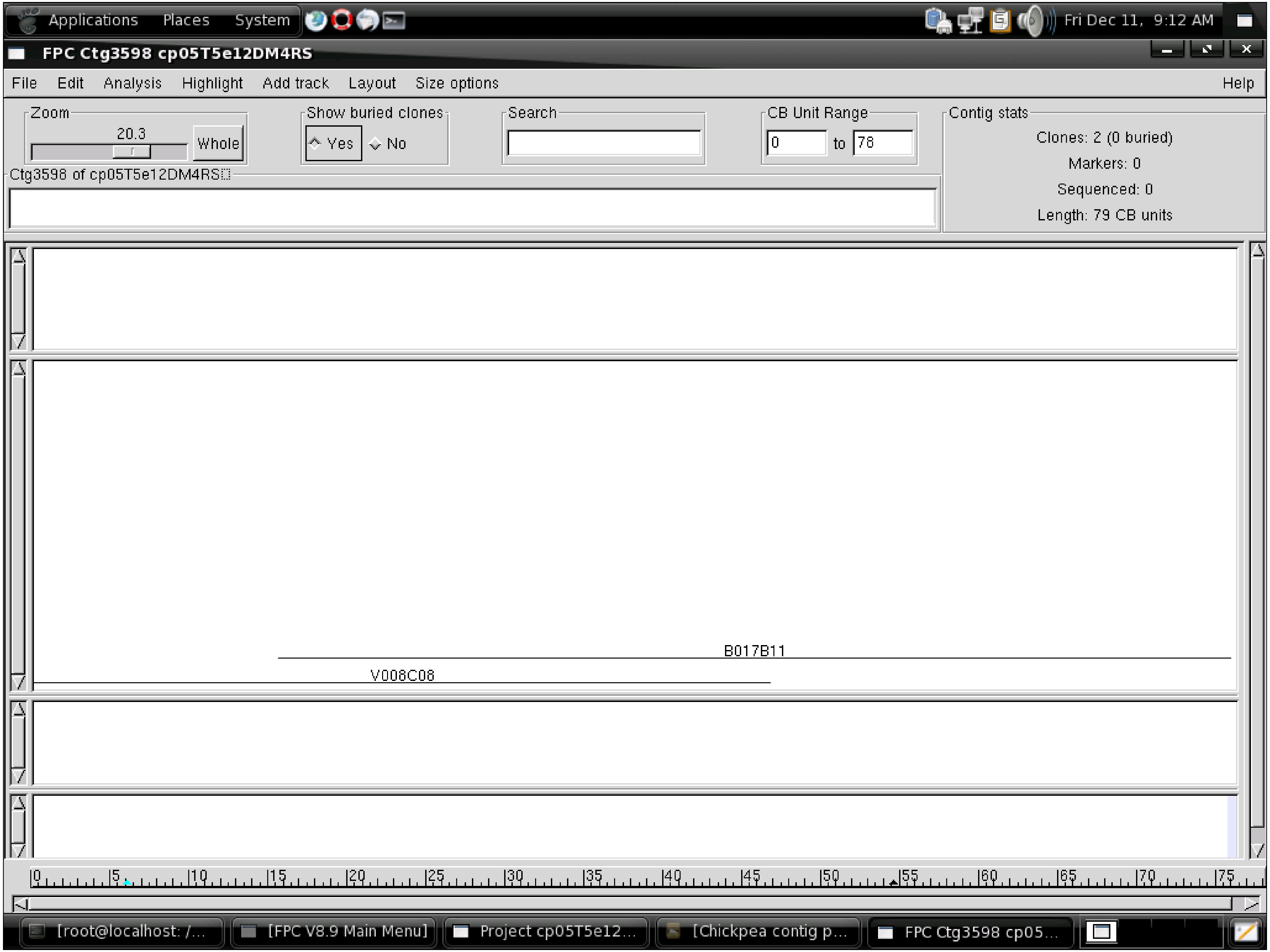
Applications Places System
Fri Dec 11, 9:12 AM
FPC Ctg3598 cp05T5e12DM4RS
File Edit Analysis Highlight Add track Layout Size options Help
Zoom
20.3
Whole
Show buried clones
Yes
No
Search
CB Unit Range
0
to
78
Contig stats
Clones: 2 (0 buried)
Markers: 0
Sequenced: 0
Length: 79 CB units
Ctg3598 of cp05T5e12DM4RS
B017B11
V008C08
0 5 10 15 20 25 30 35 40 45 50 55 60 65 70 75
[root@localhost: /...
[FPC V8.9 Main Menu]
Project cp05T5e12...
[Chickpea contig p...
FPC Ctg3598 cp05...

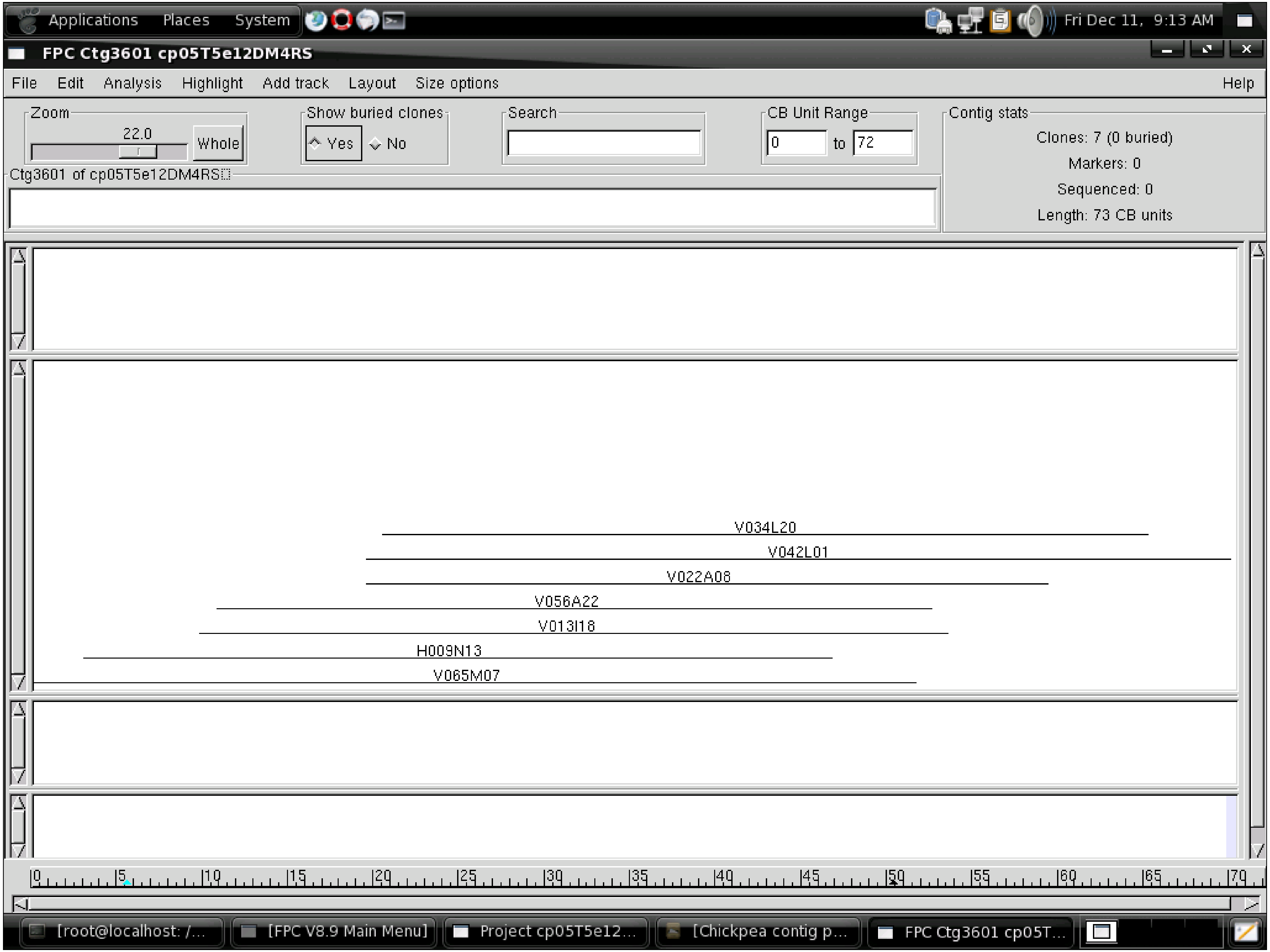
Applications Places System
Fri Dec 11, 9:13 AM
FPC Ctg3601 cp05T5e12DM4RS
File Edit Analysis Highlight Add track Layout Size options Help
Zoom
22.0
Whole
Show buried clones
Yes
No
Search
CB Unit Range
0
to
72
Contig stats
Clones: 7 (0 buried)
Markers: 0
Sequenced: 0
Length: 73 CB units
Ctg3601 of cp05T5e12DM4RS
V034L20
V042L01
V022A08
V056A22
V013I18
H009N13
V065M07
0 5 10 15 20 25 30 35 40 45 50 55 60 65 70
[root@localhost: /...
[FPC V8.9 Main Menu]
Project cp05T5e12...
[Chickpea contig p...
FPC Ctg3601 cp05T...

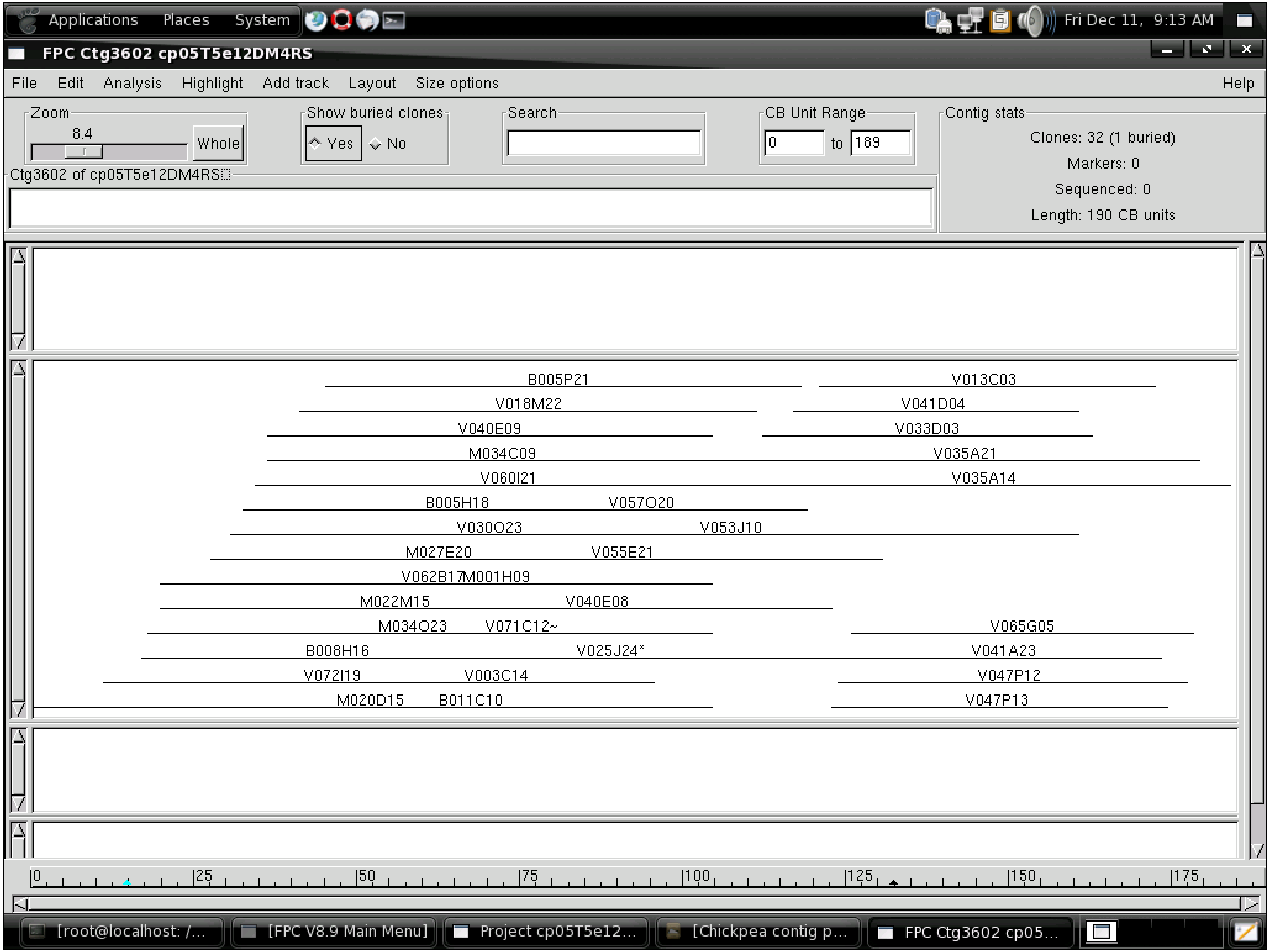
Applications Places System
Fri Dec 11, 9:13 AM
FPC Ctg3602 cp05T5e12DM4RS
File Edit Analysis Highlight Add track Layout Size options Help
Zoom
8.4
Whole
Show buried clones
Yes No
Search
CB Unit Range
0 to 189
Contig stats
Clones: 32 (1 buried)
Markers: 0
Sequenced: 0
Length: 190 CB units
Ctg3602 of cp05T5e12DM4RS
B005P21
V013C03
V018M22
V041D04
V040E09
V033D03
M034C09
V035A21
V060I21
V035A14
B005H18
V057O20
V030O23
V053J10
M027E20
V055E21
V062B17M001H09
M022M15
V040E08
M034O23
V071C12~
V065G05
B008H16
V025J24*
V041A23
V072I19
V003C14
V047P12
M020D15
B011C10
V047P13
0 25 50 75 100 125 150 175
[root@localhost: /...
[FPC V8.9 Main Menu]
Project cp05T5e12...
[Chickpea contig p...
FPC Ctg3602 cp05...

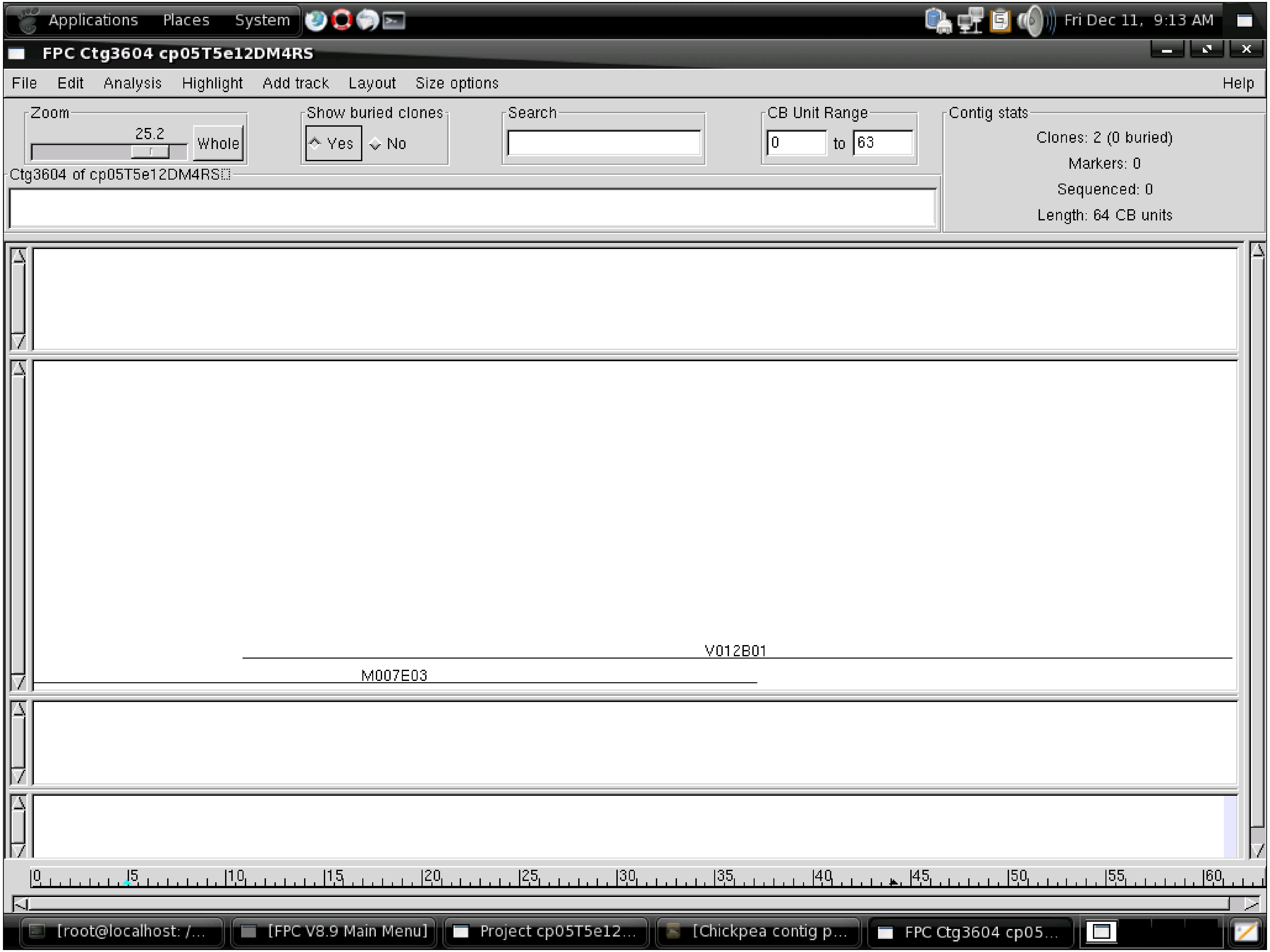
Applications Places System
Fri Dec 11, 9:13 AM
FPC Ctg3604 cp05T5e12DM4RS
File Edit Analysis Highlight Add track Layout Size options Help
Zoom
25.2
Whole
Show buried clones
Yes No
Search
CB Unit Range
0 to 63
Contig stats
Clones: 2 (0 buried)
Markers: 0
Sequenced: 0
Length: 64 CB units
Ctg3604 of cp05T5e12DM4RS
V012B01
M007E03
0 5 10 15 20 25 30 35 40 45 50 55 60
[root@localhost: /...
[FPC V8.9 Main Menu]
Project cp05T5e12...
[Chickpea contig p...
FPC Ctg3604 cp05...

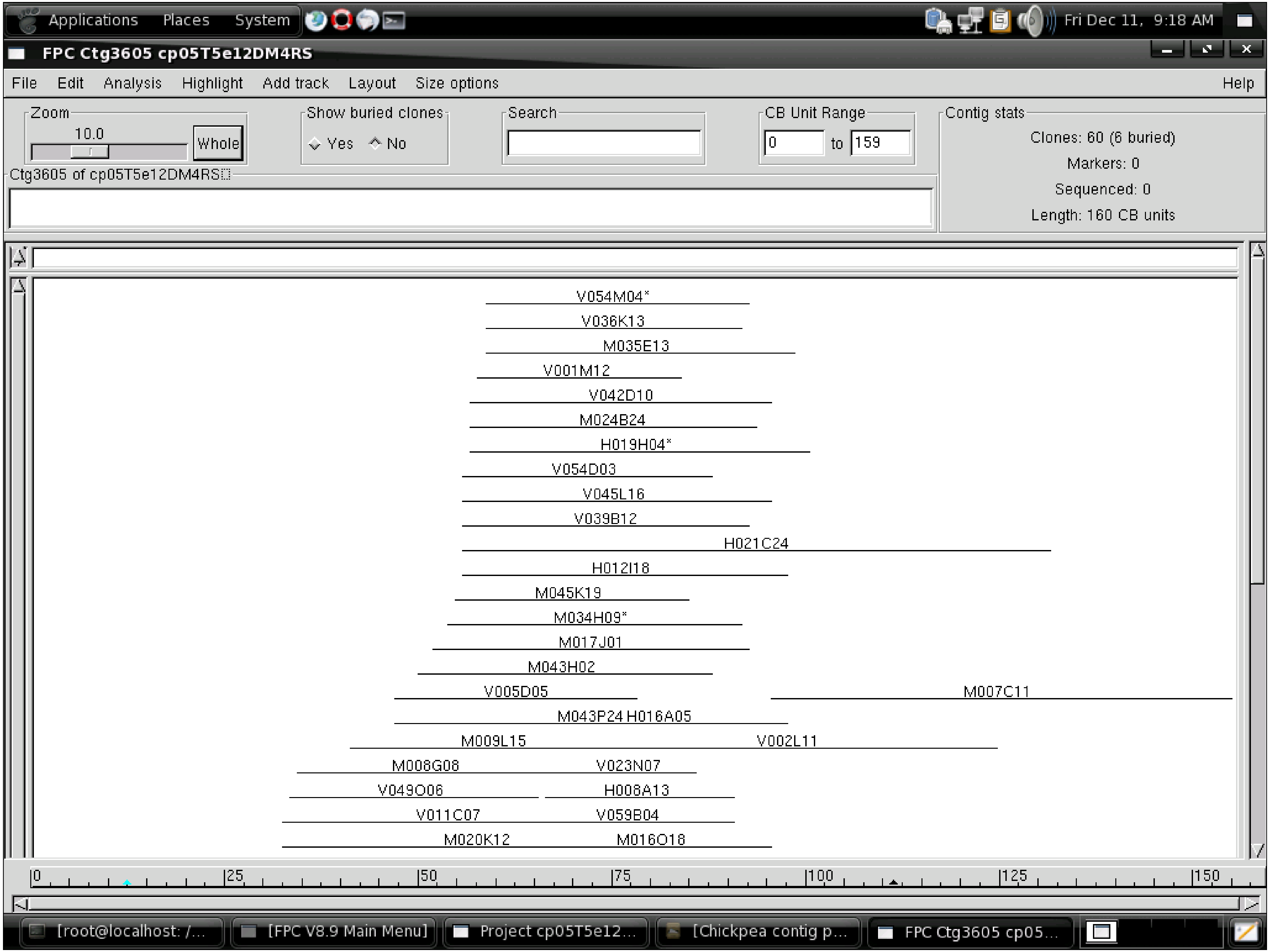
Applications Places System
Fri Dec 11, 9:18 AM
FPC Ctg3605 cp05T5e12DM4RS
File Edit Analysis Highlight Add track Layout Size options Help
Zoom
10.0
Whole
Show buried clones
Yes No
Search
CB Unit Range
0 to 159
Contig stats
Clones: 60 (6 buried)
Markers: 0
Sequenced: 0
Length: 160 CB units
Ctg3605 of cp05T5e12DM4RS
V054M04*
V036K13
M035E13
V001M12
V042D10
M024B24
H019H04*
V054D03
V045L16
V039B12
H021C24
H012I18
M045K19
M034H09*
M017J01
M043H02
V005D05
M007C11
M043P24 H016A05
M009L15
V002L11
M008G08
V023N07
V049O06
H008A13
V011C07
V059B04
M020K12
M016O18
0
25
50
75
100
125
150
[root@localhost: /...
[FPC V8.9 Main Menu]
Project cp05T5e12...
[Chickpea contig p...
FPC Ctg3605 cp05...

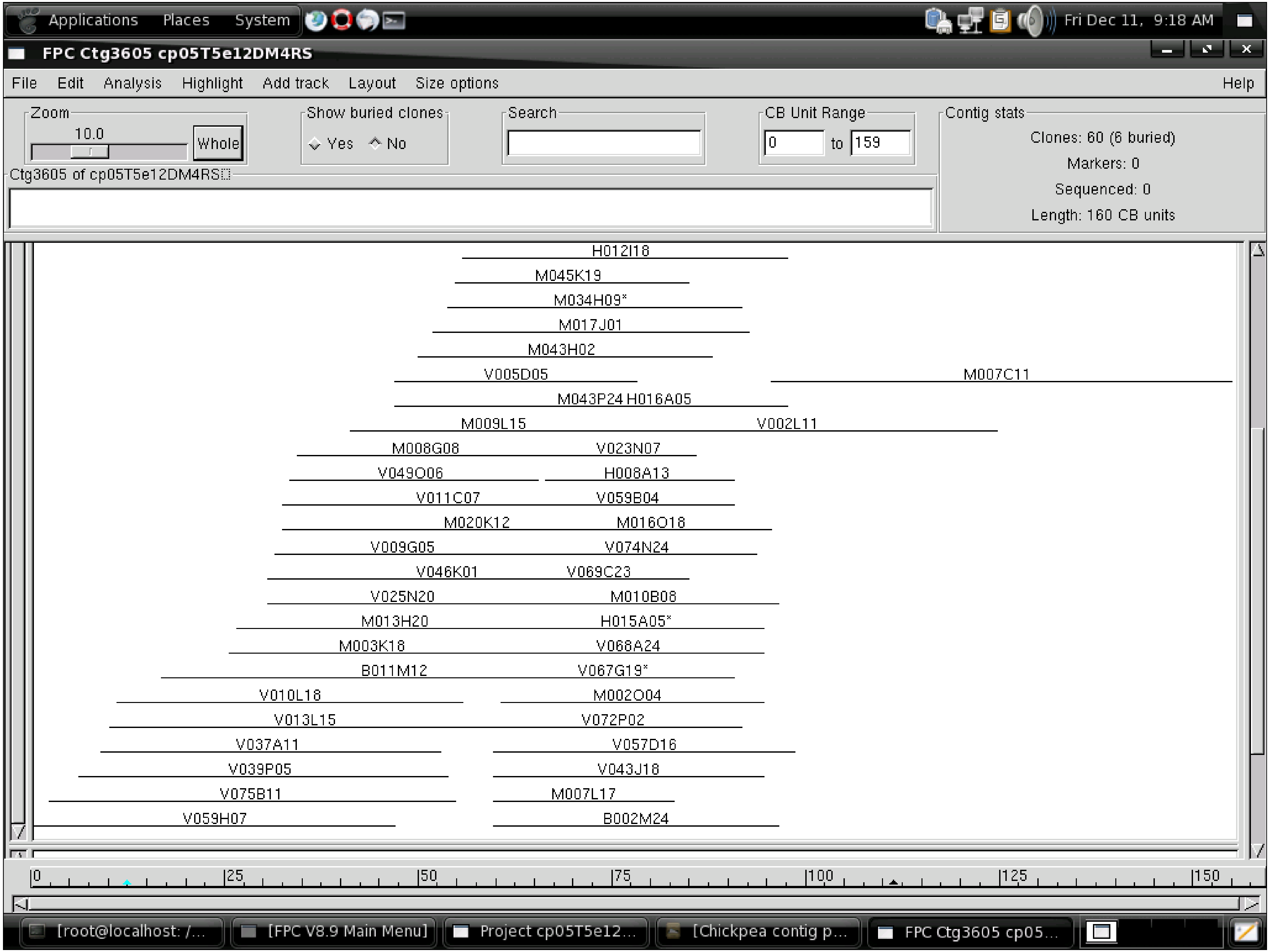

Applications Places System
Fri Dec 11, 9:18 AM
FPC Ctg3605 cp05T5e12DM4RS
File Edit Analysis Highlight Add track Layout Size options Help
Zoom
10.0
Whole
Show buried clones
Yes No
Search
CB Unit Range
0 to 159
Contig stats
Clones: 60 (6 buried)
Markers: 0
Sequenced: 0
Length: 160 CB units
Ctg3605 of cp05T5e12DM4RS
H012I18
M045K19
M034H09*
M017J01
M043H02
V005D05
M007C11
M043P24 H016A05
M009L15
V002L11
M008G08
V023N07
V049O06
H008A13
V011C07
V059B04
M020K12
M016O18
V009G05
V074N24
V046K01
V069C23
V025N20
M010B08
M013H20
H015A05*
M003K18
V068A24
B011M12
V067G19*
V010L18
M002O04
V013L15
V072P02
V037A11
V057D16
V039P05
V043J18
V075B11
M007L17
V059H07
B002M24
0 25 50 75 100 125 150
[root@localhost: /...
[FPC V8.9 Main Menu]
Project cp05T5e12...
[Chickpea contig p...
FPC Ctg3605 cp05...

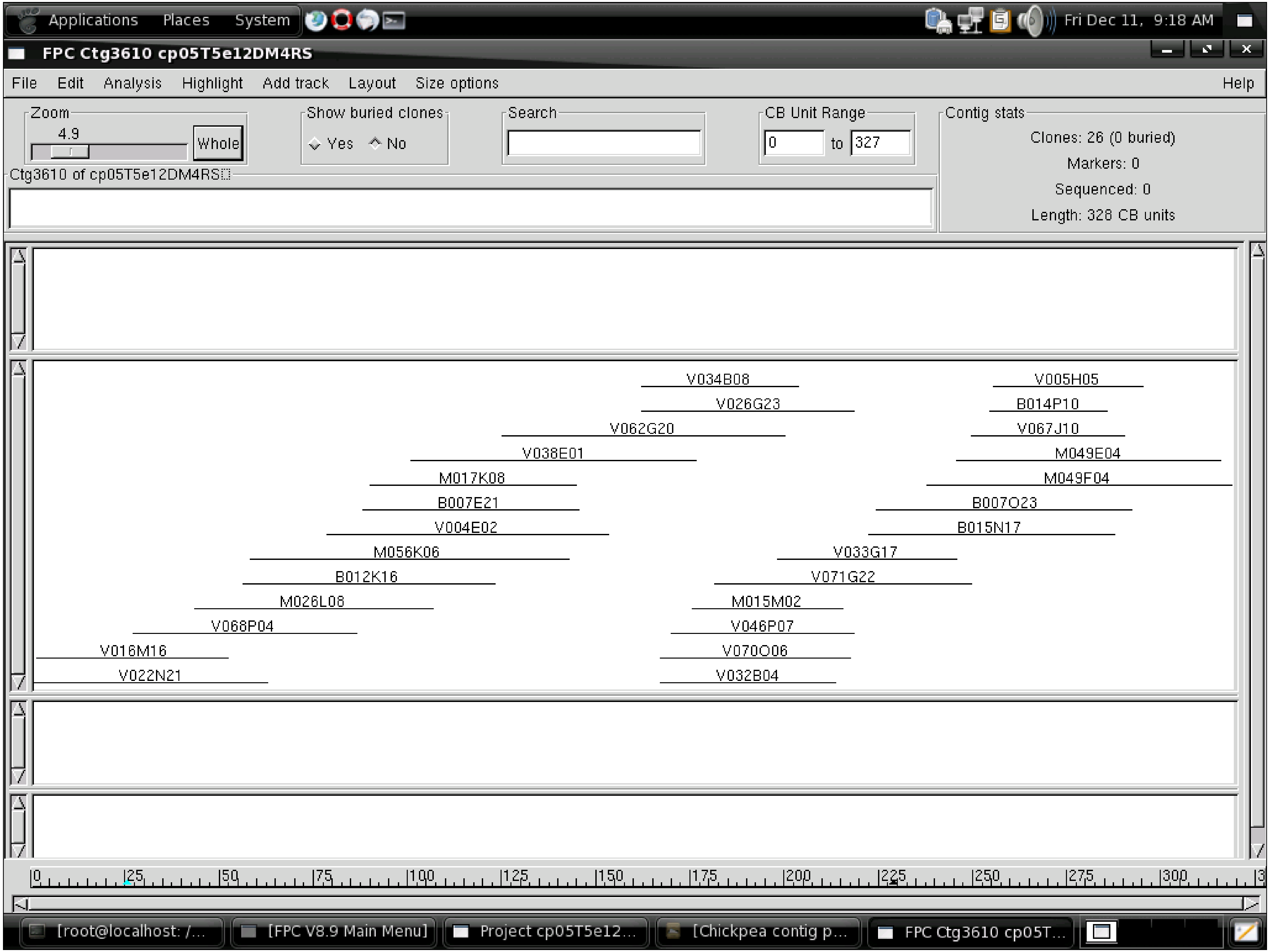

Applications Places System
Fri Dec 11, 9:18 AM
FPC Ctg3610 cp05T5e12DM4RS
File Edit Analysis Highlight Add track Layout Size options Help
Zoom
4.9
Whole
Show buried clones
Yes No
Search
CB Unit Range
0 to 327
Contig stats
Clones: 26 (0 buried)
Markers: 0
Sequenced: 0
Length: 328 CB units
Ctg3610 of cp05T5e12DM4RS
V034B08
V005H05
V026G23
B014P10
V062G20
V067J10
V038E01
M049E04
M017K08
M049F04
B007E21
B007O23
V004E02
B015N17
M056K06
V033G17
B012K16
V071G22
M026L08
M015M02
V068P04
V046P07
V016M16
V070O06
V022N21
V032B04
0 25 50 75 100 125 150 175 200 225 250 275 300
[root@localhost: /...
[FPC V8.9 Main Menu]
Project cp05T5e12...
[Chickpea contig p...
FPC Ctg3610 cp05T...

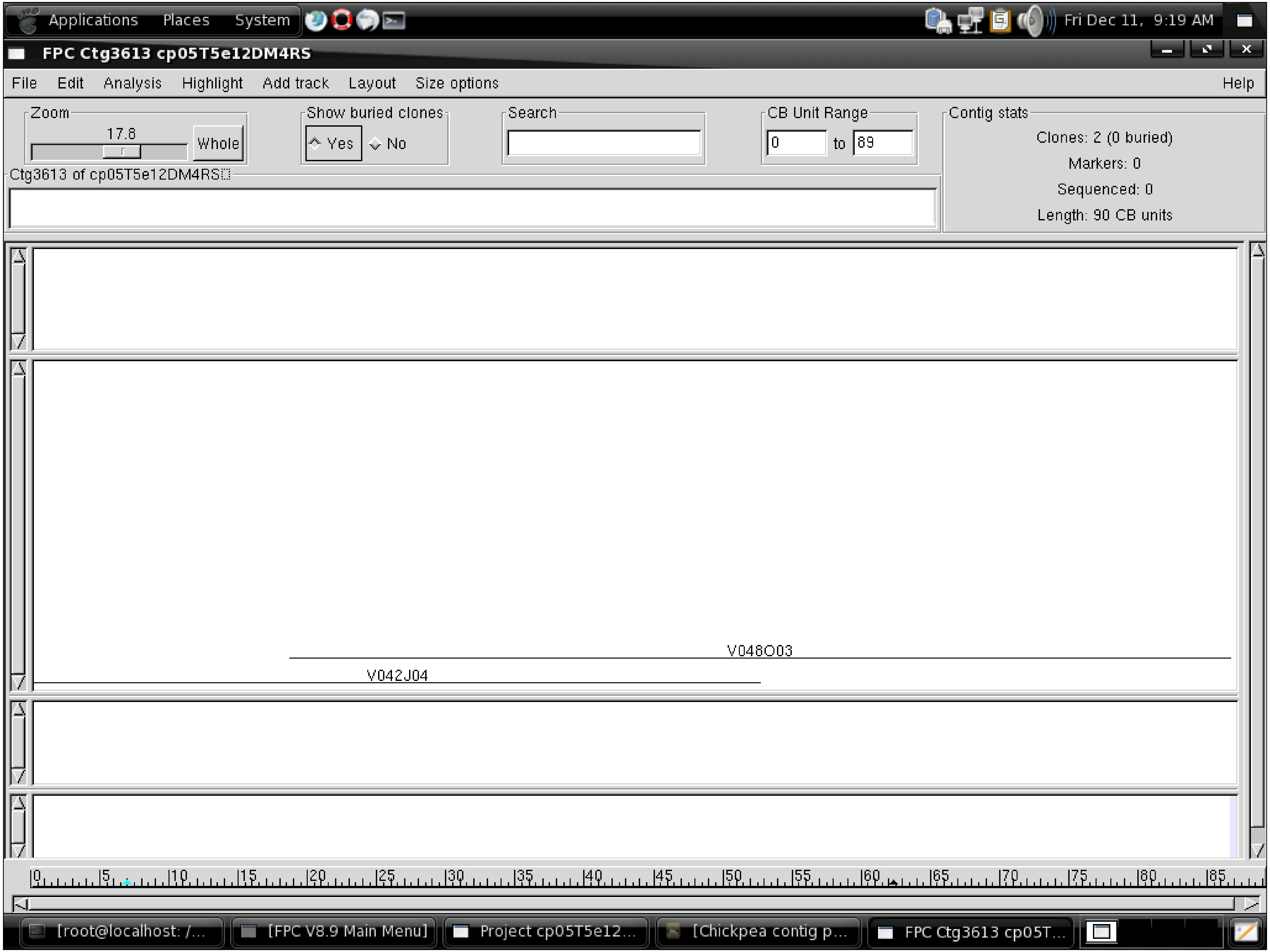
Applications
Places
System
Fri Dec 11, 9:19 AM
FPC Ctg3613 cp05T5e12DM4RS
File
Edit
Analysis
Highlight
Add track
Layout
Size options
Help
Zoom
17.8
Whole
Show buried clones
Yes
No
Search
CB Unit Range
0
to
89
Contig stats
Clones: 2 (0 buried)
Markers: 0
Sequenced: 0
Length: 90 CB units
Ctg3613 of cp05T5e12DM4RS
V048O03
V042J04
0 5 10 15 20 25 30 35 40 45 50 55 60 65 70 75 80 85
[root@localhost: /...
[FPC V8.9 Main Menu]
Project cp05T5e12...
[Chickpea contig p...
FPC Ctg3613 cp05T...

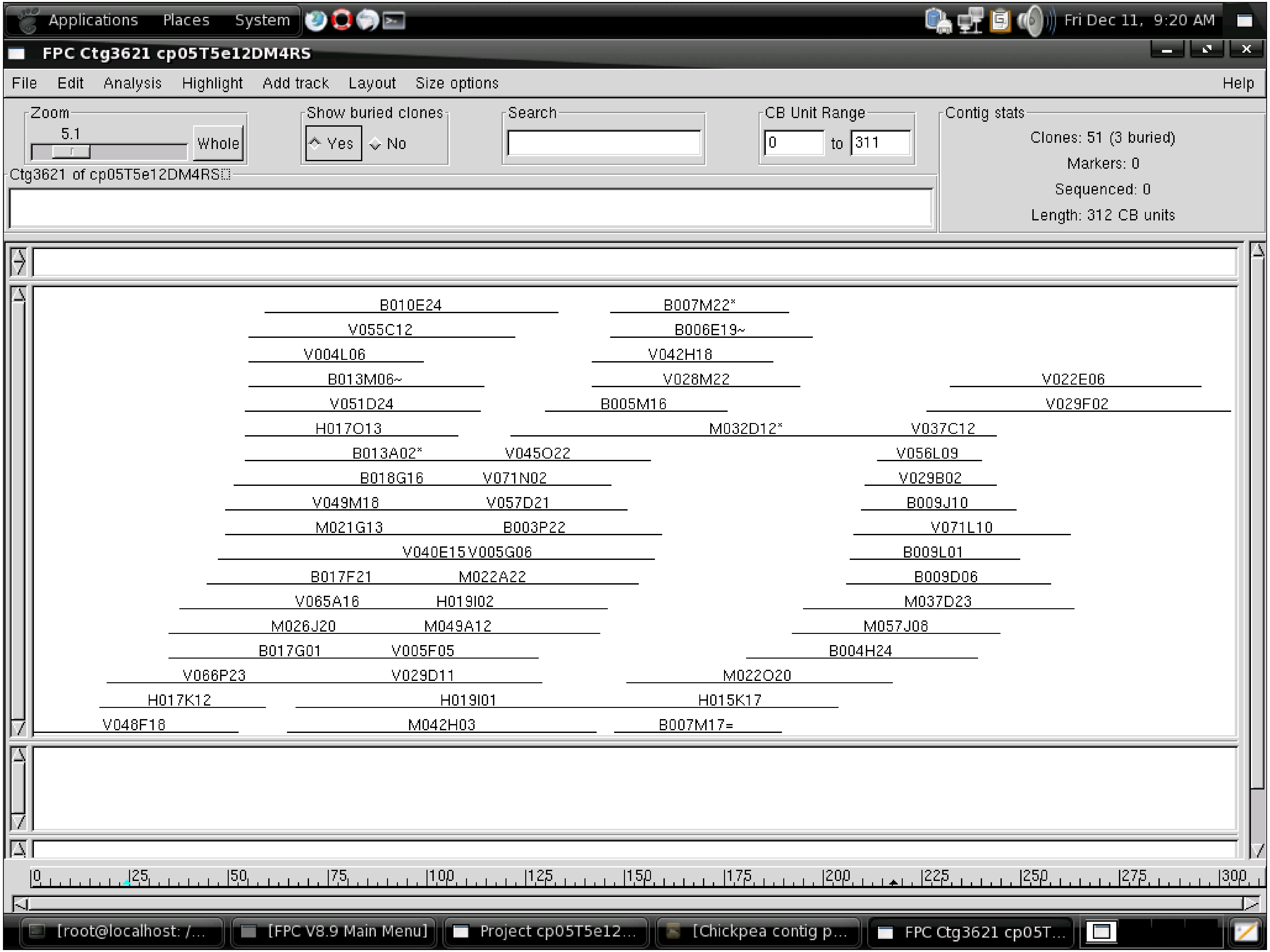

Applications Places System
Fri Dec 11, 9:20 AM
FPC Ctg3621 cp05T5e12DM4RS
File Edit Analysis Highlight Add track Layout Size options Help
Zoom 5.1 Whole
Show buried clones Yes No
Search
CB Unit Range 0 to 311
Contig stats
Clones: 51 (3 buried)
Markers: 0
Sequenced: 0
Length: 312 CB units
Ctg3621 of cp05T5e12DM4RS
B010E24
B007M22*
V055C12
B006E19~
V004L06
V042H18
B013M06~
V028M22
V022E06
V051D24
B005M16
V029F02
H017O13
M032D12*
V037C12
B013A02*
V045O22
V056L09
B018G16
V071N02
V029B02
V049M18
V057D21
B009J10
M021G13
B003P22
V071L10
V040E15
V005G06
B009L01
B017F21
M022A22
B009D06
V065A16
H019I02
M037D23
M026J20
M049A12
M057J08
B017G01
V005F05
B004H24
V066P23
V029D11
M022O20
H017K12
H019I01
H015K17
V048F18
M042H03
B007M17=
0 25 50 75 100 125 150 175 200 225 250 275 300
[root@localhost: /...
[FPC V8.9 Main Menu]
Project cp05T5e12...
[Chickpea contig p...
FPC Ctg3621 cp05T...

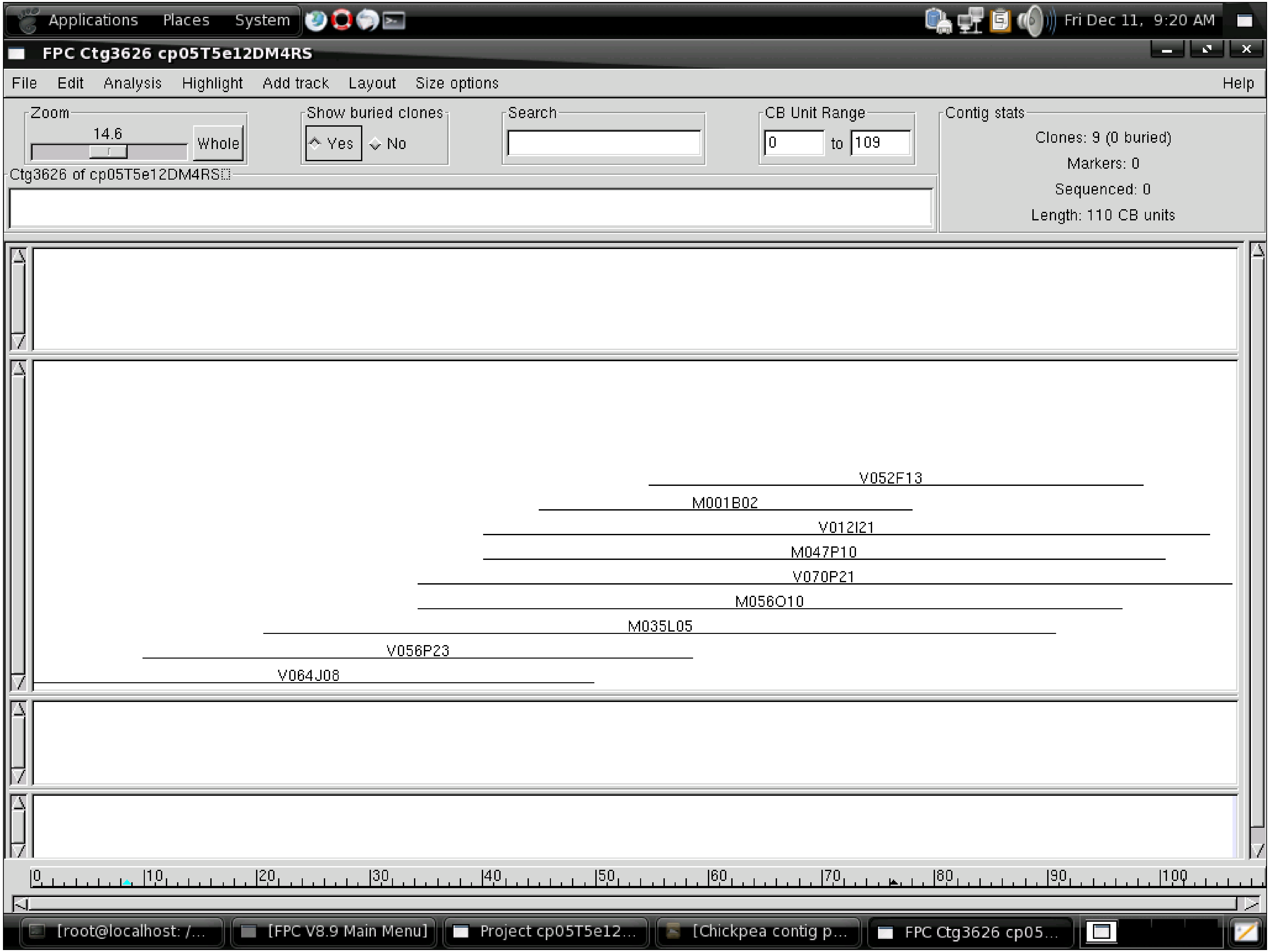
Applications
Places
System
Fri Dec 11, 9:20 AM
FPC Ctg3626 cp05T5e12DM4RS
File
Edit
Analysis
Highlight
Add track
Layout
Size options
Help
Zoom
14.6
Whole
Show buried clones
Yes
No
Search
CB Unit Range
0
to
109
Contig stats
Clones: 9 (0 buried)
Markers: 0
Sequenced: 0
Length: 110 CB units
Ctg3626 of cp05T5e12DM4RS
V052F13
M001B02
V012I21
M047P10
V070P21
M056O10
M035L05
V056P23
V064J08
0
10
20
30
40
50
60
70
80
90
100
[root@localhost: /...
[FPC V8.9 Main Menu]
Project cp05T5e12...
[Chickpea contig p...
FPC Ctg3626 cp05...

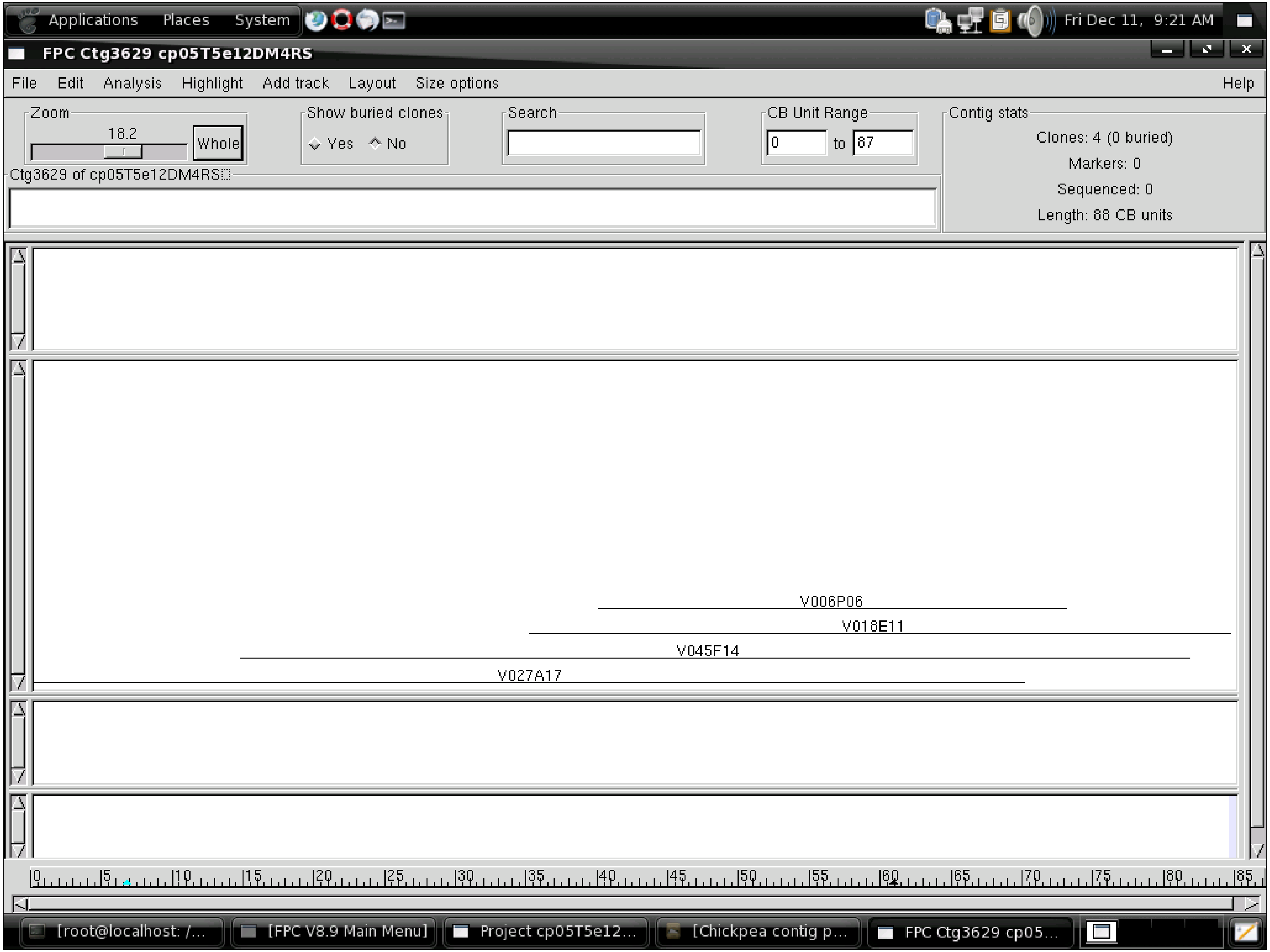
Applications Places System
Fri Dec 11, 9:21 AM
FPC Ctg3629 cp05T5e12DM4RS
File Edit Analysis Highlight Add track Layout Size options Help
Zoom
18.2
Whole
Show buried clones
Yes No
Search
CB Unit Range
0 to 87
Contig stats
Clones: 4 (0 buried)
Markers: 0
Sequenced: 0
Length: 88 CB units
Ctg3629 of cp05T5e12DM4RS
V006P06
V018E11
V045F14
V027A17
0 5 10 15 20 25 30 35 40 45 50 55 60 65 70 75 80 85
[root@localhost: /...
[FPC V8.9 Main Menu]
Project cp05T5e12...
[Chickpea contig p...
FPC Ctg3629 cp05...

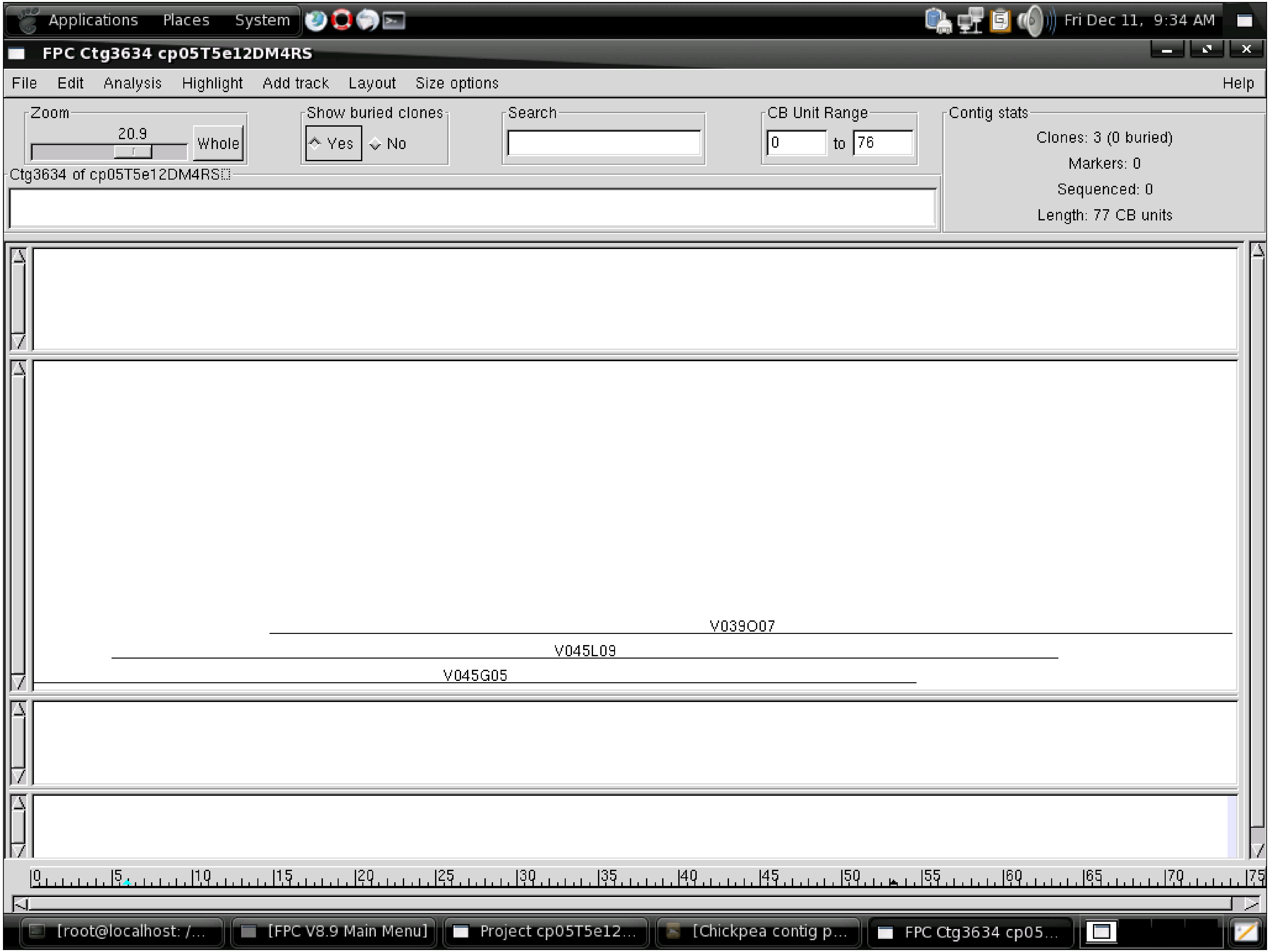
Applications Places System
Fri Dec 11, 9:34 AM
FPC Ctg3634 cp05T5e12DM4RS
File Edit Analysis Highlight Add track Layout Size options Help
Zoom
20.9
Whole
Show buried clones
Yes
No
Search
CB Unit Range
0
to
76
Contig stats
Clones: 3 (0 buried)
Markers: 0
Sequenced: 0
Length: 77 CB units
Ctg3634 of cp05T5e12DM4RS
V039O07
V045L09
V045G05
0 5 10 15 20 25 30 35 40 45 50 55 60 65 70 75
[root@localhost: /...
[FPC V8.9 Main Menu]
Project cp05T5e12...
[Chickpea contig p...
FPC Ctg3634 cp05...

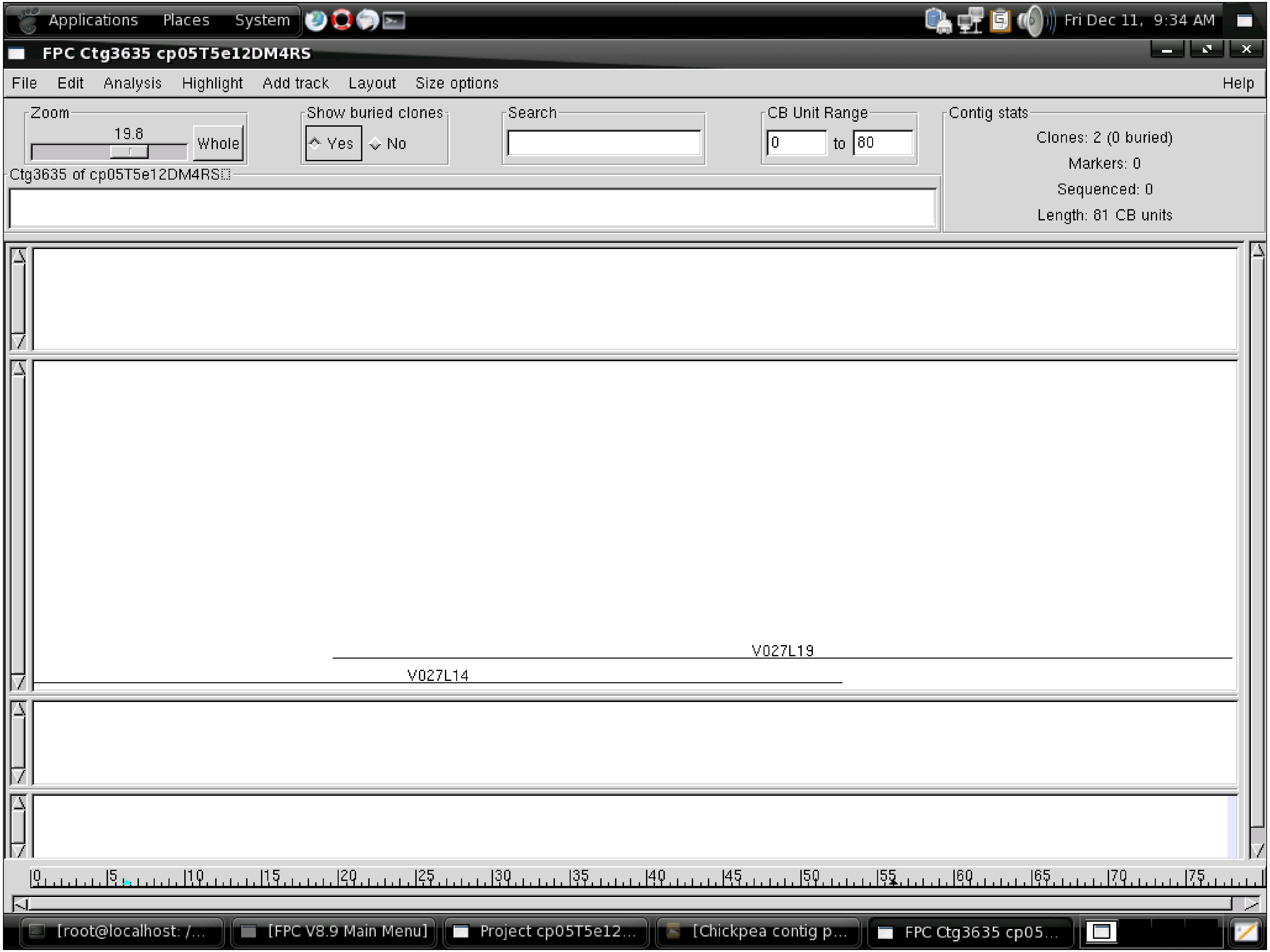
Applications
Places
System
Fri Dec 11, 9:34 AM
FPC Ctg3635 cp05T5e12DM4RS
File
Edit
Analysis
Highlight
Add track
Layout
Size options
Help
Zoom
19.8
Whole
Show buried clones
Yes
No
Search
CB Unit Range
0
to
80
Contig stats
Clones: 2 (0 buried)
Markers: 0
Sequenced: 0
Length: 81 CB units
Ctg3635 of cp05T5e12DM4RS
V027L19
V027L14
0
5
10
15
20
25
30
35
40
45
50
55
60
65
70
75
[root@localhost: /...
[FPC V8.9 Main Menu]
Project cp05T5e12...
[Chickpea contig p...
FPC Ctg3635 cp05...

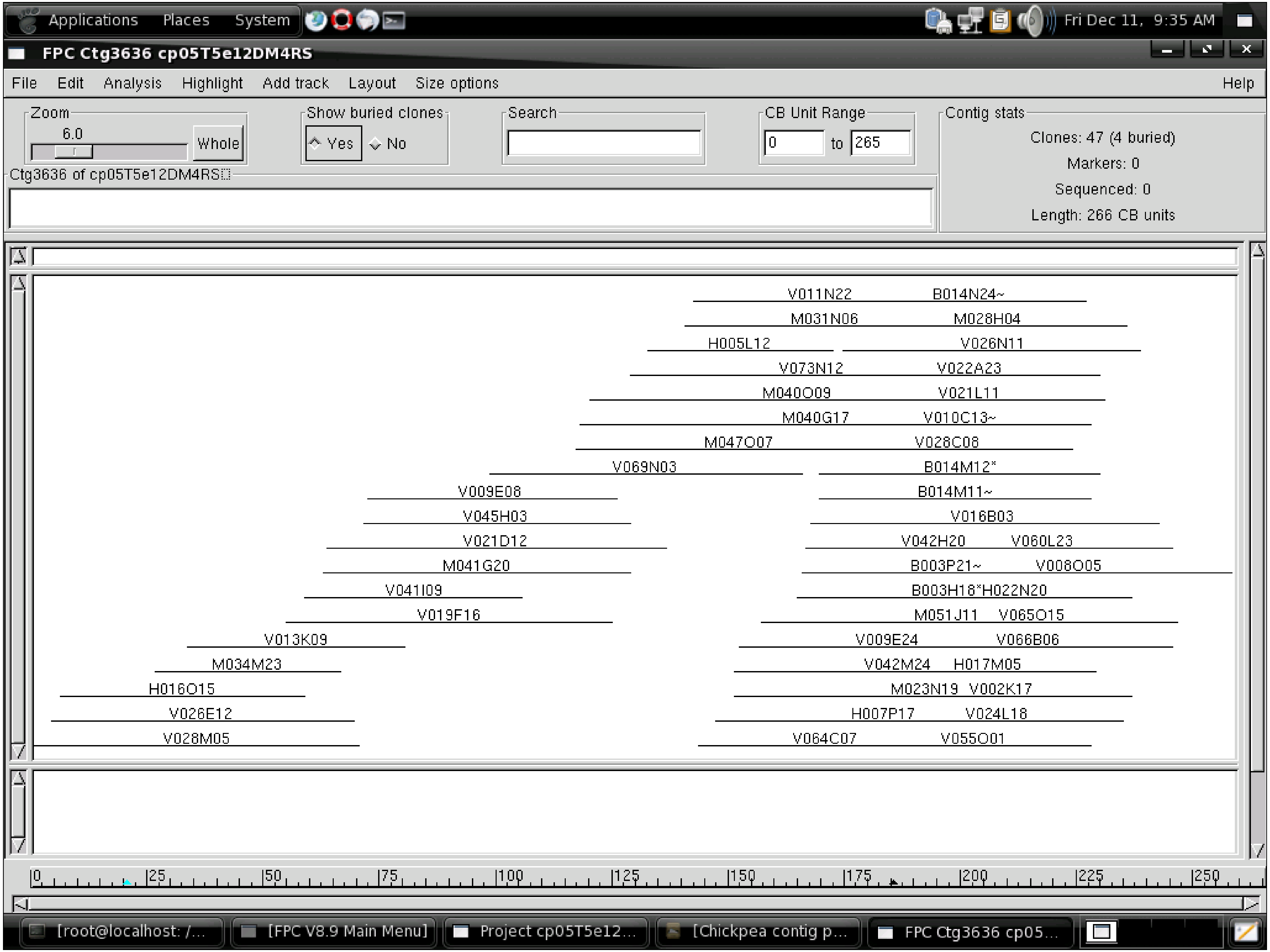
Applications Places System
Fri Dec 11, 9:35 AM
FPC Ctg3636 cp05T5e12DM4RS
File Edit Analysis Highlight Add track Layout Size options Help
Zoom
6.0
Whole
Show buried clones
Yes
No
Search
CB Unit Range
0
to
265
Contig stats
Clones: 47 (4 buried)
Markers: 0
Sequenced: 0
Length: 266 CB units
Ctg3636 of cp05T5e12DM4RS
V011N22
B014N24~
M031N06
M028H04
H005L12
V026N11
V073N12
V022A23
M040O09
V021L11
M040G17
V010C13~
M047O07
V028C08
V069N03
B014M12*
V009E08
B014M11~
V045H03
V016B03
V021D12
V042H20
V060L23
M041G20
B003P21~
V008O05
V041I09
B003H18*H022N20
V019F16
M051J11
V065O15
V013K09
V009E24
V066B06
M034M23
V042M24
H017M05
H016O15
M023N19
V002K17
V026E12
H007P17
V024L18
V028M05
V064C07
V055O01
0 25 50 75 100 125 150 175 200 225 250
[root@localhost: /...
[FPC V8.9 Main Menu]
Project cp05T5e12...
[Chickpea contig p...
FPC Ctg3636 cp05...

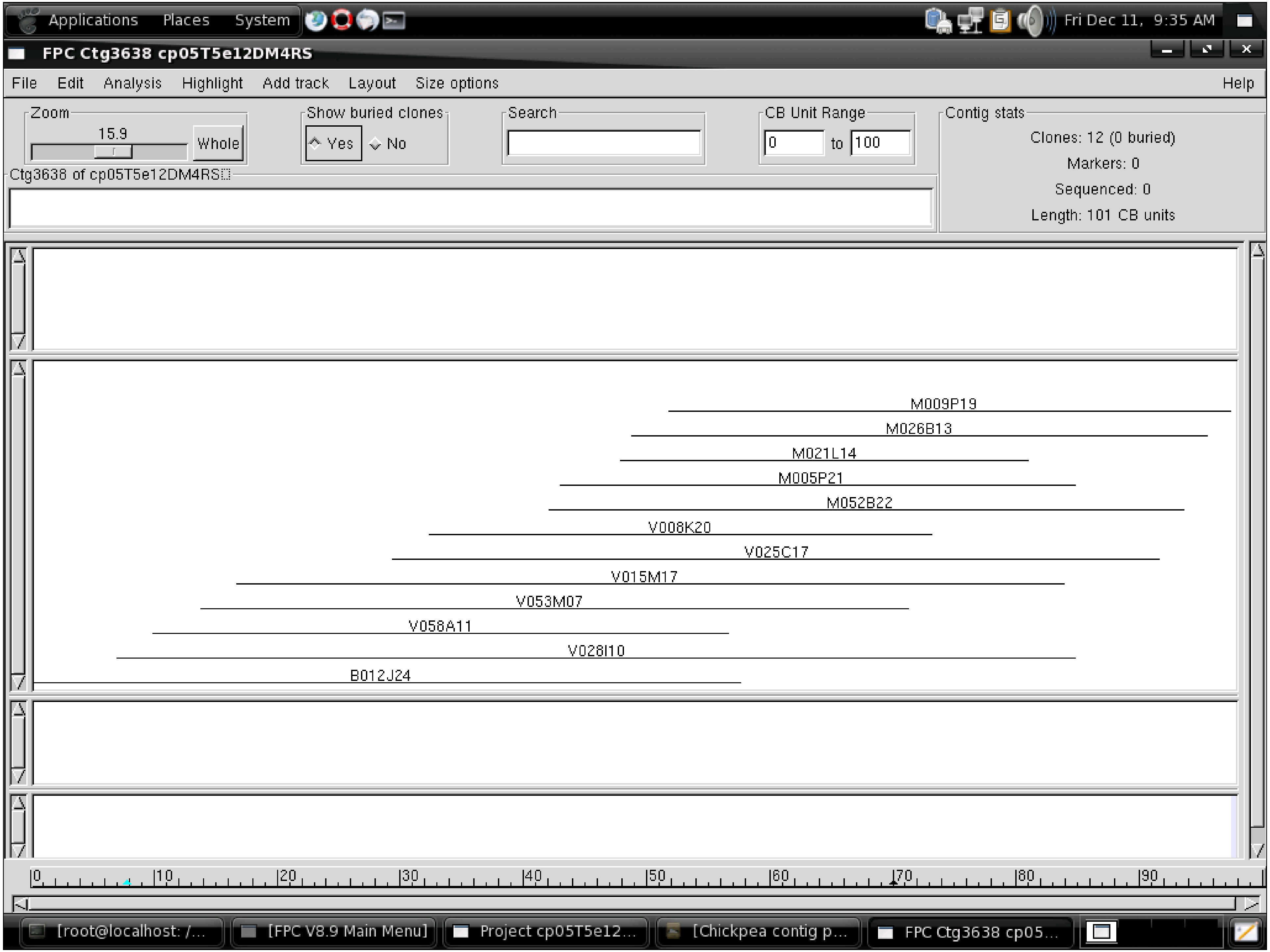
Applications Places System
Fri Dec 11, 9:35 AM
FPC Ctg3638 cp05T5e12DM4RS
File Edit Analysis Highlight Add track Layout Size options Help
Zoom
15.9
Whole
Show buried clones
Yes
No
Search
CB Unit Range
0
to
100
Contig stats
Clones: 12 (0 buried)
Markers: 0
Sequenced: 0
Length: 101 CB units
Ctg3638 of cp05T5e12DM4RS
M009P19
M026B13
M021L14
M005P21
M052B22
V008K20
V025C17
V015M17
V053M07
V058A11
V028I10
B012J24
0 10 20 30 40 50 60 70 80 90
[root@localhost: /...
[FPC V8.9 Main Menu]
Project cp05T5e12...
[Chickpea contig p...
FPC Ctg3638 cp05...

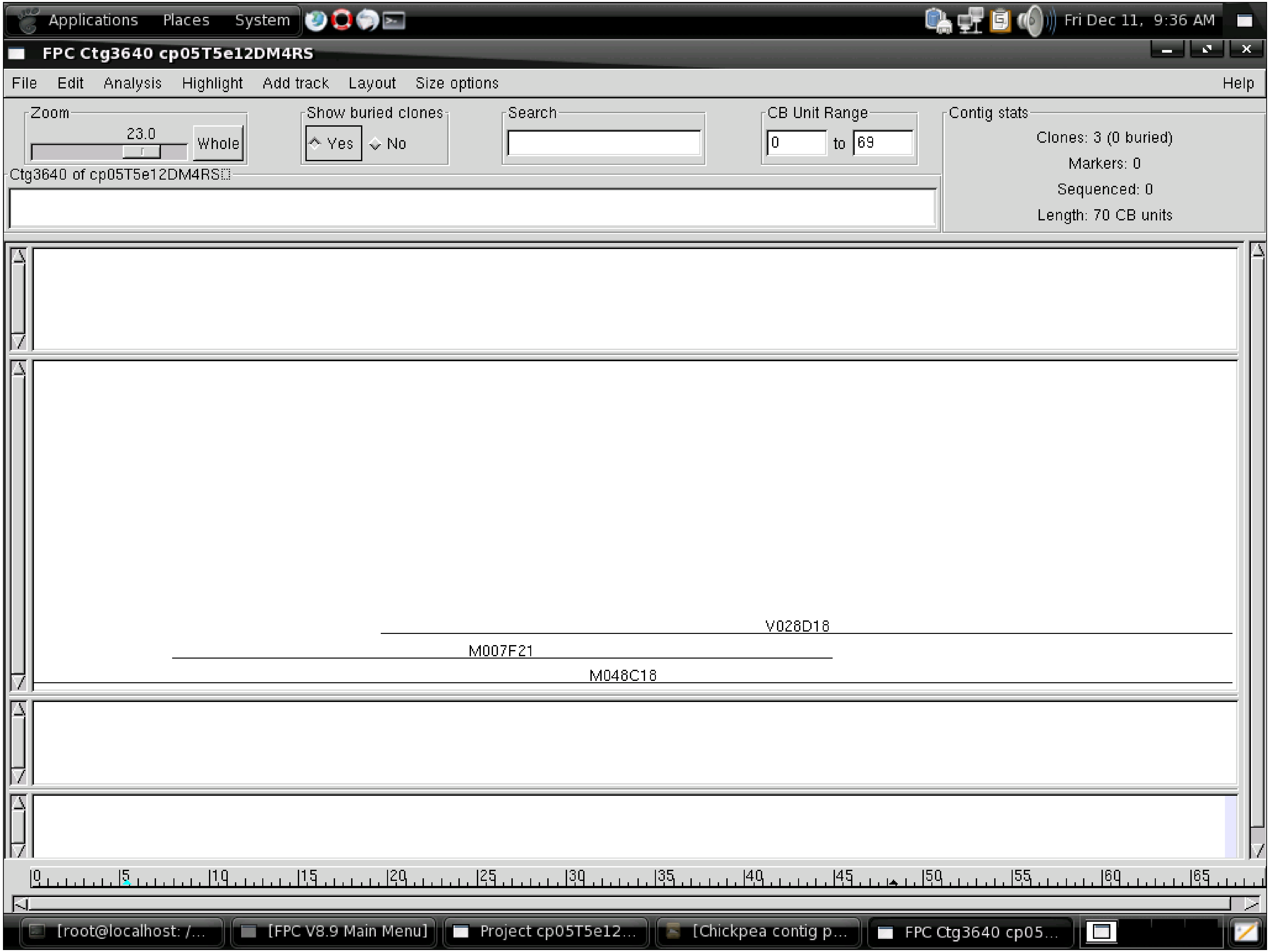
Applications
Places
System
Fri Dec 11, 9:36 AM
FPC Ctg3640 cp05T5e12DM4RS
File
Edit
Analysis
Highlight
Add track
Layout
Size options
Help
Zoom
23.0
Whole
Show buried clones
Yes
No
Search
CB Unit Range
0
to
69
Contig stats
Clones: 3 (0 buried)
Markers: 0
Sequenced: 0
Length: 70 CB units
Ctg3640 of cp05T5e12DM4RS
V028D18
M007F21
M048C18
0 5 10 15 20 25 30 35 40 45 50 55 60 65
[root@localhost: /...
[FPC V8.9 Main Menu]
Project cp05T5e12...
[Chickpea contig p...
FPC Ctg3640 cp05...

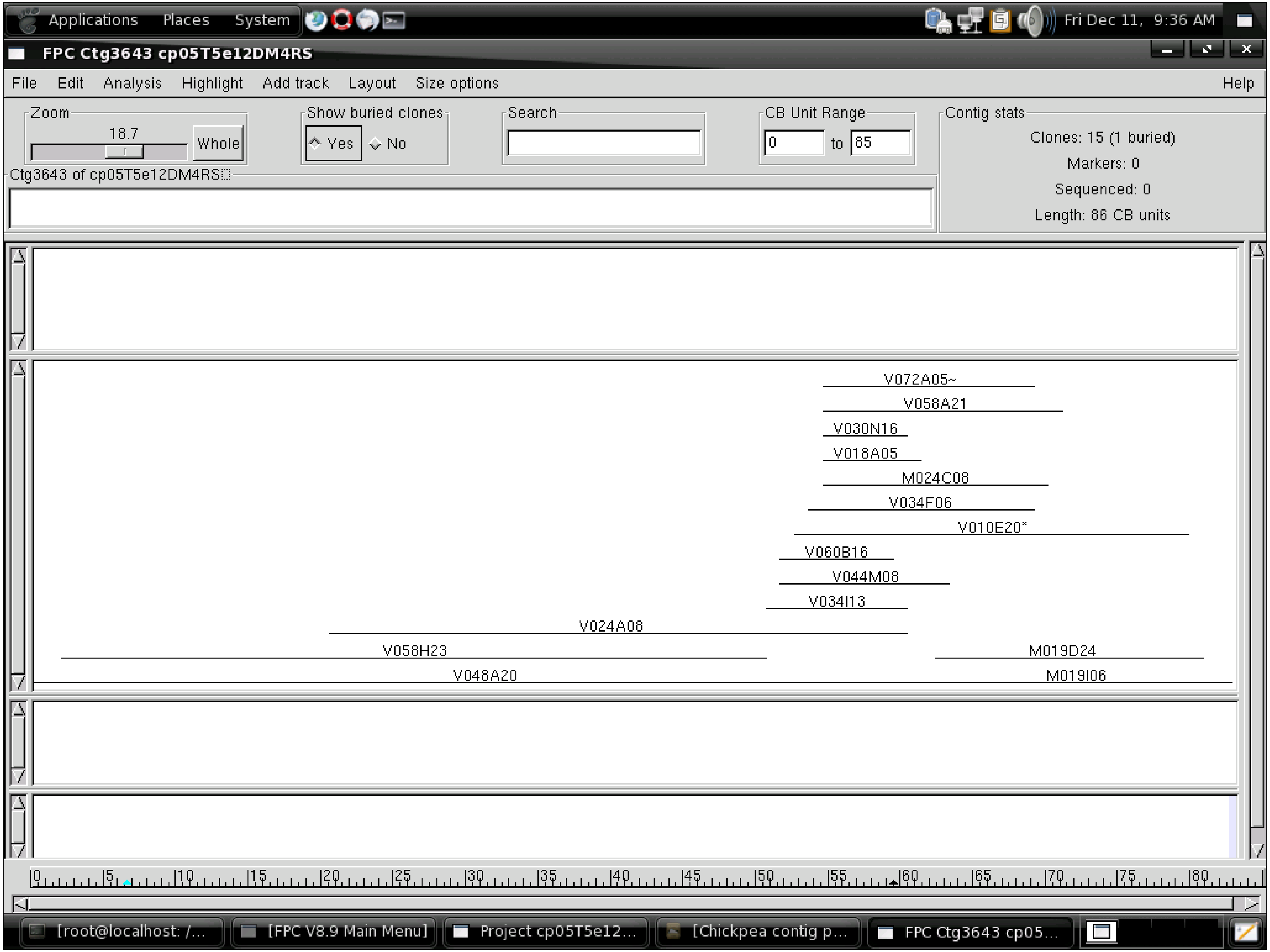
Applications Places System
Fri Dec 11, 9:36 AM
FPC Ctg3643 cp05T5e12DM4RS
File Edit Analysis Highlight Add track Layout Size options Help
Zoom
18.7
Whole
Show buried clones
Yes No
Search
CB Unit Range
0 to 85
Contig stats
Clones: 15 (1 buried)
Markers: 0
Sequenced: 0
Length: 86 CB units
Ctg3643 of cp05T5e12DM4RS
V072A05~
V058A21
V030N16
V018A05
M024C08
V034F06
V010E20*
V060B16
V044M08
V034I13
V024A08
V058H23
M019D24
V048A20
M019I06
0 5 10 15 20 25 30 35 40 45 50 55 60 65 70 75 80
[root@localhost: /...
[FPC V8.9 Main Menu]
Project cp05T5e12...
[Chickpea contig p...
FPC Ctg3643 cp05...

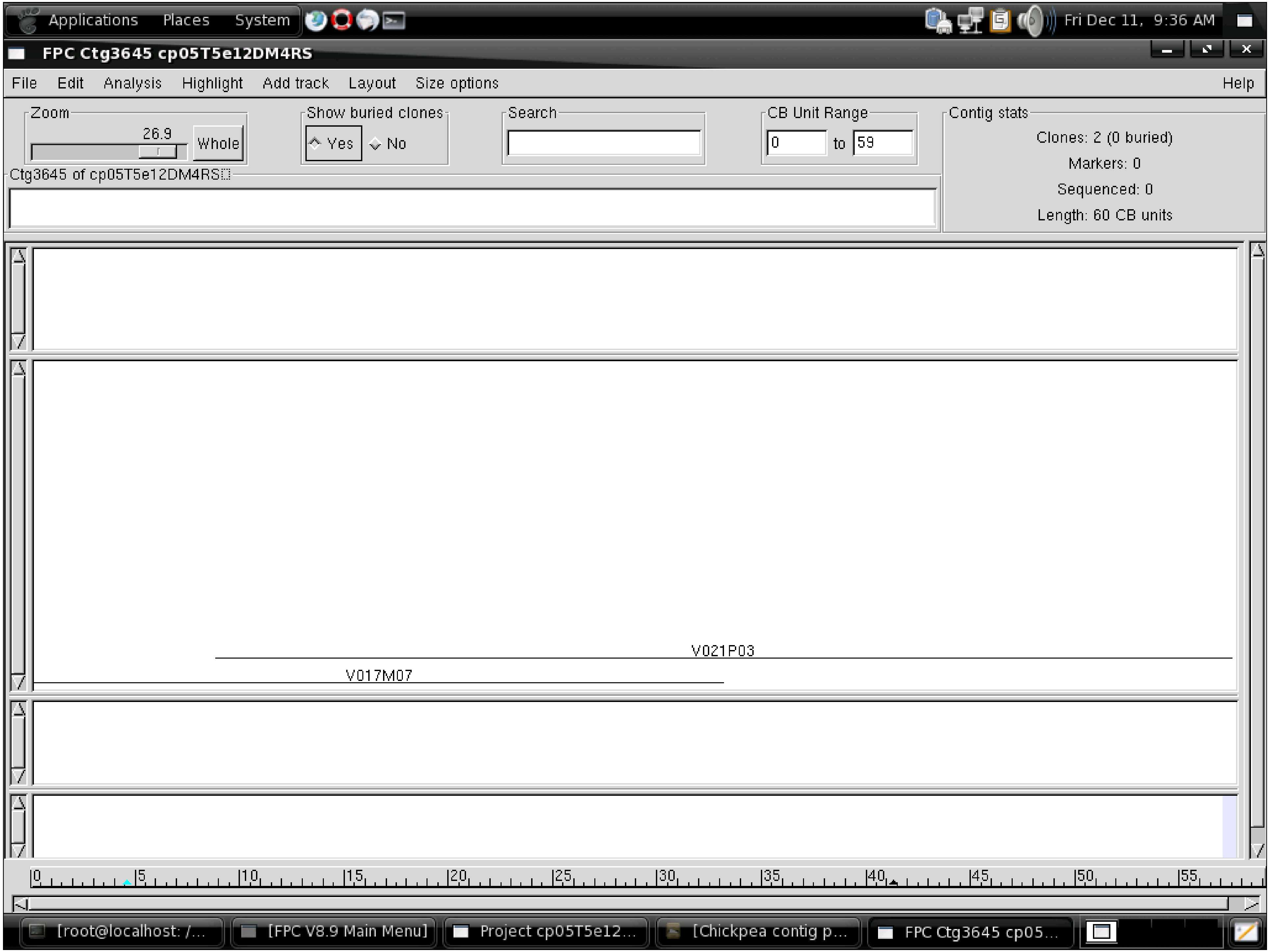
Applications Places System
Fri Dec 11, 9:36 AM
FPC Ctg3645 cp05T5e12DM4RS
File Edit Analysis Highlight Add track Layout Size options Help
Zoom
26.9
Whole
Show buried clones
Yes
No
Search
CB Unit Range
0 to 59
Contig stats
Clones: 2 (0 buried)
Markers: 0
Sequenced: 0
Length: 60 CB units
Ctg3645 of cp05T5e12DM4RS
V021P03
V017M07
0 5 10 15 20 25 30 35 40 45 50 55
[root@localhost: /...
[FPC V8.9 Main Menu]
Project cp05T5e12...
[Chickpea contig p...
FPC Ctg3645 cp05...

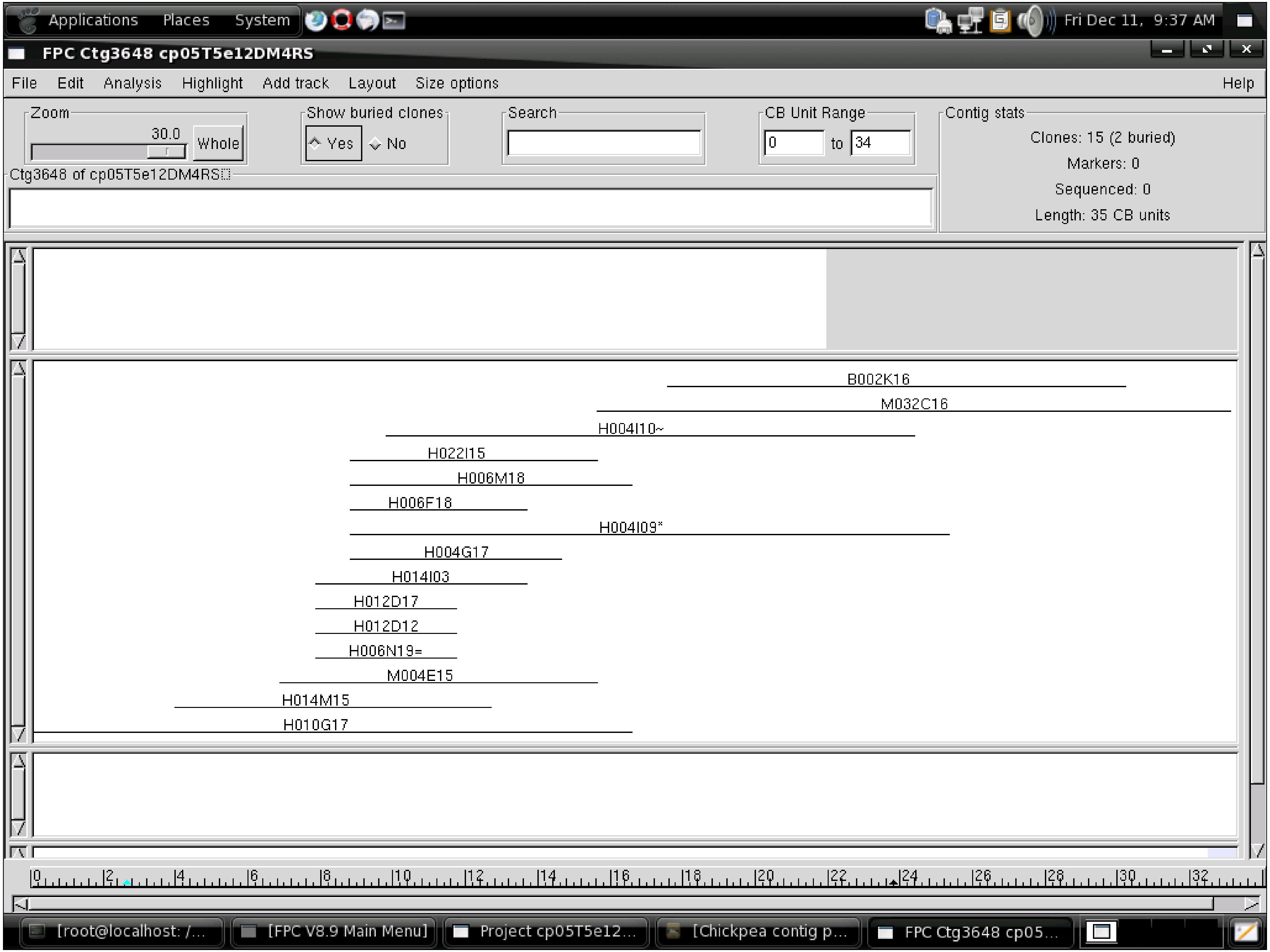
Applications Places System
Fri Dec 11, 9:37 AM
FPC Ctg3648 cp05T5e12DM4RS
File Edit Analysis Highlight Add track Layout Size options Help
Zoom
30.0
Whole
Show buried clones
Yes
No
Search
CB Unit Range
0
to
34
Contig stats
Clones: 15 (2 buried)
Markers: 0
Sequenced: 0
Length: 35 CB units
Ctg3648 of cp05T5e12DM4RS
B002K16
M032C16
H004I10~
H022I15
H006M18
H006F18
H004I09*
H004G17
H014I03
H012D17
H012D12
H006N19=
M004E15
H014M15
H010G17
0 2 4 6 8 10 12 14 16 18 20 22 24 26 28 30 32
[root@localhost: /...
[FPC V8.9 Main Menu]
Project cp05T5e12...
[Chickpea contig p...
FPC Ctg3648 cp05...

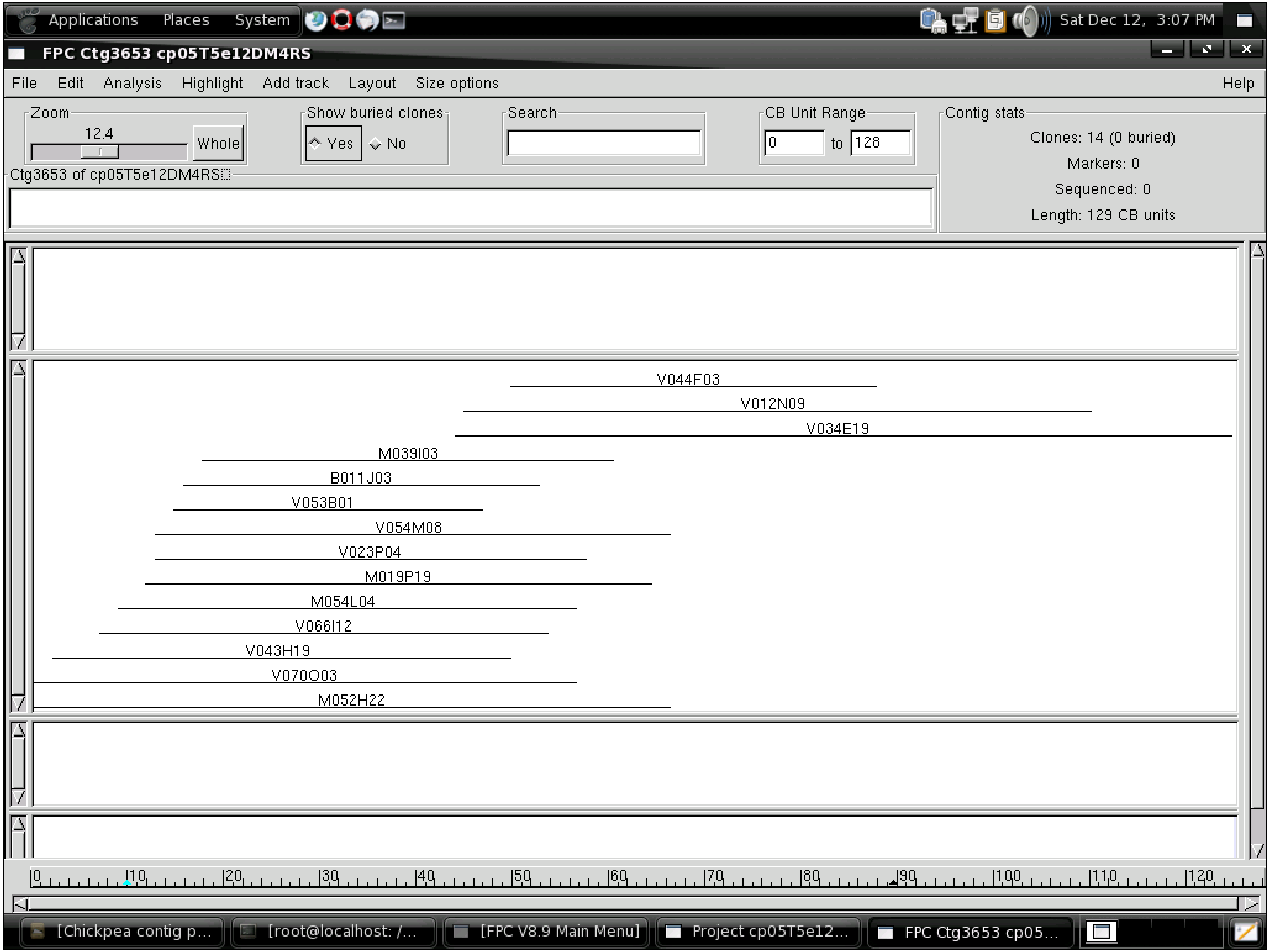
Applications Places System
Sat Dec 12, 3:07 PM
FPC Ctg3653 cp05T5e12DM4RS
File Edit Analysis Highlight Add track Layout Size options Help
Zoom
12.4
Whole
Show buried clones
Yes No
Search
CB Unit Range
0 to 128
Contig stats
Clones: 14 (0 buried)
Markers: 0
Sequenced: 0
Length: 129 CB units
Ctg3653 of cp05T5e12DM4RS
V044F03
V012N09
V034E19
M039I03
B011J03
V053B01
V054M08
V023P04
M019P19
M054L04
V066I12
V043H19
V070O03
M052H22
0 10 20 30 40 50 60 70 80 90 100 110 120
[Chickpea contig p...
[root@localhost: /...
[FPC V8.9 Main Menu]
Project cp05T5e12...
FPC Ctg3653 cp05...

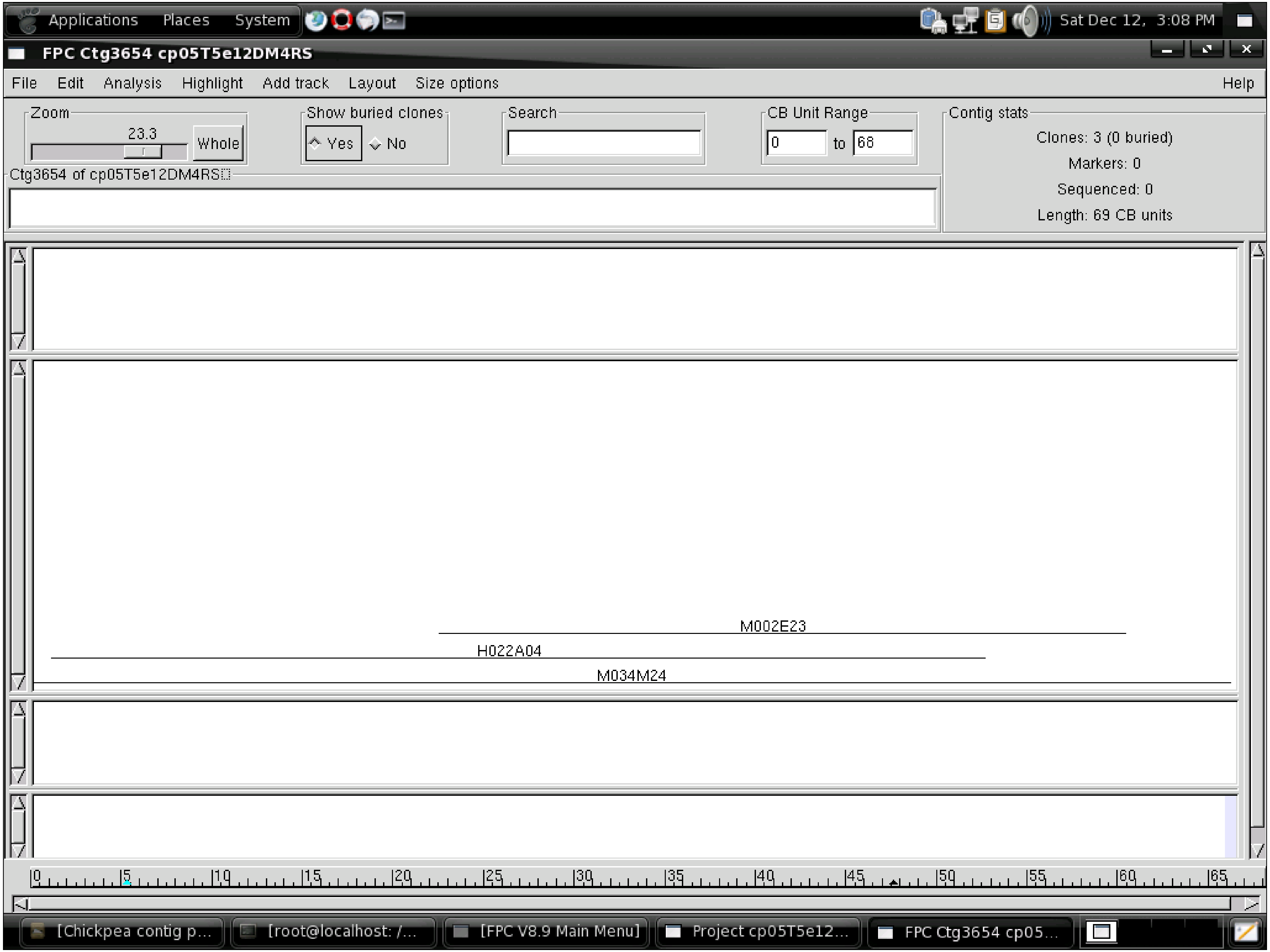
Applications Places System
Sat Dec 12, 3:08 PM
FPC Ctg3654 cp05T5e12DM4RS
File Edit Analysis Highlight Add track Layout Size options Help
Zoom
23.3
Whole
Show buried clones
Yes No
Search
CB Unit Range
0 to 68
Contig stats
Clones: 3 (0 buried)
Markers: 0
Sequenced: 0
Length: 69 CB units
Ctg3654 of cp05T5e12DM4RS
M002E23
H022A04
M034M24
0 5 10 15 20 25 30 35 40 45 50 55 60 65
[Chickpea contig p...
[root@localhost: /...
[FPC V8.9 Main Menu]
Project cp05T5e12...
FPC Ctg3654 cp05...

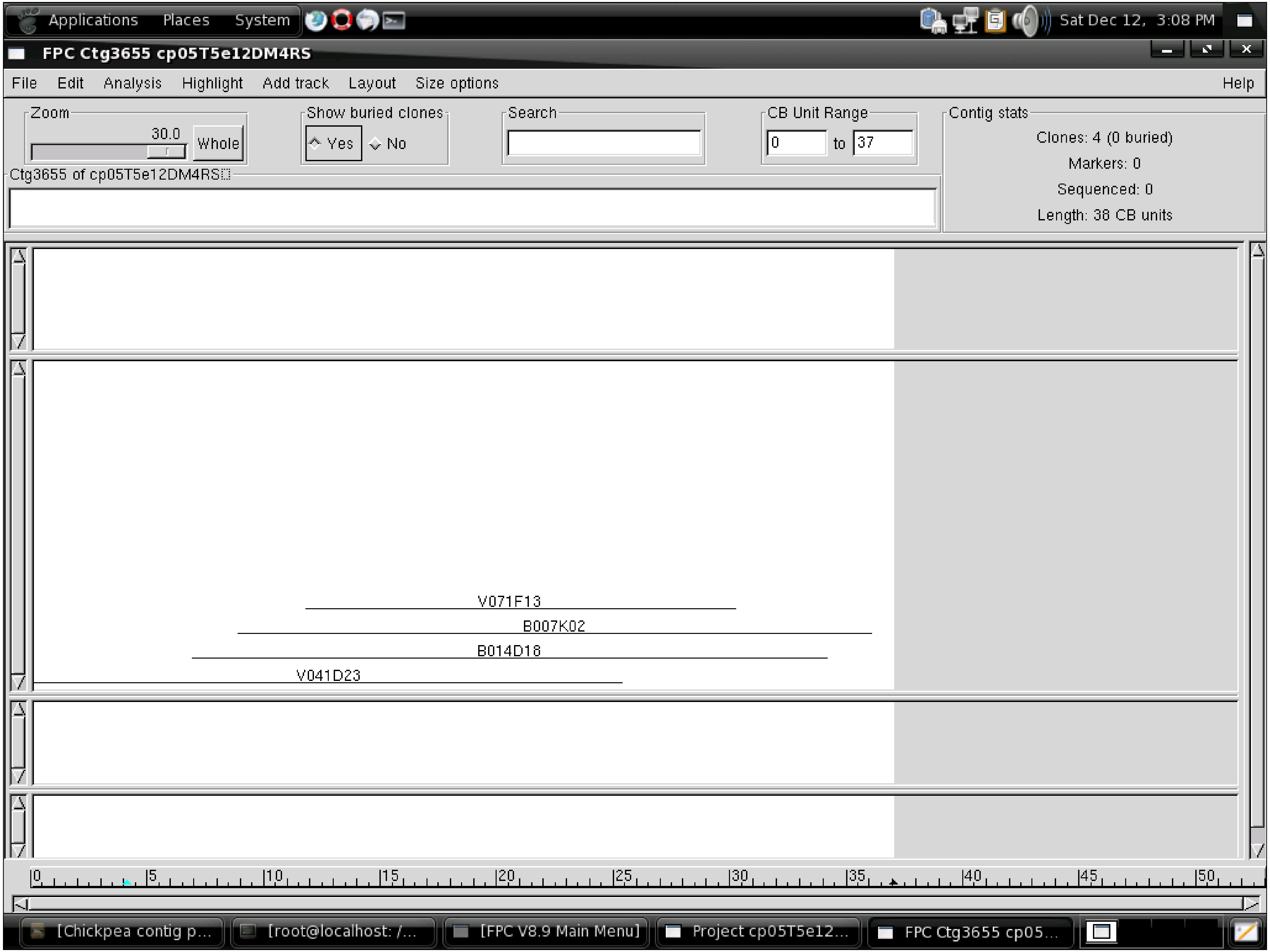
Applications Places System
Sat Dec 12, 3:08 PM
FPC Ctg3655 cp05T5e12DM4RS
File Edit Analysis Highlight Add track Layout Size options Help
Zoom
30.0
Whole
Show buried clones
Yes
No
Search
CB Unit Range
0
to
37
Contig stats
Clones: 4 (0 buried)
Markers: 0
Sequenced: 0
Length: 38 CB units
Ctg3655 of cp05T5e12DM4RS
V071F13
B007K02
B014D18
V041D23
0 5 10 15 20 25 30 35 40 45 50
[Chickpea contig p...
[root@localhost: /...
[FPC V8.9 Main Menu]
Project cp05T5e12...
FPC Ctg3655 cp05...

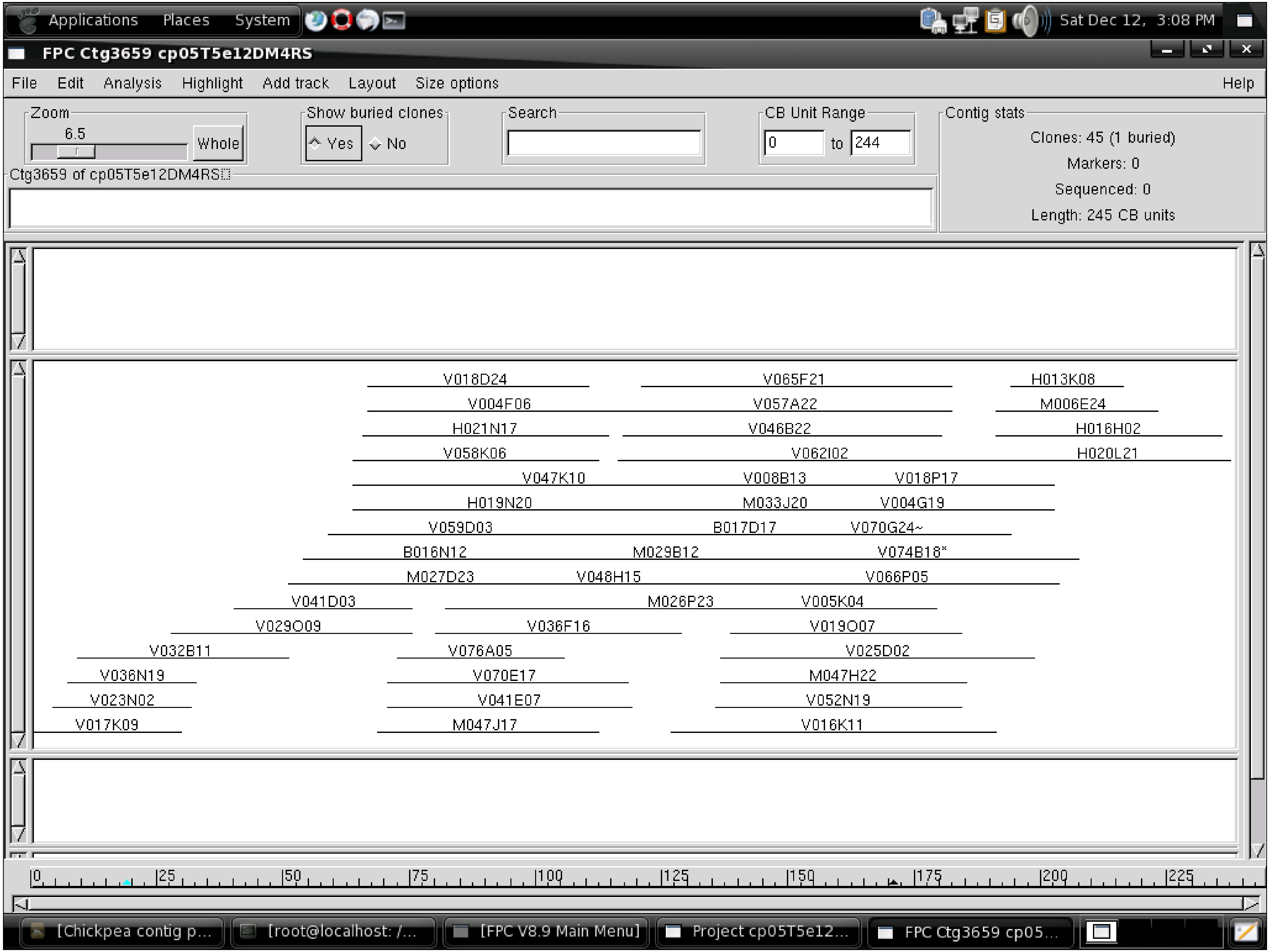
Applications Places System
Sat Dec 12, 3:08 PM
FPC Ctg3659 cp05T5e12DM4RS
File Edit Analysis Highlight Add track Layout Size options Help
Zoom
6.5
Whole
Show buried clones
Yes No
Search
CB Unit Range
0 to 244
Contig stats
Clones: 45 (1 buried)
Markers: 0
Sequenced: 0
Length: 245 CB units
Ctg3659 of cp05T5e12DM4RS
V018D24 V065F21 H013K08
V004F06 V057A22 M006E24
H021N17 V046B22 H016H02
V058K06 V062I02 H020L21
V047K10 V008B13 V018P17
H019N20 M033J20 V004G19
V059D03 B017D17 V070G24~
B016N12 M029B12 V074B18*
M027D23 V048H15 V066P05
V041D03 M026P23 V005K04
V029O09 V036F16 V019O07
V032B11 V076A05 V025D02
V036N19 V070E17 M047H22
V023N02 V041E07 V052N19
V017K09 M047J17 V016K11
0 25 50 75 100 125 150 175 200 225
[Chickpea contig p...
[root@localhost: /...
[FPC V8.9 Main Menu]
Project cp05T5e12...
FPC Ctg3659 cp05...

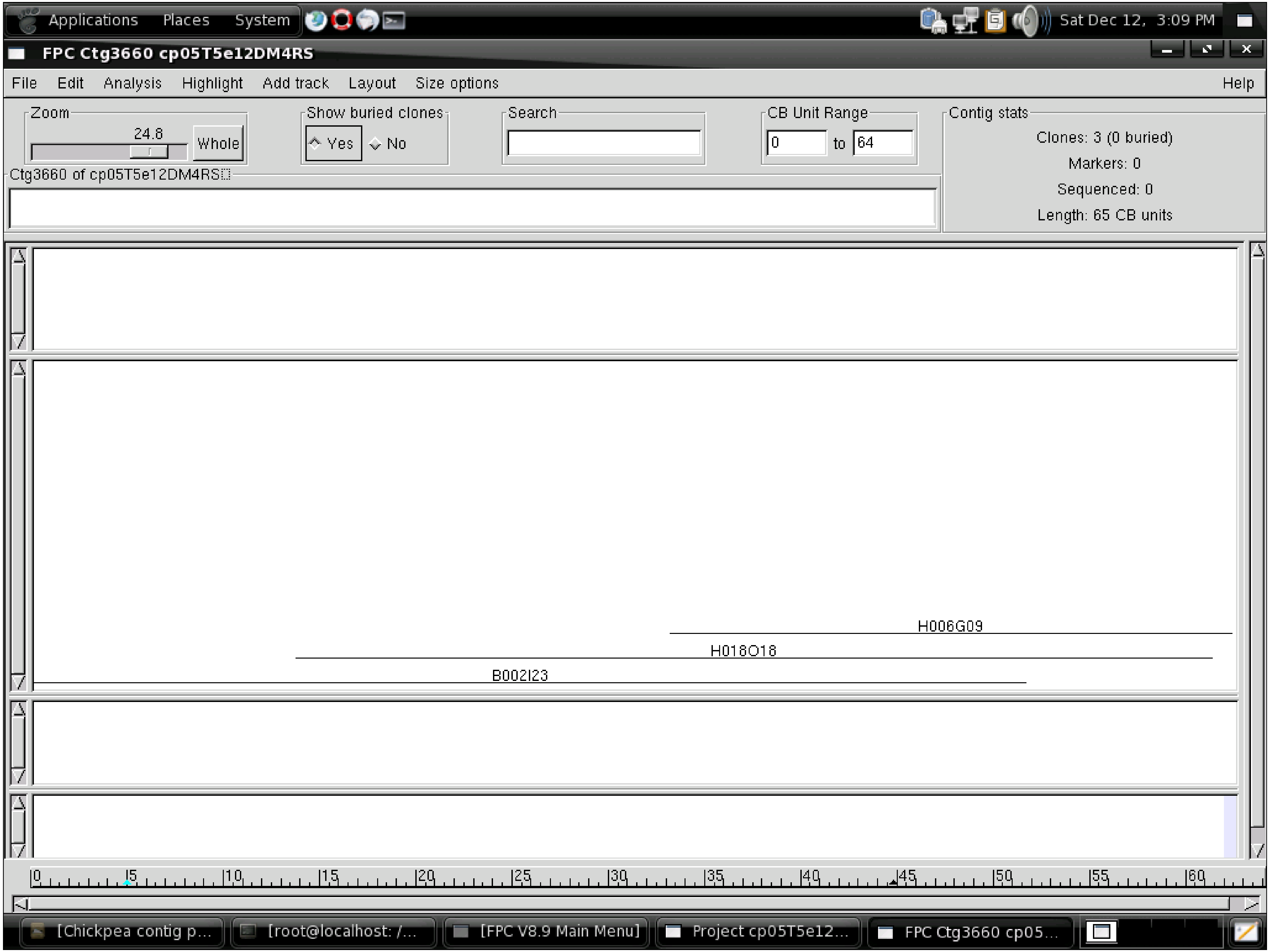
Applications Places System
Sat Dec 12, 3:09 PM
FPC Ctg3660 cp05T5e12DM4RS
File Edit Analysis Highlight Add track Layout Size options Help
Zoom
24.8
Whole
Show buried clones
Yes
No
Search
CB Unit Range
0
to
64
Contig stats
Clones: 3 (0 buried)
Markers: 0
Sequenced: 0
Length: 65 CB units
Ctg3660 of cp05T5e12DM4RS
H006G09
H018O18
B002I23
0 5 10 15 20 25 30 35 40 45 50 55 60
[Chickpea contig p...
[root@localhost: /...
[FPC V8.9 Main Menu]
Project cp05T5e12...
FPC Ctg3660 cp05...

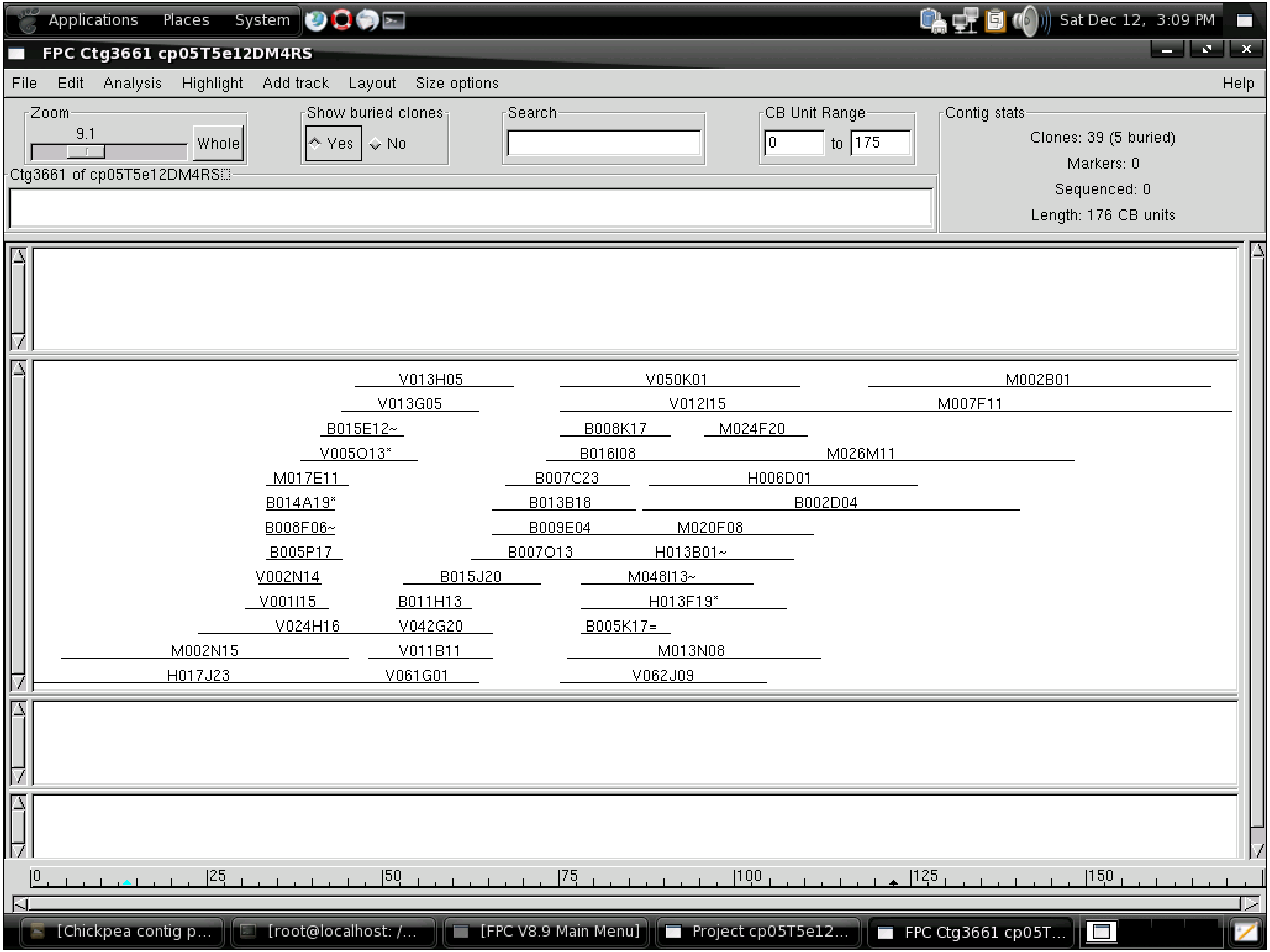
Applications Places System
Sat Dec 12, 3:09 PM
FPC Ctg3661 cp05T5e12DM4RS
File Edit Analysis Highlight Add track Layout Size options Help
Zoom
9.1
Whole
Show buried clones
Yes
No
Search
CB Unit Range
0
to
175
Contig stats
Clones: 39 (5 buried)
Markers: 0
Sequenced: 0
Length: 176 CB units
Ctg3661 of cp05T5e12DM4RS
V013H05
V050K01
M002B01
V013G05
V012I15
M007F11
B015E12~
B008K17
M024F20
V005O13*
B016I08
M026M11
M017E11
B007C23
H006D01
B014A19*
B013B18
B002D04
B008F06~
B009E04
M020F08
B005P17
B007O13
H013B01~
V002N14
B015J20
M048I13~
V001I15
B011H13
H013F19*
V024H16
V042G20
B005K17=
M002N15
V011B11
M013N08
H017J23
V061G01
V062J09
0
25
50
75
100
125
150
[Chickpea contig p...
[root@localhost: /...
[FPC V8.9 Main Menu]
Project cp05T5e12...
FPC Ctg3661 cp05T...

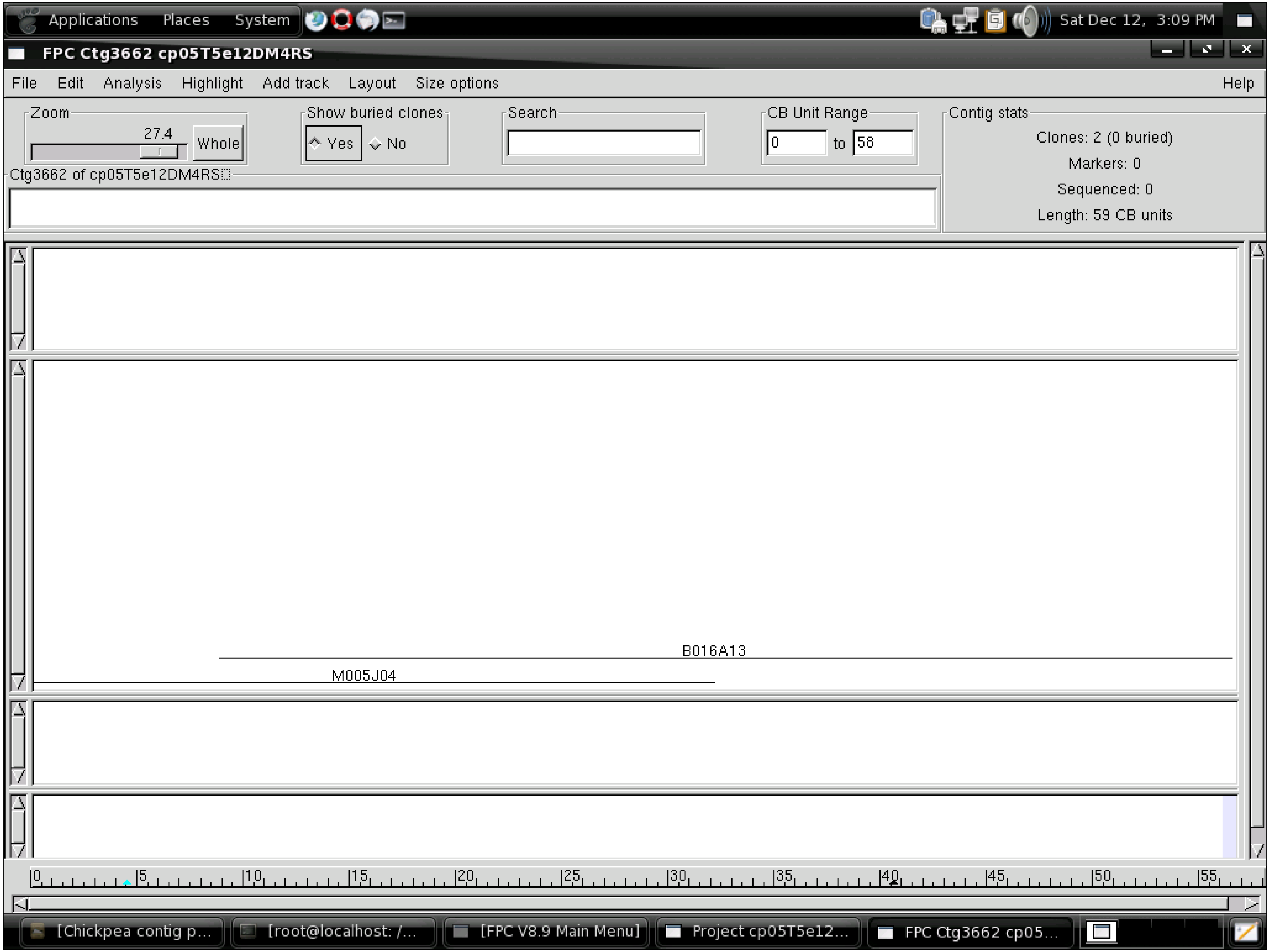
Applications Places System
Sat Dec 12, 3:09 PM
FPC Ctg3662 cp05T5e12DM4RS
File Edit Analysis Highlight Add track Layout Size options Help
Zoom
27.4
Whole
Show buried clones
Yes
No
Search
CB Unit Range
0 to 58
Contig stats
Clones: 2 (0 buried)
Markers: 0
Sequenced: 0
Length: 59 CB units
Ctg3662 of cp05T5e12DM4RS
B016A13
M005J04
0 5 10 15 20 25 30 35 40 45 50 55
[Chickpea contig p...
[root@localhost: /...
[FPC V8.9 Main Menu]
Project cp05T5e12...
FPC Ctg3662 cp05...

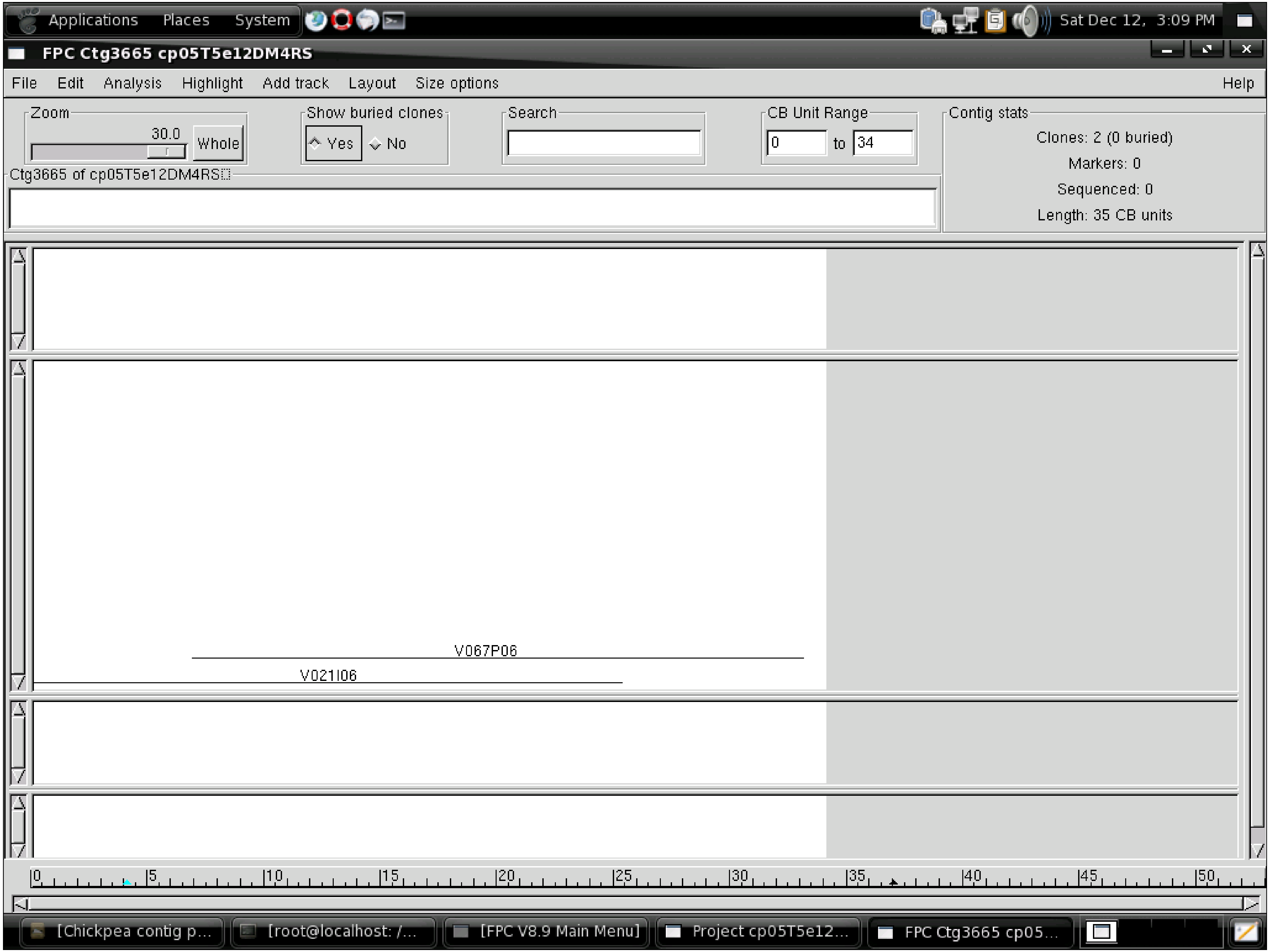
Applications Places System
Sat Dec 12, 3:09 PM
FPC Ctg3665 cp05T5e12DM4RS
File Edit Analysis Highlight Add track Layout Size options Help
Zoom
30.0
Whole
Show buried clones
Yes No
Search
CB Unit Range
0 to 34
Contig stats
Clones: 2 (0 buried)
Markers: 0
Sequenced: 0
Length: 35 CB units
Ctg3665 of cp05T5e12DM4RS
V067P06
V021I06
0 5 10 15 20 25 30 35 40 45 50
[Chickpea contig p...
[root@localhost: /...
[FPC V8.9 Main Menu]
Project cp05T5e12...
FPC Ctg3665 cp05...

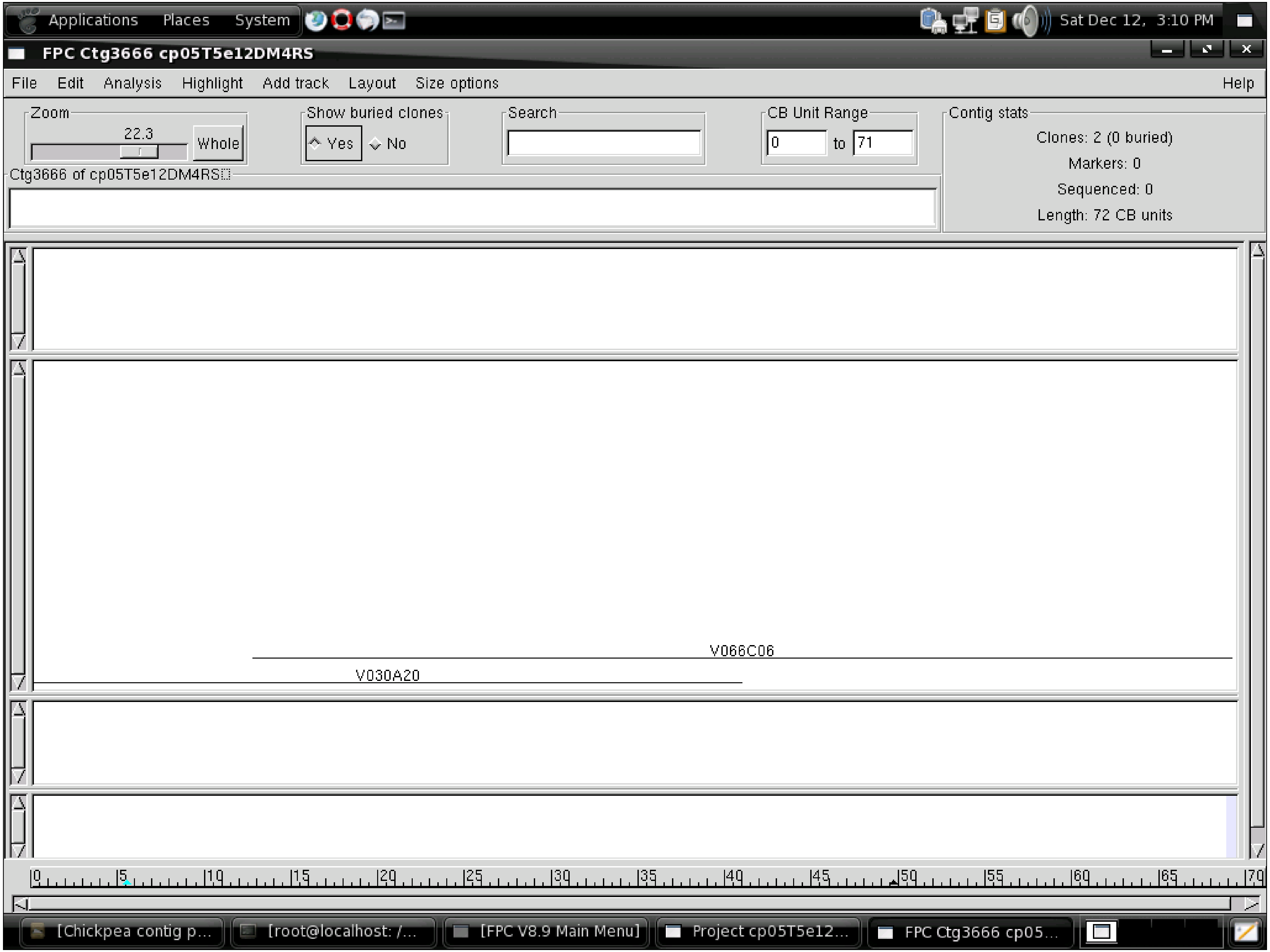
Applications Places System
Sat Dec 12, 3:10 PM
FPC Ctg3666 cp05T5e12DM4RS
File Edit Analysis Highlight Add track Layout Size options
Help
Zoom
22.3
Whole
Show buried clones
Yes
No
Search
CB Unit Range
0
to
71
Contig stats
Clones: 2 (0 buried)
Markers: 0
Sequenced: 0
Length: 72 CB units
Ctg3666 of cp05T5e12DM4RS
V066C06
V030A20
0 5 10 15 20 25 30 35 40 45 50 55 60 65 70
[Chickpea contig p...
[root@localhost: /...
[FPC V8.9 Main Menu]
Project cp05T5e12...
FPC Ctg3666 cp05...

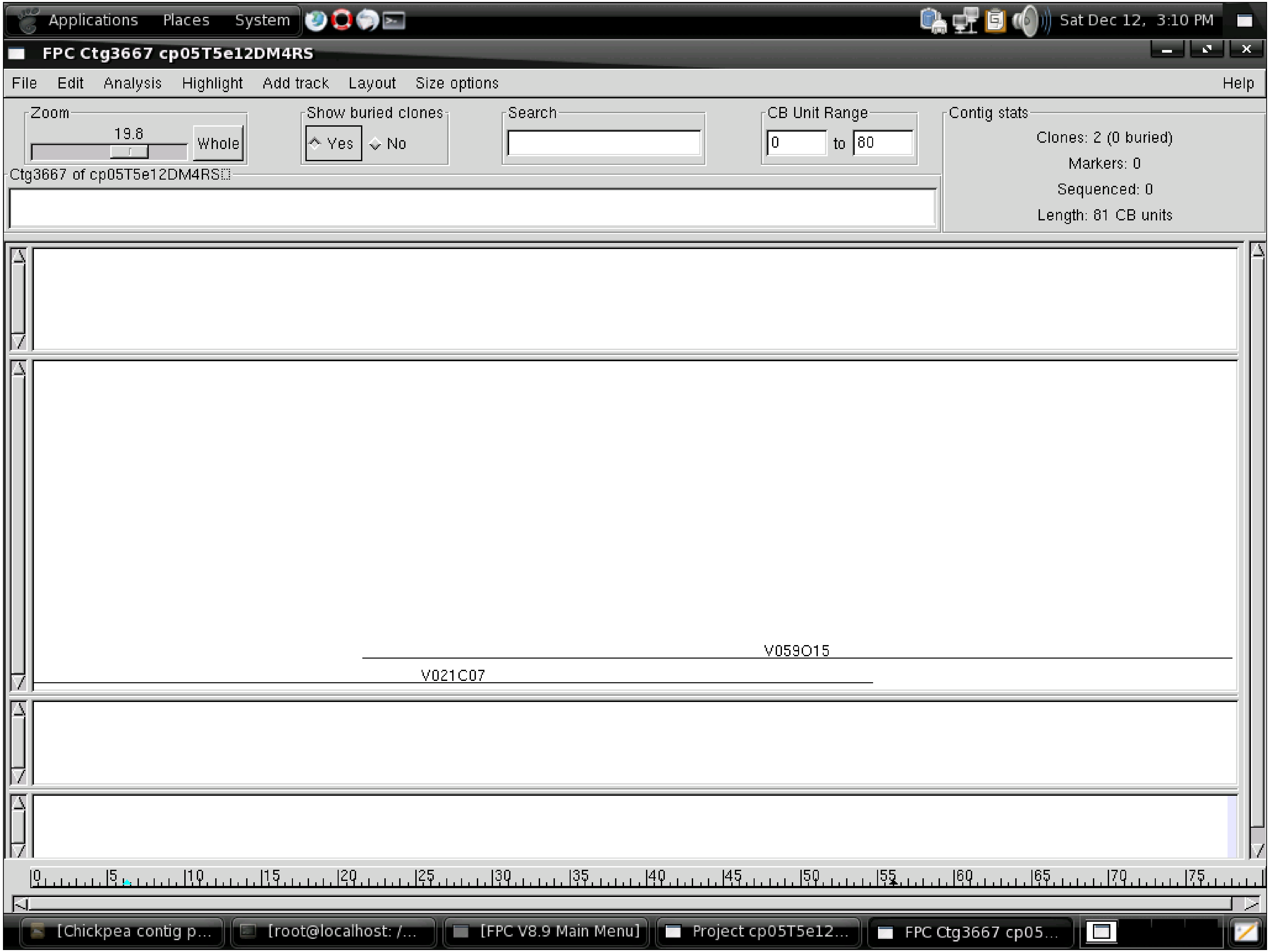
Applications Places System
Sat Dec 12, 3:10 PM
FPC Ctg3667 cp05T5e12DM4RS
File Edit Analysis Highlight Add track Layout Size options Help
Zoom
19.8
Whole
Show buried clones
Yes
No
Search
CB Unit Range
0 to 80
Contig stats
Clones: 2 (0 buried)
Markers: 0
Sequenced: 0
Length: 81 CB units
Ctg3667 of cp05T5e12DM4RS
V059O15
V021C07
0 5 10 15 20 25 30 35 40 45 50 55 60 65 70 75
[Chickpea contig p...
[root@localhost: /...
[FPC V8.9 Main Menu]
Project cp05T5e12...
FPC Ctg3667 cp05...

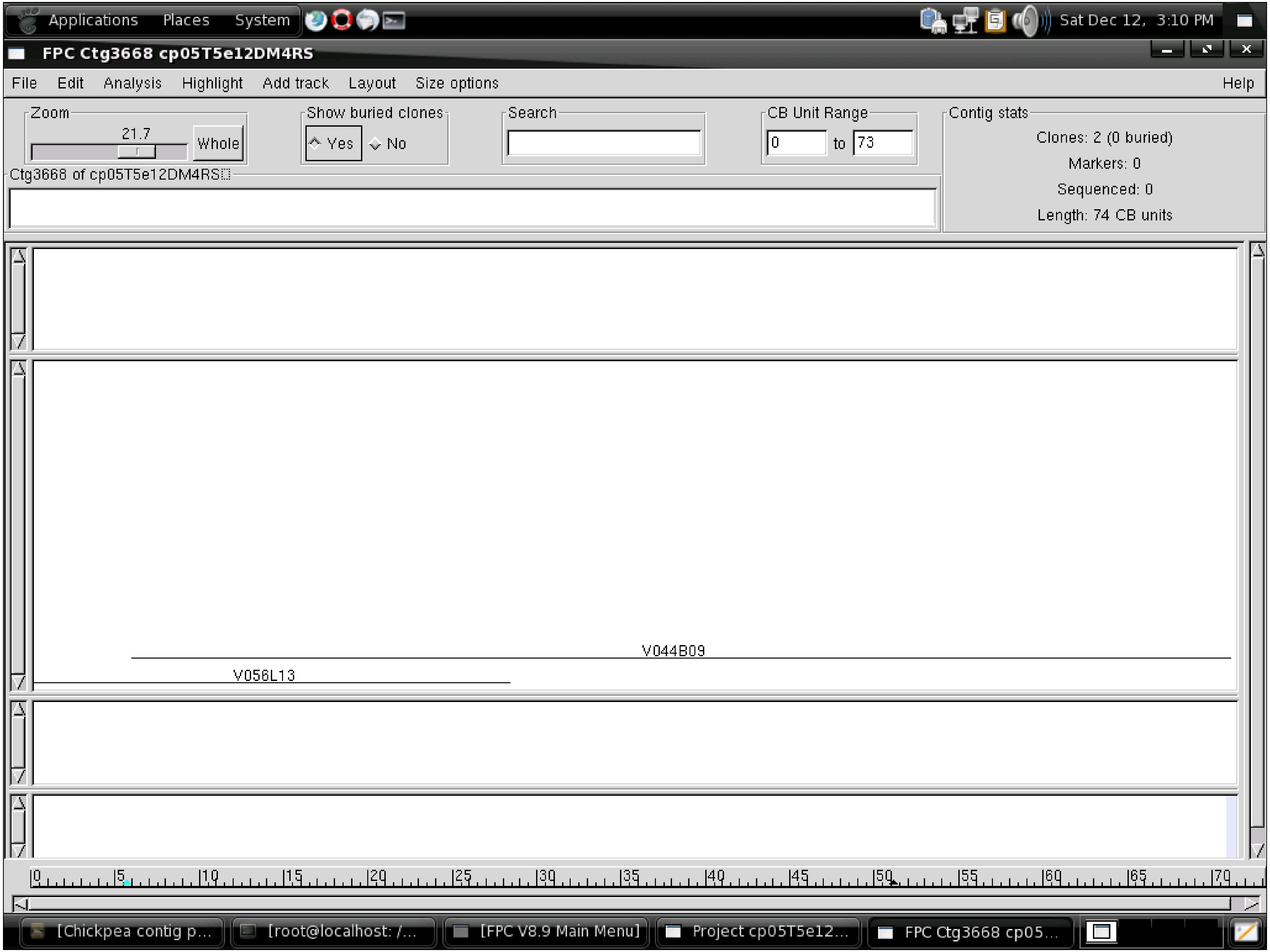
Applications Places System
Sat Dec 12, 3:10 PM
FPC Ctg3668 cp05T5e12DM4RS
File Edit Analysis Highlight Add track Layout Size options Help
Zoom
21.7
Whole
Show buried clones
Yes
No
Search
CB Unit Range
0 to 73
Contig stats
Clones: 2 (0 buried)
Markers: 0
Sequenced: 0
Length: 74 CB units
Ctg3668 of cp05T5e12DM4RS
V044B09
V056L13
0 5 10 15 20 25 30 35 40 45 50 55 60 65 70
[Chickpea contig p...
[root@localhost: /...
[FPC V8.9 Main Menu]
Project cp05T5e12...
FPC Ctg3668 cp05...

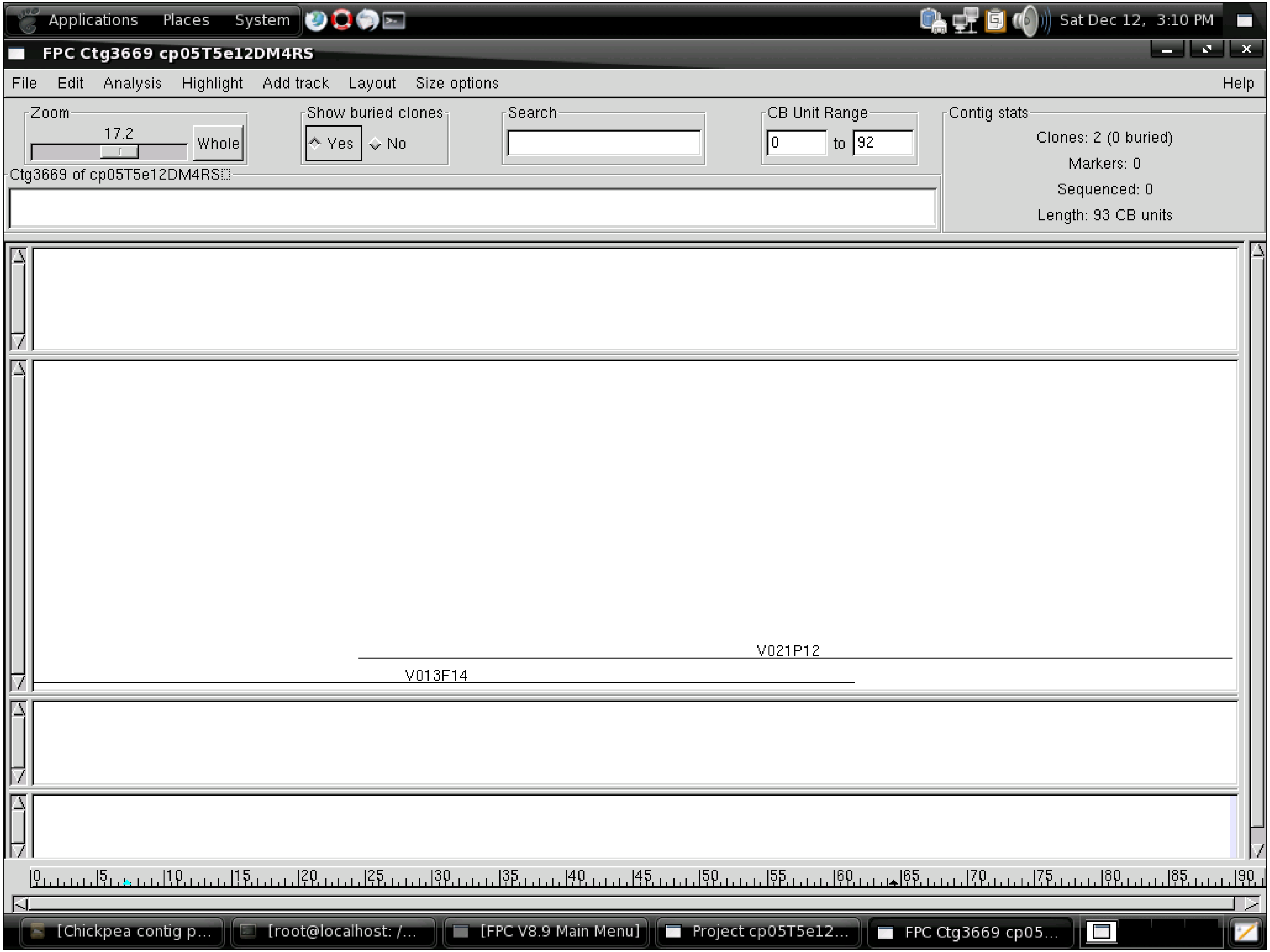
Applications Places System
Sat Dec 12, 3:10 PM
FPC Ctg3669 cp05T5e12DM4RS
File Edit Analysis Highlight Add track Layout Size options Help
Zoom
17.2
Whole
Show buried clones
Yes No
Search
CB Unit Range
0 to 92
Contig stats
Clones: 2 (0 buried)
Markers: 0
Sequenced: 0
Length: 93 CB units
Ctg3669 of cp05T5e12DM4RS
V021P12
V013F14
0 5 10 15 20 25 30 35 40 45 50 55 60 65 70 75 80 85 90
[Chickpea contig p...
[root@localhost: /...
[FPC V8.9 Main Menu]
Project cp05T5e12...
FPC Ctg3669 cp05...

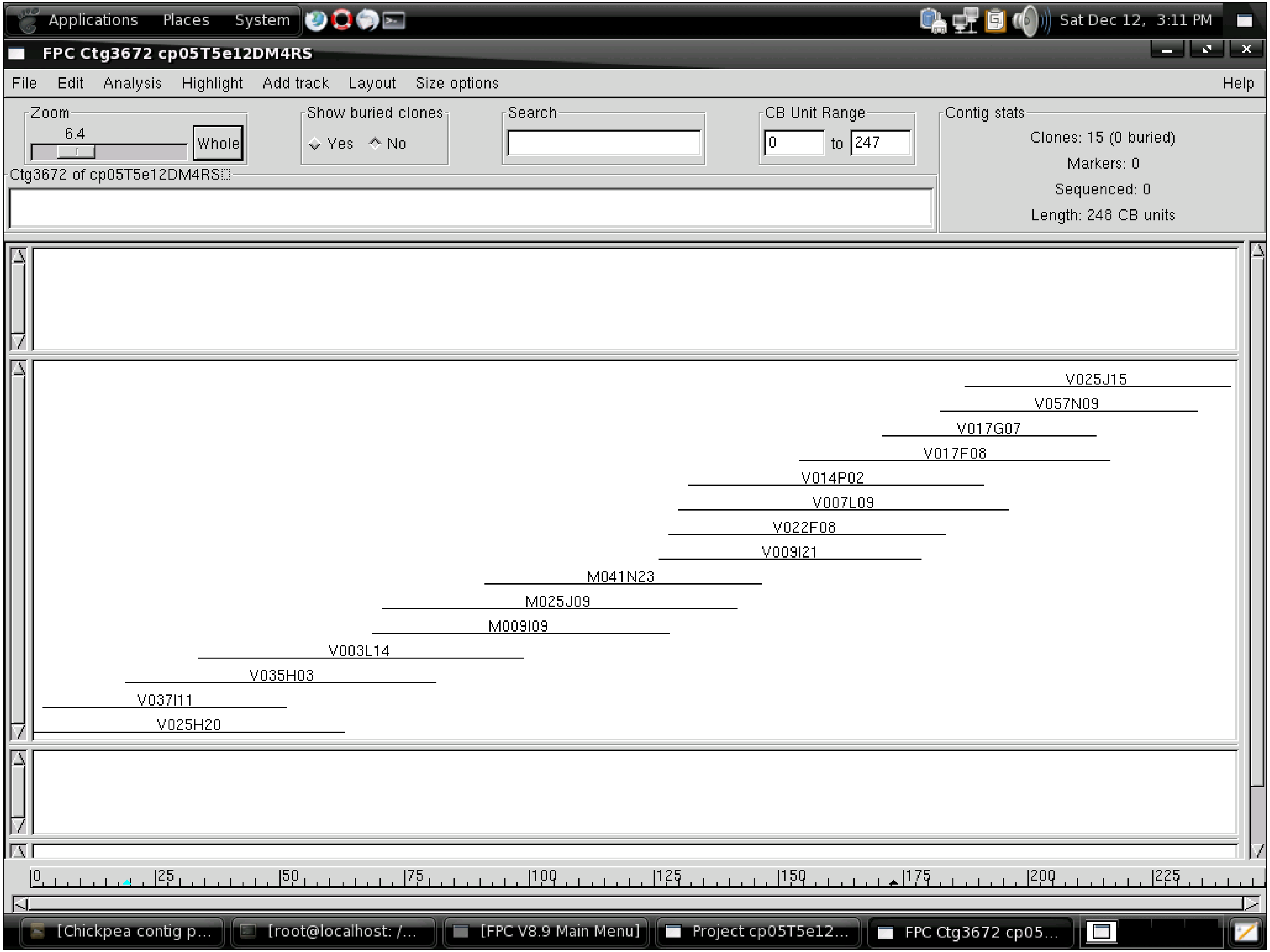
Applications Places System
Sat Dec 12, 3:11 PM
FPC Ctg3672 cp05T5e12DM4RS
File Edit Analysis Highlight Add track Layout Size options Help
Zoom
6.4
Whole
Show buried clones
Yes No
Search
CB Unit Range
0 to 247
Contig stats
Clones: 15 (0 buried)
Markers: 0
Sequenced: 0
Length: 248 CB units
Ctg3672 of cp05T5e12DM4RS
V025J15
V057N09
V017G07
V017F08
V014P02
V007L09
V022F08
V009I21
M041N23
M025J09
M009I09
V003L14
V035H03
V037I11
V025H20
0 25 50 75 100 125 150 175 200 225
[Chickpea contig p...
[root@localhost: /...
[FPC V8.9 Main Menu]
Project cp05T5e12...
FPC Ctg3672 cp05...

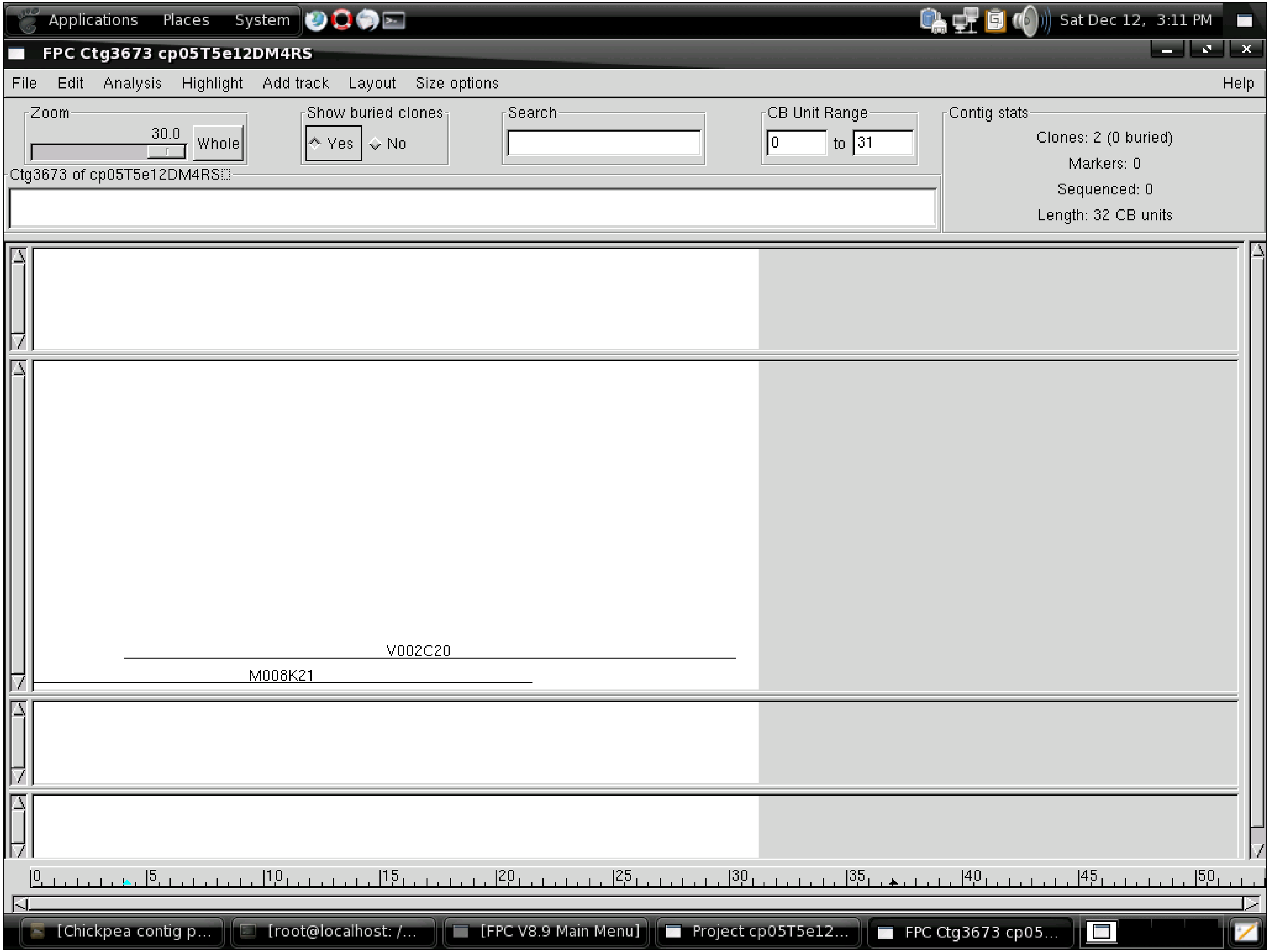
Applications
Places
System
Sat Dec 12, 3:11 PM
FPC Ctg3673 cp05T5e12DM4RS
File
Edit
Analysis
Highlight
Add track
Layout
Size options
Help
Zoom
30.0
Whole
Show buried clones
Yes
No
Search
CB Unit Range
0
to
31
Contig stats
Clones: 2 (0 buried)
Markers: 0
Sequenced: 0
Length: 32 CB units
Ctg3673 of cp05T5e12DM4RS
V002C20
M008K21
0 5 10 15 20 25 30 35 40 45 50
[Chickpea contig p...
[root@localhost: /...
[FPC V8.9 Main Menu]
Project cp05T5e12...
FPC Ctg3673 cp05...

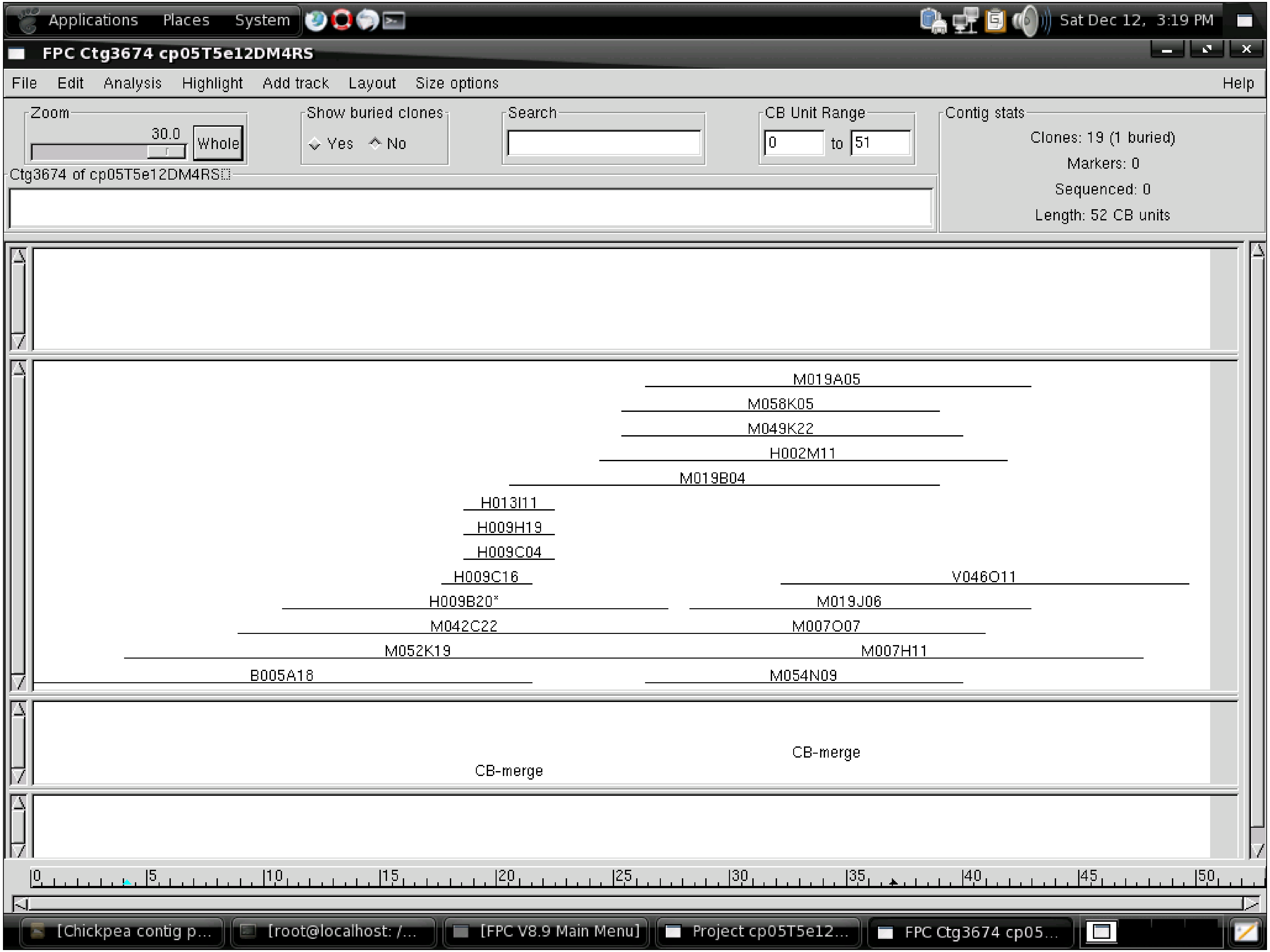
Applications Places System
Sat Dec 12, 3:19 PM
FPC Ctg3674 cp05T5e12DM4RS
File Edit Analysis Highlight Add track Layout Size options Help
Zoom
30.0
Whole
Show buried clones
Yes No
Search
CB Unit Range
0 to 51
Contig stats
Clones: 19 (1 buried)
Markers: 0
Sequenced: 0
Length: 52 CB units
Ctg3674 of cp05T5e12DM4RS
M019A05
M058K05
M049K22
H002M11
M019B04
H013I11
H009H19
H009C04
H009C16
V046O11
H009B20*
M019J06
M042C22
M007O07
M052K19
M007H11
B005A18
M054N09
CB-merge
CB-merge
0 5 10 15 20 25 30 35 40 45 50
[Chickpea contig p...
[root@localhost: /...
[FPC V8.9 Main Menu]
Project cp05T5e12...
FPC Ctg3674 cp05...

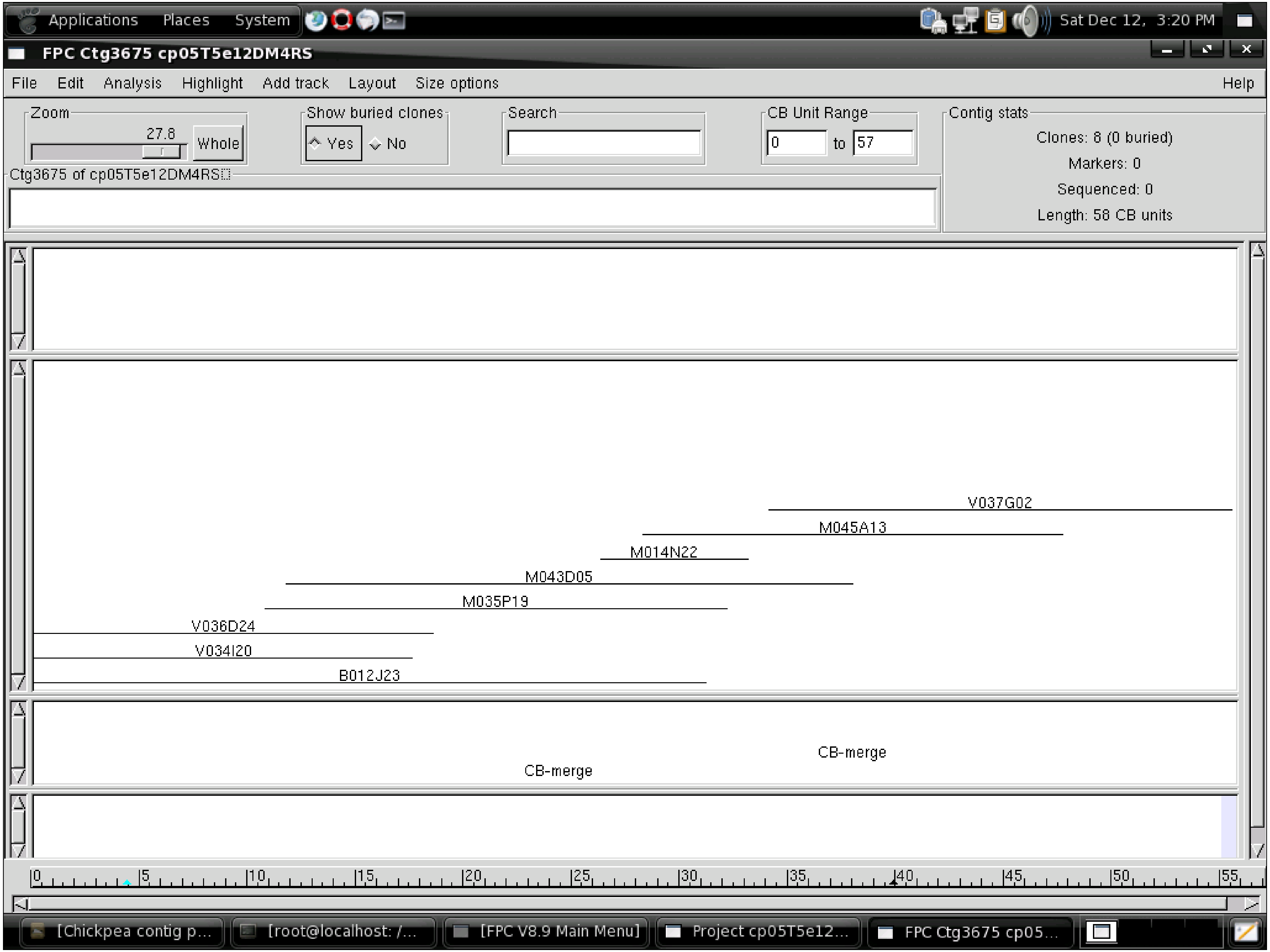
Applications Places System
Sat Dec 12, 3:20 PM
FPC Ctg3675 cp05T5e12DM4RS
File Edit Analysis Highlight Add track Layout Size options Help
Zoom
27.8
Whole
Show buried clones
Yes
No
Search
CB Unit Range
0
to
57
Contig stats
Clones: 8 (0 buried)
Markers: 0
Sequenced: 0
Length: 58 CB units
Ctg3675 of cp05T5e12DM4RS
V037G02
M045A13
M014N22
M043D05
M035P19
V036D24
V034I20
B012J23
CB-merge
CB-merge
0 5 10 15 20 25 30 35 40 45 50 55
[Chickpea contig p...
[root@localhost: /...
[FPC V8.9 Main Menu]
Project cp05T5e12...
FPC Ctg3675 cp05...

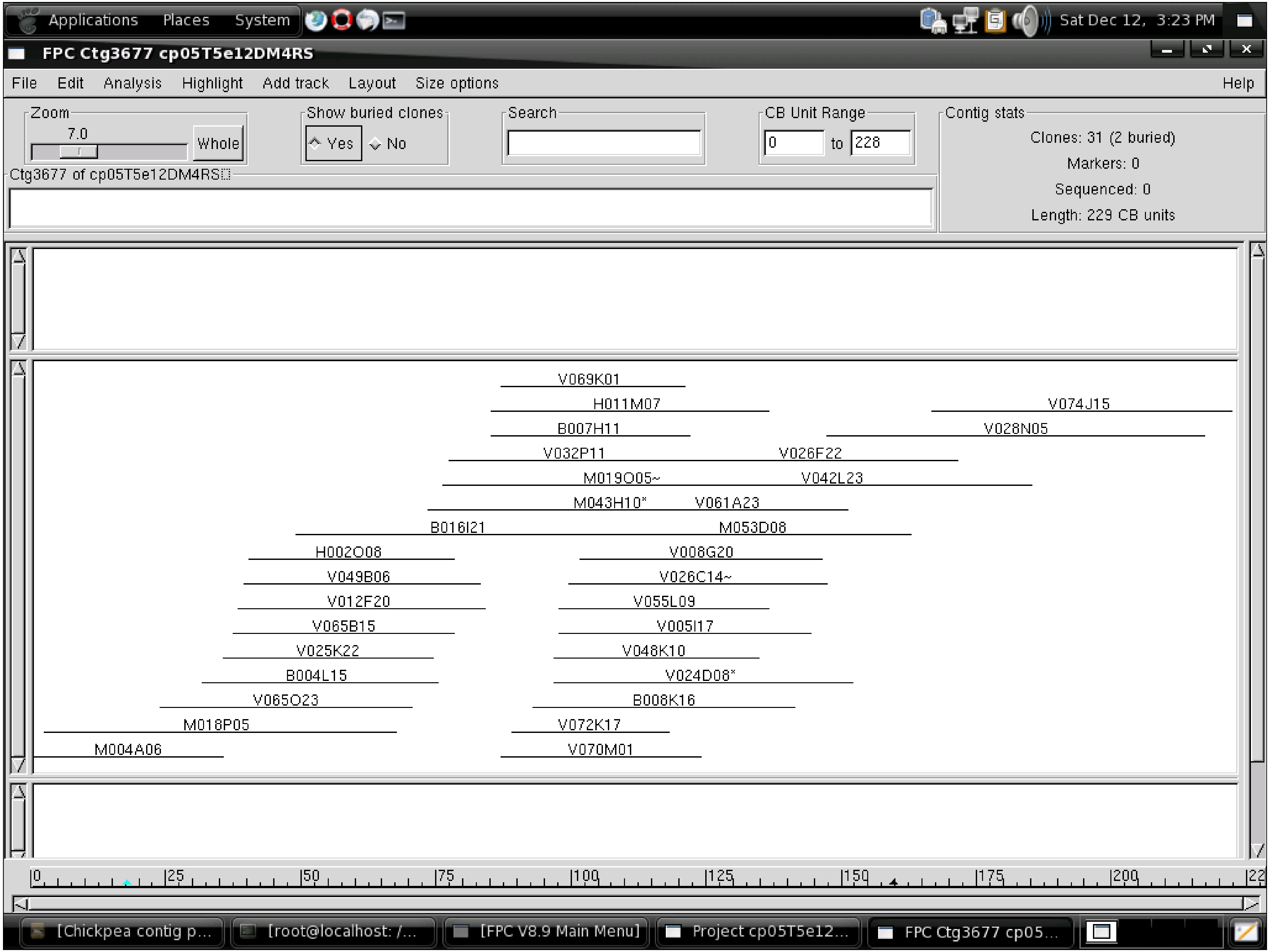

Applications
Places
System
Sat Dec 12, 3:23 PM
FPC Ctg3677 cp05T5e12DM4RS
File
Edit
Analysis
Highlight
Add track
Layout
Size options
Help
Zoom
7.0
Whole
Show buried clones
Yes
No
Search
CB Unit Range
0
to
228
Contig stats
Clones: 31 (2 buried)
Markers: 0
Sequenced: 0
Length: 229 CB units
Ctg3677 of cp05T5e12DM4RS
V069K01
H011M07
V074J15
B007H11
V028N05
V032P11
V026F22
M019O05~
V042L23
M043H10*
V061A23
B016I21
M053D08
H002O08
V008G20
V049B06
V026C14~
V012F20
V055L09
V065B15
V005I17
V025K22
V048K10
B004L15
V024D08*
V065O23
B008K16
M018P05
V072K17
M004A06
V070M01
0
25
50
75
100
125
150
175
200
22
[Chickpea contig p...
[root@localhost: /...
[FPC V8.9 Main Menu]
Project cp05T5e12...
FPC Ctg3677 cp05...

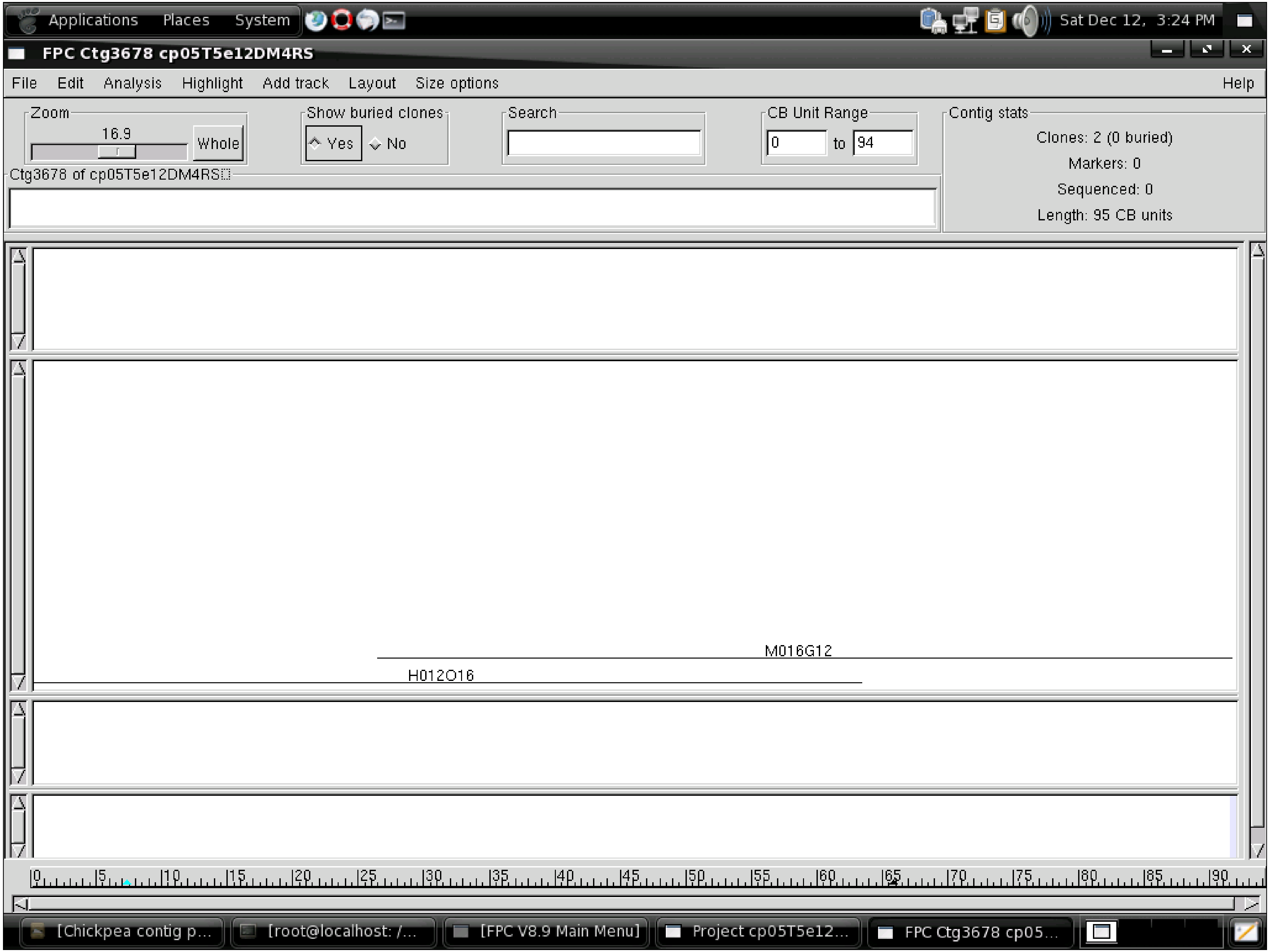
Applications Places System
Sat Dec 12, 3:24 PM
FPC Ctg3678 cp05T5e12DM4RS
File Edit Analysis Highlight Add track Layout Size options Help
Zoom
16.9
Whole
Show buried clones
Yes No
Search
CB Unit Range
0 to 94
Contig stats
Clones: 2 (0 buried)
Markers: 0
Sequenced: 0
Length: 95 CB units
Ctg3678 of cp05T5e12DM4RS
M016G12
H012O16
0 5 10 15 20 25 30 35 40 45 50 55 60 65 70 75 80 85 90
[Chickpea contig p...
[root@localhost: /...
[FPC V8.9 Main Menu]
Project cp05T5e12...
FPC Ctg3678 cp05...

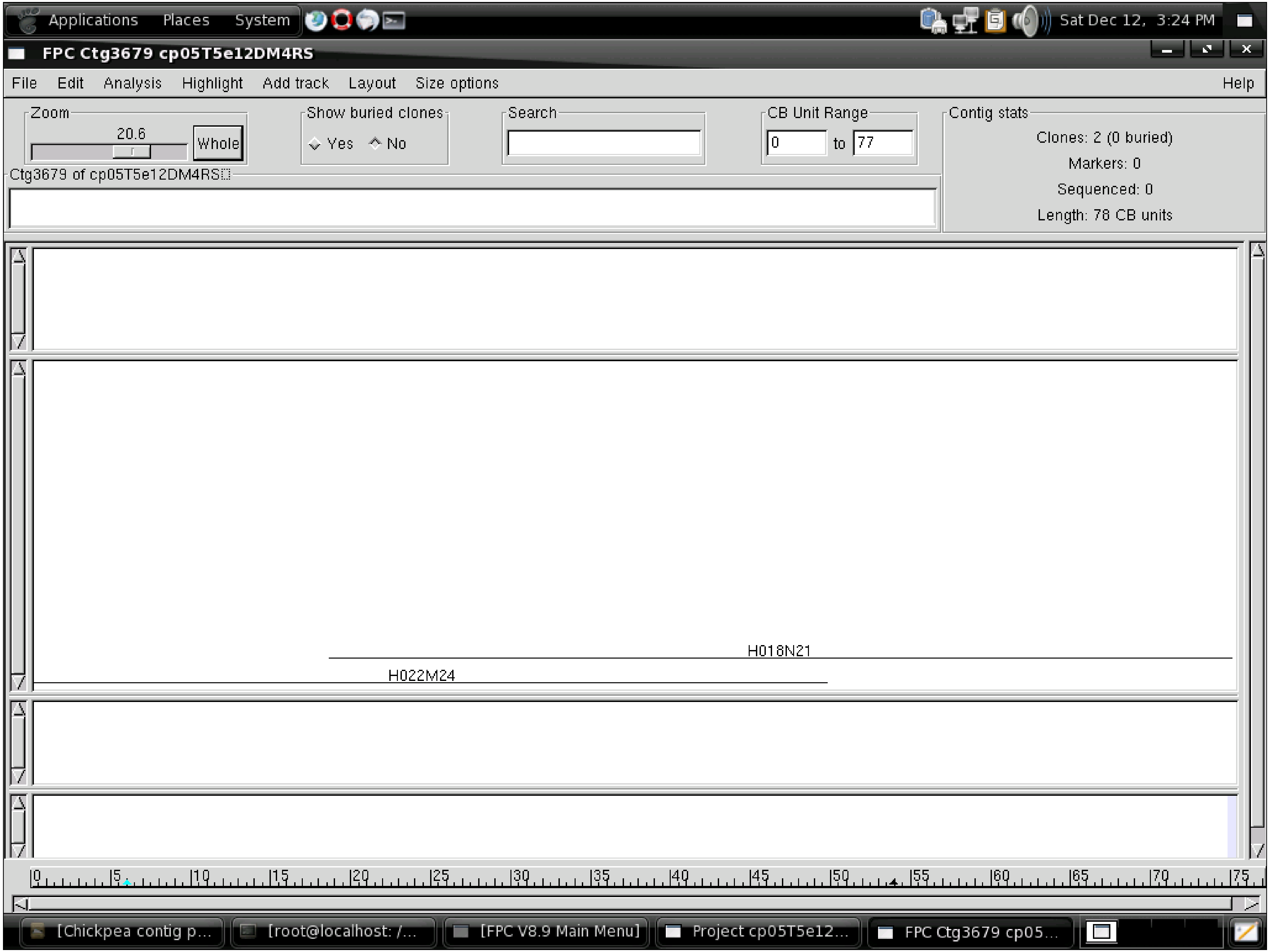
Applications Places System
Sat Dec 12, 3:24 PM
FPC Ctg3679 cp05T5e12DM4RS
File Edit Analysis Highlight Add track Layout Size options Help
Zoom
20.6
Whole
Show buried clones
Yes No
Search
CB Unit Range
0 to 77
Contig stats
Clones: 2 (0 buried)
Markers: 0
Sequenced: 0
Length: 78 CB units
Ctg3679 of cp05T5e12DM4RS
H018N21
H022M24
0 5 10 15 20 25 30 35 40 45 50 55 60 65 70 75
[Chickpea contig p...
[root@localhost: /...
[FPC V8.9 Main Menu]
Project cp05T5e12...
FPC Ctg3679 cp05...

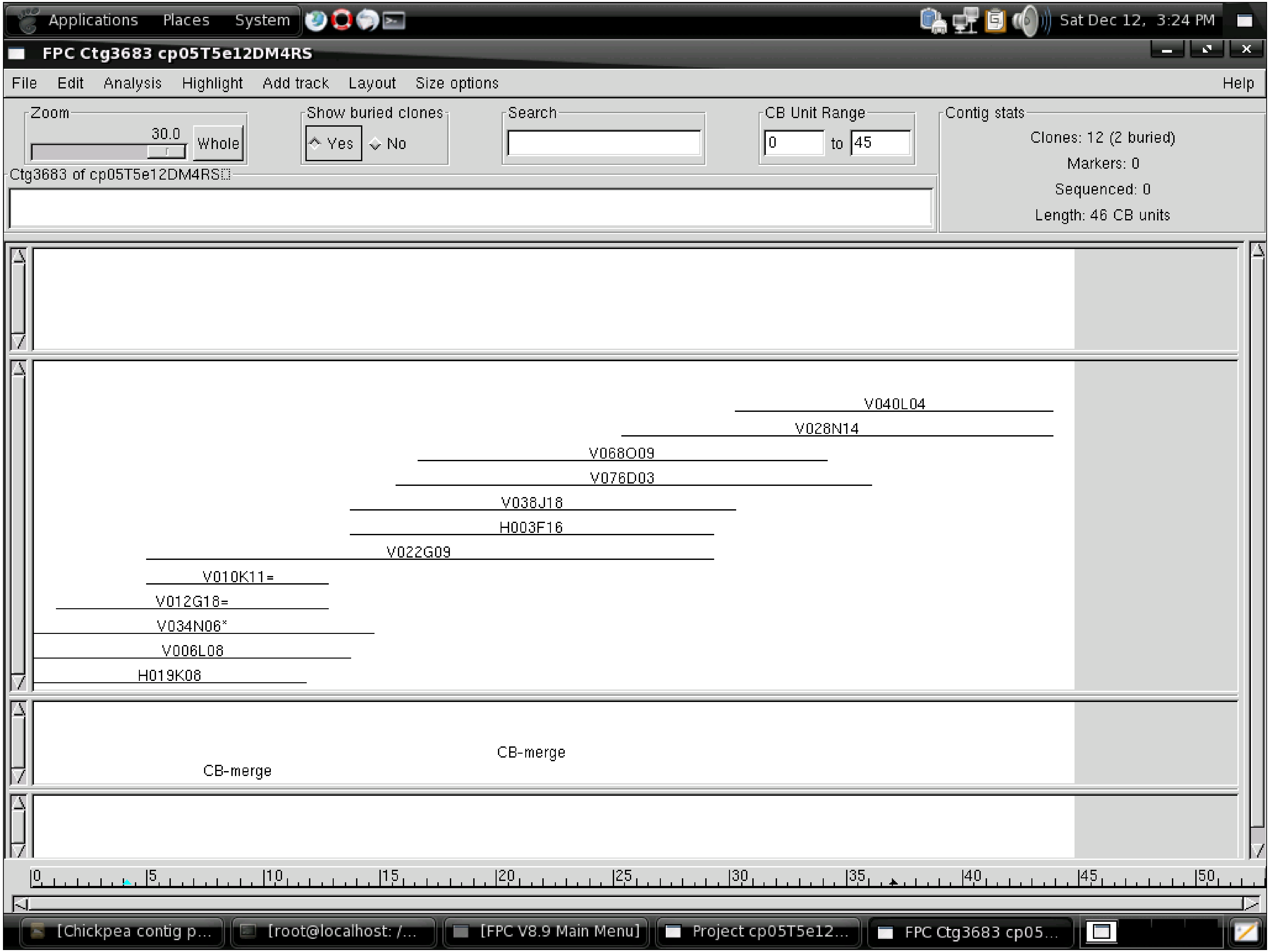
Applications Places System
Sat Dec 12, 3:24 PM
FPC Ctg3683 cp05T5e12DM4RS
File Edit Analysis Highlight Add track Layout Size options Help
Zoom
30.0
Whole
Show buried clones
Yes No
Search
CB Unit Range
0 to 45
Contig stats
Clones: 12 (2 buried)
Markers: 0
Sequenced: 0
Length: 46 CB units
Ctg3683 of cp05T5e12DM4RS
V040L04
V028N14
V068O09
V076D03
V038J18
H003F16
V022G09
V010K11=
V012G18=
V034N06*
V006L08
H019K08
CB-merge
CB-merge
0 5 10 15 20 25 30 35 40 45 50
[Chickpea contig p...
[root@localhost: /...
[FPC V8.9 Main Menu]
Project cp05T5e12...
FPC Ctg3683 cp05...

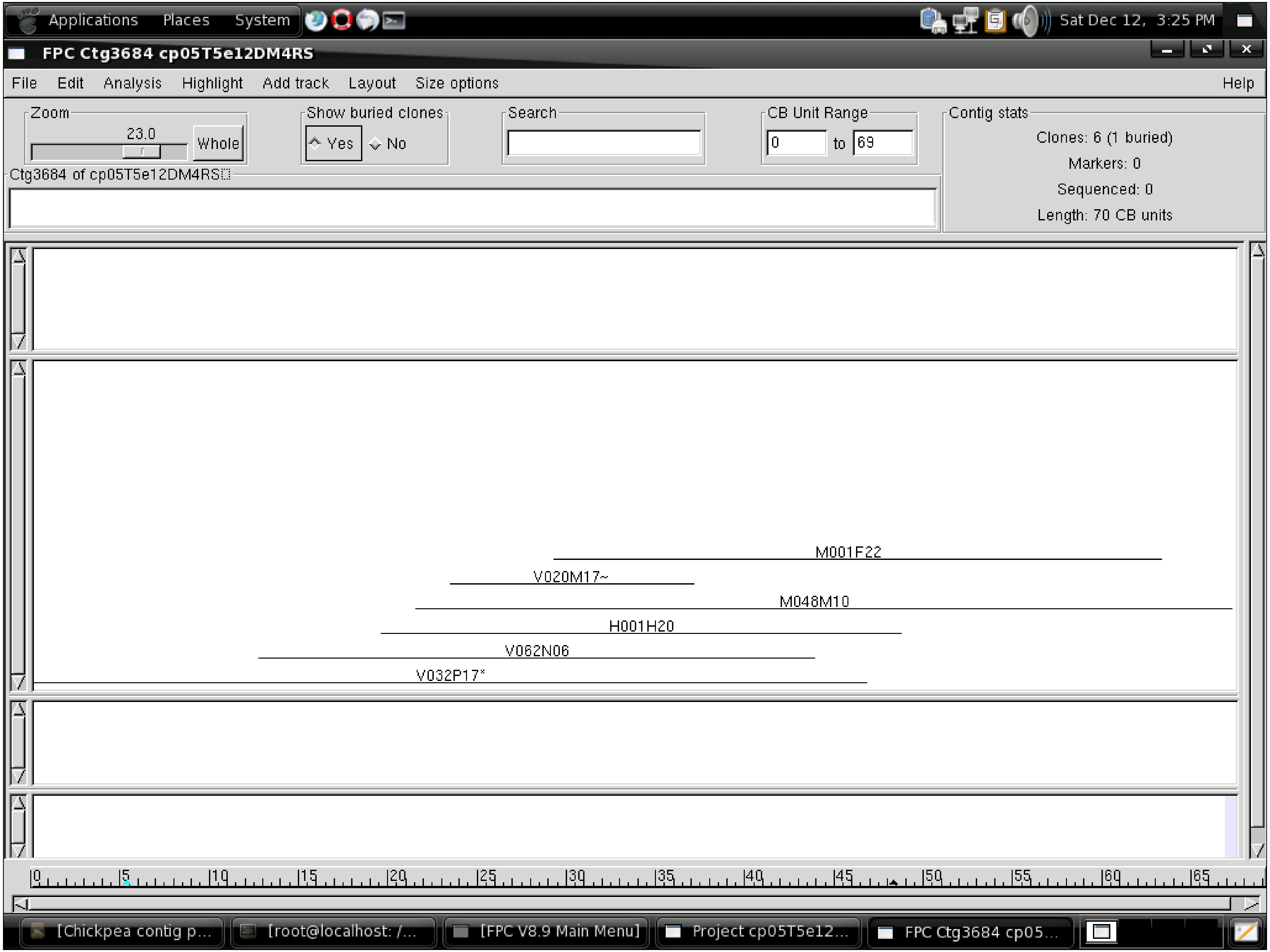
Applications
Places
System
Sat Dec 12, 3:25 PM
FPC Ctg3684 cp05T5e12DM4RS
File
Edit
Analysis
Highlight
Add track
Layout
Size options
Help
Zoom
23.0
Whole
Show buried clones
Yes
No
Search
CB Unit Range
0
to
69
Contig stats
Clones: 6 (1 buried)
Markers: 0
Sequenced: 0
Length: 70 CB units
Ctg3684 of cp05T5e12DM4RS
M001F22
V020M17~
M048M10
H001H20
V062N06
V032P17*
0 5 10 15 20 25 30 35 40 45 50 55 60 65
[Chickpea contig p...
[root@localhost: /...
[FPC V8.9 Main Menu]
Project cp05T5e12...
FPC Ctg3684 cp05...

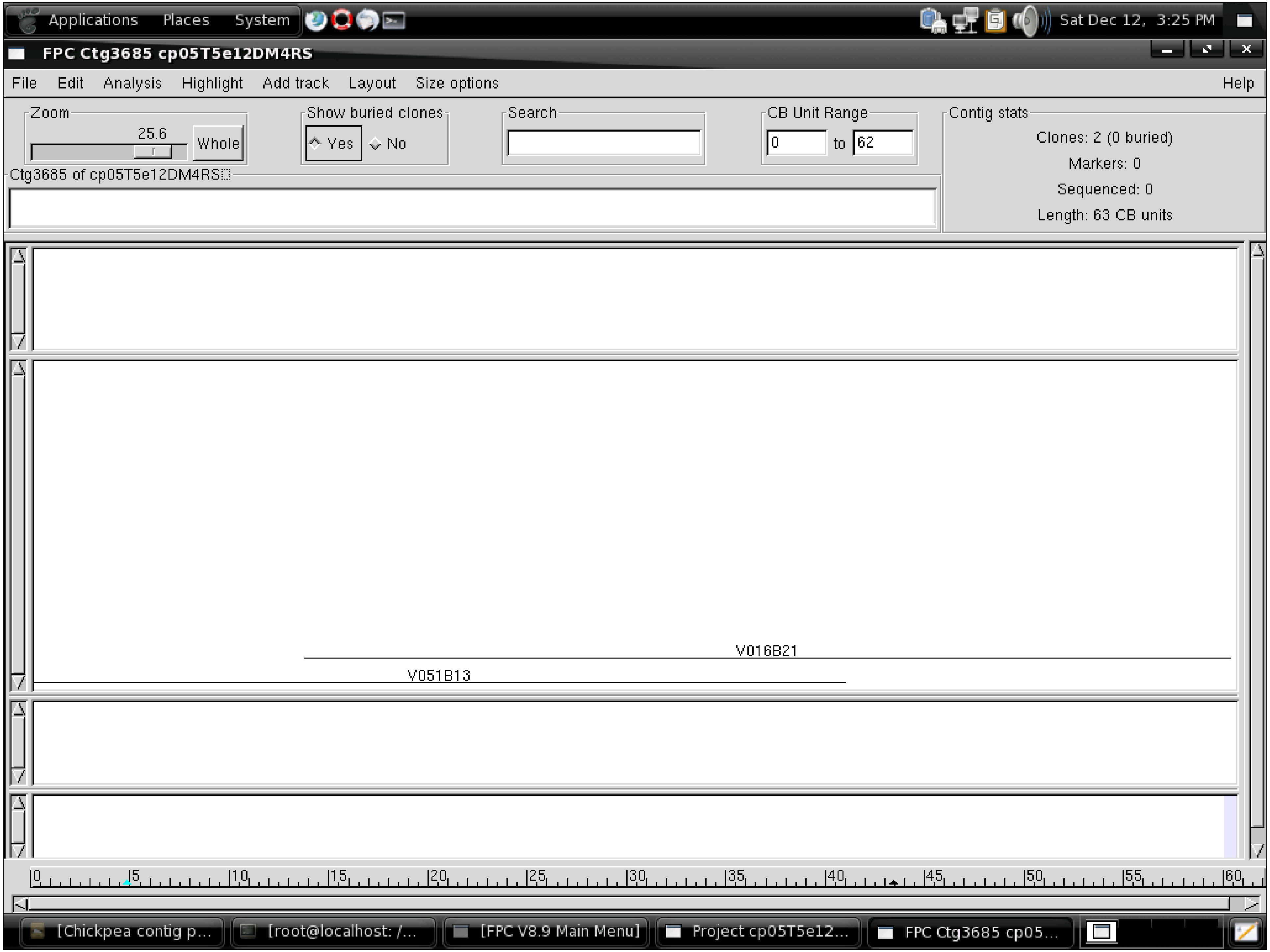
Applications
Places
System
Sat Dec 12, 3:25 PM
FPC Ctg3685 cp05T5e12DM4RS
File
Edit
Analysis
Highlight
Add track
Layout
Size options
Help
Zoom
25.6
Whole
Show buried clones
Yes
No
Search
CB Unit Range
0
to
62
Contig stats
Clones: 2 (0 buried)
Markers: 0
Sequenced: 0
Length: 63 CB units
Ctg3685 of cp05T5e12DM4RS
V016B21
V051B13
0
5
10
15
20
25
30
35
40
45
50
55
60
[Chickpea contig p...
[root@localhost: /...
[FPC V8.9 Main Menu]
Project cp05T5e12...
FPC Ctg3685 cp05...

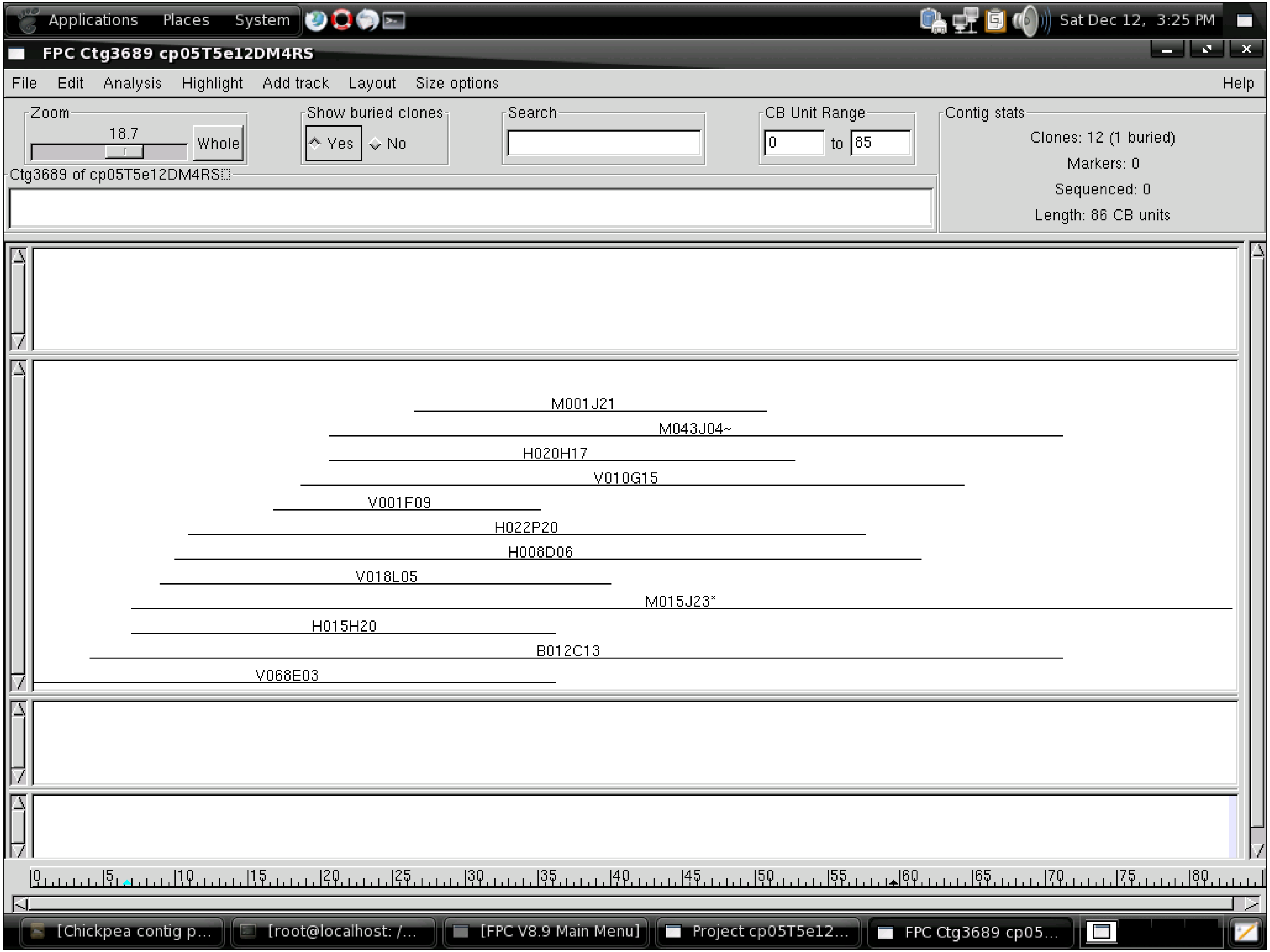
Applications Places System
Sat Dec 12, 3:25 PM
FPC Ctg3689 cp05T5e12DM4RS
File Edit Analysis Highlight Add track Layout Size options Help
Zoom
18.7
Whole
Show buried clones
Yes No
Search
CB Unit Range
0 to 85
Contig stats
Clones: 12 (1 buried)
Markers: 0
Sequenced: 0
Length: 86 CB units
Ctg3689 of cp05T5e12DM4RS
M001J21
M043J04~
H020H17
V010G15
V001F09
H022P20
H008D06
V018L05
M015J23*
H015H20
B012C13
V068E03
0 5 10 15 20 25 30 35 40 45 50 55 60 65 70 75 80
[Chickpea contig p...
[root@localhost: /...
[FPC V8.9 Main Menu]
Project cp05T5e12...
FPC Ctg3689 cp05...

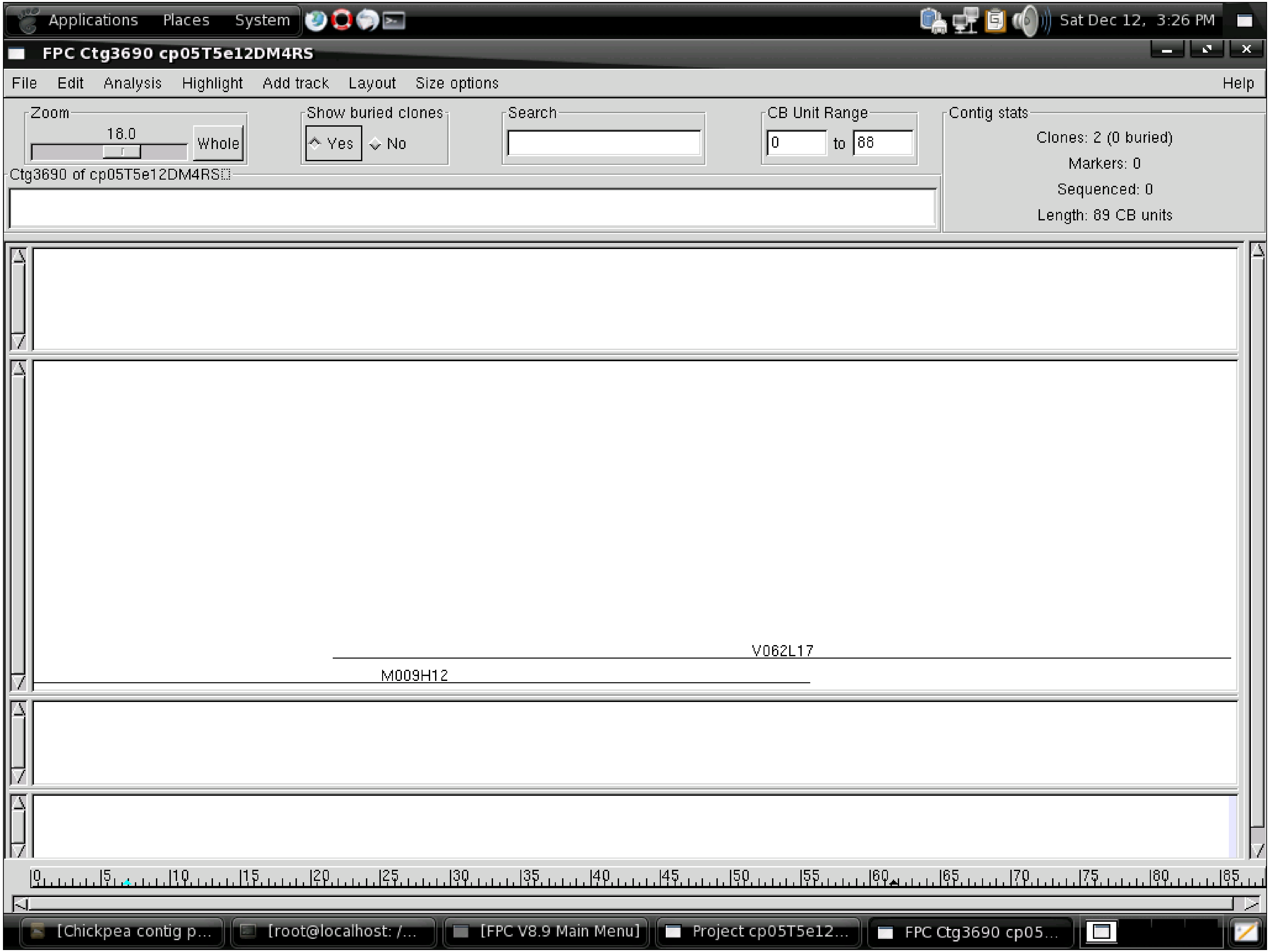
Applications Places System
Sat Dec 12, 3:26 PM
FPC Ctg3690 cp05T5e12DM4RS
File Edit Analysis Highlight Add track Layout Size options
Help
Zoom
18.0
Whole
Show buried clones
Yes
No
Search
CB Unit Range
0
to
88
Contig stats
Clones: 2 (0 buried)
Markers: 0
Sequenced: 0
Length: 89 CB units
Ctg3690 of cp05T5e12DM4RS
V062L17
M009H12
0 5 10 15 20 25 30 35 40 45 50 55 60 65 70 75 80 85
[Chickpea contig p...
[root@localhost: /...
[FPC V8.9 Main Menu]
Project cp05T5e12...
FPC Ctg3690 cp05...

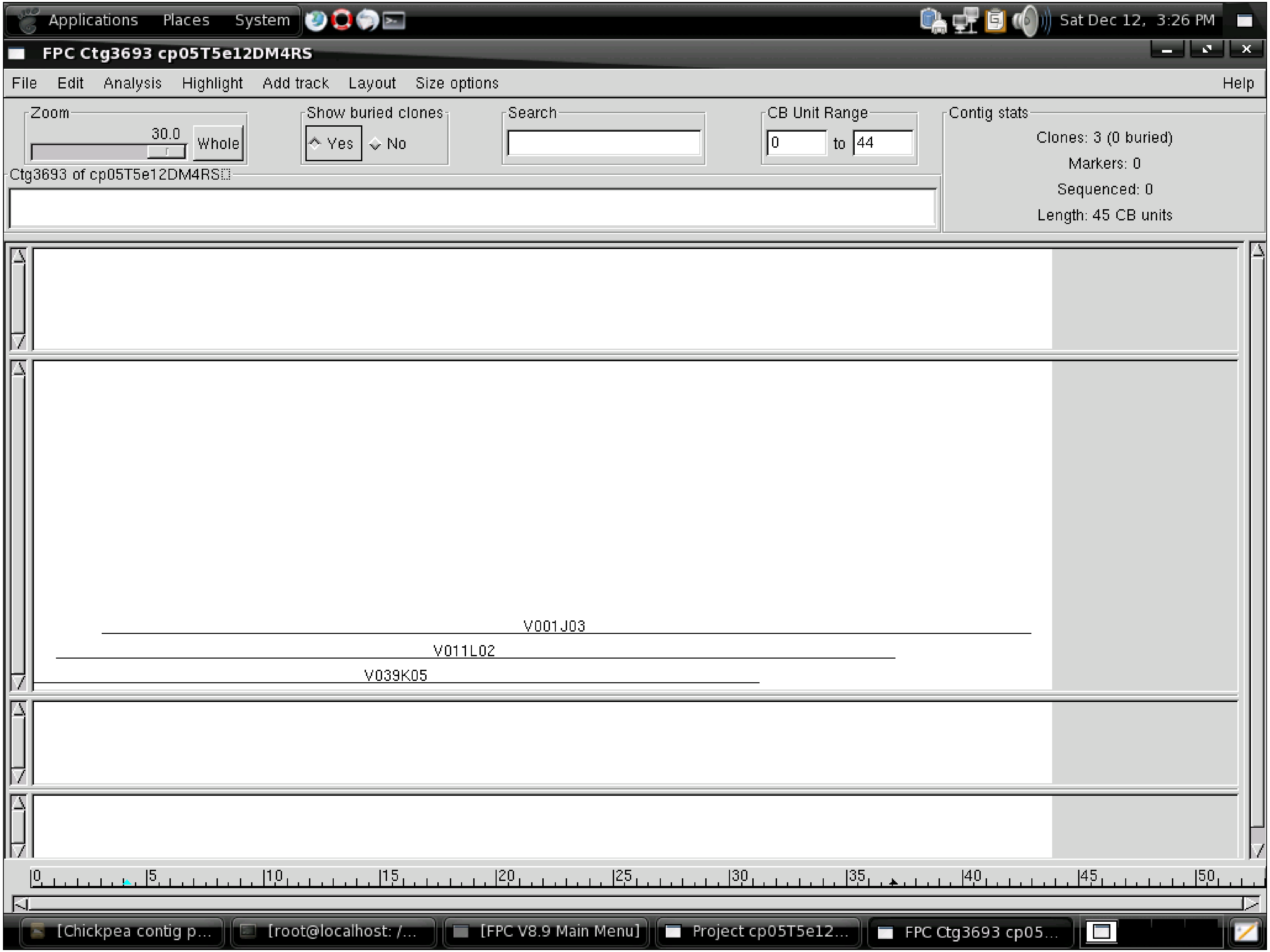
Applications Places System
Sat Dec 12, 3:26 PM
FPC Ctg3693 cp05T5e12DM4RS
File Edit Analysis Highlight Add track Layout Size options Help
Zoom
30.0
Whole
Show buried clones
Yes No
Search
CB Unit Range
0 to 44
Contig stats
Clones: 3 (0 buried)
Markers: 0
Sequenced: 0
Length: 45 CB units
Ctg3693 of cp05T5e12DM4RS
V001J03
V011L02
V039K05
0 5 10 15 20 25 30 35 40 45 50
[Chickpea contig p...
[root@localhost: /...
[FPC V8.9 Main Menu]
Project cp05T5e12...
FPC Ctg3693 cp05...

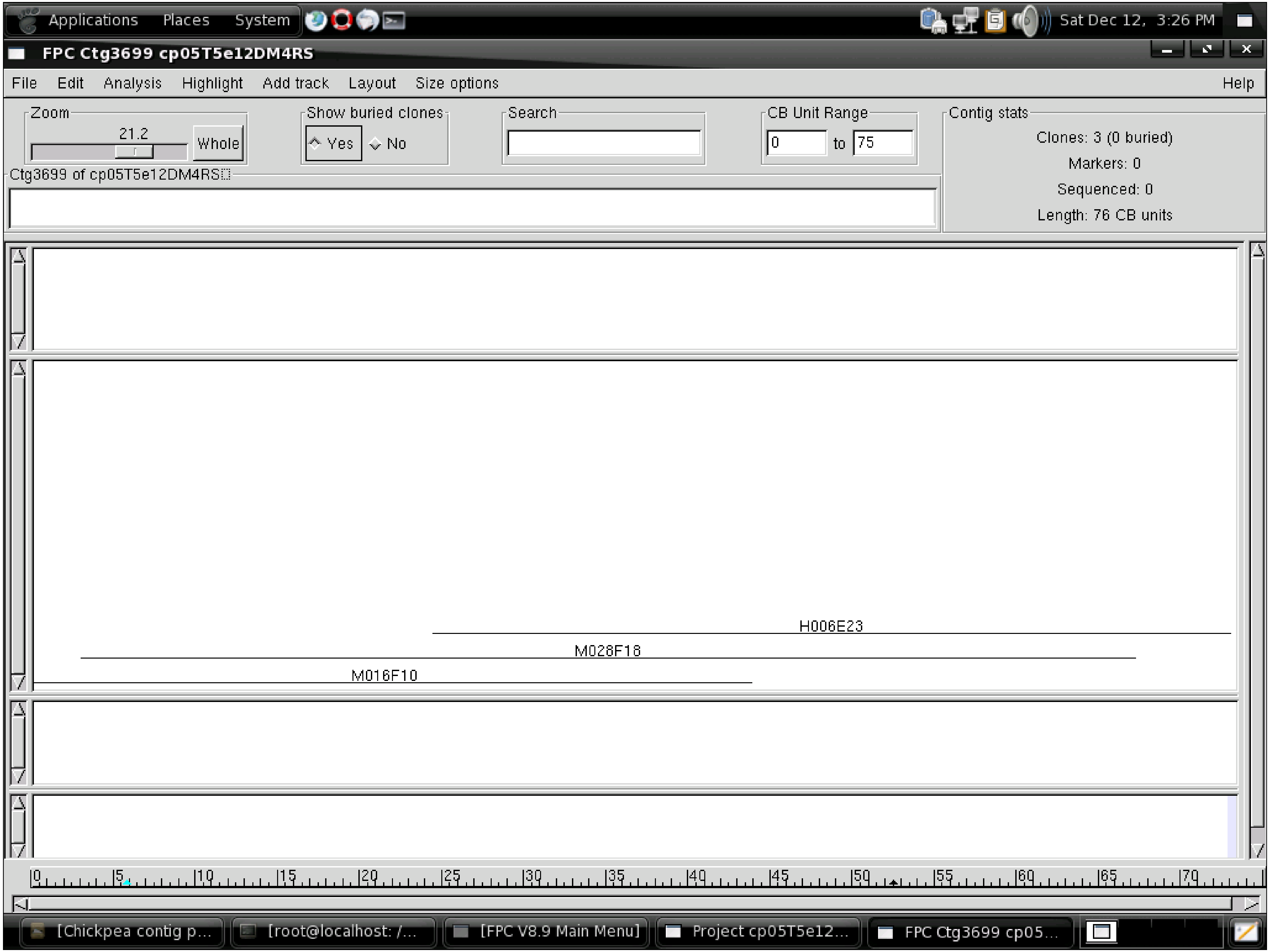
Applications Places System
Sat Dec 12, 3:26 PM
FPC Ctg3699 cp05T5e12DM4RS
File Edit Analysis Highlight Add track Layout Size options Help
Zoom
21.2
Whole
Show buried clones
Yes
No
Search
CB Unit Range
0 to 75
Contig stats
Clones: 3 (0 buried)
Markers: 0
Sequenced: 0
Length: 76 CB units
Ctg3699 of cp05T5e12DM4RS
H006E23
M028F18
M016F10
0 5 10 15 20 25 30 35 40 45 50 55 60 65 70
[Chickpea contig p...
[root@localhost: /...
[FPC V8.9 Main Menu]
Project cp05T5e12...
FPC Ctg3699 cp05...

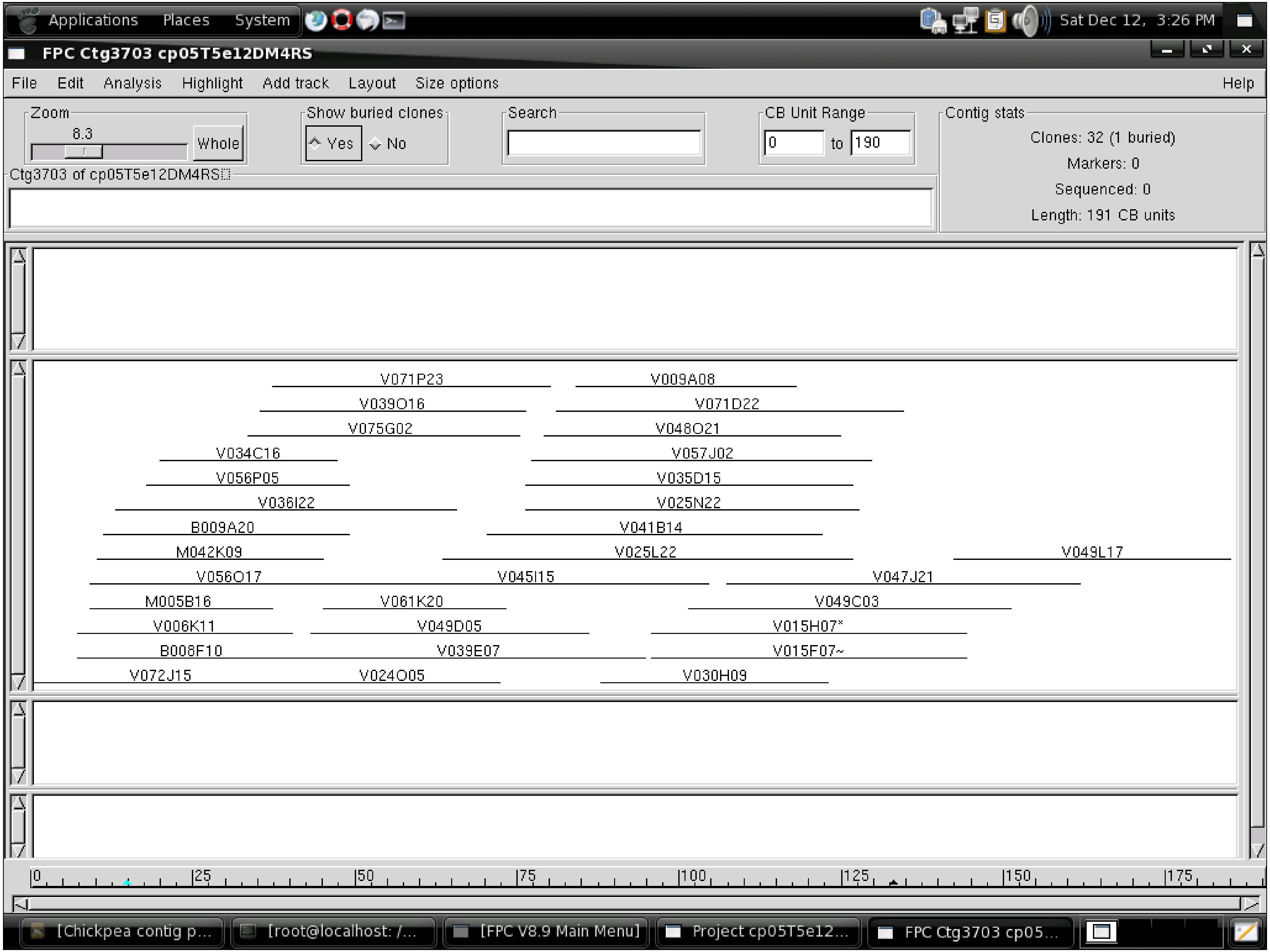

Applications
Places
System
Sat Dec 12, 3:26 PM
FPC Ctg3703 cp05T5e12DM4RS
File
Edit
Analysis
Highlight
Add track
Layout
Size options
Help
Zoom
8.3
Whole
Show buried clones
Yes
No
Search
CB Unit Range
0
to
190
Contig stats
Clones: 32 (1 buried)
Markers: 0
Sequenced: 0
Length: 191 CB units
Ctg3703 of cp05T5e12DM4RS
V071P23
V009A08
V039O16
V071D22
V075G02
V048O21
V034C16
V057J02
V056P05
V035D15
V036I22
V025N22
B009A20
V041B14
M042K09
V025L22
V049L17
V056O17
V045I15
V047J21
M005B16
V061K20
V049C03
V006K11
V049D05
V015H07*
B008F10
V039E07
V015F07~
V072J15
V024O05
V030H09
0
25
50
75
100
125
150
175
[Chickpea contig p...
[root@localhost: /...
[FPC V8.9 Main Menu]
Project cp05T5e12...
FPC Ctg3703 cp05...

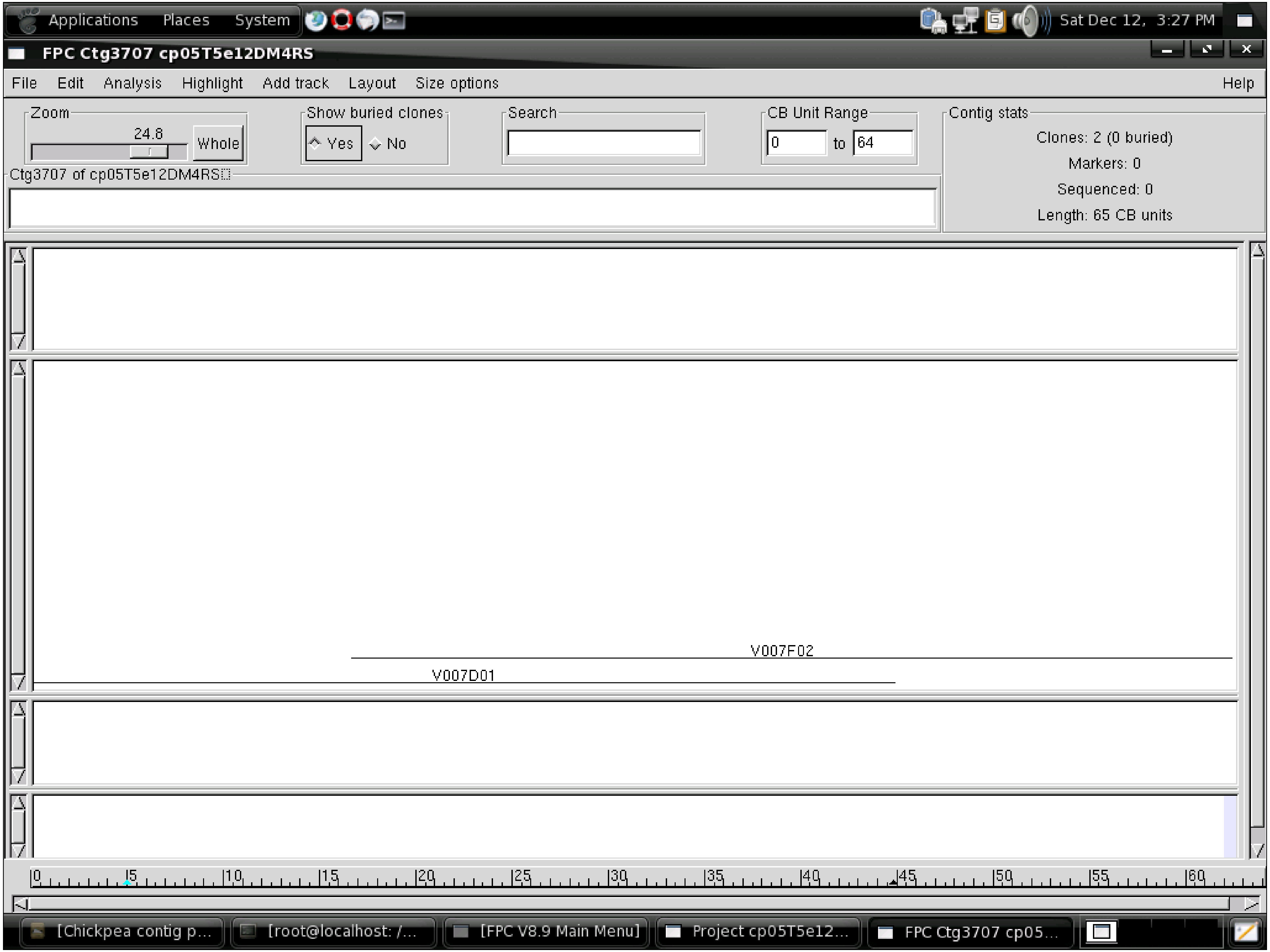
Applications
Places
System
Sat Dec 12, 3:27 PM
FPC Ctg3707 cp05T5e12DM4RS
File
Edit
Analysis
Highlight
Add track
Layout
Size options
Help
Zoom
24.8
Whole
Show buried clones
Yes
No
Search
CB Unit Range
0
to
64
Contig stats
Clones: 2 (0 buried)
Markers: 0
Sequenced: 0
Length: 65 CB units
Ctg3707 of cp05T5e12DM4RS
V007F02
V007D01
0 5 10 15 20 25 30 35 40 45 50 55 60
[Chickpea contig p...
[root@localhost: /...
[FPC V8.9 Main Menu]
Project cp05T5e12...
FPC Ctg3707 cp05...

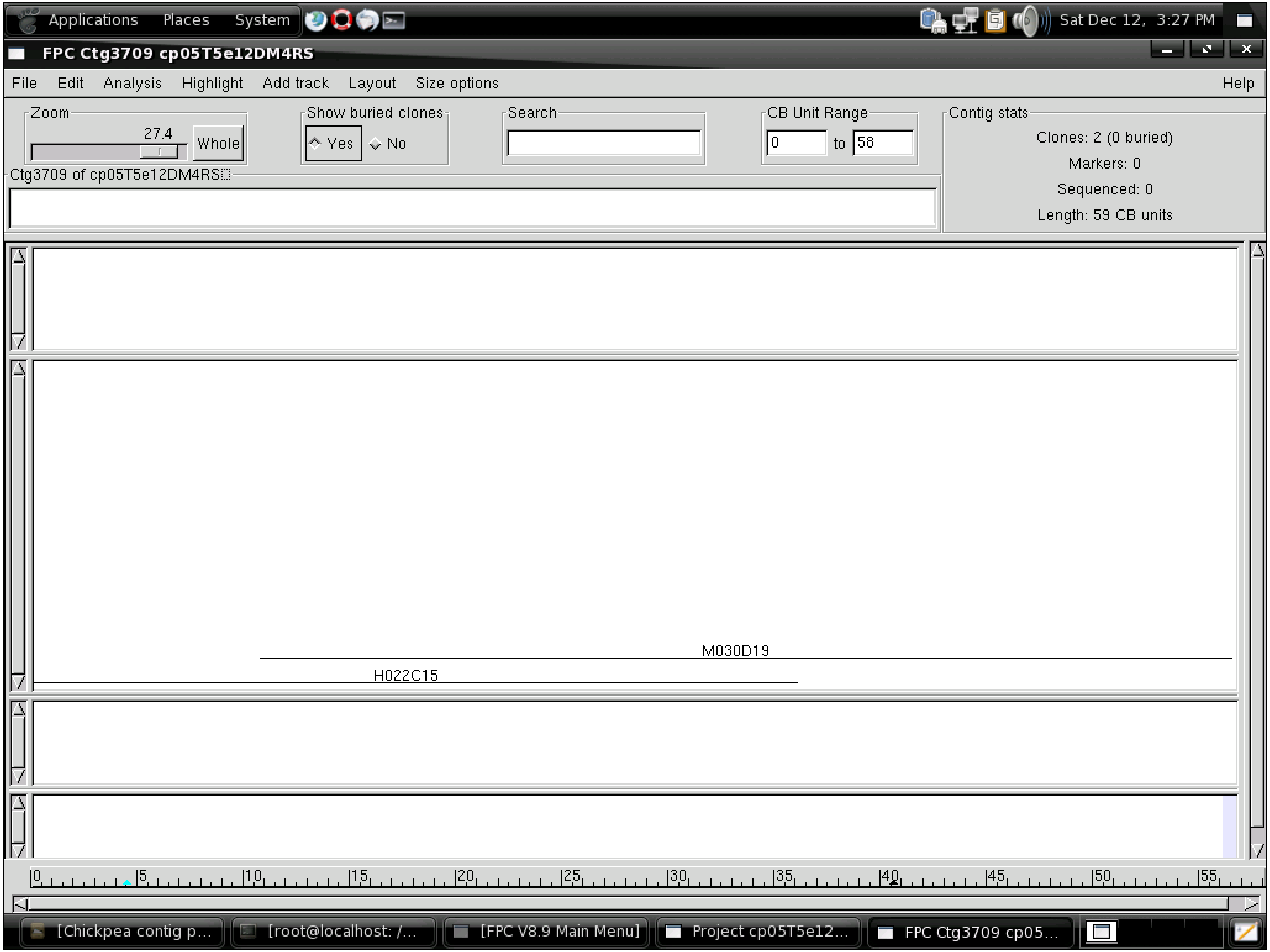
Applications Places System
Sat Dec 12, 3:27 PM
FPC Ctg3709 cp05T5e12DM4RS
File Edit Analysis Highlight Add track Layout Size options Help
Zoom
27.4
Whole
Show buried clones
Yes No
Search
CB Unit Range
0 to 58
Contig stats
Clones: 2 (0 buried)
Markers: 0
Sequenced: 0
Length: 59 CB units
Ctg3709 of cp05T5e12DM4RS
M030D19
H022C15
0 5 10 15 20 25 30 35 40 45 50 55
[Chickpea contig p...
[root@localhost: /...
[FPC V8.9 Main Menu]
Project cp05T5e12...
FPC Ctg3709 cp05...

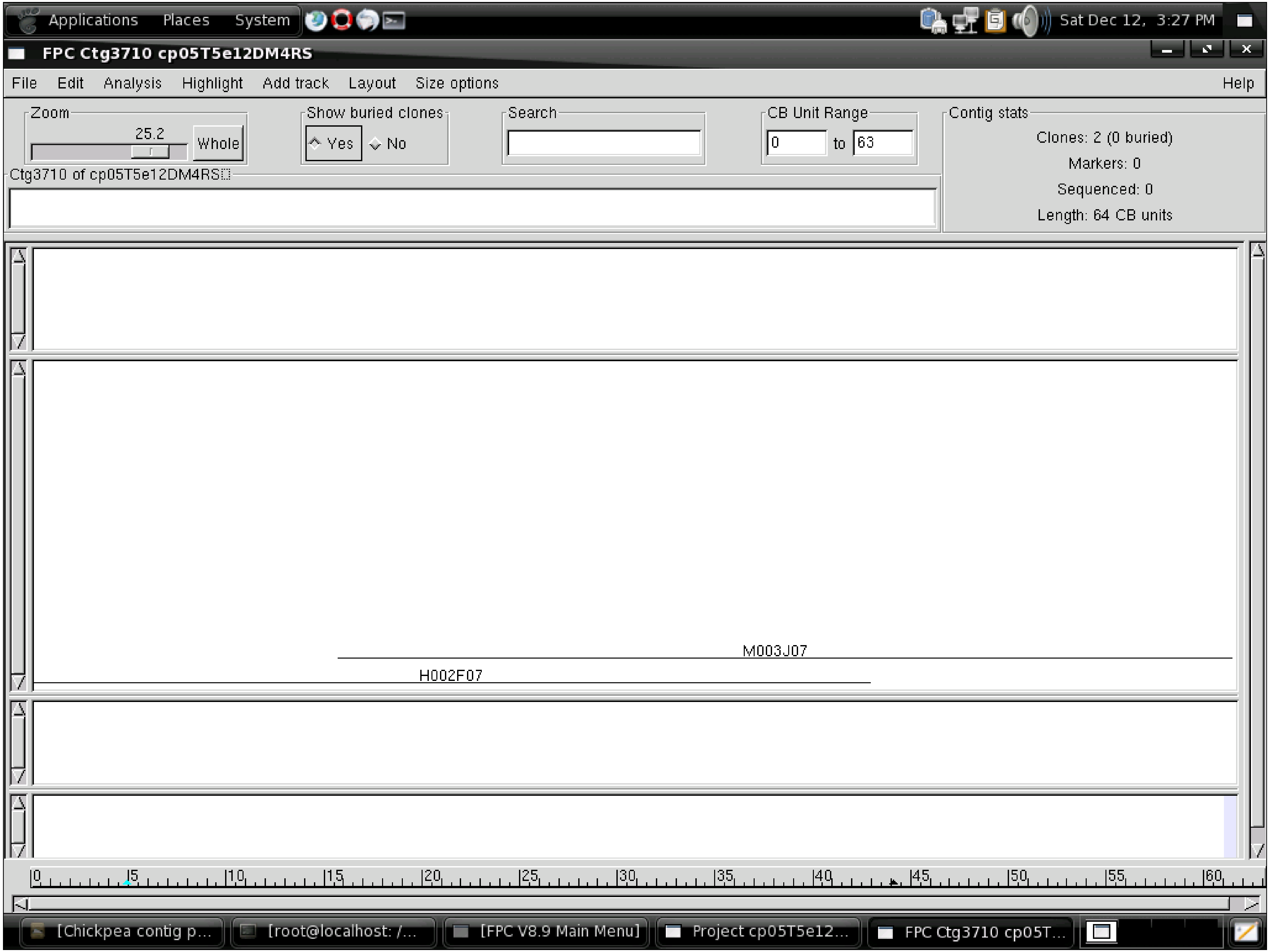
Applications
Places
System
Sat Dec 12, 3:27 PM
FPC Ctg3710 cp05T5e12DM4RS
File
Edit
Analysis
Highlight
Add track
Layout
Size options
Help
Zoom
25.2
Whole
Show buried clones
Yes
No
Search
CB Unit Range
0
to
63
Contig stats
Clones: 2 (0 buried)
Markers: 0
Sequenced: 0
Length: 64 CB units
Ctg3710 of cp05T5e12DM4RS
M003J07
H002F07
0
5
10
15
20
25
30
35
40
45
50
55
60
[Chickpea contig p...
[root@localhost: /...
[FPC V8.9 Main Menu]
Project cp05T5e12...
FPC Ctg3710 cp05T...

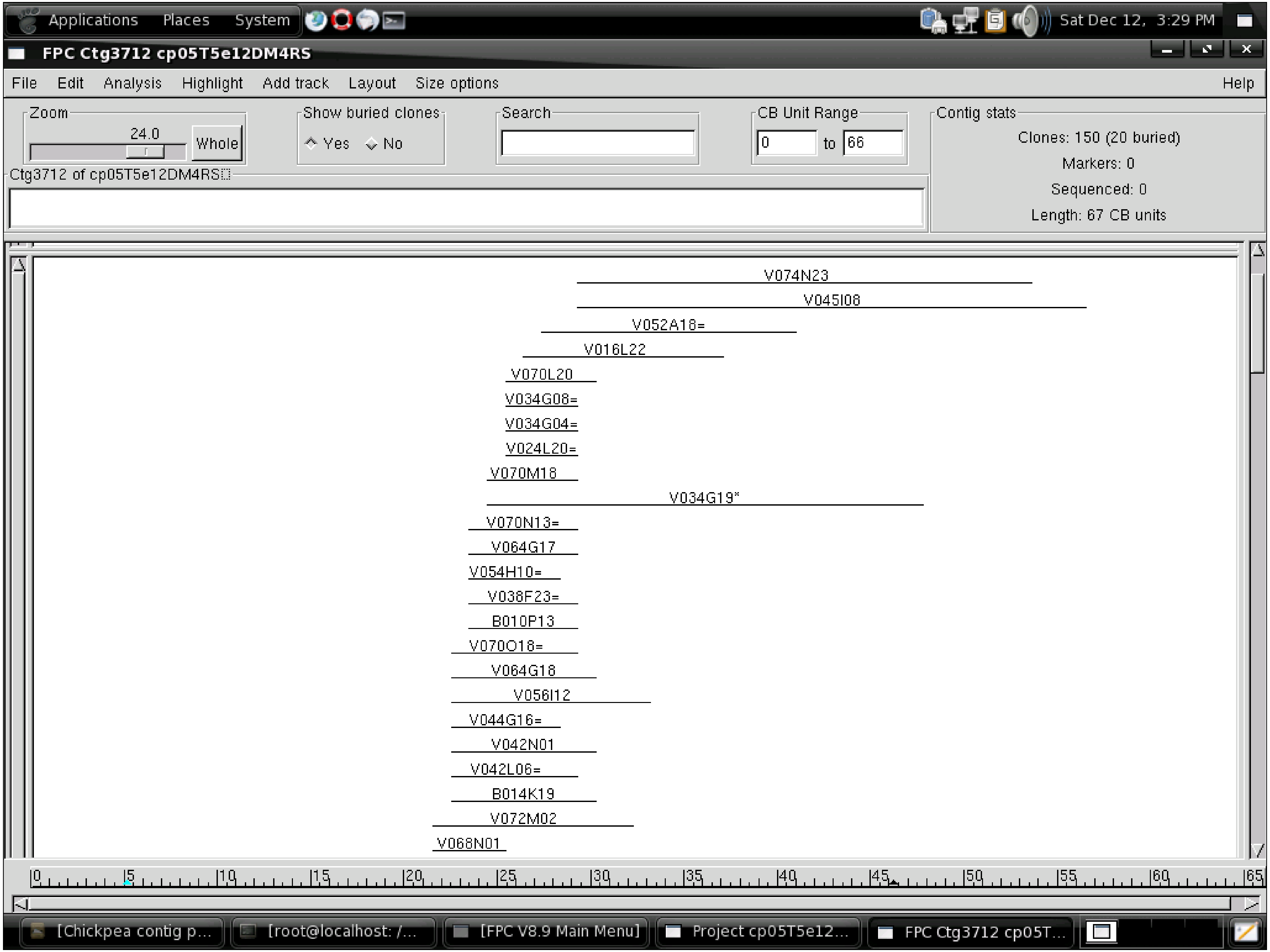
Applications Places System
Sat Dec 12, 3:29 PM
FPC Ctg3712 cp05T5e12DM4RS
File Edit Analysis Highlight Add track Layout Size options Help
Zoom
24.0
Whole
Show buried clones
Yes No
Search
CB Unit Range
0 to 66
Contig stats
Clones: 150 (20 buried)
Markers: 0
Sequenced: 0
Length: 67 CB units
Ctg3712 of cp05T5e12DM4RS
V074N23
V045I08
V052A18=
V016L22
V070L20
V034G08=
V034G04=
V024L20=
V070M18
V034G19*
V070N13=
V064G17
V054H10=
V038F23=
B010P13
V070O18=
V064G18
V056I12
V044G16=
V042N01
V042L06=
B014K19
V072M02
V068N01
0 5 10 15 20 25 30 35 40 45 50 55 60 65
[Chickpea contig p...
[root@localhost: /...
[FPC V8.9 Main Menu]
Project cp05T5e12...
FPC Ctg3712 cp05T...

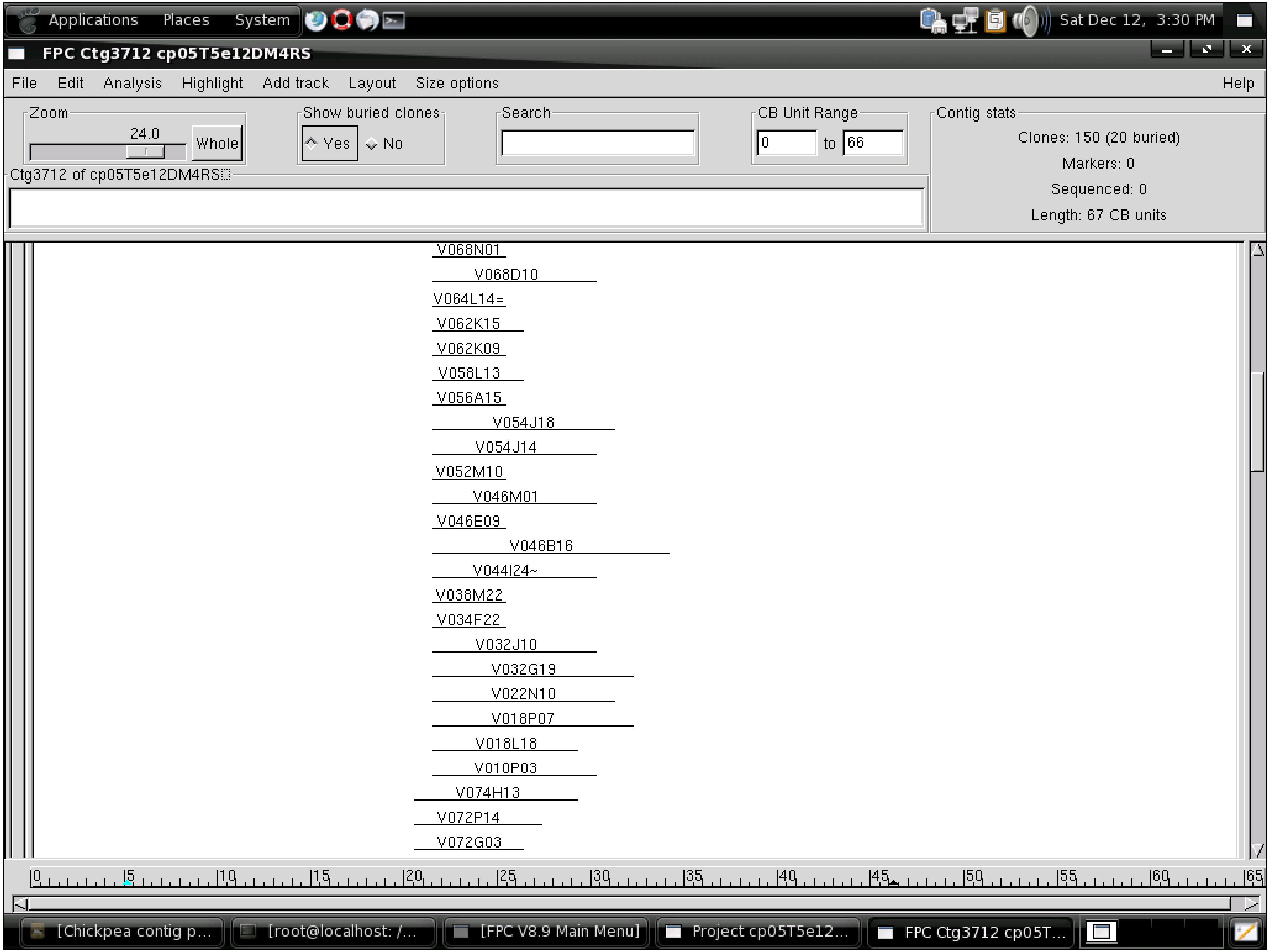
Applications Places System
Sat Dec 12, 3:30 PM
FPC Ctg3712 cp05T5e12DM4RS
File Edit Analysis Highlight Add track Layout Size options Help
Zoom
24.0
Whole
Show buried clones
Yes No
Search
CB Unit Range
0 to 66
Contig stats
Clones: 150 (20 buried)
Markers: 0
Sequenced: 0
Length: 67 CB units
Ctg3712 of cp05T5e12DM4RS
V068N01
V068D10
V064L14=
V062K15
V062K09
V058L13
V056A15
V054J18
V054J14
V052M10
V046M01
V046E09
V046B16
V044I24~
V038M22
V034F22
V032J10
V032G19
V022N10
V018P07
V018L18
V010P03
V074H13
V072P14
V072G03
0 5 10 15 20 25 30 35 40 45 50 55 60 65
[Chickpea contig p...
[root@localhost: /...
[FPC V8.9 Main Menu]
Project cp05T5e12...
FPC Ctg3712 cp05T...

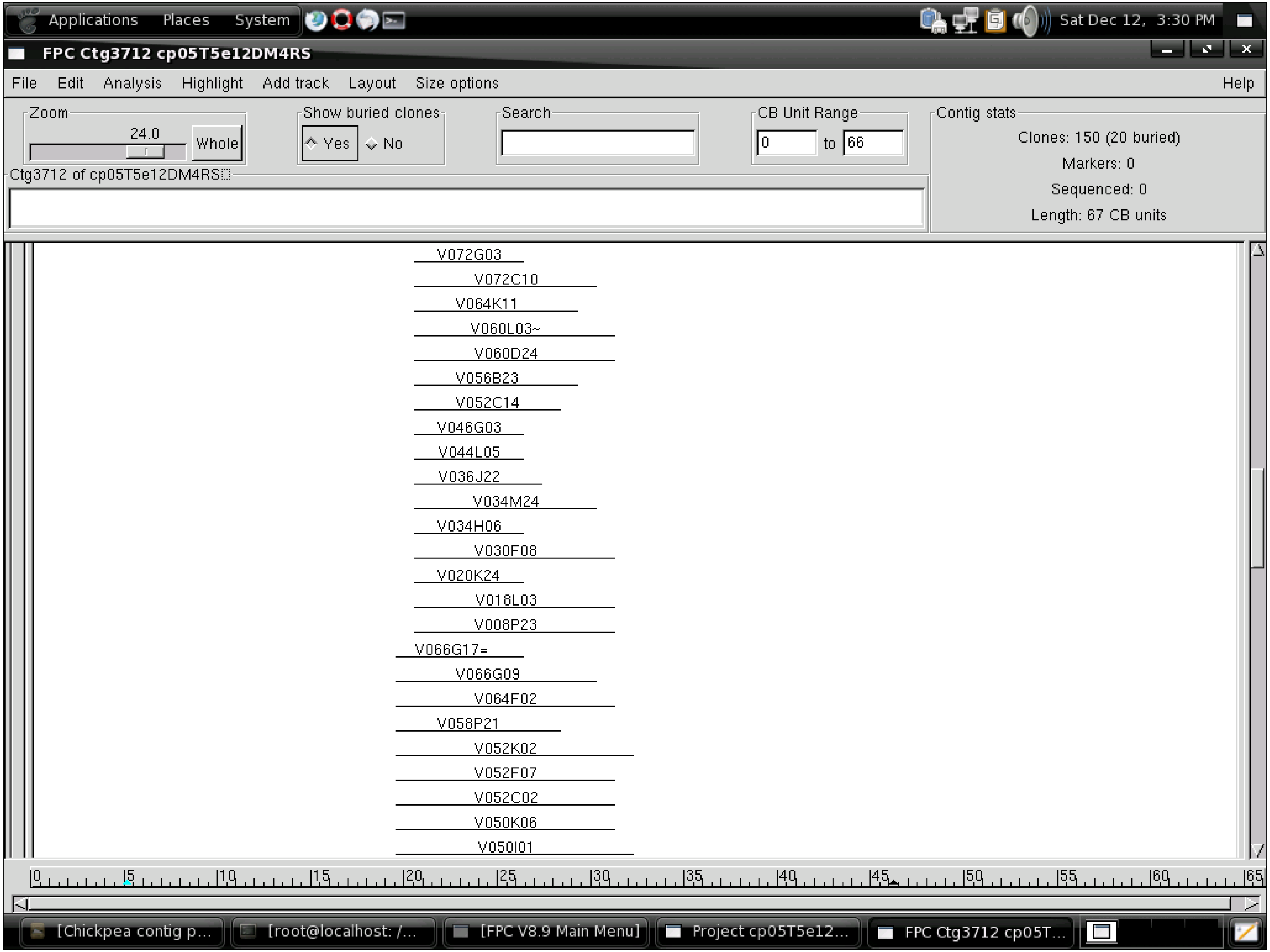
Applications Places System
Sat Dec 12, 3:30 PM
FPC Ctg3712 cp05T5e12DM4RS
File Edit Analysis Highlight Add track Layout Size options Help
Zoom
24.0
Whole
Show buried clones
Yes
No
Search
CB Unit Range
0
to
66
Contig stats
Clones: 150 (20 buried)
Markers: 0
Sequenced: 0
Length: 67 CB units
Ctg3712 of cp05T5e12DM4RS
V072G03
V072C10
V064K11
V060L03~
V060D24
V056B23
V052C14
V046G03
V044L05
V036J22
V034M24
V034H06
V030F08
V020K24
V018L03
V008P23
V066G17=
V066G09
V064F02
V058P21
V052K02
V052F07
V052C02
V050K06
V050I01
0 5 10 15 20 25 30 35 40 45 50 55 60 65
[Chickpea contig p...
[root@localhost: /...
[FPC V8.9 Main Menu]
Project cp05T5e12...
FPC Ctg3712 cp05T...

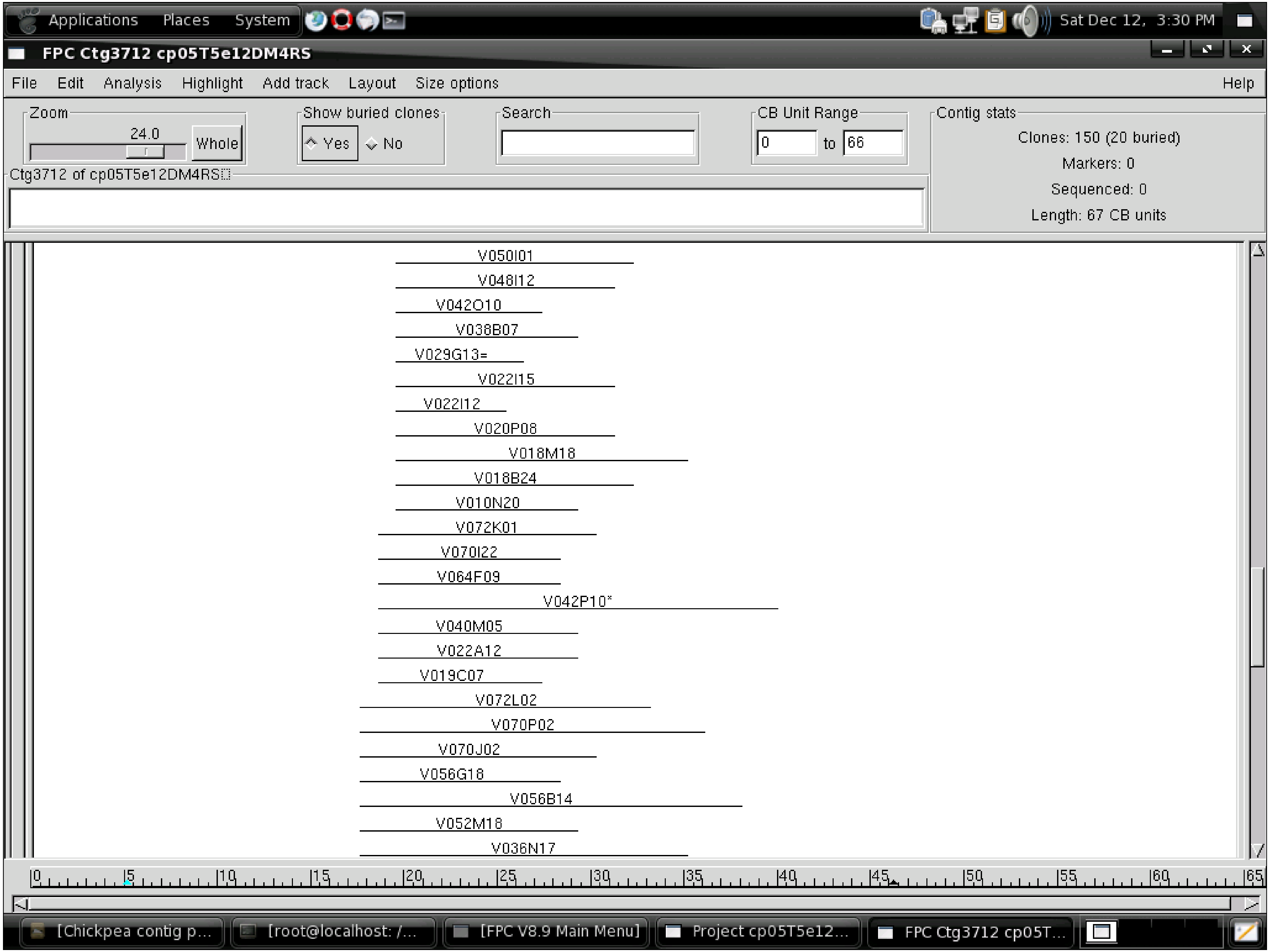
Applications Places System
Sat Dec 12, 3:30 PM
FPC Ctg3712 cp05T5e12DM4RS
File Edit Analysis Highlight Add track Layout Size options Help
Zoom
24.0
Whole
Show buried clones
Yes
No
Search
CB Unit Range
0
to
66
Contig stats
Clones: 150 (20 buried)
Markers: 0
Sequenced: 0
Length: 67 CB units
Ctg3712 of cp05T5e12DM4RS
V050I01
V048I12
V042O10
V038B07
V029G13=
V022I15
V022I12
V020P08
V018M18
V018B24
V010N20
V072K01
V070I22
V064F09
V042P10*
V040M05
V022A12
V019C07
V072L02
V070P02
V070J02
V056G18
V056B14
V052M18
V036N17
0
5
10
15
20
25
30
35
40
45
50
55
60
65
[Chickpea contig p...
[root@localhost: /...
[FPC V8.9 Main Menu]
Project cp05T5e12...
FPC Ctg3712 cp05T...

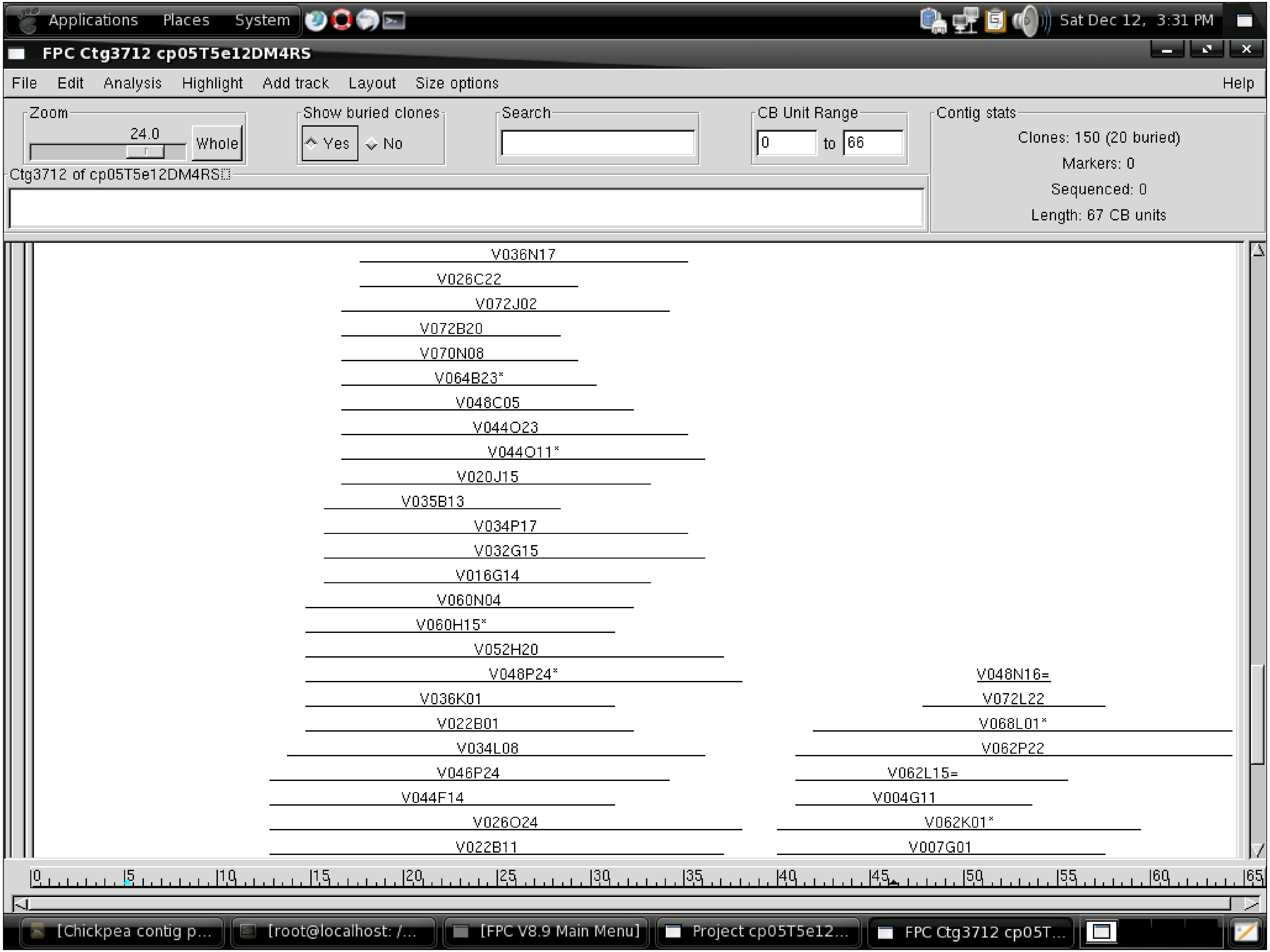
Applications Places System
Sat Dec 12, 3:31 PM
FPC Ctg3712 cp05T5e12DM4RS
File Edit Analysis Highlight Add track Layout Size options Help
Zoom
24.0
Whole
Show buried clones
Yes No
Search
CB Unit Range
0 to 66
Contig stats
Clones: 150 (20 buried)
Markers: 0
Sequenced: 0
Length: 67 CB units
Ctg3712 of cp05T5e12DM4RS
V036N17
V026C22
V072J02
V072B20
V070N08
V064B23*
V048C05
V044O23
V044O11*
V020J15
V035B13
V034P17
V032G15
V016G14
V060N04
V060H15*
V052H20
V048P24*
V048N16=
V036K01
V072L22
V022B01
V068L01*
V034L08
V062P22
V046P24
V062L15=
V044F14
V004G11
V026O24
V062K01*
V022B11
V007G01
0 5 10 15 20 25 30 35 40 45 50 55 60 65
[Chickpea contig p...
[root@localhost: /...
[FPC V8.9 Main Menu]
Project cp05T5e12...
FPC Ctg3712 cp05T...

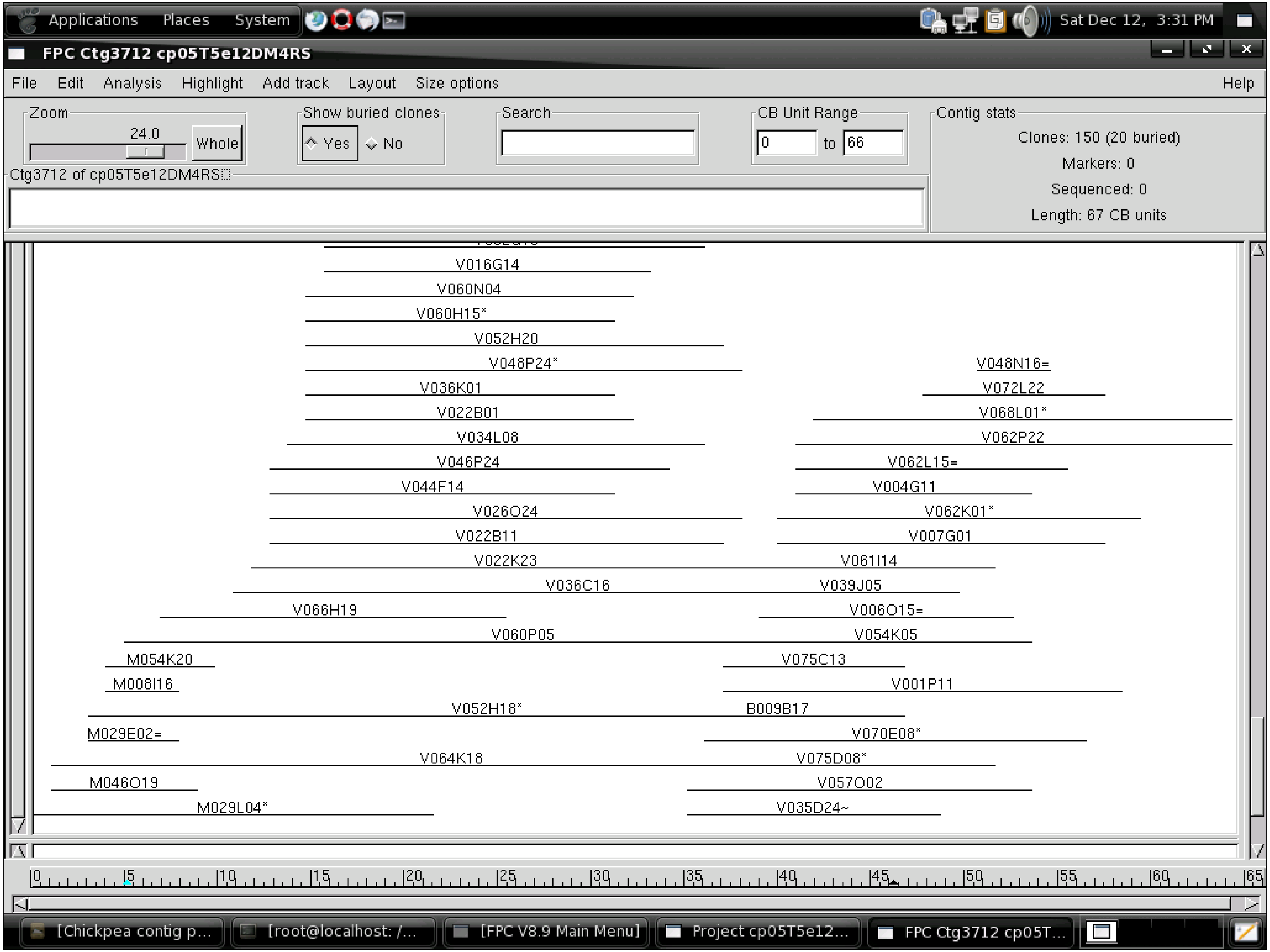
Applications Places System
Sat Dec 12, 3:31 PM
FPC Ctg3712 cp05T5e12DM4RS
File Edit Analysis Highlight Add track Layout Size options Help
Zoom
24.0
Whole
Show buried clones
Yes No
Search
CB Unit Range
0 to 66
Contig stats
Clones: 150 (20 buried)
Markers: 0
Sequenced: 0
Length: 67 CB units
Ctg3712 of cp05T5e12DM4RS
V016G14
V060N04
V060H15*
V052H20
V048P24*
V048N16=
V036K01
V072L22
V022B01
V068L01*
V034L08
V062P22
V046P24
V062L15=
V044F14
V004G11
V026O24
V062K01*
V022B11
V007G01
V022K23
V061I14
V036C16
V039J05
V066H19
V006O15=
V060P05
V054K05
M054K20
V075C13
M008I16
V001P11
V052H18*
B009B17
M029E02=
V070E08*
V064K18
V075D08*
M046O19
V057O02
M029L04*
V035D24~
0 5 10 15 20 25 30 35 40 45 50 55 60 65
[Chickpea contig p...
[root@localhost: /...
[FPC V8.9 Main Menu]
Project cp05T5e12...
FPC Ctg3712 cp05T...

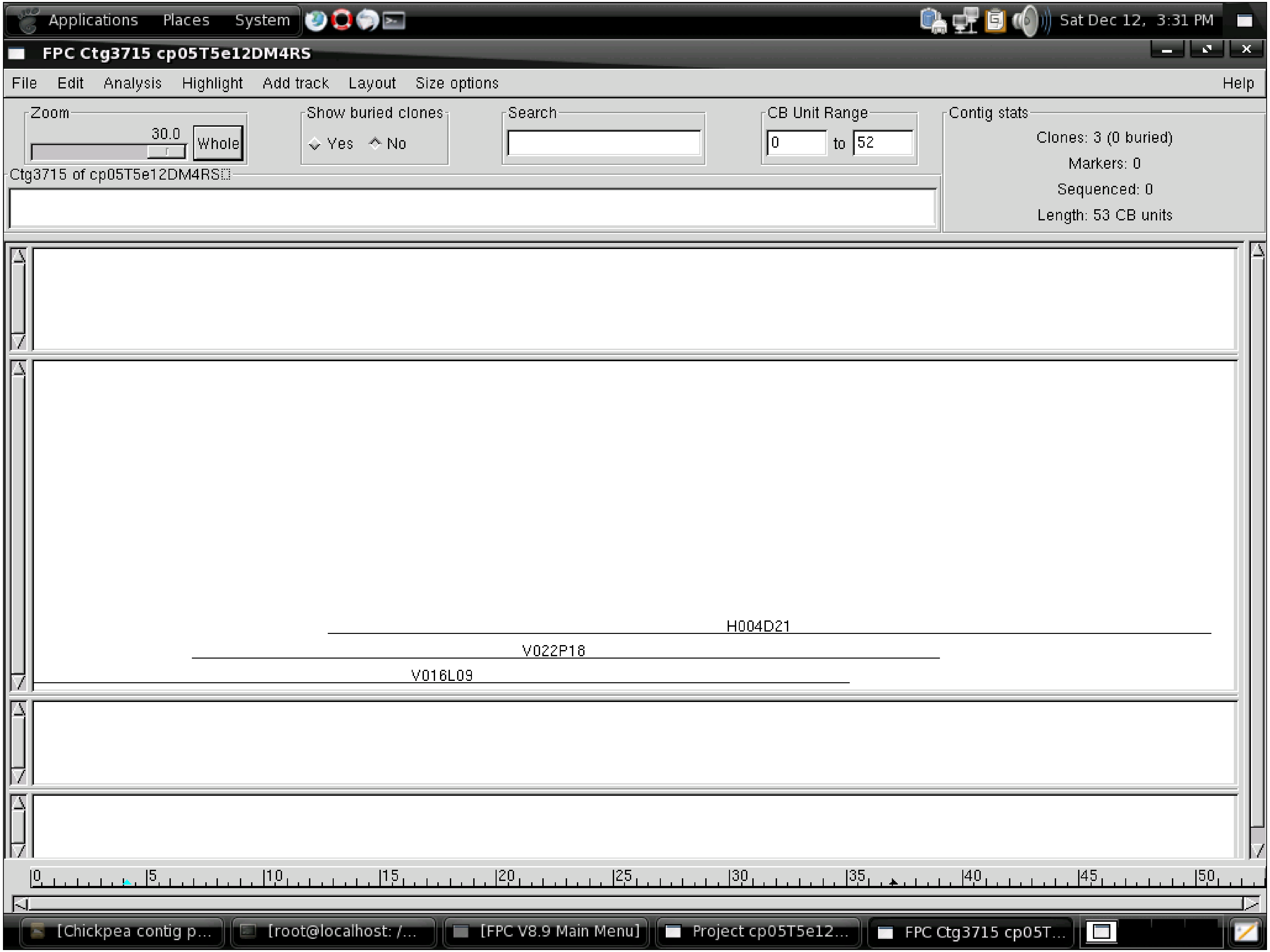
Applications Places System
Sat Dec 12, 3:31 PM
FPC Ctg3715 cp05T5e12DM4RS
File Edit Analysis Highlight Add track Layout Size options Help
Zoom
30.0
Whole
Show buried clones
Yes No
Search
CB Unit Range
0 to 52
Contig stats
Clones: 3 (0 buried)
Markers: 0
Sequenced: 0
Length: 53 CB units
Ctg3715 of cp05T5e12DM4RS
H004D21
V022P18
V016L09
0 5 10 15 20 25 30 35 40 45 50
[Chickpea contig p...
[root@localhost: /...
[FPC V8.9 Main Menu]
Project cp05T5e12...
FPC Ctg3715 cp05T...

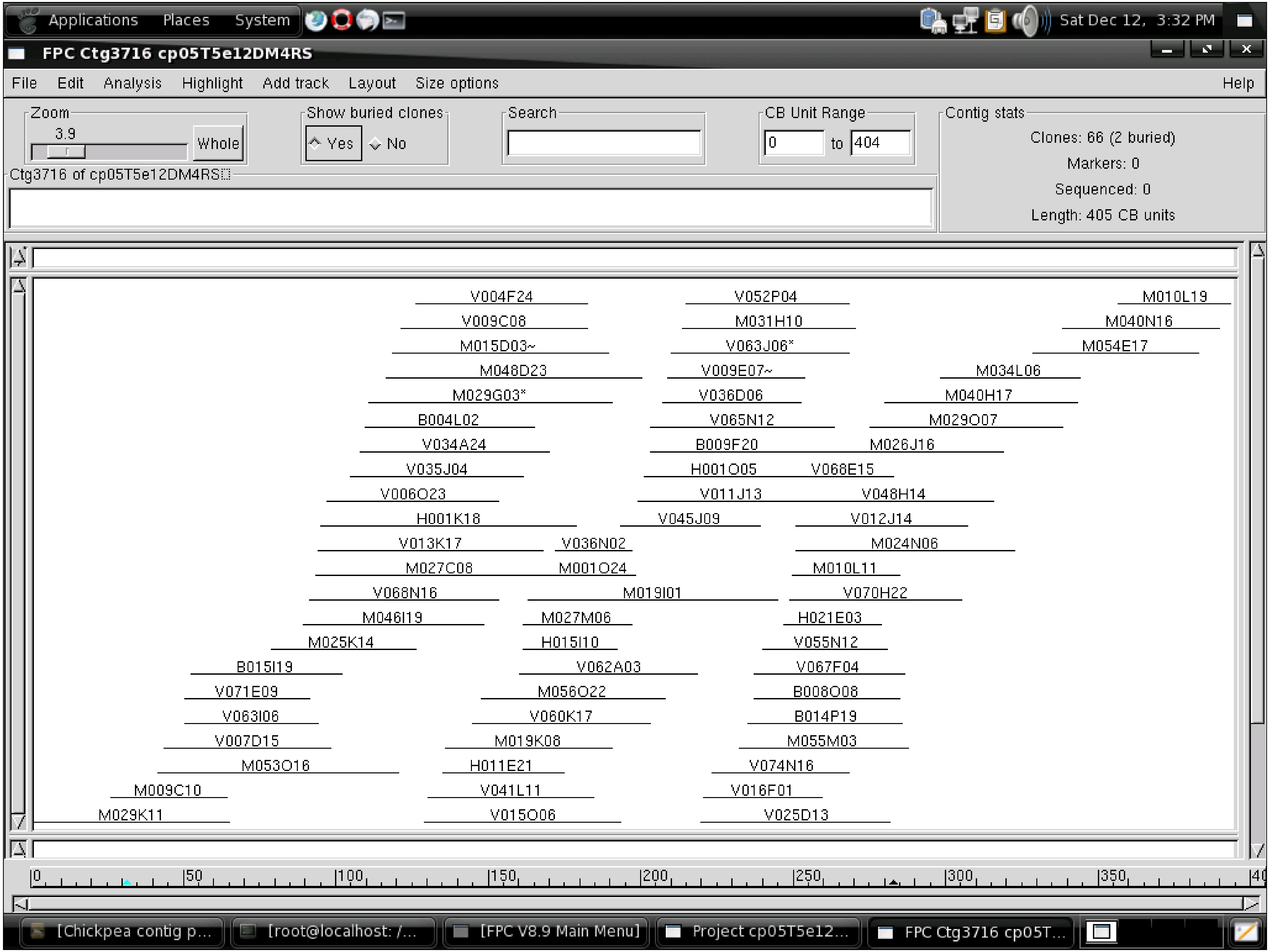
Applications Places System
Sat Dec 12, 3:32 PM
FPC Ctg3716 cp05T5e12DM4RS
File Edit Analysis Highlight Add track Layout Size options Help
Zoom
3.9
Whole
Show buried clones
Yes
No
Search
CB Unit Range
0
to
404
Contig stats
Clones: 66 (2 buried)
Markers: 0
Sequenced: 0
Length: 405 CB units
Ctg3716 of cp05T5e12DM4RS
V004F24
V052P04
M010L19
V009C08
M031H10
M040N16
M015D03~
V063J06*
M054E17
M048D23
V009E07~
M034L06
M029G03*
V036D06
M040H17
B004L02
V065N12
M029O07
V034A24
B009F20
M026J16
V035J04
H001O05
V068E15
V006O23
V011J13
V048H14
H001K18
V045J09
V012J14
V013K17
V036N02
M024N06
M027C08
M001O24
M010L11
V068N16
M019I01
V070H22
M046I19
M027M06
H021E03
M025K14
H015I10
V055N12
B015I19
V062A03
V067F04
V071E09
M056O22
B008O08
V063I06
V060K17
B014P19
V007D15
M019K08
M055M03
M053O16
H011E21
V074N16
M009C10
V041L11
V016F01
M029K11
V015O06
V025D13
0
50
100
150
200
250
300
350
[Chickpea contig p...
[root@localhost: /...
[FPC V8.9 Main Menu]
Project cp05T5e12...
FPC Ctg3716 cp05T...

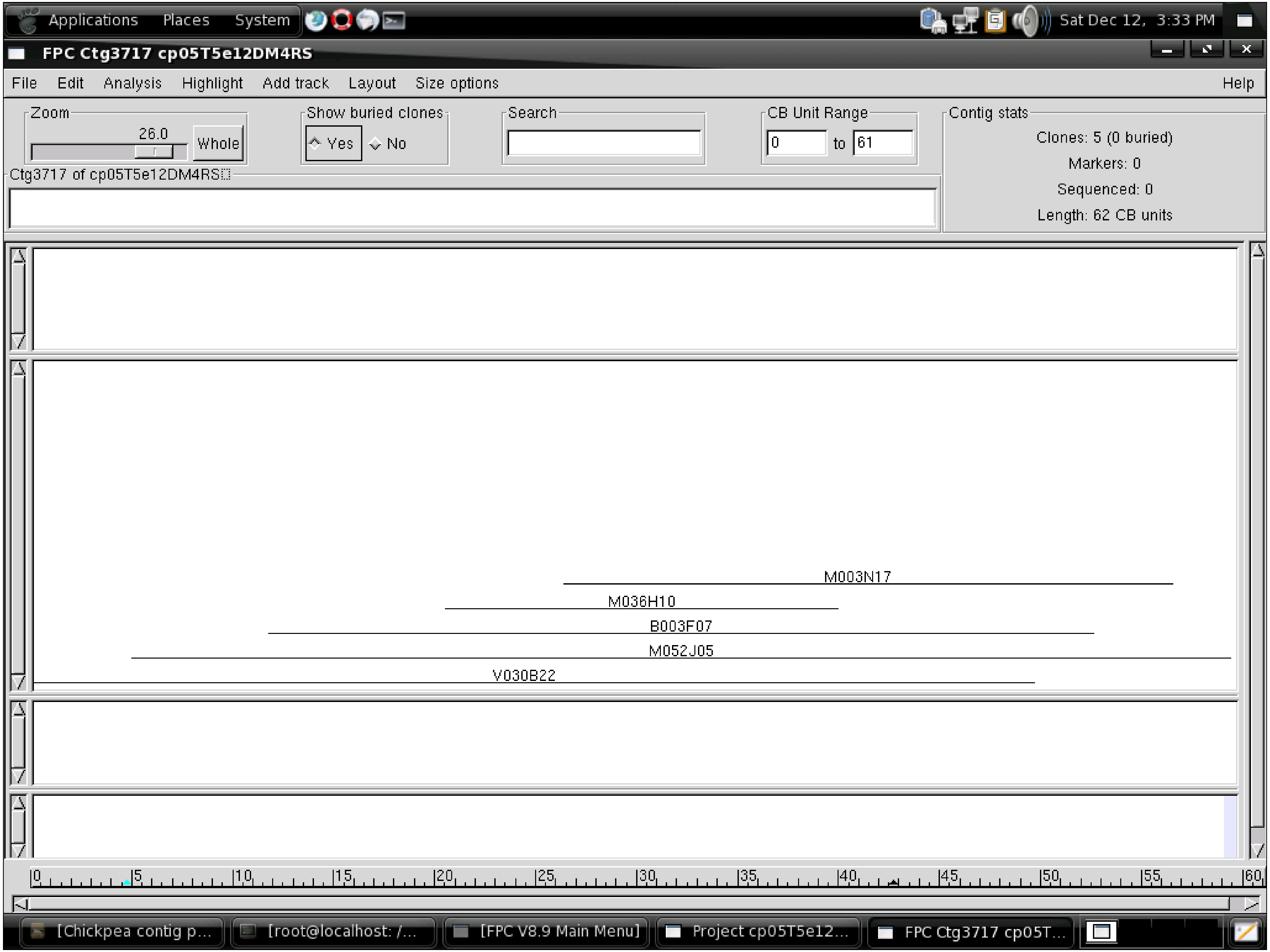
Applications Places System
Sat Dec 12, 3:33 PM
FPC Ctg3717 cp05T5e12DM4RS
File Edit Analysis Highlight Add track Layout Size options Help
Zoom
26.0
Whole
Show buried clones
Yes
No
Search
CB Unit Range
0 to 61
Contig stats
Clones: 5 (0 buried)
Markers: 0
Sequenced: 0
Length: 62 CB units
Ctg3717 of cp05T5e12DM4RS
M003N17
M036H10
B003F07
M052J05
V030B22
0 5 10 15 20 25 30 35 40 45 50 55 60
[Chickpea contig p...
[root@localhost: /...
[FPC V8.9 Main Menu]
Project cp05T5e12...
FPC Ctg3717 cp05T...

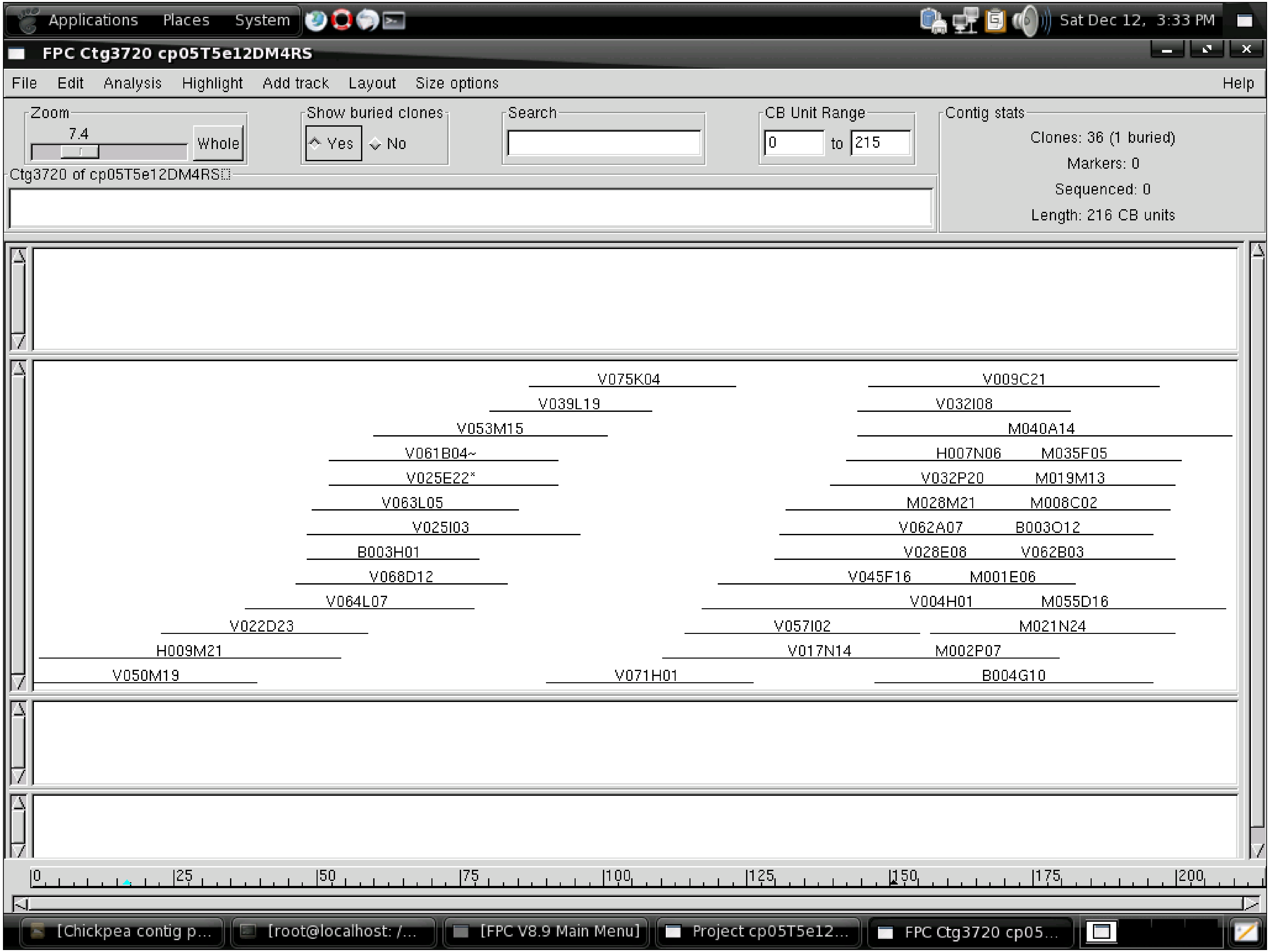
Applications Places System
Sat Dec 12, 3:33 PM
FPC Ctg3720 cp05T5e12DM4RS
File Edit Analysis Highlight Add track Layout Size options Help
Zoom
7.4
Whole
Show buried clones
Yes
No
Search
CB Unit Range
0
to
215
Contig stats
Clones: 36 (1 buried)
Markers: 0
Sequenced: 0
Length: 216 CB units
Ctg3720 of cp05T5e12DM4RS
V075K04
V009C21
V039L19
V032I08
V053M15
M040A14
V061B04~
H007N06
M035F05
V025E22*
V032P20
M019M13
V063L05
M028M21
M008C02
V025I03
V062A07
B003O12
B003H01
V028E08
V062B03
V068D12
V045F16
M001E06
V064L07
V004H01
M055D16
V022D23
V057I02
M021N24
H009M21
V017N14
M002P07
V050M19
V071H01
B004G10
0 25 50 75 100 125 150 175 200
[Chickpea contig p...
[root@localhost: /...
[FPC V8.9 Main Menu]
Project cp05T5e12...
FPC Ctg3720 cp05...

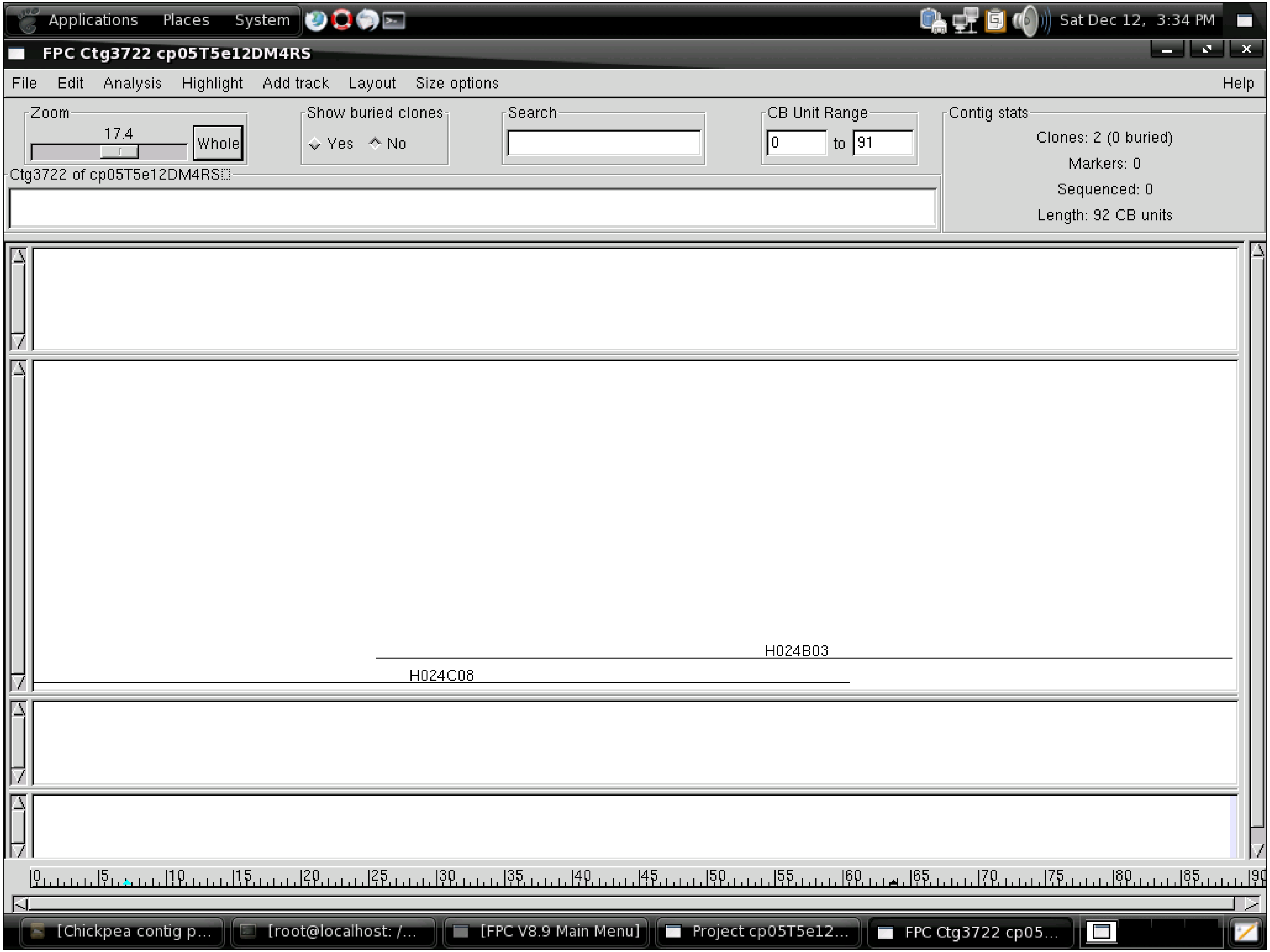
Applications Places System
Sat Dec 12, 3:34 PM
FPC Ctg3722 cp05T5e12DM4RS
File Edit Analysis Highlight Add track Layout Size options Help
Zoom
17.4
Whole
Show buried clones
Yes No
Search
CB Unit Range
0 to 91
Contig stats
Clones: 2 (0 buried)
Markers: 0
Sequenced: 0
Length: 92 CB units
Ctg3722 of cp05T5e12DM4RS
H024B03
H024C08
0 5 10 15 20 25 30 35 40 45 50 55 60 65 70 75 80 85 90
[Chickpea contig p...
[root@localhost: /...
[FPC V8.9 Main Menu]
Project cp05T5e12...
FPC Ctg3722 cp05...

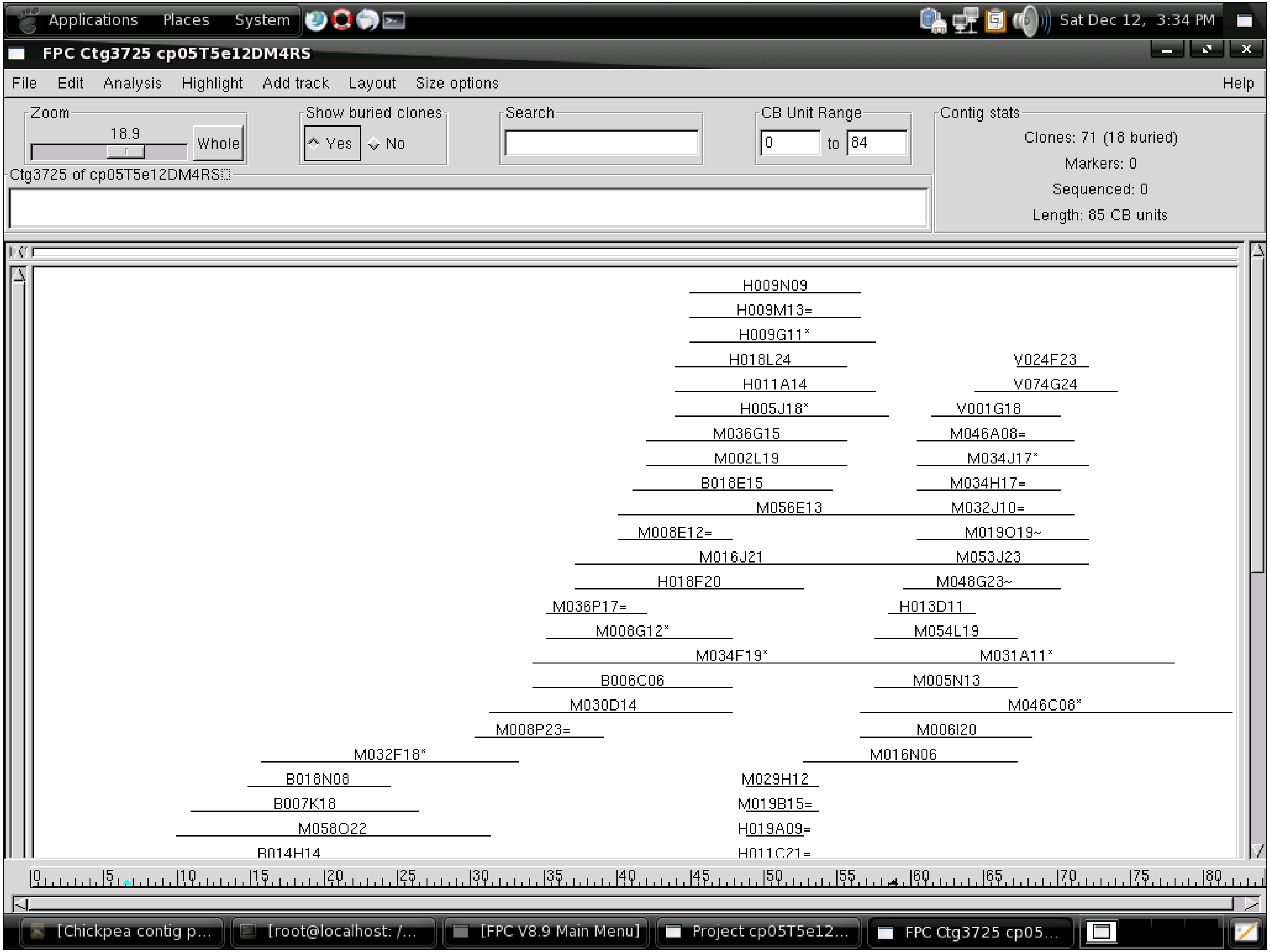
Applications
Places
System
Sat Dec 12, 3:34 PM
FPC Ctg3725 cp05T5e12DM4RS
File
Edit
Analysis
Highlight
Add track
Layout
Size options
Help
Zoom
18.9
Whole
Show buried clones
Yes
No
Search
CB Unit Range
0
to
84
Contig stats
Clones: 71 (18 buried)
Markers: 0
Sequenced: 0
Length: 85 CB units
Ctg3725 of cp05T5e12DM4RS
H009N09
H009M13=
H009G11*
H018L24
H011A14
H005J18*
M036G15
M002L19
B018E15
M056E13
M008E12=
M016J21
H018F20
M036P17=
M008G12*
M034F19*
B006C06
M030D14
M008P23=
M032F18*
B018N08
B007K18
M058O22
B014H14
V024F23
V074G24
V001G18
M046A08=
M034J17*
M034H17=
M032J10=
M019O19~
M053J23
M048G23~
H013D11
M054L19
M031A11*
M005N13
M046C08*
M006I20
M016N06
M029H12
M019B15=
H019A09=
H011C21=
0
5
10
15
20
25
30
35
40
45
50
55
60
65
70
75
80
[Chickpea contig p...
[root@localhost: /...
[FPC V8.9 Main Menu]
Project cp05T5e12...
FPC Ctg3725 cp05...

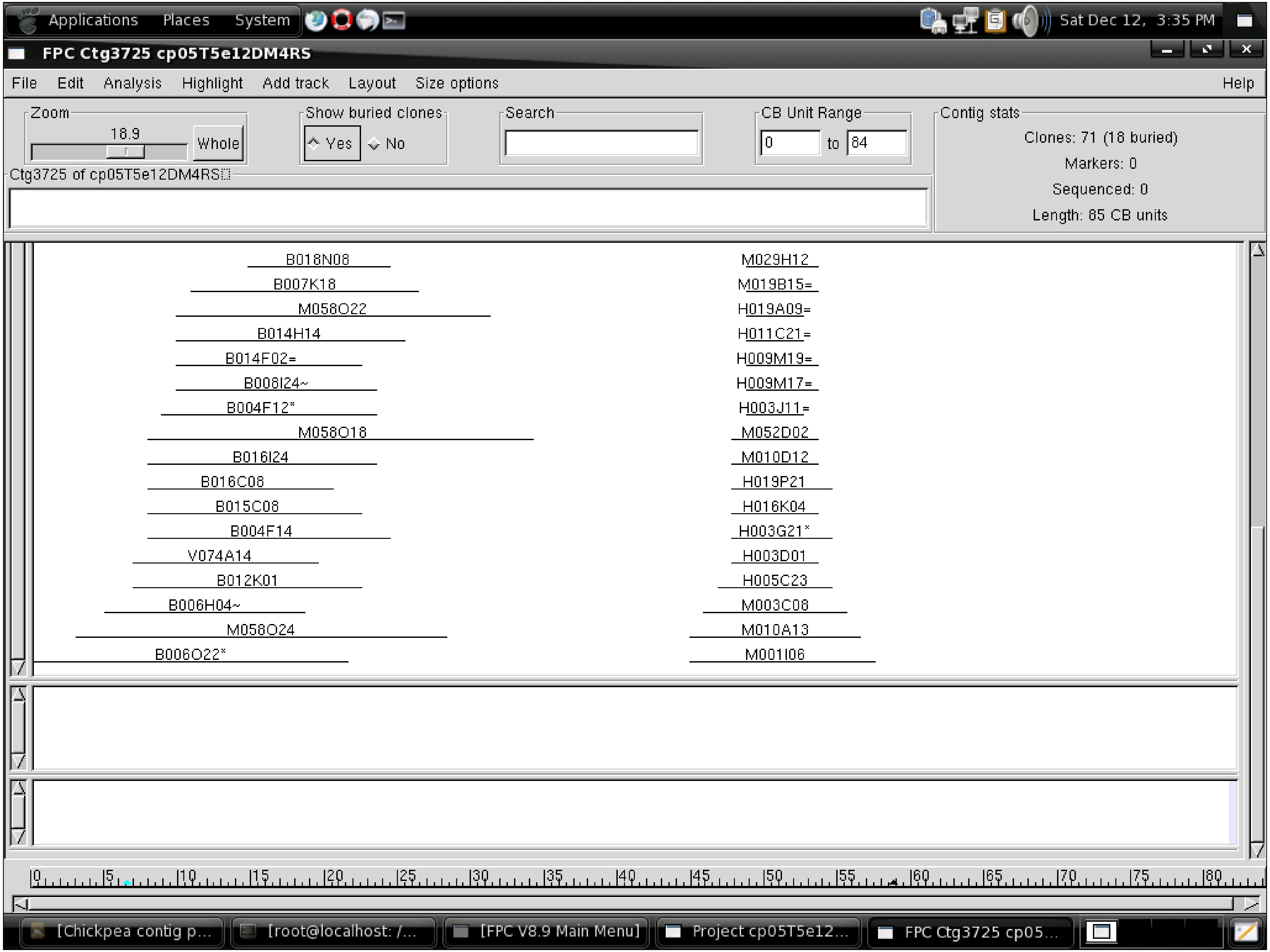
Applications Places System
Sat Dec 12, 3:35 PM
FPC Ctg3725 cp05T5e12DM4RS
File Edit Analysis Highlight Add track Layout Size options Help
Zoom
18.9
Whole
Show buried clones
Yes No
Search
CB Unit Range
0 to 84
Contig stats
Clones: 71 (18 buried)
Markers: 0
Sequenced: 0
Length: 85 CB units
Ctg3725 of cp05T5e12DM4RS
B018N08
B007K18
M058O22
B014H14
B014F02=
B008I24~
B004F12*
M058O18
B016I24
B016C08
B015C08
B004F14
V074A14
B012K01
B006H04~
M058O24
B006O22*
M029H12
M019B15=
H019A09=
H011C21=
H009M19=
H009M17=
H003J11=
M052D02
M010D12
H019P21
H016K04
H003G21*
H003D01
H005C23
M003C08
M010A13
M001I06
0 5 10 15 20 25 30 35 40 45 50 55 60 65 70 75 80
[Chickpea contig p...
[root@localhost: /...
[FPC V8.9 Main Menu]
Project cp05T5e12...
FPC Ctg3725 cp05...

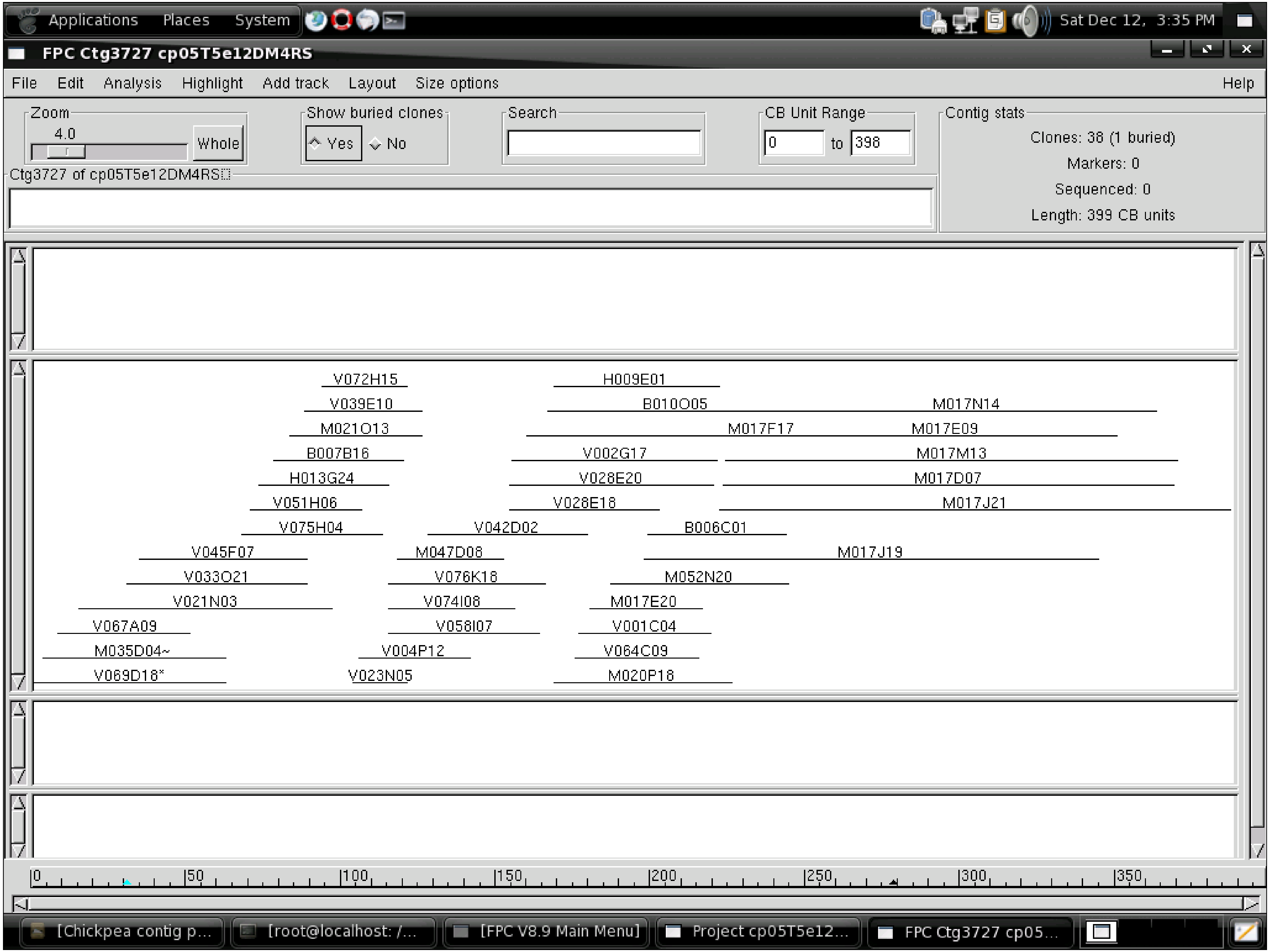
Applications Places System
Sat Dec 12, 3:35 PM
FPC Ctg3727 cp05T5e12DM4RS
File Edit Analysis Highlight Add track Layout Size options Help
Zoom
4.0
Whole
Show buried clones
Yes No
Search
CB Unit Range
0 to 398
Contig stats
Clones: 38 (1 buried)
Markers: 0
Sequenced: 0
Length: 399 CB units
Ctg3727 of cp05T5e12DM4RS
V072H15
H009E01
V039E10
B010O05
M017N14
M021O13
M017F17
M017E09
B007B16
V002G17
M017M13
H013G24
V028E20
M017D07
V051H06
V028E18
M017J21
V075H04
V042D02
B006C01
V045F07
M047D08
M017J19
V033O21
V076K18
M052N20
V021N03
V074I08
M017E20
V067A09
V058I07
V001C04
M035D04~
V004P12
V064C09
V069D18*
V023N05
M020P18
0 50 100 150 200 250 300 350
[Chickpea contig p...
[root@localhost: /...
[FPC V8.9 Main Menu]
Project cp05T5e12...
FPC Ctg3727 cp05...
