# Supplementary material for: A BAC/BIBAC-based physical map of chickpea, Cicer arietinum L
Source: BMC Genomics. 2010 Sep 17;11:501. doi: 10.1186/1471-2164-11-501 (PMC2996997; doi:10.1186/1471-2164-11-501)
Supplement: Additional file 5 — Shows the contigs constituting the physical map of the chickpea genome 5 (ctg3731-5052). [file 1471-2164-11-501-S5.PDF]

# A BAC/BIBAC-based physical map of chickpea, *Cicer arietinum* L.

Xiaojun Zhang[1,6], Chantel F Scheuring[1], Meiping Zhang[1,2], Jennifer J Dong[1], Yang Zhang[1], James J Huang[1], Mi-Kyung Lee[1], Shahal Abbo[3], Amir Sherman[4], Dani Shtienberg[4], Weidong Chen[5], Fred Muehlbauer[5], Hong-Bin Zhang[1,*]

[1] Department of Soil and Crop Sciences, Texas A&M University, College Station, Texas 77843-2474, USA
[2] College of Life Science, Jilin Agricultural University, Changchun, Jilin 130118, China
[3] Institute of Plant Science and Genetics in Agriculture, The Hebrew University of Jerusalem, Rehovot, 76100, Israel
[4] The Volcani Center, P.O. Box 6, Bet-Dagan, 50250, Israel
[5] USDA-ARS and Department of Crop and Soil Sciences, Washington State University, Pullman, WA 99164-6434, USA
[6] The Key Laboratory of Experimental Marine Biology, Institute of Oceanology, Chinese Academy of Sciences, Qingdao 266071, China

***Corresponding author:** H.-B. Zhang, Tel.: +1-979-862-2244; Fax: +1-979-845-0456; E-mail: hbz7049@tamu.edu

**Additional file 5.** The contigs constituting the physical map of the chickpea genome 5(ctg3731-5052)

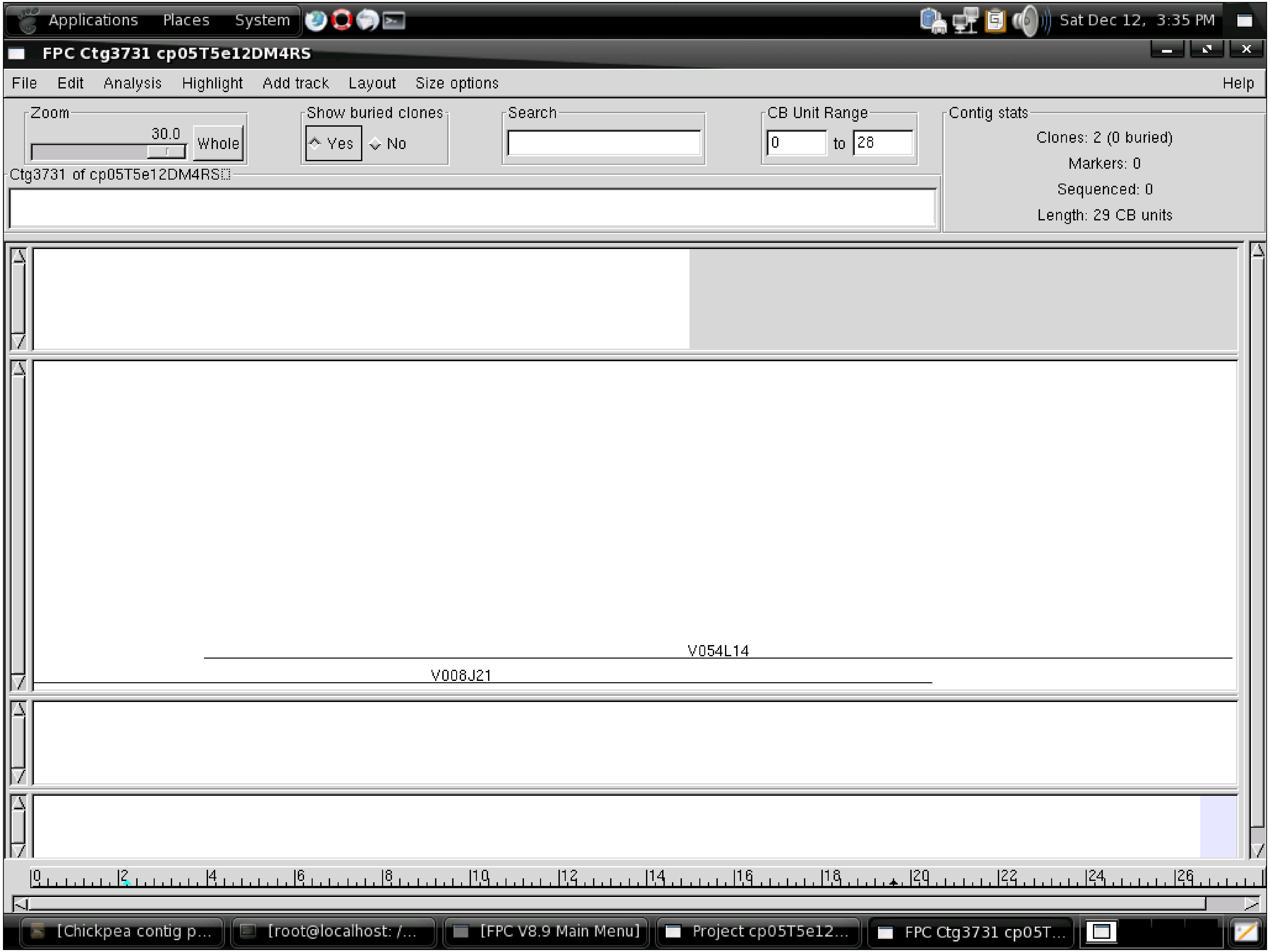
Applications Places System
Sat Dec 12, 3:35 PM
FPC Ctg3731 cp05T5e12DM4RS
File Edit Analysis Highlight Add track Layout Size options Help
Zoom
30.0
Whole
Show buried clones
Yes No
Search
CB Unit Range
0 to 28
Contig stats
Clones: 2 (0 buried)
Markers: 0
Sequenced: 0
Length: 29 CB units
Ctg3731 of cp05T5e12DM4RS
V054L14
V008J21
0 2 4 6 8 10 12 14 16 18 20 22 24 26
[Chickpea contig p...
[root@localhost: /...
[FPC V8.9 Main Menu]
Project cp05T5e12...
FPC Ctg3731 cp05T...

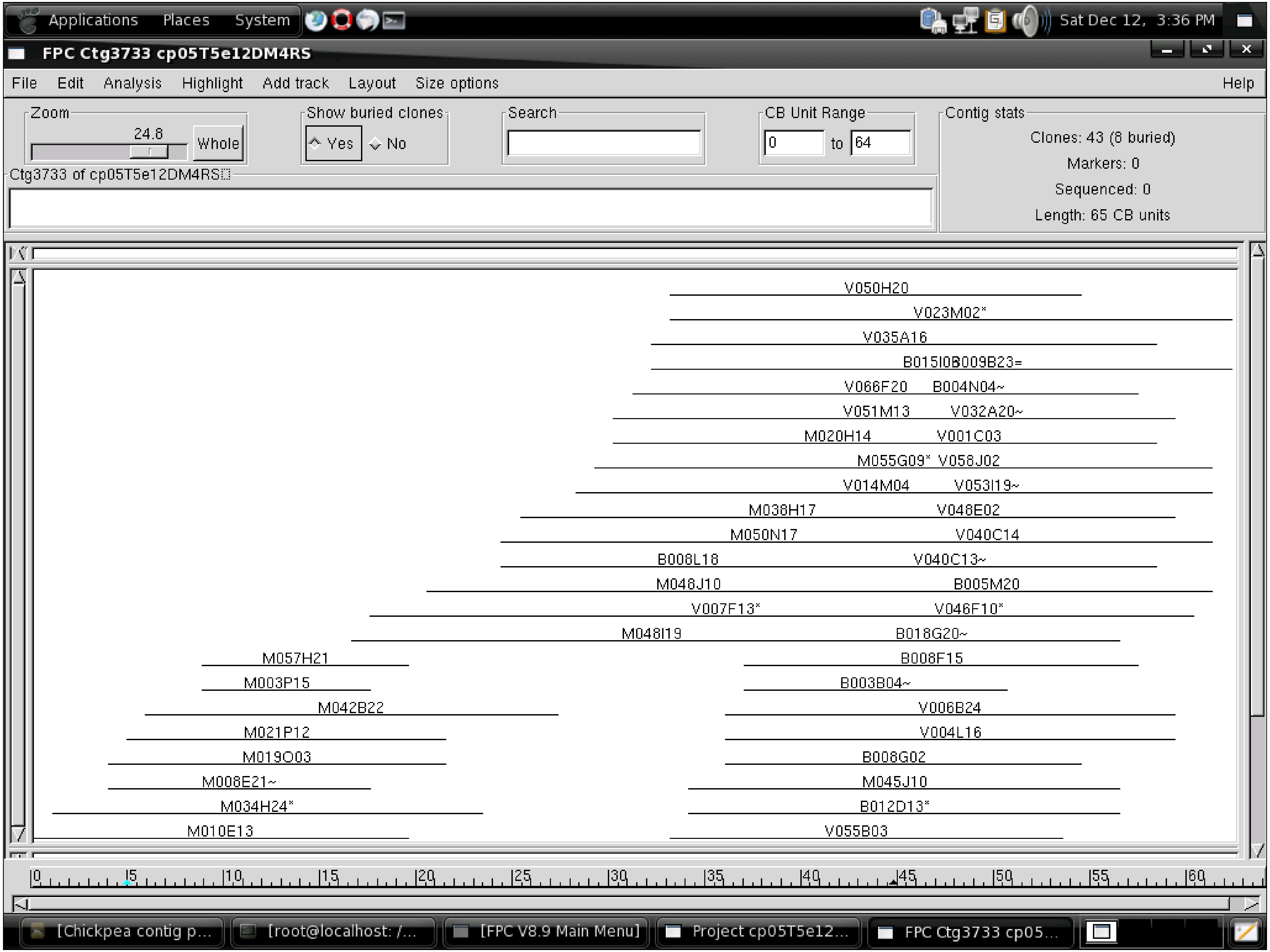
Applications Places System
Sat Dec 12, 3:36 PM
FPC Ctg3733 cp05T5e12DM4RS
File Edit Analysis Highlight Add track Layout Size options Help
Zoom
24.8
Whole
Show buried clones
Yes
No
Search
CB Unit Range
0 to 64
Contig stats
Clones: 43 (8 buried)
Markers: 0
Sequenced: 0
Length: 65 CB units
Ctg3733 of cp05T5e12DM4RS
V050H20
V023M02*
V035A16
B015I0B009B23=
V066F20 B004N04~
V051M13 V032A20~
M020H14 V001C03
M055G09* V058J02
V014M04 V053I19~
M038H17 V048E02
M050N17 V040C14
B008L18 V040C13~
M048J10 B005M20
V007F13* V046F10*
M048I19 B018G20~
M057H21 B008F15
M003P15 B003B04~
M042B22 V006B24
M021P12 V004L16
M019O03 B008G02
M008E21~ M045J10
M034H24* B012D13*
M010E13 V055B03
0 5 10 15 20 25 30 35 40 45 50 55 60
[Chickpea contig p...
[root@localhost: /...
[FPC V8.9 Main Menu]
Project cp05T5e12...
FPC Ctg3733 cp05...

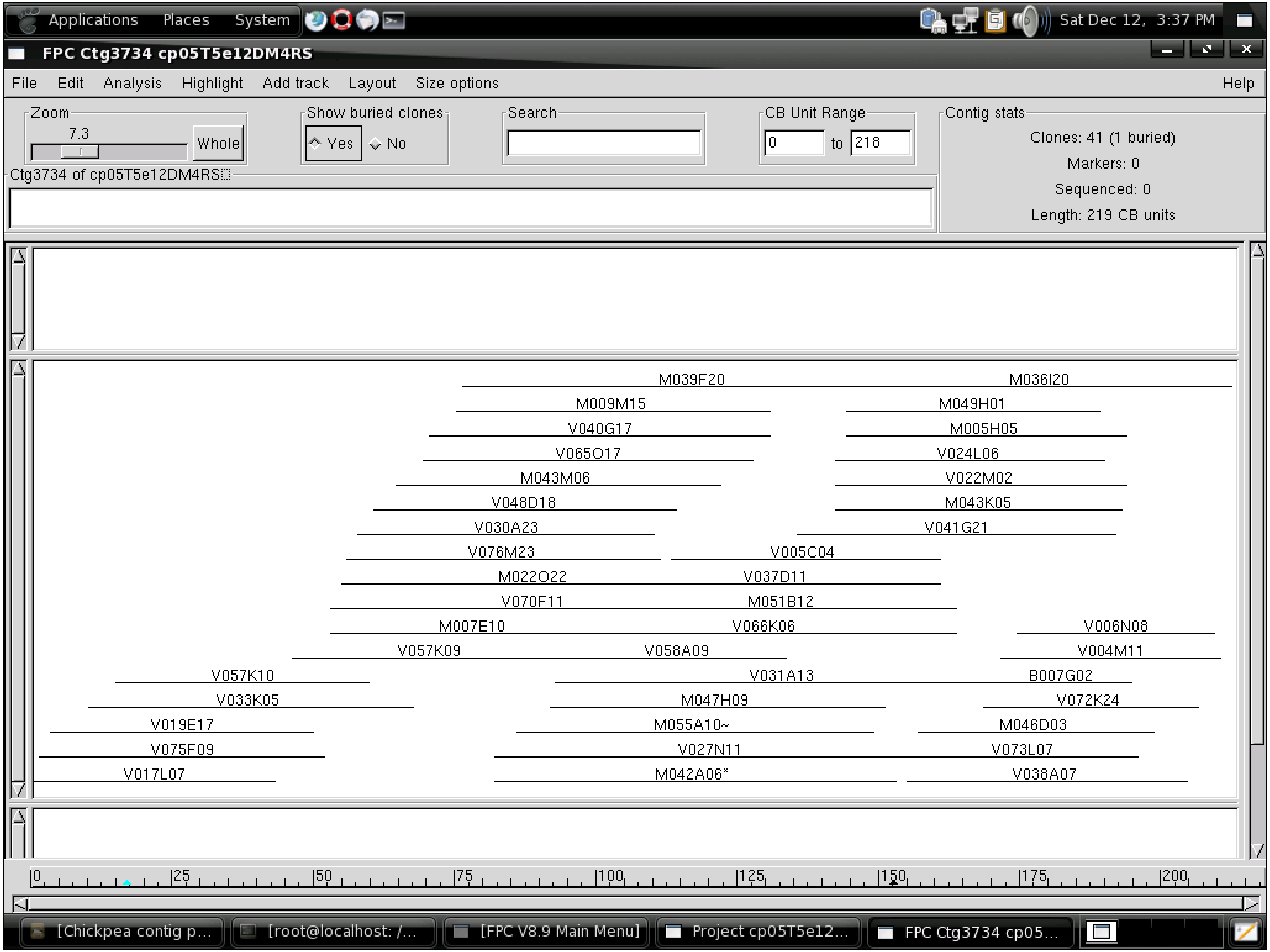
Applications Places System
Sat Dec 12, 3:37 PM
FPC Ctg3734 cp05T5e12DM4RS
File Edit Analysis Highlight Add track Layout Size options Help
Zoom
7.3
Whole
Show buried clones
Yes No
Search
CB Unit Range
0 to 218
Contig stats
Clones: 41 (1 buried)
Markers: 0
Sequenced: 0
Length: 219 CB units
Ctg3734 of cp05T5e12DM4RS
M039F20
M036I20
M009M15
M049H01
V040G17
M005H05
V065O17
V024L06
M043M06
V022M02
V048D18
M043K05
V030A23
V041G21
V076M23
V005C04
M022O22
V037D11
V070F11
M051B12
M007E10
V066K06
V006N08
V057K09
V058A09
V004M11
V057K10
V031A13
B007G02
V033K05
M047H09
V072K24
V019E17
M055A10~
M046D03
V075F09
V027N11
V073L07
V017L07
M042A06*
V038A07
0 25 50 75 100 125 150 175 200
[Chickpea contig p...
[root@localhost: /...
[FPC V8.9 Main Menu]
Project cp05T5e12...
FPC Ctg3734 cp05...

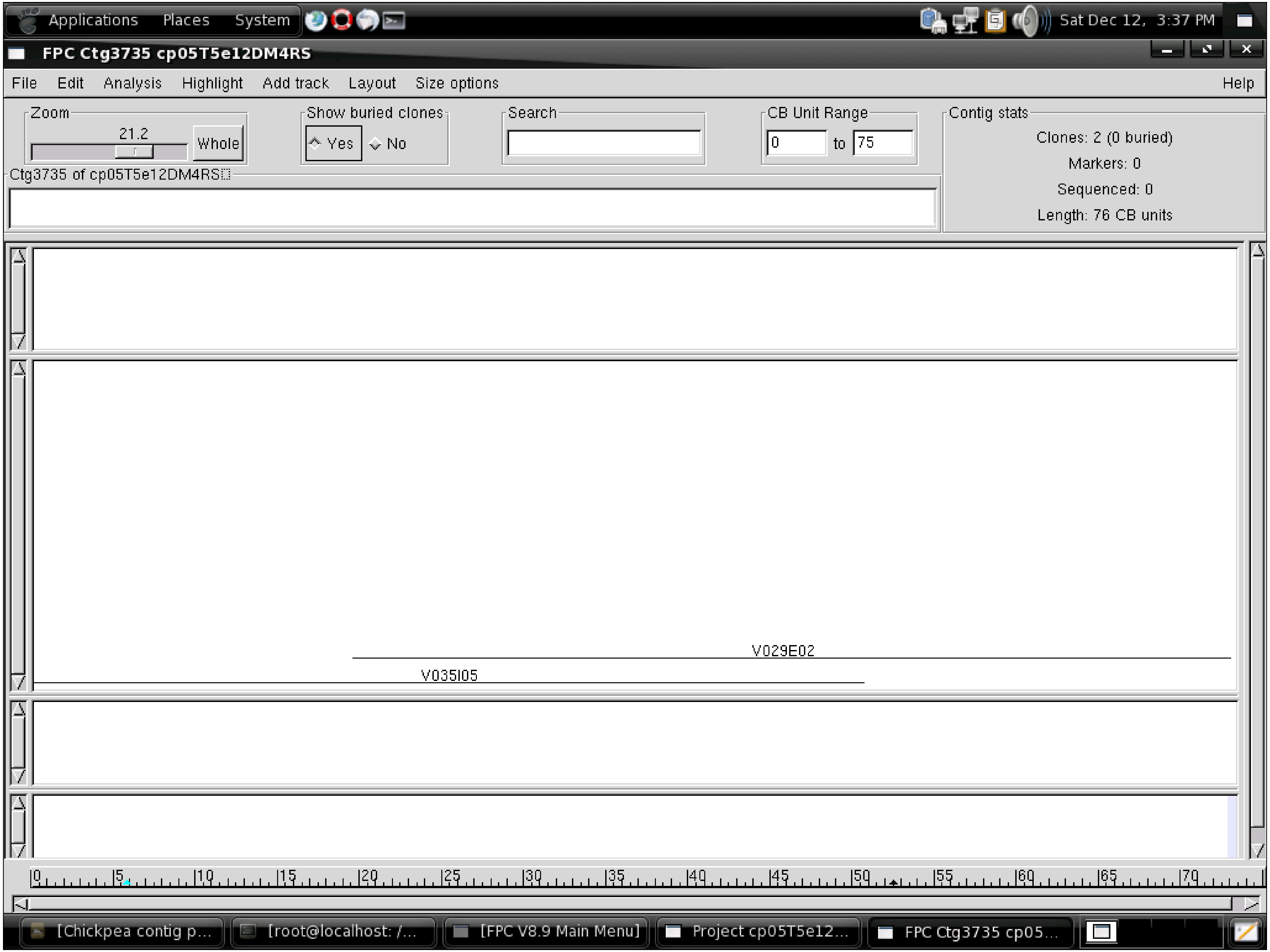
Applications Places System
Sat Dec 12, 3:37 PM
FPC Ctg3735 cp05T5e12DM4RS
File Edit Analysis Highlight Add track Layout Size options Help
Zoom
21.2
Whole
Show buried clones
Yes No
Search
CB Unit Range
0 to 75
Contig stats
Clones: 2 (0 buried)
Markers: 0
Sequenced: 0
Length: 76 CB units
Ctg3735 of cp05T5e12DM4RS
V029E02
V035I05
0 5 10 15 20 25 30 35 40 45 50 55 60 65 70
[Chickpea contig p...
[root@localhost: /...
[FPC V8.9 Main Menu]
Project cp05T5e12...
FPC Ctg3735 cp05...

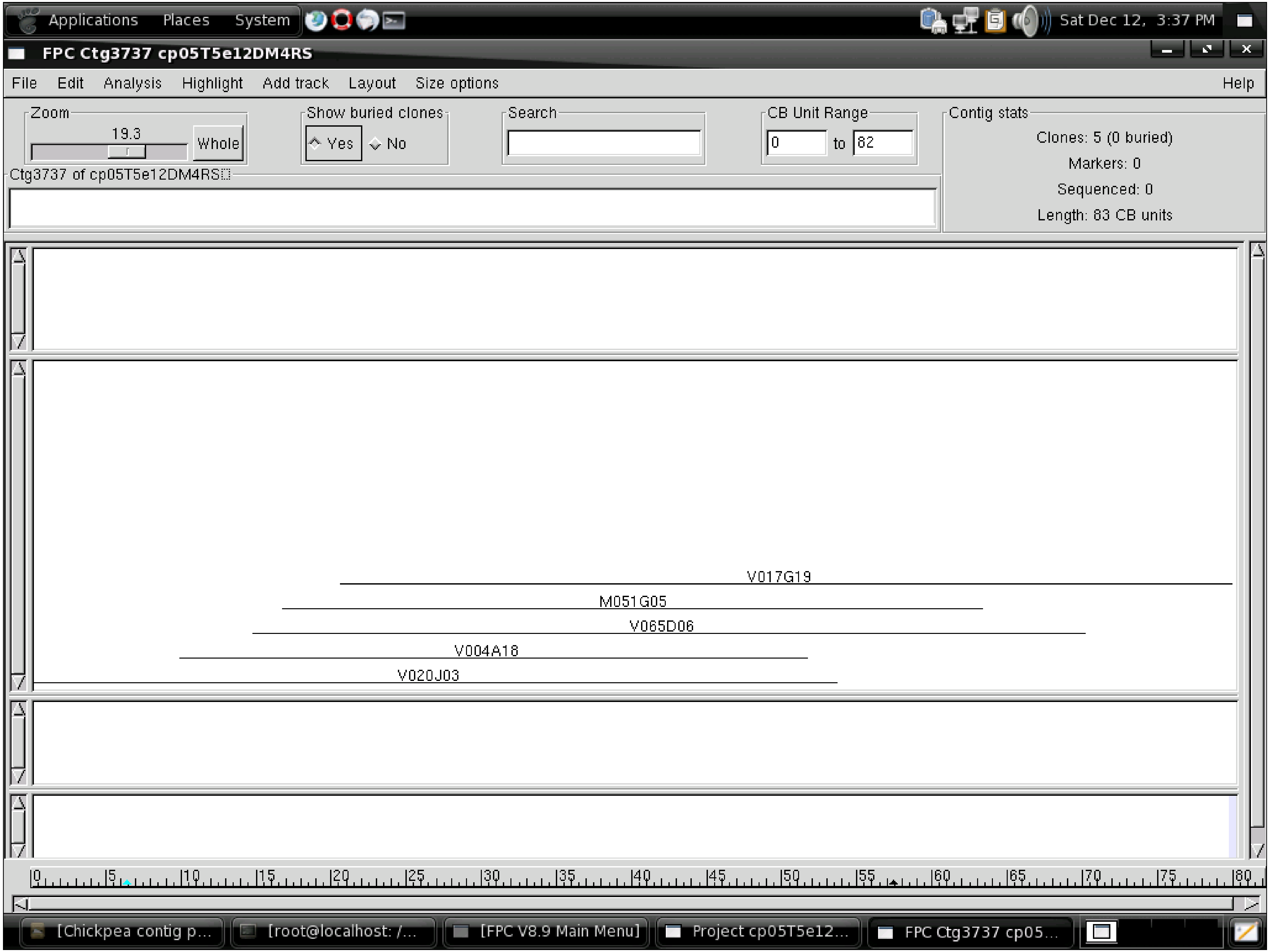
Applications Places System
Sat Dec 12, 3:37 PM
FPC Ctg3737 cp05T5e12DM4RS
File Edit Analysis Highlight Add track Layout Size options Help
Zoom
19.3
Whole
Show buried clones
Yes No
Search
CB Unit Range
0 to 82
Contig stats
Clones: 5 (0 buried)
Markers: 0
Sequenced: 0
Length: 83 CB units
Ctg3737 of cp05T5e12DM4RS
V017G19
M051G05
V065D06
V004A18
V020J03
0 5 10 15 20 25 30 35 40 45 50 55 60 65 70 75 80
[Chickpea contig p...
[root@localhost: /...
[FPC V8.9 Main Menu]
Project cp05T5e12...
FPC Ctg3737 cp05...

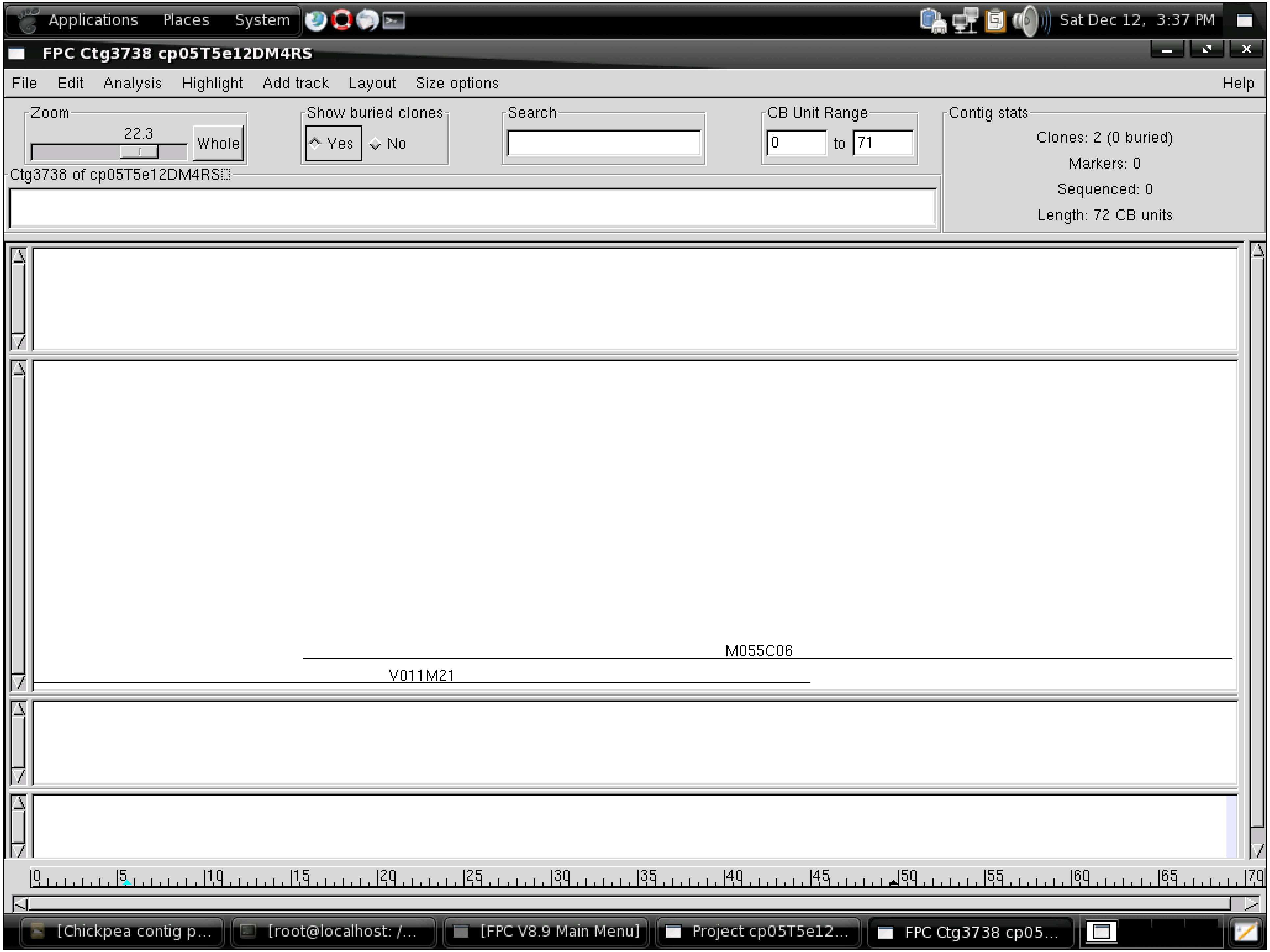
Applications Places System
Sat Dec 12, 3:37 PM
FPC Ctg3738 cp05T5e12DM4RS
File Edit Analysis Highlight Add track Layout Size options Help
Zoom
22.3
Whole
Show buried clones
Yes No
Search
CB Unit Range
0 to 71
Contig stats
Clones: 2 (0 buried)
Markers: 0
Sequenced: 0
Length: 72 CB units
Ctg3738 of cp05T5e12DM4RS
M055C06
V011M21
0 5 10 15 20 25 30 35 40 45 50 55 60 65 70
[Chickpea contig p...
[root@localhost: /...
[FPC V8.9 Main Menu]
Project cp05T5e12...
FPC Ctg3738 cp05...

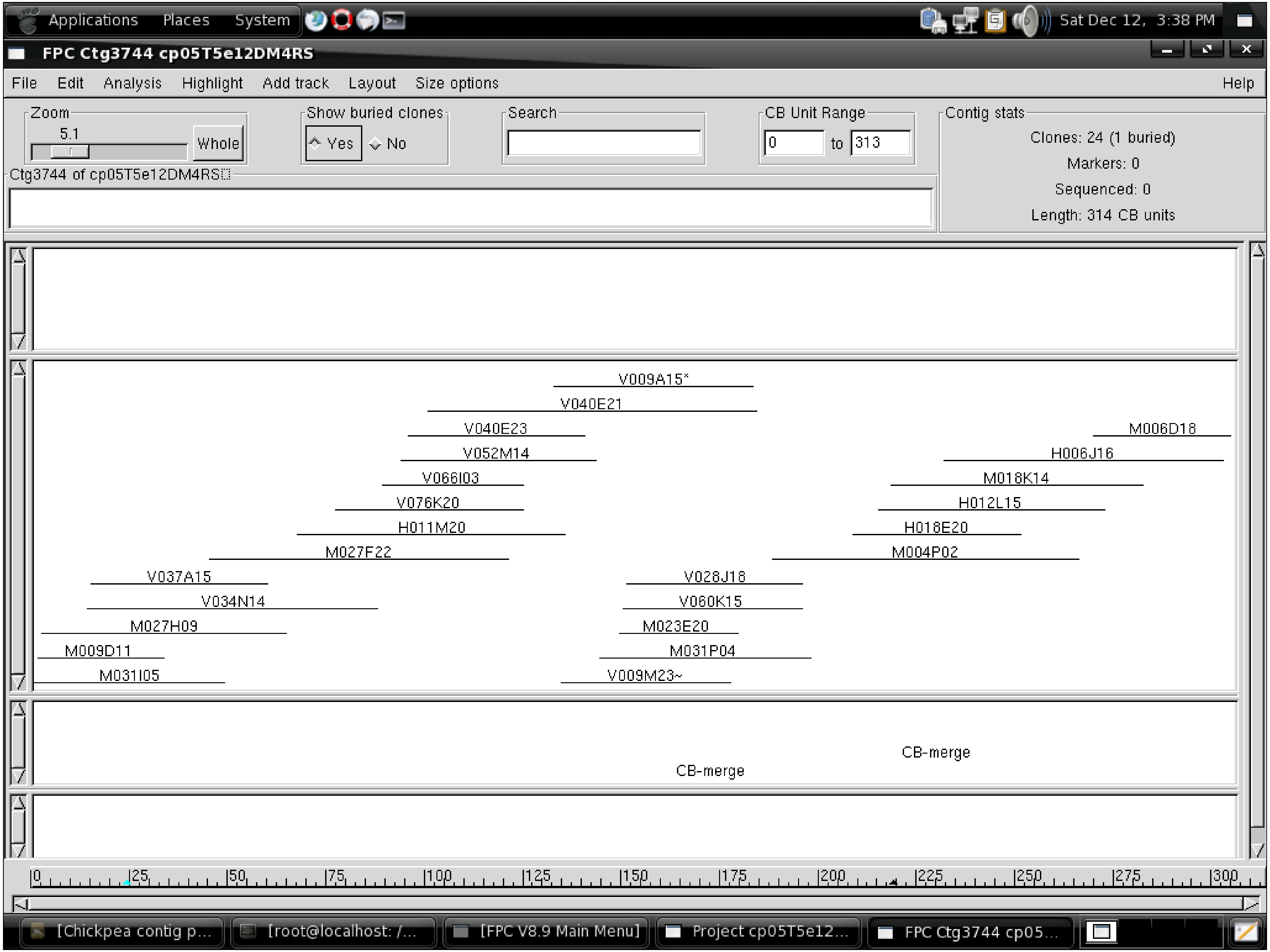
Applications Places System
Sat Dec 12, 3:38 PM
FPC Ctg3744 cp05T5e12DM4RS
File Edit Analysis Highlight Add track Layout Size options Help
Zoom
5.1
Whole
Show buried clones
Yes No
Search
CB Unit Range
0 to 313
Contig stats
Clones: 24 (1 buried)
Markers: 0
Sequenced: 0
Length: 314 CB units
Ctg3744 of cp05T5e12DM4RS
V009A15*
V040E21
V040E23
M006D18
V052M14
H006J16
V066I03
M018K14
V076K20
H012L15
H011M20
H018E20
M027F22
M004P02
V037A15
V028J18
V034N14
V060K15
M027H09
M023E20
M009D11
M031P04
M031I05
V009M23~
CB-merge
CB-merge
0 25 50 75 100 125 150 175 200 225 250 275 300
[Chickpea contig p...
[root@localhost: /...
[FPC V8.9 Main Menu]
Project cp05T5e12...
FPC Ctg3744 cp05...

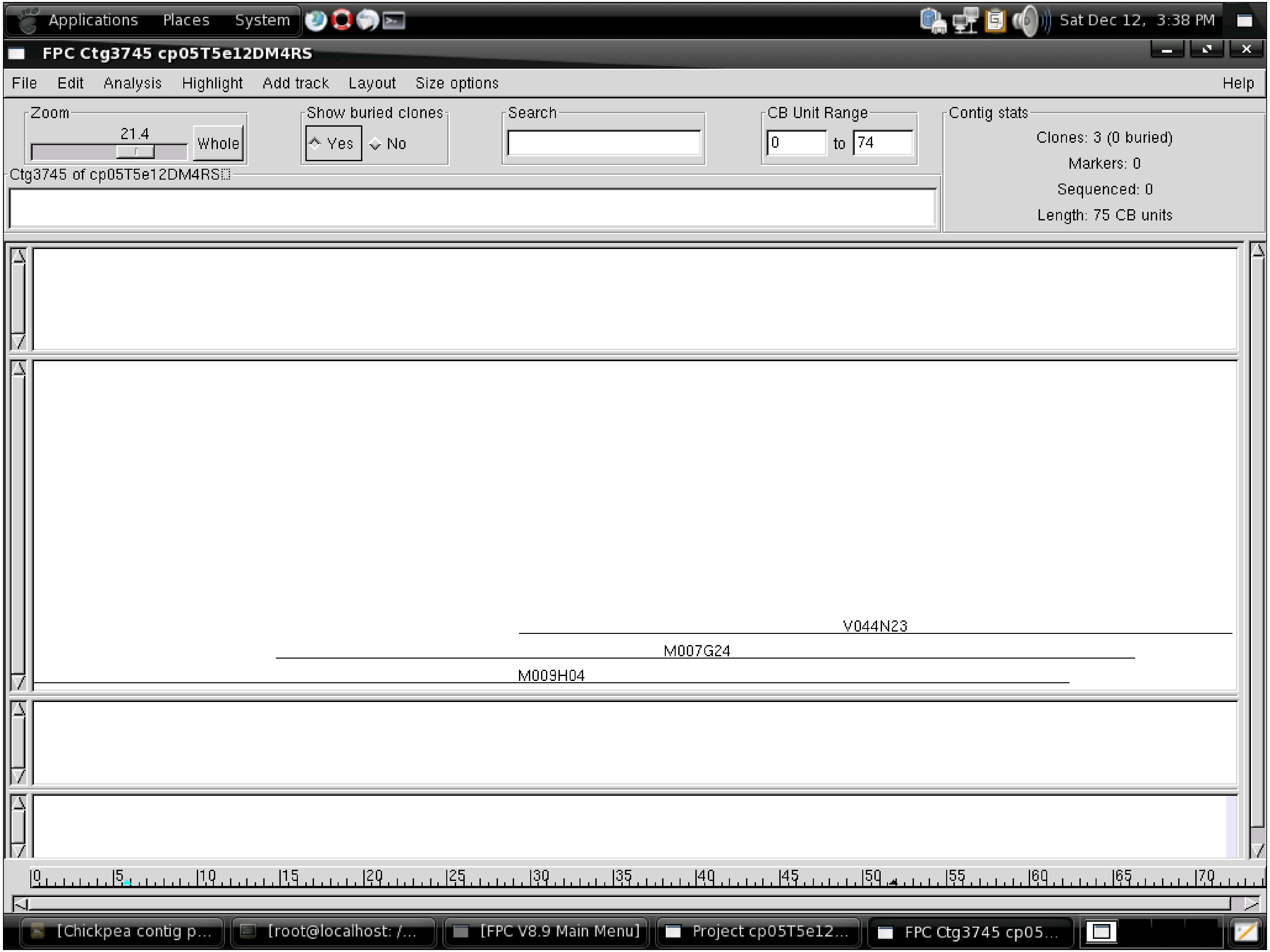
Applications Places System
Sat Dec 12, 3:38 PM
FPC Ctg3745 cp05T5e12DM4RS
File Edit Analysis Highlight Add track Layout Size options
Help
Zoom
21.4
Whole
Show buried clones
Yes
No
Search
CB Unit Range
0
to
74
Contig stats
Clones: 3 (0 buried)
Markers: 0
Sequenced: 0
Length: 75 CB units
Ctg3745 of cp05T5e12DM4RS
V044N23
M007G24
M009H04
0 5 10 15 20 25 30 35 40 45 50 55 60 65 70
[Chickpea contig p...
[root@localhost: /...
[FPC V8.9 Main Menu]
Project cp05T5e12...
FPC Ctg3745 cp05...

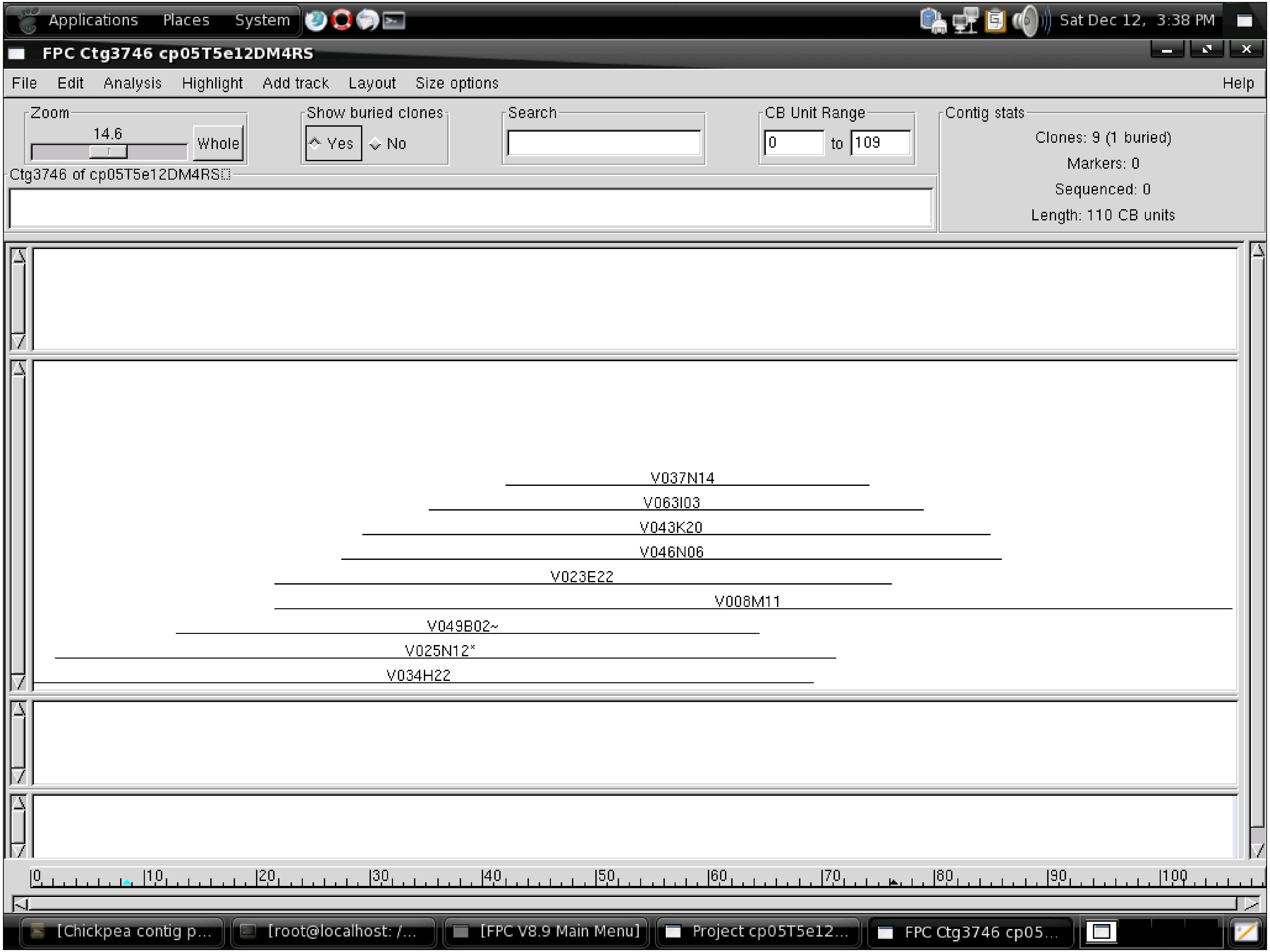
Applications Places System
Sat Dec 12, 3:38 PM
FPC Ctg3746 cp05T5e12DM4RS
File Edit Analysis Highlight Add track Layout Size options Help
Zoom
14.6
Whole
Show buried clones
Yes
No
Search
CB Unit Range
0
to
109
Contig stats
Clones: 9 (1 buried)
Markers: 0
Sequenced: 0
Length: 110 CB units
Ctg3746 of cp05T5e12DM4RS
V037N14
V063I03
V043K20
V046N06
V023E22
V008M11
V049B02~
V025N12*
V034H22
0 10 20 30 40 50 60 70 80 90 100
[Chickpea contig p...
[root@localhost: /...
[FPC V8.9 Main Menu]
Project cp05T5e12...
FPC Ctg3746 cp05...

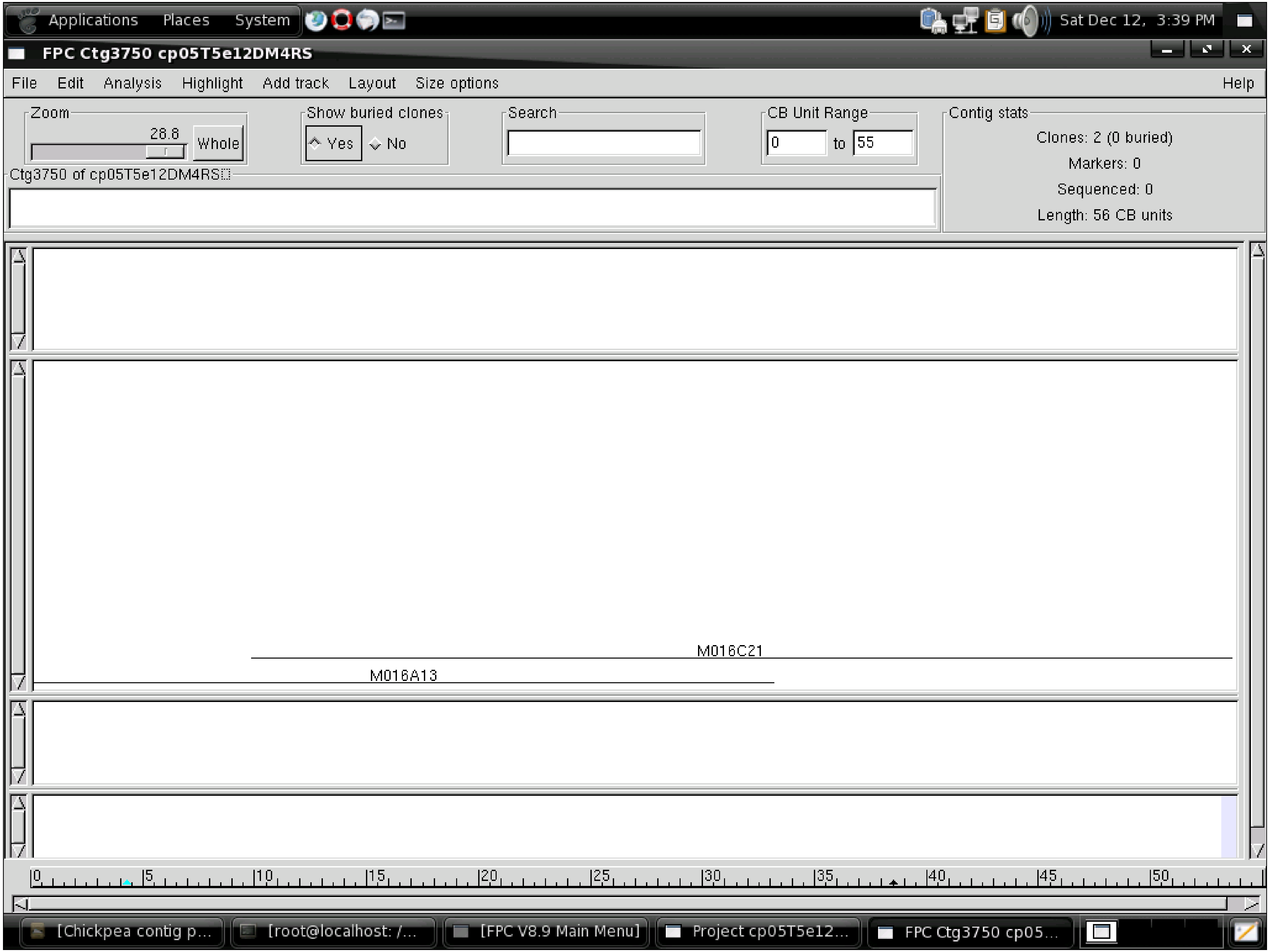
Applications Places System
Sat Dec 12, 3:39 PM
FPC Ctg3750 cp05T5e12DM4RS
File Edit Analysis Highlight Add track Layout Size options Help
Zoom
28.8
Whole
Show buried clones
Yes
No
Search
CB Unit Range
0 to 55
Contig stats
Clones: 2 (0 buried)
Markers: 0
Sequenced: 0
Length: 56 CB units
Ctg3750 of cp05T5e12DM4RS
M016C21
M016A13
0 5 10 15 20 25 30 35 40 45 50
[Chickpea contig p...
[root@localhost: /...
[FPC V8.9 Main Menu]
Project cp05T5e12...
FPC Ctg3750 cp05...

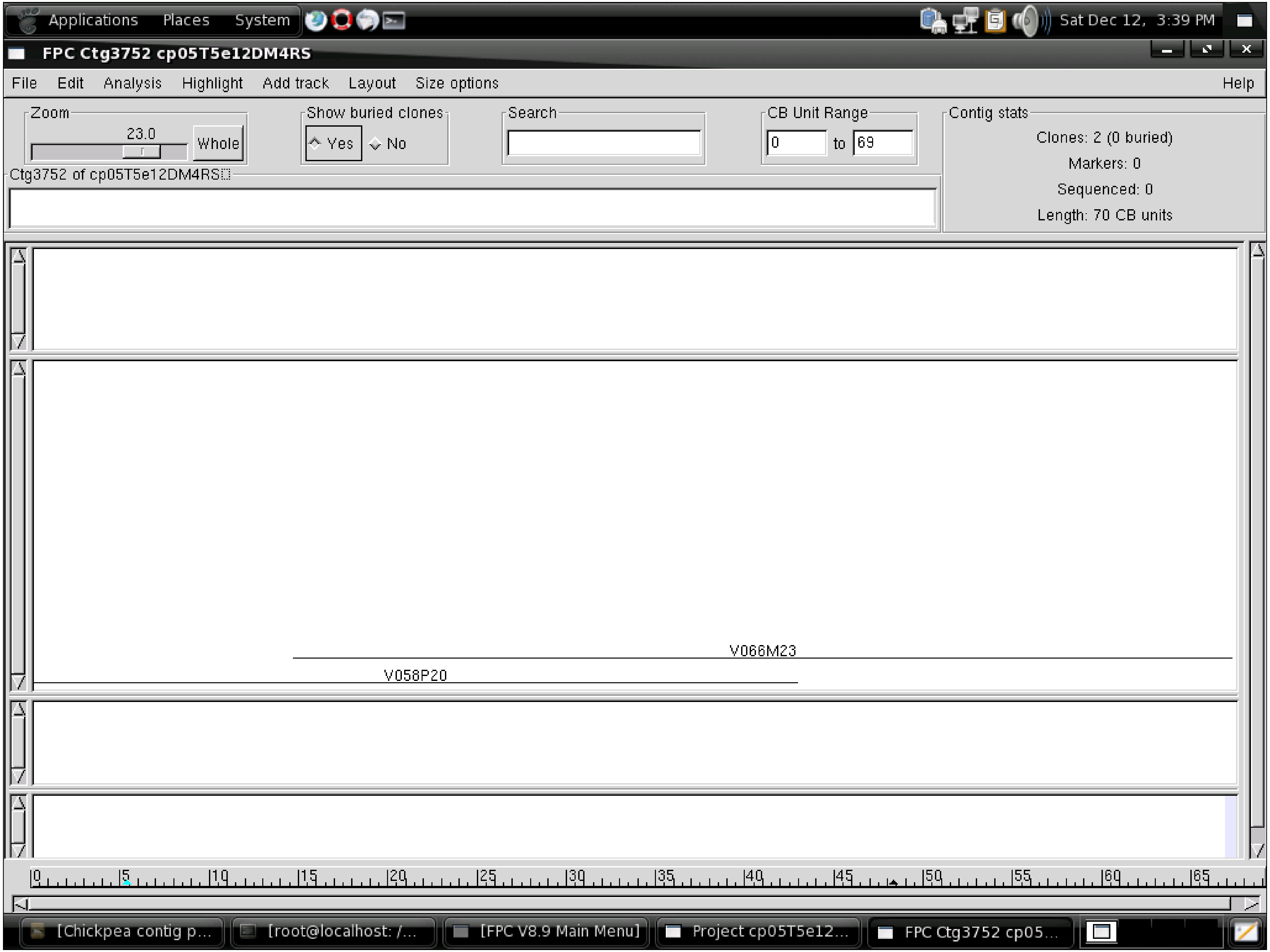
Applications Places System
Sat Dec 12, 3:39 PM
FPC Ctg3752 cp05T5e12DM4RS
File Edit Analysis Highlight Add track Layout Size options Help
Zoom
23.0
Whole
Show buried clones
Yes No
Search
CB Unit Range
0 to 69
Contig stats
Clones: 2 (0 buried)
Markers: 0
Sequenced: 0
Length: 70 CB units
Ctg3752 of cp05T5e12DM4RS
V066M23
V058P20
0 5 10 15 20 25 30 35 40 45 50 55 60 65
[Chickpea contig p...
[root@localhost: /...
[FPC V8.9 Main Menu]
Project cp05T5e12...
FPC Ctg3752 cp05...

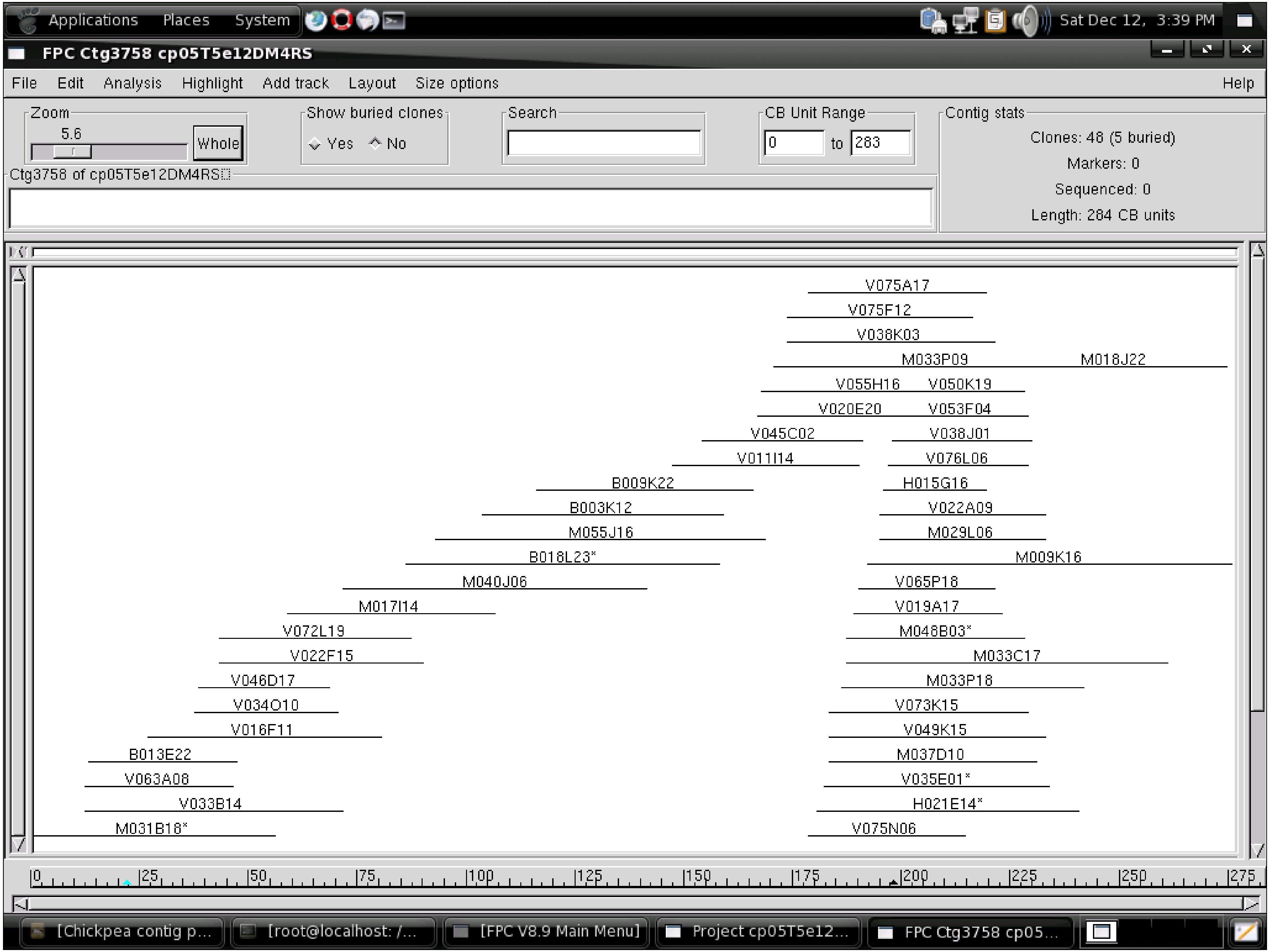

Applications Places System
Sat Dec 12, 3:39 PM
FPC Ctg3758 cp05T5e12DM4RS
File Edit Analysis Highlight Add track Layout Size options
Help
Zoom
5.6
Whole
Show buried clones
Yes No
Search
CB Unit Range
0 to 283
Contig stats
Clones: 48 (5 buried)
Markers: 0
Sequenced: 0
Length: 284 CB units
Ctg3758 of cp05T5e12DM4RS
V075A17
V075F12
V038K03
M033P09
M018J22
V055H16
V050K19
V020E20
V053F04
V045C02
V038J01
V011I14
V076L06
B009K22
H015G16
B003K12
V022A09
M055J16
M029L06
B018L23*
M009K16
M040J06
V065P18
M017I14
V019A17
V072L19
M048B03*
V022F15
M033C17
V046D17
M033P18
V034O10
V073K15
V016F11
V049K15
B013E22
M037D10
V063A08
V035E01*
V033B14
H021E14*
M031B18*
V075N06
0 25 50 75 100 125 150 175 200 225 250 275
[Chickpea contig p...
[root@localhost: /...
[FPC V8.9 Main Menu]
Project cp05T5e12...
FPC Ctg3758 cp05...

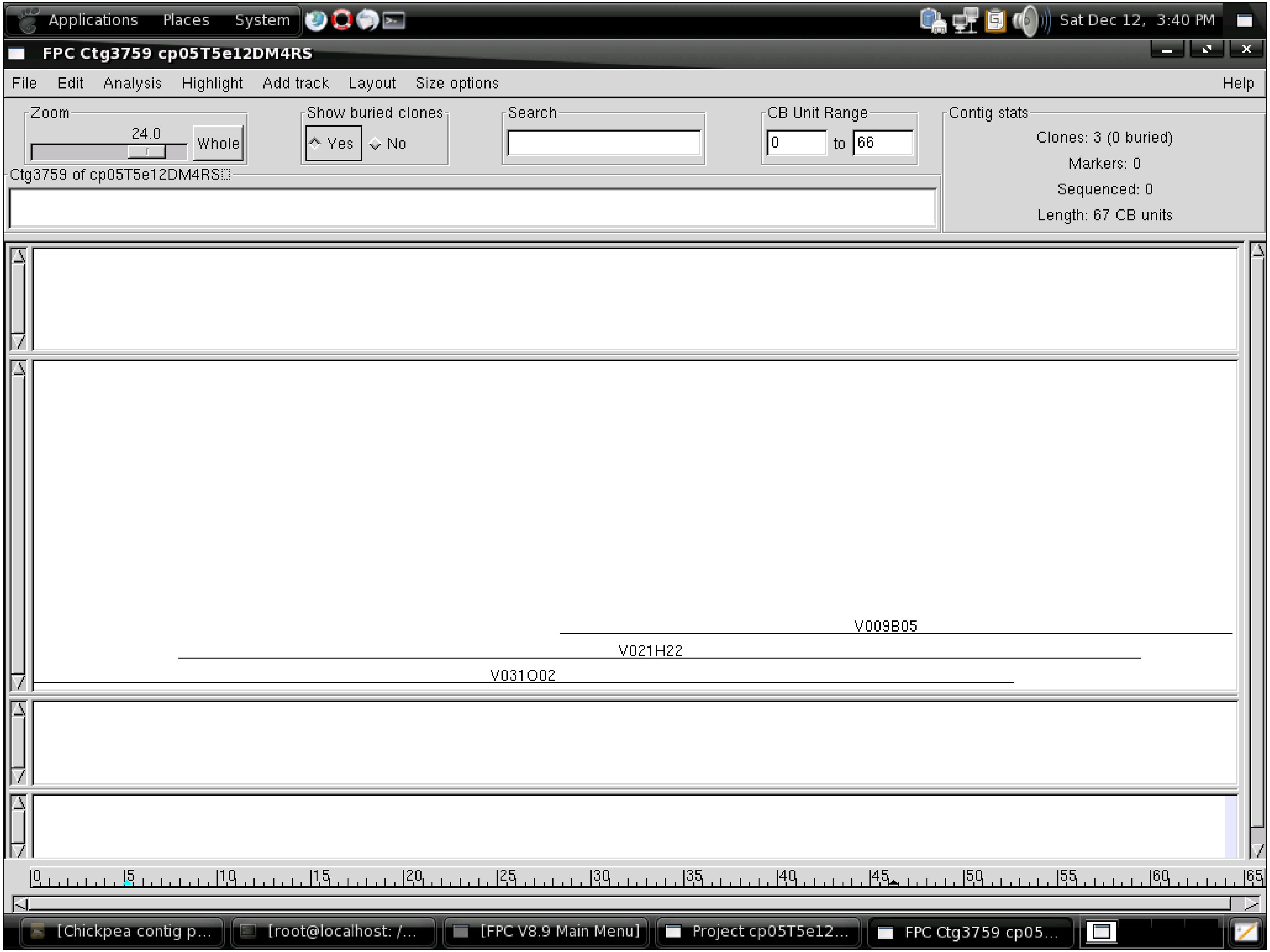
Applications Places System
Sat Dec 12, 3:40 PM
FPC Ctg3759 cp05T5e12DM4RS
File Edit Analysis Highlight Add track Layout Size options Help
Zoom
24.0
Whole
Show buried clones
Yes
No
Search
CB Unit Range
0 to 66
Contig stats
Clones: 3 (0 buried)
Markers: 0
Sequenced: 0
Length: 67 CB units
Ctg3759 of cp05T5e12DM4RS
V009B05
V021H22
V031O02
0 5 10 15 20 25 30 35 40 45 50 55 60 65
[Chickpea contig p...
[root@localhost: /...
[FPC V8.9 Main Menu]
Project cp05T5e12...
FPC Ctg3759 cp05...

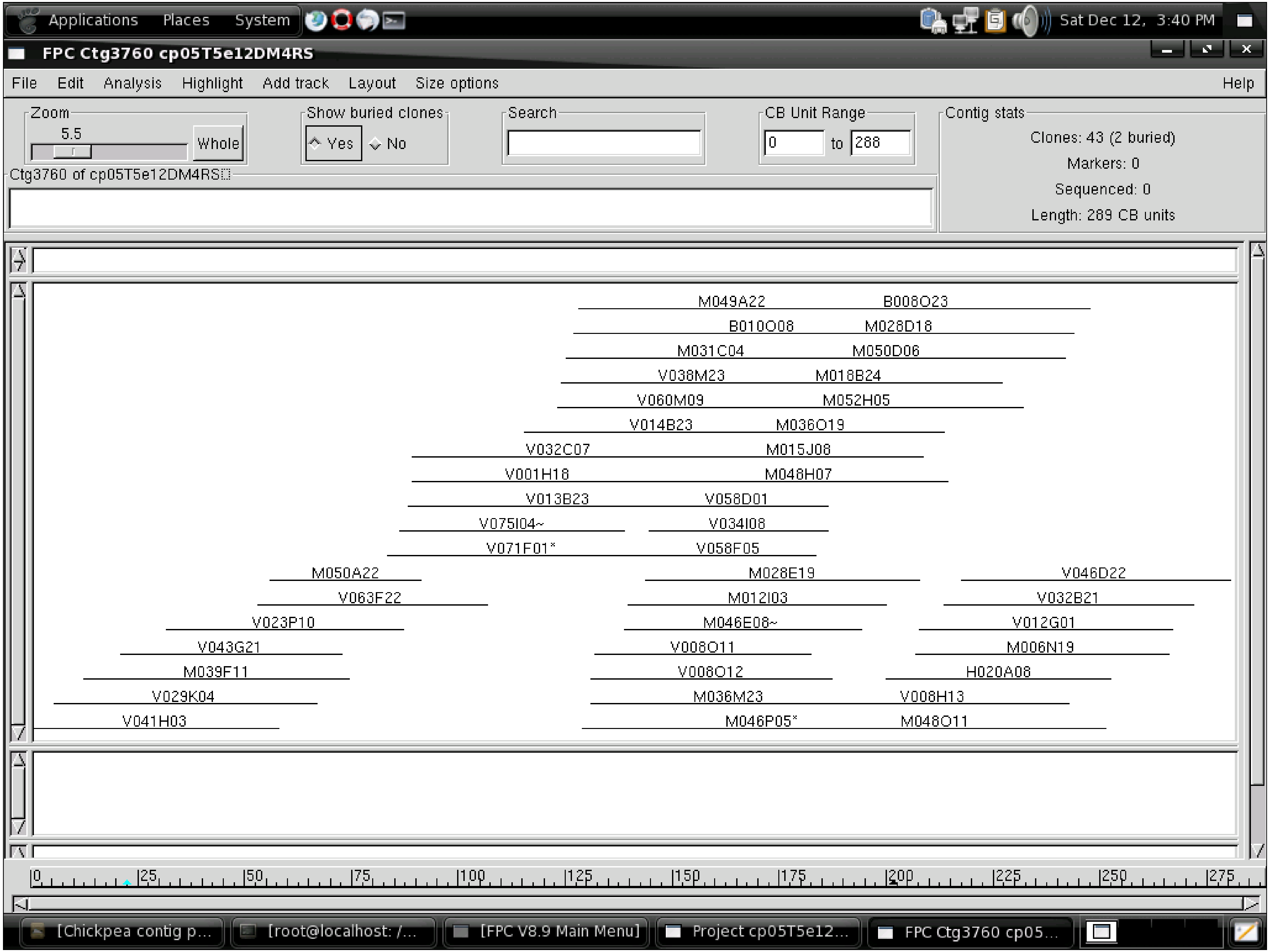
Applications Places System
Sat Dec 12, 3:40 PM
FPC Ctg3760 cp05T5e12DM4RS
File Edit Analysis Highlight Add track Layout Size options Help
Zoom 5.5 Whole
Show buried clones Yes No
Search
CB Unit Range 0 to 288
Contig stats
Clones: 43 (2 buried)
Markers: 0
Sequenced: 0
Length: 289 CB units
Ctg3760 of cp05T5e12DM4RS
M049A22 B008O23
B010O08 M028D18
M031C04 M050D06
V038M23 M018B24
V060M09 M052H05
V014B23 M036O19
V032C07 M015J08
V001H18 M048H07
V013B23 V058D01
V075I04~ V034I08
V071F01* V058F05
M050A22 M028E19 V046D22
V063F22 M012I03 V032B21
V023P10 M046E08~ V012G01
V043G21 V008O11 M006N19
M039F11 V008O12 H020A08
V029K04 M036M23 V008H13
V041H03 M046P05* M048O11
0 25 50 75 100 125 150 175 200 225 250 275
[Chickpea contig p... [root@localhost: /... [FPC V8.9 Main Menu] Project cp05T5e12... FPC Ctg3760 cp05...

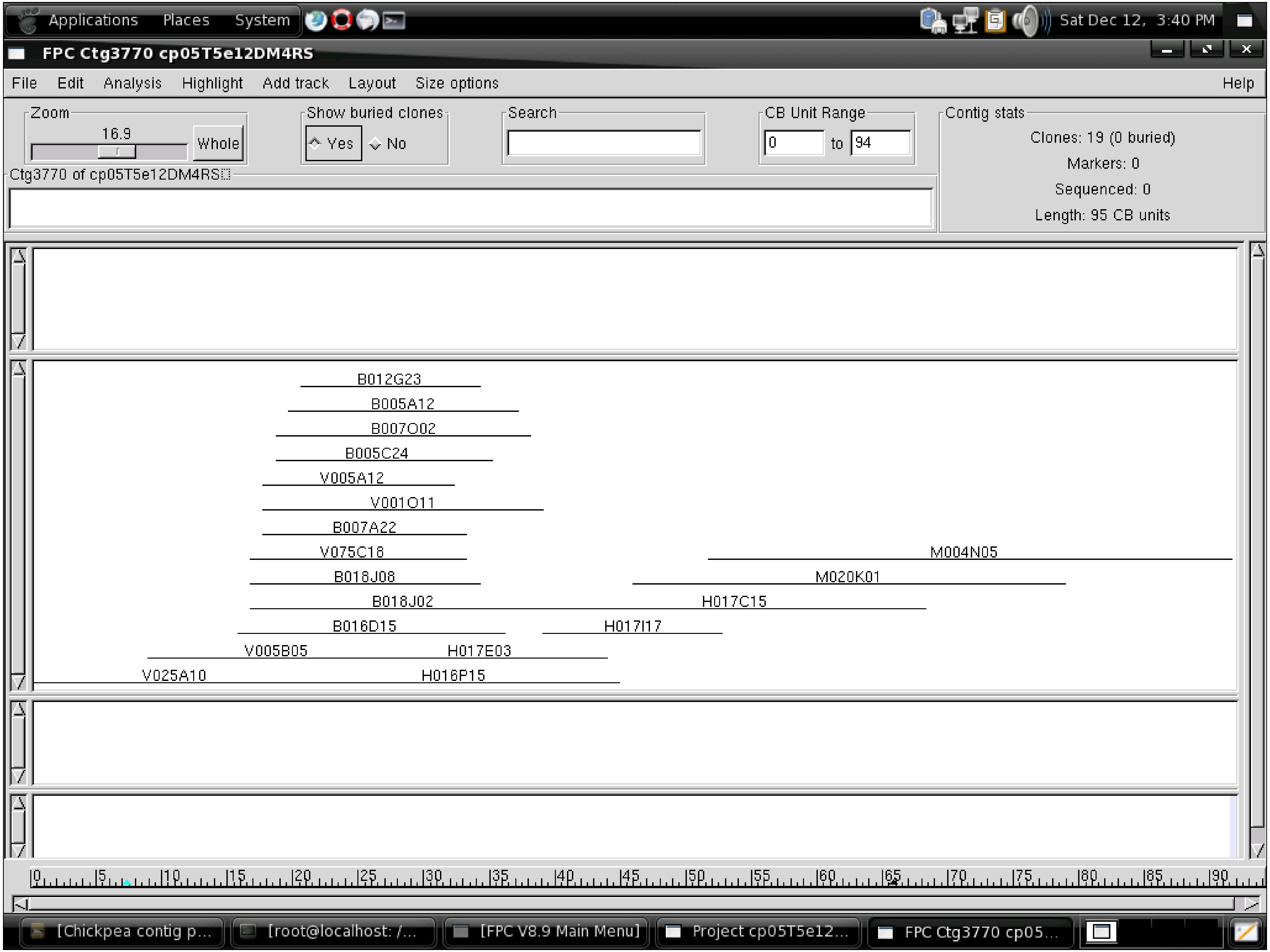
Applications Places System
Sat Dec 12, 3:40 PM
FPC Ctg3770 cp05T5e12DM4RS
File Edit Analysis Highlight Add track Layout Size options Help
Zoom
16.9
Whole
Show buried clones
Yes
No
Search
CB Unit Range
0 to 94
Contig stats
Clones: 19 (0 buried)
Markers: 0
Sequenced: 0
Length: 95 CB units
Ctg3770 of cp05T5e12DM4RS
B012G23
B005A12
B007O02
B005C24
V005A12
V001O11
B007A22
V075C18
M004N05
B018J08
M020K01
B018J02
H017C15
B016D15
H017I17
V005B05
H017E03
V025A10
H016P15
0 5 10 15 20 25 30 35 40 45 50 55 60 65 70 75 80 85 90
[Chickpea contig p...
[root@localhost: /...
[FPC V8.9 Main Menu]
Project cp05T5e12...
FPC Ctg3770 cp05...

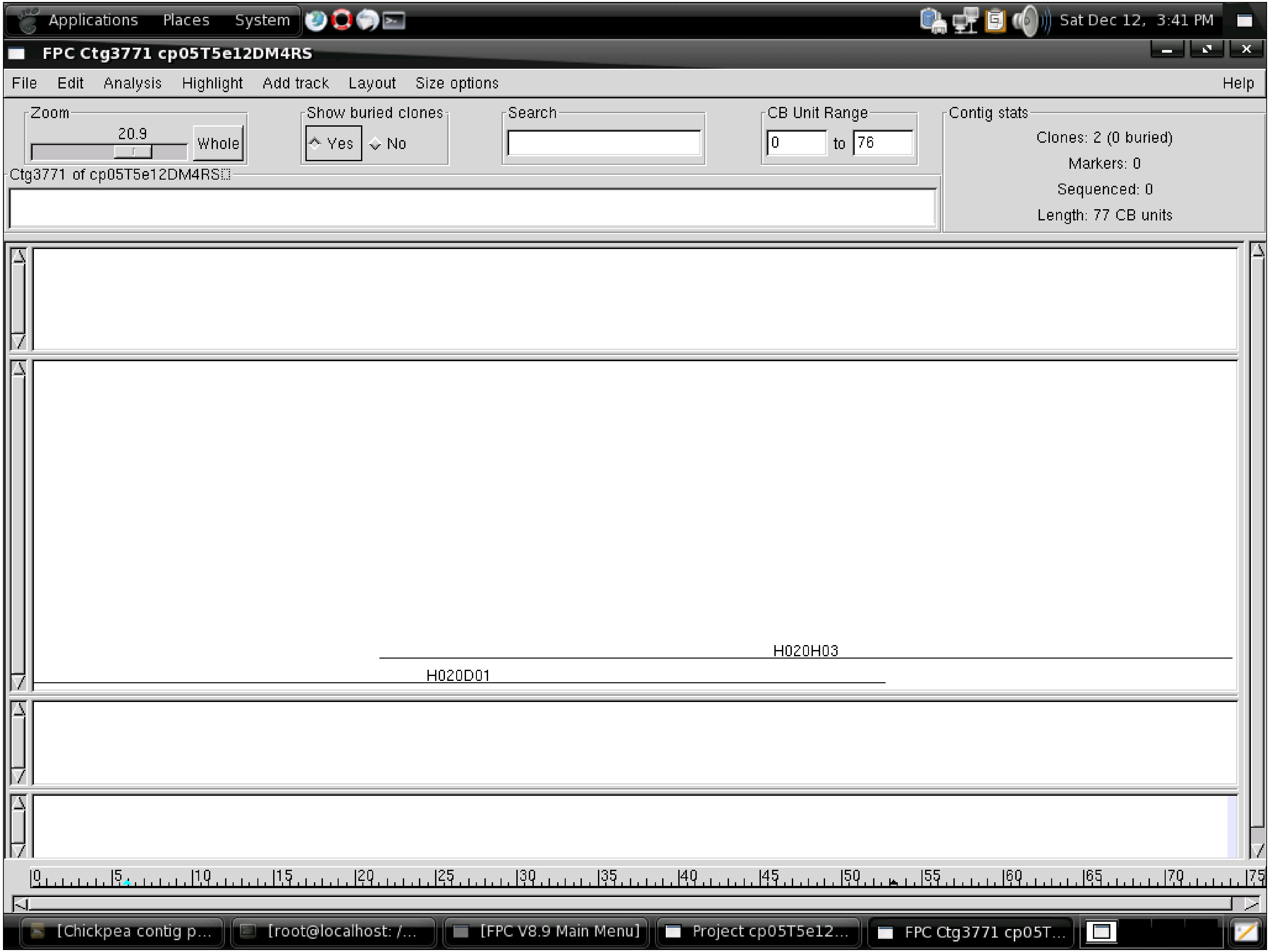

Applications
Places
System
Sat Dec 12, 3:41 PM
FPC Ctg3771 cp05T5e12DM4RS
File
Edit
Analysis
Highlight
Add track
Layout
Size options
Help
Zoom
20.9
Whole
Show buried clones
Yes
No
Search
CB Unit Range
0
to
76
Contig stats
Clones: 2 (0 buried)
Markers: 0
Sequenced: 0
Length: 77 CB units
Ctg3771 of cp05T5e12DM4RS
H020H03
H020D01
0 5 10 15 20 25 30 35 40 45 50 55 60 65 70 75
[Chickpea contig p...
[root@localhost: /...
[FPC V8.9 Main Menu]
Project cp05T5e12...
FPC Ctg3771 cp05T...

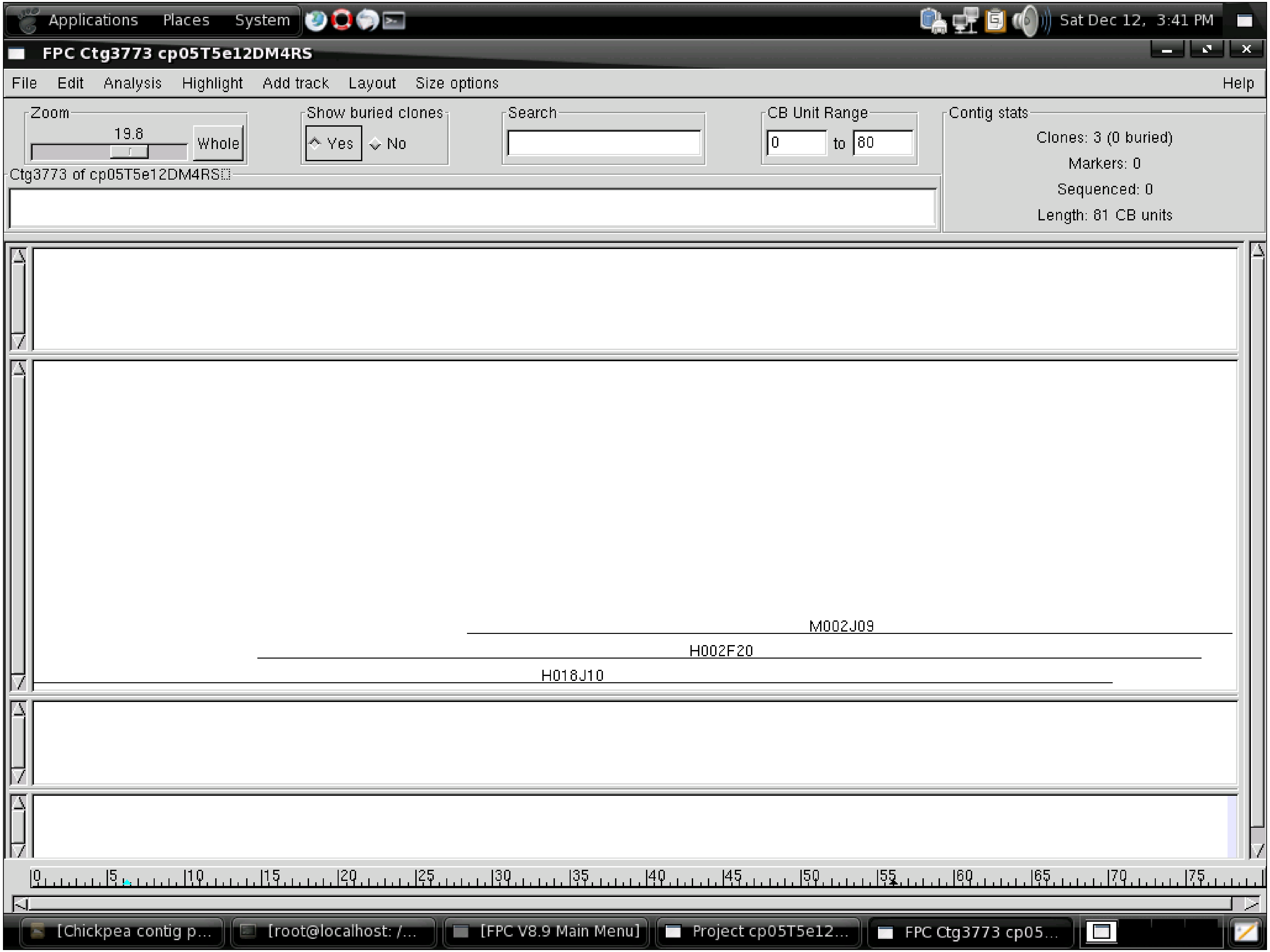
Applications Places System
Sat Dec 12, 3:41 PM
FPC Ctg3773 cp05T5e12DM4RS
File Edit Analysis Highlight Add track Layout Size options Help
Zoom
19.8
Whole
Show buried clones
Yes
No
Search
CB Unit Range
0
to
80
Contig stats
Clones: 3 (0 buried)
Markers: 0
Sequenced: 0
Length: 81 CB units
Ctg3773 of cp05T5e12DM4RS
M002J09
H002F20
H018J10
0 5 10 15 20 25 30 35 40 45 50 55 60 65 70 75
[Chickpea contig p...
[root@localhost: /...
[FPC V8.9 Main Menu]
Project cp05T5e12...
FPC Ctg3773 cp05...

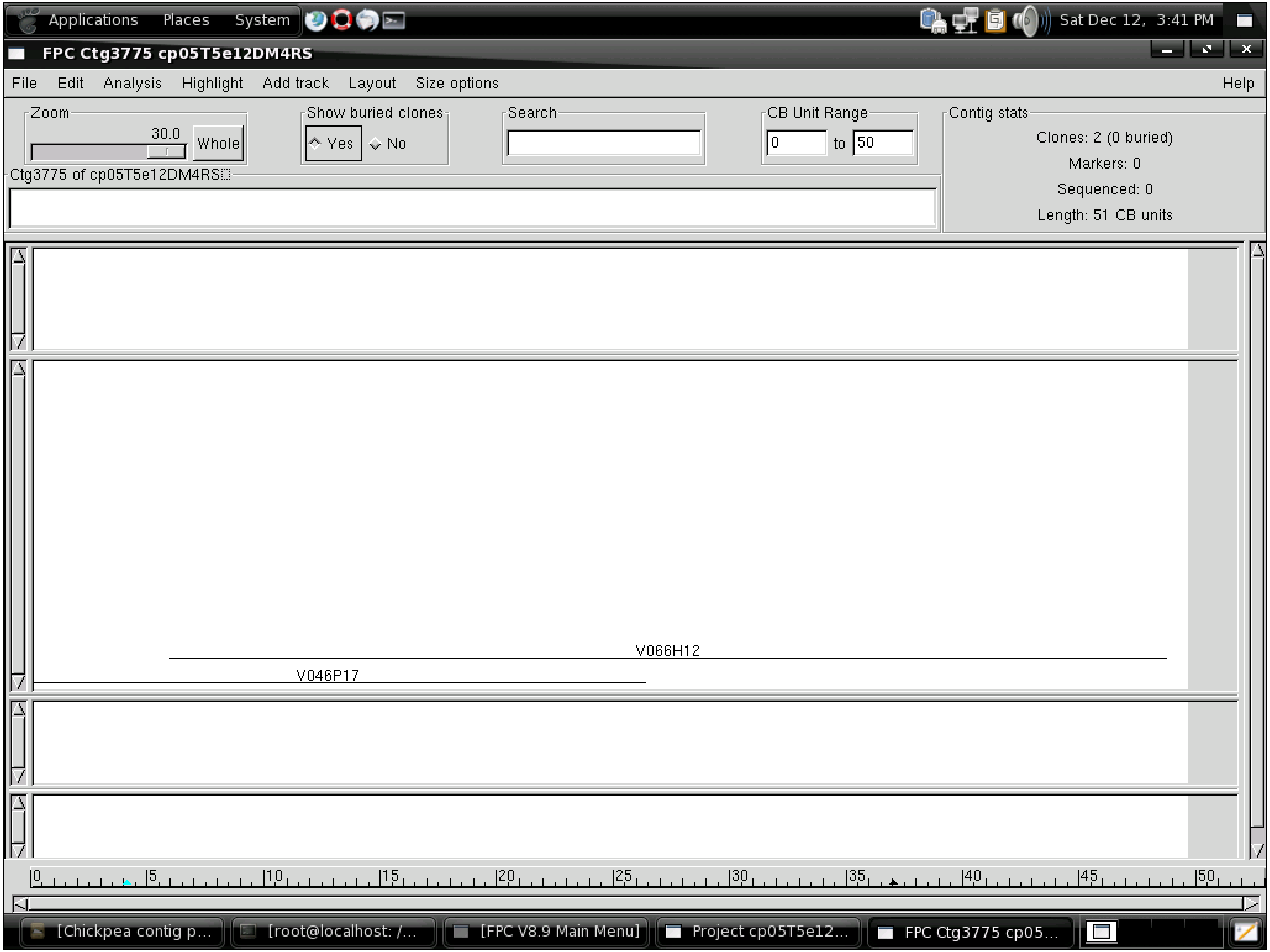
Applications Places System
Sat Dec 12, 3:41 PM
FPC Ctg3775 cp05T5e12DM4RS
File Edit Analysis Highlight Add track Layout Size options Help
Zoom
30.0
Whole
Show buried clones
Yes
No
Search
CB Unit Range
0 to 50
Contig stats
Clones: 2 (0 buried)
Markers: 0
Sequenced: 0
Length: 51 CB units
Ctg3775 of cp05T5e12DM4RS
V066H12
V046P17
0 5 10 15 20 25 30 35 40 45 50
[Chickpea contig p...
[root@localhost: /...
[FPC V8.9 Main Menu]
Project cp05T5e12...
FPC Ctg3775 cp05...

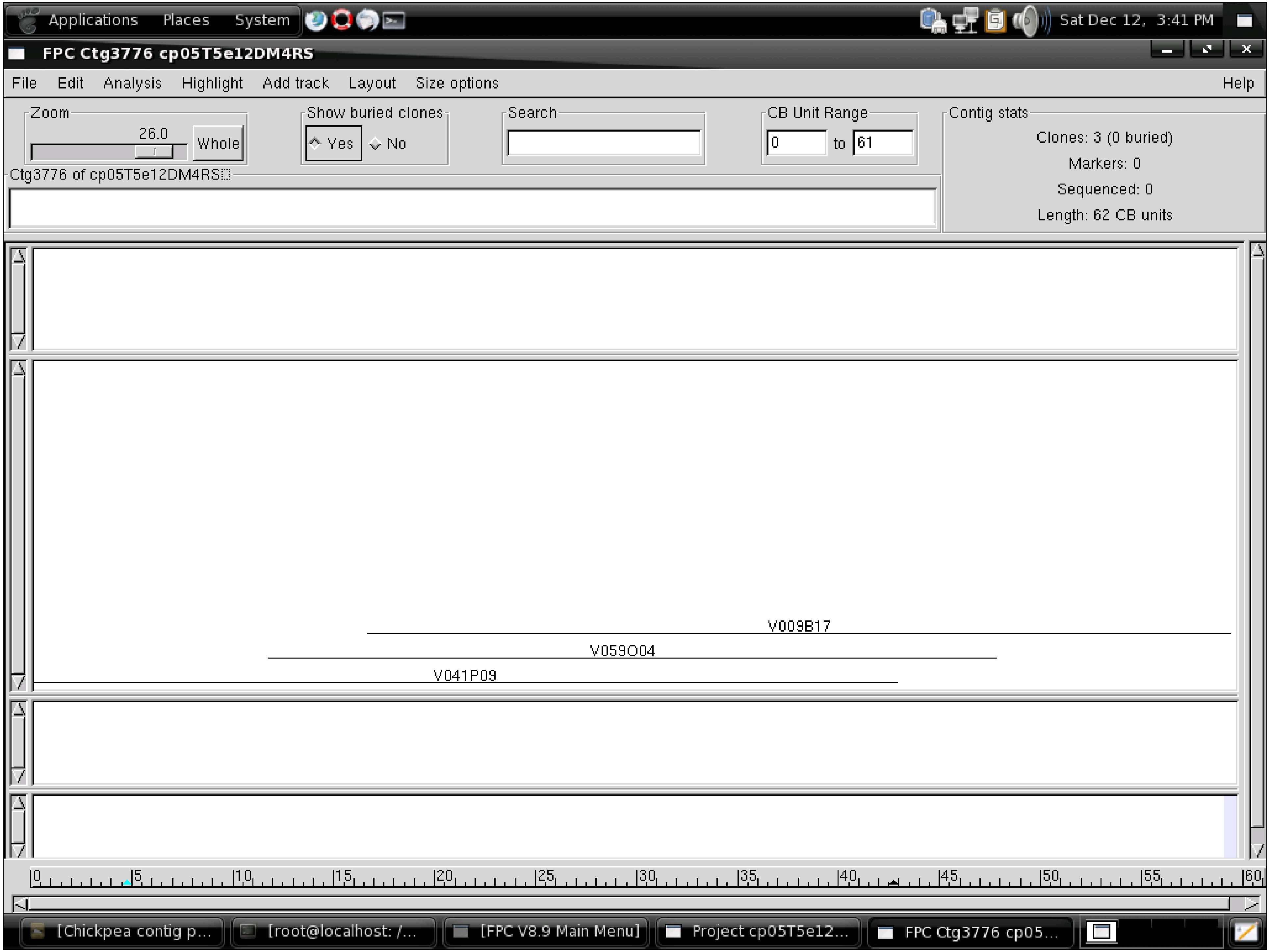
Applications
Places
System
Sat Dec 12, 3:41 PM
FPC Ctg3776 cp05T5e12DM4RS
File
Edit
Analysis
Highlight
Add track
Layout
Size options
Help
Zoom
26.0
Whole
Show buried clones
Yes
No
Search
CB Unit Range
0
to
61
Contig stats
Clones: 3 (0 buried)
Markers: 0
Sequenced: 0
Length: 62 CB units
Ctg3776 of cp05T5e12DM4RS
V009B17
V059O04
V041P09
0
5
10
15
20
25
30
35
40
45
50
55
60
[Chickpea contig p...
[root@localhost: /...
[FPC V8.9 Main Menu]
Project cp05T5e12...
FPC Ctg3776 cp05...

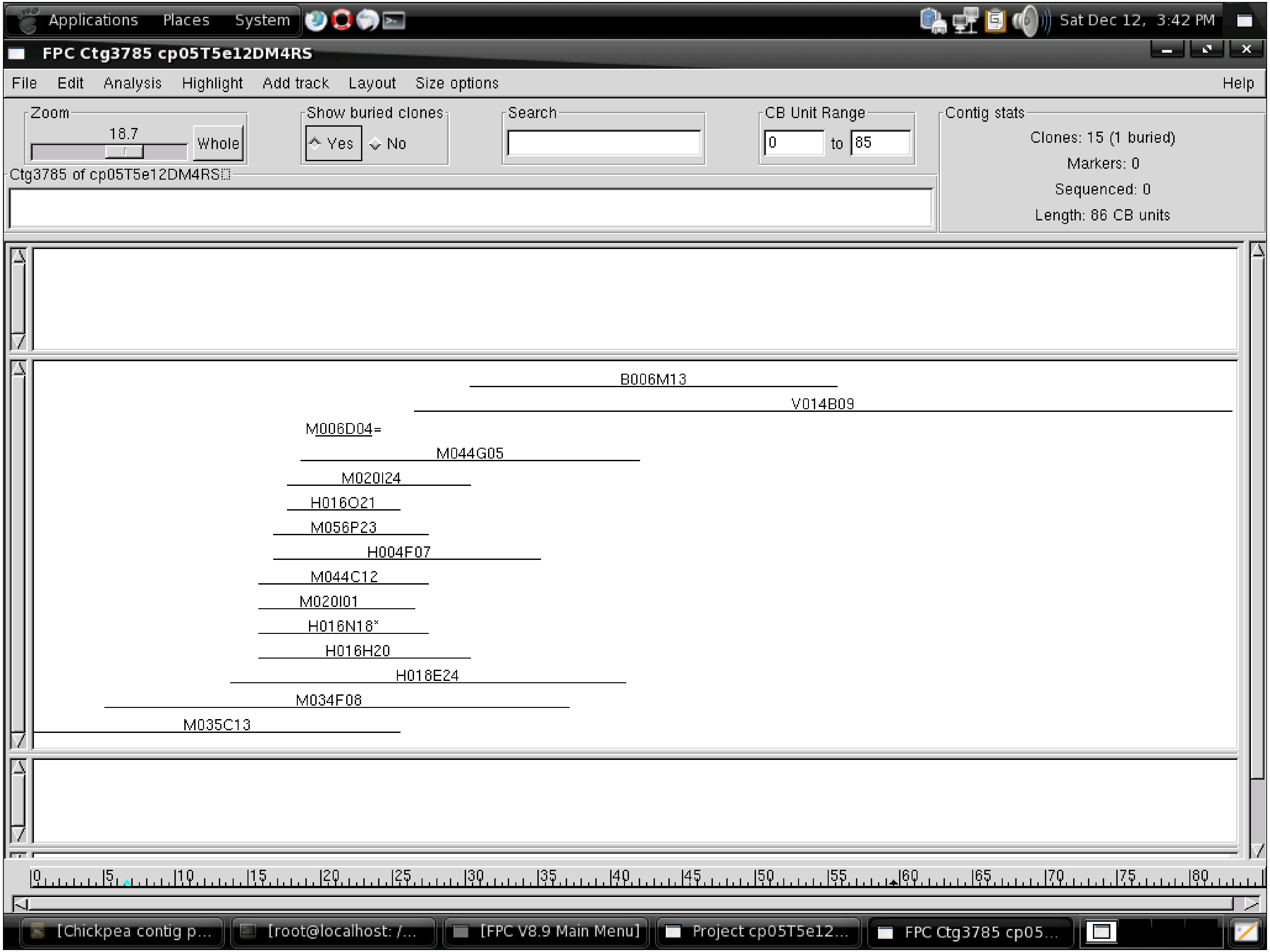

Applications Places System
Sat Dec 12, 3:42 PM
FPC Ctg3785 cp05T5e12DM4RS
File Edit Analysis Highlight Add track Layout Size options Help
Zoom
18.7
Whole
Show buried clones
Yes No
Search
CB Unit Range
0 to 85
Contig stats
Clones: 15 (1 buried)
Markers: 0
Sequenced: 0
Length: 86 CB units
Ctg3785 of cp05T5e12DM4RS
B006M13
V014B09
M006D04=
M044G05
M020I24
H016O21
M056P23
H004F07
M044C12
M020I01
H016N18*
H016H20
H018E24
M034F08
M035C13
0 5 10 15 20 25 30 35 40 45 50 55 60 65 70 75 80
[Chickpea contig p...
[root@localhost: /...
[FPC V8.9 Main Menu]
Project cp05T5e12...
FPC Ctg3785 cp05...

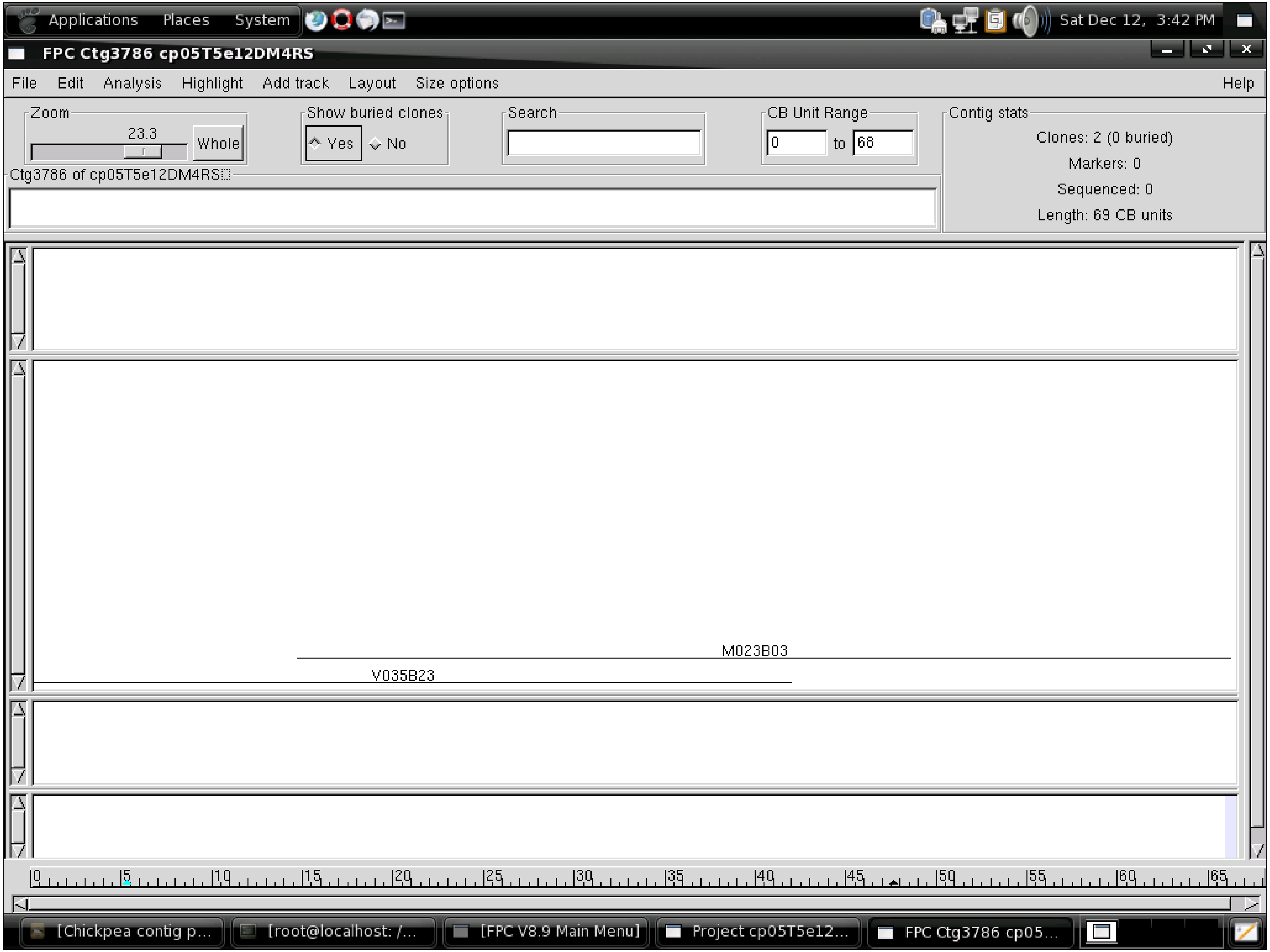
Applications
Places
System
Sat Dec 12, 3:42 PM
FPC Ctg3786 cp05T5e12DM4RS
File
Edit
Analysis
Highlight
Add track
Layout
Size options
Help
Zoom
23.3
Whole
Show buried clones
Yes
No
Search
CB Unit Range
0
to
68
Contig stats
Clones: 2 (0 buried)
Markers: 0
Sequenced: 0
Length: 69 CB units
Ctg3786 of cp05T5e12DM4RS
M023B03
V035B23
0 5 10 15 20 25 30 35 40 45 50 55 60 65
[Chickpea contig p...
[root@localhost: /...
[FPC V8.9 Main Menu]
Project cp05T5e12...
FPC Ctg3786 cp05...

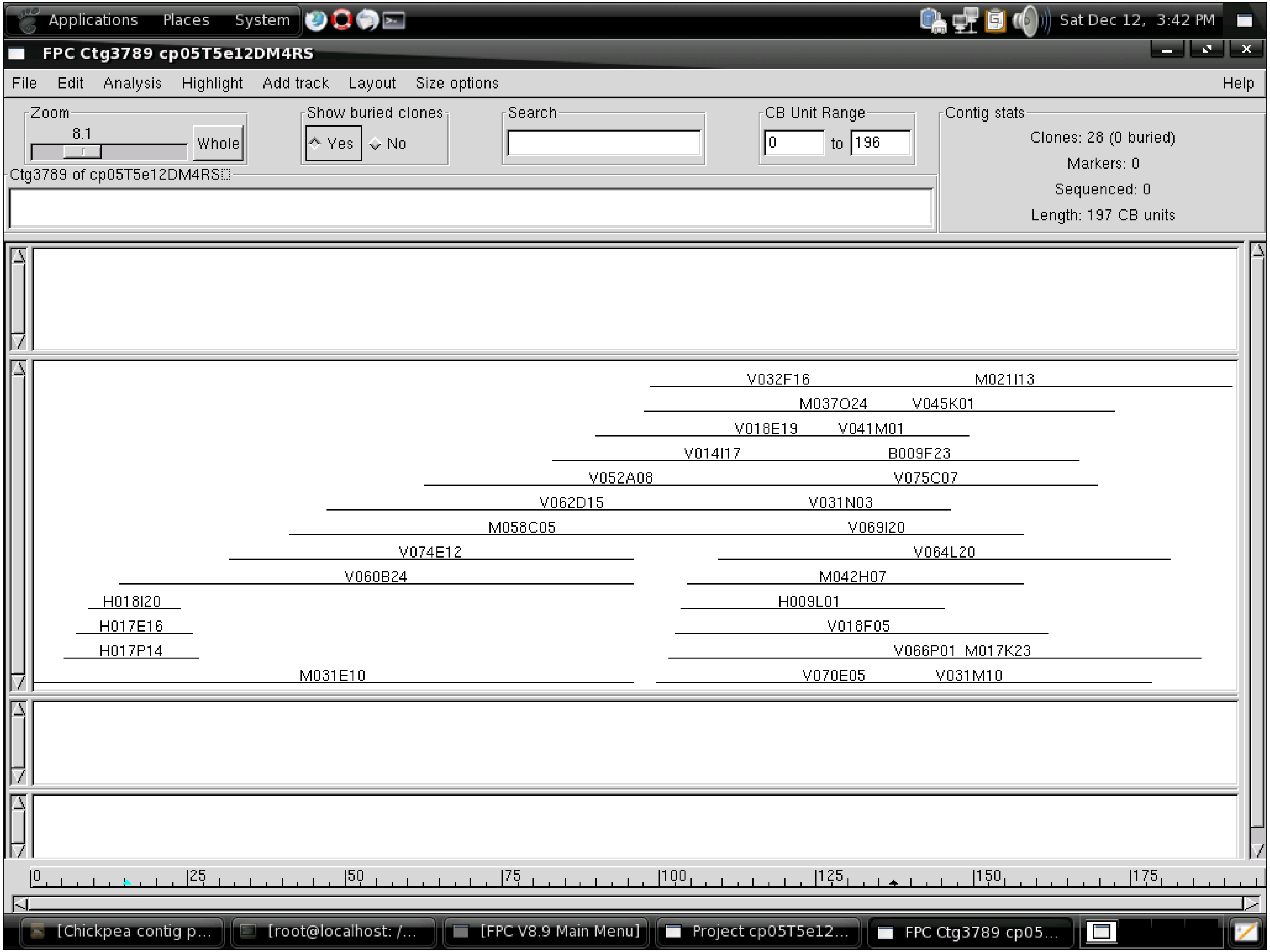
Applications Places System
Sat Dec 12, 3:42 PM
FPC Ctg3789 cp05T5e12DM4RS
File Edit Analysis Highlight Add track Layout Size options Help
Zoom
8.1
Whole
Show buried clones
Yes No
Search
CB Unit Range
0 to 196
Contig stats
Clones: 28 (0 buried)
Markers: 0
Sequenced: 0
Length: 197 CB units
Ctg3789 of cp05T5e12DM4RS
V032F16 M021I13
M037O24 V045K01
V018E19 V041M01
V014I17 B009F23
V052A08 V075C07
V062D15 V031N03
M058C05 V069I20
V074E12 V064L20
V060B24 M042H07
H018I20 H009L01
H017E16 V018F05
H017P14 V066P01 M017K23
M031E10 V070E05 V031M10
0 25 50 75 100 125 150 175
[Chickpea contig p...
[root@localhost: /...
[FPC V8.9 Main Menu]
Project cp05T5e12...
FPC Ctg3789 cp05...

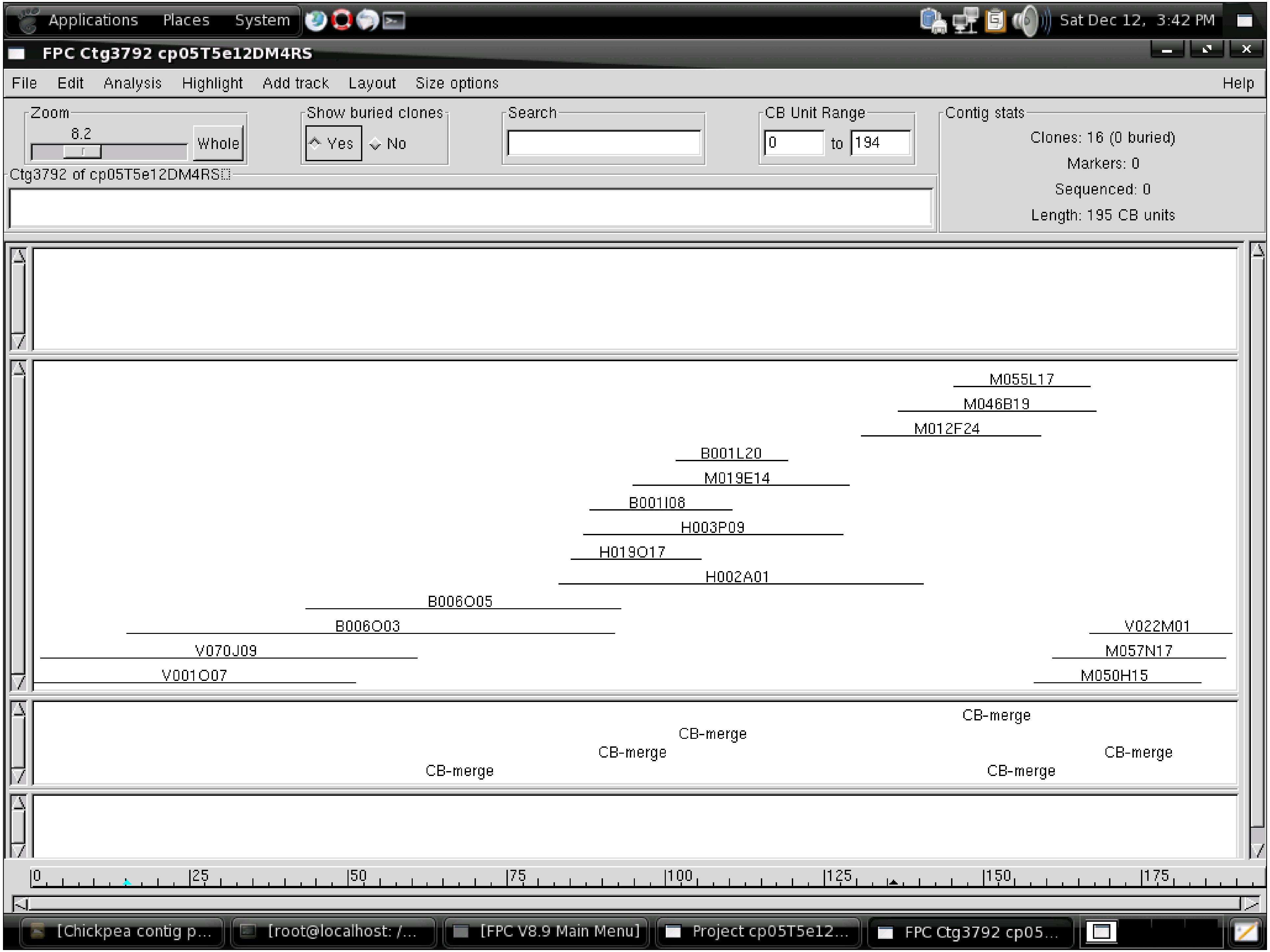
Applications Places System
Sat Dec 12, 3:42 PM
FPC Ctg3792 cp05T5e12DM4RS
File Edit Analysis Highlight Add track Layout Size options Help
Zoom
8.2
Whole
Show buried clones
Yes
No
Search
CB Unit Range
0
to
194
Contig stats
Clones: 16 (0 buried)
Markers: 0
Sequenced: 0
Length: 195 CB units
Ctg3792 of cp05T5e12DM4RS
M055L17
M046B19
M012F24
B001L20
M019E14
B001I08
H003P09
H019O17
H002A01
B006O05
B006O03
V022M01
V070J09
M057N17
V001O07
M050H15
CB-merge
CB-merge
CB-merge
CB-merge
CB-merge
CB-merge
0
25
50
75
100
125
150
175
[Chickpea contig p...
[root@localhost: /...
[FPC V8.9 Main Menu]
Project cp05T5e12...
FPC Ctg3792 cp05...

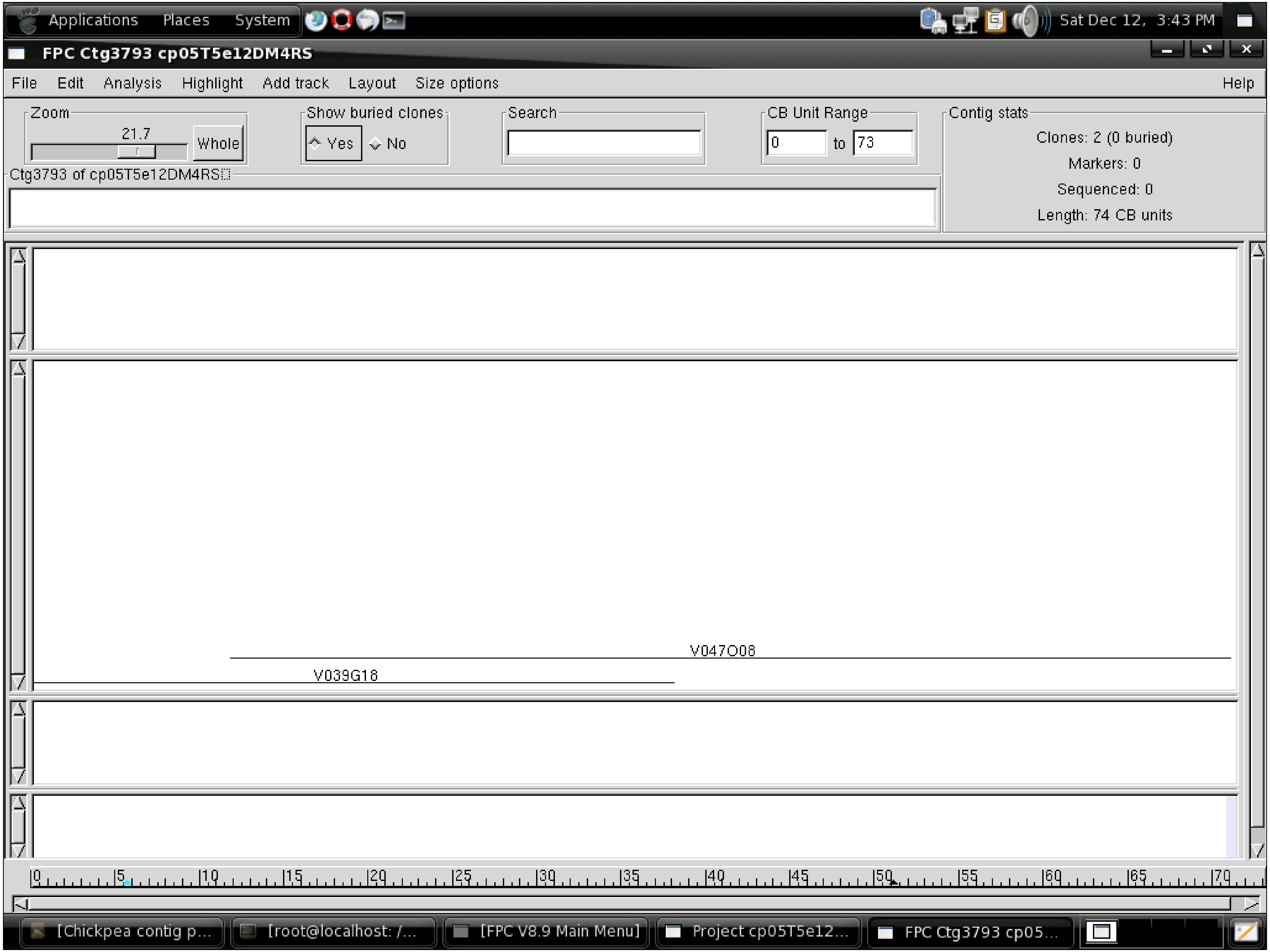
Applications Places System
Sat Dec 12, 3:43 PM
FPC Ctg3793 cp05T5e12DM4RS
File Edit Analysis Highlight Add track Layout Size options Help
Zoom
21.7
Whole
Show buried clones
Yes
No
Search
CB Unit Range
0 to 73
Contig stats
Clones: 2 (0 buried)
Markers: 0
Sequenced: 0
Length: 74 CB units
Ctg3793 of cp05T5e12DM4RS
V047O08
V039G18
0 5 10 15 20 25 30 35 40 45 50 55 60 65 70
[Chickpea contig p...
[root@localhost: /...
[FPC V8.9 Main Menu]
Project cp05T5e12...
FPC Ctg3793 cp05...

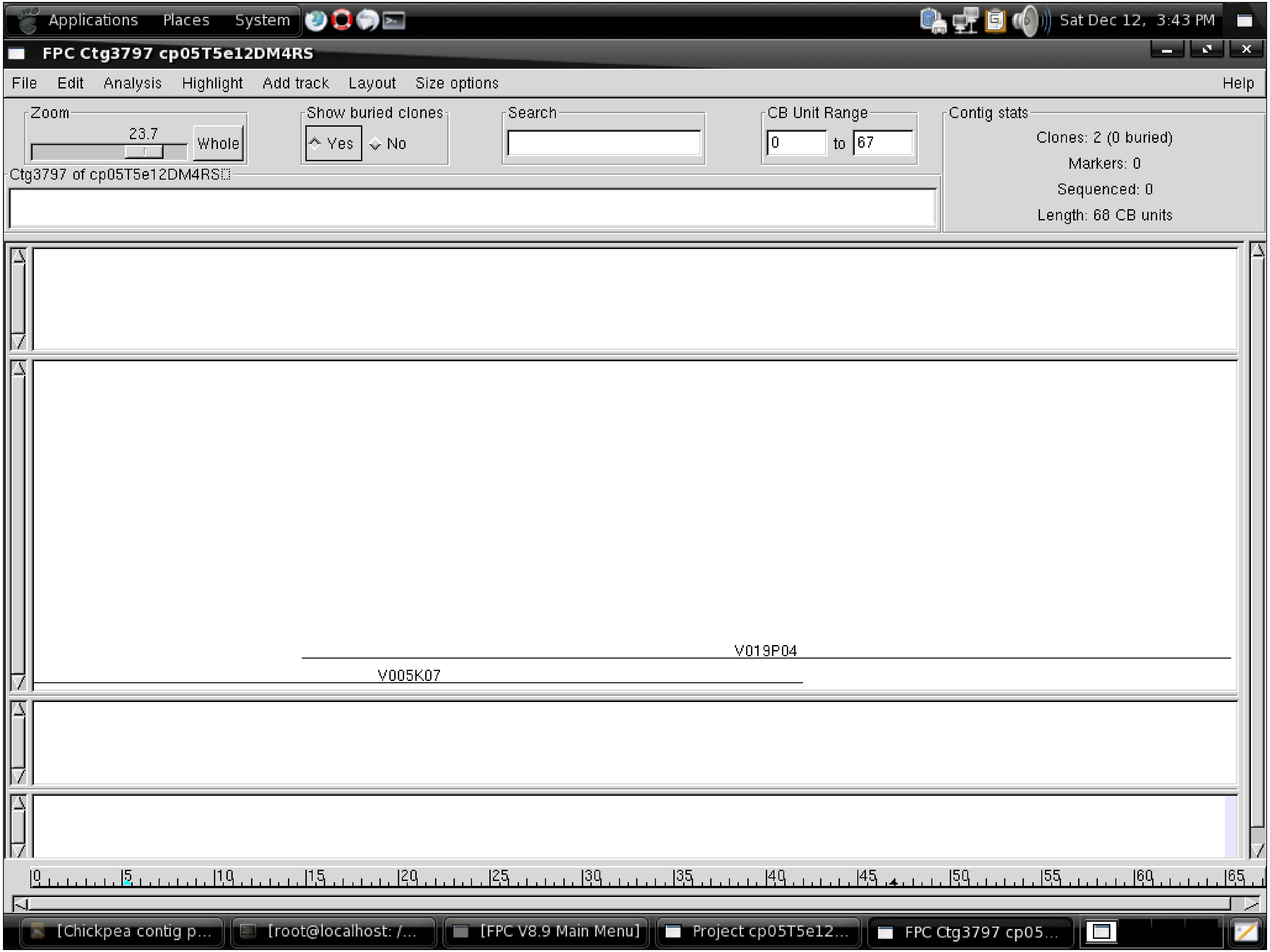
Applications
Places
System
Sat Dec 12, 3:43 PM
FPC Ctg3797 cp05T5e12DM4RS
File
Edit
Analysis
Highlight
Add track
Layout
Size options
Help
Zoom
23.7
Whole
Show buried clones
Yes
No
Search
CB Unit Range
0
to
67
Contig stats
Clones: 2 (0 buried)
Markers: 0
Sequenced: 0
Length: 68 CB units
Ctg3797 of cp05T5e12DM4RS
V019P04
V005K07
0
5
10
15
20
25
30
35
40
45
50
55
60
65
[Chickpea contig p...
[root@localhost: /...
[FPC V8.9 Main Menu]
Project cp05T5e12...
FPC Ctg3797 cp05...

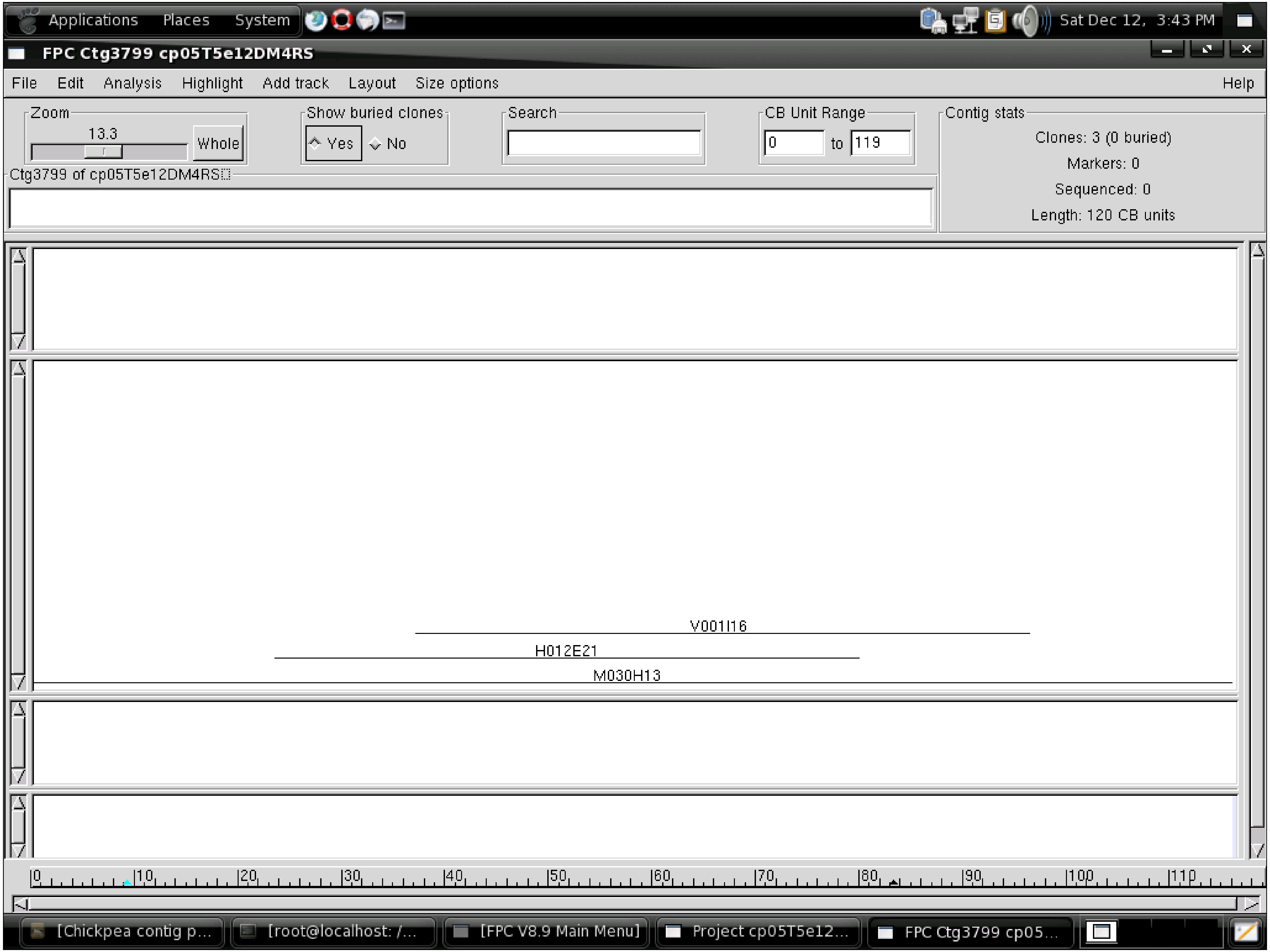
Applications
Places
System
Sat Dec 12, 3:43 PM
FPC Ctg3799 cp05T5e12DM4RS
File
Edit
Analysis
Highlight
Add track
Layout
Size options
Help
Zoom
13.3
Whole
Show buried clones
Yes
No
Search
CB Unit Range
0
to
119
Contig stats
Clones: 3 (0 buried)
Markers: 0
Sequenced: 0
Length: 120 CB units
Ctg3799 of cp05T5e12DM4RS
V001I16
H012E21
M030H13
0 10 20 30 40 50 60 70 80 90 100 110
[Chickpea contig p...
[root@localhost: /...
[FPC V8.9 Main Menu]
Project cp05T5e12...
FPC Ctg3799 cp05...

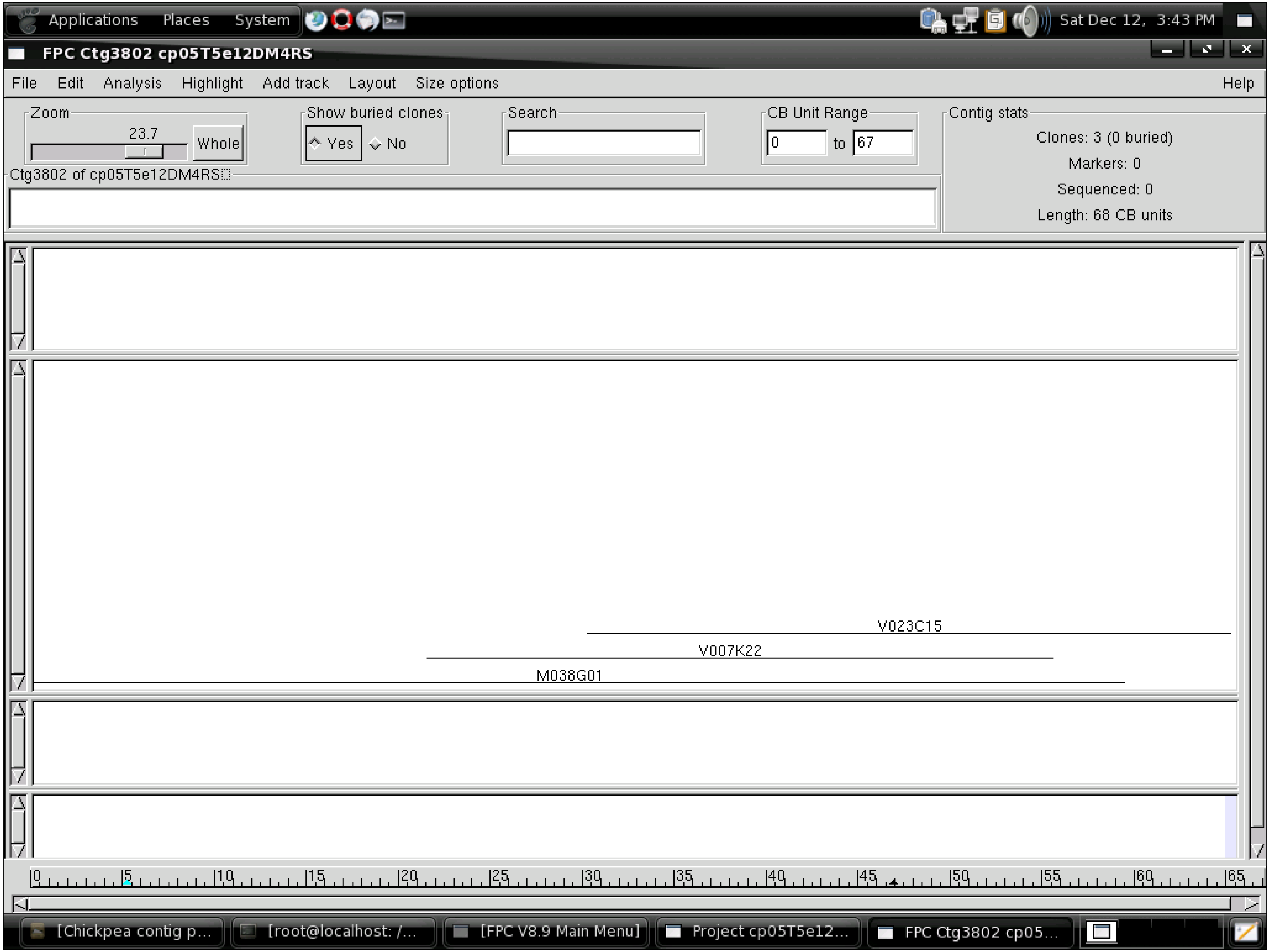
Applications Places System
Sat Dec 12, 3:43 PM
FPC Ctg3802 cp05T5e12DM4RS
File Edit Analysis Highlight Add track Layout Size options Help
Zoom
23.7
Whole
Show buried clones
Yes No
Search
CB Unit Range
0 to 67
Contig stats
Clones: 3 (0 buried)
Markers: 0
Sequenced: 0
Length: 68 CB units
Ctg3802 of cp05T5e12DM4RS
V023C15
V007K22
M038G01
0 5 10 15 20 25 30 35 40 45 50 55 60 65
[Chickpea contig p...
[root@localhost: /...
[FPC V8.9 Main Menu]
Project cp05T5e12...
FPC Ctg3802 cp05...

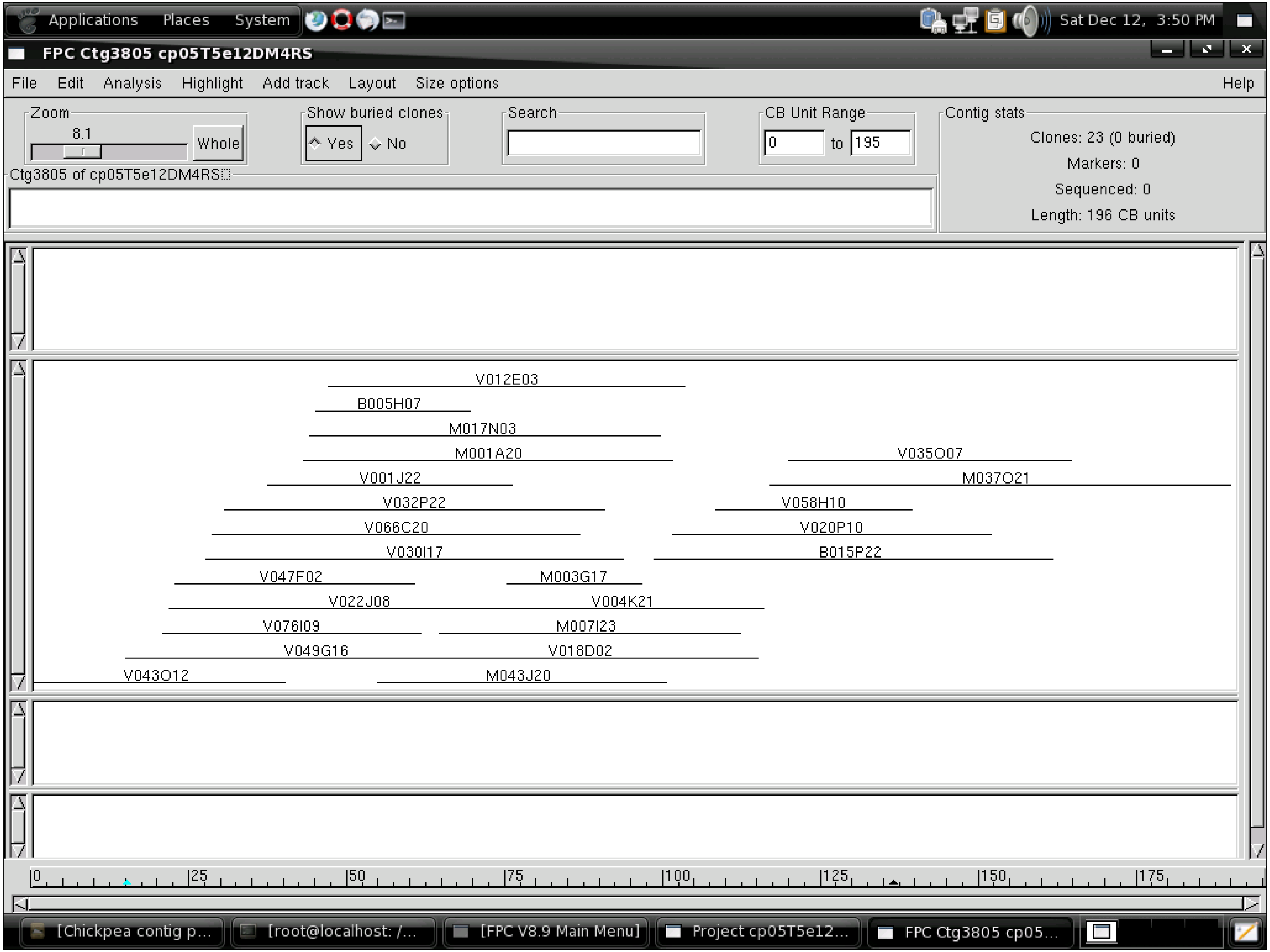
Applications Places System
Sat Dec 12, 3:50 PM
FPC Ctg3805 cp05T5e12DM4RS
File Edit Analysis Highlight Add track Layout Size options Help
Zoom
8.1
Whole
Show buried clones
Yes No
Search
CB Unit Range
0 to 195
Contig stats
Clones: 23 (0 buried)
Markers: 0
Sequenced: 0
Length: 196 CB units
Ctg3805 of cp05T5e12DM4RS
V012E03
B005H07
M017N03
M001A20
V035O07
V001J22
M037O21
V032P22
V058H10
V066C20
V020P10
V030I17
B015P22
V047F02
M003G17
V022J08
V004K21
V076I09
M007I23
V049G16
V018D02
V043O12
M043J20
0 25 50 75 100 125 150 175
[Chickpea contig p...
[root@localhost: /...
[FPC V8.9 Main Menu]
Project cp05T5e12...
FPC Ctg3805 cp05...

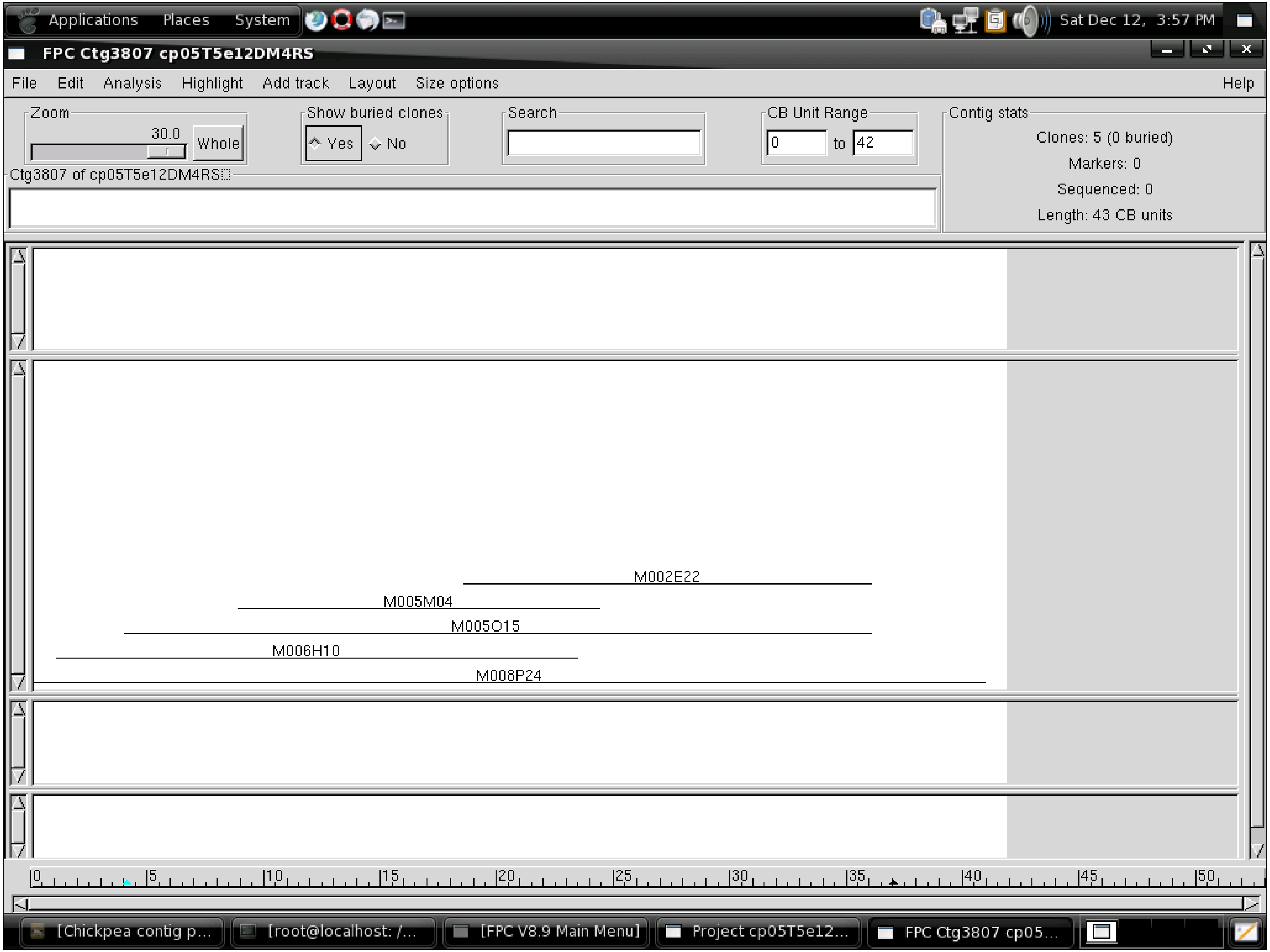
Applications Places System
Sat Dec 12, 3:57 PM
FPC Ctg3807 cp05T5e12DM4RS
File Edit Analysis Highlight Add track Layout Size options Help
Zoom
30.0
Whole
Show buried clones
Yes No
Search
CB Unit Range
0 to 42
Contig stats
Clones: 5 (0 buried)
Markers: 0
Sequenced: 0
Length: 43 CB units
Ctg3807 of cp05T5e12DM4RS
M002E22
M005M04
M005O15
M006H10
M008P24
0 5 10 15 20 25 30 35 40 45 50
[Chickpea contig p...
[root@localhost: /...
[FPC V8.9 Main Menu]
Project cp05T5e12...
FPC Ctg3807 cp05...

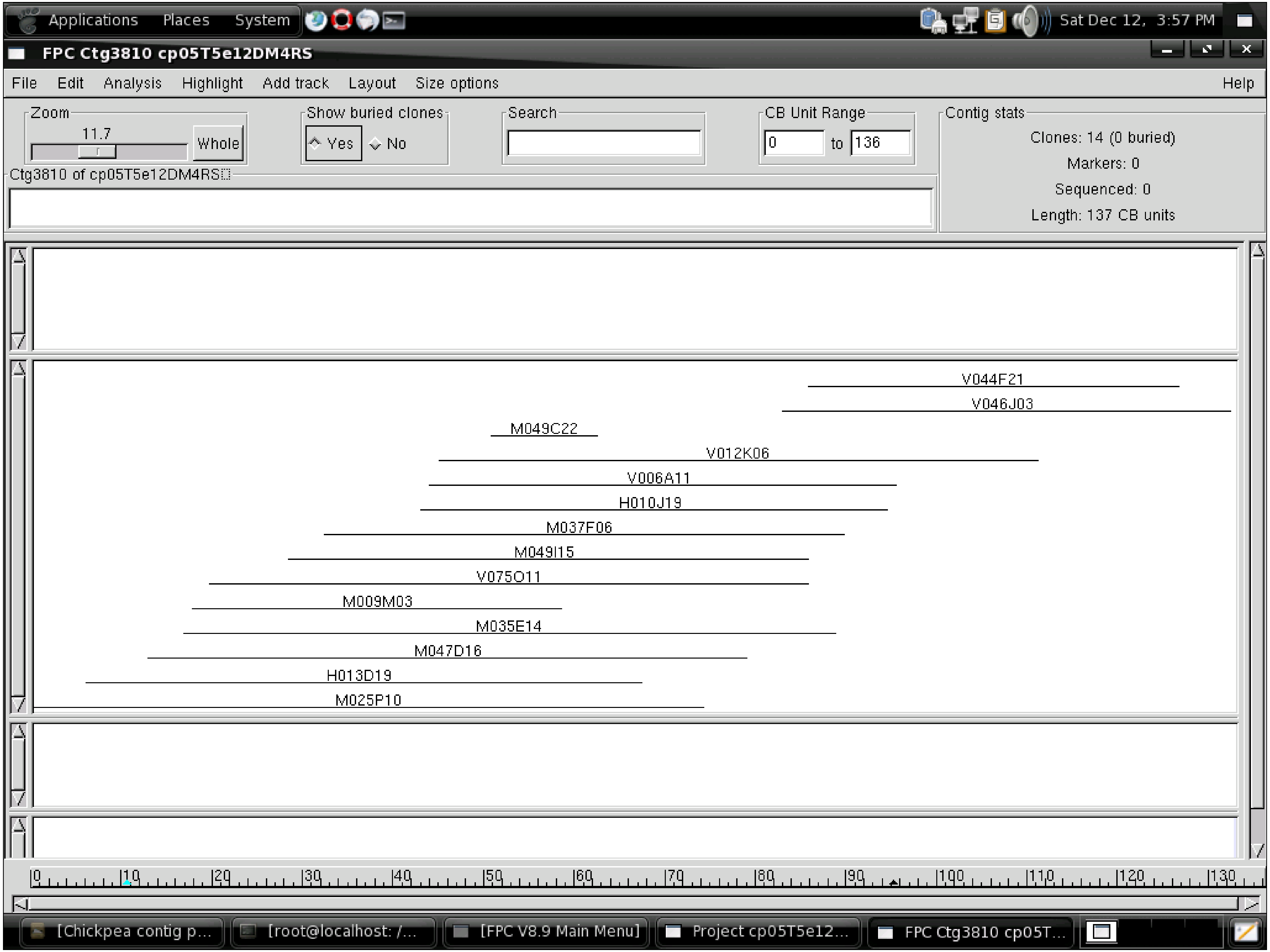
Applications Places System
Sat Dec 12, 3:57 PM
FPC Ctg3810 cp05T5e12DM4RS
File Edit Analysis Highlight Add track Layout Size options Help
Zoom
11.7
Whole
Show buried clones
Yes No
Search
CB Unit Range
0 to 136
Contig stats
Clones: 14 (0 buried)
Markers: 0
Sequenced: 0
Length: 137 CB units
Ctg3810 of cp05T5e12DM4RS
V044F21
V046J03
M049C22
V012K06
V006A11
H010J19
M037F06
M049I15
V075O11
M009M03
M035E14
M047D16
H013D19
M025P10
0 10 20 30 40 50 60 70 80 90 100 110 120 130
[Chickpea contig p...
[root@localhost: /...
[FPC V8.9 Main Menu]
Project cp05T5e12...
FPC Ctg3810 cp05T...

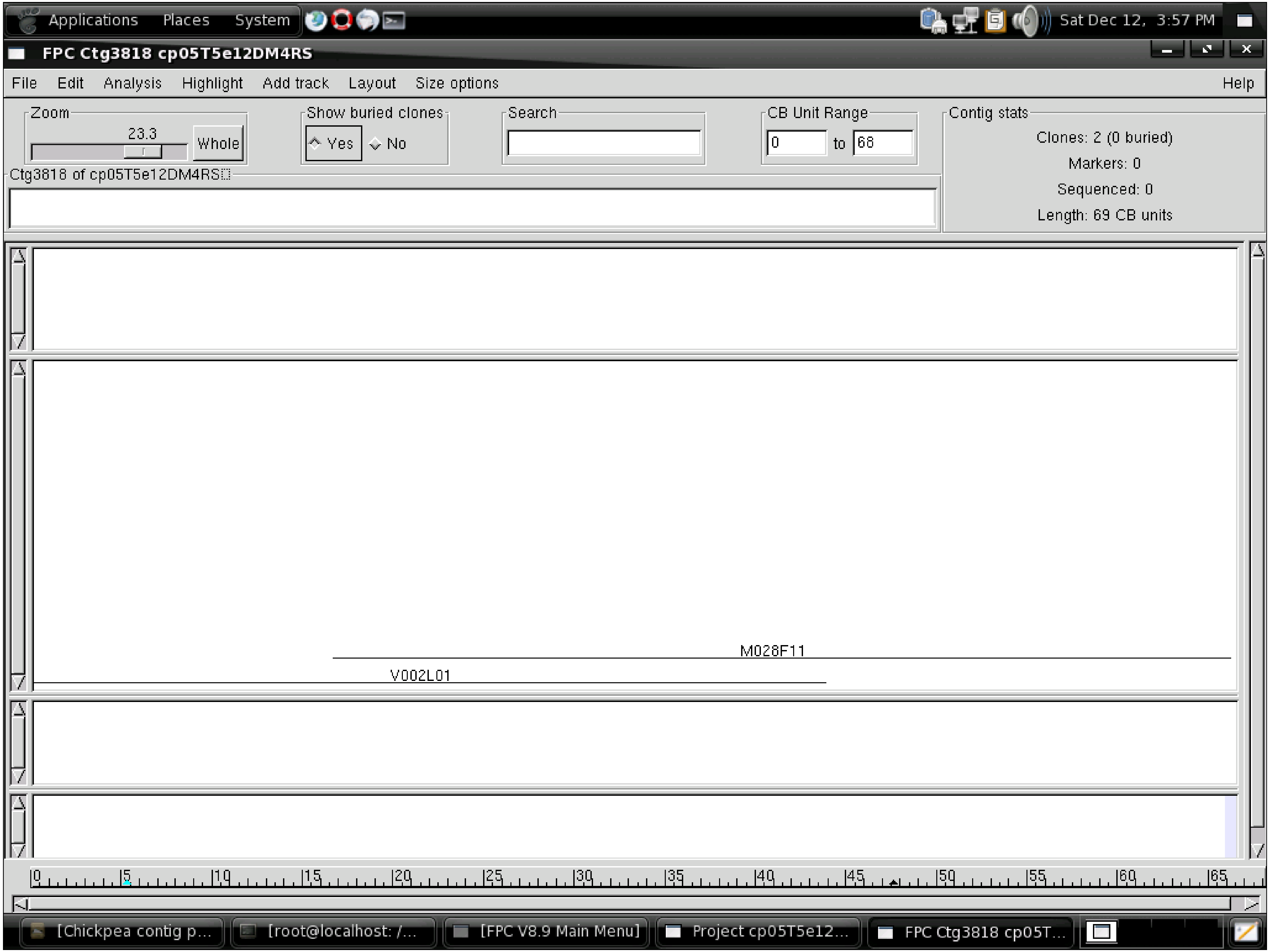

Applications Places System
Sat Dec 12, 3:57 PM
FPC Ctg3818 cp05T5e12DM4RS
File Edit Analysis Highlight Add track Layout Size options
Help
Zoom
23.3
Whole
Show buried clones
Yes
No
Search
CB Unit Range
0
to
68
Contig stats
Clones: 2 (0 buried)
Markers: 0
Sequenced: 0
Length: 69 CB units
Ctg3818 of cp05T5e12DM4RS
M028F11
V002L01
0 5 10 15 20 25 30 35 40 45 50 55 60 65
[Chickpea contig p...
[root@localhost: /...
[FPC V8.9 Main Menu]
Project cp05T5e12...
FPC Ctg3818 cp05T...

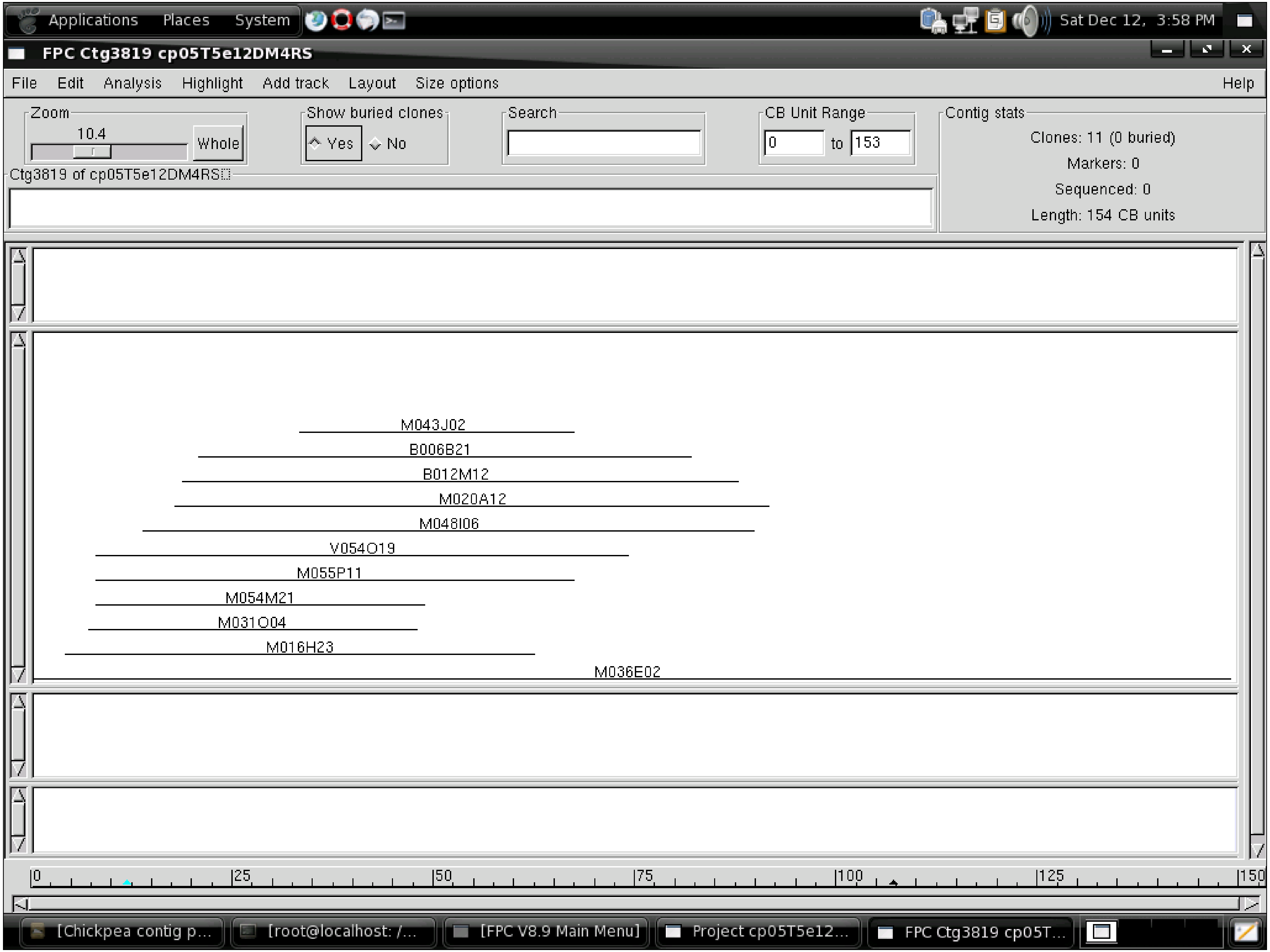

Applications Places System
Sat Dec 12, 3:58 PM
FPC Ctg3819 cp05T5e12DM4RS
File Edit Analysis Highlight Add track Layout Size options Help
Zoom
10.4
Whole
Show buried clones
Yes No
Search
CB Unit Range
0 to 153
Contig stats
Clones: 11 (0 buried)
Markers: 0
Sequenced: 0
Length: 154 CB units
Ctg3819 of cp05T5e12DM4RS
M043J02
B006B21
B012M12
M020A12
M048I06
V054O19
M055P11
M054M21
M031O04
M016H23
M036E02
0 25 50 75 100 125 150
[Chickpea contig p...
[root@localhost: /...
[FPC V8.9 Main Menu]
Project cp05T5e12...
FPC Ctg3819 cp05T...

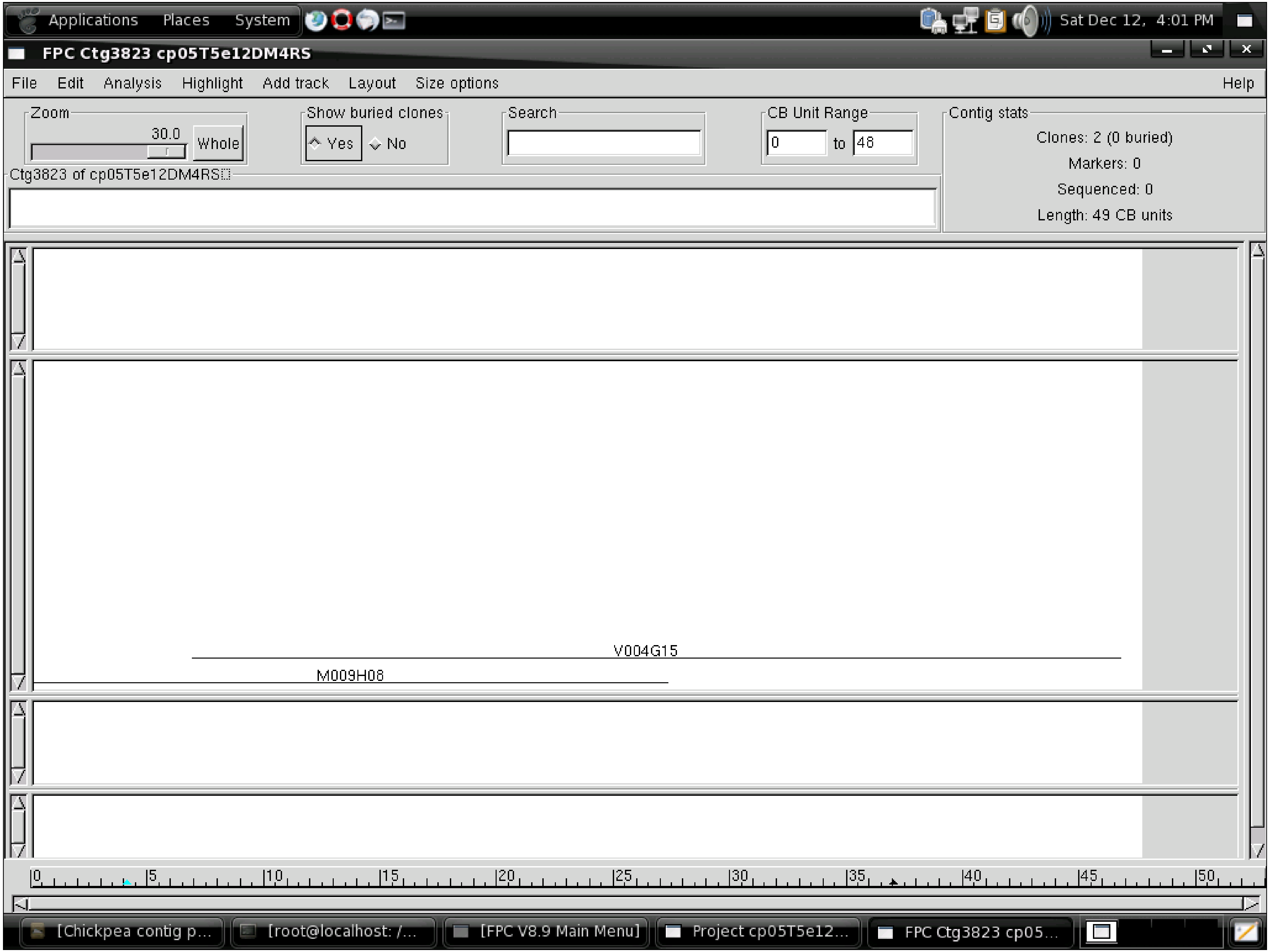
Applications Places System
Sat Dec 12, 4:01 PM
FPC Ctg3823 cp05T5e12DM4RS
File Edit Analysis Highlight Add track Layout Size options Help
Zoom
30.0
Whole
Show buried clones
Yes No
Search
CB Unit Range
0 to 48
Contig stats
Clones: 2 (0 buried)
Markers: 0
Sequenced: 0
Length: 49 CB units
Ctg3823 of cp05T5e12DM4RS
V004G15
M009H08
0 5 10 15 20 25 30 35 40 45 50
[Chickpea contig p...
[root@localhost: /...
[FPC V8.9 Main Menu]
Project cp05T5e12...
FPC Ctg3823 cp05...

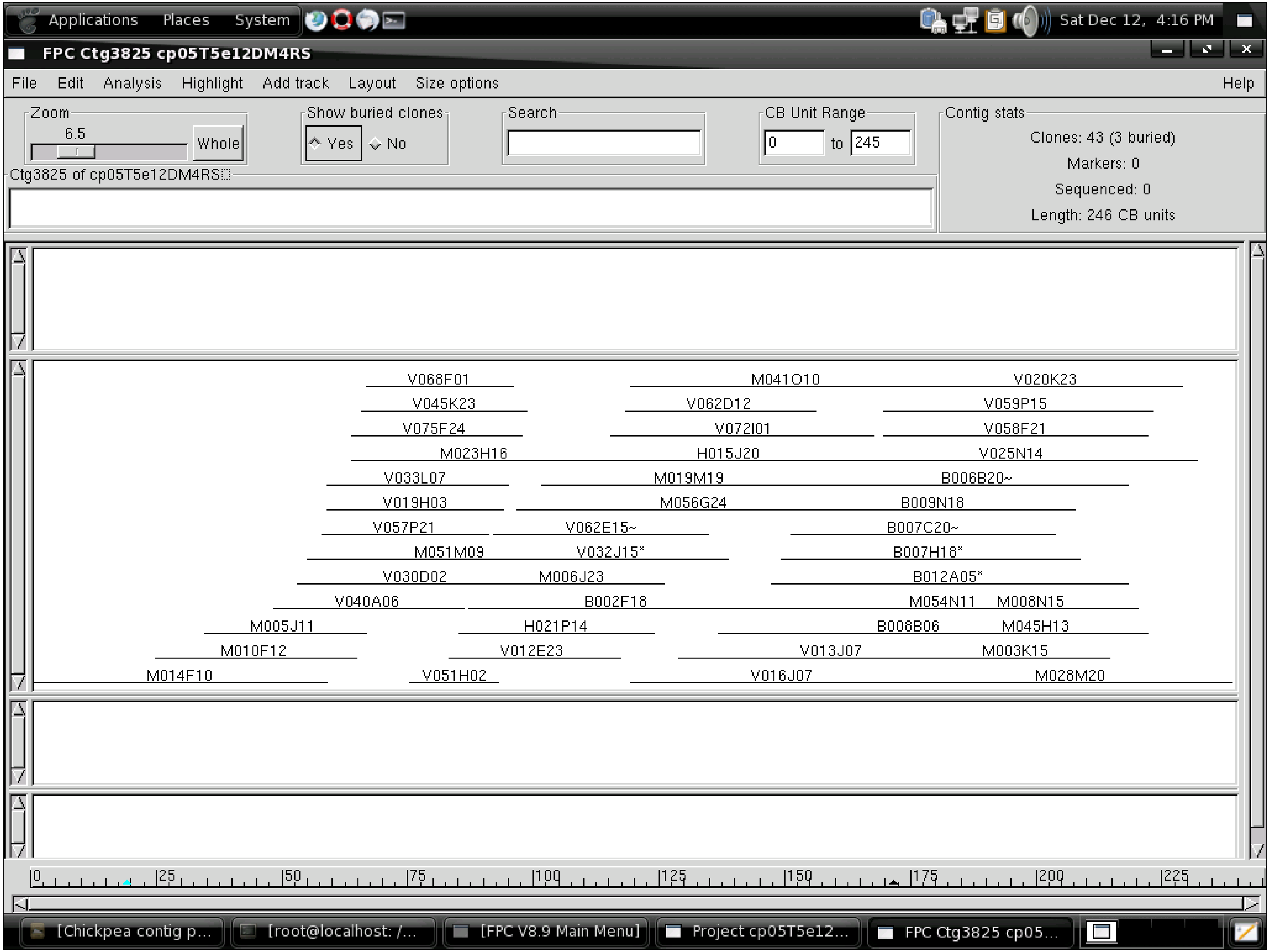
Applications Places System
Sat Dec 12, 4:16 PM
FPC Ctg3825 cp05T5e12DM4RS
File Edit Analysis Highlight Add track Layout Size options Help
Zoom 6.5 Whole
Show buried clones Yes No
Search
CB Unit Range 0 to 245
Contig stats
Clones: 43 (3 buried)
Markers: 0
Sequenced: 0
Length: 246 CB units
Ctg3825 of cp05T5e12DM4RS
V068F01 M041O10 V020K23
V045K23 V062D12 V059P15
V075F24 V072I01 V058F21
M023H16 H015J20 V025N14
V033L07 M019M19 B006B20~
V019H03 M056G24 B009N18
V057P21 V062E15~ B007C20~
M051M09 V032J15* B007H18*
V030D02 M006J23 B012A05*
V040A06 B002F18 M054N11 M008N15
M005J11 H021P14 B008B06 M045H13
M010F12 V012E23 V013J07 M003K15
M014F10 V051H02 V016J07 M028M20
0 25 50 75 100 125 150 175 200 225
[Chickpea contig p... [root@localhost: /... [FPC V8.9 Main Menu] Project cp05T5e12... FPC Ctg3825 cp05...

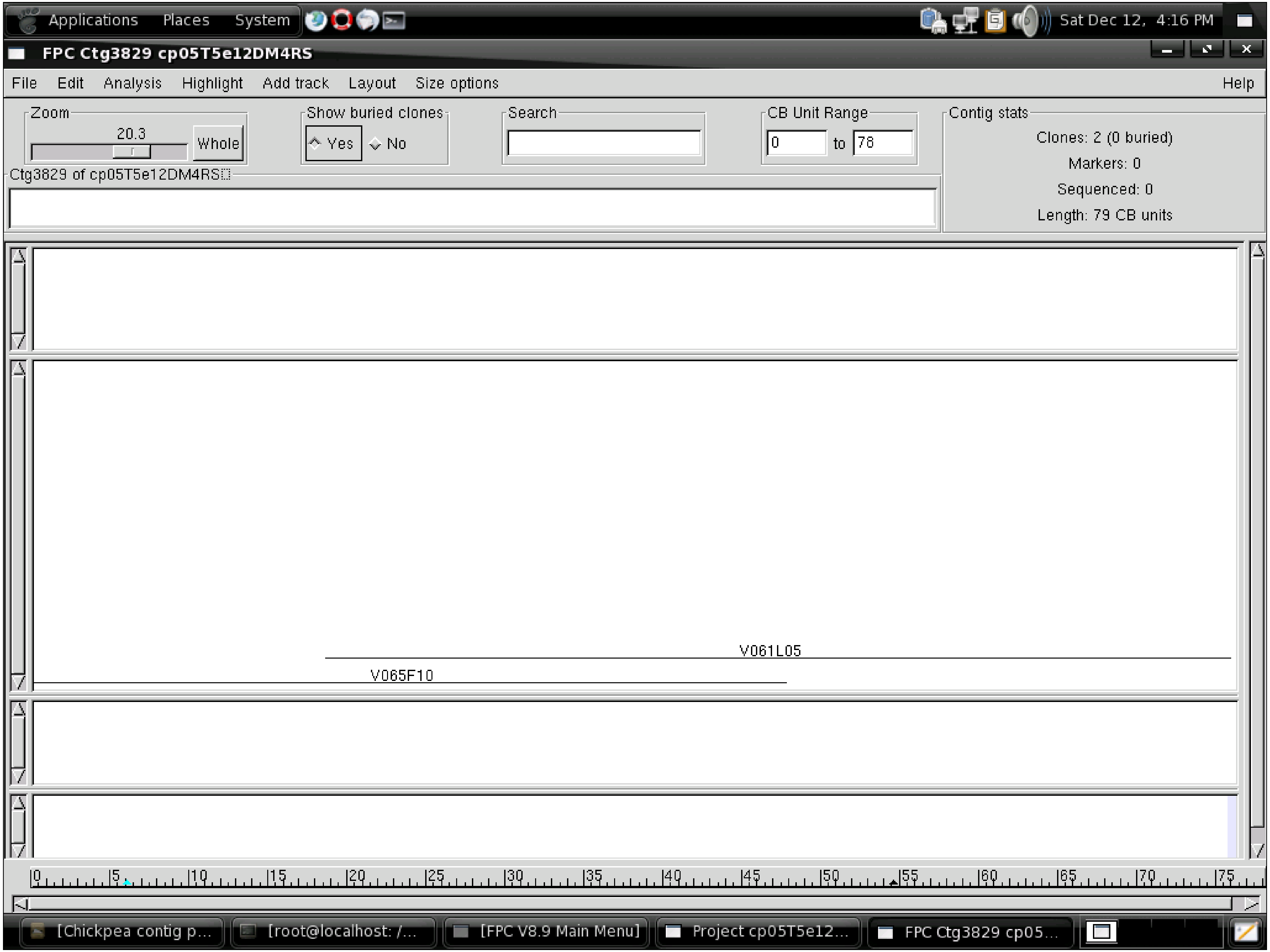
Applications
Places
System
Sat Dec 12, 4:16 PM
FPC Ctg3829 cp05T5e12DM4RS
File
Edit
Analysis
Highlight
Add track
Layout
Size options
Help
Zoom
20.3
Whole
Show buried clones
Yes
No
Search
CB Unit Range
0
to
78
Contig stats
Clones: 2 (0 buried)
Markers: 0
Sequenced: 0
Length: 79 CB units
Ctg3829 of cp05T5e12DM4RS
V061L05
V065F10
0
5
10
15
20
25
30
35
40
45
50
55
60
65
70
75
[Chickpea contig p...
[root@localhost: /...
[FPC V8.9 Main Menu]
Project cp05T5e12...
FPC Ctg3829 cp05...

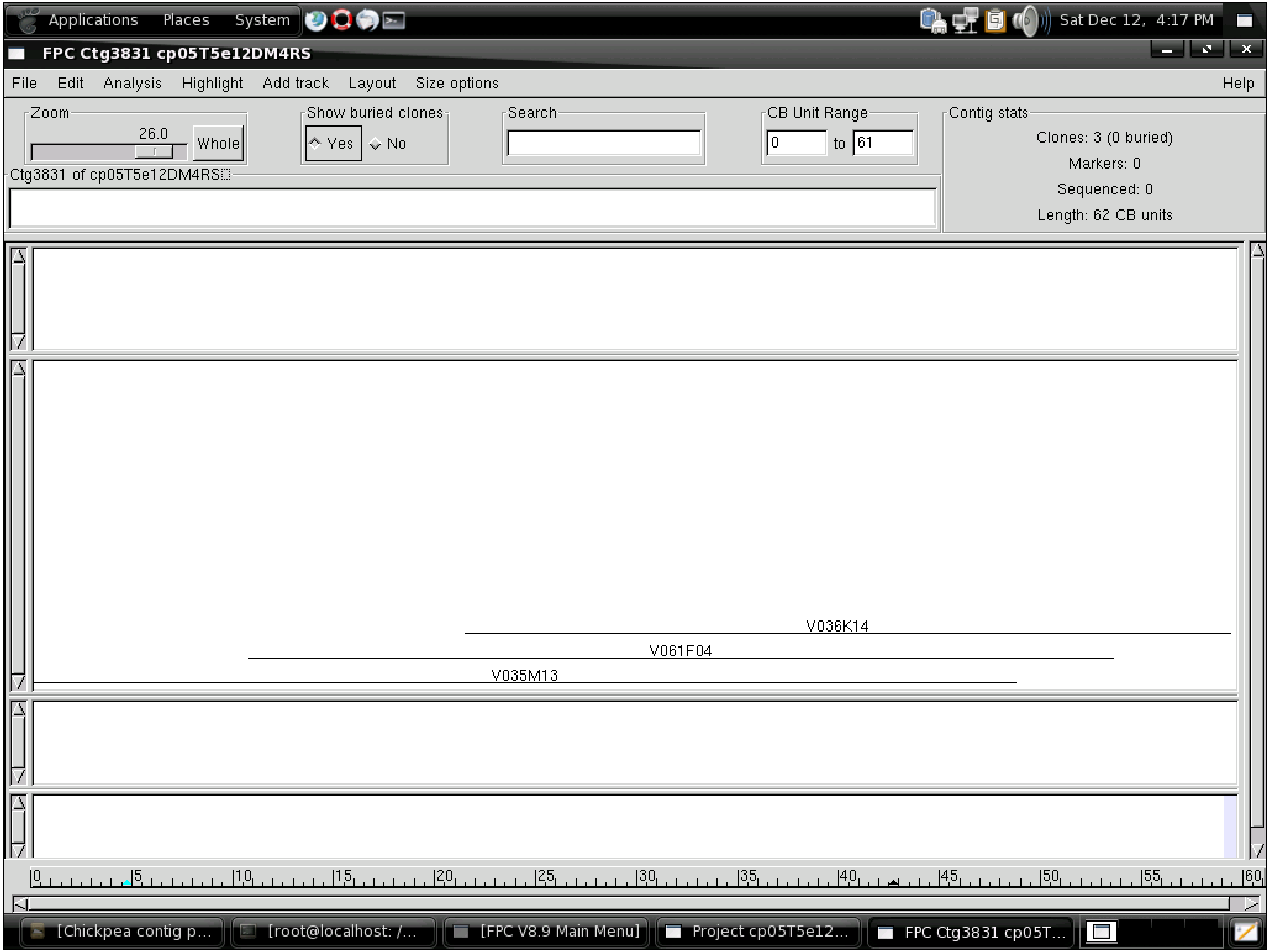
Applications Places System
Sat Dec 12, 4:17 PM
FPC Ctg3831 cp05T5e12DM4RS
File Edit Analysis Highlight Add track Layout Size options Help
Zoom
26.0
Whole
Show buried clones
Yes
No
Search
CB Unit Range
0 to 61
Contig stats
Clones: 3 (0 buried)
Markers: 0
Sequenced: 0
Length: 62 CB units
Ctg3831 of cp05T5e12DM4RS
V036K14
V061F04
V035M13
0 5 10 15 20 25 30 35 40 45 50 55 60
[Chickpea contig p...
[root@localhost: /...
[FPC V8.9 Main Menu]
Project cp05T5e12...
FPC Ctg3831 cp05T...

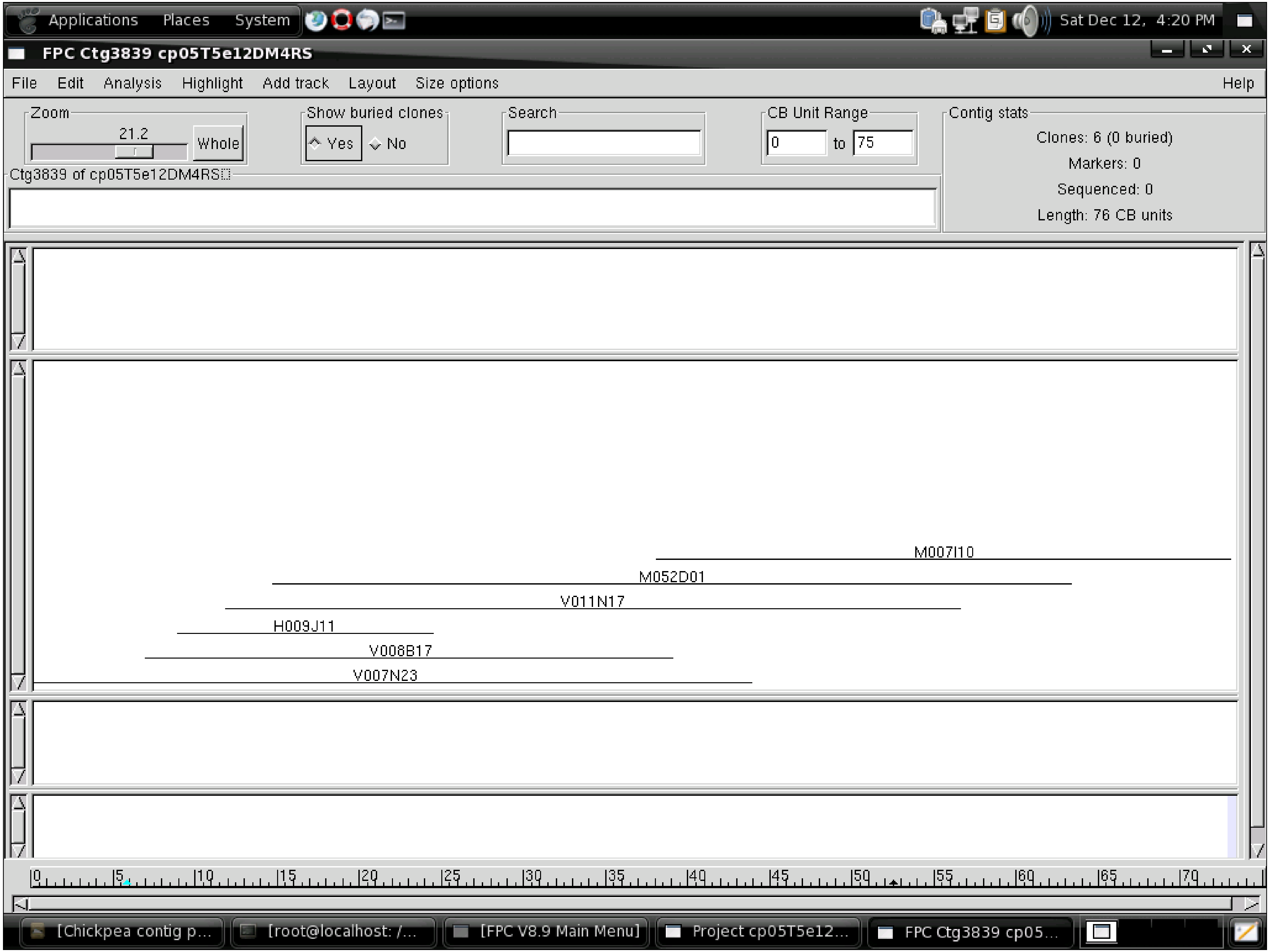
Applications Places System
Sat Dec 12, 4:20 PM
FPC Ctg3839 cp05T5e12DM4RS
File Edit Analysis Highlight Add track Layout Size options Help
Zoom
21.2
Whole
Show buried clones
Yes No
Search
CB Unit Range
0 to 75
Contig stats
Clones: 6 (0 buried)
Markers: 0
Sequenced: 0
Length: 76 CB units
Ctg3839 of cp05T5e12DM4RS
M007I10
M052D01
V011N17
H009J11
V008B17
V007N23
0 5 10 15 20 25 30 35 40 45 50 55 60 65 70
[Chickpea contig p...
[root@localhost: /...
[FPC V8.9 Main Menu]
Project cp05T5e12...
FPC Ctg3839 cp05...

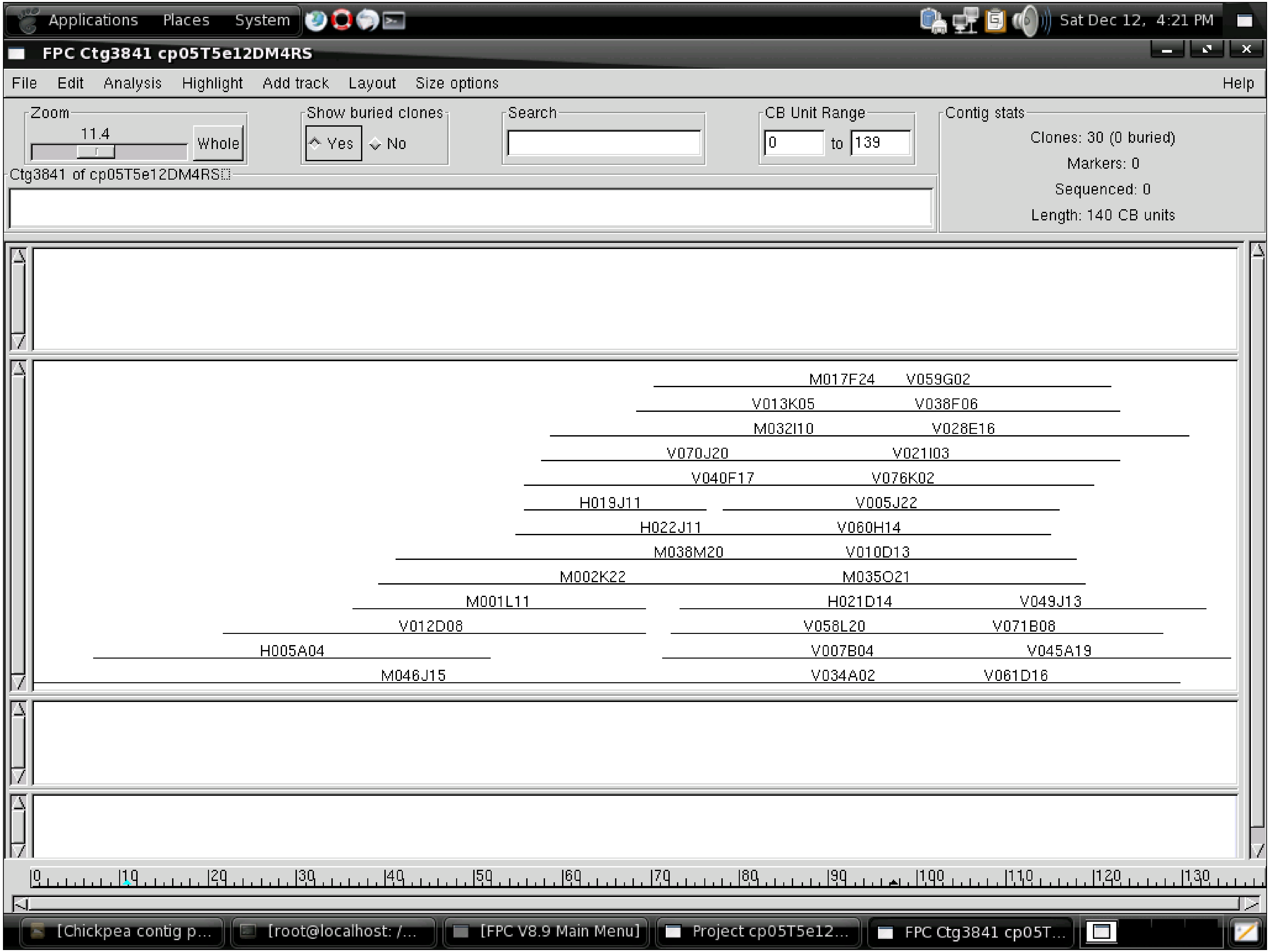
Applications Places System
Sat Dec 12, 4:21 PM
FPC Ctg3841 cp05T5e12DM4RS
File Edit Analysis Highlight Add track Layout Size options Help
Zoom
11.4
Whole
Show buried clones
Yes No
Search
CB Unit Range
0 to 139
Contig stats
Clones: 30 (0 buried)
Markers: 0
Sequenced: 0
Length: 140 CB units
Ctg3841 of cp05T5e12DM4RS
M017F24 V059G02
V013K05 V038F06
M032I10 V028E16
V070J20 V021I03
V040F17 V076K02
H019J11 V005J22
H022J11 V060H14
M038M20 V010D13
M002K22 M035O21
M001L11 H021D14 V049J13
V012D08 V058L20 V071B08
H005A04 V007B04 V045A19
M046J15 V034A02 V061D16
0 10 20 30 40 50 60 70 80 90 100 110 120 130
[Chickpea contig p...
[root@localhost: /...
[FPC V8.9 Main Menu]
Project cp05T5e12...
FPC Ctg3841 cp05T...

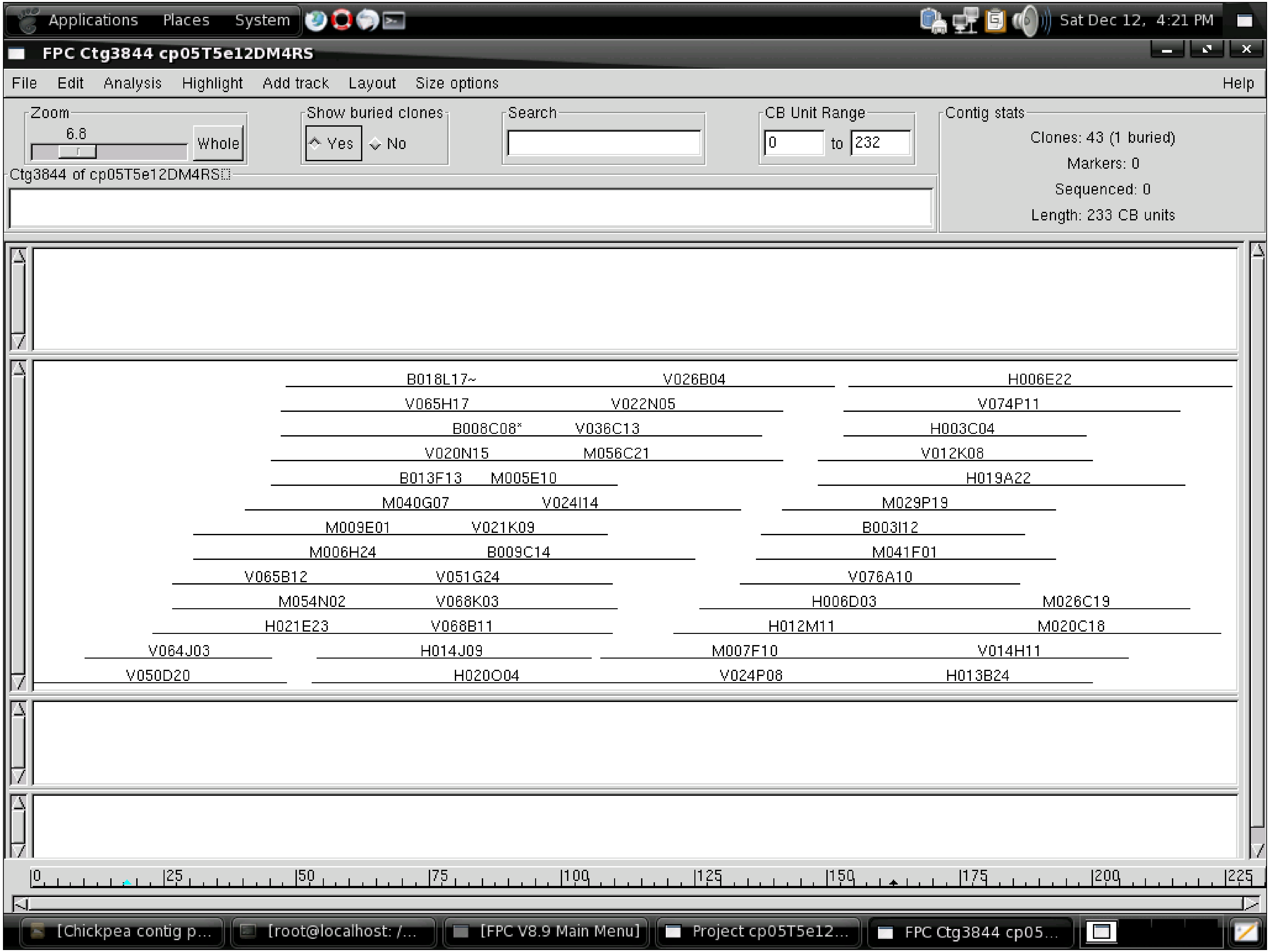
Applications Places System
Sat Dec 12, 4:21 PM
FPC Ctg3844 cp05T5e12DM4RS
File Edit Analysis Highlight Add track Layout Size options Help
Zoom
6.8
Whole
Show buried clones
Yes No
Search
CB Unit Range
0 to 232
Contig stats
Clones: 43 (1 buried)
Markers: 0
Sequenced: 0
Length: 233 CB units
Ctg3844 of cp05T5e12DM4RS
B018L17~ V026B04 H006E22
V065H17 V022N05 V074P11
B008C08* V036C13 H003C04
V020N15 M056C21 V012K08
B013F13 M005E10 H019A22
M040G07 V024I14 M029P19
M009E01 V021K09 B003I12
M006H24 B009C14 M041F01
V065B12 V051G24 V076A10
M054N02 V068K03 H006D03 M026C19
H021E23 V068B11 H012M11 M020C18
V064J03 H014J09 M007F10 V014H11
V050D20 H020O04 V024P08 H013B24
0 25 50 75 100 125 150 175 200 225
[Chickpea contig p...
[root@localhost: /...
[FPC V8.9 Main Menu]
Project cp05T5e12...
FPC Ctg3844 cp05...

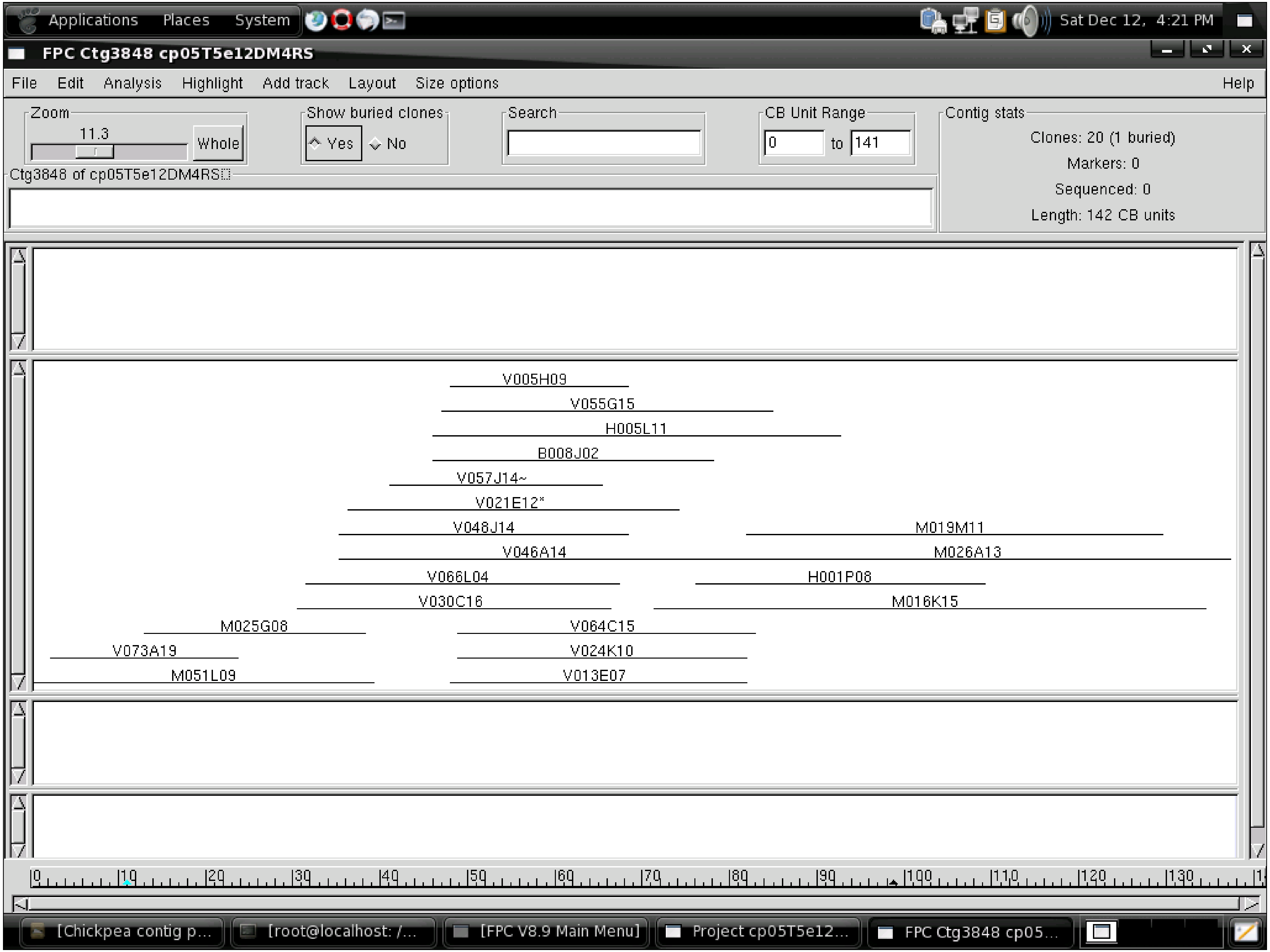
Applications Places System
Sat Dec 12, 4:21 PM
FPC Ctg3848 cp05T5e12DM4RS
File Edit Analysis Highlight Add track Layout Size options Help
Zoom
11.3
Whole
Show buried clones
Yes No
Search
CB Unit Range
0 to 141
Contig stats
Clones: 20 (1 buried)
Markers: 0
Sequenced: 0
Length: 142 CB units
Ctg3848 of cp05T5e12DM4RS
V005H09
V055G15
H005L11
B008J02
V057J14~
V021E12*
V048J14
M019M11
V046A14
M026A13
V066L04
H001P08
V030C16
M016K15
M025G08
V064C15
V073A19
V024K10
M051L09
V013E07
0 10 20 30 40 50 60 70 80 90 100 110 120 130
[Chickpea contig p...
[root@localhost: /...
[FPC V8.9 Main Menu]
Project cp05T5e12...
FPC Ctg3848 cp05...

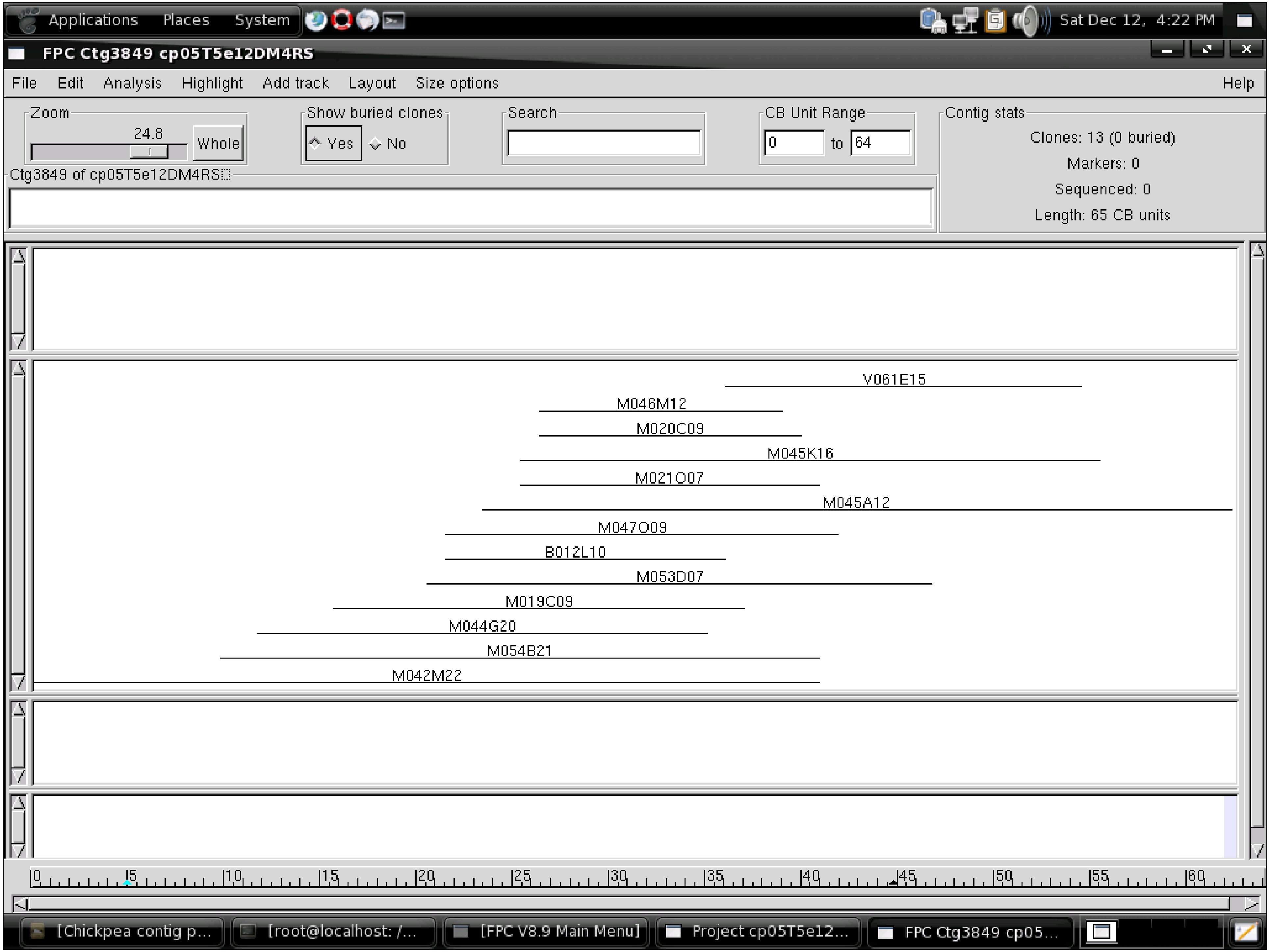
Applications
Places
System
Sat Dec 12, 4:22 PM
FPC Ctg3849 cp05T5e12DM4RS
File
Edit
Analysis
Highlight
Add track
Layout
Size options
Help
Zoom
24.8
Whole
Show buried clones
Yes
No
Search
CB Unit Range
0
to
64
Contig stats
Clones: 13 (0 buried)
Markers: 0
Sequenced: 0
Length: 65 CB units
Ctg3849 of cp05T5e12DM4RS
V061E15
M046M12
M020C09
M045K16
M021O07
M045A12
M047O09
B012L10
M053D07
M019C09
M044G20
M054B21
M042M22
0 5 10 15 20 25 30 35 40 45 50 55 60
[Chickpea contig p...
[root@localhost: /...
[FPC V8.9 Main Menu]
Project cp05T5e12...
FPC Ctg3849 cp05...

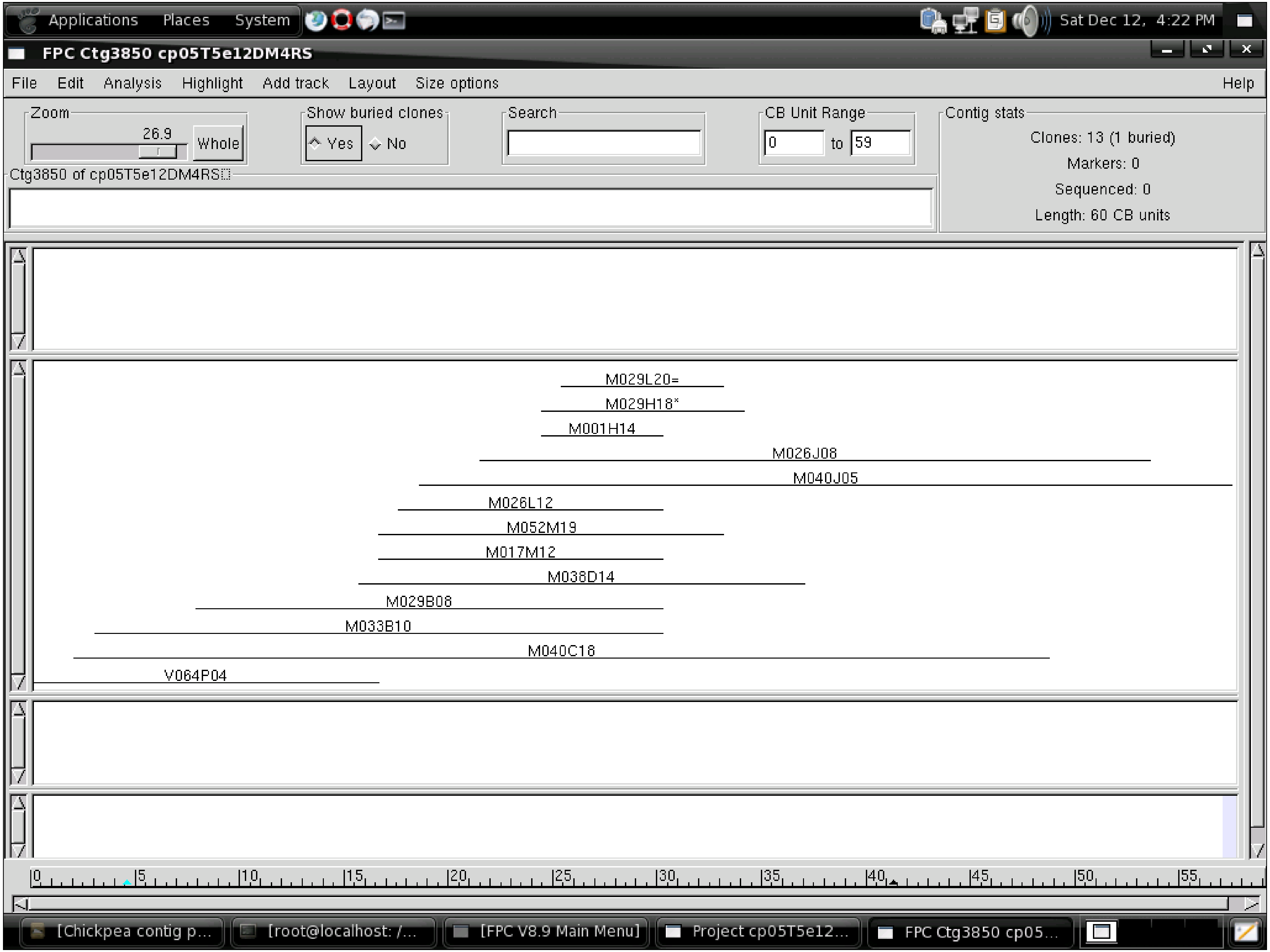
Applications
Places
System
Sat Dec 12, 4:22 PM
FPC Ctg3850 cp05T5e12DM4RS
File
Edit
Analysis
Highlight
Add track
Layout
Size options
Help
Zoom
26.9
Whole
Show buried clones
Yes
No
Search
CB Unit Range
0
to
59
Contig stats
Clones: 13 (1 buried)
Markers: 0
Sequenced: 0
Length: 60 CB units
Ctg3850 of cp05T5e12DM4RS
M029L20=
M029H18*
M001H14
M026J08
M040J05
M026L12
M052M19
M017M12
M038D14
M029B08
M033B10
M040C18
V064P04
0
5
10
15
20
25
30
35
40
45
50
55
[Chickpea contig p...
[root@localhost: /...
[FPC V8.9 Main Menu]
Project cp05T5e12...
FPC Ctg3850 cp05...

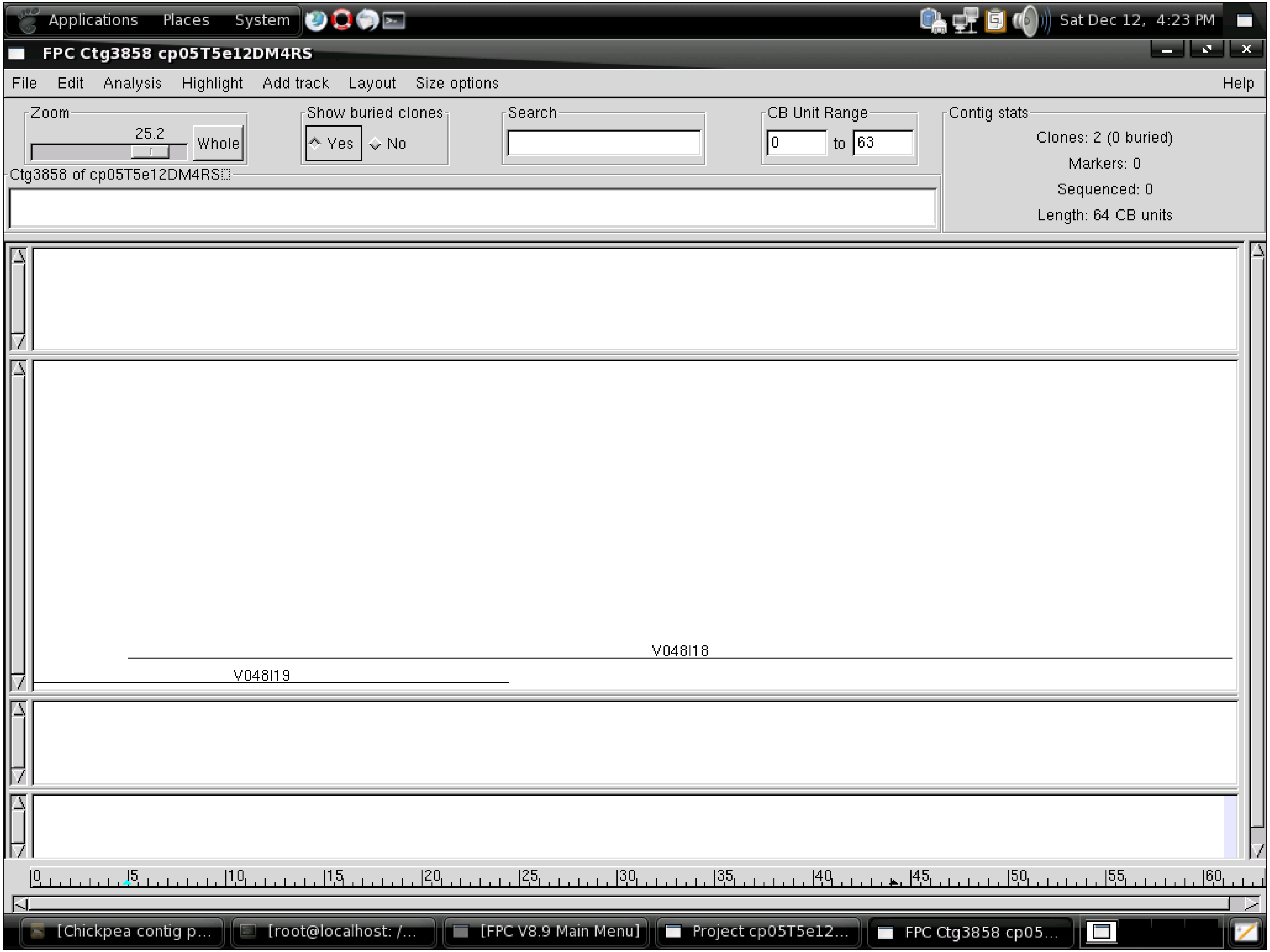
Applications Places System
Sat Dec 12, 4:23 PM
FPC Ctg3858 cp05T5e12DM4RS
File Edit Analysis Highlight Add track Layout Size options Help
Zoom
25.2
Whole
Show buried clones
Yes No
Search
CB Unit Range
0 to 63
Contig stats
Clones: 2 (0 buried)
Markers: 0
Sequenced: 0
Length: 64 CB units
Ctg3858 of cp05T5e12DM4RS
V048I18
V048I19
0 5 10 15 20 25 30 35 40 45 50 55 60
[Chickpea contig p...
[root@localhost: /...
[FPC V8.9 Main Menu]
Project cp05T5e12...
FPC Ctg3858 cp05...

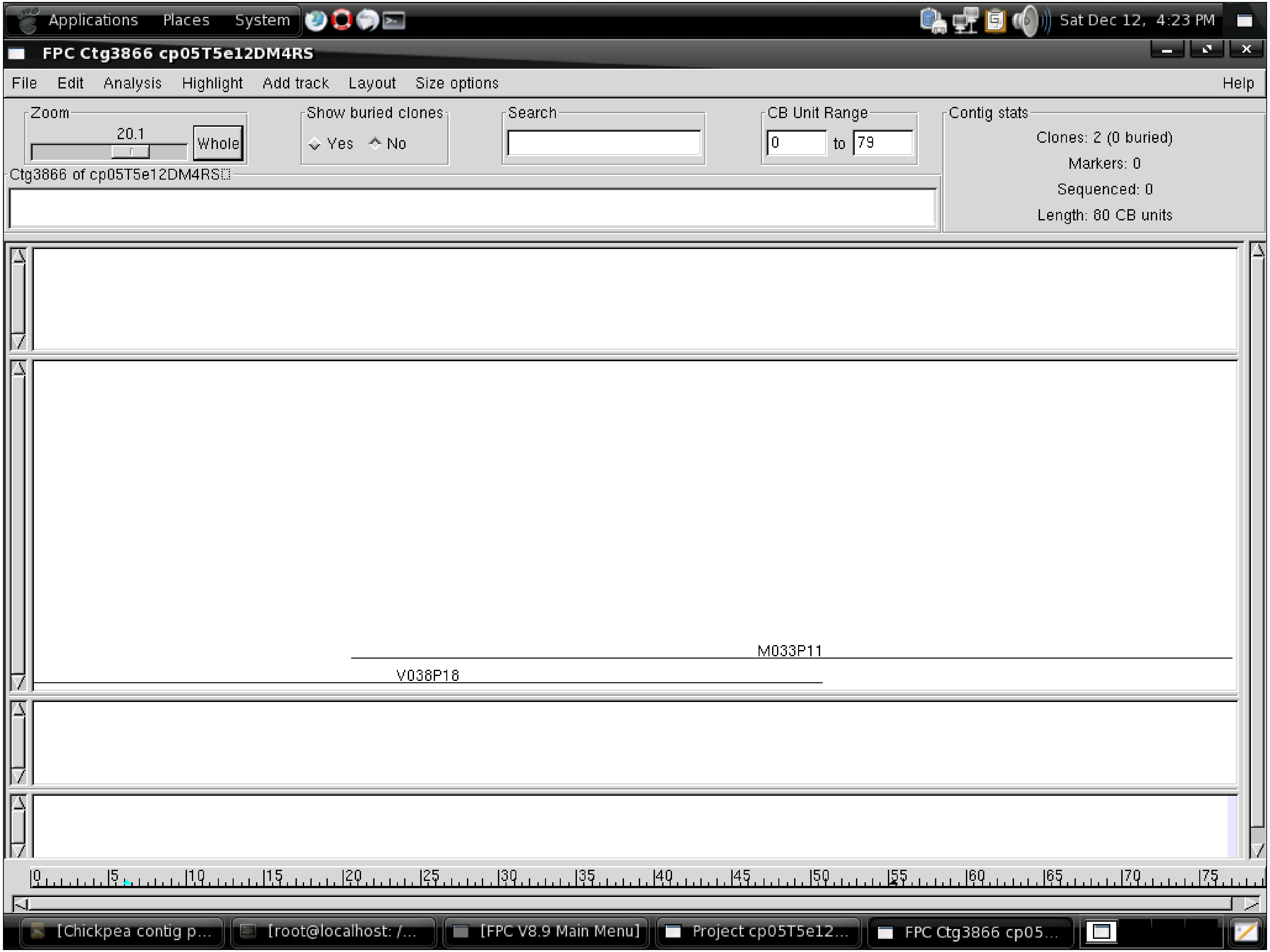
Applications Places System
Sat Dec 12, 4:23 PM
FPC Ctg3866 cp05T5e12DM4RS
File Edit Analysis Highlight Add track Layout Size options Help
Zoom
20.1
Whole
Show buried clones
Yes No
Search
CB Unit Range
0 to 79
Contig stats
Clones: 2 (0 buried)
Markers: 0
Sequenced: 0
Length: 80 CB units
Ctg3866 of cp05T5e12DM4RS
M033P11
V038P18
0 5 10 15 20 25 30 35 40 45 50 55 60 65 70 75
[Chickpea contig p...
[root@localhost: /...
[FPC V8.9 Main Menu]
Project cp05T5e12...
FPC Ctg3866 cp05...

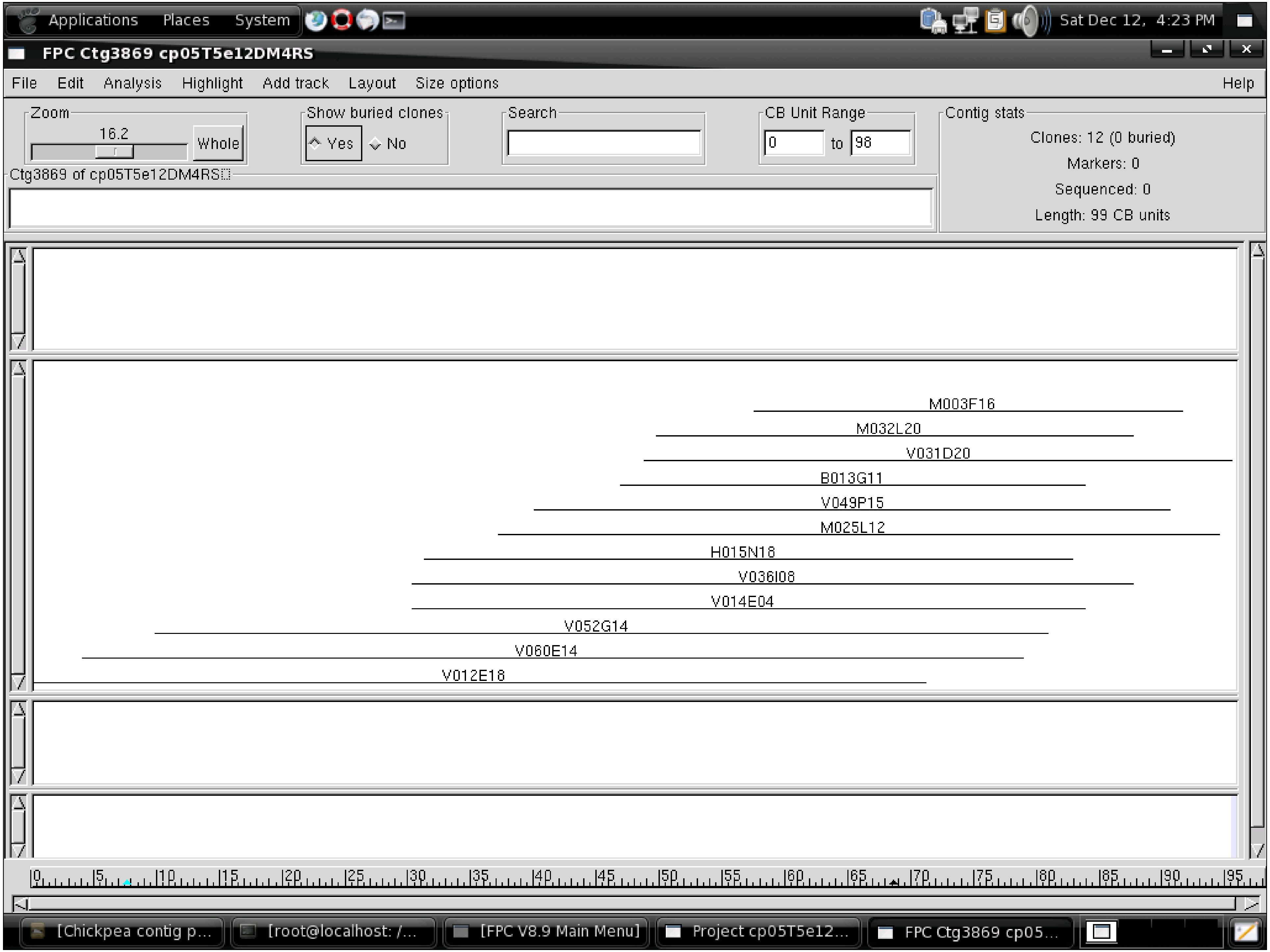

Applications Places System
Sat Dec 12, 4:23 PM
FPC Ctg3869 cp05T5e12DM4RS
File Edit Analysis Highlight Add track Layout Size options Help
Zoom
16.2
Whole
Show buried clones
Yes No
Search
CB Unit Range
0 to 98
Contig stats
Clones: 12 (0 buried)
Markers: 0
Sequenced: 0
Length: 99 CB units
Ctg3869 of cp05T5e12DM4RS
M003F16
M032L20
V031D20
B013G11
V049P15
M025L12
H015N18
V036I08
V014E04
V052G14
V060E14
V012E18
0 5 10 15 20 25 30 35 40 45 50 55 60 65 70 75 80 85 90 95
[Chickpea contig p...
[root@localhost: /...
[FPC V8.9 Main Menu]
Project cp05T5e12...
FPC Ctg3869 cp05...

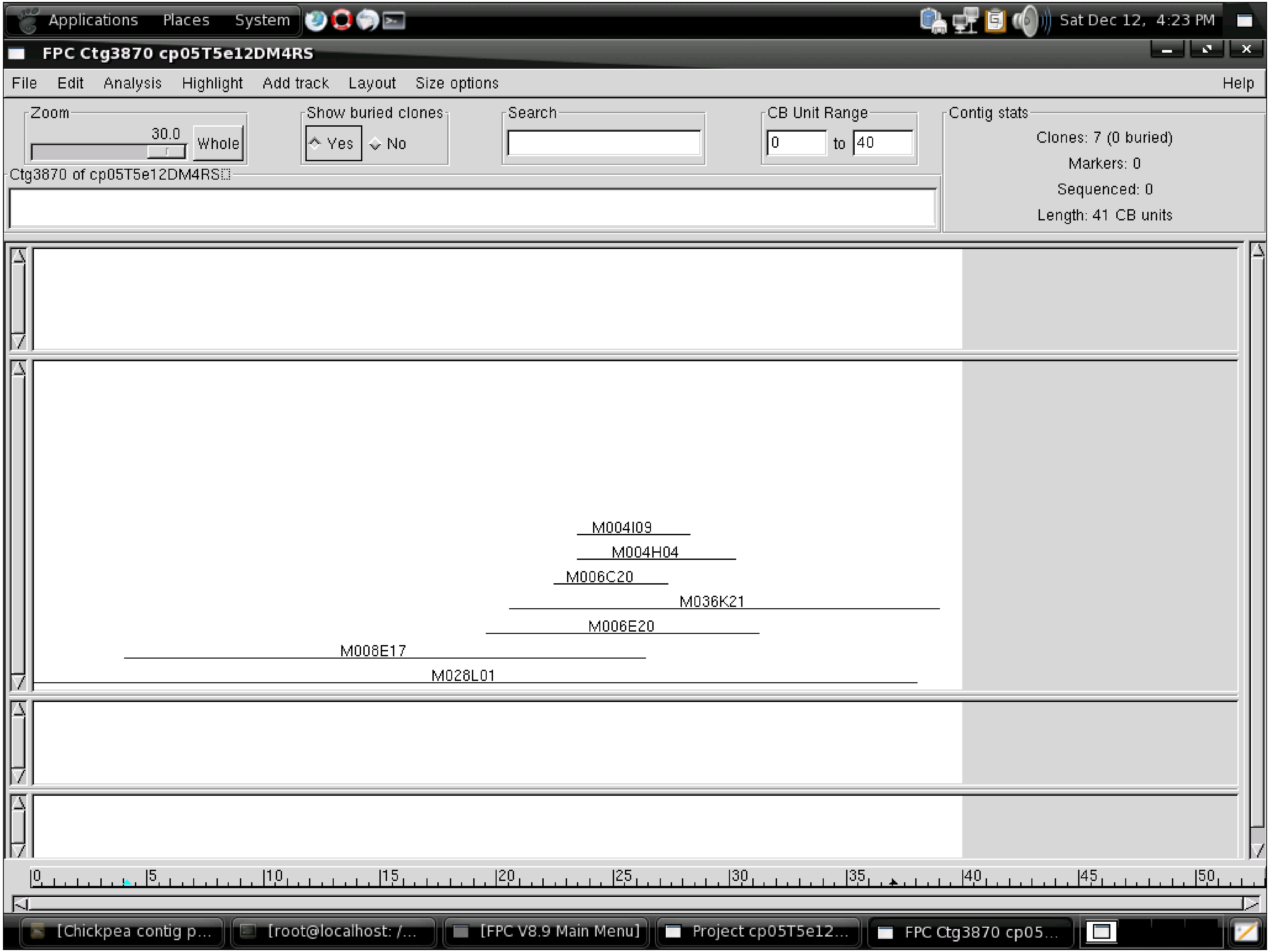
Applications Places System
Sat Dec 12, 4:23 PM
FPC Ctg3870 cp05T5e12DM4RS
File Edit Analysis Highlight Add track Layout Size options Help
Zoom
30.0
Whole
Show buried clones
Yes
No
Search
CB Unit Range
0
to
40
Contig stats
Clones: 7 (0 buried)
Markers: 0
Sequenced: 0
Length: 41 CB units
Ctg3870 of cp05T5e12DM4RS
M004I09
M004H04
M006C20
M036K21
M006E20
M008E17
M028L01
0 5 10 15 20 25 30 35 40 45 50
[Chickpea contig p...
[root@localhost: /...
[FPC V8.9 Main Menu]
Project cp05T5e12...
FPC Ctg3870 cp05...

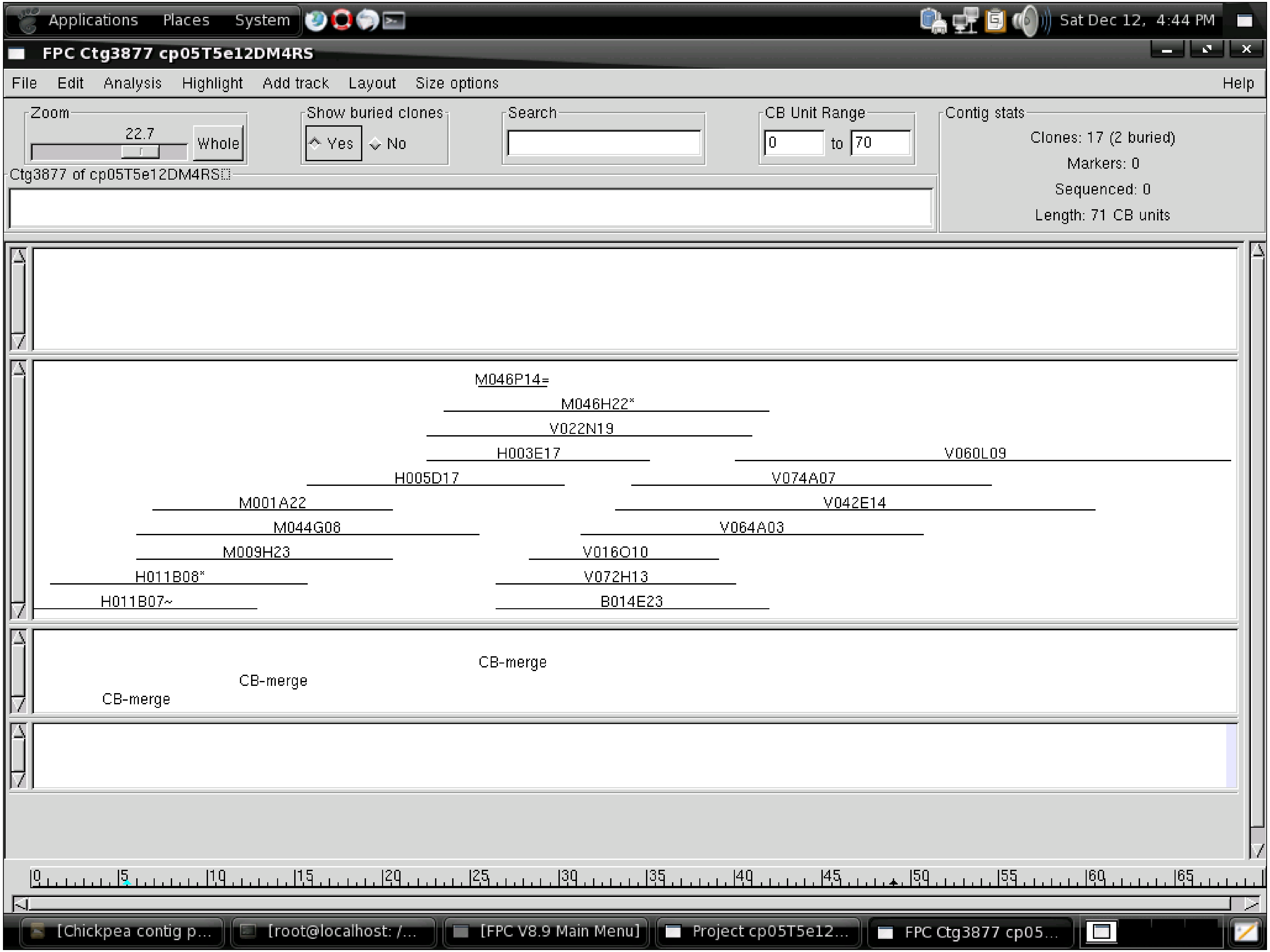
Applications Places System
Sat Dec 12, 4:44 PM
FPC Ctg3877 cp05T5e12DM4RS
File Edit Analysis Highlight Add track Layout Size options Help
Zoom
22.7
Whole
Show buried clones
Yes
No
Search
CB Unit Range
0 to 70
Contig stats
Clones: 17 (2 buried)
Markers: 0
Sequenced: 0
Length: 71 CB units
Ctg3877 of cp05T5e12DM4RS
M046P14=
M046H22*
V022N19
H003E17
V060L09
H005D17
V074A07
M001A22
V042E14
M044G08
V064A03
M009H23
V016O10
H011B08*
V072H13
H011B07~
B014E23
CB-merge
CB-merge
CB-merge
0 5 10 15 20 25 30 35 40 45 50 55 60 65
[Chickpea contig p...
[root@localhost: /...
[FPC V8.9 Main Menu]
Project cp05T5e12...
FPC Ctg3877 cp05...

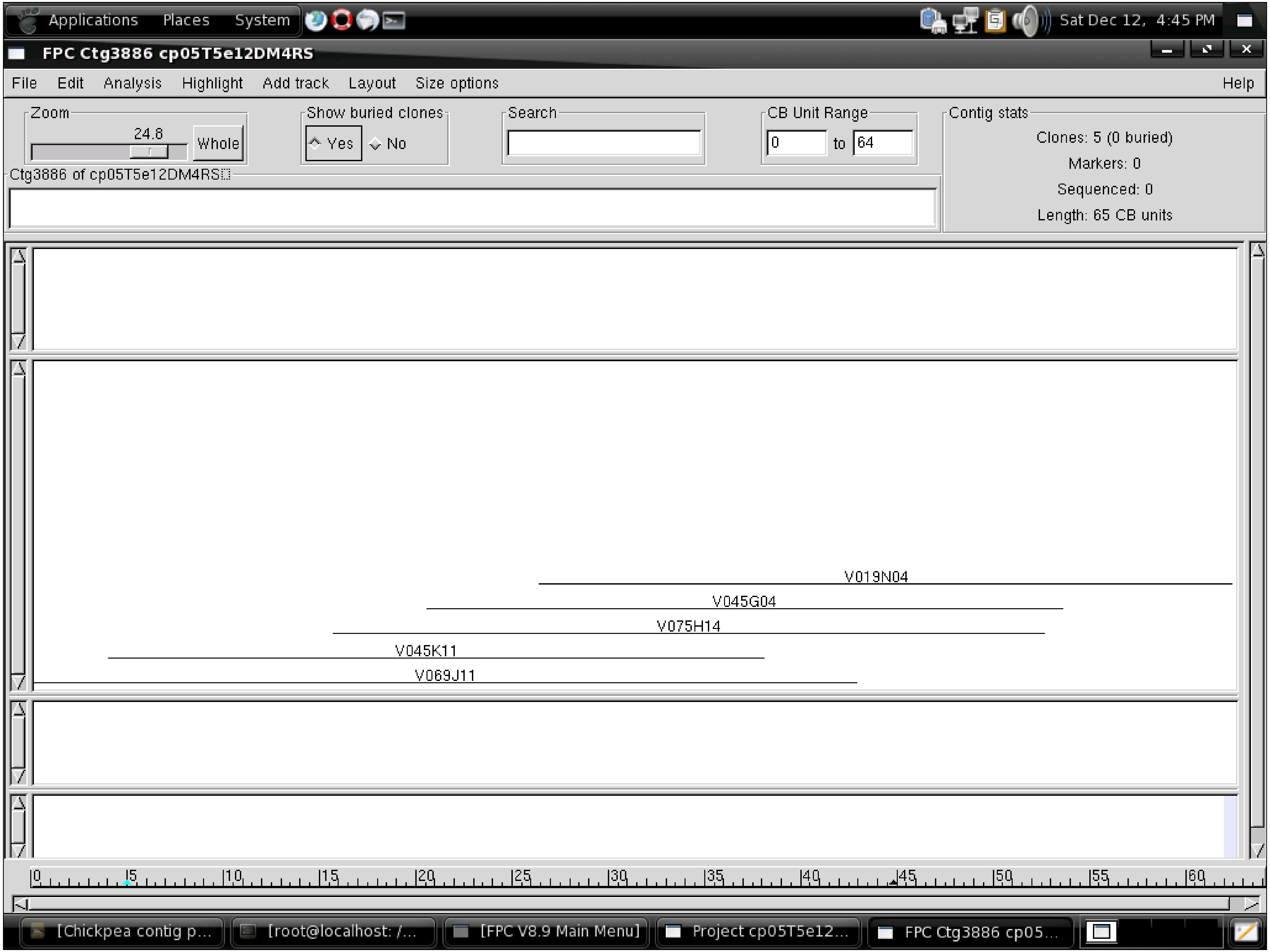
Applications Places System
Sat Dec 12, 4:45 PM
FPC Ctg3886 cp05T5e12DM4RS
File Edit Analysis Highlight Add track Layout Size options Help
Zoom
24.8
Whole
Show buried clones
Yes
No
Search
CB Unit Range
0
to
64
Contig stats
Clones: 5 (0 buried)
Markers: 0
Sequenced: 0
Length: 65 CB units
Ctg3886 of cp05T5e12DM4RS
V019N04
V045G04
V075H14
V045K11
V069J11
0 5 10 15 20 25 30 35 40 45 50 55 60
[Chickpea contig p...
[root@localhost: /...
[FPC V8.9 Main Menu]
Project cp05T5e12...
FPC Ctg3886 cp05...

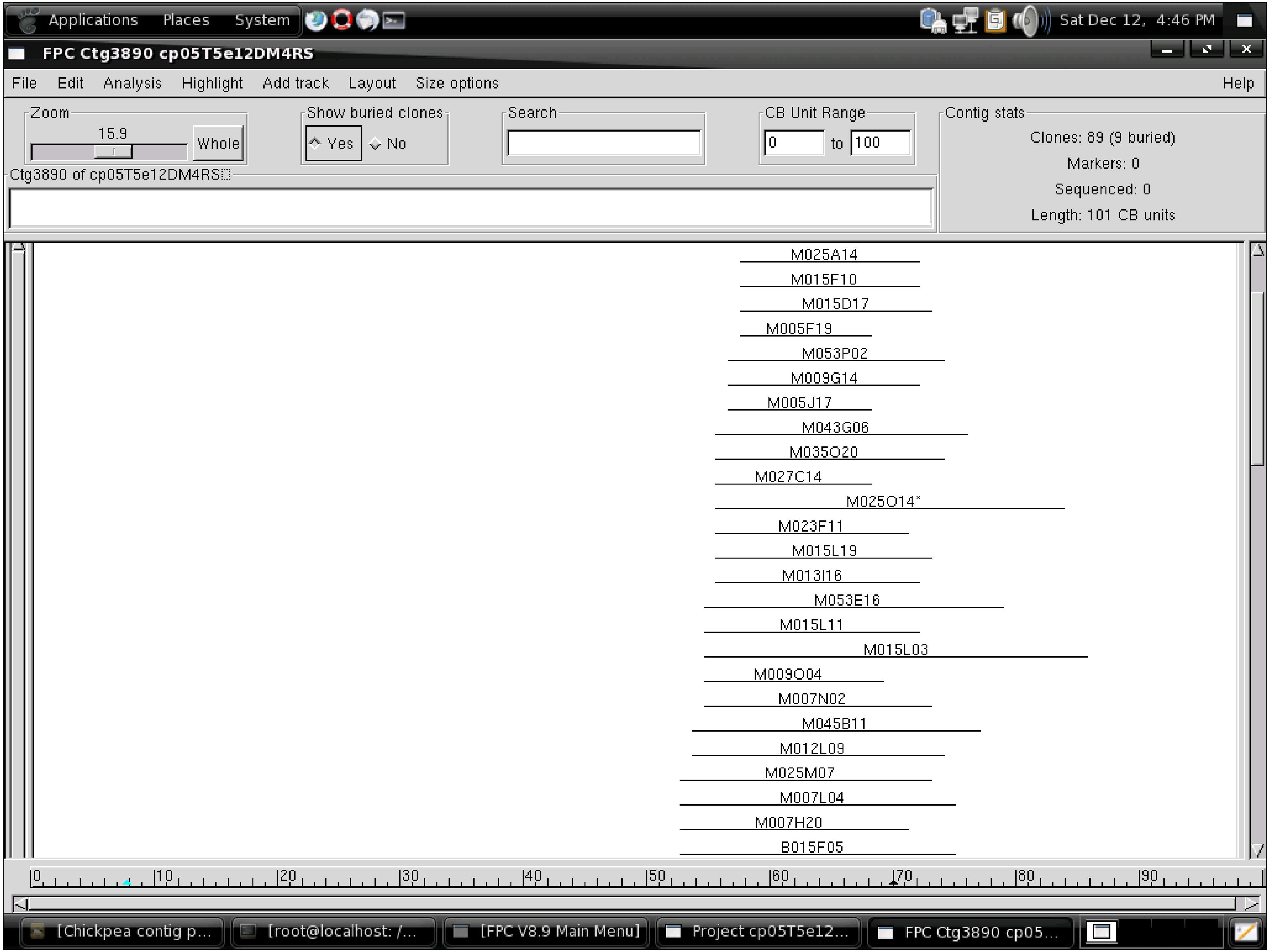
Applications Places System
Sat Dec 12, 4:46 PM
FPC Ctg3890 cp05T5e12DM4RS
File Edit Analysis Highlight Add track Layout Size options Help
Zoom
15.9
Whole
Show buried clones
Yes No
Search
CB Unit Range
0 to 100
Contig stats
Clones: 89 (9 buried)
Markers: 0
Sequenced: 0
Length: 101 CB units
Ctg3890 of cp05T5e12DM4RS
M025A14
M015F10
M015D17
M005F19
M053P02
M009G14
M005J17
M043G06
M035O20
M027C14
M025O14*
M023F11
M015L19
M013I16
M053E16
M015L11
M015L03
M009O04
M007N02
M045B11
M012L09
M025M07
M007L04
M007H20
B015F05
0 10 20 30 40 50 60 70 80 90
[Chickpea contig p...
[root@localhost: /...
[FPC V8.9 Main Menu]
Project cp05T5e12...
FPC Ctg3890 cp05...

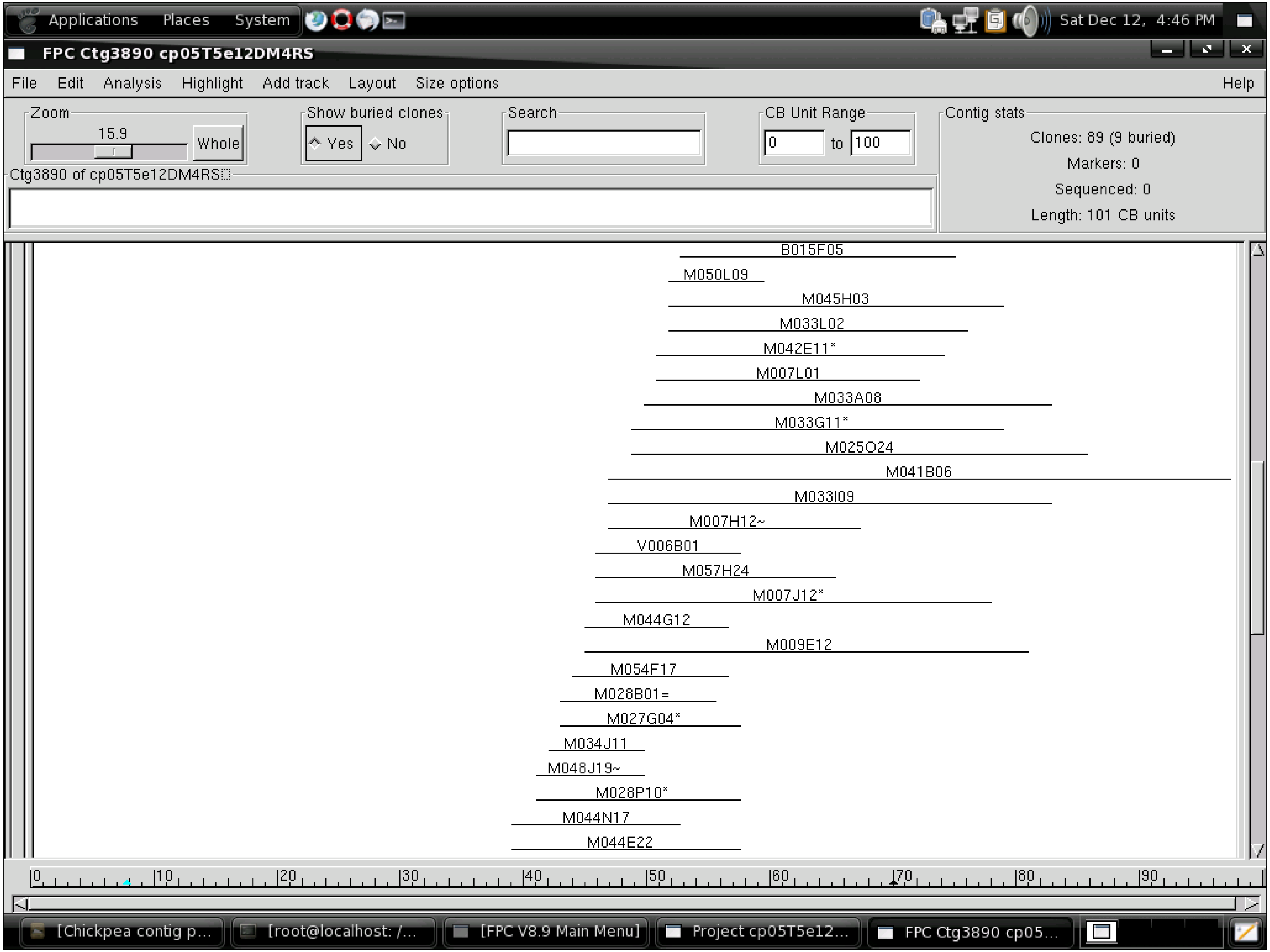
Applications Places System
Sat Dec 12, 4:46 PM
FPC Ctg3890 cp05T5e12DM4RS
File Edit Analysis Highlight Add track Layout Size options Help
Zoom
15.9
Whole
Show buried clones
Yes
No
Search
CB Unit Range
0
to
100
Contig stats
Clones: 89 (9 buried)
Markers: 0
Sequenced: 0
Length: 101 CB units
Ctg3890 of cp05T5e12DM4RS
B015F05
M050L09
M045H03
M033L02
M042E11*
M007L01
M033A08
M033G11*
M025O24
M041B06
M033I09
M007H12~
V006B01
M057H24
M007J12*
M044G12
M009E12
M054F17
M028B01=
M027G04*
M034J11
M048J19~
M028P10*
M044N17
M044E22
0 10 20 30 40 50 60 70 80 90
[Chickpea contig p...
[root@localhost: /...
[FPC V8.9 Main Menu]
Project cp05T5e12...
FPC Ctg3890 cp05...

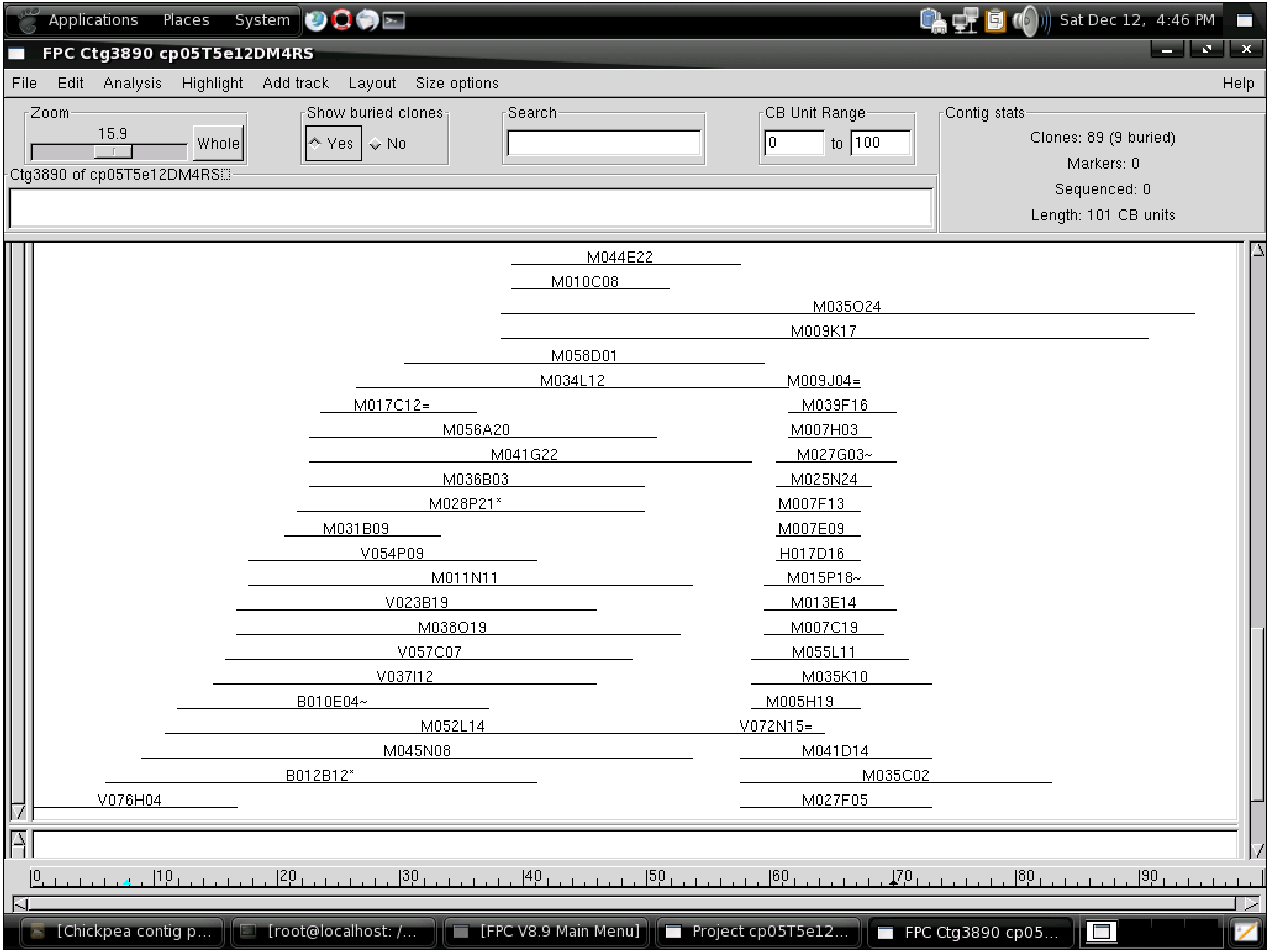

Applications Places System
Sat Dec 12, 4:46 PM
FPC Ctg3890 cp05T5e12DM4RS
File Edit Analysis Highlight Add track Layout Size options Help
Zoom
15.9
Whole
Show buried clones
Yes
No
Search
CB Unit Range
0
to
100
Contig stats
Clones: 89 (9 buried)
Markers: 0
Sequenced: 0
Length: 101 CB units
Ctg3890 of cp05T5e12DM4RS
M044E22
M010C08
M035O24
M009K17
M058D01
M034L12
M009J04=
M017C12=
M039F16
M056A20
M007H03
M041G22
M027G03~
M036B03
M025N24
M028P21*
M007F13
M031B09
M007E09
V054P09
H017D16
M011N11
M015P18~
V023B19
M013E14
M038O19
M007C19
V057C07
M055L11
V037I12
M035K10
B010E04~
M005H19
M052L14
V072N15=
M045N08
M041D14
B012B12*
M035C02
V076H04
M027F05
0 10 20 30 40 50 60 70 80 90
[Chickpea contig p...
[root@localhost: /...
[FPC V8.9 Main Menu]
Project cp05T5e12...
FPC Ctg3890 cp05...

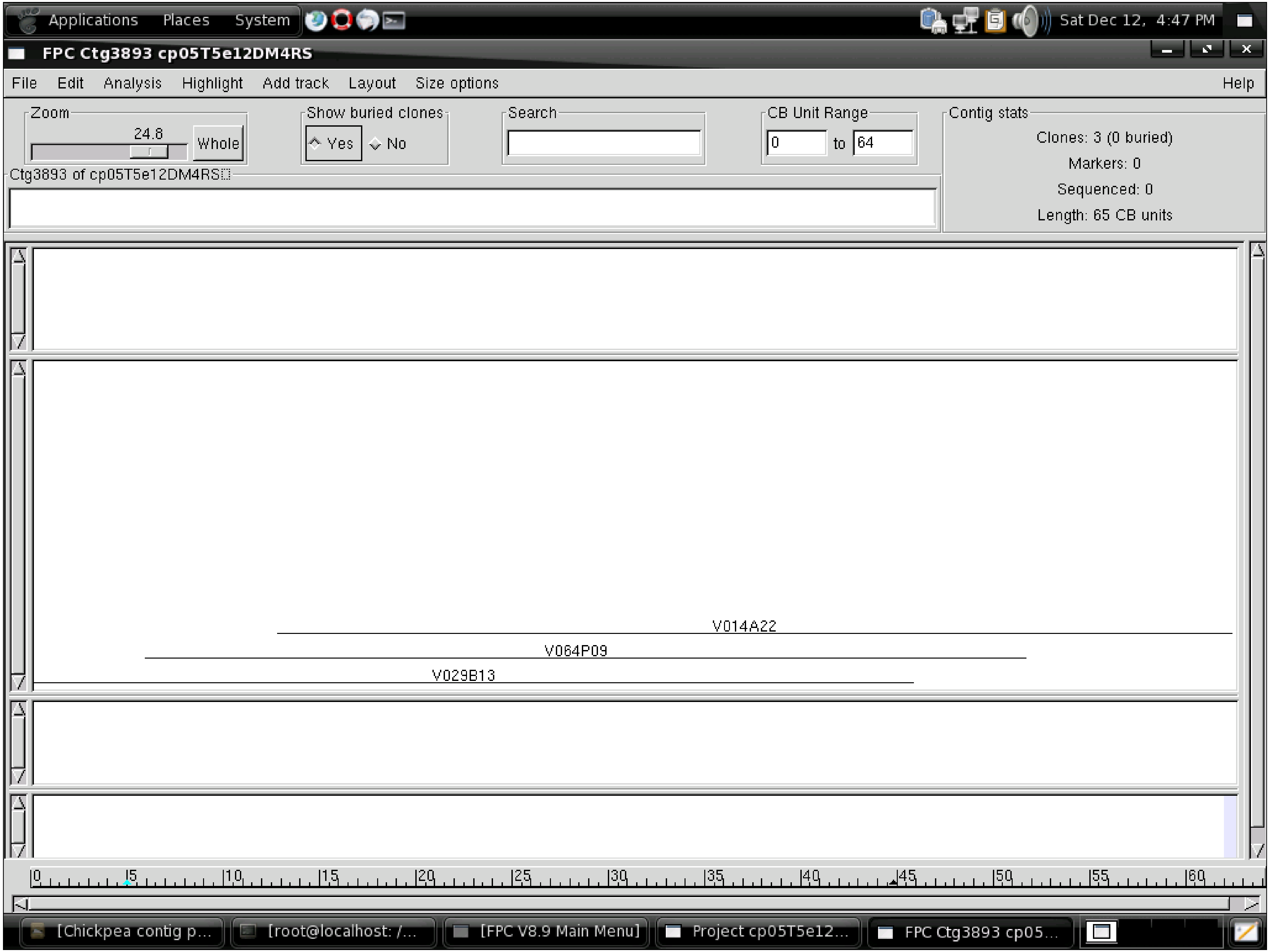
Applications Places System
Sat Dec 12, 4:47 PM
FPC Ctg3893 cp05T5e12DM4RS
File Edit Analysis Highlight Add track Layout Size options Help
Zoom
24.8
Whole
Show buried clones
Yes
No
Search
CB Unit Range
0 to 64
Contig stats
Clones: 3 (0 buried)
Markers: 0
Sequenced: 0
Length: 65 CB units
Ctg3893 of cp05T5e12DM4RS
V014A22
V064P09
V029B13
0 5 10 15 20 25 30 35 40 45 50 55 60
[Chickpea contig p...
[root@localhost: /...
[FPC V8.9 Main Menu]
Project cp05T5e12...
FPC Ctg3893 cp05...

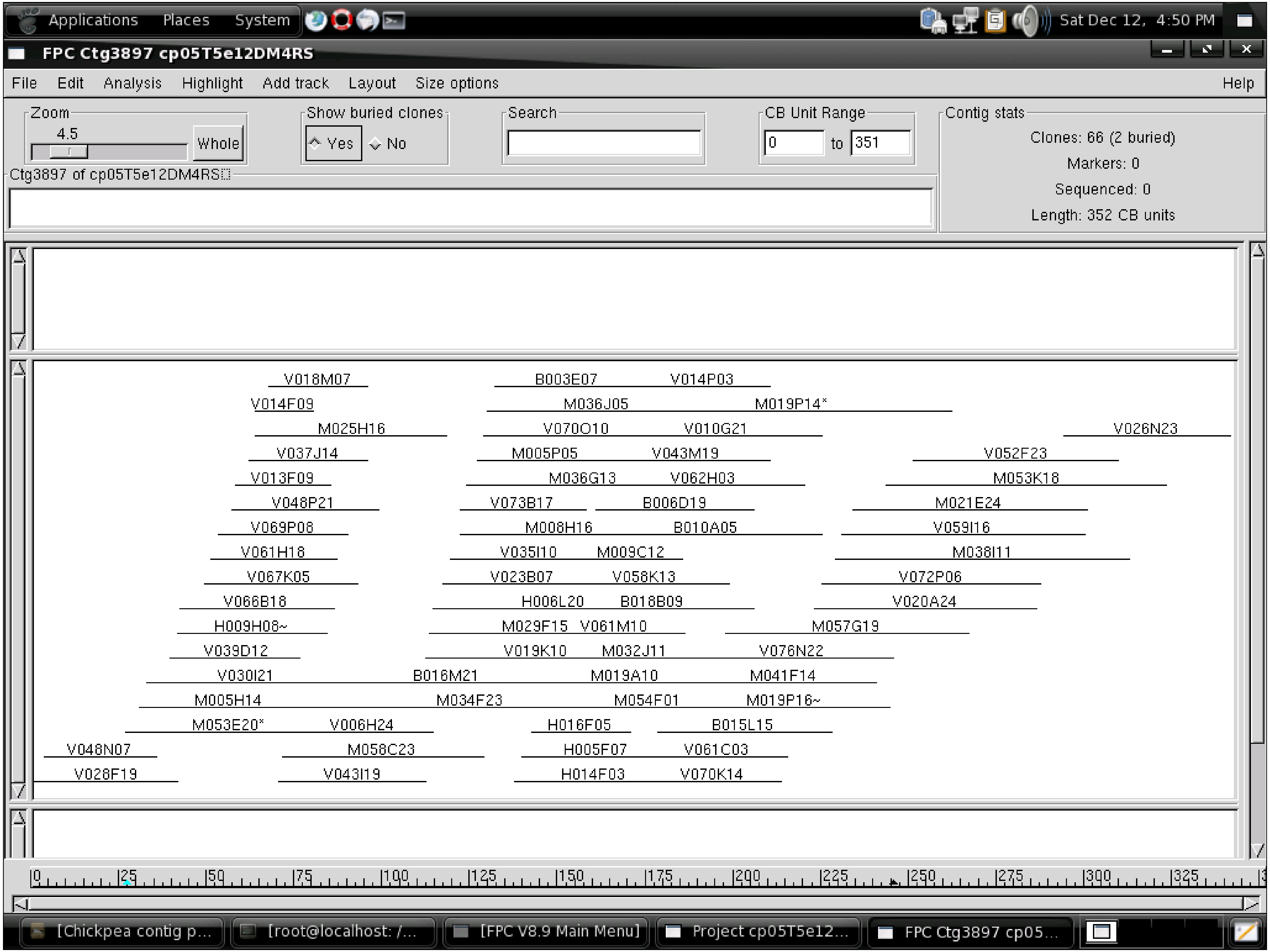
Applications Places System
Sat Dec 12, 4:50 PM
FPC Ctg3897 cp05T5e12DM4RS
File Edit Analysis Highlight Add track Layout Size options Help
Zoom
4.5
Whole
Show buried clones
Yes No
Search
CB Unit Range
0 to 351
Contig stats
Clones: 66 (2 buried)
Markers: 0
Sequenced: 0
Length: 352 CB units
Ctg3897 of cp05T5e12DM4RS
V018M07
B003E07
V014P03
V014F09
M036J05
M019P14*
M025H16
V070O10
V010G21
V026N23
V037J14
M005P05
V043M19
V052F23
V013F09
M036G13
V062H03
M053K18
V048P21
V073B17
B006D19
M021E24
V069P08
M008H16
B010A05
V059I16
V061H18
V035I10
M009C12
M038I11
V067K05
V023B07
V058K13
V072P06
V066B18
H006L20
B018B09
V020A24
H009H08~
M029F15
V061M10
M057G19
V039D12
V019K10
M032J11
V076N22
V030I21
B016M21
M019A10
M041F14
M005H14
M034F23
M054F01
M019P16~
M053E20*
V006H24
H016F05
B015L15
V048N07
M058C23
H005F07
V061C03
V028F19
V043I19
H014F03
V070K14
0 25 50 75 100 125 150 175 200 225 250 275 300 325
[Chickpea contig p...
[root@localhost: /...
[FPC V8.9 Main Menu]
Project cp05T5e12...
FPC Ctg3897 cp05...

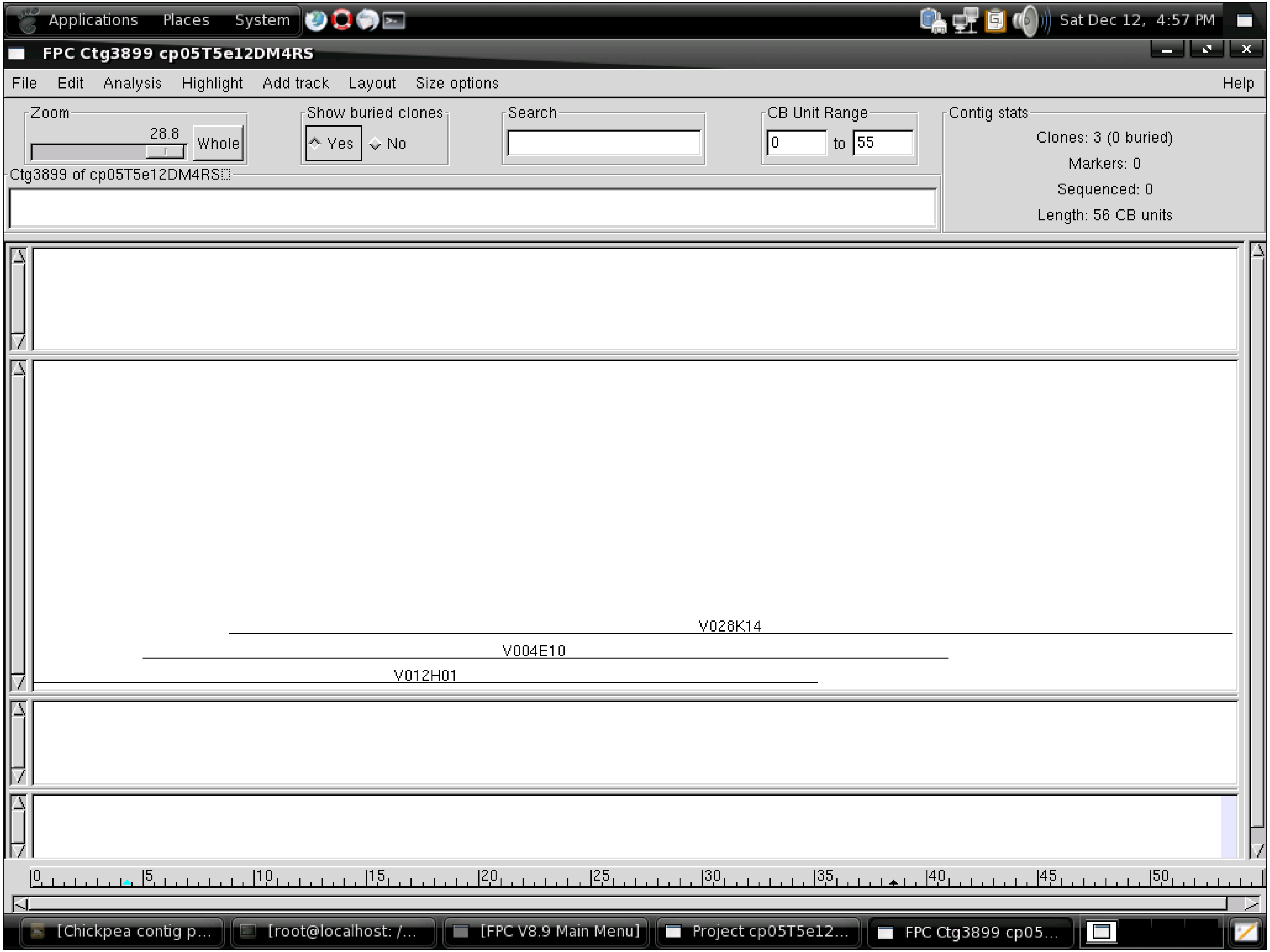
Applications
Places
System
Sat Dec 12, 4:57 PM
FPC Ctg3899 cp05T5e12DM4RS
File
Edit
Analysis
Highlight
Add track
Layout
Size options
Help
Zoom
28.8
Whole
Show buried clones
Yes
No
Search
CB Unit Range
0
to
55
Contig stats
Clones: 3 (0 buried)
Markers: 0
Sequenced: 0
Length: 56 CB units
Ctg3899 of cp05T5e12DM4RS
V028K14
V004E10
V012H01
0
5
10
15
20
25
30
35
40
45
50
[Chickpea contig p...
[root@localhost: /...
[FPC V8.9 Main Menu]
Project cp05T5e12...
FPC Ctg3899 cp05...

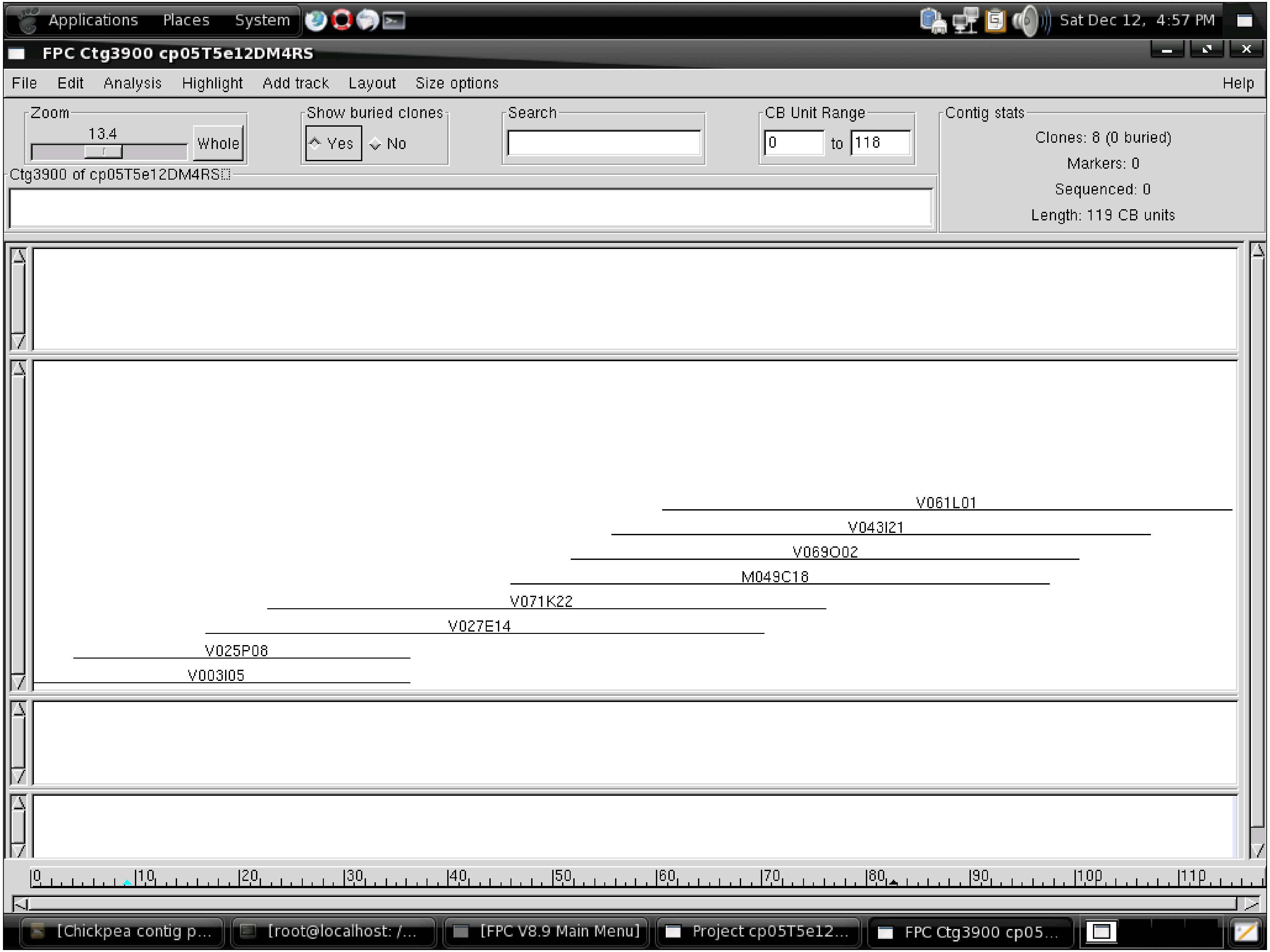
Applications Places System
Sat Dec 12, 4:57 PM
FPC Ctg3900 cp05T5e12DM4RS
File Edit Analysis Highlight Add track Layout Size options Help
Zoom
13.4
Whole
Show buried clones
Yes No
Search
CB Unit Range
0 to 118
Contig stats
Clones: 8 (0 buried)
Markers: 0
Sequenced: 0
Length: 119 CB units
Ctg3900 of cp05T5e12DM4RS
V061L01
V043I21
V069O02
M049C18
V071K22
V027E14
V025P08
V003I05
0 10 20 30 40 50 60 70 80 90 100 110
[Chickpea contig p...
[root@localhost: /...
[FPC V8.9 Main Menu]
Project cp05T5e12...
FPC Ctg3900 cp05...

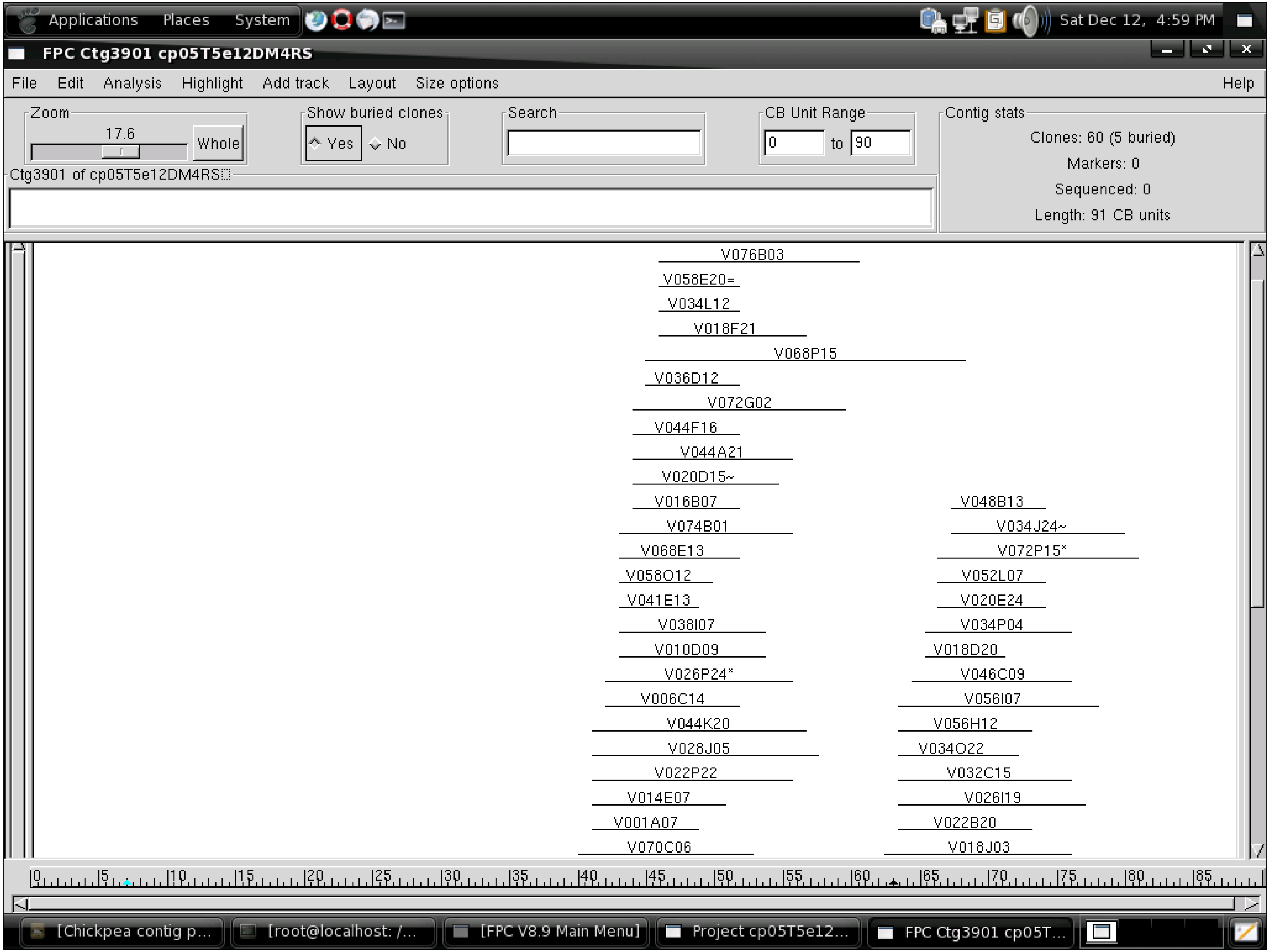
Applications Places System
Sat Dec 12, 4:59 PM
FPC Ctg3901 cp05T5e12DM4RS
File Edit Analysis Highlight Add track Layout Size options Help
Zoom
17.6
Whole
Show buried clones
Yes
No
Search
CB Unit Range
0 to 90
Contig stats
Clones: 60 (5 buried)
Markers: 0
Sequenced: 0
Length: 91 CB units
Ctg3901 of cp05T5e12DM4RS
V076B03
V058E20=
V034L12
V018F21
V068P15
V036D12
V072G02
V044F16
V044A21
V020D15~
V016B07
V074B01
V068E13
V058O12
V041E13
V038I07
V010D09
V026P24*
V006C14
V044K20
V028J05
V022P22
V014E07
V001A07
V070C06
V048B13
V034J24~
V072P15*
V052L07
V020E24
V034P04
V018D20
V046C09
V056I07
V056H12
V034O22
V032C15
V026I19
V022B20
V018J03
0 5 10 15 20 25 30 35 40 45 50 55 60 65 70 75 80 85
[Chickpea contig p...
[root@localhost: /...
[FPC V8.9 Main Menu]
Project cp05T5e12...
FPC Ctg3901 cp05T...

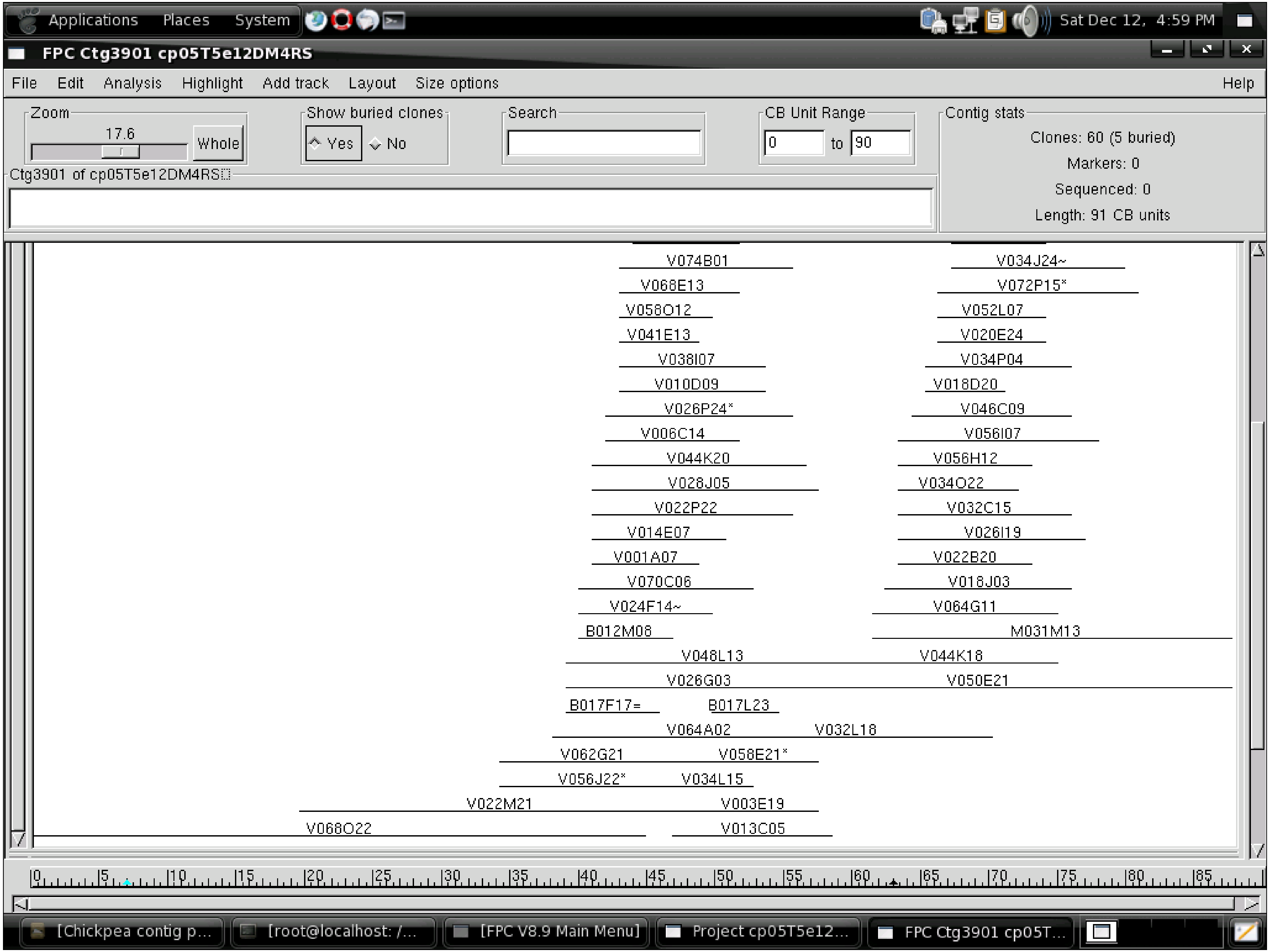
Applications Places System
Sat Dec 12, 4:59 PM
FPC Ctg3901 cp05T5e12DM4RS
File Edit Analysis Highlight Add track Layout Size options Help
Zoom
17.6
Whole
Show buried clones
Yes
No
Search
CB Unit Range
0 to 90
Contig stats
Clones: 60 (5 buried)
Markers: 0
Sequenced: 0
Length: 91 CB units
Ctg3901 of cp05T5e12DM4RS
V074B01
V034J24~
V068E13
V072P15*
V058O12
V052L07
V041E13
V020E24
V038I07
V034P04
V010D09
V018D20
V026P24*
V046C09
V006C14
V056I07
V044K20
V056H12
V028J05
V034O22
V022P22
V032C15
V014E07
V026I19
V001A07
V022B20
V070C06
V018J03
V024F14~
V064G11
B012M08
M031M13
V048L13
V044K18
V026G03
V050E21
B017F17=
B017L23
V064A02
V032L18
V062G21
V058E21*
V056J22*
V034L15
V022M21
V003E19
V068O22
V013C05
0 5 10 15 20 25 30 35 40 45 50 55 60 65 70 75 80 85
[Chickpea contig p...
[root@localhost: /...
[FPC V8.9 Main Menu]
Project cp05T5e12...
FPC Ctg3901 cp05T...

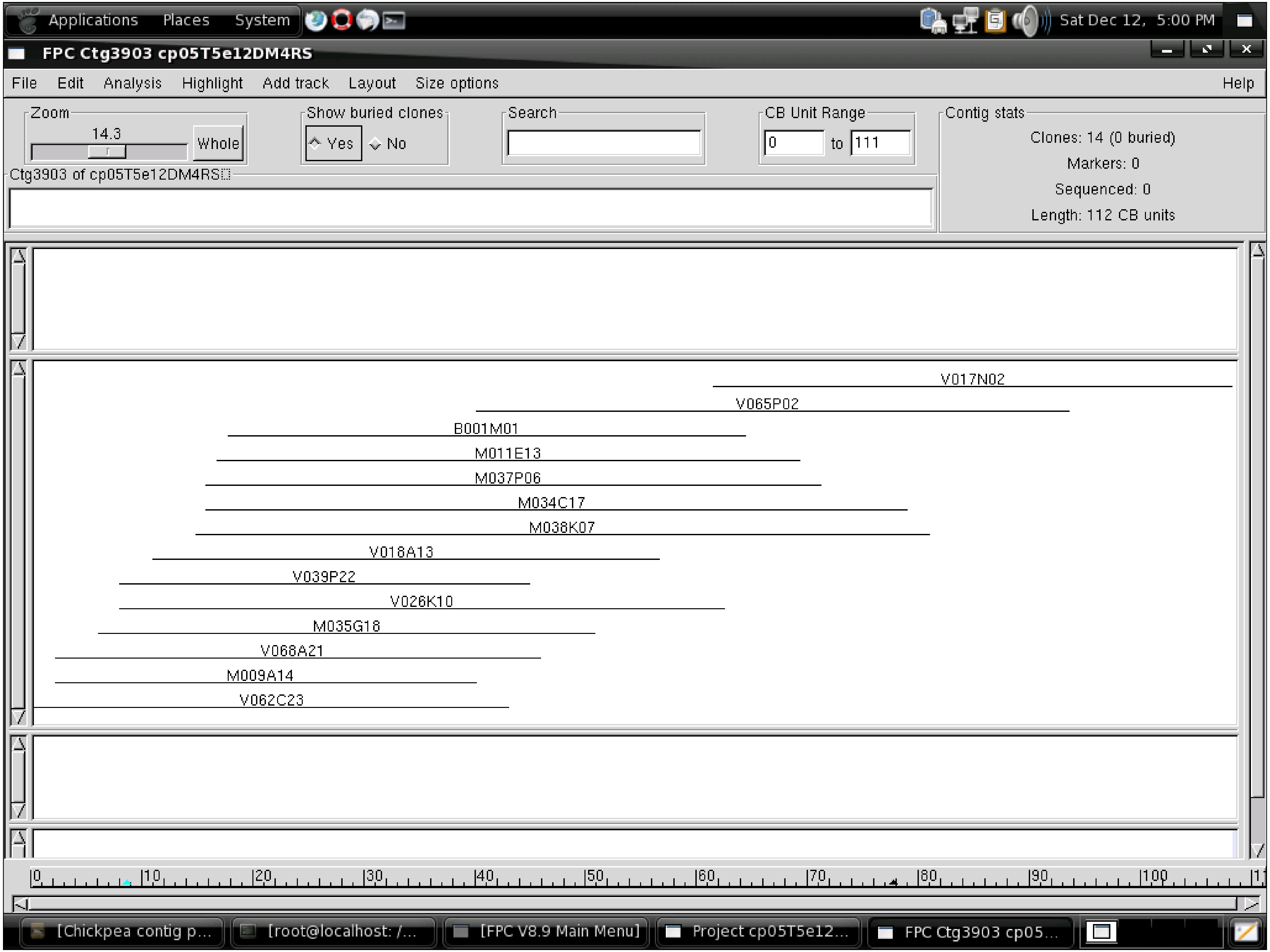
Applications Places System
Sat Dec 12, 5:00 PM
FPC Ctg3903 cp05T5e12DM4RS
File Edit Analysis Highlight Add track Layout Size options Help
Zoom
14.3
Whole
Show buried clones
Yes No
Search
CB Unit Range
0 to 111
Contig stats
Clones: 14 (0 buried)
Markers: 0
Sequenced: 0
Length: 112 CB units
Ctg3903 of cp05T5e12DM4RS
V017N02
V065P02
B001M01
M011E13
M037P06
M034C17
M038K07
V018A13
V039P22
V026K10
M035G18
V068A21
M009A14
V062C23
0 10 20 30 40 50 60 70 80 90 100
[Chickpea contig p...
[root@localhost: /...
[FPC V8.9 Main Menu]
Project cp05T5e12...
FPC Ctg3903 cp05...

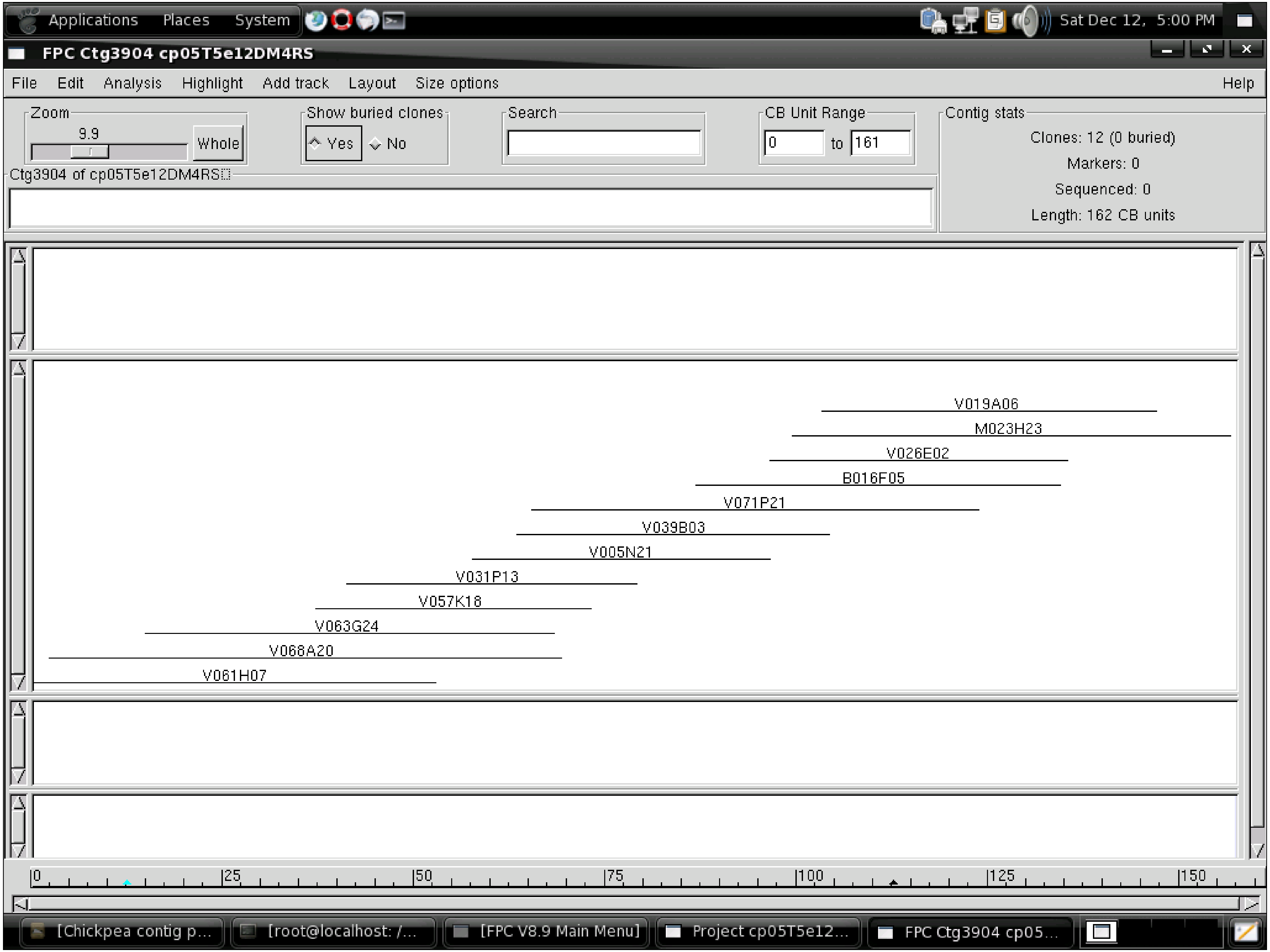

Applications Places System
Sat Dec 12, 5:00 PM
FPC Ctg3904 cp05T5e12DM4RS
File Edit Analysis Highlight Add track Layout Size options Help
Zoom
9.9
Whole
Show buried clones
Yes No
Search
CB Unit Range
0 to 161
Contig stats
Clones: 12 (0 buried)
Markers: 0
Sequenced: 0
Length: 162 CB units
Ctg3904 of cp05T5e12DM4RS
V019A06
M023H23
V026E02
B016F05
V071P21
V039B03
V005N21
V031P13
V057K18
V063G24
V068A20
V061H07
0 25 50 75 100 125 150
[Chickpea contig p...
[root@localhost: /...
[FPC V8.9 Main Menu]
Project cp05T5e12...
FPC Ctg3904 cp05...

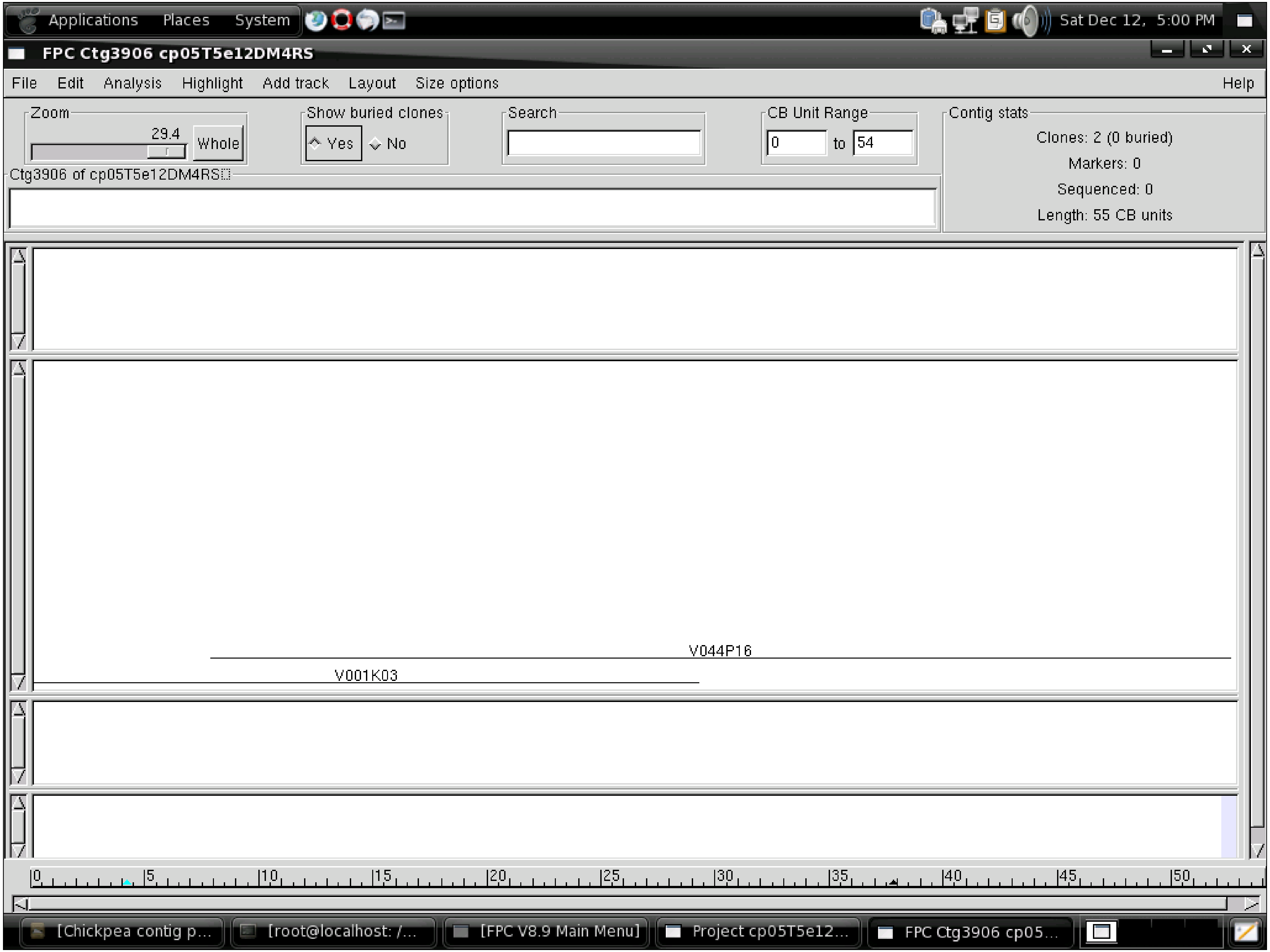
Applications Places System
Sat Dec 12, 5:00 PM
FPC Ctg3906 cp05T5e12DM4RS
File Edit Analysis Highlight Add track Layout Size options Help
Zoom
29.4
Whole
Show buried clones
Yes
No
Search
CB Unit Range
0 to 54
Contig stats
Clones: 2 (0 buried)
Markers: 0
Sequenced: 0
Length: 55 CB units
Ctg3906 of cp05T5e12DM4RS
V044P16
V001K03
0 5 10 15 20 25 30 35 40 45 50
[Chickpea contig p...
[root@localhost: /...
[FPC V8.9 Main Menu]
Project cp05T5e12...
FPC Ctg3906 cp05...

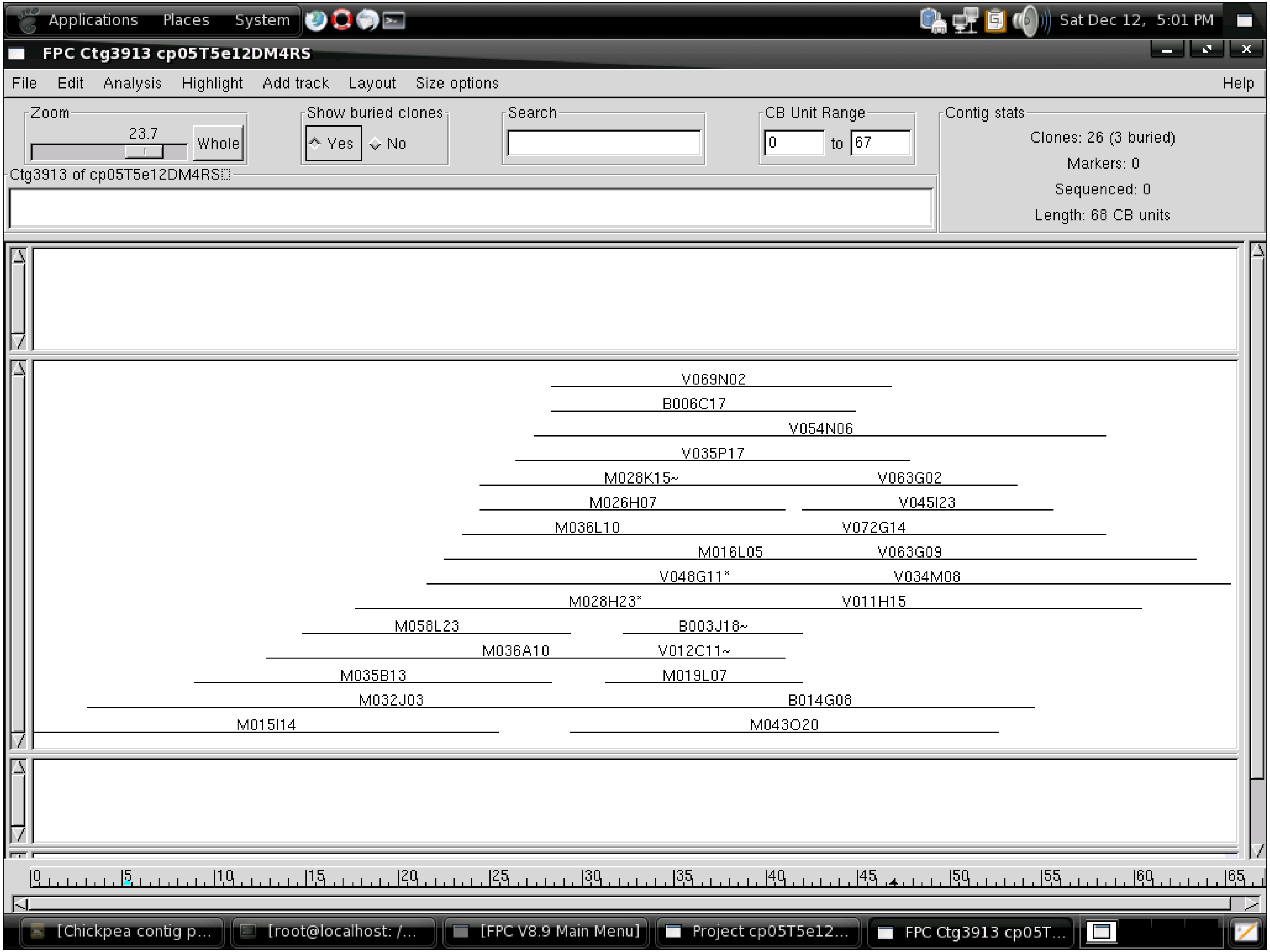
Applications Places System
Sat Dec 12, 5:01 PM
FPC Ctg3913 cp05T5e12DM4RS
File Edit Analysis Highlight Add track Layout Size options Help
Zoom
23.7
Whole
Show buried clones
Yes No
Search
CB Unit Range
0 to 67
Contig stats
Clones: 26 (3 buried)
Markers: 0
Sequenced: 0
Length: 68 CB units
Ctg3913 of cp05T5e12DM4RS
V069N02
B006C17
V054N06
V035P17
M028K15~
V063G02
M026H07
V045I23
M036L10
V072G14
M016L05
V063G09
V048G11*
V034M08
M028H23*
V011H15
M058L23
B003J18~
M036A10
V012C11~
M035B13
M019L07
M032J03
B014G08
M015I14
M043O20
0 5 10 15 20 25 30 35 40 45 50 55 60 65
[Chickpea contig p...
[root@localhost: /...
[FPC V8.9 Main Menu]
Project cp05T5e12...
FPC Ctg3913 cp05T...

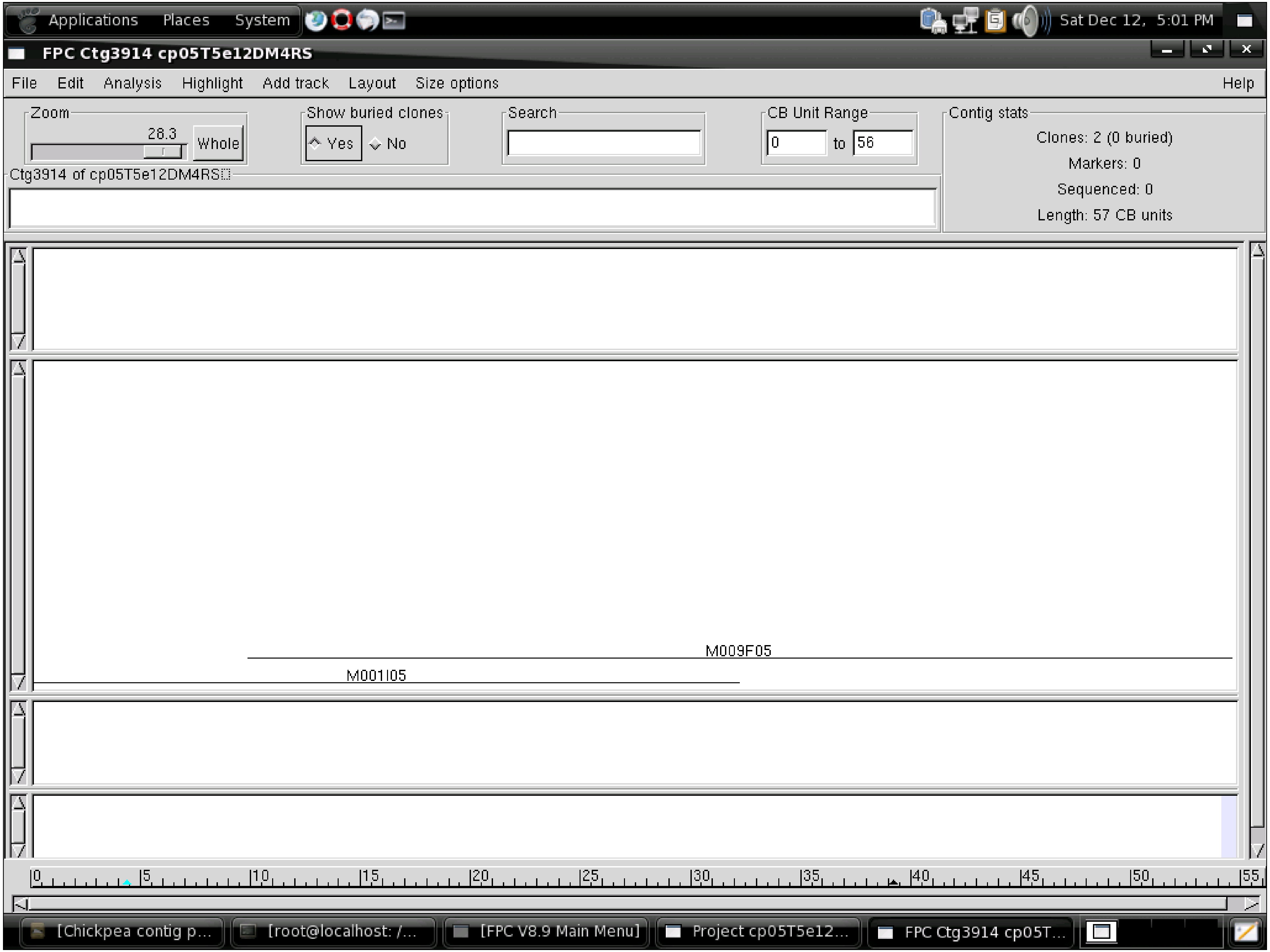
Applications
Places
System
Sat Dec 12, 5:01 PM
FPC Ctg3914 cp05T5e12DM4RS
File
Edit
Analysis
Highlight
Add track
Layout
Size options
Help
Zoom
28.3
Whole
Show buried clones
Yes
No
Search
CB Unit Range
0
to
56
Contig stats
Clones: 2 (0 buried)
Markers: 0
Sequenced: 0
Length: 57 CB units
Ctg3914 of cp05T5e12DM4RS
M009F05
M001I05
0 5 10 15 20 25 30 35 40 45 50 55
[Chickpea contig p...
[root@localhost: /...
[FPC V8.9 Main Menu]
Project cp05T5e12...
FPC Ctg3914 cp05T...

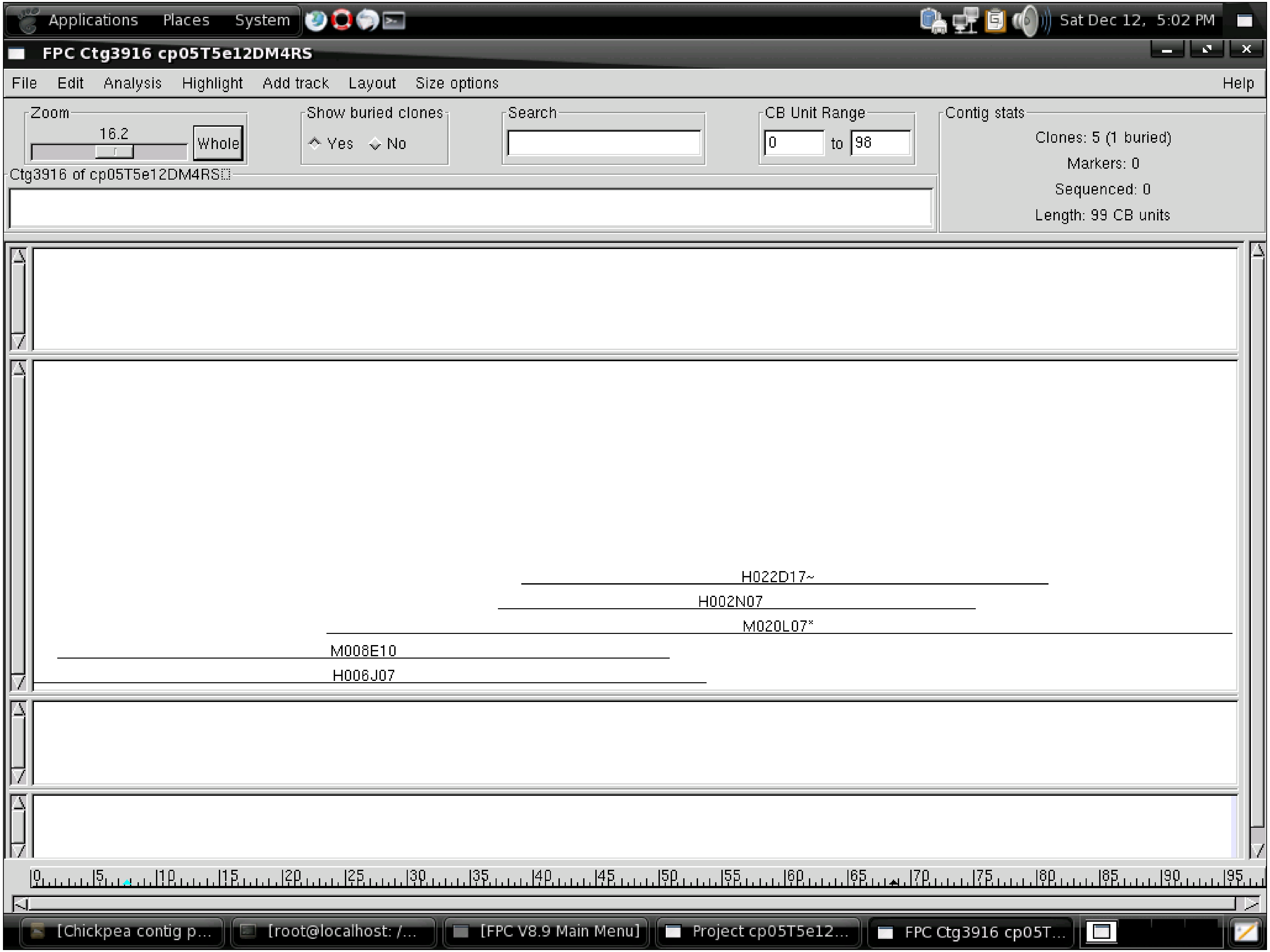
Applications
Places
System
Sat Dec 12, 5:02 PM
FPC Ctg3916 cp05T5e12DM4RS
File
Edit
Analysis
Highlight
Add track
Layout
Size options
Help
Zoom
16.2
Whole
Show buried clones
Yes
No
Search
CB Unit Range
0
to
98
Contig stats
Clones: 5 (1 buried)
Markers: 0
Sequenced: 0
Length: 99 CB units
Ctg3916 of cp05T5e12DM4RS
H022D17~
H002N07
M020L07*
M008E10
H006J07
0 5 10 15 20 25 30 35 40 45 50 55 60 65 70 75 80 85 90 95
[Chickpea contig p...
[root@localhost: /...
[FPC V8.9 Main Menu]
Project cp05T5e12...
FPC Ctg3916 cp05T...

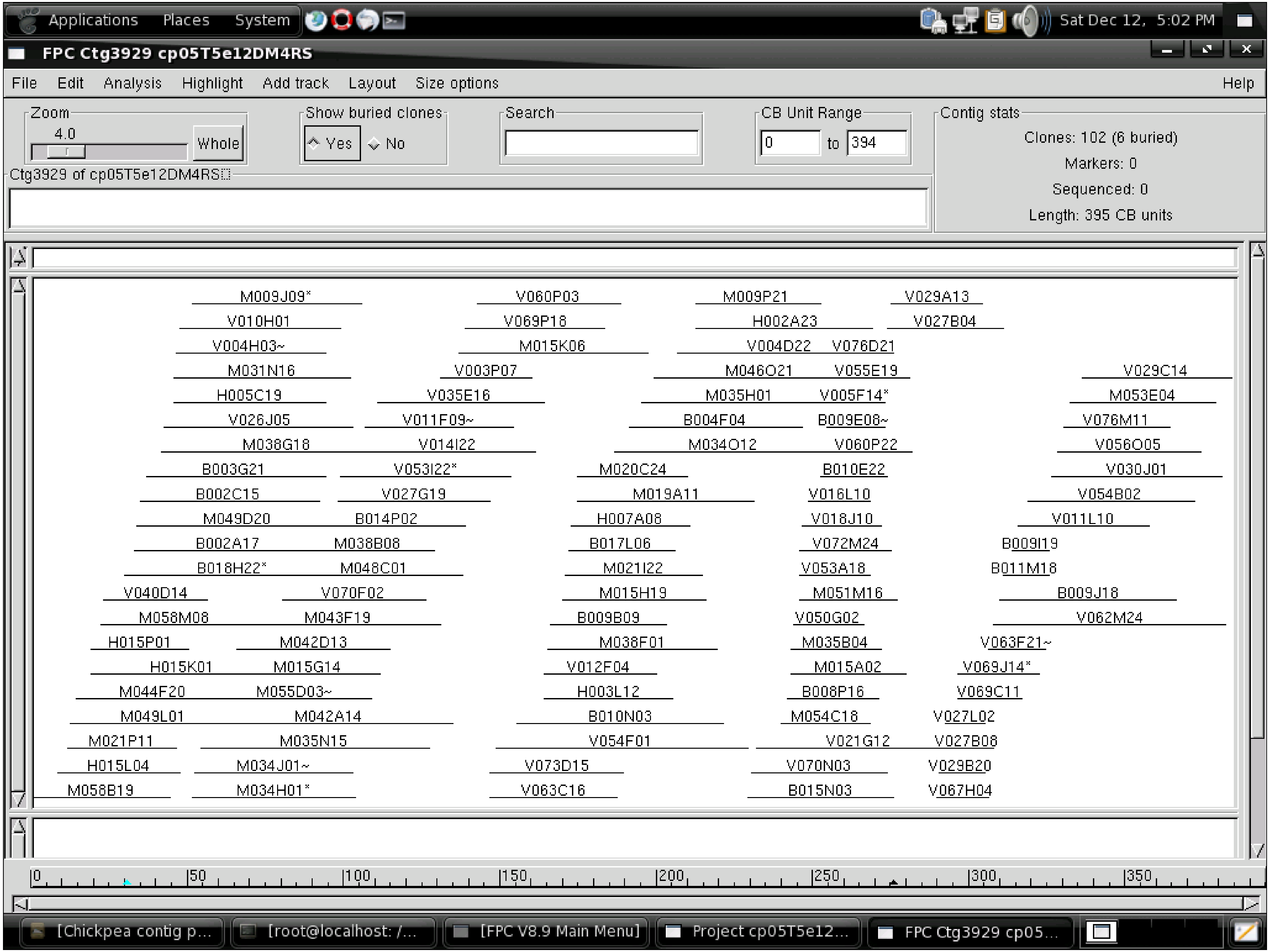
Applications Places System
Sat Dec 12, 5:02 PM
FPC Ctg3929 cp05T5e12DM4RS
File Edit Analysis Highlight Add track Layout Size options Help
Zoom
4.0
Whole
Show buried clones
Yes
No
Search
CB Unit Range
0 to 394
Contig stats
Clones: 102 (6 buried)
Markers: 0
Sequenced: 0
Length: 395 CB units
Ctg3929 of cp05T5e12DM4RS
M009J09* V060P03 M009P21 V029A13
V010H01 V069P18 H002A23 V027B04
V004H03~ M015K06 V004D22 V076D21
M031N16 V003P07 M046O21 V055E19 V029C14
H005C19 V035E16 M035H01 V005F14* M053E04
V026J05 V011F09~ B004F04 B009E08~ V076M11
M038G18 V014I22 M034O12 V060P22 V056O05
B003G21 V053I22* M020C24 B010E22 V030J01
B002C15 V027G19 M019A11 V016L10 V054B02
M049D20 B014P02 H007A08 V018J10 V011L10
B002A17 M038B08 B017L06 V072M24 B009I19
B018H22* M048C01 M021I22 V053A18 B011M18
V040D14 V070F02 M015H19 M051M16 B009J18
M058M08 M043F19 B009B09 V050G02 V062M24
H015P01 M042D13 M038F01 M035B04 V063F21~
H015K01 M015G14 V012F04 M015A02 V069J14*
M044F20 M055D03~ H003L12 B008P16 V069C11
M049L01 M042A14 B010N03 M054C18 V027L02
M021P11 M035N15 V054F01 V021G12 V027B08
H015L04 M034J01~ V073D15 V070N03 V029B20
M058B19 M034H01* V063C16 B015N03 V067H04
0 50 100 150 200 250 300 350
[Chickpea contig p...
[root@localhost: /...
[FPC V8.9 Main Menu]
Project cp05T5e12...
FPC Ctg3929 cp05...

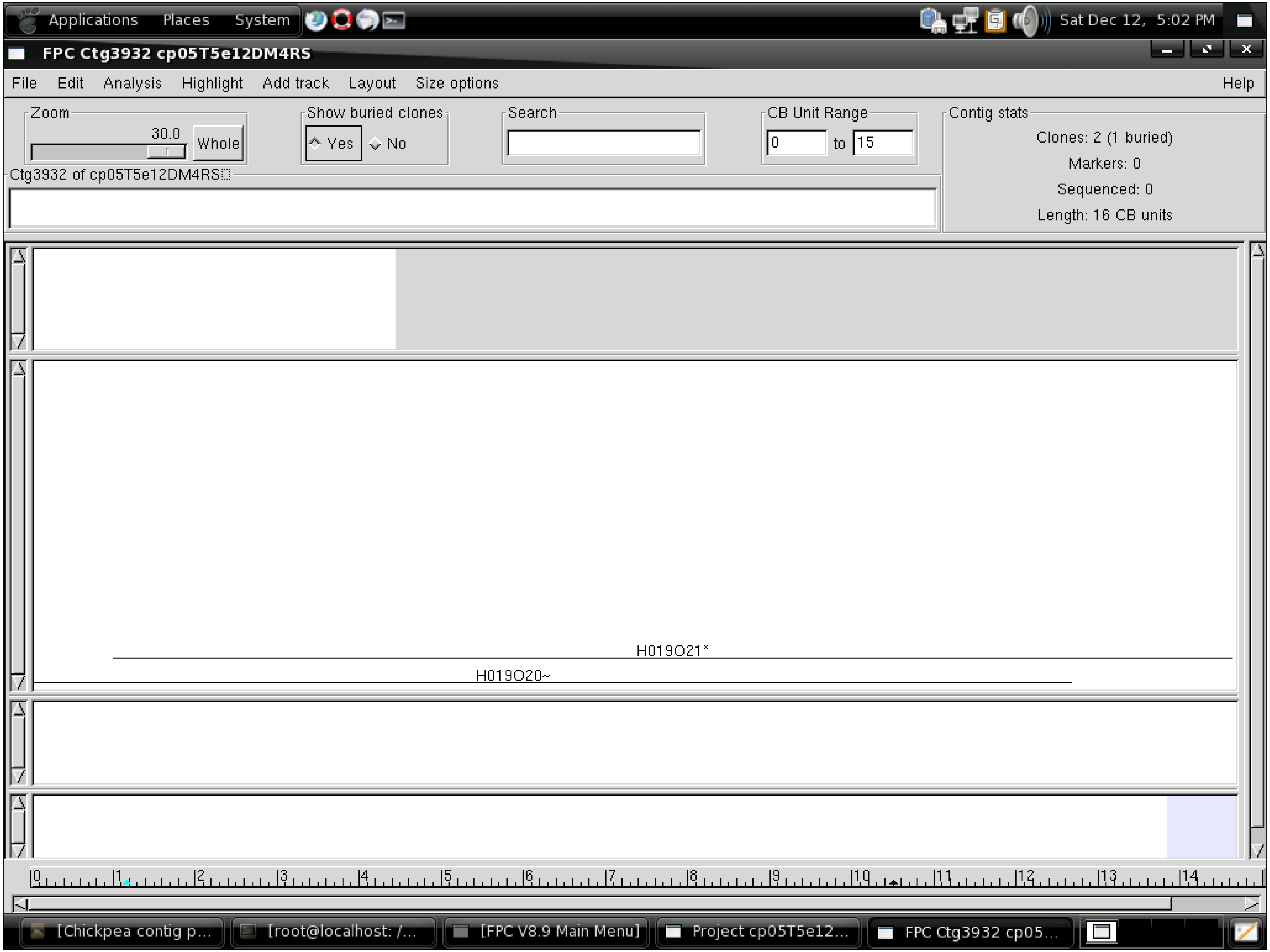
Applications Places System
Sat Dec 12, 5:02 PM
FPC Ctg3932 cp05T5e12DM4RS
File Edit Analysis Highlight Add track Layout Size options
Help
Zoom
30.0
Whole
Show buried clones
Yes
No
Search
CB Unit Range
0
to
15
Contig stats
Clones: 2 (1 buried)
Markers: 0
Sequenced: 0
Length: 16 CB units
Ctg3932 of cp05T5e12DM4RS
H019O21*
H019O20~
0 1 2 3 4 5 6 7 8 9 10 11 12 13 14
[Chickpea contig p...
[root@localhost: /...
[FPC V8.9 Main Menu]
Project cp05T5e12...
FPC Ctg3932 cp05...

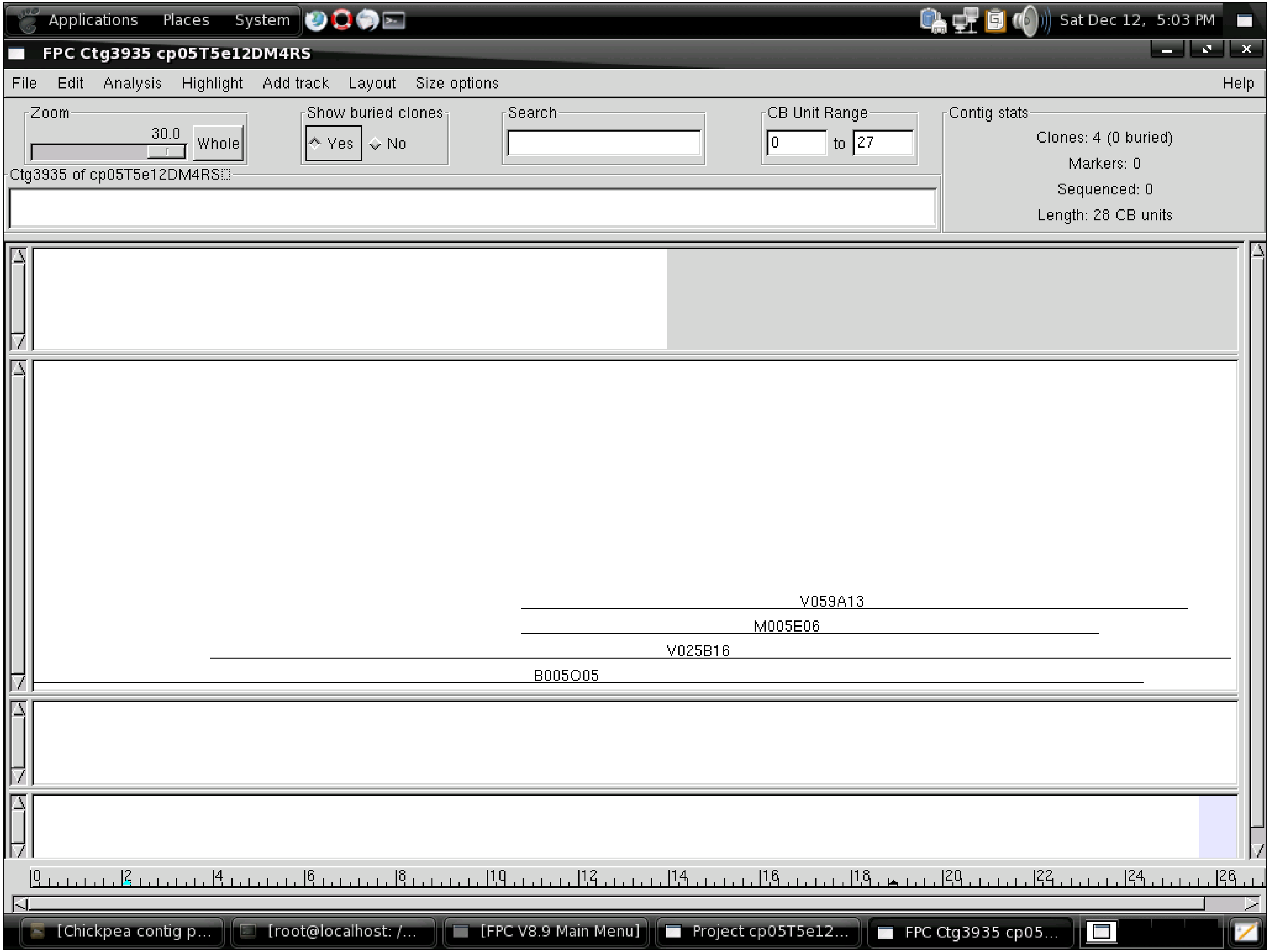
Applications Places System
Sat Dec 12, 5:03 PM
FPC Ctg3935 cp05T5e12DM4RS
File Edit Analysis Highlight Add track Layout Size options Help
Zoom
30.0
Whole
Show buried clones
Yes
No
Search
CB Unit Range
0
to
27
Contig stats
Clones: 4 (0 buried)
Markers: 0
Sequenced: 0
Length: 28 CB units
Ctg3935 of cp05T5e12DM4RS
V059A13
M005E06
V025B16
B005O05
0 2 4 6 8 10 12 14 16 18 20 22 24 26
[Chickpea contig p...
[root@localhost: /...
[FPC V8.9 Main Menu]
Project cp05T5e12...
FPC Ctg3935 cp05...

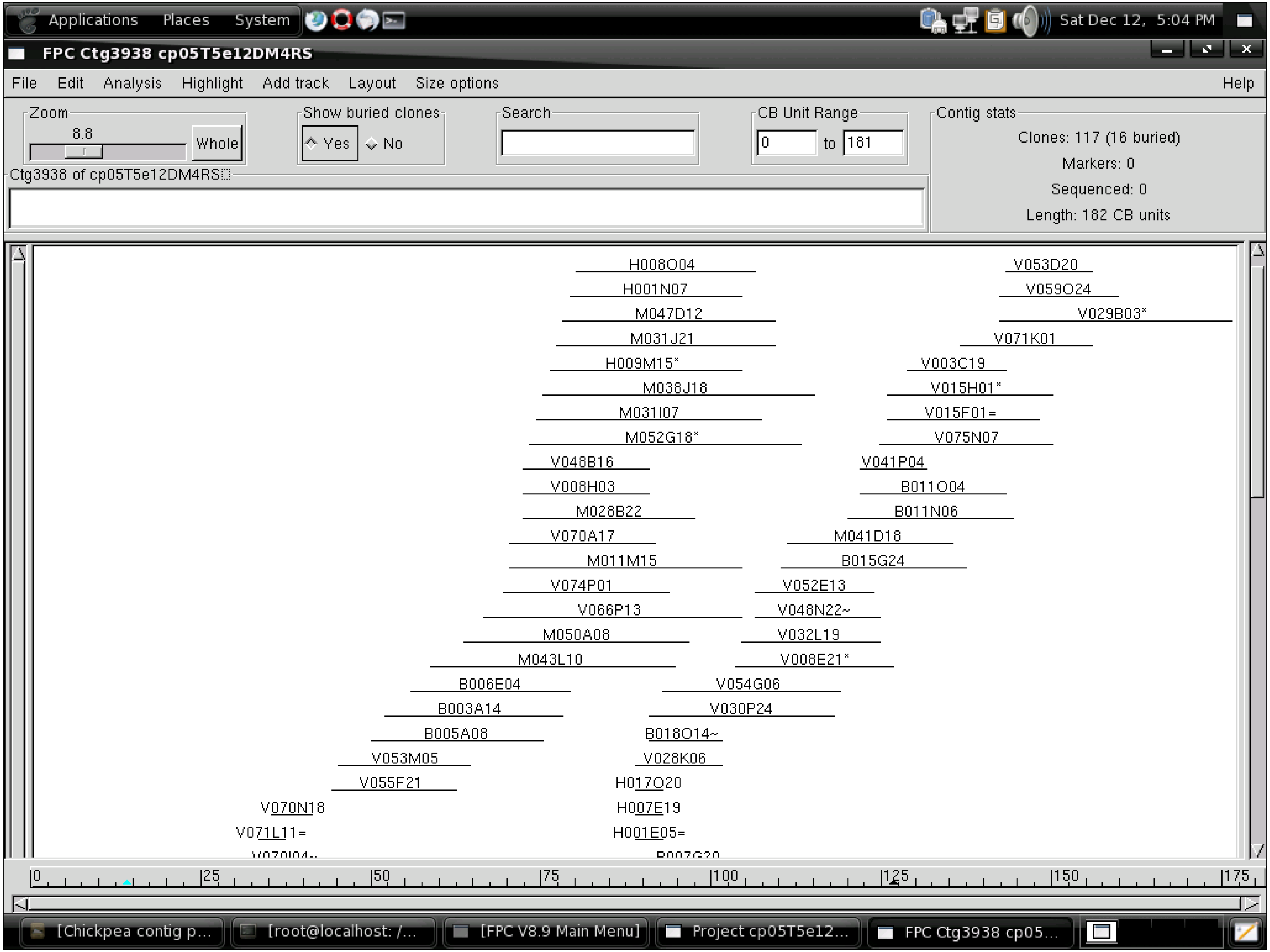

Applications
Places
System
Sat Dec 12, 5:04 PM
FPC Ctg3938 cp05T5e12DM4RS
File
Edit
Analysis
Highlight
Add track
Layout
Size options
Help
Zoom
8.8
Whole
Show buried clones
Yes
No
Search
CB Unit Range
0
to
181
Contig stats
Clones: 117 (16 buried)
Markers: 0
Sequenced: 0
Length: 182 CB units
Ctg3938 of cp05T5e12DM4RS
H008O04
H001N07
M047D12
M031J21
H009M15*
M038J18
M031I07
M052G18*
V048B16
V008H03
M028B22
V070A17
M011M15
V074P01
V066P13
M050A08
M043L10
B006E04
B003A14
B005A08
V053M05
V055F21
V070N18
V071L11=
V053D20
V059O24
V029B03*
V071K01
V003C19
V015H01*
V015F01=
V075N07
V041P04
B011O04
B011N06
M041D18
B015G24
V052E13
V048N22~
V032L19
V008E21*
V054G06
V030P24
B018O14~
V028K06
H017O20
H007E19
H001E05=
0
25
50
75
100
125
150
175
[Chickpea contig p...
[root@localhost: /...
[FPC V8.9 Main Menu]
Project cp05T5e12...
FPC Ctg3938 cp05...

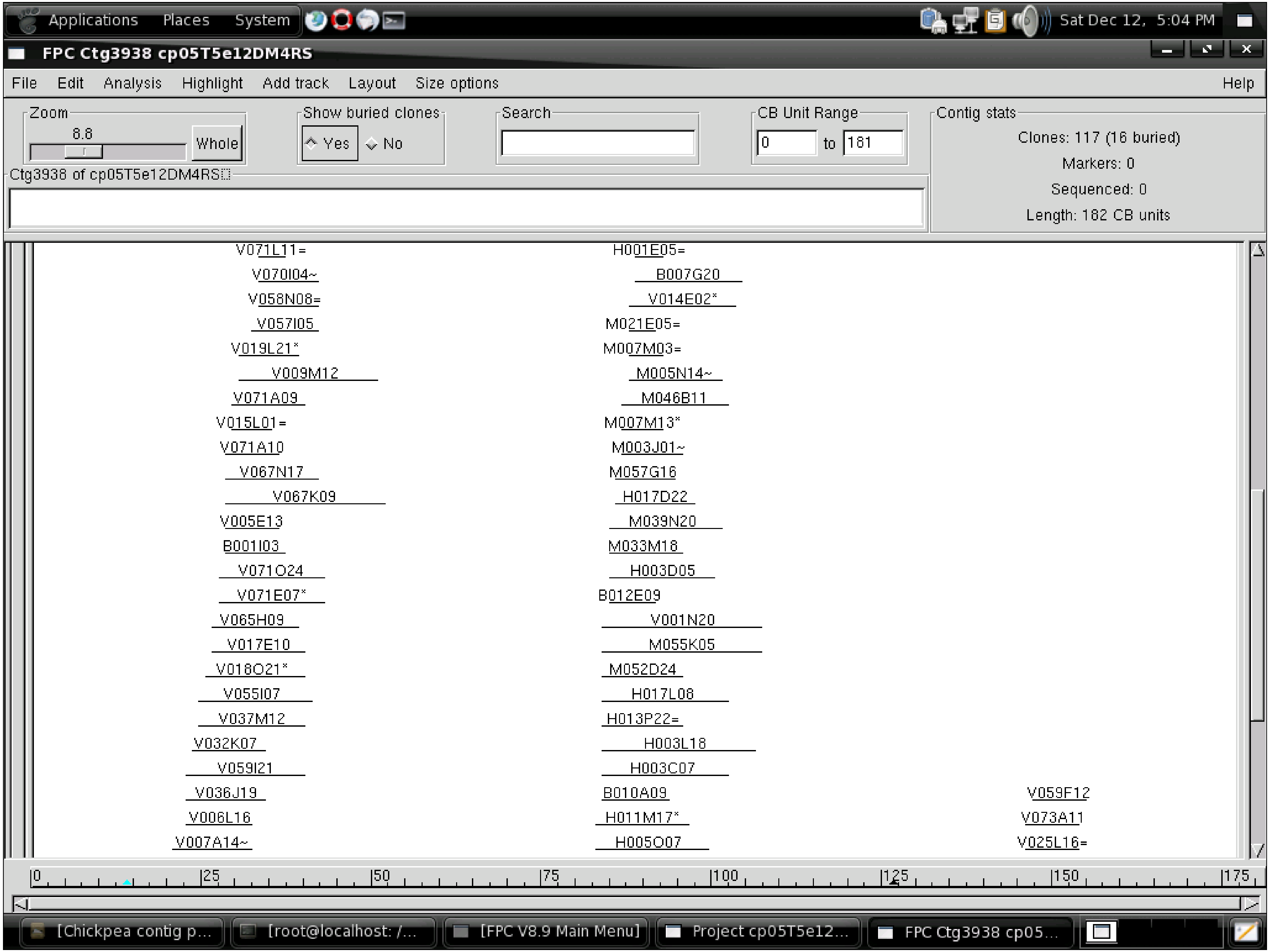
Applications Places System
Sat Dec 12, 5:04 PM
FPC Ctg3938 cp05T5e12DM4RS
File Edit Analysis Highlight Add track Layout Size options Help
Zoom
8.8
Whole
Show buried clones
Yes No
Search
CB Unit Range
0 to 181
Contig stats
Clones: 117 (16 buried)
Markers: 0
Sequenced: 0
Length: 182 CB units
Ctg3938 of cp05T5e12DM4RS
V071L11=
V070I04~
V058N08=
V057I05
V019L21*
V009M12
V071A09
V015L01=
V071A10
V067N17
V067K09
V005E13
B001I03
V071O24
V071E07*
V065H09
V017E10
V018O21*
V055I07
V037M12
V032K07
V059I21
V036J19
V006L16
V007A14~
H001E05=
B007G20
V014E02*
M021E05=
M007M03=
M005N14~
M046B11
M007M13*
M003J01~
M057G16
H017D22
M039N20
M033M18
H003D05
B012E09
V001N20
M055K05
M052D24
H017L08
H013P22=
H003L18
H003C07
B010A09
H011M17*
H005O07
V059F12
V073A11
V025L16=
0 25 50 75 100 125 150 175
[Chickpea contig p...
[root@localhost: /...
[FPC V8.9 Main Menu]
Project cp05T5e12...
FPC Ctg3938 cp05...

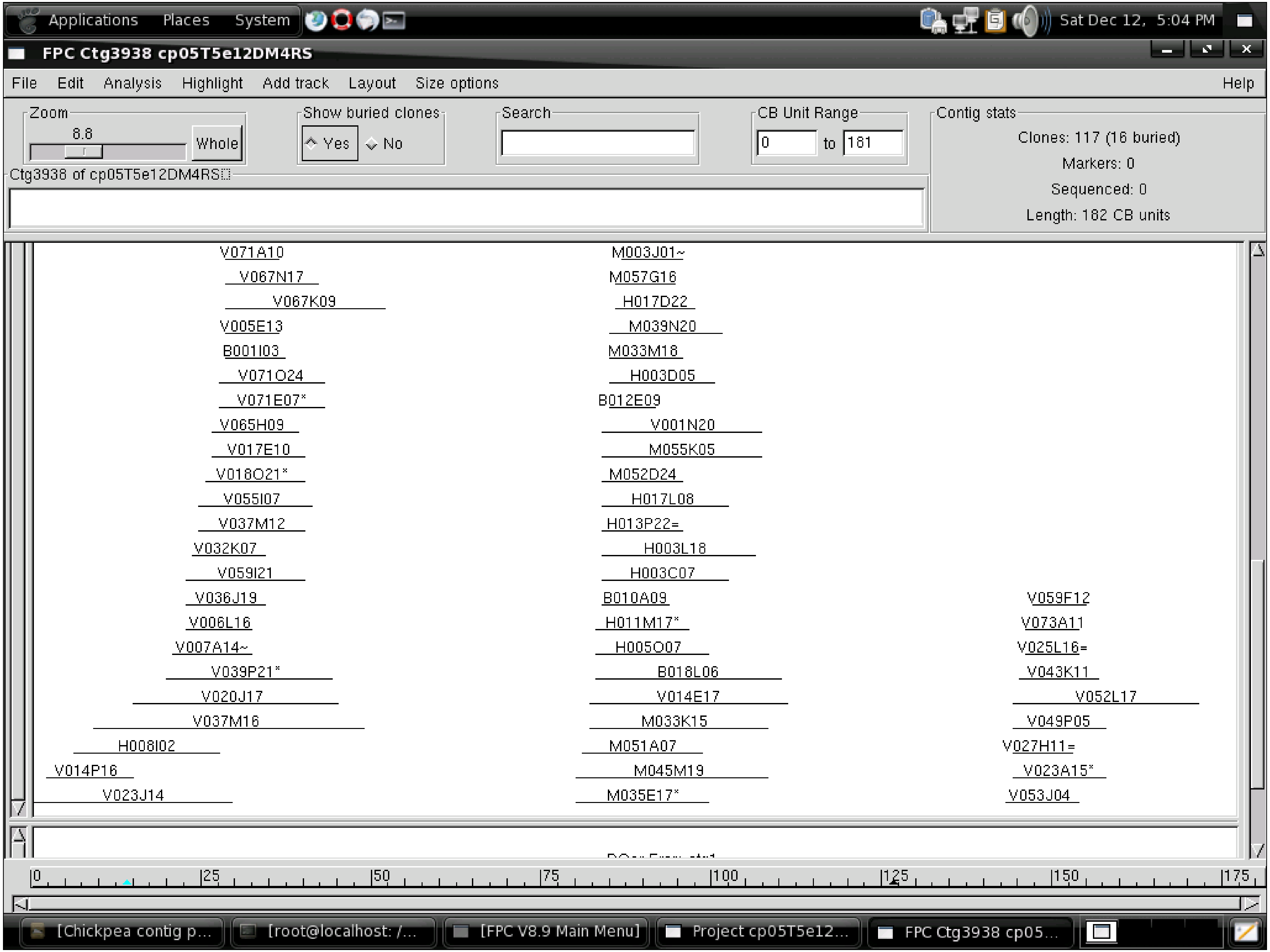
Applications Places System
Sat Dec 12, 5:04 PM
FPC Ctg3938 cp05T5e12DM4RS
File Edit Analysis Highlight Add track Layout Size options Help
Zoom
8.8
Whole
Show buried clones
Yes No
Search
CB Unit Range
0 to 181
Contig stats
Clones: 117 (16 buried)
Markers: 0
Sequenced: 0
Length: 182 CB units
Ctg3938 of cp05T5e12DM4RS
V071A10
V067N17
V067K09
V005E13
B001I03
V071O24
V071E07*
V065H09
V017E10
V018O21*
V055I07
V037M12
V032K07
V059I21
V036J19
V006L16
V007A14~
V039P21*
V020J17
V037M16
H008I02
V014P16
V023J14
M003J01~
M057G16
H017D22
M039N20
M033M18
H003D05
B012E09
V001N20
M055K05
M052D24
H017L08
H013P22=
H003L18
H003C07
B010A09
H011M17*
H005O07
B018L06
V014E17
M033K15
M051A07
M045M19
M035E17*
V059F12
V073A11
V025L16=
V043K11
V052L17
V049P05
V027H11=
V023A15*
V053J04
0 25 50 75 100 125 150 175
[Chickpea contig p...
[root@localhost: /...
[FPC V8.9 Main Menu]
Project cp05T5e12...
FPC Ctg3938 cp05...

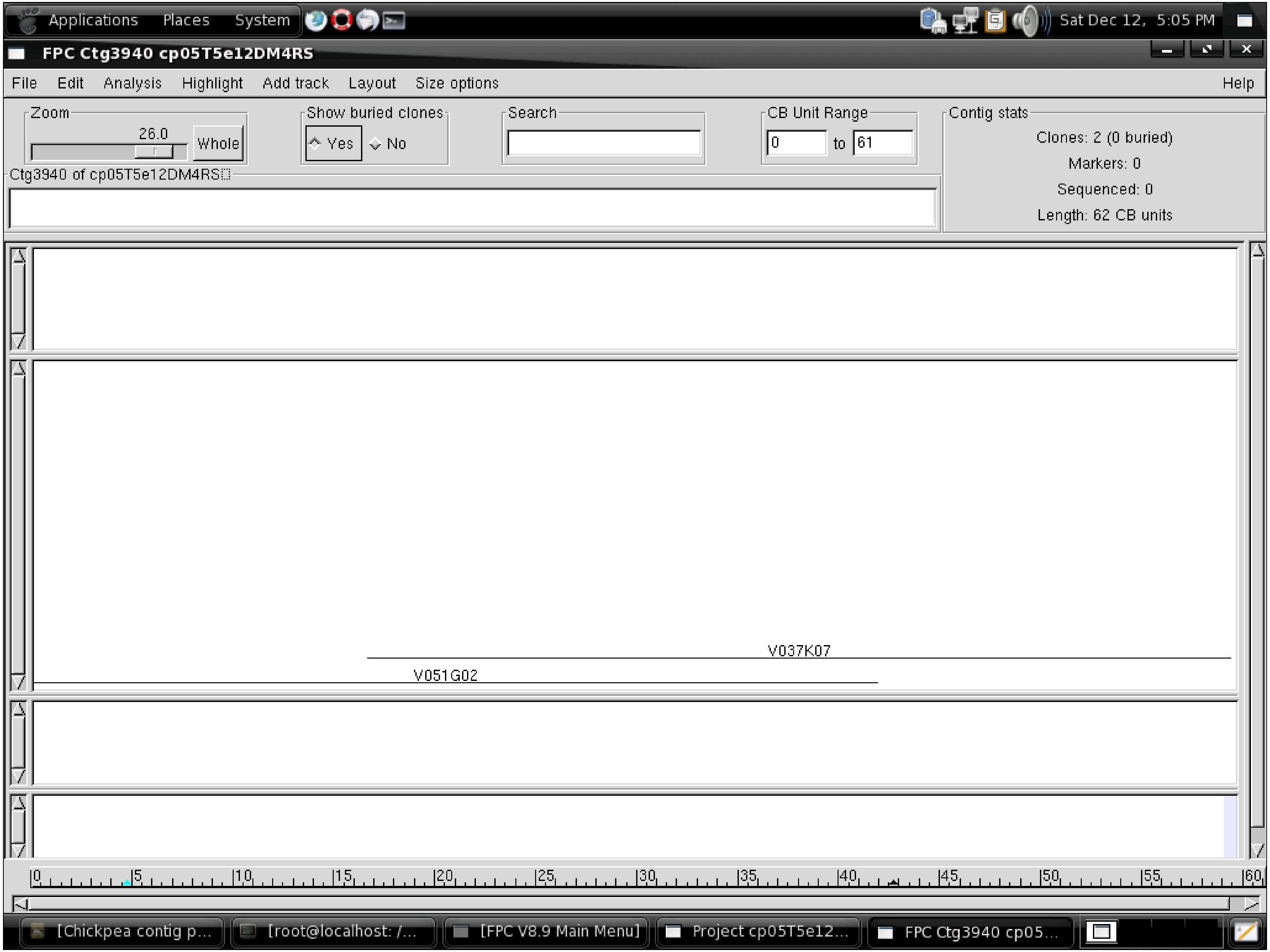
Applications Places System
Sat Dec 12, 5:05 PM
FPC Ctg3940 cp05T5e12DM4RS
File Edit Analysis Highlight Add track Layout Size options Help
Zoom
26.0
Whole
Show buried clones
Yes
No
Search
CB Unit Range
0
to
61
Contig stats
Clones: 2 (0 buried)
Markers: 0
Sequenced: 0
Length: 62 CB units
Ctg3940 of cp05T5e12DM4RS
V037K07
V051G02
0 5 10 15 20 25 30 35 40 45 50 55 60
[Chickpea contig p...
[root@localhost: /...
[FPC V8.9 Main Menu]
Project cp05T5e12...
FPC Ctg3940 cp05...

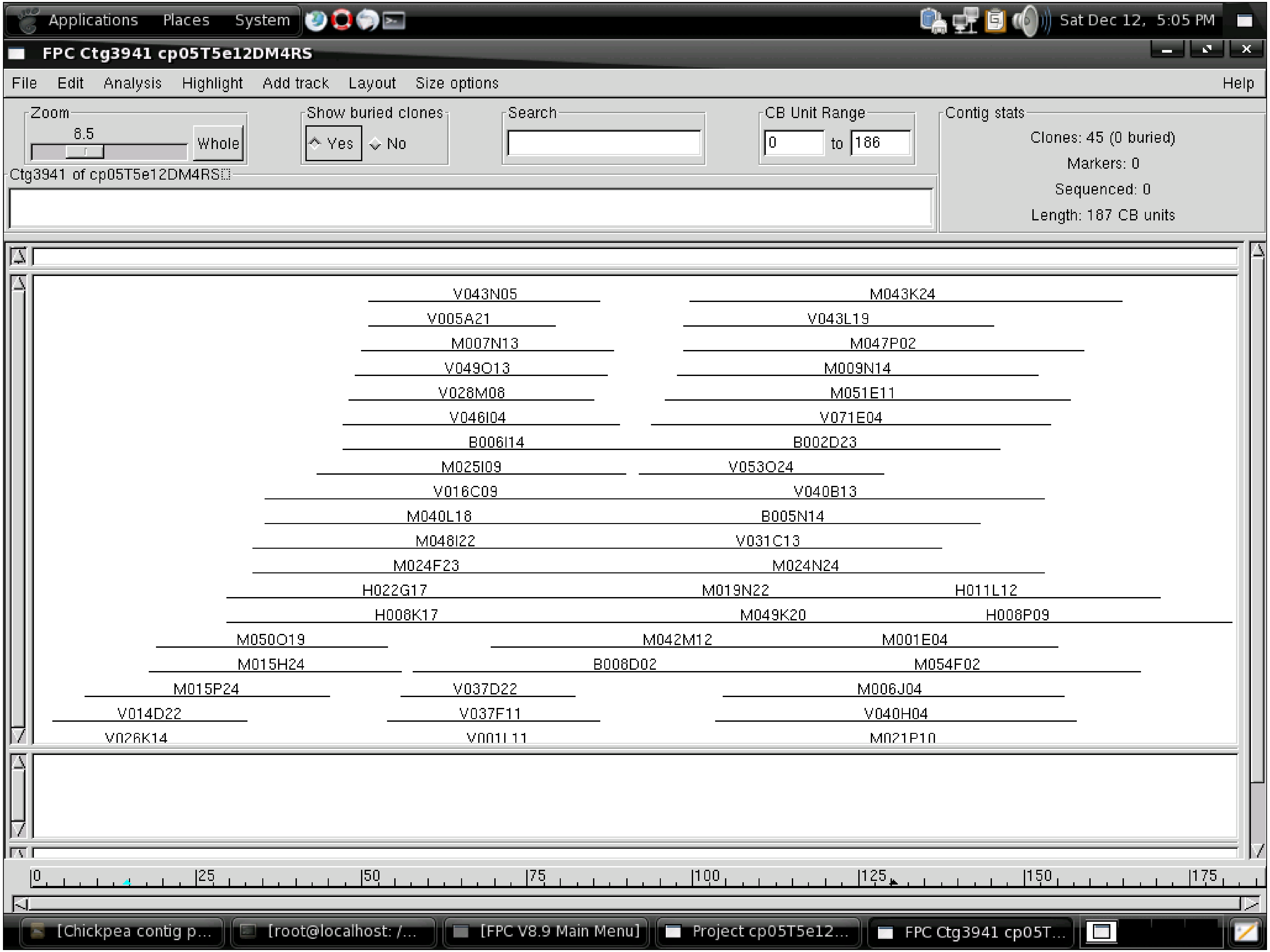

Applications Places System
Sat Dec 12, 5:05 PM
FPC Ctg3941 cp05T5e12DM4RS
File Edit Analysis Highlight Add track Layout Size options Help
Zoom
8.5
Whole
Show buried clones
Yes No
Search
CB Unit Range
0 to 186
Contig stats
Clones: 45 (0 buried)
Markers: 0
Sequenced: 0
Length: 187 CB units
Ctg3941 of cp05T5e12DM4RS
V043N05
M043K24
V005A21
V043L19
M007N13
M047P02
V049O13
M009N14
V028M08
M051E11
V046I04
V071E04
B006I14
B002D23
M025I09
V053O24
V016C09
V040B13
M040L18
B005N14
M048I22
V031C13
M024F23
M024N24
H022G17
M019N22
H011L12
H008K17
M049K20
H008P09
M050O19
M042M12
M001E04
M015H24
B008D02
M054F02
M015P24
V037D22
M006J04
V014D22
V037F11
V040H04
0 25 50 75 100 125 150 175
[Chickpea contig p...
[root@localhost: /...
[FPC V8.9 Main Menu]
Project cp05T5e12...
FPC Ctg3941 cp05T...

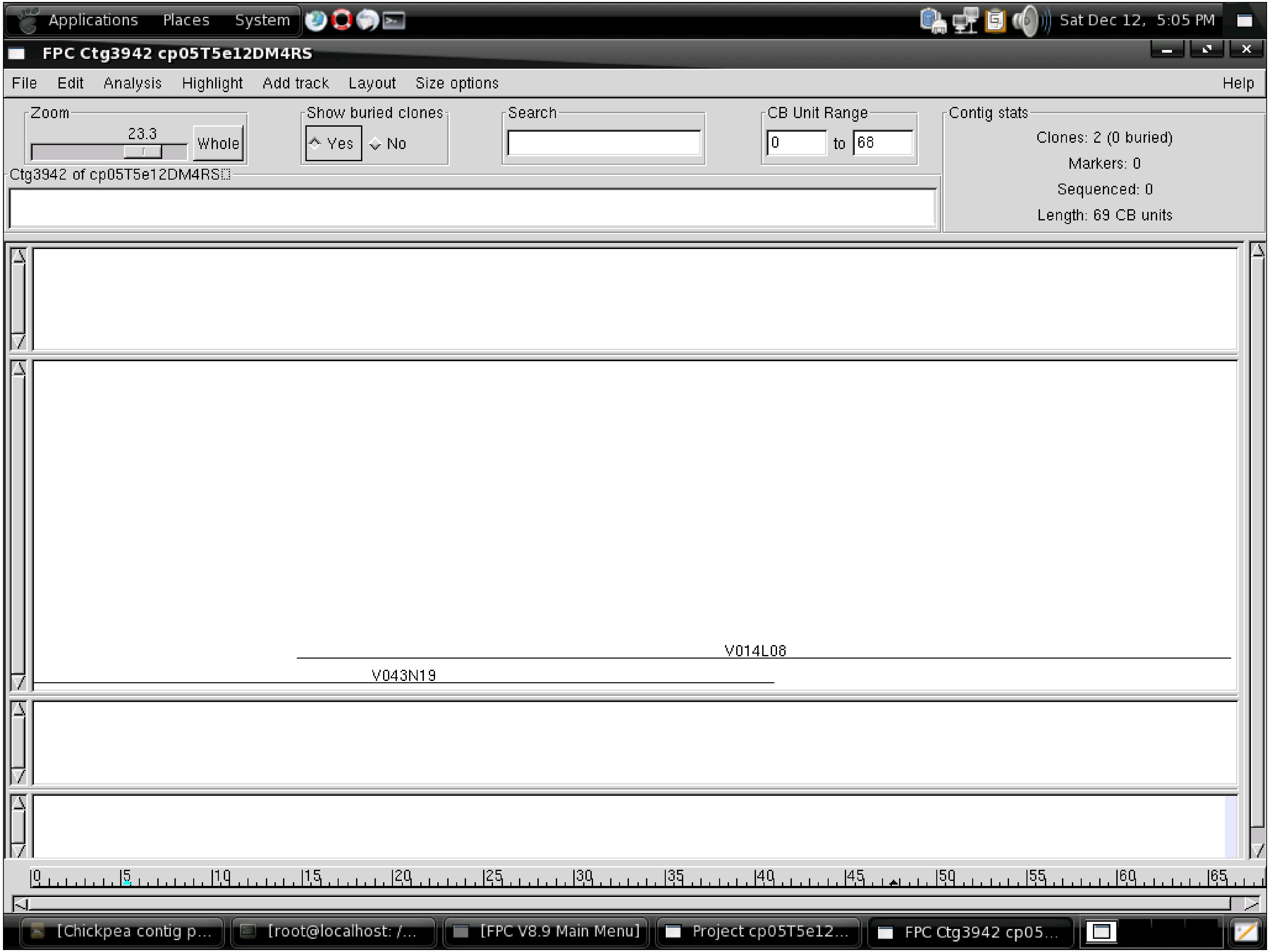
Applications Places System
Sat Dec 12, 5:05 PM
FPC Ctg3942 cp05T5e12DM4RS
File Edit Analysis Highlight Add track Layout Size options Help
Zoom
23.3
Whole
Show buried clones
Yes
No
Search
CB Unit Range
0
to
68
Contig stats
Clones: 2 (0 buried)
Markers: 0
Sequenced: 0
Length: 69 CB units
Ctg3942 of cp05T5e12DM4RS
V014L08
V043N19
0 5 10 15 20 25 30 35 40 45 50 55 60 65
[Chickpea contig p...
[root@localhost: /...
[FPC V8.9 Main Menu]
Project cp05T5e12...
FPC Ctg3942 cp05...

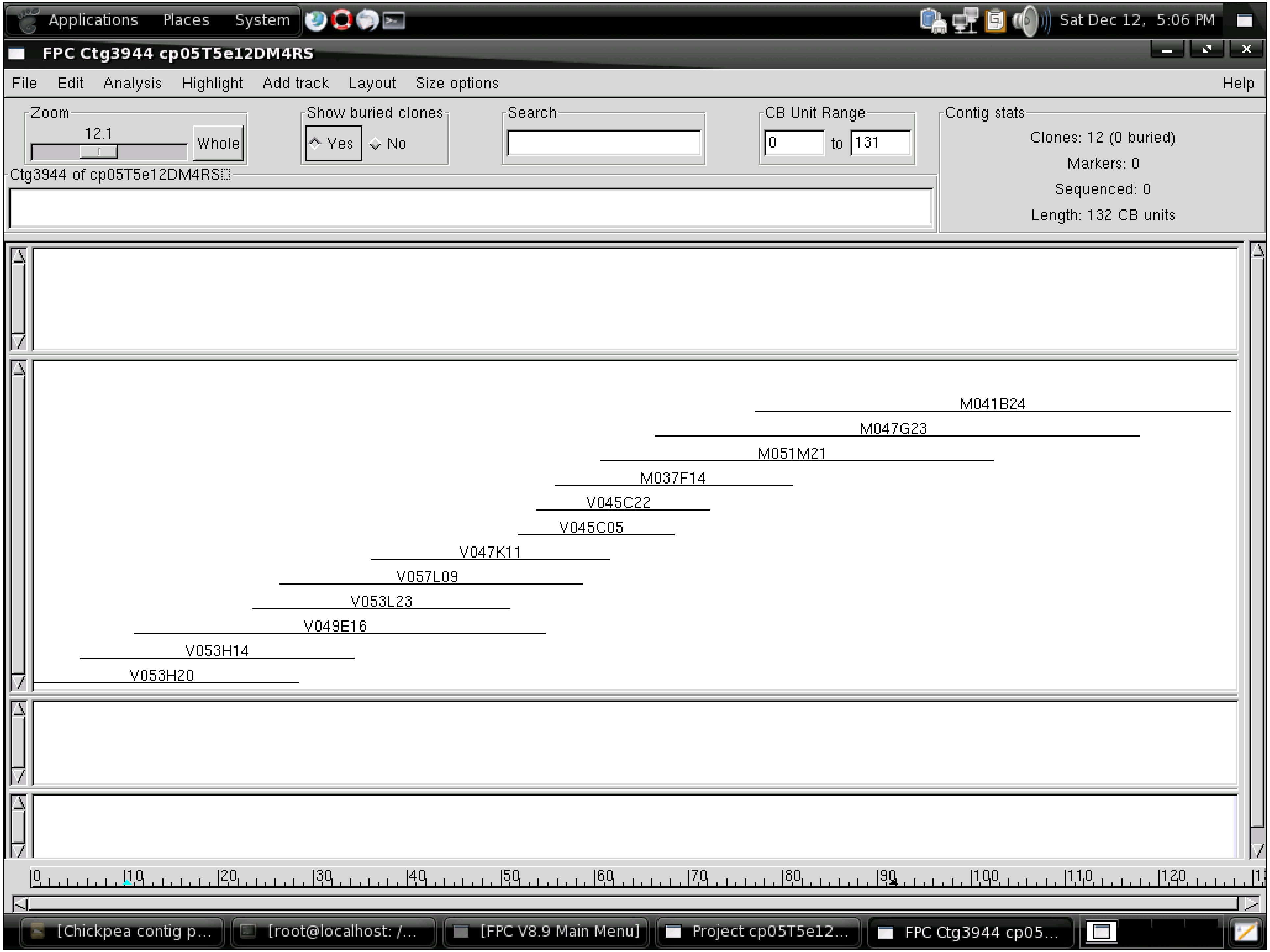
Applications Places System
Sat Dec 12, 5:06 PM
FPC Ctg3944 cp05T5e12DM4RS
File Edit Analysis Highlight Add track Layout Size options Help
Zoom
12.1
Whole
Show buried clones
Yes
No
Search
CB Unit Range
0
to
131
Contig stats
Clones: 12 (0 buried)
Markers: 0
Sequenced: 0
Length: 132 CB units
Ctg3944 of cp05T5e12DM4RS
M041B24
M047G23
M051M21
M037F14
V045C22
V045C05
V047K11
V057L09
V053L23
V049E16
V053H14
V053H20
0
10
20
30
40
50
60
70
80
90
100
110
120
[Chickpea contig p...
[root@localhost: /...
[FPC V8.9 Main Menu]
Project cp05T5e12...
FPC Ctg3944 cp05...

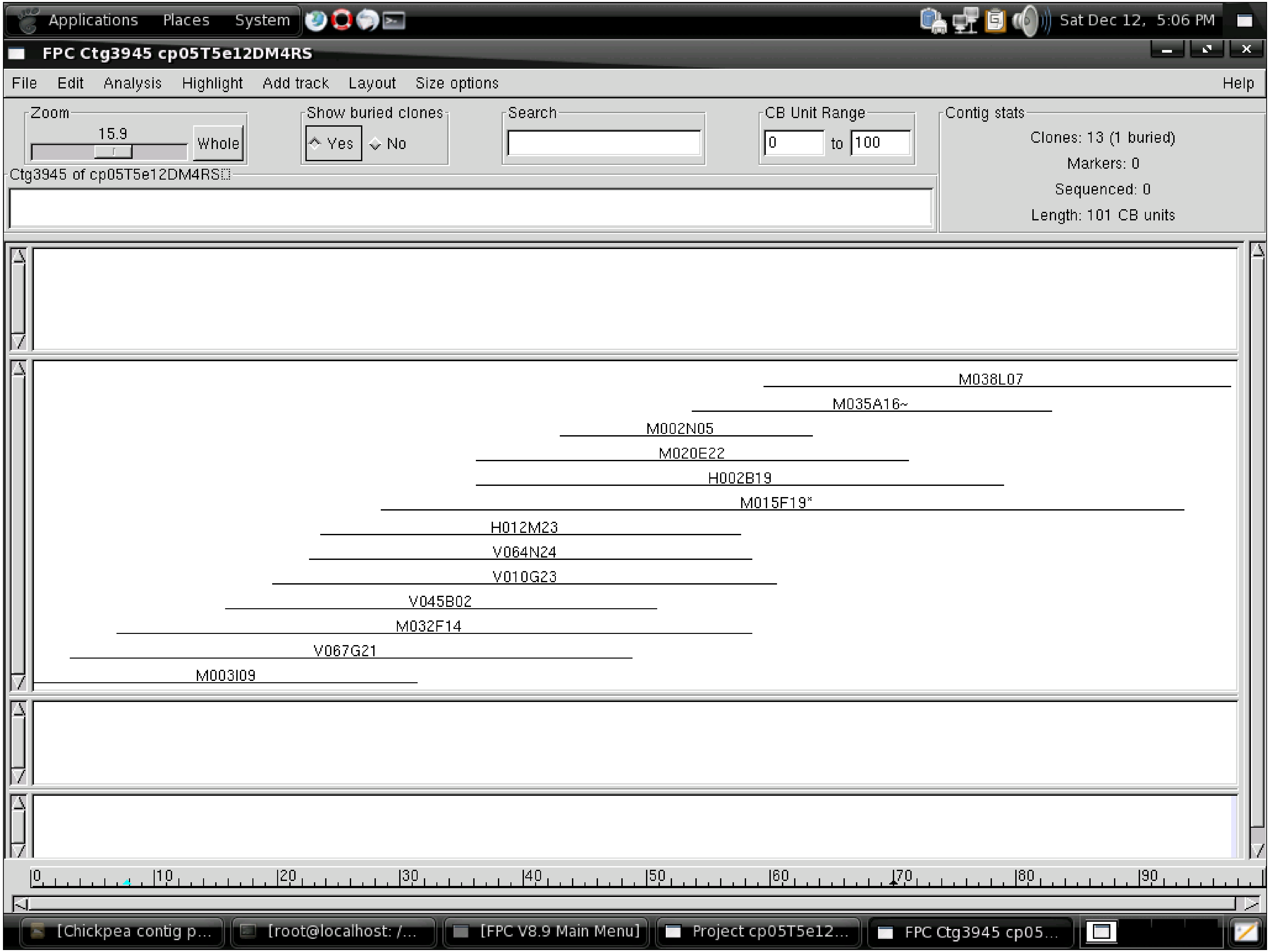
Applications Places System
Sat Dec 12, 5:06 PM
FPC Ctg3945 cp05T5e12DM4RS
File Edit Analysis Highlight Add track Layout Size options Help
Zoom
15.9
Whole
Show buried clones
Yes
No
Search
CB Unit Range
0 to 100
Contig stats
Clones: 13 (1 buried)
Markers: 0
Sequenced: 0
Length: 101 CB units
Ctg3945 of cp05T5e12DM4RS
M038L07
M035A16~
M002N05
M020E22
H002B19
M015F19*
H012M23
V064N24
V010G23
V045B02
M032F14
V067G21
M003I09
0 10 20 30 40 50 60 70 80 90
[Chickpea contig p...
[root@localhost: /...
[FPC V8.9 Main Menu]
Project cp05T5e12...
FPC Ctg3945 cp05...

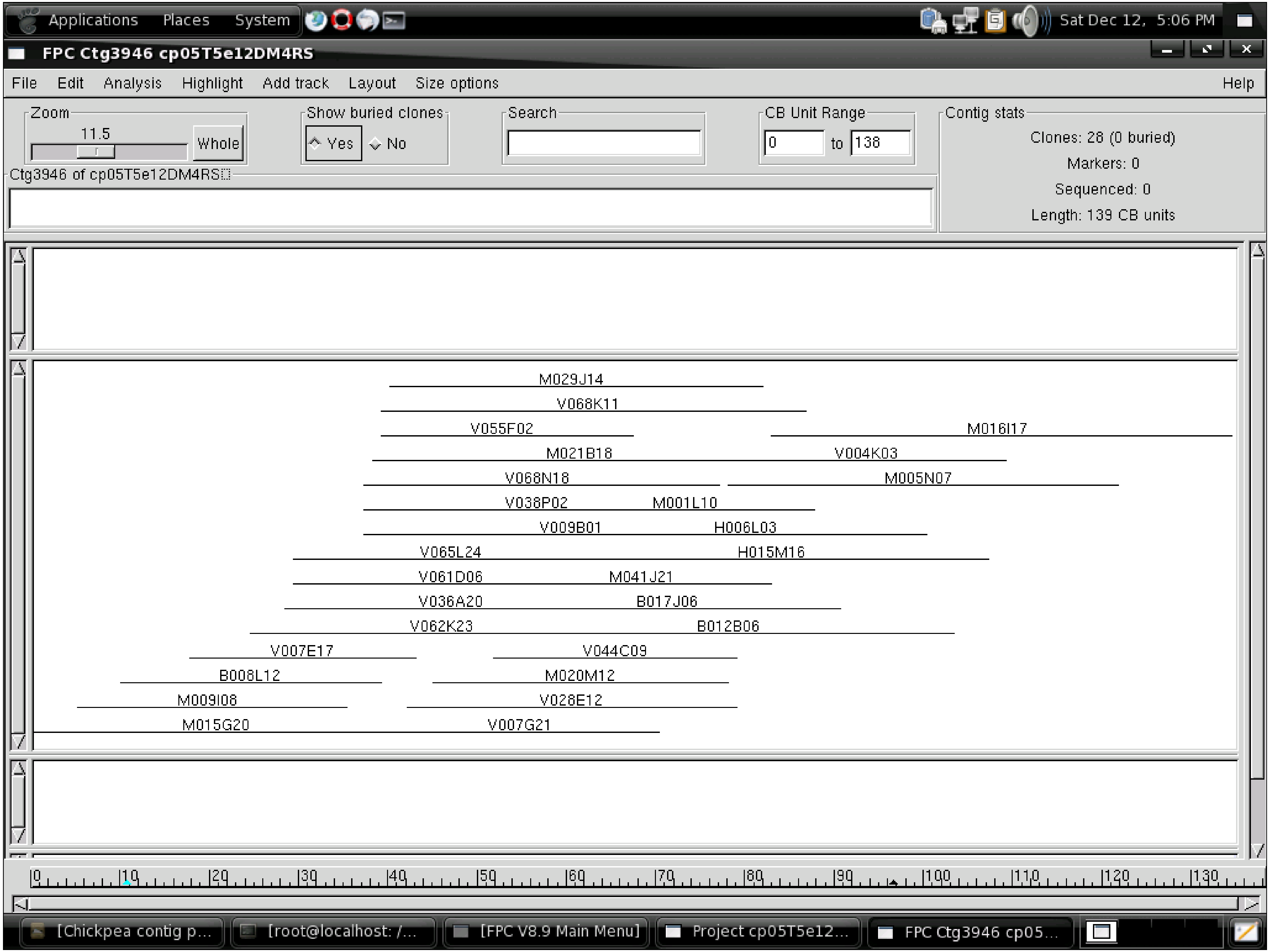
Applications Places System
Sat Dec 12, 5:06 PM
FPC Ctg3946 cp05T5e12DM4RS
File Edit Analysis Highlight Add track Layout Size options Help
Zoom
11.5
Whole
Show buried clones
Yes
No
Search
CB Unit Range
0
to
138
Contig stats
Clones: 28 (0 buried)
Markers: 0
Sequenced: 0
Length: 139 CB units
Ctg3946 of cp05T5e12DM4RS
M029J14
V068K11
V055F02
M016I17
M021B18
V004K03
V068N18
M005N07
V038P02
M001L10
V009B01
H006L03
V065L24
H015M16
V061D06
M041J21
V036A20
B017J06
V062K23
B012B06
V007E17
V044C09
B008L12
M020M12
M009I08
V028E12
M015G20
V007G21
0 10 20 30 40 50 60 70 80 90 100 110 120 130
[Chickpea contig p...
[root@localhost: /...
[FPC V8.9 Main Menu]
Project cp05T5e12...
FPC Ctg3946 cp05...

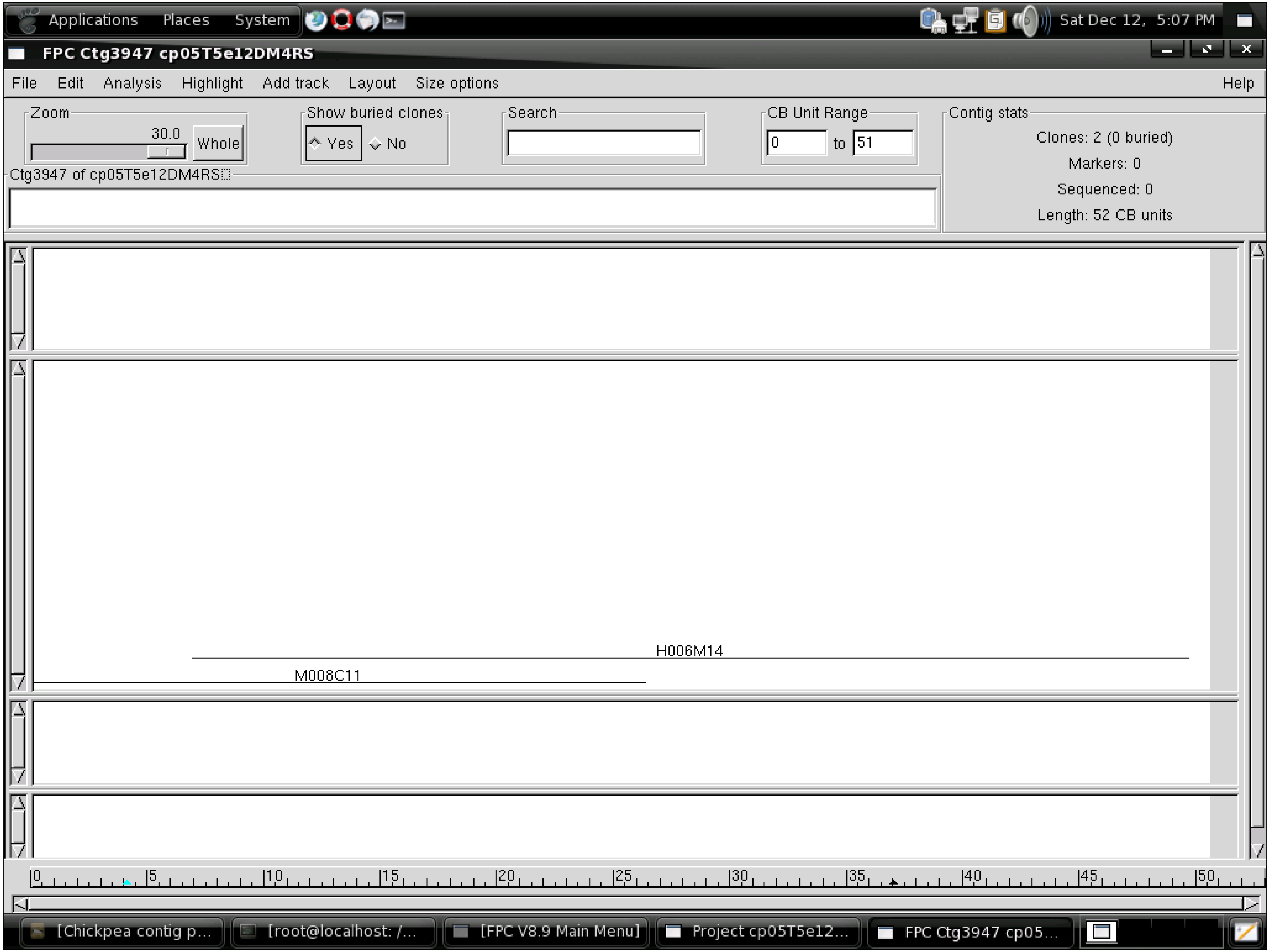
Applications Places System
Sat Dec 12, 5:07 PM
FPC Ctg3947 cp05T5e12DM4RS
File Edit Analysis Highlight Add track Layout Size options Help
Zoom
30.0
Whole
Show buried clones
Yes
No
Search
CB Unit Range
0
to
51
Contig stats
Clones: 2 (0 buried)
Markers: 0
Sequenced: 0
Length: 52 CB units
Ctg3947 of cp05T5e12DM4RS
H006M14
M008C11
0 5 10 15 20 25 30 35 40 45 50
[Chickpea contig p...
[root@localhost: /...
[FPC V8.9 Main Menu]
Project cp05T5e12...
FPC Ctg3947 cp05...

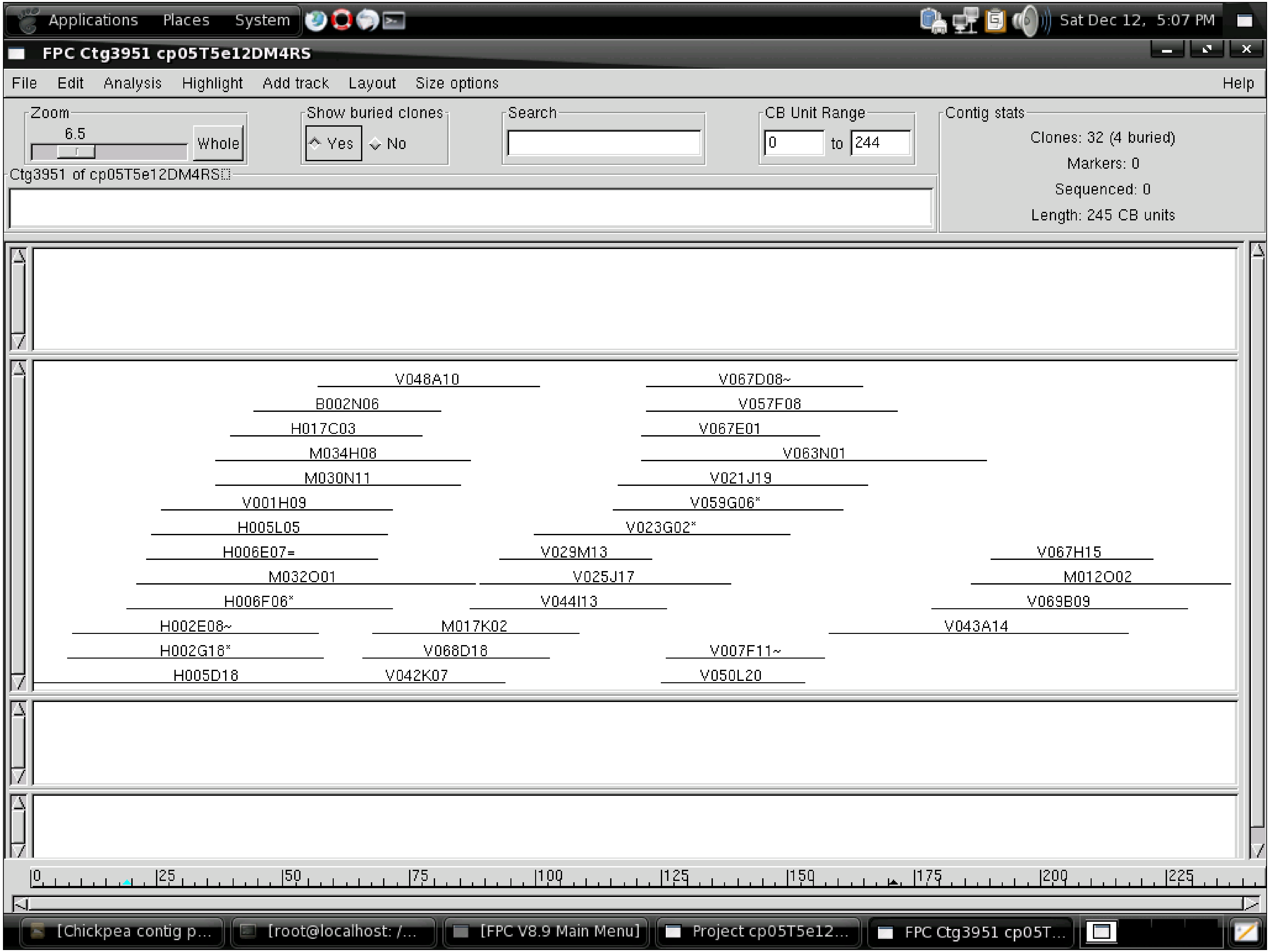
Applications Places System
Sat Dec 12, 5:07 PM
FPC Ctg3951 cp05T5e12DM4RS
File Edit Analysis Highlight Add track Layout Size options Help
Zoom
6.5
Whole
Show buried clones
Yes No
Search
CB Unit Range
0 to 244
Contig stats
Clones: 32 (4 buried)
Markers: 0
Sequenced: 0
Length: 245 CB units
Ctg3951 of cp05T5e12DM4RS
V048A10
V067D08~
B002N06
V057F08
H017C03
V067E01
M034H08
V063N01
M030N11
V021J19
V001H09
V059G06*
H005L05
V023G02*
H006E07=
V029M13
V067H15
M032O01
V025J17
M012O02
H006F06*
V044I13
V069B09
H002E08~
M017K02
V043A14
H002G18*
V068D18
V007F11~
H005D18
V042K07
V050L20
0 25 50 75 100 125 150 175 200 225
[Chickpea contig p...
[root@localhost: /...
[FPC V8.9 Main Menu]
Project cp05T5e12...
FPC Ctg3951 cp05T...

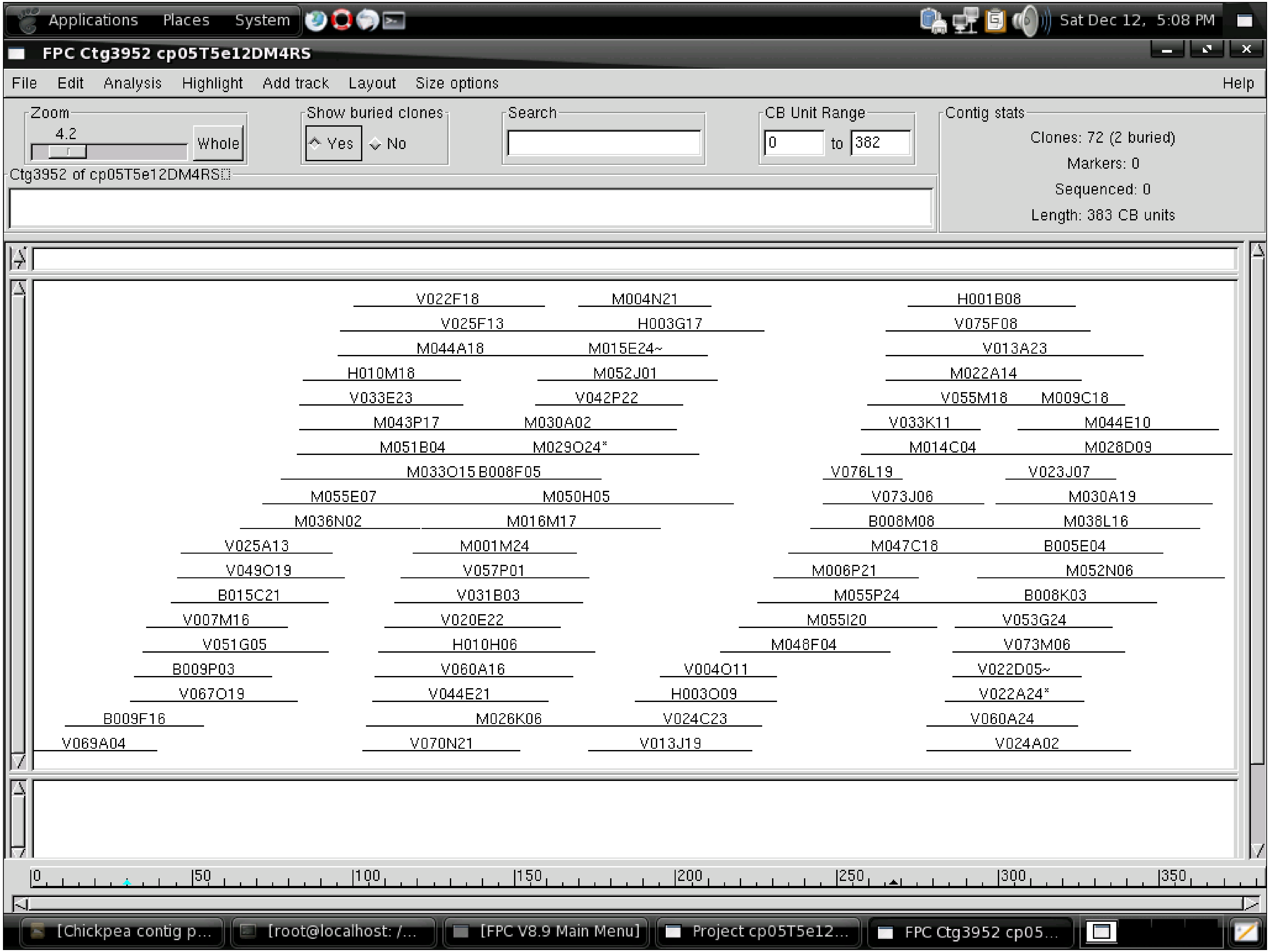
Applications Places System
Sat Dec 12, 5:08 PM
FPC Ctg3952 cp05T5e12DM4RS
File Edit Analysis Highlight Add track Layout Size options Help
Zoom 4.2 Whole
Show buried clones Yes No
Search
CB Unit Range 0 to 382
Contig stats
Clones: 72 (2 buried)
Markers: 0
Sequenced: 0
Length: 383 CB units
Ctg3952 of cp05T5e12DM4RS
V022F18 M004N21 H001B08
V025F13 H003G17 V075F08
M044A18 M015E24~ V013A23
H010M18 M052J01 M022A14
V033E23 V042P22 V055M18 M009C18
M043P17 M030A02 V033K11 M044E10
M051B04 M029O24* M014C04 M028D09
M033O15 B008F05 V076L19 V023J07
M055E07 M050H05 V073J06 M030A19
M036N02 M016M17 B008M08 M038L16
V025A13 M001M24 M047C18 B005E04
V049O19 V057P01 M006P21 M052N06
B015C21 V031B03 M055P24 B008K03
V007M16 V020E22 M055I20 V053G24
V051G05 H010H06 M048F04 V073M06
B009P03 V060A16 V004O11 V022D05~
V067O19 V044E21 H003O09 V022A24*
B009F16 M026K06 V024C23 V060A24
V069A04 V070N21 V013J19 V024A02
0 50 100 150 200 250 300 350
[Chickpea contig p... [root@localhost: /... [FPC V8.9 Main Menu] Project cp05T5e12... FPC Ctg3952 cp05...

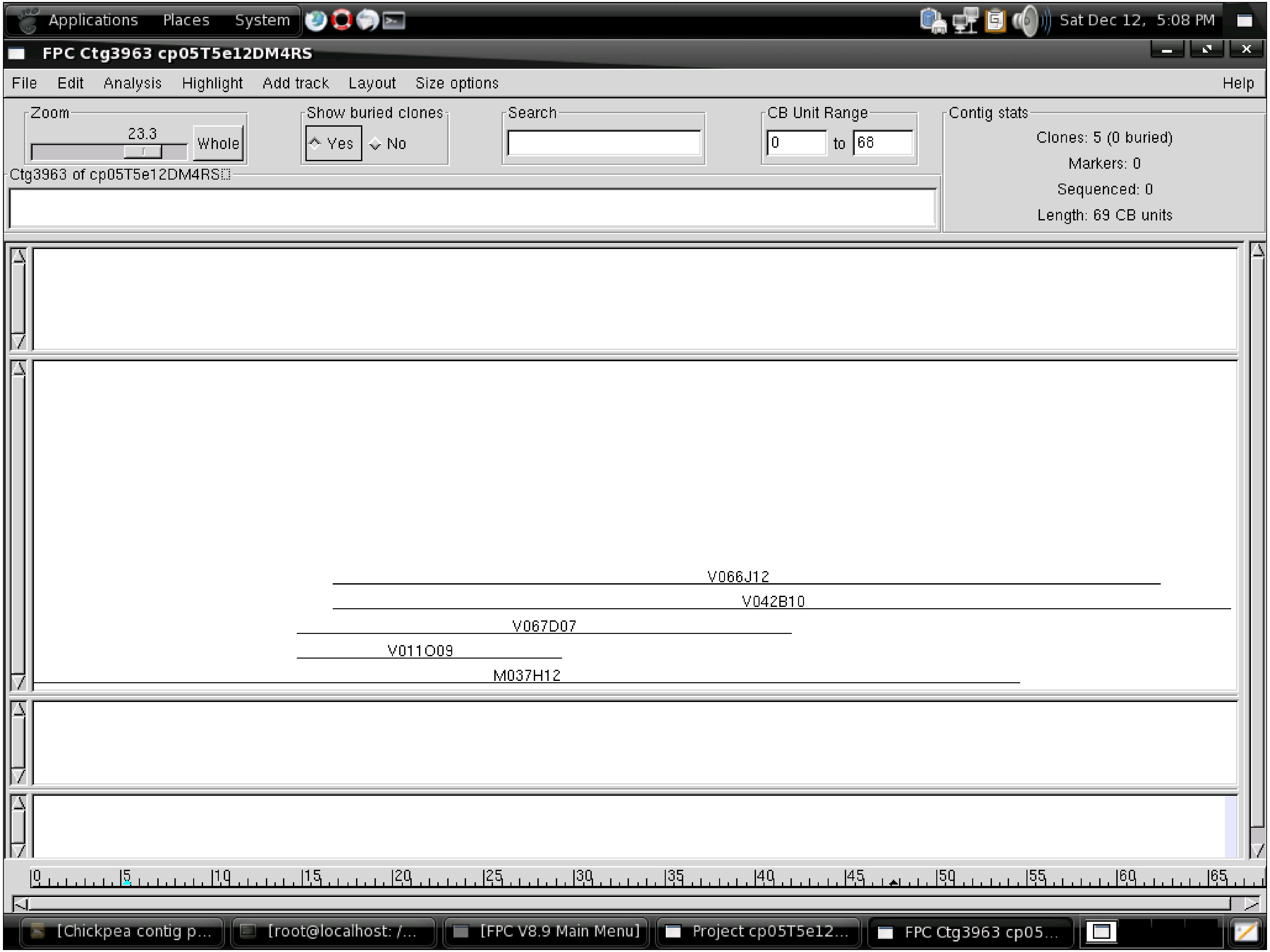

Applications Places System
Sat Dec 12, 5:08 PM
FPC Ctg3963 cp05T5e12DM4RS
File Edit Analysis Highlight Add track Layout Size options
Help
Zoom
23.3
Whole
Show buried clones
Yes
No
Search
CB Unit Range
0
to
68
Contig stats
Clones: 5 (0 buried)
Markers: 0
Sequenced: 0
Length: 69 CB units
Ctg3963 of cp05T5e12DM4RS
V066J12
V042B10
V067D07
V011O09
M037H12
0 5 10 15 20 25 30 35 40 45 50 55 60 65
[Chickpea contig p...
[root@localhost: /...
[FPC V8.9 Main Menu]
Project cp05T5e12...
FPC Ctg3963 cp05...

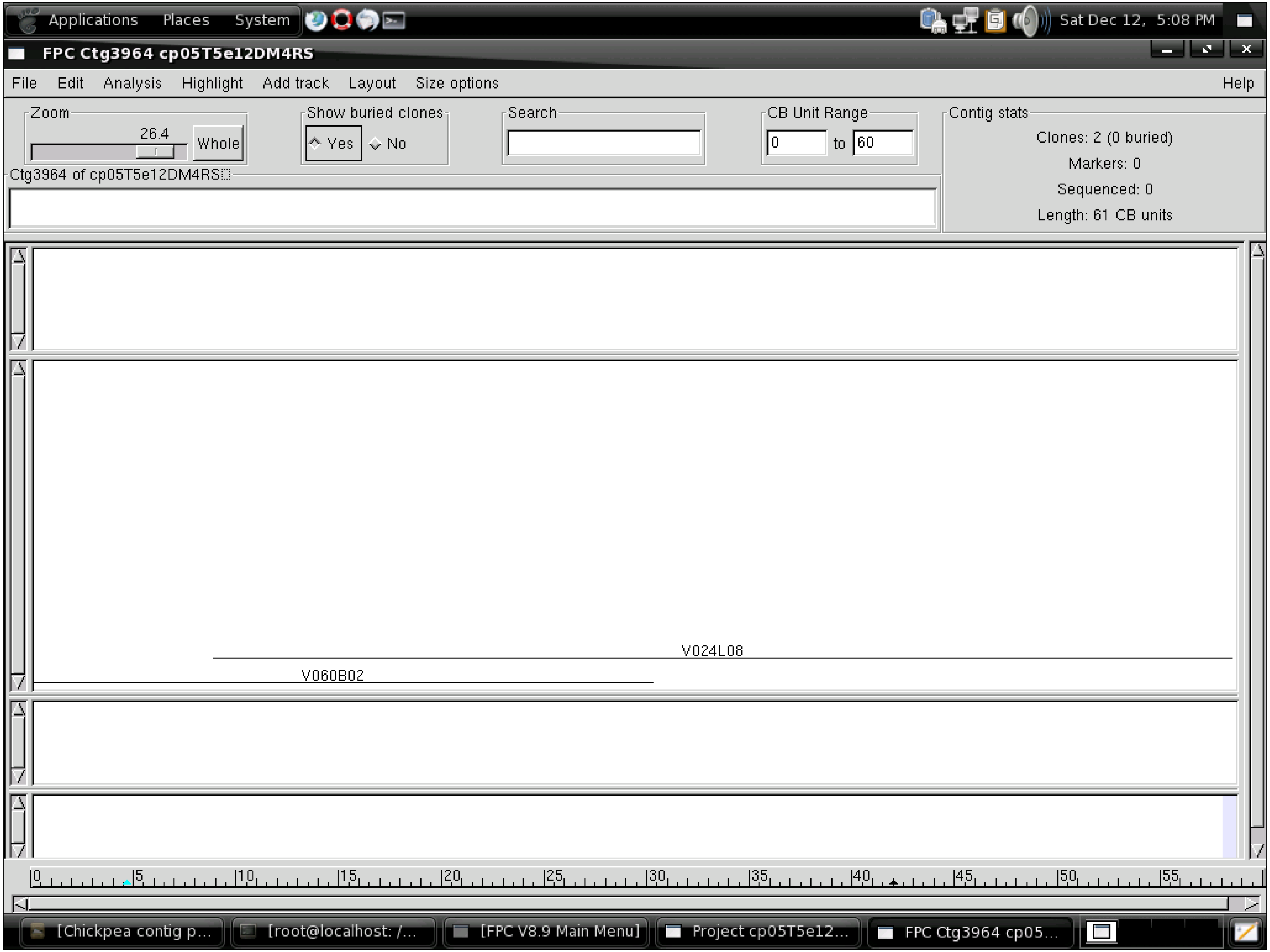
Applications Places System
Sat Dec 12, 5:08 PM
FPC Ctg3964 cp05T5e12DM4RS
File Edit Analysis Highlight Add track Layout Size options Help
Zoom
26.4
Whole
Show buried clones
Yes
No
Search
CB Unit Range
0 to 60
Contig stats
Clones: 2 (0 buried)
Markers: 0
Sequenced: 0
Length: 61 CB units
Ctg3964 of cp05T5e12DM4RS
V024L08
V060B02
0 5 10 15 20 25 30 35 40 45 50 55
[Chickpea contig p...
[root@localhost: /...
[FPC V8.9 Main Menu]
Project cp05T5e12...
FPC Ctg3964 cp05...

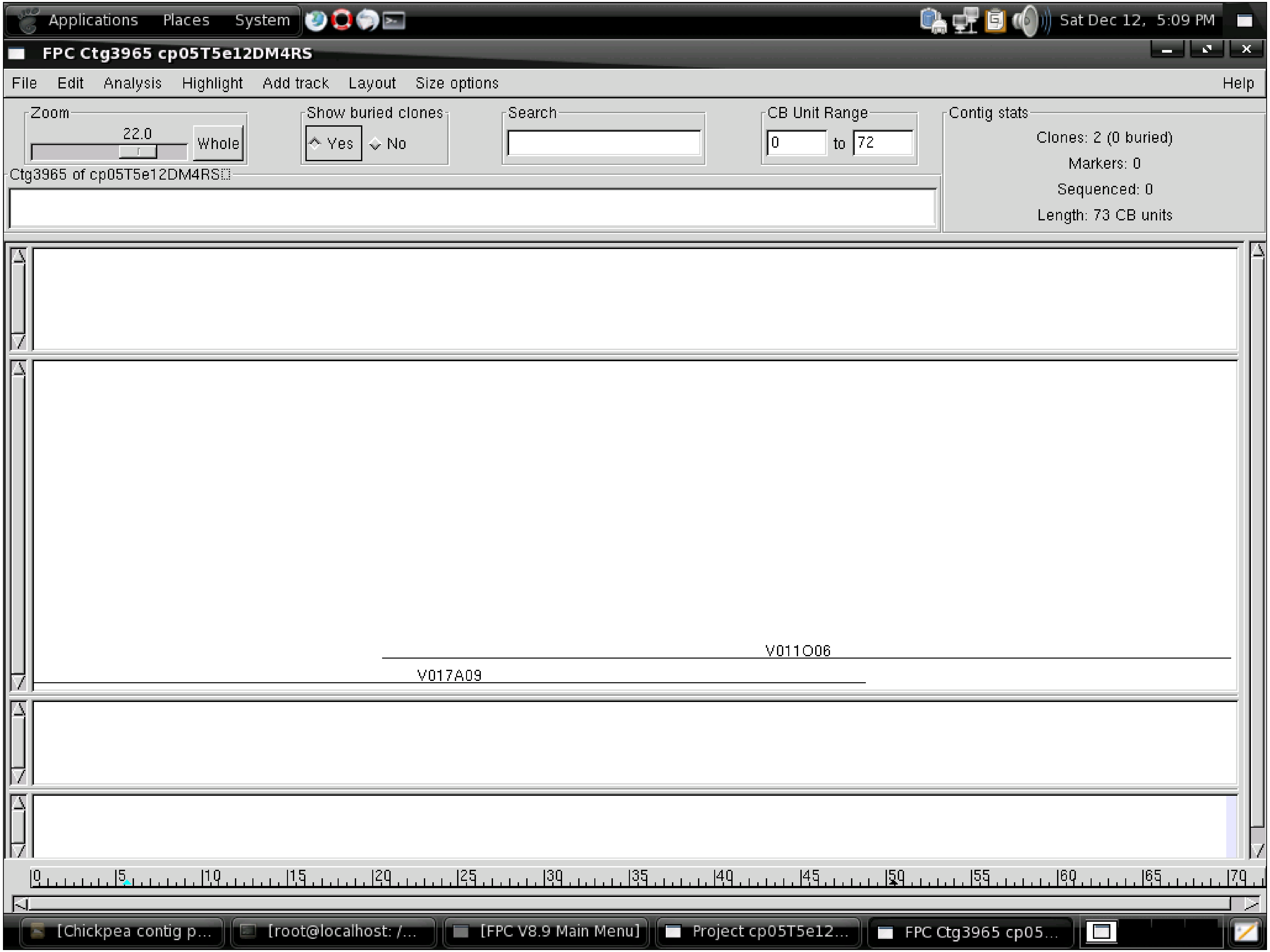
Applications Places System
Sat Dec 12, 5:09 PM
FPC Ctg3965 cp05T5e12DM4RS
File Edit Analysis Highlight Add track Layout Size options Help
Zoom
22.0
Whole
Show buried clones
Yes No
Search
CB Unit Range
0 to 72
Contig stats
Clones: 2 (0 buried)
Markers: 0
Sequenced: 0
Length: 73 CB units
Ctg3965 of cp05T5e12DM4RS
V011O06
V017A09
0 5 10 15 20 25 30 35 40 45 50 55 60 65 70
[Chickpea contig p...
[root@localhost: /...
[FPC V8.9 Main Menu]
Project cp05T5e12...
FPC Ctg3965 cp05...

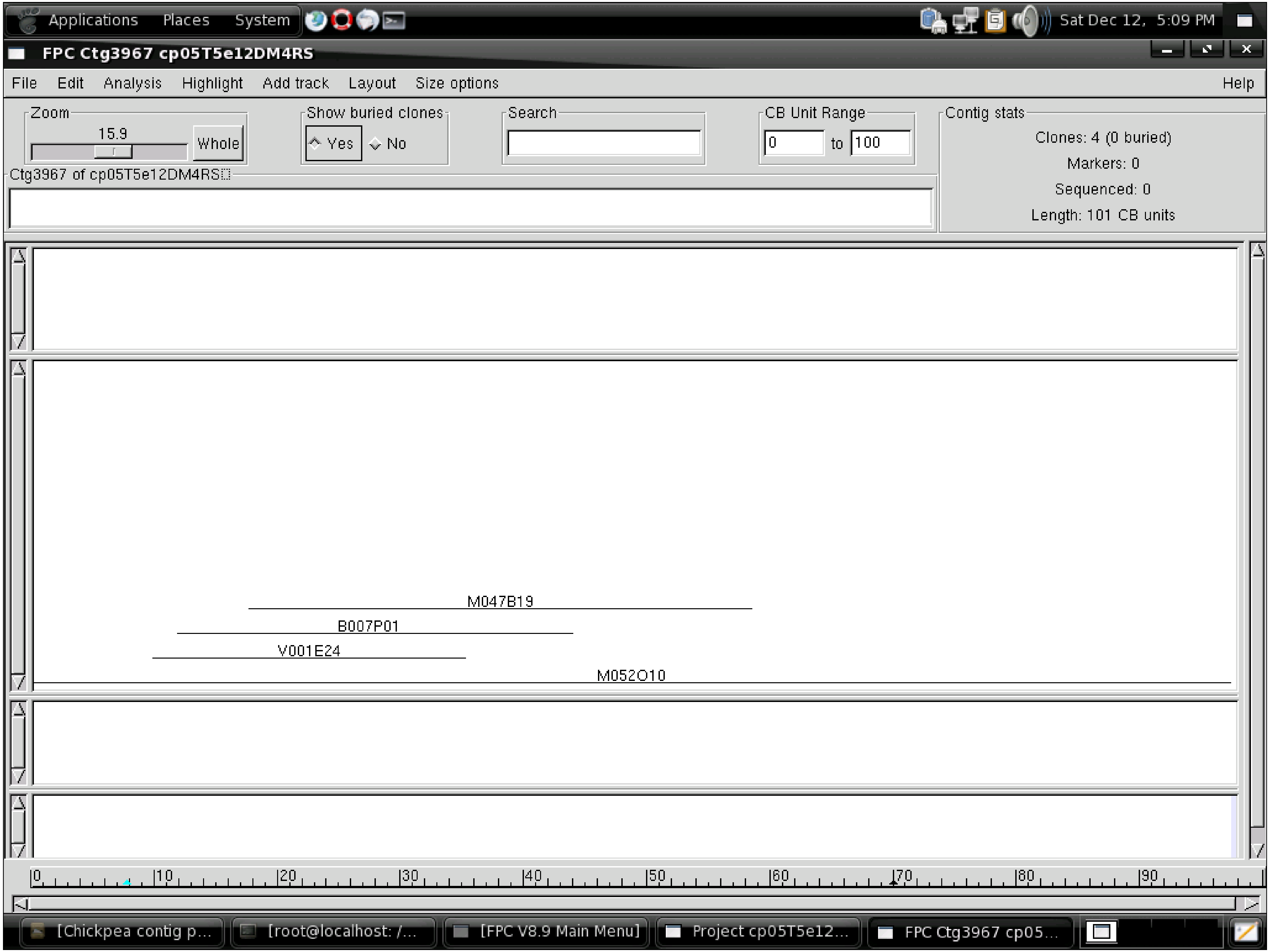
Applications
Places
System
Sat Dec 12, 5:09 PM
FPC Ctg3967 cp05T5e12DM4RS
File
Edit
Analysis
Highlight
Add track
Layout
Size options
Help
Zoom
15.9
Whole
Show buried clones
Yes
No
Search
CB Unit Range
0
to
100
Contig stats
Clones: 4 (0 buried)
Markers: 0
Sequenced: 0
Length: 101 CB units
Ctg3967 of cp05T5e12DM4RS
M047B19
B007P01
V001E24
M052O10
0
10
20
30
40
50
60
70
80
90
[Chickpea contig p...
[root@localhost: /...
[FPC V8.9 Main Menu]
Project cp05T5e12...
FPC Ctg3967 cp05...

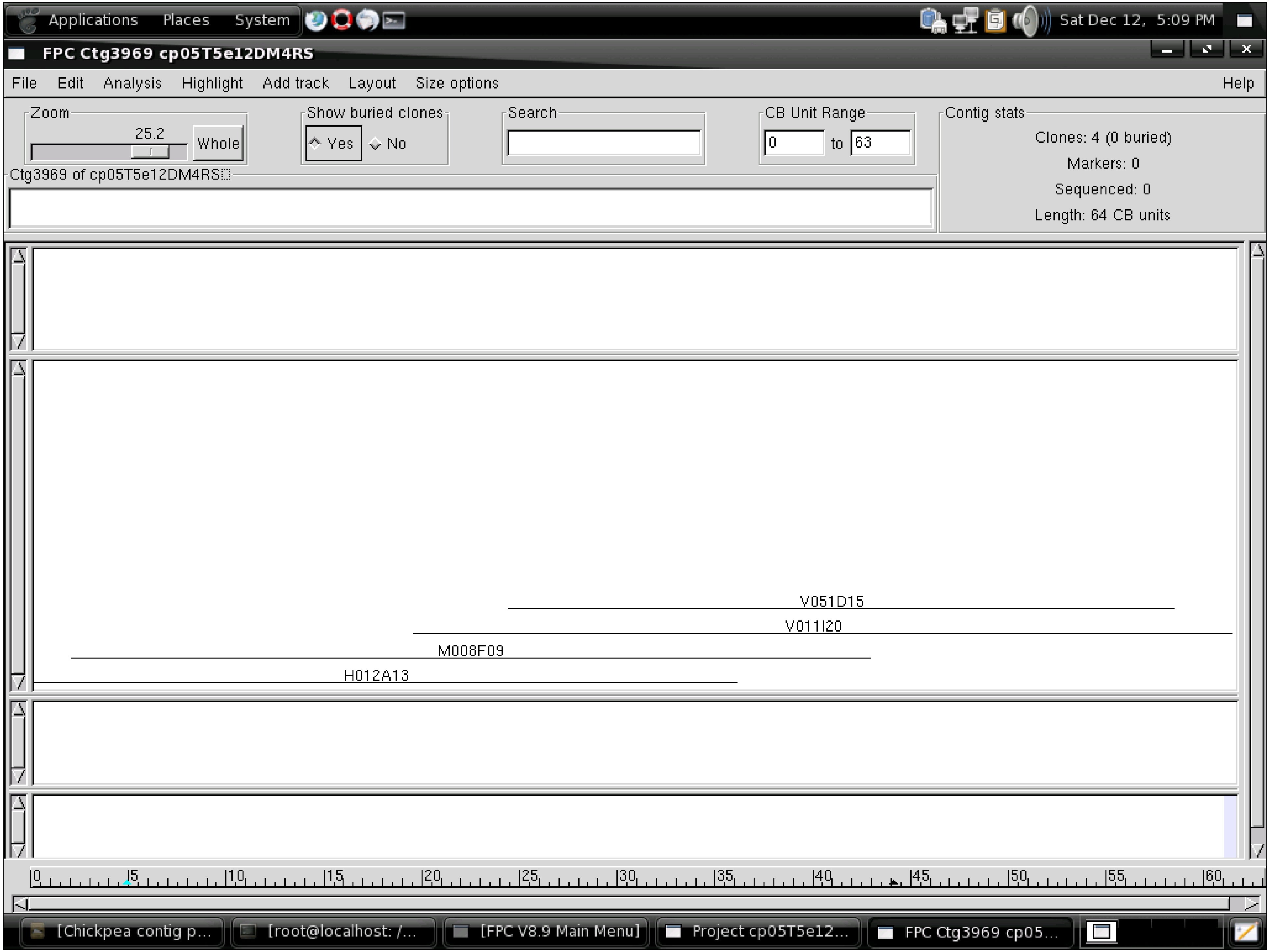
Applications Places System
Sat Dec 12, 5:09 PM
FPC Ctg3969 cp05T5e12DM4RS
File Edit Analysis Highlight Add track Layout Size options Help
Zoom
25.2
Whole
Show buried clones
Yes
No
Search
CB Unit Range
0
to
63
Contig stats
Clones: 4 (0 buried)
Markers: 0
Sequenced: 0
Length: 64 CB units
Ctg3969 of cp05T5e12DM4RS
V051D15
V011I20
M008F09
H012A13
0 5 10 15 20 25 30 35 40 45 50 55 60
[Chickpea contig p...
[root@localhost: /...
[FPC V8.9 Main Menu]
Project cp05T5e12...
FPC Ctg3969 cp05...

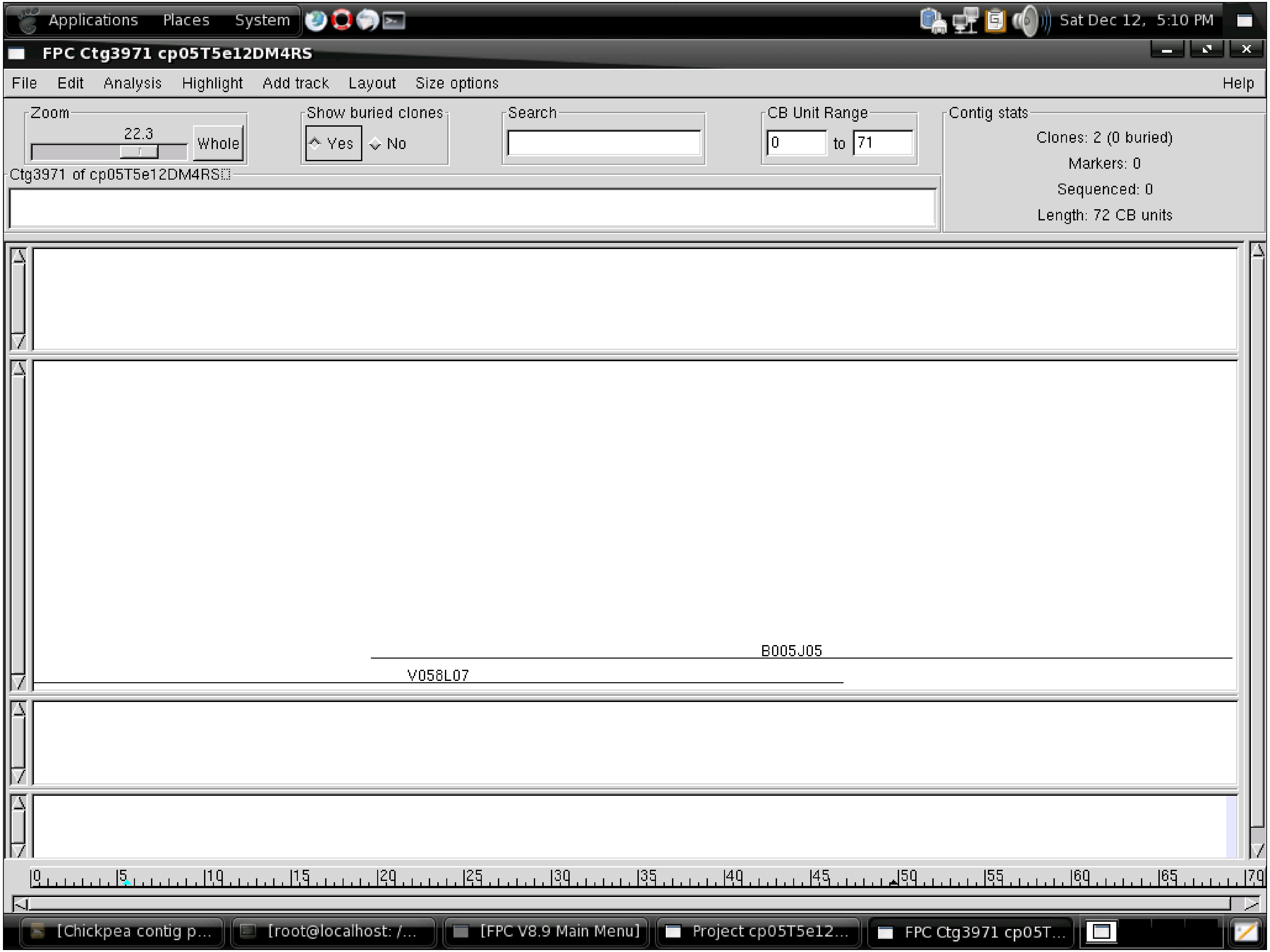
Applications Places System
Sat Dec 12, 5:10 PM
FPC Ctg3971 cp05T5e12DM4RS
File Edit Analysis Highlight Add track Layout Size options Help
Zoom
22.3
Whole
Show buried clones
Yes No
Search
CB Unit Range
0 to 71
Contig stats
Clones: 2 (0 buried)
Markers: 0
Sequenced: 0
Length: 72 CB units
Ctg3971 of cp05T5e12DM4RS
B005J05
V058L07
0 5 10 15 20 25 30 35 40 45 50 55 60 65 70
[Chickpea contig p...
[root@localhost: /...
[FPC V8.9 Main Menu]
Project cp05T5e12...
FPC Ctg3971 cp05T...

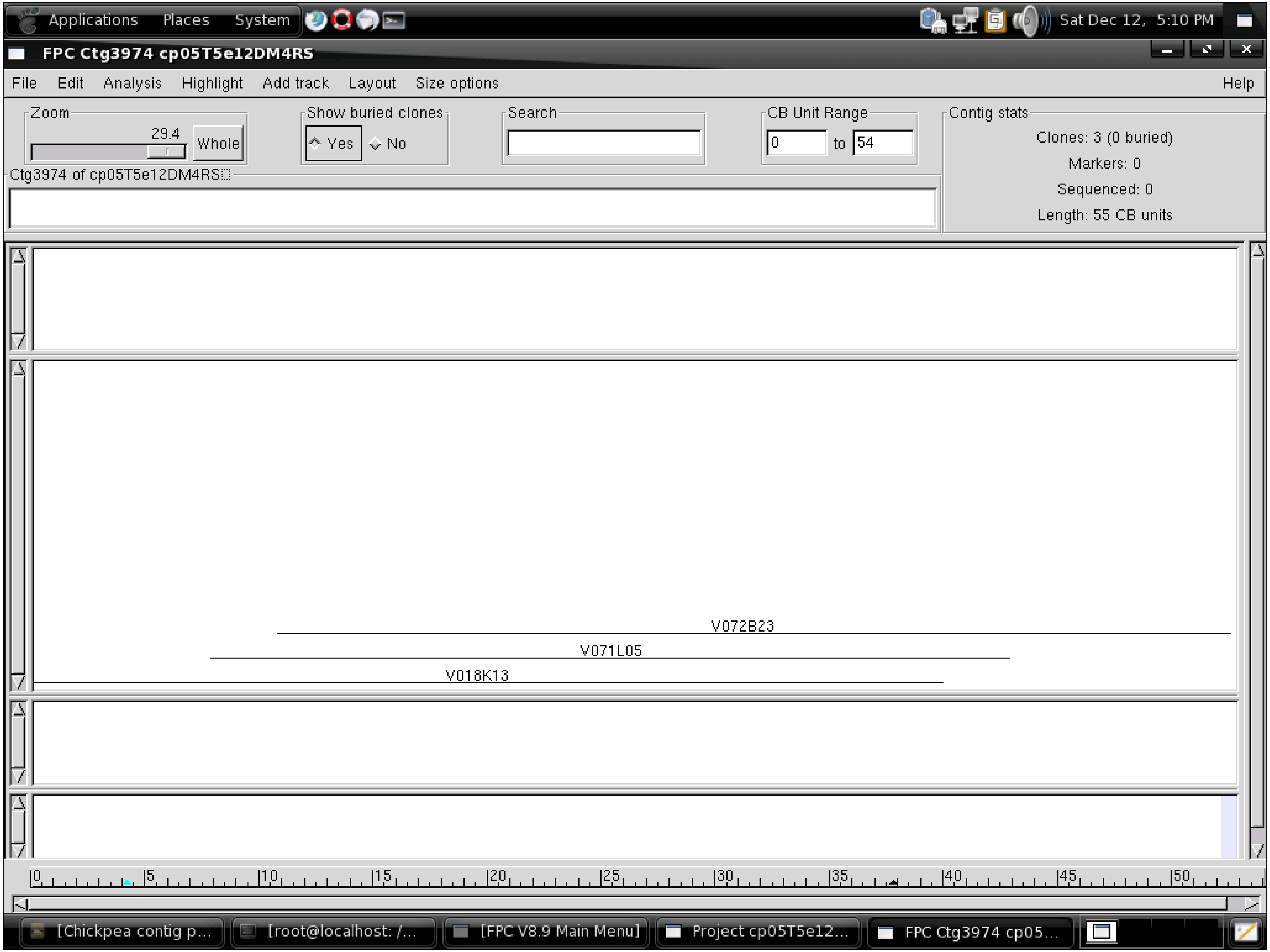
Applications Places System
Sat Dec 12, 5:10 PM
FPC Ctg3974 cp05T5e12DM4RS
File Edit Analysis Highlight Add track Layout Size options Help
Zoom
29.4
Whole
Show buried clones
Yes
No
Search
CB Unit Range
0
to
54
Contig stats
Clones: 3 (0 buried)
Markers: 0
Sequenced: 0
Length: 55 CB units
Ctg3974 of cp05T5e12DM4RS
V072B23
V071L05
V018K13
0 5 10 15 20 25 30 35 40 45 50
[Chickpea contig p...
[root@localhost: /...
[FPC V8.9 Main Menu]
Project cp05T5e12...
FPC Ctg3974 cp05...

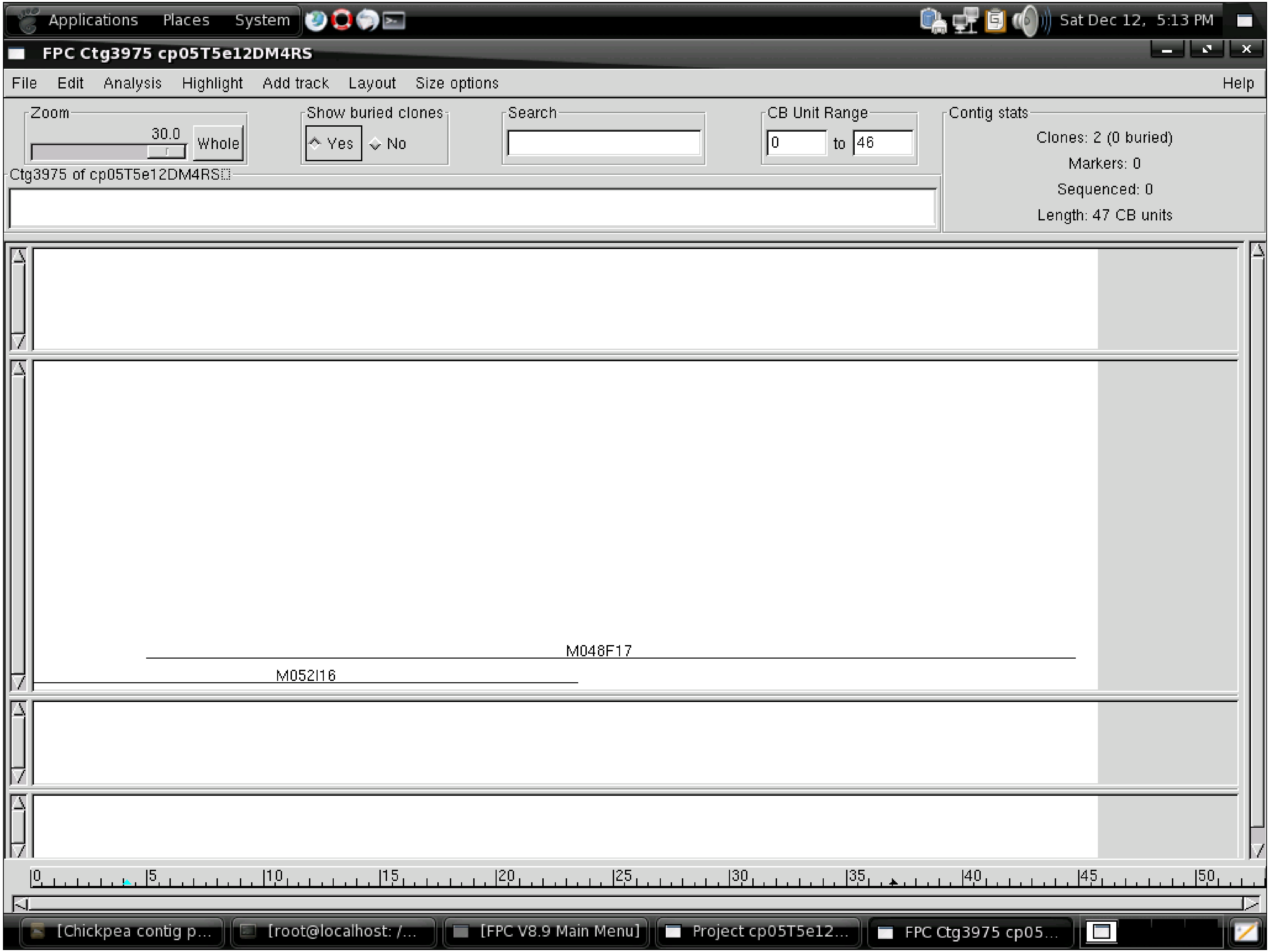
Applications Places System
Sat Dec 12, 5:13 PM
FPC Ctg3975 cp05T5e12DM4RS
File Edit Analysis Highlight Add track Layout Size options Help
Zoom
30.0
Whole
Show buried clones
Yes No
Search
CB Unit Range
0 to 46
Contig stats
Clones: 2 (0 buried)
Markers: 0
Sequenced: 0
Length: 47 CB units
Ctg3975 of cp05T5e12DM4RS
M048F17
M052I16
0 5 10 15 20 25 30 35 40 45 50
[Chickpea contig p...
[root@localhost: /...
[FPC V8.9 Main Menu]
Project cp05T5e12...
FPC Ctg3975 cp05...

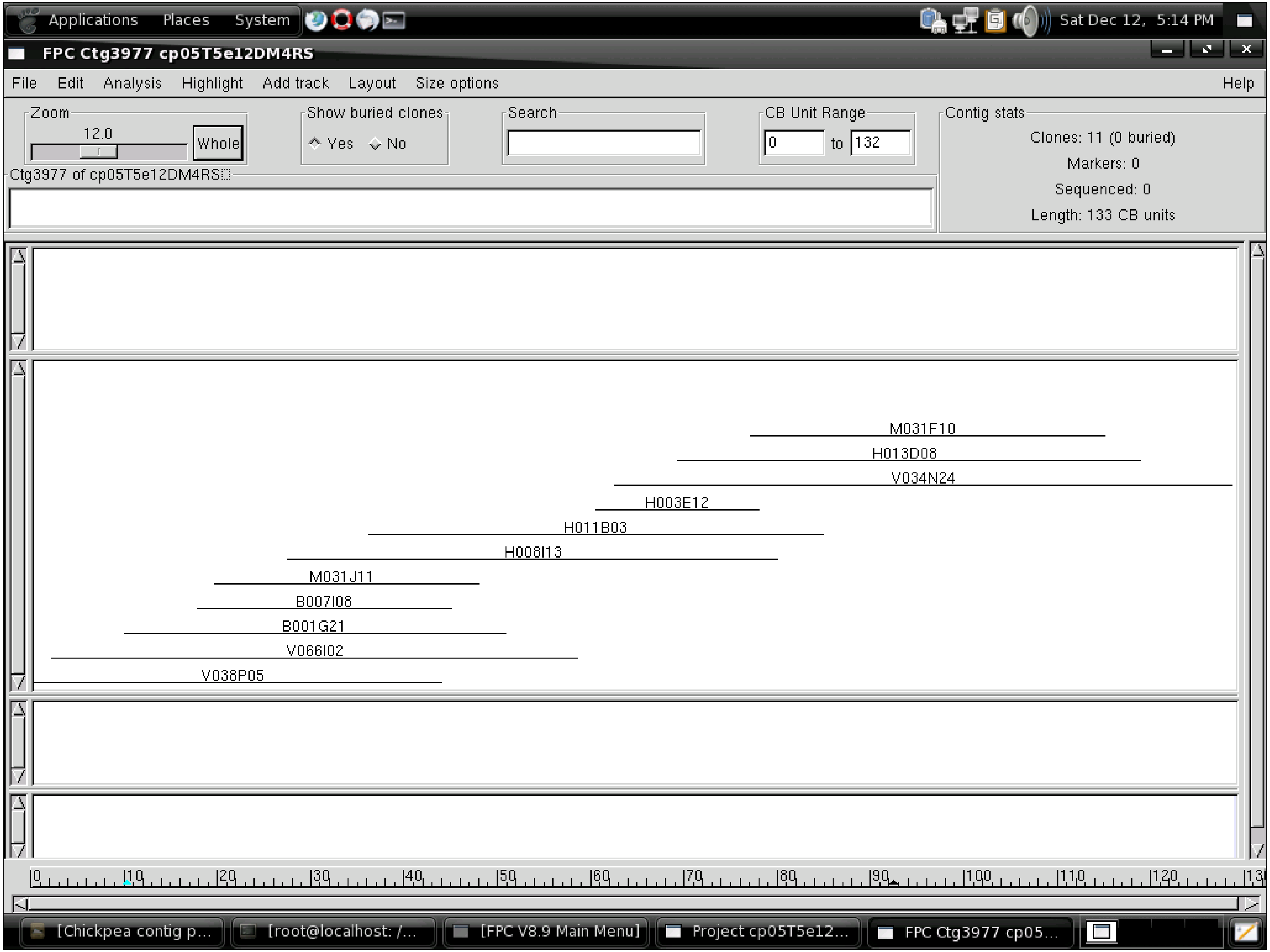
Applications Places System
Sat Dec 12, 5:14 PM
FPC Ctg3977 cp05T5e12DM4RS
File Edit Analysis Highlight Add track Layout Size options Help
Zoom
12.0
Whole
Show buried clones
Yes No
Search
CB Unit Range
0 to 132
Contig stats
Clones: 11 (0 buried)
Markers: 0
Sequenced: 0
Length: 133 CB units
Ctg3977 of cp05T5e12DM4RS
M031F10
H013D08
V034N24
H003E12
H011B03
H008I13
M031J11
B007I08
B001G21
V066I02
V038P05
0 10 20 30 40 50 60 70 80 90 100 110 120 13
[Chickpea contig p...
[root@localhost: /...
[FPC V8.9 Main Menu]
Project cp05T5e12...
FPC Ctg3977 cp05...

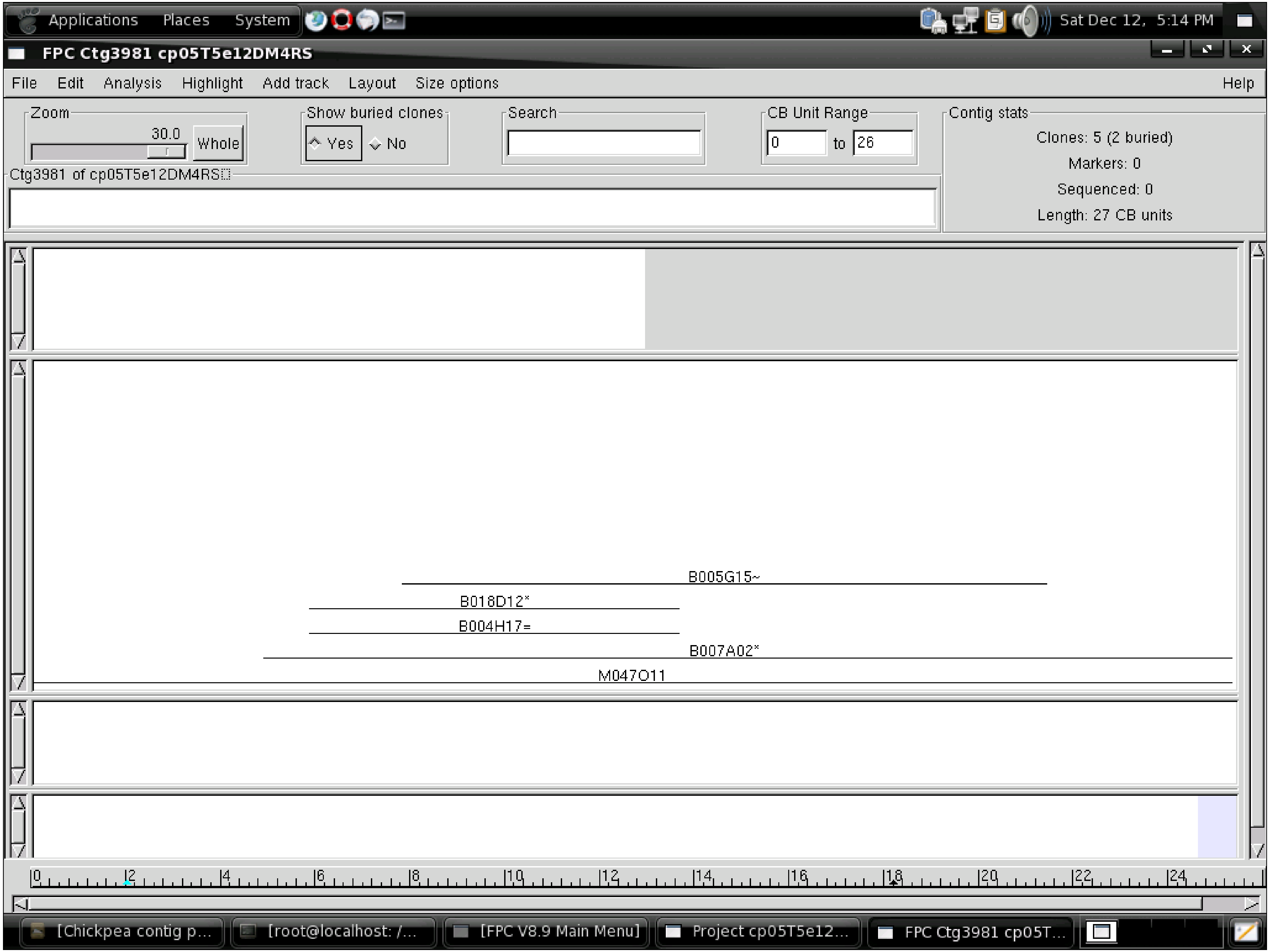
Applications Places System
Sat Dec 12, 5:14 PM
FPC Ctg3981 cp05T5e12DM4RS
File Edit Analysis Highlight Add track Layout Size options Help
Zoom
30.0
Whole
Show buried clones
Yes No
Search
CB Unit Range
0 to 26
Contig stats
Clones: 5 (2 buried)
Markers: 0
Sequenced: 0
Length: 27 CB units
Ctg3981 of cp05T5e12DM4RS
B005G15~
B018D12*
B004H17=
B007A02*
M047O11
0 2 4 6 8 10 12 14 16 18 20 22 24
[Chickpea contig p...
[root@localhost: /...
[FPC V8.9 Main Menu]
Project cp05T5e12...
FPC Ctg3981 cp05T...

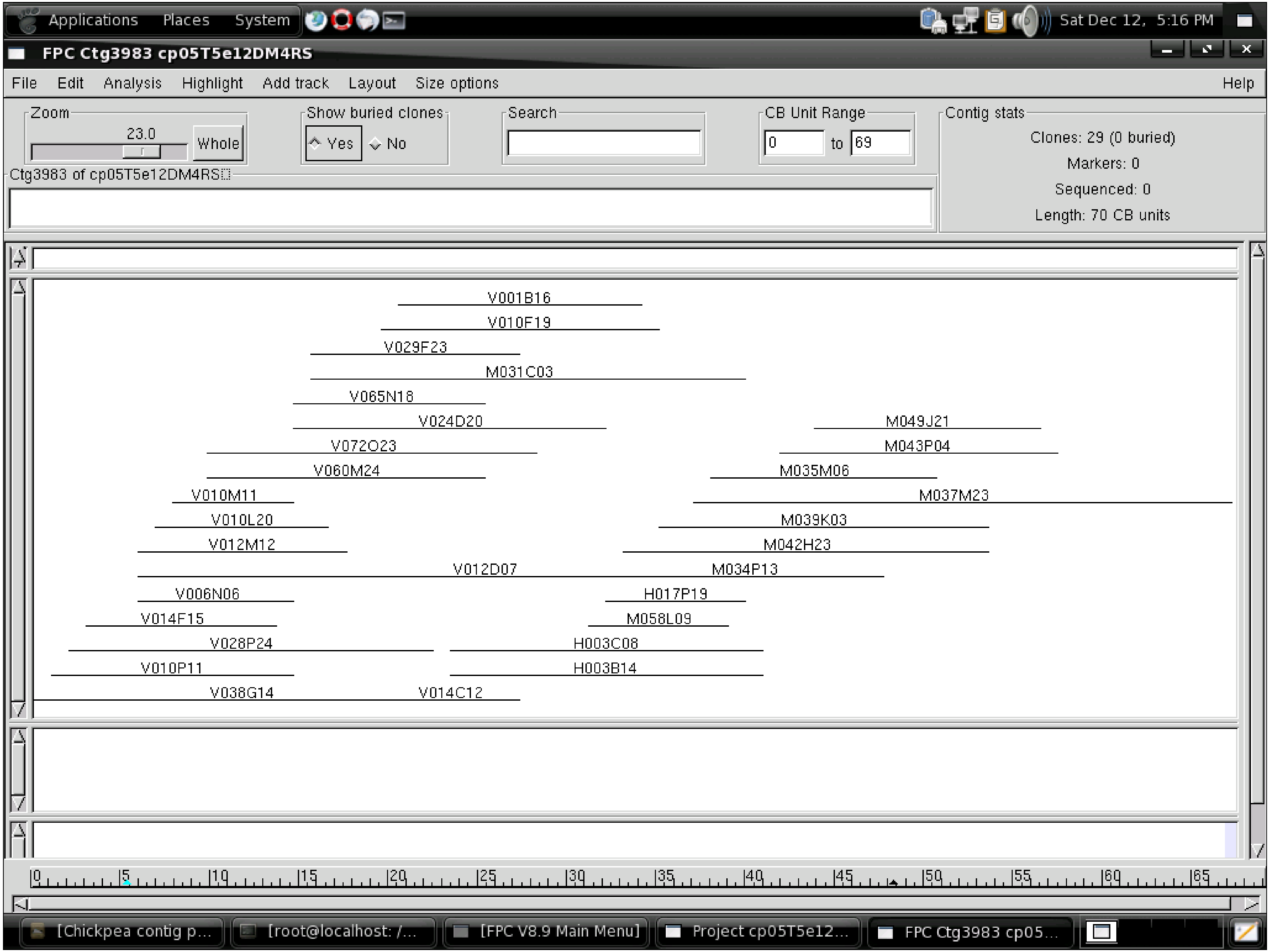
Applications Places System
Sat Dec 12, 5:16 PM
FPC Ctg3983 cp05T5e12DM4RS
File Edit Analysis Highlight Add track Layout Size options Help
Zoom
23.0
Whole
Show buried clones
Yes
No
Search
CB Unit Range
0 to 69
Contig stats
Clones: 29 (0 buried)
Markers: 0
Sequenced: 0
Length: 70 CB units
Ctg3983 of cp05T5e12DM4RS
V001B16
V010F19
V029F23
M031C03
V065N18
V024D20
M049J21
V072O23
M043P04
V060M24
M035M06
V010M11
M037M23
V010L20
M039K03
V012M12
M042H23
V012D07
M034P13
V006N06
H017P19
V014F15
M058L09
V028P24
H003C08
V010P11
H003B14
V038G14
V014C12
0 5 10 15 20 25 30 35 40 45 50 55 60 65
[Chickpea contig p...
[root@localhost: /...
[FPC V8.9 Main Menu]
Project cp05T5e12...
FPC Ctg3983 cp05...

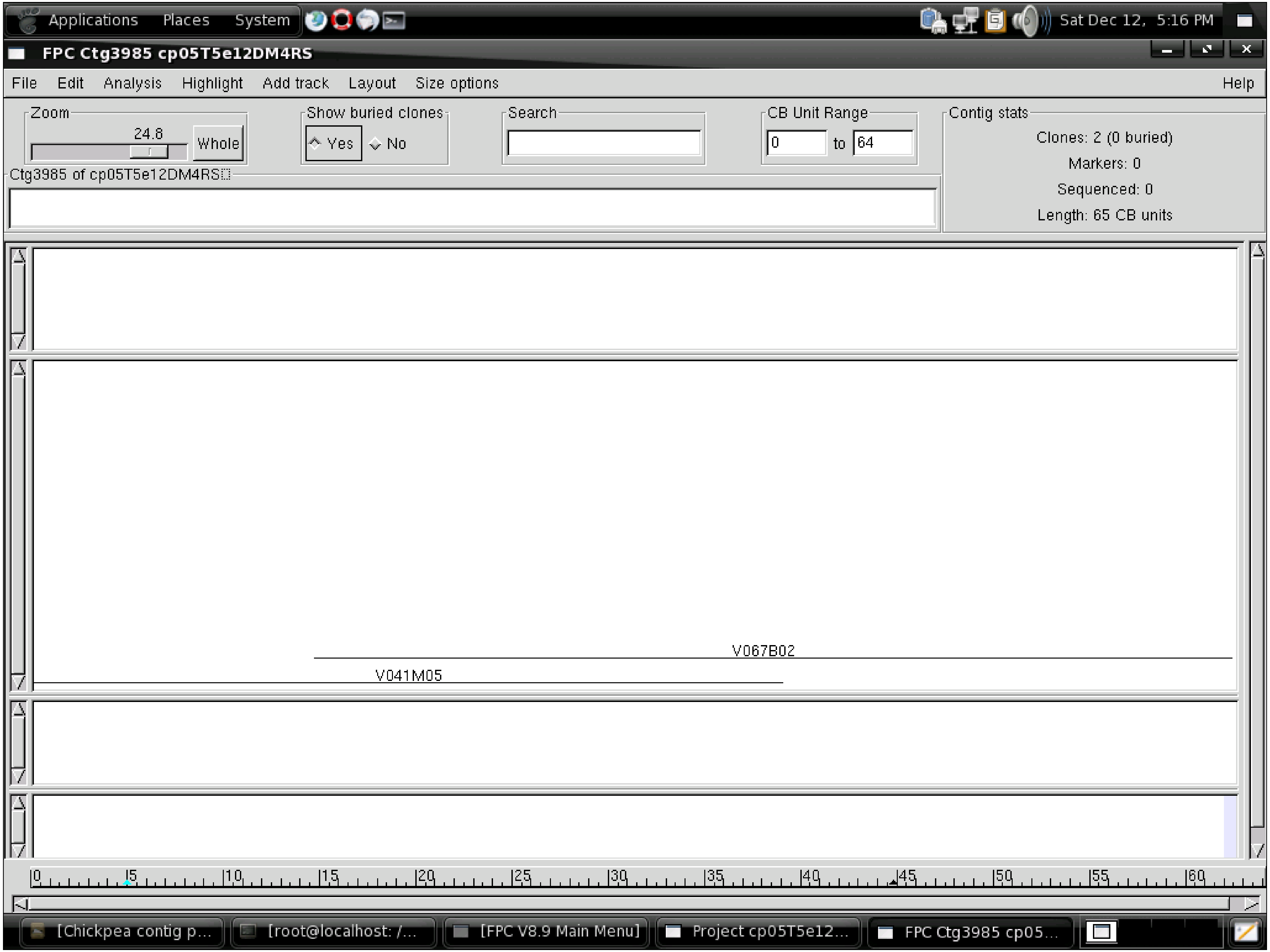
Applications Places System
Sat Dec 12, 5:16 PM
FPC Ctg3985 cp05T5e12DM4RS
File Edit Analysis Highlight Add track Layout Size options Help
Zoom
24.8
Whole
Show buried clones
Yes
No
Search
CB Unit Range
0 to 64
Contig stats
Clones: 2 (0 buried)
Markers: 0
Sequenced: 0
Length: 65 CB units
Ctg3985 of cp05T5e12DM4RS
V067B02
V041M05
0 5 10 15 20 25 30 35 40 45 50 55 60
[Chickpea contig p...
[root@localhost: /...
[FPC V8.9 Main Menu]
Project cp05T5e12...
FPC Ctg3985 cp05...

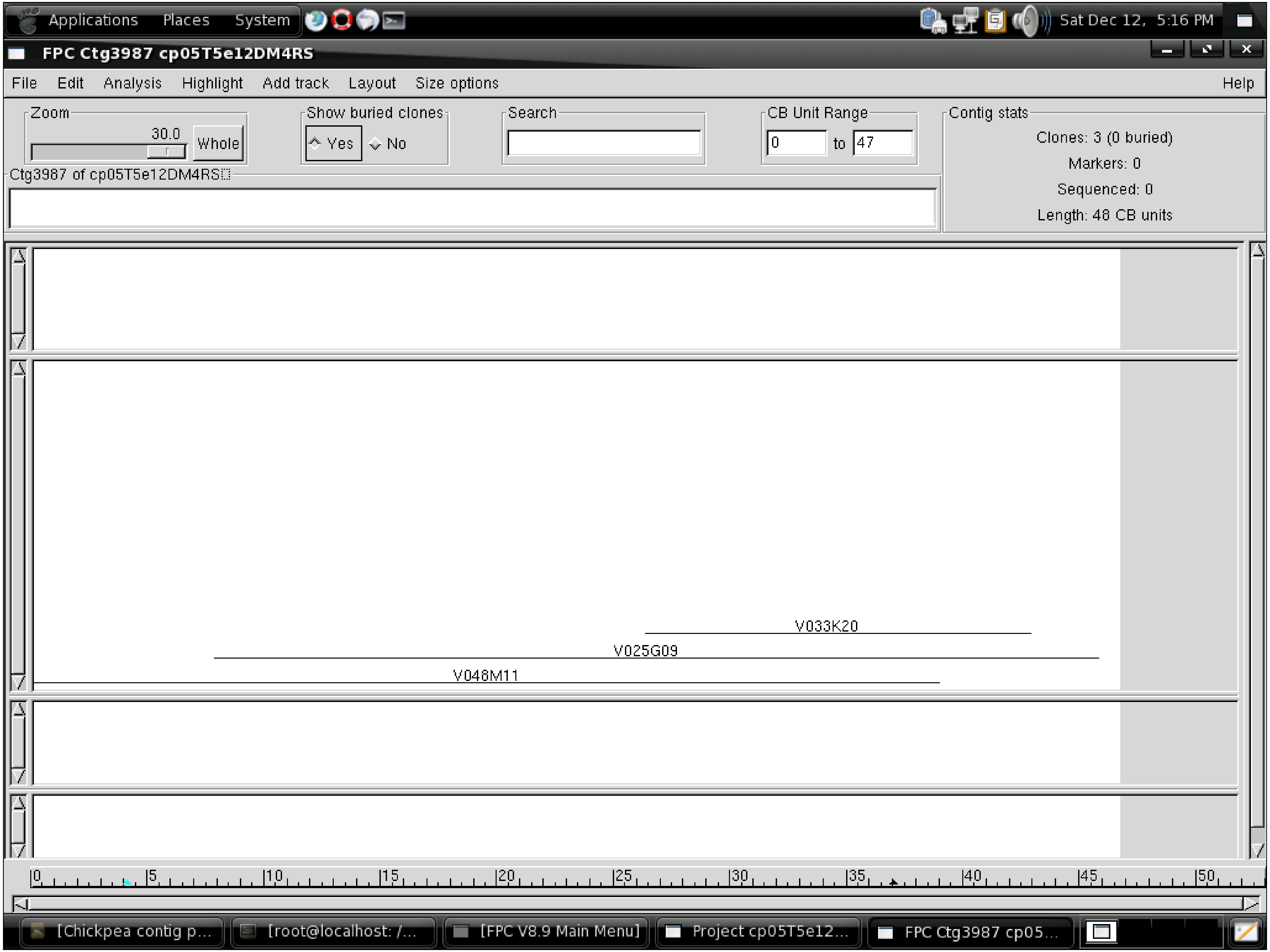
Applications Places System
Sat Dec 12, 5:16 PM
FPC Ctg3987 cp05T5e12DM4RS
File Edit Analysis Highlight Add track Layout Size options Help
Zoom
30.0
Whole
Show buried clones
Yes
No
Search
CB Unit Range
0
to
47
Contig stats
Clones: 3 (0 buried)
Markers: 0
Sequenced: 0
Length: 48 CB units
Ctg3987 of cp05T5e12DM4RS
V033K20
V025G09
V048M11
0 5 10 15 20 25 30 35 40 45 50
[Chickpea contig p...
[root@localhost: /...
[FPC V8.9 Main Menu]
Project cp05T5e12...
FPC Ctg3987 cp05...

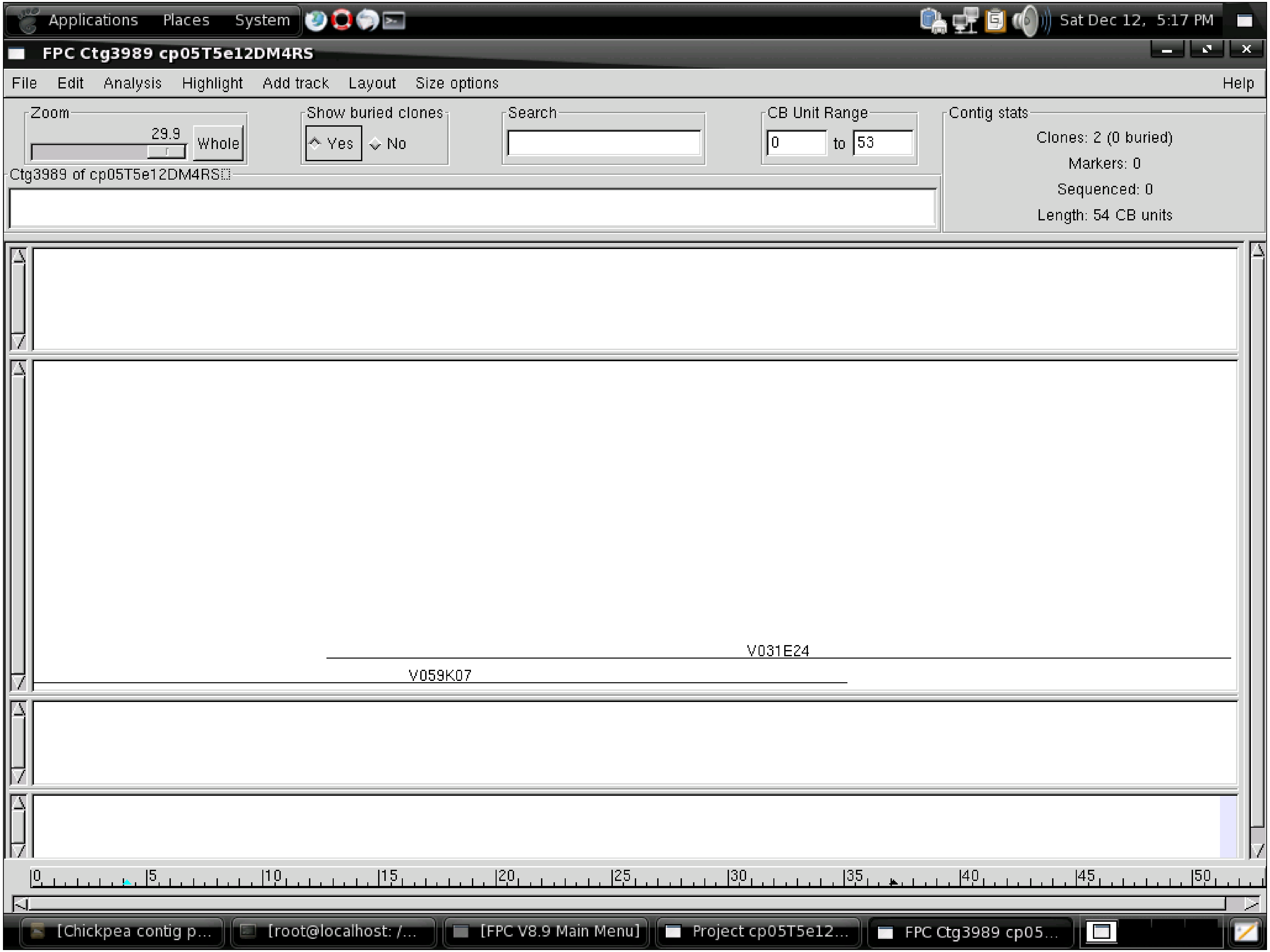
Applications Places System
Sat Dec 12, 5:17 PM
FPC Ctg3989 cp05T5e12DM4RS
File Edit Analysis Highlight Add track Layout Size options Help
Zoom
29.9
Whole
Show buried clones
Yes No
Search
CB Unit Range
0 to 53
Contig stats
Clones: 2 (0 buried)
Markers: 0
Sequenced: 0
Length: 54 CB units
Ctg3989 of cp05T5e12DM4RS
V031E24
V059K07
0 5 10 15 20 25 30 35 40 45 50
[Chickpea contig p...
[root@localhost: /...
[FPC V8.9 Main Menu]
Project cp05T5e12...
FPC Ctg3989 cp05...

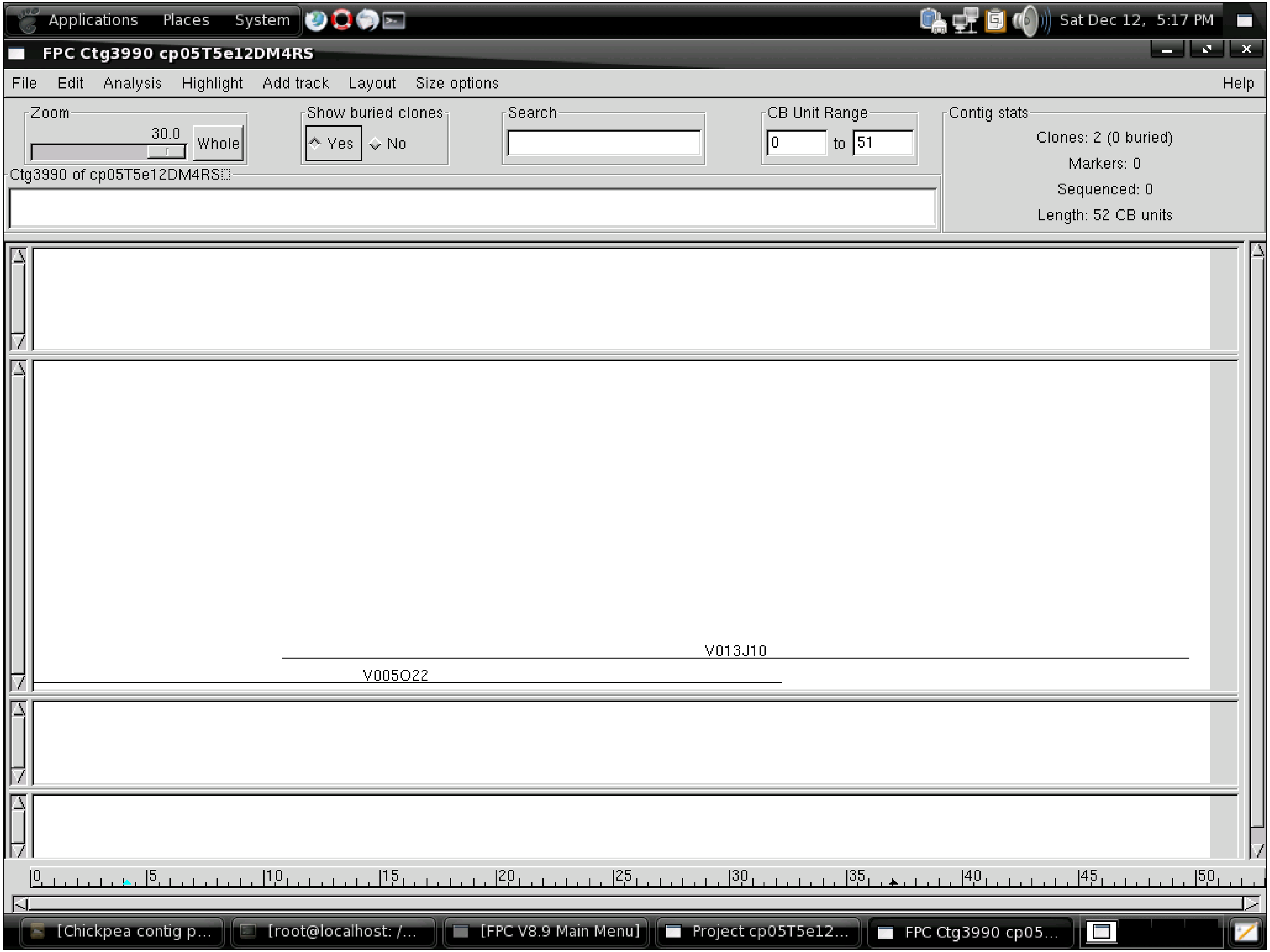
Applications Places System
Sat Dec 12, 5:17 PM
FPC Ctg3990 cp05T5e12DM4RS
File Edit Analysis Highlight Add track Layout Size options Help
Zoom
30.0
Whole
Show buried clones
Yes No
Search
CB Unit Range
0 to 51
Contig stats
Clones: 2 (0 buried)
Markers: 0
Sequenced: 0
Length: 52 CB units
Ctg3990 of cp05T5e12DM4RS
V013J10
V005O22
0 5 10 15 20 25 30 35 40 45 50
[Chickpea contig p...
[root@localhost: /...
[FPC V8.9 Main Menu]
Project cp05T5e12...
FPC Ctg3990 cp05...

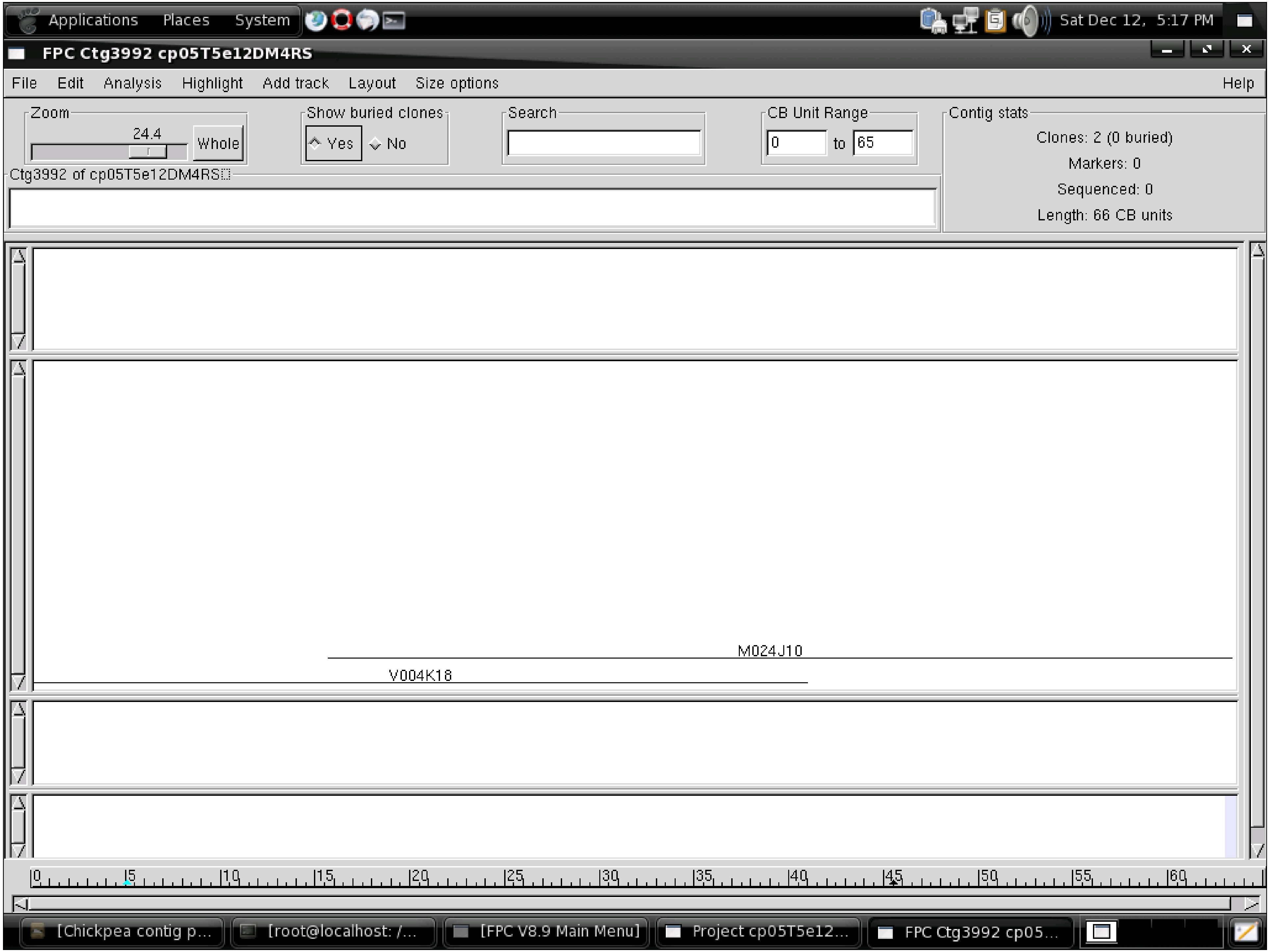
Applications Places System
Sat Dec 12, 5:17 PM
FPC Ctg3992 cp05T5e12DM4RS
File Edit Analysis Highlight Add track Layout Size options Help
Zoom
24.4
Whole
Show buried clones
Yes No
Search
CB Unit Range
0 to 65
Contig stats
Clones: 2 (0 buried)
Markers: 0
Sequenced: 0
Length: 66 CB units
Ctg3992 of cp05T5e12DM4RS
M024J10
V004K18
0 5 10 15 20 25 30 35 40 45 50 55 60
[Chickpea contig p...
[root@localhost: /...
[FPC V8.9 Main Menu]
Project cp05T5e12...
FPC Ctg3992 cp05...

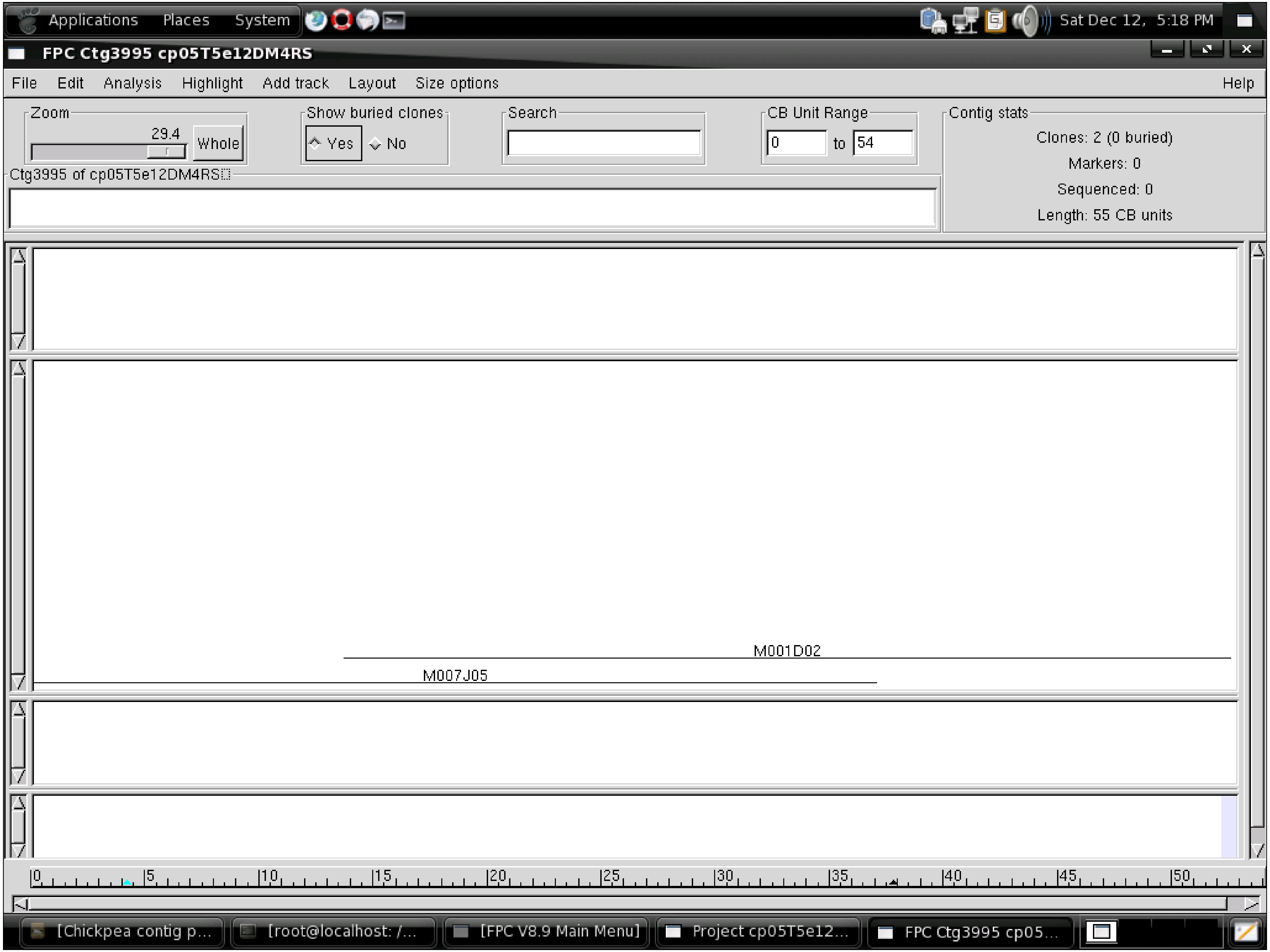
Applications Places System
Sat Dec 12, 5:18 PM
FPC Ctg3995 cp05T5e12DM4RS
File Edit Analysis Highlight Add track Layout Size options Help
Zoom
29.4
Whole
Show buried clones
Yes
No
Search
CB Unit Range
0 to 54
Contig stats
Clones: 2 (0 buried)
Markers: 0
Sequenced: 0
Length: 55 CB units
Ctg3995 of cp05T5e12DM4RS
M001D02
M007J05
0 5 10 15 20 25 30 35 40 45 50
[Chickpea contig p...
[root@localhost: /...
[FPC V8.9 Main Menu]
Project cp05T5e12...
FPC Ctg3995 cp05...

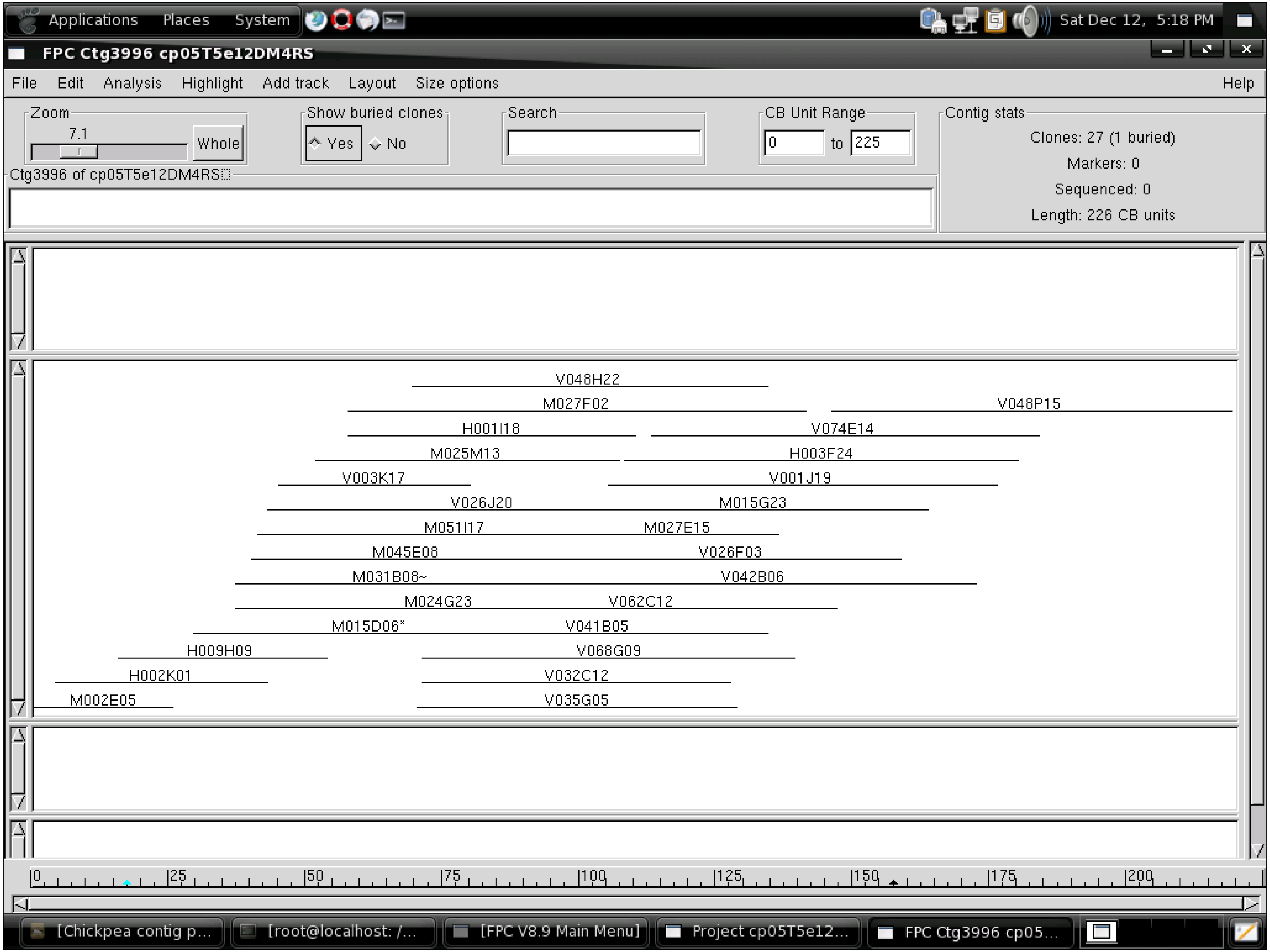
Applications Places System
Sat Dec 12, 5:18 PM
FPC Ctg3996 cp05T5e12DM4RS
File Edit Analysis Highlight Add track Layout Size options Help
Zoom 7.1 Whole
Show buried clones Yes No
Search
CB Unit Range 0 to 225
Contig stats
Clones: 27 (1 buried)
Markers: 0
Sequenced: 0
Length: 226 CB units
Ctg3996 of cp05T5e12DM4RS
V048H22
M027F02
V048P15
H001I18
V074E14
M025M13
H003F24
V003K17
V001J19
V026J20
M015G23
M051I17
M027E15
M045E08
V026F03
M031B08~
V042B06
M024G23
V062C12
M015D06*
V041B05
H009H09
V068G09
H002K01
V032C12
M002E05
V035G05
0 25 50 75 100 125 150 175 200
[Chickpea contig p...
[root@localhost: /...
[FPC V8.9 Main Menu]
Project cp05T5e12...
FPC Ctg3996 cp05...

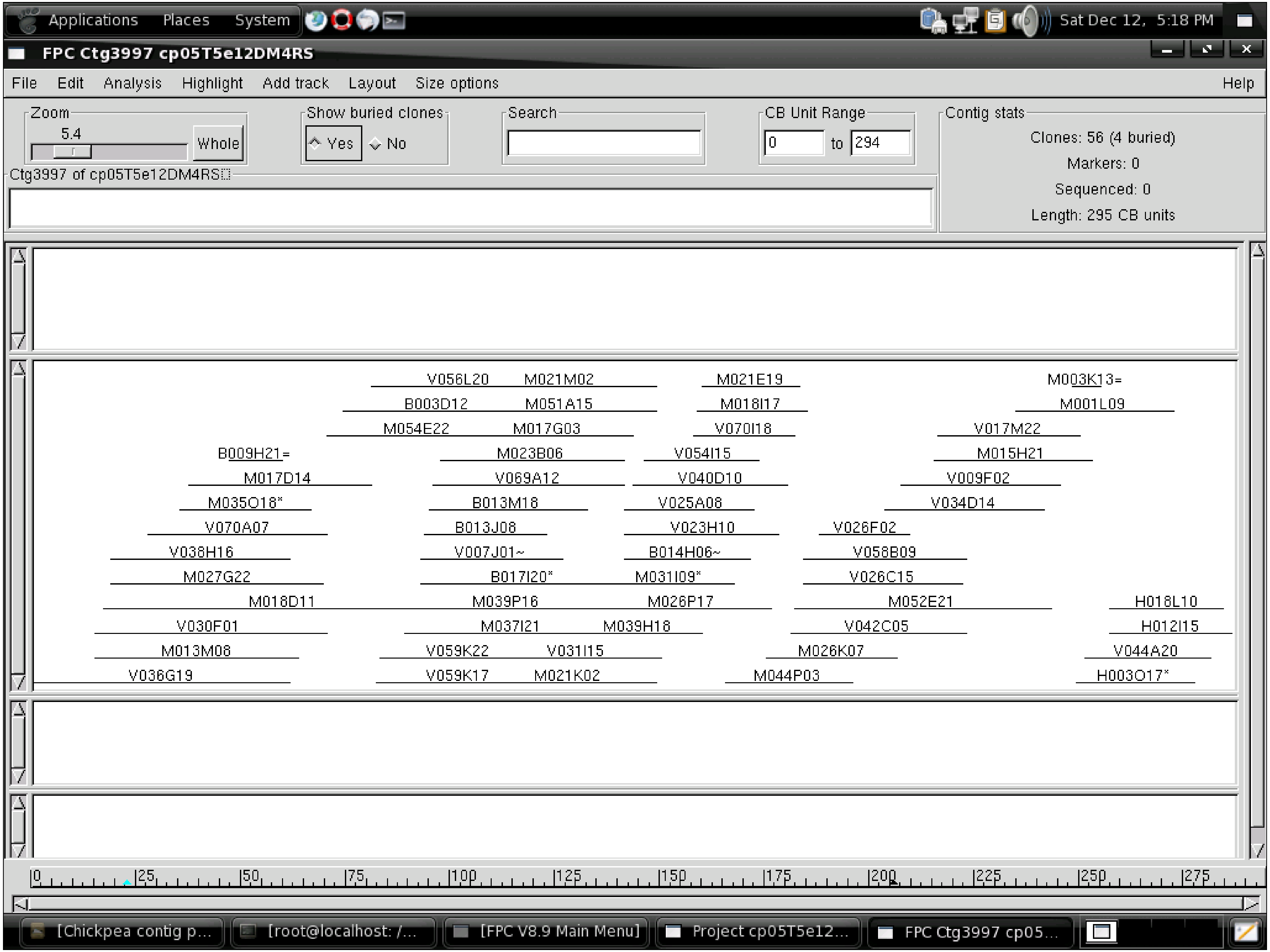
Applications Places System
Sat Dec 12, 5:18 PM
FPC Ctg3997 cp05T5e12DM4RS
File Edit Analysis Highlight Add track Layout Size options Help
Zoom 5.4 Whole
Show buried clones Yes No
Search
CB Unit Range 0 to 294
Contig stats
Clones: 56 (4 buried)
Markers: 0
Sequenced: 0
Length: 295 CB units
Ctg3997 of cp05T5e12DM4RS
V056L20 M021M02 M021E19 M003K13=
B003D12 M051A15 M018I17 M001L09
M054E22 M017G03 V070I18 V017M22
B009H21= M023B06 V054I15 M015H21
M017D14 V069A12 V040D10 V009F02
M035O18* B013M18 V025A08 V034D14
V070A07 B013J08 V023H10 V026F02
V038H16 V007J01~ B014H06~ V058B09
M027G22 B017I20* M031I09* V026C15
M018D11 M039P16 M026P17 M052E21 H018L10
V030F01 M037I21 M039H18 V042C05 H012I15
M013M08 V059K22 V031I15 M026K07 V044A20
V036G19 V059K17 M021K02 M044P03 H003O17*
0 25 50 75 100 125 150 175 200 225 250 275
[Chickpea contig p... [root@localhost: /... [FPC V8.9 Main Menu] Project cp05T5e12... FPC Ctg3997 cp05...

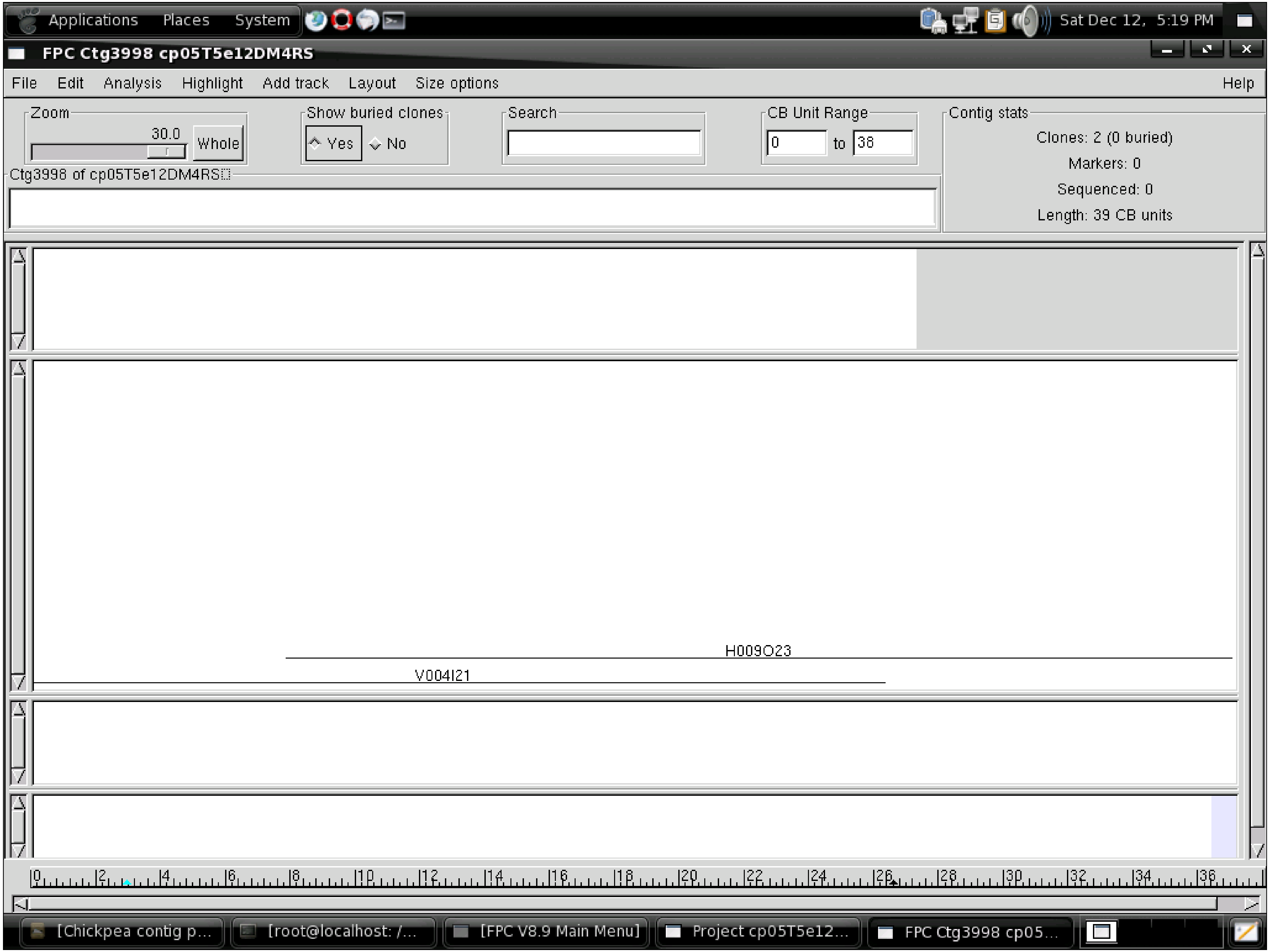
Applications Places System
Sat Dec 12, 5:19 PM
FPC Ctg3998 cp05T5e12DM4RS
File Edit Analysis Highlight Add track Layout Size options Help
Zoom
30.0
Whole
Show buried clones
Yes
No
Search
CB Unit Range
0
to
38
Contig stats
Clones: 2 (0 buried)
Markers: 0
Sequenced: 0
Length: 39 CB units
Ctg3998 of cp05T5e12DM4RS
H009O23
V004I21
0 2 4 6 8 10 12 14 16 18 20 22 24 26 28 30 32 34 36
[Chickpea contig p...
[root@localhost: /...
[FPC V8.9 Main Menu]
Project cp05T5e12...
FPC Ctg3998 cp05...

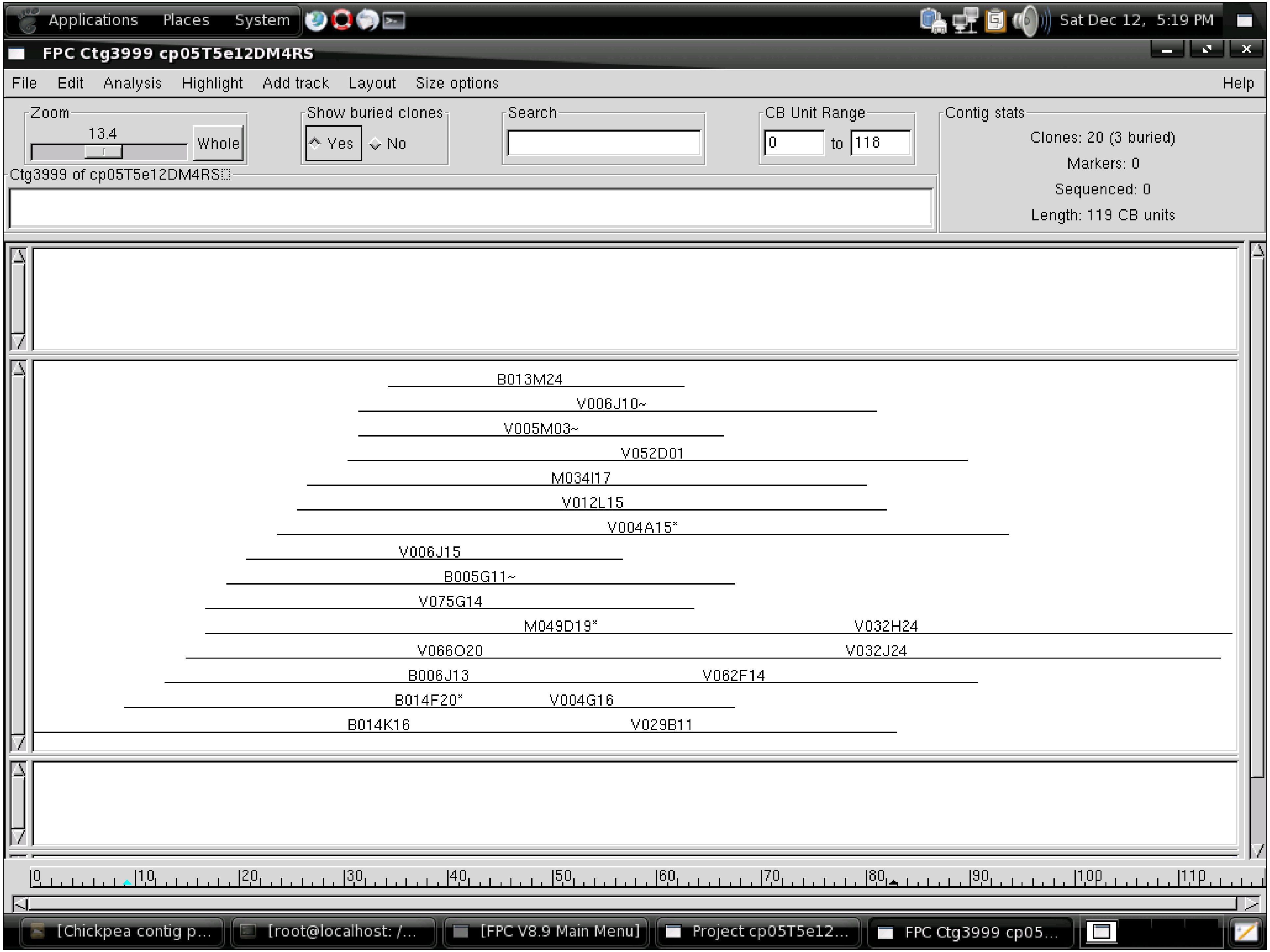
Applications Places System
Sat Dec 12, 5:19 PM
FPC Ctg3999 cp05T5e12DM4RS
File Edit Analysis Highlight Add track Layout Size options Help
Zoom
13.4
Whole
Show buried clones
Yes
No
Search
CB Unit Range
0
to
118
Contig stats
Clones: 20 (3 buried)
Markers: 0
Sequenced: 0
Length: 119 CB units
Ctg3999 of cp05T5e12DM4RS
B013M24
V006J10~
V005M03~
V052D01
M034I17
V012L15
V004A15*
V006J15
B005G11~
V075G14
M049D19*
V032H24
V066O20
V032J24
B006J13
V062F14
B014F20*
V004G16
B014K16
V029B11
0 10 20 30 40 50 60 70 80 90 100 110
[Chickpea contig p...
[root@localhost: /...
[FPC V8.9 Main Menu]
Project cp05T5e12...
FPC Ctg3999 cp05...

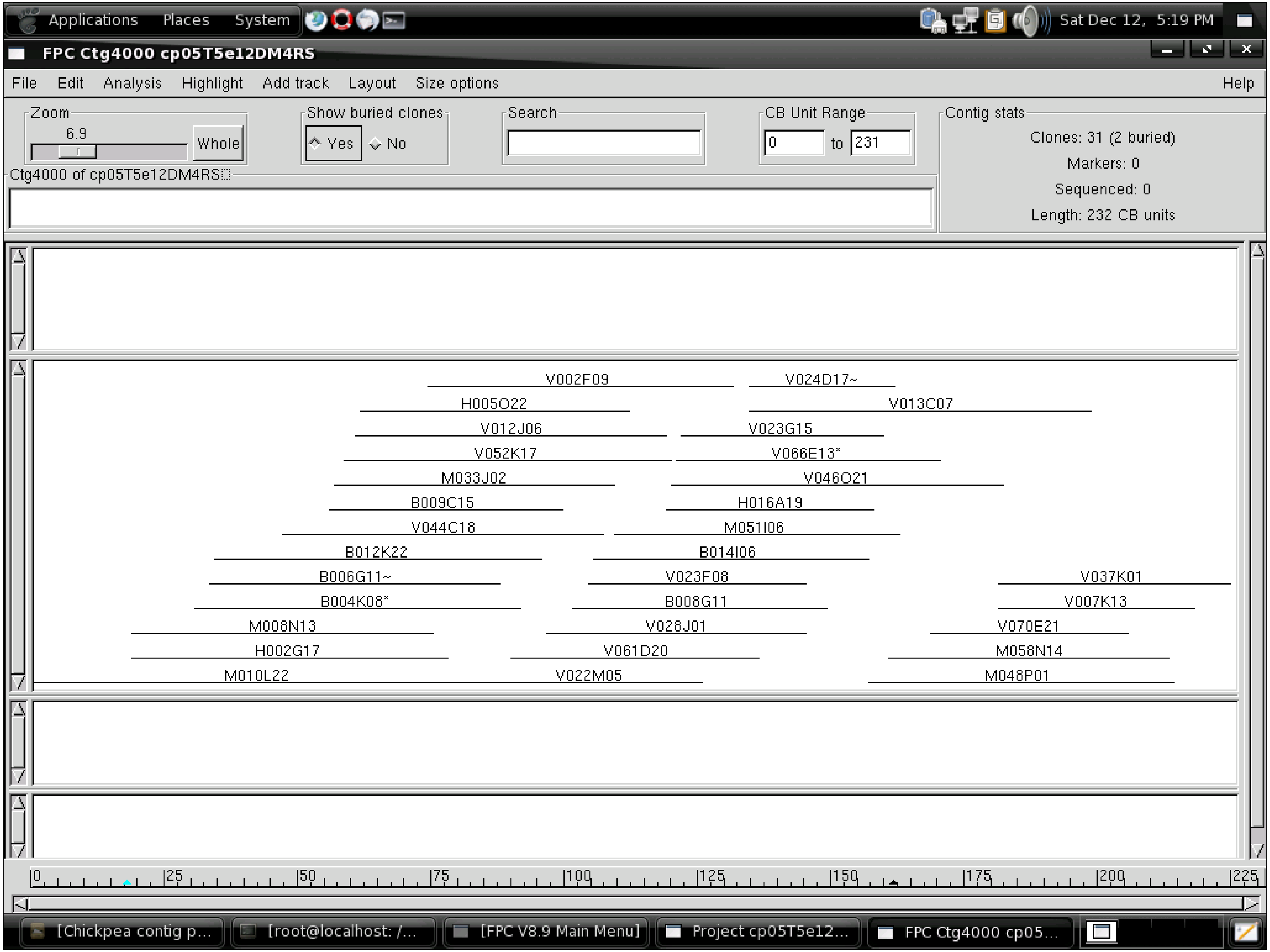
Applications Places System
Sat Dec 12, 5:19 PM
FPC Ctg4000 cp05T5e12DM4RS
File Edit Analysis Highlight Add track Layout Size options Help
Zoom
6.9
Whole
Show buried clones
Yes No
Search
CB Unit Range
0 to 231
Contig stats
Clones: 31 (2 buried)
Markers: 0
Sequenced: 0
Length: 232 CB units
Ctg4000 of cp05T5e12DM4RS
V002F09
V024D17~
H005O22
V013C07
V012J06
V023G15
V052K17
V066E13*
M033J02
V046O21
B009C15
H016A19
V044C18
M051I06
B012K22
B014I06
B006G11~
V023F08
V037K01
B004K08*
B008G11
V007K13
M008N13
V028J01
V070E21
H002G17
V061D20
M058N14
M010L22
V022M05
M048P01
0 25 50 75 100 125 150 175 200 225
[Chickpea contig p...
[root@localhost: /...
[FPC V8.9 Main Menu]
Project cp05T5e12...
FPC Ctg4000 cp05...

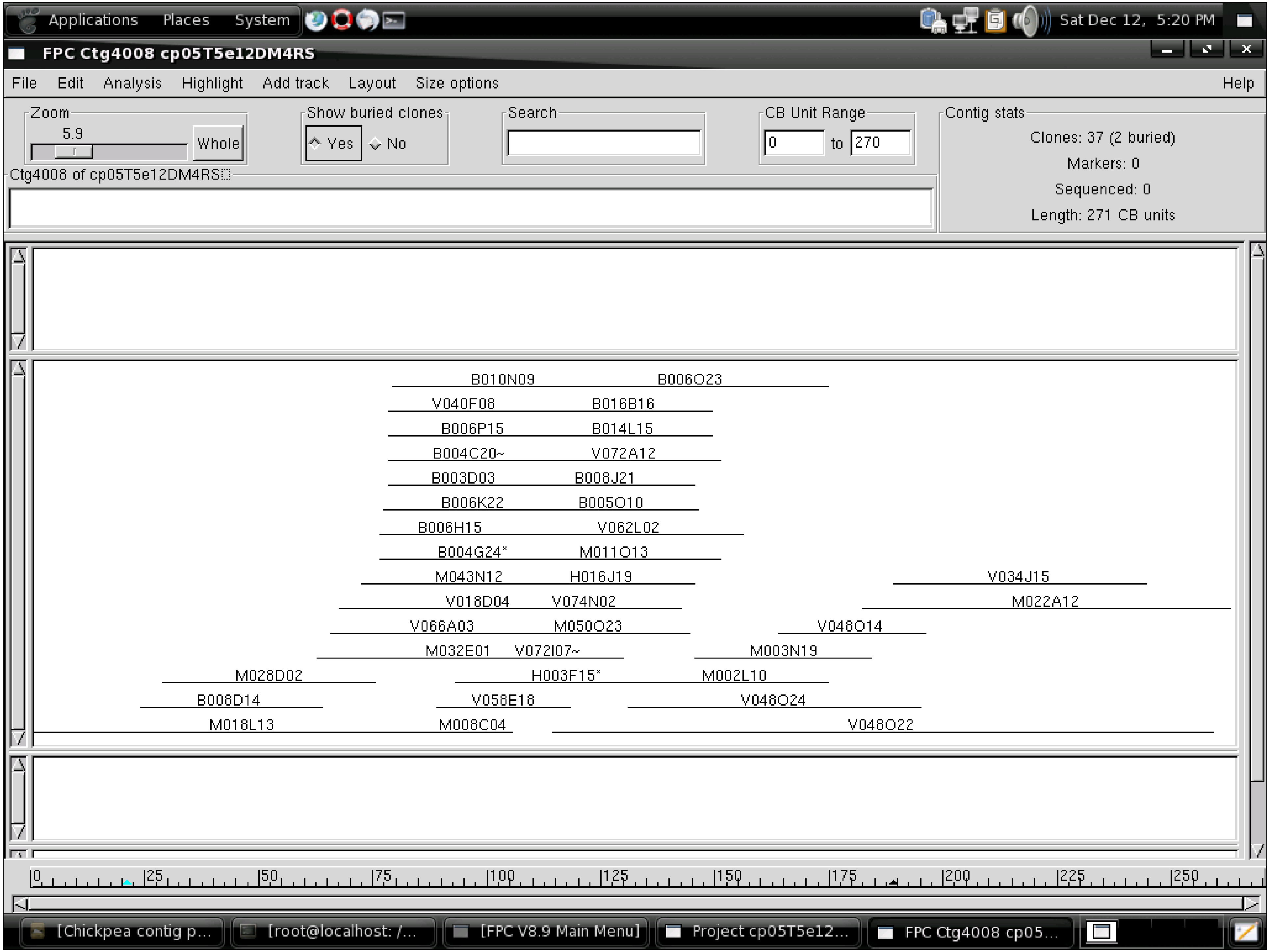
Applications Places System
Sat Dec 12, 5:20 PM
FPC Ctg4008 cp05T5e12DM4RS
File Edit Analysis Highlight Add track Layout Size options Help
Zoom
5.9
Whole
Show buried clones
Yes No
Search
CB Unit Range
0 to 270
Contig stats
Clones: 37 (2 buried)
Markers: 0
Sequenced: 0
Length: 271 CB units
Ctg4008 of cp05T5e12DM4RS
B010N09 B006O23
V040F08 B016B16
B006P15 B014L15
B004C20~ V072A12
B003D03 B008J21
B006K22 B005O10
B006H15 V062L02
B004G24* M011O13
M043N12 H016J19 V034J15
V018D04 V074N02 M022A12
V066A03 M050O23 V048O14
M032E01 V072I07~ M003N19
M028D02 H003F15* M002L10
B008D14 V058E18 V048O24
M018L13 M008C04 V048O22
0 25 50 75 100 125 150 175 200 225 250
[Chickpea contig p... [root@localhost: /... [FPC V8.9 Main Menu] Project cp05T5e12... FPC Ctg4008 cp05...

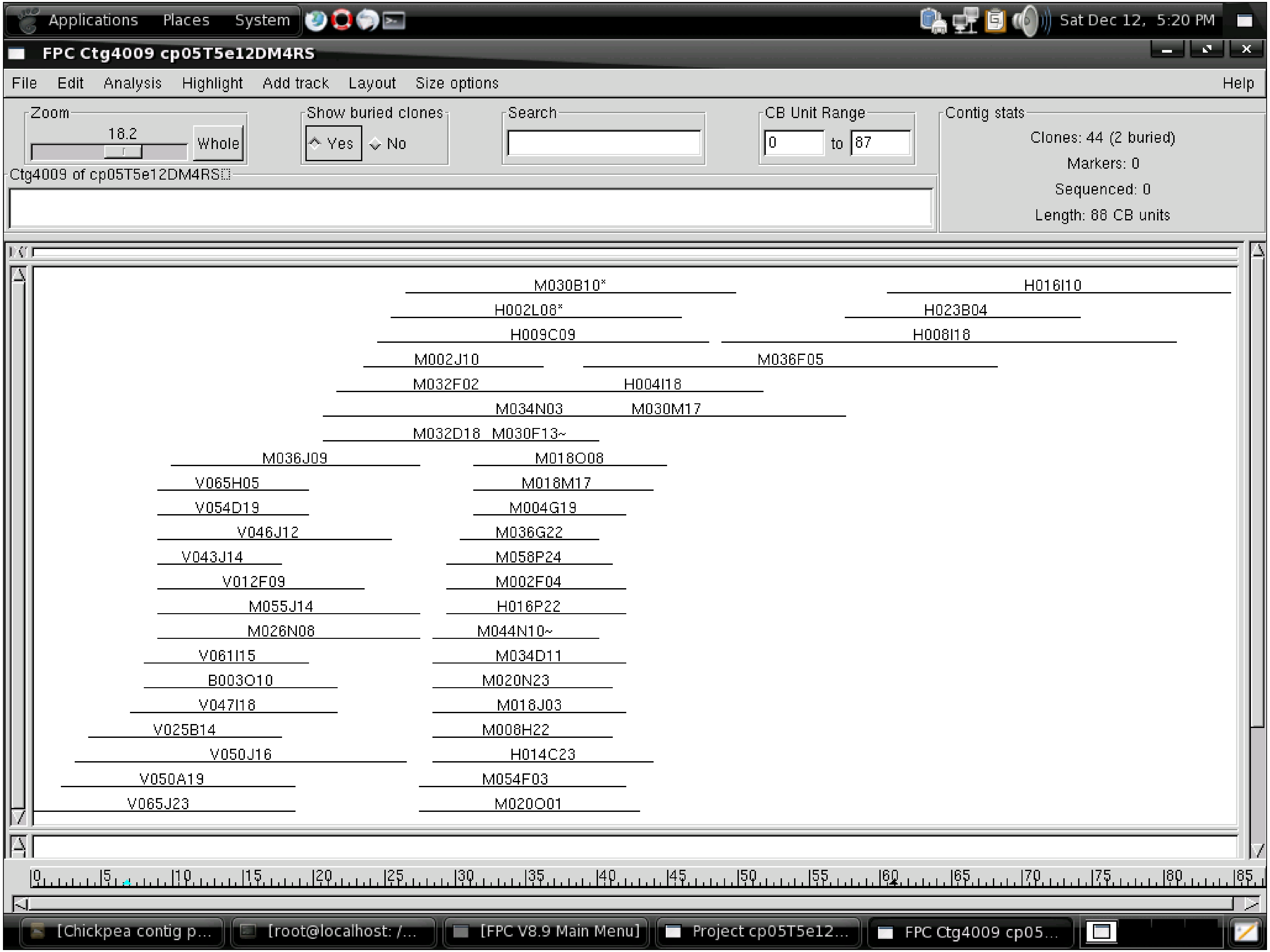
Applications Places System
Sat Dec 12, 5:20 PM
FPC Ctg4009 cp05T5e12DM4RS
File Edit Analysis Highlight Add track Layout Size options Help
Zoom
18.2
Whole
Show buried clones
Yes
No
Search
CB Unit Range
0
to
87
Contig stats
Clones: 44 (2 buried)
Markers: 0
Sequenced: 0
Length: 88 CB units
Ctg4009 of cp05T5e12DM4RS
M030B10*
H016I10
H002L08*
H023B04
H009C09
H008I18
M002J10
M036F05
M032F02
H004I18
M034N03
M030M17
M032D18
M030F13~
M036J09
M018O08
V065H05
M018M17
V054D19
M004G19
V046J12
M036G22
V043J14
M058P24
V012F09
M002F04
M055J14
H016P22
M026N08
M044N10~
V061I15
M034D11
B003O10
M020N23
V047I18
M018J03
V025B14
M008H22
V050J16
H014C23
V050A19
M054F03
V065J23
M020O01
0 5 10 15 20 25 30 35 40 45 50 55 60 65 70 75 80 85
[Chickpea contig p...
[root@localhost: /...
[FPC V8.9 Main Menu]
Project cp05T5e12...
FPC Ctg4009 cp05...

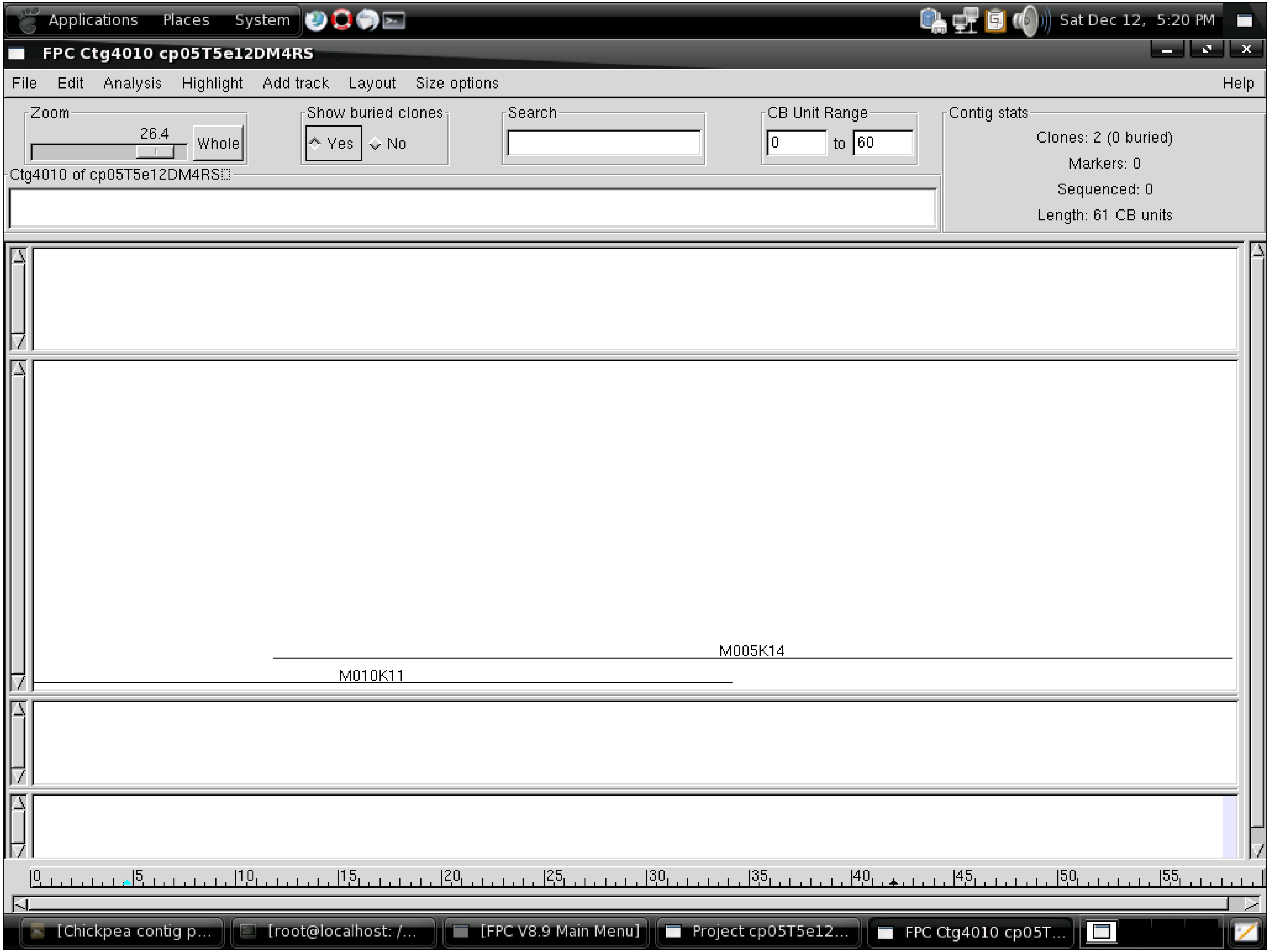
Applications Places System
Sat Dec 12, 5:20 PM
FPC Ctg4010 cp05T5e12DM4RS
File Edit Analysis Highlight Add track Layout Size options Help
Zoom
26.4
Whole
Show buried clones
Yes No
Search
CB Unit Range
0 to 60
Contig stats
Clones: 2 (0 buried)
Markers: 0
Sequenced: 0
Length: 61 CB units
Ctg4010 of cp05T5e12DM4RS
M005K14
M010K11
0 5 10 15 20 25 30 35 40 45 50 55
[Chickpea contig p...
[root@localhost: /...
[FPC V8.9 Main Menu]
Project cp05T5e12...
FPC Ctg4010 cp05T...

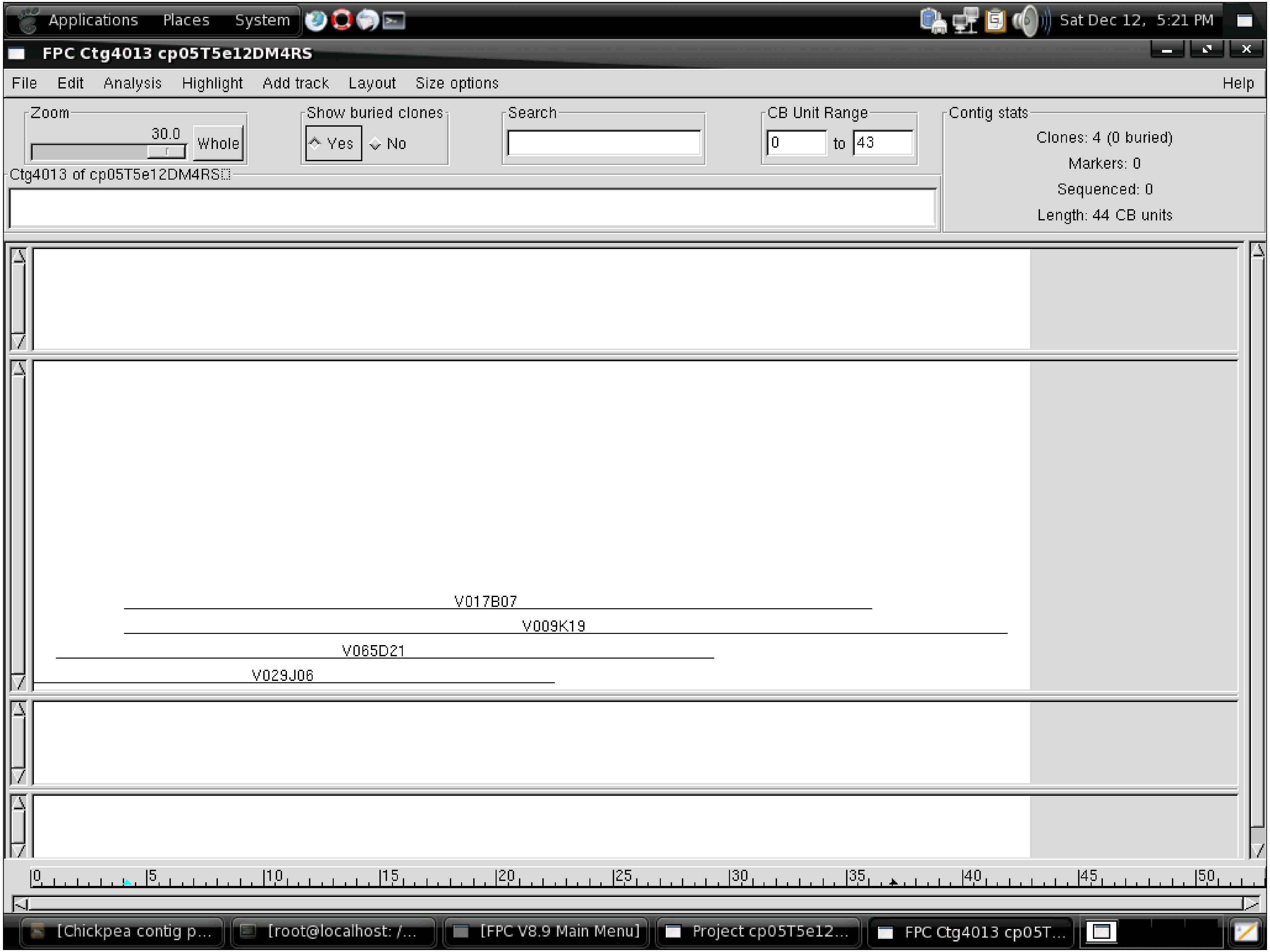
Applications
Places
System
Sat Dec 12, 5:21 PM
FPC Ctg4013 cp05T5e12DM4RS
File
Edit
Analysis
Highlight
Add track
Layout
Size options
Help
Zoom
30.0
Whole
Show buried clones
Yes
No
Search
CB Unit Range
0
to
43
Contig stats
Clones: 4 (0 buried)
Markers: 0
Sequenced: 0
Length: 44 CB units
Ctg4013 of cp05T5e12DM4RS
V017B07
V009K19
V065D21
V029J06
0
5
10
15
20
25
30
35
40
45
50
[Chickpea contig p...
[root@localhost: /...
[FPC V8.9 Main Menu]
Project cp05T5e12...
FPC Ctg4013 cp05T...

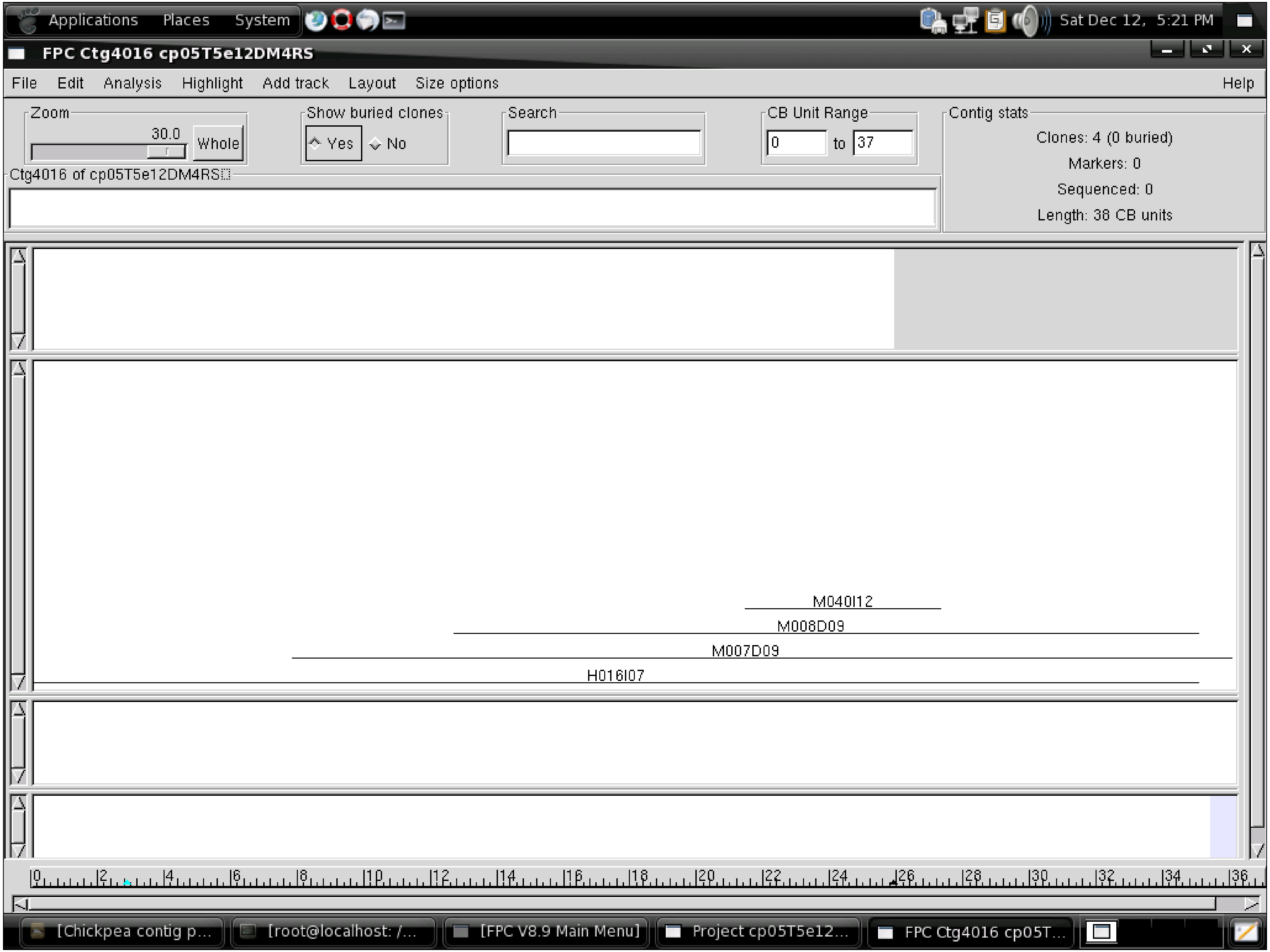
Applications
Places
System
Sat Dec 12, 5:21 PM
FPC Ctg4016 cp05T5e12DM4RS
File
Edit
Analysis
Highlight
Add track
Layout
Size options
Help
Zoom
30.0
Whole
Show buried clones
Yes
No
Search
CB Unit Range
0
to
37
Contig stats
Clones: 4 (0 buried)
Markers: 0
Sequenced: 0
Length: 38 CB units
Ctg4016 of cp05T5e12DM4RS
M040I12
M008D09
M007D09
H016I07
0 2 4 6 8 10 12 14 16 18 20 22 24 26 28 30 32 34 36
[Chickpea contig p...
[root@localhost: /...
[FPC V8.9 Main Menu]
Project cp05T5e12...
FPC Ctg4016 cp05T...

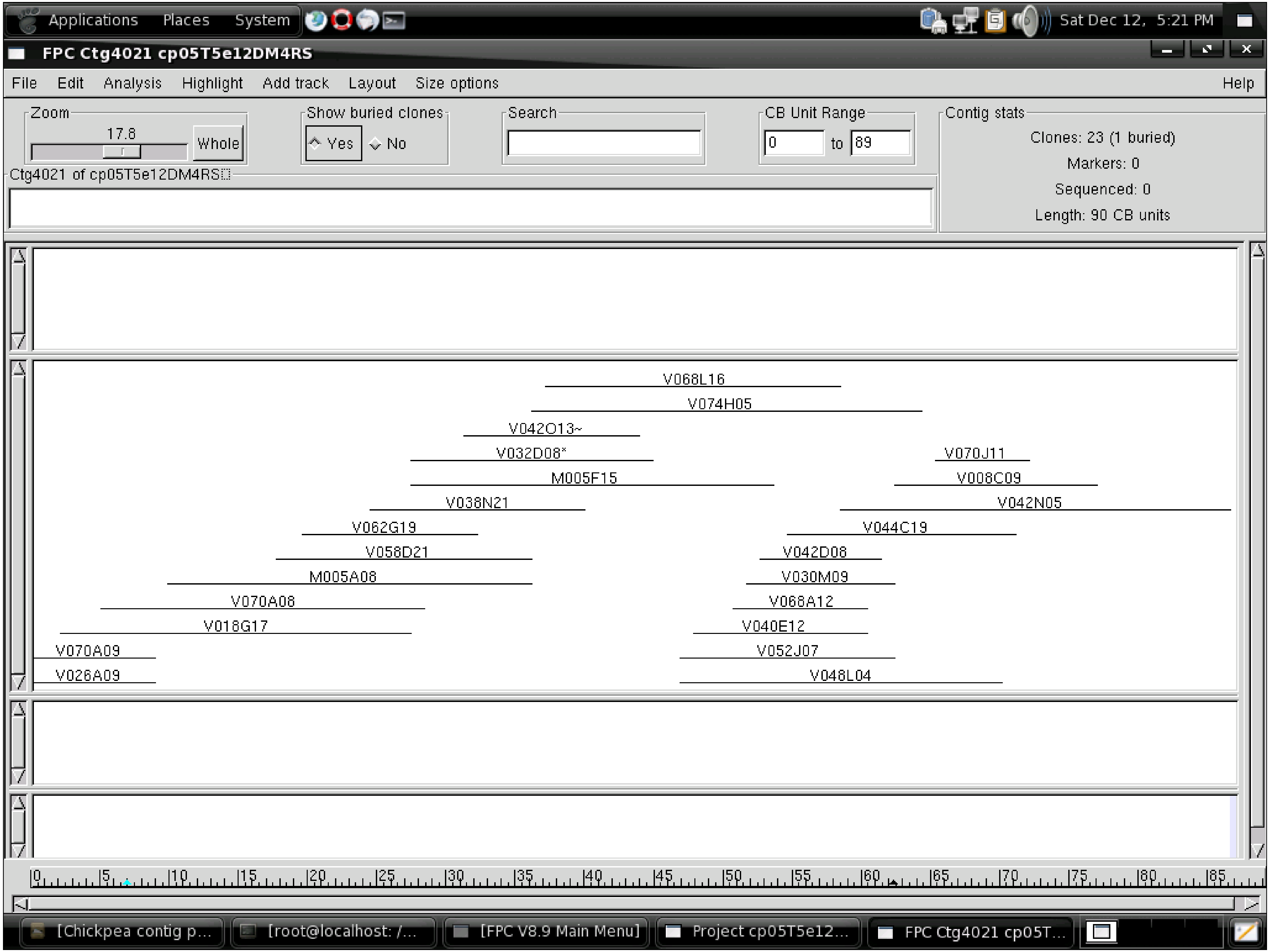
Applications Places System
Sat Dec 12, 5:21 PM
FPC Ctg4021 cp05T5e12DM4RS
File Edit Analysis Highlight Add track Layout Size options Help
Zoom
17.8
Whole
Show buried clones
Yes
No
Search
CB Unit Range
0
to
89
Contig stats
Clones: 23 (1 buried)
Markers: 0
Sequenced: 0
Length: 90 CB units
Ctg4021 of cp05T5e12DM4RS
V068L16
V074H05
V042O13~
V032D08*
V070J11
M005F15
V008C09
V038N21
V042N05
V062G19
V044C19
V058D21
V042D08
M005A08
V030M09
V070A08
V068A12
V018G17
V040E12
V070A09
V052J07
V026A09
V048L04
0 5 10 15 20 25 30 35 40 45 50 55 60 65 70 75 80 85
[Chickpea contig p...
[root@localhost: /...
[FPC V8.9 Main Menu]
Project cp05T5e12...
FPC Ctg4021 cp05T...

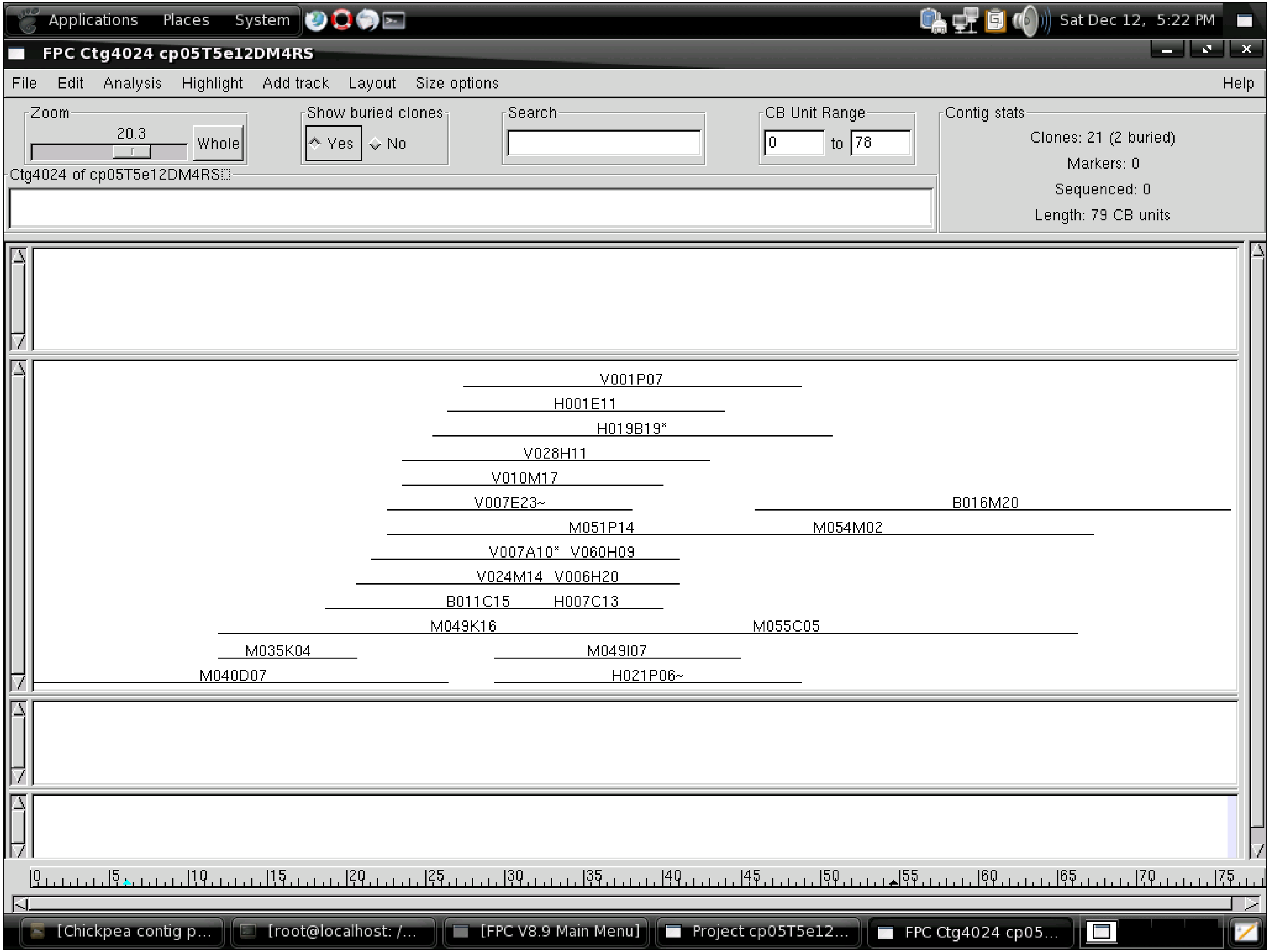
Applications Places System
Sat Dec 12, 5:22 PM
FPC Ctg4024 cp05T5e12DM4RS
File Edit Analysis Highlight Add track Layout Size options Help
Zoom
20.3
Whole
Show buried clones
Yes No
Search
CB Unit Range
0 to 78
Contig stats
Clones: 21 (2 buried)
Markers: 0
Sequenced: 0
Length: 79 CB units
Ctg4024 of cp05T5e12DM4RS
V001P07
H001E11
H019B19*
V028H11
V010M17
V007E23~
B016M20
M051P14
M054M02
V007A10* V060H09
V024M14 V006H20
B011C15 H007C13
M049K16
M055C05
M035K04
M049I07
M040D07
H021P06~
0 5 10 15 20 25 30 35 40 45 50 55 60 65 70 75
[Chickpea contig p...
[root@localhost: /...
[FPC V8.9 Main Menu]
Project cp05T5e12...
FPC Ctg4024 cp05...

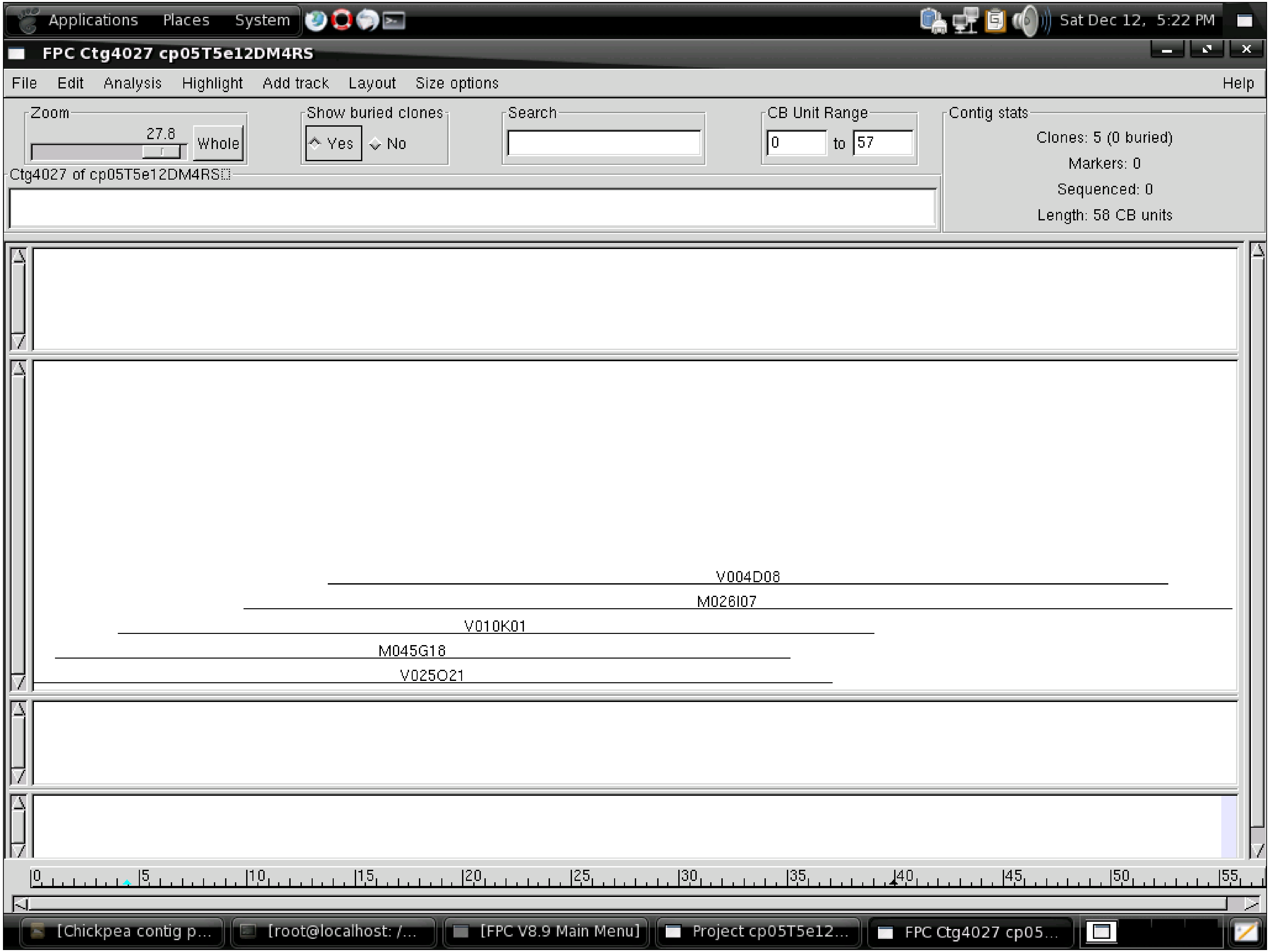
Applications
Places
System
Sat Dec 12, 5:22 PM
FPC Ctg4027 cp05T5e12DM4RS
File
Edit
Analysis
Highlight
Add track
Layout
Size options
Help
Zoom
27.8
Whole
Show buried clones
Yes
No
Search
CB Unit Range
0
to
57
Contig stats
Clones: 5 (0 buried)
Markers: 0
Sequenced: 0
Length: 58 CB units
Ctg4027 of cp05T5e12DM4RS
V004D08
M026I07
V010K01
M045G18
V025O21
0
5
10
15
20
25
30
35
40
45
50
55
[Chickpea contig p...
[root@localhost: /...
[FPC V8.9 Main Menu]
Project cp05T5e12...
FPC Ctg4027 cp05...

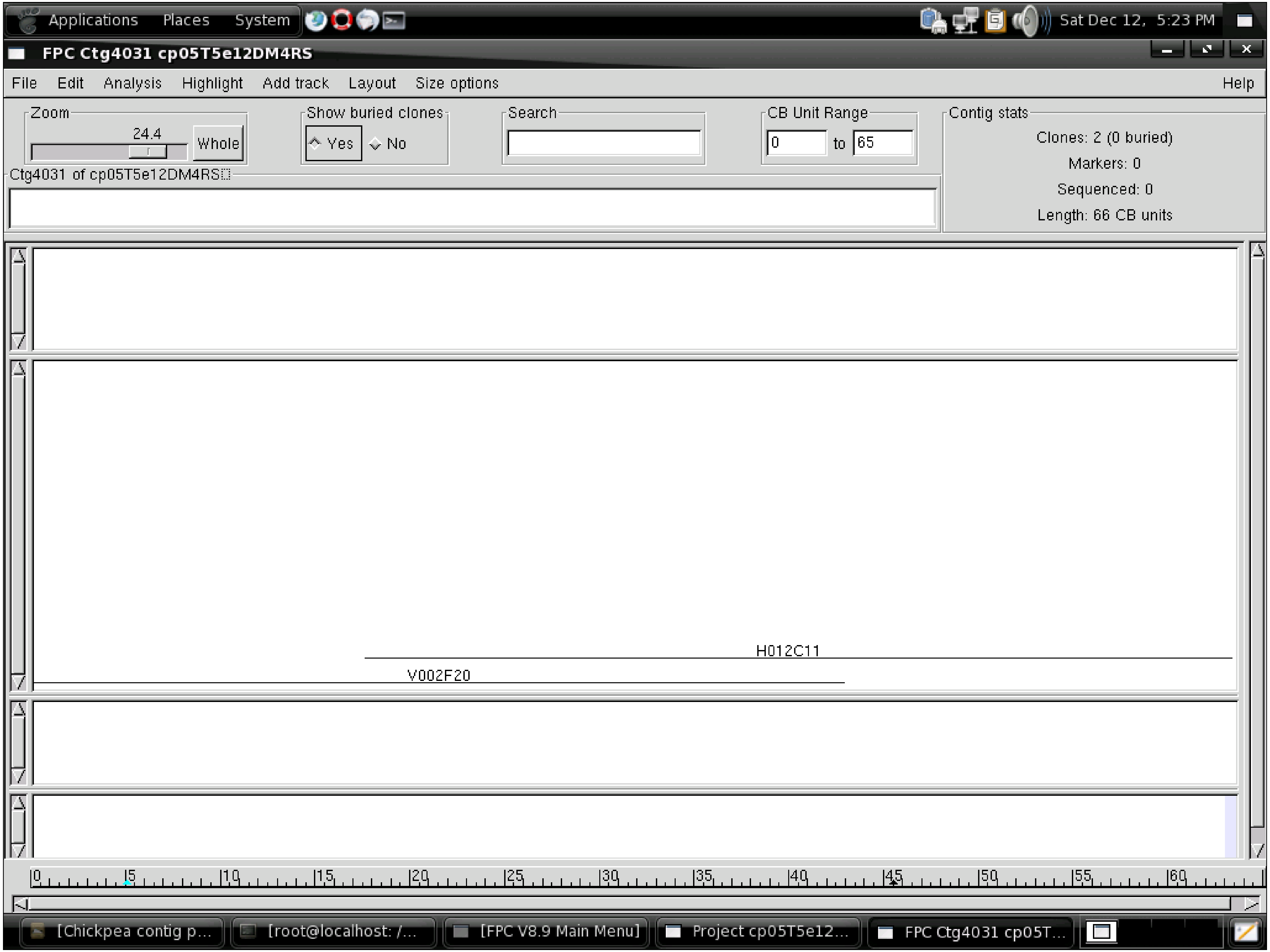
Applications Places System
Sat Dec 12, 5:23 PM
FPC Ctg4031 cp05T5e12DM4RS
File Edit Analysis Highlight Add track Layout Size options Help
Zoom
24.4
Whole
Show buried clones
Yes No
Search
CB Unit Range
0 to 65
Contig stats
Clones: 2 (0 buried)
Markers: 0
Sequenced: 0
Length: 66 CB units
Ctg4031 of cp05T5e12DM4RS
H012C11
V002F20
0 5 10 15 20 25 30 35 40 45 50 55 60
[Chickpea contig p...
[root@localhost: /...
[FPC V8.9 Main Menu]
Project cp05T5e12...
FPC Ctg4031 cp05T...

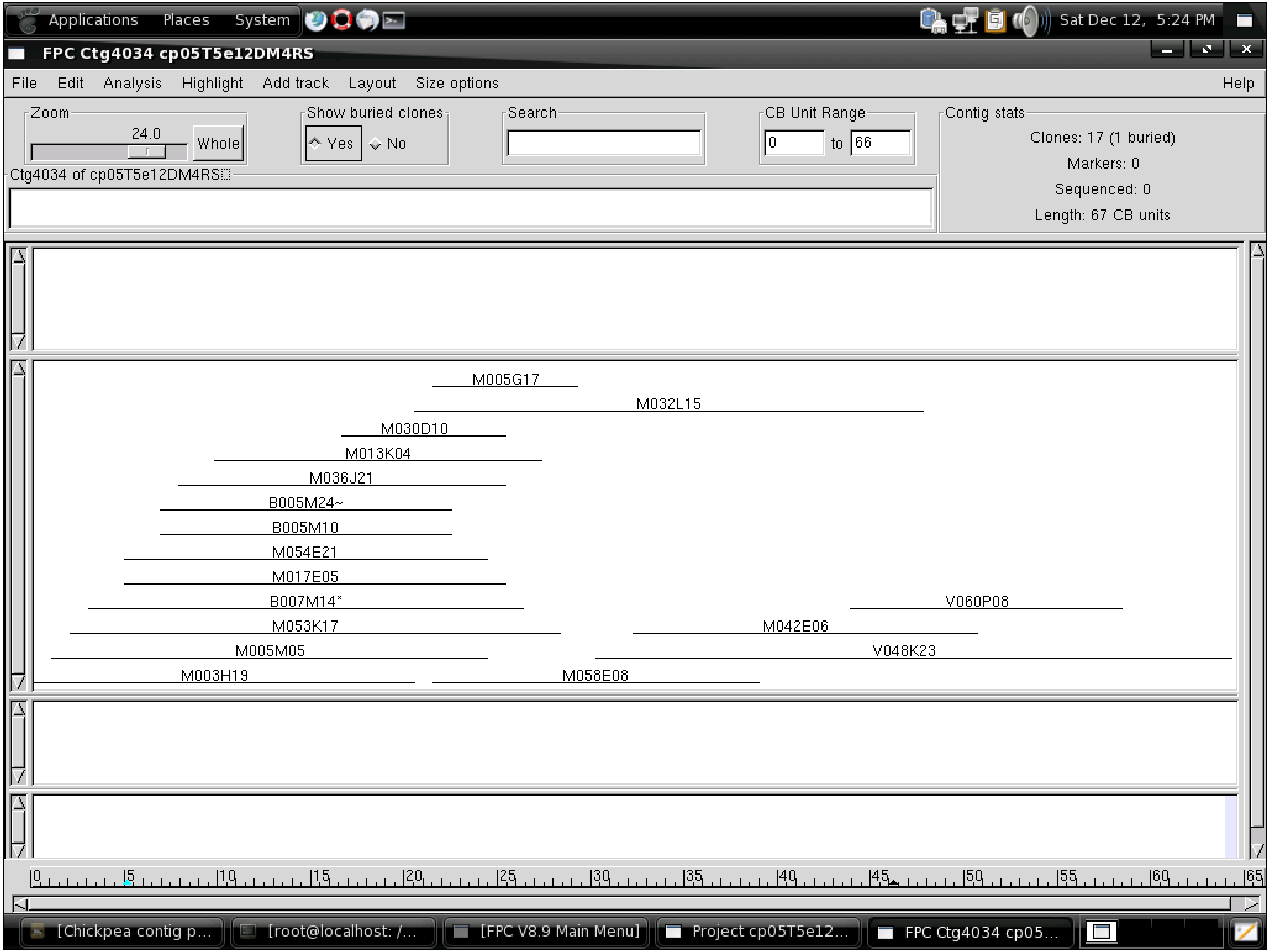
Applications Places System
Sat Dec 12, 5:24 PM
FPC Ctg4034 cp05T5e12DM4RS
File Edit Analysis Highlight Add track Layout Size options Help
Zoom
24.0
Whole
Show buried clones
Yes No
Search
CB Unit Range
0 to 66
Contig stats
Clones: 17 (1 buried)
Markers: 0
Sequenced: 0
Length: 67 CB units
Ctg4034 of cp05T5e12DM4RS
M005G17
M032L15
M030D10
M013K04
M036J21
B005M24~
B005M10
M054E21
M017E05
B007M14*
V060P08
M053K17
M042E06
M005M05
V048K23
M003H19
M058E08
0 5 10 15 20 25 30 35 40 45 50 55 60 65
[Chickpea contig p...
[root@localhost: /...
[FPC V8.9 Main Menu]
Project cp05T5e12...
FPC Ctg4034 cp05...

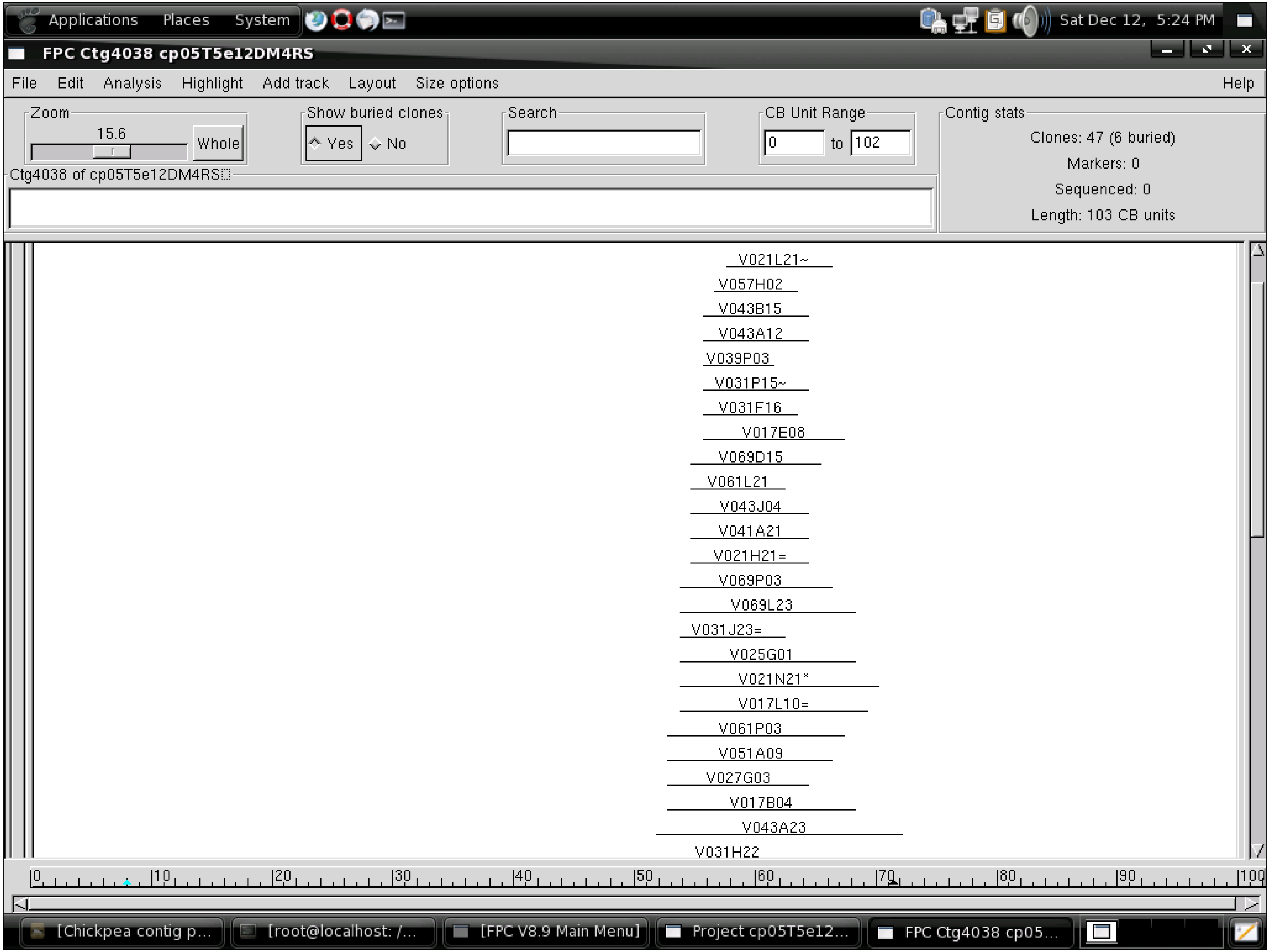
Applications Places System
Sat Dec 12, 5:24 PM
FPC Ctg4038 cp05T5e12DM4RS
File Edit Analysis Highlight Add track Layout Size options Help
Zoom
15.6
Whole
Show buried clones
Yes
No
Search
CB Unit Range
0 to 102
Contig stats
Clones: 47 (6 buried)
Markers: 0
Sequenced: 0
Length: 103 CB units
Ctg4038 of cp05T5e12DM4RS
V021L21~
V057H02
V043B15
V043A12
V039P03
V031P15~
V031F16
V017E08
V069D15
V061L21
V043J04
V041A21
V021H21=
V069P03
V069L23
V031J23=
V025G01
V021N21*
V017L10=
V061P03
V051A09
V027G03
V017B04
V043A23
V031H22
0 10 20 30 40 50 60 70 80 90 100
[Chickpea contig p...
[root@localhost: /...
[FPC V8.9 Main Menu]
Project cp05T5e12...
FPC Ctg4038 cp05...

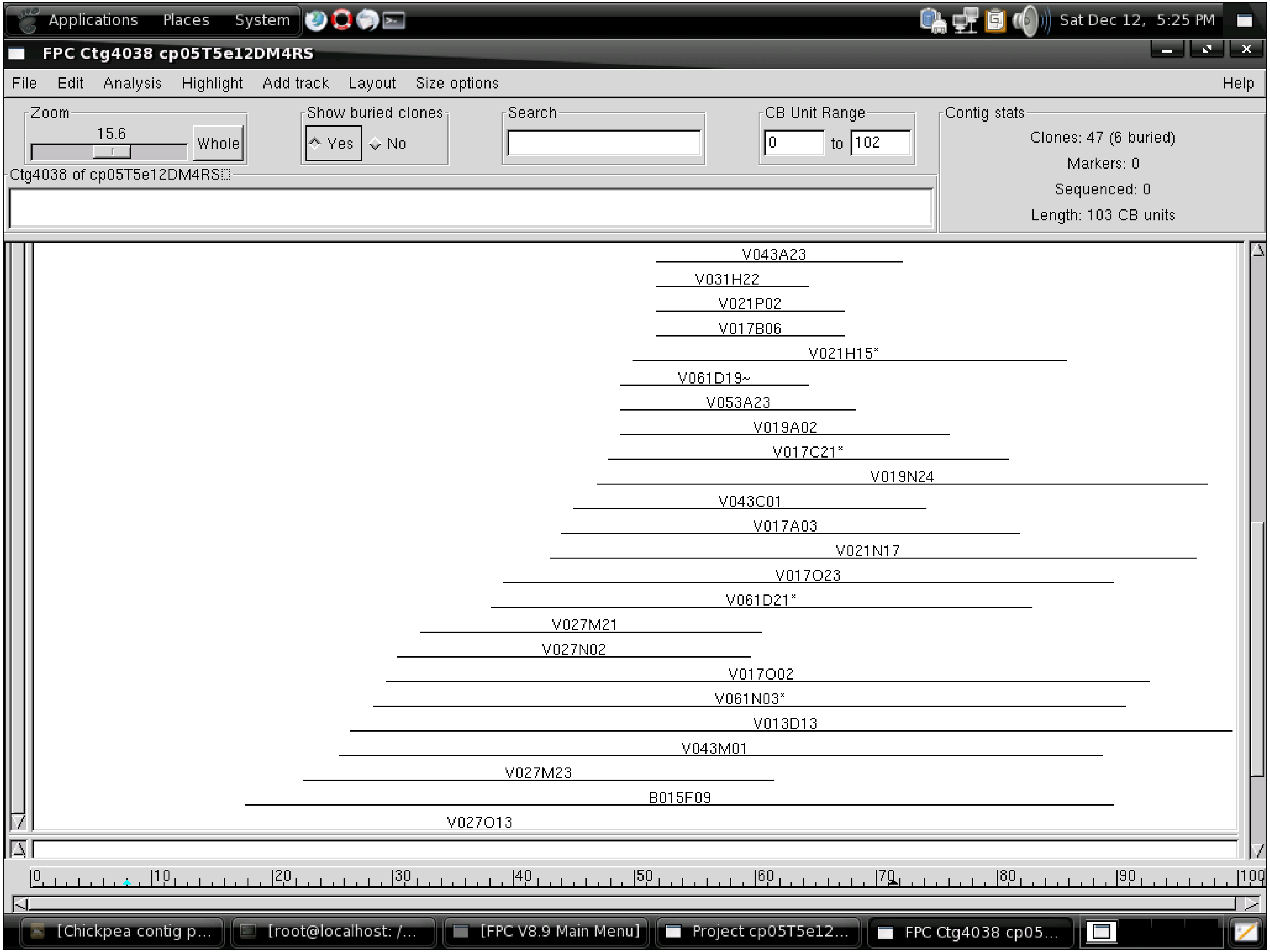
Applications
Places
System
Sat Dec 12, 5:25 PM
FPC Ctg4038 cp05T5e12DM4RS
File
Edit
Analysis
Highlight
Add track
Layout
Size options
Help
Zoom
15.6
Whole
Show buried clones
Yes
No
Search
CB Unit Range
0
to
102
Contig stats
Clones: 47 (6 buried)
Markers: 0
Sequenced: 0
Length: 103 CB units
Ctg4038 of cp05T5e12DM4RS
V043A23
V031H22
V021P02
V017B06
V021H15*
V061D19~
V053A23
V019A02
V017C21*
V019N24
V043C01
V017A03
V021N17
V017O23
V061D21*
V027M21
V027N02
V017O02
V061N03*
V013D13
V043M01
V027M23
B015F09
V027O13
0
10
20
30
40
50
60
70
80
90
100
[Chickpea contig p...
[root@localhost: /...
[FPC V8.9 Main Menu]
Project cp05T5e12...
FPC Ctg4038 cp05...

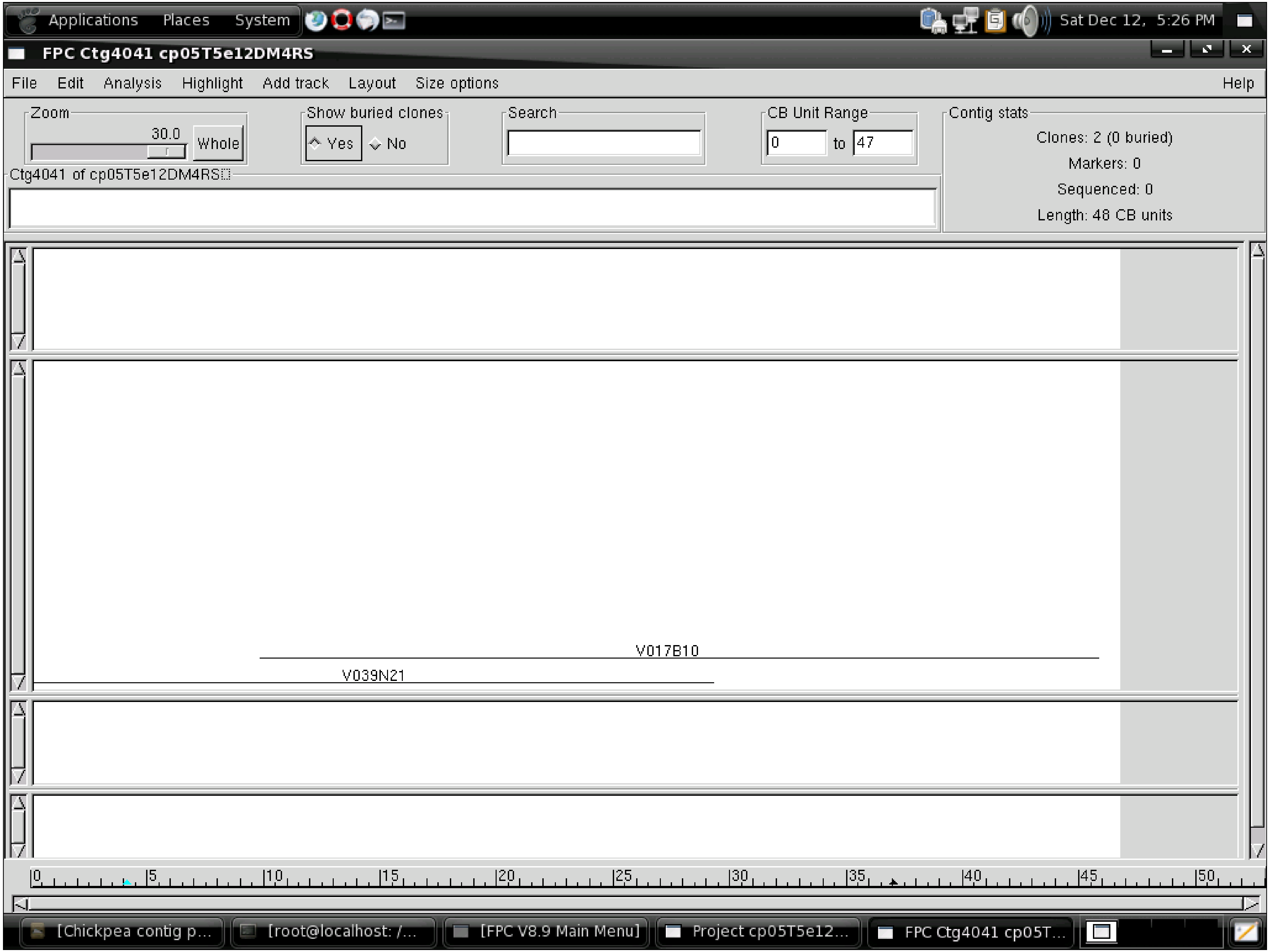

Applications Places System
Sat Dec 12, 5:26 PM
FPC Ctg4041 cp05T5e12DM4RS
File Edit Analysis Highlight Add track Layout Size options Help
Zoom
30.0
Whole
Show buried clones
Yes
No
Search
CB Unit Range
0
to
47
Contig stats
Clones: 2 (0 buried)
Markers: 0
Sequenced: 0
Length: 48 CB units
Ctg4041 of cp05T5e12DM4RS
V017B10
V039N21
0 5 10 15 20 25 30 35 40 45 50
[Chickpea contig p...
[root@localhost: /...
[FPC V8.9 Main Menu]
Project cp05T5e12...
FPC Ctg4041 cp05T...

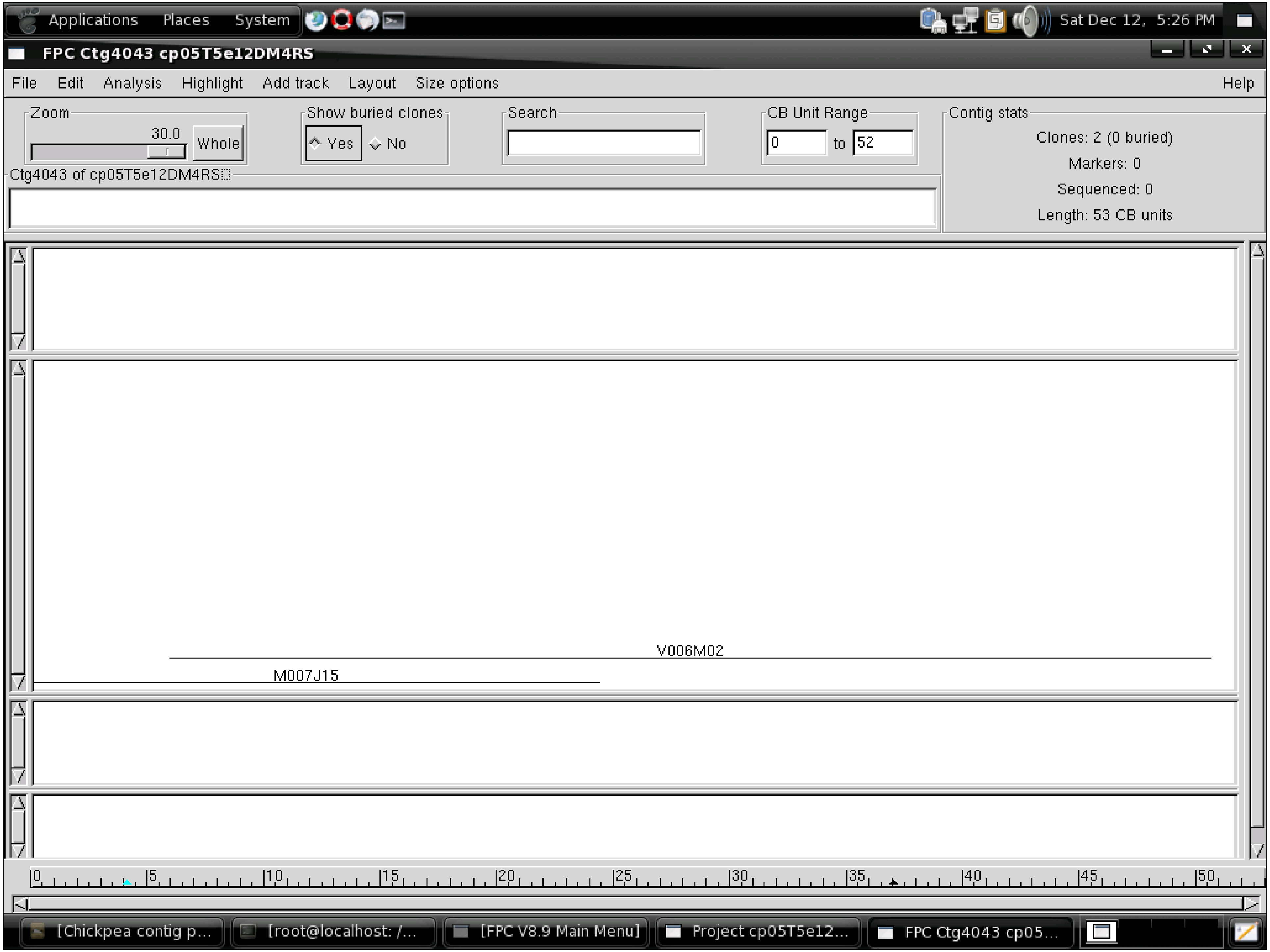
Applications
Places
System
Sat Dec 12, 5:26 PM
FPC Ctg4043 cp05T5e12DM4RS
File
Edit
Analysis
Highlight
Add track
Layout
Size options
Help
Zoom
30.0
Whole
Show buried clones
Yes
No
Search
CB Unit Range
0
to
52
Contig stats
Clones: 2 (0 buried)
Markers: 0
Sequenced: 0
Length: 53 CB units
Ctg4043 of cp05T5e12DM4RS
V006M02
M007J15
0
5
10
15
20
25
30
35
40
45
50
[Chickpea contig p...
[root@localhost: /...
[FPC V8.9 Main Menu]
Project cp05T5e12...
FPC Ctg4043 cp05...

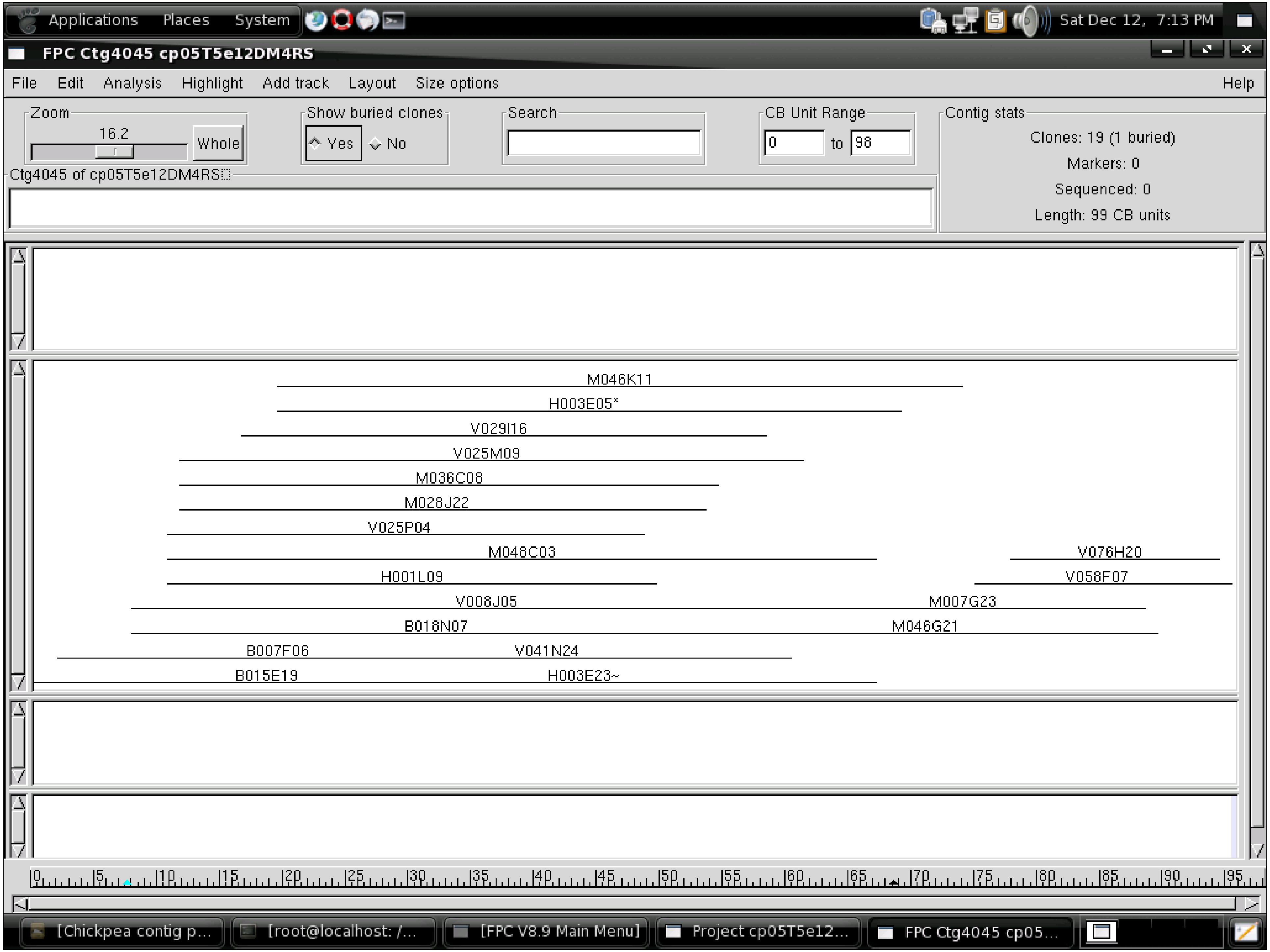
Applications Places System
Sat Dec 12, 7:13 PM
FPC Ctg4045 cp05T5e12DM4RS
File Edit Analysis Highlight Add track Layout Size options Help
Zoom
16.2
Whole
Show buried clones
Yes
No
Search
CB Unit Range
0 to 98
Contig stats
Clones: 19 (1 buried)
Markers: 0
Sequenced: 0
Length: 99 CB units
Ctg4045 of cp05T5e12DM4RS
M046K11
H003E05*
V029I16
V025M09
M036C08
M028J22
V025P04
M048C03
V076H20
H001L09
V058F07
V008J05
M007G23
B018N07
M046G21
B007F06
V041N24
B015E19
H003E23~
0 5 10 15 20 25 30 35 40 45 50 55 60 65 70 75 80 85 90 95
[Chickpea contig p...
[root@localhost: /...
[FPC V8.9 Main Menu]
Project cp05T5e12...
FPC Ctg4045 cp05...

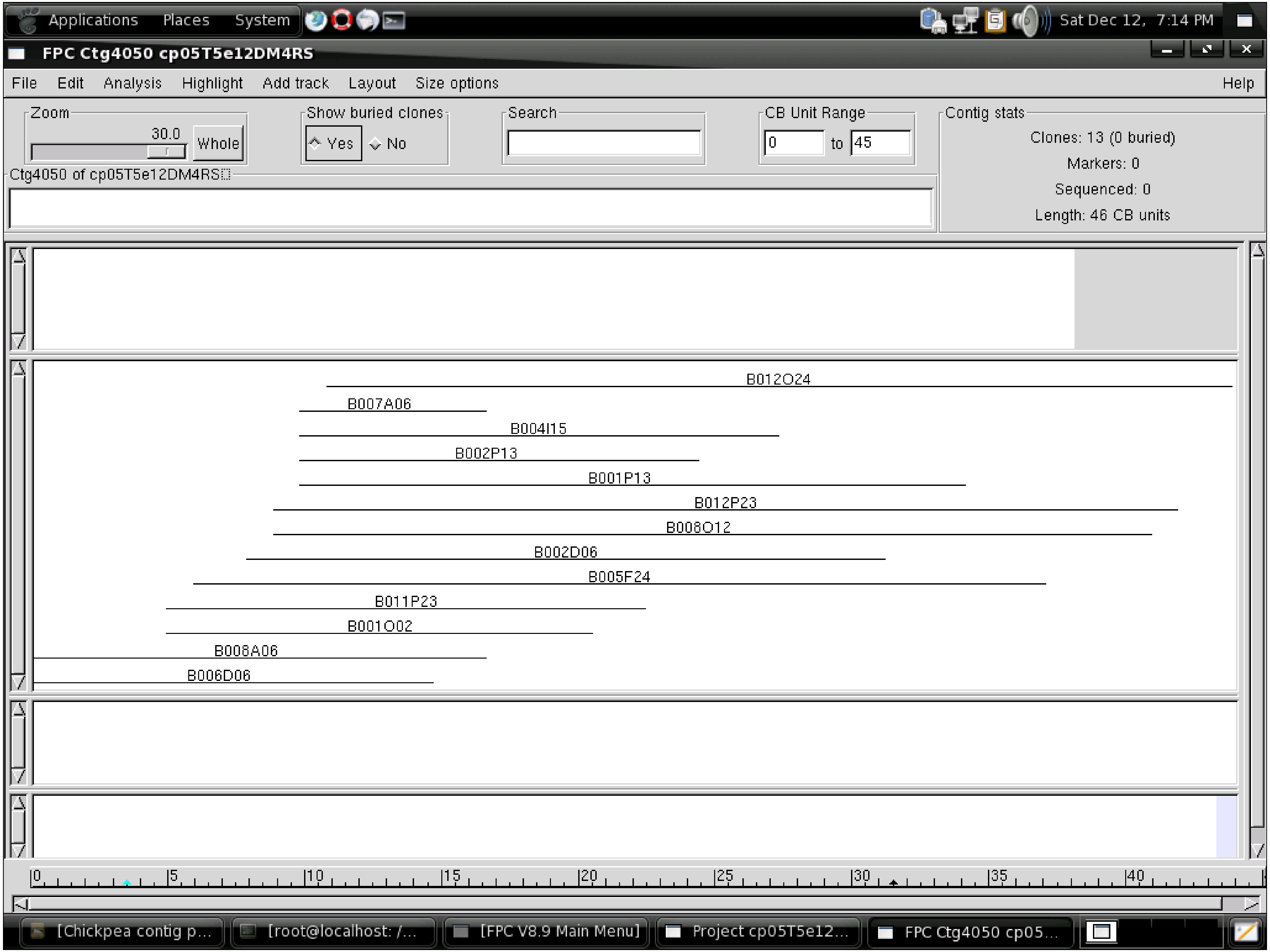
Applications
Places
System
Sat Dec 12, 7:14 PM
FPC Ctg4050 cp05T5e12DM4RS
File
Edit
Analysis
Highlight
Add track
Layout
Size options
Help
Zoom
30.0
Whole
Show buried clones
Yes
No
Search
CB Unit Range
0
to
45
Contig stats
Clones: 13 (0 buried)
Markers: 0
Sequenced: 0
Length: 46 CB units
Ctg4050 of cp05T5e12DM4RS
B012O24
B007A06
B004I15
B002P13
B001P13
B012P23
B008O12
B002D06
B005F24
B011P23
B001O02
B008A06
B006D06
0
5
10
15
20
25
30
35
40
[Chickpea contig p...
[root@localhost: /...
[FPC V8.9 Main Menu]
Project cp05T5e12...
FPC Ctg4050 cp05...

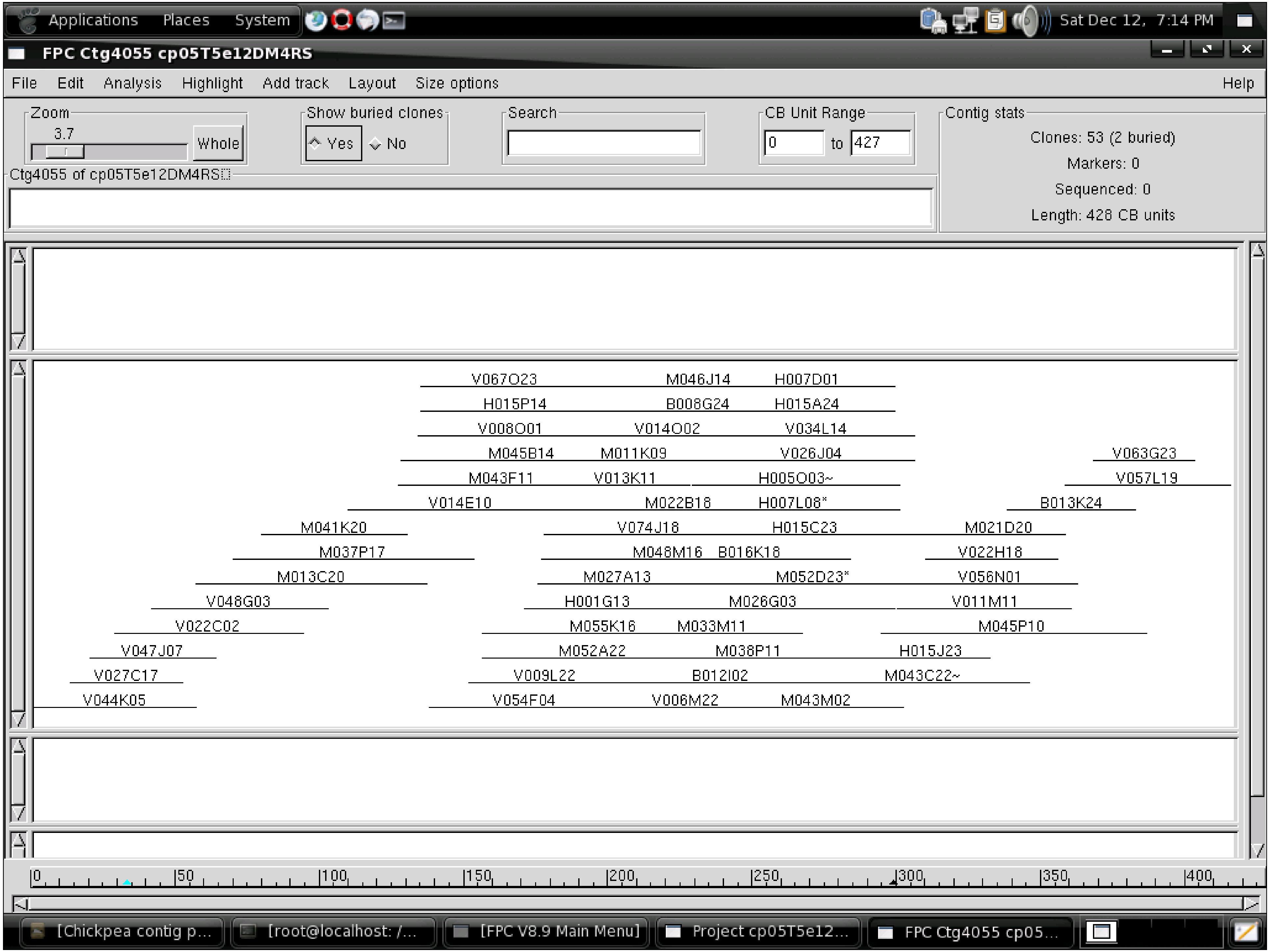
Applications Places System
Sat Dec 12, 7:14 PM
FPC Ctg4055 cp05T5e12DM4RS
File Edit Analysis Highlight Add track Layout Size options Help
Zoom
3.7
Whole
Show buried clones
Yes
No
Search
CB Unit Range
0
to
427
Contig stats
Clones: 53 (2 buried)
Markers: 0
Sequenced: 0
Length: 428 CB units
Ctg4055 of cp05T5e12DM4RS
V067O23
M046J14
H007D01
H015P14
B008G24
H015A24
V008O01
V014O02
V034L14
M045B14
M011K09
V026J04
V063G23
M043F11
V013K11
H005O03~
V057L19
V014E10
M022B18
H007L08*
B013K24
M041K20
V074J18
H015C23
M021D20
M037P17
M048M16
B016K18
V022H18
M013C20
M027A13
M052D23*
V056N01
V048G03
H001G13
M026G03
V011M11
V022C02
M055K16
M033M11
M045P10
V047J07
M052A22
M038P11
H015J23
V027C17
V009L22
B012I02
M043C22~
V044K05
V054F04
V006M22
M043M02
0
50
100
150
200
250
300
350
400
[Chickpea contig p...
[root@localhost: /...
[FPC V8.9 Main Menu]
Project cp05T5e12...
FPC Ctg4055 cp05...

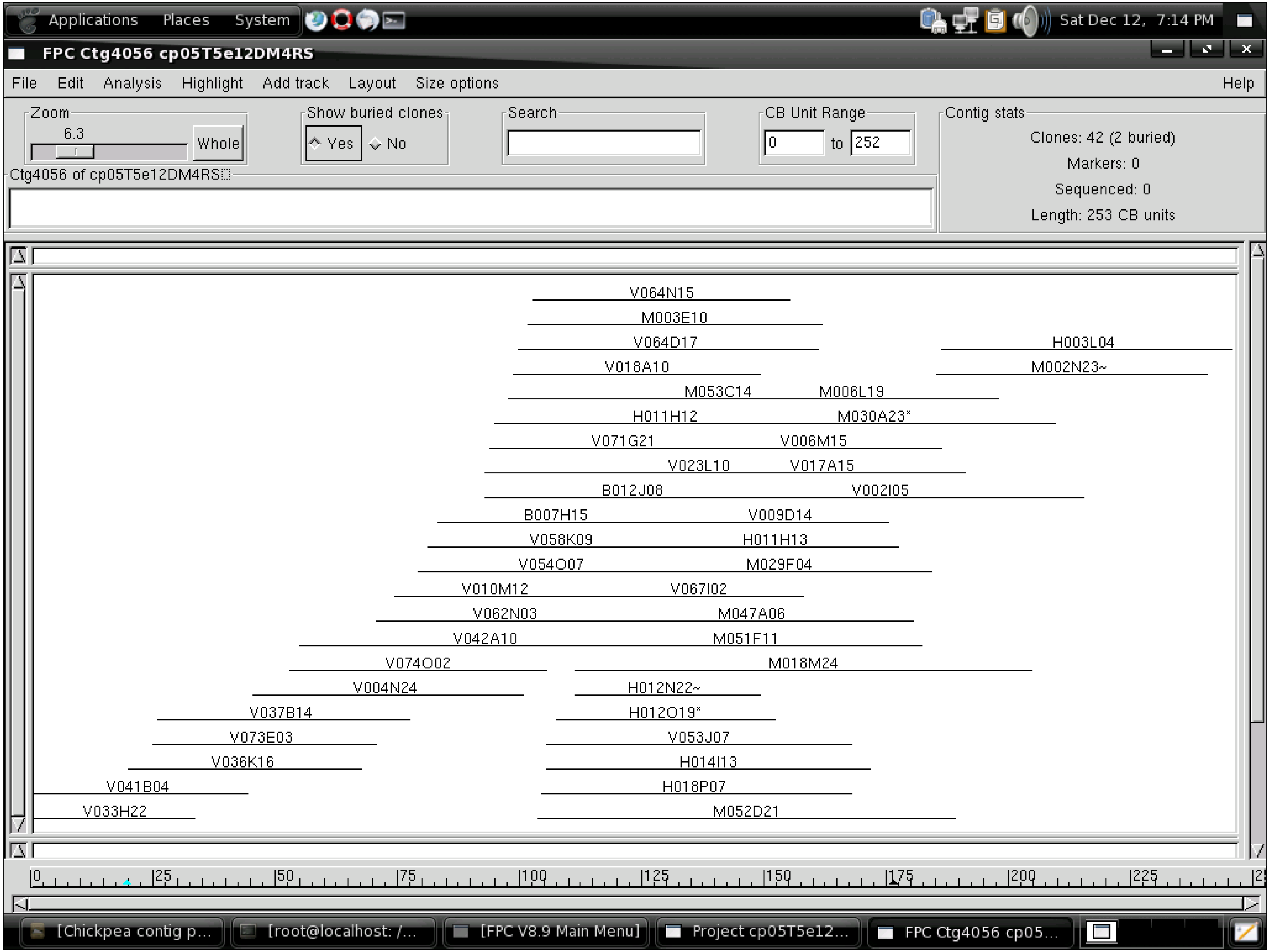
Applications Places System
Sat Dec 12, 7:14 PM
FPC Ctg4056 cp05T5e12DM4RS
File Edit Analysis Highlight Add track Layout Size options Help
Zoom
6.3
Whole
Show buried clones
Yes No
Search
CB Unit Range
0 to 252
Contig stats
Clones: 42 (2 buried)
Markers: 0
Sequenced: 0
Length: 253 CB units
Ctg4056 of cp05T5e12DM4RS
V064N15
M003E10
V064D17
H003L04
V018A10
M002N23~
M053C14
M006L19
H011H12
M030A23*
V071G21
V006M15
V023L10
V017A15
B012J08
V002I05
B007H15
V009D14
V058K09
H011H13
V054O07
M029F04
V010M12
V067I02
V062N03
M047A06
V042A10
M051F11
V074O02
M018M24
V004N24
H012N22~
V037B14
H012O19*
V073E03
V053J07
V036K16
H014I13
V041B04
H018P07
V033H22
M052D21
0 25 50 75 100 125 150 175 200 225
[Chickpea contig p...
[root@localhost: /...
[FPC V8.9 Main Menu]
Project cp05T5e12...
FPC Ctg4056 cp05...

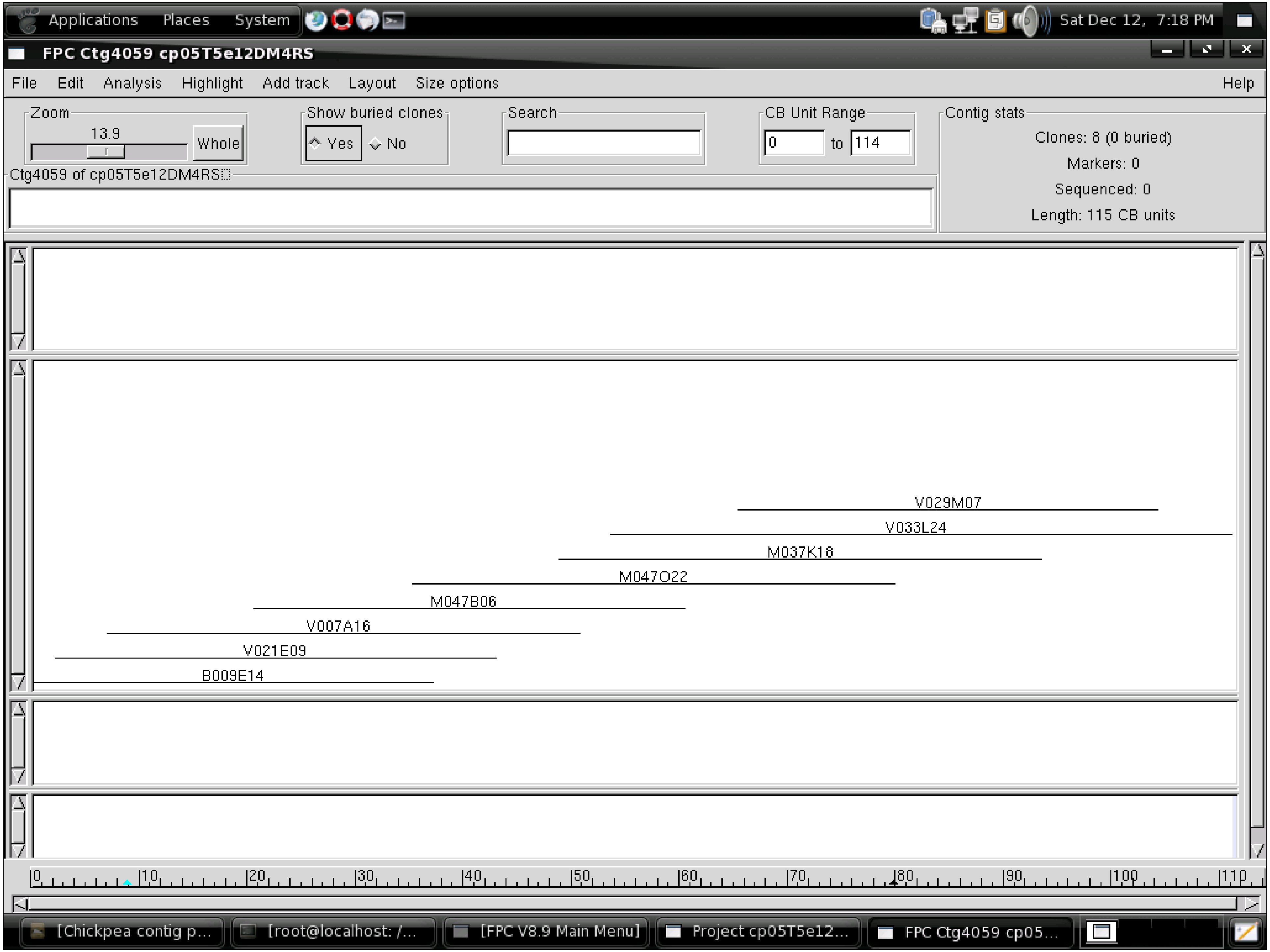
Applications Places System
Sat Dec 12, 7:18 PM
FPC Ctg4059 cp05T5e12DM4RS
File Edit Analysis Highlight Add track Layout Size options Help
Zoom
13.9
Whole
Show buried clones
Yes No
Search
CB Unit Range
0 to 114
Contig stats
Clones: 8 (0 buried)
Markers: 0
Sequenced: 0
Length: 115 CB units
Ctg4059 of cp05T5e12DM4RS
V029M07
V033L24
M037K18
M047O22
M047B06
V007A16
V021E09
B009E14
0 10 20 30 40 50 60 70 80 90 100 110
[Chickpea contig p...
[root@localhost: /...
[FPC V8.9 Main Menu]
Project cp05T5e12...
FPC Ctg4059 cp05...

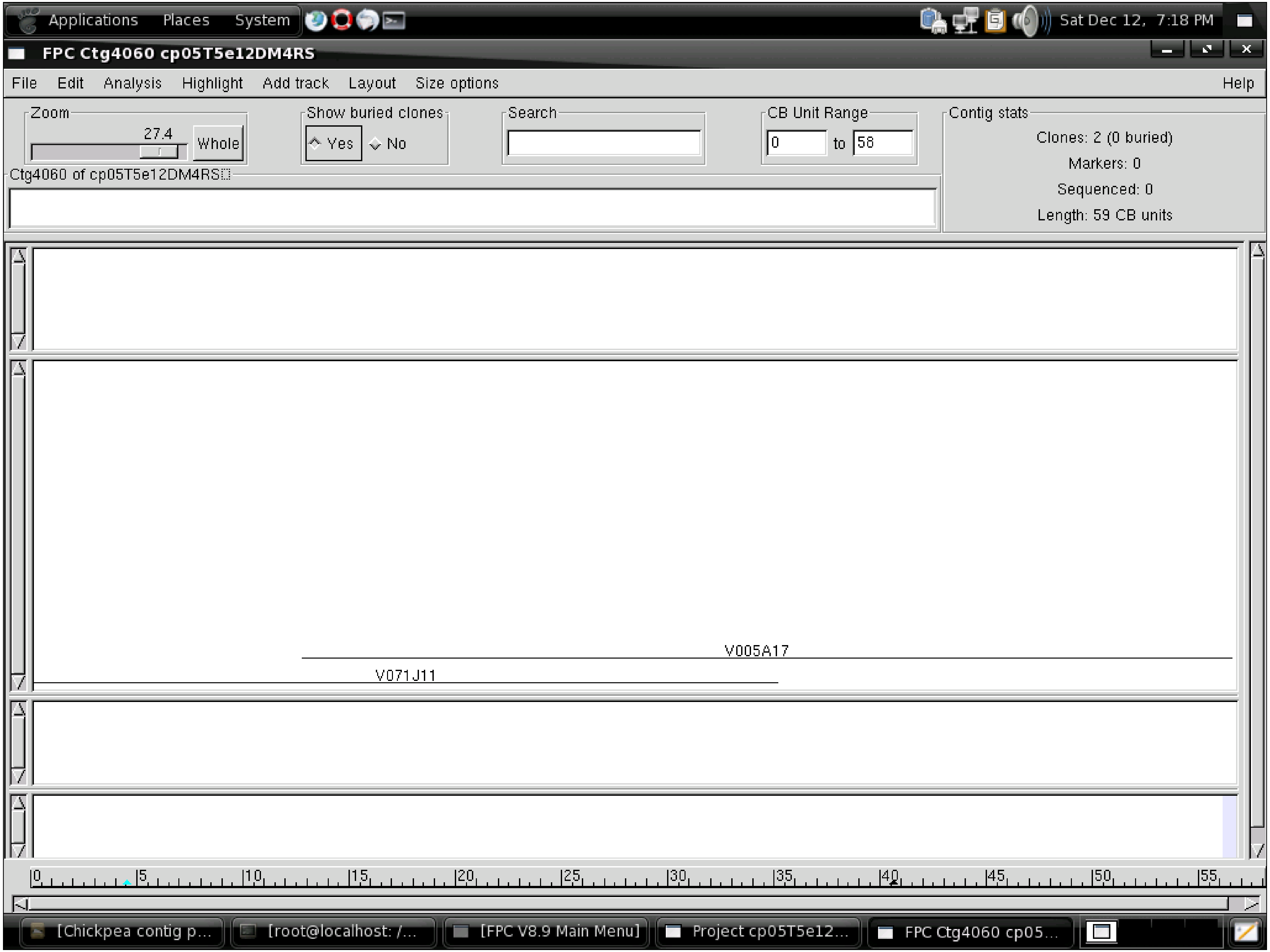
Applications Places System
Sat Dec 12, 7:18 PM
FPC Ctg4060 cp05T5e12DM4RS
File Edit Analysis Highlight Add track Layout Size options Help
Zoom
27.4
Whole
Show buried clones
Yes
No
Search
CB Unit Range
0
to
58
Contig stats
Clones: 2 (0 buried)
Markers: 0
Sequenced: 0
Length: 59 CB units
Ctg4060 of cp05T5e12DM4RS
V005A17
V071J11
0 5 10 15 20 25 30 35 40 45 50 55
[Chickpea contig p...
[root@localhost: /...
[FPC V8.9 Main Menu]
Project cp05T5e12...
FPC Ctg4060 cp05...

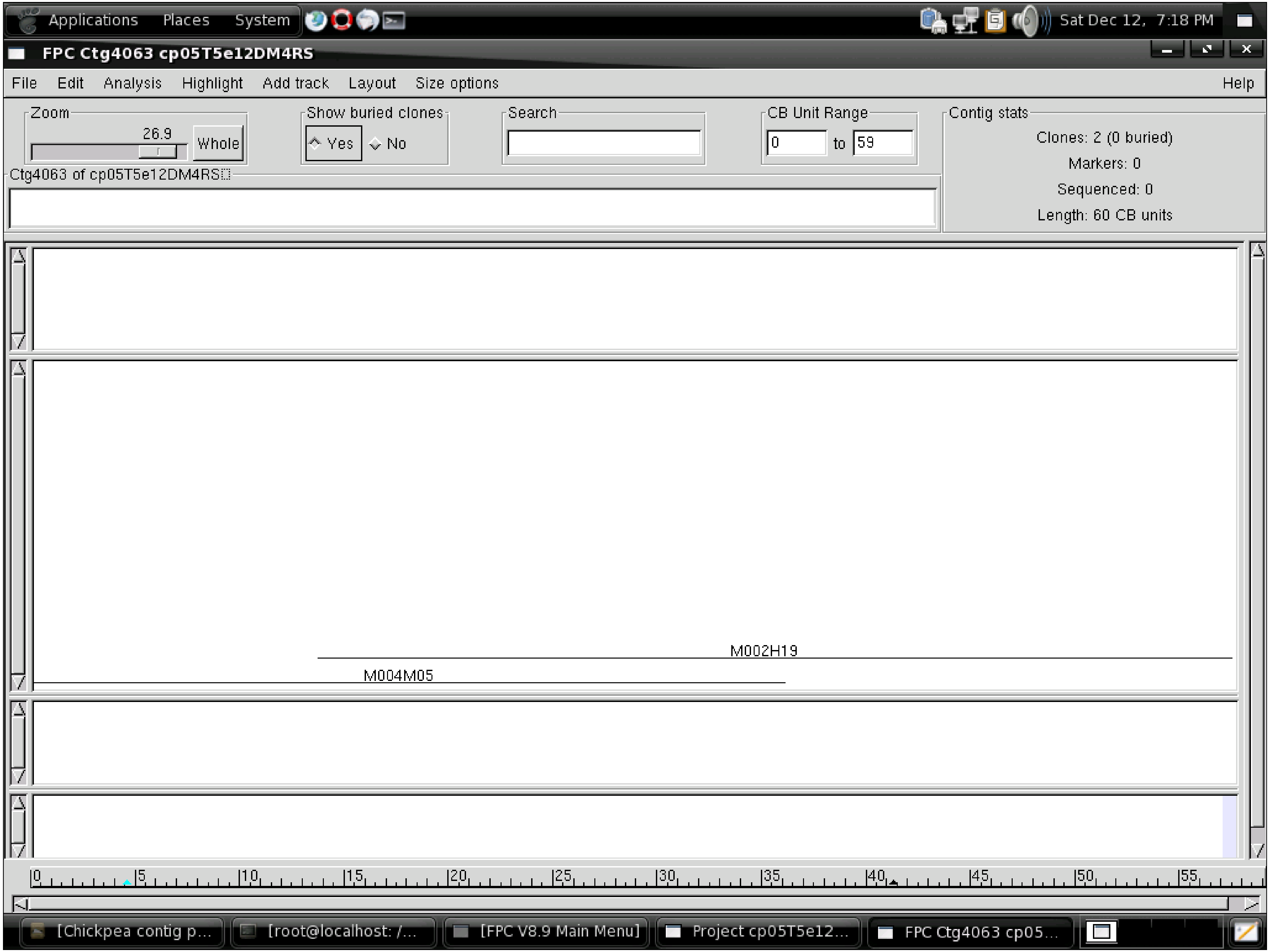
Applications Places System
Sat Dec 12, 7:18 PM
FPC Ctg4063 cp05T5e12DM4RS
File Edit Analysis Highlight Add track Layout Size options Help
Zoom
26.9
Whole
Show buried clones
Yes
No
Search
CB Unit Range
0
to
59
Contig stats
Clones: 2 (0 buried)
Markers: 0
Sequenced: 0
Length: 60 CB units
Ctg4063 of cp05T5e12DM4RS
M002H19
M004M05
0 5 10 15 20 25 30 35 40 45 50 55
[Chickpea contig p...
[root@localhost: /...
[FPC V8.9 Main Menu]
Project cp05T5e12...
FPC Ctg4063 cp05...

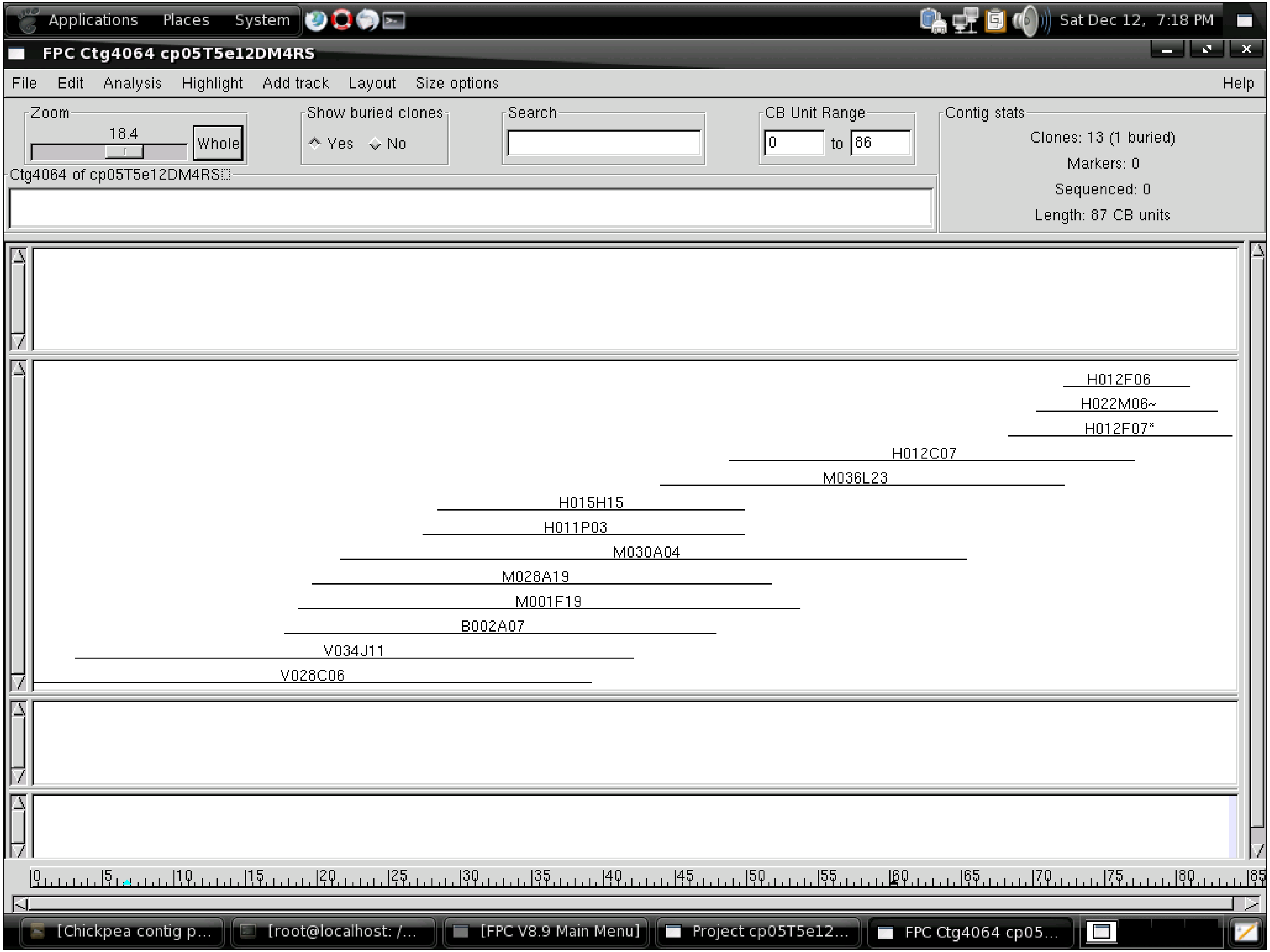
Applications Places System
Sat Dec 12, 7:18 PM
FPC Ctg4064 cp05T5e12DM4RS
File Edit Analysis Highlight Add track Layout Size options Help
Zoom
18.4
Whole
Show buried clones
Yes No
Search
CB Unit Range
0 to 86
Contig stats
Clones: 13 (1 buried)
Markers: 0
Sequenced: 0
Length: 87 CB units
Ctg4064 of cp05T5e12DM4RS
H012F06
H022M06~
H012F07*
H012C07
M036L23
H015H15
H011P03
M030A04
M028A19
M001F19
B002A07
V034J11
V028C06
0 5 10 15 20 25 30 35 40 45 50 55 60 65 70 75 80 85
[Chickpea contig p...
[root@localhost: /...
[FPC V8.9 Main Menu]
Project cp05T5e12...
FPC Ctg4064 cp05...

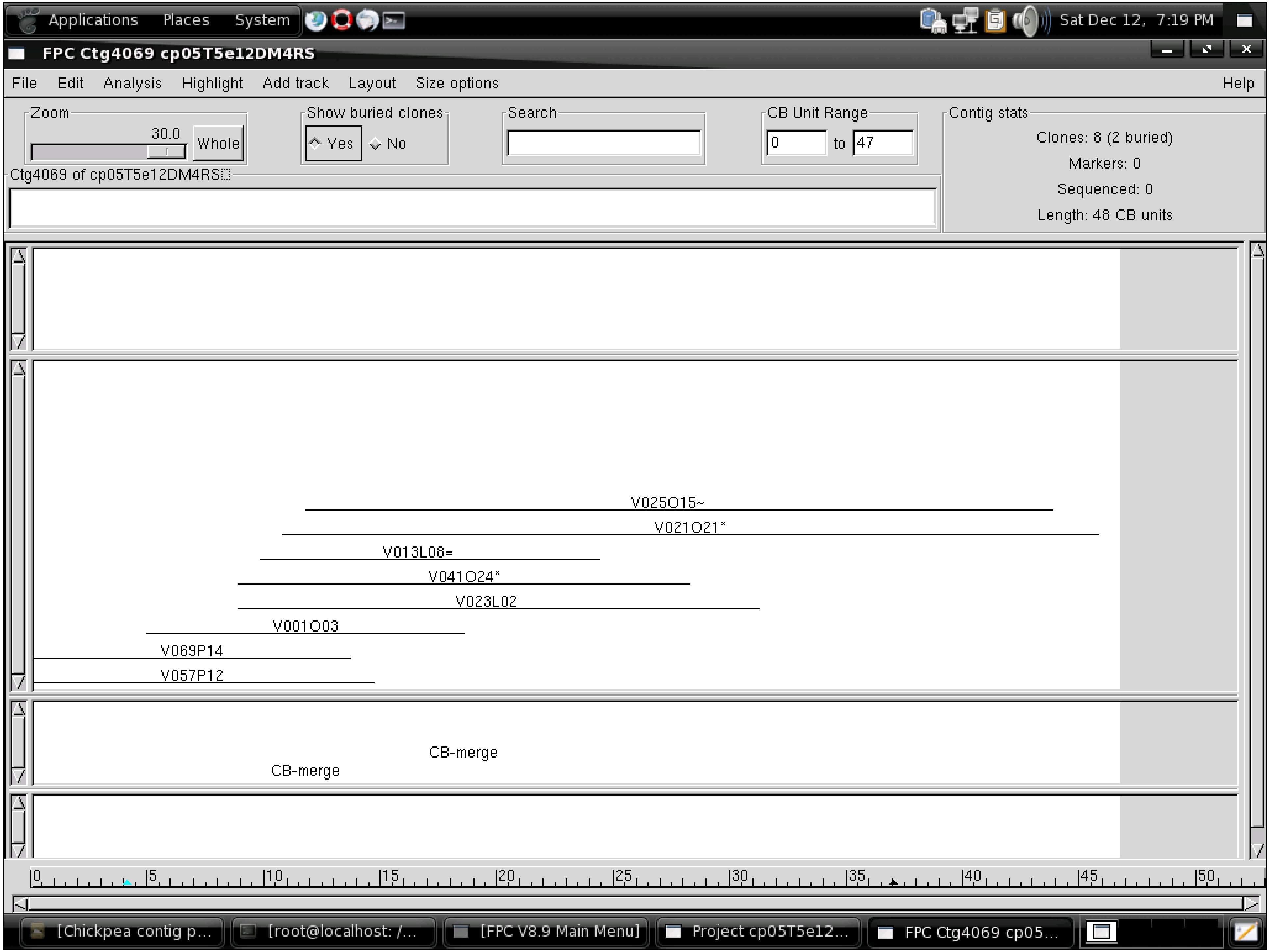
Applications Places System
Sat Dec 12, 7:19 PM
FPC Ctg4069 cp05T5e12DM4RS
File Edit Analysis Highlight Add track Layout Size options Help
Zoom
30.0
Whole
Show buried clones
Yes No
Search
CB Unit Range
0 to 47
Contig stats
Clones: 8 (2 buried)
Markers: 0
Sequenced: 0
Length: 48 CB units
Ctg4069 of cp05T5e12DM4RS
V025O15~
V021O21*
V013L08=
V041O24*
V023L02
V001O03
V069P14
V057P12
CB-merge
CB-merge
0 5 10 15 20 25 30 35 40 45 50
[Chickpea contig p...
[root@localhost: /...
[FPC V8.9 Main Menu]
Project cp05T5e12...
FPC Ctg4069 cp05...

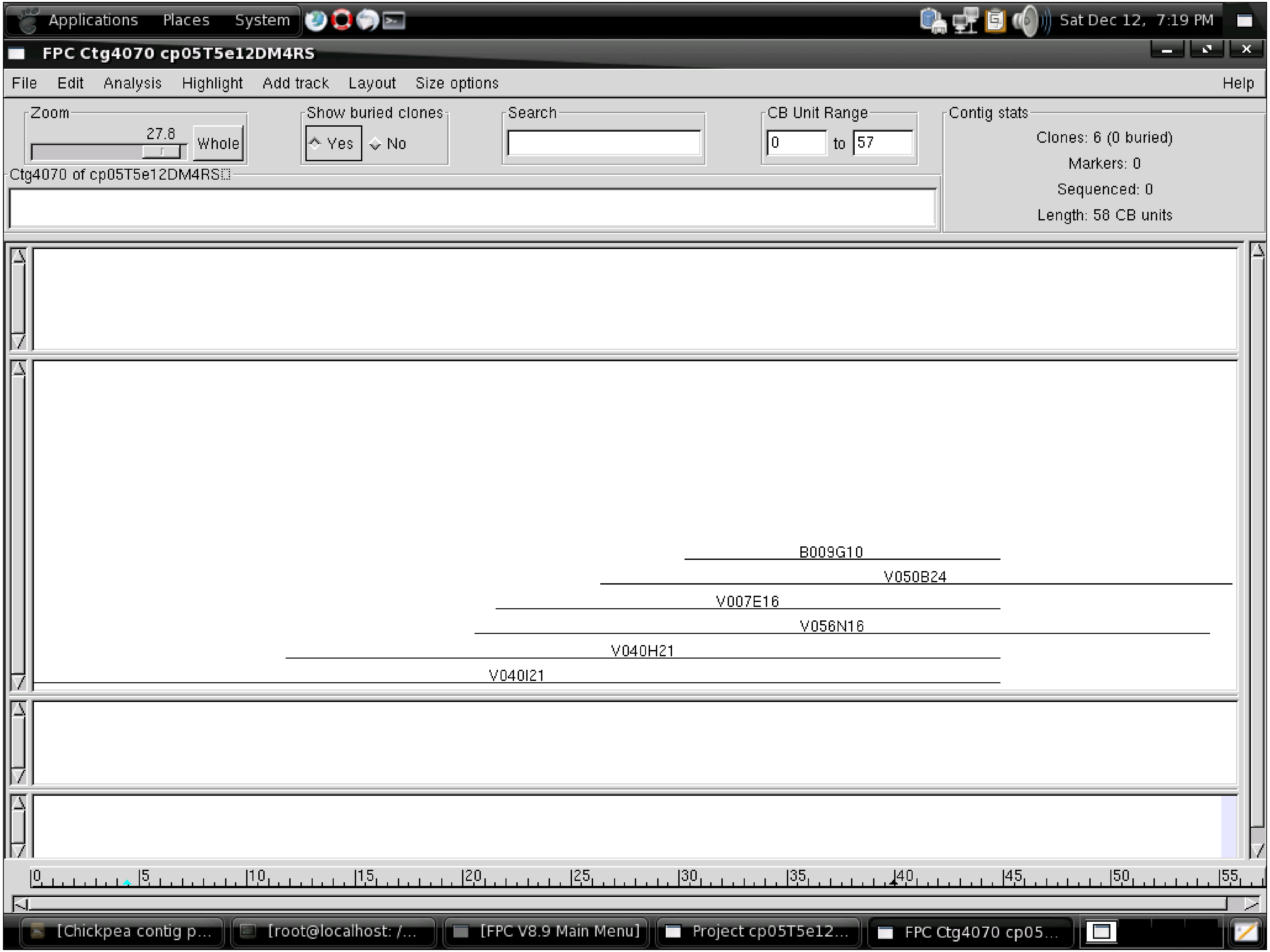
Applications Places System
Sat Dec 12, 7:19 PM
FPC Ctg4070 cp05T5e12DM4RS
File Edit Analysis Highlight Add track Layout Size options Help
Zoom
27.8
Whole
Show buried clones
Yes
No
Search
CB Unit Range
0 to 57
Contig stats
Clones: 6 (0 buried)
Markers: 0
Sequenced: 0
Length: 58 CB units
Ctg4070 of cp05T5e12DM4RS
B009G10
V050B24
V007E16
V056N16
V040H21
V040I21
0 5 10 15 20 25 30 35 40 45 50 55
[Chickpea contig p...
[root@localhost: /...
[FPC V8.9 Main Menu]
Project cp05T5e12...
FPC Ctg4070 cp05...

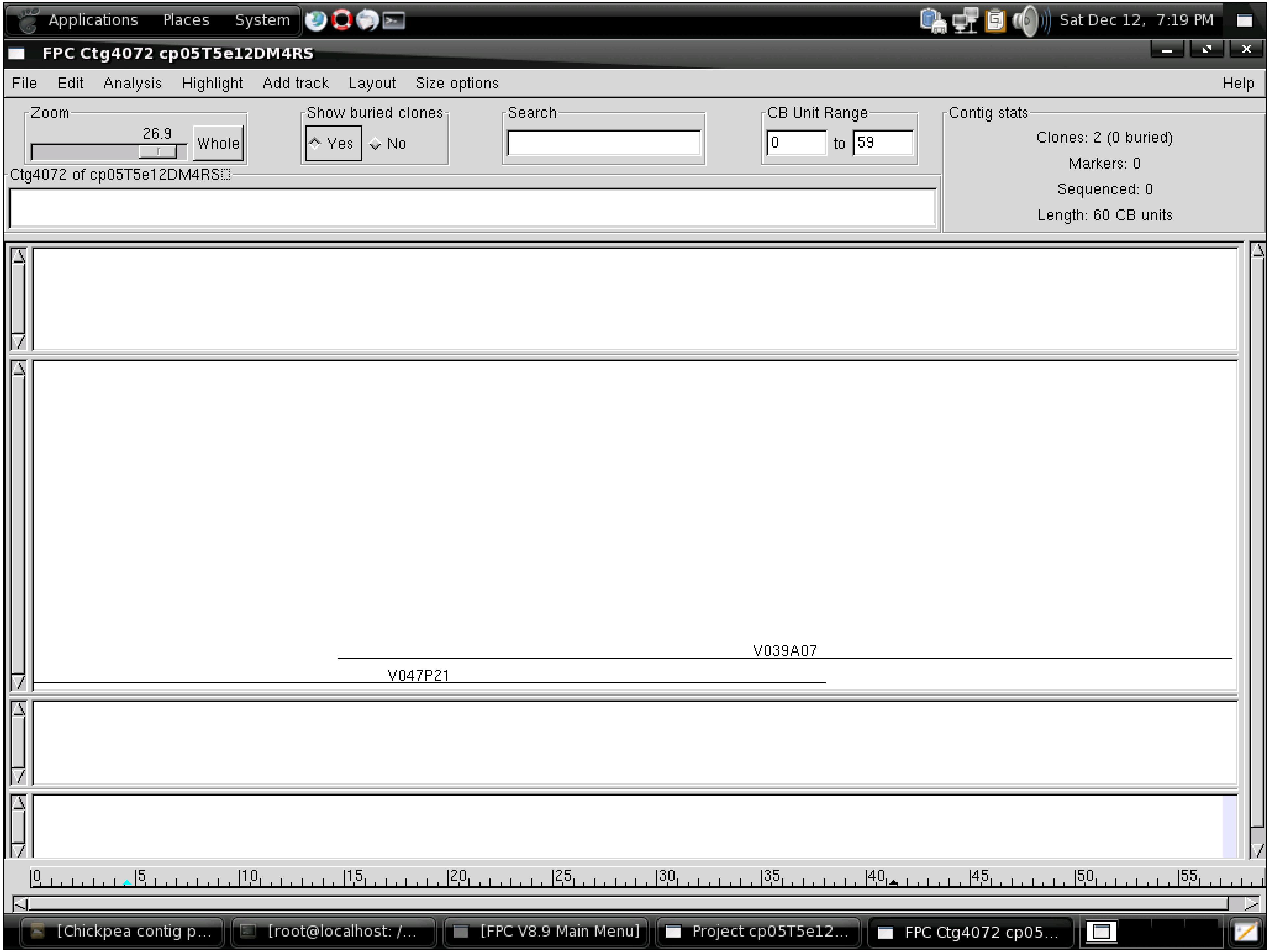

Applications
Places
System
Sat Dec 12, 7:19 PM
FPC Ctg4072 cp05T5e12DM4RS
File
Edit
Analysis
Highlight
Add track
Layout
Size options
Help
Zoom
26.9
Whole
Show buried clones
Yes
No
Search
CB Unit Range
0
to
59
Contig stats
Clones: 2 (0 buried)
Markers: 0
Sequenced: 0
Length: 60 CB units
Ctg4072 of cp05T5e12DM4RS
V039A07
V047P21
0
5
10
15
20
25
30
35
40
45
50
55
[Chickpea contig p...
[root@localhost: /...
[FPC V8.9 Main Menu]
Project cp05T5e12...
FPC Ctg4072 cp05...

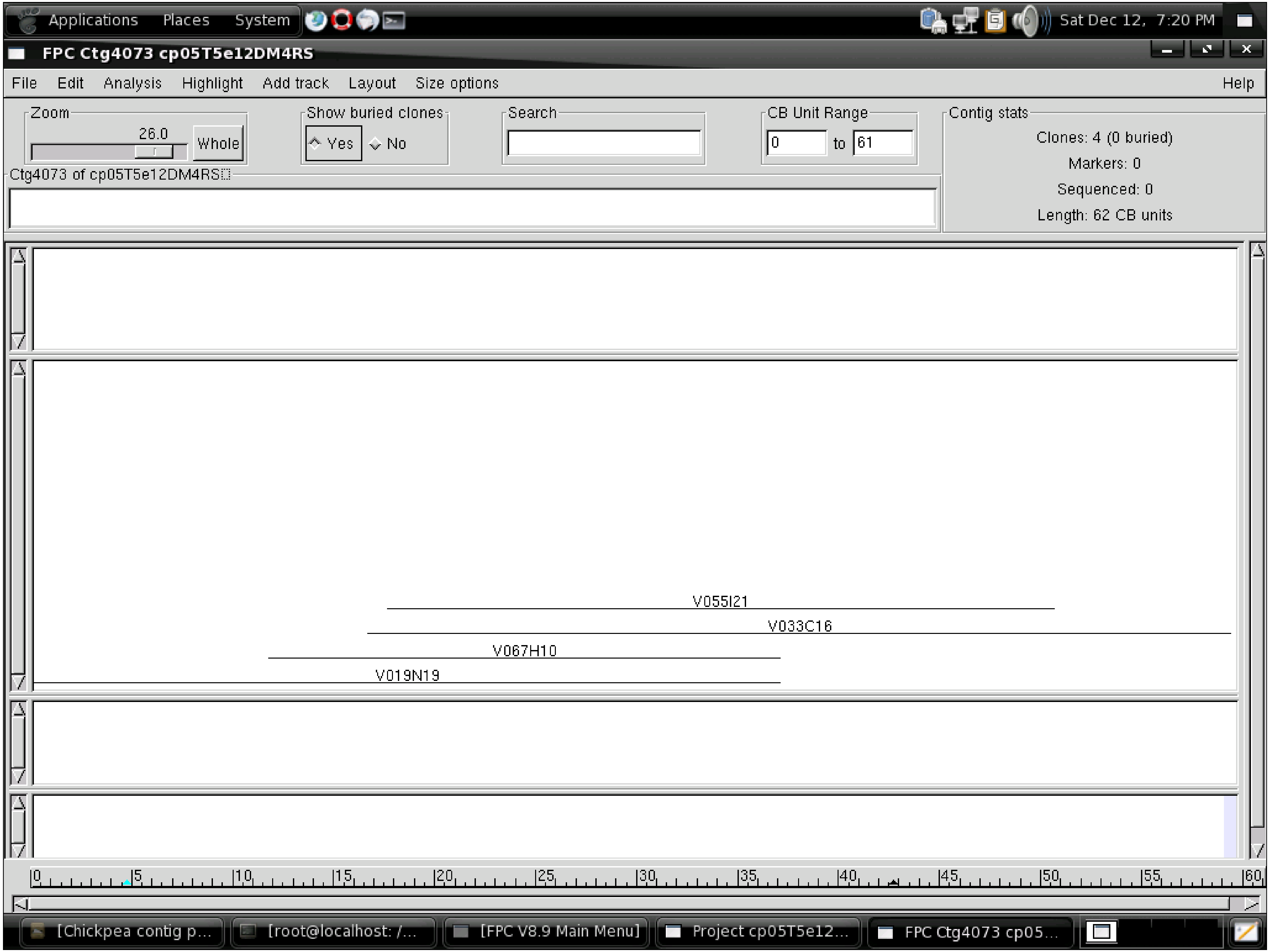
Applications Places System
Sat Dec 12, 7:20 PM
FPC Ctg4073 cp05T5e12DM4RS
File Edit Analysis Highlight Add track Layout Size options Help
Zoom
26.0
Whole
Show buried clones
Yes
No
Search
CB Unit Range
0
to
61
Contig stats
Clones: 4 (0 buried)
Markers: 0
Sequenced: 0
Length: 62 CB units
Ctg4073 of cp05T5e12DM4RS
V055I21
V033C16
V067H10
V019N19
0 5 10 15 20 25 30 35 40 45 50 55 60
[Chickpea contig p...
[root@localhost: /...
[FPC V8.9 Main Menu]
Project cp05T5e12...
FPC Ctg4073 cp05...

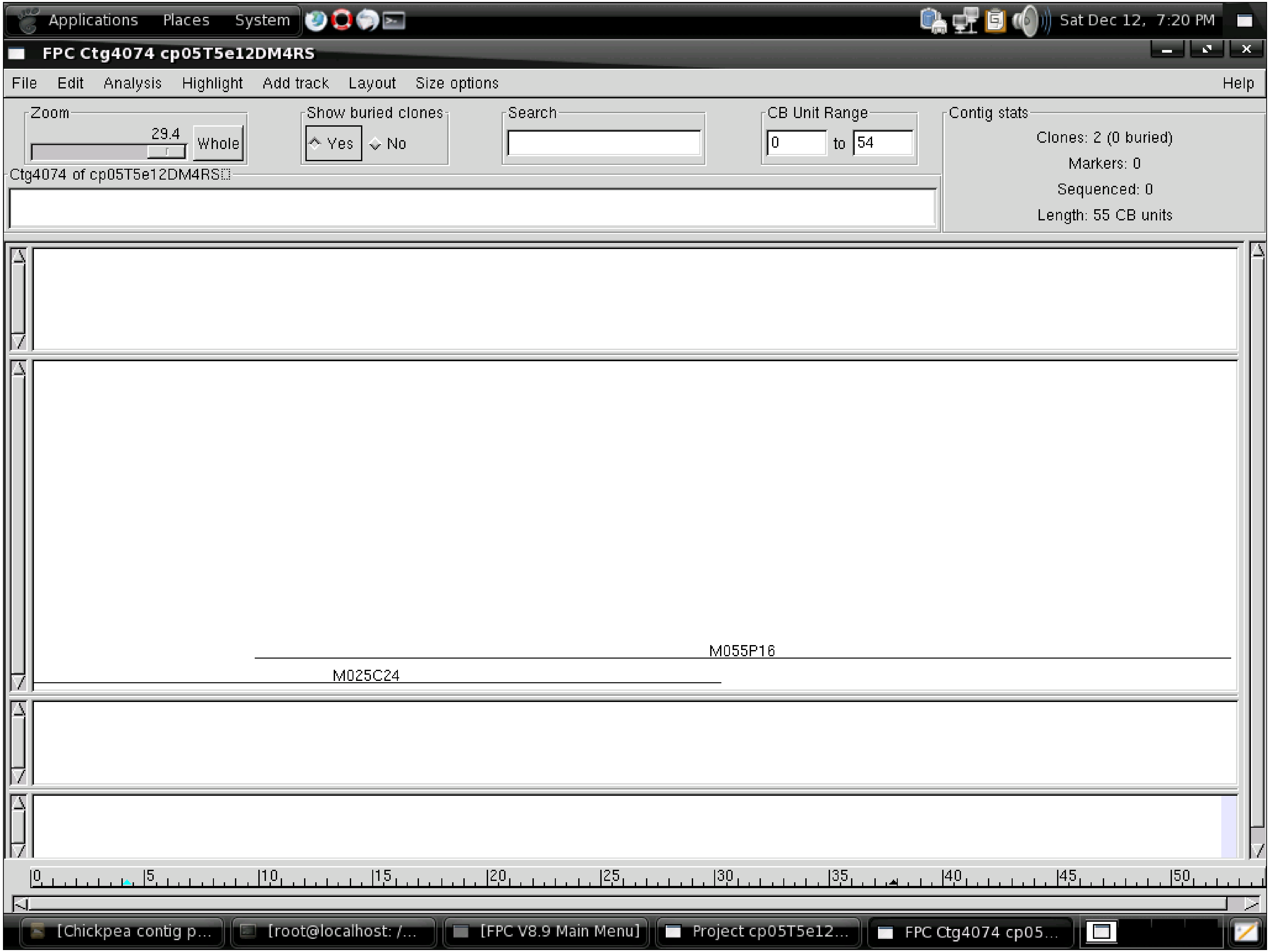
Applications Places System
Sat Dec 12, 7:20 PM
FPC Ctg4074 cp05T5e12DM4RS
File Edit Analysis Highlight Add track Layout Size options Help
Zoom
29.4
Whole
Show buried clones
Yes
No
Search
CB Unit Range
0
to
54
Contig stats
Clones: 2 (0 buried)
Markers: 0
Sequenced: 0
Length: 55 CB units
Ctg4074 of cp05T5e12DM4RS
M055P16
M025C24
0 5 10 15 20 25 30 35 40 45 50
[Chickpea contig p...
[root@localhost: /...
[FPC V8.9 Main Menu]
Project cp05T5e12...
FPC Ctg4074 cp05...

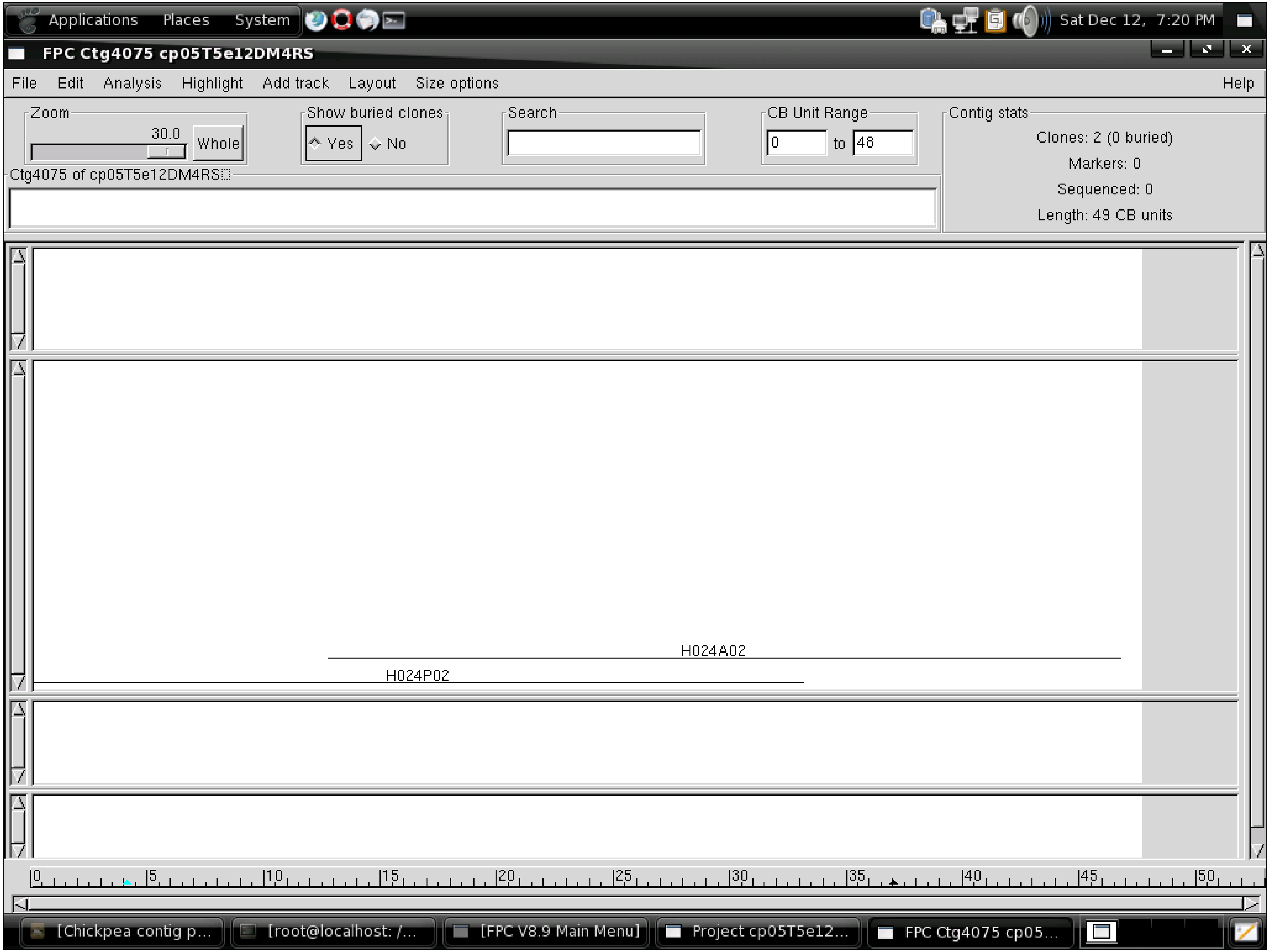
Applications Places System
Sat Dec 12, 7:20 PM
FPC Ctg4075 cp05T5e12DM4RS
File Edit Analysis Highlight Add track Layout Size options Help
Zoom
30.0
Whole
Show buried clones
Yes
No
Search
CB Unit Range
0
to
48
Contig stats
Clones: 2 (0 buried)
Markers: 0
Sequenced: 0
Length: 49 CB units
Ctg4075 of cp05T5e12DM4RS
H024A02
H024P02
0 5 10 15 20 25 30 35 40 45 50
[Chickpea contig p...
[root@localhost: /...
[FPC V8.9 Main Menu]
Project cp05T5e12...
FPC Ctg4075 cp05...

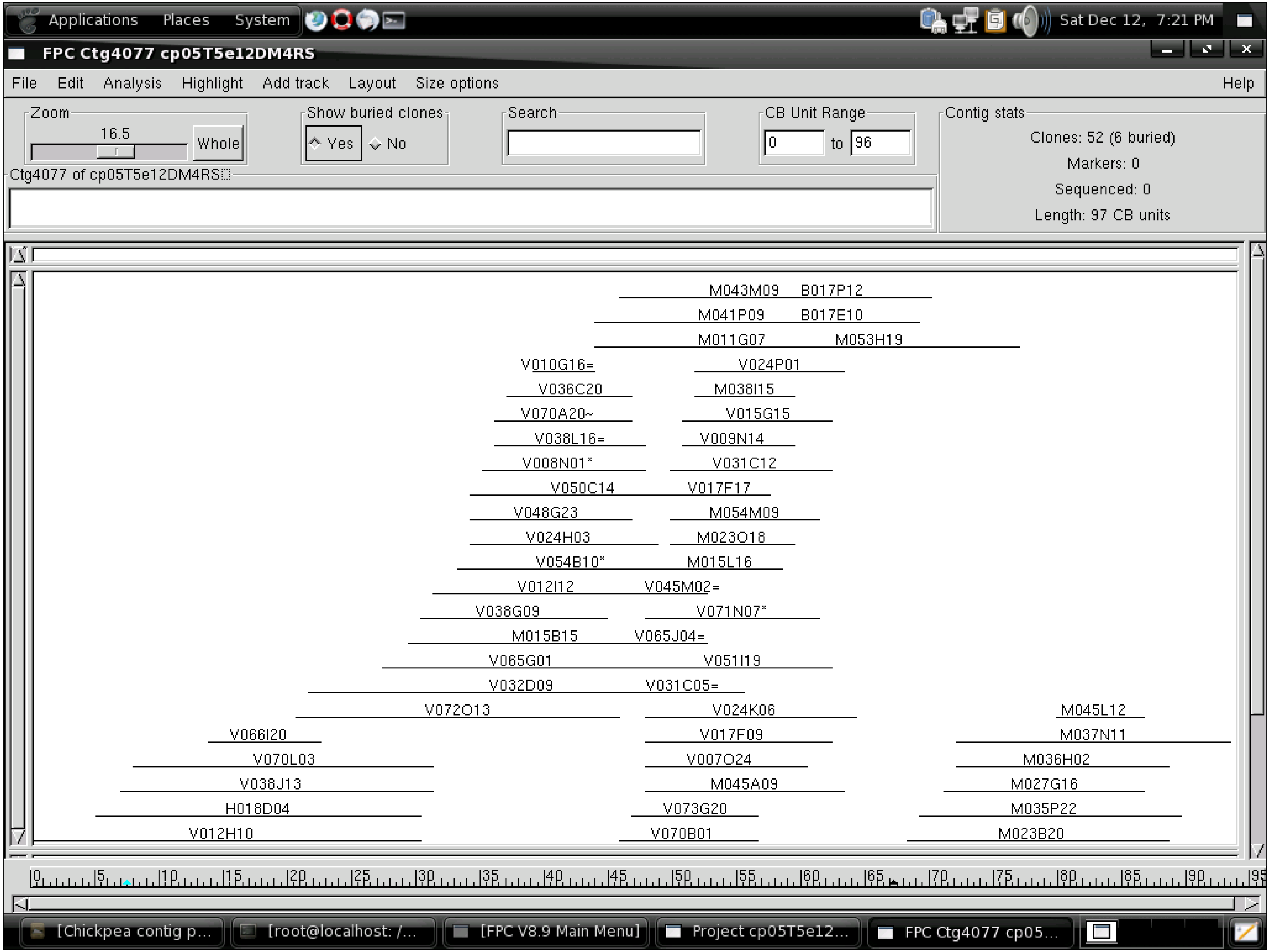

Applications Places System
Sat Dec 12, 7:21 PM
FPC Ctg4077 cp05T5e12DM4RS
File Edit Analysis Highlight Add track Layout Size options Help
Zoom
16.5
Whole
Show buried clones
Yes
No
Search
CB Unit Range
0
to
96
Contig stats
Clones: 52 (6 buried)
Markers: 0
Sequenced: 0
Length: 97 CB units
Ctg4077 of cp05T5e12DM4RS
M043M09 B017P12
M041P09 B017E10
M011G07 M053H19
V010G16= V024P01
V036C20 M038I15
V070A20~ V015G15
V038L16= V009N14
V008N01* V031C12
V050C14 V017F17
V048G23 M054M09
V024H03 M023O18
V054B10* M015L16
V012I12 V045M02=
V038G09 V071N07*
M015B15 V065J04=
V065G01 V051I19
V032D09 V031C05=
V072O13 V024K06 M045L12
V066I20 V017F09 M037N11
V070L03 V007O24 M036H02
V038J13 M045A09 M027G16
H018D04 V073G20 M035P22
V012H10 V070B01 M023B20
0 5 10 15 20 25 30 35 40 45 50 55 60 65 70 75 80 85 90 95
[Chickpea contig p...
[root@localhost: /...
[FPC V8.9 Main Menu]
Project cp05T5e12...
FPC Ctg4077 cp05...

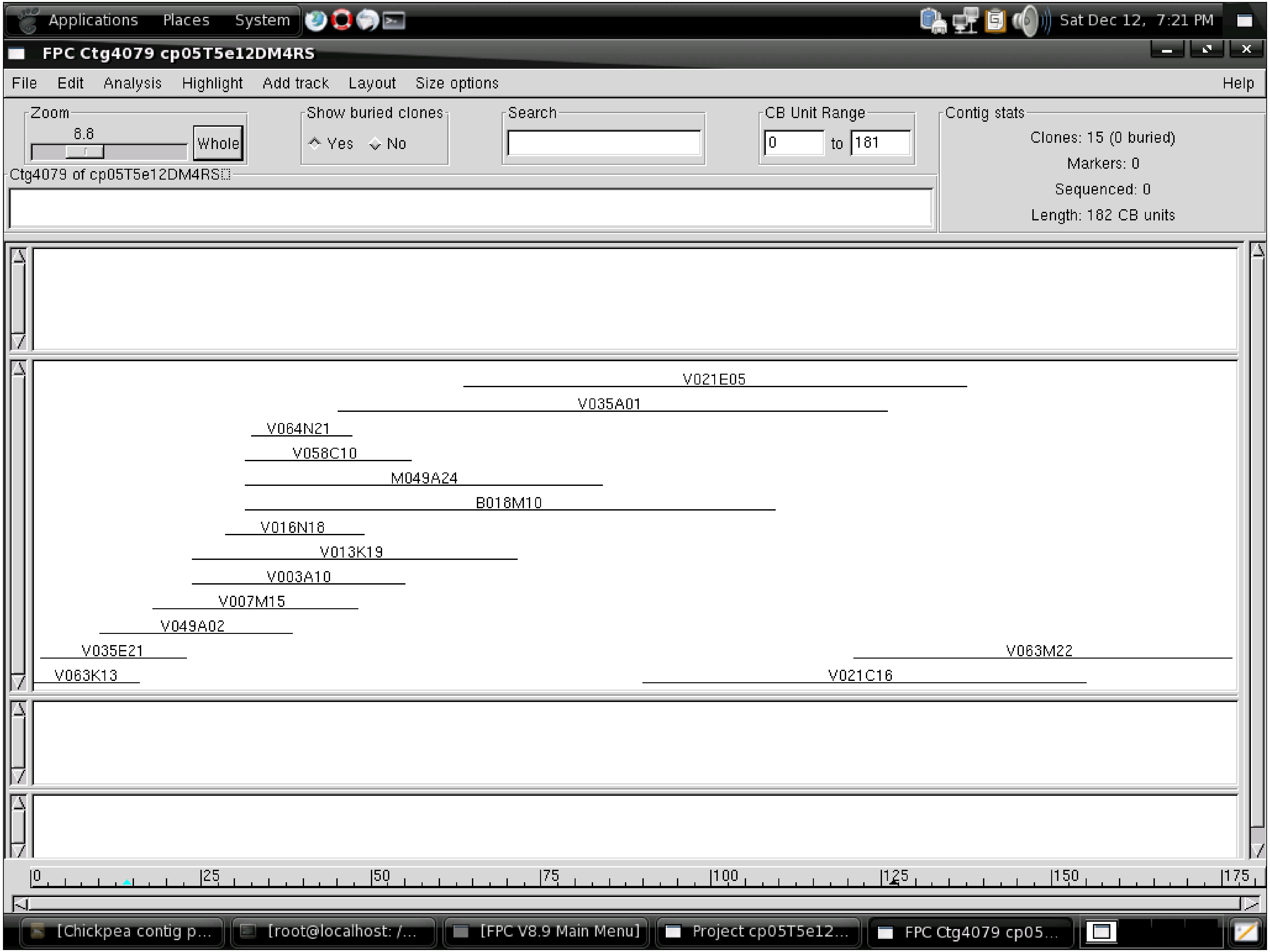
Applications Places System
Sat Dec 12, 7:21 PM
FPC Ctg4079 cp05T5e12DM4RS
File Edit Analysis Highlight Add track Layout Size options Help
Zoom
8.8
Whole
Show buried clones
Yes No
Search
CB Unit Range
0 to 181
Contig stats
Clones: 15 (0 buried)
Markers: 0
Sequenced: 0
Length: 182 CB units
Ctg4079 of cp05T5e12DM4RS
V021E05
V035A01
V064N21
V058C10
M049A24
B018M10
V016N18
V013K19
V003A10
V007M15
V049A02
V035E21
V063M22
V063K13
V021C16
0
25
50
75
100
125
150
175
[Chickpea contig p...
[root@localhost: /...
[FPC V8.9 Main Menu]
Project cp05T5e12...
FPC Ctg4079 cp05...

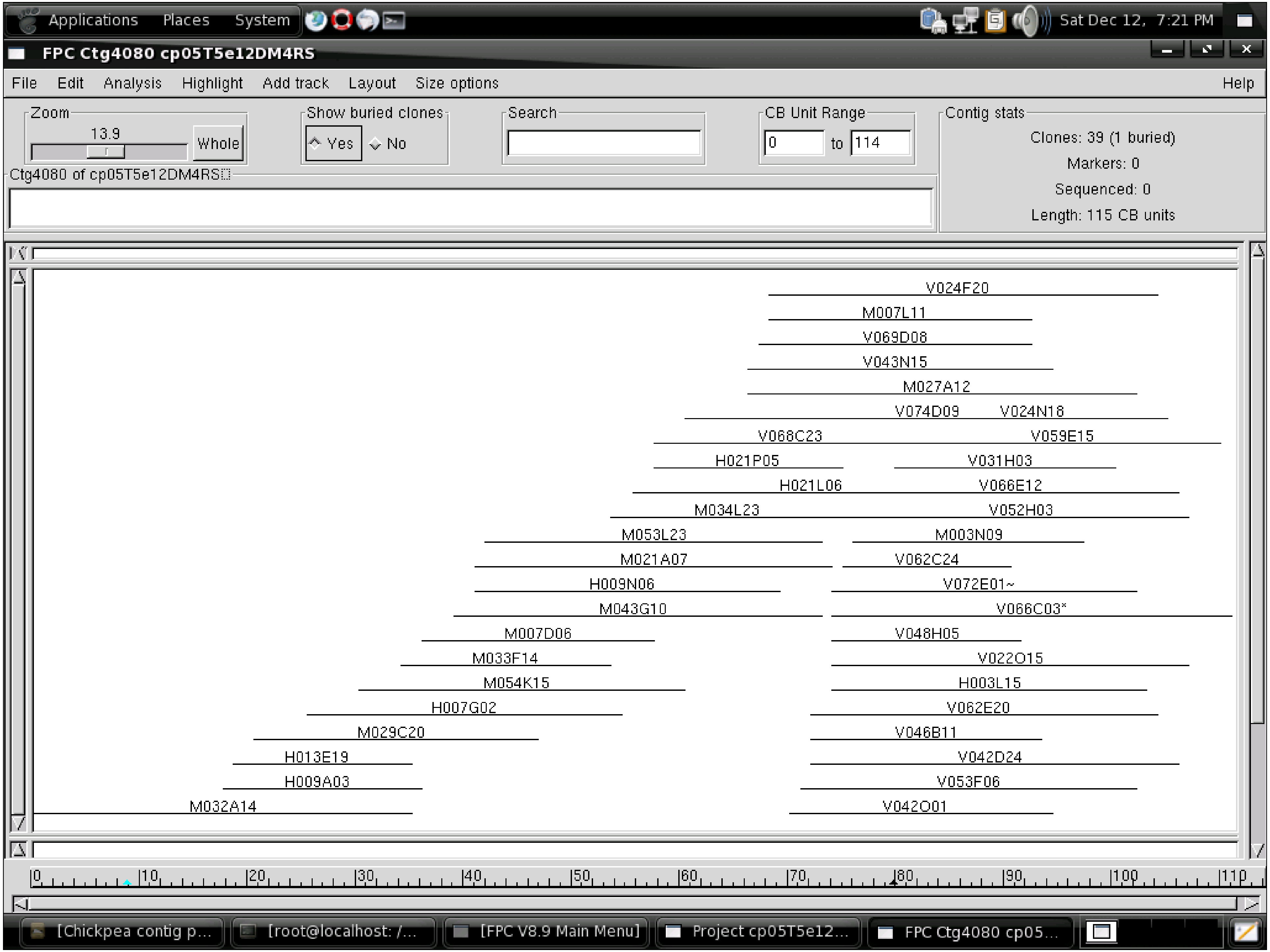
Applications Places System
Sat Dec 12, 7:21 PM
FPC Ctg4080 cp05T5e12DM4RS
File Edit Analysis Highlight Add track Layout Size options Help
Zoom
13.9
Whole
Show buried clones
Yes
No
Search
CB Unit Range
0 to 114
Contig stats
Clones: 39 (1 buried)
Markers: 0
Sequenced: 0
Length: 115 CB units
Ctg4080 of cp05T5e12DM4RS
V024F20
M007L11
V069D08
V043N15
M027A12
V074D09
V024N18
V068C23
V059E15
H021P05
V031H03
H021L06
V066E12
M034L23
V052H03
M053L23
M003N09
M021A07
V062C24
H009N06
V072E01~
M043G10
V066C03*
M007D06
V048H05
M033F14
V022O15
M054K15
H003L15
H007G02
V062E20
M029C20
V046B11
H013E19
V042D24
H009A03
V053F06
M032A14
V042O01
0 10 20 30 40 50 60 70 80 90 100 110
[Chickpea contig p...
[root@localhost: /...
[FPC V8.9 Main Menu]
Project cp05T5e12...
FPC Ctg4080 cp05...

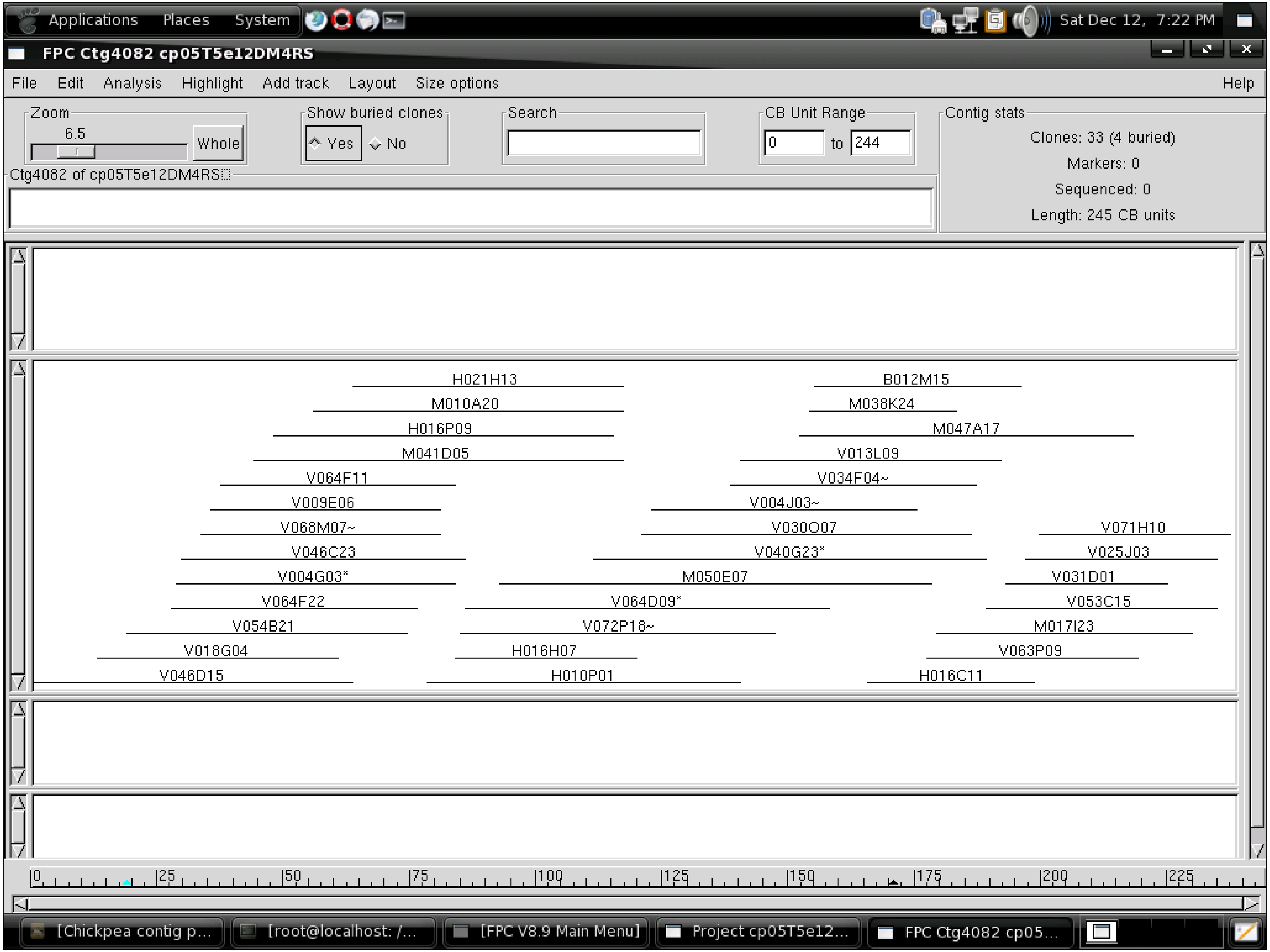
Applications Places System
Sat Dec 12, 7:22 PM
FPC Ctg4082 cp05T5e12DM4RS
File Edit Analysis Highlight Add track Layout Size options Help
Zoom
6.5
Whole
Show buried clones
Yes No
Search
CB Unit Range
0 to 244
Contig stats
Clones: 33 (4 buried)
Markers: 0
Sequenced: 0
Length: 245 CB units
Ctg4082 of cp05T5e12DM4RS
H021H13
B012M15
M010A20
M038K24
H016P09
M047A17
M041D05
V013L09
V064F11
V034F04~
V009E06
V004J03~
V068M07~
V030O07
V071H10
V046C23
V040G23*
V025J03
V004G03*
M050E07
V031D01
V064F22
V064D09*
V053C15
V054B21
V072P18~
M017I23
V018G04
H016H07
V063P09
V046D15
H010P01
H016C11
0 25 50 75 100 125 150 175 200 225
[Chickpea contig p...
[root@localhost: /...
[FPC V8.9 Main Menu]
Project cp05T5e12...
FPC Ctg4082 cp05...

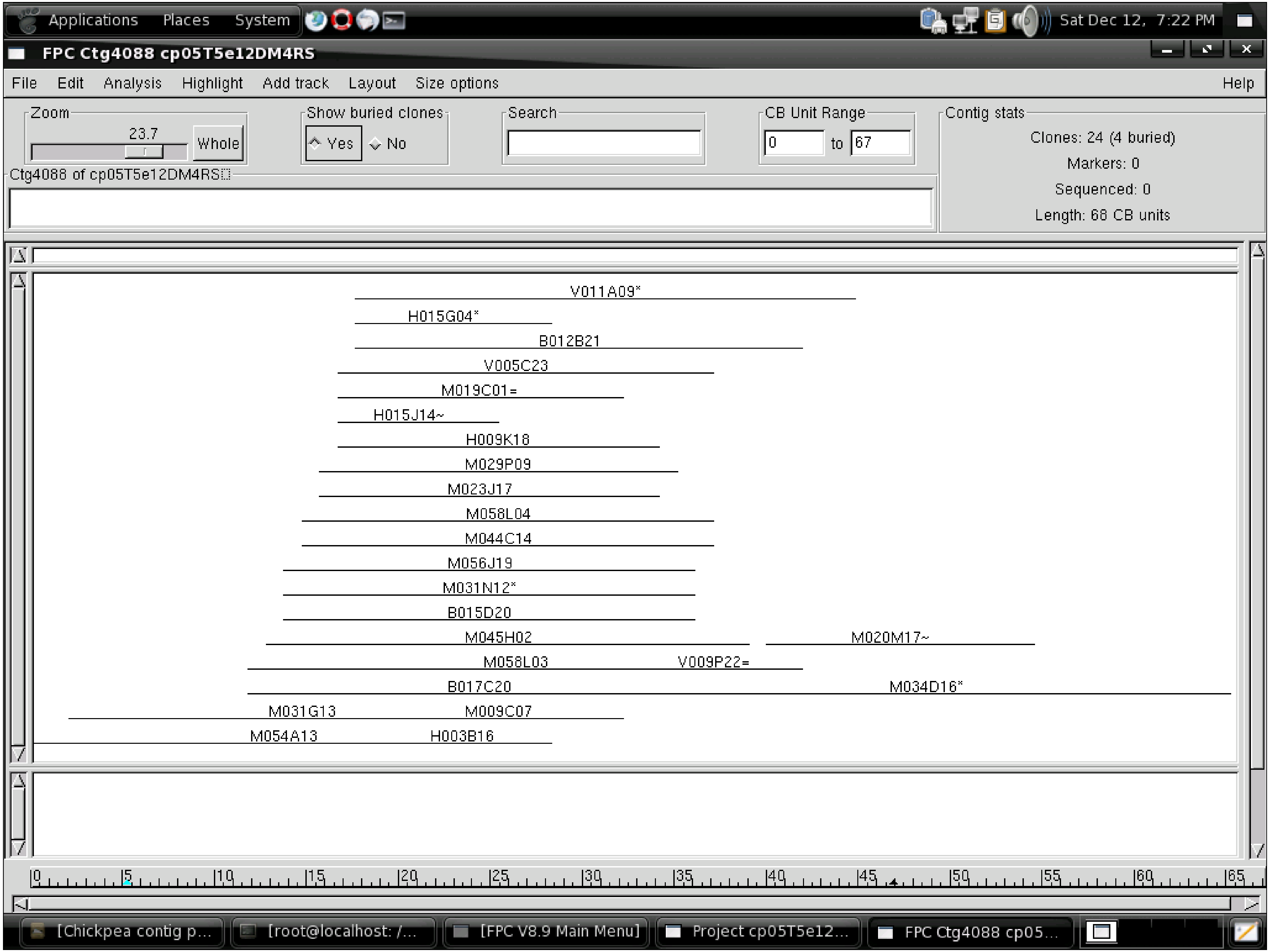
Applications Places System
Sat Dec 12, 7:22 PM
FPC Ctg4088 cp05T5e12DM4RS
File Edit Analysis Highlight Add track Layout Size options Help
Zoom
23.7
Whole
Show buried clones
Yes No
Search
CB Unit Range
0 to 67
Contig stats
Clones: 24 (4 buried)
Markers: 0
Sequenced: 0
Length: 68 CB units
Ctg4088 of cp05T5e12DM4RS
V011A09*
H015G04*
B012B21
V005C23
M019C01=
H015J14~
H009K18
M029P09
M023J17
M058L04
M044C14
M056J19
M031N12*
B015D20
M045H02
M020M17~
M058L03
V009P22=
B017C20
M034D16*
M031G13
M009C07
M054A13
H003B16
0 5 10 15 20 25 30 35 40 45 50 55 60 65
[Chickpea contig p...
[root@localhost: /...
[FPC V8.9 Main Menu]
Project cp05T5e12...
FPC Ctg4088 cp05...

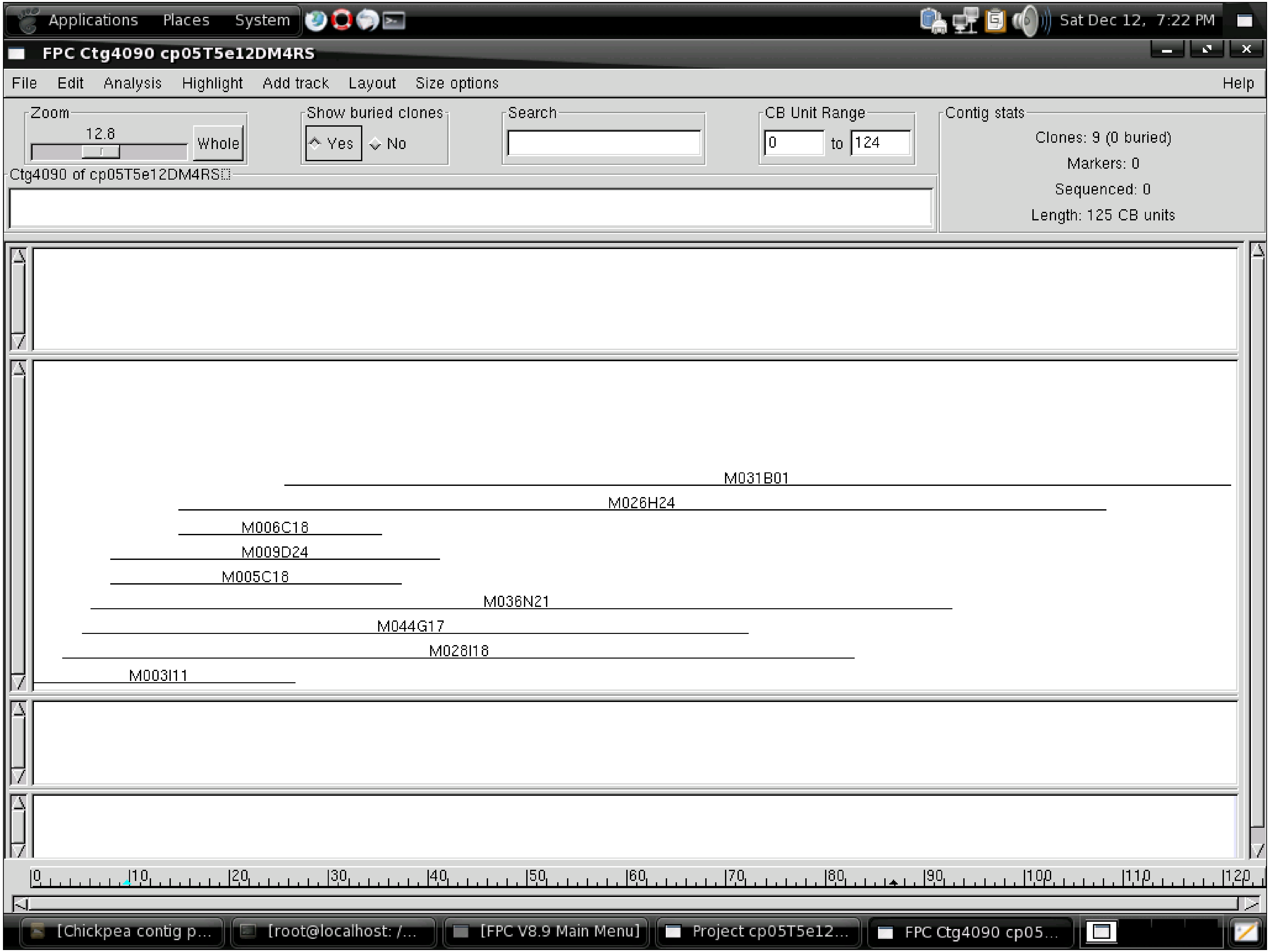
Applications Places System
Sat Dec 12, 7:22 PM
FPC Ctg4090 cp05T5e12DM4RS
File Edit Analysis Highlight Add track Layout Size options Help
Zoom
12.8
Whole
Show buried clones
Yes No
Search
CB Unit Range
0 to 124
Contig stats
Clones: 9 (0 buried)
Markers: 0
Sequenced: 0
Length: 125 CB units
Ctg4090 of cp05T5e12DM4RS
M031B01
M026H24
M006C18
M009D24
M005C18
M036N21
M044G17
M028I18
M003I11
0 10 20 30 40 50 60 70 80 90 100 110 120
[Chickpea contig p...
[root@localhost: /...
[FPC V8.9 Main Menu]
Project cp05T5e12...
FPC Ctg4090 cp05...

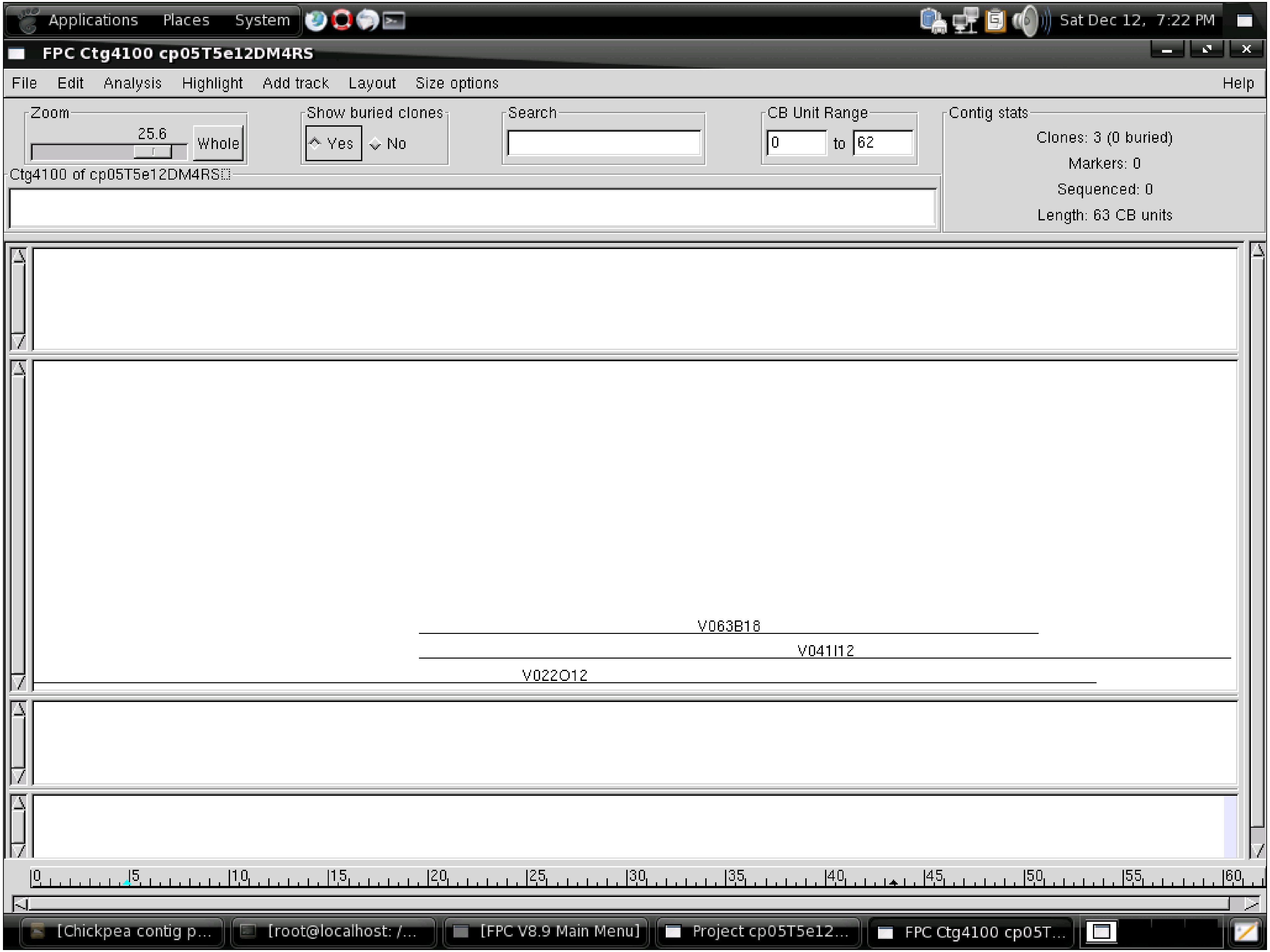
Applications Places System
Sat Dec 12, 7:22 PM
FPC Ctg4100 cp05T5e12DM4RS
File Edit Analysis Highlight Add track Layout Size options Help
Zoom
25.6
Whole
Show buried clones
Yes
No
Search
CB Unit Range
0
to
62
Contig stats
Clones: 3 (0 buried)
Markers: 0
Sequenced: 0
Length: 63 CB units
Ctg4100 of cp05T5e12DM4RS
V063B18
V041I12
V022O12
0 5 10 15 20 25 30 35 40 45 50 55 60
[Chickpea contig p...
[root@localhost: /...
[FPC V8.9 Main Menu]
Project cp05T5e12...
FPC Ctg4100 cp05T...

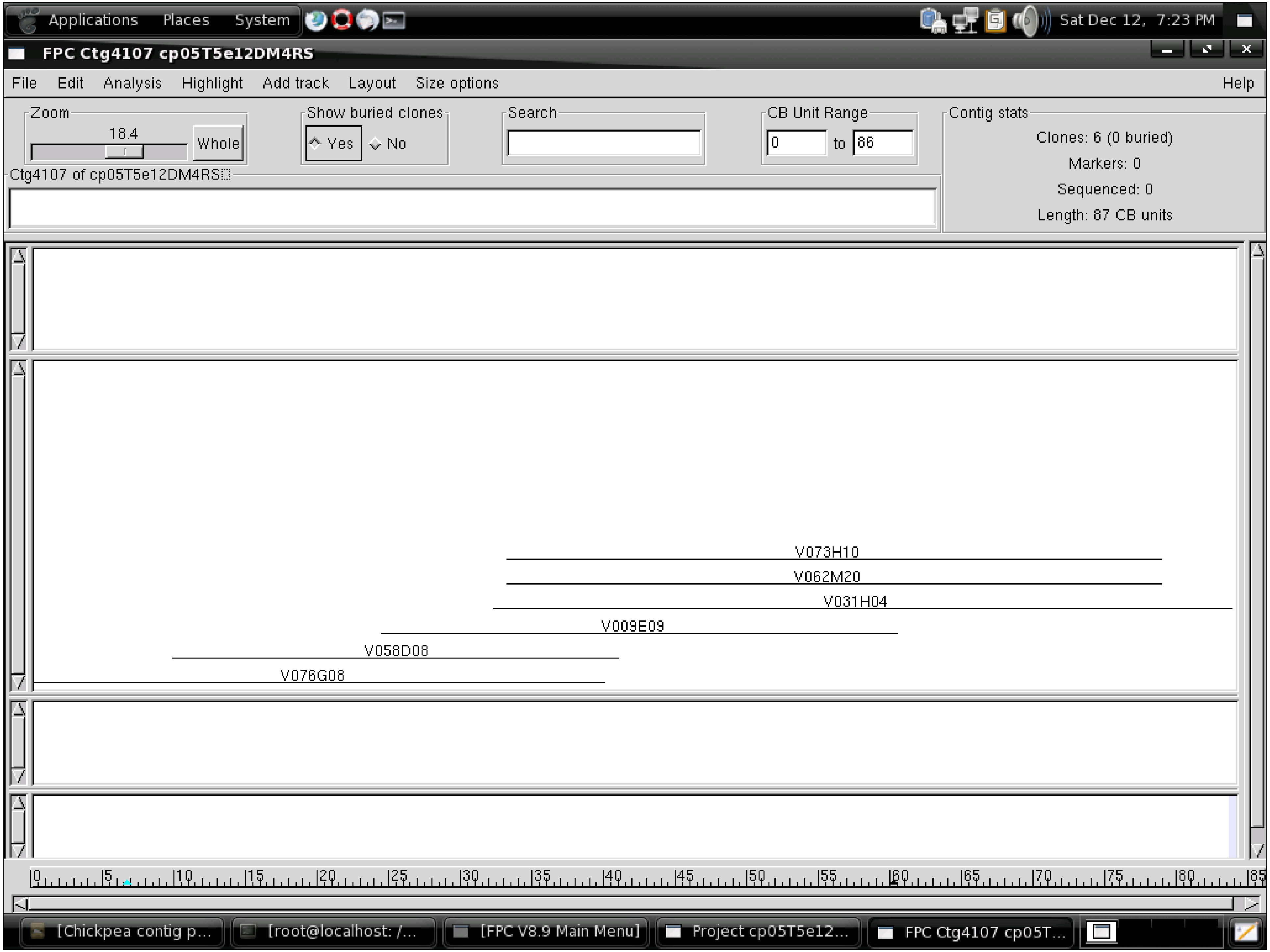
Applications
Places
System
Sat Dec 12, 7:23 PM
FPC Ctg4107 cp05T5e12DM4RS
File
Edit
Analysis
Highlight
Add track
Layout
Size options
Help
Zoom
18.4
Whole
Show buried clones
Yes
No
Search
CB Unit Range
0
to
86
Contig stats
Clones: 6 (0 buried)
Markers: 0
Sequenced: 0
Length: 87 CB units
Ctg4107 of cp05T5e12DM4RS
V073H10
V062M20
V031H04
V009E09
V058D08
V076G08
0 5 10 15 20 25 30 35 40 45 50 55 60 65 70 75 80 85
[Chickpea contig p...
[root@localhost: /...
[FPC V8.9 Main Menu]
Project cp05T5e12...
FPC Ctg4107 cp05T...

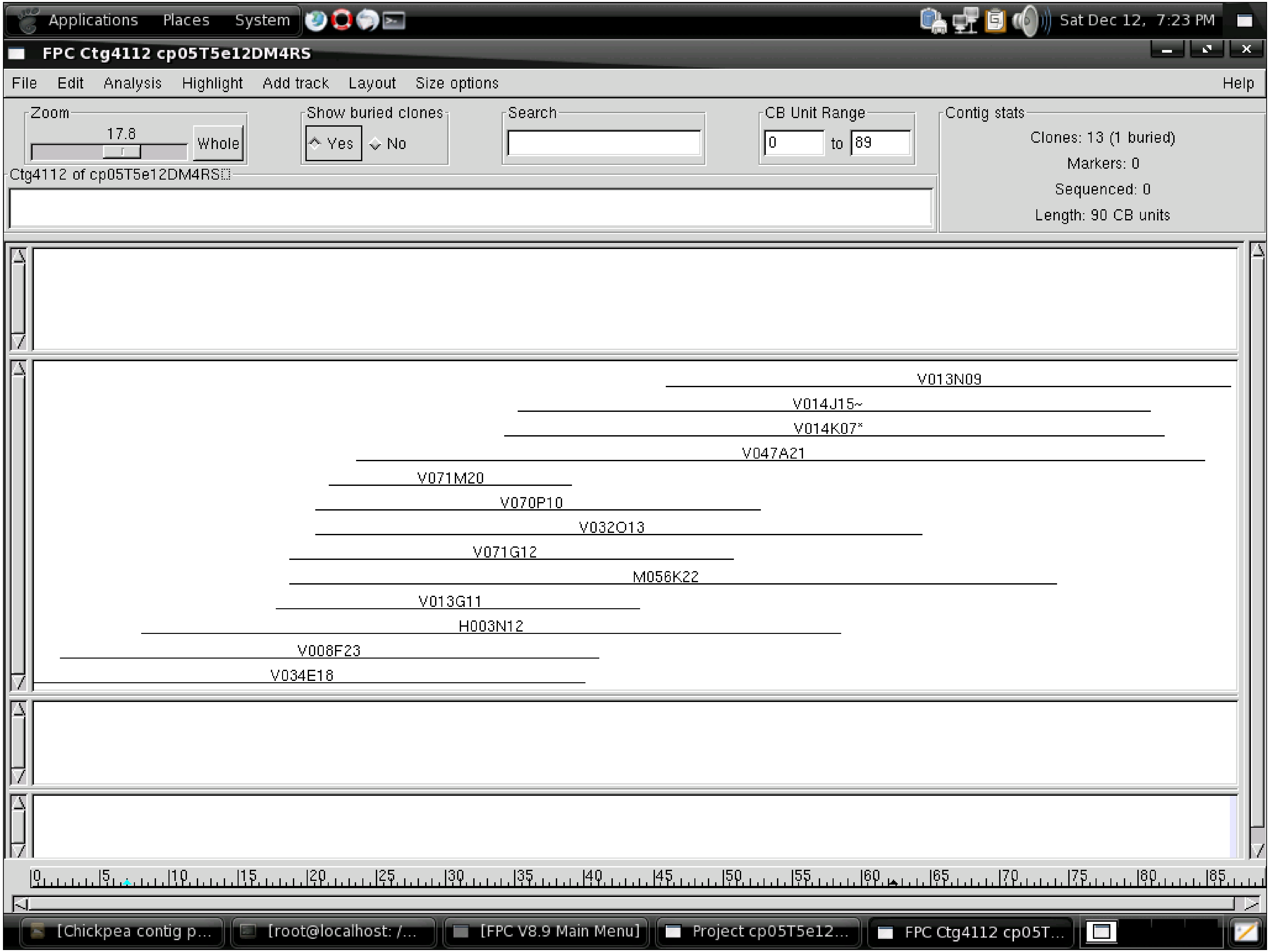
Applications
Places
System
Sat Dec 12, 7:23 PM
FPC Ctg4112 cp05T5e12DM4RS
File
Edit
Analysis
Highlight
Add track
Layout
Size options
Help
Zoom
17.8
Whole
Show buried clones
Yes
No
Search
CB Unit Range
0
to
89
Contig stats
Clones: 13 (1 buried)
Markers: 0
Sequenced: 0
Length: 90 CB units
Ctg4112 of cp05T5e12DM4RS
V013N09
V014J15~
V014K07*
V047A21
V071M20
V070P10
V032O13
V071G12
M056K22
V013G11
H003N12
V008F23
V034E18
0 5 10 15 20 25 30 35 40 45 50 55 60 65 70 75 80 85
[Chickpea contig p...
[root@localhost: /...
[FPC V8.9 Main Menu]
Project cp05T5e12...
FPC Ctg4112 cp05T...

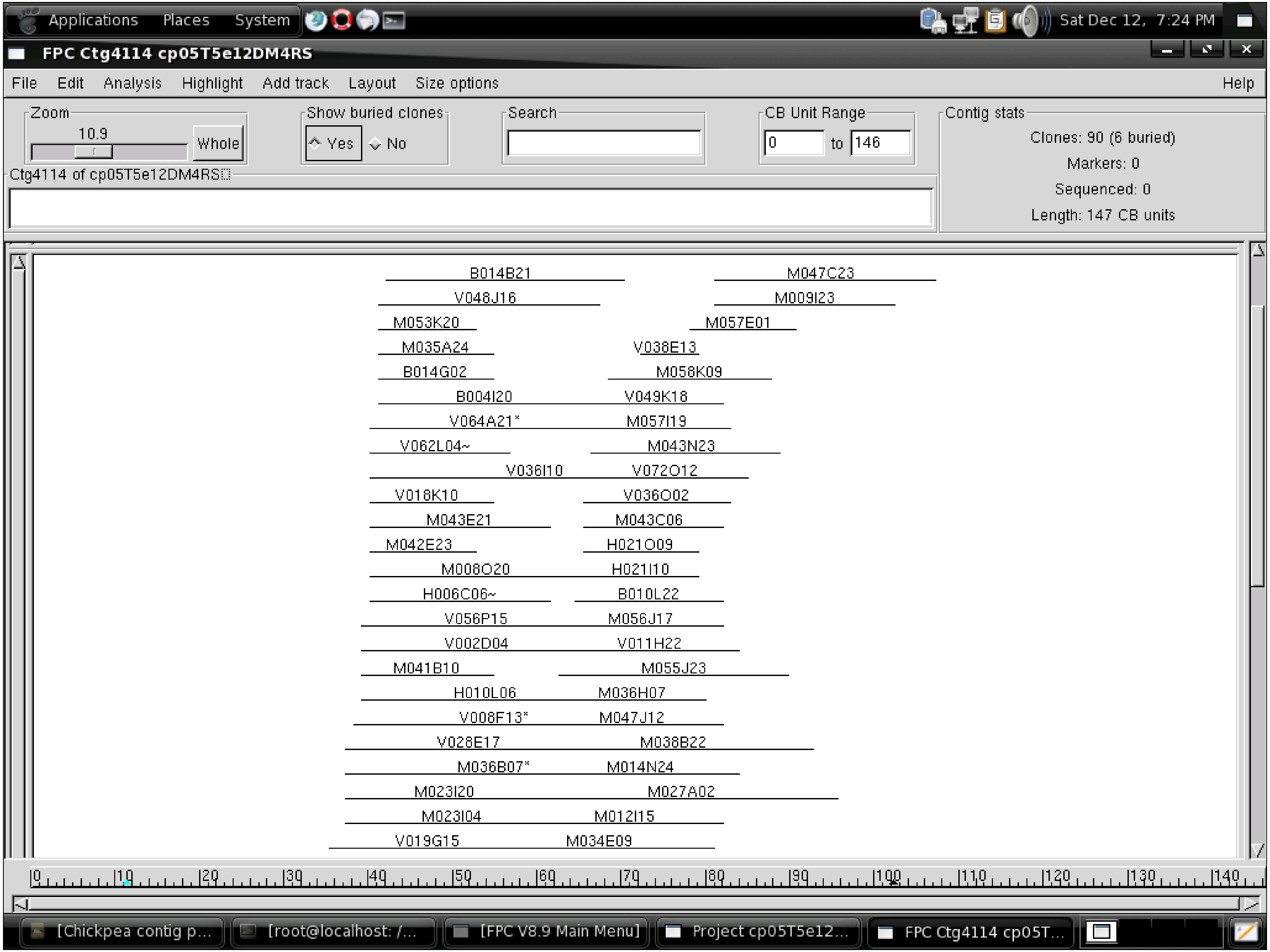

Applications Places System
Sat Dec 12, 7:24 PM
FPC Ctg4114 cp05T5e12DM4RS
File Edit Analysis Highlight Add track Layout Size options Help
Zoom
10.9
Whole
Show buried clones
Yes No
Search
CB Unit Range
0 to 146
Contig stats
Clones: 90 (6 buried)
Markers: 0
Sequenced: 0
Length: 147 CB units
Ctg4114 of cp05T5e12DM4RS
B014B21
M047C23
V048J16
M009I23
M053K20
M057E01
M035A24
V038E13
B014G02
M058K09
B004I20
V049K18
V064A21*
M057I19
V062L04~
M043N23
V036I10
V072O12
V018K10
V036O02
M043E21
M043C06
M042E23
H021O09
M008O20
H021I10
H006C06~
B010L22
V056P15
M056J17
V002D04
V011H22
M041B10
M055J23
H010L06
M036H07
V008F13*
M047J12
V028E17
M038B22
M036B07*
M014N24
M023I20
M027A02
M023I04
M012I15
V019G15
M034E09
0 10 20 30 40 50 60 70 80 90 100 110 120 130 140
[Chickpea contig p...
[root@localhost: /...
[FPC V8.9 Main Menu]
Project cp05T5e12...
FPC Ctg4114 cp05T...

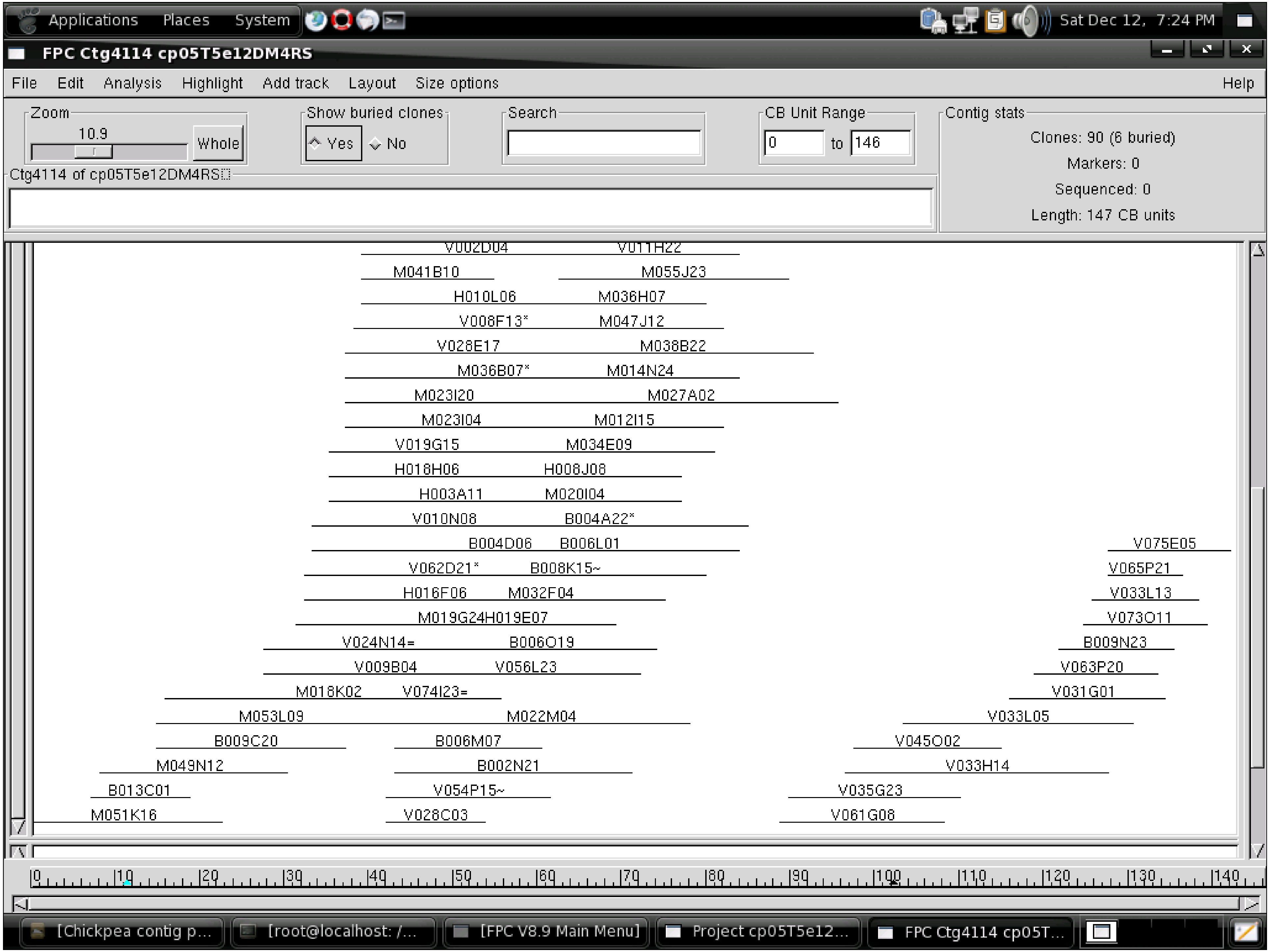
Applications Places System
Sat Dec 12, 7:24 PM
FPC Ctg4114 cp05T5e12DM4RS
File Edit Analysis Highlight Add track Layout Size options Help
Zoom
10.9
Whole
Show buried clones
Yes No
Search
CB Unit Range
0 to 146
Contig stats
Clones: 90 (6 buried)
Markers: 0
Sequenced: 0
Length: 147 CB units
Ctg4114 of cp05T5e12DM4RS
V002D04 V011H22
M041B10 M055J23
H010L06 M036H07
V008F13* M047J12
V028E17 M038B22
M036B07* M014N24
M023I20 M027A02
M023I04 M012I15
V019G15 M034E09
H018H06 H008J08
H003A11 M020I04
V010N08 B004A22*
B004D06 B006L01 V075E05
V062D21* B008K15~ V065P21
H016F06 M032F04 V033L13
M019G24H019E07 V073O11
V024N14= B006O19 B009N23
V009B04 V056L23 V063P20
M018K02 V074I23= V031G01
M053L09 M022M04 V033L05
B009C20 B006M07 V045O02
M049N12 B002N21 V033H14
B013C01 V054P15~ V035G23
M051K16 V028C03 V061G08
0 10 20 30 40 50 60 70 80 90 100 110 120 130 140
[Chickpea contig p... [root@localhost: /... [FPC V8.9 Main Menu] Project cp05T5e12... FPC Ctg4114 cp05T...

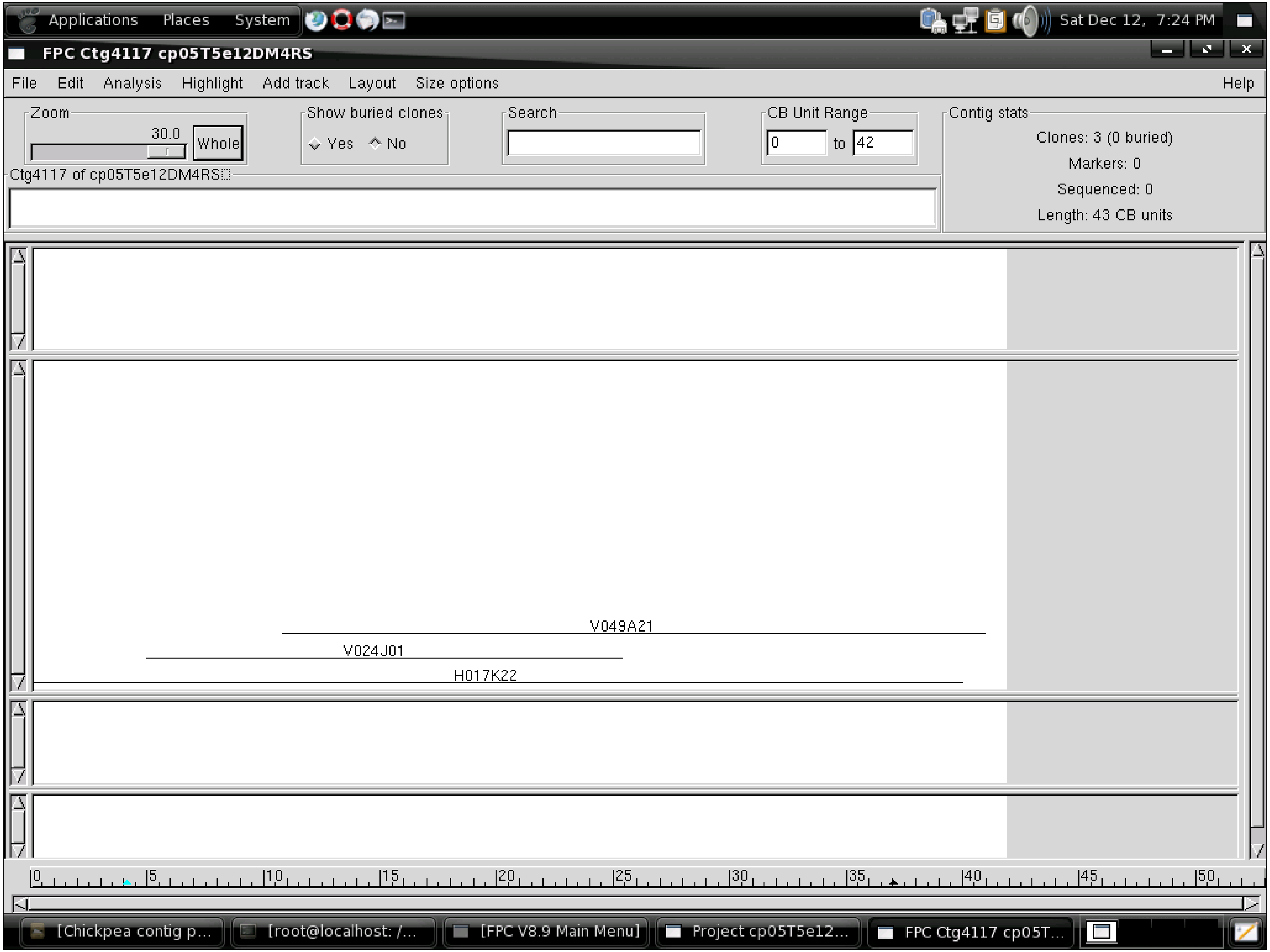

Applications Places System
Sat Dec 12, 7:24 PM
FPC Ctg4117 cp05T5e12DM4RS
File Edit Analysis Highlight Add track Layout Size options Help
Zoom
30.0
Whole
Show buried clones
Yes No
Search
CB Unit Range
0 to 42
Contig stats
Clones: 3 (0 buried)
Markers: 0
Sequenced: 0
Length: 43 CB units
Ctg4117 of cp05T5e12DM4RS
V049A21
V024J01
H017K22
0 5 10 15 20 25 30 35 40 45 50
[Chickpea contig p...
[root@localhost: /...
[FPC V8.9 Main Menu]
Project cp05T5e12...
FPC Ctg4117 cp05T...

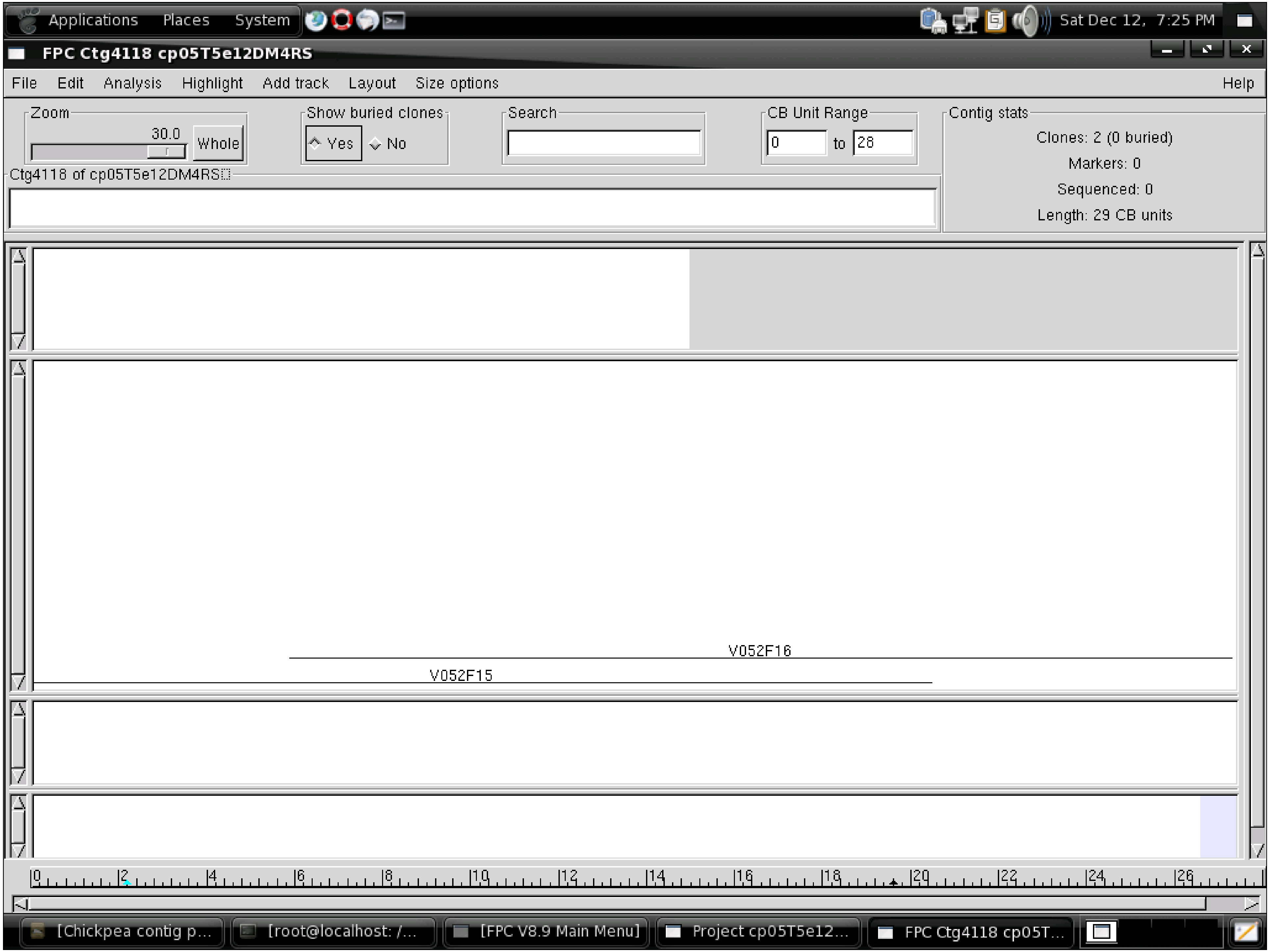
Applications Places System
Sat Dec 12, 7:25 PM
FPC Ctg4118 cp05T5e12DM4RS
File Edit Analysis Highlight Add track Layout Size options Help
Zoom
30.0
Whole
Show buried clones
Yes No
Search
CB Unit Range
0 to 28
Contig stats
Clones: 2 (0 buried)
Markers: 0
Sequenced: 0
Length: 29 CB units
Ctg4118 of cp05T5e12DM4RS
V052F16
V052F15
0 2 4 6 8 10 12 14 16 18 20 22 24 26
[Chickpea contig p...
[root@localhost: /...
[FPC V8.9 Main Menu]
Project cp05T5e12...
FPC Ctg4118 cp05T...

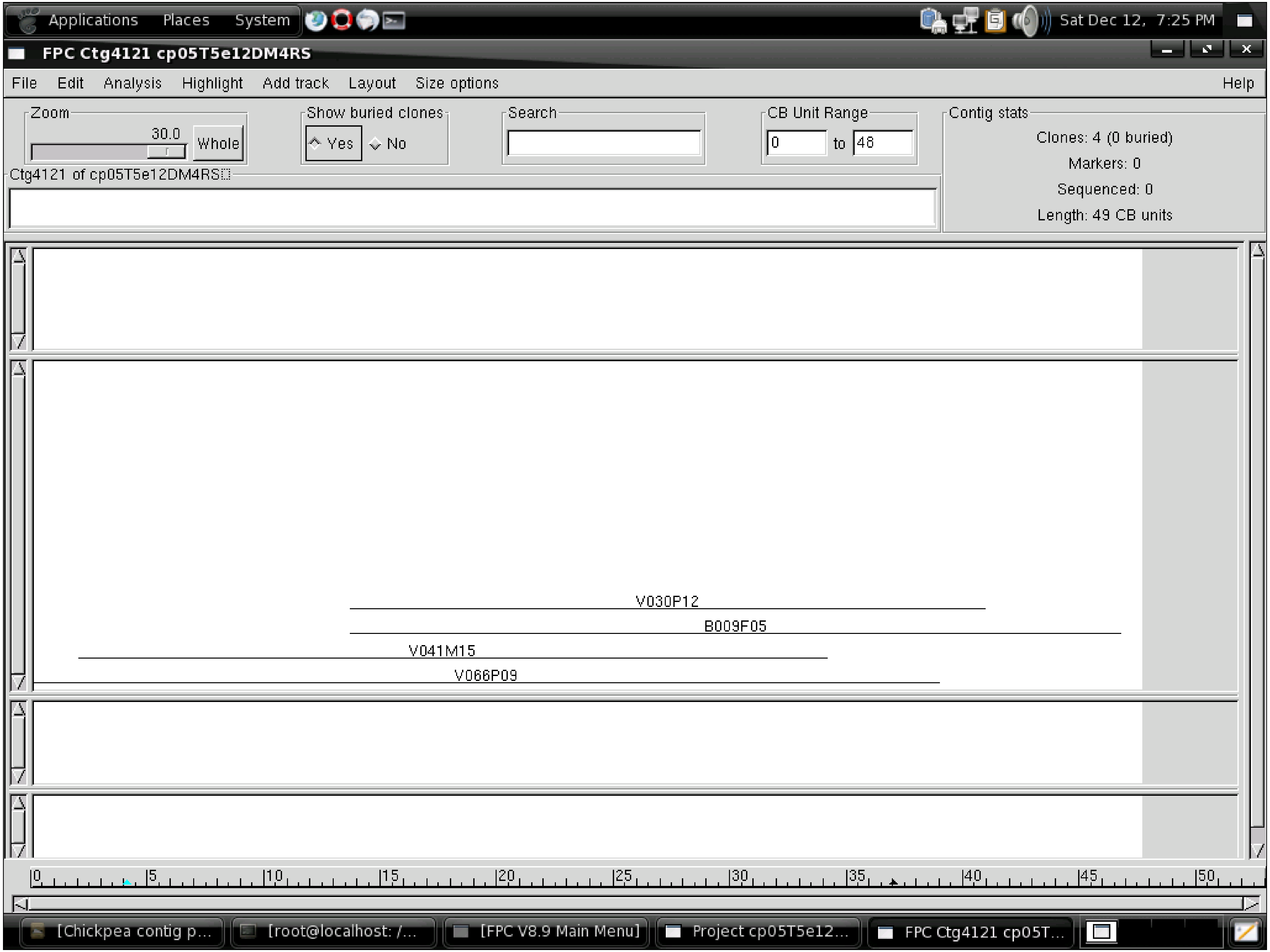
Applications Places System
Sat Dec 12, 7:25 PM
FPC Ctg4121 cp05T5e12DM4RS
File Edit Analysis Highlight Add track Layout Size options Help
Zoom
30.0
Whole
Show buried clones
Yes
No
Search
CB Unit Range
0 to 48
Contig stats
Clones: 4 (0 buried)
Markers: 0
Sequenced: 0
Length: 49 CB units
Ctg4121 of cp05T5e12DM4RS
V030P12
B009F05
V041M15
V066P09
0 5 10 15 20 25 30 35 40 45 50
[Chickpea contig p...
[root@localhost: /...
[FPC V8.9 Main Menu]
Project cp05T5e12...
FPC Ctg4121 cp05T...

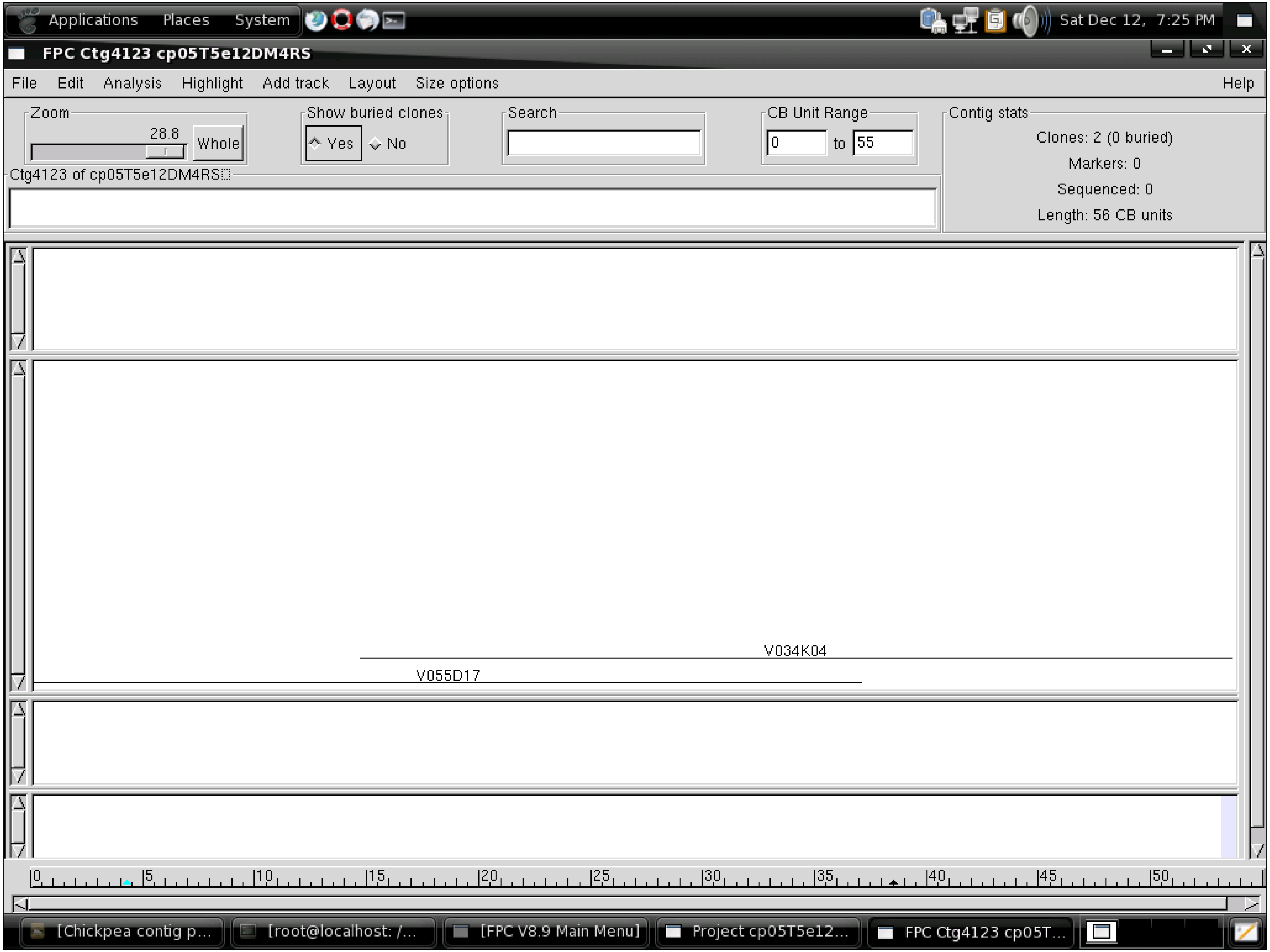
Applications
Places
System
Sat Dec 12, 7:25 PM
FPC Ctg4123 cp05T5e12DM4RS
File
Edit
Analysis
Highlight
Add track
Layout
Size options
Help
Zoom
28.8
Whole
Show buried clones
Yes
No
Search
CB Unit Range
0
to
55
Contig stats
Clones: 2 (0 buried)
Markers: 0
Sequenced: 0
Length: 56 CB units
Ctg4123 of cp05T5e12DM4RS
V034K04
V055D17
0
5
10
15
20
25
30
35
40
45
50
[Chickpea contig p...
[root@localhost: /...
[FPC V8.9 Main Menu]
Project cp05T5e12...
FPC Ctg4123 cp05T...

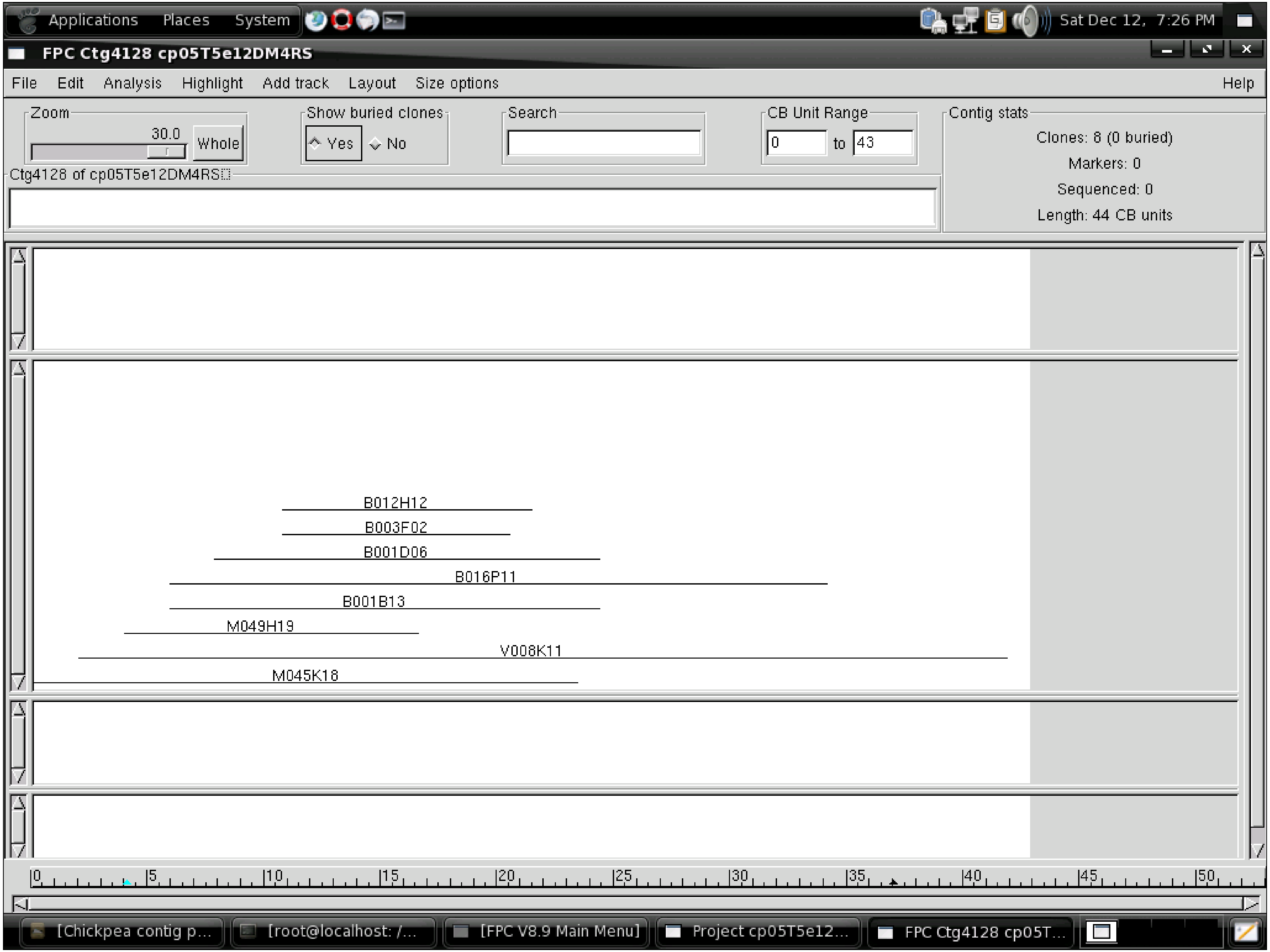
Applications Places System
Sat Dec 12, 7:26 PM
FPC Ctg4128 cp05T5e12DM4RS
File Edit Analysis Highlight Add track Layout Size options Help
Zoom
30.0
Whole
Show buried clones
Yes
No
Search
CB Unit Range
0
to
43
Contig stats
Clones: 8 (0 buried)
Markers: 0
Sequenced: 0
Length: 44 CB units
Ctg4128 of cp05T5e12DM4RS
B012H12
B003F02
B001D06
B016P11
B001B13
M049H19
V008K11
M045K18
0 5 10 15 20 25 30 35 40 45 50
[Chickpea contig p...
[root@localhost: /...
[FPC V8.9 Main Menu]
Project cp05T5e12...
FPC Ctg4128 cp05T...

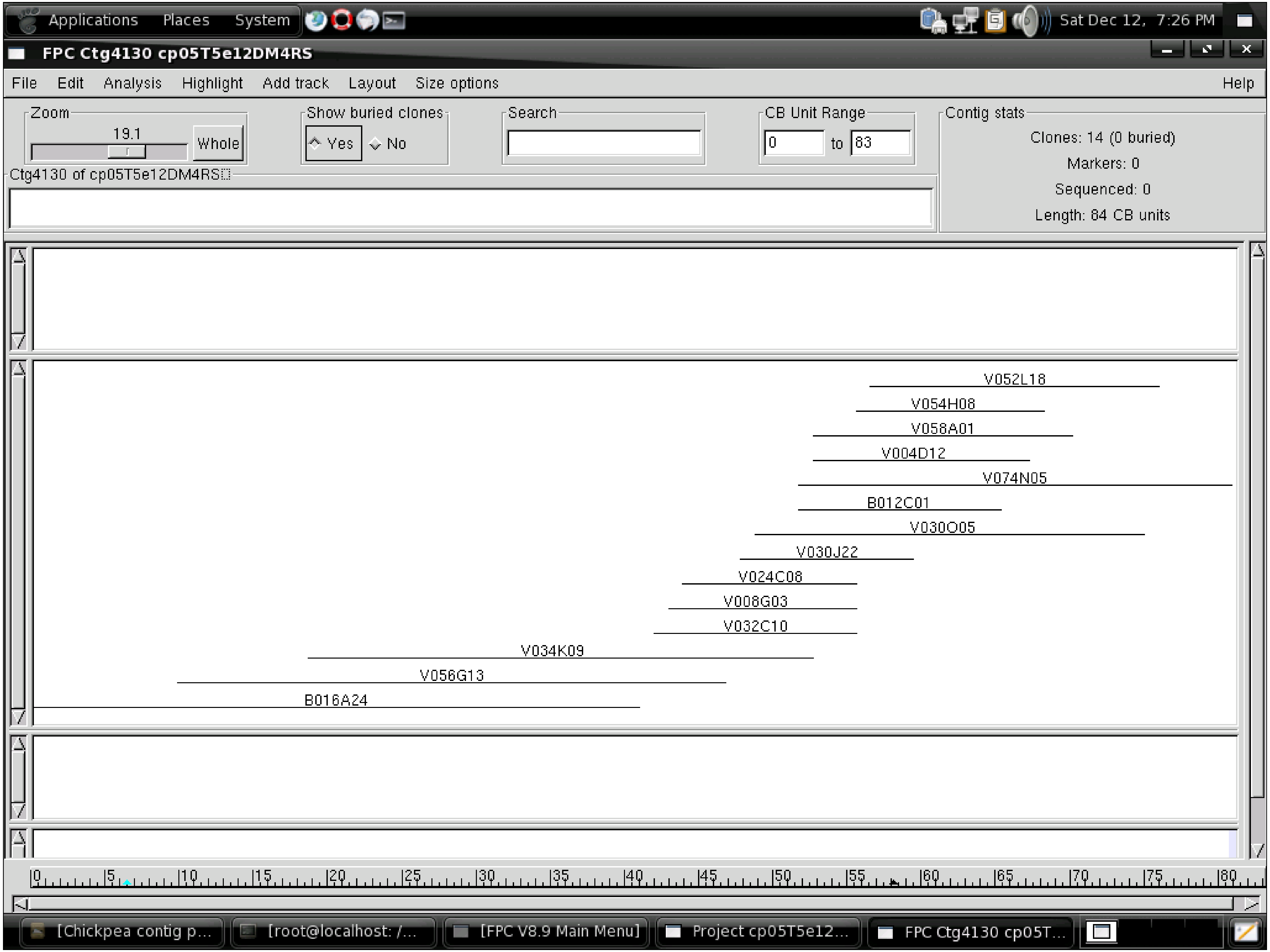
Applications Places System
Sat Dec 12, 7:26 PM
FPC Ctg4130 cp05T5e12DM4RS
File Edit Analysis Highlight Add track Layout Size options Help
Zoom
19.1
Whole
Show buried clones
Yes No
Search
CB Unit Range
0 to 83
Contig stats
Clones: 14 (0 buried)
Markers: 0
Sequenced: 0
Length: 84 CB units
Ctg4130 of cp05T5e12DM4RS
V052L18
V054H08
V058A01
V004D12
V074N05
B012C01
V030O05
V030J22
V024C08
V008G03
V032C10
V034K09
V056G13
B016A24
0 5 10 15 20 25 30 35 40 45 50 55 60 65 70 75 80
[Chickpea contig p...
[root@localhost: /...
[FPC V8.9 Main Menu]
Project cp05T5e12...
FPC Ctg4130 cp05T...

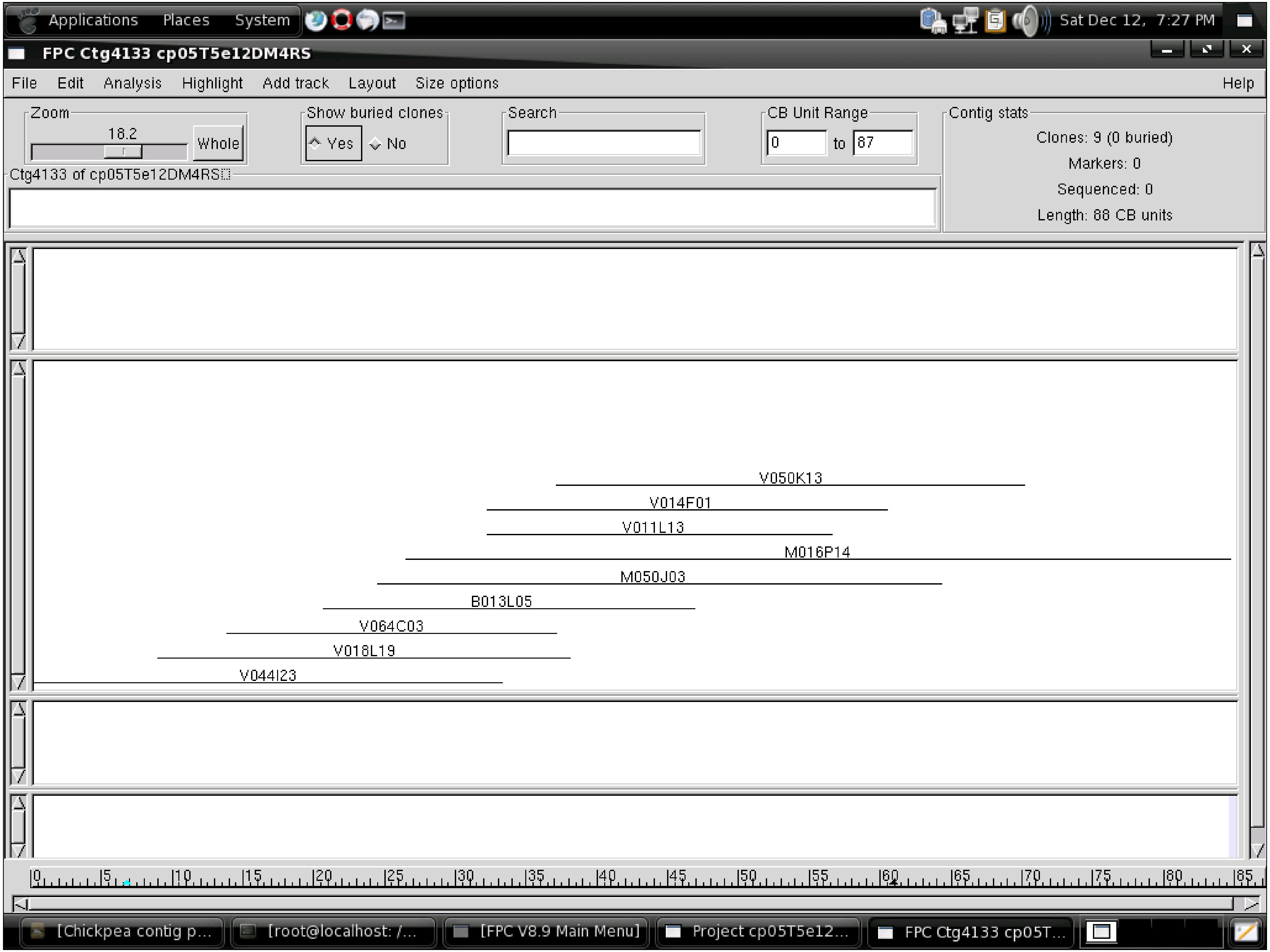
Applications
Places
System
Sat Dec 12, 7:27 PM
FPC Ctg4133 cp05T5e12DM4RS
File
Edit
Analysis
Highlight
Add track
Layout
Size options
Help
Zoom
18.2
Whole
Show buried clones
Yes
No
Search
CB Unit Range
0
to
87
Contig stats
Clones: 9 (0 buried)
Markers: 0
Sequenced: 0
Length: 88 CB units
Ctg4133 of cp05T5e12DM4RS
V050K13
V014F01
V011L13
M016P14
M050J03
B013L05
V064C03
V018L19
V044I23
0 5 10 15 20 25 30 35 40 45 50 55 60 65 70 75 80 85
[Chickpea contig p...
[root@localhost: /...
[FPC V8.9 Main Menu]
Project cp05T5e12...
FPC Ctg4133 cp05T...

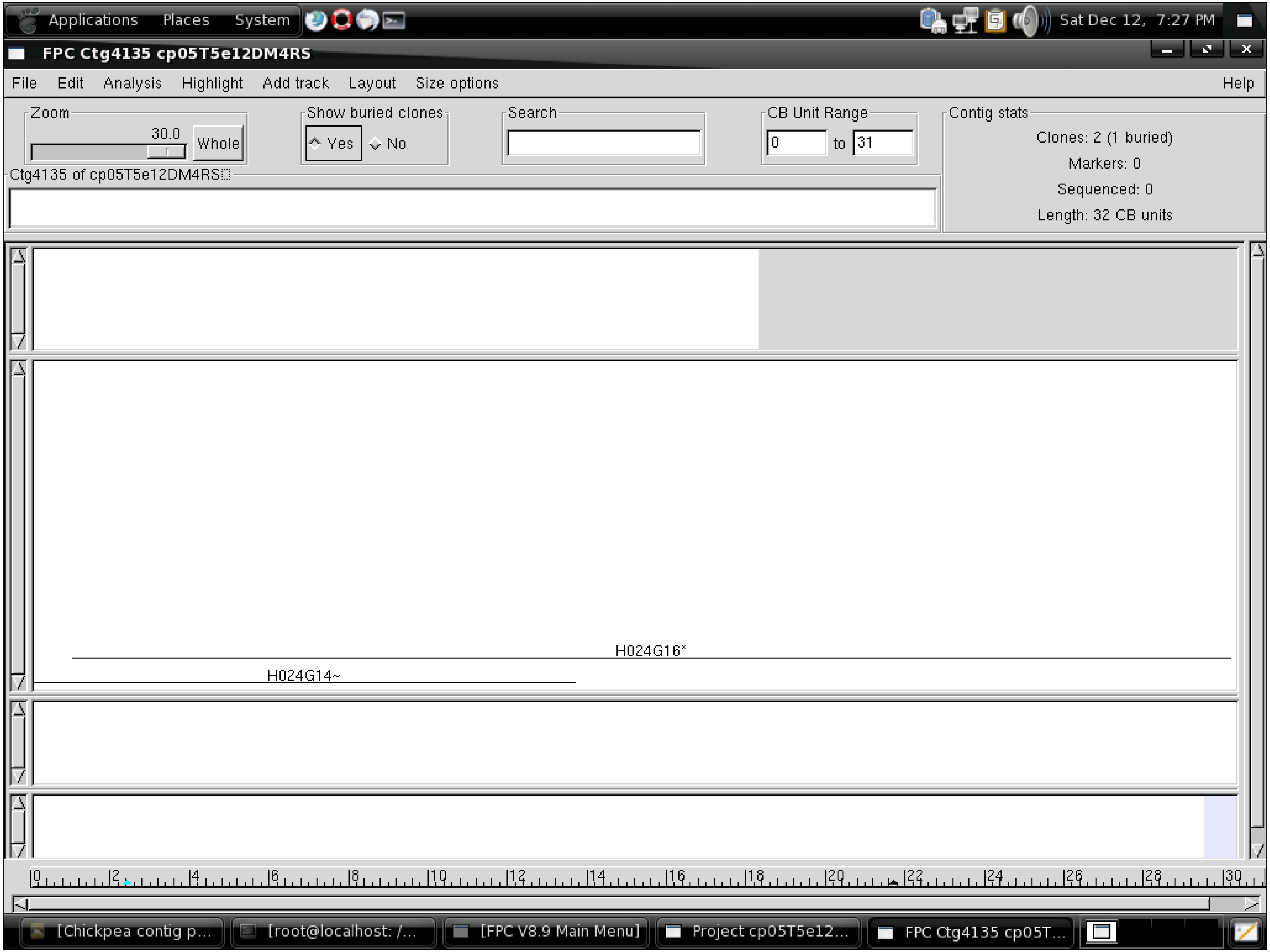
Applications Places System
Sat Dec 12, 7:27 PM
FPC Ctg4135 cp05T5e12DM4RS
File Edit Analysis Highlight Add track Layout Size options Help
Zoom
30.0
Whole
Show buried clones
Yes
No
Search
CB Unit Range
0
to
31
Contig stats
Clones: 2 (1 buried)
Markers: 0
Sequenced: 0
Length: 32 CB units
Ctg4135 of cp05T5e12DM4RS
H024G16*
H024G14~
0 2 4 6 8 10 12 14 16 18 20 22 24 26 28 30
[Chickpea contig p...
[root@localhost: /...
[FPC V8.9 Main Menu]
Project cp05T5e12...
FPC Ctg4135 cp05T...

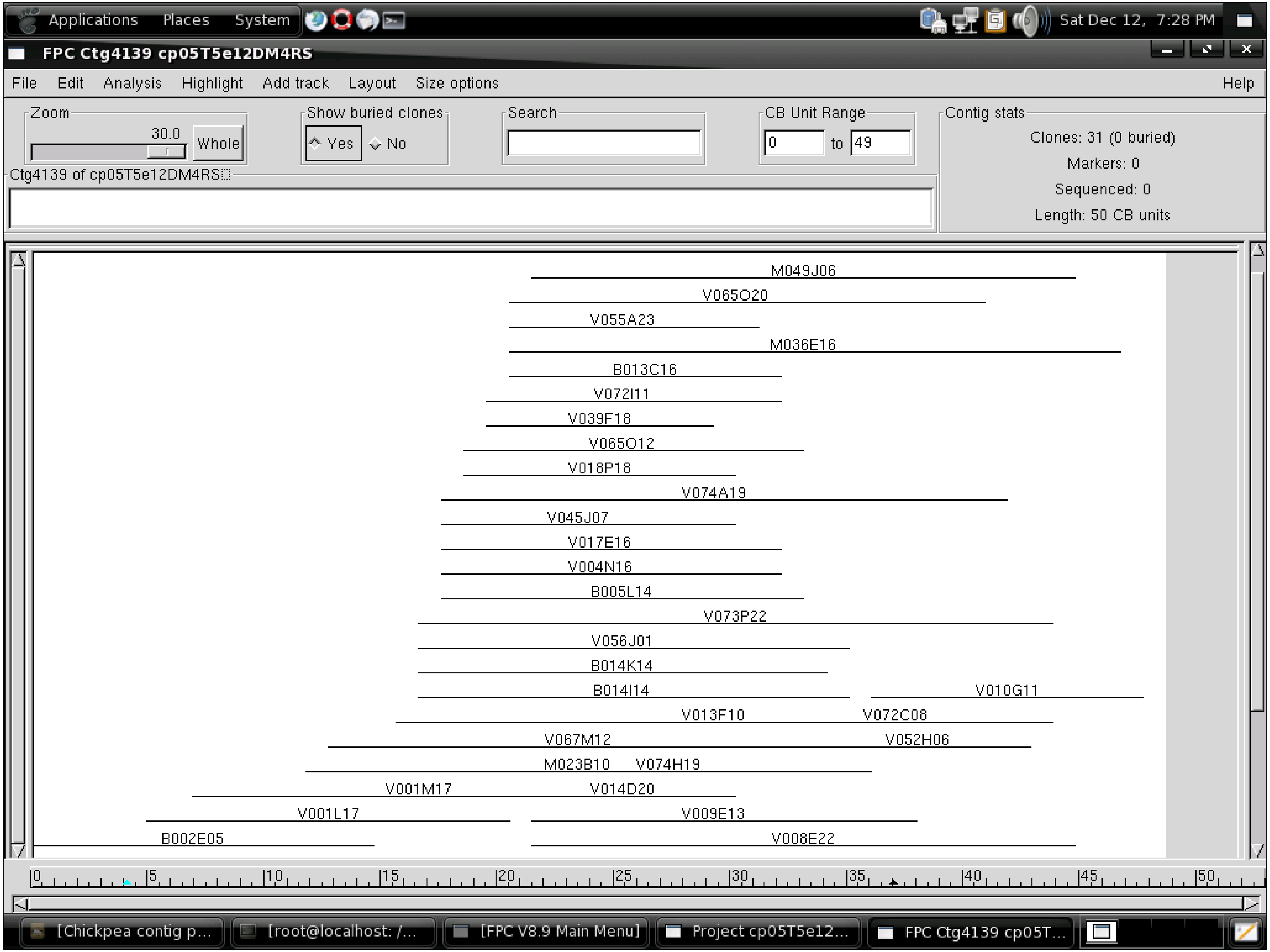
Applications Places System
Sat Dec 12, 7:28 PM
FPC Ctg4139 cp05T5e12DM4RS
File Edit Analysis Highlight Add track Layout Size options Help
Zoom
30.0
Whole
Show buried clones
Yes
No
Search
CB Unit Range
0
to
49
Contig stats
Clones: 31 (0 buried)
Markers: 0
Sequenced: 0
Length: 50 CB units
Ctg4139 of cp05T5e12DM4RS
M049J06
V065O20
V055A23
M036E16
B013C16
V072I11
V039F18
V065O12
V018P18
V074A19
V045J07
V017E16
V004N16
B005L14
V073P22
V056J01
B014K14
B014I14
V010G11
V013F10
V072C08
V067M12
V052H06
M023B10
V074H19
V001M17
V014D20
V001L17
V009E13
B002E05
V008E22
0 5 10 15 20 25 30 35 40 45 50
[Chickpea contig p...
[root@localhost: /...
[FPC V8.9 Main Menu]
Project cp05T5e12...
FPC Ctg4139 cp05T...

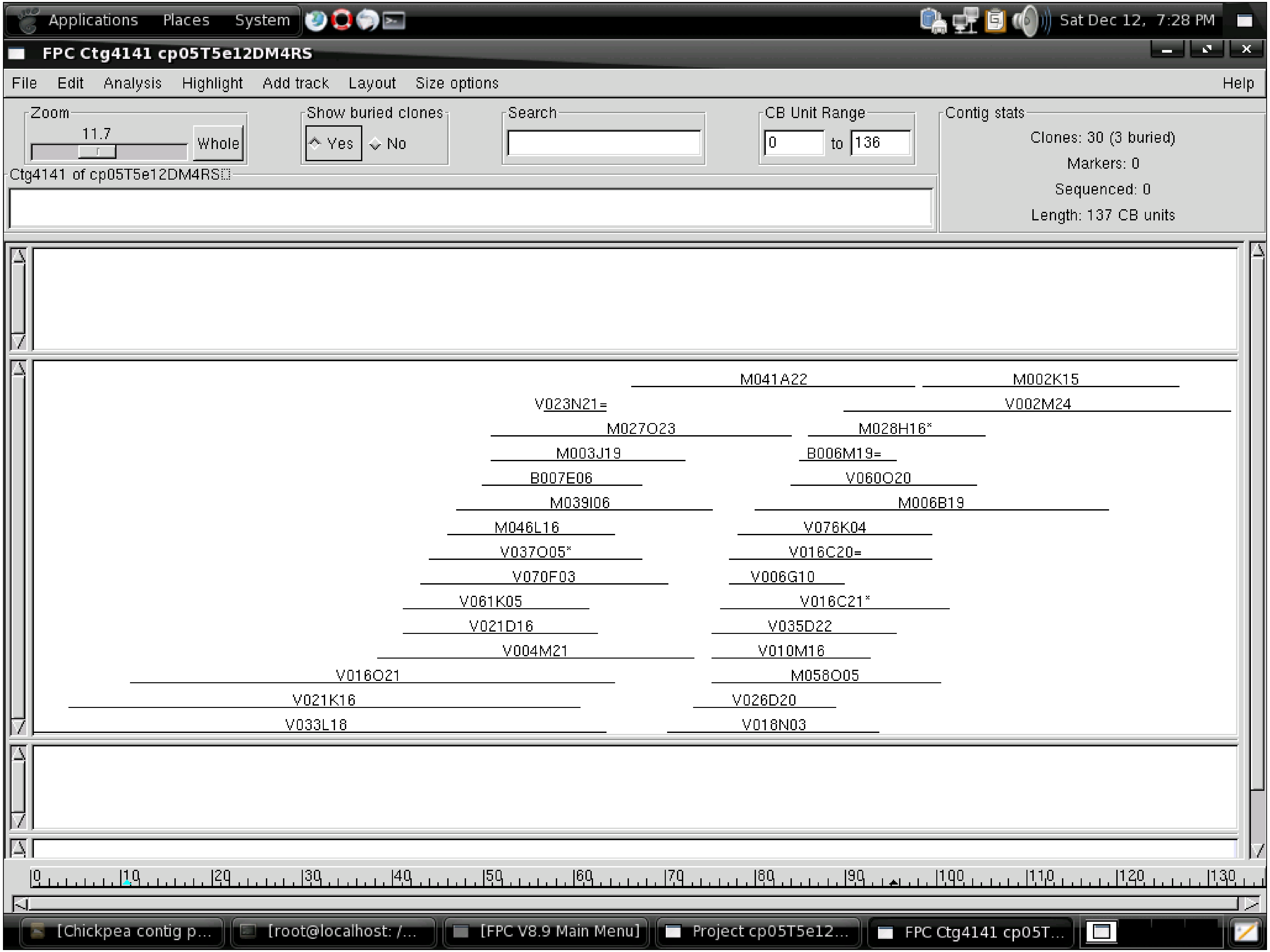
Applications Places System
Sat Dec 12, 7:28 PM
FPC Ctg4141 cp05T5e12DM4RS
File Edit Analysis Highlight Add track Layout Size options Help
Zoom
11.7
Whole
Show buried clones
Yes No
Search
CB Unit Range
0 to 136
Contig stats
Clones: 30 (3 buried)
Markers: 0
Sequenced: 0
Length: 137 CB units
Ctg4141 of cp05T5e12DM4RS
M041A22
M002K15
V023N21=
V002M24
M027O23
M028H16*
M003J19
B006M19=
B007E06
V060O20
M039I06
M006B19
M046L16
V076K04
V037O05*
V016C20=
V070F03
V006G10
V061K05
V016C21*
V021D16
V035D22
V004M21
V010M16
V016O21
M058O05
V021K16
V026D20
V033L18
V018N03
0 10 20 30 40 50 60 70 80 90 100 110 120 130
[Chickpea contig p...
[root@localhost: /...
[FPC V8.9 Main Menu]
Project cp05T5e12...
FPC Ctg4141 cp05T...

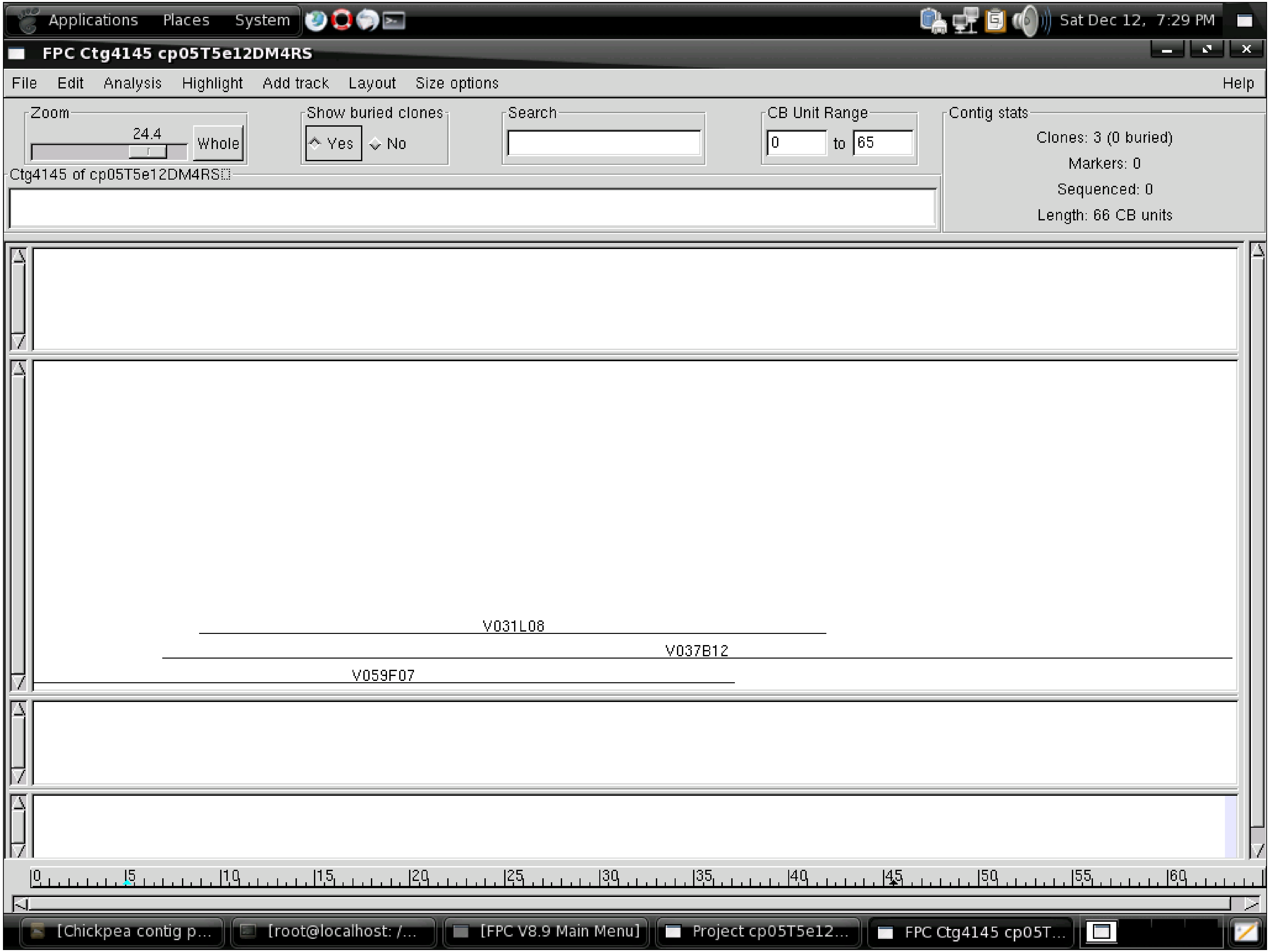
Applications Places System
Sat Dec 12, 7:29 PM
FPC Ctg4145 cp05T5e12DM4RS
File Edit Analysis Highlight Add track Layout Size options Help
Zoom
24.4
Whole
Show buried clones
Yes
No
Search
CB Unit Range
0
to
65
Contig stats
Clones: 3 (0 buried)
Markers: 0
Sequenced: 0
Length: 66 CB units
Ctg4145 of cp05T5e12DM4RS
V031L08
V037B12
V059F07
0 5 10 15 20 25 30 35 40 45 50 55 60
[Chickpea contig p...
[root@localhost: /...
[FPC V8.9 Main Menu]
Project cp05T5e12...
FPC Ctg4145 cp05T...

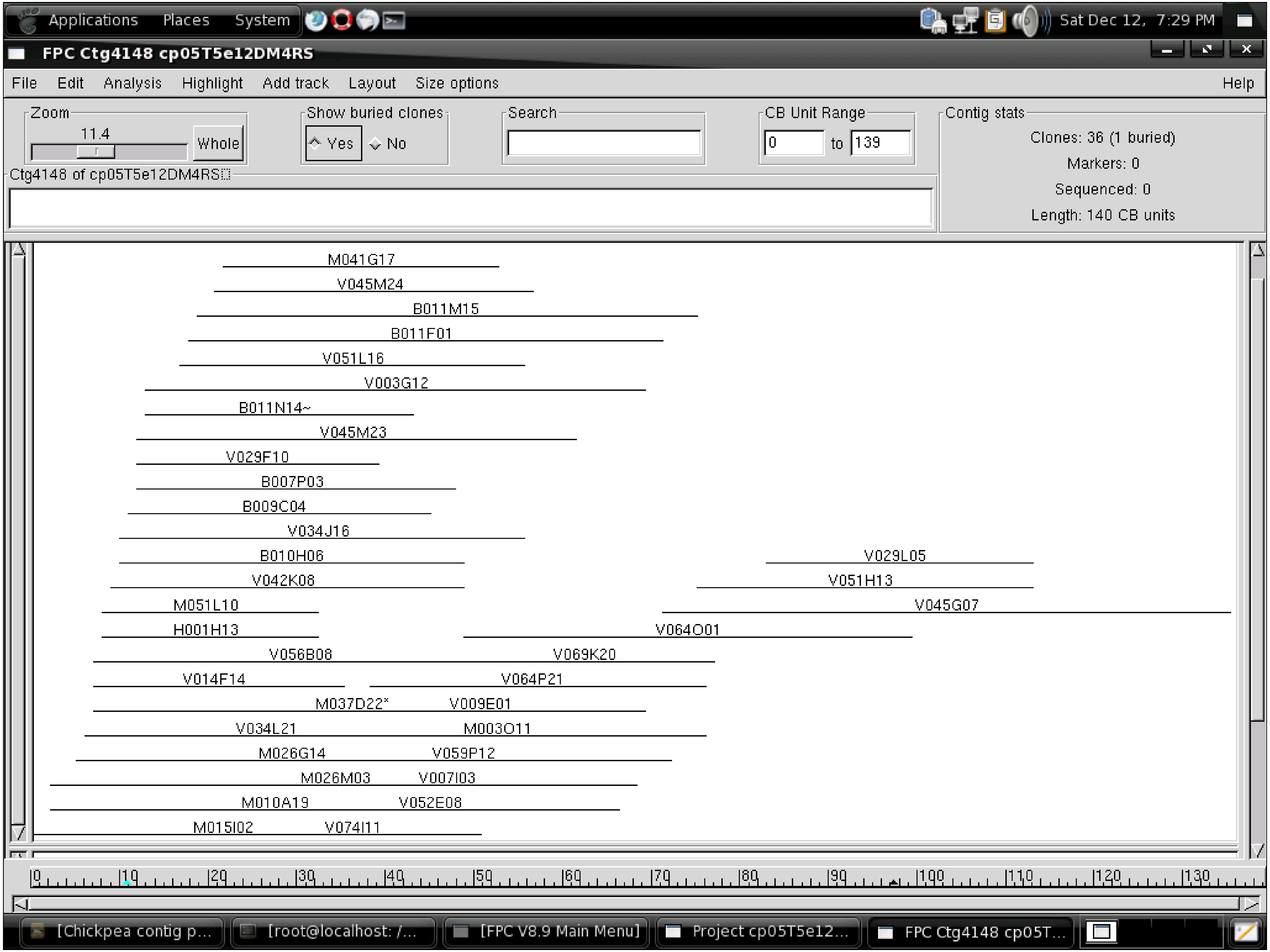
Applications Places System
Sat Dec 12, 7:29 PM
FPC Ctg4148 cp05T5e12DM4RS
File Edit Analysis Highlight Add track Layout Size options Help
Zoom
11.4
Whole
Show buried clones
Yes No
Search
CB Unit Range
0 to 139
Contig stats
Clones: 36 (1 buried)
Markers: 0
Sequenced: 0
Length: 140 CB units
Ctg4148 of cp05T5e12DM4RS
M041G17
V045M24
B011M15
B011F01
V051L16
V003G12
B011N14~
V045M23
V029F10
B007P03
B009C04
V034J16
B010H06
V029L05
V042K08
V051H13
M051L10
V045G07
H001H13
V064O01
V056B08
V069K20
V014F14
V064P21
M037D22*
V009E01
V034L21
M003O11
M026G14
V059P12
M026M03
V007I03
M010A19
V052E08
M015I02
V074I11
0 10 20 30 40 50 60 70 80 90 100 110 120 130
[Chickpea contig p...
[root@localhost: /...
[FPC V8.9 Main Menu]
Project cp05T5e12...
FPC Ctg4148 cp05T...

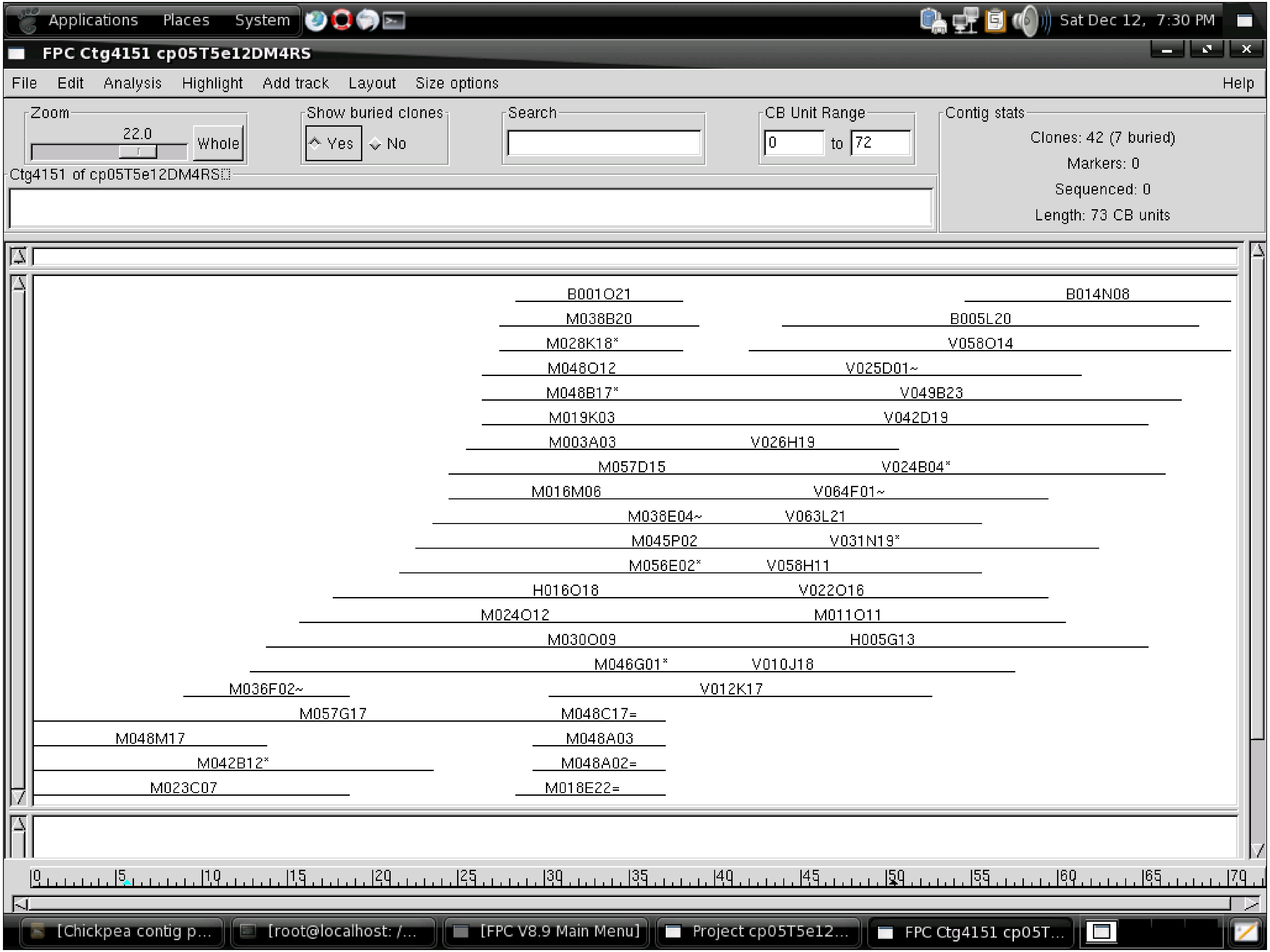
Applications Places System
Sat Dec 12, 7:30 PM
FPC Ctg4151 cp05T5e12DM4RS
File Edit Analysis Highlight Add track Layout Size options Help
Zoom
22.0
Whole
Show buried clones
Yes
No
Search
CB Unit Range
0
to
72
Contig stats
Clones: 42 (7 buried)
Markers: 0
Sequenced: 0
Length: 73 CB units
Ctg4151 of cp05T5e12DM4RS
B001O21
B014N08
M038B20
B005L20
M028K18*
V058O14
M048O12
V025D01~
M048B17*
V049B23
M019K03
V042D19
M003A03
V026H19
M057D15
V024B04*
M016M06
V064F01~
M038E04~
V063L21
M045P02
V031N19*
M056E02*
V058H11
H016O18
V022O16
M024O12
M011O11
M030O09
H005G13
M046G01*
V010J18
M036F02~
V012K17
M057G17
M048C17=
M048M17
M048A03
M042B12*
M048A02=
M023C07
M018E22=
0 5 10 15 20 25 30 35 40 45 50 55 60 65 70
[Chickpea contig p...
[root@localhost: /...
[FPC V8.9 Main Menu]
Project cp05T5e12...
FPC Ctg4151 cp05T...

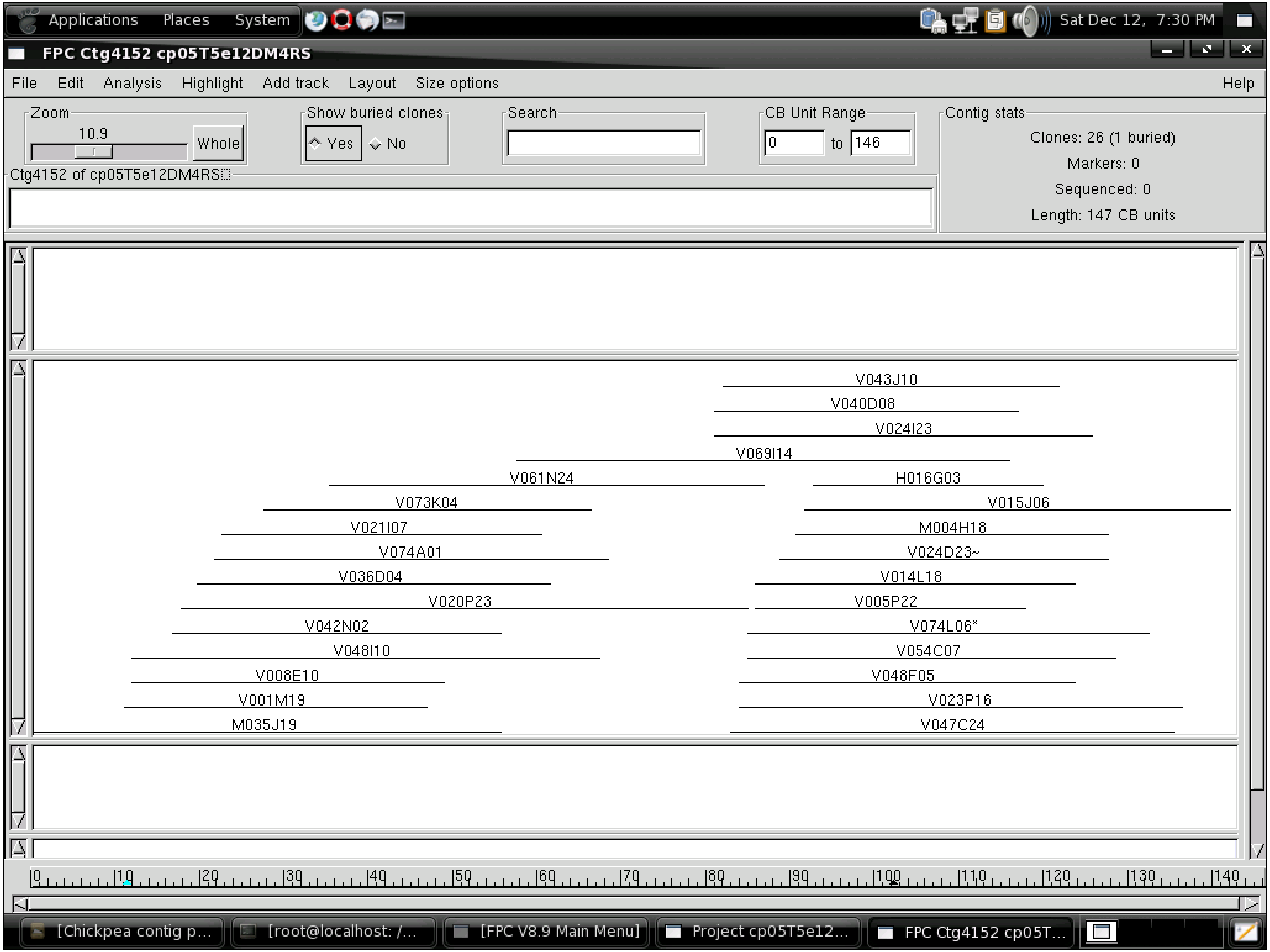
Applications Places System
Sat Dec 12, 7:30 PM
FPC Ctg4152 cp05T5e12DM4RS
File Edit Analysis Highlight Add track Layout Size options Help
Zoom
10.9
Whole
Show buried clones
Yes No
Search
CB Unit Range
0 to 146
Contig stats
Clones: 26 (1 buried)
Markers: 0
Sequenced: 0
Length: 147 CB units
Ctg4152 of cp05T5e12DM4RS
V043J10
V040D08
V024I23
V069I14
V061N24
H016G03
V073K04
V015J06
V021I07
M004H18
V074A01
V024D23~
V036D04
V014L18
V020P23
V005P22
V042N02
V074L06*
V048I10
V054C07
V008E10
V048F05
V001M19
V023P16
M035J19
V047C24
0 10 20 30 40 50 60 70 80 90 100 110 120 130 140
[Chickpea contig p...
[root@localhost: /...
[FPC V8.9 Main Menu]
Project cp05T5e12...
FPC Ctg4152 cp05T...

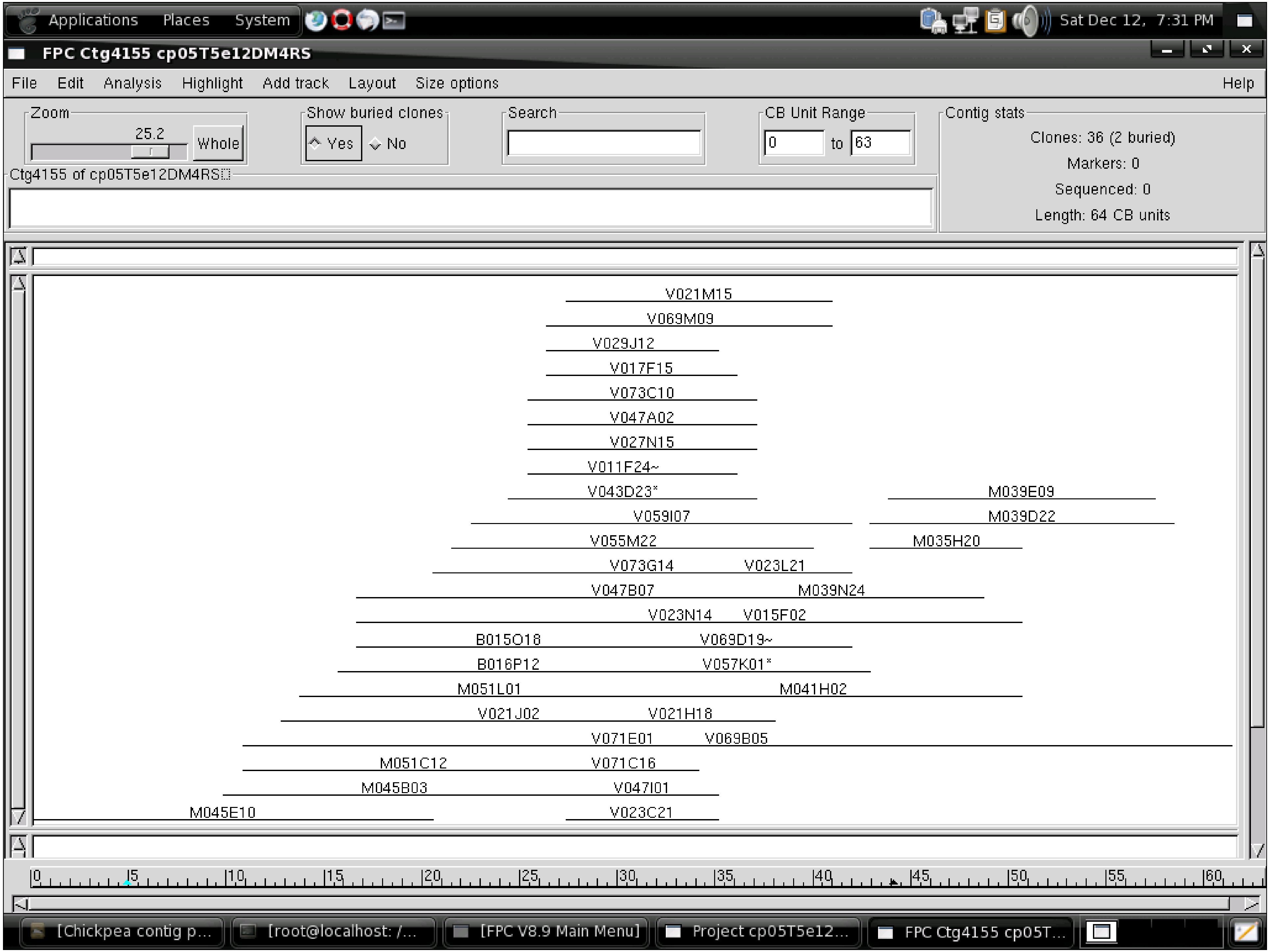
Applications Places System
Sat Dec 12, 7:31 PM
FPC Ctg4155 cp05T5e12DM4RS
File Edit Analysis Highlight Add track Layout Size options Help
Zoom
25.2
Whole
Show buried clones
Yes
No
Search
CB Unit Range
0 to 63
Contig stats
Clones: 36 (2 buried)
Markers: 0
Sequenced: 0
Length: 64 CB units
Ctg4155 of cp05T5e12DM4RS
V021M15
V069M09
V029J12
V017F15
V073C10
V047A02
V027N15
V011F24~
V043D23*
M039E09
V059I07
M039D22
V055M22
M035H20
V073G14
V023L21
V047B07
M039N24
V023N14
V015F02
B015O18
V069D19~
B016P12
V057K01*
M051L01
M041H02
V021J02
V021H18
V071E01
V069B05
M051C12
V071C16
M045B03
V047I01
M045E10
V023C21
0 5 10 15 20 25 30 35 40 45 50 55 60
[Chickpea contig p...
[root@localhost: /...
[FPC V8.9 Main Menu]
Project cp05T5e12...
FPC Ctg4155 cp05T...

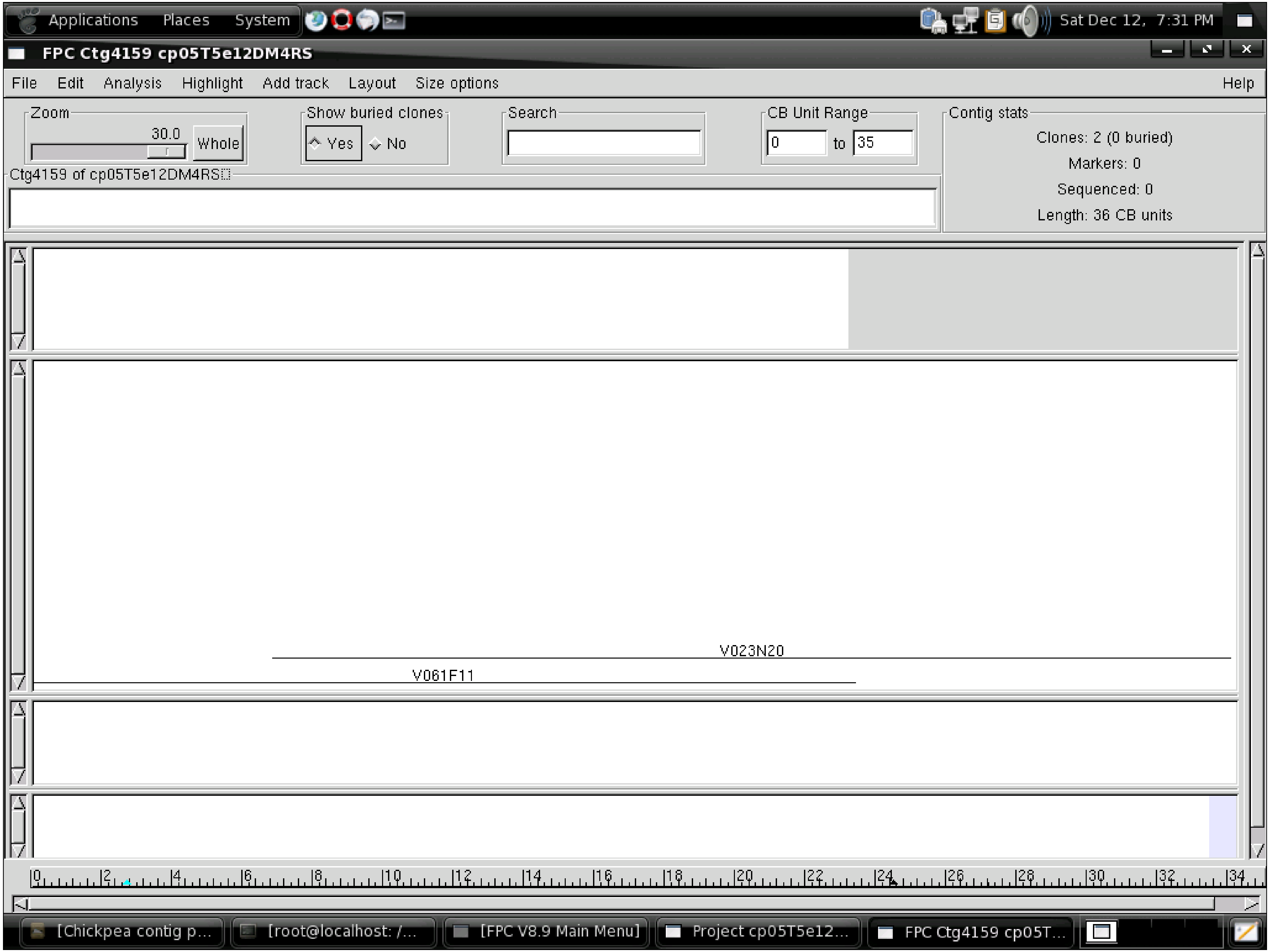

Applications Places System
Sat Dec 12, 7:31 PM
FPC Ctg4159 cp05T5e12DM4RS
File Edit Analysis Highlight Add track Layout Size options Help
Zoom
30.0
Whole
Show buried clones
Yes
No
Search
CB Unit Range
0 to 35
Contig stats
Clones: 2 (0 buried)
Markers: 0
Sequenced: 0
Length: 36 CB units
Ctg4159 of cp05T5e12DM4RS
V023N20
V061F11
0 2 4 6 8 10 12 14 16 18 20 22 24 26 28 30 32 34
[Chickpea contig p...
[root@localhost: /...
[FPC V8.9 Main Menu]
Project cp05T5e12...
FPC Ctg4159 cp05T...

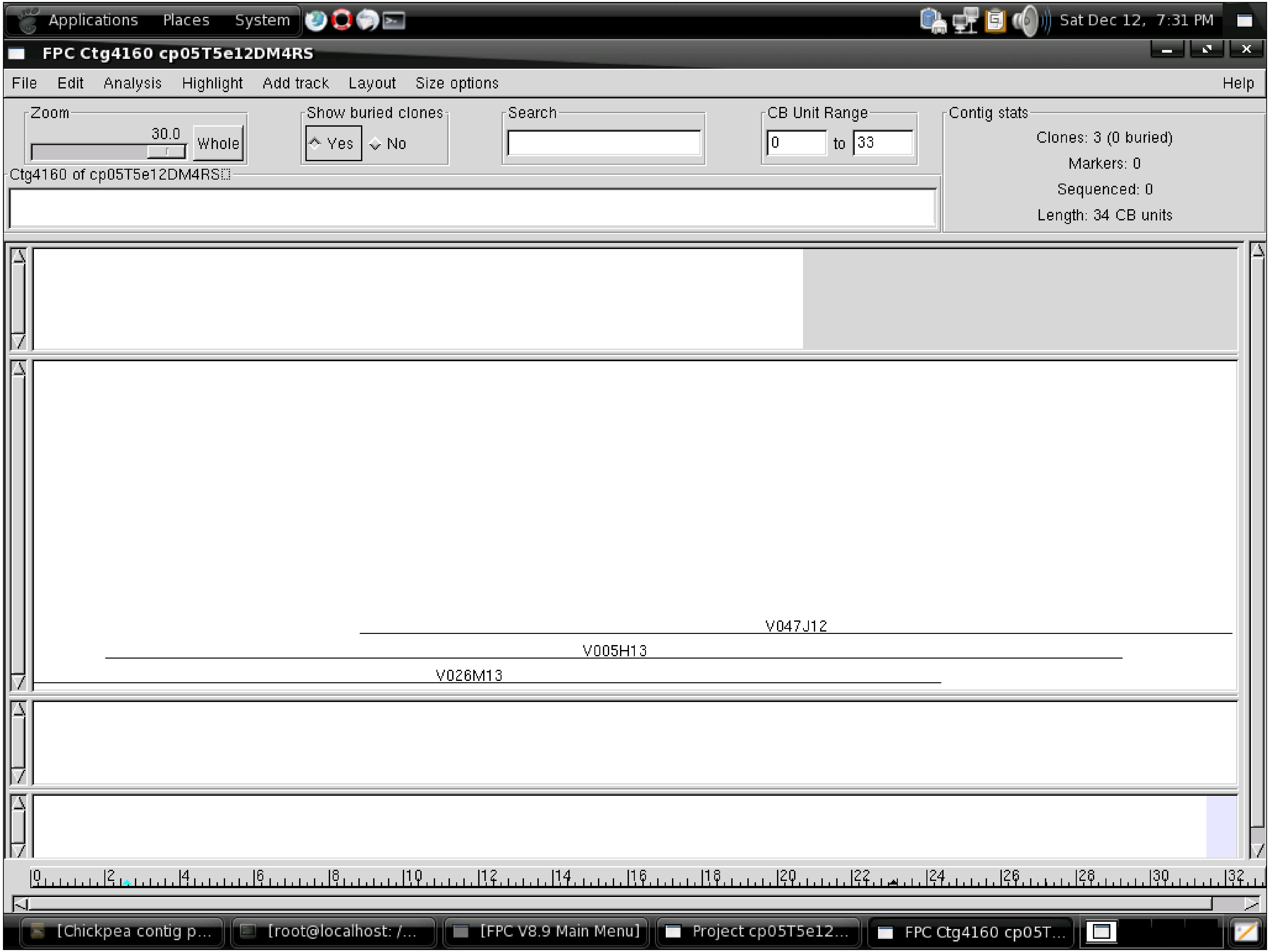
Applications Places System
Sat Dec 12, 7:31 PM
FPC Ctg4160 cp05T5e12DM4RS
File Edit Analysis Highlight Add track Layout Size options Help
Zoom
30.0
Whole
Show buried clones
Yes
No
Search
CB Unit Range
0
to
33
Contig stats
Clones: 3 (0 buried)
Markers: 0
Sequenced: 0
Length: 34 CB units
Ctg4160 of cp05T5e12DM4RS
V047J12
V005H13
V026M13
0 2 4 6 8 10 12 14 16 18 20 22 24 26 28 30 32
[Chickpea contig p...
[root@localhost: /...
[FPC V8.9 Main Menu]
Project cp05T5e12...
FPC Ctg4160 cp05T...

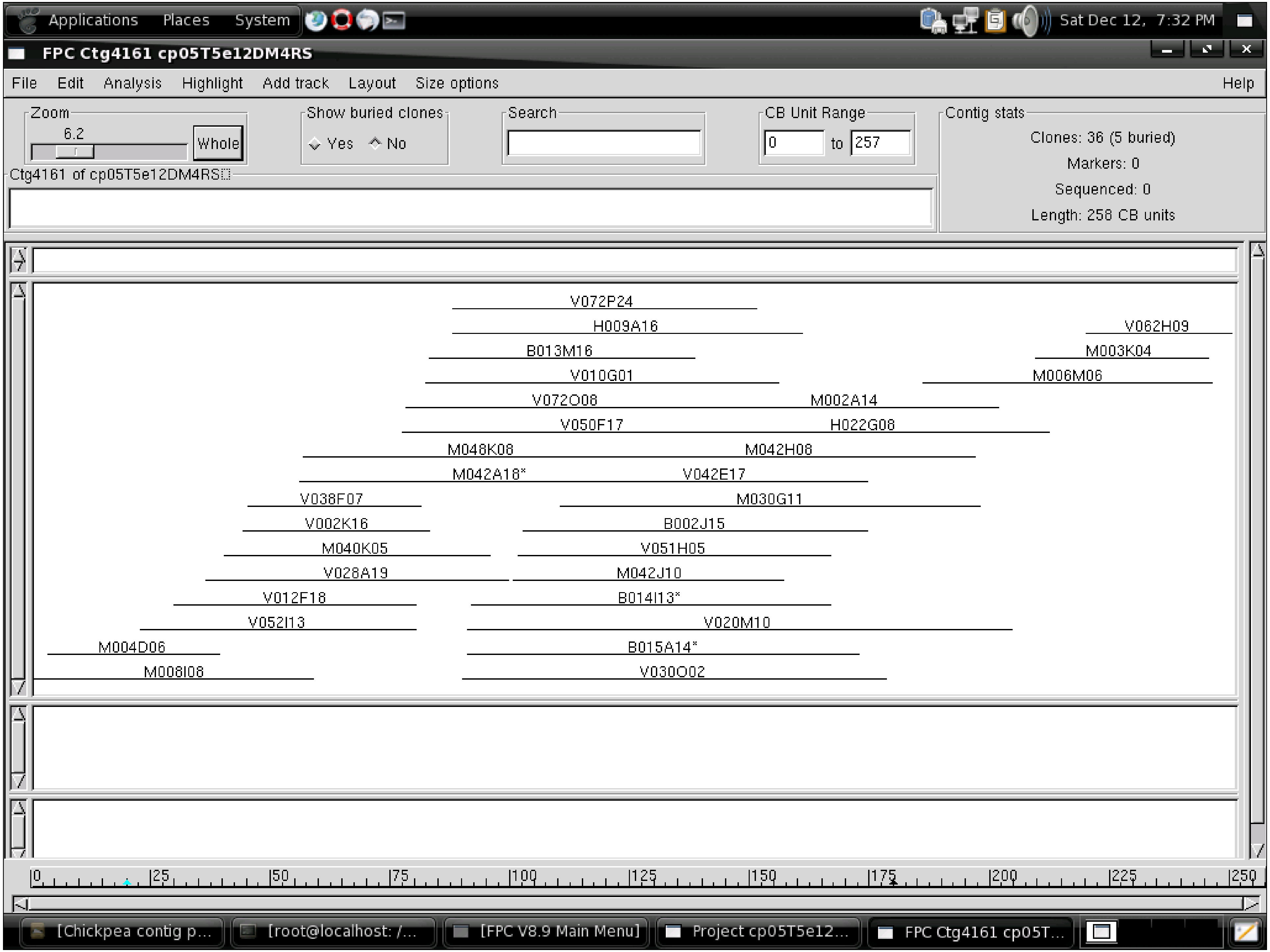
Applications Places System
Sat Dec 12, 7:32 PM
FPC Ctg4161 cp05T5e12DM4RS
File Edit Analysis Highlight Add track Layout Size options Help
Zoom
6.2
Whole
Show buried clones
Yes No
Search
CB Unit Range
0 to 257
Contig stats
Clones: 36 (5 buried)
Markers: 0
Sequenced: 0
Length: 258 CB units
Ctg4161 of cp05T5e12DM4RS
V072P24
H009A16
V062H09
B013M16
M003K04
V010G01
M006M06
V072O08
M002A14
V050F17
H022G08
M048K08
M042H08
M042A18*
V042E17
V038F07
M030G11
V002K16
B002J15
M040K05
V051H05
V028A19
M042J10
V012F18
B014I13*
V052I13
V020M10
M004D06
B015A14*
M008I08
V030O02
0 25 50 75 100 125 150 175 200 225 250
[Chickpea contig p...
[root@localhost: /...
[FPC V8.9 Main Menu]
Project cp05T5e12...
FPC Ctg4161 cp05T...

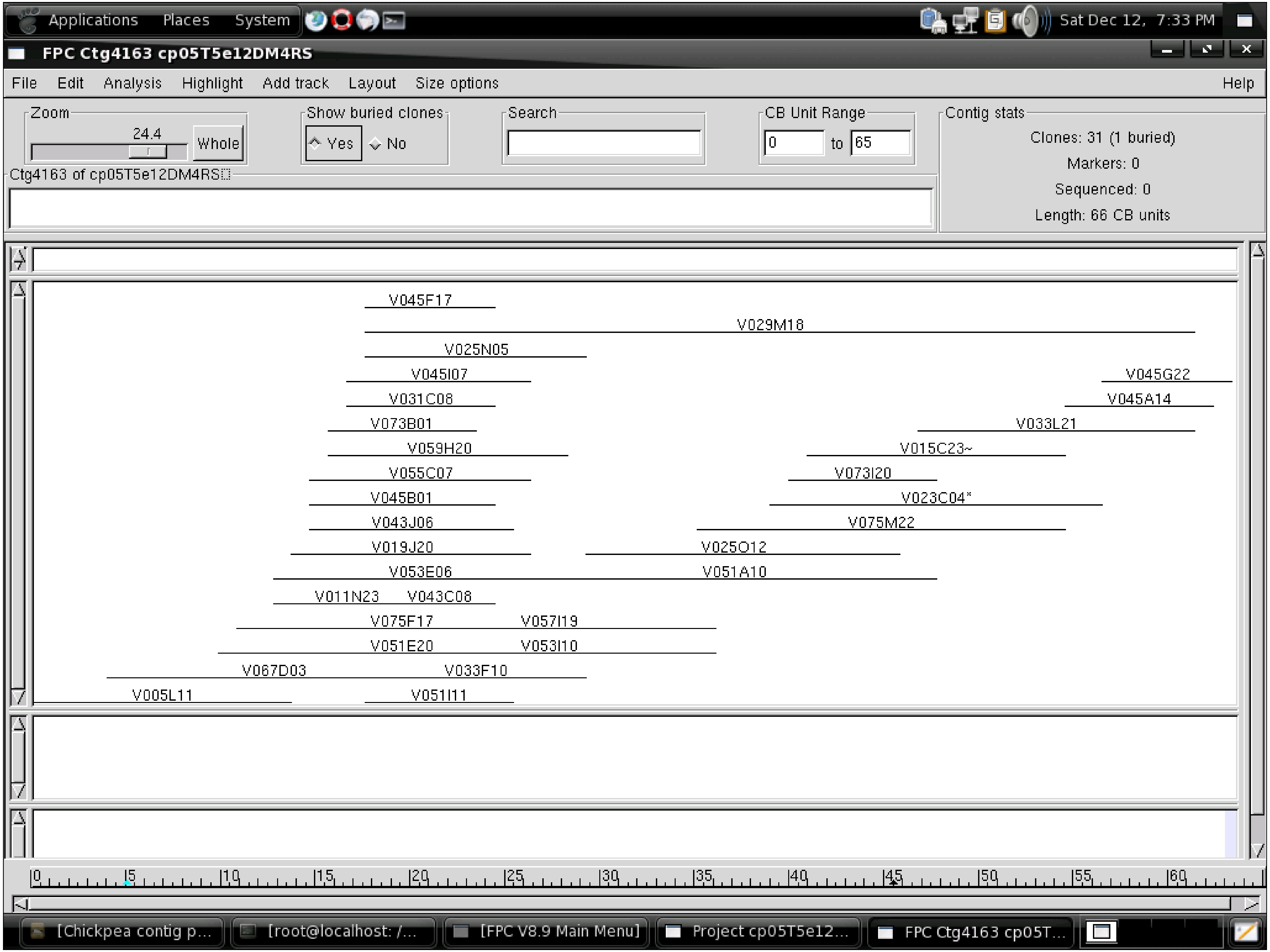
Applications Places System
Sat Dec 12, 7:33 PM
FPC Ctg4163 cp05T5e12DM4RS
File Edit Analysis Highlight Add track Layout Size options Help
Zoom
24.4
Whole
Show buried clones
Yes No
Search
CB Unit Range
0 to 65
Contig stats
Clones: 31 (1 buried)
Markers: 0
Sequenced: 0
Length: 66 CB units
Ctg4163 of cp05T5e12DM4RS
V045F17
V029M18
V025N05
V045I07
V045G22
V031C08
V045A14
V073B01
V033L21
V059H20
V015C23~
V055C07
V073I20
V045B01
V023C04*
V043J06
V075M22
V019J20
V025O12
V053E06
V051A10
V011N23
V043C08
V075F17
V057I19
V051E20
V053I10
V067D03
V033F10
V005L11
V051I11
0 5 10 15 20 25 30 35 40 45 50 55 60
[Chickpea contig p...
[root@localhost: /...
[FPC V8.9 Main Menu]
Project cp05T5e12...
FPC Ctg4163 cp05T...

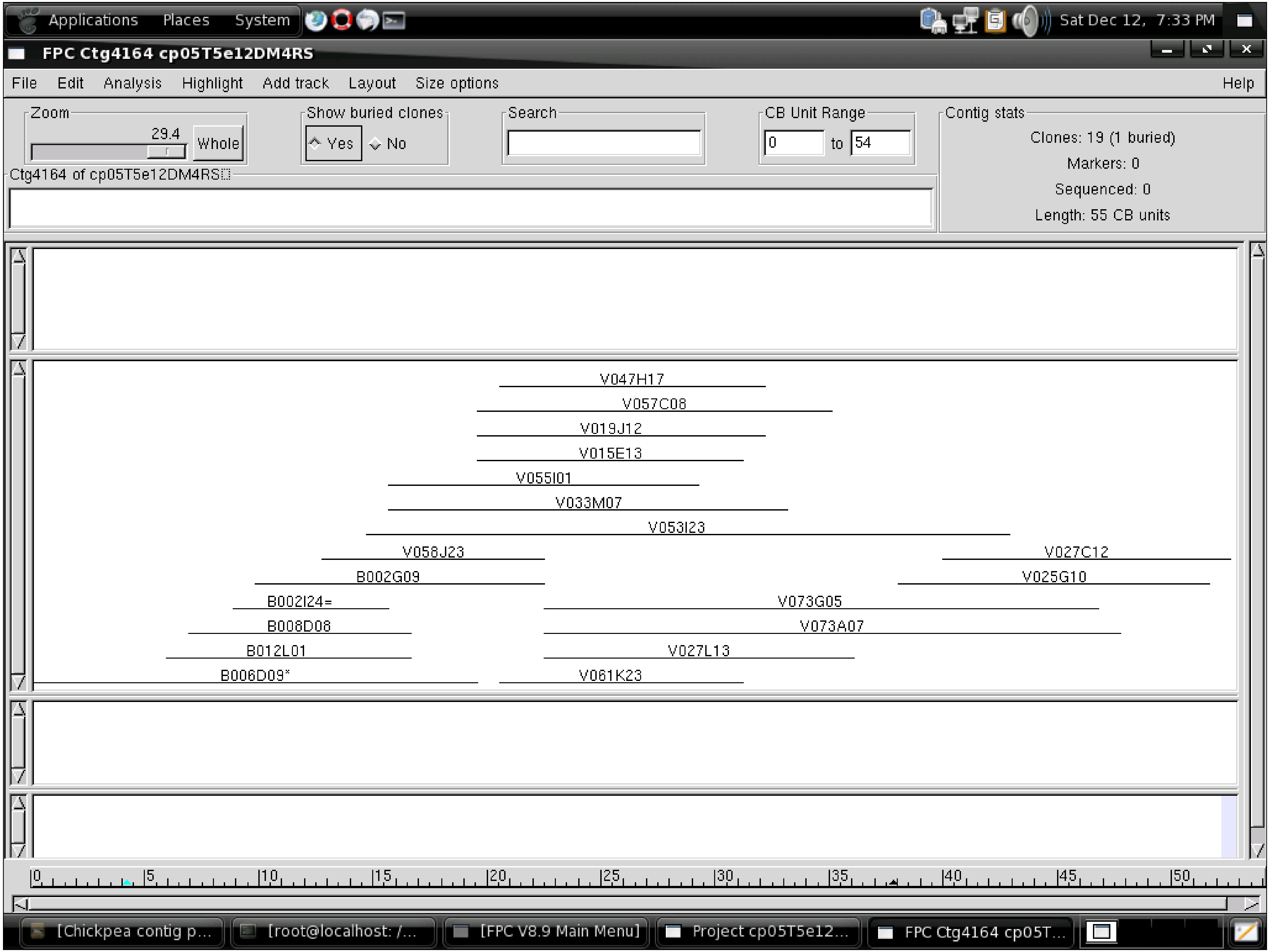
Applications Places System
Sat Dec 12, 7:33 PM
FPC Ctg4164 cp05T5e12DM4RS
File Edit Analysis Highlight Add track Layout Size options Help
Zoom
29.4
Whole
Show buried clones
Yes No
Search
CB Unit Range
0 to 54
Contig stats
Clones: 19 (1 buried)
Markers: 0
Sequenced: 0
Length: 55 CB units
Ctg4164 of cp05T5e12DM4RS
V047H17
V057C08
V019J12
V015E13
V055I01
V033M07
V053I23
V058J23
V027C12
B002G09
V025G10
B002I24=
V073G05
B008D08
V073A07
B012L01
V027L13
B006D09*
V061K23
0 5 10 15 20 25 30 35 40 45 50
[Chickpea contig p...
[root@localhost: /...
[FPC V8.9 Main Menu]
Project cp05T5e12...
FPC Ctg4164 cp05T...

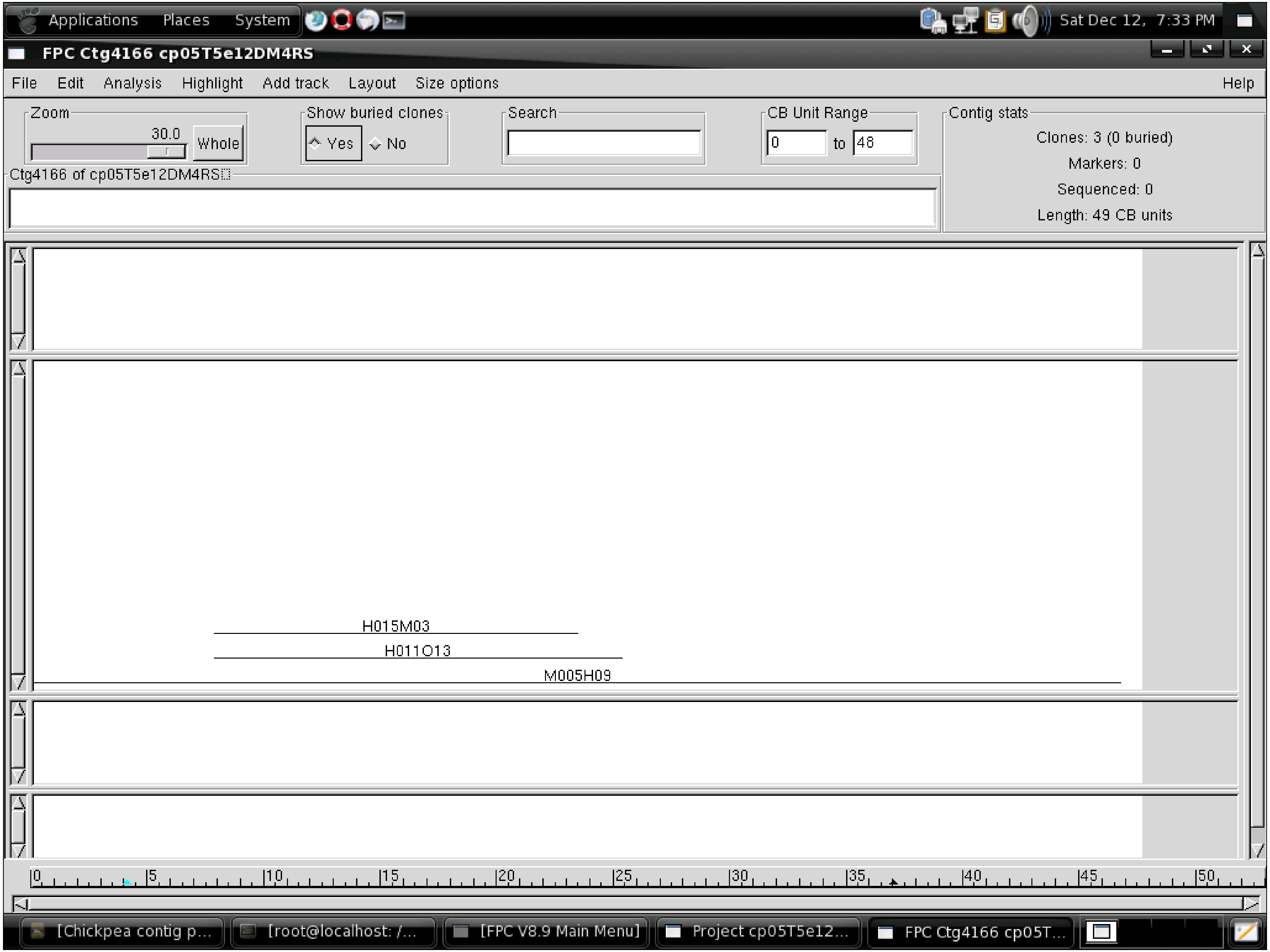

Applications Places System
Sat Dec 12, 7:33 PM
FPC Ctg4166 cp05T5e12DM4RS
File Edit Analysis Highlight Add track Layout Size options Help
Zoom
30.0
Whole
Show buried clones
Yes
No
Search
CB Unit Range
0 to 48
Contig stats
Clones: 3 (0 buried)
Markers: 0
Sequenced: 0
Length: 49 CB units
Ctg4166 of cp05T5e12DM4RS
H015M03
H011O13
M005H09
0 5 10 15 20 25 30 35 40 45 50
[Chickpea contig p...
[root@localhost: /...
[FPC V8.9 Main Menu]
Project cp05T5e12...
FPC Ctg4166 cp05T...

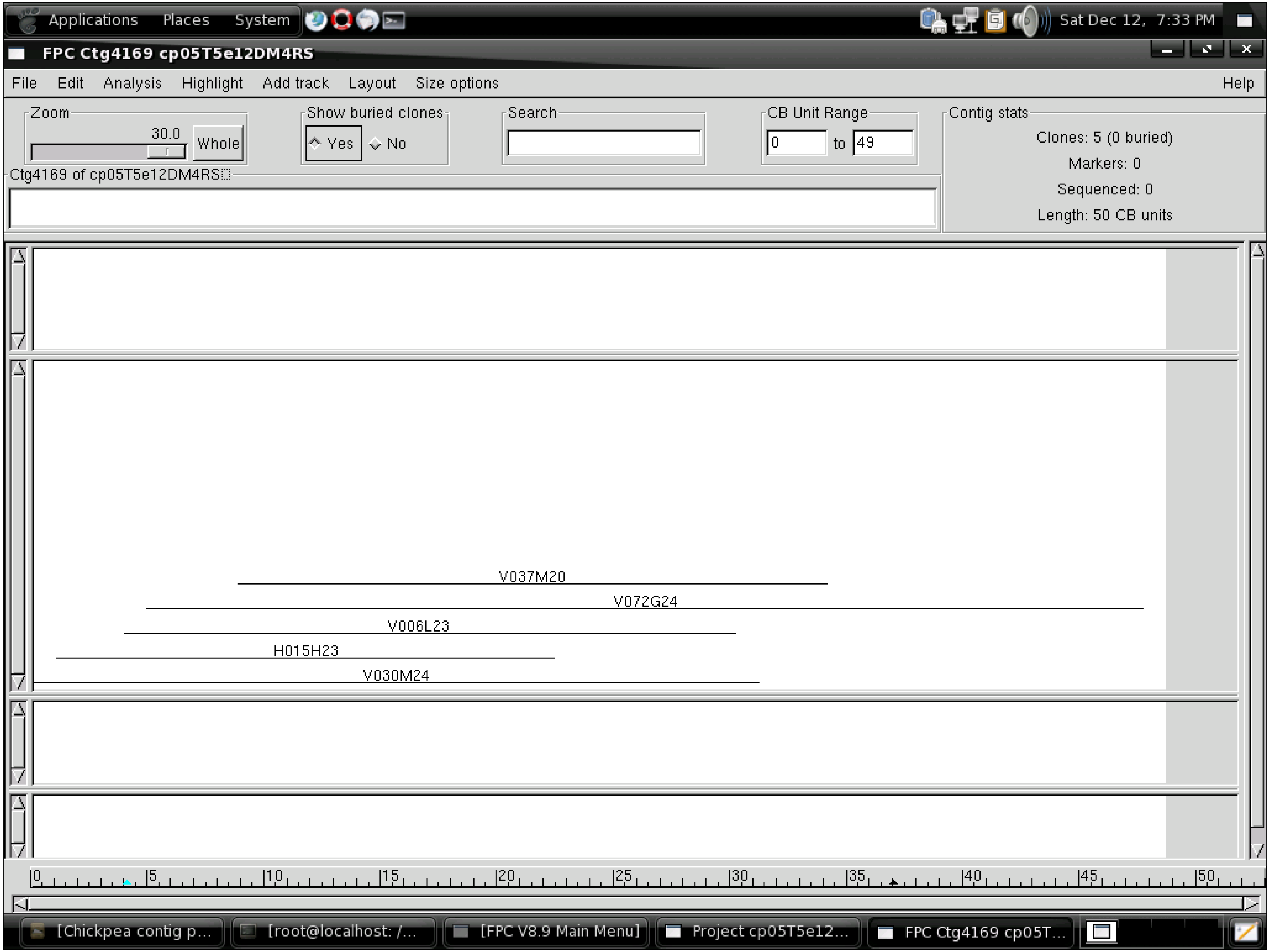
Applications
Places
System
Sat Dec 12, 7:33 PM
FPC Ctg4169 cp05T5e12DM4RS
File
Edit
Analysis
Highlight
Add track
Layout
Size options
Help
Zoom
30.0
Whole
Show buried clones
Yes
No
Search
CB Unit Range
0
to
49
Contig stats
Clones: 5 (0 buried)
Markers: 0
Sequenced: 0
Length: 50 CB units
Ctg4169 of cp05T5e12DM4RS
V037M20
V072G24
V006L23
H015H23
V030M24
0
5
10
15
20
25
30
35
40
45
50
[Chickpea contig p...
[root@localhost: /...
[FPC V8.9 Main Menu]
Project cp05T5e12...
FPC Ctg4169 cp05T...

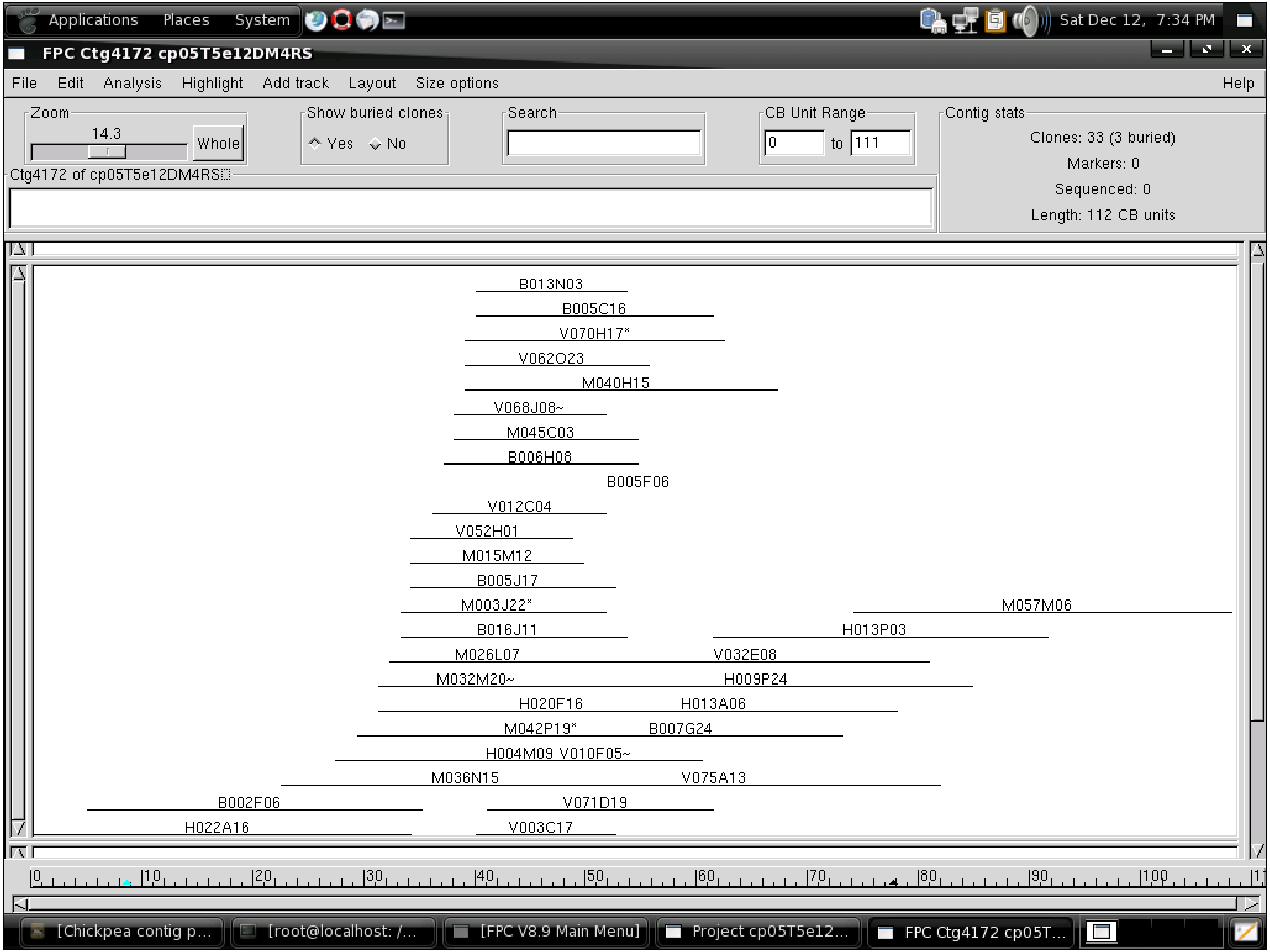
Applications Places System
Sat Dec 12, 7:34 PM
FPC Ctg4172 cp05T5e12DM4RS
File Edit Analysis Highlight Add track Layout Size options Help
Zoom
14.3
Whole
Show buried clones
Yes No
Search
CB Unit Range
0 to 111
Contig stats
Clones: 33 (3 buried)
Markers: 0
Sequenced: 0
Length: 112 CB units
Ctg4172 of cp05T5e12DM4RS
B013N03
B005C16
V070H17*
V062O23
M040H15
V068J08~
M045C03
B006H08
B005F06
V012C04
V052H01
M015M12
B005J17
M003J22*
M057M06
B016J11
H013P03
M026L07
V032E08
M032M20~
H009P24
H020F16
H013A06
M042P19*
B007G24
H004M09 V010F05~
M036N15
V075A13
B002F06
V071D19
H022A16
V003C17
0 10 20 30 40 50 60 70 80 90 100
[Chickpea contig p...
[root@localhost: /...
[FPC V8.9 Main Menu]
Project cp05T5e12...
FPC Ctg4172 cp05T...

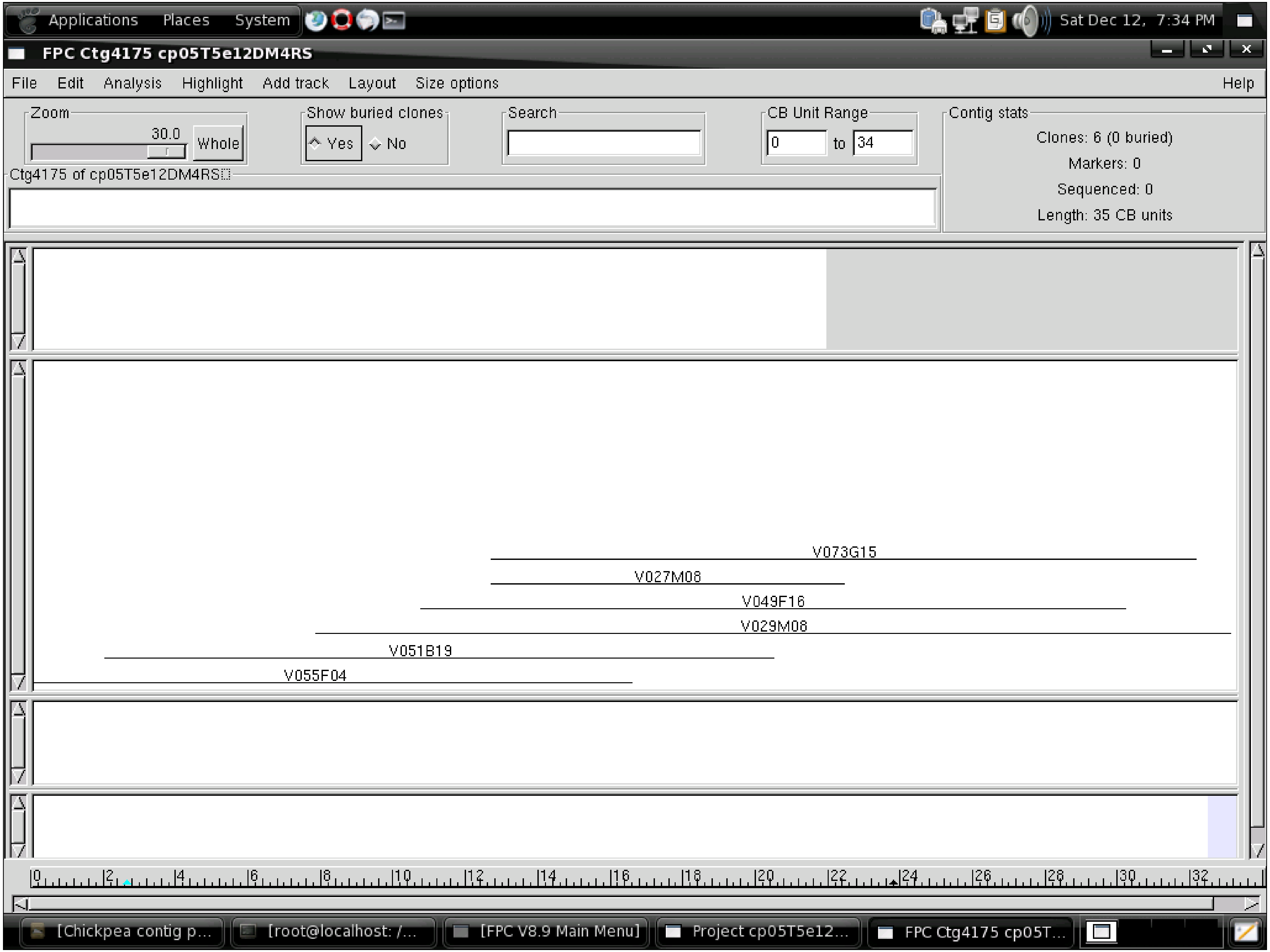
Applications Places System
Sat Dec 12, 7:34 PM
FPC Ctg4175 cp05T5e12DM4RS
File Edit Analysis Highlight Add track Layout Size options Help
Zoom 30.0 Whole
Show buried clones Yes No
Search
CB Unit Range 0 to 34
Contig stats
Clones: 6 (0 buried)
Markers: 0
Sequenced: 0
Length: 35 CB units
Ctg4175 of cp05T5e12DM4RS
V073G15
V027M08
V049F16
V029M08
V051B19
V055F04
0 2 4 6 8 10 12 14 16 18 20 22 24 26 28 30 32
[Chickpea contig p...
[root@localhost: /...
[FPC V8.9 Main Menu]
Project cp05T5e12...
FPC Ctg4175 cp05T...

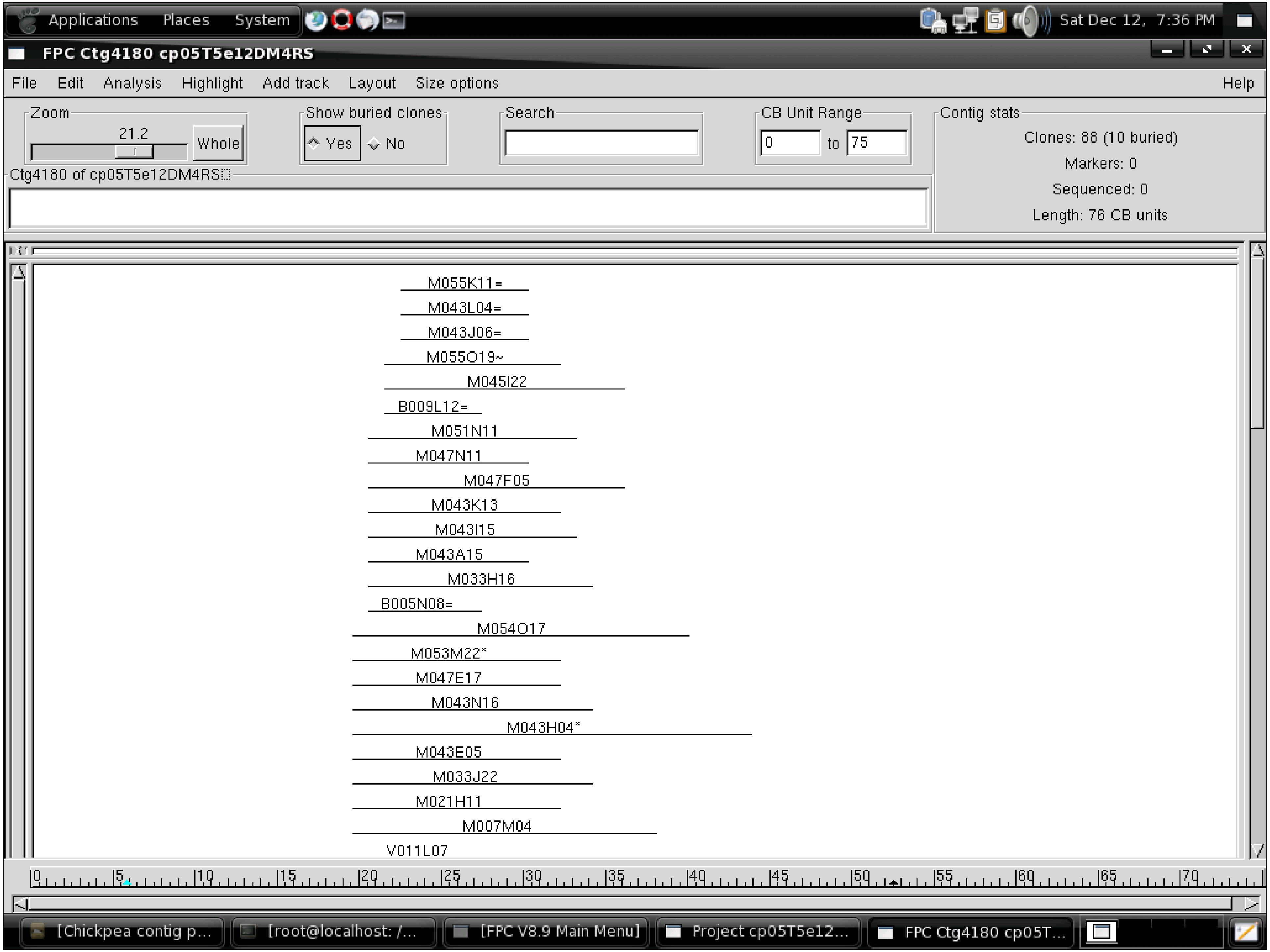
Applications Places System
Sat Dec 12, 7:36 PM
FPC Ctg4180 cp05T5e12DM4RS
File Edit Analysis Highlight Add track Layout Size options Help
Zoom
21.2
Whole
Show buried clones
Yes No
Search
CB Unit Range
0 to 75
Contig stats
Clones: 88 (10 buried)
Markers: 0
Sequenced: 0
Length: 76 CB units
Ctg4180 of cp05T5e12DM4RS
M055K11=
M043L04=
M043J06=
M055O19~
M045I22
B009L12=
M051N11
M047N11
M047F05
M043K13
M043I15
M043A15
M033H16
B005N08=
M054O17
M053M22*
M047E17
M043N16
M043H04*
M043E05
M033J22
M021H11
M007M04
V011L07
0 5 10 15 20 25 30 35 40 45 50 55 60 65 70
[Chickpea contig p...
[root@localhost: /...
[FPC V8.9 Main Menu]
Project cp05T5e12...
FPC Ctg4180 cp05T...

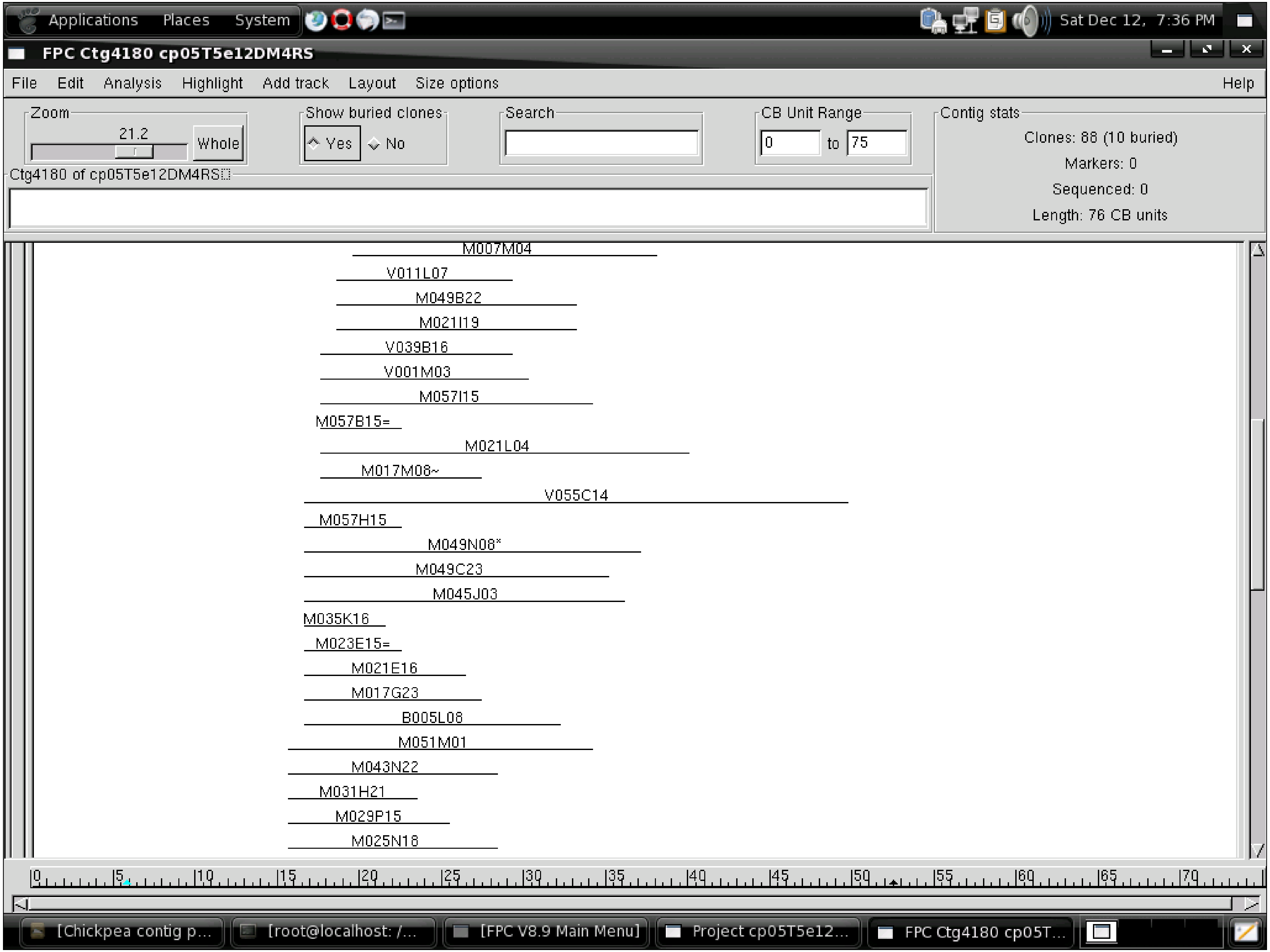
Applications Places System
Sat Dec 12, 7:36 PM
FPC Ctg4180 cp05T5e12DM4RS
File Edit Analysis Highlight Add track Layout Size options Help
Zoom
21.2
Whole
Show buried clones
Yes No
Search
CB Unit Range
0 to 75
Contig stats
Clones: 88 (10 buried)
Markers: 0
Sequenced: 0
Length: 76 CB units
Ctg4180 of cp05T5e12DM4RS
M007M04
V011L07
M049B22
M021I19
V039B16
V001M03
M057I15
M057B15=
M021L04
M017M08~
V055C14
M057H15
M049N08*
M049C23
M045J03
M035K16
M023E15=
M021E16
M017G23
B005L08
M051M01
M043N22
M031H21
M029P15
M025N18
0 5 10 15 20 25 30 35 40 45 50 55 60 65 70
[Chickpea contig p...
[root@localhost: /...
[FPC V8.9 Main Menu]
Project cp05T5e12...
FPC Ctg4180 cp05T...

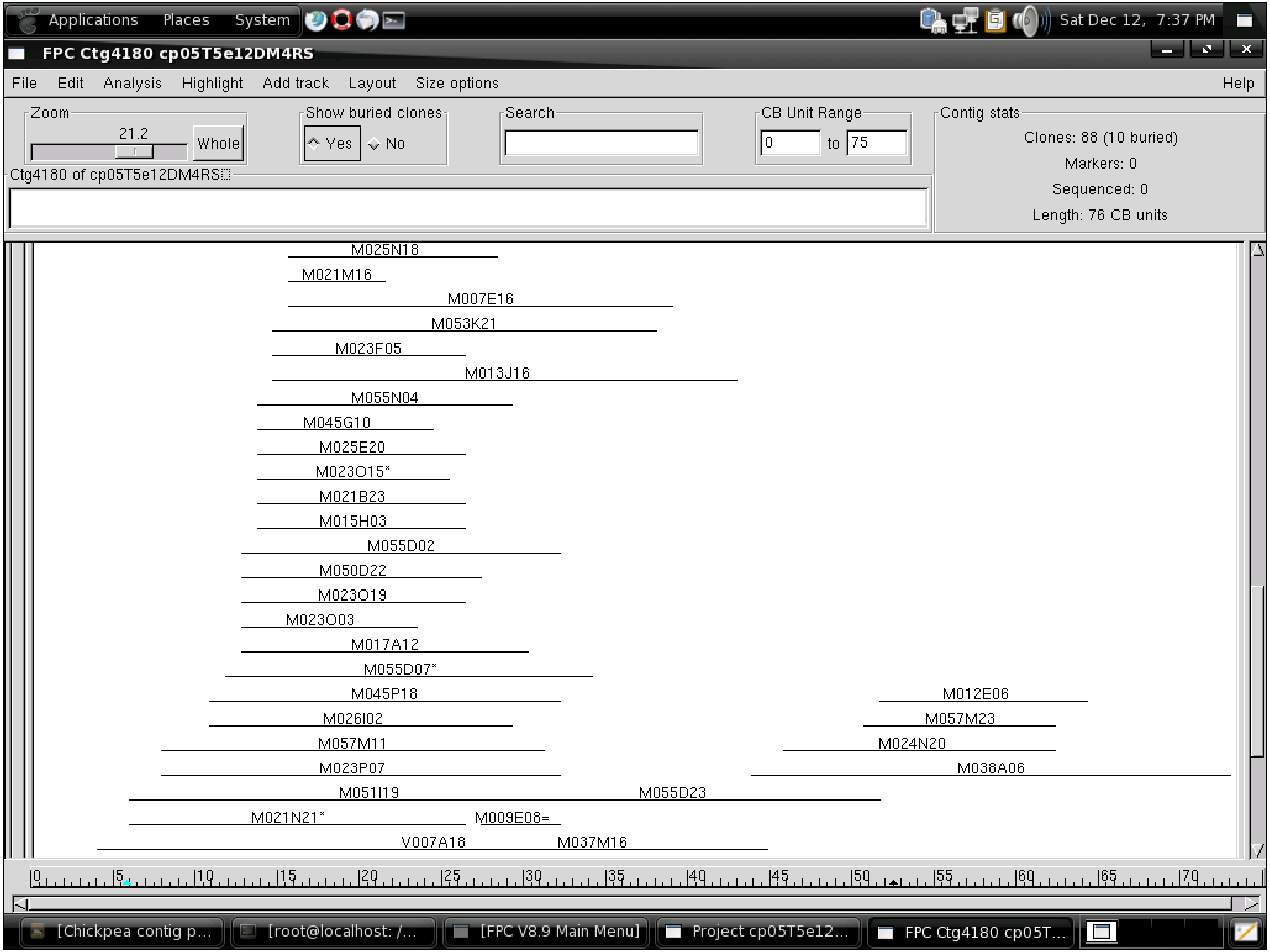
Applications Places System
Sat Dec 12, 7:37 PM
FPC Ctg4180 cp05T5e12DM4RS
File Edit Analysis Highlight Add track Layout Size options Help
Zoom
21.2
Whole
Show buried clones
Yes No
Search
CB Unit Range
0 to 75
Contig stats
Clones: 88 (10 buried)
Markers: 0
Sequenced: 0
Length: 76 CB units
Ctg4180 of cp05T5e12DM4RS
M025N18
M021M16
M007E16
M053K21
M023F05
M013J16
M055N04
M045G10
M025E20
M023O15*
M021B23
M015H03
M055D02
M050D22
M023O19
M023O03
M017A12
M055D07*
M045P18
M012E06
M026I02
M057M23
M057M11
M024N20
M023P07
M038A06
M051I19
M055D23
M021N21*
M009E08=
V007A18
M037M16
0 5 10 15 20 25 30 35 40 45 50 55 60 65 70
[Chickpea contig p...
[root@localhost: /...
[FPC V8.9 Main Menu]
Project cp05T5e12...
FPC Ctg4180 cp05T...

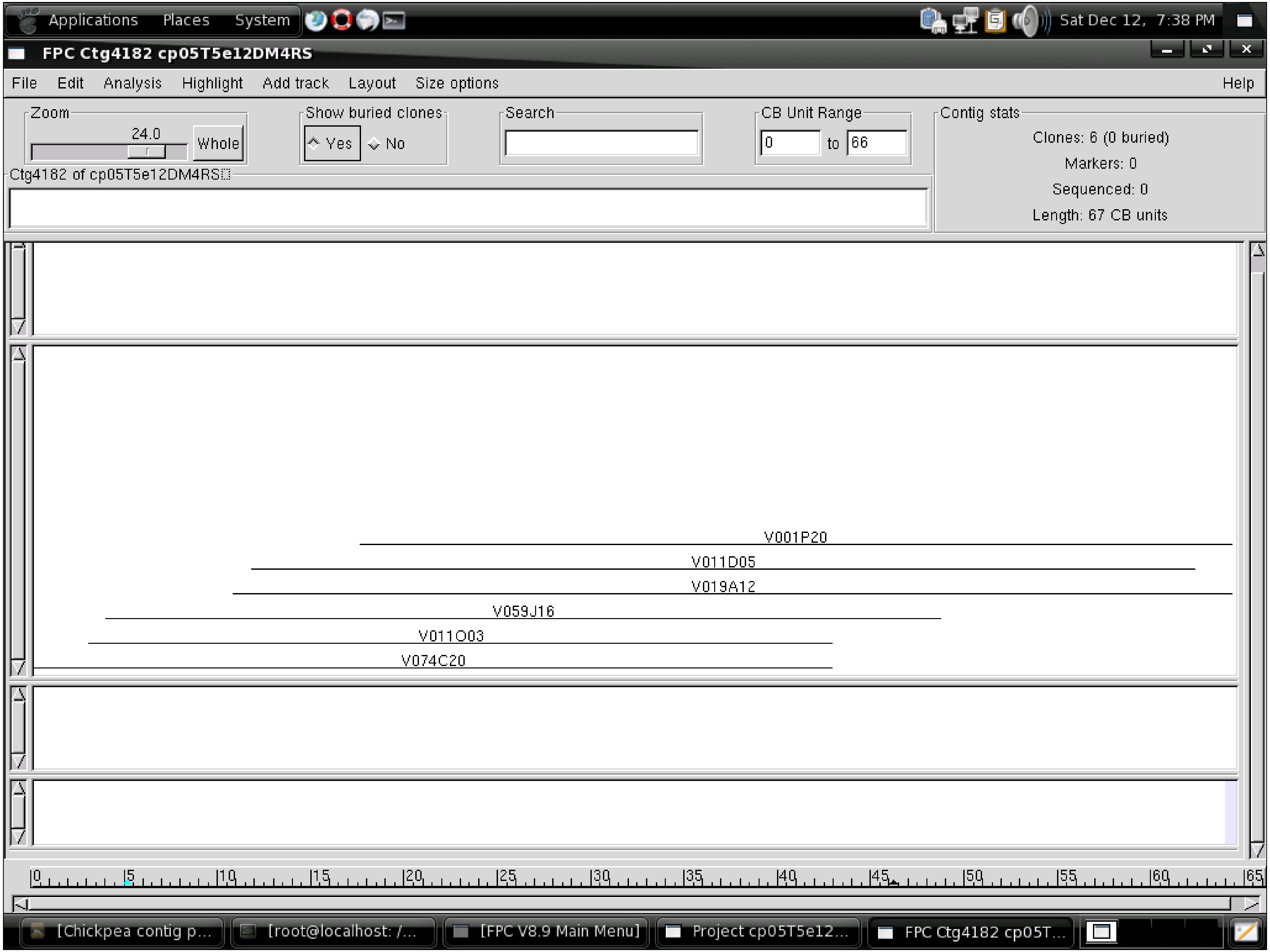
Applications Places System
Sat Dec 12, 7:38 PM
FPC Ctg4182 cp05T5e12DM4RS
File Edit Analysis Highlight Add track Layout Size options Help
Zoom
24.0
Whole
Show buried clones
Yes No
Search
CB Unit Range
0 to 66
Contig stats
Clones: 6 (0 buried)
Markers: 0
Sequenced: 0
Length: 67 CB units
Ctg4182 of cp05T5e12DM4RS
V001P20
V011D05
V019A12
V059J16
V011O03
V074C20
0 5 10 15 20 25 30 35 40 45 50 55 60 65
[Chickpea contig p...
[root@localhost: /...
[FPC V8.9 Main Menu]
Project cp05T5e12...
FPC Ctg4182 cp05T...

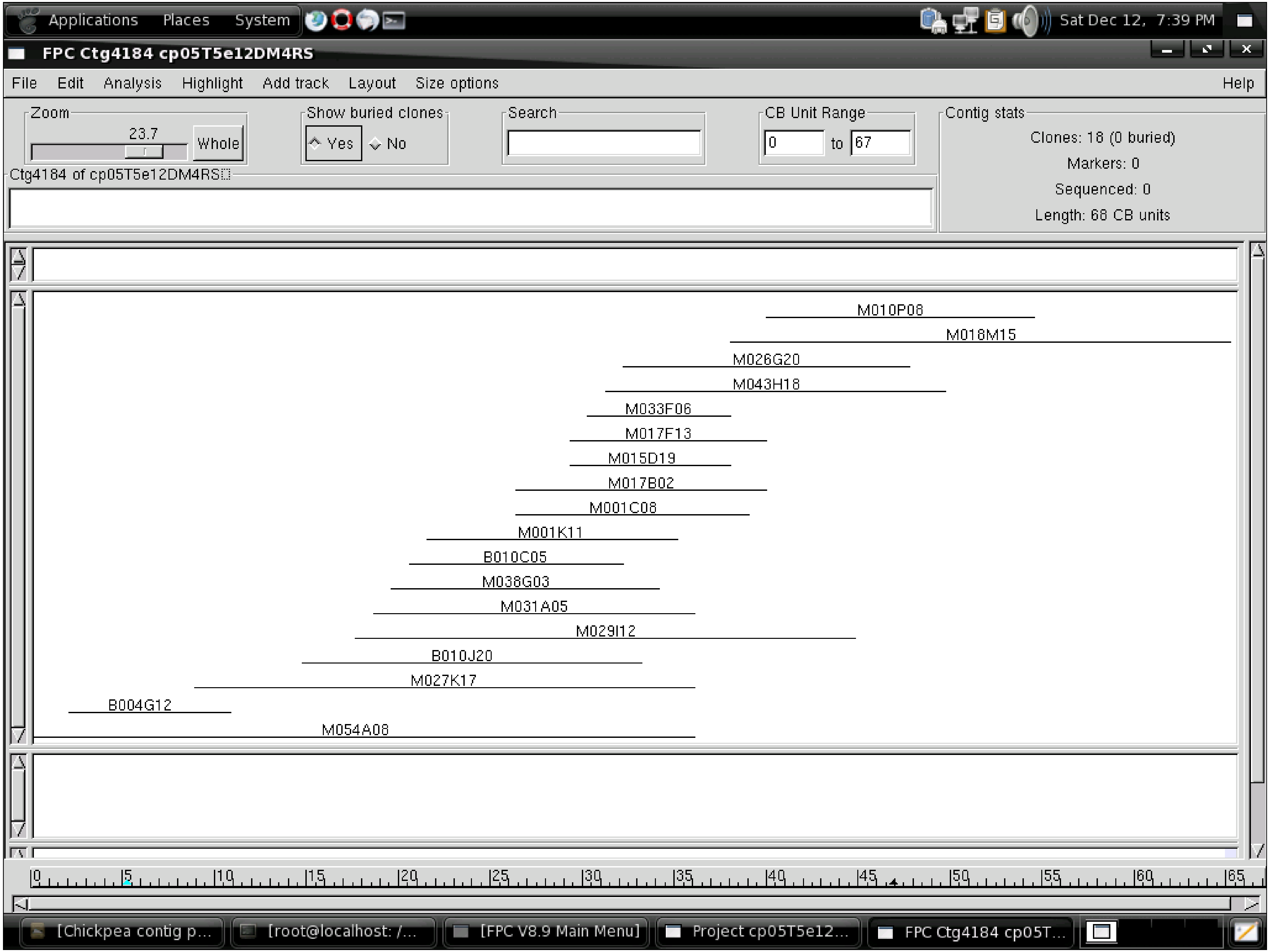
Applications Places System
Sat Dec 12, 7:39 PM
FPC Ctg4184 cp05T5e12DM4RS
File Edit Analysis Highlight Add track Layout Size options Help
Zoom
23.7
Whole
Show buried clones
Yes
No
Search
CB Unit Range
0 to 67
Contig stats
Clones: 18 (0 buried)
Markers: 0
Sequenced: 0
Length: 68 CB units
Ctg4184 of cp05T5e12DM4RS
M010P08
M018M15
M026G20
M043H18
M033F06
M017F13
M015D19
M017B02
M001C08
M001K11
B010C05
M038G03
M031A05
M029I12
B010J20
M027K17
B004G12
M054A08
0 5 10 15 20 25 30 35 40 45 50 55 60 65
[Chickpea contig p...
[root@localhost: /...
[FPC V8.9 Main Menu]
Project cp05T5e12...
FPC Ctg4184 cp05T...

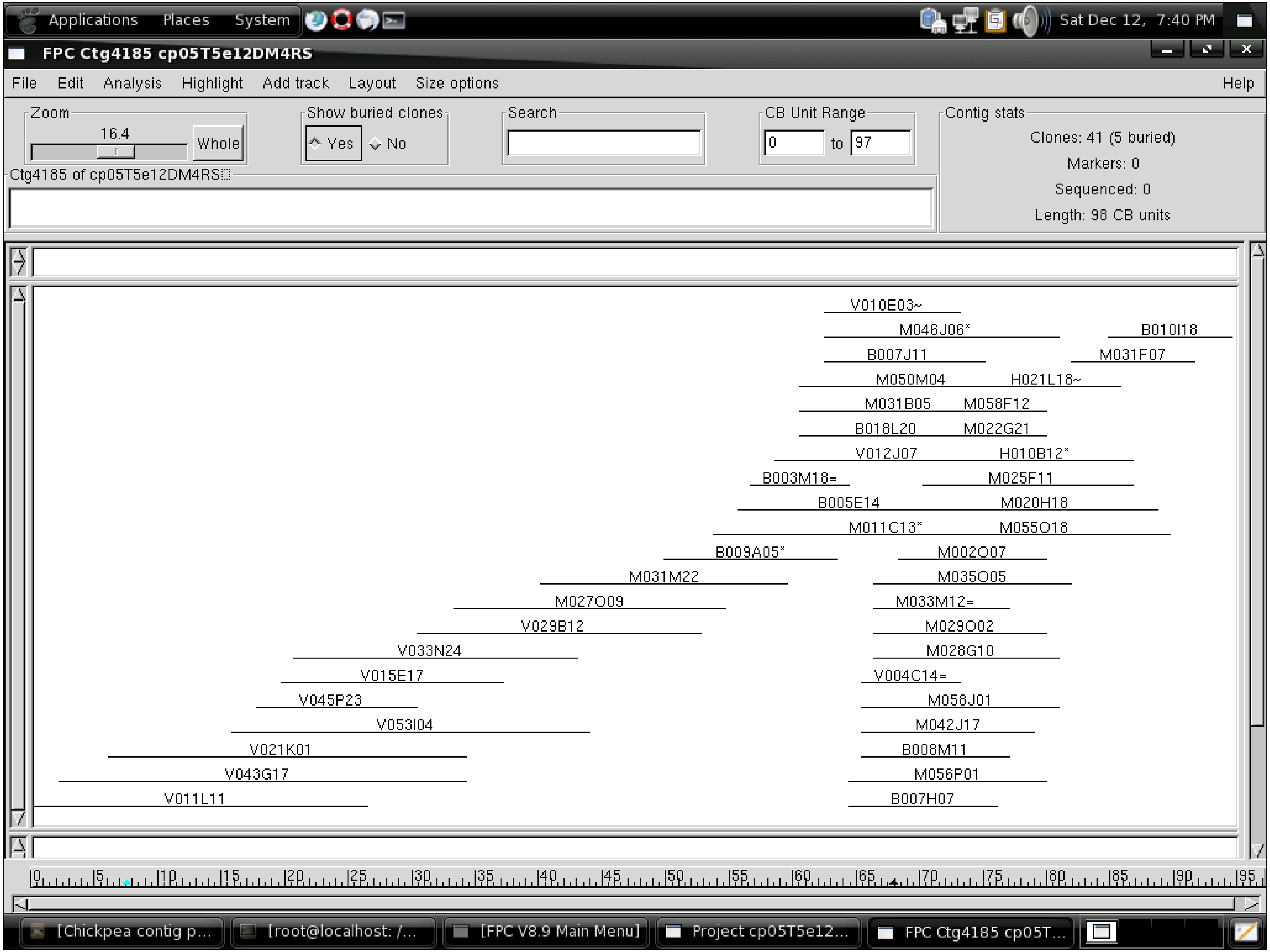
Applications Places System
Sat Dec 12, 7:40 PM
FPC Ctg4185 cp05T5e12DM4RS
File Edit Analysis Highlight Add track Layout Size options Help
Zoom
16.4
Whole
Show buried clones
Yes No
Search
CB Unit Range
0 to 97
Contig stats
Clones: 41 (5 buried)
Markers: 0
Sequenced: 0
Length: 98 CB units
Ctg4185 of cp05T5e12DM4RS
V010E03~
M046J06*
B010I18
B007J11
M031F07
M050M04
H021L18~
M031B05
M058F12
B018L20
M022G21
V012J07
H010B12*
B003M18=
M025F11
B005E14
M020H18
M011C13*
M055O18
B009A05*
M002O07
M031M22
M035O05
M027O09
M033M12=
V029B12
M029O02
V033N24
M028G10
V015E17
V004C14=
V045P23
M058J01
V053I04
M042J17
V021K01
B008M11
V043G17
M056P01
V011L11
B007H07
0 5 10 15 20 25 30 35 40 45 50 55 60 65 70 75 80 85 90 95
[Chickpea contig p...
[root@localhost: /...
[FPC V8.9 Main Menu]
Project cp05T5e12...
FPC Ctg4185 cp05T...

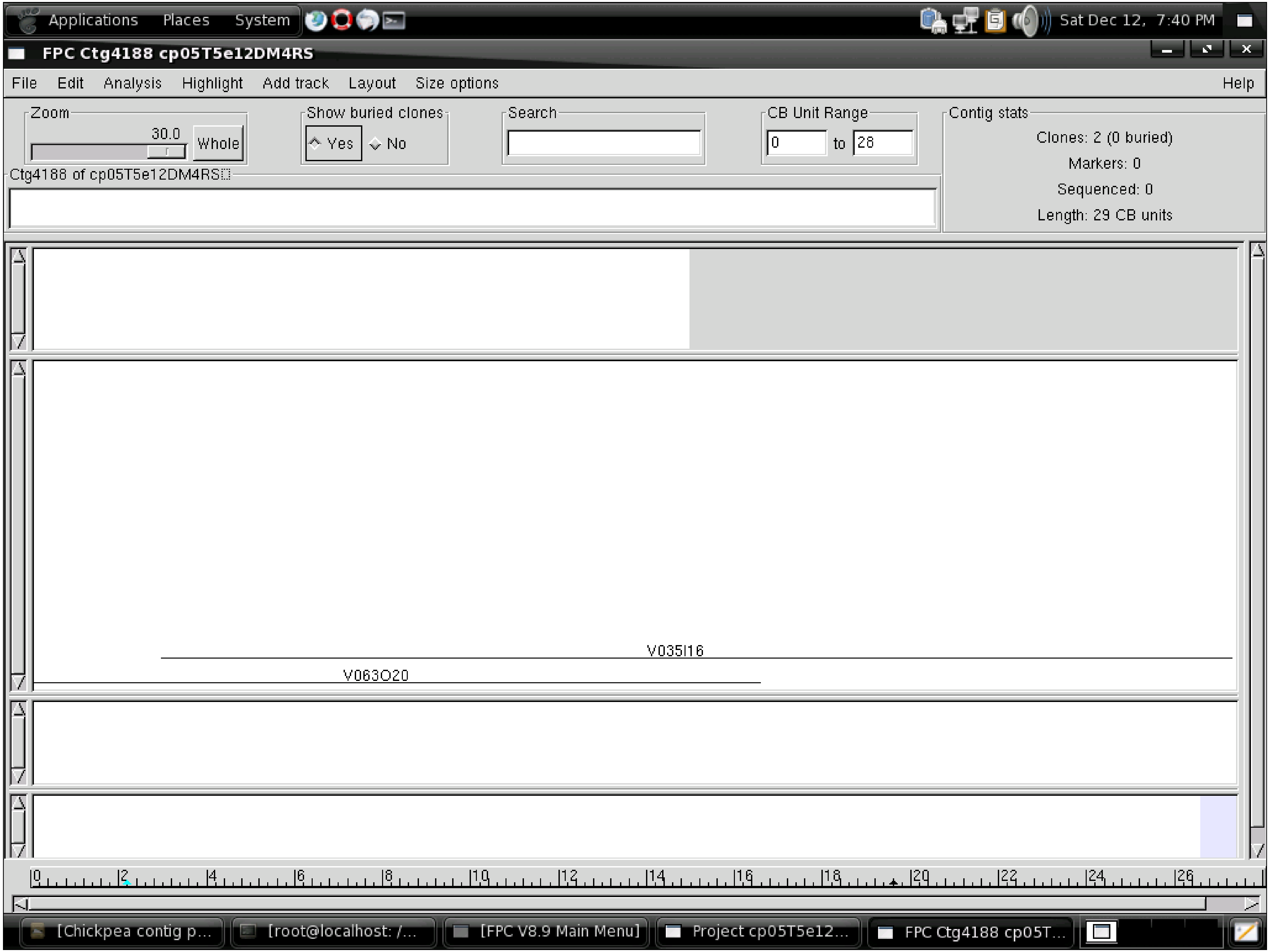
Applications Places System
Sat Dec 12, 7:40 PM
FPC Ctg4188 cp05T5e12DM4RS
File Edit Analysis Highlight Add track Layout Size options Help
Zoom
30.0
Whole
Show buried clones
Yes
No
Search
CB Unit Range
0 to 28
Contig stats
Clones: 2 (0 buried)
Markers: 0
Sequenced: 0
Length: 29 CB units
Ctg4188 of cp05T5e12DM4RS
V035I16
V063O20
0 2 4 6 8 10 12 14 16 18 20 22 24 26
[Chickpea contig p...
[root@localhost: /...
[FPC V8.9 Main Menu]
Project cp05T5e12...
FPC Ctg4188 cp05T...

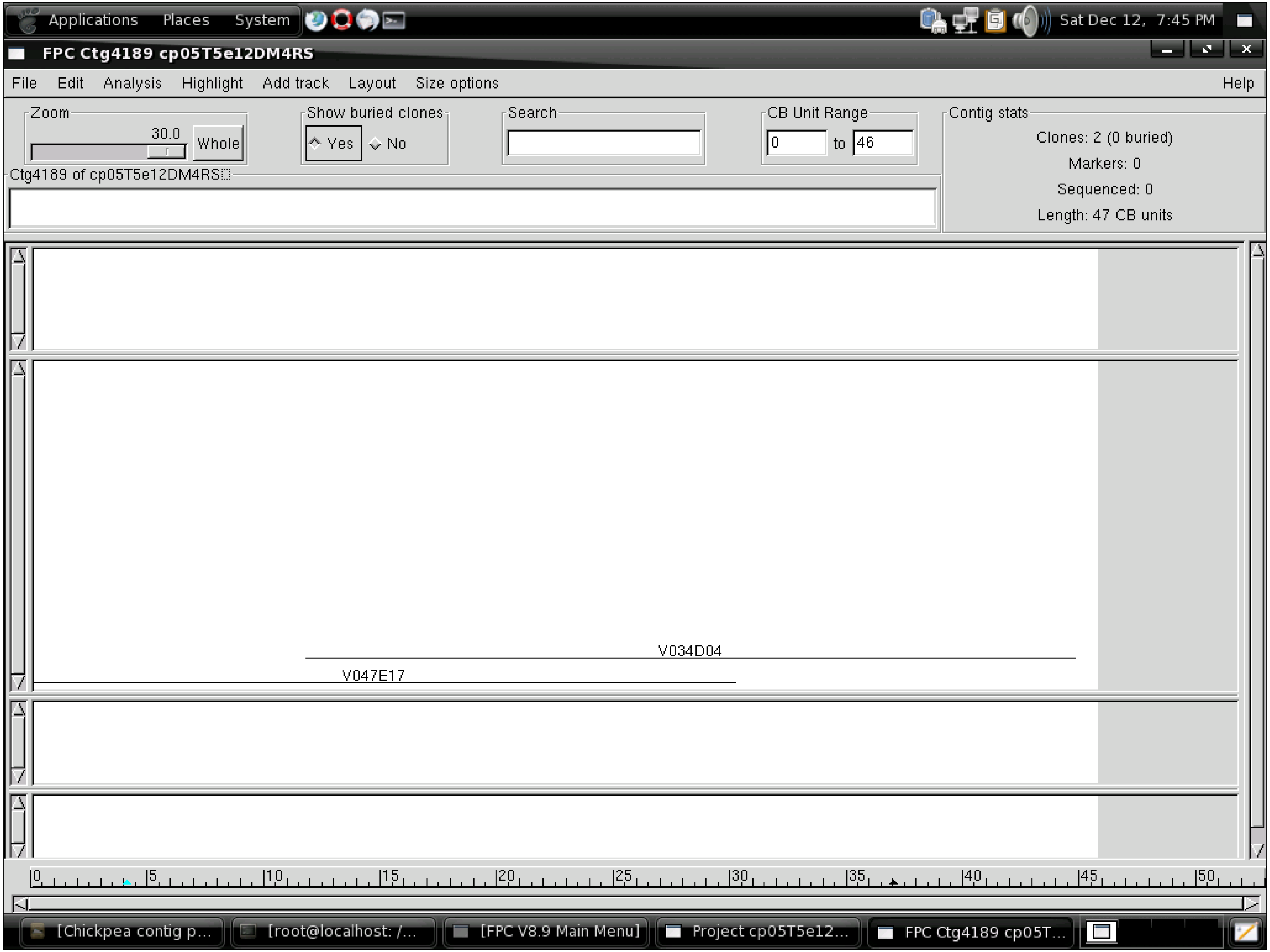

Applications Places System
Sat Dec 12, 7:45 PM
FPC Ctg4189 cp05T5e12DM4RS
File Edit Analysis Highlight Add track Layout Size options Help
Zoom
30.0
Whole
Show buried clones
Yes No
Search
CB Unit Range
0 to 46
Contig stats
Clones: 2 (0 buried)
Markers: 0
Sequenced: 0
Length: 47 CB units
Ctg4189 of cp05T5e12DM4RS
V034D04
V047E17
0 5 10 15 20 25 30 35 40 45 50
[Chickpea contig p...
[root@localhost: /...
[FPC V8.9 Main Menu]
Project cp05T5e12...
FPC Ctg4189 cp05T...

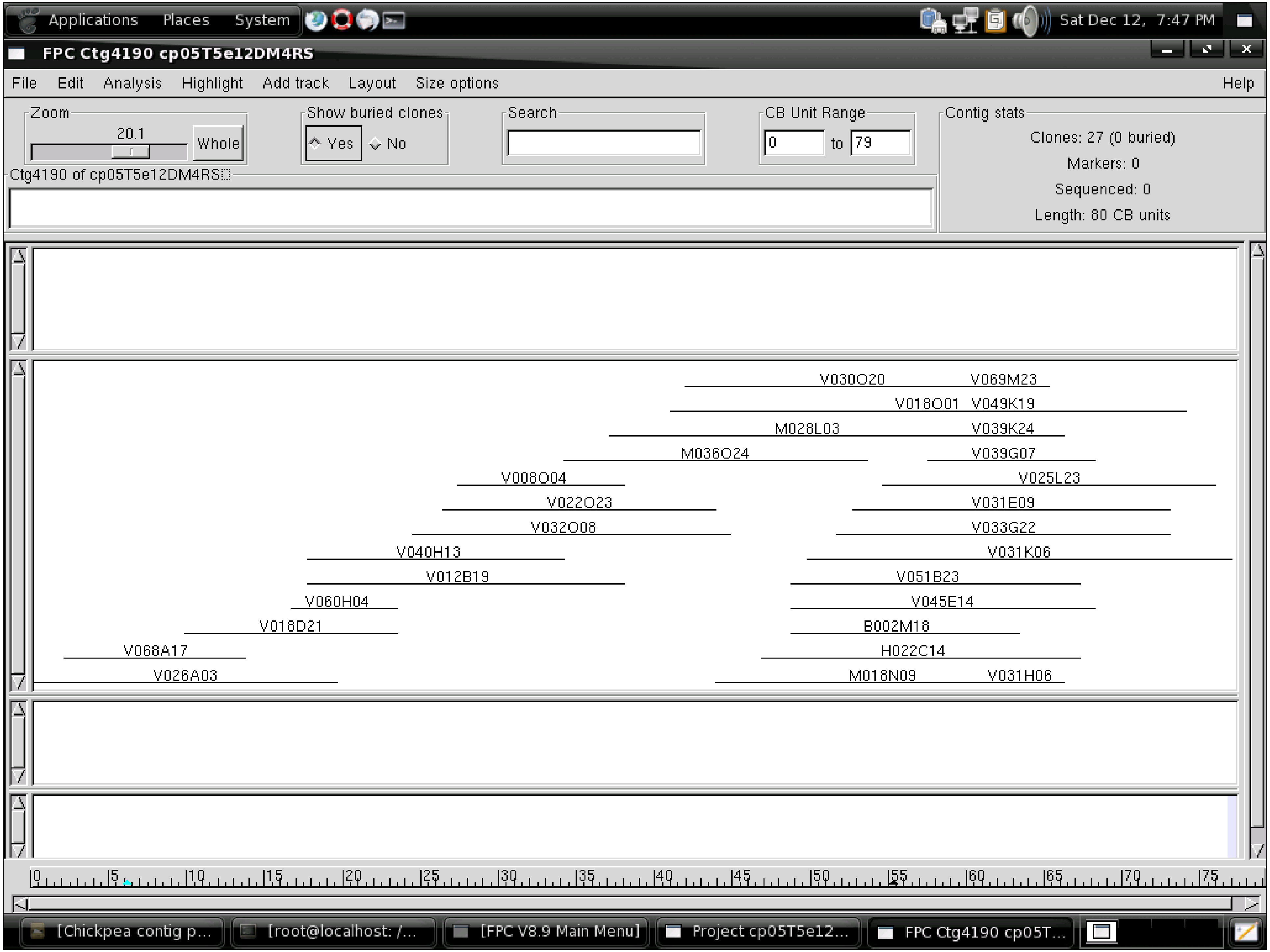
Applications Places System
Sat Dec 12, 7:47 PM
FPC Ctg4190 cp05T5e12DM4RS
File Edit Analysis Highlight Add track Layout Size options Help
Zoom
20.1
Whole
Show buried clones
Yes
No
Search
CB Unit Range
0
to
79
Contig stats
Clones: 27 (0 buried)
Markers: 0
Sequenced: 0
Length: 80 CB units
Ctg4190 of cp05T5e12DM4RS
V030O20
V069M23
V018O01
V049K19
M028L03
V039K24
M036O24
V039G07
V008O04
V025L23
V022O23
V031E09
V032O08
V033G22
V040H13
V031K06
V012B19
V051B23
V060H04
V045E14
V018D21
B002M18
V068A17
H022C14
V026A03
M018N09
V031H06
0 5 10 15 20 25 30 35 40 45 50 55 60 65 70 75
[Chickpea contig p...
[root@localhost: /...
[FPC V8.9 Main Menu]
Project cp05T5e12...
FPC Ctg4190 cp05T...

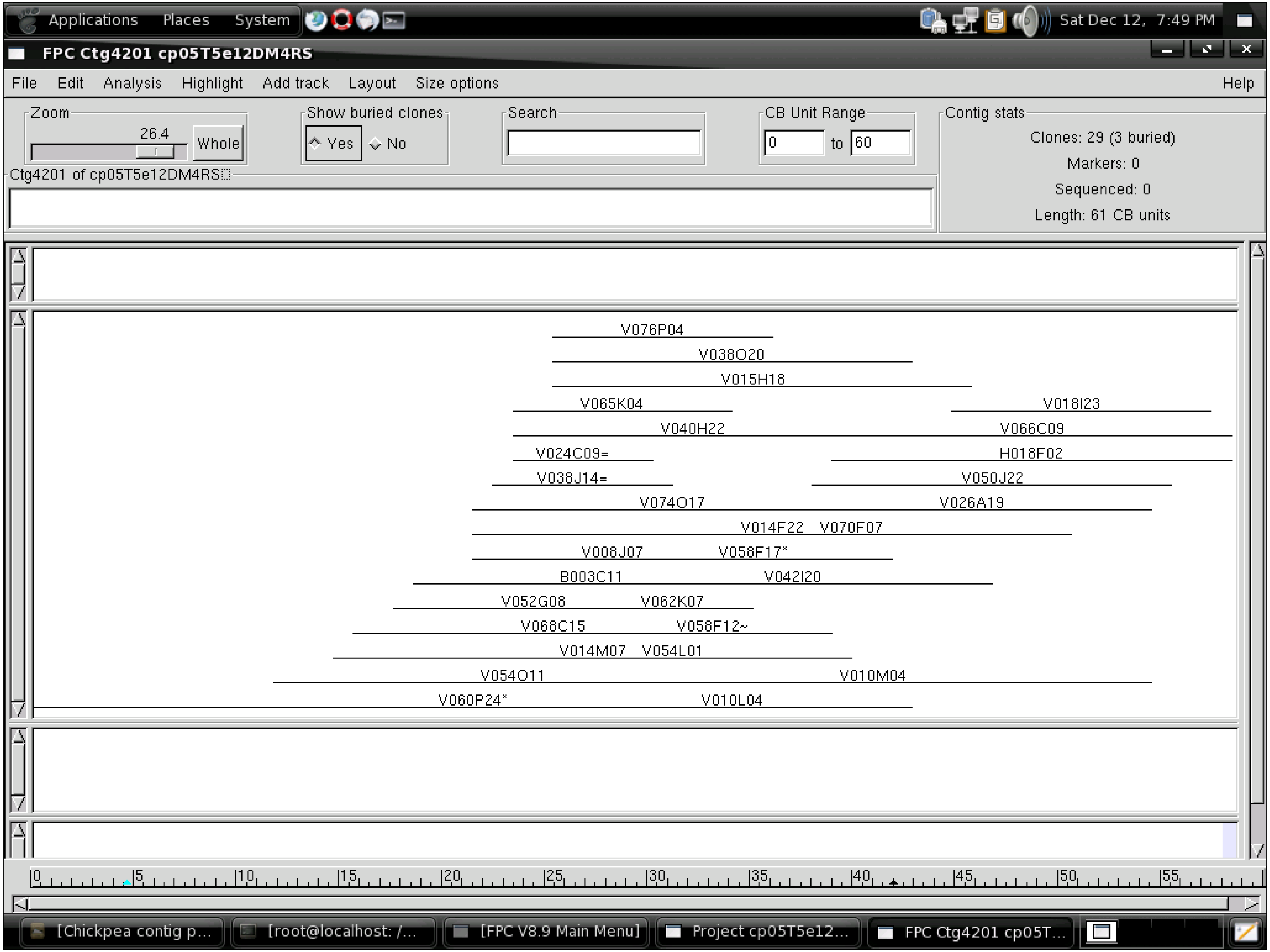

Applications Places System
Sat Dec 12, 7:49 PM
FPC Ctg4201 cp05T5e12DM4RS
File Edit Analysis Highlight Add track Layout Size options Help
Zoom
26.4
Whole
Show buried clones
Yes No
Search
CB Unit Range
0 to 60
Contig stats
Clones: 29 (3 buried)
Markers: 0
Sequenced: 0
Length: 61 CB units
Ctg4201 of cp05T5e12DM4RS
V076P04
V038O20
V015H18
V065K04
V018I23
V040H22
V066C09
V024C09=
H018F02
V038J14=
V050J22
V074O17
V026A19
V014F22
V070F07
V008J07
V058F17*
B003C11
V042I20
V052G08
V062K07
V068C15
V058F12~
V014M07
V054L01
V054O11
V010M04
V060P24*
V010L04
0 5 10 15 20 25 30 35 40 45 50 55
[Chickpea contig p...
[root@localhost: /...
[FPC V8.9 Main Menu]
Project cp05T5e12...
FPC Ctg4201 cp05T...

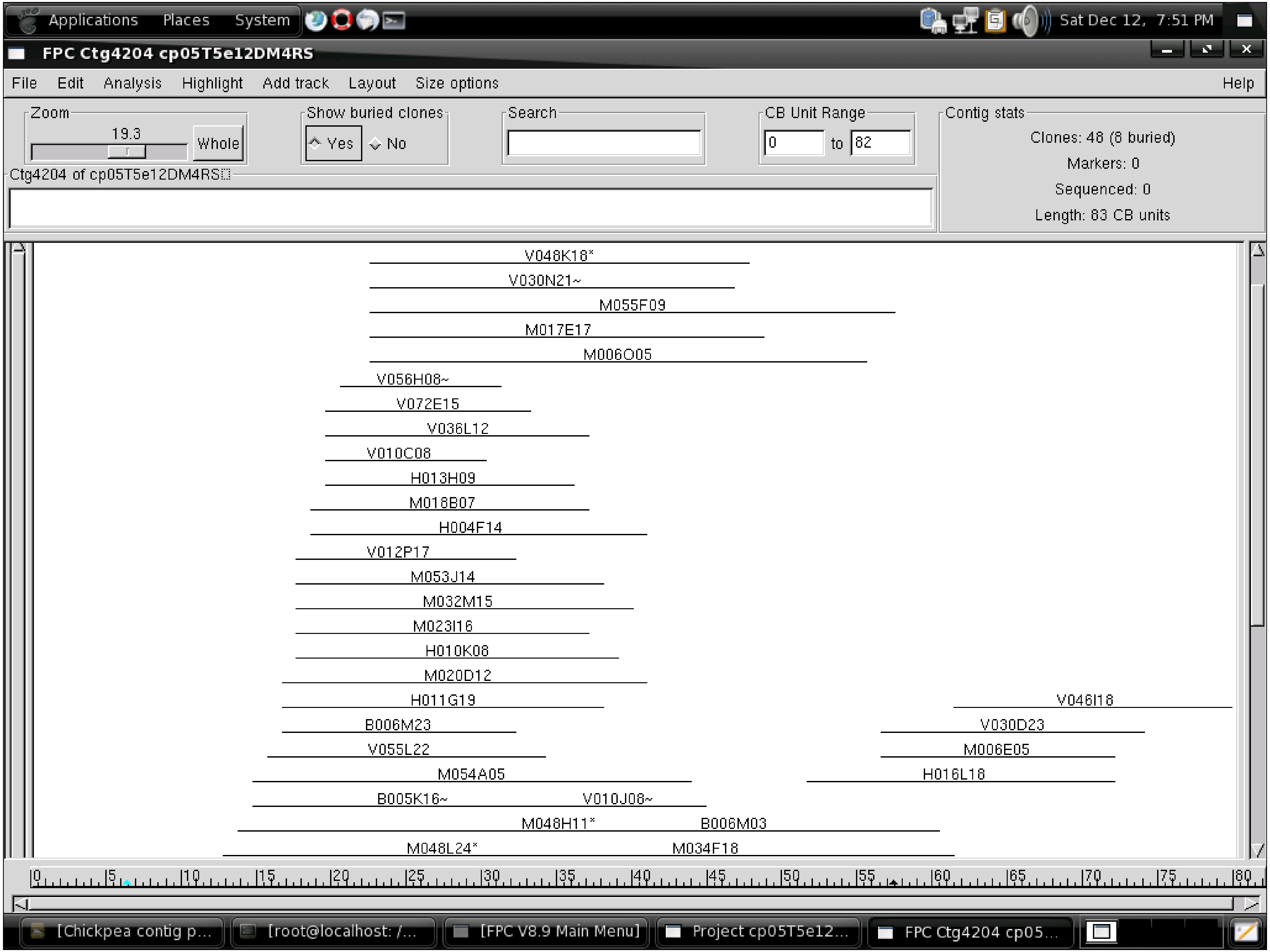
Applications Places System
Sat Dec 12, 7:51 PM
FPC Ctg4204 cp05T5e12DM4RS
File Edit Analysis Highlight Add track Layout Size options Help
Zoom
19.3
Whole
Show buried clones
Yes
No
Search
CB Unit Range
0 to 82
Contig stats
Clones: 48 (8 buried)
Markers: 0
Sequenced: 0
Length: 83 CB units
Ctg4204 of cp05T5e12DM4RS
V048K18*
V030N21~
M055F09
M017E17
M006O05
V056H08~
V072E15
V036L12
V010C08
H013H09
M018B07
H004F14
V012P17
M053J14
M032M15
M023I16
H010K08
M020D12
H011G19
V046I18
B006M23
V030D23
V055L22
M006E05
M054A05
H016L18
B005K16~
V010J08~
M048H11*
B006M03
M048L24*
M034F18
0 5 10 15 20 25 30 35 40 45 50 55 60 65 70 75 80
[Chickpea contig p...
[root@localhost: /...
[FPC V8.9 Main Menu]
Project cp05T5e12...
FPC Ctg4204 cp05...

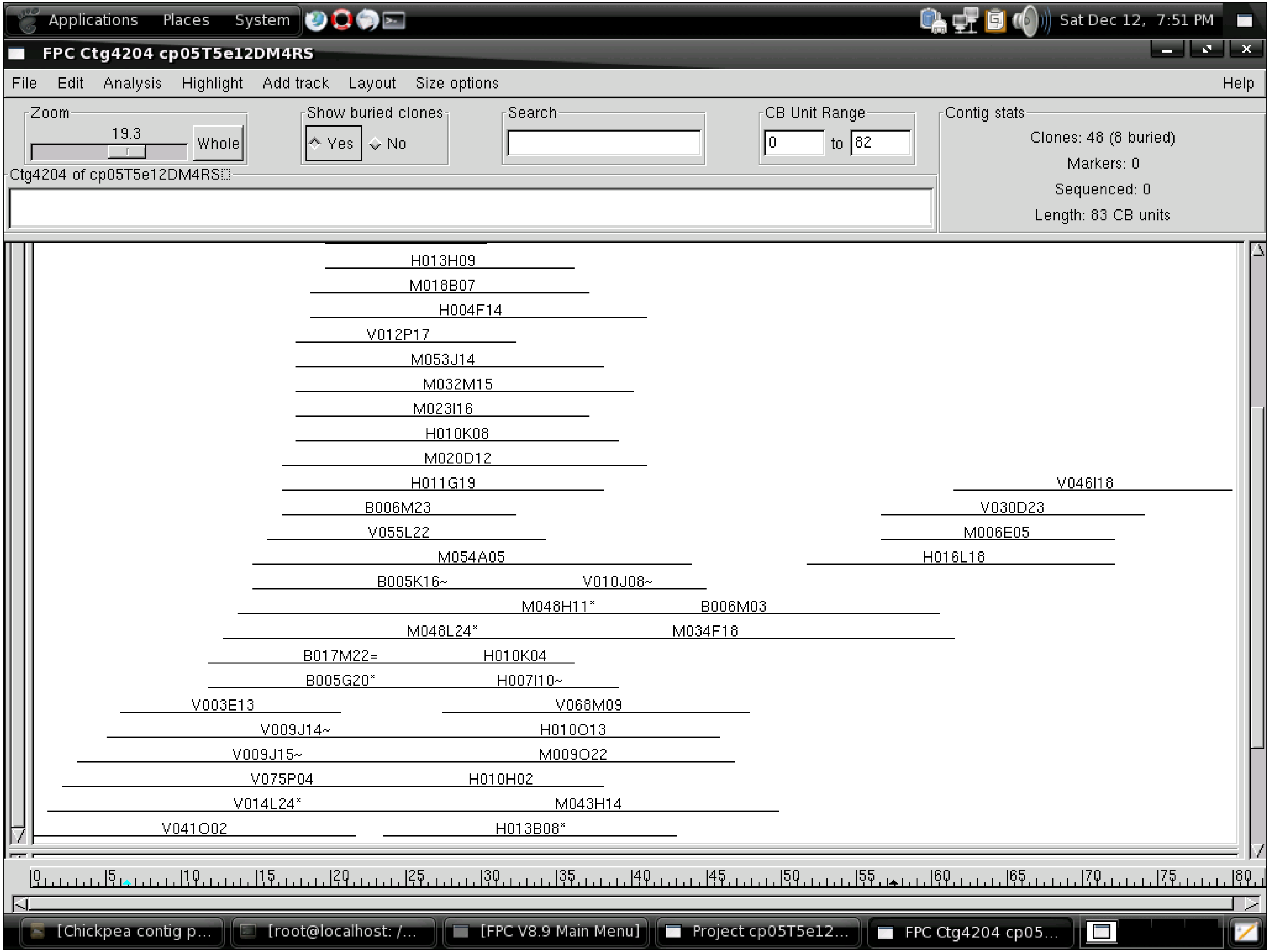
Applications Places System
Sat Dec 12, 7:51 PM
FPC Ctg4204 cp05T5e12DM4RS
File Edit Analysis Highlight Add track Layout Size options Help
Zoom
19.3
Whole
Show buried clones
Yes No
Search
CB Unit Range
0 to 82
Contig stats
Clones: 48 (8 buried)
Markers: 0
Sequenced: 0
Length: 83 CB units
Ctg4204 of cp05T5e12DM4RS
H013H09
M018B07
H004F14
V012P17
M053J14
M032M15
M023I16
H010K08
M020D12
H011G19
V046I18
B006M23
V030D23
V055L22
M006E05
M054A05
H016L18
B005K16~
V010J08~
M048H11*
B006M03
M048L24*
M034F18
B017M22=
H010K04
B005G20*
H007I10~
V003E13
V068M09
V009J14~
H010O13
V009J15~
M009O22
V075P04
H010H02
V014L24*
M043H14
V041O02
H013B08*
0 5 10 15 20 25 30 35 40 45 50 55 60 65 70 75 80
[Chickpea contig p...
[root@localhost: /...
[FPC V8.9 Main Menu]
Project cp05T5e12...
FPC Ctg4204 cp05...

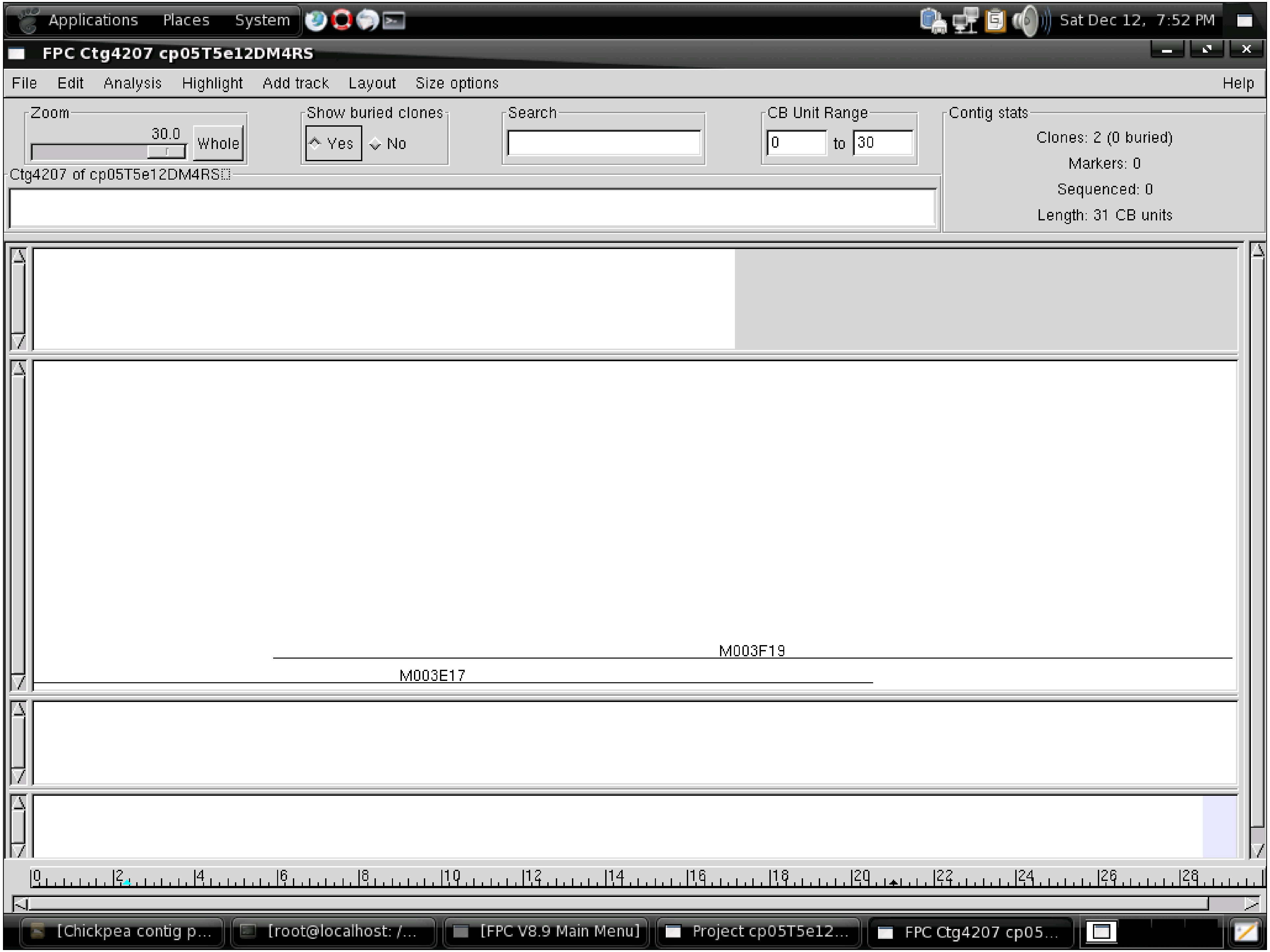
Applications Places System
Sat Dec 12, 7:52 PM
FPC Ctg4207 cp05T5e12DM4RS
File Edit Analysis Highlight Add track Layout Size options Help
Zoom
30.0
Whole
Show buried clones
Yes
No
Search
CB Unit Range
0 to 30
Contig stats
Clones: 2 (0 buried)
Markers: 0
Sequenced: 0
Length: 31 CB units
Ctg4207 of cp05T5e12DM4RS
M003F19
M003E17
0 2 4 6 8 10 12 14 16 18 20 22 24 26 28
[Chickpea contig p...
[root@localhost: /...
[FPC V8.9 Main Menu]
Project cp05T5e12...
FPC Ctg4207 cp05...

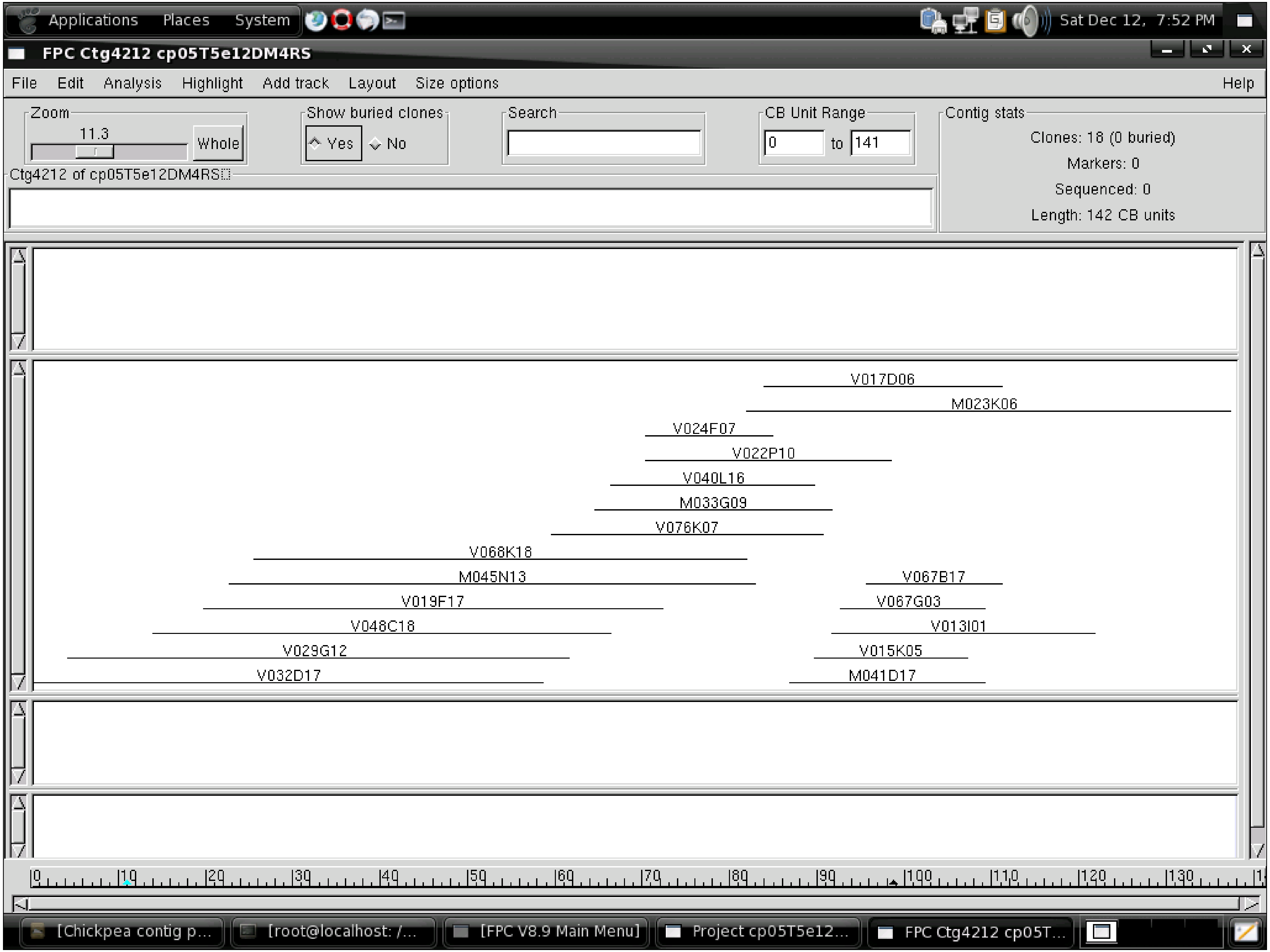
Applications Places System
Sat Dec 12, 7:52 PM
FPC Ctg4212 cp05T5e12DM4RS
File Edit Analysis Highlight Add track Layout Size options Help
Zoom
11.3
Whole
Show buried clones
Yes
No
Search
CB Unit Range
0
to
141
Contig stats
Clones: 18 (0 buried)
Markers: 0
Sequenced: 0
Length: 142 CB units
Ctg4212 of cp05T5e12DM4RS
V017D06
M023K06
V024F07
V022P10
V040L16
M033G09
V076K07
V068K18
M045N13
V067B17
V019F17
V067G03
V048C18
V013I01
V029G12
V015K05
V032D17
M041D17
0 10 20 30 40 50 60 70 80 90 100 110 120 130
[Chickpea contig p...
[root@localhost: /...
[FPC V8.9 Main Menu]
Project cp05T5e12...
FPC Ctg4212 cp05T...

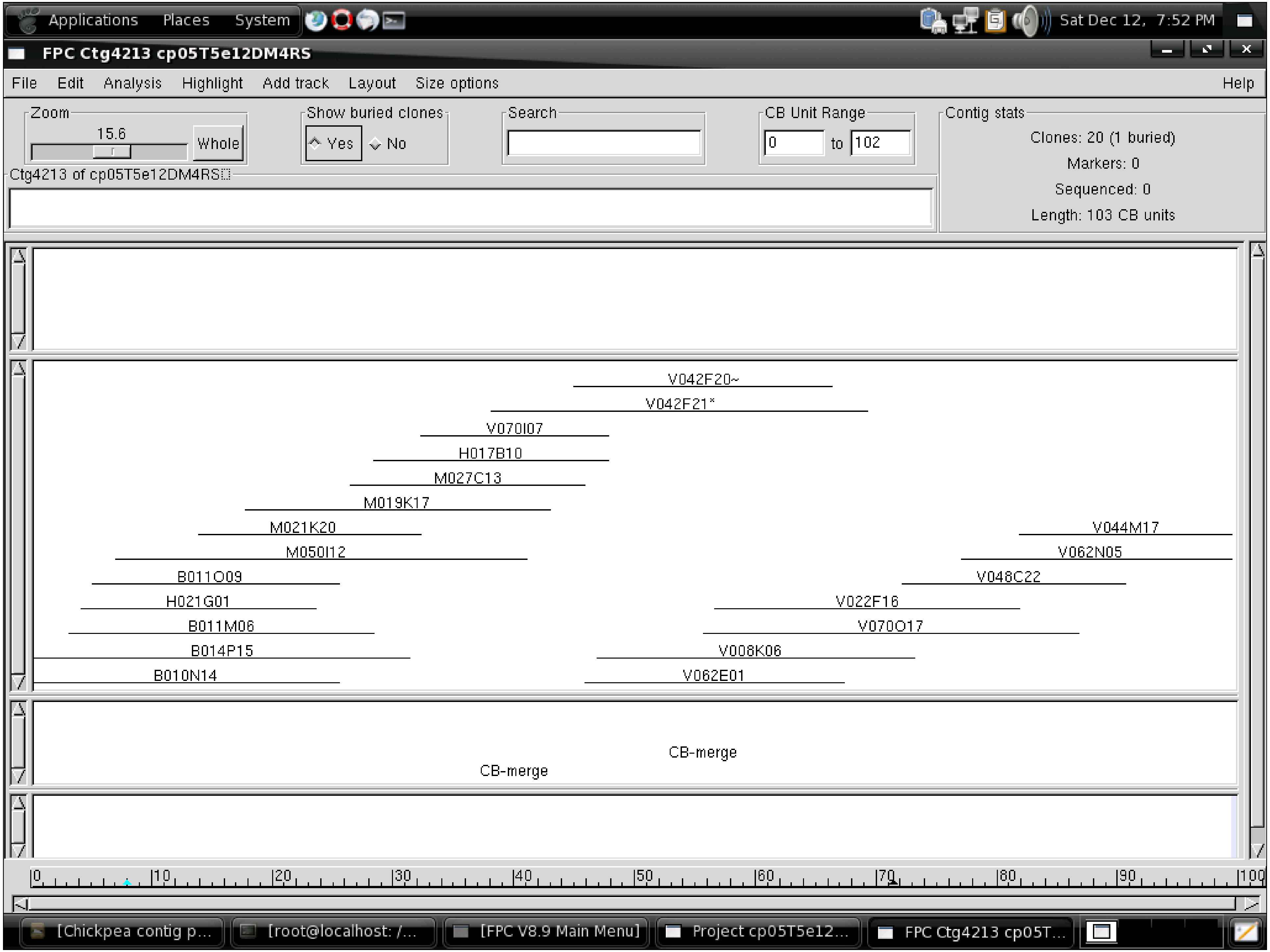
Applications Places System
Sat Dec 12, 7:52 PM
FPC Ctg4213 cp05T5e12DM4RS
File Edit Analysis Highlight Add track Layout Size options Help
Zoom
15.6
Whole
Show buried clones
Yes No
Search
CB Unit Range
0 to 102
Contig stats
Clones: 20 (1 buried)
Markers: 0
Sequenced: 0
Length: 103 CB units
Ctg4213 of cp05T5e12DM4RS
V042F20~
V042F21*
V070I07
H017B10
M027C13
M019K17
M021K20
M050I12
B011O09
H021G01
B011M06
B014P15
B010N14
V044M17
V062N05
V048C22
V022F16
V070O17
V008K06
V062E01
CB-merge
CB-merge
0 10 20 30 40 50 60 70 80 90 100
[Chickpea contig p...
[root@localhost: /...
[FPC V8.9 Main Menu]
Project cp05T5e12...
FPC Ctg4213 cp05T...

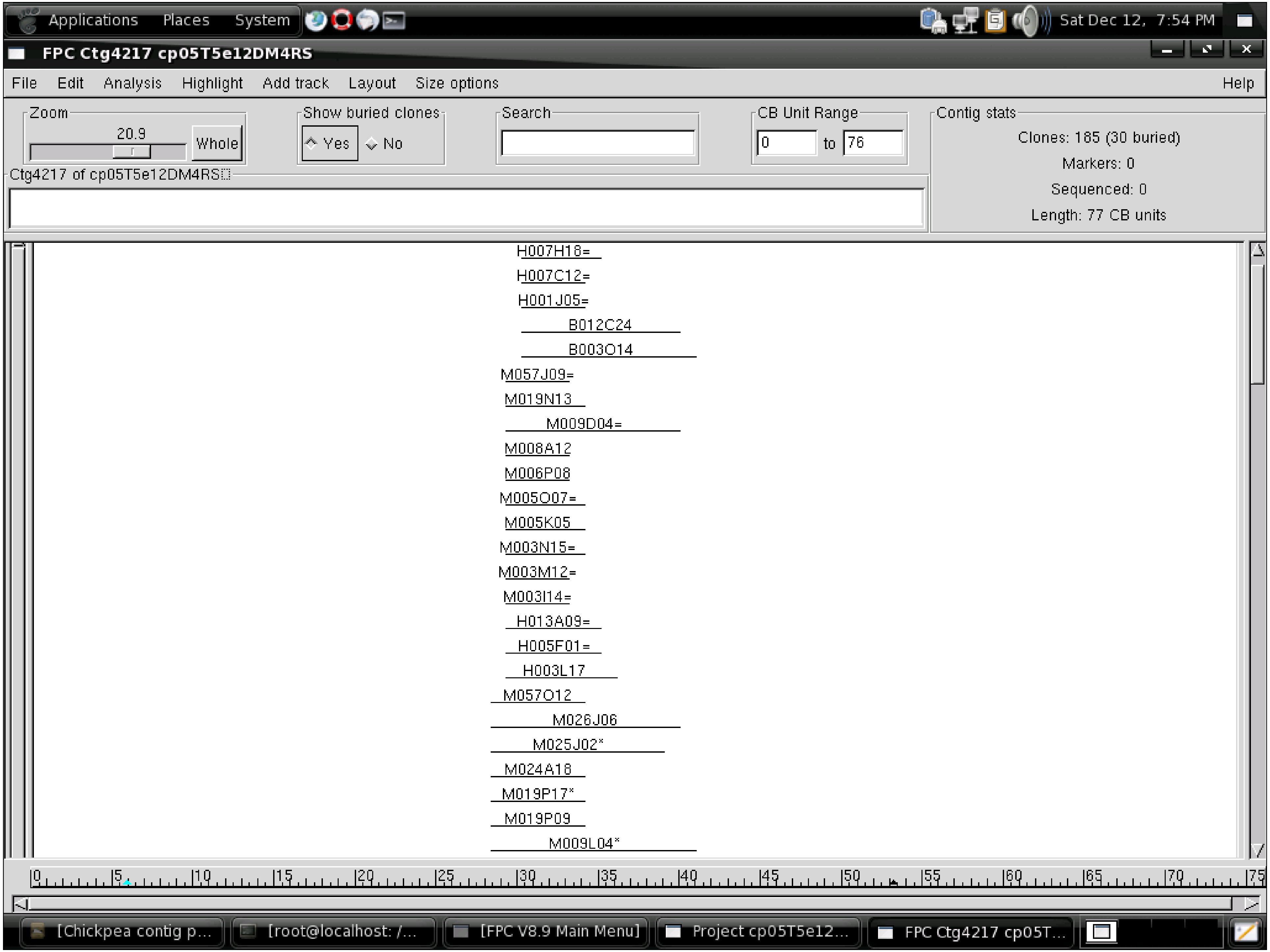
Applications Places System
Sat Dec 12, 7:54 PM
FPC Ctg4217 cp05T5e12DM4RS
File Edit Analysis Highlight Add track Layout Size options Help
Zoom
20.9
Whole
Show buried clones
Yes No
Search
CB Unit Range
0 to 76
Contig stats
Clones: 185 (30 buried)
Markers: 0
Sequenced: 0
Length: 77 CB units
Ctg4217 of cp05T5e12DM4RS
H007H18=
H007C12=
H001J05=
B012C24
B003O14
M057J09=
M019N13
M009D04=
M008A12
M006P08
M005O07=
M005K05
M003N15=
M003M12=
M003I14=
H013A09=
H005F01=
H003L17
M057O12
M026J06
M025J02*
M024A18
M019P17*
M019P09
M009L04*
0 5 10 15 20 25 30 35 40 45 50 55 60 65 70 75
[Chickpea contig p...
[root@localhost: /...
[FPC V8.9 Main Menu]
Project cp05T5e12...
FPC Ctg4217 cp05T...

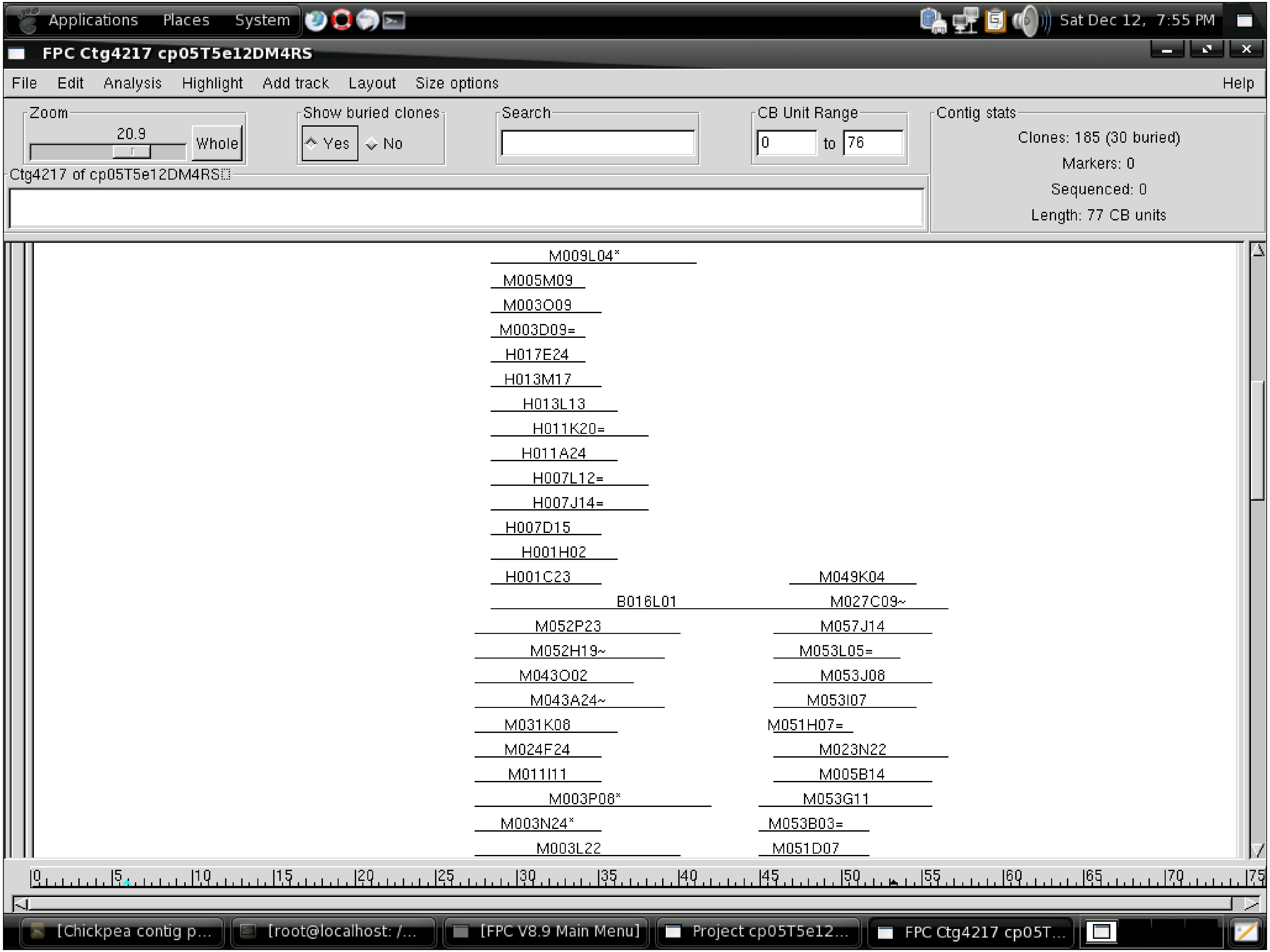

Applications Places System
Sat Dec 12, 7:55 PM
FPC Ctg4217 cp05T5e12DM4RS
File Edit Analysis Highlight Add track Layout Size options Help
Zoom
20.9
Whole
Show buried clones
Yes No
Search
CB Unit Range
0 to 76
Contig stats
Clones: 185 (30 buried)
Markers: 0
Sequenced: 0
Length: 77 CB units
Ctg4217 of cp05T5e12DM4RS
M009L04*
M005M09
M003O09
M003D09=
H017E24
H013M17
H013L13
H011K20=
H011A24
H007L12=
H007J14=
H007D15
H001H02
H001C23
B016L01
M052P23
M052H19~
M043O02
M043A24~
M031K08
M024F24
M011I11
M003P08*
M003N24*
M003L22
M049K04
M027C09~
M057J14
M053L05=
M053J08
M053I07
M051H07=
M023N22
M005B14
M053G11
M053B03=
M051D07
0 5 10 15 20 25 30 35 40 45 50 55 60 65 70 75
[Chickpea contig p...
[root@localhost: /...
[FPC V8.9 Main Menu]
Project cp05T5e12...
FPC Ctg4217 cp05T...

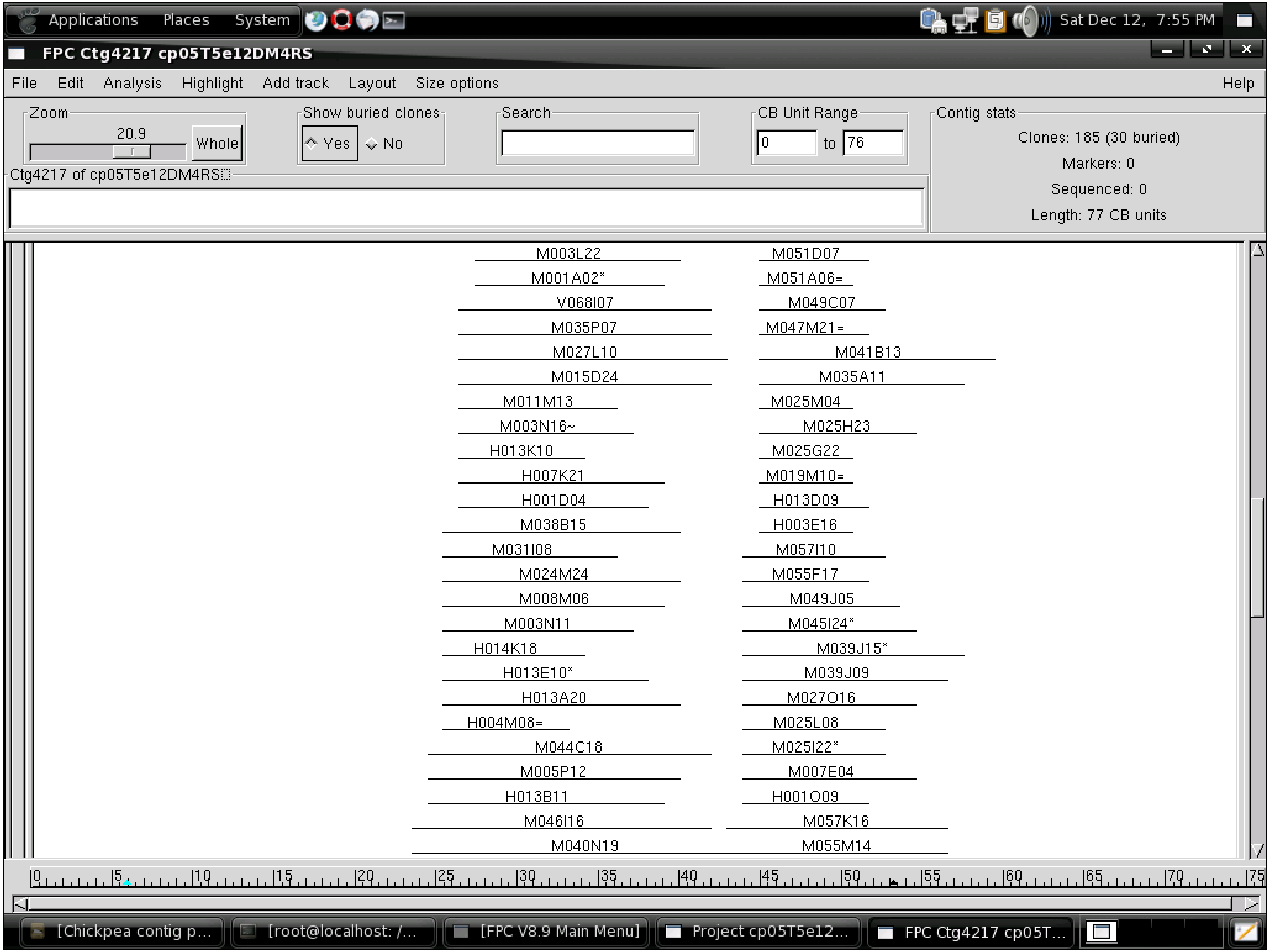
Applications Places System
Sat Dec 12, 7:55 PM
FPC Ctg4217 cp05T5e12DM4RS
File Edit Analysis Highlight Add track Layout Size options Help
Zoom
20.9
Whole
Show buried clones
Yes No
Search
CB Unit Range
0 to 76
Contig stats
Clones: 185 (30 buried)
Markers: 0
Sequenced: 0
Length: 77 CB units
Ctg4217 of cp05T5e12DM4RS
M003L22
M001A02*
V068I07
M035P07
M027L10
M015D24
M011M13
M003N16~
H013K10
H007K21
H001D04
M038B15
M031I08
M024M24
M008M06
M003N11
H014K18
H013E10*
H013A20
H004M08=
M044C18
M005P12
H013B11
M046I16
M040N19
M051D07
M051A06=
M049C07
M047M21=
M041B13
M035A11
M025M04
M025H23
M025G22
M019M10=
H013D09
H003E16
M057I10
M055F17
M049J05
M045I24*
M039J15*
M039J09
M027O16
M025L08
M025I22*
M007E04
H001O09
M057K16
M055M14
0 5 10 15 20 25 30 35 40 45 50 55 60 65 70 75
[Chickpea contig p...
[root@localhost: /...
[FPC V8.9 Main Menu]
Project cp05T5e12...
FPC Ctg4217 cp05T...

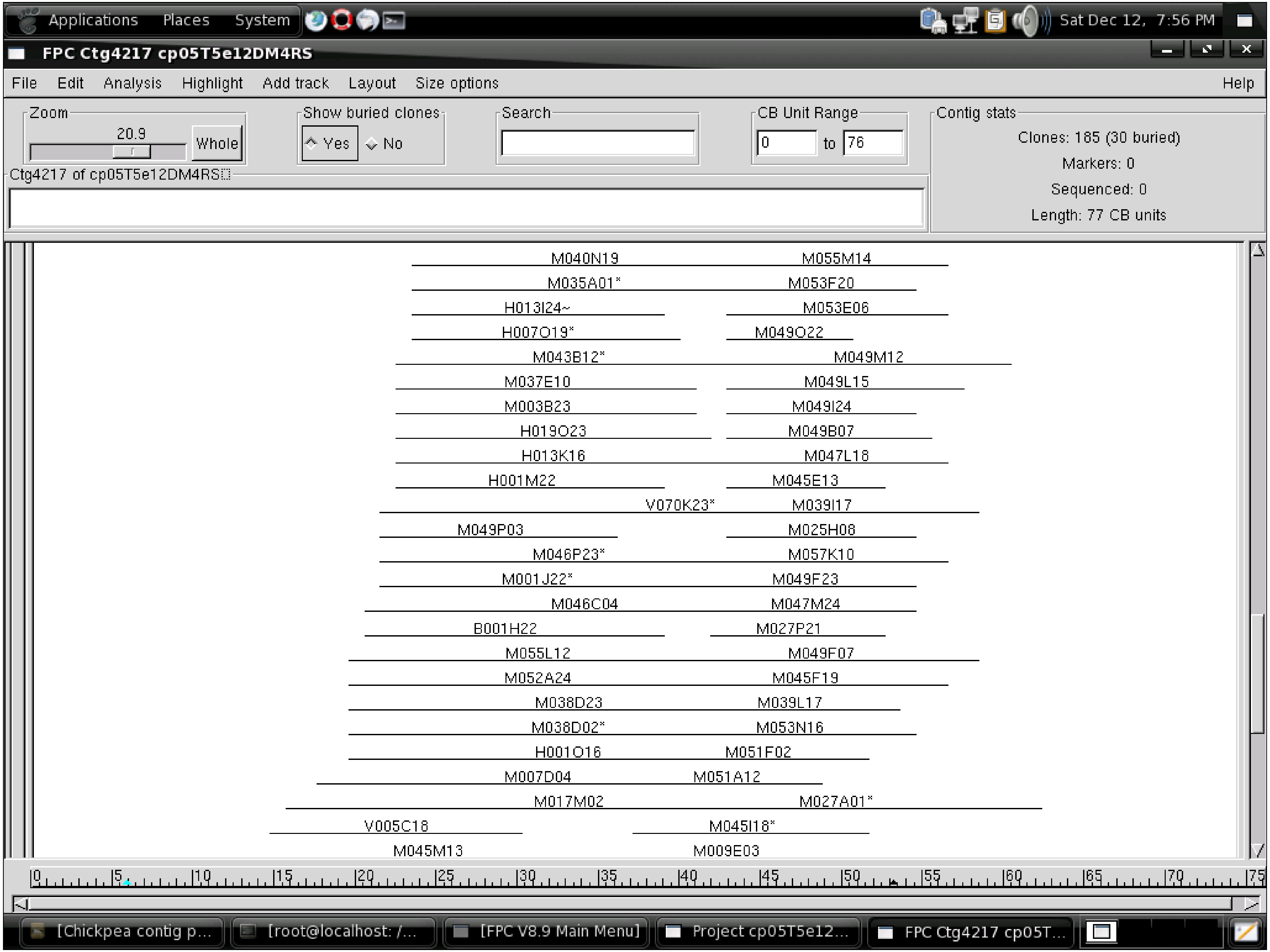
Applications Places System
Sat Dec 12, 7:56 PM
FPC Ctg4217 cp05T5e12DM4RS
File Edit Analysis Highlight Add track Layout Size options Help
Zoom
20.9
Whole
Show buried clones
Yes No
Search
CB Unit Range
0 to 76
Contig stats
Clones: 185 (30 buried)
Markers: 0
Sequenced: 0
Length: 77 CB units
Ctg4217 of cp05T5e12DM4RS
M040N19
M055M14
M035A01*
M053F20
H013I24~
M053E06
H007O19*
M049O22
M043B12*
M049M12
M037E10
M049L15
M003B23
M049I24
H019O23
M049B07
H013K16
M047L18
H001M22
M045E13
V070K23*
M039I17
M049P03
M025H08
M046P23*
M057K10
M001J22*
M049F23
M046C04
M047M24
B001H22
M027P21
M055L12
M049F07
M052A24
M045F19
M038D23
M039L17
M038D02*
M053N16
H001O16
M051F02
M007D04
M051A12
M017M02
M027A01*
V005C18
M045I18*
M045M13
M009E03
0 5 10 15 20 25 30 35 40 45 50 55 60 65 70 75
[Chickpea contig p...
[root@localhost: /...
[FPC V8.9 Main Menu]
Project cp05T5e12...
FPC Ctg4217 cp05T...

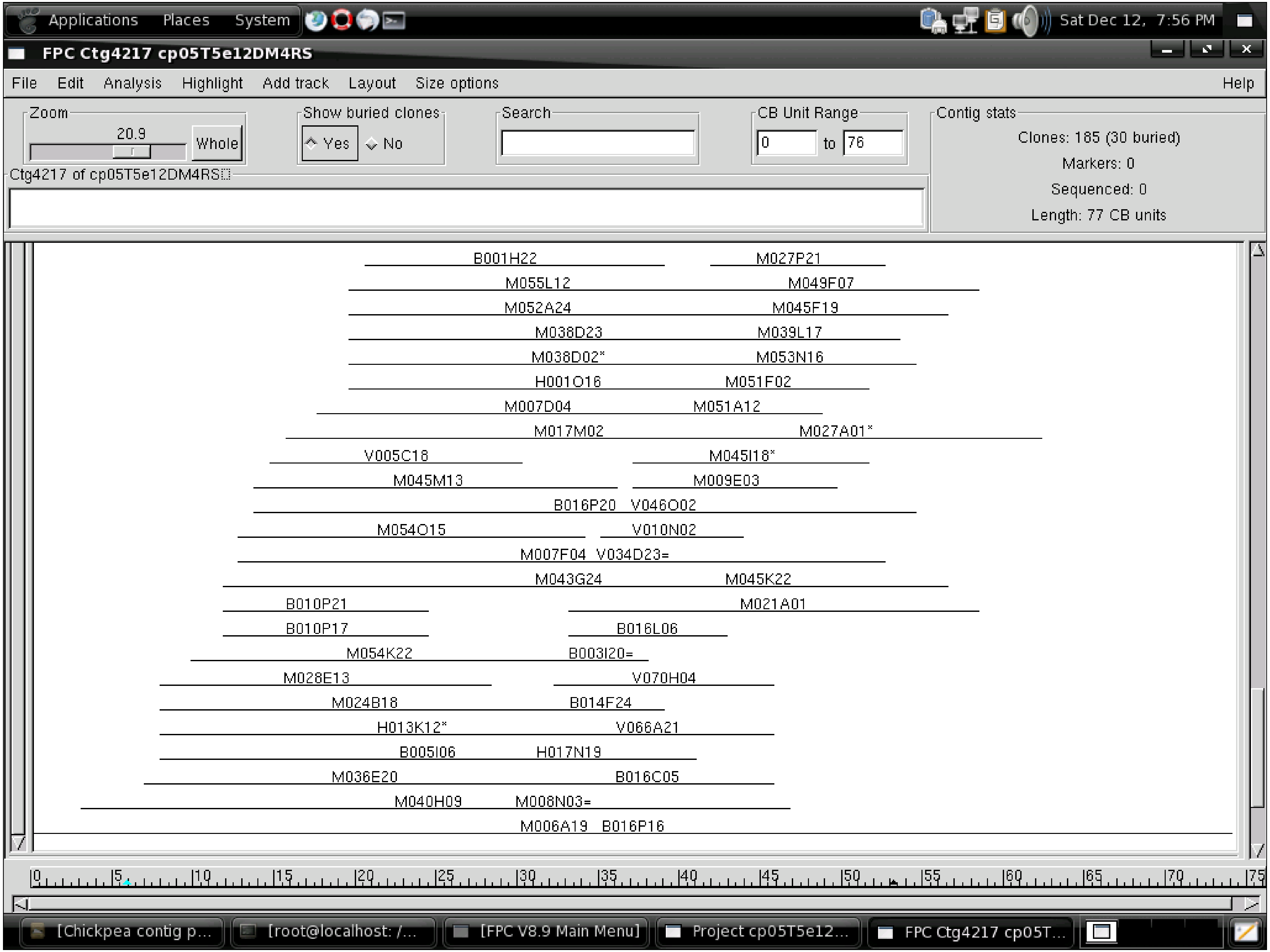
Applications Places System
Sat Dec 12, 7:56 PM
FPC Ctg4217 cp05T5e12DM4RS
File Edit Analysis Highlight Add track Layout Size options Help
Zoom
20.9
Whole
Show buried clones
Yes No
Search
CB Unit Range
0 to 76
Contig stats
Clones: 185 (30 buried)
Markers: 0
Sequenced: 0
Length: 77 CB units
Ctg4217 of cp05T5e12DM4RS
B001H22 M027P21
M055L12 M049F07
M052A24 M045F19
M038D23 M039L17
M038D02* M053N16
H001O16 M051F02
M007D04 M051A12
M017M02 M027A01*
V005C18 M045I18*
M045M13 M009E03
B016P20 V046O02
M054O15 V010N02
M007F04 V034D23=
M043G24 M045K22
B010P21 M021A01
B010P17 B016L06
M054K22 B003I20=
M028E13 V070H04
M024B18 B014F24
H013K12* V066A21
B005I06 H017N19
M036E20 B016C05
M040H09 M008N03=
M006A19 B016P16
0 5 10 15 20 25 30 35 40 45 50 55 60 65 70 75
[Chickpea contig p...
[root@localhost: /...
[FPC V8.9 Main Menu]
Project cp05T5e12...
FPC Ctg4217 cp05T...

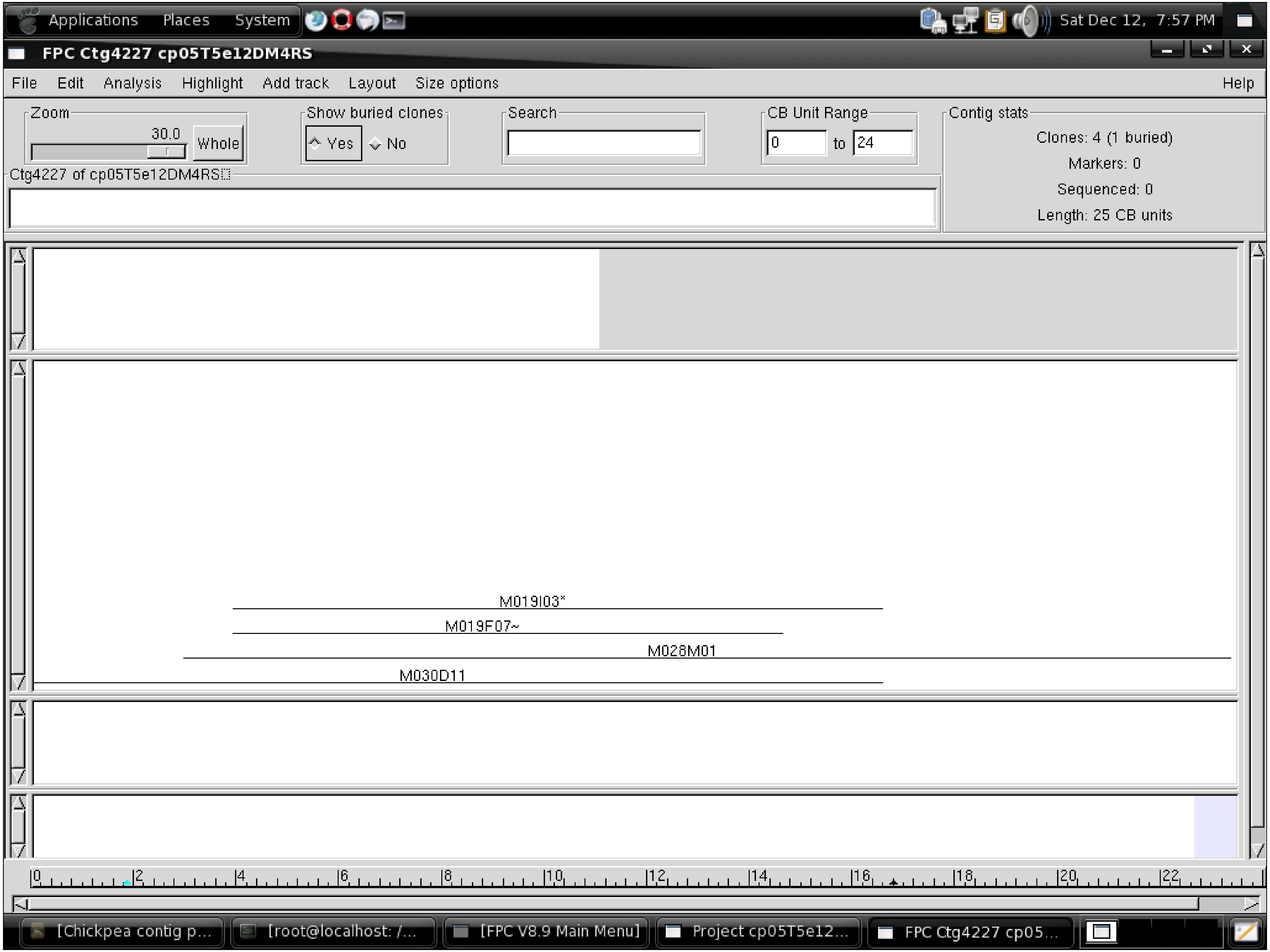
Applications Places System
Sat Dec 12, 7:57 PM
FPC Ctg4227 cp05T5e12DM4RS
File Edit Analysis Highlight Add track Layout Size options Help
Zoom
30.0
Whole
Show buried clones
Yes No
Search
CB Unit Range
0 to 24
Contig stats
Clones: 4 (1 buried)
Markers: 0
Sequenced: 0
Length: 25 CB units
Ctg4227 of cp05T5e12DM4RS
M019I03*
M019F07~
M028M01
M030D11
0 2 4 6 8 10 12 14 16 18 20 22
[Chickpea contig p...
[root@localhost: /...
[FPC V8.9 Main Menu]
Project cp05T5e12...
FPC Ctg4227 cp05...

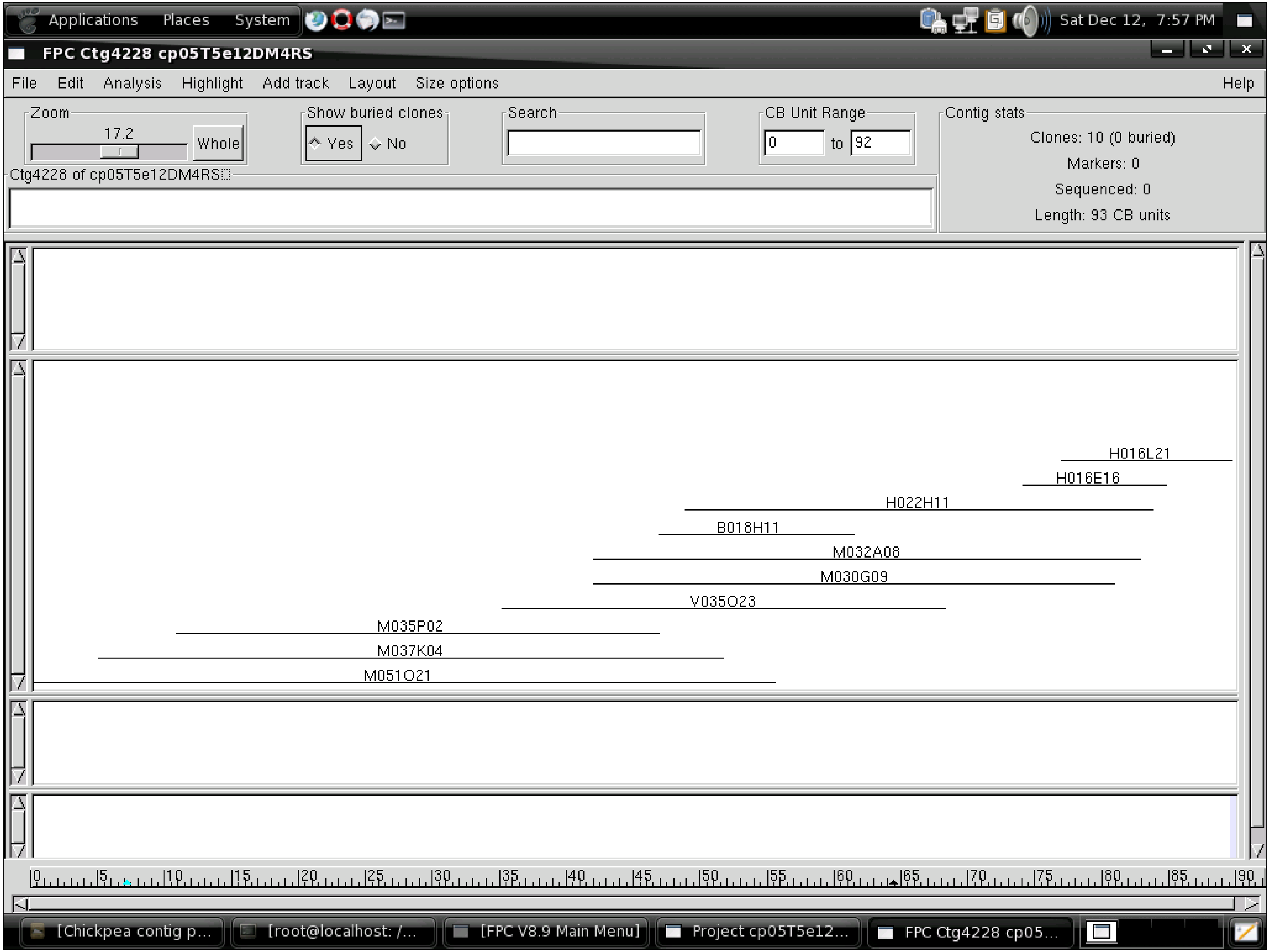
Applications Places System
Sat Dec 12, 7:57 PM
FPC Ctg4228 cp05T5e12DM4RS
File Edit Analysis Highlight Add track Layout Size options Help
Zoom
17.2
Whole
Show buried clones
Yes
No
Search
CB Unit Range
0
to
92
Contig stats
Clones: 10 (0 buried)
Markers: 0
Sequenced: 0
Length: 93 CB units
Ctg4228 of cp05T5e12DM4RS
H016L21
H016E16
H022H11
B018H11
M032A08
M030G09
V035O23
M035P02
M037K04
M051O21
0 5 10 15 20 25 30 35 40 45 50 55 60 65 70 75 80 85 90
[Chickpea contig p...
[root@localhost: /...
[FPC V8.9 Main Menu]
Project cp05T5e12...
FPC Ctg4228 cp05...

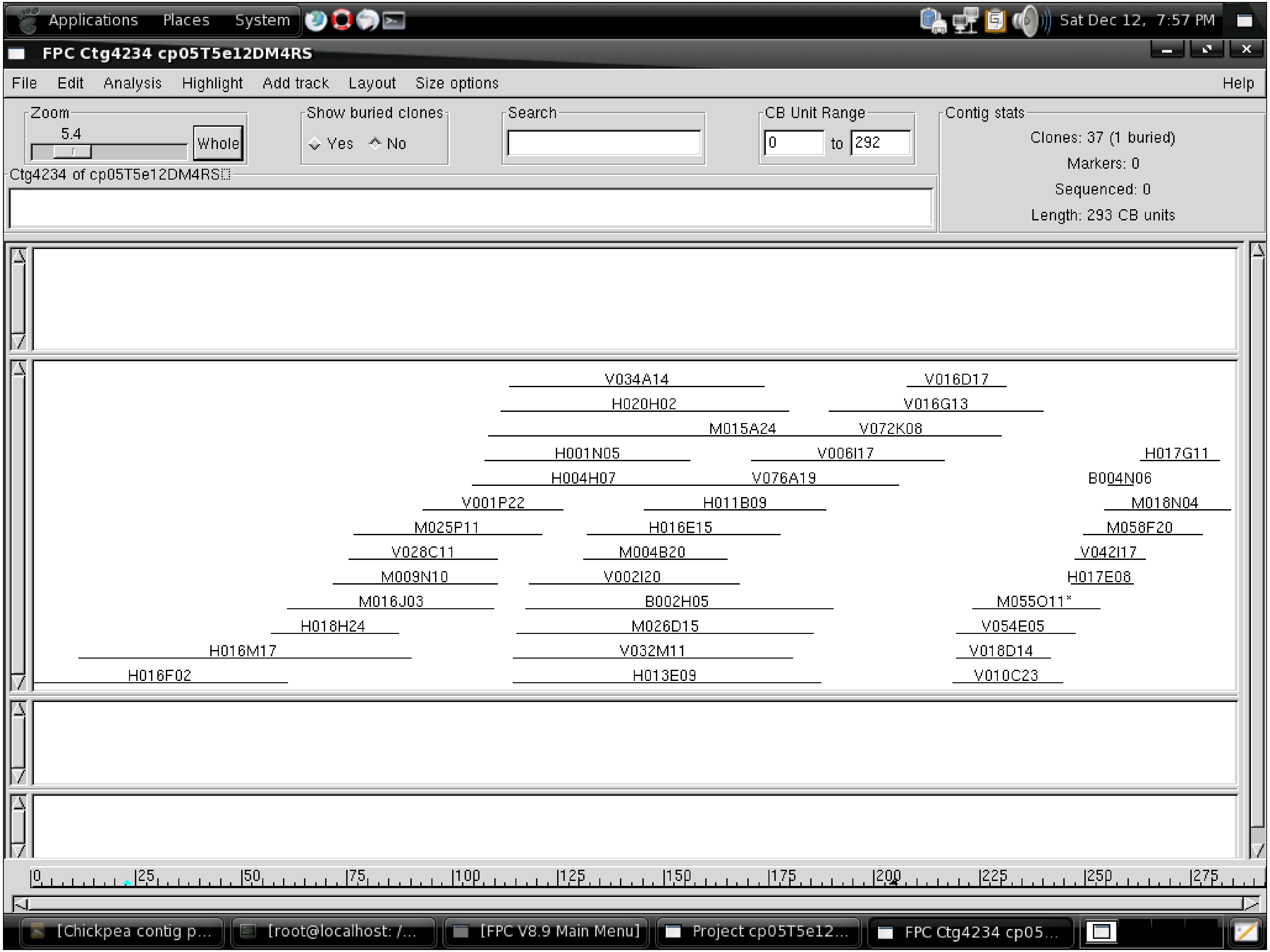
Applications Places System
Sat Dec 12, 7:57 PM
FPC Ctg4234 cp05T5e12DM4RS
File Edit Analysis Highlight Add track Layout Size options Help
Zoom
5.4
Whole
Show buried clones
Yes No
Search
CB Unit Range
0 to 292
Contig stats
Clones: 37 (1 buried)
Markers: 0
Sequenced: 0
Length: 293 CB units
Ctg4234 of cp05T5e12DM4RS
V034A14
V016D17
H020H02
V016G13
M015A24
V072K08
H001N05
V006I17
H017G11
H004H07
V076A19
B004N06
V001P22
H011B09
M018N04
M025P11
H016E15
M058F20
V028C11
M004B20
V042I17
M009N10
V002I20
H017E08
M016J03
B002H05
M055O11*
H018H24
M026D15
V054E05
H016M17
V032M11
V018D14
H016F02
H013E09
V010C23
0 25 50 75 100 125 150 175 200 225 250 275
[Chickpea contig p...
[root@localhost: /...
[FPC V8.9 Main Menu]
Project cp05T5e12...
FPC Ctg4234 cp05...

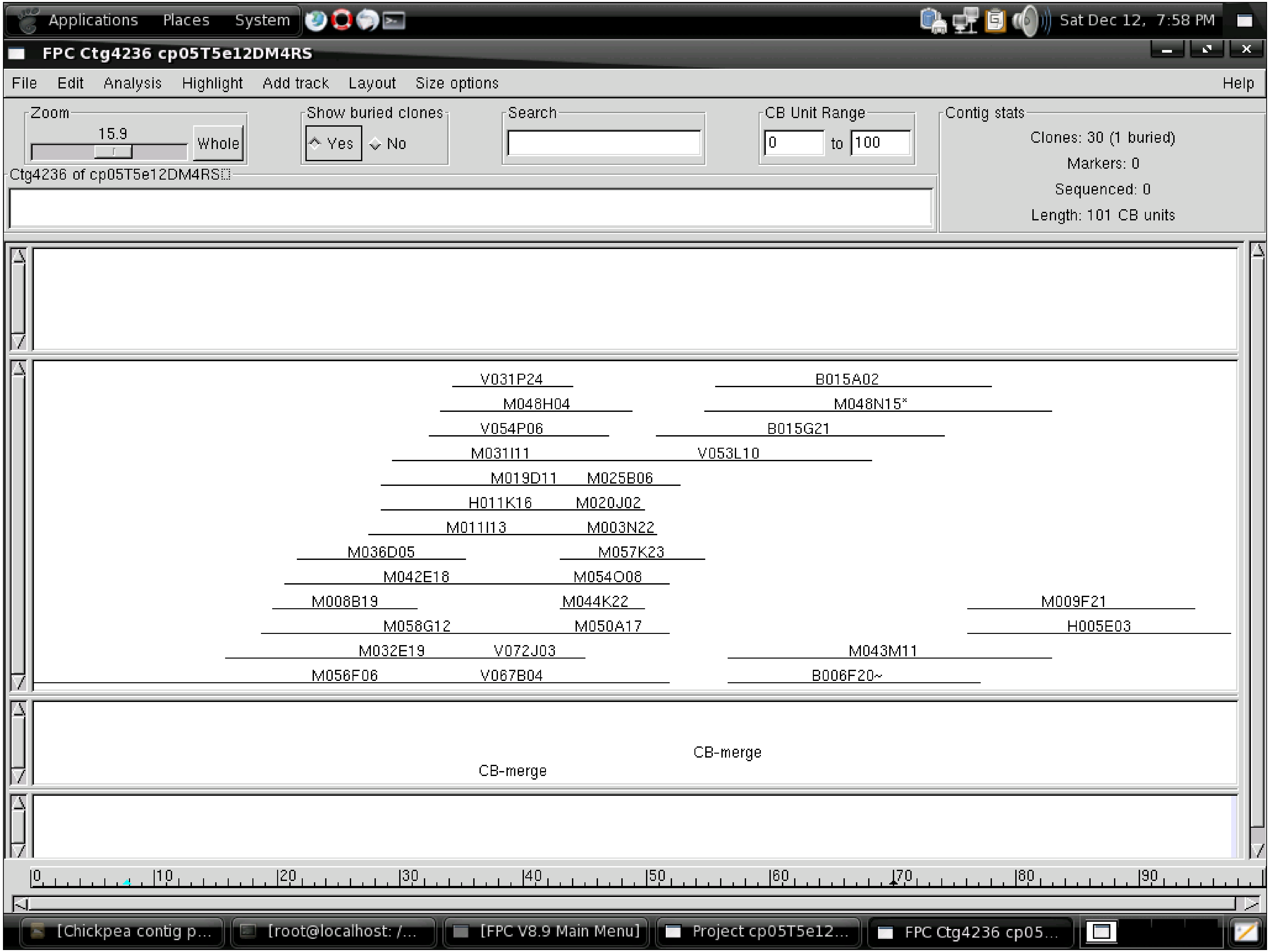
Applications Places System
Sat Dec 12, 7:58 PM
FPC Ctg4236 cp05T5e12DM4RS
File Edit Analysis Highlight Add track Layout Size options Help
Zoom
15.9
Whole
Show buried clones
Yes No
Search
CB Unit Range
0 to 100
Contig stats
Clones: 30 (1 buried)
Markers: 0
Sequenced: 0
Length: 101 CB units
Ctg4236 of cp05T5e12DM4RS
V031P24
B015A02
M048H04
M048N15*
V054P06
B015G21
M031I11
V053L10
M019D11
M025B06
H011K16
M020J02
M011I13
M003N22
M036D05
M057K23
M042E18
M054O08
M008B19
M044K22
M009F21
M058G12
M050A17
H005E03
M032E19
V072J03
M043M11
M056F06
V067B04
B006F20~
CB-merge
CB-merge
0 10 20 30 40 50 60 70 80 90
[Chickpea contig p...
[root@localhost: /...
[FPC V8.9 Main Menu]
Project cp05T5e12...
FPC Ctg4236 cp05...

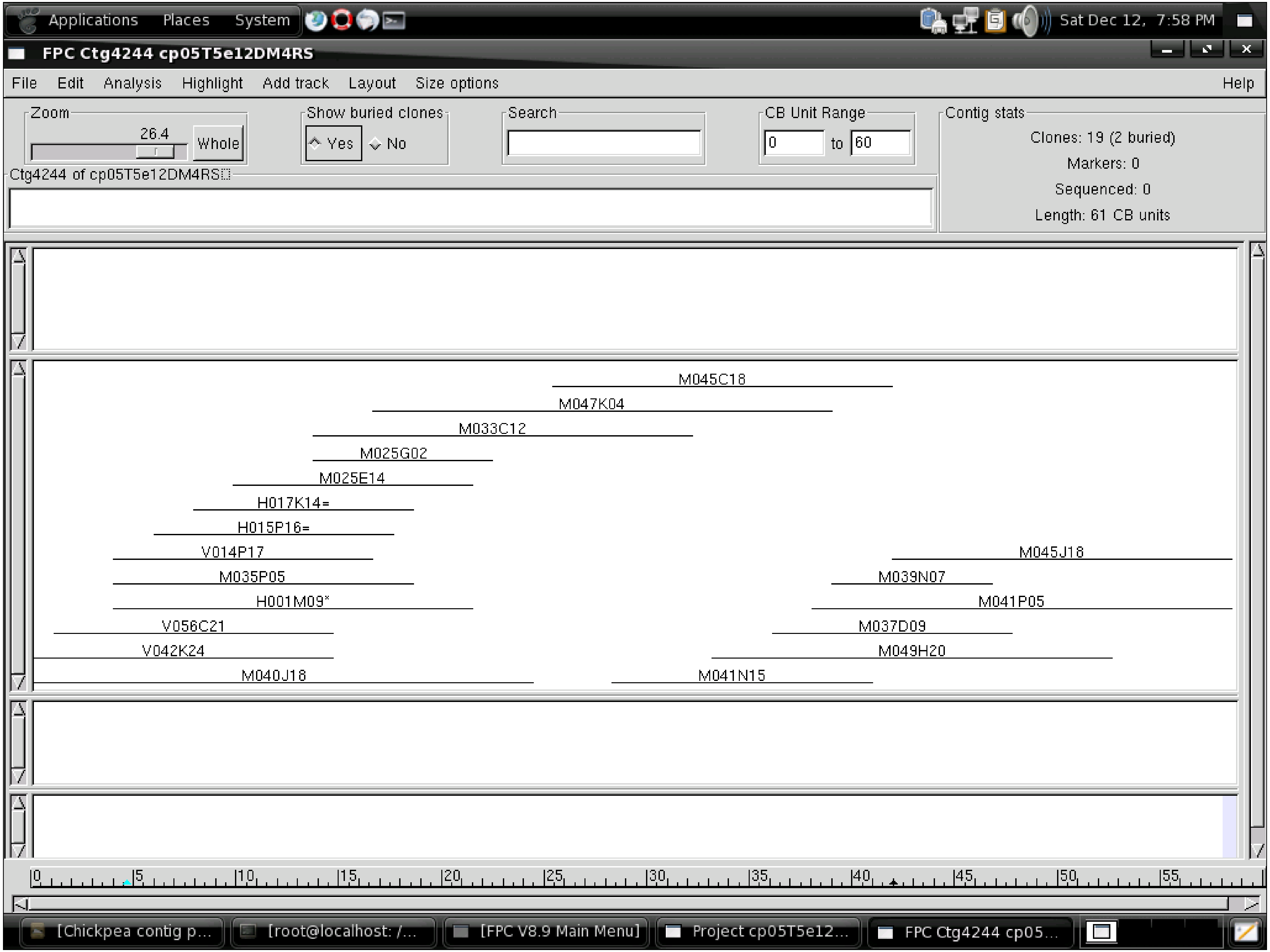
Applications Places System
Sat Dec 12, 7:58 PM
FPC Ctg4244 cp05T5e12DM4RS
File Edit Analysis Highlight Add track Layout Size options Help
Zoom
26.4
Whole
Show buried clones
Yes
No
Search
CB Unit Range
0
to
60
Contig stats
Clones: 19 (2 buried)
Markers: 0
Sequenced: 0
Length: 61 CB units
Ctg4244 of cp05T5e12DM4RS
M045C18
M047K04
M033C12
M025G02
M025E14
H017K14=
H015P16=
V014P17
M035P05
H001M09*
V056C21
V042K24
M040J18
M045J18
M039N07
M041P05
M037D09
M049H20
M041N15
0 5 10 15 20 25 30 35 40 45 50 55
[Chickpea contig p...
[root@localhost: /...
[FPC V8.9 Main Menu]
Project cp05T5e12...
FPC Ctg4244 cp05...

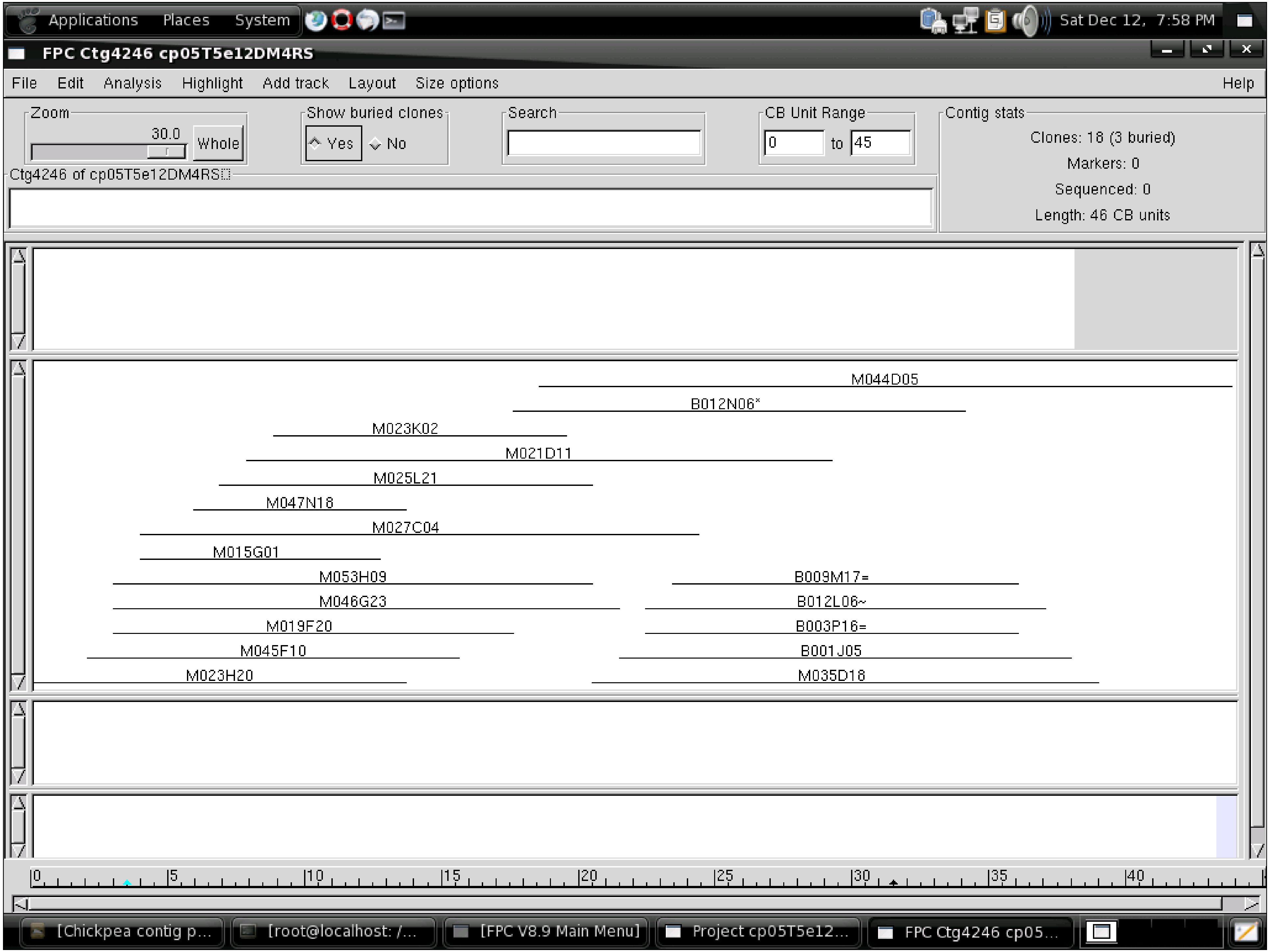
Applications Places System
Sat Dec 12, 7:58 PM
FPC Ctg4246 cp05T5e12DM4RS
File Edit Analysis Highlight Add track Layout Size options Help
Zoom
30.0
Whole
Show buried clones
Yes No
Search
CB Unit Range
0 to 45
Contig stats
Clones: 18 (3 buried)
Markers: 0
Sequenced: 0
Length: 46 CB units
Ctg4246 of cp05T5e12DM4RS
M044D05
B012N06*
M023K02
M021D11
M025L21
M047N18
M027C04
M015G01
M053H09
B009M17=
M046G23
B012L06~
M019F20
B003P16=
M045F10
B001J05
M023H20
M035D18
0 5 10 15 20 25 30 35 40
[Chickpea contig p...
[root@localhost: /...
[FPC V8.9 Main Menu]
Project cp05T5e12...
FPC Ctg4246 cp05...

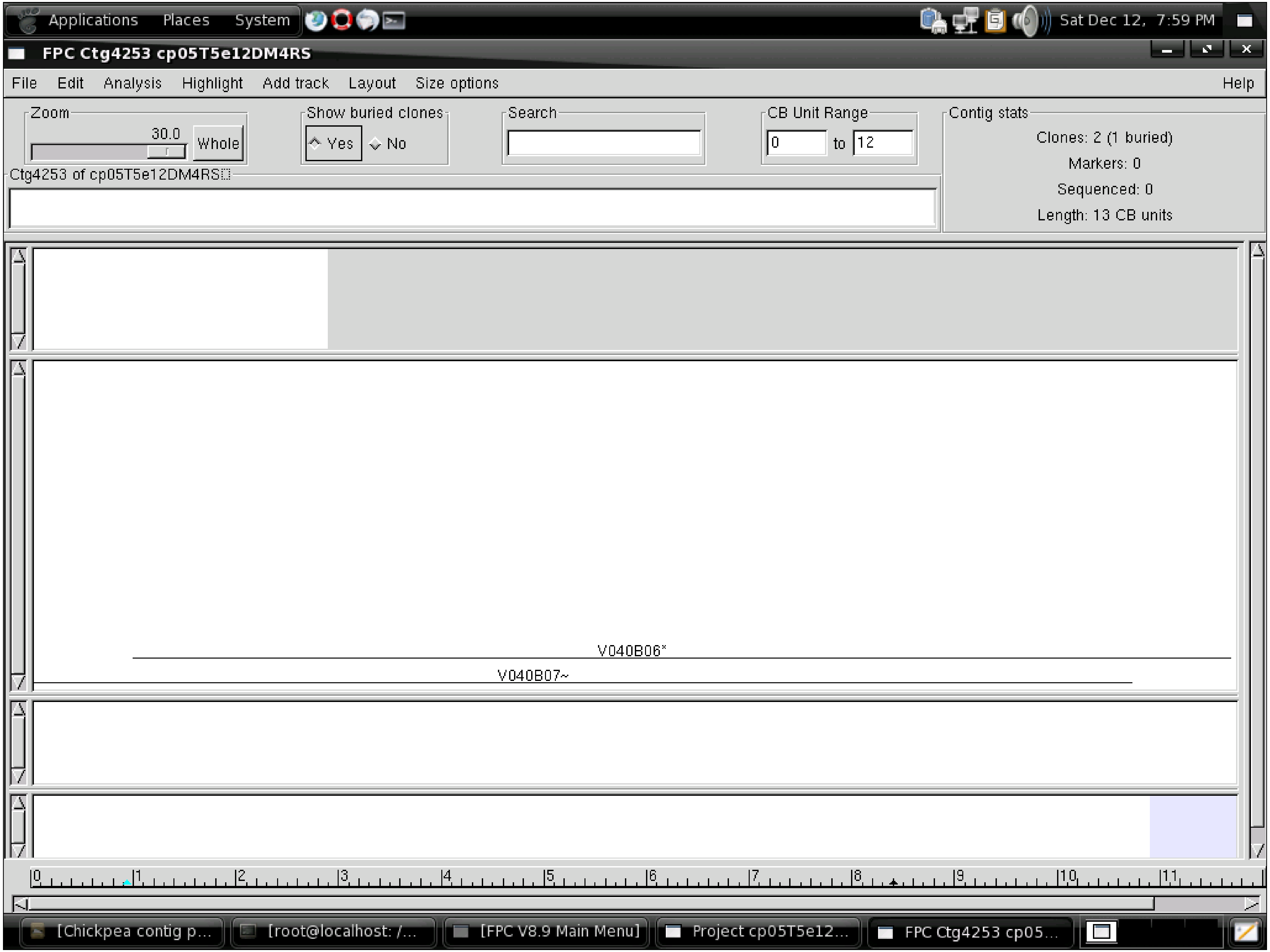
Applications Places System
Sat Dec 12, 7:59 PM
FPC Ctg4253 cp05T5e12DM4RS
File Edit Analysis Highlight Add track Layout Size options
Help
Zoom
30.0
Whole
Show buried clones
Yes
No
Search
CB Unit Range
0
to
12
Contig stats
Clones: 2 (1 buried)
Markers: 0
Sequenced: 0
Length: 13 CB units
Ctg4253 of cp05T5e12DM4RS
V040B06*
V040B07~
0 1 2 3 4 5 6 7 8 9 10 11
[Chickpea contig p...
[root@localhost: /...
[FPC V8.9 Main Menu]
Project cp05T5e12...
FPC Ctg4253 cp05...

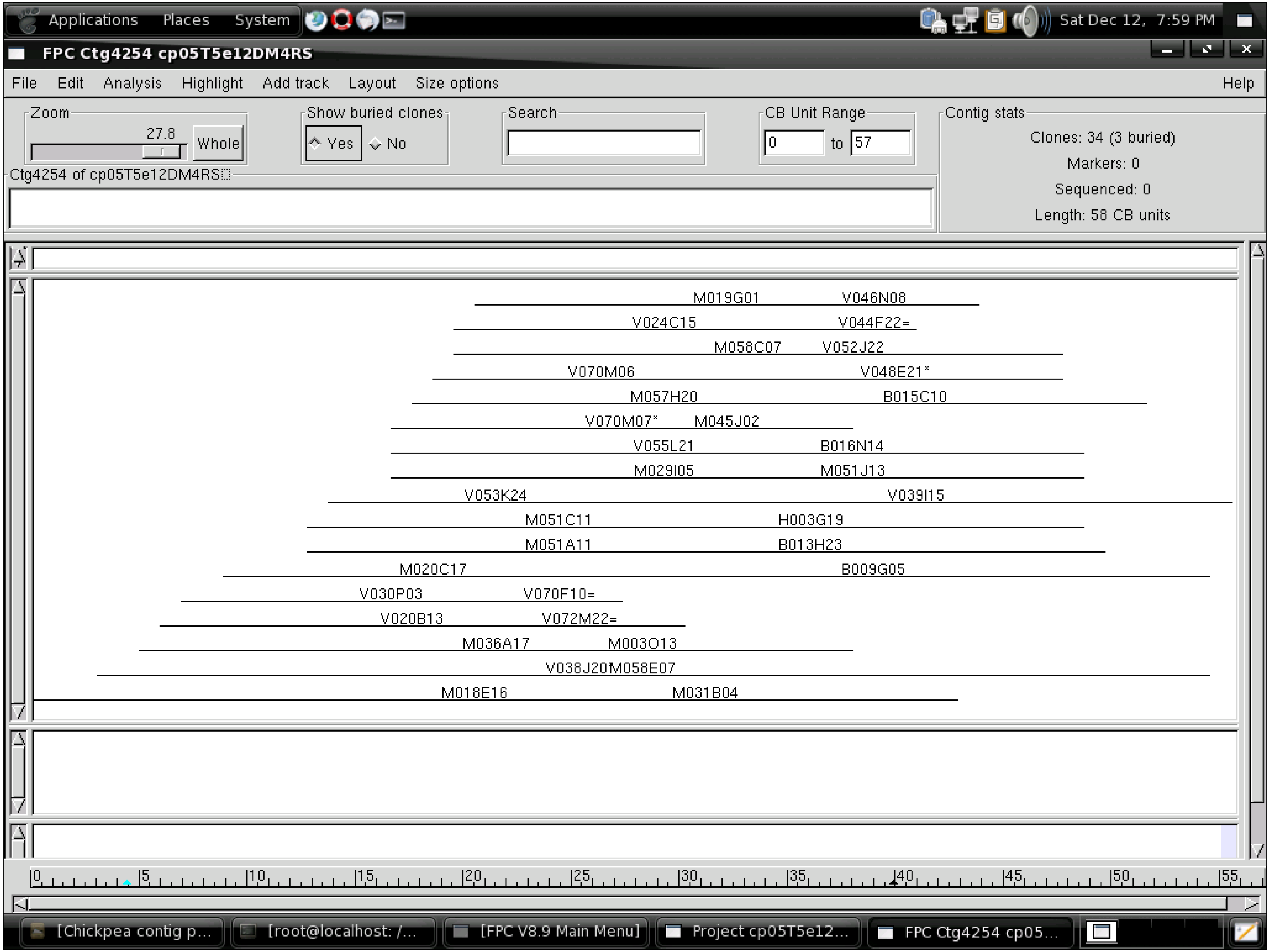
Applications Places System
Sat Dec 12, 7:59 PM
FPC Ctg4254 cp05T5e12DM4RS
File Edit Analysis Highlight Add track Layout Size options Help
Zoom 27.8 Whole
Show buried clones Yes No
Search
CB Unit Range 0 to 57
Contig stats
Clones: 34 (3 buried)
Markers: 0
Sequenced: 0
Length: 58 CB units
Ctg4254 of cp05T5e12DM4RS
M019G01 V046N08
V024C15 V044F22=
M058C07 V052J22
V070M06 V048E21*
M057H20 B015C10
V070M07* M045J02
V055L21 B016N14
M029I05 M051J13
V053K24 V039I15
M051C11 H003G19
M051A11 B013H23
M020C17 B009G05
V030P03 V070F10=
V020B13 V072M22=
M036A17 M003O13
V038J20 M058E07
M018E16 M031B04
0 5 10 15 20 25 30 35 40 45 50 55
[Chickpea contig p...
[root@localhost: /...
[FPC V8.9 Main Menu]
Project cp05T5e12...
FPC Ctg4254 cp05...

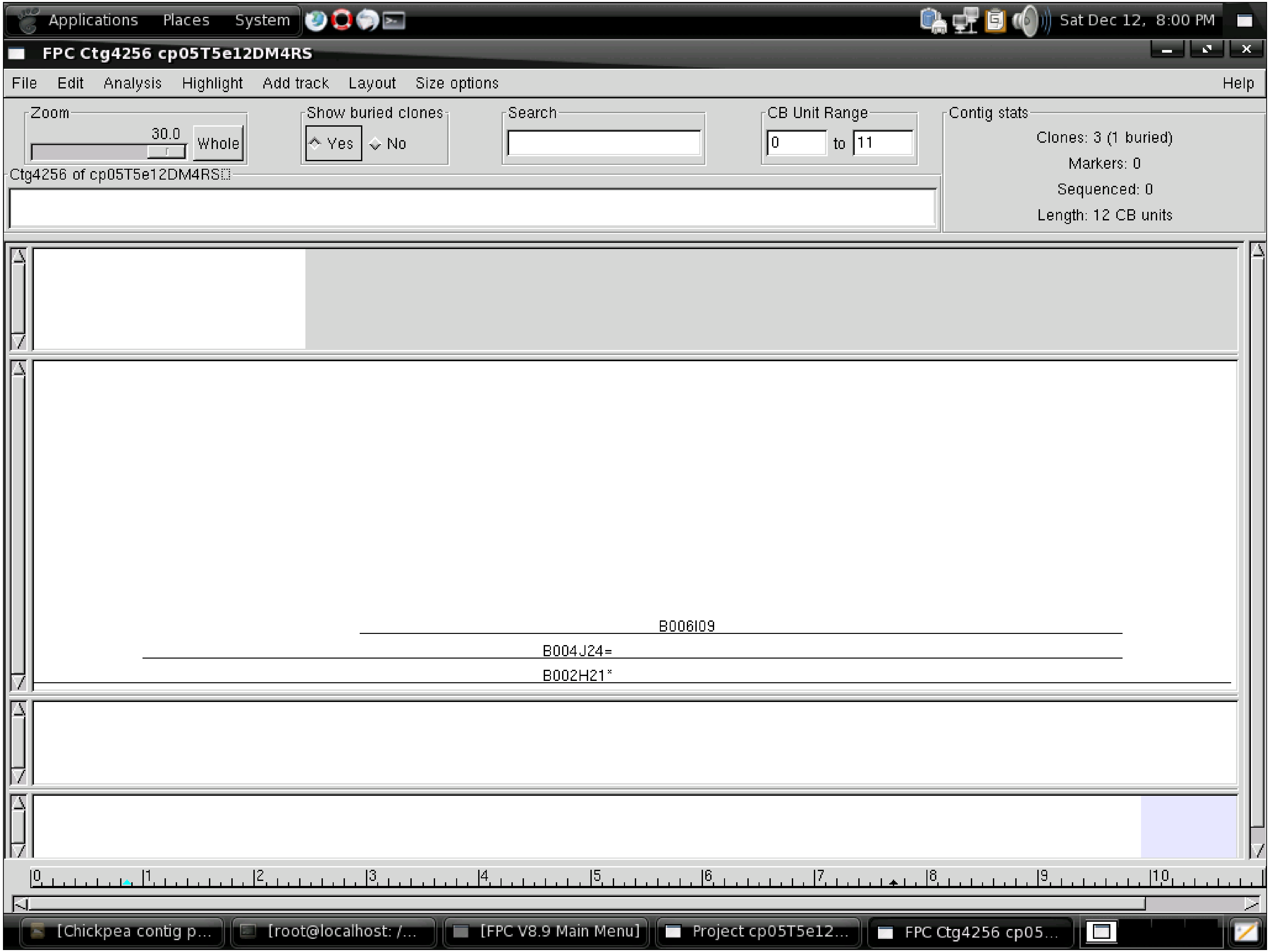
Applications Places System
Sat Dec 12, 8:00 PM
FPC Ctg4256 cp05T5e12DM4RS
File Edit Analysis Highlight Add track Layout Size options Help
Zoom
30.0
Whole
Show buried clones
Yes No
Search
CB Unit Range
0 to 11
Contig stats
Clones: 3 (1 buried)
Markers: 0
Sequenced: 0
Length: 12 CB units
Ctg4256 of cp05T5e12DM4RS
B006I09
B004J24=
B002H21*
0 1 2 3 4 5 6 7 8 9 10
[Chickpea contig p...
[root@localhost: /...
[FPC V8.9 Main Menu]
Project cp05T5e12...
FPC Ctg4256 cp05...

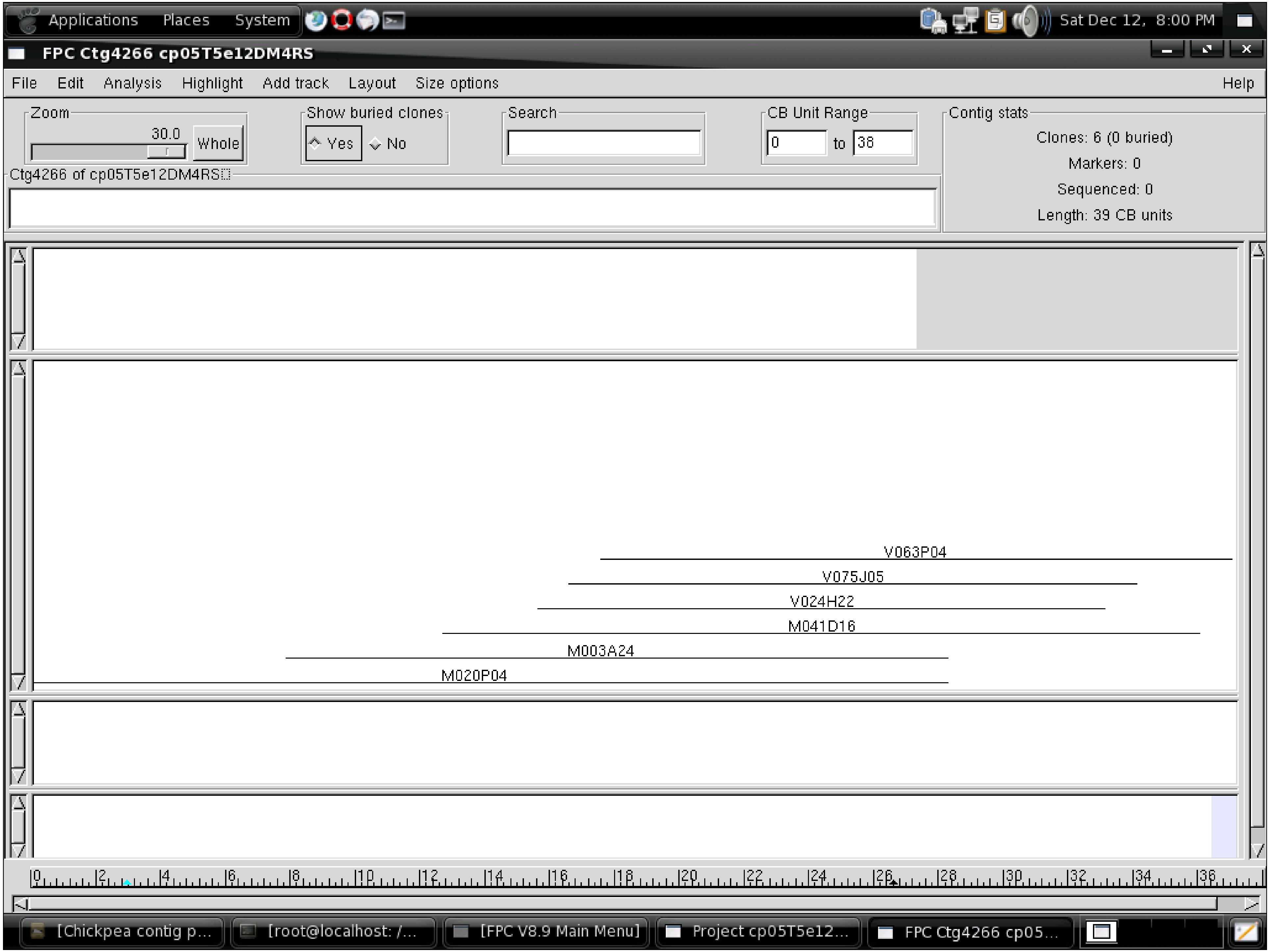
Applications
Places
System
Sat Dec 12, 8:00 PM
FPC Ctg4266 cp05T5e12DM4RS
File
Edit
Analysis
Highlight
Add track
Layout
Size options
Help
Zoom
30.0
Whole
Show buried clones
Yes
No
Search
CB Unit Range
0
to
38
Contig stats
Clones: 6 (0 buried)
Markers: 0
Sequenced: 0
Length: 39 CB units
Ctg4266 of cp05T5e12DM4RS
V063P04
V075J05
V024H22
M041D16
M003A24
M020P04
0 2 4 6 8 10 12 14 16 18 20 22 24 26 28 30 32 34 36
[Chickpea contig p...
[root@localhost: /...
[FPC V8.9 Main Menu]
Project cp05T5e12...
FPC Ctg4266 cp05...

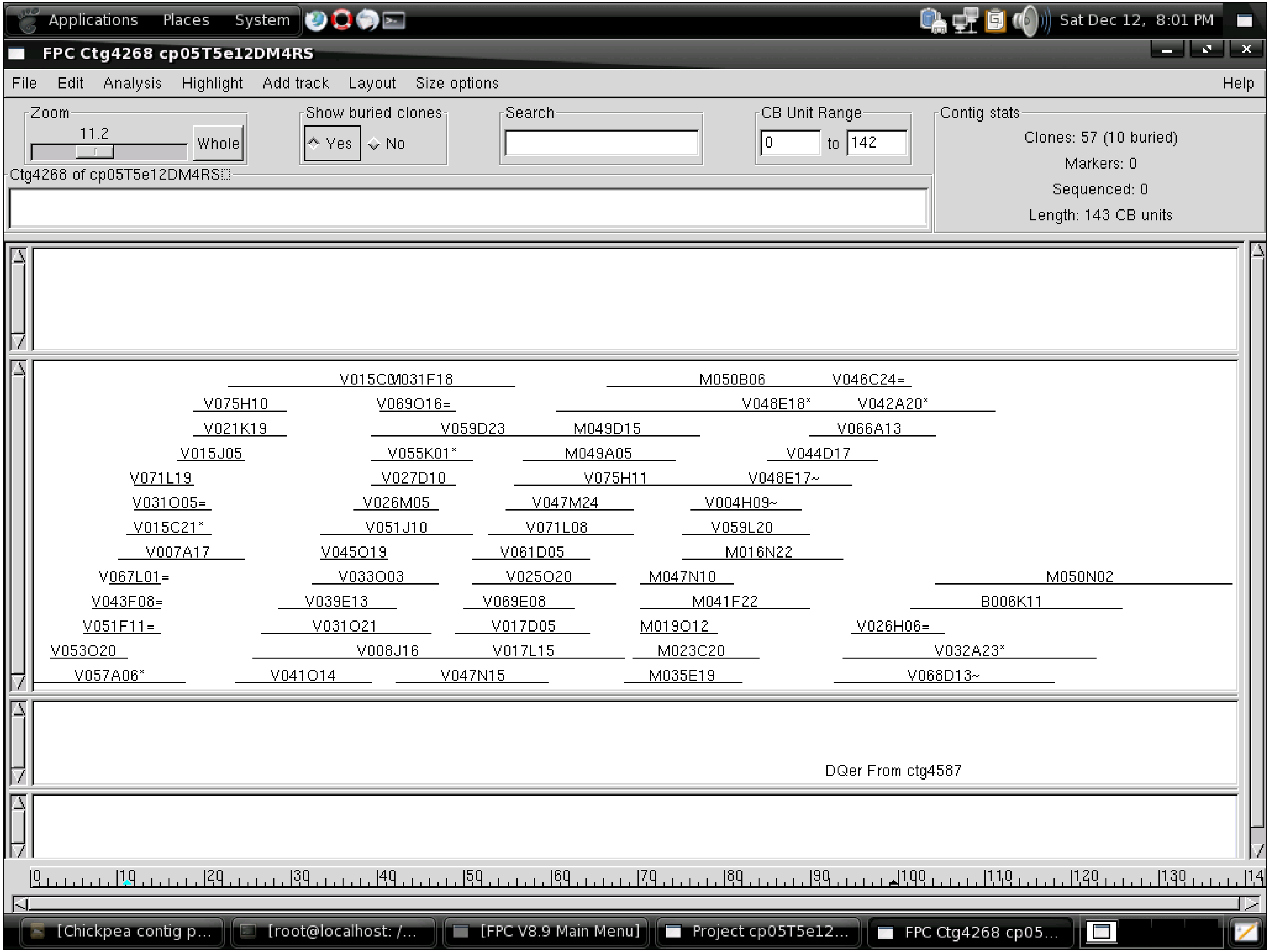
Applications Places System
Sat Dec 12, 8:01 PM
FPC Ctg4268 cp05T5e12DM4RS
File Edit Analysis Highlight Add track Layout Size options Help
Zoom
11.2
Whole
Show buried clones
Yes
No
Search
CB Unit Range
0
to
142
Contig stats
Clones: 57 (10 buried)
Markers: 0
Sequenced: 0
Length: 143 CB units
Ctg4268 of cp05T5e12DM4RS
V015C0M031F18
M050B06
V046C24=
V075H10
V069O16=
V048E18*
V042A20*
V021K19
V059D23
M049D15
V066A13
V015J05
V055K01*
M049A05
V044D17
V071L19
V027D10
V075H11
V048E17~
V031O05=
V026M05
V047M24
V004H09~
V015C21*
V051J10
V071L08
V059L20
V007A17
V045O19
V061D05
M016N22
V067L01=
V033O03
V025O20
M047N10
M050N02
V043F08=
V039E13
V069E08
M041F22
B006K11
V051F11=
V031O21
V017D05
M019O12
V026H06=
V053O20
V008J16
V017L15
M023C20
V032A23*
V057A06*
V041O14
V047N15
M035E19
V068D13~
DQer From ctg4587
0 10 20 30 40 50 60 70 80 90 100 110 120 130 14
[Chickpea contig p...
[root@localhost: /...
[FPC V8.9 Main Menu]
Project cp05T5e12...
FPC Ctg4268 cp05...

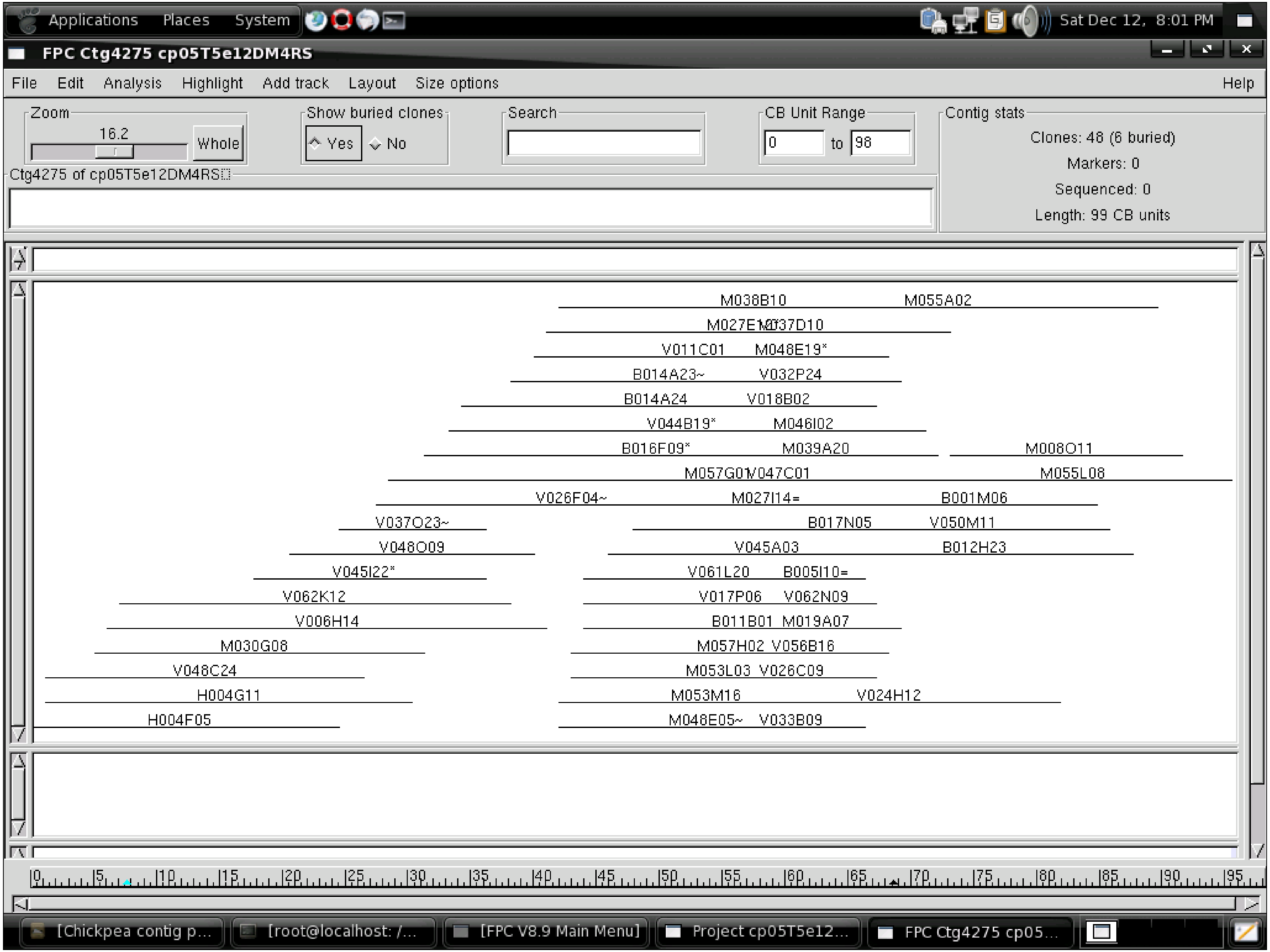
Applications Places System
Sat Dec 12, 8:01 PM
FPC Ctg4275 cp05T5e12DM4RS
File Edit Analysis Highlight Add track Layout Size options Help
Zoom
16.2
Whole
Show buried clones
Yes
No
Search
CB Unit Range
0
to
98
Contig stats
Clones: 48 (6 buried)
Markers: 0
Sequenced: 0
Length: 99 CB units
Ctg4275 of cp05T5e12DM4RS
M038B10 M055A02
M027E1M037D10
V011C01 M048E19*
B014A23~ V032P24
B014A24 V018B02
V044B19* M046I02
B016F09* M039A20 M008O11
M057G01V047C01 M055L08
V026F04~ M027I14= B001M06
V037O23~ B017N05 V050M11
V048O09 V045A03 B012H23
V045I22* V061L20 B005I10=
V062K12 V017P06 V062N09
V006H14 B011B01 M019A07
M030G08 M057H02 V056B16
V048C24 M053L03 V026C09
H004G11 M053M16 V024H12
H004F05 M048E05~ V033B09
0 5 10 15 20 25 30 35 40 45 50 55 60 65 70 75 80 85 90 95
[Chickpea contig p...
[root@localhost: /...
[FPC V8.9 Main Menu]
Project cp05T5e12...
FPC Ctg4275 cp05...

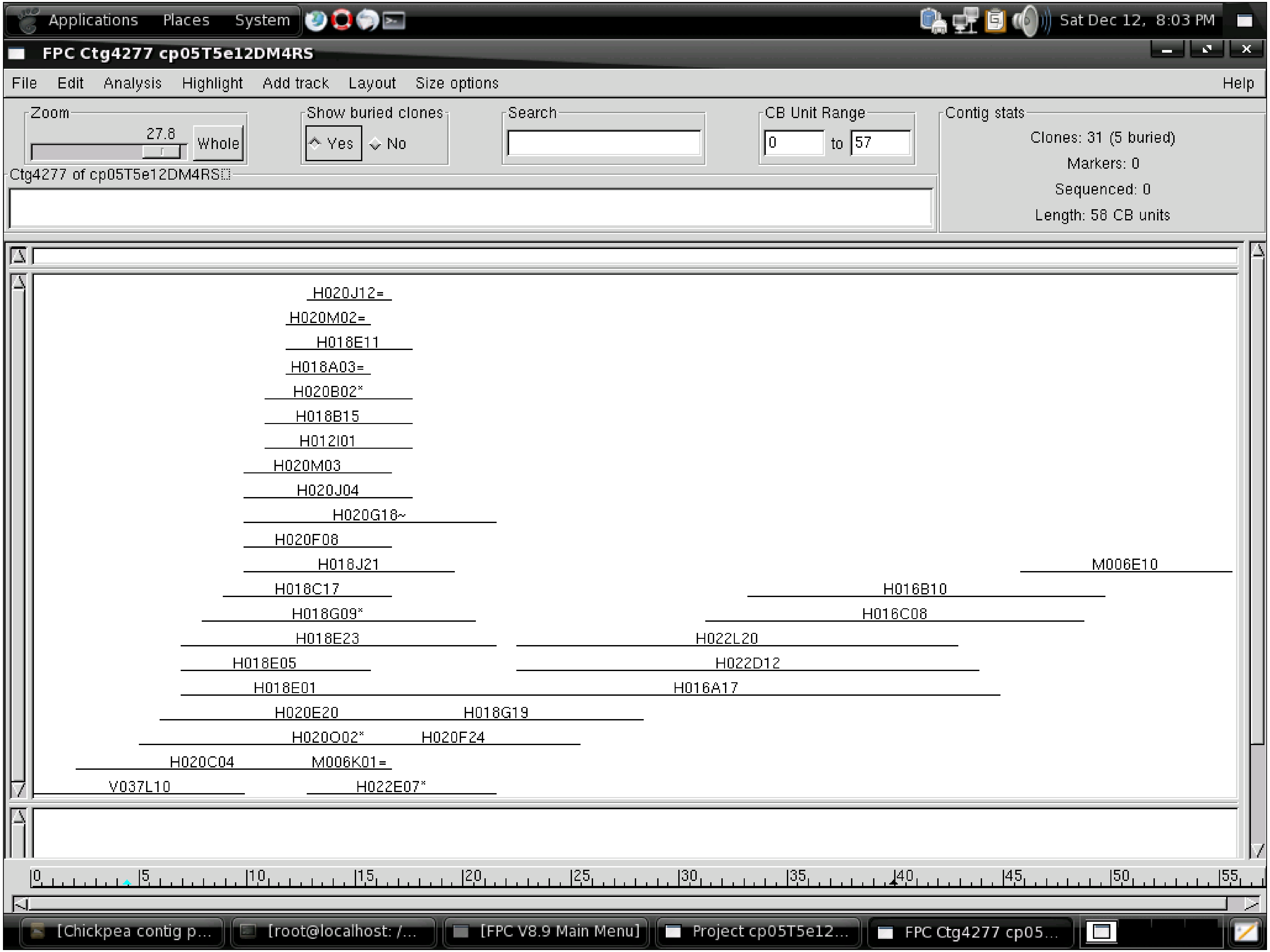
Applications Places System
Sat Dec 12, 8:03 PM
FPC Ctg4277 cp05T5e12DM4RS
File Edit Analysis Highlight Add track Layout Size options Help
Zoom
27.8
Whole
Show buried clones
Yes
No
Search
CB Unit Range
0
to
57
Contig stats
Clones: 31 (5 buried)
Markers: 0
Sequenced: 0
Length: 58 CB units
Ctg4277 of cp05T5e12DM4RS
H020J12=
H020M02=
H018E11
H018A03=
H020B02*
H018B15
H012I01
H020M03
H020J04
H020G18~
H020F08
H018J21
M006E10
H018C17
H016B10
H018G09*
H016C08
H018E23
H022L20
H018E05
H022D12
H018E01
H016A17
H020E20
H018G19
H020O02*
H020F24
H020C04
M006K01=
V037L10
H022E07*
0 5 10 15 20 25 30 35 40 45 50 55
[Chickpea contig p...
[root@localhost: /...
[FPC V8.9 Main Menu]
Project cp05T5e12...
FPC Ctg4277 cp05...

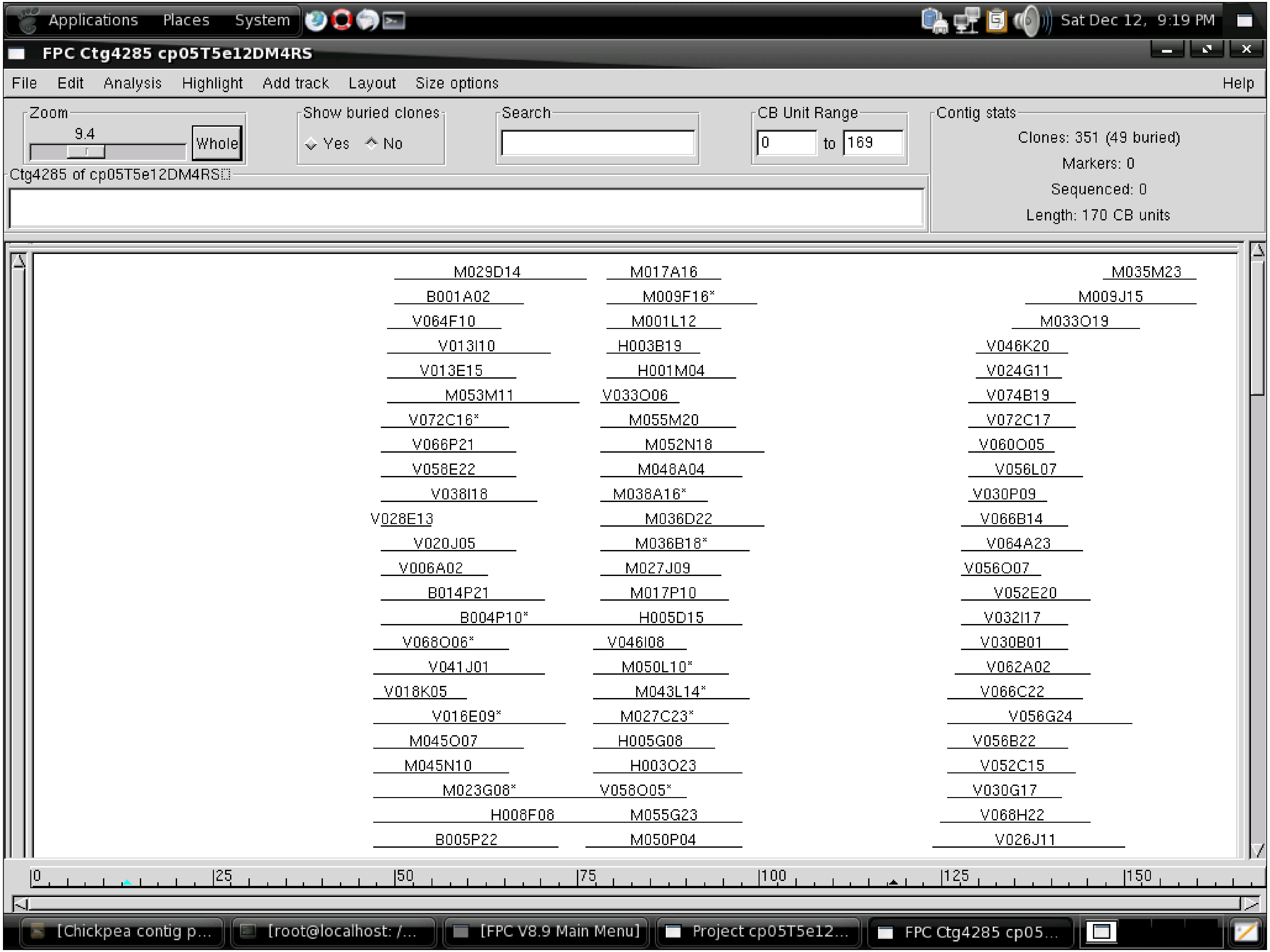
Applications Places System
Sat Dec 12, 9:19 PM
FPC Ctg4285 cp05T5e12DM4RS
File Edit Analysis Highlight Add track Layout Size options Help
Zoom
9.4
Whole
Show buried clones
Yes No
Search
CB Unit Range
0 to 169
Contig stats
Clones: 351 (49 buried)
Markers: 0
Sequenced: 0
Length: 170 CB units
Ctg4285 of cp05T5e12DM4RS
M029D14
B001A02
V064F10
V013I10
V013E15
M053M11
V072C16*
V066P21
V058E22
V038I18
V028E13
V020J05
V006A02
B014P21
B004P10*
V068O06*
V041J01
V018K05
V016E09*
M045O07
M045N10
M023G08*
H008F08
B005P22
M017A16
M009F16*
M001L12
H003B19
H001M04
V033O06
M055M20
M052N18
M048A04
M038A16*
M036D22
M036B18*
M027J09
M017P10
H005D15
V046I08
M050L10*
M043L14*
M027C23*
H005G08
H003O23
V058O05*
M055G23
M050P04
M035M23
M009J15
M033O19
V046K20
V024G11
V074B19
V072C17
V060O05
V056L07
V030P09
V066B14
V064A23
V056O07
V052E20
V032I17
V030B01
V062A02
V066C22
V056G24
V056B22
V052C15
V030G17
V068H22
V026J11
0 25 50 75 100 125 150
[Chickpea contig p...
[root@localhost: /...
[FPC V8.9 Main Menu]
Project cp05T5e12...
FPC Ctg4285 cp05...

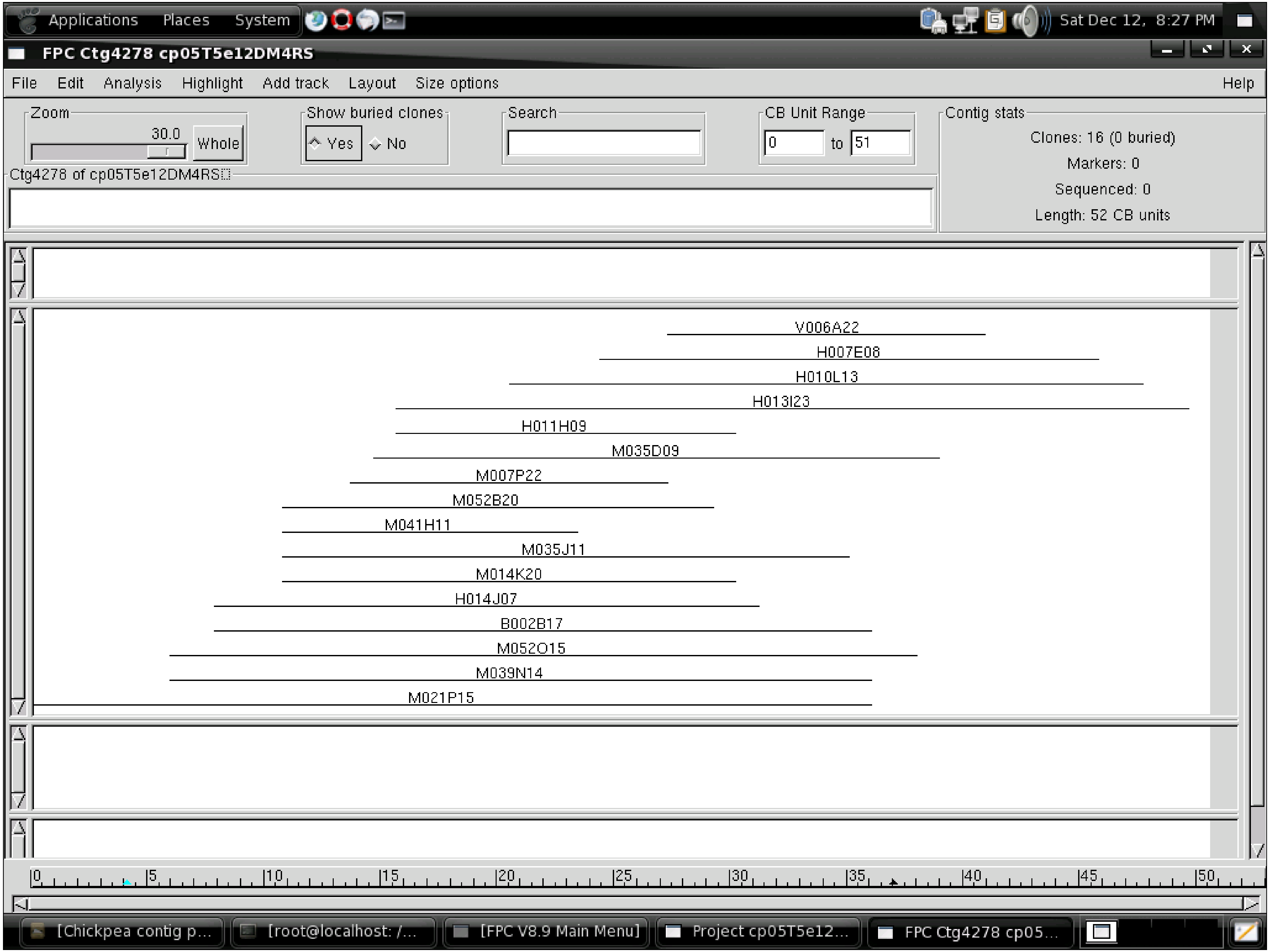
Applications Places System
Sat Dec 12, 8:27 PM
FPC Ctg4278 cp05T5e12DM4RS
File Edit Analysis Highlight Add track Layout Size options Help
Zoom
30.0
Whole
Show buried clones
Yes
No
Search
CB Unit Range
0
to
51
Contig stats
Clones: 16 (0 buried)
Markers: 0
Sequenced: 0
Length: 52 CB units
Ctg4278 of cp05T5e12DM4RS
V006A22
H007E08
H010L13
H013I23
H011H09
M035D09
M007P22
M052B20
M041H11
M035J11
M014K20
H014J07
B002B17
M052O15
M039N14
M021P15
0 5 10 15 20 25 30 35 40 45 50
[Chickpea contig p...
[root@localhost: /...
[FPC V8.9 Main Menu]
Project cp05T5e12...
FPC Ctg4278 cp05...

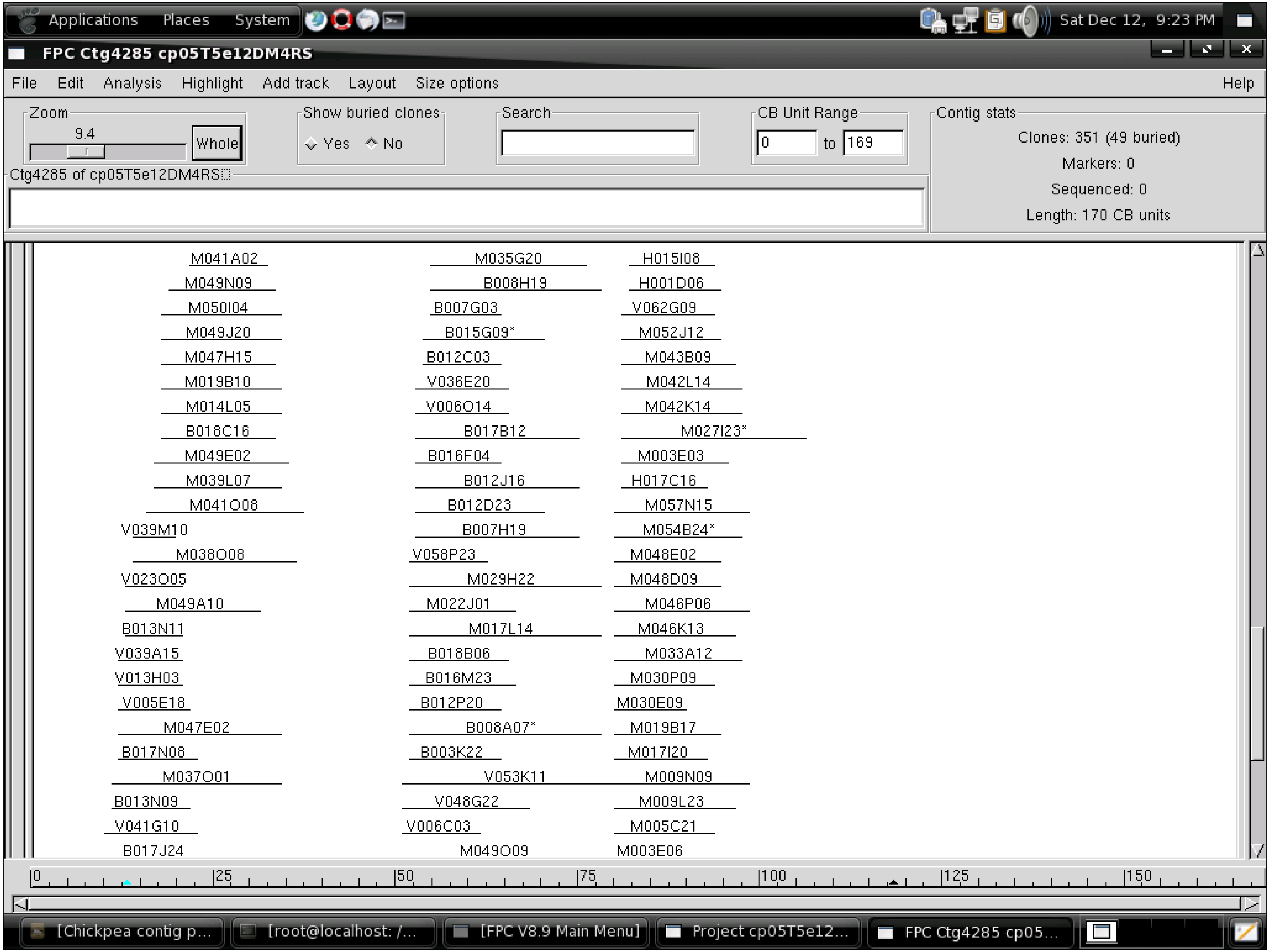
Applications Places System
Sat Dec 12, 9:23 PM
FPC Ctg4285 cp05T5e12DM4RS
File Edit Analysis Highlight Add track Layout Size options Help
Zoom 9.4 Whole
Show buried clones Yes No
Search
CB Unit Range 0 to 169
Contig stats
Clones: 351 (49 buried)
Markers: 0
Sequenced: 0
Length: 170 CB units
Ctg4285 of cp05T5e12DM4RS
M041A02
M049N09
M050I04
M049J20
M047H15
M019B10
M014L05
B018C16
M049E02
M039L07
M041O08
V039M10
M038O08
V023O05
M049A10
B013N11
V039A15
V013H03
V005E18
M047E02
B017N08
M037O01
B013N09
V041G10
B017J24
M035G20
B008H19
B007G03
B015G09*
B012C03
V036E20
V006O14
B017B12
B016F04
B012J16
B012D23
B007H19
V058P23
M029H22
M022J01
M017L14
B018B06
B016M23
B012P20
B008A07*
B003K22
V053K11
V048G22
V006C03
M049O09
H015I08
H001D06
V062G09
M052J12
M043B09
M042L14
M042K14
M027I23*
M003E03
H017C16
M057N15
M054B24*
M048E02
M048D09
M046P06
M046K13
M033A12
M030P09
M030E09
M019B17
M017I20
M009N09
M009L23
M005C21
M003E06
0 25 50 75 100 125 150
[Chickpea contig p...
[root@localhost: /...
[FPC V8.9 Main Menu]
Project cp05T5e12...
FPC Ctg4285 cp05...

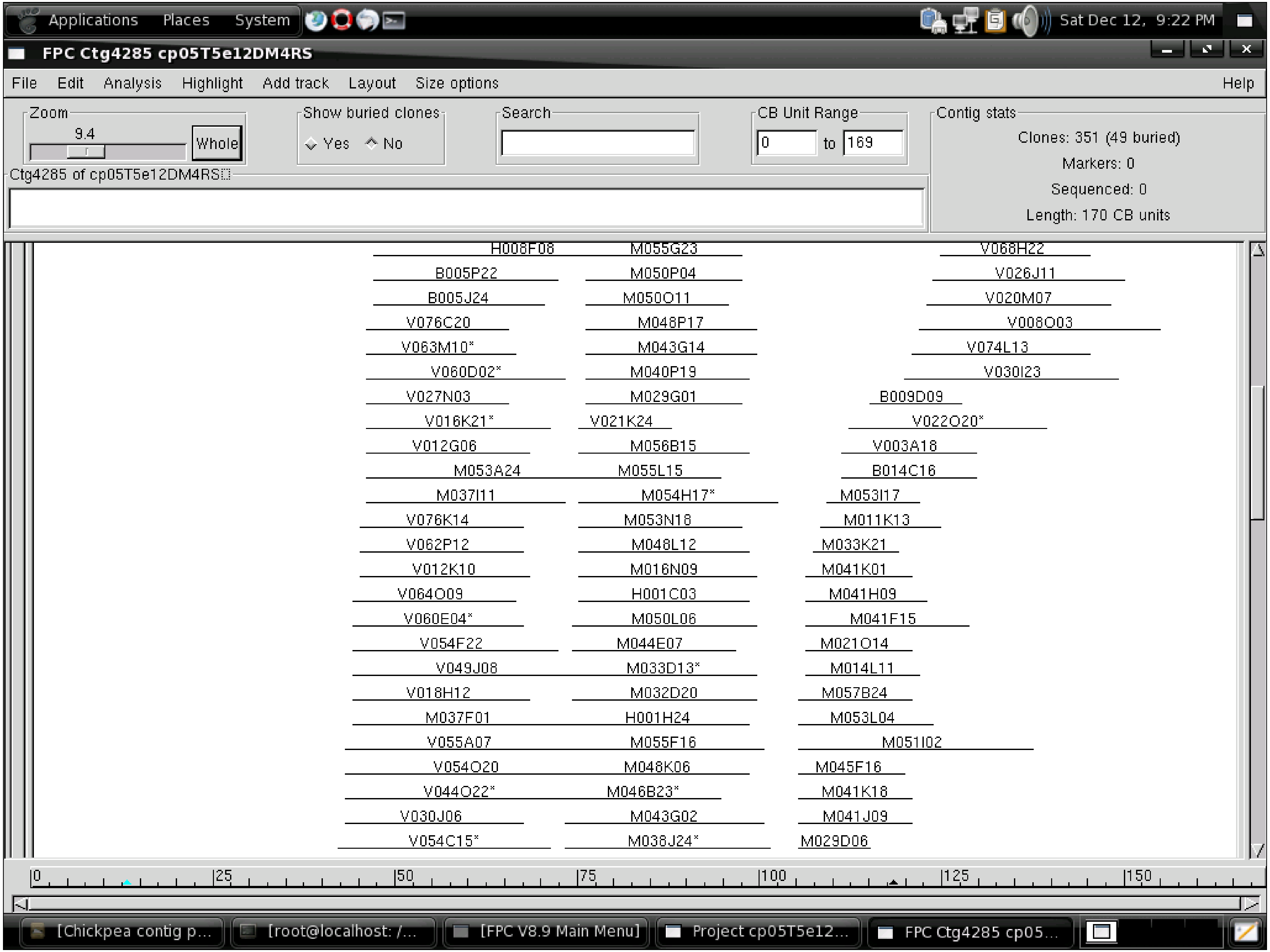
Applications Places System
Sat Dec 12, 9:22 PM
FPC Ctg4285 cp05T5e12DM4RS
File Edit Analysis Highlight Add track Layout Size options Help
Zoom
9.4
Whole
Show buried clones
Yes No
Search
CB Unit Range
0 to 169
Contig stats
Clones: 351 (49 buried)
Markers: 0
Sequenced: 0
Length: 170 CB units
Ctg4285 of cp05T5e12DM4RS
H008F08
M055G23
V068H22
B005P22
M050P04
V026J11
B005J24
M050O11
V020M07
V076C20
M048P17
V008O03
V063M10*
M043G14
V074L13
V060D02*
M040P19
V030I23
V027N03
M029G01
B009D09
V016K21*
V021K24
V022O20*
V012G06
M056B15
V003A18
M053A24
M055L15
B014C16
M037I11
M054H17*
M053I17
V076K14
M053N18
M011K13
V062P12
M048L12
M033K21
V012K10
M016N09
M041K01
V064O09
H001C03
M041H09
V060E04*
M050L06
M041F15
V054F22
M044E07
M021O14
V049J08
M033D13*
M014L11
V018H12
M032D20
M057B24
M037F01
H001H24
M053L04
V055A07
M055F16
M051I02
V054O20
M048K06
M045F16
V044O22*
M046B23*
M041K18
V030J06
M043G02
M041J09
V054C15*
M038J24*
M029D06
0 25 50 75 100 125 150
[Chickpea contig p...
[root@localhost: /...
[FPC V8.9 Main Menu]
Project cp05T5e12...
FPC Ctg4285 cp05...

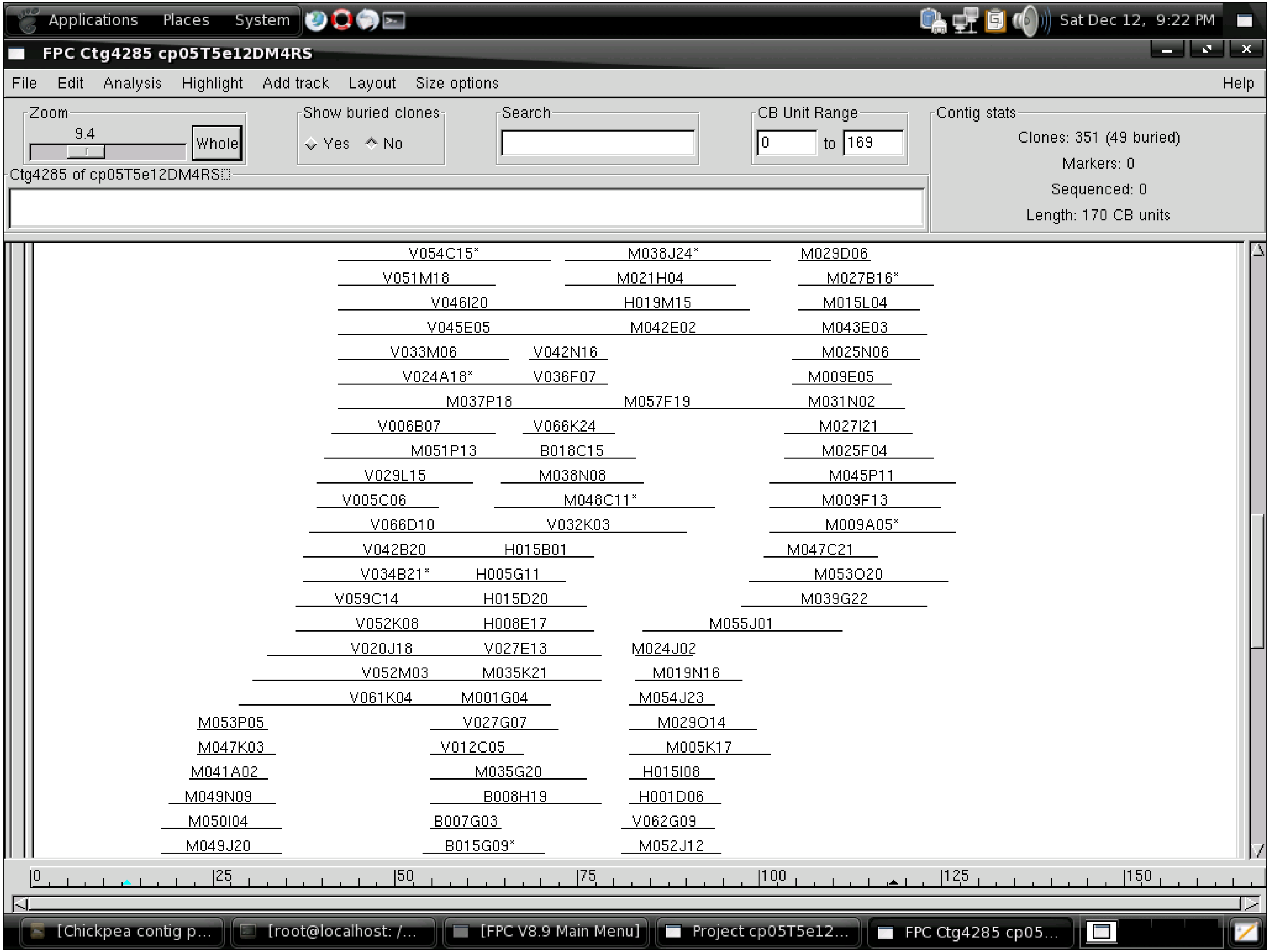
Applications Places System
Sat Dec 12, 9:22 PM
FPC Ctg4285 cp05T5e12DM4RS
File Edit Analysis Highlight Add track Layout Size options Help
Zoom
9.4
Whole
Show buried clones
Yes No
Search
CB Unit Range
0 to 169
Contig stats
Clones: 351 (49 buried)
Markers: 0
Sequenced: 0
Length: 170 CB units
Ctg4285 of cp05T5e12DM4RS
V054C15* M038J24* M029D06
V051M18 M021H04 M027B16*
V046I20 H019M15 M015L04
V045E05 M042E02 M043E03
V033M06 V042N16 M025N06
V024A18* V036F07 M009E05
M037P18 M057F19 M031N02
V006B07 V066K24 M027I21
M051P13 B018C15 M025F04
V029L15 M038N08 M045P11
V005C06 M048C11* M009F13
V066D10 V032K03 M009A05*
V042B20 H015B01 M047C21
V034B21* H005G11 M053O20
V059C14 H015D20 M039G22
V052K08 H008E17 M055J01
V020J18 V027E13 M024J02
V052M03 M035K21 M019N16
V061K04 M001G04 M054J23
M053P05 V027G07 M029O14
M047K03 V012C05 M005K17
M041A02 M035G20 H015I08
M049N09 B008H19 H001D06
M050I04 B007G03 V062G09
M049J20 B015G09* M052J12
0 25 50 75 100 125 150
[Chickpea contig p...
[root@localhost: /...
[FPC V8.9 Main Menu]
Project cp05T5e12...
FPC Ctg4285 cp05...

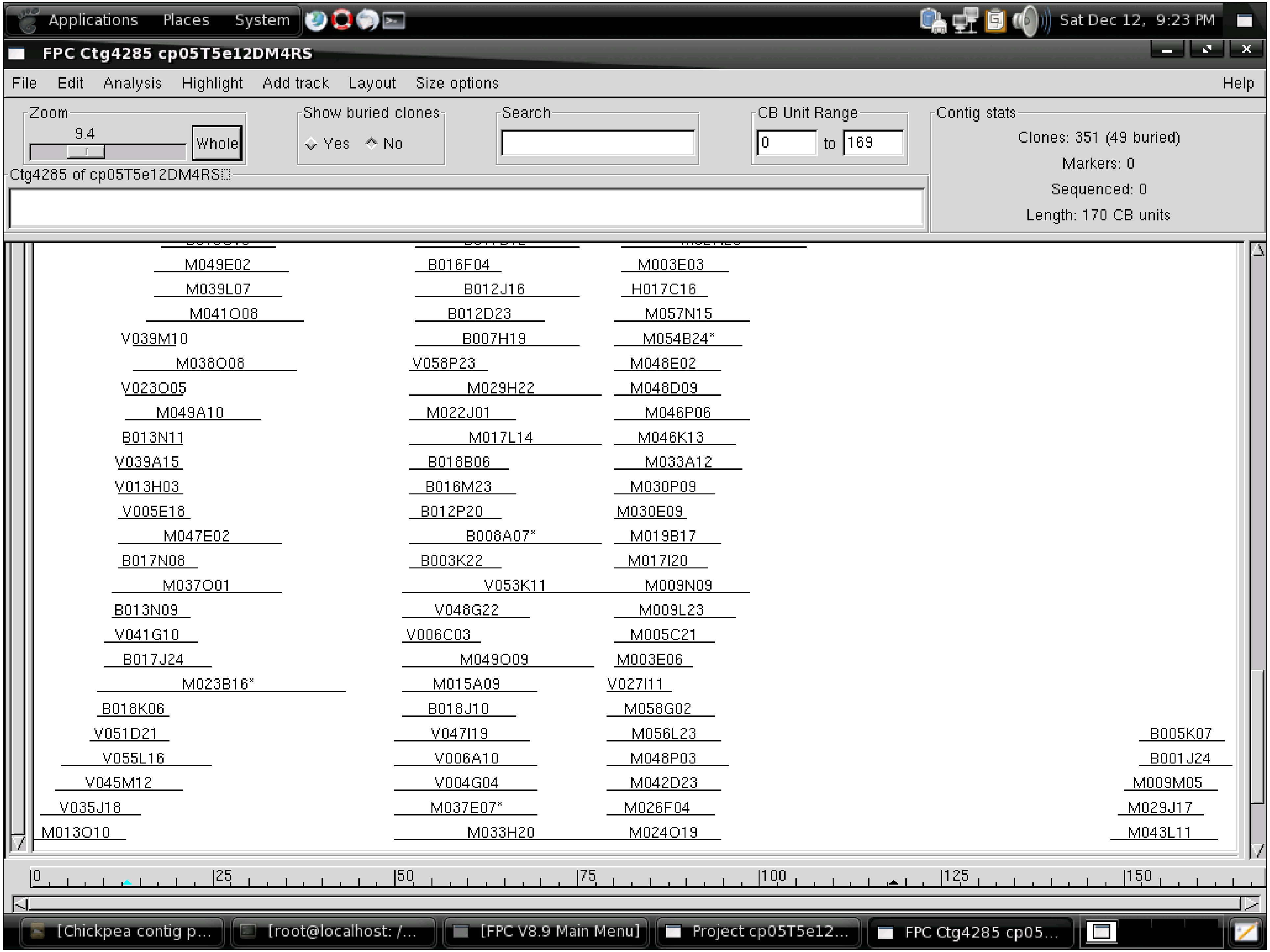
Applications Places System
Sat Dec 12, 9:23 PM
FPC Ctg4285 cp05T5e12DM4RS
File Edit Analysis Highlight Add track Layout Size options Help
Zoom
9.4
Whole
Show buried clones
Yes No
Search
CB Unit Range
0 to 169
Contig stats
Clones: 351 (49 buried)
Markers: 0
Sequenced: 0
Length: 170 CB units
Ctg4285 of cp05T5e12DM4RS
M049E02
B016F04
M003E03
M039L07
B012J16
H017C16
M041O08
B012D23
M057N15
V039M10
B007H19
M054B24*
M038O08
V058P23
M048E02
V023O05
M029H22
M048D09
M049A10
M022J01
M046P06
B013N11
M017L14
M046K13
V039A15
B018B06
M033A12
V013H03
B016M23
M030P09
V005E18
B012P20
M030E09
M047E02
B008A07*
M019B17
B017N08
B003K22
M017I20
M037O01
V053K11
M009N09
B013N09
V048G22
M009L23
V041G10
V006C03
M005C21
B017J24
M049O09
M003E06
M023B16*
M015A09
V027I11
B018K06
B018J10
M058G02
V051D21
V047I19
M056L23
B005K07
V055L16
V006A10
M048P03
B001J24
V045M12
V004G04
M042D23
M009M05
V035J18
M037E07*
M026F04
M029J17
M013O10
M033H20
M024O19
M043L11
0 25 50 75 100 125 150
[Chickpea contig p...
[root@localhost: /...
[FPC V8.9 Main Menu]
Project cp05T5e12...
FPC Ctg4285 cp05...

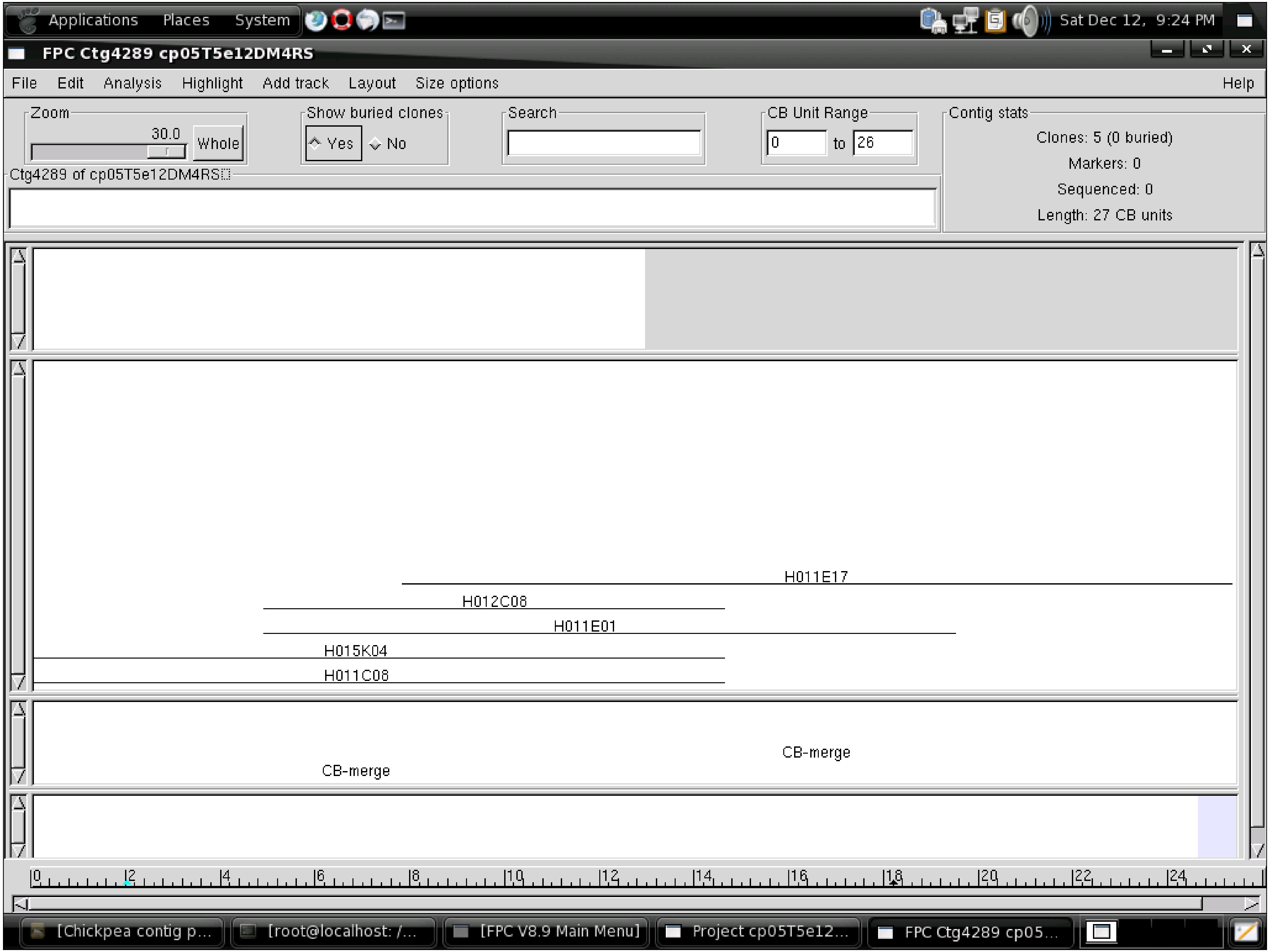
Applications Places System
Sat Dec 12, 9:24 PM
FPC Ctg4289 cp05T5e12DM4RS
File Edit Analysis Highlight Add track Layout Size options Help
Zoom
30.0
Whole
Show buried clones
Yes
No
Search
CB Unit Range
0 to 26
Contig stats
Clones: 5 (0 buried)
Markers: 0
Sequenced: 0
Length: 27 CB units
Ctg4289 of cp05T5e12DM4RS
H011E17
H012C08
H011E01
H015K04
H011C08
CB-merge
CB-merge
0 2 4 6 8 10 12 14 16 18 20 22 24
[Chickpea contig p...
[root@localhost: /...
[FPC V8.9 Main Menu]
Project cp05T5e12...
FPC Ctg4289 cp05...

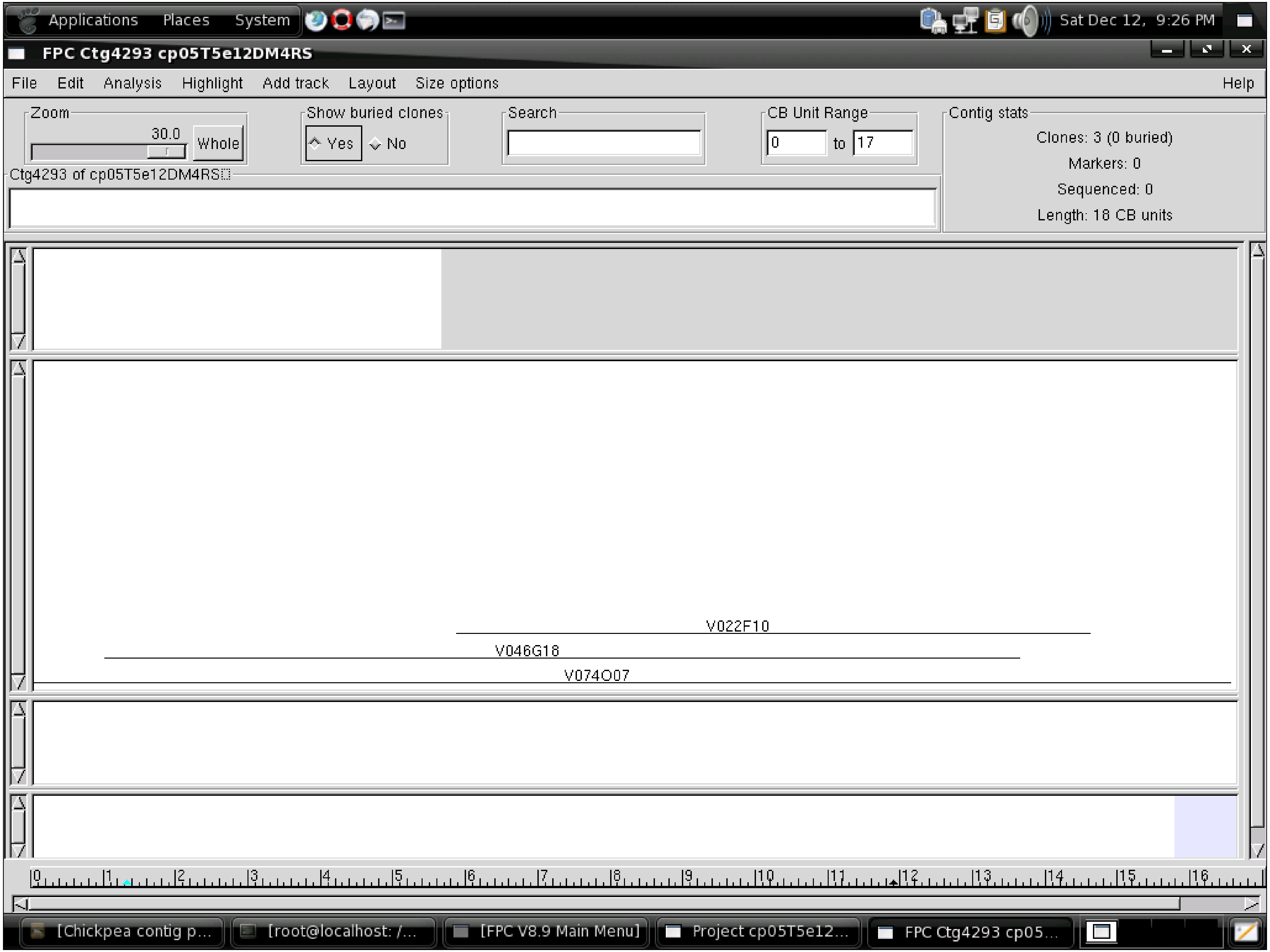
Applications Places System
Sat Dec 12, 9:26 PM
FPC Ctg4293 cp05T5e12DM4RS
File Edit Analysis Highlight Add track Layout Size options
Help
Zoom
30.0
Whole
Show buried clones
Yes
No
Search
CB Unit Range
0
to
17
Contig stats
Clones: 3 (0 buried)
Markers: 0
Sequenced: 0
Length: 18 CB units
Ctg4293 of cp05T5e12DM4RS
V022F10
V046G18
V074O07
0 1 2 3 4 5 6 7 8 9 10 11 12 13 14 15 16
[Chickpea contig p...
[root@localhost: /...
[FPC V8.9 Main Menu]
Project cp05T5e12...
FPC Ctg4293 cp05...

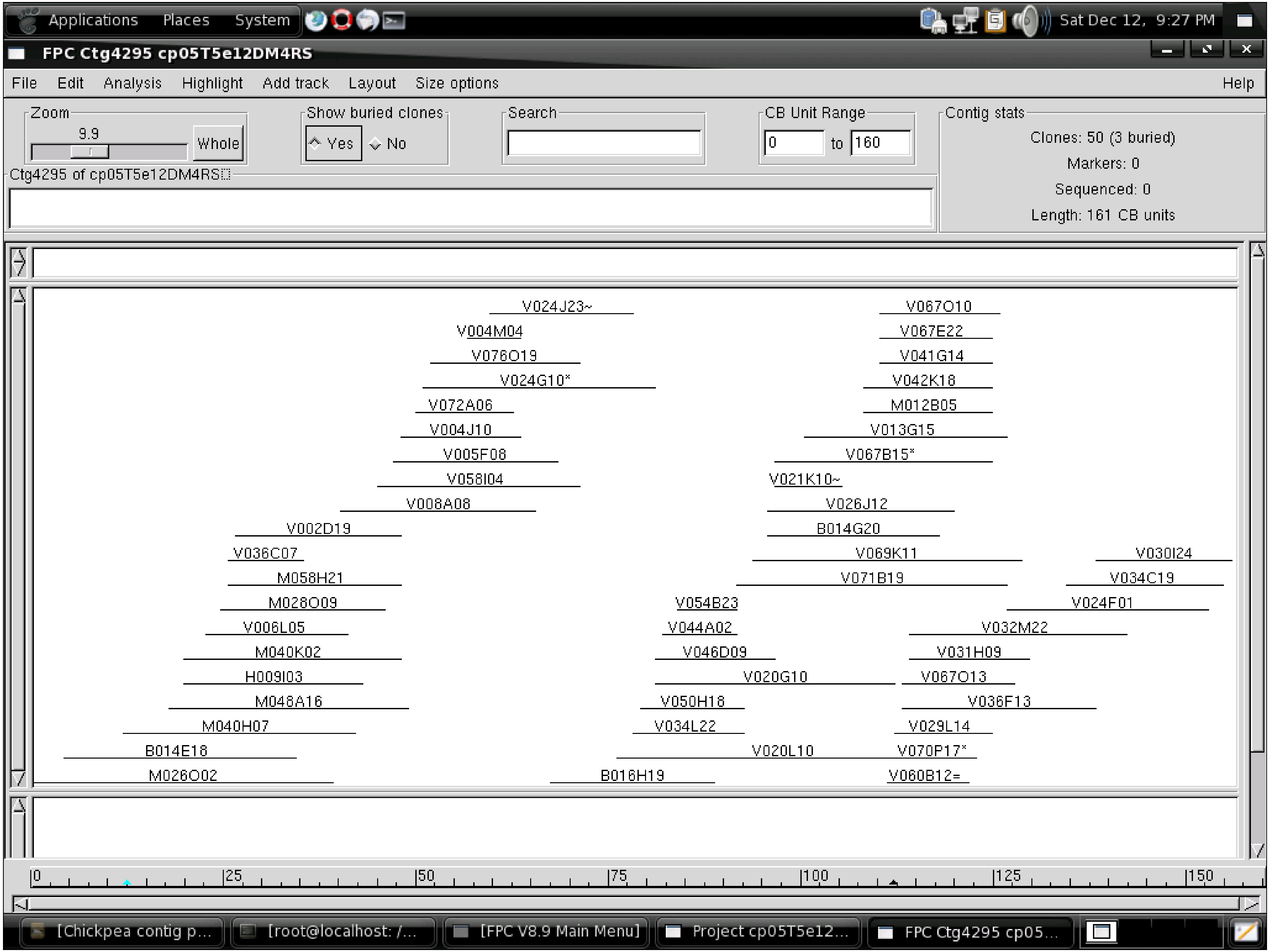
Applications Places System
Sat Dec 12, 9:27 PM
FPC Ctg4295 cp05T5e12DM4RS
File Edit Analysis Highlight Add track Layout Size options Help
Zoom
9.9
Whole
Show buried clones
Yes No
Search
CB Unit Range
0 to 160
Contig stats
Clones: 50 (3 buried)
Markers: 0
Sequenced: 0
Length: 161 CB units
Ctg4295 of cp05T5e12DM4RS
V024J23~
V004M04
V076O19
V024G10*
V072A06
V004J10
V005F08
V058I04
V008A08
V002D19
V036C07
M058H21
M028O09
V006L05
M040K02
H009I03
M048A16
M040H07
B014E18
M026O02
B016H19
V067O10
V067E22
V041G14
V042K18
M012B05
V013G15
V067B15*
V021K10~
V026J12
B014G20
V069K11
V071B19
V054B23
V044A02
V046D09
V020G10
V050H18
V034L22
V020L10
V030I24
V034C19
V024F01
V032M22
V031H09
V067O13
V036F13
V029L14
V070P17*
V060B12=
0
25
50
75
100
125
150
[Chickpea contig p...
[root@localhost: /...
[FPC V8.9 Main Menu]
Project cp05T5e12...
FPC Ctg4295 cp05...

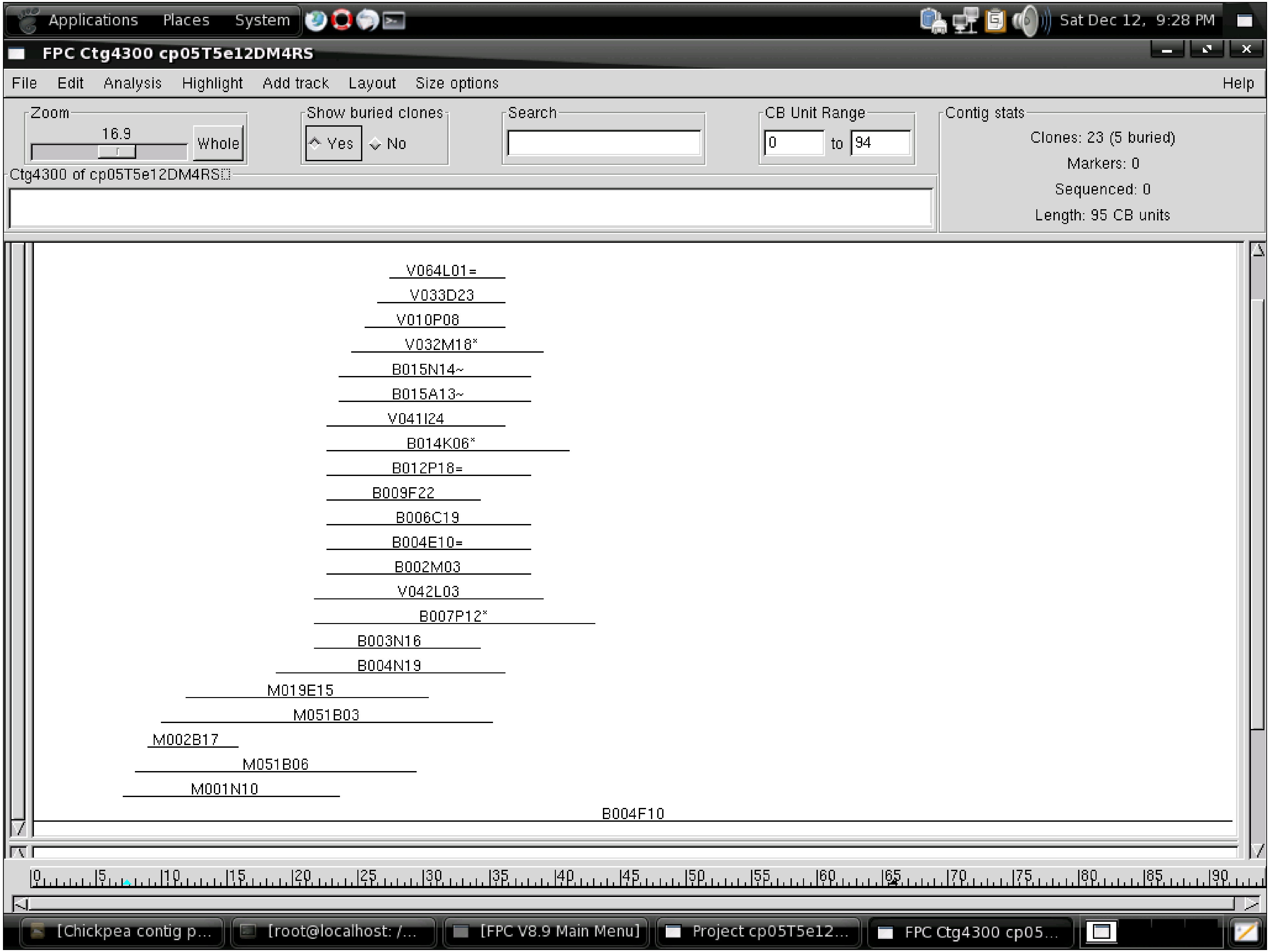
Applications Places System
Sat Dec 12, 9:28 PM
FPC Ctg4300 cp05T5e12DM4RS
File Edit Analysis Highlight Add track Layout Size options Help
Zoom
16.9
Whole
Show buried clones
Yes No
Search
CB Unit Range
0 to 94
Contig stats
Clones: 23 (5 buried)
Markers: 0
Sequenced: 0
Length: 95 CB units
Ctg4300 of cp05T5e12DM4RS
V064L01=
V033D23
V010P08
V032M18*
B015N14~
B015A13~
V041I24
B014K06*
B012P18=
B009F22
B006C19
B004E10=
B002M03
V042L03
B007P12*
B003N16
B004N19
M019E15
M051B03
M002B17
M051B06
M001N10
B004F10
0 5 10 15 20 25 30 35 40 45 50 55 60 65 70 75 80 85 90
[Chickpea contig p...
[root@localhost: /...
[FPC V8.9 Main Menu]
Project cp05T5e12...
FPC Ctg4300 cp05...

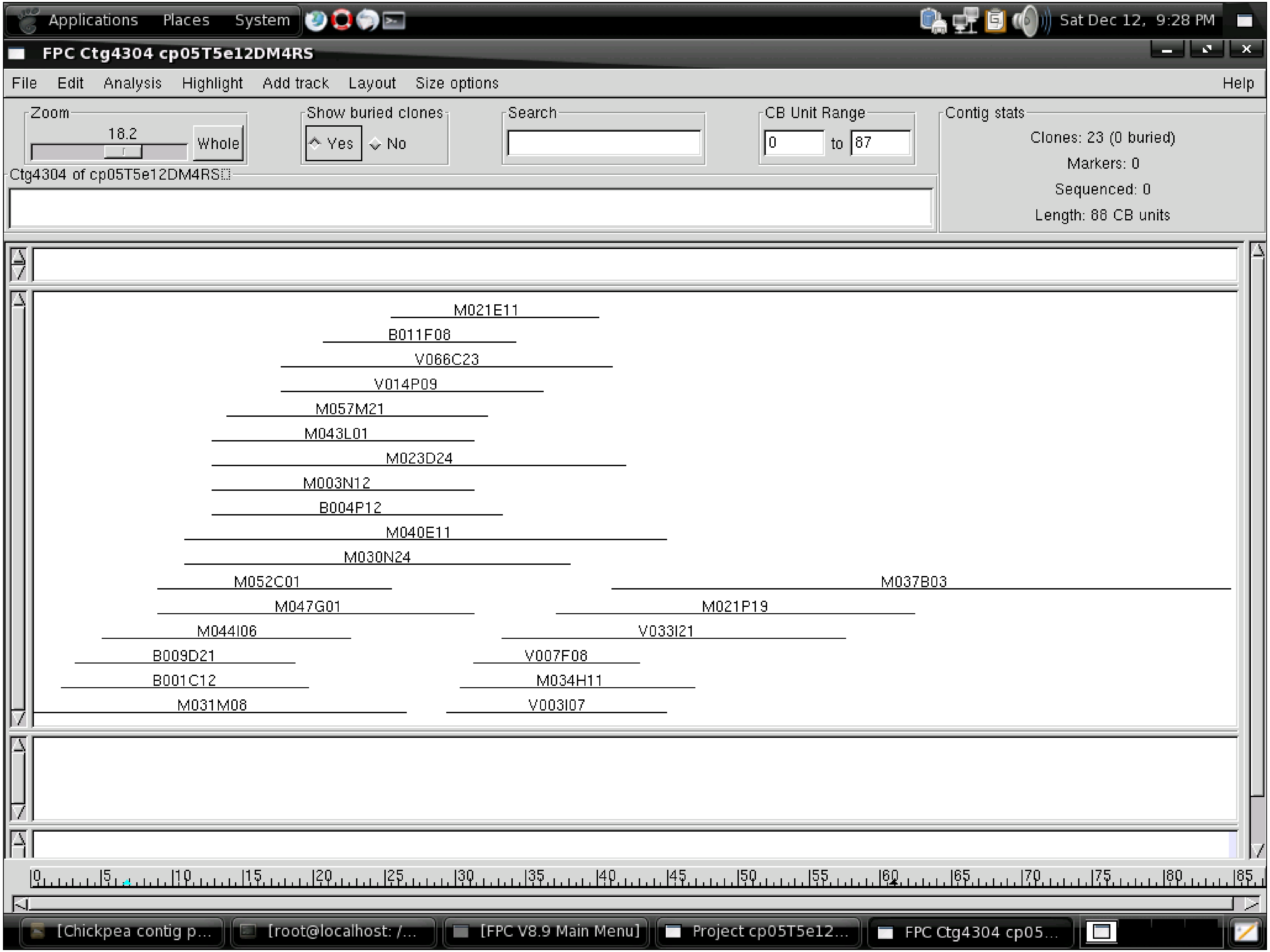
Applications
Places
System
Sat Dec 12, 9:28 PM
FPC Ctg4304 cp05T5e12DM4RS
File
Edit
Analysis
Highlight
Add track
Layout
Size options
Help
Zoom
18.2
Whole
Show buried clones
Yes
No
Search
CB Unit Range
0
to
87
Contig stats
Clones: 23 (0 buried)
Markers: 0
Sequenced: 0
Length: 88 CB units
Ctg4304 of cp05T5e12DM4RS
M021E11
B011F08
V066C23
V014P09
M057M21
M043L01
M023D24
M003N12
B004P12
M040E11
M030N24
M052C01
M037B03
M047G01
M021P19
M044I06
V033I21
B009D21
V007F08
B001C12
M034H11
M031M08
V003I07
0
5
10
15
20
25
30
35
40
45
50
55
60
65
70
75
80
85
[Chickpea contig p...
[root@localhost: /...
[FPC V8.9 Main Menu]
Project cp05T5e12...
FPC Ctg4304 cp05...

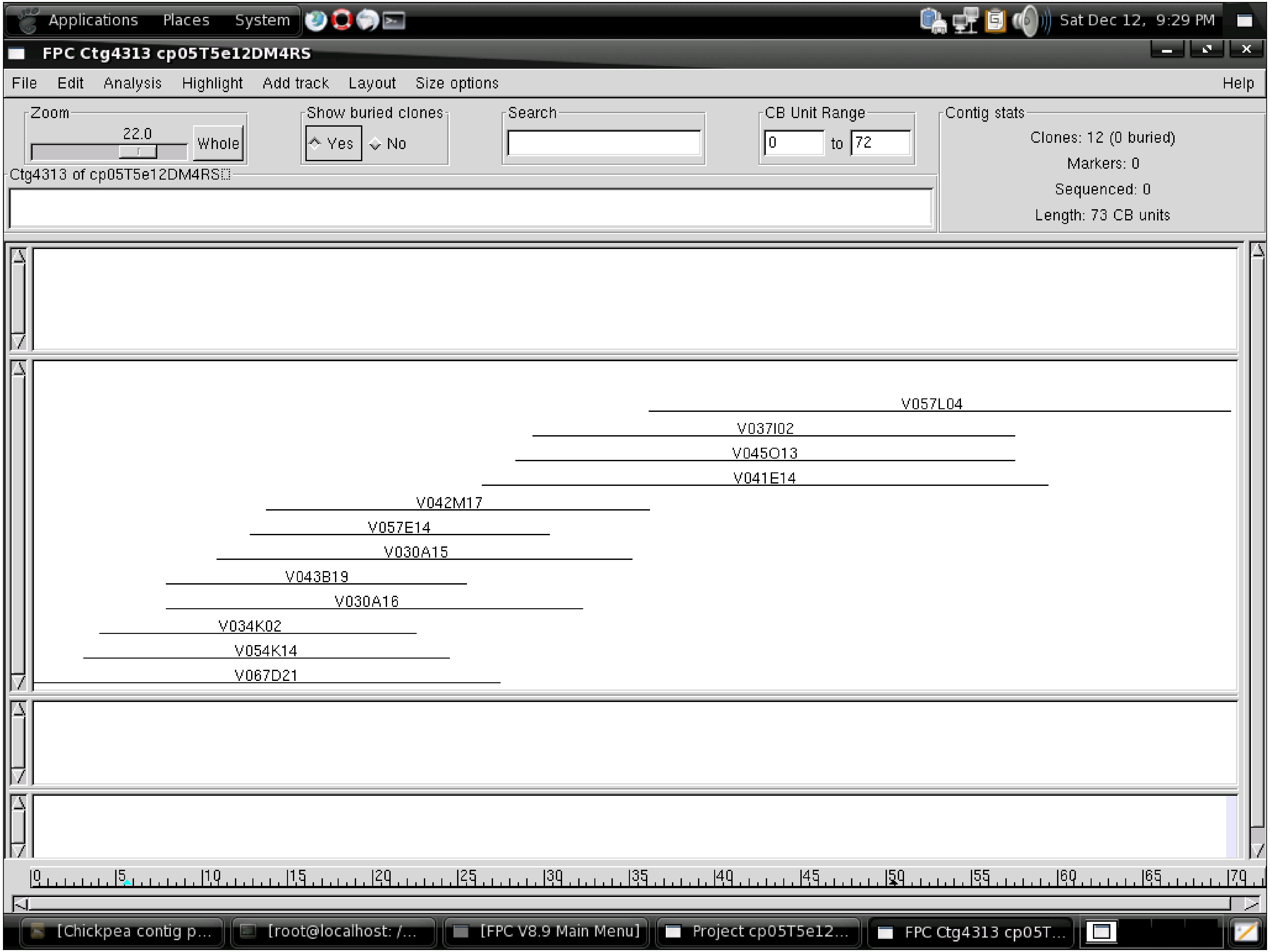
Applications Places System
Sat Dec 12, 9:29 PM
FPC Ctg4313 cp05T5e12DM4RS
File Edit Analysis Highlight Add track Layout Size options Help
Zoom
22.0
Whole
Show buried clones
Yes No
Search
CB Unit Range
0 to 72
Contig stats
Clones: 12 (0 buried)
Markers: 0
Sequenced: 0
Length: 73 CB units
Ctg4313 of cp05T5e12DM4RS
V057L04
V037I02
V045O13
V041E14
V042M17
V057E14
V030A15
V043B19
V030A16
V034K02
V054K14
V067D21
0 5 10 15 20 25 30 35 40 45 50 55 60 65 70
[Chickpea contig p...
[root@localhost: /...
[FPC V8.9 Main Menu]
Project cp05T5e12...
FPC Ctg4313 cp05T...

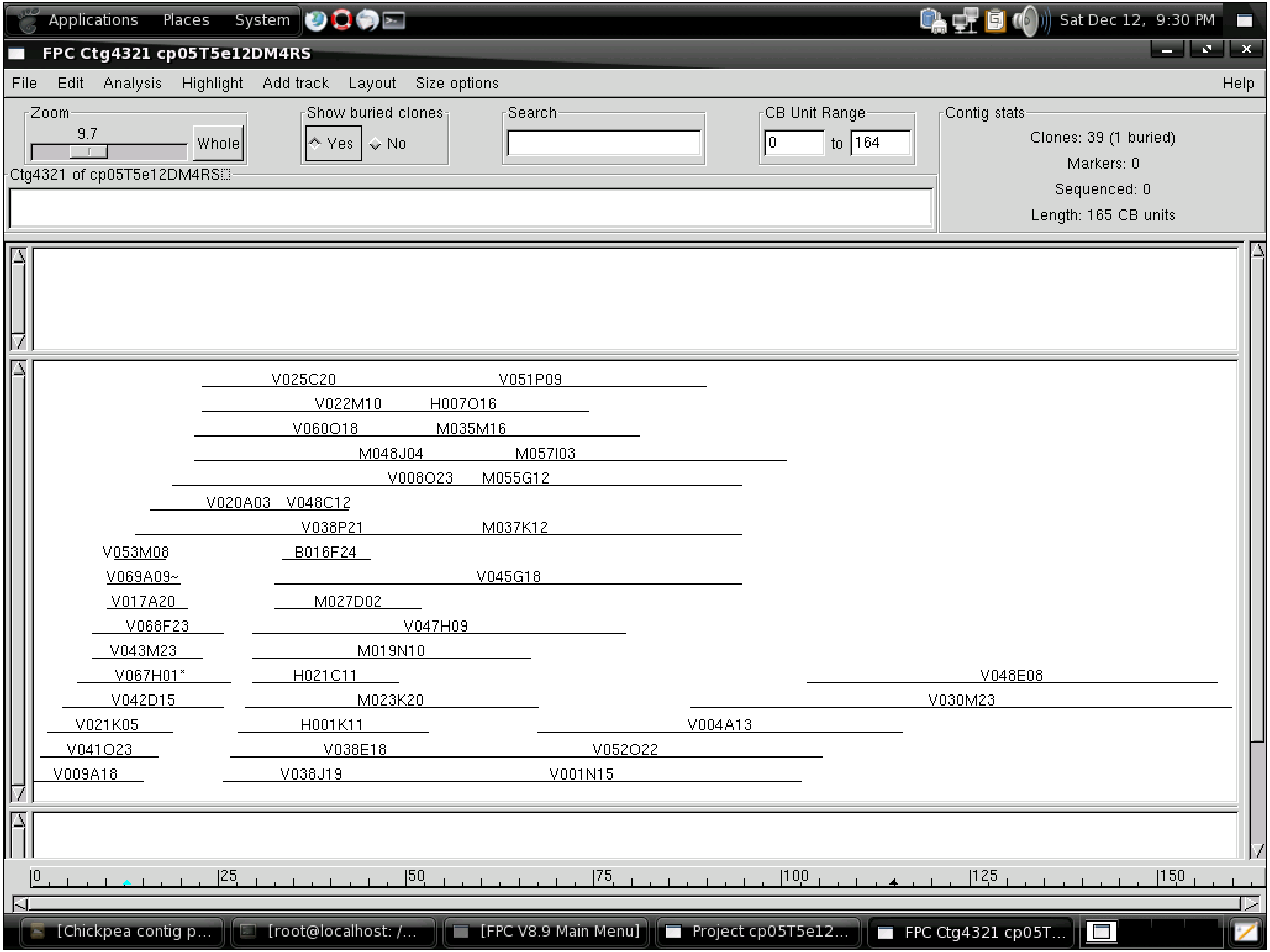
Applications Places System
Sat Dec 12, 9:30 PM
FPC Ctg4321 cp05T5e12DM4RS
File Edit Analysis Highlight Add track Layout Size options Help
Zoom
9.7
Whole
Show buried clones
Yes No
Search
CB Unit Range
0 to 164
Contig stats
Clones: 39 (1 buried)
Markers: 0
Sequenced: 0
Length: 165 CB units
Ctg4321 of cp05T5e12DM4RS
V025C20 V051P09
V022M10 H007O16
V060O18 M035M16
M048J04 M057I03
V008O23 M055G12
V020A03 V048C12
V038P21 M037K12
V053M08 B016F24
V069A09~ V045G18
V017A20 M027D02
V068F23 V047H09
V043M23 M019N10
V067H01* H021C11 V048E08
V042D15 M023K20 V030M23
V021K05 H001K11 V004A13
V041O23 V038E18 V052O22
V009A18 V038J19 V001N15
0 25 50 75 100 125 150
[Chickpea contig p...
[root@localhost: /...
[FPC V8.9 Main Menu]
Project cp05T5e12...
FPC Ctg4321 cp05T...

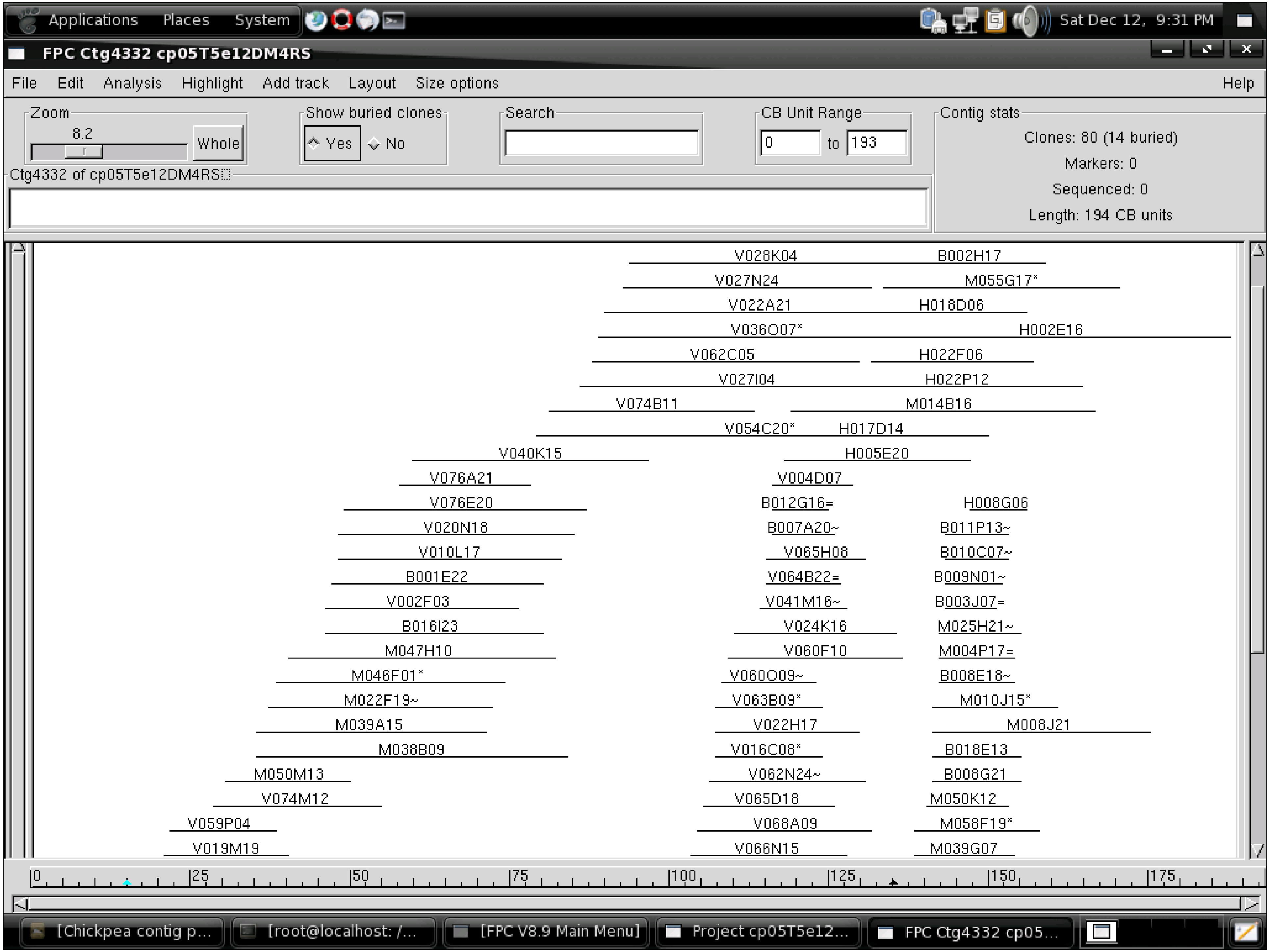
Applications Places System
Sat Dec 12, 9:31 PM
FPC Ctg4332 cp05T5e12DM4RS
File Edit Analysis Highlight Add track Layout Size options Help
Zoom
8.2
Whole
Show buried clones
Yes No
Search
CB Unit Range
0 to 193
Contig stats
Clones: 80 (14 buried)
Markers: 0
Sequenced: 0
Length: 194 CB units
Ctg4332 of cp05T5e12DM4RS
V028K04
B002H17
V027N24
M055G17*
V022A21
H018D06
V036O07*
H002E16
V062C05
H022F06
V027I04
H022P12
V074B11
M014B16
V054C20*
H017D14
V040K15
H005E20
V076A21
V004D07
V076E20
B012G16=
H008G06
V020N18
B007A20~
B011P13~
V010L17
V065H08
B010C07~
B001E22
V064B22=
B009N01~
V002F03
V041M16~
B003J07=
B016I23
V024K16
M025H21~
M047H10
V060F10
M004P17=
M046F01*
V060O09~
B008E18~
M022F19~
V063B09*
M010J15*
M039A15
V022H17
M008J21
M038B09
V016C08*
B018E13
M050M13
V062N24~
B008G21
V074M12
V065D18
M050K12
V059P04
V068A09
M058F19*
V019M19
V066N15
M039G07
0 25 50 75 100 125 150 175
[Chickpea contig p...
[root@localhost: /...
[FPC V8.9 Main Menu]
Project cp05T5e12...
FPC Ctg4332 cp05...

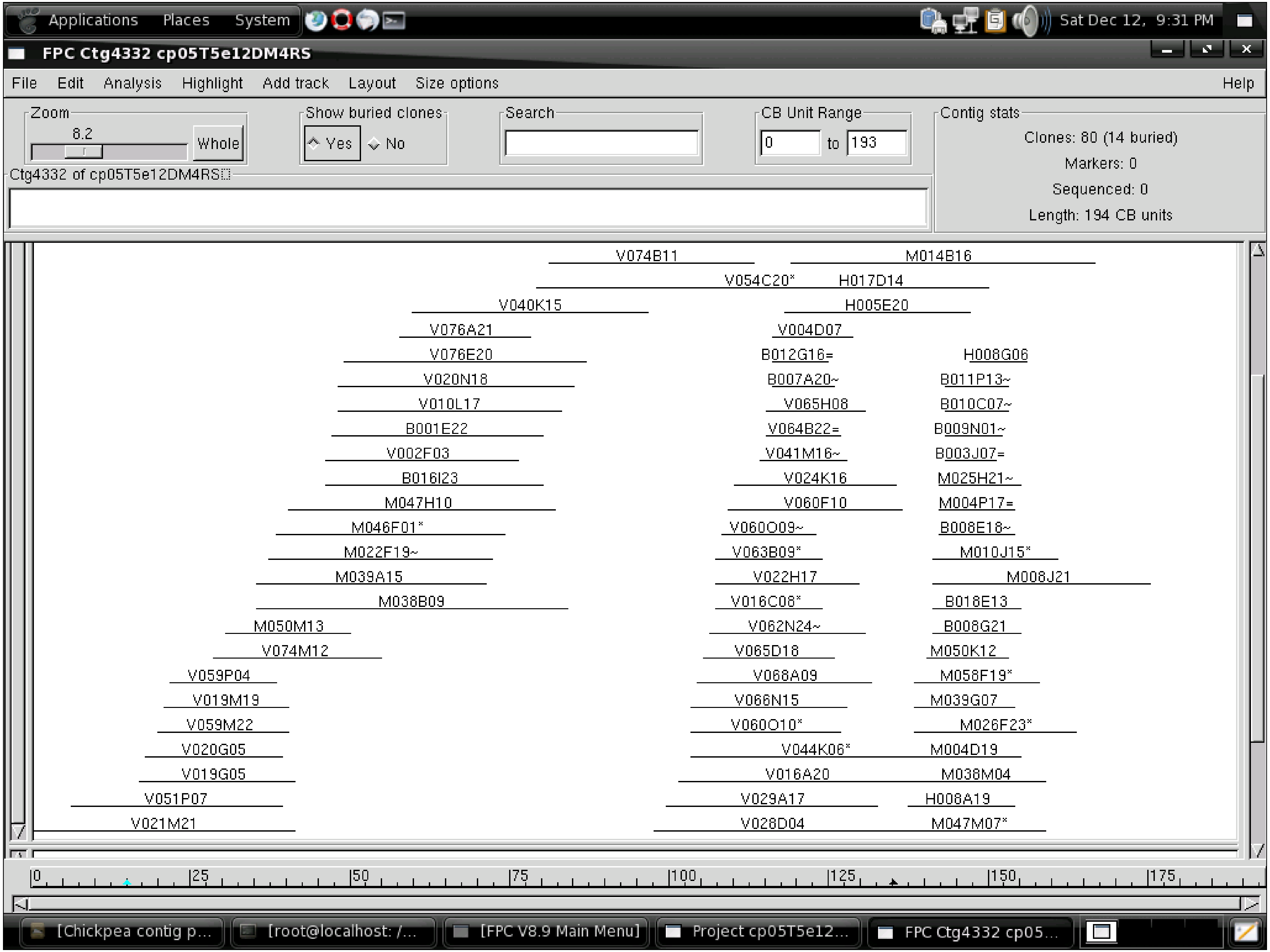
Applications Places System
Sat Dec 12, 9:31 PM
FPC Ctg4332 cp05T5e12DM4RS
File Edit Analysis Highlight Add track Layout Size options Help
Zoom
8.2
Whole
Show buried clones
Yes No
Search
CB Unit Range
0 to 193
Contig stats
Clones: 80 (14 buried)
Markers: 0
Sequenced: 0
Length: 194 CB units
Ctg4332 of cp05T5e12DM4RS
V074B11
M014B16
V054C20*
H017D14
V040K15
H005E20
V076A21
V004D07
V076E20
B012G16=
H008G06
V020N18
B007A20~
B011P13~
V010L17
V065H08
B010C07~
B001E22
V064B22=
B009N01~
V002F03
V041M16~
B003J07=
B016I23
V024K16
M025H21~
M047H10
V060F10
M004P17=
M046F01*
V060O09~
B008E18~
M022F19~
V063B09*
M010J15*
M039A15
V022H17
M008J21
M038B09
V016C08*
B018E13
M050M13
V062N24~
B008G21
V074M12
V065D18
M050K12
V059P04
V068A09
M058F19*
V019M19
V066N15
M039G07
V059M22
V060O10*
M026F23*
V020G05
V044K06*
M004D19
V019G05
V016A20
M038M04
V051P07
V029A17
H008A19
V021M21
V028D04
M047M07*
0 25 50 75 100 125 150 175
[Chickpea contig p...
[root@localhost: /...
[FPC V8.9 Main Menu]
Project cp05T5e12...
FPC Ctg4332 cp05...

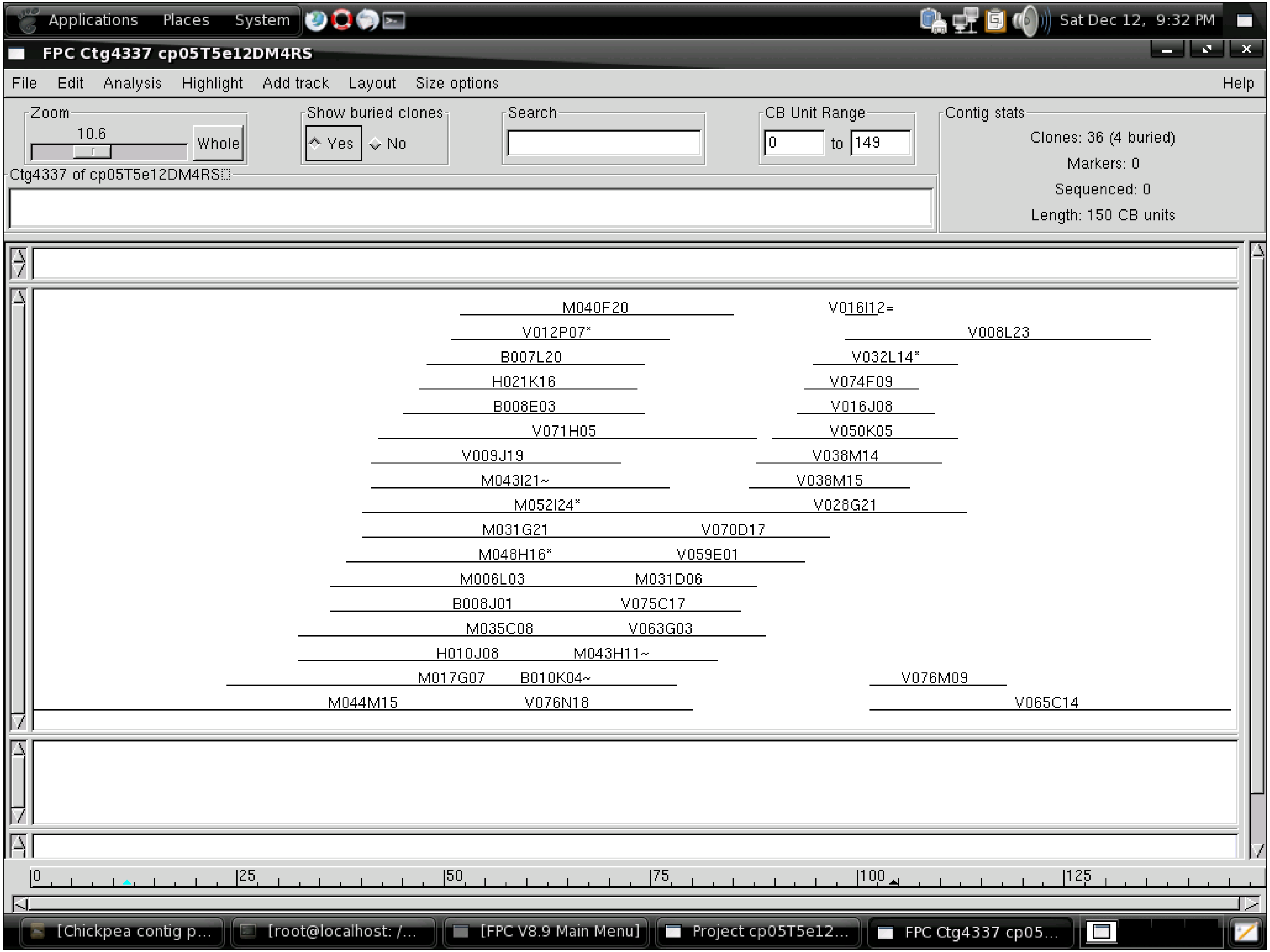
Applications Places System
Sat Dec 12, 9:32 PM
FPC Ctg4337 cp05T5e12DM4RS
File Edit Analysis Highlight Add track Layout Size options Help
Zoom
10.6
Whole
Show buried clones
Yes
No
Search
CB Unit Range
0 to 149
Contig stats
Clones: 36 (4 buried)
Markers: 0
Sequenced: 0
Length: 150 CB units
Ctg4337 of cp05T5e12DM4RS
M040F20
V016I12=
V012P07*
V008L23
B007L20
V032L14*
H021K16
V074F09
B008E03
V016J08
V071H05
V050K05
V009J19
V038M14
M043I21~
V038M15
M052I24*
V028G21
M031G21
V070D17
M048H16*
V059E01
M006L03
M031D06
B008J01
V075C17
M035C08
V063G03
H010J08
M043H11~
M017G07
B010K04~
V076M09
M044M15
V076N18
V065C14
0
25
50
75
100
125
[Chickpea contig p...
[root@localhost: /...
[FPC V8.9 Main Menu]
Project cp05T5e12...
FPC Ctg4337 cp05...

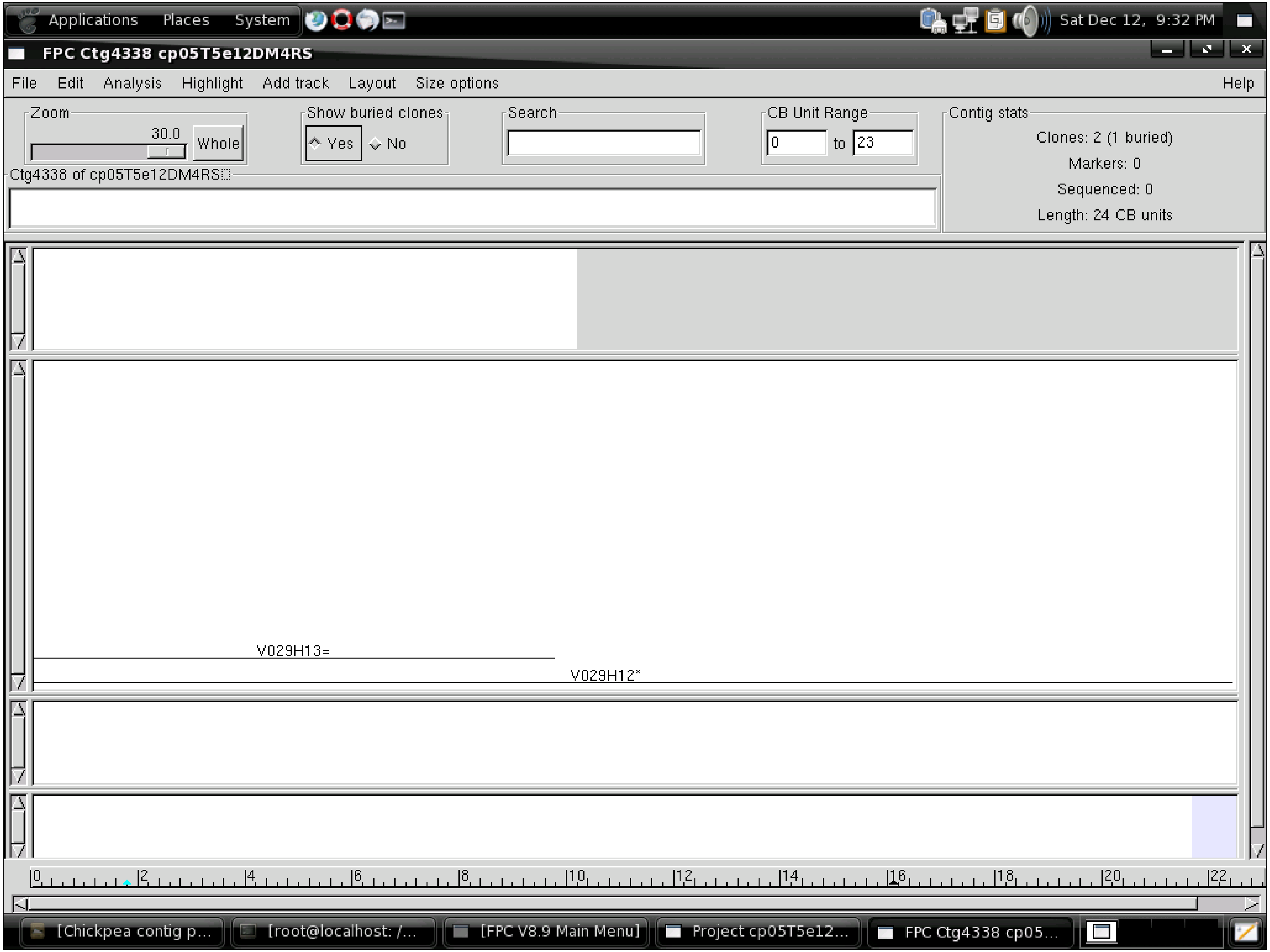
Applications Places System
Sat Dec 12, 9:32 PM
FPC Ctg4338 cp05T5e12DM4RS
File Edit Analysis Highlight Add track Layout Size options Help
Zoom
30.0
Whole
Show buried clones
Yes No
Search
CB Unit Range
0 to 23
Contig stats
Clones: 2 (1 buried)
Markers: 0
Sequenced: 0
Length: 24 CB units
Ctg4338 of cp05T5e12DM4RS
V029H13=
V029H12*
0 2 4 6 8 10 12 14 16 18 20 22
[Chickpea contig p...
[root@localhost: /...
[FPC V8.9 Main Menu]
Project cp05T5e12...
FPC Ctg4338 cp05...

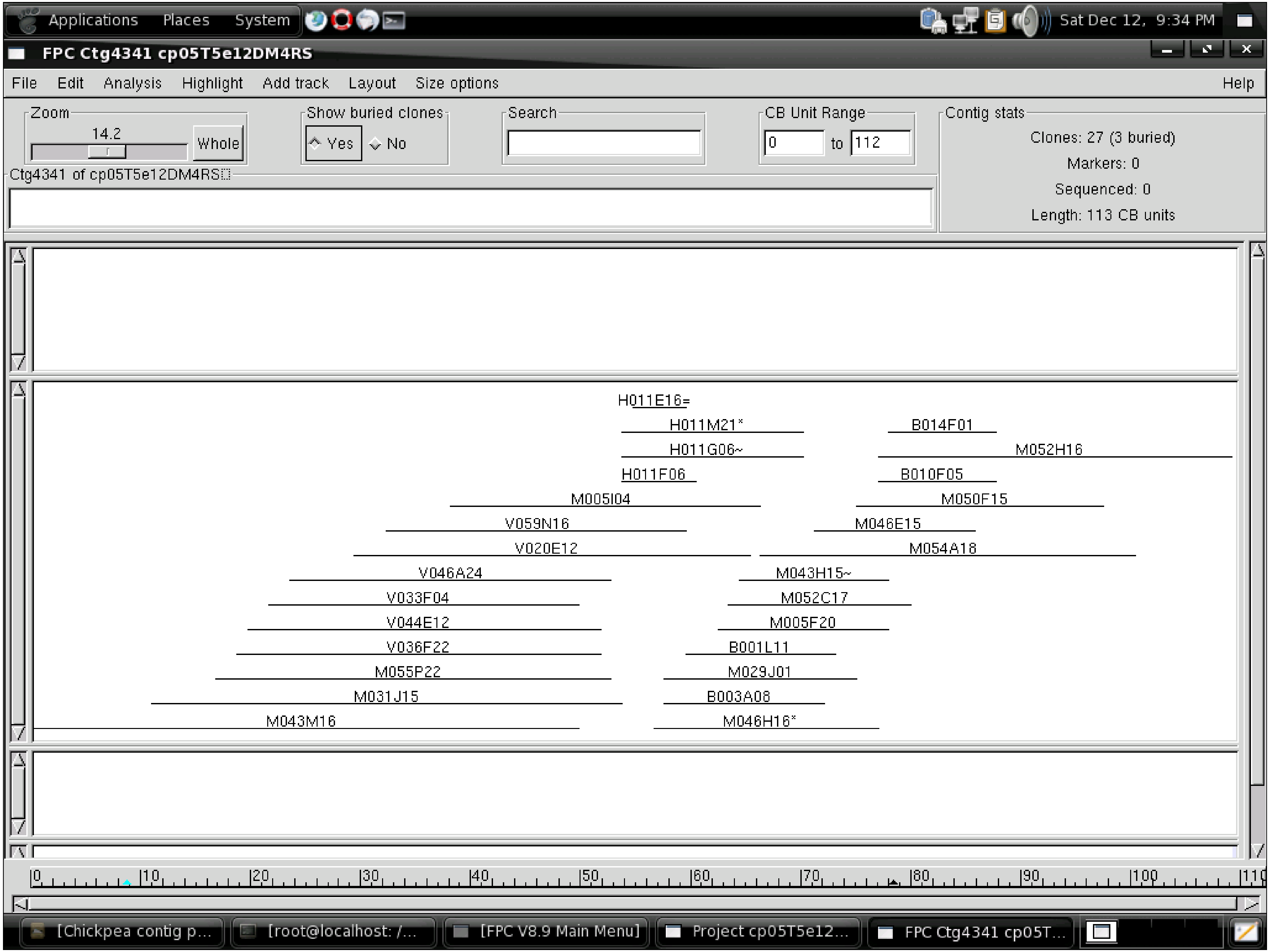
Applications Places System
Sat Dec 12, 9:34 PM
FPC Ctg4341 cp05T5e12DM4RS
File Edit Analysis Highlight Add track Layout Size options Help
Zoom
14.2
Whole
Show buried clones
Yes
No
Search
CB Unit Range
0 to 112
Contig stats
Clones: 27 (3 buried)
Markers: 0
Sequenced: 0
Length: 113 CB units
Ctg4341 of cp05T5e12DM4RS
H011E16=
H011M21*
B014F01
H011G06~
M052H16
H011F06
B010F05
M005I04
M050F15
V059N16
M046E15
V020E12
M054A18
V046A24
M043H15~
V033F04
M052C17
V044E12
M005F20
V036F22
B001L11
M055P22
M029J01
M031J15
B003A08
M043M16
M046H16*
0 10 20 30 40 50 60 70 80 90 100 110
[Chickpea contig p...
[root@localhost: /...
[FPC V8.9 Main Menu]
Project cp05T5e12...
FPC Ctg4341 cp05T...

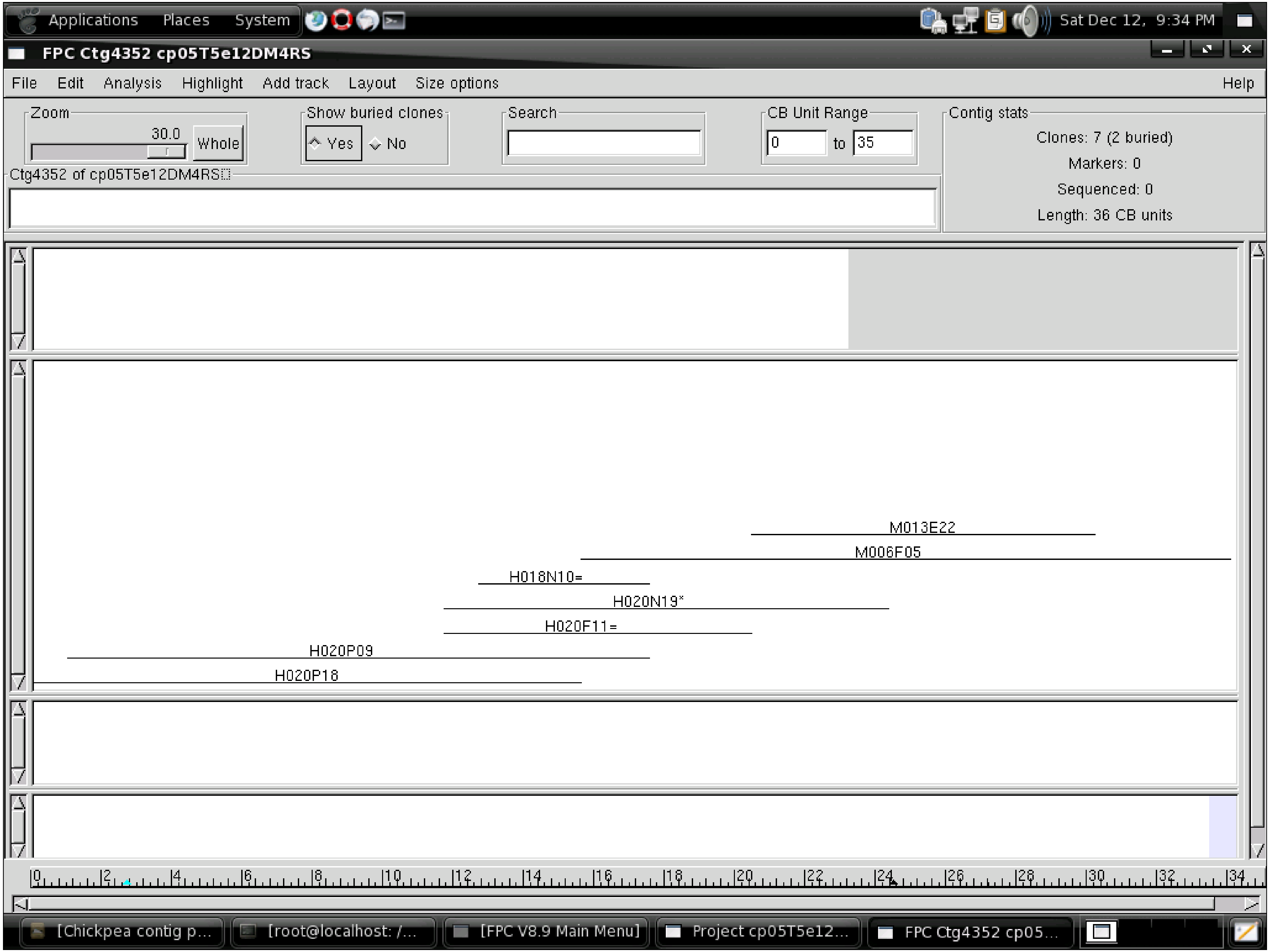
Applications Places System
Sat Dec 12, 9:34 PM
FPC Ctg4352 cp05T5e12DM4RS
File Edit Analysis Highlight Add track Layout Size options Help
Zoom
30.0
Whole
Show buried clones
Yes
No
Search
CB Unit Range
0 to 35
Contig stats
Clones: 7 (2 buried)
Markers: 0
Sequenced: 0
Length: 36 CB units
Ctg4352 of cp05T5e12DM4RS
M013E22
M006F05
H018N10=
H020N19*
H020F11=
H020P09
H020P18
0 2 4 6 8 10 12 14 16 18 20 22 24 26 28 30 32 34
[Chickpea contig p...
[root@localhost: /...
[FPC V8.9 Main Menu]
Project cp05T5e12...
FPC Ctg4352 cp05...

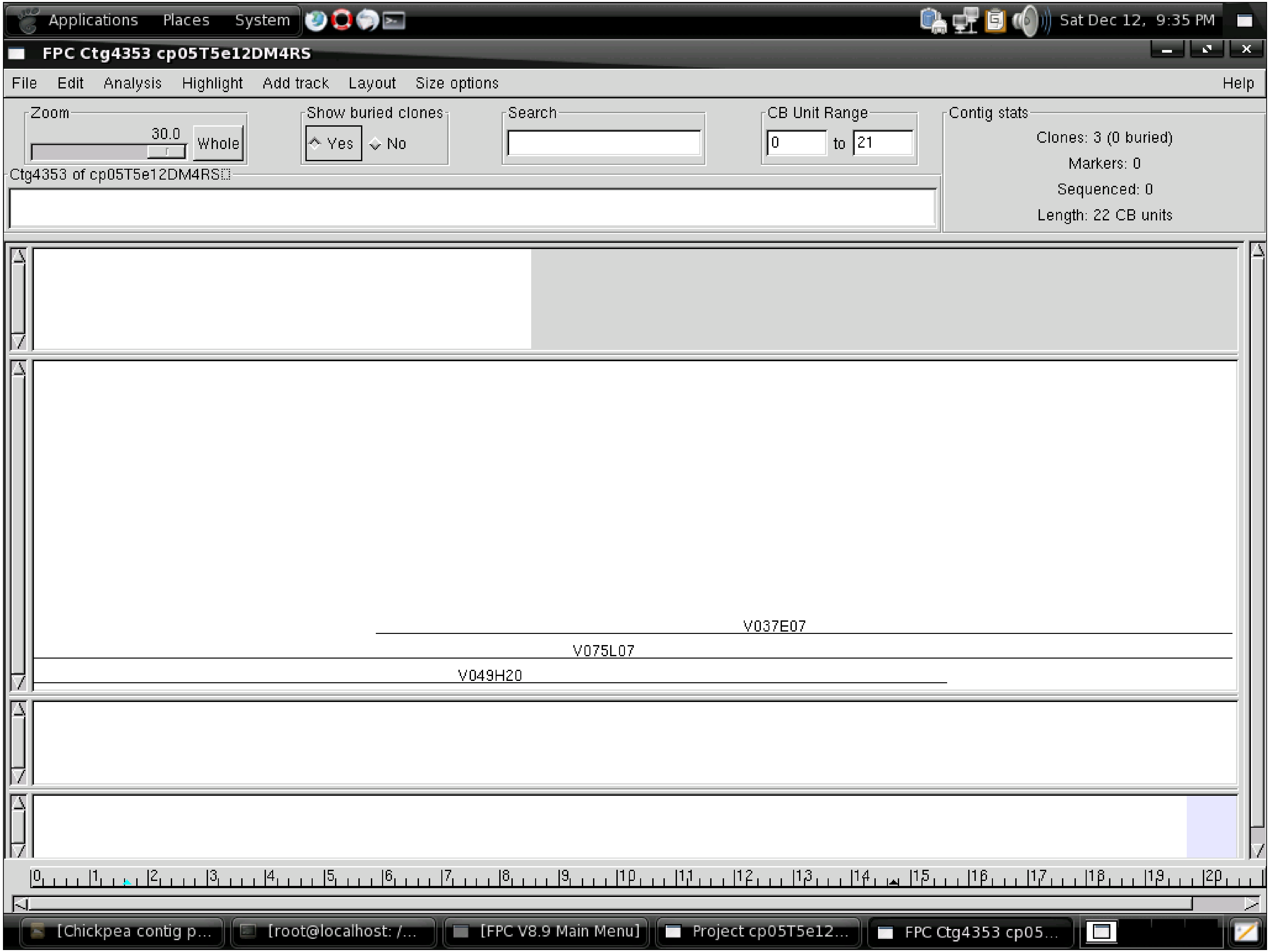
Applications Places System
Sat Dec 12, 9:35 PM
FPC Ctg4353 cp05T5e12DM4RS
File Edit Analysis Highlight Add track Layout Size options Help
Zoom
30.0
Whole
Show buried clones
Yes
No
Search
CB Unit Range
0
to
21
Contig stats
Clones: 3 (0 buried)
Markers: 0
Sequenced: 0
Length: 22 CB units
Ctg4353 of cp05T5e12DM4RS
V037E07
V075L07
V049H20
0 1 2 3 4 5 6 7 8 9 10 11 12 13 14 15 16 17 18 19 20
[Chickpea contig p...
[root@localhost: /...
[FPC V8.9 Main Menu]
Project cp05T5e12...
FPC Ctg4353 cp05...

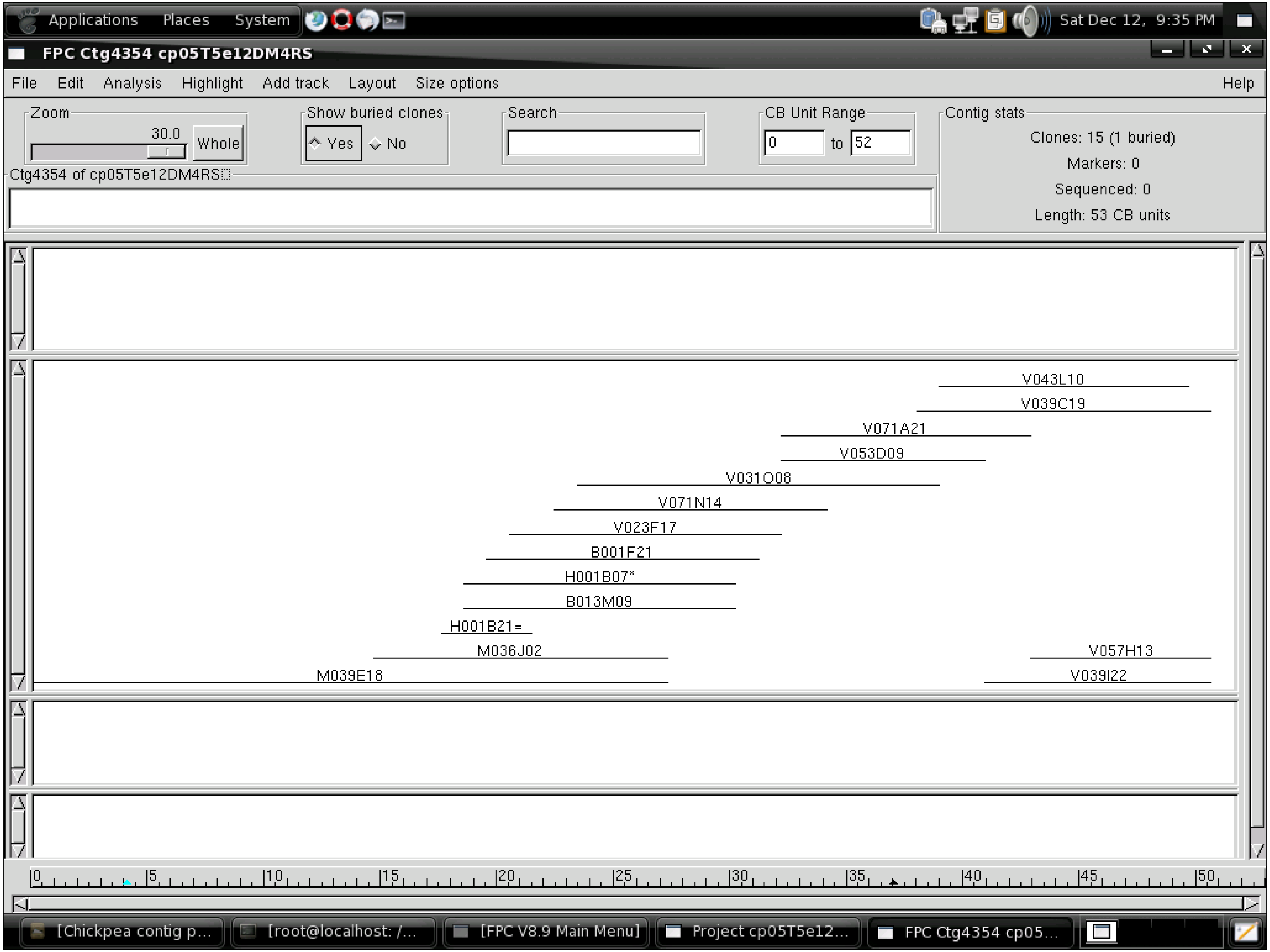
Applications Places System
Sat Dec 12, 9:35 PM
FPC Ctg4354 cp05T5e12DM4RS
File Edit Analysis Highlight Add track Layout Size options Help
Zoom 30.0 Whole
Show buried clones Yes No
Search
CB Unit Range 0 to 52
Contig stats
Clones: 15 (1 buried)
Markers: 0
Sequenced: 0
Length: 53 CB units
Ctg4354 of cp05T5e12DM4RS
V043L10
V039C19
V071A21
V053D09
V031O08
V071N14
V023F17
B001F21
H001B07*
B013M09
H001B21=
M036J02
V057H13
M039E18
V039I22
0 5 10 15 20 25 30 35 40 45 50
[Chickpea contig p...
[root@localhost: /...
[FPC V8.9 Main Menu]
Project cp05T5e12...
FPC Ctg4354 cp05...

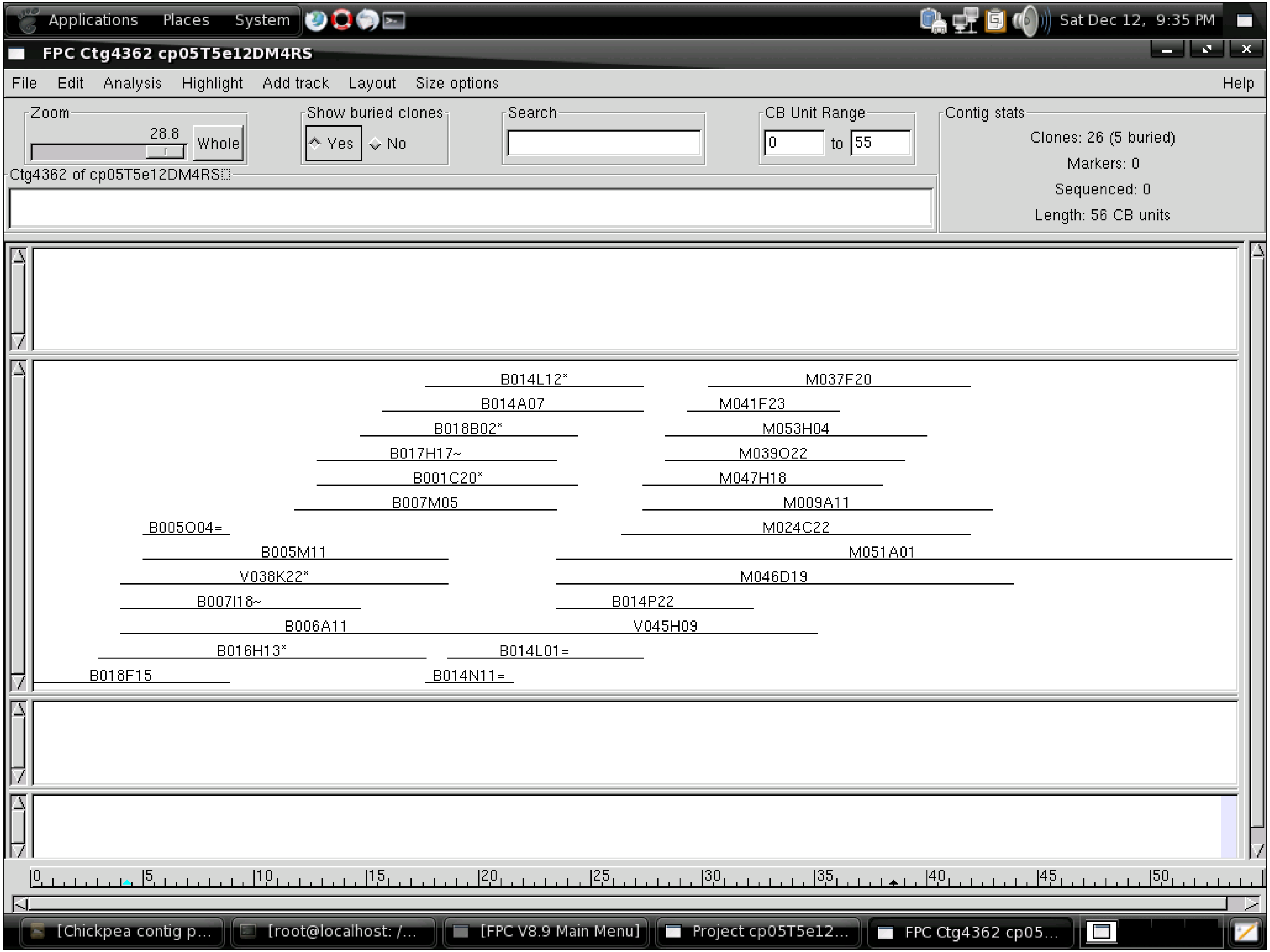
Applications Places System
Sat Dec 12, 9:35 PM
FPC Ctg4362 cp05T5e12DM4RS
File Edit Analysis Highlight Add track Layout Size options Help
Zoom
28.8
Whole
Show buried clones
Yes No
Search
CB Unit Range
0 to 55
Contig stats
Clones: 26 (5 buried)
Markers: 0
Sequenced: 0
Length: 56 CB units
Ctg4362 of cp05T5e12DM4RS
B014L12*
M037F20
B014A07
M041F23
B018B02*
M053H04
B017H17~
M039O22
B001C20*
M047H18
B007M05
M009A11
B005O04=
M024C22
B005M11
M051A01
V038K22*
M046D19
B007I18~
B014P22
B006A11
V045H09
B016H13*
B014L01=
B018F15
B014N11=
0 5 10 15 20 25 30 35 40 45 50
[Chickpea contig p...
[root@localhost: /...
[FPC V8.9 Main Menu]
Project cp05T5e12...
FPC Ctg4362 cp05...

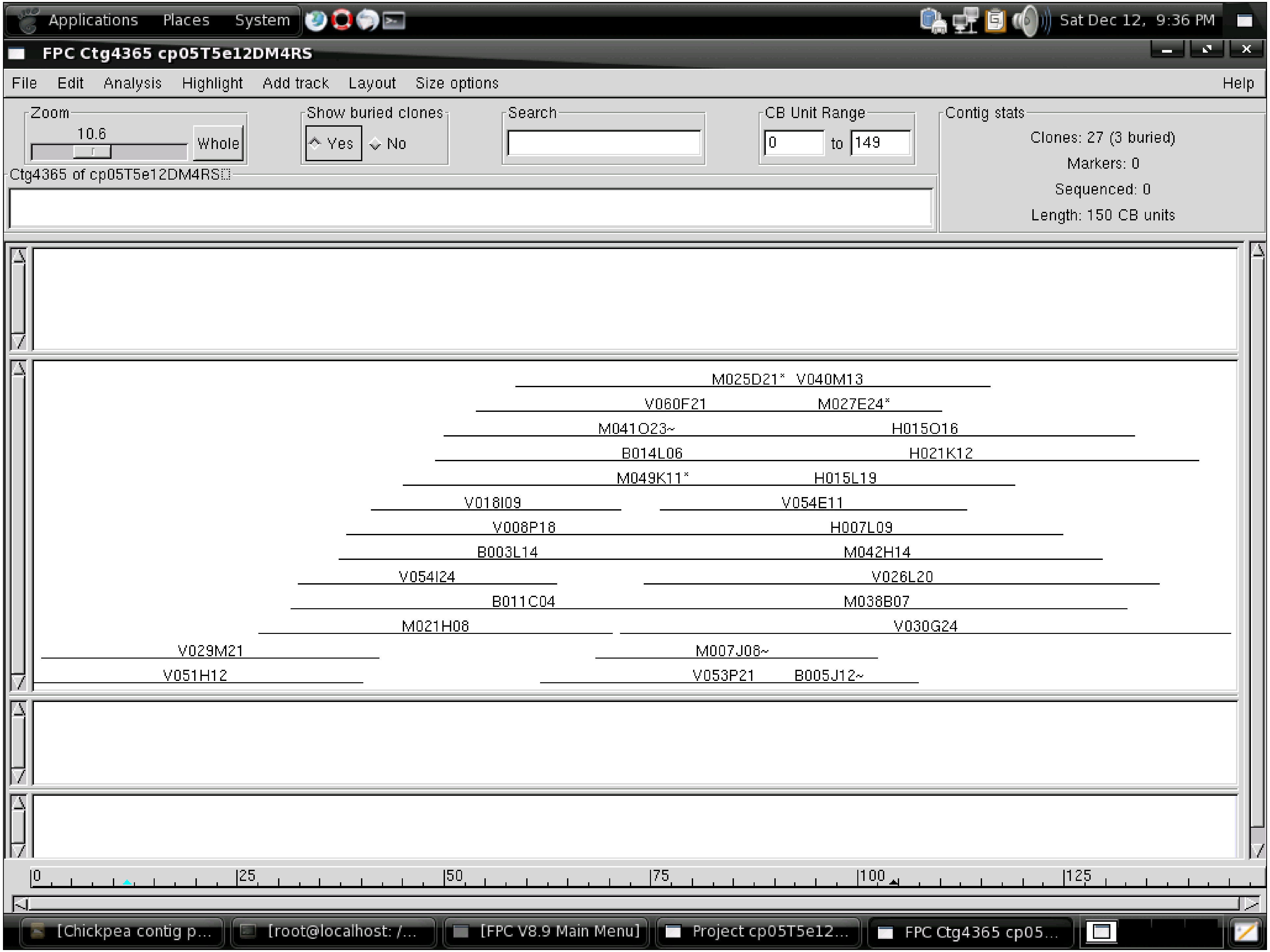
Applications Places System
Sat Dec 12, 9:36 PM
FPC Ctg4365 cp05T5e12DM4RS
File Edit Analysis Highlight Add track Layout Size options Help
Zoom
10.6
Whole
Show buried clones
Yes
No
Search
CB Unit Range
0
to
149
Contig stats
Clones: 27 (3 buried)
Markers: 0
Sequenced: 0
Length: 150 CB units
Ctg4365 of cp05T5e12DM4RS
M025D21* V040M13
V060F21 M027E24*
M041O23~ H015O16
B014L06 H021K12
M049K11* H015L19
V018I09 V054E11
V008P18 H007L09
B003L14 M042H14
V054I24 V026L20
B011C04 M038B07
M021H08 V030G24
V029M21 M007J08~
V051H12 V053P21 B005J12~
0 25 50 75 100 125
[Chickpea contig p...
[root@localhost: /...
[FPC V8.9 Main Menu]
Project cp05T5e12...
FPC Ctg4365 cp05...

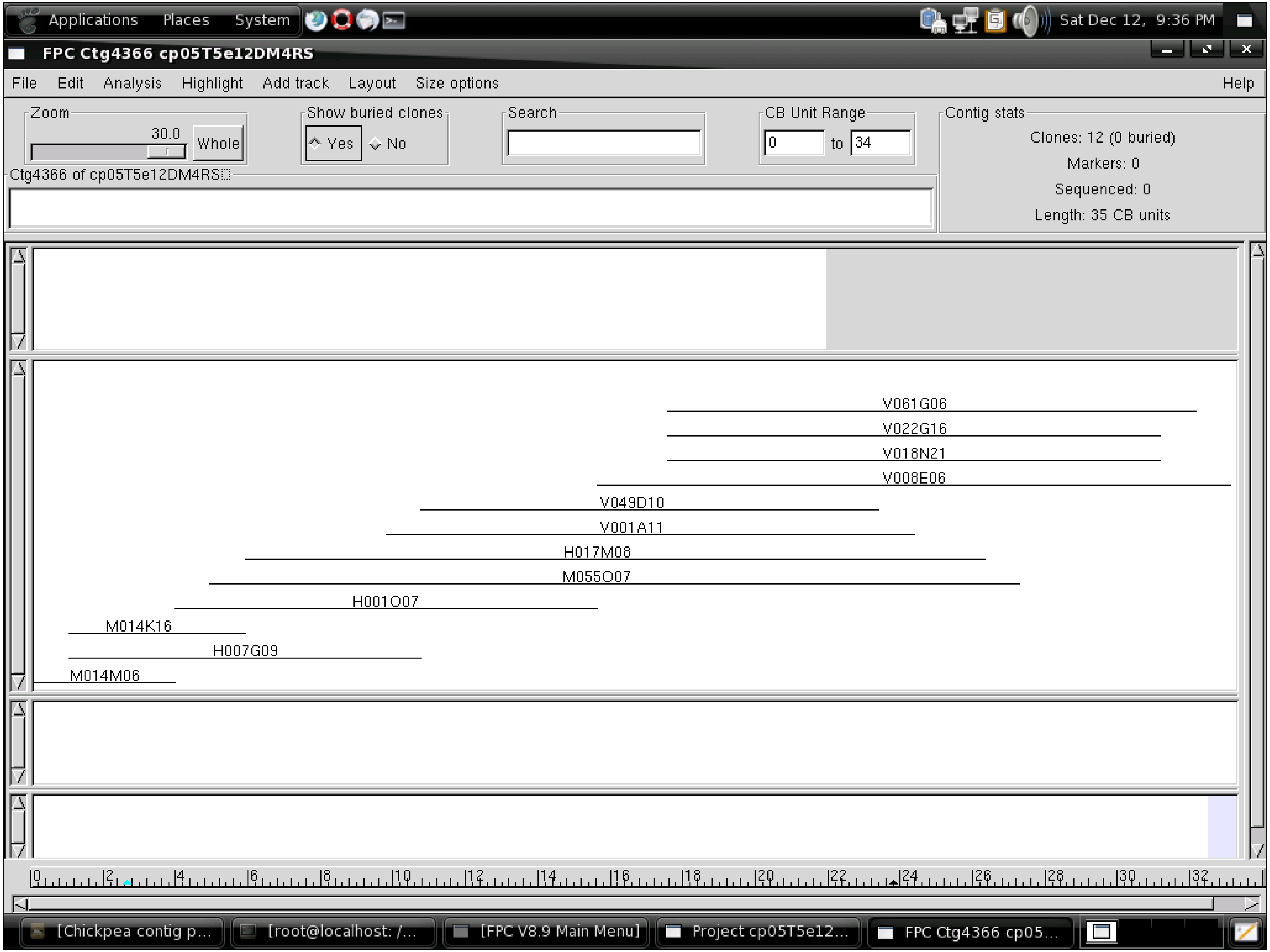

Applications Places System
Sat Dec 12, 9:36 PM
FPC Ctg4366 cp05T5e12DM4RS
File Edit Analysis Highlight Add track Layout Size options Help
Zoom
30.0
Whole
Show buried clones
Yes
No
Search
CB Unit Range
0
to
34
Contig stats
Clones: 12 (0 buried)
Markers: 0
Sequenced: 0
Length: 35 CB units
Ctg4366 of cp05T5e12DM4RS
V061G06
V022G16
V018N21
V008E06
V049D10
V001A11
H017M08
M055O07
H001O07
M014K16
H007G09
M014M06
0 2 4 6 8 10 12 14 16 18 20 22 24 26 28 30 32
[Chickpea contig p...
[root@localhost: /...
[FPC V8.9 Main Menu]
Project cp05T5e12...
FPC Ctg4366 cp05...

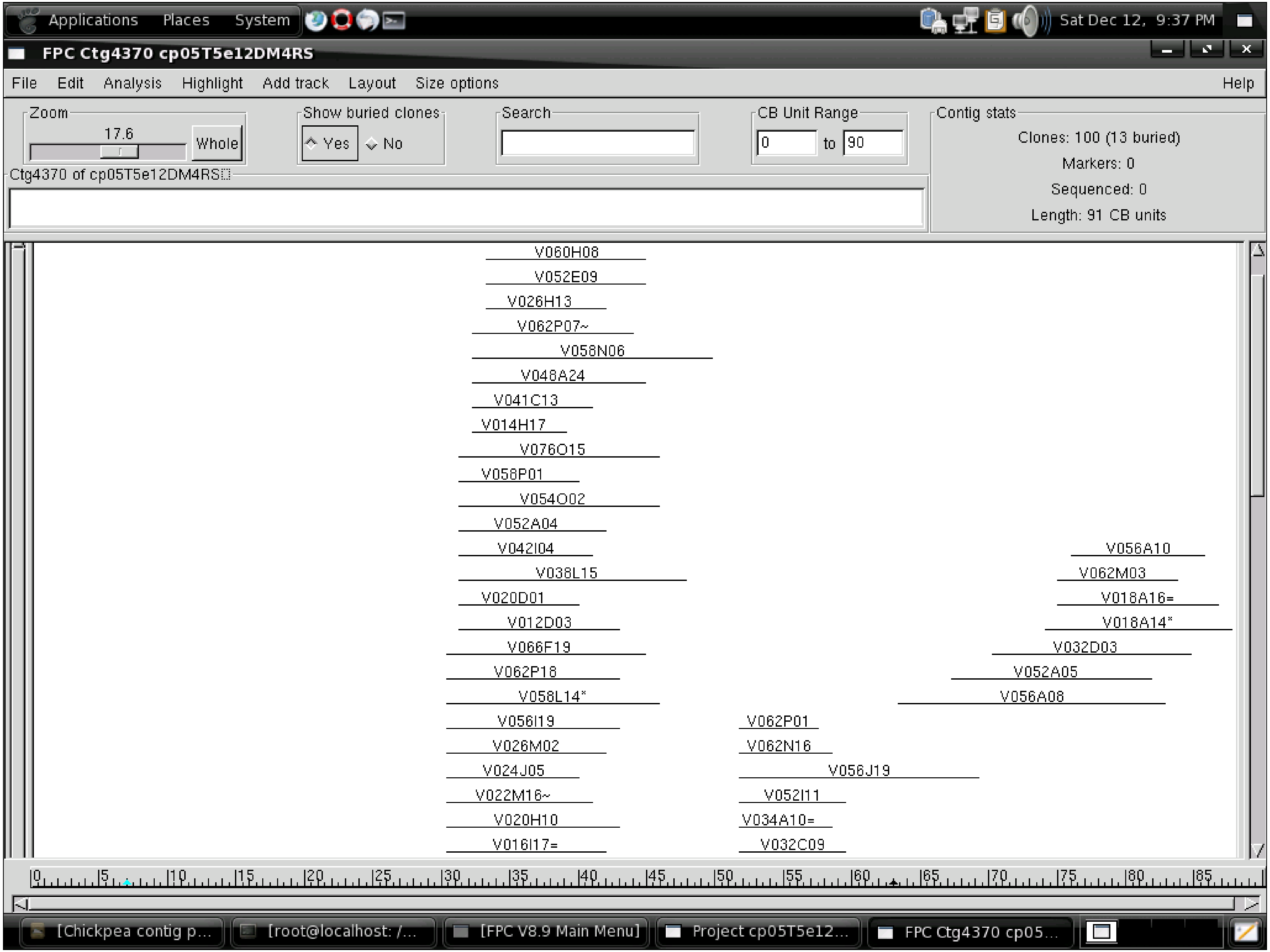
Applications Places System
Sat Dec 12, 9:37 PM
FPC Ctg4370 cp05T5e12DM4RS
File Edit Analysis Highlight Add track Layout Size options Help
Zoom
17.6
Whole
Show buried clones
Yes No
Search
CB Unit Range
0 to 90
Contig stats
Clones: 100 (13 buried)
Markers: 0
Sequenced: 0
Length: 91 CB units
Ctg4370 of cp05T5e12DM4RS
V060H08
V052E09
V026H13
V062P07~
V058N06
V048A24
V041C13
V014H17
V076O15
V058P01
V054O02
V052A04
V042I04
V038L15
V020D01
V012D03
V066F19
V062P18
V058L14*
V056I19
V026M02
V024J05
V022M16~
V020H10
V016I17=
V062P01
V062N16
V056J19
V052I11
V034A10=
V032C09
V056A10
V062M03
V018A16=
V018A14*
V032D03
V052A05
V056A08
0 5 10 15 20 25 30 35 40 45 50 55 60 65 70 75 80 85
[Chickpea contig p...
[root@localhost: /...
[FPC V8.9 Main Menu]
Project cp05T5e12...
FPC Ctg4370 cp05...

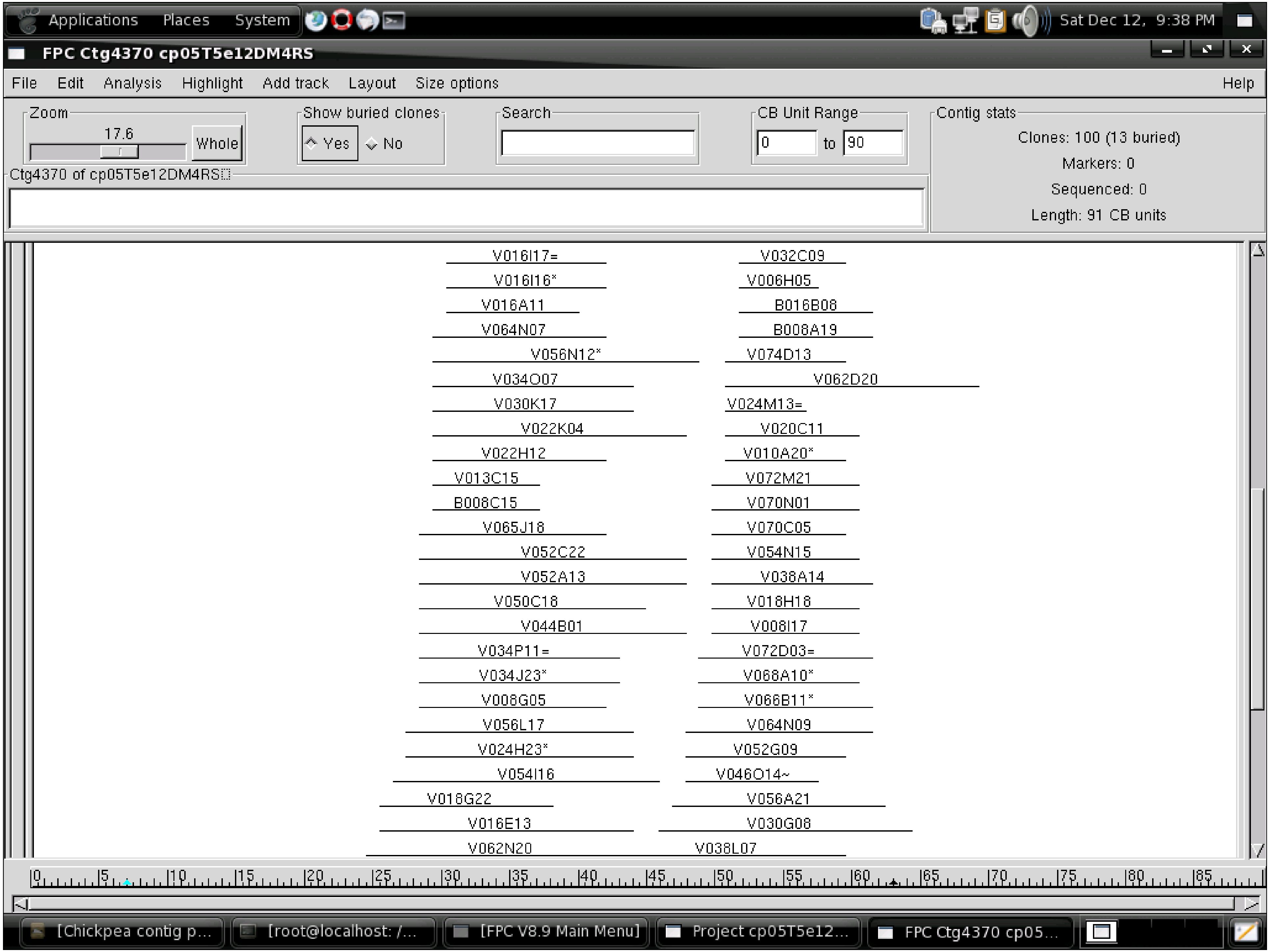
Applications Places System
Sat Dec 12, 9:38 PM
FPC Ctg4370 cp05T5e12DM4RS
File Edit Analysis Highlight Add track Layout Size options Help
Zoom
17.6
Whole
Show buried clones
Yes No
Search
CB Unit Range
0 to 90
Contig stats
Clones: 100 (13 buried)
Markers: 0
Sequenced: 0
Length: 91 CB units
Ctg4370 of cp05T5e12DM4RS
V016I17=
V032C09
V016I16*
V006H05
V016A11
B016B08
V064N07
B008A19
V056N12*
V074D13
V034O07
V062D20
V030K17
V024M13=
V022K04
V020C11
V022H12
V010A20*
V013C15
V072M21
B008C15
V070N01
V065J18
V070C05
V052C22
V054N15
V052A13
V038A14
V050C18
V018H18
V044B01
V008I17
V034P11=
V072D03=
V034J23*
V068A10*
V008G05
V066B11*
V056L17
V064N09
V024H23*
V052G09
V054I16
V046O14~
V018G22
V056A21
V016E13
V030G08
V062N20
V038L07
0 5 10 15 20 25 30 35 40 45 50 55 60 65 70 75 80 85
[Chickpea contig p...
[root@localhost: /...
[FPC V8.9 Main Menu]
Project cp05T5e12...
FPC Ctg4370 cp05...

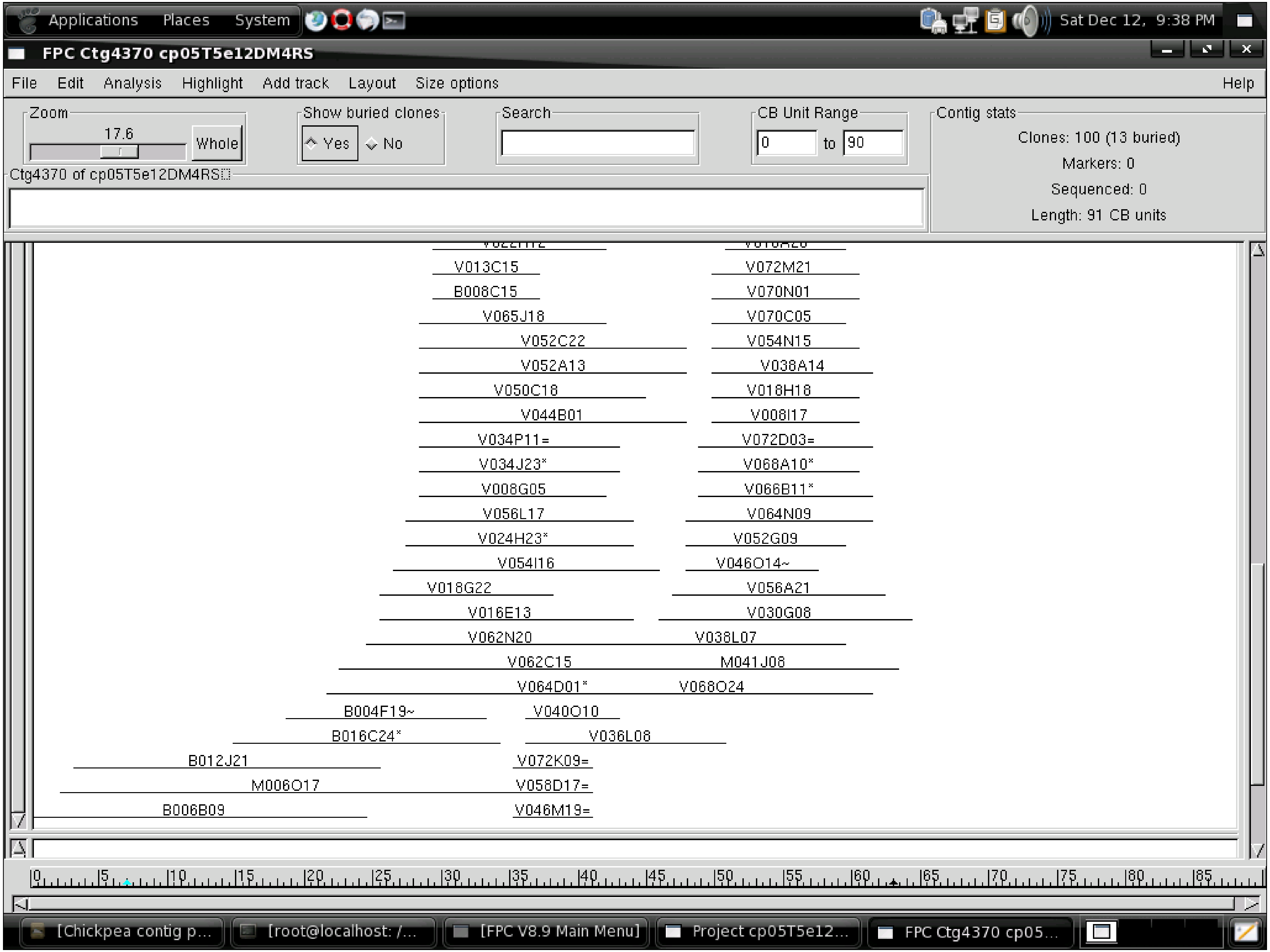
Applications Places System
Sat Dec 12, 9:38 PM
FPC Ctg4370 cp05T5e12DM4RS
File Edit Analysis Highlight Add track Layout Size options Help
Zoom
17.6
Whole
Show buried clones
Yes No
Search
CB Unit Range
0 to 90
Contig stats
Clones: 100 (13 buried)
Markers: 0
Sequenced: 0
Length: 91 CB units
Ctg4370 of cp05T5e12DM4RS
V013C15
V072M21
B008C15
V070N01
V065J18
V070C05
V052C22
V054N15
V052A13
V038A14
V050C18
V018H18
V044B01
V008I17
V034P11=
V072D03=
V034J23*
V068A10*
V008G05
V066B11*
V056L17
V064N09
V024H23*
V052G09
V054I16
V046O14~
V018G22
V056A21
V016E13
V030G08
V062N20
V038L07
V062C15
M041J08
V064D01*
V068O24
B004F19~
V040O10
B016C24*
V036L08
B012J21
V072K09=
M006O17
V058D17=
B006B09
V046M19=
0 5 10 15 20 25 30 35 40 45 50 55 60 65 70 75 80 85
[Chickpea contig p...
[root@localhost: /...
[FPC V8.9 Main Menu]
Project cp05T5e12...
FPC Ctg4370 cp05...

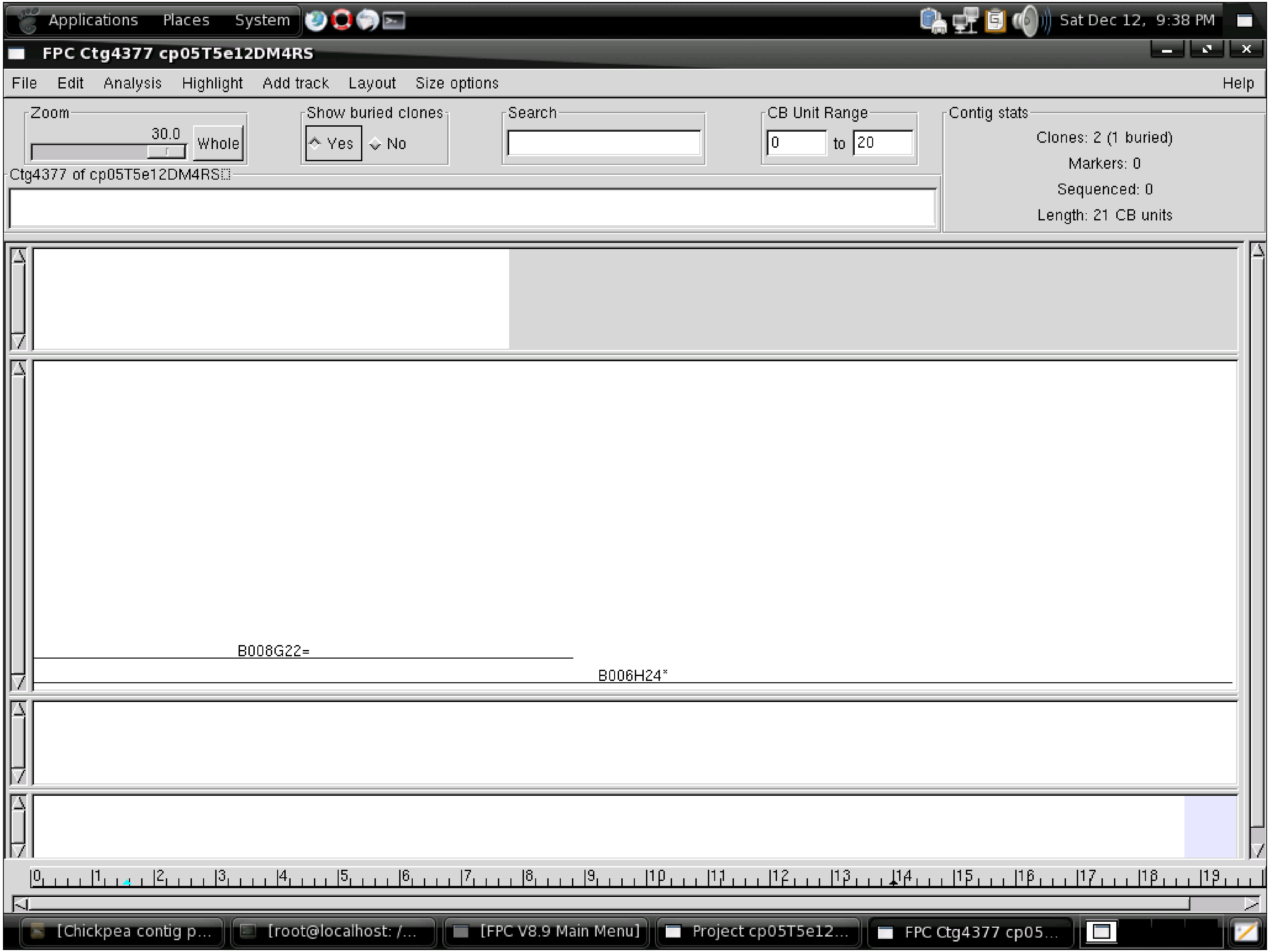
Applications
Places
System
Sat Dec 12, 9:38 PM
FPC Ctg4377 cp05T5e12DM4RS
File
Edit
Analysis
Highlight
Add track
Layout
Size options
Help
Zoom
30.0
Whole
Show buried clones
Yes
No
Search
CB Unit Range
0
to
20
Contig stats
Clones: 2 (1 buried)
Markers: 0
Sequenced: 0
Length: 21 CB units
Ctg4377 of cp05T5e12DM4RS
B008G22=
B006H24*
0 1 2 3 4 5 6 7 8 9 10 11 12 13 14 15 16 17 18 19
[Chickpea contig p...
[root@localhost: /...
[FPC V8.9 Main Menu]
Project cp05T5e12...
FPC Ctg4377 cp05...

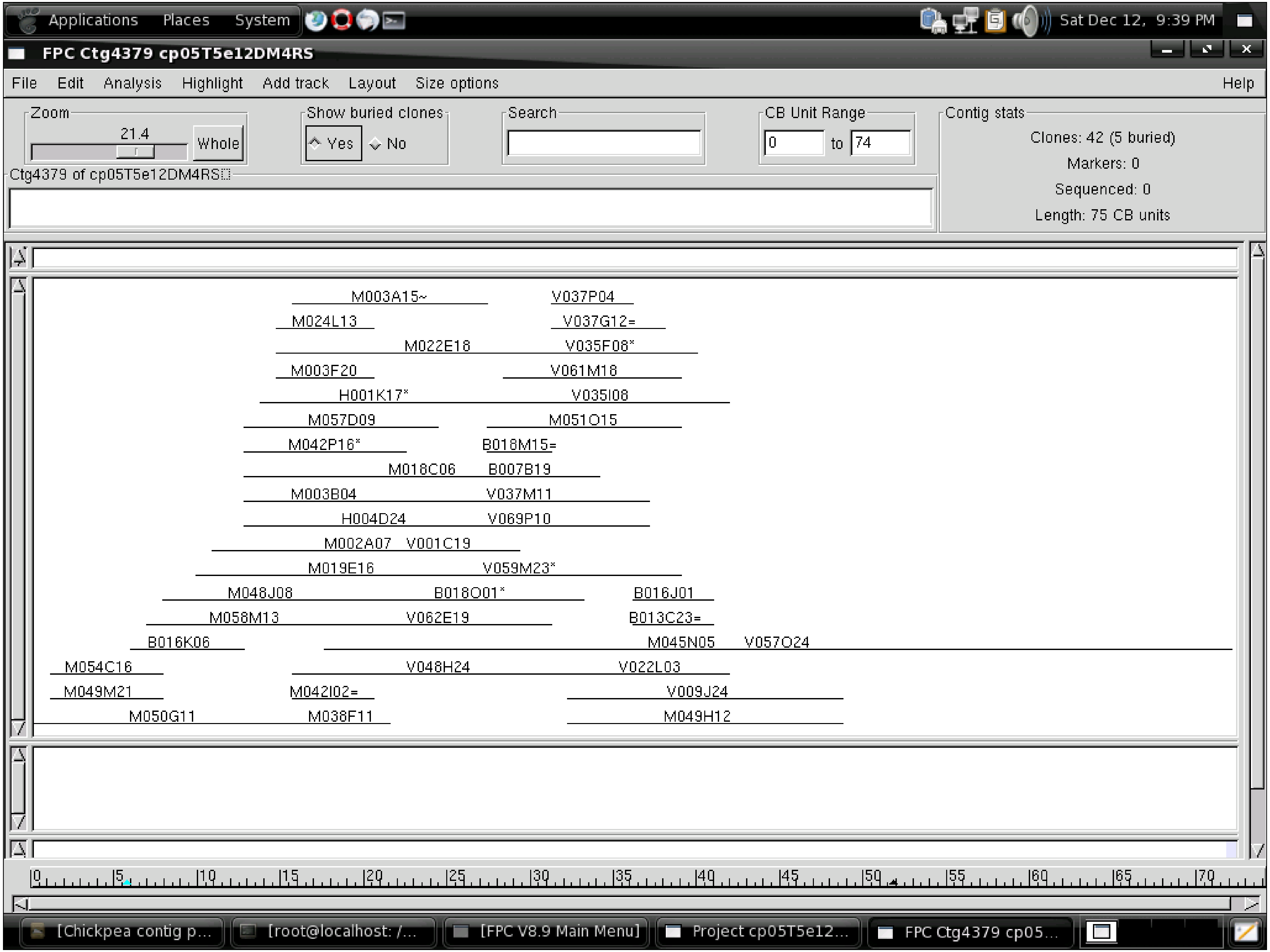
Applications Places System
Sat Dec 12, 9:39 PM
FPC Ctg4379 cp05T5e12DM4RS
File Edit Analysis Highlight Add track Layout Size options Help
Zoom
21.4
Whole
Show buried clones
Yes No
Search
CB Unit Range
0 to 74
Contig stats
Clones: 42 (5 buried)
Markers: 0
Sequenced: 0
Length: 75 CB units
Ctg4379 of cp05T5e12DM4RS
M003A15~ V037P04
M024L13 V037G12=
M022E18 V035F08*
M003F20 V061M18
H001K17* V035I08
M057D09 M051O15
M042P16* B018M15=
M018C06 B007B19
M003B04 V037M11
H004D24 V069P10
M002A07 V001C19
M019E16 V059M23*
M048J08 B018O01* B016J01
M058M13 V062E19 B013C23=
B016K06 M045N05 V057O24
M054C16 V048H24 V022L03
M049M21 M042I02= V009J24
M050G11 M038F11 M049H12
0 5 10 15 20 25 30 35 40 45 50 55 60 65 70
[Chickpea contig p...
[root@localhost: /...
[FPC V8.9 Main Menu]
Project cp05T5e12...
FPC Ctg4379 cp05...

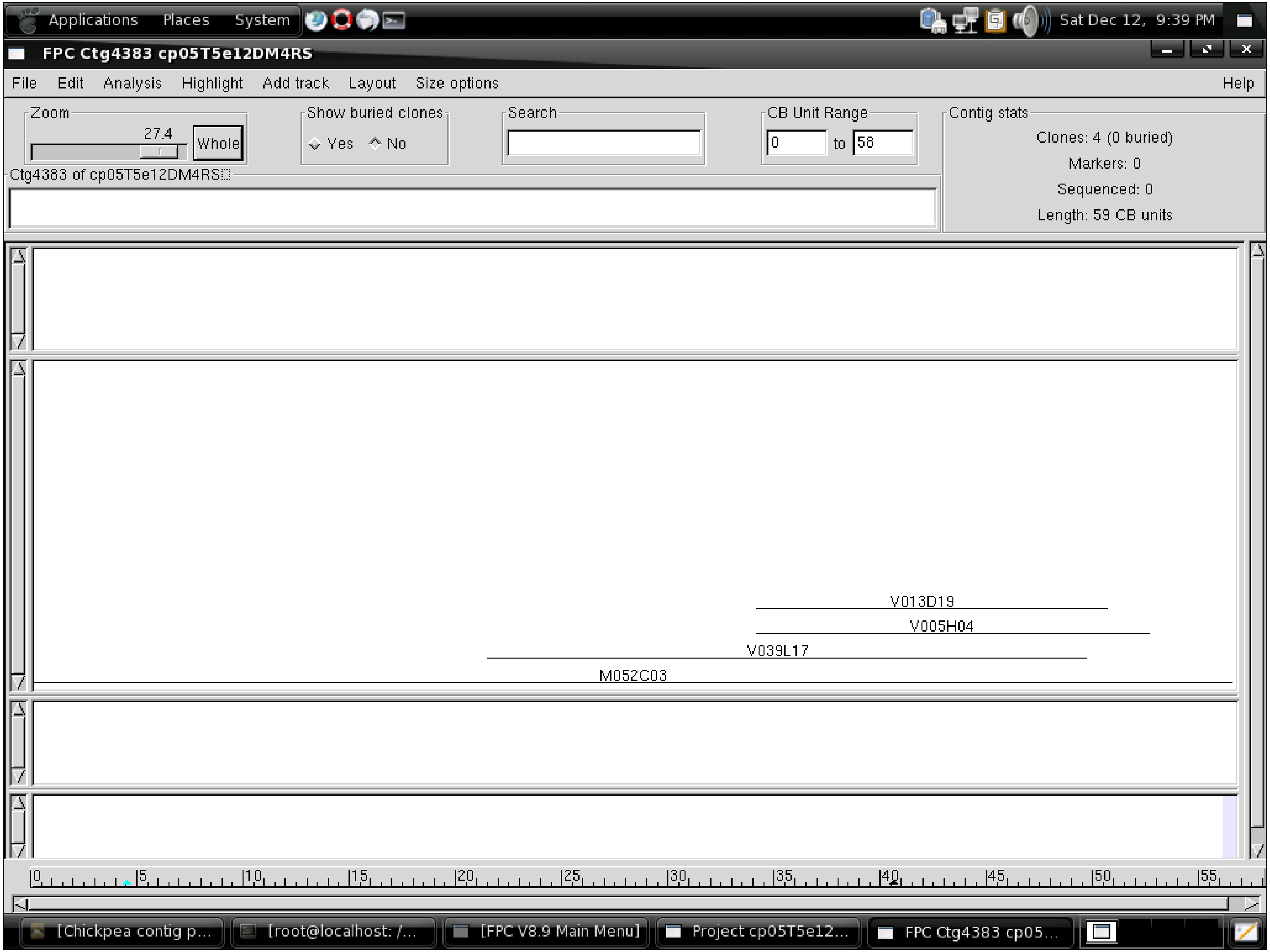
Applications Places System
Sat Dec 12, 9:39 PM
FPC Ctg4383 cp05T5e12DM4RS
File Edit Analysis Highlight Add track Layout Size options Help
Zoom
27.4
Whole
Show buried clones
Yes No
Search
CB Unit Range
0 to 58
Contig stats
Clones: 4 (0 buried)
Markers: 0
Sequenced: 0
Length: 59 CB units
Ctg4383 of cp05T5e12DM4RS
V013D19
V005H04
V039L17
M052C03
0 5 10 15 20 25 30 35 40 45 50 55
[Chickpea contig p...
[root@localhost: /...
[FPC V8.9 Main Menu]
Project cp05T5e12...
FPC Ctg4383 cp05...

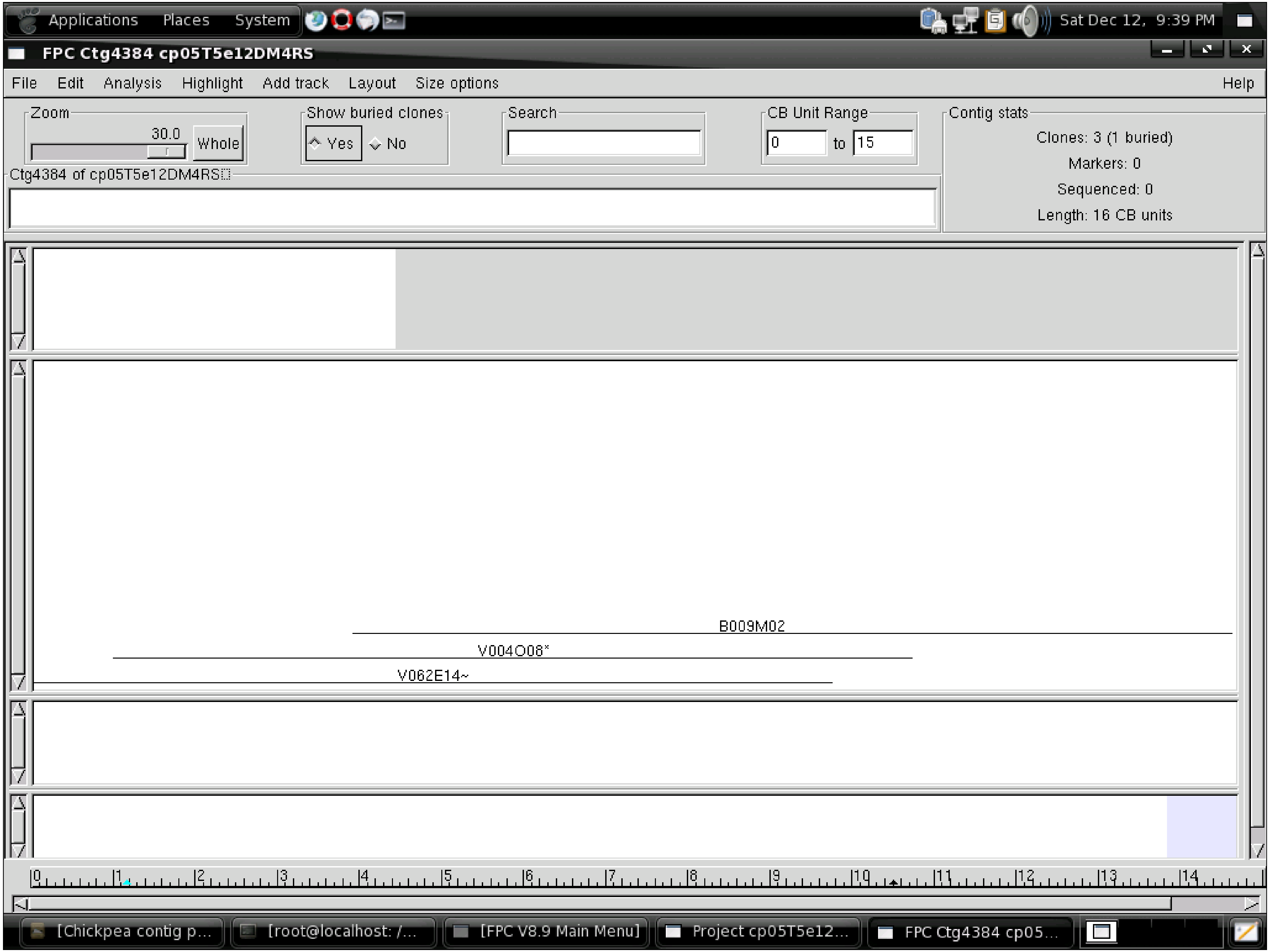
Applications Places System
Sat Dec 12, 9:39 PM
FPC Ctg4384 cp05T5e12DM4RS
File Edit Analysis Highlight Add track Layout Size options
Help
Zoom
30.0
Whole
Show buried clones
Yes
No
Search
CB Unit Range
0
to
15
Contig stats
Clones: 3 (1 buried)
Markers: 0
Sequenced: 0
Length: 16 CB units
Ctg4384 of cp05T5e12DM4RS
B009M02
V004O08*
V062E14~
0 1 2 3 4 5 6 7 8 9 10 11 12 13 14
[Chickpea contig p...
[root@localhost: /...
[FPC V8.9 Main Menu]
Project cp05T5e12...
FPC Ctg4384 cp05...

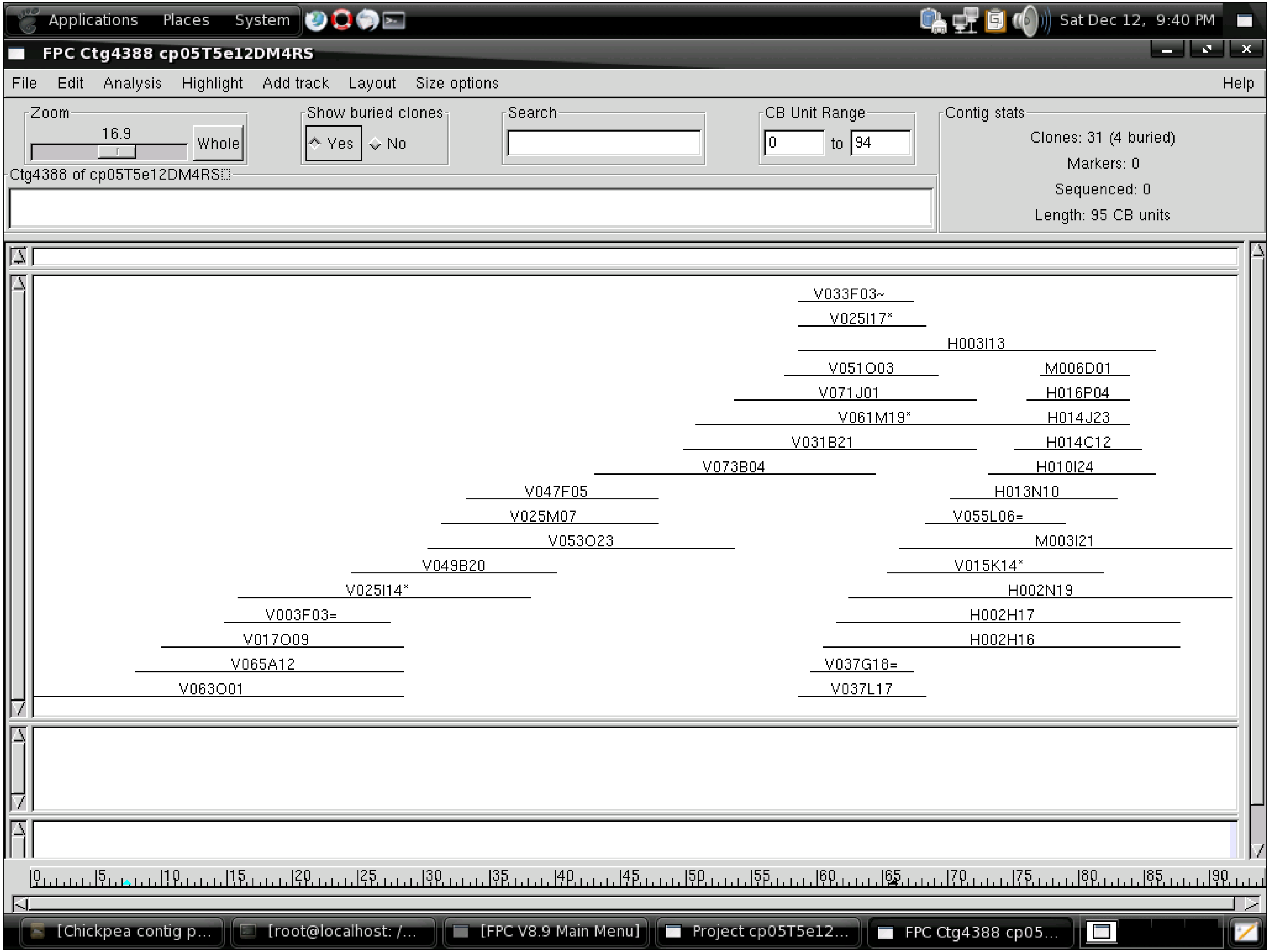
Applications Places System
Sat Dec 12, 9:40 PM
FPC Ctg4388 cp05T5e12DM4RS
File Edit Analysis Highlight Add track Layout Size options Help
Zoom
16.9
Whole
Show buried clones
Yes No
Search
CB Unit Range
0 to 94
Contig stats
Clones: 31 (4 buried)
Markers: 0
Sequenced: 0
Length: 95 CB units
Ctg4388 of cp05T5e12DM4RS
V033F03~
V025I17*
H003I13
V051O03
M006D01
V071J01
H016P04
V061M19*
H014J23
V031B21
H014C12
V073B04
H010I24
V047F05
H013N10
V025M07
V055L06=
V053O23
M003I21
V049B20
V015K14*
V025I14*
H002N19
V003F03=
H002H17
V017O09
H002H16
V065A12
V037G18=
V063O01
V037L17
0 5 10 15 20 25 30 35 40 45 50 55 60 65 70 75 80 85 90
[Chickpea contig p...
[root@localhost: /...
[FPC V8.9 Main Menu]
Project cp05T5e12...
FPC Ctg4388 cp05...

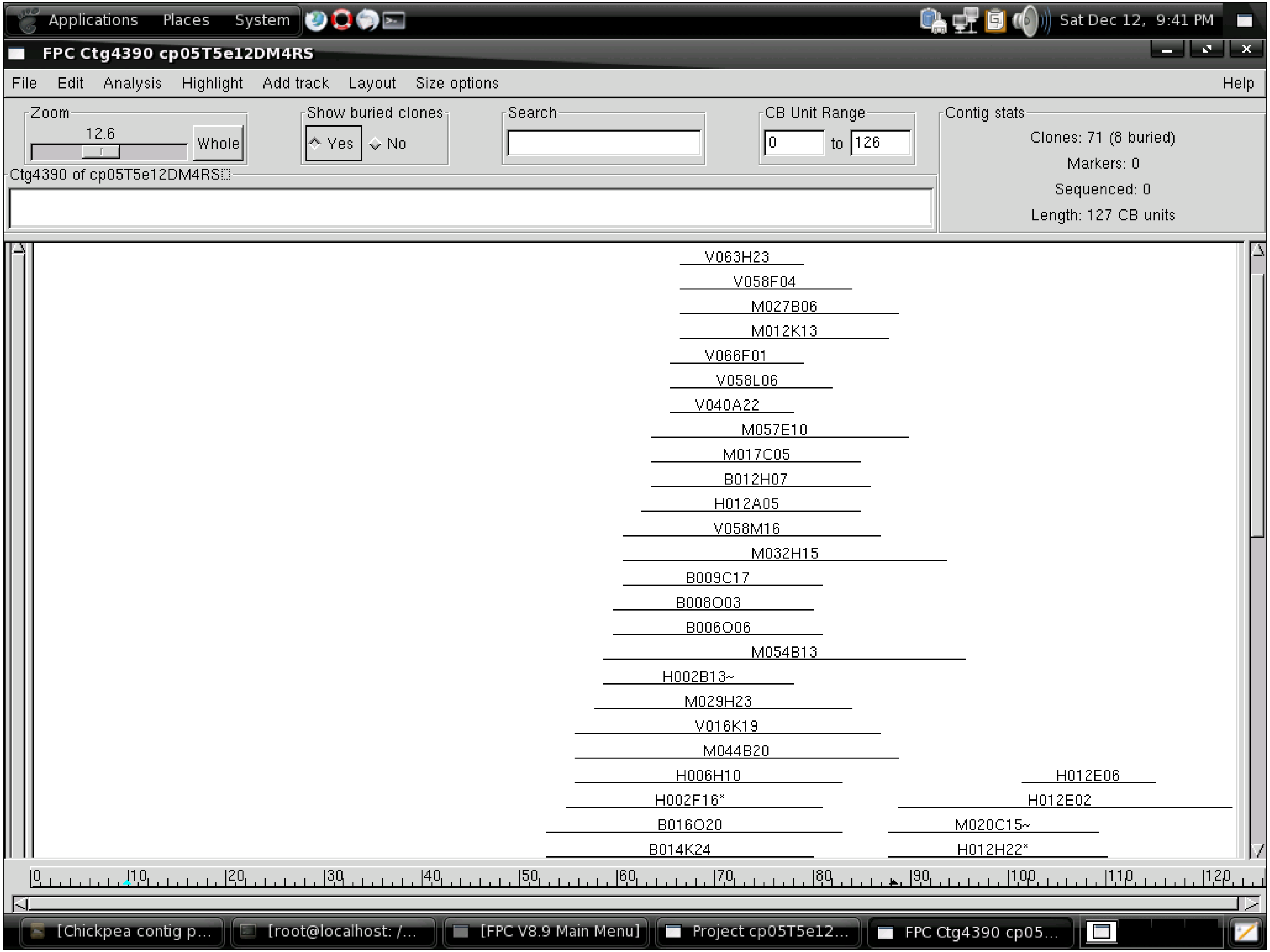
Applications Places System
Sat Dec 12, 9:41 PM
FPC Ctg4390 cp05T5e12DM4RS
File Edit Analysis Highlight Add track Layout Size options Help
Zoom
12.6
Whole
Show buried clones
Yes
No
Search
CB Unit Range
0 to 126
Contig stats
Clones: 71 (8 buried)
Markers: 0
Sequenced: 0
Length: 127 CB units
Ctg4390 of cp05T5e12DM4RS
V063H23
V058F04
M027B06
M012K13
V066F01
V058L06
V040A22
M057E10
M017C05
B012H07
H012A05
V058M16
M032H15
B009C17
B008O03
B006O06
M054B13
H002B13~
M029H23
V016K19
M044B20
H006H10
H012E06
H002F16*
H012E02
B016O20
M020C15~
B014K24
H012H22*
0 10 20 30 40 50 60 70 80 90 100 110 120
[Chickpea contig p...
[root@localhost: /...
[FPC V8.9 Main Menu]
Project cp05T5e12...
FPC Ctg4390 cp05...

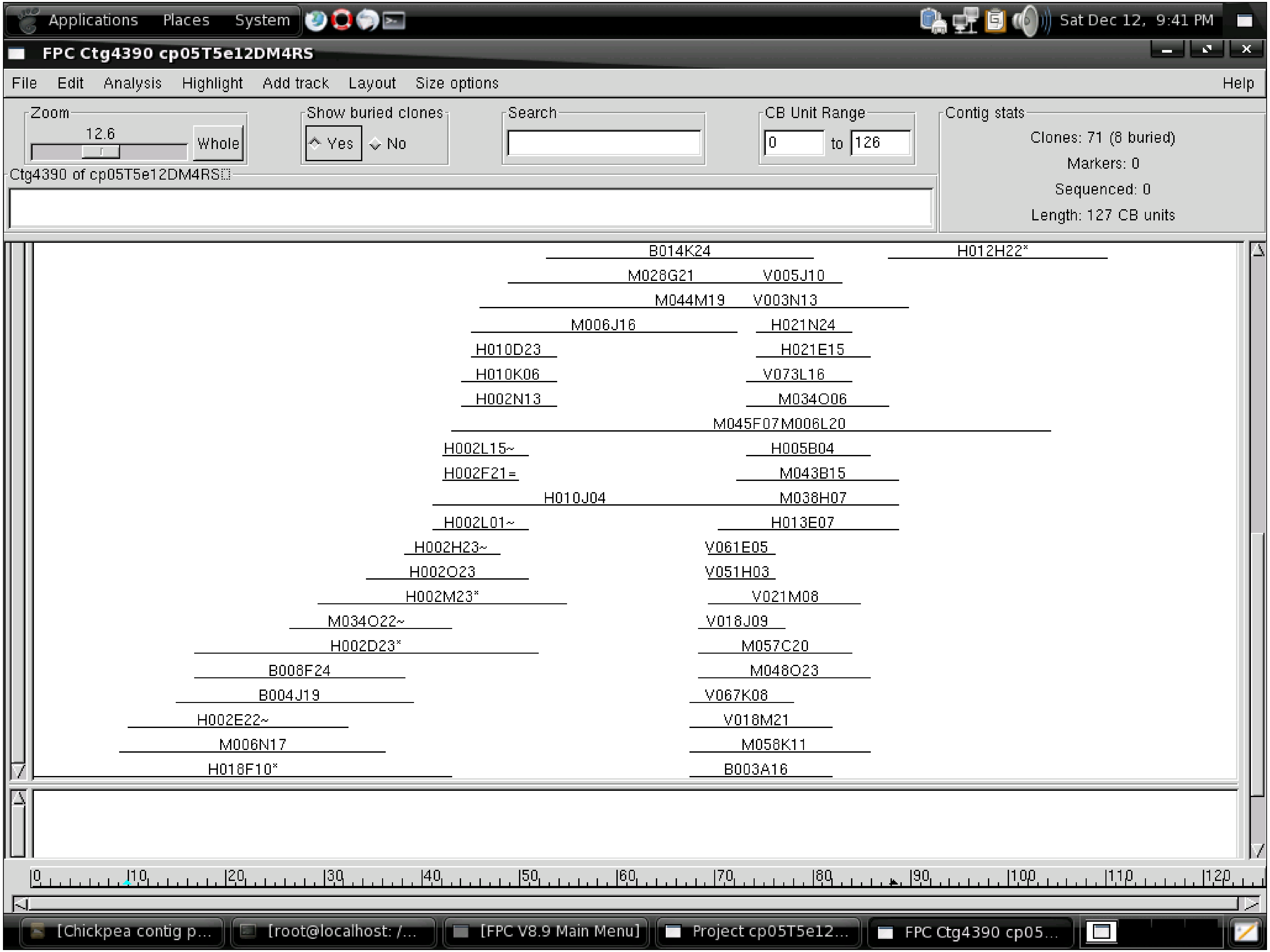
Applications Places System
Sat Dec 12, 9:41 PM
FPC Ctg4390 cp05T5e12DM4RS
File Edit Analysis Highlight Add track Layout Size options Help
Zoom
12.6
Whole
Show buried clones
Yes No
Search
CB Unit Range
0 to 126
Contig stats
Clones: 71 (8 buried)
Markers: 0
Sequenced: 0
Length: 127 CB units
Ctg4390 of cp05T5e12DM4RS
B014K24
H012H22*
M028G21
V005J10
M044M19
V003N13
M006J16
H021N24
H010D23
H021E15
H010K06
V073L16
H002N13
M034O06
M045F07
M006L20
H002L15~
H005B04
H002F21=
M043B15
H010J04
M038H07
H002L01~
H013E07
H002H23~
V061E05
H002O23
V051H03
H002M23*
V021M08
M034O22~
V018J09
H002D23*
M057C20
B008F24
M048O23
B004J19
V067K08
H002E22~
V018M21
M006N17
M058K11
H018F10*
B003A16
0 10 20 30 40 50 60 70 80 90 100 110 120
[Chickpea contig p...
[root@localhost: /...
[FPC V8.9 Main Menu]
Project cp05T5e12...
FPC Ctg4390 cp05...

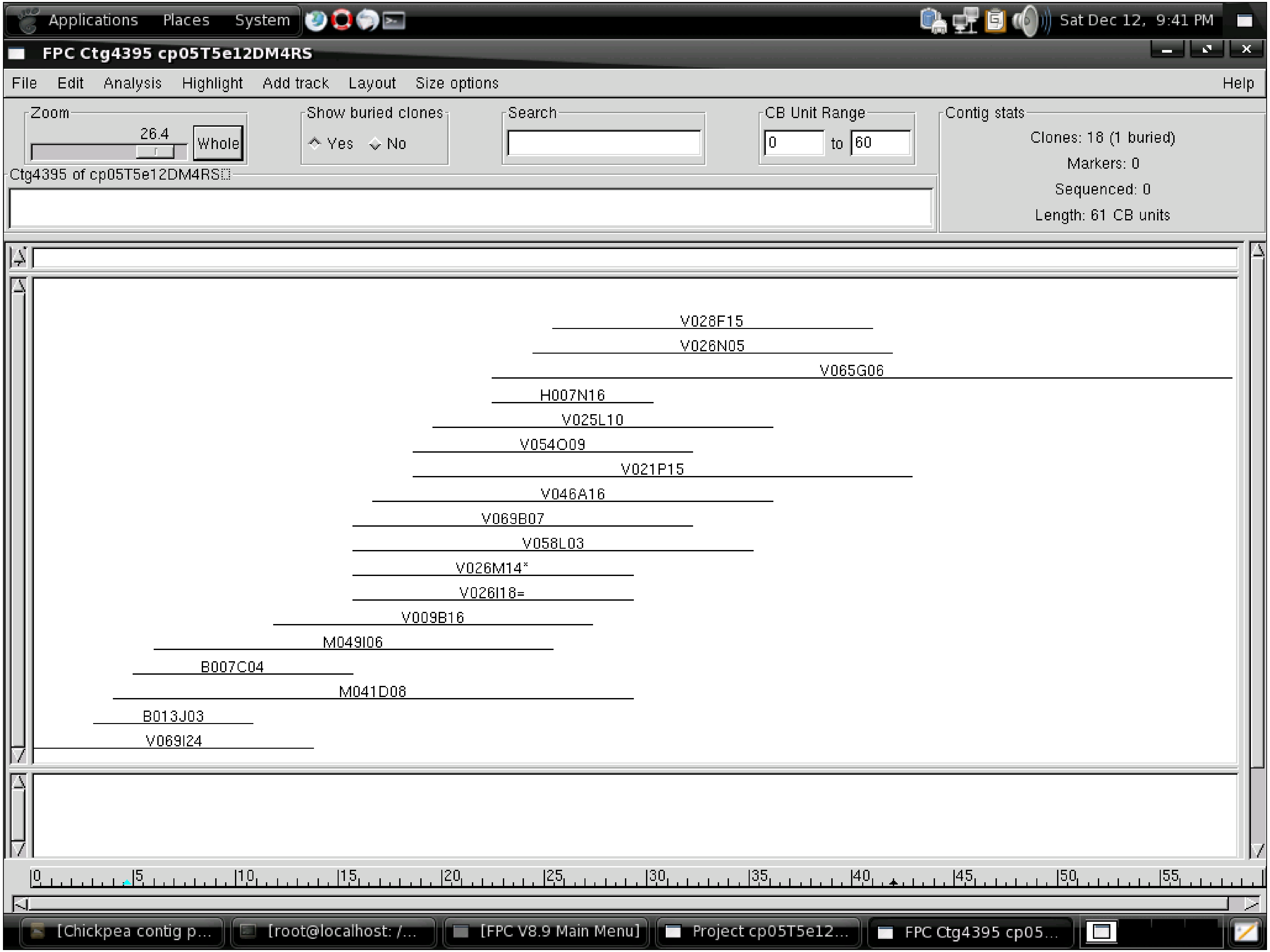
Applications Places System
Sat Dec 12, 9:41 PM
FPC Ctg4395 cp05T5e12DM4RS
File Edit Analysis Highlight Add track Layout Size options Help
Zoom
26.4
Whole
Show buried clones
Yes No
Search
CB Unit Range
0 to 60
Contig stats
Clones: 18 (1 buried)
Markers: 0
Sequenced: 0
Length: 61 CB units
Ctg4395 of cp05T5e12DM4RS
V028F15
V026N05
V065G06
H007N16
V025L10
V054O09
V021P15
V046A16
V069B07
V058L03
V026M14*
V026I18=
V009B16
M049I06
B007C04
M041D08
B013J03
V069I24
0 5 10 15 20 25 30 35 40 45 50 55
[Chickpea contig p...
[root@localhost: /...
[FPC V8.9 Main Menu]
Project cp05T5e12...
FPC Ctg4395 cp05...

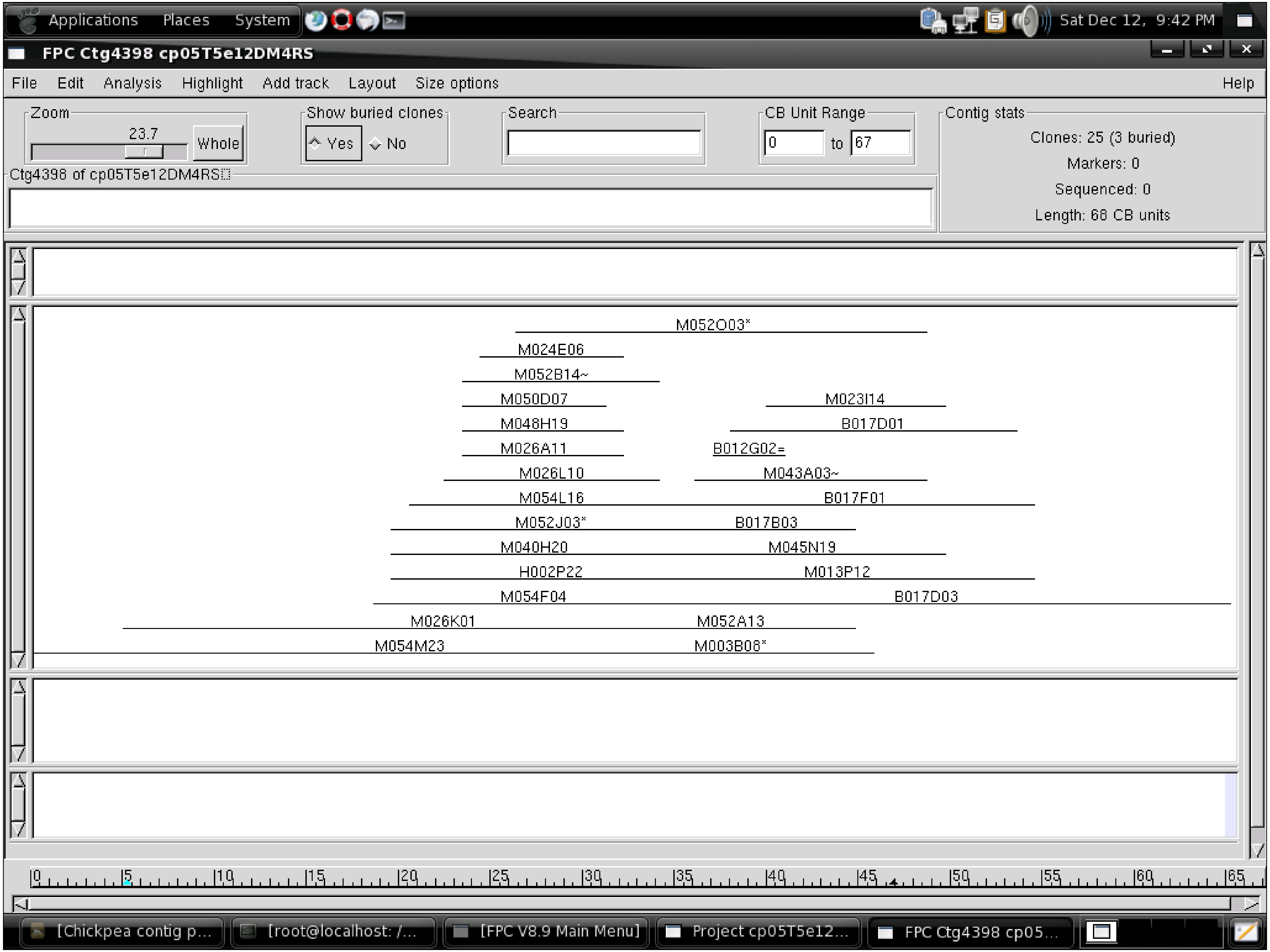
Applications Places System
Sat Dec 12, 9:42 PM
FPC Ctg4398 cp05T5e12DM4RS
File Edit Analysis Highlight Add track Layout Size options Help
Zoom
23.7
Whole
Show buried clones
Yes No
Search
CB Unit Range
0 to 67
Contig stats
Clones: 25 (3 buried)
Markers: 0
Sequenced: 0
Length: 68 CB units
Ctg4398 of cp05T5e12DM4RS
M052O03*
M024E06
M052B14~
M050D07
M023I14
M048H19
B017D01
M026A11
B012G02=
M026L10
M043A03~
M054L16
B017F01
M052J03*
B017B03
M040H20
M045N19
H002P22
M013P12
M054F04
B017D03
M026K01
M052A13
M054M23
M003B08*
0 5 10 15 20 25 30 35 40 45 50 55 60 65
[Chickpea contig p...
[root@localhost: /...
[FPC V8.9 Main Menu]
Project cp05T5e12...
FPC Ctg4398 cp05...

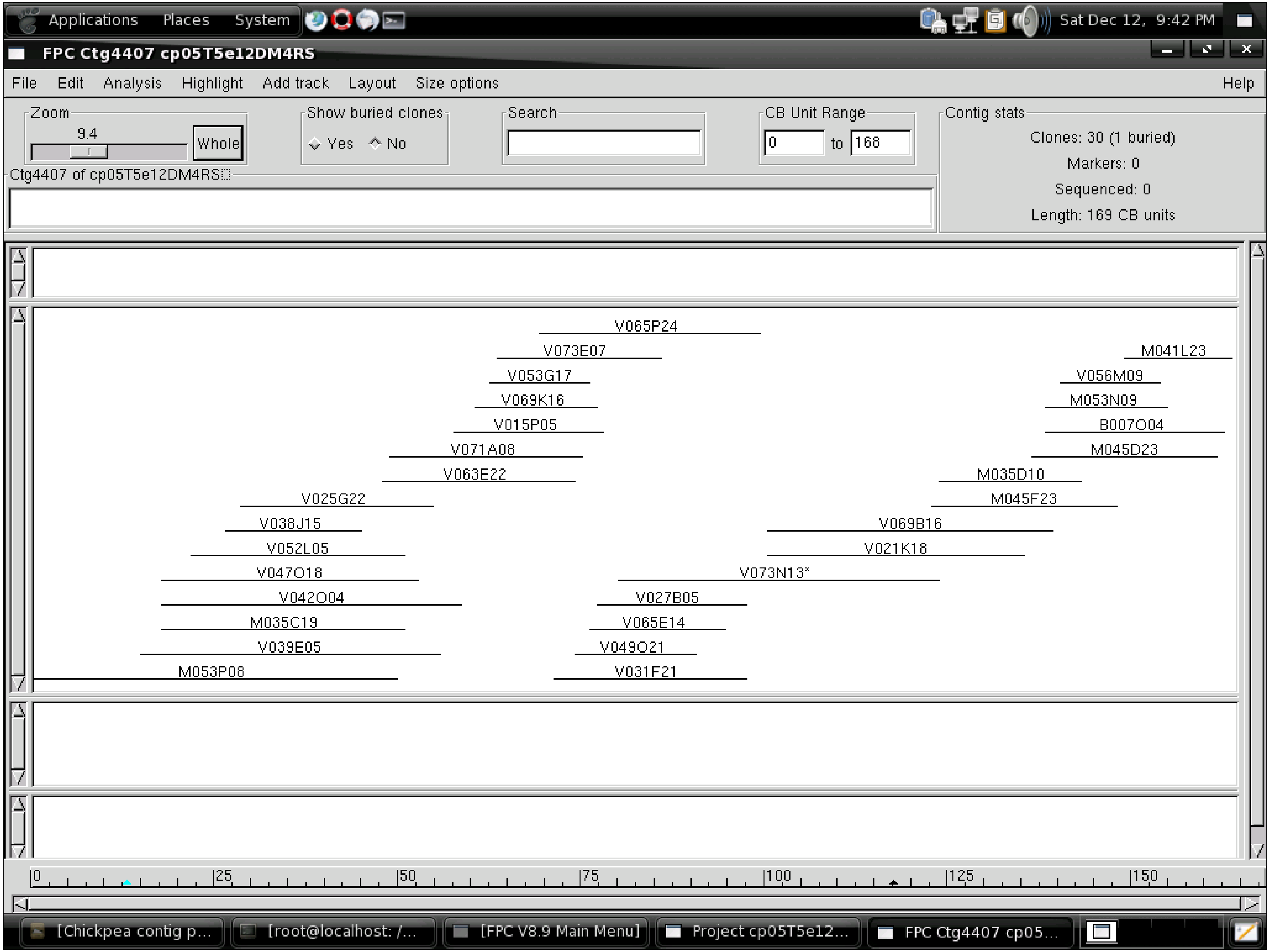

Applications Places System
Sat Dec 12, 9:42 PM
FPC Ctg4407 cp05T5e12DM4RS
File Edit Analysis Highlight Add track Layout Size options Help
Zoom
9.4
Whole
Show buried clones
Yes No
Search
CB Unit Range
0 to 168
Contig stats
Clones: 30 (1 buried)
Markers: 0
Sequenced: 0
Length: 169 CB units
Ctg4407 of cp05T5e12DM4RS
V065P24
V073E07
M041L23
V053G17
V056M09
V069K16
M053N09
V015P05
B007O04
V071A08
M045D23
V063E22
M035D10
V025G22
M045F23
V038J15
V069B16
V052L05
V021K18
V047O18
V073N13*
V042O04
V027B05
M035C19
V065E14
V039E05
V049O21
M053P08
V031F21
0 25 50 75 100 125 150
[Chickpea contig p...
[root@localhost: /...
[FPC V8.9 Main Menu]
Project cp05T5e12...
FPC Ctg4407 cp05...

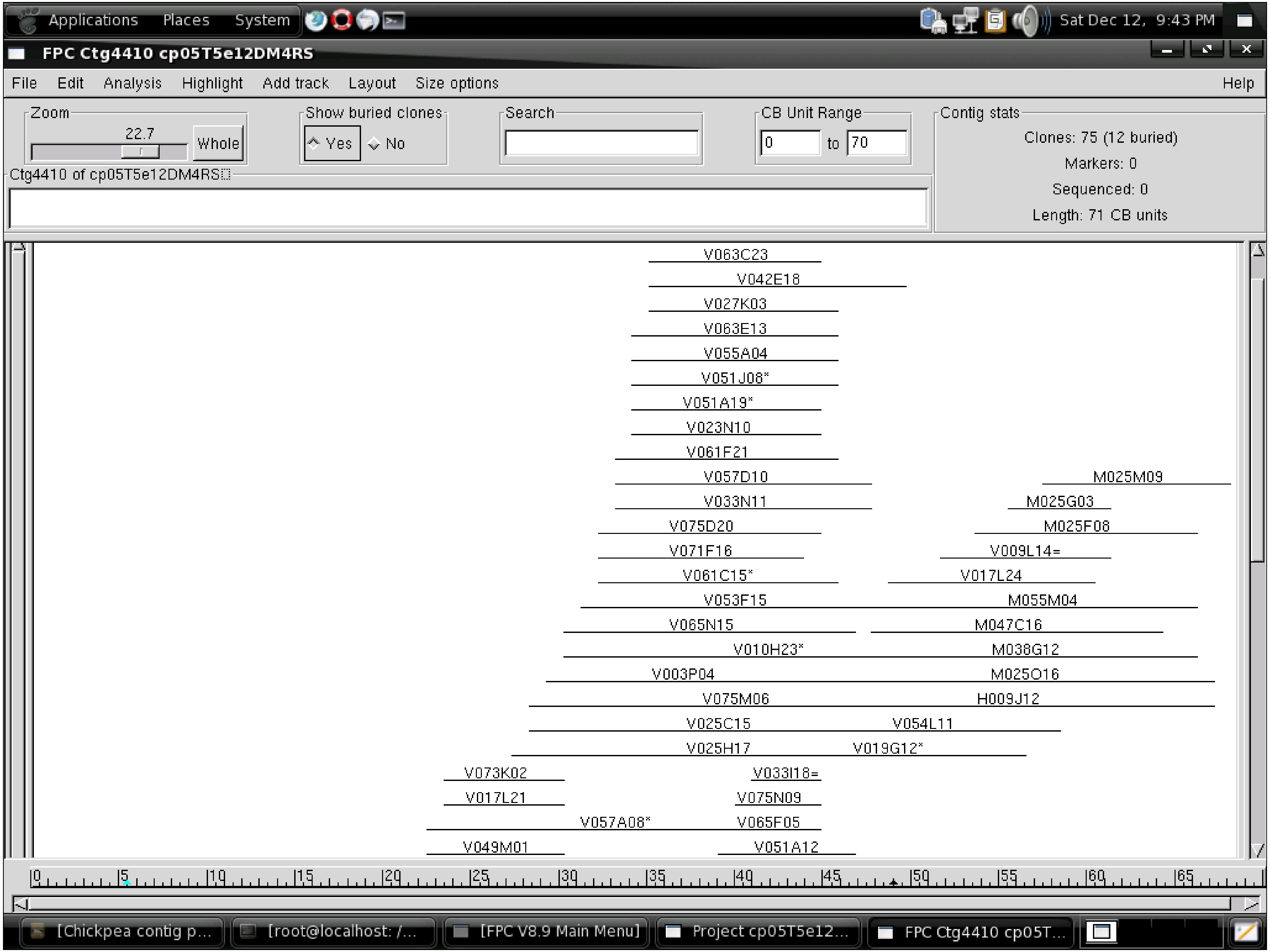

Applications
Places
System
Sat Dec 12, 9:43 PM
FPC Ctg4410 cp05T5e12DM4RS
File
Edit
Analysis
Highlight
Add track
Layout
Size options
Help
Zoom
22.7
Whole
Show buried clones
Yes
No
Search
CB Unit Range
0
to
70
Contig stats
Clones: 75 (12 buried)
Markers: 0
Sequenced: 0
Length: 71 CB units
Ctg4410 of cp05T5e12DM4RS
V063C23
V042E18
V027K03
V063E13
V055A04
V051J08*
V051A19*
V023N10
V061F21
V057D10
M025M09
V033N11
M025G03
V075D20
M025F08
V071F16
V009L14=
V061C15*
V017L24
V053F15
M055M04
V065N15
M047C16
V010H23*
M038G12
V003P04
M025O16
V075M06
H009J12
V025C15
V054L11
V025H17
V019G12*
V073K02
V033I18=
V017L21
V075N09
V057A08*
V065F05
V049M01
V051A12
0 5 10 15 20 25 30 35 40 45 50 55 60 65
[Chickpea contig p...
[root@localhost: /...
[FPC V8.9 Main Menu]
Project cp05T5e12...
FPC Ctg4410 cp05T...

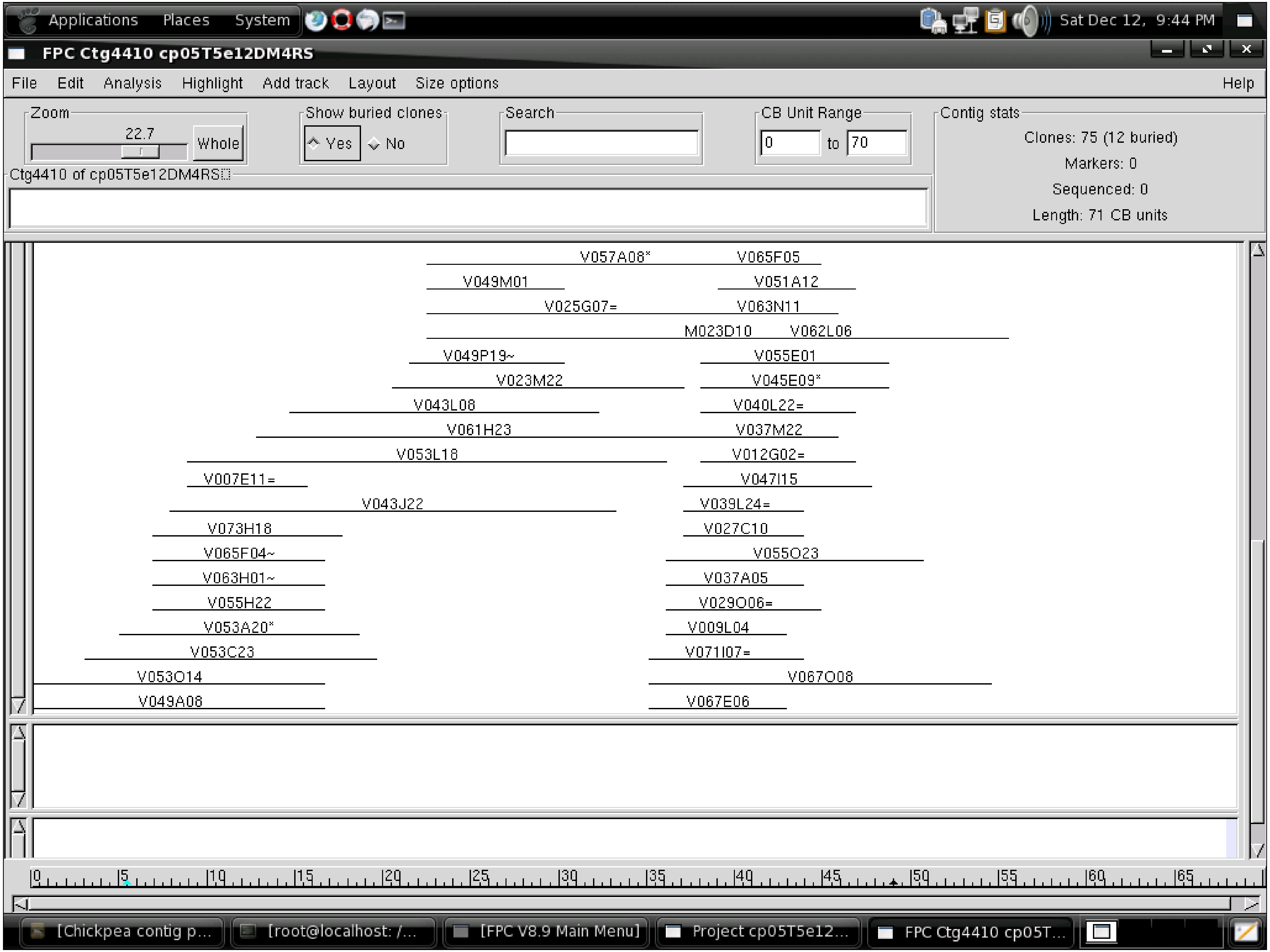
Applications Places System
Sat Dec 12, 9:44 PM
FPC Ctg4410 cp05T5e12DM4RS
File Edit Analysis Highlight Add track Layout Size options Help
Zoom
22.7
Whole
Show buried clones
Yes No
Search
CB Unit Range
0 to 70
Contig stats
Clones: 75 (12 buried)
Markers: 0
Sequenced: 0
Length: 71 CB units
Ctg4410 of cp05T5e12DM4RS
V057A08*
V065F05
V049M01
V051A12
V025G07=
V063N11
M023D10
V062L06
V049P19~
V055E01
V023M22
V045E09*
V043L08
V040L22=
V061H23
V037M22
V053L18
V012G02=
V007E11=
V047I15
V043J22
V039L24=
V073H18
V027C10
V065F04~
V055O23
V063H01~
V037A05
V055H22
V029O06=
V053A20*
V009L04
V053C23
V071I07=
V053O14
V067O08
V049A08
V067E06
0 5 10 15 20 25 30 35 40 45 50 55 60 65
[Chickpea contig p...
[root@localhost: /...
[FPC V8.9 Main Menu]
Project cp05T5e12...
FPC Ctg4410 cp05T...

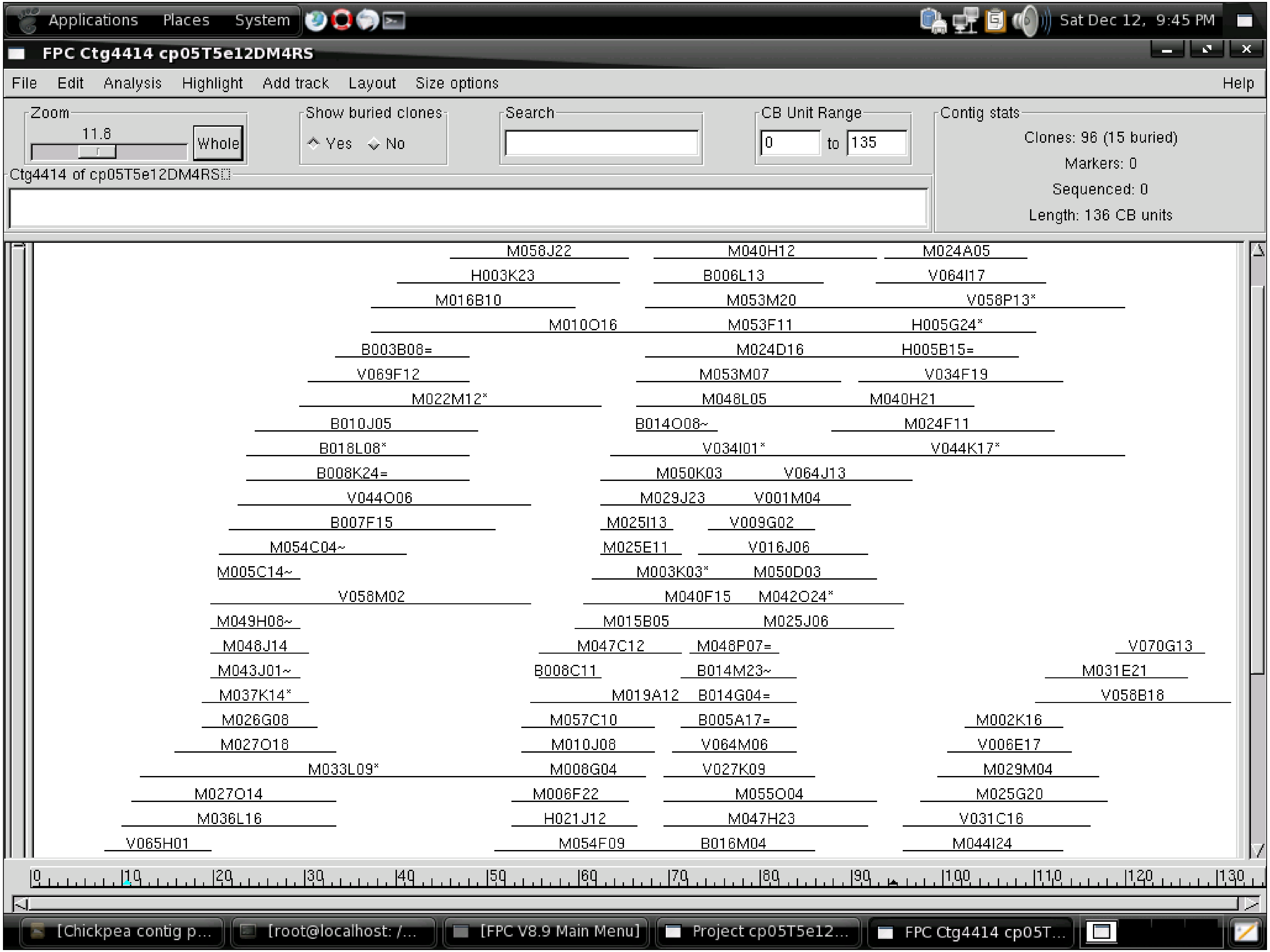
Applications Places System
Sat Dec 12, 9:45 PM
FPC Ctg4414 cp05T5e12DM4RS
File Edit Analysis Highlight Add track Layout Size options Help
Zoom
11.8
Whole
Show buried clones
Yes No
Search
CB Unit Range
0 to 135
Contig stats
Clones: 96 (15 buried)
Markers: 0
Sequenced: 0
Length: 136 CB units
Ctg4414 of cp05T5e12DM4RS
M058J22 M040H12 M024A05
H003K23 B006L13 V064I17
M016B10 M053M20 V058P13*
M010O16 M053F11 H005G24*
B003B08= M024D16 H005B15=
V069F12 M053M07 V034F19
M022M12* M048L05 M040H21
B010J05 B014O08~ M024F11
B018L08* V034I01* V044K17*
B008K24= M050K03 V064J13
V044O06 M029J23 V001M04
B007F15 M025I13 V009G02
M054C04~ M025E11 V016J06
M005C14~ M003K03* M050D03
V058M02 M040F15 M042O24*
M049H08~ M015B05 M025J06
M048J14 M047C12 M048P07= V070G13
M043J01~ B008C11 B014M23~ M031E21
M037K14* M019A12 B014G04= V058B18
M026G08 M057C10 B005A17= M002K16
M027O18 M010J08 V064M06 V006E17
M033L09* M008G04 V027K09 M029M04
M027O14 M006F22 M055O04 M025G20
M036L16 H021J12 M047H23 V031C16
V065H01 M054F09 B016M04 M044I24
0 10 20 30 40 50 60 70 80 90 100 110 120 130
[Chickpea contig p... [root@localhost: /... [FPC V8.9 Main Menu] Project cp05T5e12... FPC Ctg4414 cp05T...

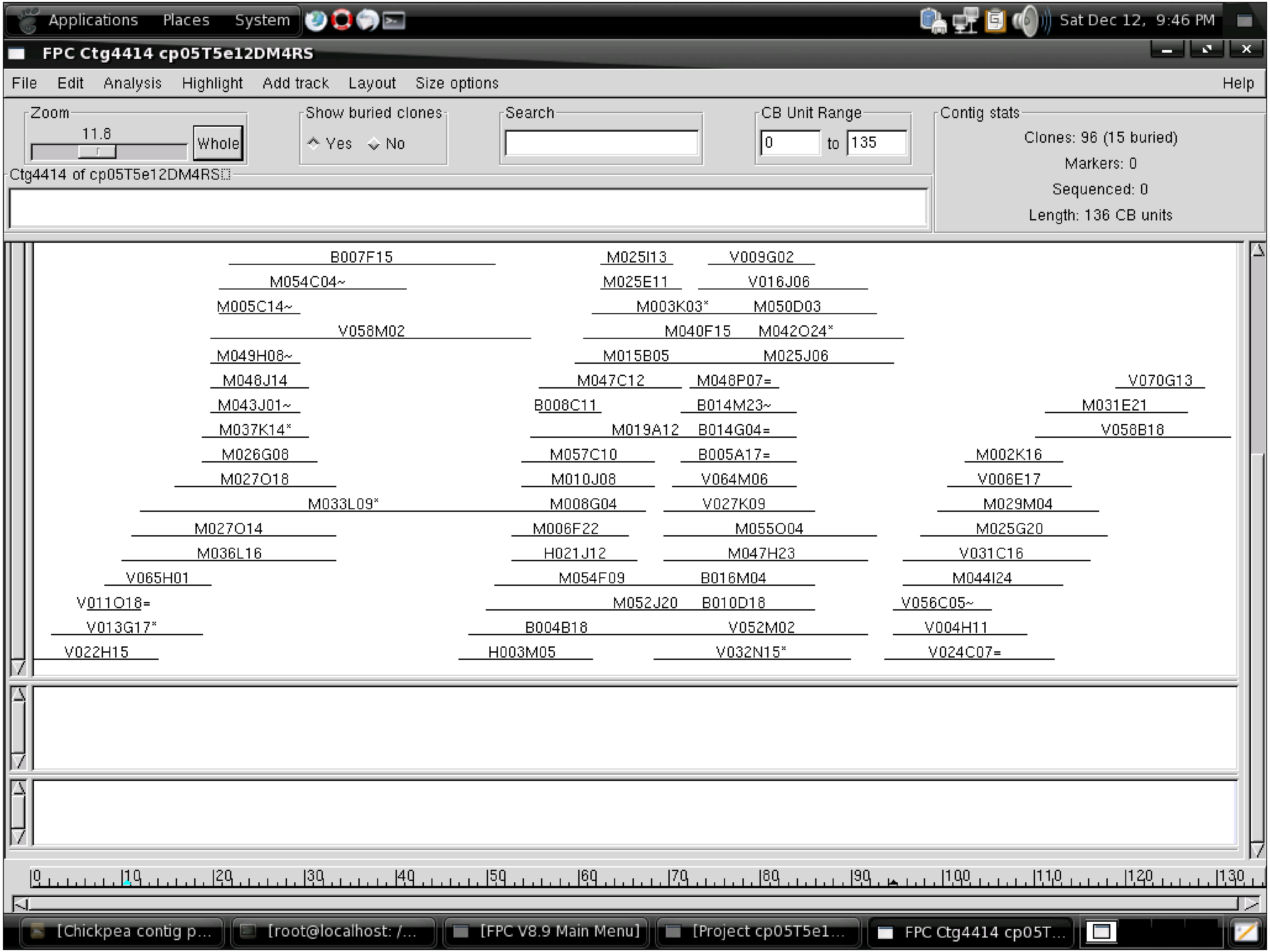
Applications Places System
Sat Dec 12, 9:46 PM
FPC Ctg4414 cp05T5e12DM4RS
File Edit Analysis Highlight Add track Layout Size options Help
Zoom
11.8
Whole
Show buried clones
Yes No
Search
CB Unit Range
0 to 135
Contig stats
Clones: 96 (15 buried)
Markers: 0
Sequenced: 0
Length: 136 CB units
Ctg4414 of cp05T5e12DM4RS
B007F15
M054C04~
M005C14~
V058M02
M049H08~
M048J14
M043J01~
M037K14*
M026G08
M027O18
M033L09*
M027O14
M036L16
V065H01
V011O18=
V013G17*
V022H15
M025I13
M025E11
M003K03*
M040F15
M015B05
M047C12
B008C11
M019A12
M057C10
M010J08
M008G04
M006F22
H021J12
M054F09
M052J20
B004B18
H003M05
V009G02
V016J06
M050D03
M042O24*
M025J06
M048P07=
B014M23~
B014G04=
B005A17=
V064M06
V027K09
M055O04
M047H23
B016M04
B010D18
V052M02
V032N15*
V070G13
M031E21
V058B18
M002K16
V006E17
M029M04
M025G20
V031C16
M044I24
V056C05~
V004H11
V024C07=
0 10 20 30 40 50 60 70 80 90 100 110 120 130
[Chickpea contig p...
[root@localhost: /...
[FPC V8.9 Main Menu]
[Project cp05T5e1...
FPC Ctg4414 cp05T...

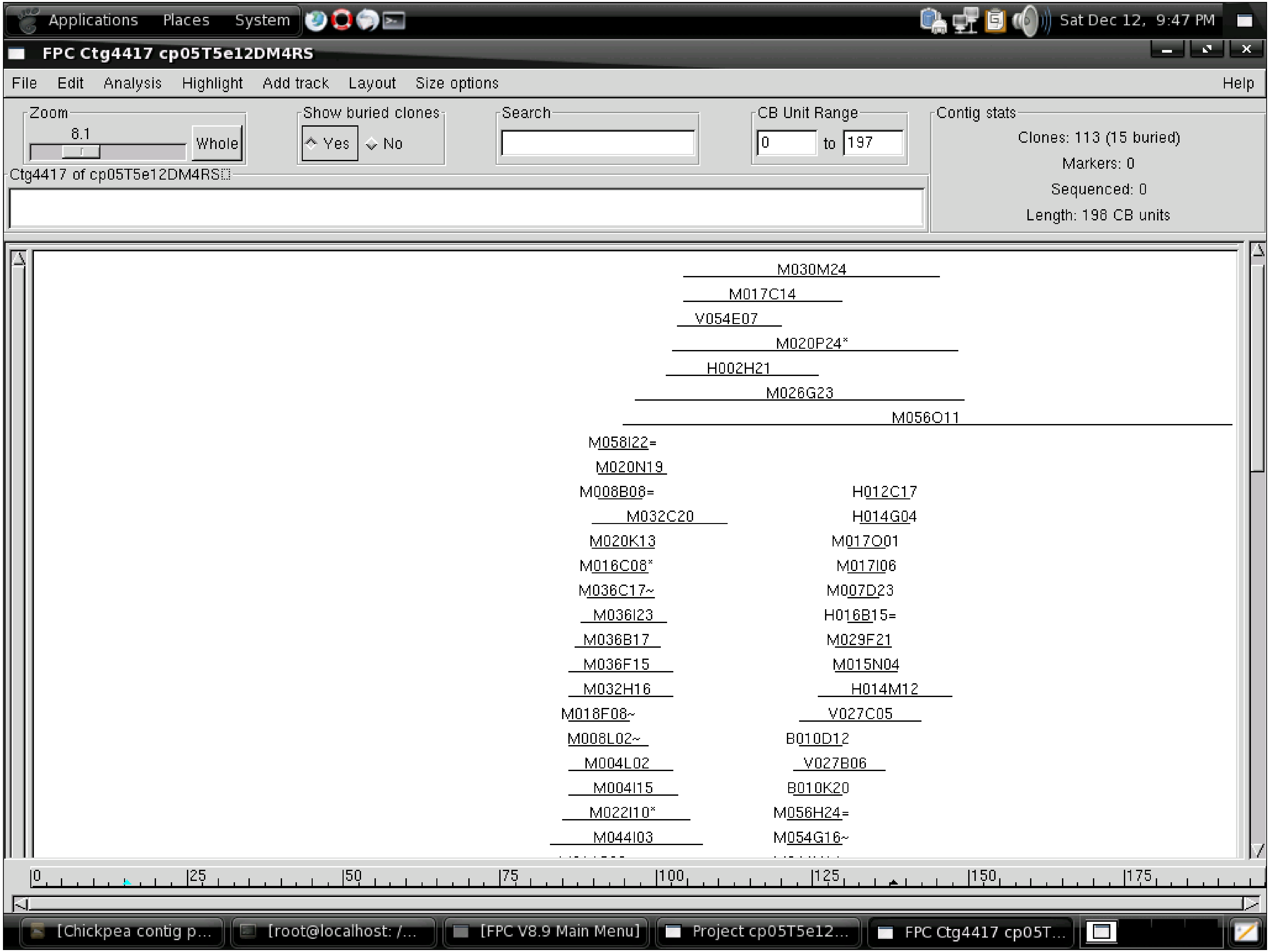
Applications Places System
Sat Dec 12, 9:47 PM
FPC Ctg4417 cp05T5e12DM4RS
File Edit Analysis Highlight Add track Layout Size options Help
Zoom
8.1
Whole
Show buried clones
Yes
No
Search
CB Unit Range
0 to 197
Contig stats
Clones: 113 (15 buried)
Markers: 0
Sequenced: 0
Length: 198 CB units
Ctg4417 of cp05T5e12DM4RS
M030M24
M017C14
V054E07
M020P24*
H002H21
M026G23
M056O11
M058I22=
M020N19
M008B08=
H012C17
M032C20
H014G04
M020K13
M017O01
M016C08*
M017I06
M036C17~
M007D23
M036I23
H016B15=
M036B17
M029F21
M036F15
M015N04
M032H16
H014M12
M018F08~
V027C05
M008L02~
B010D12
M004L02
V027B06
M004I15
B010K20
M022I10*
M056H24=
M044I03
M054G16~
0 25 50 75 100 125 150 175
[Chickpea contig p...
[root@localhost: /...
[FPC V8.9 Main Menu]
Project cp05T5e12...
FPC Ctg4417 cp05T...

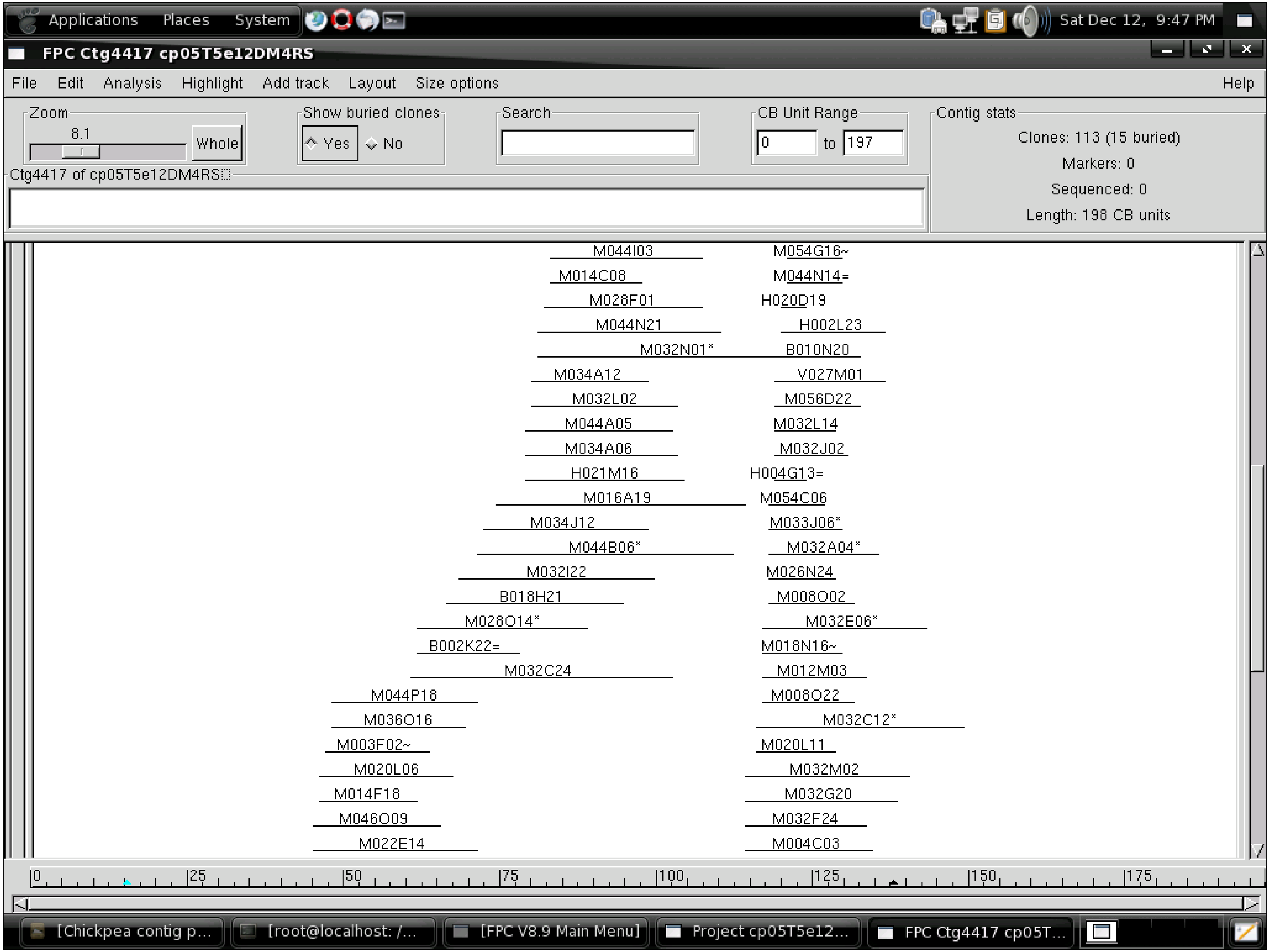
Applications Places System
Sat Dec 12, 9:47 PM
FPC Ctg4417 cp05T5e12DM4RS
File Edit Analysis Highlight Add track Layout Size options Help
Zoom
8.1
Whole
Show buried clones
Yes
No
Search
CB Unit Range
0
to
197
Contig stats
Clones: 113 (15 buried)
Markers: 0
Sequenced: 0
Length: 198 CB units
Ctg4417 of cp05T5e12DM4RS
M044I03
M054G16~
M014C08
M044N14=
M028F01
H020D19
M044N21
H002L23
M032N01*
B010N20
M034A12
V027M01
M032L02
M056D22
M044A05
M032L14
M034A06
M032J02
H021M16
H004G13=
M016A19
M054C06
M034J12
M033J06*
M044B06*
M032A04*
M032I22
M026N24
B018H21
M008O02
M028O14*
M032E06*
B002K22=
M018N16~
M032C24
M012M03
M044P18
M008O22
M036O16
M032C12*
M003F02~
M020L11
M020L06
M032M02
M014F18
M032G20
M046O09
M032F24
M022E14
M004C03
0
25
50
75
100
125
150
175
[Chickpea contig p...
[root@localhost: /...
[FPC V8.9 Main Menu]
Project cp05T5e12...
FPC Ctg4417 cp05T...

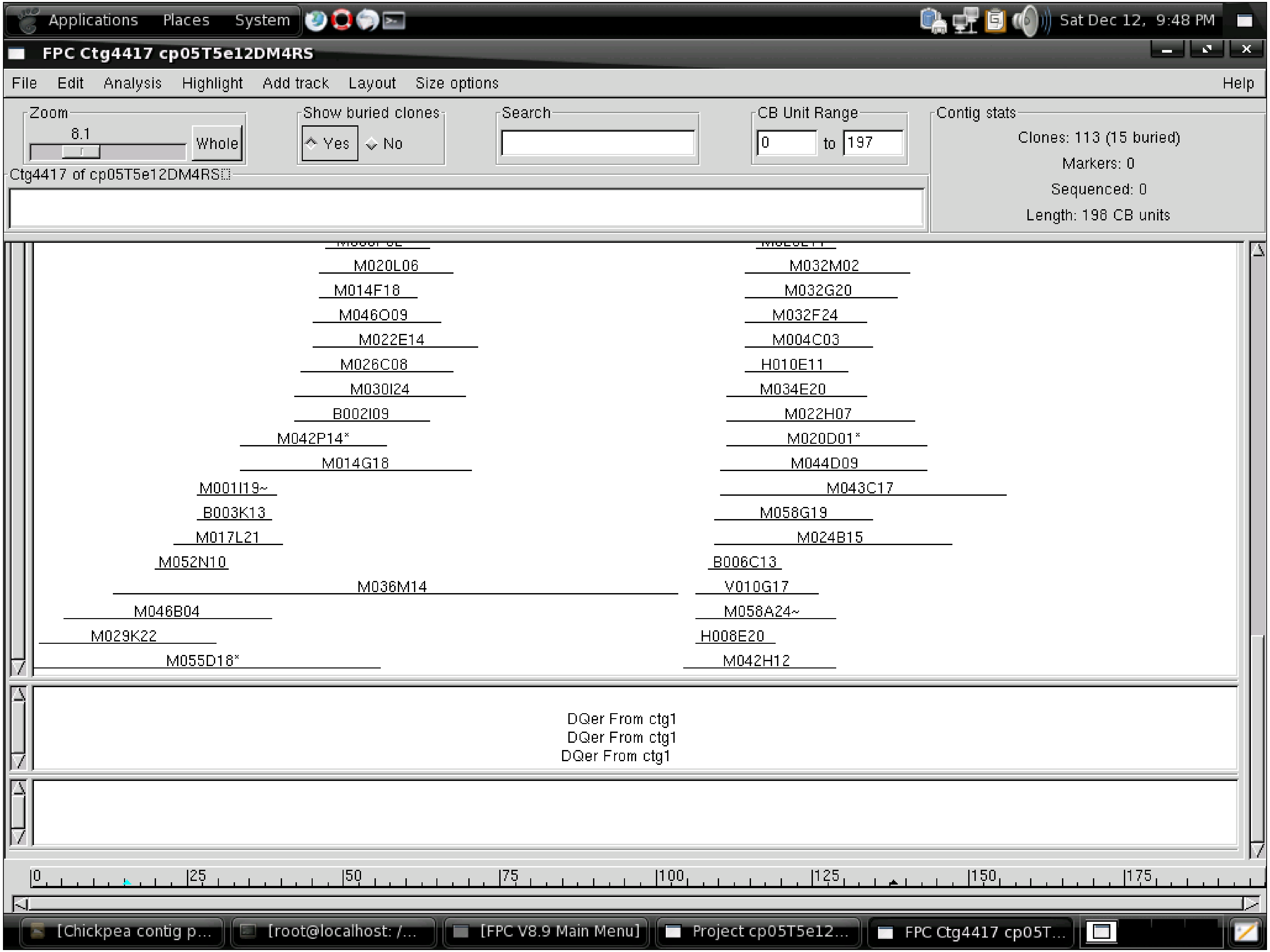
Applications
Places
System
Sat Dec 12, 9:48 PM
FPC Ctg4417 cp05T5e12DM4RS
File
Edit
Analysis
Highlight
Add track
Layout
Size options
Help
Zoom
8.1
Whole
Show buried clones
Yes
No
Search
CB Unit Range
0
to
197
Contig stats
Clones: 113 (15 buried)
Markers: 0
Sequenced: 0
Length: 198 CB units
Ctg4417 of cp05T5e12DM4RS
M020L06
M014F18
M046O09
M022E14
M026C08
M030I24
B002I09
M042P14*
M014G18
M001I19~
B003K13
M017L21
M052N10
M036M14
M046B04
M029K22
M055D18*
M032M02
M032G20
M032F24
M004C03
H010E11
M034E20
M022H07
M020D01*
M044D09
M043C17
M058G19
M024B15
B006C13
V010G17
M058A24~
H008E20
M042H12
DQer From ctg1
DQer From ctg1
DQer From ctg1
0
25
50
75
100
125
150
175
[Chickpea contig p...
[root@localhost: /...
[FPC V8.9 Main Menu]
Project cp05T5e12...
FPC Ctg4417 cp05T...

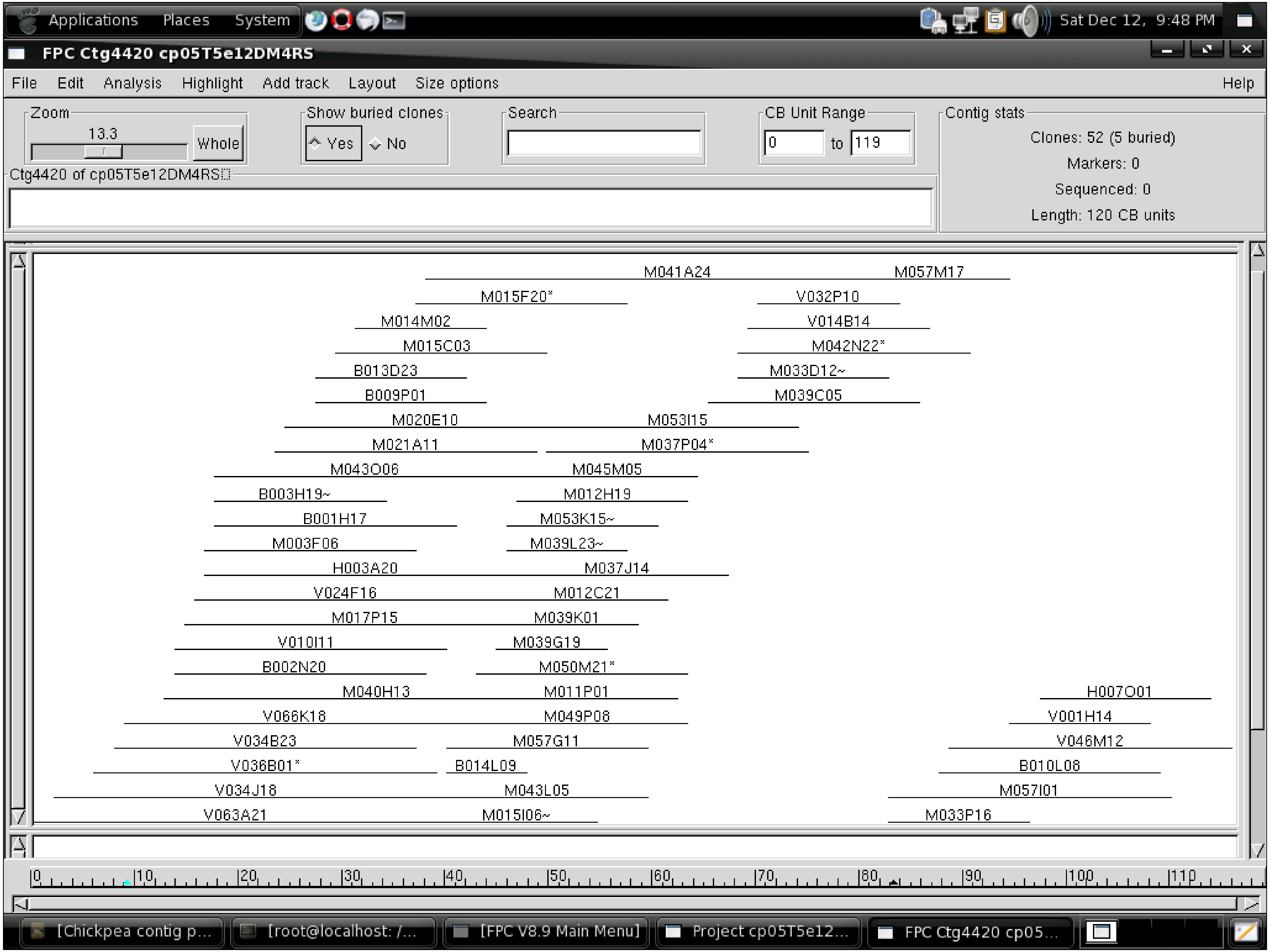
Applications Places System
Sat Dec 12, 9:48 PM
FPC Ctg4420 cp05T5e12DM4RS
File Edit Analysis Highlight Add track Layout Size options Help
Zoom
13.3
Whole
Show buried clones
Yes
No
Search
CB Unit Range
0 to 119
Contig stats
Clones: 52 (5 buried)
Markers: 0
Sequenced: 0
Length: 120 CB units
Ctg4420 of cp05T5e12DM4RS
M041A24
M057M17
M015F20*
V032P10
M014M02
V014B14
M015C03
M042N22*
B013D23
M033D12~
B009P01
M039C05
M020E10
M053I15
M021A11
M037P04*
M043O06
M045M05
B003H19~
M012H19
B001H17
M053K15~
M003F06
M039L23~
H003A20
M037J14
V024F16
M012C21
M017P15
M039K01
V010I11
M039G19
B002N20
M050M21*
M040H13
M011P01
H007O01
V066K18
M049P08
V001H14
V034B23
M057G11
V046M12
V036B01*
B014L09
B010L08
V034J18
M043L05
M057I01
V063A21
M015I06~
M033P16
0 10 20 30 40 50 60 70 80 90 100 110
[Chickpea contig p...
[root@localhost: /...
[FPC V8.9 Main Menu]
Project cp05T5e12...
FPC Ctg4420 cp05...

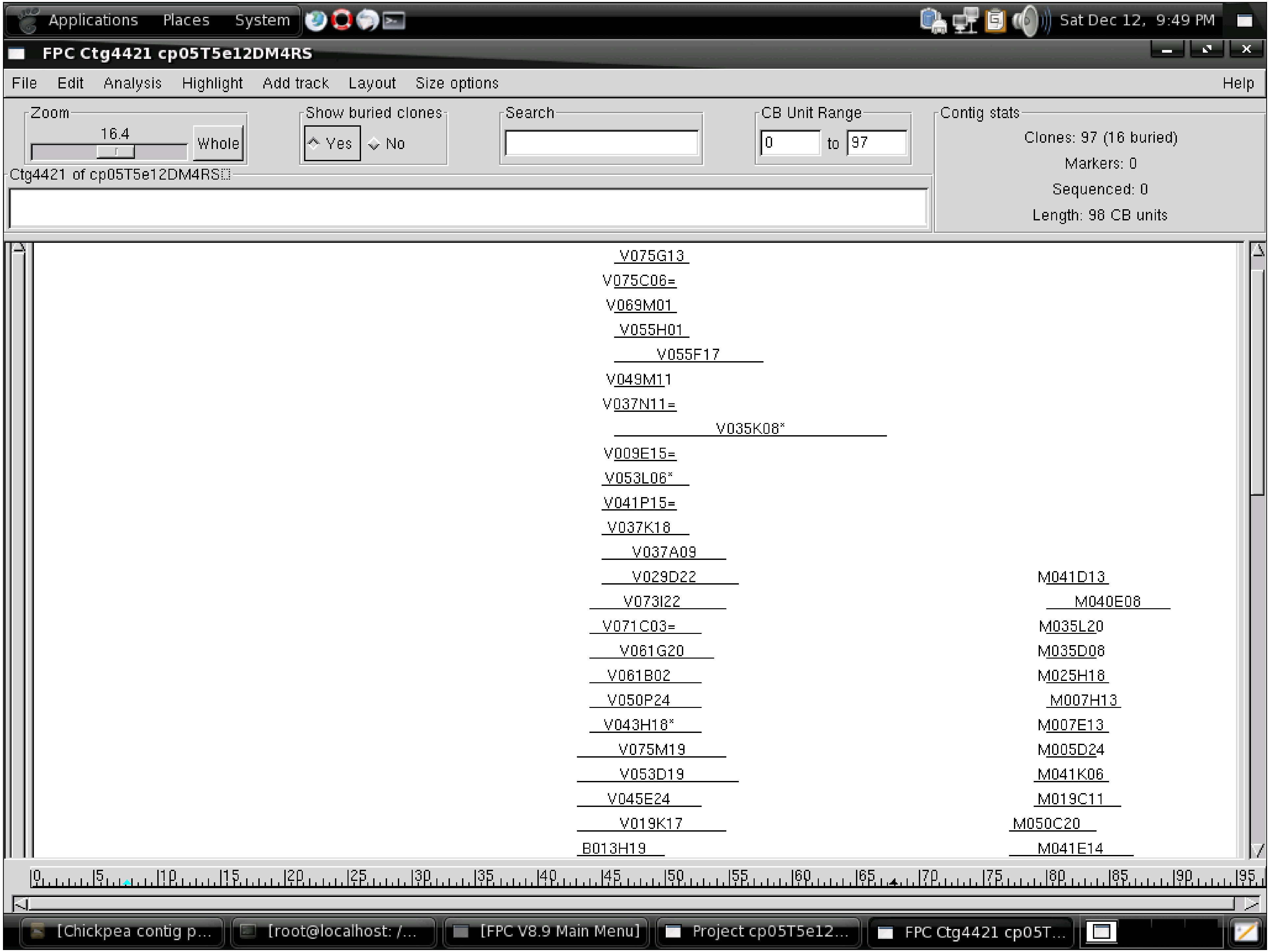
Applications Places System
Sat Dec 12, 9:49 PM
FPC Ctg4421 cp05T5e12DM4RS
File Edit Analysis Highlight Add track Layout Size options Help
Zoom
16.4
Whole
Show buried clones
Yes No
Search
CB Unit Range
0 to 97
Contig stats
Clones: 97 (16 buried)
Markers: 0
Sequenced: 0
Length: 98 CB units
Ctg4421 of cp05T5e12DM4RS
V075G13
V075C06=
V069M01
V055H01
V055F17
V049M11
V037N11=
V035K08*
V009E15=
V053L06*
V041P15=
V037K18
V037A09
V029D22
V073I22
V071C03=
V061G20
V061B02
V050P24
V043H18*
V075M19
V053D19
V045E24
V019K17
B013H19
M041D13
M040E08
M035L20
M035D08
M025H18
M007H13
M007E13
M005D24
M041K06
M019C11
M050C20
M041E14
0 5 10 15 20 25 30 35 40 45 50 55 60 65 70 75 80 85 90 95
[Chickpea contig p...
[root@localhost: /...
[FPC V8.9 Main Menu]
Project cp05T5e12...
FPC Ctg4421 cp05T...

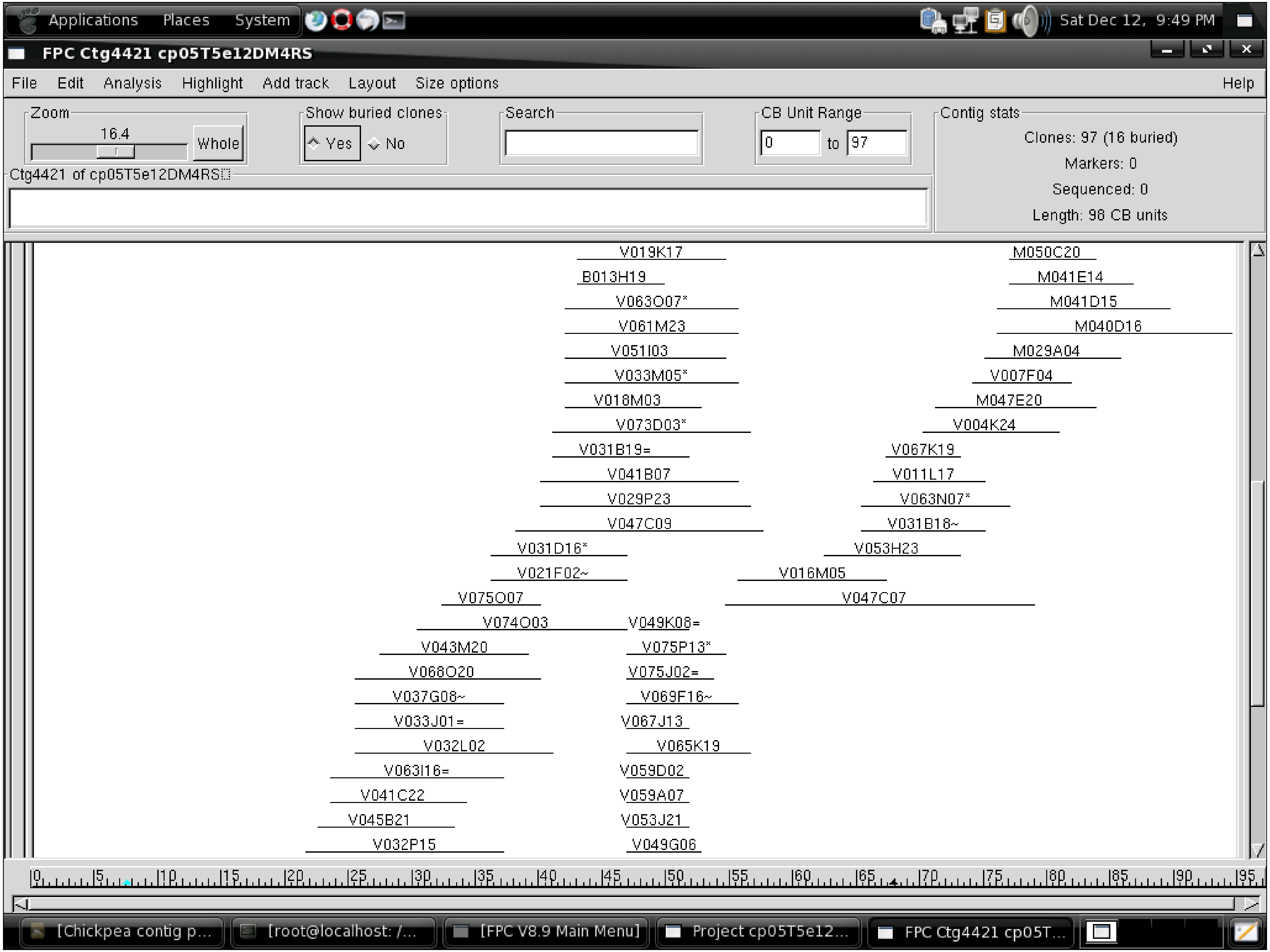
Applications Places System
Sat Dec 12, 9:49 PM
FPC Ctg4421 cp05T5e12DM4RS
File Edit Analysis Highlight Add track Layout Size options Help
Zoom
16.4
Whole
Show buried clones
Yes No
Search
CB Unit Range
0 to 97
Contig stats
Clones: 97 (16 buried)
Markers: 0
Sequenced: 0
Length: 98 CB units
Ctg4421 of cp05T5e12DM4RS
V019K17
B013H19
V063O07*
V061M23
V051I03
V033M05*
V018M03
V073D03*
V031B19=
V041B07
V029P23
V047C09
V031D16*
V021F02~
V075O07
V074O03
V043M20
V068O20
V037G08~
V033J01=
V032L02
V063I16=
V041C22
V045B21
V032P15
V049K08=
V075P13*
V075J02=
V069F16~
V067J13
V065K19
V059D02
V059A07
V053J21
V049G06
M050C20
M041E14
M041D15
M040D16
M029A04
V007F04
M047E20
V004K24
V067K19
V011L17
V063N07*
V031B18~
V053H23
V016M05
V047C07
0 5 10 15 20 25 30 35 40 45 50 55 60 65 70 75 80 85 90 95
[Chickpea contig p...
[root@localhost: /...
[FPC V8.9 Main Menu]
Project cp05T5e12...
FPC Ctg4421 cp05T...

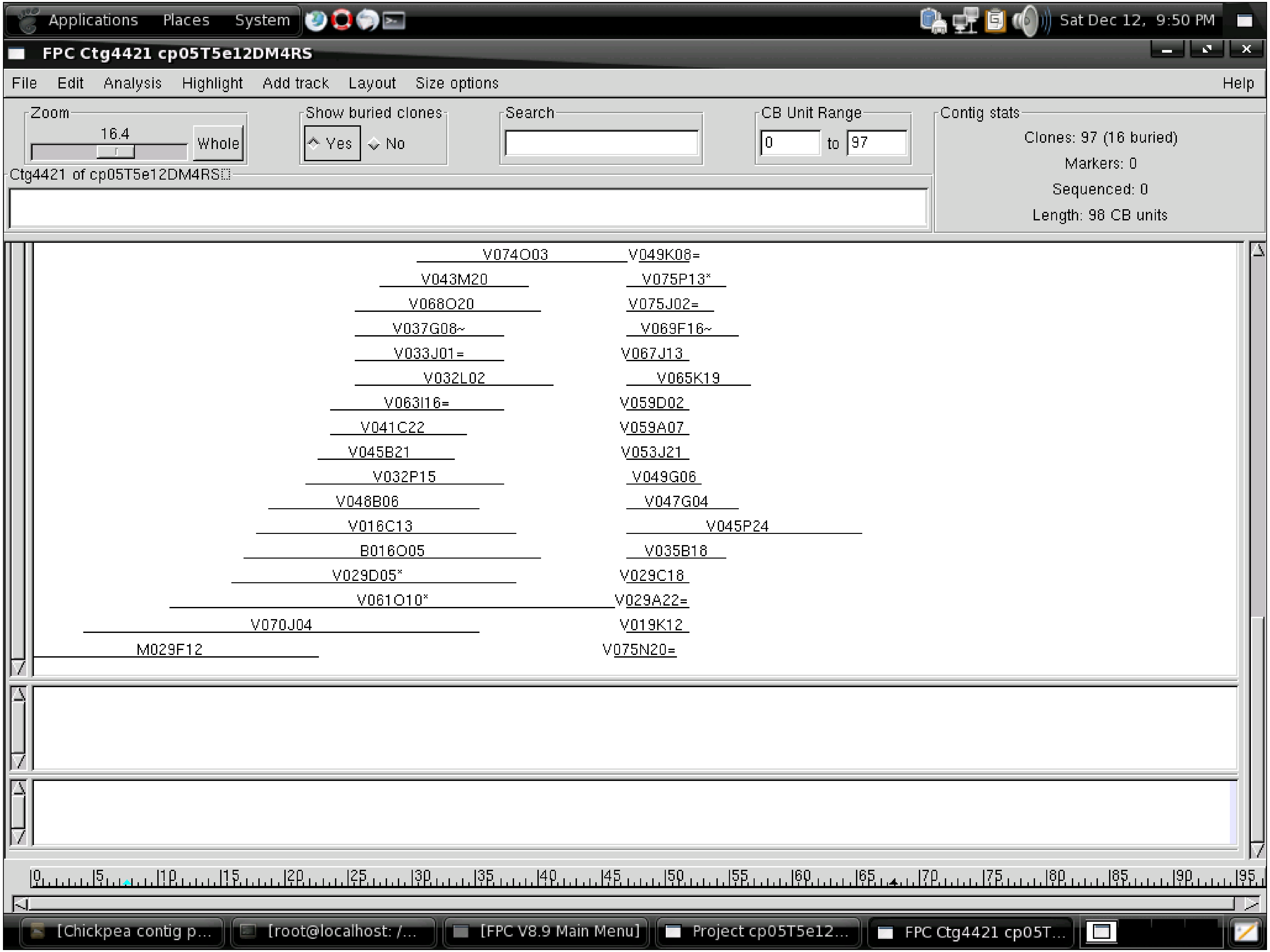
Applications Places System
Sat Dec 12, 9:50 PM
FPC Ctg4421 cp05T5e12DM4RS
File Edit Analysis Highlight Add track Layout Size options Help
Zoom
16.4
Whole
Show buried clones
Yes No
Search
CB Unit Range
0 to 97
Contig stats
Clones: 97 (16 buried)
Markers: 0
Sequenced: 0
Length: 98 CB units
Ctg4421 of cp05T5e12DM4RS
V074O03
V049K08=
V043M20
V075P13*
V068O20
V075J02=
V037G08~
V069F16~
V033J01=
V067J13
V032L02
V065K19
V063I16=
V059D02
V041C22
V059A07
V045B21
V053J21
V032P15
V049G06
V048B06
V047G04
V016C13
V045P24
B016O05
V035B18
V029D05*
V029C18
V061O10*
V029A22=
V070J04
V019K12
M029F12
V075N20=
0 5 10 15 20 25 30 35 40 45 50 55 60 65 70 75 80 85 90 95
[Chickpea contig p...
[root@localhost: /...
[FPC V8.9 Main Menu]
Project cp05T5e12...
FPC Ctg4421 cp05T...

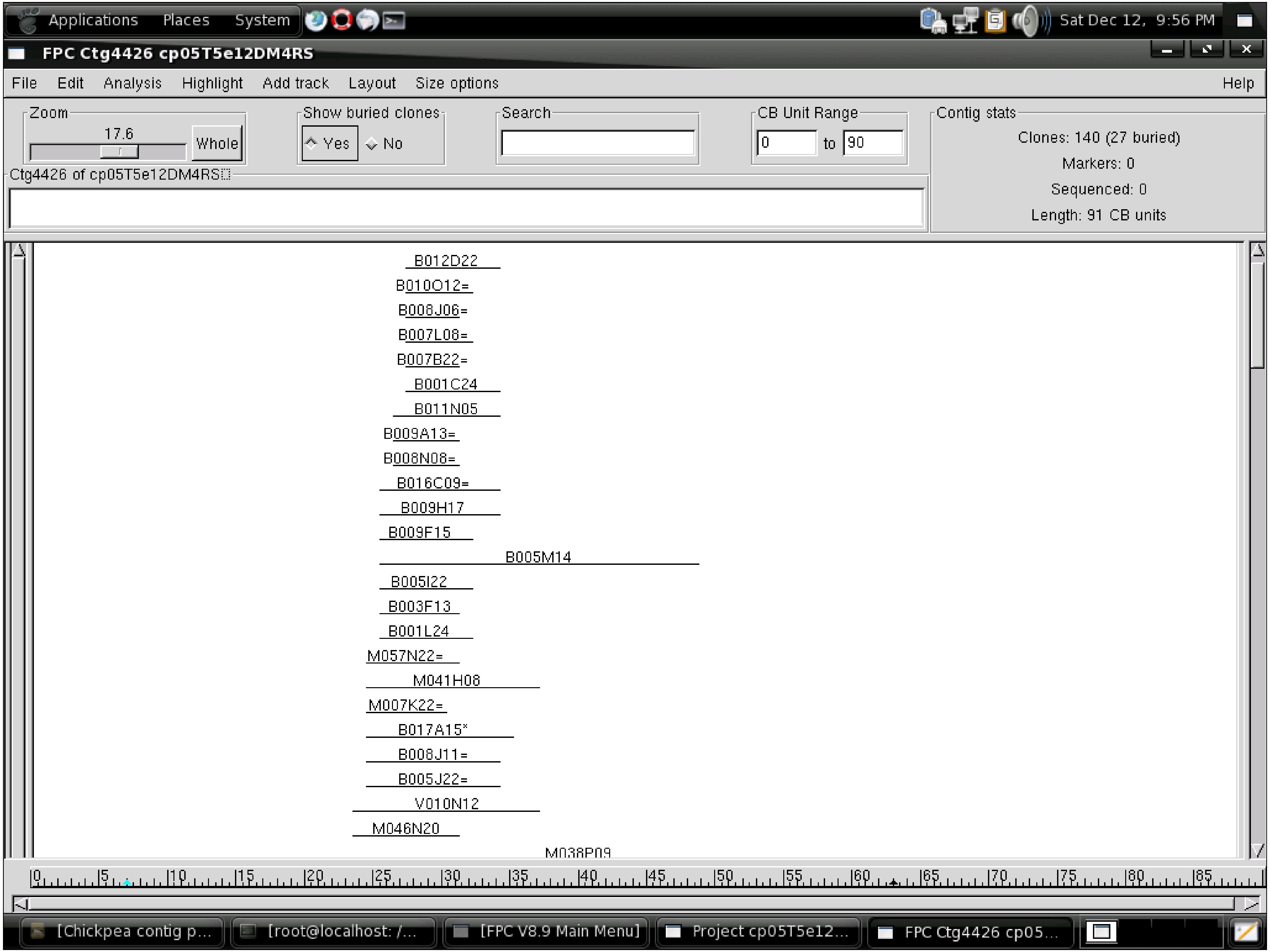
Applications
Places
System
Sat Dec 12, 9:56 PM
FPC Ctg4426 cp05T5e12DM4RS
File
Edit
Analysis
Highlight
Add track
Layout
Size options
Help
Zoom
17.6
Whole
Show buried clones
Yes
No
Search
CB Unit Range
0
to
90
Contig stats
Clones: 140 (27 buried)
Markers: 0
Sequenced: 0
Length: 91 CB units
Ctg4426 of cp05T5e12DM4RS
B012D22
B010O12=
B008J06=
B007L08=
B007B22=
B001C24
B011N05
B009A13=
B008N08=
B016C09=
B009H17
B009F15
B005M14
B005I22
B003F13
B001L24
M057N22=
M041H08
M007K22=
B017A15*
B008J11=
B005J22=
V010N12
M046N20
M038P09
0
5
10
15
20
25
30
35
40
45
50
55
60
65
70
75
80
85
[Chickpea contig p...
[root@localhost: /...
[FPC V8.9 Main Menu]
Project cp05T5e12...
FPC Ctg4426 cp05...

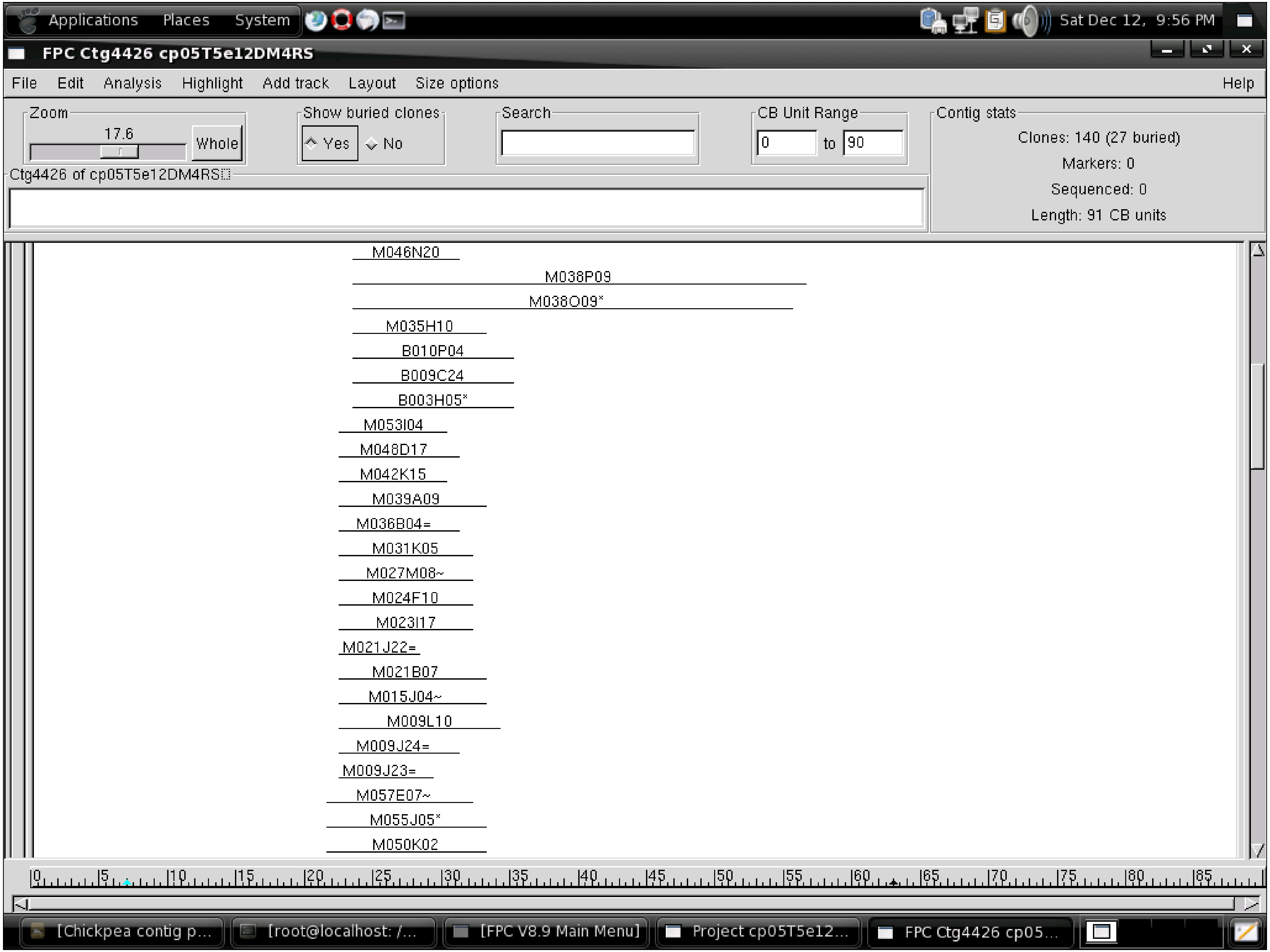
Applications Places System
Sat Dec 12, 9:56 PM
FPC Ctg4426 cp05T5e12DM4RS
File Edit Analysis Highlight Add track Layout Size options Help
Zoom
17.6
Whole
Show buried clones
Yes
No
Search
CB Unit Range
0
to
90
Contig stats
Clones: 140 (27 buried)
Markers: 0
Sequenced: 0
Length: 91 CB units
Ctg4426 of cp05T5e12DM4RS
M046N20
M038P09
M038O09*
M035H10
B010P04
B009C24
B003H05*
M053I04
M048D17
M042K15
M039A09
M036B04=
M031K05
M027M08~
M024F10
M023I17
M021J22=
M021B07
M015J04~
M009L10
M009J24=
M009J23=
M057E07~
M055J05*
M050K02
0 5 10 15 20 25 30 35 40 45 50 55 60 65 70 75 80 85
[Chickpea contig p...
[root@localhost: /...
[FPC V8.9 Main Menu]
Project cp05T5e12...
FPC Ctg4426 cp05...

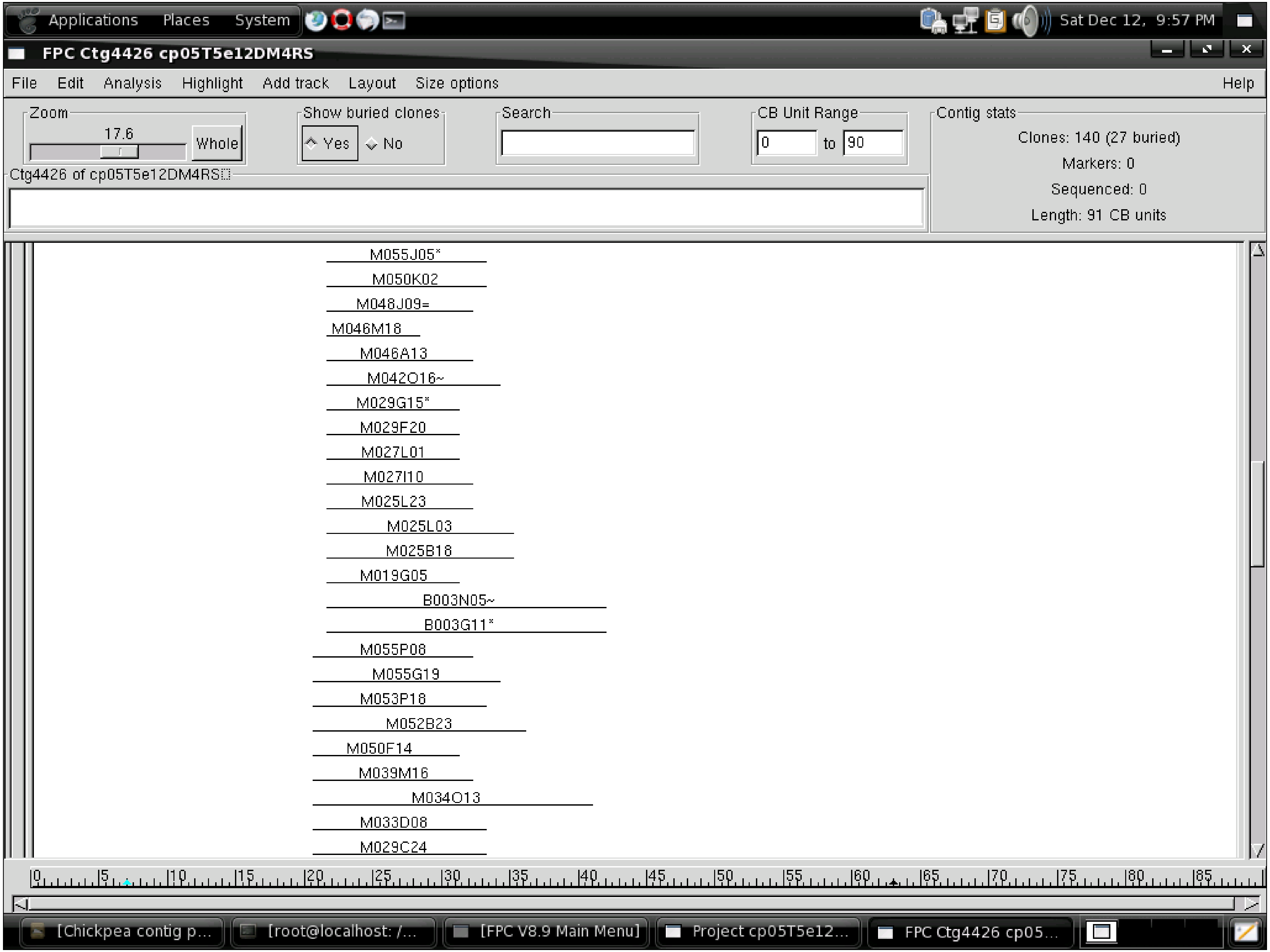
Applications
Places
System
Sat Dec 12, 9:57 PM
FPC Ctg4426 cp05T5e12DM4RS
File
Edit
Analysis
Highlight
Add track
Layout
Size options
Help
Zoom
17.6
Whole
Show buried clones
Yes
No
Search
CB Unit Range
0
to
90
Contig stats
Clones: 140 (27 buried)
Markers: 0
Sequenced: 0
Length: 91 CB units
Ctg4426 of cp05T5e12DM4RS
M055J05*
M050K02
M048J09=
M046M18
M046A13
M042O16~
M029G15*
M029F20
M027L01
M027I10
M025L23
M025L03
M025B18
M019G05
B003N05~
B003G11*
M055P08
M055G19
M053P18
M052B23
M050F14
M039M16
M034O13
M033D08
M029C24
0
5
10
15
20
25
30
35
40
45
50
55
60
65
70
75
80
85
[Chickpea contig p...
[root@localhost: /...
[FPC V8.9 Main Menu]
Project cp05T5e12...
FPC Ctg4426 cp05...

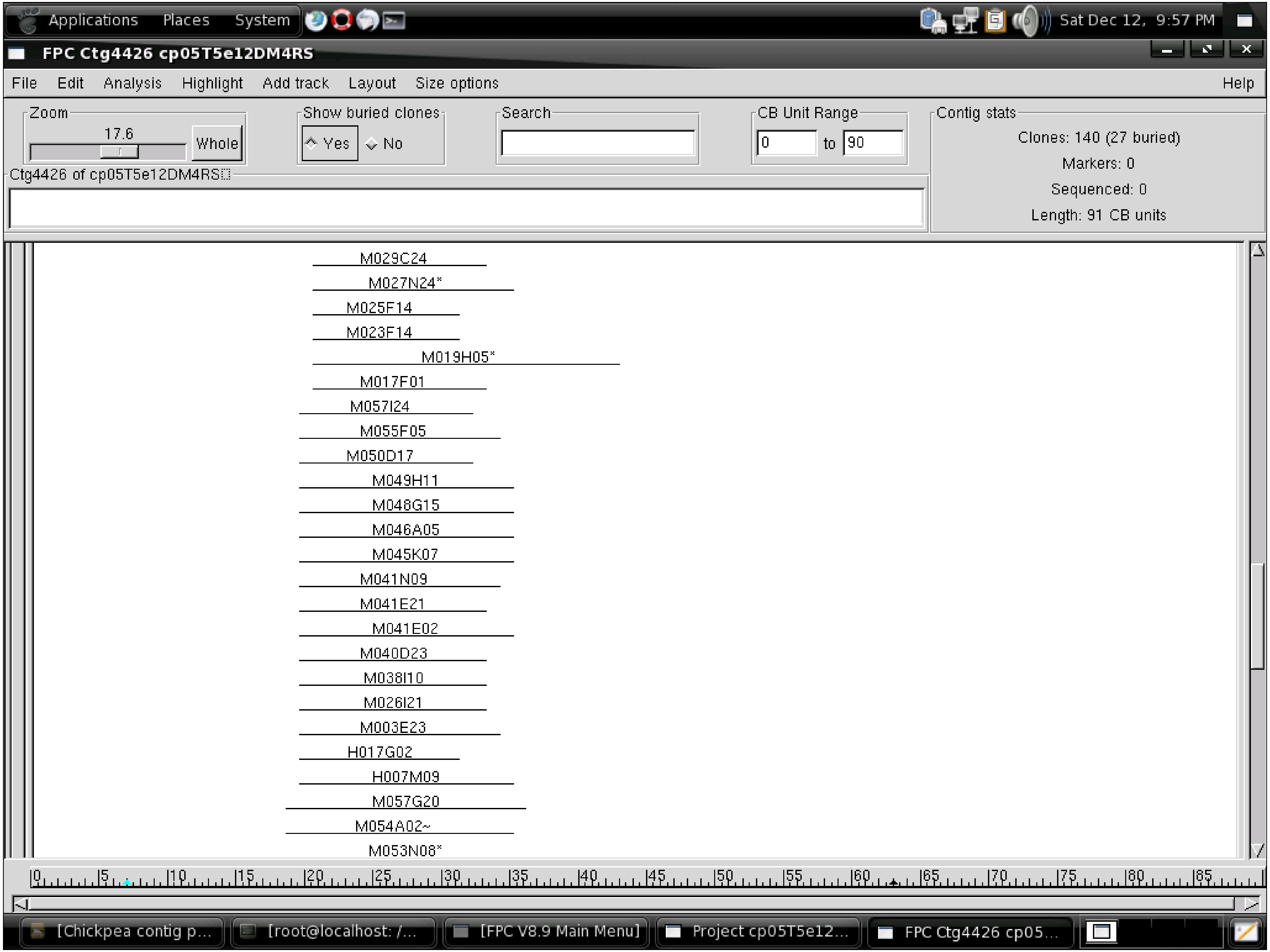
Applications Places System
Sat Dec 12, 9:57 PM
FPC Ctg4426 cp05T5e12DM4RS
File Edit Analysis Highlight Add track Layout Size options Help
Zoom
17.6
Whole
Show buried clones
Yes No
Search
CB Unit Range
0 to 90
Contig stats
Clones: 140 (27 buried)
Markers: 0
Sequenced: 0
Length: 91 CB units
Ctg4426 of cp05T5e12DM4RS
M029C24
M027N24*
M025F14
M023F14
M019H05*
M017F01
M057I24
M055F05
M050D17
M049H11
M048G15
M046A05
M045K07
M041N09
M041E21
M041E02
M040D23
M038I10
M026I21
M003E23
H017G02
H007M09
M057G20
M054A02~
M053N08*
0 5 10 15 20 25 30 35 40 45 50 55 60 65 70 75 80 85
[Chickpea contig p...
[root@localhost: /...
[FPC V8.9 Main Menu]
Project cp05T5e12...
FPC Ctg4426 cp05...

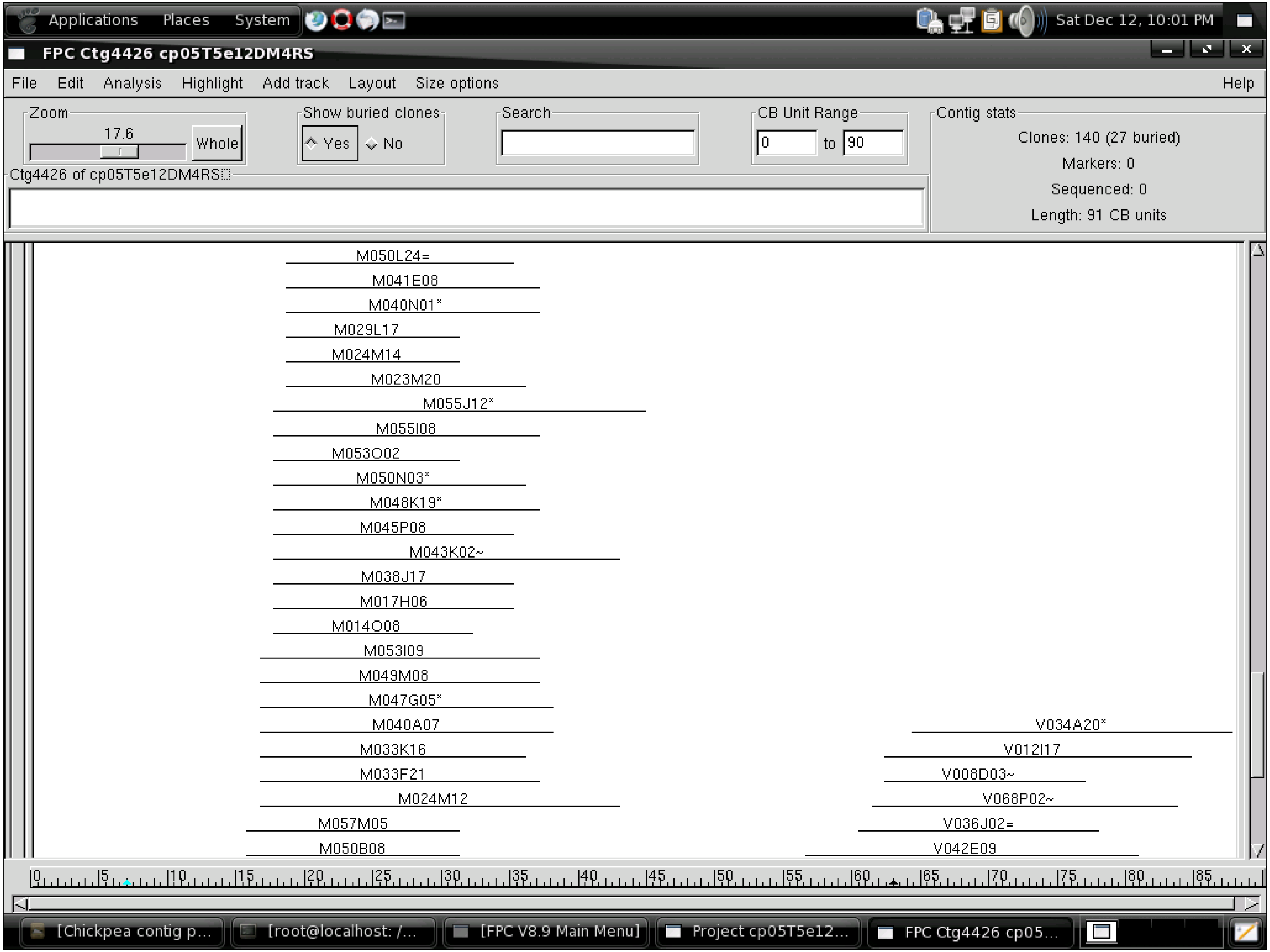
Applications Places System
Sat Dec 12, 10:01 PM
FPC Ctg4426 cp05T5e12DM4RS
File Edit Analysis Highlight Add track Layout Size options Help
Zoom
17.6
Whole
Show buried clones
Yes No
Search
CB Unit Range
0 to 90
Contig stats
Clones: 140 (27 buried)
Markers: 0
Sequenced: 0
Length: 91 CB units
Ctg4426 of cp05T5e12DM4RS
M050L24=
M041E08
M040N01*
M029L17
M024M14
M023M20
M055J12*
M055I08
M053O02
M050N03*
M048K19*
M045P08
M043K02~
M038J17
M017H06
M014O08
M053I09
M049M08
M047G05*
M040A07
M033K16
M033F21
M024M12
M057M05
M050B08
V034A20*
V012I17
V008D03~
V068P02~
V036J02=
V042E09
0 5 10 15 20 25 30 35 40 45 50 55 60 65 70 75 80 85
[Chickpea contig p...
[root@localhost: /...
[FPC V8.9 Main Menu]
Project cp05T5e12...
FPC Ctg4426 cp05...

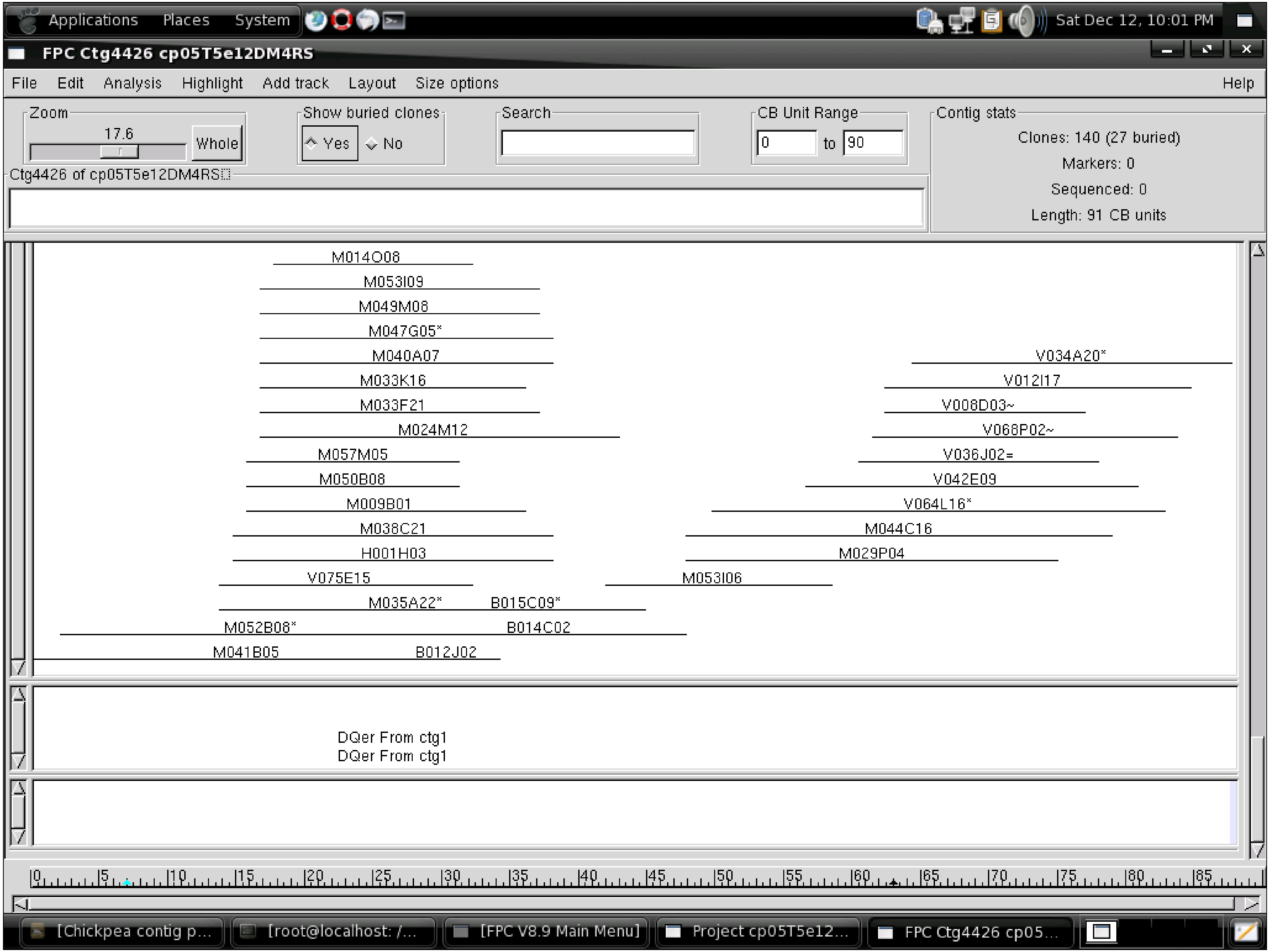
Applications Places System
Sat Dec 12, 10:01 PM
FPC Ctg4426 cp05T5e12DM4RS
File Edit Analysis Highlight Add track Layout Size options Help
Zoom
17.6
Whole
Show buried clones
Yes No
Search
CB Unit Range
0 to 90
Contig stats
Clones: 140 (27 buried)
Markers: 0
Sequenced: 0
Length: 91 CB units
Ctg4426 of cp05T5e12DM4RS
M014O08
M053I09
M049M08
M047G05*
M040A07
V034A20*
M033K16
V012I17
M033F21
V008D03~
M024M12
V068P02~
M057M05
V036J02=
M050B08
V042E09
M009B01
V064L16*
M038C21
M044C16
H001H03
M029P04
V075E15
M053I06
M035A22*
B015C09*
M052B08*
B014C02
M041B05
B012J02
DQer From ctg1
DQer From ctg1
0 5 10 15 20 25 30 35 40 45 50 55 60 65 70 75 80 85
[Chickpea contig p...
[root@localhost: /...
[FPC V8.9 Main Menu]
Project cp05T5e12...
FPC Ctg4426 cp05...

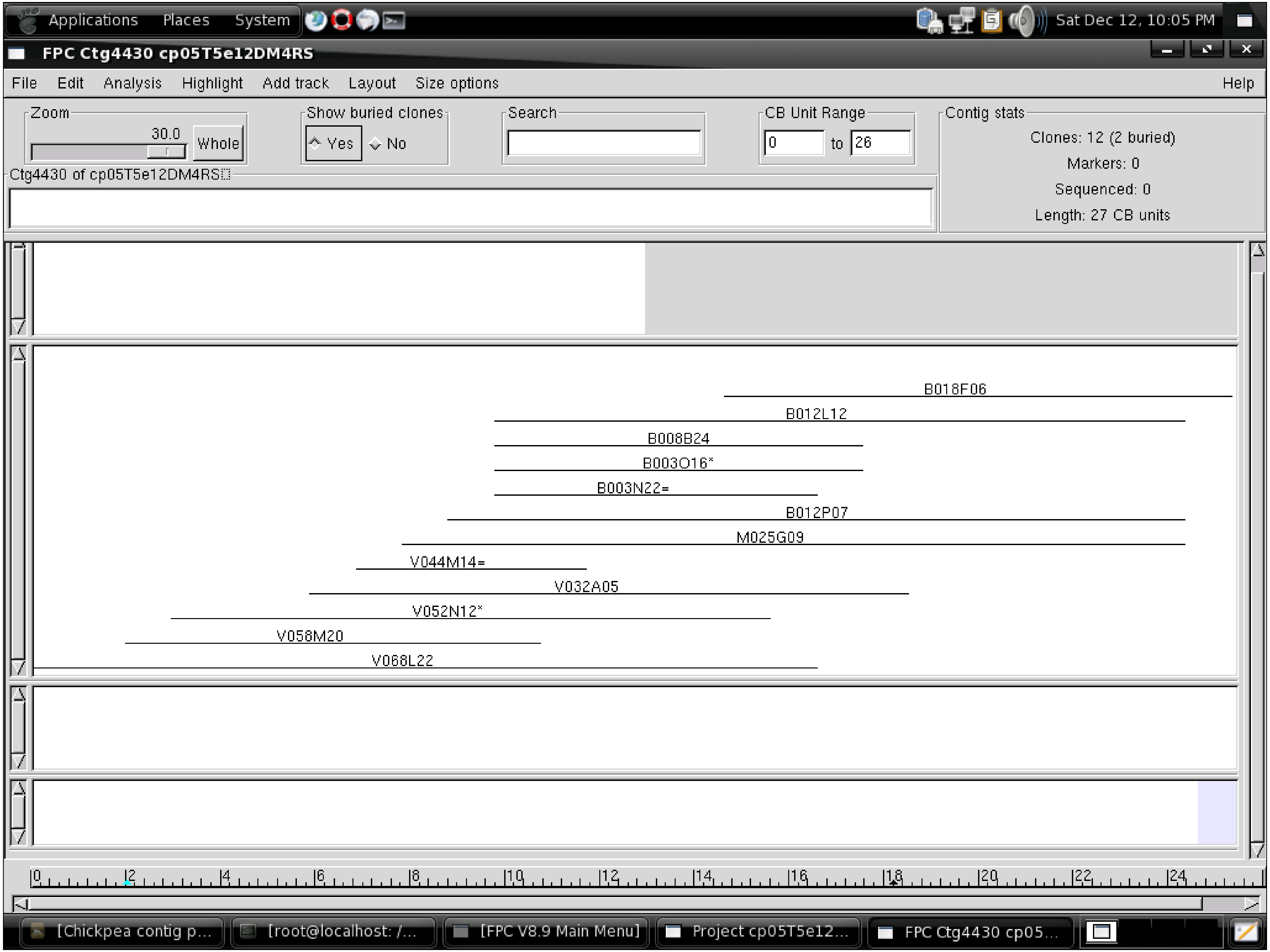
Applications Places System
Sat Dec 12, 10:05 PM
FPC Ctg4430 cp05T5e12DM4RS
File Edit Analysis Highlight Add track Layout Size options Help
Zoom
30.0
Whole
Show buried clones
Yes
No
Search
CB Unit Range
0
to
26
Contig stats
Clones: 12 (2 buried)
Markers: 0
Sequenced: 0
Length: 27 CB units
Ctg4430 of cp05T5e12DM4RS
B018F06
B012L12
B008B24
B003O16*
B003N22=
B012P07
M025G09
V044M14=
V032A05
V052N12*
V058M20
V068L22
0 2 4 6 8 10 12 14 16 18 20 22 24
[Chickpea contig p...
[root@localhost: /...
[FPC V8.9 Main Menu]
Project cp05T5e12...
FPC Ctg4430 cp05...

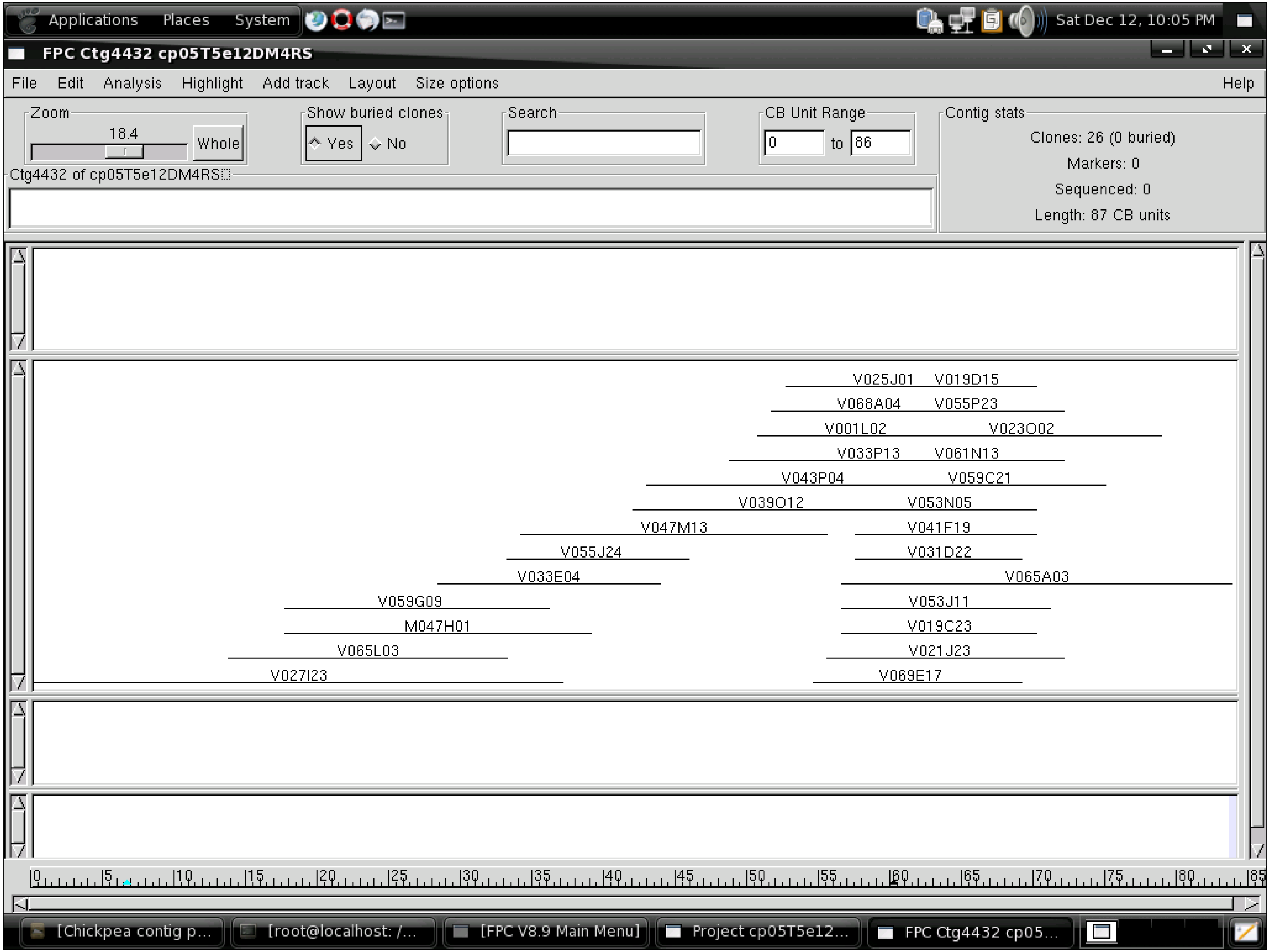
Applications
Places
System
Sat Dec 12, 10:05 PM
FPC Ctg4432 cp05T5e12DM4RS
File
Edit
Analysis
Highlight
Add track
Layout
Size options
Help
Zoom
18.4
Whole
Show buried clones
Yes
No
Search
CB Unit Range
0
to
86
Contig stats
Clones: 26 (0 buried)
Markers: 0
Sequenced: 0
Length: 87 CB units
Ctg4432 of cp05T5e12DM4RS
V025J01
V019D15
V068A04
V055P23
V001L02
V023O02
V033P13
V061N13
V043P04
V059C21
V039O12
V053N05
V047M13
V041F19
V055J24
V031D22
V033E04
V065A03
V059G09
V053J11
M047H01
V019C23
V065L03
V021J23
V027I23
V069E17
0 5 10 15 20 25 30 35 40 45 50 55 60 65 70 75 80 85
[Chickpea contig p...
[root@localhost: /...
[FPC V8.9 Main Menu]
Project cp05T5e12...
FPC Ctg4432 cp05...

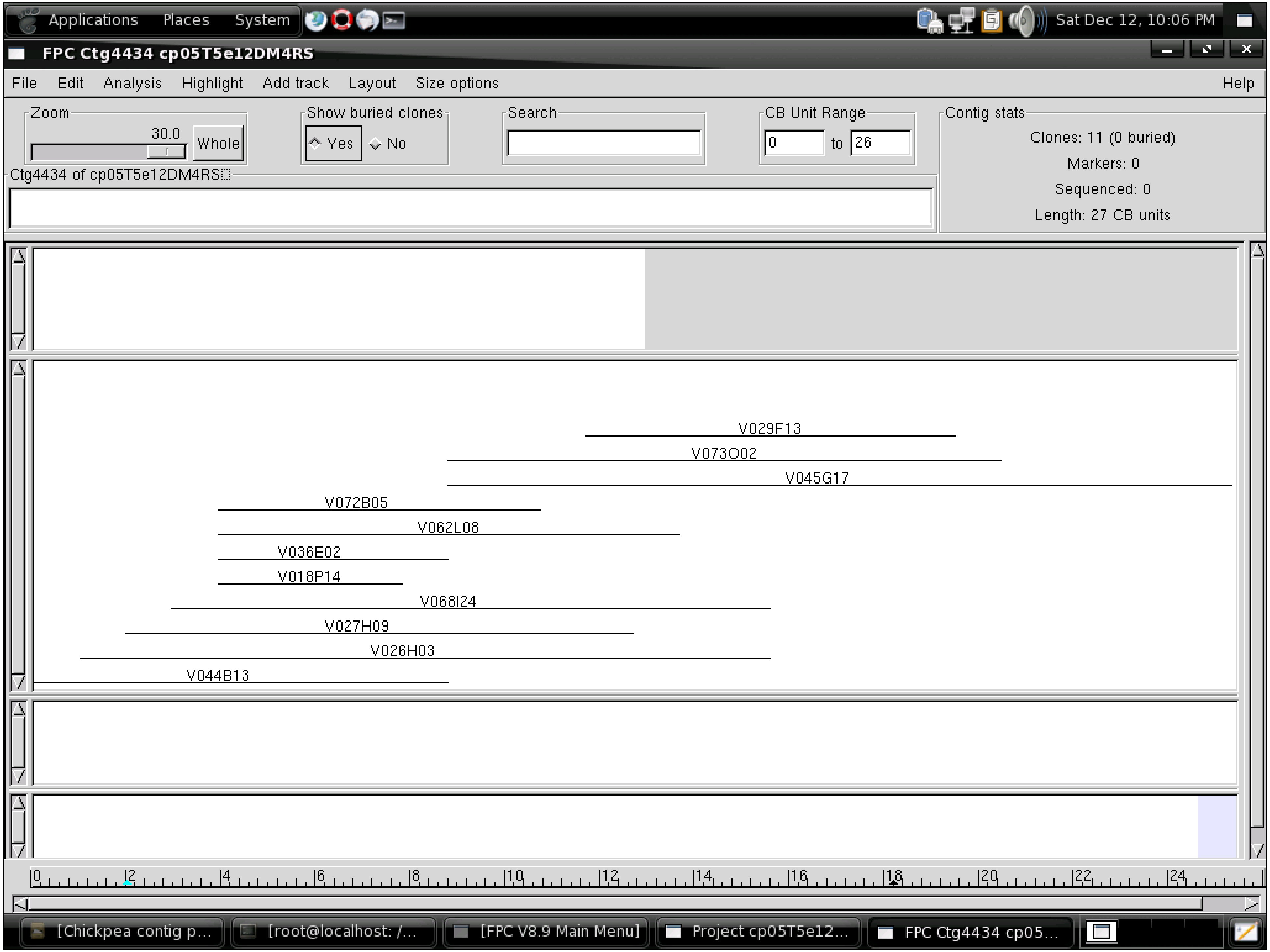
Applications Places System
Sat Dec 12, 10:06 PM
FPC Ctg4434 cp05T5e12DM4RS
File Edit Analysis Highlight Add track Layout Size options
Help
Zoom
30.0
Whole
Show buried clones
Yes
No
Search
CB Unit Range
0
to
26
Contig stats
Clones: 11 (0 buried)
Markers: 0
Sequenced: 0
Length: 27 CB units
Ctg4434 of cp05T5e12DM4RS
V029F13
V073O02
V045G17
V072B05
V062L08
V036E02
V018P14
V068I24
V027H09
V026H03
V044B13
0 2 4 6 8 10 12 14 16 18 20 22 24
[Chickpea contig p...
[root@localhost: /...
[FPC V8.9 Main Menu]
Project cp05T5e12...
FPC Ctg4434 cp05...

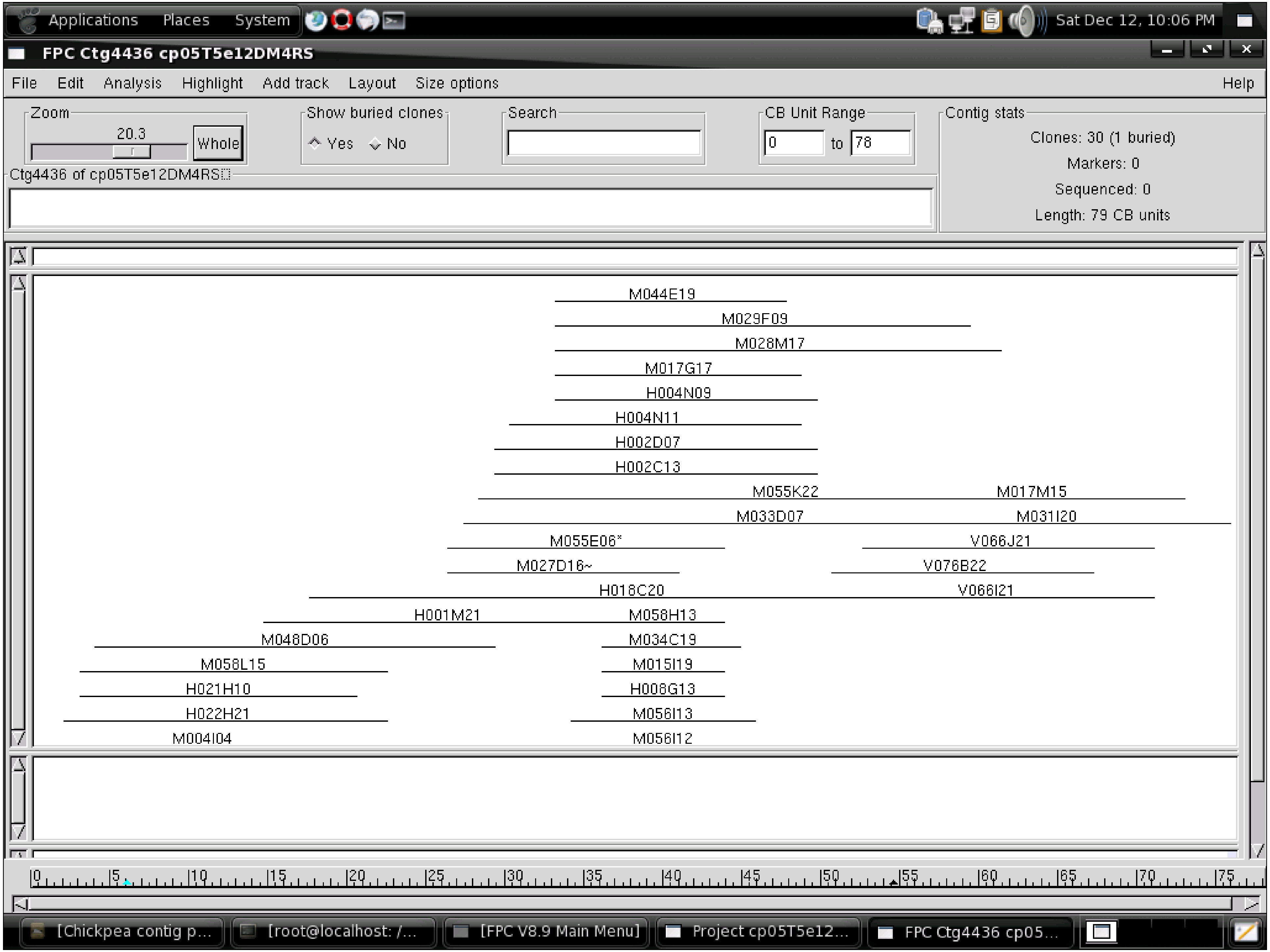
Applications Places System
Sat Dec 12, 10:06 PM
FPC Ctg4436 cp05T5e12DM4RS
File Edit Analysis Highlight Add track Layout Size options Help
Zoom
20.3
Whole
Show buried clones
Yes No
Search
CB Unit Range
0 to 78
Contig stats
Clones: 30 (1 buried)
Markers: 0
Sequenced: 0
Length: 79 CB units
Ctg4436 of cp05T5e12DM4RS
M044E19
M029F09
M028M17
M017G17
H004N09
H004N11
H002D07
H002C13
M055K22
M017M15
M033D07
M031I20
M055E06*
V066J21
M027D16~
V076B22
H018C20
V066I21
H001M21
M058H13
M048D06
M034C19
M058L15
M015I19
H021H10
H008G13
H022H21
M056I13
M004I04
M056I12
0 5 10 15 20 25 30 35 40 45 50 55 60 65 70 75
[Chickpea contig p...
[root@localhost: /...
[FPC V8.9 Main Menu]
Project cp05T5e12...
FPC Ctg4436 cp05...

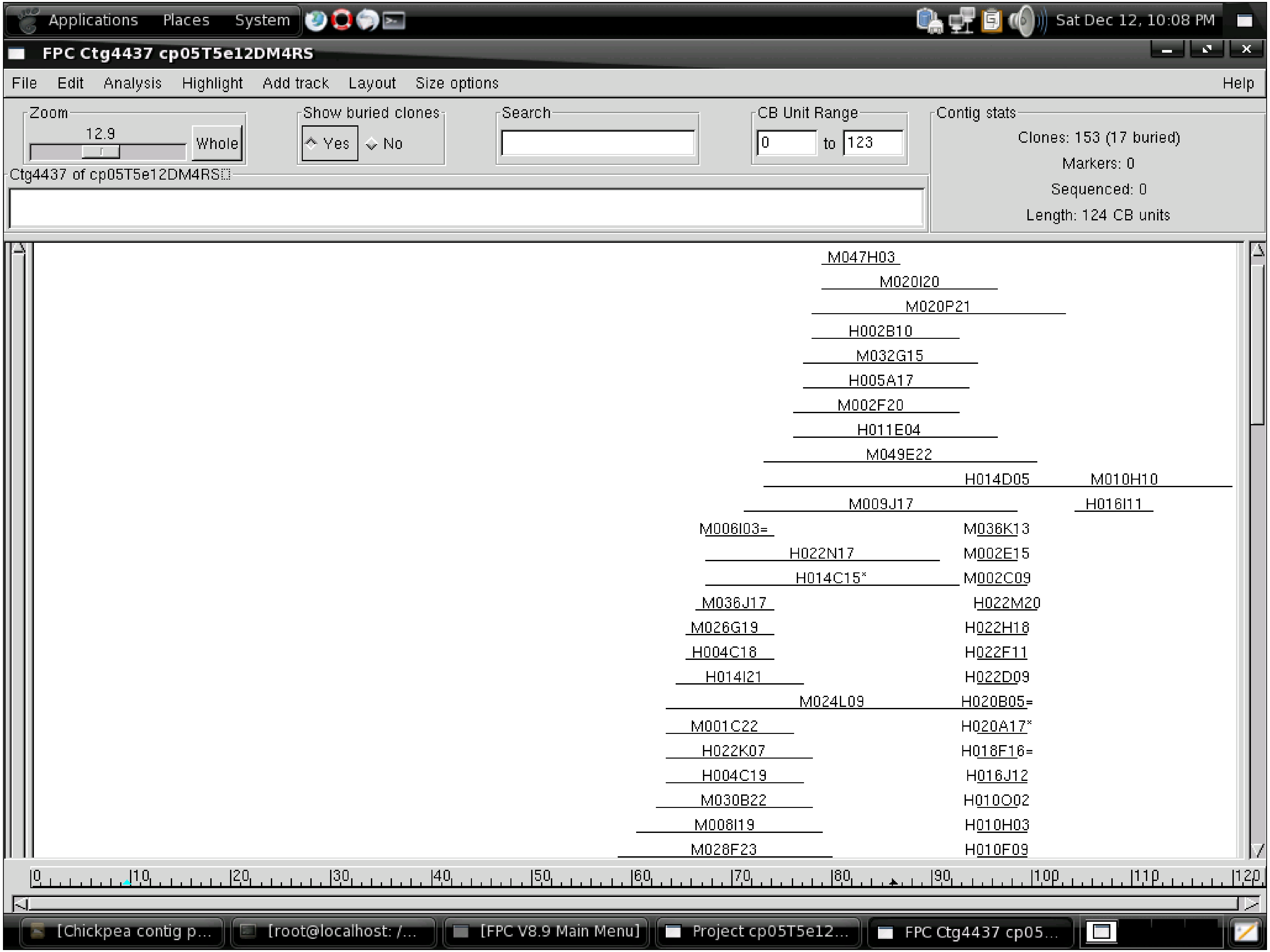
Applications Places System
Sat Dec 12, 10:08 PM
FPC Ctg4437 cp05T5e12DM4RS
File Edit Analysis Highlight Add track Layout Size options Help
Zoom
12.9
Whole
Show buried clones
Yes No
Search
CB Unit Range
0 to 123
Contig stats
Clones: 153 (17 buried)
Markers: 0
Sequenced: 0
Length: 124 CB units
Ctg4437 of cp05T5e12DM4RS
M047H03
M020I20
M020P21
H002B10
M032G15
H005A17
M002F20
H011E04
M049E22
H014D05
M010H10
M009J17
H016I11
M006I03=
M036K13
H022N17
M002E15
H014C15*
M002C09
M036J17
H022M20
M026G19
H022H18
H004C18
H022F11
H014I21
H022D09
M024L09
H020B05=
M001C22
H020A17*
H022K07
H018F16=
H004C19
H016J12
M030B22
H010O02
M008I19
H010H03
M028F23
H010F09
0 10 20 30 40 50 60 70 80 90 100 110 120
[Chickpea contig p...
[root@localhost: /...
[FPC V8.9 Main Menu]
Project cp05T5e12...
FPC Ctg4437 cp05...

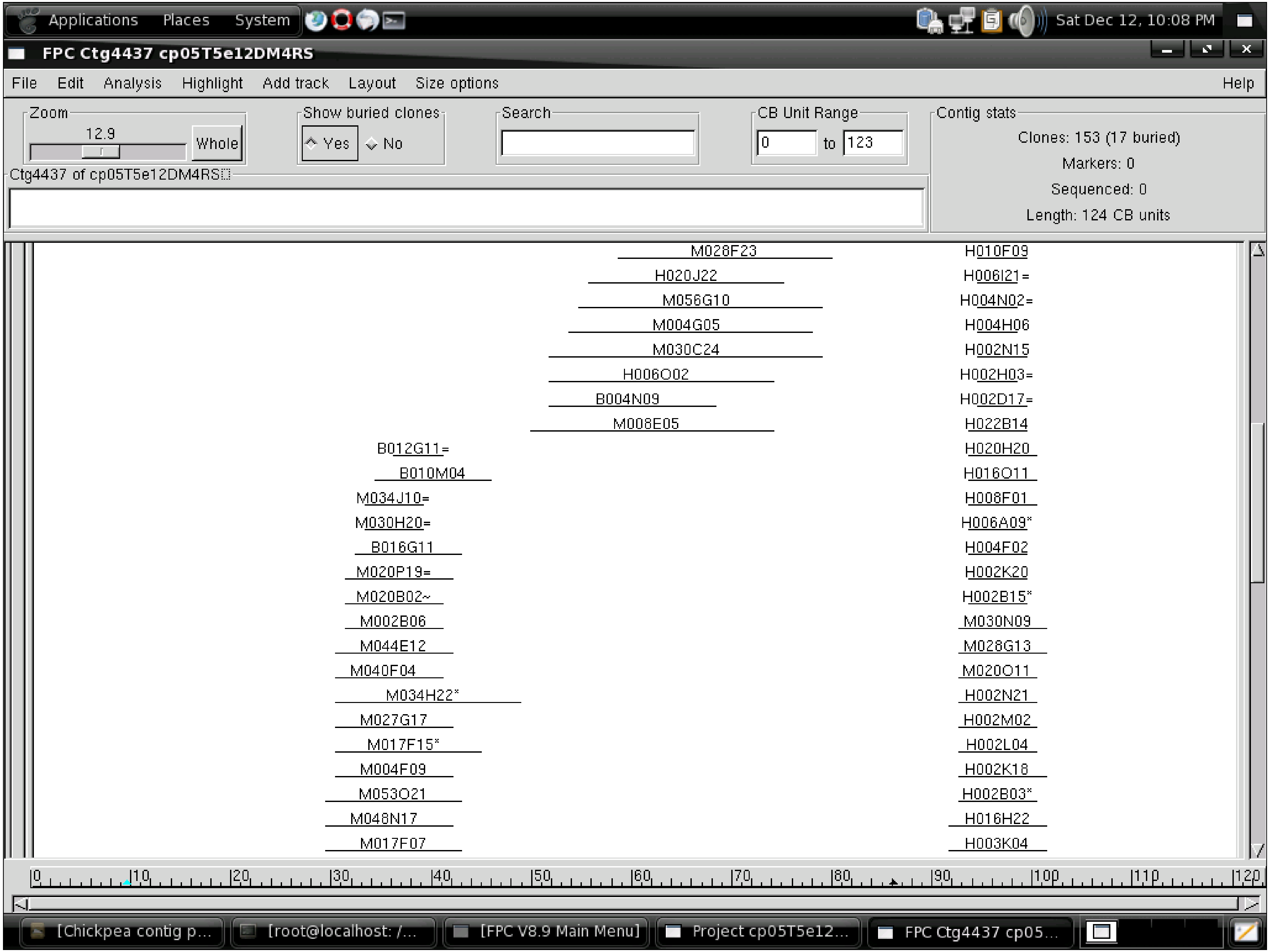
Applications Places System
Sat Dec 12, 10:08 PM
FPC Ctg4437 cp05T5e12DM4RS
File Edit Analysis Highlight Add track Layout Size options Help
Zoom 12.9 Whole
Show buried clones Yes No
Search
CB Unit Range 0 to 123
Contig stats
Clones: 153 (17 buried)
Markers: 0
Sequenced: 0
Length: 124 CB units
Ctg4437 of cp05T5e12DM4RS
M028F23
H020J22
M056G10
M004G05
M030C24
H006O02
B004N09
M008E05
B012G11=
B010M04
M034J10=
M030H20=
B016G11
M020P19=
M020B02~
M002B06
M044E12
M040F04
M034H22*
M027G17
M017F15*
M004F09
M053O21
M048N17
M017F07
H010F09
H006I21=
H004N02=
H004H06
H002N15
H002H03=
H002D17=
H022B14
H020H20
H016O11
H008F01
H006A09*
H004F02
H002K20
H002B15*
M030N09
M028G13
M020O11
H002N21
H002M02
H002L04
H002K18
H002B03*
H016H22
H003K04
0 10 20 30 40 50 60 70 80 90 100 110 120
[Chickpea contig p...
[root@localhost: /...
[FPC V8.9 Main Menu]
Project cp05T5e12...
FPC Ctg4437 cp05...

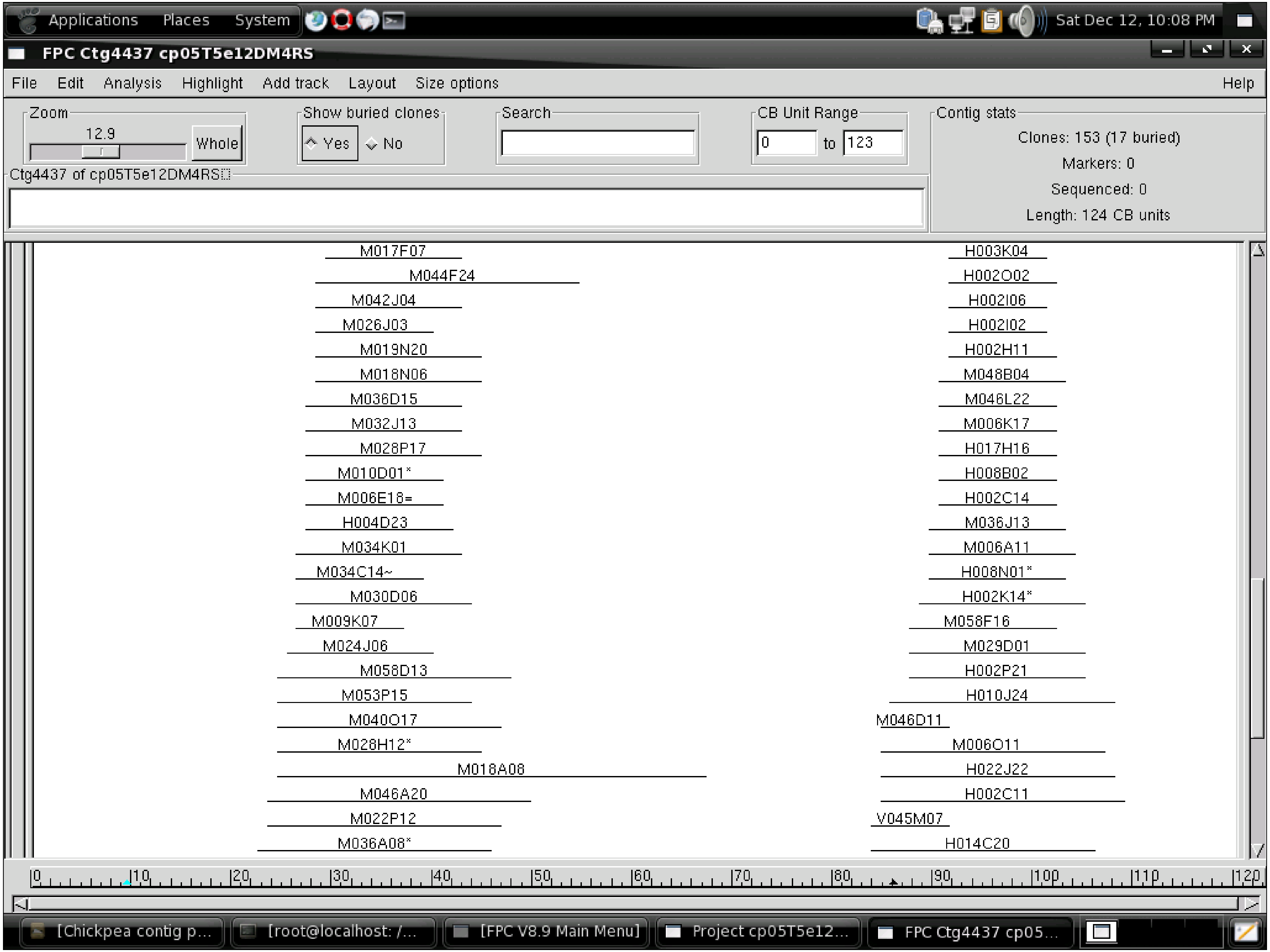
Applications Places System
Sat Dec 12, 10:08 PM
FPC Ctg4437 cp05T5e12DM4RS
File Edit Analysis Highlight Add track Layout Size options Help
Zoom
12.9
Whole
Show buried clones
Yes No
Search
CB Unit Range
0 to 123
Contig stats
Clones: 153 (17 buried)
Markers: 0
Sequenced: 0
Length: 124 CB units
Ctg4437 of cp05T5e12DM4RS
M017F07
M044F24
M042J04
M026J03
M019N20
M018N06
M036D15
M032J13
M028P17
M010D01*
M006E18=
H004D23
M034K01
M034C14~
M030D06
M009K07
M024J06
M058D13
M053P15
M040O17
M028H12*
M018A08
M046A20
M022P12
M036A08*
H003K04
H002O02
H002I06
H002I02
H002H11
M048B04
M046L22
M006K17
H017H16
H008B02
H002C14
M036J13
M006A11
H008N01*
H002K14*
M058F16
M029D01
H002P21
H010J24
M046D11
M006O11
H022J22
H002C11
V045M07
H014C20
0 10 20 30 40 50 60 70 80 90 100 110 120
[Chickpea contig p...
[root@localhost: /...
[FPC V8.9 Main Menu]
Project cp05T5e12...
FPC Ctg4437 cp05...

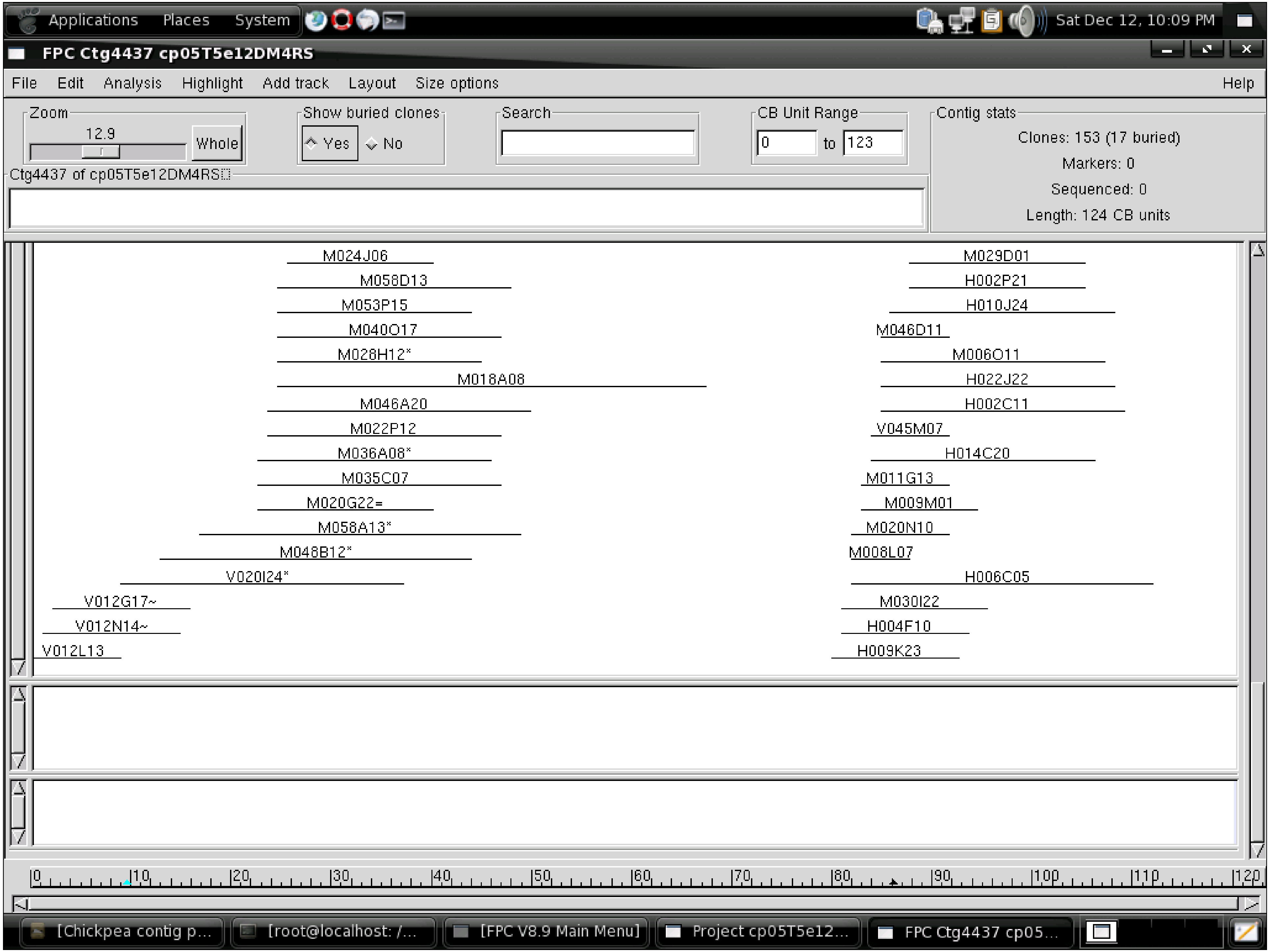
Applications Places System
Sat Dec 12, 10:09 PM
FPC Ctg4437 cp05T5e12DM4RS
File Edit Analysis Highlight Add track Layout Size options Help
Zoom
12.9
Whole
Show buried clones
Yes No
Search
CB Unit Range
0 to 123
Contig stats
Clones: 153 (17 buried)
Markers: 0
Sequenced: 0
Length: 124 CB units
Ctg4437 of cp05T5e12DM4RS
M024J06
M058D13
M053P15
M040O17
M028H12*
M018A08
M046A20
M022P12
M036A08*
M035C07
M020G22=
M058A13*
M048B12*
V020I24*
V012G17~
V012N14~
V012L13
M029D01
H002P21
H010J24
M046D11
M006O11
H022J22
H002C11
V045M07
H014C20
M011G13
M009M01
M020N10
M008L07
H006C05
M030I22
H004F10
H009K23
0 10 20 30 40 50 60 70 80 90 100 110 120
[Chickpea contig p...
[root@localhost: /...
[FPC V8.9 Main Menu]
Project cp05T5e12...
FPC Ctg4437 cp05...

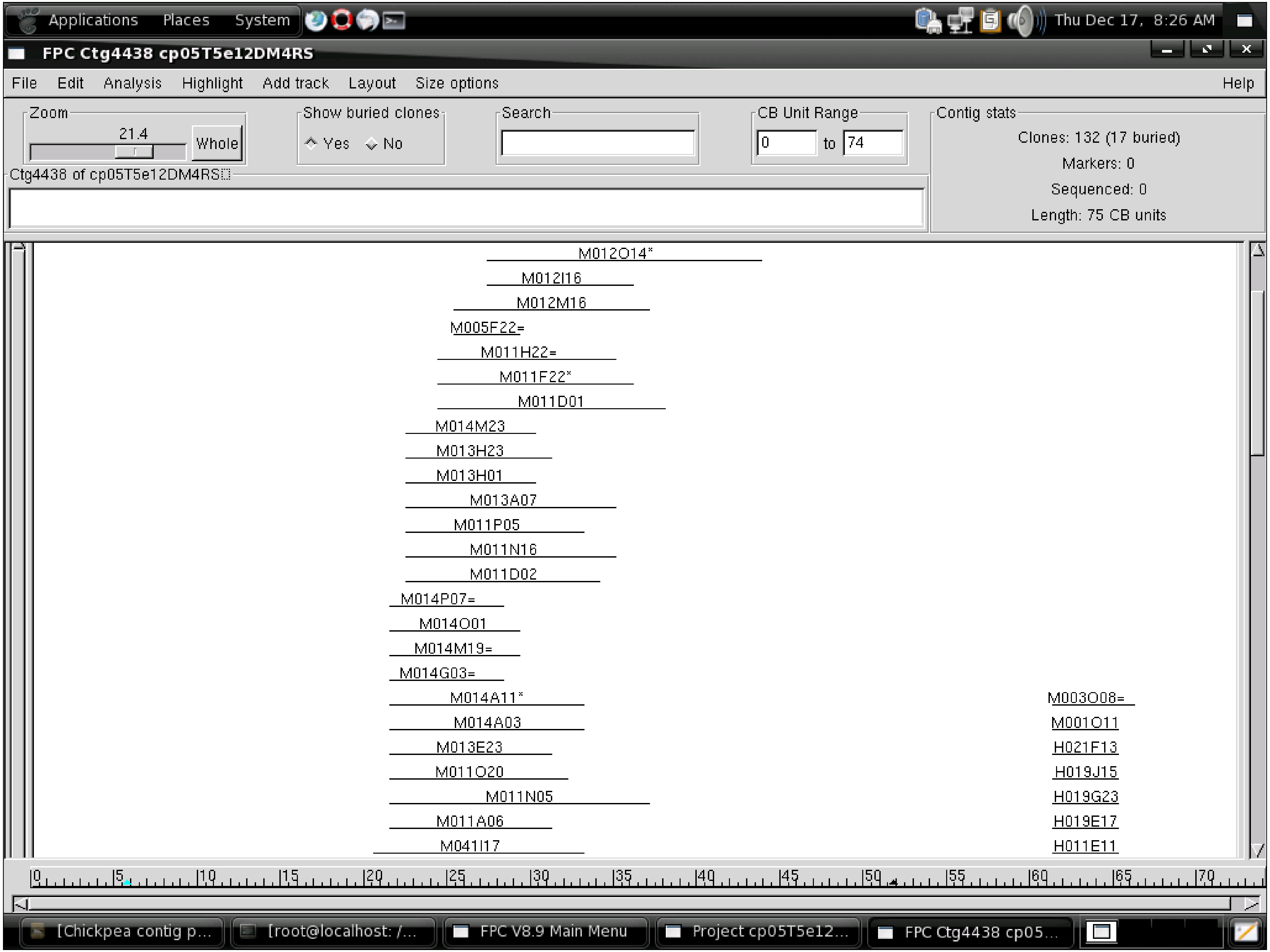
Applications Places System
Thu Dec 17, 8:26 AM
FPC Ctg4438 cp05T5e12DM4RS
File Edit Analysis Highlight Add track Layout Size options Help
Zoom
21.4
Whole
Show buried clones
Yes No
Search
CB Unit Range
0 to 74
Contig stats
Clones: 132 (17 buried)
Markers: 0
Sequenced: 0
Length: 75 CB units
Ctg4438 of cp05T5e12DM4RS
M012O14*
M012I16
M012M16
M005F22=
M011H22=
M011F22*
M011D01
M014M23
M013H23
M013H01
M013A07
M011P05
M011N16
M011D02
M014P07=
M014O01
M014M19=
M014G03=
M014A11*
M014A03
M013E23
M011O20
M011N05
M011A06
M041I17
M003O08=
M001O11
H021F13
H019J15
H019G23
H019E17
H011E11
0 5 10 15 20 25 30 35 40 45 50 55 60 65 70
[Chickpea contig p...
[root@localhost: /...
FPC V8.9 Main Menu
Project cp05T5e12...
FPC Ctg4438 cp05...

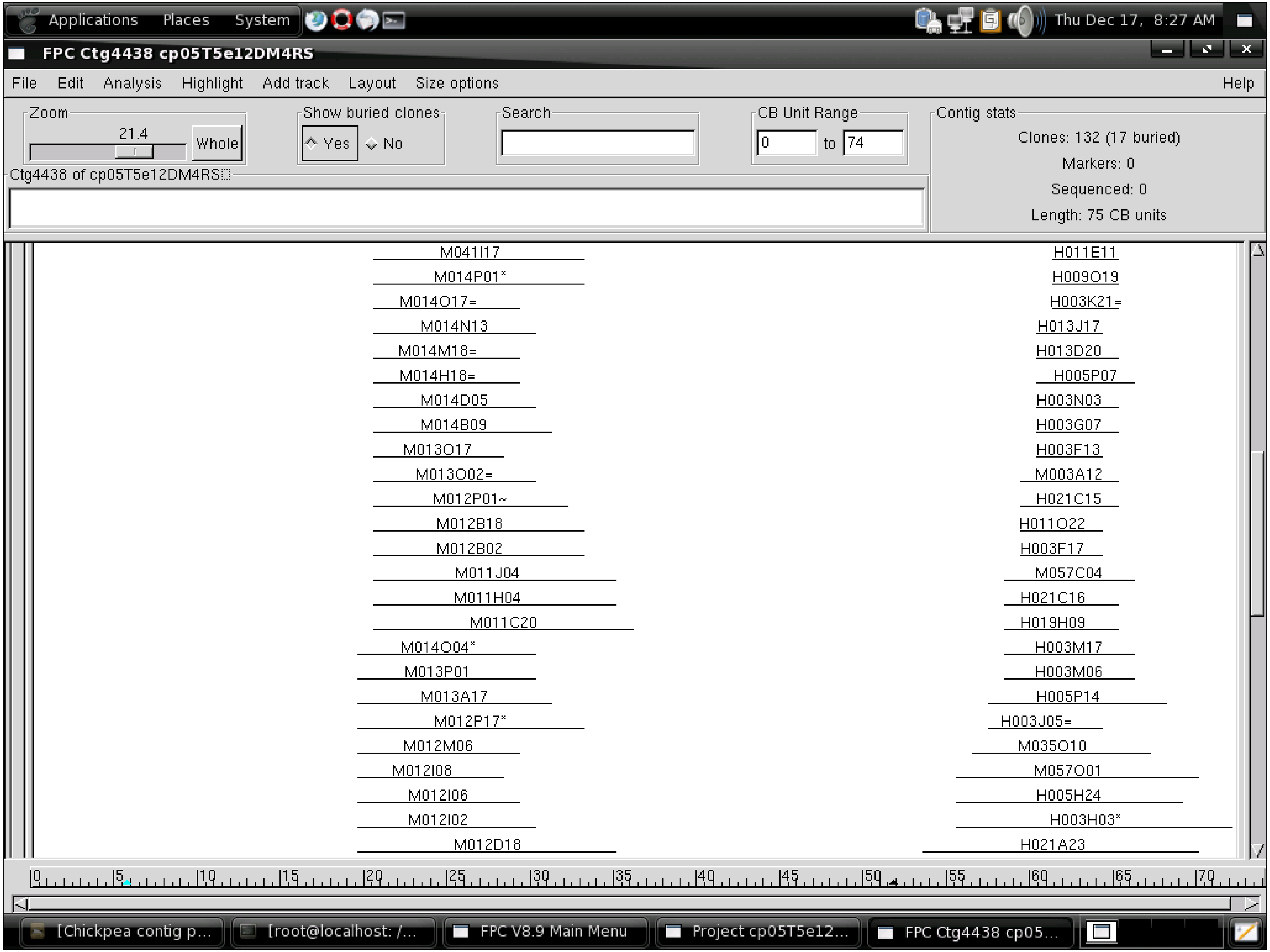
Applications Places System
Thu Dec 17, 8:27 AM
FPC Ctg4438 cp05T5e12DM4RS
File Edit Analysis Highlight Add track Layout Size options Help
Zoom
21.4
Whole
Show buried clones
Yes No
Search
CB Unit Range
0 to 74
Contig stats
Clones: 132 (17 buried)
Markers: 0
Sequenced: 0
Length: 75 CB units
Ctg4438 of cp05T5e12DM4RS
M041I17
M014P01*
M014O17=
M014N13
M014M18=
M014H18=
M014D05
M014B09
M013O17
M013O02=
M012P01~
M012B18
M012B02
M011J04
M011H04
M011C20
M014O04*
M013P01
M013A17
M012P17*
M012M06
M012I08
M012I06
M012I02
M012D18
H011E11
H009O19
H003K21=
H013J17
H013D20
H005P07
H003N03
H003G07
H003F13
M003A12
H021C15
H011O22
H003F17
M057C04
H021C16
H019H09
H003M17
H003M06
H005P14
H003J05=
M035O10
M057O01
H005H24
H003H03*
H021A23
0 5 10 15 20 25 30 35 40 45 50 55 60 65 70
[Chickpea contig p...
[root@localhost: /...
FPC V8.9 Main Menu
Project cp05T5e12...
FPC Ctg4438 cp05...

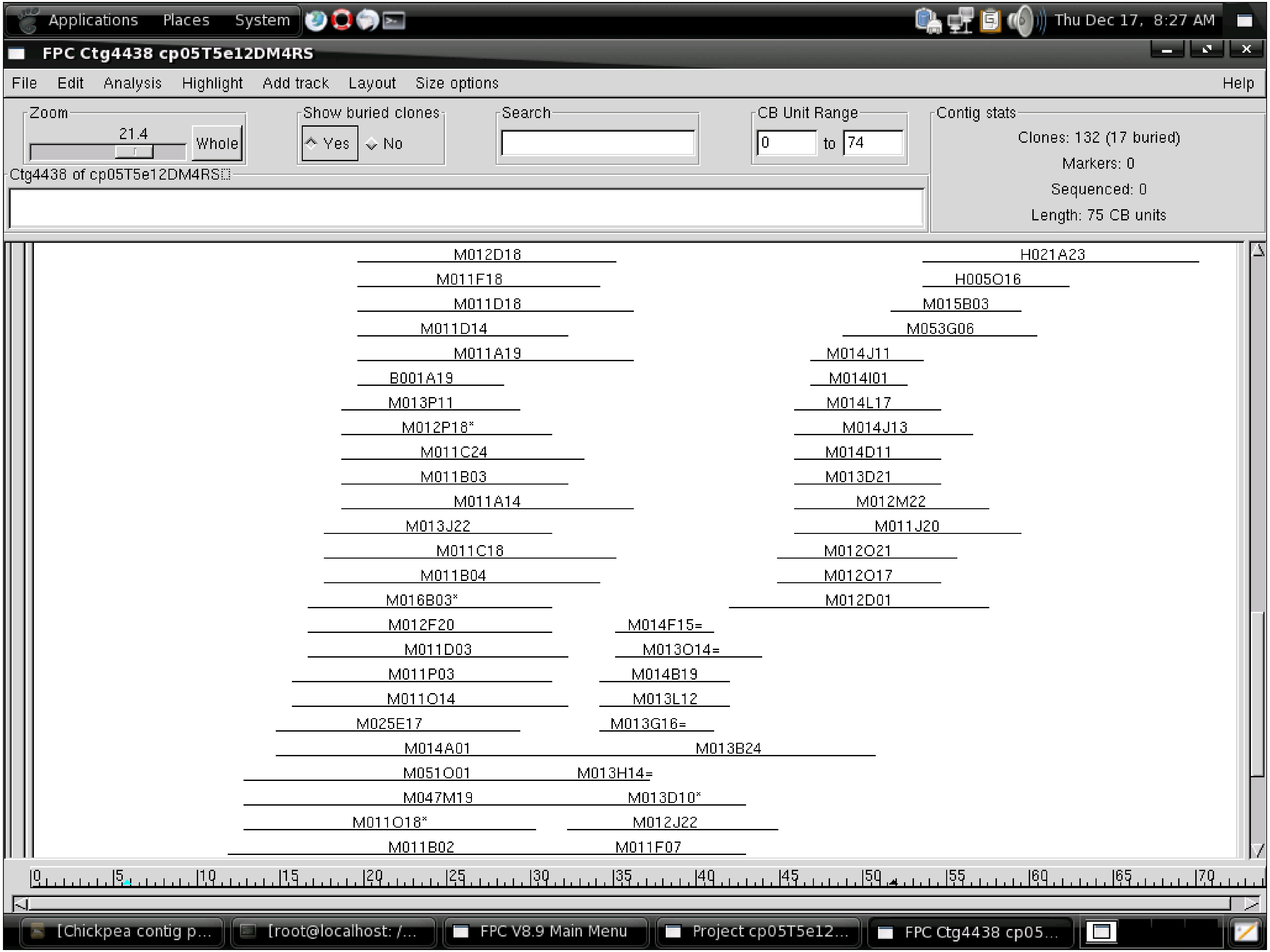
Applications Places System
Thu Dec 17, 8:27 AM
FPC Ctg4438 cp05T5e12DM4RS
File Edit Analysis Highlight Add track Layout Size options Help
Zoom
21.4
Whole
Show buried clones
Yes No
Search
CB Unit Range
0 to 74
Contig stats
Clones: 132 (17 buried)
Markers: 0
Sequenced: 0
Length: 75 CB units
Ctg4438 of cp05T5e12DM4RS
M012D18
H021A23
M011F18
H005O16
M011D18
M015B03
M011D14
M053G06
M011A19
M014J11
B001A19
M014I01
M013P11
M014L17
M012P18*
M014J13
M011C24
M014D11
M011B03
M013D21
M011A14
M012M22
M013J22
M011J20
M011C18
M012O21
M011B04
M012O17
M016B03*
M012D01
M012F20
M014F15=
M011D03
M013O14=
M011P03
M014B19
M011O14
M013L12
M025E17
M013G16=
M014A01
M013B24
M051O01
M013H14=
M047M19
M013D10*
M011O18*
M012J22
M011B02
M011F07
0 5 10 15 20 25 30 35 40 45 50 55 60 65 70
[Chickpea contig p...
[root@localhost: /...
FPC V8.9 Main Menu
Project cp05T5e12...
FPC Ctg4438 cp05...

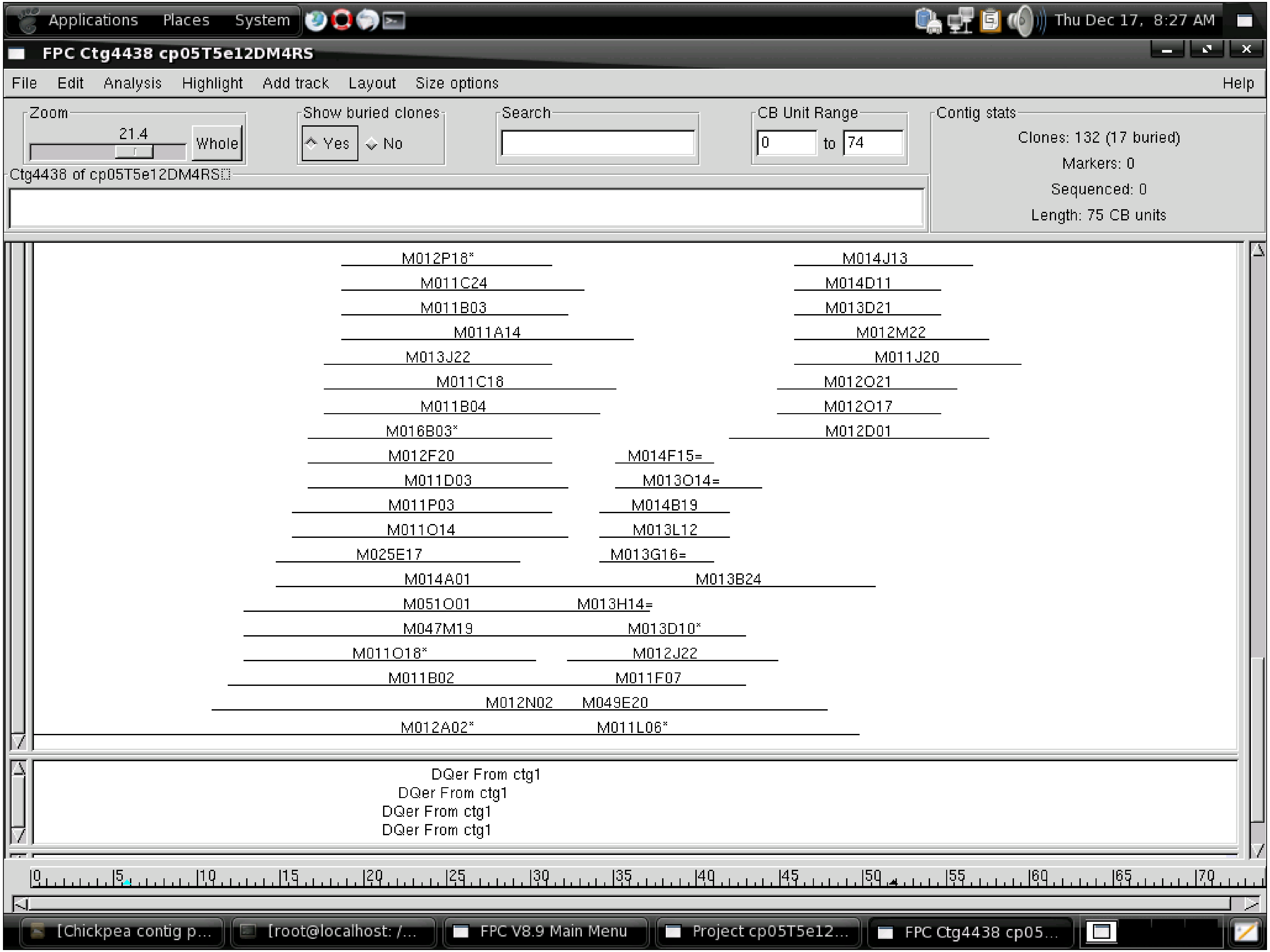
Applications Places System
Thu Dec 17, 8:27 AM
FPC Ctg4438 cp05T5e12DM4RS
File Edit Analysis Highlight Add track Layout Size options Help
Zoom
21.4
Whole
Show buried clones
Yes
No
Search
CB Unit Range
0 to 74
Contig stats
Clones: 132 (17 buried)
Markers: 0
Sequenced: 0
Length: 75 CB units
Ctg4438 of cp05T5e12DM4RS
M012P18*
M014J13
M011C24
M014D11
M011B03
M013D21
M011A14
M012M22
M013J22
M011J20
M011C18
M012O21
M011B04
M012O17
M016B03*
M012D01
M012F20
M014F15=
M011D03
M013O14=
M011P03
M014B19
M011O14
M013L12
M025E17
M013G16=
M014A01
M013B24
M051O01
M013H14=
M047M19
M013D10*
M011O18*
M012J22
M011B02
M011F07
M012N02
M049E20
M012A02*
M011L06*
DQer From ctg1
DQer From ctg1
DQer From ctg1
DQer From ctg1
0 5 10 15 20 25 30 35 40 45 50 55 60 65 70
[Chickpea contig p...
[root@localhost: /...
FPC V8.9 Main Menu
Project cp05T5e12...
FPC Ctg4438 cp05...

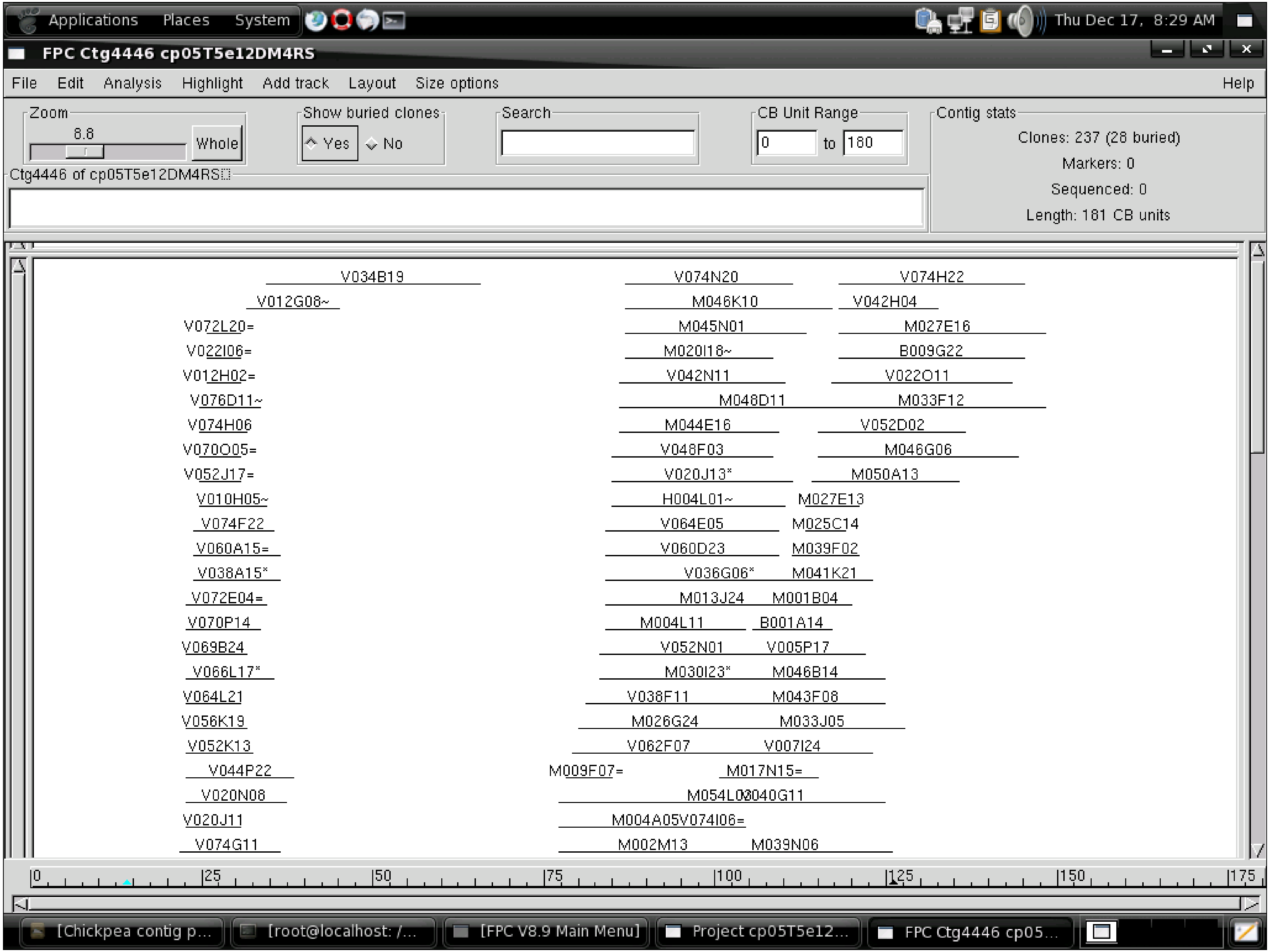
Applications Places System
Thu Dec 17, 8:29 AM
FPC Ctg4446 cp05T5e12DM4RS
File Edit Analysis Highlight Add track Layout Size options Help
Zoom
8.8
Whole
Show buried clones
Yes No
Search
CB Unit Range
0 to 180
Contig stats
Clones: 237 (28 buried)
Markers: 0
Sequenced: 0
Length: 181 CB units
Ctg4446 of cp05T5e12DM4RS
V034B19
V074N20
V074H22
V012G08~
M046K10
V042H04
V072L20=
M045N01
M027E16
V022I06=
M020I18~
B009G22
V012H02=
V042N11
V022O11
V076D11~
M048D11
M033F12
V074H06
M044E16
V052D02
V070O05=
V048F03
M046G06
V052J17=
V020J13*
M050A13
V010H05~
H004L01~
M027E13
V074F22
V064E05
M025C14
V060A15=
V060D23
M039F02
V038A15*
V036G06*
M041K21
V072E04=
M013J24
M001B04
V070P14
M004L11
B001A14
V069B24
V052N01
V005P17
V066L17*
M030I23*
M046B14
V064L21
V038F11
M043F08
V056K19
M026G24
M033J05
V052K13
V062F07
V007I24
V044P22
M009F07=
M017N15=
V020N08
M054L0040G11
V020J11
M004A05V074I06=
V074G11
M002M13
M039N06
0 25 50 75 100 125 150 175
[Chickpea contig p...
[root@localhost: /...
[FPC V8.9 Main Menu]
Project cp05T5e12...
FPC Ctg4446 cp05...

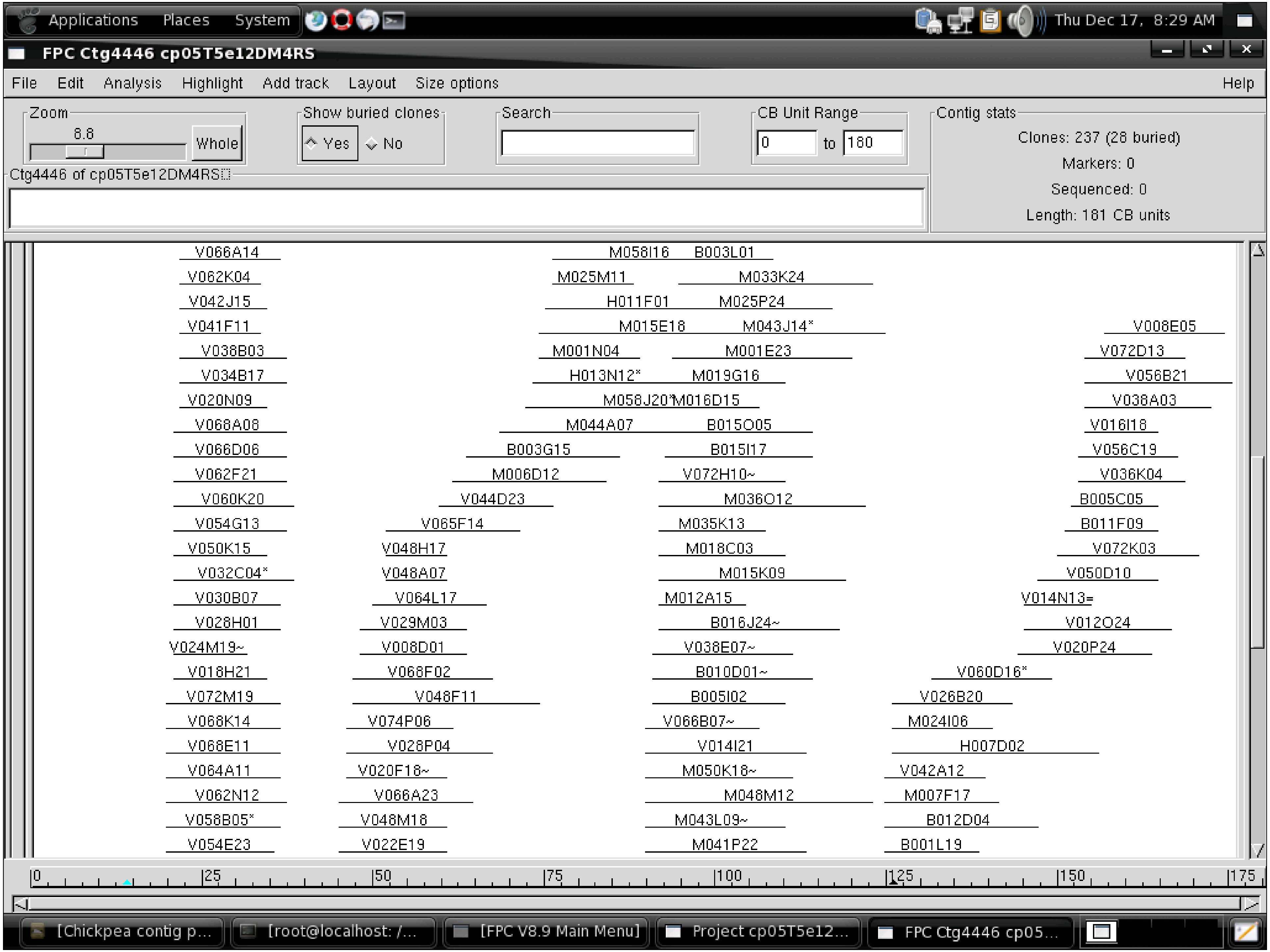
Applications Places System
Thu Dec 17, 8:29 AM
FPC Ctg4446 cp05T5e12DM4RS
File Edit Analysis Highlight Add track Layout Size options Help
Zoom
8.8
Whole
Show buried clones
Yes No
Search
CB Unit Range
0 to 180
Contig stats
Clones: 237 (28 buried)
Markers: 0
Sequenced: 0
Length: 181 CB units
Ctg4446 of cp05T5e12DM4RS
V066A14 M058I16 B003L01
V062K04 M025M11 M033K24
V042J15 H011F01 M025P24
V041F11 M015E18 M043J14* V008E05
V038B03 M001N04 M001E23 V072D13
V034B17 H013N12* M019G16 V056B21
V020N09 M058J20*M016D15 V038A03
V068A08 M044A07 B015O05 V016I18
V066D06 B003G15 B015I17 V056C19
V062F21 M006D12 V072H10~ V036K04
V060K20 V044D23 M036O12 B005C05
V054G13 V065F14 M035K13 B011F09
V050K15 V048H17 M018C03 V072K03
V032C04* V048A07 M015K09 V050D10
V030B07 V064L17 M012A15 V014N13=
V028H01 V029M03 B016J24~ V012O24
V024M19~ V008D01 V038E07~ V020P24
V018H21 V068F02 B010D01~ V060D16*
V072M19 V048F11 B005I02 V026B20
V068K14 V074P06 V066B07~ M024I06
V068E11 V028P04 V014I21 H007D02
V064A11 V020F18~ M050K18~ V042A12
V062N12 V066A23 M048M12 M007F17
V058B05* V048M18 M043L09~ B012D04
V054E23 V022E19 M041P22 B001L19
0 25 50 75 100 125 150 175
[Chickpea contig p... [root@localhost: /... [FPC V8.9 Main Menu] Project cp05T5e12... FPC Ctg4446 cp05...

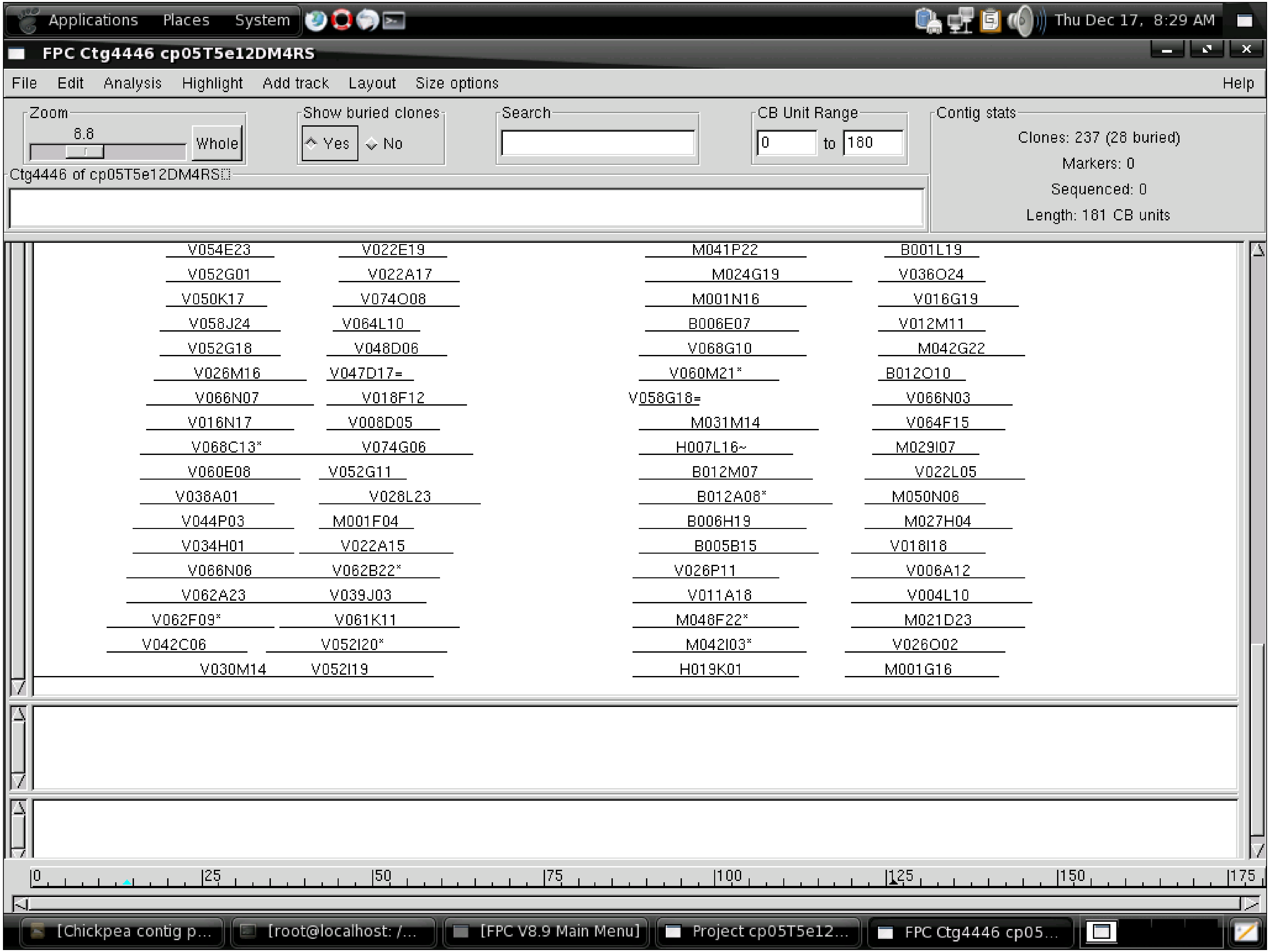

Applications Places System
Thu Dec 17, 8:29 AM
FPC Ctg4446 cp05T5e12DM4RS
File Edit Analysis Highlight Add track Layout Size options Help
Zoom
8.8
Whole
Show buried clones
Yes No
Search
CB Unit Range
0 to 180
Contig stats
Clones: 237 (28 buried)
Markers: 0
Sequenced: 0
Length: 181 CB units
Ctg4446 of cp05T5e12DM4RS
V054E23 V022E19 M041P22 B001L19
V052G01 V022A17 M024G19 V036O24
V050K17 V074O08 M001N16 V016G19
V058J24 V064L10 B006E07 V012M11
V052G18 V048D06 V068G10 M042G22
V026M16 V047D17= V060M21* B012O10
V066N07 V018F12 V058G18= V066N03
V016N17 V008D05 M031M14 V064F15
V068C13* V074G06 H007L16~ M029I07
V060E08 V052G11 B012M07 V022L05
V038A01 V028L23 B012A08* M050N06
V044P03 M001F04 B006H19 M027H04
V034H01 V022A15 B005B15 V018I18
V066N06 V062B22* V026P11 V006A12
V062A23 V039J03 V011A18 V004L10
V062F09* V061K11 M048F22* M021D23
V042C06 V052I20* M042I03* V026O02
V030M14 V052I19 H019K01 M001G16
0 25 50 75 100 125 150 175
[Chickpea contig p... [root@localhost: /... [FPC V8.9 Main Menu] Project cp05T5e12... FPC Ctg4446 cp05...

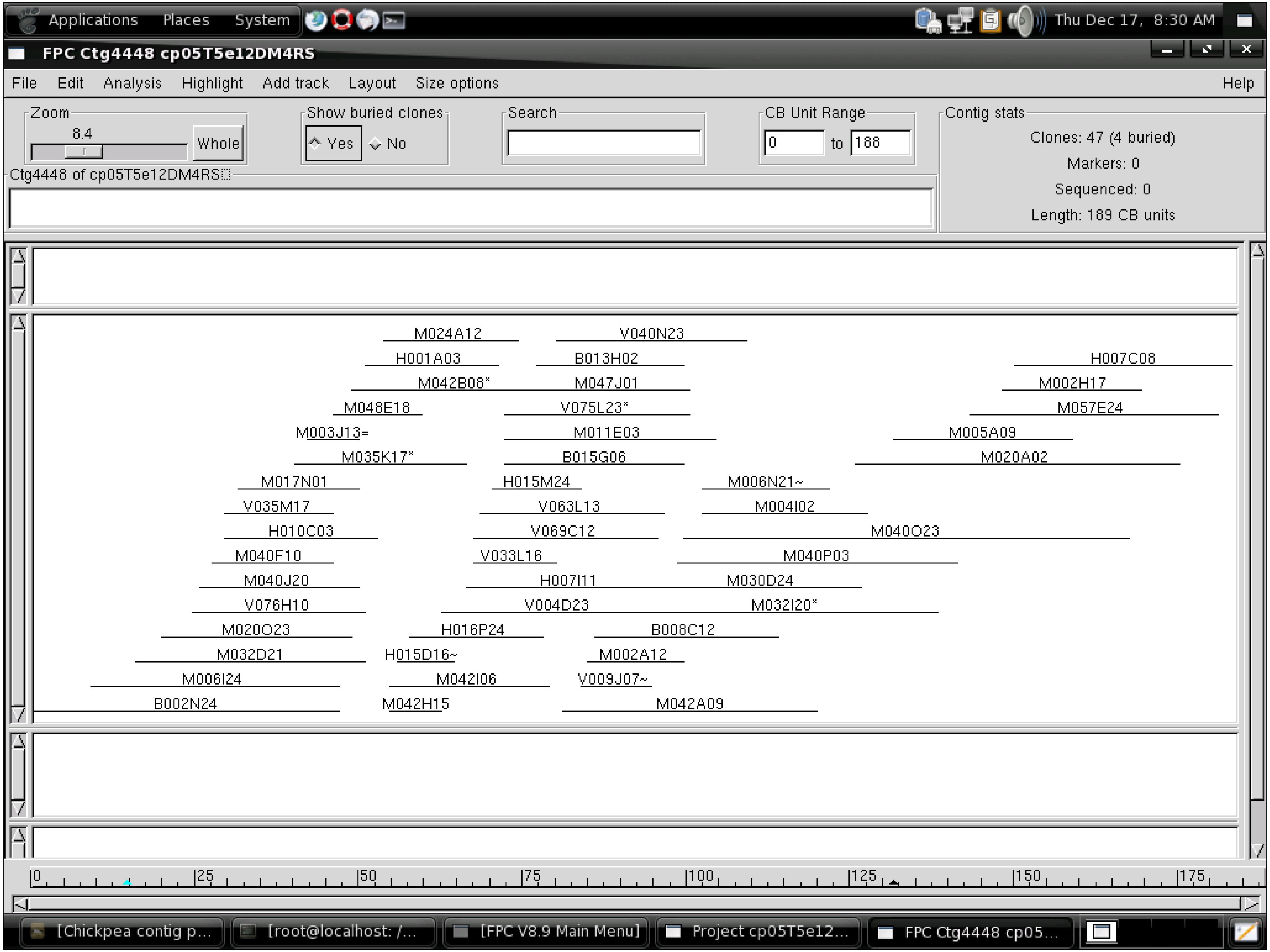
Applications Places System
Thu Dec 17, 8:30 AM
FPC Ctg4448 cp05T5e12DM4RS
File Edit Analysis Highlight Add track Layout Size options Help
Zoom
8.4
Whole
Show buried clones
Yes
No
Search
CB Unit Range
0 to 188
Contig stats
Clones: 47 (4 buried)
Markers: 0
Sequenced: 0
Length: 189 CB units
Ctg4448 of cp05T5e12DM4RS
M024A12
V040N23
H001A03
B013H02
H007C08
M042B08*
M047J01
M002H17
M048E18
V075L23*
M057E24
M003J13=
M011E03
M005A09
M035K17*
B015G06
M020A02
M017N01
H015M24
M006N21~
V035M17
V063L13
M004I02
H010C03
V069C12
M040O23
M040F10
V033L16
M040P03
M040J20
H007I11
M030D24
V076H10
V004D23
M032I20*
M020O23
H016P24
B008C12
M032D21
H015D16~
M002A12
M006I24
M042I06
V009J07~
B002N24
M042H15
M042A09
0 25 50 75 100 125 150 175
[Chickpea contig p...
[root@localhost: /...
[FPC V8.9 Main Menu]
Project cp05T5e12...
FPC Ctg4448 cp05...

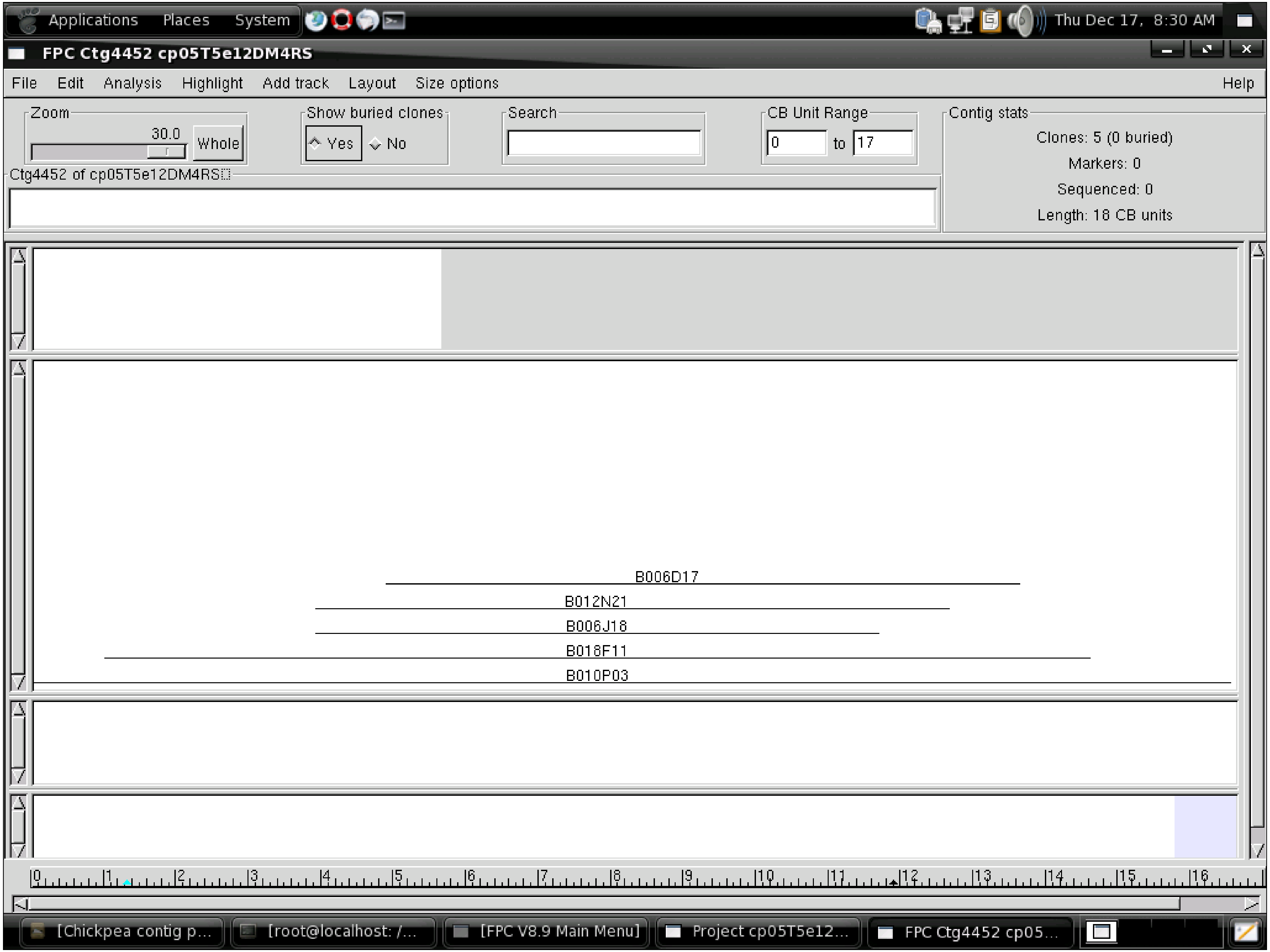
Applications Places System
Thu Dec 17, 8:30 AM
FPC Ctg4452 cp05T5e12DM4RS
File Edit Analysis Highlight Add track Layout Size options Help
Zoom
30.0
Whole
Show buried clones
Yes
No
Search
CB Unit Range
0
to
17
Contig stats
Clones: 5 (0 buried)
Markers: 0
Sequenced: 0
Length: 18 CB units
Ctg4452 of cp05T5e12DM4RS
B006D17
B012N21
B006J18
B018F11
B010P03
0 1 2 3 4 5 6 7 8 9 10 11 12 13 14 15 16
[Chickpea contig p...
[root@localhost: /...
[FPC V8.9 Main Menu]
Project cp05T5e12...
FPC Ctg4452 cp05...

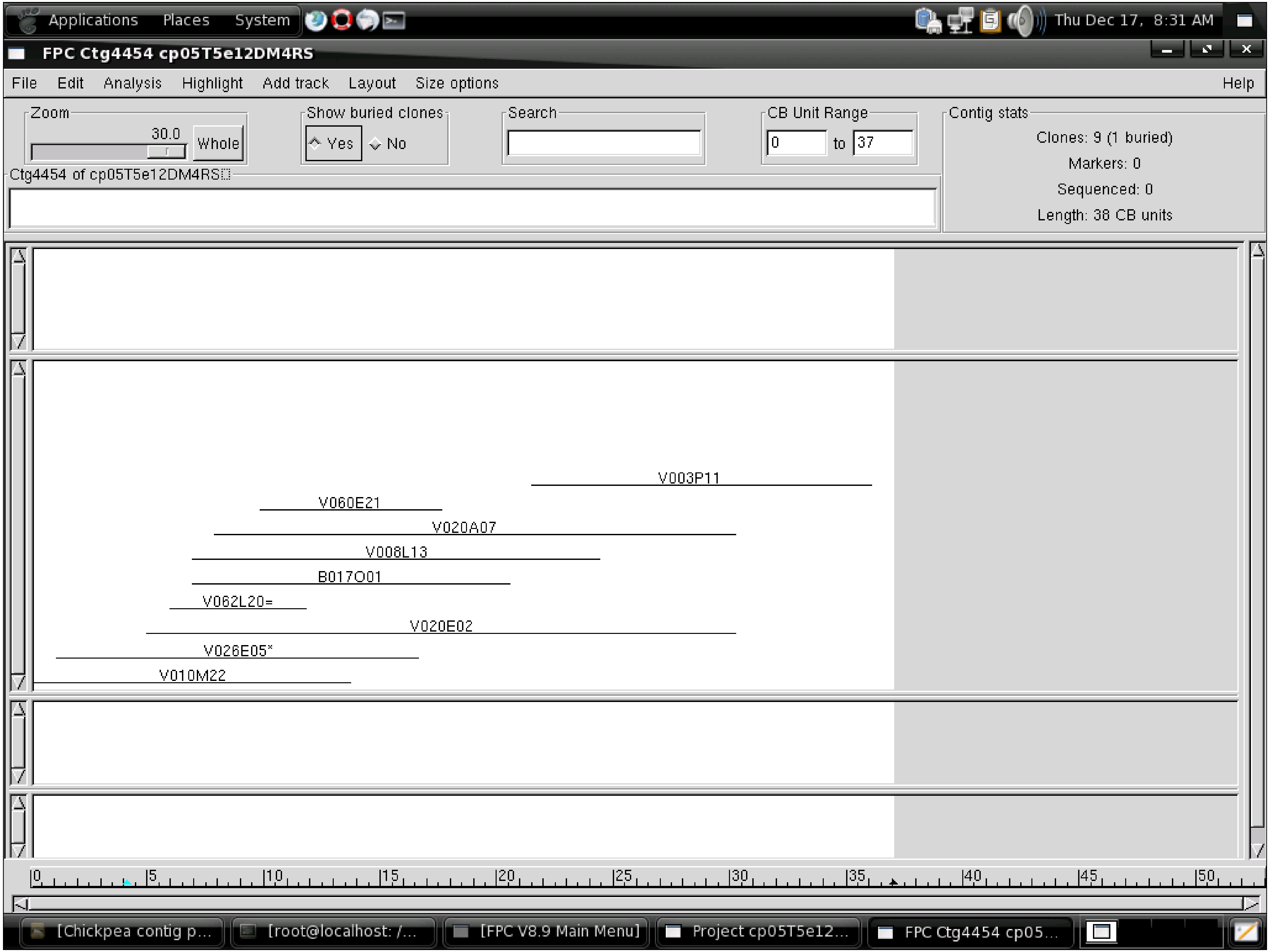

Applications Places System
Thu Dec 17, 8:31 AM
FPC Ctg4454 cp05T5e12DM4RS
File Edit Analysis Highlight Add track Layout Size options Help
Zoom
30.0
Whole
Show buried clones
Yes No
Search
CB Unit Range
0 to 37
Contig stats
Clones: 9 (1 buried)
Markers: 0
Sequenced: 0
Length: 38 CB units
Ctg4454 of cp05T5e12DM4RS
V003P11
V060E21
V020A07
V008L13
B017O01
V062L20=
V020E02
V026E05*
V010M22
0 5 10 15 20 25 30 35 40 45 50
[Chickpea contig p...
[root@localhost: /...
[FPC V8.9 Main Menu]
Project cp05T5e12...
FPC Ctg4454 cp05...

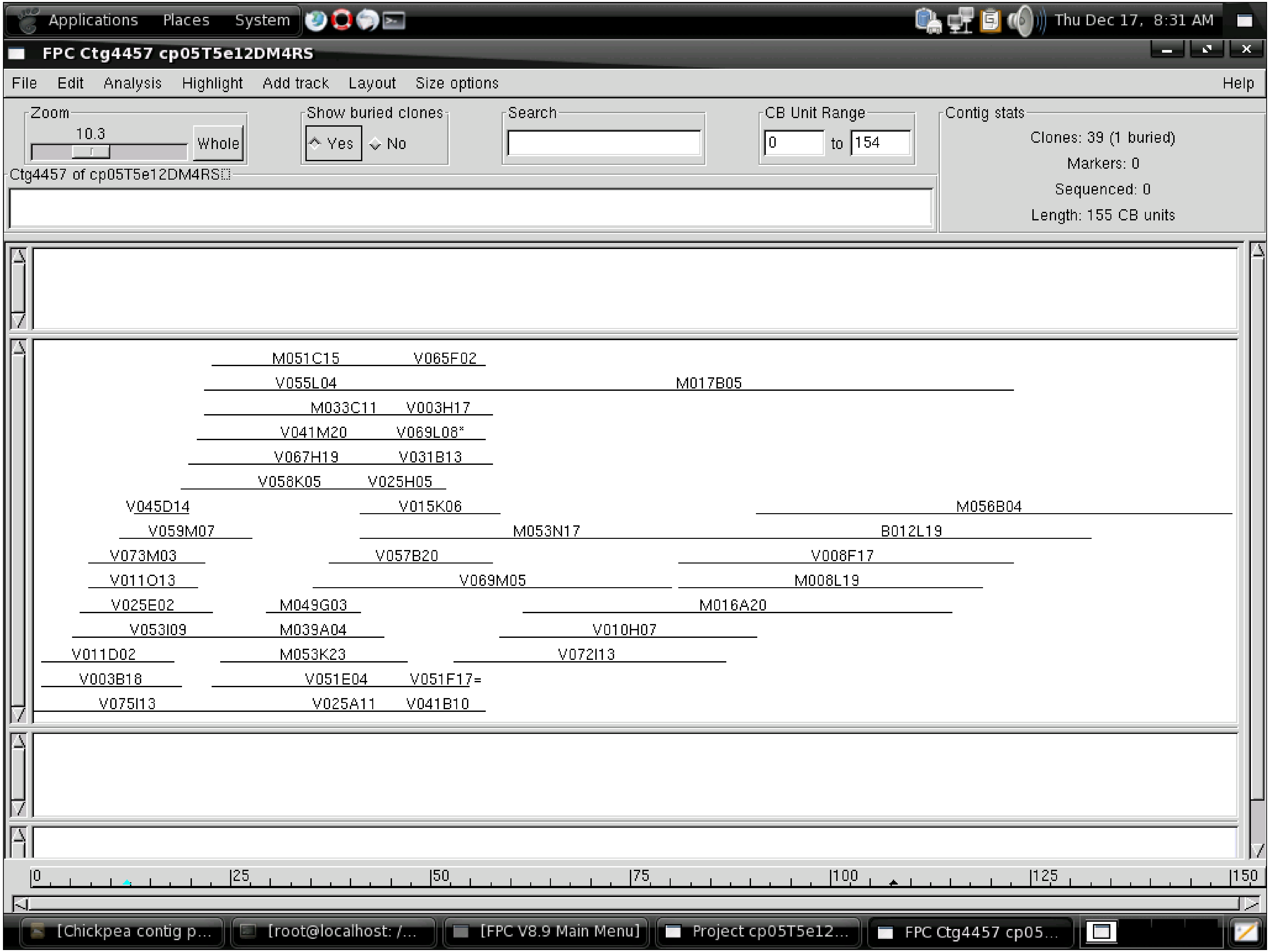
Applications Places System
Thu Dec 17, 8:31 AM
FPC Ctg4457 cp05T5e12DM4RS
File Edit Analysis Highlight Add track Layout Size options Help
Zoom
10.3
Whole
Show buried clones
Yes No
Search
CB Unit Range
0 to 154
Contig stats
Clones: 39 (1 buried)
Markers: 0
Sequenced: 0
Length: 155 CB units
Ctg4457 of cp05T5e12DM4RS
M051C15 V065F02
V055L04 M017B05
M033C11 V003H17
V041M20 V069L08*
V067H19 V031B13
V058K05 V025H05
V045D14 V015K06 M056B04
V059M07 M053N17 B012L19
V073M03 V057B20 V008F17
V011O13 V069M05 M008L19
V025E02 M049G03 M016A20
V053I09 M039A04 V010H07
V011D02 M053K23 V072I13
V003B18 V051E04 V051F17=
V075I13 V025A11 V041B10
0 25 50 75 100 125 150
[Chickpea contig p... [root@localhost: /... [FPC V8.9 Main Menu] Project cp05T5e12... FPC Ctg4457 cp05...

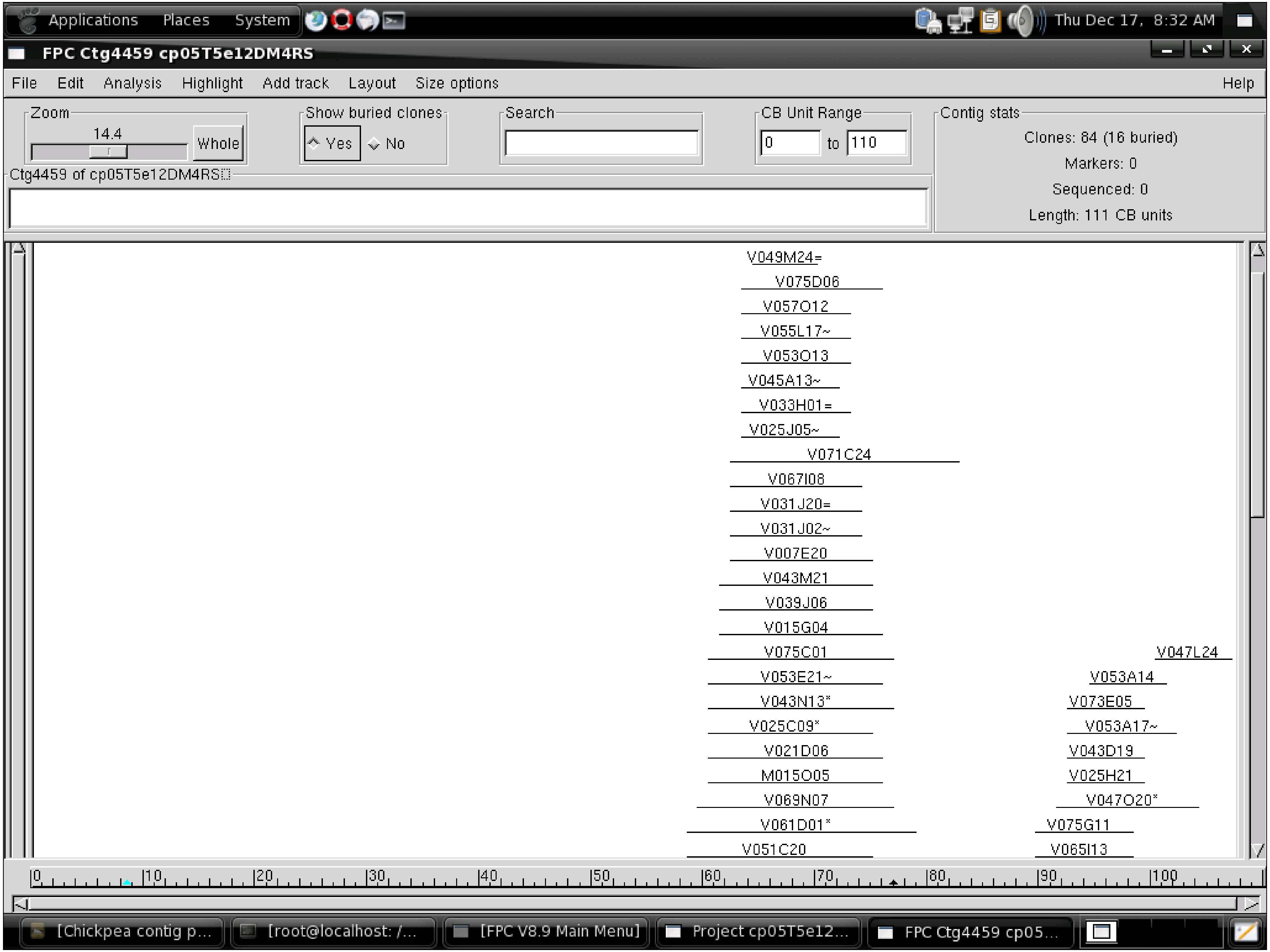
Applications Places System
Thu Dec 17, 8:32 AM
FPC Ctg4459 cp05T5e12DM4RS
File Edit Analysis Highlight Add track Layout Size options Help
Zoom
14.4
Whole
Show buried clones
Yes
No
Search
CB Unit Range
0 to 110
Contig stats
Clones: 84 (16 buried)
Markers: 0
Sequenced: 0
Length: 111 CB units
Ctg4459 of cp05T5e12DM4RS
V049M24=
V075D06
V057O12
V055L17~
V053O13
V045A13~
V033H01=
V025J05~
V071C24
V067I08
V031J20=
V031J02~
V007E20
V043M21
V039J06
V015G04
V075C01
V047L24
V053E21~
V053A14
V043N13*
V073E05
V025C09*
V053A17~
V021D06
V043D19
M015O05
V025H21
V069N07
V047O20*
V061D01*
V075G11
V051C20
V065I13
0 10 20 30 40 50 60 70 80 90 100
[Chickpea contig p...
[root@localhost: /...
[FPC V8.9 Main Menu]
Project cp05T5e12...
FPC Ctg4459 cp05...

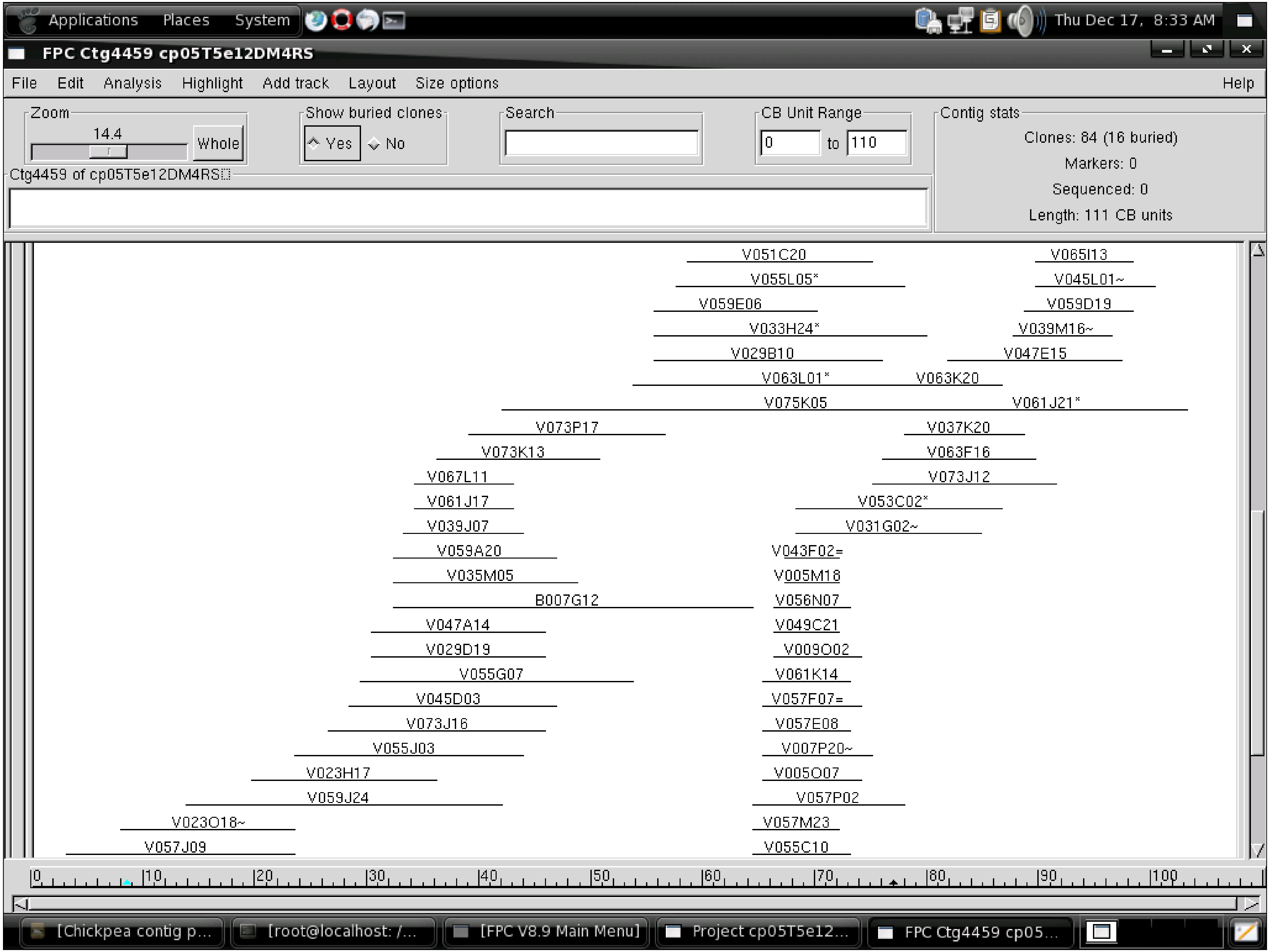
Applications Places System
Thu Dec 17, 8:33 AM
FPC Ctg4459 cp05T5e12DM4RS
File Edit Analysis Highlight Add track Layout Size options Help
Zoom
14.4
Whole
Show buried clones
Yes No
Search
CB Unit Range
0 to 110
Contig stats
Clones: 84 (16 buried)
Markers: 0
Sequenced: 0
Length: 111 CB units
Ctg4459 of cp05T5e12DM4RS
V051C20
V065I13
V055L05*
V045L01~
V059E06
V059D19
V033H24*
V039M16~
V029B10
V047E15
V063L01*
V063K20
V075K05
V061J21*
V073P17
V037K20
V073K13
V063F16
V067L11
V073J12
V061J17
V053C02*
V039J07
V031G02~
V059A20
V043F02=
V035M05
V005M18
B007G12
V056N07
V047A14
V049C21
V029D19
V009O02
V055G07
V061K14
V045D03
V057F07=
V073J16
V057E08
V055J03
V007P20~
V023H17
V005O07
V059J24
V057P02
V023O18~
V057M23
V057J09
V055C10
0 10 20 30 40 50 60 70 80 90 100
[Chickpea contig p...
[root@localhost: /...
[FPC V8.9 Main Menu]
Project cp05T5e12...
FPC Ctg4459 cp05...

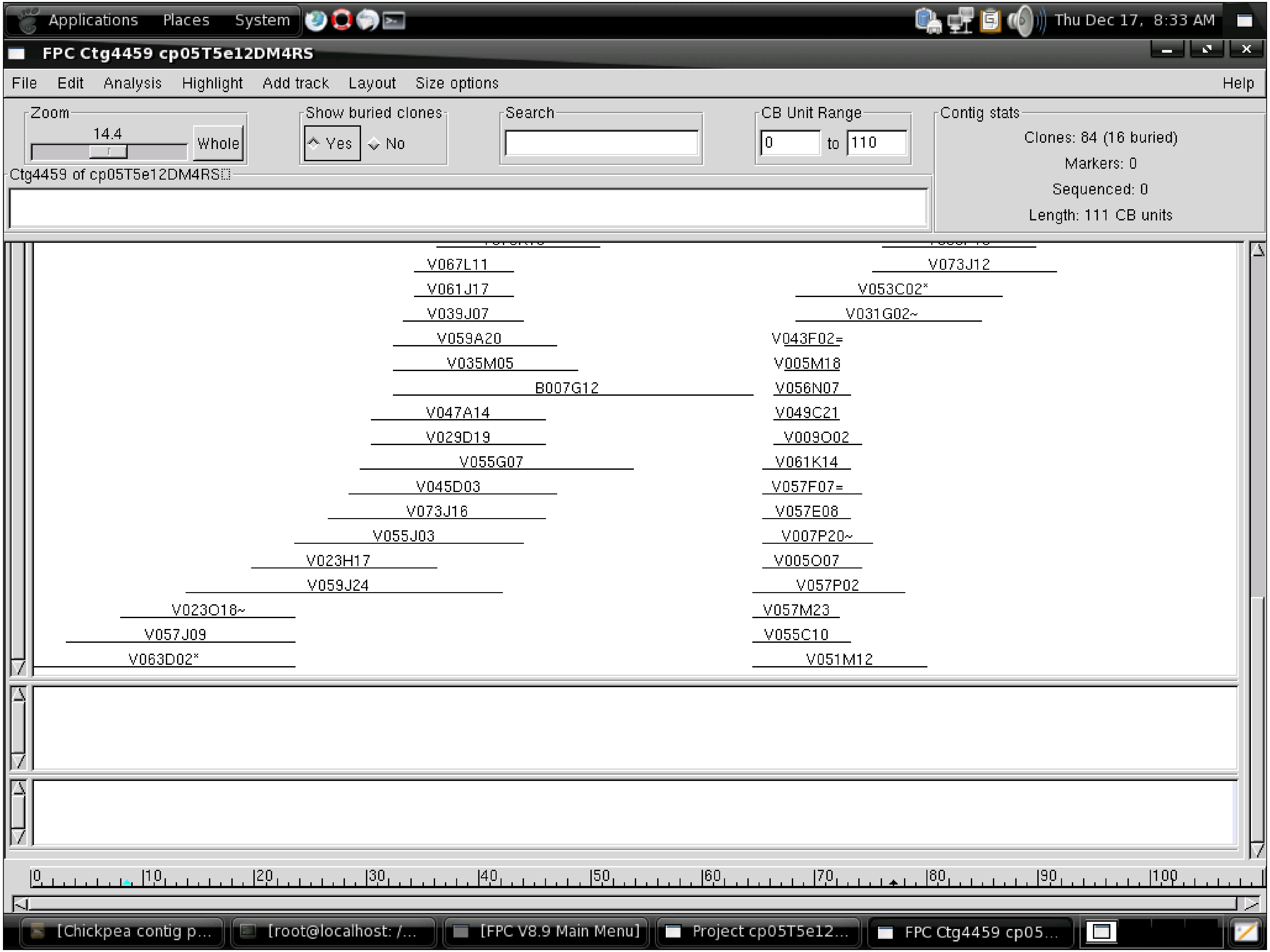
Applications Places System
Thu Dec 17, 8:33 AM
FPC Ctg4459 cp05T5e12DM4RS
File Edit Analysis Highlight Add track Layout Size options Help
Zoom
14.4
Whole
Show buried clones
Yes No
Search
CB Unit Range
0 to 110
Contig stats
Clones: 84 (16 buried)
Markers: 0
Sequenced: 0
Length: 111 CB units
Ctg4459 of cp05T5e12DM4RS
V067L11
V061J17
V039J07
V059A20
V035M05
B007G12
V047A14
V029D19
V055G07
V045D03
V073J16
V055J03
V023H17
V059J24
V023O18~
V057J09
V063D02*
V073J12
V053C02*
V031G02~
V043F02=
V005M18
V056N07
V049C21
V009O02
V061K14
V057F07=
V057E08
V007P20~
V005O07
V057P02
V057M23
V055C10
V051M12
0 10 20 30 40 50 60 70 80 90 100
[Chickpea contig p...
[root@localhost: /...
[FPC V8.9 Main Menu]
Project cp05T5e12...
FPC Ctg4459 cp05...

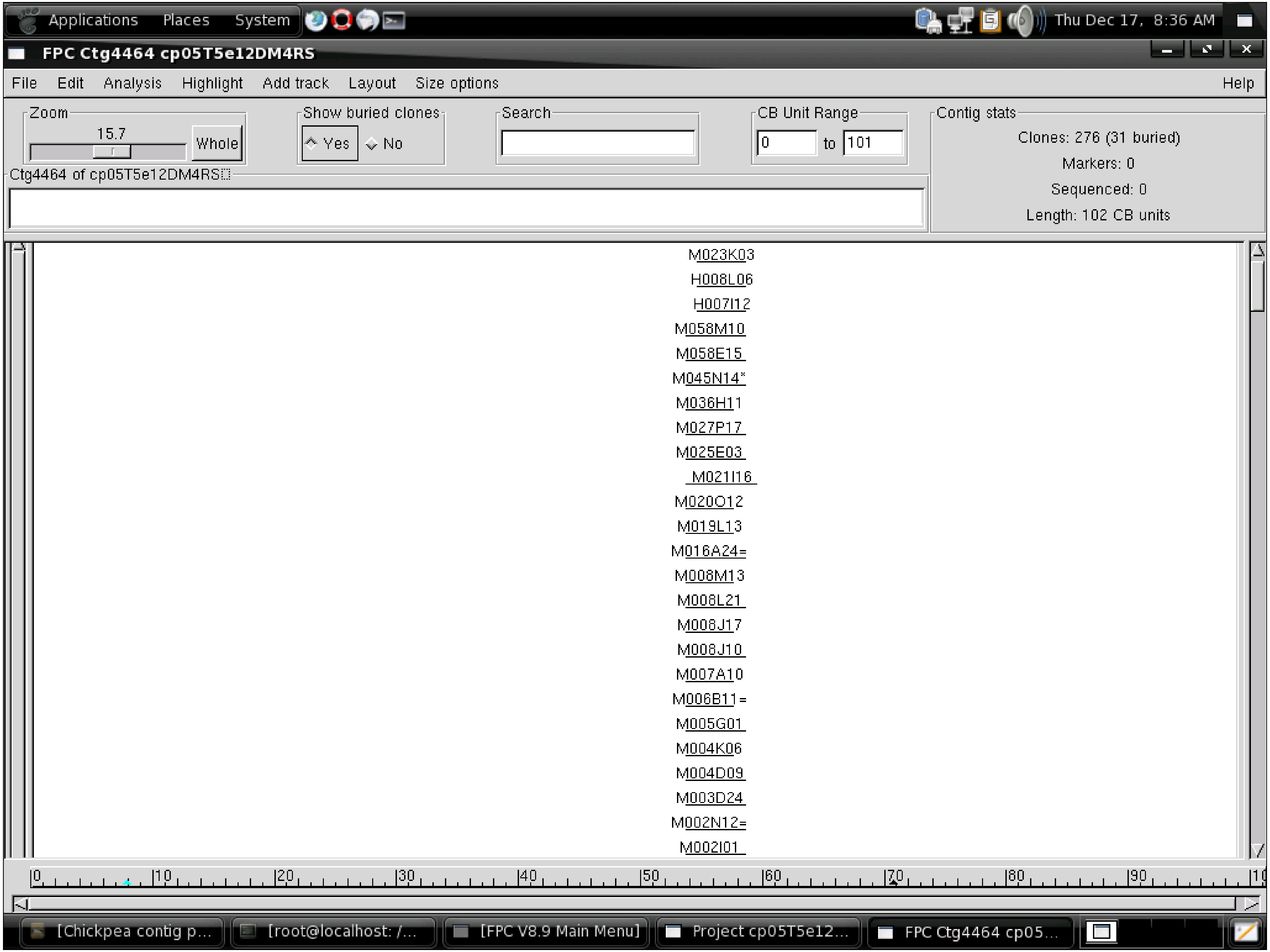

Applications Places System
Thu Dec 17, 8:36 AM
FPC Ctg4464 cp05T5e12DM4RS
File Edit Analysis Highlight Add track Layout Size options Help
Zoom
15.7
Whole
Show buried clones
Yes No
Search
CB Unit Range
0 to 101
Contig stats
Clones: 276 (31 buried)
Markers: 0
Sequenced: 0
Length: 102 CB units
Ctg4464 of cp05T5e12DM4RS
M023K03
H008L06
H007I12
M058M10
M058E15
M045N14*
M036H11
M027P17
M025E03
M021I16
M020O12
M019L13
M016A24=
M008M13
M008L21
M008J17
M008J10
M007A10
M006B11=
M005G01
M004K06
M004D09
M003D24
M002N12=
M002I01
0 10 20 30 40 50 60 70 80 90
[Chickpea contig p...
[root@localhost: /...
[FPC V8.9 Main Menu]
Project cp05T5e12...
FPC Ctg4464 cp05...

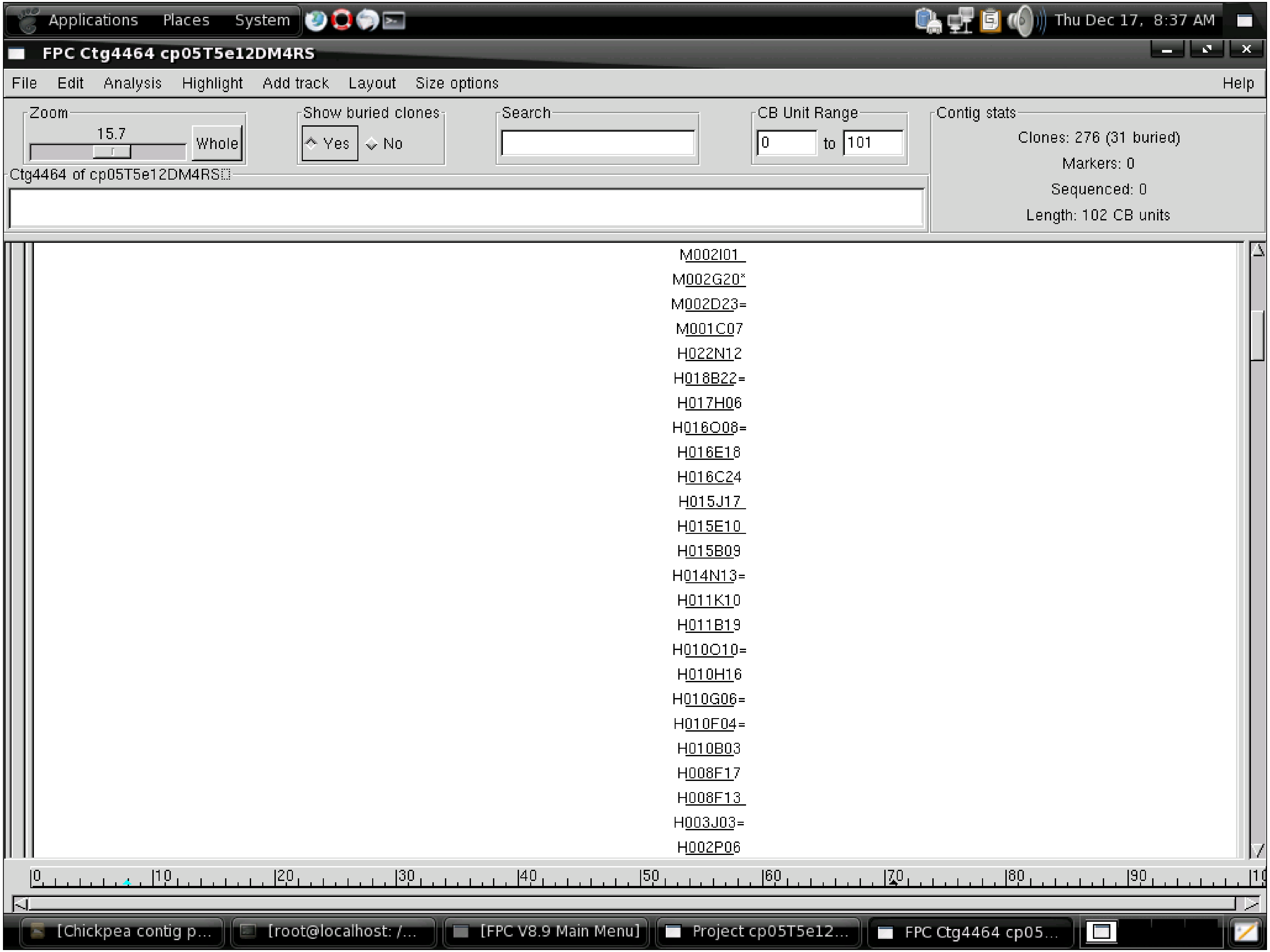
Applications Places System
Thu Dec 17, 8:37 AM
FPC Ctg4464 cp05T5e12DM4RS
File Edit Analysis Highlight Add track Layout Size options Help
Zoom
15.7
Whole
Show buried clones
Yes
No
Search
CB Unit Range
0 to 101
Contig stats
Clones: 276 (31 buried)
Markers: 0
Sequenced: 0
Length: 102 CB units
Ctg4464 of cp05T5e12DM4RS
M002I01
M002G20*
M002D23=
M001C07
H022N12
H018B22=
H017H06
H016O08=
H016E18
H016C24
H015J17
H015E10
H015B09
H014N13=
H011K10
H011B19
H010O10=
H010H16
H010G06=
H010F04=
H010B03
H008F17
H008F13
H003J03=
H002P06
0 10 20 30 40 50 60 70 80 90
[Chickpea contig p...
[root@localhost: /...
[FPC V8.9 Main Menu]
Project cp05T5e12...
FPC Ctg4464 cp05...

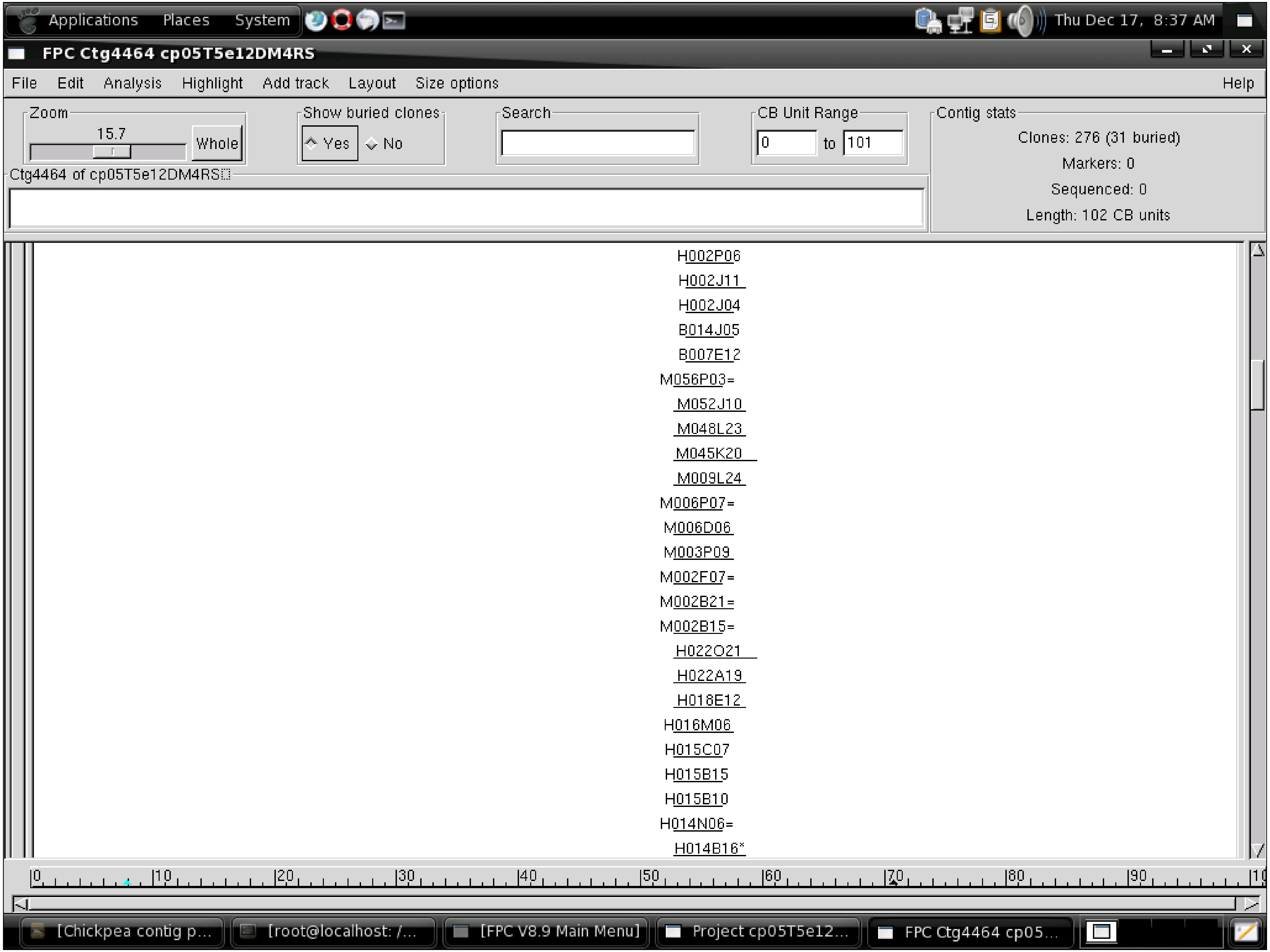
Applications
Places
System
Thu Dec 17, 8:37 AM
FPC Ctg4464 cp05T5e12DM4RS
File
Edit
Analysis
Highlight
Add track
Layout
Size options
Help
Zoom
15.7
Whole
Show buried clones
Yes
No
Search
CB Unit Range
0
to
101
Contig stats
Clones: 276 (31 buried)
Markers: 0
Sequenced: 0
Length: 102 CB units
Ctg4464 of cp05T5e12DM4RS
H002P06
H002J11
H002J04
B014J05
B007E12
M056P03=
M052J10
M048L23
M045K20
M009L24
M006P07=
M006D06
M003P09
M002F07=
M002B21=
M002B15=
H022O21
H022A19
H018E12
H016M06
H015C07
H015B15
H015B10
H014N06=
H014B16*
0
10
20
30
40
50
60
70
80
90
[Chickpea contig p...
[root@localhost: /...
[FPC V8.9 Main Menu]
Project cp05T5e12...
FPC Ctg4464 cp05...

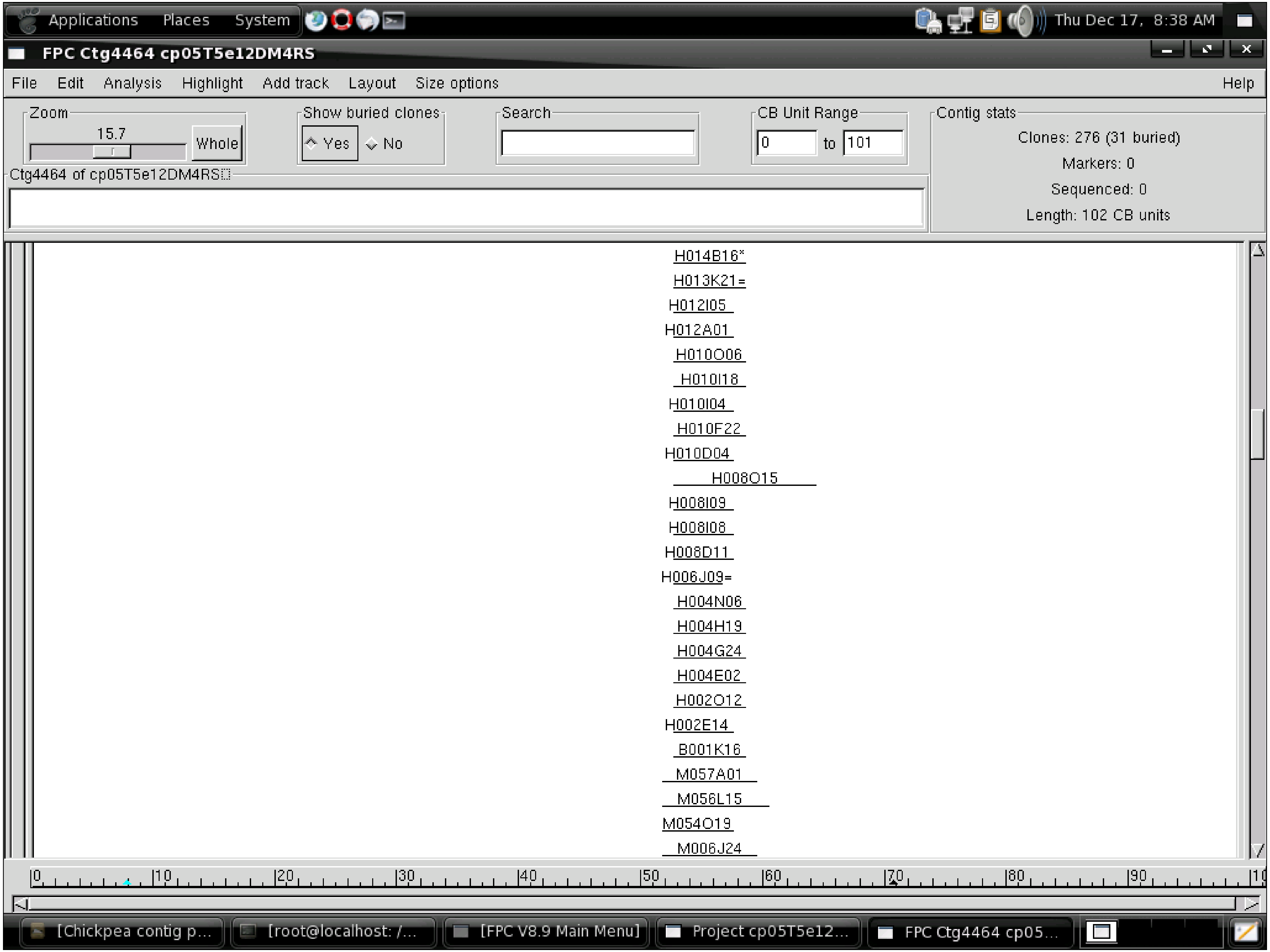
Applications Places System
Thu Dec 17, 8:38 AM
FPC Ctg4464 cp05T5e12DM4RS
File Edit Analysis Highlight Add track Layout Size options Help
Zoom
15.7
Whole
Show buried clones
Yes No
Search
CB Unit Range
0 to 101
Contig stats
Clones: 276 (31 buried)
Markers: 0
Sequenced: 0
Length: 102 CB units
Ctg4464 of cp05T5e12DM4RS
H014B16*
H013K21=
H012I05
H012A01
H010O06
H010I18
H010I04
H010F22
H010D04
H008O15
H008I09
H008I08
H008D11
H006J09=
H004N06
H004H19
H004G24
H004E02
H002O12
H002E14
B001K16
M057A01
M056L15
M054O19
M006J24
0 10 20 30 40 50 60 70 80 90
[Chickpea contig p...
[root@localhost: /...
[FPC V8.9 Main Menu]
Project cp05T5e12...
FPC Ctg4464 cp05...

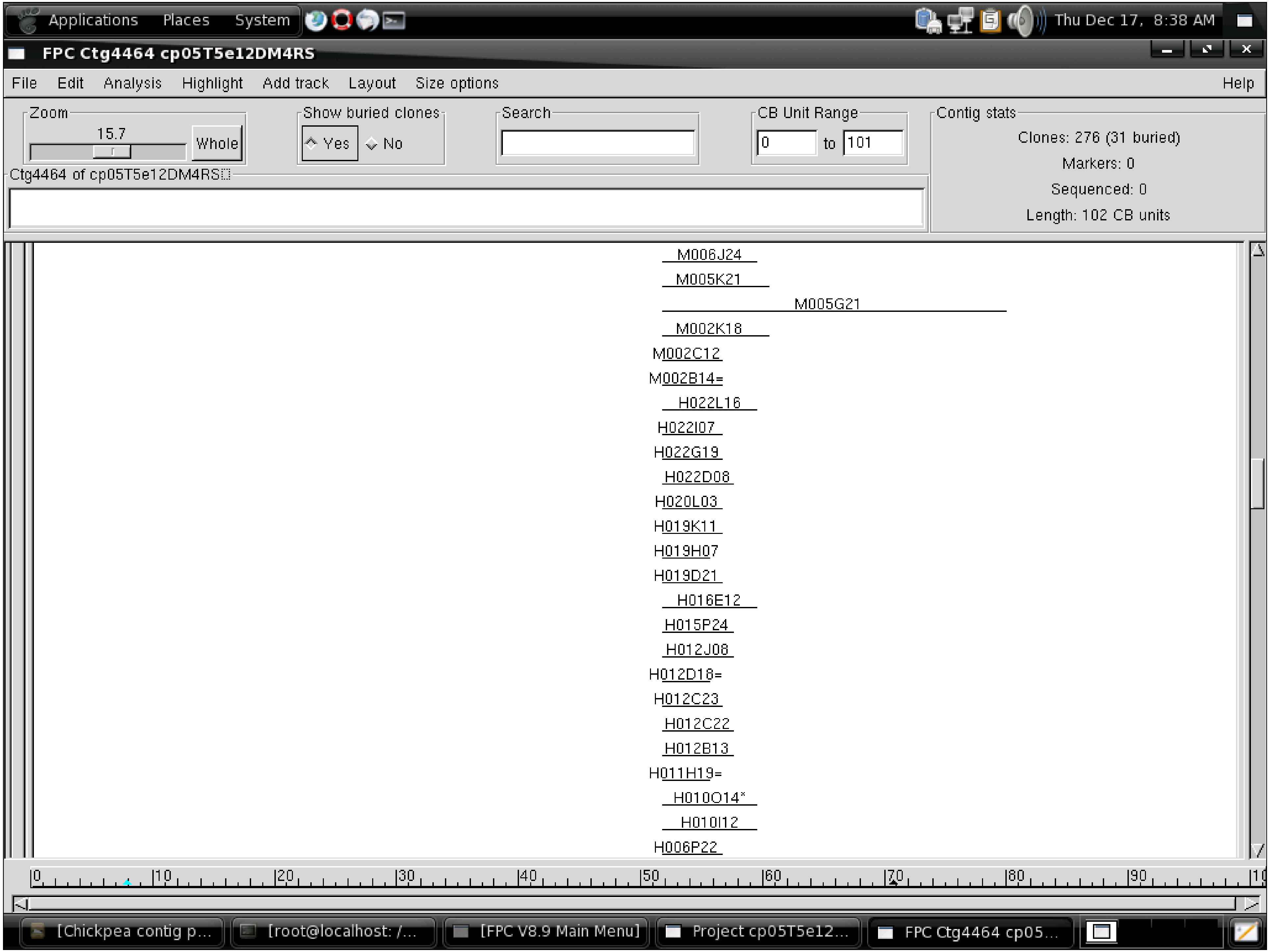
Applications Places System
Thu Dec 17, 8:38 AM
FPC Ctg4464 cp05T5e12DM4RS
File Edit Analysis Highlight Add track Layout Size options Help
Zoom
15.7
Whole
Show buried clones
Yes No
Search
CB Unit Range
0 to 101
Contig stats
Clones: 276 (31 buried)
Markers: 0
Sequenced: 0
Length: 102 CB units
Ctg4464 of cp05T5e12DM4RS
M006J24
M005K21
M005G21
M002K18
M002C12
M002B14=
H022L16
H022I07
H022G19
H022D08
H020L03
H019K11
H019H07
H019D21
H016E12
H015P24
H012J08
H012D18=
H012C23
H012C22
H012B13
H011H19=
H010O14*
H010I12
H006P22
0 10 20 30 40 50 60 70 80 90 10
[Chickpea contig p...
[root@localhost: /...
[FPC V8.9 Main Menu]
Project cp05T5e12...
FPC Ctg4464 cp05...

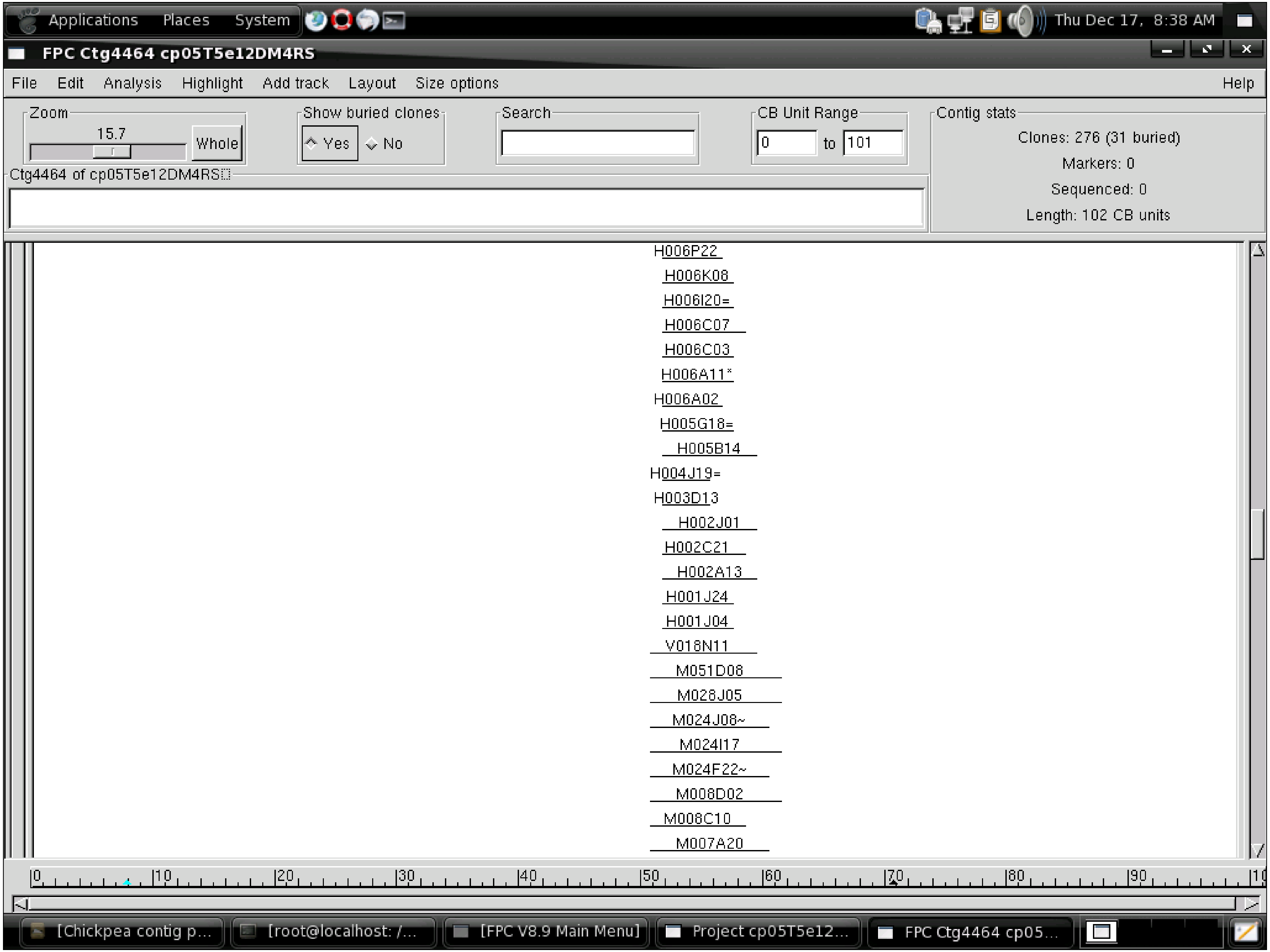
Applications Places System
Thu Dec 17, 8:38 AM
FPC Ctg4464 cp05T5e12DM4RS
File Edit Analysis Highlight Add track Layout Size options Help
Zoom
15.7
Whole
Show buried clones
Yes No
Search
CB Unit Range
0 to 101
Contig stats
Clones: 276 (31 buried)
Markers: 0
Sequenced: 0
Length: 102 CB units
Ctg4464 of cp05T5e12DM4RS
H006P22
H006K08
H006I20=
H006C07
H006C03
H006A11*
H006A02
H005G18=
H005B14
H004J19=
H003D13
H002J01
H002C21
H002A13
H001J24
H001J04
V018N11
M051D08
M028J05
M024J08~
M024I17
M024F22~
M008D02
M008C10
M007A20
0 10 20 30 40 50 60 70 80 90
[Chickpea contig p...
[root@localhost: /...
[FPC V8.9 Main Menu]
Project cp05T5e12...
FPC Ctg4464 cp05...

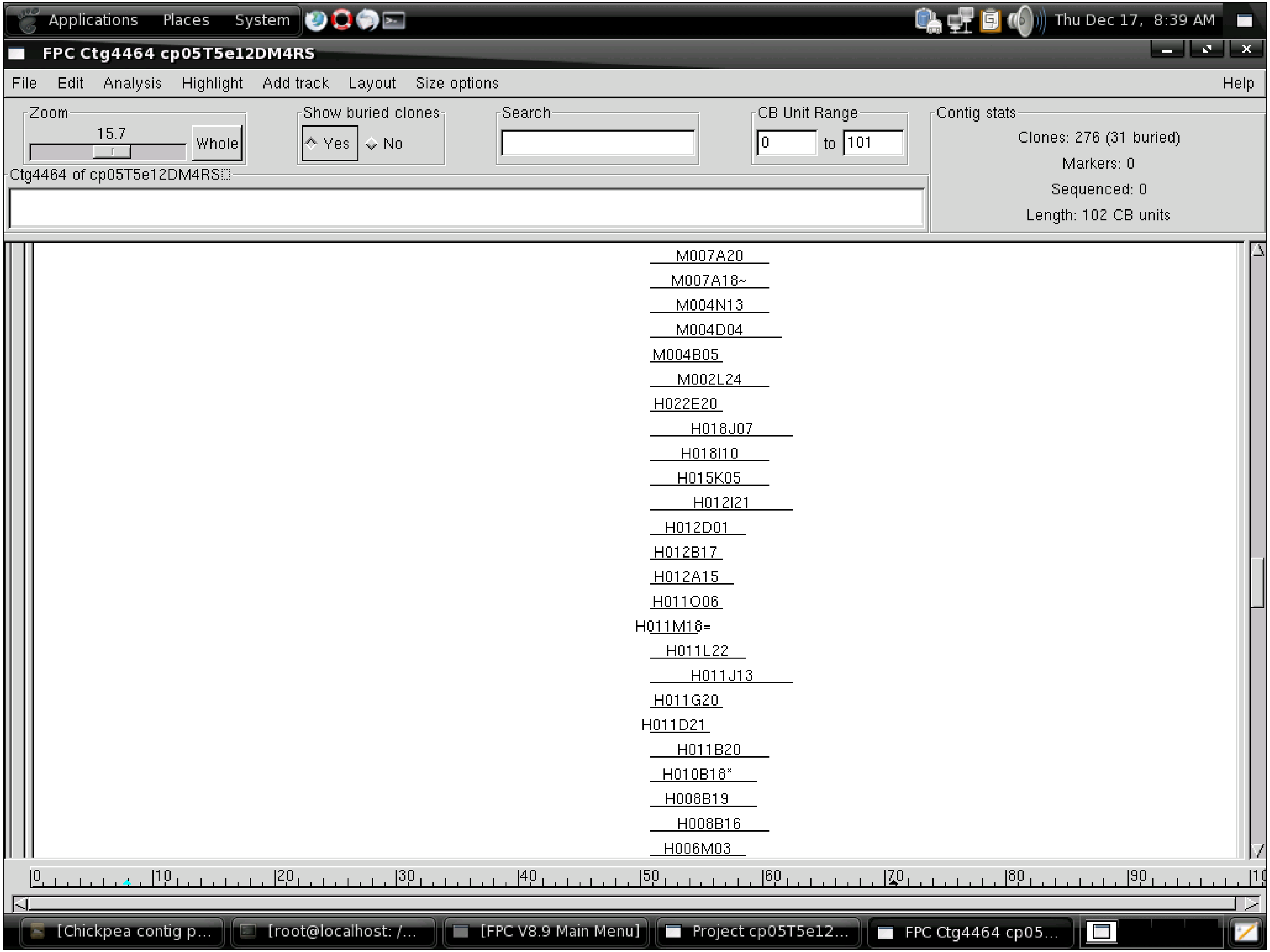

Applications
Places
System
Thu Dec 17, 8:39 AM
FPC Ctg4464 cp05T5e12DM4RS
File
Edit
Analysis
Highlight
Add track
Layout
Size options
Help
Zoom
15.7
Whole
Show buried clones
Yes
No
Search
CB Unit Range
0
to
101
Contig stats
Clones: 276 (31 buried)
Markers: 0
Sequenced: 0
Length: 102 CB units
Ctg4464 of cp05T5e12DM4RS
M007A20
M007A18~
M004N13
M004D04
M004B05
M002L24
H022E20
H018J07
H018I10
H015K05
H012I21
H012D01
H012B17
H012A15
H011O06
H011M18=
H011L22
H011J13
H011G20
H011D21
H011B20
H010B18*
H008B19
H008B16
H006M03
0
10
20
30
40
50
60
70
80
90
[Chickpea contig p...
[root@localhost: /...
[FPC V8.9 Main Menu]
Project cp05T5e12...
FPC Ctg4464 cp05...

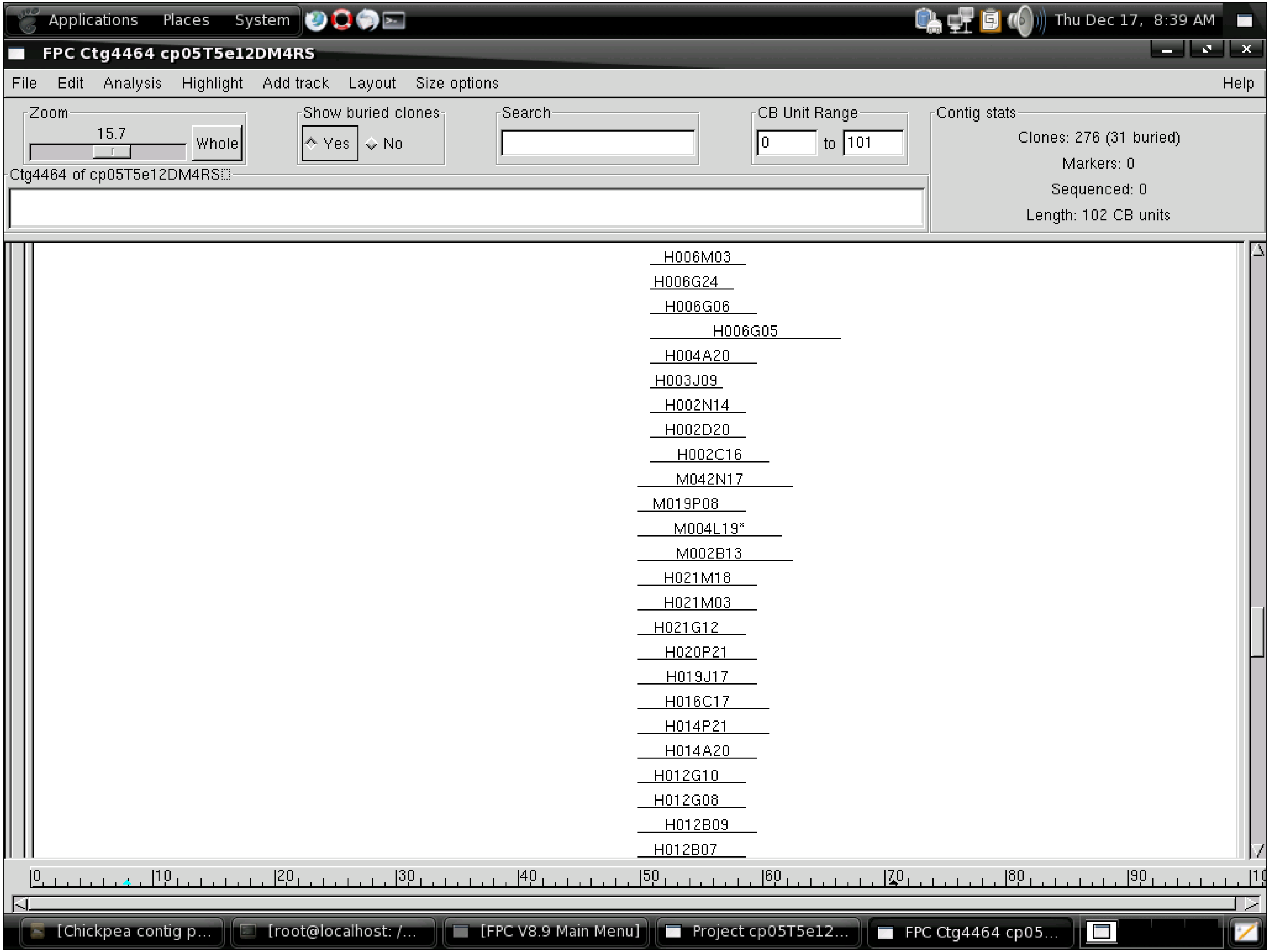

Applications Places System
Thu Dec 17, 8:39 AM
FPC Ctg4464 cp05T5e12DM4RS
File Edit Analysis Highlight Add track Layout Size options Help
Zoom
15.7
Whole
Show buried clones
Yes No
Search
CB Unit Range
0 to 101
Contig stats
Clones: 276 (31 buried)
Markers: 0
Sequenced: 0
Length: 102 CB units
Ctg4464 of cp05T5e12DM4RS
H006M03
H006G24
H006G06
H006G05
H004A20
H003J09
H002N14
H002D20
H002C16
M042N17
M019P08
M004L19*
M002B13
H021M18
H021M03
H021G12
H020P21
H019J17
H016C17
H014P21
H014A20
H012G10
H012G08
H012B09
H012B07
0 10 20 30 40 50 60 70 80 90
[Chickpea contig p...
[root@localhost: /...
[FPC V8.9 Main Menu]
Project cp05T5e12...
FPC Ctg4464 cp05...

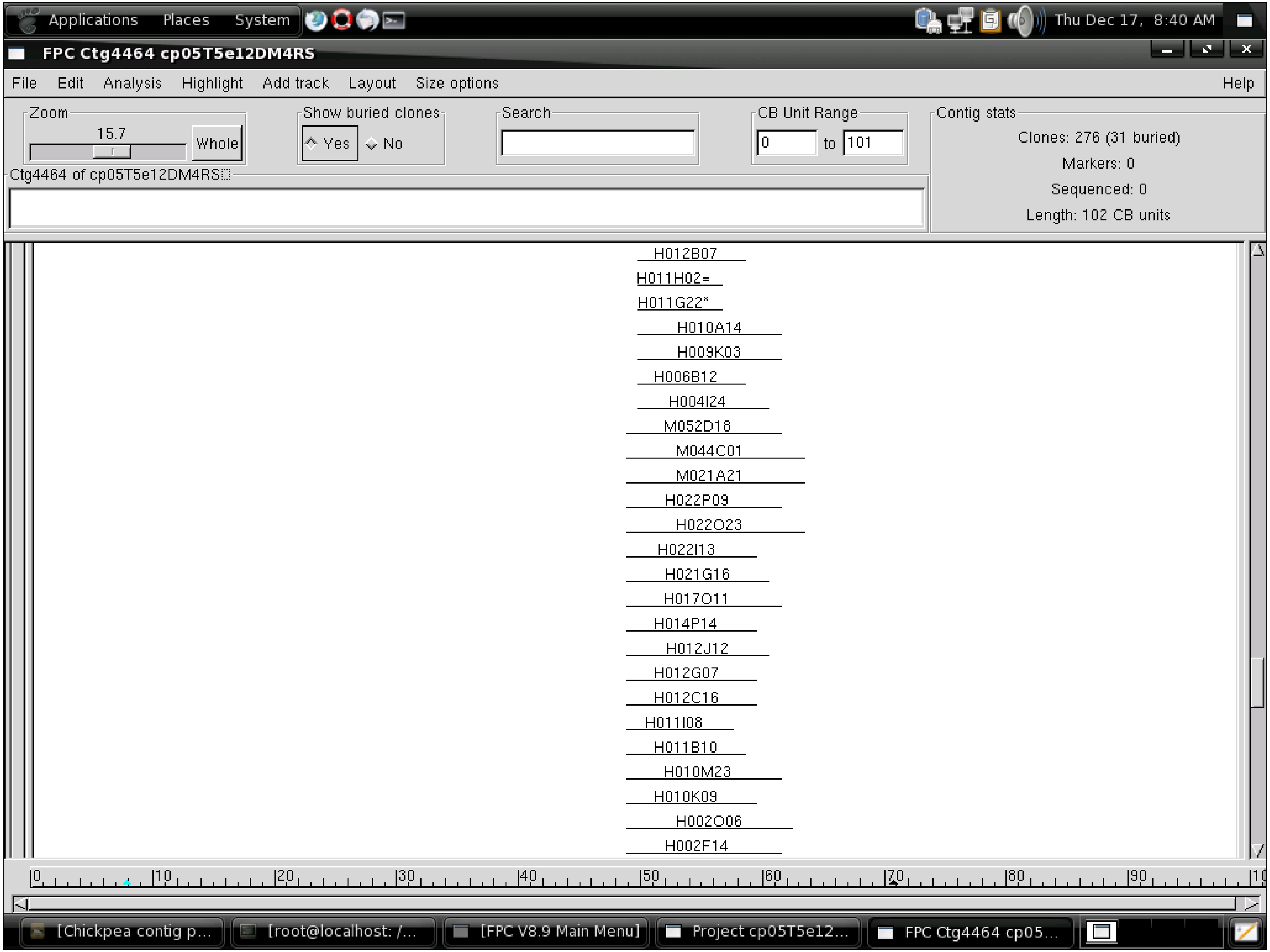
Applications Places System
Thu Dec 17, 8:40 AM
FPC Ctg4464 cp05T5e12DM4RS
File Edit Analysis Highlight Add track Layout Size options Help
Zoom
15.7
Whole
Show buried clones
Yes No
Search
CB Unit Range
0 to 101
Contig stats
Clones: 276 (31 buried)
Markers: 0
Sequenced: 0
Length: 102 CB units
Ctg4464 of cp05T5e12DM4RS
H012B07
H011H02=
H011G22*
H010A14
H009K03
H006B12
H004I24
M052D18
M044C01
M021A21
H022P09
H022O23
H022I13
H021G16
H017O11
H014P14
H012J12
H012G07
H012C16
H011I08
H011B10
H010M23
H010K09
H002O06
H002F14
0 10 20 30 40 50 60 70 80 90
[Chickpea contig p...
[root@localhost: /...
[FPC V8.9 Main Menu]
Project cp05T5e12...
FPC Ctg4464 cp05...

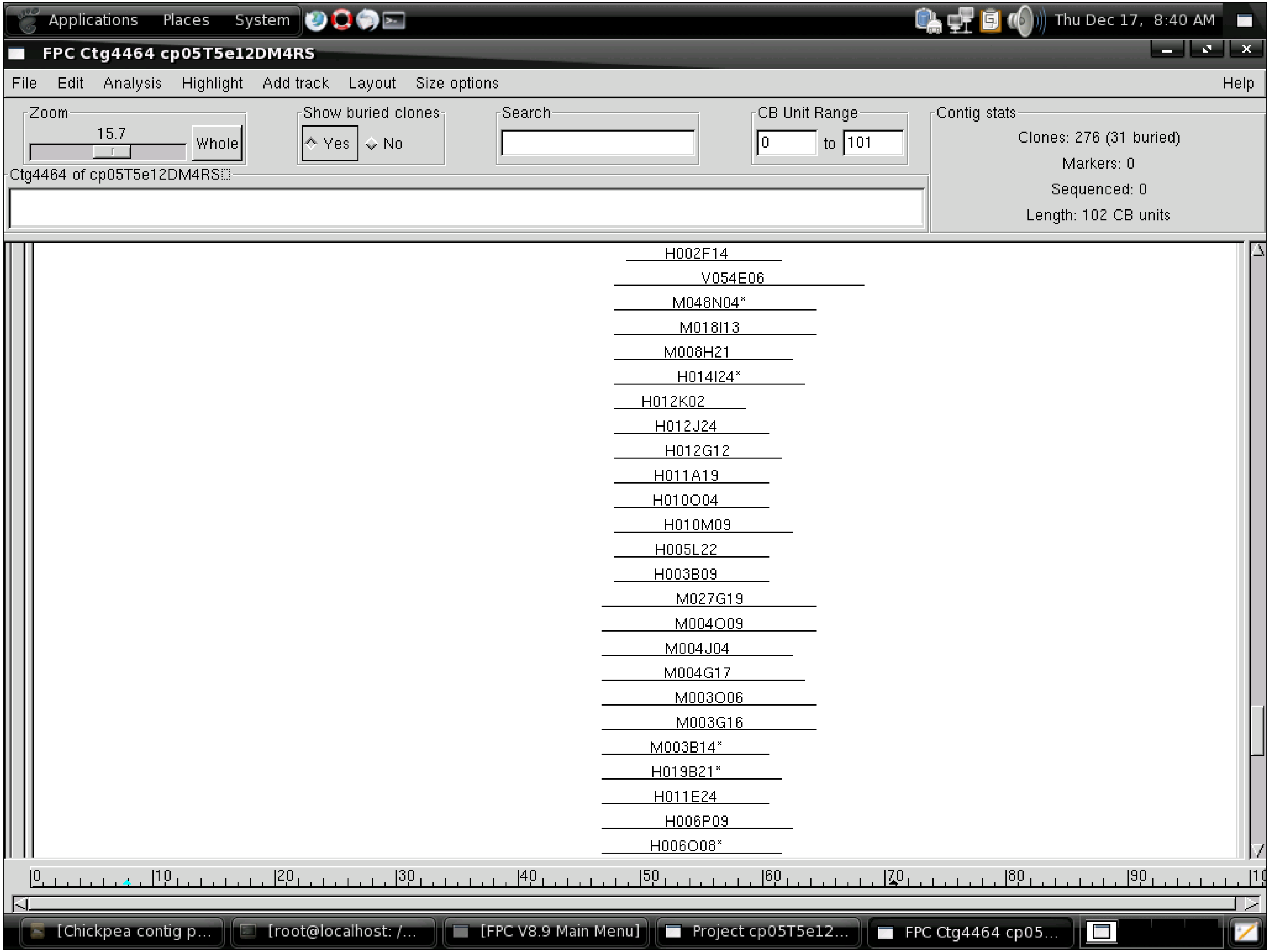
Applications Places System
Thu Dec 17, 8:40 AM
FPC Ctg4464 cp05T5e12DM4RS
File Edit Analysis Highlight Add track Layout Size options
Help
Zoom
15.7
Whole
Show buried clones
Yes
No
Search
CB Unit Range
0
to
101
Contig stats
Clones: 276 (31 buried)
Markers: 0
Sequenced: 0
Length: 102 CB units
Ctg4464 of cp05T5e12DM4RS
H002F14
V054E06
M048N04*
M018I13
M008H21
H014I24*
H012K02
H012J24
H012G12
H011A19
H010O04
H010M09
H005L22
H003B09
M027G19
M004O09
M004J04
M004G17
M003O06
M003G16
M003B14*
H019B21*
H011E24
H006P09
H006O08*
0 10 20 30 40 50 60 70 80 90
[Chickpea contig p...
[root@localhost: /...
[FPC V8.9 Main Menu]
Project cp05T5e12...
FPC Ctg4464 cp05...

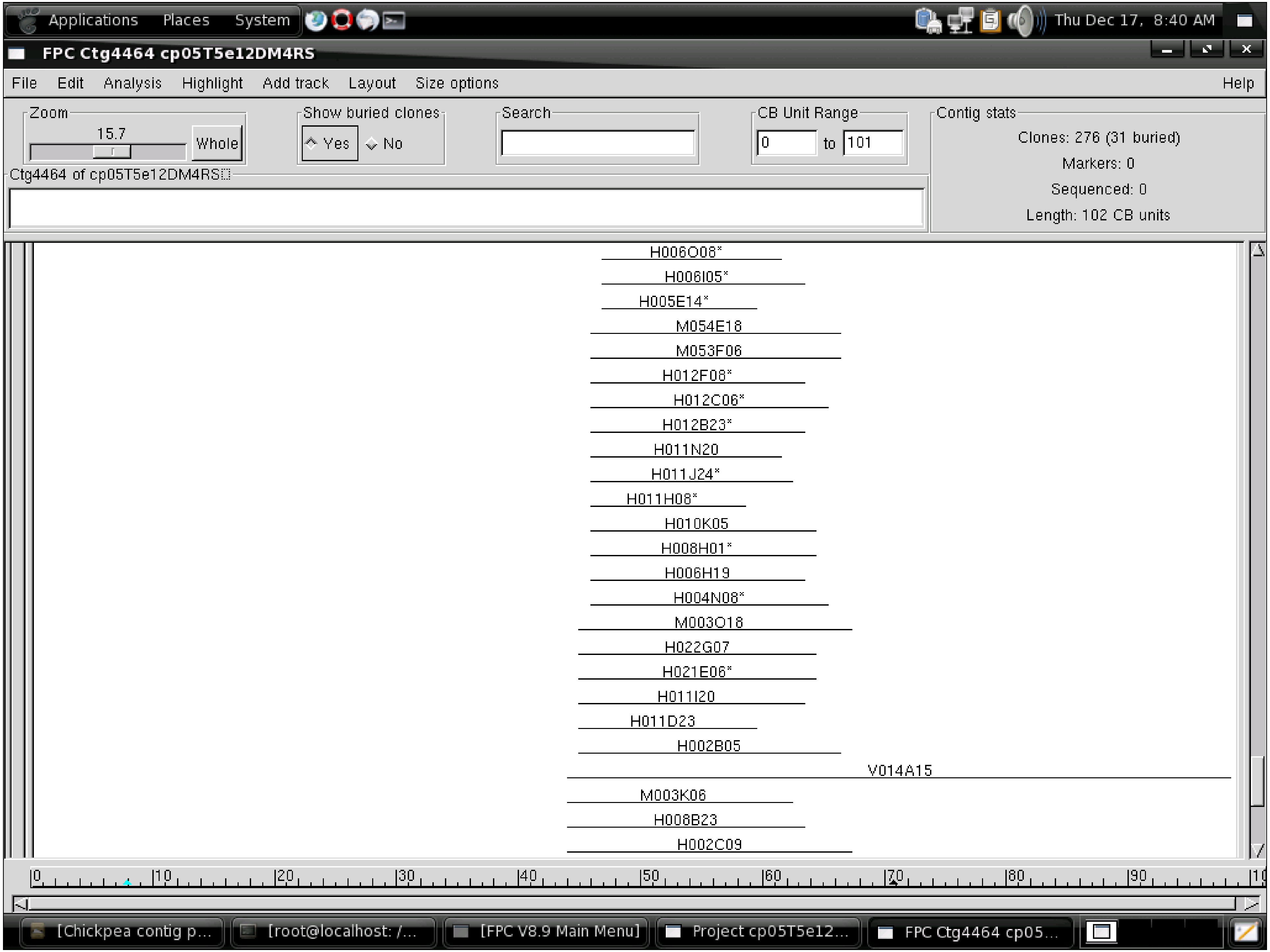
Applications Places System
Thu Dec 17, 8:40 AM
FPC Ctg4464 cp05T5e12DM4RS
File Edit Analysis Highlight Add track Layout Size options Help
Zoom
15.7
Whole
Show buried clones
Yes No
Search
CB Unit Range
0 to 101
Contig stats
Clones: 276 (31 buried)
Markers: 0
Sequenced: 0
Length: 102 CB units
Ctg4464 of cp05T5e12DM4RS
H006O08*
H006I05*
H005E14*
M054E18
M053F06
H012F08*
H012C06*
H012B23*
H011N20
H011J24*
H011H08*
H010K05
H008H01*
H006H19
H004N08*
M003O18
H022G07
H021E06*
H011I20
H011D23
H002B05
V014A15
M003K06
H008B23
H002C09
0 10 20 30 40 50 60 70 80 90
[Chickpea contig p...
[root@localhost: /...
[FPC V8.9 Main Menu]
Project cp05T5e12...
FPC Ctg4464 cp05...

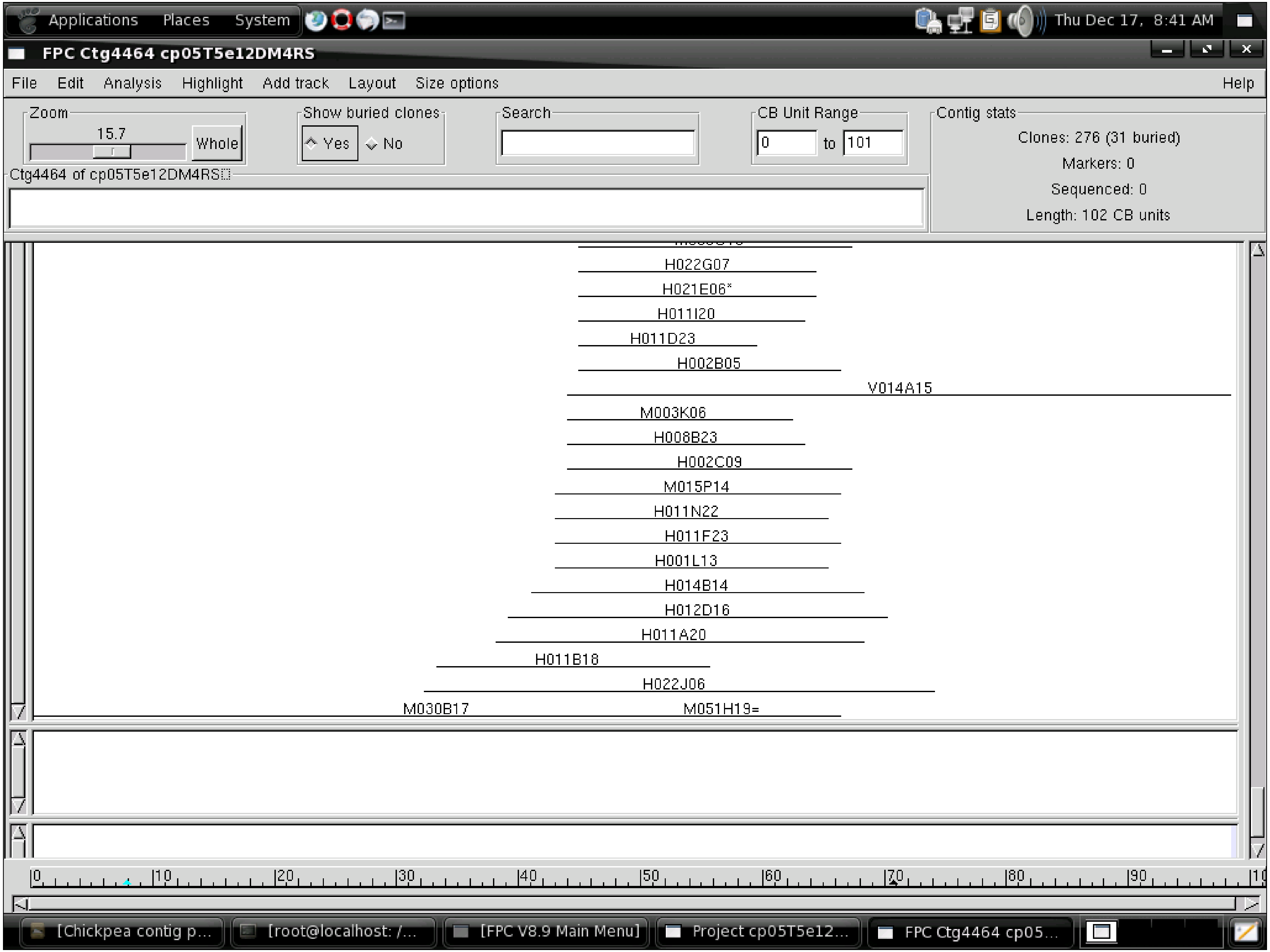
Applications Places System
Thu Dec 17, 8:41 AM
FPC Ctg4464 cp05T5e12DM4RS
File Edit Analysis Highlight Add track Layout Size options Help
Zoom
15.7
Whole
Show buried clones
Yes
No
Search
CB Unit Range
0
to
101
Contig stats
Clones: 276 (31 buried)
Markers: 0
Sequenced: 0
Length: 102 CB units
Ctg4464 of cp05T5e12DM4RS
H022G07
H021E06*
H011I20
H011D23
H002B05
V014A15
M003K06
H008B23
H002C09
M015P14
H011N22
H011F23
H001L13
H014B14
H012D16
H011A20
H011B18
H022J06
M030B17
M051H19=
0
10
20
30
40
50
60
70
80
90
[Chickpea contig p...
[root@localhost: /...
[FPC V8.9 Main Menu]
Project cp05T5e12...
FPC Ctg4464 cp05...

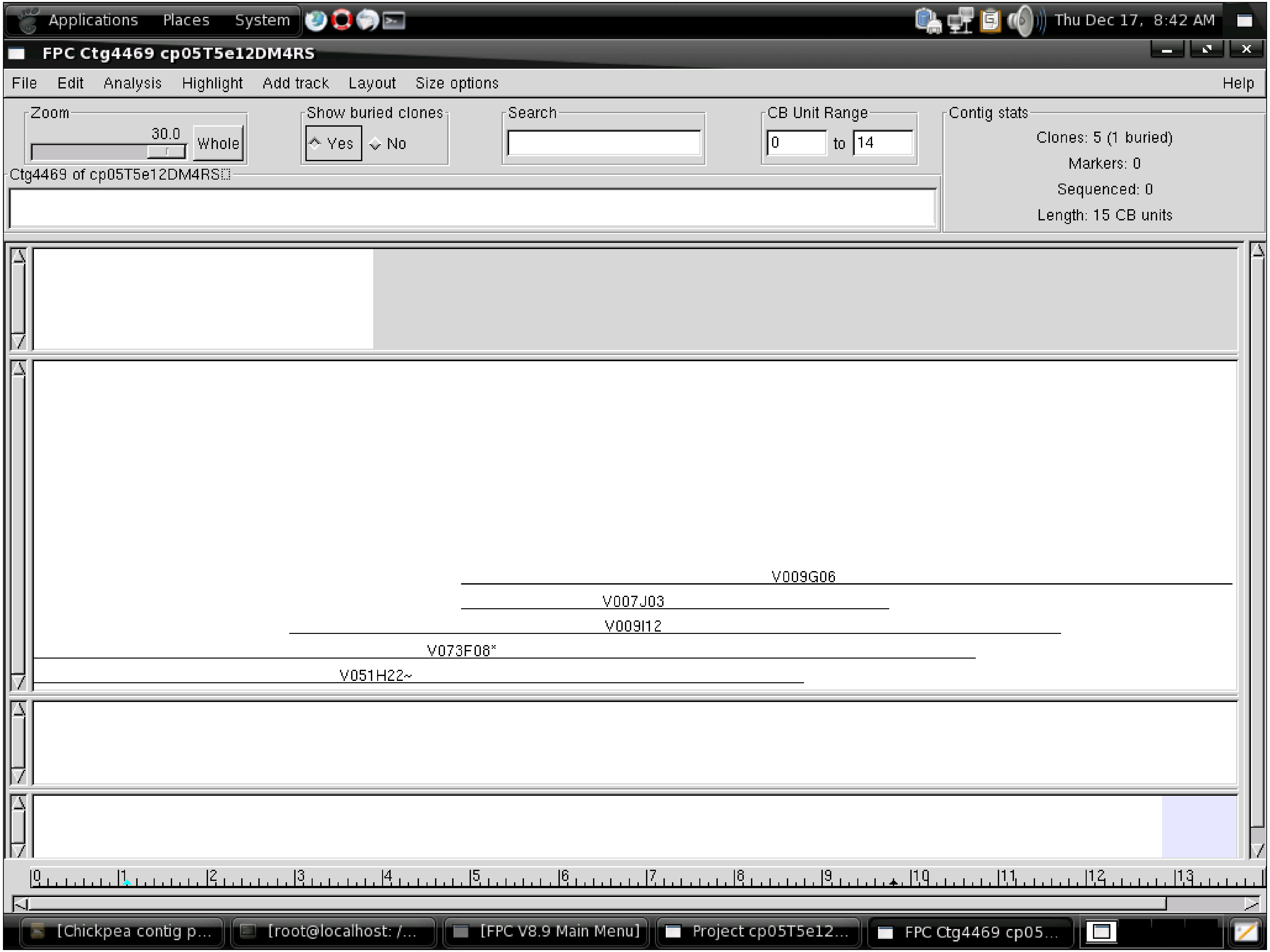
Applications Places System
Thu Dec 17, 8:42 AM
FPC Ctg4469 cp05T5e12DM4RS
File Edit Analysis Highlight Add track Layout Size options Help
Zoom
30.0
Whole
Show buried clones
Yes No
Search
CB Unit Range
0 to 14
Contig stats
Clones: 5 (1 buried)
Markers: 0
Sequenced: 0
Length: 15 CB units
Ctg4469 of cp05T5e12DM4RS
V009G06
V007J03
V009I12
V073F08*
V051H22~
0 1 2 3 4 5 6 7 8 9 10 11 12 13
[Chickpea contig p...
[root@localhost: /...
[FPC V8.9 Main Menu]
Project cp05T5e12...
FPC Ctg4469 cp05...

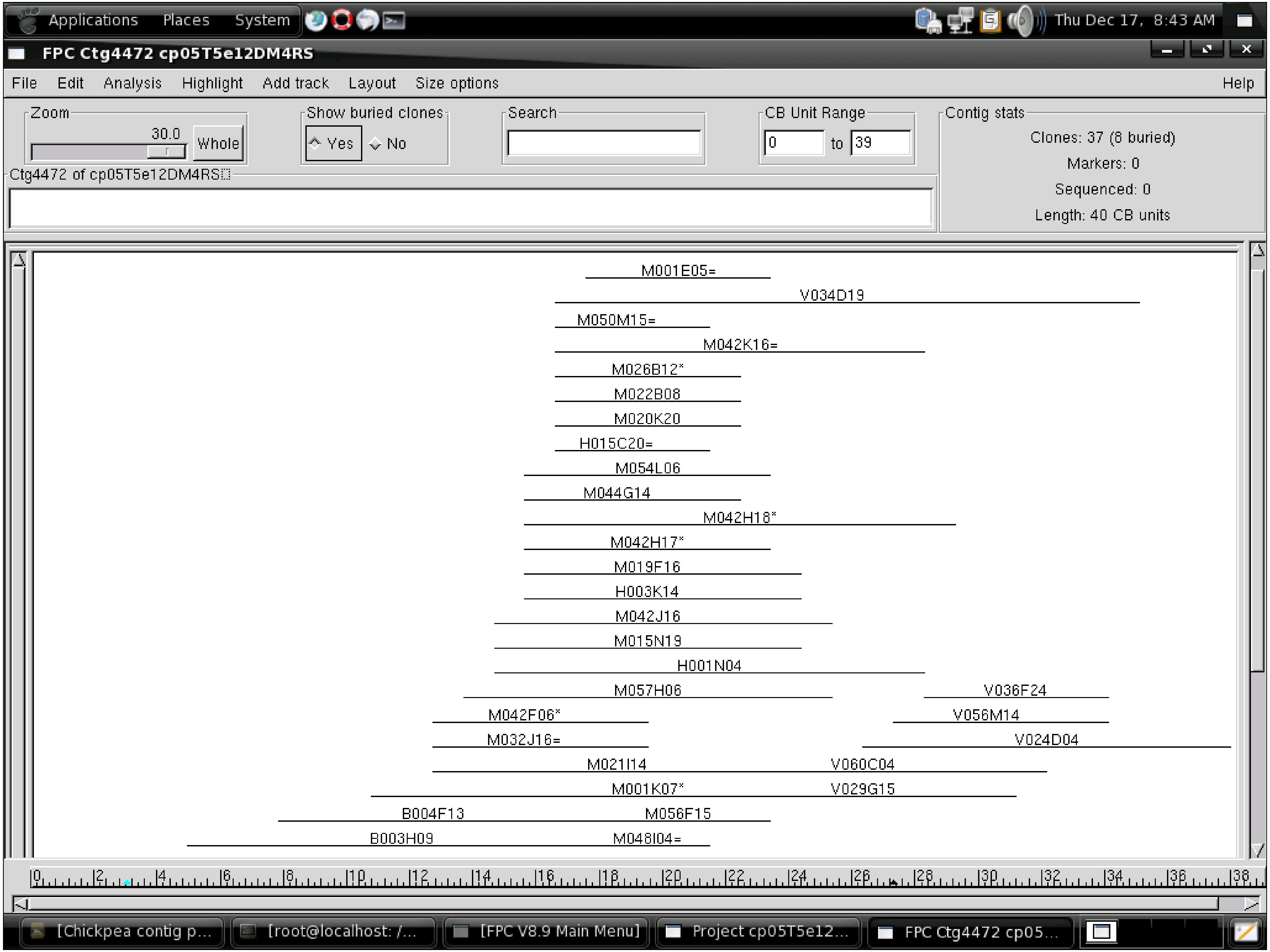

Applications Places System
Thu Dec 17, 8:43 AM
FPC Ctg4472 cp05T5e12DM4RS
File Edit Analysis Highlight Add track Layout Size options Help
Zoom
30.0
Whole
Show buried clones
Yes No
Search
CB Unit Range
0 to 39
Contig stats
Clones: 37 (8 buried)
Markers: 0
Sequenced: 0
Length: 40 CB units
Ctg4472 of cp05T5e12DM4RS
M001E05=
V034D19
M050M15=
M042K16=
M026B12*
M022B08
M020K20
H015C20=
M054L06
M044G14
M042H18*
M042H17*
M019F16
H003K14
M042J16
M015N19
H001N04
M057H06
V036F24
M042F06*
V056M14
M032J16=
V024D04
M021I14
V060C04
M001K07*
V029G15
B004F13
M056F15
B003H09
M048I04=
0 2 4 6 8 10 12 14 16 18 20 22 24 26 28 30 32 34 36 38
[Chickpea contig p...
[root@localhost: /...
[FPC V8.9 Main Menu]
Project cp05T5e12...
FPC Ctg4472 cp05...

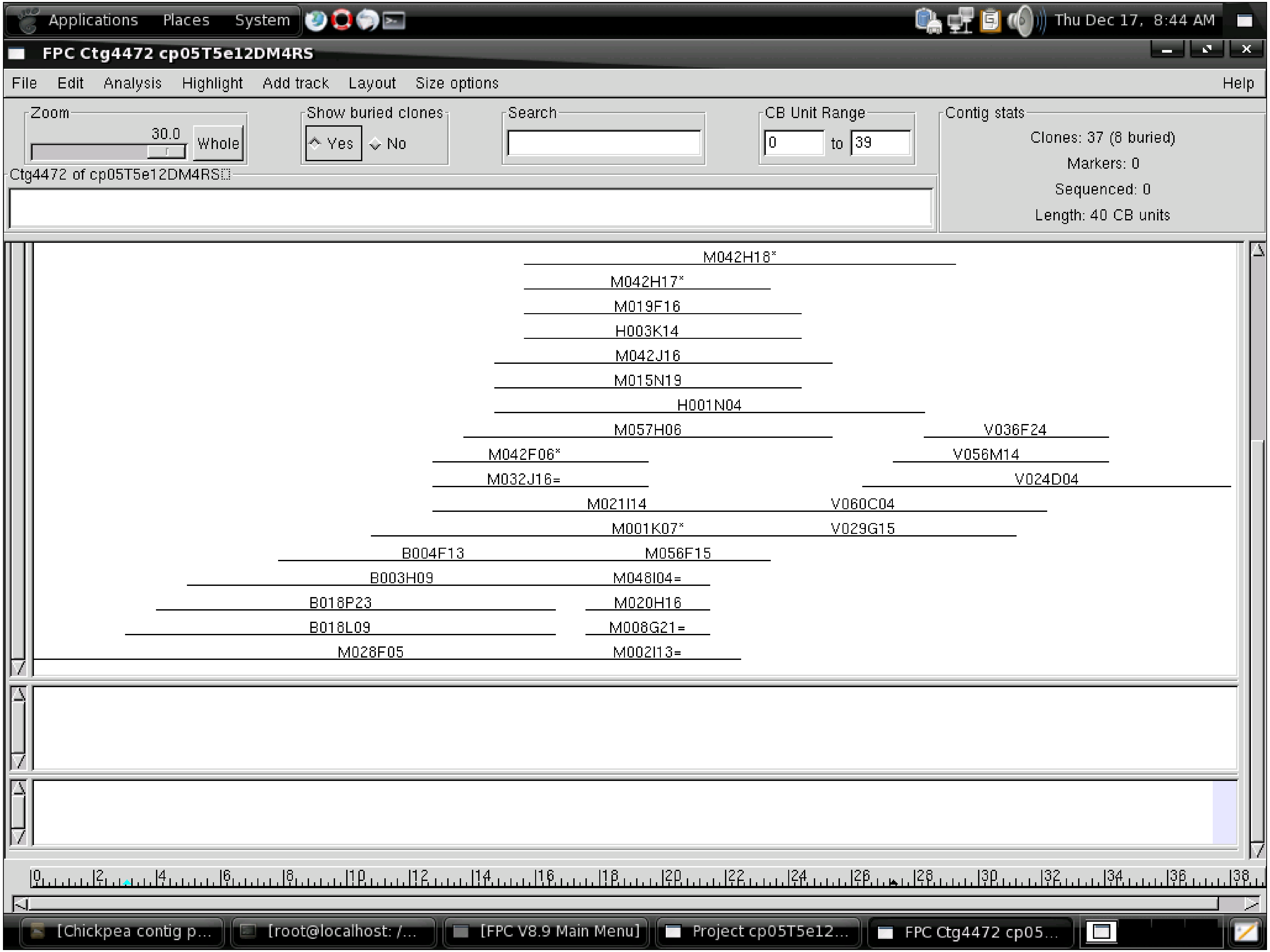
Applications Places System
Thu Dec 17, 8:44 AM
FPC Ctg4472 cp05T5e12DM4RS
File Edit Analysis Highlight Add track Layout Size options Help
Zoom
30.0
Whole
Show buried clones
Yes No
Search
CB Unit Range
0 to 39
Contig stats
Clones: 37 (8 buried)
Markers: 0
Sequenced: 0
Length: 40 CB units
Ctg4472 of cp05T5e12DM4RS
M042H18*
M042H17*
M019F16
H003K14
M042J16
M015N19
H001N04
M057H06
V036F24
M042F06*
V056M14
M032J16=
V024D04
M021I14
V060C04
M001K07*
V029G15
B004F13
M056F15
B003H09
M048I04=
B018P23
M020H16
B018L09
M008G21=
M028F05
M002I13=
0 2 4 6 8 10 12 14 16 18 20 22 24 26 28 30 32 34 36 38
[Chickpea contig p...
[root@localhost: /...
[FPC V8.9 Main Menu]
Project cp05T5e12...
FPC Ctg4472 cp05...

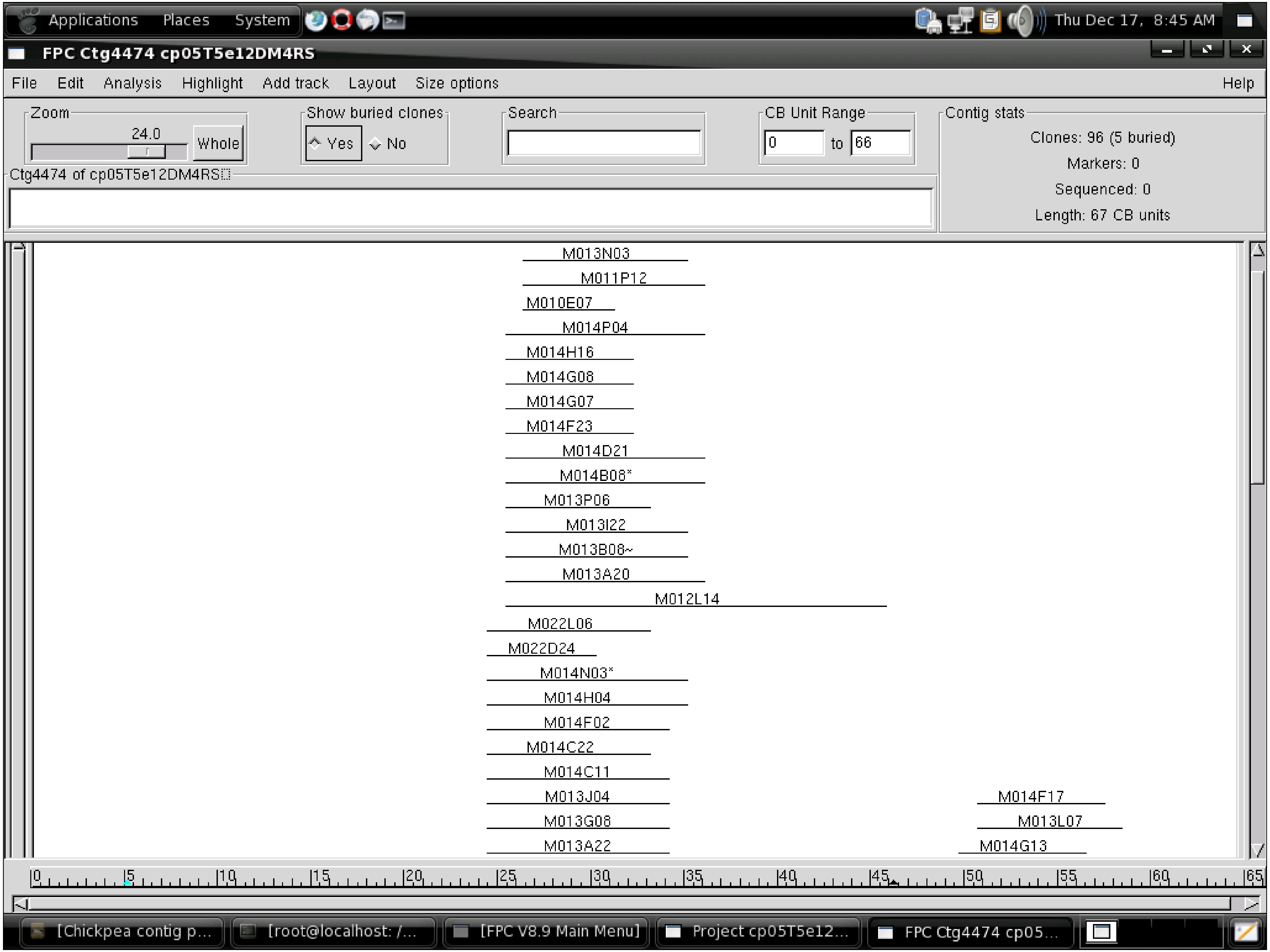

Applications Places System
Thu Dec 17, 8:45 AM
FPC Ctg4474 cp05T5e12DM4RS
File Edit Analysis Highlight Add track Layout Size options Help
Zoom 24.0 Whole
Show buried clones Yes No
Search
CB Unit Range 0 to 66
Contig stats
Clones: 96 (5 buried)
Markers: 0
Sequenced: 0
Length: 67 CB units
Ctg4474 of cp05T5e12DM4RS
M013N03
M011P12
M010E07
M014P04
M014H16
M014G08
M014G07
M014F23
M014D21
M014B08*
M013P06
M013I22
M013B08~
M013A20
M012L14
M022L06
M022D24
M014N03*
M014H04
M014F02
M014C22
M014C11
M013J04
M013G08
M013A22
M014F17
M013L07
M014G13
0 5 10 15 20 25 30 35 40 45 50 55 60 65
[Chickpea contig p...
[root@localhost: /...
[FPC V8.9 Main Menu]
Project cp05T5e12...
FPC Ctg4474 cp05...

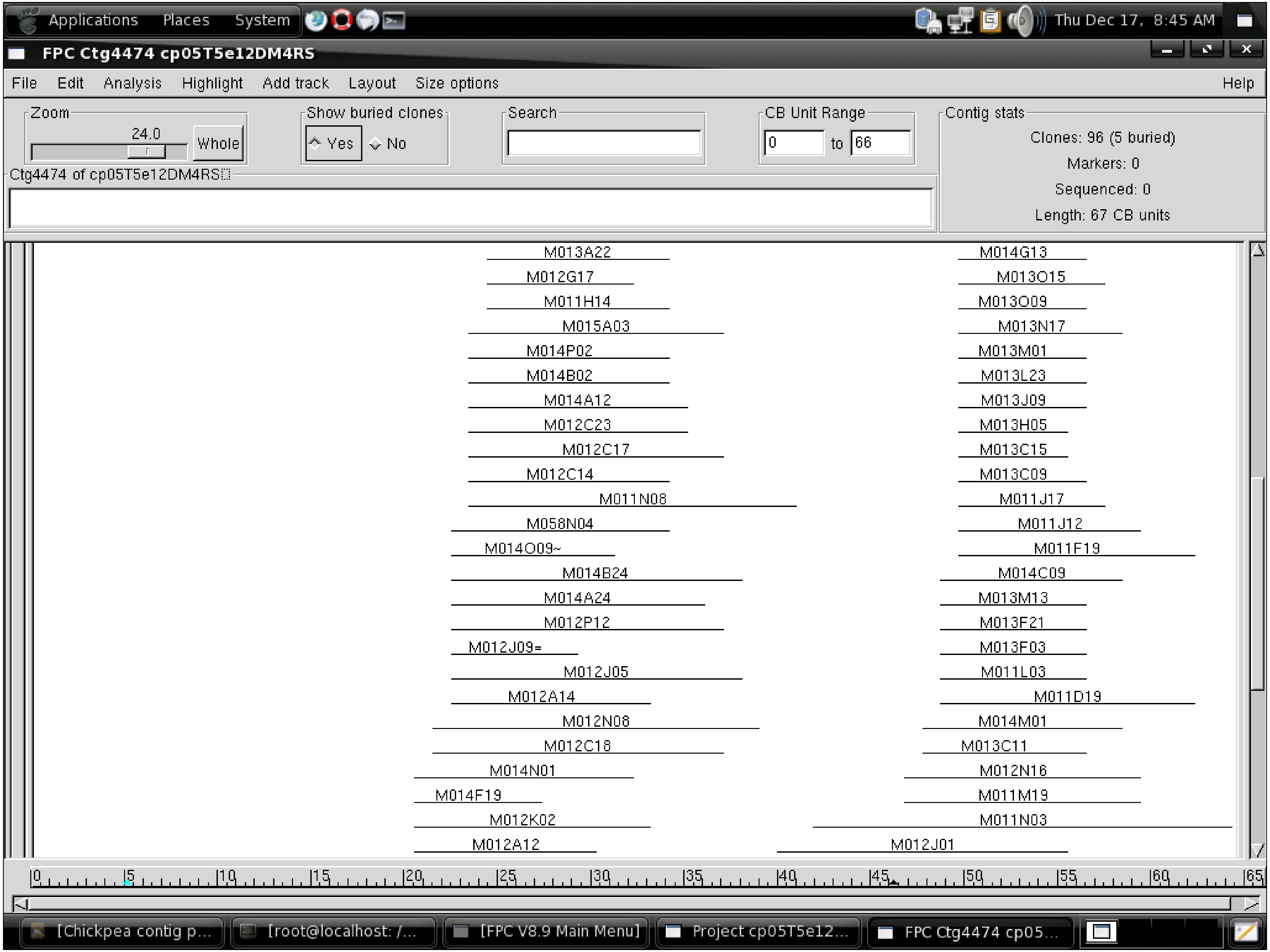
Applications Places System
Thu Dec 17, 8:45 AM
FPC Ctg4474 cp05T5e12DM4RS
File Edit Analysis Highlight Add track Layout Size options Help
Zoom
24.0
Whole
Show buried clones
Yes No
Search
CB Unit Range
0 to 66
Contig stats
Clones: 96 (5 buried)
Markers: 0
Sequenced: 0
Length: 67 CB units
Ctg4474 of cp05T5e12DM4RS
M013A22
M012G17
M011H14
M015A03
M014P02
M014B02
M014A12
M012C23
M012C17
M012C14
M011N08
M058N04
M014O09~
M014B24
M014A24
M012P12
M012J09=
M012J05
M012A14
M012N08
M012C18
M014N01
M014F19
M012K02
M012A12
M014G13
M013O15
M013O09
M013N17
M013M01
M013L23
M013J09
M013H05
M013C15
M013C09
M011J17
M011J12
M011F19
M014C09
M013M13
M013F21
M013F03
M011L03
M011D19
M014M01
M013C11
M012N16
M011M19
M011N03
M012J01
0 5 10 15 20 25 30 35 40 45 50 55 60 65
[Chickpea contig p...
[root@localhost: /...
[FPC V8.9 Main Menu]
Project cp05T5e12...
FPC Ctg4474 cp05...

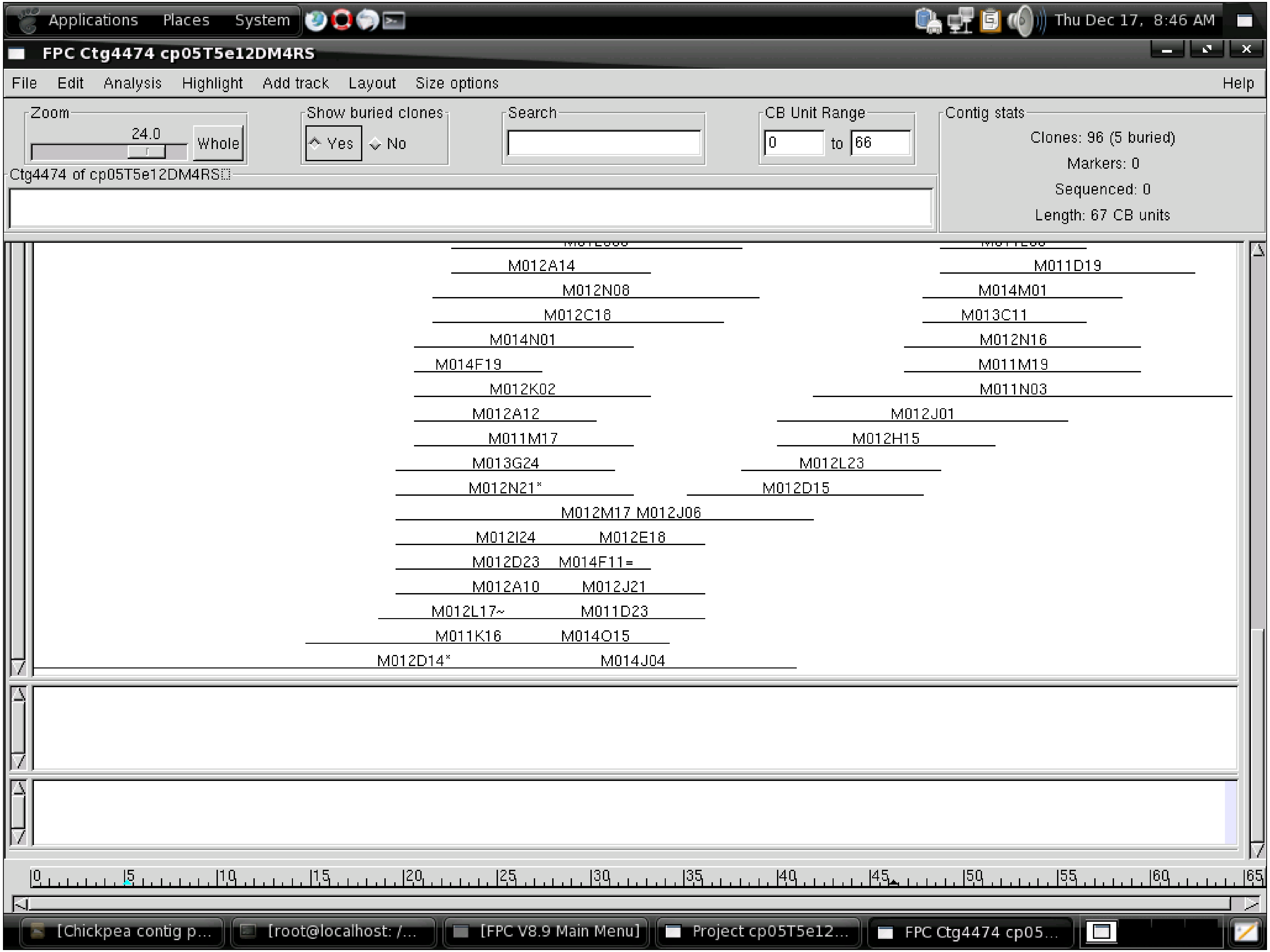
Applications Places System
Thu Dec 17, 8:46 AM
FPC Ctg4474 cp05T5e12DM4RS
File Edit Analysis Highlight Add track Layout Size options Help
Zoom
24.0
Whole
Show buried clones
Yes
No
Search
CB Unit Range
0 to 66
Contig stats
Clones: 96 (5 buried)
Markers: 0
Sequenced: 0
Length: 67 CB units
Ctg4474 of cp05T5e12DM4RS
M012A14
M011D19
M012N08
M014M01
M012C18
M013C11
M014N01
M012N16
M014F19
M011M19
M012K02
M011N03
M012A12
M012J01
M011M17
M012H15
M013G24
M012L23
M012N21*
M012D15
M012M17 M012J06
M012I24
M012E18
M012D23
M014F11=
M012A10
M012J21
M012L17~
M011D23
M011K16
M014O15
M012D14*
M014J04
0 5 10 15 20 25 30 35 40 45 50 55 60 65
[Chickpea contig p...
[root@localhost: /...
[FPC V8.9 Main Menu]
Project cp05T5e12...
FPC Ctg4474 cp05...

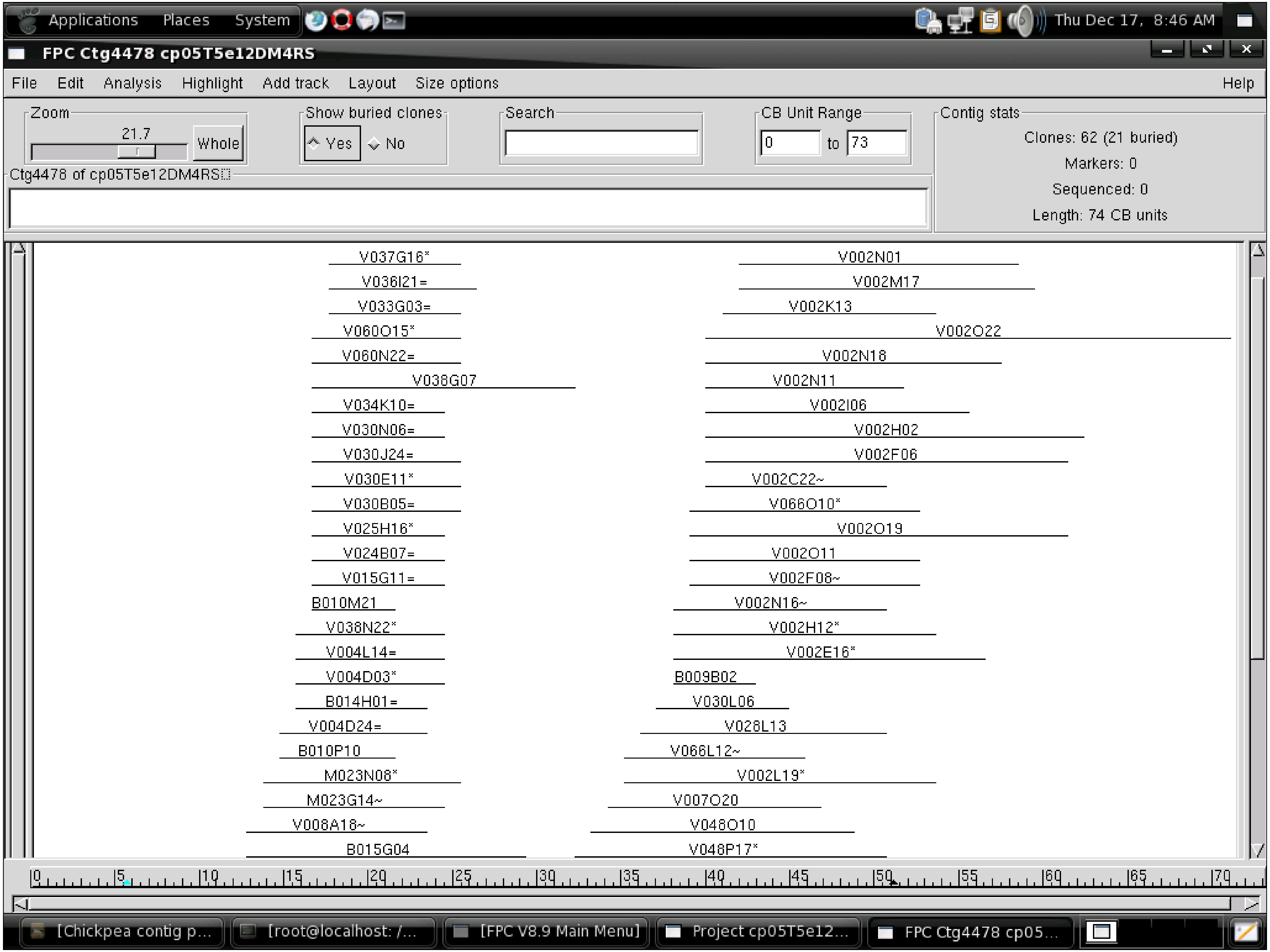

Applications
Places
System
Thu Dec 17, 8:46 AM
FPC Ctg4478 cp05T5e12DM4RS
File
Edit
Analysis
Highlight
Add track
Layout
Size options
Help
Zoom
21.7
Whole
Show buried clones
Yes
No
Search
CB Unit Range
0
to
73
Contig stats
Clones: 62 (21 buried)
Markers: 0
Sequenced: 0
Length: 74 CB units
Ctg4478 of cp05T5e12DM4RS
V037G16*
V036I21=
V033G03=
V060O15*
V060N22=
V038G07
V034K10=
V030N06=
V030J24=
V030E11*
V030B05=
V025H16*
V024B07=
V015G11=
B010M21
V038N22*
V004L14=
V004D03*
B014H01=
V004D24=
B010P10
M023N08*
M023G14~
V008A18~
B015G04
V002N01
V002M17
V002K13
V002O22
V002N18
V002N11
V002I06
V002H02
V002F06
V002C22~
V066O10*
V002O19
V002O11
V002F08~
V002N16~
V002H12*
V002E16*
B009B02
V030L06
V028L13
V066L12~
V002L19*
V007O20
V048O10
V048P17*
0
5
10
15
20
25
30
35
40
45
50
55
60
65
70
[Chickpea contig p...
[root@localhost: /...
[FPC V8.9 Main Menu]
Project cp05T5e12...
FPC Ctg4478 cp05...

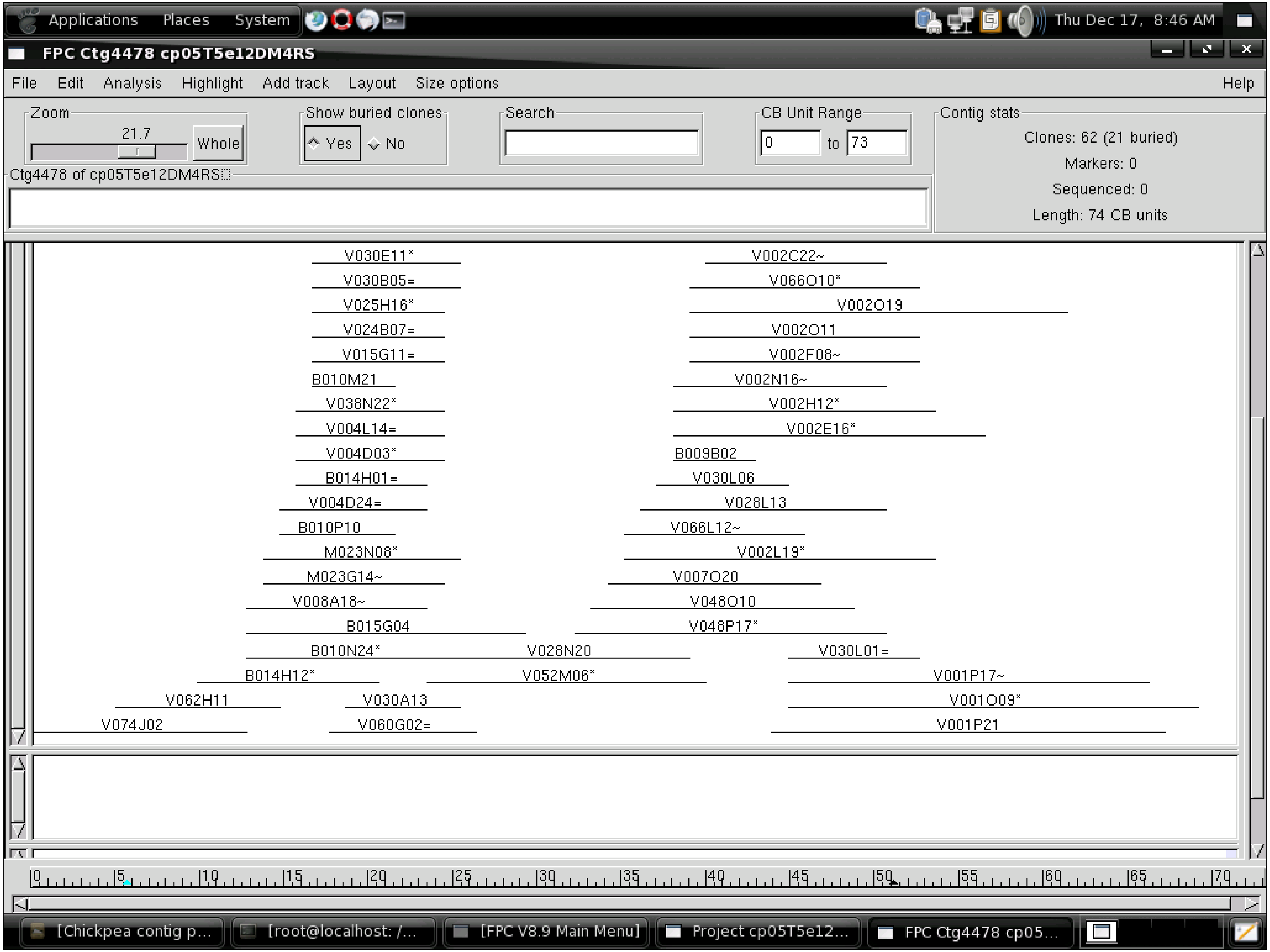
Applications Places System
Thu Dec 17, 8:46 AM
FPC Ctg4478 cp05T5e12DM4RS
File Edit Analysis Highlight Add track Layout Size options Help
Zoom
21.7
Whole
Show buried clones
Yes
No
Search
CB Unit Range
0 to 73
Contig stats
Clones: 62 (21 buried)
Markers: 0
Sequenced: 0
Length: 74 CB units
Ctg4478 of cp05T5e12DM4RS
V030E11*
V030B05=
V025H16*
V024B07=
V015G11=
B010M21
V038N22*
V004L14=
V004D03*
B014H01=
V004D24=
B010P10
M023N08*
M023G14~
V008A18~
B015G04
B010N24*
V028N20
B014H12*
V052M06*
V062H11
V030A13
V074J02
V060G02=
V002C22~
V066O10*
V002O19
V002O11
V002F08~
V002N16~
V002H12*
V002E16*
B009B02
V030L06
V028L13
V066L12~
V002L19*
V007O20
V048O10
V048P17*
V030L01=
V001P17~
V001O09*
V001P21
0 5 10 15 20 25 30 35 40 45 50 55 60 65 70
[Chickpea contig p...
[root@localhost: /...
[FPC V8.9 Main Menu]
Project cp05T5e12...
FPC Ctg4478 cp05...

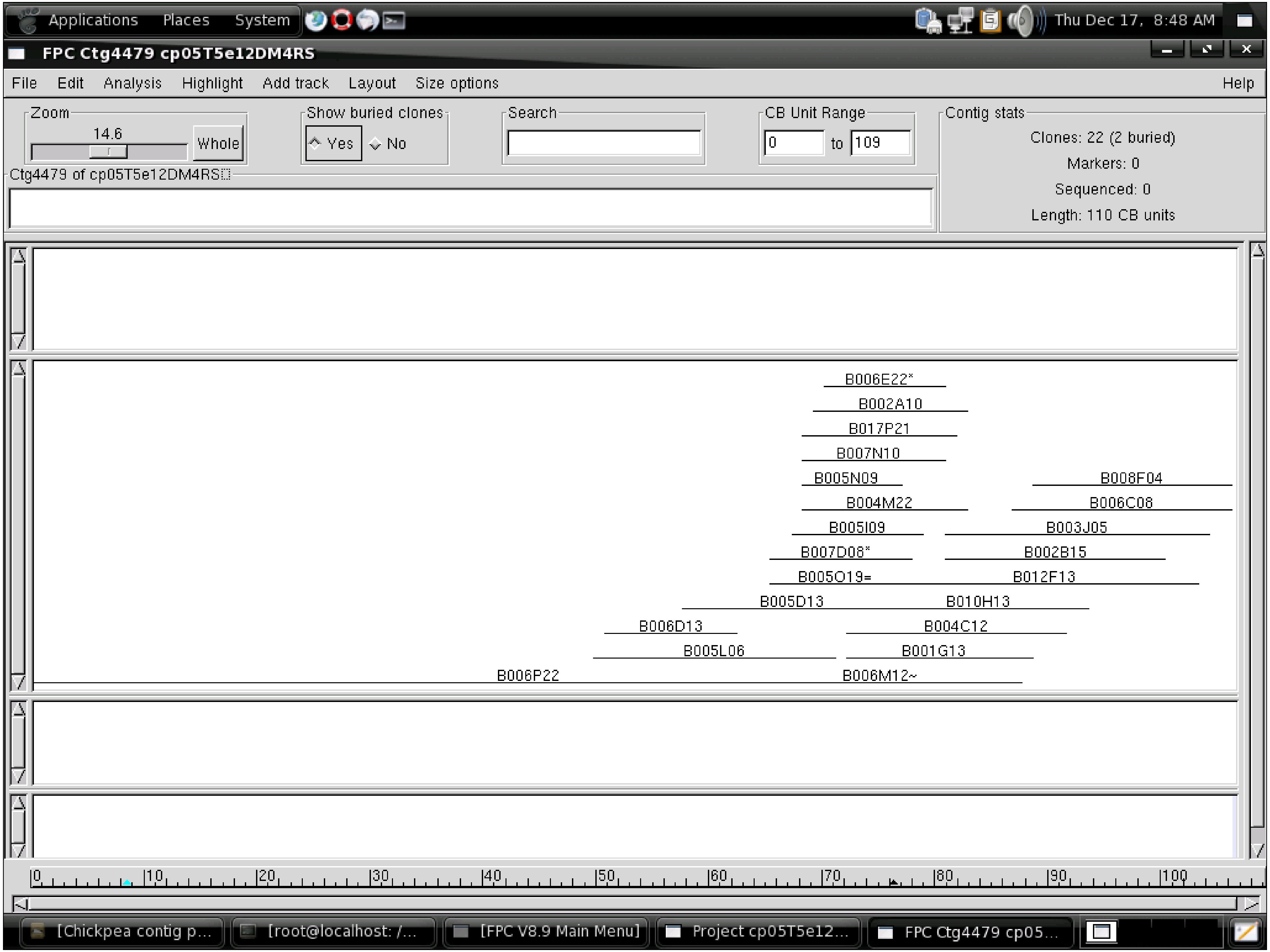
Applications Places System
Thu Dec 17, 8:48 AM
FPC Ctg4479 cp05T5e12DM4RS
File Edit Analysis Highlight Add track Layout Size options Help
Zoom
14.6
Whole
Show buried clones
Yes No
Search
CB Unit Range
0 to 109
Contig stats
Clones: 22 (2 buried)
Markers: 0
Sequenced: 0
Length: 110 CB units
Ctg4479 of cp05T5e12DM4RS
B006E22*
B002A10
B017P21
B007N10
B005N09
B008F04
B004M22
B006C08
B005I09
B003J05
B007D08*
B002B15
B005O19=
B012F13
B005D13
B010H13
B006D13
B004C12
B005L06
B001G13
B006P22
B006M12~
0 10 20 30 40 50 60 70 80 90 100
[Chickpea contig p...
[root@localhost: /...
[FPC V8.9 Main Menu]
Project cp05T5e12...
FPC Ctg4479 cp05...

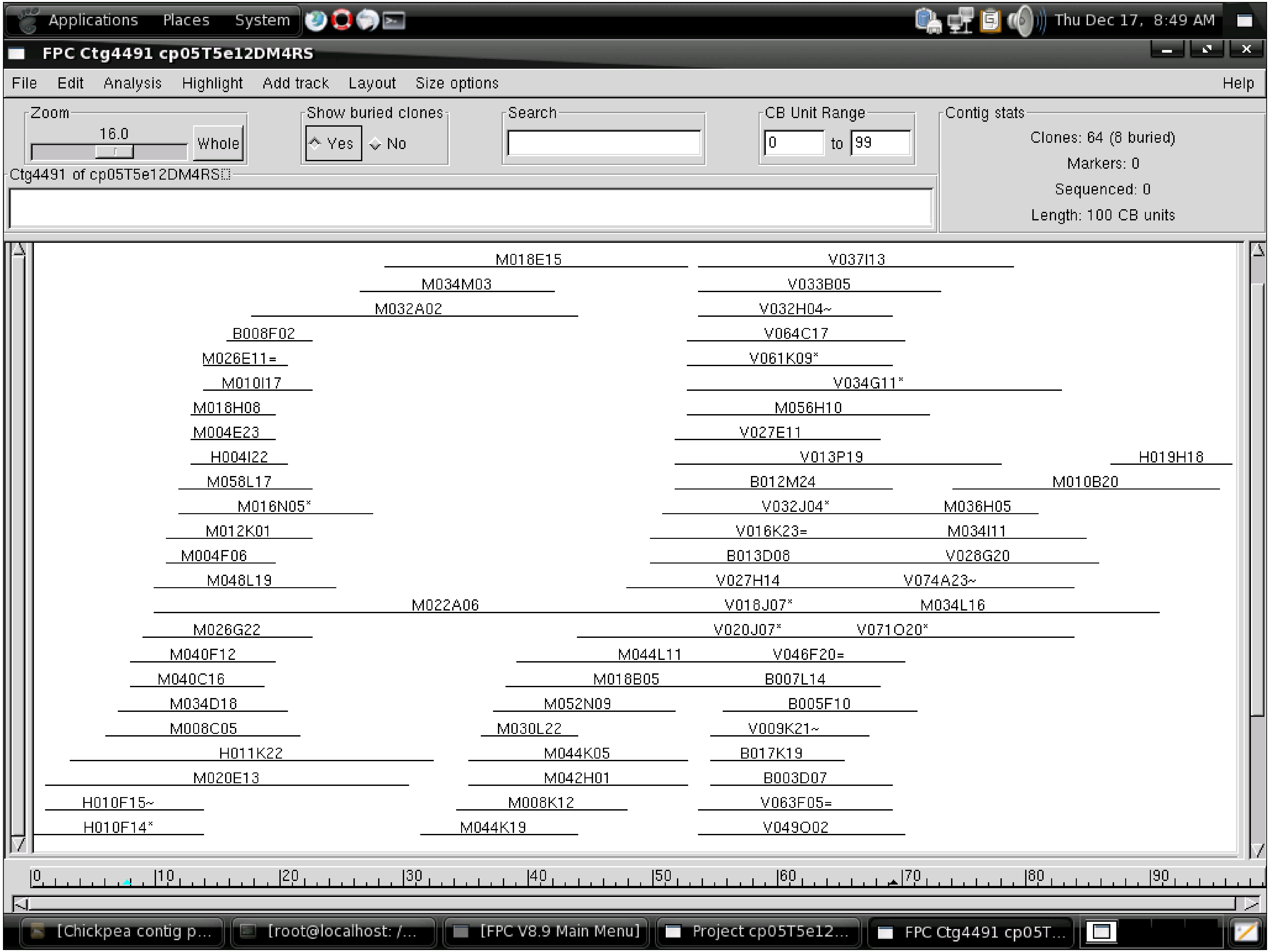
Applications Places System
Thu Dec 17, 8:49 AM
FPC Ctg4491 cp05T5e12DM4RS
File Edit Analysis Highlight Add track Layout Size options Help
Zoom
16.0
Whole
Show buried clones
Yes No
Search
CB Unit Range
0 to 99
Contig stats
Clones: 64 (8 buried)
Markers: 0
Sequenced: 0
Length: 100 CB units
Ctg4491 of cp05T5e12DM4RS
M018E15
V037I13
M034M03
V033B05
M032A02
V032H04~
B008F02
V064C17
M026E11=
V061K09*
M010I17
V034G11*
M018H08
M056H10
M004E23
V027E11
H004I22
V013P19
H019H18
M058L17
B012M24
M010B20
M016N05*
V032J04*
M036H05
M012K01
V016K23=
M034I11
M004F06
B013D08
V028G20
M048L19
V027H14
V074A23~
M022A06
V018J07*
M034L16
M026G22
V020J07*
V071O20*
M040F12
M044L11
V046F20=
M040C16
M018B05
B007L14
M034D18
M052N09
B005F10
M008C05
M030L22
V009K21~
H011K22
M044K05
B017K19
M020E13
M042H01
B003D07
H010F15~
M008K12
V063F05=
H010F14*
M044K19
V049O02
0 10 20 30 40 50 60 70 80 90
[Chickpea contig p...
[root@localhost: /...
[FPC V8.9 Main Menu]
Project cp05T5e12...
FPC Ctg4491 cp05T...

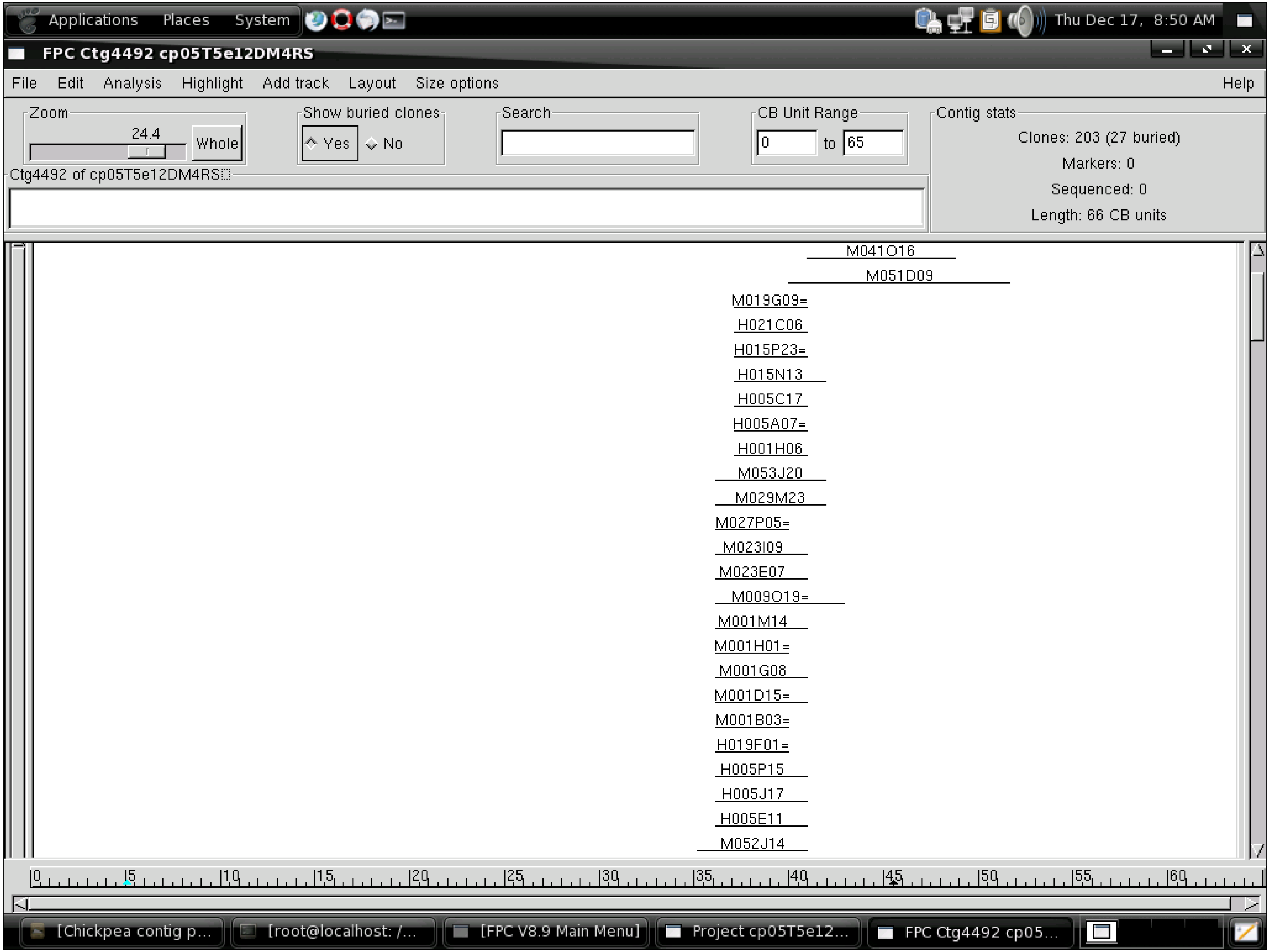
Applications Places System
Thu Dec 17, 8:50 AM
FPC Ctg4492 cp05T5e12DM4RS
File Edit Analysis Highlight Add track Layout Size options Help
Zoom
24.4
Whole
Show buried clones
Yes No
Search
CB Unit Range
0 to 65
Contig stats
Clones: 203 (27 buried)
Markers: 0
Sequenced: 0
Length: 66 CB units
Ctg4492 of cp05T5e12DM4RS
M041O16
M051D09
M019G09=
H021C06
H015P23=
H015N13
H005C17
H005A07=
H001H06
M053J20
M029M23
M027P05=
M023I09
M023E07
M009O19=
M001M14
M001H01=
M001G08
M001D15=
M001B03=
H019F01=
H005P15
H005J17
H005E11
M052J14
0 5 10 15 20 25 30 35 40 45 50 55 60
[Chickpea contig p...
[root@localhost: /...
[FPC V8.9 Main Menu]
Project cp05T5e12...
FPC Ctg4492 cp05...

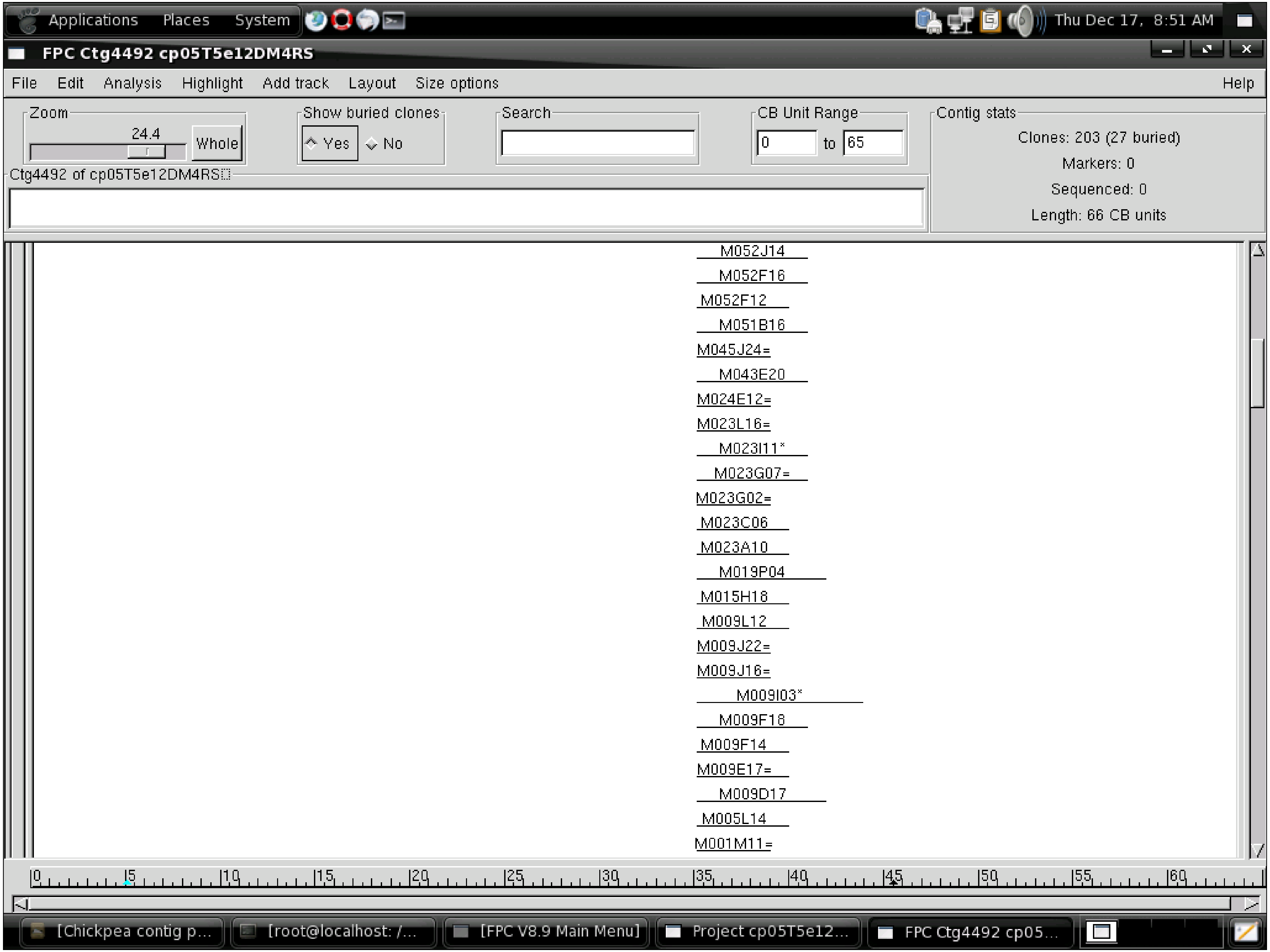

Applications Places System
Thu Dec 17, 8:51 AM
FPC Ctg4492 cp05T5e12DM4RS
File Edit Analysis Highlight Add track Layout Size options Help
Zoom
24.4
Whole
Show buried clones
Yes No
Search
CB Unit Range
0 to 65
Contig stats
Clones: 203 (27 buried)
Markers: 0
Sequenced: 0
Length: 66 CB units
Ctg4492 of cp05T5e12DM4RS
M052J14
M052F16
M052F12
M051B16
M045J24=
M043E20
M024E12=
M023L16=
M023I11*
M023G07=
M023G02=
M023C06
M023A10
M019P04
M015H18
M009L12
M009J22=
M009J16=
M009I03*
M009F18
M009F14
M009E17=
M009D17
M005L14
M001M11=
0 5 10 15 20 25 30 35 40 45 50 55 60
[Chickpea contig p...
[root@localhost: /...
[FPC V8.9 Main Menu]
Project cp05T5e12...
FPC Ctg4492 cp05...

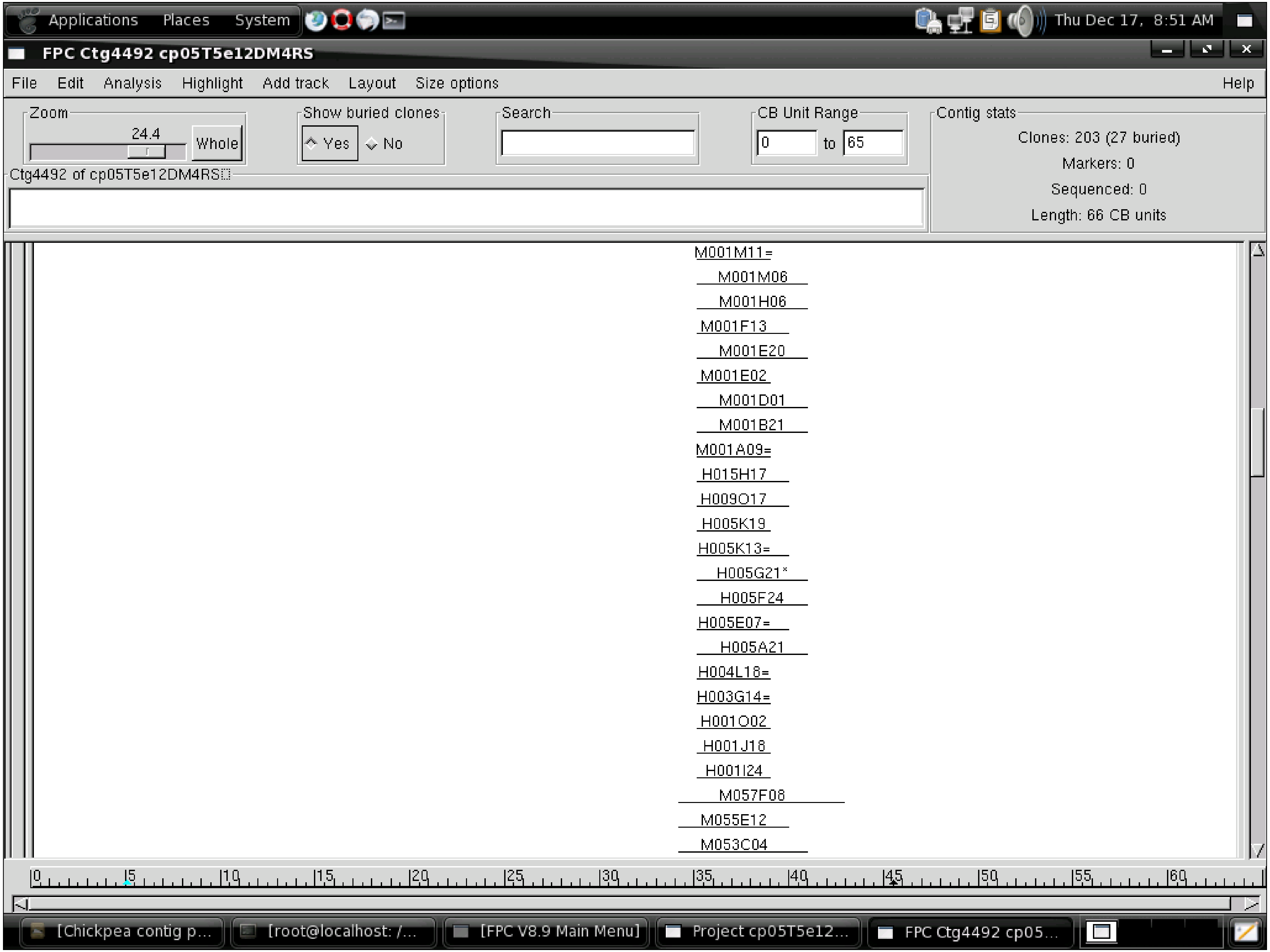
Applications Places System
Thu Dec 17, 8:51 AM
FPC Ctg4492 cp05T5e12DM4RS
File Edit Analysis Highlight Add track Layout Size options Help
Zoom
24.4
Whole
Show buried clones
Yes No
Search
CB Unit Range
0 to 65
Contig stats
Clones: 203 (27 buried)
Markers: 0
Sequenced: 0
Length: 66 CB units
Ctg4492 of cp05T5e12DM4RS
M001M11=
M001M06
M001H06
M001F13
M001E20
M001E02
M001D01
M001B21
M001A09=
H015H17
H009O17
H005K19
H005K13=
H005G21*
H005F24
H005E07=
H005A21
H004L18=
H003G14=
H001O02
H001J18
H001I24
M057F08
M055E12
M053C04
0 5 10 15 20 25 30 35 40 45 50 55 60
[Chickpea contig p...
[root@localhost: /...
[FPC V8.9 Main Menu]
Project cp05T5e12...
FPC Ctg4492 cp05...

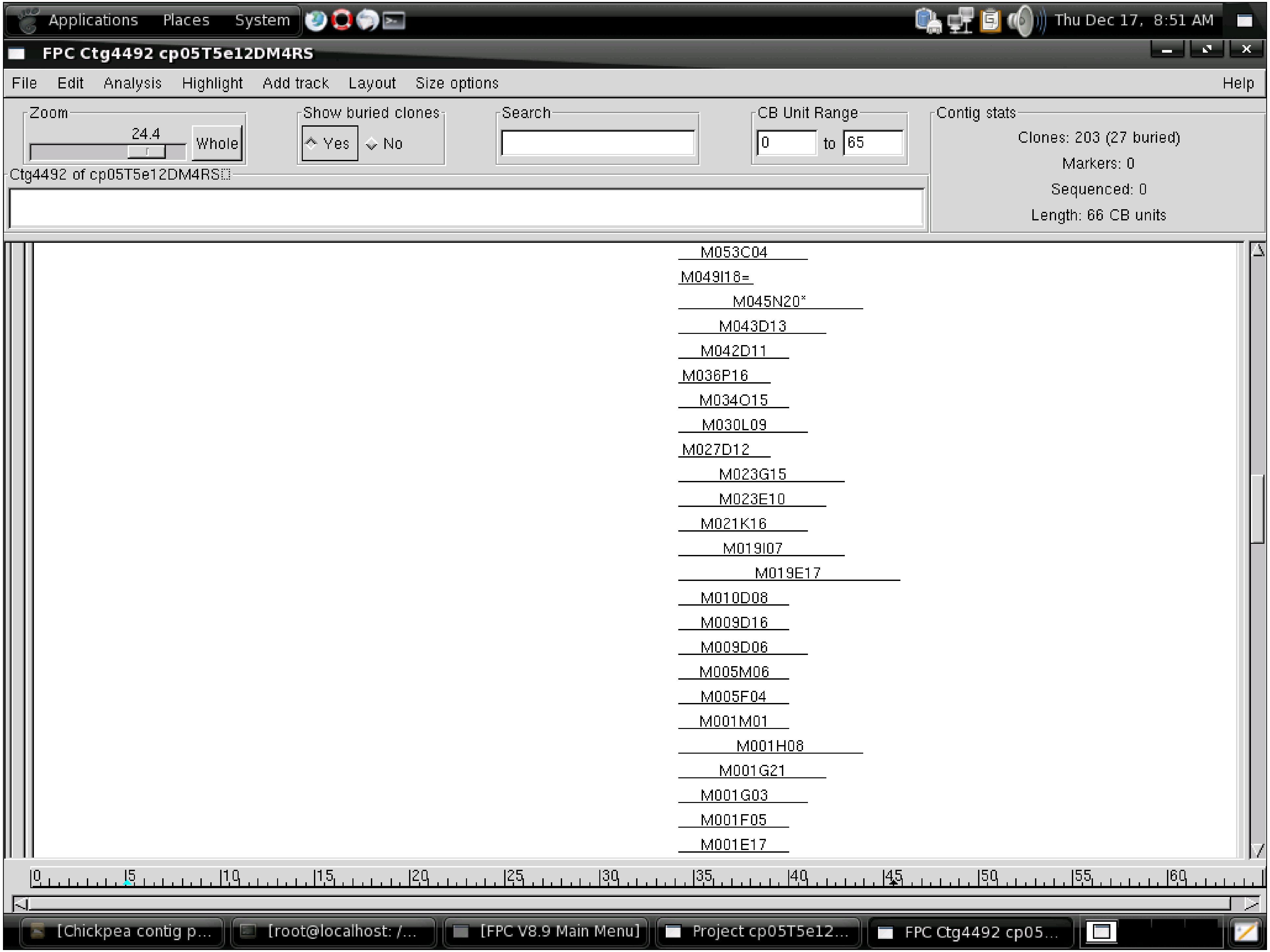
Applications Places System
Thu Dec 17, 8:51 AM
FPC Ctg4492 cp05T5e12DM4RS
File Edit Analysis Highlight Add track Layout Size options Help
Zoom
24.4
Whole
Show buried clones
Yes No
Search
CB Unit Range
0 to 65
Contig stats
Clones: 203 (27 buried)
Markers: 0
Sequenced: 0
Length: 66 CB units
Ctg4492 of cp05T5e12DM4RS
M053C04
M049I18=
M045N20*
M043D13
M042D11
M036P16
M034O15
M030L09
M027D12
M023G15
M023E10
M021K16
M019I07
M019E17
M010D08
M009D16
M009D06
M005M06
M005F04
M001M01
M001H08
M001G21
M001G03
M001F05
M001E17
0 5 10 15 20 25 30 35 40 45 50 55 60
[Chickpea contig p...
[root@localhost: /...
[FPC V8.9 Main Menu]
Project cp05T5e12...
FPC Ctg4492 cp05...

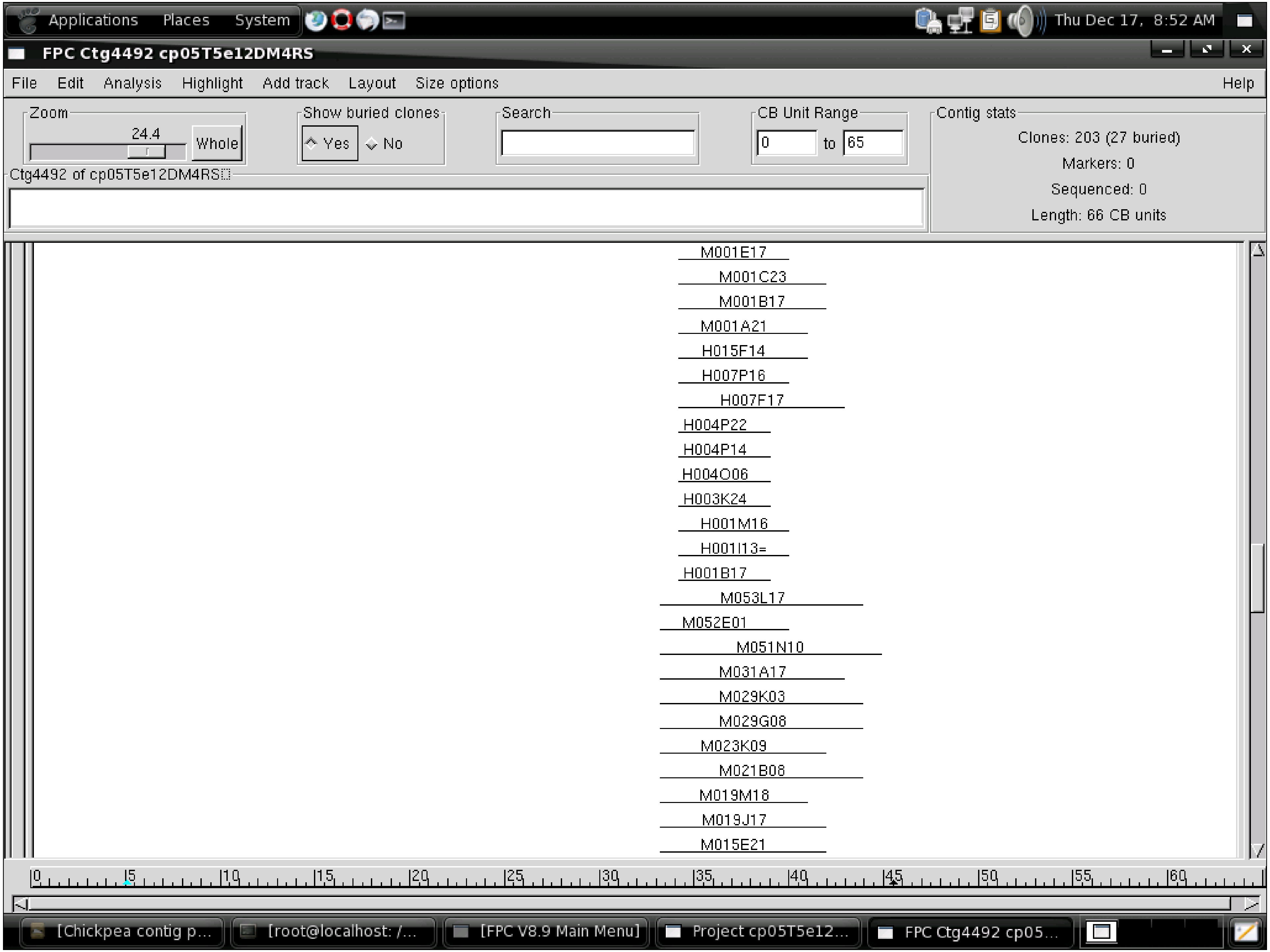
Applications Places System
Thu Dec 17, 8:52 AM
FPC Ctg4492 cp05T5e12DM4RS
File Edit Analysis Highlight Add track Layout Size options Help
Zoom
24.4
Whole
Show buried clones
Yes No
Search
CB Unit Range
0 to 65
Contig stats
Clones: 203 (27 buried)
Markers: 0
Sequenced: 0
Length: 66 CB units
Ctg4492 of cp05T5e12DM4RS
M001E17
M001C23
M001B17
M001A21
H015F14
H007P16
H007F17
H004P22
H004P14
H004O06
H003K24
H001M16
H001I13=
H001B17
M053L17
M052E01
M051N10
M031A17
M029K03
M029G08
M023K09
M021B08
M019M18
M019J17
M015E21
0 5 10 15 20 25 30 35 40 45 50 55 60
[Chickpea contig p...
[root@localhost: /...
[FPC V8.9 Main Menu]
Project cp05T5e12...
FPC Ctg4492 cp05...

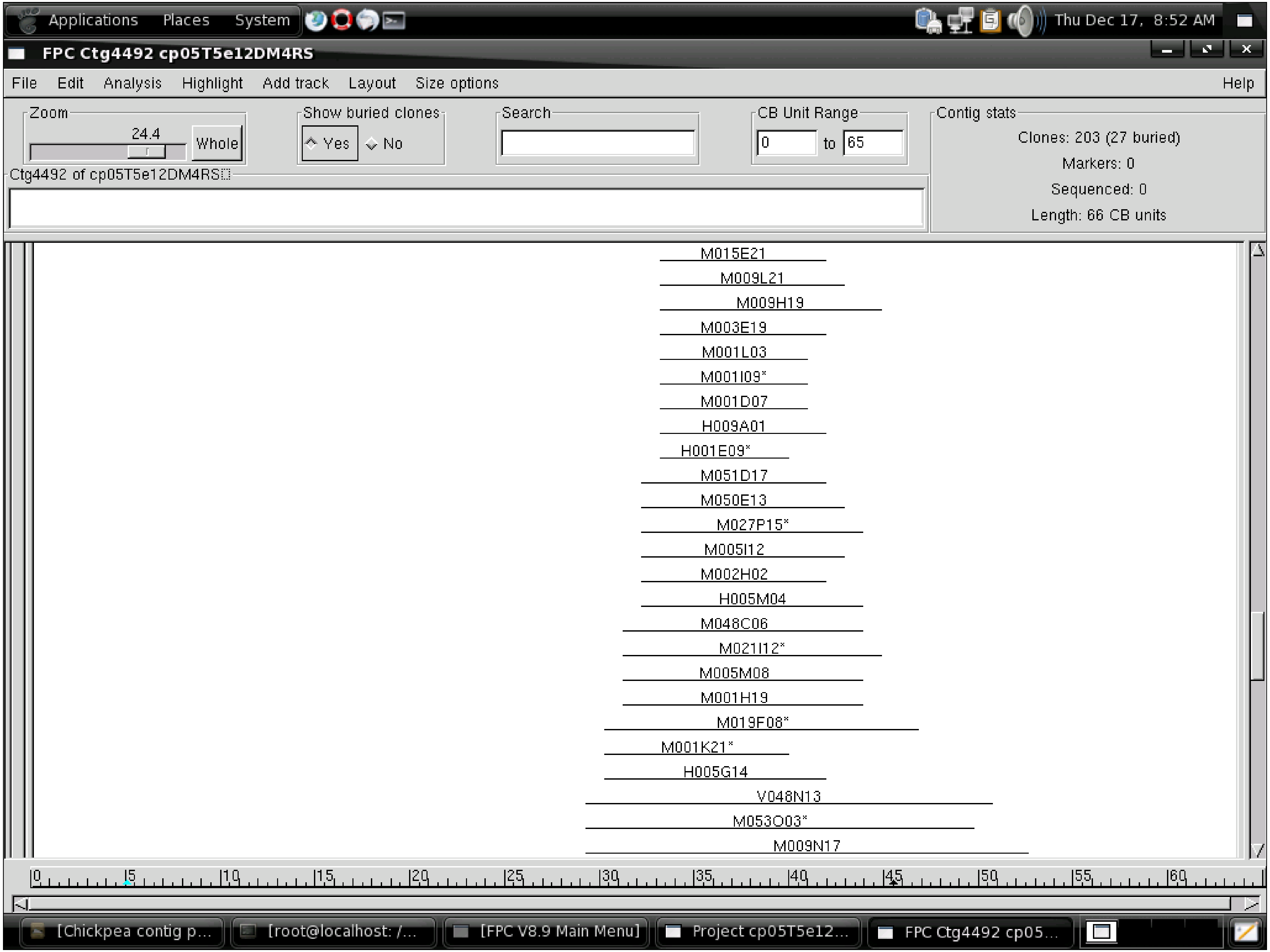
Applications Places System
Thu Dec 17, 8:52 AM
FPC Ctg4492 cp05T5e12DM4RS
File Edit Analysis Highlight Add track Layout Size options Help
Zoom
24.4
Whole
Show buried clones
Yes No
Search
CB Unit Range
0 to 65
Contig stats
Clones: 203 (27 buried)
Markers: 0
Sequenced: 0
Length: 66 CB units
Ctg4492 of cp05T5e12DM4RS
M015E21
M009L21
M009H19
M003E19
M001L03
M001I09*
M001D07
H009A01
H001E09*
M051D17
M050E13
M027P15*
M005I12
M002H02
H005M04
M048C06
M021I12*
M005M08
M001H19
M019F08*
M001K21*
H005G14
V048N13
M053O03*
M009N17
0 5 10 15 20 25 30 35 40 45 50 55 60
[Chickpea contig p...
[root@localhost: /...
[FPC V8.9 Main Menu]
Project cp05T5e12...
FPC Ctg4492 cp05...

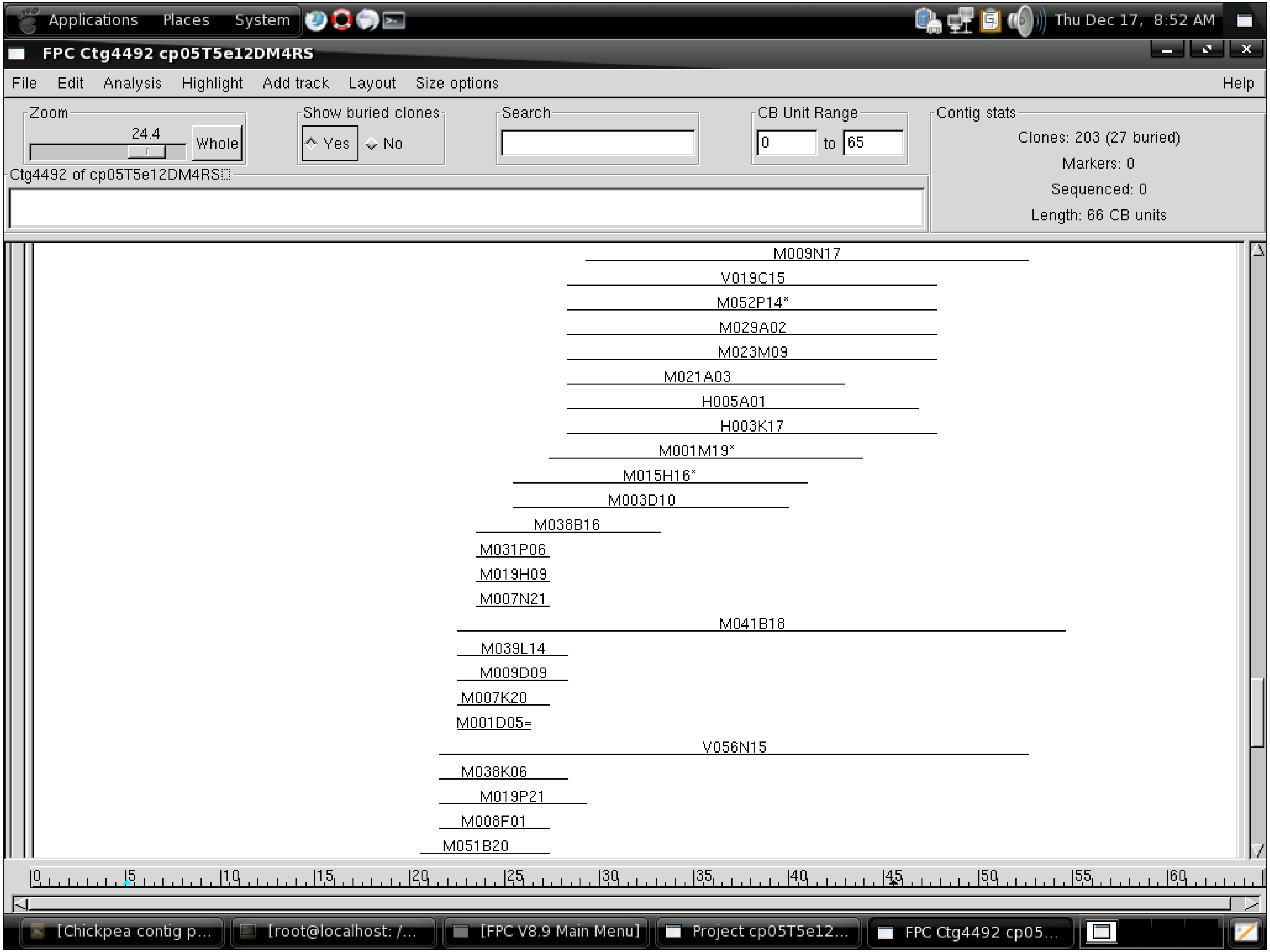
Applications Places System
Thu Dec 17, 8:52 AM
FPC Ctg4492 cp05T5e12DM4RS
File Edit Analysis Highlight Add track Layout Size options Help
Zoom
24.4
Whole
Show buried clones
Yes No
Search
CB Unit Range
0 to 65
Contig stats
Clones: 203 (27 buried)
Markers: 0
Sequenced: 0
Length: 66 CB units
Ctg4492 of cp05T5e12DM4RS
M009N17
V019C15
M052P14*
M029A02
M023M09
M021A03
H005A01
H003K17
M001M19*
M015H16*
M003D10
M038B16
M031P06
M019H09
M007N21
M041B18
M039L14
M009D09
M007K20
M001D05=
V056N15
M038K06
M019P21
M008F01
M051B20
0 5 10 15 20 25 30 35 40 45 50 55 60
[Chickpea contig p...
[root@localhost: /...
[FPC V8.9 Main Menu]
Project cp05T5e12...
FPC Ctg4492 cp05...

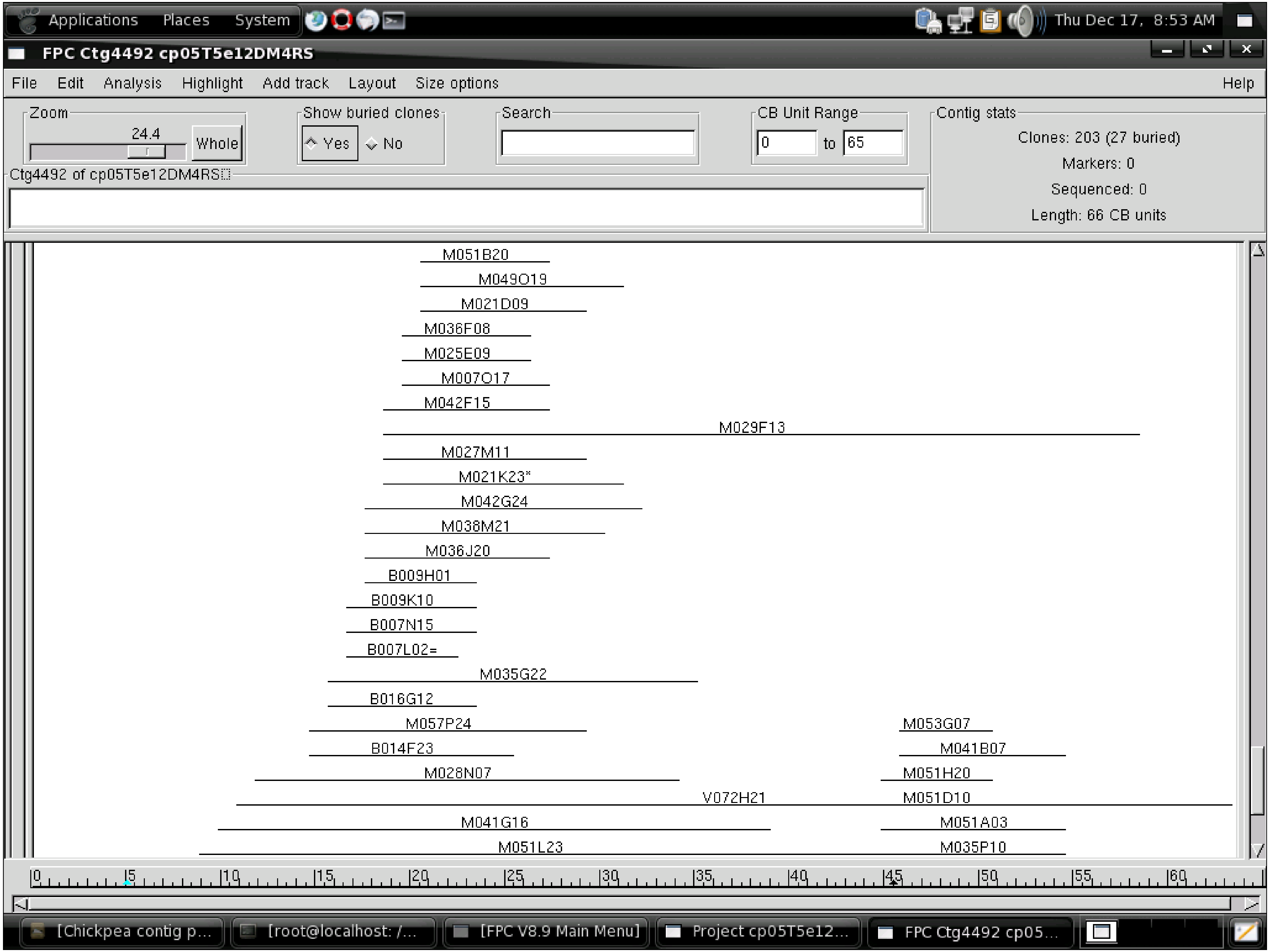
Applications Places System
Thu Dec 17, 8:53 AM
FPC Ctg4492 cp05T5e12DM4RS
File Edit Analysis Highlight Add track Layout Size options Help
Zoom
24.4
Whole
Show buried clones
Yes No
Search
CB Unit Range
0 to 65
Contig stats
Clones: 203 (27 buried)
Markers: 0
Sequenced: 0
Length: 66 CB units
Ctg4492 of cp05T5e12DM4RS
M051B20
M049O19
M021D09
M036F08
M025E09
M007O17
M042F15
M029F13
M027M11
M021K23*
M042G24
M038M21
M036J20
B009H01
B009K10
B007N15
B007L02=
M035G22
B016G12
M057P24
M053G07
B014F23
M041B07
M028N07
M051H20
V072H21
M051D10
M041G16
M051A03
M051L23
M035P10
0 5 10 15 20 25 30 35 40 45 50 55 60
[Chickpea contig p...
[root@localhost: /...
[FPC V8.9 Main Menu]
Project cp05T5e12...
FPC Ctg4492 cp05...

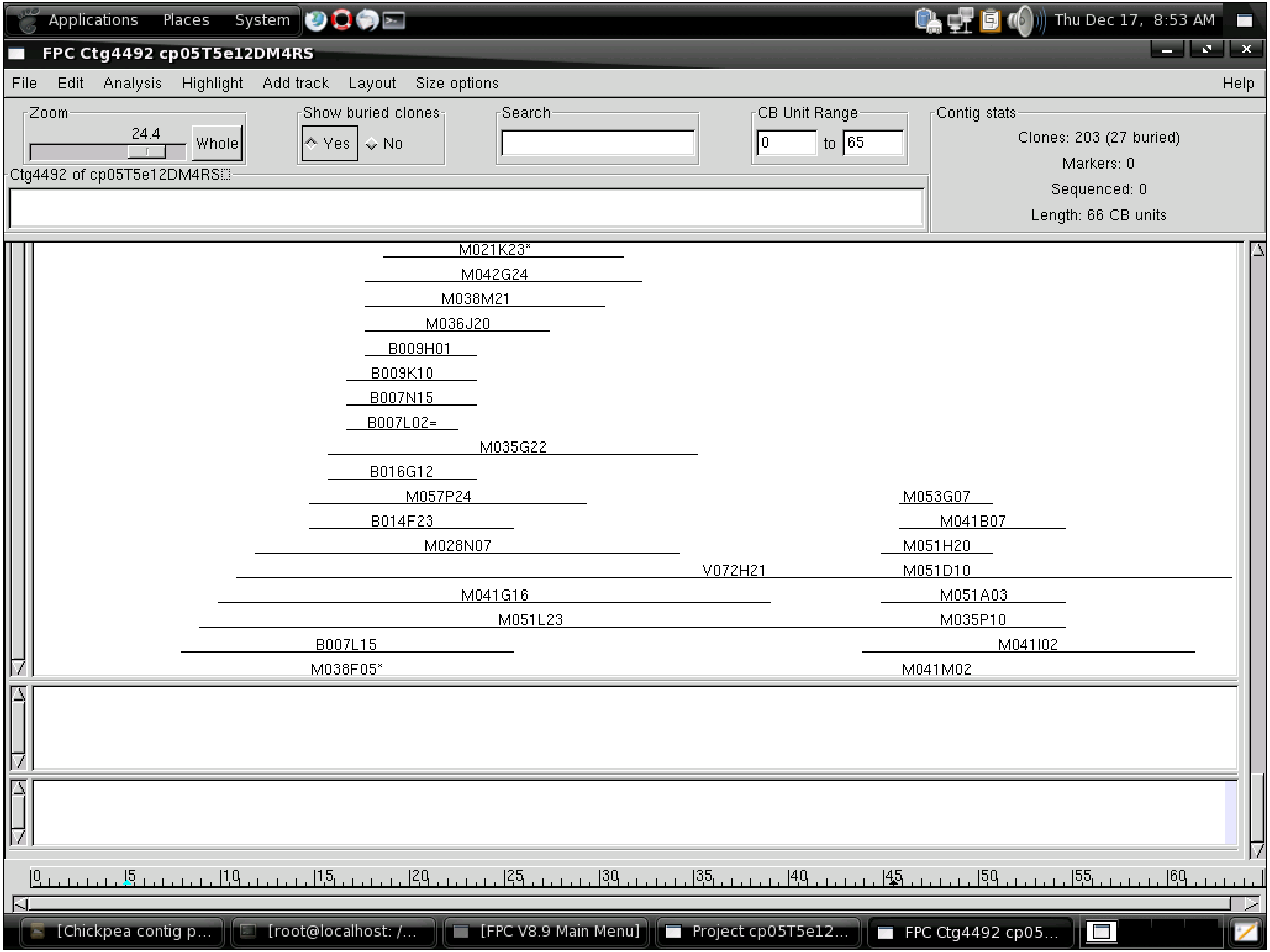
Applications Places System
Thu Dec 17, 8:53 AM
FPC Ctg4492 cp05T5e12DM4RS
File Edit Analysis Highlight Add track Layout Size options Help
Zoom
24.4
Whole
Show buried clones
Yes No
Search
CB Unit Range
0 to 65
Contig stats
Clones: 203 (27 buried)
Markers: 0
Sequenced: 0
Length: 66 CB units
Ctg4492 of cp05T5e12DM4RS
M021K23*
M042G24
M038M21
M036J20
B009H01
B009K10
B007N15
B007L02=
M035G22
B016G12
M057P24
M053G07
B014F23
M041B07
M028N07
M051H20
V072H21
M051D10
M041G16
M051A03
M051L23
M035P10
B007L15
M041I02
M038F05*
M041M02
0 5 10 15 20 25 30 35 40 45 50 55 60
[Chickpea contig p...
[root@localhost: /...
[FPC V8.9 Main Menu]
Project cp05T5e12...
FPC Ctg4492 cp05...

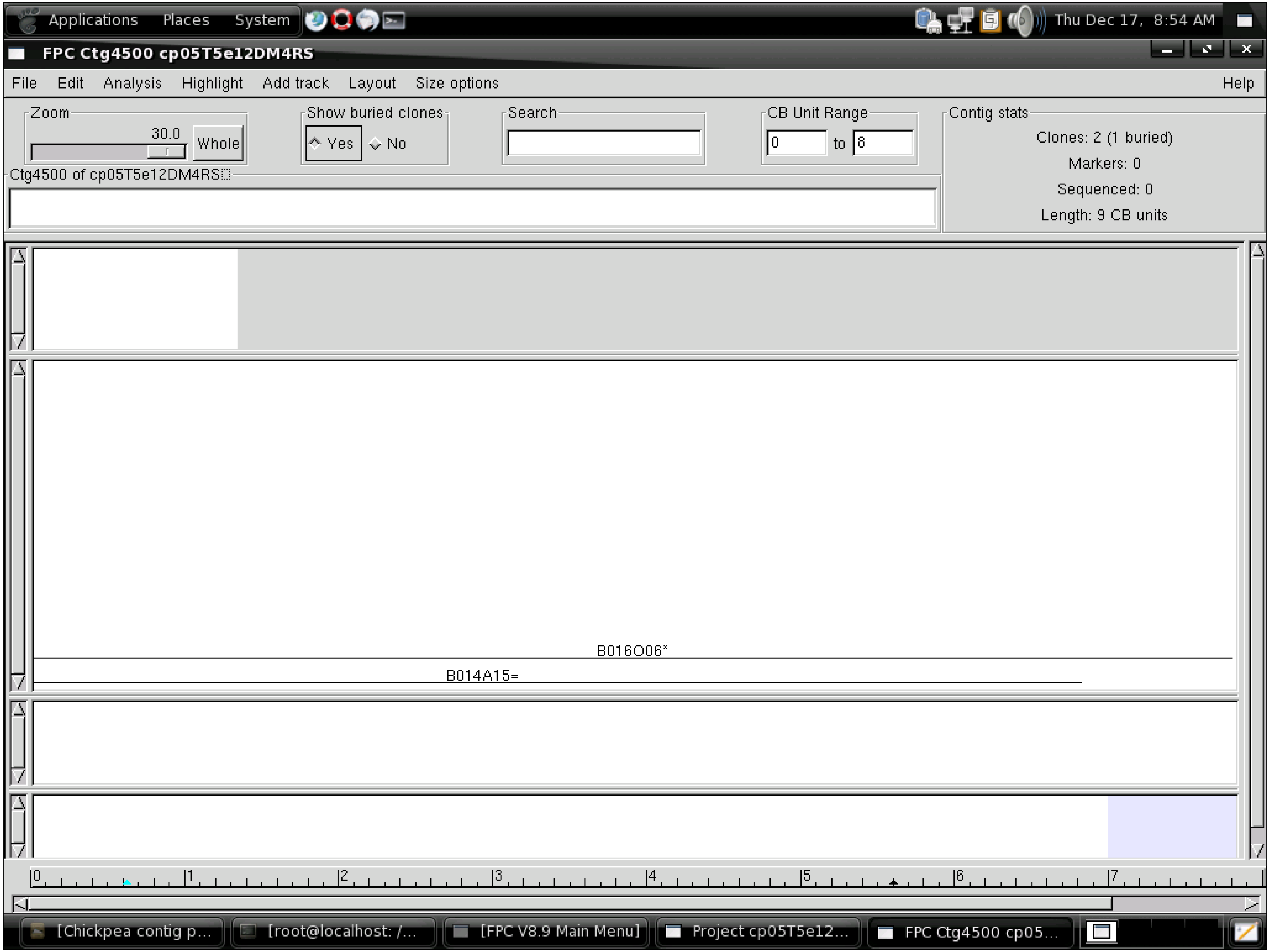
Applications Places System
Thu Dec 17, 8:54 AM
FPC Ctg4500 cp05T5e12DM4RS
File Edit Analysis Highlight Add track Layout Size options Help
Zoom
30.0
Whole
Show buried clones
Yes
No
Search
CB Unit Range
0
to
8
Contig stats
Clones: 2 (1 buried)
Markers: 0
Sequenced: 0
Length: 9 CB units
Ctg4500 of cp05T5e12DM4RS
B016O06*
B014A15=
0 1 2 3 4 5 6 7
[Chickpea contig p...
[root@localhost: /...
[FPC V8.9 Main Menu]
Project cp05T5e12...
FPC Ctg4500 cp05...

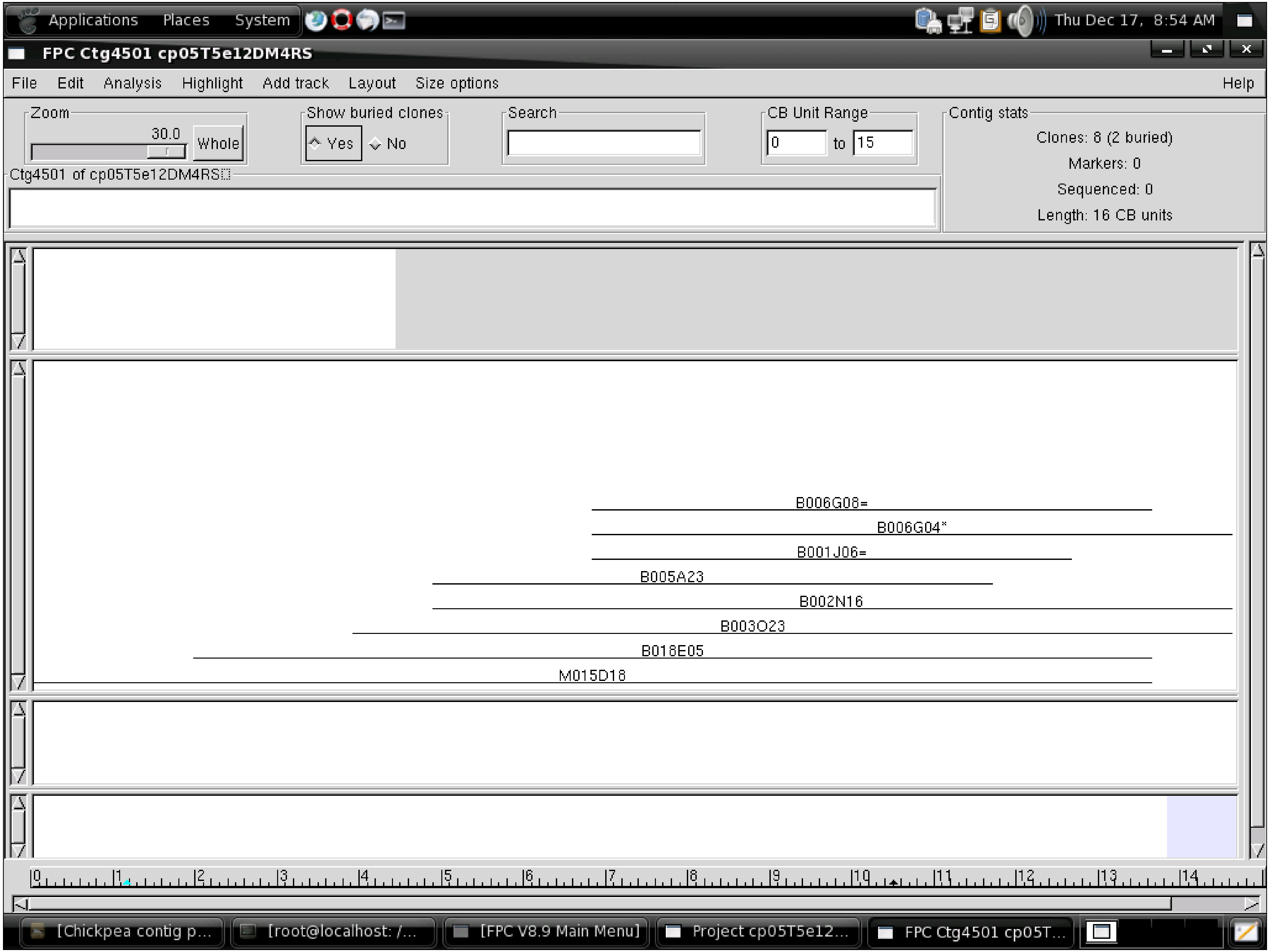
Applications Places System
Thu Dec 17, 8:54 AM
FPC Ctg4501 cp05T5e12DM4RS
File Edit Analysis Highlight Add track Layout Size options Help
Zoom
30.0
Whole
Show buried clones
Yes
No
Search
CB Unit Range
0 to 15
Contig stats
Clones: 8 (2 buried)
Markers: 0
Sequenced: 0
Length: 16 CB units
Ctg4501 of cp05T5e12DM4RS
B006G08=
B006G04*
B001J06=
B005A23
B002N16
B003O23
B018E05
M015D18
0 1 2 3 4 5 6 7 8 9 10 11 12 13 14
[Chickpea contig p...
[root@localhost: /...
[FPC V8.9 Main Menu]
Project cp05T5e12...
FPC Ctg4501 cp05T...

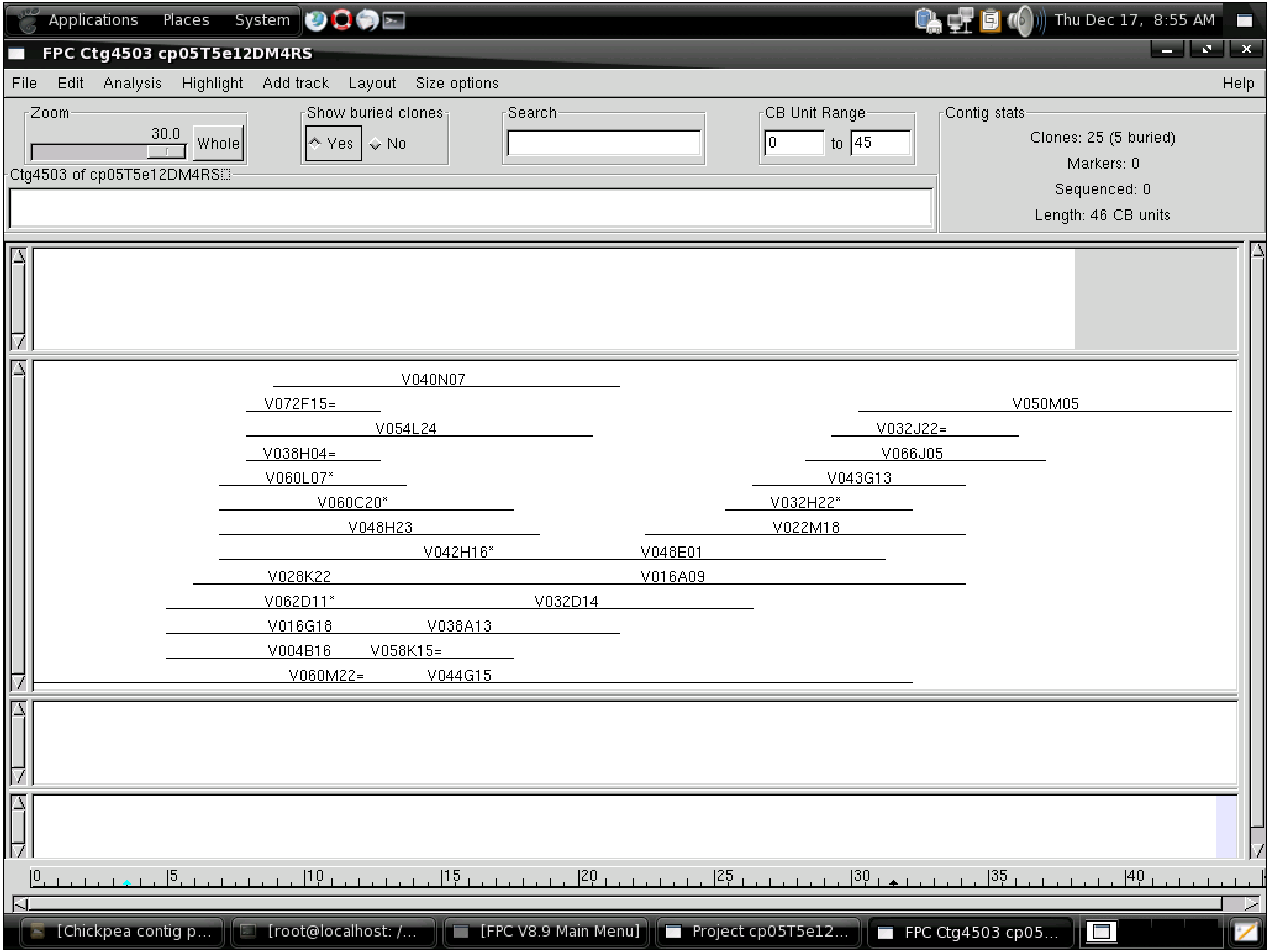
Applications Places System
Thu Dec 17, 8:55 AM
FPC Ctg4503 cp05T5e12DM4RS
File Edit Analysis Highlight Add track Layout Size options Help
Zoom
30.0
Whole
Show buried clones
Yes No
Search
CB Unit Range
0 to 45
Contig stats
Clones: 25 (5 buried)
Markers: 0
Sequenced: 0
Length: 46 CB units
Ctg4503 of cp05T5e12DM4RS
V040N07
V072F15=
V050M05
V054L24
V032J22=
V038H04=
V066J05
V060L07*
V043G13
V060C20*
V032H22*
V048H23
V022M18
V042H16*
V048E01
V028K22
V016A09
V062D11*
V032D14
V016G18
V038A13
V004B16
V058K15=
V060M22=
V044G15
0 5 10 15 20 25 30 35 40
[Chickpea contig p...
[root@localhost: /...
[FPC V8.9 Main Menu]
Project cp05T5e12...
FPC Ctg4503 cp05...

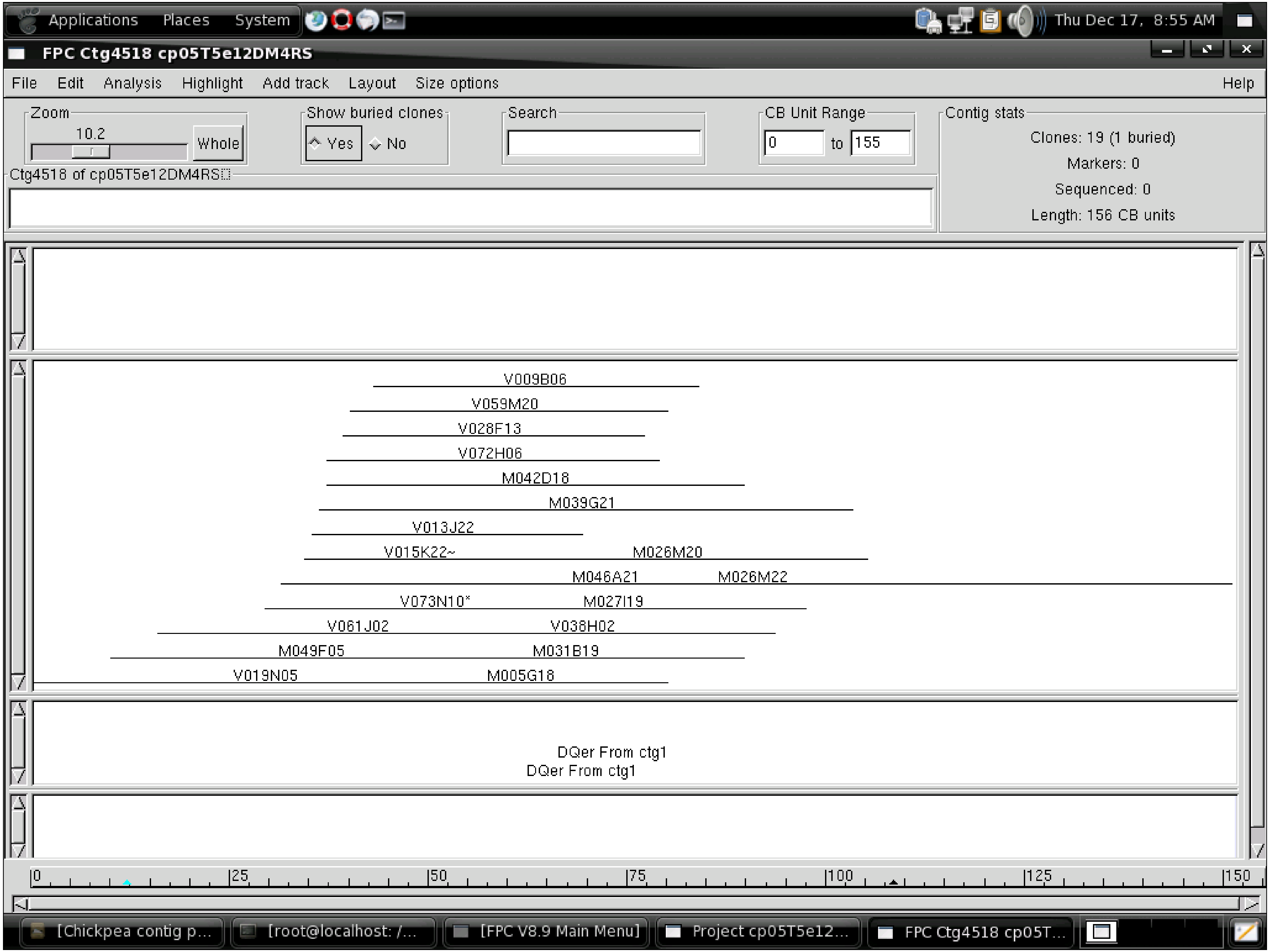
Applications Places System
Thu Dec 17, 8:55 AM
FPC Ctg4518 cp05T5e12DM4RS
File Edit Analysis Highlight Add track Layout Size options Help
Zoom
10.2
Whole
Show buried clones
Yes No
Search
CB Unit Range
0 to 155
Contig stats
Clones: 19 (1 buried)
Markers: 0
Sequenced: 0
Length: 156 CB units
Ctg4518 of cp05T5e12DM4RS
V009B06
V059M20
V028F13
V072H06
M042D18
M039G21
V013J22
V015K22~
M026M20
M046A21
M026M22
V073N10*
M027I19
V061J02
V038H02
M049F05
M031B19
V019N05
M005G18
DQer From ctg1
DQer From ctg1
0 25 50 75 100 125 150
[Chickpea contig p...
[root@localhost: /...
[FPC V8.9 Main Menu]
Project cp05T5e12...
FPC Ctg4518 cp05T...

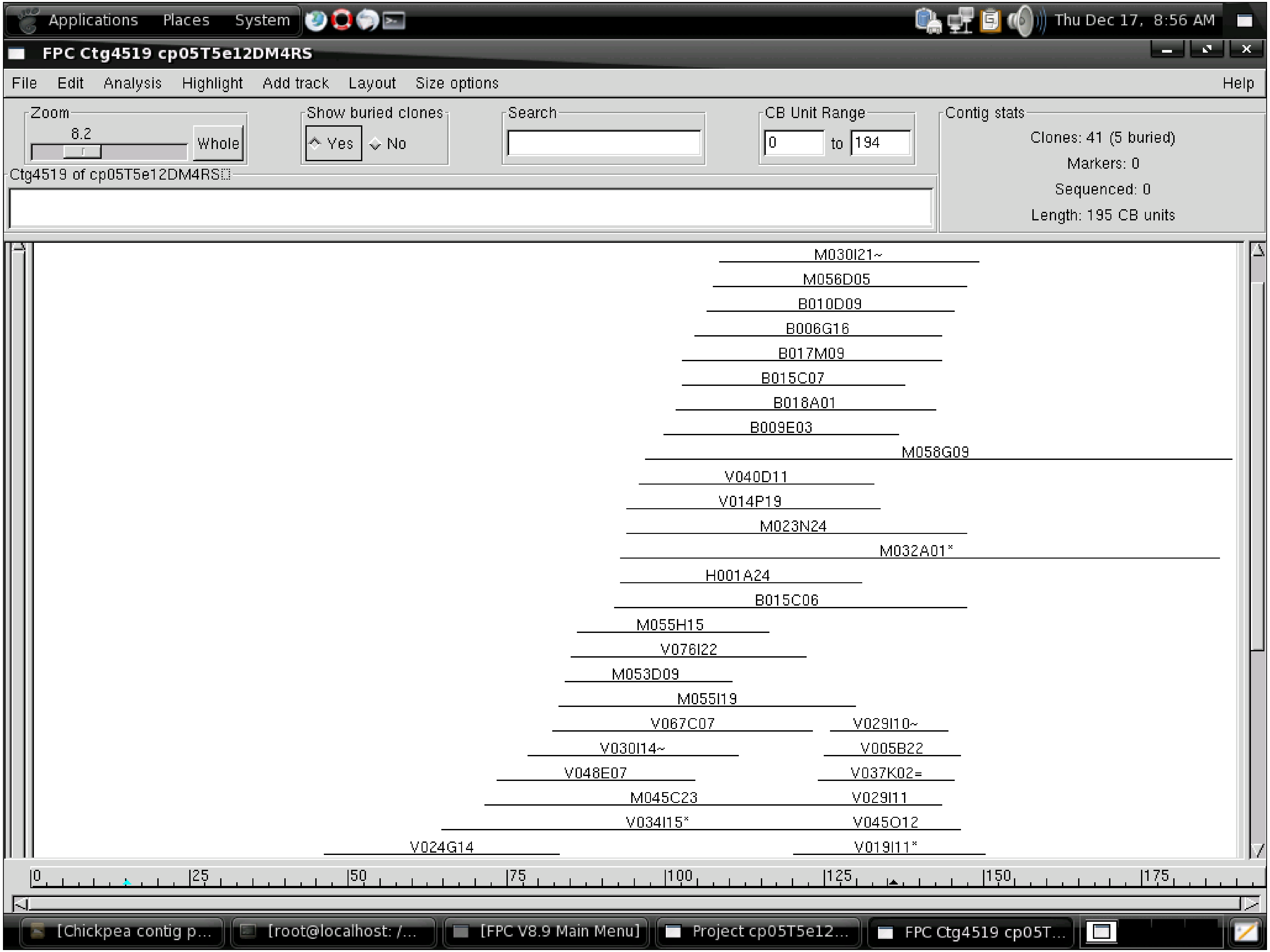
Applications Places System
Thu Dec 17, 8:56 AM
FPC Ctg4519 cp05T5e12DM4RS
File Edit Analysis Highlight Add track Layout Size options Help
Zoom
8.2
Whole
Show buried clones
Yes No
Search
CB Unit Range
0 to 194
Contig stats
Clones: 41 (5 buried)
Markers: 0
Sequenced: 0
Length: 195 CB units
Ctg4519 of cp05T5e12DM4RS
M030I21~
M056D05
B010D09
B006G16
B017M09
B015C07
B018A01
B009E03
M058G09
V040D11
V014P19
M023N24
M032A01*
H001A24
B015C06
M055H15
V076I22
M053D09
M055I19
V067C07
V029I10~
V030I14~
V005B22
V048E07
V037K02=
M045C23
V029I11
V034I15*
V045O12
V024G14
V019I11*
0 25 50 75 100 125 150 175
[Chickpea contig p...
[root@localhost: /...
[FPC V8.9 Main Menu]
Project cp05T5e12...
FPC Ctg4519 cp05T...

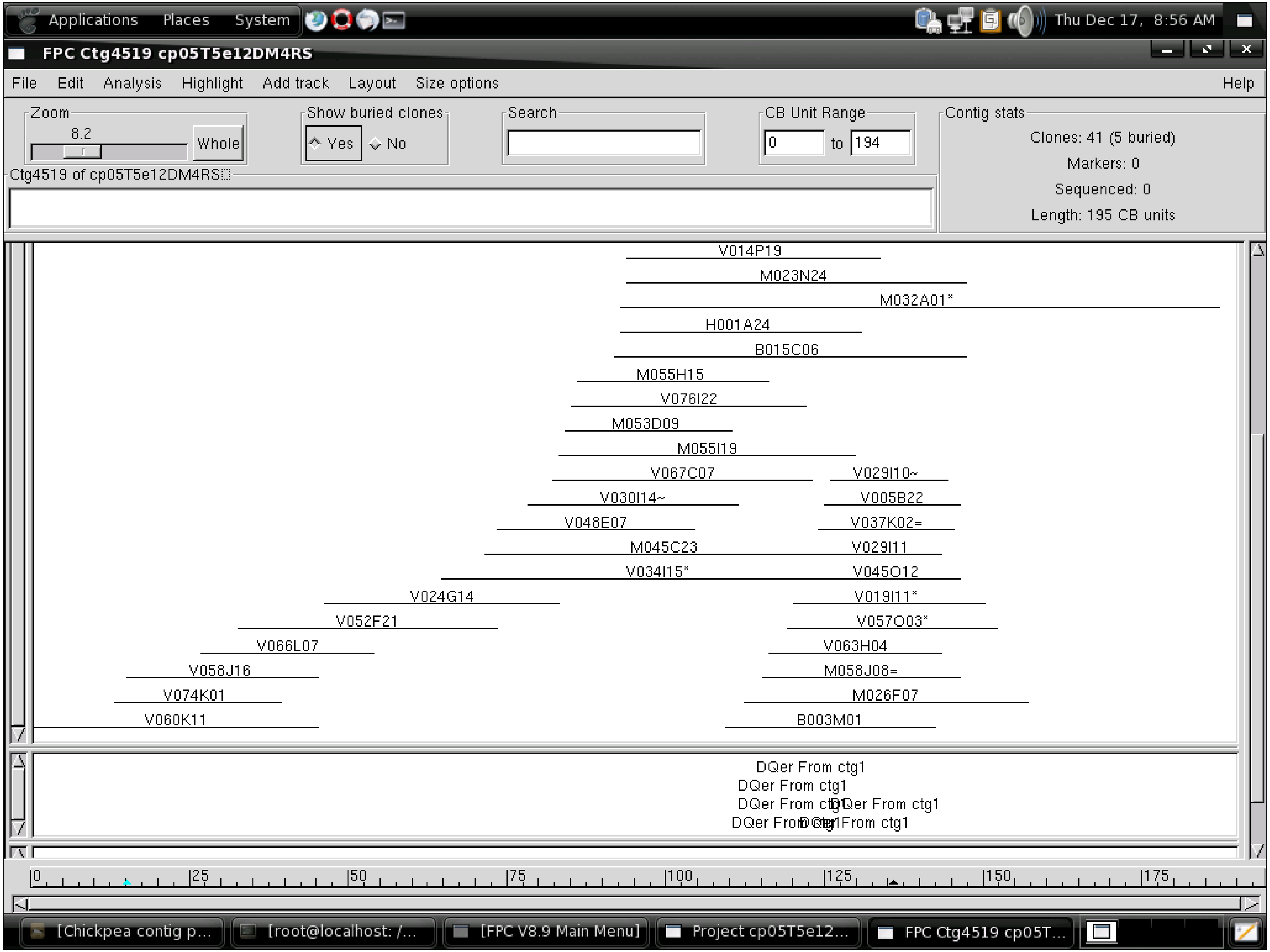
Applications Places System
Thu Dec 17, 8:56 AM
FPC Ctg4519 cp05T5e12DM4RS
File Edit Analysis Highlight Add track Layout Size options Help
Zoom
8.2
Whole
Show buried clones
Yes No
Search
CB Unit Range
0 to 194
Contig stats
Clones: 41 (5 buried)
Markers: 0
Sequenced: 0
Length: 195 CB units
Ctg4519 of cp05T5e12DM4RS
V014P19
M023N24
M032A01*
H001A24
B015C06
M055H15
V076I22
M053D09
M055I19
V067C07
V029I10~
V030I14~
V005B22
V048E07
V037K02=
M045C23
V029I11
V034I15*
V045O12
V024G14
V019I11*
V052F21
V057O03*
V066L07
V063H04
V058J16
M058J08=
V074K01
M026F07
V060K11
B003M01
DQer From ctg1
DQer From ctg1
DQer From ctg1
DQer From ctg1
DQer From ctg1
DQer From ctg1
0 25 50 75 100 125 150 175
[Chickpea contig p...
[root@localhost: /...
[FPC V8.9 Main Menu]
Project cp05T5e12...
FPC Ctg4519 cp05T...

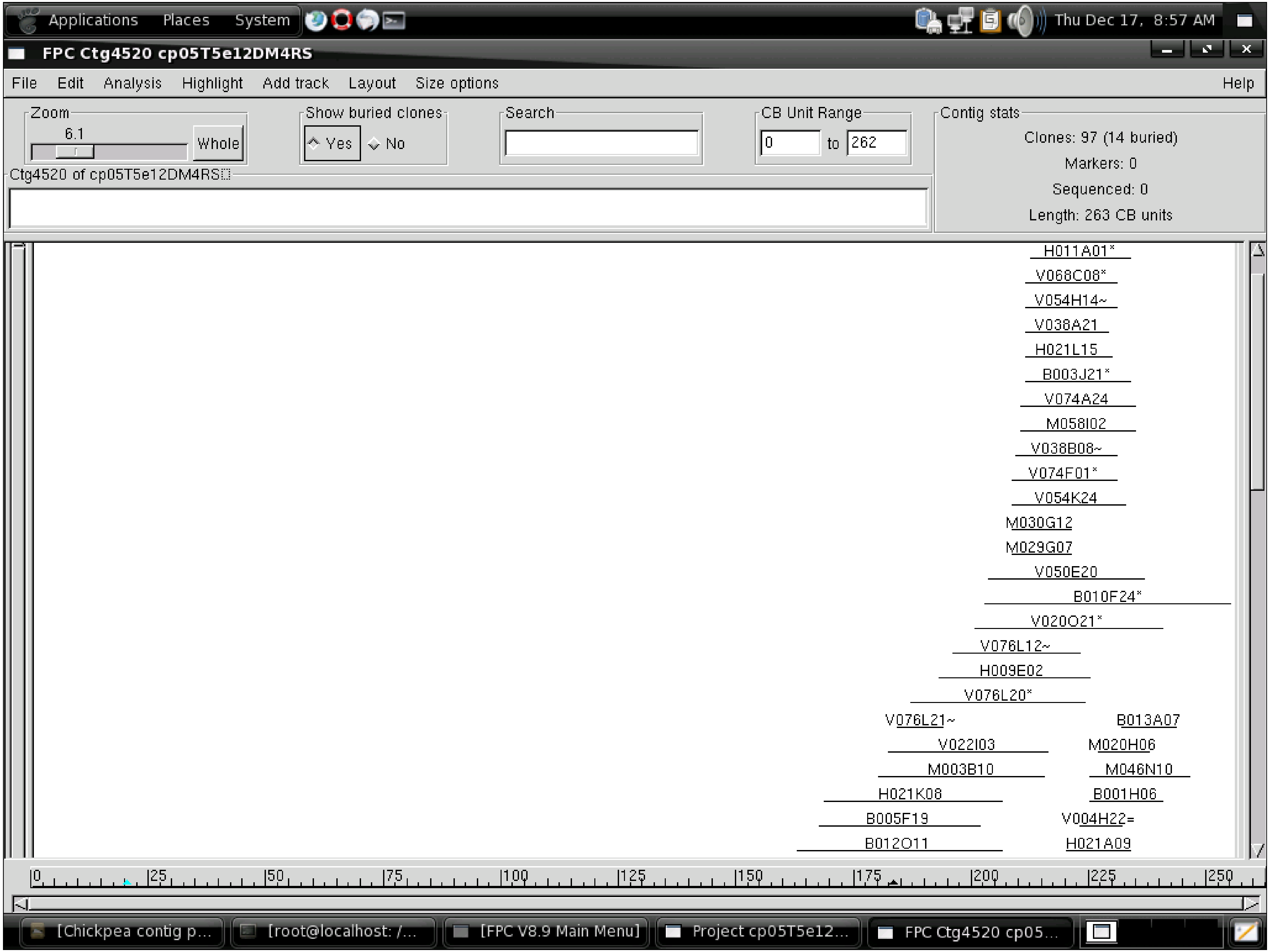
Applications Places System
Thu Dec 17, 8:57 AM
FPC Ctg4520 cp05T5e12DM4RS
File Edit Analysis Highlight Add track Layout Size options Help
Zoom
6.1
Whole
Show buried clones
Yes No
Search
CB Unit Range
0 to 262
Contig stats
Clones: 97 (14 buried)
Markers: 0
Sequenced: 0
Length: 263 CB units
Ctg4520 of cp05T5e12DM4RS
H011A01*
V068C08*
V054H14~
V038A21
H021L15
B003J21*
V074A24
M058I02
V038B08~
V074F01*
V054K24
M030G12
M029G07
V050E20
B010F24*
V020O21*
V076L12~
H009E02
V076L20*
V076L21~
B013A07
V022I03
M020H06
M003B10
M046N10
H021K08
B001H06
B005F19
V004H22=
B012O11
H021A09
0 25 50 75 100 125 150 175 200 225 250
[Chickpea contig p...
[root@localhost: /...
[FPC V8.9 Main Menu]
Project cp05T5e12...
FPC Ctg4520 cp05...

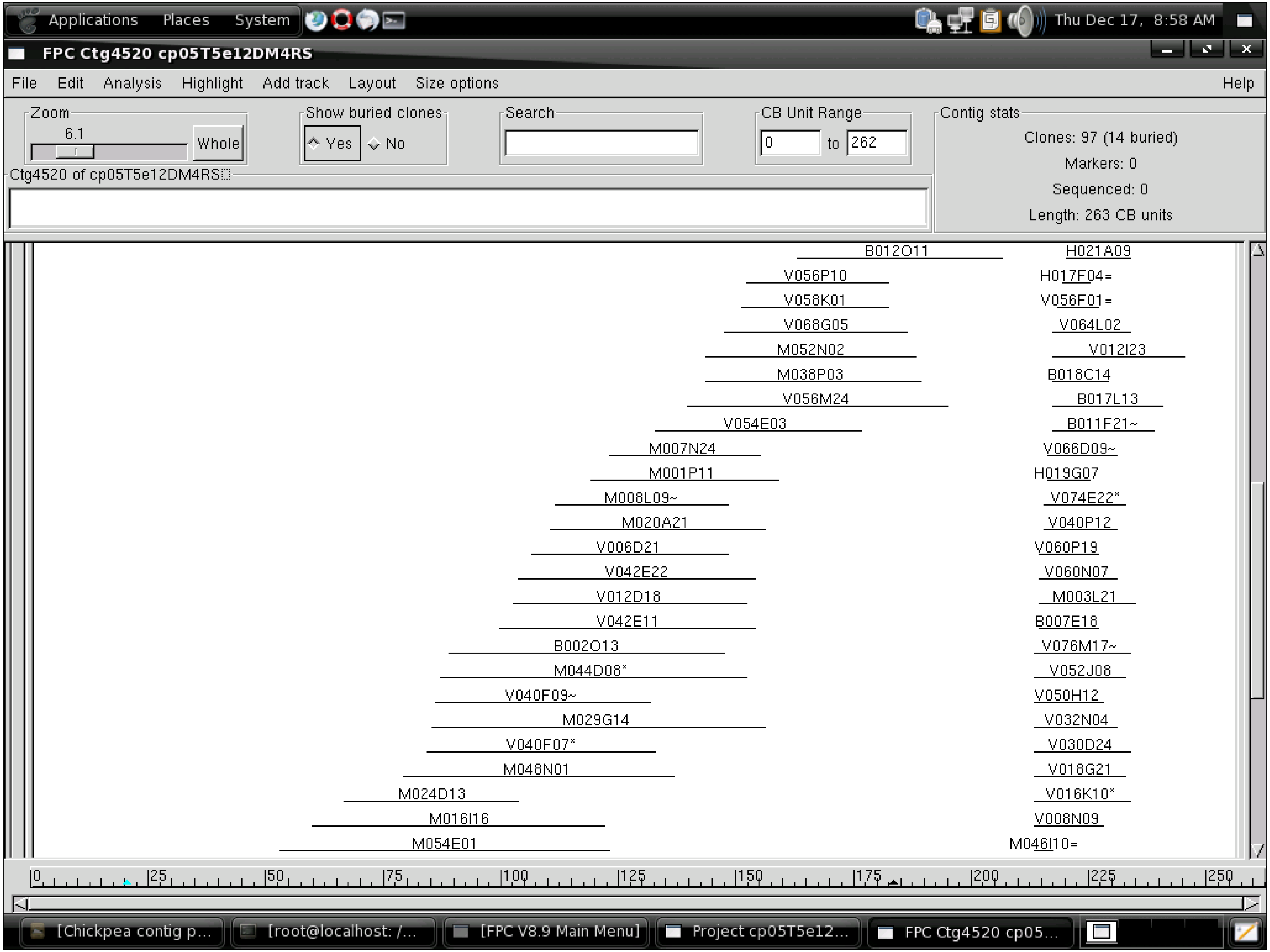

Applications Places System
Thu Dec 17, 8:58 AM
FPC Ctg4520 cp05T5e12DM4RS
File Edit Analysis Highlight Add track Layout Size options Help
Zoom 6.1 Whole
Show buried clones Yes No
Search
CB Unit Range 0 to 262
Contig stats
Clones: 97 (14 buried)
Markers: 0
Sequenced: 0
Length: 263 CB units
Ctg4520 of cp05T5e12DM4RS
B012O11
V056P10
V058K01
V068G05
M052N02
M038P03
V056M24
V054E03
M007N24
M001P11
M008L09~
M020A21
V006D21
V042E22
V012D18
V042E11
B002O13
M044D08*
V040F09~
V029G14
V040F07*
M048N01
M024D13
M016I16
M054E01
H021A09
H017F04=
V056F01=
V064L02
V012I23
B018C14
B017L13
B011F21~
V066D09~
H019G07
V074E22*
V040P12
V060P19
V060N07
M003L21
B007E18
V076M17~
V052J08
V050H12
V032N04
V030D24
V018G21
V016K10*
V008N09
M046I10=
0 25 50 75 100 125 150 175 200 225 250
[Chickpea contig p...
[root@localhost: /...
[FPC V8.9 Main Menu]
Project cp05T5e12...
FPC Ctg4520 cp05...

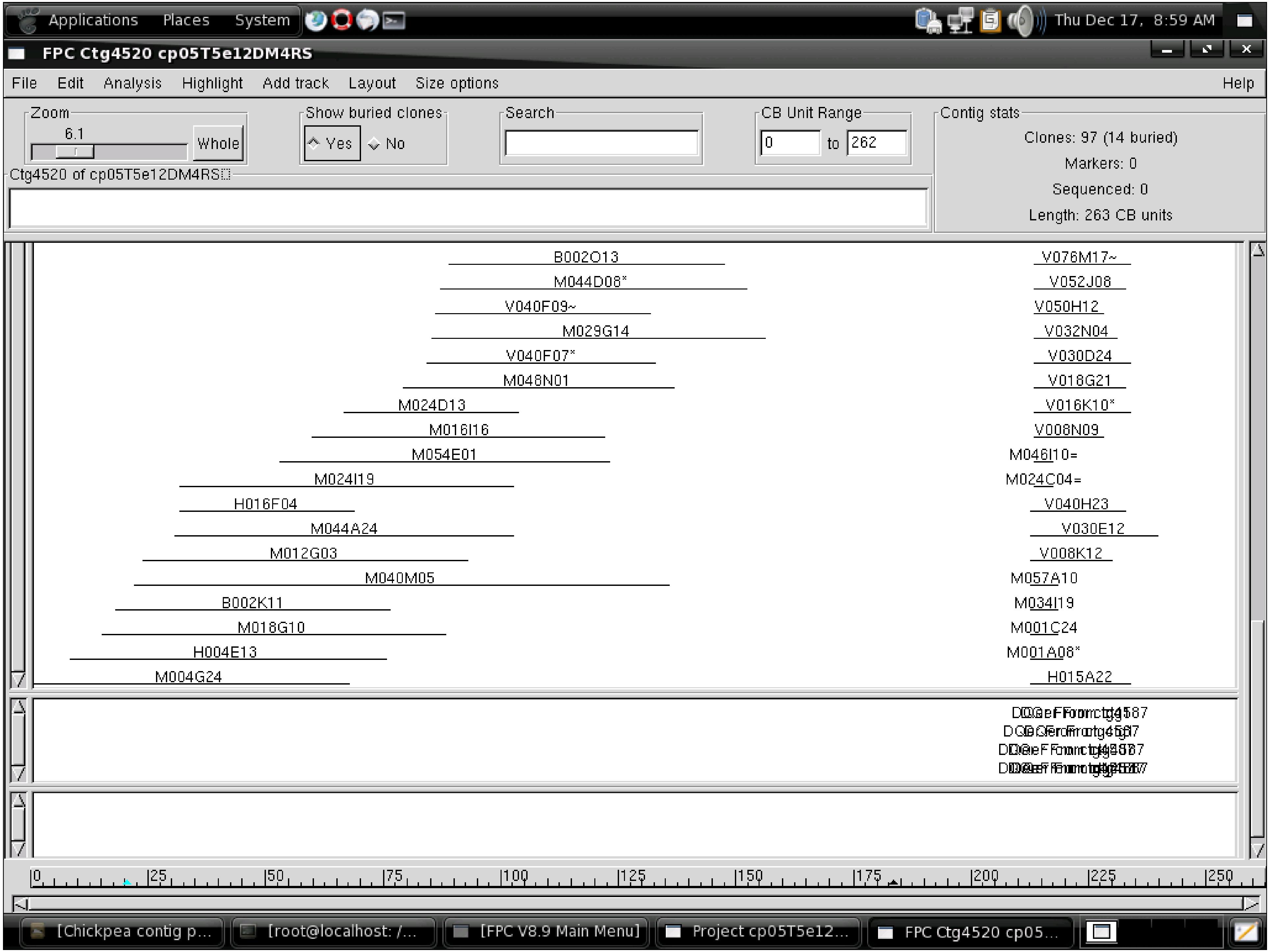

Applications Places System
Thu Dec 17, 8:59 AM
FPC Ctg4520 cp05T5e12DM4RS
File Edit Analysis Highlight Add track Layout Size options Help
Zoom
6.1
Whole
Show buried clones
Yes No
Search
CB Unit Range
0 to 262
Contig stats
Clones: 97 (14 buried)
Markers: 0
Sequenced: 0
Length: 263 CB units
Ctg4520 of cp05T5e12DM4RS
B002O13
M044D08*
V040F09~
M029G14
V040F07*
M048N01
M024D13
M016I16
M054E01
M024I19
H016F04
M044A24
M012G03
M040M05
B002K11
M018G10
H004E13
M004G24
V076M17~
V052J08
V050H12
V032N04
V030D24
V018G21
V016K10*
V008N09
M046I10=
M024C04=
V040H23
V030E12
V008K12
M057A10
M034I19
M001C24
M001A08*
H015A22
0 25 50 75 100 125 150 175 200 225 250
[Chickpea contig p...
[root@localhost: /...
[FPC V8.9 Main Menu]
Project cp05T5e12...
FPC Ctg4520 cp05...

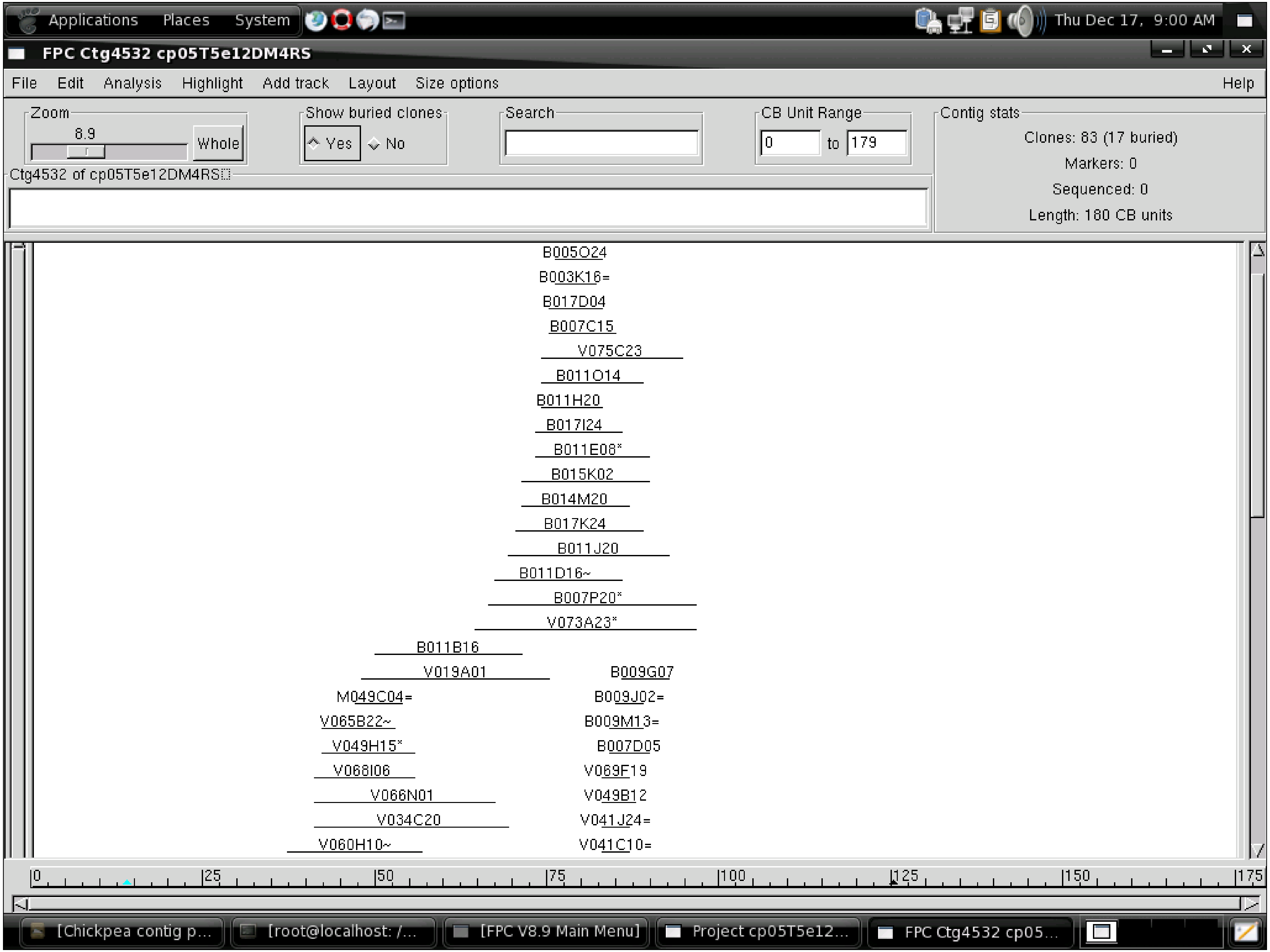
Applications Places System
Thu Dec 17, 9:00 AM
FPC Ctg4532 cp05T5e12DM4RS
File Edit Analysis Highlight Add track Layout Size options Help
Zoom
8.9
Whole
Show buried clones
Yes
No
Search
CB Unit Range
0 to 179
Contig stats
Clones: 83 (17 buried)
Markers: 0
Sequenced: 0
Length: 180 CB units
Ctg4532 of cp05T5e12DM4RS
B005O24
B003K16=
B017D04
B007C15
V075C23
B011O14
B011H20
B017I24
B011E08*
B015K02
B014M20
B017K24
B011J20
B011D16~
B007P20*
V073A23*
B011B16
V019A01
B009G07
M049C04=
B009J02=
V065B22~
B009M13=
V049H15*
B007D05
V068I06
V069F19
V066N01
V049B12
V034C20
V041J24=
V060H10~
V041C10=
0 25 50 75 100 125 150 175
[Chickpea contig p...
[root@localhost: /...
[FPC V8.9 Main Menu]
Project cp05T5e12...
FPC Ctg4532 cp05...

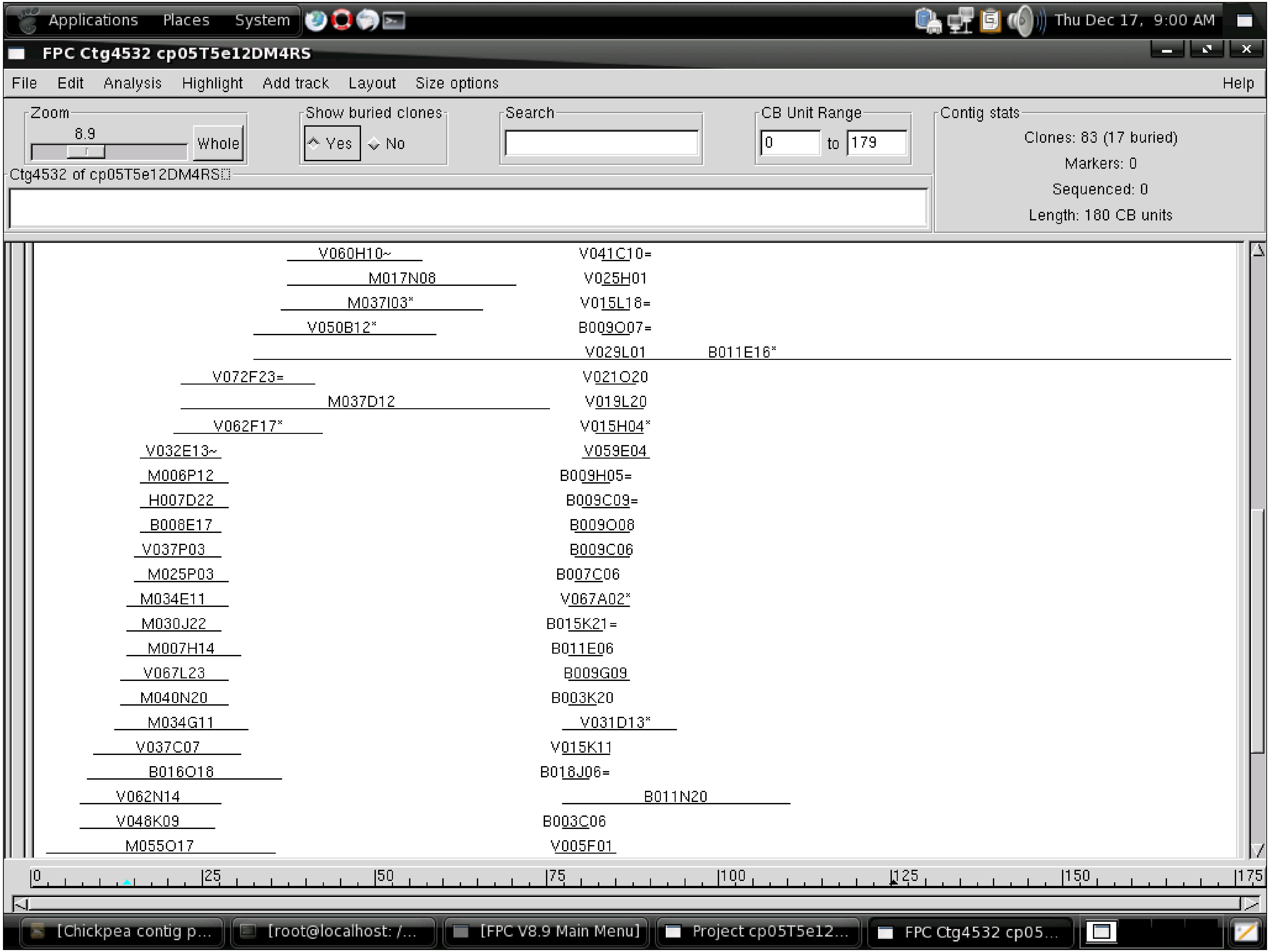
Applications Places System
Thu Dec 17, 9:00 AM
FPC Ctg4532 cp05T5e12DM4RS
File Edit Analysis Highlight Add track Layout Size options Help
Zoom
8.9
Whole
Show buried clones
Yes No
Search
CB Unit Range
0 to 179
Contig stats
Clones: 83 (17 buried)
Markers: 0
Sequenced: 0
Length: 180 CB units
Ctg4532 of cp05T5e12DM4RS
V060H10~
V041C10=
M017N08
V025H01
M037I03*
V015L18=
V050B12*
B009O07=
V029L01
B011E16*
V072F23=
V021O20
M037D12
V019L20
V062F17*
V015H04*
V032E13~
V059E04
M006P12
B009H05=
H007D22
B009C09=
B008E17
B009O08
V037P03
B009C06
M025P03
B007C06
M034E11
V067A02*
M030J22
B015K21=
M007H14
B011E06
V067L23
B009G09
M040N20
B003K20
M034G11
V031D13*
V037C07
V015K11
B016O18
B018J06=
V062N14
B011N20
V048K09
B003C06
M055O17
V005F01
0 25 50 75 100 125 150 175
[Chickpea contig p...
[root@localhost: /...
[FPC V8.9 Main Menu]
Project cp05T5e12...
FPC Ctg4532 cp05...

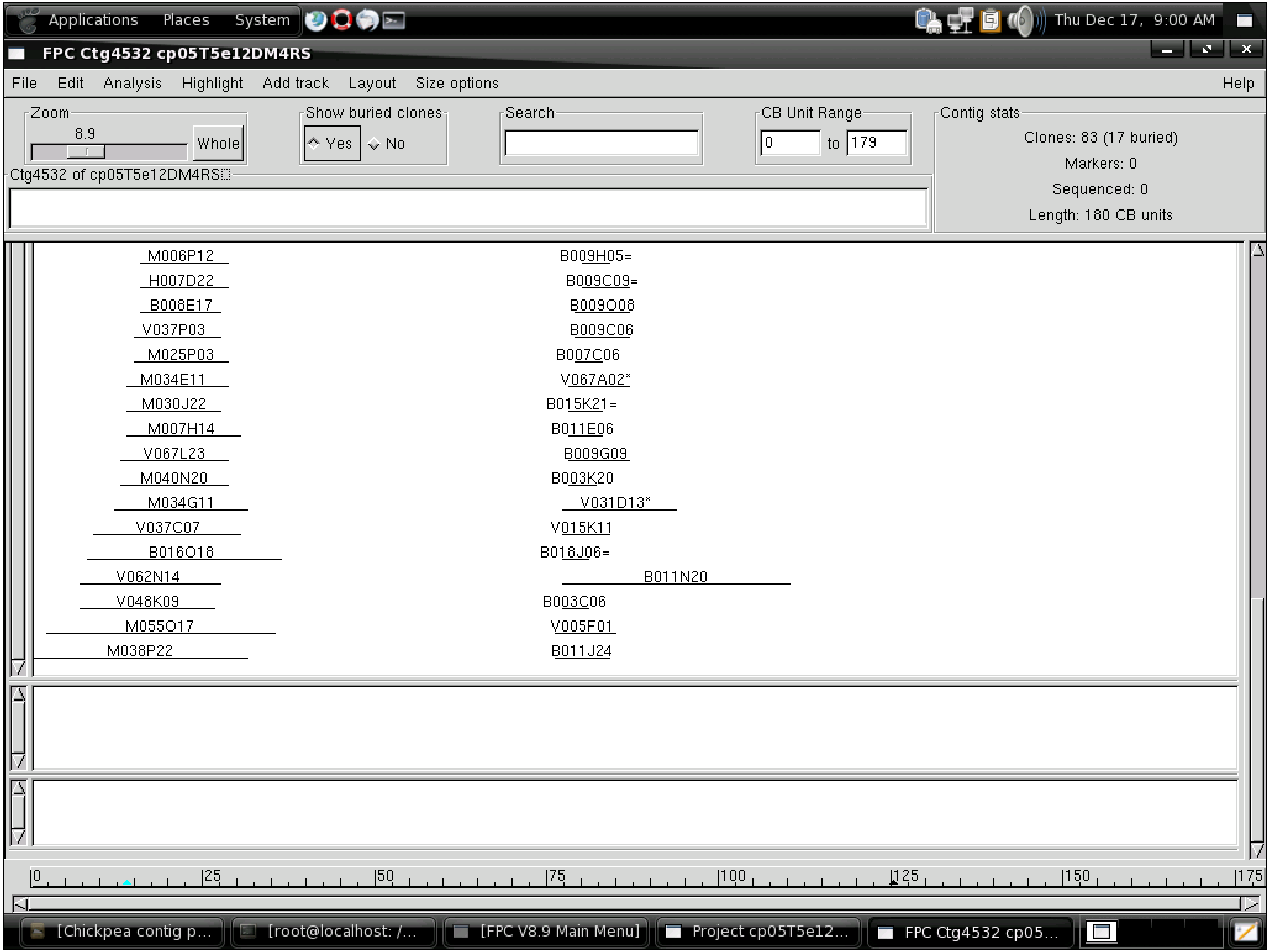

Applications Places System
Thu Dec 17, 9:00 AM
FPC Ctg4532 cp05T5e12DM4RS
File Edit Analysis Highlight Add track Layout Size options Help
Zoom
8.9
Whole
Show buried clones
Yes No
Search
CB Unit Range
0 to 179
Contig stats
Clones: 83 (17 buried)
Markers: 0
Sequenced: 0
Length: 180 CB units
Ctg4532 of cp05T5e12DM4RS
M006P12
H007D22
B008E17
V037P03
M025P03
M034E11
M030J22
M007H14
V067L23
M040N20
M034G11
V037C07
B016O18
V062N14
V048K09
M055O17
M038P22
B009H05=
B009C09=
B009O08
B009C06
B007C06
V067A02*
B015K21=
B011E06
B009G09
B003K20
V031D13*
V015K11
B018J06=
B011N20
B003C06
V005F01
B011J24
0 25 50 75 100 125 150 175
[Chickpea contig p...
[root@localhost: /...
[FPC V8.9 Main Menu]
Project cp05T5e12...
FPC Ctg4532 cp05...

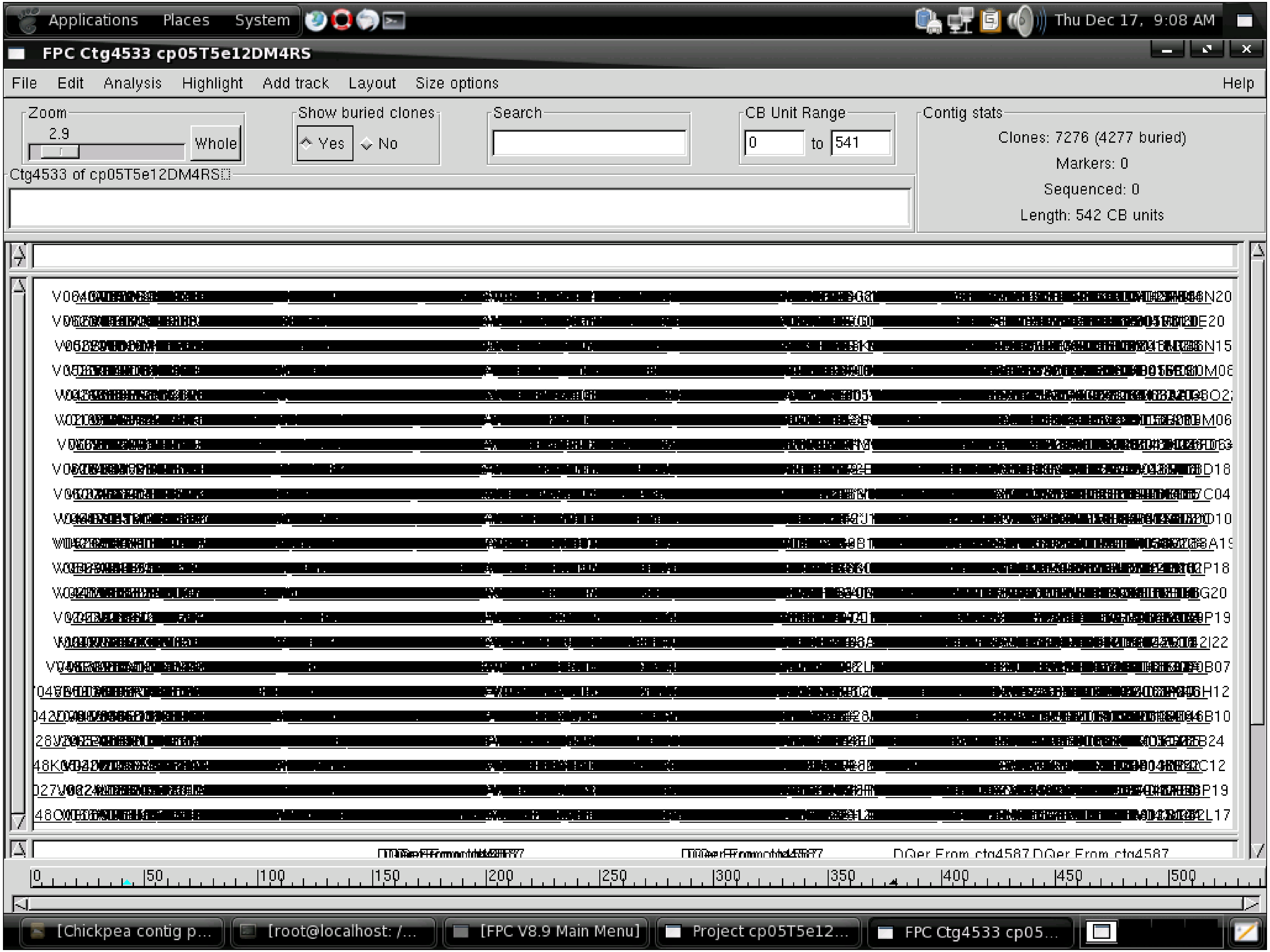
Applications Places System
Thu Dec 17, 9:08 AM
FPC Ctg4533 cp05T5e12DM4RS
File Edit Analysis Highlight Add track Layout Size options Help
Zoom
2.9
Whole
Show buried clones
Yes
No
Search
CB Unit Range
0 to 541
Contig stats
Clones: 7276 (4277 buried)
Markers: 0
Sequenced: 0
Length: 542 CB units
Ctg4533 of cp05T5e12DM4RS
DQer From ctg4587 DQer From ctg4587
0 50 100 150 200 250 300 350 400 450 500
[Chickpea contig p...
[root@localhost: /...
[FPC V8.9 Main Menu]
Project cp05T5e12...
FPC Ctg4533 cp05...

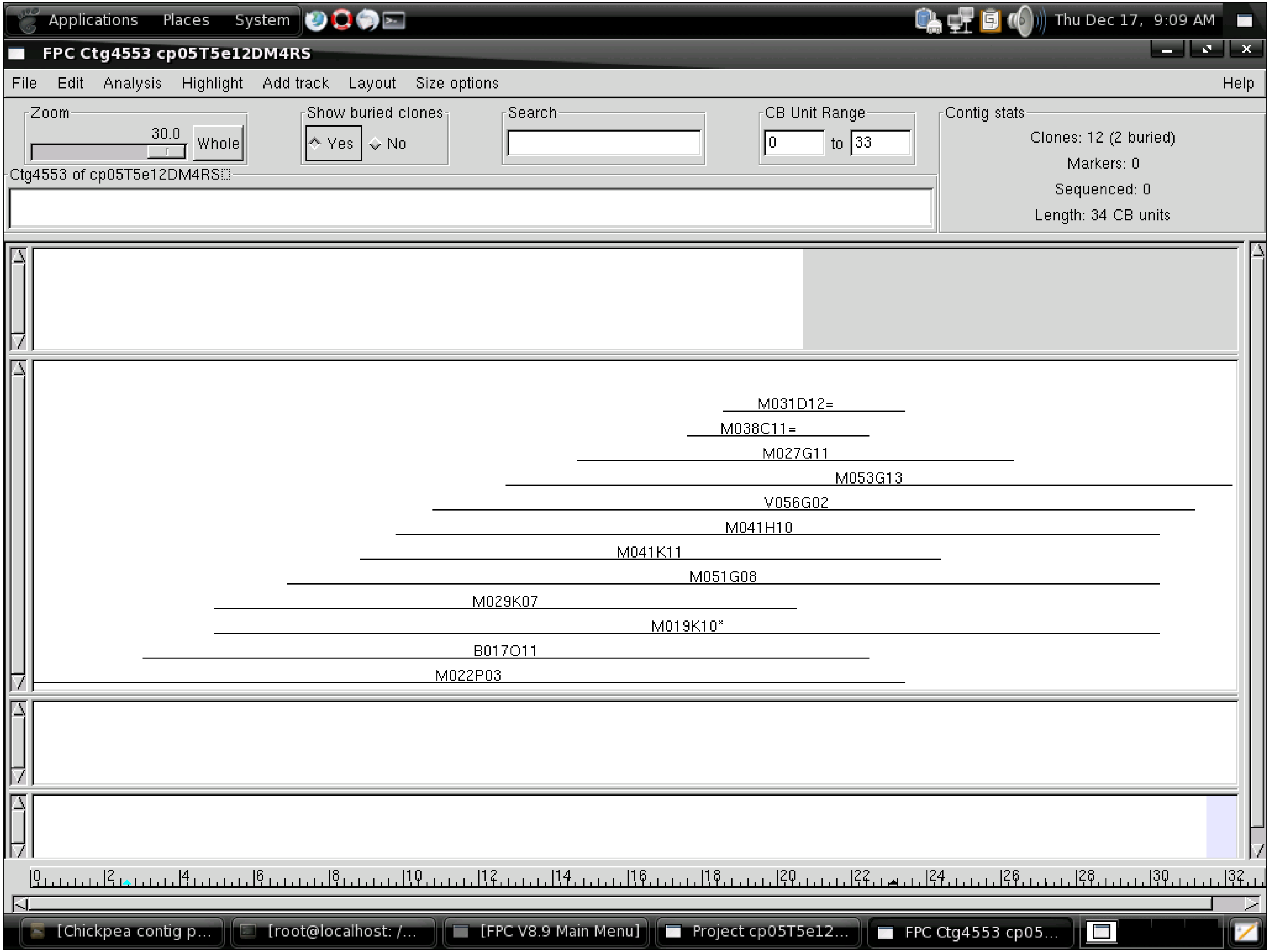
Applications Places System
Thu Dec 17, 9:09 AM
FPC Ctg4553 cp05T5e12DM4RS
File Edit Analysis Highlight Add track Layout Size options Help
Zoom
30.0
Whole
Show buried clones
Yes
No
Search
CB Unit Range
0
to
33
Contig stats
Clones: 12 (2 buried)
Markers: 0
Sequenced: 0
Length: 34 CB units
Ctg4553 of cp05T5e12DM4RS
M031D12=
M038C11=
M027G11
M053G13
V056G02
M041H10
M041K11
M051G08
M029K07
M019K10*
B017O11
M022P03
0 2 4 6 8 10 12 14 16 18 20 22 24 26 28 30 32
[Chickpea contig p...
[root@localhost: /...
[FPC V8.9 Main Menu]
Project cp05T5e12...
FPC Ctg4553 cp05...

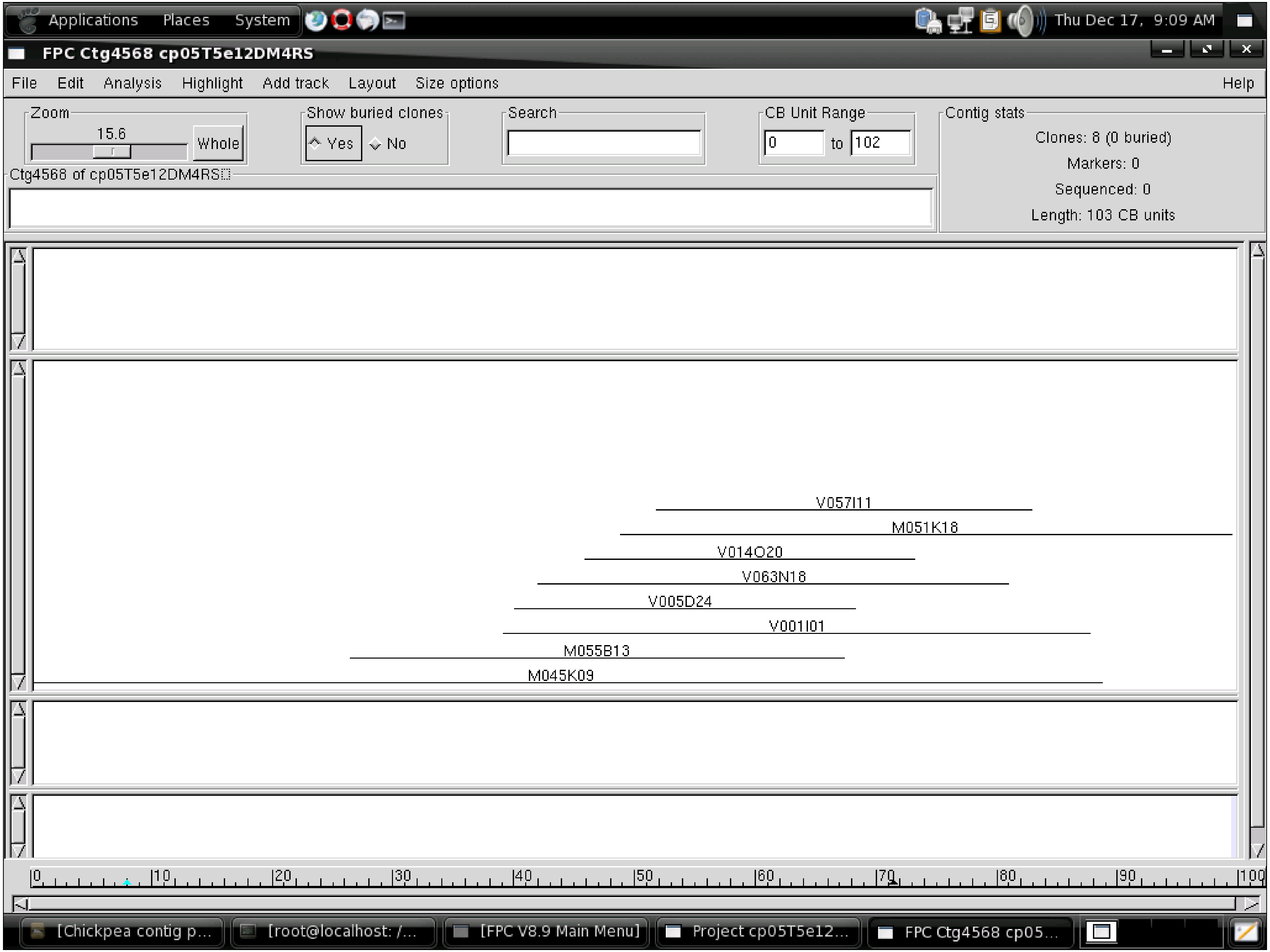
Applications Places System
Thu Dec 17, 9:09 AM
FPC Ctg4568 cp05T5e12DM4RS
File Edit Analysis Highlight Add track Layout Size options Help
Zoom
15.6
Whole
Show buried clones
Yes
No
Search
CB Unit Range
0 to 102
Contig stats
Clones: 8 (0 buried)
Markers: 0
Sequenced: 0
Length: 103 CB units
Ctg4568 of cp05T5e12DM4RS
V057I11
M051K18
V014O20
V063N18
V005D24
V001I01
M055B13
M045K09
0 10 20 30 40 50 60 70 80 90 100
[Chickpea contig p...
[root@localhost: /...
[FPC V8.9 Main Menu]
Project cp05T5e12...
FPC Ctg4568 cp05...

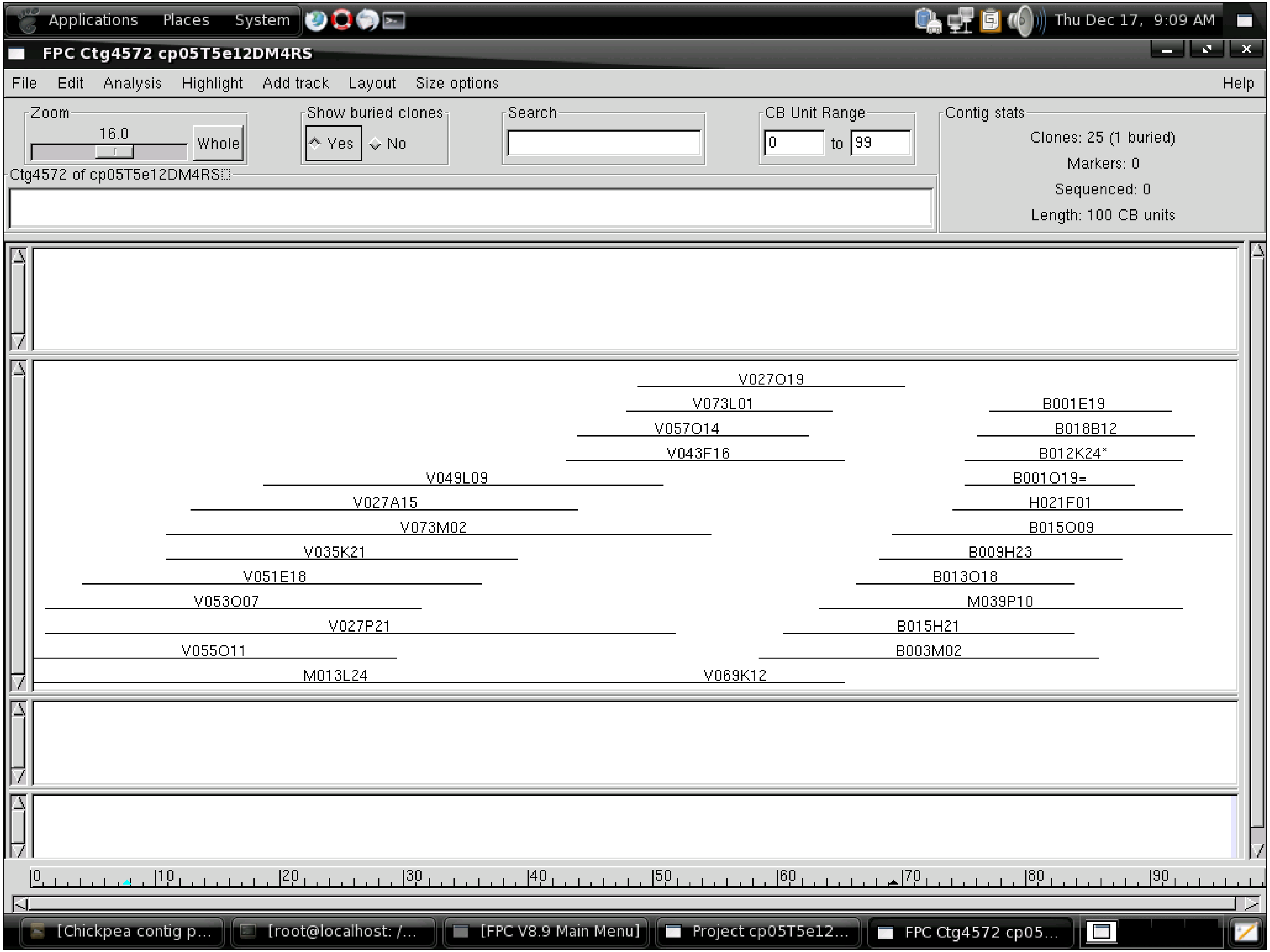
Applications Places System
Thu Dec 17, 9:09 AM
FPC Ctg4572 cp05T5e12DM4RS
File Edit Analysis Highlight Add track Layout Size options Help
Zoom
16.0
Whole
Show buried clones
Yes No
Search
CB Unit Range
0 to 99
Contig stats
Clones: 25 (1 buried)
Markers: 0
Sequenced: 0
Length: 100 CB units
Ctg4572 of cp05T5e12DM4RS
V027O19
V073L01
B001E19
V057O14
B018B12
V043F16
B012K24*
V049L09
B001O19=
V027A15
H021F01
V073M02
B015O09
V035K21
B009H23
V051E18
B013O18
V053O07
M039P10
V027P21
B015H21
V055O11
B003M02
M013L24
V069K12
0 10 20 30 40 50 60 70 80 90
[Chickpea contig p...
[root@localhost: /...
[FPC V8.9 Main Menu]
Project cp05T5e12...
FPC Ctg4572 cp05...

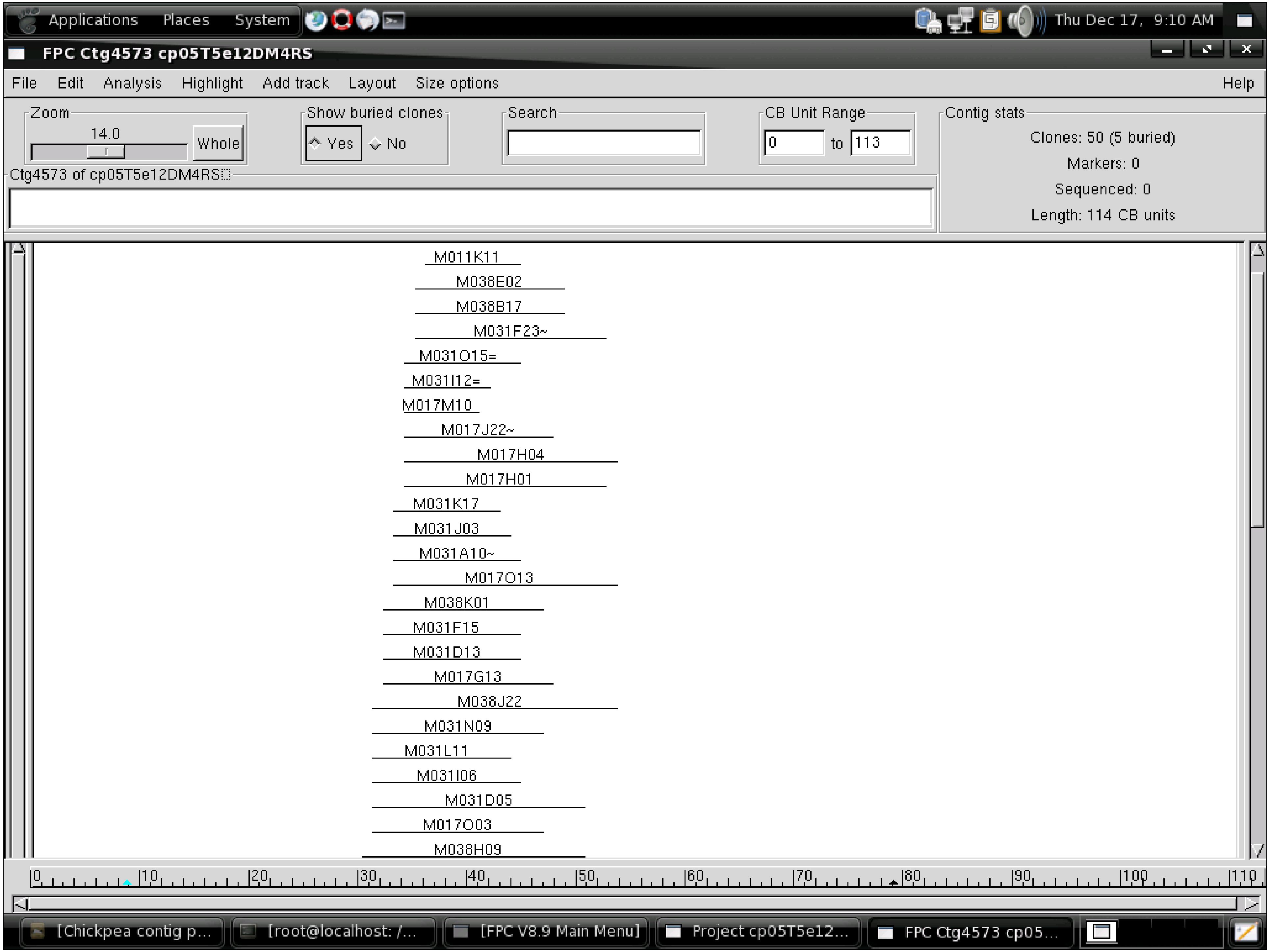

Applications Places System
Thu Dec 17, 9:10 AM
FPC Ctg4573 cp05T5e12DM4RS
File Edit Analysis Highlight Add track Layout Size options Help
Zoom
14.0
Whole
Show buried clones
Yes
No
Search
CB Unit Range
0
to
113
Contig stats
Clones: 50 (5 buried)
Markers: 0
Sequenced: 0
Length: 114 CB units
Ctg4573 of cp05T5e12DM4RS
M011K11
M038E02
M038B17
M031F23~
M031O15=
M031I12=
M017M10
M017J22~
M017H04
M017H01
M031K17
M031J03
M031A10~
M017O13
M038K01
M031F15
M031D13
M017G13
M038J22
M031N09
M031L11
M031I06
M031D05
M017O03
M038H09
0 10 20 30 40 50 60 70 80 90 100 110
[Chickpea contig p...
[root@localhost: /...
[FPC V8.9 Main Menu]
Project cp05T5e12...
FPC Ctg4573 cp05...

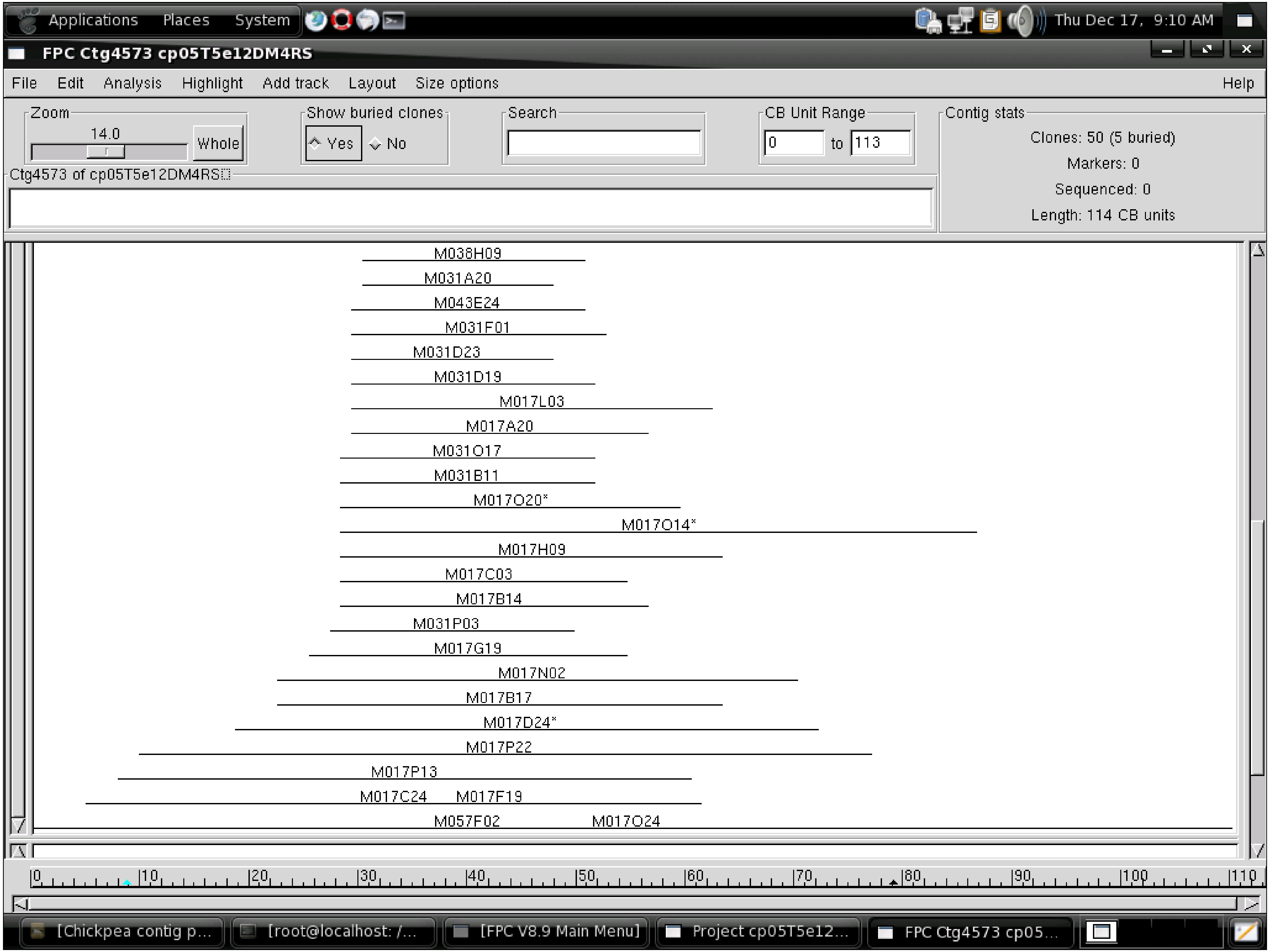
Applications Places System
Thu Dec 17, 9:10 AM
FPC Ctg4573 cp05T5e12DM4RS
File Edit Analysis Highlight Add track Layout Size options Help
Zoom
14.0
Whole
Show buried clones
Yes No
Search
CB Unit Range
0 to 113
Contig stats
Clones: 50 (5 buried)
Markers: 0
Sequenced: 0
Length: 114 CB units
Ctg4573 of cp05T5e12DM4RS
M038H09
M031A20
M043E24
M031F01
M031D23
M031D19
M017L03
M017A20
M031O17
M031B11
M017O20*
M017O14*
M017H09
M017C03
M017B14
M031P03
M017G19
M017N02
M017B17
M017D24*
M017P22
M017P13
M017C24
M017F19
M057F02
M017O24
0 10 20 30 40 50 60 70 80 90 100 110
[Chickpea contig p...
[root@localhost: /...
[FPC V8.9 Main Menu]
Project cp05T5e12...
FPC Ctg4573 cp05...

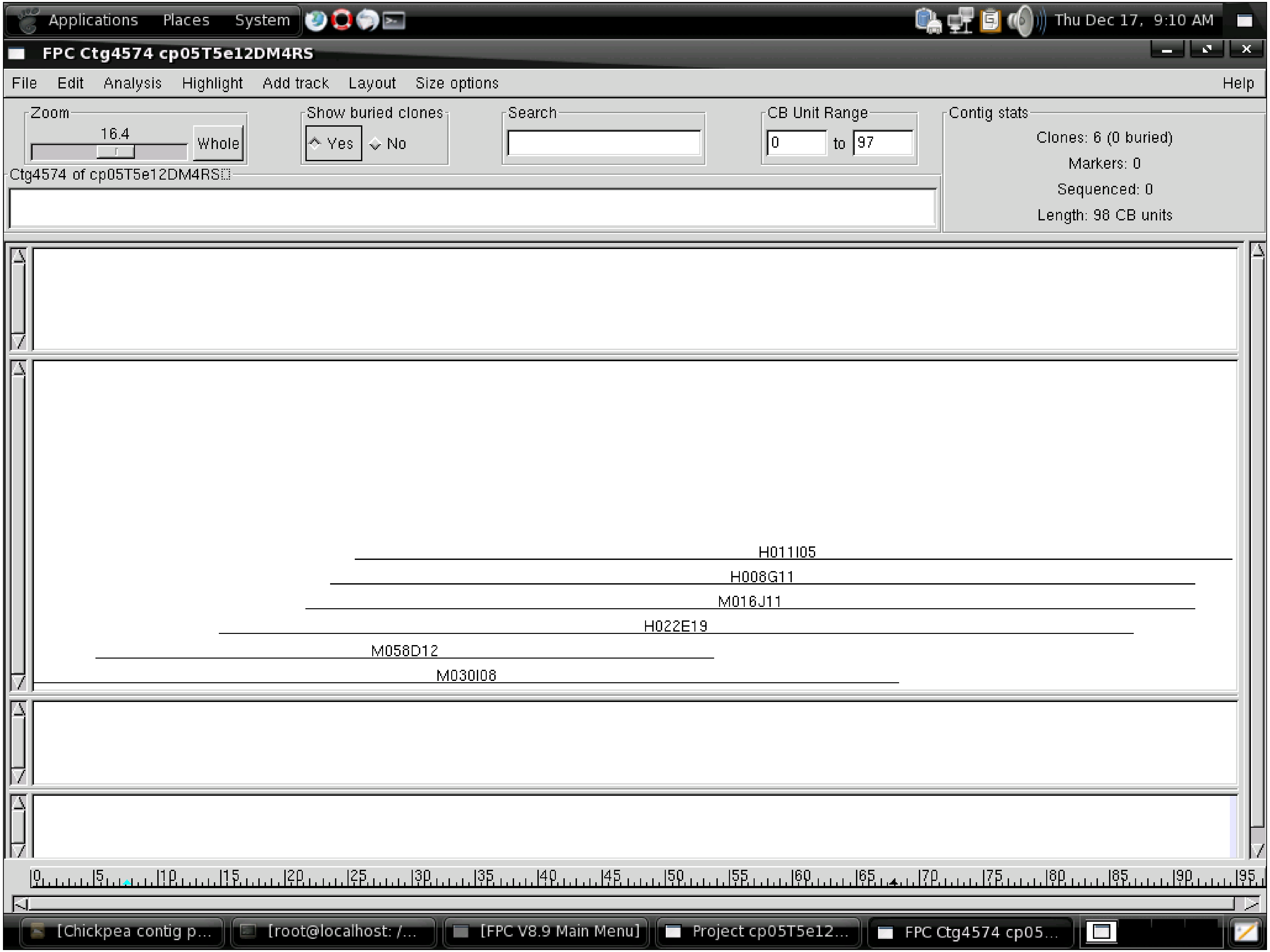
Applications
Places
System
Thu Dec 17, 9:10 AM
FPC Ctg4574 cp05T5e12DM4RS
File
Edit
Analysis
Highlight
Add track
Layout
Size options
Help
Zoom
16.4
Whole
Show buried clones
Yes
No
Search
CB Unit Range
0
to
97
Contig stats
Clones: 6 (0 buried)
Markers: 0
Sequenced: 0
Length: 98 CB units
Ctg4574 of cp05T5e12DM4RS
H011I05
H008G11
M016J11
H022E19
M058D12
M030I08
0
5
10
15
20
25
30
35
40
45
50
55
60
65
70
75
80
85
90
95
[Chickpea contig p...
[root@localhost: /...
[FPC V8.9 Main Menu]
Project cp05T5e12...
FPC Ctg4574 cp05...

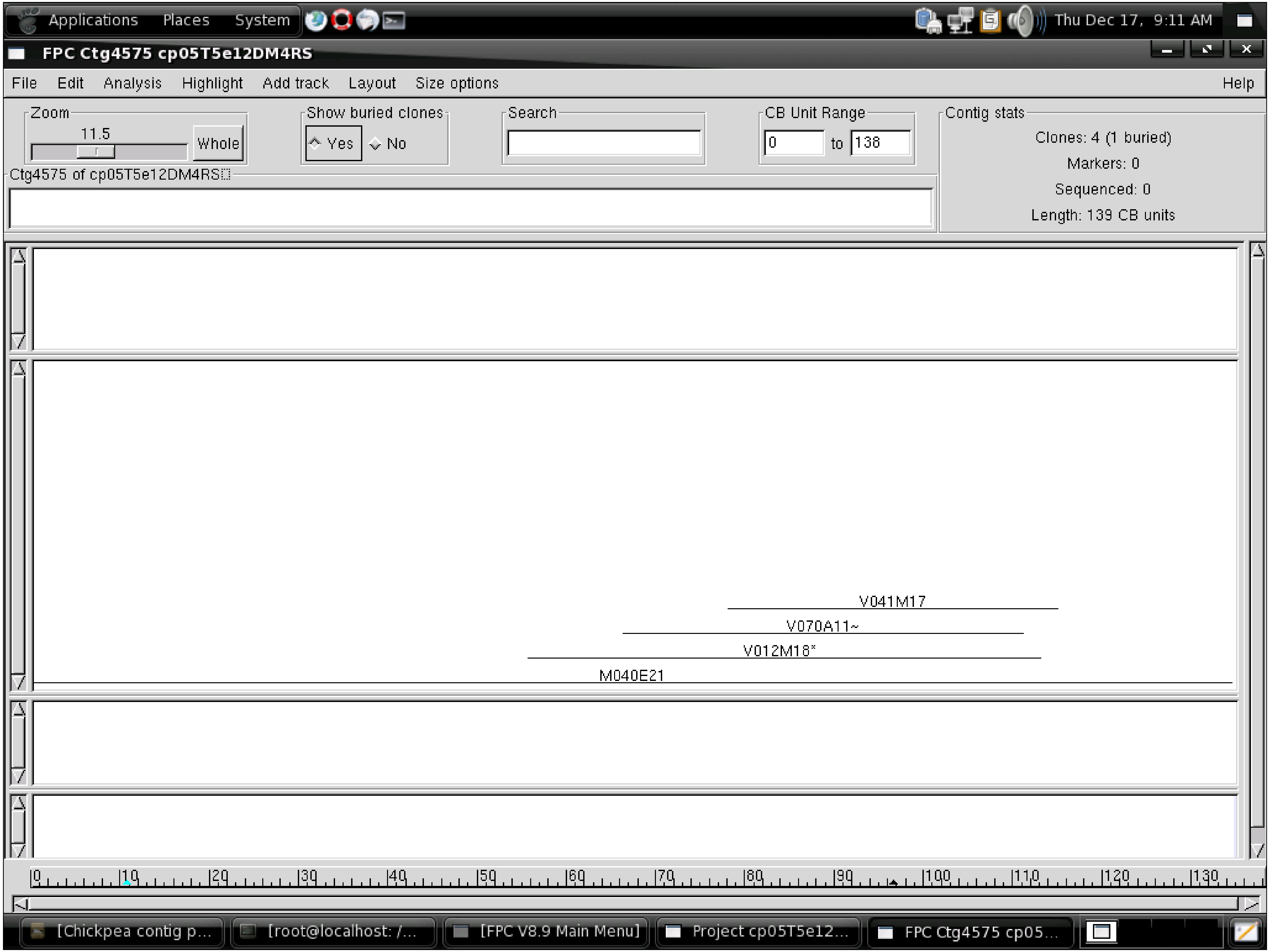
Applications Places System
Thu Dec 17, 9:11 AM
FPC Ctg4575 cp05T5e12DM4RS
File Edit Analysis Highlight Add track Layout Size options Help
Zoom
11.5
Whole
Show buried clones
Yes
No
Search
CB Unit Range
0
to
138
Contig stats
Clones: 4 (1 buried)
Markers: 0
Sequenced: 0
Length: 139 CB units
Ctg4575 of cp05T5e12DM4RS
V041M17
V070A11~
V012M18*
M040E21
0 10 20 30 40 50 60 70 80 90 100 110 120 130
[Chickpea contig p...
[root@localhost: /...
[FPC V8.9 Main Menu]
Project cp05T5e12...
FPC Ctg4575 cp05...

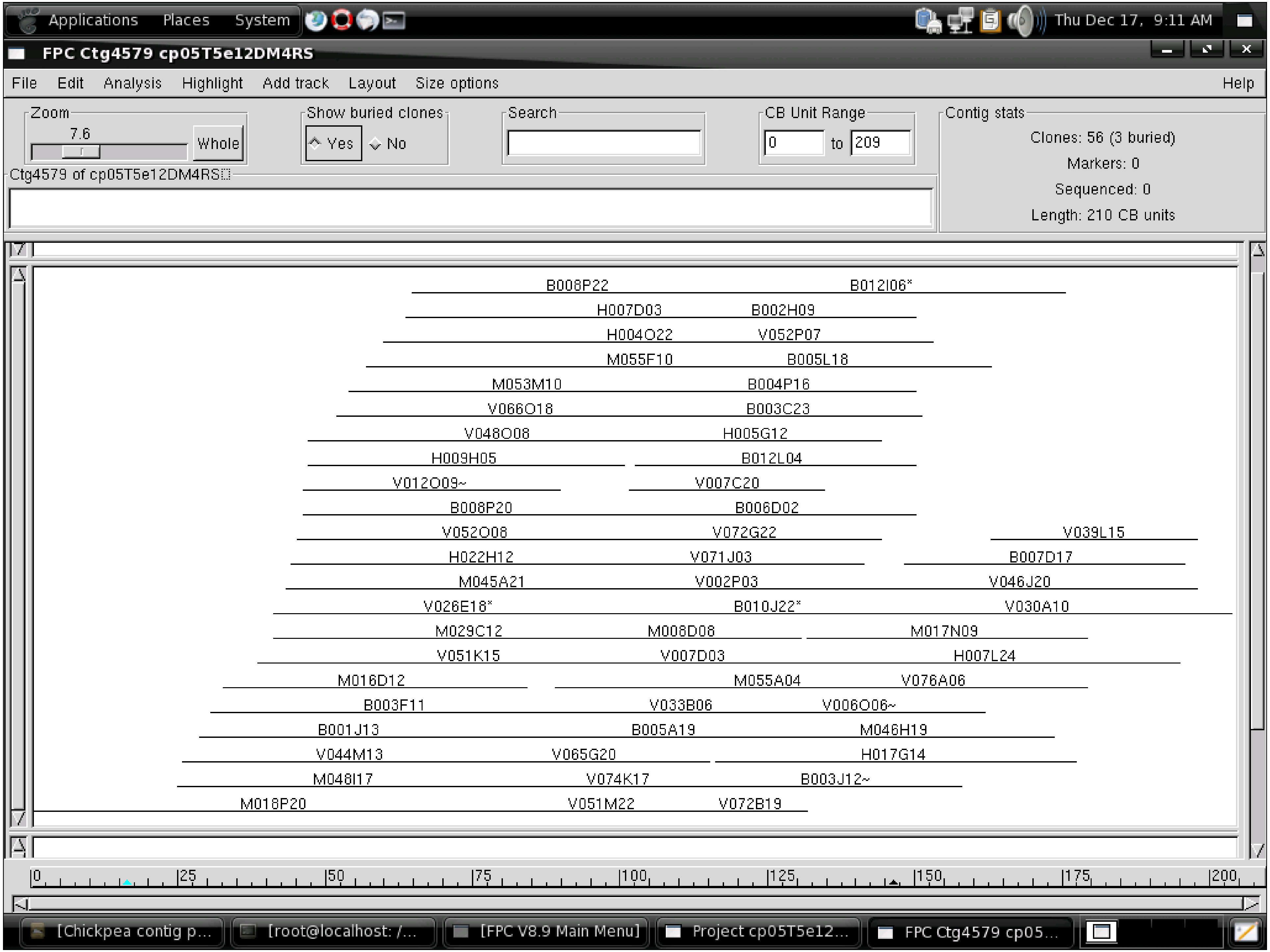
Applications Places System
Thu Dec 17, 9:11 AM
FPC Ctg4579 cp05T5e12DM4RS
File Edit Analysis Highlight Add track Layout Size options Help
Zoom
7.6
Whole
Show buried clones
Yes No
Search
CB Unit Range
0 to 209
Contig stats
Clones: 56 (3 buried)
Markers: 0
Sequenced: 0
Length: 210 CB units
Ctg4579 of cp05T5e12DM4RS
B008P22 B012I06*
H007D03 B002H09
H004O22 V052P07
M055F10 B005L18
M053M10 B004P16
V066O18 B003C23
V048O08 H005G12
H009H05 B012L04
V012O09~ V007C20
B008P20 B006D02
V052O08 V072G22 V039L15
H022H12 V071J03 B007D17
M045A21 V002P03 V046J20
V026E18* B010J22* V030A10
M029C12 M008D08 M017N09
V051K15 V007D03 H007L24
M016D12 M055A04 V076A06
B003F11 V033B06 V006O06~
B001J13 B005A19 M046H19
V044M13 V065G20 H017G14
M048I17 V074K17 B003J12~
M018P20 V051M22 V072B19
0 25 50 75 100 125 150 175 200
[Chickpea contig p... [root@localhost: /... [FPC V8.9 Main Menu] Project cp05T5e12... FPC Ctg4579 cp05...

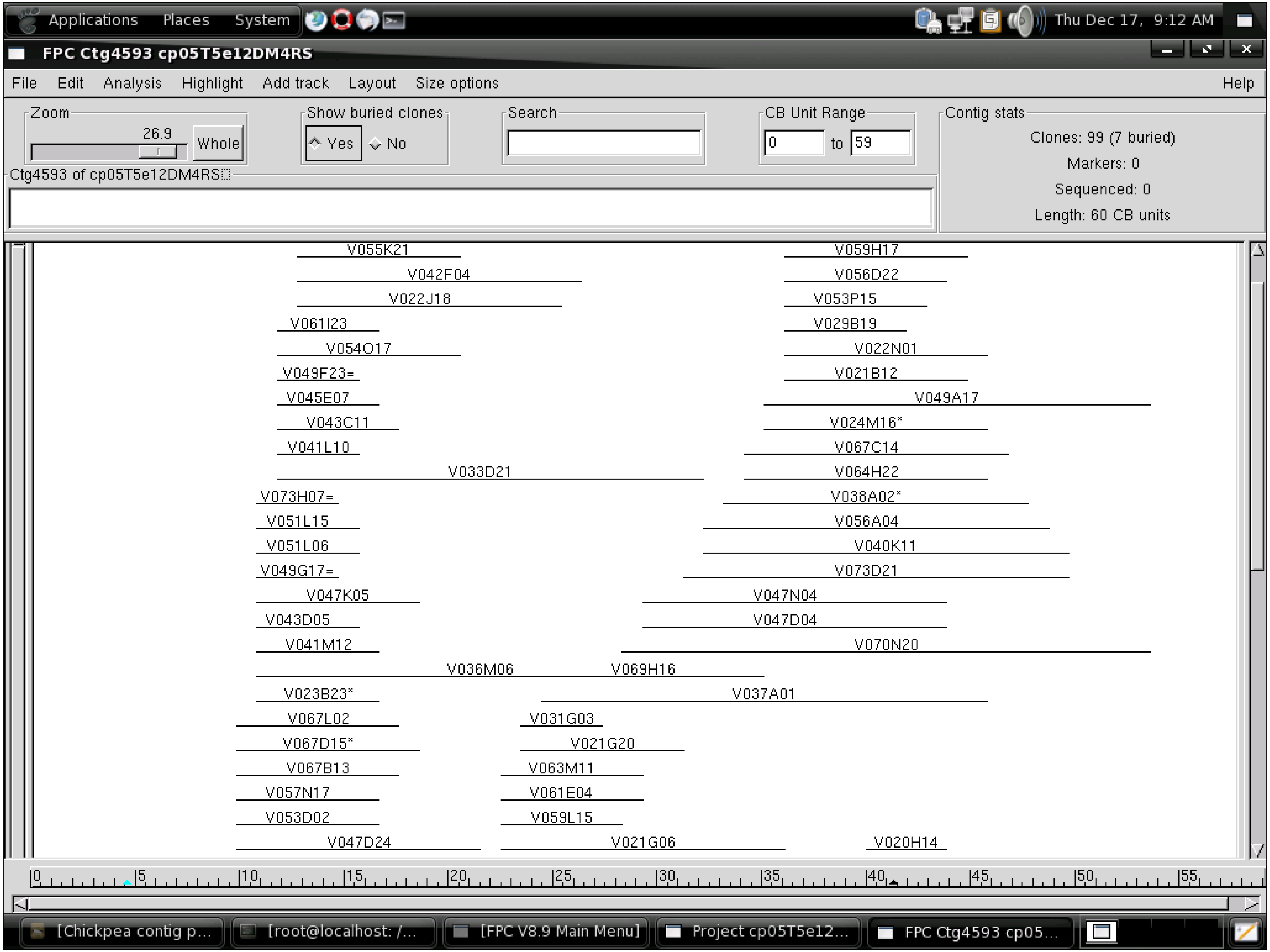
Applications Places System
Thu Dec 17, 9:12 AM
FPC Ctg4593 cp05T5e12DM4RS
File Edit Analysis Highlight Add track Layout Size options Help
Zoom
26.9
Whole
Show buried clones
Yes No
Search
CB Unit Range
0 to 59
Contig stats
Clones: 99 (7 buried)
Markers: 0
Sequenced: 0
Length: 60 CB units
Ctg4593 of cp05T5e12DM4RS
V055K21
V059H17
V042F04
V056D22
V022J18
V053P15
V061I23
V029B19
V054O17
V022N01
V049F23=
V021B12
V045E07
V049A17
V043C11
V024M16*
V041L10
V067C14
V033D21
V064H22
V073H07=
V038A02*
V051L15
V056A04
V051L06
V040K11
V049G17=
V073D21
V047K05
V047N04
V043D05
V047D04
V041M12
V070N20
V036M06
V069H16
V023B23*
V037A01
V067L02
V031G03
V067D15*
V021G20
V067B13
V063M11
V057N17
V061E04
V053D02
V059L15
V047D24
V021G06
V020H14
0 5 10 15 20 25 30 35 40 45 50 55
[Chickpea contig p...
[root@localhost: /...
[FPC V8.9 Main Menu]
Project cp05T5e12...
FPC Ctg4593 cp05...

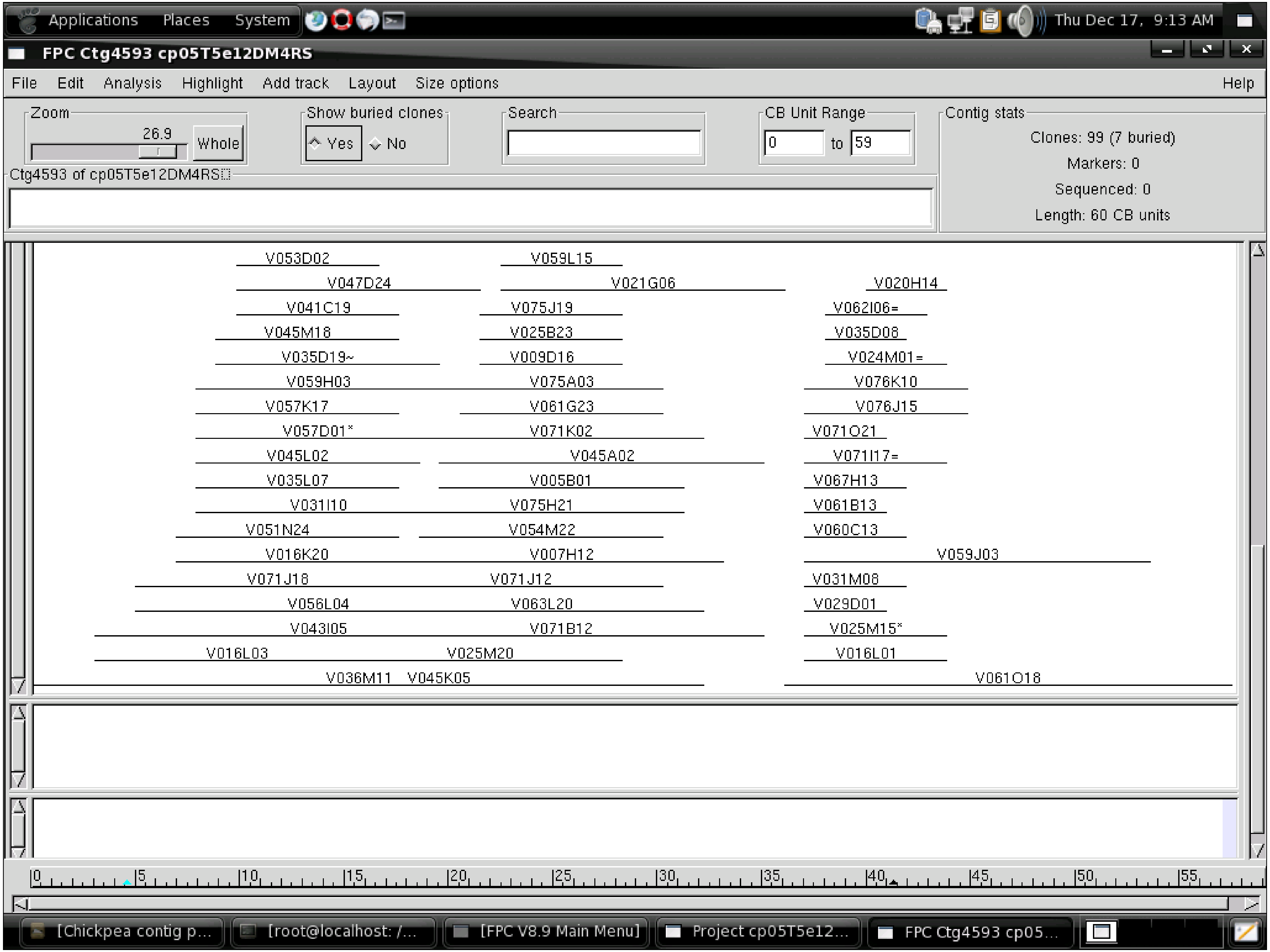
Applications Places System
Thu Dec 17, 9:13 AM
FPC Ctg4593 cp05T5e12DM4RS
File Edit Analysis Highlight Add track Layout Size options Help
Zoom
26.9
Whole
Show buried clones
Yes No
Search
CB Unit Range
0 to 59
Contig stats
Clones: 99 (7 buried)
Markers: 0
Sequenced: 0
Length: 60 CB units
Ctg4593 of cp05T5e12DM4RS
V053D02 V059L15
V047D24 V021G06 V020H14
V041C19 V075J19 V062I06=
V045M18 V025B23 V035D08
V035D19~ V009D16 V024M01=
V059H03 V075A03 V076K10
V057K17 V061G23 V076J15
V057D01* V071K02 V071O21
V045L02 V045A02 V071I17=
V035L07 V005B01 V067H13
V031I10 V075H21 V061B13
V051N24 V054M22 V060C13
V016K20 V007H12 V059J03
V071J18 V071J12 V031M08
V056L04 V063L20 V029D01
V043I05 V071B12 V025M15*
V016L03 V025M20 V016L01
V036M11 V045K05 V061O18
0 5 10 15 20 25 30 35 40 45 50 55
[Chickpea contig p... [root@localhost: /... [FPC V8.9 Main Menu] Project cp05T5e12... FPC Ctg4593 cp05...

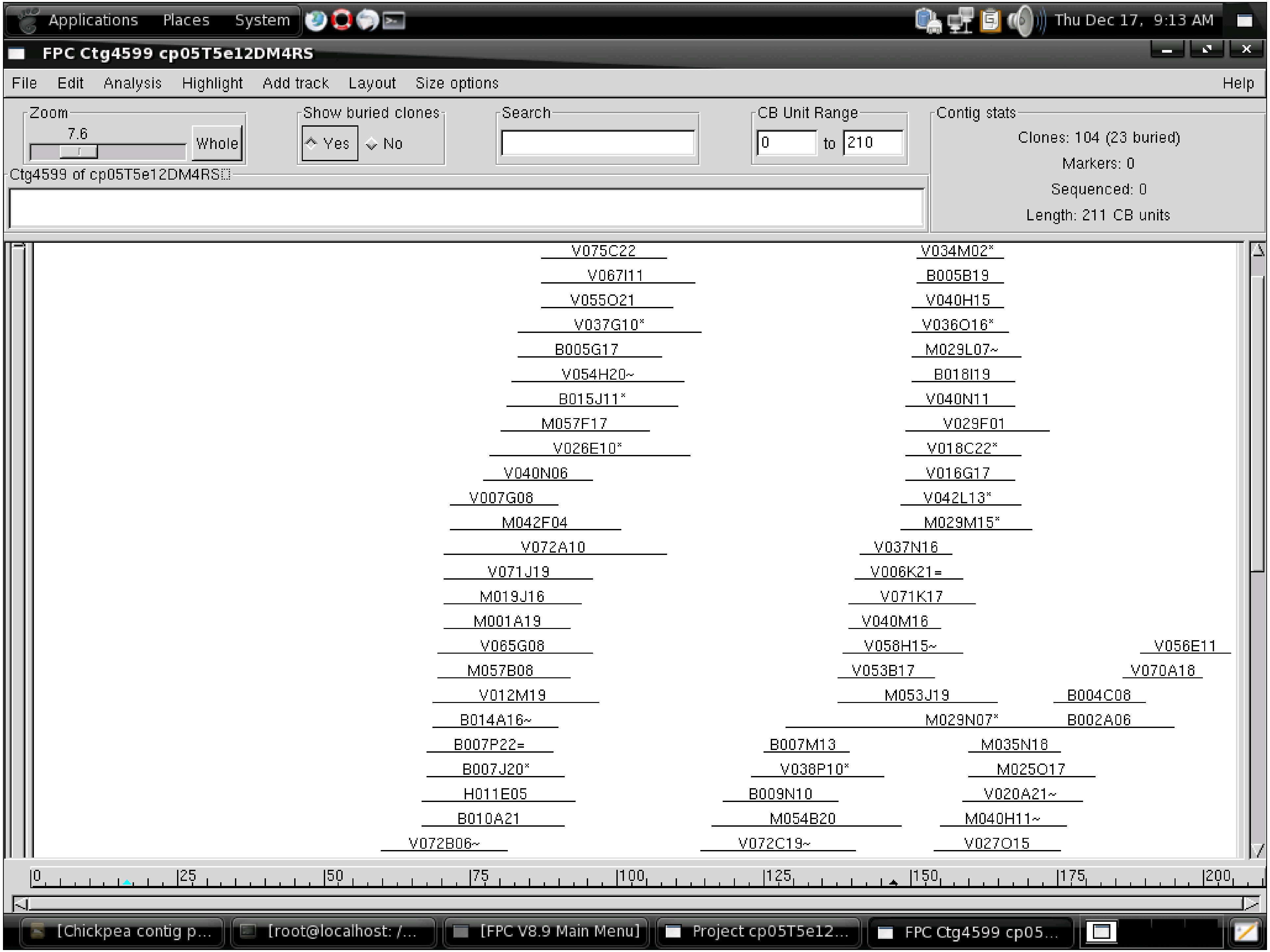
Applications Places System
Thu Dec 17, 9:13 AM
FPC Ctg4599 cp05T5e12DM4RS
File Edit Analysis Highlight Add track Layout Size options Help
Zoom
7.6
Whole
Show buried clones
Yes No
Search
CB Unit Range
0 to 210
Contig stats
Clones: 104 (23 buried)
Markers: 0
Sequenced: 0
Length: 211 CB units
Ctg4599 of cp05T5e12DM4RS
V075C22
V067I11
V055O21
V037G10*
B005G17
V054H20~
B015J11*
M057F17
V026E10*
V040N06
V007G08
M042F04
V072A10
V071J19
M019J16
M001A19
V065G08
M057B08
V012M19
B014A16~
B007P22=
B007J20*
H011E05
B010A21
V072B06~
V034M02*
B005B19
V040H15
V036O16*
M029L07~
B018I19
V040N11
V029F01
V018C22*
V016G17
V042L13*
M029M15*
V037N16
V006K21=
V071K17
V040M16
V058H15~
V053B17
M053J19
M029N07*
B007M13
V038P10*
B009N10
M054B20
V072C19~
V056E11
V070A18
B004C08
B002A06
M035N18
M025O17
V020A21~
M040H11~
V027O15
0 25 50 75 100 125 150 175 200
[Chickpea contig p...
[root@localhost: /...
[FPC V8.9 Main Menu]
Project cp05T5e12...
FPC Ctg4599 cp05...

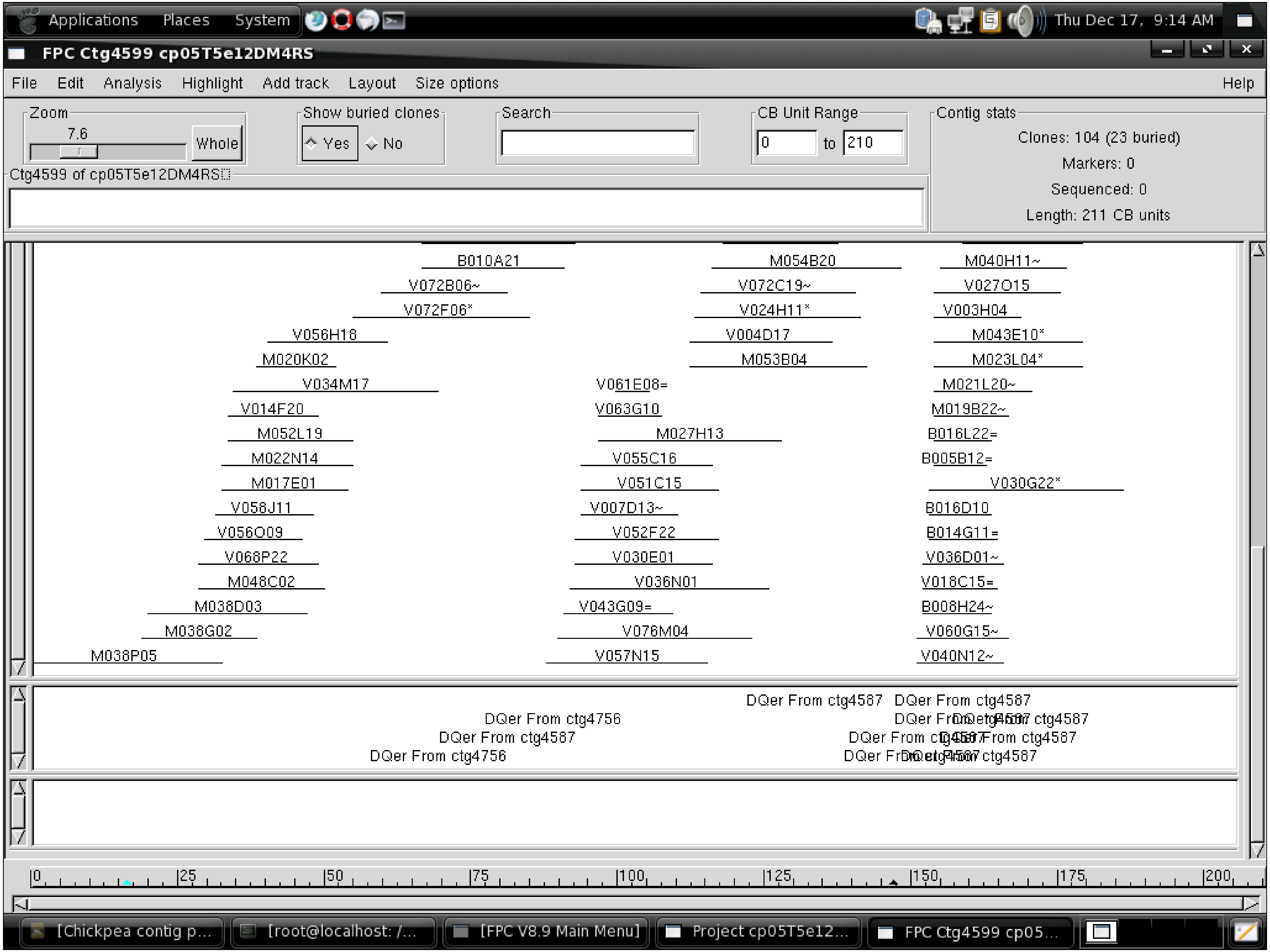
Applications Places System
Thu Dec 17, 9:14 AM
FPC Ctg4599 cp05T5e12DM4RS
File Edit Analysis Highlight Add track Layout Size options Help
Zoom
7.6
Whole
Show buried clones
Yes No
Search
CB Unit Range
0 to 210
Contig stats
Clones: 104 (23 buried)
Markers: 0
Sequenced: 0
Length: 211 CB units
Ctg4599 of cp05T5e12DM4RS
B010A21
V072B06~
V072F06*
V056H18
M020K02
V034M17
V014F20
M052L19
M022N14
M017E01
V058J11
V056O09
V068P22
M048C02
M038D03
M038G02
M038P05
M054B20
V072C19~
V024H11*
V004D17
M053B04
V061E08=
V063G10
M027H13
V055C16
V051C15
V007D13~
V052F22
V030E01
V036N01
V043G09=
V076M04
V057N15
M040H11~
V027O15
V003H04
M043E10*
M023L04*
M021L20~
M019B22~
B016L22=
B005B12=
V030G22*
B016D10
B014G11=
V036D01~
V018C15=
B008H24~
V060G15~
V040N12~
DQer From ctg4587
DQer From ctg4587
DQer From ctg4756
DQer From ctg4587
DQer From ctg4756
0 25 50 75 100 125 150 175 200
[Chickpea contig p...
[root@localhost: /...
[FPC V8.9 Main Menu]
Project cp05T5e12...
FPC Ctg4599 cp05...

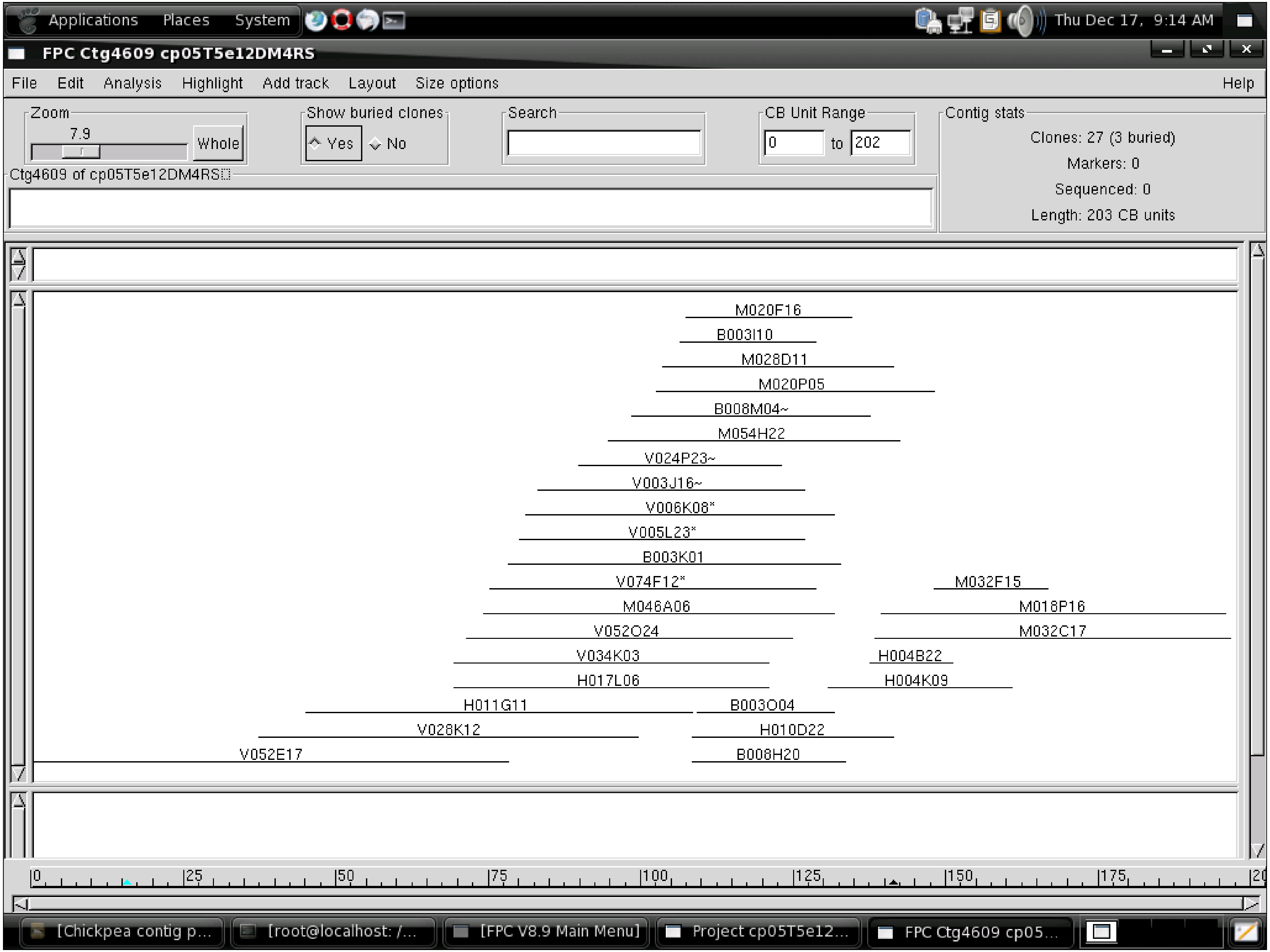
Applications Places System
Thu Dec 17, 9:14 AM
FPC Ctg4609 cp05T5e12DM4RS
File Edit Analysis Highlight Add track Layout Size options Help
Zoom
7.9
Whole
Show buried clones
Yes No
Search
CB Unit Range
0 to 202
Contig stats
Clones: 27 (3 buried)
Markers: 0
Sequenced: 0
Length: 203 CB units
Ctg4609 of cp05T5e12DM4RS
M020F16
B003I10
M028D11
M020P05
B008M04~
M054H22
V024P23~
V003J16~
V006K08*
V005L23*
B003K01
V074F12*
M032F15
M046A06
M018P16
V052O24
M032C17
V034K03
H004B22
H017L06
H004K09
H011G11
B003O04
V028K12
H010D22
V052E17
B008H20
0 25 50 75 100 125 150 175
[Chickpea contig p...
[root@localhost: /...
[FPC V8.9 Main Menu]
Project cp05T5e12...
FPC Ctg4609 cp05...

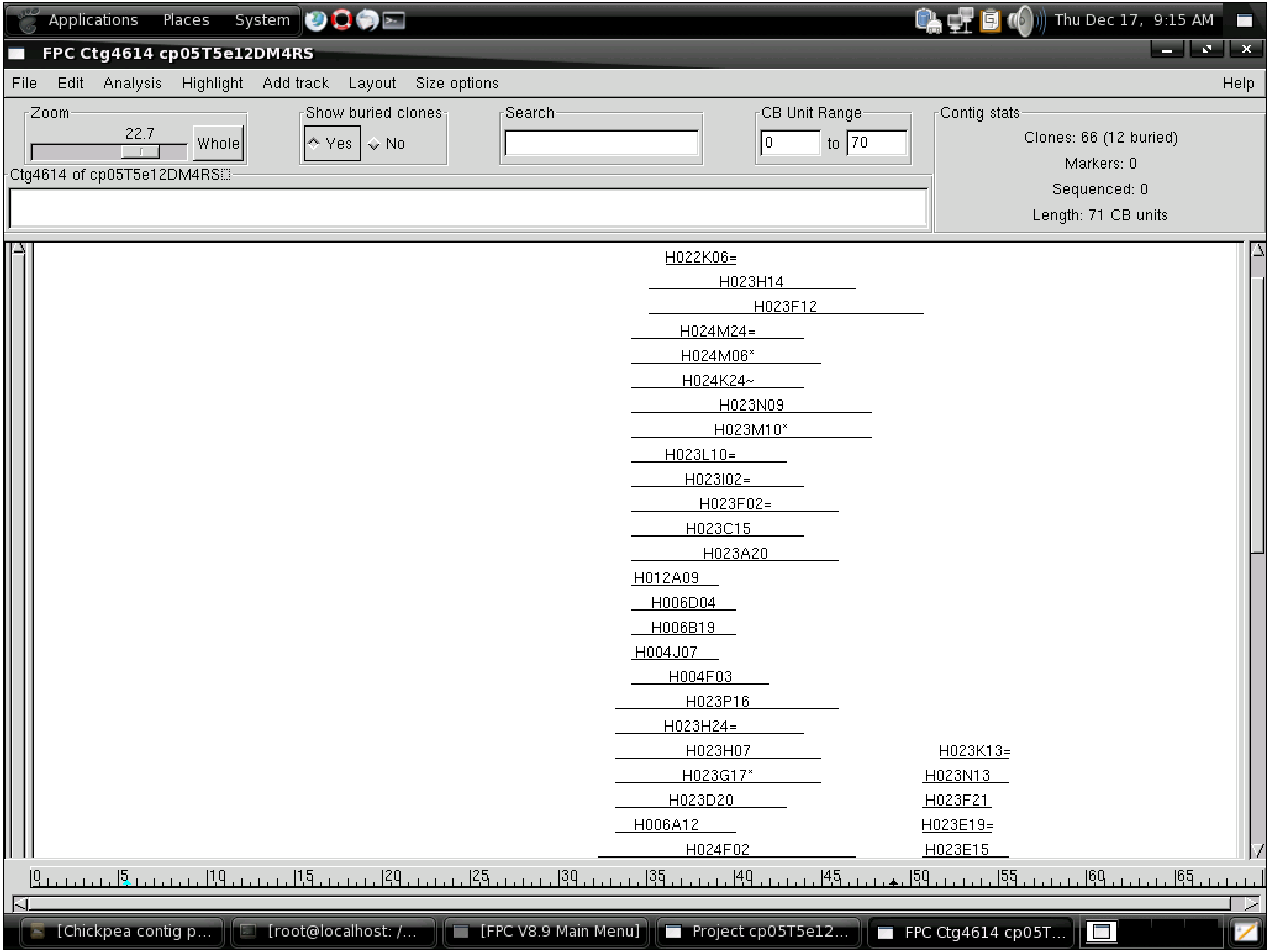
Applications Places System
Thu Dec 17, 9:15 AM
FPC Ctg4614 cp05T5e12DM4RS
File Edit Analysis Highlight Add track Layout Size options Help
Zoom
22.7
Whole
Show buried clones
Yes No
Search
CB Unit Range
0 to 70
Contig stats
Clones: 66 (12 buried)
Markers: 0
Sequenced: 0
Length: 71 CB units
Ctg4614 of cp05T5e12DM4RS
H022K06=
H023H14
H023F12
H024M24=
H024M06*
H024K24~
H023N09
H023M10*
H023L10=
H023I02=
H023F02=
H023C15
H023A20
H012A09
H006D04
H006B19
H004J07
H004F03
H023P16
H023H24=
H023H07
H023K13=
H023G17*
H023N13
H023D20
H023F21
H006A12
H023E19=
H024F02
H023E15
0 5 10 15 20 25 30 35 40 45 50 55 60 65
[Chickpea contig p...
[root@localhost: /...
[FPC V8.9 Main Menu]
Project cp05T5e12...
FPC Ctg4614 cp05T...

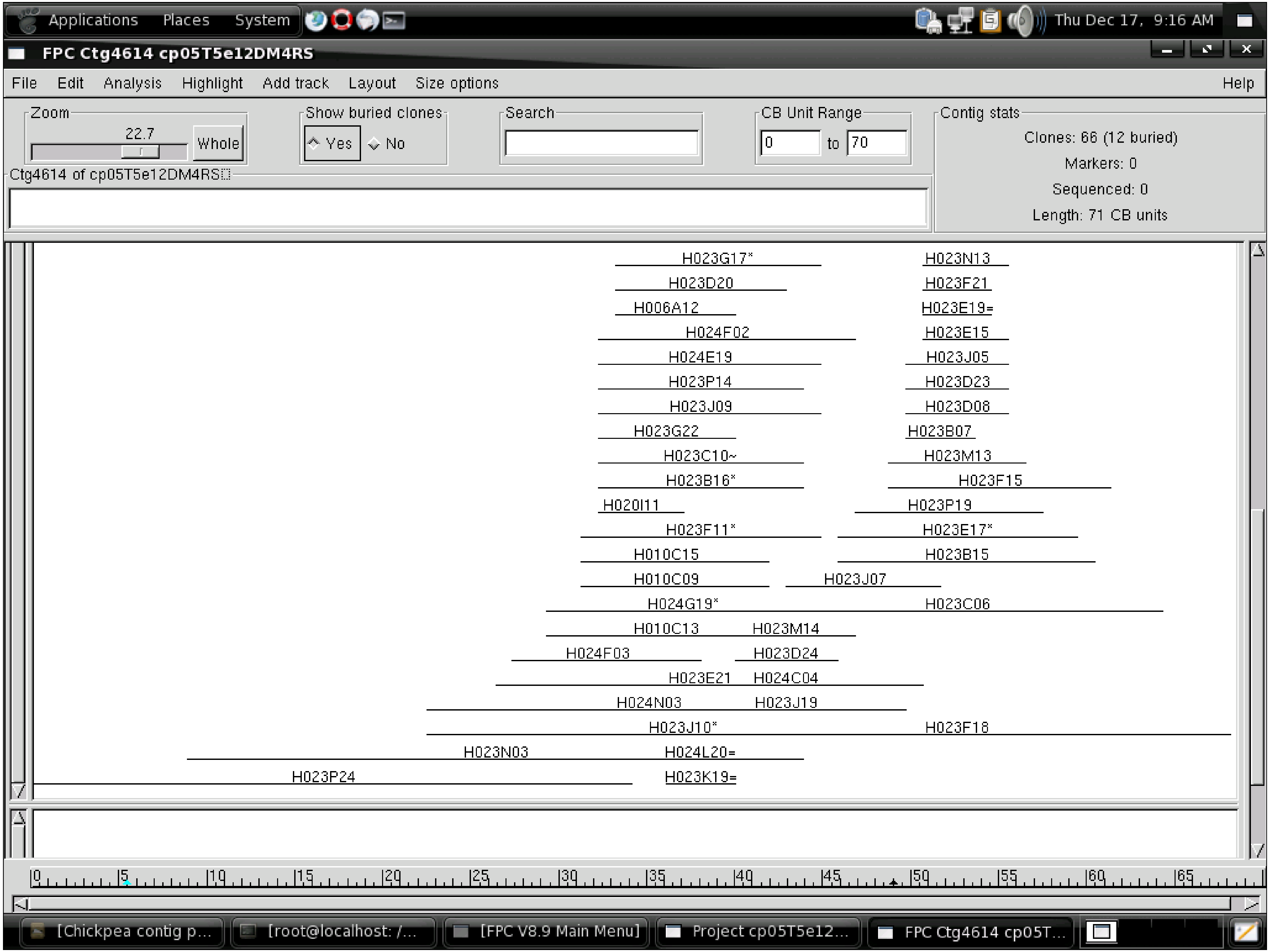
Applications Places System
Thu Dec 17, 9:16 AM
FPC Ctg4614 cp05T5e12DM4RS
File Edit Analysis Highlight Add track Layout Size options Help
Zoom
22.7
Whole
Show buried clones
Yes
No
Search
CB Unit Range
0
to
70
Contig stats
Clones: 66 (12 buried)
Markers: 0
Sequenced: 0
Length: 71 CB units
Ctg4614 of cp05T5e12DM4RS
H023G17*
H023N13
H023D20
H023F21
H006A12
H023E19=
H024F02
H023E15
H024E19
H023J05
H023P14
H023D23
H023J09
H023D08
H023G22
H023B07
H023C10~
H023M13
H023B16*
H023F15
H020I11
H023P19
H023F11*
H023E17*
H010C15
H023B15
H010C09
H023J07
H024G19*
H023C06
H010C13
H023M14
H024F03
H023D24
H023E21
H024C04
H024N03
H023J19
H023J10*
H023F18
H023N03
H024L20=
H023P24
H023K19=
0 5 10 15 20 25 30 35 40 45 50 55 60 65
[Chickpea contig p...
[root@localhost: /...
[FPC V8.9 Main Menu]
Project cp05T5e12...
FPC Ctg4614 cp05T...

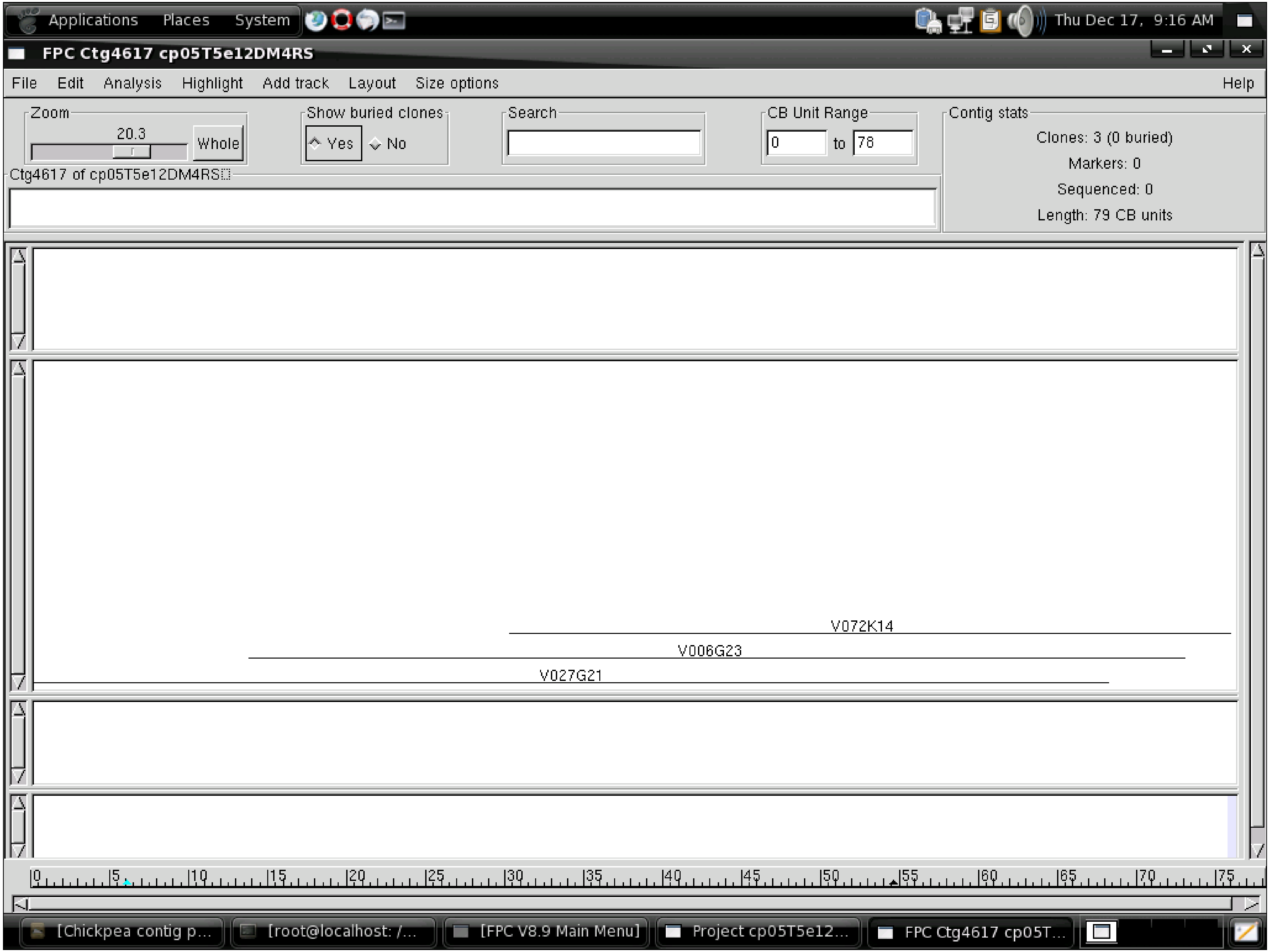
Applications Places System
Thu Dec 17, 9:16 AM
FPC Ctg4617 cp05T5e12DM4RS
File Edit Analysis Highlight Add track Layout Size options Help
Zoom
20.3
Whole
Show buried clones
Yes No
Search
CB Unit Range
0 to 78
Contig stats
Clones: 3 (0 buried)
Markers: 0
Sequenced: 0
Length: 79 CB units
Ctg4617 of cp05T5e12DM4RS
V072K14
V006G23
V027G21
0 5 10 15 20 25 30 35 40 45 50 55 60 65 70 75
[Chickpea contig p...
[root@localhost: /...
[FPC V8.9 Main Menu]
Project cp05T5e12...
FPC Ctg4617 cp05T...

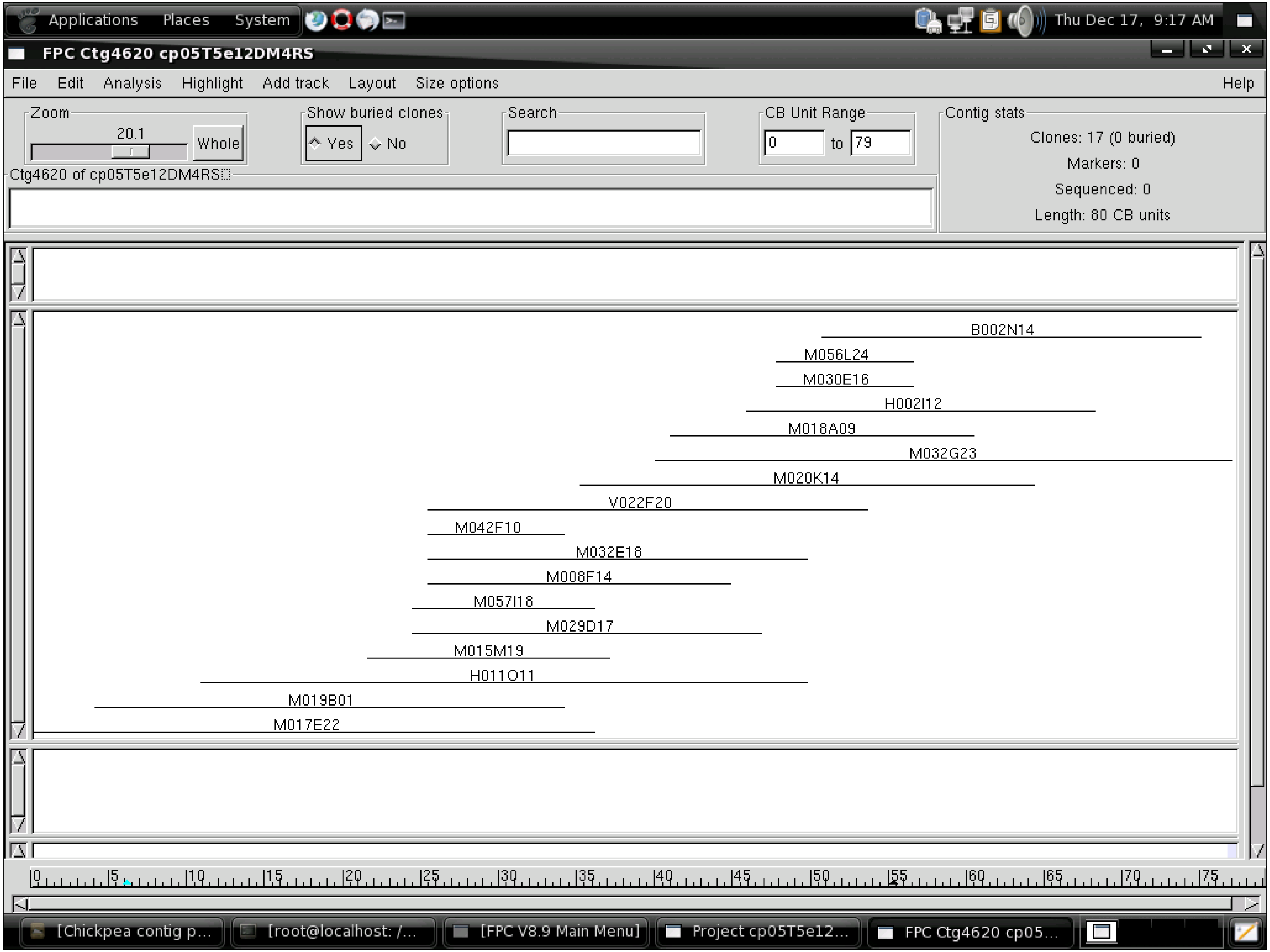
Applications Places System
Thu Dec 17, 9:17 AM
FPC Ctg4620 cp05T5e12DM4RS
File Edit Analysis Highlight Add track Layout Size options Help
Zoom
20.1
Whole
Show buried clones
Yes No
Search
CB Unit Range
0 to 79
Contig stats
Clones: 17 (0 buried)
Markers: 0
Sequenced: 0
Length: 80 CB units
Ctg4620 of cp05T5e12DM4RS
B002N14
M056L24
M030E16
H002I12
M018A09
M032G23
M020K14
V022F20
M042F10
M032E18
M008F14
M057I18
M029D17
M015M19
H011O11
M019B01
M017E22
0 5 10 15 20 25 30 35 40 45 50 55 60 65 70 75
[Chickpea contig p...
[root@localhost: /...
[FPC V8.9 Main Menu]
Project cp05T5e12...
FPC Ctg4620 cp05...

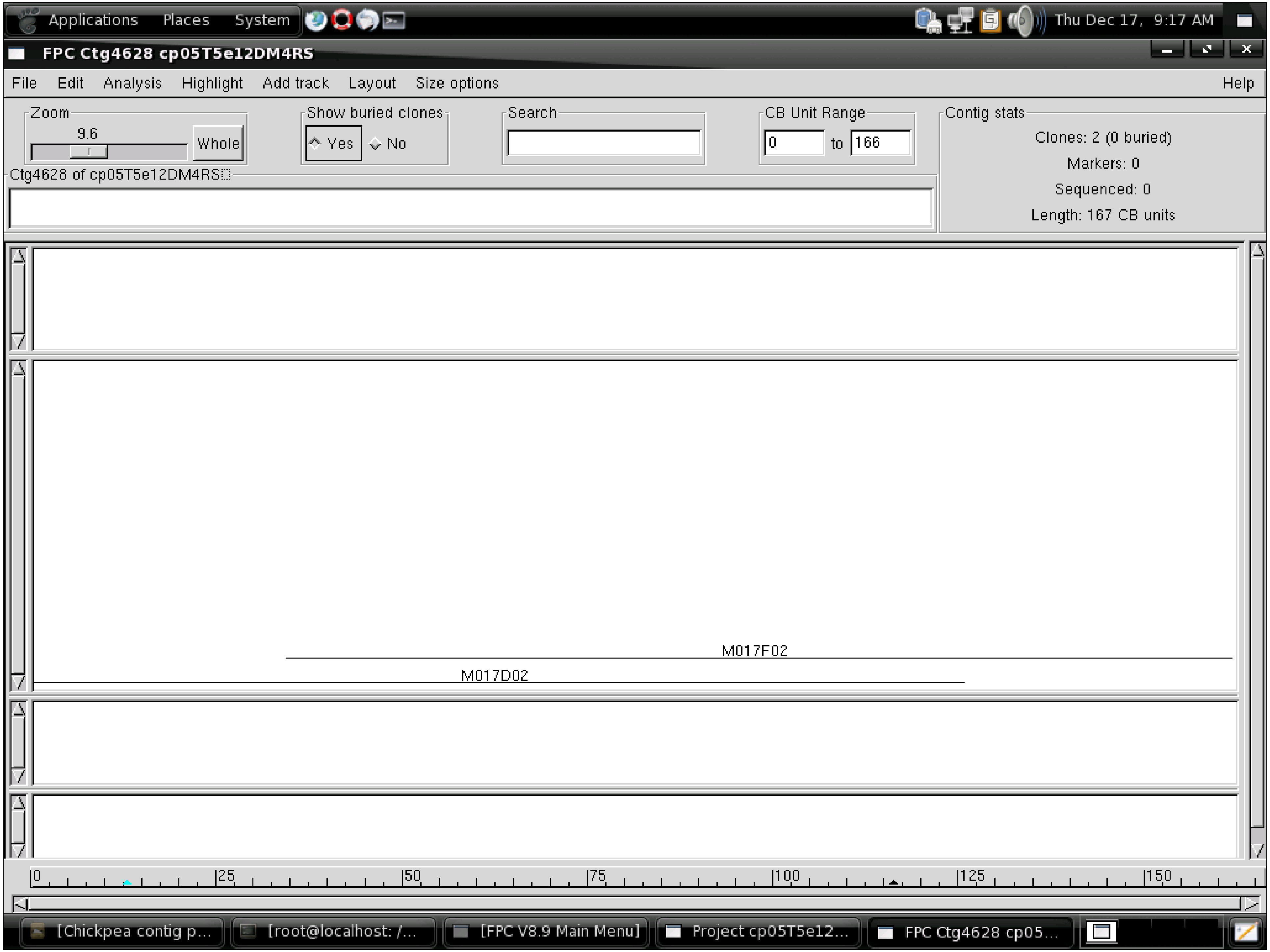
Applications Places System
Thu Dec 17, 9:17 AM
FPC Ctg4628 cp05T5e12DM4RS
File Edit Analysis Highlight Add track Layout Size options Help
Zoom
9.6
Whole
Show buried clones
Yes No
Search
CB Unit Range
0 to 166
Contig stats
Clones: 2 (0 buried)
Markers: 0
Sequenced: 0
Length: 167 CB units
Ctg4628 of cp05T5e12DM4RS
M017F02
M017D02
0 25 50 75 100 125 150
[Chickpea contig p...
[root@localhost: /...
[FPC V8.9 Main Menu]
Project cp05T5e12...
FPC Ctg4628 cp05...

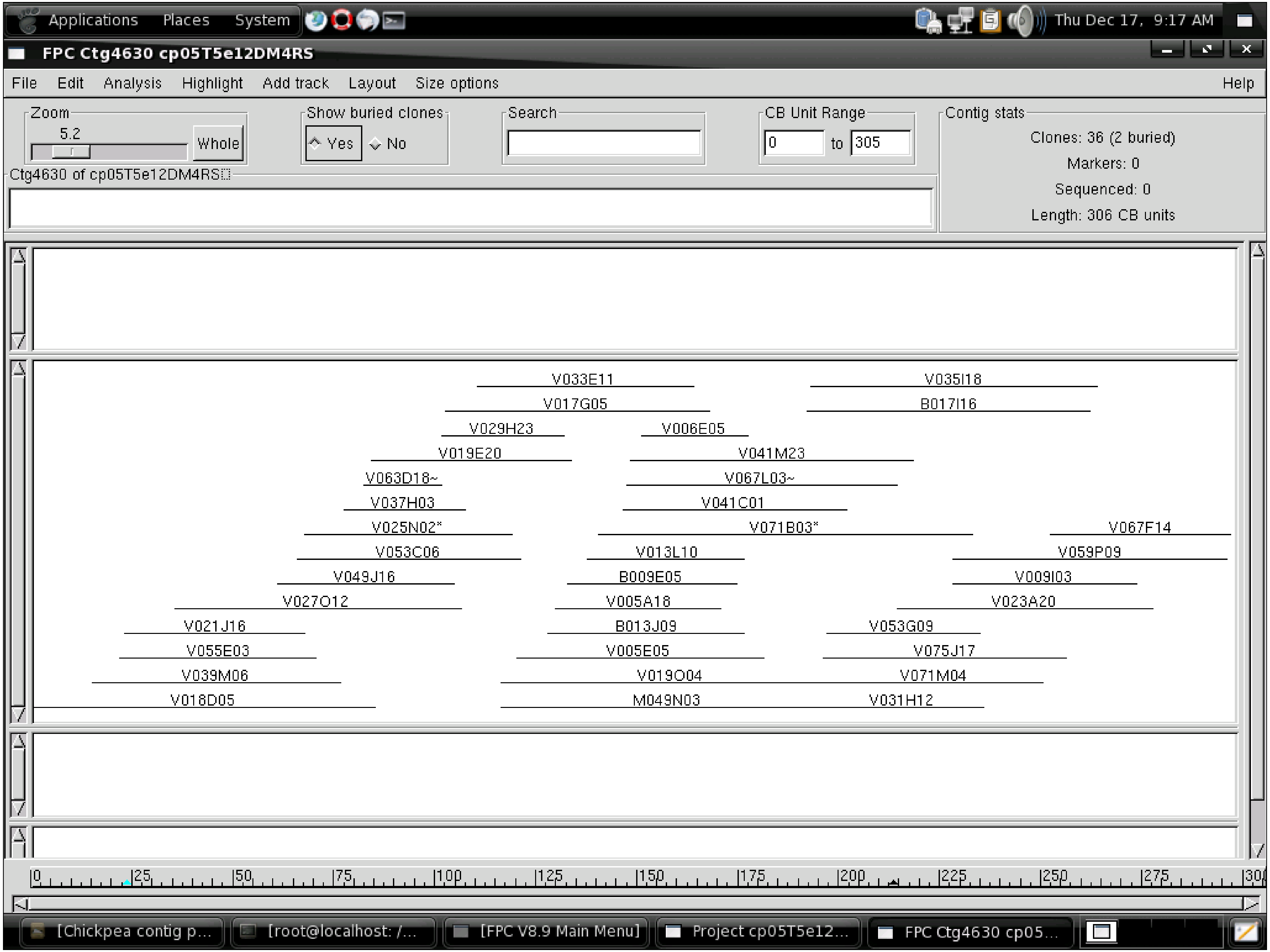
Applications Places System
Thu Dec 17, 9:17 AM
FPC Ctg4630 cp05T5e12DM4RS
File Edit Analysis Highlight Add track Layout Size options Help
Zoom
5.2
Whole
Show buried clones
Yes
No
Search
CB Unit Range
0
to
305
Contig stats
Clones: 36 (2 buried)
Markers: 0
Sequenced: 0
Length: 306 CB units
Ctg4630 of cp05T5e12DM4RS
V033E11
V035I18
V017G05
B017I16
V029H23
V006E05
V019E20
V041M23
V063D18~
V067L03~
V037H03
V041C01
V025N02*
V071B03*
V067F14
V053C06
V013L10
V059P09
V049J16
B009E05
V009I03
V027O12
V005A18
V023A20
V021J16
B013J09
V053G09
V055E03
V005E05
V075J17
V039M06
V019O04
V071M04
V018D05
M049N03
V031H12
0 25 50 75 100 125 150 175 200 225 250 275 30
[Chickpea contig p...
[root@localhost: /...
[FPC V8.9 Main Menu]
Project cp05T5e12...
FPC Ctg4630 cp05...

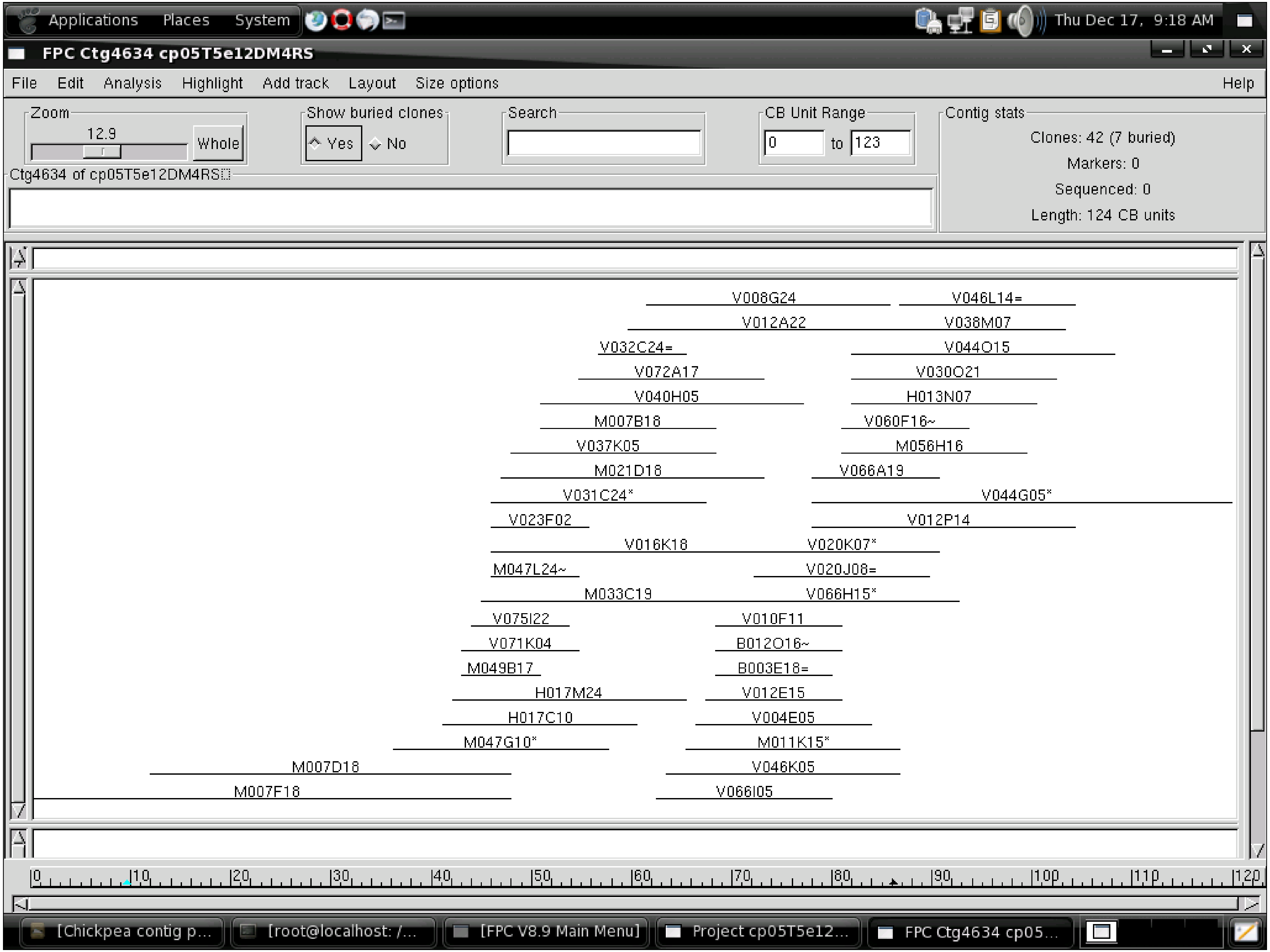
Applications Places System
Thu Dec 17, 9:18 AM
FPC Ctg4634 cp05T5e12DM4RS
File Edit Analysis Highlight Add track Layout Size options Help
Zoom
12.9
Whole
Show buried clones
Yes No
Search
CB Unit Range
0 to 123
Contig stats
Clones: 42 (7 buried)
Markers: 0
Sequenced: 0
Length: 124 CB units
Ctg4634 of cp05T5e12DM4RS
V008G24
V046L14=
V012A22
V038M07
V032C24=
V044O15
V072A17
V030O21
V040H05
H013N07
M007B18
V060F16~
V037K05
M056H16
M021D18
V066A19
V031C24*
V044G05*
V023F02
V012P14
V016K18
V020K07*
M047L24~
V020J08=
M033C19
V066H15*
V075I22
V010F11
V071K04
B012O16~
M049B17
B003E18=
H017M24
V012E15
H017C10
V004E05
M047G10*
M011K15*
M007D18
V046K05
M007F18
V066I05
0 10 20 30 40 50 60 70 80 90 100 110 120
[Chickpea contig p...
[root@localhost: /...
[FPC V8.9 Main Menu]
Project cp05T5e12...
FPC Ctg4634 cp05...

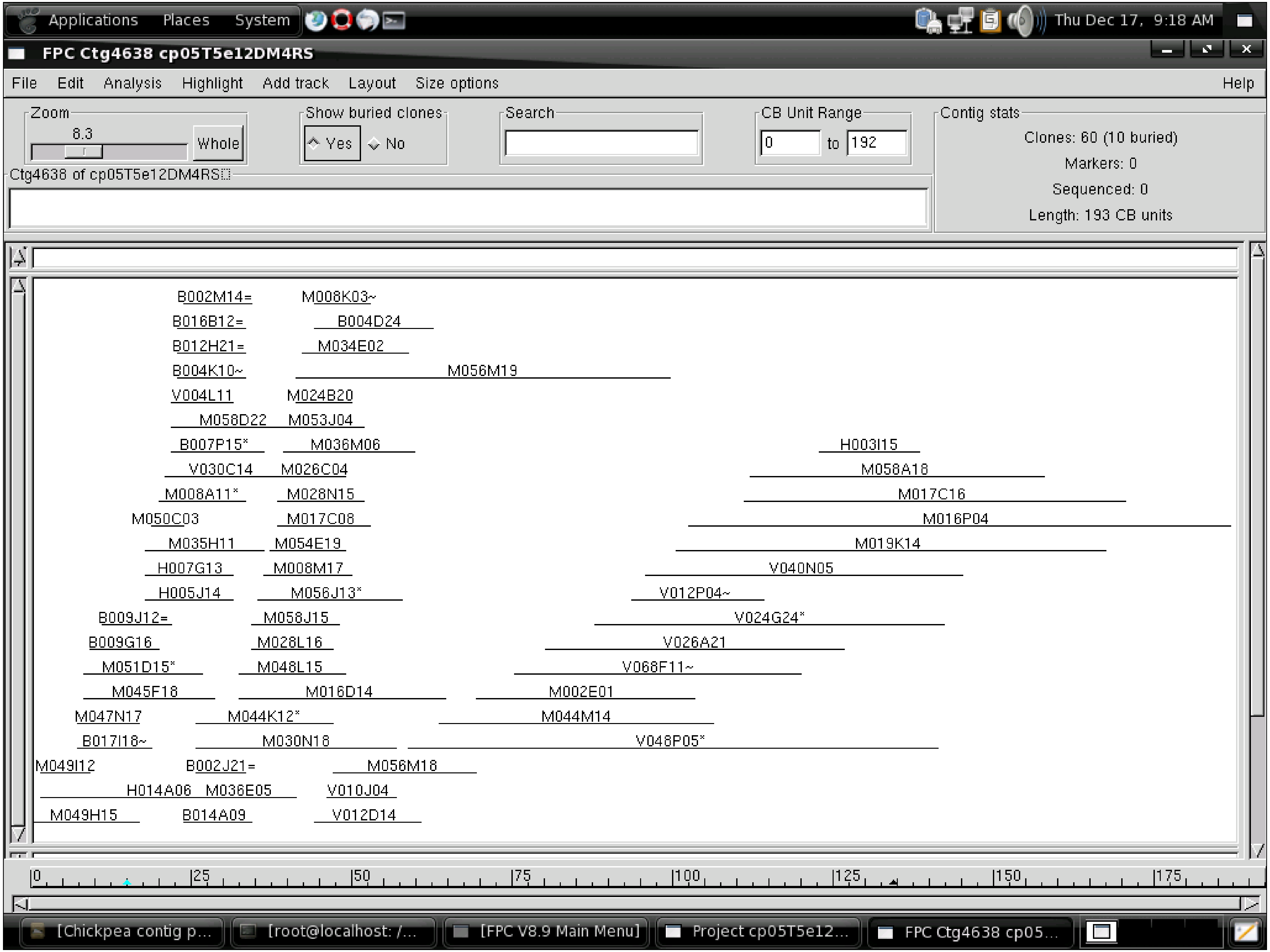
Applications Places System
Thu Dec 17, 9:18 AM
FPC Ctg4638 cp05T5e12DM4RS
File Edit Analysis Highlight Add track Layout Size options Help
Zoom
8.3
Whole
Show buried clones
Yes No
Search
CB Unit Range
0 to 192
Contig stats
Clones: 60 (10 buried)
Markers: 0
Sequenced: 0
Length: 193 CB units
Ctg4638 of cp05T5e12DM4RS
B002M14= M008K03~
B016B12= B004D24
B012H21= M034E02
B004K10~ M056M19
V004L11 M024B20
M058D22 M053J04
B007P15* M036M06 H003I15
V030C14 M026C04 M058A18
M008A11* M028N15 M017C16
M050C03 M017C08 M016P04
M035H11 M054E19 M019K14
H007G13 M008M17 V040N05
H005J14 M056J13* V012P04~
B009J12= M058J15 V024G24*
B009G16 M028L16 V026A21
M051D15* M048L15 V068F11~
M045F18 M016D14 M002E01
M047N17 M044K12* M044M14
B017I18~ M030N18 V048P05*
M049I12 B002J21= M056M18
H014A06 M036E05 V010J04
M049H15 B014A09 V012D14
0 25 50 75 100 125 150 175
[Chickpea contig p...
[root@localhost: /...
[FPC V8.9 Main Menu]
Project cp05T5e12...
FPC Ctg4638 cp05...

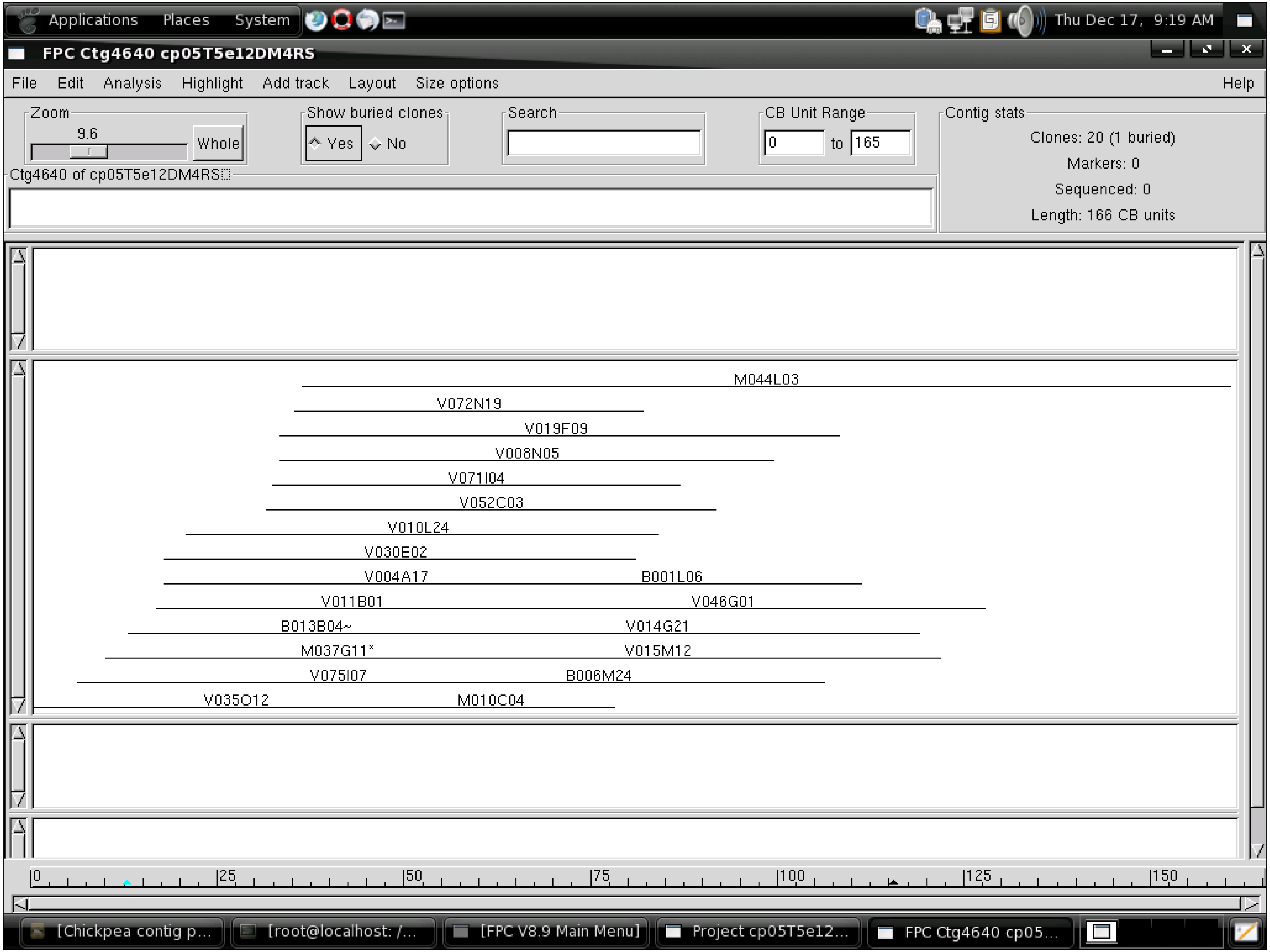

Applications Places System
Thu Dec 17, 9:19 AM
FPC Ctg4640 cp05T5e12DM4RS
File Edit Analysis Highlight Add track Layout Size options Help
Zoom
9.6
Whole
Show buried clones
Yes No
Search
CB Unit Range
0 to 165
Contig stats
Clones: 20 (1 buried)
Markers: 0
Sequenced: 0
Length: 166 CB units
Ctg4640 of cp05T5e12DM4RS
M044L03
V072N19
V019F09
V008N05
V071I04
V052C03
V010L24
V030E02
V004A17
B001L06
V011B01
V046G01
B013B04~
V014G21
M037G11*
V015M12
V075I07
B006M24
V035O12
M010C04
0 25 50 75 100 125 150
[Chickpea contig p...
[root@localhost: /...
[FPC V8.9 Main Menu]
Project cp05T5e12...
FPC Ctg4640 cp05...

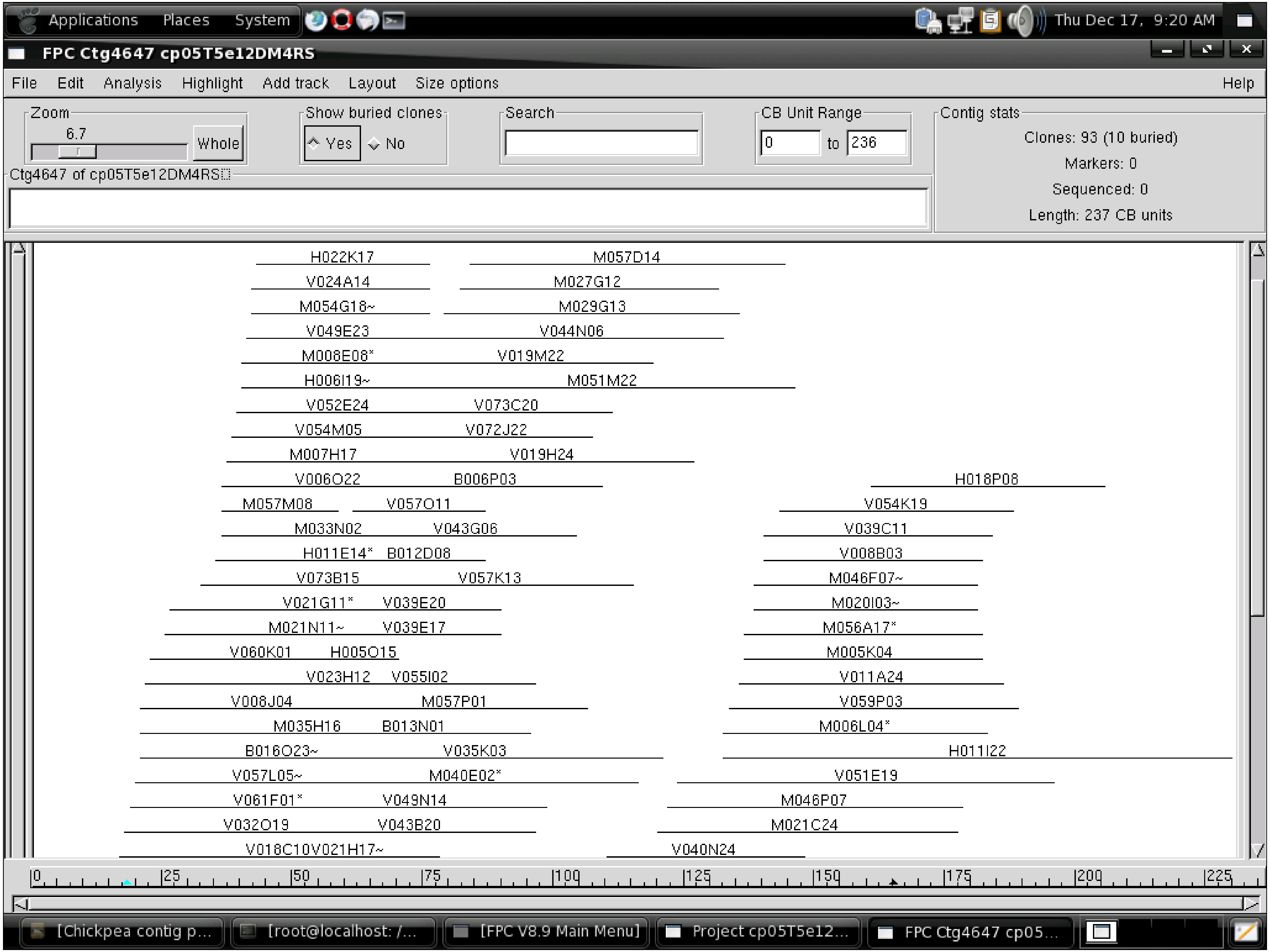
Applications Places System
Thu Dec 17, 9:20 AM
FPC Ctg4647 cp05T5e12DM4RS
File Edit Analysis Highlight Add track Layout Size options Help
Zoom
6.7
Whole
Show buried clones
Yes No
Search
CB Unit Range
0 to 236
Contig stats
Clones: 93 (10 buried)
Markers: 0
Sequenced: 0
Length: 237 CB units
Ctg4647 of cp05T5e12DM4RS
H022K17 M057D14
V024A14 M027G12
M054G18~ M029G13
V049E23 V044N06
M008E08* V019M22
H006I19~ M051M22
V052E24 V073C20
V054M05 V072J22
M007H17 V019H24
V006O22 B006P03 H018P08
M057M08 V057O11 V054K19
M033N02 V043G06 V039C11
H011E14* B012D08 V008B03
V073B15 V057K13 M046F07~
V021G11* V039E20 M020I03~
M021N11~ V039E17 M056A17*
V060K01 H005O15 M005K04
V023H12 V055I02 V011A24
V008J04 M057P01 V059P03
M035H16 B013N01 M006L04*
B016O23~ V035K03 H011I22
V057L05~ M040E02* V051E19
V061F01* V049N14 M046P07
V032O19 V043B20 M021C24
V018C10V021H17~ V040N24
0 25 50 75 100 125 150 175 200 225
[Chickpea contig p... [root@localhost: /... [FPC V8.9 Main Menu] Project cp05T5e12... FPC Ctg4647 cp05...

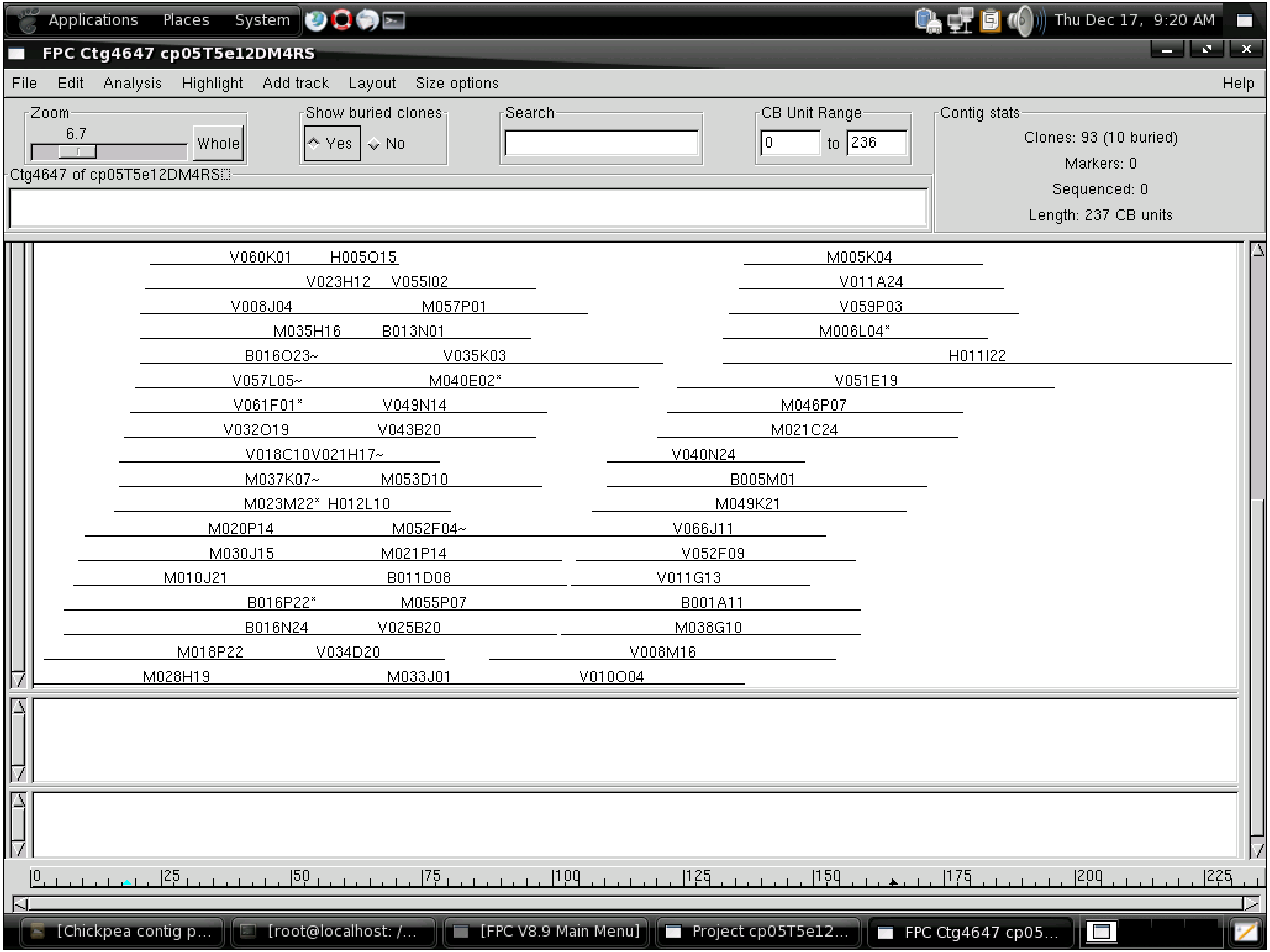
Applications Places System
Thu Dec 17, 9:20 AM
FPC Ctg4647 cp05T5e12DM4RS
File Edit Analysis Highlight Add track Layout Size options Help
Zoom
6.7
Whole
Show buried clones
Yes No
Search
CB Unit Range
0 to 236
Contig stats
Clones: 93 (10 buried)
Markers: 0
Sequenced: 0
Length: 237 CB units
Ctg4647 of cp05T5e12DM4RS
V060K01 H005O15 M005K04
V023H12 V055I02 V011A24
V008J04 M057P01 V059P03
M035H16 B013N01 M006L04*
B016O23~ V035K03 H011I22
V057L05~ M040E02* V051E19
V061F01* V049N14 M046P07
V032O19 V043B20 M021C24
V018C10V021H17~ V040N24
M037K07~ M053D10 B005M01
M023M22* H012L10 M049K21
M020P14 M052F04~ V066J11
M030J15 M021P14 V052F09
M010J21 B011D08 V011G13
B016P22* M055P07 B001A11
B016N24 V025B20 M038G10
M018P22 V034D20 V008M16
M028H19 M033J01 V010O04
0 25 50 75 100 125 150 175 200 225
[Chickpea contig p...
[root@localhost: /...
[FPC V8.9 Main Menu]
Project cp05T5e12...
FPC Ctg4647 cp05...

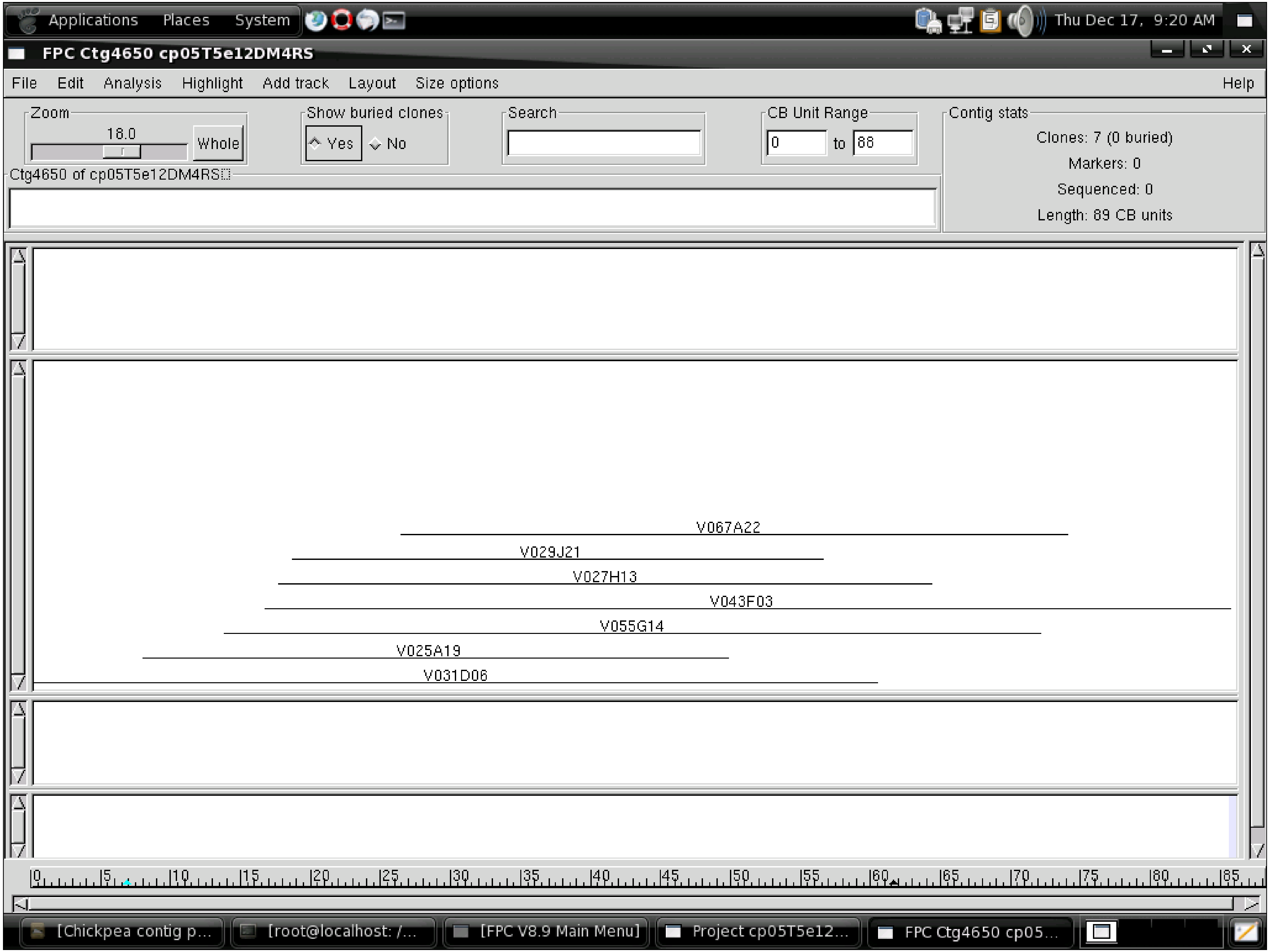
Applications Places System
Thu Dec 17, 9:20 AM
FPC Ctg4650 cp05T5e12DM4RS
File Edit Analysis Highlight Add track Layout Size options Help
Zoom
18.0
Whole
Show buried clones
Yes
No
Search
CB Unit Range
0
to
88
Contig stats
Clones: 7 (0 buried)
Markers: 0
Sequenced: 0
Length: 89 CB units
Ctg4650 of cp05T5e12DM4RS
V067A22
V029J21
V027H13
V043F03
V055G14
V025A19
V031D06
0 5 10 15 20 25 30 35 40 45 50 55 60 65 70 75 80 85
[Chickpea contig p...
[root@localhost: /...
[FPC V8.9 Main Menu]
Project cp05T5e12...
FPC Ctg4650 cp05...

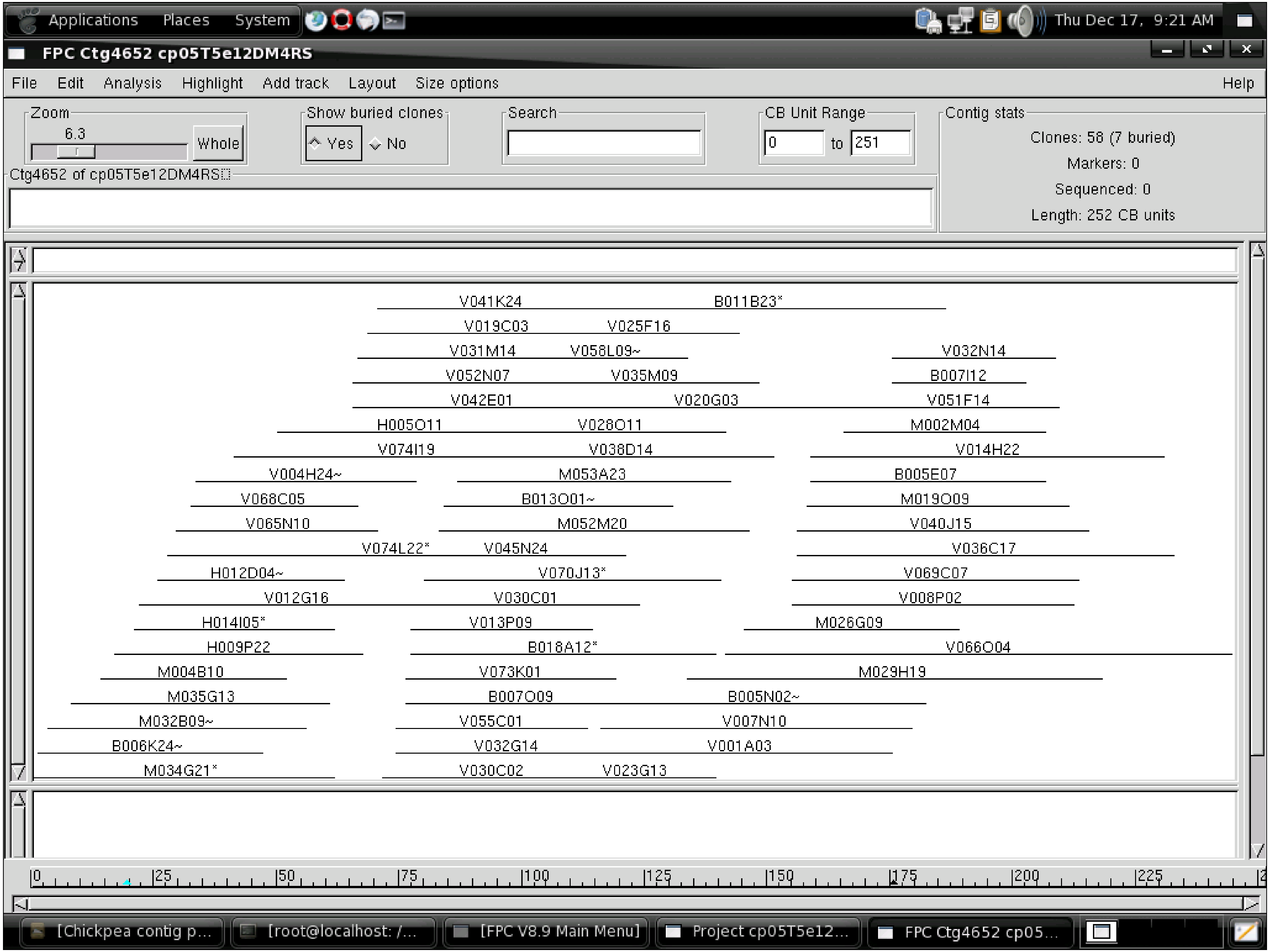
Applications Places System
Thu Dec 17, 9:21 AM
FPC Ctg4652 cp05T5e12DM4RS
File Edit Analysis Highlight Add track Layout Size options Help
Zoom
6.3
Whole
Show buried clones
Yes No
Search
CB Unit Range
0 to 251
Contig stats
Clones: 58 (7 buried)
Markers: 0
Sequenced: 0
Length: 252 CB units
Ctg4652 of cp05T5e12DM4RS
V041K24
B011B23*
V019C03
V025F16
V031M14
V058L09~
V032N14
V052N07
V035M09
B007I12
V042E01
V020G03
V051F14
H005O11
V028O11
M002M04
V074I19
V038D14
V014H22
V004H24~
M053A23
B005E07
V068C05
B013O01~
M019O09
V065N10
M052M20
V040J15
V074L22*
V045N24
V036C17
H012D04~
V070J13*
V069C07
V012G16
V030C01
V008P02
H014I05*
V013P09
M026G09
H009P22
B018A12*
V066O04
M004B10
V073K01
M029H19
M035G13
B007O09
B005N02~
M032B09~
V055C01
V007N10
B006K24~
V032G14
V001A03
M034G21*
V030C02
V023G13
0 25 50 75 100 125 150 175 200 225
[Chickpea contig p...
[root@localhost: /...
[FPC V8.9 Main Menu]
Project cp05T5e12...
FPC Ctg4652 cp05...

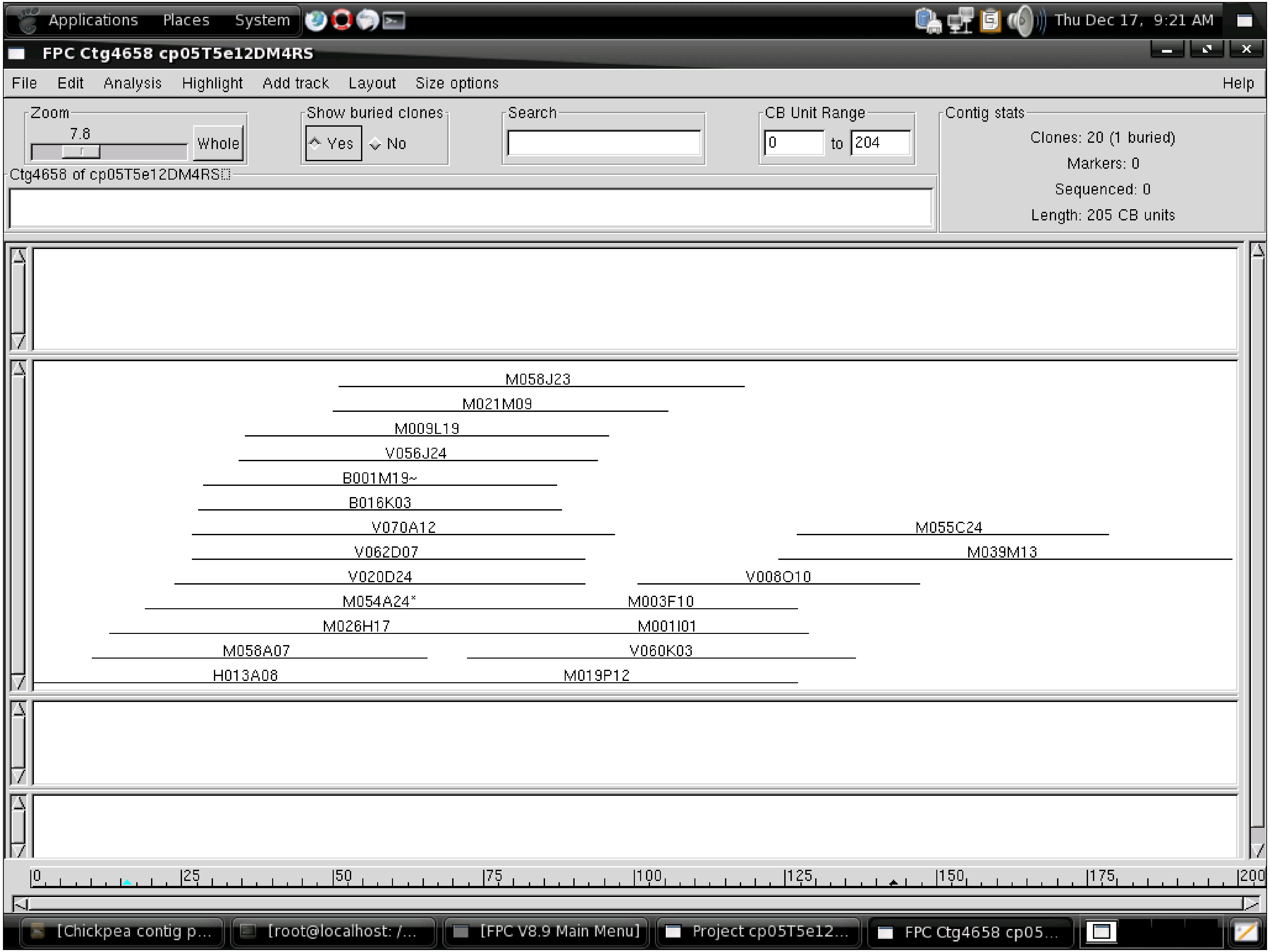
Applications Places System
Thu Dec 17, 9:21 AM
FPC Ctg4658 cp05T5e12DM4RS
File Edit Analysis Highlight Add track Layout Size options Help
Zoom
7.8
Whole
Show buried clones
Yes
No
Search
CB Unit Range
0 to 204
Contig stats
Clones: 20 (1 buried)
Markers: 0
Sequenced: 0
Length: 205 CB units
Ctg4658 of cp05T5e12DM4RS
M058J23
M021M09
M009L19
V056J24
B001M19~
B016K03
V070A12
M055C24
V062D07
M039M13
V020D24
V008O10
M054A24*
M003F10
M026H17
M001I01
M058A07
V060K03
H013A08
M019P12
0 25 50 75 100 125 150 175 200
[Chickpea contig p...
[root@localhost: /...
[FPC V8.9 Main Menu]
Project cp05T5e12...
FPC Ctg4658 cp05...

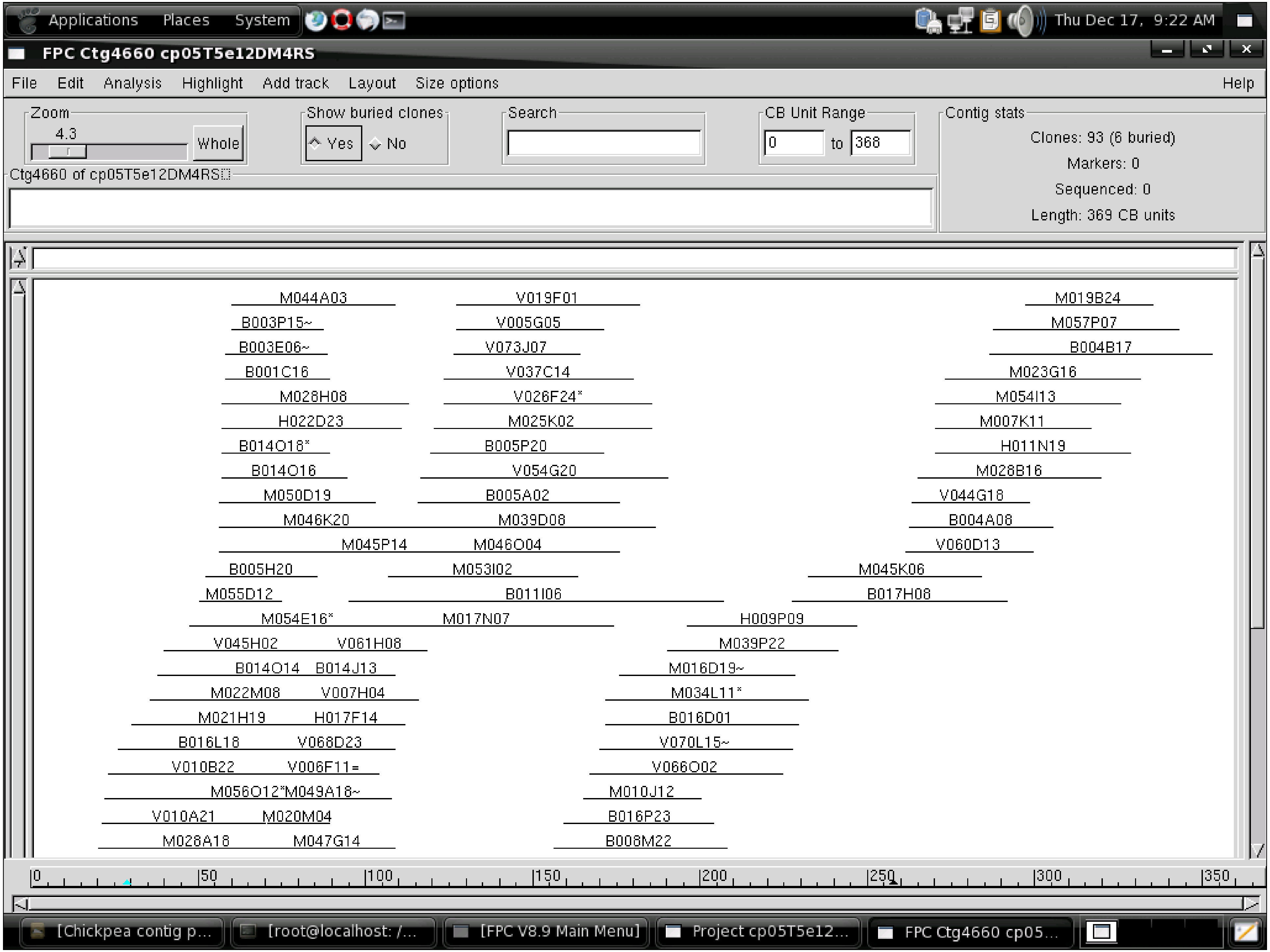
Applications Places System
Thu Dec 17, 9:22 AM
FPC Ctg4660 cp05T5e12DM4RS
File Edit Analysis Highlight Add track Layout Size options Help
Zoom
4.3
Whole
Show buried clones
Yes No
Search
CB Unit Range
0 to 368
Contig stats
Clones: 93 (6 buried)
Markers: 0
Sequenced: 0
Length: 369 CB units
Ctg4660 of cp05T5e12DM4RS
M044A03 V019F01 M019B24
B003P15~ V005G05 M057P07
B003E06~ V073J07 B004B17
B001C16 V037C14 M023G16
M028H08 V026F24* M054I13
H022D23 M025K02 M007K11
B014O18* B005P20 H011N19
B014O16 V054G20 M028B16
M050D19 B005A02 V044G18
M046K20 M039D08 B004A08
M045P14 M046O04 V060D13
B005H20 M053I02 M045K06
M055D12 B011I06 B017H08
M054E16* M017N07 H009P09
V045H02 V061H08 M039P22
B014O14 B014J13 M016D19~
M022M08 V007H04 M034L11*
M021H19 H017F14 B016D01
B016L18 V068D23 V070L15~
V010B22 V006F11= V066O02
M056O12*M049A18~ M010J12
V010A21 M020M04 B016P23
M028A18 M047G14 B008M22
0 50 100 150 200 250 300 350
[Chickpea contig p...
[root@localhost: /...
[FPC V8.9 Main Menu]
Project cp05T5e12...
FPC Ctg4660 cp05...

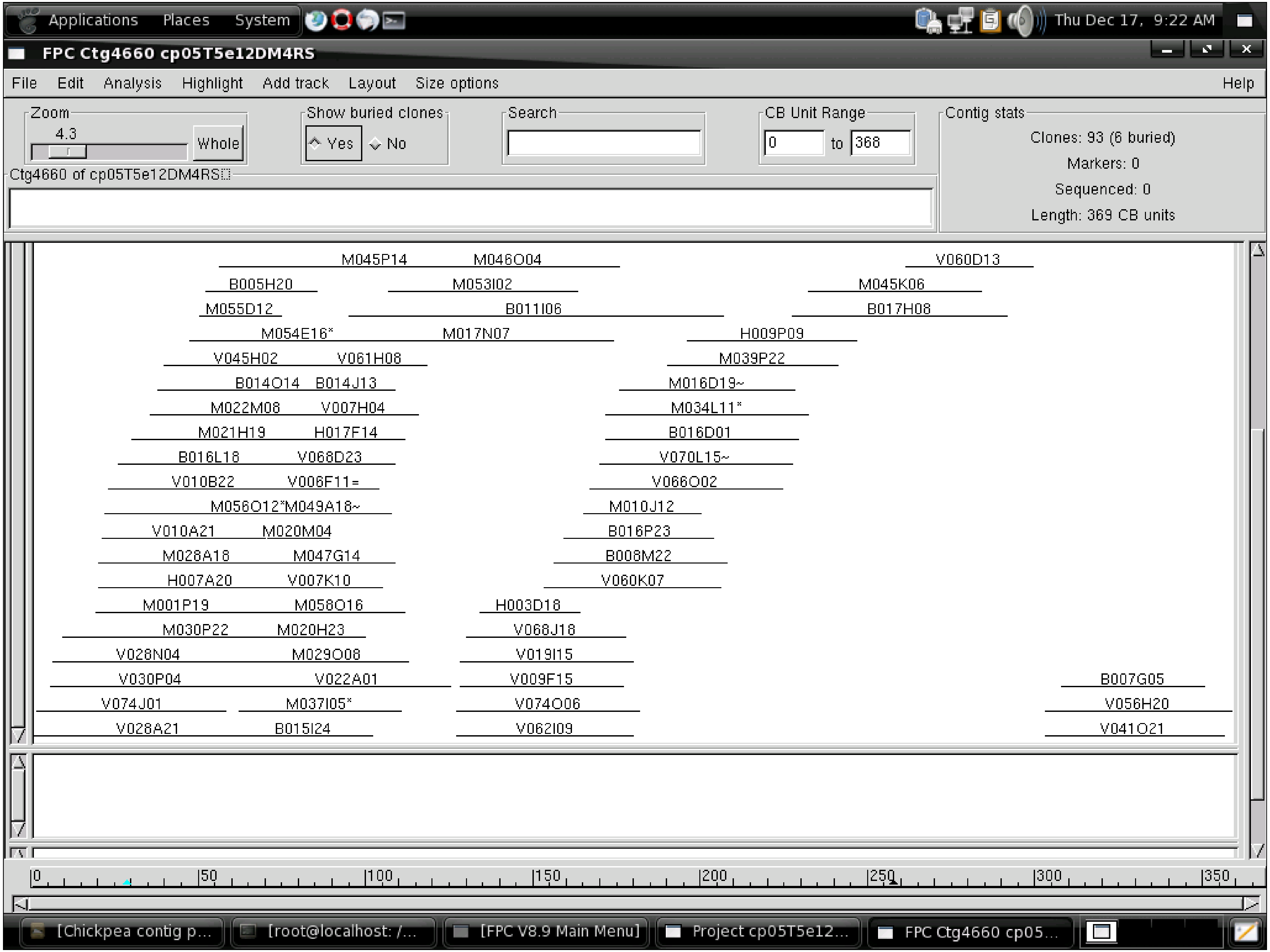
Applications Places System
Thu Dec 17, 9:22 AM
FPC Ctg4660 cp05T5e12DM4RS
File Edit Analysis Highlight Add track Layout Size options Help
Zoom
4.3
Whole
Show buried clones
Yes No
Search
CB Unit Range
0 to 368
Contig stats
Clones: 93 (6 buried)
Markers: 0
Sequenced: 0
Length: 369 CB units
Ctg4660 of cp05T5e12DM4RS
M045P14 M046O04 V060D13
B005H20 M053I02 M045K06
M055D12 B011I06 B017H08
M054E16* M017N07 H009P09
V045H02 V061H08 M039P22
B014O14 B014J13 M016D19~
M022M08 V007H04 M034L11*
M021H19 H017F14 B016D01
B016L18 V068D23 V070L15~
V010B22 V006F11= V066O02
M056O12*M049A18~ M010J12
V010A21 M020M04 B016P23
M028A18 M047G14 B008M22
H007A20 V007K10 V060K07
M001P19 M058O16 H003D18
M030P22 M020H23 V068J18
V028N04 M029O08 V019I15
V030P04 V022A01 V009F15 B007G05
V074J01 M037I05* V074O06 V056H20
V028A21 B015I24 V062I09 V041O21
0 50 100 150 200 250 300 350
[Chickpea contig p...
[root@localhost: /...
[FPC V8.9 Main Menu]
Project cp05T5e12...
FPC Ctg4660 cp05...

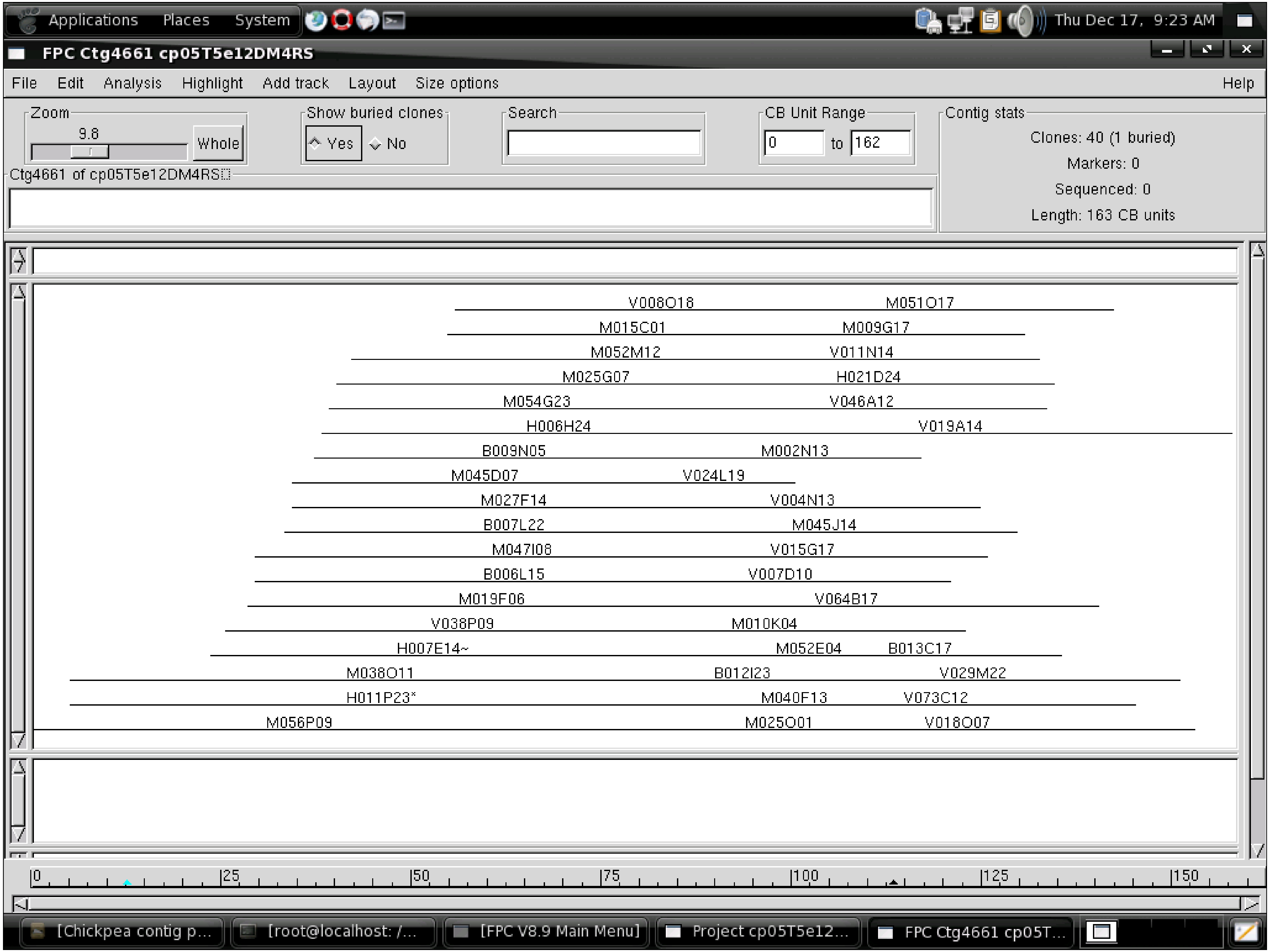
Applications Places System
Thu Dec 17, 9:23 AM
FPC Ctg4661 cp05T5e12DM4RS
File Edit Analysis Highlight Add track Layout Size options Help
Zoom
9.8
Whole
Show buried clones
Yes
No
Search
CB Unit Range
0 to 162
Contig stats
Clones: 40 (1 buried)
Markers: 0
Sequenced: 0
Length: 163 CB units
Ctg4661 of cp05T5e12DM4RS
V008O18 M051O17
M015C01 M009G17
M052M12 V011N14
M025G07 H021D24
M054G23 V046A12
H006H24 V019A14
B009N05 M002N13
M045D07 V024L19
M027F14 V004N13
B007L22 M045J14
M047I08 V015G17
B006L15 V007D10
M019F06 V064B17
V038P09 M010K04
H007E14~ M052E04 B013C17
M038O11 B012I23 V029M22
H011P23* M040F13 V073C12
M056P09 M025O01 V018O07
0 25 50 75 100 125 150
[Chickpea contig p...
[root@localhost: /...
[FPC V8.9 Main Menu]
Project cp05T5e12...
FPC Ctg4661 cp05T...

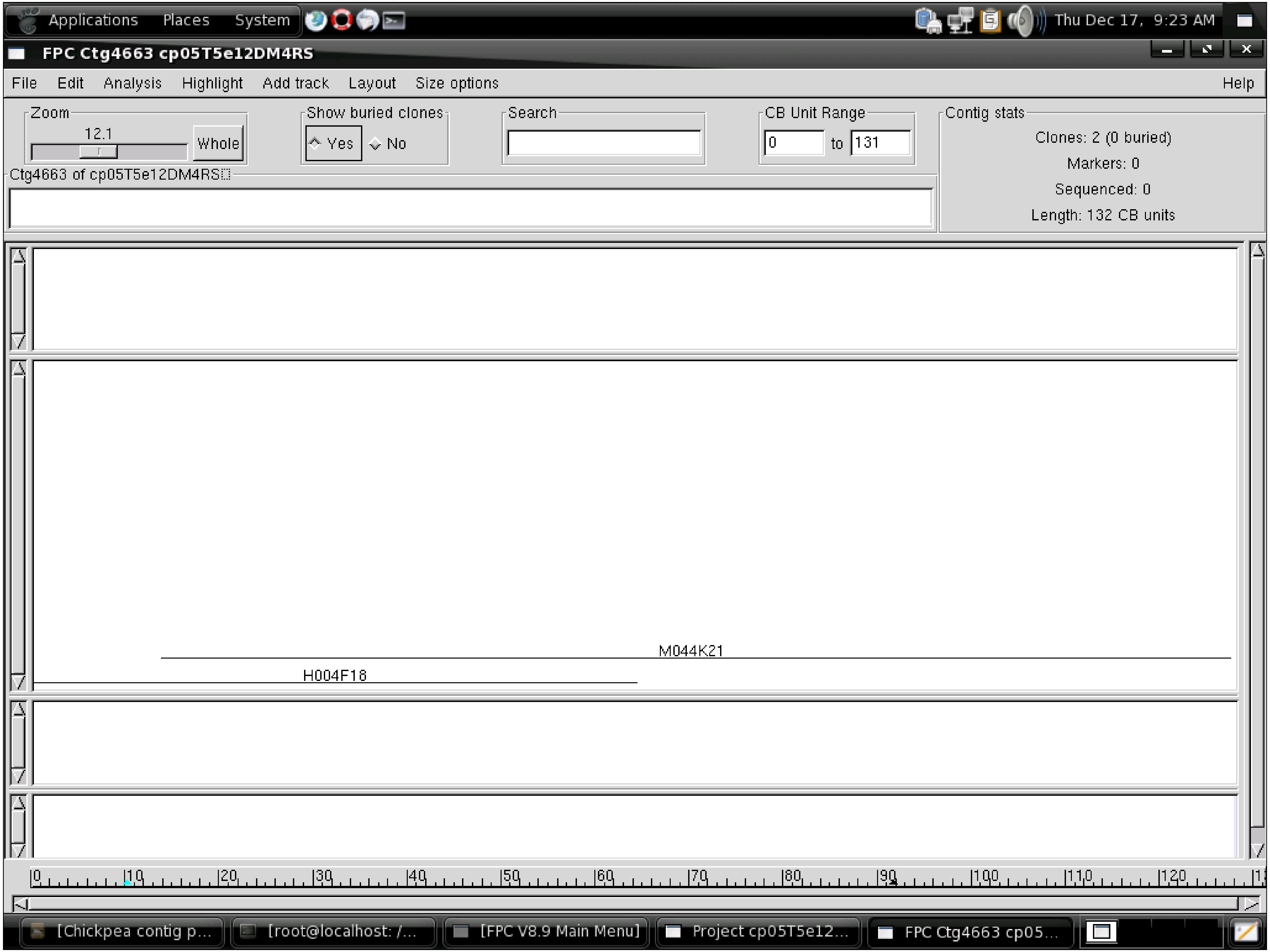
Applications Places System
Thu Dec 17, 9:23 AM
FPC Ctg4663 cp05T5e12DM4RS
File Edit Analysis Highlight Add track Layout Size options Help
Zoom
12.1
Whole
Show buried clones
Yes
No
Search
CB Unit Range
0 to 131
Contig stats
Clones: 2 (0 buried)
Markers: 0
Sequenced: 0
Length: 132 CB units
Ctg4663 of cp05T5e12DM4RS
M044K21
H004F18
0 10 20 30 40 50 60 70 80 90 100 110 120
[Chickpea contig p...
[root@localhost: /...
[FPC V8.9 Main Menu]
Project cp05T5e12...
FPC Ctg4663 cp05...

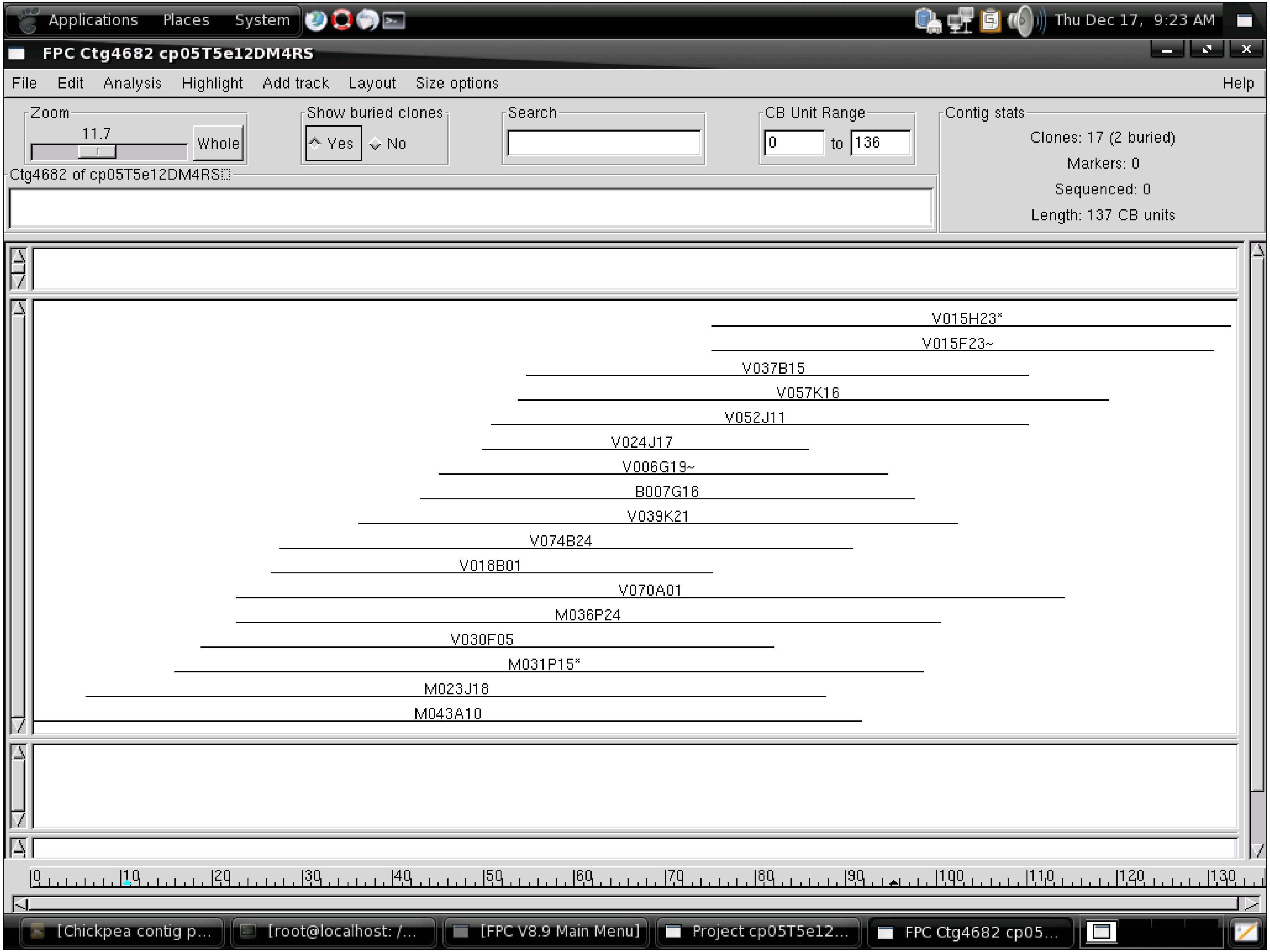
Applications Places System
Thu Dec 17, 9:23 AM
FPC Ctg4682 cp05T5e12DM4RS
File Edit Analysis Highlight Add track Layout Size options Help
Zoom
11.7
Whole
Show buried clones
Yes No
Search
CB Unit Range
0 to 136
Contig stats
Clones: 17 (2 buried)
Markers: 0
Sequenced: 0
Length: 137 CB units
Ctg4682 of cp05T5e12DM4RS
V015H23*
V015F23~
V037B15
V057K16
V052J11
V024J17
V006G19~
B007G16
V039K21
V074B24
V018B01
V070A01
M036P24
V030F05
M031P15*
M023J18
M043A10
0 10 20 30 40 50 60 70 80 90 100 110 120 130
[Chickpea contig p...
[root@localhost: /...
[FPC V8.9 Main Menu]
Project cp05T5e12...
FPC Ctg4682 cp05...

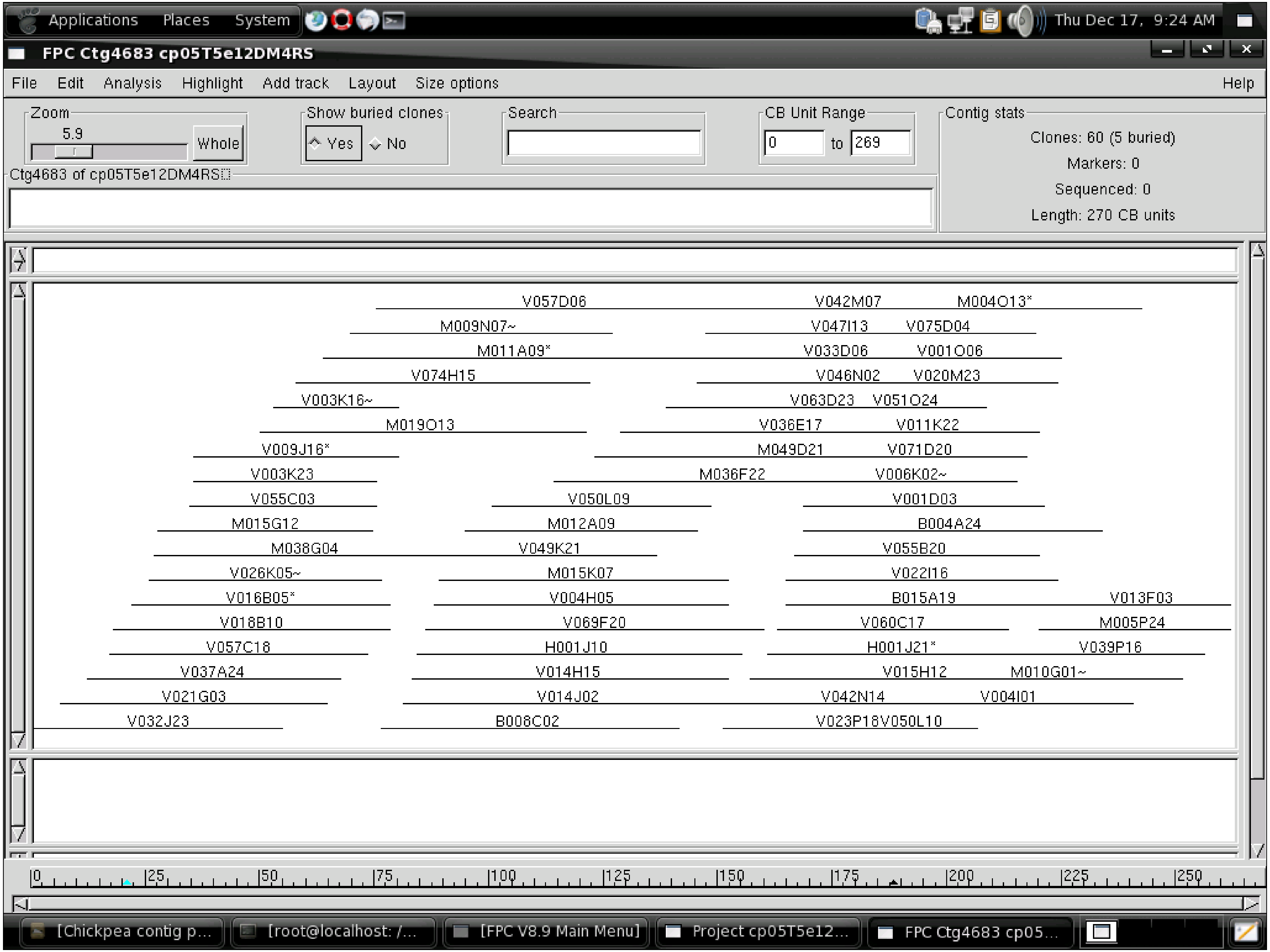
Applications Places System
Thu Dec 17, 9:24 AM
FPC Ctg4683 cp05T5e12DM4RS
File Edit Analysis Highlight Add track Layout Size options Help
Zoom
5.9
Whole
Show buried clones
Yes
No
Search
CB Unit Range
0
to
269
Contig stats
Clones: 60 (5 buried)
Markers: 0
Sequenced: 0
Length: 270 CB units
Ctg4683 of cp05T5e12DM4RS
V057D06
V042M07
M004O13*
M009N07~
V047I13
V075D04
M011A09*
V033D06
V001O06
V074H15
V046N02
V020M23
V003K16~
V063D23
V051O24
M019O13
V036E17
V011K22
V009J16*
M049D21
V071D20
V003K23
M036F22
V006K02~
V055C03
V050L09
V001D03
M015G12
M012A09
B004A24
M038G04
V049K21
V055B20
V026K05~
M015K07
V022I16
V016B05*
V004H05
B015A19
V013F03
V018B10
V069F20
V060C17
M005P24
V057C18
H001J10
H001J21*
V039P16
V037A24
V014H15
V015H12
M010G01~
V021G03
V014J02
V042N14
V004I01
V032J23
B008C02
V023P18V050L10
0
25
50
75
100
125
150
175
200
225
250
[Chickpea contig p...
[root@localhost: /...
[FPC V8.9 Main Menu]
Project cp05T5e12...
FPC Ctg4683 cp05...

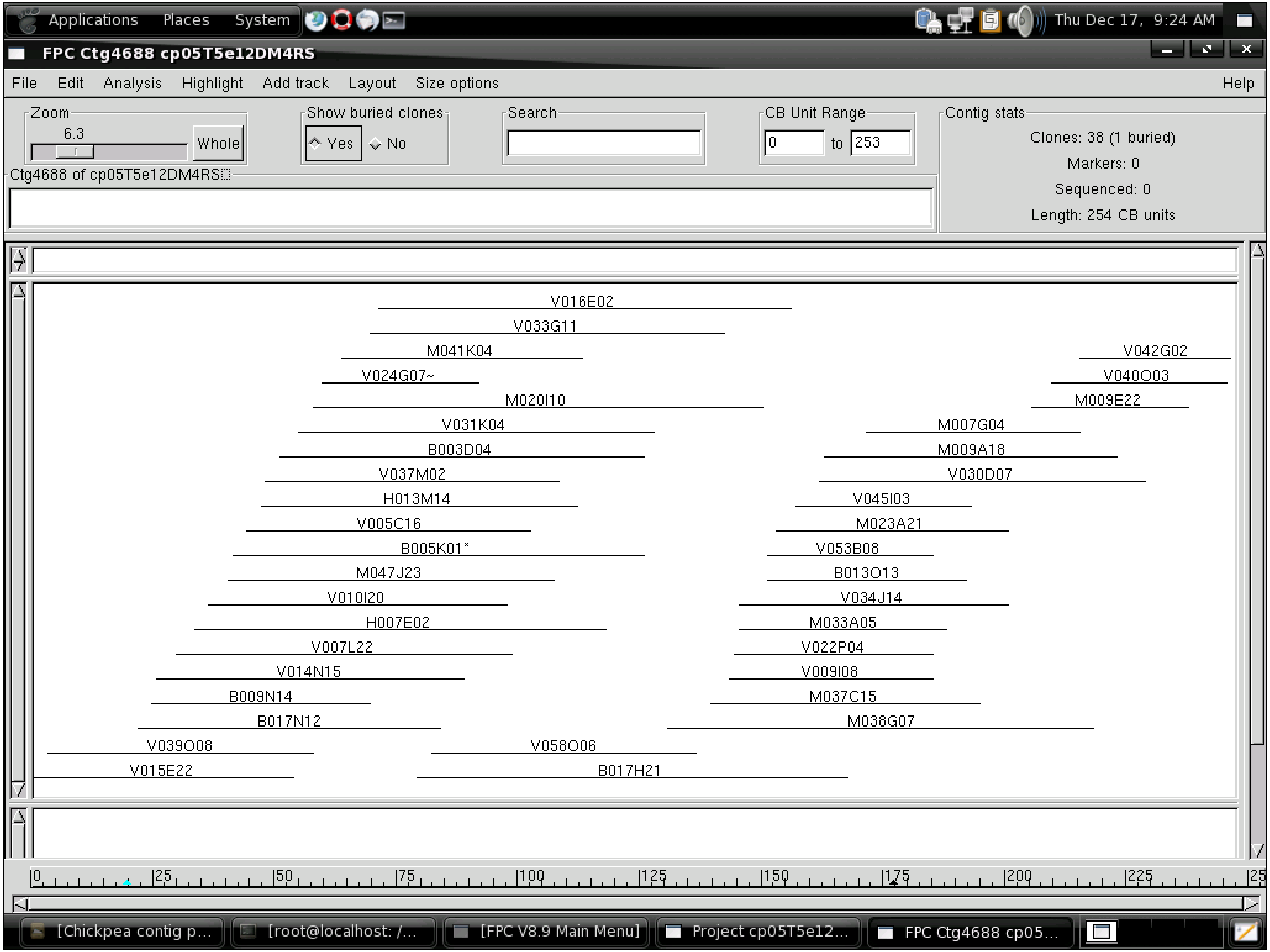
Applications Places System
Thu Dec 17, 9:24 AM
FPC Ctg4688 cp05T5e12DM4RS
File Edit Analysis Highlight Add track Layout Size options Help
Zoom
6.3
Whole
Show buried clones
Yes No
Search
CB Unit Range
0 to 253
Contig stats
Clones: 38 (1 buried)
Markers: 0
Sequenced: 0
Length: 254 CB units
Ctg4688 of cp05T5e12DM4RS
V016E02
V033G11
M041K04
V042G02
V024G07~
V040O03
M020I10
M009E22
V031K04
M007G04
B003D04
M009A18
V037M02
V030D07
H013M14
V045I03
V005C16
M023A21
B005K01*
V053B08
M047J23
B013O13
V010I20
V034J14
H007E02
M033A05
V007L22
V022P04
V014N15
V009I08
B009N14
M037C15
B017N12
M038G07
V039O08
V058O06
V015E22
B017H21
0 25 50 75 100 125 150 175 200 225 25
[Chickpea contig p...
[root@localhost: /...
[FPC V8.9 Main Menu]
Project cp05T5e12...
FPC Ctg4688 cp05...

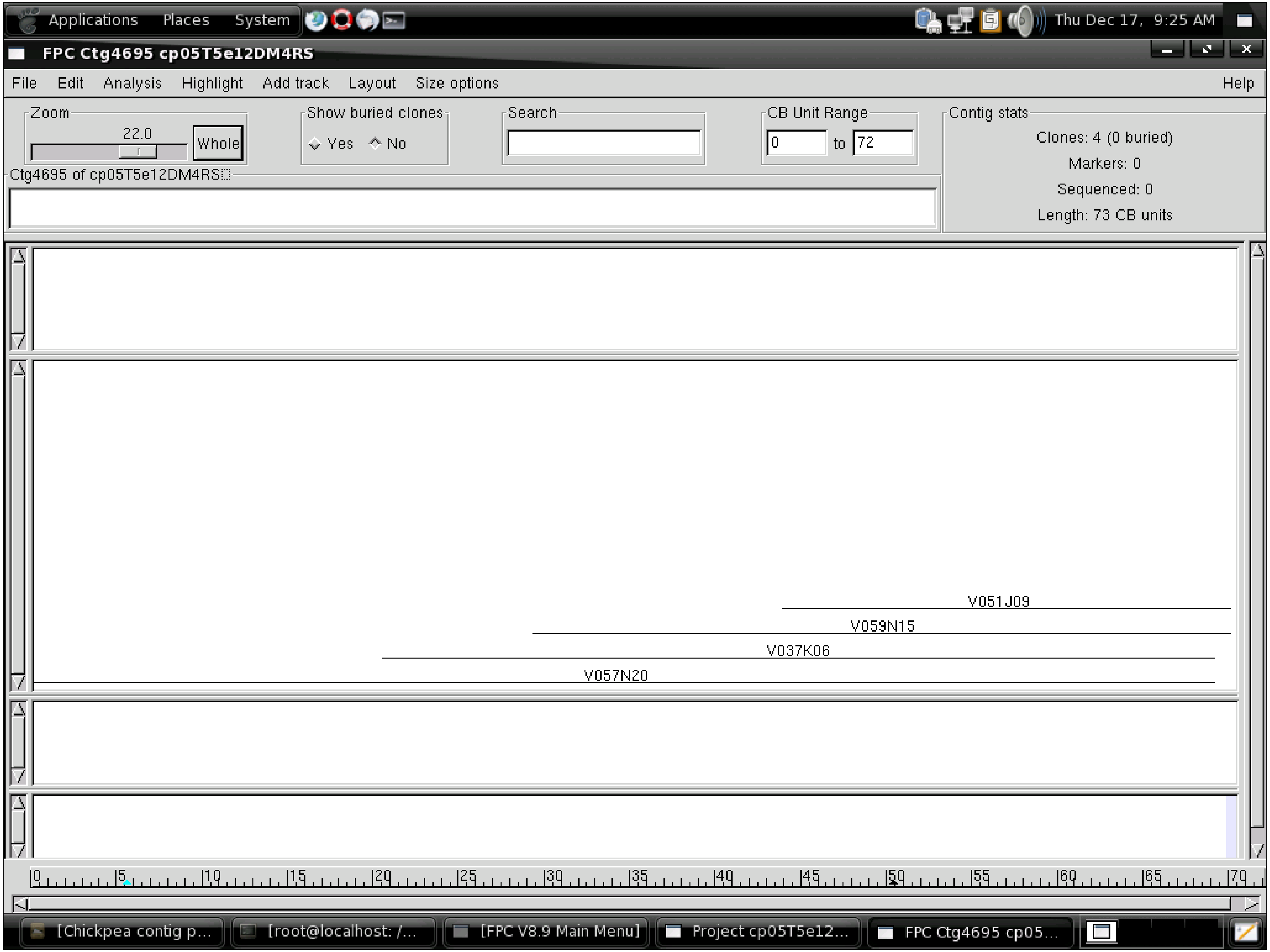
Applications Places System
Thu Dec 17, 9:25 AM
FPC Ctg4695 cp05T5e12DM4RS
File Edit Analysis Highlight Add track Layout Size options Help
Zoom
22.0
Whole
Show buried clones
Yes No
Search
CB Unit Range
0 to 72
Contig stats
Clones: 4 (0 buried)
Markers: 0
Sequenced: 0
Length: 73 CB units
Ctg4695 of cp05T5e12DM4RS
V051J09
V059N15
V037K06
V057N20
0 5 10 15 20 25 30 35 40 45 50 55 60 65 70
[Chickpea contig p...
[root@localhost: /...
[FPC V8.9 Main Menu]
Project cp05T5e12...
FPC Ctg4695 cp05...

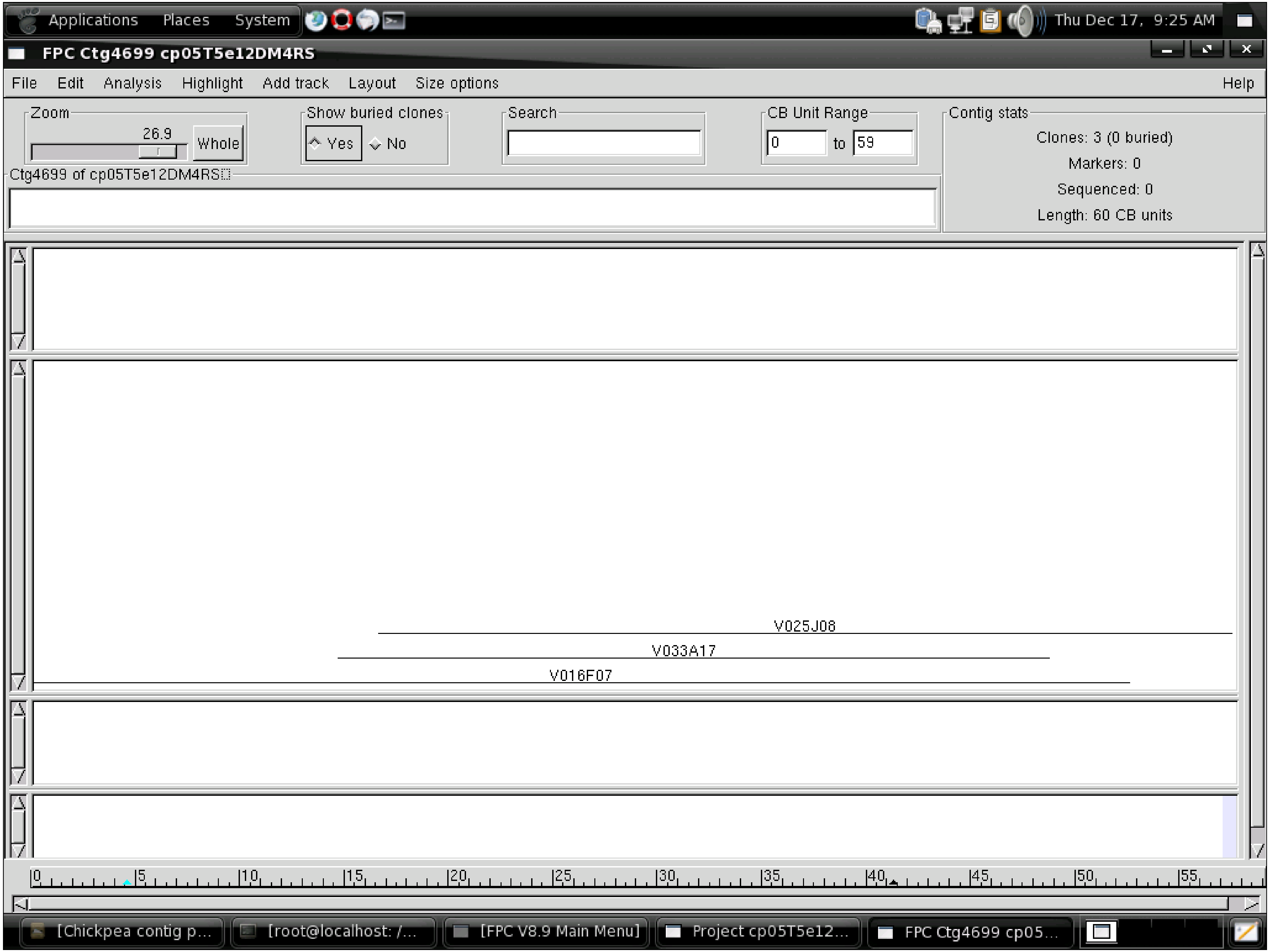
Applications
Places
System
Thu Dec 17, 9:25 AM
FPC Ctg4699 cp05T5e12DM4RS
File
Edit
Analysis
Highlight
Add track
Layout
Size options
Help
Zoom
26.9
Whole
Show buried clones
Yes
No
Search
CB Unit Range
0
to
59
Contig stats
Clones: 3 (0 buried)
Markers: 0
Sequenced: 0
Length: 60 CB units
Ctg4699 of cp05T5e12DM4RS
V025J08
V033A17
V016F07
0
5
10
15
20
25
30
35
40
45
50
55
[Chickpea contig p...
[root@localhost: /...
[FPC V8.9 Main Menu]
Project cp05T5e12...
FPC Ctg4699 cp05...

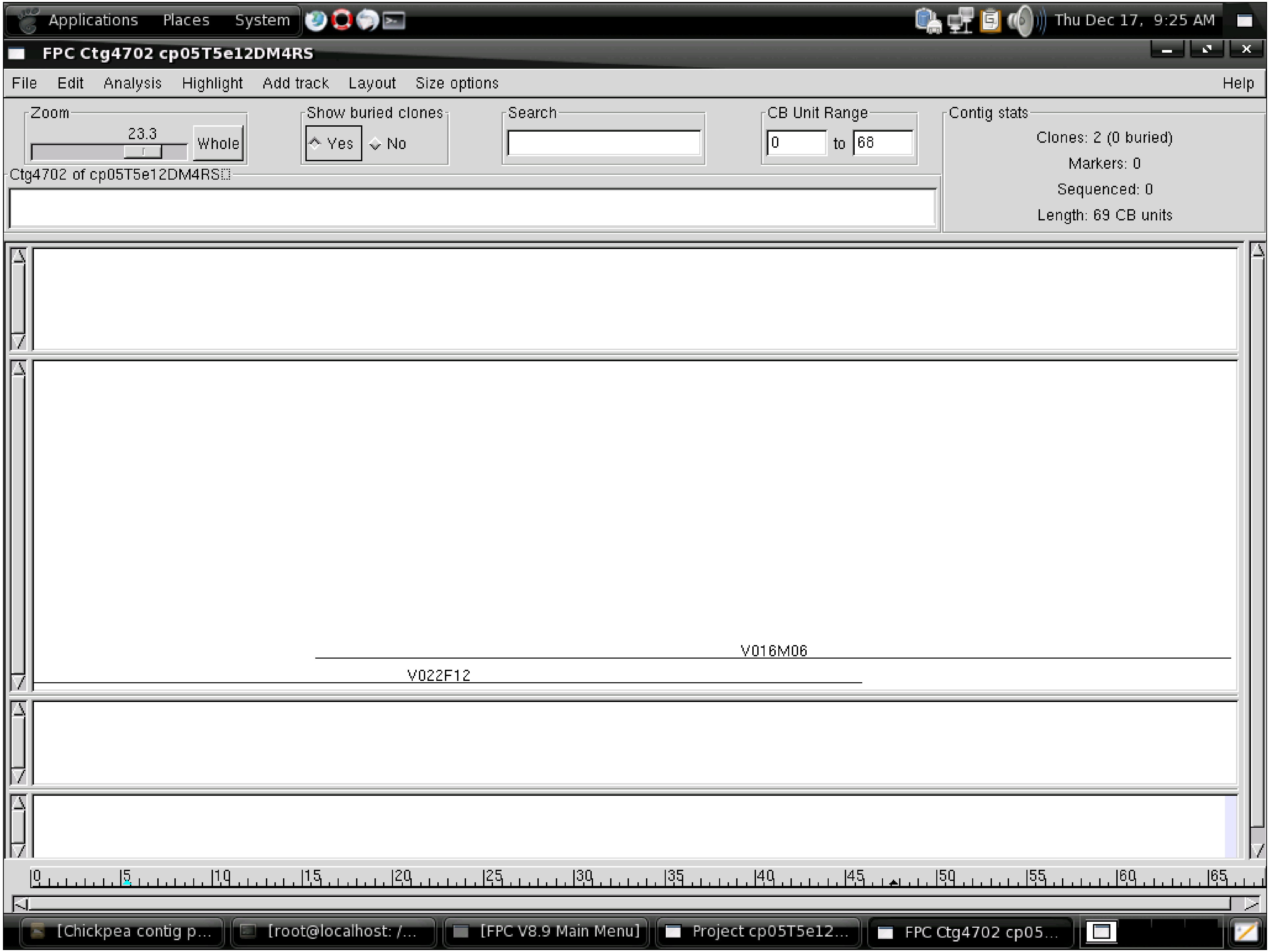
Applications Places System
Thu Dec 17, 9:25 AM
FPC Ctg4702 cp05T5e12DM4RS
File Edit Analysis Highlight Add track Layout Size options Help
Zoom
23.3
Whole
Show buried clones
Yes
No
Search
CB Unit Range
0
to
68
Contig stats
Clones: 2 (0 buried)
Markers: 0
Sequenced: 0
Length: 69 CB units
Ctg4702 of cp05T5e12DM4RS
V016M06
V022F12
0 5 10 15 20 25 30 35 40 45 50 55 60 65
[Chickpea contig p...
[root@localhost: /...
[FPC V8.9 Main Menu]
Project cp05T5e12...
FPC Ctg4702 cp05...

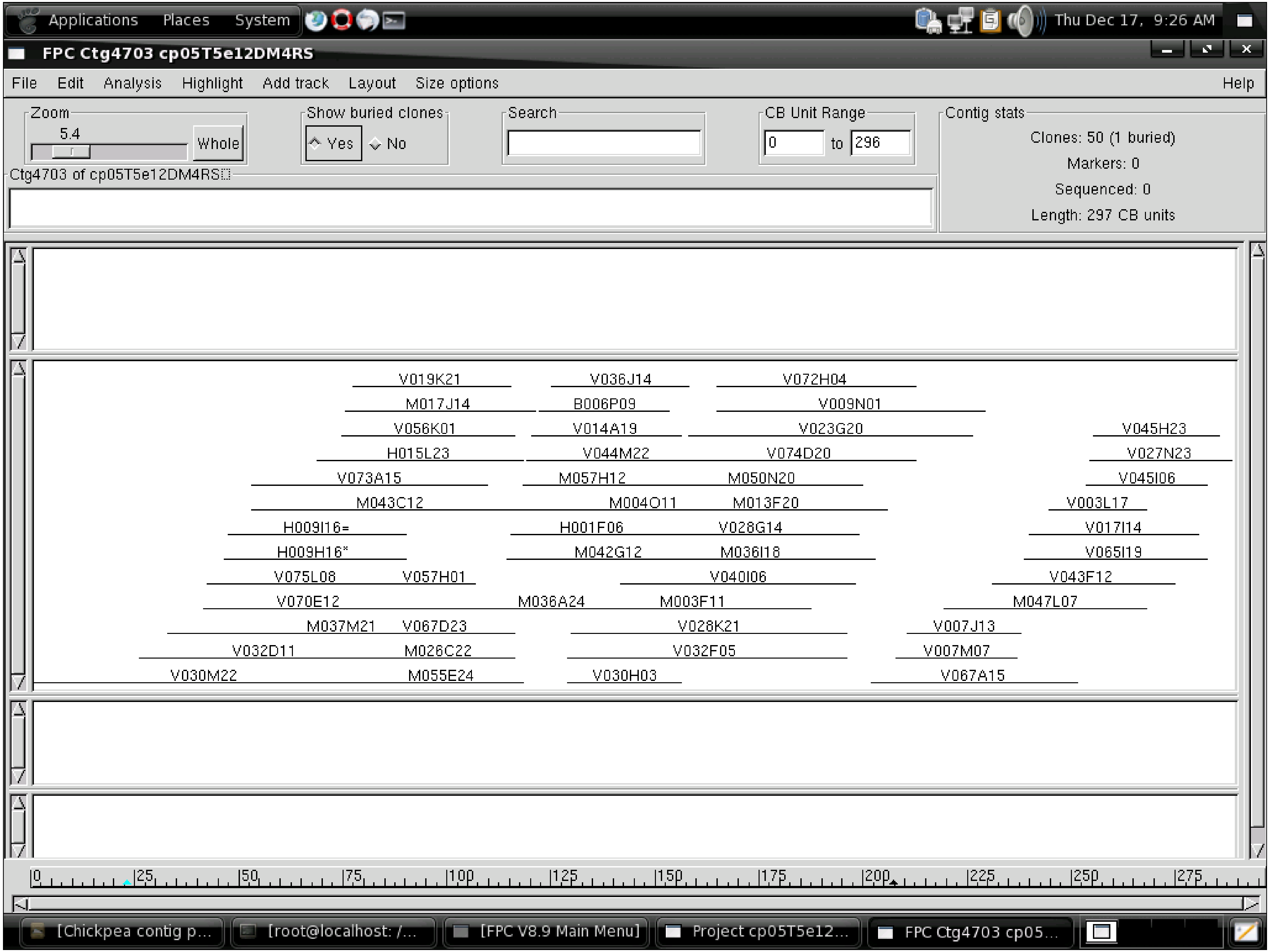
Applications Places System
Thu Dec 17, 9:26 AM
FPC Ctg4703 cp05T5e12DM4RS
File Edit Analysis Highlight Add track Layout Size options Help
Zoom
5.4
Whole
Show buried clones
Yes No
Search
CB Unit Range
0 to 296
Contig stats
Clones: 50 (1 buried)
Markers: 0
Sequenced: 0
Length: 297 CB units
Ctg4703 of cp05T5e12DM4RS
V019K21 V036J14 V072H04
M017J14 B006P09 V009N01
V056K01 V014A19 V023G20 V045H23
H015L23 V044M22 V074D20 V027N23
V073A15 M057H12 M050N20 V045I06
M043C12 M004O11 M013F20 V003L17
H009I16= H001F06 V028G14 V017I14
H009H16* M042G12 M036I18 V065I19
V075L08 V057H01 V040I06 V043F12
V070E12 M036A24 M003F11 M047L07
M037M21 V067D23 V028K21 V007J13
V032D11 M026C22 V032F05 V007M07
V030M22 M055E24 V030H03 V067A15
0 25 50 75 100 125 150 175 200 225 250 275
[Chickpea contig p... [root@localhost: /... [FPC V8.9 Main Menu] Project cp05T5e12... FPC Ctg4703 cp05...

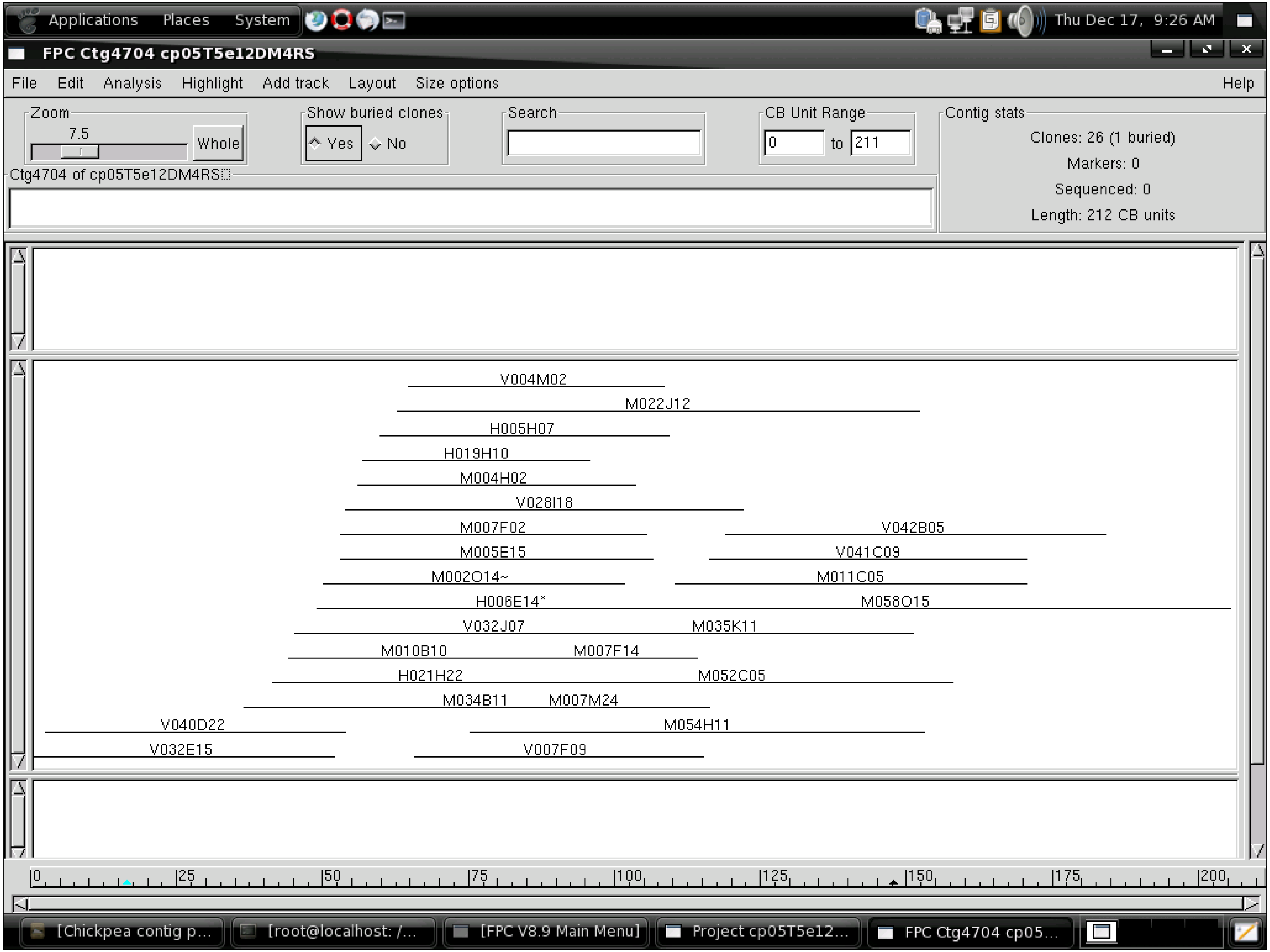
Applications Places System
Thu Dec 17, 9:26 AM
FPC Ctg4704 cp05T5e12DM4RS
File Edit Analysis Highlight Add track Layout Size options Help
Zoom
7.5
Whole
Show buried clones
Yes
No
Search
CB Unit Range
0
to
211
Contig stats
Clones: 26 (1 buried)
Markers: 0
Sequenced: 0
Length: 212 CB units
Ctg4704 of cp05T5e12DM4RS
V004M02
M022J12
H005H07
H019H10
M004H02
V028I18
M007F02
V042B05
M005E15
V041C09
M002O14~
M011C05
H006E14*
M058O15
V032J07
M035K11
M010B10
M007F14
H021H22
M052C05
M034B11
M007M24
V040D22
M054H11
V032E15
V007F09
0 25 50 75 100 125 150 175 200
[Chickpea contig p...
[root@localhost: /...
[FPC V8.9 Main Menu]
Project cp05T5e12...
FPC Ctg4704 cp05...

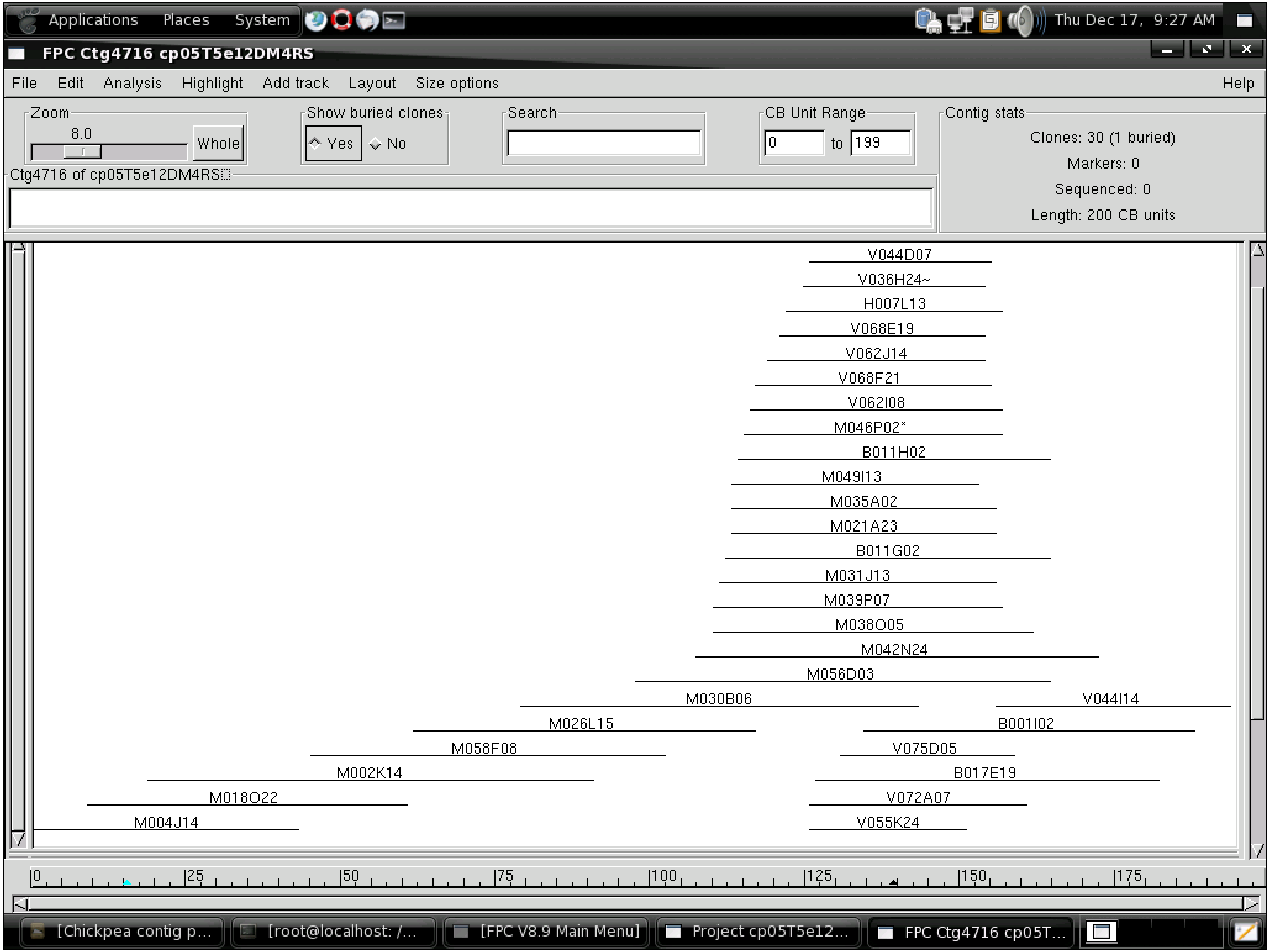
Applications Places System
Thu Dec 17, 9:27 AM
FPC Ctg4716 cp05T5e12DM4RS
File Edit Analysis Highlight Add track Layout Size options Help
Zoom
8.0
Whole
Show buried clones
Yes
No
Search
CB Unit Range
0
to
199
Contig stats
Clones: 30 (1 buried)
Markers: 0
Sequenced: 0
Length: 200 CB units
Ctg4716 of cp05T5e12DM4RS
V044D07
V036H24~
H007L13
V068E19
V062J14
V068F21
V062I08
M046P02*
B011H02
M049I13
M035A02
M021A23
B011G02
M031J13
M039P07
M038O05
M042N24
M056D03
M030B06
V044I14
M026L15
B001I02
M058F08
V075D05
M002K14
B017E19
M018O22
V072A07
M004J14
V055K24
0 25 50 75 100 125 150 175
[Chickpea contig p...
[root@localhost: /...
[FPC V8.9 Main Menu]
Project cp05T5e12...
FPC Ctg4716 cp05T...

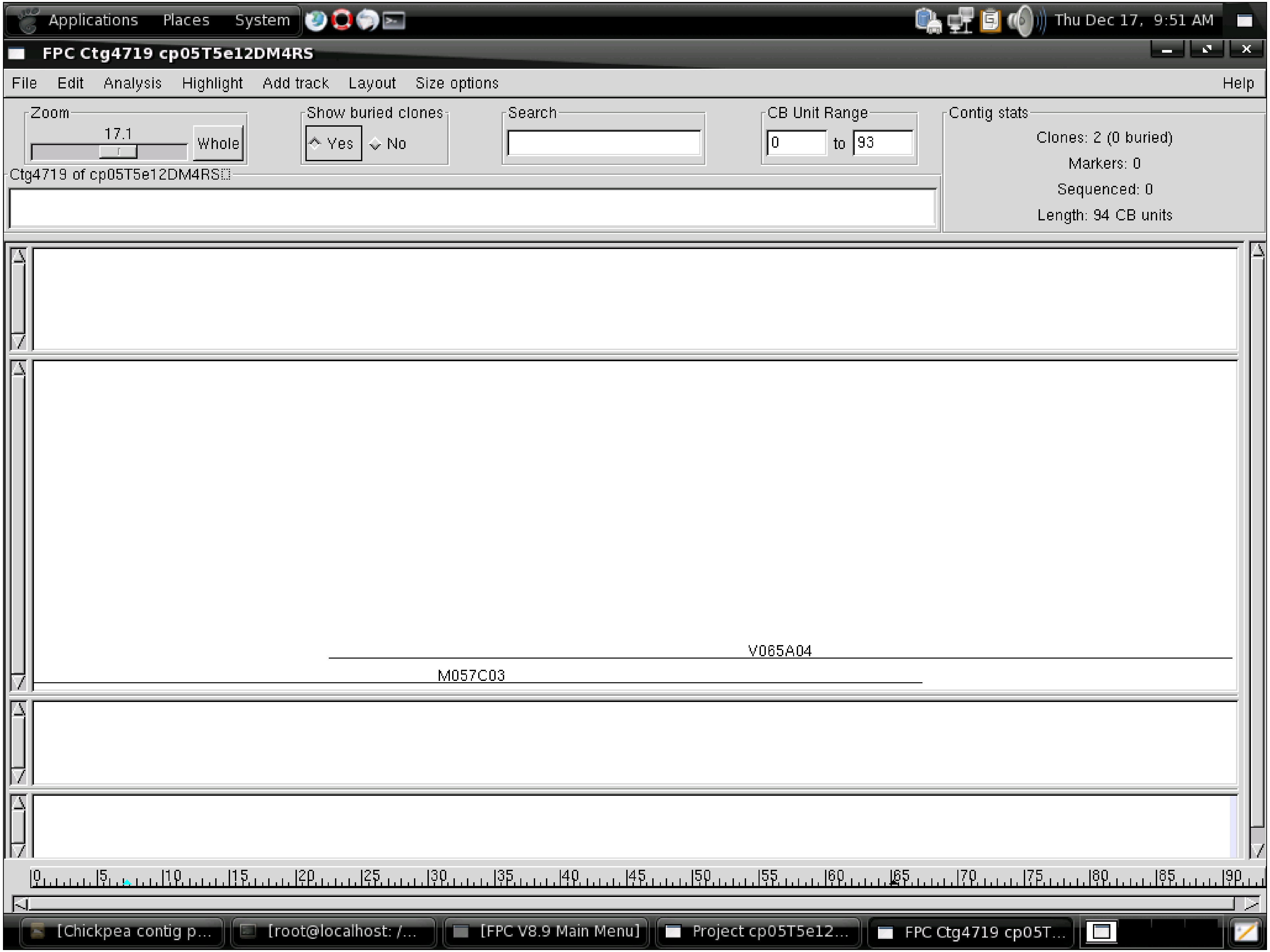
Applications Places System
Thu Dec 17, 9:51 AM
FPC Ctg4719 cp05T5e12DM4RS
File Edit Analysis Highlight Add track Layout Size options Help
Zoom
17.1
Whole
Show buried clones
Yes No
Search
CB Unit Range
0 to 93
Contig stats
Clones: 2 (0 buried)
Markers: 0
Sequenced: 0
Length: 94 CB units
Ctg4719 of cp05T5e12DM4RS
V065A04
M057C03
0 5 10 15 20 25 30 35 40 45 50 55 60 65 70 75 80 85 90
[Chickpea contig p...
[root@localhost: /...
[FPC V8.9 Main Menu]
Project cp05T5e12...
FPC Ctg4719 cp05T...

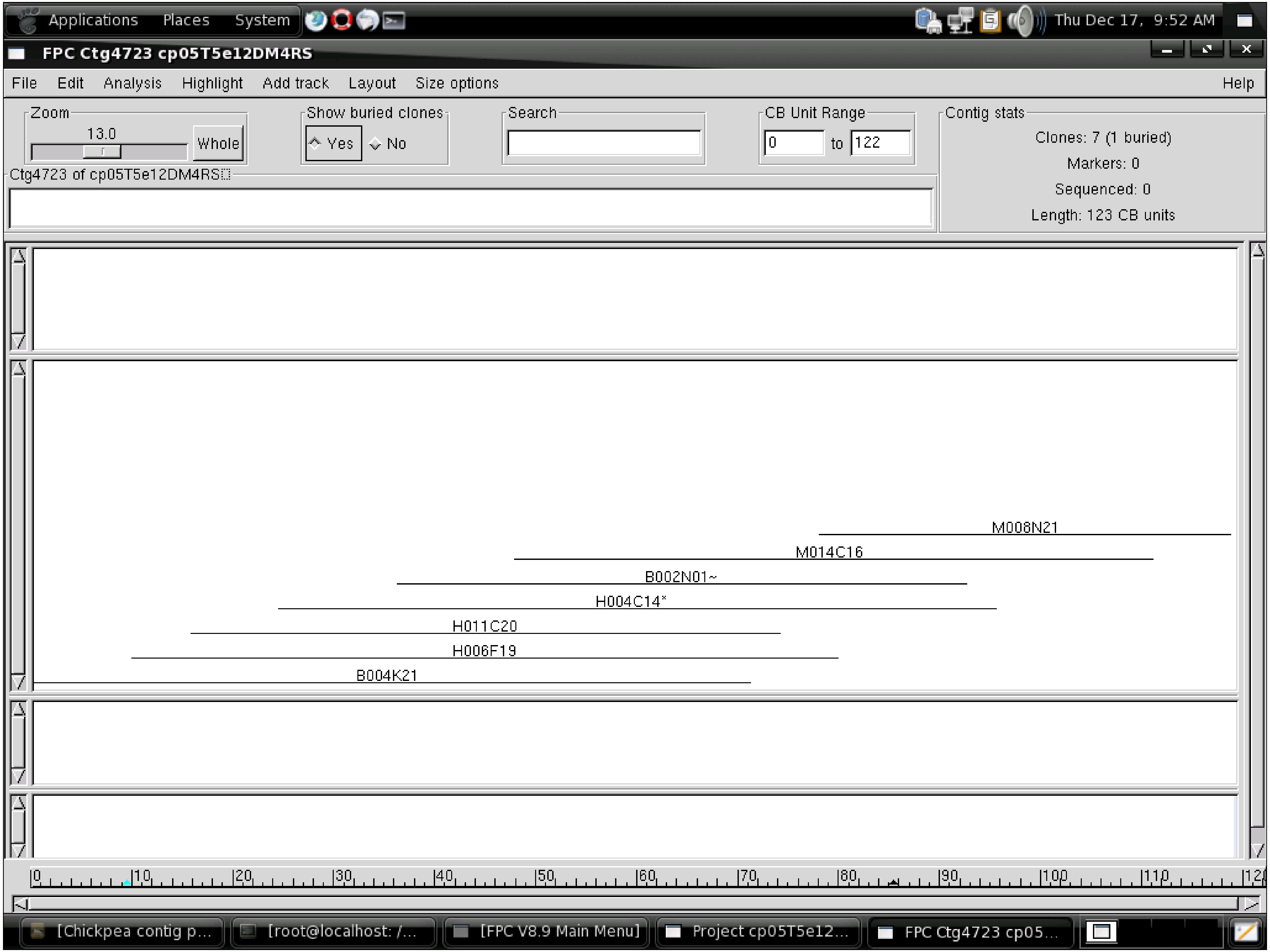

Applications Places System
Thu Dec 17, 9:52 AM
FPC Ctg4723 cp05T5e12DM4RS
File Edit Analysis Highlight Add track Layout Size options Help
Zoom
13.0
Whole
Show buried clones
Yes No
Search
CB Unit Range
0 to 122
Contig stats
Clones: 7 (1 buried)
Markers: 0
Sequenced: 0
Length: 123 CB units
Ctg4723 of cp05T5e12DM4RS
M008N21
M014C16
B002N01~
H004C14*
H011C20
H006F19
B004K21
0 10 20 30 40 50 60 70 80 90 100 110 12
[Chickpea contig p...
[root@localhost: /...
[FPC V8.9 Main Menu]
Project cp05T5e12...
FPC Ctg4723 cp05...

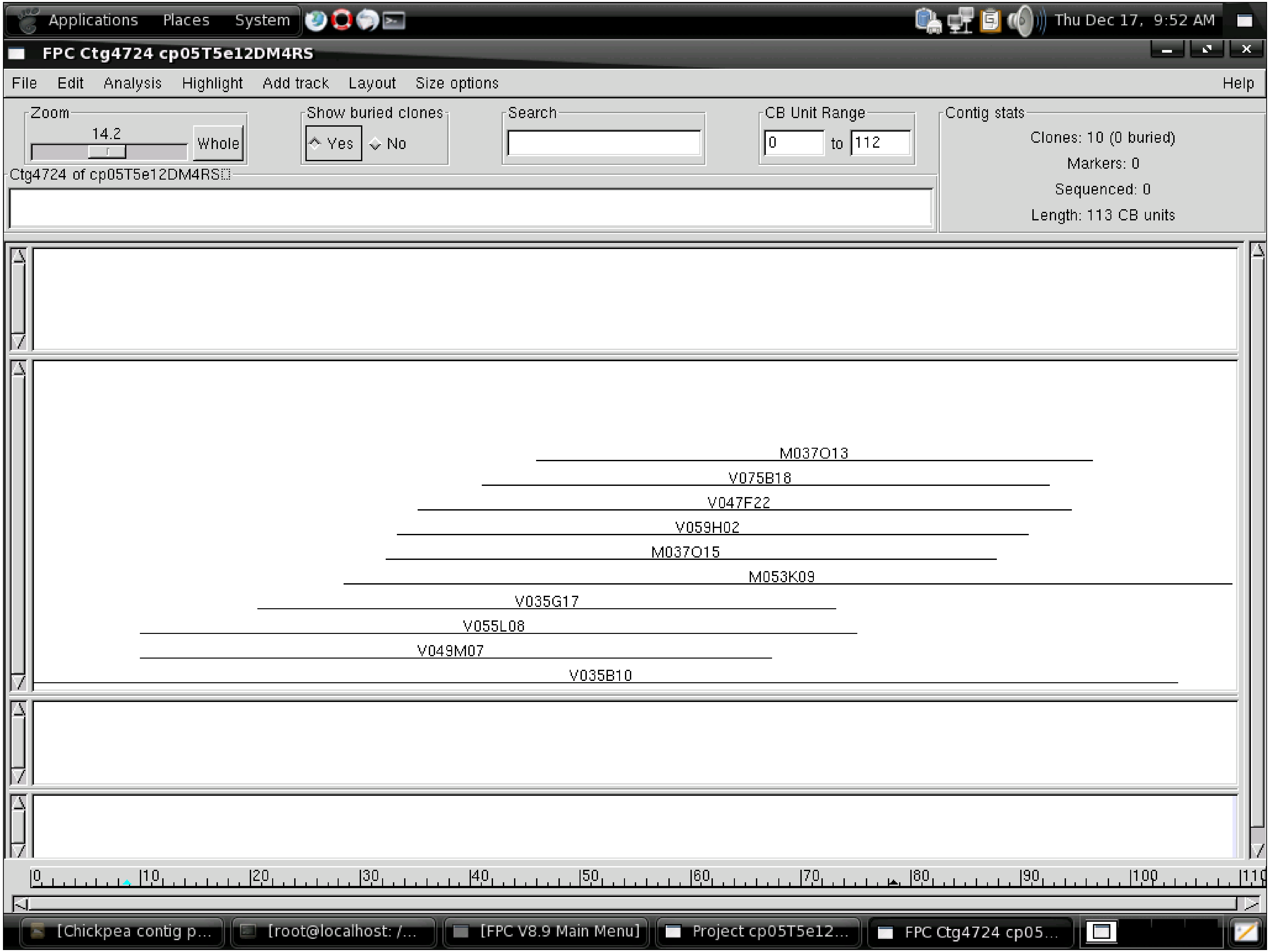
Applications
Places
System
Thu Dec 17, 9:52 AM
FPC Ctg4724 cp05T5e12DM4RS
File
Edit
Analysis
Highlight
Add track
Layout
Size options
Help
Zoom
14.2
Whole
Show buried clones
Yes
No
Search
CB Unit Range
0
to
112
Contig stats
Clones: 10 (0 buried)
Markers: 0
Sequenced: 0
Length: 113 CB units
Ctg4724 of cp05T5e12DM4RS
M037O13
V075B18
V047F22
V059H02
M037O15
M053K09
V035G17
V055L08
V049M07
V035B10
0
10
20
30
40
50
60
70
80
90
100
[Chickpea contig p...
[root@localhost: /...
[FPC V8.9 Main Menu]
Project cp05T5e12...
FPC Ctg4724 cp05...

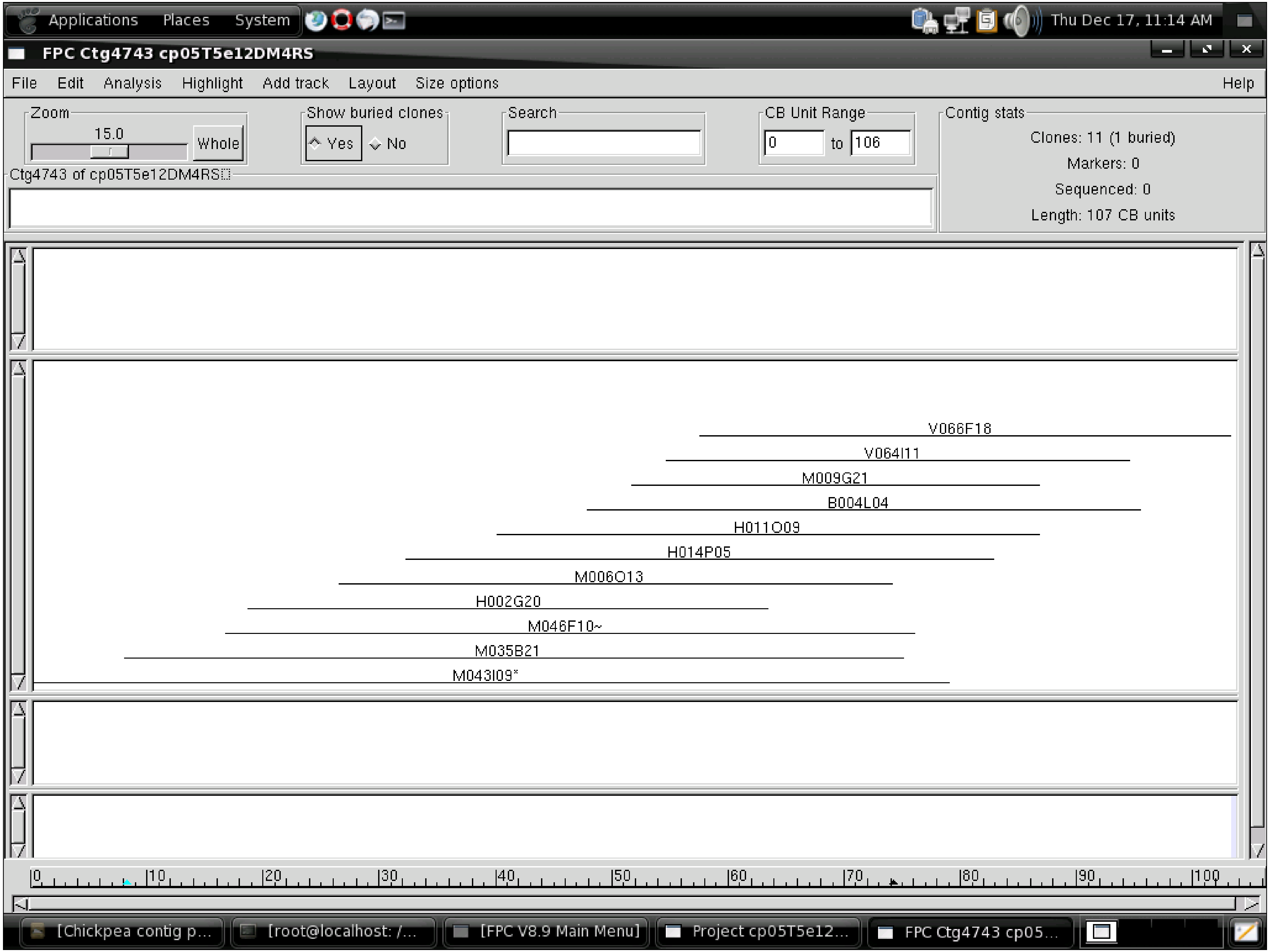
Applications Places System
Thu Dec 17, 11:14 AM
FPC Ctg4743 cp05T5e12DM4RS
File Edit Analysis Highlight Add track Layout Size options Help
Zoom
15.0
Whole
Show buried clones
Yes No
Search
CB Unit Range
0 to 106
Contig stats
Clones: 11 (1 buried)
Markers: 0
Sequenced: 0
Length: 107 CB units
Ctg4743 of cp05T5e12DM4RS
V066F18
V064I11
M009G21
B004L04
H011O09
H014P05
M006O13
H002G20
M046F10~
M035B21
M043I09*
0 10 20 30 40 50 60 70 80 90 100
[Chickpea contig p...
[root@localhost: /...
[FPC V8.9 Main Menu]
Project cp05T5e12...
FPC Ctg4743 cp05...

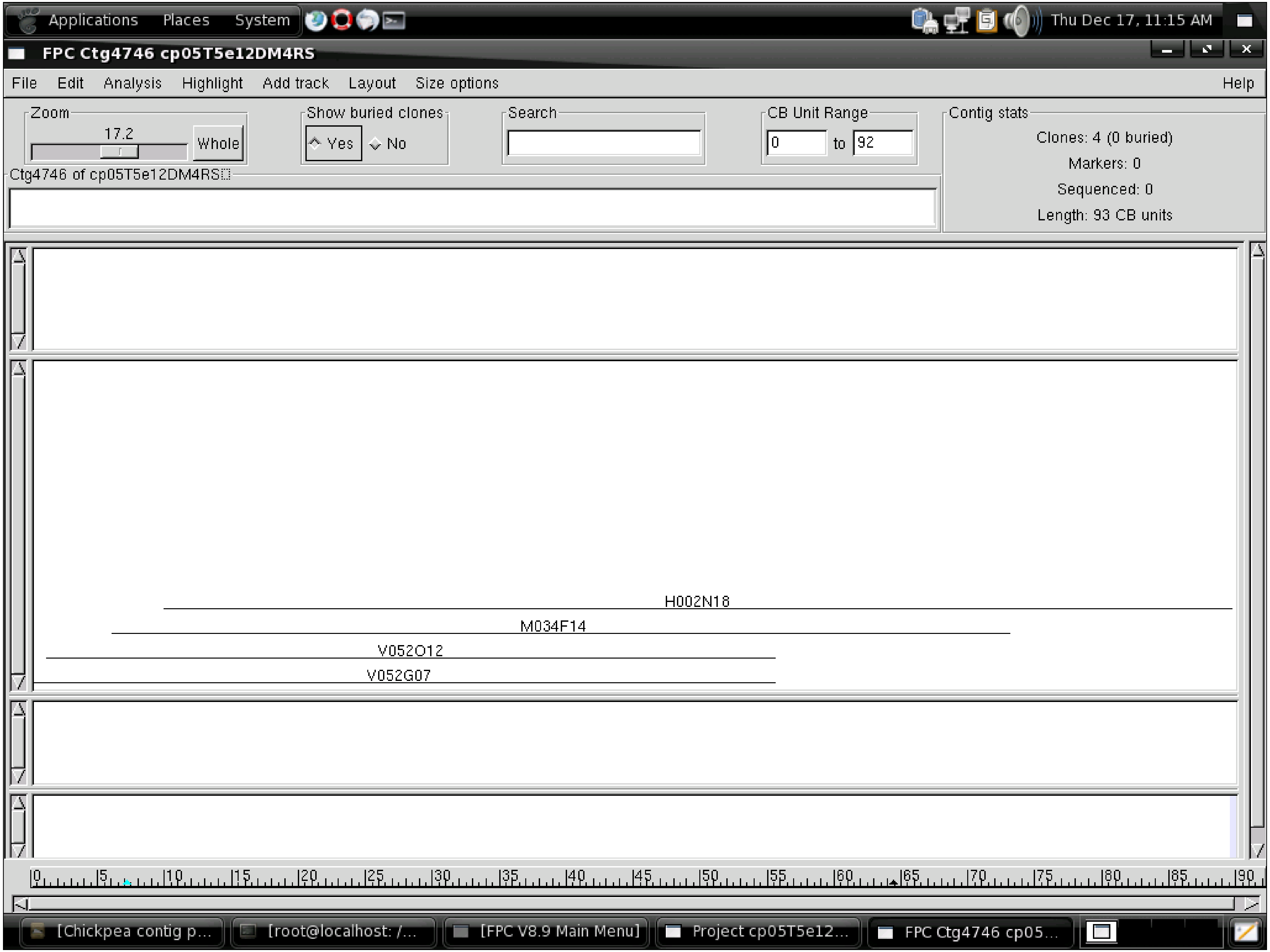
Applications Places System
Thu Dec 17, 11:15 AM
FPC Ctg4746 cp05T5e12DM4RS
File Edit Analysis Highlight Add track Layout Size options Help
Zoom
17.2
Whole
Show buried clones
Yes No
Search
CB Unit Range
0 to 92
Contig stats
Clones: 4 (0 buried)
Markers: 0
Sequenced: 0
Length: 93 CB units
Ctg4746 of cp05T5e12DM4RS
H002N18
M034F14
V052O12
V052G07
0 5 10 15 20 25 30 35 40 45 50 55 60 65 70 75 80 85 90
[Chickpea contig p...
[root@localhost: /...
[FPC V8.9 Main Menu]
Project cp05T5e12...
FPC Ctg4746 cp05...

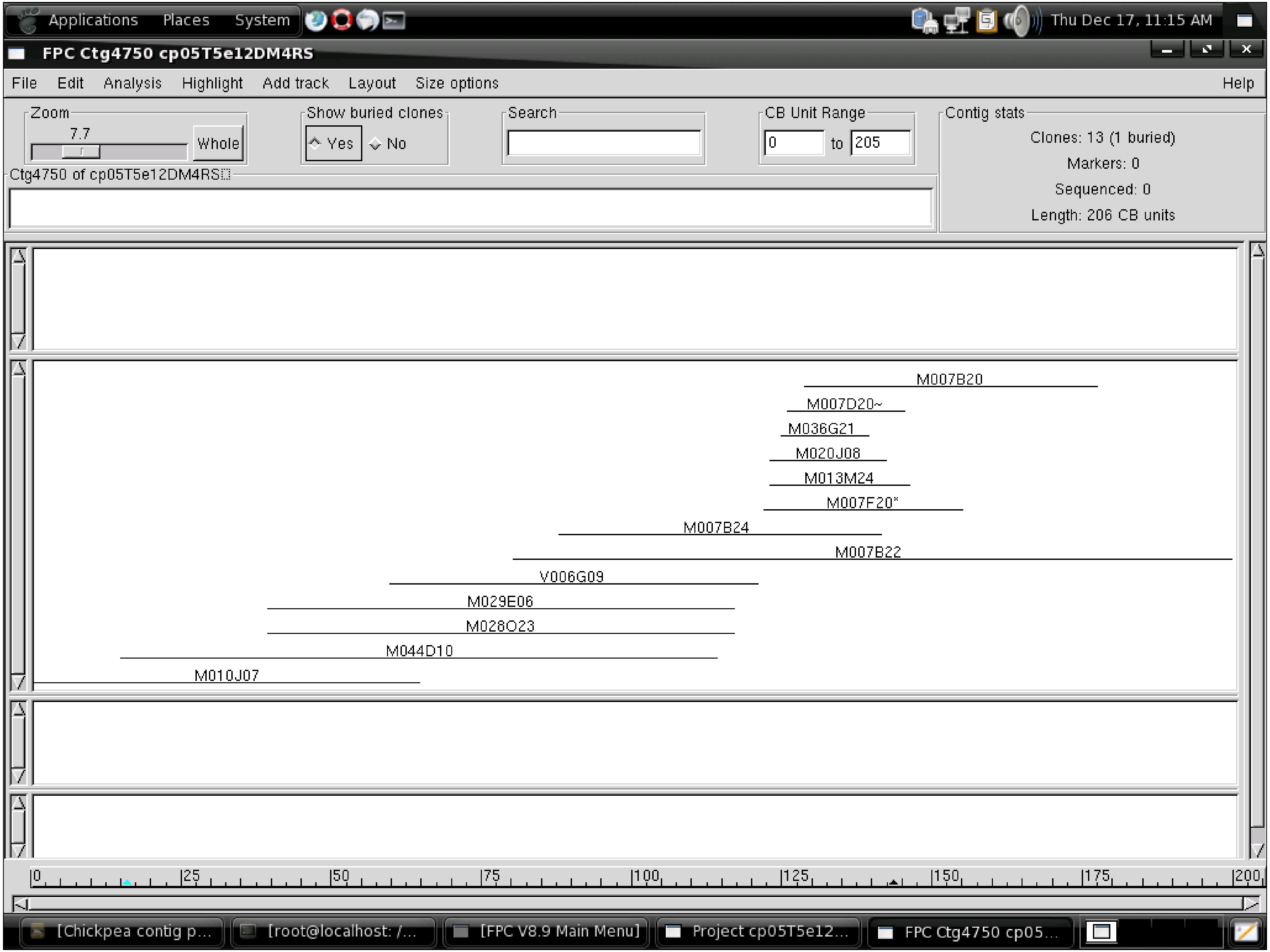

Applications Places System
Thu Dec 17, 11:15 AM
FPC Ctg4750 cp05T5e12DM4RS
File Edit Analysis Highlight Add track Layout Size options Help
Zoom
7.7
Whole
Show buried clones
Yes No
Search
CB Unit Range
0 to 205
Contig stats
Clones: 13 (1 buried)
Markers: 0
Sequenced: 0
Length: 206 CB units
Ctg4750 of cp05T5e12DM4RS
M007B20
M007D20~
M036G21
M020J08
M013M24
M007F20*
M007B24
M007B22
V006G09
M029E06
M028O23
M044D10
M010J07
0 25 50 75 100 125 150 175 200
[Chickpea contig p...
[root@localhost: /...
[FPC V8.9 Main Menu]
Project cp05T5e12...
FPC Ctg4750 cp05...

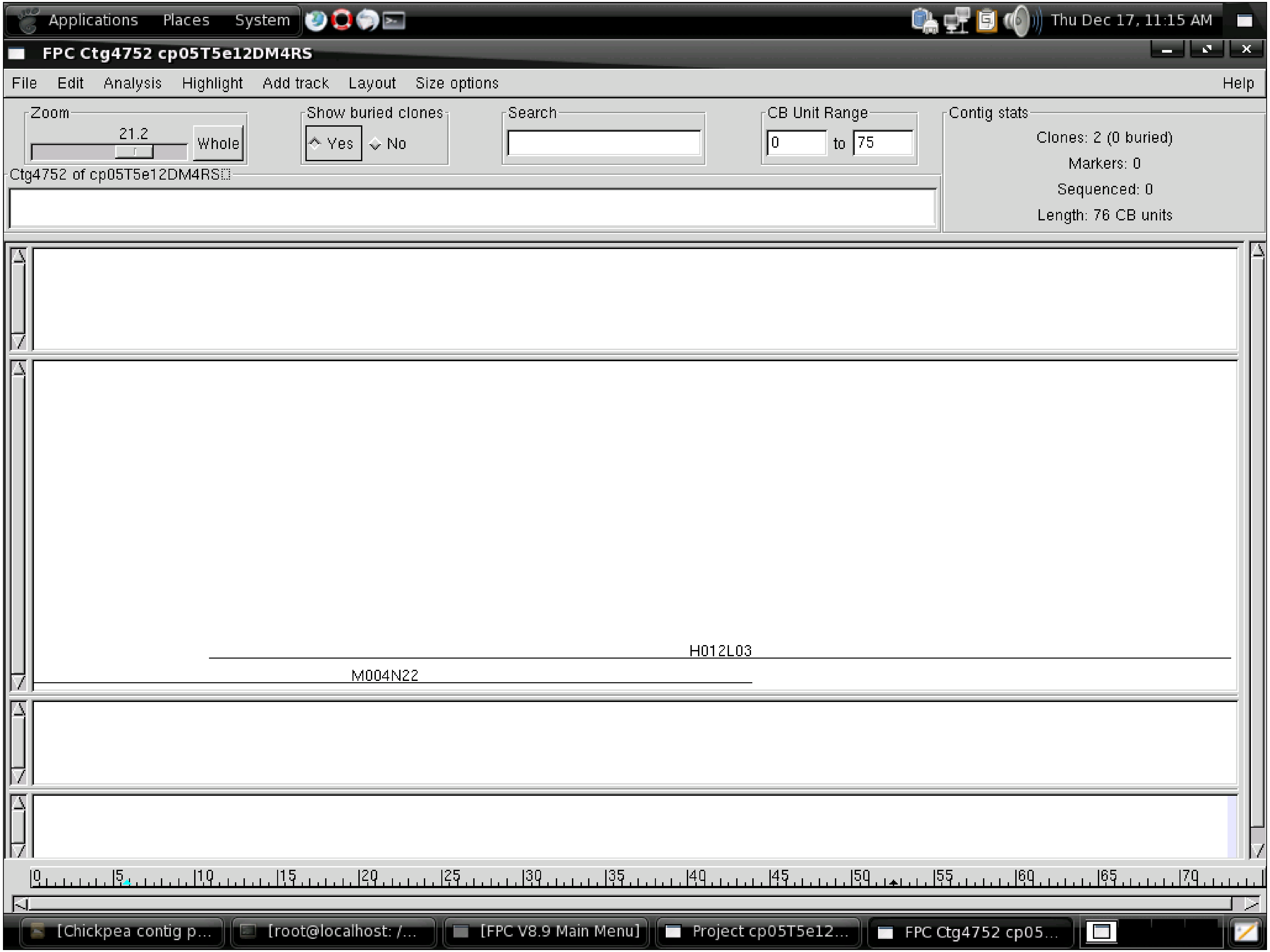
Applications Places System
Thu Dec 17, 11:15 AM
FPC Ctg4752 cp05T5e12DM4RS
File Edit Analysis Highlight Add track Layout Size options Help
Zoom
21.2
Whole
Show buried clones
Yes No
Search
CB Unit Range
0 to 75
Contig stats
Clones: 2 (0 buried)
Markers: 0
Sequenced: 0
Length: 76 CB units
Ctg4752 of cp05T5e12DM4RS
H012L03
M004N22
0 5 10 15 20 25 30 35 40 45 50 55 60 65 70
[Chickpea contig p...
[root@localhost: /...
[FPC V8.9 Main Menu]
Project cp05T5e12...
FPC Ctg4752 cp05...

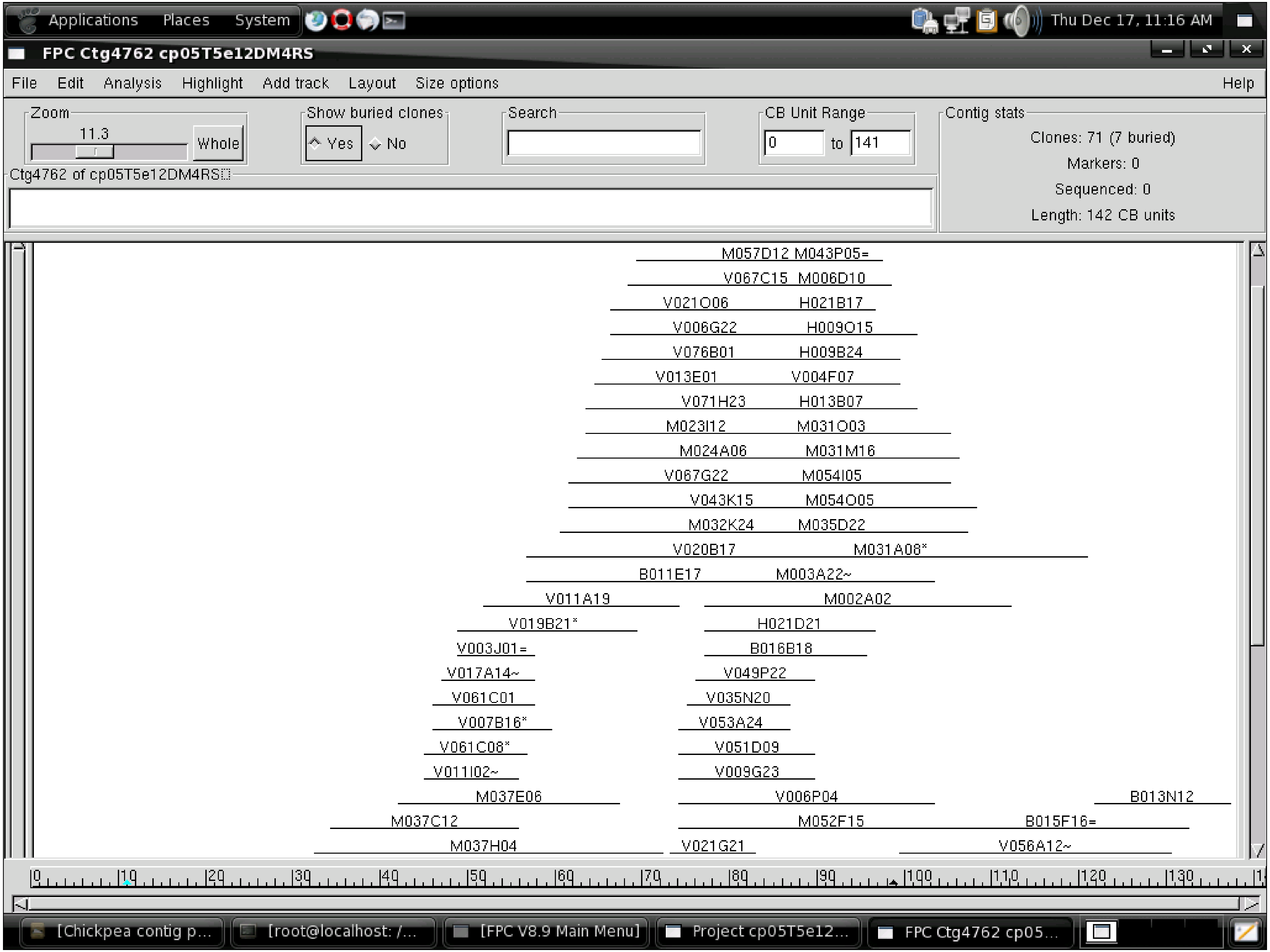
Applications Places System
Thu Dec 17, 11:16 AM
FPC Ctg4762 cp05T5e12DM4RS
File Edit Analysis Highlight Add track Layout Size options Help
Zoom
11.3
Whole
Show buried clones
Yes
No
Search
CB Unit Range
0 to 141
Contig stats
Clones: 71 (7 buried)
Markers: 0
Sequenced: 0
Length: 142 CB units
Ctg4762 of cp05T5e12DM4RS
M057D12 M043P05=
V067C15 M006D10
V021O06 H021B17
V006G22 H009O15
V076B01 H009B24
V013E01 V004F07
V071H23 H013B07
M023I12 M031O03
M024A06 M031M16
V067G22 M054I05
V043K15 M054O05
M032K24 M035D22
V020B17 M031A08*
B011E17 M003A22~
V011A19 M002A02
V019B21* H021D21
V003J01= B016B18
V017A14~ V049P22
V061C01 V035N20
V007B16* V053A24
V061C08* V051D09
V011I02~ V009G23
M037E06 V006P04 B013N12
M037C12 M052F15 B015F16=
M037H04 V021G21 V056A12~
0 10 20 30 40 50 60 70 80 90 100 110 120 130
[Chickpea contig p...
[root@localhost: /...
[FPC V8.9 Main Menu]
Project cp05T5e12...
FPC Ctg4762 cp05...

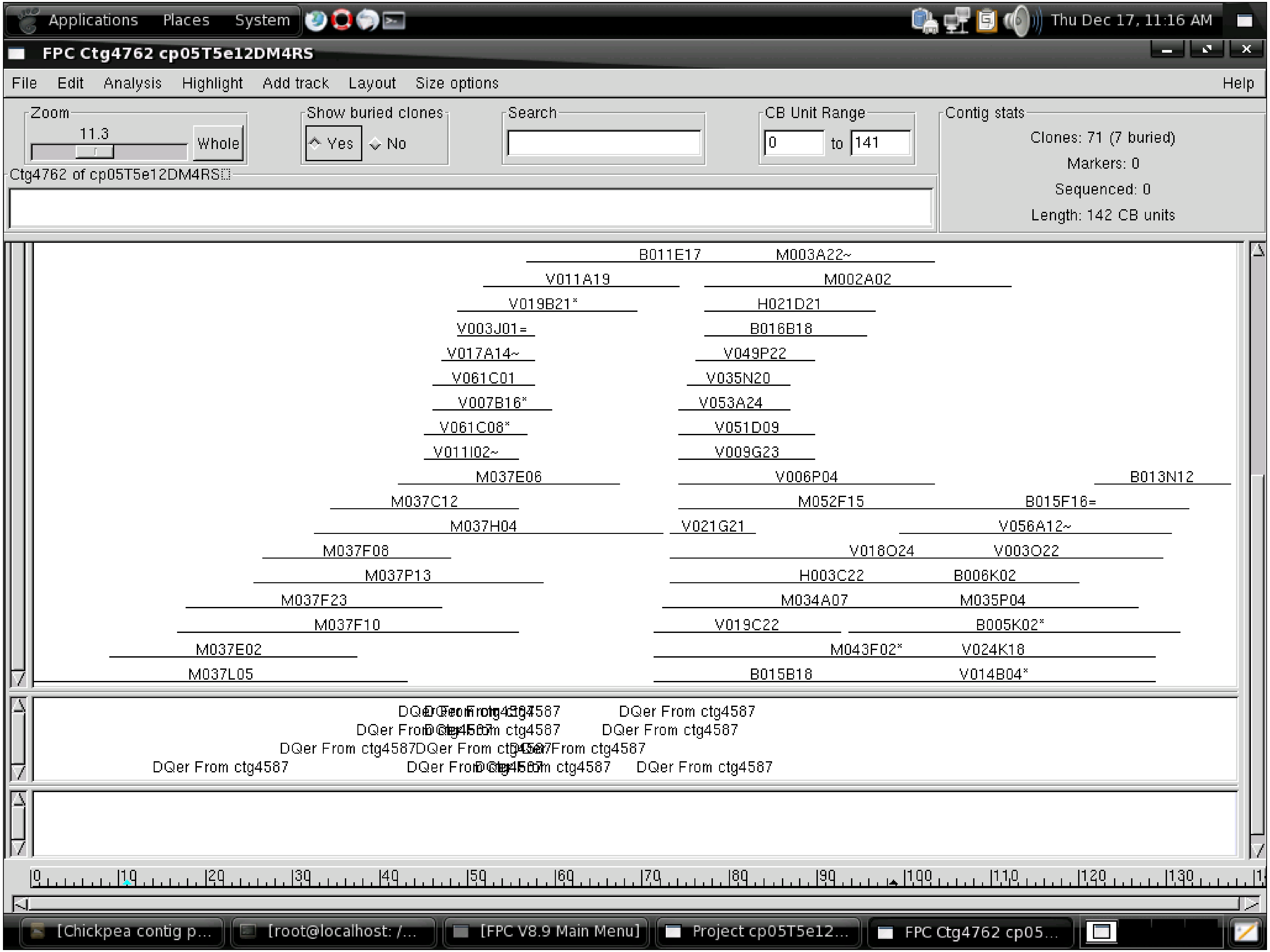
Applications Places System
Thu Dec 17, 11:16 AM
FPC Ctg4762 cp05T5e12DM4RS
File Edit Analysis Highlight Add track Layout Size options Help
Zoom
11.3
Whole
Show buried clones
Yes
No
Search
CB Unit Range
0 to 141
Contig stats
Clones: 71 (7 buried)
Markers: 0
Sequenced: 0
Length: 142 CB units
Ctg4762 of cp05T5e12DM4RS
B011E17
M003A22~
V011A19
M002A02
V019B21*
H021D21
V003J01=
B016B18
V017A14~
V049P22
V061C01
V035N20
V007B16*
V053A24
V061C08*
V051D09
V011I02~
V009G23
M037E06
V006P04
B013N12
M037C12
M052F15
B015F16=
M037H04
V021G21
V056A12~
M037F08
V018O24
V003O22
M037P13
H003C22
B006K02
M037F23
M034A07
M035P04
M037F10
V019C22
B005K02*
M037E02
M043F02*
V024K18
M037L05
B015B18
V014B04*
DQer From ctg4587
0 10 20 30 40 50 60 70 80 90 100 110 120 130
[Chickpea contig p...
[root@localhost: /...
[FPC V8.9 Main Menu]
Project cp05T5e12...
FPC Ctg4762 cp05...

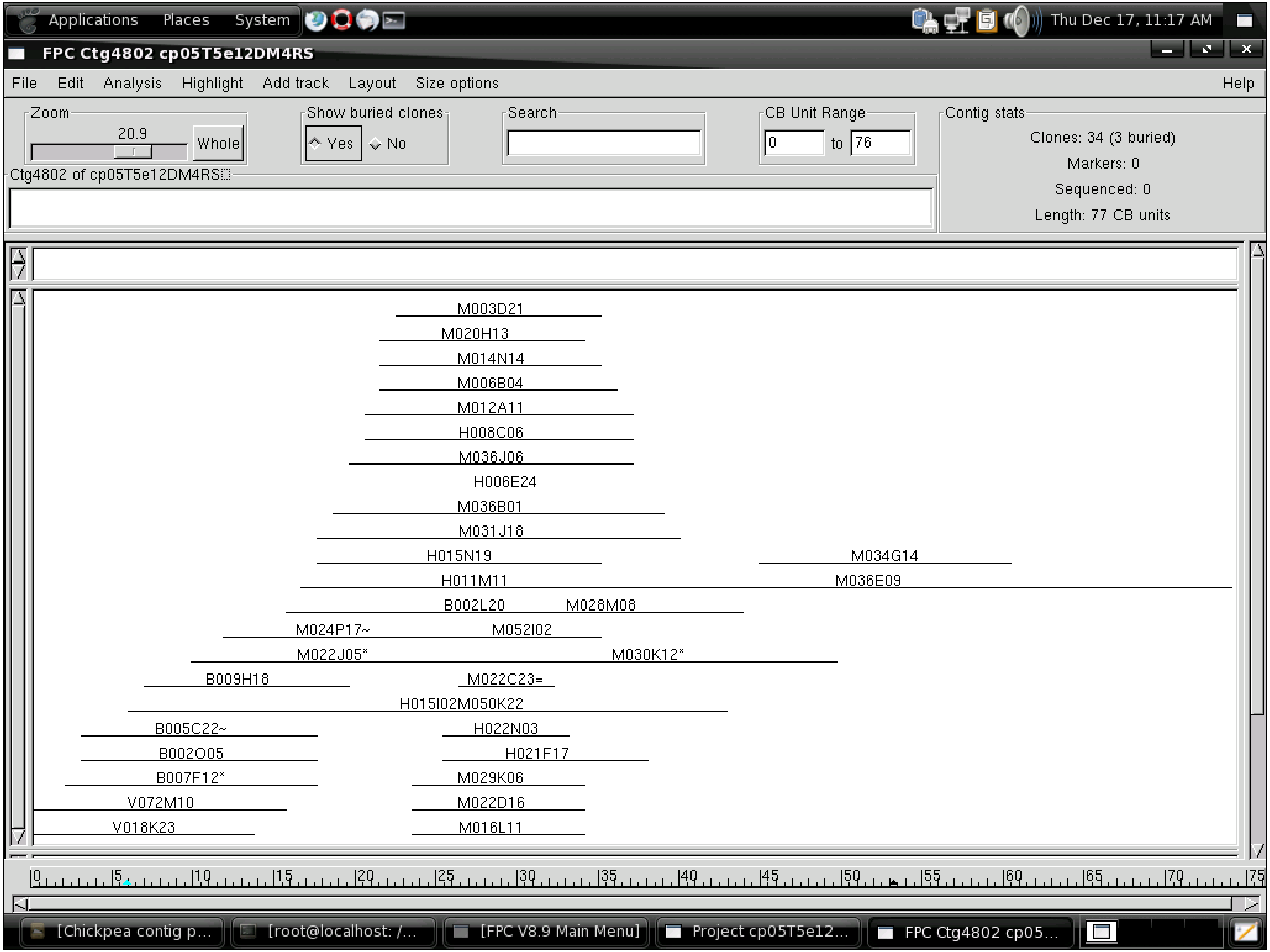
Applications Places System
Thu Dec 17, 11:17 AM
FPC Ctg4802 cp05T5e12DM4RS
File Edit Analysis Highlight Add track Layout Size options Help
Zoom
20.9
Whole
Show buried clones
Yes
No
Search
CB Unit Range
0 to 76
Contig stats
Clones: 34 (3 buried)
Markers: 0
Sequenced: 0
Length: 77 CB units
Ctg4802 of cp05T5e12DM4RS
M003D21
M020H13
M014N14
M006B04
M012A11
H008C06
M036J06
H006E24
M036B01
M031J18
H015N19
M034G14
H011M11
M036E09
B002L20
M028M08
M024P17~
M052I02
M022J05*
M030K12*
B009H18
M022C23=
H015I02M050K22
B005C22~
H022N03
B002O05
H021F17
B007F12*
M029K06
V072M10
M022D16
V018K23
M016L11
0 5 10 15 20 25 30 35 40 45 50 55 60 65 70 75
[Chickpea contig p...
[root@localhost: /...
[FPC V8.9 Main Menu]
Project cp05T5e12...
FPC Ctg4802 cp05...

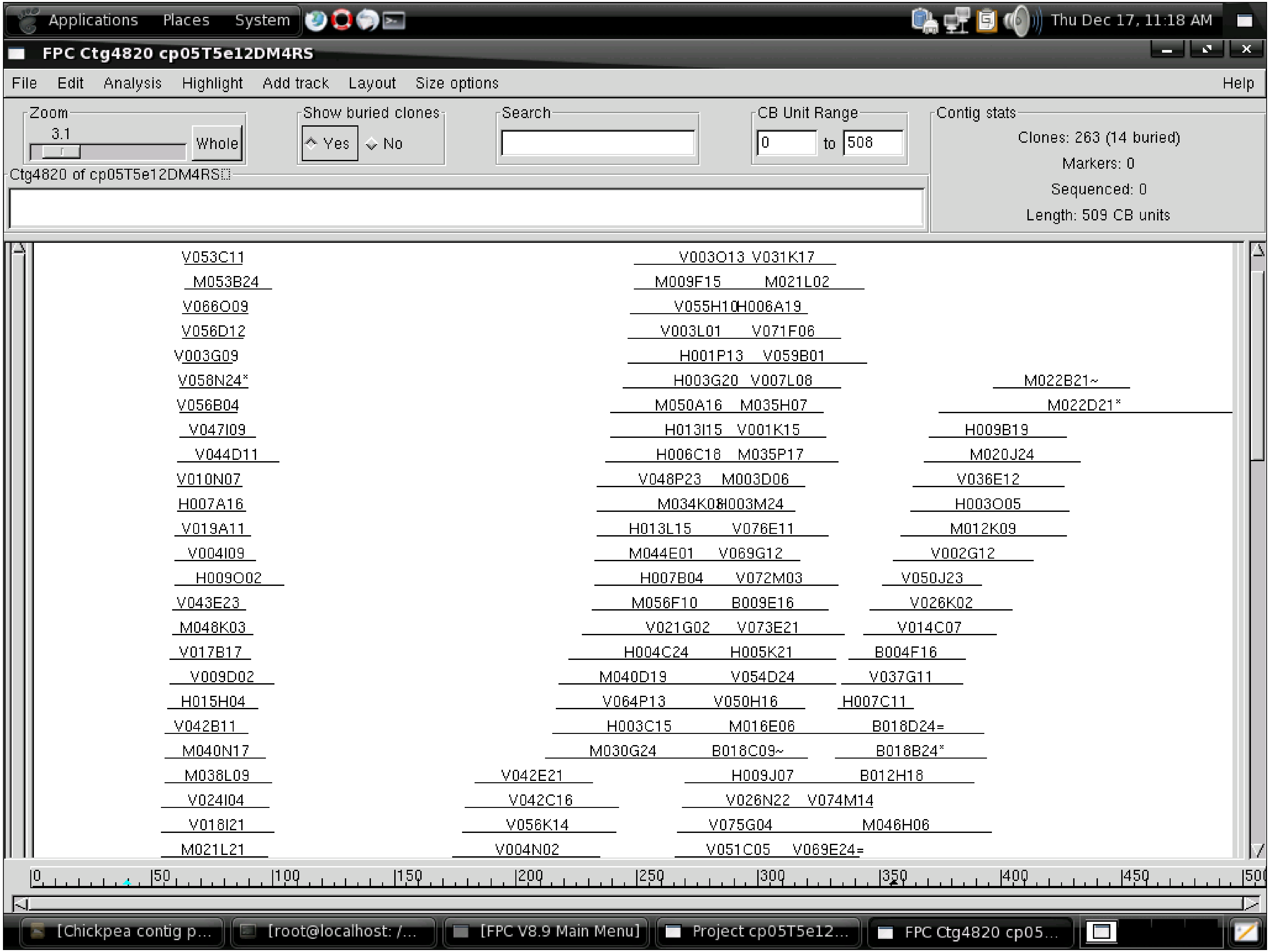

Applications Places System
Thu Dec 17, 11:18 AM
FPC Ctg4820 cp05T5e12DM4RS
File Edit Analysis Highlight Add track Layout Size options Help
Zoom
3.1
Whole
Show buried clones
Yes
No
Search
CB Unit Range
0 to 508
Contig stats
Clones: 263 (14 buried)
Markers: 0
Sequenced: 0
Length: 509 CB units
Ctg4820 of cp05T5e12DM4RS
V053C11 V003O13 V031K17
M053B24 M009F15 M021L02
V066O09 V055H10 H006A19
V056D12 V003L01 V071F06
V003G09 H001P13 V059B01
V058N24* H003G20 V007L08 M022B21~
V056B04 M050A16 M035H07 M022D21*
V047I09 H013I15 V001K15 H009B19
V044D11 H006C18 M035P17 M020J24
V010N07 V048P23 M003D06 V036E12
H007A16 M034K08 H003M24 H003O05
V019A11 H013L15 V076E11 M012K09
V004I09 M044E01 V069G12 V002G12
H009O02 H007B04 V072M03 V050J23
V043E23 M056F10 B009E16 V026K02
M048K03 V021G02 V073E21 V014C07
V017B17 H004C24 H005K21 B004F16
V009D02 M040D19 V054D24 V037G11
H015H04 V064P13 V050H16 H007C11
V042B11 H003C15 M016E06 B018D24=
M040N17 M030G24 B018C09~ B018B24*
M038L09 V042E21 H009J07 B012H18
V024I04 V042C16 V026N22 V074M14
V018I21 V056K14 V075G04 M046H06
M021L21 V004N02 V051C05 V069E24=
0 50 100 150 200 250 300 350 400 450 500
[Chickpea contig p...
[root@localhost: /...
[FPC V8.9 Main Menu]
Project cp05T5e12...
FPC Ctg4820 cp05...

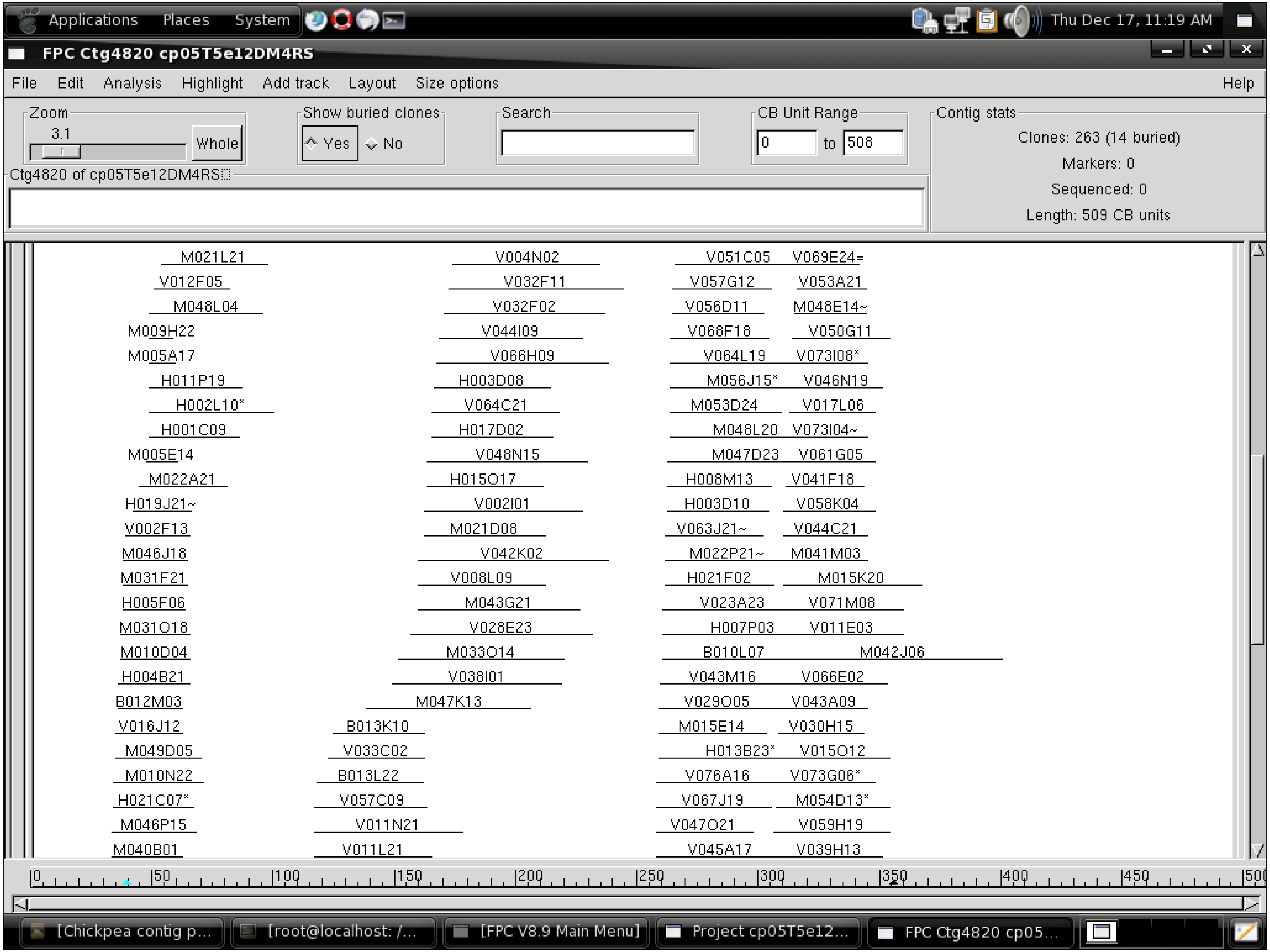
Applications Places System
Thu Dec 17, 11:19 AM
FPC Ctg4820 cp05T5e12DM4RS
File Edit Analysis Highlight Add track Layout Size options Help
Zoom
3.1
Whole
Show buried clones
Yes No
Search
CB Unit Range
0 to 508
Contig stats
Clones: 263 (14 buried)
Markers: 0
Sequenced: 0
Length: 509 CB units
Ctg4820 of cp05T5e12DM4RS
M021L21 V004N02 V051C05 V069E24=
V012F05 V032F11 V057G12 V053A21
M048L04 V032F02 V056D11 M048E14~
M009H22 V044I09 V068F18 V050G11
M005A17 V066H09 V064L19 V073I08*
H011P19 H003D08 M056J15* V046N19
H002L10* V064C21 M053D24 V017L06
H001C09 H017D02 M048L20 V073I04~
M005E14 V048N15 M047D23 V061G05
M022A21 H015O17 H008M13 V041F18
H019J21~ V002I01 H003D10 V058K04
V002F13 M021D08 V063J21~ V044C21
M046J18 V042K02 M022P21~ M041M03
M031F21 V008L09 H021F02 M015K20
H005F06 M043G21 V023A23 V071M08
M031O18 V028E23 H007P03 V011E03
M010D04 M033O14 B010L07 M042J06
H004B21 V038I01 V043M16 V066E02
B012M03 M047K13 V029O05 V043A09
V016J12 B013K10 M015E14 V030H15
M049D05 V033C02 H013B23* V015O12
M010N22 B013L22 V076A16 V073G06*
H021C07* V057C09 V067J19 M054D13*
M046P15 V011N21 V047O21 V059H19
M040B01 V011L21 V045A17 V039H13
0 50 100 150 200 250 300 350 400 450 500
[Chickpea contig p... [root@localhost: /... [FPC V8.9 Main Menu] Project cp05T5e12... FPC Ctg4820 cp05...

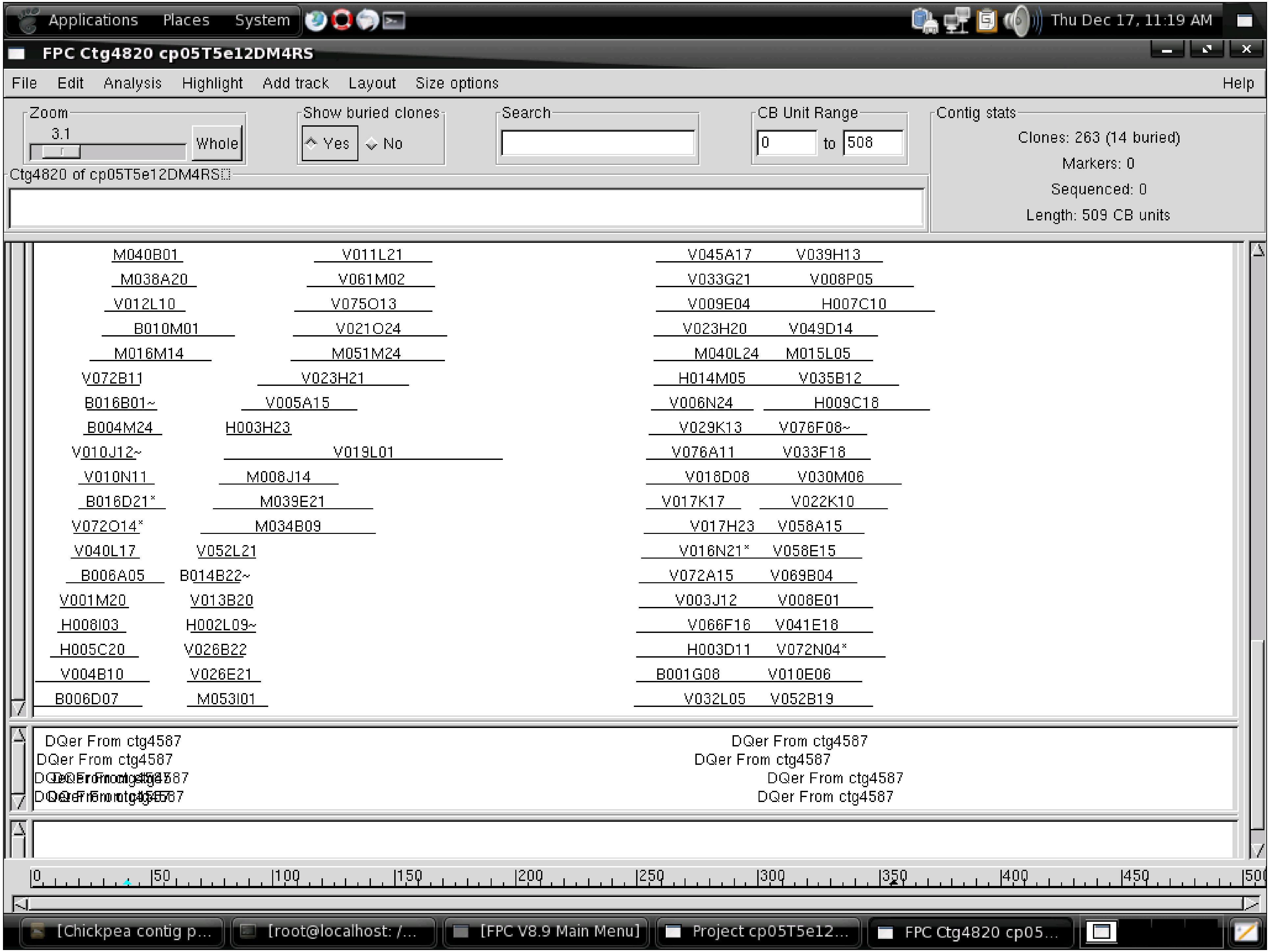

Applications Places System
Thu Dec 17, 11:19 AM
FPC Ctg4820 cp05T5e12DM4RS
File Edit Analysis Highlight Add track Layout Size options Help
Zoom
3.1
Whole
Show buried clones
Yes No
Search
CB Unit Range
0 to 508
Contig stats
Clones: 263 (14 buried)
Markers: 0
Sequenced: 0
Length: 509 CB units
Ctg4820 of cp05T5e12DM4RS
M040B01
M038A20
V012L10
B010M01
M016M14
V072B11
B016B01~
B004M24
V010J12~
V010N11
B016D21*
V072O14*
V040L17
B006A05
V001M20
H008I03
H005C20
V004B10
B006D07
V011L21
V061M02
V075O13
V021O24
M051M24
V023H21
V005A15
H003H23
V019L01
M008J14
M039E21
M034B09
V052L21
B014B22~
V013B20
H002L09~
V026B22
V026E21
M053I01
V045A17 V039H13
V033G21 V008P05
V009E04 H007C10
V023H20 V049D14
M040L24 M015L05
H014M05 V035B12
V006N24 H009C18
V029K13 V076F08~
V076A11 V033F18
V018D08 V030M06
V017K17 V022K10
V017H23 V058A15
V016N21* V058E15
V072A15 V069B04
V003J12 V008E01
V066F16 V041E18
H003D11 V072N04*
B001G08 V010E06
V032L05 V052B19
DQer From ctg4587
DQer From ctg4587
DQer From ctg4587
DQer From ctg4587
DQer From ctg4587
DQer From ctg4587
0 50 100 150 200 250 300 350 400 450 500
[Chickpea contig p...
[root@localhost: /...
[FPC V8.9 Main Menu]
Project cp05T5e12...
FPC Ctg4820 cp05...

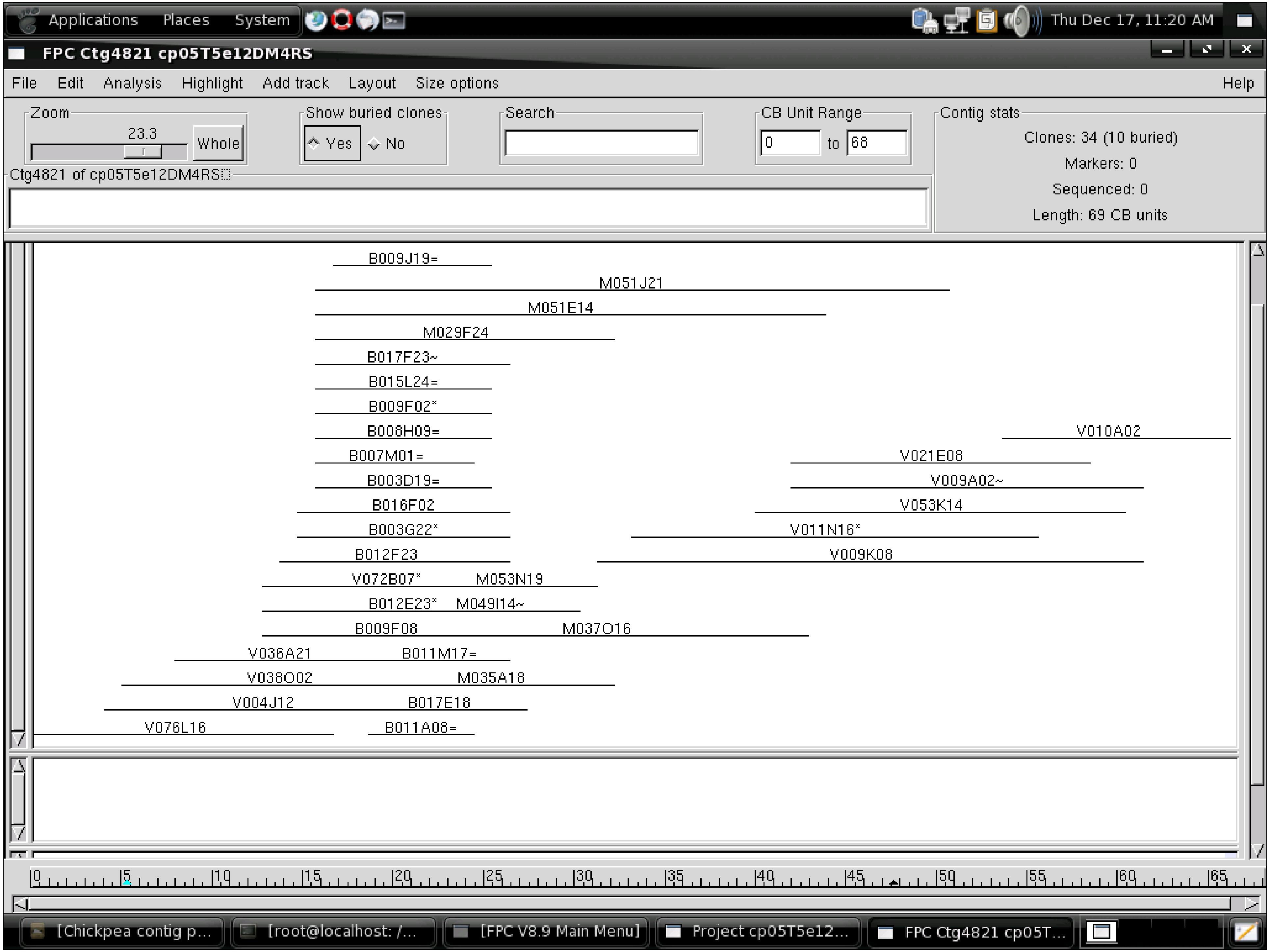
Applications Places System
Thu Dec 17, 11:20 AM
FPC Ctg4821 cp05T5e12DM4RS
File Edit Analysis Highlight Add track Layout Size options Help
Zoom
23.3
Whole
Show buried clones
Yes No
Search
CB Unit Range
0 to 68
Contig stats
Clones: 34 (10 buried)
Markers: 0
Sequenced: 0
Length: 69 CB units
Ctg4821 of cp05T5e12DM4RS
B009J19=
M051J21
M051E14
M029F24
B017F23~
B015L24=
B009F02*
B008H09=
V010A02
B007M01=
V021E08
B003D19=
V009A02~
B016F02
V053K14
B003G22*
V011N16*
B012F23
V009K08
V072B07*
M053N19
B012E23*
M049I14~
B009F08
M037O16
V036A21
B011M17=
V038O02
M035A18
V004J12
B017E18
V076L16
B011A08=
0 5 10 15 20 25 30 35 40 45 50 55 60 65
[Chickpea contig p...
[root@localhost: /...
[FPC V8.9 Main Menu]
Project cp05T5e12...
FPC Ctg4821 cp05T...

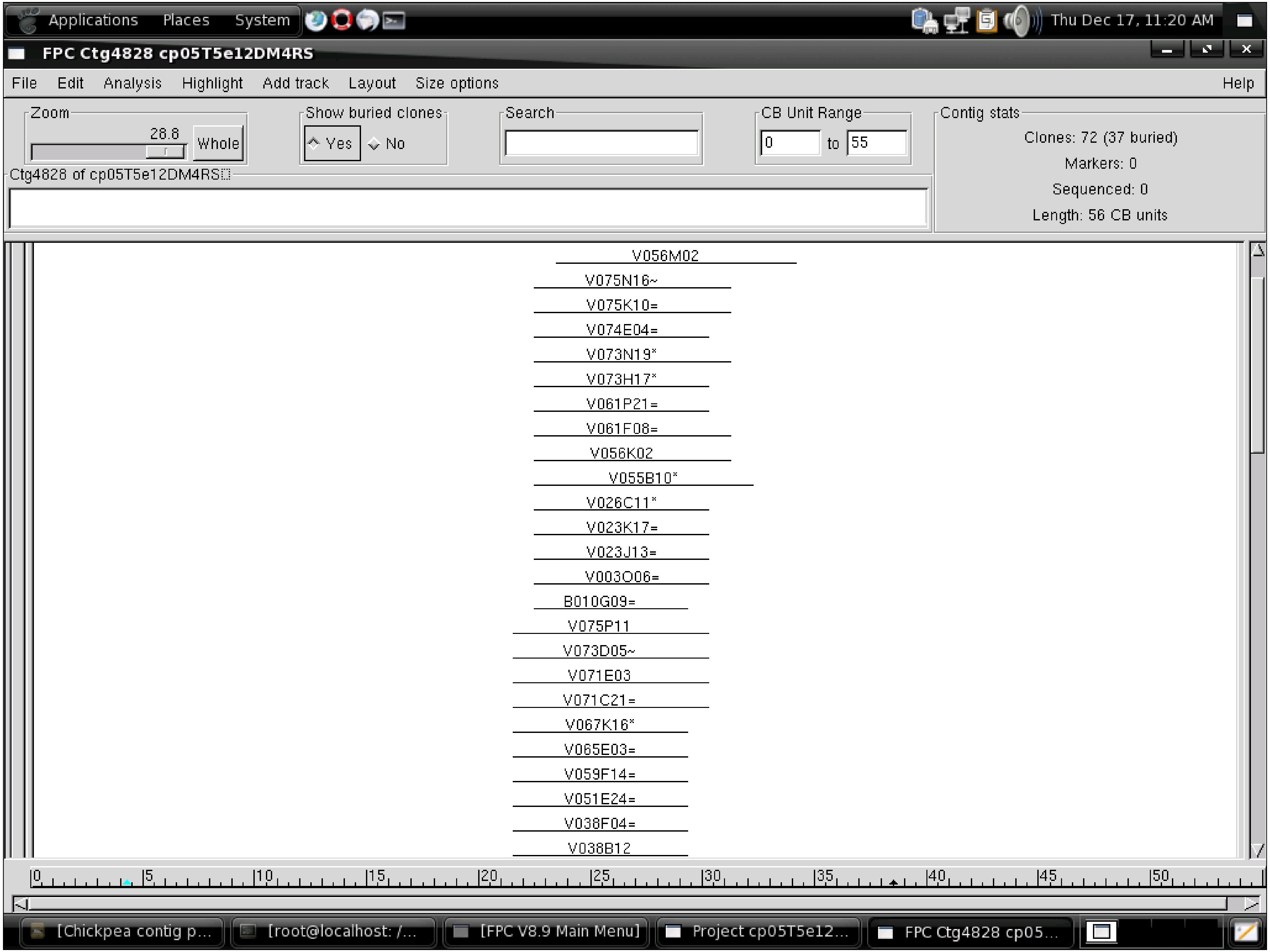
Applications Places System
Thu Dec 17, 11:20 AM
FPC Ctg4828 cp05T5e12DM4RS
File Edit Analysis Highlight Add track Layout Size options Help
Zoom
28.8
Whole
Show buried clones
Yes
No
Search
CB Unit Range
0 to 55
Contig stats
Clones: 72 (37 buried)
Markers: 0
Sequenced: 0
Length: 56 CB units
Ctg4828 of cp05T5e12DM4RS
V056M02
V075N16~
V075K10=
V074E04=
V073N19*
V073H17*
V061P21=
V061F08=
V056K02
V055B10*
V026C11*
V023K17=
V023J13=
V003O06=
B010G09=
V075P11
V073D05~
V071E03
V071C21=
V067K16*
V065E03=
V059F14=
V051E24=
V038F04=
V038B12
0 5 10 15 20 25 30 35 40 45 50
[Chickpea contig p...
[root@localhost: /...
[FPC V8.9 Main Menu]
Project cp05T5e12...
FPC Ctg4828 cp05...

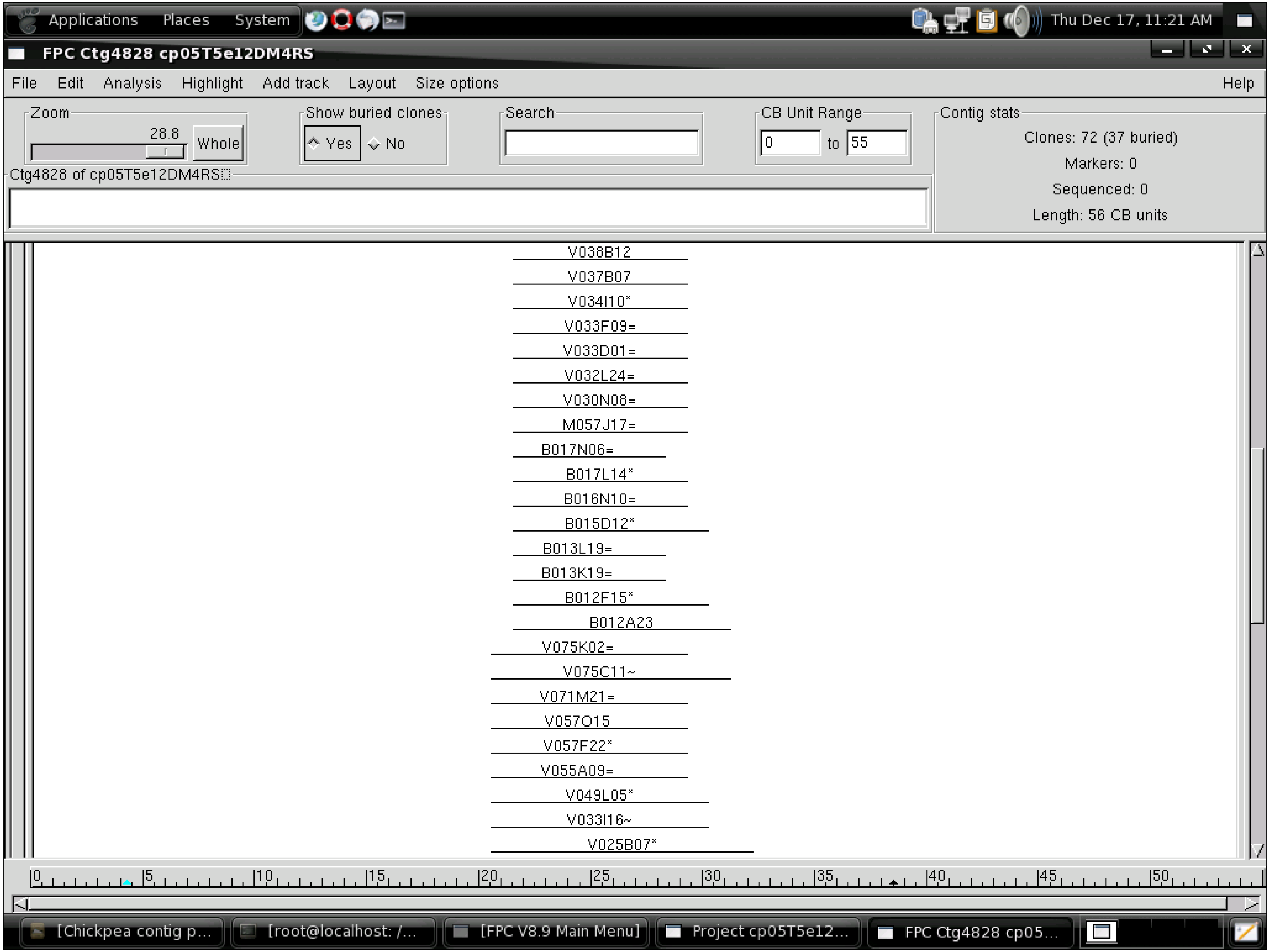
Applications Places System
Thu Dec 17, 11:21 AM
FPC Ctg4828 cp05T5e12DM4RS
File Edit Analysis Highlight Add track Layout Size options Help
Zoom
28.8
Whole
Show buried clones
Yes
No
Search
CB Unit Range
0
to
55
Contig stats
Clones: 72 (37 buried)
Markers: 0
Sequenced: 0
Length: 56 CB units
Ctg4828 of cp05T5e12DM4RS
V038B12
V037B07
V034I10*
V033F09=
V033D01=
V032L24=
V030N08=
M057J17=
B017N06=
B017L14*
B016N10=
B015D12*
B013L19=
B013K19=
B012F15*
B012A23
V075K02=
V075C11~
V071M21=
V057O15
V057F22*
V055A09=
V049L05*
V033I16~
V025B07*
0
5
10
15
20
25
30
35
40
45
50
[Chickpea contig p...
[root@localhost: /...
[FPC V8.9 Main Menu]
Project cp05T5e12...
FPC Ctg4828 cp05...

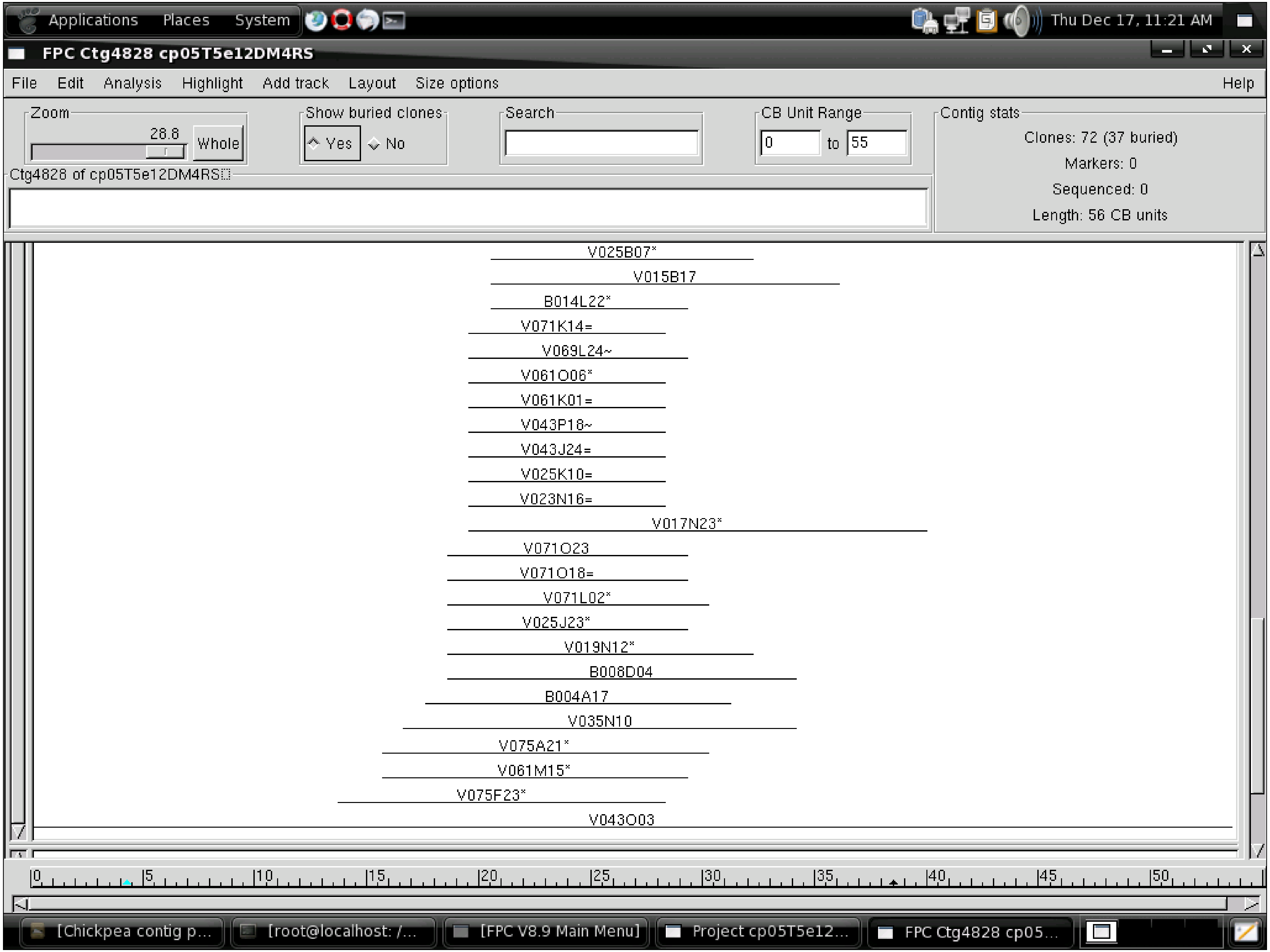
Applications Places System
Thu Dec 17, 11:21 AM
FPC Ctg4828 cp05T5e12DM4RS
File Edit Analysis Highlight Add track Layout Size options Help
Zoom
28.8
Whole
Show buried clones
Yes No
Search
CB Unit Range
0 to 55
Contig stats
Clones: 72 (37 buried)
Markers: 0
Sequenced: 0
Length: 56 CB units
Ctg4828 of cp05T5e12DM4RS
V025B07*
V015B17
B014L22*
V071K14=
V069L24~
V061O06*
V061K01=
V043P18~
V043J24=
V025K10=
V023N16=
V017N23*
V071O23
V071O18=
V071L02*
V025J23*
V019N12*
B008D04
B004A17
V035N10
V075A21*
V061M15*
V075F23*
V043O03
0 5 10 15 20 25 30 35 40 45 50
[Chickpea contig p...
[root@localhost: /...
[FPC V8.9 Main Menu]
Project cp05T5e12...
FPC Ctg4828 cp05...

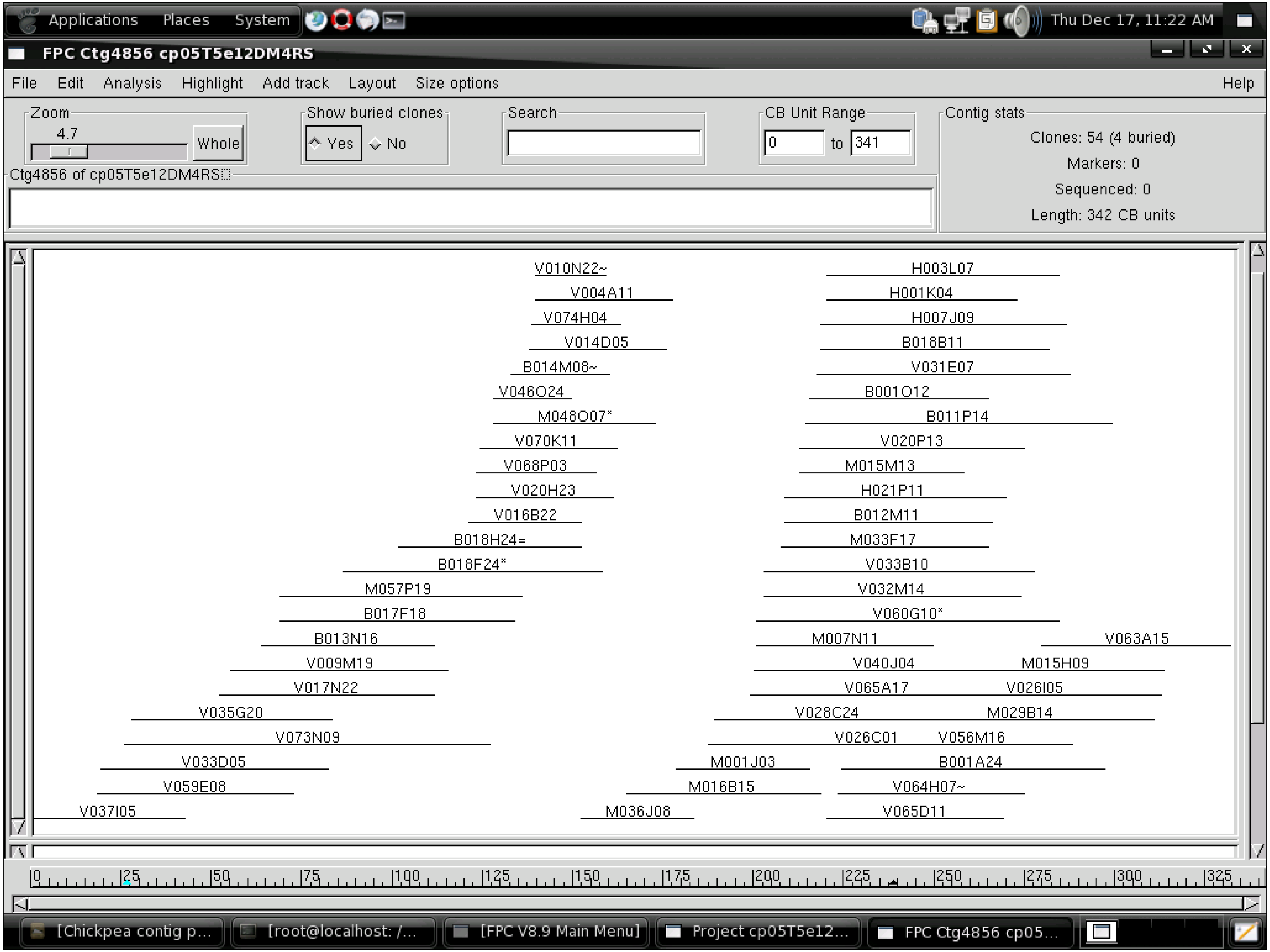
Applications Places System
Thu Dec 17, 11:22 AM
FPC Ctg4856 cp05T5e12DM4RS
File Edit Analysis Highlight Add track Layout Size options Help
Zoom
4.7
Whole
Show buried clones
Yes
No
Search
CB Unit Range
0 to 341
Contig stats
Clones: 54 (4 buried)
Markers: 0
Sequenced: 0
Length: 342 CB units
Ctg4856 of cp05T5e12DM4RS
V010N22~
V004A11
V074H04
V014D05
B014M08~
V046O24
M048O07*
V070K11
V068P03
V020H23
V016B22
B018H24=
B018F24*
M057P19
B017F18
B013N16
V009M19
V017N22
V035G20
V073N09
V033D05
V059E08
V037I05
H003L07
H001K04
H007J09
B018B11
V031E07
B001O12
B011P14
V020P13
M015M13
H021P11
B012M11
M033F17
V033B10
V032M14
V060G10*
M007N11
V063A15
V040J04
M015H09
V065A17
V026I05
V028C24
M029B14
V026C01
V056M16
M001J03
B001A24
M016B15
V064H07~
M036J08
V065D11
0 25 50 75 100 125 150 175 200 225 250 275 300 325
[Chickpea contig p...
[root@localhost: /...
[FPC V8.9 Main Menu]
Project cp05T5e12...
FPC Ctg4856 cp05...

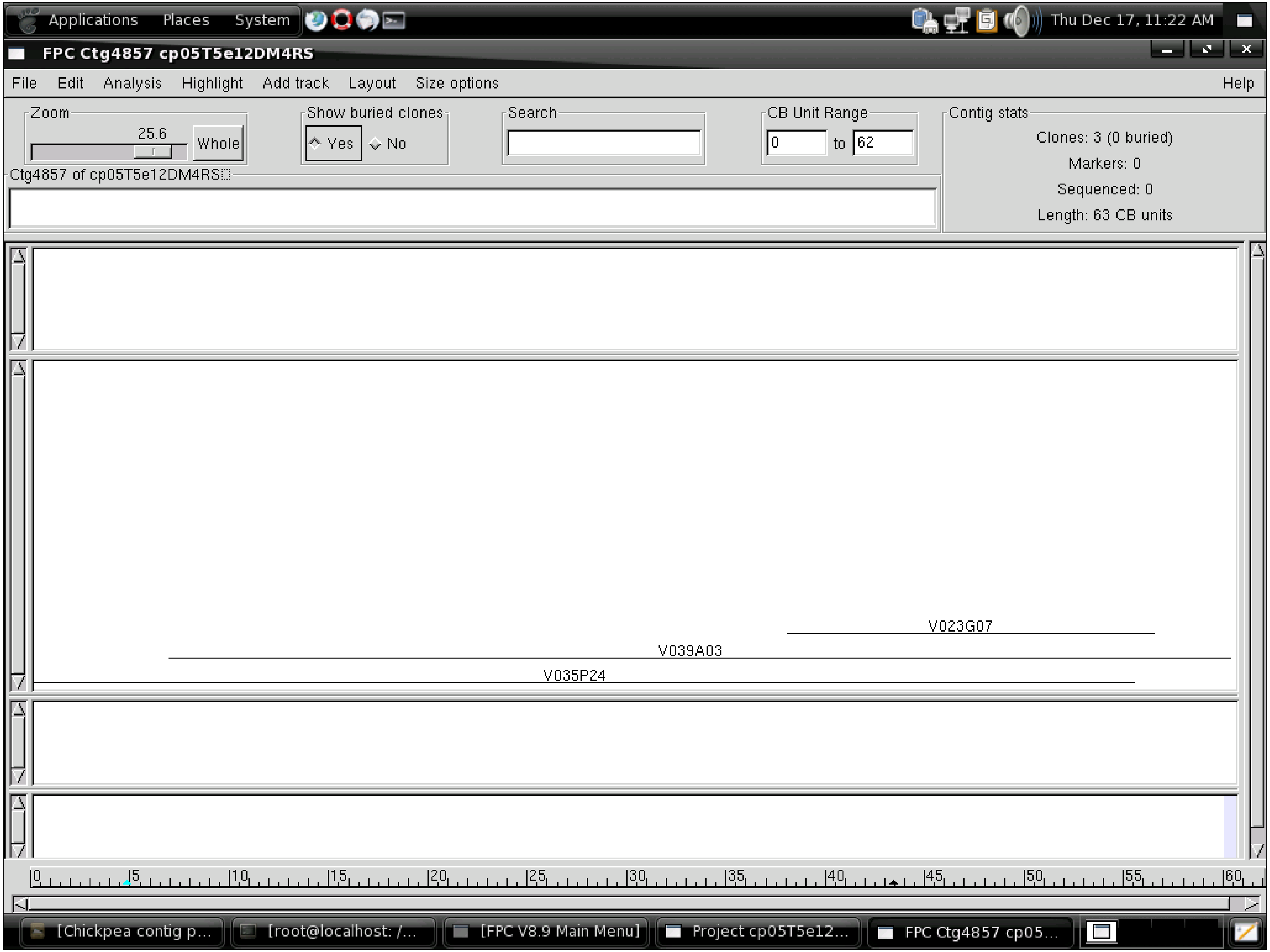
Applications
Places
System
Thu Dec 17, 11:22 AM
FPC Ctg4857 cp05T5e12DM4RS
File
Edit
Analysis
Highlight
Add track
Layout
Size options
Help
Zoom
25.6
Whole
Show buried clones
Yes
No
Search
CB Unit Range
0
to
62
Contig stats
Clones: 3 (0 buried)
Markers: 0
Sequenced: 0
Length: 63 CB units
Ctg4857 of cp05T5e12DM4RS
V023G07
V039A03
V035P24
0 5 10 15 20 25 30 35 40 45 50 55 60
[Chickpea contig p...
[root@localhost: /...
[FPC V8.9 Main Menu]
Project cp05T5e12...
FPC Ctg4857 cp05...

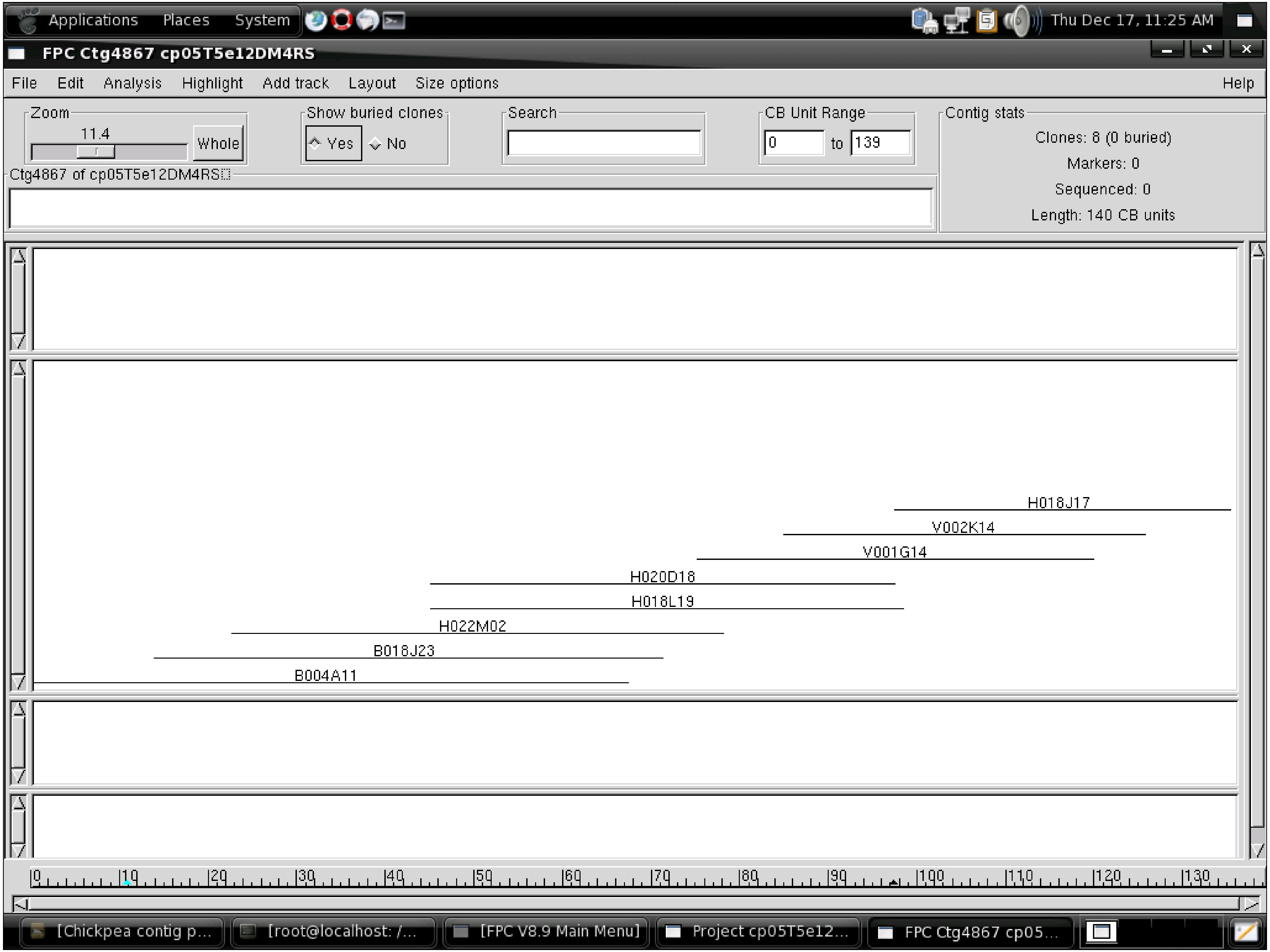
Applications Places System
Thu Dec 17, 11:25 AM
FPC Ctg4867 cp05T5e12DM4RS
File Edit Analysis Highlight Add track Layout Size options Help
Zoom
11.4
Whole
Show buried clones
Yes
No
Search
CB Unit Range
0
to
139
Contig stats
Clones: 8 (0 buried)
Markers: 0
Sequenced: 0
Length: 140 CB units
Ctg4867 of cp05T5e12DM4RS
H018J17
V002K14
V001G14
H020D18
H018L19
H022M02
B018J23
B004A11
0 10 20 30 40 50 60 70 80 90 100 110 120 130
[Chickpea contig p...
[root@localhost: /...
[FPC V8.9 Main Menu]
Project cp05T5e12...
FPC Ctg4867 cp05...

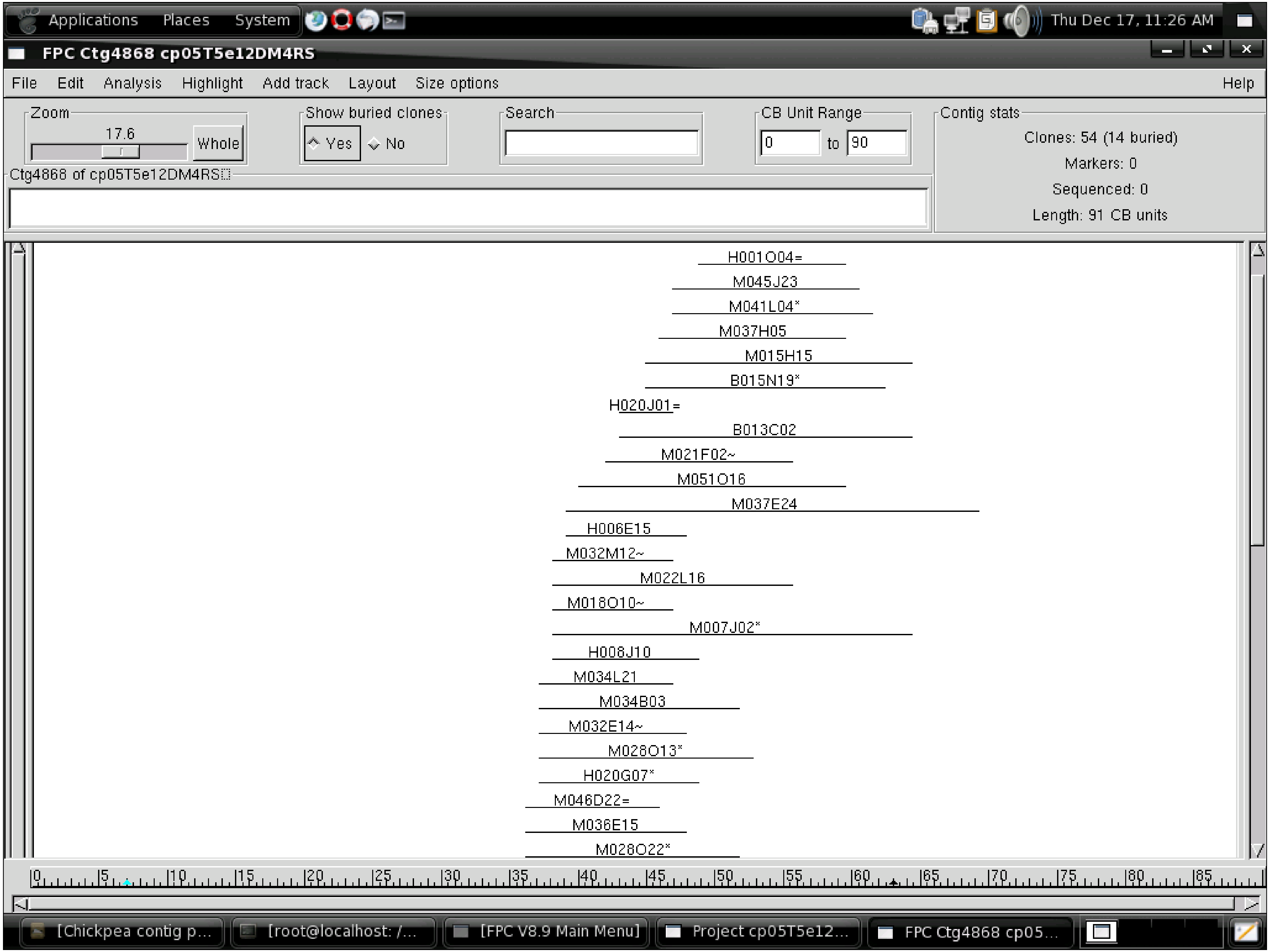

Applications Places System
Thu Dec 17, 11:26 AM
FPC Ctg4868 cp05T5e12DM4RS
File Edit Analysis Highlight Add track Layout Size options Help
Zoom
17.6
Whole
Show buried clones
Yes
No
Search
CB Unit Range
0 to 90
Contig stats
Clones: 54 (14 buried)
Markers: 0
Sequenced: 0
Length: 91 CB units
Ctg4868 of cp05T5e12DM4RS
H001O04=
M045J23
M041L04*
M037H05
M015H15
B015N19*
H020J01=
B013C02
M021F02~
M051O16
M037E24
H006E15
M032M12~
M022L16
M018O10~
M007J02*
H008J10
M034L21
M034B03
M032E14~
M028O13*
H020G07*
M046D22=
M036E15
M028O22*
0 5 10 15 20 25 30 35 40 45 50 55 60 65 70 75 80 85
[Chickpea contig p...
[root@localhost: /...
[FPC V8.9 Main Menu]
Project cp05T5e12...
FPC Ctg4868 cp05...

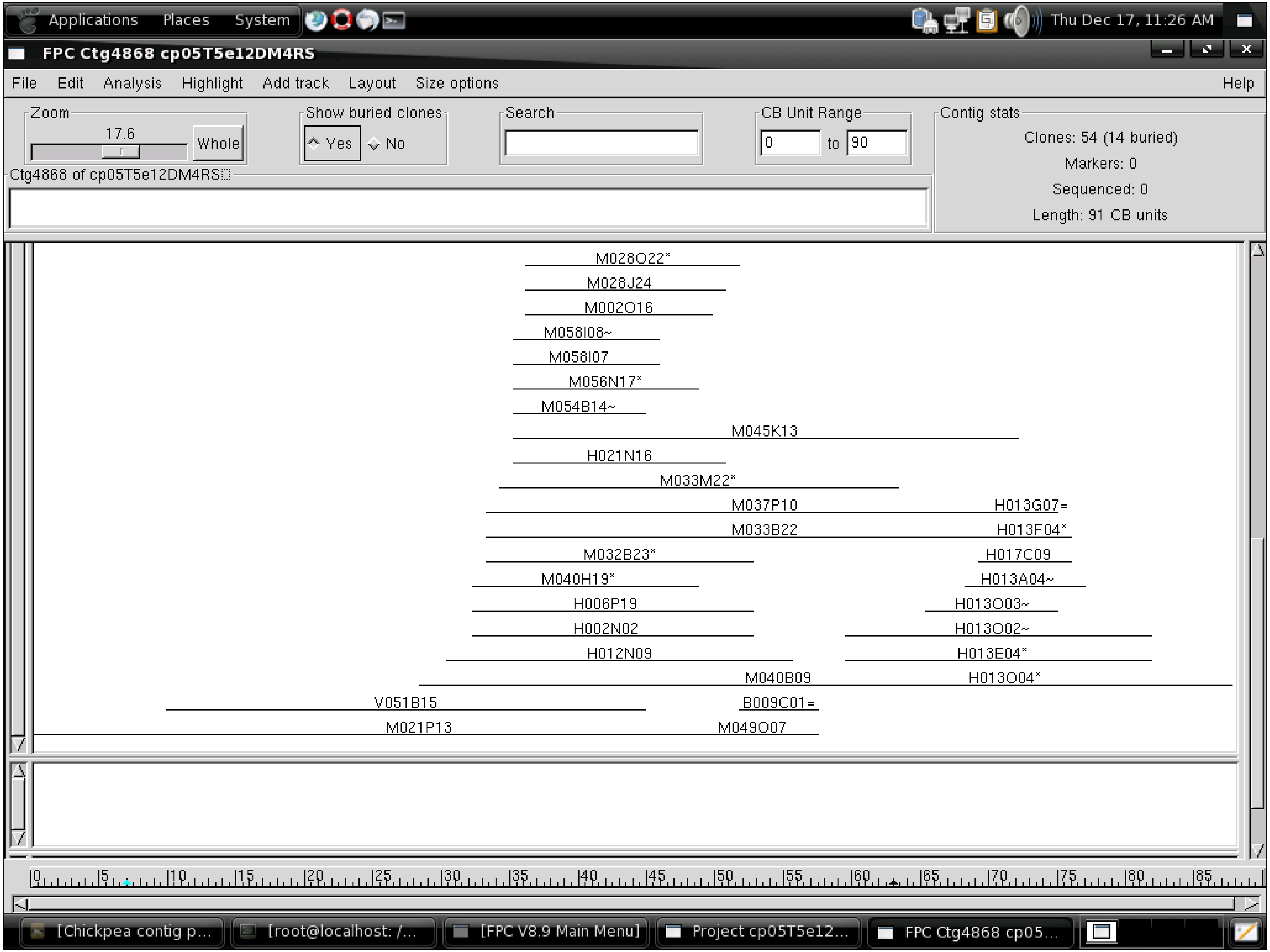
Applications Places System
Thu Dec 17, 11:26 AM
FPC Ctg4868 cp05T5e12DM4RS
File Edit Analysis Highlight Add track Layout Size options Help
Zoom
17.6
Whole
Show buried clones
Yes No
Search
CB Unit Range
0 to 90
Contig stats
Clones: 54 (14 buried)
Markers: 0
Sequenced: 0
Length: 91 CB units
Ctg4868 of cp05T5e12DM4RS
M028O22*
M028J24
M002O16
M058I08~
M058I07
M056N17*
M054B14~
M045K13
H021N16
M033M22*
M037P10
H013G07=
M033B22
H013F04*
M032B23*
H017C09
M040H19*
H013A04~
H006P19
H013O03~
H002N02
H013O02~
H012N09
H013E04*
M040B09
H013O04*
V051B15
B009C01=
M021P13
M049O07
0 5 10 15 20 25 30 35 40 45 50 55 60 65 70 75 80 85
[Chickpea contig p...
[root@localhost: /...
[FPC V8.9 Main Menu]
Project cp05T5e12...
FPC Ctg4868 cp05...

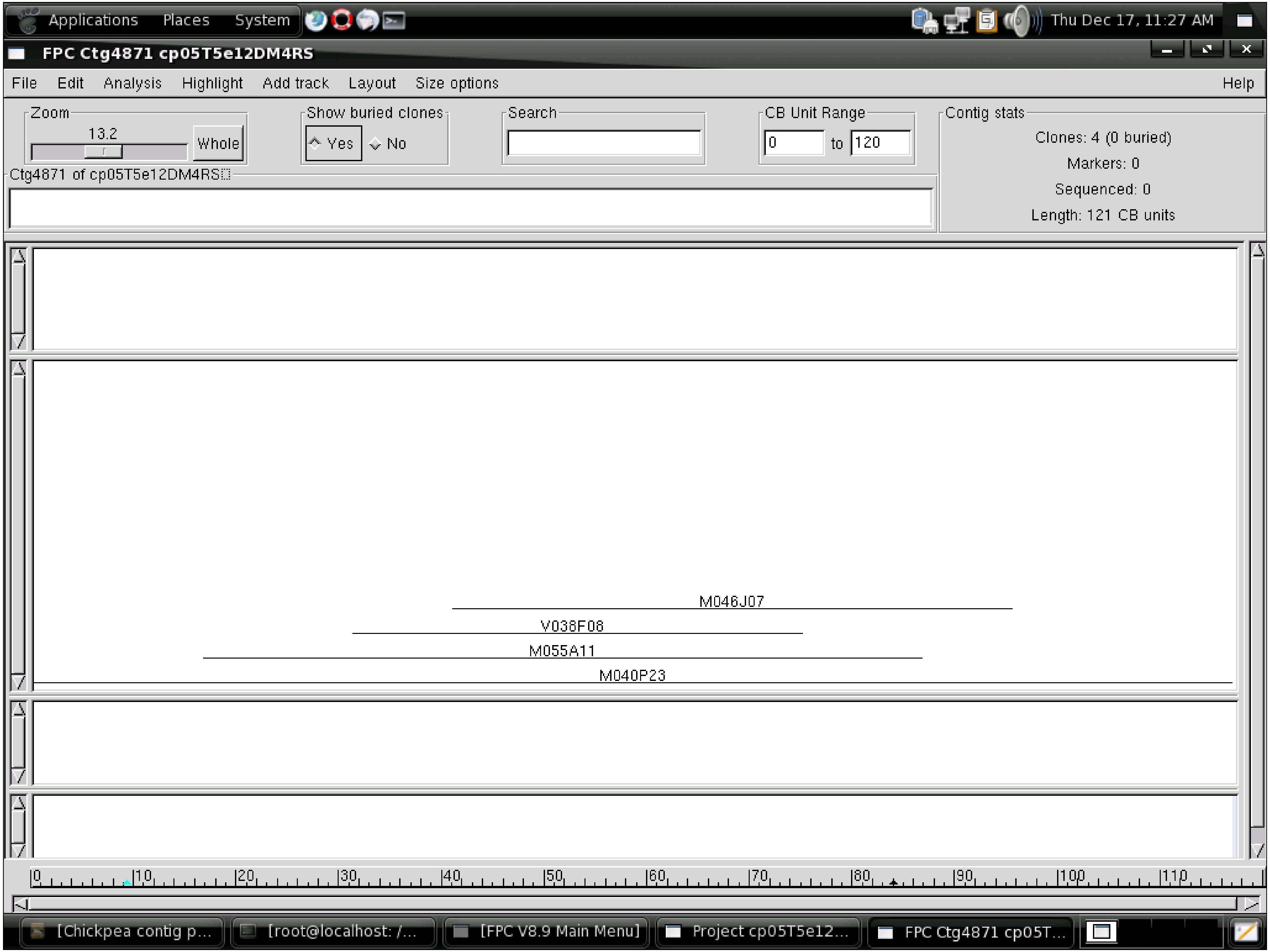
Applications Places System
Thu Dec 17, 11:27 AM
FPC Ctg4871 cp05T5e12DM4RS
File Edit Analysis Highlight Add track Layout Size options Help
Zoom
13.2
Whole
Show buried clones
Yes
No
Search
CB Unit Range
0
to
120
Contig stats
Clones: 4 (0 buried)
Markers: 0
Sequenced: 0
Length: 121 CB units
Ctg4871 of cp05T5e12DM4RS
M046J07
V038F08
M055A11
M040P23
0 10 20 30 40 50 60 70 80 90 100 110
[Chickpea contig p...
[root@localhost: /...
[FPC V8.9 Main Menu]
Project cp05T5e12...
FPC Ctg4871 cp05T...

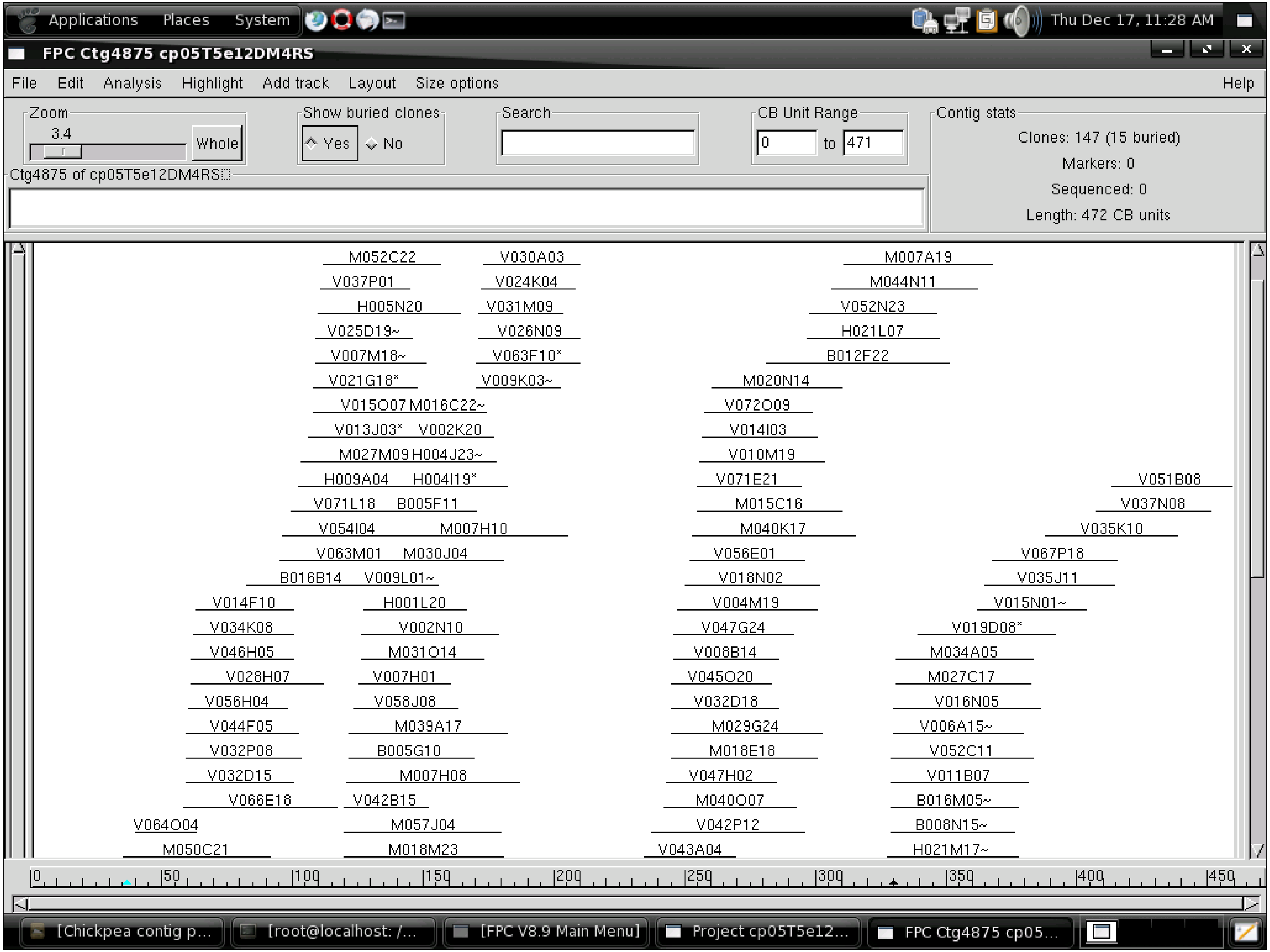

Applications Places System
Thu Dec 17, 11:28 AM
FPC Ctg4875 cp05T5e12DM4RS
File Edit Analysis Highlight Add track Layout Size options Help
Zoom
3.4
Whole
Show buried clones
Yes No
Search
CB Unit Range
0 to 471
Contig stats
Clones: 147 (15 buried)
Markers: 0
Sequenced: 0
Length: 472 CB units
Ctg4875 of cp05T5e12DM4RS
M052C22 V030A03 M007A19
V037P01 V024K04 M044N11
H005N20 V031M09 V052N23
V025D19~ V026N09 H021L07
V007M18~ V063F10* B012F22
V021G18* V009K03~ M020N14
V015O07 M016C22~ V072O09
V013J03* V002K20 V014I03
M027M09 H004J23~ V010M19
H009A04 H004I19* V071E21 V051B08
V071L18 B005F11 M015C16 V037N08
V054I04 M007H10 M040K17 V035K10
V063M01 M030J04 V056E01 V067P18
B016B14 V009L01~ V018N02 V035J11
V014F10 H001L20 V004M19 V015N01~
V034K08 V002N10 V047G24 V019D08*
V046H05 M031O14 V008B14 M034A05
V028H07 V007H01 V045O20 M027C17
V056H04 V058J08 V032D18 V016N05
V044F05 M039A17 M029G24 V006A15~
V032P08 B005G10 M018E18 V052C11
V032D15 M007H08 V047H02 V011B07
V066E18 V042B15 M040O07 B016M05~
V064O04 M057J04 V042P12 B008N15~
M050C21 M018M23 V043A04 H021M17~
0 50 100 150 200 250 300 350 400 450
[Chickpea contig p... [root@localhost: /... [FPC V8.9 Main Menu] Project cp05T5e12... FPC Ctg4875 cp05...

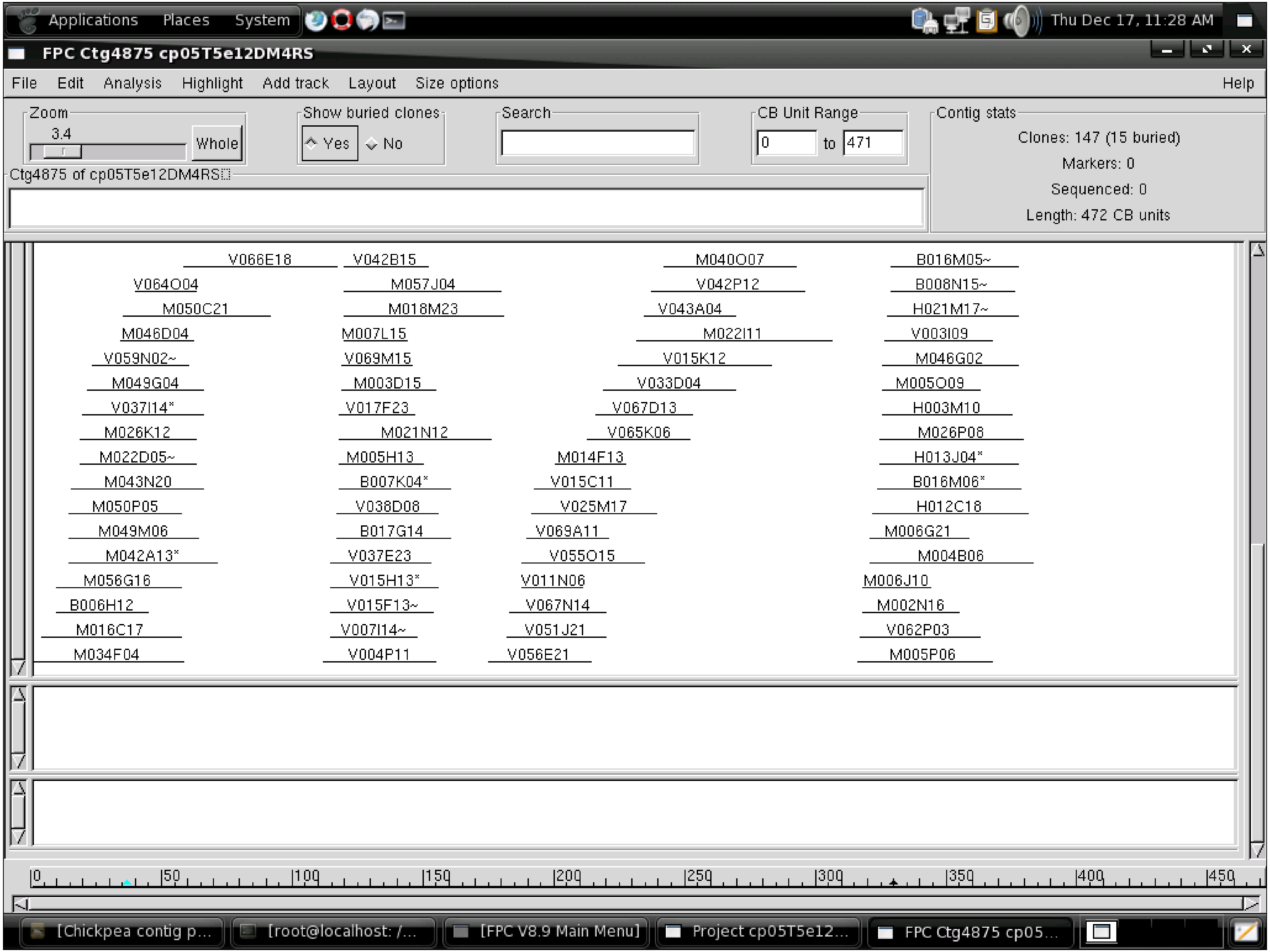
Applications Places System
Thu Dec 17, 11:28 AM
FPC Ctg4875 cp05T5e12DM4RS
File Edit Analysis Highlight Add track Layout Size options Help
Zoom
3.4
Whole
Show buried clones
Yes No
Search
CB Unit Range
0 to 471
Contig stats
Clones: 147 (15 buried)
Markers: 0
Sequenced: 0
Length: 472 CB units
Ctg4875 of cp05T5e12DM4RS
V066E18 V042B15 M040O07 B016M05~
V064O04 M057J04 V042P12 B008N15~
M050C21 M018M23 V043A04 H021M17~
M046D04 M007L15 M022I11 V003I09
V059N02~ V069M15 V015K12 M046G02
M049G04 M003D15 V033D04 M005O09
V037I14* V017F23 V067D13 H003M10
M026K12 M021N12 V065K06 M026P08
M022D05~ M005H13 M014F13 H013J04*
M043N20 B007K04* V015C11 B016M06*
M050P05 V038D08 V025M17 H012C18
M049M06 B017G14 V069A11 M006G21
M042A13* V037E23 V055O15 M004B06
M056G16 V015H13* V011N06 M006J10
B006H12 V015F13~ V067N14 M002N16
M016C17 V007I14~ V051J21 V062P03
M034F04 V004P11 V056E21 M005P06
0 50 100 150 200 250 300 350 400 450
[Chickpea contig p...
[root@localhost: /...
[FPC V8.9 Main Menu]
Project cp05T5e12...
FPC Ctg4875 cp05...

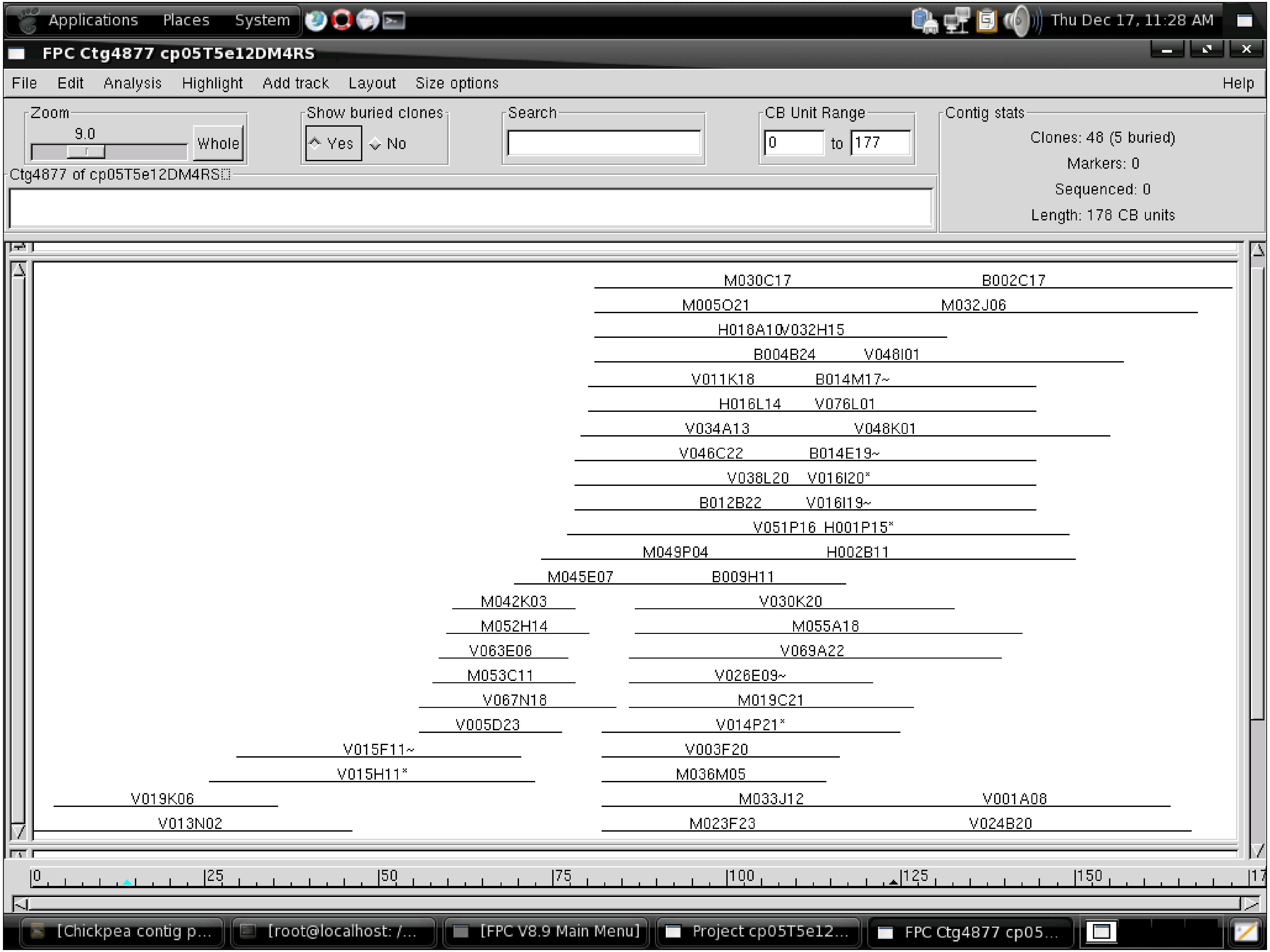

Applications Places System
Thu Dec 17, 11:28 AM
FPC Ctg4877 cp05T5e12DM4RS
File Edit Analysis Highlight Add track Layout Size options Help
Zoom
9.0
Whole
Show buried clones
Yes
No
Search
CB Unit Range
0
to
177
Contig stats
Clones: 48 (5 buried)
Markers: 0
Sequenced: 0
Length: 178 CB units
Ctg4877 of cp05T5e12DM4RS
M030C17 B002C17
M005O21 M032J06
H018A10V032H15
B004B24 V048I01
V011K18 B014M17~
H016L14 V076L01
V034A13 V048K01
V046C22 B014E19~
V038L20 V016I20*
B012B22 V016I19~
V051P16 H001P15*
M049P04 H002B11
M045E07 B009H11
M042K03 V030K20
M052H14 M055A18
V063E06 V069A22
M053C11 V026E09~
V067N18 M019C21
V005D23 V014P21*
V015F11~ V003F20
V015H11* M036M05
V019K06 M033J12 V001A08
V013N02 M023F23 V024B20
0 25 50 75 100 125 150
[Chickpea contig p...
[root@localhost: /...
[FPC V8.9 Main Menu]
Project cp05T5e12...
FPC Ctg4877 cp05...

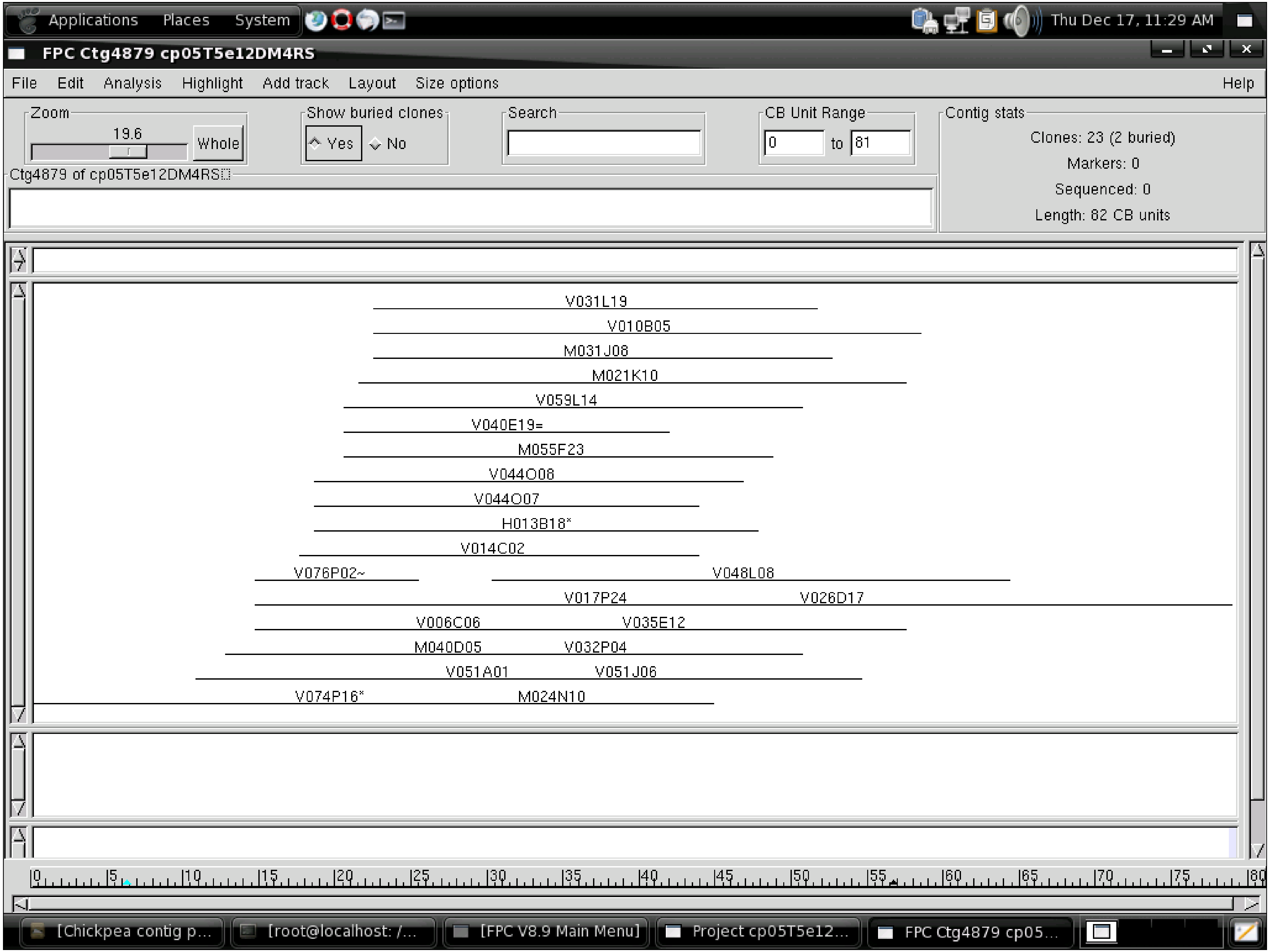
Applications
Places
System
Thu Dec 17, 11:29 AM
FPC Ctg4879 cp05T5e12DM4RS
File
Edit
Analysis
Highlight
Add track
Layout
Size options
Help
Zoom
19.6
Whole
Show buried clones
Yes
No
Search
CB Unit Range
0
to
81
Contig stats
Clones: 23 (2 buried)
Markers: 0
Sequenced: 0
Length: 82 CB units
Ctg4879 of cp05T5e12DM4RS
V031L19
V010B05
M031J08
M021K10
V059L14
V040E19=
M055F23
V044O08
V044O07
H013B18*
V014C02
V076P02~
V048L08
V017P24
V026D17
V006C06
V035E12
M040D05
V032P04
V051A01
V051J06
V074P16*
M024N10
0
5
10
15
20
25
30
35
40
45
50
55
60
65
70
75
80
[Chickpea contig p...
[root@localhost: /...
[FPC V8.9 Main Menu]
Project cp05T5e12...
FPC Ctg4879 cp05...

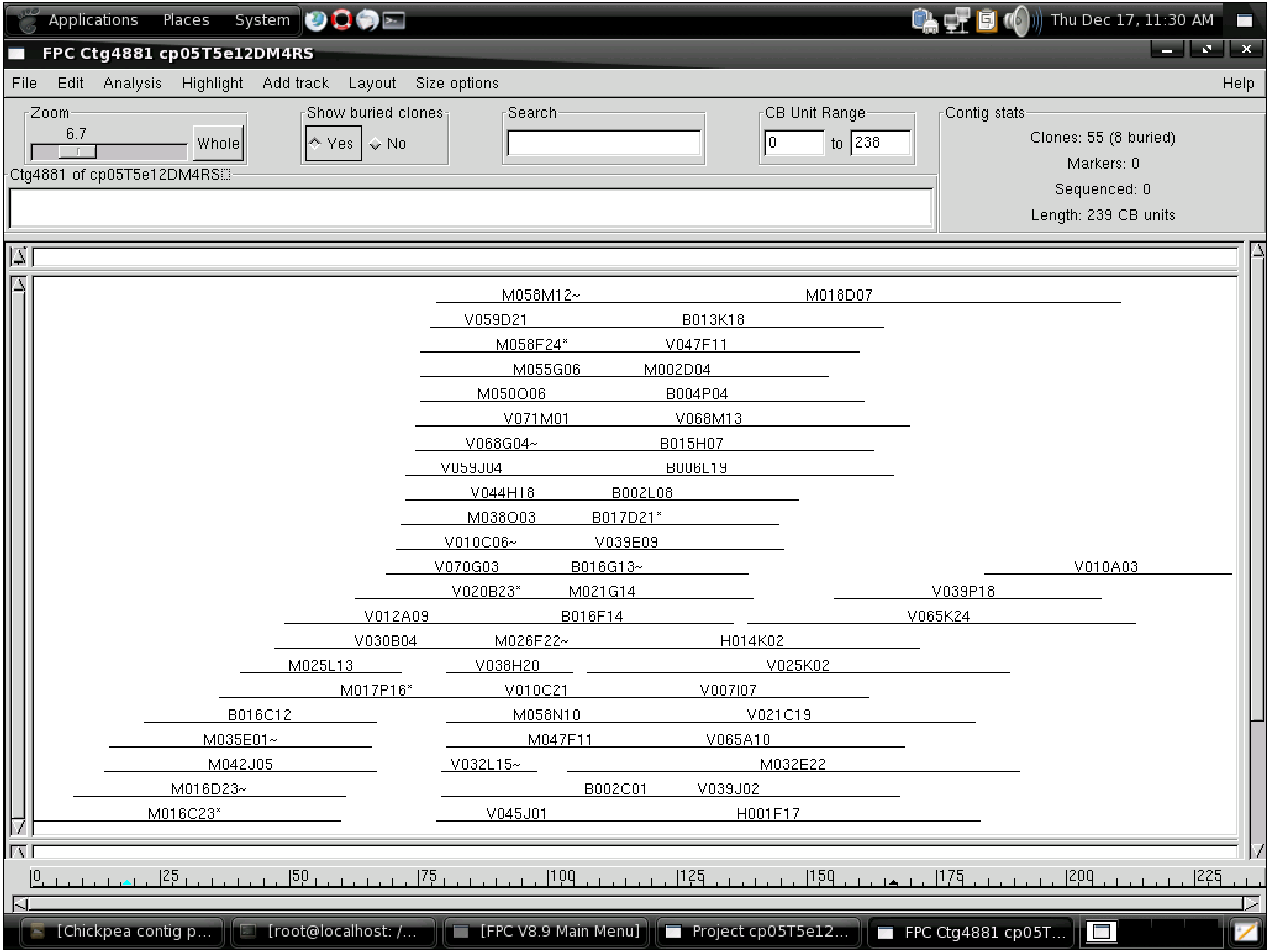
Applications Places System
Thu Dec 17, 11:30 AM
FPC Ctg4881 cp05T5e12DM4RS
File Edit Analysis Highlight Add track Layout Size options Help
Zoom
6.7
Whole
Show buried clones
Yes
No
Search
CB Unit Range
0 to 238
Contig stats
Clones: 55 (8 buried)
Markers: 0
Sequenced: 0
Length: 239 CB units
Ctg4881 of cp05T5e12DM4RS
M058M12~
M018D07
V059D21
B013K18
M058F24*
V047F11
M055G06
M002D04
M050O06
B004P04
V071M01
V068M13
V068G04~
B015H07
V059J04
B006L19
V044H18
B002L08
M038O03
B017D21*
V010C06~
V039E09
V070G03
B016G13~
V010A03
V020B23*
M021G14
V039P18
V012A09
B016F14
V065K24
V030B04
M026F22~
H014K02
M025L13
V038H20
V025K02
M017P16*
V010C21
V007I07
B016C12
M058N10
V021C19
M035E01~
M047F11
V065A10
M042J05
V032L15~
M032E22
M016D23~
B002C01
V039J02
M016C23*
V045J01
H001F17
0 25 50 75 100 125 150 175 200 225
[Chickpea contig p...
[root@localhost: /...
[FPC V8.9 Main Menu]
Project cp05T5e12...
FPC Ctg4881 cp05T...

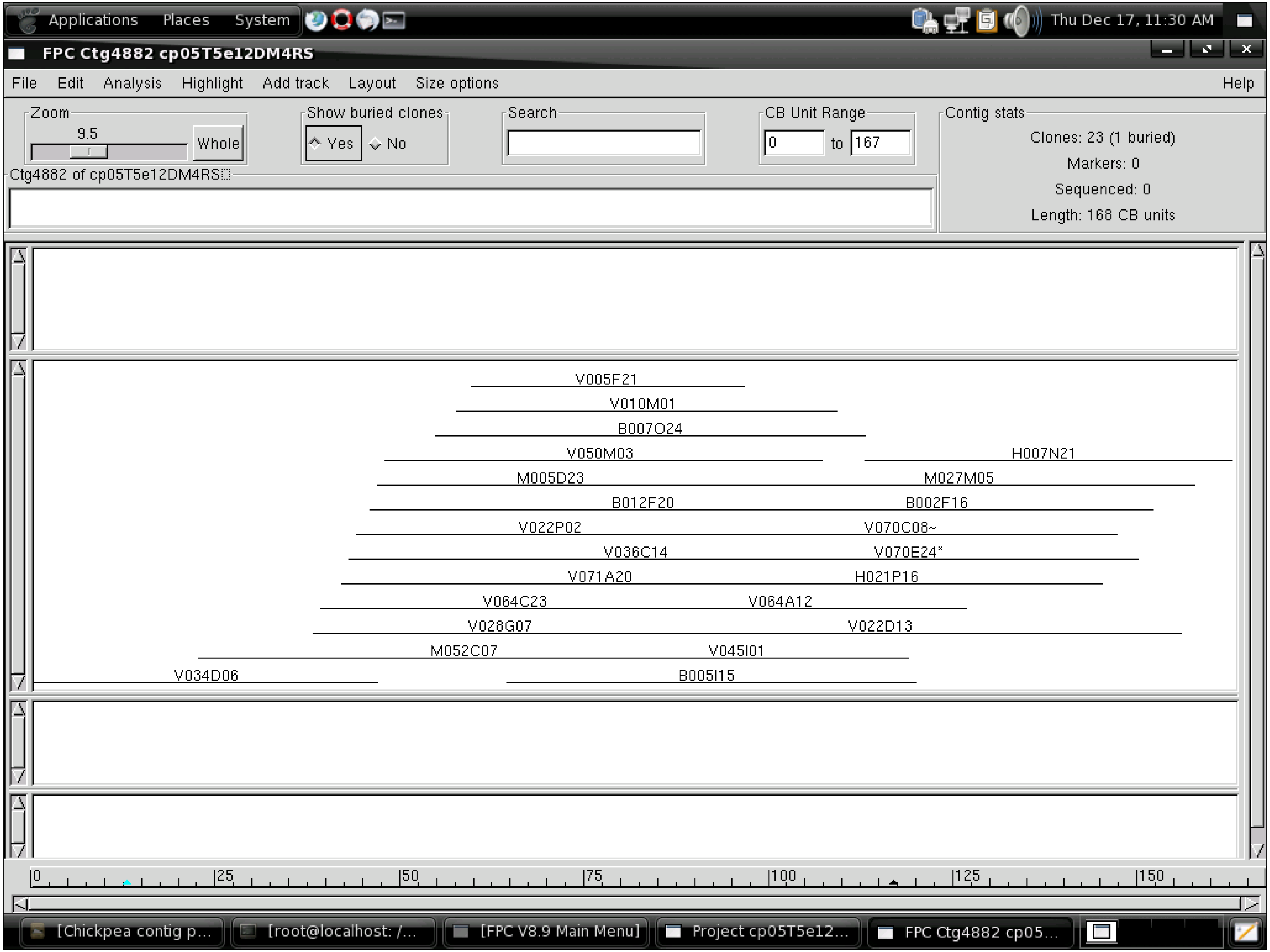
Applications Places System
Thu Dec 17, 11:30 AM
FPC Ctg4882 cp05T5e12DM4RS
File Edit Analysis Highlight Add track Layout Size options Help
Zoom
9.5
Whole
Show buried clones
Yes No
Search
CB Unit Range
0 to 167
Contig stats
Clones: 23 (1 buried)
Markers: 0
Sequenced: 0
Length: 168 CB units
Ctg4882 of cp05T5e12DM4RS
V005F21
V010M01
B007O24
V050M03
H007N21
M005D23
M027M05
B012F20
B002F16
V022P02
V070C08~
V036C14
V070E24*
V071A20
H021P16
V064C23
V064A12
V028G07
V022D13
M052C07
V045I01
V034D06
B005I15
0 25 50 75 100 125 150
[Chickpea contig p...
[root@localhost: /...
[FPC V8.9 Main Menu]
Project cp05T5e12...
FPC Ctg4882 cp05...

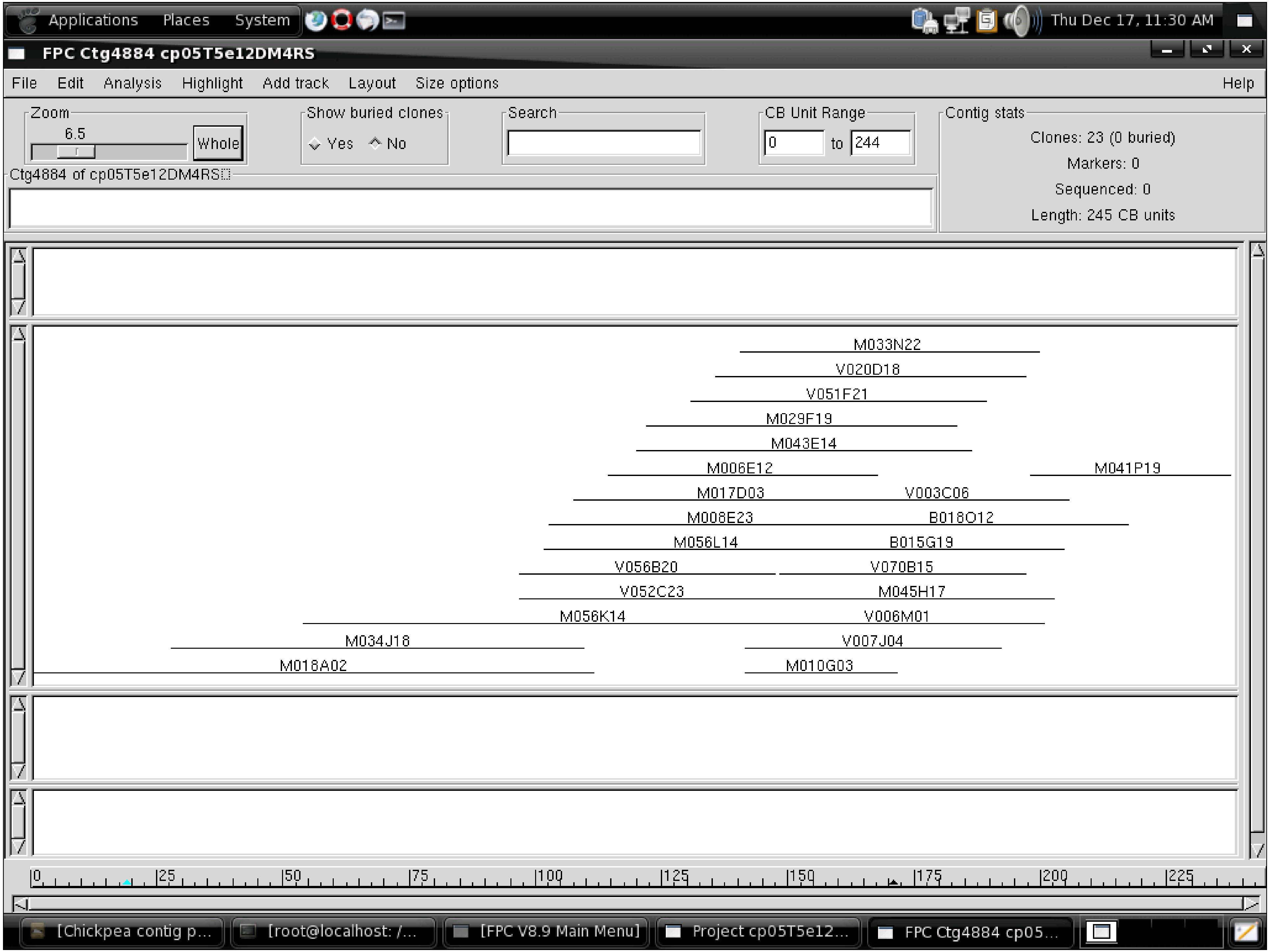
Applications Places System
Thu Dec 17, 11:30 AM
FPC Ctg4884 cp05T5e12DM4RS
File Edit Analysis Highlight Add track Layout Size options Help
Zoom
6.5
Whole
Show buried clones
Yes No
Search
CB Unit Range
0 to 244
Contig stats
Clones: 23 (0 buried)
Markers: 0
Sequenced: 0
Length: 245 CB units
Ctg4884 of cp05T5e12DM4RS
M033N22
V020D18
V051F21
M029F19
M043E14
M006E12
M041P19
M017D03
V003C06
M008E23
B018O12
M056L14
B015G19
V056B20
V070B15
V052C23
M045H17
M056K14
V006M01
M034J18
V007J04
M018A02
M010G03
0 25 50 75 100 125 150 175 200 225
[Chickpea contig p...
[root@localhost: /...
[FPC V8.9 Main Menu]
Project cp05T5e12...
FPC Ctg4884 cp05...

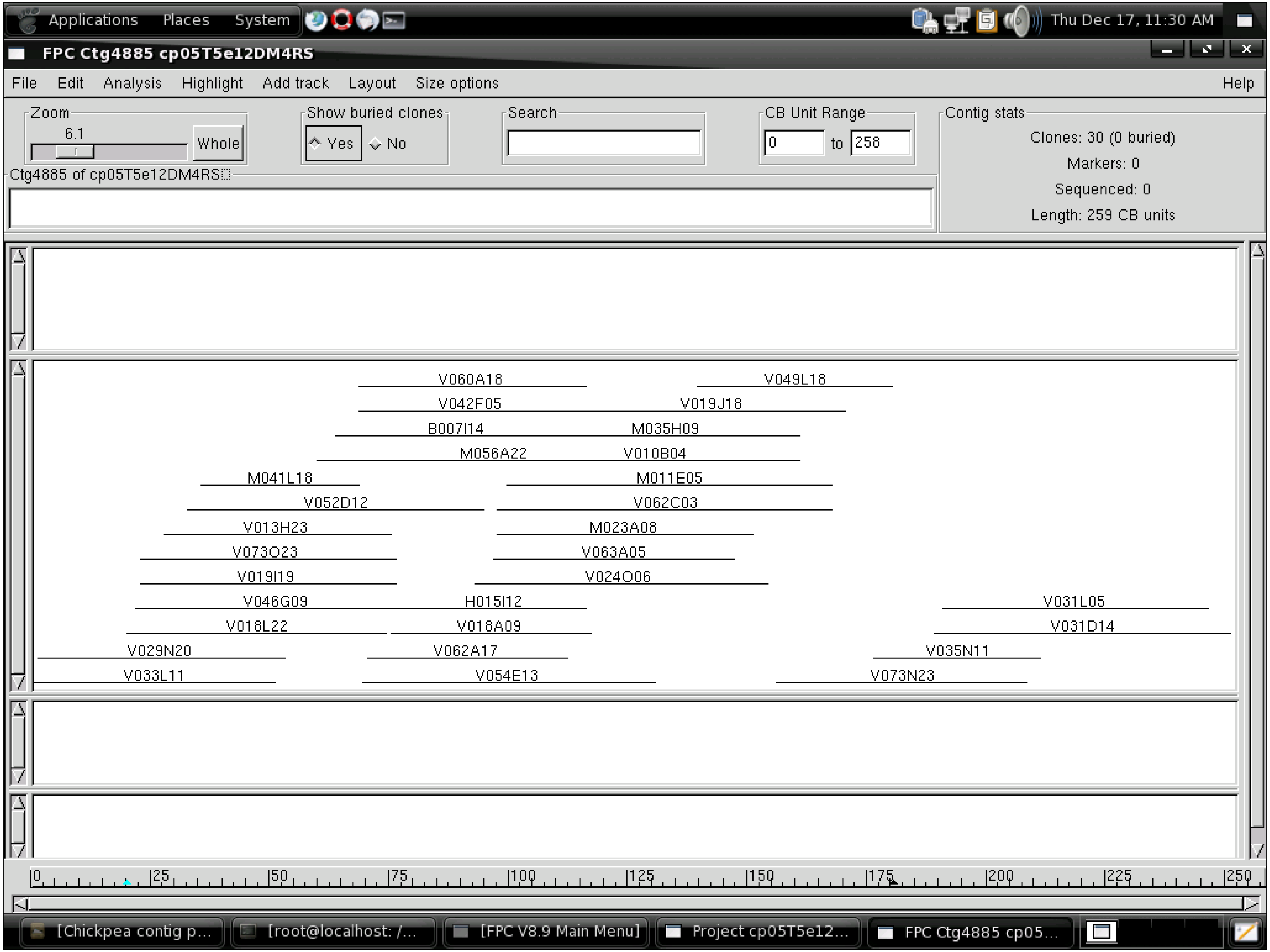
Applications Places System
Thu Dec 17, 11:30 AM
FPC Ctg4885 cp05T5e12DM4RS
File Edit Analysis Highlight Add track Layout Size options Help
Zoom
6.1
Whole
Show buried clones
Yes No
Search
CB Unit Range
0 to 258
Contig stats
Clones: 30 (0 buried)
Markers: 0
Sequenced: 0
Length: 259 CB units
Ctg4885 of cp05T5e12DM4RS
V060A18
V049L18
V042F05
V019J18
B007I14
M035H09
M056A22
V010B04
M041L18
M011E05
V052D12
V062C03
V013H23
M023A08
V073O23
V063A05
V019I19
V024O06
V046G09
H015I12
V031L05
V018L22
V018A09
V031D14
V029N20
V062A17
V035N11
V033L11
V054E13
V073N23
0 25 50 75 100 125 150 175 200 225 250
[Chickpea contig p...
[root@localhost: /...
[FPC V8.9 Main Menu]
Project cp05T5e12...
FPC Ctg4885 cp05...

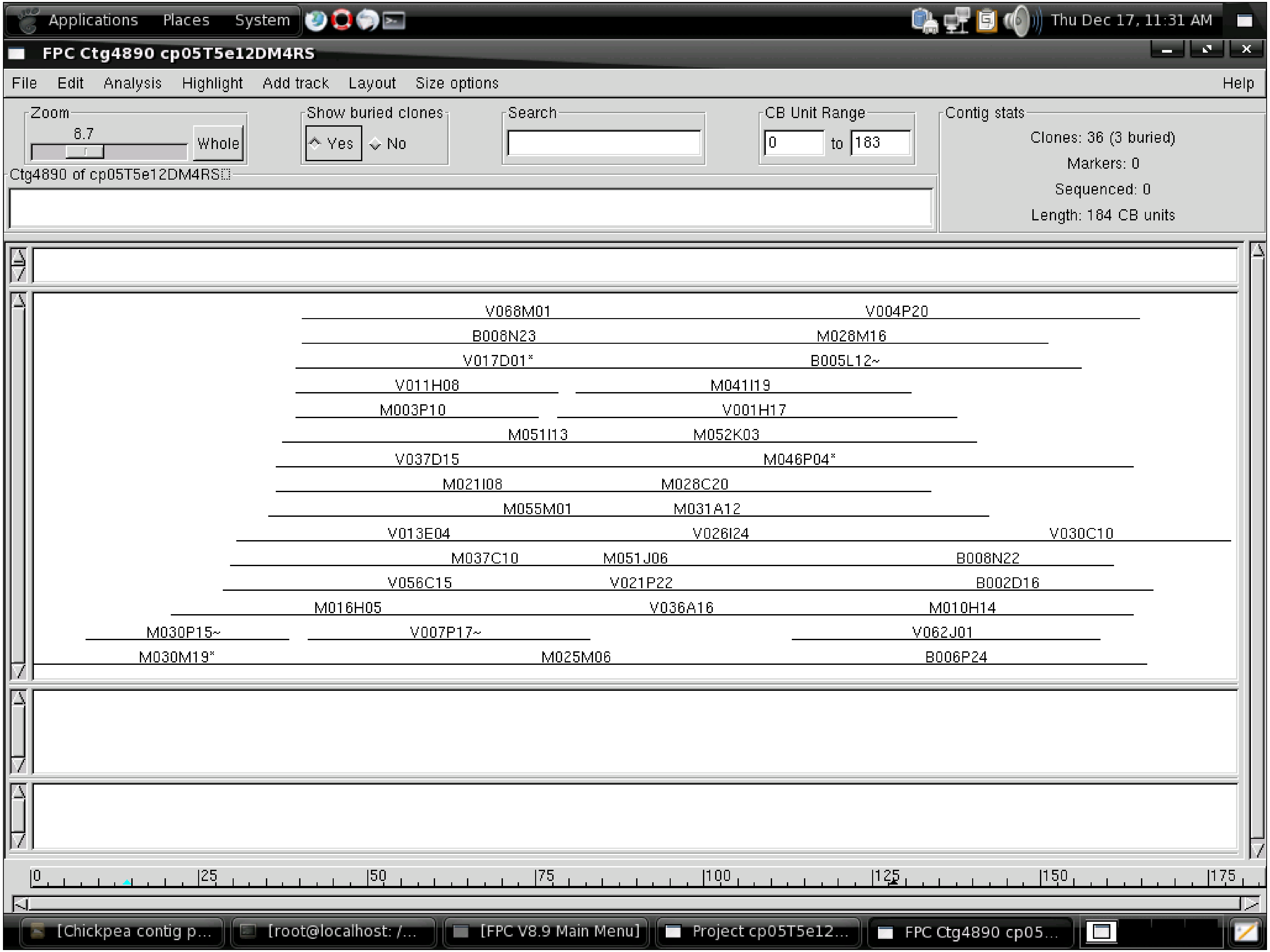

Applications Places System
Thu Dec 17, 11:31 AM
FPC Ctg4890 cp05T5e12DM4RS
File Edit Analysis Highlight Add track Layout Size options Help
Zoom
8.7
Whole
Show buried clones
Yes No
Search
CB Unit Range
0 to 183
Contig stats
Clones: 36 (3 buried)
Markers: 0
Sequenced: 0
Length: 184 CB units
Ctg4890 of cp05T5e12DM4RS
V068M01
V004P20
B008N23
M028M16
V017D01*
B005L12~
V011H08
M041I19
M003P10
V001H17
M051I13
M052K03
V037D15
M046P04*
M021I08
M028C20
M055M01
M031A12
V013E04
V026I24
V030C10
M037C10
M051J06
B008N22
V056C15
V021P22
B002D16
M016H05
V036A16
M010H14
M030P15~
V007P17~
V062J01
M030M19*
M025M06
B006P24
0
25
50
75
100
125
150
175
[Chickpea contig p...
[root@localhost: /...
[FPC V8.9 Main Menu]
Project cp05T5e12...
FPC Ctg4890 cp05...

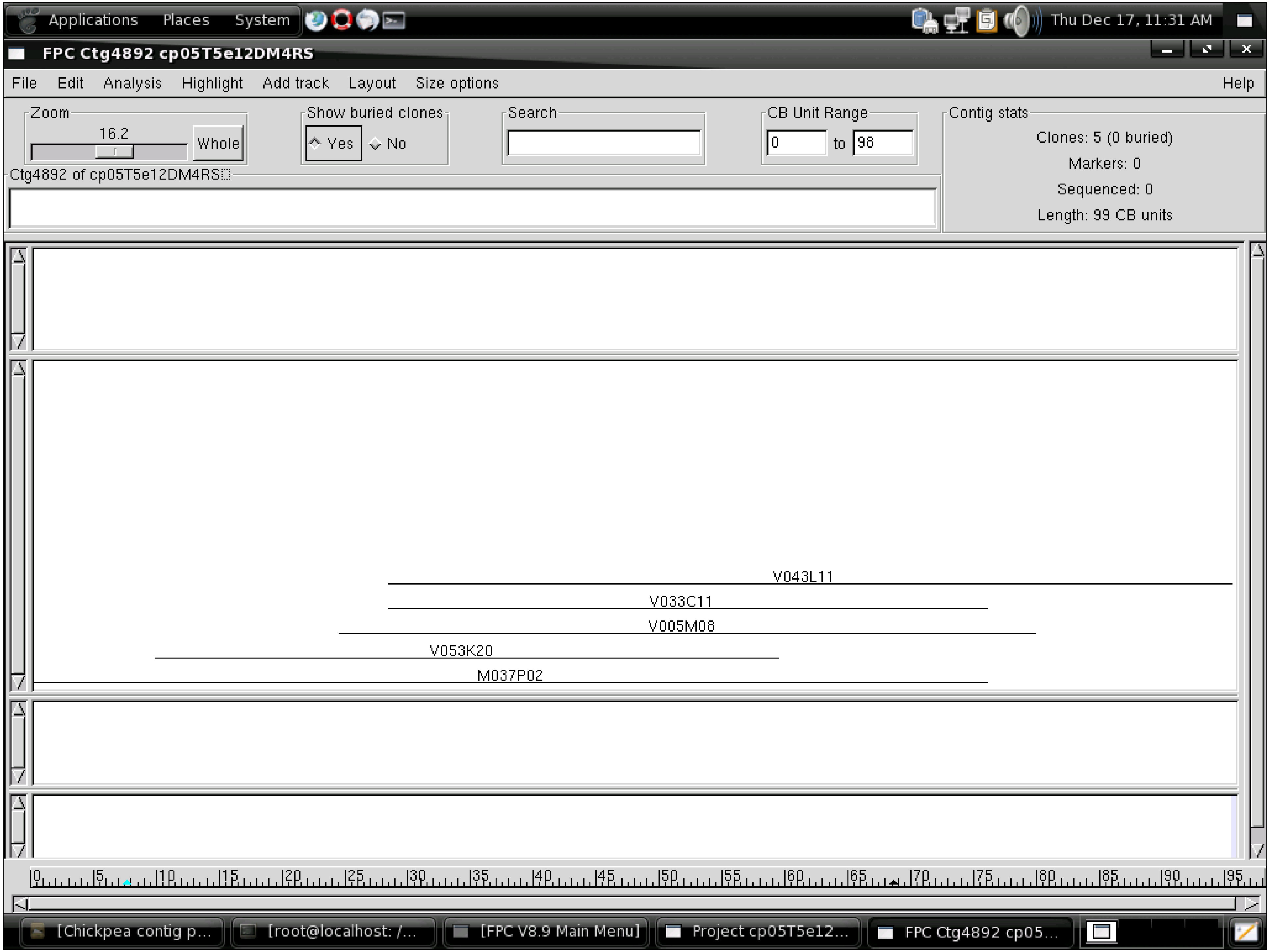
Applications
Places
System
Thu Dec 17, 11:31 AM
FPC Ctg4892 cp05T5e12DM4RS
File
Edit
Analysis
Highlight
Add track
Layout
Size options
Help
Zoom
16.2
Whole
Show buried clones
Yes
No
Search
CB Unit Range
0
to
98
Contig stats
Clones: 5 (0 buried)
Markers: 0
Sequenced: 0
Length: 99 CB units
Ctg4892 of cp05T5e12DM4RS
V043L11
V033C11
V005M08
V053K20
M037P02
0 5 10 15 20 25 30 35 40 45 50 55 60 65 70 75 80 85 90 95
[Chickpea contig p...
[root@localhost: /...
[FPC V8.9 Main Menu]
Project cp05T5e12...
FPC Ctg4892 cp05...

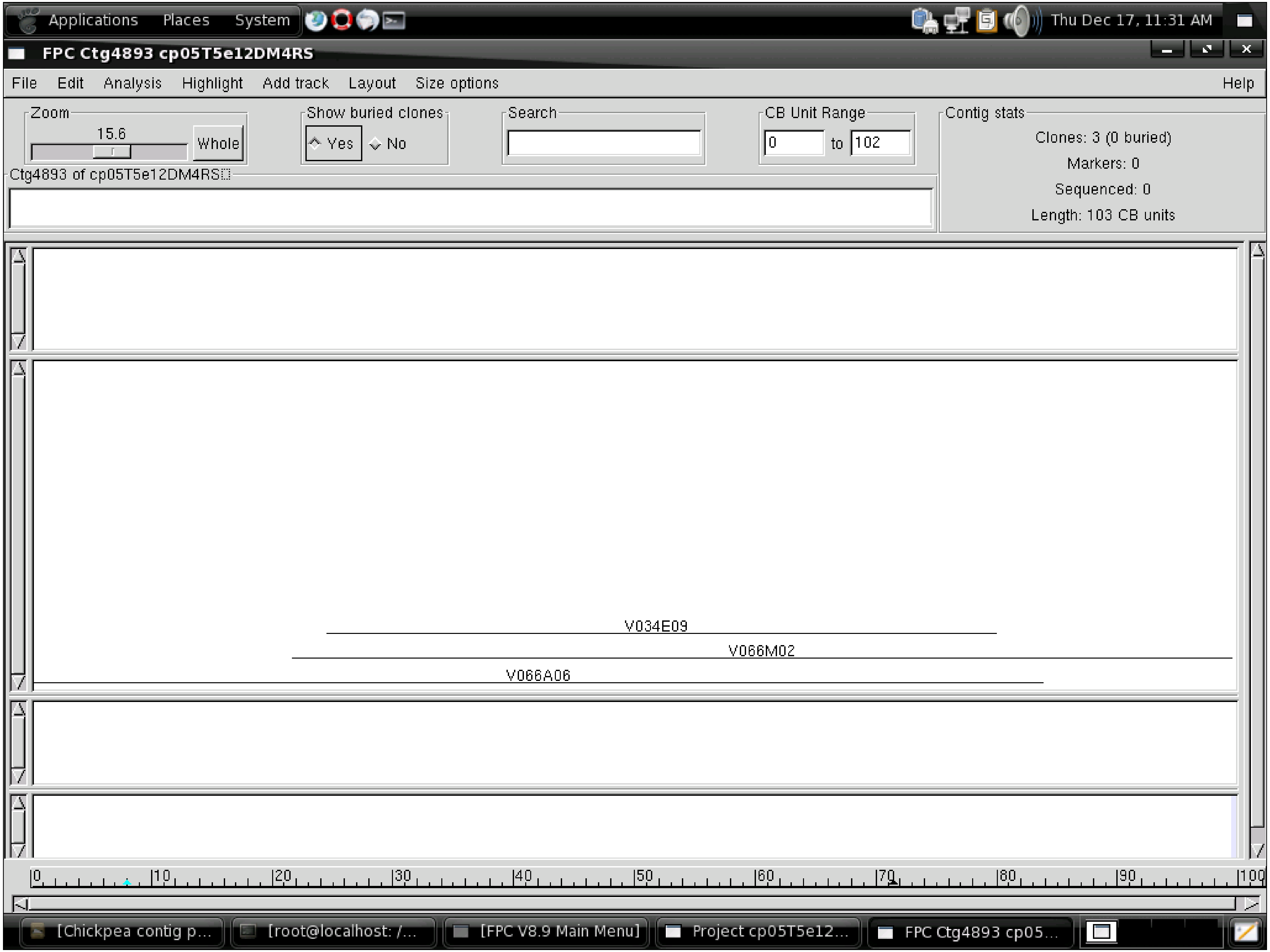
Applications Places System
Thu Dec 17, 11:31 AM
FPC Ctg4893 cp05T5e12DM4RS
File Edit Analysis Highlight Add track Layout Size options Help
Zoom
15.6
Whole
Show buried clones
Yes
No
Search
CB Unit Range
0 to 102
Contig stats
Clones: 3 (0 buried)
Markers: 0
Sequenced: 0
Length: 103 CB units
Ctg4893 of cp05T5e12DM4RS
V034E09
V066M02
V066A06
0 10 20 30 40 50 60 70 80 90 100
[Chickpea contig p...
[root@localhost: /...
[FPC V8.9 Main Menu]
Project cp05T5e12...
FPC Ctg4893 cp05...

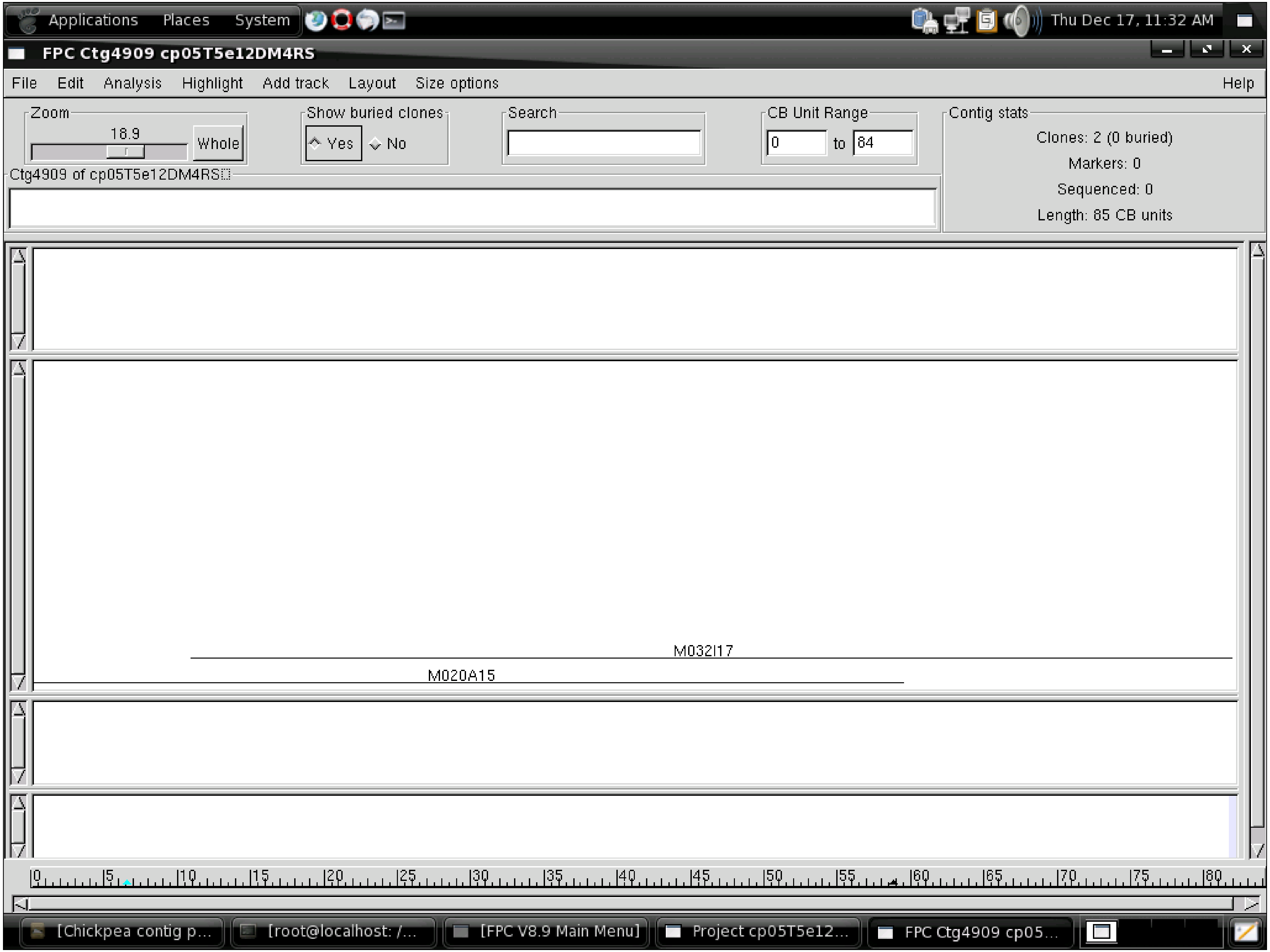
Applications
Places
System
Thu Dec 17, 11:32 AM
FPC Ctg4909 cp05T5e12DM4RS
File
Edit
Analysis
Highlight
Add track
Layout
Size options
Help
Zoom
18.9
Whole
Show buried clones
Yes
No
Search
CB Unit Range
0
to
84
Contig stats
Clones: 2 (0 buried)
Markers: 0
Sequenced: 0
Length: 85 CB units
Ctg4909 of cp05T5e12DM4RS
M032I17
M020A15
0 5 10 15 20 25 30 35 40 45 50 55 60 65 70 75 80
[Chickpea contig p...
[root@localhost: /...
[FPC V8.9 Main Menu]
Project cp05T5e12...
FPC Ctg4909 cp05...

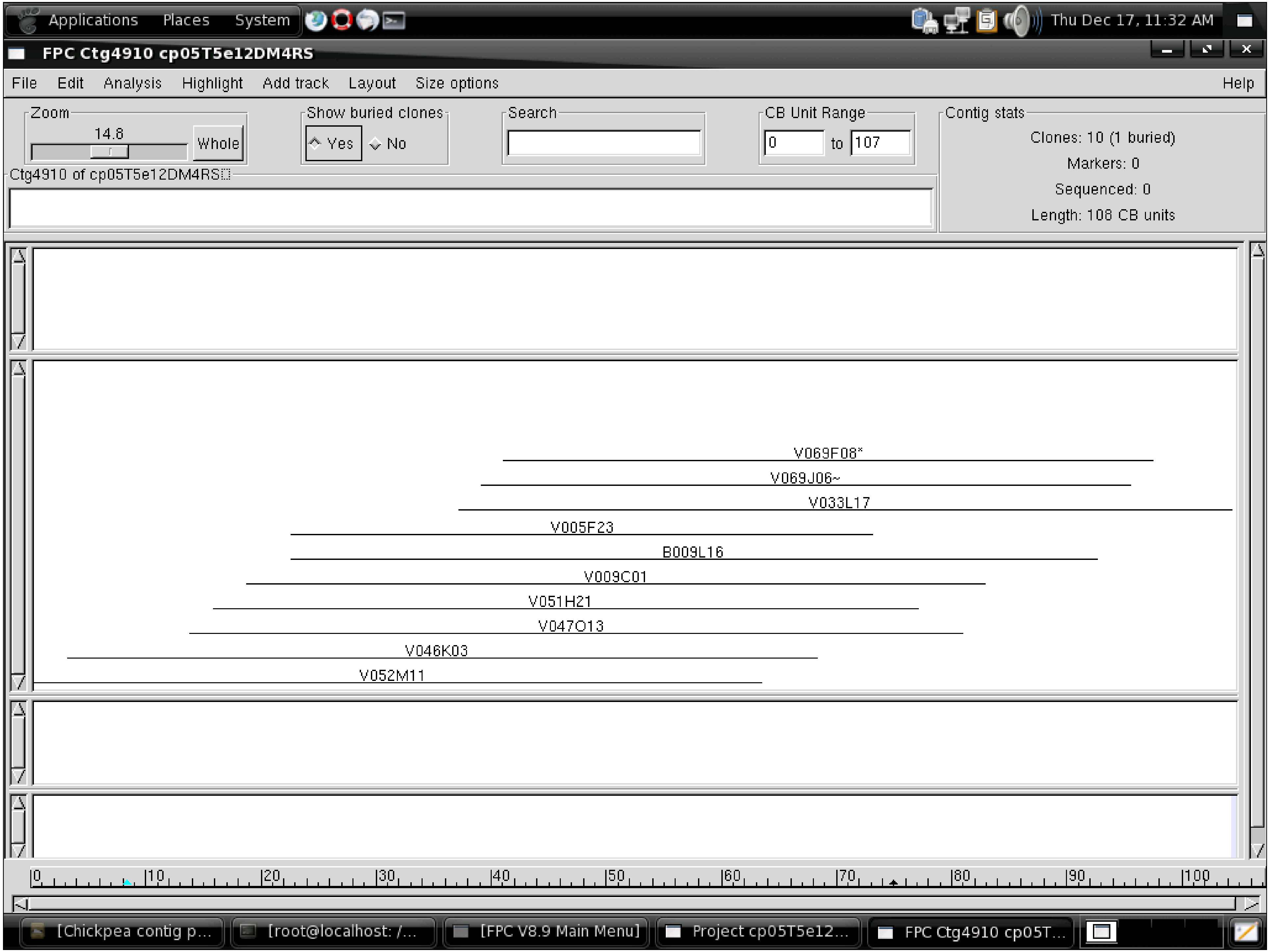
Applications
Places
System
Thu Dec 17, 11:32 AM
FPC Ctg4910 cp05T5e12DM4RS
File
Edit
Analysis
Highlight
Add track
Layout
Size options
Help
Zoom
14.8
Whole
Show buried clones
Yes
No
Search
CB Unit Range
0
to
107
Contig stats
Clones: 10 (1 buried)
Markers: 0
Sequenced: 0
Length: 108 CB units
Ctg4910 of cp05T5e12DM4RS
V069F08*
V069J06~
V033L17
V005F23
B009L16
V009C01
V051H21
V047O13
V046K03
V052M11
0
10
20
30
40
50
60
70
80
90
100
[Chickpea contig p...
[root@localhost: /...
[FPC V8.9 Main Menu]
Project cp05T5e12...
FPC Ctg4910 cp05T...

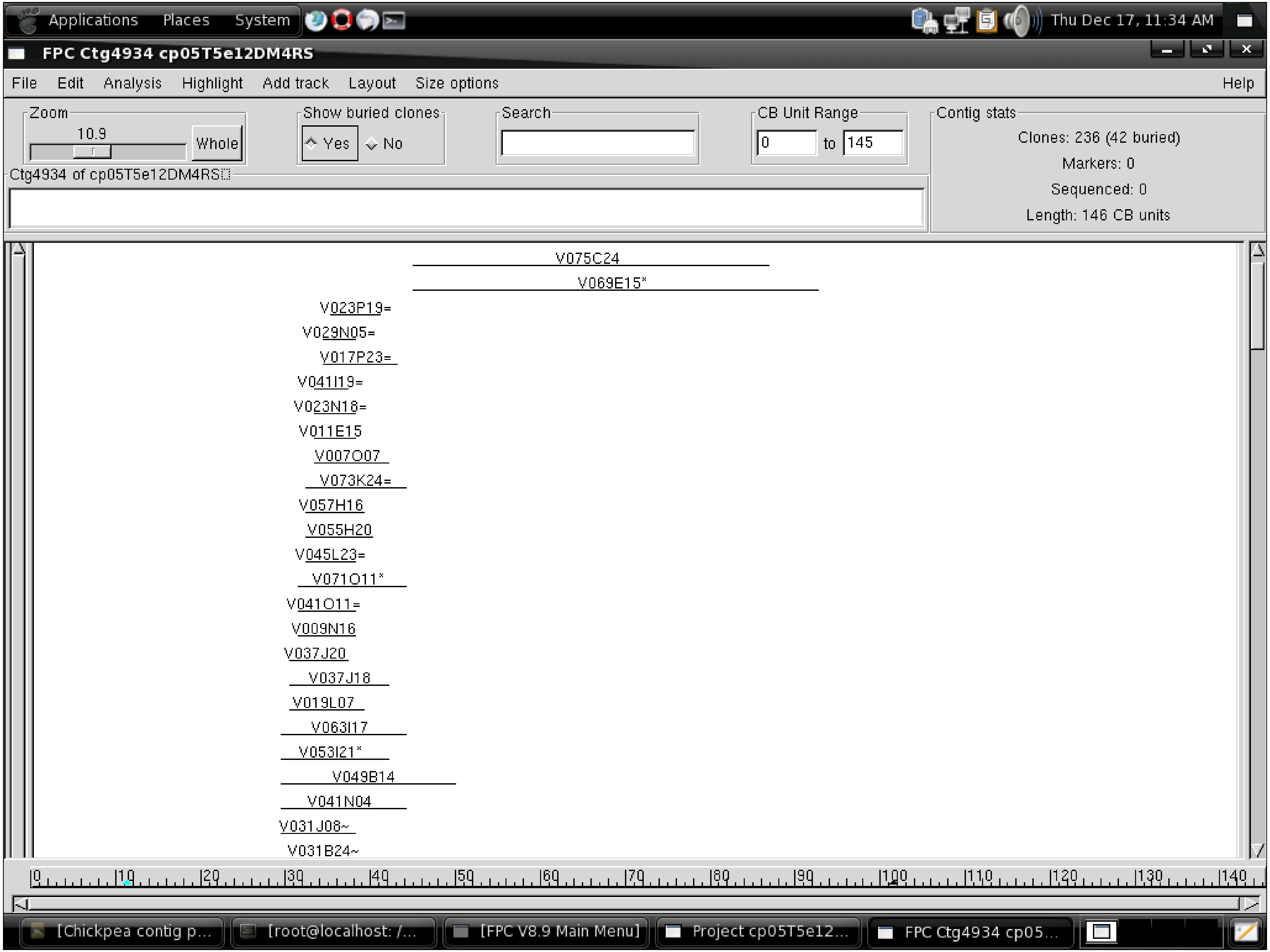
Applications Places System
Thu Dec 17, 11:34 AM
FPC Ctg4934 cp05T5e12DM4RS
File Edit Analysis Highlight Add track Layout Size options Help
Zoom
10.9
Whole
Show buried clones
Yes No
Search
CB Unit Range
0 to 145
Contig stats
Clones: 236 (42 buried)
Markers: 0
Sequenced: 0
Length: 146 CB units
Ctg4934 of cp05T5e12DM4RS
V075C24
V069E15*
V023P19=
V029N05=
V017P23=
V041I19=
V023N18=
V011E15
V007O07
V073K24=
V057H16
V055H20
V045L23=
V071O11*
V041O11=
V009N16
V037J20
V037J18
V019L07
V063I17
V053I21*
V049B14
V041N04
V031J08~
V031B24~
0 10 20 30 40 50 60 70 80 90 100 110 120 130 140
[Chickpea contig p...
[root@localhost: /...
[FPC V8.9 Main Menu]
Project cp05T5e12...
FPC Ctg4934 cp05...

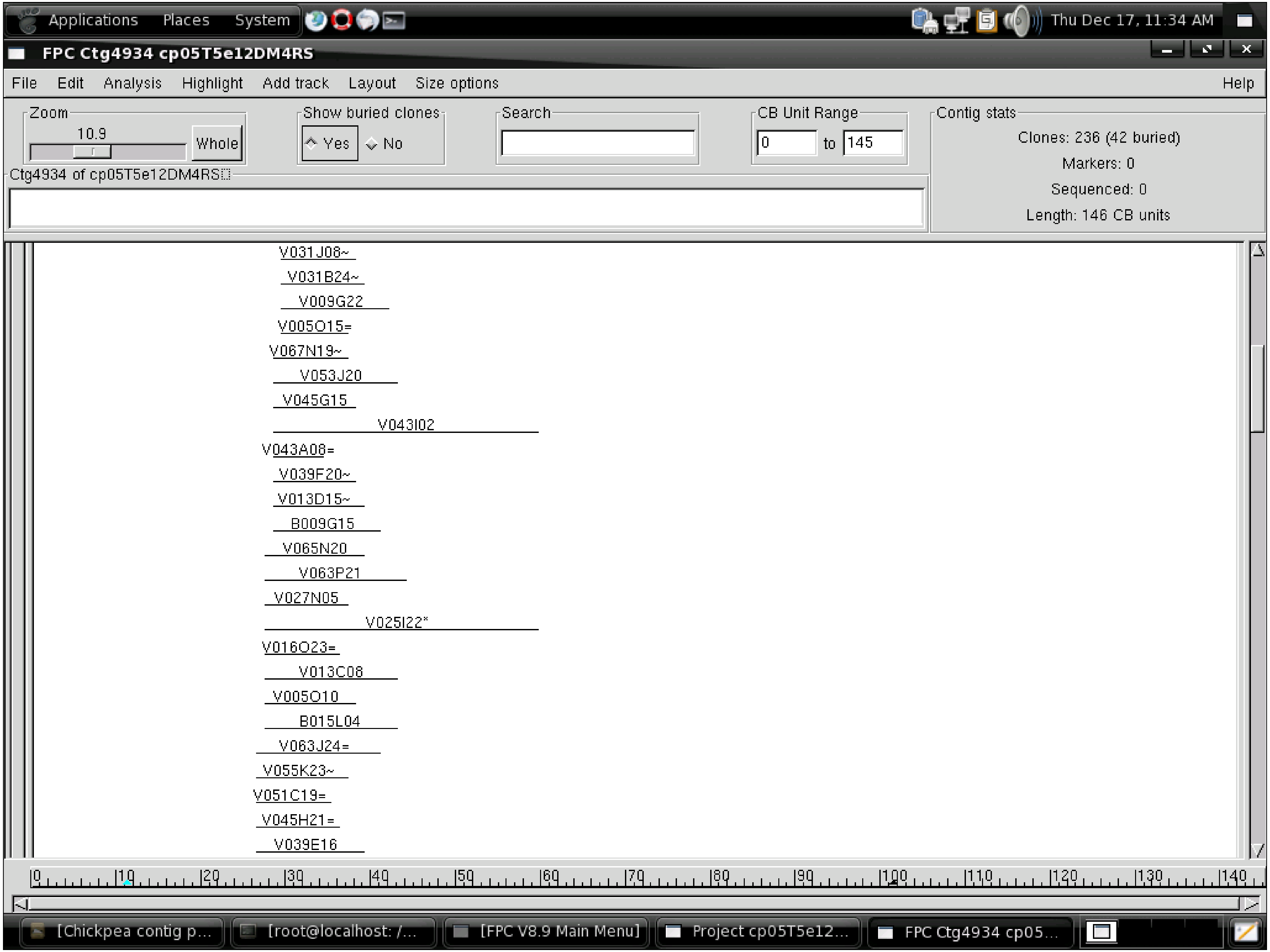
Applications Places System
Thu Dec 17, 11:34 AM
FPC Ctg4934 cp05T5e12DM4RS
File Edit Analysis Highlight Add track Layout Size options
Help
Zoom
10.9
Whole
Show buried clones
Yes
No
Search
CB Unit Range
0
to
145
Contig stats
Clones: 236 (42 buried)
Markers: 0
Sequenced: 0
Length: 146 CB units
Ctg4934 of cp05T5e12DM4RS
V031J08~
V031B24~
V009G22
V005O15=
V067N19~
V053J20
V045G15
V043I02
V043A08=
V039F20~
V013D15~
B009G15
V065N20
V063P21
V027N05
V025I22*
V016O23=
V013C08
V005O10
B015L04
V063J24=
V055K23~
V051C19=
V045H21=
V039E16
0 10 20 30 40 50 60 70 80 90 100 110 120 130 140
[Chickpea contig p...
[root@localhost: /...
[FPC V8.9 Main Menu]
Project cp05T5e12...
FPC Ctg4934 cp05...

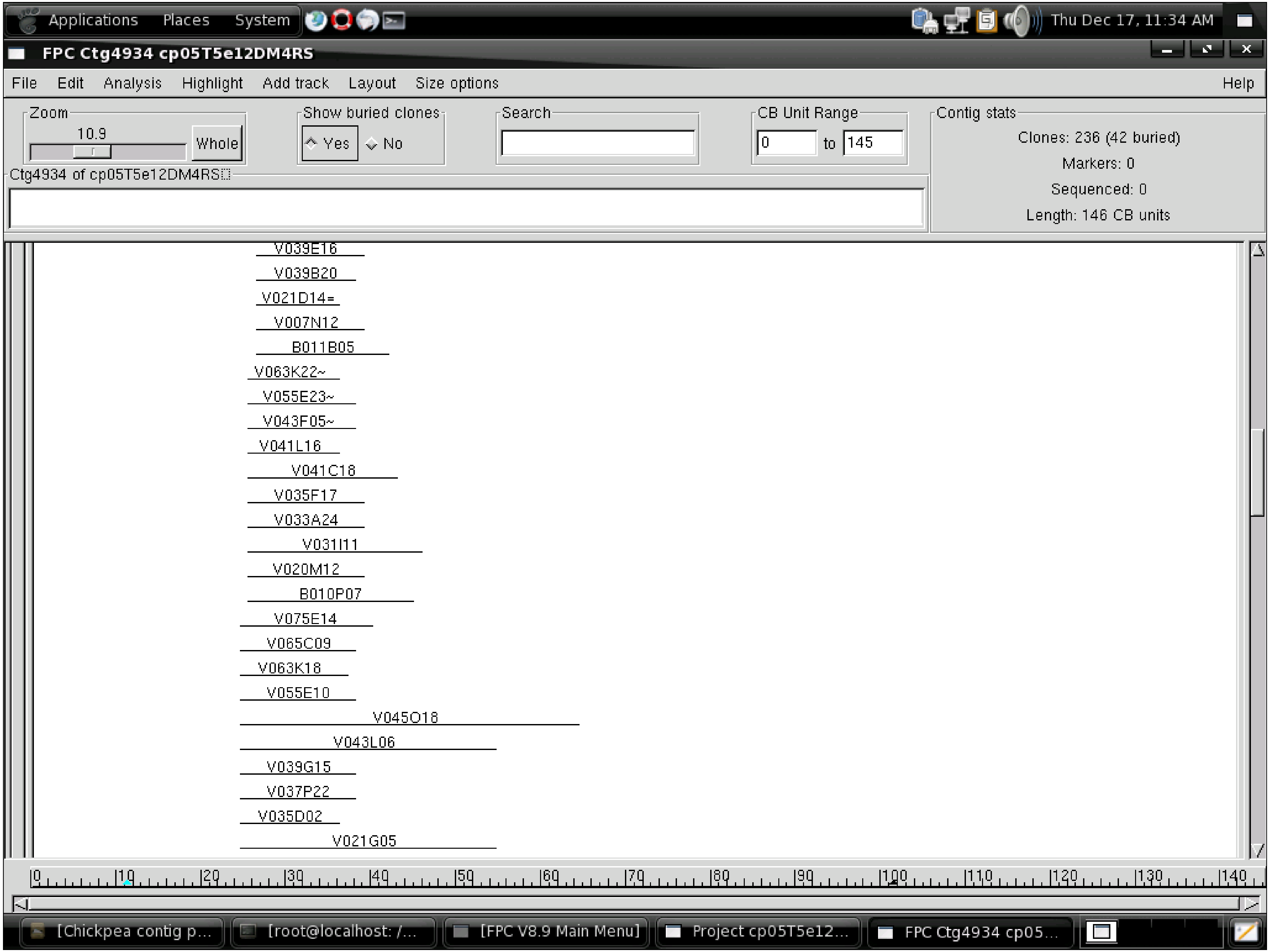
Applications Places System
Thu Dec 17, 11:34 AM
FPC Ctg4934 cp05T5e12DM4RS
File Edit Analysis Highlight Add track Layout Size options Help
Zoom
10.9
Whole
Show buried clones
Yes No
Search
CB Unit Range
0 to 145
Contig stats
Clones: 236 (42 buried)
Markers: 0
Sequenced: 0
Length: 146 CB units
Ctg4934 of cp05T5e12DM4RS
V039E16
V039B20
V021D14=
V007N12
B011B05
V063K22~
V055E23~
V043F05~
V041L16
V041C18
V035F17
V033A24
V031I11
V020M12
B010P07
V075E14
V065C09
V063K18
V055E10
V045O18
V043L06
V039G15
V037P22
V035D02
V021G05
0 10 20 30 40 50 60 70 80 90 100 110 120 130 140
[Chickpea contig p...
[root@localhost: /...
[FPC V8.9 Main Menu]
Project cp05T5e12...
FPC Ctg4934 cp05...

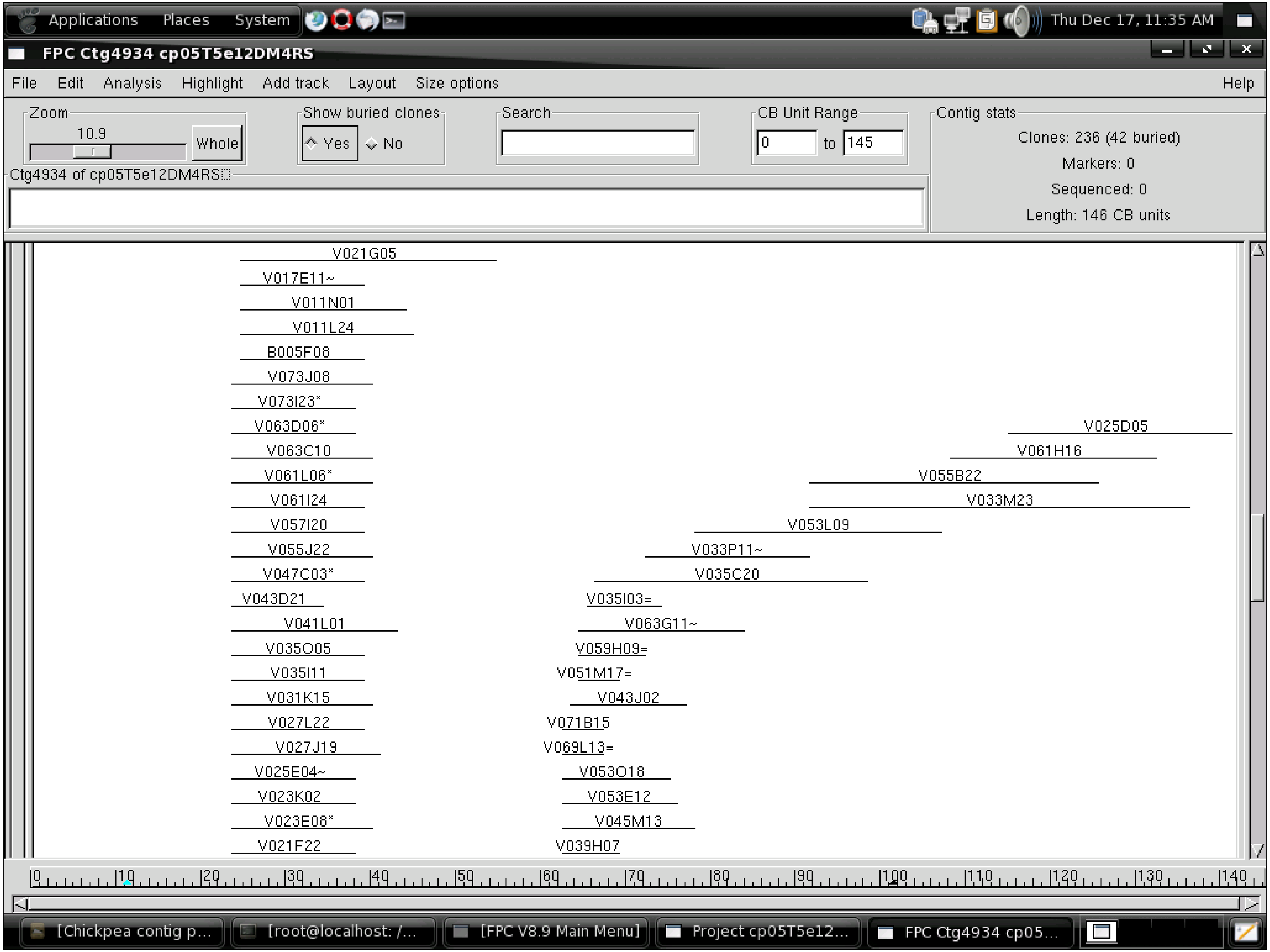
Applications Places System
Thu Dec 17, 11:35 AM
FPC Ctg4934 cp05T5e12DM4RS
File Edit Analysis Highlight Add track Layout Size options Help
Zoom
10.9
Whole
Show buried clones
Yes No
Search
CB Unit Range
0 to 145
Contig stats
Clones: 236 (42 buried)
Markers: 0
Sequenced: 0
Length: 146 CB units
Ctg4934 of cp05T5e12DM4RS
V021G05
V017E11~
V011N01
V011L24
B005F08
V073J08
V073I23*
V063D06*
V063C10
V061L06*
V061I24
V057I20
V055J22
V047C03*
V043D21
V041L01
V035O05
V035I11
V031K15
V027L22
V027J19
V025E04~
V023K02
V023E08*
V021F22
V025D05
V061H16
V055B22
V033M23
V053L09
V033P11~
V035C20
V035I03=
V063G11~
V059H09=
V051M17=
V043J02
V071B15
V069L13=
V053O18
V053E12
V045M13
V039H07
0 10 20 30 40 50 60 70 80 90 100 110 120 130 140
[Chickpea contig p...
[root@localhost: /...
[FPC V8.9 Main Menu]
Project cp05T5e12...
FPC Ctg4934 cp05...

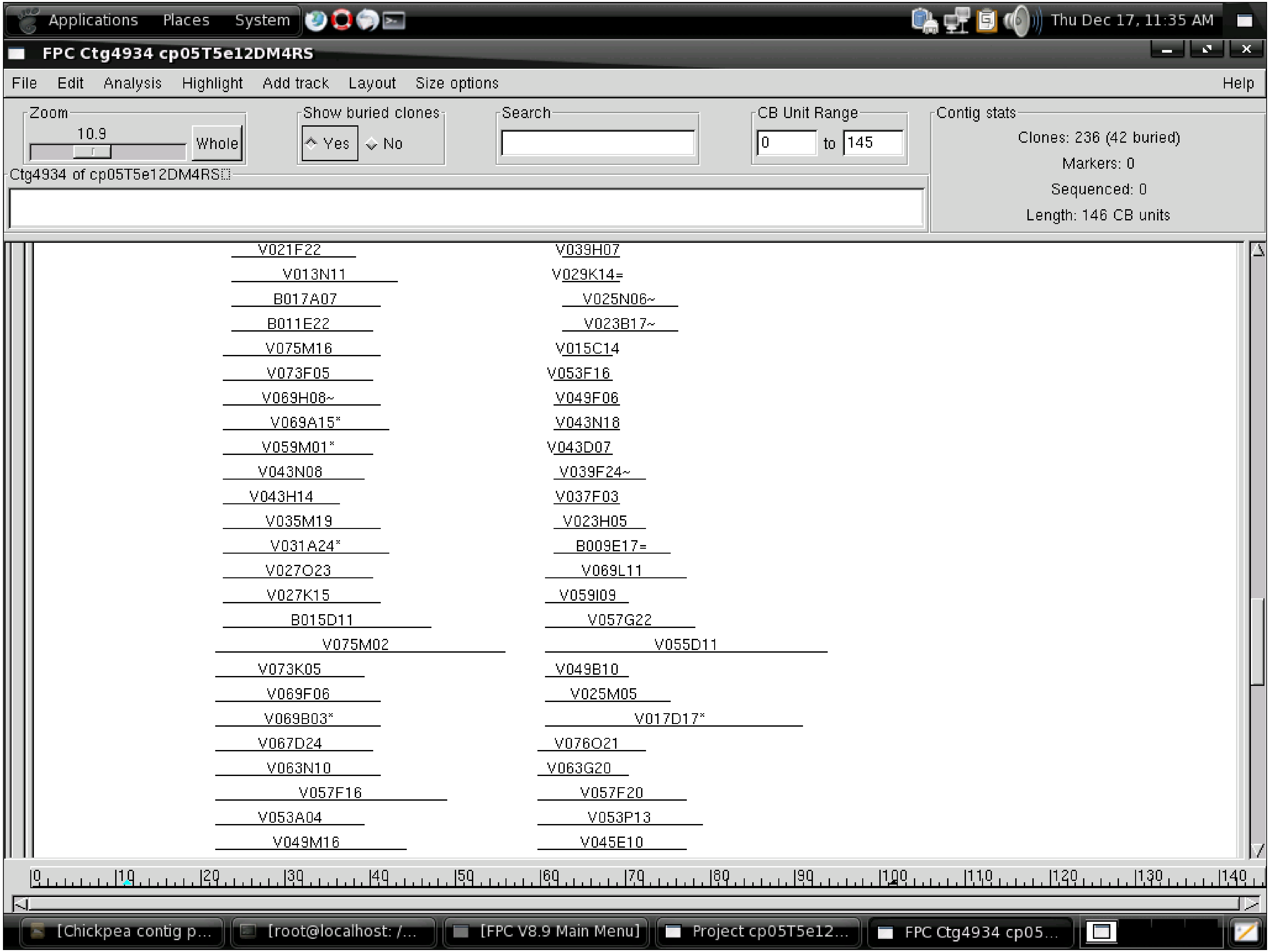
Applications Places System
Thu Dec 17, 11:35 AM
FPC Ctg4934 cp05T5e12DM4RS
File Edit Analysis Highlight Add track Layout Size options Help
Zoom
10.9
Whole
Show buried clones
Yes No
Search
CB Unit Range
0 to 145
Contig stats
Clones: 236 (42 buried)
Markers: 0
Sequenced: 0
Length: 146 CB units
Ctg4934 of cp05T5e12DM4RS
V021F22
V013N11
B017A07
B011E22
V075M16
V073F05
V069H08~
V069A15*
V059M01*
V043N08
V043H14
V035M19
V031A24*
V027O23
V027K15
B015D11
V075M02
V073K05
V069F06
V069B03*
V067D24
V063N10
V057F16
V053A04
V049M16
V039H07
V029K14=
V025N06~
V023B17~
V015C14
V053F16
V049F06
V043N18
V043D07
V039F24~
V037F03
V023H05
B009E17=
V069L11
V059I09
V057G22
V055D11
V049B10
V025M05
V017D17*
V076O21
V063G20
V057F20
V053P13
V045E10
0 10 20 30 40 50 60 70 80 90 100 110 120 130 140
[Chickpea contig p...
[root@localhost: /...
[FPC V8.9 Main Menu]
Project cp05T5e12...
FPC Ctg4934 cp05...

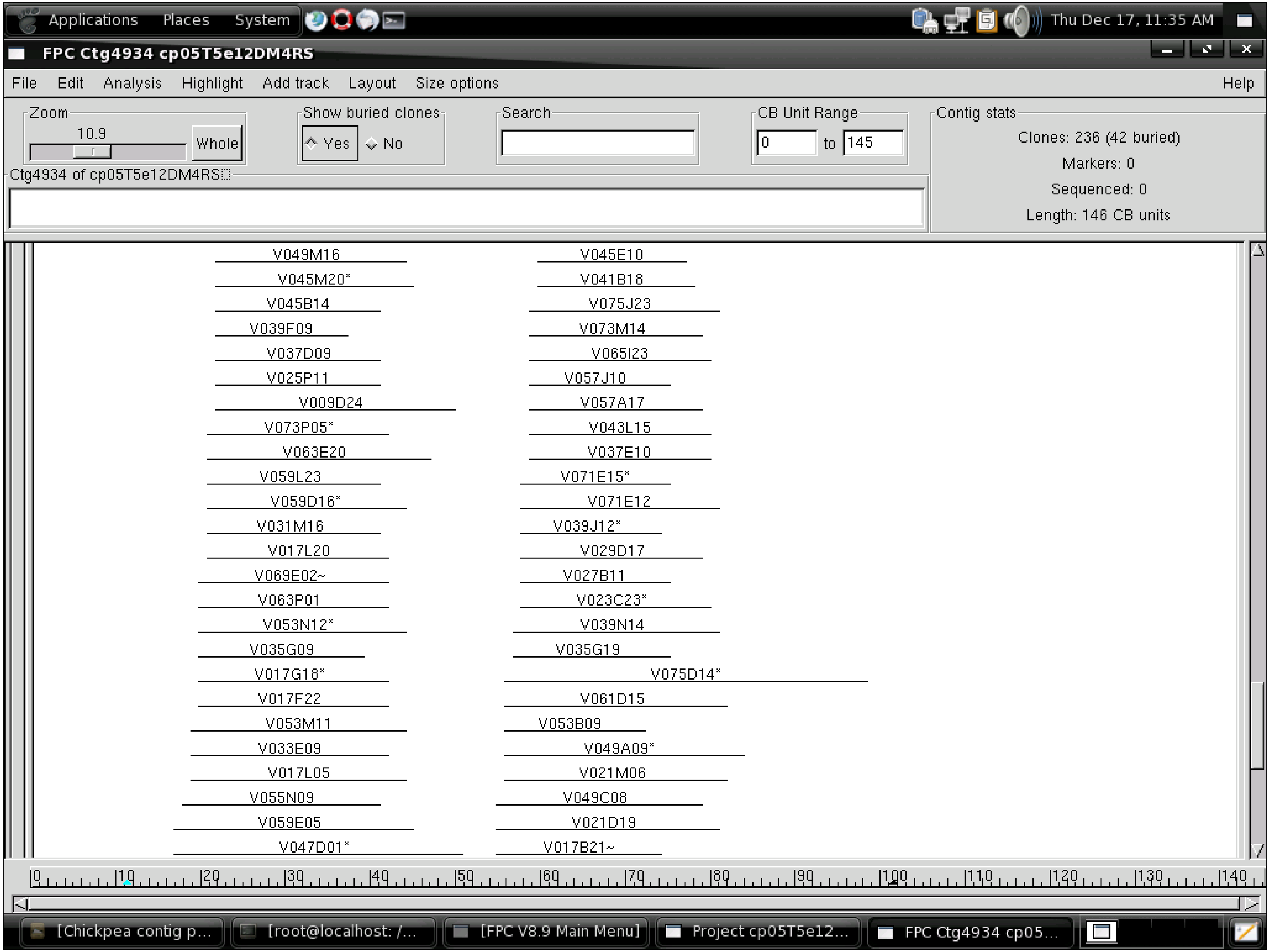
Applications Places System
Thu Dec 17, 11:35 AM
FPC Ctg4934 cp05T5e12DM4RS
File Edit Analysis Highlight Add track Layout Size options Help
Zoom
10.9
Whole
Show buried clones
Yes No
Search
CB Unit Range
0 to 145
Contig stats
Clones: 236 (42 buried)
Markers: 0
Sequenced: 0
Length: 146 CB units
Ctg4934 of cp05T5e12DM4RS
V049M16
V045M20*
V045B14
V039F09
V037D09
V025P11
V009D24
V073P05*
V063E20
V059L23
V059D16*
V031M16
V017L20
V069E02~
V063P01
V053N12*
V035G09
V017G18*
V017F22
V053M11
V033E09
V017L05
V055N09
V059E05
V047D01*
V045E10
V041B18
V075J23
V073M14
V065I23
V057J10
V057A17
V043L15
V037E10
V071E15*
V071E12
V039J12*
V029D17
V027B11
V023C23*
V039N14
V035G19
V075D14*
V061D15
V053B09
V049A09*
V021M06
V049C08
V021D19
V017B21~
0 10 20 30 40 50 60 70 80 90 100 110 120 130 140
[Chickpea contig p...
[root@localhost: /...
[FPC V8.9 Main Menu]
Project cp05T5e12...
FPC Ctg4934 cp05...

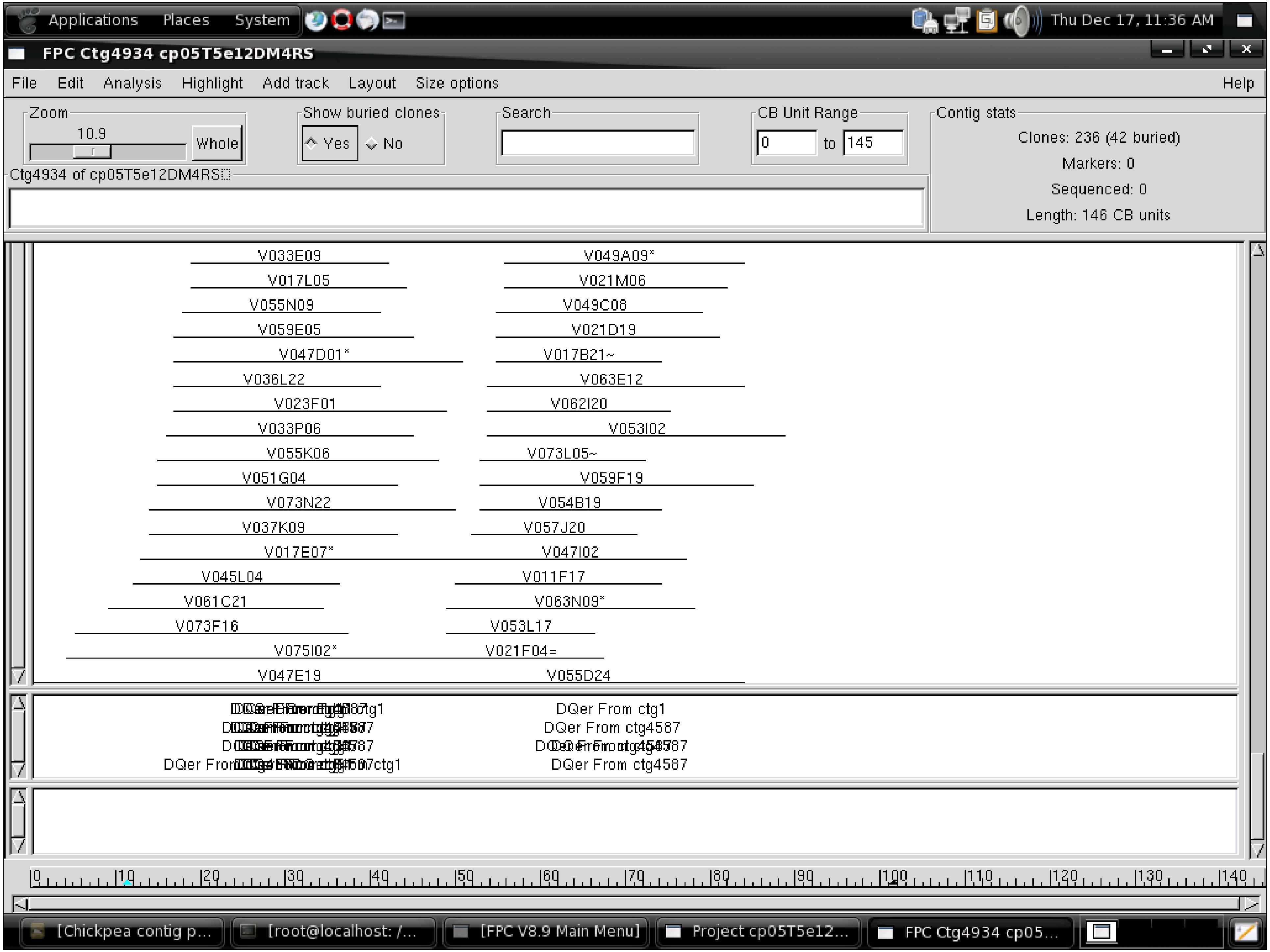
Applications Places System
Thu Dec 17, 11:36 AM
FPC Ctg4934 cp05T5e12DM4RS
File Edit Analysis Highlight Add track Layout Size options Help
Zoom
10.9
Whole
Show buried clones
Yes No
Search
CB Unit Range
0 to 145
Contig stats
Clones: 236 (42 buried)
Markers: 0
Sequenced: 0
Length: 146 CB units
Ctg4934 of cp05T5e12DM4RS
V033E09
V049A09*
V017L05
V021M06
V055N09
V049C08
V059E05
V021D19
V047D01*
V017B21~
V036L22
V063E12
V023F01
V062I20
V033P06
V053I02
V055K06
V073L05~
V051G04
V059F19
V073N22
V054B19
V037K09
V057J20
V017E07*
V047I02
V045L04
V011F17
V061C21
V063N09*
V073F16
V053L17
V075I02*
V021F04=
V047E19
V055D24
DQer From ctg1
DQer From ctg4587
DQer From ctg4587
DQer From ctg4587
0 10 20 30 40 50 60 70 80 90 100 110 120 130 140
[Chickpea contig p...
[root@localhost: /...
[FPC V8.9 Main Menu]
Project cp05T5e12...
FPC Ctg4934 cp05...

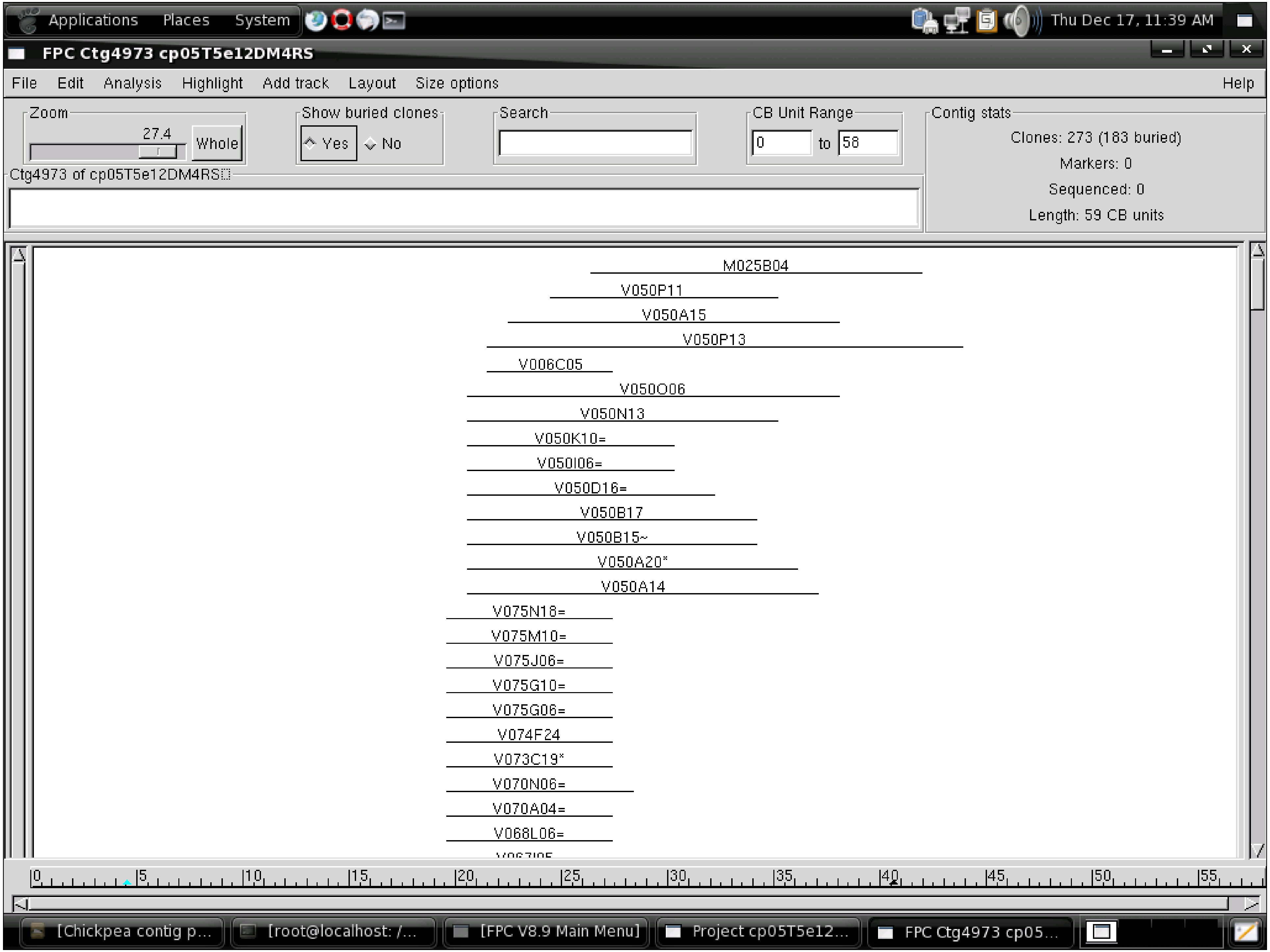
Applications Places System
Thu Dec 17, 11:39 AM
FPC Ctg4973 cp05T5e12DM4RS
File Edit Analysis Highlight Add track Layout Size options Help
Zoom
27.4
Whole
Show buried clones
Yes No
Search
CB Unit Range
0 to 58
Contig stats
Clones: 273 (183 buried)
Markers: 0
Sequenced: 0
Length: 59 CB units
Ctg4973 of cp05T5e12DM4RS
M025B04
V050P11
V050A15
V050P13
V006C05
V050O06
V050N13
V050K10=
V050I06=
V050D16=
V050B17
V050B15~
V050A20*
V050A14
V075N18=
V075M10=
V075J06=
V075G10=
V075G06=
V074F24
V073C19*
V070N06=
V070A04=
V068L06=
0 5 10 15 20 25 30 35 40 45 50 55
[Chickpea contig p...
[root@localhost: /...
[FPC V8.9 Main Menu]
Project cp05T5e12...
FPC Ctg4973 cp05...

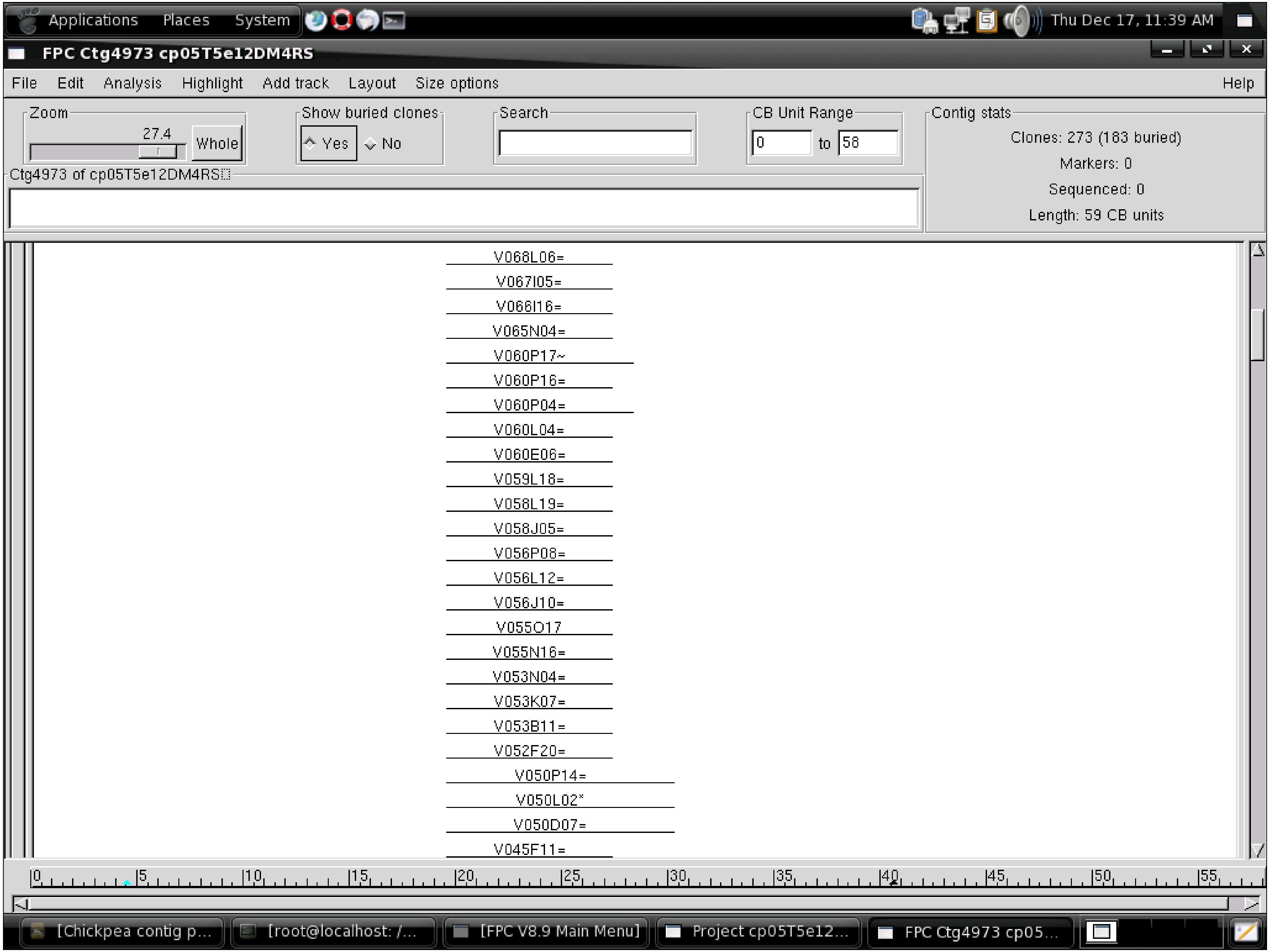
Applications Places System
Thu Dec 17, 11:39 AM
FPC Ctg4973 cp05T5e12DM4RS
File Edit Analysis Highlight Add track Layout Size options Help
Zoom
27.4
Whole
Show buried clones
Yes No
Search
CB Unit Range
0 to 58
Contig stats
Clones: 273 (183 buried)
Markers: 0
Sequenced: 0
Length: 59 CB units
Ctg4973 of cp05T5e12DM4RS
V068L06=
V067I05=
V066I16=
V065N04=
V060P17~
V060P16=
V060P04=
V060L04=
V060E06=
V059L18=
V058L19=
V058J05=
V056P08=
V056L12=
V056J10=
V055O17
V055N16=
V053N04=
V053K07=
V053B11=
V052F20=
V050P14=
V050L02*
V050D07=
V045F11=
0 5 10 15 20 25 30 35 40 45 50 55
[Chickpea contig p...
[root@localhost: /...
[FPC V8.9 Main Menu]
Project cp05T5e12...
FPC Ctg4973 cp05...

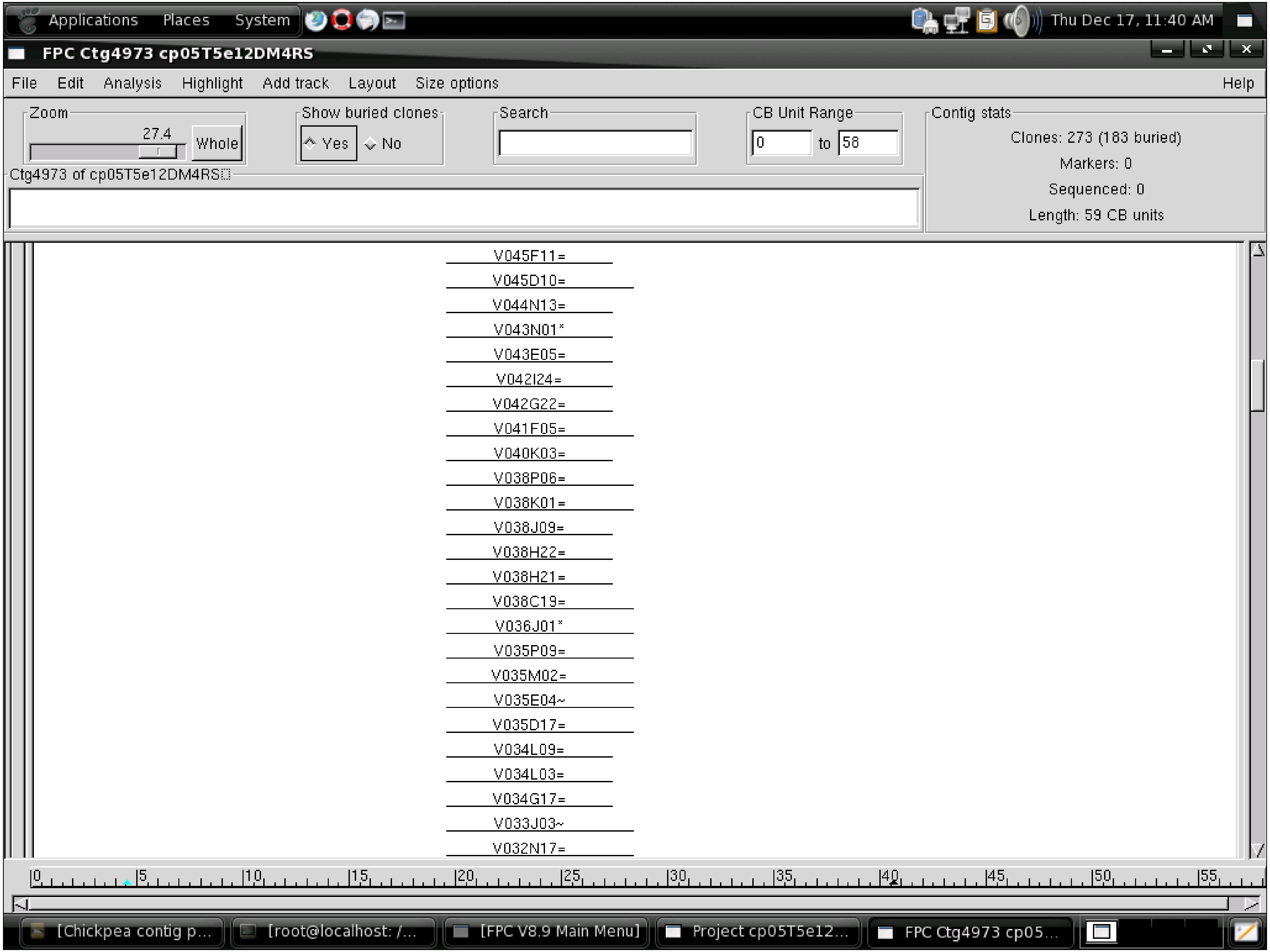
Applications Places System
Thu Dec 17, 11:40 AM
FPC Ctg4973 cp05T5e12DM4RS
File Edit Analysis Highlight Add track Layout Size options Help
Zoom
27.4
Whole
Show buried clones
Yes No
Search
CB Unit Range
0 to 58
Contig stats
Clones: 273 (183 buried)
Markers: 0
Sequenced: 0
Length: 59 CB units
Ctg4973 of cp05T5e12DM4RS
V045F11=
V045D10=
V044N13=
V043N01*
V043E05=
V042I24=
V042G22=
V041F05=
V040K03=
V038P06=
V038K01=
V038J09=
V038H22=
V038H21=
V038C19=
V036J01*
V035P09=
V035M02=
V035E04~
V035D17=
V034L09=
V034L03=
V034G17=
V033J03~
V032N17=
0 5 10 15 20 25 30 35 40 45 50 55
[Chickpea contig p...
[root@localhost: /...
[FPC V8.9 Main Menu]
Project cp05T5e12...
FPC Ctg4973 cp05...

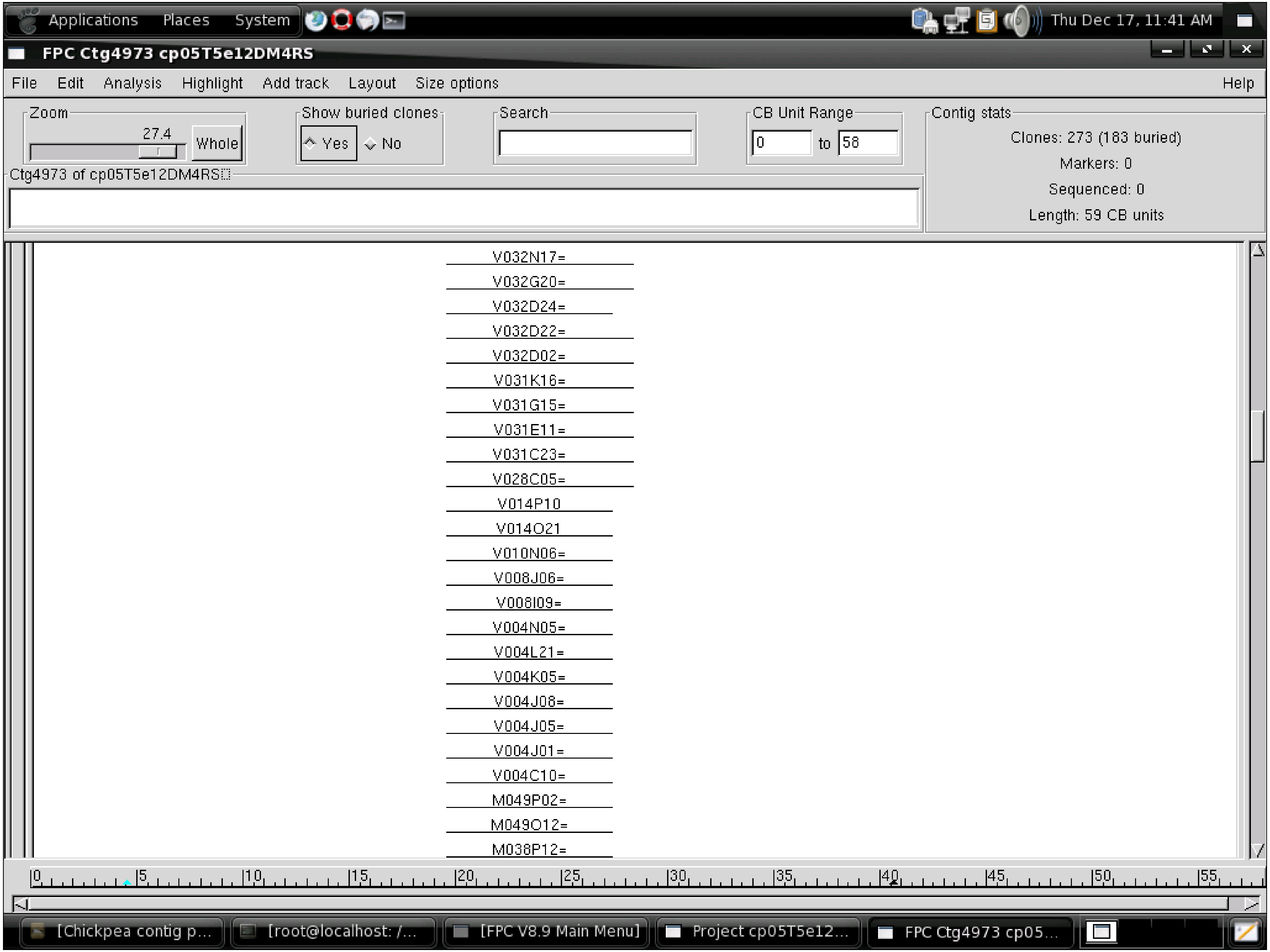
Applications Places System
Thu Dec 17, 11:41 AM
FPC Ctg4973 cp05T5e12DM4RS
File Edit Analysis Highlight Add track Layout Size options Help
Zoom
27.4
Whole
Show buried clones
Yes No
Search
CB Unit Range
0 to 58
Contig stats
Clones: 273 (183 buried)
Markers: 0
Sequenced: 0
Length: 59 CB units
Ctg4973 of cp05T5e12DM4RS
V032N17=
V032G20=
V032D24=
V032D22=
V032D02=
V031K16=
V031G15=
V031E11=
V031C23=
V028C05=
V014P10
V014O21
V010N06=
V008J06=
V008I09=
V004N05=
V004L21=
V004K05=
V004J08=
V004J05=
V004J01=
V004C10=
M049P02=
M049O12=
M038P12=
0 5 10 15 20 25 30 35 40 45 50 55
[Chickpea contig p...
[root@localhost: /...
[FPC V8.9 Main Menu]
Project cp05T5e12...
FPC Ctg4973 cp05...

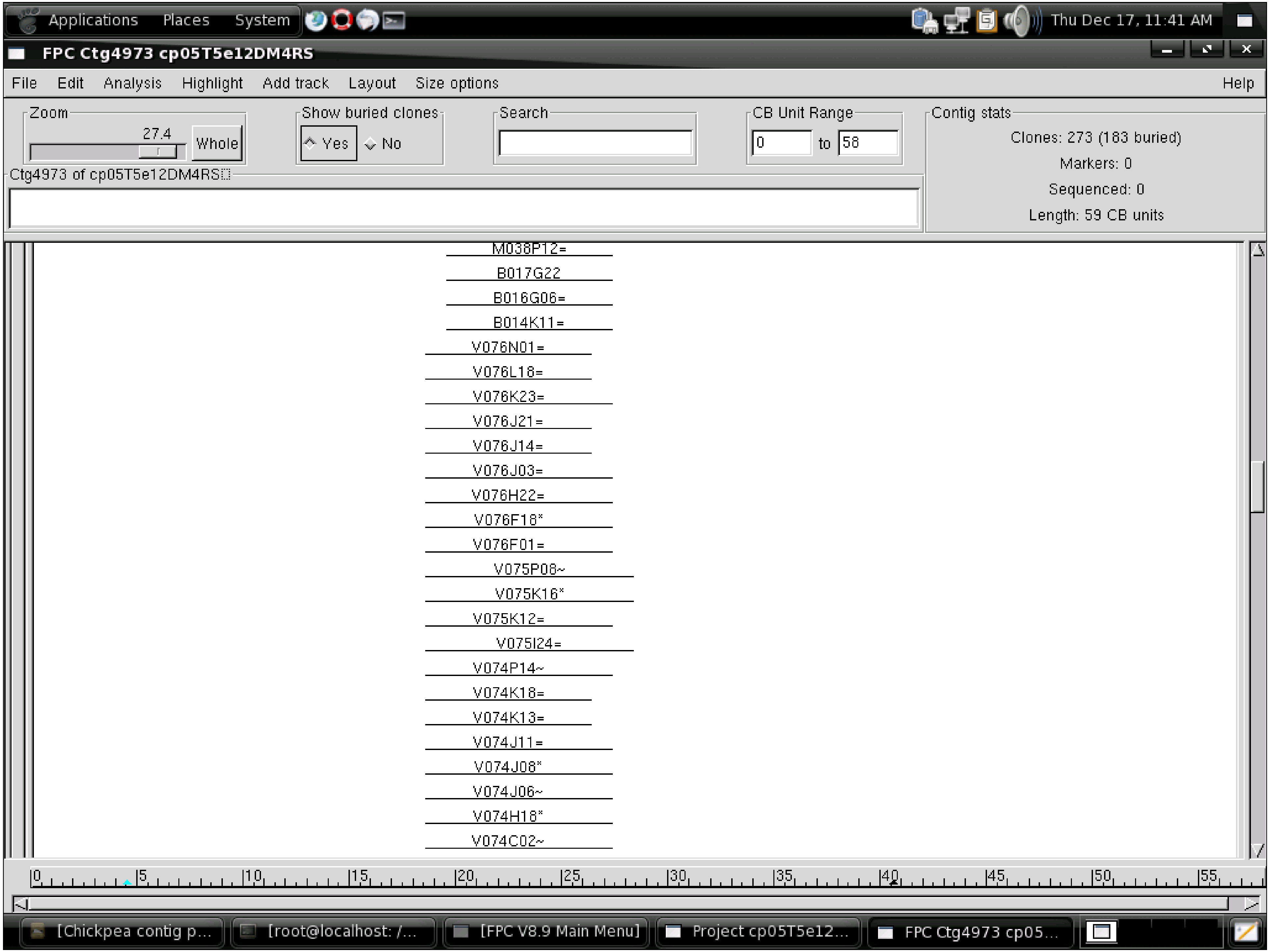
Applications Places System
Thu Dec 17, 11:41 AM
FPC Ctg4973 cp05T5e12DM4RS
File Edit Analysis Highlight Add track Layout Size options Help
Zoom
27.4
Whole
Show buried clones
Yes No
Search
CB Unit Range
0 to 58
Contig stats
Clones: 273 (183 buried)
Markers: 0
Sequenced: 0
Length: 59 CB units
Ctg4973 of cp05T5e12DM4RS
M038P12=
B017G22
B016G06=
B014K11=
V076N01=
V076L18=
V076K23=
V076J21=
V076J14=
V076J03=
V076H22=
V076F18*
V076F01=
V075P08~
V075K16*
V075K12=
V075I24=
V074P14~
V074K18=
V074K13=
V074J11=
V074J08*
V074J06~
V074H18*
V074C02~
0 5 10 15 20 25 30 35 40 45 50 55
[Chickpea contig p...
[root@localhost: /...
[FPC V8.9 Main Menu]
Project cp05T5e12...
FPC Ctg4973 cp05...

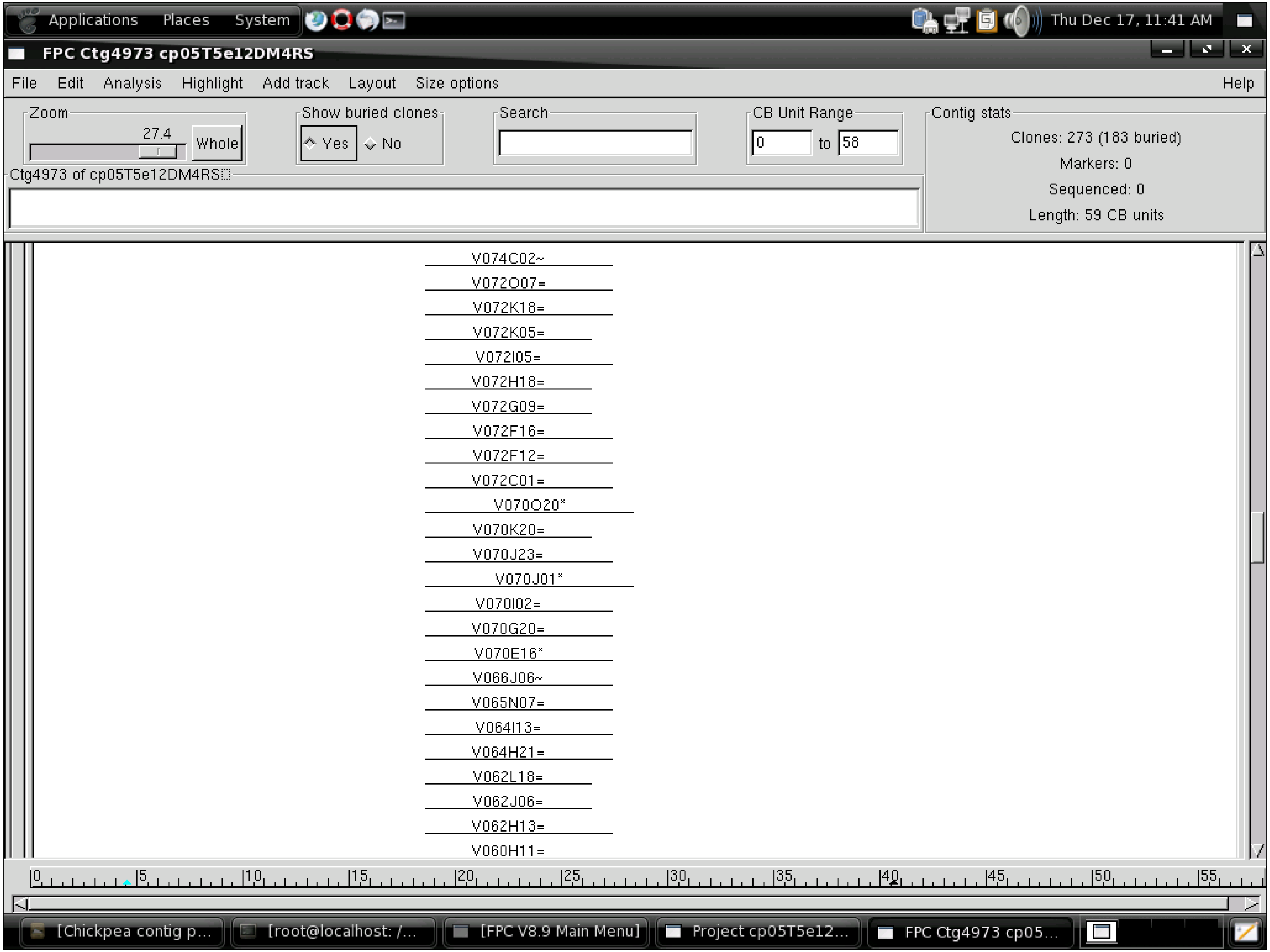
Applications Places System
Thu Dec 17, 11:41 AM
FPC Ctg4973 cp05T5e12DM4RS
File Edit Analysis Highlight Add track Layout Size options Help
Zoom
27.4
Whole
Show buried clones
Yes No
Search
CB Unit Range
0 to 58
Contig stats
Clones: 273 (183 buried)
Markers: 0
Sequenced: 0
Length: 59 CB units
Ctg4973 of cp05T5e12DM4RS
V074C02~
V072O07=
V072K18=
V072K05=
V072I05=
V072H18=
V072G09=
V072F16=
V072F12=
V072C01=
V070O20*
V070K20=
V070J23=
V070J01*
V070I02=
V070G20=
V070E16*
V066J06~
V065N07=
V064I13=
V064H21=
V062L18=
V062J06=
V062H13=
V060H11=
0 5 10 15 20 25 30 35 40 45 50 55
[Chickpea contig p...
[root@localhost: /...
[FPC V8.9 Main Menu]
Project cp05T5e12...
FPC Ctg4973 cp05...

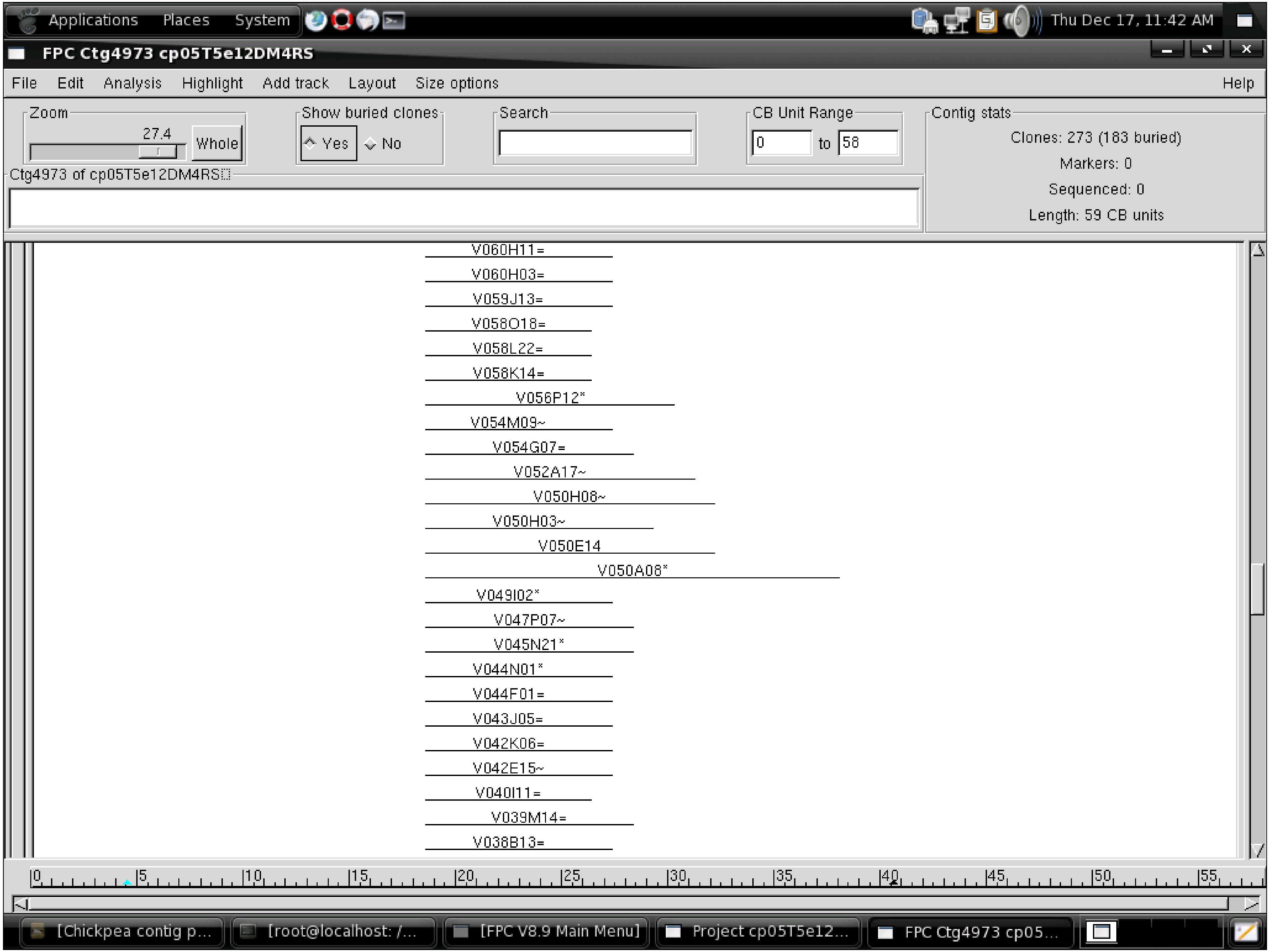
Applications Places System
Thu Dec 17, 11:42 AM
FPC Ctg4973 cp05T5e12DM4RS
File Edit Analysis Highlight Add track Layout Size options Help
Zoom
27.4
Whole
Show buried clones
Yes No
Search
CB Unit Range
0 to 58
Contig stats
Clones: 273 (183 buried)
Markers: 0
Sequenced: 0
Length: 59 CB units
Ctg4973 of cp05T5e12DM4RS
V060H11=
V060H03=
V059J13=
V058O18=
V058L22=
V058K14=
V056P12*
V054M09~
V054G07=
V052A17~
V050H08~
V050H03~
V050E14
V050A08*
V049I02*
V047P07~
V045N21*
V044N01*
V044F01=
V043J05=
V042K06=
V042E15~
V040I11=
V039M14=
V038B13=
0 5 10 15 20 25 30 35 40 45 50 55
[Chickpea contig p...
[root@localhost: /...
[FPC V8.9 Main Menu]
Project cp05T5e12...
FPC Ctg4973 cp05...

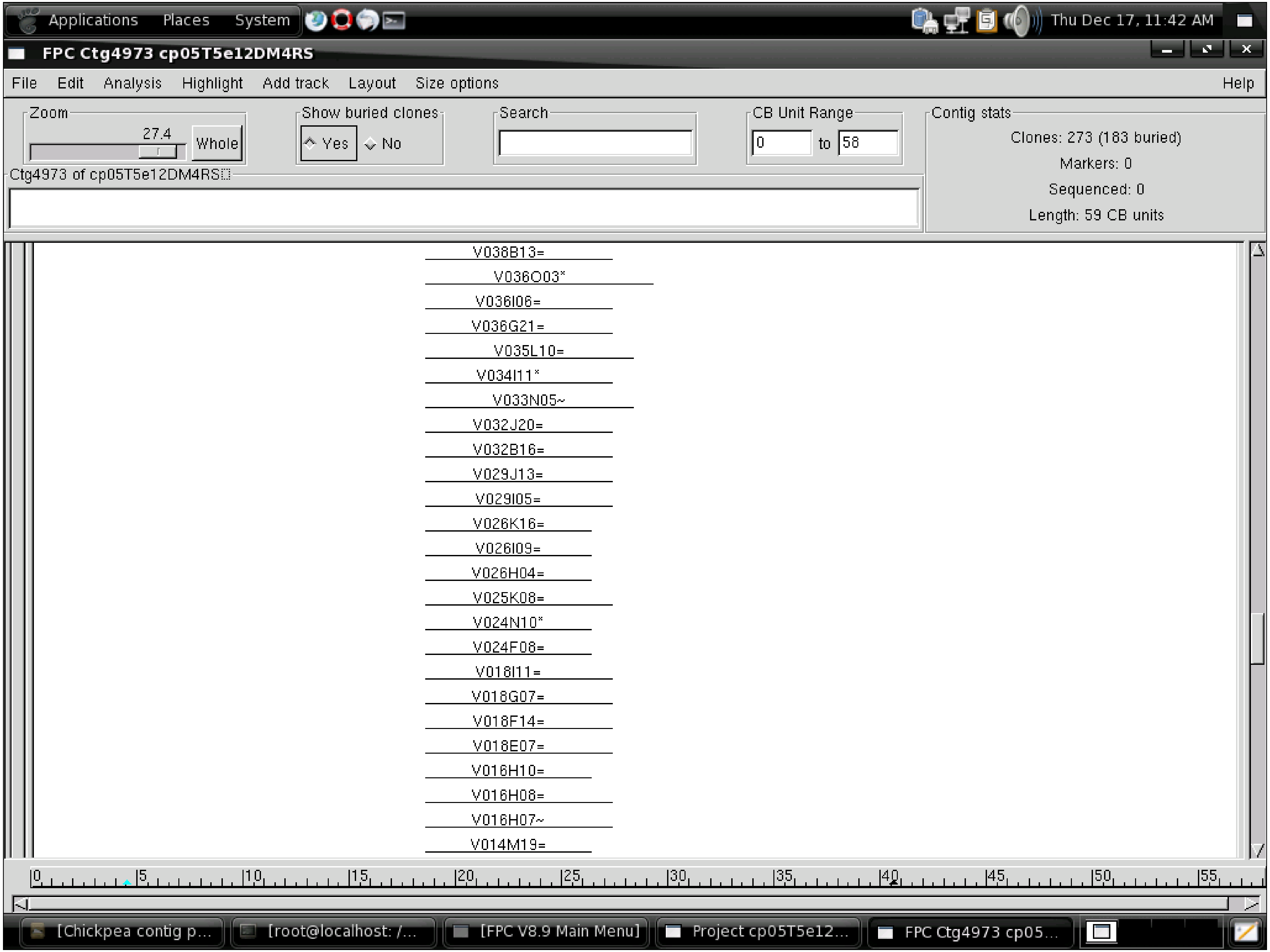
Applications
Places
System
Thu Dec 17, 11:42 AM
FPC Ctg4973 cp05T5e12DM4RS
File
Edit
Analysis
Highlight
Add track
Layout
Size options
Help
Zoom
27.4
Whole
Show buried clones
Yes
No
Search
CB Unit Range
0
to
58
Contig stats
Clones: 273 (183 buried)
Markers: 0
Sequenced: 0
Length: 59 CB units
Ctg4973 of cp05T5e12DM4RS
V038B13=
V036O03*
V036I06=
V036G21=
V035L10=
V034I11*
V033N05~
V032J20=
V032B16=
V029J13=
V029I05=
V026K16=
V026I09=
V026H04=
V025K08=
V024N10*
V024F08=
V018I11=
V018G07=
V018F14=
V018E07=
V016H10=
V016H08=
V016H07~
V014M19=
0
5
10
15
20
25
30
35
40
45
50
55
[Chickpea contig p...
[root@localhost: /...
[FPC V8.9 Main Menu]
Project cp05T5e12...
FPC Ctg4973 cp05...

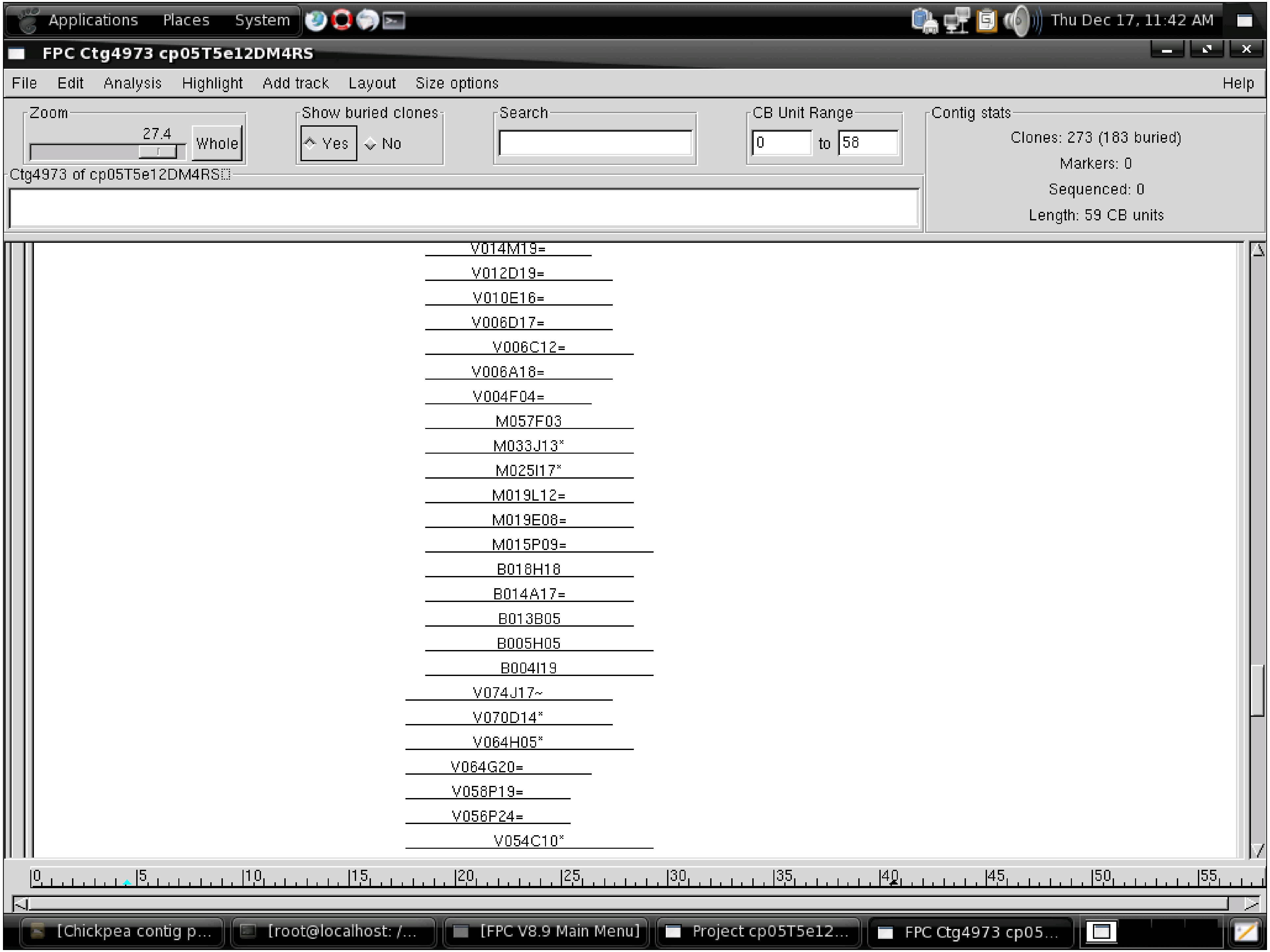
Applications Places System
Thu Dec 17, 11:42 AM
FPC Ctg4973 cp05T5e12DM4RS
File Edit Analysis Highlight Add track Layout Size options Help
Zoom
27.4
Whole
Show buried clones
Yes No
Search
CB Unit Range
0 to 58
Contig stats
Clones: 273 (183 buried)
Markers: 0
Sequenced: 0
Length: 59 CB units
Ctg4973 of cp05T5e12DM4RS
V014M19=
V012D19=
V010E16=
V006D17=
V006C12=
V006A18=
V004F04=
M057F03
M033J13*
M025I17*
M019L12=
M019E08=
M015P09=
B018H18
B014A17=
B013B05
B005H05
B004I19
V074J17~
V070D14*
V064H05*
V064G20=
V058P19=
V056P24=
V054C10*
0 5 10 15 20 25 30 35 40 45 50 55
[Chickpea contig p...
[root@localhost: /...
[FPC V8.9 Main Menu]
Project cp05T5e12...
FPC Ctg4973 cp05...

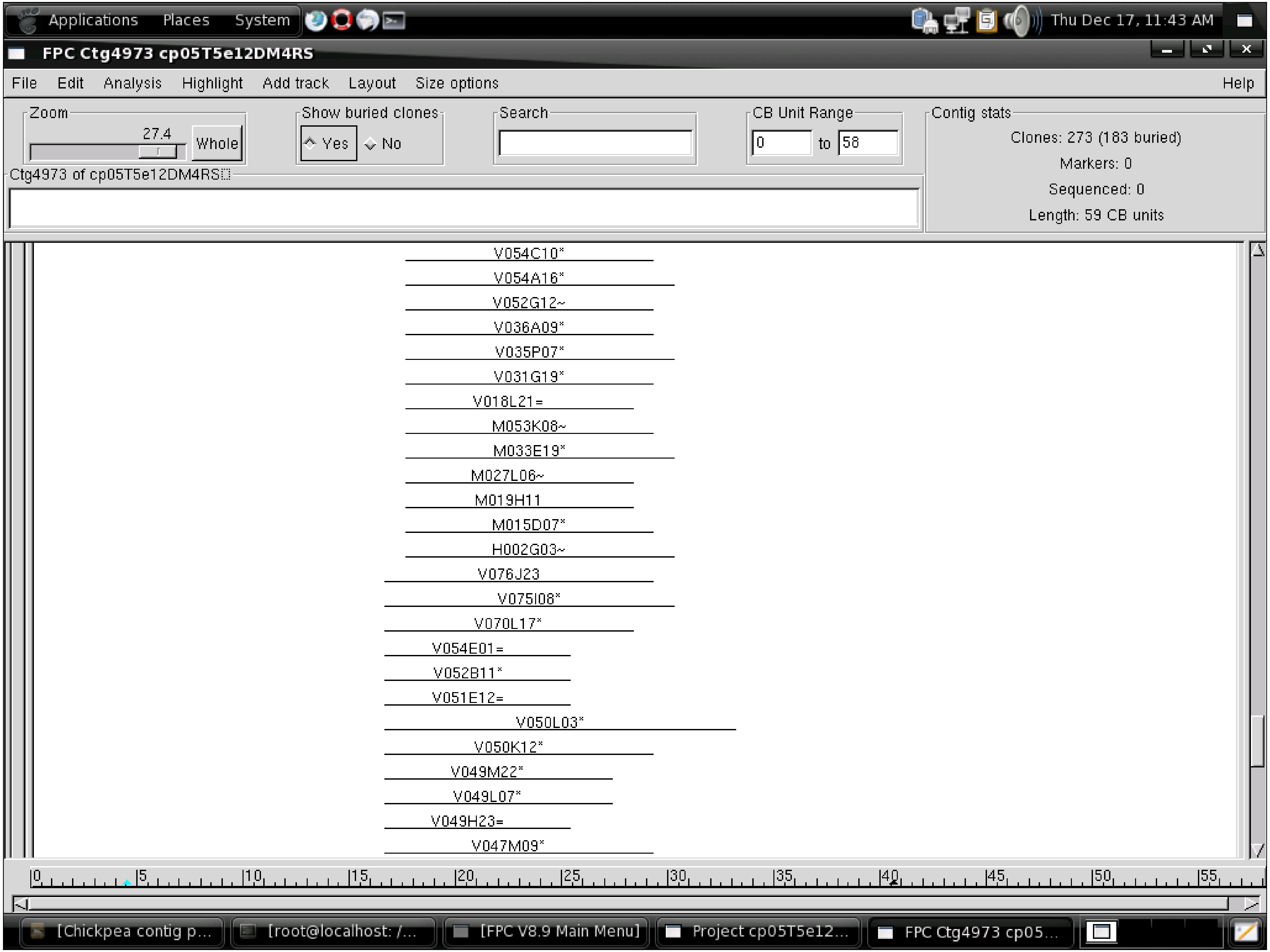
Applications Places System
Thu Dec 17, 11:43 AM
FPC Ctg4973 cp05T5e12DM4RS
File Edit Analysis Highlight Add track Layout Size options Help
Zoom
27.4
Whole
Show buried clones
Yes No
Search
CB Unit Range
0 to 58
Contig stats
Clones: 273 (183 buried)
Markers: 0
Sequenced: 0
Length: 59 CB units
Ctg4973 of cp05T5e12DM4RS
V054C10*
V054A16*
V052G12~
V036A09*
V035P07*
V031G19*
V018L21=
M053K08~
M033E19*
M027L06~
M019H11
M015D07*
H002G03~
V076J23
V075I08*
V070L17*
V054E01=
V052B11*
V051E12=
V050L03*
V050K12*
V049M22*
V049L07*
V049H23=
V047M09*
0 5 10 15 20 25 30 35 40 45 50 55
[Chickpea contig p...
[root@localhost: /...
[FPC V8.9 Main Menu]
Project cp05T5e12...
FPC Ctg4973 cp05...

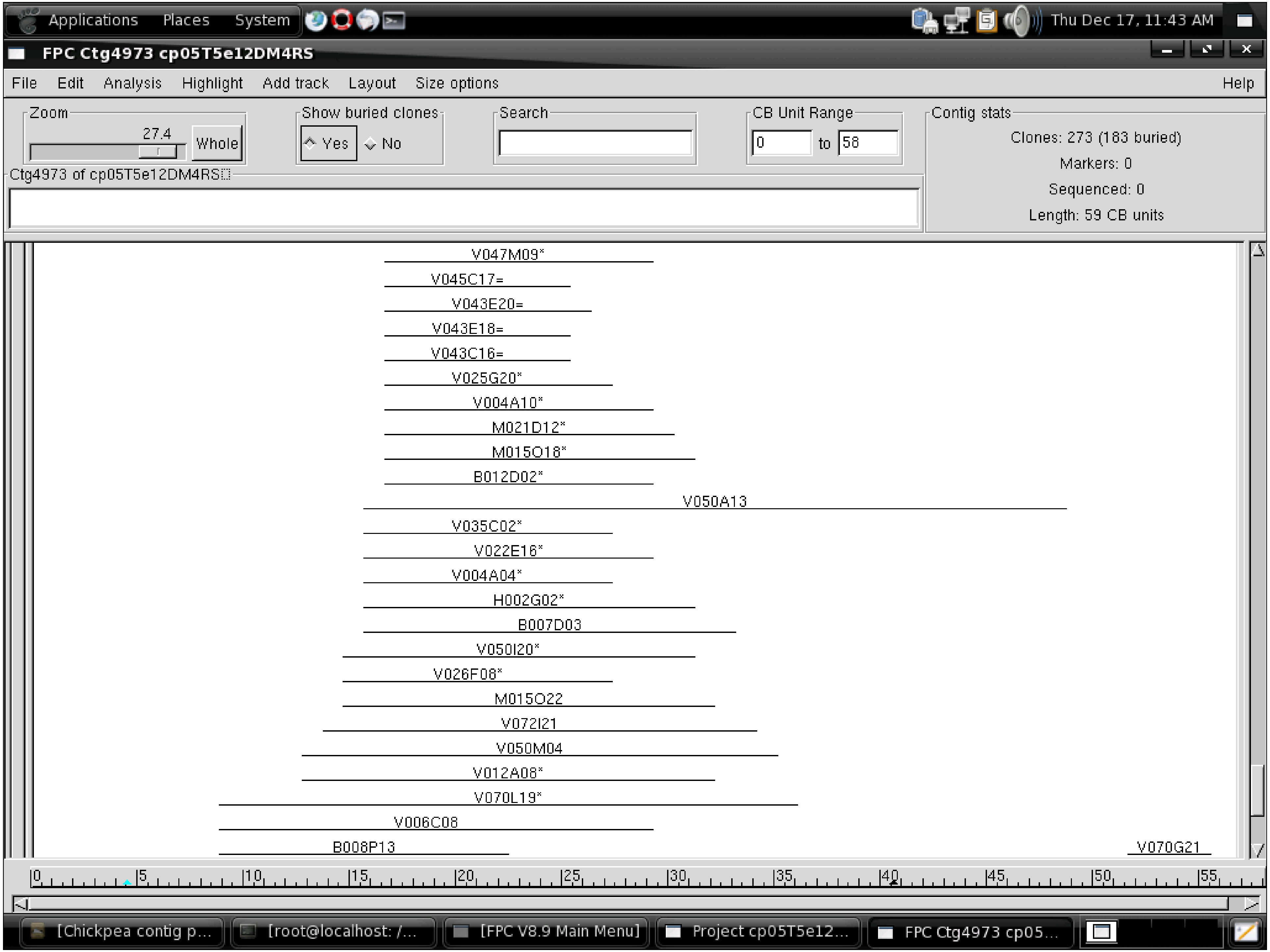
Applications Places System
Thu Dec 17, 11:43 AM
FPC Ctg4973 cp05T5e12DM4RS
File Edit Analysis Highlight Add track Layout Size options Help
Zoom
27.4
Whole
Show buried clones
Yes
No
Search
CB Unit Range
0
to
58
Contig stats
Clones: 273 (183 buried)
Markers: 0
Sequenced: 0
Length: 59 CB units
Ctg4973 of cp05T5e12DM4RS
V047M09*
V045C17=
V043E20=
V043E18=
V043C16=
V025G20*
V004A10*
M021D12*
M015O18*
B012D02*
V050A13
V035C02*
V022E16*
V004A04*
H002G02*
B007D03
V050I20*
V026F08*
M015O22
V072I21
V050M04
V012A08*
V070L19*
V006C08
B008P13
V070G21
0 5 10 15 20 25 30 35 40 45 50 55
[Chickpea contig p...
[root@localhost: /...
[FPC V8.9 Main Menu]
Project cp05T5e12...
FPC Ctg4973 cp05...

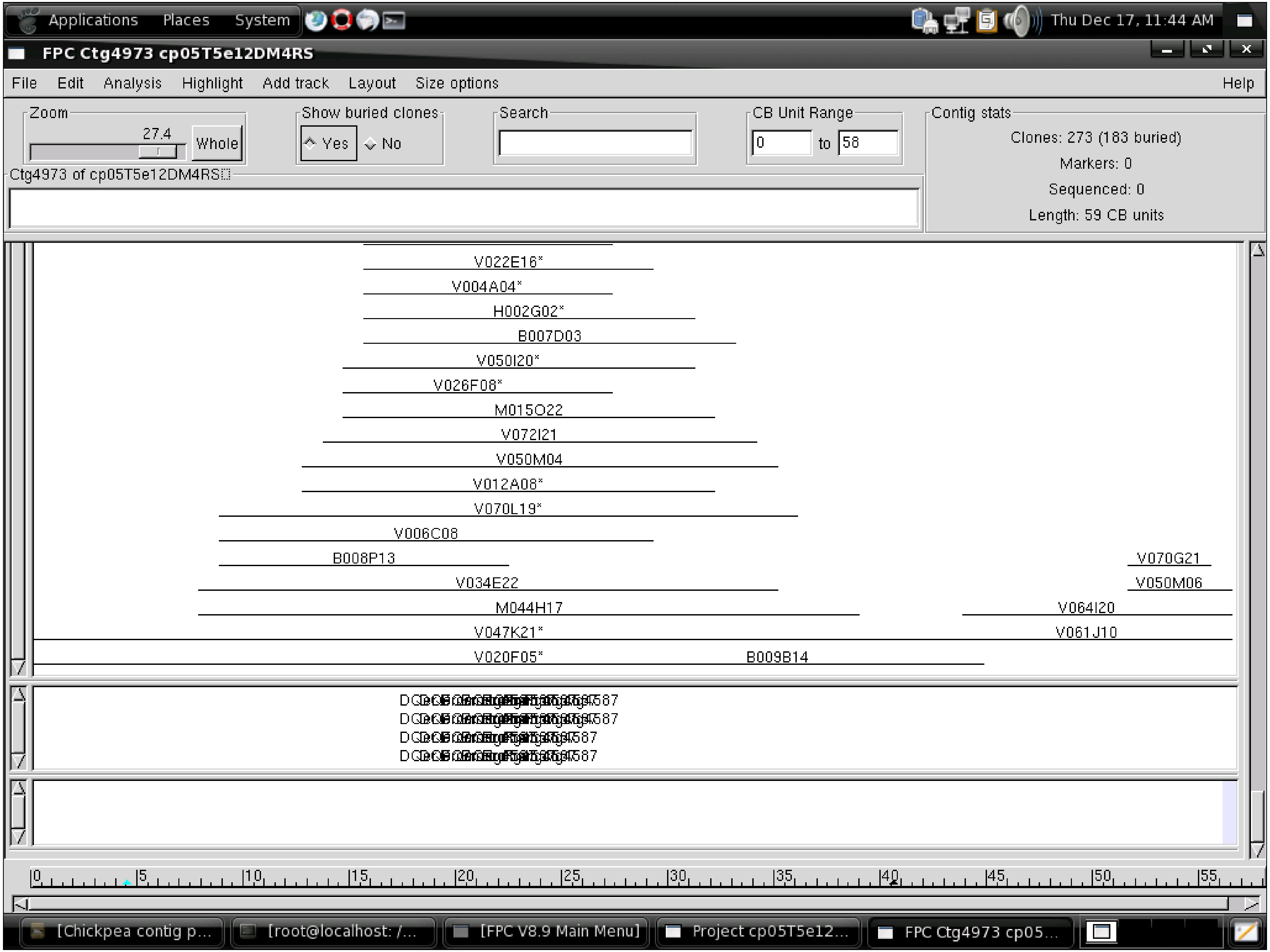
Applications Places System
Thu Dec 17, 11:44 AM
FPC Ctg4973 cp05T5e12DM4RS
File Edit Analysis Highlight Add track Layout Size options Help
Zoom
27.4
Whole
Show buried clones
Yes
No
Search
CB Unit Range
0 to 58
Contig stats
Clones: 273 (183 buried)
Markers: 0
Sequenced: 0
Length: 59 CB units
Ctg4973 of cp05T5e12DM4RS
V022E16*
V004A04*
H002G02*
B007D03
V050I20*
V026F08*
M015O22
V072I21
V050M04
V012A08*
V070L19*
V006C08
B008P13
V070G21
V034E22
V050M06
M044H17
V064I20
V047K21*
V061J10
V020F05*
B009B14
0 5 10 15 20 25 30 35 40 45 50 55
[Chickpea contig p...
[root@localhost: /...
[FPC V8.9 Main Menu]
Project cp05T5e12...
FPC Ctg4973 cp05...

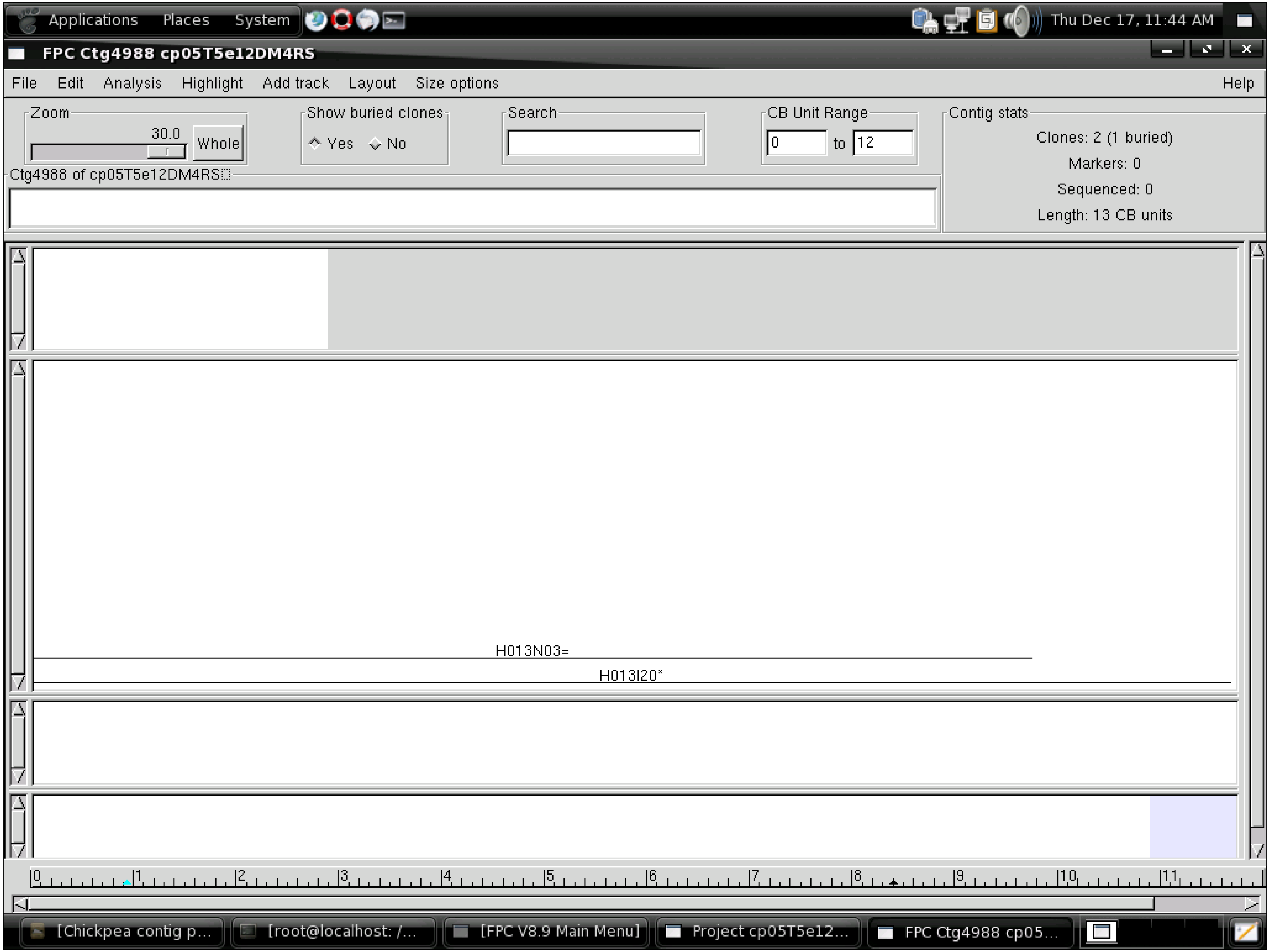
Applications Places System
Thu Dec 17, 11:44 AM
FPC Ctg4988 cp05T5e12DM4RS
File Edit Analysis Highlight Add track Layout Size options Help
Zoom
30.0
Whole
Show buried clones
Yes No
Search
CB Unit Range
0 to 12
Contig stats
Clones: 2 (1 buried)
Markers: 0
Sequenced: 0
Length: 13 CB units
Ctg4988 of cp05T5e12DM4RS
H013N03=
H013I20*
0 1 2 3 4 5 6 7 8 9 10 11
[Chickpea contig p...
[root@localhost: /...
[FPC V8.9 Main Menu]
Project cp05T5e12...
FPC Ctg4988 cp05...

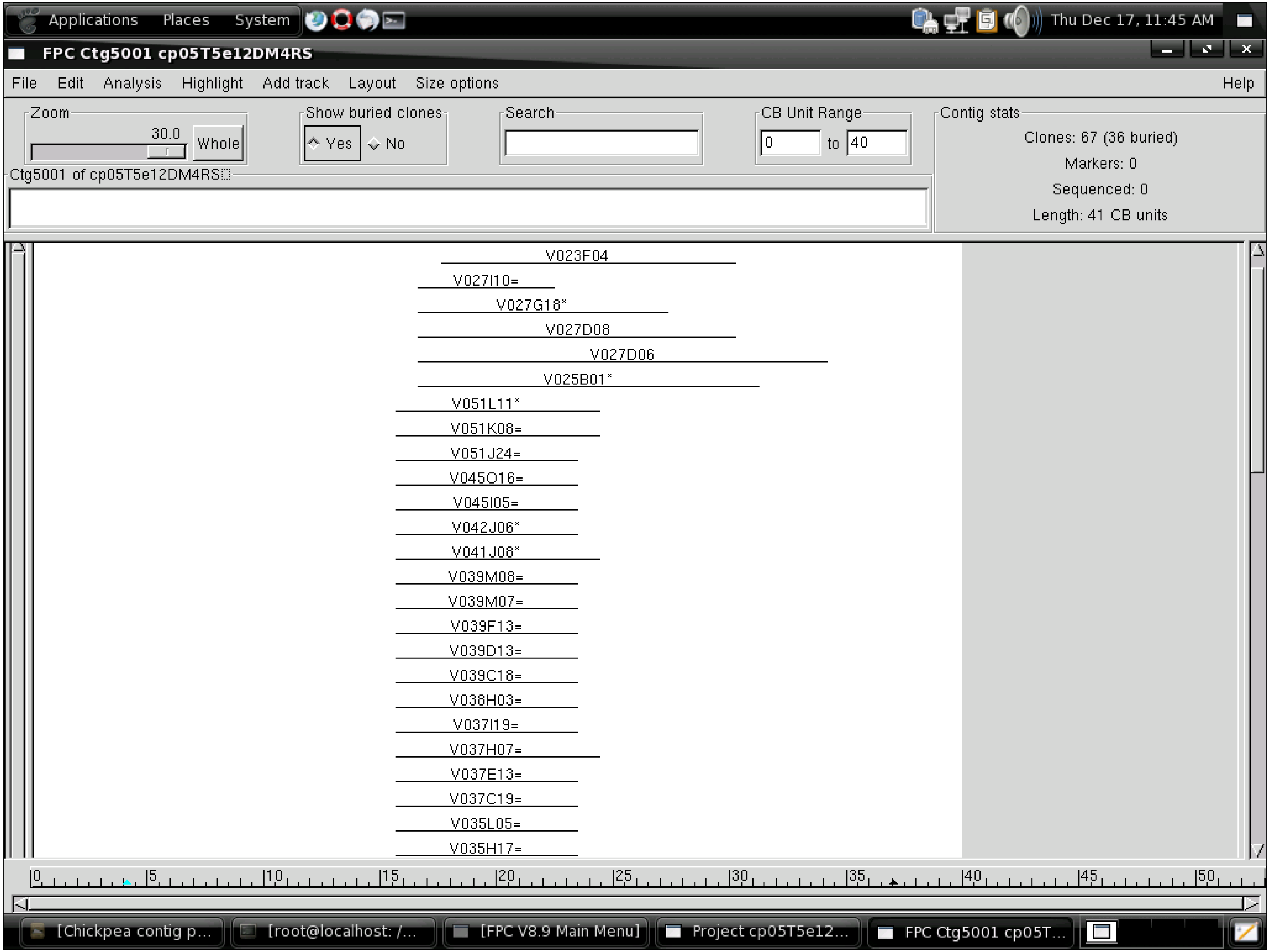
Applications Places System
Thu Dec 17, 11:45 AM
FPC Ctg5001 cp05T5e12DM4RS
File Edit Analysis Highlight Add track Layout Size options Help
Zoom
30.0
Whole
Show buried clones
Yes
No
Search
CB Unit Range
0
to
40
Contig stats
Clones: 67 (36 buried)
Markers: 0
Sequenced: 0
Length: 41 CB units
Ctg5001 of cp05T5e12DM4RS
V023F04
V027I10=
V027G18*
V027D08
V027D06
V025B01*
V051L11*
V051K08=
V051J24=
V045O16=
V045I05=
V042J06*
V041J08*
V039M08=
V039M07=
V039F13=
V039D13=
V039C18=
V038H03=
V037I19=
V037H07=
V037E13=
V037C19=
V035L05=
V035H17=
0
5
10
15
20
25
30
35
40
45
50
[Chickpea contig p...
[root@localhost: /...
[FPC V8.9 Main Menu]
Project cp05T5e12...
FPC Ctg5001 cp05T...

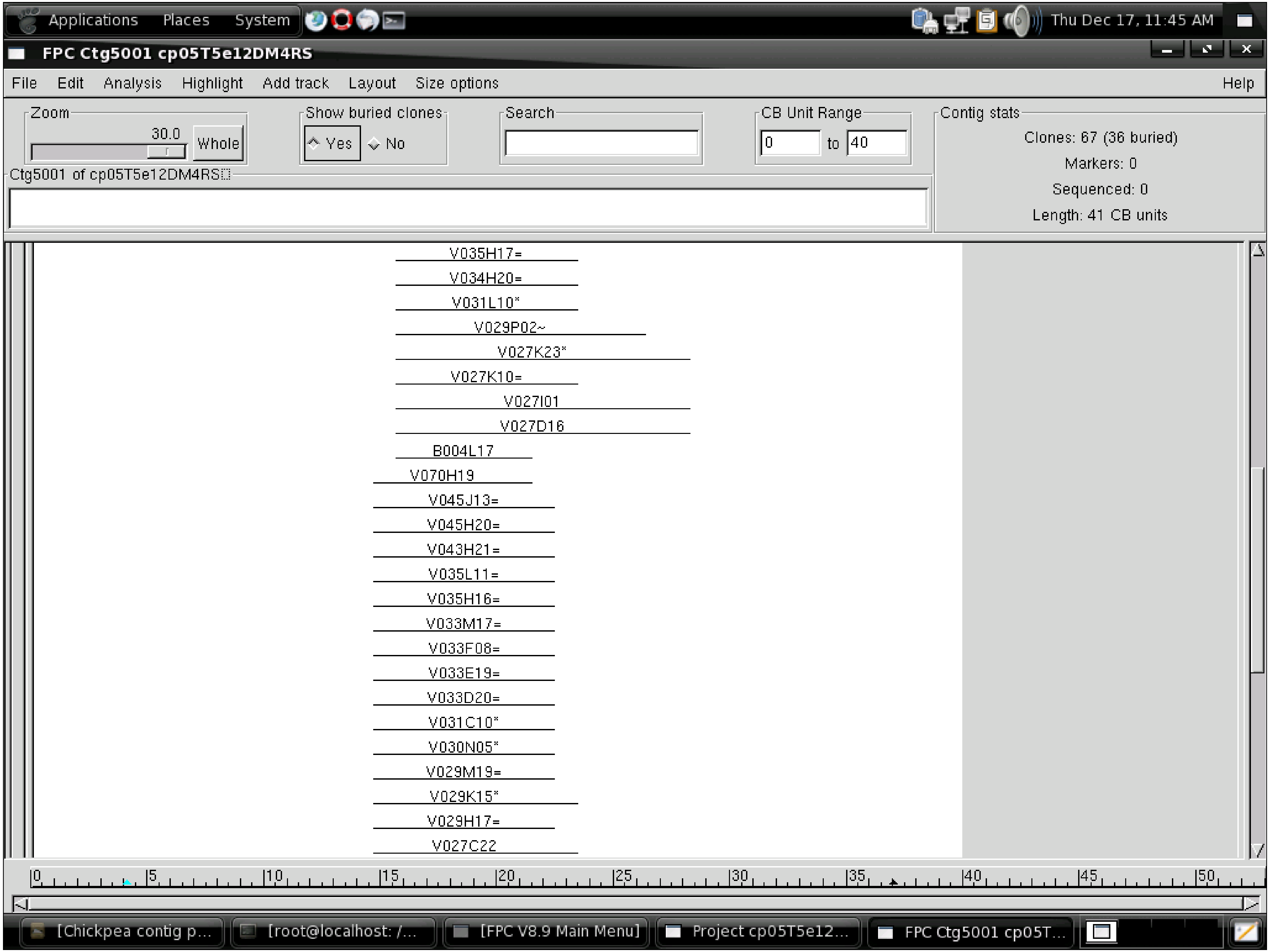
Applications Places System
Thu Dec 17, 11:45 AM
FPC Ctg5001 cp05T5e12DM4RS
File Edit Analysis Highlight Add track Layout Size options Help
Zoom
30.0
Whole
Show buried clones
Yes No
Search
CB Unit Range
0 to 40
Contig stats
Clones: 67 (36 buried)
Markers: 0
Sequenced: 0
Length: 41 CB units
Ctg5001 of cp05T5e12DM4RS
V035H17=
V034H20=
V031L10*
V029P02~
V027K23*
V027K10=
V027I01
V027D16
B004L17
V070H19
V045J13=
V045H20=
V043H21=
V035L11=
V035H16=
V033M17=
V033F08=
V033E19=
V033D20=
V031C10*
V030N05*
V029M19=
V029K15*
V029H17=
V027C22
0 5 10 15 20 25 30 35 40 45 50
[Chickpea contig p...
[root@localhost: /...
[FPC V8.9 Main Menu]
Project cp05T5e12...
FPC Ctg5001 cp05T...

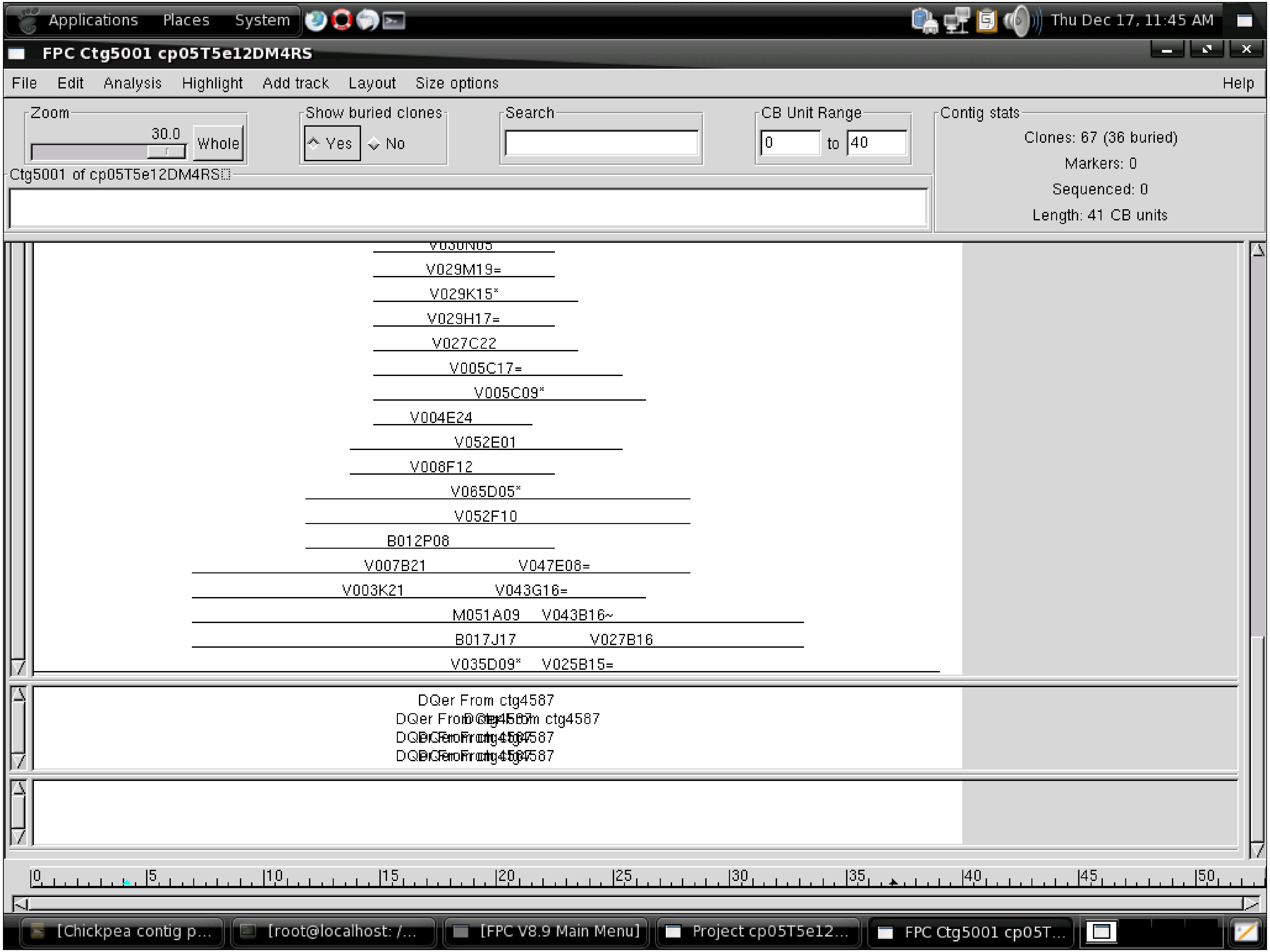
Applications Places System
Thu Dec 17, 11:45 AM
FPC Ctg5001 cp05T5e12DM4RS
File Edit Analysis Highlight Add track Layout Size options Help
Zoom
30.0
Whole
Show buried clones
Yes No
Search
CB Unit Range
0 to 40
Contig stats
Clones: 67 (36 buried)
Markers: 0
Sequenced: 0
Length: 41 CB units
Ctg5001 of cp05T5e12DM4RS
V029M19=
V029K15*
V029H17=
V027C22
V005C17=
V005C09*
V004E24
V052E01
V008F12
V065D05*
V052F10
B012P08
V007B21 V047E08=
V003K21 V043G16=
M051A09 V043B16~
B017J17 V027B16
V035D09* V025B15=
DQer From ctg4587
0 5 10 15 20 25 30 35 40 45 50
[Chickpea contig p...
[root@localhost: /...
[FPC V8.9 Main Menu]
Project cp05T5e12...
FPC Ctg5001 cp05T...

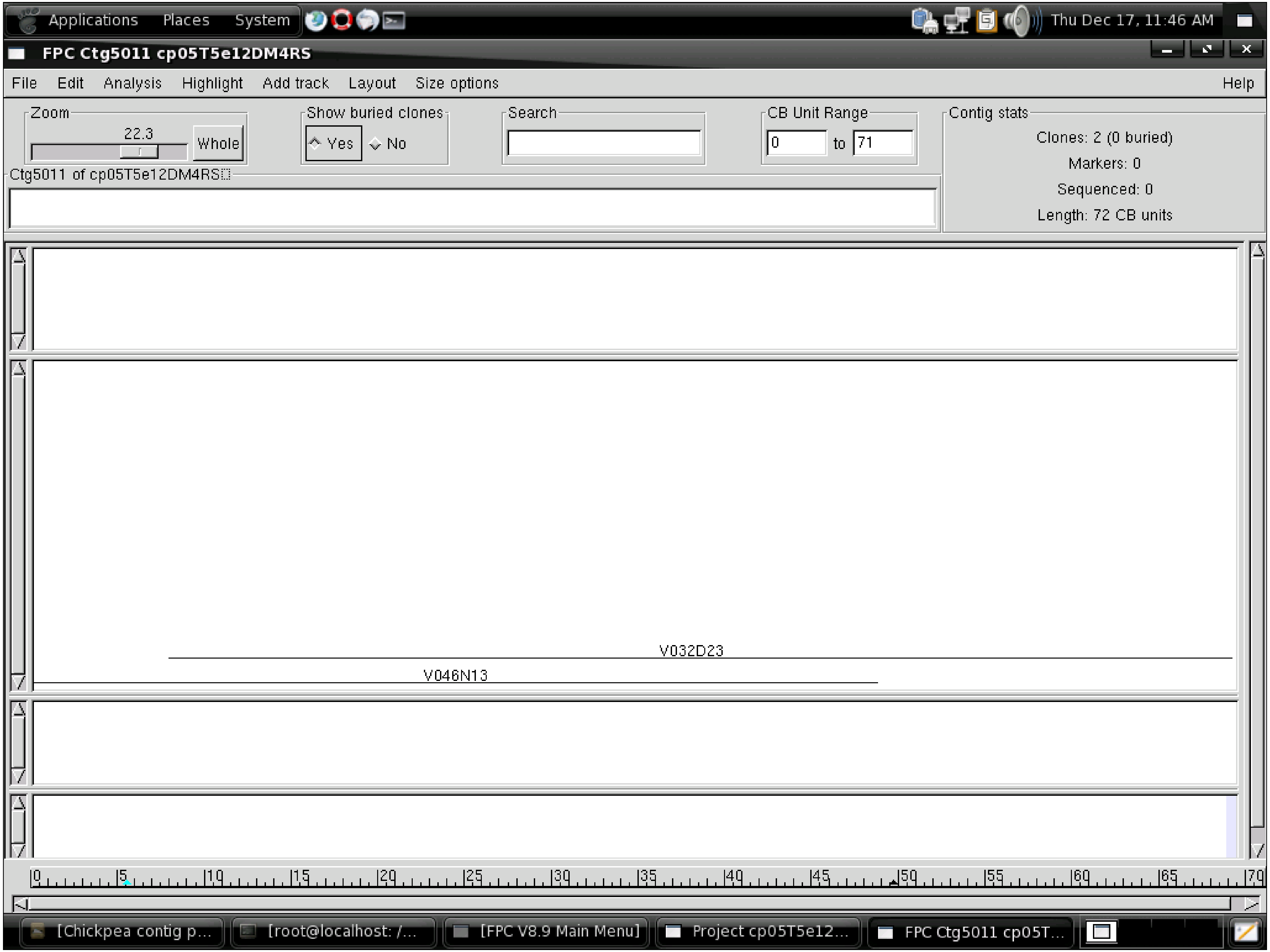
Applications Places System
Thu Dec 17, 11:46 AM
FPC Ctg5011 cp05T5e12DM4RS
File Edit Analysis Highlight Add track Layout Size options Help
Zoom
22.3
Whole
Show buried clones
Yes
No
Search
CB Unit Range
0 to 71
Contig stats
Clones: 2 (0 buried)
Markers: 0
Sequenced: 0
Length: 72 CB units
Ctg5011 of cp05T5e12DM4RS
V032D23
V046N13
0 5 10 15 20 25 30 35 40 45 50 55 60 65 70
[Chickpea contig p...
[root@localhost: /...
[FPC V8.9 Main Menu]
Project cp05T5e12...
FPC Ctg5011 cp05T...

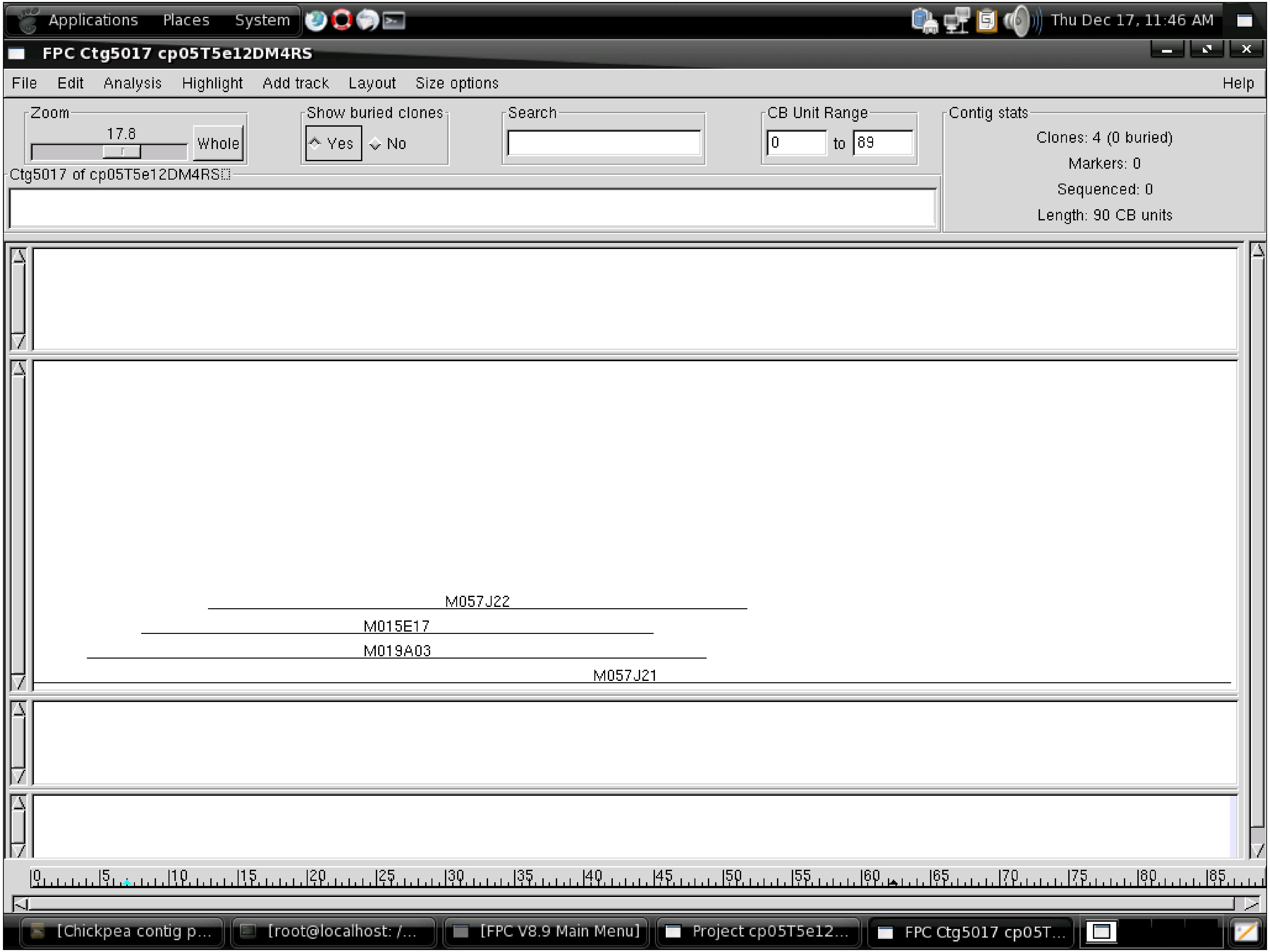
Applications Places System
Thu Dec 17, 11:46 AM
FPC Ctg5017 cp05T5e12DM4RS
File Edit Analysis Highlight Add track Layout Size options Help
Zoom
17.8
Whole
Show buried clones
Yes
No
Search
CB Unit Range
0 to 89
Contig stats
Clones: 4 (0 buried)
Markers: 0
Sequenced: 0
Length: 90 CB units
Ctg5017 of cp05T5e12DM4RS
M057J22
M015E17
M019A03
M057J21
0 5 10 15 20 25 30 35 40 45 50 55 60 65 70 75 80 85
[Chickpea contig p...
[root@localhost: /...
[FPC V8.9 Main Menu]
Project cp05T5e12...
FPC Ctg5017 cp05T...

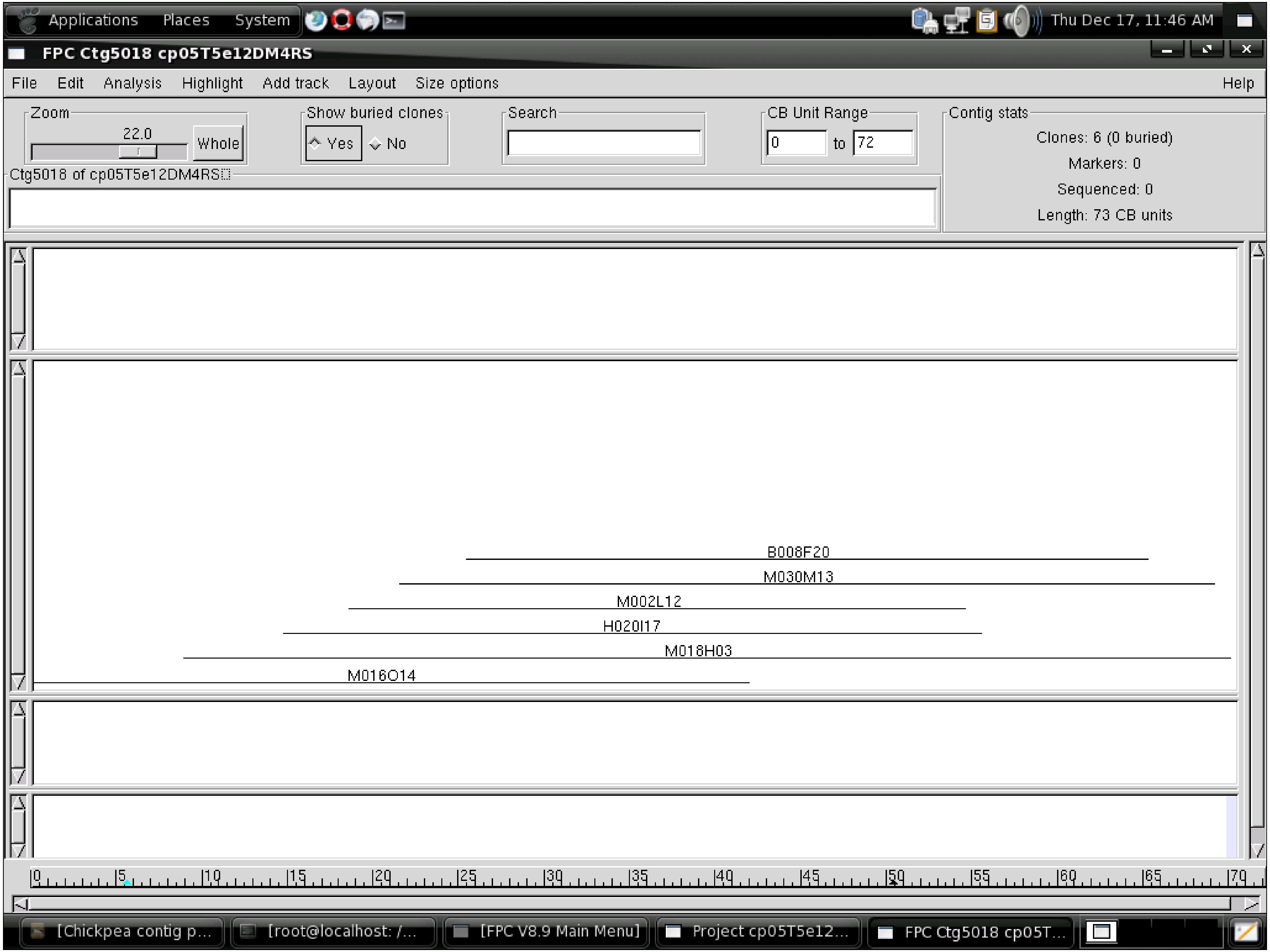
Applications Places System
Thu Dec 17, 11:46 AM
FPC Ctg5018 cp05T5e12DM4RS
File Edit Analysis Highlight Add track Layout Size options Help
Zoom
22.0
Whole
Show buried clones
Yes
No
Search
CB Unit Range
0 to 72
Contig stats
Clones: 6 (0 buried)
Markers: 0
Sequenced: 0
Length: 73 CB units
Ctg5018 of cp05T5e12DM4RS
B008F20
M030M13
M002L12
H020I17
M018H03
M016O14
0 5 10 15 20 25 30 35 40 45 50 55 60 65 70
[Chickpea contig p...
[root@localhost: /...
[FPC V8.9 Main Menu]
Project cp05T5e12...
FPC Ctg5018 cp05T...

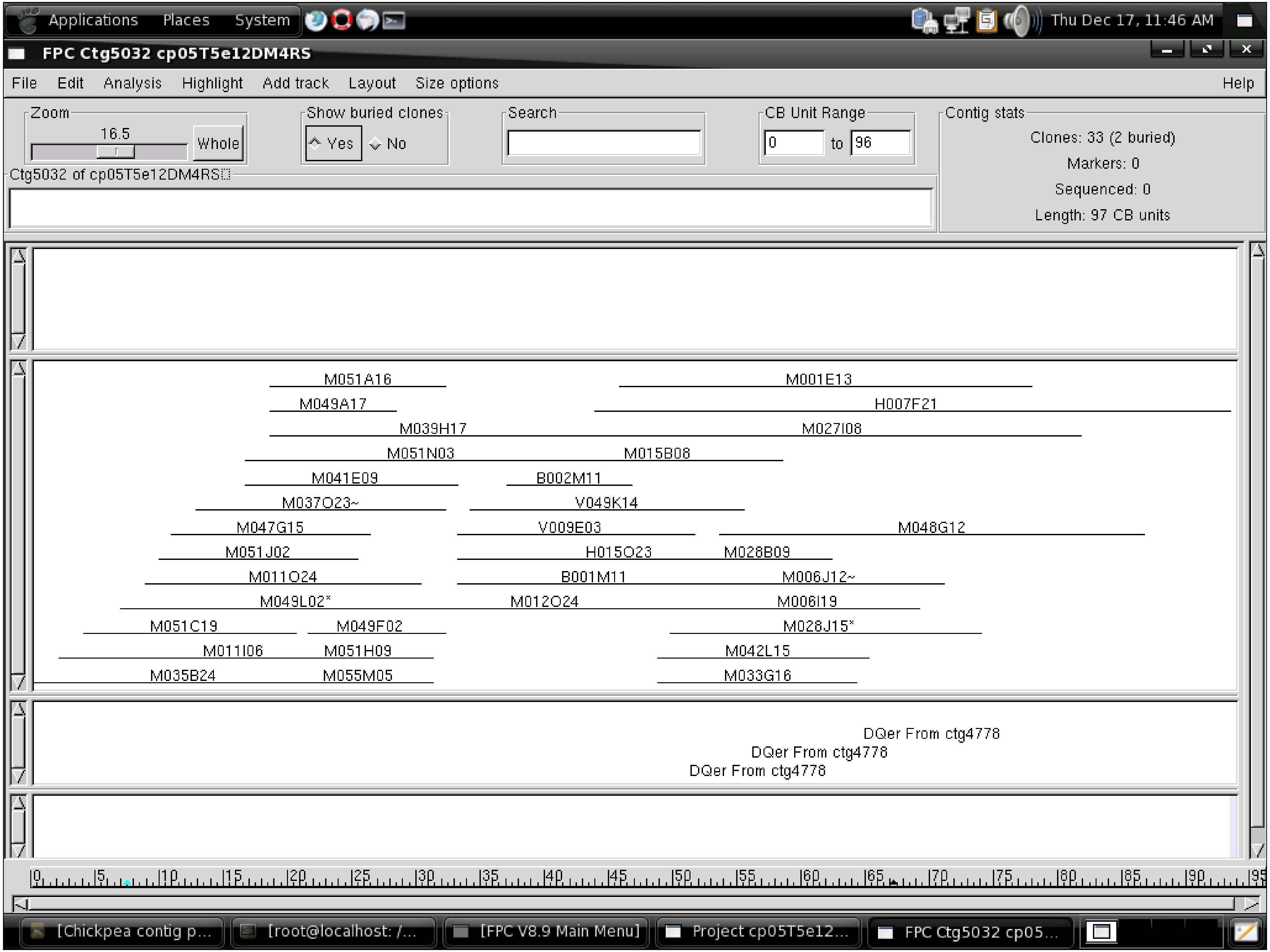
Applications
Places
System
Thu Dec 17, 11:46 AM
FPC Ctg5032 cp05T5e12DM4RS
File
Edit
Analysis
Highlight
Add track
Layout
Size options
Help
Zoom
16.5
Whole
Show buried clones
Yes
No
Search
CB Unit Range
0
to
96
Contig stats
Clones: 33 (2 buried)
Markers: 0
Sequenced: 0
Length: 97 CB units
Ctg5032 of cp05T5e12DM4RS
M051A16
M001E13
M049A17
H007F21
M039H17
M027I08
M051N03
M015B08
M041E09
B002M11
M037O23~
V049K14
M047G15
V009E03
M048G12
M051J02
H015O23
M028B09
M011O24
B001M11
M006J12~
M049L02*
M012O24
M006I19
M051C19
M049F02
M028J15*
M011I06
M051H09
M042L15
M035B24
M055M05
M033G16
DQer From ctg4778
DQer From ctg4778
DQer From ctg4778
0 5 10 15 20 25 30 35 40 45 50 55 60 65 70 75 80 85 90 95
[Chickpea contig p...
[root@localhost: /...
[FPC V8.9 Main Menu]
Project cp05T5e12...
FPC Ctg5032 cp05...

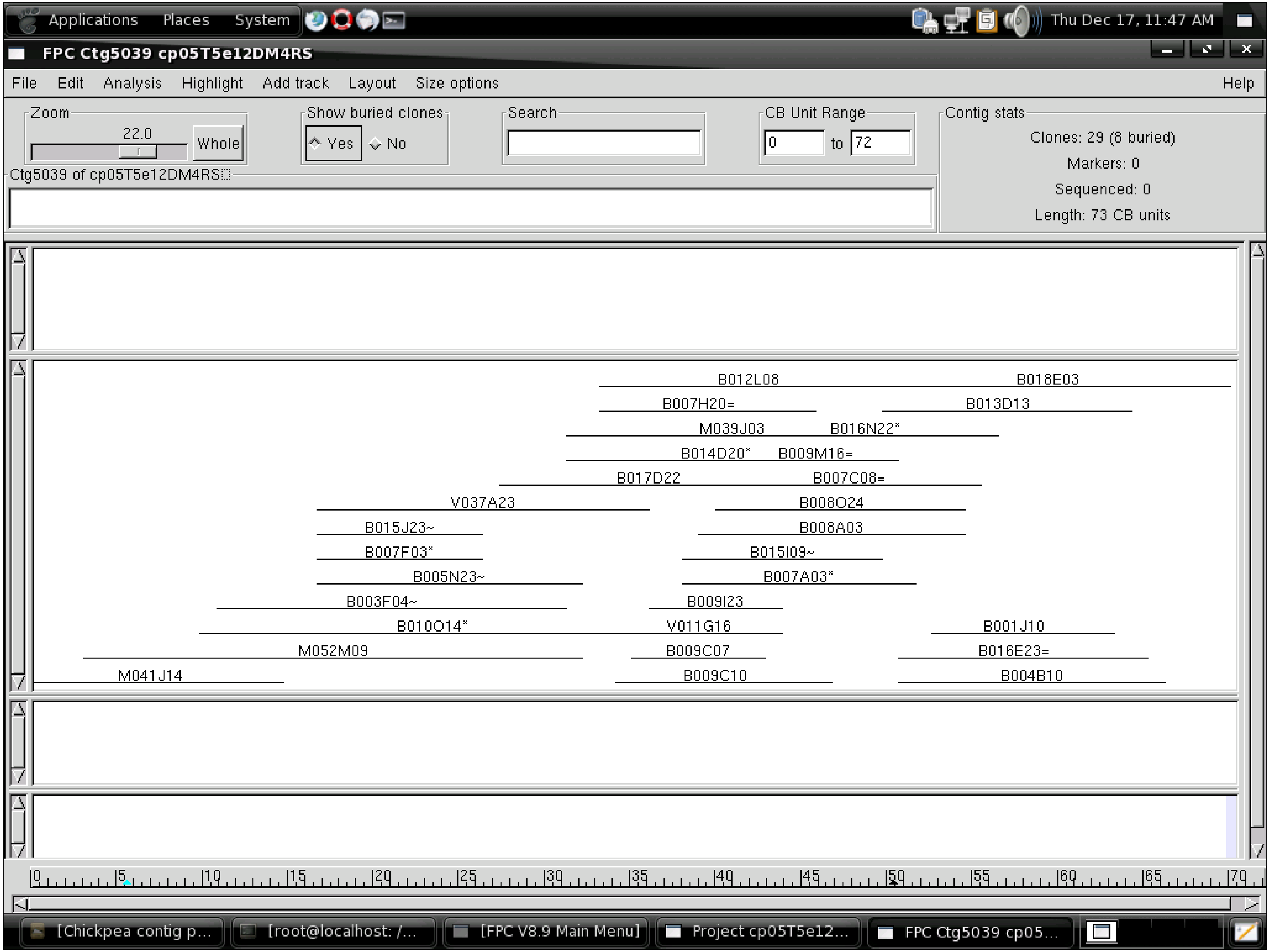
Applications
Places
System
Thu Dec 17, 11:47 AM
FPC Ctg5039 cp05T5e12DM4RS
File
Edit
Analysis
Highlight
Add track
Layout
Size options
Help
Zoom
22.0
Whole
Show buried clones
Yes
No
Search
CB Unit Range
0
to
72
Contig stats
Clones: 29 (8 buried)
Markers: 0
Sequenced: 0
Length: 73 CB units
Ctg5039 of cp05T5e12DM4RS
B012L08
B018E03
B007H20=
B013D13
M039J03
B016N22*
B014D20*
B009M16=
B017D22
B007C08=
V037A23
B008O24
B015J23~
B008A03
B007F03*
B015I09~
B005N23~
B007A03*
B003F04~
B009I23
B010O14*
V011G16
B001J10
M052M09
B009C07
B016E23=
M041J14
B009C10
B004B10
0
5
10
15
20
25
30
35
40
45
50
55
60
65
70
[Chickpea contig p...
[root@localhost: /...
[FPC V8.9 Main Menu]
Project cp05T5e12...
FPC Ctg5039 cp05...

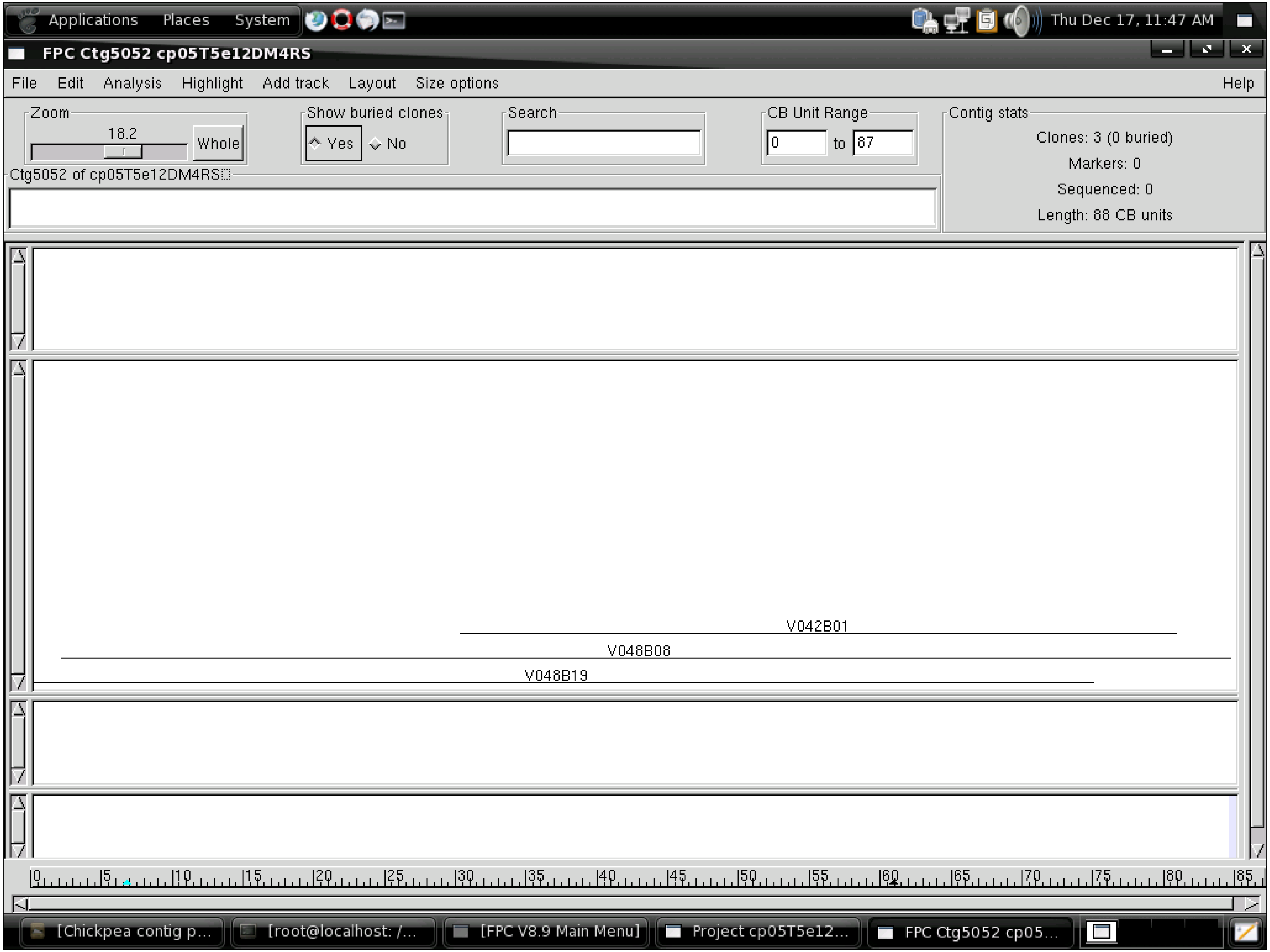
Applications Places System
Thu Dec 17, 11:47 AM
FPC Ctg5052 cp05T5e12DM4RS
File Edit Analysis Highlight Add track Layout Size options Help
Zoom
18.2
Whole
Show buried clones
Yes No
Search
CB Unit Range
0 to 87
Contig stats
Clones: 3 (0 buried)
Markers: 0
Sequenced: 0
Length: 88 CB units
Ctg5052 of cp05T5e12DM4RS
V042B01
V048B08
V048B19
0 5 10 15 20 25 30 35 40 45 50 55 60 65 70 75 80 85
[Chickpea contig p...
[root@localhost: /...
[FPC V8.9 Main Menu]
Project cp05T5e12...
FPC Ctg5052 cp05...
